# Genome-wide classification, evolutionary analysis and gene expression patterns of the kinome in *Gossypium*

**DOI:** 10.1371/journal.pone.0197392

**Published:** 2018-05-16

**Authors:** Jun Yan, Guilin Li, Xingqi Guo, Yang Li, Xuecheng Cao

**Affiliations:** 1 College of Information Science and Engineering, Shandong Agricultural University, Tai’an, Shandong, PR China; 2 College of Life Sciences, Shandong Agricultural University, Tai’an, Shandong, PR China; USDA-ARS Southern Regional Research Center, UNITED STATES

## Abstract

The protein kinase (PK, kinome) family is one of the largest families in plants and regulates almost all aspects of plant processes, including plant development and stress responses. Despite their important functions, comprehensive functional classification, evolutionary analysis and expression patterns of the cotton PK gene family has yet to be performed on PK genes. In this study, we identified the cotton kinomes in the *Gossypium raimondii*, *Gossypium arboretum*, *Gossypium hirsutum* and *Gossypium barbadense* genomes and classified them into 7 groups and 122–24 subfamilies using software HMMER v3.0 scanning and neighbor-joining (NJ) phylogenetic analysis. Some conserved exon-intron structures were identified not only in cotton species but also in primitive plants, ferns and moss, suggesting the significant function and ancient origination of these PK genes. Collinearity analysis revealed that 16.6 million years ago (Mya) cotton-specific whole genome duplication (WGD) events may have played a partial role in the expansion of the cotton kinomes, whereas tandem duplication (TD) events mainly contributed to the expansion of the cotton RLK group. Synteny analysis revealed that tetraploidization of *G*. *hirsutum* and *G*. *barbadense* contributed to the expansion of *G*. *hirsutum* and *G*. *barbadense* PKs. Global expression analysis of cotton PKs revealed stress-specific and fiber development-related expression patterns, suggesting that many cotton PKs might be involved in the regulation of the stress response and fiber development processes. This study provides foundational information for further studies on the evolution and molecular function of cotton PKs.

## Introduction

The protein kinase (PK, kinome) family is one of the largest families in plants and regulates many signaling pathways that are triggered during stress and development processes [1]. The common structural feature of PKs is their 250–300 amino acid catalytic domain, which uses the γ-phosphate of adenosine triphosphate (ATP) to phosphorylate serine, threonine or tyrosine residues of proteins [2]. Phylogenetic analyses of the PK family were initially conducted using the conserved feature of the catalytic domain [3]. Based on analysis of the hundreds of known kinase domain sequences at that moment, the PK family was classified into five major groups: (1) AGC, which mainly includes PKA (cyclic AMP-dependent protein kinase), PKG (cyclic GMP-dependent protein kinase) and PKC (protein kinase C); (2) CAMK, which mainly includes CDPK (calcium- and calmodulin-regulated kinase) and Snfl/AMPK (adenosine 5'-monophosphate (AMP)-activated protein kinase); (3) CMGC, which mainly includes CDKs (cyclin-dependent kinases), MAPK (mitogen-activated protein kinases), GSK3 (glycogen synthase kinase) and Clk (cyclin-dependent-like kinases); (4) PTK (protein-tyrosine kinase); (5) others [2]. In 2002, Manning analyzed the evolution of sequences from yeast to human and further divided Metazoan PKs into eight major groups: AGC, CAMK, CK1 (casein kinase 1), CMGC, STE (sterile), TK (tyrosine kinase), TKL (tyrosine kinase-like kinase) and others [4]. Recently, PKs from 25 plant genomes were identified and classified into nine main groups: AGC, CMGC, CAMK, CMGC, CK1, STE, TKL, RLK (receptor-like kinase) and others [5]. *Arabidopsis* PKs were identified and classified into six large groups: RLK (620), CMGC (65), CaMK (89), AGC (43), STE (67) and Raf (52) [6]. Gene duplication events and functional diversification of the *Arabidopsis* kinome were further studied [7]. Identification, functional classification and expression analysis of the soybean kinome were performed by Liu et al. [8]. They demonstrated that whole genome duplication (WGD) events might play a vital role in soybean PK expansion, whereas tandem duplication (TD) events might only contribute to the expansion of certain soybean PK subfamilies.

Numerous studies demonstrated that the expansion of plant PKs is mainly due to the expansions of certain RLK subfamilies [9–12]. Lehti-Shiu and Shiu also demonstrated that each flower plant kinome is significantly larger than other eukaryotes’ kinomes mainly due to the expansion of certain RLK subfamilies [5]. In 2001, Shiu and Bleecker performed a genome-wide identification and phylogenetic analysis of *Arabidopsis* RLKs [13]. Then, they analyzed the expansion mechanism of *Arabidopsis* RLKs and found that the RLKs ancient origin could be attributed to a more recent plant-specific expansion [9]. By comparing rice (*Oryza sativa*) RLKs with *Arabidopsis* RLKs, they found that *Os*RLKs involved in development have rarely undergone duplications after the rice-*Arabidopsis* split, whereas *Os*RLKs involved in defense resistance have undergone more duplications [10]. In 2008, Vij et al. performed identification and phylogenetic analysis of *Os*RLCKs (receptor-like cytoplasmic kinase, subfamily of RLKs) and analyzed the expression patterns of certain *Os*RLCKs involved in development and stress [14]. The expression analysis of rice RLKs was studied in different tissues, with the emphasis on seed development and abiotic stress response [11]. Evolutionary analysis indicated that the expansion of RLK family coincided with the establishment of land plants, and expression analysis of *Arabidopsis* RLKs supported the importance of RLKs in biotic stress response [15]. In addition, phylogenetic analysis and expression profile analysis of the LRRs (leucine-rich repeat, subfamily of RLKs) were performed in *Populus trichocarpa* [16], *Platanus × acerifolia* [17], *Brassica rapa* [18], *Arabidopsis* [19], tomato [20] and cotton [21]. Based on phylogenetic analysis of the RLK-LRR genes from 31 sequenced flower plants, Fischer et al. found that subgroup- and species-specific expansion rates differ significantly due to the complex history of expansion-retention-loss cycles and whole genome multiplication [22].

Functional characterization studies of cotton PKs indicated that cotton mitogen-activated protein kinases (MAPKs) are involved in abiotic and biotic stress responses [23–26], and cotton RLKs function in fiber development [27], anther development [28], disease resistance [29] and drought-stress tolerance [30]. The completion of cotton genome sequencing provides an opportunity to perform genome-wide research on cotton PKs. In this study, we identified the putative PK genes in *Gossypium raimondii*, *Gossypium arboretum*, *Gossypium hirsutum* and *Gossypium barbadense* and classified them into groups, families and subfamilies. Some conserved exon-intron structures were identified not only in cotton species but also in primitive plants fern and moss, suggesting that these subfamilies may play important and conserved roles during plant evolutionary processes. Analyses of chromosome location and collinearity events were combined to explore the relationship between expansion and duplication events (WGD, TD and synteny analysis). Expression profiles in developing fibers and under abiotic and biotic stresses were assessed in *G*. *hirsutum* and *G*. *barbadense* PKs. This work will help to understand the cotton PK evolution mechanism and provide a starting point for further experimental research.

## Materials and methods

### Identification and classification of cotton PKs

The genome and proteome sequences of *G*. *raimondii* were downloaded from Phytozome V10.0 (http://phytozome.jgi.doe.gov/pz/portal.html). The genome and proteome sequences of *G*. *arboretum* (V2.0) and *G*. *hirsutum* (V1.0) were downloaded from the Cotton Genome Project (CGP: http://cgp.genomics.org.cn/). The genome and proteome sequences of *G*. *barbadense* were downloaded from the website CHGC (http://database.chgc.sh.cn/cotton/index.html). HMMER v3.0 [31] in our local server and Pfam 28.0 in batch (http://pfam.xfam.org/) with an E-value of less than 0.01 were performed against all proteomes. The Pfam profiles PF00069 (Pkinase domain) and PF07714 (Pkinase_Tyr domain) were used in HMMER. After the initial screen, putative typical PKs were retained only if Pkinase or Pkinase_Tyr domain alignments covered greater than 50%, as previously described by Legti-Shiu and Shui [5]. Classifications of "typical" cotton PKs were performed using the HMM models developed by Legti-Shiu and Shui [5]. The classifications were further confirmed by phylogenetic analysis using the neighbor-joining (NJ) method. The truncated sequences of the Pkinase or Pkinase_Tyr domain were aligned using ClustalW (v2.0) [32]. The NJ phylogenetic analysis with the p-distance model and 1000 bootstrap repetitions was constructed by MEGAcc 7.0 [33] in our local server. The classification information of *Vitis vinifera*, *Arabidopsis thaliana*, *Carica papaya*, *Zea mays* and *Oryza sativa* PKs was extracted from the supplemental files of Legti-Shiu and Shui [5].

### Chromosome location and synteny analysis of cotton PKs

The chromosome location information of *G*. *raimondii*, *G*. *arboretum*, *G*. *hirsutum* and *G*. *barbadense* was extracted from GFF files of Phytozome, CGP and CHGC using our perl script. A BLAST search was performed against all the cotton PKs with an E-value of 1e-100. Based on the BLAST and GFF results, the segmental/tandem duplication events of *G*. *raimondii*, *G*. *arboretum*, *G*. *hirsutum* and *G*. *barbadense* were determined using MCScanX software [34]. The "add_ka_and_ks_to_collinearity.pl" of MCScanX was used to calculate the synonymous (*K*_*s*_) and non-synonymous substitution (*K*_*a*_) rates. The collinearity visualization of duplicated PKs was performed using GenomePixelizer [35]. The chromosomal location of identified tandem PKs was mapped on each chromosome with Mapchart v2.3 (http://www.wageningenur.nl/en/show/mapchart.htm). The synteny blocks between *G*. *arboretum* and *G*. *hirsutum* At-subgenome, *G*. *raimondii* and *G*. *hirsutum* Dt-subgenome; *G*. *arboretum* and *G*. *barbadense* At-subgenome; *G*. *raimondii* and *G*. *barbadense* Dt-subgenome; and *G*. *raimondii* and *G*. *arboretum* were identified by MCScanX [34]. All paralogous and orthologous PK pairs were visualized using the Circos program [36].

### Domain and exon-intron structure search of cotton PKs

The classifications of *V*. *vinifera*, *A*. *thaliana*, *O*. *sativa*, *Selaginella moellendorffii*, *Physcomitrella patens* and *Chlamydomonas reinhardtii* PKs were extracted from Legti-Shiu and Shui [5]. Exon-intron structure diagrams of these six plants and four cotton species were generated by our perl and R scripts based on extracting information from Phytozome, CGP and CHGC GFF files. The domain information was obtained from pfam 28.0 in batch (http://pfam.xfam.org/).

### Microarray expression data analysis

Public cotton expression datasets were retrieved from the Gene Expression Omnibus (GEO) of NCBI. Microarray datasets for stress treatments and fiber development stages were selected from the Affymetrix Cotton Genome Array platform GPL8672. (1) Abiotic stresses: ① Five abiotic stresses: GSE50770, cotton microarray-based datasets under ABA, cold, drought, salinity and alkalinity treatments [37]. ② Heat stress: GSE41725, *G*. *hirsutum* (cultivar: Sicala 45 and Sicot 53) in heat tolerance (32°C and 42°C). ③ Waterlog and drought stresses: GSE16467, flooded-stress *G*. *hirsutum* root and leaf tissues [38]. GSE29810 (This SuperSeries is composed of GSE29566 and GSE29567), *G*. *hirsutum* under drought stress in leaf tissue and during fiber development stages (0, 5, 10, 15, and 20 dpa) [39]. (2) Biotic stresses: ① *Alternaria alternata* disease: GSE74412, *G*. *hirsutum* leaf tissues under *A*. *alternata* infected stress with or without chilling pre-treatment. ② Bollworm infection: GSE55511, *G*. *hirsutum* during boll development stages (0, 2, 5 and 10 dpa (days post anthesis) under bollworm-infected biotic stress. (3) Fiber development: GSE36228, cotton fiber at different developmental stages (0, 6, 9, 12, 19 and 25 dpa) from five *G*. *hirsutum* varieties (JKC 725 and JKC 777 were superior in fiber traits compared with JKC 703, JKC 737 and JKC 783) [40]. GSE36021, *GhHD-1*-silenced, *GhHD-1-*over-expression and wild-type cotton lines [41]. GSE38490, fiber bearing and fuzzless-lintless mutant *G*. *hirsutum* during fiber development stages (0, 5, 10, 15 and 20 dpa) [42]. The raw data were normalized using RMAexpress v1.2.0 (http://www.rmaexpress.bmbolstad.com/). The normalization of raw data and quality control were performed by RMAexpress v1.2.0 (http://www.rmaexpress.bmbolstad.com/). The expression levels (treatment vs. control, log2 value of fold change) of cotton PKs for stress treatments and fiber development were calculated using R software and R package limma. Heat maps of cotton PK expression levels were generated by Mev4.9 [43].

### RNA-seq expression data analysis

Public cotton expression datasets were retrieved from the Sequence Read Archive (SRA) of NCBI. The RNA-seq data of two commercial cotton species, *G*. *barbadense* and *G*. *hirsutum*, in fiber developmental stages (10, 15, 18, 21, and 28 dpa) were used (PRJNA263926). The RNA-seq data of *G*. *hirsutum* Li1 mutant and wild type in leaf tissue and ovules of different fiber developmental stages (1, 3, and 8dpa) were used (PRJNA301536). Quality control assessment of raw data was performed using FastQC. High-quality RNA-seq reads were aligned to reference cotton genomes by software Hisat2. The counts of expression genes were performed using Samtools and HTseq software. The reads per kilobase of exon model per million mapped reads (RPKM) algorithm was used to normalize the data. The expression levels (PRJNA301536, Li1 mutant vs. wild type, log2 value of fold change) of cotton PKs for fiber development was calculated using R software and R package DESeq2. Heat maps of cotton PK expression levels were generated using Mev4.9 [43].

## Results

### Genome-wide identification and classification of cotton PKs

**Fig 1:**
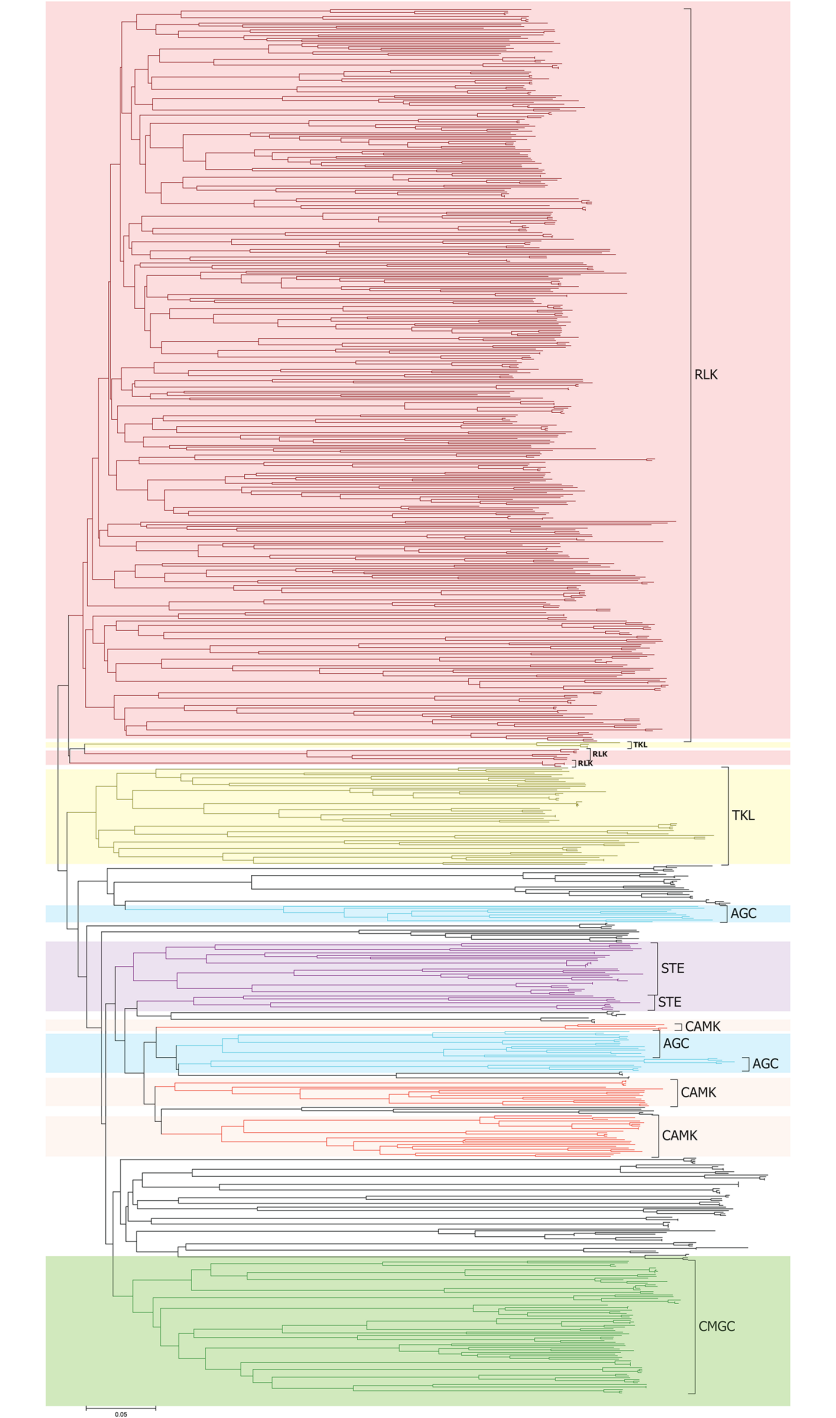
Classification and phylogenetic relationships of the cotton PK subfamilies. The Neighbor-Joining tree was built by the amino acid sequences of the kinase domain using the MEGAcc 7.0 with the p-distance model. Random representative PKs of each subfamily are selected by following criteria: members ≤ 6, 1 PK; 6 < members ≤ 30, 2 PKs; members > 30, 3 PKs. Six major groups are labeled with different colors, including AGC, CAMK, CMGC, RLK, STE, TKL. Detailed information is provided in S1A Fig.

We identified 1517, 1407, 2508 and 2745 PK genes with typical kinase domains (S1 Table) (see Method) and excluded 70, 147, 363 and 390 sequences with atypical kinase domains (domain alignments covered less than 50% of Pfam domain models) in *G*. *raimondii*, *G*. *arboretum*, *G*. *hirsutum* and *G*. *barbadense*, respectively (S2 Table). The classifications of these cotton PKs were performed against HMM models developed by Legti-Shiu and Shui [5]. These genes were classified into 122–124 subfamilies (S1 Table). To validate the classification by HMMER, we selected 1–3 random genes from each subfamily as representatives to construct a NJ phylogenetic tree using the truncated kinase domain sequences with the p-distance model and 1000 bootstraps (Fig 1, Figure A in S1 File). Interestingly, almost all the cotton PKs belonging to the same clade indicated the same subfamily classification as identified by HMMER. We also constructed the NJ tree based on all the cotton PK members (Figure B in S1 File). In summary, cotton PKs were classified into seven groups: RLK (*G*. *raimondii* 1019, *G*. *arboretum* 913, and *G*. *hirsutum* 1681, and *G*. *barbadense* 1855), AGC (52, 55, 81, 96), CAMK (114, 113, 189, 207), CMGC (105, 100, 180, 182), STE (60, 59, 103, 112), TKL (80, 80, 130, 143), and others (87, 87, 144, 150). These seven groups were further classified into 123, 123, 124 and 123 subfamilies in *G*. *raimondii*, *G*. *arboretum*, *G*. *hirsutum* and *G*. *barbadense*, respectively. However, each subfamily member size was highly variable. The RLK group contained 57–58 subfamilies and formed two subgroup clusters, which are leucine-rich repeat (LRR) and receptor-like cytoplasmic kinase (RLCK). The RLK-DLSV subfamily contained the largest members (129, 93, 205, 225) among the cotton PK subfamilies.

To comprehensively study the cotton PK classifications, we compared the PK distributions of four cotton species with three eudicots (*V*. *vinifera*, *A*. *thaliana*, and *C*. *papaya*) and two monocots (*Z*. *mays* and *O*. *sativa*) (S3 Table). First, the "one-two-four" pattern was found to be existed in cotton PK subfamily member size comparing with grape. For instance, the RLK/Pelle_RLCK-XI subfamily of *V*. *vinifera*, *G*. *raimondii*, *G*. *arboretum*, *G*. *hirsutum* and *G*. *barbadense* contained 2, 4, 4, 8 and 6 members, respectively. Similar examples were also found in other PK subfamilies, such as AGC_Pl, AGC_RSK-2, CAMK_CAMKL-CHK1 and CAMK_CDPK. This expansion pattern could be explained by the report indicating that cotton has experienced only one cotton-specific WGD event after it split from the resemble-grape ancestor [44]. Moreover, tetraploidization of *G*. *hirsutum* and *G*. *barbadense* also contributed to the "one-two-four" expansion pattern [45–46]. Second, some species-specific expansions were identified in some PK subfamilies. For example, cotton RLK/Pelle_CrRLK1L-1 (*G*. *raimondii* 55, *G*. *arboretum* 56, *G*. *hirsutum* 93 and *G*. *barbadense* 103) contained more members compared with *V*. *vinifera* (9), suggesting that it potentially experienced cotton-specific expansion during the evolutionary process. Similar examples were also identified in RLK/Pelle_L-LEC, RLK/Pelle_LRK10L-2 and RLK/Pelle_WAK. One monocot-specific PK subfamily, CMGC_CDKL-Os, was absent in all the six investigated eudicots but was present in the two monocots (*Z*. *mays* and *O*. *sativa*). RLK/Pelle_URK-4 had only one member in *V*. *vinifera*, *A*. *thaliana*, and *C*. *papaya*, separately, but was absent in four cottons and two monocots (*Z*. *mays* and *O*. *sativa*). These results suggest that it might arise in an eudicot ancestor after the monocot-eudicot split but was lost in cotton evolution. RLK/Pelle_LRR-I-1 contained more members in in *O*. *sativa* (34) and *A*. *thaliana* (48) compared with other investigated plants (8–22), implying that it might experience rice- and *Arabidopsis*-specific expansions. RLK/Pelle_DLSV contained the largest members among PK subfamilies in all the nine investigated plants.

### Conserved domains and intron-exon structures of cotton PKs

Based on the Pfam information, we diagrammed the domain distributions of all the putative cotton PKs (*G*. *raimondii* 1517, *G*. *arboretum* 1407, *G*. *hirsutum* 2508 and *G*. *barbadense* 2745) (S2 Fig). Our results indicated that approximately half of cotton PKs exclusively contained the kinase domain, including *G*. *raimondii* (662, 43.6%), *G*. *arboretum* (669, 47.5%), *G*. *hirsutum* (1217, 48.5%) and *G*. *barbadense* (1247 45.4%). The other PKs contained additional domains in addition to the kinase domain. We calculated the proportion of PKs with additional domains in PK groups, including AGC (*G*. *raimondii* 36.5%, *G*. *arboretum* 38.2%, *G*. *hirsutum* 33.3% and *G*. *barbadense* 38.5%); CAMK (*G*. *raimondii* 72.8%, *G*. *arboretum* 73.5%, *G*. *hirsutum* 74.6% and *G*. *barbadense* 69.6%); CMGC (*G*. *raimondii* 1.9%, *G*. *arboretum* 2%, *G*. *hirsutum* 1.7% and *G*. *barbadense* 10.4%); RLK-Pelle (*G*. *raimondii* 68.5%, *G*. *arboretum* 63.3%, *G*. *hirsutum* 61.3% and *G*. *barbadense* 64.4%); STE (*G*. *raimondii* 0%, *G*. *arboretum* 1.7%, *G*. *hirsutum* 1% and *G*. *barbadense* 3.6%) and TKL (*G*. *raimondii* 58.8%, *G*. *arboretum* 57.5%, *G*. *hirsutum* 58.5% and *G*. *barbadense* 53.8%). These results demonstrate that various subfamilies contained various domain compositions. By contrast, PKs of the same subfamily shared similar domain arrangements, suggesting that they might originate from a common ancestor through domain fusion between the kinase domain and additional domains. We identified some special PKs containing 2–4 PK kinase domains (*G*. *raimondii* 58, *G*. *arboretum* 63, *G*. *hirsutum* 115 and *G*. *barbadense* 175, S4 Table). Most of these genes belonged to the AGC_NDR, AGC_RSK-2, and CMGC_SRPK subfamilies and RLK group.

To gain further insights into the cotton PKs, we generated the exon-intron structures within the kinase domain in four cotton species (S3 Fig). Interestingly, cotton PKs belonging to the same subfamily contained similar exon-intron structures within the conserved exon phases, especially in the kinase domain. For example, AGC_ NDR sequences in *G*. *raimondii* (Gorai.011G175100.1), *G*. *arboretum* (Cotton_A_39141), *G*. *hirsutum* (CotAD_71296) and *G*. *barbadense* (GOBAR_DD23594) shared the same exon-intron structure within "0020–200" exon phases in the kinase domain.

**Fig 2:**
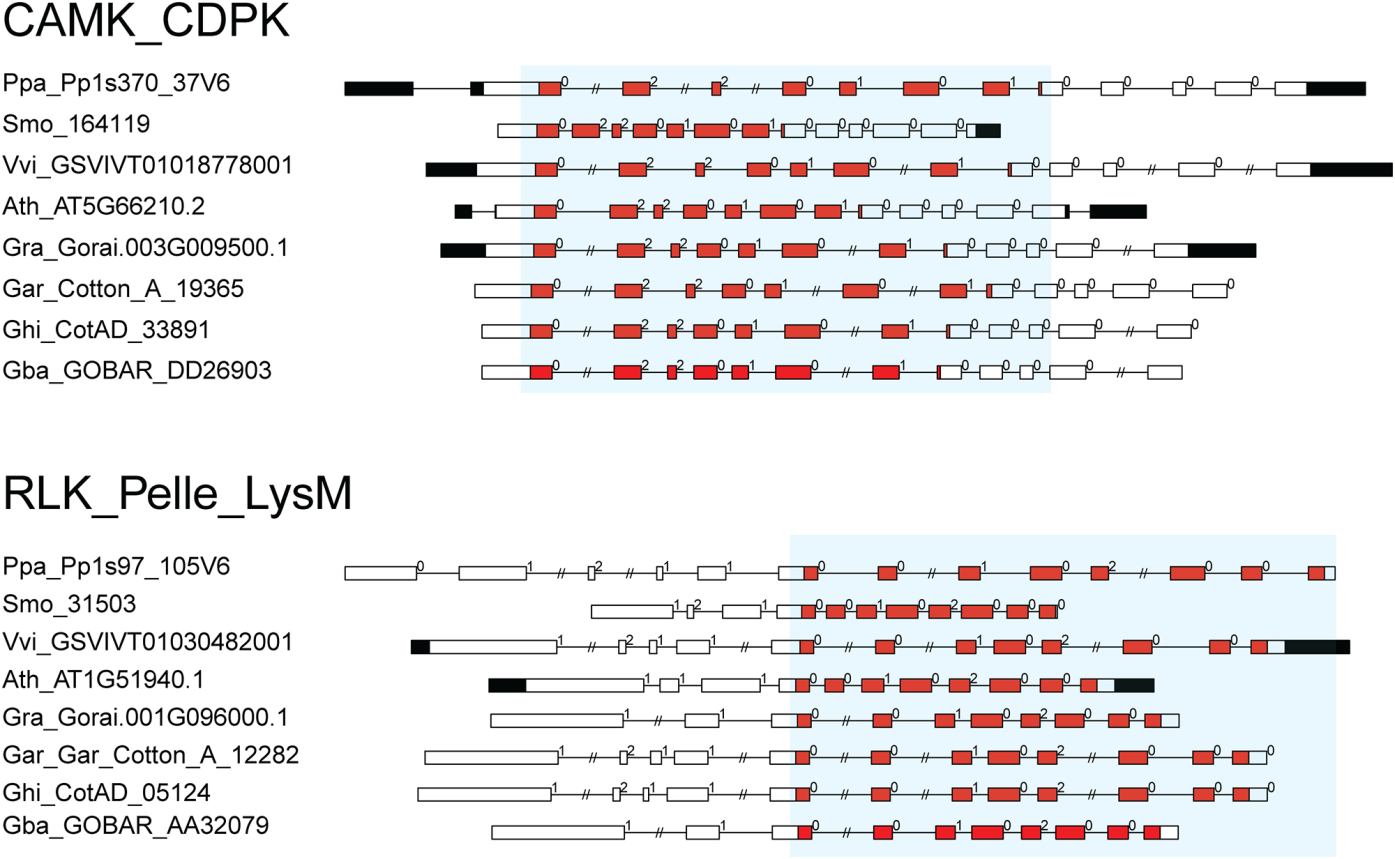
Two examples of conserved exon–intron structure. This diagram indicated that conserved exon–intron structure with conserved exon phases existed in kinase domain. Filled boxes: red represents Pkinase or Pkinase_Tyr domain; black boxes: untranslated regions (UTRs); white boxes: other exon regions; lines: introns. Numbers 0, 1, and 2: exon phases. The lengths of the boxes and lines are scaled based on the lengths of genes. The long introns are shorted by “//”. (A) non-RLK example: CAMK_CDPK. (B) RLK example: RLK_Pelle_LysM.

Our other research on wheat PKs showed that these exon-intron structures were conserved across the plant evolution [47]. To test it in cotton PKs, we also diagramed the PK exon-intron structures in *V*. *vinifera* (grape), *A*. *thaliana*, *O*. *sativa* (rice), *S*. *moellendorffii* (a fern), *P*. *patens* (moss) and *C*. *reinhardtii* (a green alga) (S3 Fig). Similarly, our results revealed that these conserved exon-intron structures were also identified in investigated plants, especially in the primitive plants *S*. *moellendorffii* and *P*. *patens* but not in *C*. *reinhardtii*. These findings suggest that these conserved exon-intron structures may have occurred during the emergence of Pteridophytes or Bryophytes. For instance, the CAMK_CDPK subfamily sequences from *P*. *patens* (Pp1s370_37V6), *S*. *moellendorffii* (164119), *O*. *sativa* (LOC_Os02g03410.1), *V*. *vinifera* (GSVIVT01018778001) *A*. *thaliana* (AT5G66210.2), *G*. *raimondii* (Gorai.003G009500.1), *G*. *arboretum* (Cotton_A_19365), *G*. *hirsutum* (CotAD_33891) and *G*. *barbadense* (GOBAR_DD26903) shared the same exon-intron structure of the kinase domain within the exon phases "022–0101" (Fig 2A). Similarly, RLK_Pelle_LysM members from moss to cotton also shared the conserved exon-intron structure with exon phases "001–0200" in the kinase domain (Fig 2B). Based on the above analysis, we summarized all the conserved exon-intron structures of some PK subfamilies in *P*. *patens*, *S*. *moellendorffii*, *V*. *vinifera* and *G*. *raimondii* (S4 Fig). These results suggested that these conserved exon-intron structures might have important biological functions for plants during the evolutionary process.

### Chromosome location and duplication events of cotton PKs

We mapped the 1501 *G*. *raimondii* PKs (Figure A in S5 File, excluding 16 genes from scaffold), 1407 *G*. *arboretum* PKs (Figure B in S5 File), 1893 *G*. *hirsutum* PKs (Figure C in S5 File, excluding 615 genes from scaffolds) and 2477 *G*. *barbadense* PKs (Figure D in S5 File, excluding 268 genes from scaffolds) to chromosome positions (S5 Table). The distributions of cotton PKs were assessed in *G*. *raimondii*, *G*. *arboretum*, *G*. *hirsutum* and *G*. *barbadense* (Table 1). The results showed that cotton PKs were unequally distributed across 13 or 26 chromosomes. *G*. *raimondii* chromosome 9 contained the most PKs (205), whereas *G*. *arboretum* chromosome 10, *G*. *hirsutum* chromosome 9Dt and *G*. *barbadense* chromosome 5At/5Dt contained the most PKs (145, 207, and 175, respectively).

**Table 1:** The PK distributions among the chromosomes in of *G*. *raimondii*, *G*. *arboretum*, *G*. *hirsutum* and *G*. *barbadense*.

Chromosome	*G*. *arboretum*	*G*. *hirsutum* (At)	*G*. *barbadense* (At)	*G*. *raimondii*	*G*. *hirsutum* (Dt)	*G*. *barbadense* (Dt)
1	127	71	72	104	105	89
2	59	56	78	120	34	110
3	73	59	60	63	32	45
4	135	8	46	79	15	60
5	73	43	175	115	99	175
6	122	71	91	134	116	108
7	123	44	88	171	102	90
8	119	67	60	102	78	67
9	113	183	108	205	207	113
10	145	63	119	85	70	131
11	130	78	125	155	101	141
12	98	42	91	81	49	87
13	90	43	68	88	57	80

**Fig 3:**
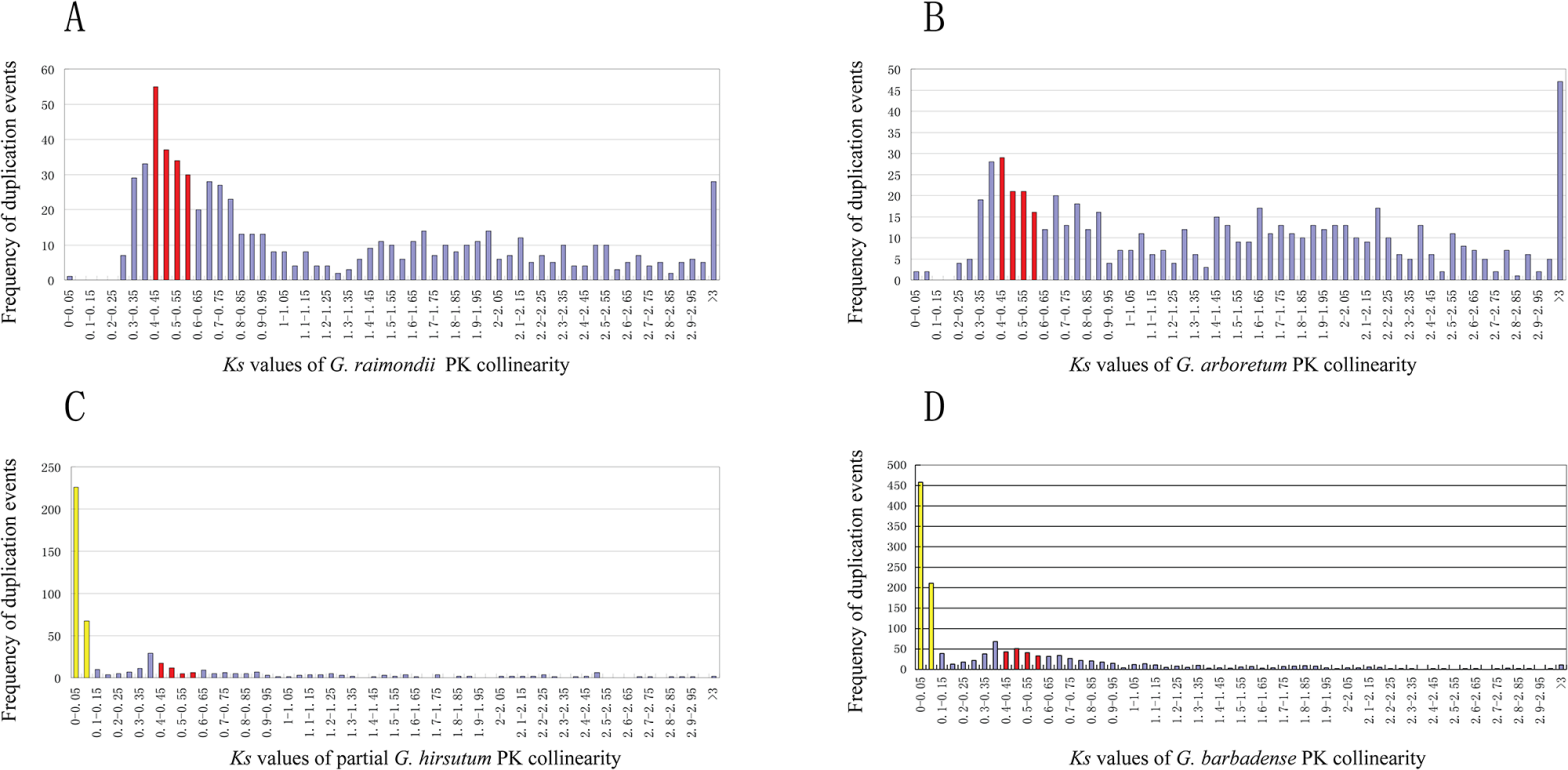
*Ks* values of PK collinearity in *G*. *raimondii*, *G*. *arboretum*, *G*. *hirsutum* and *G*. *barbadense*. This diagram indicated that the *Ks* values of PK collinearity in *G*. *raimondii* (A), *G*. *arboretum* (B), *G*. *hirsutum* (C) and *G*. *barbadense* (D). The red bars denote the collinearity events resulting from 16.6 Mya WGD (*Ks* values 0.4–0.6) in *G*. *raimondii*, *G*. *arboretum*, *G*. *hirsutum* and *G*. *barbadense*. The yellow bars denote the collinearity events contributed by tetraploidization in *G*. *hirsutum* and *G*. *barbadense*. The blue bars denote the other collinearity events.

*Gossypium* lineages experienced two WGD events, which occurred 16.6 and 130.8 million years ago (Mya) [48–50]. Therefore, we decided to investigate the contributions of WGD events to cotton PK expansions. We identified 681, 643, 510 and 1391 collinearity events in *G*. *raimondii*, *G*. *arboretum*, *G*. *hirsutum* and *G*. *barbadense*, respectively (S6 Table). These results suggest that segmental duplication events might play important roles in the cotton PK expansions. The WGD event appeared after cotton speciation 16.6 Mya, and its synonymous distance (*Ks* value) peak ranged from 0.4 to 0.6 [48]. Our results were consistent with a paper suggesting that cotton PK collinearity events also formed peaks of *Ks* 0.4–0.6 (Fig 3A–3D). We detected the chromosome positions of these collinearity events with *Ks* 0.4–0.6 in *G*. *raimondii*, *G*. *arboretum*, *G*. *hirsutum* and *G*. *barbadense*, respectively (Figure A-N in S6 File). The results indicated that 271, 158, 72 and 264 PKs of *G*. *raimondii*, *G*. *arboretum*, *G*. *hirsutum* and *G*. *barbadense*, respectively, were involved in the collinearity events (*Ks* 0.4–0.6). These results suggest that the cotton PK expansions might be due in part to WGD 16.6 Mya. We noticed that 440 *G*. *hirsutum* PKs and 1333 *G*. *barbadense* PKs were involved in the collinearity events (*Ks* 0.0–0.1) (Fig 3C and 3D, Figure G and K in S6 File), suggesting that tetraploidization of *G*. *hirsutum* and *G*. *barbadense* might play important roles in the PK expansion.

We identified 291, 246, 264 and 455 tandem cotton PKs in *G*. *raimondii*, *G*. *arboretum*, *G*. *hirsutum* and *G*. *barbadense*, respectively. The chromosome positions of these cotton PKs were detected in *G*. *raimondii*, *G*. *arboretum*, *G*. *hirsutum* and *G*. *barbadense* (S7 Table). They formed several tandem PK clusters among 13 or 26 cotton chromosomes. The *G*. *raimondii* tandem PKs formed 83 clusters that were distributed among the 13 chromosomes (Figure A in S7 File). The largest *G*. *raimondii* cluster contained 13 RLK-Pelle_LRR-XII-1 genes located on chromosome 11. The *G*. *arboretum* tandem PKs formed 87 clusters among the 13 chromosomes (Figure B in S7 File). The largest *G*. *arboretum* cluster was identified on chromosome 1 and contained 11 RLK-Pelle_CrRLK1L-1 genes. The *G*. *hirsutum* tandem PKs formed 89 clusters among 24 chromosomes (Figure C in S7 File). Tandem PKs were not detected on chromosome Dt2 and At4 of *G*. *hirsutum* perhaps due to its incomplete chromosome location information (scaffold). The largest *G*. *hirsutum* cluster contained 8 RLK-Pelle_CrRLK1L-1 genes located on chromosome Dt7. The *G*. *barbadense* tandem PKs formed 152 clusters among 26 chromosomes (Figure D in S7 File). The largest *G*. *barbadense* cluster contained 13 RLK-Pelle_LRR-XI-1 genes located on chromosome Dt9. Most of these cotton tandem PK clusters belonged to the RLK subfamilies, suggesting that TD mainly contributed to the expansion of the cotton RLK group.

**Fig 4:**
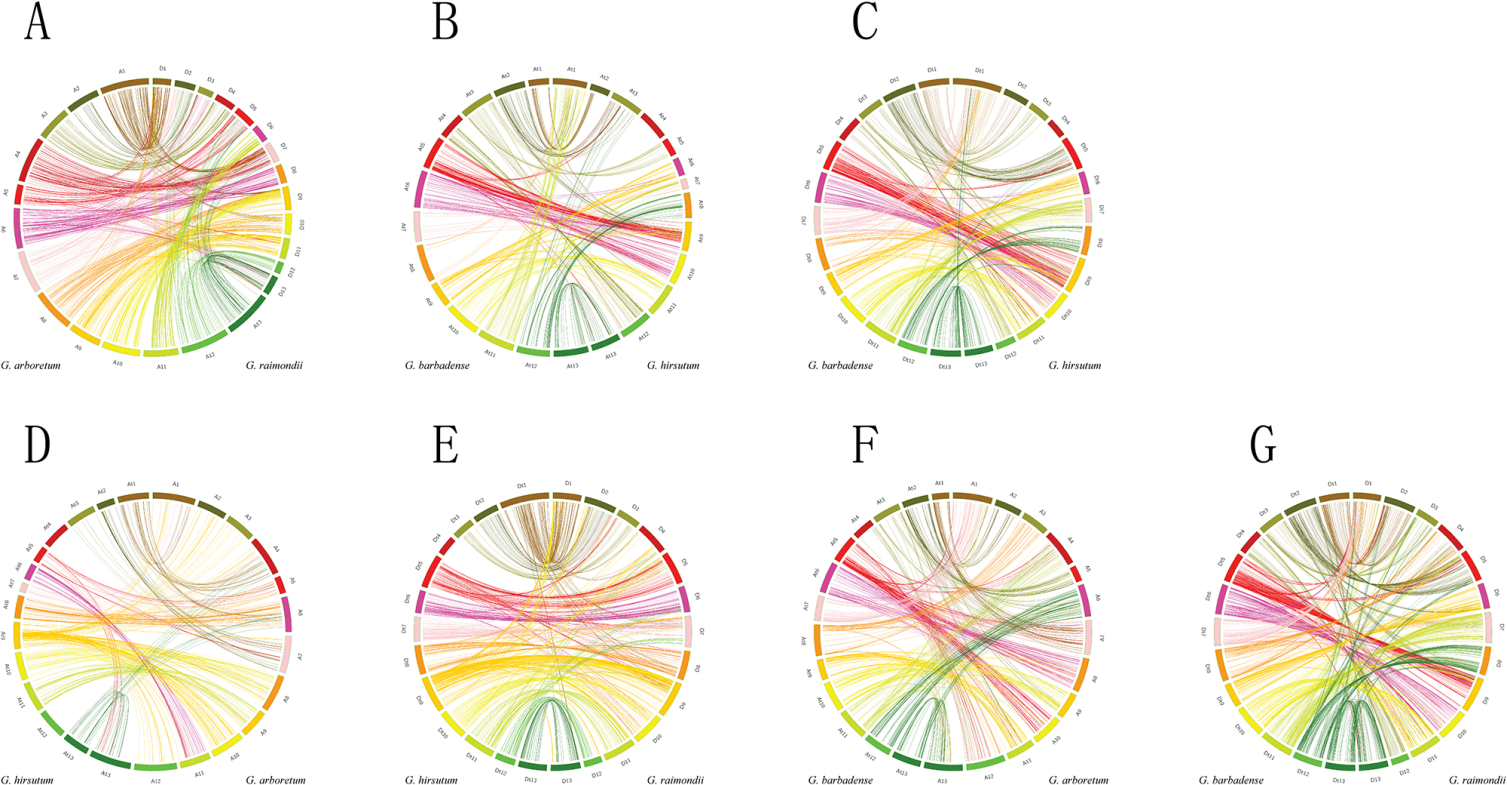
Synteny analysis of PK genes. (A) Synteny between *G*. *raimondii* (A1-A13) and *G*. *arboretum* (D1-D13). (B) Synteny between *G*. *barbadense* (At1-At13) and *G*. *hirsutum* (At1-At13). (C) Synteny between *G*. *barbadense* (Dt1-Dt13) and *G*. *hirsutum* (Dt1-Dt13). (D) Synteny between *G*. *arboretum* (A1-A13) and *G*. *hirsutum* (At1-At13). (E) Synteny between *G*. *raimondii* (D1-D13) and *G*. *hirsutum* (Dt1-Dt13). (F) Synteny between *G*. *arboretum* (A1-A13) and *G*. *barbadense* (At1-At13). (G) Synteny between *G*. *raimondii* (D1-D13) and *G*. *barbadense* (Dt1-Dt13). The different cotton chromosomes were labeled with different colors.

To further explore the relationship between PK expansions and cotton duplication events, we constructed the synteny analysis of cotton PKs between *G*. *raimondii* and *G*. *arboretum* (Fig 4A); At-subgenomes of *G*. *hirsutum* and *G*. *barbadense* (Fig 4B); and Dt-subgenomes of *G*. *hirsutum* and *G*. *barbadense* (Fig 4C). The similar synteny analysis of cotton PKs was also performed between *G*. *arboretum* and *G*. *hirsutum* At-subgenome (Fig 4D), *G*. *raimondii* and *G*. *hirsutum* Dt-subgenome (Fig 4E), *G*. *arboretum* and *G*. *barbadense* At-subgenome (Fig 4F), and *G*. *raimondii* and *G*. *barbadense* Dt-subgenome (Fig 4G). The related collinearity events were also demonstrated among *G*. *raimondii*, *G*. *arboretum*, *G*. *hirsutum* and *G*. *barbadense* (S8 Table). Our results demonstrated that approximately half of collinearity events exhibited single gene correspondence (S9 Table), indicating that these single gene pairs might exist in the common cotton ancestor and did not experience expansion after the split of cotton A- and D-subgenomes. We also noticed that some gene pairs from chromosome fragments did not locate on the corresponding chromosomes between subgenomes, suggesting that they might experience chromosome rearrangement after the split of cotton A- and D-subgenomes. For instance, eight gene pairs (from Cotton_A_10476-Gorai.013G272100.1 to Cotton_A_10556-Gorai.013G263500.1) are located in the A8 chromosome fragment of *G*. *arboretum* and D13 chromosome fragment of *G*. *raimondii*, separately (S8 Table).

### Expression pattern of cotton PKs in abiotic and biotic stresses

**Fig 5:**
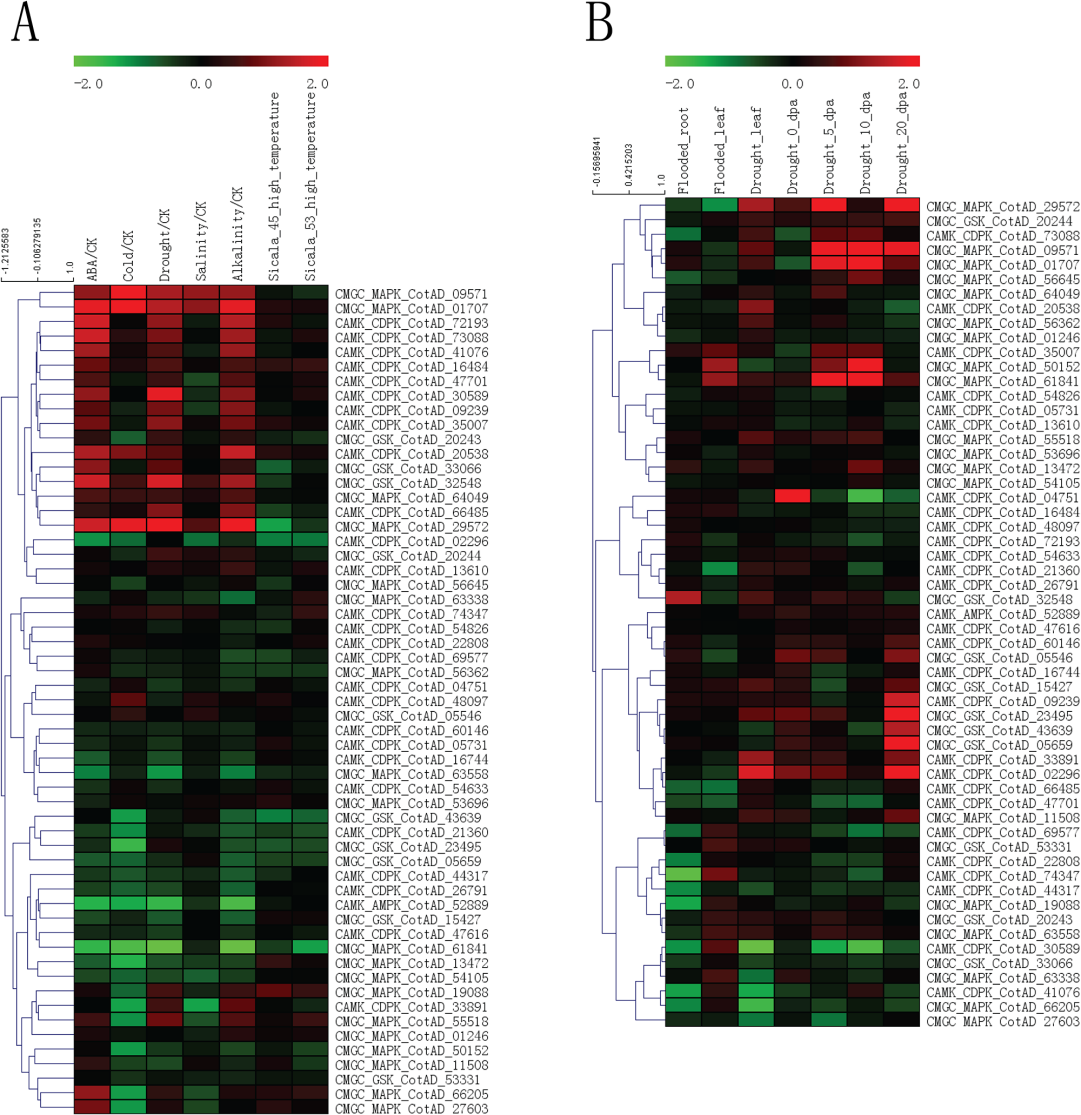
Heat map of the expression patterns of *G*. *hirsutum* PK genes from known subfamilies under various abiotic stress conditions. The expression patterns of PKs of known subfamilies, including CAMK_CDPK, CAMK_AMPK, CMGC_GSK and CMGC_MAPK. (A) The expression patterns under abiotic stress treatments. (B) The expression patterns under waterlog and drought stress treatments.

We analyzed the gene expression profiles of *G*. *hirsutum* PKs using 11 public datasets of the Affymetrix microarray GPL8672 platform. As a result, 564 of 2508 *G*. *hirsutum* PKs have probes in GPL8672. Based on the RLE and NUSE diagrams (S8 Fig), we performed quality control assessment and excluded 5 CEL files in the following analysis (S10 Table). The expression patterns of 564 *G*. *hirsutum* PKs were assessed under various abiotic stresses (S9 Fig and S11 Table). To identify PK genes differentially expressed under abiotic stresses, the PKs with |FC|>1.5 (fold change) and p<0.01 were retained (S12 Table). (1) Five stress conditions: We assessed genes in response to five abiotic stress conditions: ABA, cold, drought, salinity and alkalinity stresses. Some PKs, such as CotAD_10936 (CAMK_CAMKL-CHK1), CotAD_29572 (CMGC_MAPK) and CotAD_24426 (AGC-Pl), exhibited up-regulation in response to these five stresses. These results suggest that several common components of signaling pathways might be shared by these five abiotic stress response. In addition, certain PKs responded to particular stress treatments. For example, CotAD_51610 (CMGC_CDK-CRK7-CDK9) only exhibited up-regulation in response to ABA treatment but down-regulation to other four abiotic stress treatments. (2) Heat stress: Most PKs exhibited the same expression trend in both varieties "Sicala 45" and "Sicala 53" under high temperature treatment. However, some PKs exhibited two opposing expression trends between variety "Sicala 45" and "Sicala 53". For instance, CotAD_74347 (CAMK_CDPK) exhibited slight down-regulation in "Sicala 45" but up-regulation in "Sicala 53". (3) Waterlog and drought stresses (S10 Fig, S13 and S14 Tables): We observed that some PKs, such as CotAD_65513 (RLK-Pelle_RLCK-VIIa-2), CotAD_27962 (AGC-Pl) and CotAD_57424 (RLK-Pelle_DLSV), exhibited up-regulation in root under waterlog stress, whereas these PKs exhibited no change or down-regulation in leaf. The reason for these findings might be that the low oxygen of the cotton root influenced some energy metabolism. Under drought stress, some PKs, such as CotAD_09571 (CMGC_MAPK), CotAD_06862 (CAMK_CAMKL-CHK1) and CotAD_43021 (CAMK_OST1L), exhibited up-regulation at 5, 15 and 20 dpa (days post anthesis). Similarly, several PKs, such as CotAD_70308 (RLK-Pelle_LRK10L-2), CotAD_62253 (TKL-Pl-3) and CotAD_62987 (RLK-Pelle_DLSV), exhibited down-regulation at 5, 15 and 20 dpa. We also selected PKs of some known subfamilies, such as CMGC_MAPK, CAMK_CDPK and CAMK_AMPK, to investigate their expression patterns under abiotic stresses (Fig 5A and 5B).

**Fig 6:**
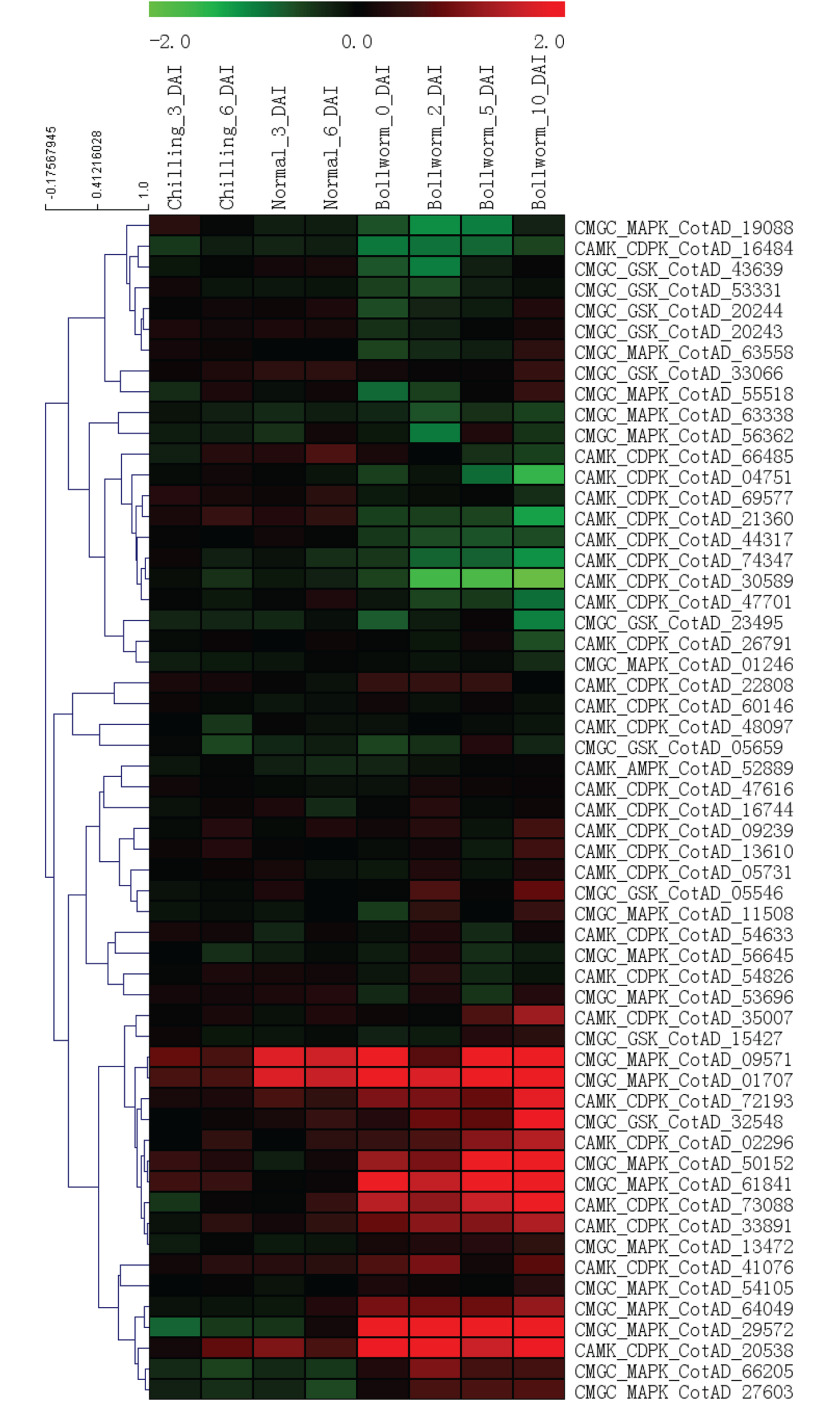
Heat map of the expression patterns of *G*. *hirsutum* PK genes from known subfamilies under various biotic stress conditions. The selected PKs of known subfamilies are same as Fig 5. The expression patterns under biotic stress treatments.

The expression patterns of 564 *G*. *hirsutum* PKs were also analyzed under biotic stresses (S11 Fig and S15 Table). The differential expression PKs with |FC|>1.5 and p<0.01 were also assessed under biotic stresses (S16 Table). (1) *A*. *alternata* disease: Some PKs, such as CotAD_29801 (WNK_NRBP), CotAD_07539 (RLK-Pelle_LRR-XI-1) and CotAD_37359 (RLK-Pelle_LRR-XII-1), exhibited up-regulation at 3 and 6 DAI (days after inoculation) under *A*. *alternata-*infected stress conditions with or without chilling pre-treatment, suggesting that these genes might participate in the *A*. *alternata* defense response. (2) Bollworm infection: Some PKs, such as CotAD_20538 (CAMK_CDPK), CotAD_75319 (RLK-Pelle_WAK) and CotAD_29572 (CMGC_MAPK), exhibited up-regulation at 0, 2, 5 and 10 dpa with *Helicoverpa armigera* infection. We also selected the same PKs of known subfamilies to investigate their expression patterns under biotic stresses (Fig 6).

### Expression pattern of cotton PKs during fiber development stages

**Fig 7:**
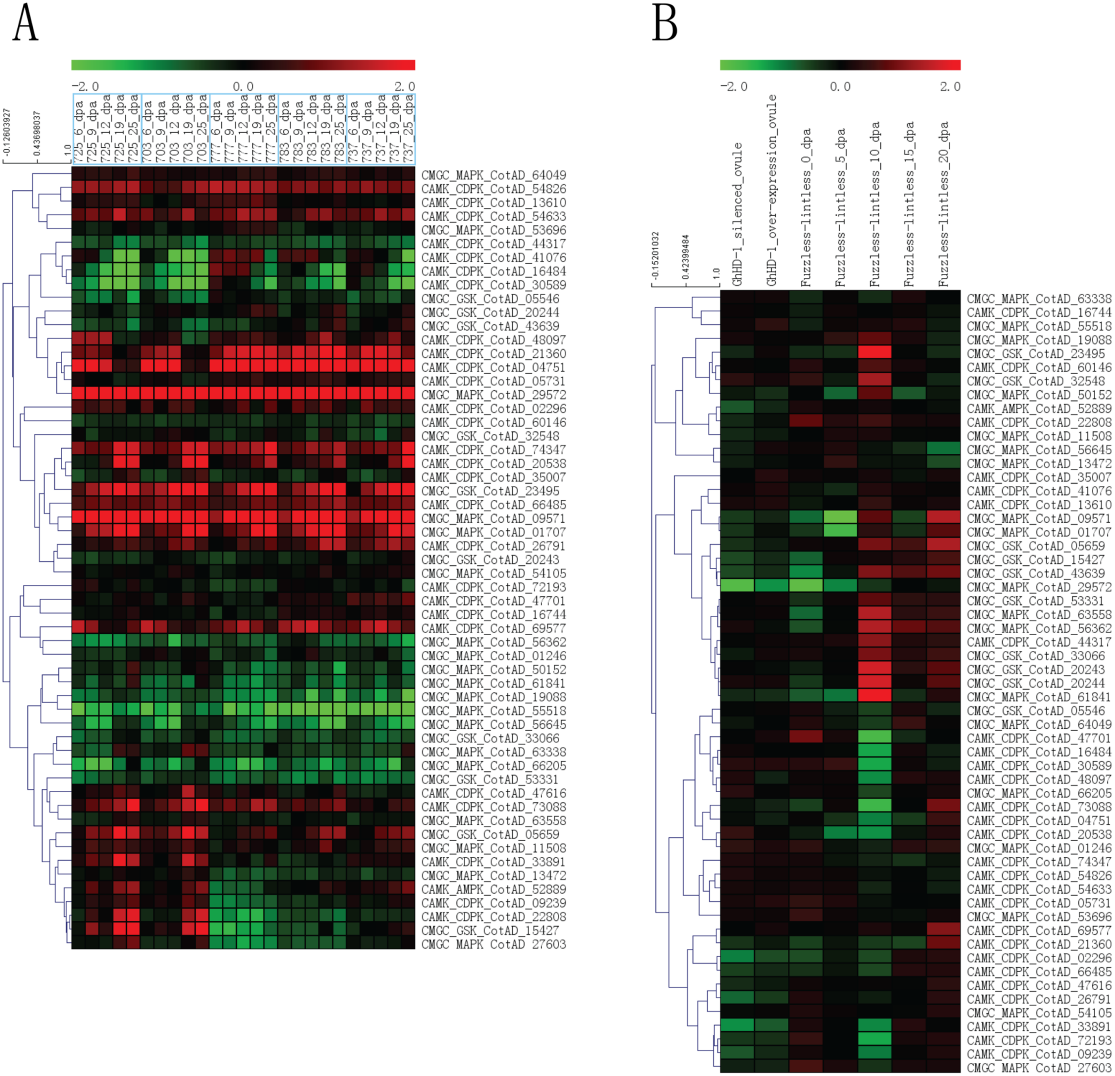
Heat map of the expression patterns of *G*. *hirsutum* PK genes from known subfamilies during fiber development stages. The selected PKs of known subfamilies are same as Fig 5. (A) The expression patterns for genotypes (725, 703, 777, 783 and 737) and fiber development stages (6, 9, 12, 19 and 25 dpa). (B) The expression patterns about *GhHD-1* and fuzzless-lintless mutant during fiber development stages.

We assessed the expression pattern of cotton PK genes in five *G*. *hirsutum* varieties during fiber development stages (6, 9, 12, 19 and 25 dpa) (S12 Fig and S17 Table). Some PKs, such as CotAD_10084 (RLK-Pelle_RLCK-IXa), CotAD_70308 (RLK-Pelle_LRK10L-2) and CotAD_29572 (CMGC_MAPK), exhibited sustained up-regulation at 6–25 dpa among the investigated five varieties. Similarly, several PKs, such as CotAD_06790 (RLK-Pelle_RLCK-VIIa-2), CotAD_54619 (CAMK_OST1L) and CotAD_17222 (RLK-Pelle_LRR-XI-1), exhibited sustained down-regulation at 6–25 dpa among the five varieties. We also noticed that some PKs exhibited different expression patterns among these five cotton varieties. For example, a few PKs, including CotAD_22808 (CAMK_CDPK), CotAD_15427 (CMGC_GSK) and CotAD_61960 (TKL-Pl-4), exhibited peak up-regulation at 19 and 25 dpa in varieties "JKC 725" and "JKC 703", respectively, but remained at the same expression level or exhibited down-regulation in the other three cotton varieties. We also assessed PKs exhibiting differential expression (|FC|>1.5 and p<0.01) (S18 Table) and selected the PKs of known subfamilies to investigate their expression patterns during fiber development stages (Fig 7A).

The cotton transcription factor *GhHD-1* plays roles in cotton fiber initiation and is expressed in trichomes and early fibers [41]. The expression patterns of cotton PKs between *GhHD-1* silenced and over-expressed lines were assessed during the early fiber development stage (S13 Fig and S19 Table). Our result revealed that a few PKs exhibited reverse expression trends between *GhHD-1* silenced and over-expressed lines, hinting that they might be the downstream of *GhHD-1* in the signaling pathway. For instance, CotAD_03134 (RLK-Pelle_LRR-XI-1) and CotAD_34129 (RLK-Pelle_CR4L) exhibited down-regulation in a *GhHD-1*-silenced line but up-regulation in a line exhibiting *GhHD-1* over-expression.

Fuzzless-lintless cotton mutants represent ideal materials to identify genes involving in fiber development by comparison with fiber-bearing wild-type cottons [42]. Our result revealed that several cotton PKs exhibited peak up- or down-regulation at 10 dpa in the fuzzless-lintless cotton lines (S13 Fig and S19 Table). For example, CotAD_67858 (CMGC_CDK-CDK7), CotAD_10699 (AGC_NDR) and CotAD_32629 (CK1_CK1-Pl) exhibited peak up-regulation at 10 dpa. Similarly, some PKs, including CotAD_75319 (RLK-Pelle_WAK), CotAD_08639 (STE_STE11) and CotAD_16484 (CAMK_CDPK), exhibited down-regulation at 10 dpa. PKs exhibiting differential expression (|FC|>1.5 and p<0.01) (S20 Table) and PKs of known subfamilies (Fig 7B) were also assessed in *GhHD-1* lines and fuzzless-lintless lines.

**Fig 8:**
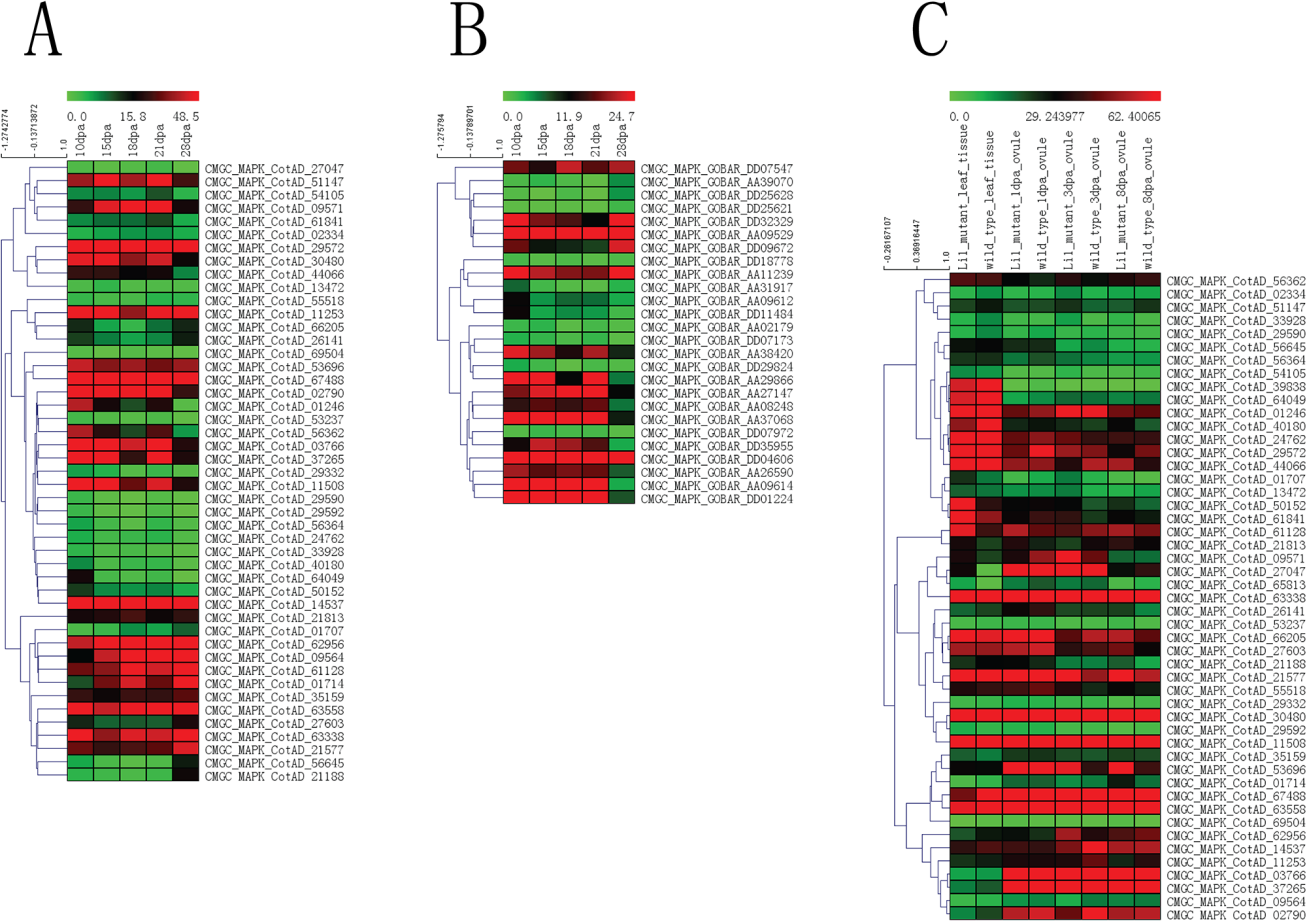
Heat map of the expression patterns of *G*. *hirsutum* and *G*. *barbadense* PK genes from MAPK subfamily during fiber development stages. (A) Normalized gene expression RPKM values of *G*. *hirsutum* MAPK genes in fiber development stages (10, 15, 18, 21 and 28 dpa). (B) Normalized gene expression RPKM values of *G*. *barbadense* MAPK genes in fiber development stages (10, 15, 18, 21 and 28 dpa). (C) Normalized gene expression RPKM values of *G*. *hirsutum* PK genes for genotypes (Li1 mutant and wild type) ahout leaf tissue and ovule tissues in fiber development stages (1, 3 and 8 dpa).

We also analyzed two public RNA-seq data of NCBI SRA about the fiber development of *G*. *hirsutum* and *G*. *barbadense*. Based on the results of FastQC, we performed quality control assessment and excluded 15 RUN files in the following analysis (S21 Table). 1619 *G*. *hirsutum* PKs and 963 *G*. *barbadense* PKs were identified in all the five runs of 10, 15, 18, 21 and 28 dpa (S14 and S15 Figs, and S22 Table). We noticed that some *G*. *hirsutum* PKs, such as CotAD_12382 (NEK) and CotAD_16078 (CAMK_CAMKL-CHK1), exhibited sustained high expression from 10 to 28 dpa. Similarly, some *G*. *hirsutum* PKs, such as CotAD_31836 (RLK-Pelle_LRR-XI-1) and CotAD_21804 (RLK-Pelle_DLSV), exhibited sustained low expression from 10 to 28 dpa. We also selected PKs of the known subfamily, CMGC_MAPK, to investigate their expression patterns of fiber development (Fig 8A and 8B).

1749 *G*. *hirsutum* PKs of two genotypes (Li1 mutant and wild type) were identified in all the eight RUN files of leaf tissue and ovule tissues in fiber development stages (1, 3 and 8 dpa) (S16 Fig and S23 Table). Most PKs exhibited the similar expression levels between genotypes Li1 mutant and wild type. We also found that some PKs, such as CotAD_21607 (RLK-Pelle_DLSV) and CotAD_58349 (RLK-Pelle_RLCK-XII-1), exhibited different expression levels in leaf tissue between the two genotypes. In order to investigate the differential expression PKs between genotypes Li1 mutant and wild type, we also caculated the log2 Fold Change (Li1 mutant vs. wild type) and padj values (S24 Table). For instance, the log2 value of CotAD_27047 (CMGC_MAPK) in leaf tissue is 5.885973619 and its padj value is 0.000776909, suggesting that Li1 mutant might greatly influence the expression of CotAD_27047 (CMGC_MAPK) in leaf tissue. We also selected PKs of the known subfamily, CMGC_MAPK, to investigate their expression patterns of fiber development between the two genotypes (Fig 8C).

## Discussion

### Phylogeny of cotton PK family

The identification and analyses of *A*. *thaliana*, wheat, soybean and maize kinomes has been reported in recent years [7–8, 12, 47]. Diversity, classification and functions of 25 plant kinomes were also assessed to determine the evolutionary history of PK subfamilies [5]. In this study, we also identified, classified, and performed phylogenetic and expression pattern analyses of the PK family in four cotton species *G*. *raimondii*, *G*. *arboretum*, *G*. *hirsutum* and *G*. *barbadense*. As reported in the other plant kinomes, cotton PKs were also classified into seven groups: RLK, AGC, CAMK, CMGC, STE, TKL and others.

Our previous research on wheat PKs revealed that some conserved exon-intron structures were present from primitive plant moss to flowering plant wheat [47]. In this study, we also revealed that some conserved exon-intron structures with conserved exon phases in the kinase domain existed in cotton PKs (S4 Fig). For instance, the CAMK_AMPK subfamily in cotton (Gorai.007G080200.1), grape (GSVIVT01011467001), fern (80443) and moss (Pp1s3_550V6) shared the same exon-intron structure within the exon phase "1000" in the kinase domain. This finding suggested that these conserved exon-intron structures might play important roles in plant development and evolution. Indeed, previous functional studies of plant PKs indicated that these PK genes within conserved exon-intron structures function as core components of various signaling pathways that control multiple plant cellular processes. (1) Mitosis: Aur [51] and ULK_ULK4 [52] function in cell division in *A*. *thaliana*. The rice and *Arabidopsis* CMGC_CDK−CDK7 homologs phosphorylate both CDKs (cyclin-dependent protein kinases) and the CTD (carboxy-terminal domain) of RNA polymerase II [53–54]. (2) Microtubule: *Arabidopsis* CK1_CK1 affects cortical microtubules organization [55]. *Arabidopsis* NEKs form homo- or heterodimers to regulate microtubule organization during epidermal cell expansion [56]. (3) Metabolism: The *Arabidopsis* CAMK_AMPK homolog SnRK1 forms the SnRK1-ADK complex and plays significant roles in energy homeostasis [57]. The *Arabidopsis* CAMK_CAMKL−LKB homologs GRIK1 and GRIK2 specifically activate SnRK1, and the GRIK-SnRK1 cascade may coordinate metabolic requirements of rapidly growing cells [58]. The *Arabidopsis* CAMK_CAMKL−CHK1 homologs SOS2 (Salt Overly Sensitive2) and SOS3 maintain ion homeostasis and confer salt tolerance [59]. (4) MAPK signaling network: Plant MAPK cascades regulate several processes, including stress response, immunity and development [60]. STE_STE11 functions as a MAPK3K [60]. Similar to animal RSK−2, the plant AGC_RSK−2 homolog is also activated by PDK1 [61], which is involved in the MAPK signaling cascade [62]. (5) Stress response and development: The plant CMGC_GSK homolog GSK3 functions in floral organs, cell expansion, and abiotic and biotic stress responses [63]. Plant RLK/Pelle members play various roles in cell specification [64], pathogen recognition [65], stress response and development [66]. The soybean WNK_NRBP member *GmWNK1* regulates root system architecture via ABA and osmotic signals [67] and modulates the osmotic stress response [68].

We observed that some cotton PKs contained two or more kinase domains (S4 Table). Interestingly, soybean and wheat also contained similar special PKs within two or three kinase domains [8, 47]. Comparing these special PKs within 2–4 kinase domains of cotton, soybean and wheat, we identified four overlapping PK subfamilies: AGC_NDR, AGC_RSK-2, CMGC_SRPK and RLK-Pelle_DLSV. Notably, the structure of AGC_RSK-2 and CMGC_SRPK within two kinase domains can be found in moss, fern, grape and cotton (S4 Fig), suggesting that the duplication event of the kinase domain in these PKs occurred during the emergence of land plants and these structures were subsequently retained during the evolutionary process. Given that some PKs form homo- or hetero-dimers [56, 69], we hypothesized that these structures within two or three kinase domains might be required for specific substrates to form PK dimers. This hypothesis is consistent with soybean PKs [8] and requires more functional research.

### Evolution, duplication and expansion of cotton PKs

The cotton genome experienced two WGD events: a cotton-specific WGD event 16.6 Mya and a WGD that occurred 130.8 Mya and was shared by eudicots [48]. Our results revealed that the cotton-specific WGD that occurred 16.6 Mya partly contributed to the expansion of cotton PKs (Fig 3). Similar to the *Ks* peak (0.4–0.6) of the *G*. *raimondii* whole genome [48], *G*. *raimondii* PKs also formed a *Ks* peak (0.4–0.6) (Fig 3A). The paleo-hexaploidy of ancient eudicot occurred after the split of Monocotyledons and Dicotyledons [70]. Considering that the WGD that occurred 130.8 Mya is shared by eudicots [48], we proposed that most of the cotton PK collinearity events (*Ks* 0.6–3) potentially attributed to the 130.8 Mya WGD. However, we did not identify the second obvious cotton PK *Ks* peak, which corresponds to the WGD that occurred 130.8 Mya. We were potentially unable to identify this peak because these duplicated PKs might exhibit low retention after the WGD that occurred 130.8 Mya. Indeed, the TD after the paleo 130.8 Mya WGD caused dosage imbalance as the gene balance hypothesis claimed [71]. Tetraploidization of *G*. *hirsutum* occurred 1.5 Mya and corresponds to a *Ks* peak [50]. In our study, we also identified *G*. *hirsutum* and *G*. *barbadense* PK *Ks* peaks (0–0.1) (Fig 3C and 3D), indicating that the tetraploidization event of *G*. *hirsutum* and *G*. *barbadense* was important for PK expansion. We also identified several PK collinearity blocks among *G*. *raimondii* (D-genome), *G*. *arboretum* (A), *G*. *hirsutum* (At and Dt) and *G*. *barbadense* (At and Dt) (Fig 4).

Most tandem cotton PKs belonged to the RLK group, suggesting that TD partly contributed to the cotton PKs expansion (S7 Table). Our result was consistent with reports demonstrating that most soybean and wheat TD PKs belonged to the RLK group [8, 47]. An A-genome ancestor resembling *G*. *arboretum* and a D-genome ancestor resembling *G*. *raimondii* diverged from a common ancestor 5–10 Mya and subsequently reunited to produce an allotetraploid *Gossypium* species [50]. Our result demonstrated that the TD PK clusters of *G*. *raimondii*, *G*. *arboretum*, *G*. *hirsutum* and *G*. *barbadense* exhibited different chromosome locations and related subfamilies, suggesting that they might experience independent PK TDs or different chromosome rearrangements after the split of cotton A- and D-subgenomes. Indeed, the *G*. *raimondii* genome underwent large-scale rearrangement on chromosomes 2 and 3 compared with *G*. *arboretum* [49].

Similar to *A*. *thaliana*, rice and soybean [8–10], cotton RLK groups were also remarkably large, ranging from 913 to 1,855. The expansion of cotton RLKs was associated with specific subfamilies or subgroups, including LRR, RLCK and DLSV.

LRR contained the highest number of members among the RLK group in cottons (*G*. *raimondii* 347, *G*. *arboretum* 323, *G*. *hirsutum* 619, and *G*. *barbadense* 660) and formed a cluster of 23 subfamilies. RLK/Pelle_LRR-XI-1 contained the highest number in LRR subfamilies in *G*. *raimondii* (82), *G*. *arboretum* (80), *G*. *hirsutum* (162) and *G*. *barbadense* (155). This finding suggested that RLK-LRRs experienced remarkable expansions and play important roles in plant growth, development and defense response. Indeed, many RLK-LRRs enhance drought resistance [72], improve drought and salt stress tolerance [73], respond to diverse abiotic stresses [74], and regulate root meristem development [75]. RLCK contained the second highest number of members among the RLK group in cottons (*G*. *raimondii* 184, *G*. *arboretum* 178, *G*. *hirsutum* 310, and *G*. *barbadense* 346) and was grouped into 17–18 subfamilies. Without the extracellular domain, RLCK interacts with RLK to form the RLCK-RLK complex, which could mediate plant growth and immune responses [76]. DLSV contained the third highest number of members among the RLK group in cottons (*G*. *raimondii* 129, *G*. *arboretum* 93, *G*. *hirsutum* 205, and *G*. *barbadense* 225).

### Expression pattern of cotton PKs

Our previous studies demonstrated that cotton PKs *GhMKK1* [24], *GhMKK3* [26] and *GhMKK5* [23] are involved in drought stress resistance. In this study, we provided some candidate cotton PKs involved in drought resistance. For example, expression pattern analysis revealed that CotAD_10106 (STE_STE7) of *G*. *hirsutum* exhibited slight up-regulation (log2 value 0.3230955 in leaf) under drought stress (S13 Table), suggesting that it exerts a positive effect in drought tolerance. Using BLAST in NCBI, we found that cotton PK CotAD_10106 is a homolog of *A*. *thaliana* MKK3. This prediction is consistent with our laboratory research demonstrating that *GhMKK3* positively regulates the drought stress response [26]. In addition, a cotton RLK *GbRLK* from *Gossypium barbadense* is involved in drought and high salinity stress pathways by participating or activating the ABA signaling pathway [30]. In this study, we provided some candidate cotton RLKs for drought resistance. For instance, CotAD_53902 (RLK-Pelle_SD-2b) and CotAD_15239 (RLK-Pelle_LRR-Xb-1) exhibited up-regulation at 5 and 10 dpa under drought stress (S13 Table).

Our previous research also demonstrated that several cotton PKs are involved in disease defense. *GhMPK2* mediates defense responses to oxidative stress and pathogen infection [77]. *GhMPK6a* interacts with the upstream MAPK kinase *GhMKK4* and negatively regulates bacterial infection and osmotic stress [78]. *GhMPK7* plays a role in SA-regulated resistance to pathogen infection [79]. *GhMPK16* displays significant resistance to fungi (*Alternaria alternata* and *Colletotrichum nicotianae*) and bacteria (*Pseudomonas solanacearum*) [80]. In this study, we also observed that some cotton MAPKs, such as CotAD_66205, CotAD_27603 and CotAD_29572, exhibited up-regulation in response to bollworm infection at 2–10 DAI (S11 Fig). Interestingly, CotAD_53696 (CMGC_MAPK, a homolog of *A*. *thaliana* MPK16) of *G*. *hirsutum* exhibited slight up-regulation at 3 and 6 DAI (log2 values 0.213498 and 0.296303333, respectively) after *A*. *alternate* infection without chilling pre-treatment (S15 Table), suggesting that it positively regulates the *A*. *alternate* defense response. This prediction is consistent with our laboratory research demonstrating that *GhMPK16* regulates *A*. *alternata* resistance [80]. In addition, cotton RLKs are also involved in disease defense. Two cotton RLK genes, *Gbve1* [29] and *GbRLK* [81], improve tolerance to *Verticillium dahliae* infection. In this study, we also found that some cotton RLKs, such as CotAD_75319 (RLK-Pelle_WAK), CotAD_53902 (RLK-Pelle_SD-2b) and CotAD_03142 (RLK-Pelle_WAK_LRK10L-1), exhibited up-regulation at 2–10 DAI after bollworm infection (S11 Fig).

Cotton PKs are also involved in fiber development. According to expression analysis, some cotton CrRLK1L proteins were predicted to be related to fiber development [21]. A cotton CDPK gene *GhCPK1* [82] and a cotton CDK gene *GhCDKA* [83] were cloned and characterized to be associated with fiber development. In this study, we also found that some cotton RLKs (CotAD_49930, CotAD_10084 and CotAD_70308), MAPKs (CotAD_29572 and CotAD_09571), CDPKs (CotAD_54826 and CotAD_04751) and CDKs (CotAD_20875 and CotAD_75205) exhibited up-regulation during fiber development (S12 Fig).

## Conclusion

In this study, we systematically identified cotton PKs and analyzed their classification, evolution and expression patterns. Some conserved exon-intron kinase domain structures were identified during plant evolution. WGD, TD and synteny PKs of three cotton species *G*. *raimondii*, *G*. *arboretum* and *G*. *hirsutum* were analyzed by MCScanX. These results suggest that cotton-specific WGD, TD and tetraploidization of *G*. *hirsutu* that occurred 16.6 Mya partially contributed to the expansion of cotton PKs. We also performed global expression pattern analysis under abiotic and biotic stress conditions and during fiber development, providing candidate PKs for further experimental research. Our results will provide clues for further research on the evolution and physiology of the cotton kinome.

## Supporting information

S1 Fig Expanded phylogenetic classification of cotton PKs using the neighbor-joining method. (A) Representatives of each subfamily; (B) All members.
(PDF)

S2 Fig Domain diagrams of cotton PKs in *G*. *raimondii*, *G*. *arboretum*, *G*. *hirsutum* and *G*. *barbadense*. Filled boxes: red represents the Pkinase or Pkinase_Tyr domain; other colors represent various domains labeled in each page. The lengths of the boxes and lines are scaled based on the length of proteins.
(PDF)

S3 Fig Exon−intron and domain diagrams of PKs in *G*. *raimondii*, *G*. *arboretum*, *G*. *hirsutum*, *G*. *barbadense*, *A*. *thaliana*, *V*. *vinifera*, *O*. *sat*iva, *S*. *moellendorffii*, *P*. *patens* and *C*. *reinhardtii*. The descriptions of domain and exon phases are the same as in Fig 2. The lengths of the boxes and lines are scaled based on the length of genes.
(PDF)

S4 Fig Conserved exon−intron and domain diagrams of PKs in *G*. *raimondii*, *V*. *vinifera*, *S*. *moellendorffii* and *P*. *patens*. The descriptions of domain and exon phases are the same as in Fig 2. The lengths of the boxes and lines are scaled based on the length of genes.
(PDF)

S5 Fig Chromosome locations of *G*. *raimondii*, *G*. *arboretum*, *G*. *hirsutum* and *G*. *barbadense* PK genes. Chromosomal locations of *G*. *raimondii* (A), *G*. *arboretum* (B), *G*. *hirsutum* (C) and *G*. *barbadense* (D) PKs. Yellow boxes denote PK genes.
(PDF)

S6 Fig Collinearity events of *G*. *raimondii*, *G*. *arboretum*, *G*. *hirsutum* and *G*. *barbadense* PK genes. (A) The collinearity events of *G*. *raimondii* PKs resulting from 16.6-Mya WGD. (B) The other collinearity events of *G*. *raimondii* PKs. (C) All of the collinearity events of *G*. *raimondii* PKs. (D) The collinearity events of *G*. *arboretum* PKs resulting from 16.6-Mya WGD. (E) The other collinearity events of *G*. *arboretum* PKs. (F) All of the collinearity events of *G*. *arboretum* PKs. (G) The collinearity events of *G*. *hirsutum* PKs contributed by tetraploidization. (H) The collinearity events of *G*. *hirsutum* PKs resulting from 16.6-Mya WGD. (I) The other collinearity events of *G*. *hirsutum* PKs. (J) All of the collinearity events of *G*. *hirsutum* PKs. (K) The collinearity events of *G*. *barbadense* PKs contributed by tetraploidization. (L) The collinearity events of *G*. *barbadense* PKs resulting from 16.6-Mya WGD. (M) The other collinearity events of *G*. *barbadense* PKs. (N) All of the collinearity events of *G*. *barbadense* PKs. Red lines denote the collinearity events resulting from 16.6-Mya WGD. Red lines denote the collinearity events resulting from 16.6-Mya WGD. Yellow lines denote the collinearity events contributed by tetraploidization. Blue lines denote other collinearity events.
(PDF)

S7 Fig Chromosomal locations of the tandemly arrayed *G*. *raimondii*, *G*. *arboretum*, *G*. *hirsutum* and *G*. *barbadense* PK genes. (A) The 291 tandemly arrayed *G*. *raimondii* PK genes were grouped in 83 clusters distributed unevenly among the 13 chromosomes. (B) The 246 tandemly arrayed *G*. *arboretum* PK genes were grouped in 87 clusters distributed unevenly among the 13 chromosomes. (C) The 264 tandemly arrayed *G*. *hirsutum* PK genes were grouped in 91 clusters distributed unevenly among the 26 chromosomes. (D) The 455 tandemly arrayed *G*. *barbadense* PK genes were grouped in 152 clusters distributed unevenly among the 26 chromosomes. Gene IDs and subfamilies are labeled in the right of each chromosome, and the chromosomal location of each cluster is in the left of each chromosome. Genes in the same cluster are highlighted in the same color.
(PDF)

S8 Fig Quality control of GEO microarray datasets. RLE (Relative log expression) and NUSE (Normalized unscaled standard errors) values of each GEO microarray dataset.
(PDF)

S9 Fig Heat map of the expression patterns of individual cotton PK genes under abiotic stress treatments. The expression patterns of 564 PK genes under abiotic stress treatments are presented. The heat maps were generated using MeV (Multiple Experiment Viewer) software, version 4.9. Red and green correspond to up-regulation and down-regulation, respectively. Normalized gene expression values are provided in Supplemental S11 Table.
(PDF)

S10 Fig Heat map of the expression patterns of cotton PKs under waterlog and drought stress treatments. Normalized gene expression values are provided in Supplemental S13 Table.
(PDF)

S11 Fig Heat map of the expression patterns of cotton PKs under biotic stress treatments. Normalized gene expression values are provided in Supplemental S15 Table.
(PDF)

S12 Fig Heat map of the expression patterns of cotton PKs for genotypes (725, 703, 777, 783 and 737) and fiber development stages (6, 9, 12, 19 and 25 dpa). Normalized gene expression values are provided in Supplemental S17 Table.
(PDF)

S13 Fig Heat map of the expression patterns of cotton PKs about *GhHD-1* and fuzzless-lintless mutant during fiber development stages. Normalized gene expression values are provided in Supplemental S19 Table.
(PDF)

S14 Fig Heat map of the expression patterns of *G*. *hirsutum* PK genes during fiber development stages. Normalized gene expression RPKM values of *G*. *hirsutum* PK genes in fiber development stages (10, 15, 18, 21 and 28 dpa). Normalized gene expression values are provided in Supplemental S22 Table.
(PDF)

S15 Fig Heat map of the expression patterns of *G*. *barbadense* PK genes during fiber development stages. Normalized gene expression RPKM values of *G*. *barbadense* PK genes in fiber development stages (10, 15, 18, 21 and 28 dpa). Normalized gene expression values are provided in Supplemental S22 Table.
(PDF)

S16 Fig Heat map of the expression patterns of PK genes in two *G*. *hirsutum* genotypes during fiber development stages. Normalized gene expression RPKM values of *G*. *hirsutum* PK genes for genotypes (Li1 mutant and wild type) ahout leaf tissue and ovule tissues in fiber development stages (1, 3 and 8 dpa). Normalized gene expression values are provided in Supplemental S23 Table.
(PNG)

S1 Table Subfamily classification of cotton PKs in *G*. *raimondii*, *G*. *arboretum*, *G*. *hirsutum* and *G*. *barbadense*. *G*. *raimondii*, *G*. *arboretum*, *G*. *hirsutum* and *G*. *barbadense* classifications are provided in sheets 1–4, respectively.
(XLS)

S2 Table List of atypical cotton kinases in *G*. *raimondii*, *G*. *arboretum*, *G*. *hirsutum* and *G*. *barbadense*. *G*. *raimondii*, *G*. *arboretum*, *G*. *hirsutum* and *G*. *barbadense* atypical kinases are provided in sheets 1–4, respectively.
(XLS)

S3 Table Comparison of four cotton species PK subfamilies size with other angiosperm species. (XLS)

S4 Table Cotton protein kinases within 2–4 Pkinase or Pkinase_Tyr domains in *G*. *raimondii*, *G*. *arboretum*, *G*. *hirsutum* and *G*. *barbadense*. *G*. *raimondii*, *G*. *arboretum*, *G*. *hirsutum* and *G*. *barbadense* protein kinases within 2–4 Pkinase or Pkinase_Tyr domains are provided in sheets 1–4, respectively.
(XLS)

S5 Table Chromosome locations of PKs in *G*. *raimondii*, *G*. *arboretum*, *G*. *hirsutum* and *G*. *barbadense*. Chromosome locations of PKs in *G*. *raimondii*, *G*. *arboretum*, *G*. *hirsutum* and *G*. *barbadense* are provided in sheets 1–4, respectively.
(XLS)

S6 Table Collinearity events and *Ka/Ks* values of cotton PKs in *G*. *raimondii*, *G*. *arboretum*, *G*. *hirsutum* and *G*. *barbadense*. *G*. *raimondii*, *G*. *arboretum*, *G*. *hirsutum* and *G*. *barbadense Ka/Ks* values of protein kinases are provided in sheets 1–4, respectively.
(XLS)

S7 Table Lists of tandemly arrayed cotton PKs in *G*. *raimondii*, *G*. *arboretum*, *G*. *hirsutum* and *G*. *barbadense*. *G*. *raimondii*, *G*. *arboretum*, *G*. *hirsutum* and *G*. *barbadense* tandemly arrayed PKs are provided in sheets 1–4, respectively.
(XLS)

S8 Table Synteny analyses of PK genes in *G*. *raimondii*, *G*. *arboretum*, *G*. *hirsutum* and *G*. *barbadense*. Synteny analyses of PK genes bewteen *G*. *arboretum* A-genome and *G*. *raimondii* D-genome; bewteen *G*. *hirsutum* At-subgenome and *G*. *barbadense* At-subgenome; bewteen *G*. *hirsutum* Dt-subgenome and *G*. *barbadense* Dt-subgenome; bewteen *G*. *hirsutum* At-subgenome and *G*. *arboretum* A-genome*;* bwtween *G*. *hirsutum* Dt-subgenome and *G*. *raimondii* D-genome; between *G*. *barbadense* At-subgenome and *G*. *arboretum* A-genome; and between *G*. *barbadense* Dt-subgenome and *G*. *raimondii* D-genome are provided in sheets 1–7, respectively.
(XLS)

S9 Table Synteny analyses of PK genes with single correspondence in *G*. *raimondii*, *G*. *arboretum*, *G*. *hirsutum* and *G*. *barbadense*. The descriptions of sheets 1–7 are same as S8 Table.
(XLS)

S10 Table Public cotton expression data to use. (XLS)

S11 Table Normalized gene expression values of 564 *G*. *hirsutum* PK genes under abiotic stress treatments. (XLS)

S12 Table The differential expression genes (p<0.01 and |FC|>1.5) of *G*. *hirsutum* PK genes under abiotic stress treatments. (XLS)

S13 Table Normalized gene expression values of 564 *G*. *hirsutum* PK genes under waterlog and drought stress treatments. (XLS)

S14 Table The differential expression genes (p<0.01 and |FC|>1.5) of *G*. *hirsutum* PK genes under waterlog and drought stress treatments. (XLS)

S15 Table Normalized gene expression values of 564 *G*. *hirsutum* PK genes under biotic stress treatments. (XLS)

S16 Table The differential expression genes (p<0.01 and |FC|>1.5) of *G*. *hirsutum* PK genes under biotic stress treatments. (XLS)

S17 Table Normalized gene expression values of 564 *G*. *hirsutum* PK genes for genotypes (725, 703, 777, 783 and 737) and fiber development stages (6, 9, 12, 19 and 25dpa). (XLS)

S18 Table The differential expression genes (p<0.01 and |FC|>1.5) of *G*. *hirsutum* PK genes for genotypes (725, 703, 777, 783 and 737) and fiber development stages (6, 9, 12, 19 and 25dpa). (XLS)

S19 Table Normalized gene expression values of 564 *G*. *hirsutum* PK genes in *GhHD-1* and fuzzless-lintless mutants during fiber development stages. (XLS)

S20 Table The differential expression genes (p<0.01 and |FC|>1.5) of *G*. *hirsutum* PK genes in *GhHD-1* and fuzzless-lintless mutants during fiber development stages. (XLS)

S21 Table Public cotton RNA-seq expression data to use. (XLS)

S22 Table Normalized gene expression RPKM values of *G*. *hirsutum* and *G*. *barbadense* PK genes in fiber development stages (10, 15, 18, 21 and 28 dpa). *G*. *hirsutum* and *G*. *barbadense* PK genes are provided in sheets 1–2, respectively.
(XLS)

S23 Table Normalized gene expression RPKM values of G. hirsutum PK genes for genotypes (Li1 mutant and wild type) ahout leaf tissue and ovule tissues in fiber development stages (1, 3 and 8 dpa). (XLS)

S24 Table Normalized gene expression log2 Fold Change (Li1 mutant/wild type) values of *G*. *hirsutum* PK genes for genotypes (Li1 mutant and wild type) ahout leaf tissue and ovule tissues in fiber development stages (1, 3 and 8 dpa). Leaf tissue, ovule tissues in 1, 3 and 8 dpa are provided in sheets 1–4, respectively.
(XLS)

## Abbreviations

AGC: PKA–PKG–PKC
CAMK: calcium- and calmodulin-regulated kinase
CDPK: calcium-dependent PKs
CK1: casein kinase 1
CMGC: cyclin-dependent kinases, mitogen-activated protein kinases, glycogen synthase kinase and cyclin-dependent-like kinases
LRR: leucine-rich repeat
PK: protein kinase
RLCK: receptor-like cytoplasmic kinase
RLK: receptor-like kinase
TK: tyrosine kinase
TKL: tyrosine kinase-like kinase
