# Supplementary material for: Genome-wide classification, evolutionary analysis and gene expression patterns of the kinome in Gossypium
Source: PLoS One. 2018 May 16;13(5):e0197392. doi: 10.1371/journal.pone.0197392 (PMC5955557; doi:10.1371/journal.pone.0197392)
Supplement: S4 Fig — The descriptions of domain and exon phases are the same as in Fig 2. The lengths of the boxes and lines are scaled based on the length of genes. (PDF) [file pone.0197392.s004.pdf]

AGC conserved exon−intron and domain diagram (part 1)

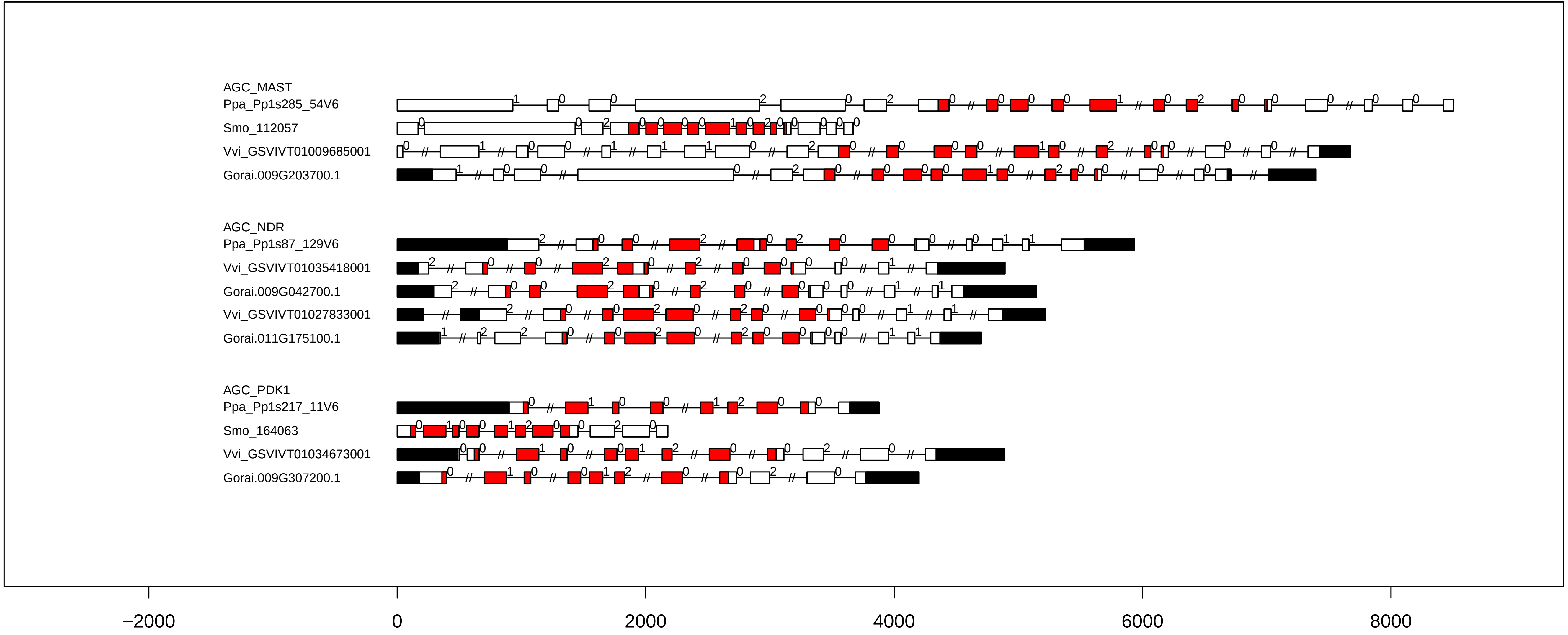

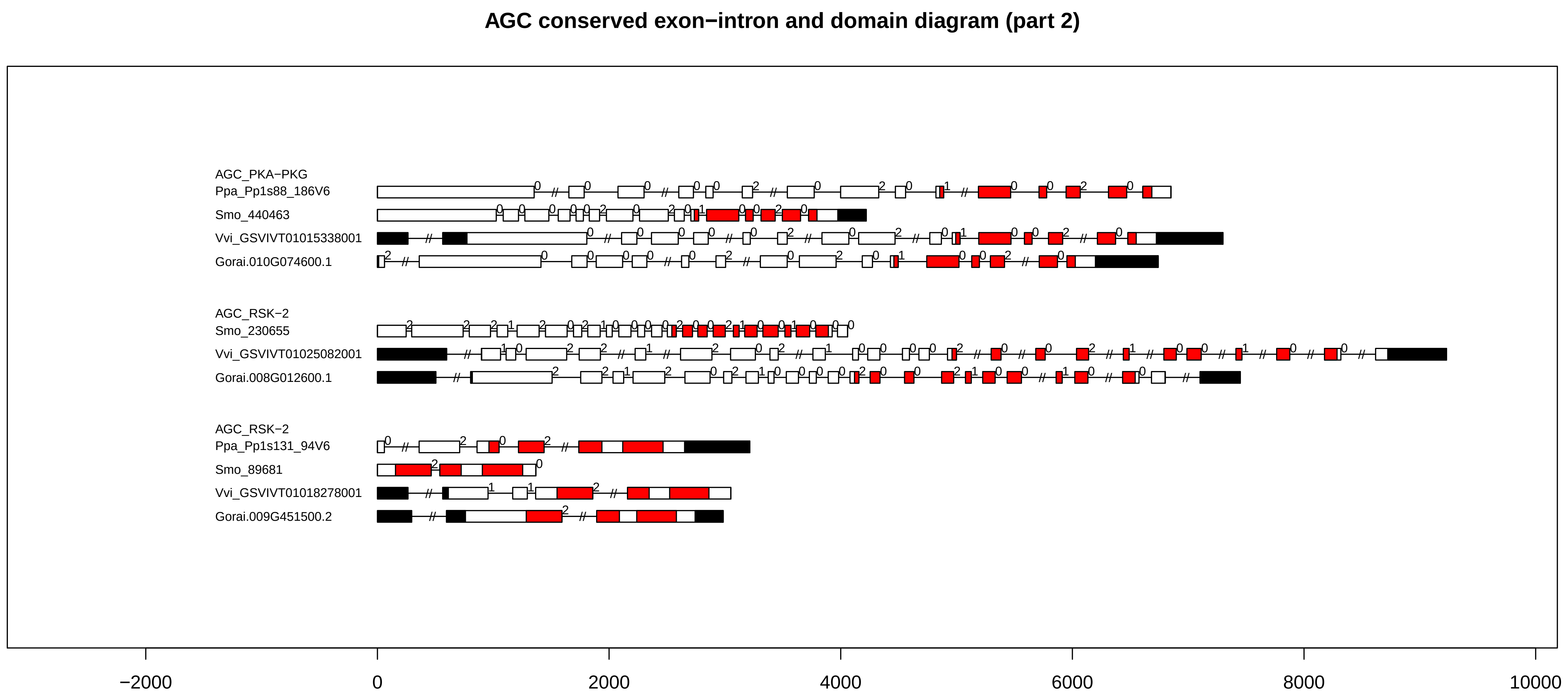
AGC conserved exon−intron and domain diagram (part 2)
AGC_PKA−PKG
Ppa_Pp1s88_186V6
Smo_440463
Vvi_GSVIVT01015338001
Gorai.010G074600.1
AGC_RSK−2
Smo_230655
Vvi_GSVIVT01025082001
Gorai.008G012600.1
AGC_RSK−2
Ppa_Pp1s131_94V6
Smo_89681
Vvi_GSVIVT01018278001
Gorai.009G451500.2
−2000
0
2000
4000
6000
8000
10000

AGC conserved exon−intron and domain diagram (part 3)

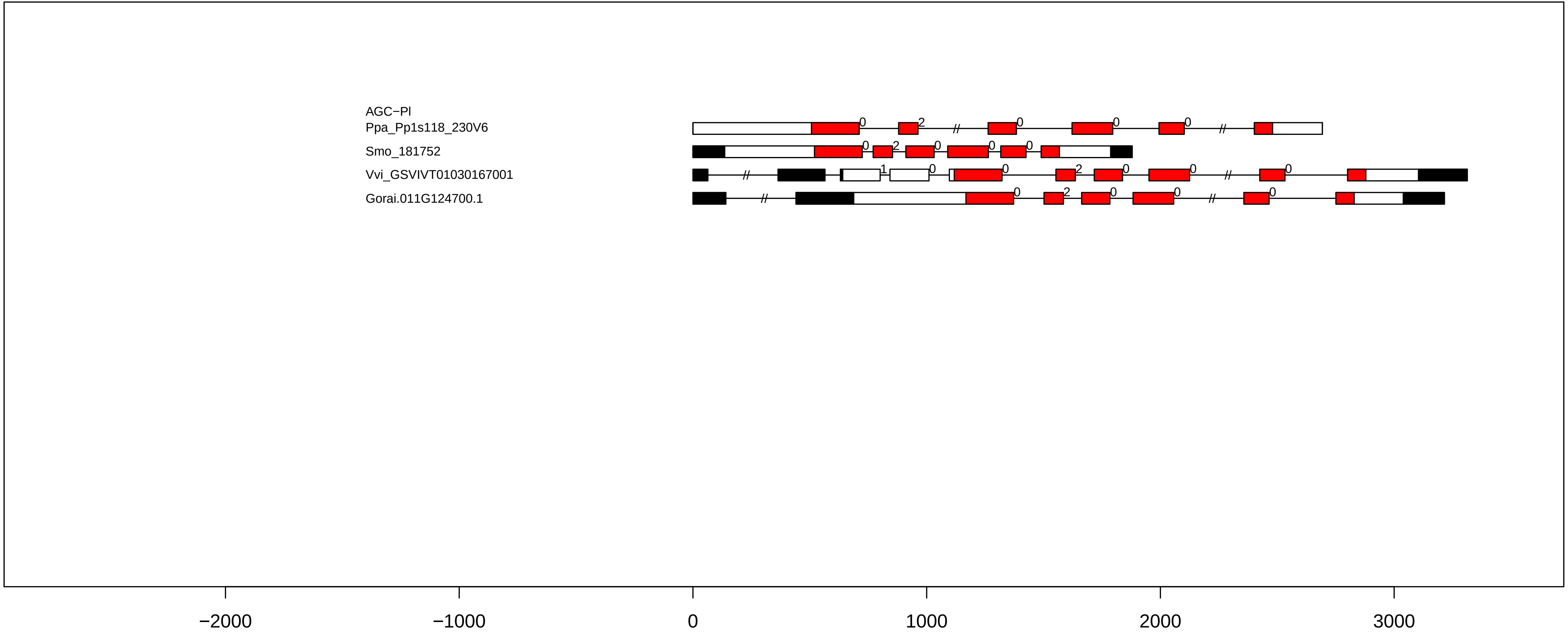

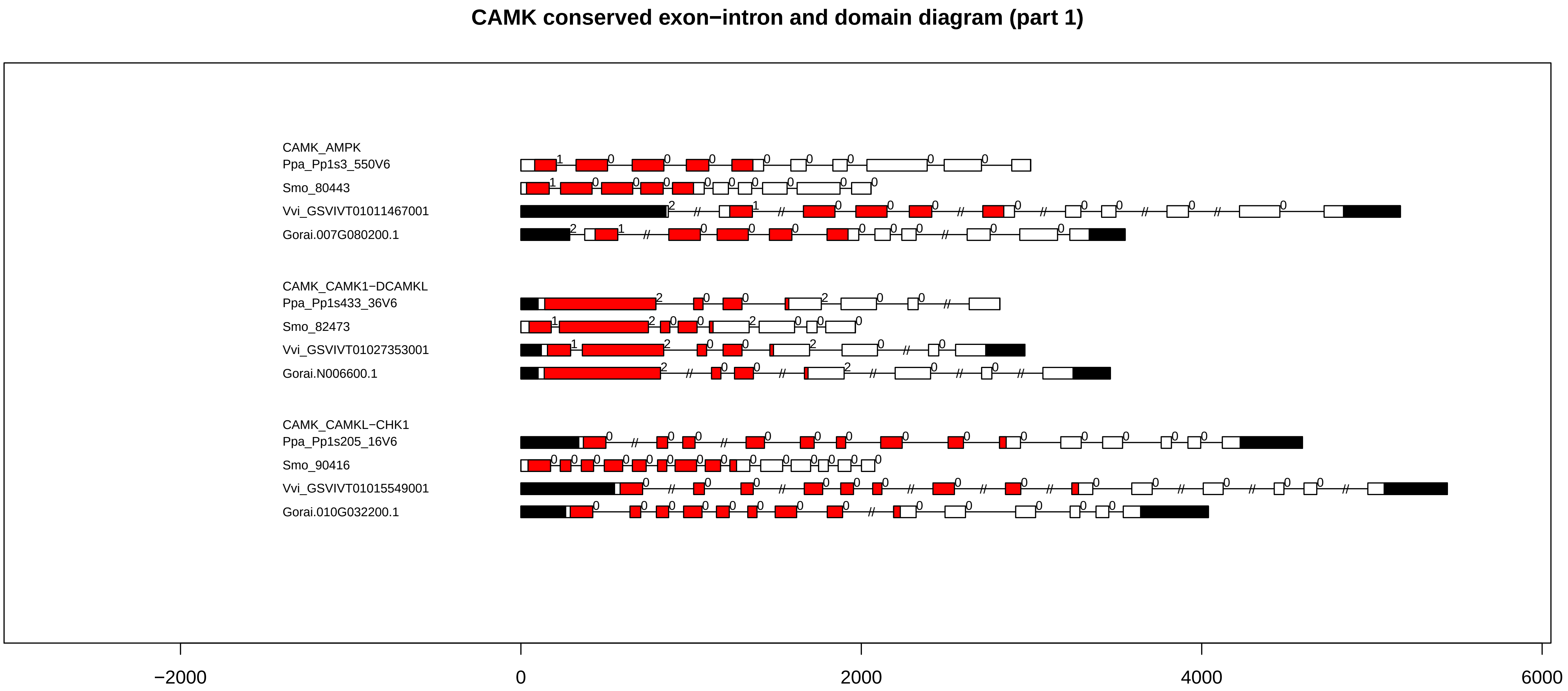

CAMK conserved exon−intron and domain diagram (part 1)
CAMK_AMPK
Ppa_Pp1s3_550V6
Smo_80443
Vvi_GSVIVT01011467001
Gorai.007G080200.1
CAMK_CAMK1−DCAMKL
Ppa_Pp1s433_36V6
Smo_82473
Vvi_GSVIVT01027353001
Gorai.N006600.1
CAMK_CAMKL−CHK1
Ppa_Pp1s205_16V6
Smo_90416
Vvi_GSVIVT01015549001
Gorai.010G032200.1
−2000
0
2000
4000
6000

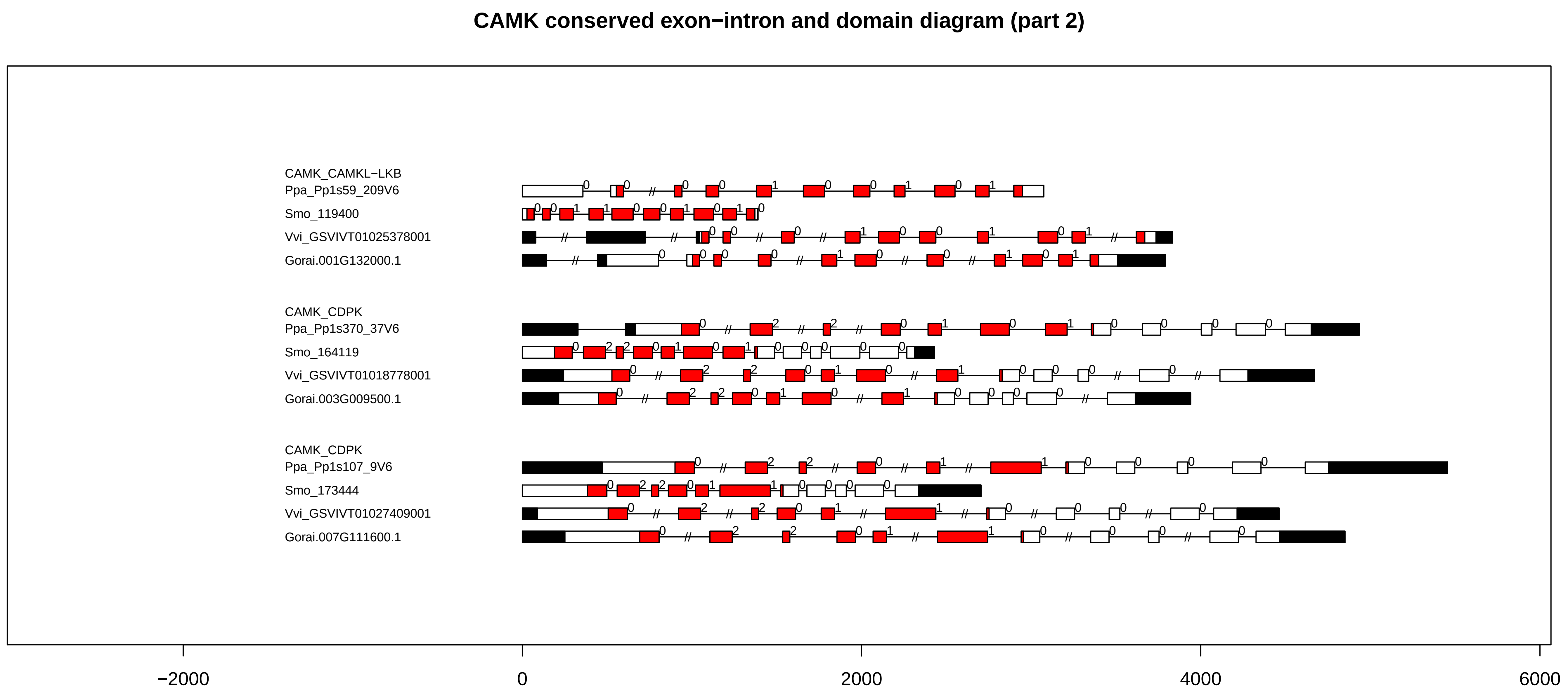

CAMK conserved exon−intron and domain diagram (part 2)
CAMK_CAMKL−LKB
Ppa_Pp1s59_209V6
Smo_119400
Vvi_GSVIVT01025378001
Gorai.001G132000.1
CAMK_CDPK
Ppa_Pp1s370_37V6
Smo_164119
Vvi_GSVIVT01018778001
Gorai.003G009500.1
CAMK_CDPK
Ppa_Pp1s107_9V6
Smo_173444
Vvi_GSVIVT01027409001
Gorai.007G111600.1
−2000
0
2000
4000
6000

CAMK conserved exon−intron and domain diagram (part 3)

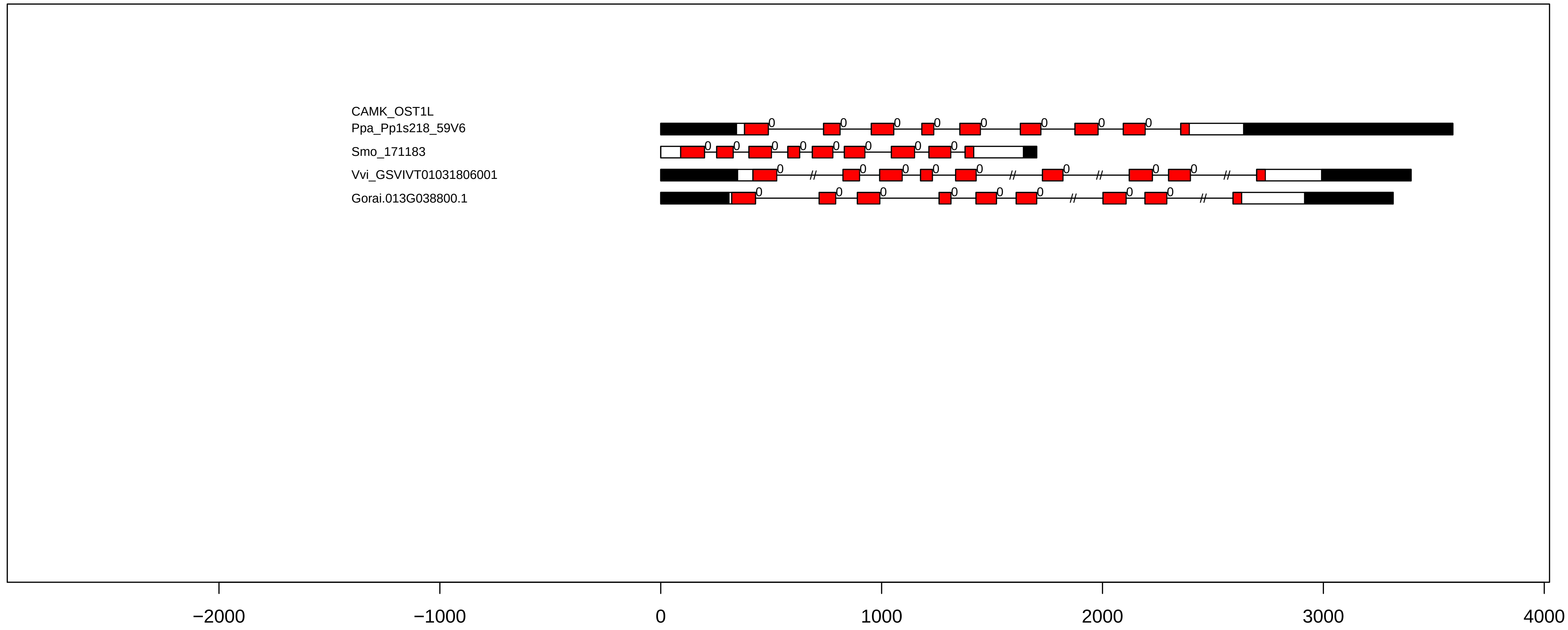

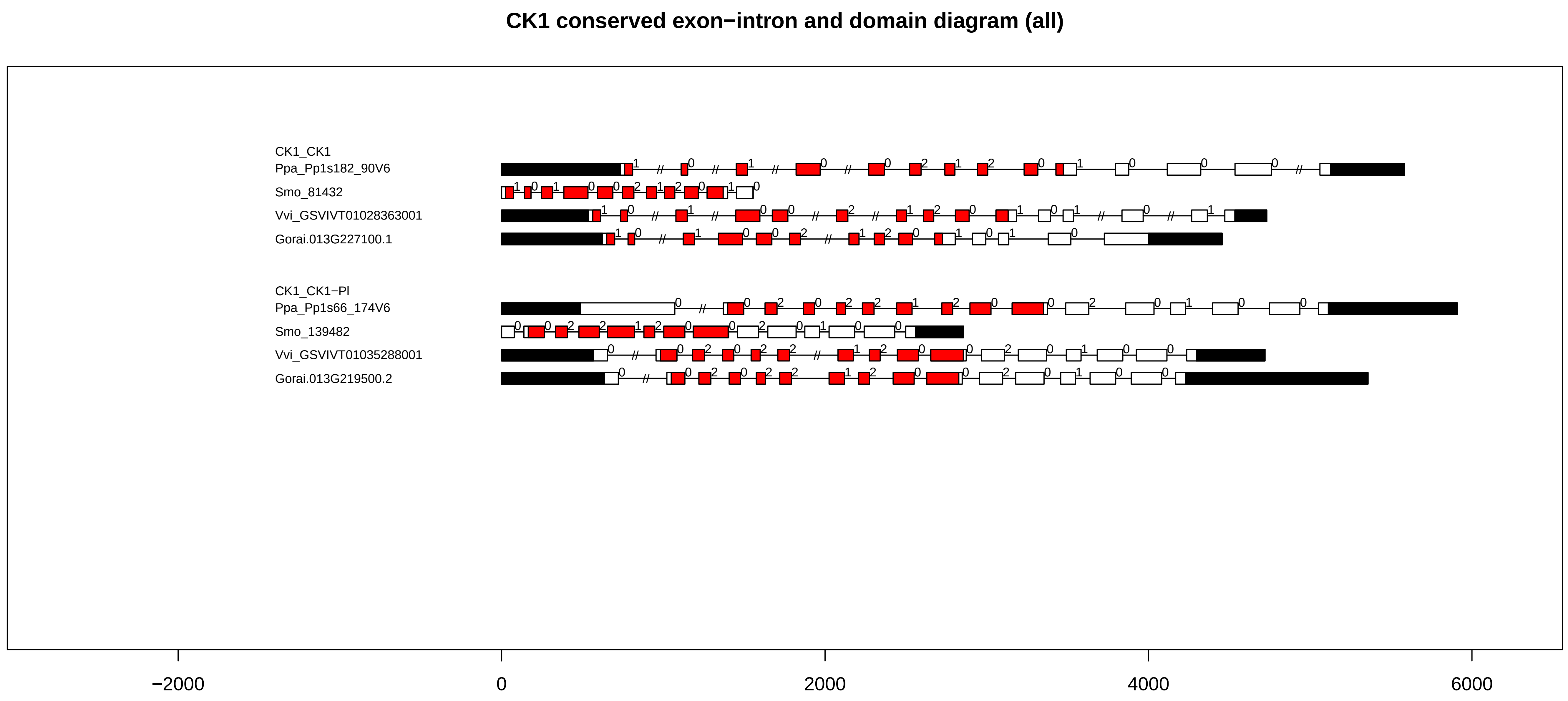
CK1 conserved exon−intron and domain diagram (all)
CK1_CK1
Ppa_Pp1s182_90V6
Smo_81432
Vvi_GSVIVT01028363001
Gorai.013G227100.1
CK1_CK1−Pl
Ppa_Pp1s66_174V6
Smo_139482
Vvi_GSVIVT01035288001
Gorai.013G219500.2
−2000
0
2000
4000
6000

CMGC conserved exon−intron and domain diagram (part 1)

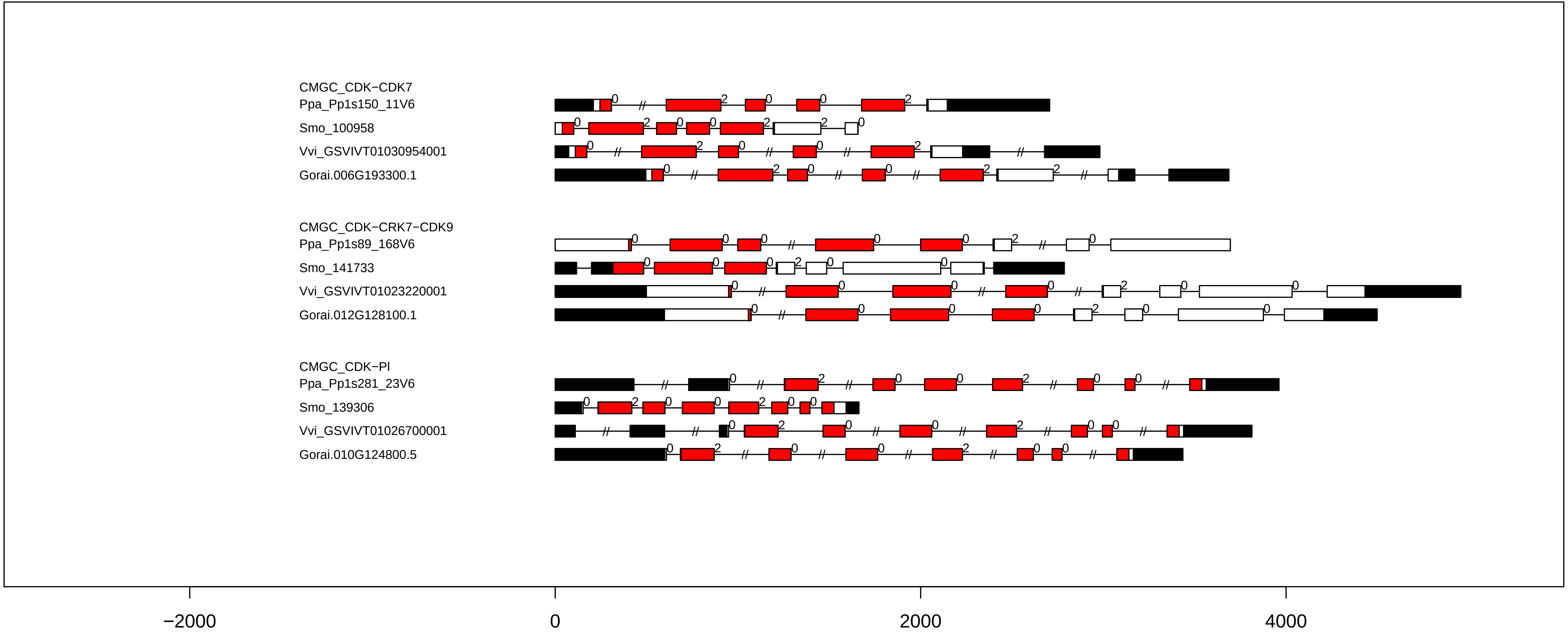

CMGC conserved exon−intron and domain diagram (part 2)

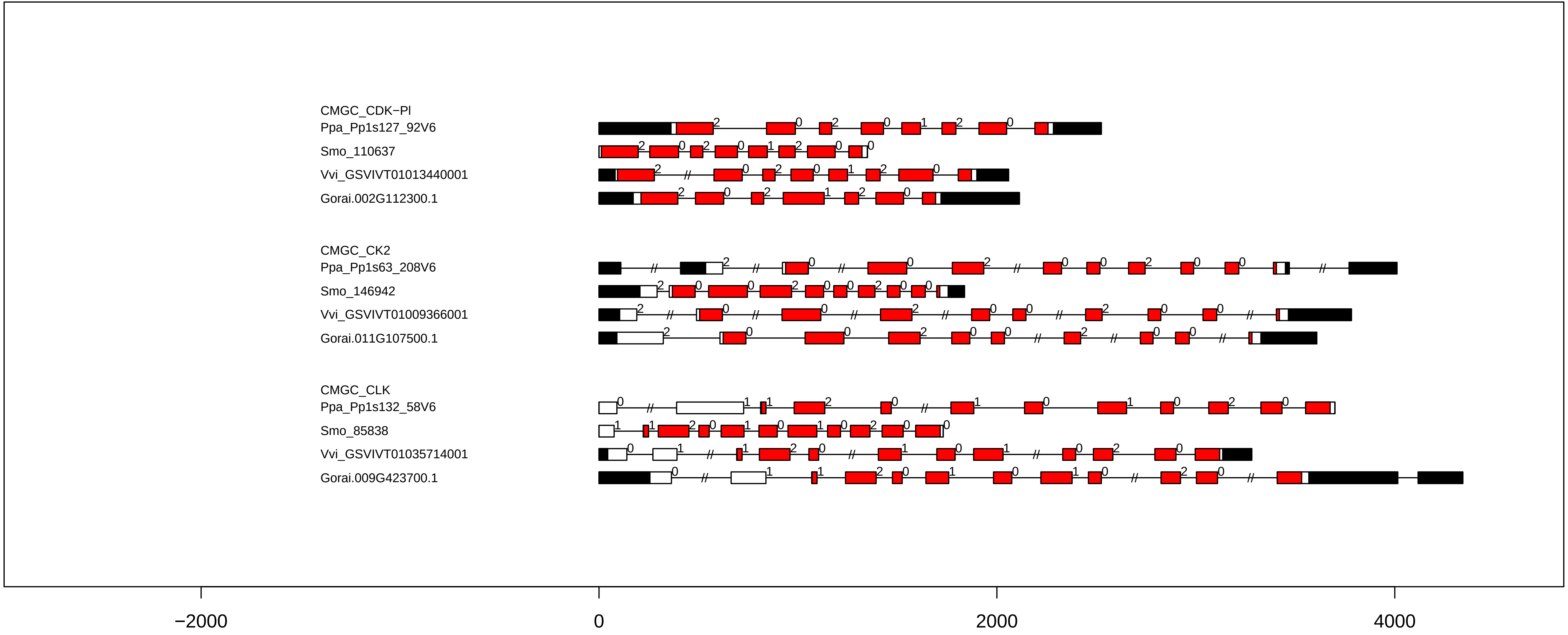

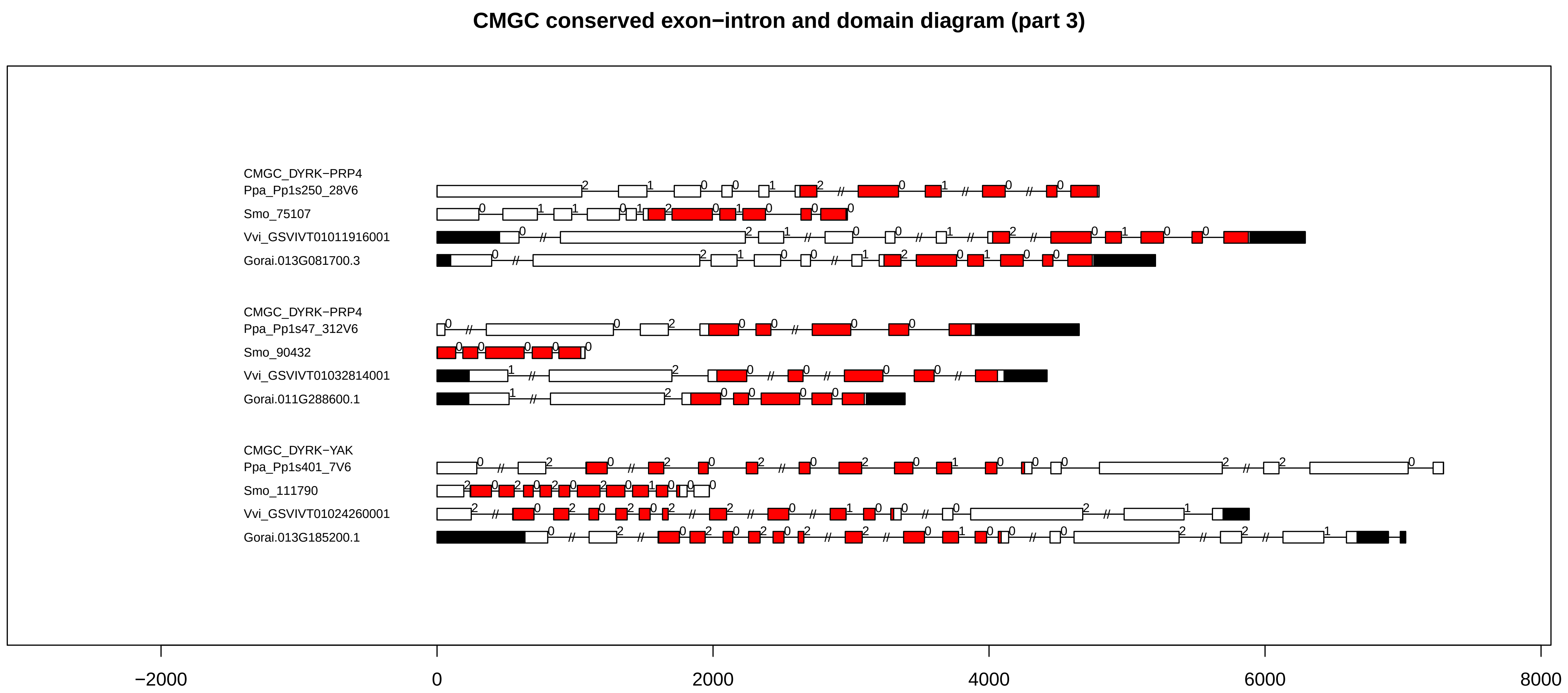
CMGC conserved exon−intron and domain diagram (part 3)
CMGC_DYRK−PRP4
Ppa_Pp1s250_28V6
Smo_75107
Vvi_GSVIVT01011916001
Gorai.013G081700.3
CMGC_DYRK−PRP4
Ppa_Pp1s47_312V6
Smo_90432
Vvi_GSVIVT01032814001
Gorai.011G288600.1
CMGC_DYRK−YAK
Ppa_Pp1s401_7V6
Smo_111790
Vvi_GSVIVT01024260001
Gorai.013G185200.1
−2000
0
2000
4000
6000
8000

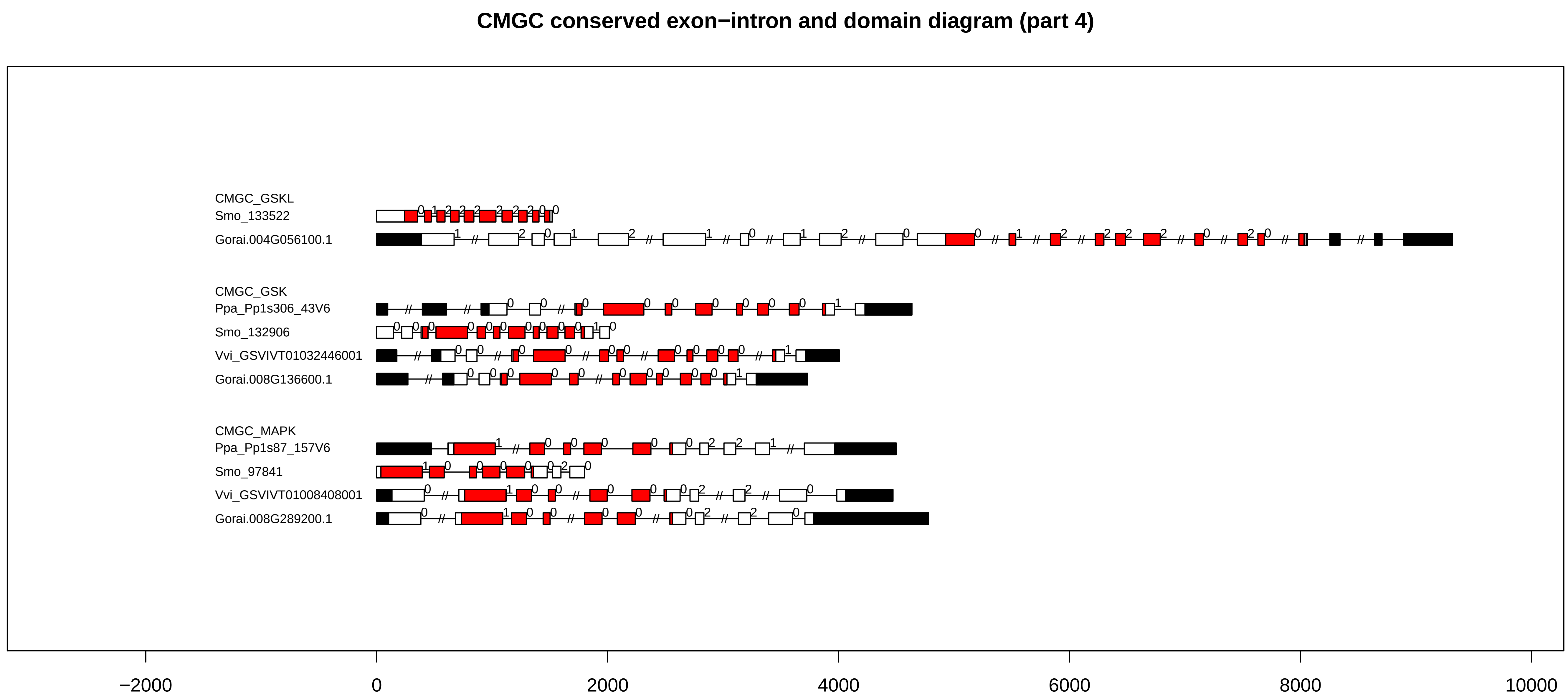
CMGC conserved exon−intron and domain diagram (part 4)
CMGC_GSKL
Smo_133522
Gorai.004G056100.1
CMGC_GSK
Ppa_Pp1s306_43V6
Smo_132906
Vvi_GSVIVT01032446001
Gorai.008G136600.1
CMGC_MAPK
Ppa_Pp1s87_157V6
Smo_97841
Vvi_GSVIVT01008408001
Gorai.008G289200.1
−2000
0
2000
4000
6000
8000
10000

CMGC conserved exon−intron and domain diagram (part 5)

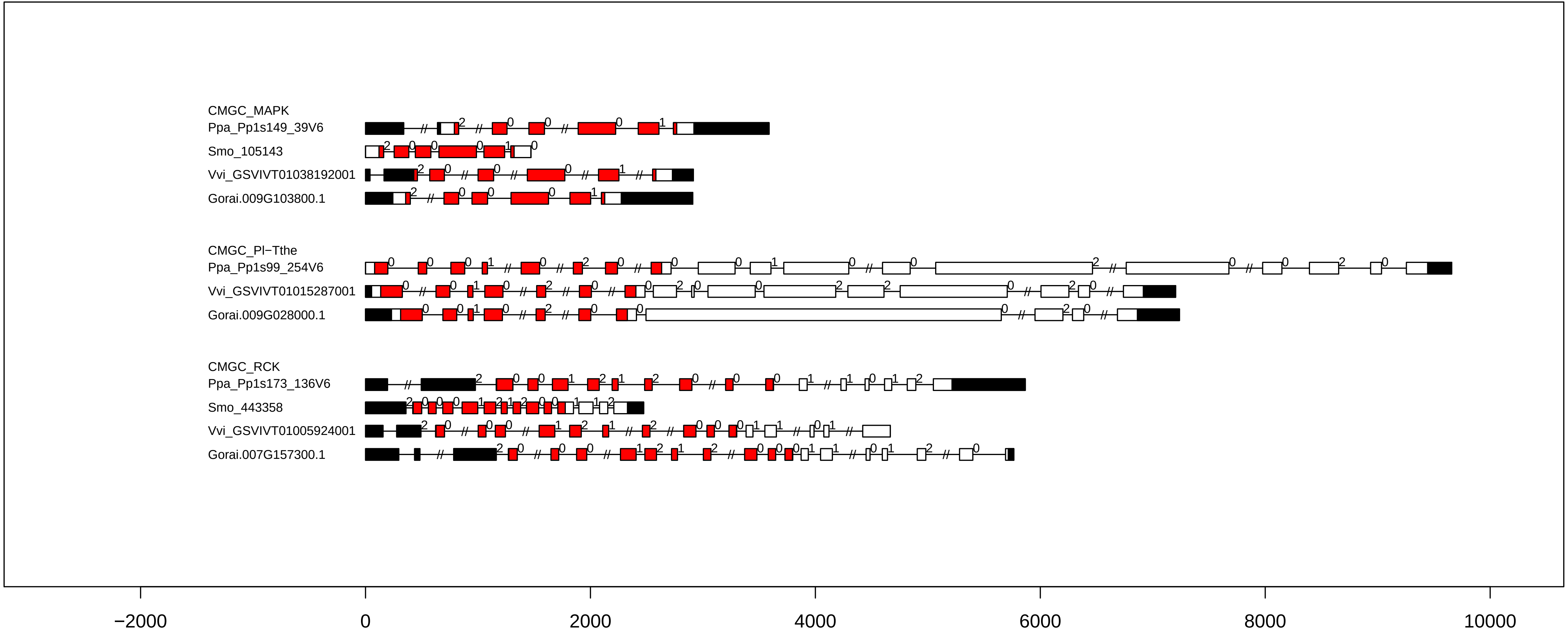

CMGC conserved exon−intron and domain diagram (part 6)

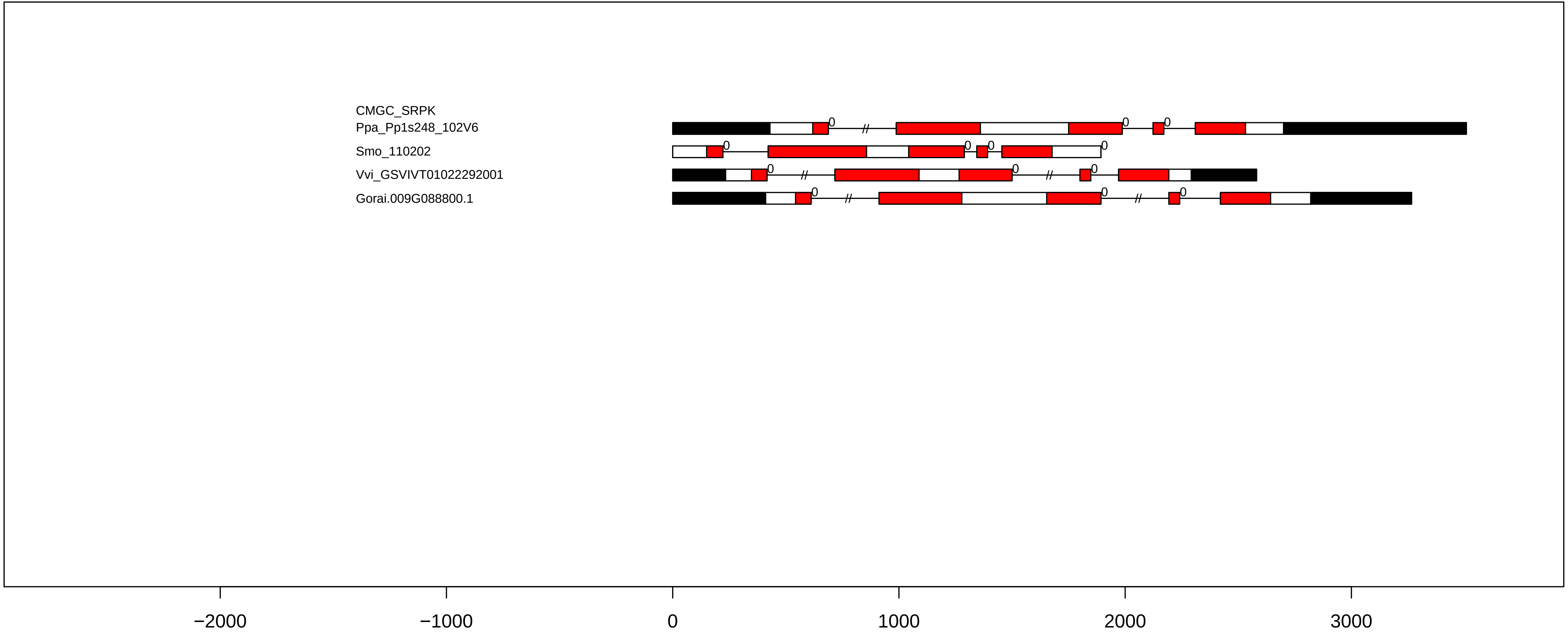

other conserved exon−intron and domain diagram (part 1)

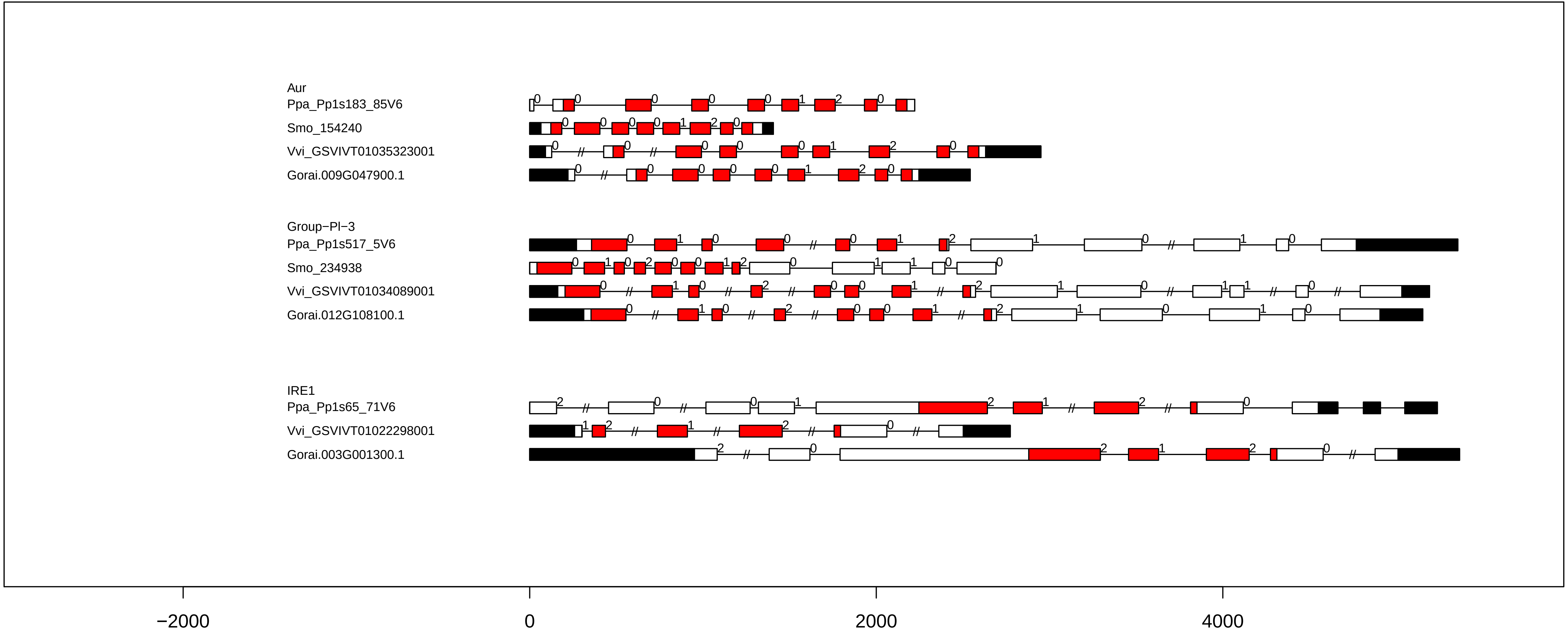

other conserved exon−intron and domain diagram (part 2)

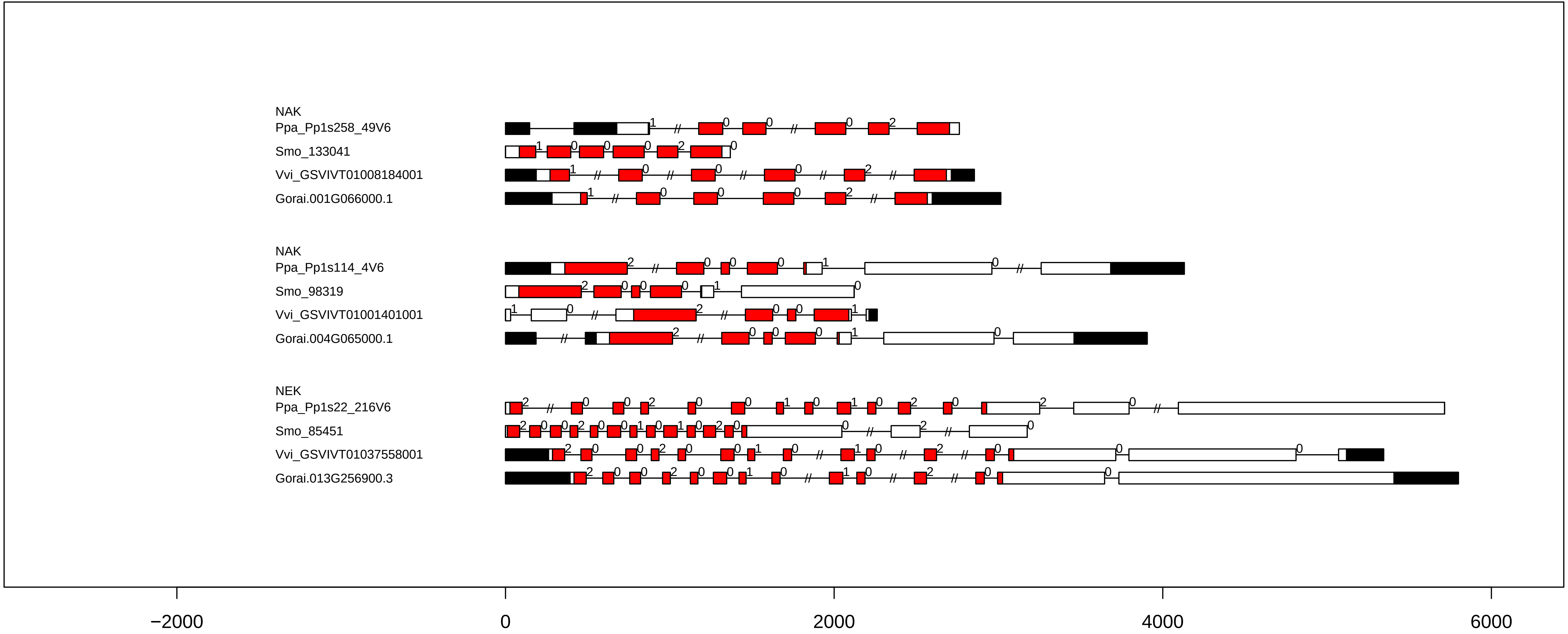

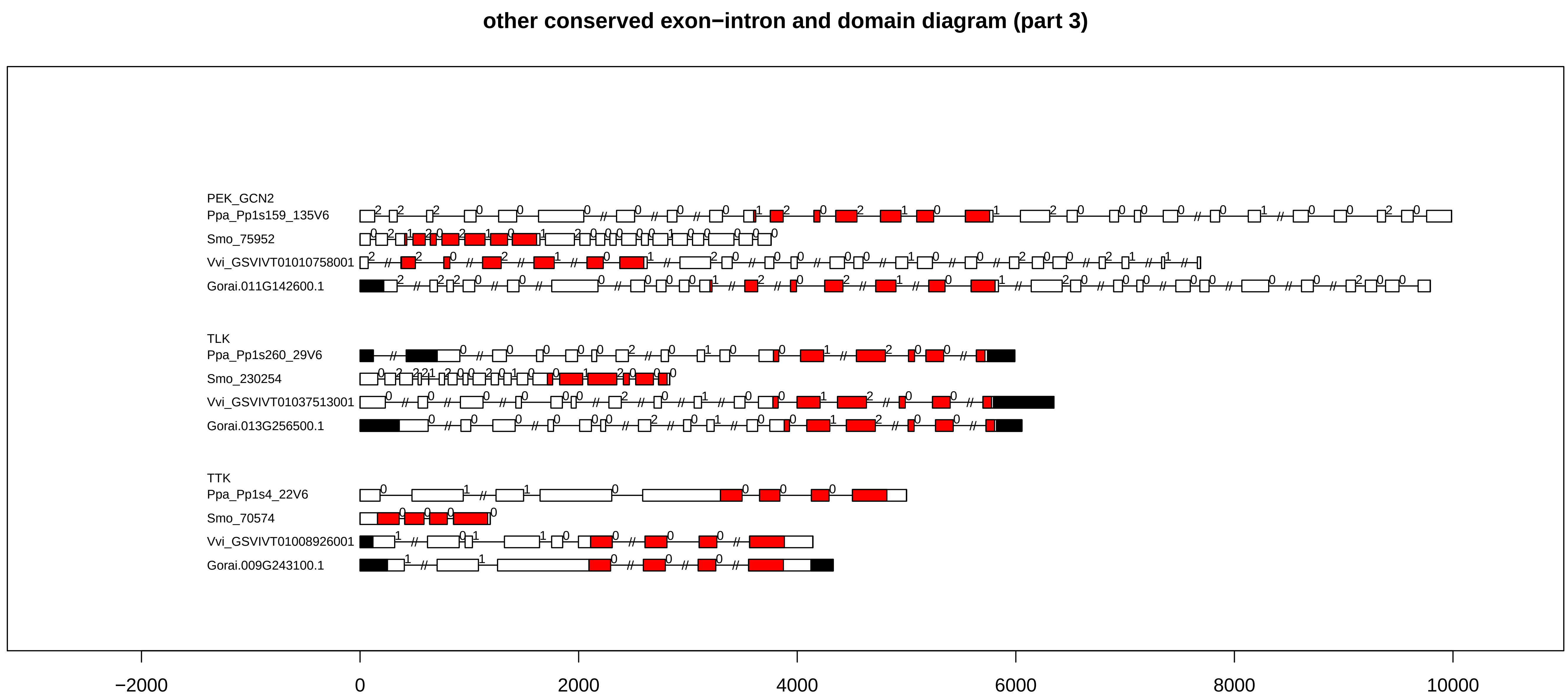
other conserved exon−intron and domain diagram (part 3)
PEK_GCN2
Ppa_Pp1s159_135V6
Smo_75952
Vvi_GSVIVT01010758001
Gorai.011G142600.1
TLK
Ppa_Pp1s260_29V6
Smo_230254
Vvi_GSVIVT01037513001
Gorai.013G256500.1
TTK
Ppa_Pp1s4_22V6
Smo_70574
Vvi_GSVIVT01008926001
Gorai.009G243100.1
−2000
0
2000
4000
6000
8000
10000

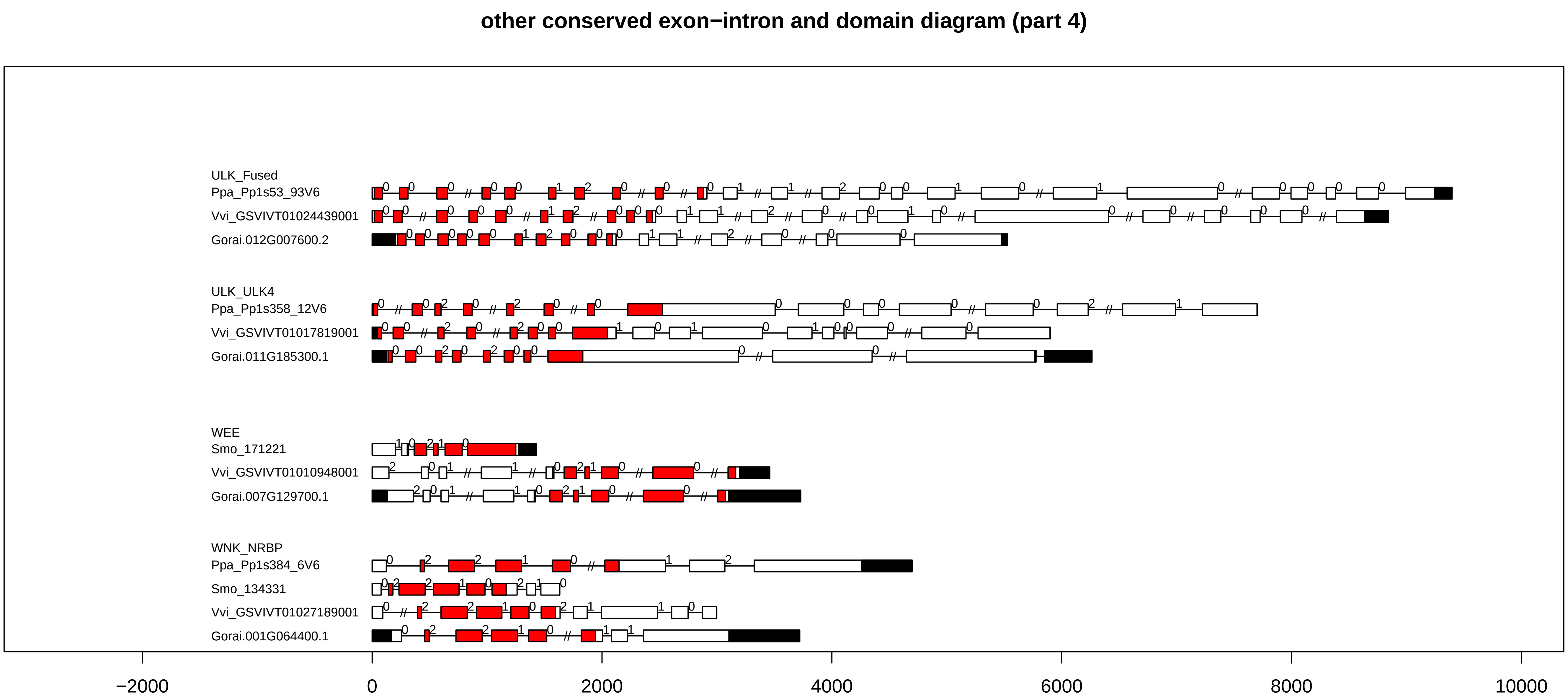
other conserved exon−intron and domain diagram (part 4)
ULK_Fused
Ppa_Pp1s53_93V6
Vvi_GSVIVT01024439001
Gorai.012G007600.2
ULK_ULK4
Ppa_Pp1s358_12V6
Vvi_GSVIVT01017819001
Gorai.011G185300.1
WEE
Smo_171221
Vvi_GSVIVT01010948001
Gorai.007G129700.1
WNK_NRBP
Ppa_Pp1s384_6V6
Smo_134331
Vvi_GSVIVT01027189001
Gorai.001G064400.1
−2000
0
2000
4000
6000
8000
10000

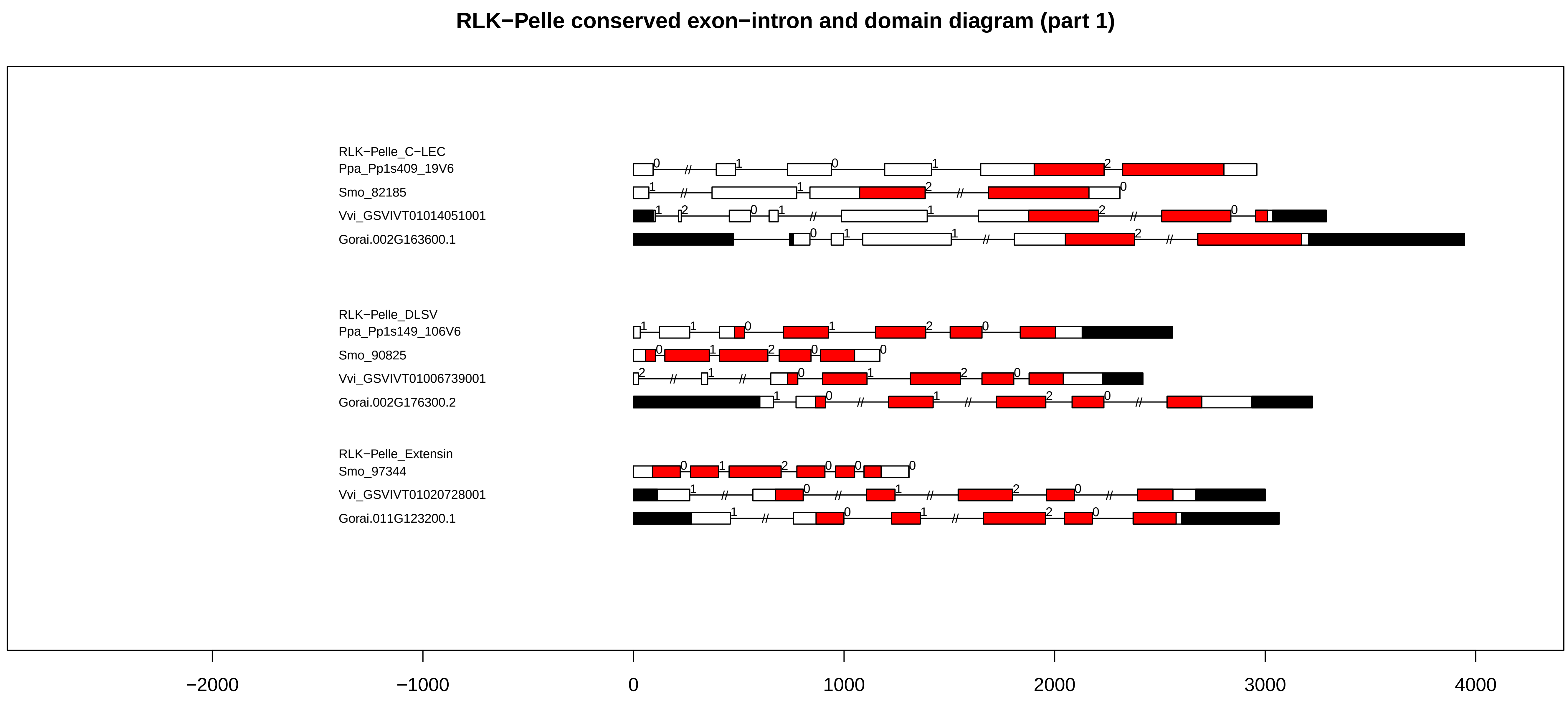
RLK−Pelle conserved exon−intron and domain diagram (part 1)
RLK−Pelle_C−LEC
Ppa_Pp1s409_19V6
Smo_82185
Vvi_GSVIVT01014051001
Gorai.002G163600.1
RLK−Pelle_DLSV
Ppa_Pp1s149_106V6
Smo_90825
Vvi_GSVIVT01006739001
Gorai.002G176300.2
RLK−Pelle_Extensin
Smo_97344
Vvi_GSVIVT01020728001
Gorai.011G123200.1
−2000
−1000
0
1000
2000
3000
4000

RLK−Pelle conserved exon−intron and domain diagram (part 2)

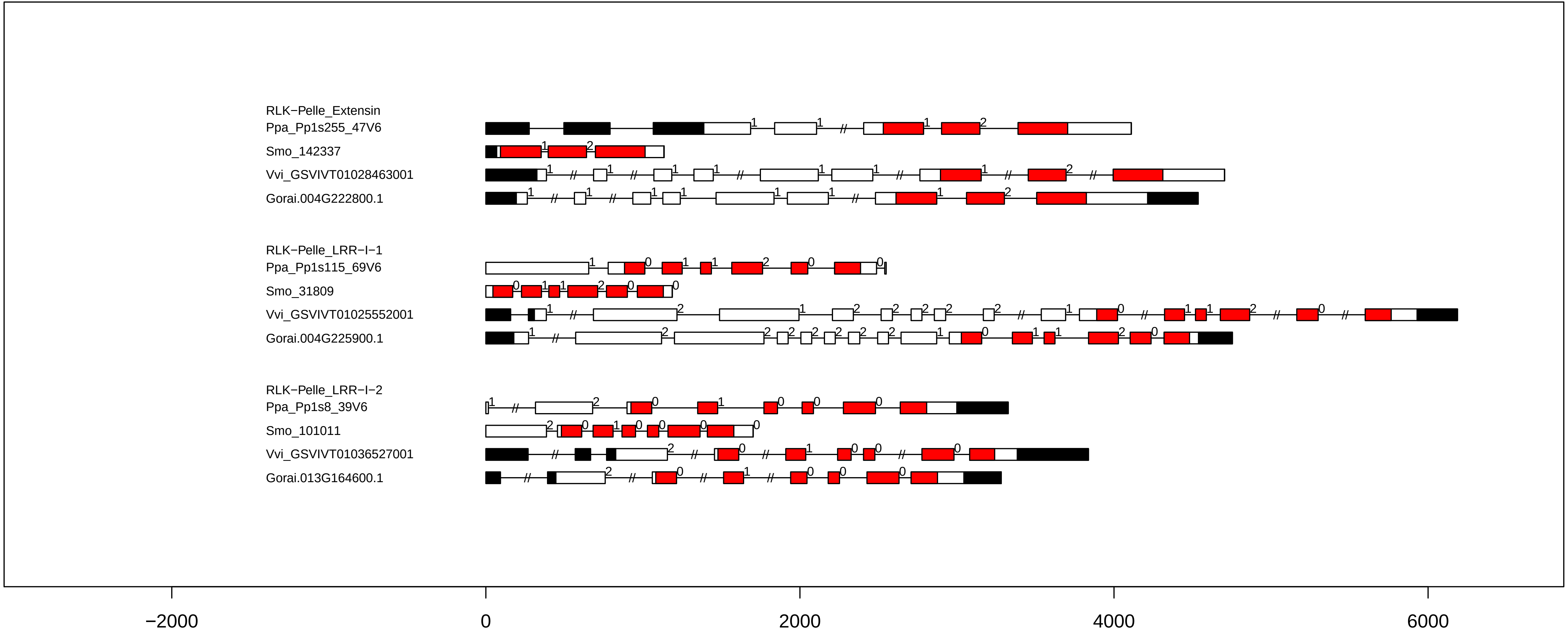

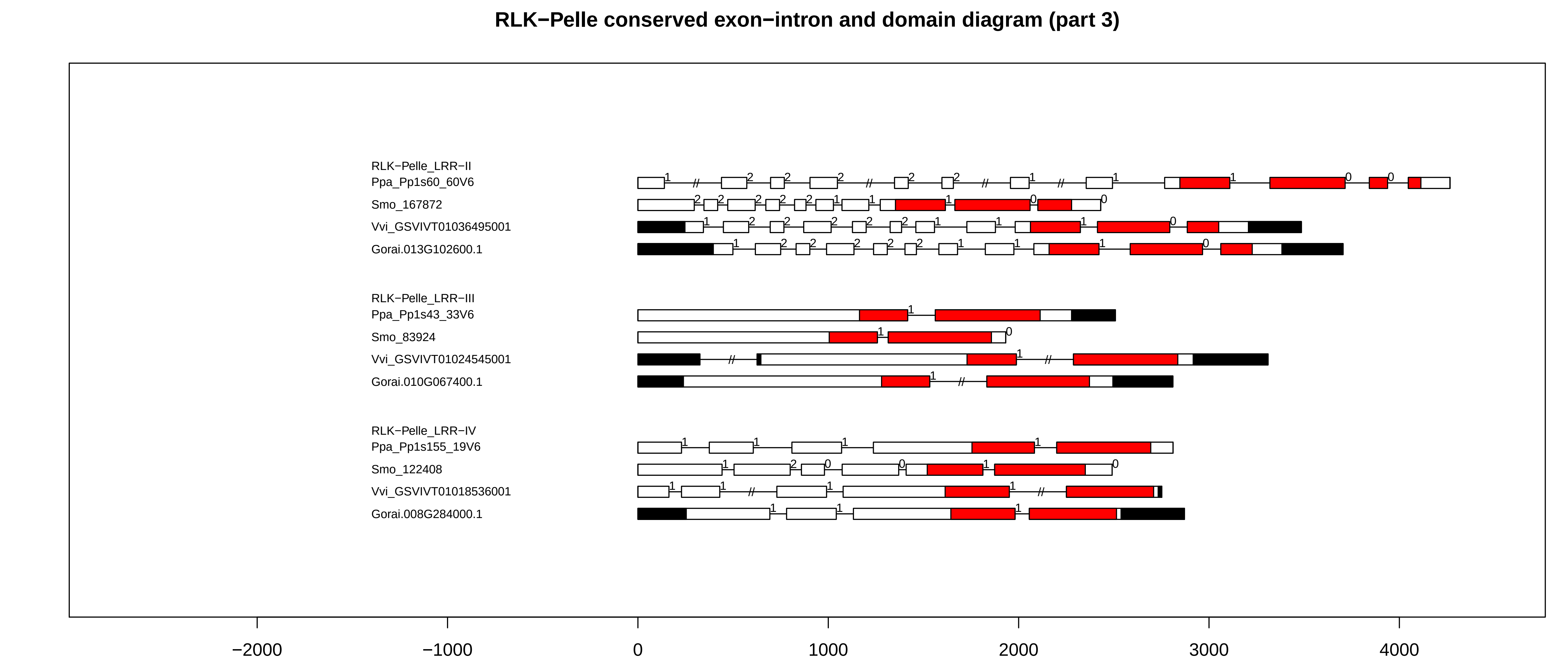
RLK−Pelle conserved exon−intron and domain diagram (part 3)
RLK−Pelle_LRR−II
Ppa_Pp1s60_60V6
Smo_167872
Vvi_GSVIVT01036495001
Gorai.013G102600.1
RLK−Pelle_LRR−III
Ppa_Pp1s43_33V6
Smo_83924
Vvi_GSVIVT01024545001
Gorai.010G067400.1
RLK−Pelle_LRR−IV
Ppa_Pp1s155_19V6
Smo_122408
Vvi_GSVIVT01018536001
Gorai.008G284000.1
−2000
−1000
0
1000
2000
3000
4000

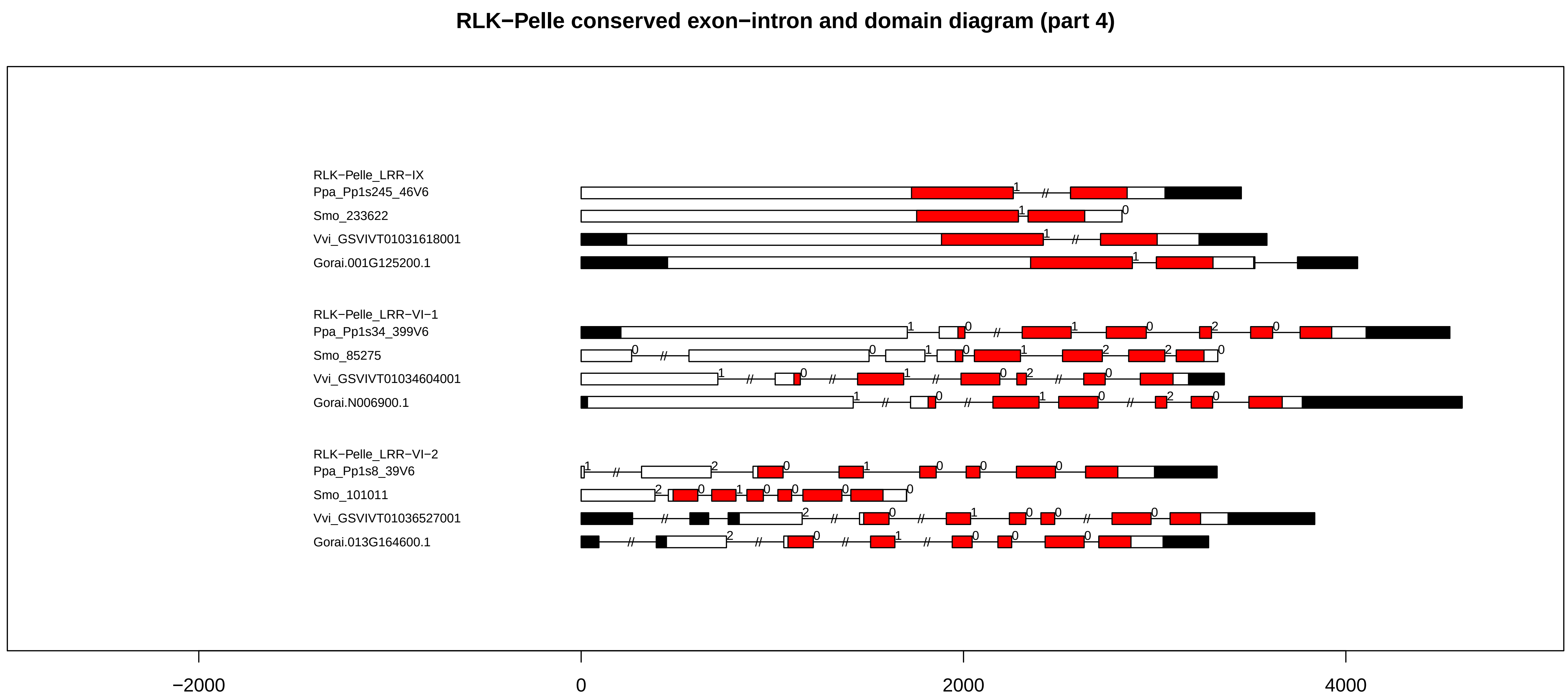
RLK−Pelle conserved exon−intron and domain diagram (part 4)
RLK−Pelle_LRR−IX
Ppa_Pp1s245_46V6
Smo_233622
Vvi_GSVIVT01031618001
Gorai.001G125200.1
RLK−Pelle_LRR−VI−1
Ppa_Pp1s34_399V6
Smo_85275
Vvi_GSVIVT01034604001
Gorai.N006900.1
RLK−Pelle_LRR−VI−2
Ppa_Pp1s8_39V6
Smo_101011
Vvi_GSVIVT01036527001
Gorai.013G164600.1
−2000
0
2000
4000

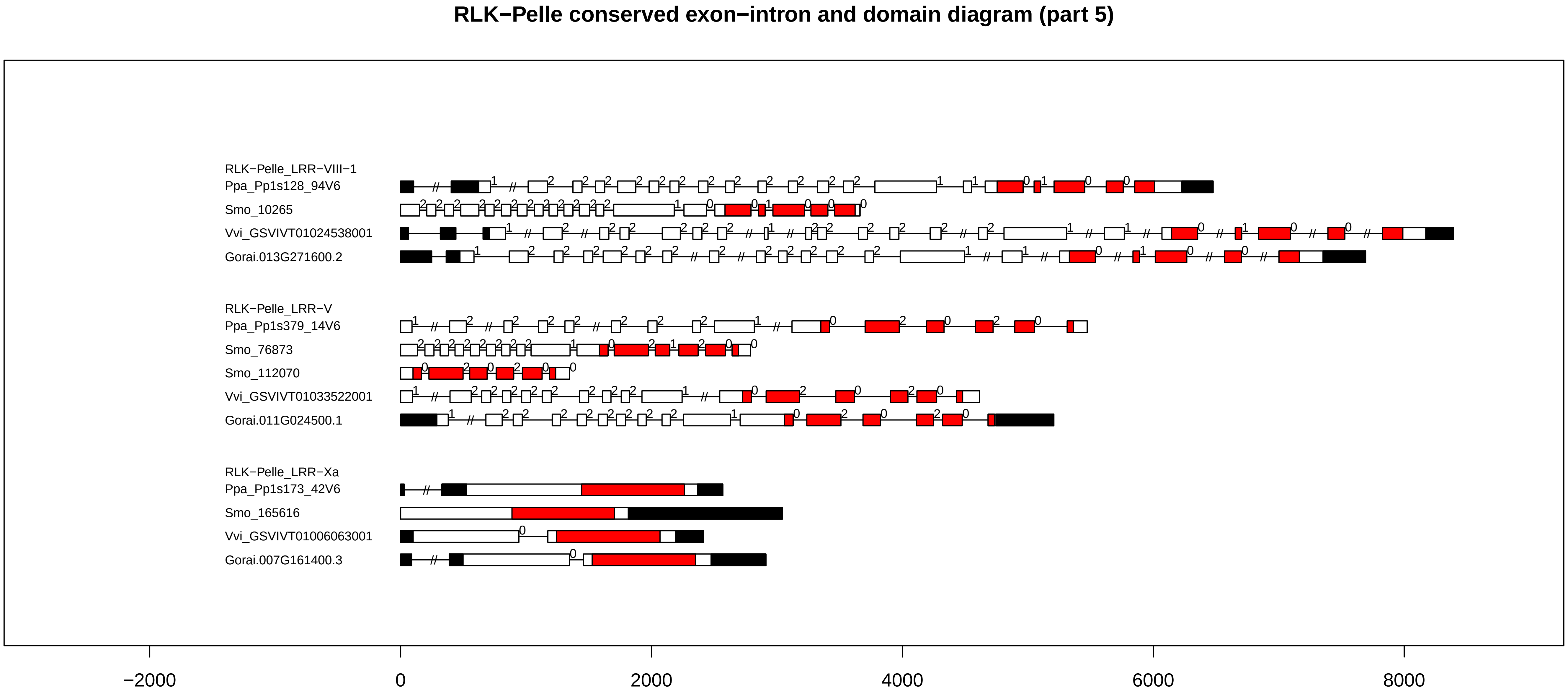
RLK−Pelle conserved exon−intron and domain diagram (part 5)
RLK−Pelle_LRR−VIII−1
Ppa_Pp1s128_94V6
Smo_10265
Vvi_GSVIVT01024538001
Gorai.013G271600.2
RLK−Pelle_LRR−V
Ppa_Pp1s379_14V6
Smo_76873
Smo_112070
Vvi_GSVIVT01033522001
Gorai.011G024500.1
RLK−Pelle_LRR−Xa
Ppa_Pp1s173_42V6
Smo_165616
Vvi_GSVIVT01006063001
Gorai.007G161400.3
−2000
0
2000
4000
6000
8000

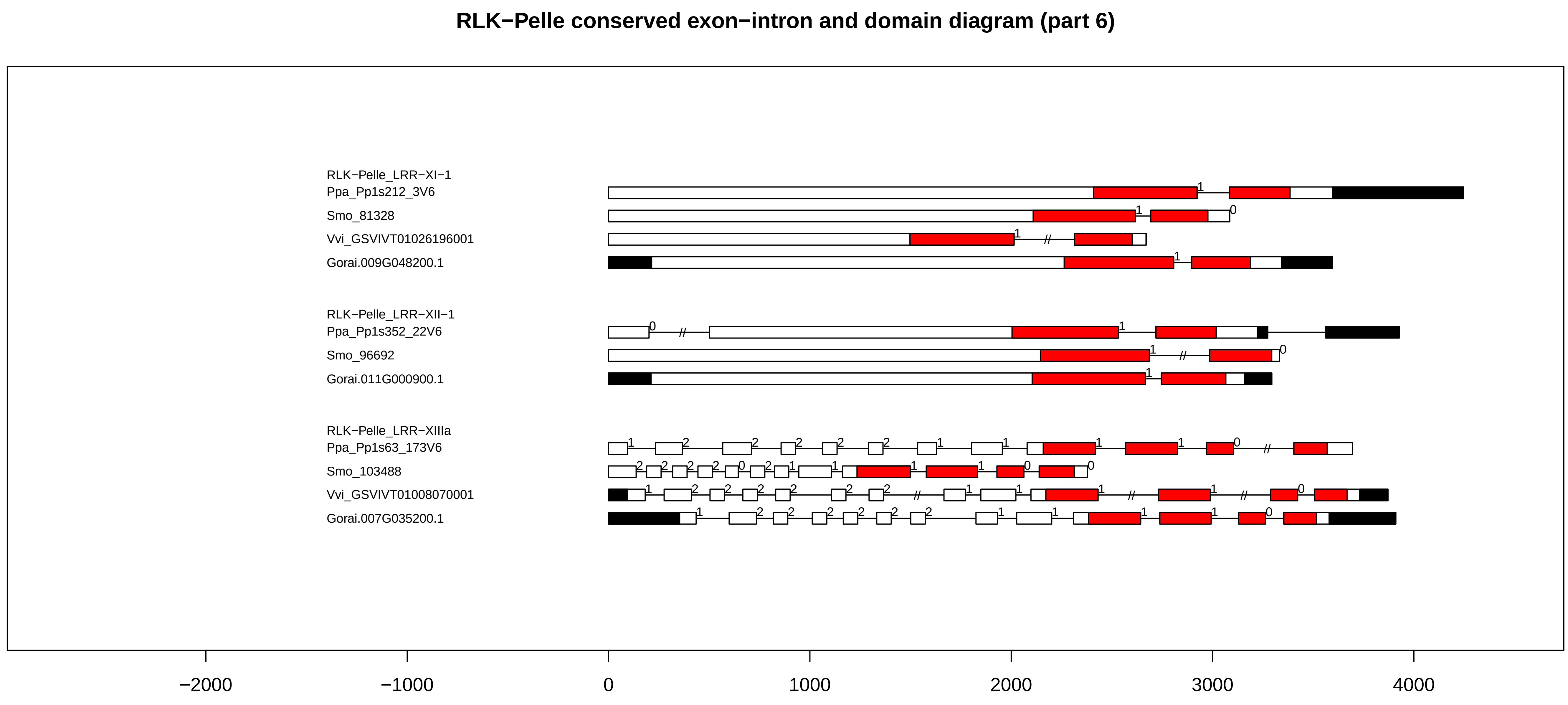
RLK−Pelle conserved exon−intron and domain diagram (part 6)
RLK−Pelle_LRR−XI−1
Ppa_Pp1s212_3V6
Smo_81328
Vvi_GSVIVT01026196001
Gorai.009G048200.1
RLK−Pelle_LRR−XII−1
Ppa_Pp1s352_22V6
Smo_96692
Gorai.011G000900.1
RLK−Pelle_LRR−XIIIa
Ppa_Pp1s63_173V6
Smo_103488
Vvi_GSVIVT01008070001
Gorai.007G035200.1
−2000
−1000
0
1000
2000
3000
4000

RLK−Pelle conserved exon−intron and domain diagram (part 7)

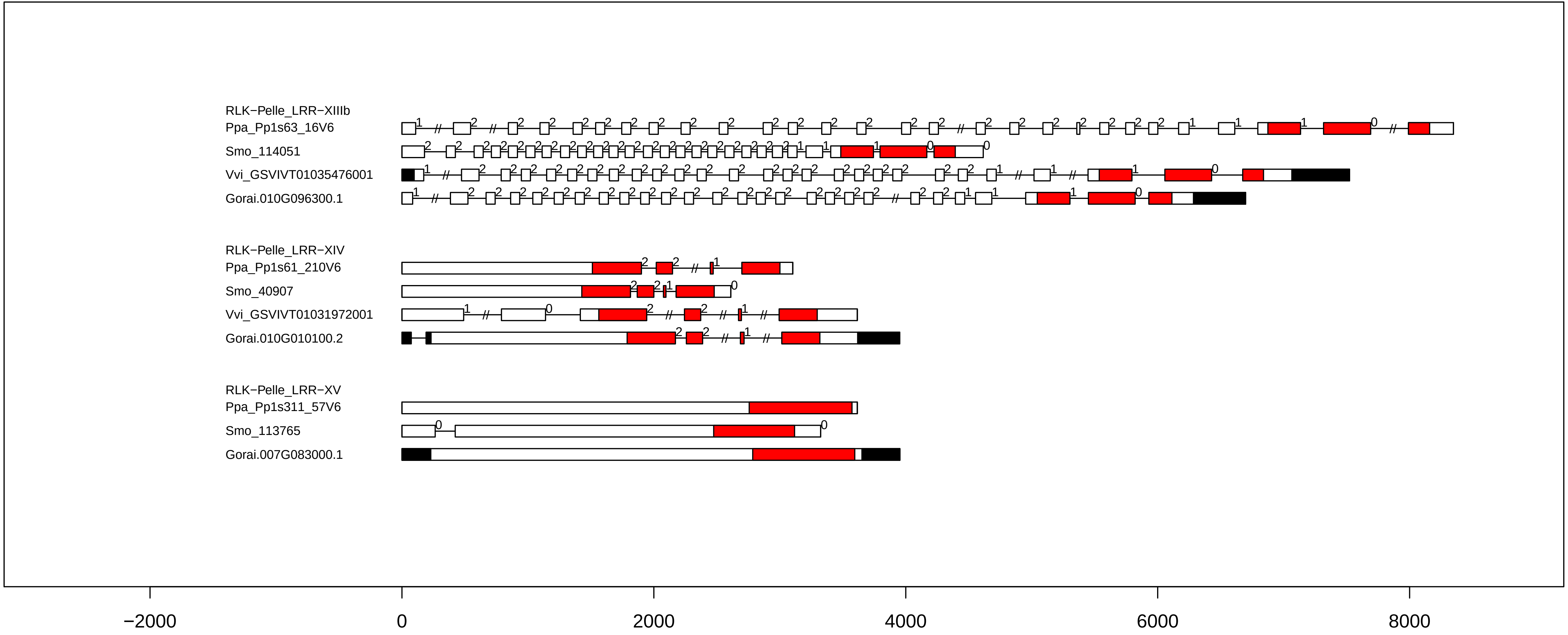

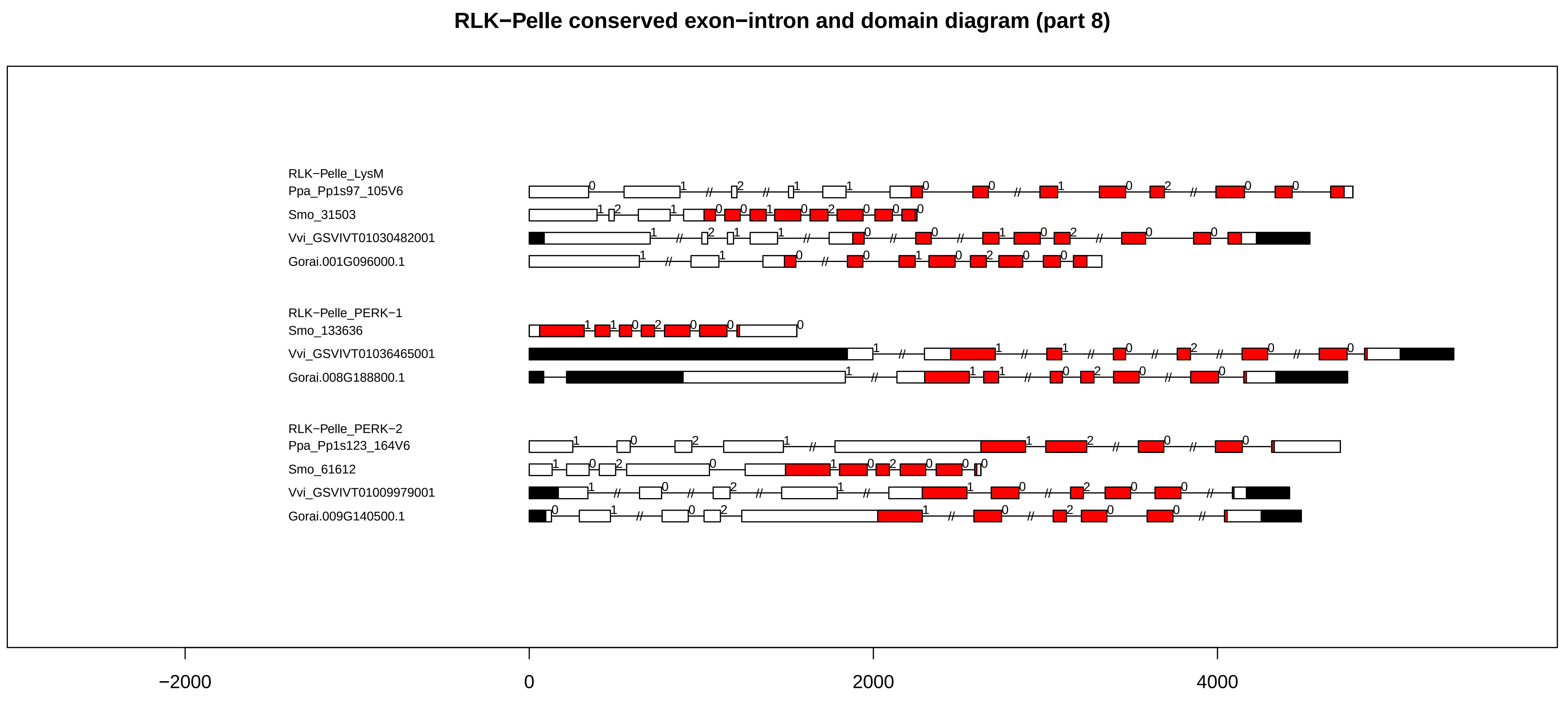

RLK−Pelle conserved exon−intron and domain diagram (part 8)
RLK−Pelle_LysM
Ppa_Pp1s97_105V6
Smo_31503
Vvi_GSVIVT01030482001
Gorai.001G096000.1
RLK−Pelle_PERK−1
Smo_133636
Vvi_GSVIVT01036465001
Gorai.008G188800.1
RLK−Pelle_PERK−2
Ppa_Pp1s123_164V6
Smo_61612
Vvi_GSVIVT01009979001
Gorai.009G140500.1
−2000
0
2000
4000

RLK−Pelle conserved exon−intron and domain diagram (part 9)

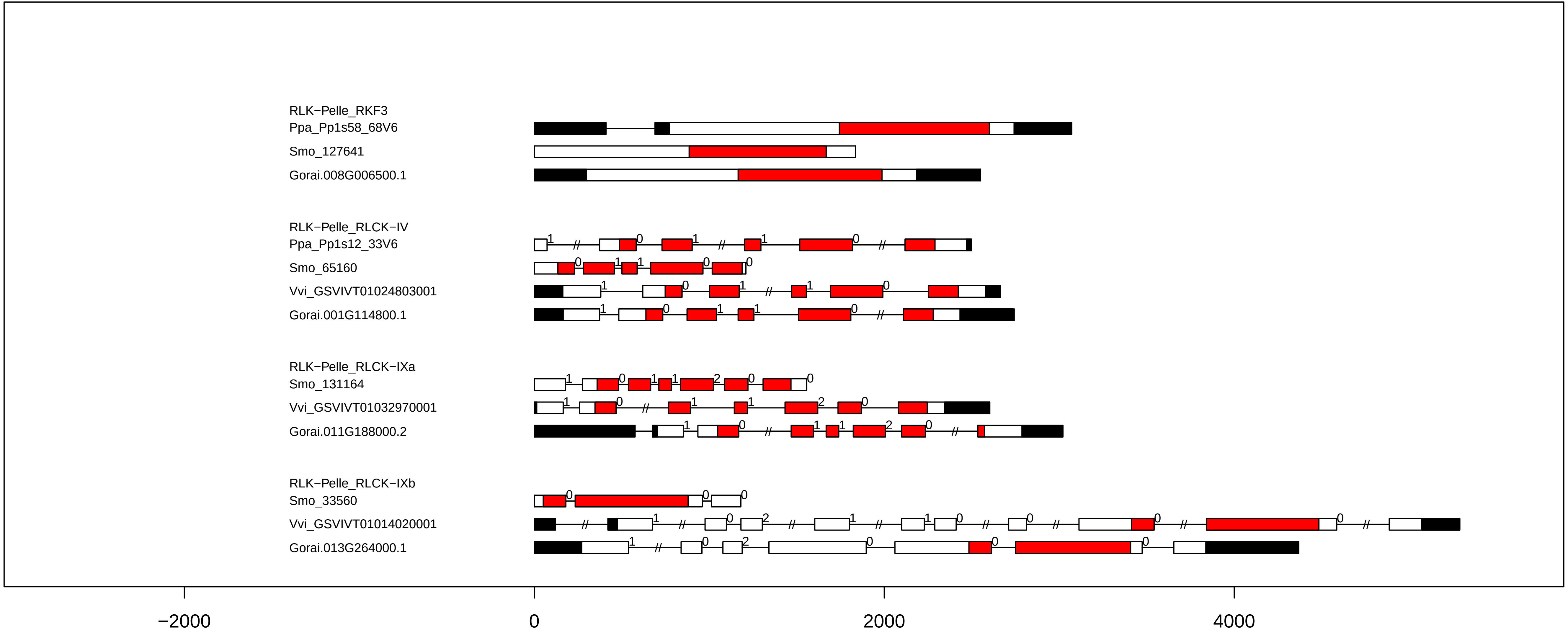

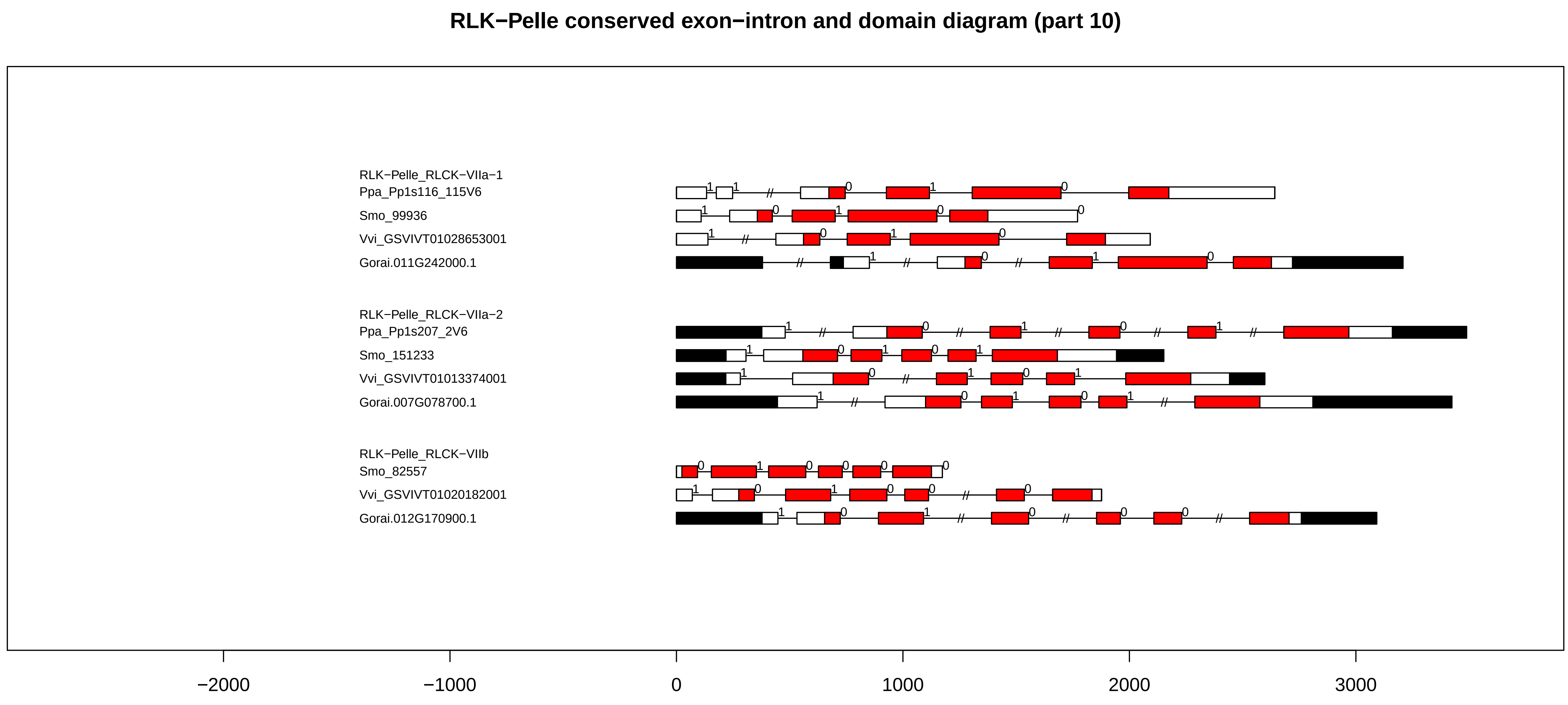

RLK−Pelle conserved exon−intron and domain diagram (part 10)
RLK−Pelle_RLCK−VIIa−1
Ppa_Pp1s116_115V6
Smo_99936
Vvi_GSVIVT01028653001
Gorai.011G242000.1
RLK−Pelle_RLCK−VIIa−2
Ppa_Pp1s207_2V6
Smo_151233
Vvi_GSVIVT01013374001
Gorai.007G078700.1
RLK−Pelle_RLCK−VIIb
Smo_82557
Vvi_GSVIVT01020182001
Gorai.012G170900.1
−2000
−1000
0
1000
2000
3000

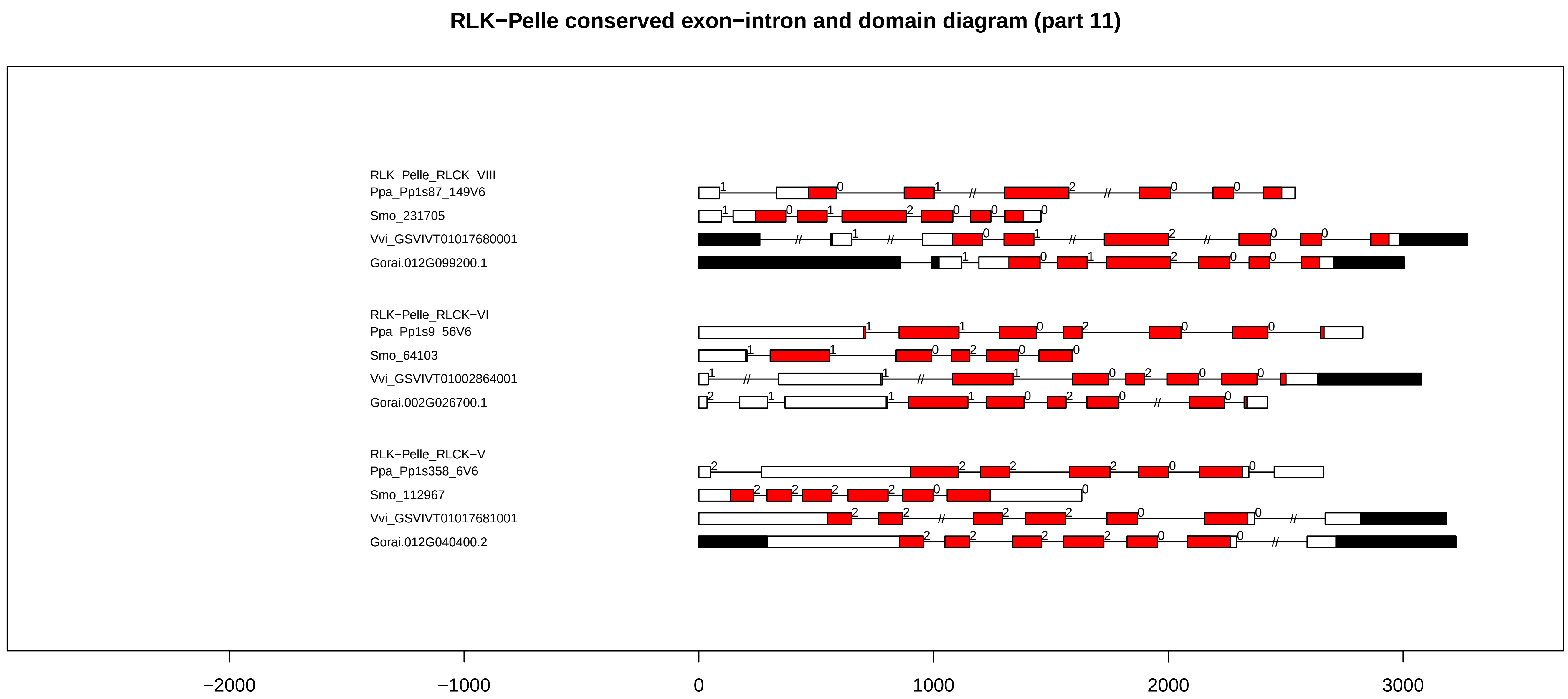
RLK−Pelle conserved exon−intron and domain diagram (part 11)
RLK−Pelle_RLCK−VIII
Ppa_Pp1s87_149V6
Smo_231705
Vvi_GSVIVT01017680001
Gorai.012G099200.1
RLK−Pelle_RLCK−VI
Ppa_Pp1s9_56V6
Smo_64103
Vvi_GSVIVT01002864001
Gorai.002G026700.1
RLK−Pelle_RLCK−V
Ppa_Pp1s358_6V6
Smo_112967
Vvi_GSVIVT01017681001
Gorai.012G040400.2
−2000
−1000
0
1000
2000
3000

RLK−Pelle conserved exon−intron and domain diagram (part 12)

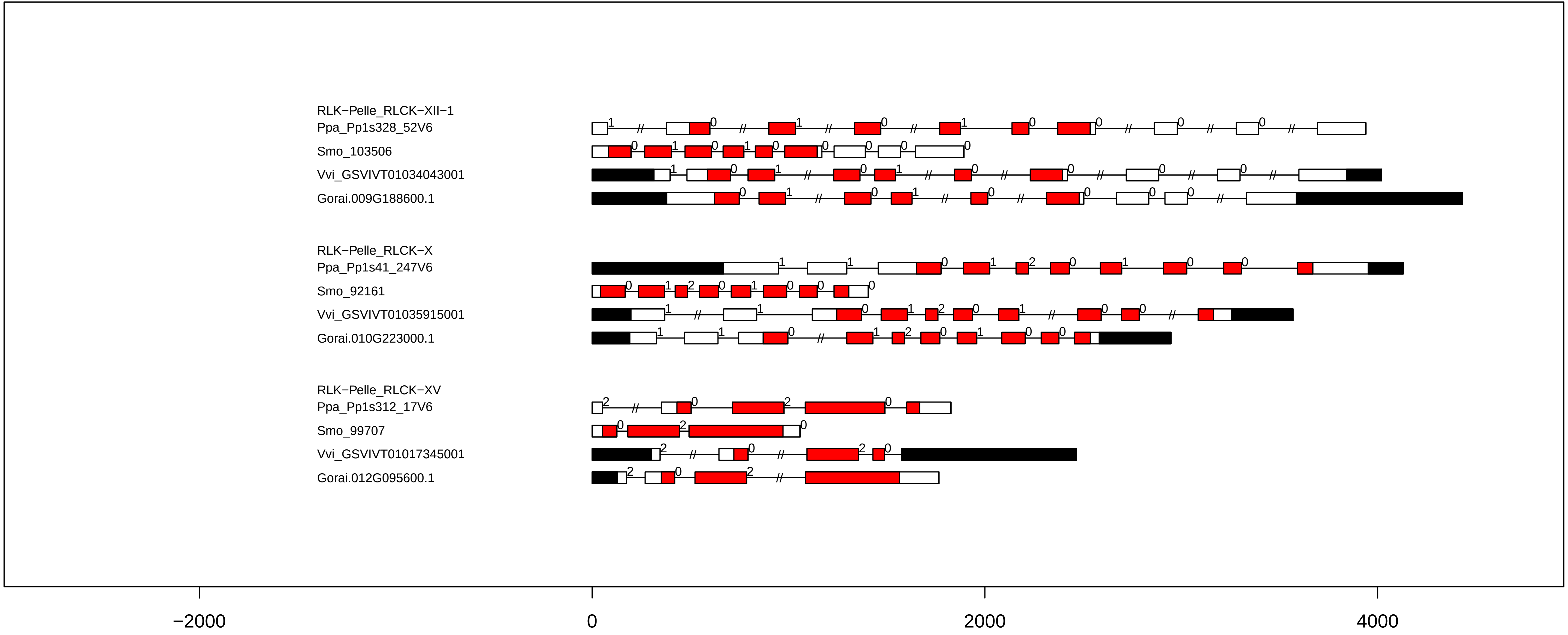

RLK−Pelle conserved exon−intron and domain diagram (part 13)

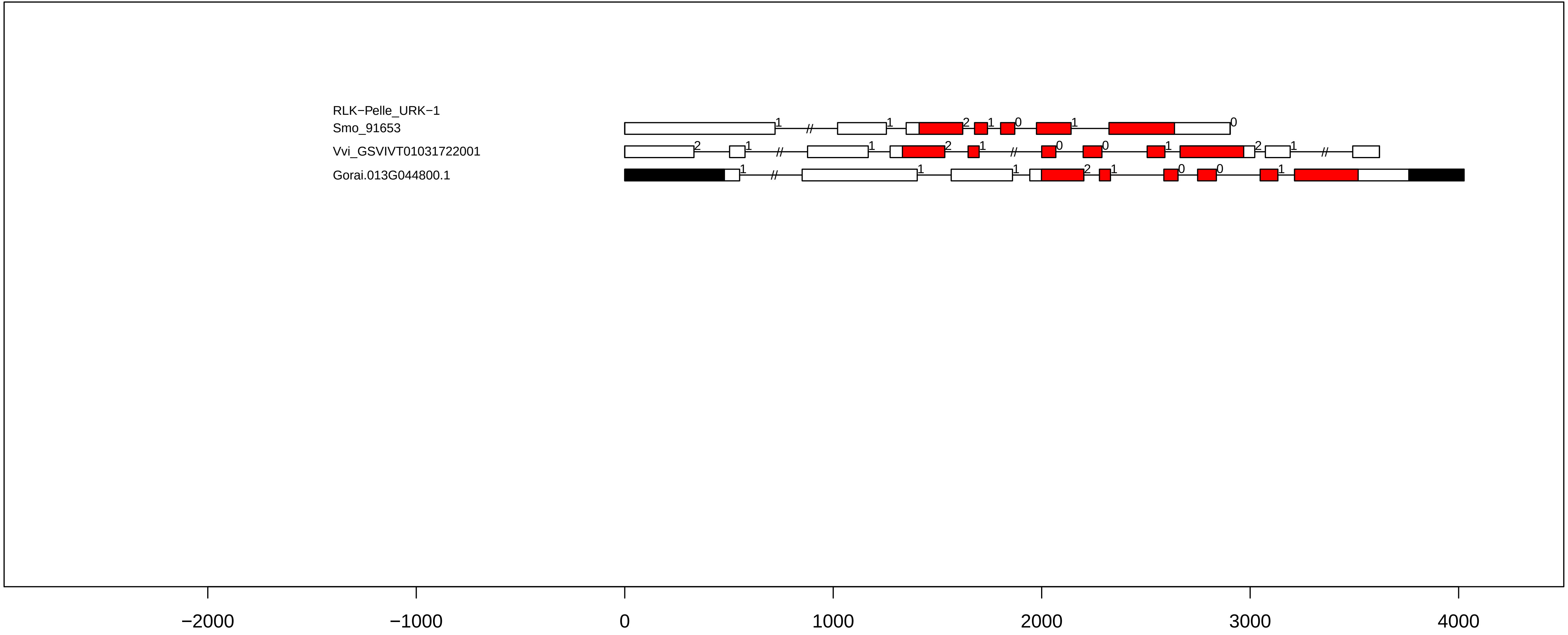

SCY1 conserved exon−intron and domain diagram (all)

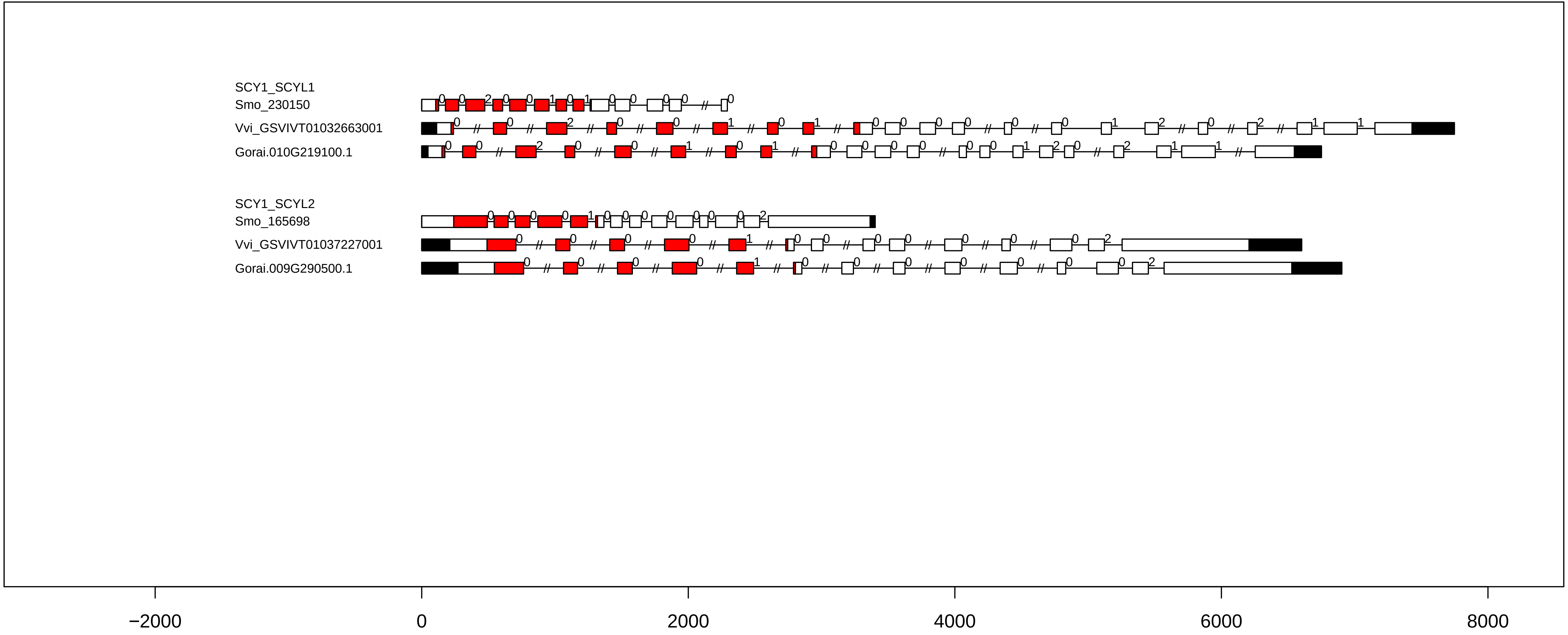

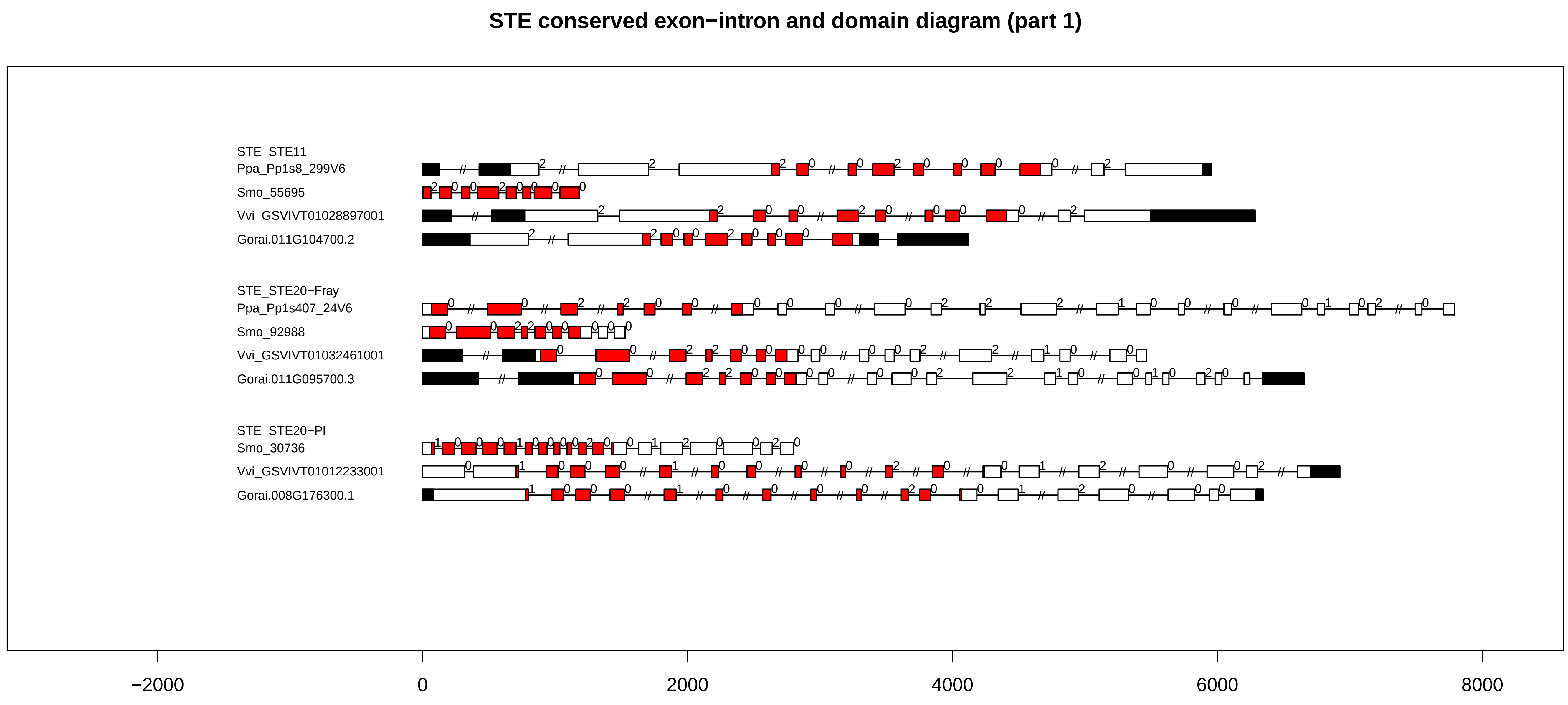

STE conserved exon−intron and domain diagram (part 1)
STE_STE11
Ppa_Pp1s8_299V6
Smo_55695
Vvi_GSVIVT01028897001
Gorai.011G104700.2
STE_STE20−Fray
Ppa_Pp1s407_24V6
Smo_92988
Vvi_GSVIVT01032461001
Gorai.011G095700.3
STE_STE20−Pl
Smo_30736
Vvi_GSVIVT01012233001
Gorai.008G176300.1
−2000
0
2000
4000
6000
8000

STE conserved exon−intron and domain diagram (part 2)

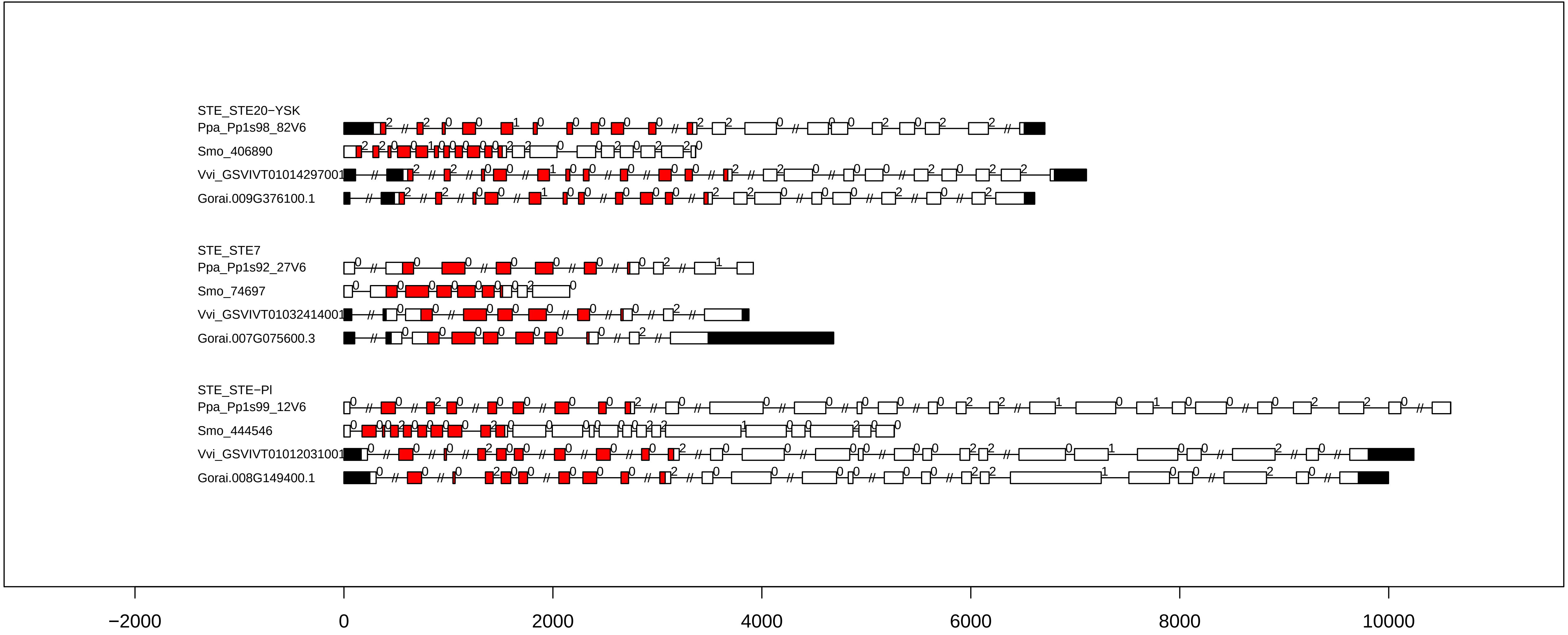

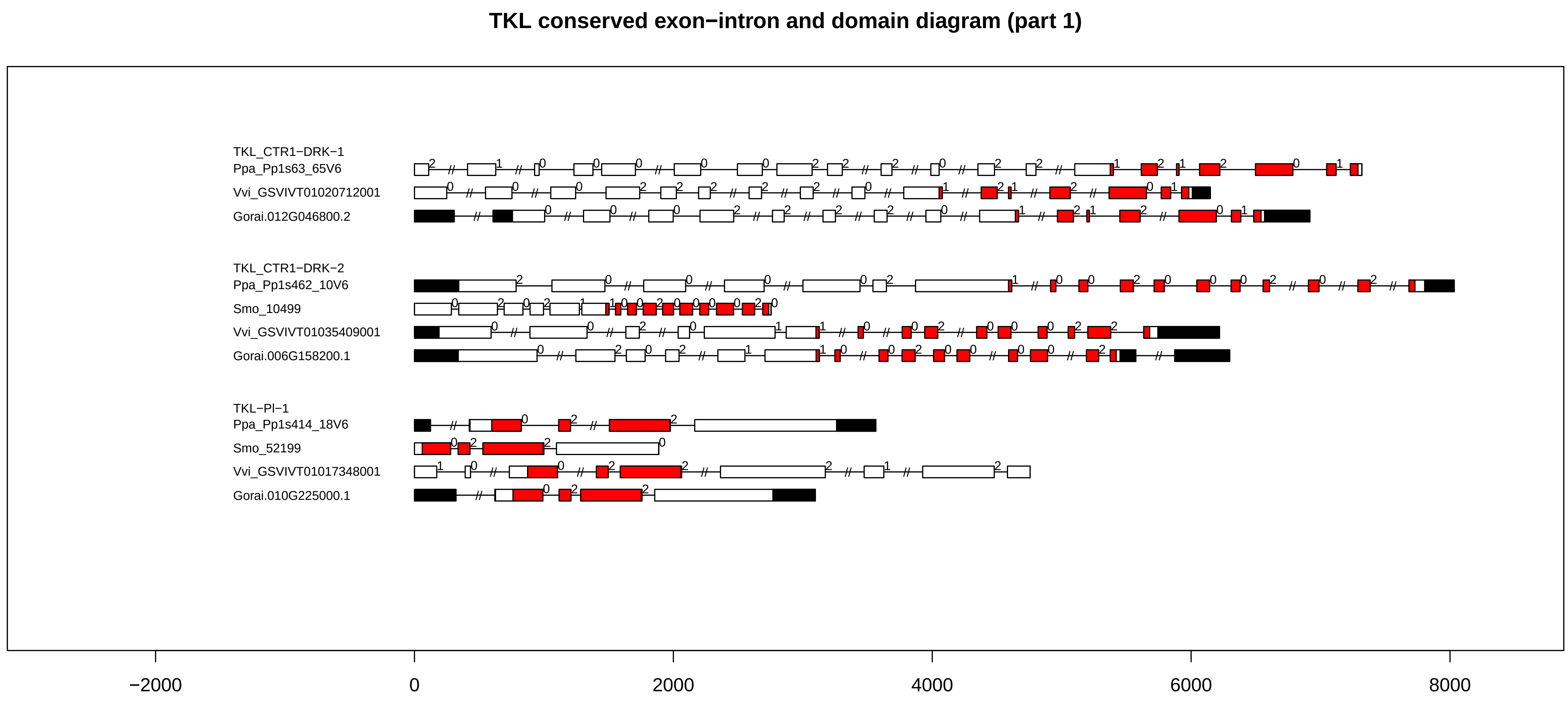
TKL conserved exon−intron and domain diagram (part 1)
TKL_CTR1−DRK−1
Ppa_Pp1s63_65V6
Vvi_GSVIVT01020712001
Gorai.012G046800.2
TKL_CTR1−DRK−2
Ppa_Pp1s462_10V6
Smo_10499
Vvi_GSVIVT01035409001
Gorai.006G158200.1
TKL−PI−1
Ppa_Pp1s414_18V6
Smo_52199
Vvi_GSVIVT01017348001
Gorai.010G225000.1
−2000
0
2000
4000
6000
8000

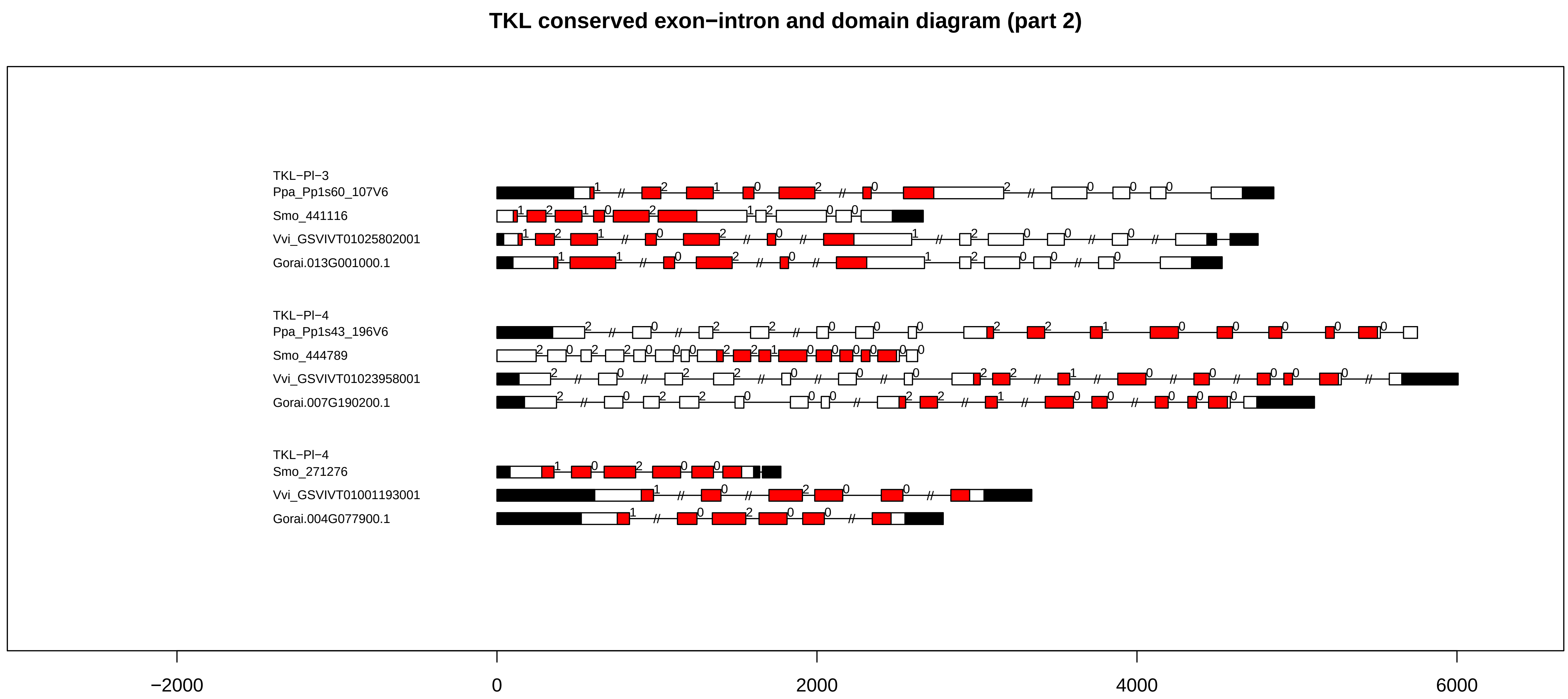

TKL conserved exon−intron and domain diagram (part 2)
TKL−Pl−3
Ppa_Pp1s60_107V6
Smo_441116
Vvi_GSVIVT01025802001
Gorai.013G001000.1
TKL−Pl−4
Ppa_Pp1s43_196V6
Smo_444789
Vvi_GSVIVT01023958001
Gorai.007G190200.1
TKL−Pl−4
Smo_271276
Vvi_GSVIVT01001193001
Gorai.004G077900.1
−2000
0
2000
4000
6000

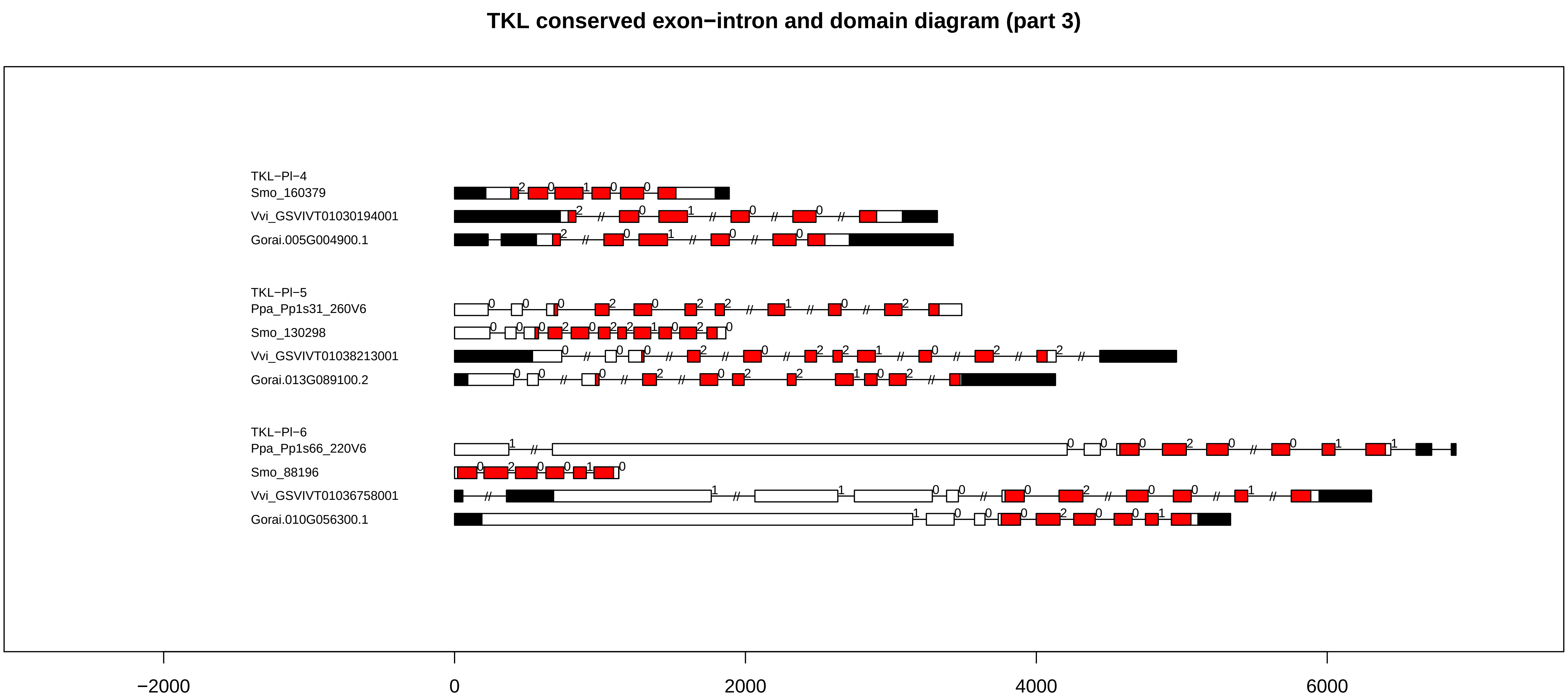
TKL conserved exon−intron and domain diagram (part 3)
TKL−Pl−4
Smo_160379
Vvi_GSVIVT01030194001
Gorai.005G004900.1
TKL−Pl−5
Ppa_Pp1s31_260V6
Smo_130298
Vvi_GSVIVT01038213001
Gorai.013G089100.2
TKL−Pl−6
Ppa_Pp1s66_220V6
Smo_88196
Vvi_GSVIVT01036758001
Gorai.010G056300.1
−2000
0
2000
4000
6000
