# Supplementary figures and images for: Genome-wide classification, evolutionary analysis and gene expression patterns of the kinome in Gossypium
Source: PLoS One. 2018 May 16;13(5):e0197392. doi: 10.1371/journal.pone.0197392 (PMC5955557; doi:10.1371/journal.pone.0197392)

A

0.0  
7.1M  
14.2M  
21.3M

G. ra\_Chr01

02

03

04

05

06

07

08

09

10

11

12

13

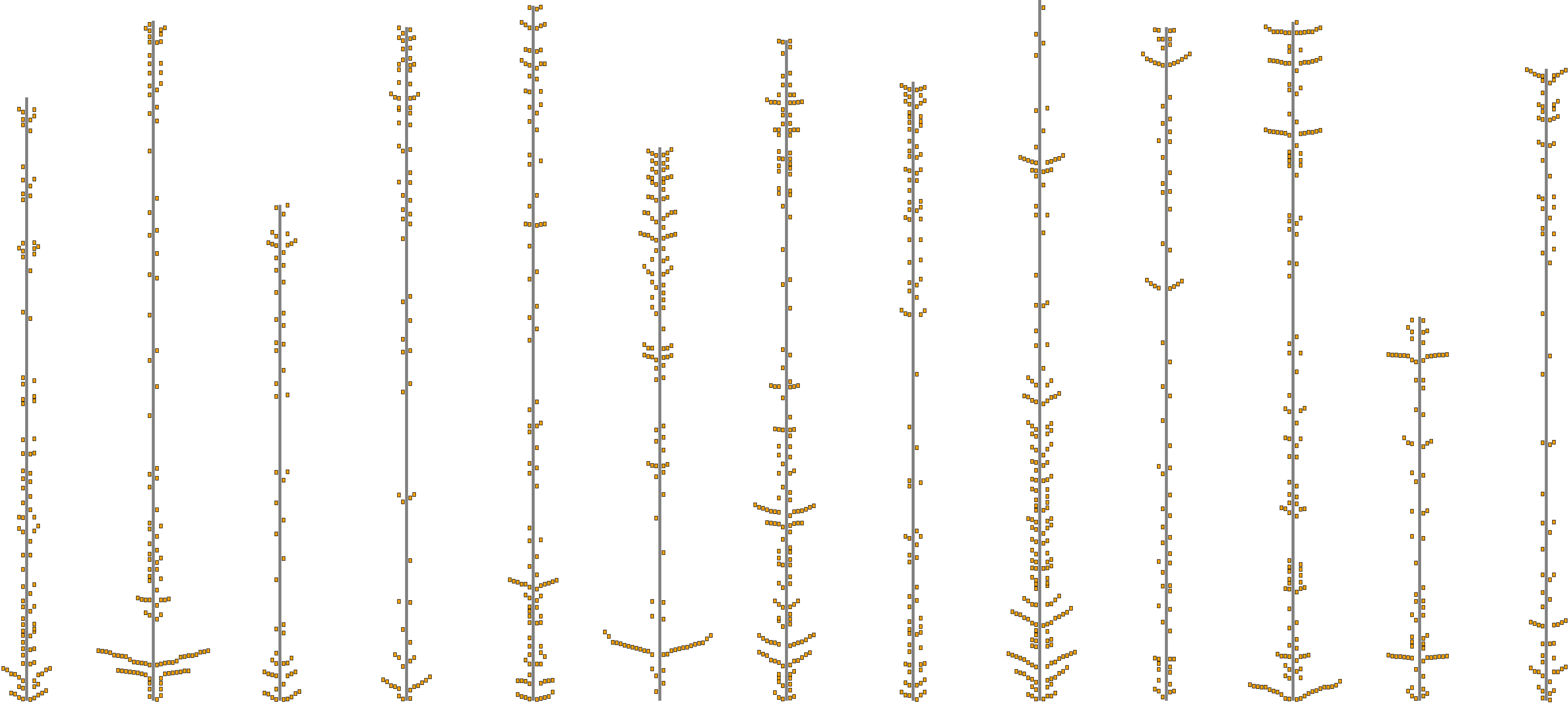

B

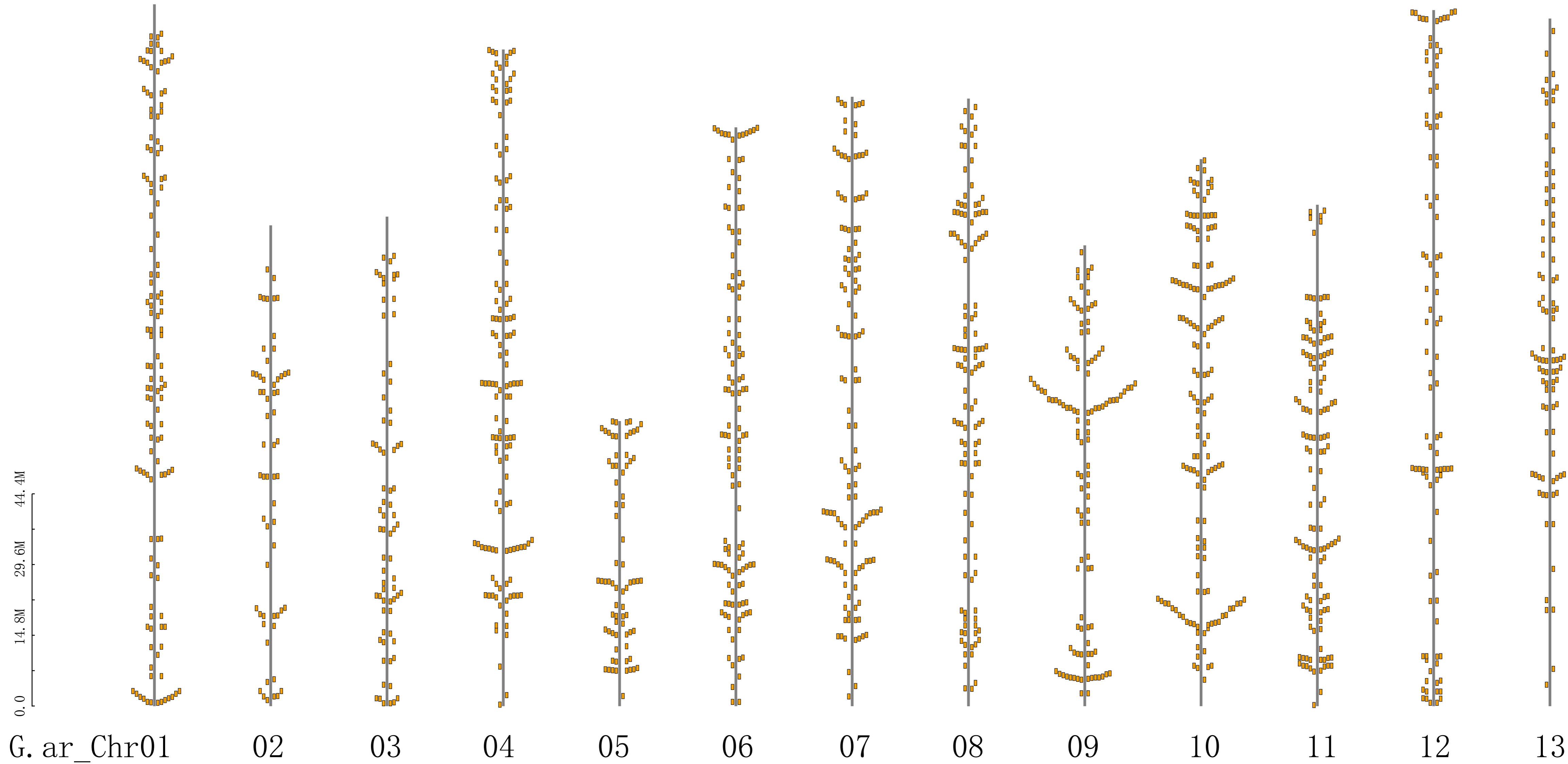

C

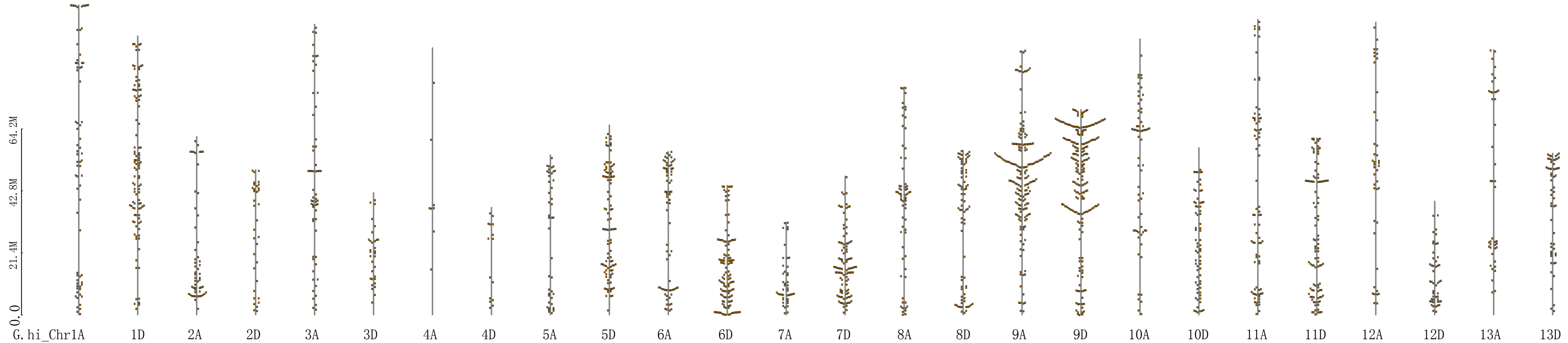

[illegible]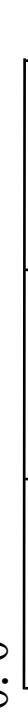

G. ba\_Chr1A

1D

2A

2D

3A

31

4.

4

5

5

6

Supplement: S5 Fig — Chromosomal locations of G. raimondii (A), G. arboretum (B), G. hirsutum (C) and G. barbadense (D) PKs. Yellow boxes denote PK genes. (PDF) [file pone.0197392.s005.pdf]

# A

G. ra

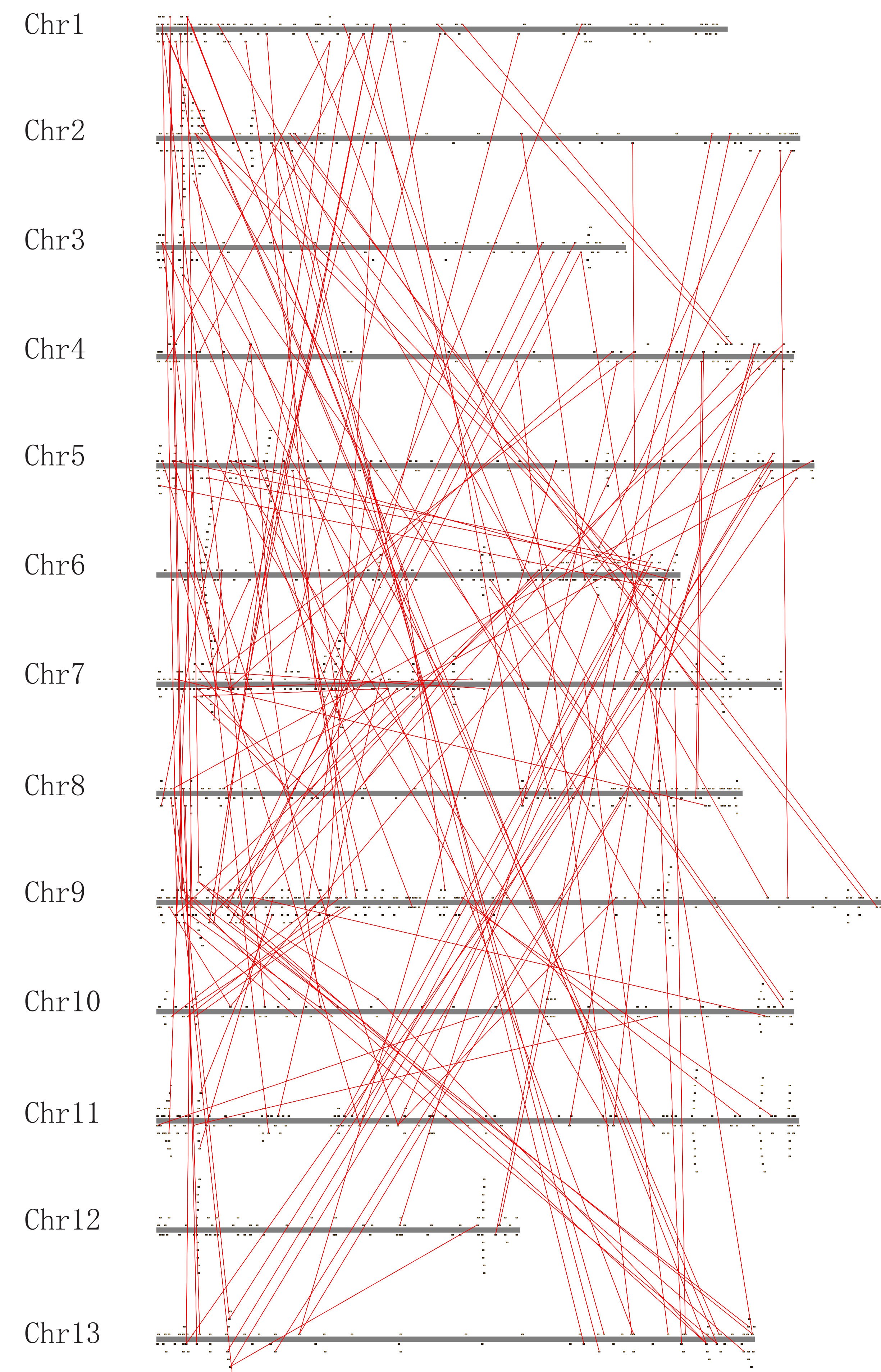

# B

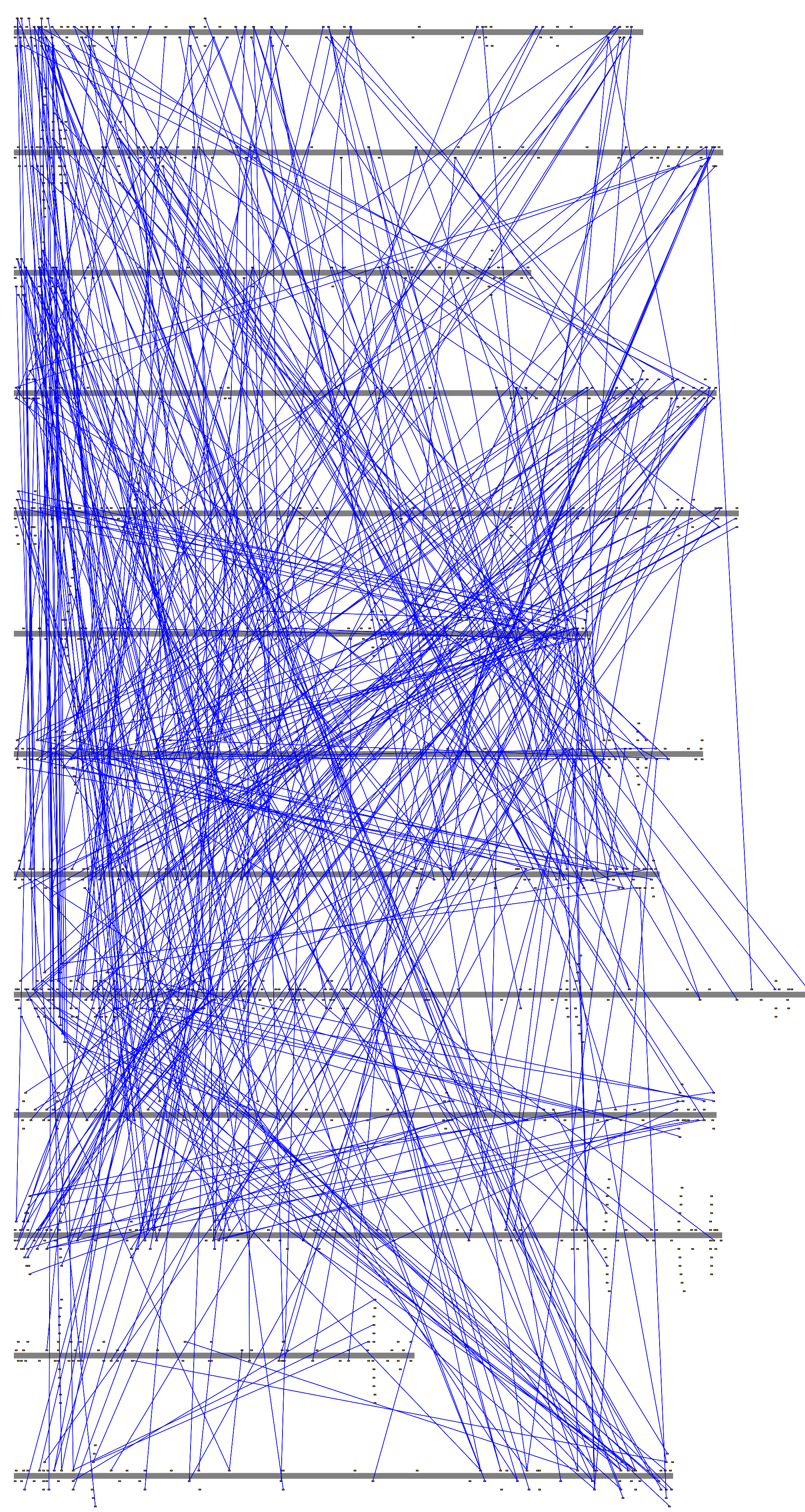

# C

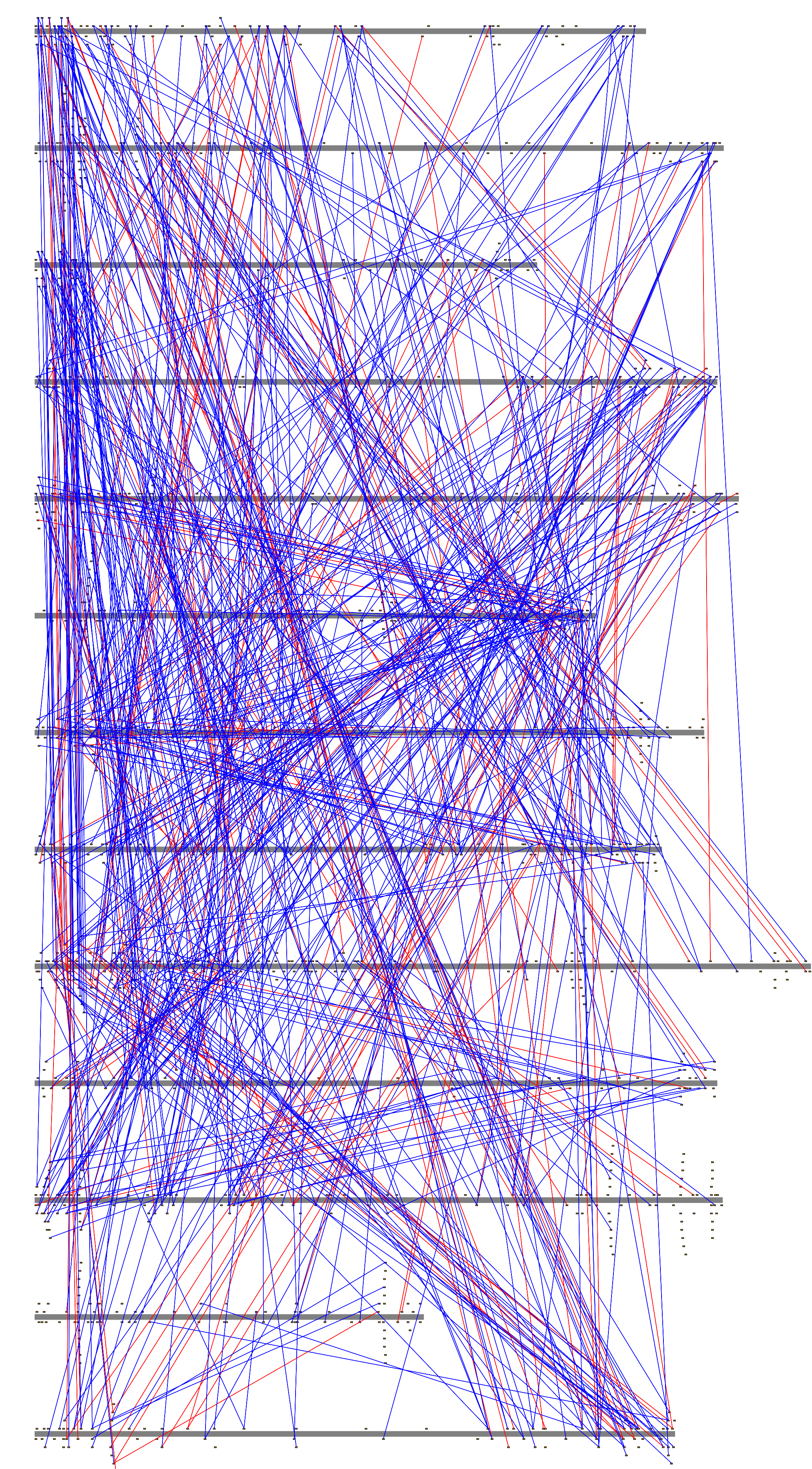

# E

G. ar

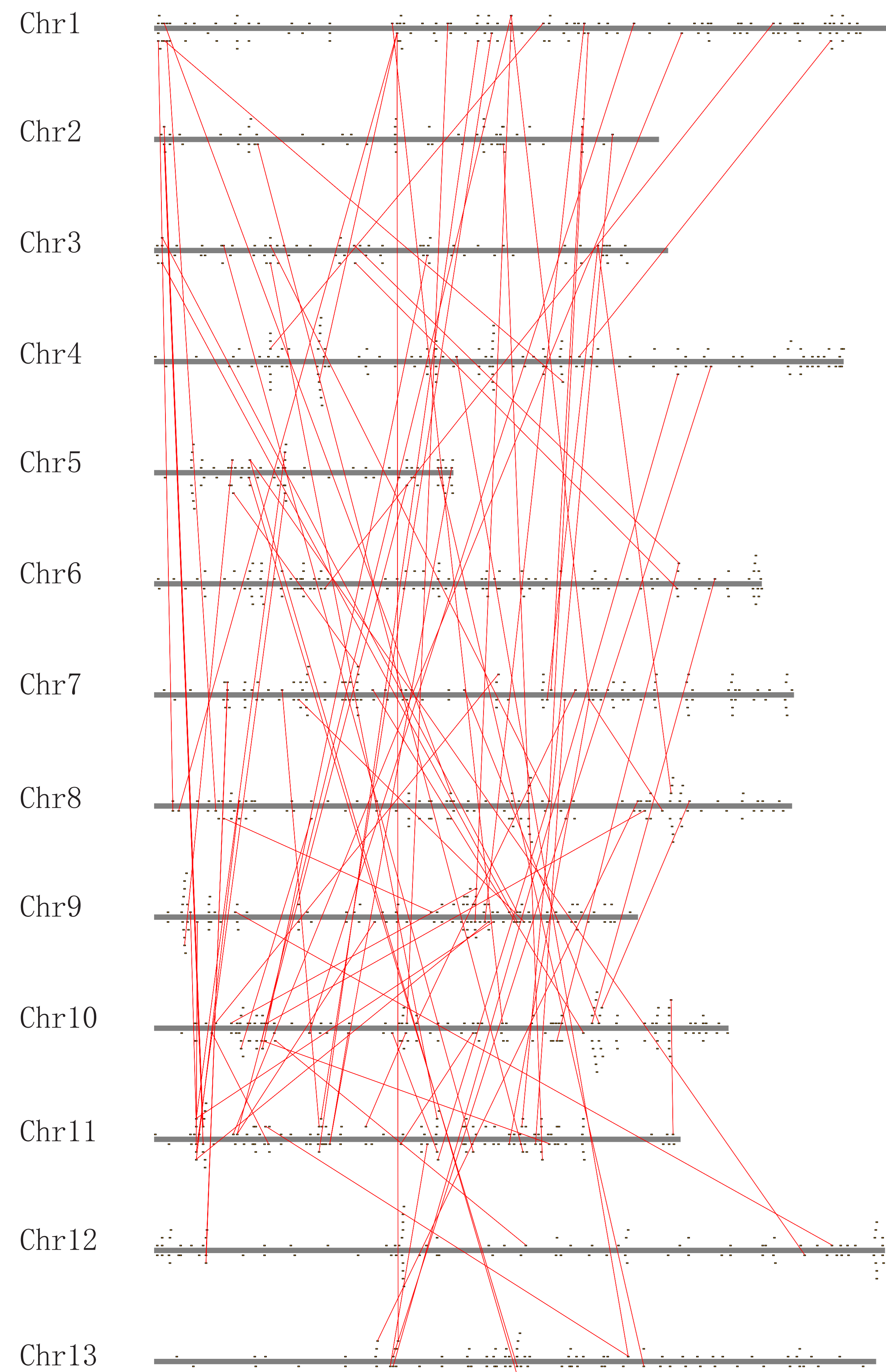

# F

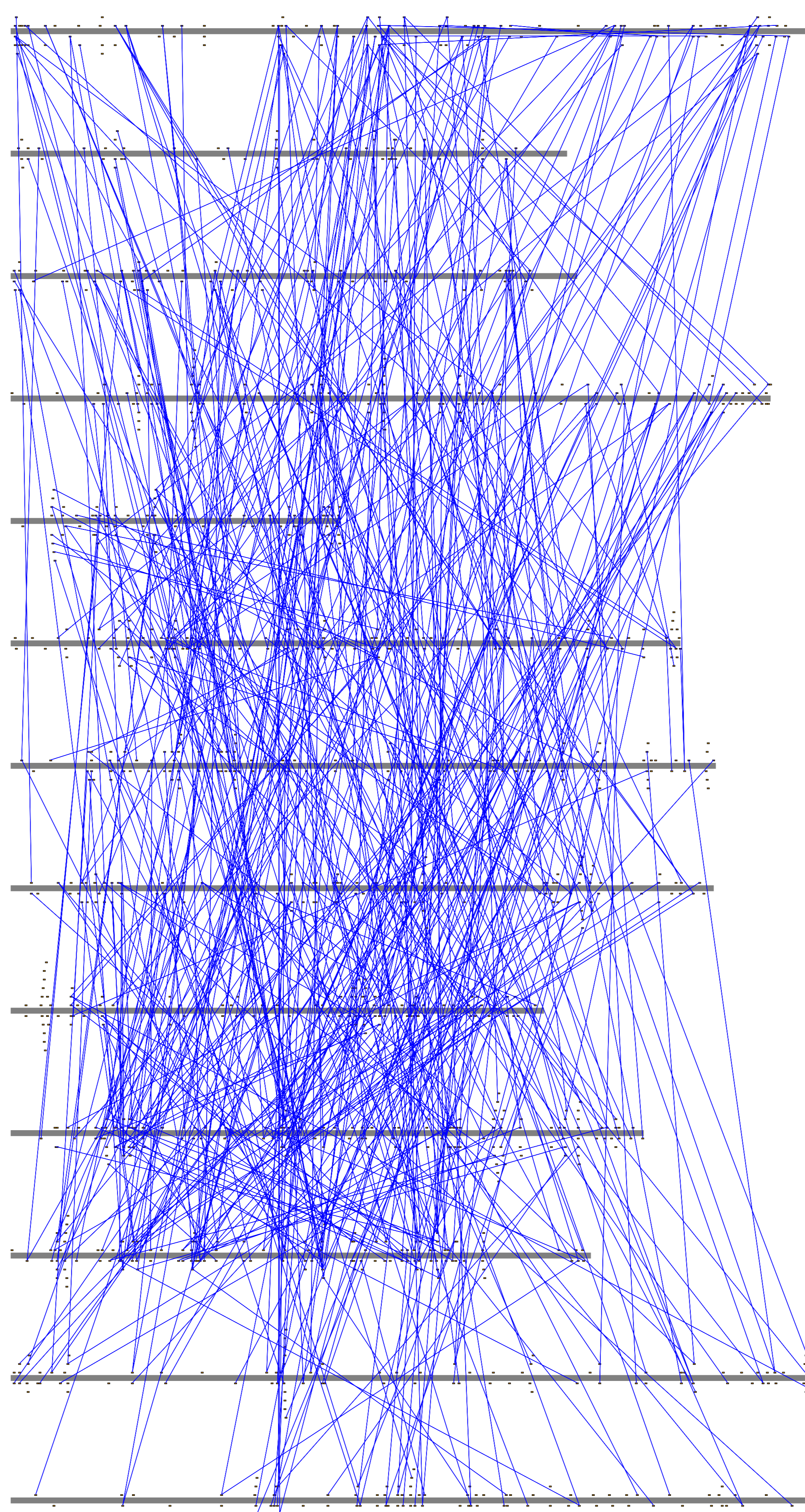

# G

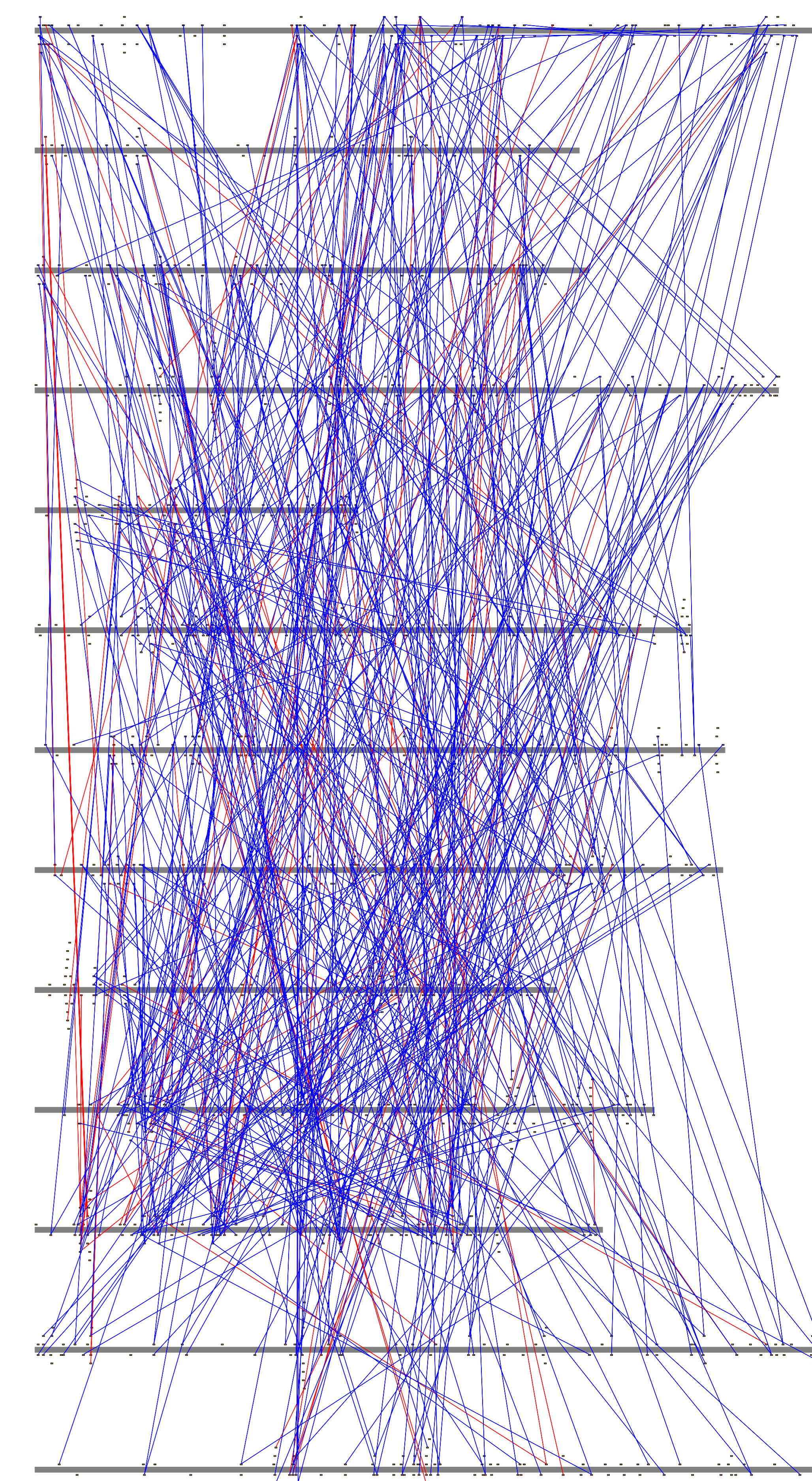

G

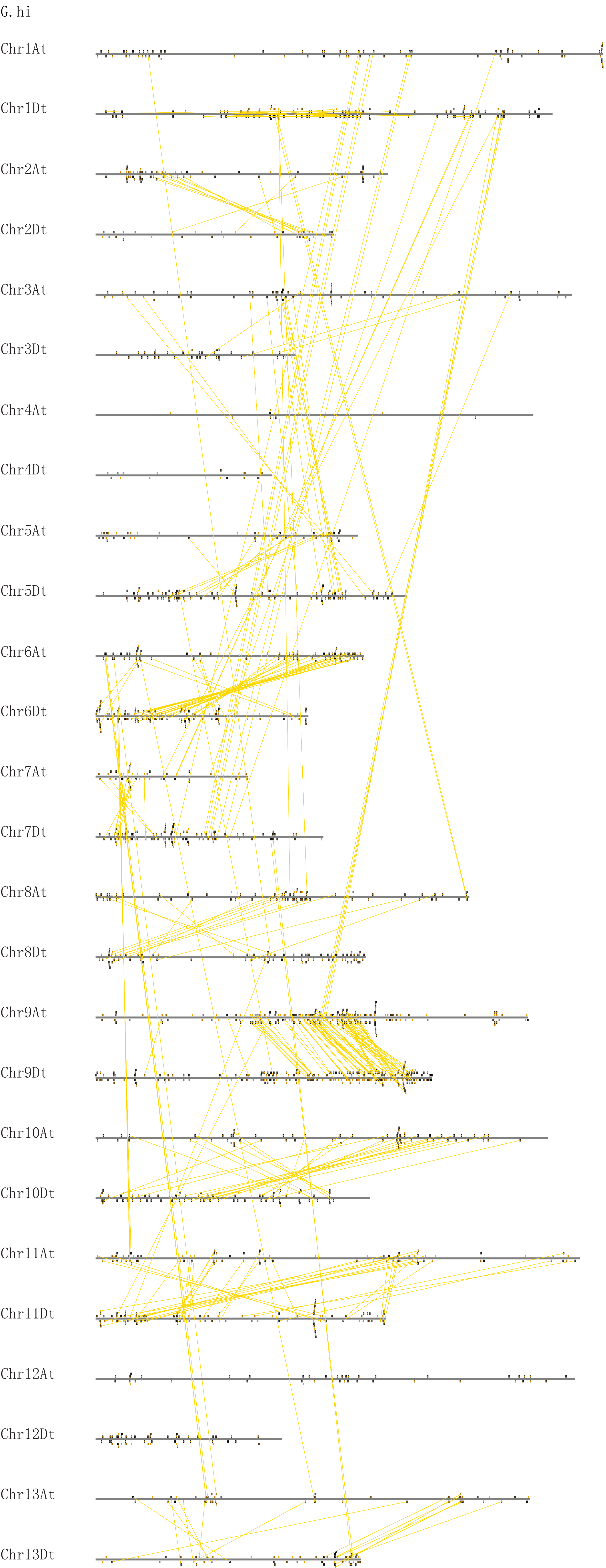

H

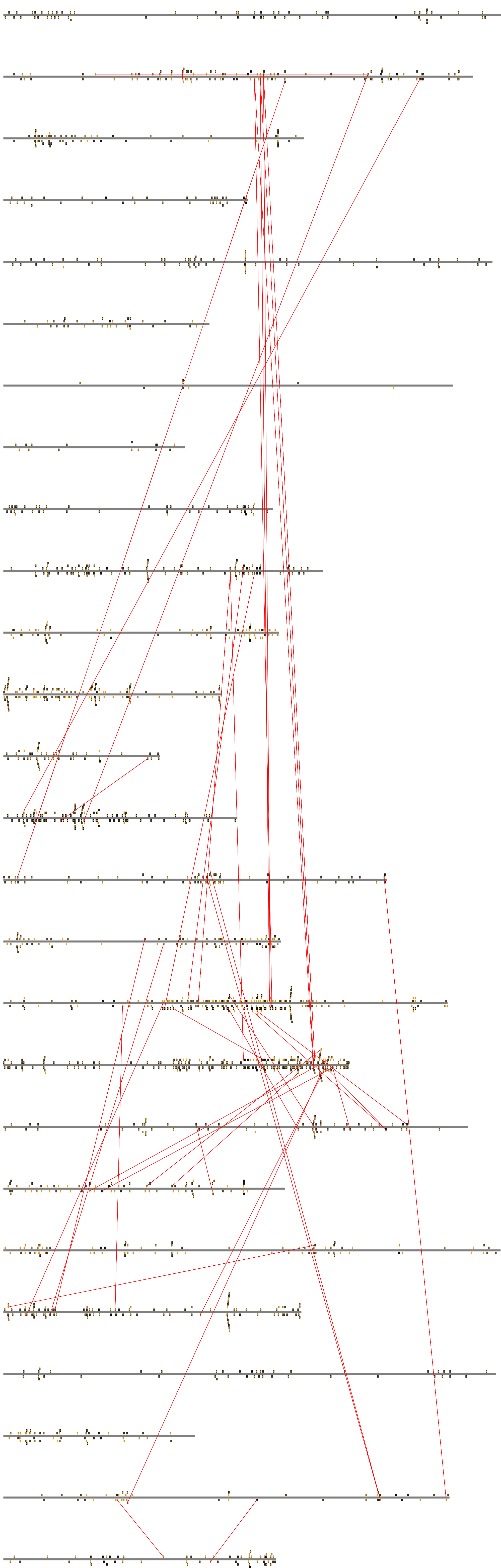

I

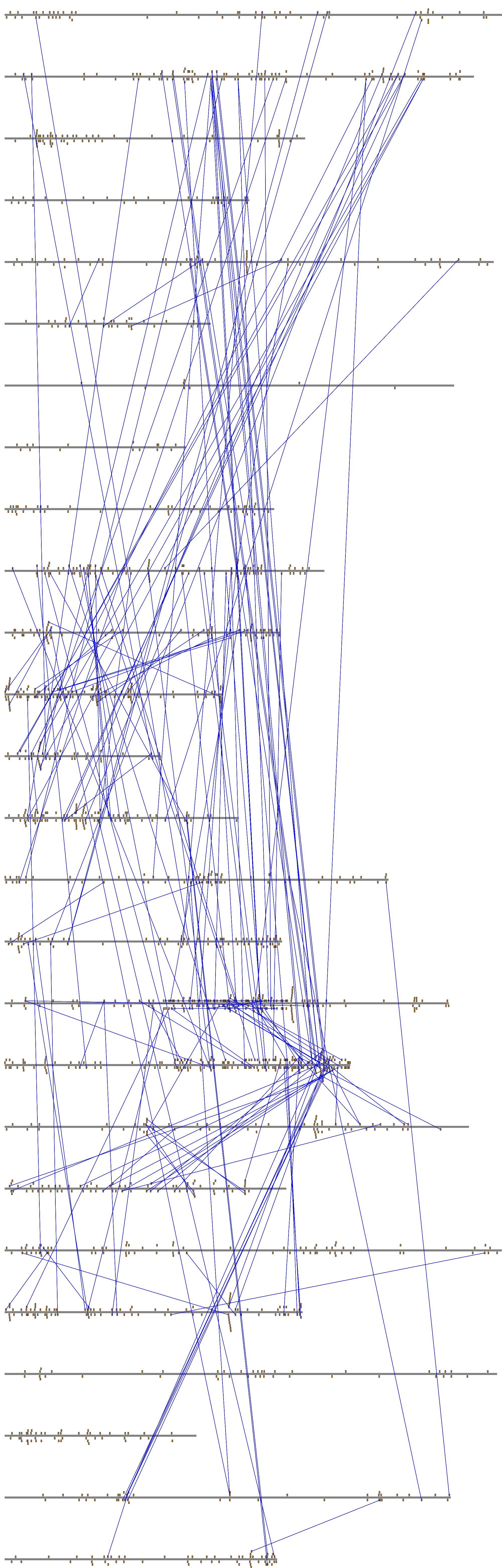

J

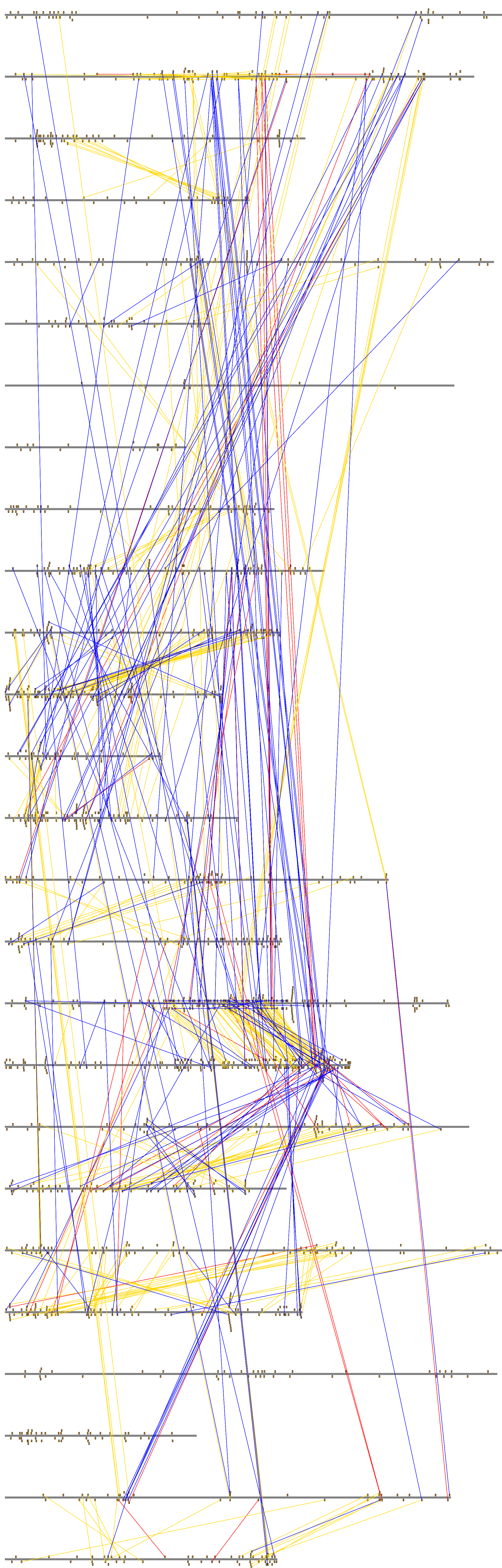

K

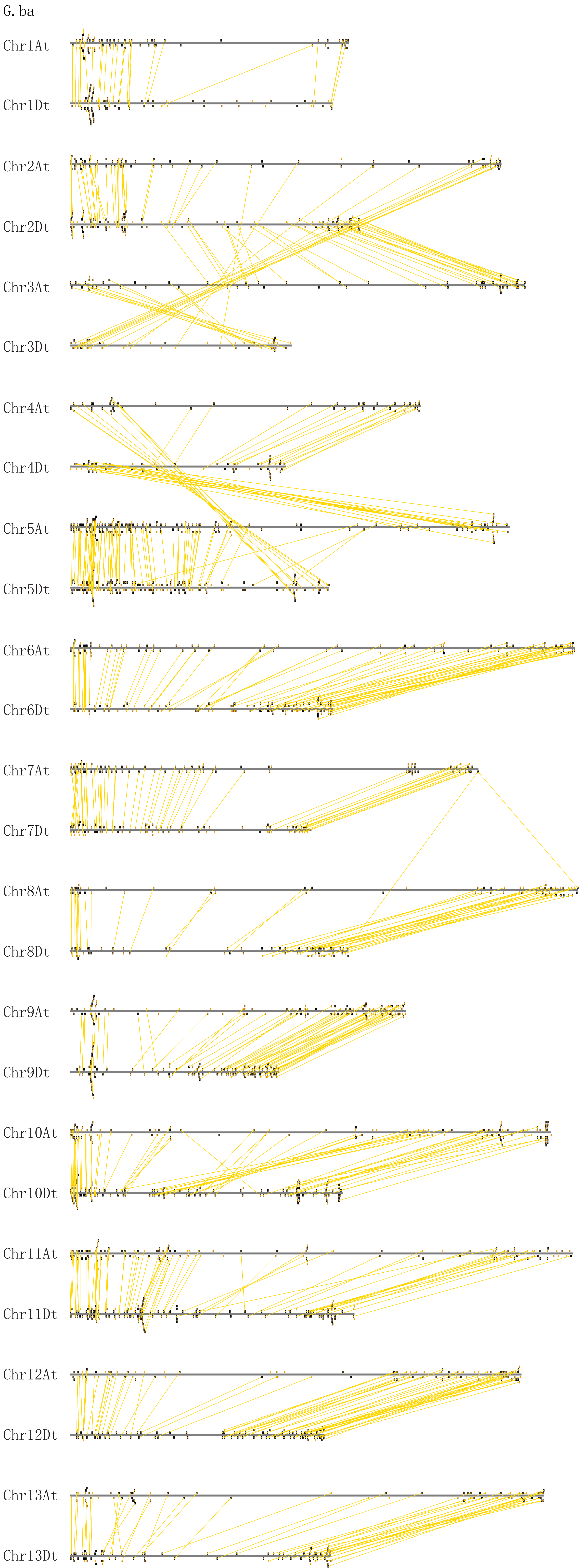

L

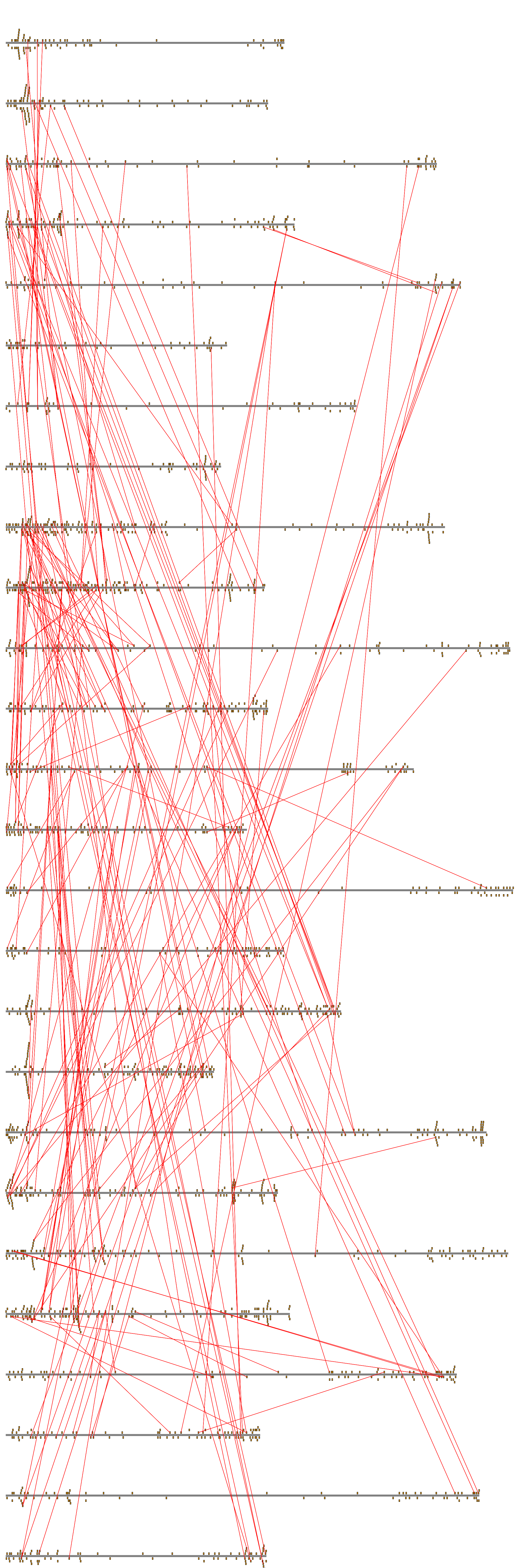

M

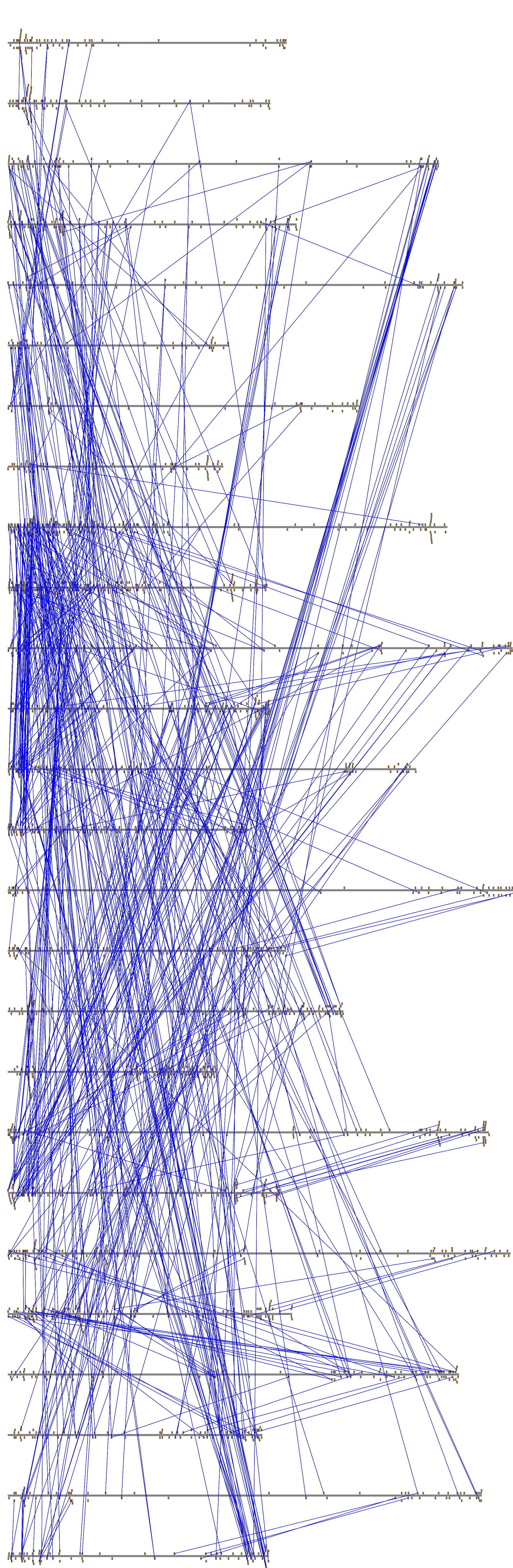

N

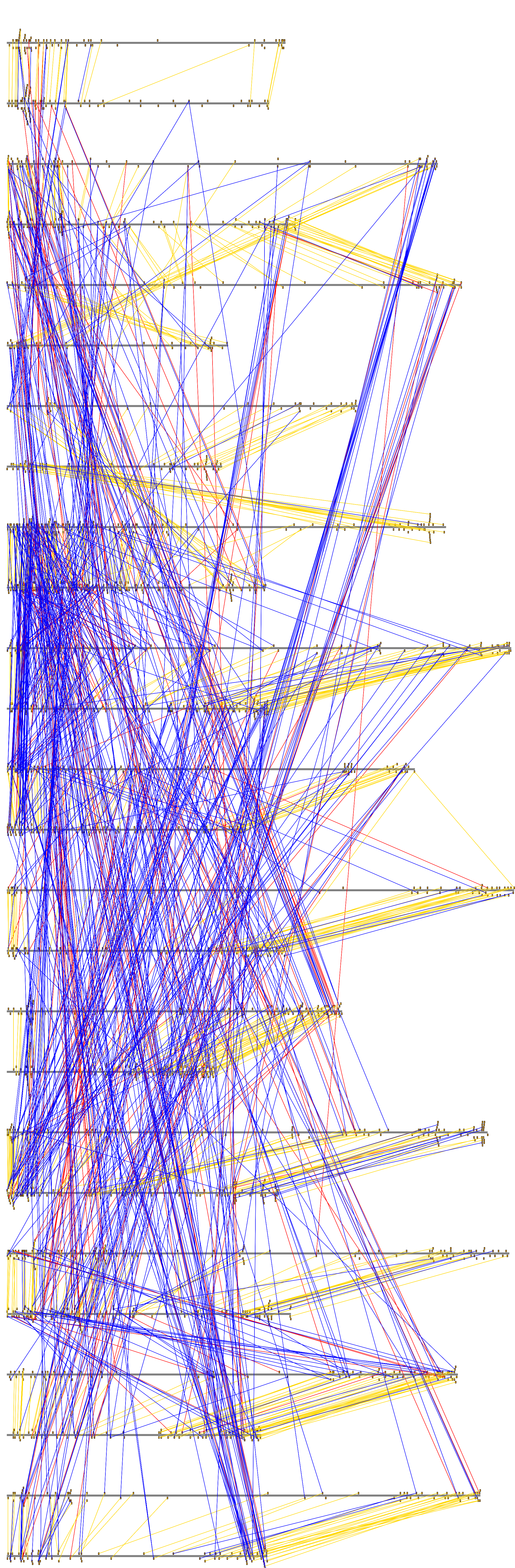

Supplement: S6 Fig — (A) The collinearity events of G. raimondii PKs resulting from 16.6-Mya WGD. (B) The other collinearity events of G. raimondii PKs. (C) All of the collinearity events of G. raimondii PKs. (D) The collinearity events of G. arboretum PKs resulting from 16.6-Mya WGD. (E) The other collinearity events of G. arboretum PKs. (F) All of the collinearity events of G. arboretum PKs. (G) The collinearity events of G. hirsutum PKs contributed by tetraploidization. (H) The collinearity events of G. hirsutum PKs resulting from 16.6-Mya WGD. (I) The other collinearity events of G. hirsutum PKs. (J) All of the collinearity events of G. hirsutum PKs. (K) The collinearity events of G. barbadense PKs contributed by tetraploidization. (L) The collinearity events of G. barbadense PKs resulting from 16.6-Mya WGD. (M) The other collinearity events of G. barbadense PKs. (N) All of the collinearity events of G. barbadense PKs. Red lines denote the collinearity events resulting from 16.6-Mya WGD. Red lines denote the collinearity events resulting from 16.6-Mya WGD. Yellow lines denote the collinearity events contributed by tetraploidization. Blue lines denote other collinearity events. (PDF) [file pone.0197392.s006.pdf]
