# Supplementary material for: Genome-wide classification, evolutionary analysis and gene expression patterns of the kinome in Gossypium
Source: PLoS One. 2018 May 16;13(5):e0197392. doi: 10.1371/journal.pone.0197392 (PMC5955557; doi:10.1371/journal.pone.0197392)
Supplement: S7 Fig — (A) The 291 tandemly arrayed G. raimondii PK genes were grouped in 83 clusters distributed unevenly among the 13 chromosomes. (B) The 246 tandemly arrayed G. arboretum PK genes were grouped in 87 clusters distributed unevenly among the 13 chromosomes. (C) The 264 tandemly arrayed G. hirsutum PK genes were grouped in 91 clusters distributed unevenly among the 26 chromosomes. (D) The 455 tandemly arrayed G. barbadense PK genes were grouped in 152 clusters distributed unevenly among the 26 chromosomes. Gene IDs and subfamilies are labeled in the right of each chromosome, and the chromosomal location of each cluster is in the left of each chromosome. Genes in the same cluster are highlighted in the same color. (PDF) [file pone.0197392.s007.pdf]

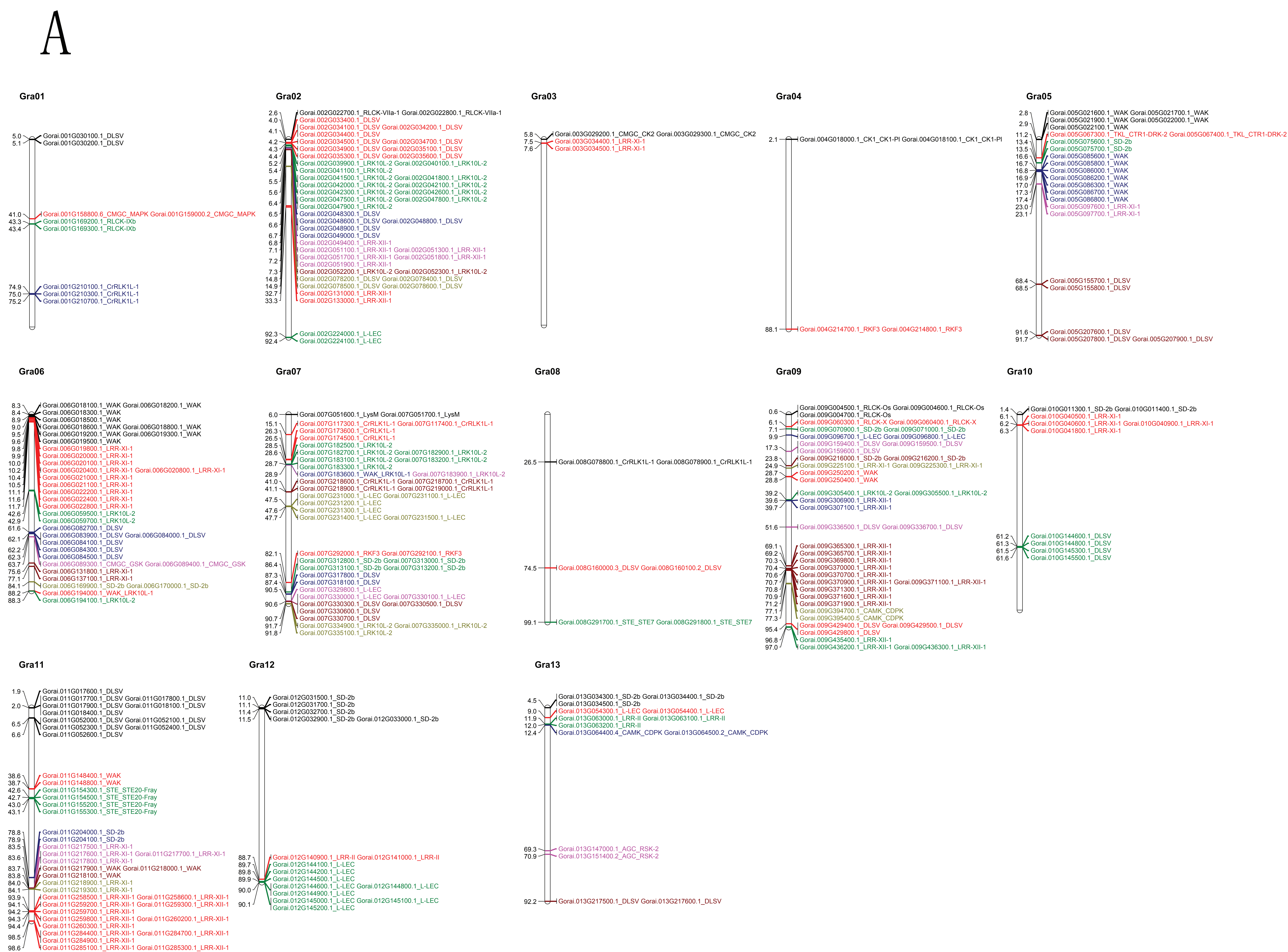

A
Gra01
5.0 Gorai.001G030100.1_DLSV
5.1 Gorai.001G030200.1_DLSV
41.0 Gorai.001G158800.6_CMGC_MAPK Gorai.001G159000.2_CMGC_MAPK
43.3 Gorai.001G169200.1_RLCK-IXb
43.4 Gorai.001G169300.1_RLCK-IXb
74.9 Gorai.001G210100.1_CrRLK1L-1
75.0 Gorai.001G210300.1_CrRLK1L-1
75.2 Gorai.001G210700.1_CrRLK1L-1
Gra02
2.6 Gorai.002G022700.1_RLCK-VIIa-1 Gorai.002G022800.1_RLCK-VIIa-1
4.0 Gorai.002G033400.1_DLSV
4.1 Gorai.002G034100.1_DLSV Gorai.002G034200.1_DLSV
4.2 Gorai.002G034400.1_DLSV
4.3 Gorai.002G034500.1_DLSV Gorai.002G034700.1_DLSV
4.4 Gorai.002G034900.1_DLSV Gorai.002G035100.1_DLSV
5.2 Gorai.002G035300.1_DLSV Gorai.002G035600.1_DLSV
5.4 Gorai.002G039900.1_LRK10L-2 Gorai.002G040100.1_LRK10L-2
5.5 Gorai.002G041100.1_LRK10L-2
5.6 Gorai.002G041500.1_LRK10L-2 Gorai.002G041800.1_LRK10L-2
6.4 Gorai.002G042000.1_LRK10L-2 Gorai.002G042100.1_LRK10L-2
6.5 Gorai.002G042300.1_LRK10L-2 Gorai.002G042600.1_LRK10L-2
6.6 Gorai.002G047500.1_LRK10L-2 Gorai.002G047800.1_LRK10L-2
6.7 Gorai.002G047900.1_LRK10L-2
6.8 Gorai.002G048300.1_DLSV
7.1 Gorai.002G048600.1_DLSV Gorai.002G048800.1_DLSV
7.2 Gorai.002G048900.1_DLSV
7.3 Gorai.002G049000.1_DLSV
14.8 Gorai.002G049400.1_LRR-XII-1
14.9 Gorai.002G051100.1_LRR-XII-1 Gorai.002G051300.1_LRR-XII-1
32.7 Gorai.002G051700.1_LRR-XII-1 Gorai.002G051800.1_LRR-XII-1
33.3 Gorai.002G051900.1_LRR-XII-1
Gorai.002G052200.1_LRK10L-2 Gorai.002G052300.1_LRK10L-2
Gorai.002G078200.1_DLSV Gorai.002G078400.1_DLSV
Gorai.002G078500.1_DLSV Gorai.002G078600.1_DLSV
Gorai.002G131000.1_LRR-XII-1
Gorai.002G133000.1_LRR-XII-1
92.3 Gorai.002G224000.1_L-LEC
92.4 Gorai.002G224100.1_L-LEC
Gra03
5.8 Gorai.003G029200.1_CMGC_CK2 Gorai.003G029300.1_CMGC_CK2
7.5 Gorai.003G034400.1_LRR-XI-1
7.6 Gorai.003G034500.1_LRR-XI-1
Gra04
2.1 Gorai.004G018000.1_CK1_CK1-Pl Gorai.004G018100.1_CK1_CK1-Pl
88.1 Gorai.004G214700.1_RKF3 Gorai.004G214800.1_RKF3
Gra05
2.8 Gorai.005G021600.1_WAK Gorai.005G021700.1_WAK
2.9 Gorai.005G021900.1_WAK Gorai.005G022000.1_WAK
11.2 Gorai.005G022100.1_WAK
13.4 Gorai.005G067300.1_TKL_CTR1-DRK-2 Gorai.005G067400.1_TKL_CTR1-DRK-2
13.5 Gorai.005G075600.1_SD-2b
16.6 Gorai.005G075700.1_SD-2b
16.7 Gorai.005G085600.1_WAK
16.8 Gorai.005G085800.1_WAK
16.9 Gorai.005G086000.1_WAK
17.0 Gorai.005G086200.1_WAK
17.3 Gorai.005G086300.1_WAK
17.4 Gorai.005G086700.1_WAK
23.0 Gorai.005G086800.1_WAK
23.1 Gorai.005G097600.1_LRR-XI-1
Gorai.005G097700.1_LRR-XI-1
68.4 Gorai.005G155700.1_DLSV
68.5 Gorai.005G155800.1_DLSV
91.6 Gorai.005G207600.1_DLSV
91.7 Gorai.005G207800.1_DLSV Gorai.005G207900.1_DLSV
Gra06
8.3 Gorai.006G018100.1_WAK Gorai.006G018200.1_WAK
8.4 Gorai.006G018300.1_WAK
8.9 Gorai.006G018500.1_WAK
9.0 Gorai.006G018600.1_WAK Gorai.006G018800.1_WAK
9.5 Gorai.006G019200.1_WAK Gorai.006G019300.1_WAK
9.6 Gorai.006G019500.1_WAK
9.8 Gorai.006G019800.1_LRR-XI-1
9.9 Gorai.006G020000.1_LRR-XI-1
10.0 Gorai.006G020100.1_LRR-XI-1
10.2 Gorai.006G020400.1_LRR-XI-1 Gorai.006G020800.1_LRR-XI-1
10.4 Gorai.006G021000.1_LRR-XI-1
10.5 Gorai.006G021100.1_LRR-XI-1
11.1 Gorai.006G022200.1_LRR-XI-1
11.6 Gorai.006G022400.1_LRR-XI-1
11.7 Gorai.006G022800.1_LRR-XI-1
42.6 Gorai.006G059500.1_LRK10L-2
42.9 Gorai.006G059700.1_LRK10L-2
61.6 Gorai.006G082700.1_DLSV
62.1 Gorai.006G083900.1_DLSV Gorai.006G084000.1_DLSV
62.2 Gorai.006G084100.1_DLSV
62.3 Gorai.006G084300.1_DLSV
63.7 Gorai.006G084500.1_DLSV
75.6 Gorai.006G089300.1_CMGC_GSK Gorai.006G089400.1_CMGC_GSK
77.1 Gorai.006G131800.1_LRR-XI-1
84.1 Gorai.006G137100.1_LRR-XI-1
88.2 Gorai.006G169900.1_SD-2b Gorai.006G170000.1_SD-2b
88.3 Gorai.006G194000.1_WAK_LRK10L-1
Gorai.006G194100.1_LRK10L-2
Gra07
6.0 Gorai.007G051600.1_LysM Gorai.007G051700.1_LysM
15.1 Gorai.007G117300.1_CrRLK1L-1 Gorai.007G117400.1_CrRLK1L-1
26.3 Gorai.007G173600.1_CrRLK1L-1
26.5 Gorai.007G174500.1_CrRLK1L-1
28.5 Gorai.007G182500.1_LRK10L-2
28.6 Gorai.007G182700.1_LRK10L-2 Gorai.007G182900.1_LRK10L-2
28.7 Gorai.007G183100.1_LRK10L-2 Gorai.007G183200.1_LRK10L-2
Gorai.007G183300.1_LRK10L-2
28.9 Gorai.007G183600.1_WAK_LRK10L-1 Gorai.007G183900.1_LRK10L-2
41.0 Gorai.007G218600.1_CrRLK1L-1 Gorai.007G218700.1_CrRLK1L-1
41.1 Gorai.007G218900.1_CrRLK1L-1 Gorai.007G219000.1_CrRLK1L-1
47.5 Gorai.007G231000.1_L-LEC Gorai.007G231100.1_L-LEC
Gorai.007G231200.1_L-LEC
47.6 Gorai.007G231300.1_L-LEC
47.7 Gorai.007G231400.1_L-LEC Gorai.007G231500.1_L-LEC
82.1 Gorai.007G292000.1_RKF3 Gorai.007G292100.1_RKF3
86.4 Gorai.007G312800.1_SD-2b Gorai.007G313000.1_SD-2b
Gorai.007G313100.1_SD-2b Gorai.007G313200.1_SD-2b
87.3 Gorai.007G317800.1_DLSV
87.4 Gorai.007G318100.1_DLSV
90.5 Gorai.007G329800.1_L-LEC
Gorai.007G330000.1_L-LEC Gorai.007G330100.1_L-LEC
90.6 Gorai.007G330300.1_DLSV Gorai.007G330500.1_DLSV
Gorai.007G330600.1_DLSV
90.7 Gorai.007G330700.1_DLSV
91.7 Gorai.007G334900.1_LRK10L-2 Gorai.007G335000.1_LRK10L-2
91.8 Gorai.007G335100.1_LRK10L-2
Gra08
26.5 Gorai.008G078800.1_CrRLK1L-1 Gorai.008G078900.1_CrRLK1L-1
74.5 Gorai.008G160000.3_DLSV Gorai.008G160100.2_DLSV
99.1 Gorai.008G291700.1_STE_STE7 Gorai.008G291800.1_STE_STE7
Gra09
0.6 Gorai.009G004500.1_RLCK-Os Gorai.009G004600.1_RLCK-Os
Gorai.009G004700.1_RLCK-Os
6.1 Gorai.009G060300.1_RLCK-X Gorai.009G060400.1_RLCK-X
7.1 Gorai.009G070900.1_SD-2b Gorai.009G071000.1_SD-2b
9.9 Gorai.009G096700.1_L-LEC Gorai.009G096800.1_L-LEC
17.3 Gorai.009G159400.1_DLSV Gorai.009G159500.1_DLSV
Gorai.009G159600.1_DLSV
23.8 Gorai.009G216000.1_SD-2b Gorai.009G216200.1_SD-2b
24.9 Gorai.009G225100.1_LRR-XI-1 Gorai.009G225300.1_LRR-XI-1
28.7 Gorai.009G250200.1_WAK
28.8 Gorai.009G250400.1_WAK
39.2 Gorai.009G305400.1_LRK10L-2 Gorai.009G305500.1_LRK10L-2
39.6 Gorai.009G306900.1_LRR-XII-1
39.7 Gorai.009G307100.1_LRR-XII-1
51.6 Gorai.009G336500.1_DLSV Gorai.009G336700.1_DLSV
69.1 Gorai.009G365300.1_LRR-XII-1
69.2 Gorai.009G365700.1_LRR-XII-1
70.3 Gorai.009G369800.1_LRR-XII-1
70.4 Gorai.009G370000.1_LRR-XII-1
70.6 Gorai.009G370700.1_LRR-XII-1
70.7 Gorai.009G370900.1_LRR-XII-1 Gorai.009G371100.1_LRR-XII-1
70.8 Gorai.009G371300.1_LRR-XII-1
70.9 Gorai.009G371600.1_LRR-XII-1
71.2 Gorai.009G371900.1_LRR-XII-1
77.1 Gorai.009G394700.1_CAMK_CDPK
77.3 Gorai.009G395400.5_CAMK_CDPK
95.4 Gorai.009G429400.1_DLSV Gorai.009G429500.1_DLSV
Gorai.009G429800.1_DLSV
96.8 Gorai.009G435400.1_LRR-XII-1
97.0 Gorai.009G436200.1_LRR-XII-1 Gorai.009G436300.1_LRR-XII-1
Gra10
1.4 Gorai.010G011300.1_SD-2b Gorai.010G011400.1_SD-2b
6.1 Gorai.010G040500.1_LRR-XI-1
6.2 Gorai.010G040600.1_LRR-XI-1 Gorai.010G040900.1_LRR-XI-1
6.3 Gorai.010G041800.1_LRR-XI-1
61.2 Gorai.010G144600.1_DLSV
61.3 Gorai.010G144800.1_DLSV
61.5 Gorai.010G145300.1_DLSV
61.6 Gorai.010G145500.1_DLSV
Gra11
1.9 Gorai.011G017600.1_DLSV
Gorai.011G017700.1_DLSV Gorai.011G017800.1_DLSV
2.0 Gorai.011G017900.1_DLSV Gorai.011G018100.1_DLSV
Gorai.011G018400.1_DLSV
6.5 Gorai.011G052000.1_DLSV Gorai.011G052100.1_DLSV
Gorai.011G052300.1_DLSV Gorai.011G052400.1_DLSV
6.6 Gorai.011G052600.1_DLSV
38.6 Gorai.011G148400.1_WAK
38.7 Gorai.011G148800.1_WAK
42.6 Gorai.011G154300.1_STE_STE20-Fray
42.7 Gorai.011G154500.1_STE_STE20-Fray
43.0 Gorai.011G155200.1_STE_STE20-Fray
43.1 Gorai.011G155300.1_STE_STE20-Fray
78.8 Gorai.011G204000.1_SD-2b
78.9 Gorai.011G204100.1_SD-2b
83.5 Gorai.011G217500.1_LRR-XI-1
83.6 Gorai.011G217600.1_LRR-XI-1 Gorai.011G217700.1_LRR-XI-1
Gorai.011G217800.1_LRR-XI-1
83.7 Gorai.011G217900.1_WAK Gorai.011G218000.1_WAK
83.8 Gorai.011G218100.1_WAK
84.0 Gorai.011G218900.1_LRR-XI-1
84.1 Gorai.011G219300.1_LRR-XI-1
93.9 Gorai.011G258500.1_LRR-XII-1 Gorai.011G258600.1_LRR-XII-1
94.1 Gorai.011G259200.1_LRR-XII-1 Gorai.011G259300.1_LRR-XII-1
94.2 Gorai.011G259700.1_LRR-XII-1
94.3 Gorai.011G259800.1_LRR-XII-1 Gorai.011G260200.1_LRR-XII-1
94.4 Gorai.011G260300.1_LRR-XII-1
98.5 Gorai.011G284400.1_LRR-XII-1 Gorai.011G284700.1_LRR-XII-1
Gorai.011G284900.1_LRR-XII-1
98.6 Gorai.011G285100.1_LRR-XII-1 Gorai.011G285300.1_LRR-XII-1
Gra12
11.0 Gorai.012G031500.1_SD-2b
11.1 Gorai.012G031700.1_SD-2b
11.4 Gorai.012G032700.1_SD-2b
11.5 Gorai.012G032900.1_SD-2b Gorai.012G033000.1_SD-2b
88.7 Gorai.012G140900.1_LRR-II Gorai.012G141000.1_LRR-II
89.7 Gorai.012G144100.1_L-LEC
89.8 Gorai.012G144200.1_L-LEC
89.9 Gorai.012G144500.1_L-LEC
90.0 Gorai.012G144600.1_L-LEC Gorai.012G144800.1_L-LEC
Gorai.012G144900.1_L-LEC
90.1 Gorai.012G145000.1_L-LEC Gorai.012G145100.1_L-LEC
Gorai.012G145200.1_L-LEC
Gra13
4.5 Gorai.013G034300.1_SD-2b Gorai.013G034400.1_SD-2b
Gorai.013G034500.1_SD-2b
9.0 Gorai.013G054300.1_L-LEC Gorai.013G054400.1_L-LEC
11.9 Gorai.013G063000.1_LRR-II Gorai.013G063100.1_LRR-II
12.0 Gorai.013G063200.1_LRR-II
12.4 Gorai.013G064400.4_CAMK_CDPK Gorai.013G064500.2_CAMK_CDPK
69.3 Gorai.013G147000.1_AGC_RSK-2
70.9 Gorai.013G151400.2_AGC_RSK-2
92.2 Gorai.013G217500.1_DLSV Gorai.013G217600.1_DLSV

B

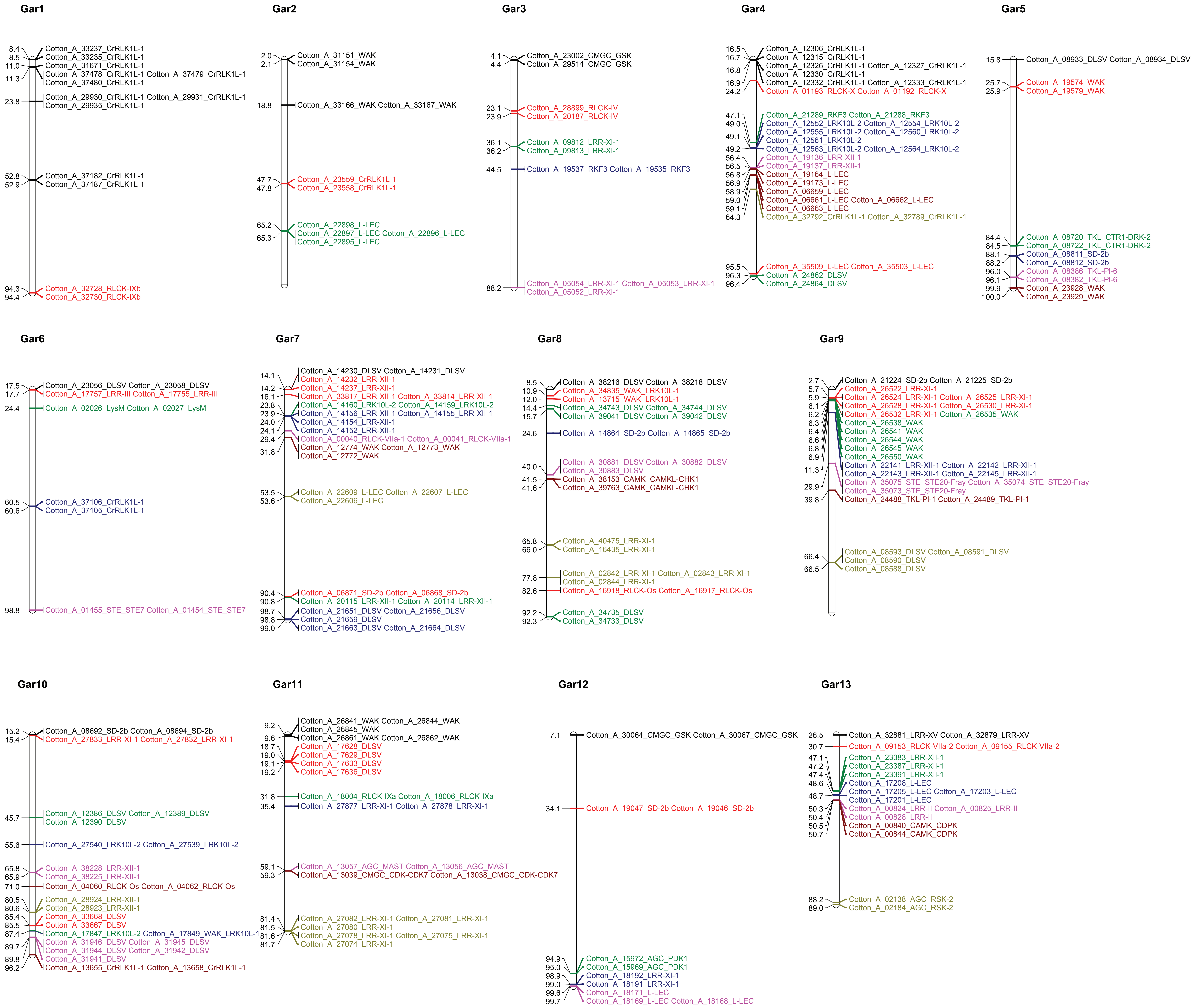
Gar1
8.4
8.5
11.0
11.3
Cotton_A_33237_CrRLK1L-1
Cotton_A_33235_CrRLK1L-1
Cotton_A_31671_CrRLK1L-1
Cotton_A_37478_CrRLK1L-1 Cotton_A_37479_CrRLK1L-1
Cotton_A_37480_CrRLK1L-1
23.8
Cotton_A_29930_CrRLK1L-1 Cotton_A_29931_CrRLK1L-1
Cotton_A_29935_CrRLK1L-1
52.8
52.9
Cotton_A_37182_CrRLK1L-1
Cotton_A_37187_CrRLK1L-1
94.3
94.4
Cotton_A_32728_RLCK-IXb
Cotton_A_32730_RLCK-IXb
Gar2
2.0
2.1
Cotton_A_31151_WAK
Cotton_A_31154_WAK
18.8
Cotton_A_33166_WAK Cotton_A_33167_WAK
47.7
47.8
Cotton_A_23559_CrRLK1L-1
Cotton_A_23558_CrRLK1L-1
65.2
65.3
Cotton_A_22898_L-LEC
Cotton_A_22897_L-LEC Cotton_A_22896_L-LEC
Cotton_A_22895_L-LEC
Gar3
4.1
4.4
Cotton_A_23002_CMGC_GSK
Cotton_A_29514_CMGC_GSK
23.1
23.9
Cotton_A_28899_RLCK-IV
Cotton_A_20187_RLCK-IV
36.1
36.2
Cotton_A_09812_LRR-XI-1
Cotton_A_09813_LRR-XI-1
44.5
Cotton_A_19537_RKF3 Cotton_A_19535_RKF3
88.2
Cotton_A_05054_LRR-XI-1 Cotton_A_05053_LRR-XI-1
Cotton_A_05052_LRR-XI-1
Gar4
16.5
16.7
16.8
16.9
24.2
Cotton_A_12306_CrRLK1L-1
Cotton_A_12315_CrRLK1L-1
Cotton_A_12326_CrRLK1L-1 Cotton_A_12327_CrRLK1L-1
Cotton_A_12330_CrRLK1L-1
Cotton_A_12332_CrRLK1L-1 Cotton_A_12333_CrRLK1L-1
Cotton_A_01193_RLCK-X Cotton_A_01192_RLCK-X
47.1
49.0
49.1
49.2
Cotton_A_21289_RKF3 Cotton_A_21288_RKF3
Cotton_A_12552_LRK10L-2 Cotton_A_12554_LRK10L-2
Cotton_A_12555_LRK10L-2 Cotton_A_12560_LRK10L-2
Cotton_A_12561_LRK10L-2
Cotton_A_12563_LRK10L-2 Cotton_A_12564_LRK10L-2
56.4
56.5
56.8
56.9
58.9
59.0
59.1
64.3
Cotton_A_19136_LRR-XII-1
Cotton_A_19137_LRR-XII-1
Cotton_A_19164_L-LEC
Cotton_A_19173_L-LEC
Cotton_A_06659_L-LEC
Cotton_A_06661_L-LEC Cotton_A_06662_L-LEC
Cotton_A_06663_L-LEC
Cotton_A_32792_CrRLK1L-1 Cotton_A_32789_CrRLK1L-1
95.5
96.3
96.4
Cotton_A_35509_L-LEC Cotton_A_35503_L-LEC
Cotton_A_24862_DLSV
Cotton_A_24864_DLSV
Gar5
15.8
Cotton_A_08933_DLSV Cotton_A_08934_DLSV
25.7
25.9
Cotton_A_19574_WAK
Cotton_A_19579_WAK
84.4
84.5
88.1
88.2
96.0
96.1
99.9
100.0
Cotton_A_08720_TKL_CTR1-DRK-2
Cotton_A_08722_TKL_CTR1-DRK-2
Cotton_A_08811_SD-2b
Cotton_A_08812_SD-2b
Cotton_A_08386_TKL-Pl-6
Cotton_A_08382_TKL-Pl-6
Cotton_A_23928_WAK
Cotton_A_23929_WAK
Gar6
17.5
17.7
Cotton_A_23056_DLSV Cotton_A_23058_DLSV
Cotton_A_17757_LRR-III Cotton_A_17755_LRR-III
24.4
Cotton_A_02026_LysM Cotton_A_02027_LysM
60.5
60.6
Cotton_A_37106_CrRLK1L-1
Cotton_A_37105_CrRLK1L-1
98.8
Cotton_A_01455_STE_STE7 Cotton_A_01454_STE_STE7
Gar7
14.1
14.2
16.1
23.8
23.9
24.0
24.1
29.4
31.8
Cotton_A_14230_DLSV Cotton_A_14231_DLSV
Cotton_A_14232_LRR-XII-1
Cotton_A_14237_LRR-XII-1
Cotton_A_33817_LRR-XII-1 Cotton_A_33814_LRR-XII-1
Cotton_A_14160_LRK10L-2 Cotton_A_14159_LRK10L-2
Cotton_A_14156_LRR-XII-1 Cotton_A_14155_LRR-XII-1
Cotton_A_14154_LRR-XII-1
Cotton_A_14152_LRR-XII-1
Cotton_A_00040_RLCK-VIIa-1 Cotton_A_00041_RLCK-VIIa-1
Cotton_A_12774_WAK Cotton_A_12773_WAK
Cotton_A_12772_WAK
53.5
53.6
Cotton_A_22609_L-LEC Cotton_A_22607_L-LEC
Cotton_A_22606_L-LEC
90.4
90.8
98.7
98.8
99.0
Cotton_A_06871_SD-2b Cotton_A_06868_SD-2b
Cotton_A_20115_LRR-XII-1 Cotton_A_20114_LRR-XII-1
Cotton_A_21651_DLSV Cotton_A_21656_DLSV
Cotton_A_21659_DLSV
Cotton_A_21663_DLSV Cotton_A_21664_DLSV
Gar8
8.5
10.9
12.0
14.4
15.7
Cotton_A_38216_DLSV Cotton_A_38218_DLSV
Cotton_A_34835_WAK_LRK10L-1
Cotton_A_13715_WAK_LRK10L-1
Cotton_A_34743_DLSV Cotton_A_34744_DLSV
Cotton_A_39041_DLSV Cotton_A_39042_DLSV
24.6
Cotton_A_14864_SD-2b Cotton_A_14865_SD-2b
40.0
41.5
41.6
Cotton_A_30881_DLSV Cotton_A_30882_DLSV
Cotton_A_30883_DLSV
Cotton_A_38153_CAMK_CAMKL-CHK1
Cotton_A_39763_CAMK_CAMKL-CHK1
65.8
66.0
Cotton_A_40475_LRR-XI-1
Cotton_A_16435_LRR-XI-1
77.8
82.6
Cotton_A_02842_LRR-XI-1 Cotton_A_02843_LRR-XI-1
Cotton_A_02844_LRR-XI-1
Cotton_A_16918_RLCK-Os Cotton_A_16917_RLCK-Os
92.2
92.3
Cotton_A_34735_DLSV
Cotton_A_34733_DLSV
Gar9
2.7
5.7
5.9
6.1
6.2
6.3
6.4
6.6
6.8
6.9
11.3
29.9
39.8
Cotton_A_21224_SD-2b Cotton_A_21225_SD-2b
Cotton_A_26522_LRR-XI-1
Cotton_A_26524_LRR-XI-1 Cotton_A_26525_LRR-XI-1
Cotton_A_26528_LRR-XI-1 Cotton_A_26530_LRR-XI-1
Cotton_A_26532_LRR-XI-1 Cotton_A_26535_WAK
Cotton_A_26538_WAK
Cotton_A_26541_WAK
Cotton_A_26544_WAK
Cotton_A_26545_WAK
Cotton_A_26550_WAK
Cotton_A_22141_LRR-XII-1 Cotton_A_22142_LRR-XII-1
Cotton_A_22143_LRR-XII-1 Cotton_A_22145_LRR-XII-1
Cotton_A_35075_STE_STE20-Fray Cotton_A_35074_STE_STE20-Fray
Cotton_A_35073_STE_STE20-Fray
Cotton_A_24488_TKL-Pl-1 Cotton_A_24489_TKL-Pl-1
66.4
66.5
Cotton_A_08593_DLSV Cotton_A_08591_DLSV
Cotton_A_08590_DLSV
Cotton_A_08588_DLSV
Gar10
15.2
15.4
Cotton_A_08692_SD-2b Cotton_A_08694_SD-2b
Cotton_A_27833_LRR-XI-1 Cotton_A_27832_LRR-XI-1
45.7
Cotton_A_12386_DLSV Cotton_A_12389_DLSV
Cotton_A_12390_DLSV
55.6
Cotton_A_27540_LRK10L-2 Cotton_A_27539_LRK10L-2
65.8
65.9
71.0
Cotton_A_38228_LRR-XII-1
Cotton_A_38225_LRR-XII-1
Cotton_A_04060_RLCK-Os Cotton_A_04062_RLCK-Os
80.5
80.6
85.4
85.5
87.4
89.7
89.8
96.2
Cotton_A_28924_LRR-XII-1
Cotton_A_28923_LRR-XII-1
Cotton_A_33668_DLSV
Cotton_A_33667_DLSV
Cotton_A_17847_LRK10L-2 Cotton_A_17849_WAK_LRK10L-1
Cotton_A_31946_DLSV Cotton_A_31945_DLSV
Cotton_A_31944_DLSV Cotton_A_31942_DLSV
Cotton_A_31941_DLSV
Cotton_A_13655_CrRLK1L-1 Cotton_A_13658_CrRLK1L-1
Gar11
9.2
9.6
18.7
19.0
19.1
19.2
Cotton_A_26841_WAK Cotton_A_26844_WAK
Cotton_A_26845_WAK
Cotton_A_26861_WAK Cotton_A_26862_WAK
Cotton_A_17628_DLSV
Cotton_A_17629_DLSV
Cotton_A_17633_DLSV
Cotton_A_17636_DLSV
31.8
35.4
Cotton_A_18004_RLCK-IXa Cotton_A_18006_RLCK-IXa
Cotton_A_27877_LRR-XI-1 Cotton_A_27878_LRR-XI-1
59.1
59.3
Cotton_A_13057_AGC_MAST Cotton_A_13056_AGC_MAST
Cotton_A_13039_CMGC_CDK-CDK7 Cotton_A_13038_CMGC_CDK-CDK7
81.4
81.5
81.6
81.7
Cotton_A_27082_LRR-XI-1 Cotton_A_27081_LRR-XI-1
Cotton_A_27080_LRR-XI-1
Cotton_A_27078_LRR-XI-1 Cotton_A_27075_LRR-XI-1
Cotton_A_27074_LRR-XI-1
Gar12
7.1
Cotton_A_30064_CMGC_GSK Cotton_A_30067_CMGC_GSK
34.1
Cotton_A_19047_SD-2b Cotton_A_19046_SD-2b
94.9
95.0
98.9
99.0
99.6
99.7
Cotton_A_15972_AGC_PDK1
Cotton_A_15969_AGC_PDK1
Cotton_A_18192_LRR-XI-1
Cotton_A_18191_LRR-XI-1
Cotton_A_18171_L-LEC
Cotton_A_18169_L-LEC Cotton_A_18168_L-LEC
Gar13
26.5
30.7
Cotton_A_32881_LRR-XV Cotton_A_32879_LRR-XV
Cotton_A_09153_RLCK-VIIa-2 Cotton_A_09155_RLCK-VIIa-2
47.1
47.2
47.4
48.6
48.7
50.3
50.4
50.5
50.7
Cotton_A_23383_LRR-XII-1
Cotton_A_23387_LRR-XII-1
Cotton_A_23391_LRR-XII-1
Cotton_A_17208_L-LEC
Cotton_A_17205_L-LEC Cotton_A_17203_L-LEC
Cotton_A_17201_L-LEC
Cotton_A_00824_LRR-II Cotton_A_00825_LRR-II
Cotton_A_00828_LRR-II
Cotton_A_00840_CAMK_CDPK
Cotton_A_00844_CAMK_CDPK
88.2
89.0
Cotton_A_02138_AGC_RSK-2
Cotton_A_02184_AGC_RSK-2

C

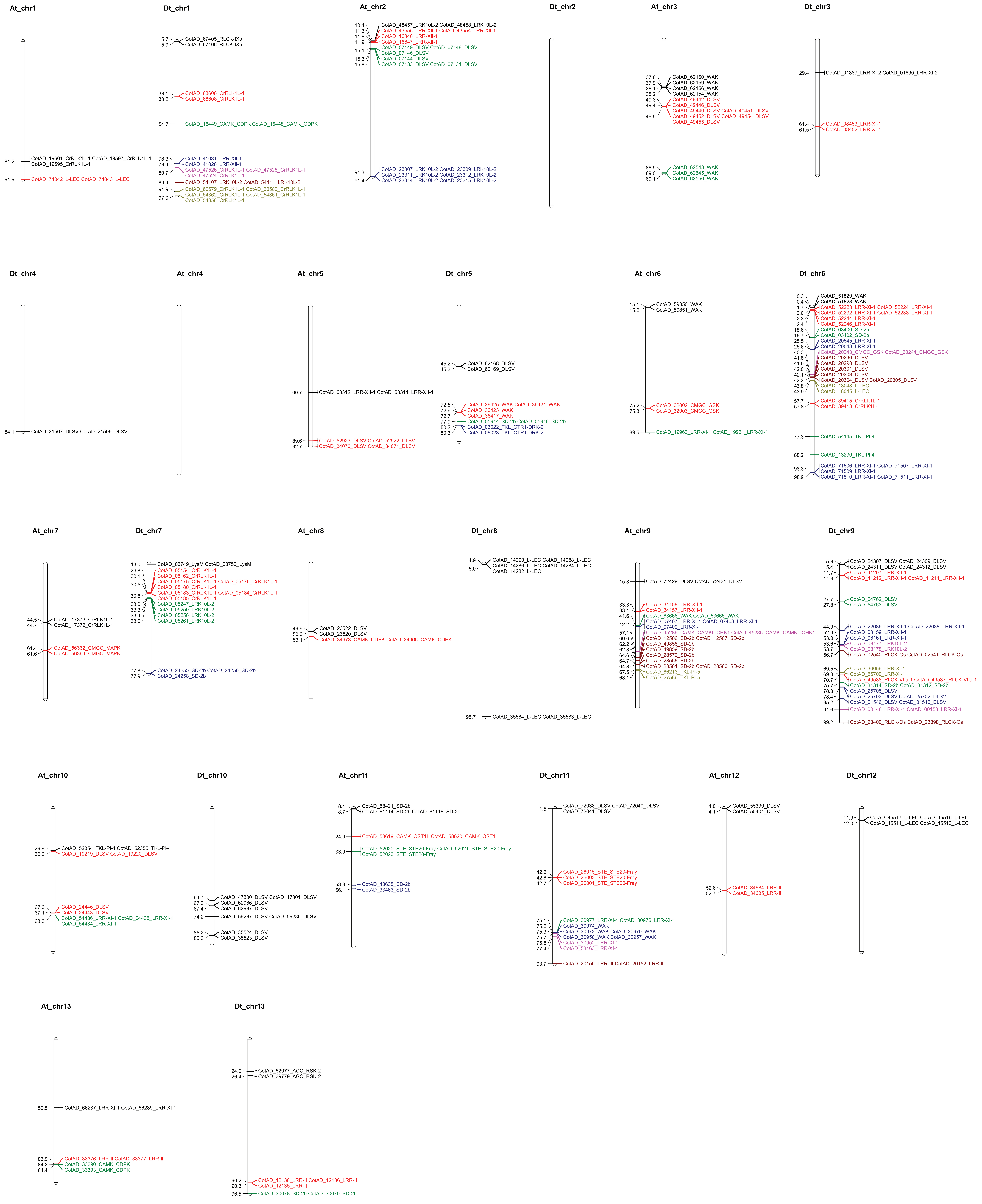
At_chr1
81.2 CotAD_19601_CrRLK1L-1 CotAD_19597_CrRLK1L-1
CotAD_19595_CrRLK1L-1
91.9 CotAD_74042_L-LEC CotAD_74043_L-LEC
Dt_chr1
5.7 CotAD_67405_RLCK-IXb
5.9 CotAD_67406_RLCK-IXb
38.1 CotAD_68606_CrRLK1L-1
38.2 CotAD_68608_CrRLK1L-1
54.7 CotAD_16449_CAMK_CDPK CotAD_16448_CAMK_CDPK
78.3 CotAD_41031_LRR-XII-1
78.4 CotAD_41028_LRR-XII-1
80.7 CotAD_47526_CrRLK1L-1 CotAD_47525_CrRLK1L-1
CotAD_47524_CrRLK1L-1
89.4 CotAD_54107_LRK10L-2 CotAD_54111_LRK10L-2
94.9 CotAD_60579_CrRLK1L-1 CotAD_60580_CrRLK1L-1
97.0 CotAD_54362_CrRLK1L-1 CotAD_54361_CrRLK1L-1
CotAD_54358_CrRLK1L-1
At_chr2
10.4 CotAD_48457_LRK10L-2 CotAD_48458_LRK10L-2
11.3 CotAD_43555_LRR-XII-1 CotAD_43554_LRR-XII-1
11.8 CotAD_16846_LRR-XII-1
11.9 CotAD_16847_LRR-XII-1
15.1 CotAD_07149_DLSV CotAD_07148_DLSV
CotAD_07146_DLSV
15.3 CotAD_07144_DLSV
15.8 CotAD_07133_DLSV CotAD_07131_DLSV
91.3 CotAD_23307_LRK10L-2 CotAD_23309_LRK10L-2
CotAD_23311_LRK10L-2 CotAD_23312_LRK10L-2
91.4 CotAD_23314_LRK10L-2 CotAD_23315_LRK10L-2
Dt_chr2
At_chr3
37.8 CotAD_62160_WAK
37.9 CotAD_62159_WAK
38.1 CotAD_62156_WAK
38.2 CotAD_62154_WAK
49.3 CotAD_49442_DLSV
49.4 CotAD_49446_DLSV
CotAD_49449_DLSV CotAD_49451_DLSV
49.5 CotAD_49452_DLSV CotAD_49454_DLSV
CotAD_49455_DLSV
88.9 CotAD_62543_WAK
89.0 CotAD_62545_WAK
89.1 CotAD_62550_WAK
Dt_chr3
29.4 CotAD_01889_LRR-XI-2 CotAD_01890_LRR-XI-2
61.4 CotAD_08453_LRR-XI-1
61.5 CotAD_08452_LRR-XI-1
Dt_chr4
84.1 CotAD_21507_DLSV CotAD_21506_DLSV
At_chr4
At_chr5
60.7 CotAD_63312_LRR-XII-1 CotAD_63311_LRR-XII-1
89.6 CotAD_52923_DLSV CotAD_52922_DLSV
92.7 CotAD_34070_DLSV CotAD_34071_DLSV
Dt_chr5
45.2 CotAD_62168_DLSV
45.3 CotAD_62169_DLSV
72.5 CotAD_36425_WAK CotAD_36424_WAK
72.6 CotAD_36423_WAK
72.7 CotAD_36417_WAK
77.9 CotAD_05914_SD-2b CotAD_05916_SD-2b
80.2 CotAD_06022_TKL_CTR1-DRK-2
80.3 CotAD_06023_TKL_CTR1-DRK-2
At_chr6
15.1 CotAD_59850_WAK
15.2 CotAD_59851_WAK
75.2 CotAD_32002_CMGC_GSK
75.3 CotAD_32003_CMGC_GSK
89.5 CotAD_19963_LRR-XI-1 CotAD_19961_LRR-XI-1
Dt_chr6
0.3 CotAD_51829_WAK
0.4 CotAD_51828_WAK
1.7 CotAD_52223_LRR-XI-1 CotAD_52224_LRR-XI-1
2.0 CotAD_52232_LRR-XI-1 CotAD_52233_LRR-XI-1
2.3 CotAD_52244_LRR-XI-1
2.4 CotAD_52246_LRR-XI-1
18.6 CotAD_03400_SD-2b
18.7 CotAD_03402_SD-2b
25.5 CotAD_20545_LRR-XI-1
25.6 CotAD_20548_LRR-XI-1
40.3 CotAD_20243_CMGC_GSK CotAD_20244_CMGC_GSK
41.8 CotAD_20296_DLSV
41.9 CotAD_20298_DLSV
42.0 CotAD_20301_DLSV
42.1 CotAD_20303_DLSV
42.2 CotAD_20304_DLSV CotAD_20305_DLSV
43.8 CotAD_18043_L-LEC
43.9 CotAD_18045_L-LEC
57.7 CotAD_39415_CrRLK1L-1
57.8 CotAD_39418_CrRLK1L-1
77.3 CotAD_54145_TKL-Pl-4
88.2 CotAD_13230_TKL-Pl-4
98.8 CotAD_71506_LRR-XI-1 CotAD_71507_LRR-XI-1
CotAD_71509_LRR-XI-1
98.9 CotAD_71510_LRR-XI-1 CotAD_71511_LRR-XI-1
At_chr7
44.5 CotAD_17373_CrRLK1L-1
44.7 CotAD_17372_CrRLK1L-1
61.4 CotAD_56362_CMGC_MAPK
61.6 CotAD_56364_CMGC_MAPK
Dt_chr7
13.0 CotAD_03749_LysM CotAD_03750_LysM
29.8 CotAD_05154_CrRLK1L-1
30.1 CotAD_05162_CrRLK1L-1
30.5 CotAD_05175_CrRLK1L-1 CotAD_05176_CrRLK1L-1
CotAD_05180_CrRLK1L-1
30.6 CotAD_05183_CrRLK1L-1 CotAD_05184_CrRLK1L-1
CotAD_05185_CrRLK1L-1
33.0 CotAD_05247_LRK10L-2
33.3 CotAD_05250_LRK10L-2
33.4 CotAD_05256_LRK10L-2
33.6 CotAD_05261_LRK10L-2
77.8 CotAD_24255_SD-2b CotAD_24256_SD-2b
77.9 CotAD_24258_SD-2b
At_chr8
49.9 CotAD_23522_DLSV
50.0 CotAD_23520_DLSV
53.1 CotAD_34973_CAMK_CDPK CotAD_34966_CAMK_CDPK
Dt_chr8
4.9 CotAD_14290_L-LEC CotAD_14288_L-LEC
5.0 CotAD_14286_L-LEC CotAD_14284_L-LEC
CotAD_14282_L-LEC
95.7 CotAD_35584_L-LEC CotAD_35583_L-LEC
At_chr9
15.3 CotAD_72429_DLSV CotAD_72431_DLSV
33.3 CotAD_34158_LRR-XII-1
33.4 CotAD_34157_LRR-XII-1
41.6 CotAD_63666_WAK CotAD_63665_WAK
42.2 CotAD_07407_LRR-XI-1 CotAD_07408_LRR-XI-1
CotAD_07409_LRR-XI-1
57.1 CotAD_45286_CAMK_CAMKL-CHK1 CotAD_45285_CAMK_CAMKL-CHK1
60.6 CotAD_12506_SD-2b CotAD_12507_SD-2b
62.2 CotAD_49858_SD-2b
62.3 CotAD_49859_SD-2b
64.6 CotAD_28570_SD-2b
64.7 CotAD_28566_SD-2b
64.8 CotAD_28561_SD-2b CotAD_28560_SD-2b
67.5 CotAD_66213_TKL-Pl-5
68.1 CotAD_27586_TKL-Pl-5
Dt_chr9
5.3 CotAD_24307_DLSV CotAD_24309_DLSV
5.4 CotAD_24311_DLSV CotAD_24312_DLSV
11.7 CotAD_41207_LRR-XII-1
11.9 CotAD_41212_LRR-XII-1 CotAD_41214_LRR-XII-1
27.7 CotAD_54762_DLSV
27.8 CotAD_54763_DLSV
44.9 CotAD_22086_LRR-XII-1 CotAD_22088_LRR-XII-1
52.9 CotAD_08159_LRR-XII-1
53.0 CotAD_08161_LRR-XII-1
53.6 CotAD_08177_LRK10L-2
53.7 CotAD_08178_LRK10L-2
56.7 CotAD_02540_RLCK-Os CotAD_02541_RLCK-Os
69.5 CotAD_36059_LRR-XI-1
69.8 CotAD_55700_LRR-XI-1
70.7 CotAD_49588_RLCK-VIIa-1 CotAD_49587_RLCK-VIIa-1
75.7 CotAD_31314_SD-2b CotAD_31312_SD-2b
78.3 CotAD_25705_DLSV
78.4 CotAD_25703_DLSV CotAD_25702_DLSV
85.2 CotAD_01546_DLSV CotAD_01545_DLSV
91.6 CotAD_00148_LRR-XI-1 CotAD_00150_LRR-XI-1
99.2 CotAD_23400_RLCK-Os CotAD_23398_RLCK-Os
At_chr10
29.9 CotAD_52354_TKL-Pl-4 CotAD_52355_TKL-Pl-4
30.6 CotAD_19219_DLSV CotAD_19220_DLSV
67.0 CotAD_24446_DLSV
67.1 CotAD_24448_DLSV
68.3 CotAD_54436_LRR-XI-1 CotAD_54435_LRR-XI-1
CotAD_54434_LRR-XI-1
Dt_chr10
64.7 CotAD_47800_DLSV CotAD_47801_DLSV
67.3 CotAD_62986_DLSV
67.4 CotAD_62987_DLSV
74.2 CotAD_59287_DLSV CotAD_59286_DLSV
85.2 CotAD_35524_DLSV
85.3 CotAD_35523_DLSV
At_chr11
8.4 CotAD_58421_SD-2b
8.7 CotAD_61114_SD-2b CotAD_61116_SD-2b
24.9 CotAD_58619_CAMK_OST1L CotAD_58620_CAMK_OST1L
33.9 CotAD_52020_STE_STE20-Fray CotAD_52021_STE_STE20-Fray
CotAD_52023_STE_STE20-Fray
53.9 CotAD_43635_SD-2b
56.1 CotAD_33463_SD-2b
Dt_chr11
1.5 CotAD_72038_DLSV CotAD_72040_DLSV
CotAD_72041_DLSV
42.2 CotAD_26015_STE_STE20-Fray
42.6 CotAD_26003_STE_STE20-Fray
42.7 CotAD_26001_STE_STE20-Fray
75.1 CotAD_30977_LRR-XI-1 CotAD_30976_LRR-XI-1
75.2 CotAD_30974_WAK
75.3 CotAD_30972_WAK CotAD_30970_WAK
75.7 CotAD_30958_WAK CotAD_30957_WAK
75.8 CotAD_30952_LRR-XI-1
77.4 CotAD_53463_LRR-XI-1
93.7 CotAD_20150_LRR-III CotAD_20152_LRR-III
At_chr12
4.0 CotAD_55399_DLSV
4.1 CotAD_55401_DLSV
52.6 CotAD_34684_LRR-II
52.7 CotAD_34685_LRR-II
Dt_chr12
11.9 CotAD_45517_L-LEC CotAD_45516_L-LEC
12.0 CotAD_45514_L-LEC CotAD_45513_L-LEC
At_chr13
50.5 CotAD_66287_LRR-XI-1 CotAD_66289_LRR-XI-1
83.9 CotAD_33376_LRR-II CotAD_33377_LRR-II
84.2 CotAD_33390_CAMK_CDPK
84.4 CotAD_33393_CAMK_CDPK
Dt_chr13
24.0 CotAD_52077_AGC_RSK-2
26.4 CotAD_39779_AGC_RSK-2
90.2 CotAD_12138_LRR-II CotAD_12136_LRR-II
90.3 CotAD_12135_LRR-II
96.5 CotAD_30678_SD-2b CotAD_30679_SD-2b

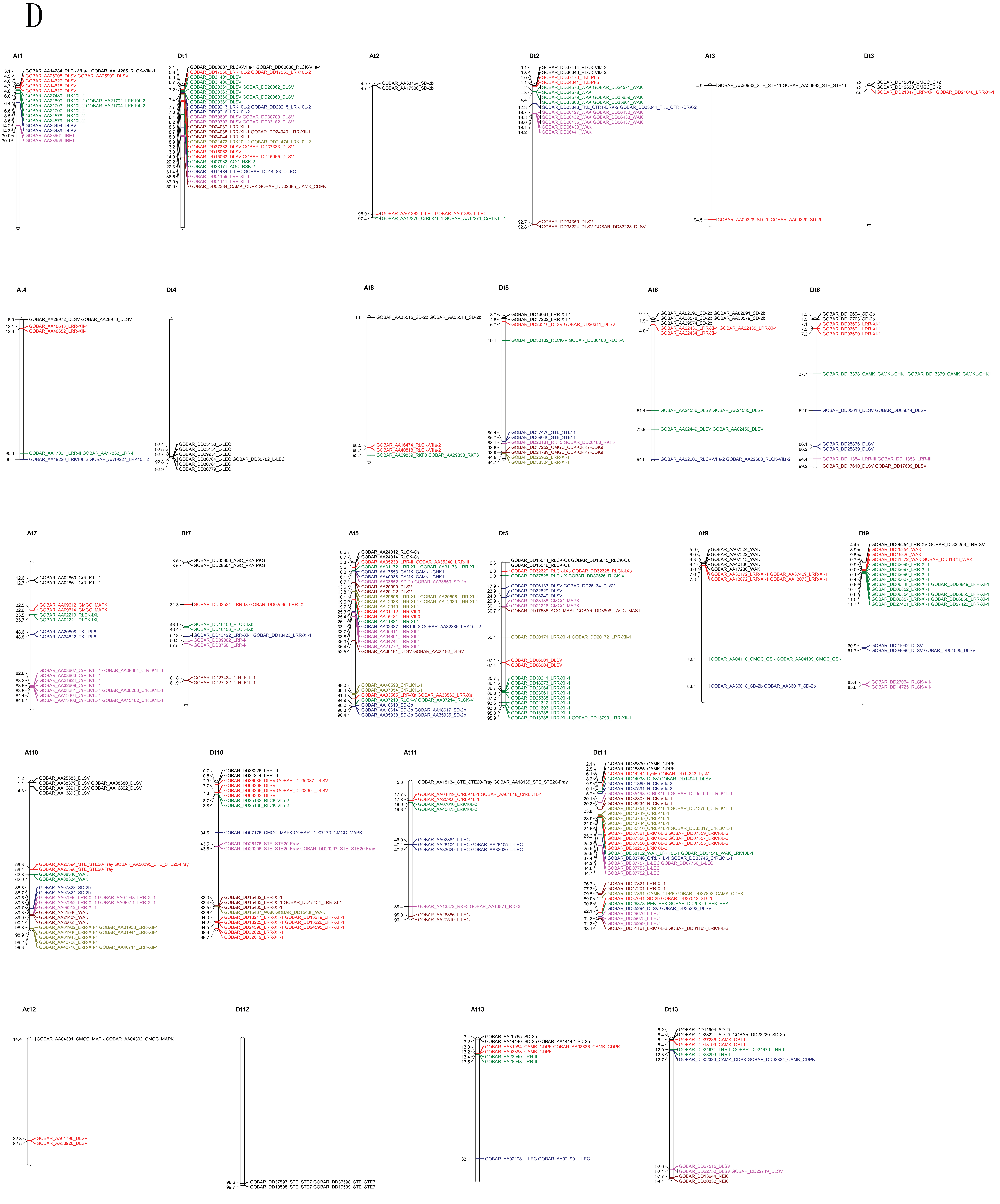
D
At1
Dt1
At2
Dt2
At3
Dt3
At4
Dt4
At8
Dt8
At6
Dt6
At7
Dt7
At5
Dt5
At9
Dt9
At10
Dt10
At11
Dt11
At12
Dt12
At13
Dt13
