# Supplementary material for: Genome-wide classification, evolutionary analysis and gene expression patterns of the kinome in Gossypium
Source: PLoS One. 2018 May 16;13(5):e0197392. doi: 10.1371/journal.pone.0197392 (PMC5955557; doi:10.1371/journal.pone.0197392)

GSE50770 (abiotic stresses)

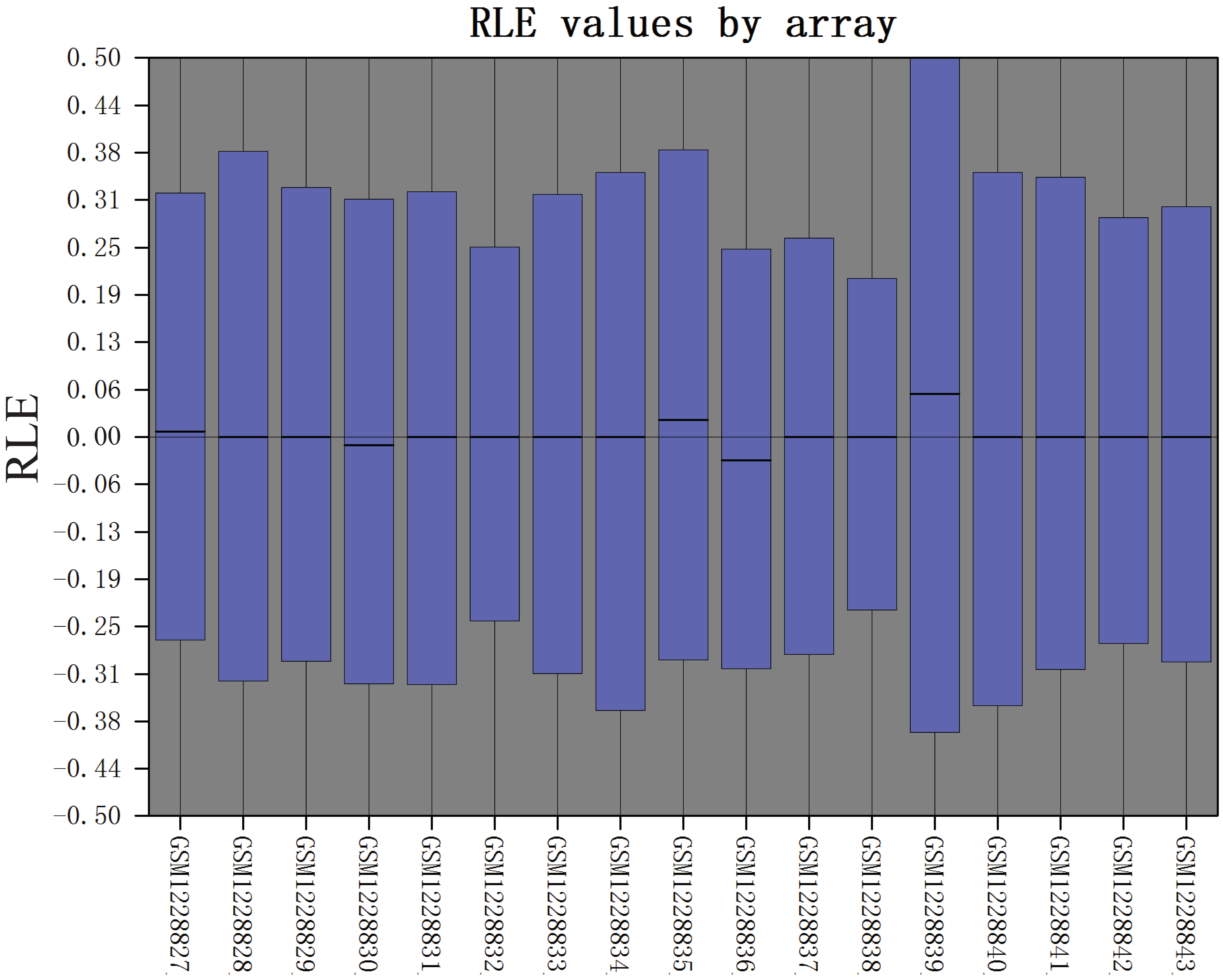

GSE50770 (abiotic stresses)

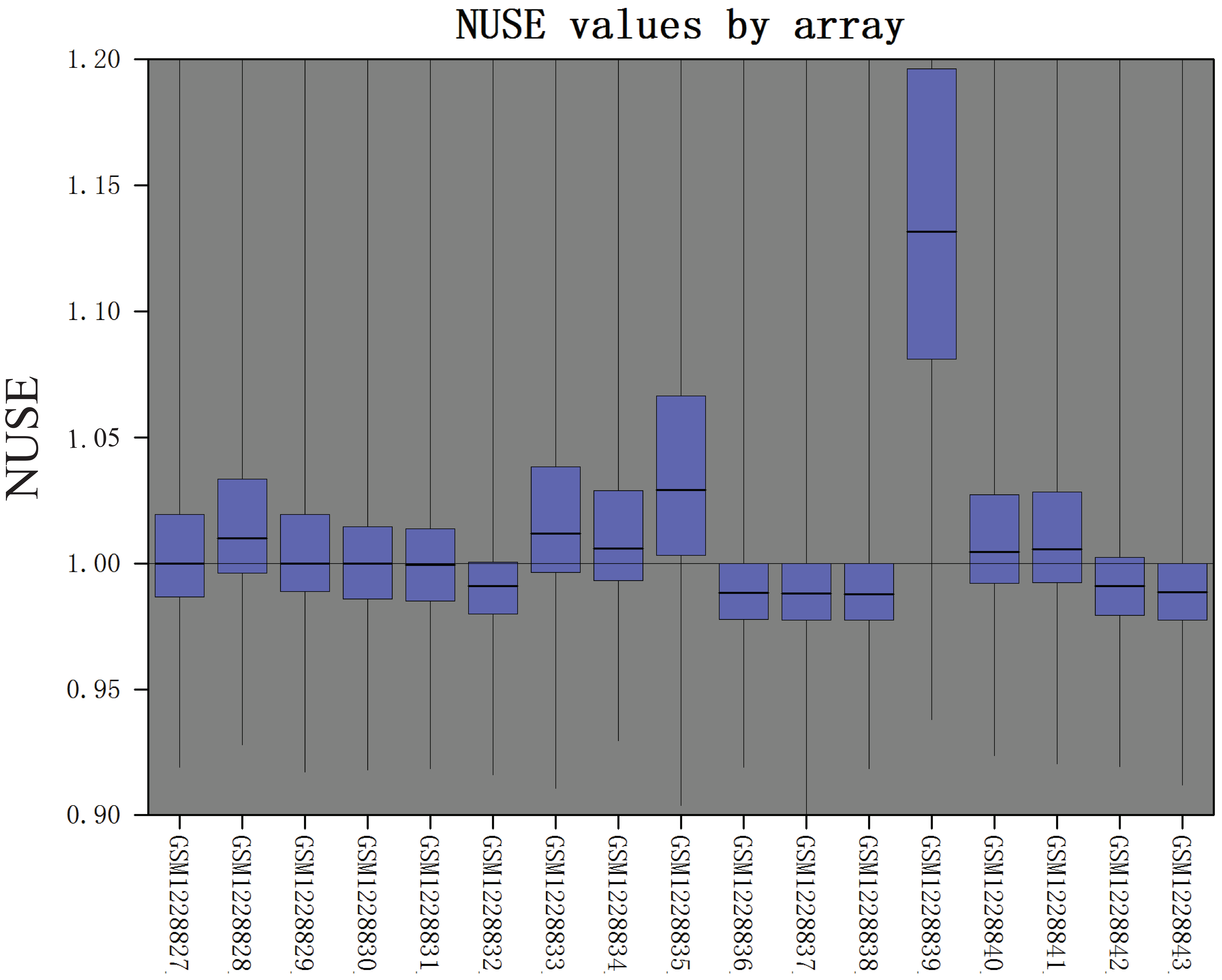

GSE41725（heat tolerance）

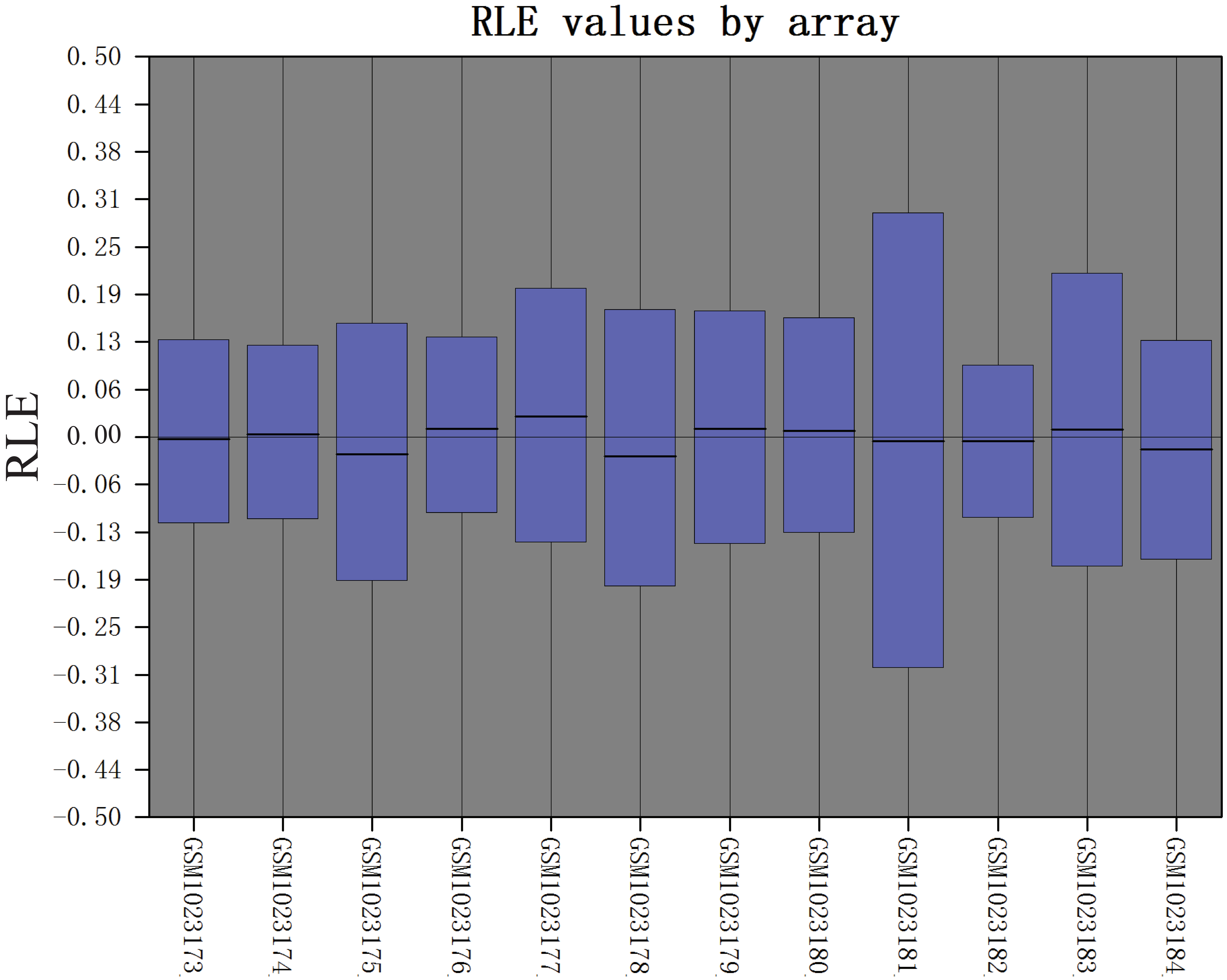

GSE41725 (heat tolerance)

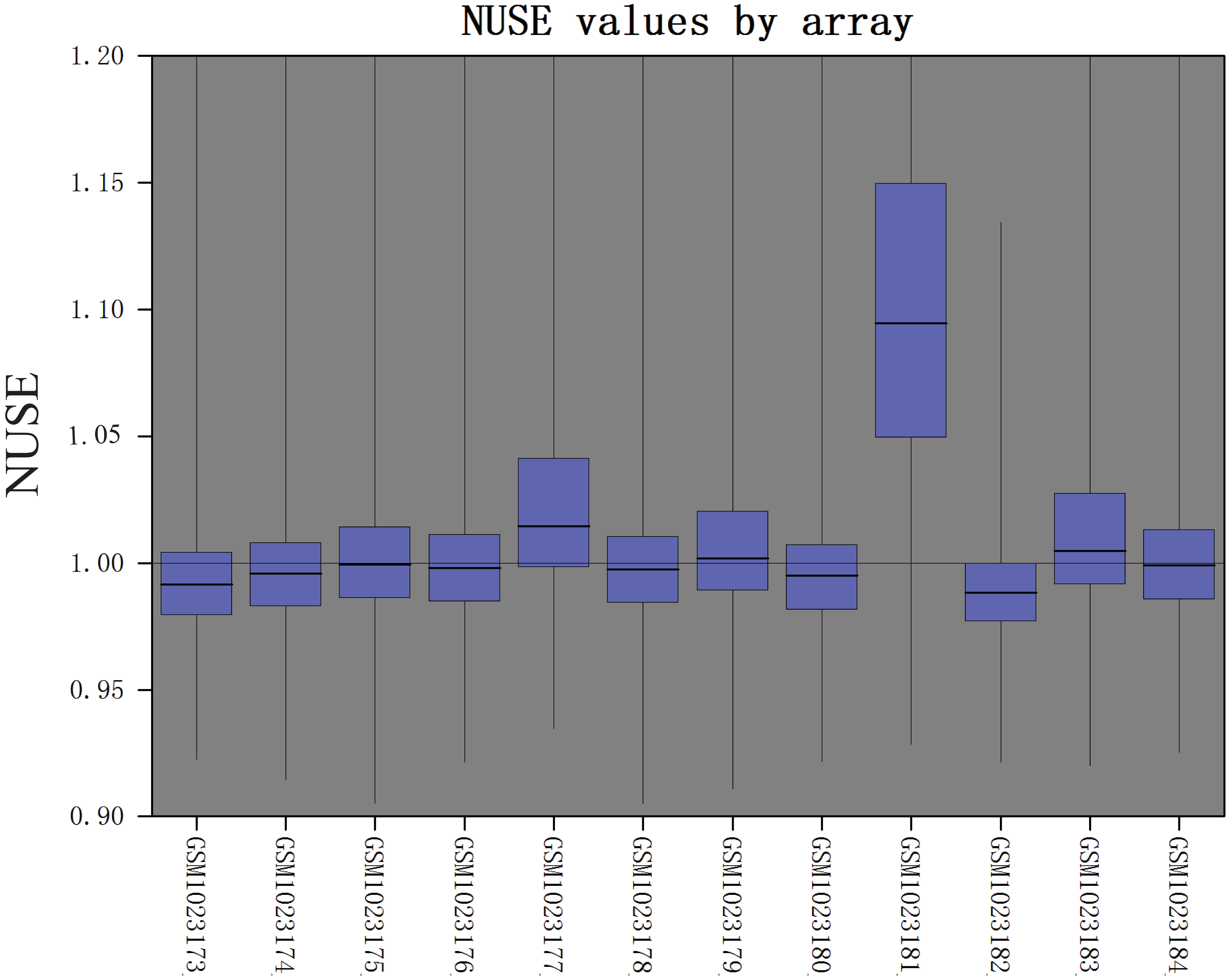

GSE16467（flooded-stress）

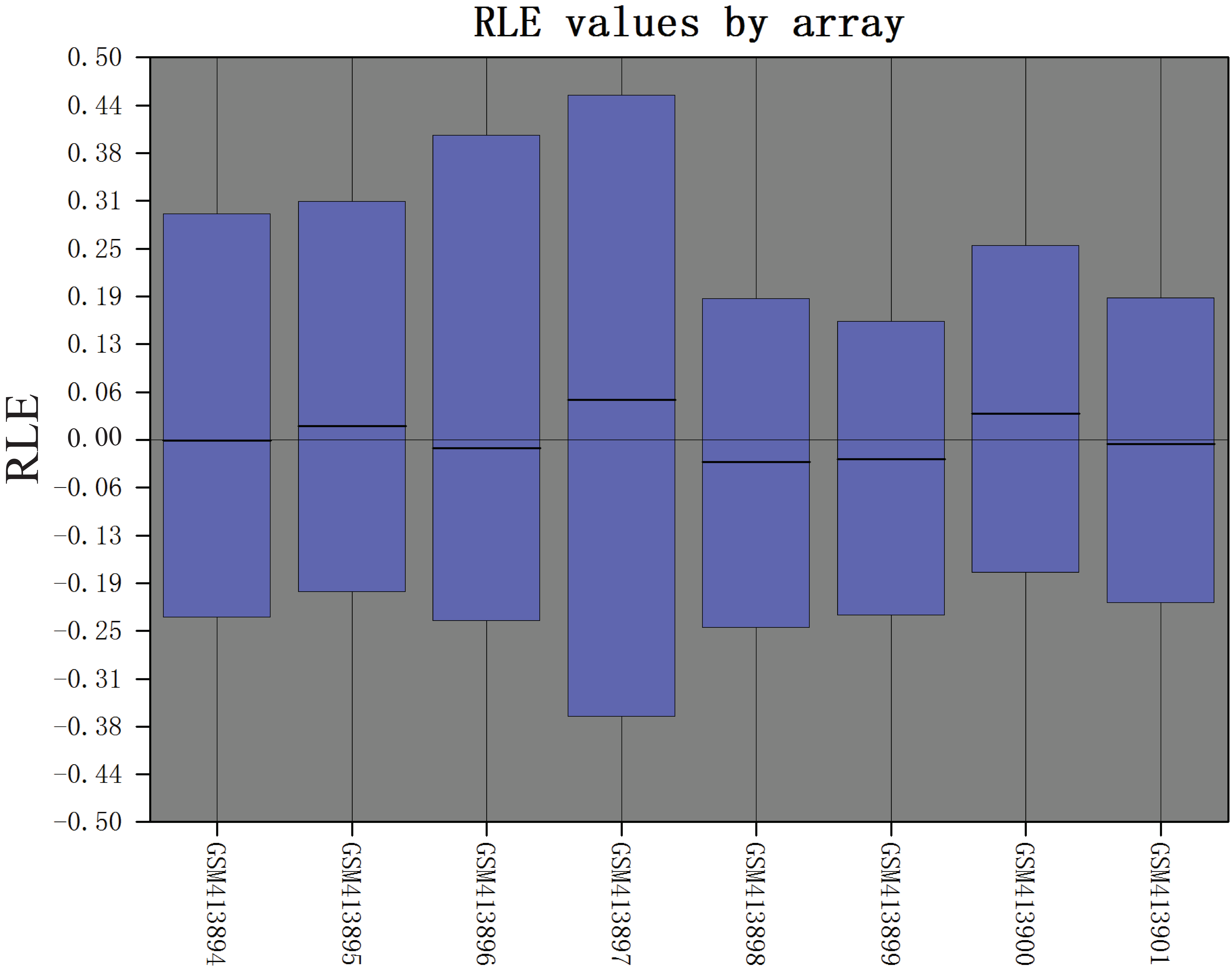

GSE16467 (flooded-stress)

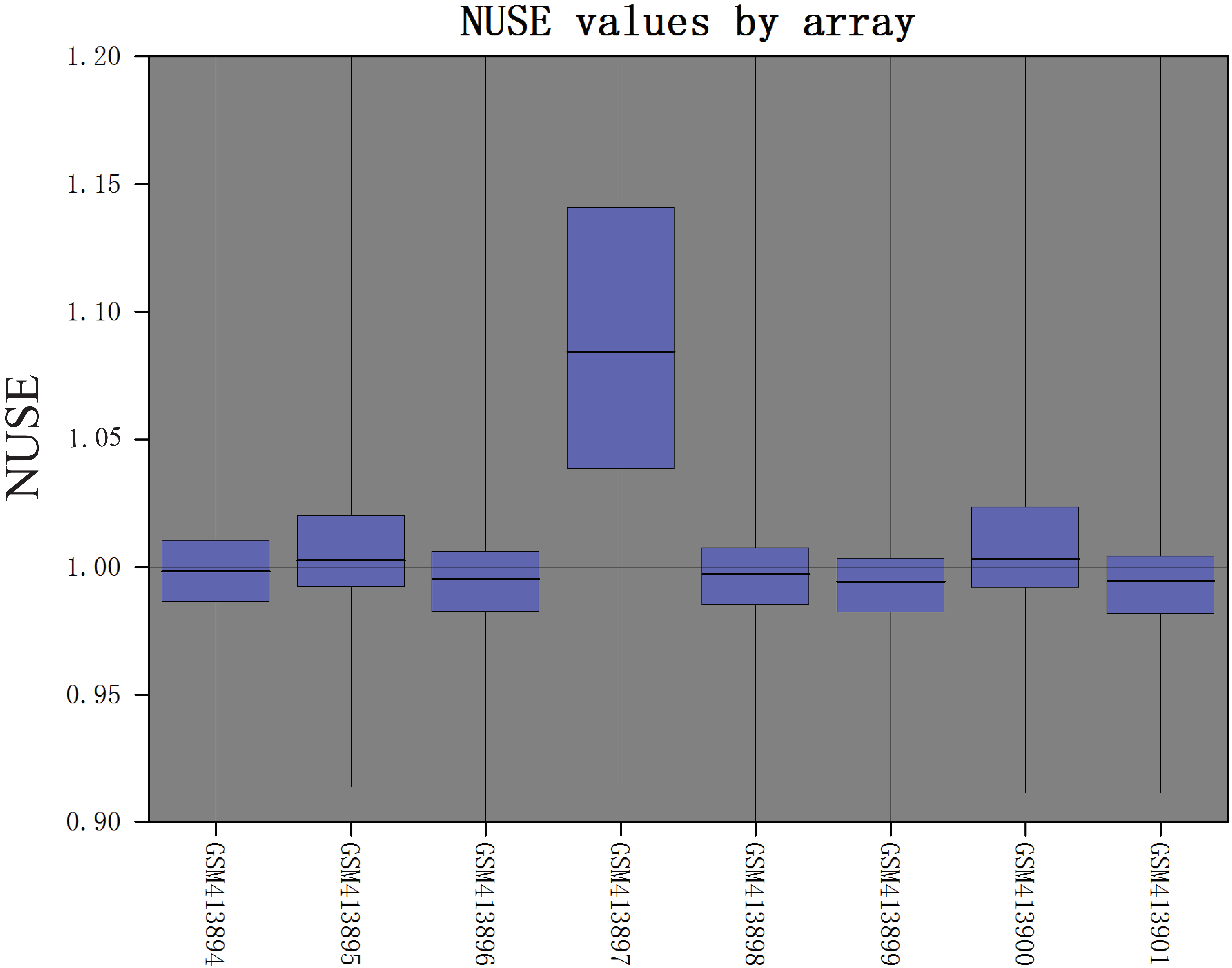

GSE29810（drought stress）

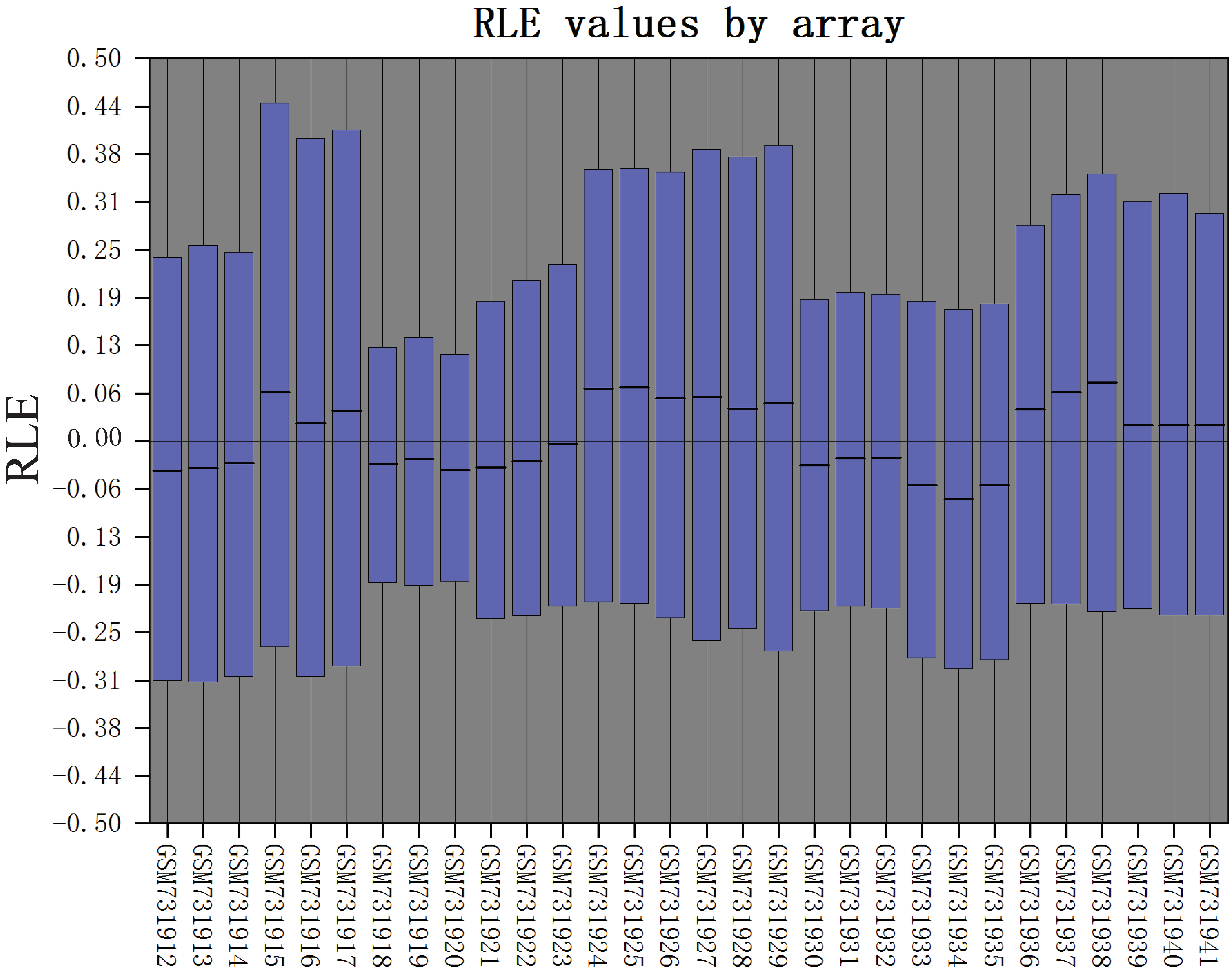

GSE29810（drought stress）

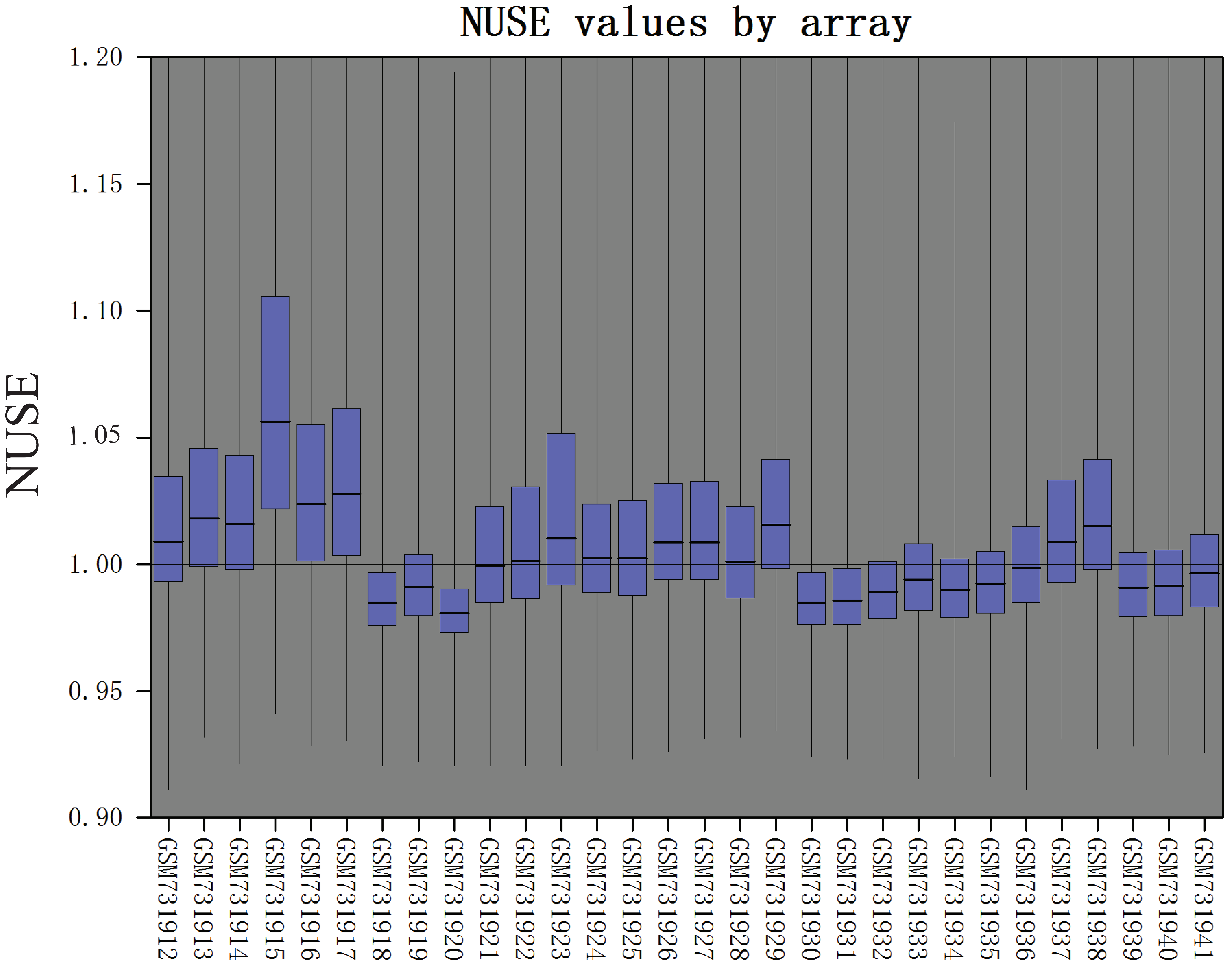

GSE74412（*Alternaria alternata* infection）

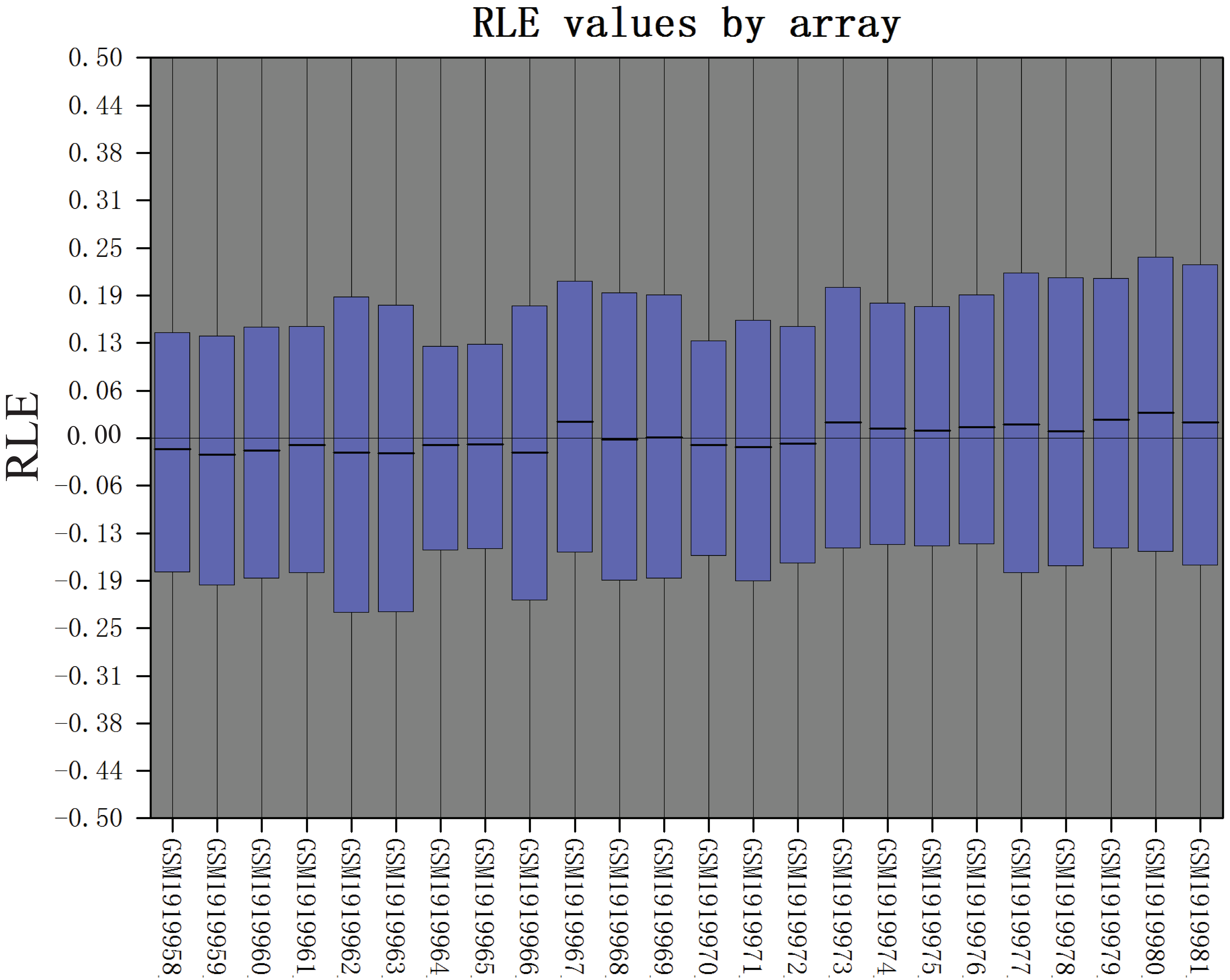

GSE74412 (*Alternaria alternata* infection)

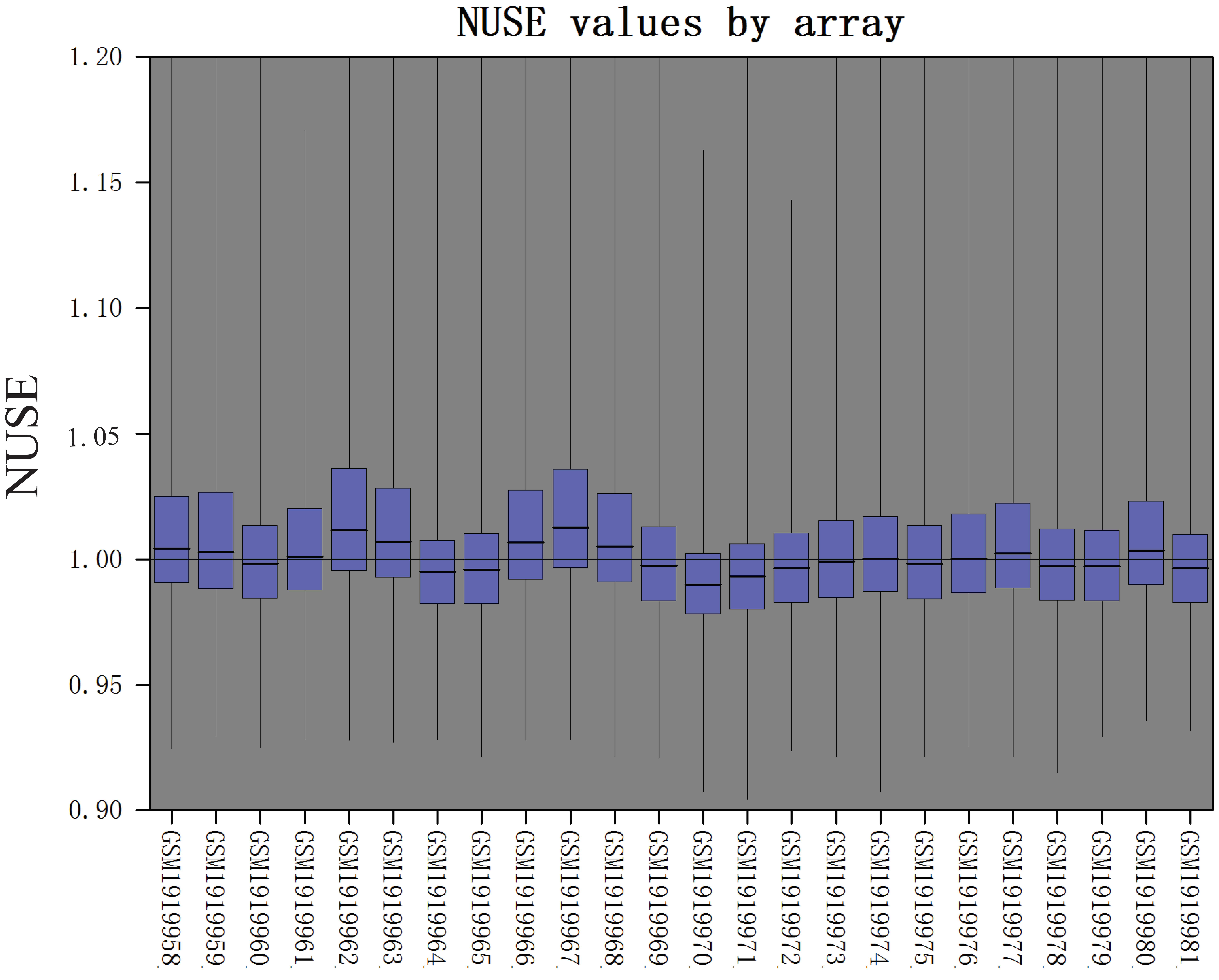

GSE55511（bollworm infection）

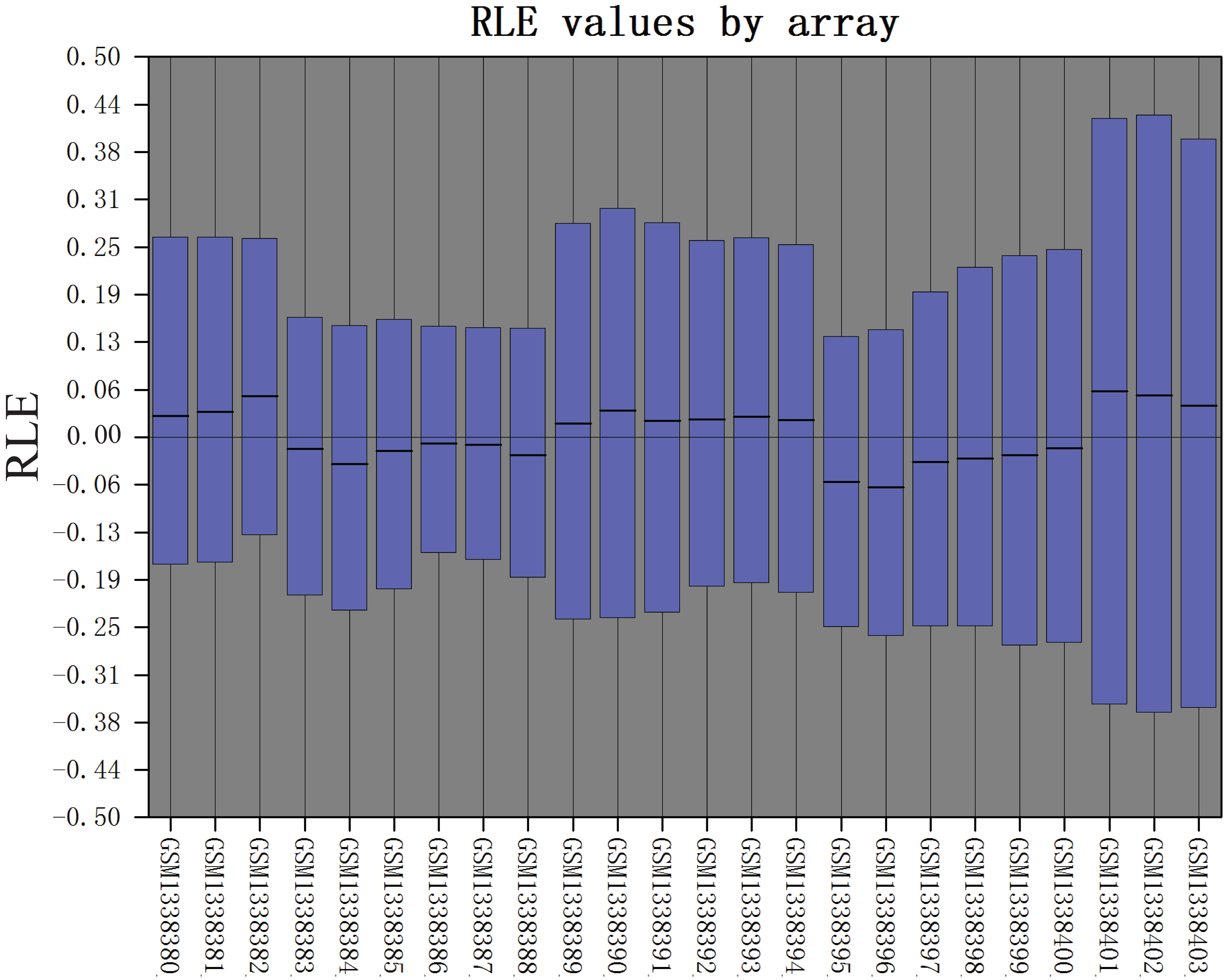

GSE55511（bollworm infection）

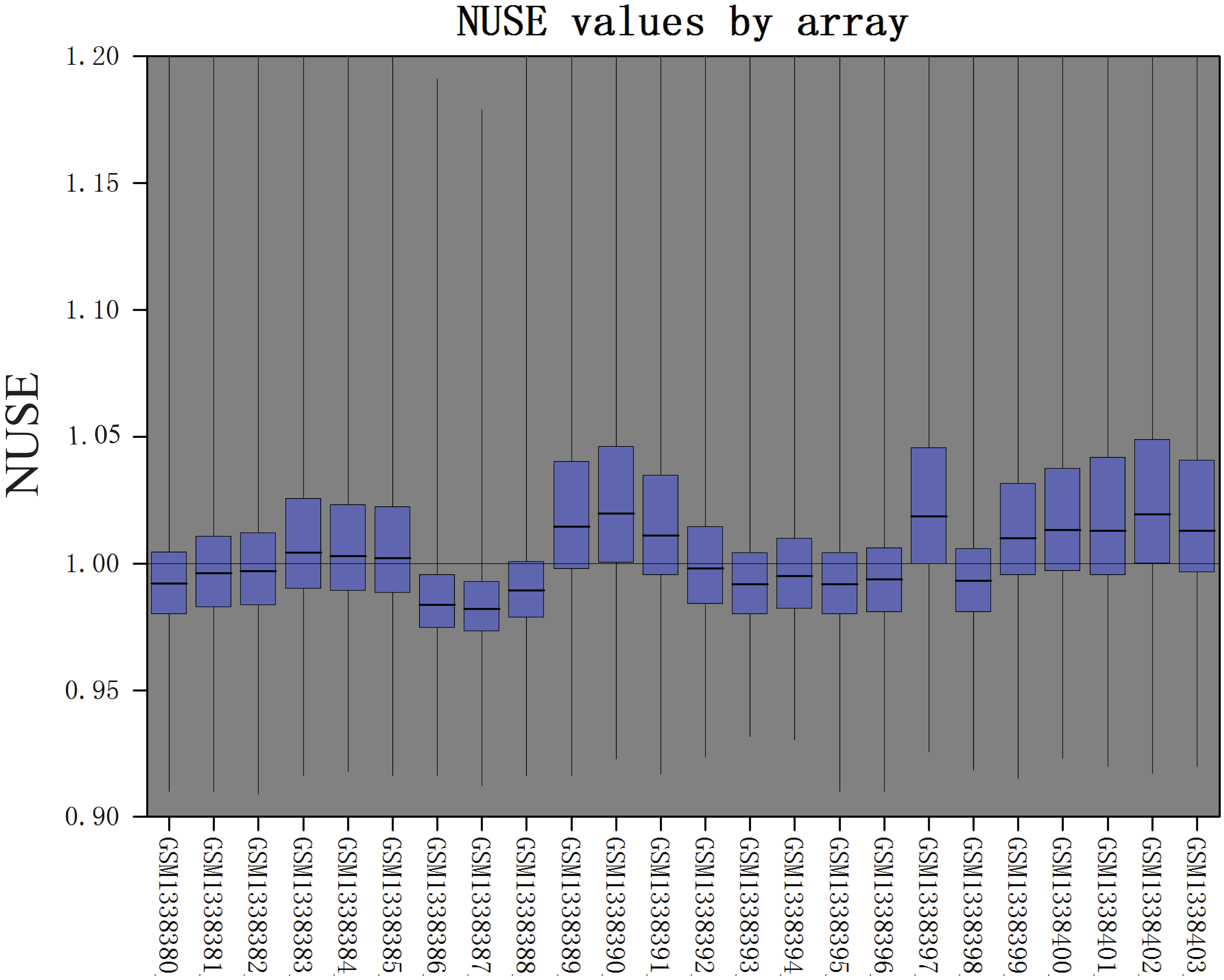

GSE36228（fiber development）part 1

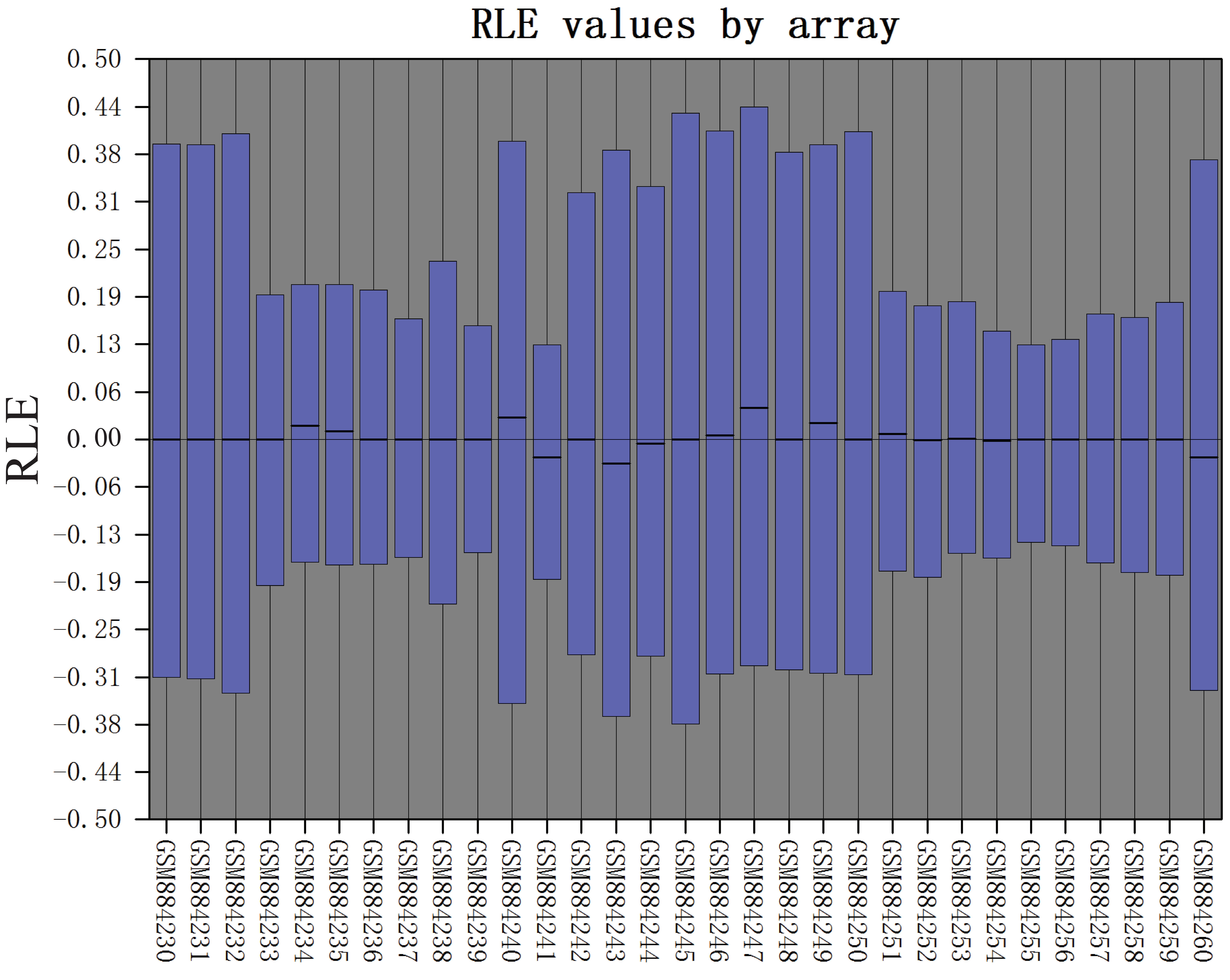

GSE36228 (fiber development) part 1

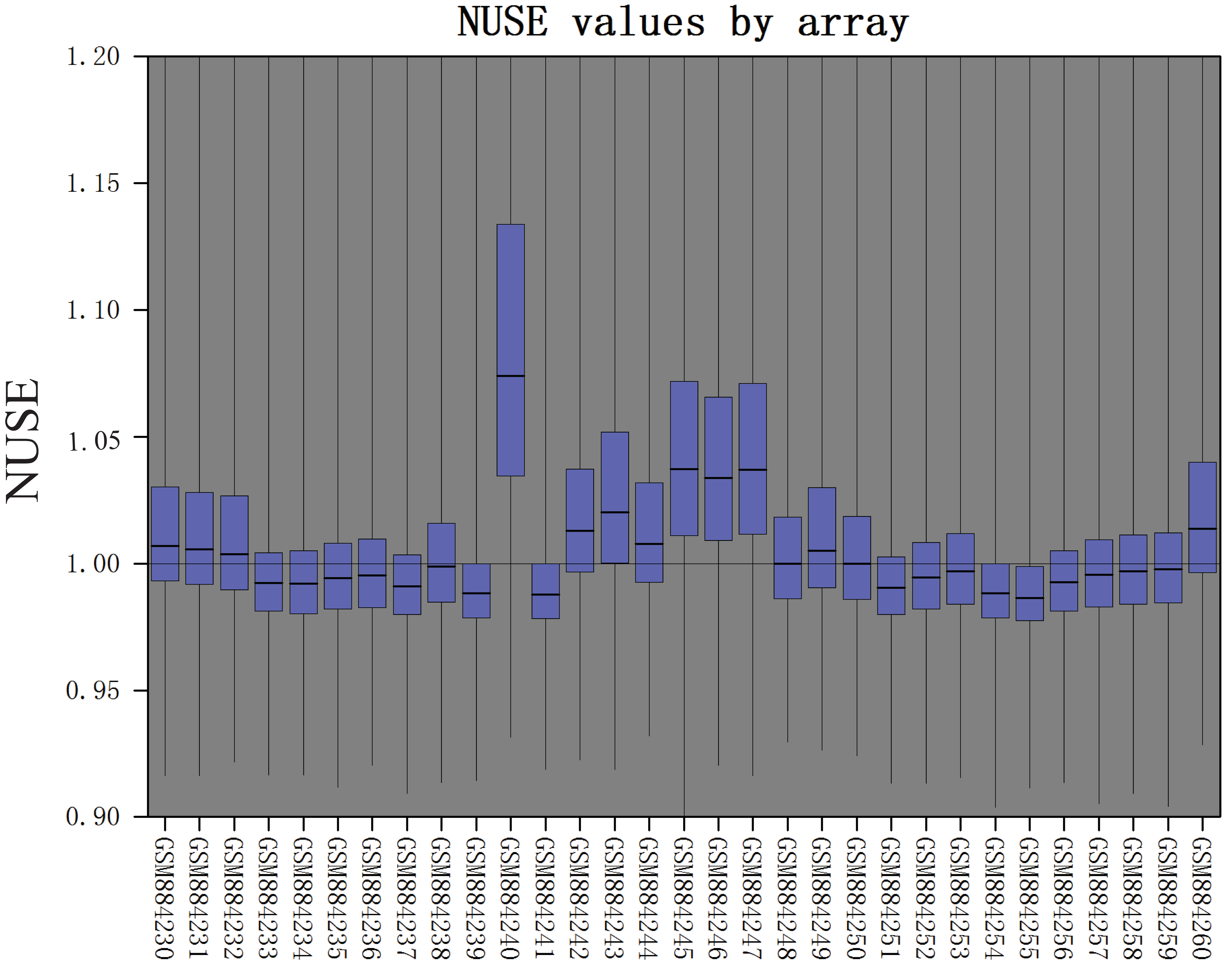

GSE36228（fiber development）part 2

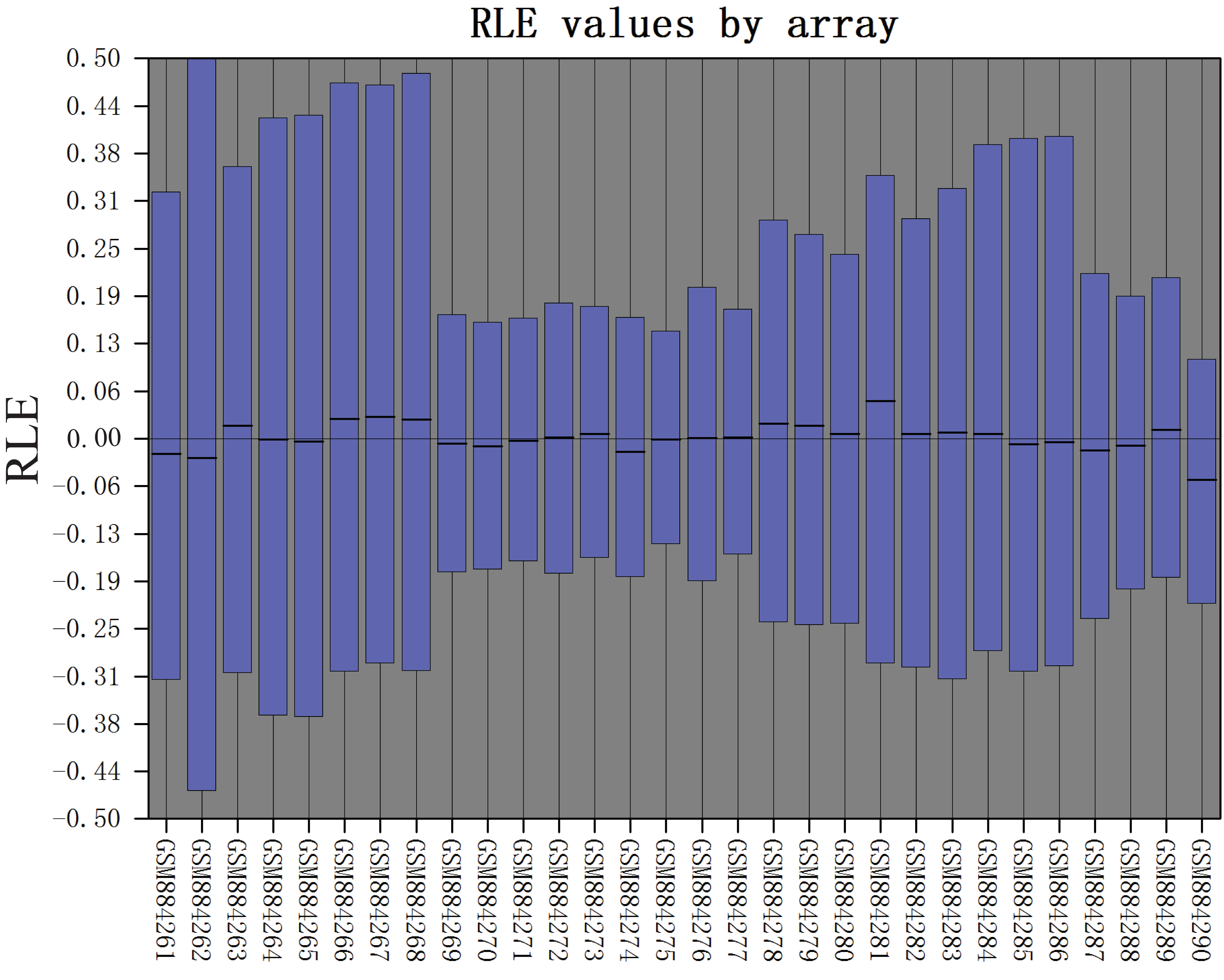

GSE36228（fiber development）part 2

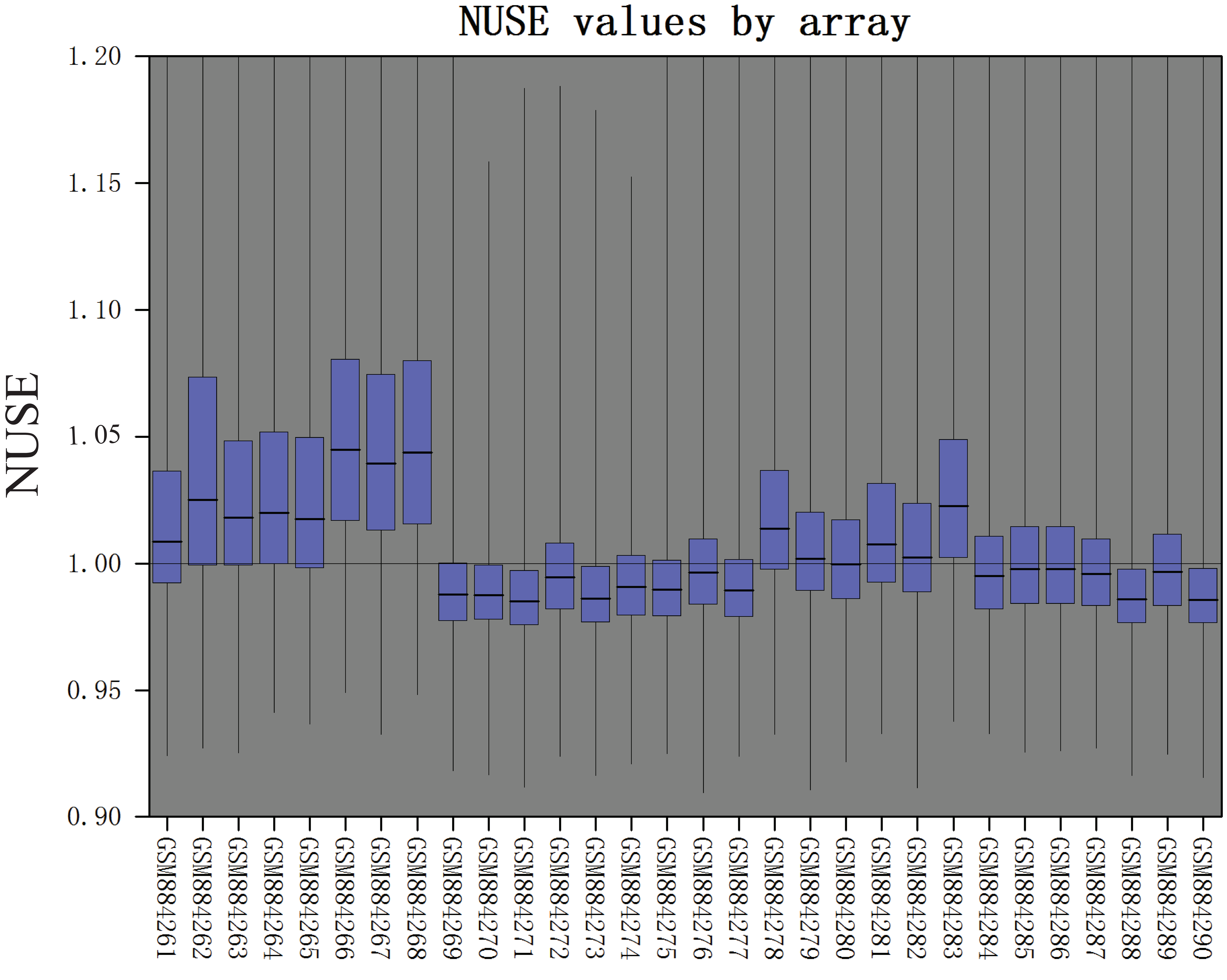

GSE36228（fiber development）part 3

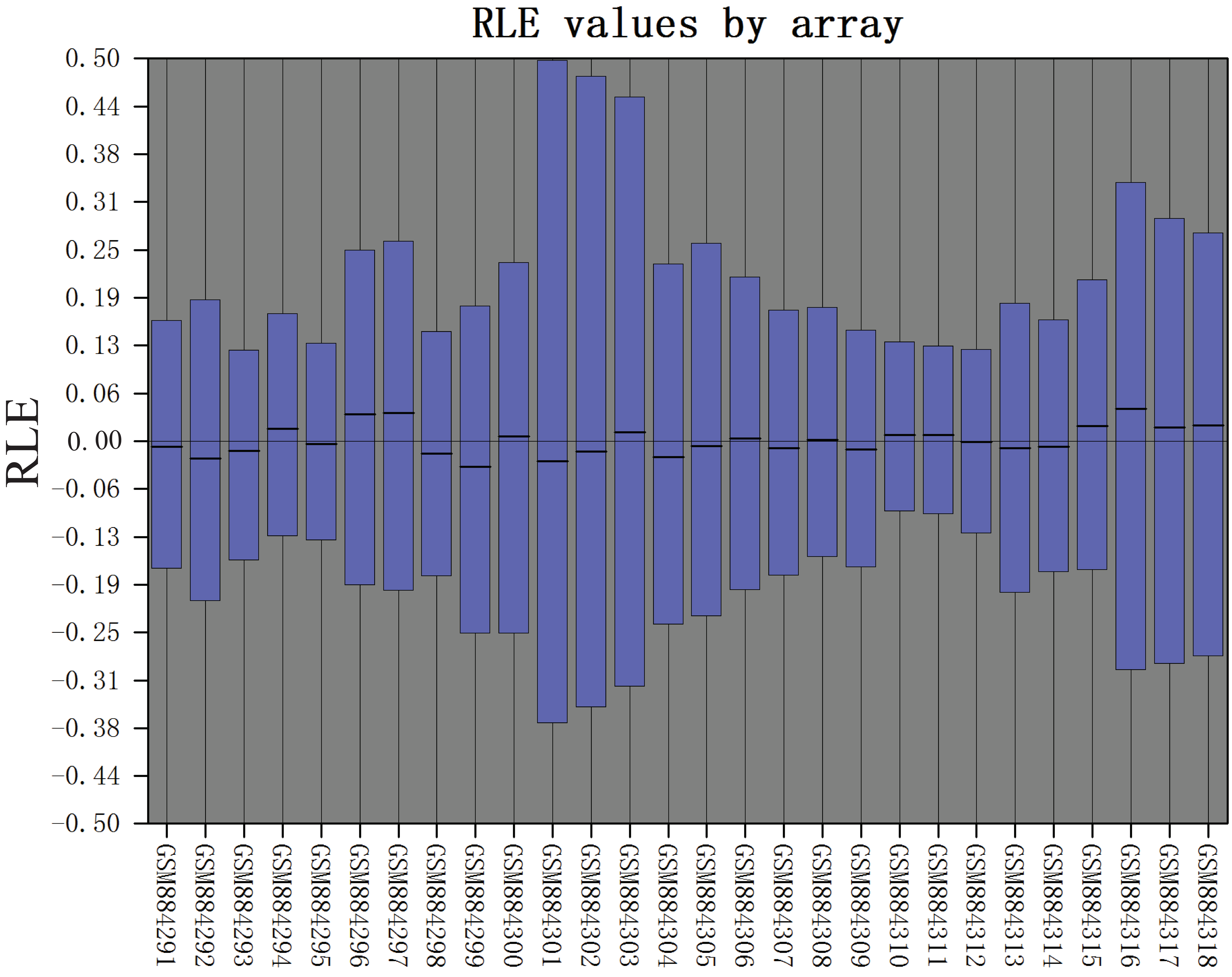

GSE36228 (fiber development) part 3

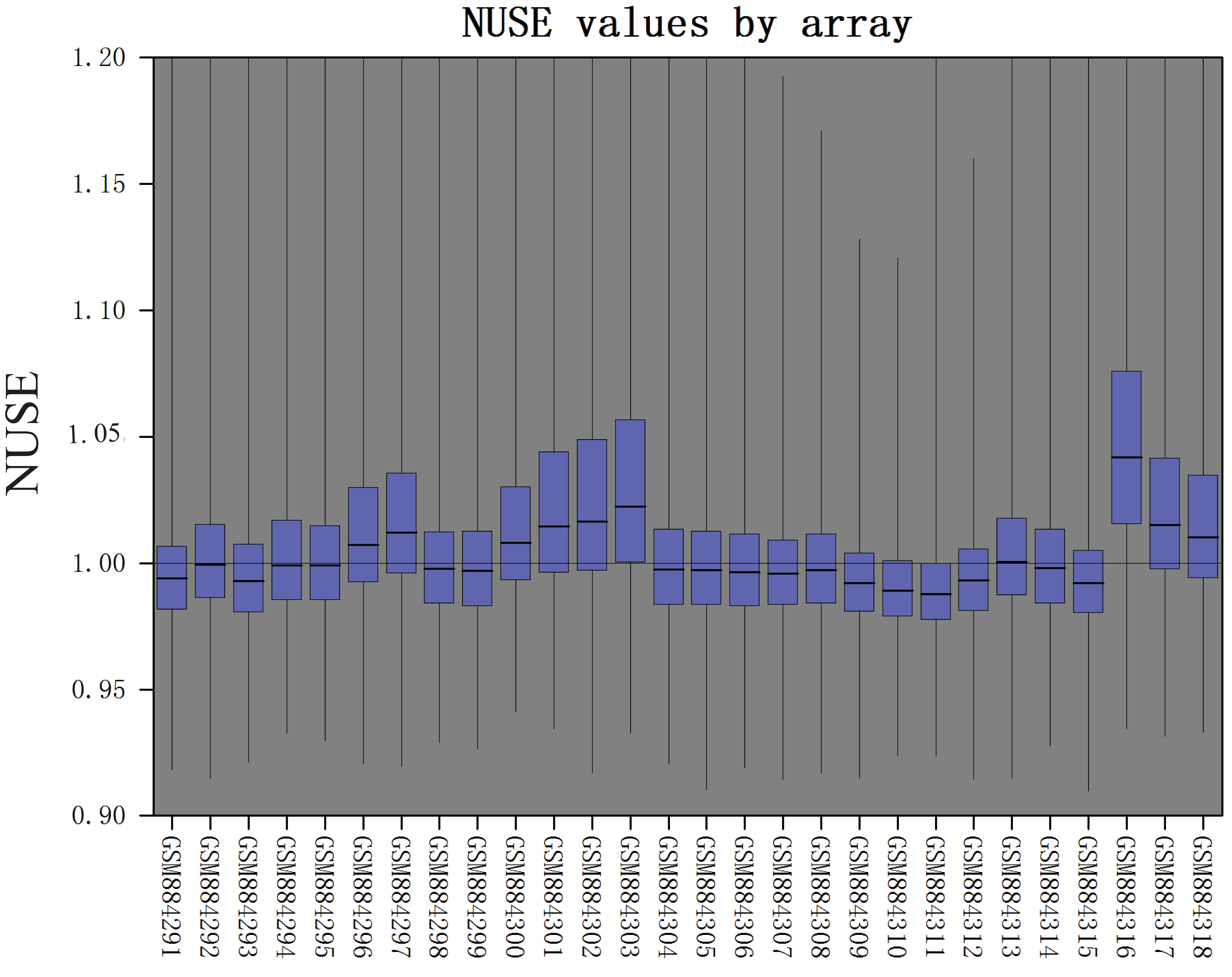

GSE36021（GhHD-1 fiber development）

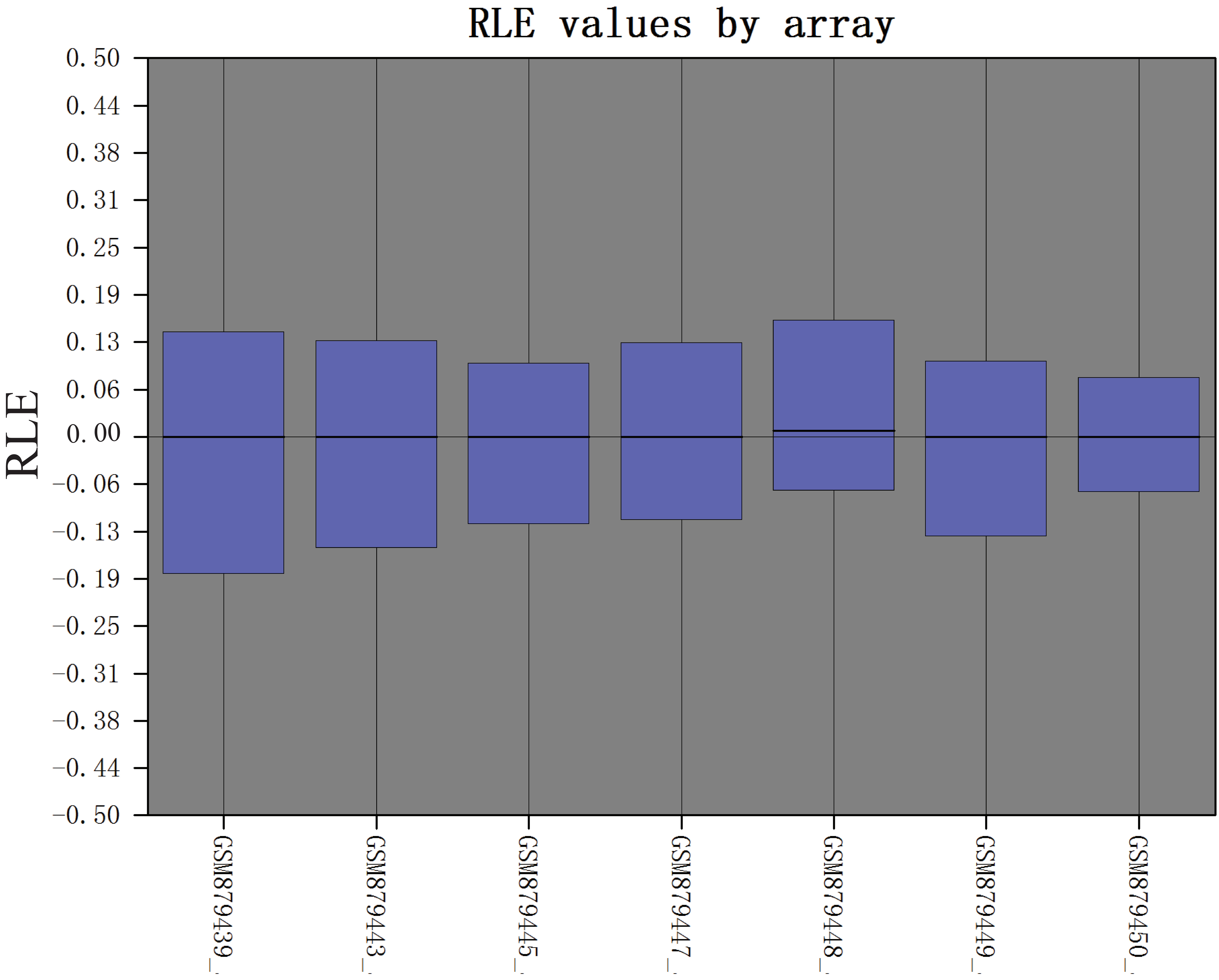

GSE36021 (GhHD-1 fiber development)

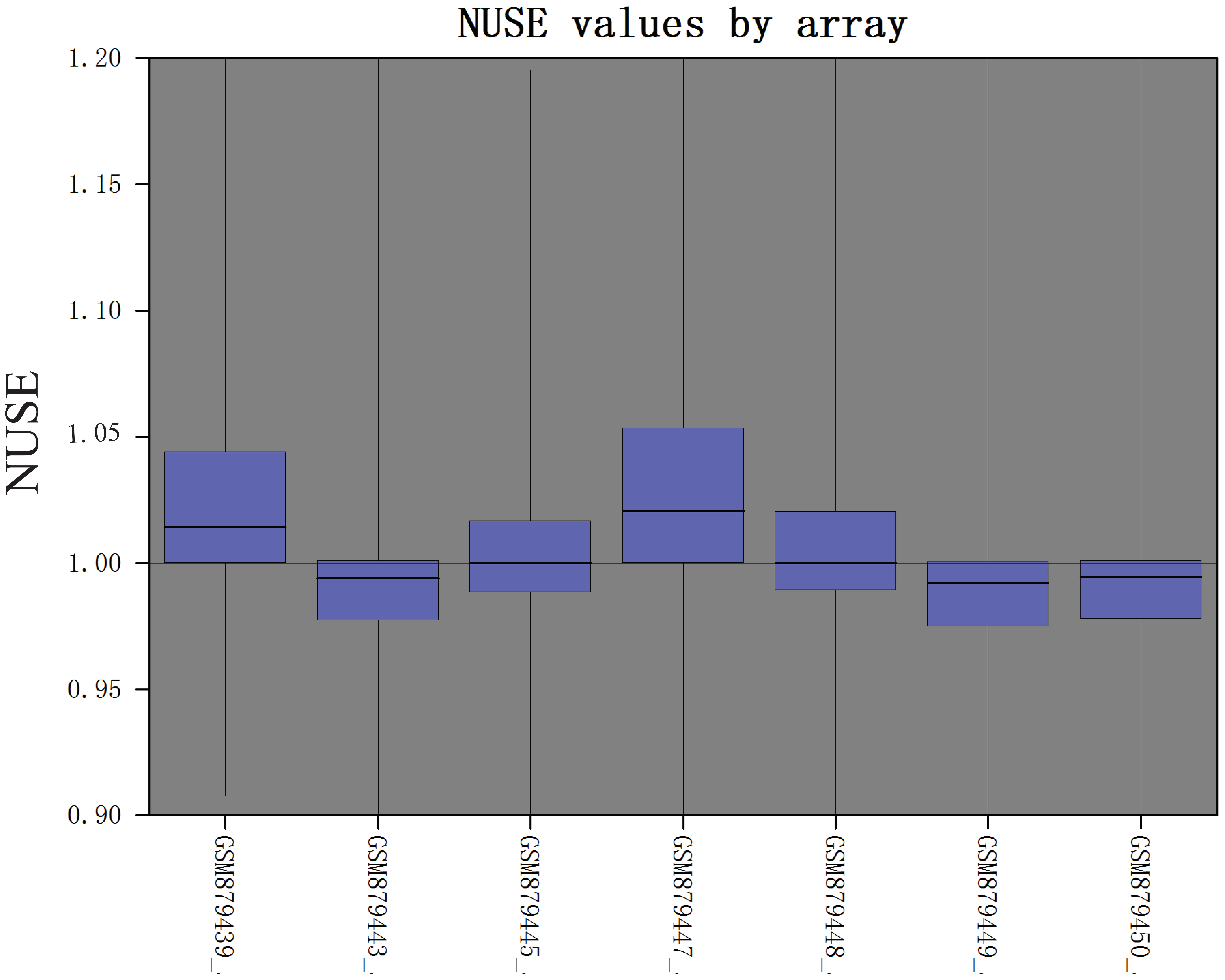

GSE38490 (fiber development)

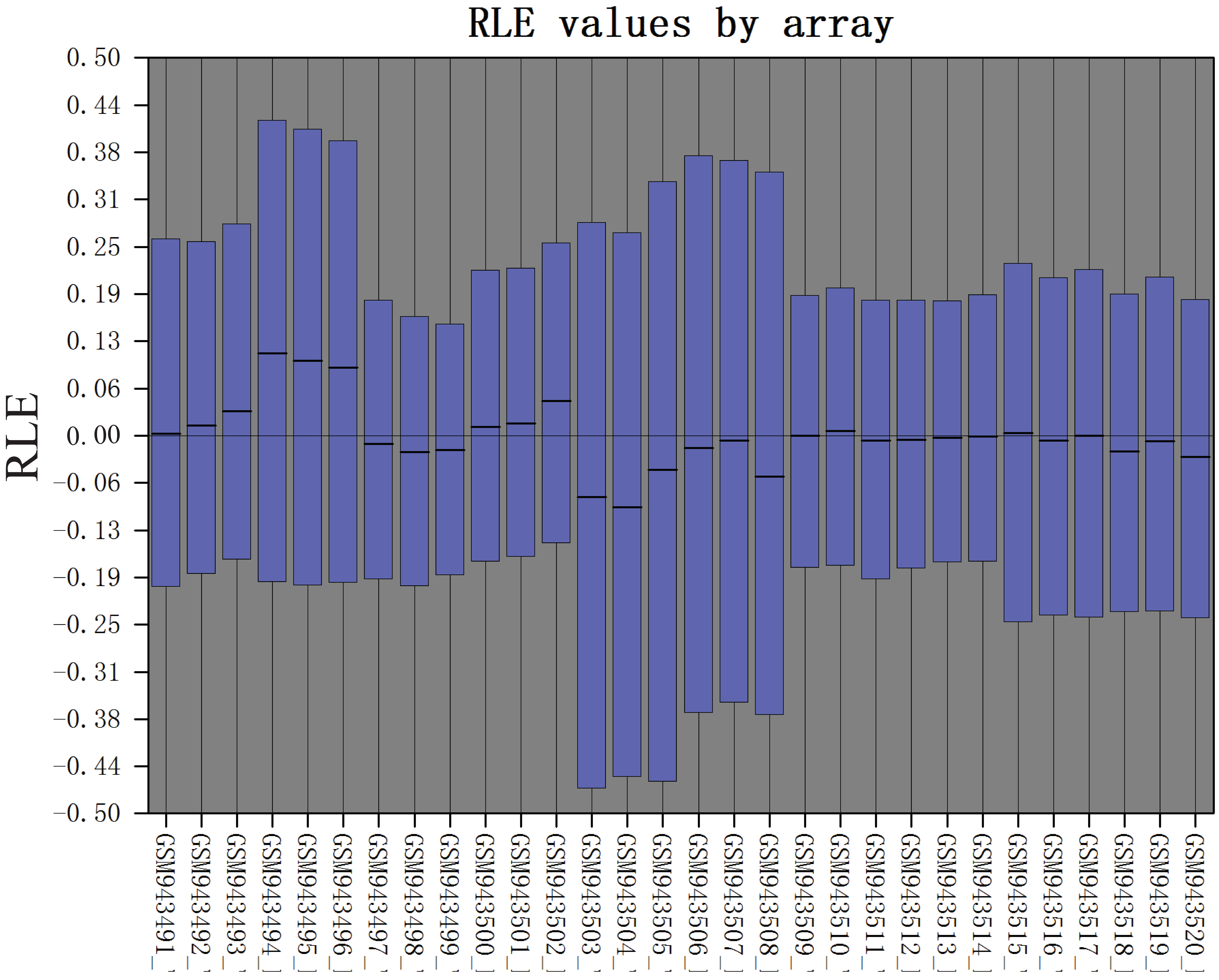

GSE38490（fiber development）

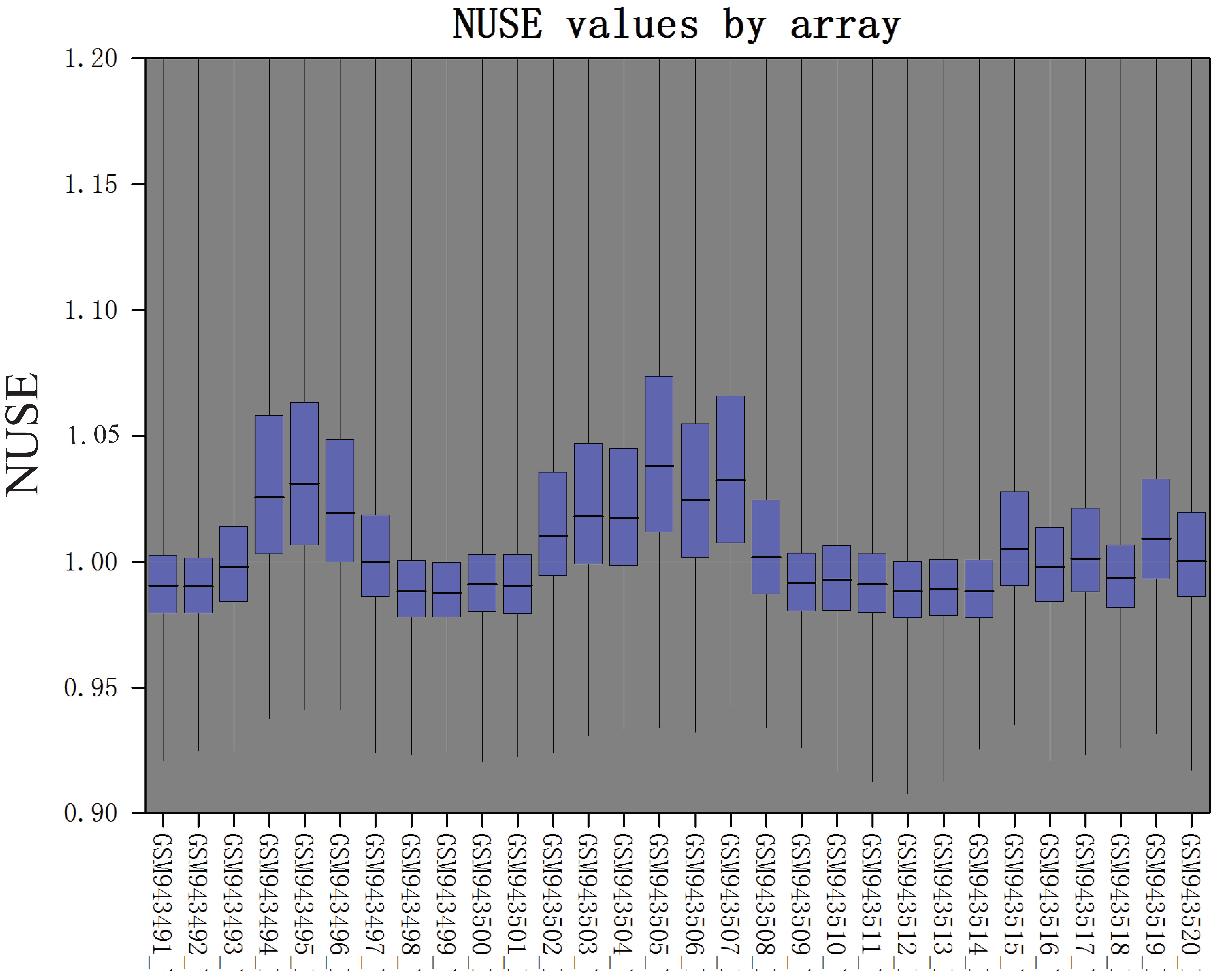

Supplement: S8 Fig — RLE (Relative log expression) and NUSE (Normalized unscaled standard errors) values of each GEO microarray dataset. (PDF) [file pone.0197392.s008.pdf]
