# Supplementary material for: Genome-wide classification, evolutionary analysis and gene expression patterns of the kinome in Gossypium
Source: PLoS One. 2018 May 16;13(5):e0197392. doi: 10.1371/journal.pone.0197392 (PMC5955557; doi:10.1371/journal.pone.0197392)
Supplement: S2 Fig — Filled boxes: red represents the Pkinase or Pkinase_Tyr domain; other colors represent various domains labeled in each page. The lengths of the boxes and lines are scaled based on the length of proteins. (PDF) [file pone.0197392.s002.pdf]

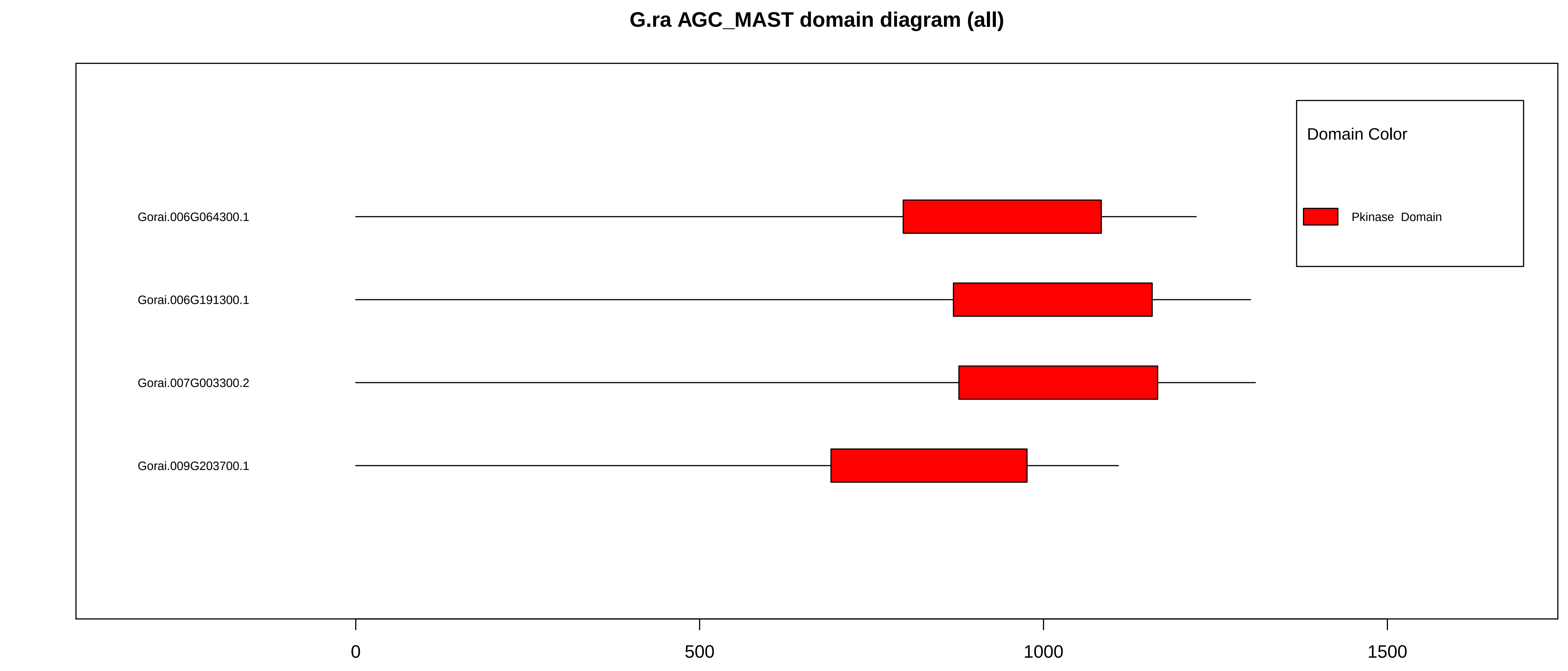
G.ra AGC_MAST domain diagram (all)
Domain Color
Pkinase Domain
Gorai.006G064300.1
Gorai.006G191300.1
Gorai.007G003300.2
Gorai.009G203700.1
0
500
1000
1500

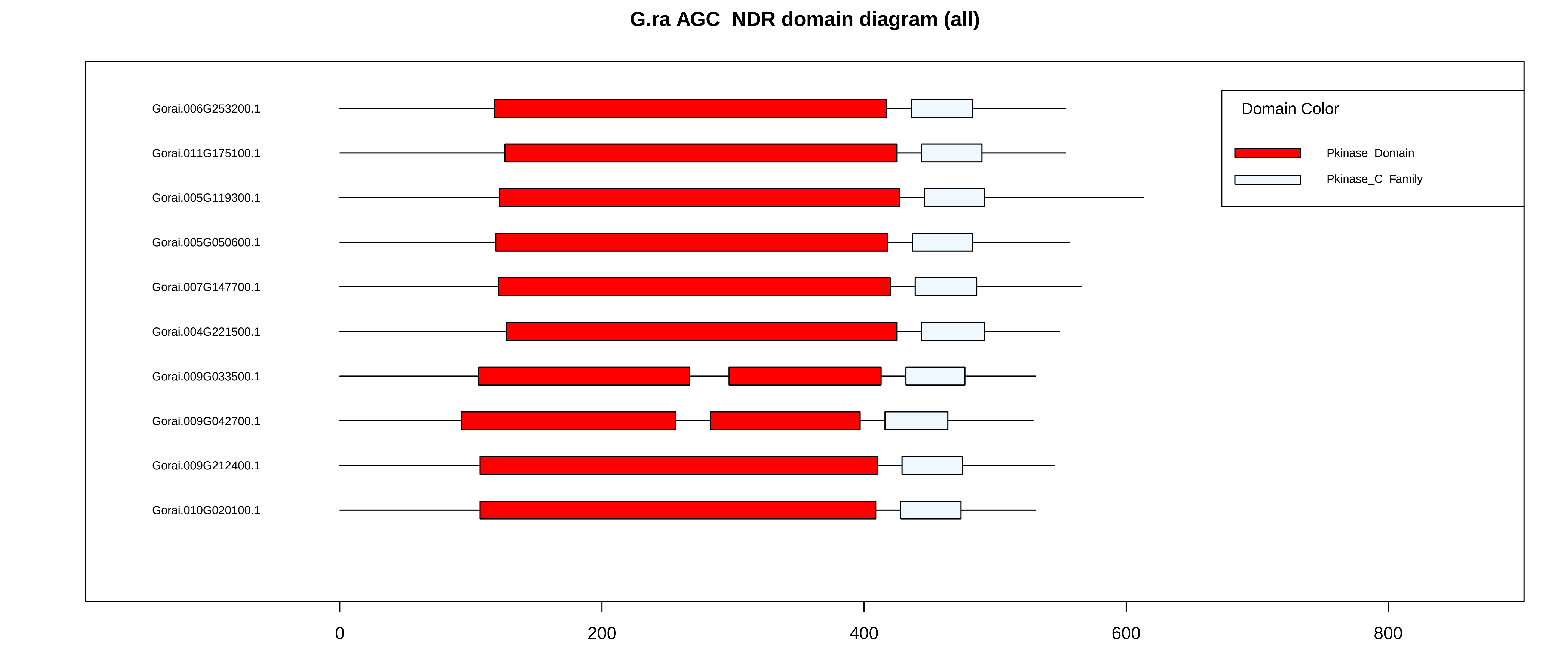

G.ra AGC_NDR domain diagram (all)
Gorai.006G253200.1
Gorai.011G175100.1
Gorai.005G119300.1
Gorai.005G050600.1
Gorai.007G147700.1
Gorai.004G221500.1
Gorai.009G033500.1
Gorai.009G042700.1
Gorai.009G212400.1
Gorai.010G020100.1
Domain Color
Pkinase Domain
Pkinase_C Family
0
200
400
600
800

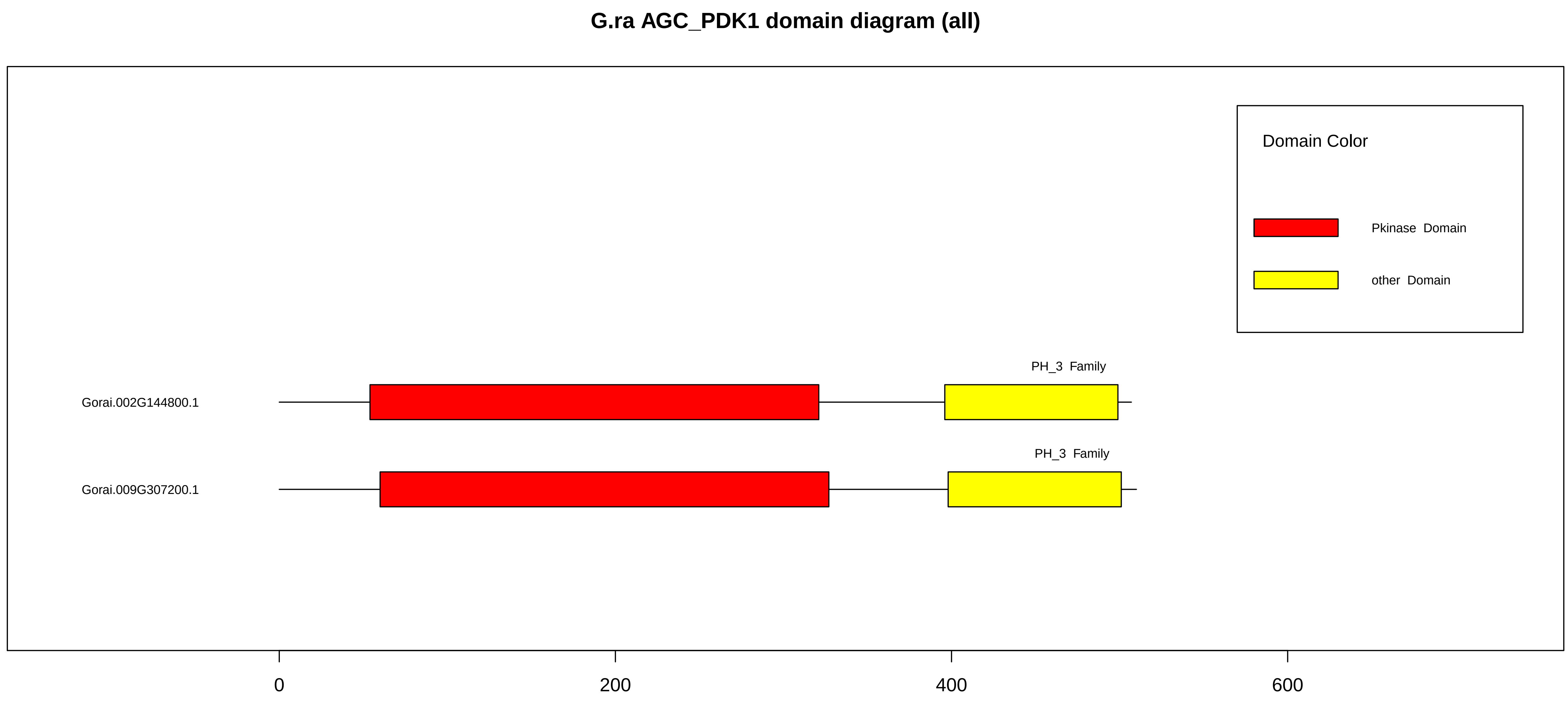
G.ra AGC_PDK1 domain diagram (all)
Domain Color
Pkinase Domain
other Domain
PH_3 Family
Gorai.002G144800.1
PH_3 Family
Gorai.009G307200.1
0
200
400
600

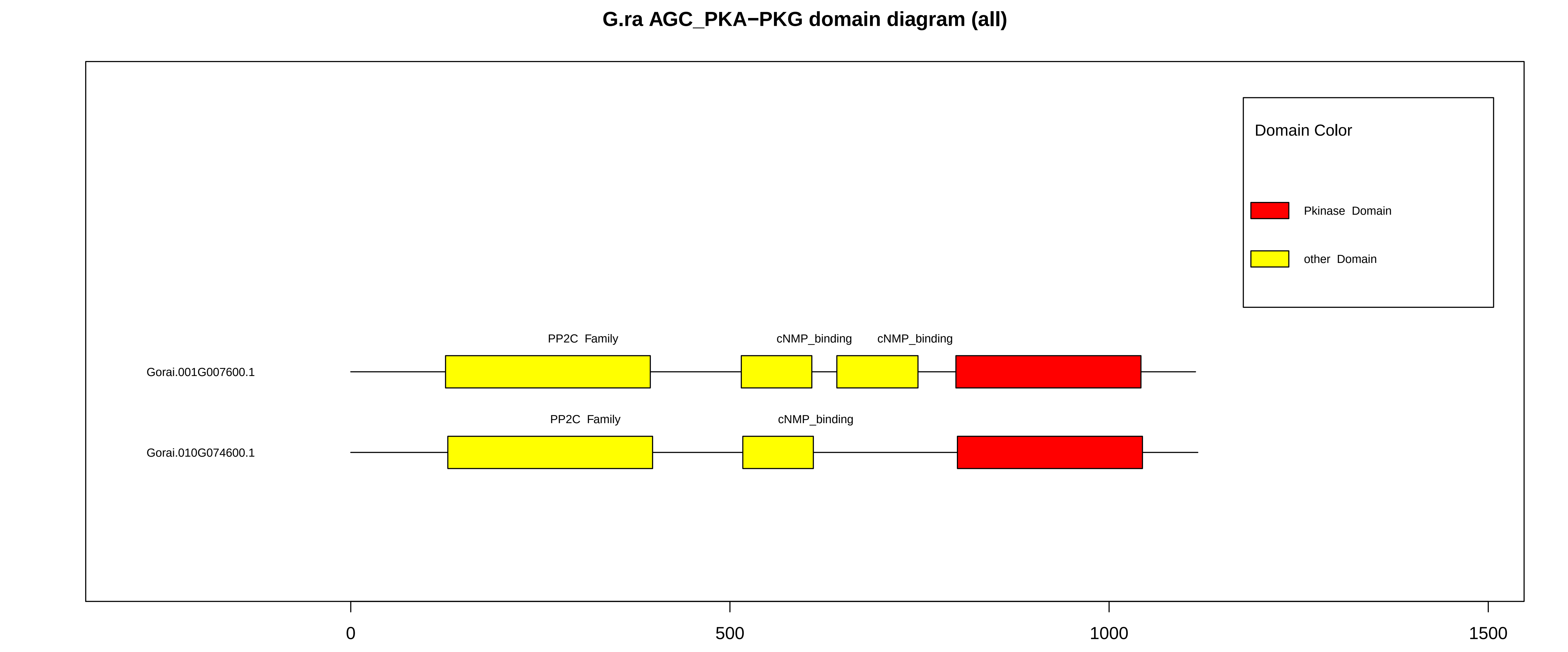
G.ra AGC_PKA−PKG domain diagram (all)
Domain Color
Pkinase Domain
other Domain
PP2C Family
cNMP_binding
cNMP_binding
Gorai.001G007600.1
PP2C Family
cNMP_binding
Gorai.010G074600.1
0
500
1000
1500

G.ra AGC_RSK−2 domain diagram (part 1)

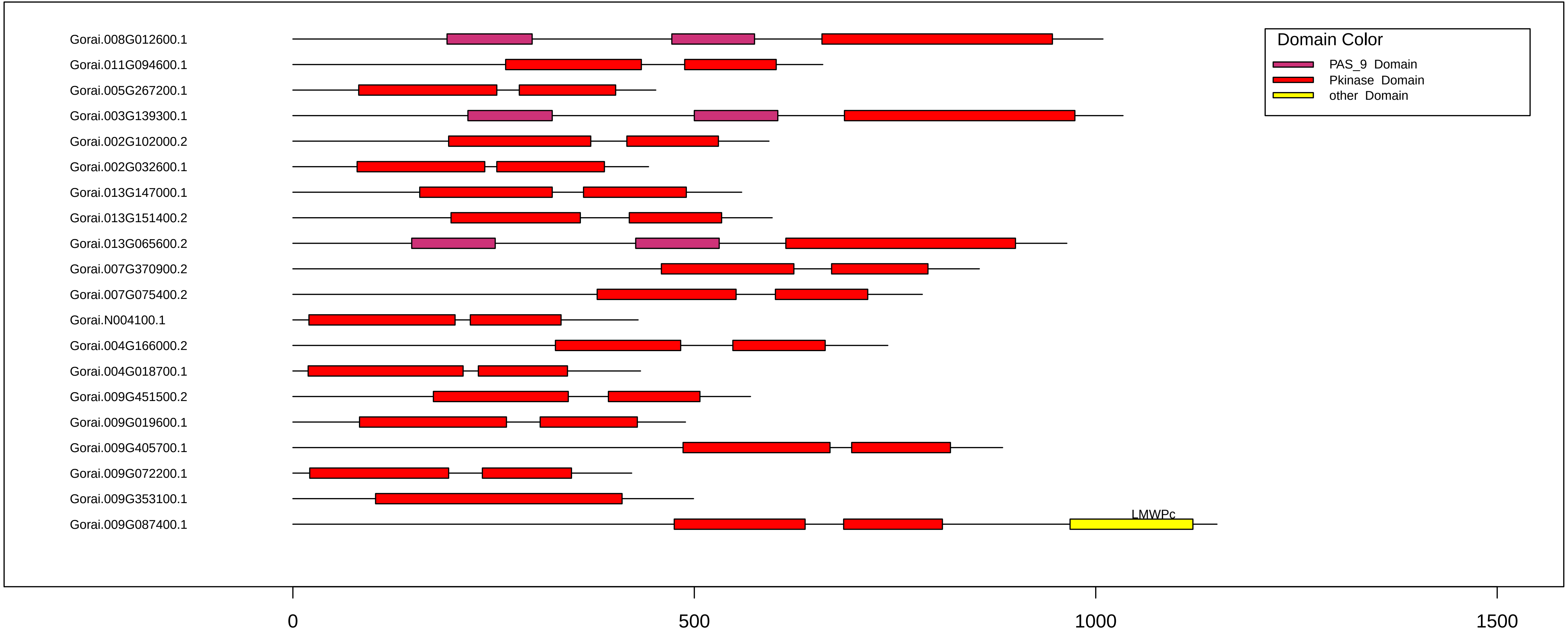

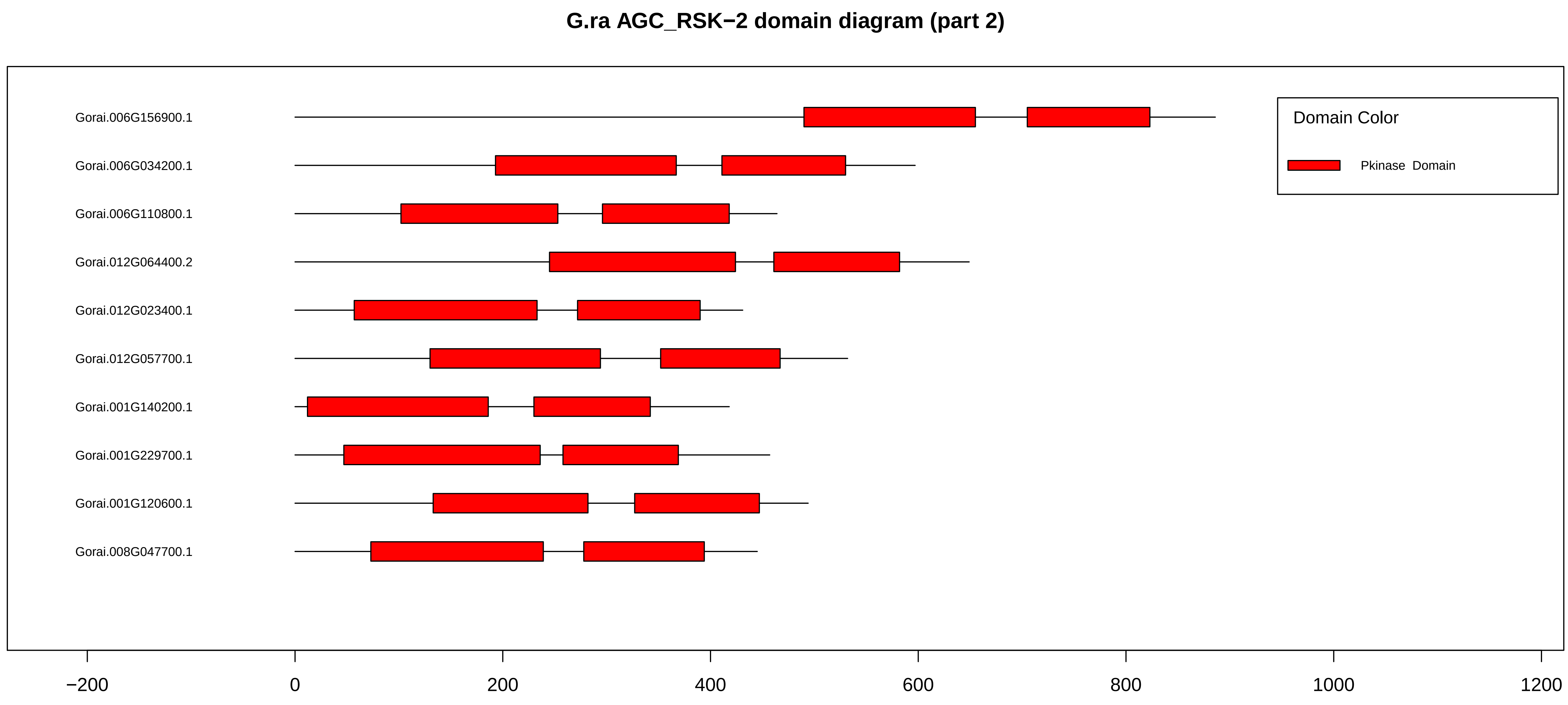
G.ra AGC_RSK−2 domain diagram (part 2)
Gorai.006G156900.1
Gorai.006G034200.1
Gorai.006G110800.1
Gorai.012G064400.2
Gorai.012G023400.1
Gorai.012G057700.1
Gorai.001G140200.1
Gorai.001G229700.1
Gorai.001G120600.1
Gorai.008G047700.1
Domain Color
Pkinase Domain
−200
0
200
400
600
800
1000
1200

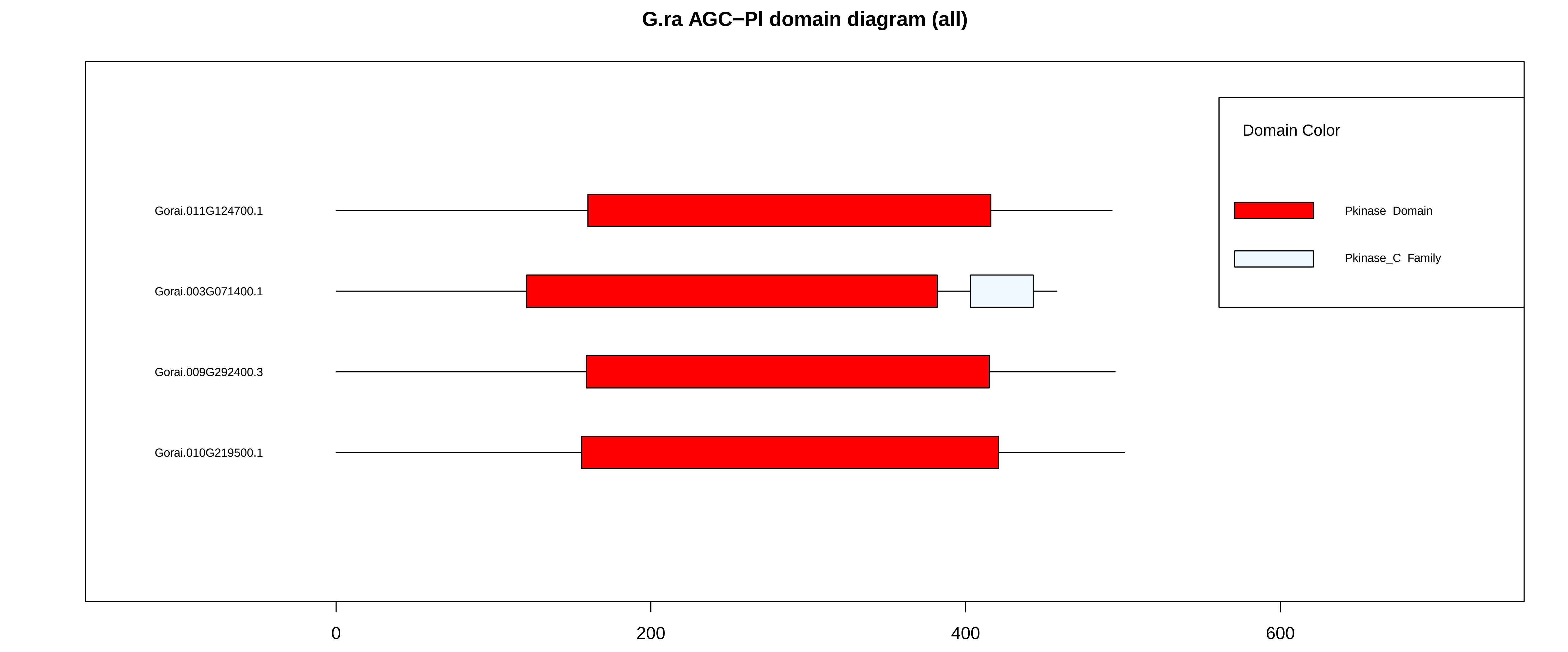
G.ra AGC−PI domain diagram (all)
Domain Color
Pkinase Domain
Pkinase_C Family
Gorai.011G124700.1
Gorai.003G071400.1
Gorai.009G292400.3
Gorai.010G219500.1
0
200
400
600

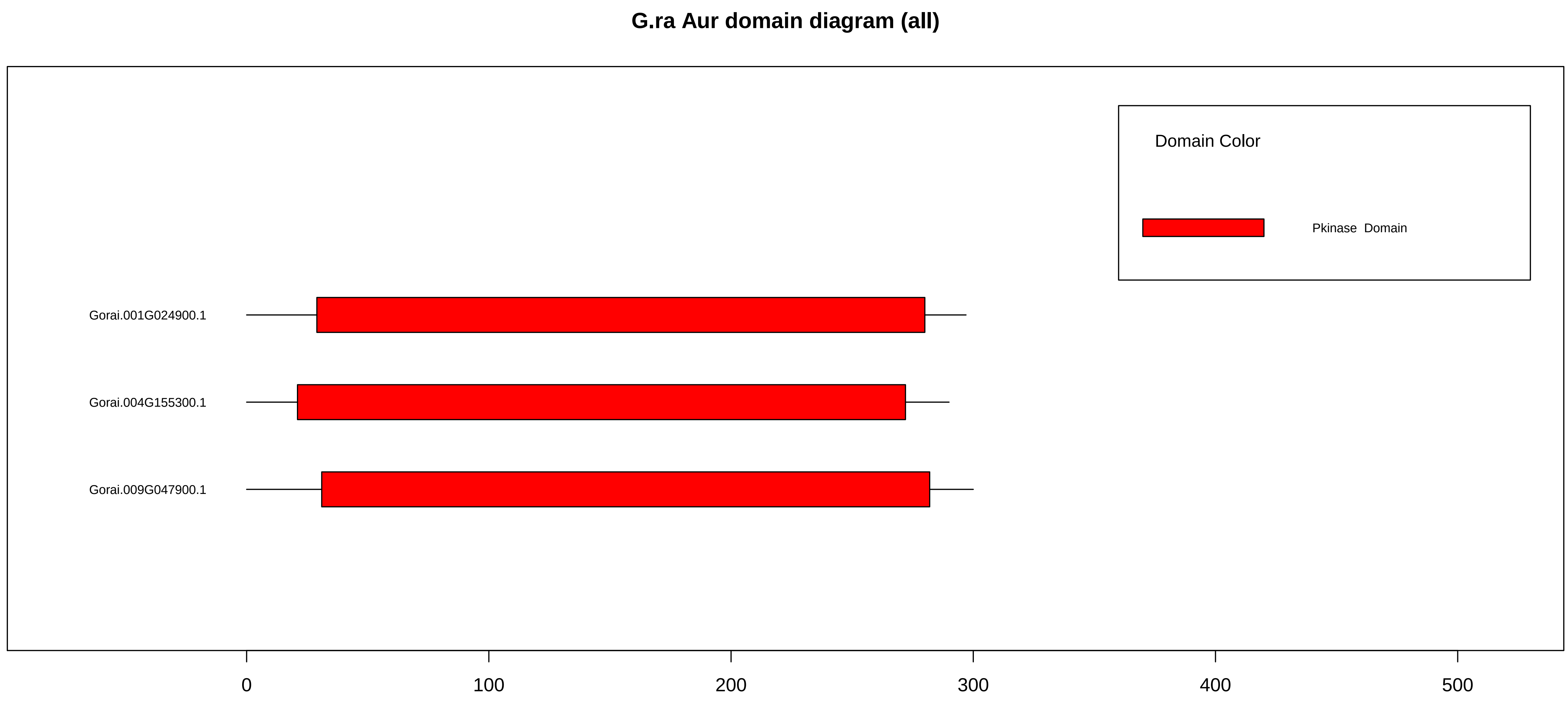
G.ra Aur domain diagram (all)
Domain Color
Pkinase Domain
Gorai.001G024900.1
Gorai.004G155300.1
Gorai.009G047900.1
0
100
200
300
400
500

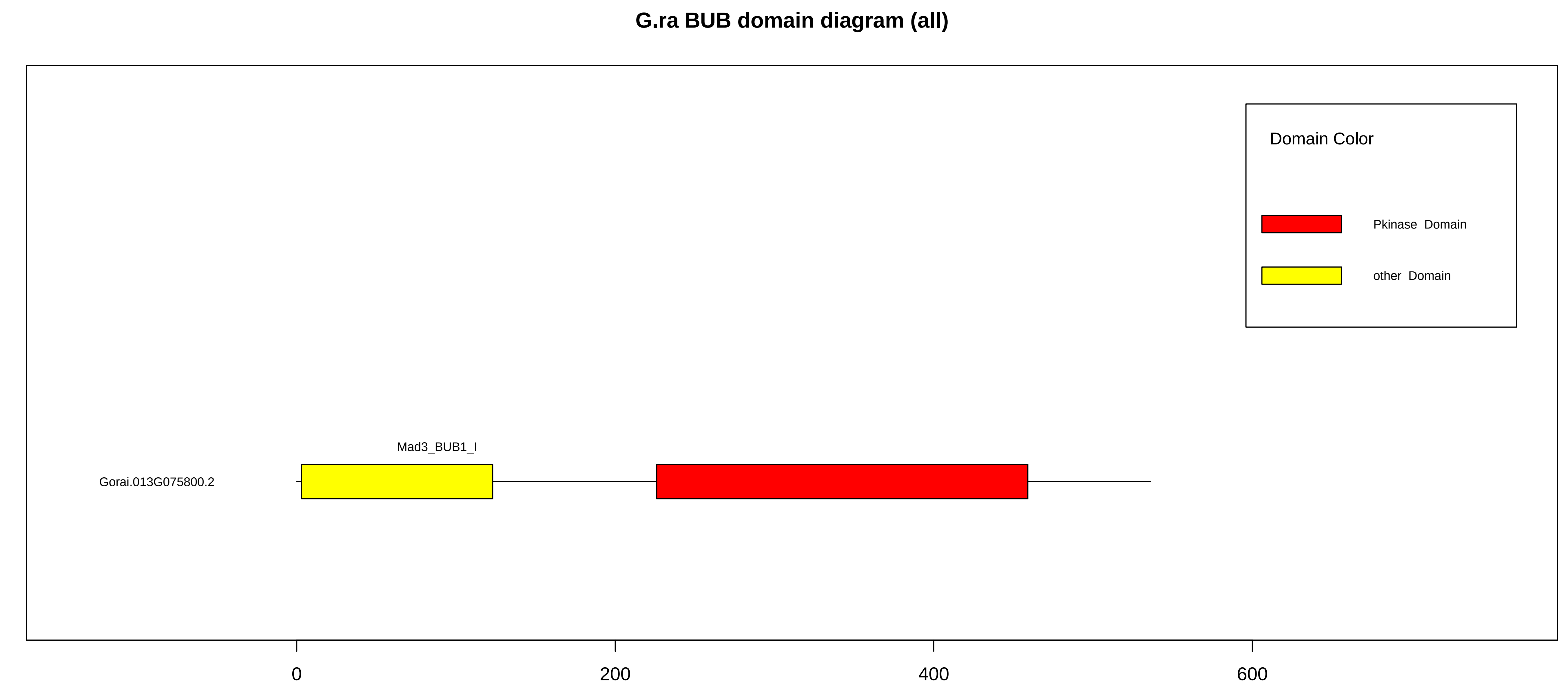

G.ra BUB domain diagram (all)
Domain Color
Pkinase Domain
other Domain
Mad3_BUB1_I
Gorai.013G075800.2
0
200
400
600

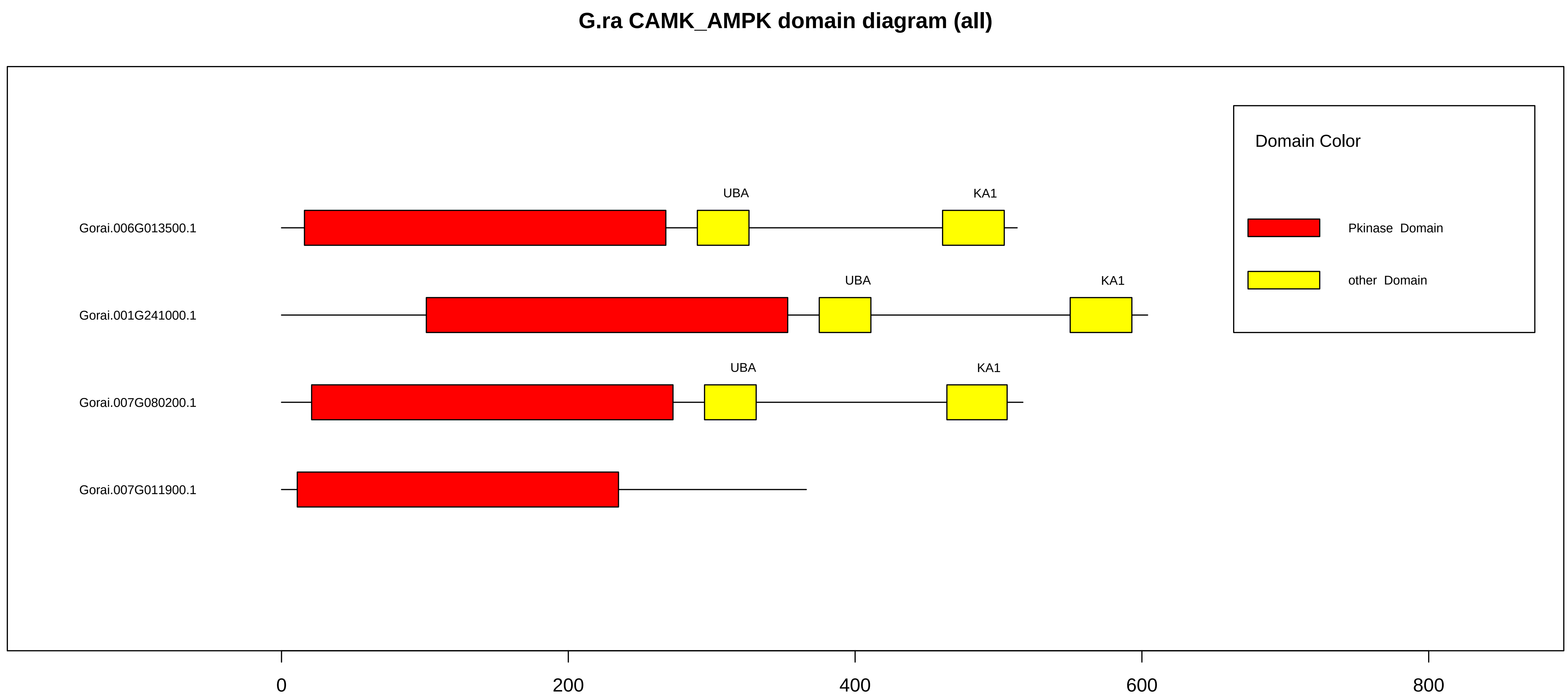

G.ra CAMK_AMPK domain diagram (all)
Domain Color
Pkinase Domain
other Domain
Gorai.006G013500.1
UBA
KA1
Gorai.001G241000.1
UBA
KA1
Gorai.007G080200.1
UBA
KA1
Gorai.007G011900.1
0
200
400
600
800

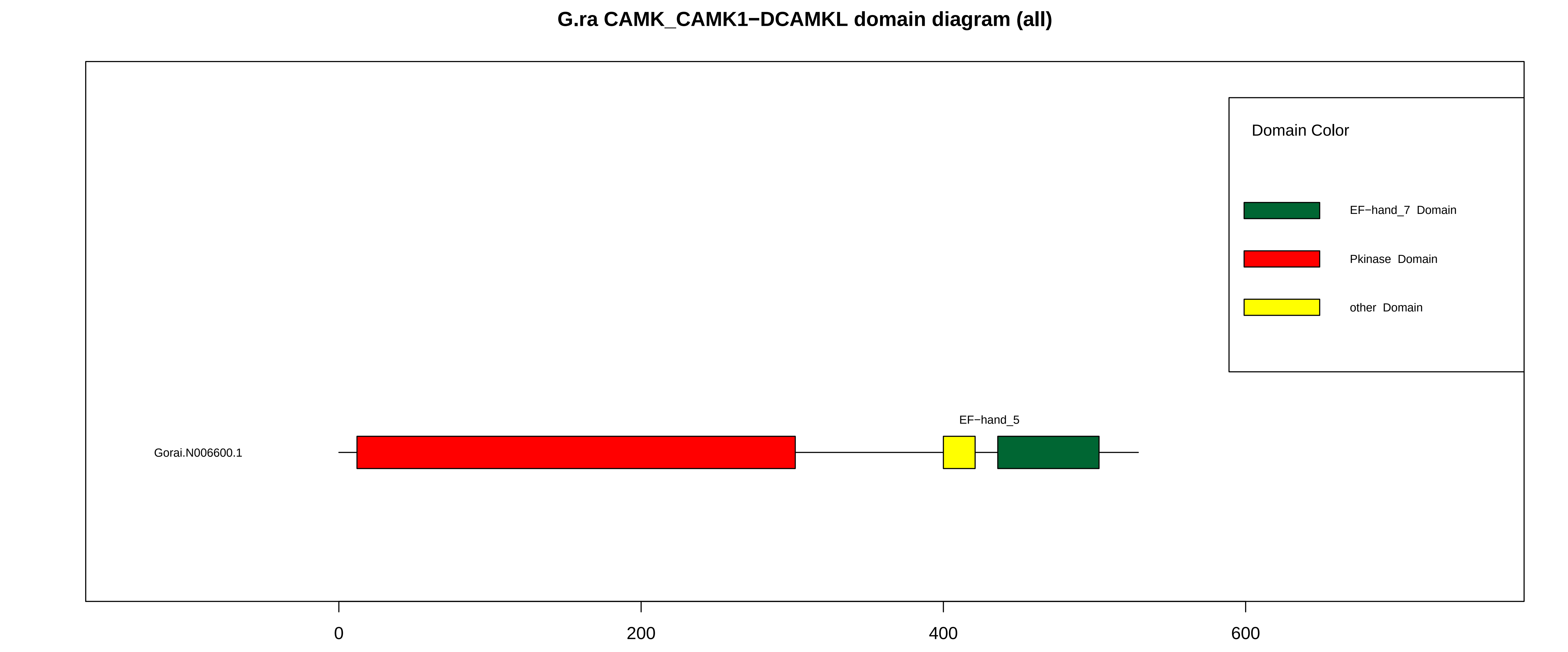
G.ra CAMK_CAMK1−DCAMKL domain diagram (all)
Domain Color
EF−hand_7 Domain
Pkinase Domain
other Domain
EF−hand_5
Gorai.N006600.1
0
200
400
600

G.ra CAMK_CAMKL−CHK1 domain diagram (part 1)

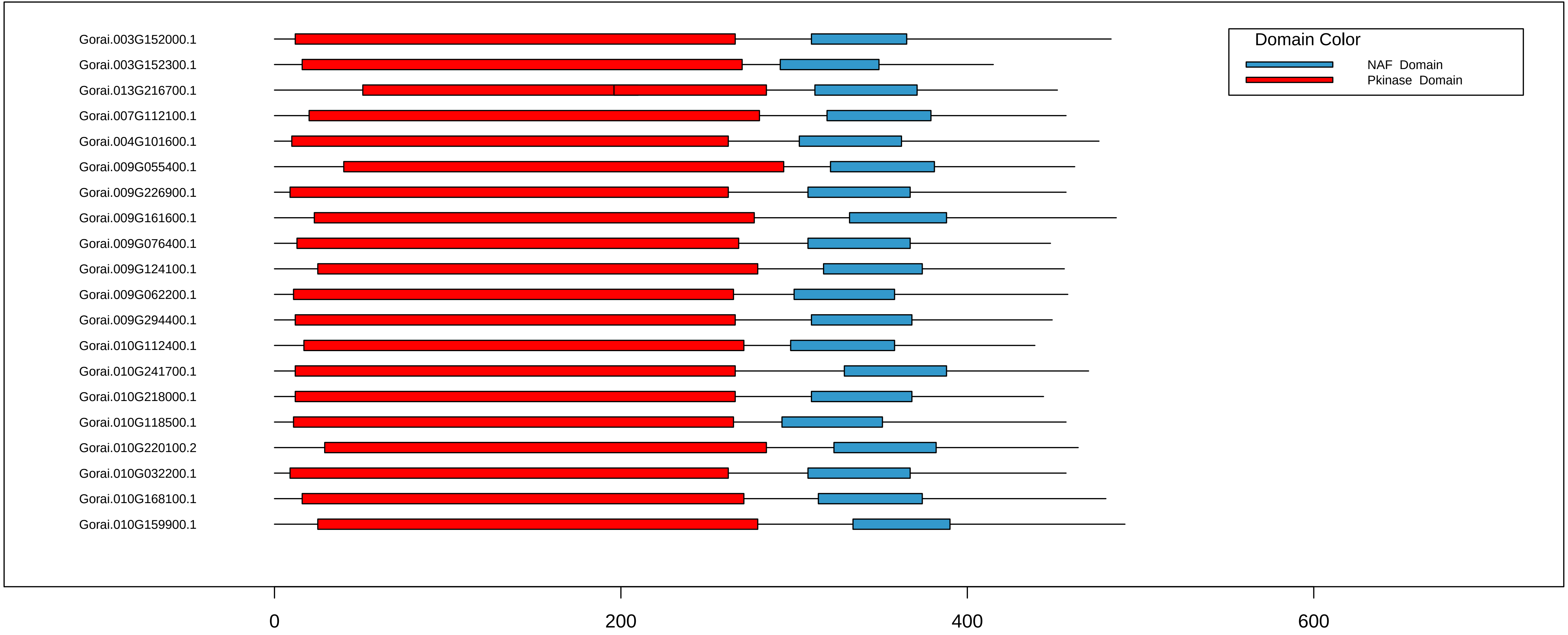

G.ra CAMK_CAMKL−CHK1 domain diagram (part 2)

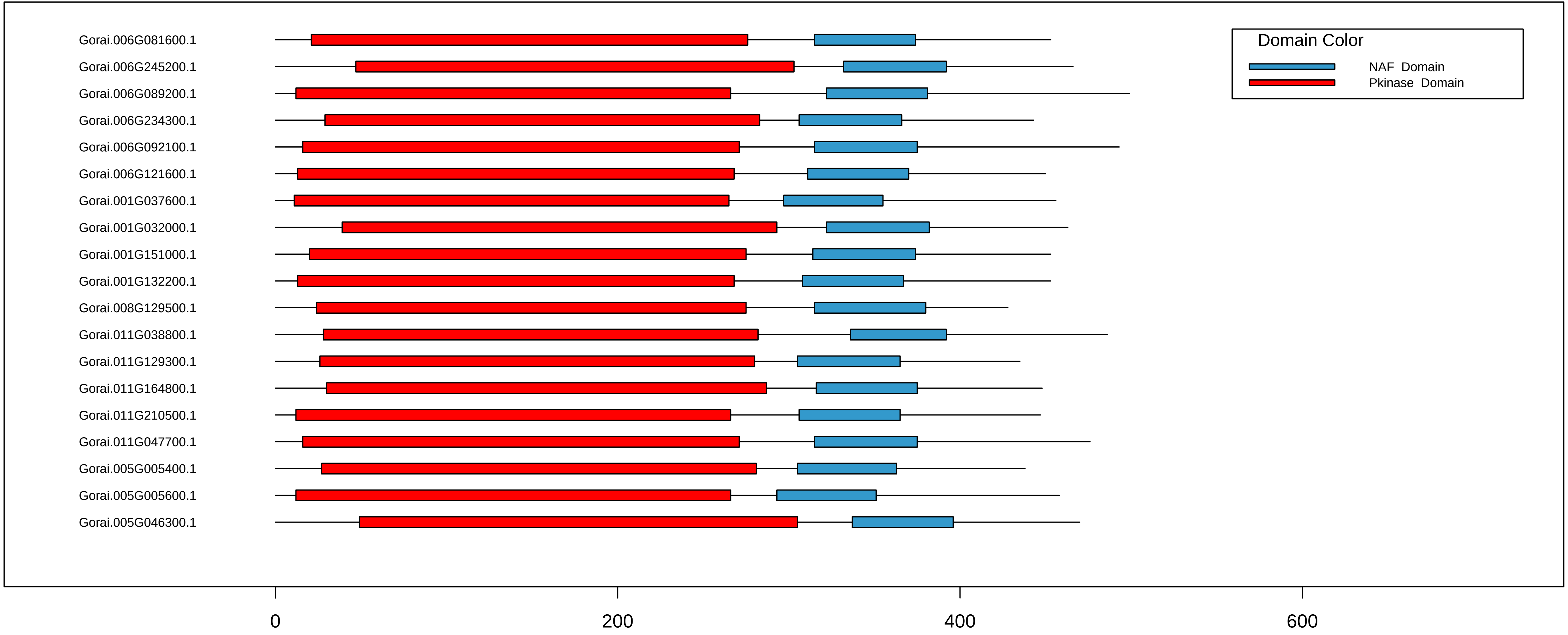

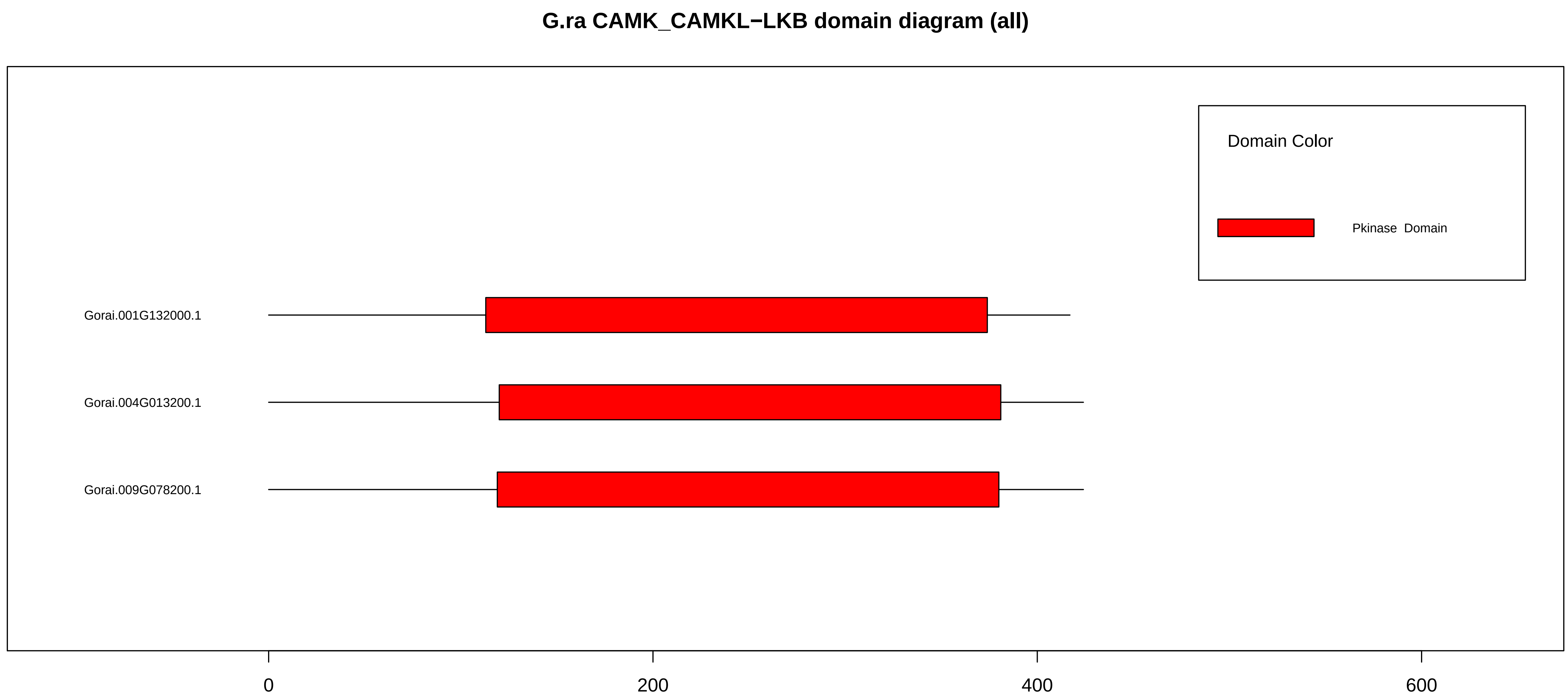
G.ra CAMK_CAMKL−LKB domain diagram (all)
Domain Color
Pkinase Domain
Gorai.001G132000.1
Gorai.004G013200.1
Gorai.009G078200.1
0
200
400
600

G.ra CAMK_CDPK domain diagram (part 1)

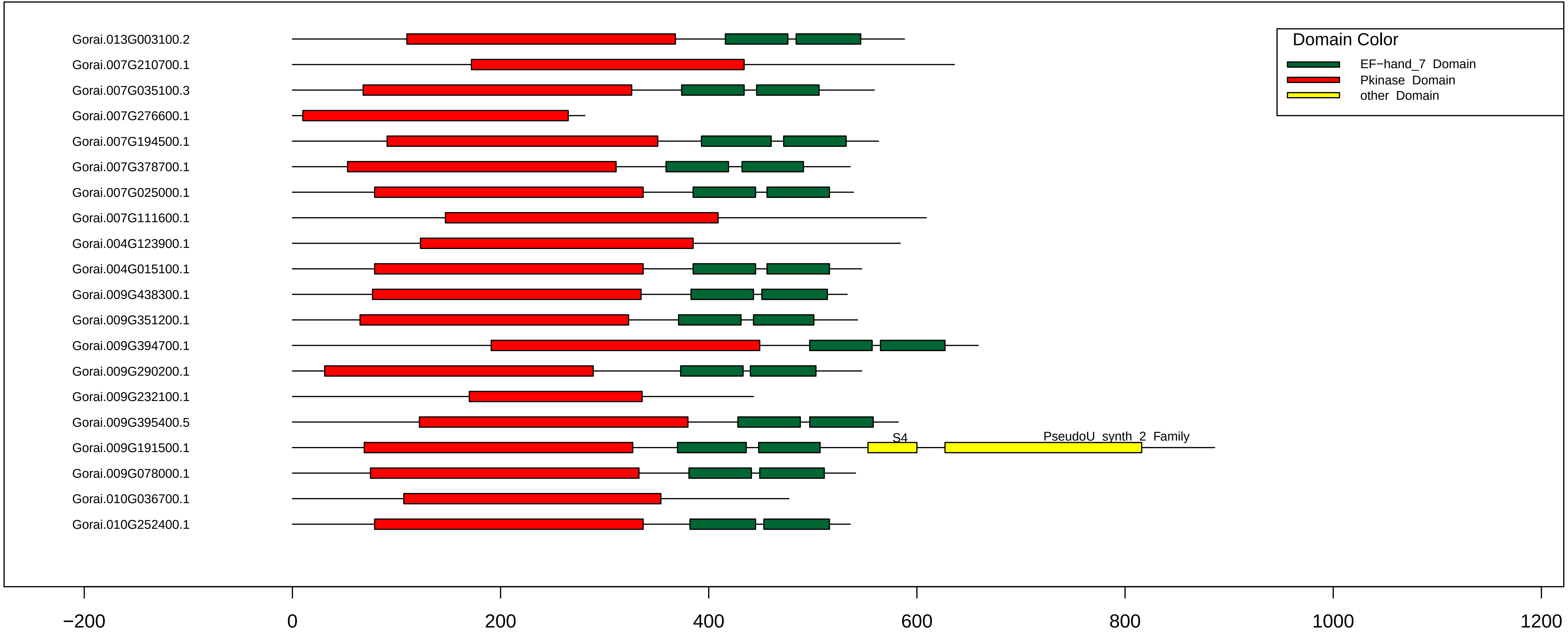

G.ra CAMK_CDPK domain diagram (part 2)

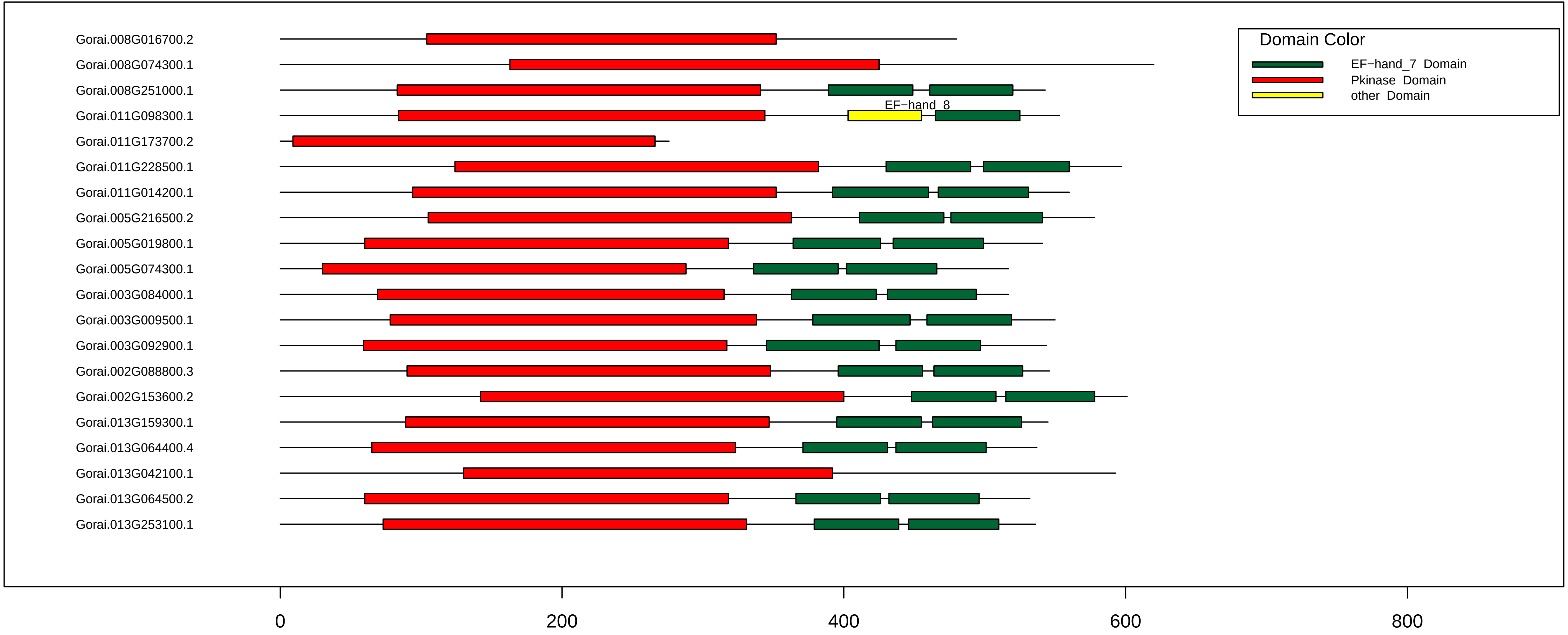

G.ra CAMK_CDPK domain diagram (part 3)

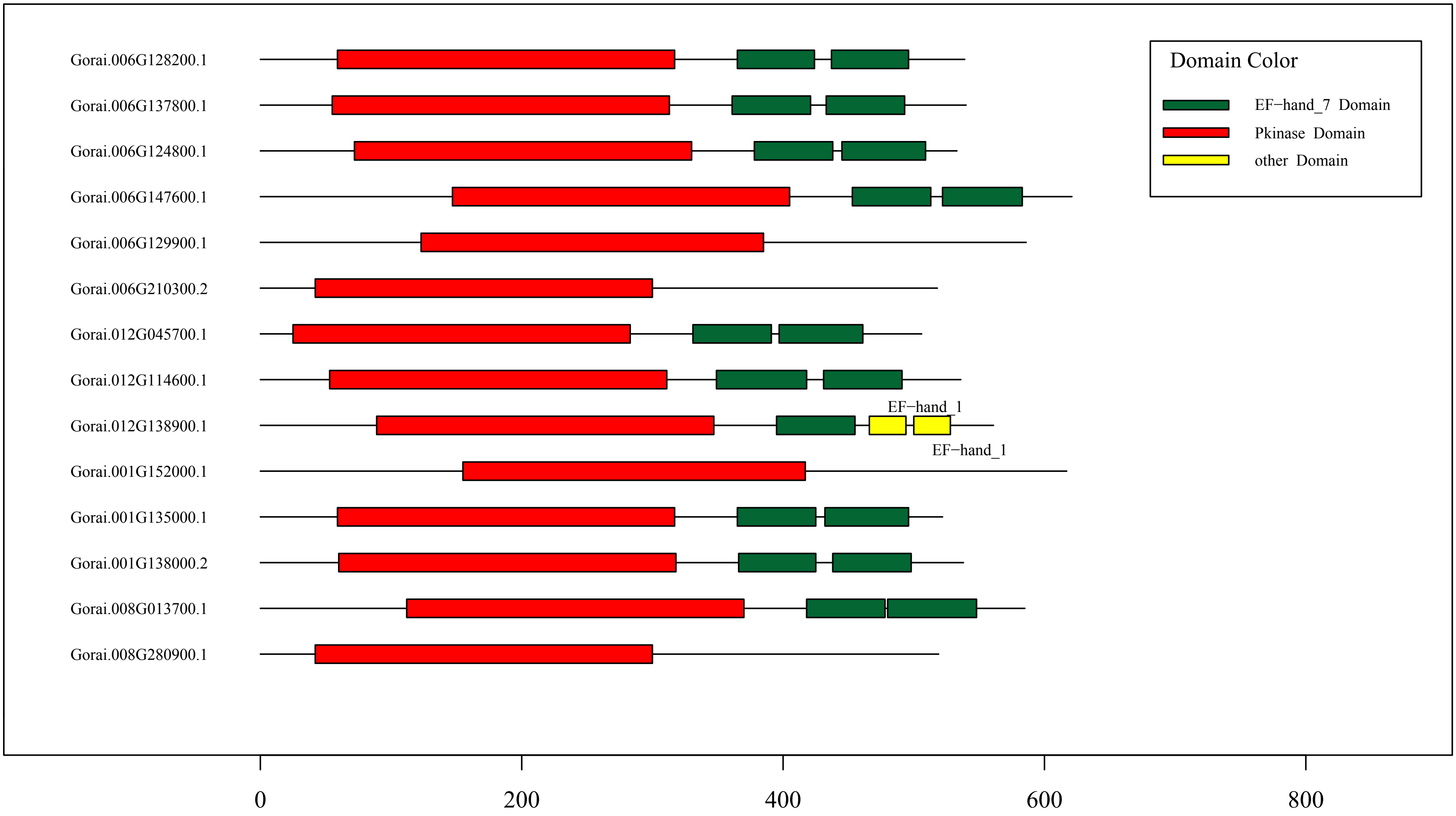

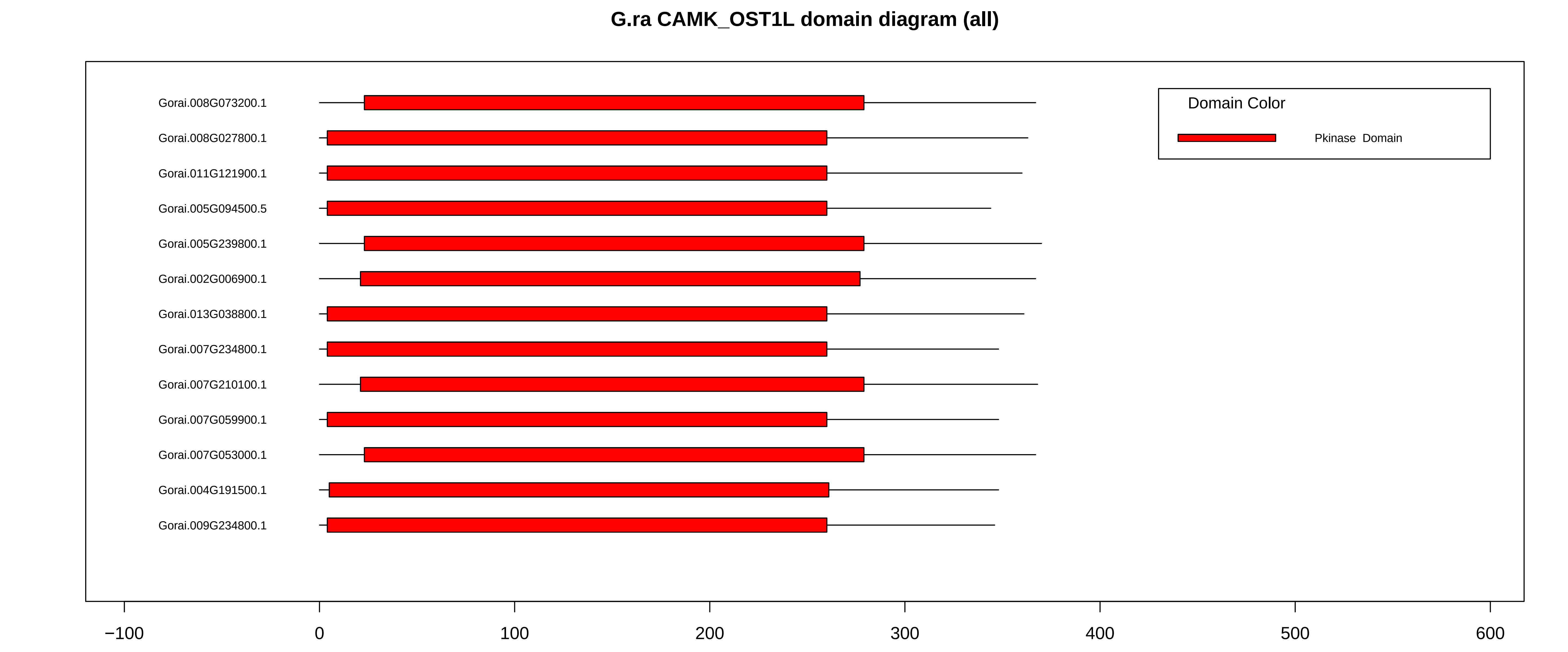
G.ra CAMK_OST1L domain diagram (all)
Gorai.008G073200.1
Gorai.008G027800.1
Gorai.011G121900.1
Gorai.005G094500.5
Gorai.005G239800.1
Gorai.002G006900.1
Gorai.013G038800.1
Gorai.007G234800.1
Gorai.007G210100.1
Gorai.007G059900.1
Gorai.007G053000.1
Gorai.004G191500.1
Gorai.009G234800.1
Domain Color
Pkinase Domain
−100
0
100
200
300
400
500
600

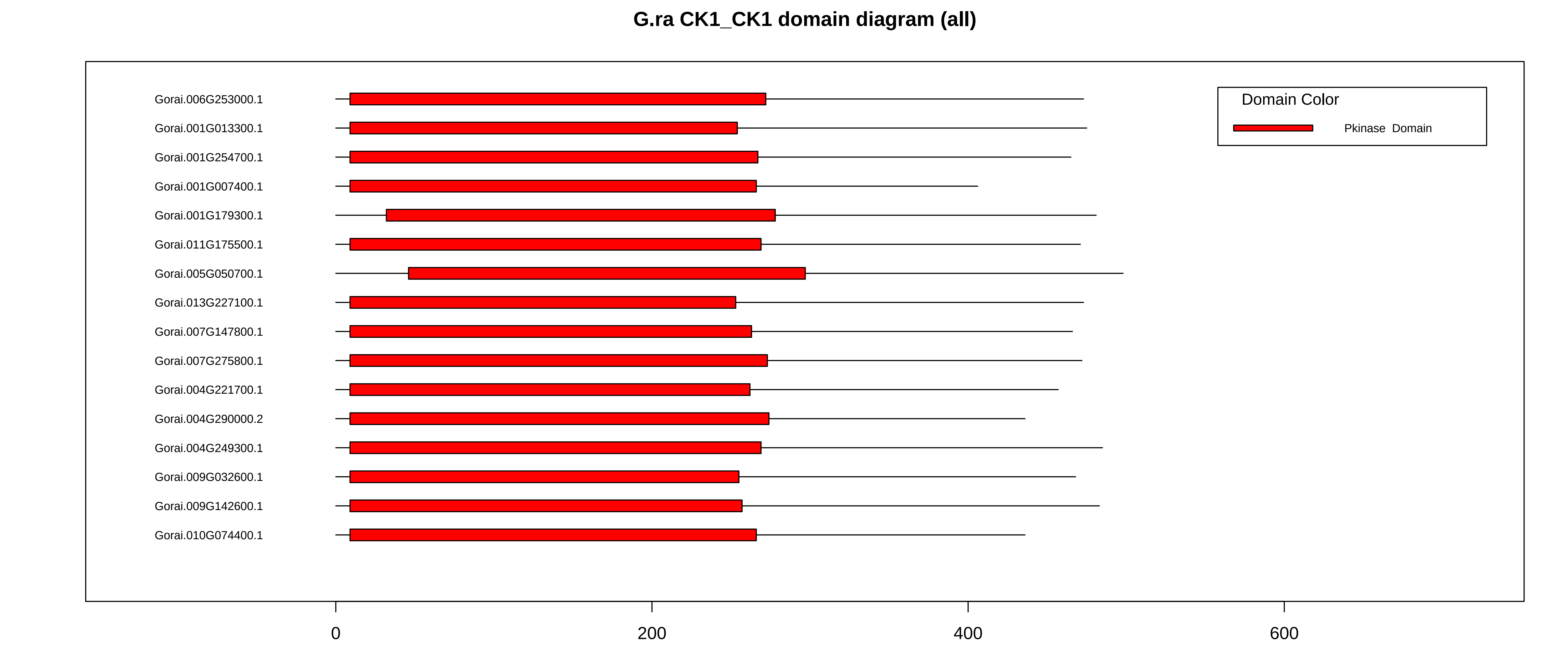
G.ra CK1_CK1 domain diagram (all)
Domain Color
Pkinase Domain
Gorai.006G253000.1
Gorai.001G013300.1
Gorai.001G254700.1
Gorai.001G007400.1
Gorai.001G179300.1
Gorai.011G175500.1
Gorai.005G050700.1
Gorai.013G227100.1
Gorai.007G147800.1
Gorai.007G275800.1
Gorai.004G221700.1
Gorai.004G290000.2
Gorai.004G249300.1
Gorai.009G032600.1
Gorai.009G142600.1
Gorai.010G074400.1
0
200
400
600

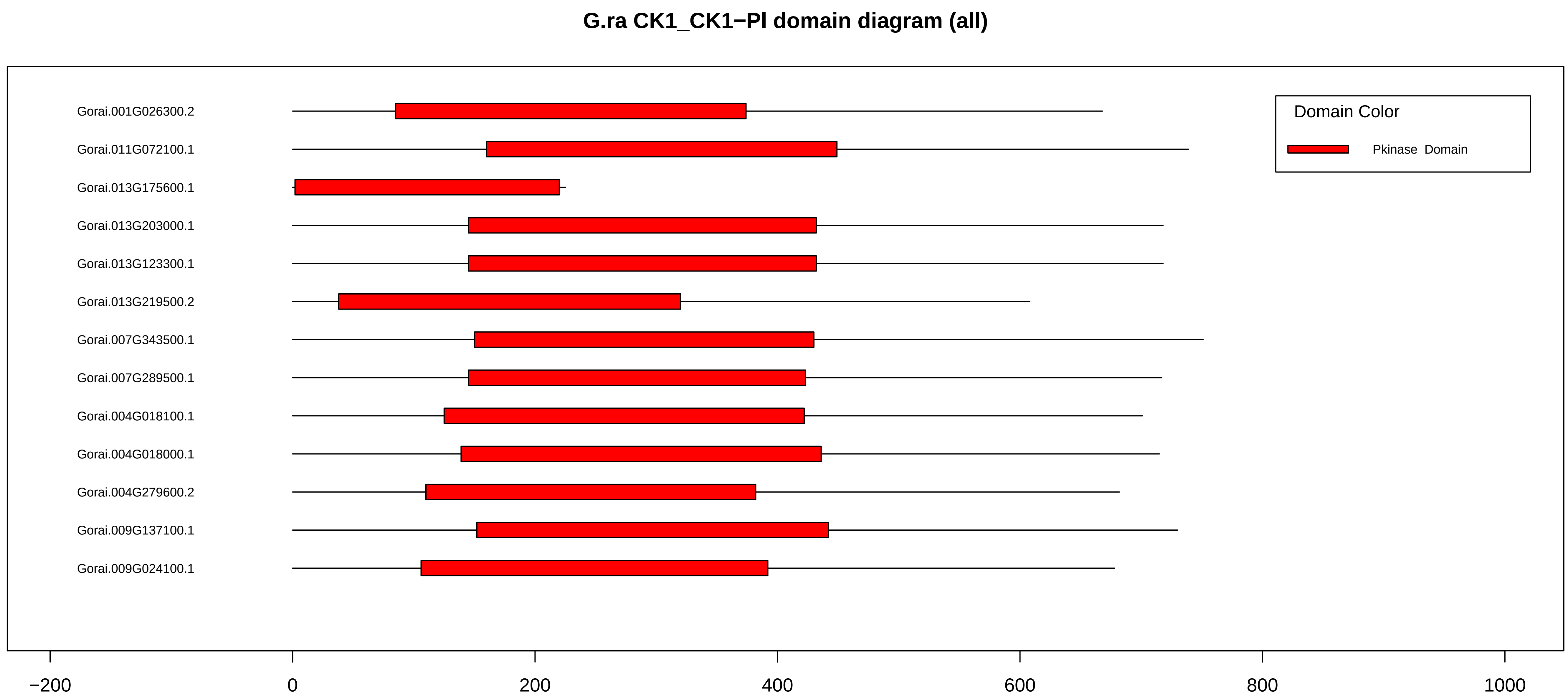

G.ra CK1_CK1−PI domain diagram (all)
Gorai.001G026300.2
Gorai.011G072100.1
Gorai.013G175600.1
Gorai.013G203000.1
Gorai.013G123300.1
Gorai.013G219500.2
Gorai.007G343500.1
Gorai.007G289500.1
Gorai.004G018100.1
Gorai.004G018000.1
Gorai.004G279600.2
Gorai.009G137100.1
Gorai.009G024100.1
Domain Color
Pkinase Domain
−200
0
200
400
600
800
1000

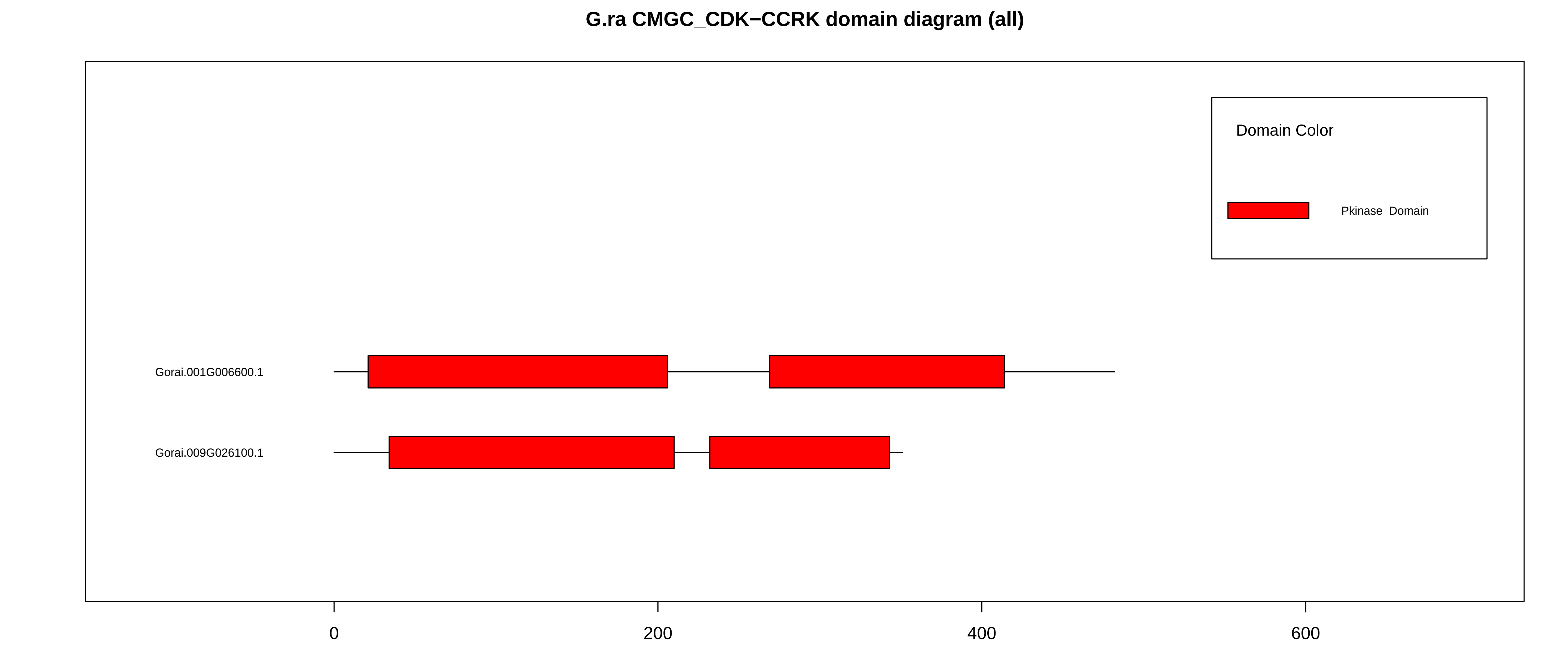
G.ra CMGC_CDK−CCRK domain diagram (all)
Domain Color
Pkinase Domain
Gorai.001G006600.1
Gorai.009G026100.1
0
200
400
600

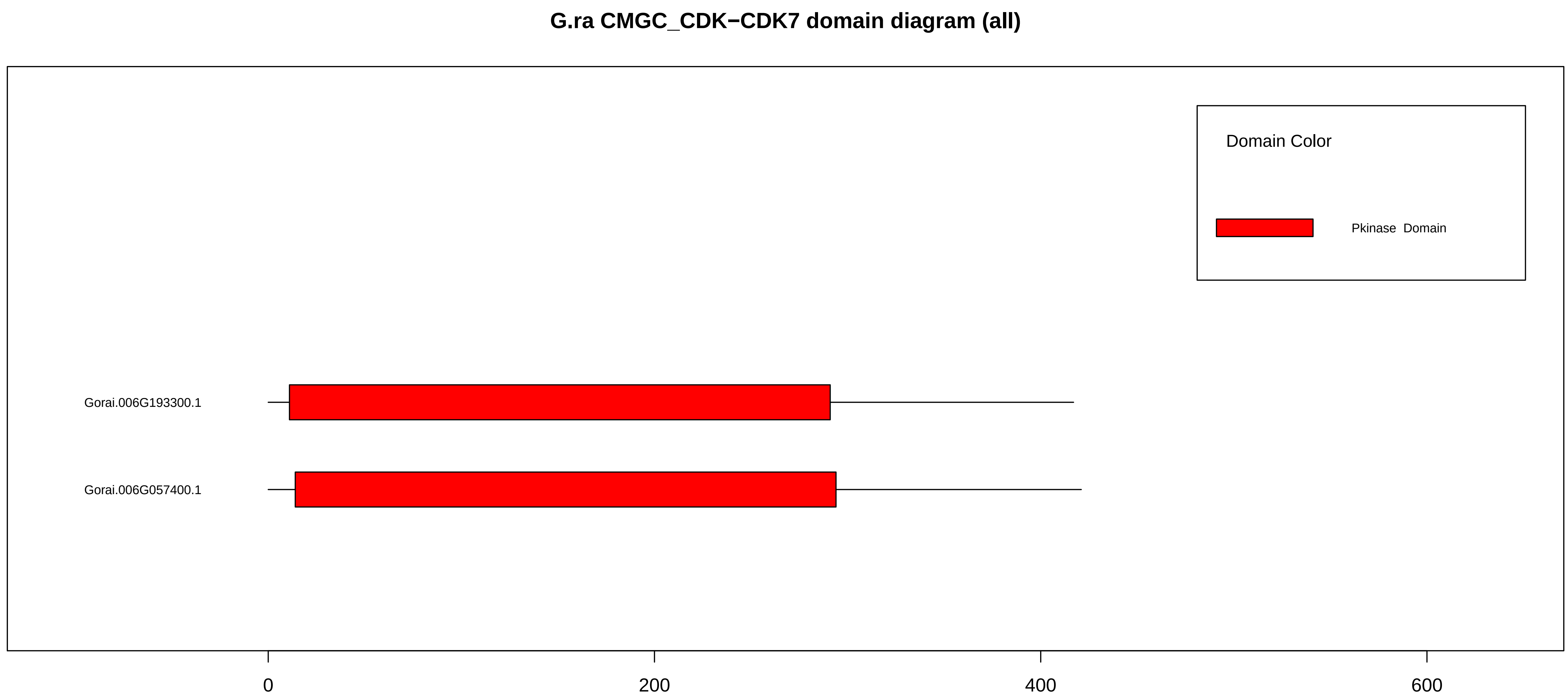
G.ra CMGC_CDK−CDK7 domain diagram (all)
Domain Color
Pkinase Domain
Gorai.006G193300.1
Gorai.006G057400.1
0
200
400
600

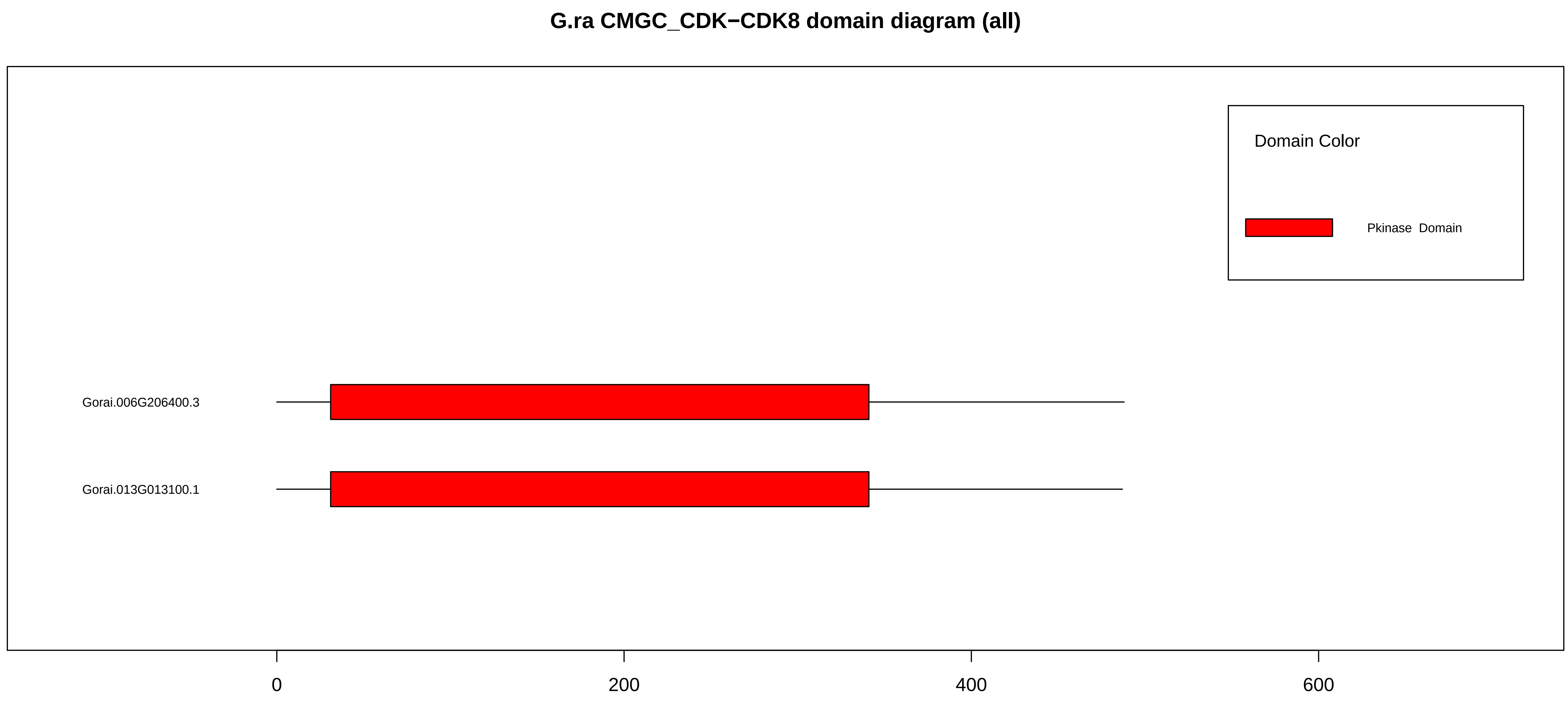
G.ra CMGC_CDK−CDK8 domain diagram (all)
Domain Color
Pkinase Domain
Gorai.006G206400.3
Gorai.013G013100.1
0
200
400
600

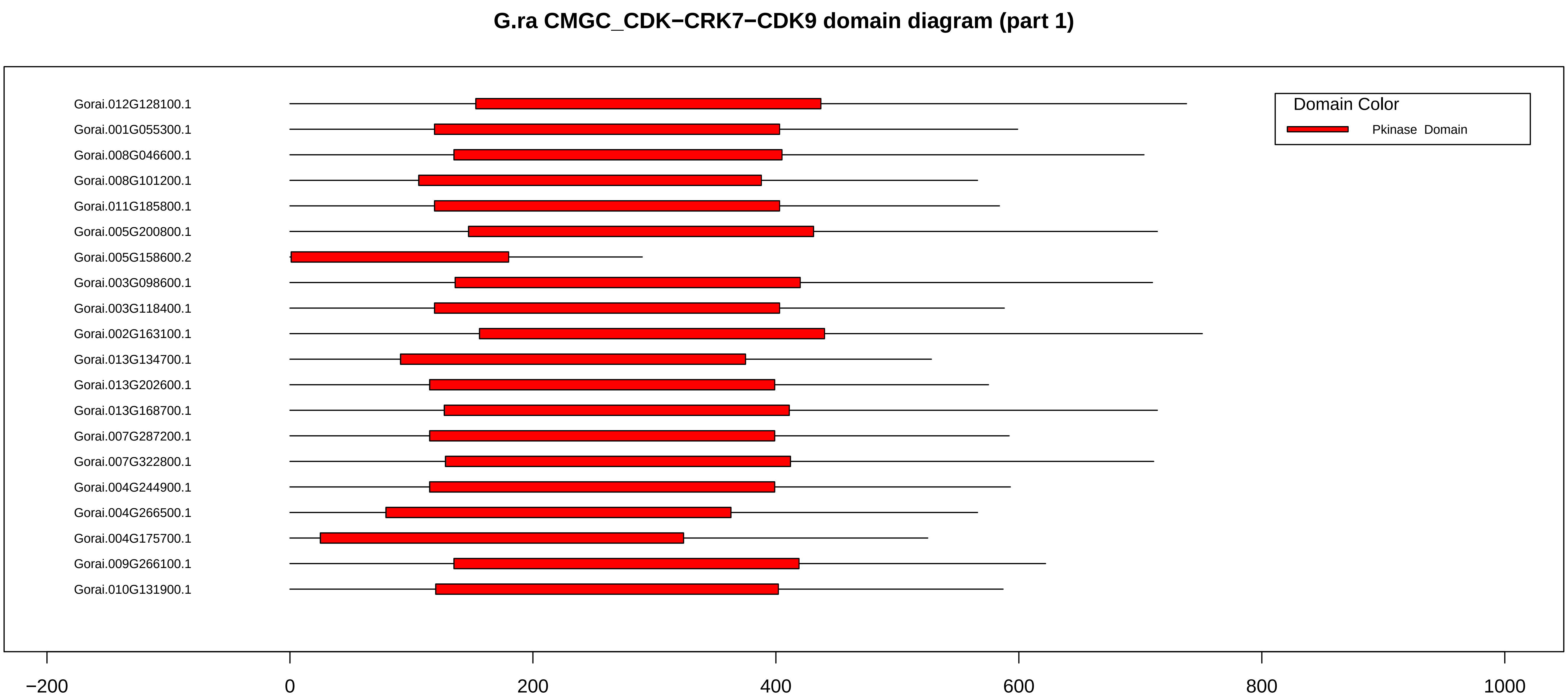
G.ra CMGC_CDK−CRK7−CDK9 domain diagram (part 1)
Domain Color
Pkinase Domain
Gorai.012G128100.1
Gorai.001G055300.1
Gorai.008G046600.1
Gorai.008G101200.1
Gorai.011G185800.1
Gorai.005G200800.1
Gorai.005G158600.2
Gorai.003G098600.1
Gorai.003G118400.1
Gorai.002G163100.1
Gorai.013G134700.1
Gorai.013G202600.1
Gorai.013G168700.1
Gorai.007G287200.1
Gorai.007G322800.1
Gorai.004G244900.1
Gorai.004G266500.1
Gorai.004G175700.1
Gorai.009G266100.1
Gorai.010G131900.1
−200
0
200
400
600
800
1000

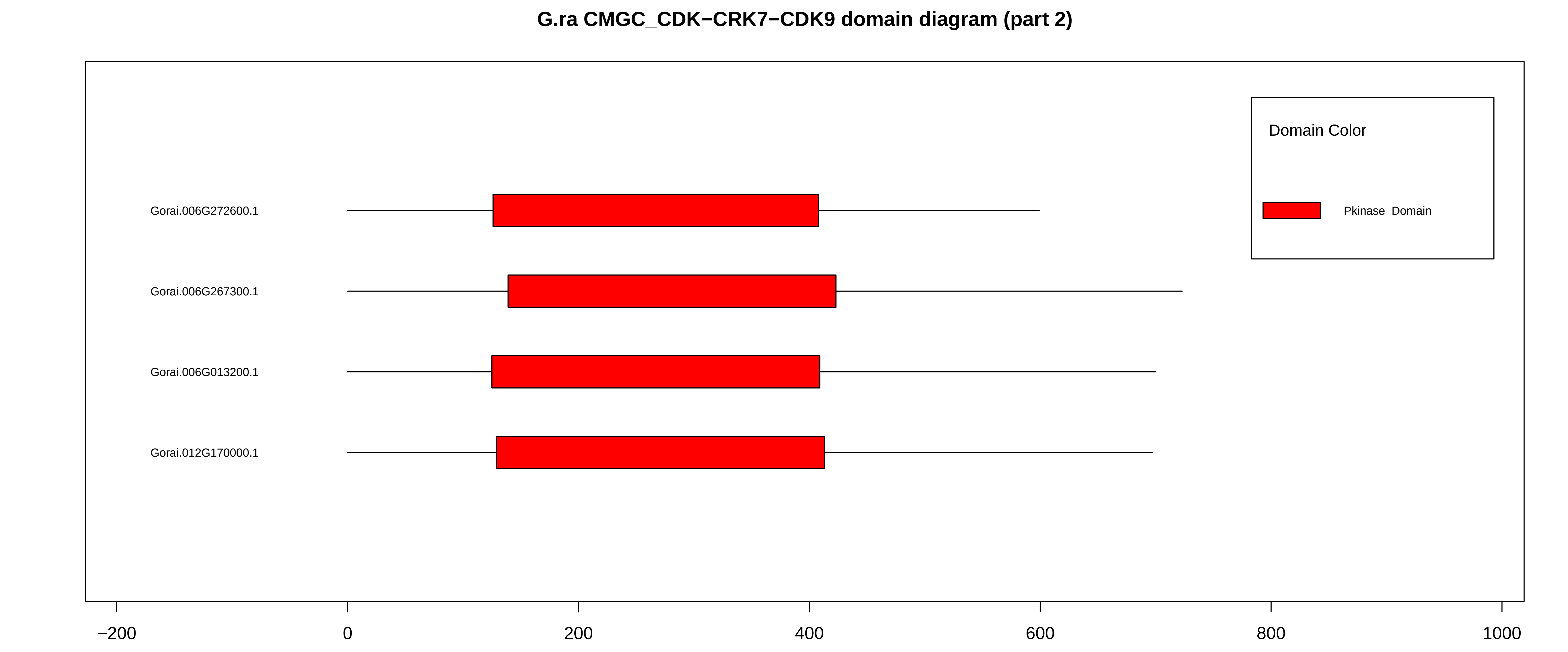
G.ra CMGC_CDK−CRK7−CDK9 domain diagram (part 2)
Domain Color
Pkinase Domain
Gorai.006G272600.1
Gorai.006G267300.1
Gorai.006G013200.1
Gorai.012G170000.1
−200
0
200
400
600
800
1000

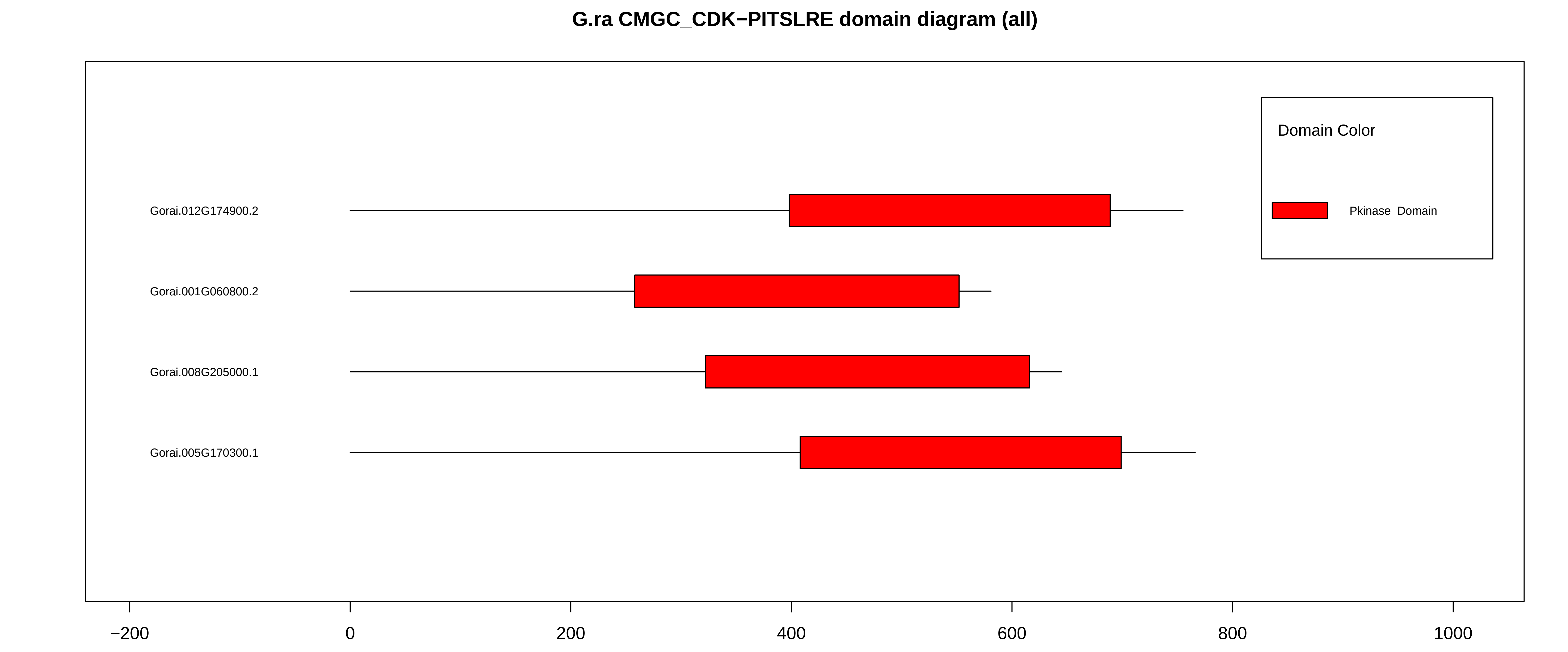

G.ra CMGC_CDK−PITSLRE domain diagram (all)
Domain Color
Pkinase Domain
Gorai.012G174900.2
Gorai.001G060800.2
Gorai.008G205000.1
Gorai.005G170300.1
−200
0
200
400
600
800
1000

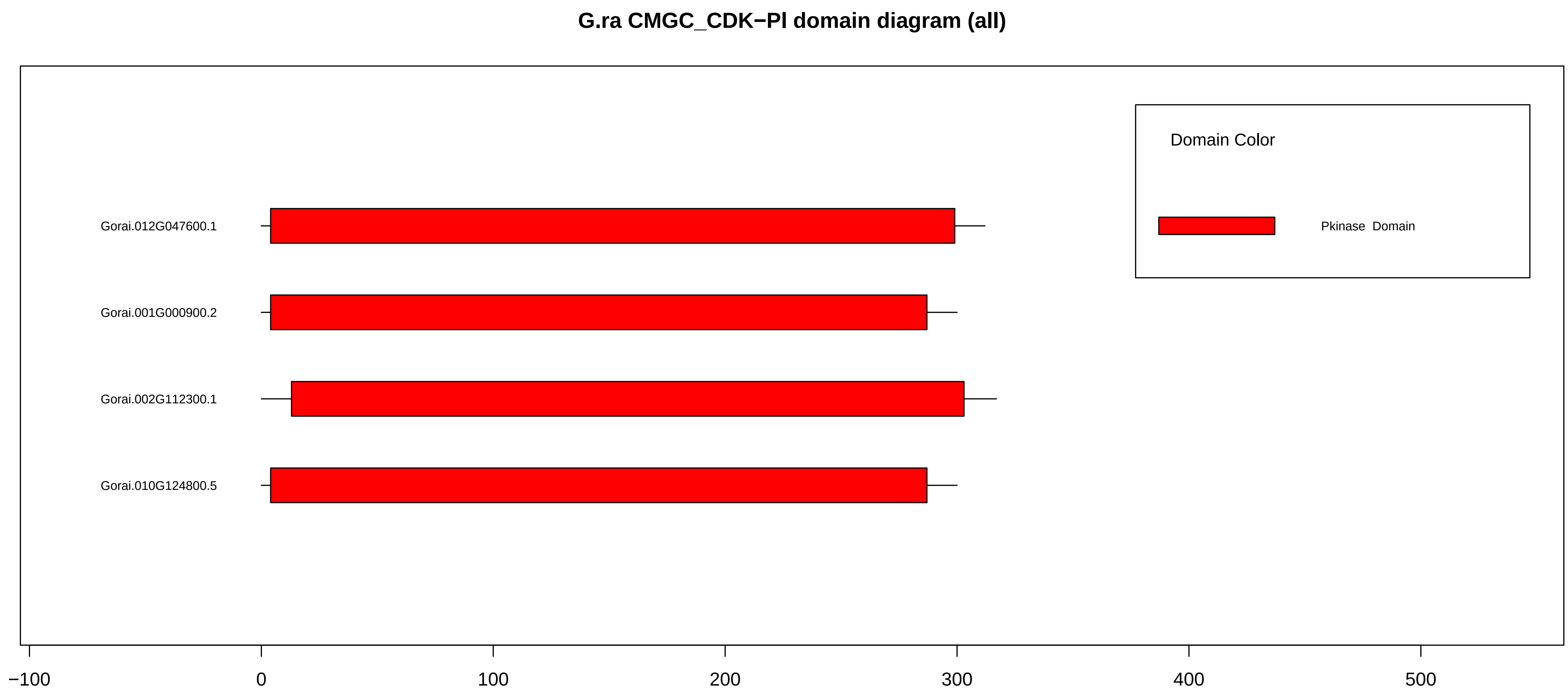
G.ra CMGC_CDK−Pl domain diagram (all)
Domain Color
Pkinase Domain
Gorai.012G047600.1
Gorai.001G000900.2
Gorai.002G112300.1
Gorai.010G124800.5
−100
0
100
200
300
400
500

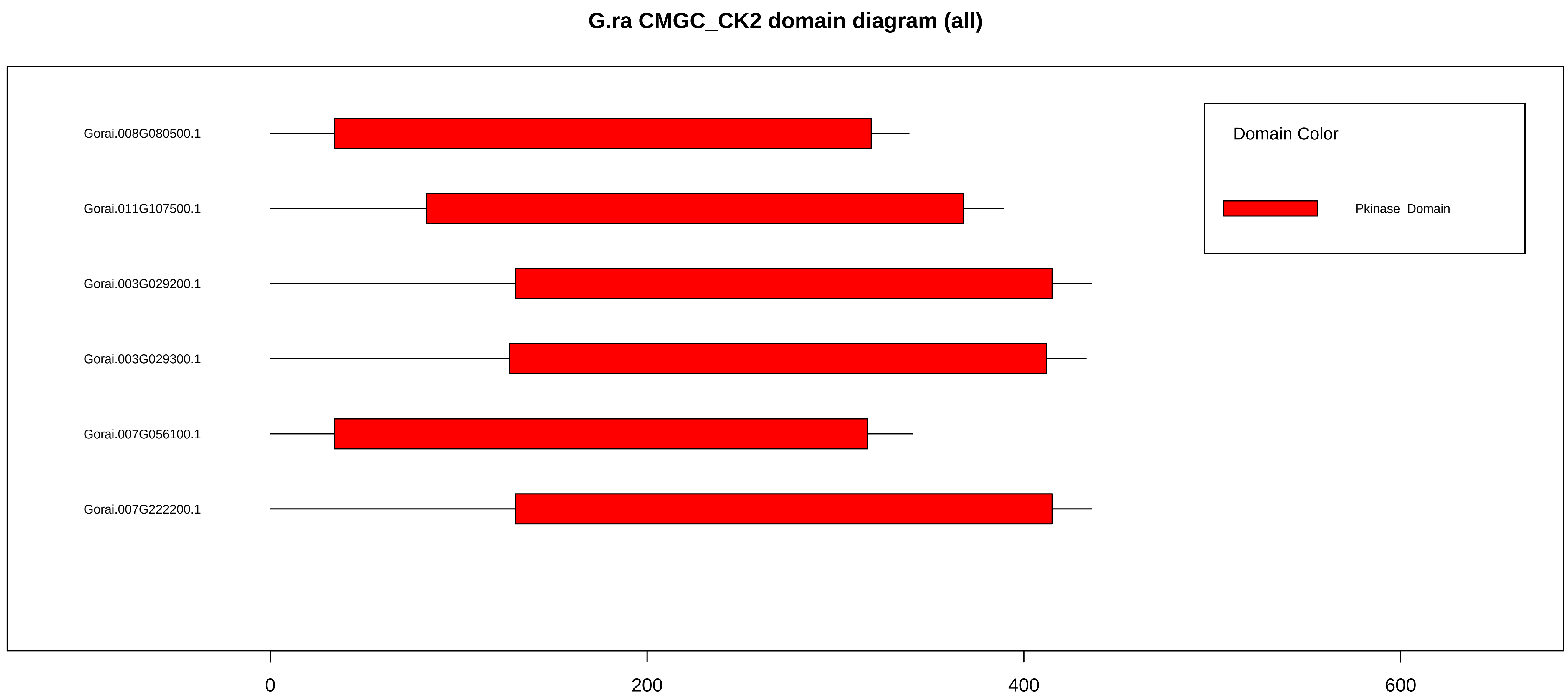

G.ra CMGC_CK2 domain diagram (all)
Gorai.008G080500.1
Gorai.011G107500.1
Gorai.003G029200.1
Gorai.003G029300.1
Gorai.007G056100.1
Gorai.007G222200.1
Domain Color
Pkinase Domain
0
200
400
600

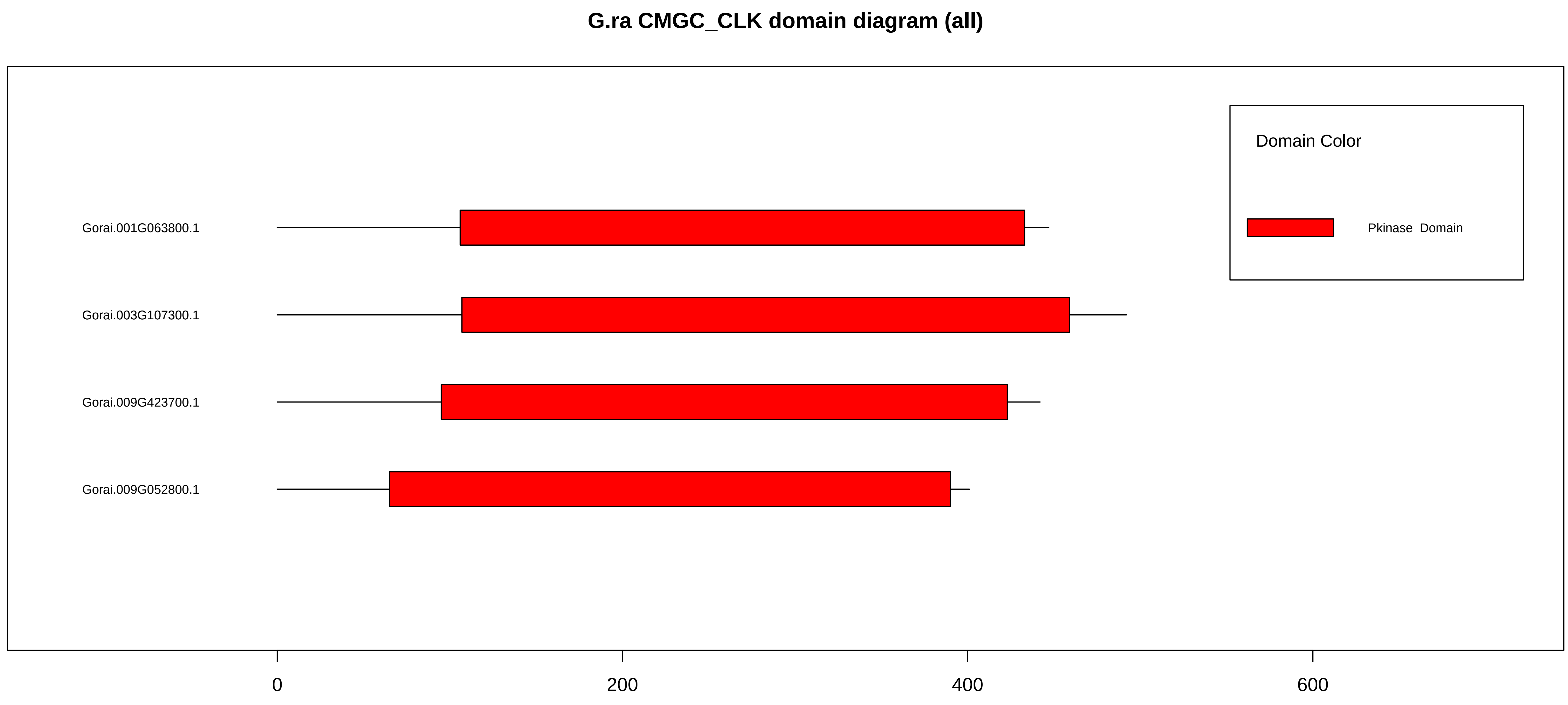
G.ra CMGC_CLK domain diagram (all)
Domain Color
Pkinase Domain
Gorai.001G063800.1
Gorai.003G107300.1
Gorai.009G423700.1
Gorai.009G052800.1
0
200
400
600

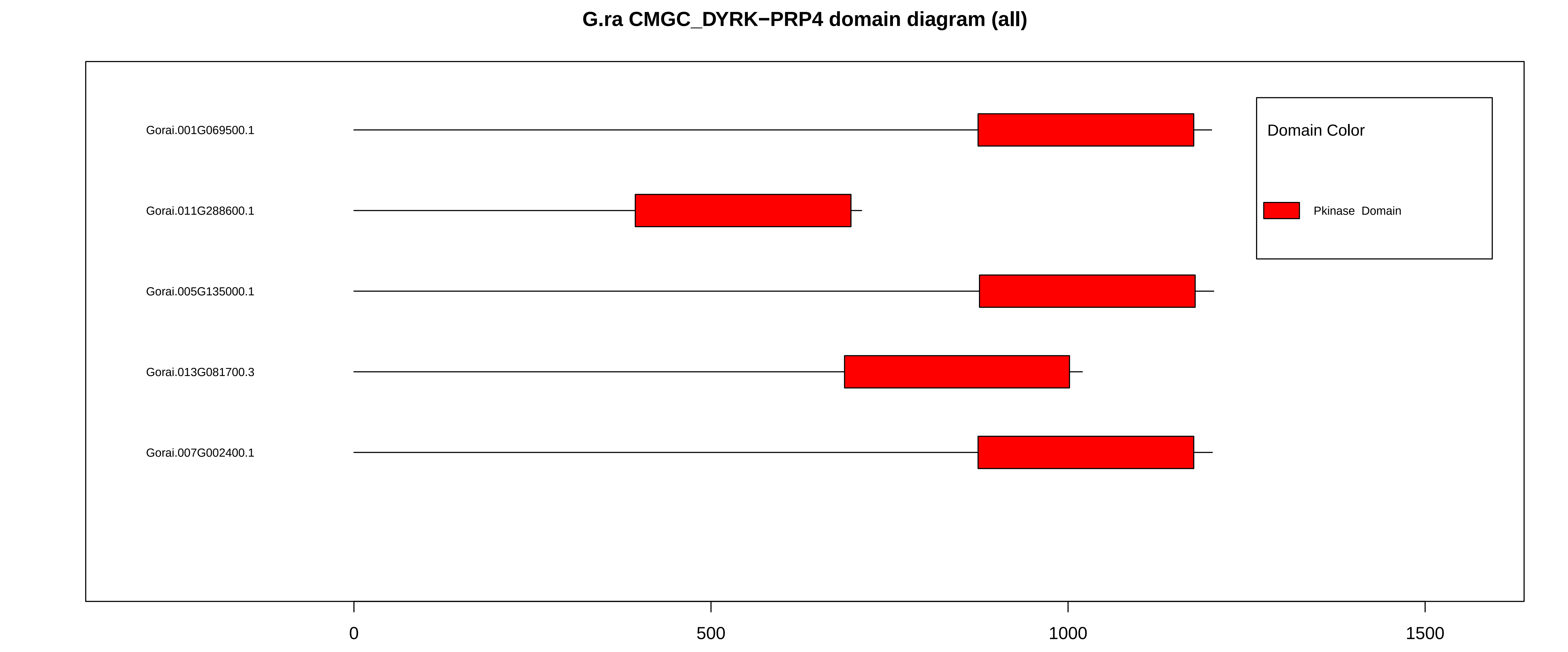
G.ra CMGC_DYRK−PRP4 domain diagram (all)
Gorai.001G069500.1
Gorai.011G288600.1
Gorai.005G135000.1
Gorai.013G081700.3
Gorai.007G002400.1
Domain Color
Pkinase Domain
0
500
1000
1500

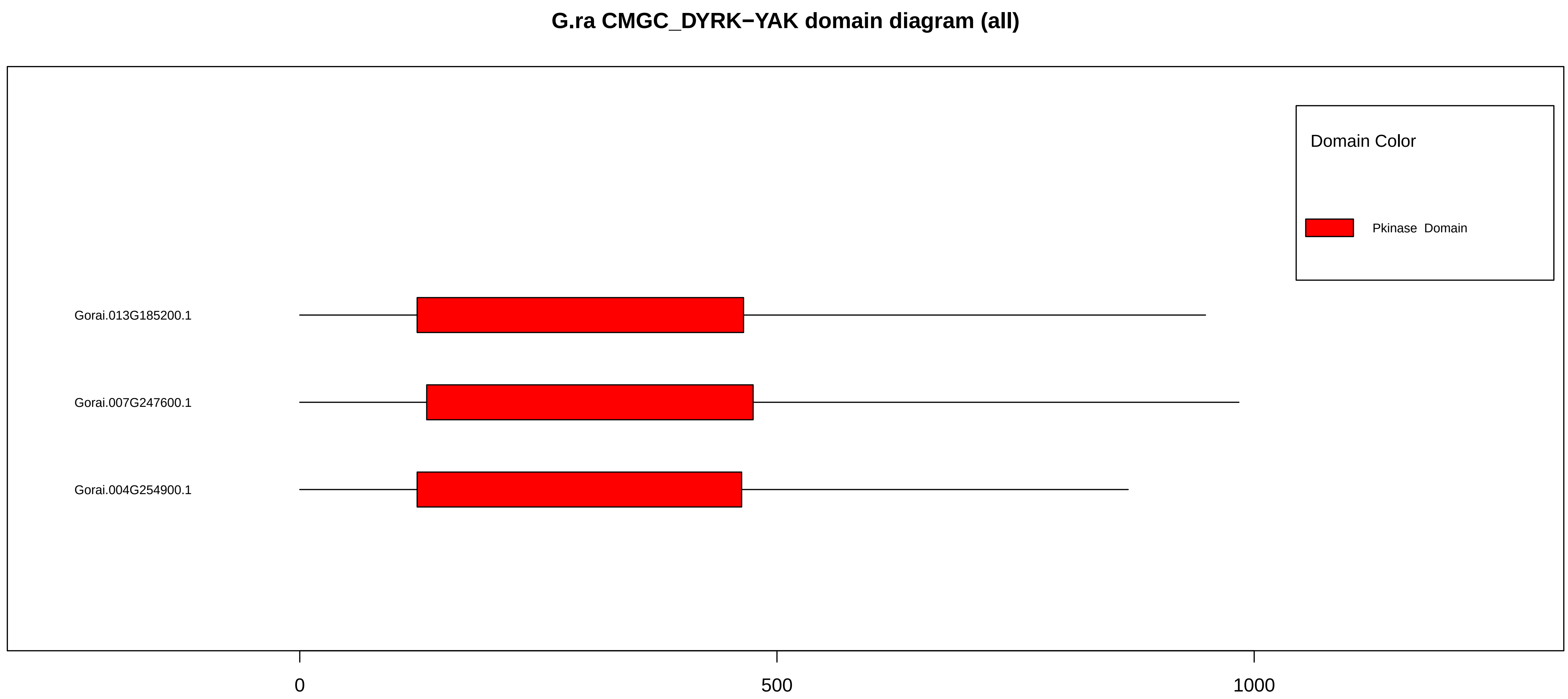
G.ra CMGC_DYRK−YAK domain diagram (all)
Domain Color
Pkinase Domain
Gorai.013G185200.1
Gorai.007G247600.1
Gorai.004G254900.1
0
500
1000

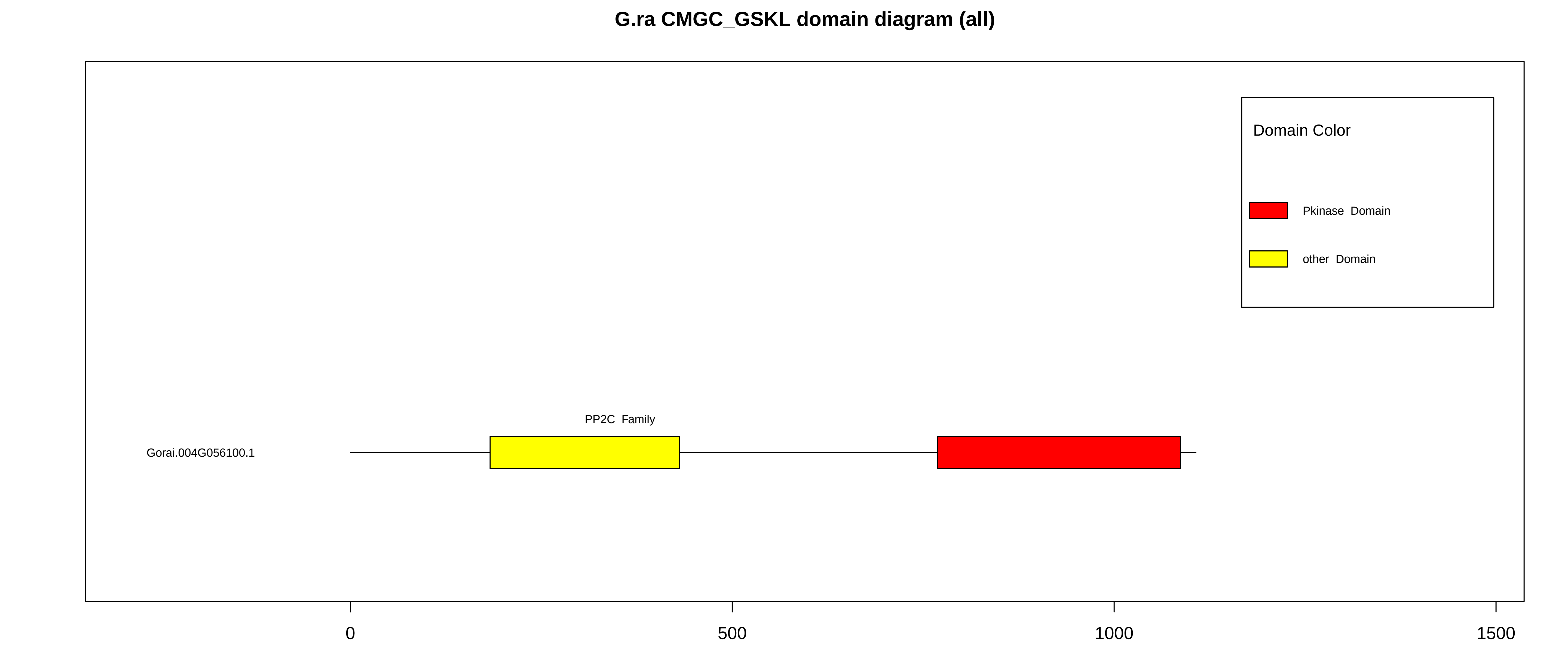
G.ra CMGC_GSKL domain diagram (all)
Domain Color
Pkinase Domain
other Domain
PP2C Family
Gorai.004G056100.1
0
500
1000
1500

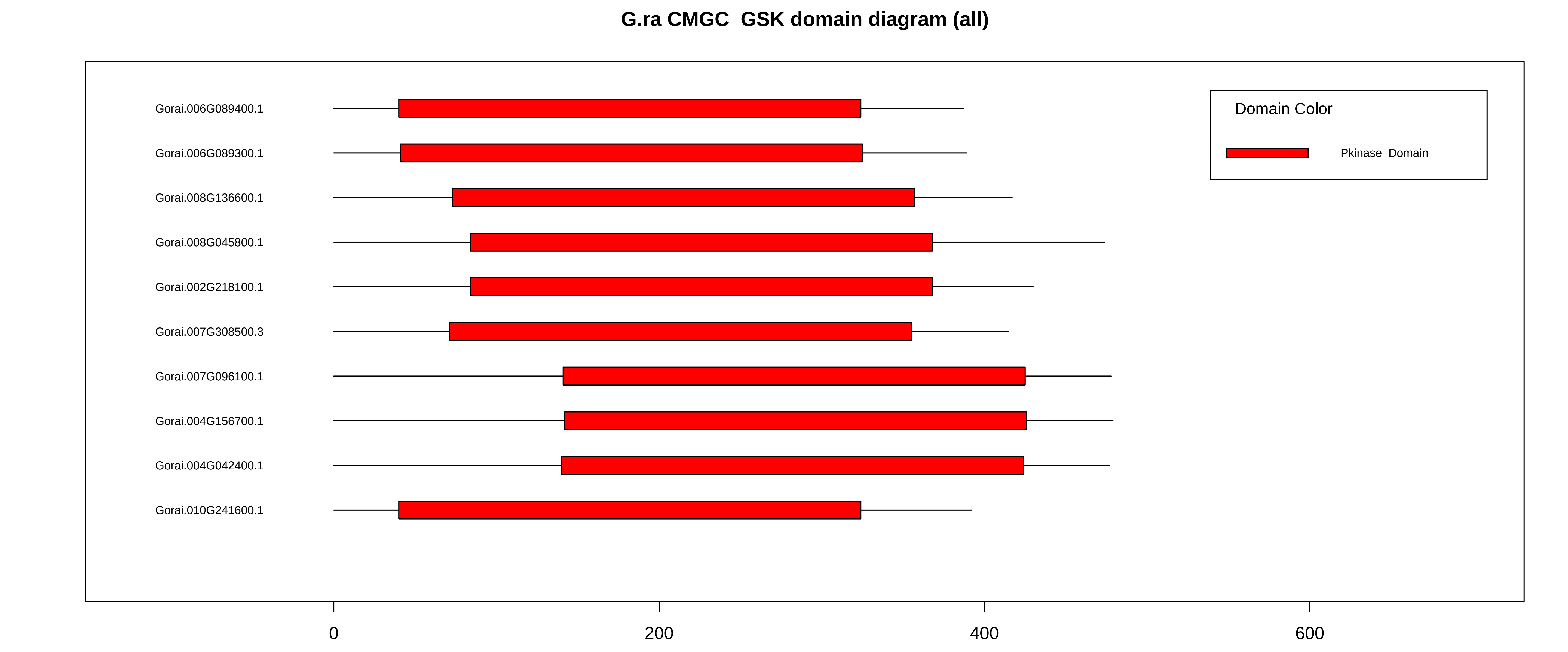
G.ra CMGC_GSK domain diagram (all)
Gorai.006G089400.1
Gorai.006G089300.1
Gorai.008G136600.1
Gorai.008G045800.1
Gorai.002G218100.1
Gorai.007G308500.3
Gorai.007G096100.1
Gorai.004G156700.1
Gorai.004G042400.1
Gorai.010G241600.1
Domain Color
Pkinase Domain
0
200
400
600

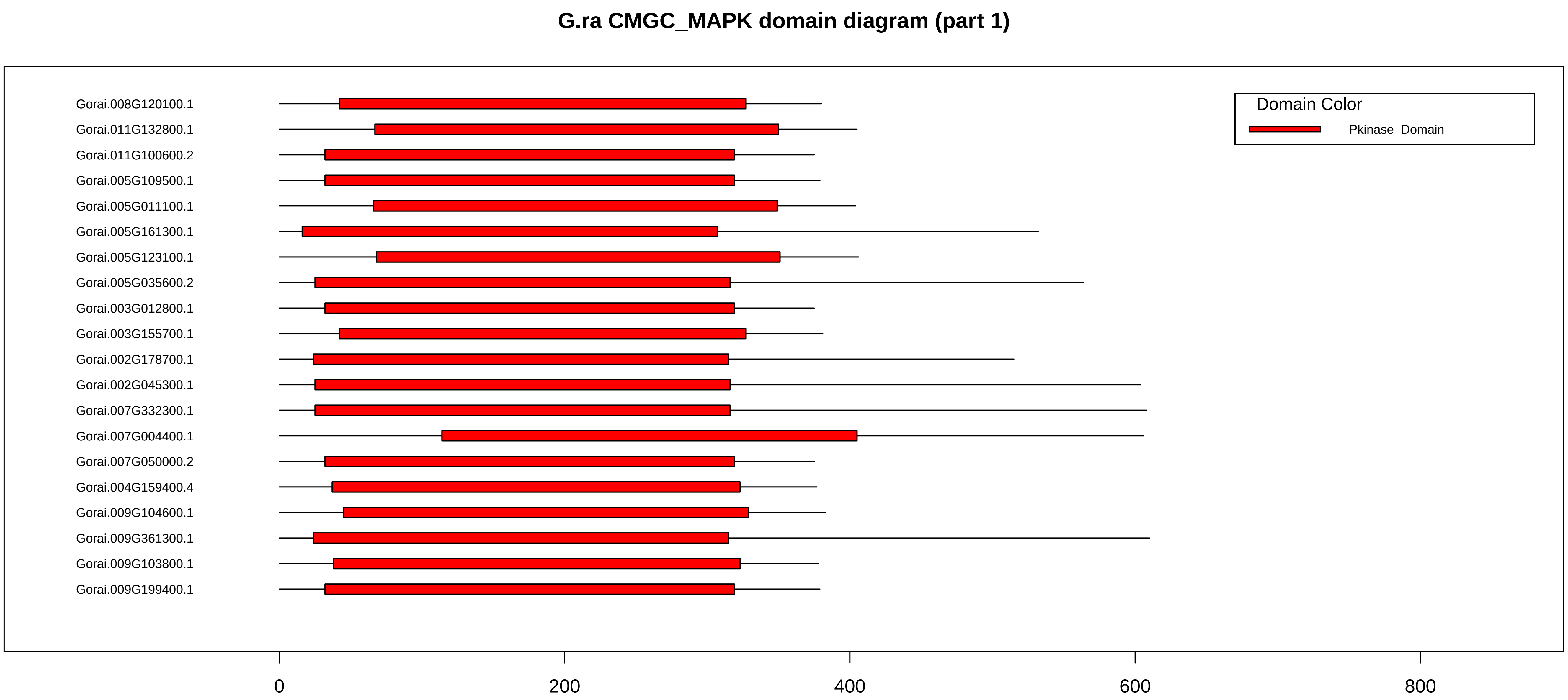
G.ra CMGC_MAPK domain diagram (part 1)
Gorai.008G120100.1
Gorai.011G132800.1
Gorai.011G100600.2
Gorai.005G109500.1
Gorai.005G011100.1
Gorai.005G161300.1
Gorai.005G123100.1
Gorai.005G035600.2
Gorai.003G012800.1
Gorai.003G155700.1
Gorai.002G178700.1
Gorai.002G045300.1
Gorai.007G332300.1
Gorai.007G004400.1
Gorai.007G050000.2
Gorai.004G159400.4
Gorai.009G104600.1
Gorai.009G361300.1
Gorai.009G103800.1
Gorai.009G199400.1
Domain Color
Pkinase Domain
0
200
400
600
800

G.ra CMGC_MAPK domain diagram (part 2)

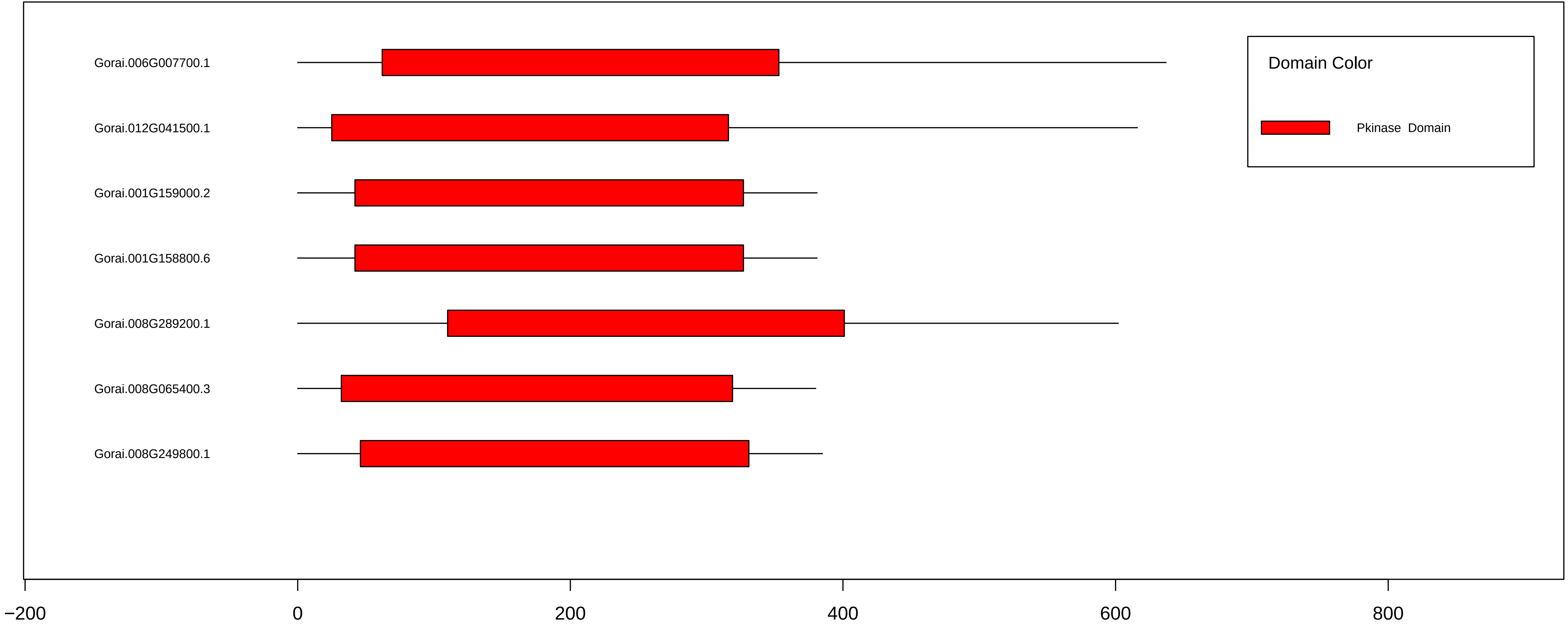

G.ra CMGC_Pl−Tthe domain diagram (all)

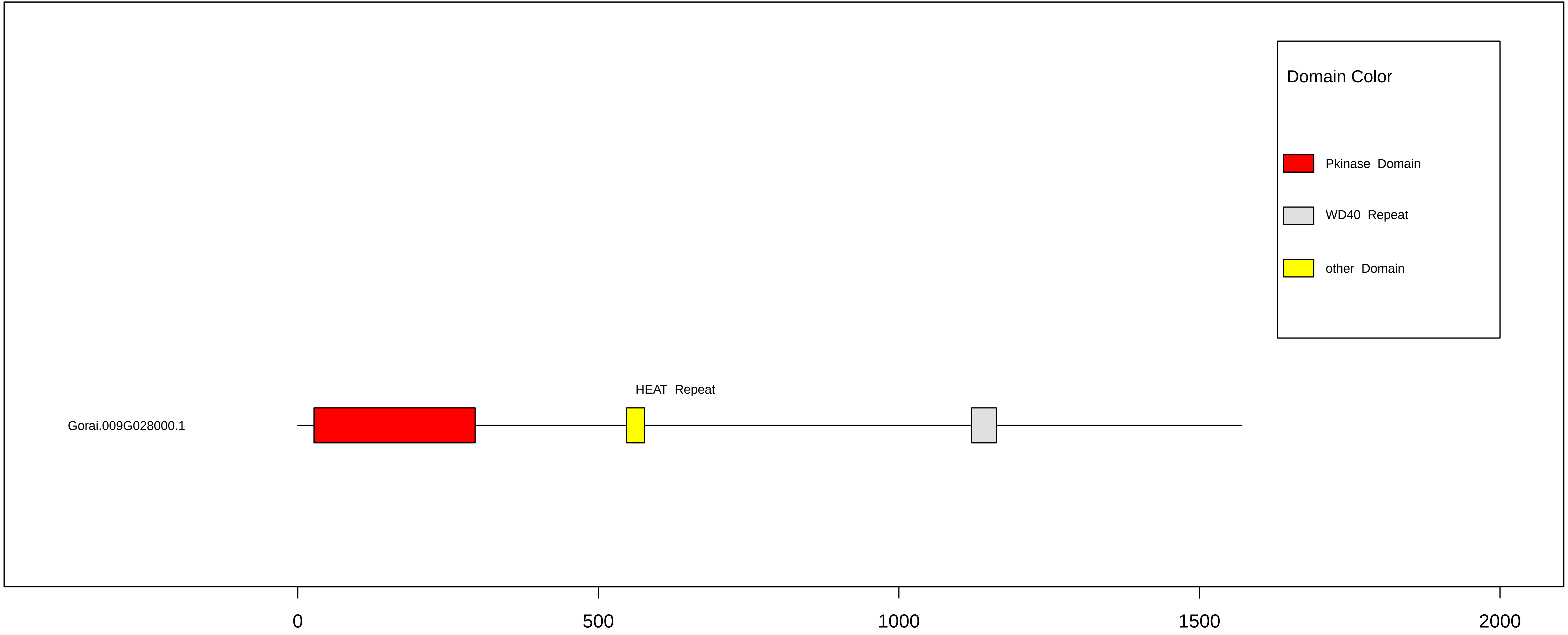

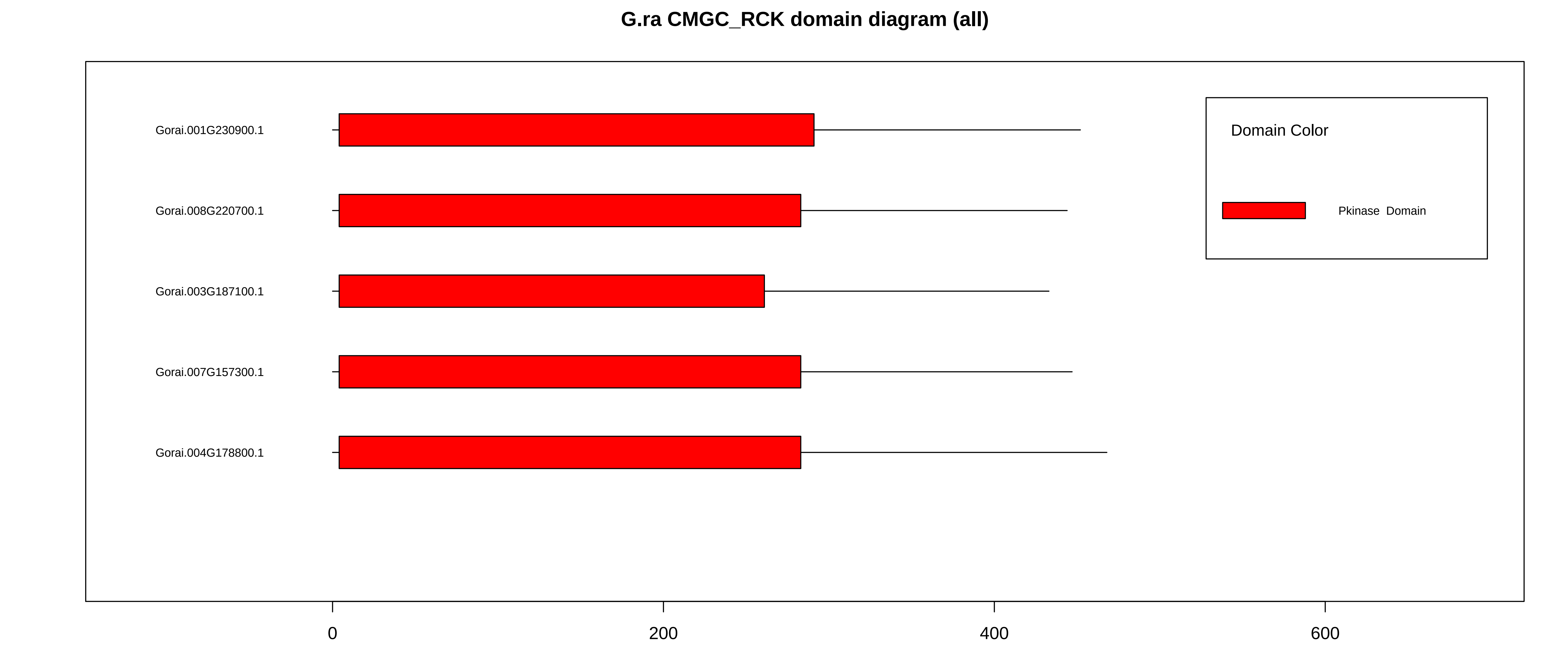

G.ra CMGC_RCK domain diagram (all)
Gorai.001G230900.1
Gorai.008G220700.1
Gorai.003G187100.1
Gorai.007G157300.1
Gorai.004G178800.1
Domain Color
Pkinase Domain
0
200
400
600

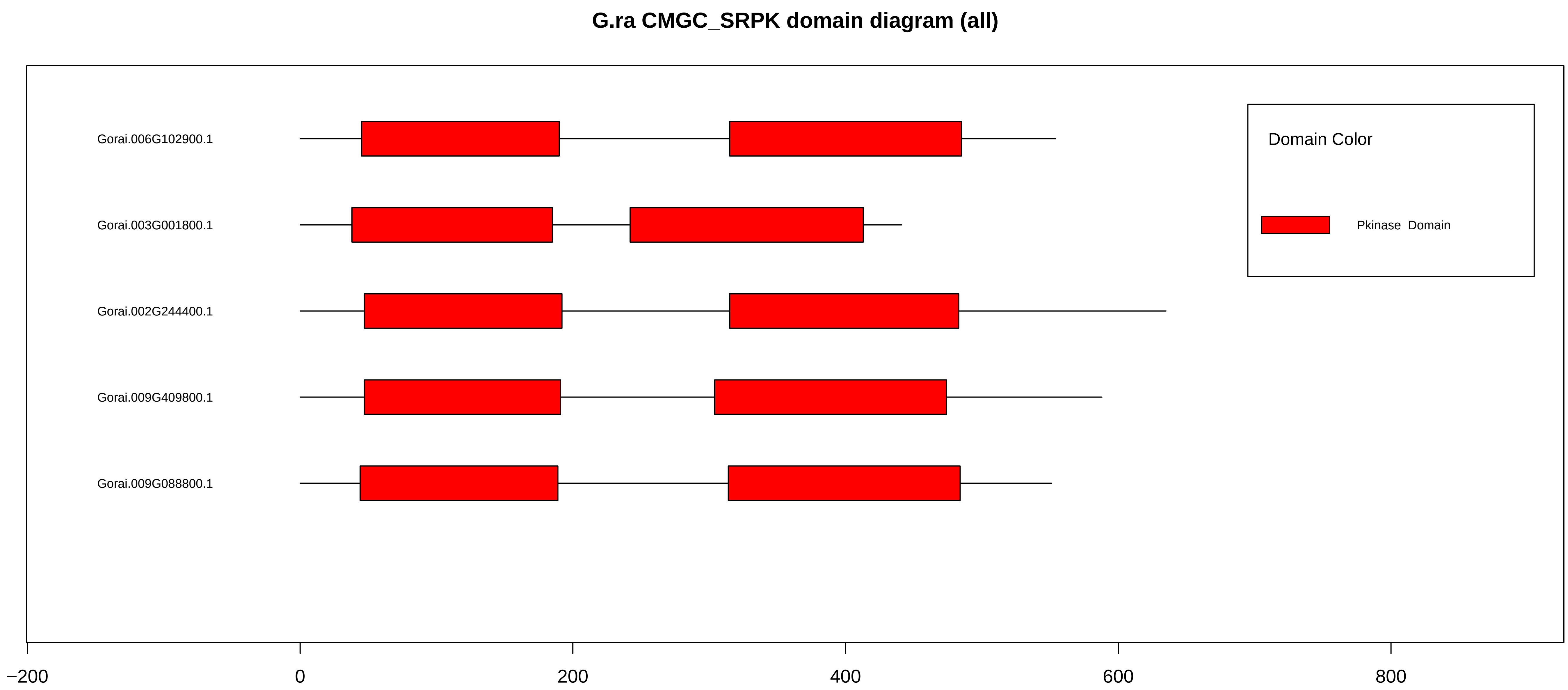
G.ra CMGC_SRPK domain diagram (all)
Gorai.006G102900.1
Gorai.003G001800.1
Gorai.002G244400.1
Gorai.009G409800.1
Gorai.009G088800.1
Domain Color
Pkinase Domain
−200
0
200
400
600
800

G.ra Group−PI−2 domain diagram (all)

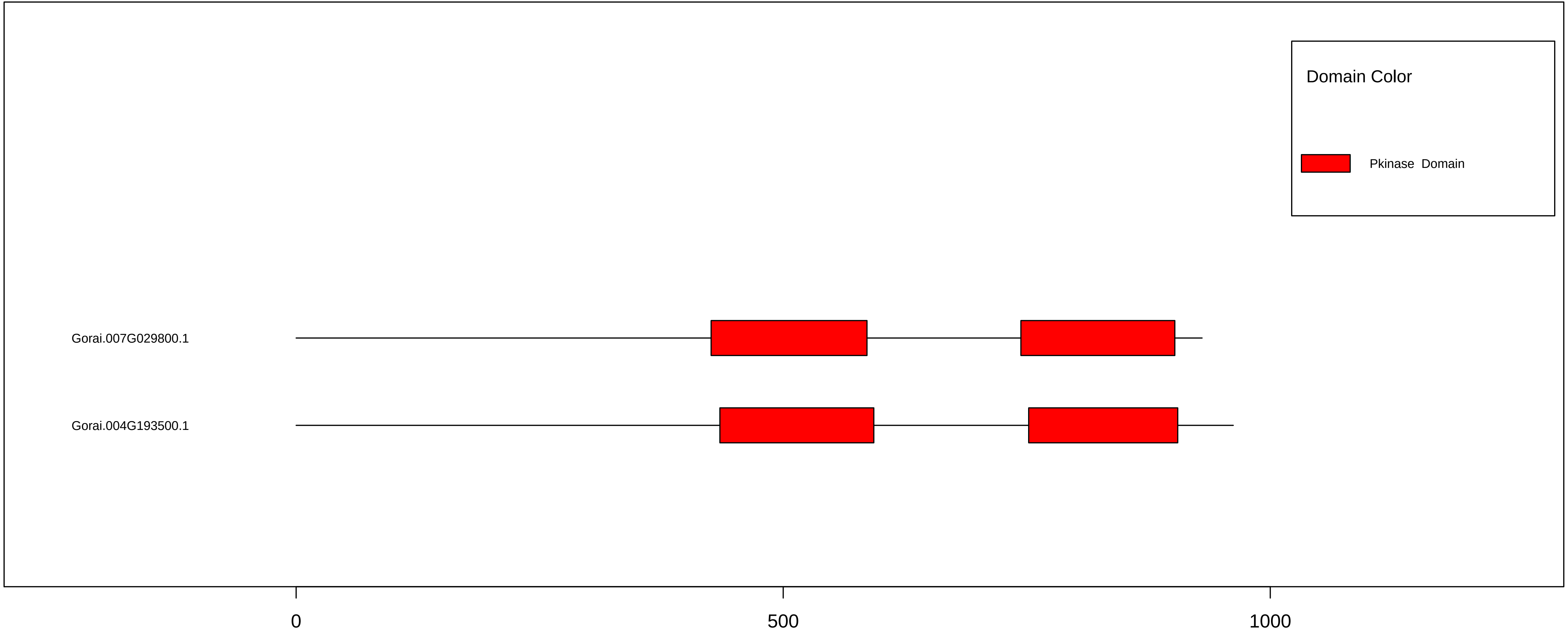

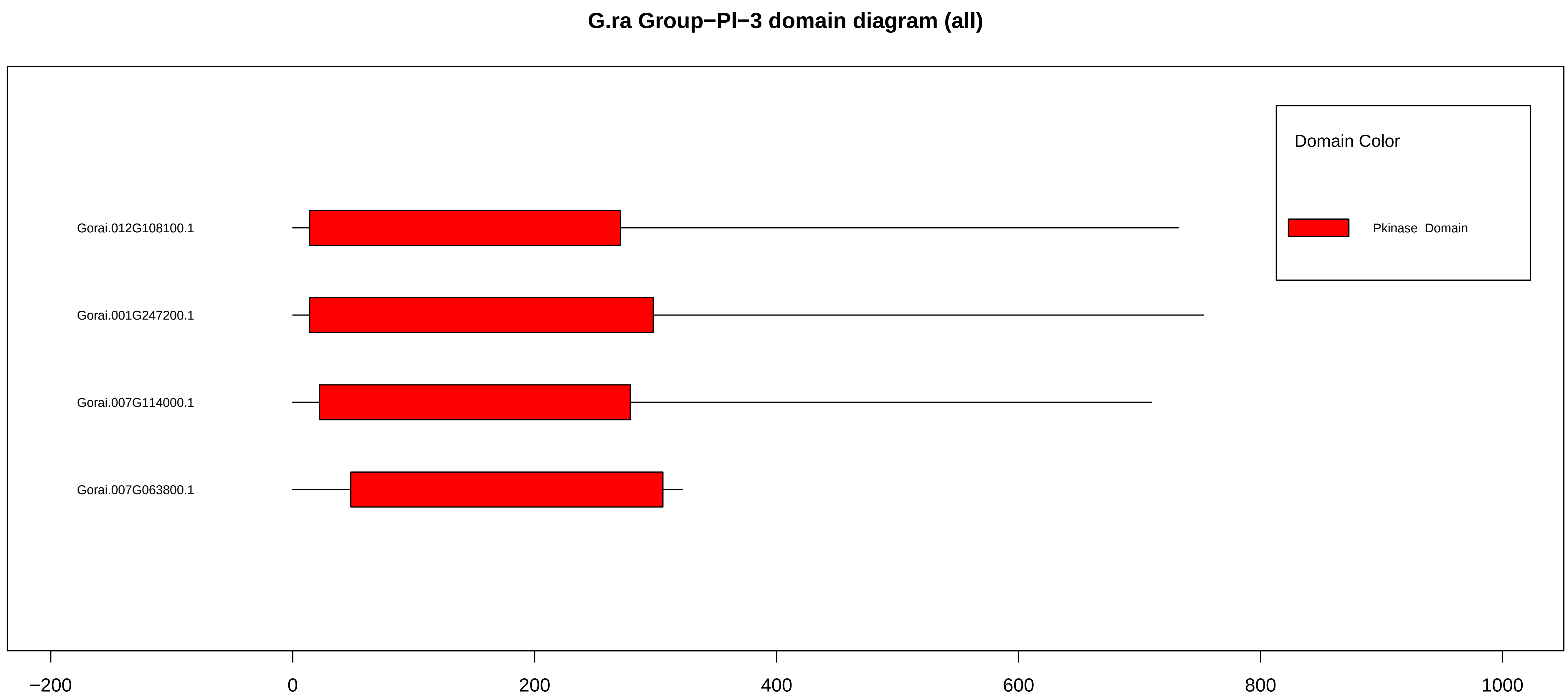
G.ra Group−PI−3 domain diagram (all)
Domain Color
Pkinase Domain
Gorai.012G108100.1
Gorai.001G247200.1
Gorai.007G114000.1
Gorai.007G063800.1
−200
0
200
400
600
800
1000

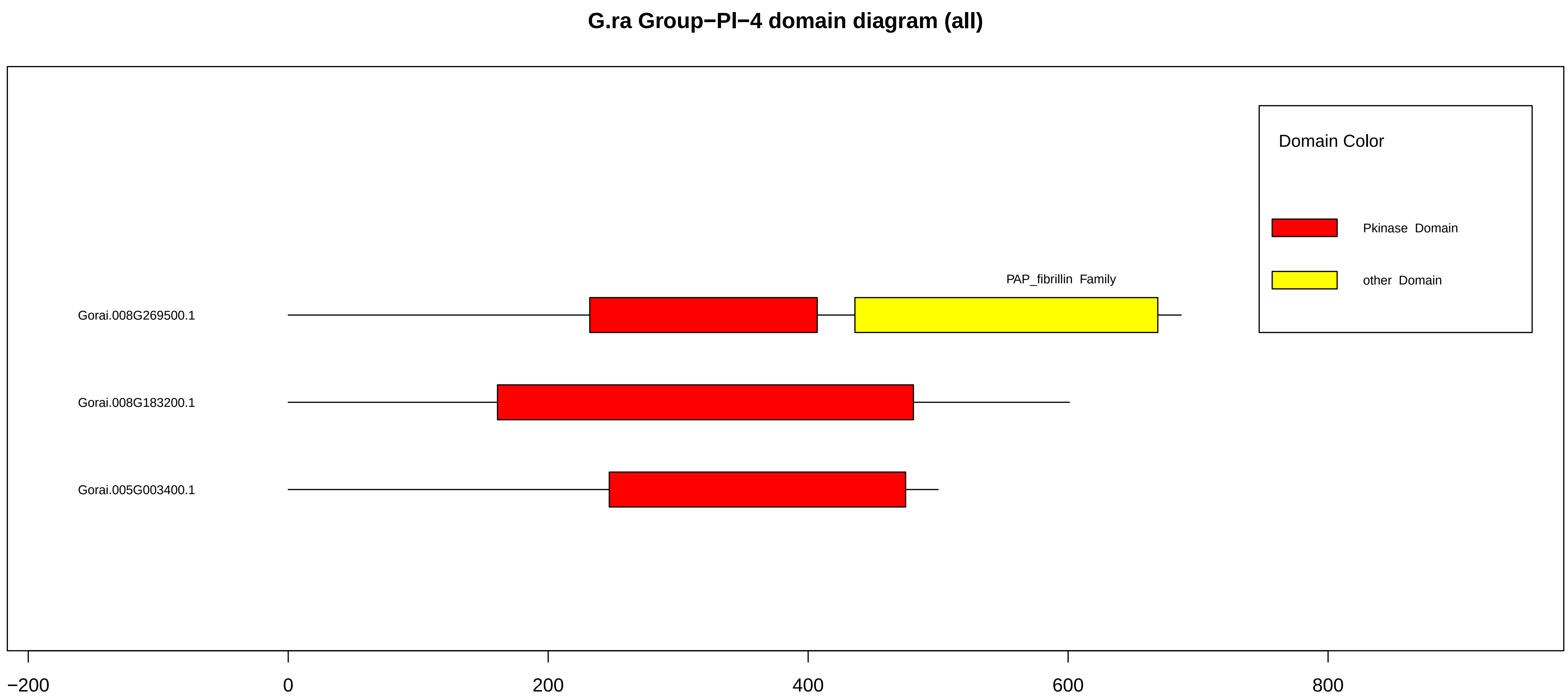
G.ra Group−PI−4 domain diagram (all)
Domain Color
Pkinase Domain
other Domain
PAP_fibrillin Family
Gorai.008G269500.1
Gorai.008G183200.1
Gorai.005G003400.1
−200
0
200
400
600
800

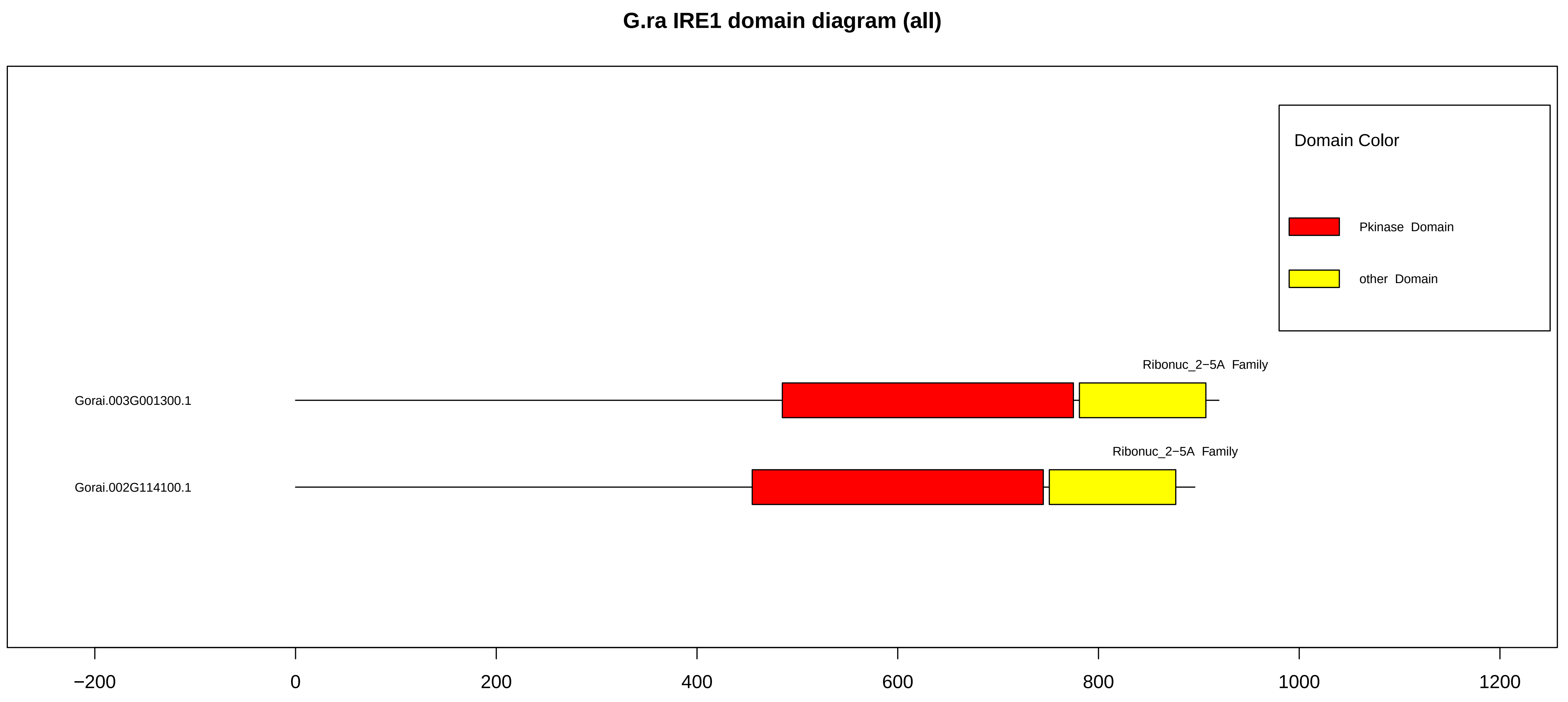

G.ra IRE1 domain diagram (all)
Domain Color
Pkinase Domain
other Domain
Ribonuc_2−5A Family
Gorai.003G001300.1
Ribonuc_2−5A Family
Gorai.002G114100.1
−200
0
200
400
600
800
1000
1200

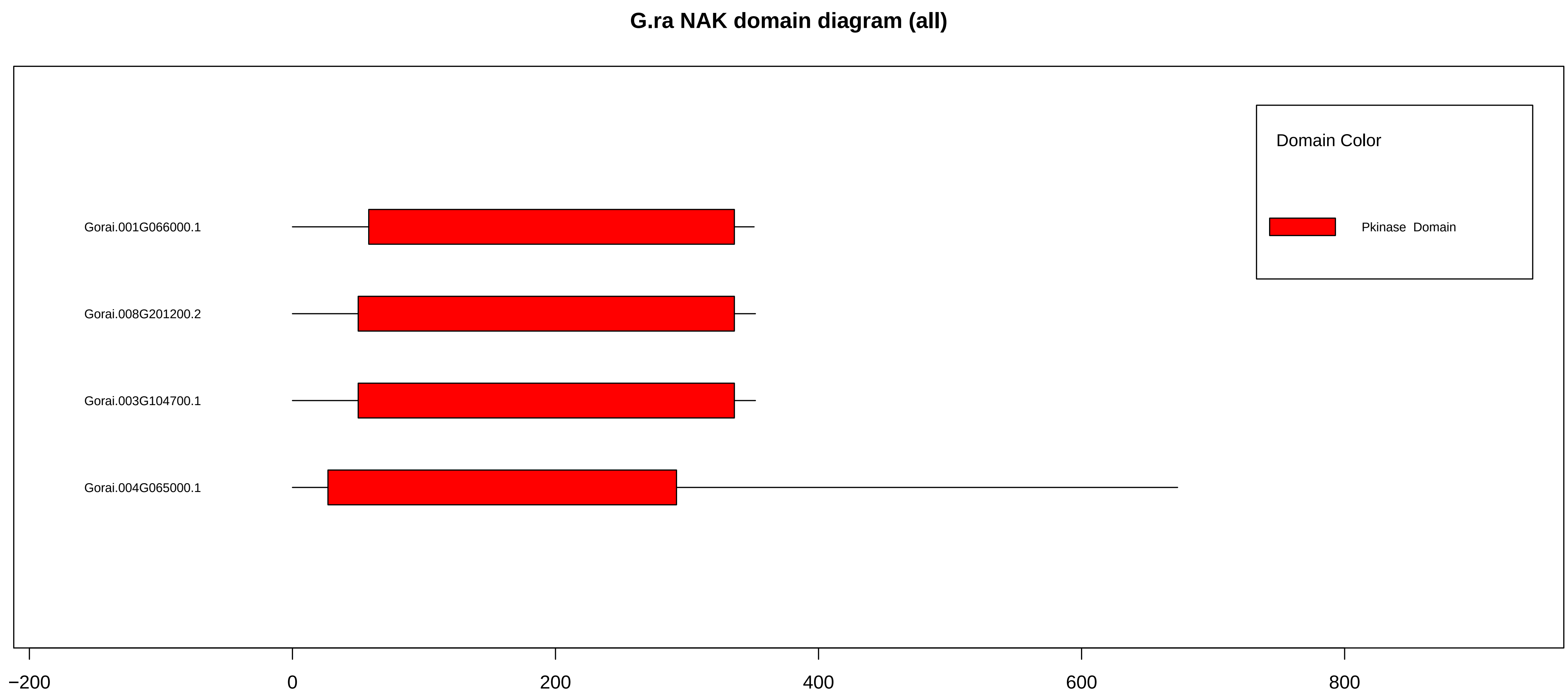
G.ra NAK domain diagram (all)
Gorai.001G066000.1
Gorai.008G201200.2
Gorai.003G104700.1
Gorai.004G065000.1
Domain Color
Pkinase Domain
−200
0
200
400
600
800

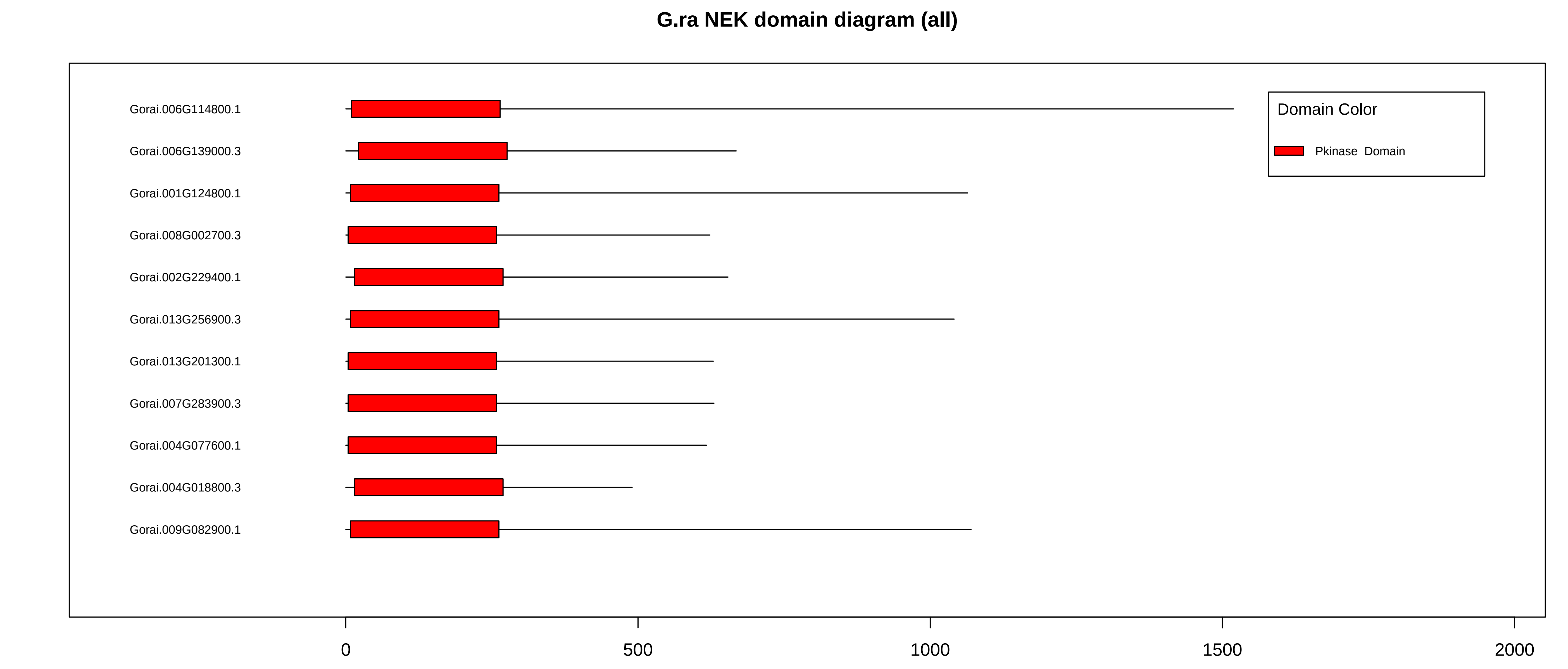
G.ra NEK domain diagram (all)
Domain Color
Pkinase Domain
Gorai.006G114800.1
Gorai.006G139000.3
Gorai.001G124800.1
Gorai.008G002700.3
Gorai.002G229400.1
Gorai.013G256900.3
Gorai.013G201300.1
Gorai.007G283900.3
Gorai.004G077600.1
Gorai.004G018800.3
Gorai.009G082900.1
0
500
1000
1500
2000

G.ra PEK_GCN2 domain diagram (all)

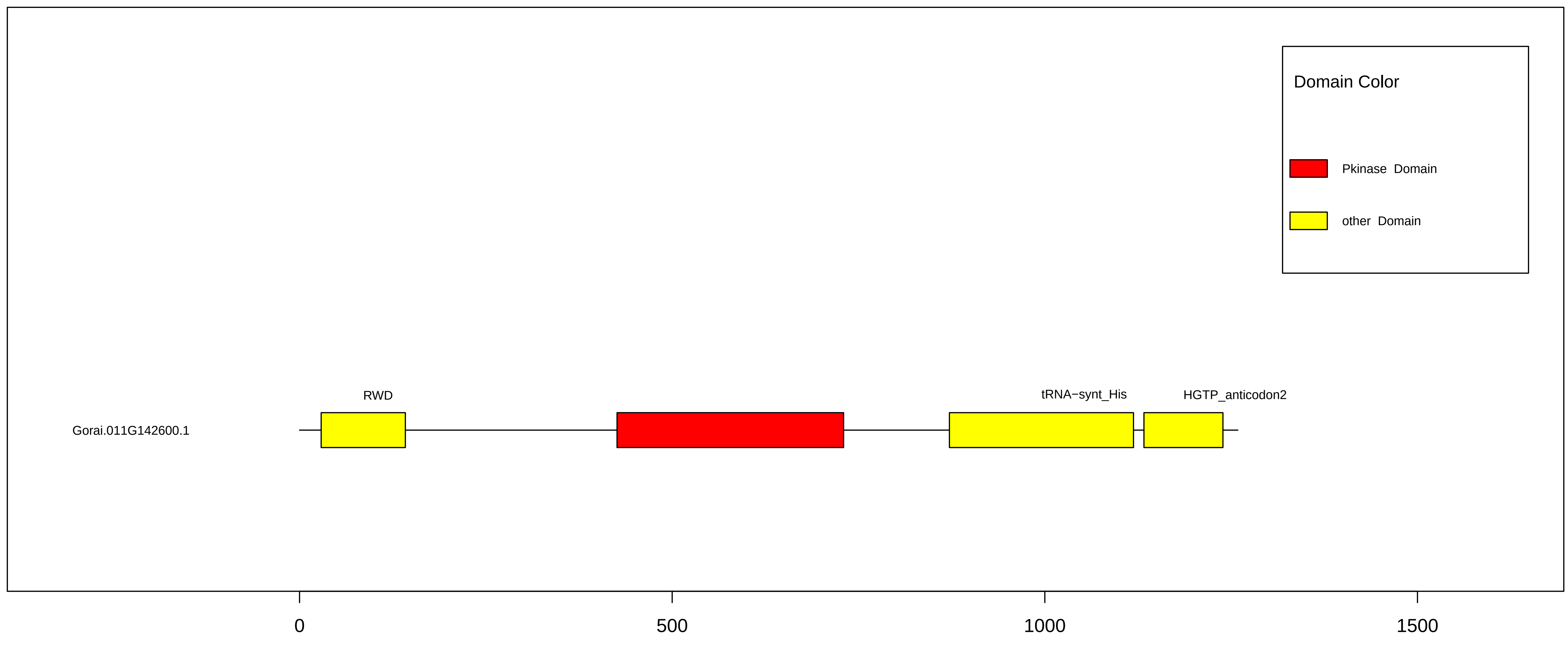

G.ra PEK_PEK domain diagram (all)

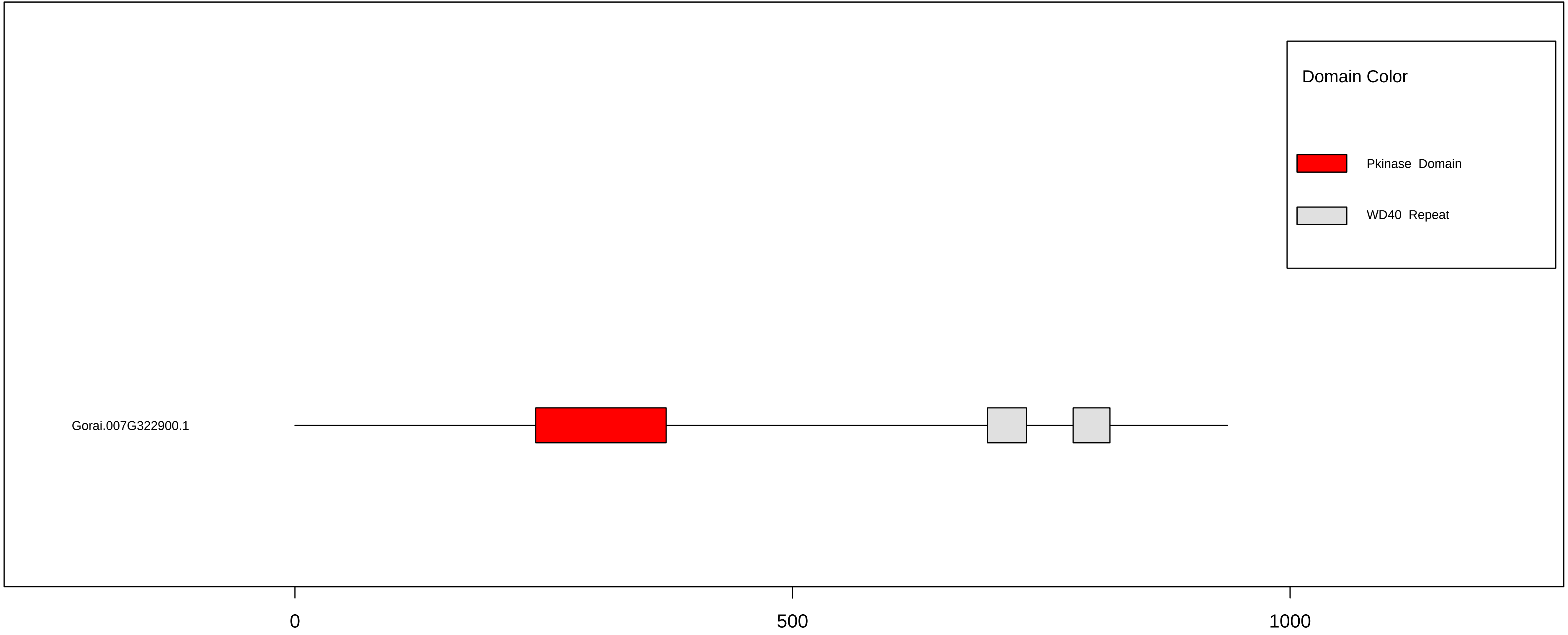

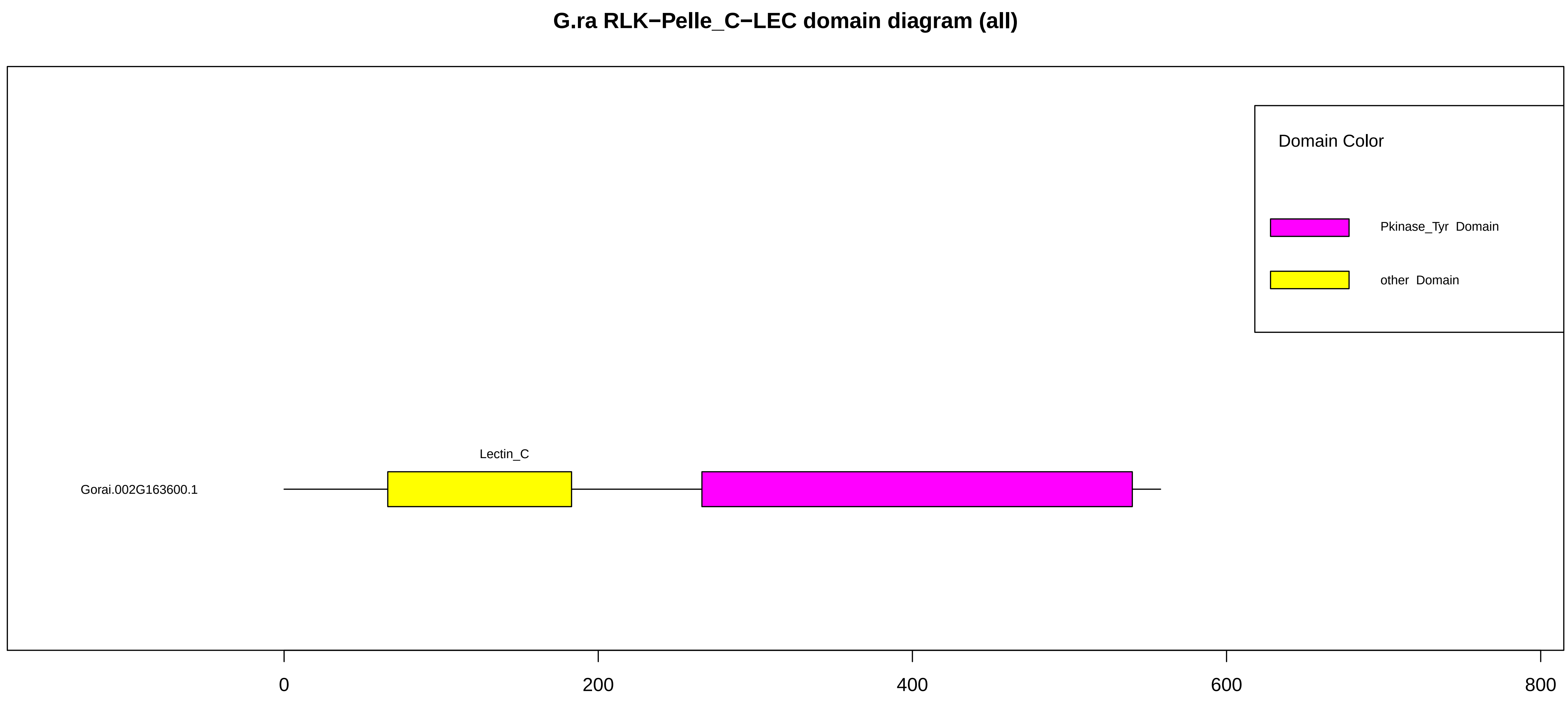
G.ra RLK−Pelle_C−LEC domain diagram (all)
Domain Color
Pkinase_Tyr Domain
other Domain
Lectin_C
Gorai.002G163600.1
0
200
400
600
800

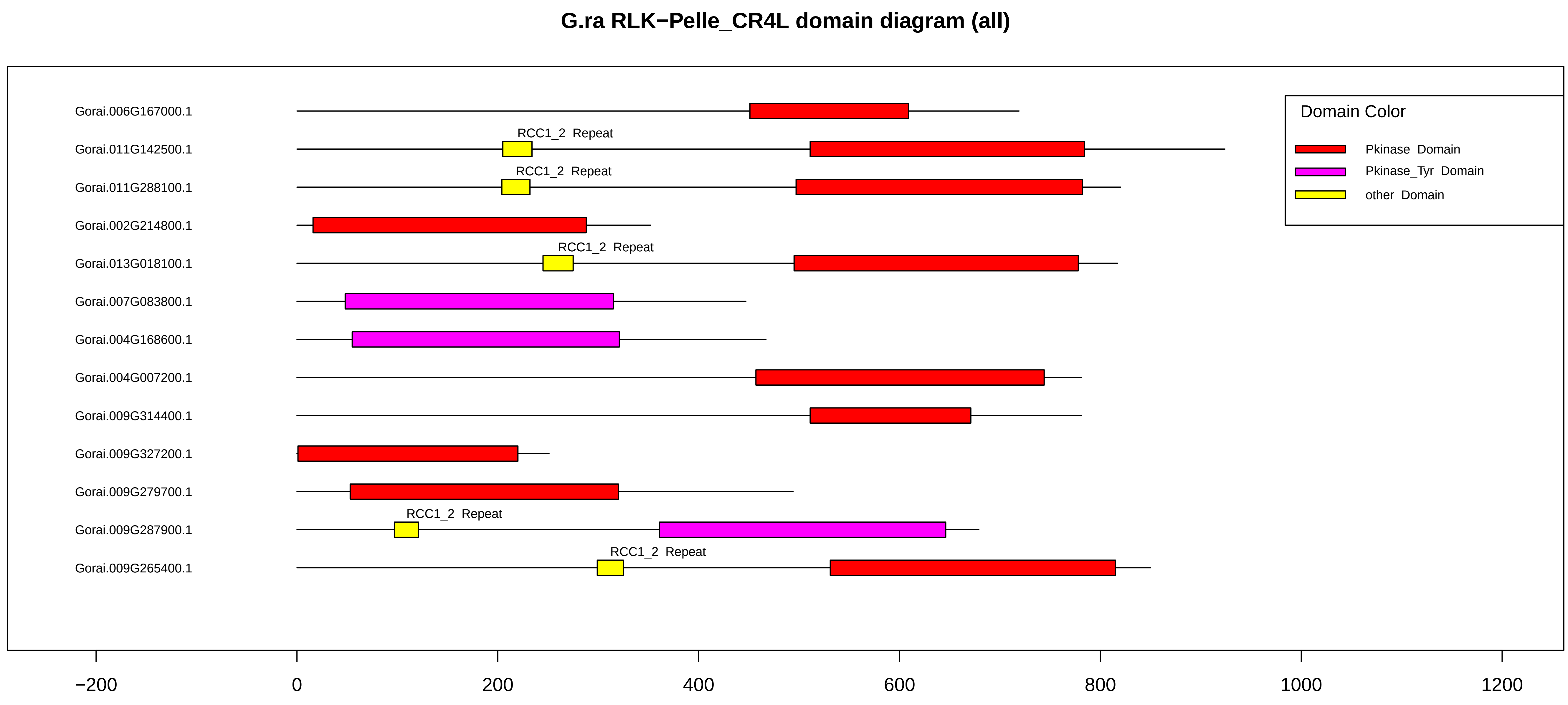
G.ra RLK−Pelle_CR4L domain diagram (all)
Gorai.006G167000.1
Gorai.011G142500.1
Gorai.011G288100.1
Gorai.002G214800.1
Gorai.013G018100.1
Gorai.007G083800.1
Gorai.004G168600.1
Gorai.004G007200.1
Gorai.009G314400.1
Gorai.009G327200.1
Gorai.009G279700.1
Gorai.009G287900.1
Gorai.009G265400.1
RCC1_2 Repeat
RCC1_2 Repeat
RCC1_2 Repeat
RCC1_2 Repeat
RCC1_2 Repeat
Domain Color
Pkinase Domain
Pkinase_Tyr Domain
other Domain
−200
0
200
400
600
800
1000
1200

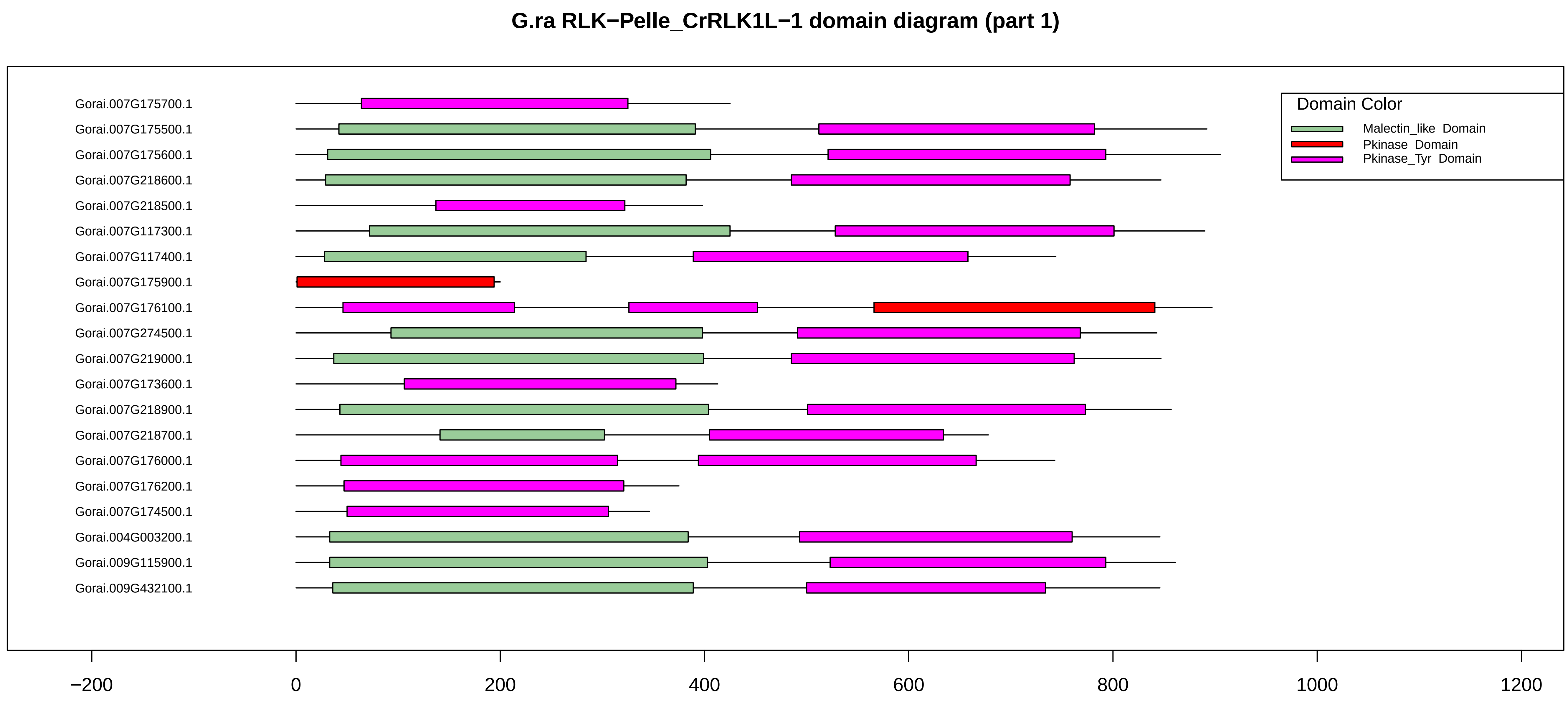
G.ra RLK−Pelle_CrRLK1L−1 domain diagram (part 1)
Domain Color
Malectin_like Domain
Pkinase Domain
Pkinase_Tyr Domain
Gorai.007G175700.1
Gorai.007G175500.1
Gorai.007G175600.1
Gorai.007G218600.1
Gorai.007G218500.1
Gorai.007G117300.1
Gorai.007G117400.1
Gorai.007G175900.1
Gorai.007G176100.1
Gorai.007G274500.1
Gorai.007G219000.1
Gorai.007G173600.1
Gorai.007G218900.1
Gorai.007G218700.1
Gorai.007G176000.1
Gorai.007G176200.1
Gorai.007G174500.1
Gorai.004G003200.1
Gorai.009G115900.1
Gorai.009G432100.1
−200
0
200
400
600
800
1000
1200

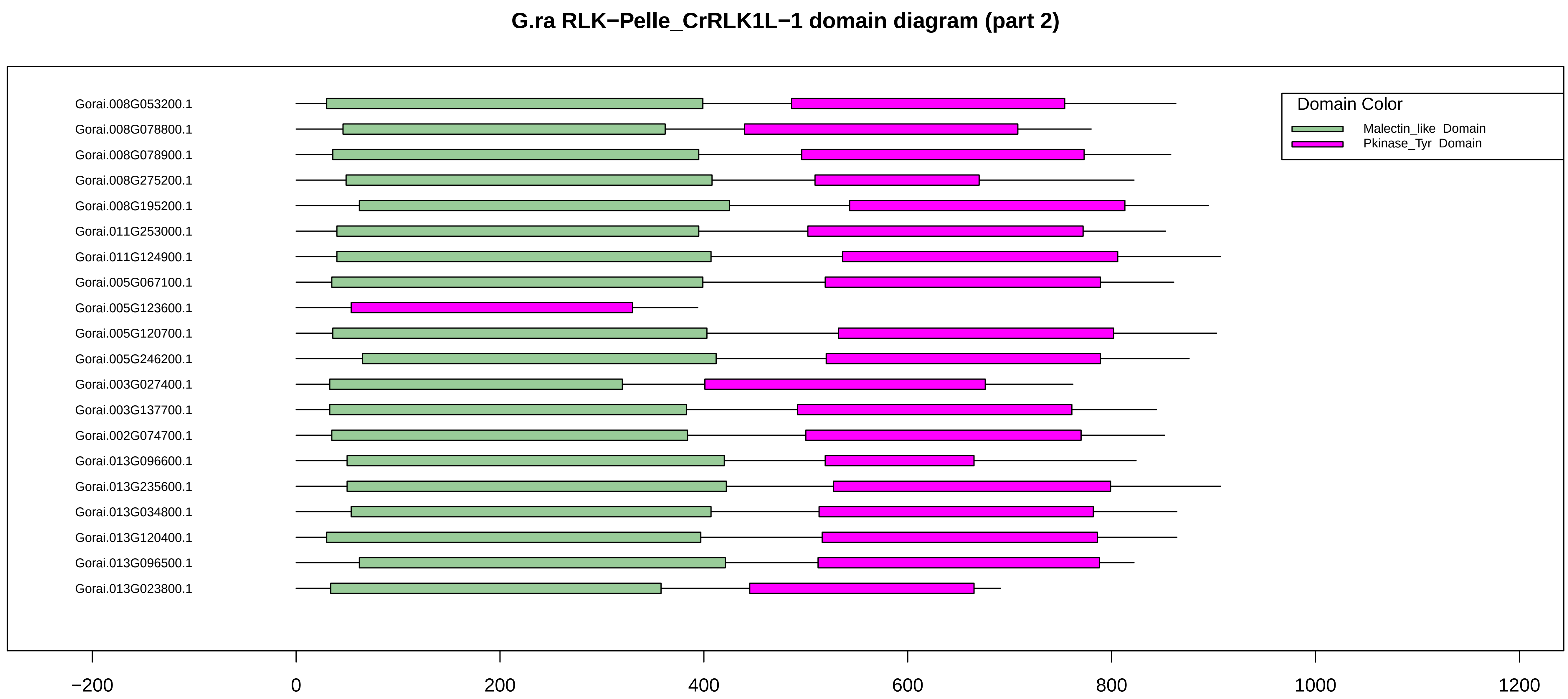
G.ra RLK−Pelle_CrRLK1L−1 domain diagram (part 2)
Gorai.008G053200.1
Gorai.008G078800.1
Gorai.008G078900.1
Gorai.008G275200.1
Gorai.008G195200.1
Gorai.011G253000.1
Gorai.011G124900.1
Gorai.005G067100.1
Gorai.005G123600.1
Gorai.005G120700.1
Gorai.005G246200.1
Gorai.003G027400.1
Gorai.003G137700.1
Gorai.002G074700.1
Gorai.013G096600.1
Gorai.013G235600.1
Gorai.013G034800.1
Gorai.013G120400.1
Gorai.013G096500.1
Gorai.013G023800.1
Domain Color
Malectin_like Domain
Pkinase_Tyr Domain
−200
0
200
400
600
800
1000
1200

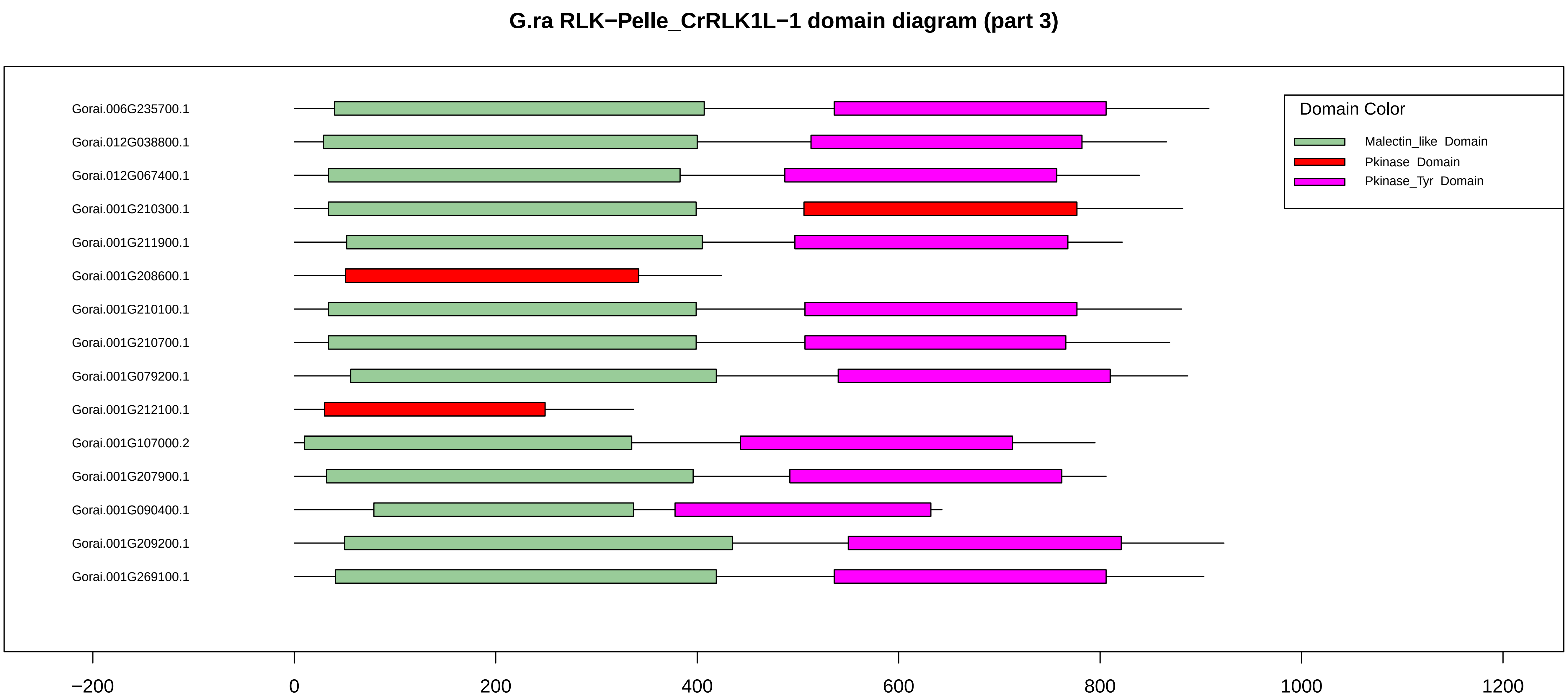

G.ra RLK−Pelle_CrRLK1L−1 domain diagram (part 3)
Gorai.006G235700.1
Gorai.012G038800.1
Gorai.012G067400.1
Gorai.001G210300.1
Gorai.001G211900.1
Gorai.001G208600.1
Gorai.001G210100.1
Gorai.001G210700.1
Gorai.001G079200.1
Gorai.001G212100.1
Gorai.001G107000.2
Gorai.001G207900.1
Gorai.001G090400.1
Gorai.001G209200.1
Gorai.001G269100.1
Domain Color
Malectin_like Domain
Pkinase Domain
Pkinase_Tyr Domain
−200
0
200
400
600
800
1000
1200

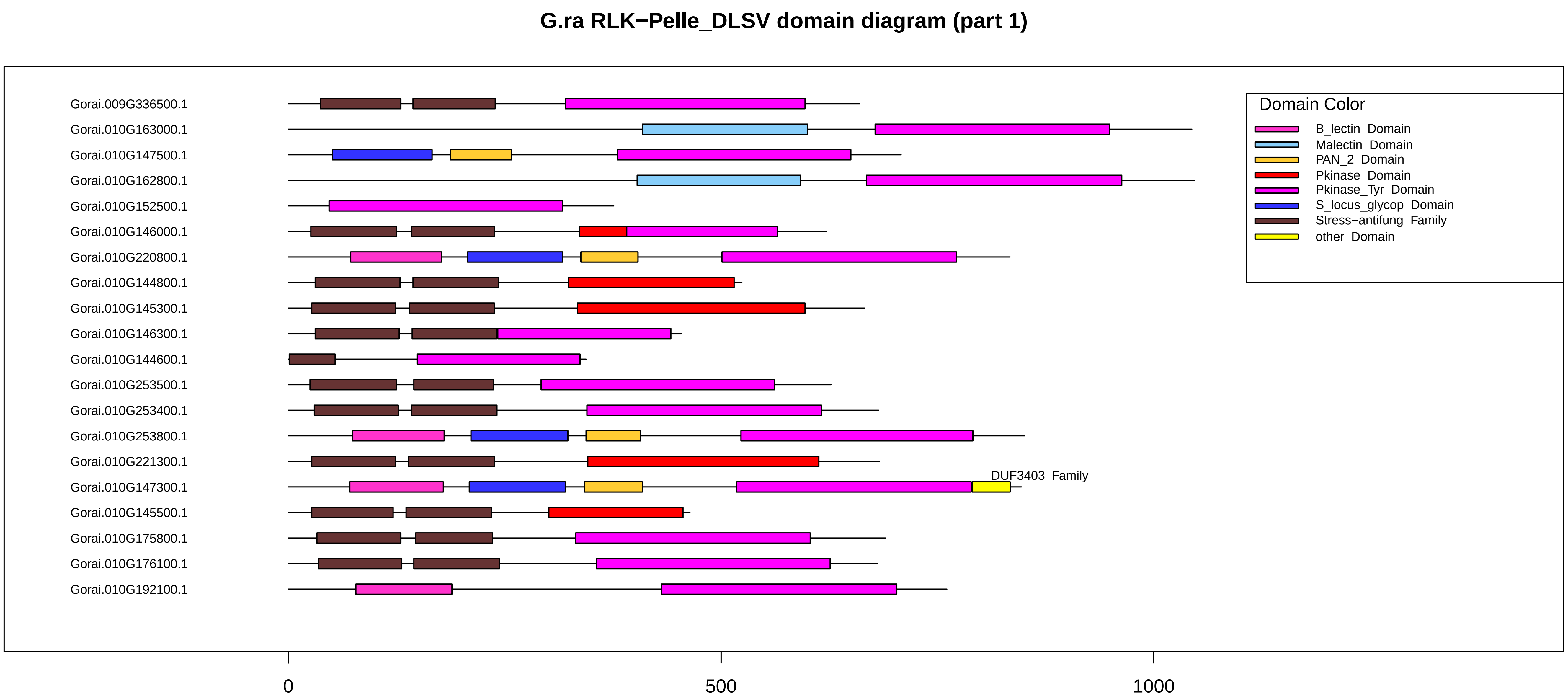
G.ra RLK−Pelle_DLSV domain diagram (part 1)
Gorai.009G336500.1
Gorai.010G163000.1
Gorai.010G147500.1
Gorai.010G162800.1
Gorai.010G152500.1
Gorai.010G146000.1
Gorai.010G220800.1
Gorai.010G144800.1
Gorai.010G145300.1
Gorai.010G146300.1
Gorai.010G144600.1
Gorai.010G253500.1
Gorai.010G253400.1
Gorai.010G253800.1
Gorai.010G221300.1
Gorai.010G147300.1
Gorai.010G145500.1
Gorai.010G175800.1
Gorai.010G176100.1
Gorai.010G192100.1
DUF3403 Family
Domain Color
B_lectin Domain
Malectin Domain
PAN_2 Domain
Pkinase Domain
Pkinase_Tyr Domain
S_locus_glycop Domain
Stress−antifung Family
other Domain
0
500
1000

G.ra RLK−Pelle_DLSV domain diagram (part 2)

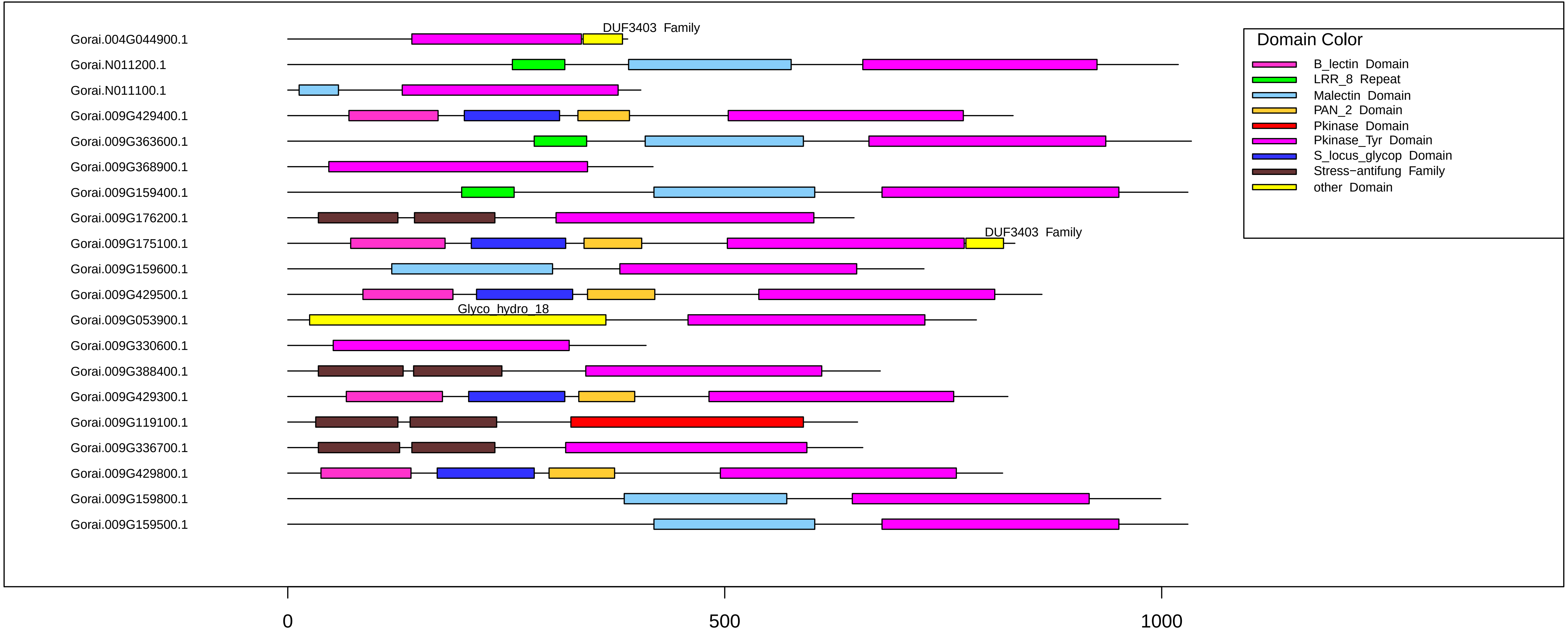

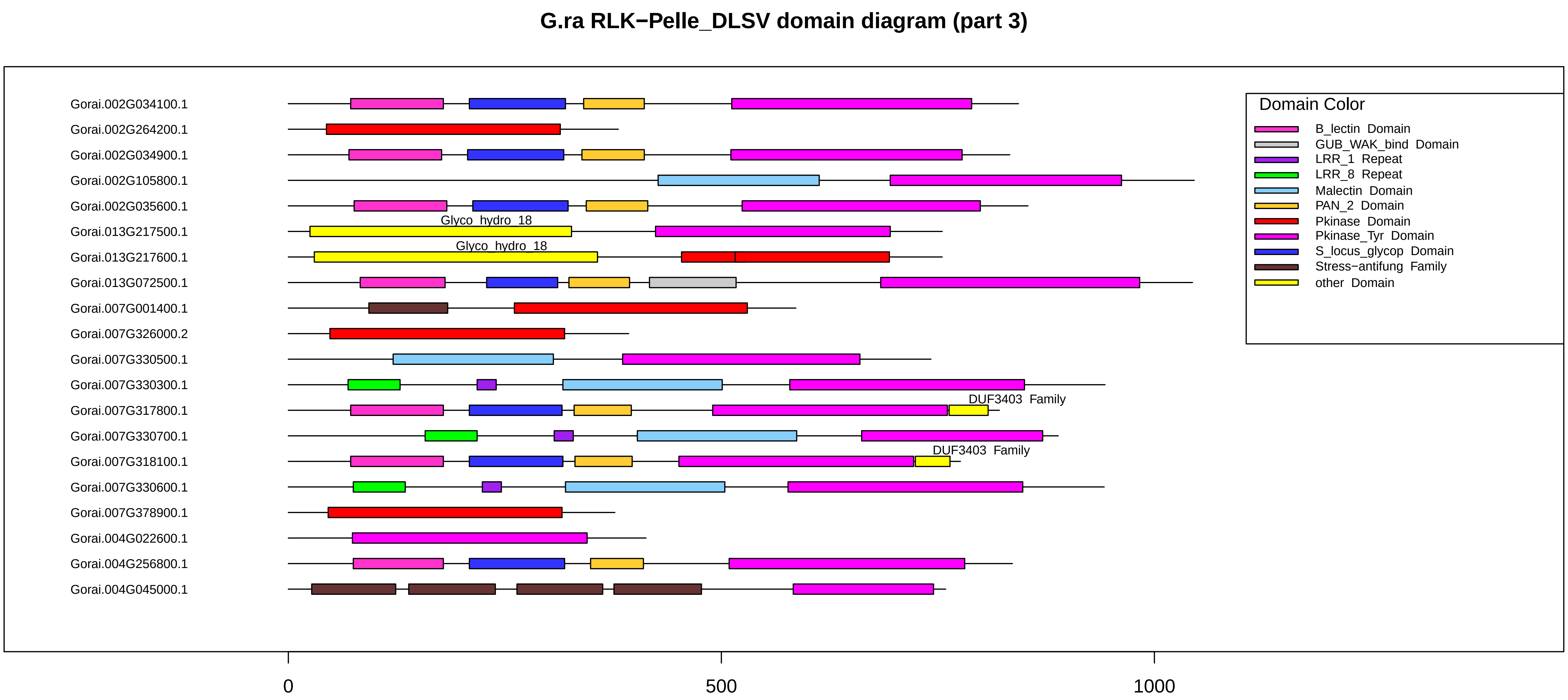
G.ra RLK−Pelle_DLSV domain diagram (part 3)
Gorai.002G034100.1
Gorai.002G264200.1
Gorai.002G034900.1
Gorai.002G105800.1
Gorai.002G035600.1
Gorai.013G217500.1
Gorai.013G217600.1
Gorai.013G072500.1
Gorai.007G001400.1
Gorai.007G326000.2
Gorai.007G330500.1
Gorai.007G330300.1
Gorai.007G317800.1
Gorai.007G330700.1
Gorai.007G318100.1
Gorai.007G330600.1
Gorai.007G378900.1
Gorai.004G022600.1
Gorai.004G256800.1
Gorai.004G045000.1
Glyco_hydro_18
Glyco_hydro_18
DUF3403 Family
DUF3403 Family
Domain Color
B_lectin Domain
GUB_WAK_bind Domain
LRR_1 Repeat
LRR_8 Repeat
Malectin Domain
PAN_2 Domain
Pkinase Domain
Pkinase_Tyr Domain
S_locus_glycop Domain
Stress−antifung Family
other Domain
0
500
1000

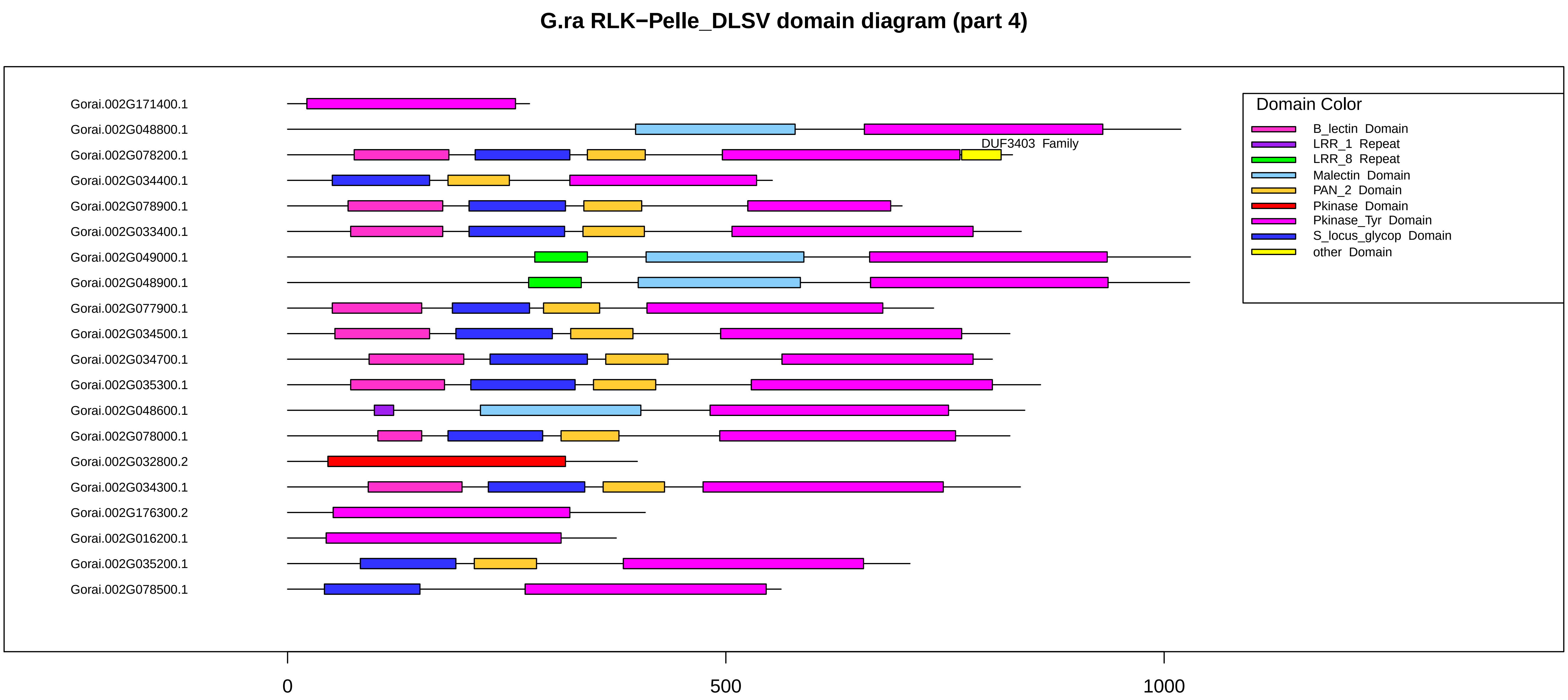
G.ra RLK−Pelle_DLSV domain diagram (part 4)
Gorai.002G171400.1
Gorai.002G048800.1
Gorai.002G078200.1
Gorai.002G034400.1
Gorai.002G078900.1
Gorai.002G033400.1
Gorai.002G049000.1
Gorai.002G048900.1
Gorai.002G077900.1
Gorai.002G034500.1
Gorai.002G034700.1
Gorai.002G035300.1
Gorai.002G048600.1
Gorai.002G078000.1
Gorai.002G032800.2
Gorai.002G034300.1
Gorai.002G176300.2
Gorai.002G016200.1
Gorai.002G035200.1
Gorai.002G078500.1
DUF3403 Family
0
500
1000
Domain Color
B_lectin Domain
LRR_1 Repeat
LRR_8 Repeat
Malectin Domain
PAN_2 Domain
Pkinase Domain
Pkinase_Tyr Domain
S_locus_glycop Domain
other Domain

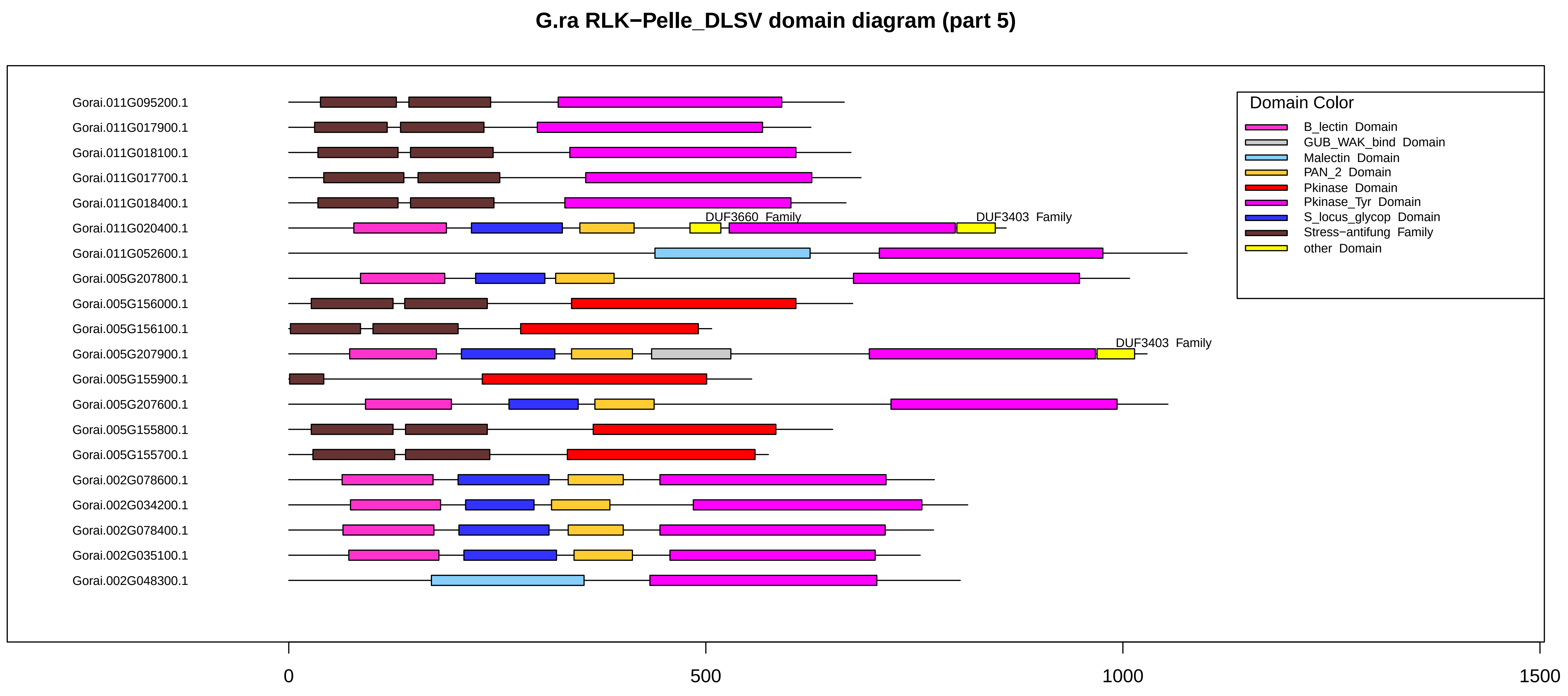
G.ra RLK−Pelle_DLSV domain diagram (part 5)
Gorai.011G095200.1
Gorai.011G017900.1
Gorai.011G018100.1
Gorai.011G017700.1
Gorai.011G018400.1
Gorai.011G020400.1
Gorai.011G052600.1
Gorai.005G207800.1
Gorai.005G156000.1
Gorai.005G156100.1
Gorai.005G207900.1
Gorai.005G155900.1
Gorai.005G207600.1
Gorai.005G155800.1
Gorai.005G155700.1
Gorai.002G078600.1
Gorai.002G034200.1
Gorai.002G078400.1
Gorai.002G035100.1
Gorai.002G048300.1
DUF3660 Family
DUF3403 Family
DUF3403 Family
Domain Color
B_lectin Domain
GUB_WAK_bind Domain
Malectin Domain
PAN_2 Domain
Pkinase Domain
Pkinase_Tyr Domain
S_locus_glycop Domain
Stress−antifung Family
other Domain
0
500
1000
1500

G.ra RLK−Pelle_DLSV domain diagram (part 6)

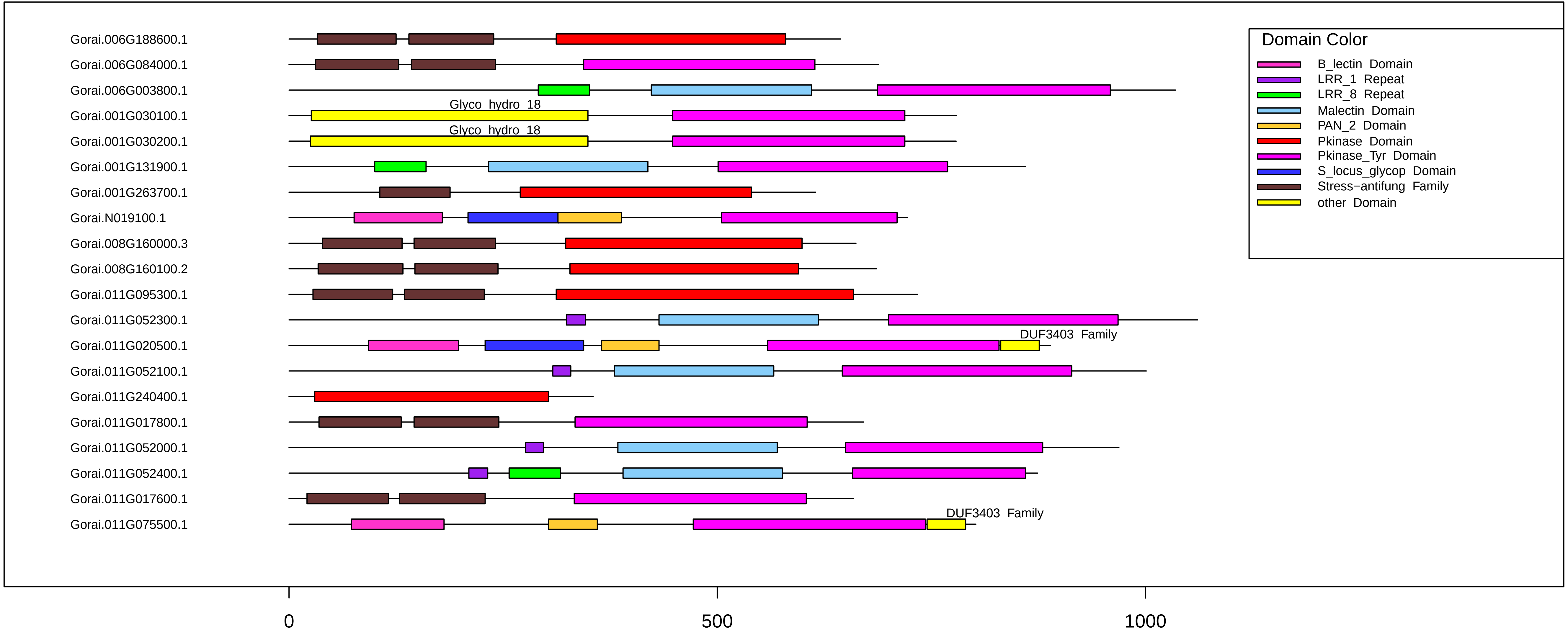

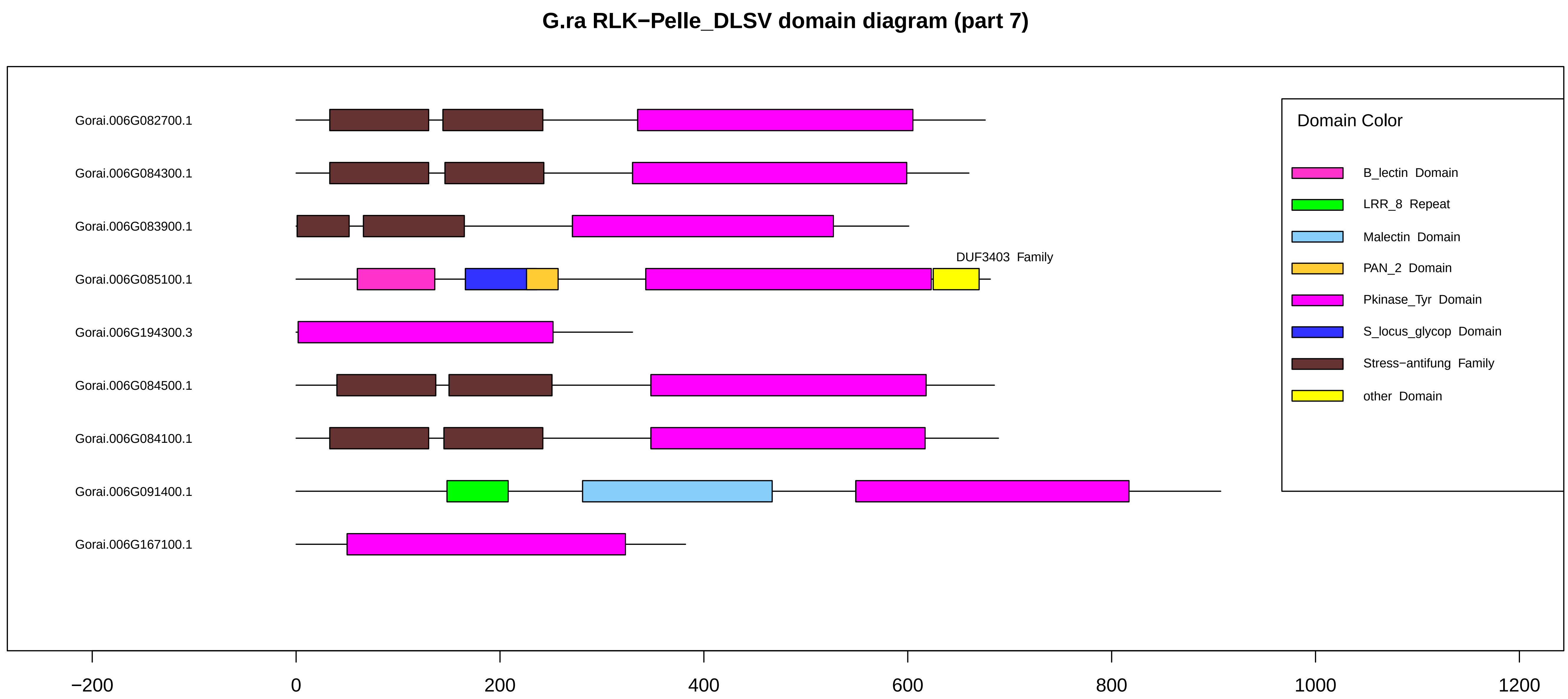
G.ra RLK−Pelle_DLSV domain diagram (part 7)
Gorai.006G082700.1
Gorai.006G084300.1
Gorai.006G083900.1
Gorai.006G085100.1
Gorai.006G194300.3
Gorai.006G084500.1
Gorai.006G084100.1
Gorai.006G091400.1
Gorai.006G167100.1
DUF3403 Family
Domain Color
B_lectin Domain
LRR_8 Repeat
Malectin Domain
PAN_2 Domain
Pkinase_Tyr Domain
S_locus_glycop Domain
Stress−antifung Family
other Domain
−200
0
200
400
600
800
1000
1200

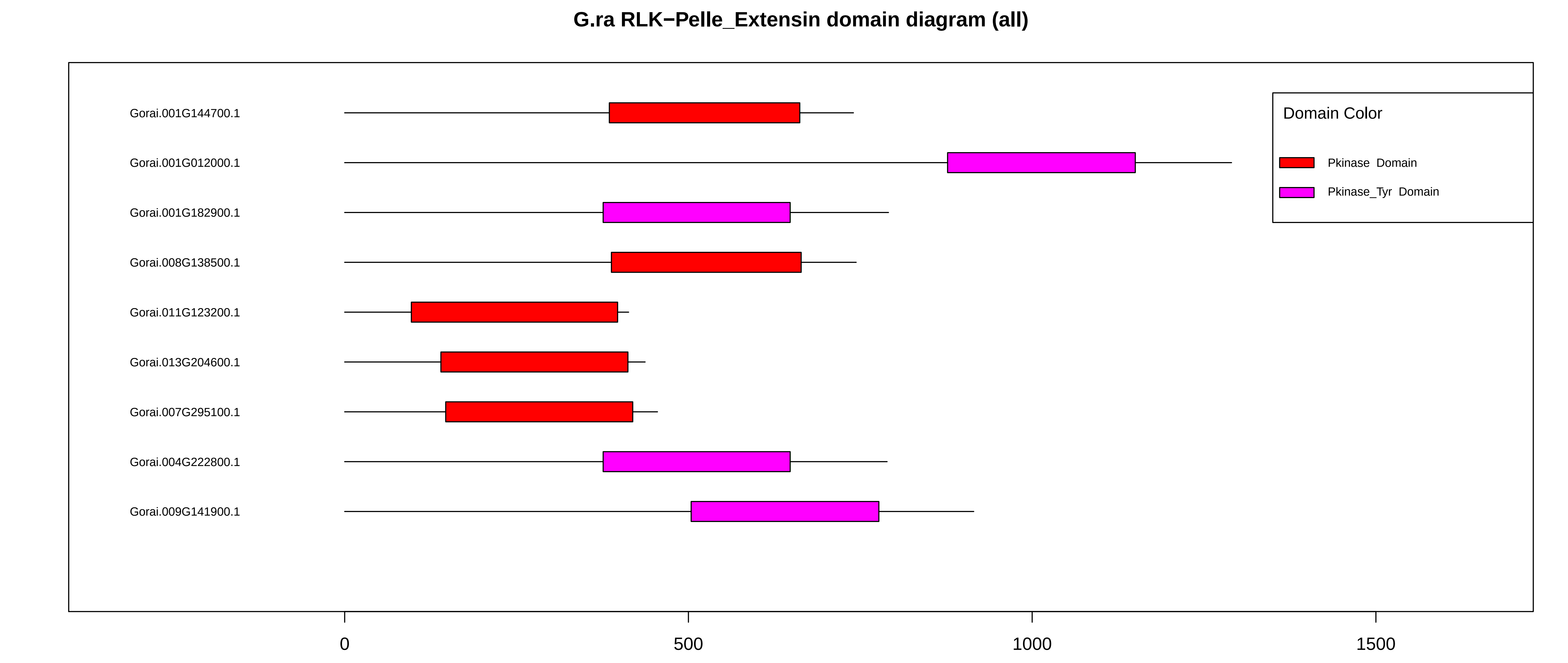

G.ra RLK−Pelle_Extensin domain diagram (all)
Gorai.001G144700.1
Gorai.001G012000.1
Gorai.001G182900.1
Gorai.008G138500.1
Gorai.011G123200.1
Gorai.013G204600.1
Gorai.007G295100.1
Gorai.004G222800.1
Gorai.009G141900.1
Domain Color
Pkinase Domain
Pkinase_Tyr Domain
0
500
1000
1500

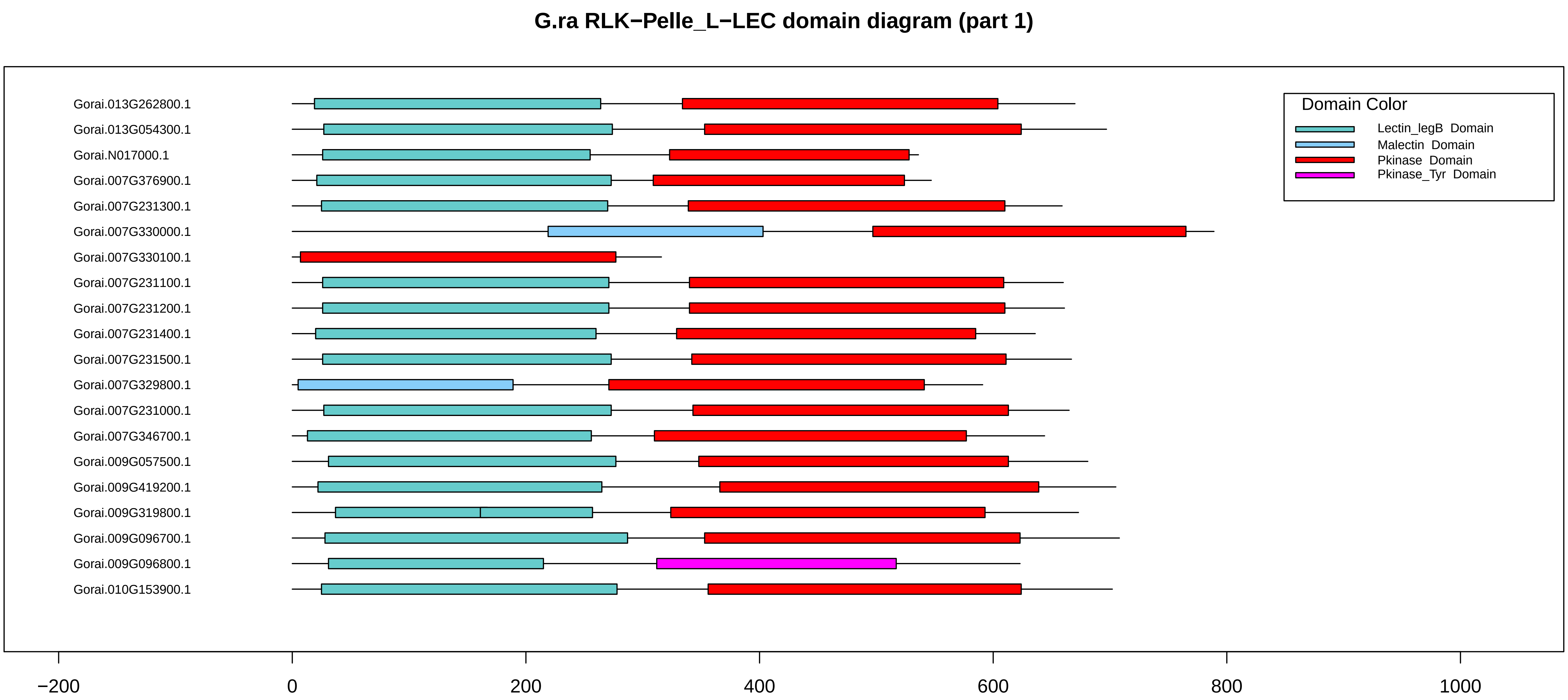
G.ra RLK−Pelle_L−LEC domain diagram (part 1)
Gorai.013G262800.1
Gorai.013G054300.1
Gorai.N017000.1
Gorai.007G376900.1
Gorai.007G231300.1
Gorai.007G330000.1
Gorai.007G330100.1
Gorai.007G231100.1
Gorai.007G231200.1
Gorai.007G231400.1
Gorai.007G231500.1
Gorai.007G329800.1
Gorai.007G231000.1
Gorai.007G346700.1
Gorai.009G057500.1
Gorai.009G419200.1
Gorai.009G319800.1
Gorai.009G096700.1
Gorai.009G096800.1
Gorai.010G153900.1
Domain Color
Lectin_legB Domain
Malectin Domain
Pkinase Domain
Pkinase_Tyr Domain
−200
0
200
400
600
800
1000

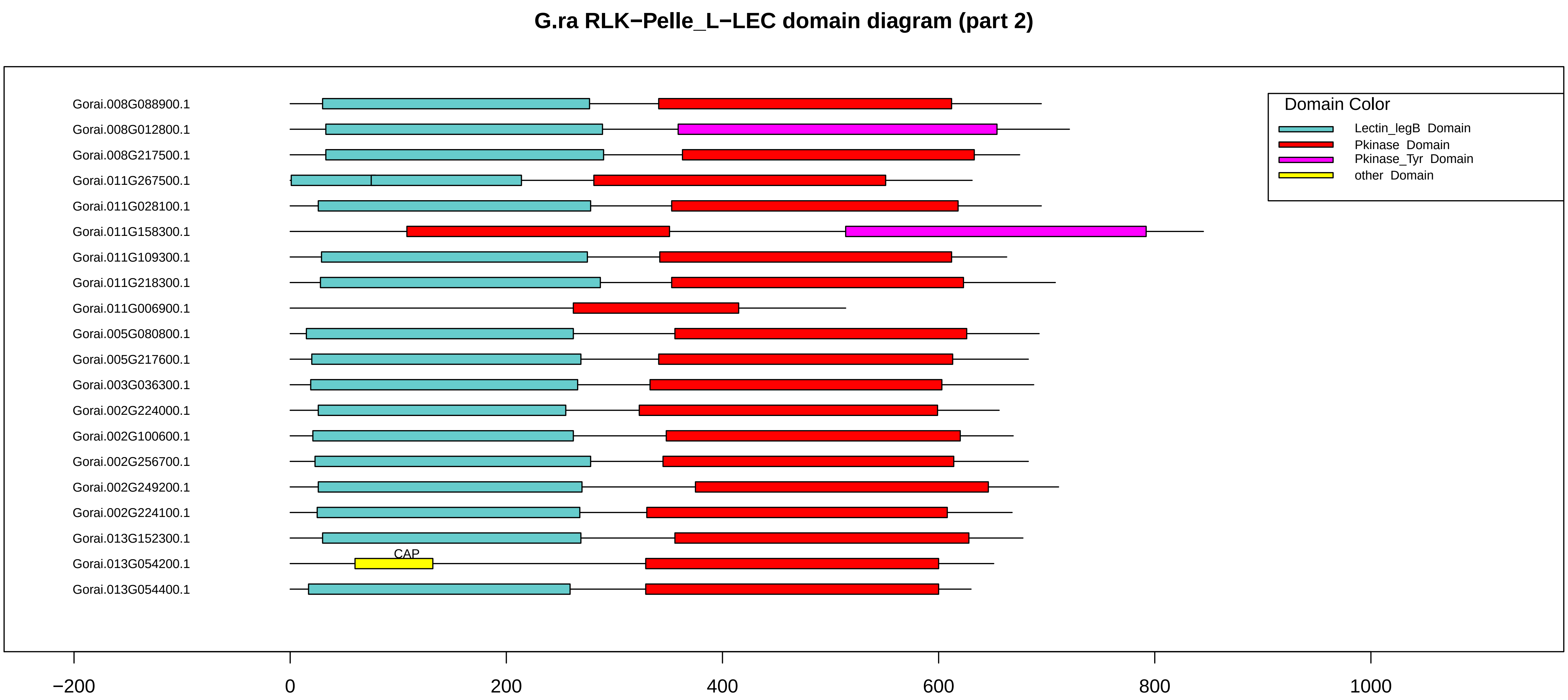
G.ra RLK−Pelle_L−LEC domain diagram (part 2)
Gorai.008G088900.1
Gorai.008G012800.1
Gorai.008G217500.1
Gorai.011G267500.1
Gorai.011G028100.1
Gorai.011G158300.1
Gorai.011G109300.1
Gorai.011G218300.1
Gorai.011G006900.1
Gorai.005G080800.1
Gorai.005G217600.1
Gorai.003G036300.1
Gorai.002G224000.1
Gorai.002G100600.1
Gorai.002G256700.1
Gorai.002G249200.1
Gorai.002G224100.1
Gorai.013G152300.1
Gorai.013G054200.1
Gorai.013G054400.1
CAP
Domain Color
Lectin_legB Domain
Pkinase Domain
Pkinase_Tyr Domain
other Domain
−200
0
200
400
600
800
1000

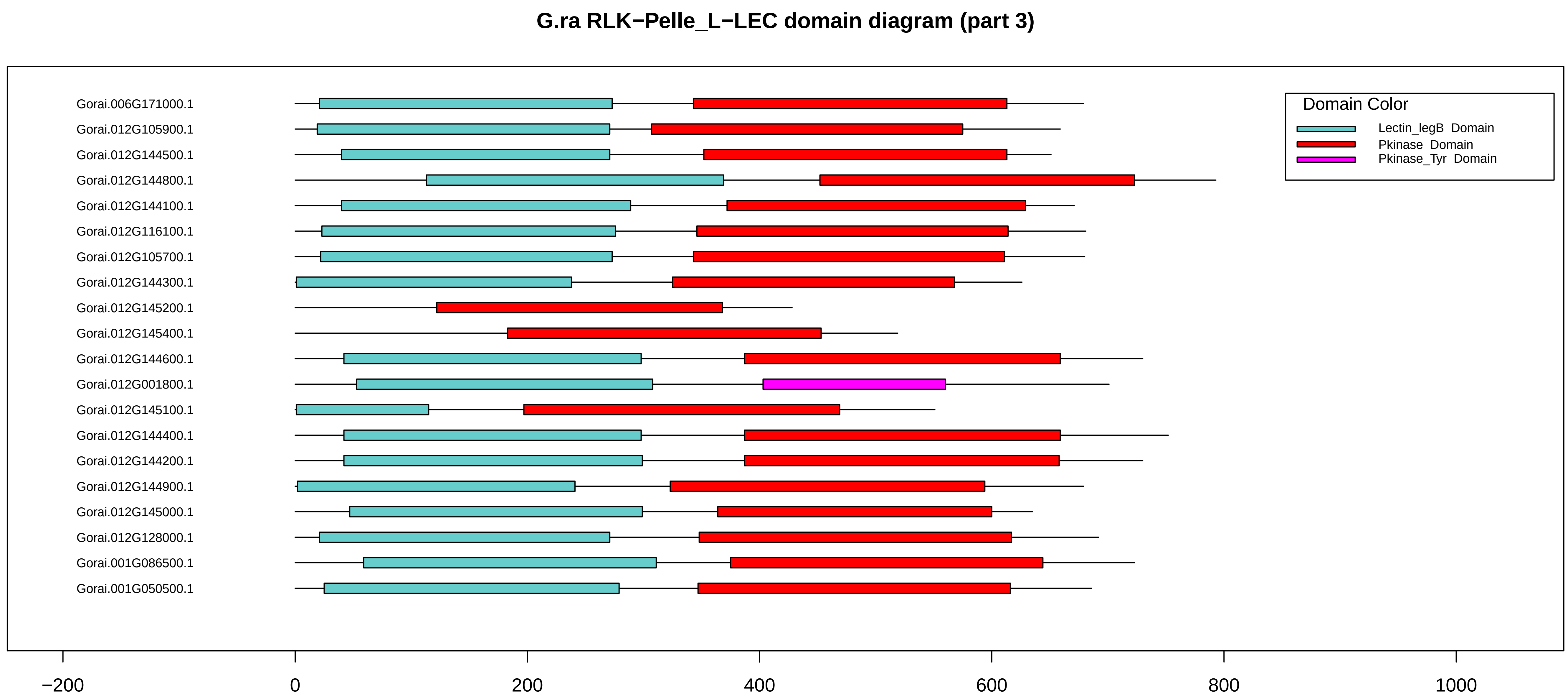
G.ra RLK−Pelle_L−LEC domain diagram (part 3)
Gorai.006G171000.1
Gorai.012G105900.1
Gorai.012G144500.1
Gorai.012G144800.1
Gorai.012G144100.1
Gorai.012G116100.1
Gorai.012G105700.1
Gorai.012G144300.1
Gorai.012G145200.1
Gorai.012G145400.1
Gorai.012G144600.1
Gorai.012G001800.1
Gorai.012G145100.1
Gorai.012G144400.1
Gorai.012G144200.1
Gorai.012G144900.1
Gorai.012G145000.1
Gorai.012G128000.1
Gorai.001G086500.1
Gorai.001G050500.1
Domain Color
Lectin_legB Domain
Pkinase Domain
Pkinase_Tyr Domain
−200
0
200
400
600
800
1000

G.ra RLK−Pelle_L−LEC domain diagram (part 4)

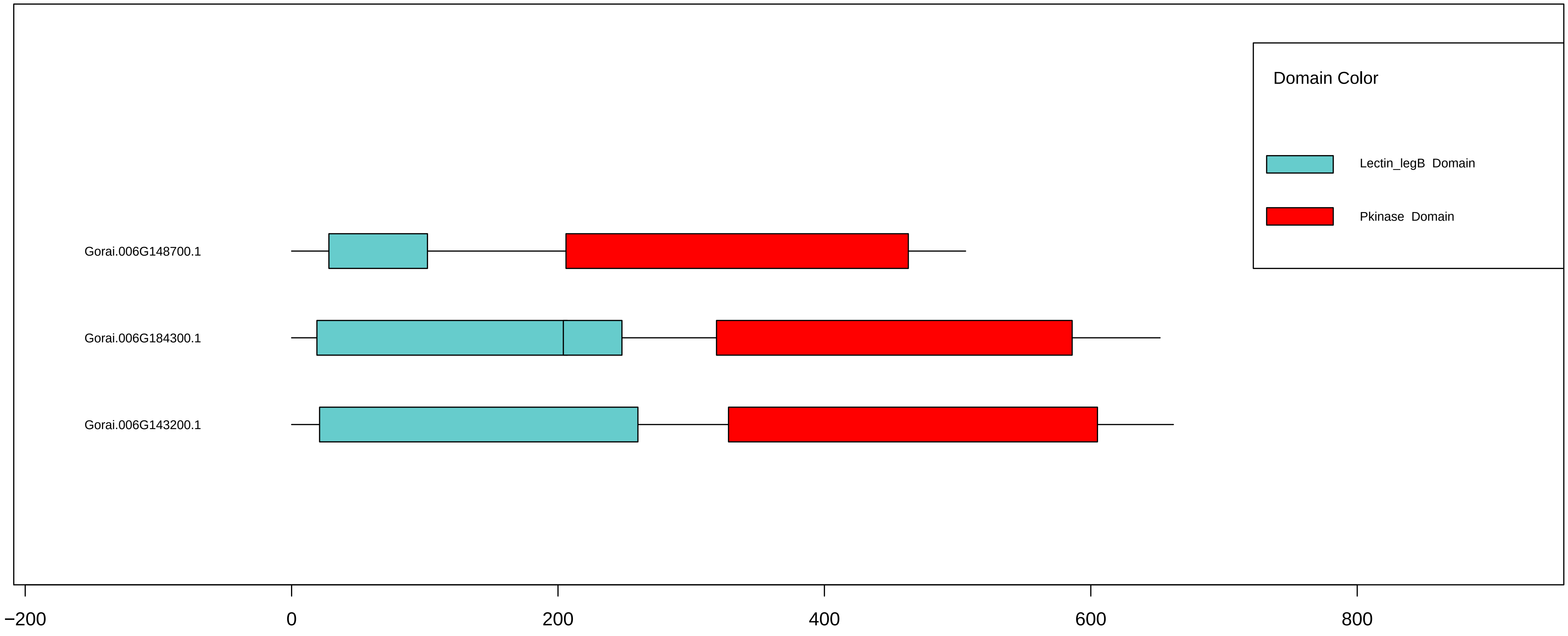

G.ra RLK−Pelle_LRK10L−2 domain diagram (part 1)

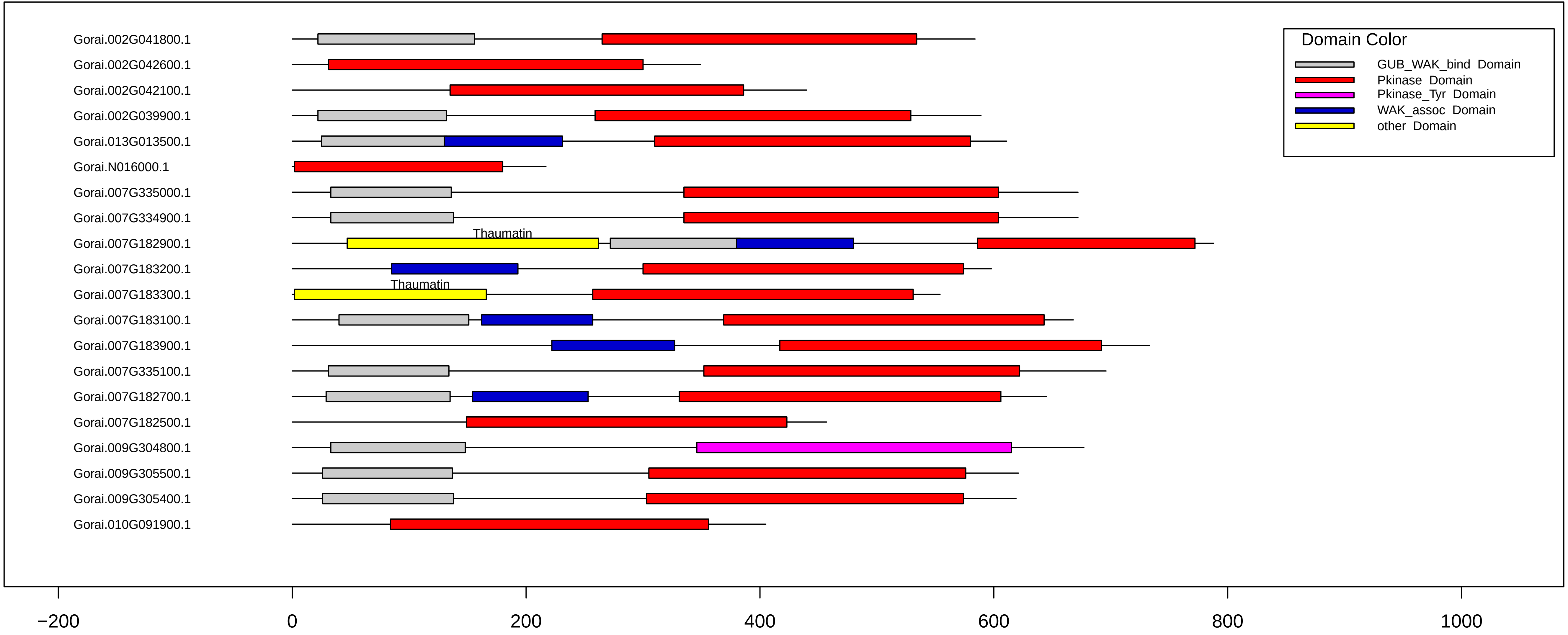

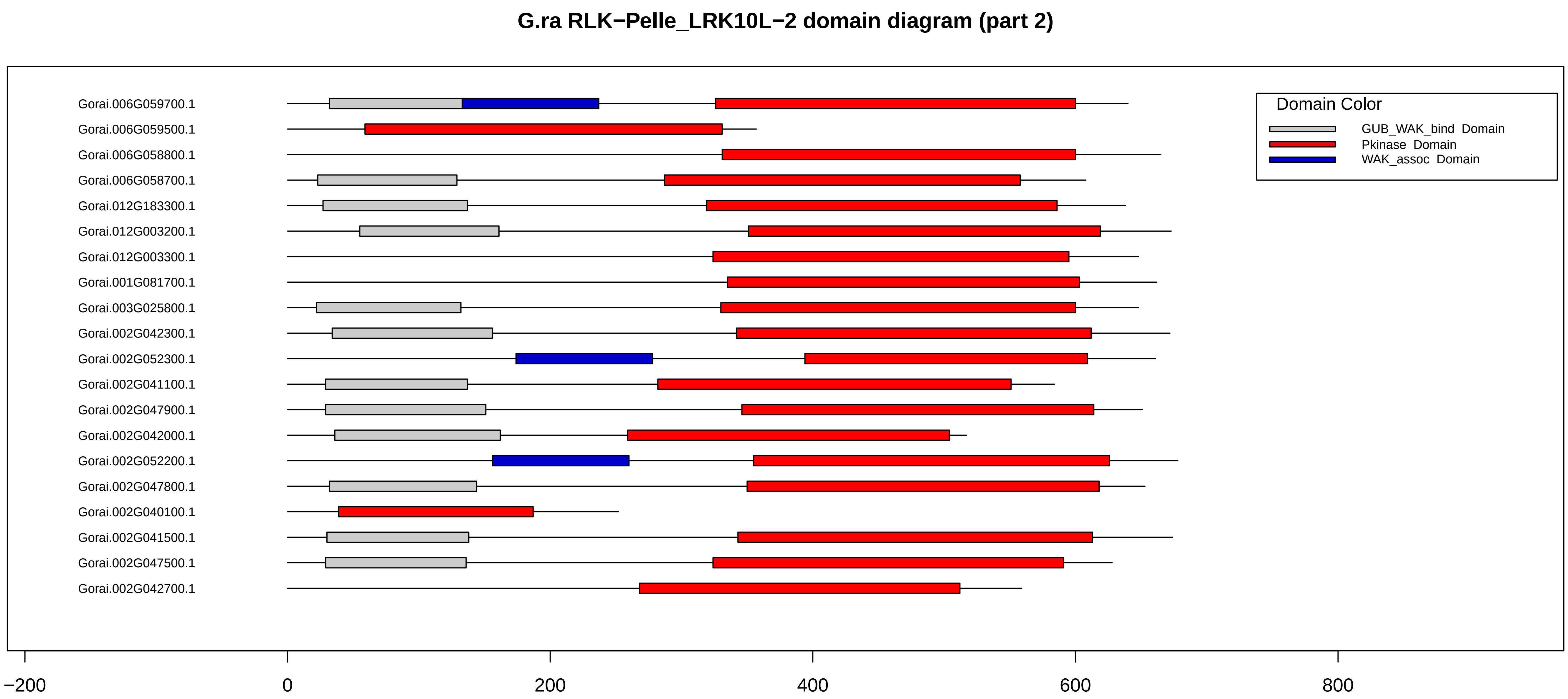

G.ra RLK−Pelle_LRK10L−2 domain diagram (part 2)
Gorai.006G059700.1
Gorai.006G059500.1
Gorai.006G058800.1
Gorai.006G058700.1
Gorai.012G183300.1
Gorai.012G003200.1
Gorai.012G003300.1
Gorai.001G081700.1
Gorai.003G025800.1
Gorai.002G042300.1
Gorai.002G052300.1
Gorai.002G041100.1
Gorai.002G047900.1
Gorai.002G042000.1
Gorai.002G052200.1
Gorai.002G047800.1
Gorai.002G040100.1
Gorai.002G041500.1
Gorai.002G047500.1
Gorai.002G042700.1
Domain Color
GUB_WAK_bind Domain
Pkinase Domain
WAK_assoc Domain
−200
0
200
400
600
800

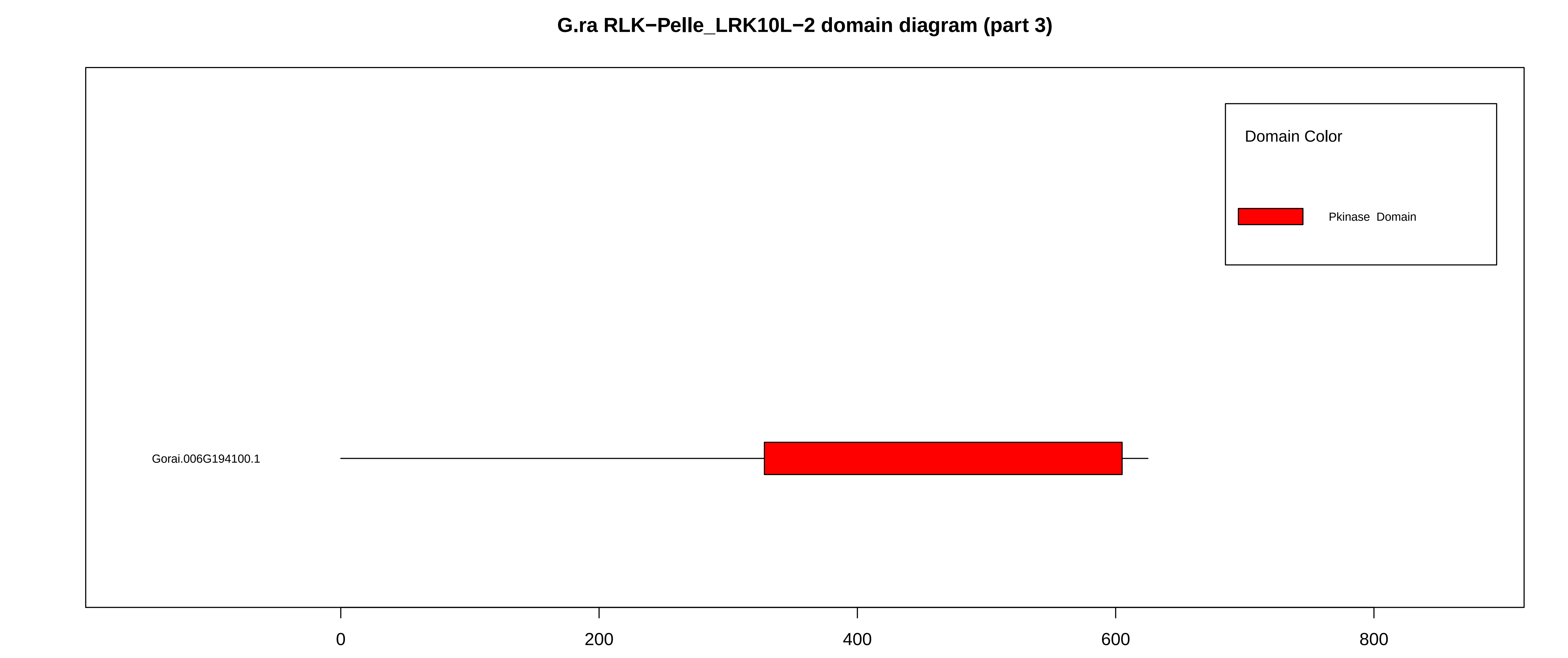
G.ra RLK−Pelle_LRK10L−2 domain diagram (part 3)
Domain Color
Pkinase Domain
Gorai.006G194100.1
0
200
400
600
800

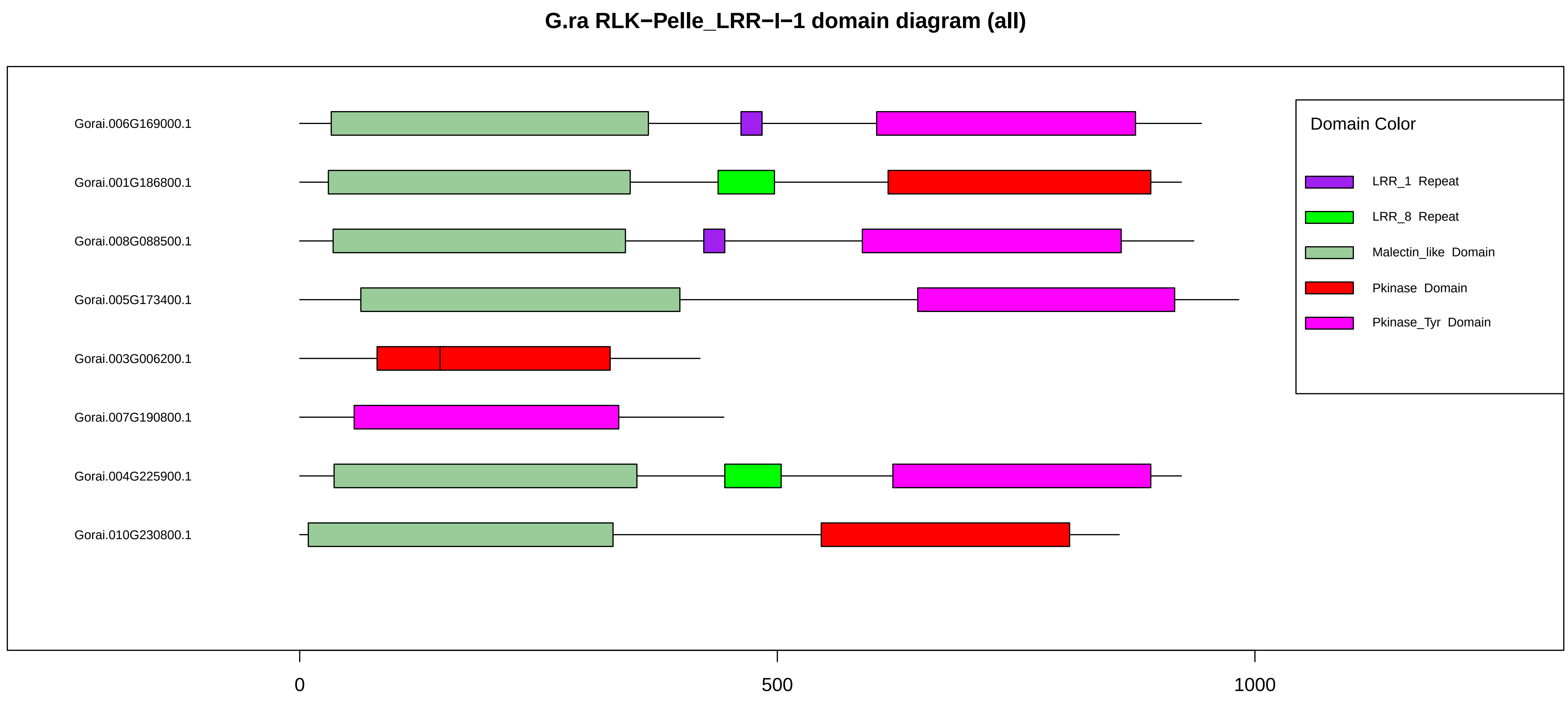
G.ra RLK−Pelle_LRR−I−1 domain diagram (all)
Gorai.006G169000.1
Gorai.001G186800.1
Gorai.008G088500.1
Gorai.005G173400.1
Gorai.003G006200.1
Gorai.007G190800.1
Gorai.004G225900.1
Gorai.010G230800.1
Domain Color
LRR_1 Repeat
LRR_8 Repeat
Malectin_like Domain
Pkinase Domain
Pkinase_Tyr Domain
0
500
1000

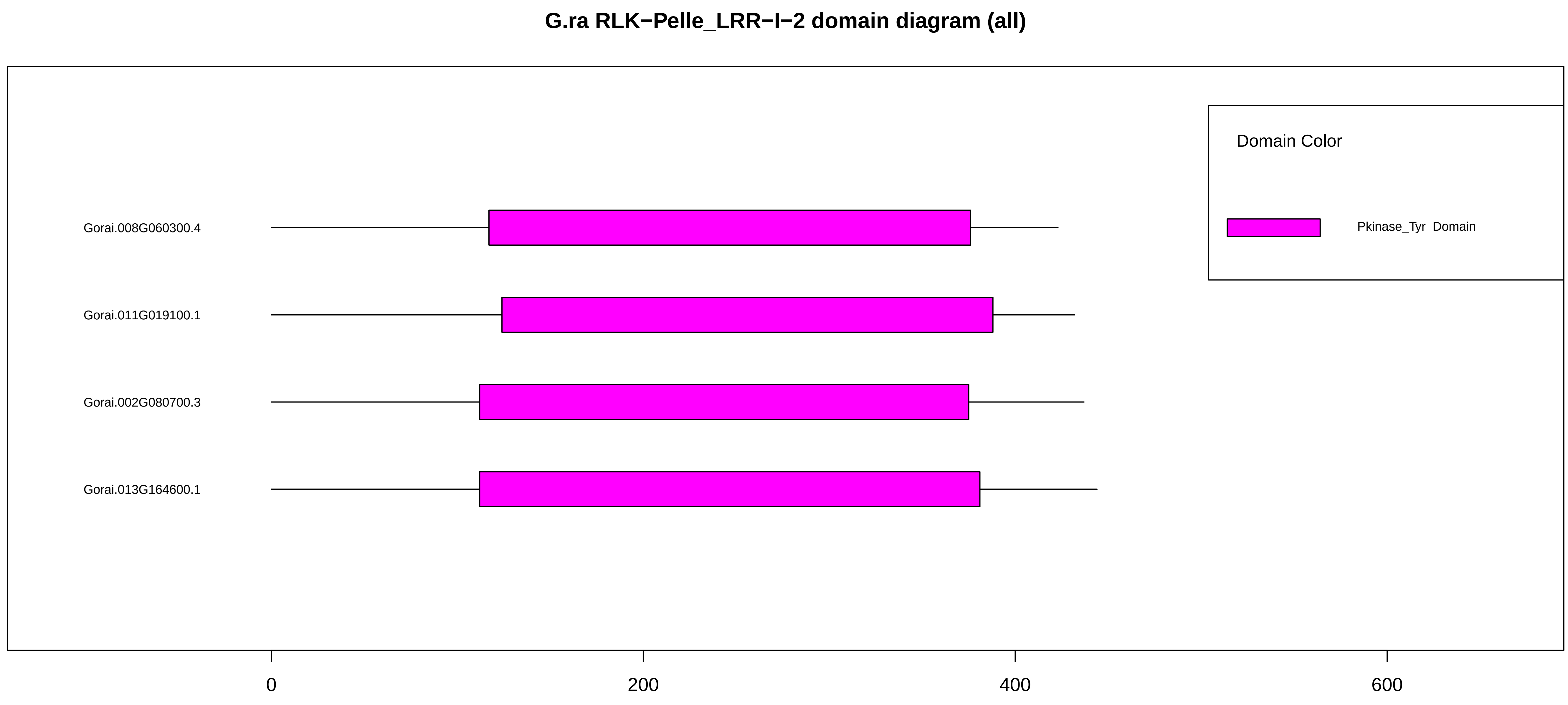
G.ra RLK−Pelle_LRR−I−2 domain diagram (all)
Domain Color
Pkinase_Tyr Domain
Gorai.008G060300.4
Gorai.011G019100.1
Gorai.002G080700.3
Gorai.013G164600.1
0
200
400
600

G.ra RLK−Pelle_LRR−III domain diagram (part 1)

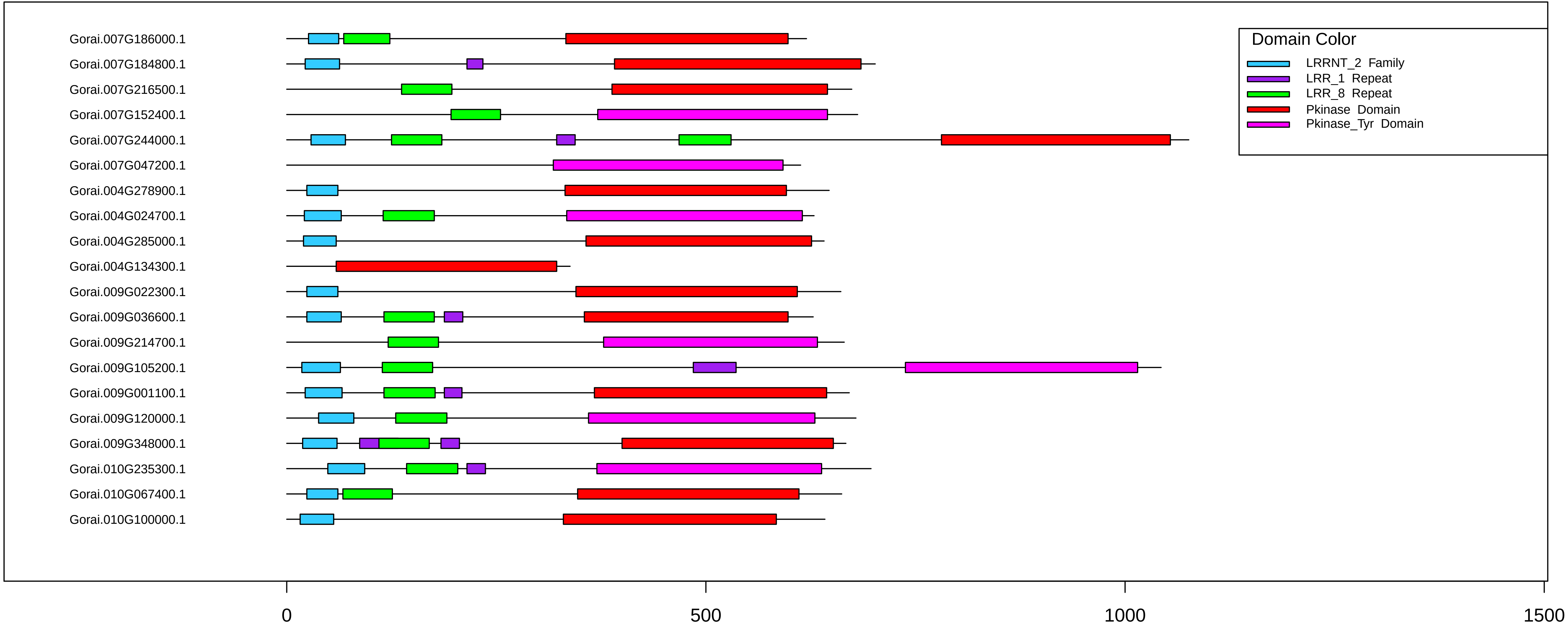

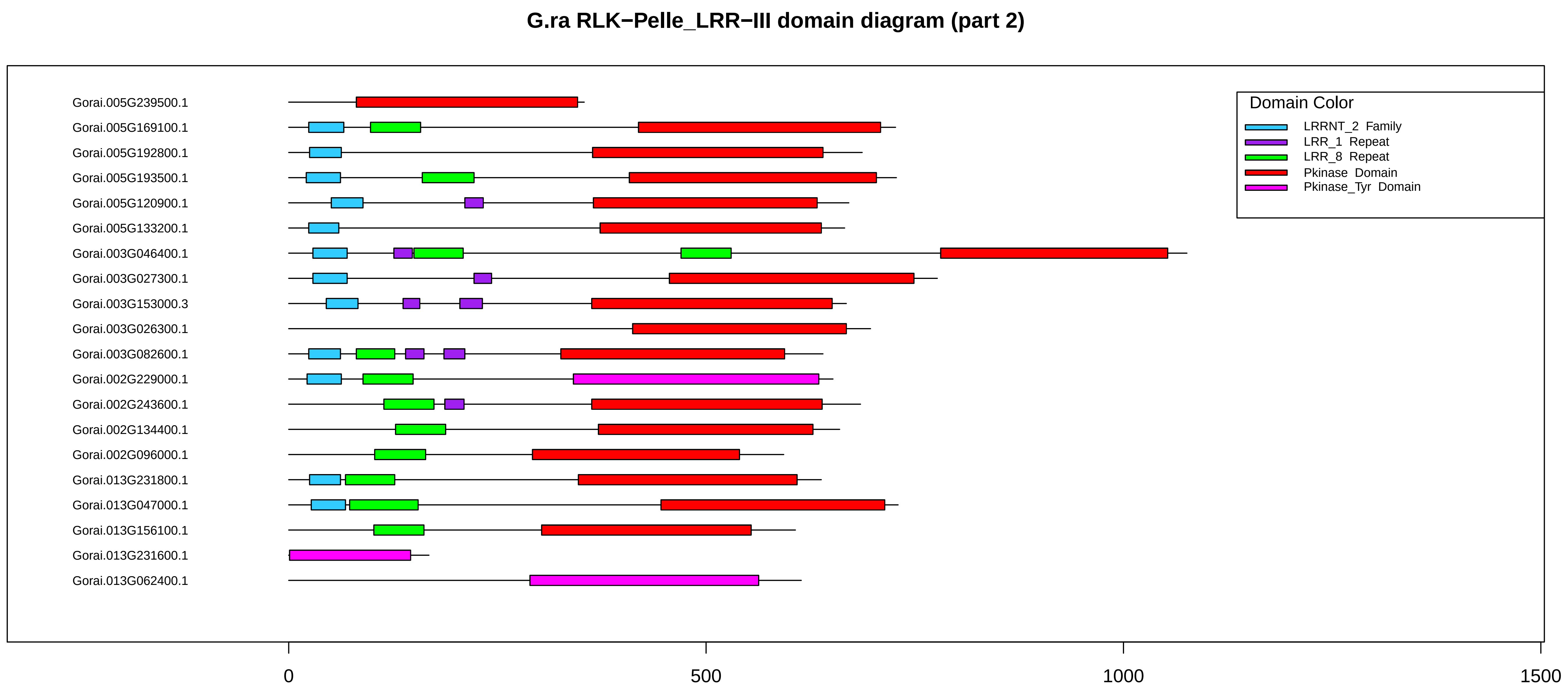
G.ra RLK−Pelle_LRR−III domain diagram (part 2)
Gorai.005G239500.1
Gorai.005G169100.1
Gorai.005G192800.1
Gorai.005G193500.1
Gorai.005G120900.1
Gorai.005G133200.1
Gorai.003G046400.1
Gorai.003G027300.1
Gorai.003G153000.3
Gorai.003G026300.1
Gorai.003G082600.1
Gorai.002G229000.1
Gorai.002G243600.1
Gorai.002G134400.1
Gorai.002G096000.1
Gorai.013G231800.1
Gorai.013G047000.1
Gorai.013G156100.1
Gorai.013G231600.1
Gorai.013G062400.1
Domain Color
LRRNT_2 Family
LRR_1 Repeat
LRR_8 Repeat
Pkinase Domain
Pkinase_Tyr Domain
0
500
1000
1500

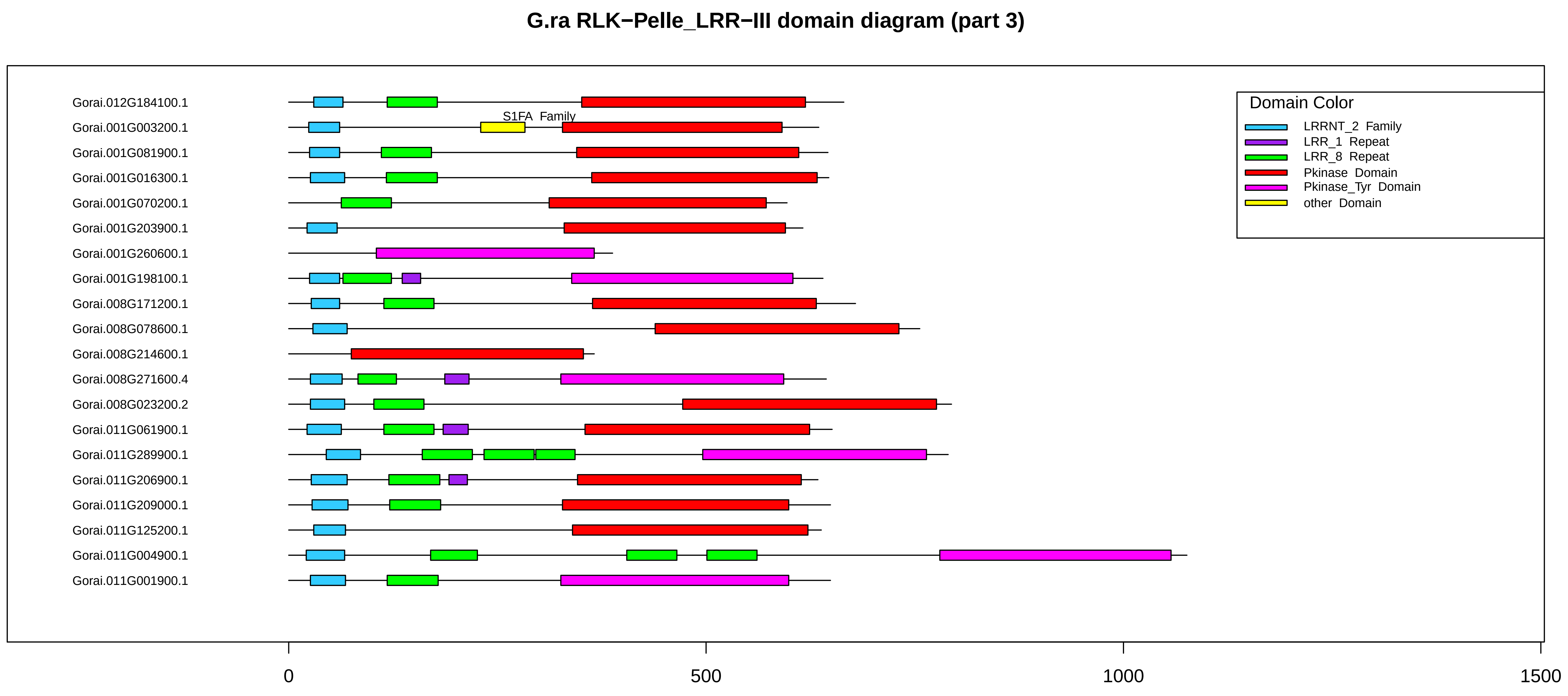
G.ra RLK−Pelle_LRR−III domain diagram (part 3)
Gorai.012G184100.1
Gorai.001G003200.1
S1FA Family
Gorai.001G081900.1
Gorai.001G016300.1
Gorai.001G070200.1
Gorai.001G203900.1
Gorai.001G260600.1
Gorai.001G198100.1
Gorai.008G171200.1
Gorai.008G078600.1
Gorai.008G214600.1
Gorai.008G271600.4
Gorai.008G023200.2
Gorai.011G061900.1
Gorai.011G289900.1
Gorai.011G206900.1
Gorai.011G209000.1
Gorai.011G125200.1
Gorai.011G004900.1
Gorai.011G001900.1
Domain Color
LRRNT_2 Family
LRR_1 Repeat
LRR_8 Repeat
Pkinase Domain
Pkinase_Tyr Domain
other Domain
0
500
1000
1500

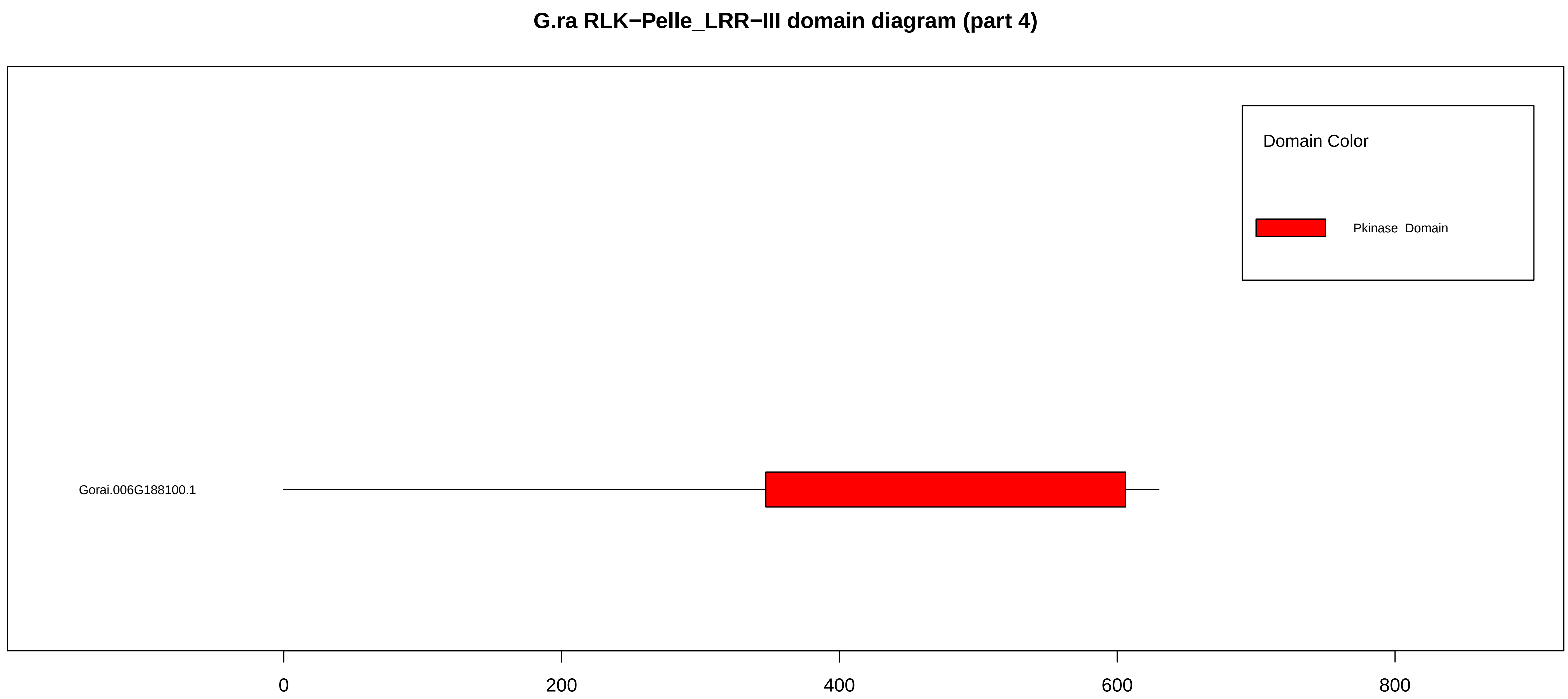

G.ra RLK−Pelle_LRR−III domain diagram (part 4)
Domain Color
Pkinase Domain
Gorai.006G188100.1
0
200
400
600
800

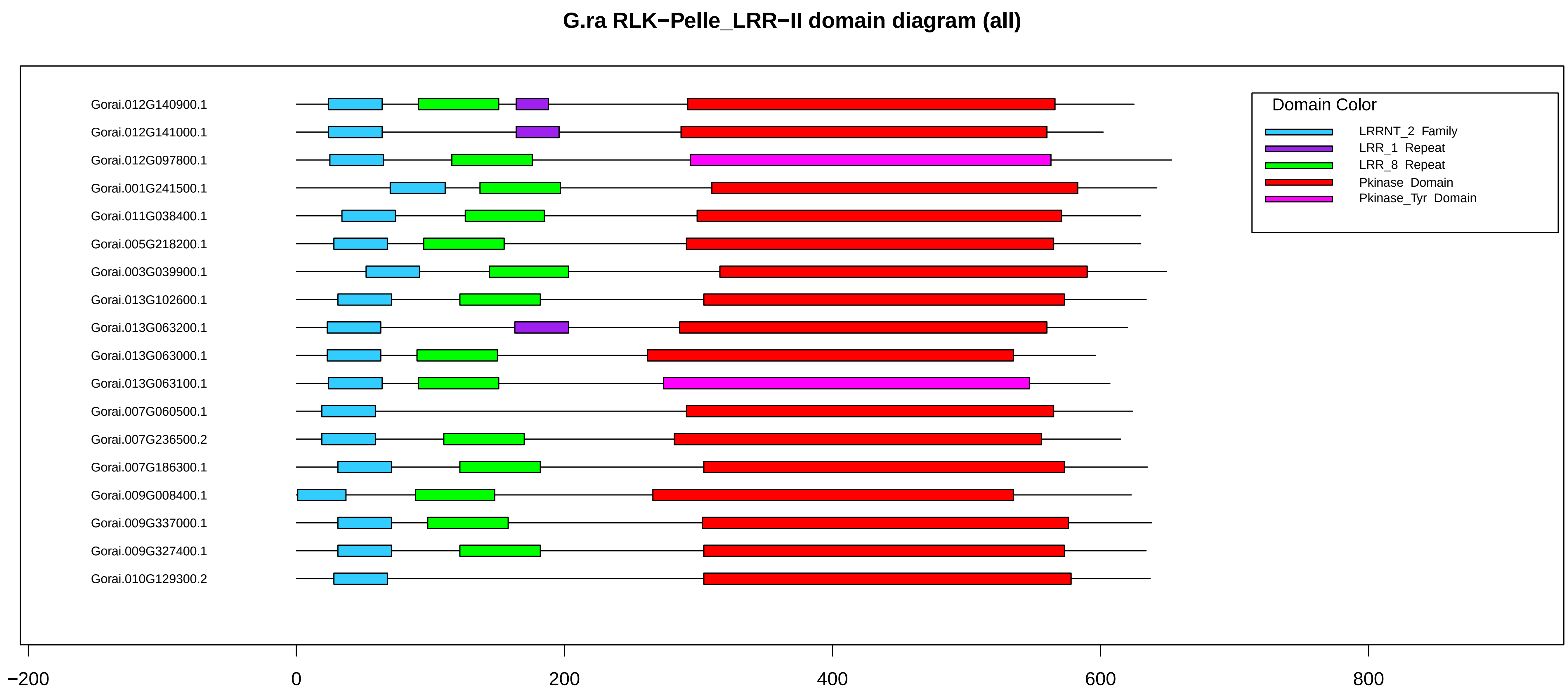
G.ra RLK−Pelle_LRR−II domain diagram (all)
Gorai.012G140900.1
Gorai.012G141000.1
Gorai.012G097800.1
Gorai.001G241500.1
Gorai.011G038400.1
Gorai.005G218200.1
Gorai.003G039900.1
Gorai.013G102600.1
Gorai.013G063200.1
Gorai.013G063000.1
Gorai.013G063100.1
Gorai.007G060500.1
Gorai.007G236500.2
Gorai.007G186300.1
Gorai.009G008400.1
Gorai.009G337000.1
Gorai.009G327400.1
Gorai.010G129300.2
Domain Color
LRRNT_2 Family
LRR_1 Repeat
LRR_8 Repeat
Pkinase Domain
Pkinase_Tyr Domain
−200
0
200
400
600
800

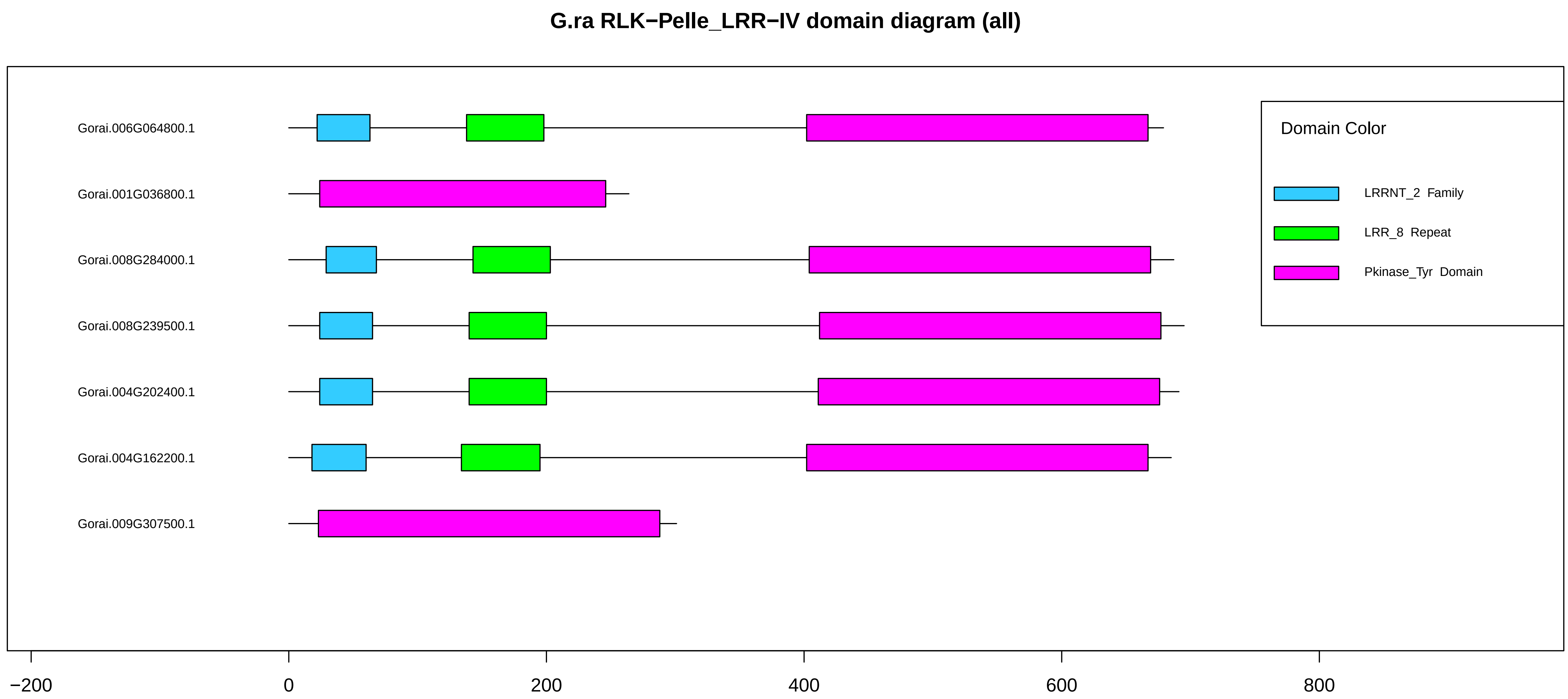
G.ra RLK−Pelle_LRR−IV domain diagram (all)
Gorai.006G064800.1
Gorai.001G036800.1
Gorai.008G284000.1
Gorai.008G239500.1
Gorai.004G202400.1
Gorai.004G162200.1
Gorai.009G307500.1
Domain Color
LRRNT_2 Family
LRR_8 Repeat
Pkinase_Tyr Domain
−200
0
200
400
600
800

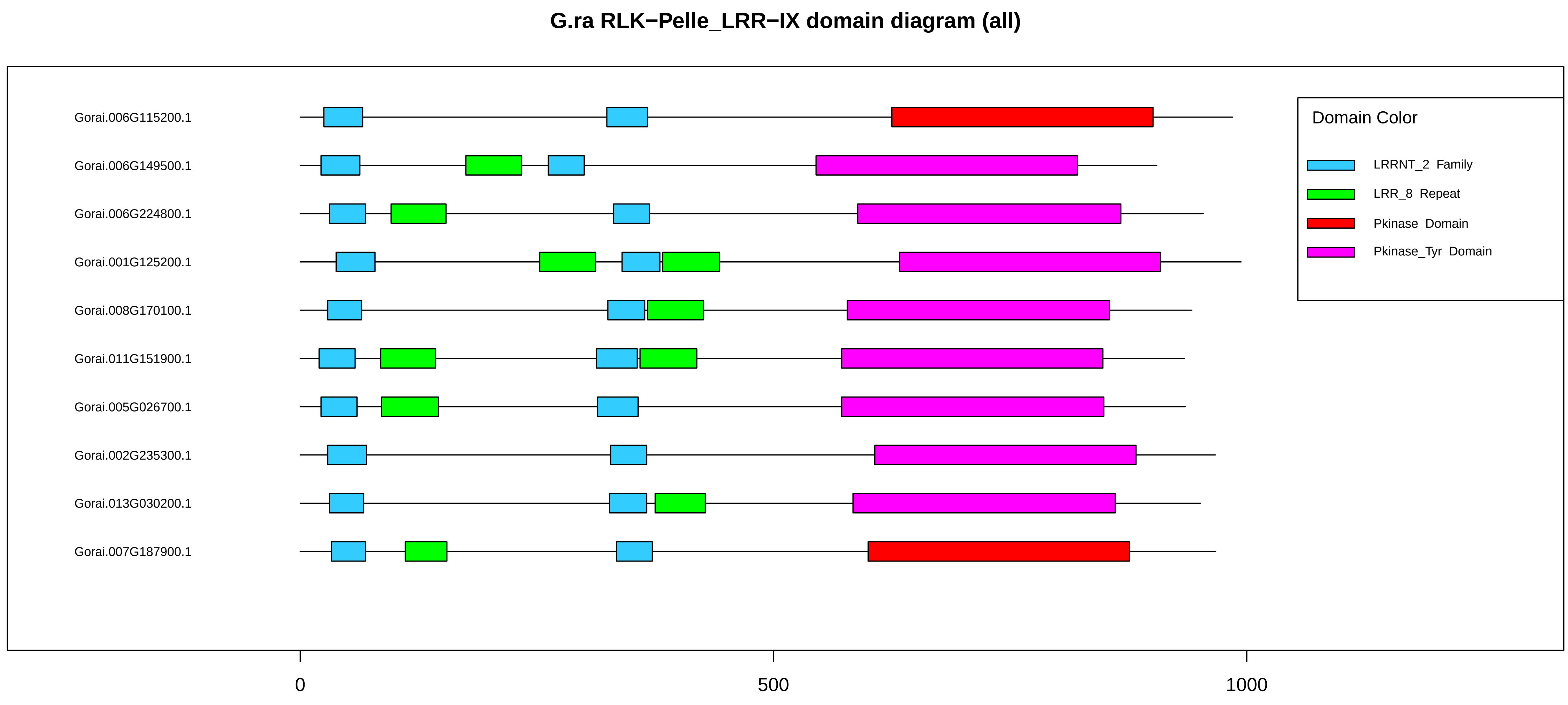
G.ra RLK−Pelle_LRR−IX domain diagram (all)
Gorai.006G115200.1
Gorai.006G149500.1
Gorai.006G224800.1
Gorai.001G125200.1
Gorai.008G170100.1
Gorai.011G151900.1
Gorai.005G026700.1
Gorai.002G235300.1
Gorai.013G030200.1
Gorai.007G187900.1
Domain Color
LRRNT_2 Family
LRR_8 Repeat
Pkinase Domain
Pkinase_Tyr Domain
0
500
1000

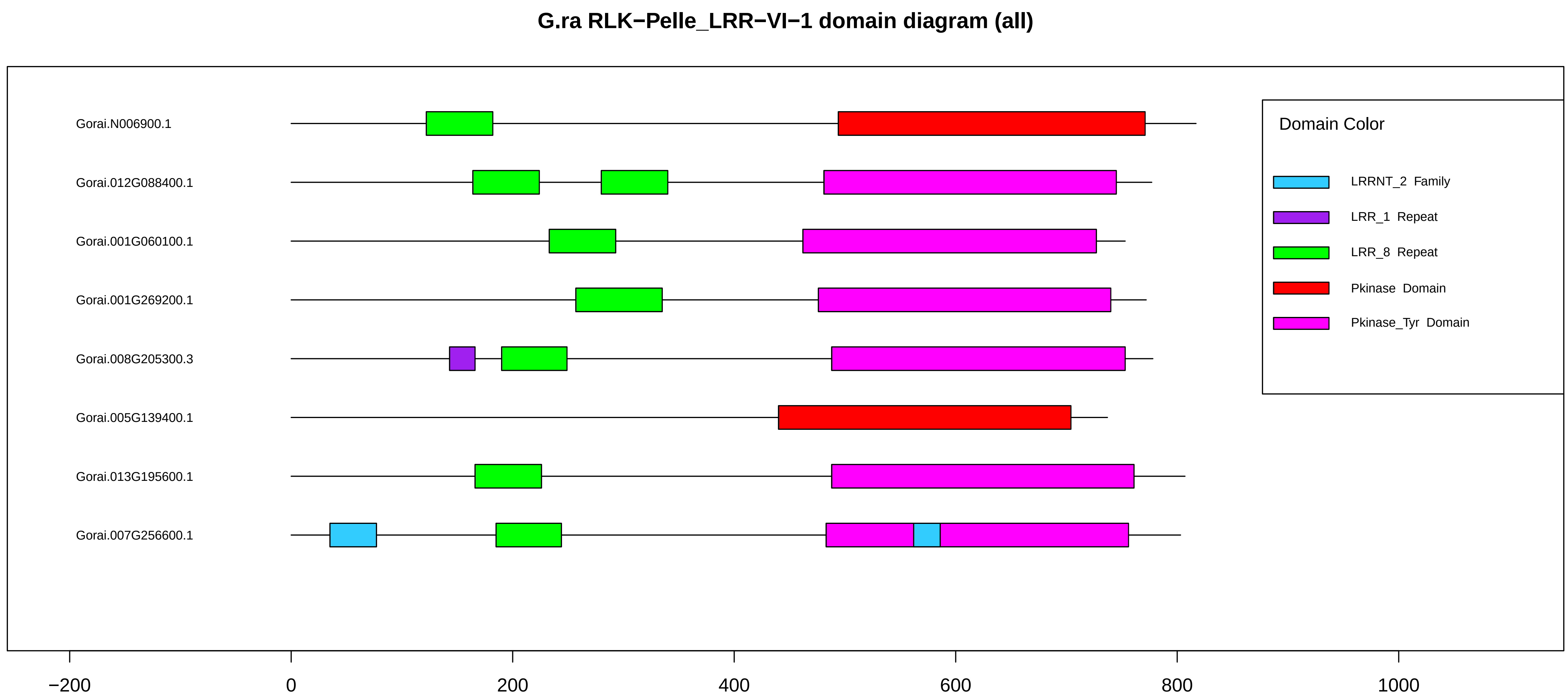

G.ra RLK−Pelle_LRR−VI−1 domain diagram (all)
Gorai.N006900.1
Gorai.012G088400.1
Gorai.001G060100.1
Gorai.001G269200.1
Gorai.008G205300.3
Gorai.005G139400.1
Gorai.013G195600.1
Gorai.007G256600.1
Domain Color
LRRNT_2 Family
LRR_1 Repeat
LRR_8 Repeat
Pkinase Domain
Pkinase_Tyr Domain
−200
0
200
400
600
800
1000

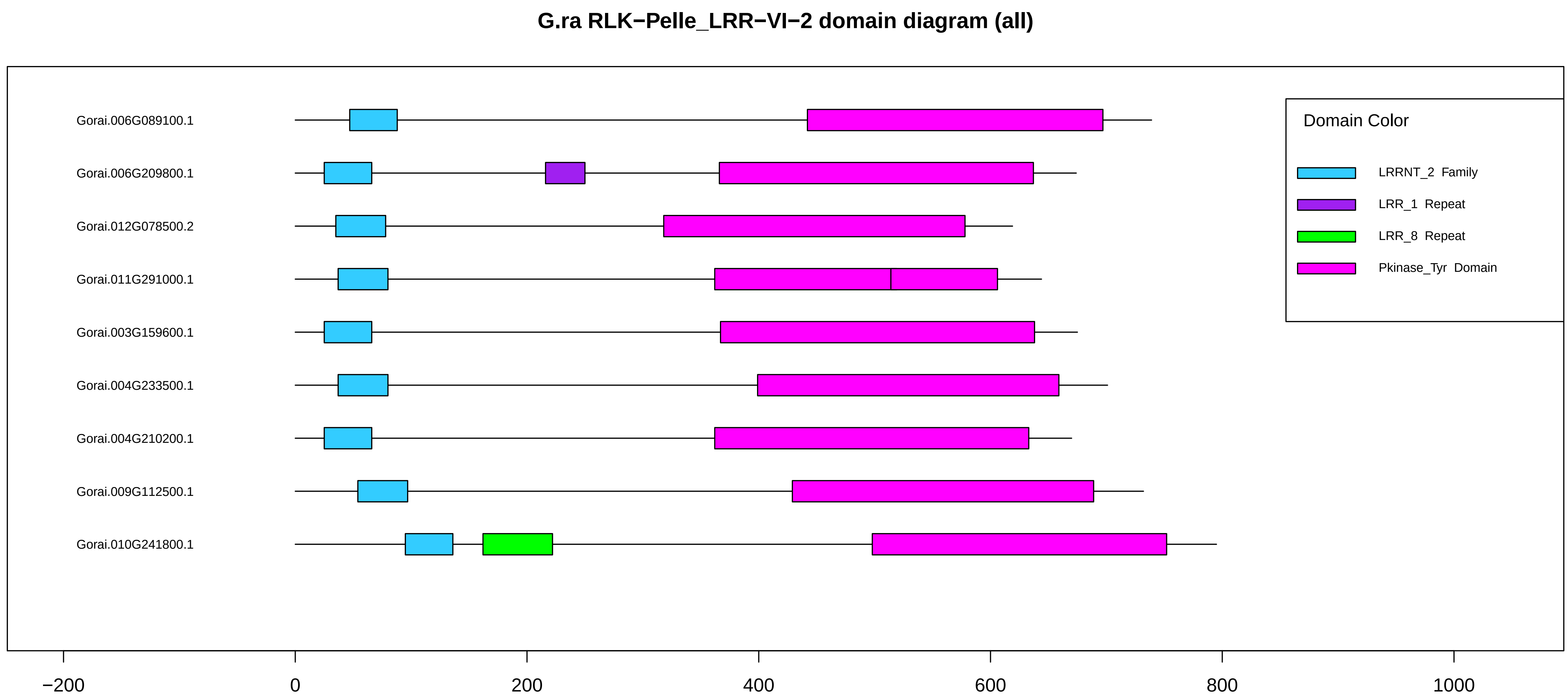

G.ra RLK−Pelle_LRR−VI−2 domain diagram (all)
Gorai.006G089100.1
Gorai.006G209800.1
Gorai.012G078500.2
Gorai.011G291000.1
Gorai.003G159600.1
Gorai.004G233500.1
Gorai.004G210200.1
Gorai.009G112500.1
Gorai.010G241800.1
Domain Color
LRRNT_2 Family
LRR_1 Repeat
LRR_8 Repeat
Pkinase_Tyr Domain
−200
0
200
400
600
800
1000

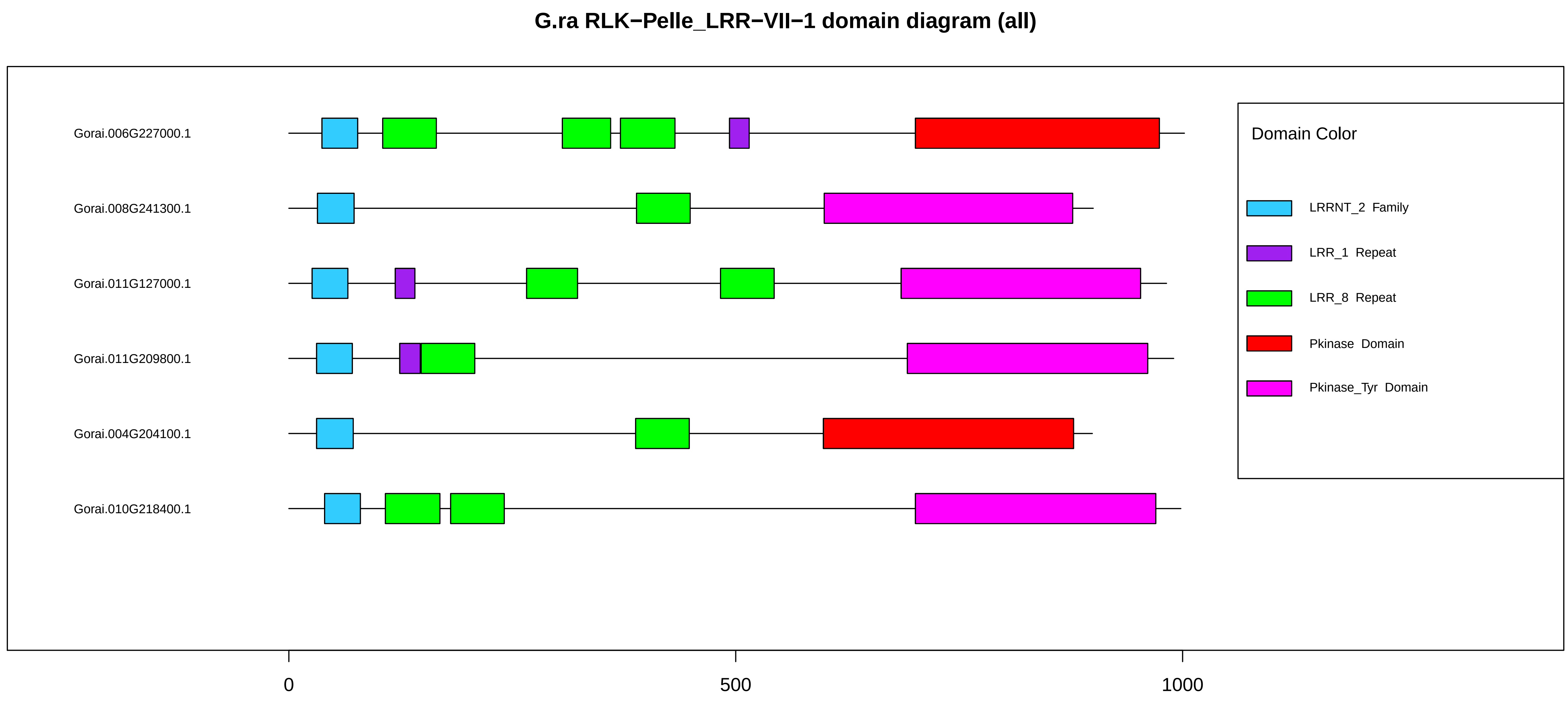

G.ra RLK−Pelle_LRR−VII−1 domain diagram (all)
Gorai.006G227000.1
Gorai.008G241300.1
Gorai.011G127000.1
Gorai.011G209800.1
Gorai.004G204100.1
Gorai.010G218400.1
Domain Color
LRRNT_2 Family
LRR_1 Repeat
LRR_8 Repeat
Pkinase Domain
Pkinase_Tyr Domain
0
500
1000

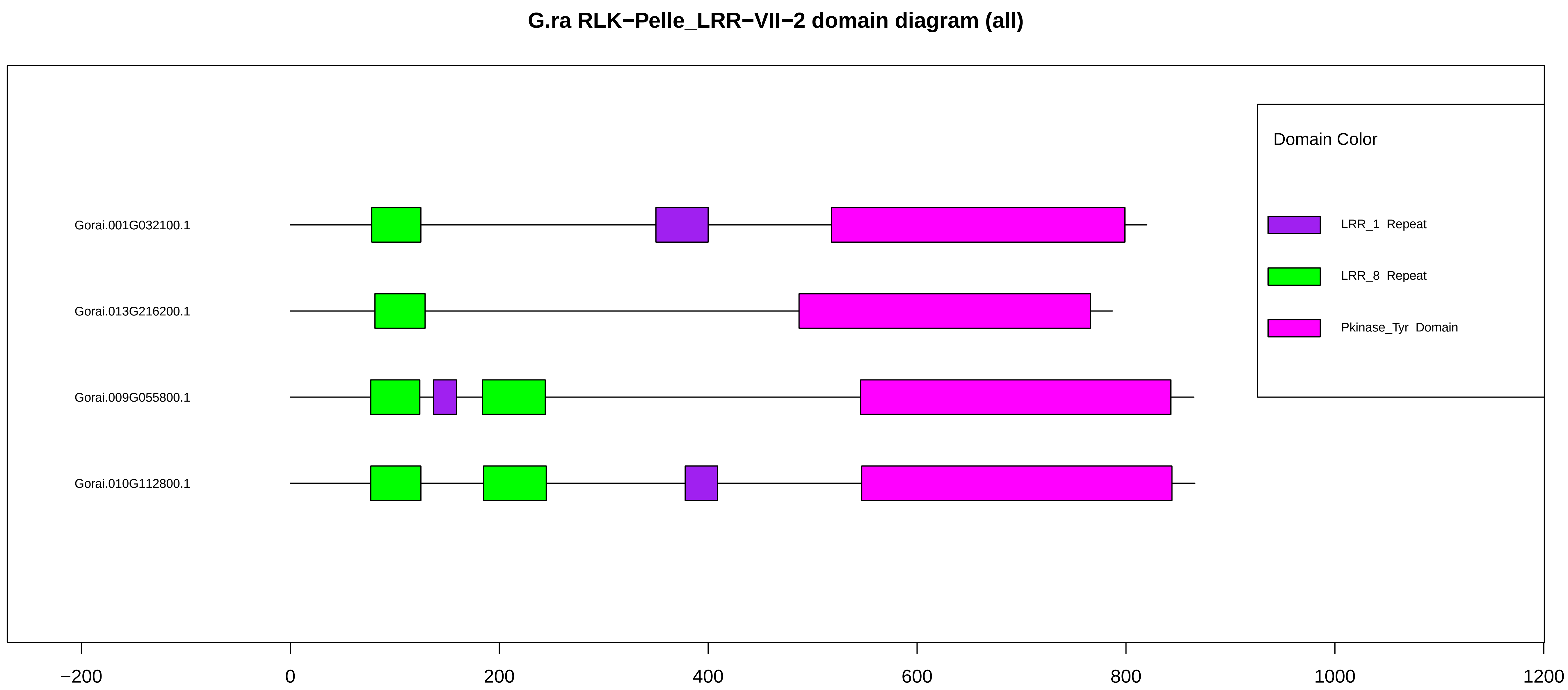
G.ra RLK−Pelle_LRR−VII−2 domain diagram (all)
Gorai.001G032100.1
Gorai.013G216200.1
Gorai.009G055800.1
Gorai.010G112800.1
Domain Color
LRR_1 Repeat
LRR_8 Repeat
Pkinase_Tyr Domain
−200
0
200
400
600
800
1000
1200

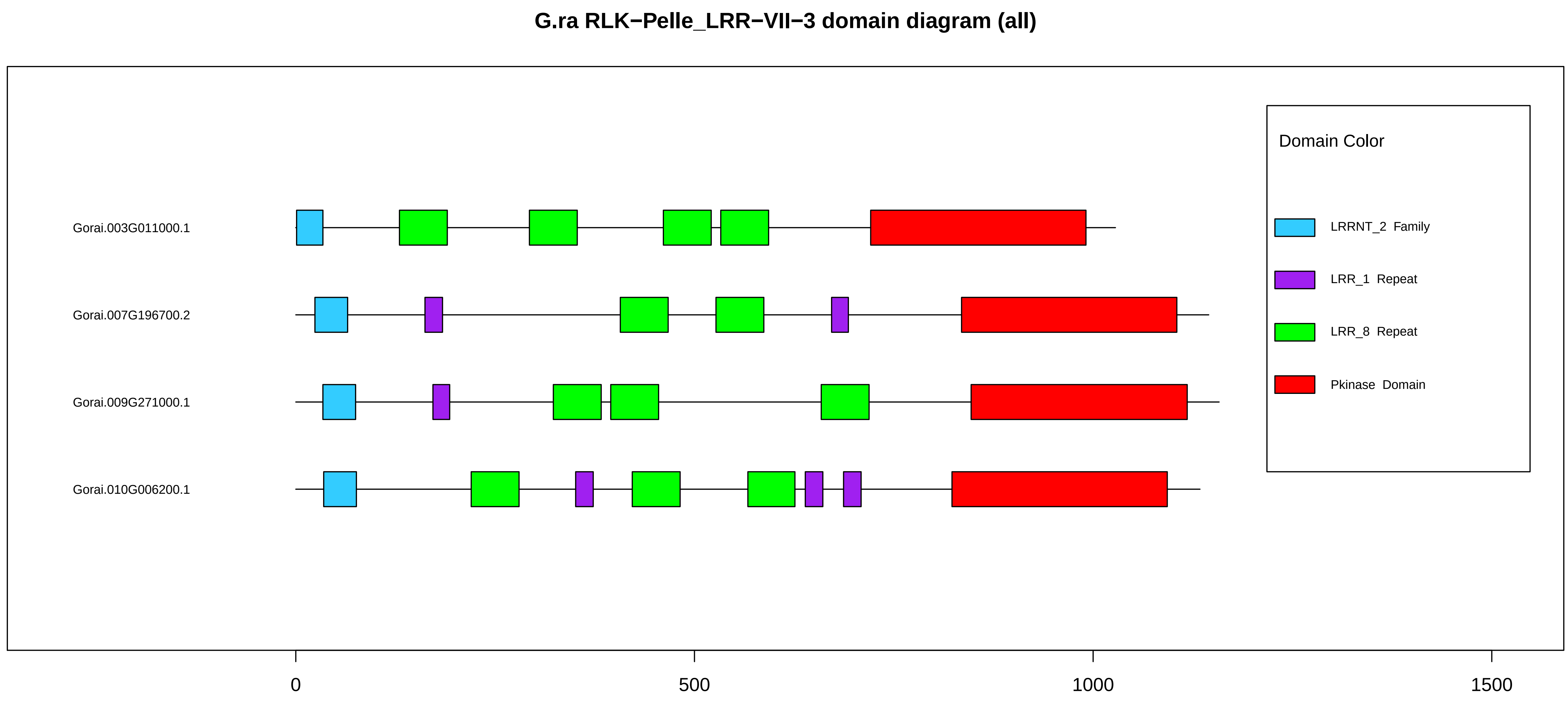

G.ra RLK−Pelle_LRR−VII−3 domain diagram (all)
Gorai.003G011000.1
Gorai.007G196700.2
Gorai.009G271000.1
Gorai.010G006200.1
Domain Color
LRRNT_2 Family
LRR_1 Repeat
LRR_8 Repeat
Pkinase Domain
0
500
1000
1500

G.ra RLK−Pelle_LRR−VIII−1 domain diagram (all)

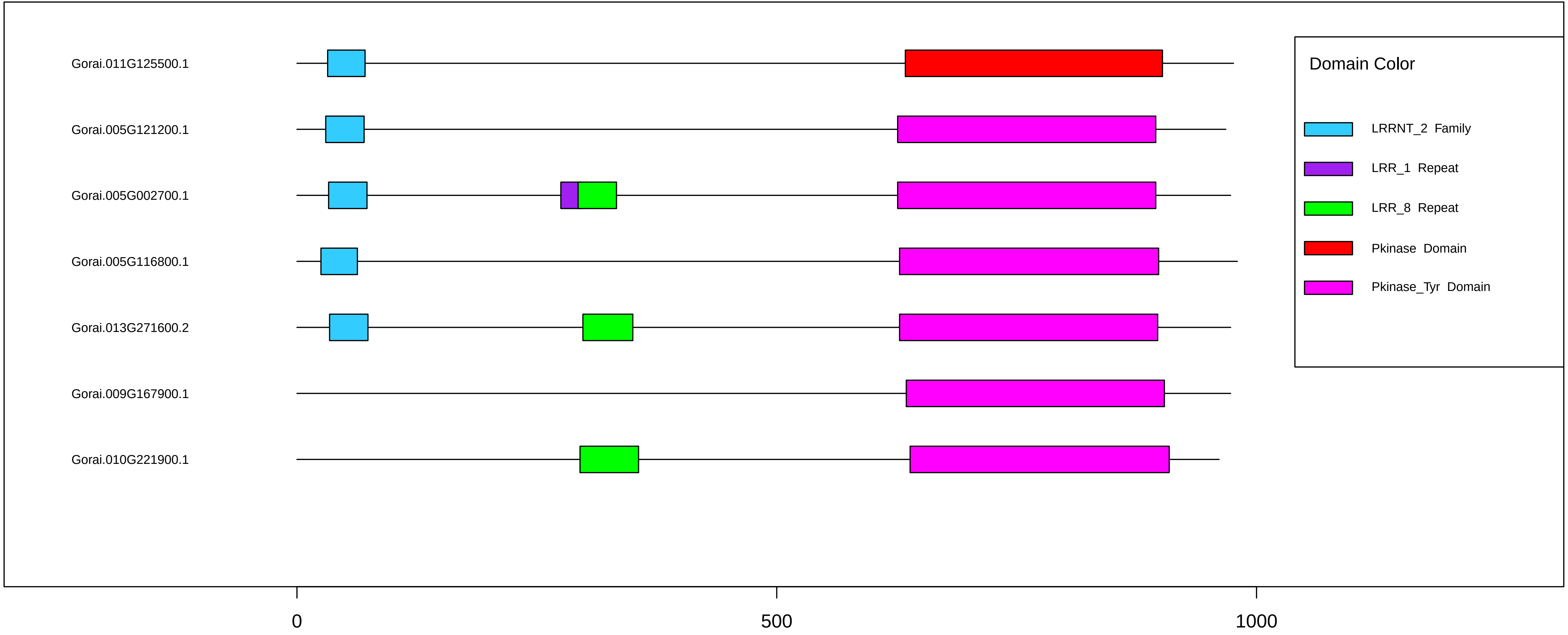

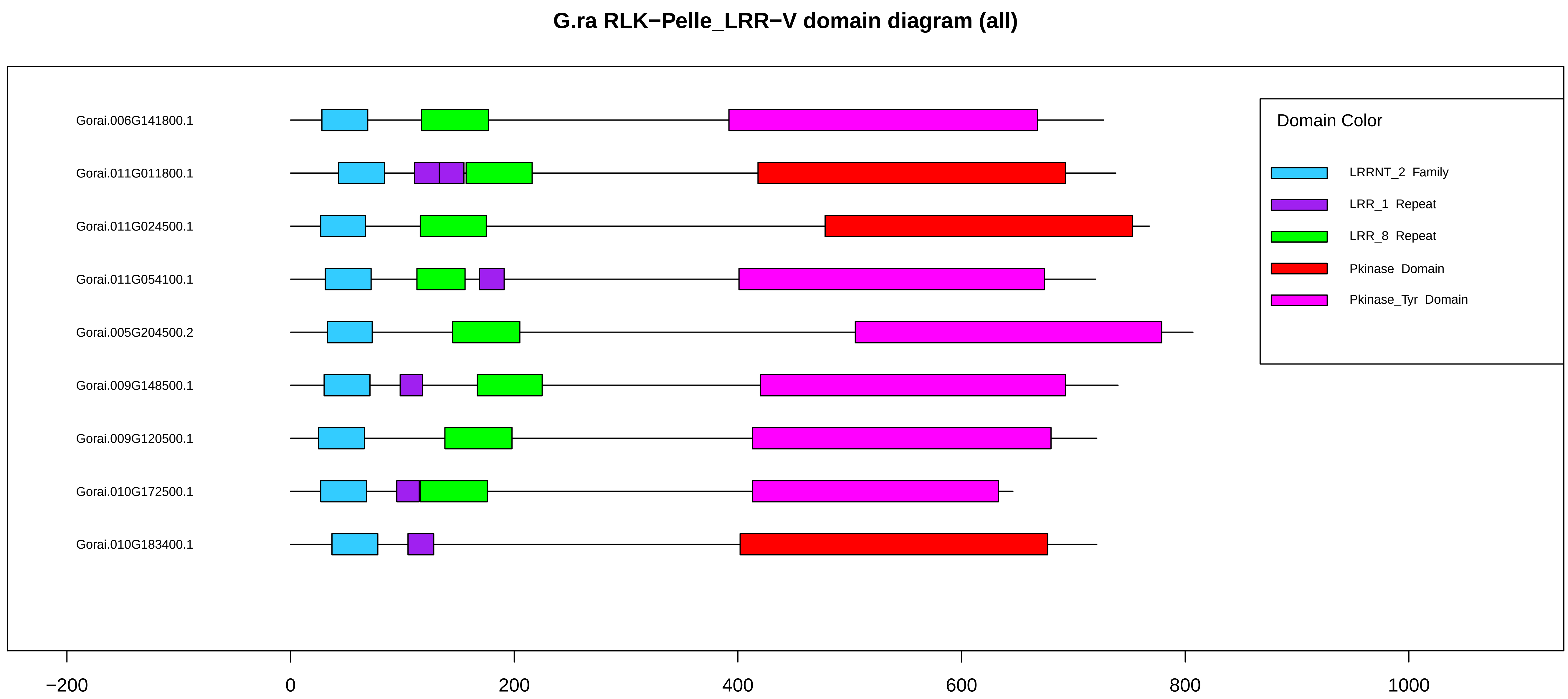
G.ra RLK−Pelle_LRR−V domain diagram (all)
Gorai.006G141800.1
Gorai.011G011800.1
Gorai.011G024500.1
Gorai.011G054100.1
Gorai.005G204500.2
Gorai.009G148500.1
Gorai.009G120500.1
Gorai.010G172500.1
Gorai.010G183400.1
Domain Color
LRRNT_2 Family
LRR_1 Repeat
LRR_8 Repeat
Pkinase Domain
Pkinase_Tyr Domain
−200
0
200
400
600
800
1000

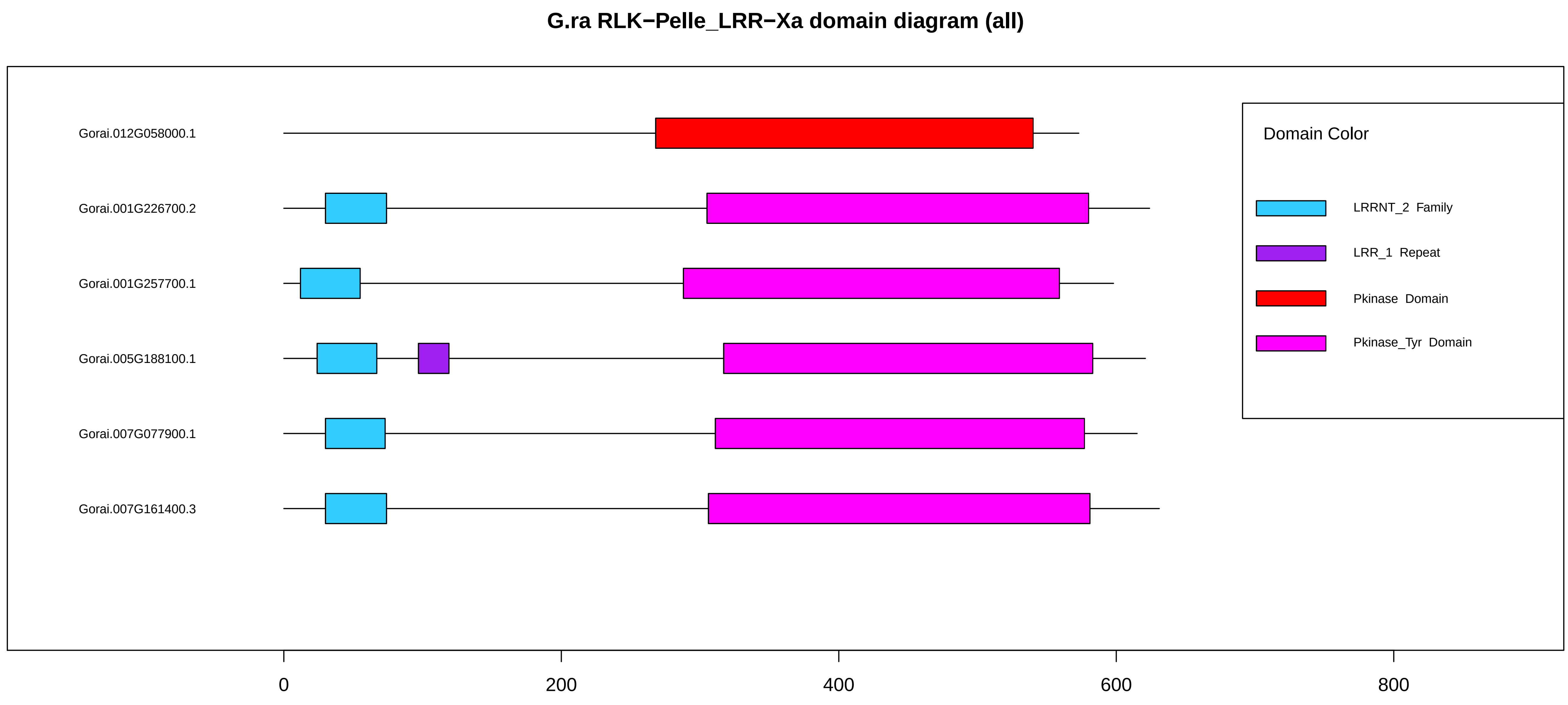
G.ra RLK−Pelle_LRR−Xa domain diagram (all)
Gorai.012G058000.1
Gorai.001G226700.2
Gorai.001G257700.1
Gorai.005G188100.1
Gorai.007G077900.1
Gorai.007G161400.3
Domain Color
LRRNT_2 Family
LRR_1 Repeat
Pkinase Domain
Pkinase_Tyr Domain
0
200
400
600
800

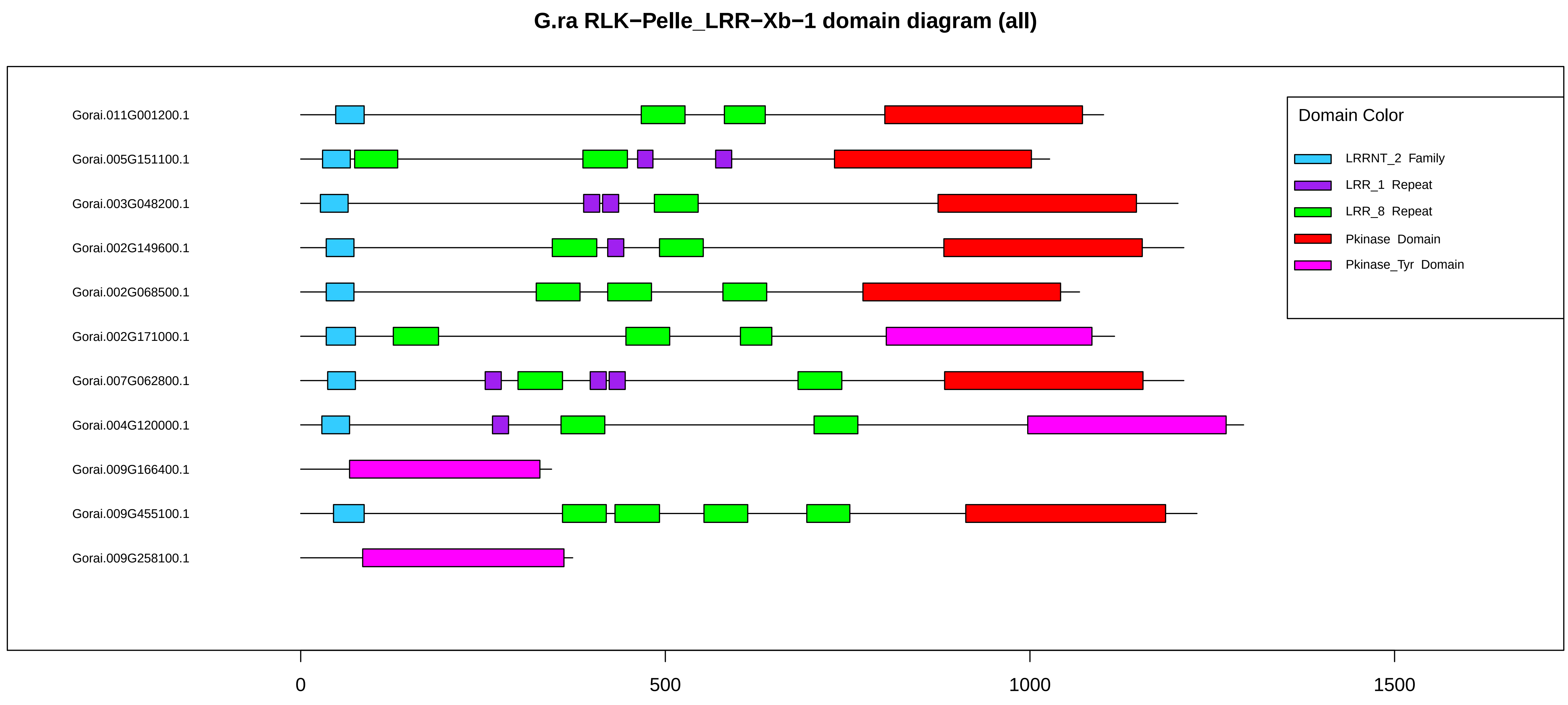
G.ra RLK−Pelle_LRR−Xb−1 domain diagram (all)
Gorai.011G001200.1
Gorai.005G151100.1
Gorai.003G048200.1
Gorai.002G149600.1
Gorai.002G068500.1
Gorai.002G171000.1
Gorai.007G062800.1
Gorai.004G120000.1
Gorai.009G166400.1
Gorai.009G455100.1
Gorai.009G258100.1
Domain Color
LRRNT_2 Family
LRR_1 Repeat
LRR_8 Repeat
Pkinase Domain
Pkinase_Tyr Domain
0
500
1000
1500

G.ra RLK−Pelle_LRR−Xb−2 domain diagram (all)

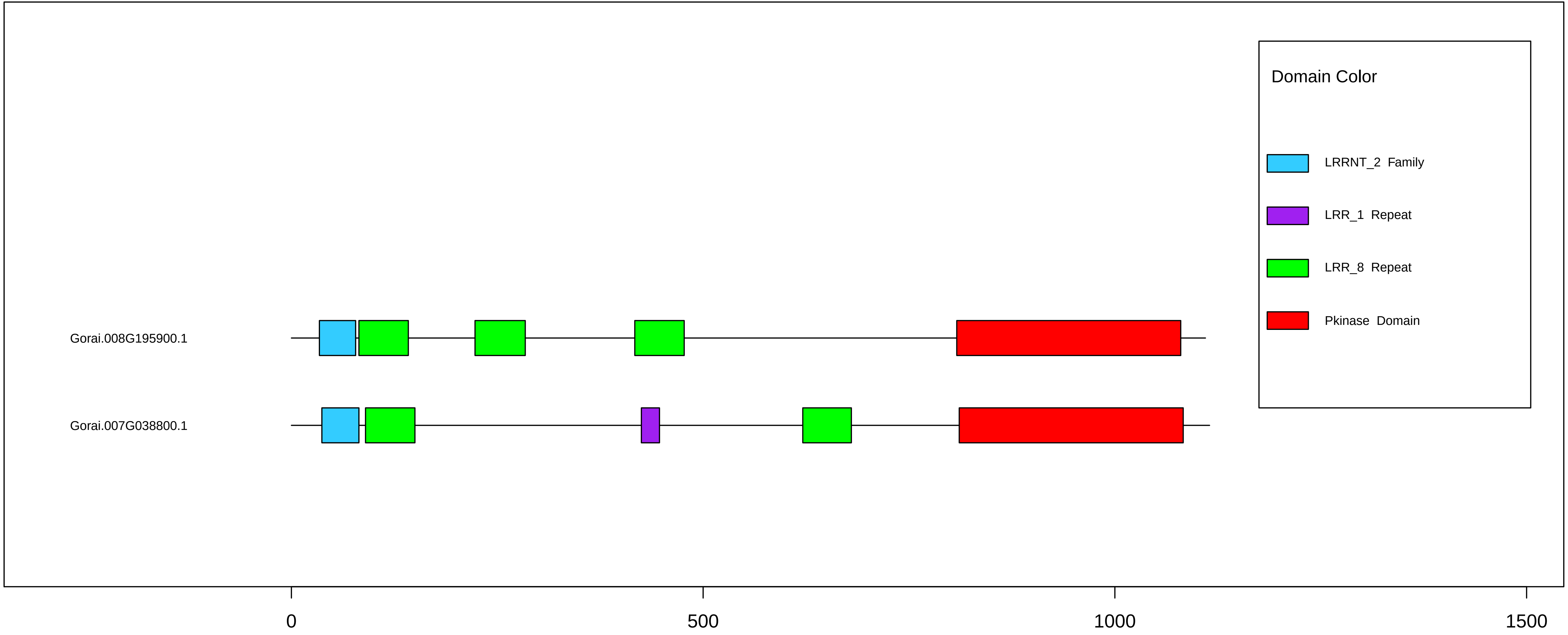

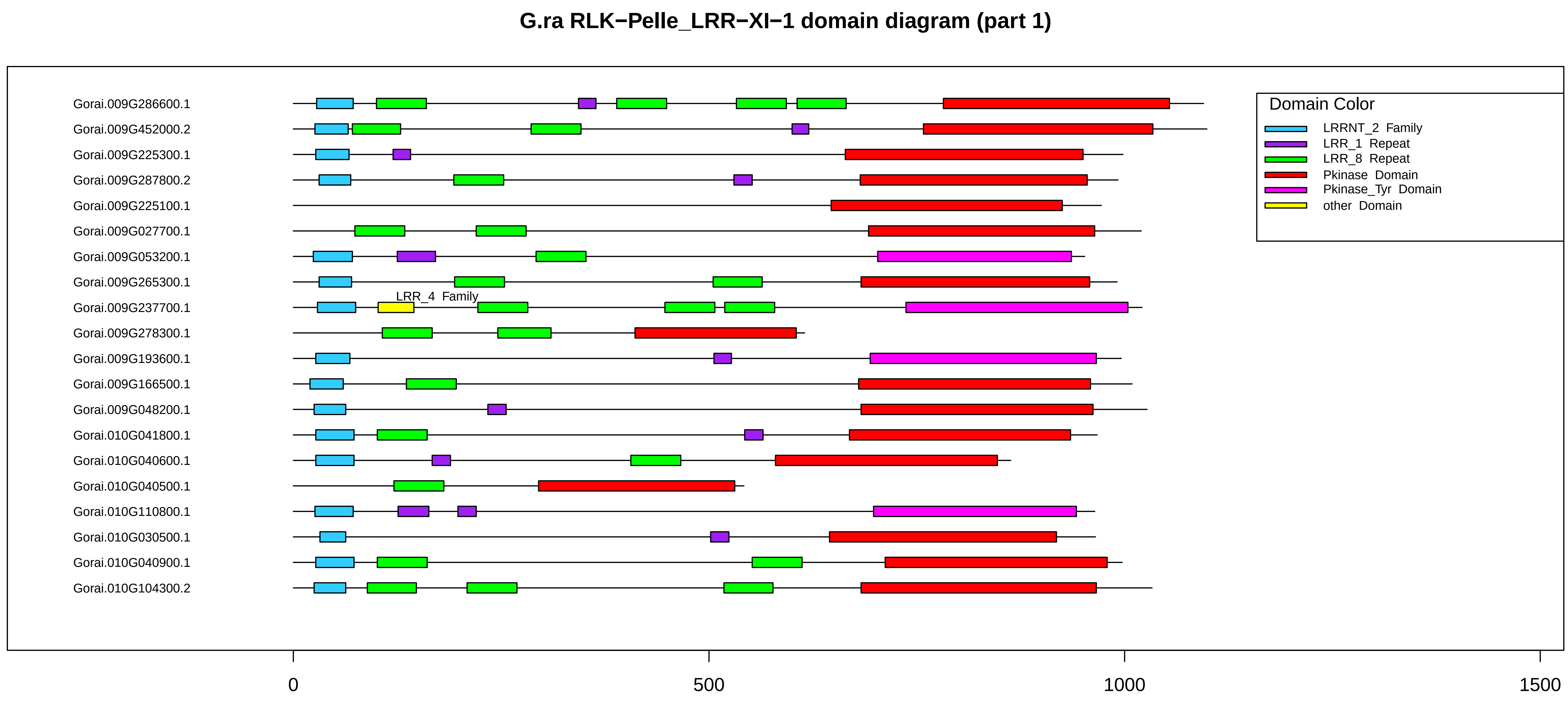
G.ra RLK−Pelle_LRR−XI−1 domain diagram (part 1)
Gorai.009G286600.1
Gorai.009G452000.2
Gorai.009G225300.1
Gorai.009G287800.2
Gorai.009G225100.1
Gorai.009G027700.1
Gorai.009G053200.1
Gorai.009G265300.1
Gorai.009G237700.1
LRR_4 Family
Gorai.009G278300.1
Gorai.009G193600.1
Gorai.009G166500.1
Gorai.009G048200.1
Gorai.010G041800.1
Gorai.010G040600.1
Gorai.010G040500.1
Gorai.010G110800.1
Gorai.010G030500.1
Gorai.010G040900.1
Gorai.010G104300.2
Domain Color
LRRNT_2 Family
LRR_1 Repeat
LRR_8 Repeat
Pkinase Domain
Pkinase_Tyr Domain
other Domain
0
500
1000
1500

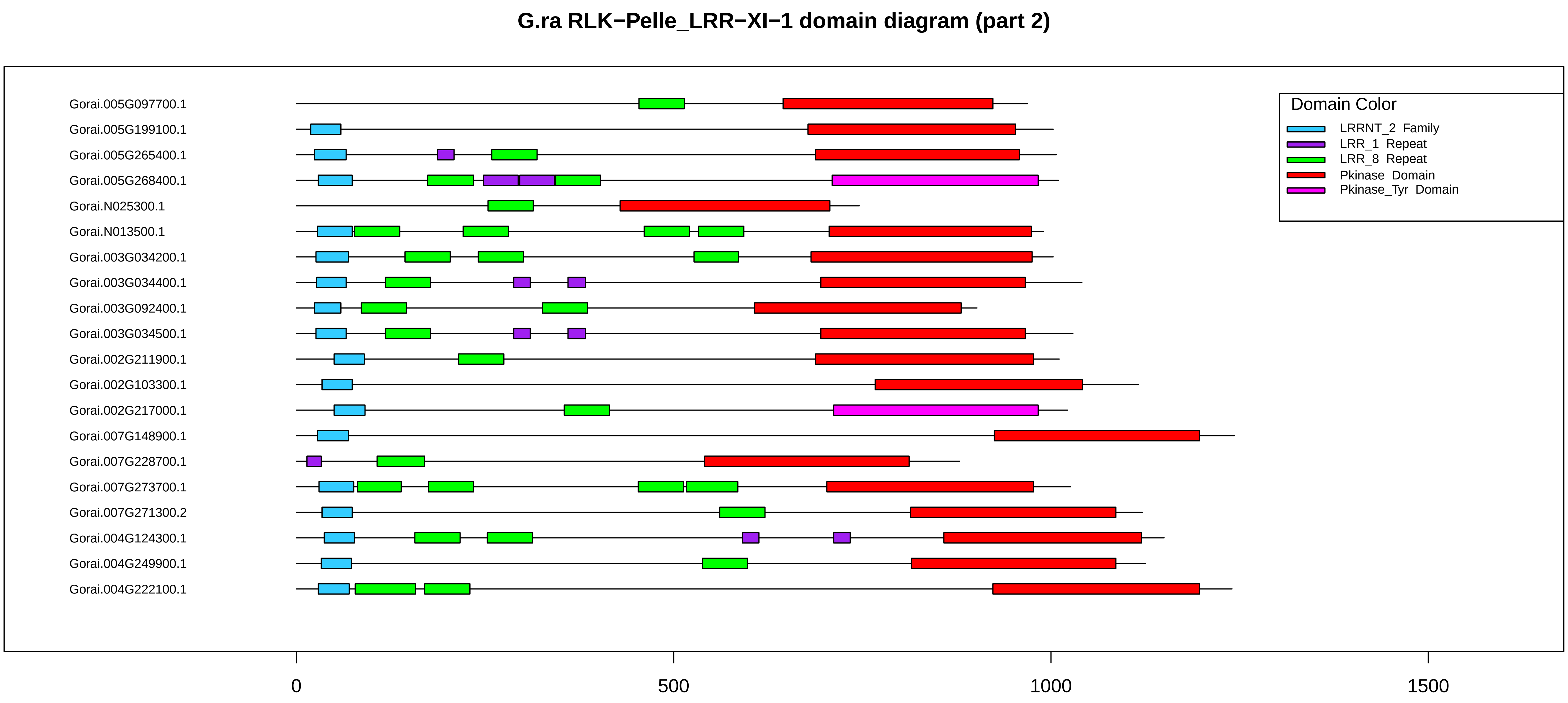
G.ra RLK−Pelle_LRR−XI−1 domain diagram (part 2)
Gorai.005G097700.1
Gorai.005G199100.1
Gorai.005G265400.1
Gorai.005G268400.1
Gorai.N025300.1
Gorai.N013500.1
Gorai.003G034200.1
Gorai.003G034400.1
Gorai.003G092400.1
Gorai.003G034500.1
Gorai.002G211900.1
Gorai.002G103300.1
Gorai.002G217000.1
Gorai.007G148900.1
Gorai.007G228700.1
Gorai.007G273700.1
Gorai.007G271300.2
Gorai.004G124300.1
Gorai.004G249900.1
Gorai.004G222100.1
Domain Color
LRRNT_2 Family
LRR_1 Repeat
LRR_8 Repeat
Pkinase Domain
Pkinase_Tyr Domain
0
500
1000
1500

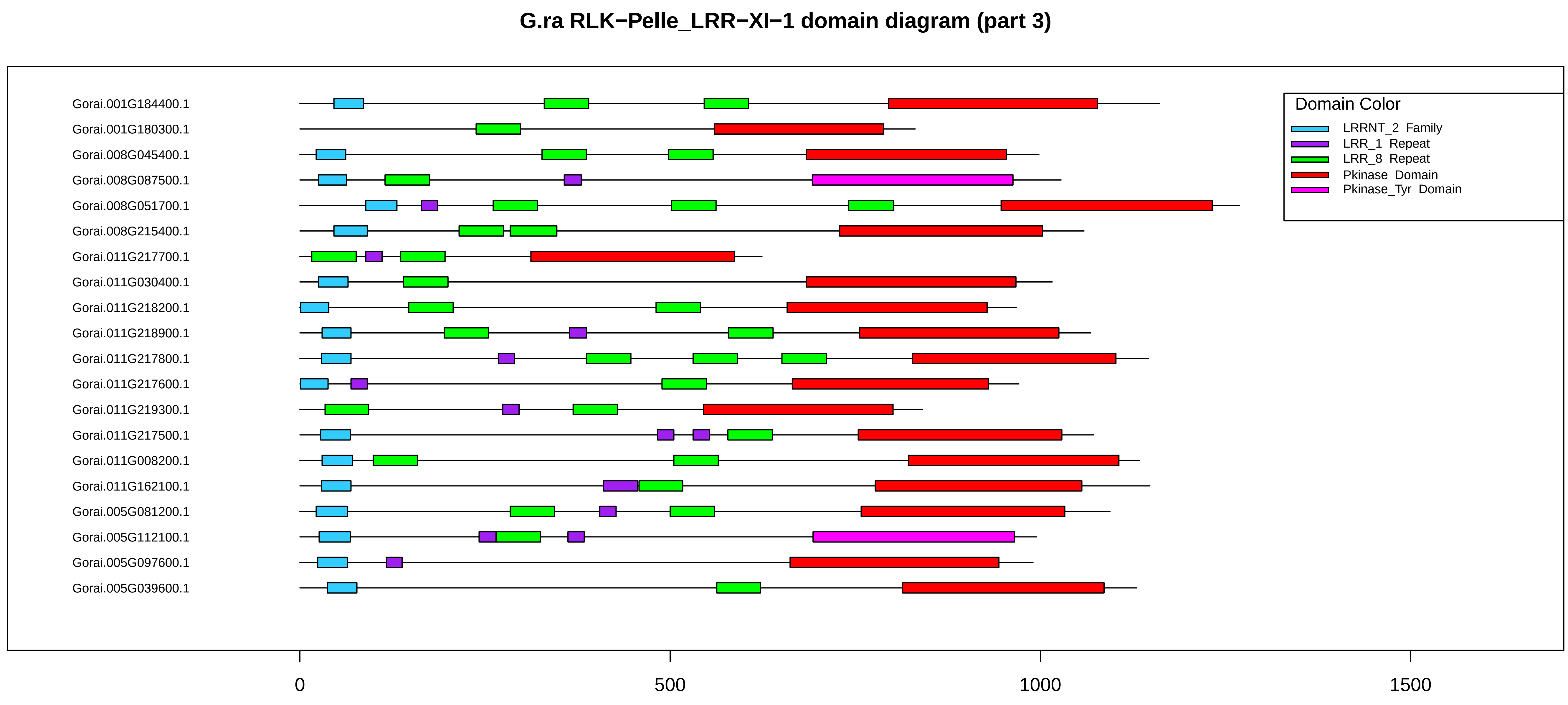

G.ra RLK−Pelle_LRR−XI−1 domain diagram (part 3)
Gorai.001G184400.1
Gorai.001G180300.1
Gorai.008G045400.1
Gorai.008G087500.1
Gorai.008G051700.1
Gorai.008G215400.1
Gorai.011G217700.1
Gorai.011G030400.1
Gorai.011G218200.1
Gorai.011G218900.1
Gorai.011G217800.1
Gorai.011G217600.1
Gorai.011G219300.1
Gorai.011G217500.1
Gorai.011G008200.1
Gorai.011G162100.1
Gorai.005G081200.1
Gorai.005G112100.1
Gorai.005G097600.1
Gorai.005G039600.1
Domain Color
LRRNT_2 Family
LRR_1 Repeat
LRR_8 Repeat
Pkinase Domain
Pkinase_Tyr Domain
0
500
1000
1500

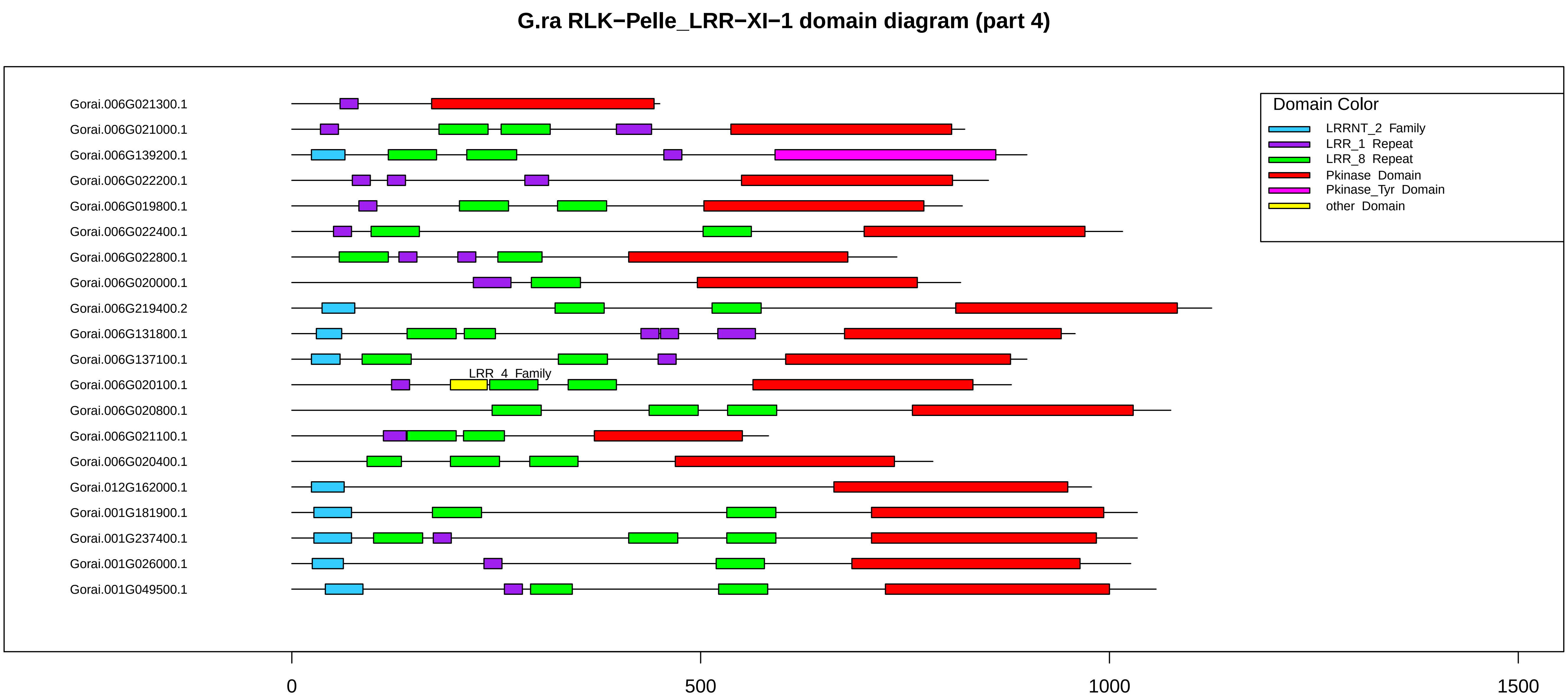

G.ra RLK−Pelle_LRR−XI−1 domain diagram (part 4)
Gorai.006G021300.1
Gorai.006G021000.1
Gorai.006G139200.1
Gorai.006G022200.1
Gorai.006G019800.1
Gorai.006G022400.1
Gorai.006G022800.1
Gorai.006G020000.1
Gorai.006G219400.2
Gorai.006G131800.1
Gorai.006G137100.1
Gorai.006G020100.1
Gorai.006G020800.1
Gorai.006G021100.1
Gorai.006G020400.1
Gorai.012G162000.1
Gorai.001G181900.1
Gorai.001G237400.1
Gorai.001G026000.1
Gorai.001G049500.1
LRR_4 Family
Domain Color
LRRNT_2 Family
LRR_1 Repeat
LRR_8 Repeat
Pkinase Domain
Pkinase_Tyr Domain
other Domain
0
500
1000
1500

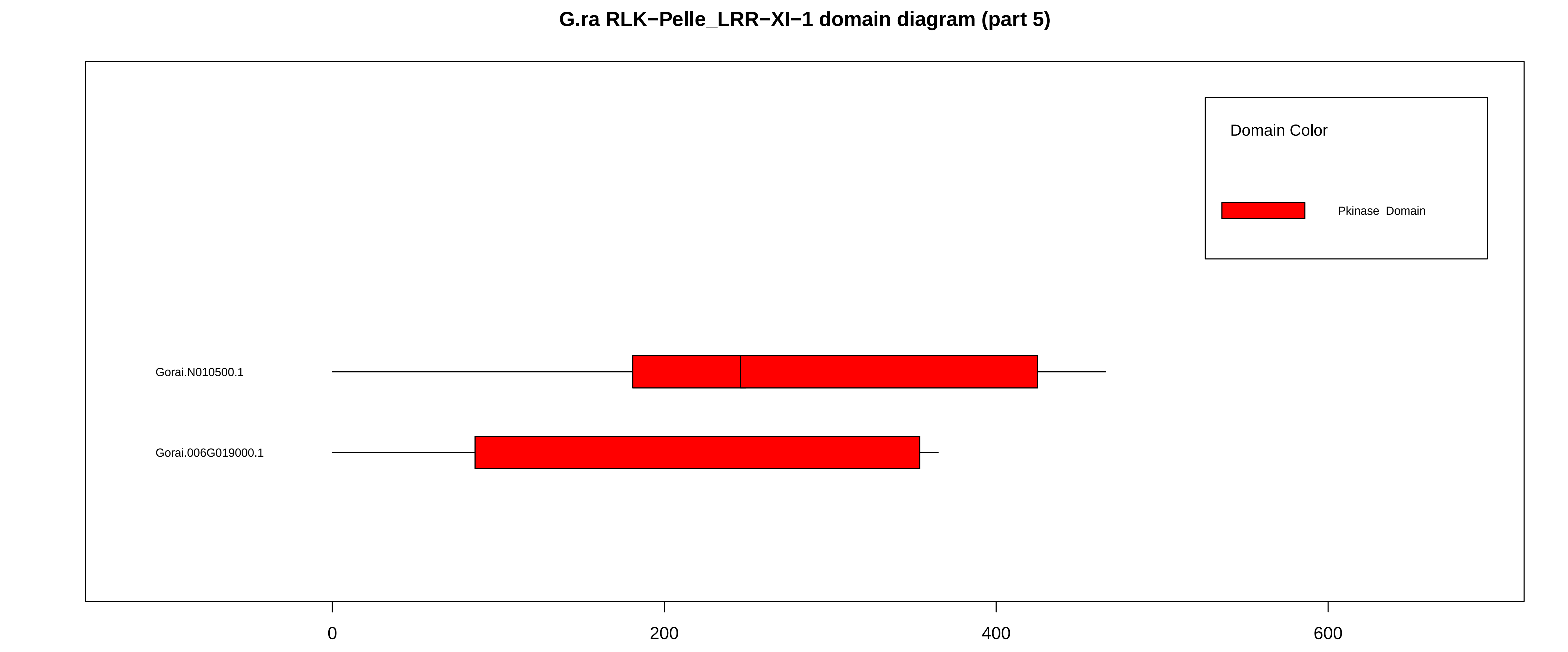
G.ra RLK−Pelle_LRR−XI−1 domain diagram (part 5)
Domain Color
Pkinase Domain
Gorai.N010500.1
Gorai.006G019000.1
0
200
400
600

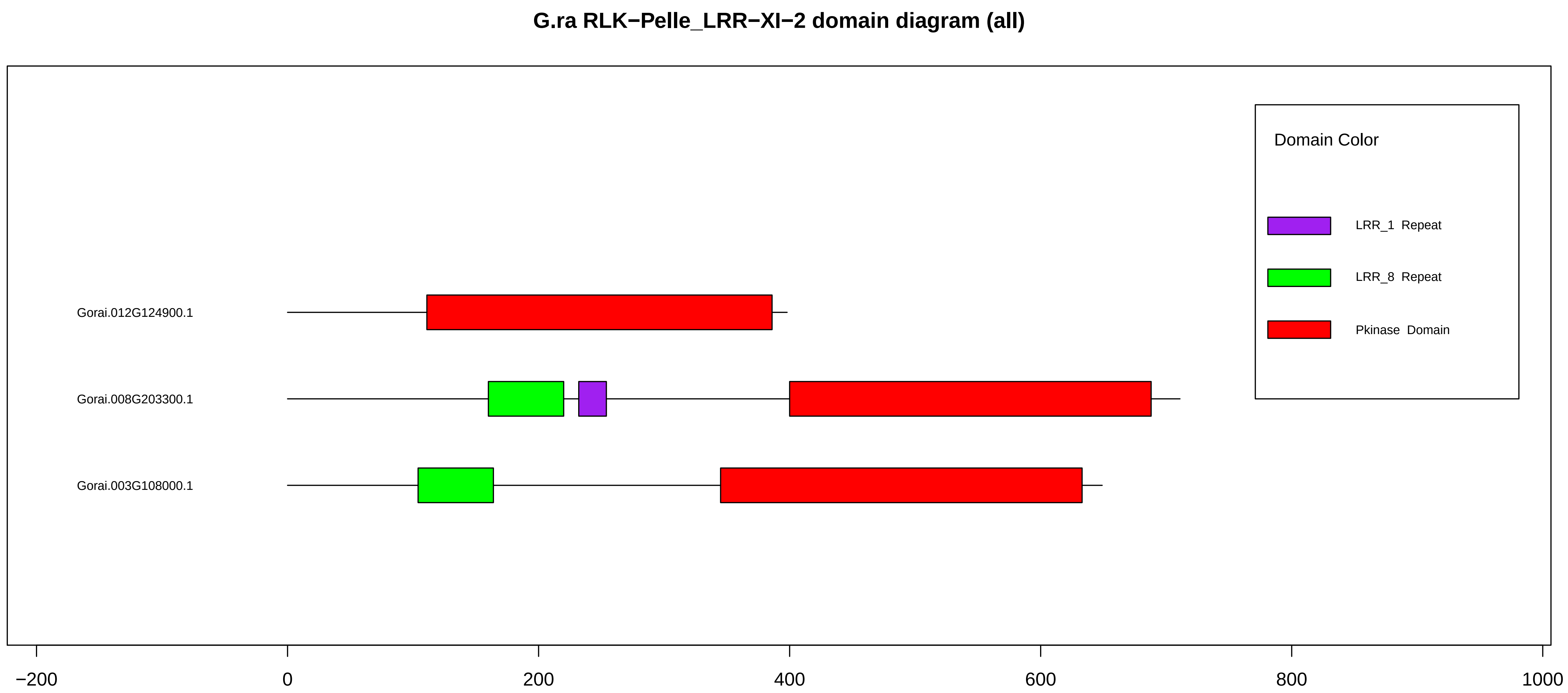
G.ra RLK−Pelle_LRR−XI−2 domain diagram (all)
Domain Color
LRR_1 Repeat
LRR_8 Repeat
Pkinase Domain
Gorai.012G124900.1
Gorai.008G203300.1
Gorai.003G108000.1
−200
0
200
400
600
800
1000

G.ra RLK−Pelle_LRR−XII−1 domain diagram (part 1)

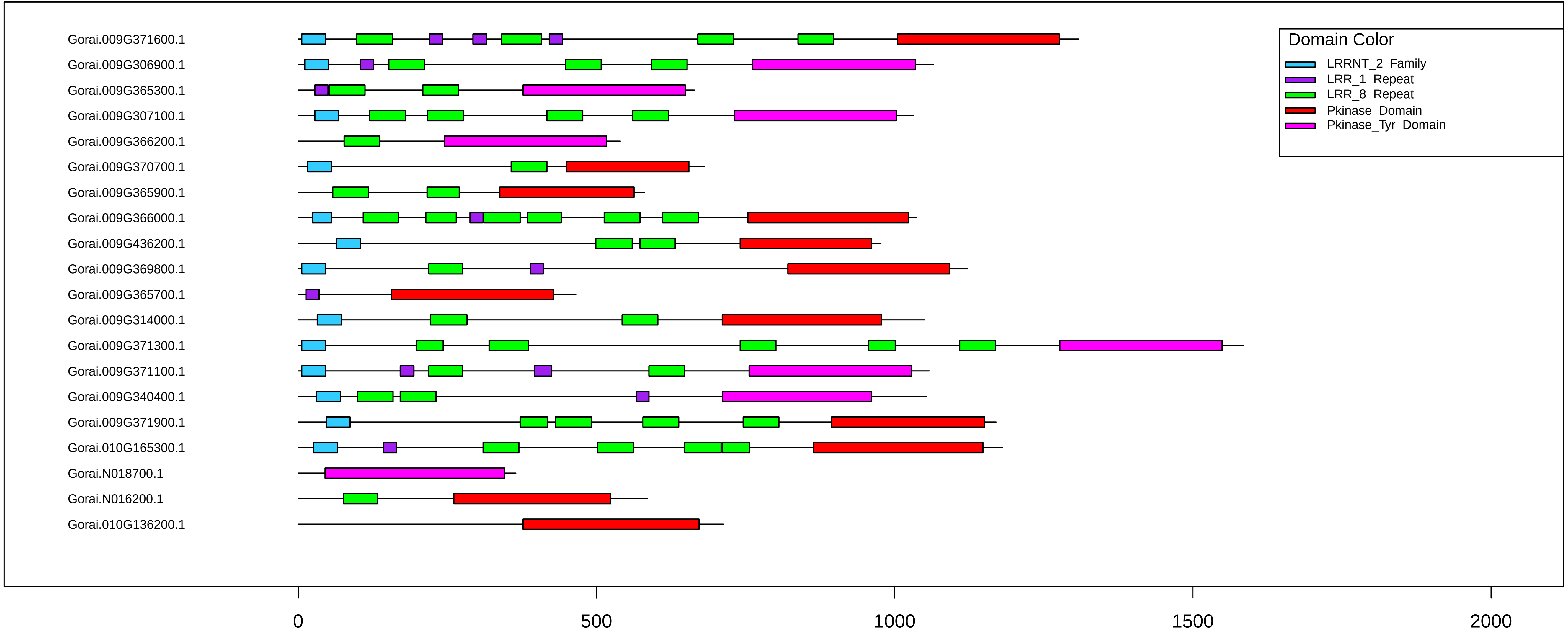

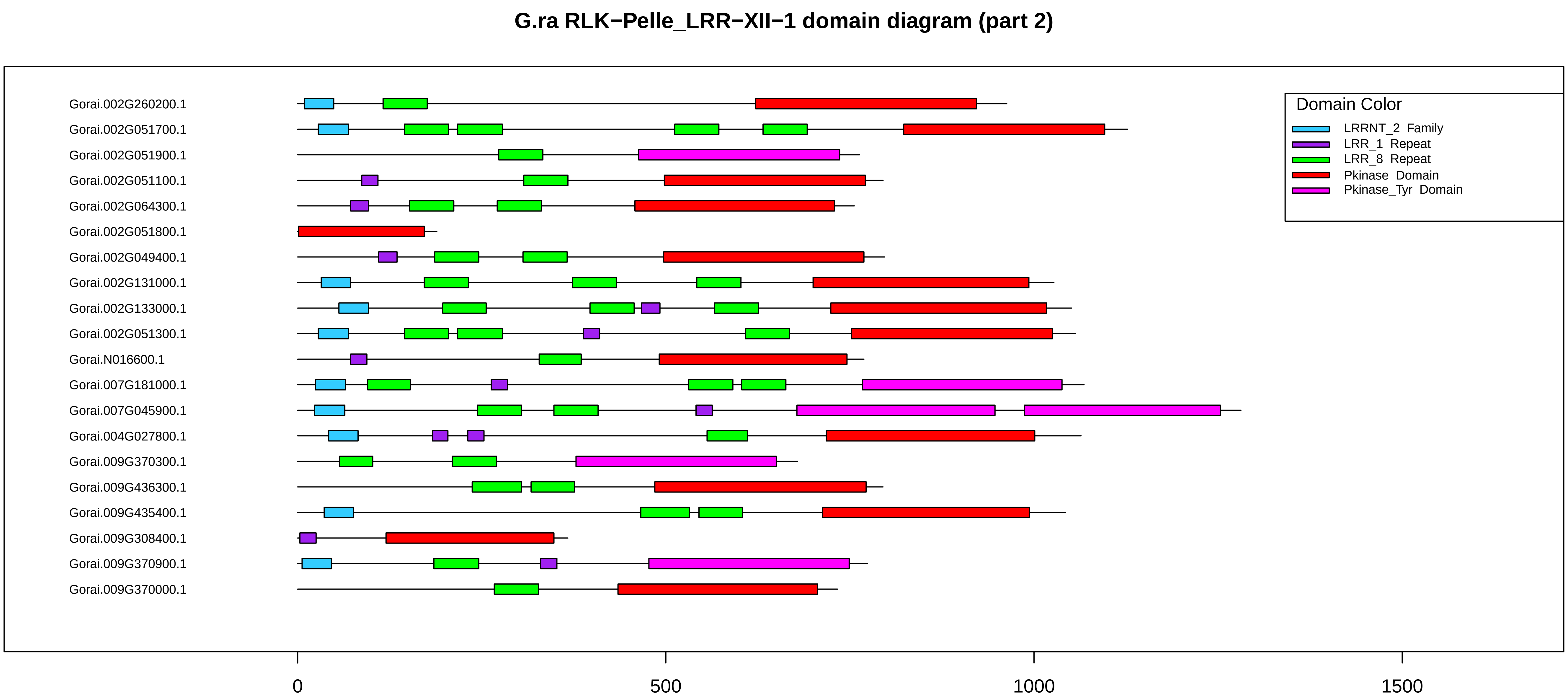
G.ra RLK−Pelle_LRR−XII−1 domain diagram (part 2)
Gorai.002G260200.1
Gorai.002G051700.1
Gorai.002G051900.1
Gorai.002G051100.1
Gorai.002G064300.1
Gorai.002G051800.1
Gorai.002G049400.1
Gorai.002G131000.1
Gorai.002G133000.1
Gorai.002G051300.1
Gorai.N016600.1
Gorai.007G181000.1
Gorai.007G045900.1
Gorai.004G027800.1
Gorai.009G370300.1
Gorai.009G436300.1
Gorai.009G435400.1
Gorai.009G308400.1
Gorai.009G370900.1
Gorai.009G370000.1
Domain Color
LRRNT_2 Family
LRR_1 Repeat
LRR_8 Repeat
Pkinase Domain
Pkinase_Tyr Domain
0
500
1000
1500

G.ra RLK−Pelle_LRR−XII−1 domain diagram (part 3)

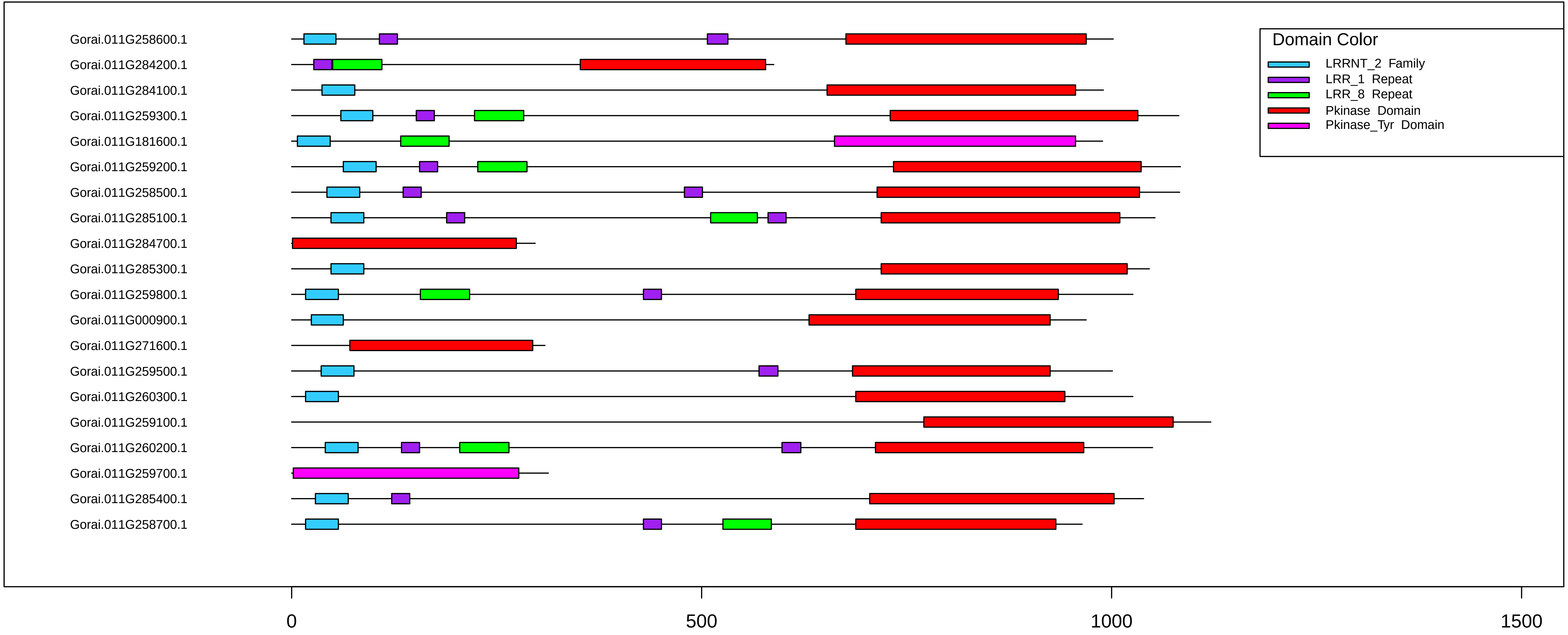

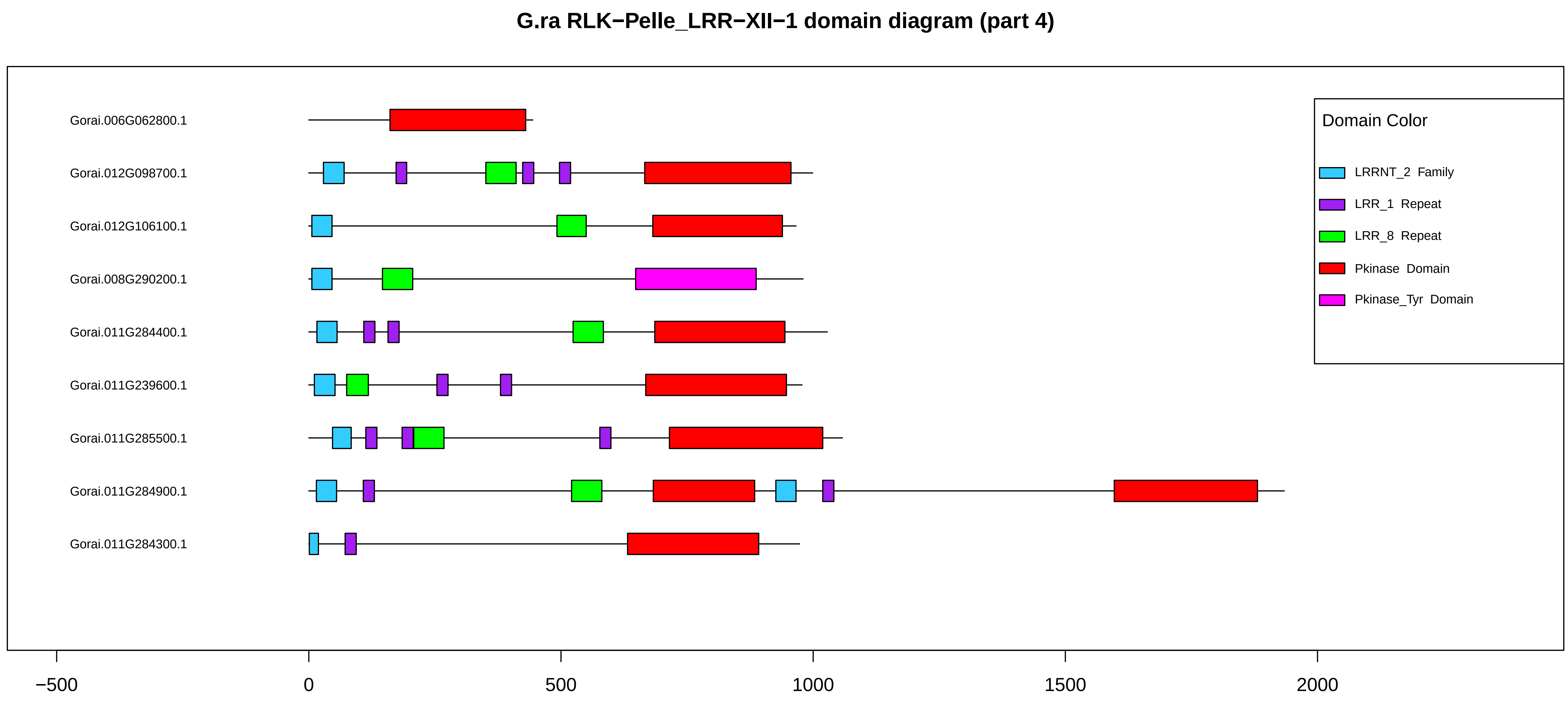
G.ra RLK−Pelle_LRR−XII−1 domain diagram (part 4)
Gorai.006G062800.1
Gorai.012G098700.1
Gorai.012G106100.1
Gorai.008G290200.1
Gorai.011G284400.1
Gorai.011G239600.1
Gorai.011G285500.1
Gorai.011G284900.1
Gorai.011G284300.1
Domain Color
LRRNT_2 Family
LRR_1 Repeat
LRR_8 Repeat
Pkinase Domain
Pkinase_Tyr Domain
−500
0
500
1000
1500
2000

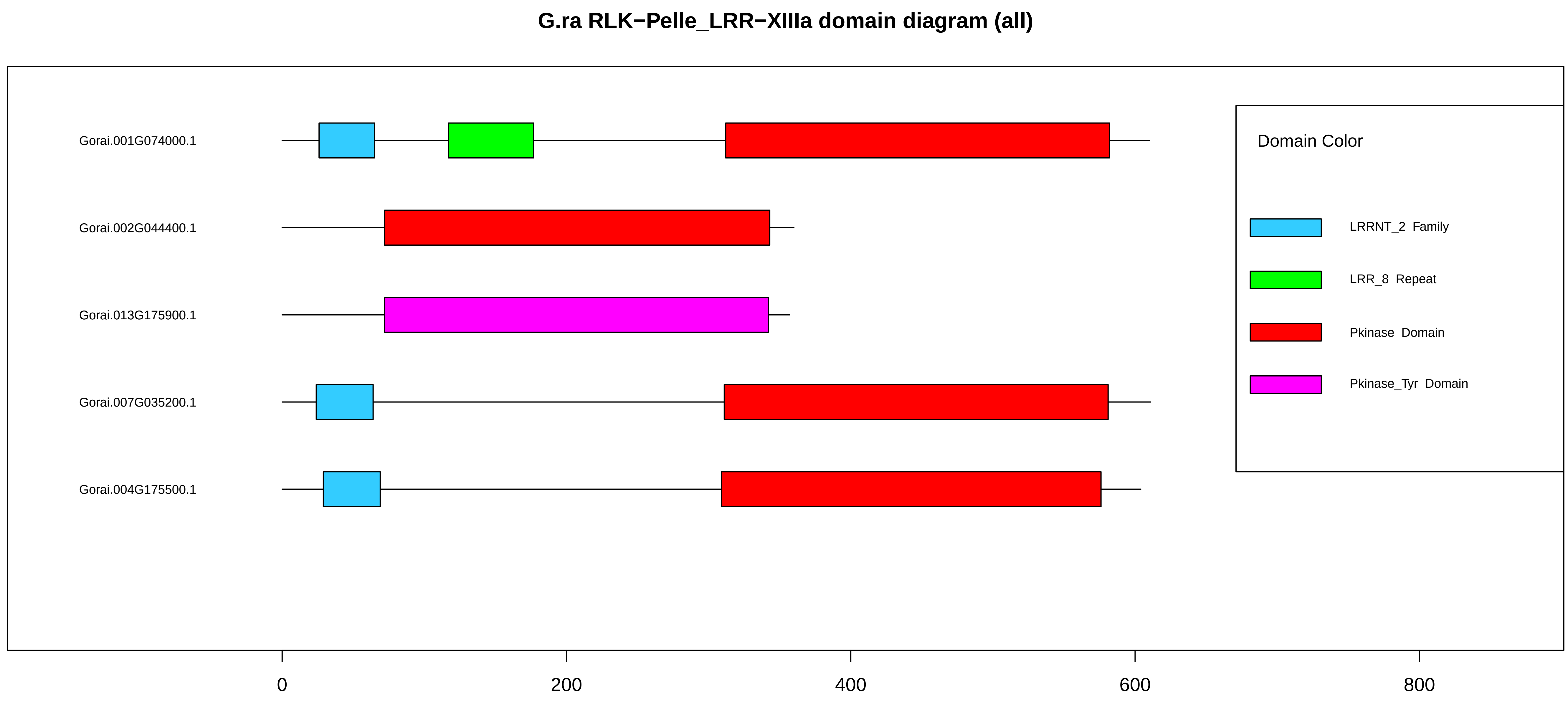
G.ra RLK−Pelle_LRR−XIIIa domain diagram (all)
Gorai.001G074000.1
Gorai.002G044400.1
Gorai.013G175900.1
Gorai.007G035200.1
Gorai.004G175500.1
Domain Color
LRRNT_2 Family
LRR_8 Repeat
Pkinase Domain
Pkinase_Tyr Domain
0
200
400
600
800

G.ra RLK−Pelle_LRR−XIIIb domain diagram (all)

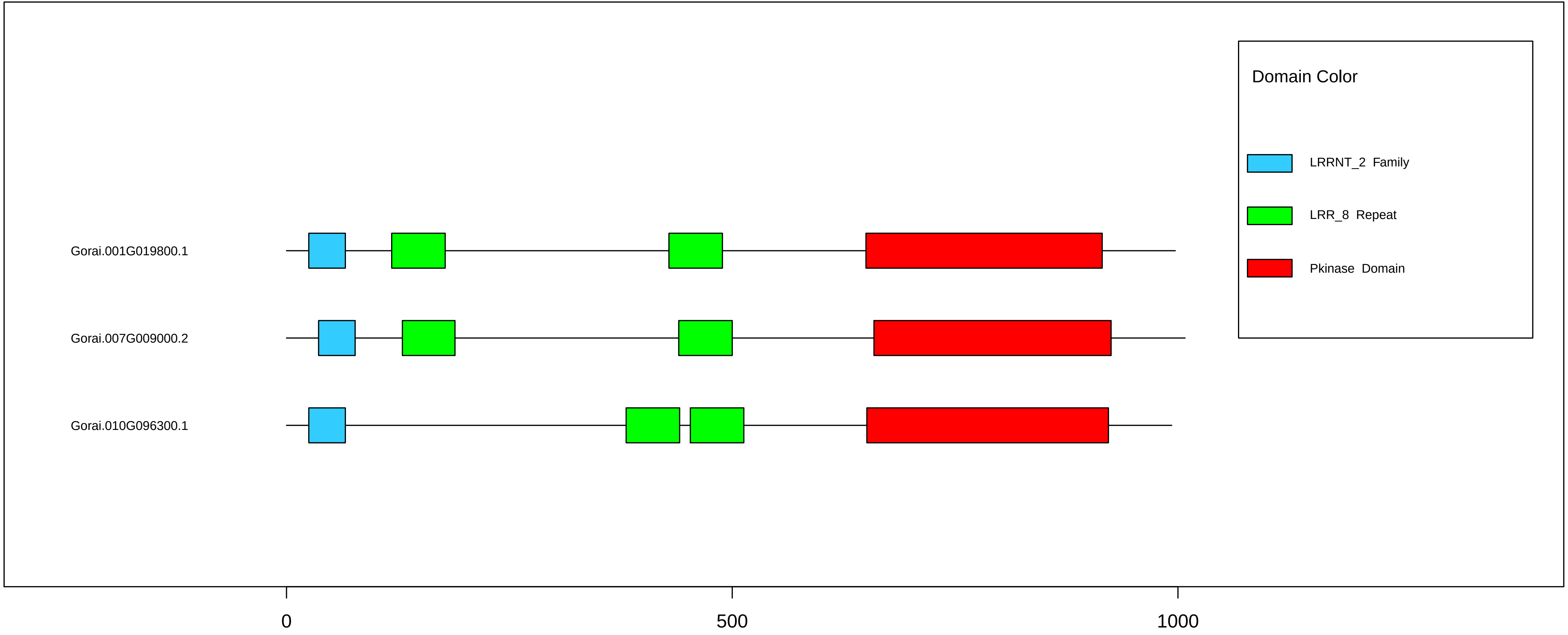

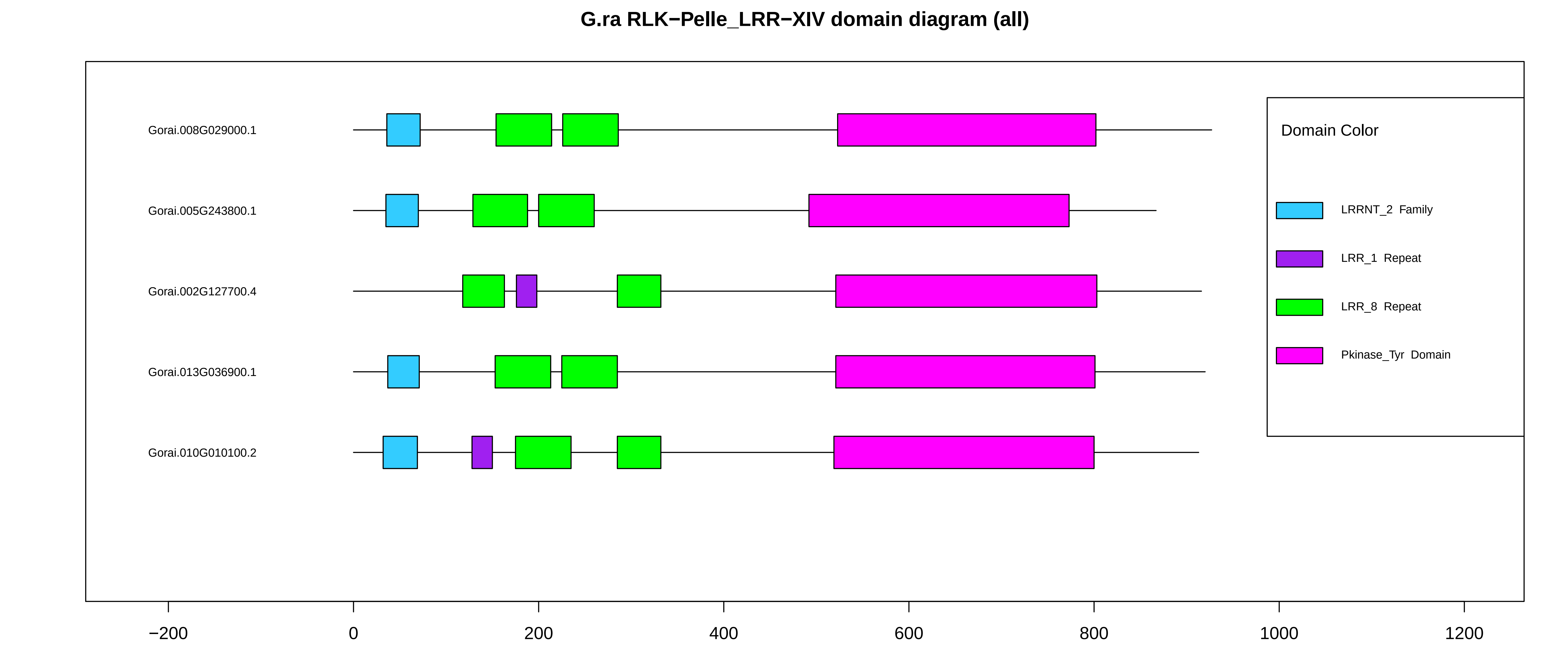

G.ra RLK−Pelle_LRR−XIV domain diagram (all)
Gorai.008G029000.1
Gorai.005G243800.1
Gorai.002G127700.4
Gorai.013G036900.1
Gorai.010G010100.2
Domain Color
LRRNT_2 Family
LRR_1 Repeat
LRR_8 Repeat
Pkinase_Tyr Domain
−200
0
200
400
600
800
1000
1200

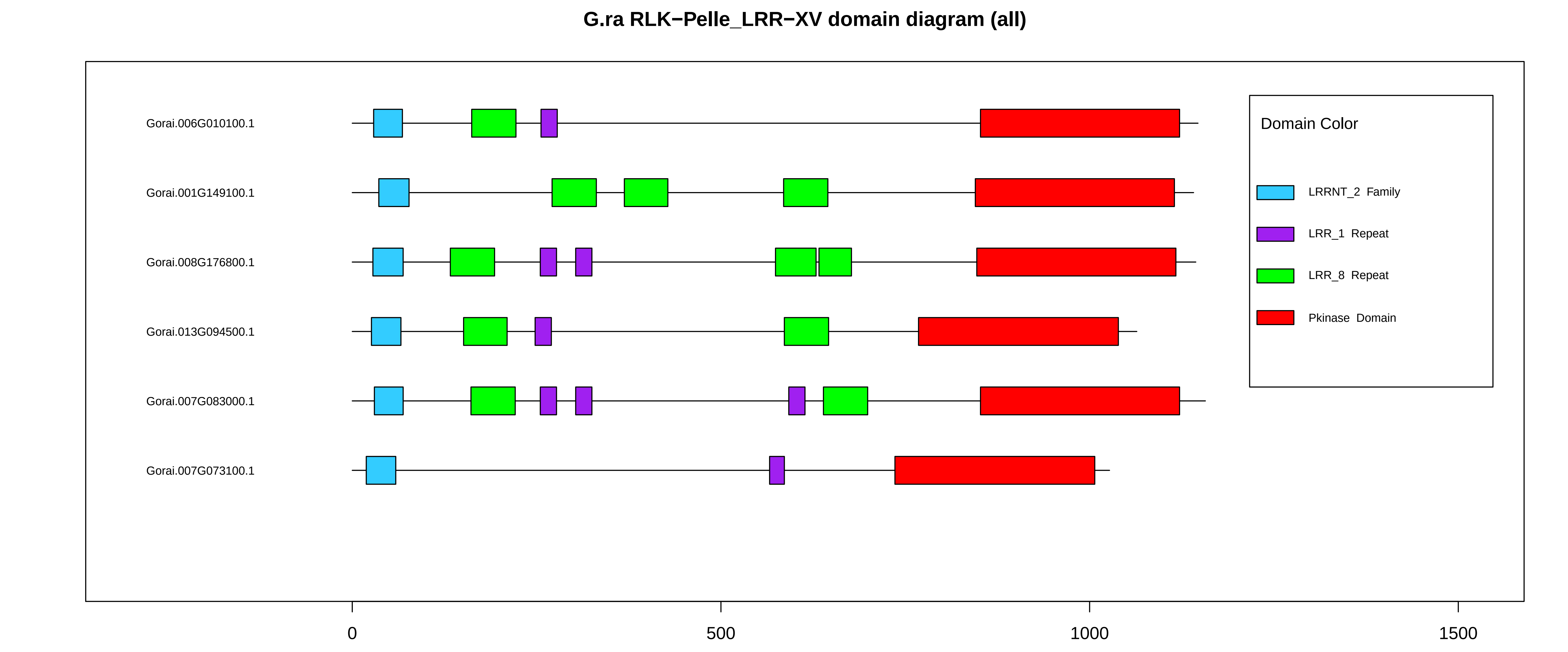

G.ra RLK−Pelle_LRR−XV domain diagram (all)
Gorai.006G010100.1
Gorai.001G149100.1
Gorai.008G176800.1
Gorai.013G094500.1
Gorai.007G083000.1
Gorai.007G073100.1
Domain Color
LRRNT_2 Family
LRR_1 Repeat
LRR_8 Repeat
Pkinase Domain
0
500
1000
1500

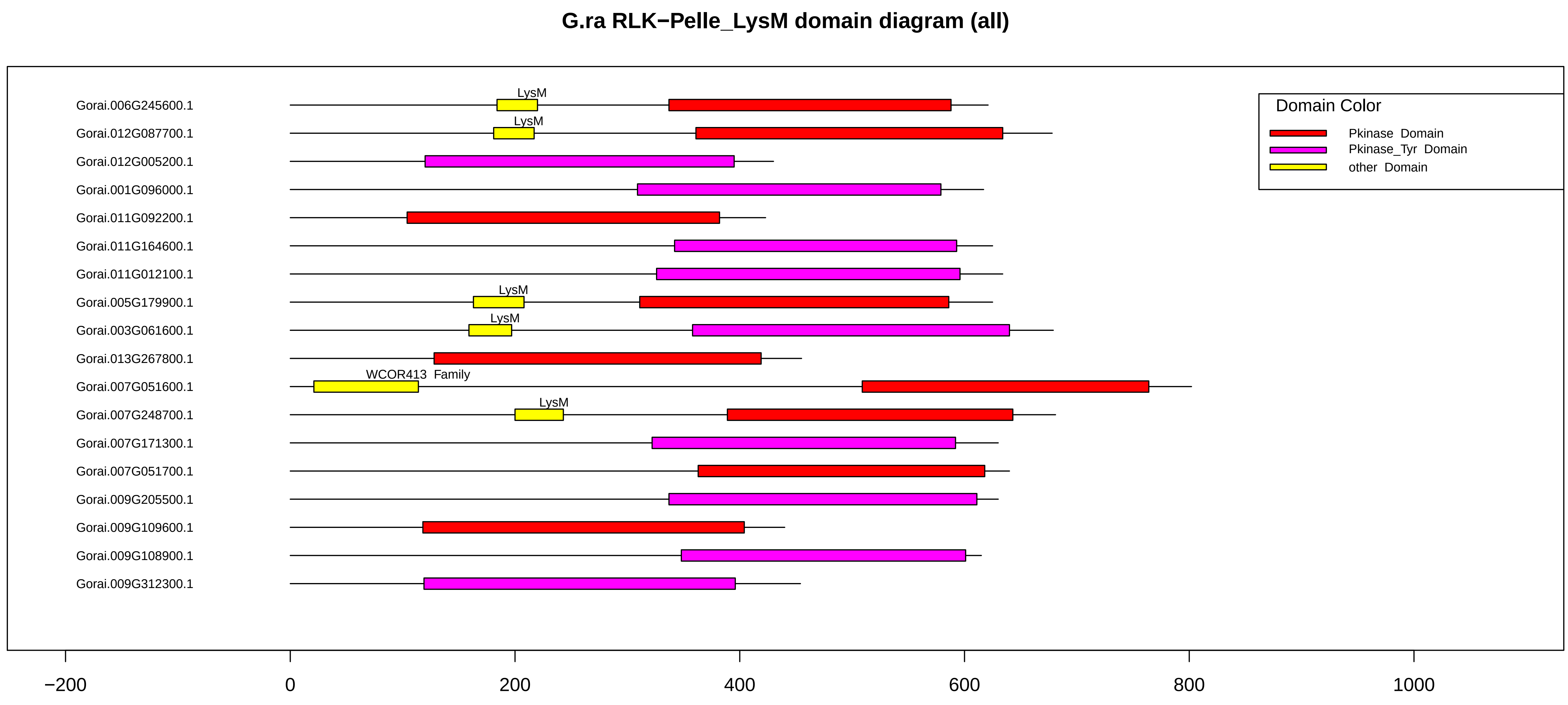
G.ra RLK−Pelle_LysM domain diagram (all)
Gorai.006G245600.1
Gorai.012G087700.1
Gorai.012G005200.1
Gorai.001G096000.1
Gorai.011G092200.1
Gorai.011G164600.1
Gorai.011G012100.1
Gorai.005G179900.1
Gorai.003G061600.1
Gorai.013G267800.1
Gorai.007G051600.1
Gorai.007G248700.1
Gorai.007G171300.1
Gorai.007G051700.1
Gorai.009G205500.1
Gorai.009G109600.1
Gorai.009G108900.1
Gorai.009G312300.1
LysM
LysM
LysM
LysM
WCOR413 Family
LysM
Domain Color
Pkinase Domain
Pkinase_Tyr Domain
other Domain
−200
0
200
400
600
800
1000

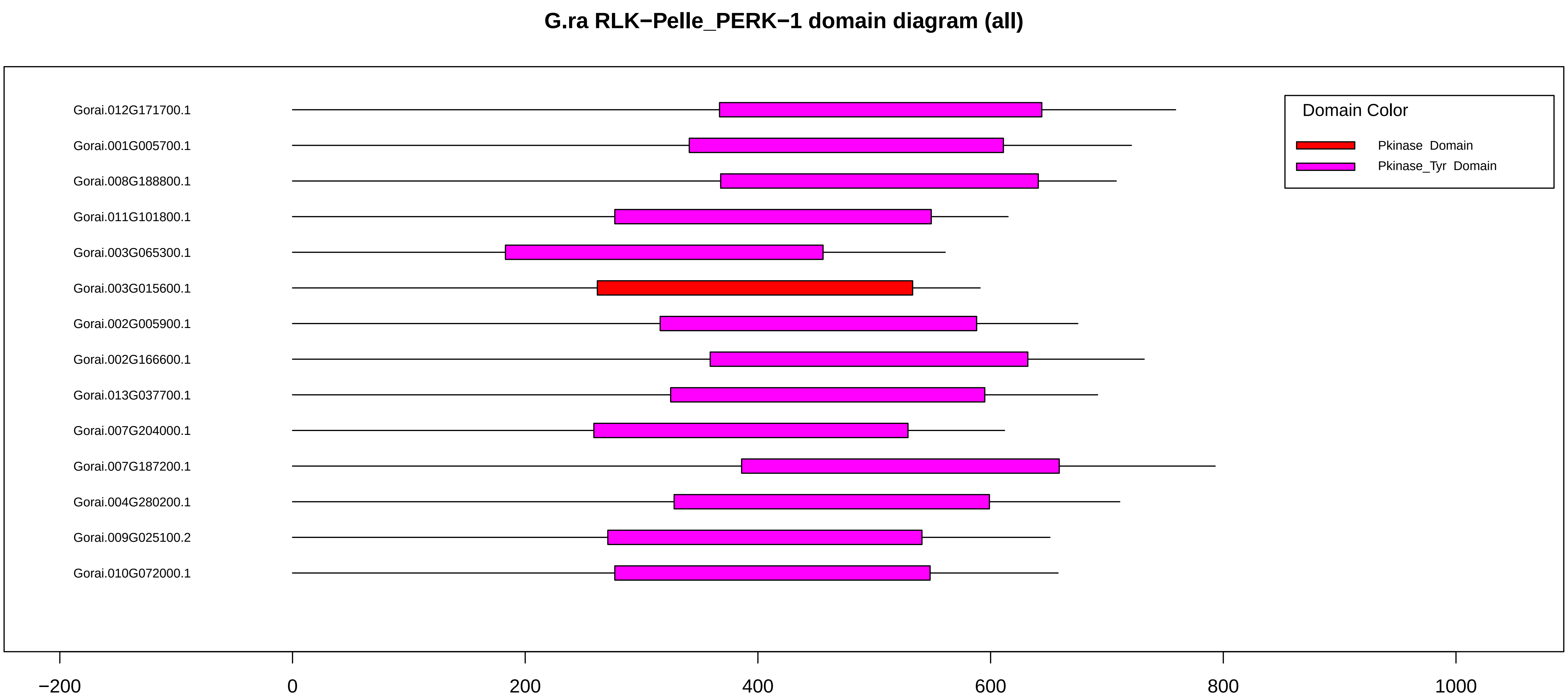
G.ra RLK−Pelle_PERK−1 domain diagram (all)
Gorai.012G171700.1
Gorai.001G005700.1
Gorai.008G188800.1
Gorai.011G101800.1
Gorai.003G065300.1
Gorai.003G015600.1
Gorai.002G005900.1
Gorai.002G166600.1
Gorai.013G037700.1
Gorai.007G204000.1
Gorai.007G187200.1
Gorai.004G280200.1
Gorai.009G025100.2
Gorai.010G072000.1
Domain Color
Pkinase Domain
Pkinase_Tyr Domain
−200
0
200
400
600
800
1000

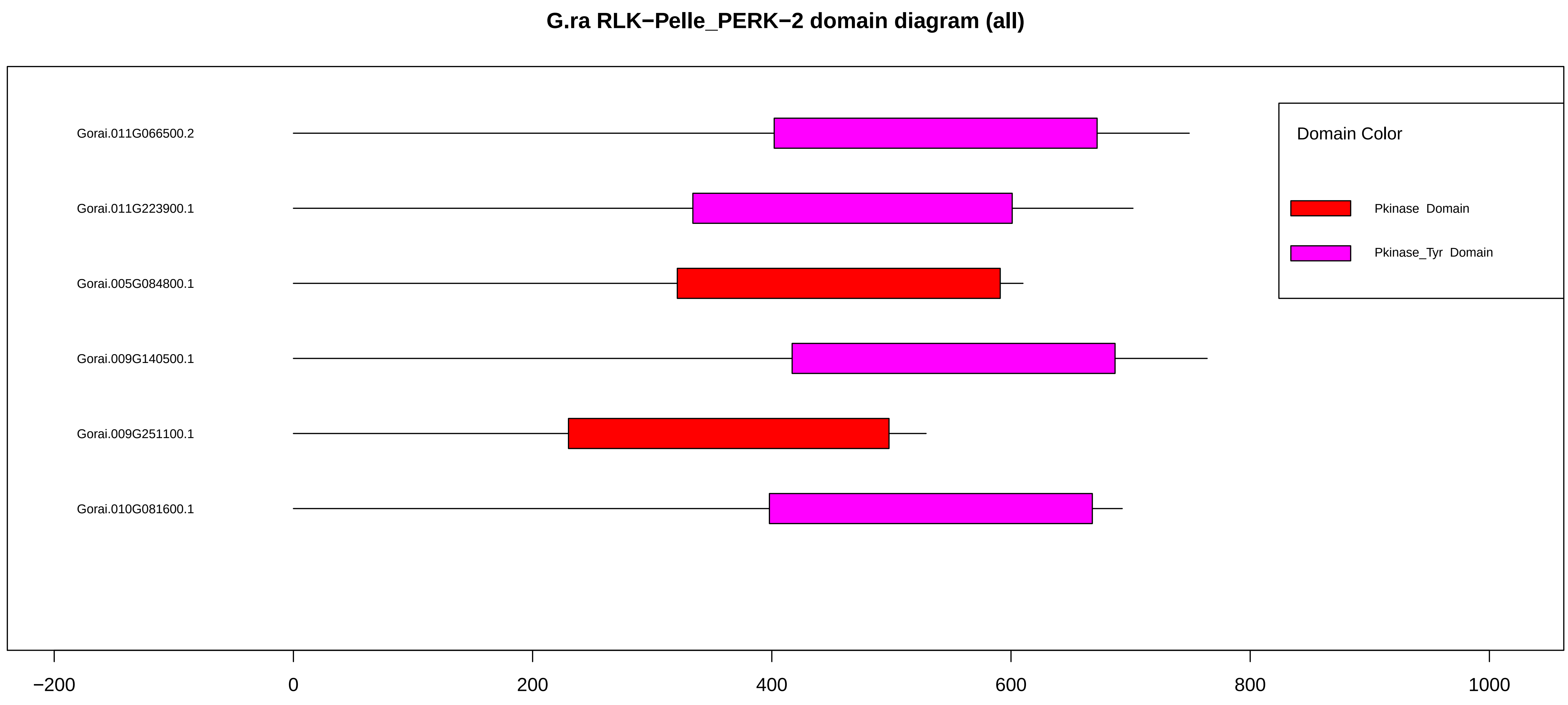
G.ra RLK−Pelle_PERK−2 domain diagram (all)
Gorai.011G066500.2
Gorai.011G223900.1
Gorai.005G084800.1
Gorai.009G140500.1
Gorai.009G251100.1
Gorai.010G081600.1
Domain Color
Pkinase Domain
Pkinase_Tyr Domain
−200
0
200
400
600
800
1000

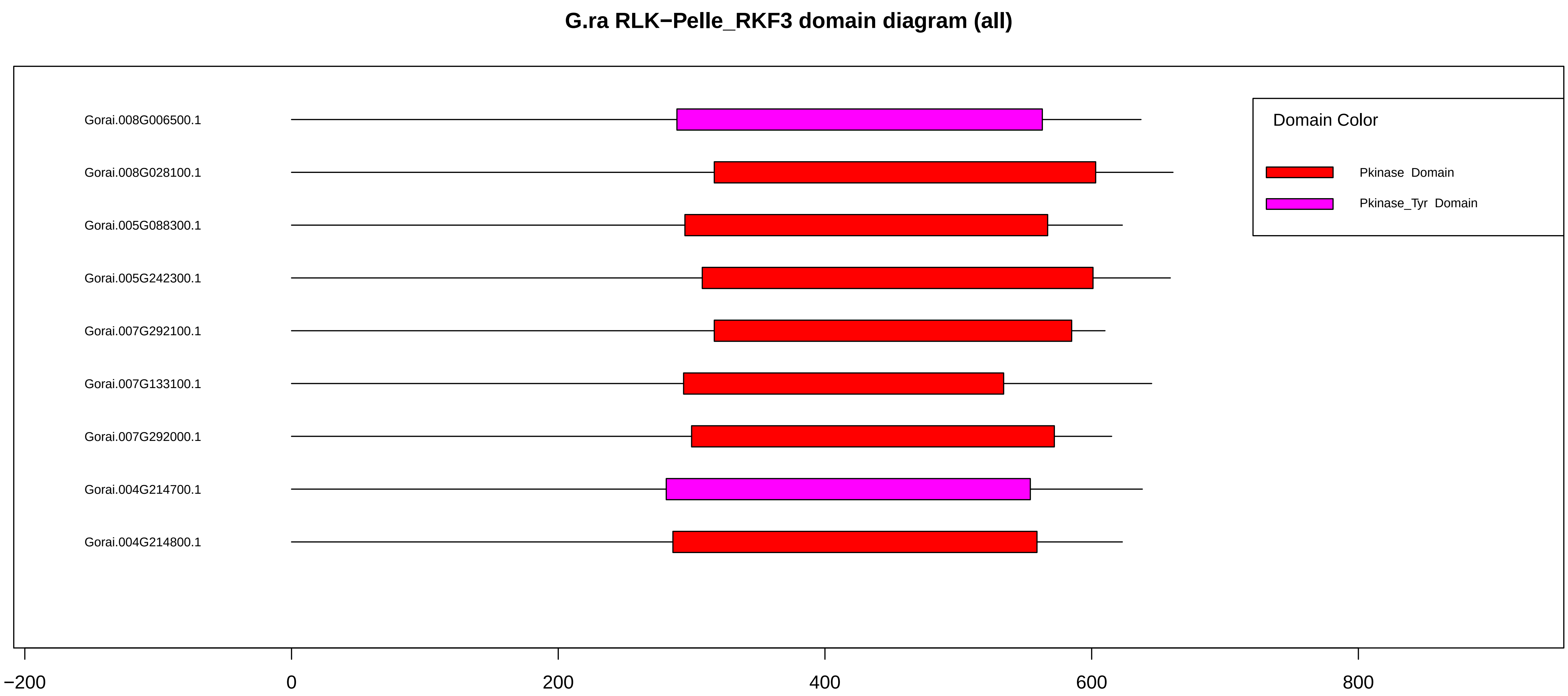
G.ra RLK−Pelle_RKF3 domain diagram (all)
Gorai.008G006500.1
Gorai.008G028100.1
Gorai.005G088300.1
Gorai.005G242300.1
Gorai.007G292100.1
Gorai.007G133100.1
Gorai.007G292000.1
Gorai.004G214700.1
Gorai.004G214800.1
Domain Color
Pkinase Domain
Pkinase_Tyr Domain
−200
0
200
400
600
800

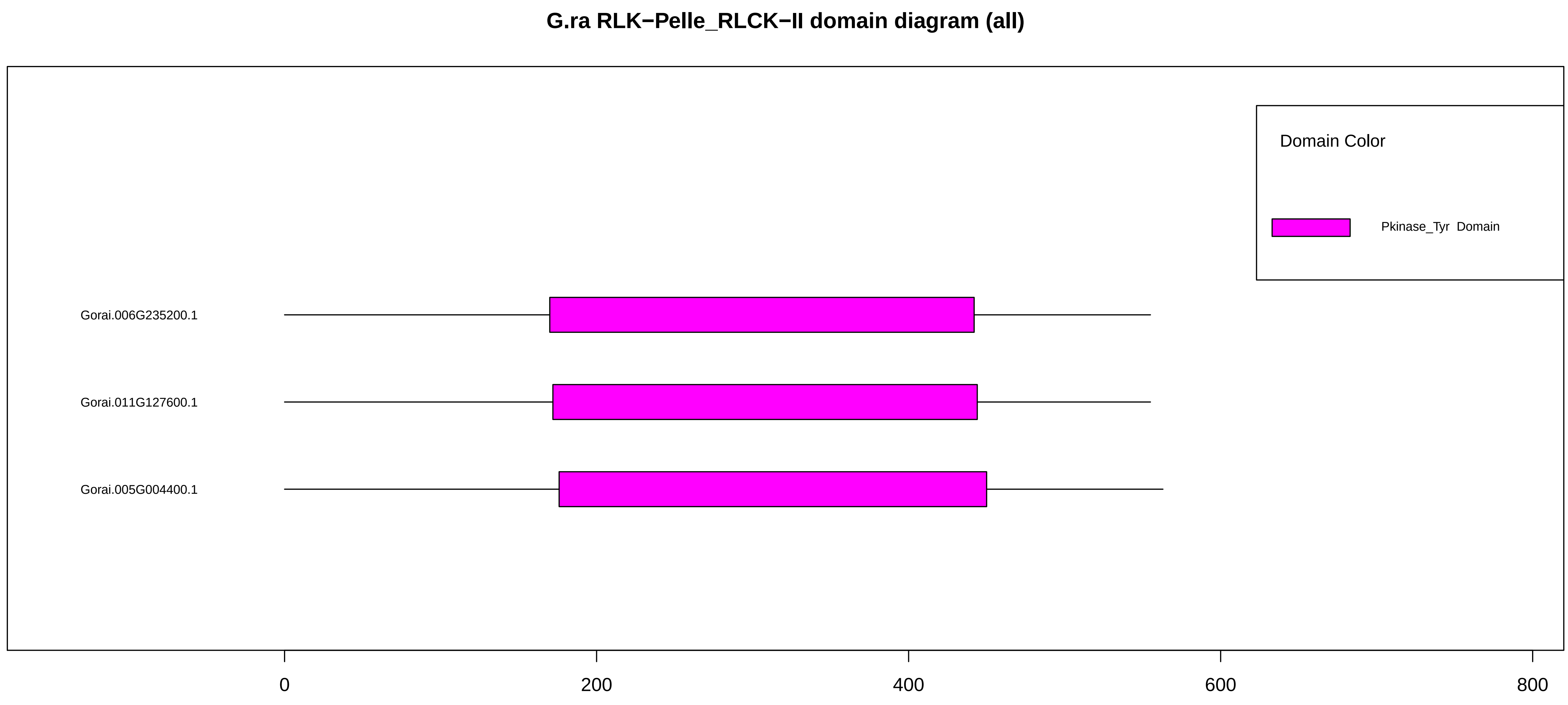
G.ra RLK−Pelle_RLCK−II domain diagram (all)
Domain Color
Pkinase_Tyr Domain
Gorai.006G235200.1
Gorai.011G127600.1
Gorai.005G004400.1
0
200
400
600
800

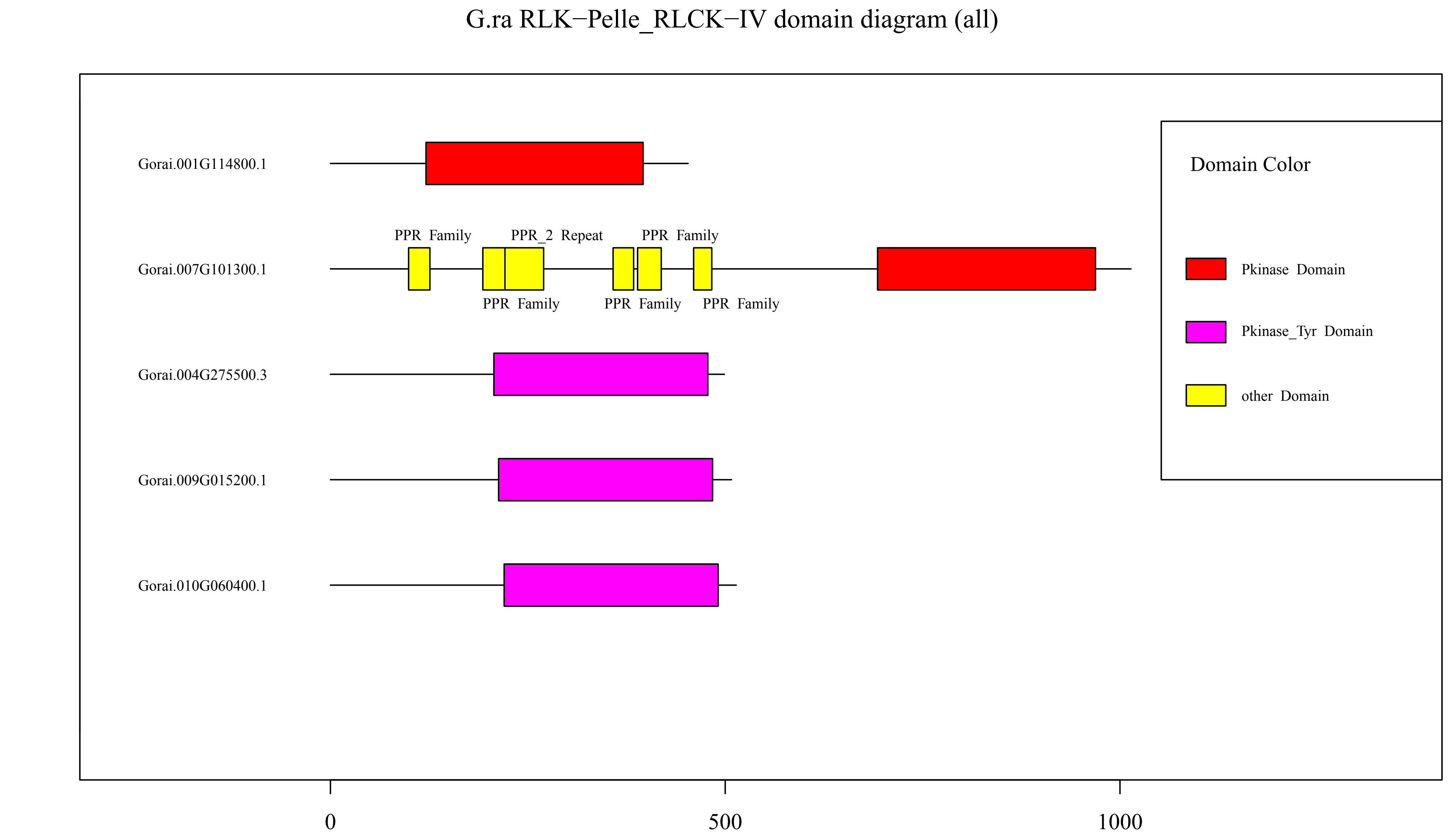
G.ra RLK−Pelle_RLCK−IV domain diagram (all)
Gorai.001G114800.1
Gorai.007G101300.1
Gorai.004G275500.3
Gorai.009G015200.1
Gorai.010G060400.1
PPR Family
PPR_2 Repeat
PPR Family
PPR Family
PPR Family
PPR Family
Domain Color
Pkinase Domain
Pkinase_Tyr Domain
other Domain
0
500
1000

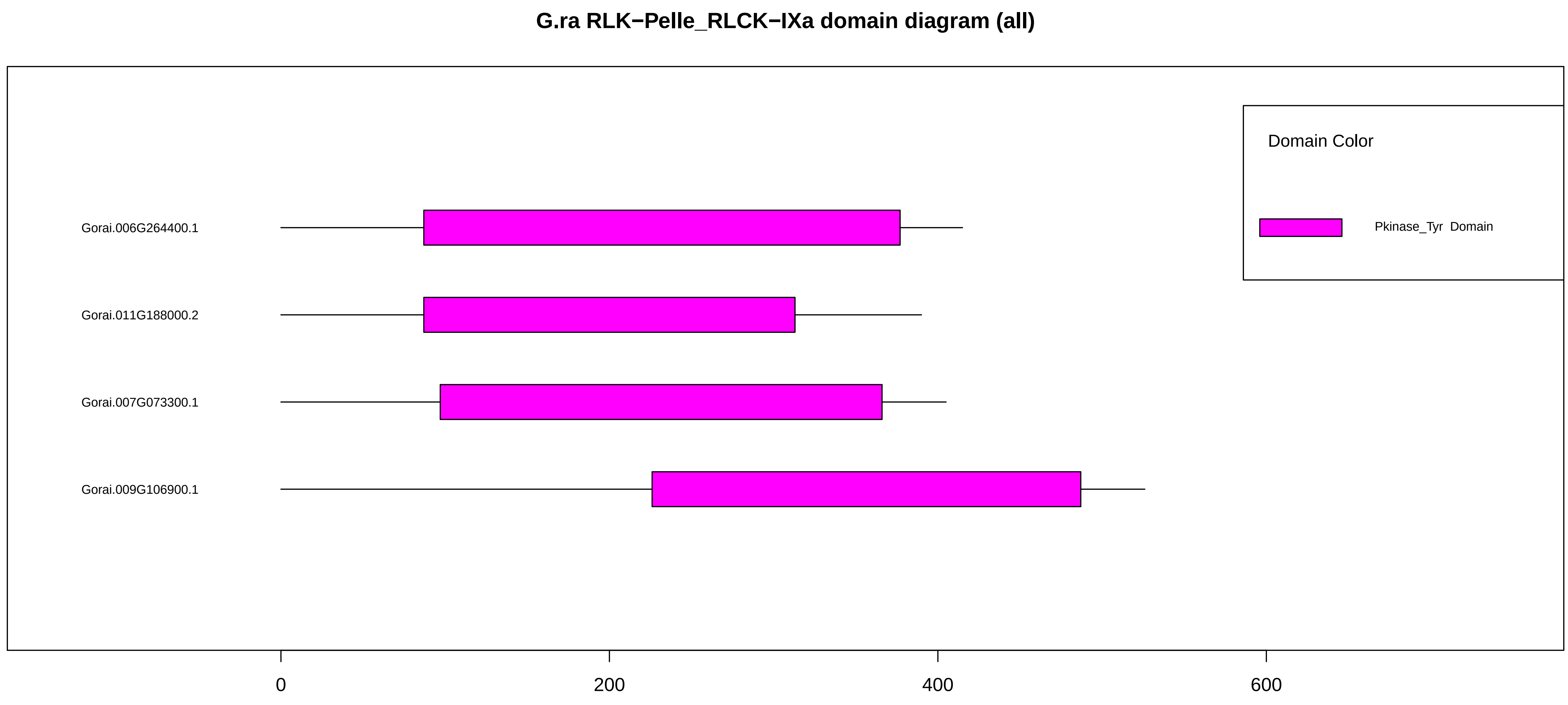

G.ra RLK−Pelle_RLCK−IXa domain diagram (all)
Domain Color
Pkinase_Tyr Domain
Gorai.006G264400.1
Gorai.011G188000.2
Gorai.007G073300.1
Gorai.009G106900.1
0
200
400
600

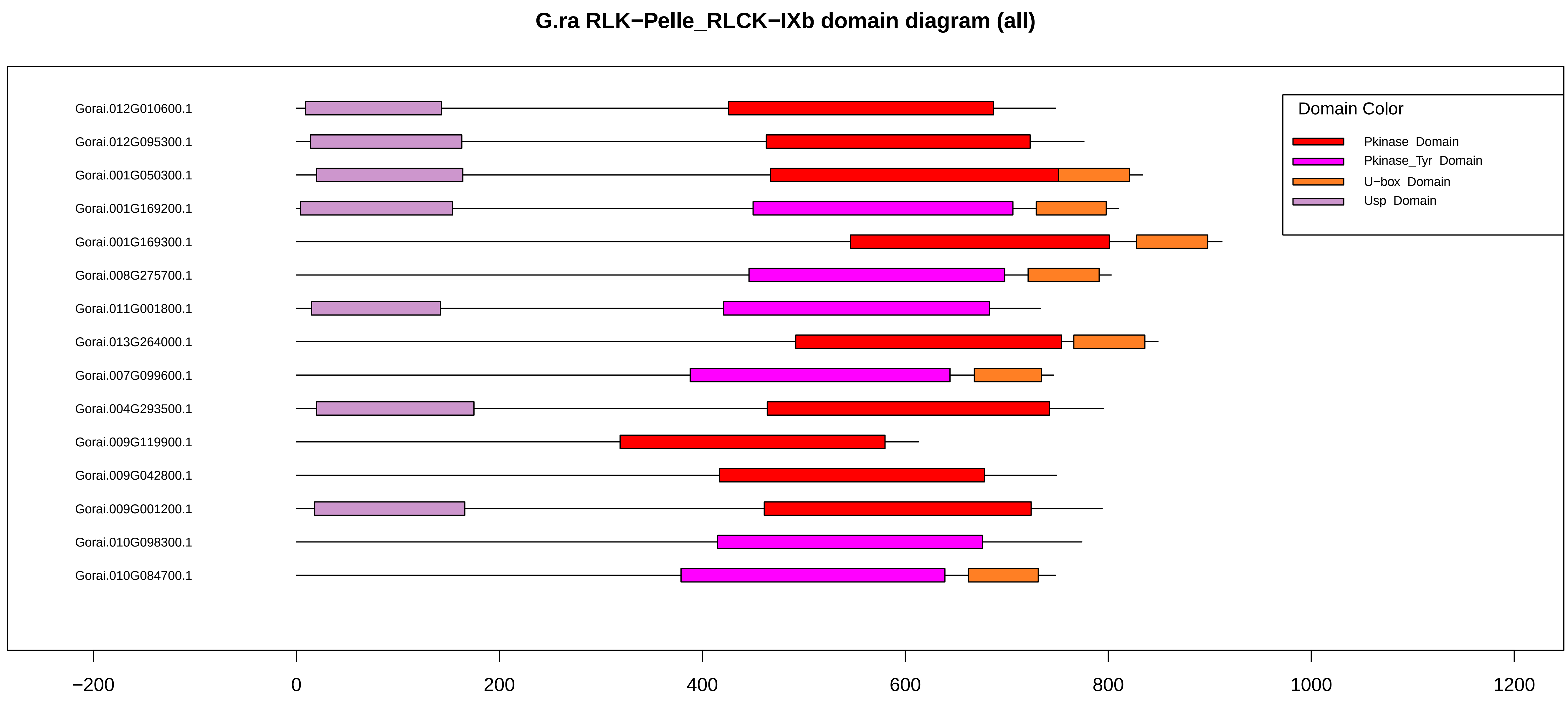

G.ra RLK−Pelle_RLCK−IXb domain diagram (all)
Gorai.012G010600.1
Gorai.012G095300.1
Gorai.001G050300.1
Gorai.001G169200.1
Gorai.001G169300.1
Gorai.008G275700.1
Gorai.011G001800.1
Gorai.013G264000.1
Gorai.007G099600.1
Gorai.004G293500.1
Gorai.009G119900.1
Gorai.009G042800.1
Gorai.009G001200.1
Gorai.010G098300.1
Gorai.010G084700.1
Domain Color
Pkinase Domain
Pkinase_Tyr Domain
U−box Domain
Usp Domain
−200
0
200
400
600
800
1000
1200

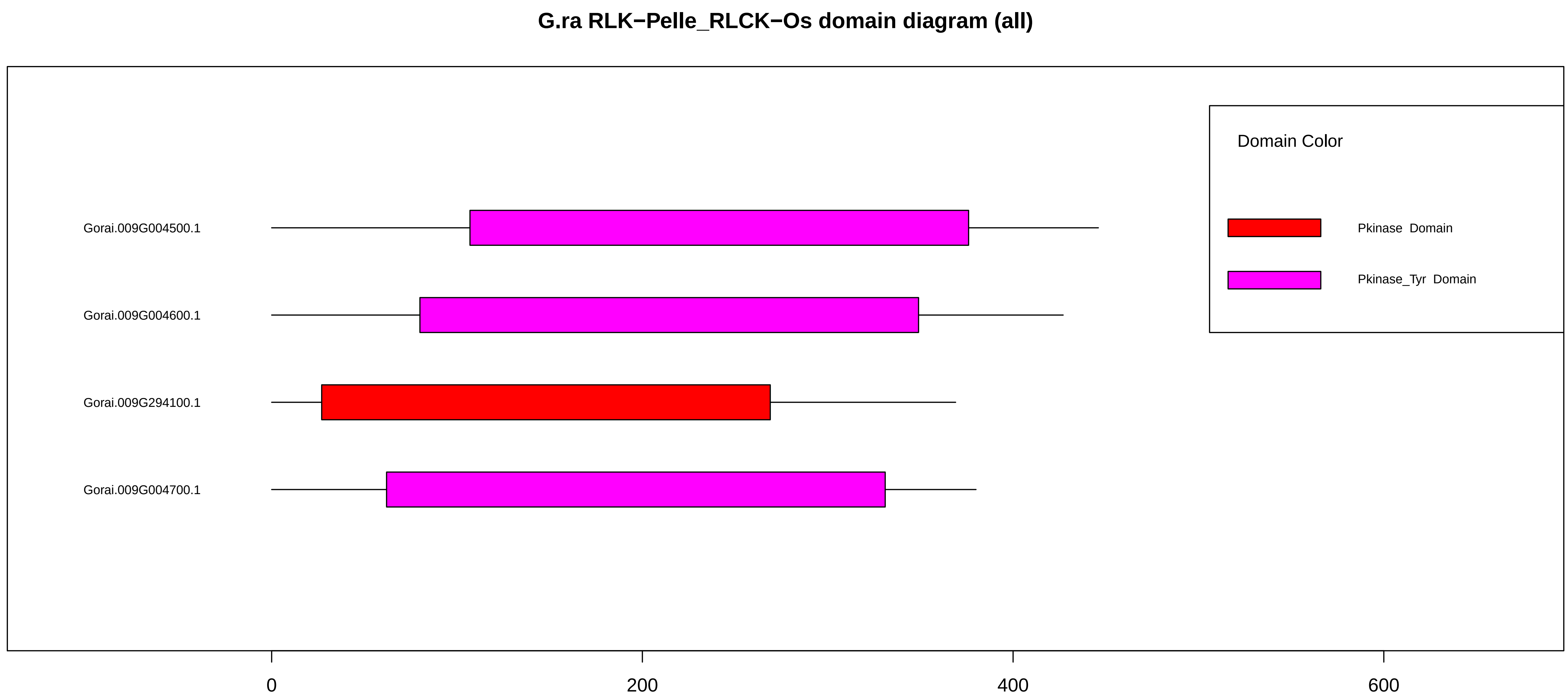
G.ra RLK−Pelle_RLCK−Os domain diagram (all)
Gorai.009G004500.1
Gorai.009G004600.1
Gorai.009G294100.1
Gorai.009G004700.1
Domain Color
Pkinase Domain
Pkinase_Tyr Domain
0
200
400
600

G.ra RLK−Pelle_RLCK−VIIa−1 domain diagram (part 1)

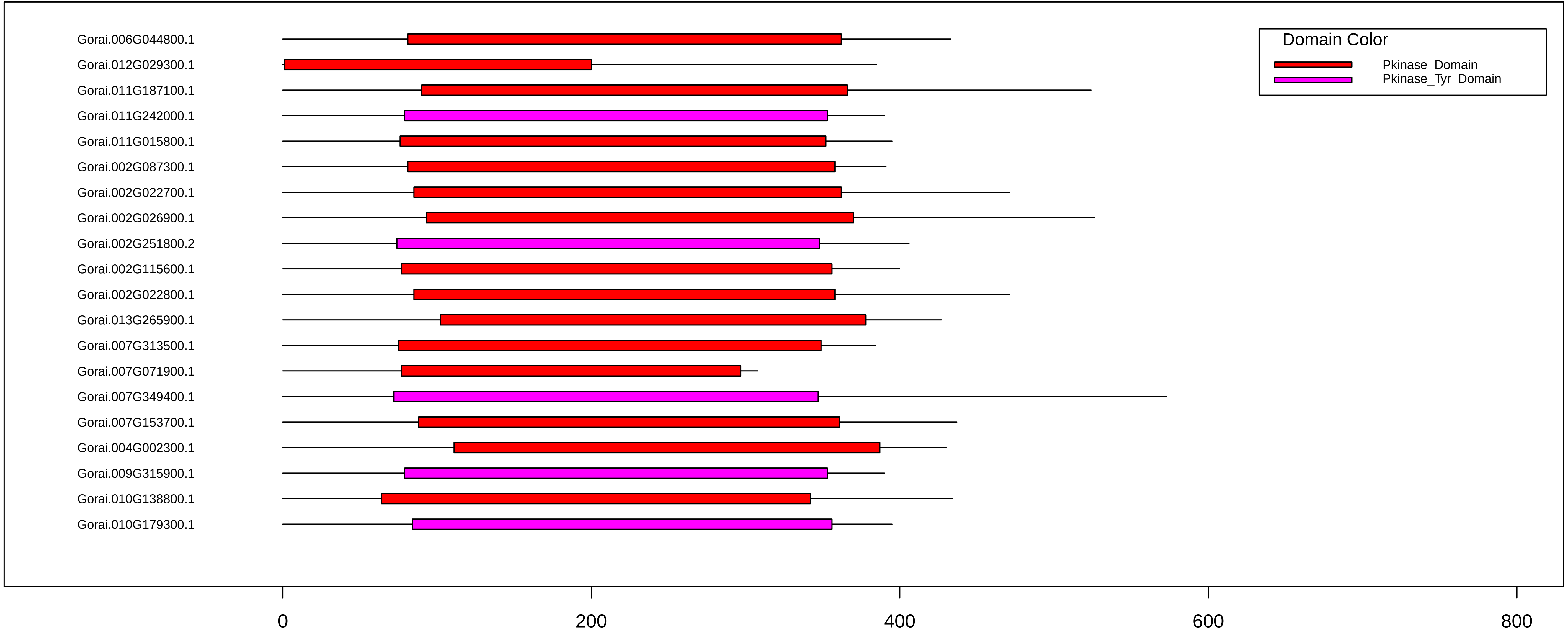

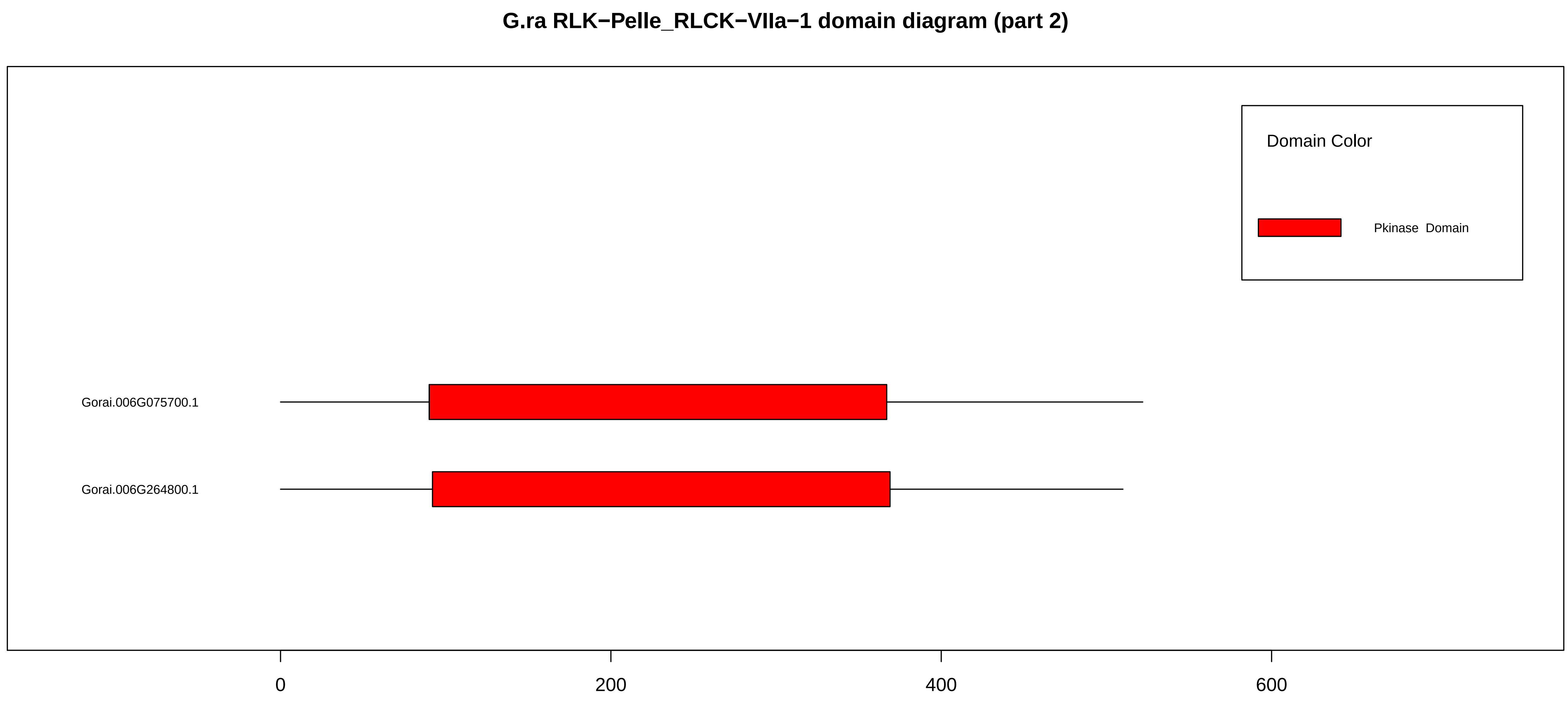
G.ra RLK−Pelle_RLCK−VIIa−1 domain diagram (part 2)
Domain Color
Pkinase Domain
Gorai.006G075700.1
Gorai.006G264800.1
0
200
400
600

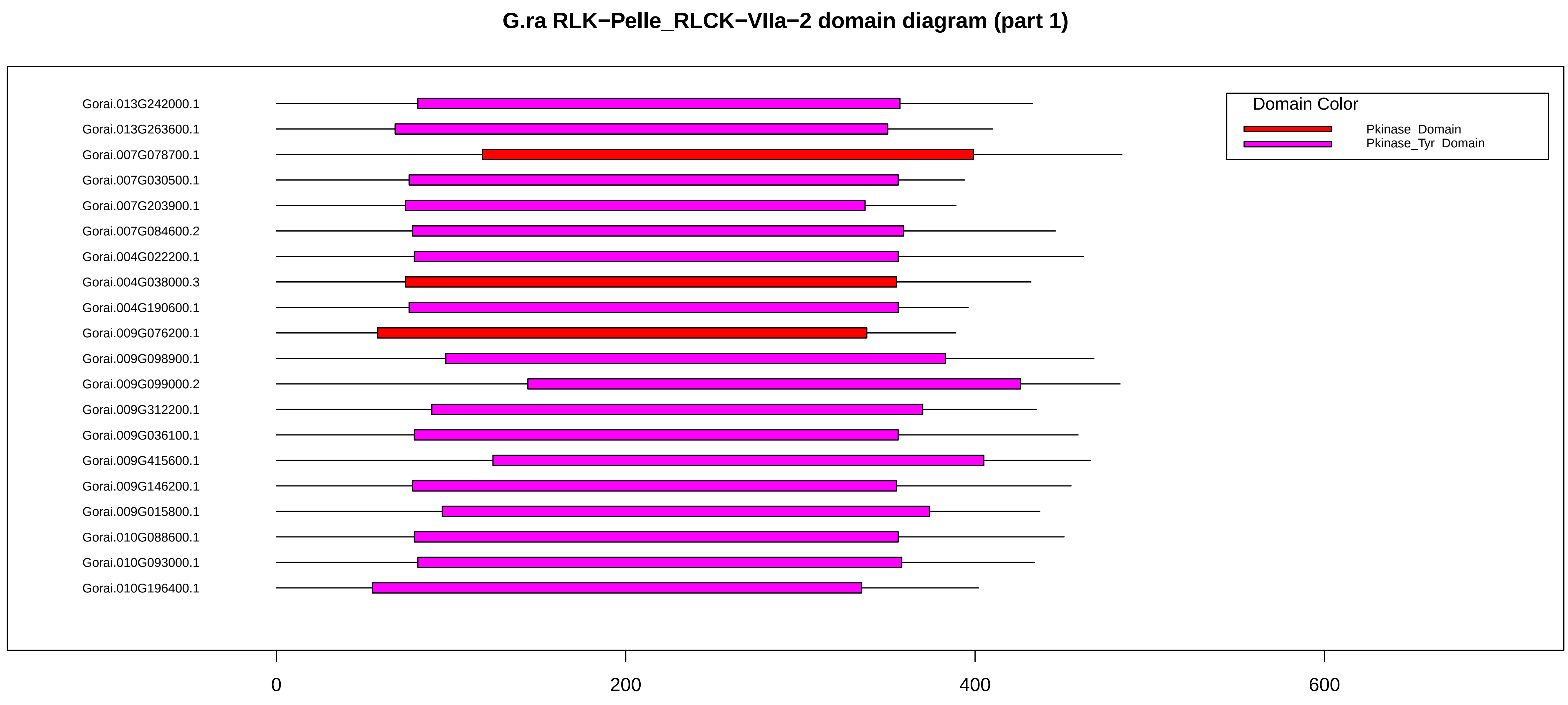
G.ra RLK−Pelle_RLCK−VIIa−2 domain diagram (part 1)
Gorai.013G242000.1
Gorai.013G263600.1
Gorai.007G078700.1
Gorai.007G030500.1
Gorai.007G203900.1
Gorai.007G084600.2
Gorai.004G022200.1
Gorai.004G038000.3
Gorai.004G190600.1
Gorai.009G076200.1
Gorai.009G098900.1
Gorai.009G099000.2
Gorai.009G312200.1
Gorai.009G036100.1
Gorai.009G415600.1
Gorai.009G146200.1
Gorai.009G015800.1
Gorai.010G088600.1
Gorai.010G093000.1
Gorai.010G196400.1
Domain Color
Pkinase Domain
Pkinase_Tyr Domain
0
200
400
600

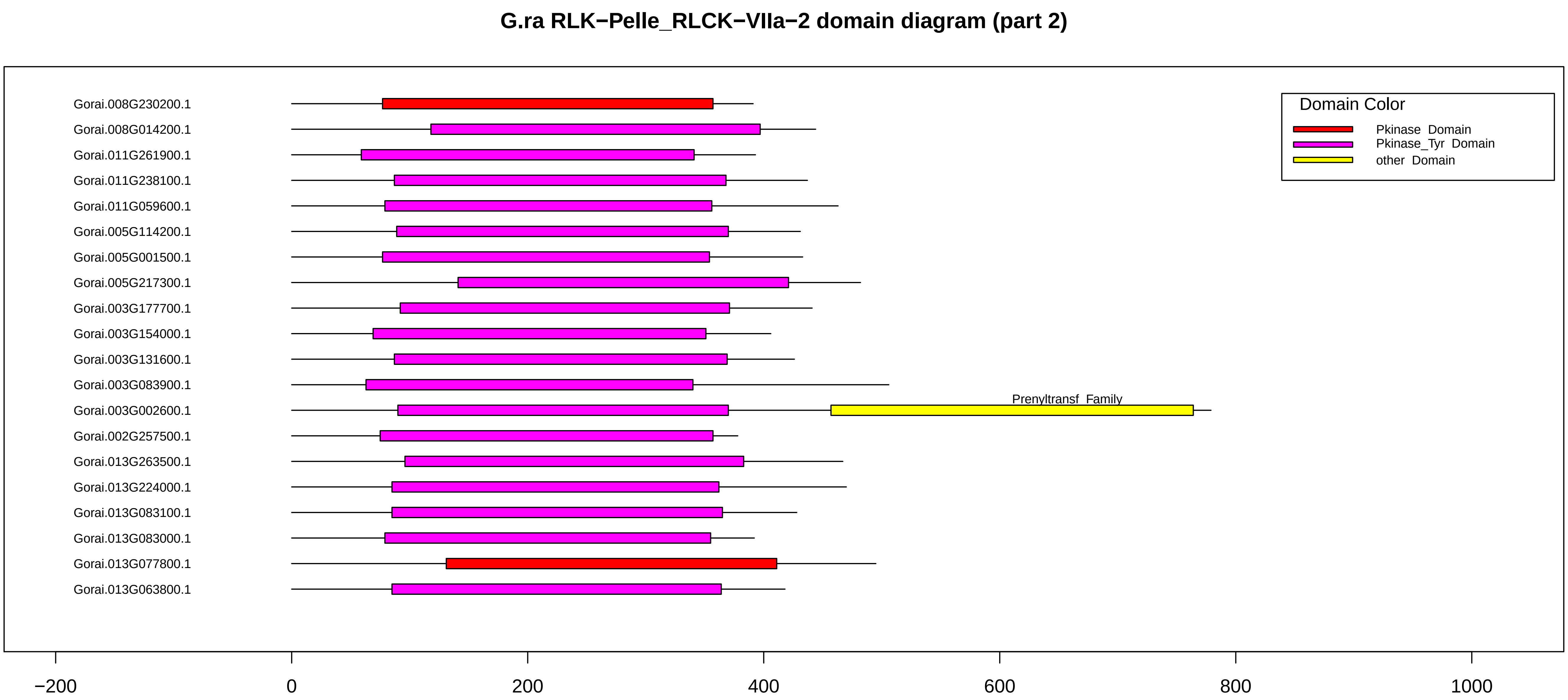
G.ra RLK−Pelle_RLCK−VIIa−2 domain diagram (part 2)
Gorai.008G230200.1
Gorai.008G014200.1
Gorai.011G261900.1
Gorai.011G238100.1
Gorai.011G059600.1
Gorai.005G114200.1
Gorai.005G001500.1
Gorai.005G217300.1
Gorai.003G177700.1
Gorai.003G154000.1
Gorai.003G131600.1
Gorai.003G083900.1
Gorai.003G002600.1
Gorai.002G257500.1
Gorai.013G263500.1
Gorai.013G224000.1
Gorai.013G083100.1
Gorai.013G083000.1
Gorai.013G077800.1
Gorai.013G063800.1
Prenyltransf_Family
Domain Color
Pkinase Domain
Pkinase_Tyr Domain
other Domain
−200
0
200
400
600
800
1000

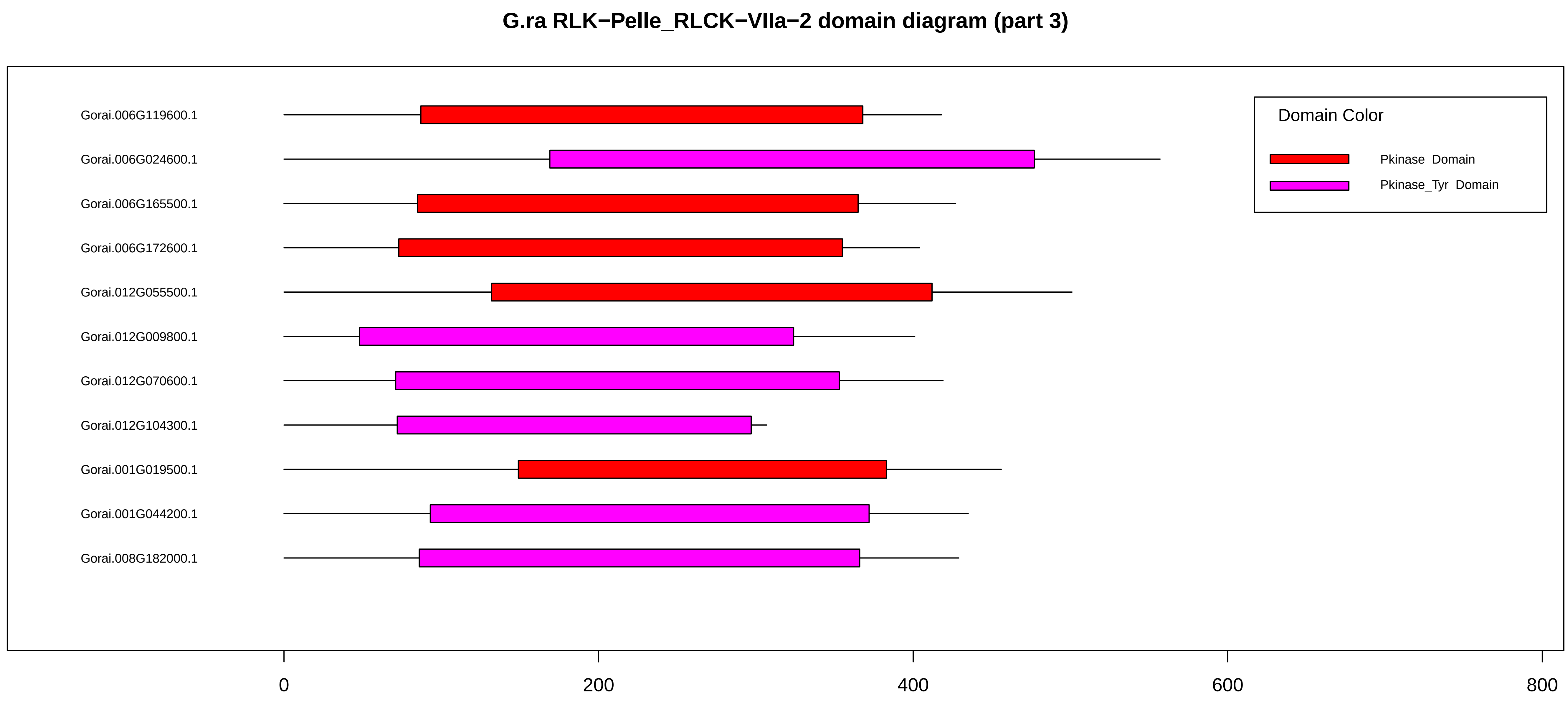

G.ra RLK−Pelle_RLCK−VIIa−2 domain diagram (part 3)
Gorai.006G119600.1
Gorai.006G024600.1
Gorai.006G165500.1
Gorai.006G172600.1
Gorai.012G055500.1
Gorai.012G009800.1
Gorai.012G070600.1
Gorai.012G104300.1
Gorai.001G019500.1
Gorai.001G044200.1
Gorai.008G182000.1
Domain Color
Pkinase Domain
Pkinase_Tyr Domain
0
200
400
600
800

G.ra RLK−Pelle_RLCK−VIIb domain diagram (all)

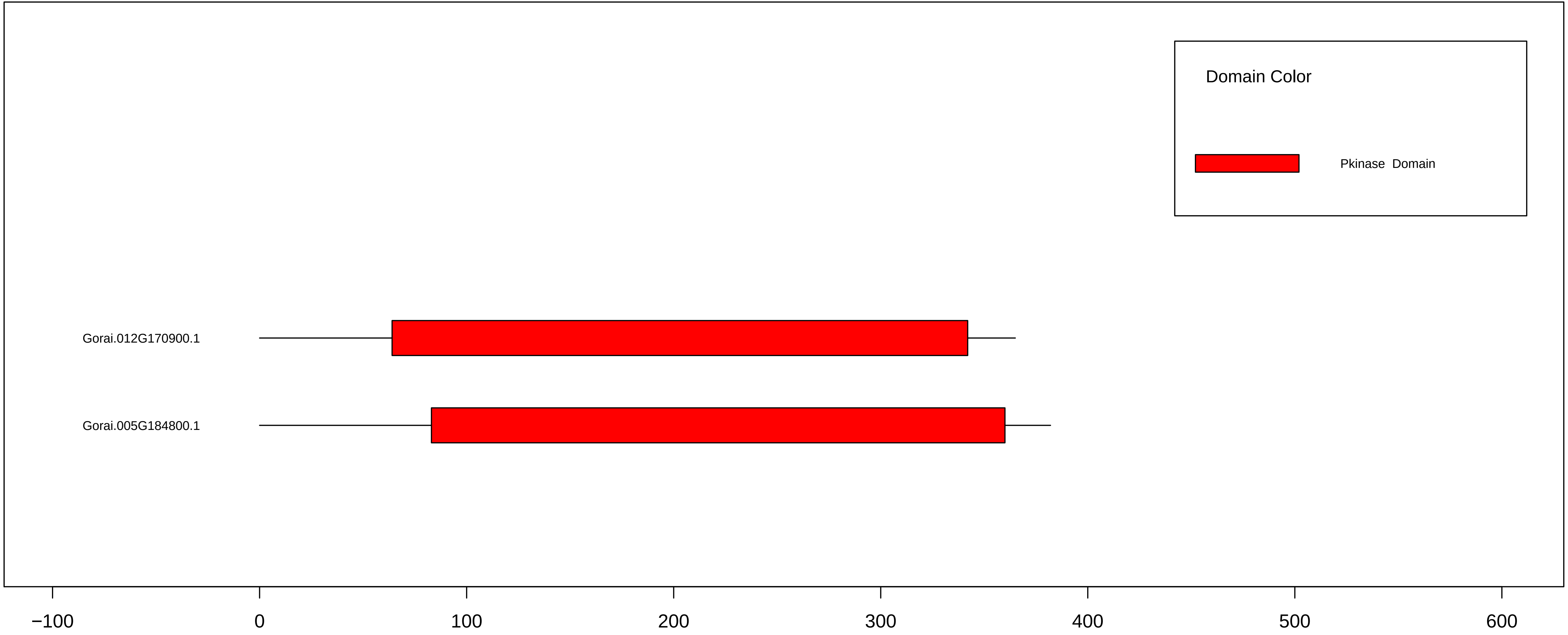

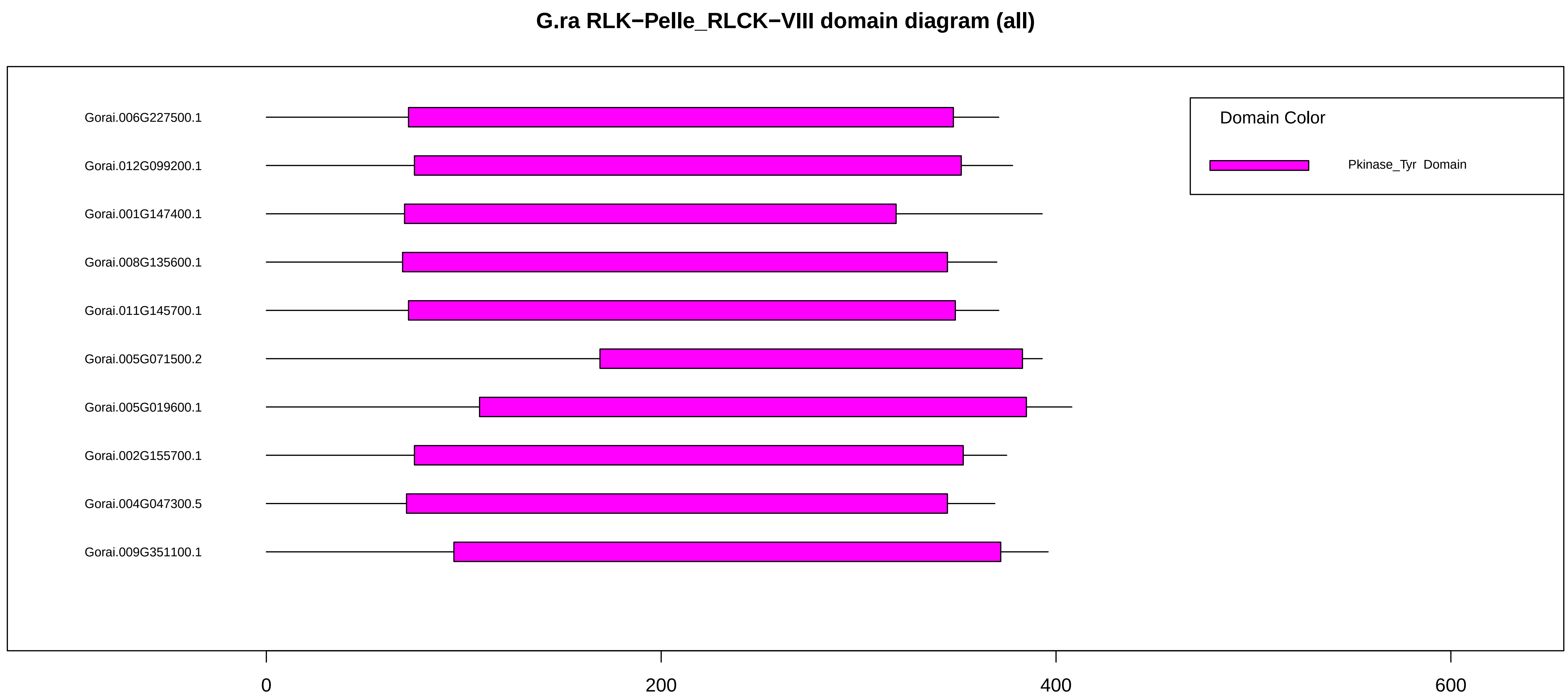
G.ra RLK−Pelle_RLCK−VIII domain diagram (all)
Gorai.006G227500.1
Gorai.012G099200.1
Gorai.001G147400.1
Gorai.008G135600.1
Gorai.011G145700.1
Gorai.005G071500.2
Gorai.005G019600.1
Gorai.002G155700.1
Gorai.004G047300.5
Gorai.009G351100.1
Domain Color
Pkinase_Tyr Domain
0
200
400
600

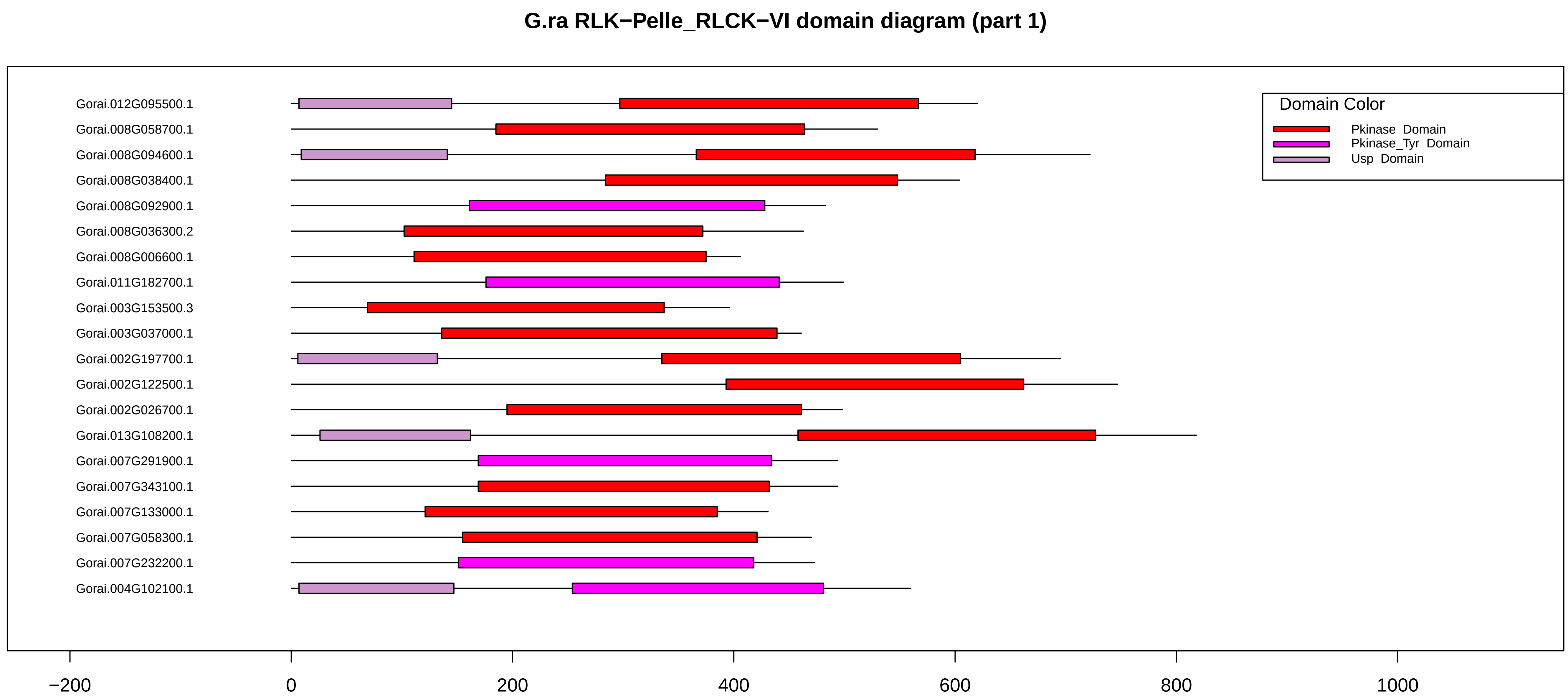
G.ra RLK−Pelle_RLCK−VI domain diagram (part 1)
Gorai.012G095500.1
Gorai.008G058700.1
Gorai.008G094600.1
Gorai.008G038400.1
Gorai.008G092900.1
Gorai.008G036300.2
Gorai.008G006600.1
Gorai.011G182700.1
Gorai.003G153500.3
Gorai.003G037000.1
Gorai.002G197700.1
Gorai.002G122500.1
Gorai.002G026700.1
Gorai.013G108200.1
Gorai.007G291900.1
Gorai.007G343100.1
Gorai.007G133000.1
Gorai.007G058300.1
Gorai.007G232200.1
Gorai.004G102100.1
Domain Color
Pkinase Domain
Pkinase_Tyr Domain
Usp Domain
−200
0
200
400
600
800
1000

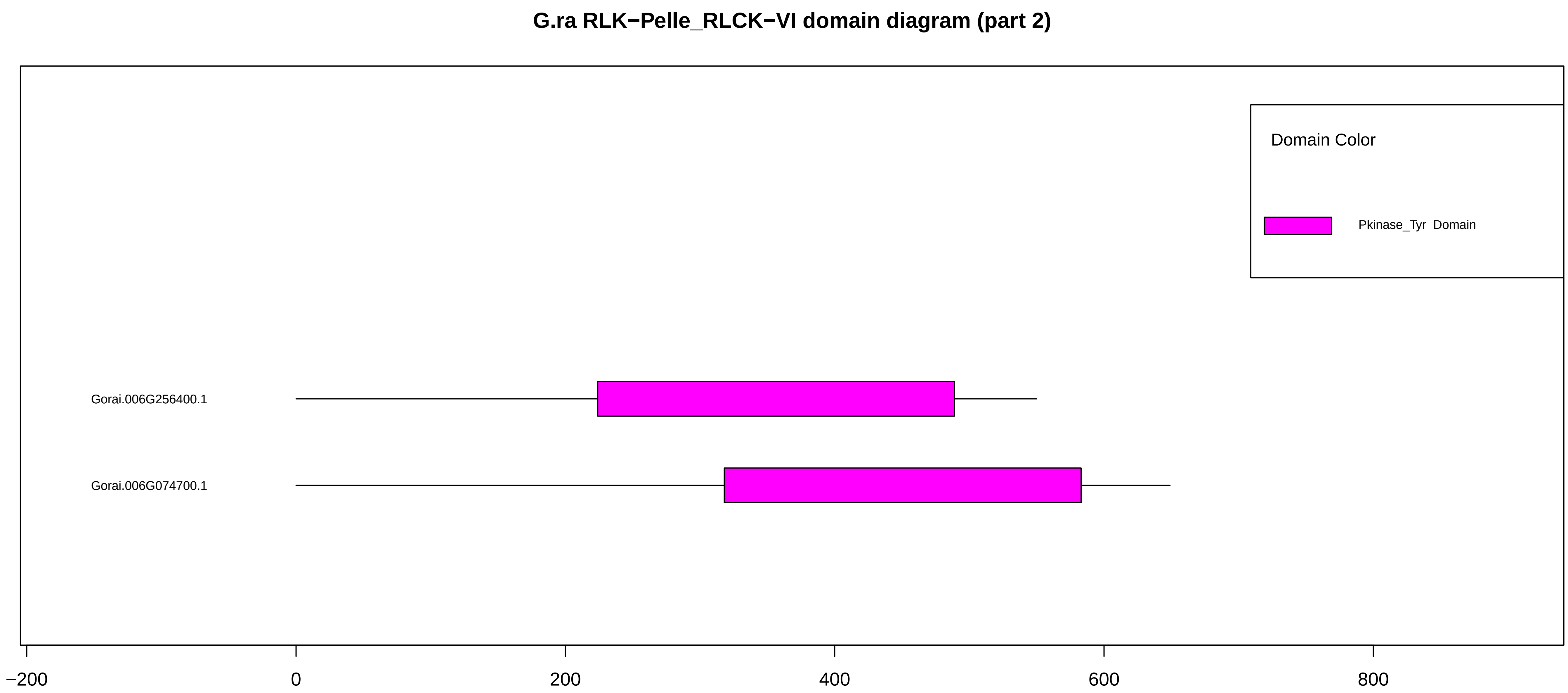
G.ra RLK−Pelle_RLCK−VI domain diagram (part 2)
Domain Color
Pkinase_Tyr Domain
Gorai.006G256400.1
Gorai.006G074700.1
−200
0
200
400
600
800

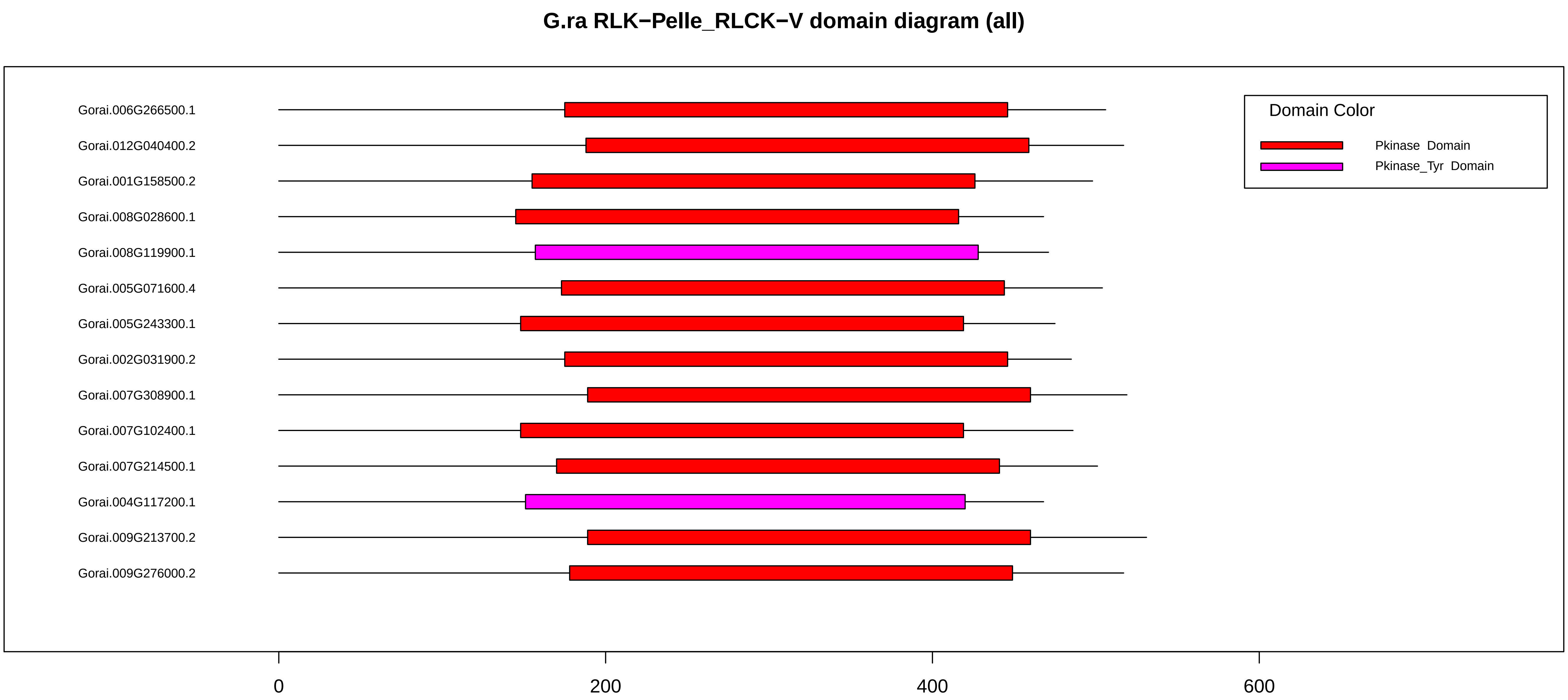
G.ra RLK−Pelle_RLCK−V domain diagram (all)
Gorai.006G266500.1
Gorai.012G040400.2
Gorai.001G158500.2
Gorai.008G028600.1
Gorai.008G119900.1
Gorai.005G071600.4
Gorai.005G243300.1
Gorai.002G031900.2
Gorai.007G308900.1
Gorai.007G102400.1
Gorai.007G214500.1
Gorai.004G117200.1
Gorai.009G213700.2
Gorai.009G276000.2
Domain Color
Pkinase Domain
Pkinase_Tyr Domain
0
200
400
600

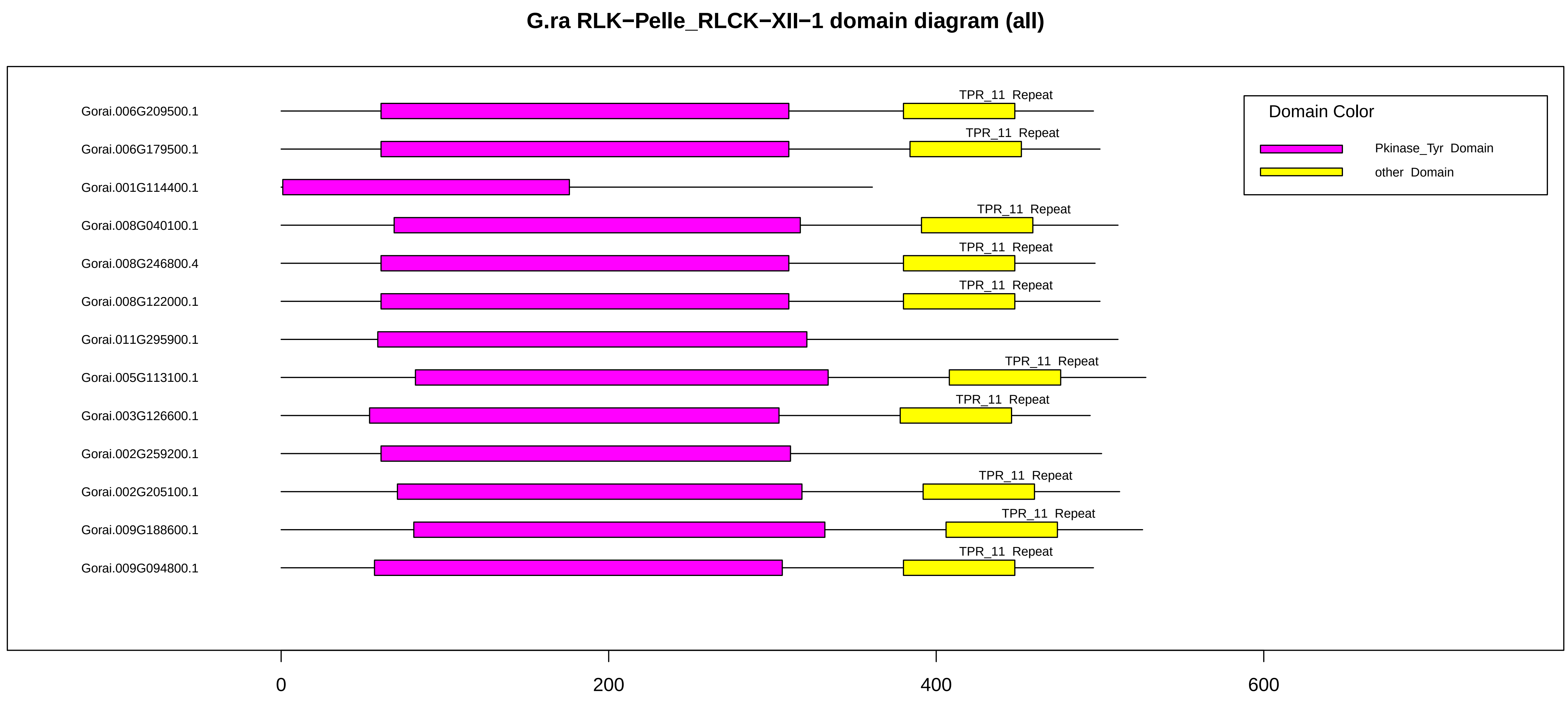
G.ra RLK−Pelle_RLCK−XII−1 domain diagram (all)
Gorai.006G209500.1
Gorai.006G179500.1
Gorai.001G114400.1
Gorai.008G040100.1
Gorai.008G246800.4
Gorai.008G122000.1
Gorai.011G295900.1
Gorai.005G113100.1
Gorai.003G126600.1
Gorai.002G259200.1
Gorai.002G205100.1
Gorai.009G188600.1
Gorai.009G094800.1
TPR_11 Repeat
Domain Color
Pkinase_Tyr Domain
other Domain
0
200
400
600

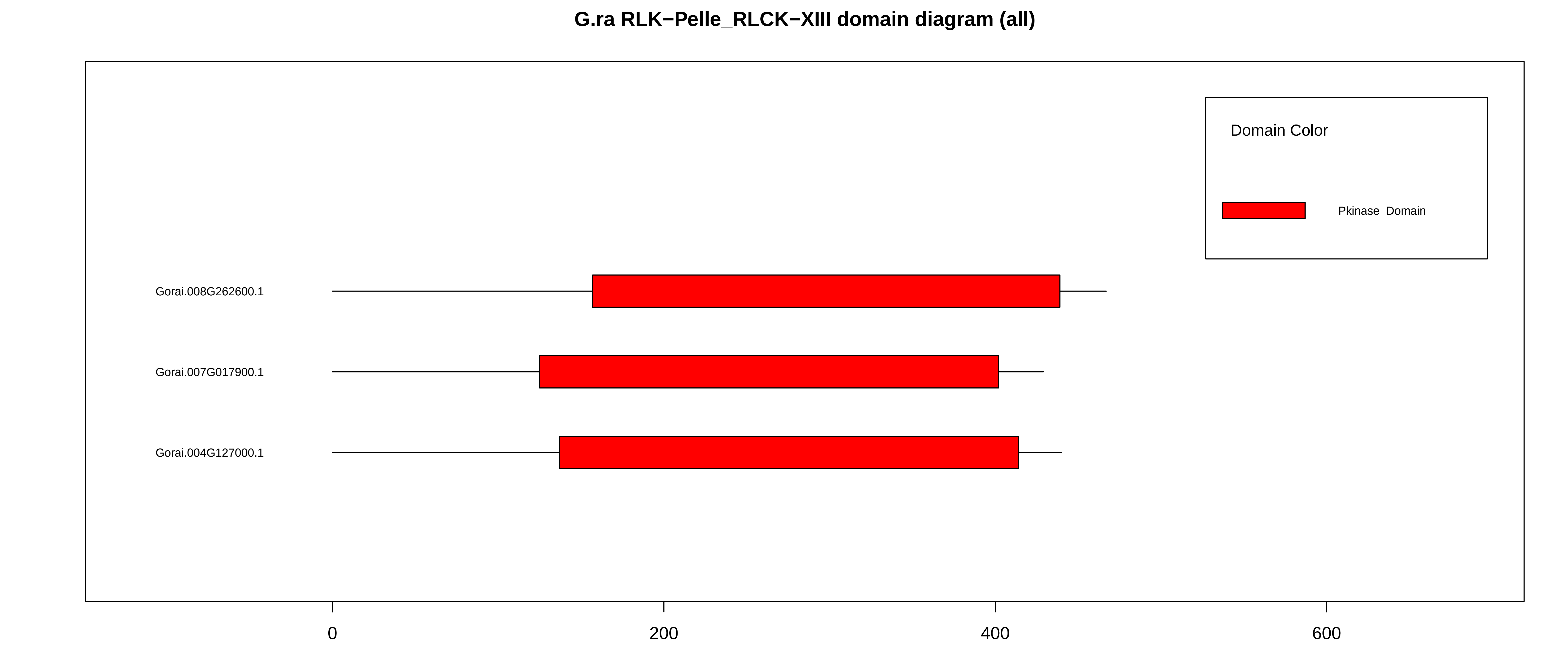
G.ra RLK−Pelle_RLCK−XIII domain diagram (all)
Domain Color
Pkinase Domain
Gorai.008G262600.1
Gorai.007G017900.1
Gorai.004G127000.1
0
200
400
600

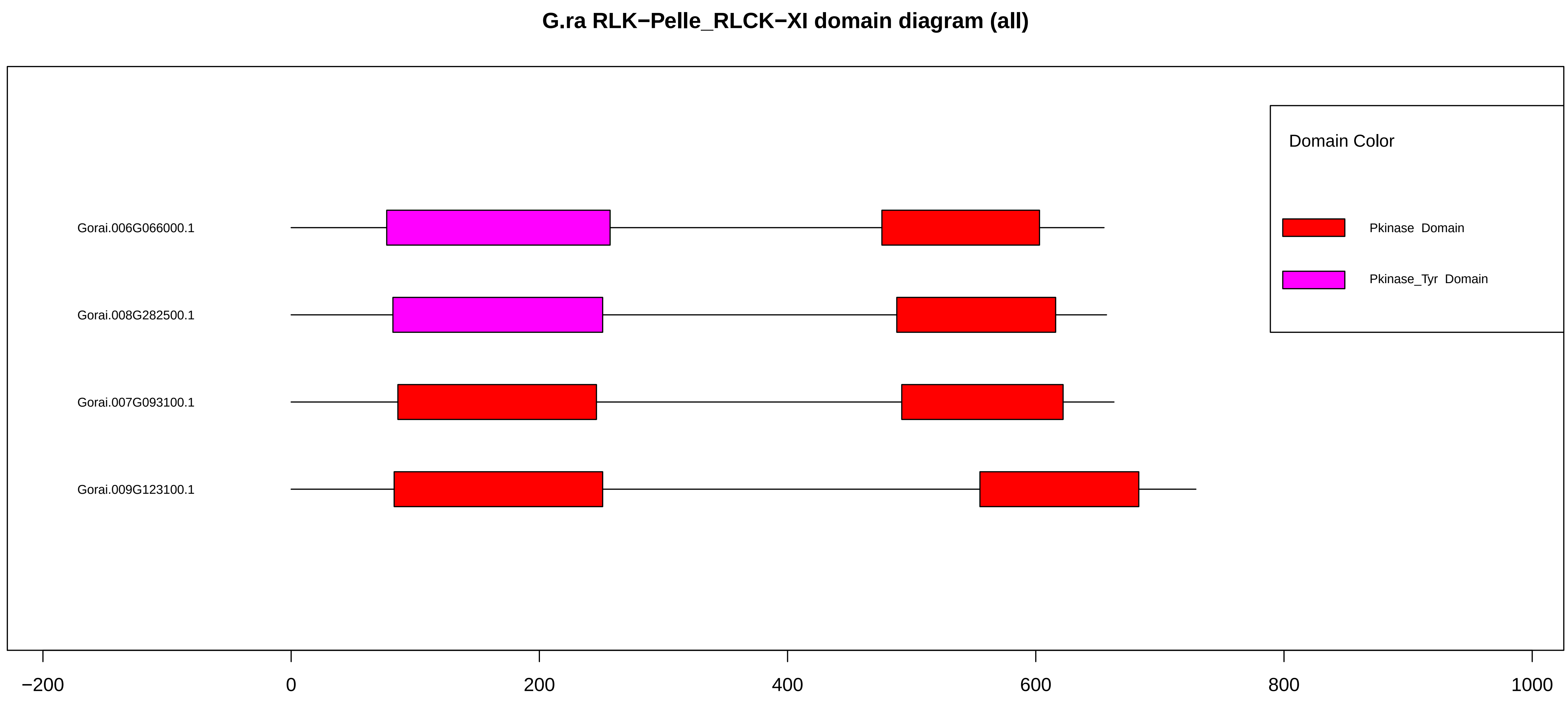
G.ra RLK−Pelle_RLCK−XI domain diagram (all)
Gorai.006G066000.1
Gorai.008G282500.1
Gorai.007G093100.1
Gorai.009G123100.1
Domain Color
Pkinase Domain
Pkinase_Tyr Domain
−200
0
200
400
600
800
1000

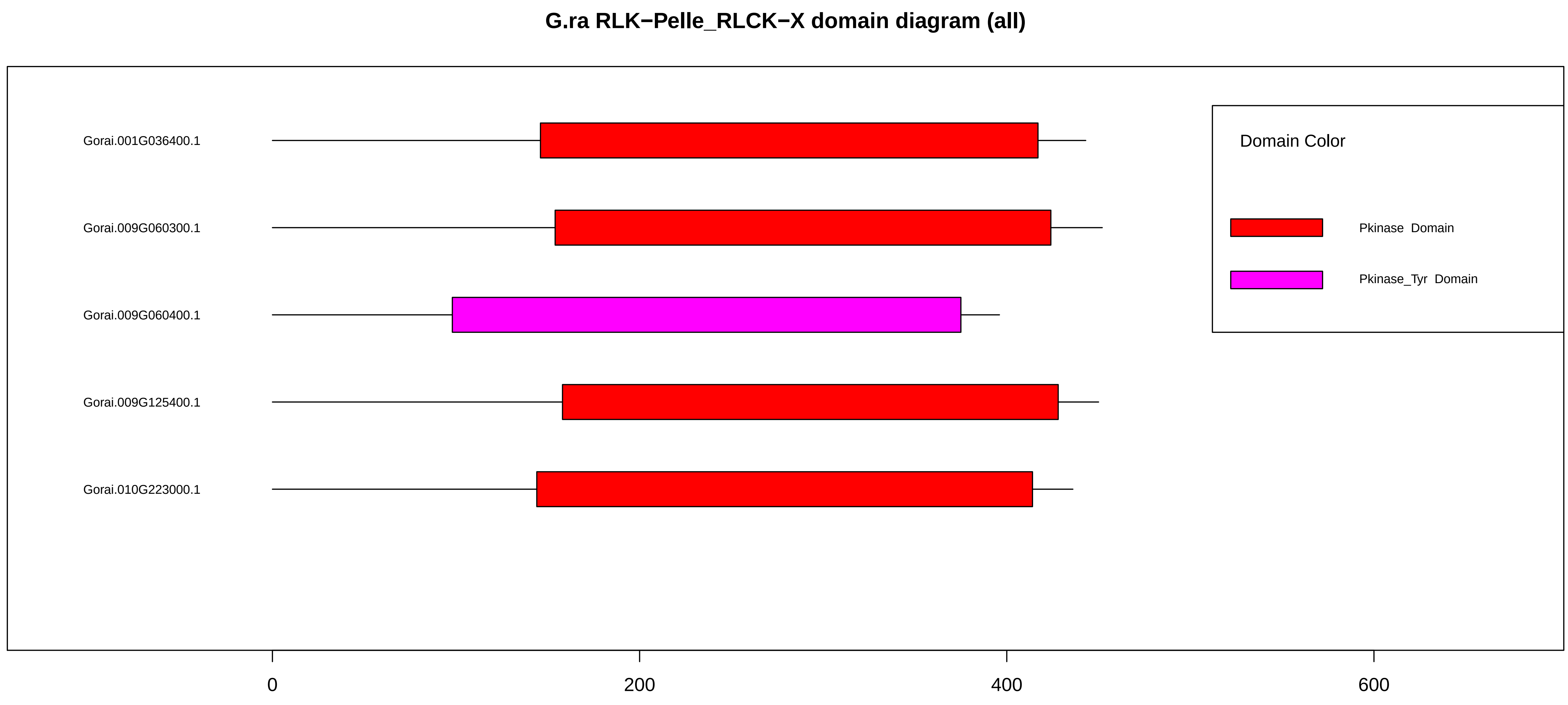
G.ra RLK−Pelle_RLCK−X domain diagram (all)
Gorai.001G036400.1
Gorai.009G060300.1
Gorai.009G060400.1
Gorai.009G125400.1
Gorai.010G223000.1
Domain Color
Pkinase Domain
Pkinase_Tyr Domain
0
200
400
600

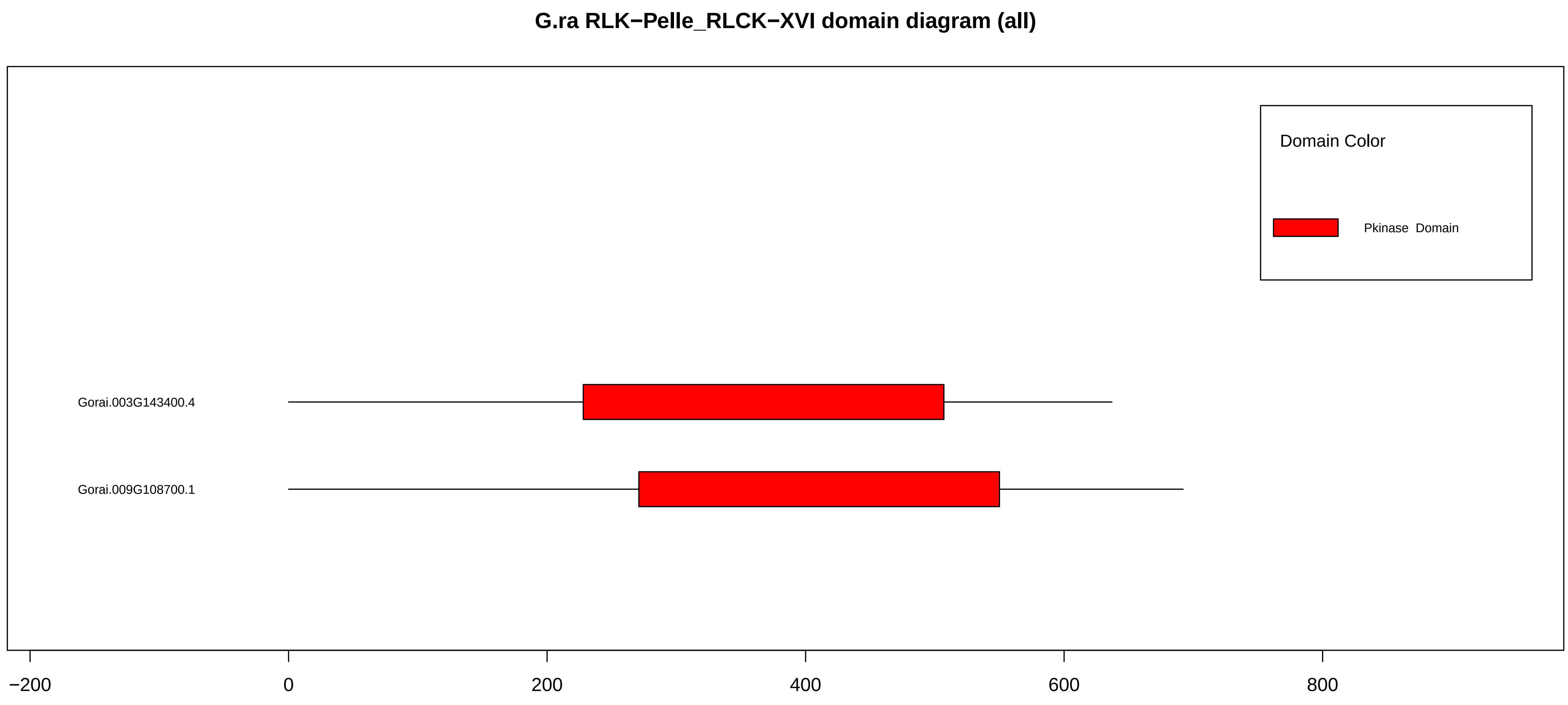

G.ra RLK−Pelle_RLCK−XVI domain diagram (all)
Domain Color
Pkinase Domain
Gorai.003G143400.4
Gorai.009G108700.1
−200
0
200
400
600
800

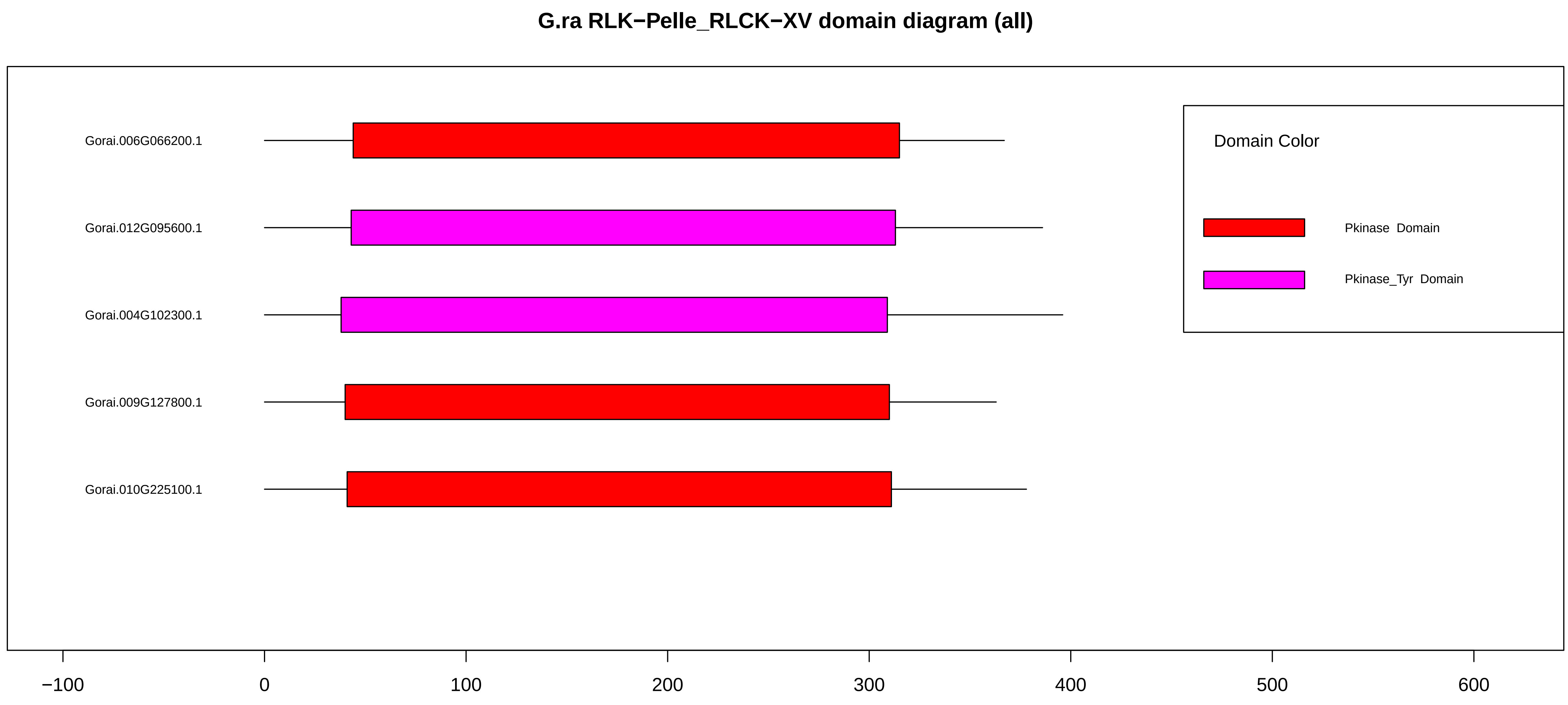
G.ra RLK−Pelle_RLCK−XV domain diagram (all)
Gorai.006G066200.1
Gorai.012G095600.1
Gorai.004G102300.1
Gorai.009G127800.1
Gorai.010G225100.1
Domain Color
Pkinase Domain
Pkinase_Tyr Domain
−100
0
100
200
300
400
500
600

G.ra RLK−Pelle_SD−2b domain diagram (part 1)

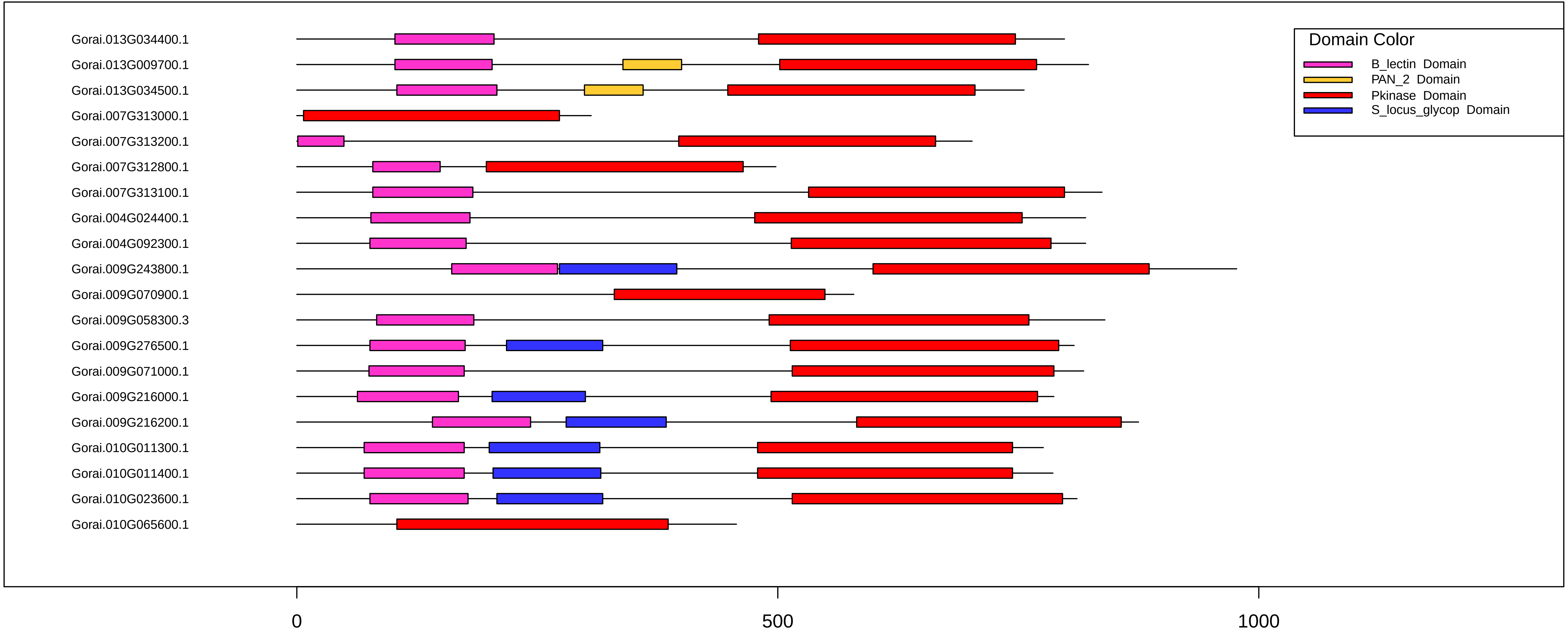

G.ra RLK−Pelle_SD−2b domain diagram (part 2)

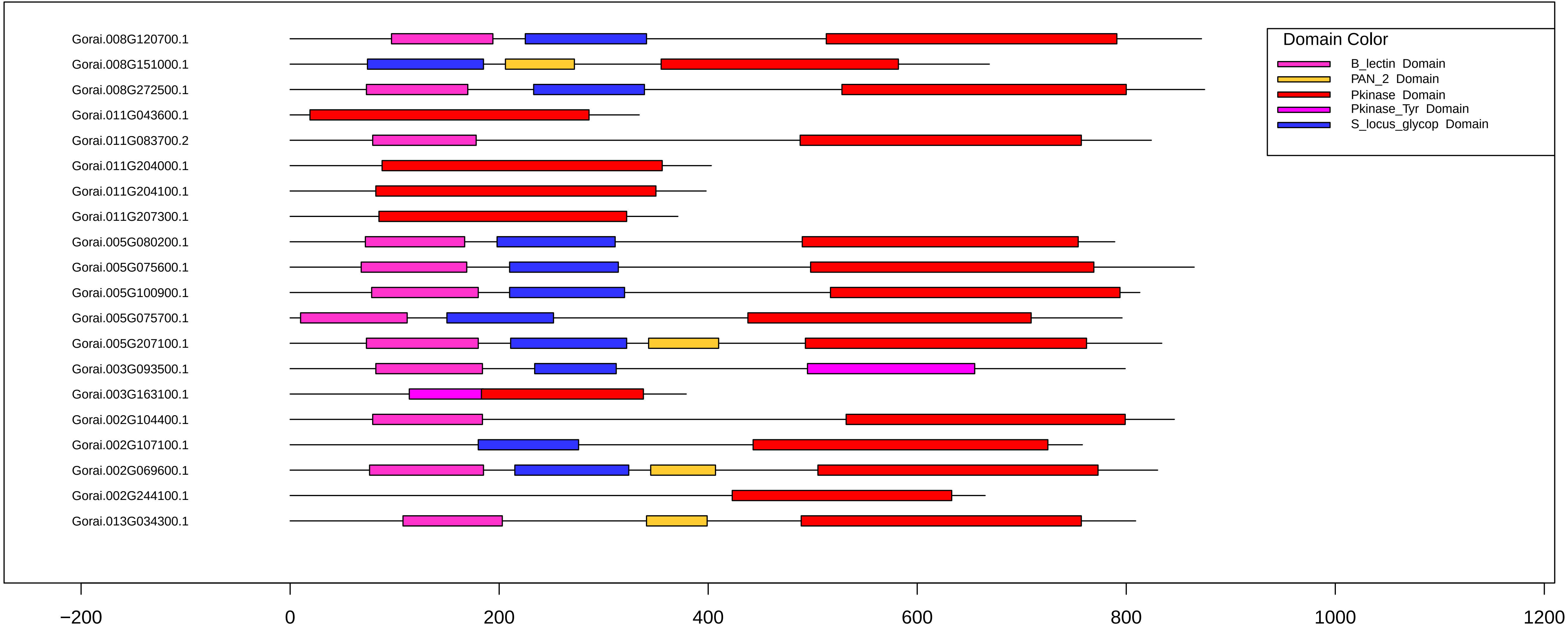

G.ra RLK−Pelle_SD−2b domain diagram (part 3)

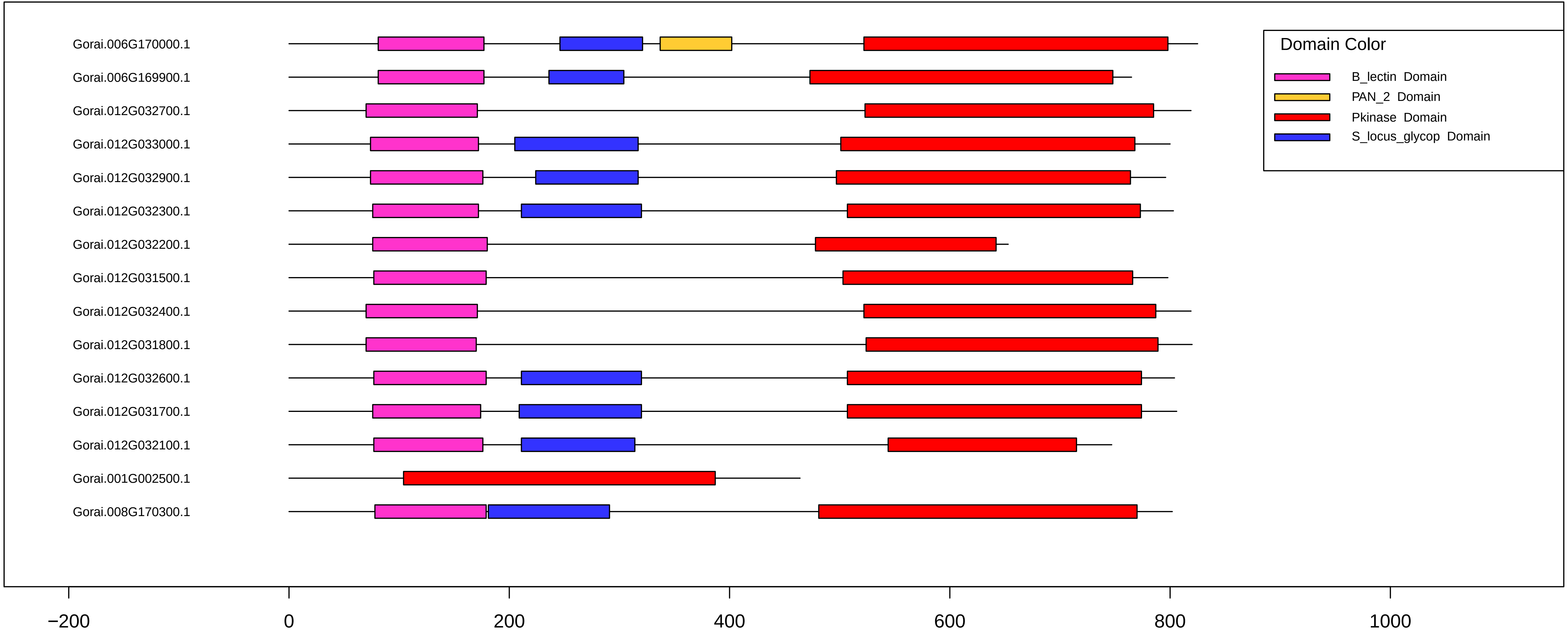

G.ra RLK−Pelle_Singleton domain diagram (all)

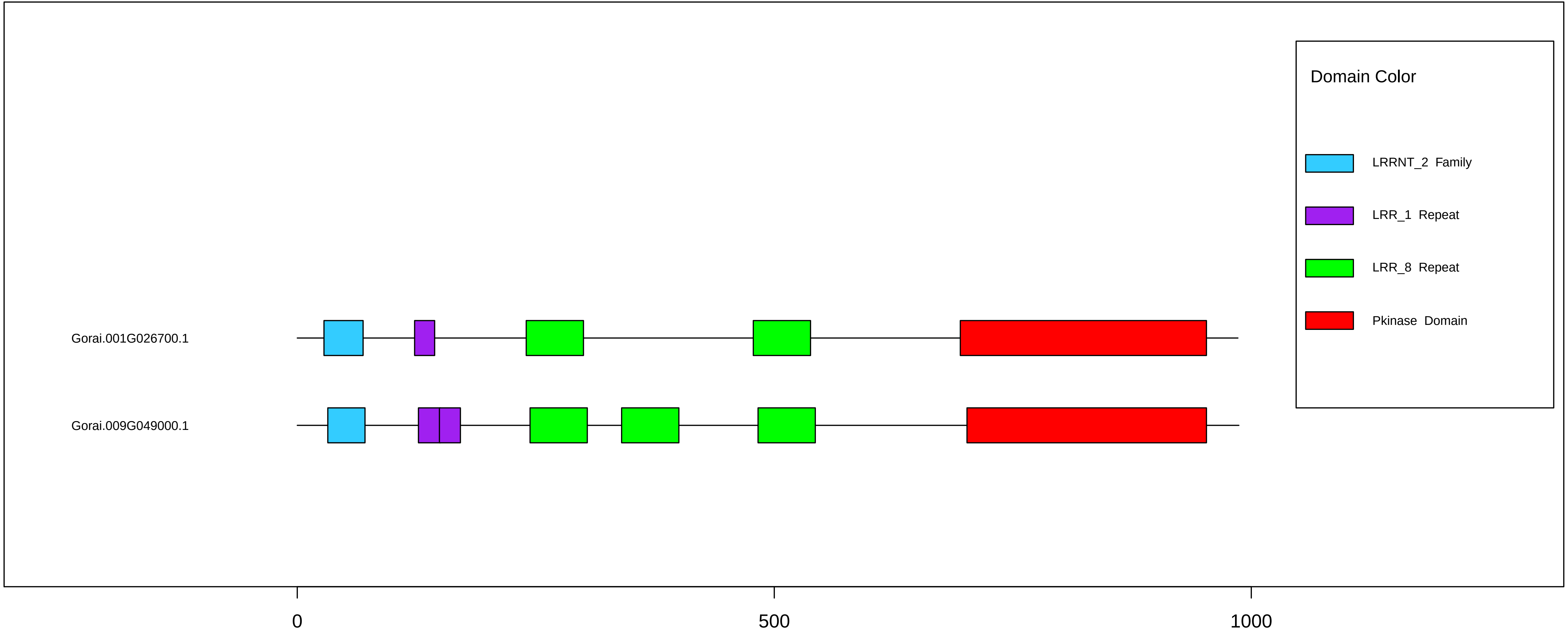

G.ra RLK−Pelle_URK−1 domain diagram (all)

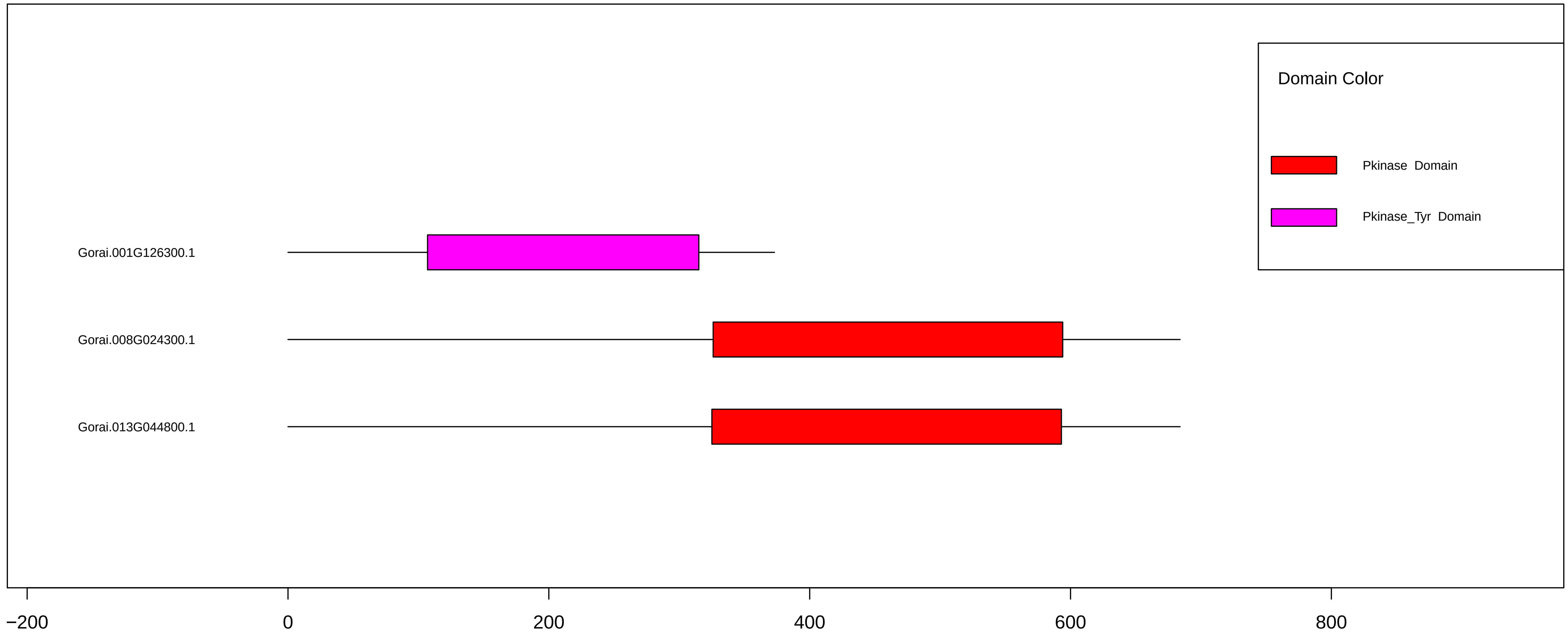

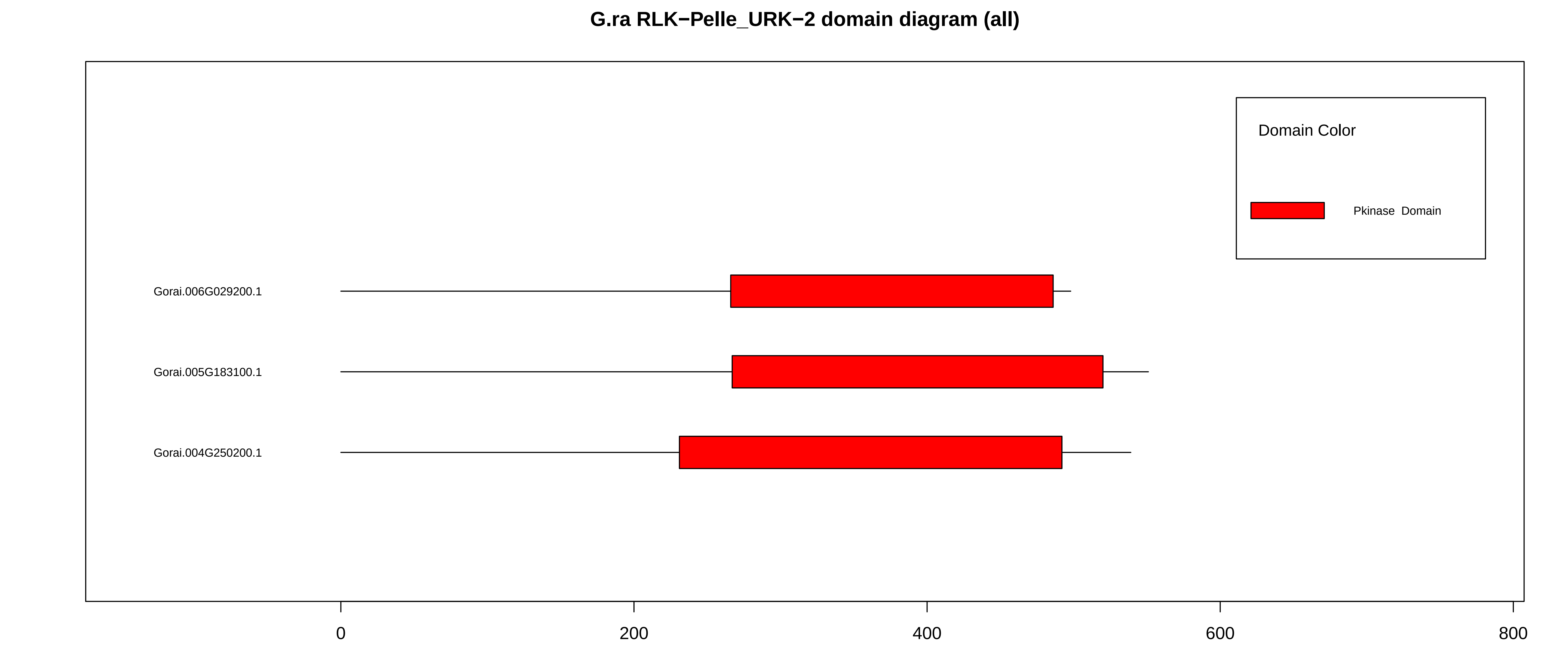

G.ra RLK−Pelle_URK−2 domain diagram (all)
Domain Color
Pkinase Domain
Gorai.006G029200.1
Gorai.005G183100.1
Gorai.004G250200.1
0
200
400
600
800

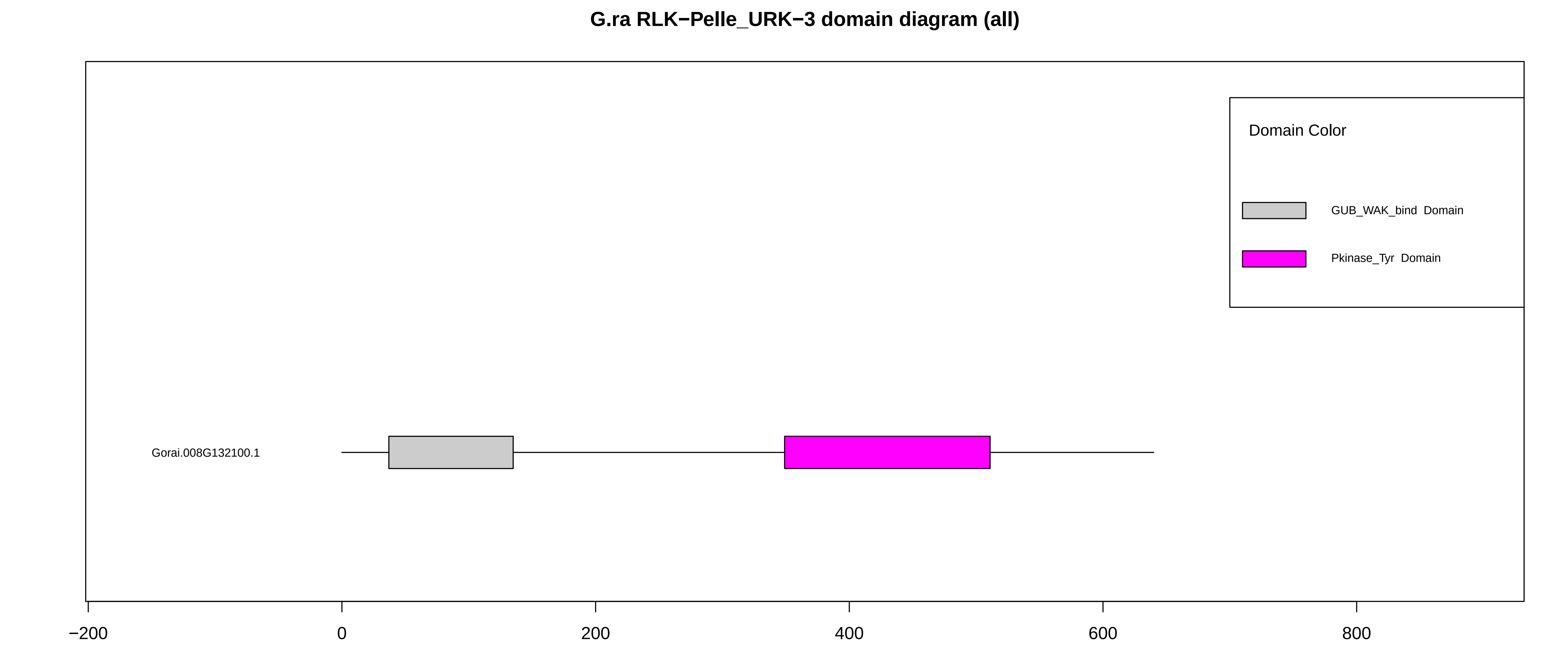
G.ra RLK−Pelle_URK−3 domain diagram (all)
Domain Color
GUB_WAK_bind Domain
Pkinase_Tyr Domain
Gorai.008G132100.1
−200
0
200
400
600
800

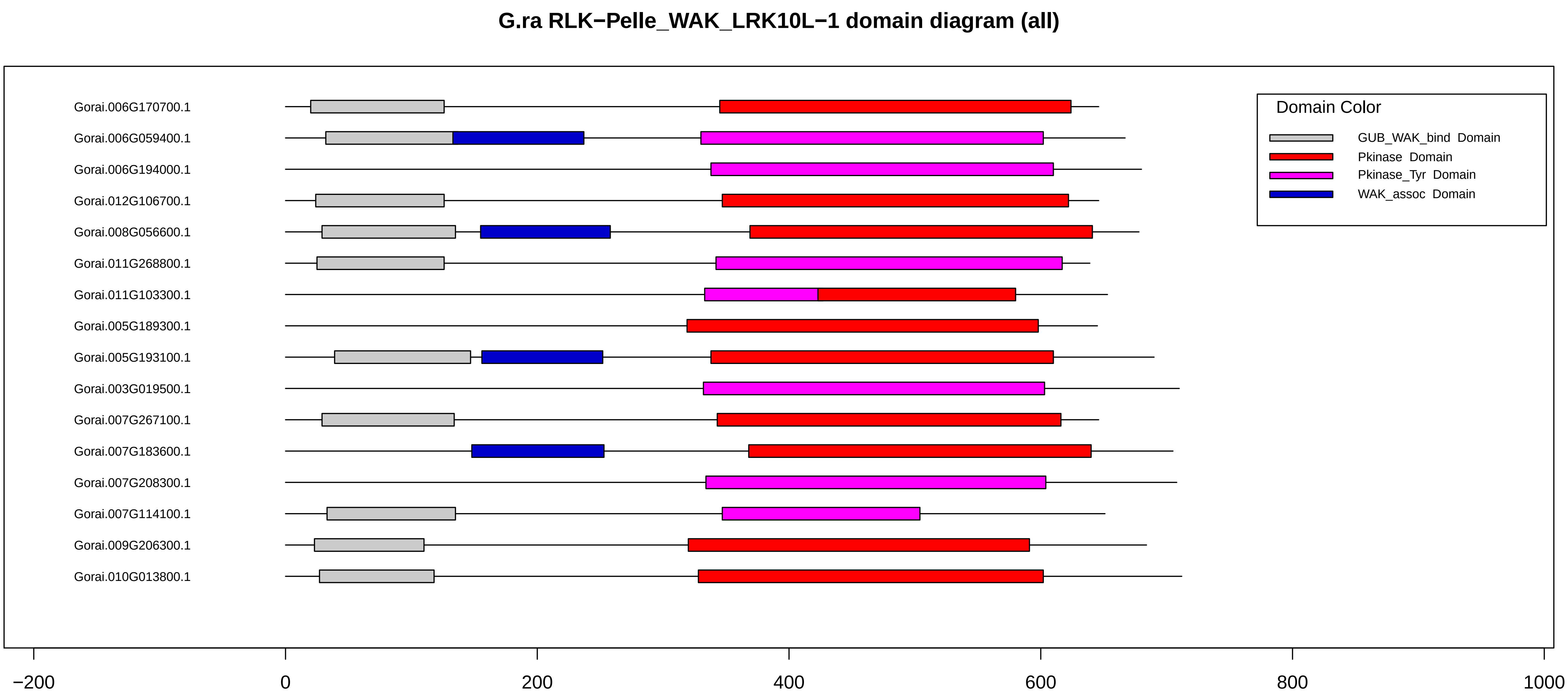

G.ra RLK−Pelle_WAK_LRK10L−1 domain diagram (all)
Gorai.006G170700.1
Gorai.006G059400.1
Gorai.006G194000.1
Gorai.012G106700.1
Gorai.008G056600.1
Gorai.011G268800.1
Gorai.011G103300.1
Gorai.005G189300.1
Gorai.005G193100.1
Gorai.003G019500.1
Gorai.007G267100.1
Gorai.007G183600.1
Gorai.007G208300.1
Gorai.007G114100.1
Gorai.009G206300.1
Gorai.010G013800.1
Domain Color
GUB_WAK_bind Domain
Pkinase Domain
Pkinase_Tyr Domain
WAK_assoc Domain
−200
0
200
400
600
800
1000

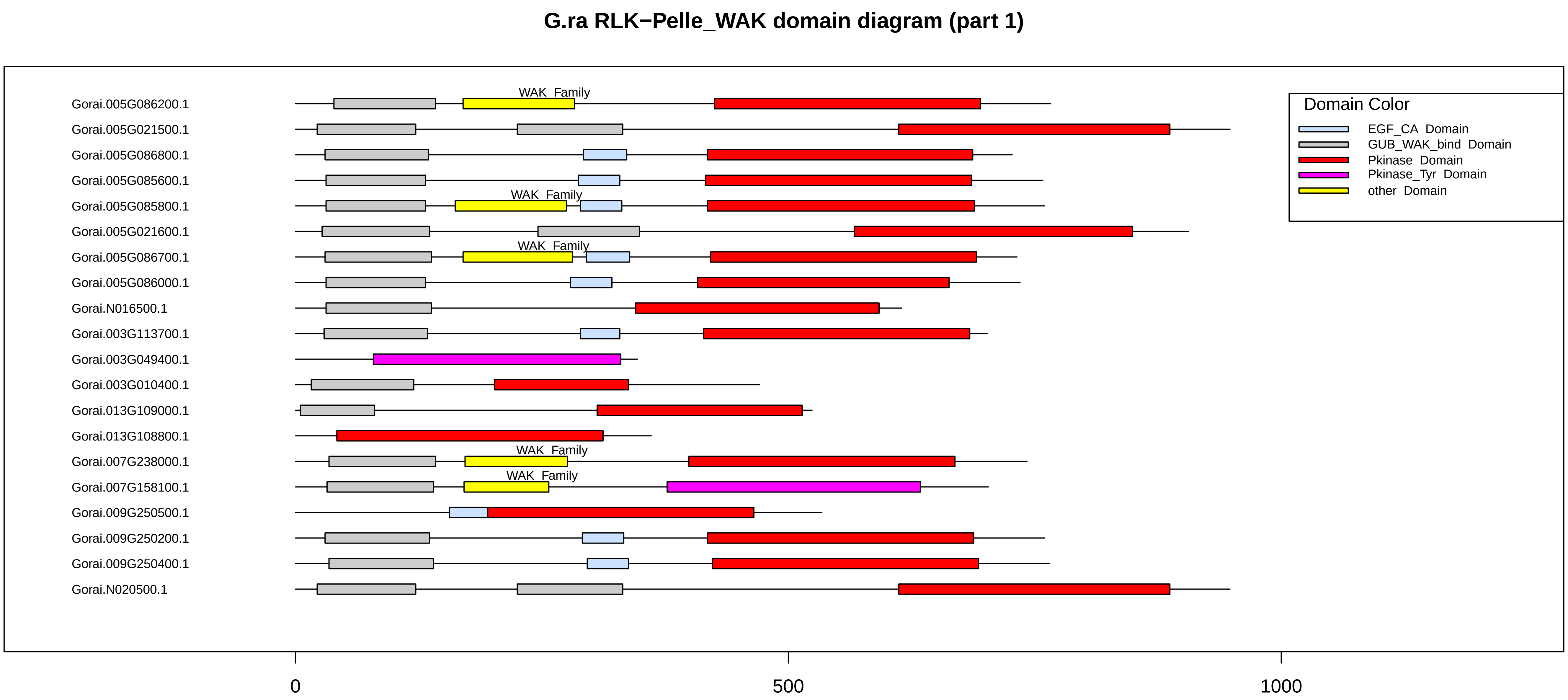

G.ra RLK−Pelle_WAK domain diagram (part 1)
Gorai.005G086200.1
Gorai.005G021500.1
Gorai.005G086800.1
Gorai.005G085600.1
Gorai.005G085800.1
Gorai.005G021600.1
Gorai.005G086700.1
Gorai.005G086000.1
Gorai.N016500.1
Gorai.003G113700.1
Gorai.003G049400.1
Gorai.003G010400.1
Gorai.013G109000.1
Gorai.013G108800.1
Gorai.007G238000.1
Gorai.007G158100.1
Gorai.009G250500.1
Gorai.009G250200.1
Gorai.009G250400.1
Gorai.N020500.1
WAK_Family
Domain Color
EGF_CA Domain
GUB_WAK_bind Domain
Pkinase Domain
Pkinase_Tyr Domain
other Domain
0
500
1000

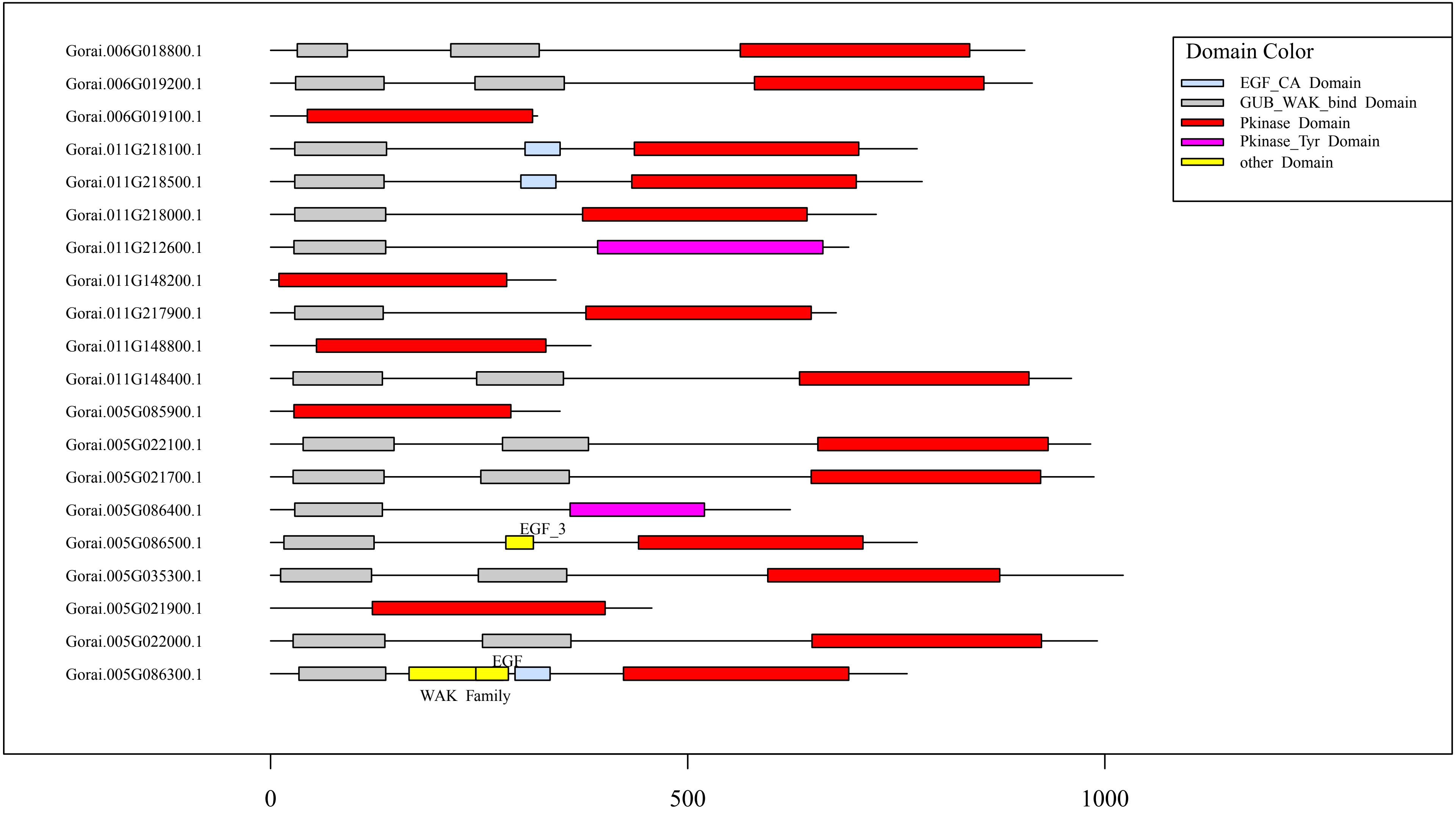
G.ra RLK−Pelle_WAK domain diagram (part 2)
Gorai.006G018800.1
Gorai.006G019200.1
Gorai.006G019100.1
Gorai.011G218100.1
Gorai.011G218500.1
Gorai.011G218000.1
Gorai.011G212600.1
Gorai.011G148200.1
Gorai.011G217900.1
Gorai.011G148800.1
Gorai.011G148400.1
Gorai.005G085900.1
Gorai.005G022100.1
Gorai.005G021700.1
Gorai.005G086400.1
Gorai.005G086500.1
Gorai.005G035300.1
Gorai.005G021900.1
Gorai.005G022000.1
Gorai.005G086300.1
EGF_3
EGF
WAK_Family
Domain Color
EGF_CA Domain
GUB_WAK_bind Domain
Pkinase Domain
Pkinase_Tyr Domain
other Domain
0
500
1000

G.ra RLK−Pelle_WAK domain diagram (part 3)

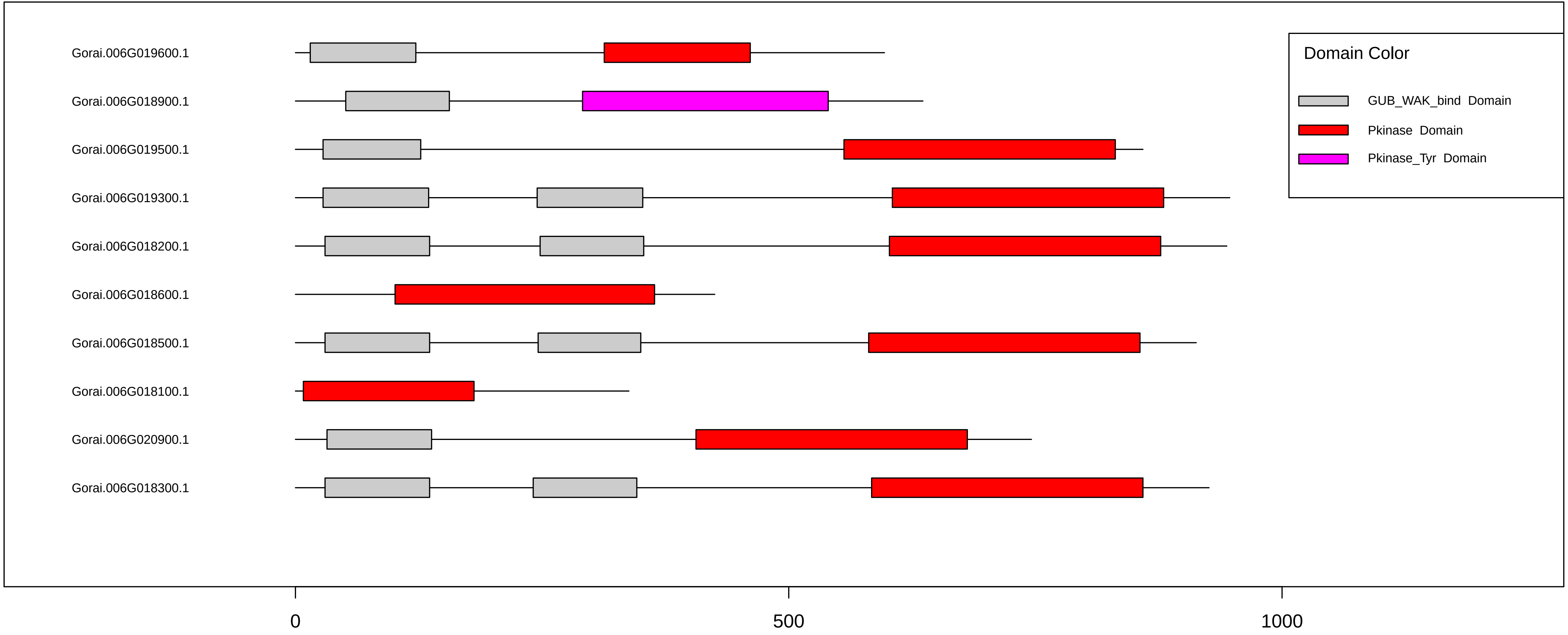

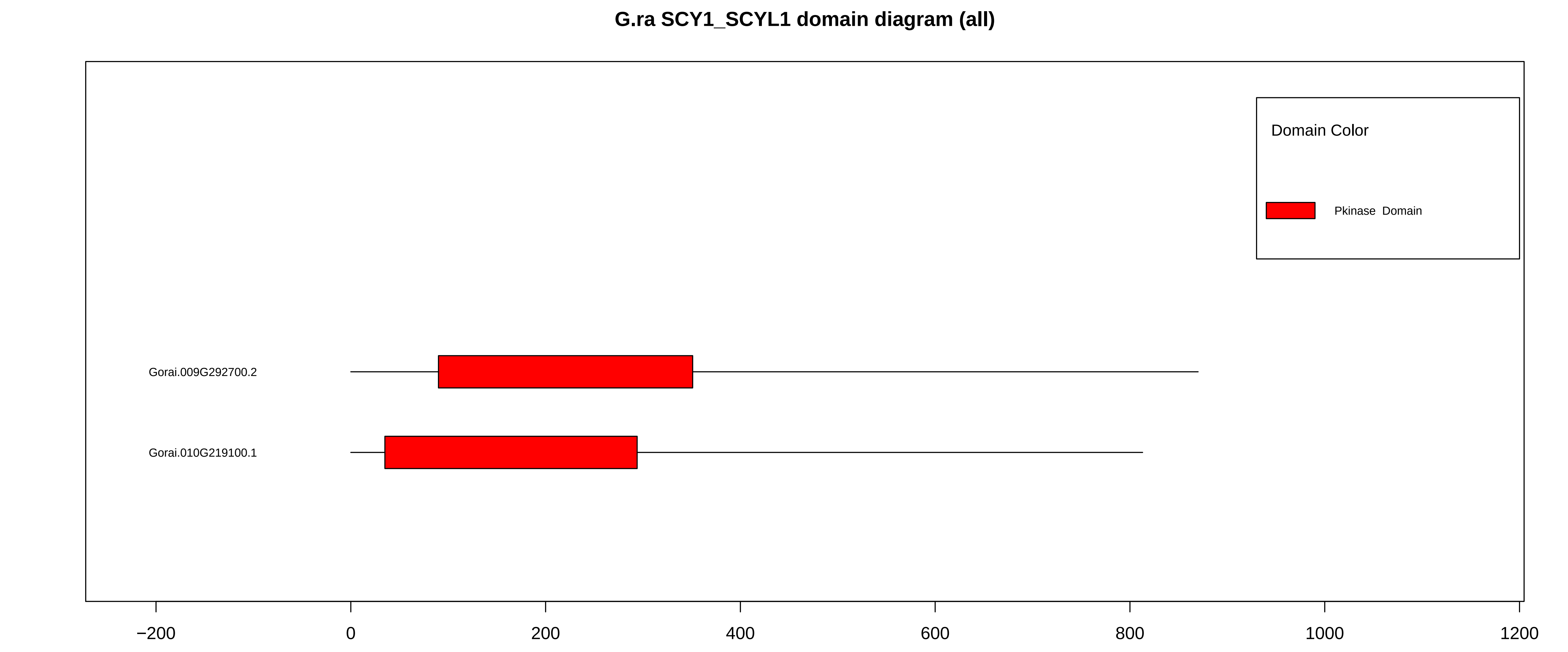

G.ra SCY1_SCYL1 domain diagram (all)
Domain Color
Pkinase Domain
Gorai.009G292700.2
Gorai.010G219100.1
−200
0
200
400
600
800
1000
1200

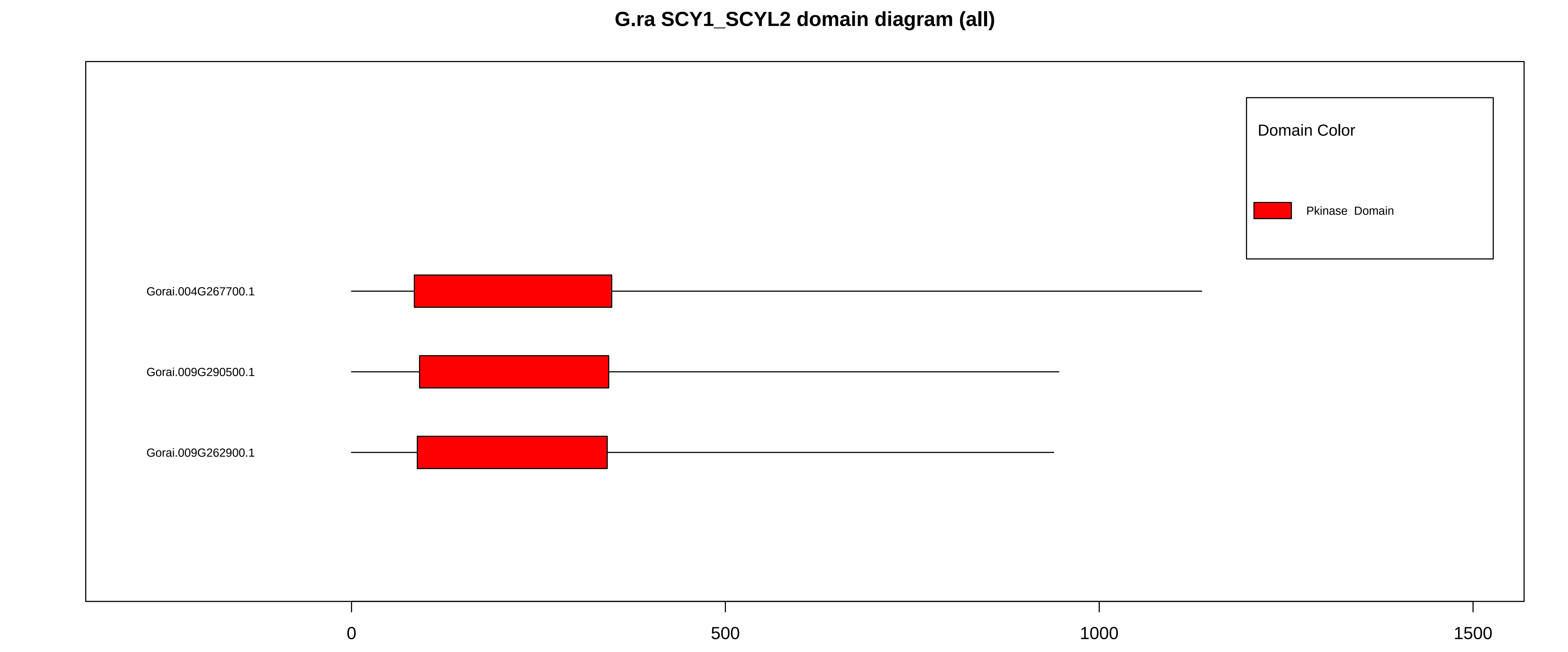
G.ra SCY1_SCYL2 domain diagram (all)
Domain Color
Pkinase Domain
Gorai.004G267700.1
Gorai.009G290500.1
Gorai.009G262900.1
0
500
1000
1500

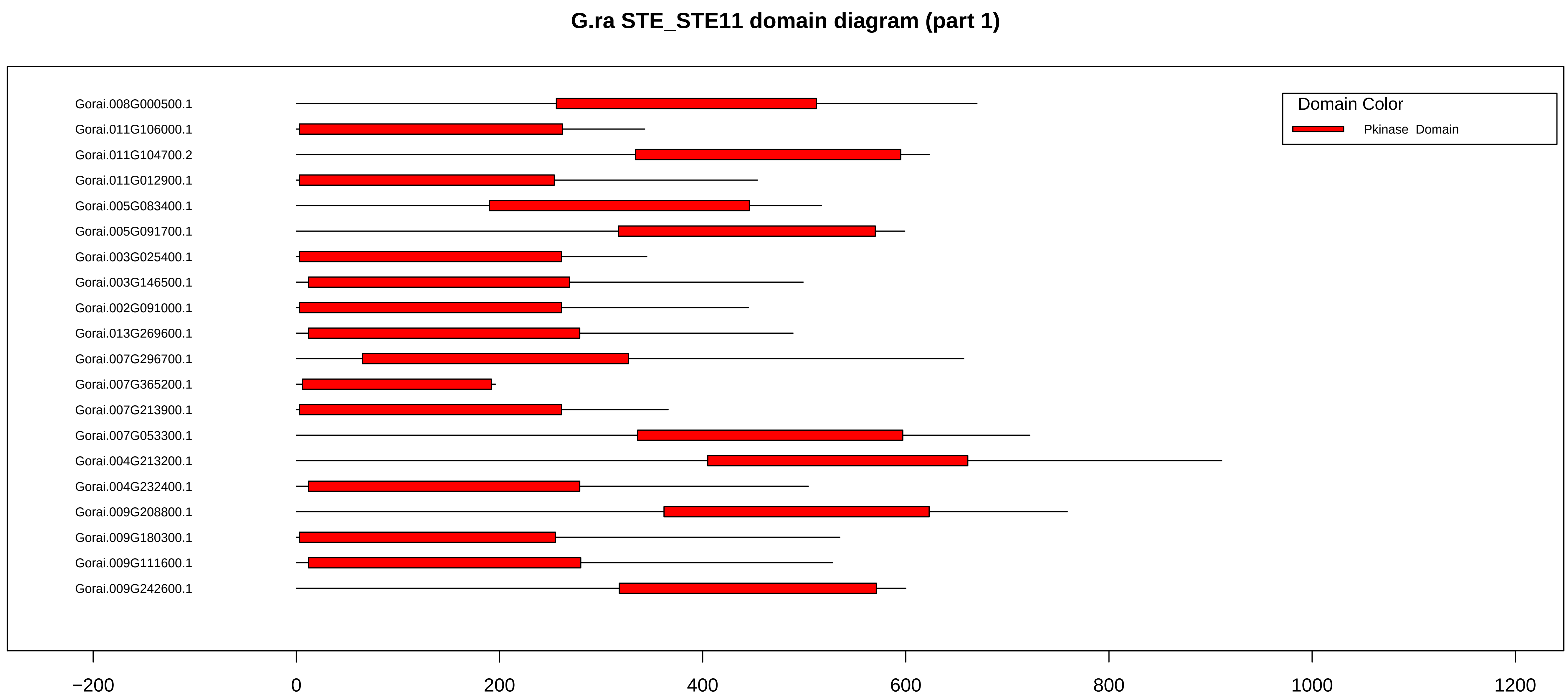
G.ra STE_STE11 domain diagram (part 1)
Gorai.008G000500.1
Gorai.011G106000.1
Gorai.011G104700.2
Gorai.011G012900.1
Gorai.005G083400.1
Gorai.005G091700.1
Gorai.003G025400.1
Gorai.003G146500.1
Gorai.002G091000.1
Gorai.013G269600.1
Gorai.007G296700.1
Gorai.007G365200.1
Gorai.007G213900.1
Gorai.007G053300.1
Gorai.004G213200.1
Gorai.004G232400.1
Gorai.009G208800.1
Gorai.009G180300.1
Gorai.009G111600.1
Gorai.009G242600.1
Domain Color
Pkinase Domain
−200
0
200
400
600
800
1000
1200

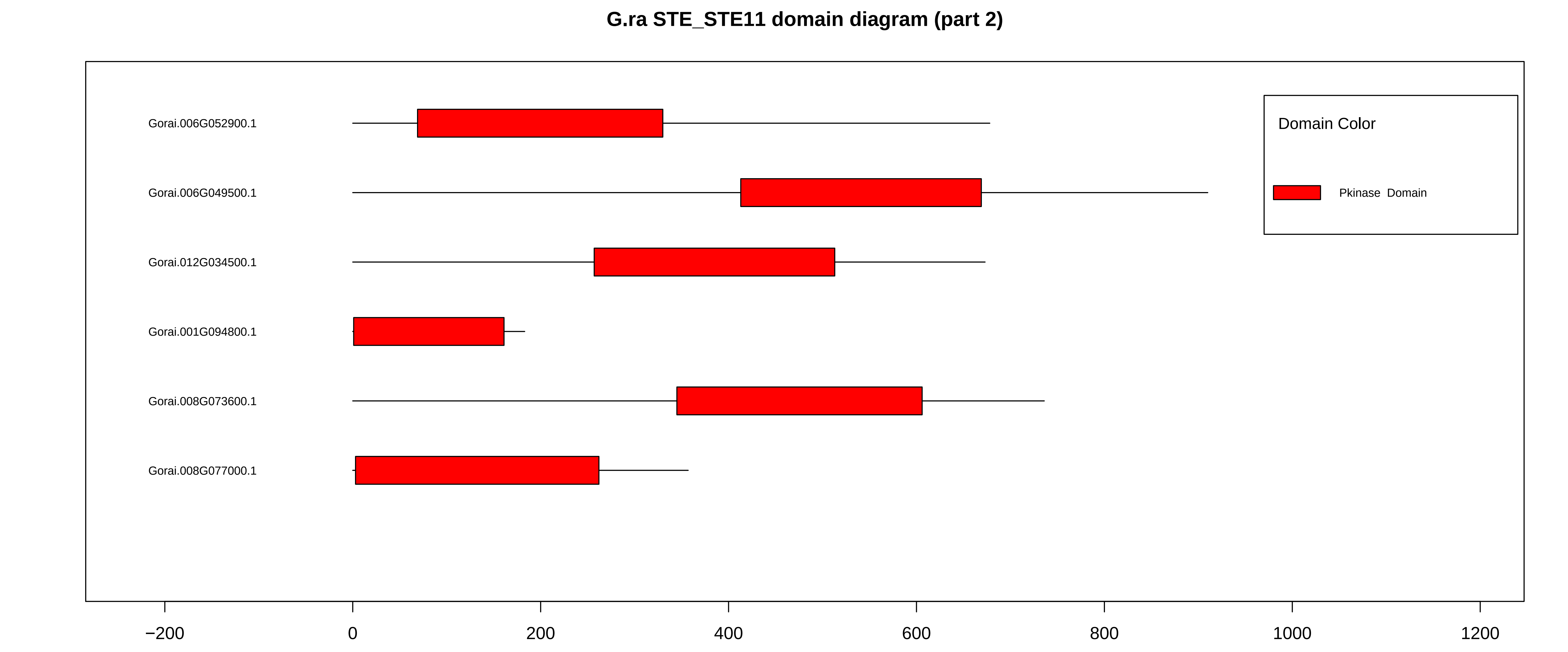

G.ra STE_STE11 domain diagram (part 2)
Gorai.006G052900.1
Gorai.006G049500.1
Gorai.012G034500.1
Gorai.001G094800.1
Gorai.008G073600.1
Gorai.008G077000.1
Domain Color
Pkinase Domain
−200
0
200
400
600
800
1000
1200

G.ra STE_STE20−Fray domain diagram (all)

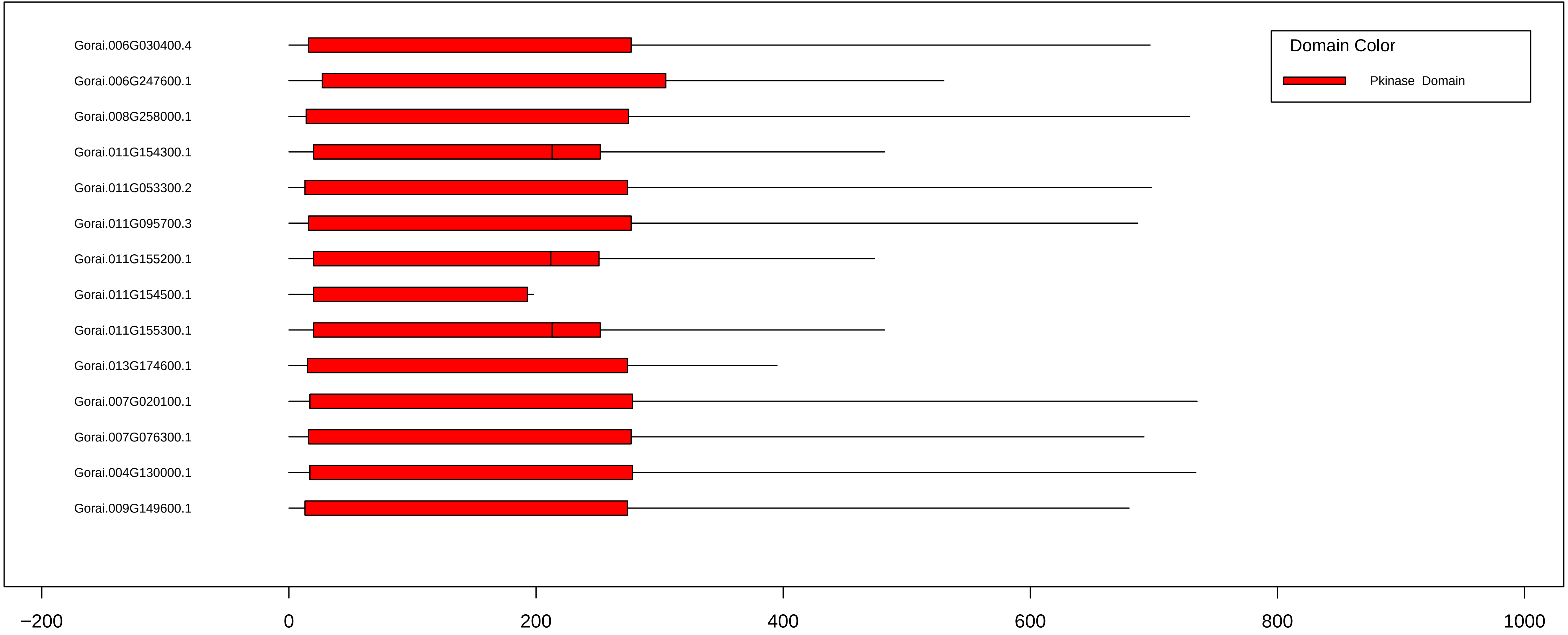

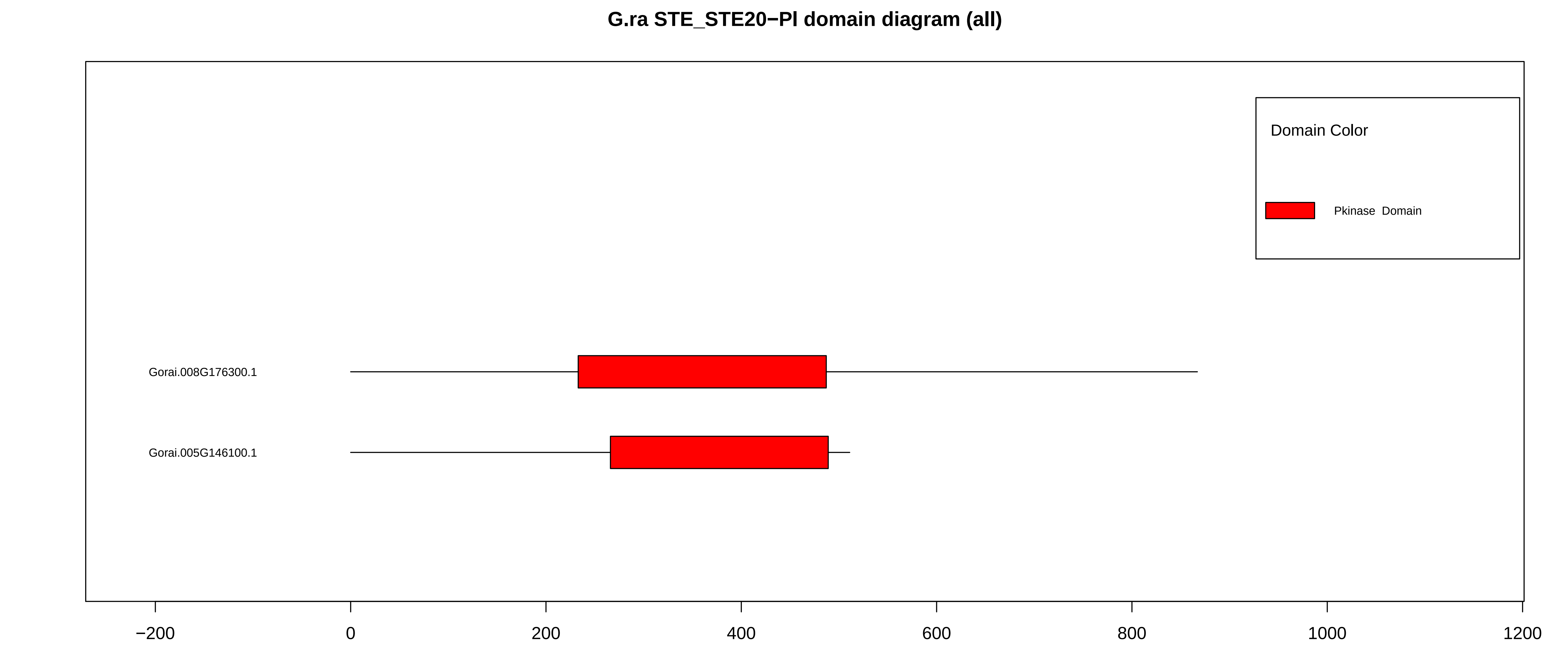

G.ra STE_STE20−Pl domain diagram (all)
Domain Color
Pkinase Domain
Gorai.008G176300.1
Gorai.005G146100.1
−200
0
200
400
600
800
1000
1200

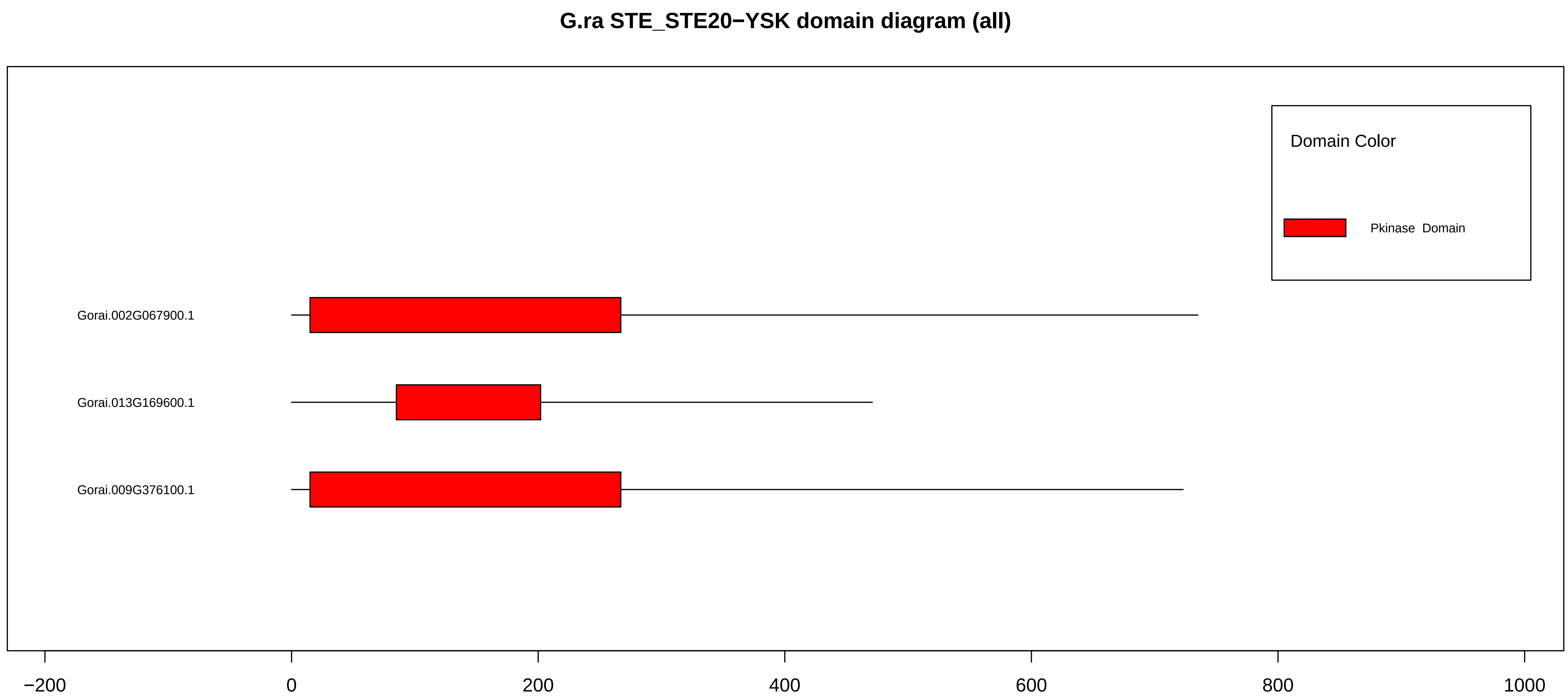
G.ra STE_STE20−YSK domain diagram (all)
Domain Color
Pkinase Domain
Gorai.002G067900.1
Gorai.013G169600.1
Gorai.009G376100.1
−200
0
200
400
600
800
1000

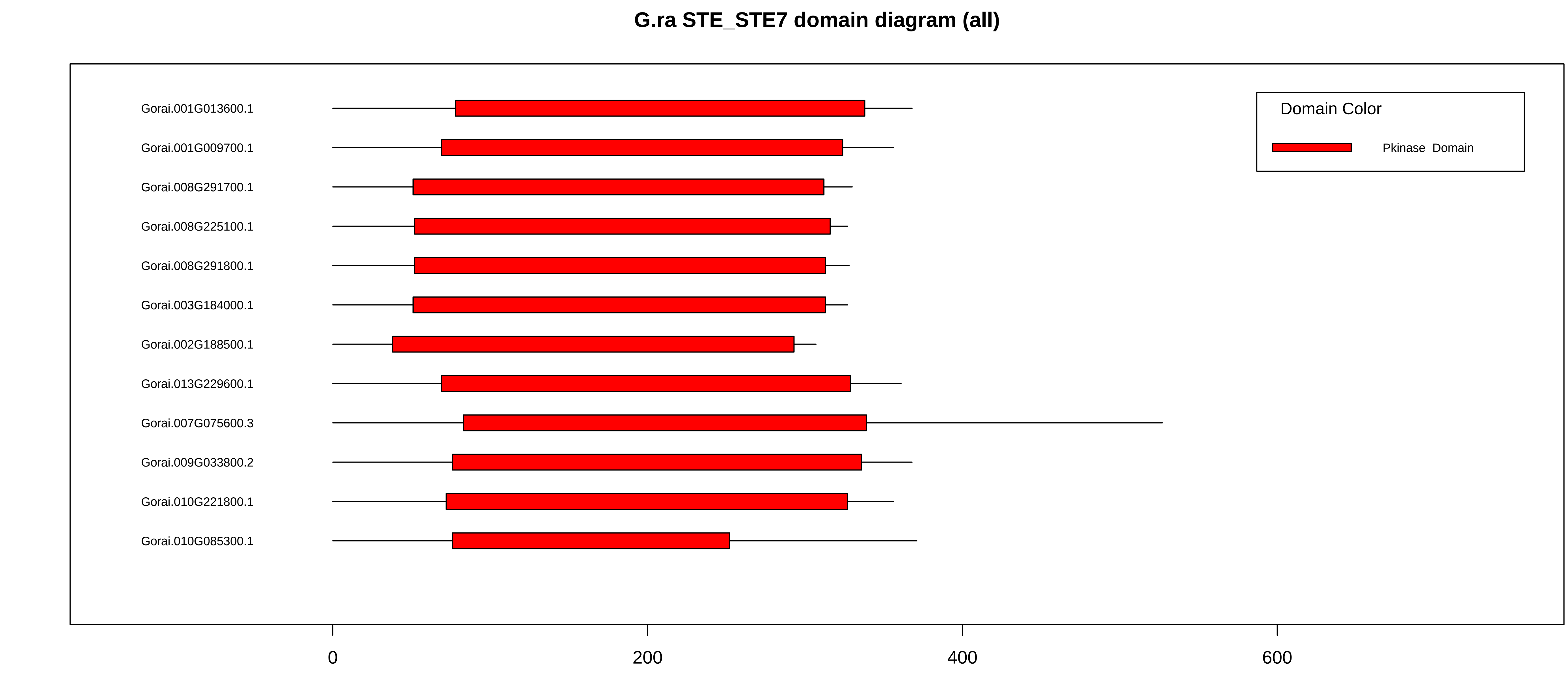
G.ra STE_STE7 domain diagram (all)
Domain Color
Pkinase Domain
Gorai.001G013600.1
Gorai.001G009700.1
Gorai.008G291700.1
Gorai.008G225100.1
Gorai.008G291800.1
Gorai.003G184000.1
Gorai.002G188500.1
Gorai.013G229600.1
Gorai.007G075600.3
Gorai.009G033800.2
Gorai.010G221800.1
Gorai.010G085300.1
0
200
400
600

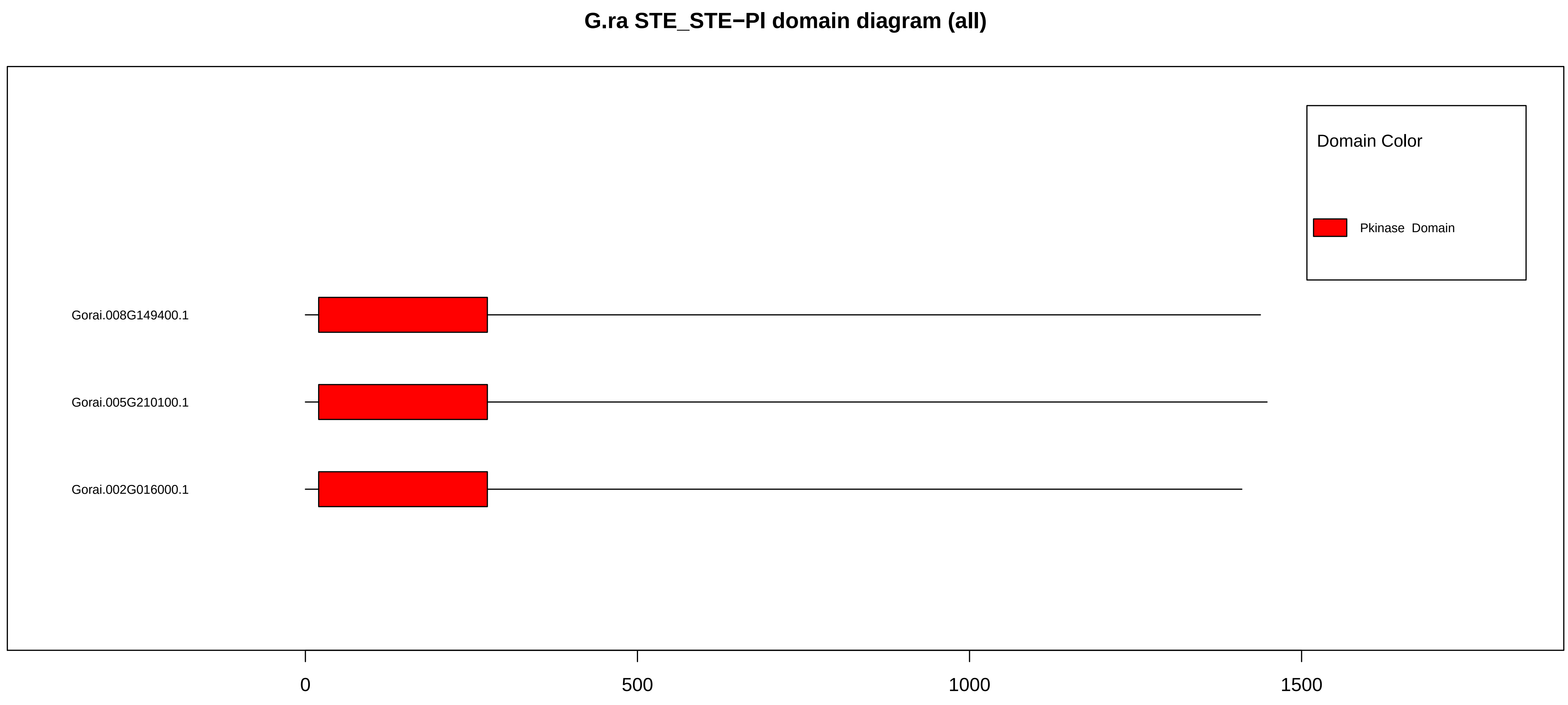
G.ra STE_STE−PI domain diagram (all)
Domain Color
Pkinase Domain
Gorai.008G149400.1
Gorai.005G210100.1
Gorai.002G016000.1
0
500
1000
1500

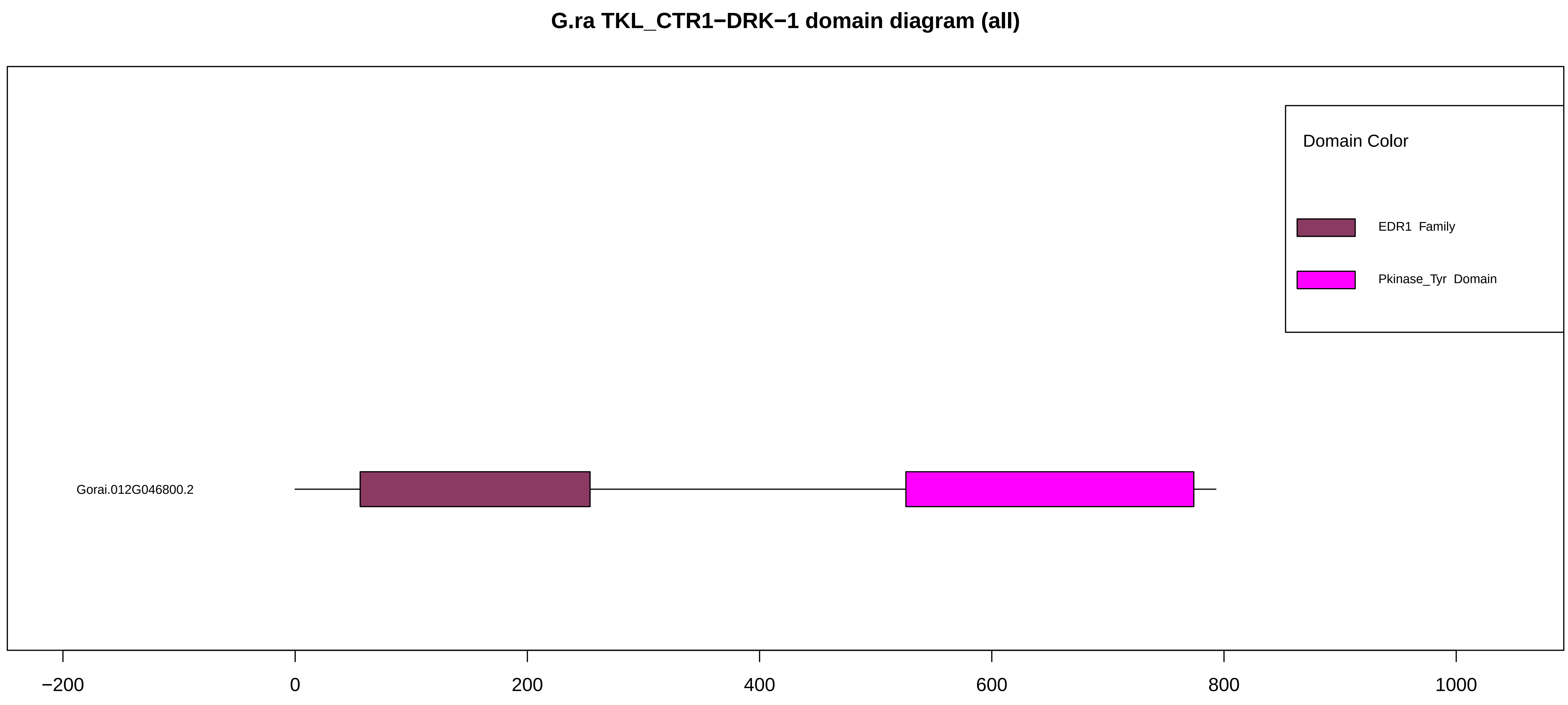
G.ra TKL_CTR1−DRK−1 domain diagram (all)
Domain Color
EDR1 Family
Pkinase_Tyr Domain
Gorai.012G046800.2
−200
0
200
400
600
800
1000

G.ra TKL_CTR1−DRK−2 domain diagram (all)

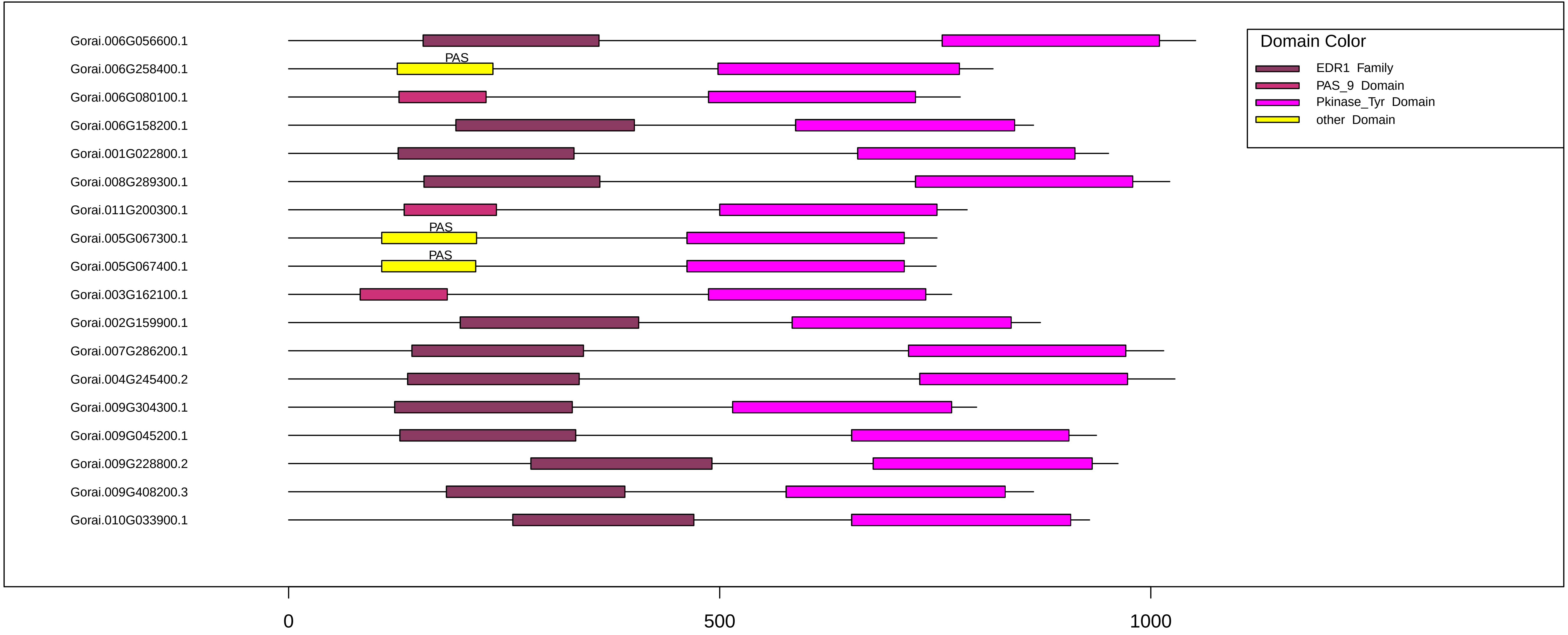

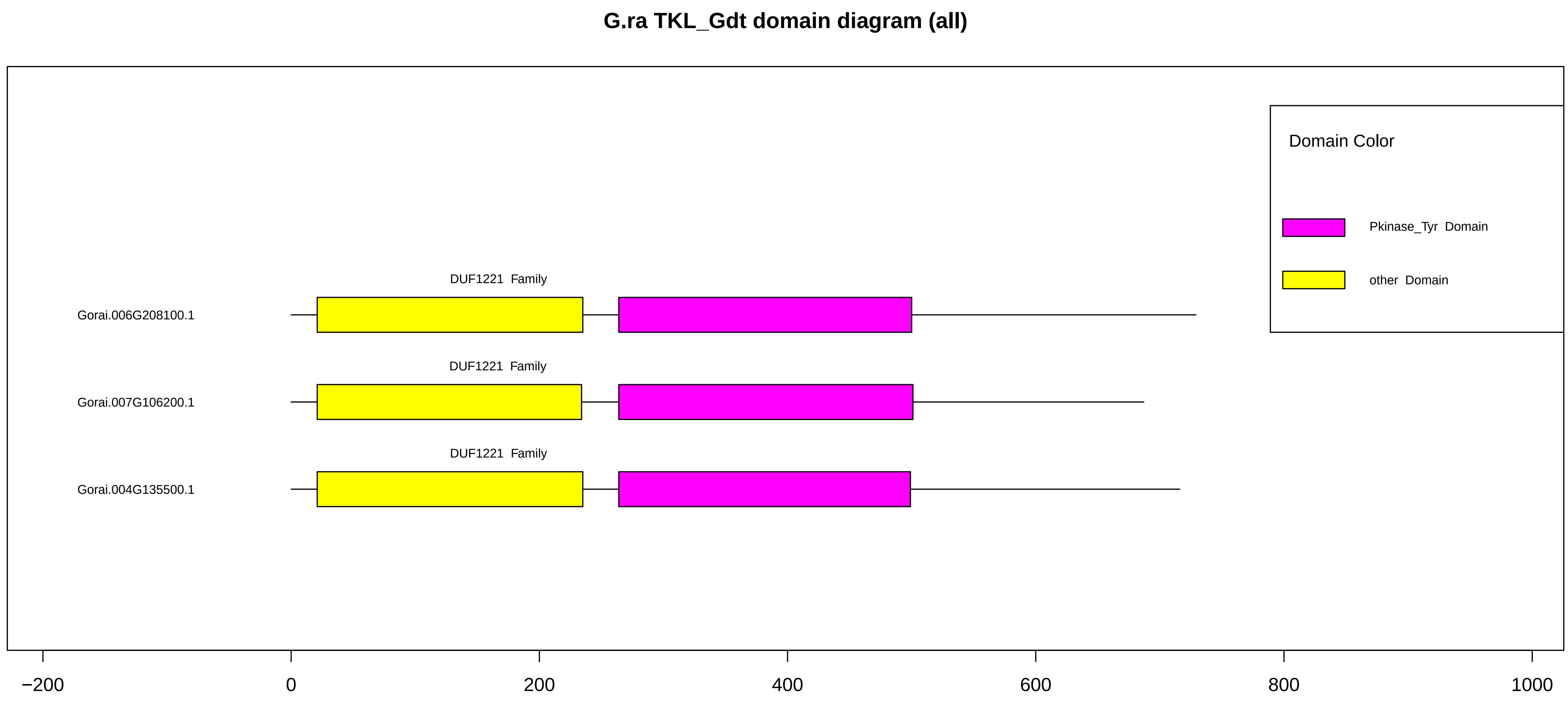

G.ra TKL_Gdt domain diagram (all)
Domain Color
Pkinase_Tyr Domain
other Domain
DUF1221 Family
Gorai.006G208100.1
DUF1221 Family
Gorai.007G106200.1
DUF1221 Family
Gorai.004G135500.1
−200
0
200
400
600
800
1000

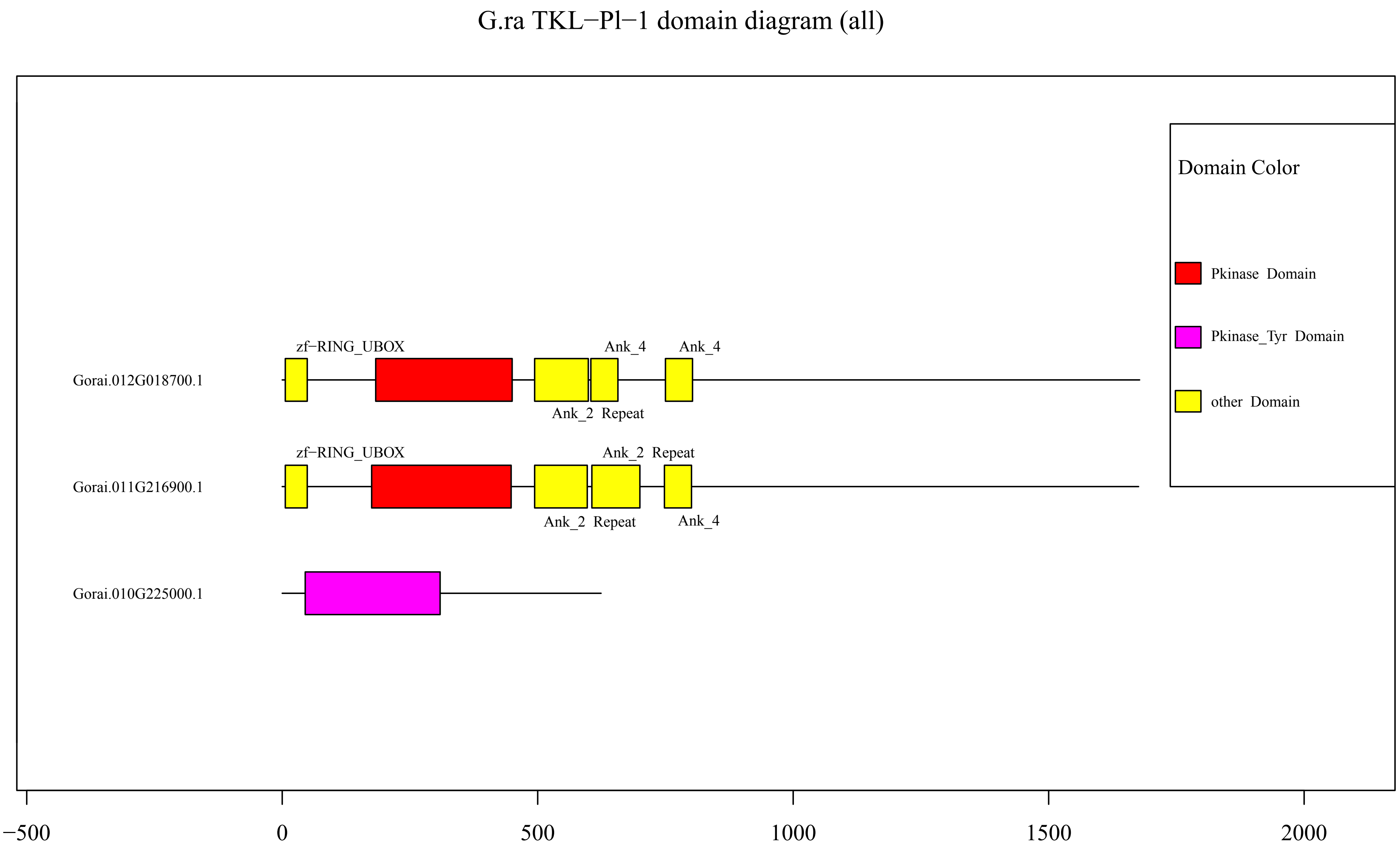

G.ra TKL−Pl−1 domain diagram (all)
Domain Color
Pkinase Domain
Pkinase_Tyr Domain
other Domain
Gorai.012G018700.1
zf−RING_UBOX
Ank_4
Ank_4
Ank_2 Repeat
Gorai.011G216900.1
zf−RING_UBOX
Ank_2 Repeat
Ank_2 Repeat
Ank_4
Gorai.010G225000.1
−500
0
500
1000
1500
2000

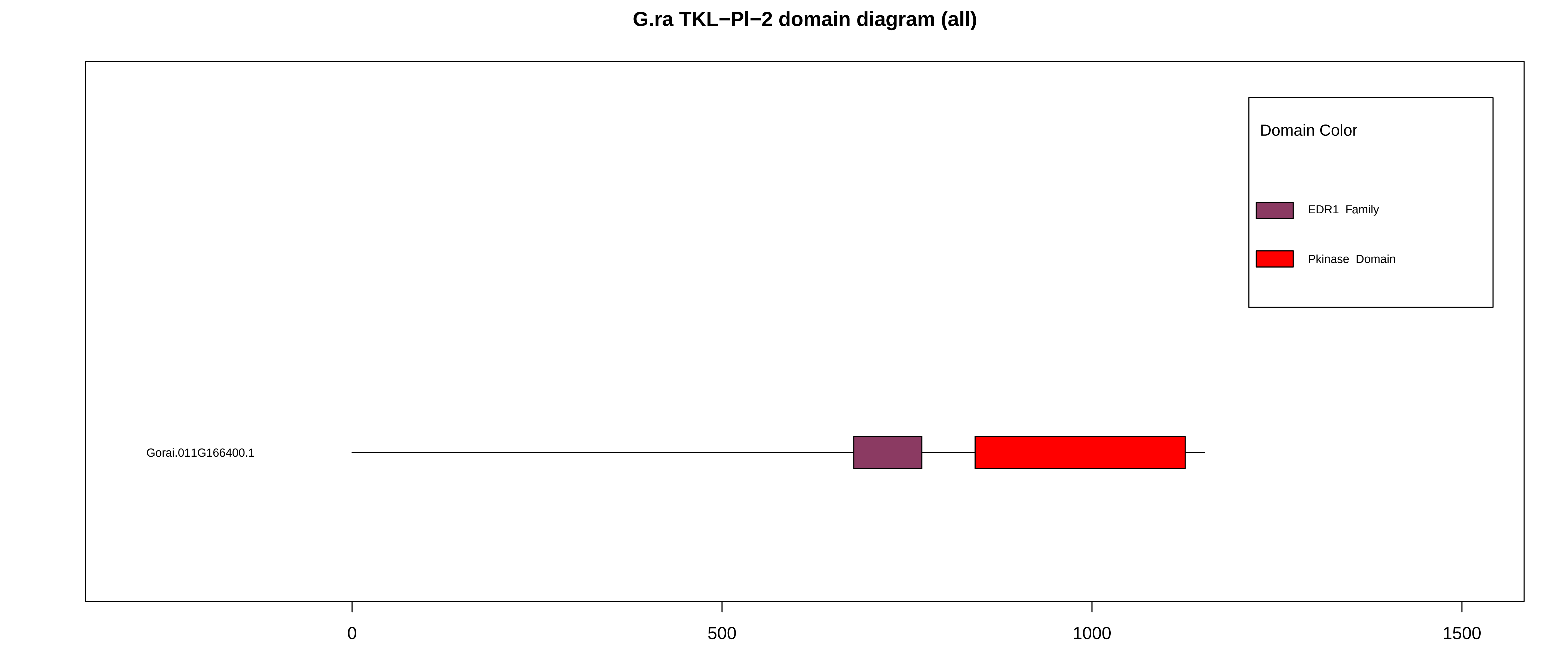
G.ra TKL−Pl−2 domain diagram (all)
Domain Color
EDR1 Family
Pkinase Domain
Gorai.011G166400.1
0
500
1000
1500

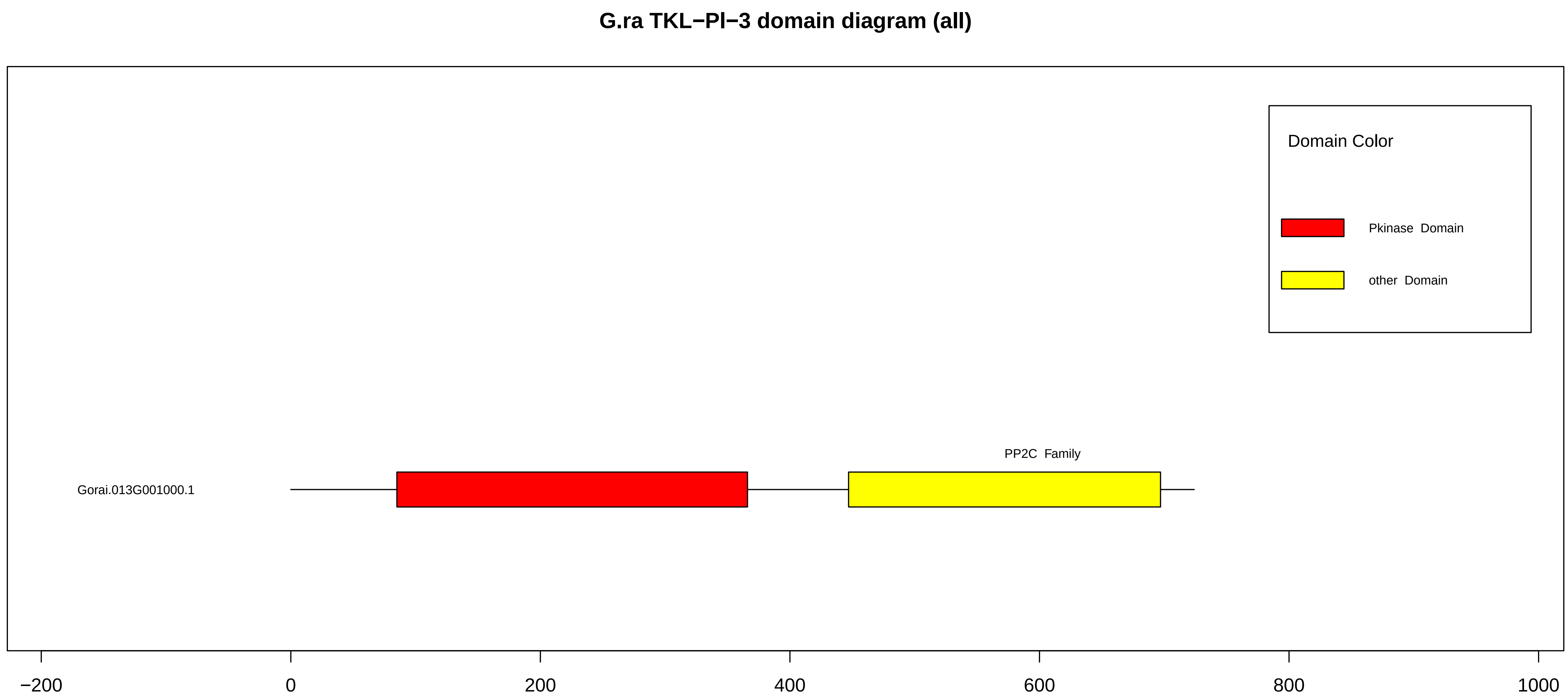
G.ra TKL−Pl−3 domain diagram (all)
Domain Color
Pkinase Domain
other Domain
PP2C Family
Gorai.013G001000.1
−200
0
200
400
600
800
1000

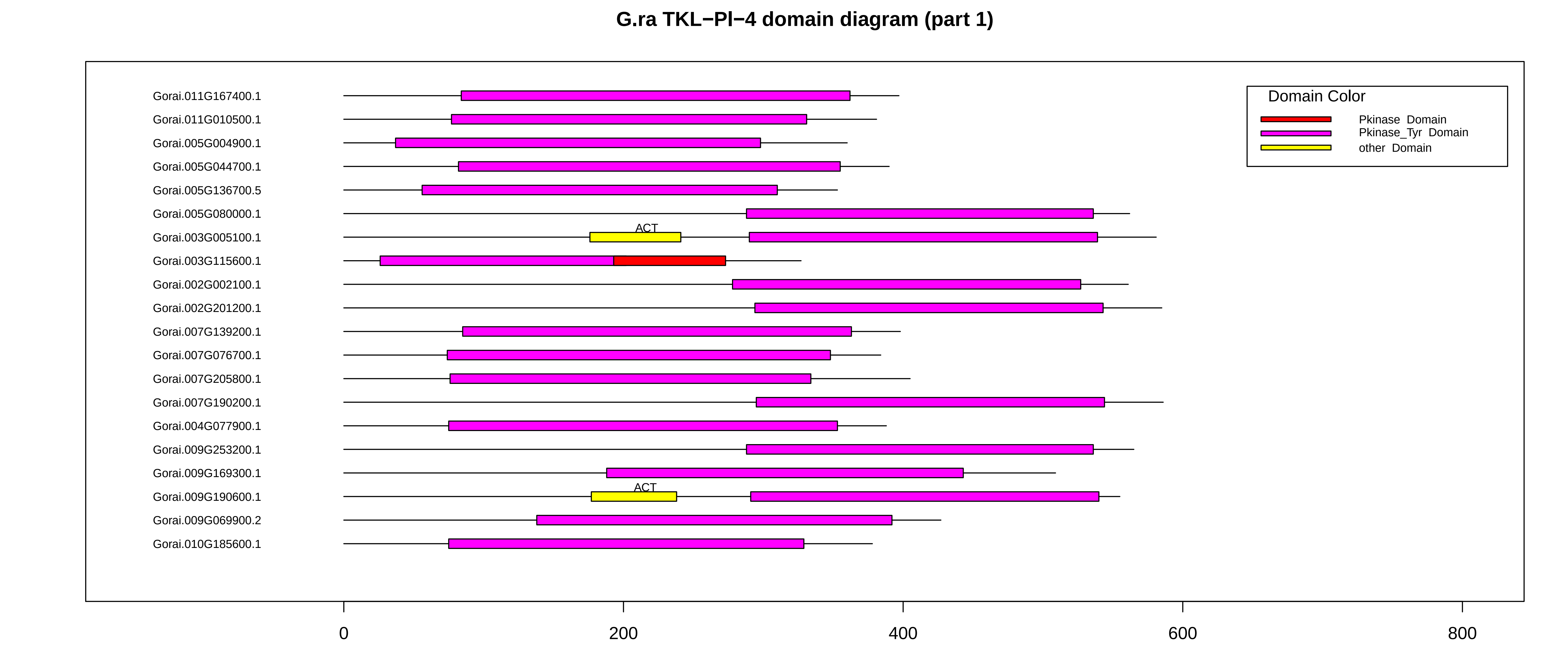
G.ra TKL−Pl−4 domain diagram (part 1)
Domain Color
Pkinase Domain
Pkinase_Tyr Domain
other Domain
Gorai.011G167400.1
Gorai.011G010500.1
Gorai.005G004900.1
Gorai.005G044700.1
Gorai.005G136700.5
Gorai.005G080000.1
ACT
Gorai.003G005100.1
Gorai.003G115600.1
Gorai.002G002100.1
Gorai.002G201200.1
Gorai.007G139200.1
Gorai.007G076700.1
Gorai.007G205800.1
Gorai.007G190200.1
Gorai.004G077900.1
Gorai.009G253200.1
Gorai.009G169300.1
ACT
Gorai.009G190600.1
Gorai.009G069900.2
Gorai.010G185600.1
0
200
400
600
800

G.ra TKL−Pl−4 domain diagram (part 2)

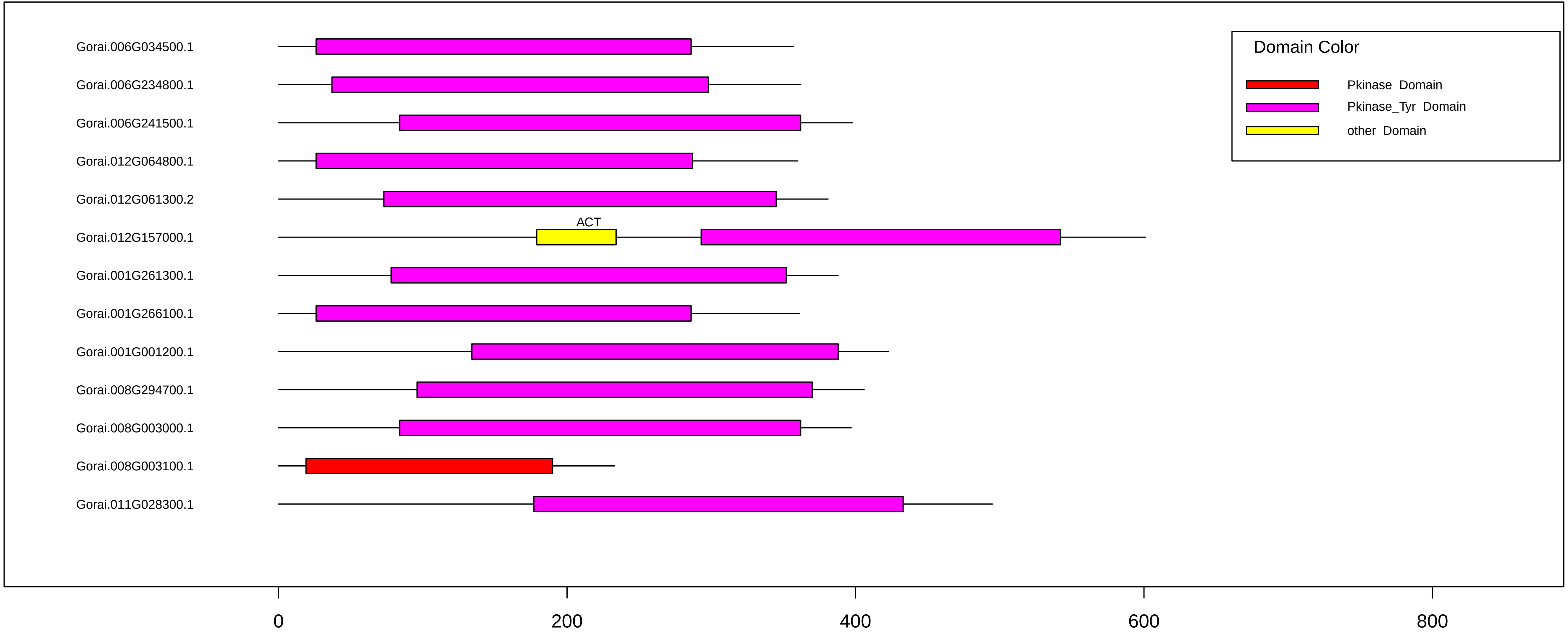

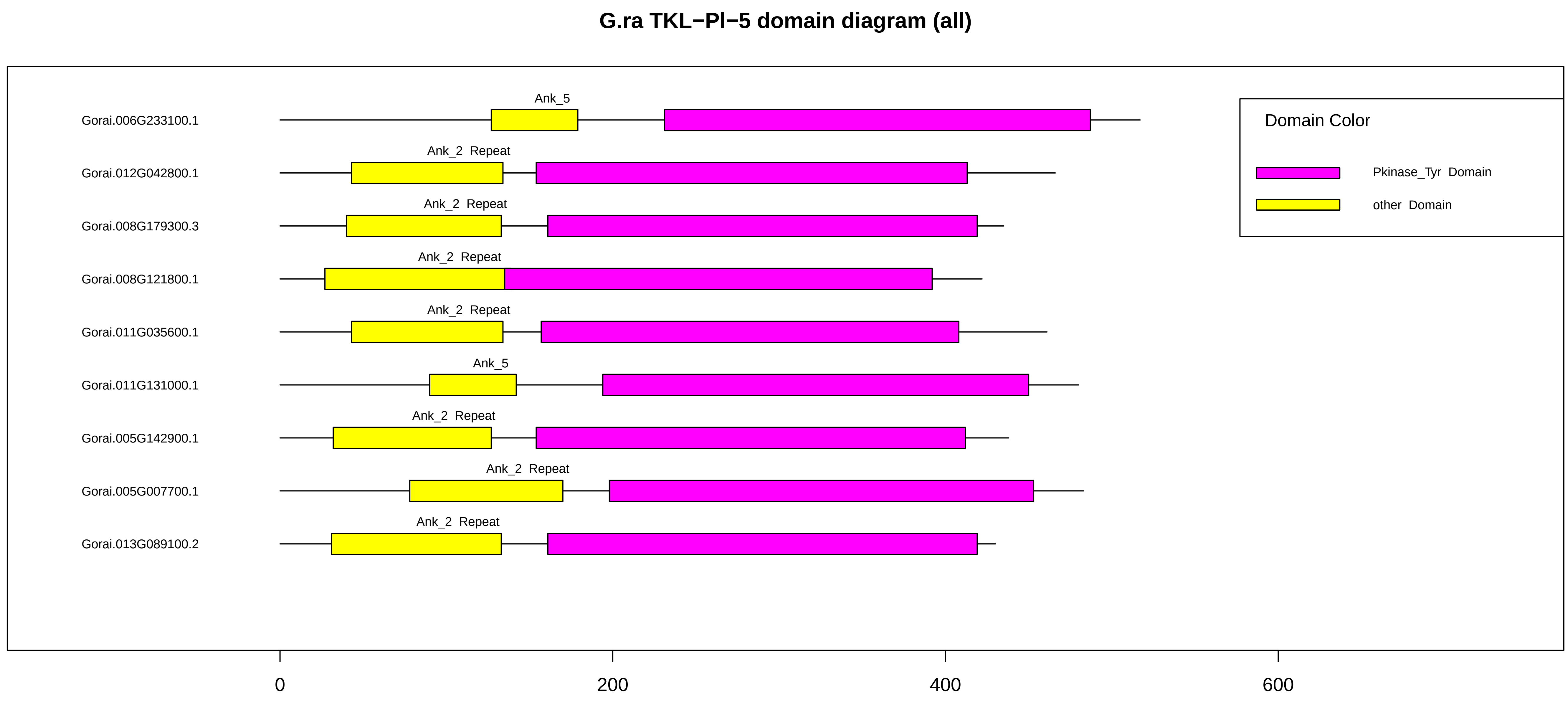
G.ra TKL−Pl−5 domain diagram (all)
Gorai.006G233100.1
Ank_5
Gorai.012G042800.1
Ank_2 Repeat
Gorai.008G179300.3
Ank_2 Repeat
Gorai.008G121800.1
Ank_2 Repeat
Gorai.011G035600.1
Ank_2 Repeat
Gorai.011G131000.1
Ank_5
Gorai.005G142900.1
Ank_2 Repeat
Gorai.005G007700.1
Ank_2 Repeat
Gorai.013G089100.2
Ank_2 Repeat
Domain Color
Pkinase_Tyr Domain
other Domain
0
200
400
600

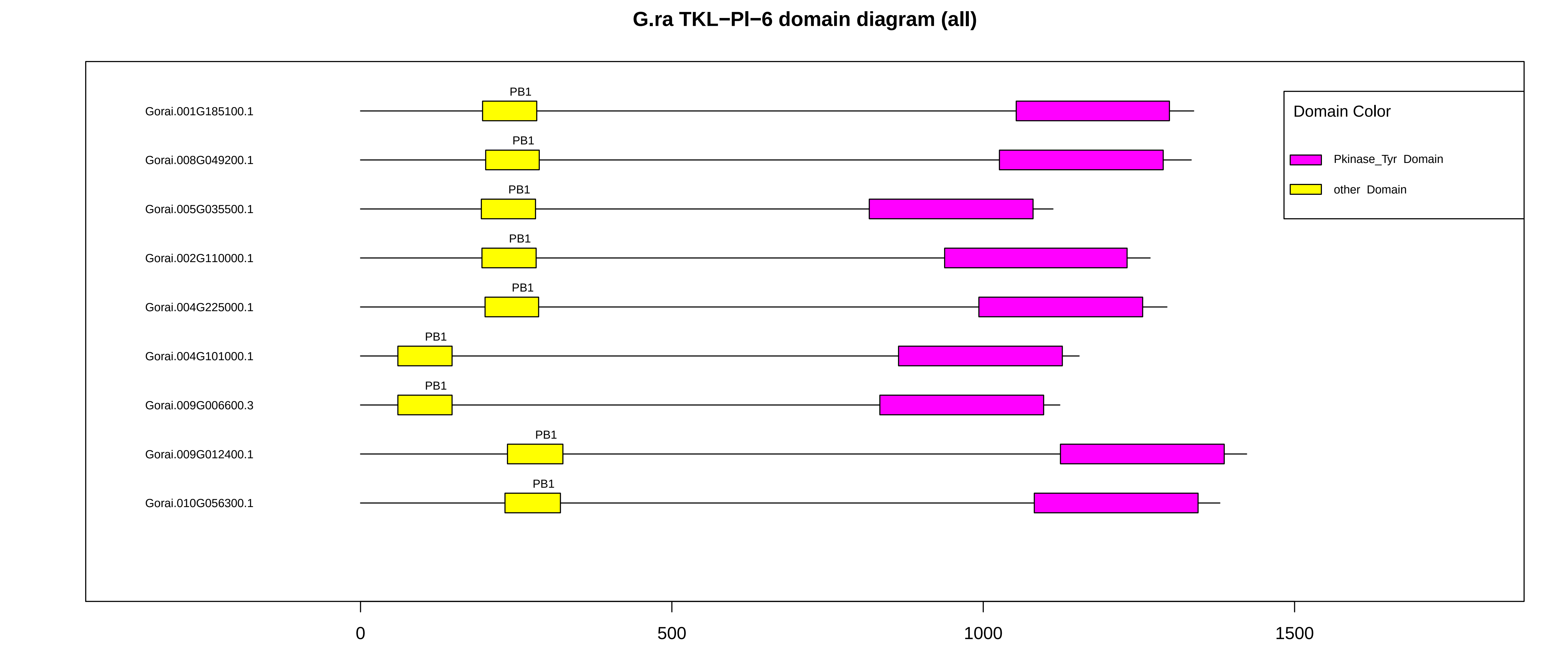
G.ra TKL−Pl−6 domain diagram (all)
Gorai.001G185100.1
Gorai.008G049200.1
Gorai.005G035500.1
Gorai.002G110000.1
Gorai.004G225000.1
Gorai.004G101000.1
Gorai.009G006600.3
Gorai.009G012400.1
Gorai.010G056300.1
PB1
Domain Color
Pkinase_Tyr Domain
other Domain
0
500
1000
1500

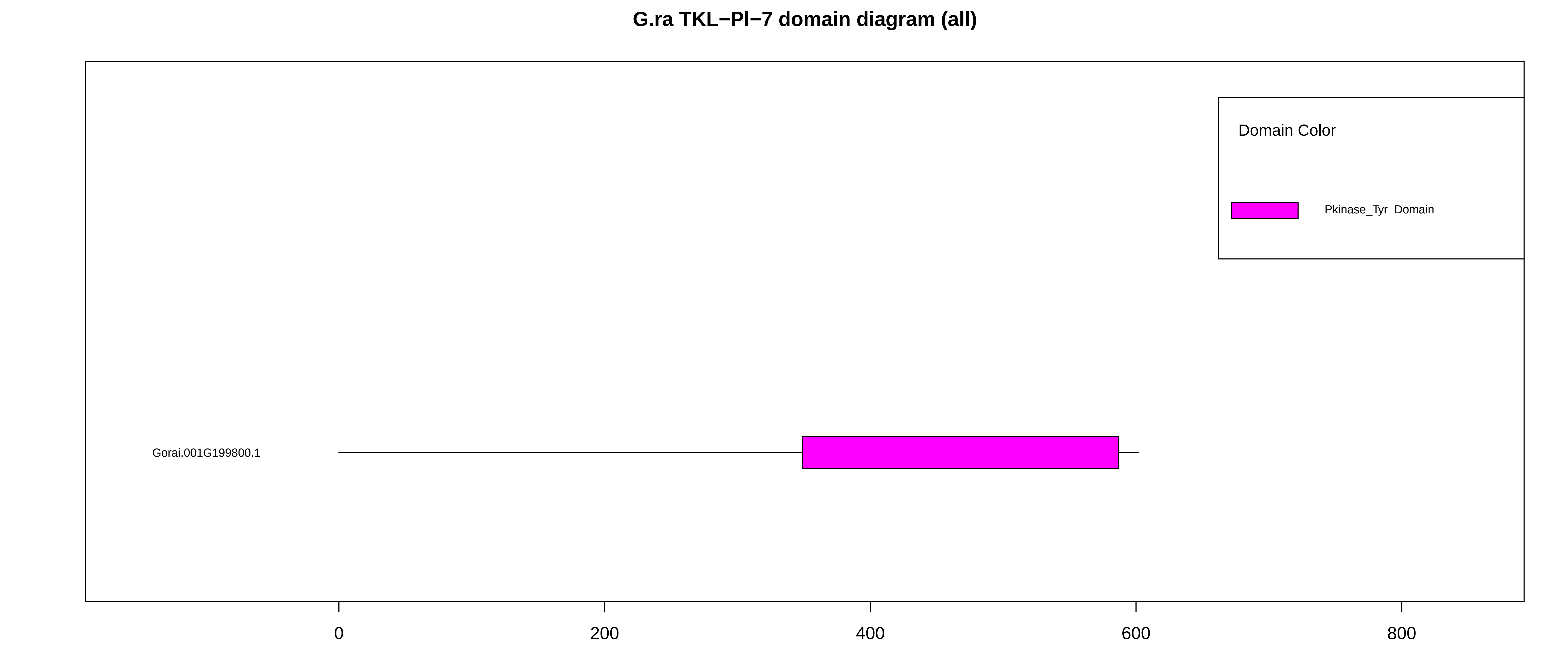
G.ra TKL−Pl−7 domain diagram (all)
Domain Color
Pkinase_Tyr Domain
Gorai.001G199800.1
0
200
400
600
800

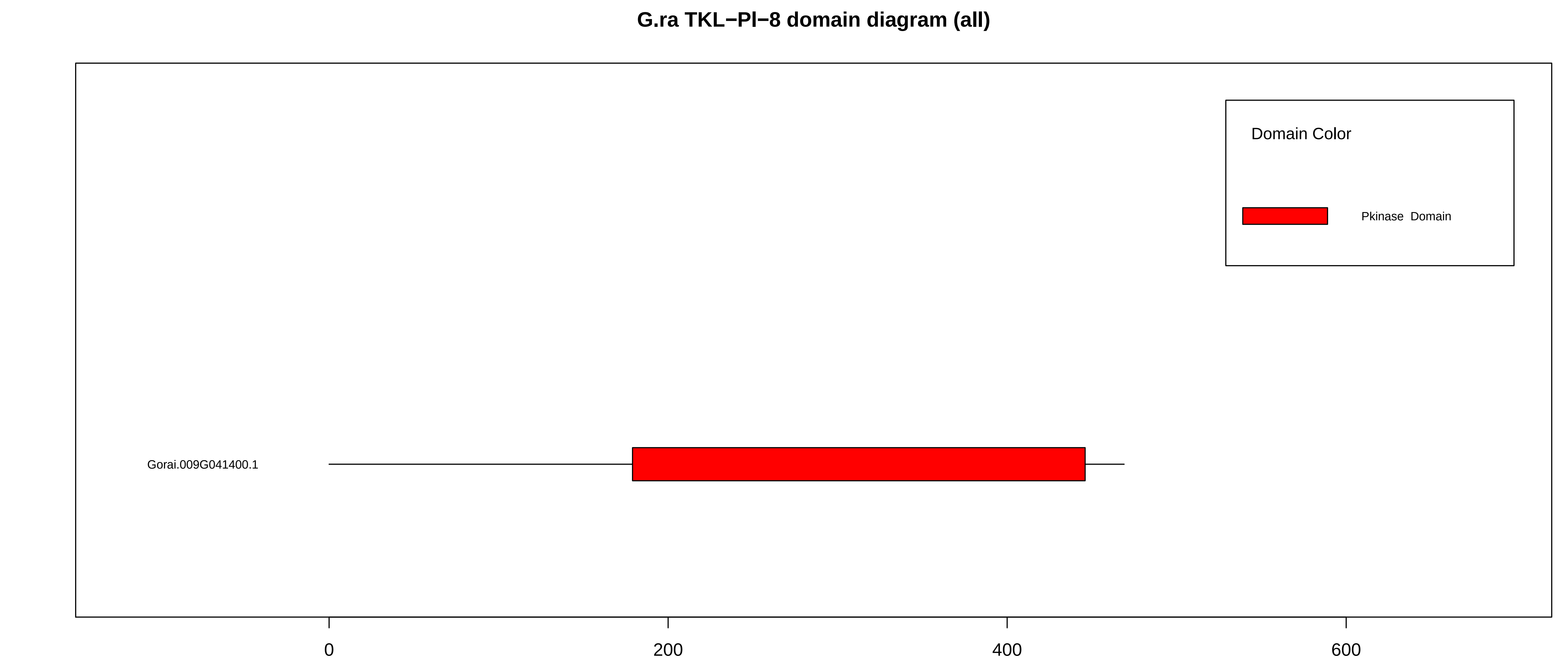

G.ra TKL−Pl−8 domain diagram (all)
Domain Color
Pkinase Domain
Gorai.009G041400.1
0
200
400
600

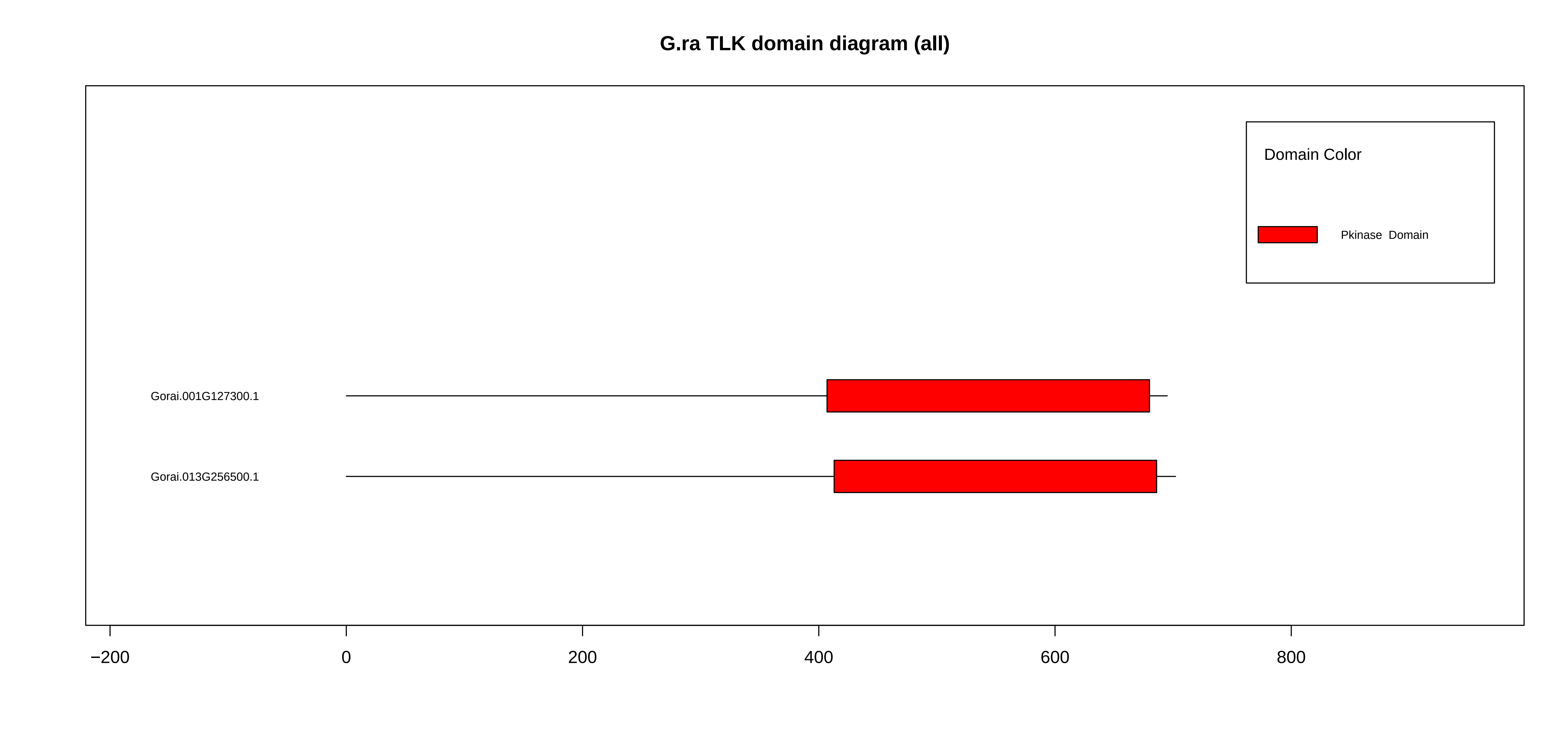
G.ra TLK domain diagram (all)
Domain Color
Pkinase Domain
Gorai.001G127300.1
Gorai.013G256500.1
−200
0
200
400
600
800

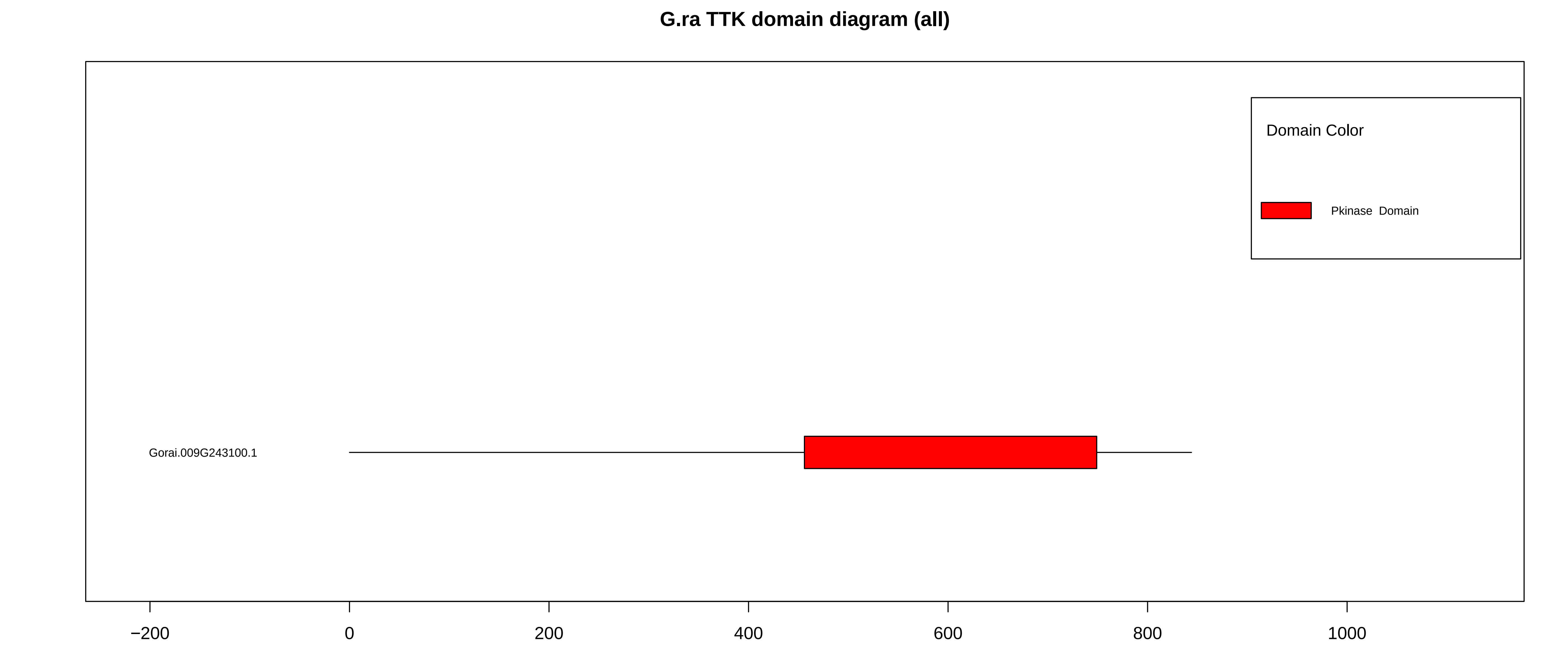
G.ra TTK domain diagram (all)
Domain Color
Pkinase Domain
Gorai.009G243100.1
−200
0
200
400
600
800
1000

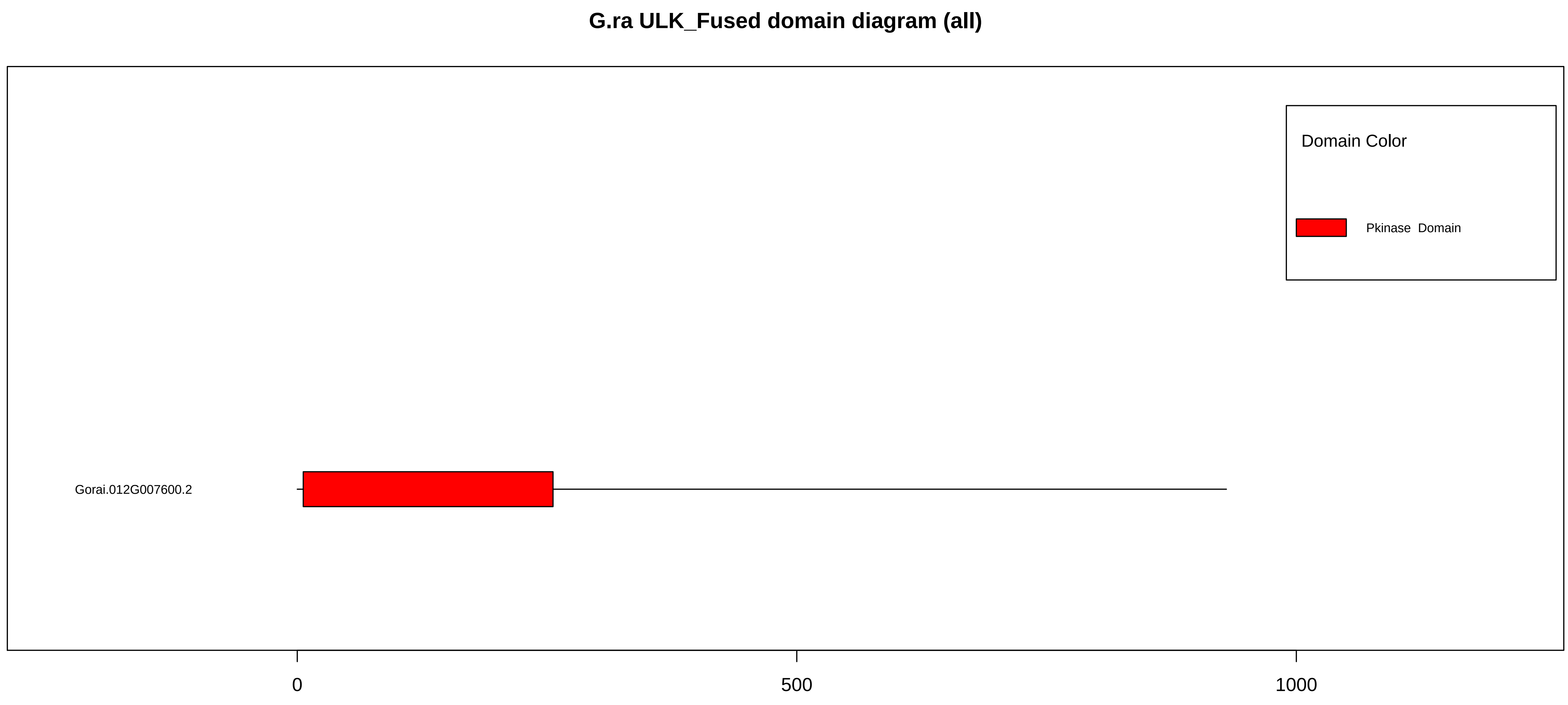
G.ra ULK_Fused domain diagram (all)
Domain Color
Pkinase Domain
Gorai.012G007600.2
0
500
1000

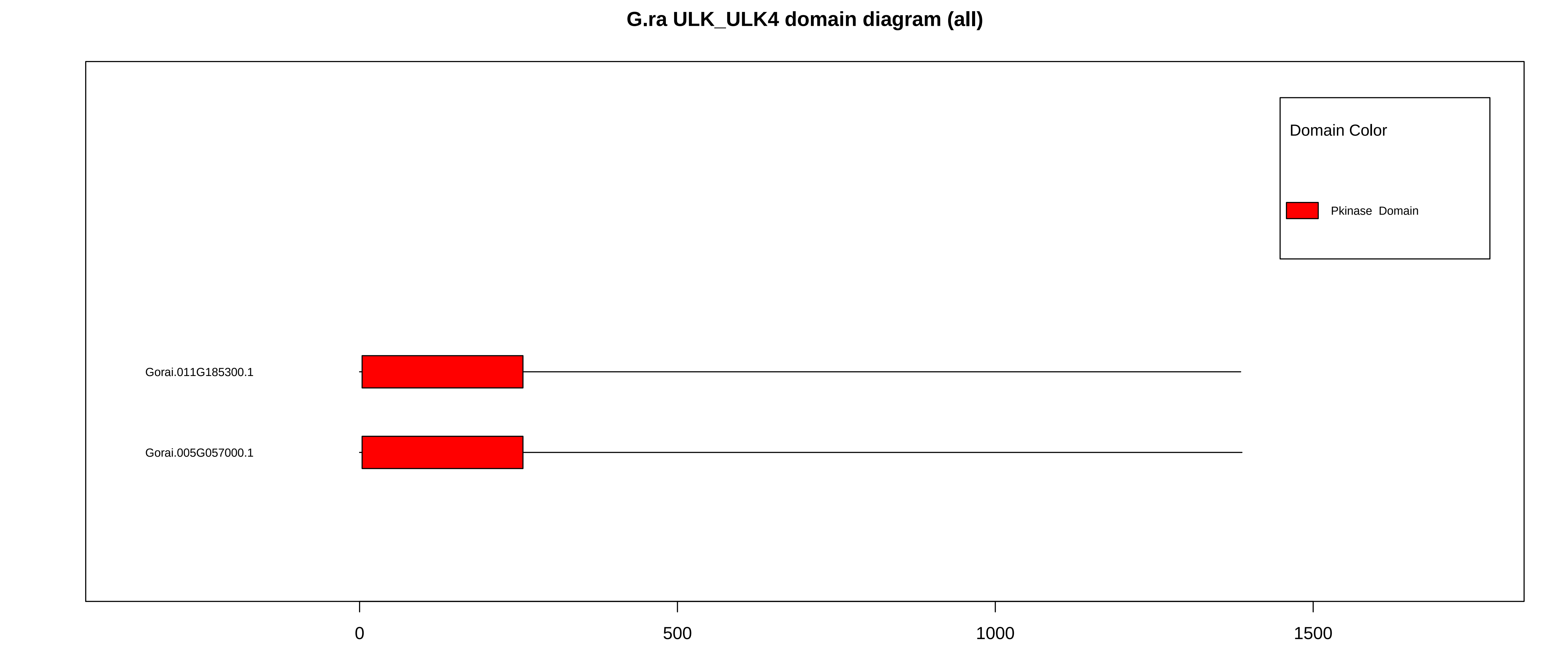
G.ra ULK_ULK4 domain diagram (all)
Domain Color
Pkinase Domain
Gorai.011G185300.1
Gorai.005G057000.1
0
500
1000
1500

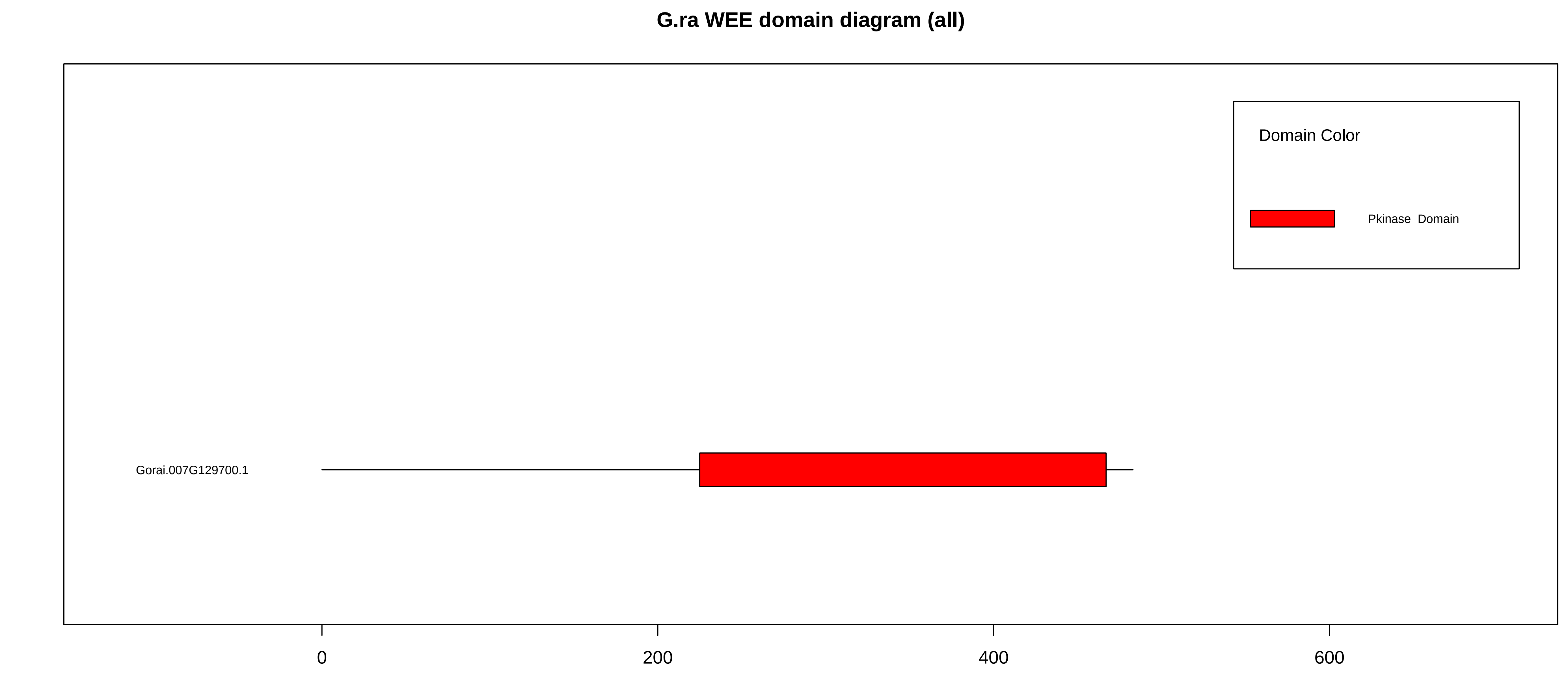

G.ra WEE domain diagram (all)
Domain Color
Pkinase Domain
Gorai.007G129700.1
0
200
400
600

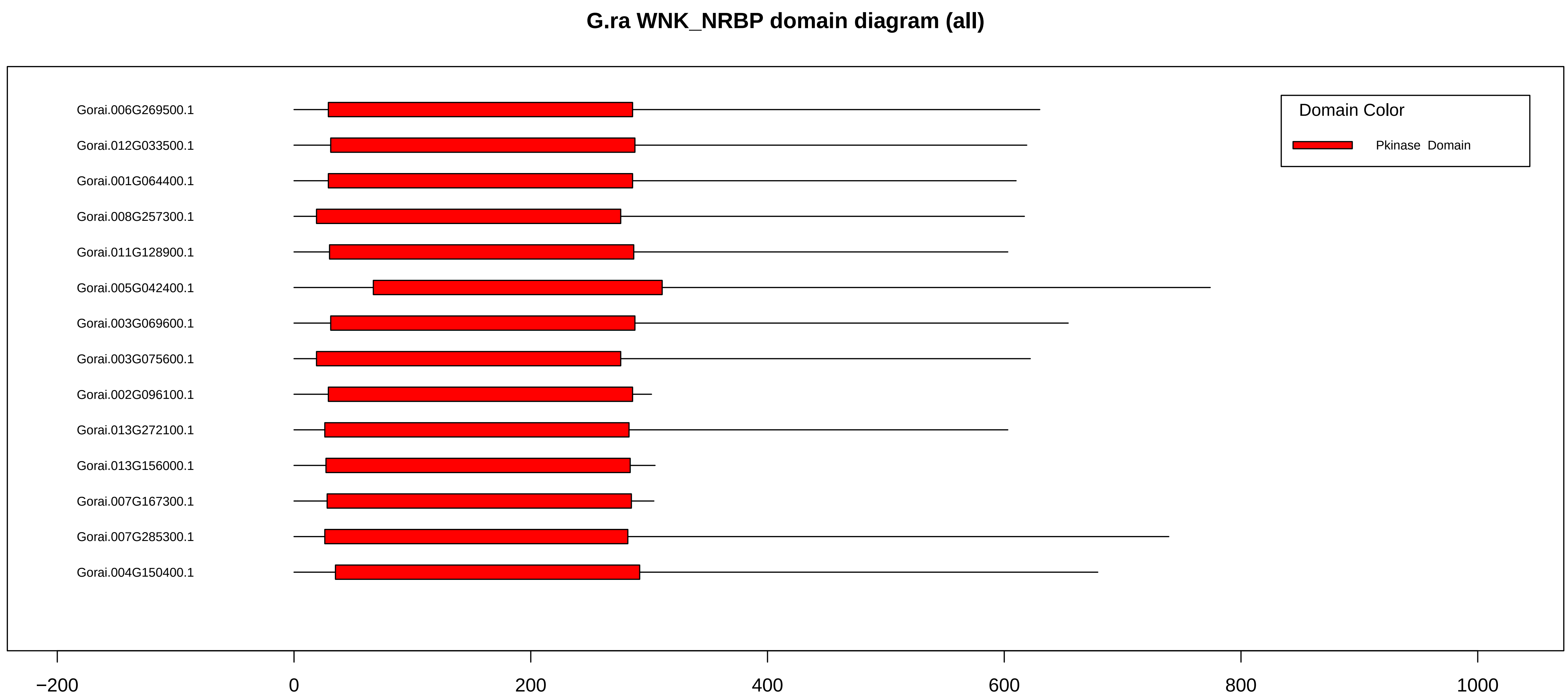
G.ra WNK_NRBP domain diagram (all)
Gorai.006G269500.1
Gorai.012G033500.1
Gorai.001G064400.1
Gorai.008G257300.1
Gorai.011G128900.1
Gorai.005G042400.1
Gorai.003G069600.1
Gorai.003G075600.1
Gorai.002G096100.1
Gorai.013G272100.1
Gorai.013G156000.1
Gorai.007G167300.1
Gorai.007G285300.1
Gorai.004G150400.1
Domain Color
Pkinase Domain
−200
0
200
400
600
800
1000

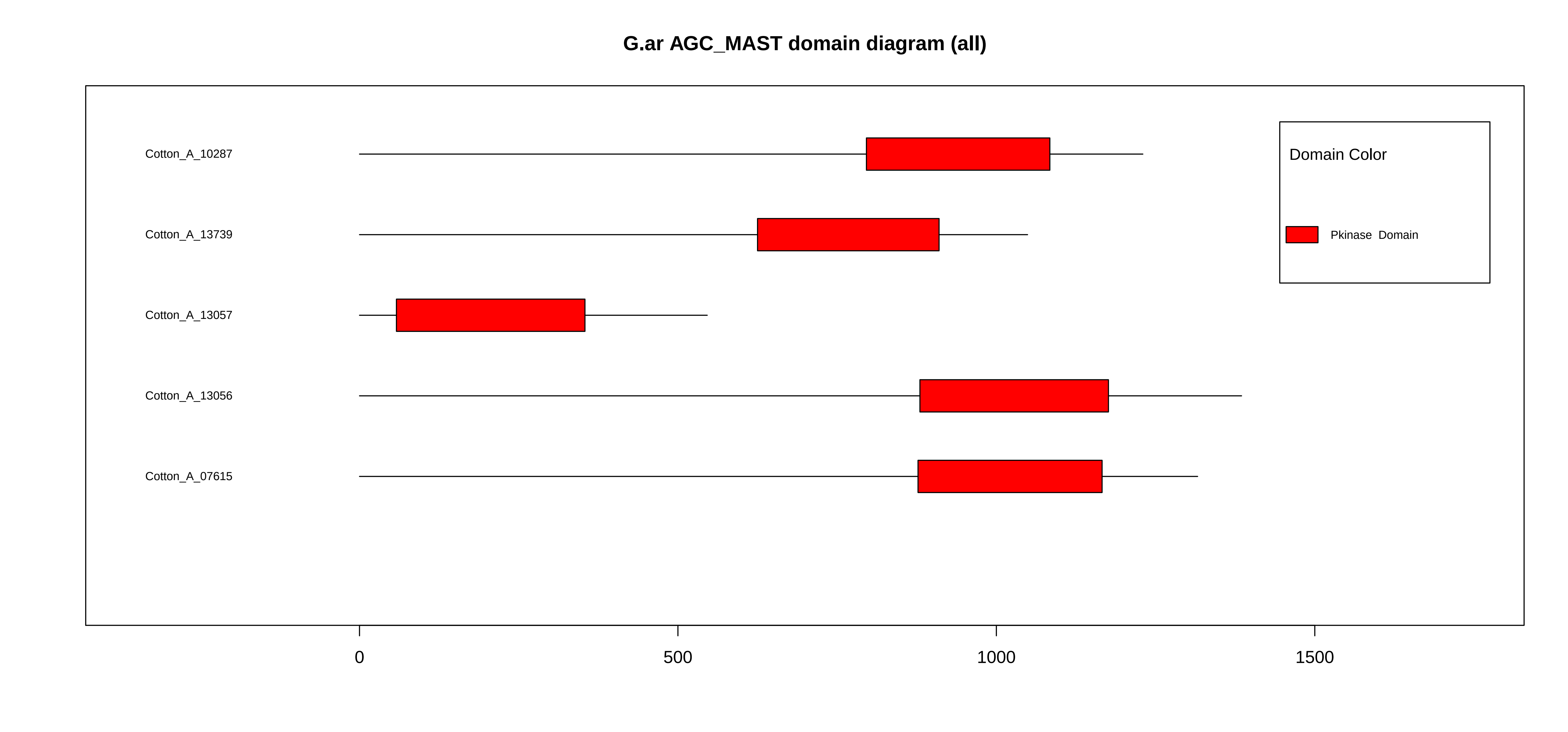

G.ar AGC_MAST domain diagram (all)
Cotton_A_10287
Cotton_A_13739
Cotton_A_13057
Cotton_A_13056
Cotton_A_07615
Domain Color
Pkinase Domain
0
500
1000
1500

G.ar AGC_NDR domain diagram (all)

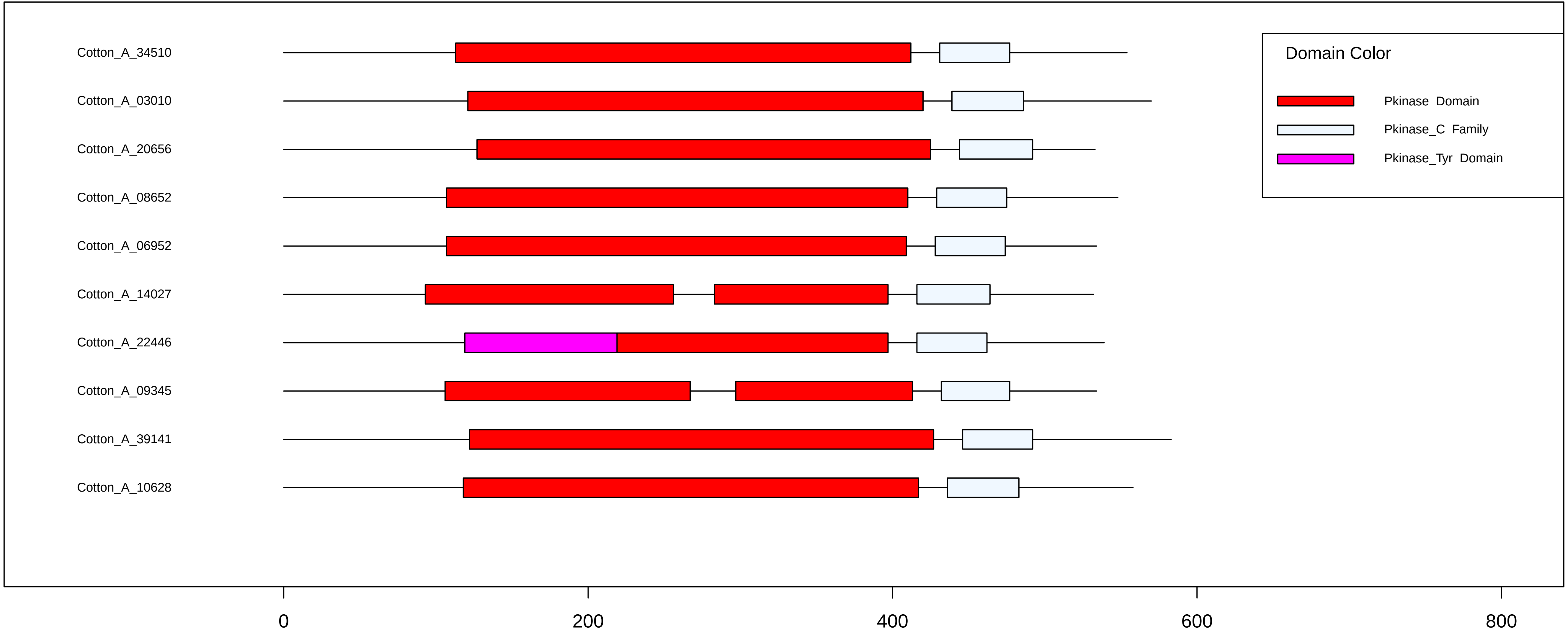

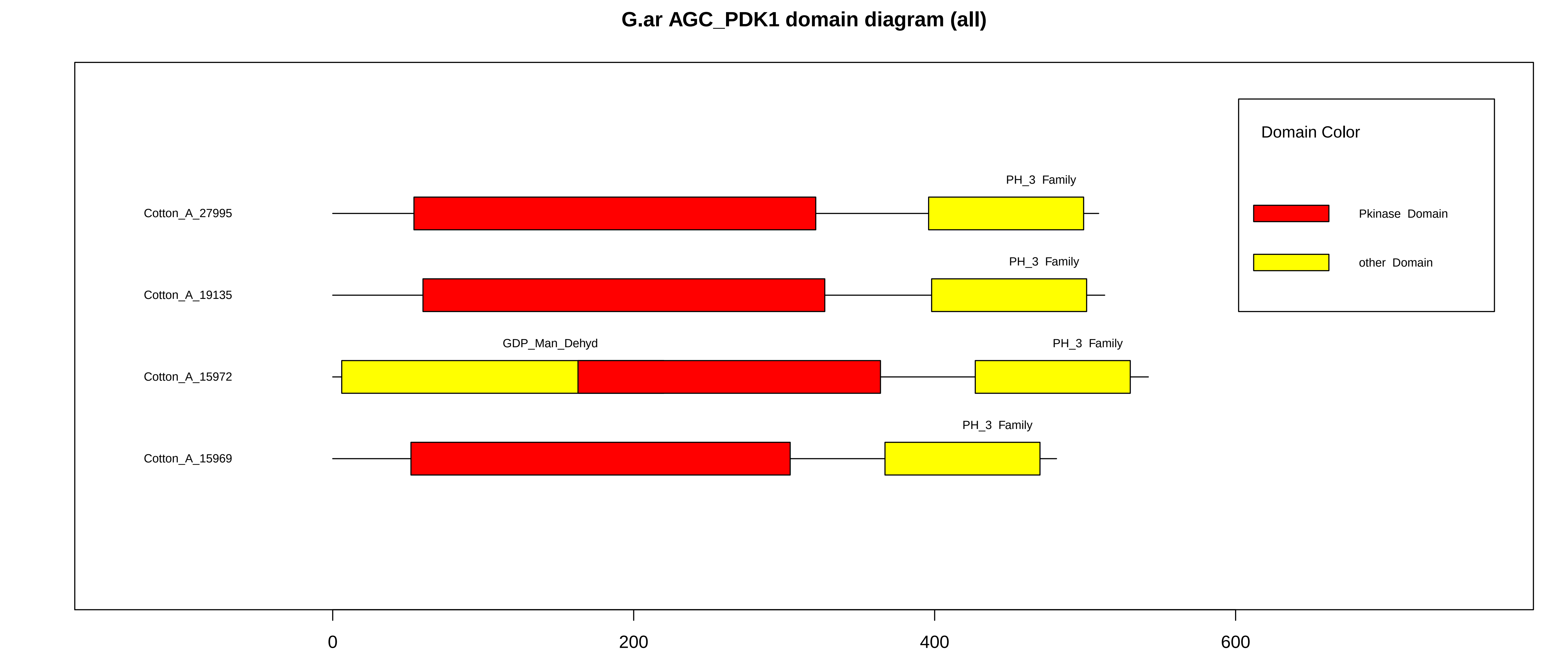
G.ar AGC_PDK1 domain diagram (all)
Domain Color
Pkinase Domain
other Domain
Cotton_A_27995
Cotton_A_19135
Cotton_A_15972
Cotton_A_15969
PH_3 Family
PH_3 Family
GDP_Man_Dehyd
PH_3 Family
PH_3 Family
0
200
400
600

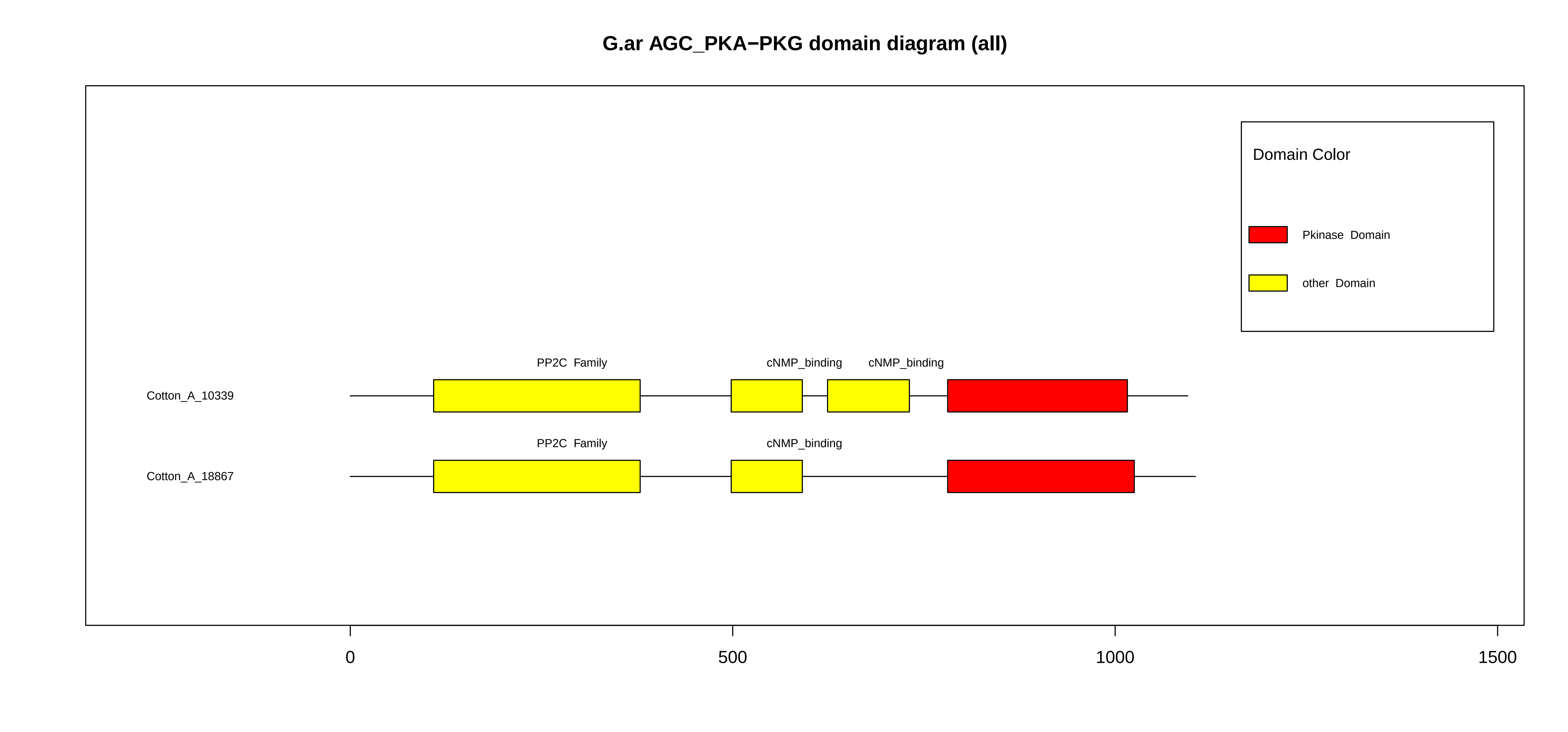
G.ar AGC_PKA−PKG domain diagram (all)
Domain Color
Pkinase Domain
other Domain
PP2C Family
cNMP_binding
cNMP_binding
Cotton_A_10339
PP2C Family
cNMP_binding
Cotton_A_18867
0
500
1000
1500

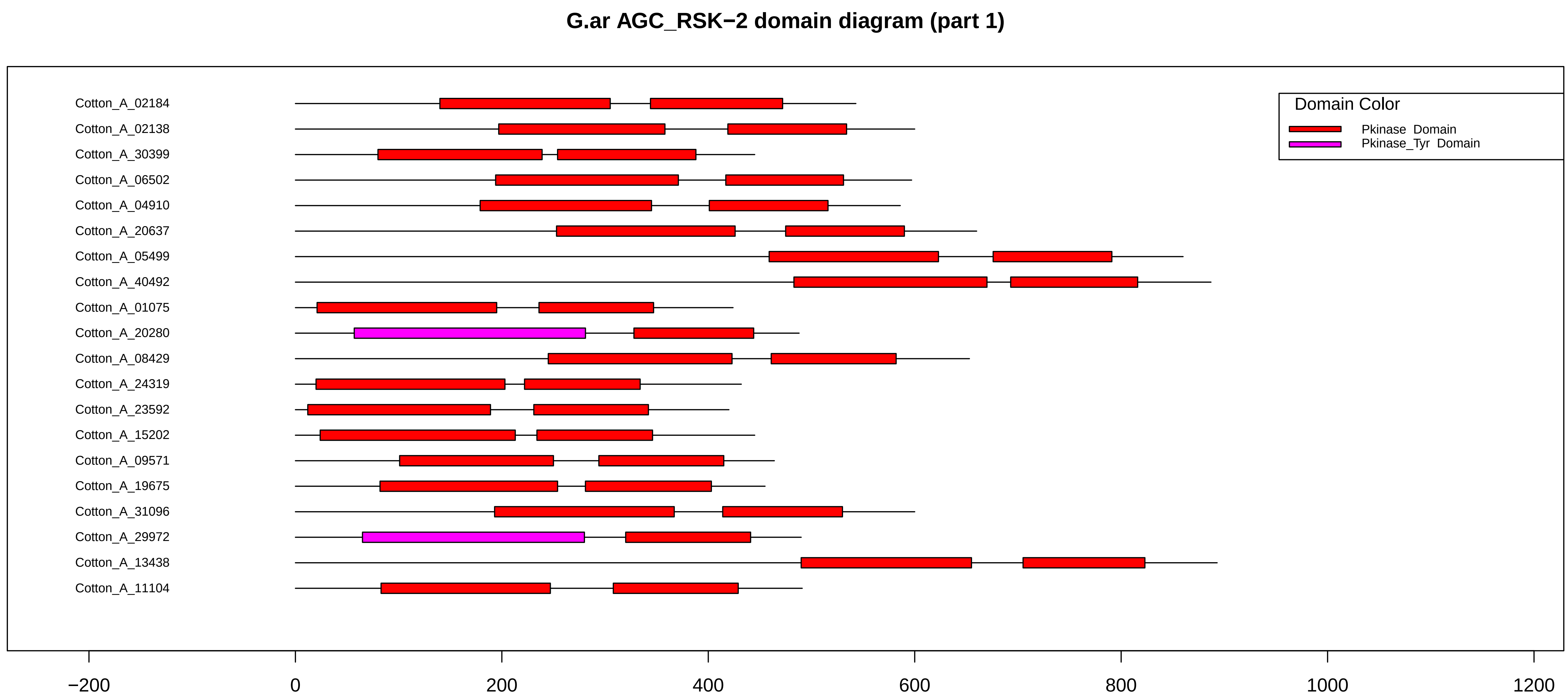

G.ar AGC_RSK−2 domain diagram (part 1)
Cotton_A_02184
Cotton_A_02138
Cotton_A_30399
Cotton_A_06502
Cotton_A_04910
Cotton_A_20637
Cotton_A_05499
Cotton_A_40492
Cotton_A_01075
Cotton_A_20280
Cotton_A_08429
Cotton_A_24319
Cotton_A_23592
Cotton_A_15202
Cotton_A_09571
Cotton_A_19675
Cotton_A_31096
Cotton_A_29972
Cotton_A_13438
Cotton_A_11104
Domain Color
Pkinase Domain
Pkinase_Tyr Domain
−200
0
200
400
600
800
1000
1200

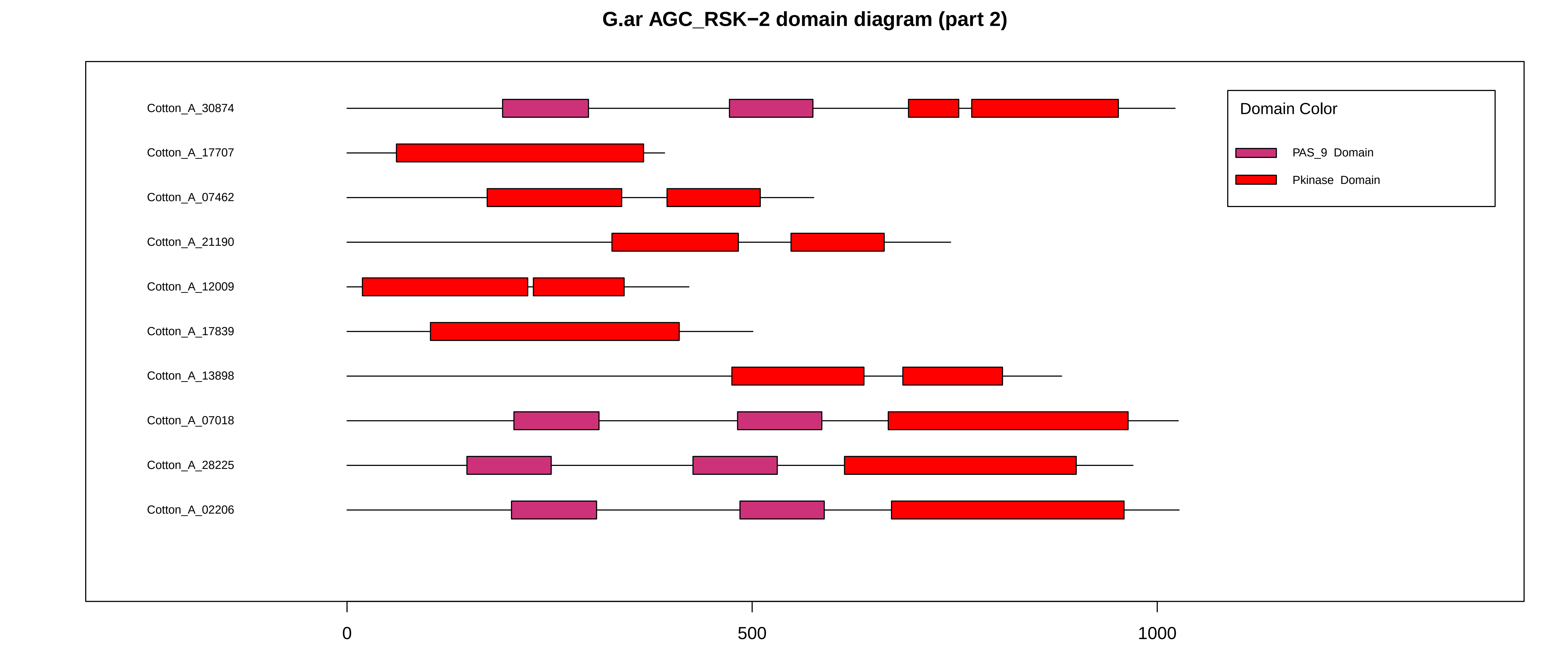
G.ar AGC_RSK−2 domain diagram (part 2)
Cotton_A_30874
Cotton_A_17707
Cotton_A_07462
Cotton_A_21190
Cotton_A_12009
Cotton_A_17839
Cotton_A_13898
Cotton_A_07018
Cotton_A_28225
Cotton_A_02206
Domain Color
PAS_9 Domain
Pkinase Domain
0
500
1000

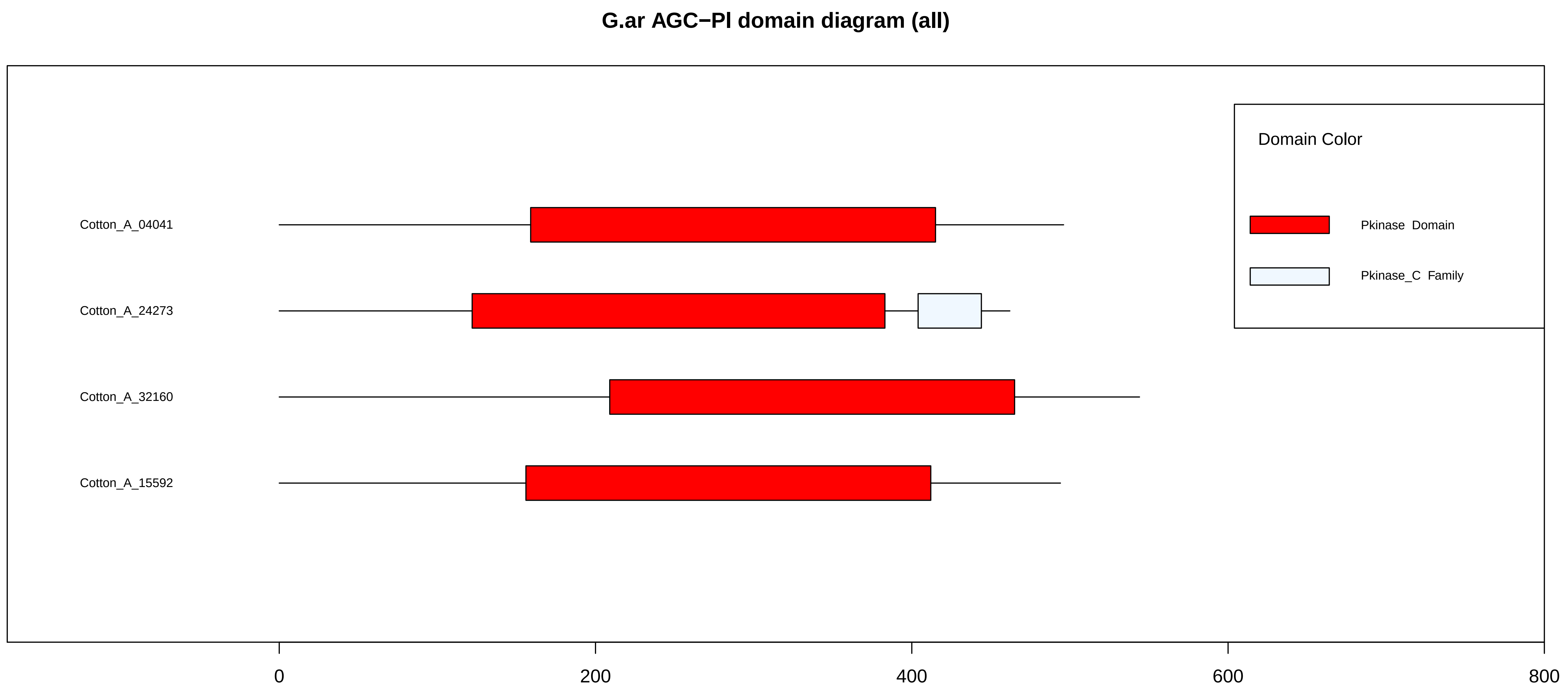
G.ar AGC−PI domain diagram (all)
Domain Color
Pkinase Domain
Pkinase_C Family
Cotton_A_04041
Cotton_A_24273
Cotton_A_32160
Cotton_A_15592
0
200
400
600
800

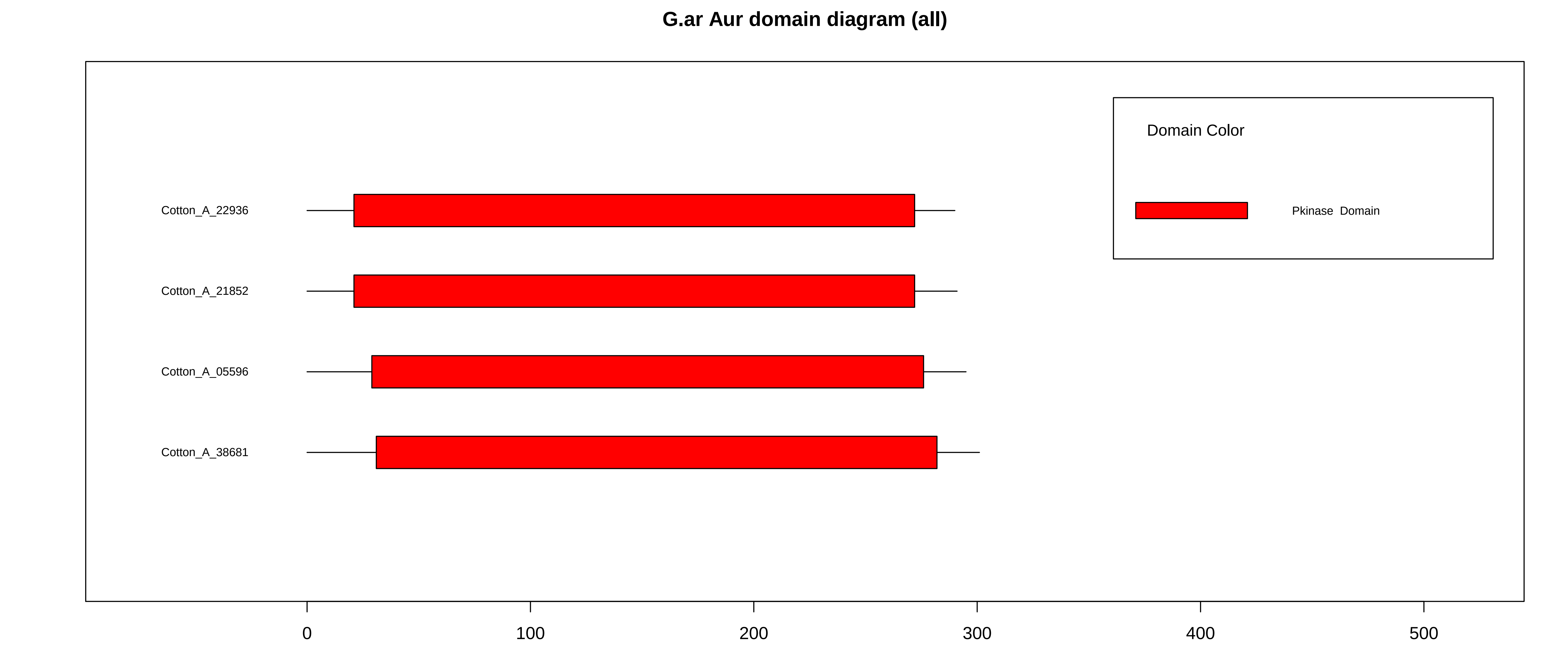
G.ar Aur domain diagram (all)
Domain Color
Pkinase Domain
Cotton_A_22936
Cotton_A_21852
Cotton_A_05596
Cotton_A_38681
0
100
200
300
400
500

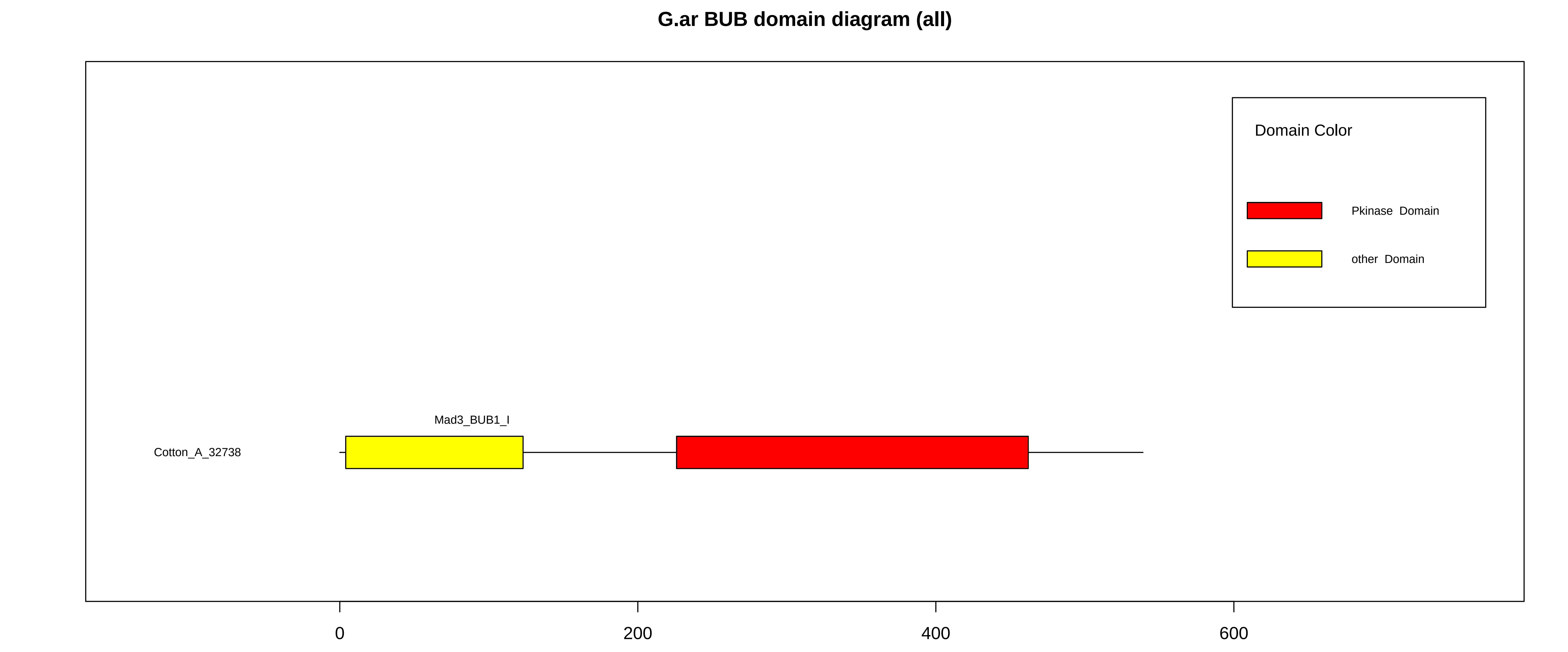
G.ar BUB domain diagram (all)
Domain Color
Pkinase Domain
other Domain
Mad3_BUB1_I
Cotton_A_32738
0
200
400
600

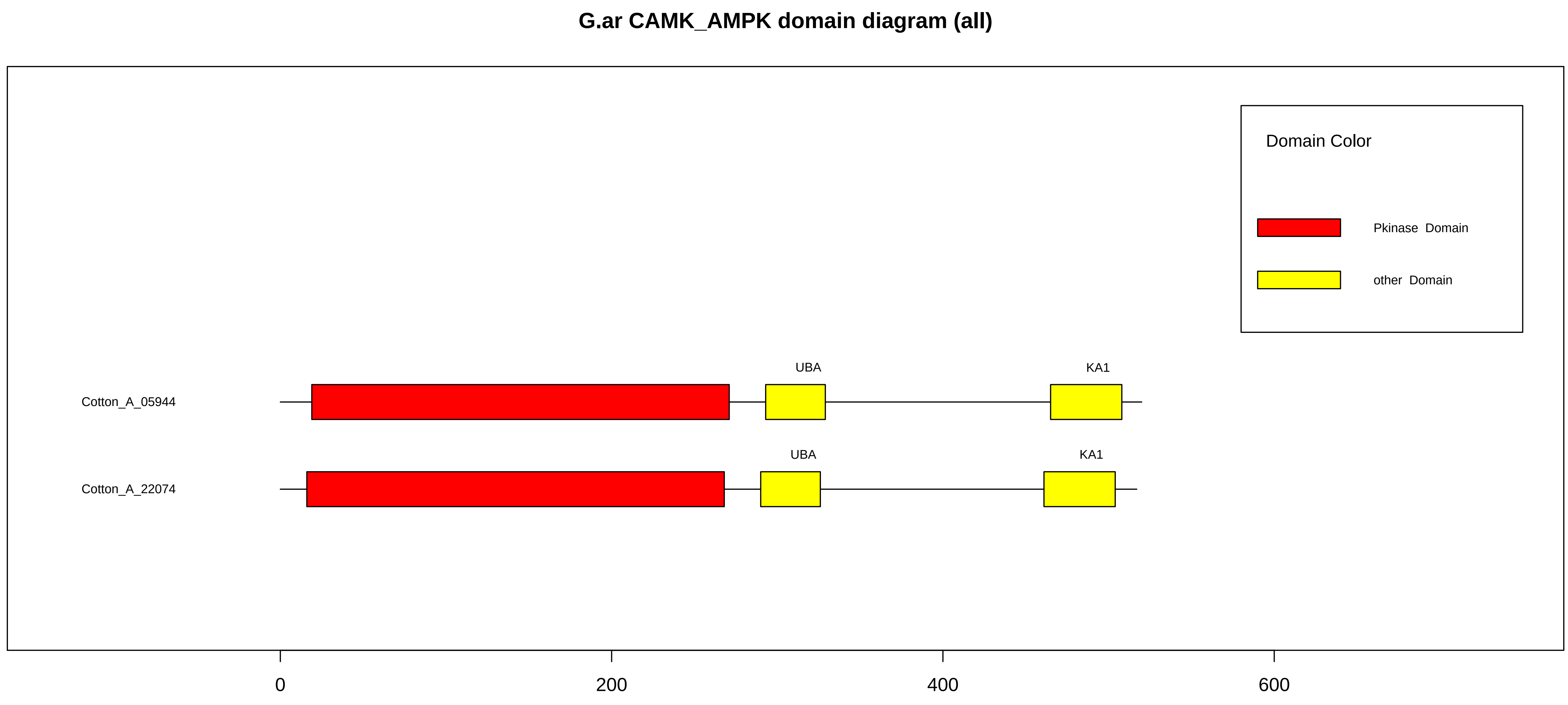
G.ar CAMK_AMPK domain diagram (all)
Domain Color
Pkinase Domain
other Domain
UBA
KA1
Cotton_A_05944
UBA
KA1
Cotton_A_22074
0
200
400
600

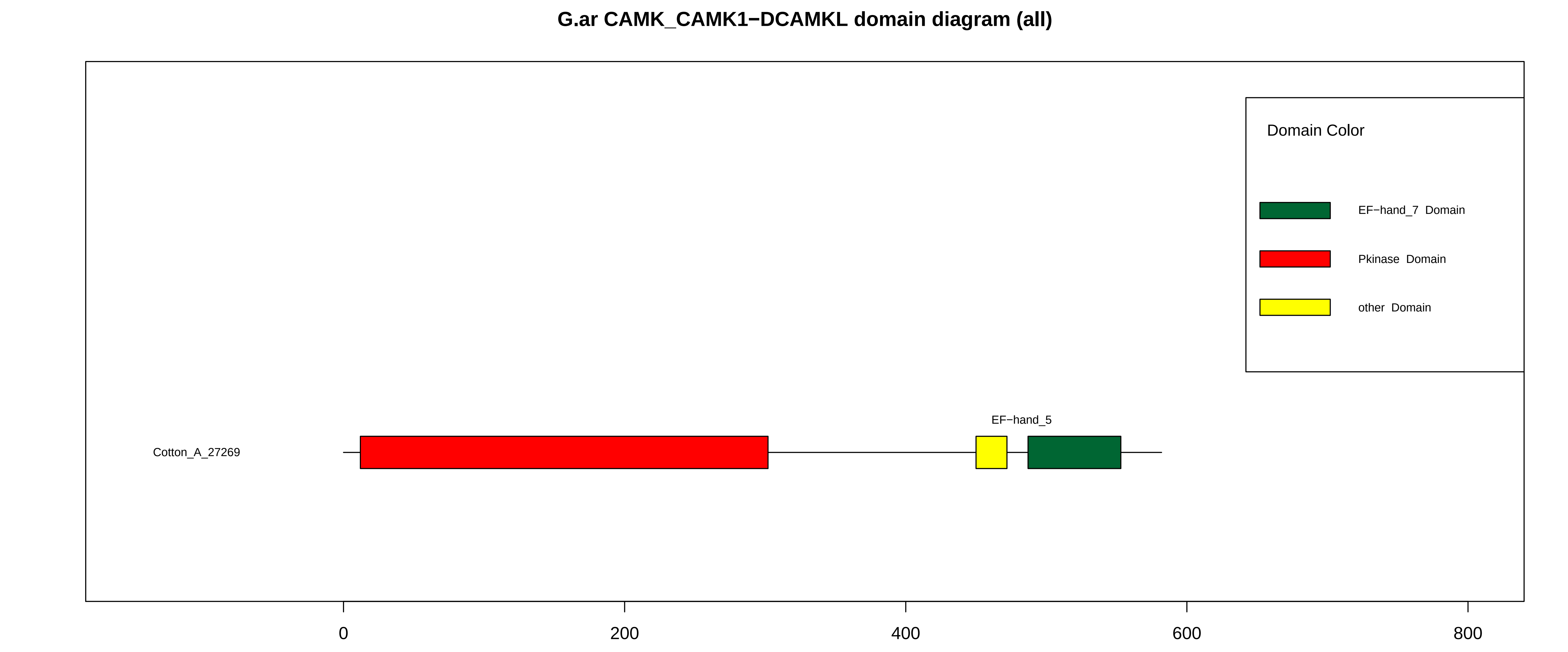
G.ar CAMK_CAMK1−DCAMKL domain diagram (all)
Domain Color
EF−hand_7 Domain
Pkinase Domain
other Domain
EF−hand_5
Cotton_A_27269
0
200
400
600
800

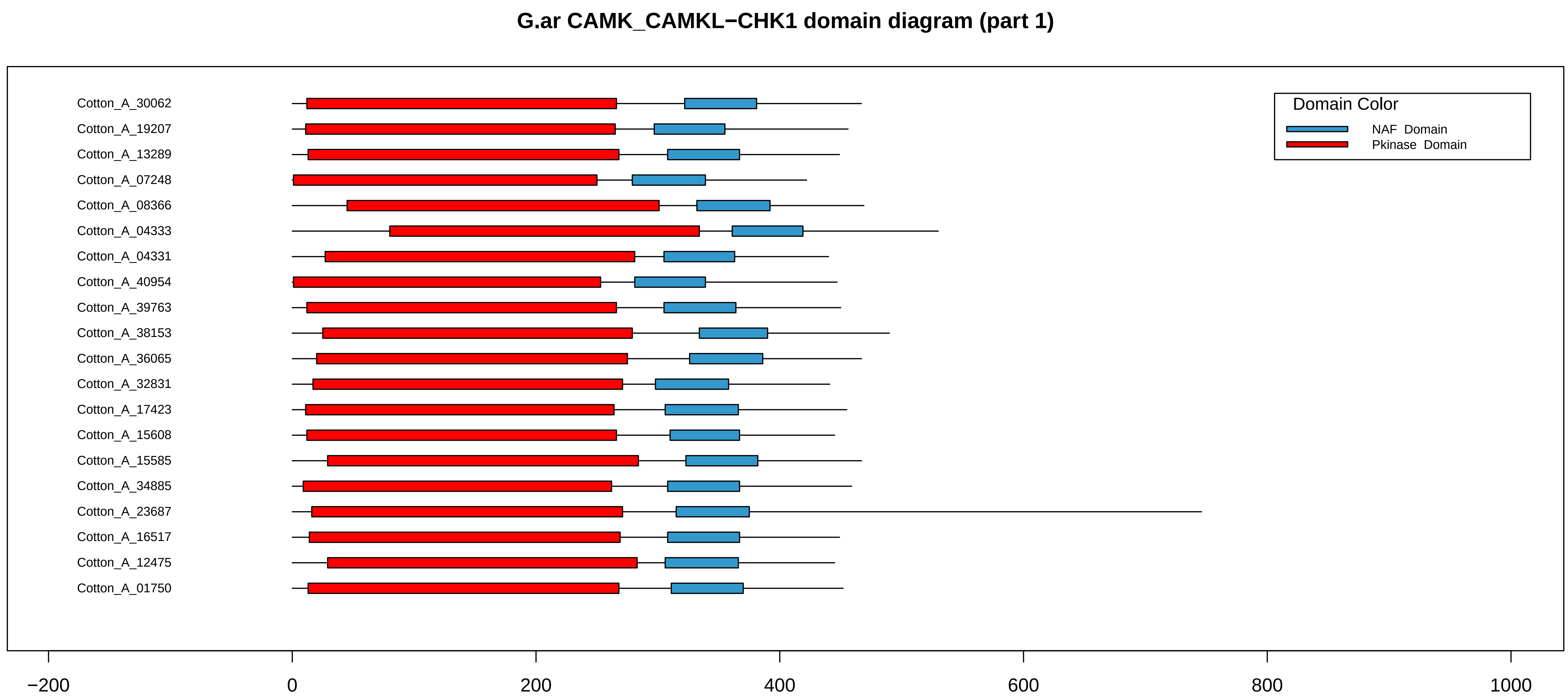

G.ar CAMK_CAMKL−CHK1 domain diagram (part 1)
Cotton_A_30062
Cotton_A_19207
Cotton_A_13289
Cotton_A_07248
Cotton_A_08366
Cotton_A_04333
Cotton_A_04331
Cotton_A_40954
Cotton_A_39763
Cotton_A_38153
Cotton_A_36065
Cotton_A_32831
Cotton_A_17423
Cotton_A_15608
Cotton_A_15585
Cotton_A_34885
Cotton_A_23687
Cotton_A_16517
Cotton_A_12475
Cotton_A_01750
Domain Color
NAF Domain
Pkinase Domain
−200
0
200
400
600
800
1000

G.ar CAMK_CAMKL−CHK1 domain diagram (part 2)

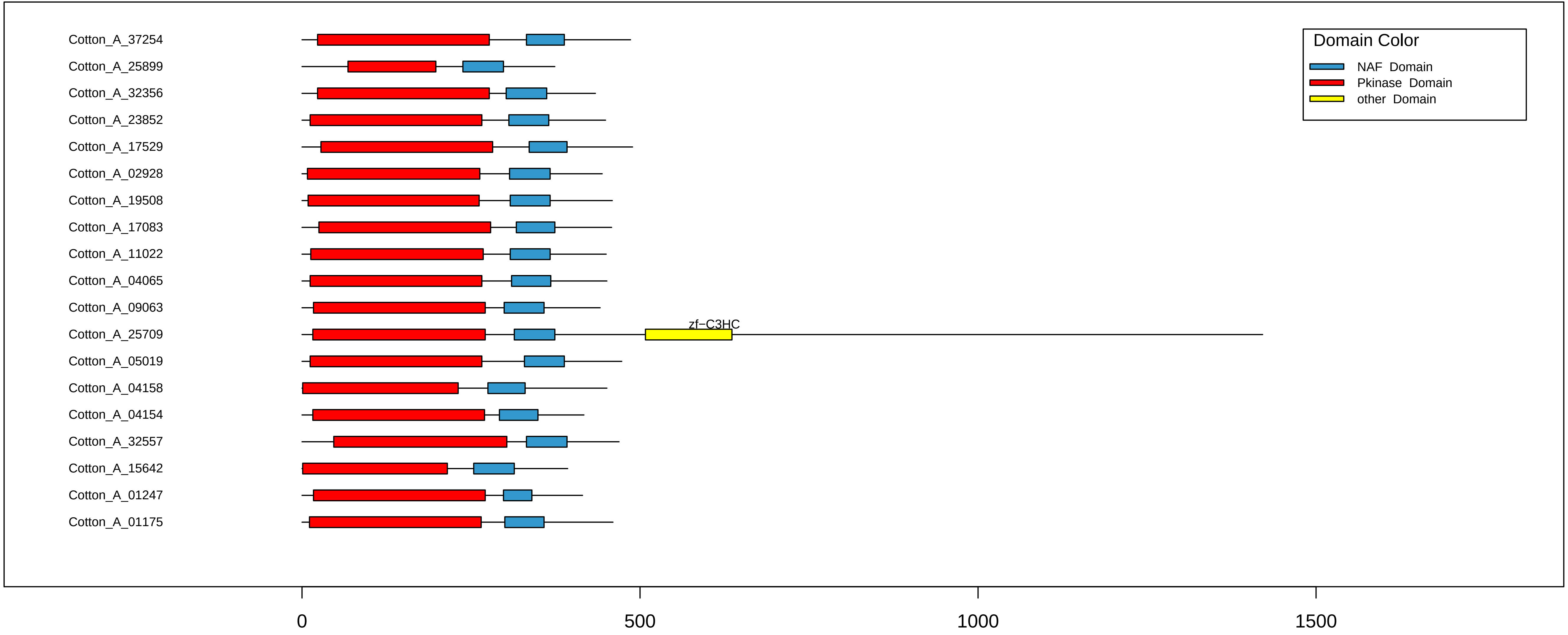

G.ar CAMK_CAMKL−LKB domain diagram (all)

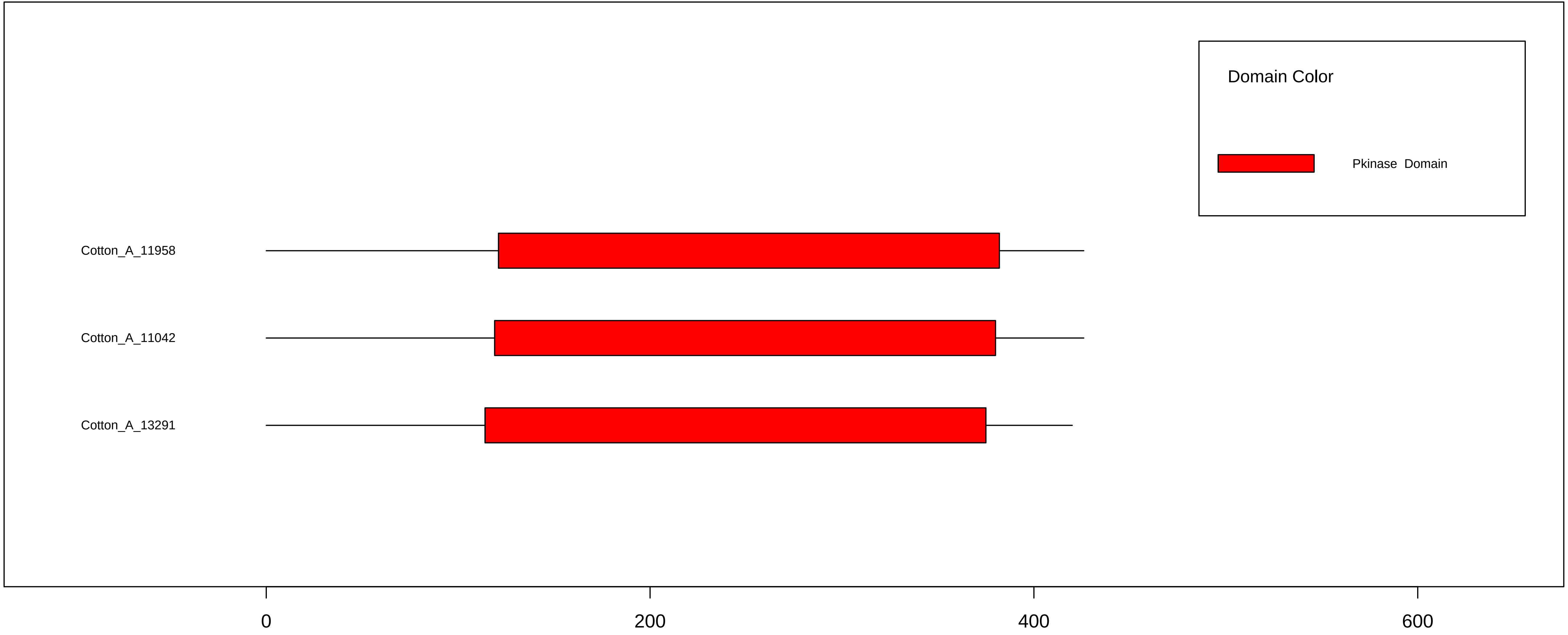

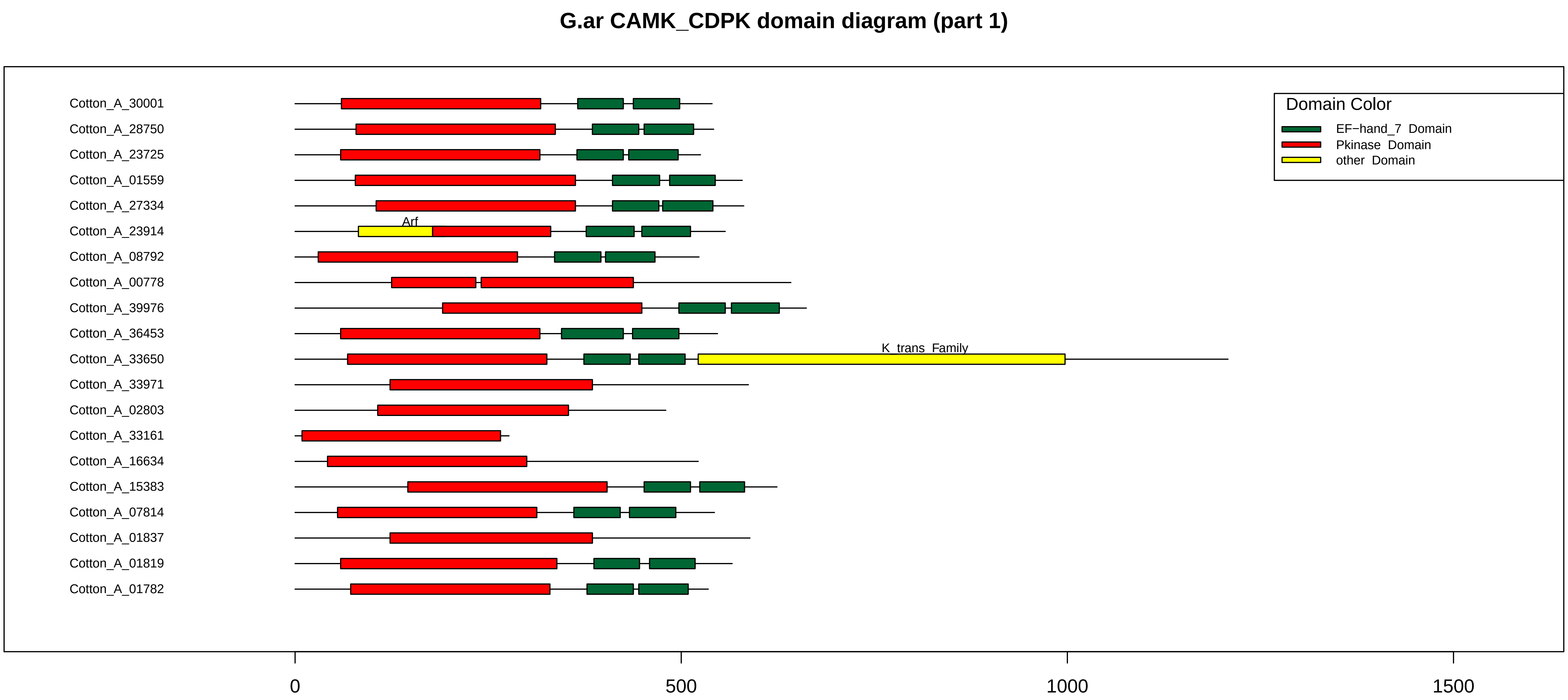
G.ar CAMK_CDPK domain diagram (part 1)
Cotton_A_30001
Cotton_A_28750
Cotton_A_23725
Cotton_A_01559
Cotton_A_27334
Cotton_A_23914
Cotton_A_08792
Cotton_A_00778
Cotton_A_39976
Cotton_A_36453
Cotton_A_33650
Cotton_A_33971
Cotton_A_02803
Cotton_A_33161
Cotton_A_16634
Cotton_A_15383
Cotton_A_07814
Cotton_A_01837
Cotton_A_01819
Cotton_A_01782
Arf
K_trans_Family
Domain Color
EF−hand_7 Domain
Pkinase Domain
other Domain
0
500
1000
1500

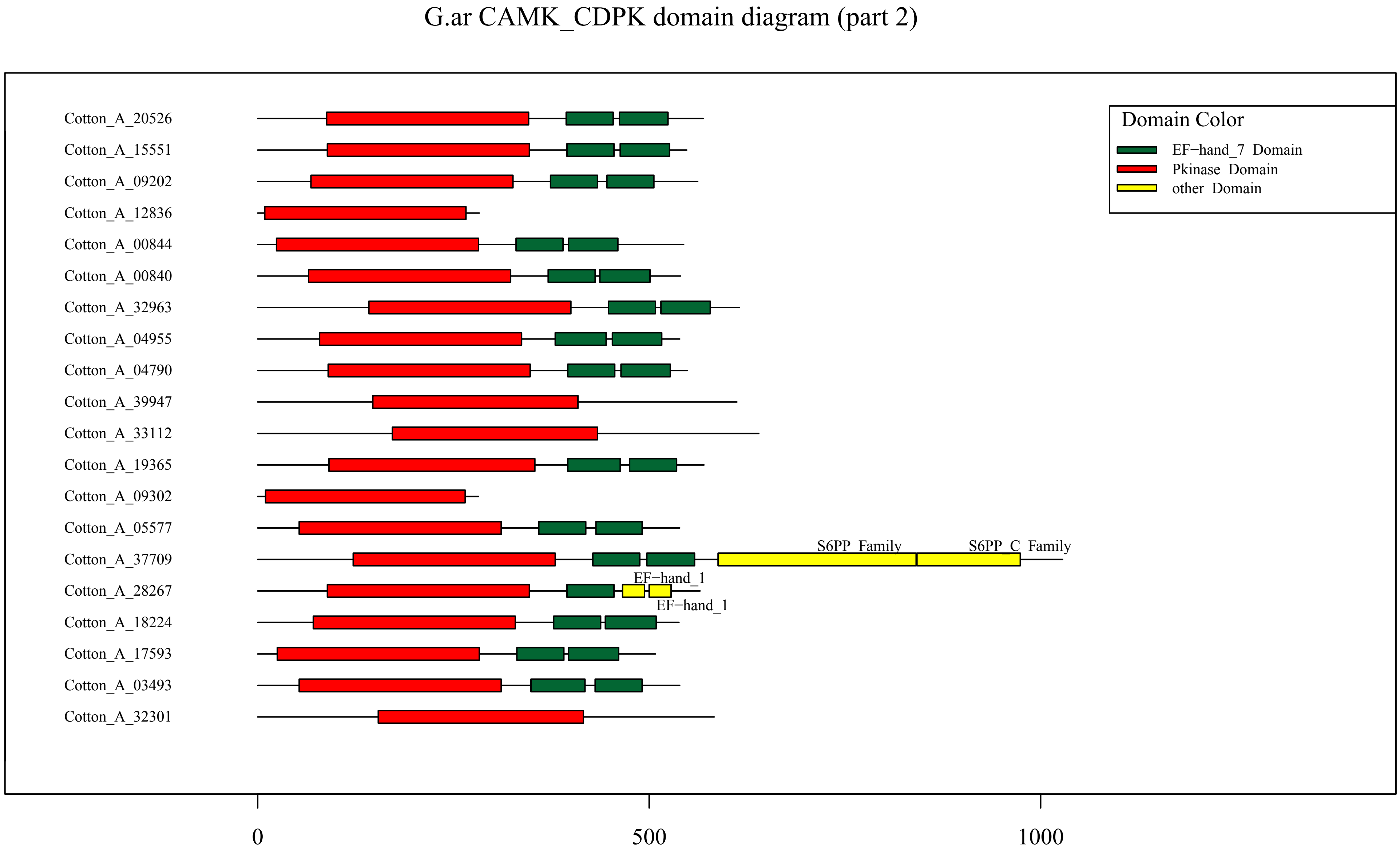

G.ar CAMK_CDPK domain diagram (part 2)
Cotton_A_20526
Cotton_A_15551
Cotton_A_09202
Cotton_A_12836
Cotton_A_00844
Cotton_A_00840
Cotton_A_32963
Cotton_A_04955
Cotton_A_04790
Cotton_A_39947
Cotton_A_33112
Cotton_A_19365
Cotton_A_09302
Cotton_A_05577
Cotton_A_37709
Cotton_A_28267
Cotton_A_18224
Cotton_A_17593
Cotton_A_03493
Cotton_A_32301
S6PP_Family
S6PP_C_Family
EF−hand_1
EF−hand_1
Domain Color
EF−hand_7 Domain
Pkinase Domain
other Domain
0
500
1000

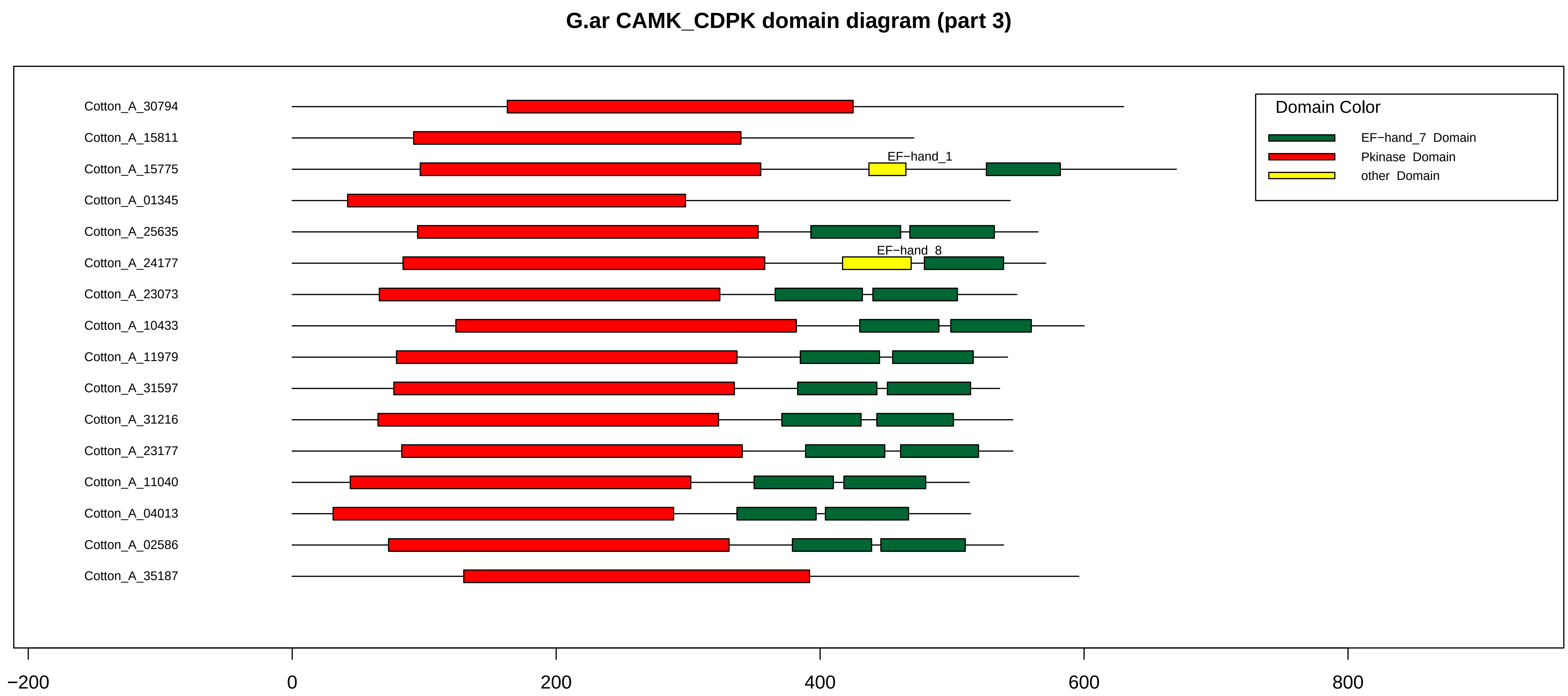
G.ar CAMK_CDPK domain diagram (part 3)
Cotton_A_30794
Cotton_A_15811
Cotton_A_15775
Cotton_A_01345
Cotton_A_25635
Cotton_A_24177
Cotton_A_23073
Cotton_A_10433
Cotton_A_11979
Cotton_A_31597
Cotton_A_31216
Cotton_A_23177
Cotton_A_11040
Cotton_A_04013
Cotton_A_02586
Cotton_A_35187
EF−hand_1
EF−hand_8
Domain Color
EF−hand_7 Domain
Pkinase Domain
other Domain
−200
0
200
400
600
800

G.ar CAMK_OST1L domain diagram (all)

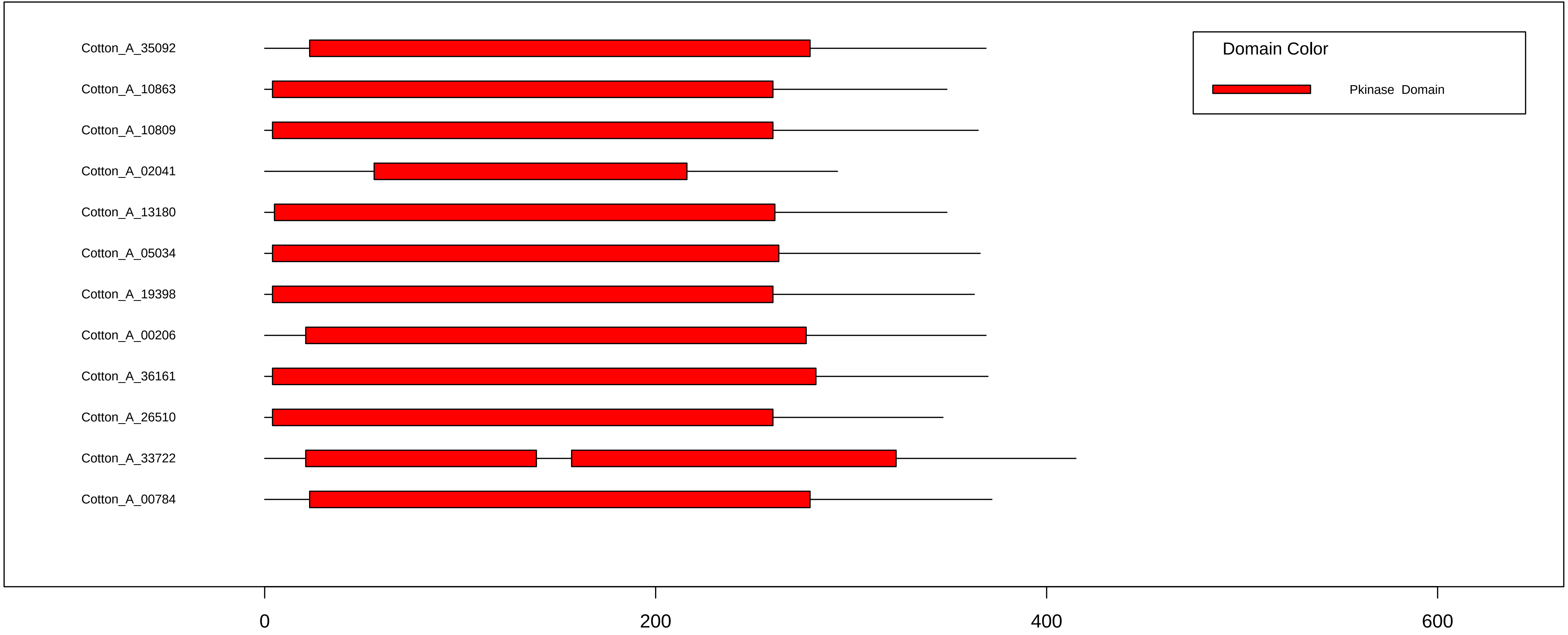

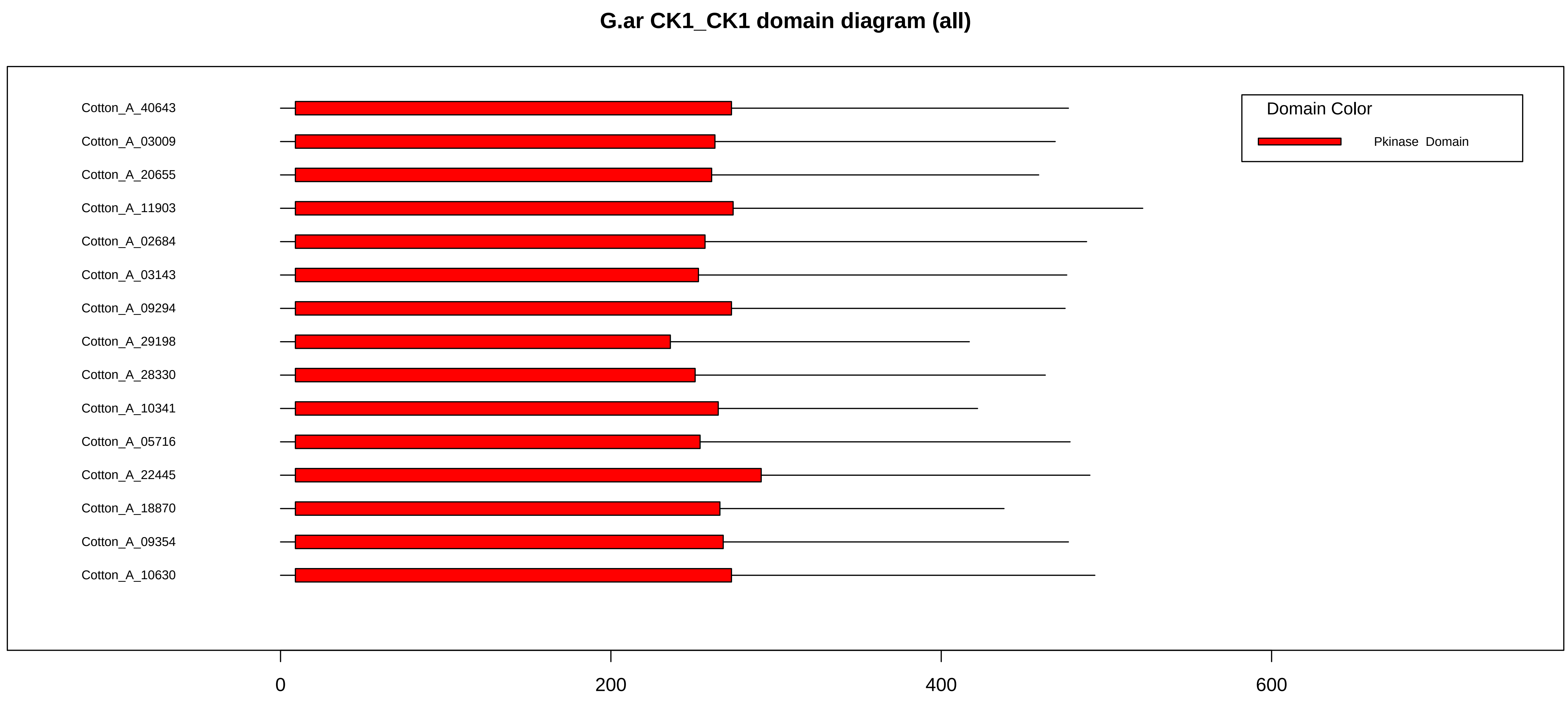
G.ar CK1_CK1 domain diagram (all)
Cotton_A_40643
Cotton_A_03009
Cotton_A_20655
Cotton_A_11903
Cotton_A_02684
Cotton_A_03143
Cotton_A_09294
Cotton_A_29198
Cotton_A_28330
Cotton_A_10341
Cotton_A_05716
Cotton_A_22445
Cotton_A_18870
Cotton_A_09354
Cotton_A_10630
Domain Color
Pkinase Domain
0
200
400
600

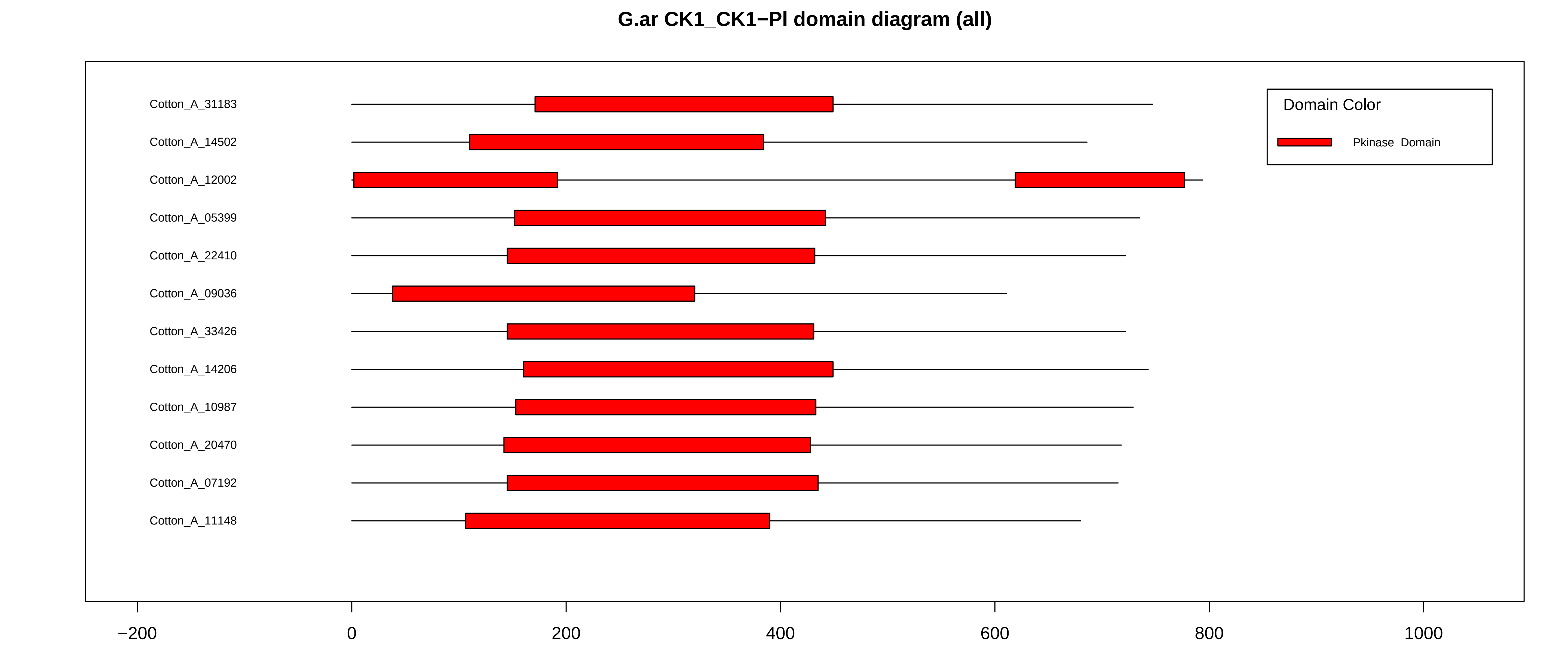
G.ar CK1_CK1−Pl domain diagram (all)
Cotton_A_31183
Cotton_A_14502
Cotton_A_12002
Cotton_A_05399
Cotton_A_22410
Cotton_A_09036
Cotton_A_33426
Cotton_A_14206
Cotton_A_10987
Cotton_A_20470
Cotton_A_07192
Cotton_A_11148
Domain Color
Pkinase Domain
−200
0
200
400
600
800
1000

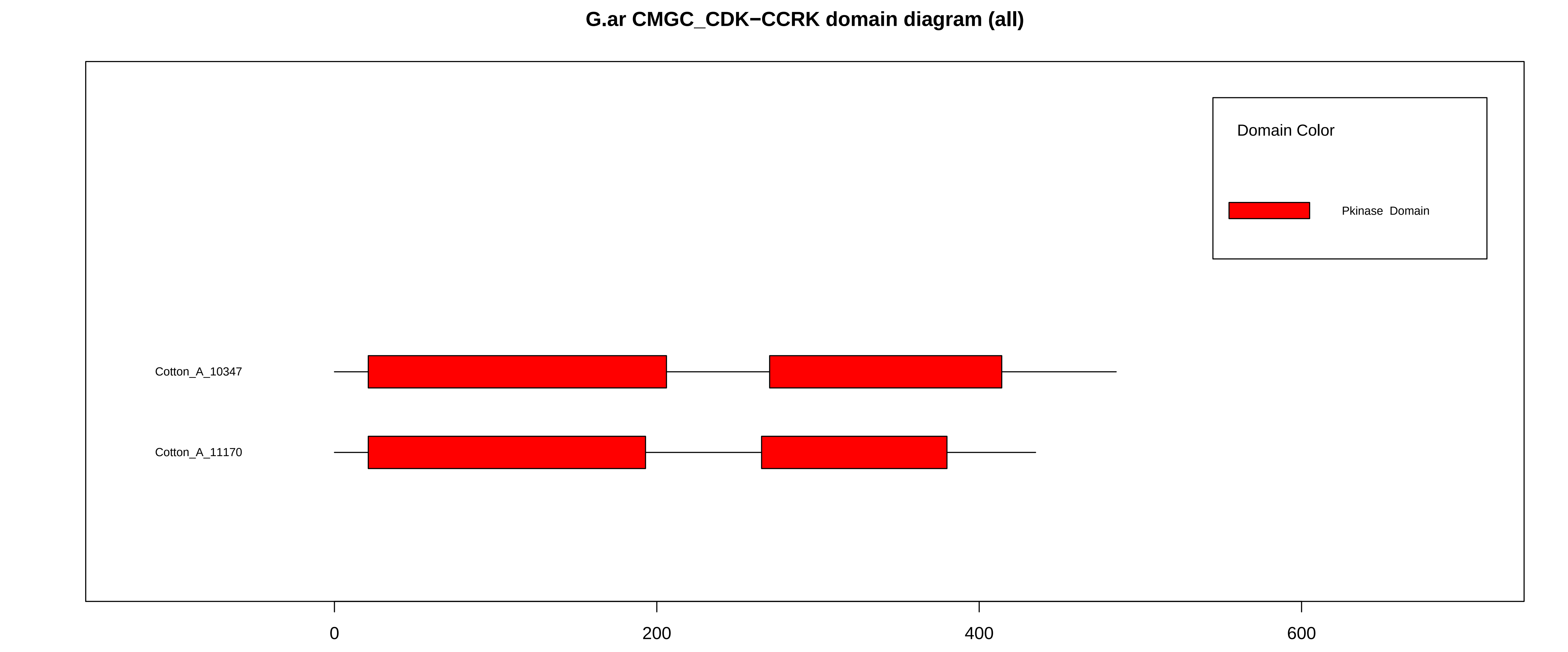
G.ar CMGC_CDK−CCRK domain diagram (all)
Domain Color
Pkinase Domain
Cotton_A_10347
Cotton_A_11170
0
200
400
600

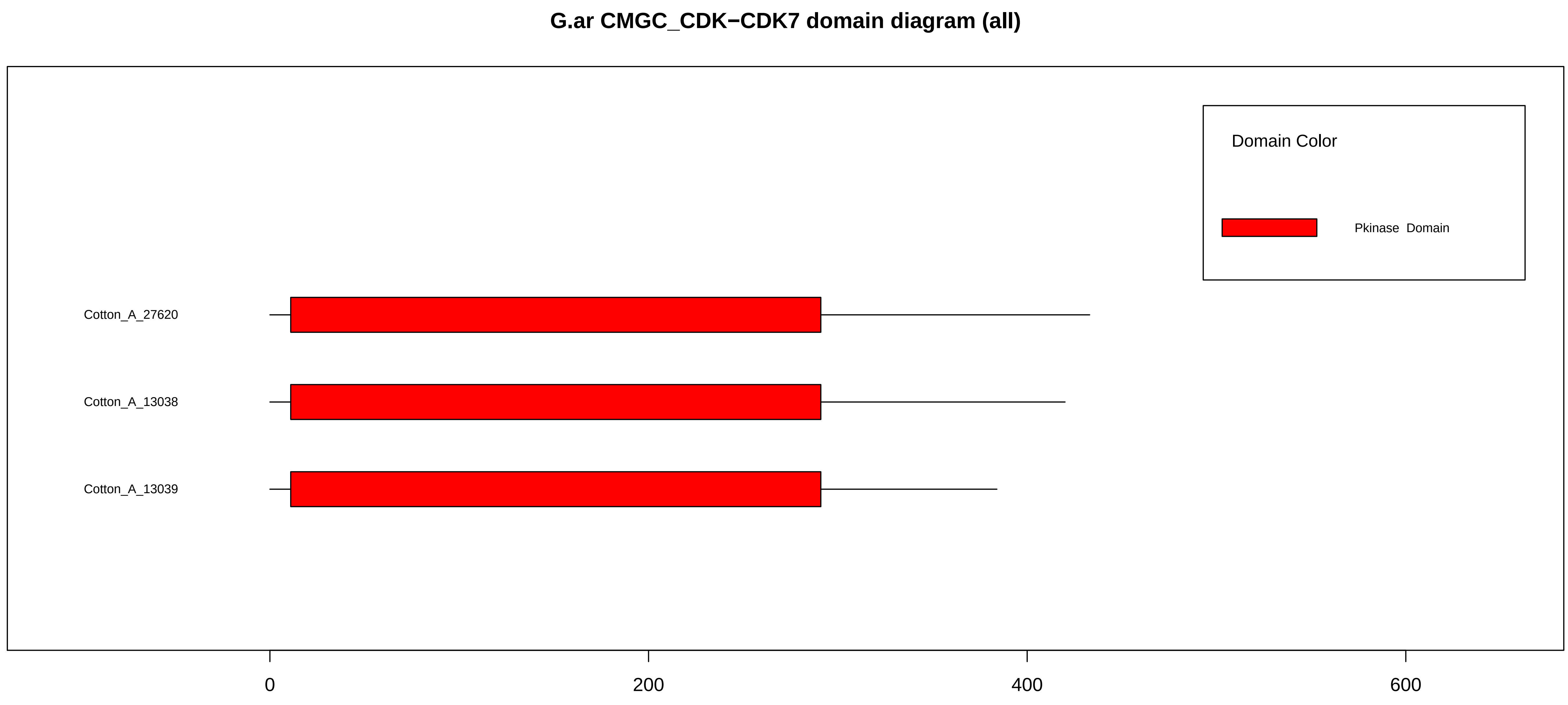
G.ar CMGC_CDK−CDK7 domain diagram (all)
Domain Color
Pkinase Domain
Cotton_A_27620
Cotton_A_13038
Cotton_A_13039
0
200
400
600

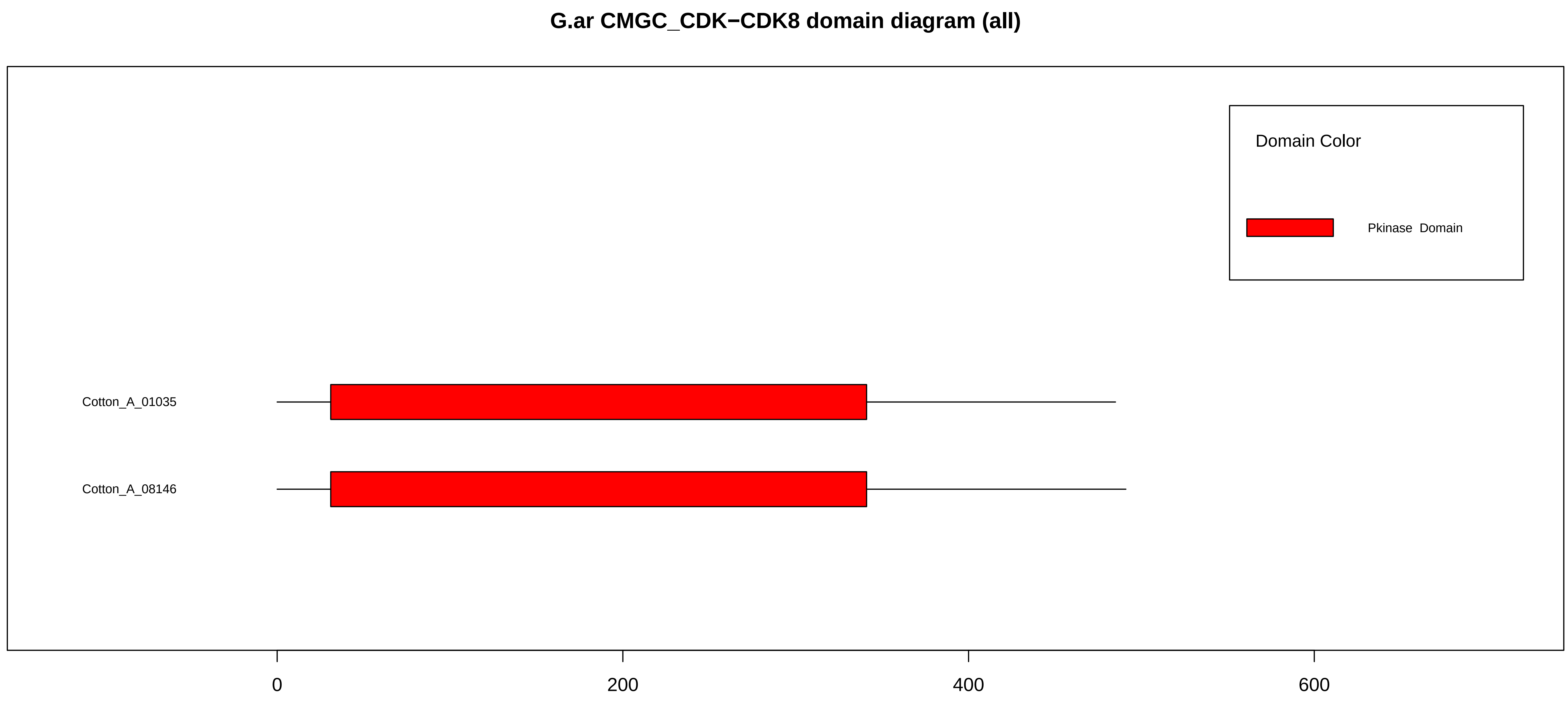
G.ar CMGC_CDK−CDK8 domain diagram (all)
Domain Color
Pkinase Domain
Cotton_A_01035
Cotton_A_08146
0
200
400
600

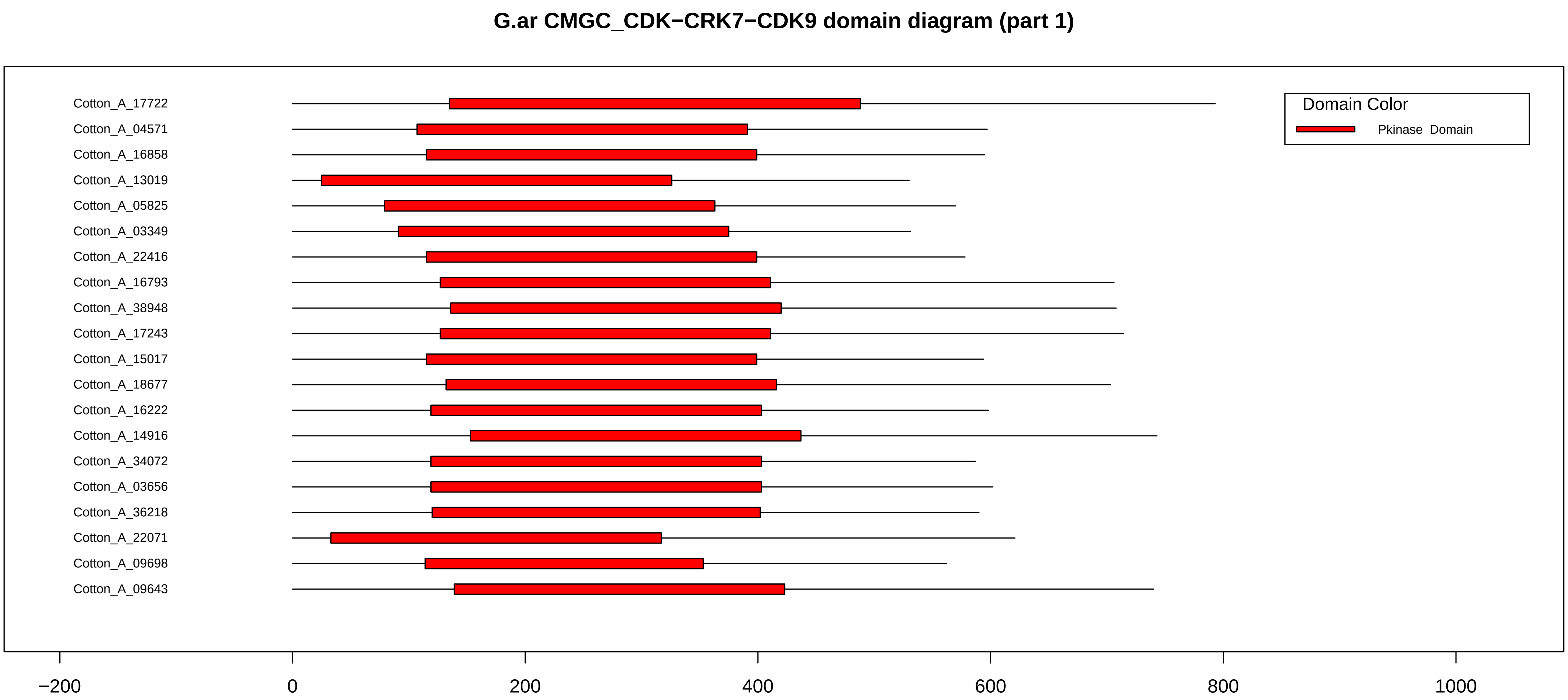
G.ar CMGC_CDK−CRK7−CDK9 domain diagram (part 1)
Cotton_A_17722
Cotton_A_04571
Cotton_A_16858
Cotton_A_13019
Cotton_A_05825
Cotton_A_03349
Cotton_A_22416
Cotton_A_16793
Cotton_A_38948
Cotton_A_17243
Cotton_A_15017
Cotton_A_18677
Cotton_A_16222
Cotton_A_14916
Cotton_A_34072
Cotton_A_03656
Cotton_A_36218
Cotton_A_22071
Cotton_A_09698
Cotton_A_09643
Domain Color
Pkinase Domain
−200
0
200
400
600
800
1000

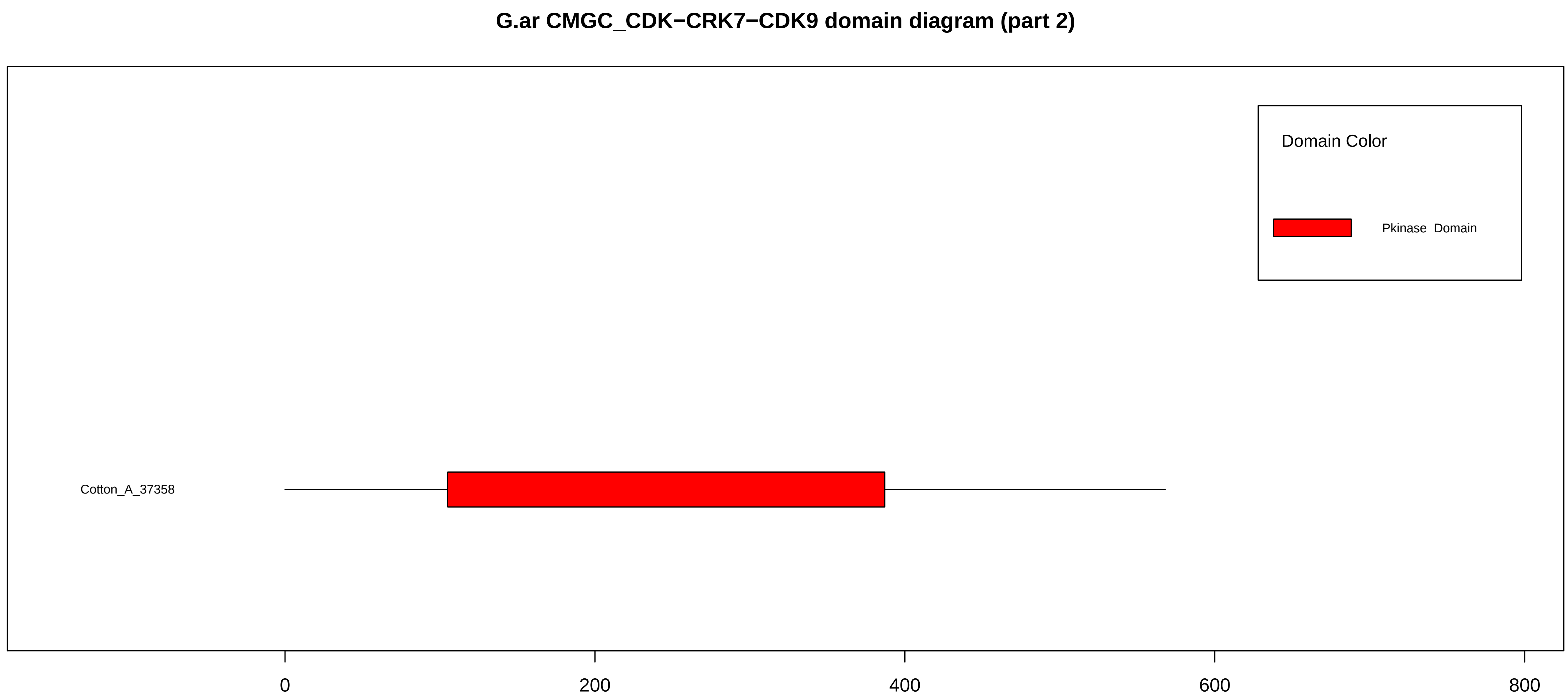
G.ar CMGC_CDK−CRK7−CDK9 domain diagram (part 2)
Domain Color
Pkinase Domain
Cotton_A_37358
0
200
400
600
800

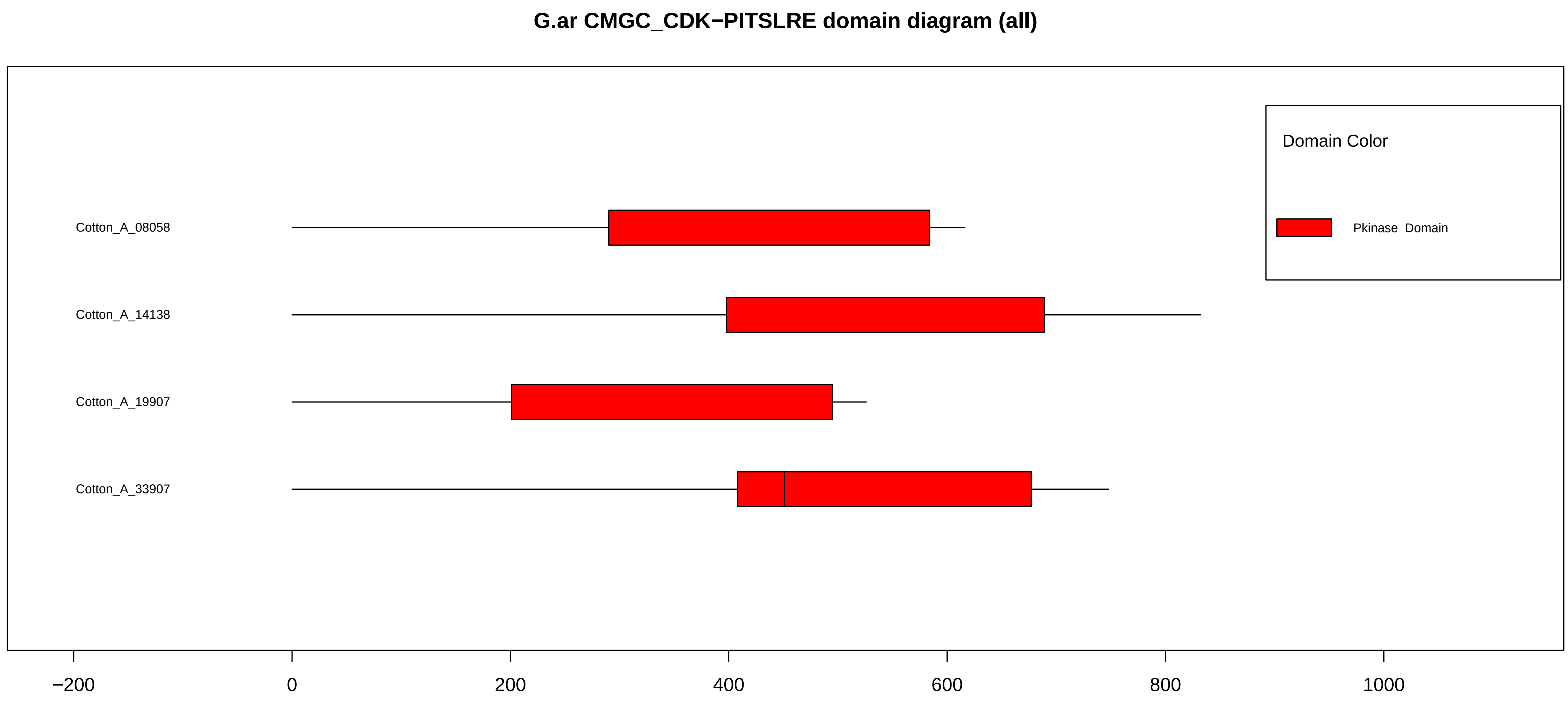
G.ar CMGC_CDK−PITSLRE domain diagram (all)
Domain Color
Pkinase Domain
Cotton_A_08058
Cotton_A_14138
Cotton_A_19907
Cotton_A_33907
−200
0
200
400
600
800
1000

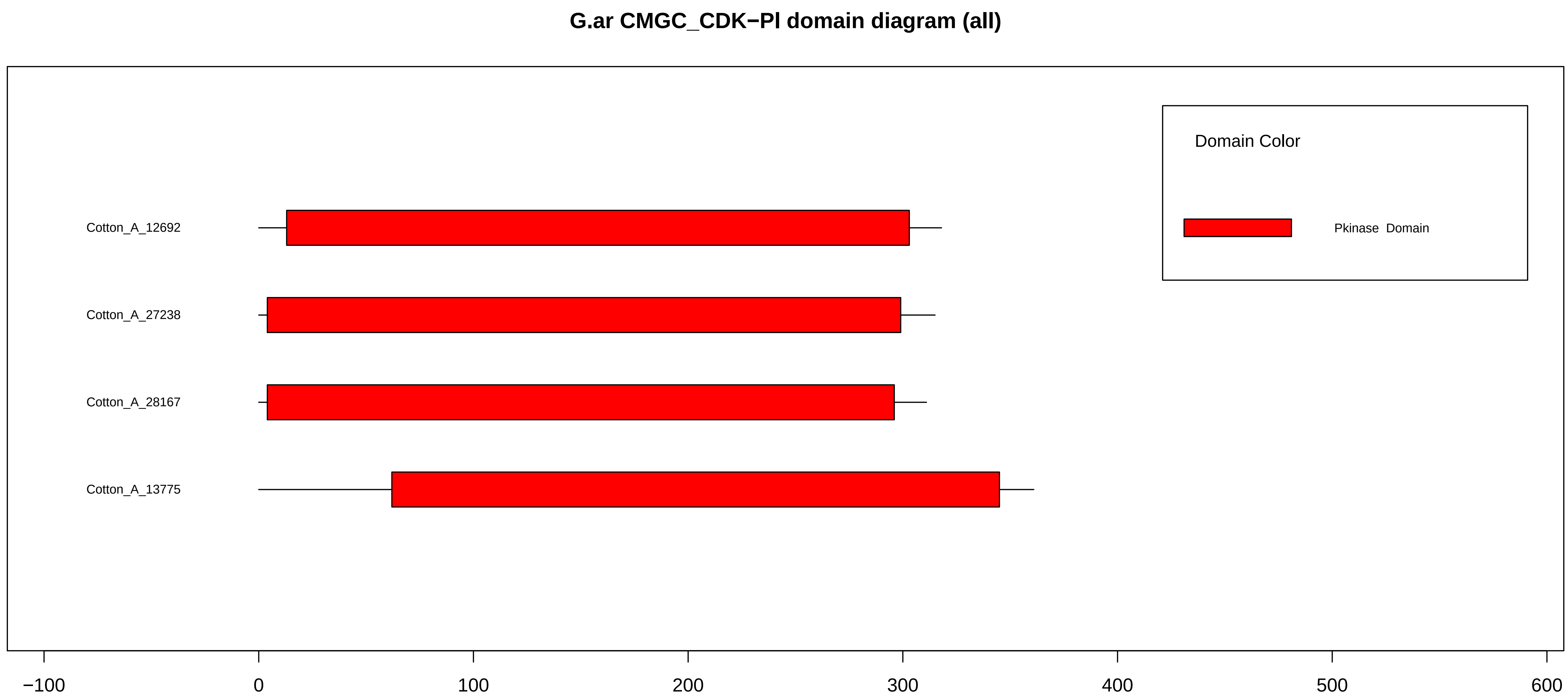

G.ar CMGC_CDK−Pl domain diagram (all)
Domain Color
Pkinase Domain
Cotton_A_12692
Cotton_A_27238
Cotton_A_28167
Cotton_A_13775
−100
0
100
200
300
400
500
600

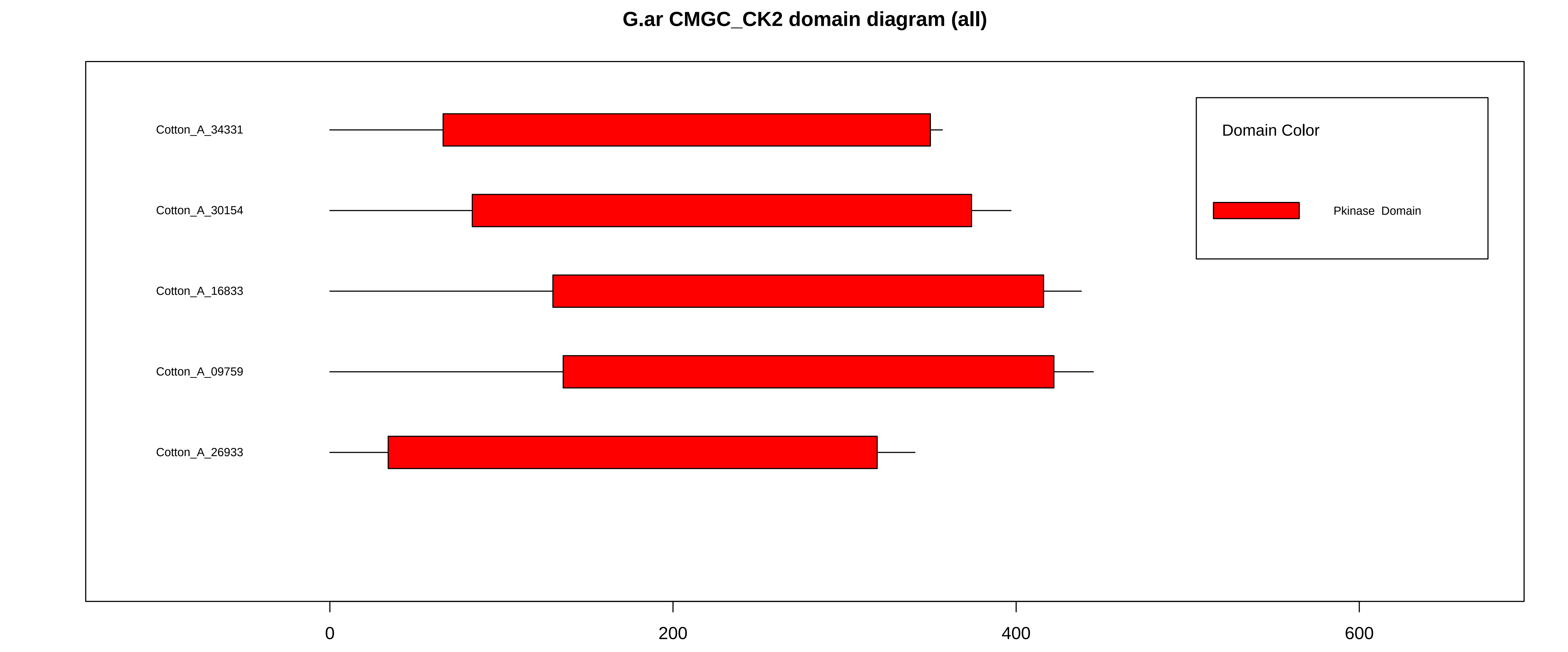
G.ar CMGC_CK2 domain diagram (all)
Cotton_A_34331
Cotton_A_30154
Cotton_A_16833
Cotton_A_09759
Cotton_A_26933
Domain Color
Pkinase Domain
0
200
400
600

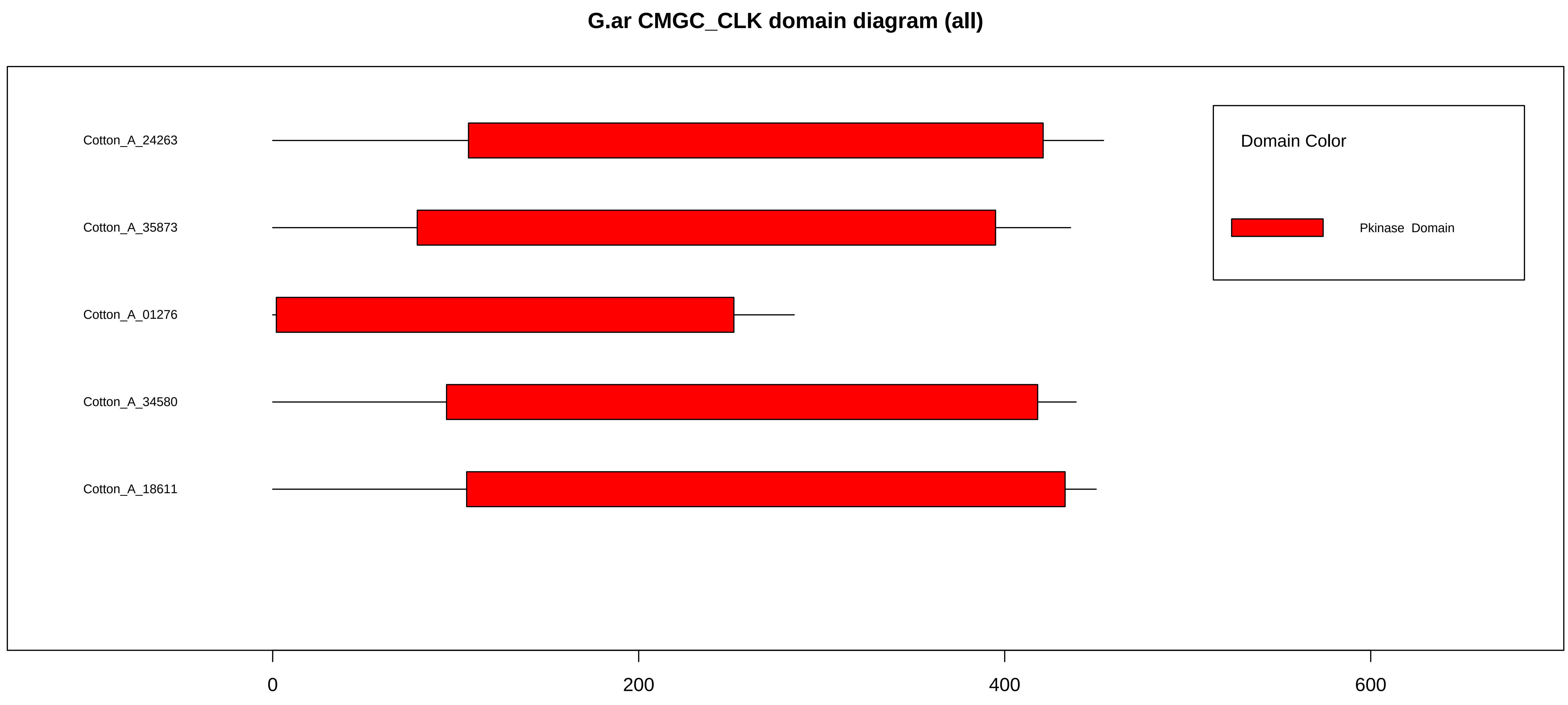
G.ar CMGC_CLK domain diagram (all)
Cotton_A_24263
Cotton_A_35873
Cotton_A_01276
Cotton_A_34580
Cotton_A_18611
Domain Color
Pkinase Domain
0
200
400
600

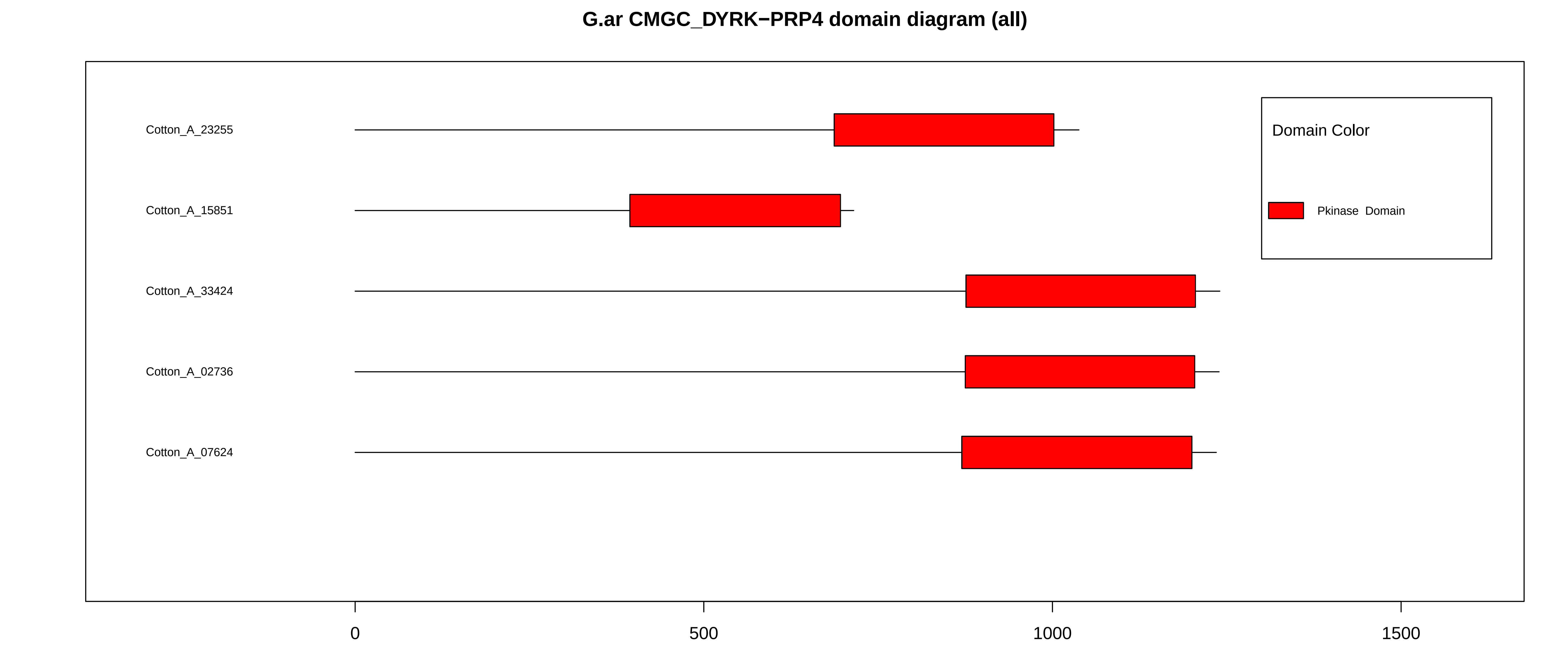
G.ar CMGC_DYRK−PRP4 domain diagram (all)
Cotton_A_23255
Cotton_A_15851
Cotton_A_33424
Cotton_A_02736
Cotton_A_07624
Domain Color
Pkinase Domain
0
500
1000
1500

G.ar CMGC_DYRK−YAK domain diagram (all)

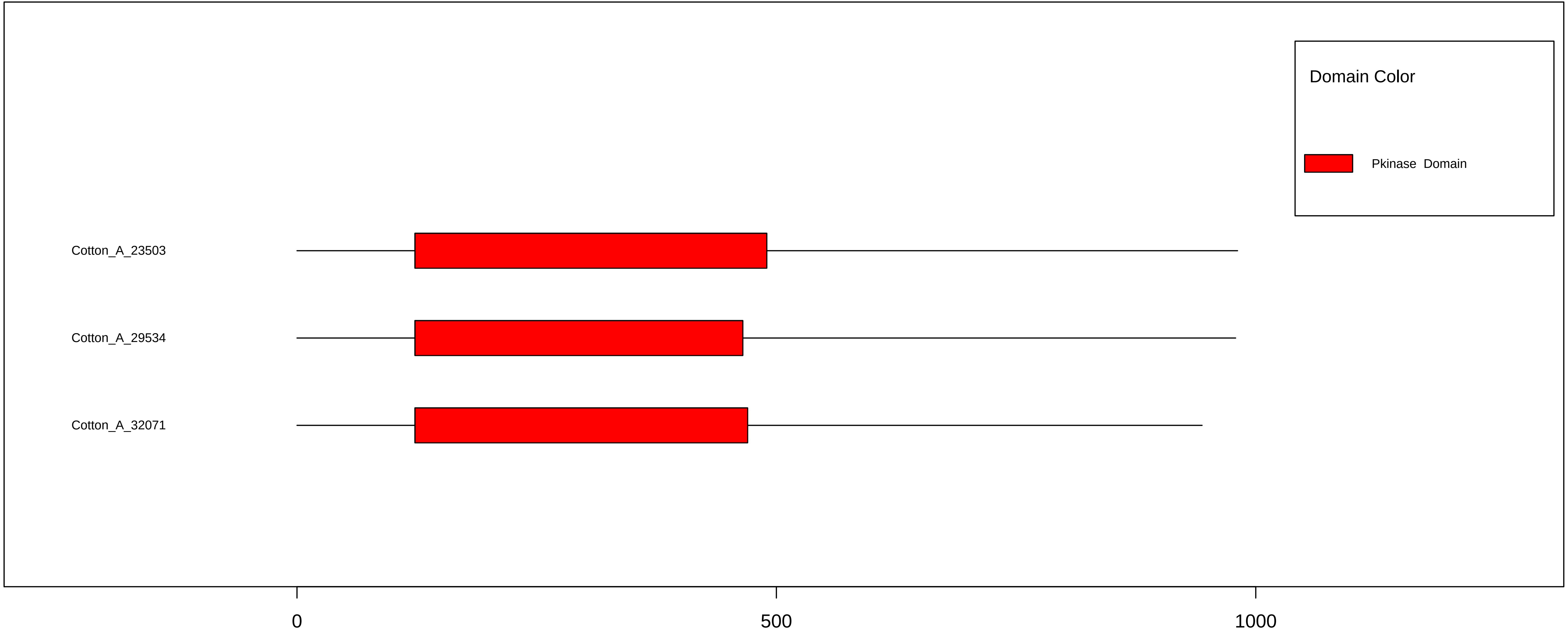

G.ar CMGC_GSKL domain diagram (all)

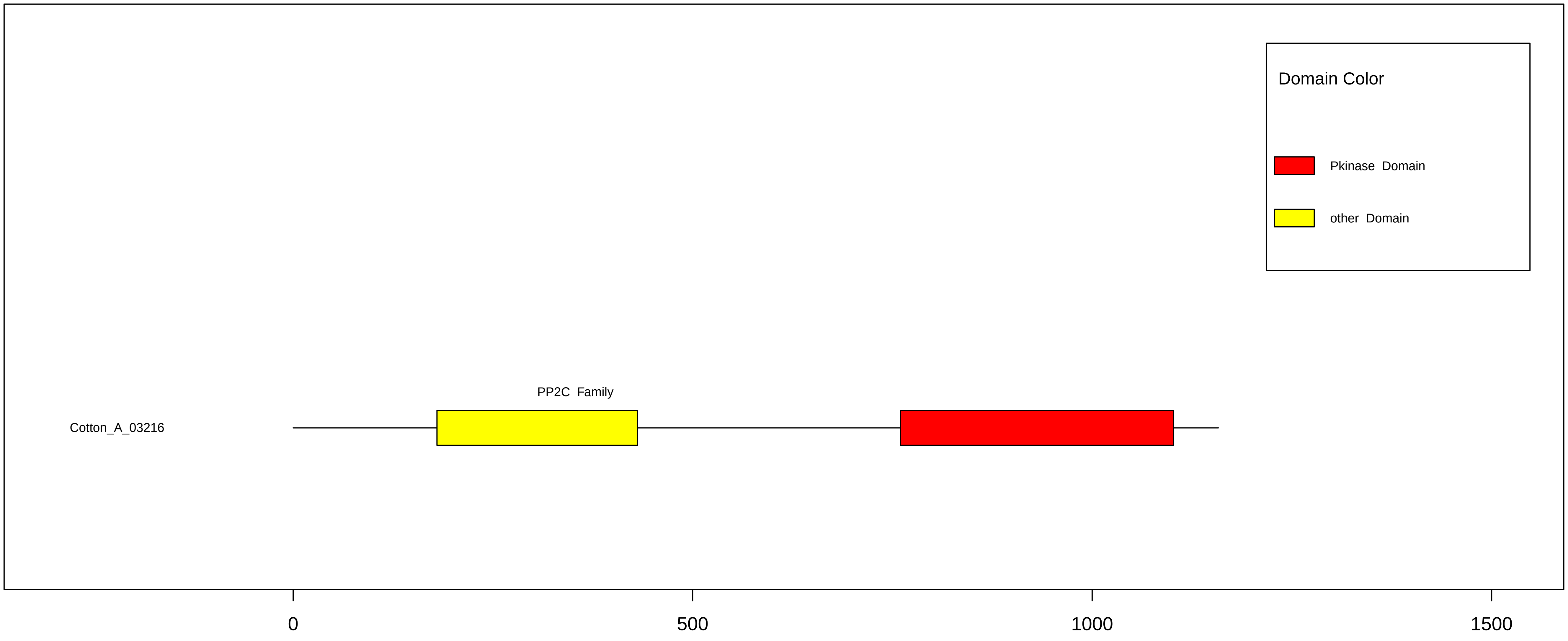

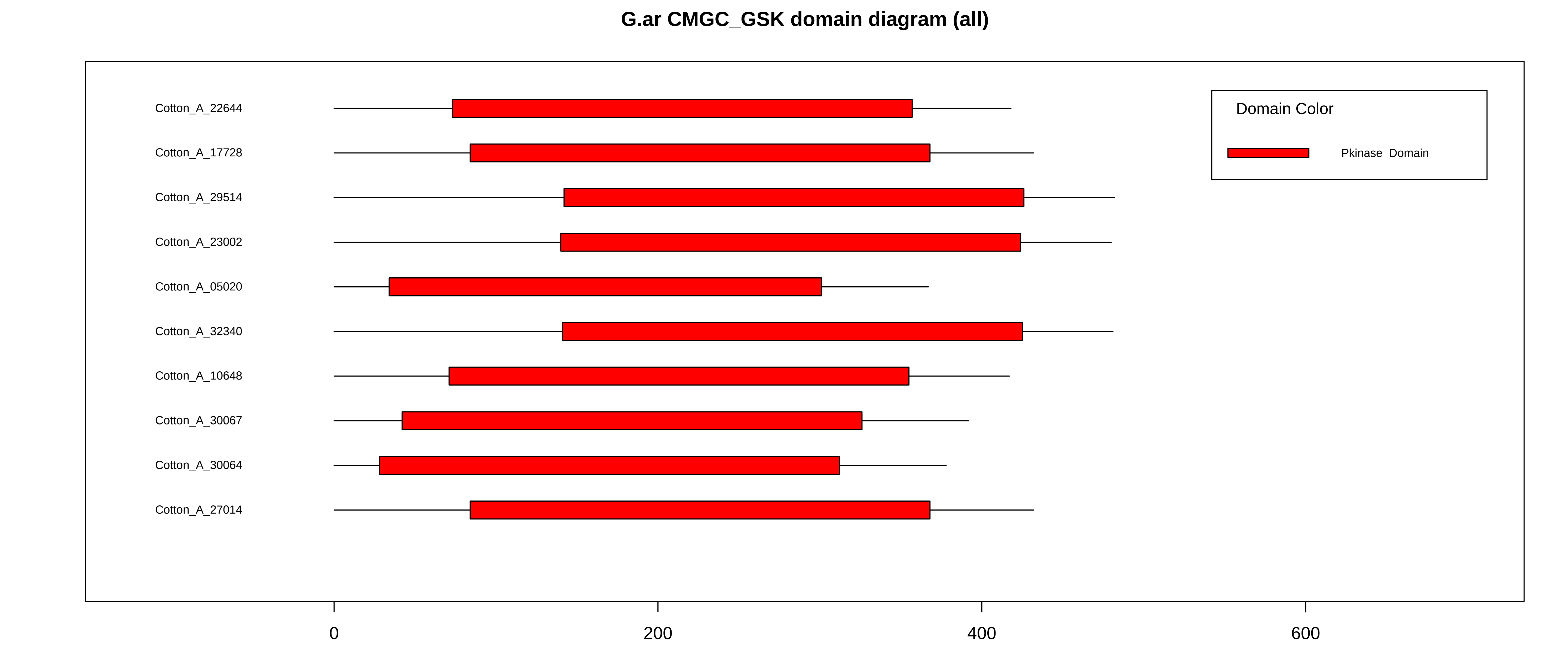
G.ar CMGC_GSK domain diagram (all)
Cotton_A_22644
Cotton_A_17728
Cotton_A_29514
Cotton_A_23002
Cotton_A_05020
Cotton_A_32340
Cotton_A_10648
Cotton_A_30067
Cotton_A_30064
Cotton_A_27014
Domain Color
Pkinase Domain
0
200
400
600

G.ar CMGC_MAPK domain diagram (part 1)

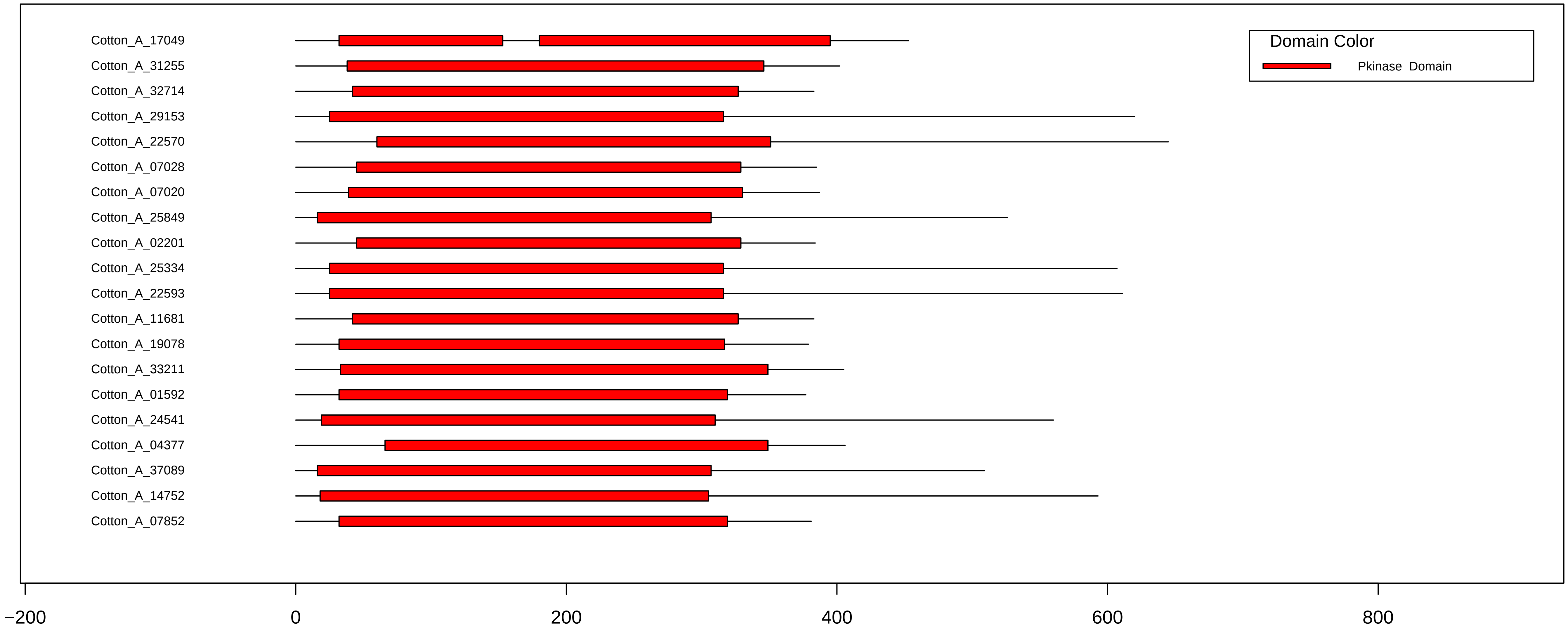

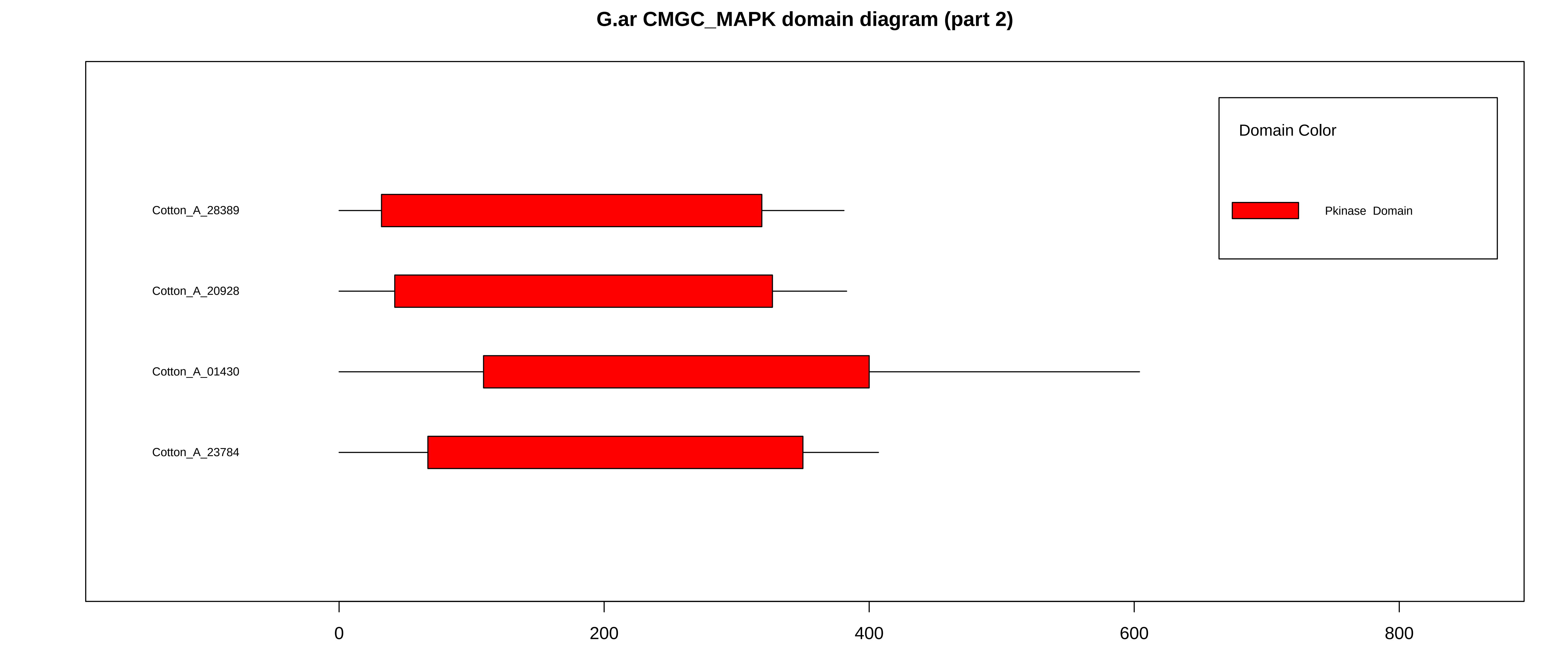
G.ar CMGC_MAPK domain diagram (part 2)
Domain Color
Pkinase Domain
Cotton_A_28389
Cotton_A_20928
Cotton_A_01430
Cotton_A_23784
0
200
400
600
800

G.ar CMGC_Pl−Tthe domain diagram (all)

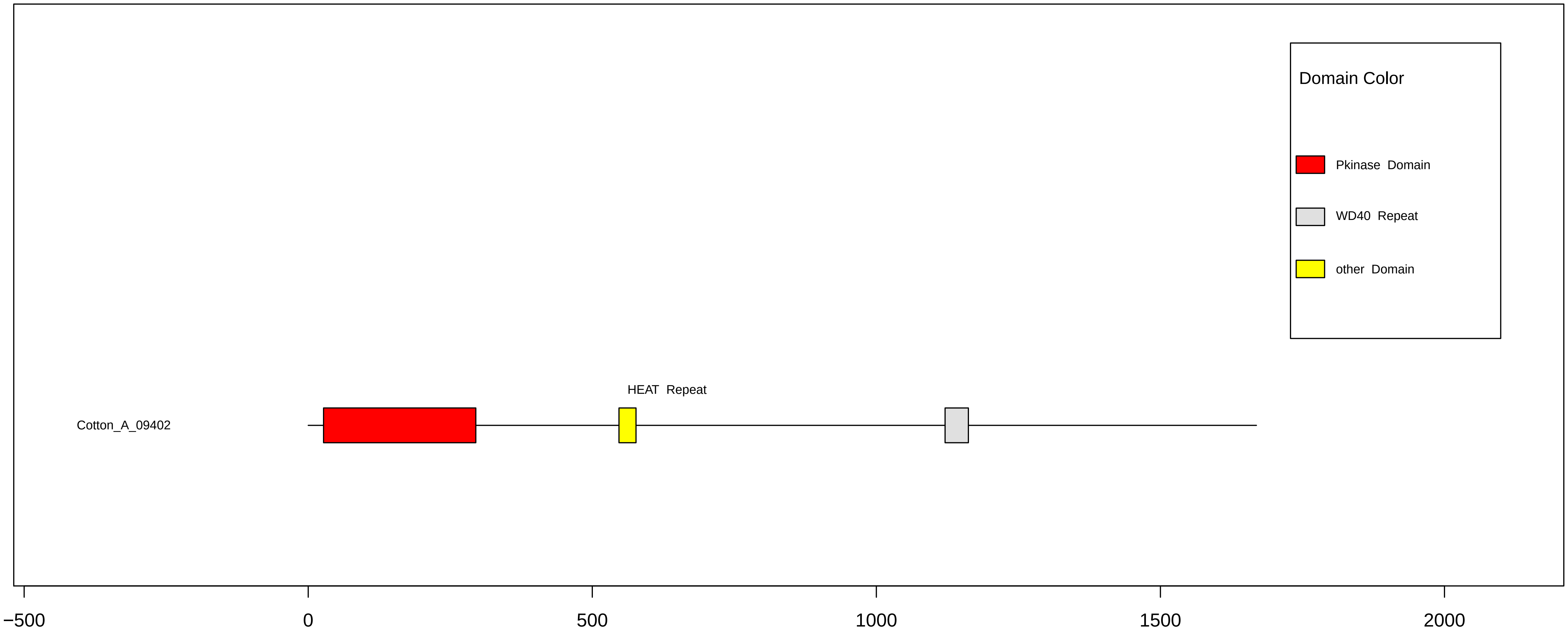

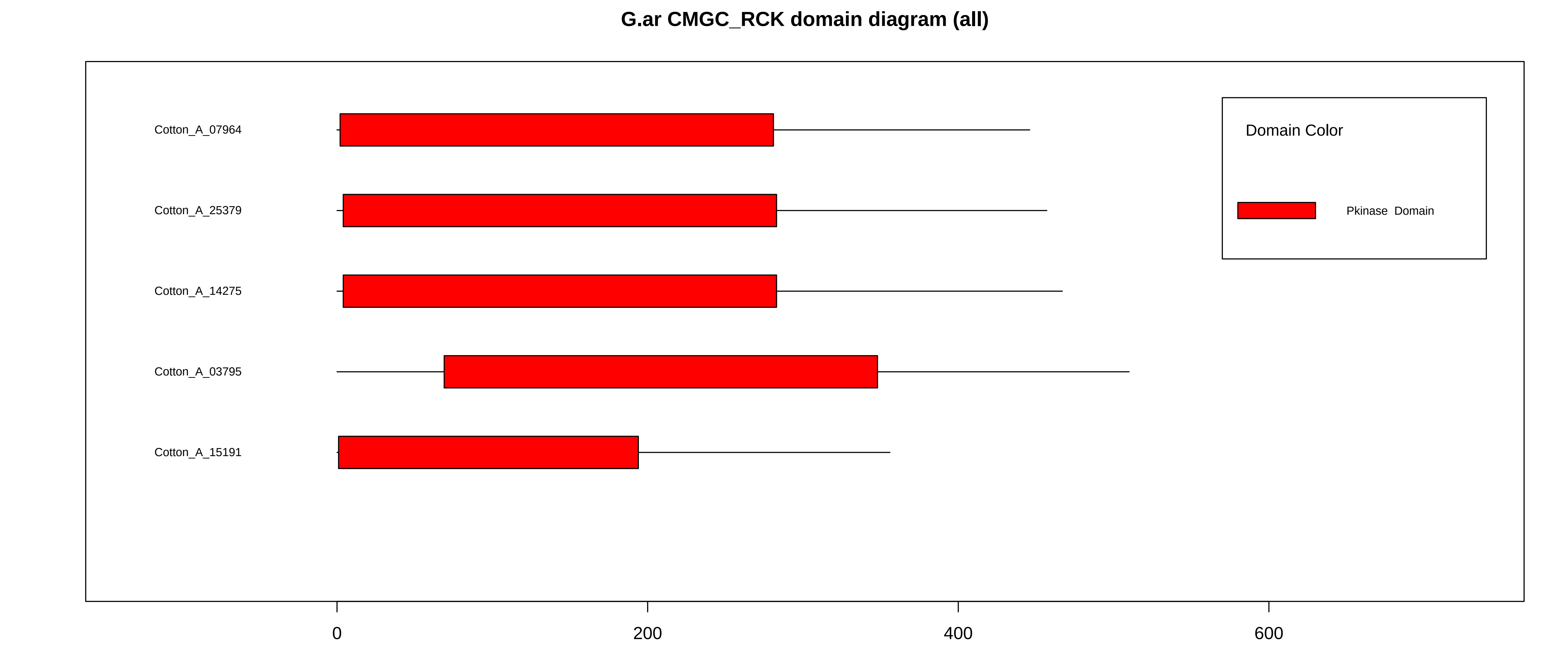
G.ar CMGC_RCK domain diagram (all)
Cotton_A_07964
Cotton_A_25379
Cotton_A_14275
Cotton_A_03795
Cotton_A_15191
Domain Color
Pkinase Domain
0
200
400
600

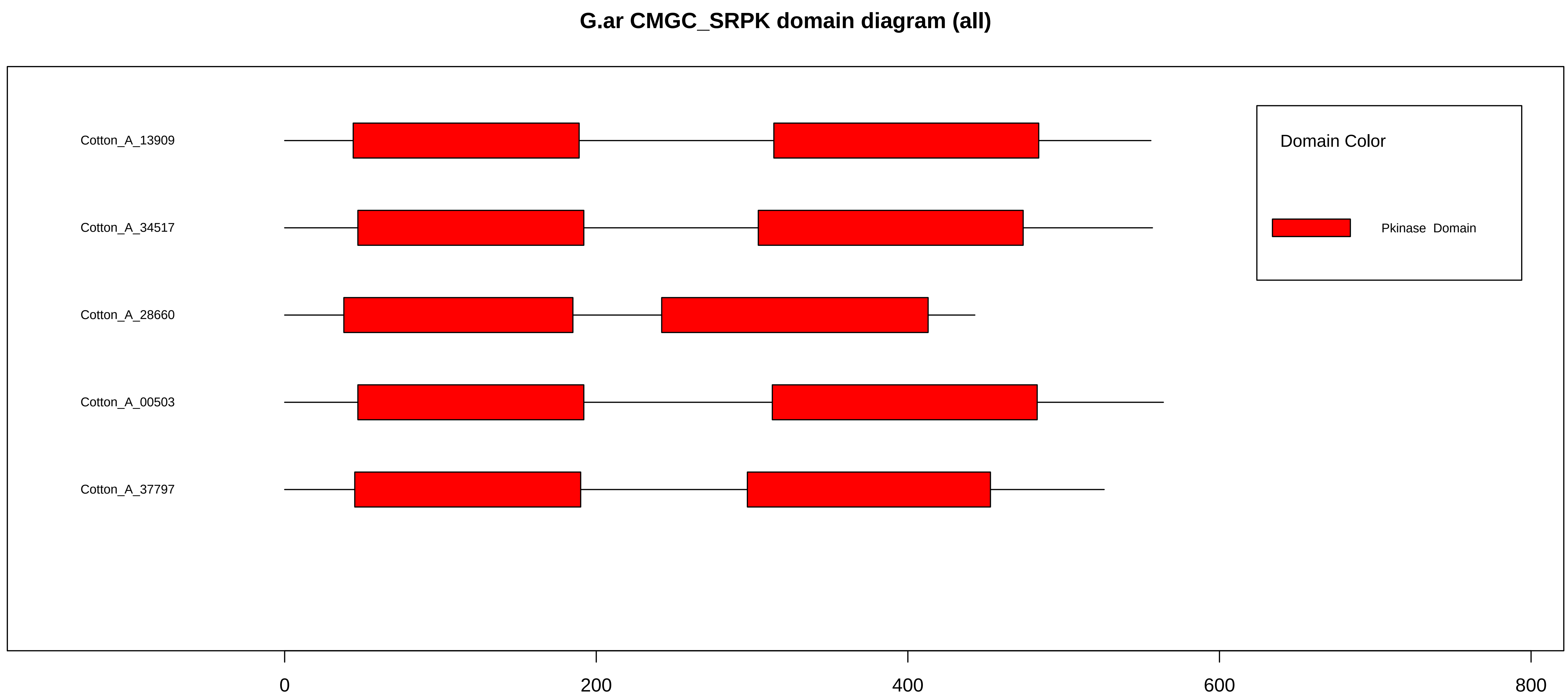
G.ar CMGC_SRPK domain diagram (all)
Cotton_A_13909
Cotton_A_34517
Cotton_A_28660
Cotton_A_00503
Cotton_A_37797
Domain Color
Pkinase Domain
0
200
400
600
800

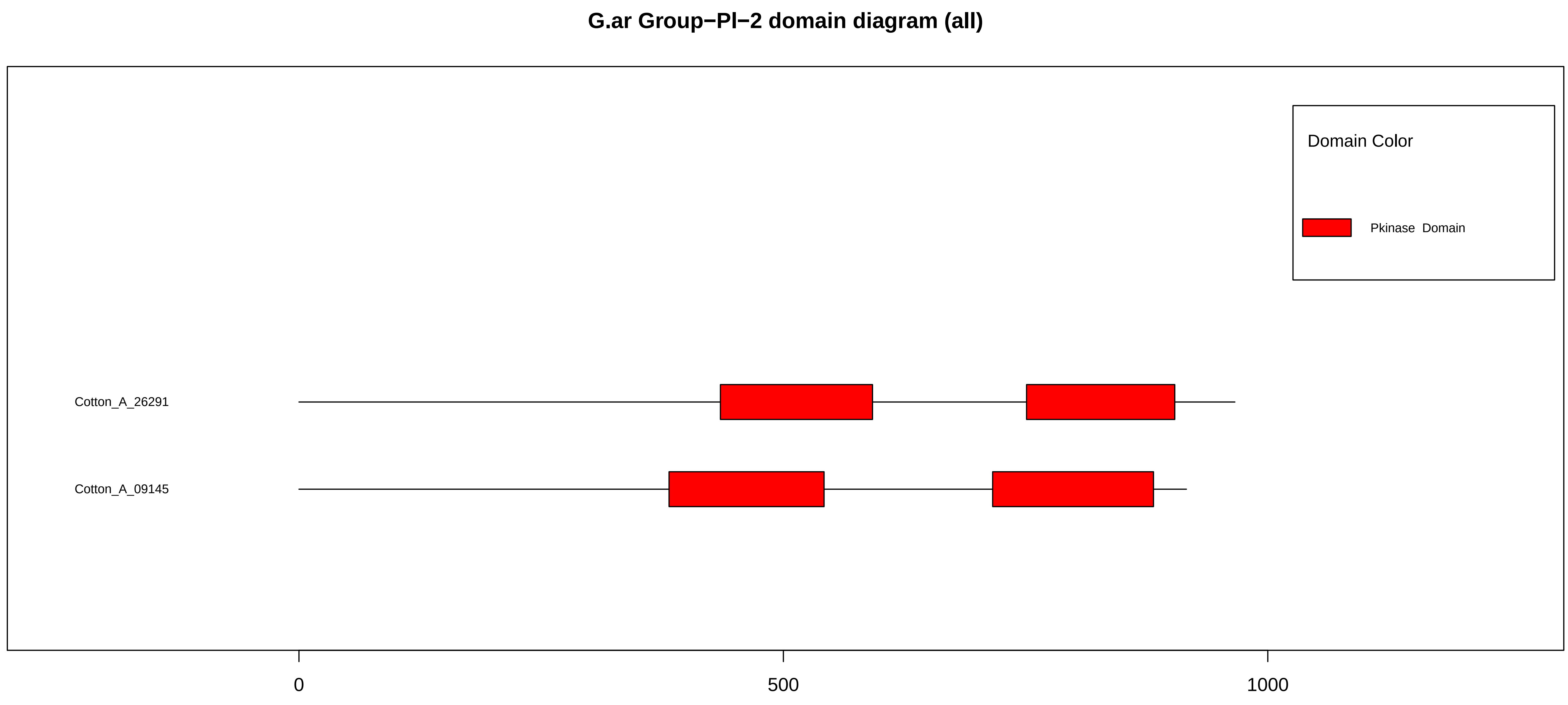
G.ar Group−PI−2 domain diagram (all)
Domain Color
Pkinase Domain
Cotton_A_26291
Cotton_A_09145
0
500
1000

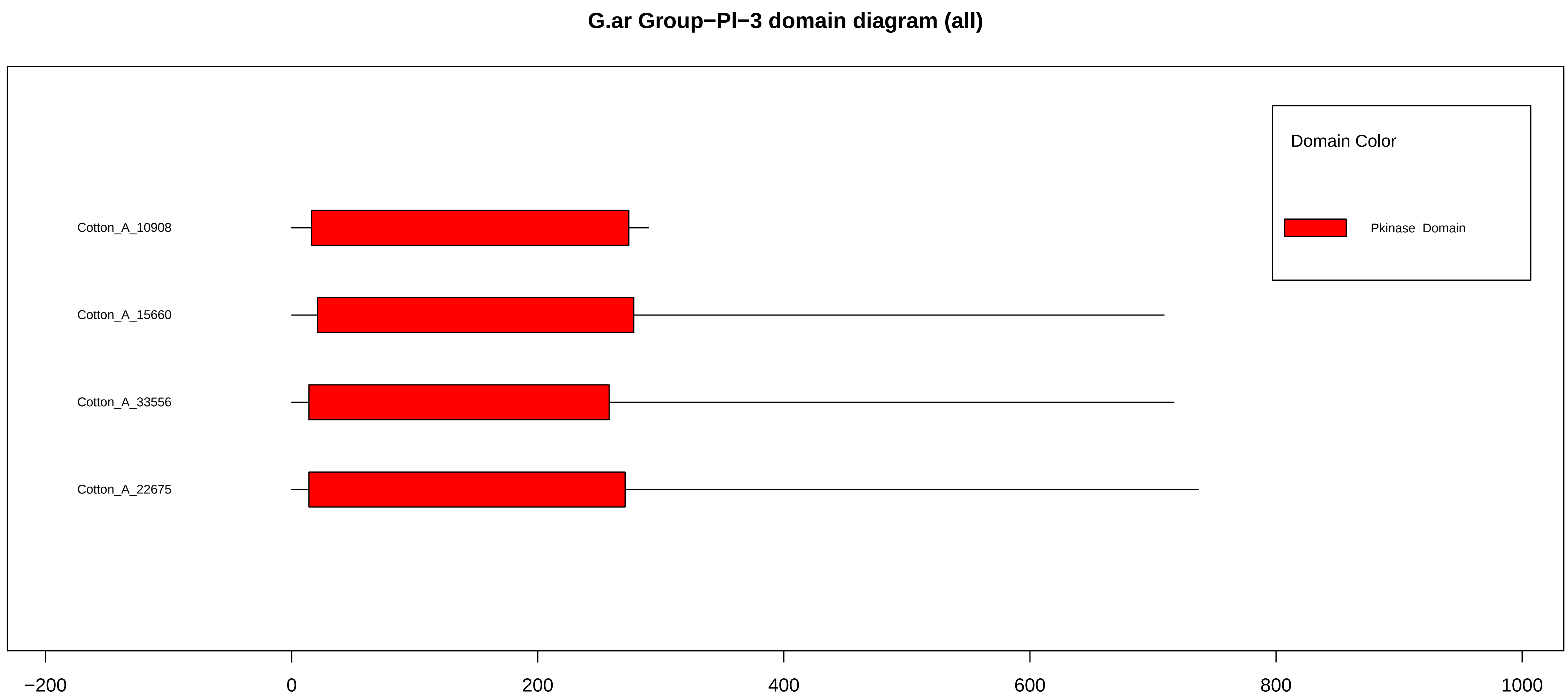

G.ar Group−PI−3 domain diagram (all)
Domain Color
Pkinase Domain
Cotton_A_10908
Cotton_A_15660
Cotton_A_33556
Cotton_A_22675
−200
0
200
400
600
800
1000

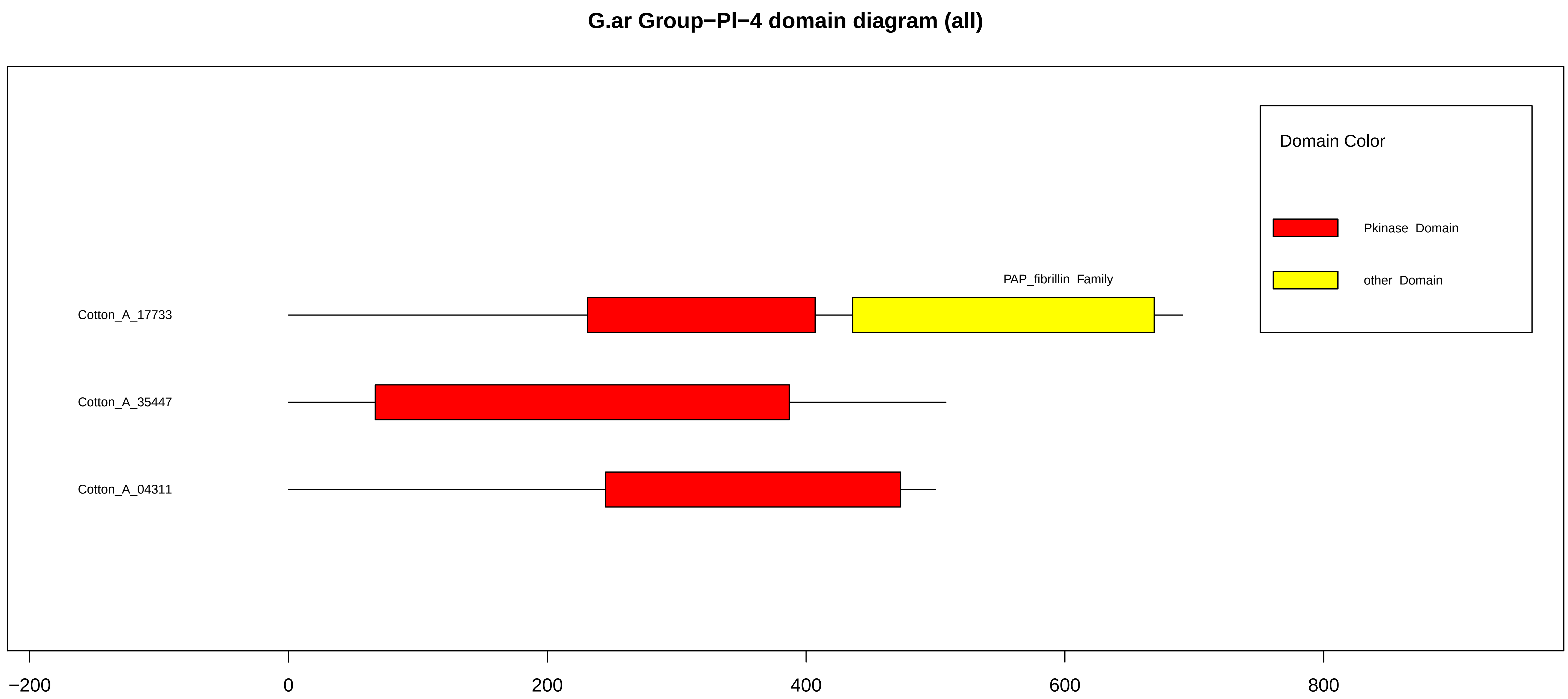
G.ar Group−PI−4 domain diagram (all)
Domain Color
Pkinase Domain
other Domain
PAP_fibrillin Family
Cotton_A_17733
Cotton_A_35447
Cotton_A_04311
−200
0
200
400
600
800

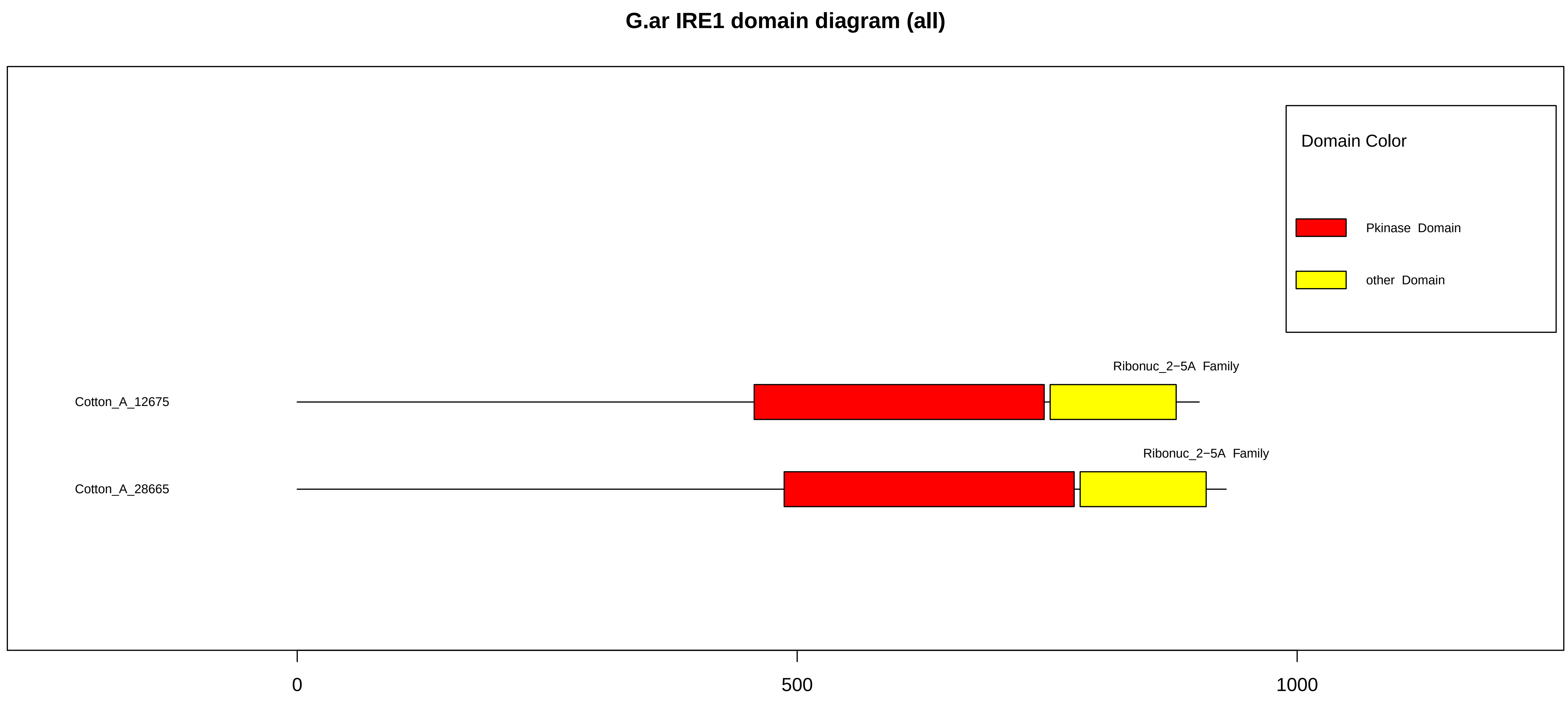
G.ar IRE1 domain diagram (all)
Domain Color
Pkinase Domain
other Domain
Ribonuc_2−5A Family
Cotton_A_12675
Ribonuc_2−5A Family
Cotton_A_28665
0
500
1000

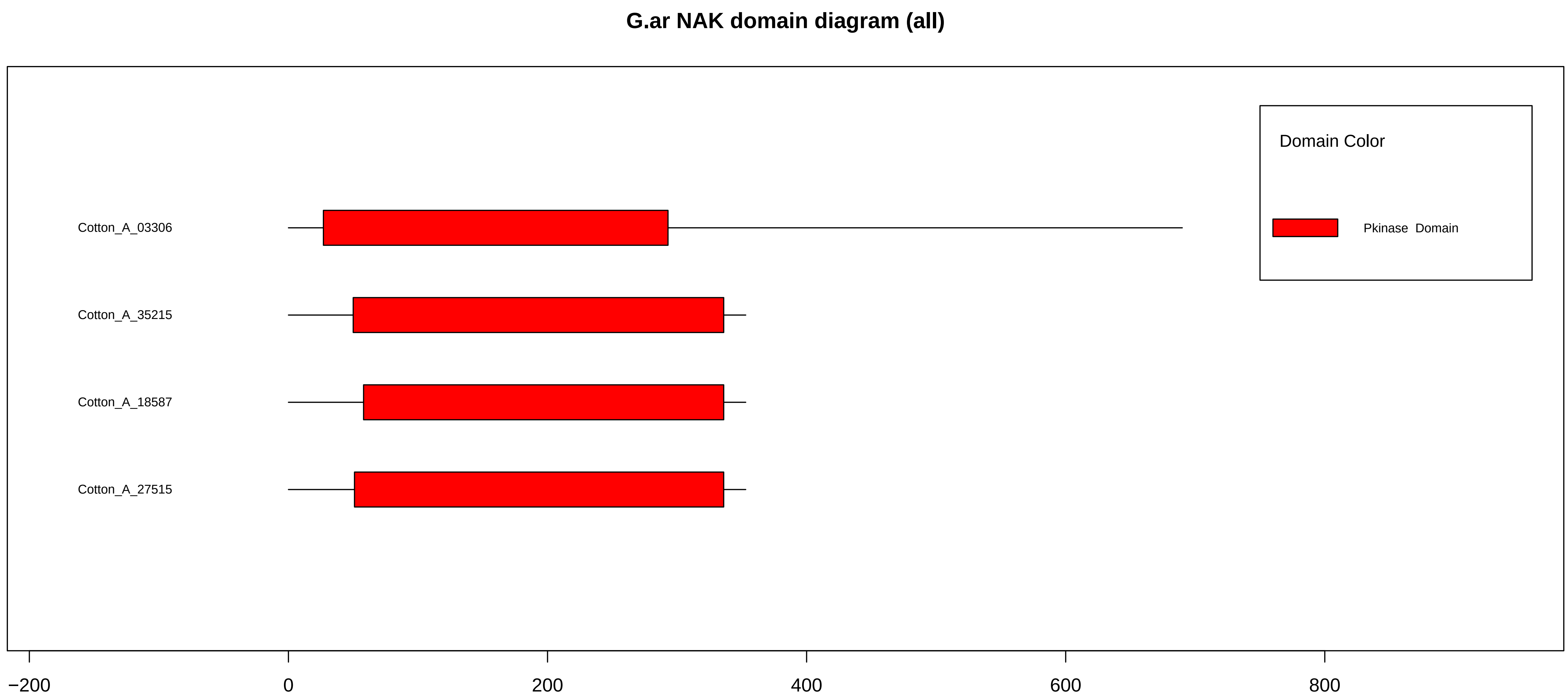

G.ar NAK domain diagram (all)
Domain Color
Pkinase Domain
Cotton_A_03306
Cotton_A_35215
Cotton_A_18587
Cotton_A_27515
−200
0
200
400
600
800

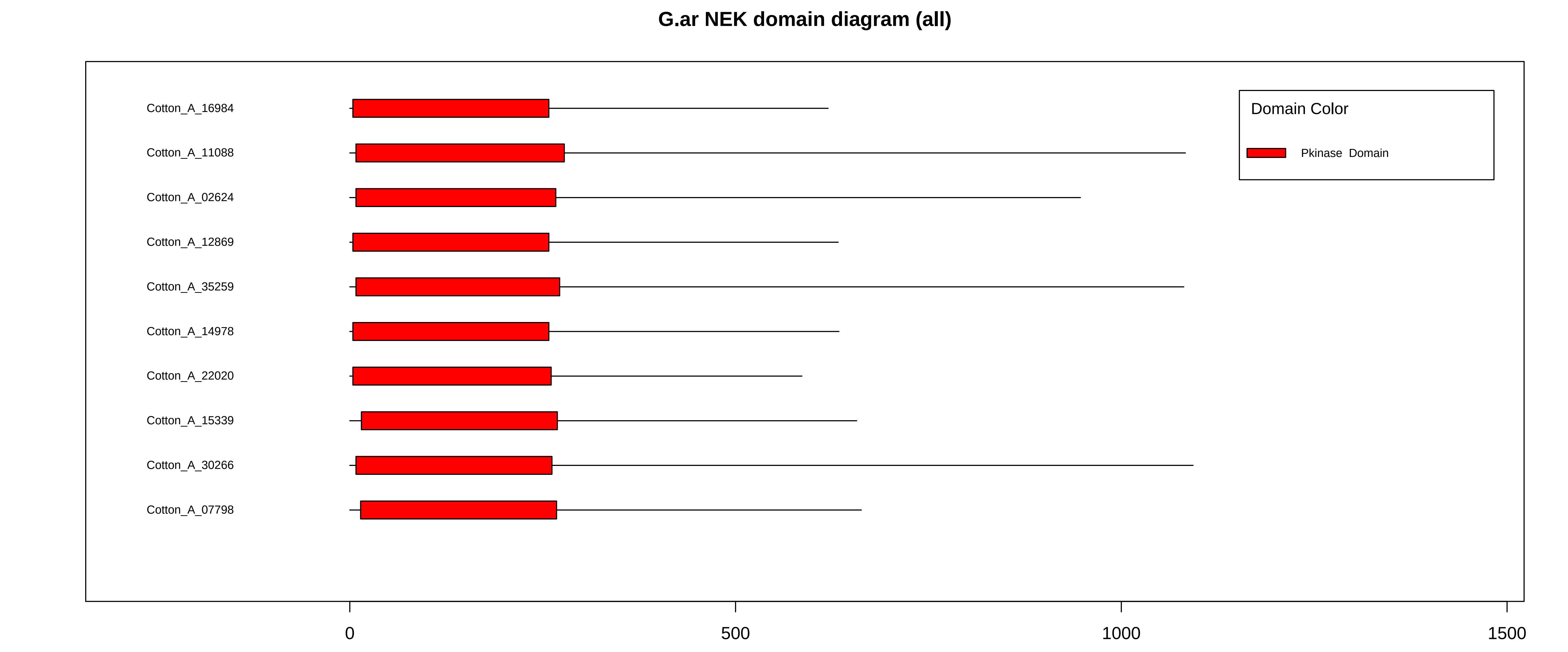

G.ar NEK domain diagram (all)
Cotton_A_16984
Cotton_A_11088
Cotton_A_02624
Cotton_A_12869
Cotton_A_35259
Cotton_A_14978
Cotton_A_22020
Cotton_A_15339
Cotton_A_30266
Cotton_A_07798
Domain Color
Pkinase Domain
0
500
1000
1500

G.ar PEK_GCN2 domain diagram (all)

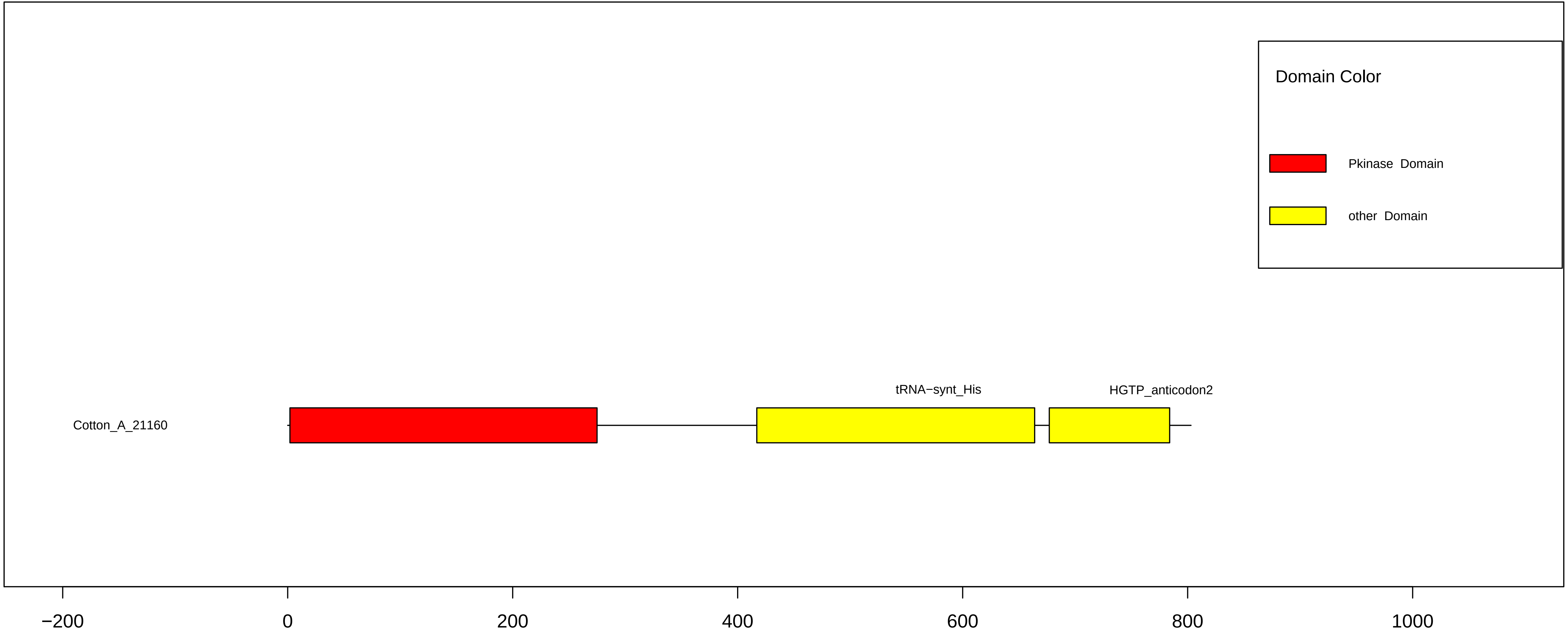

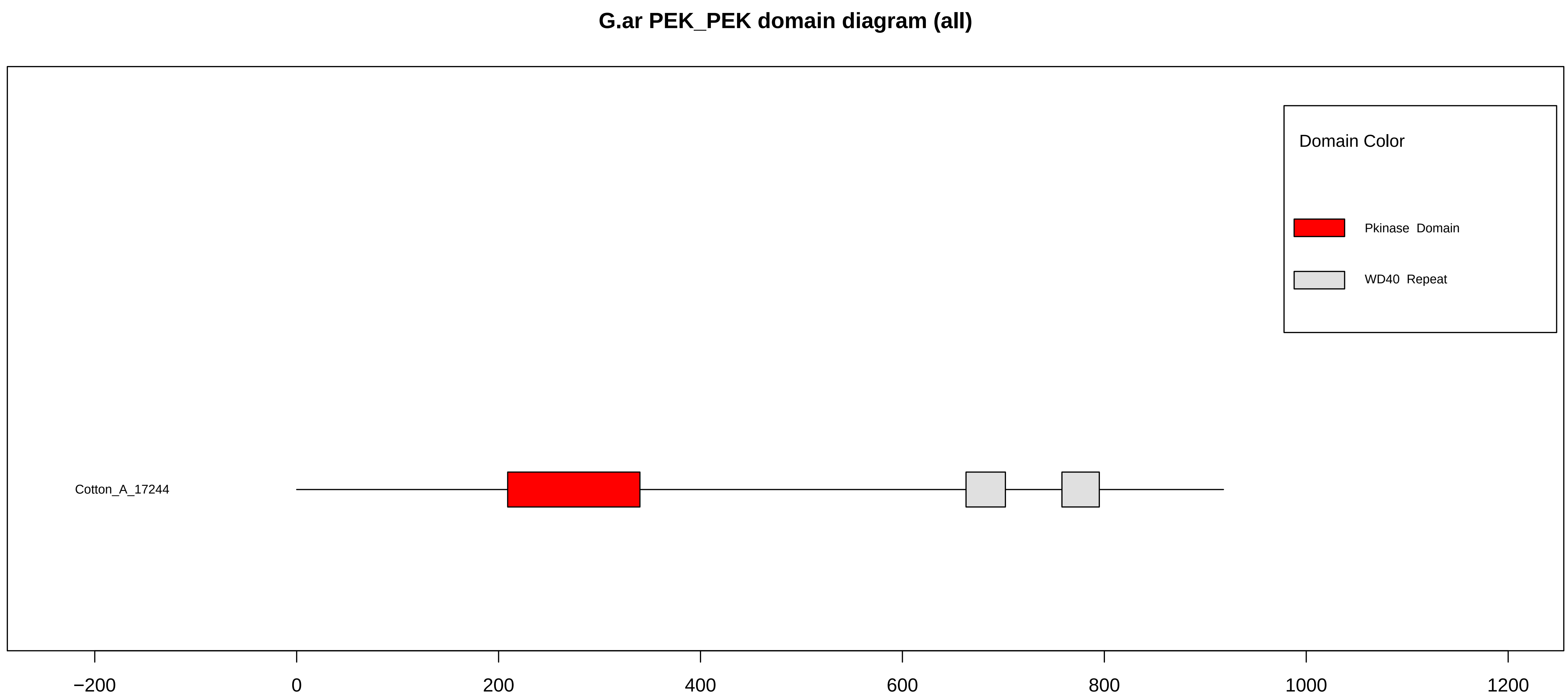
G.ar PEK_PEK domain diagram (all)
Domain Color
Pkinase Domain
WD40 Repeat
Cotton_A_17244
−200
0
200
400
600
800
1000
1200

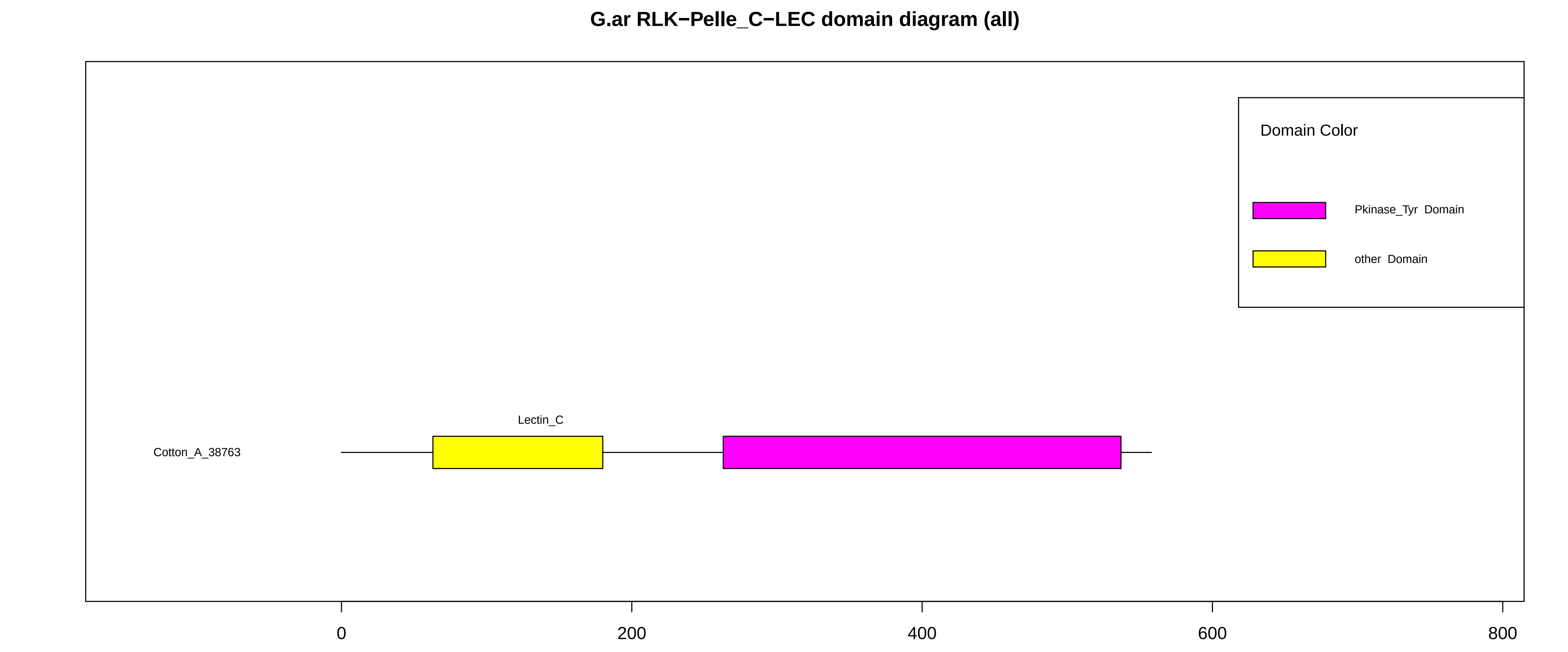
G.ar RLK−Pelle_C−LEC domain diagram (all)
Domain Color
Pkinase_Tyr Domain
other Domain
Lectin_C
Cotton_A_38763
0
200
400
600
800

G.ar RLK−Pelle_CR4L domain diagram (all)

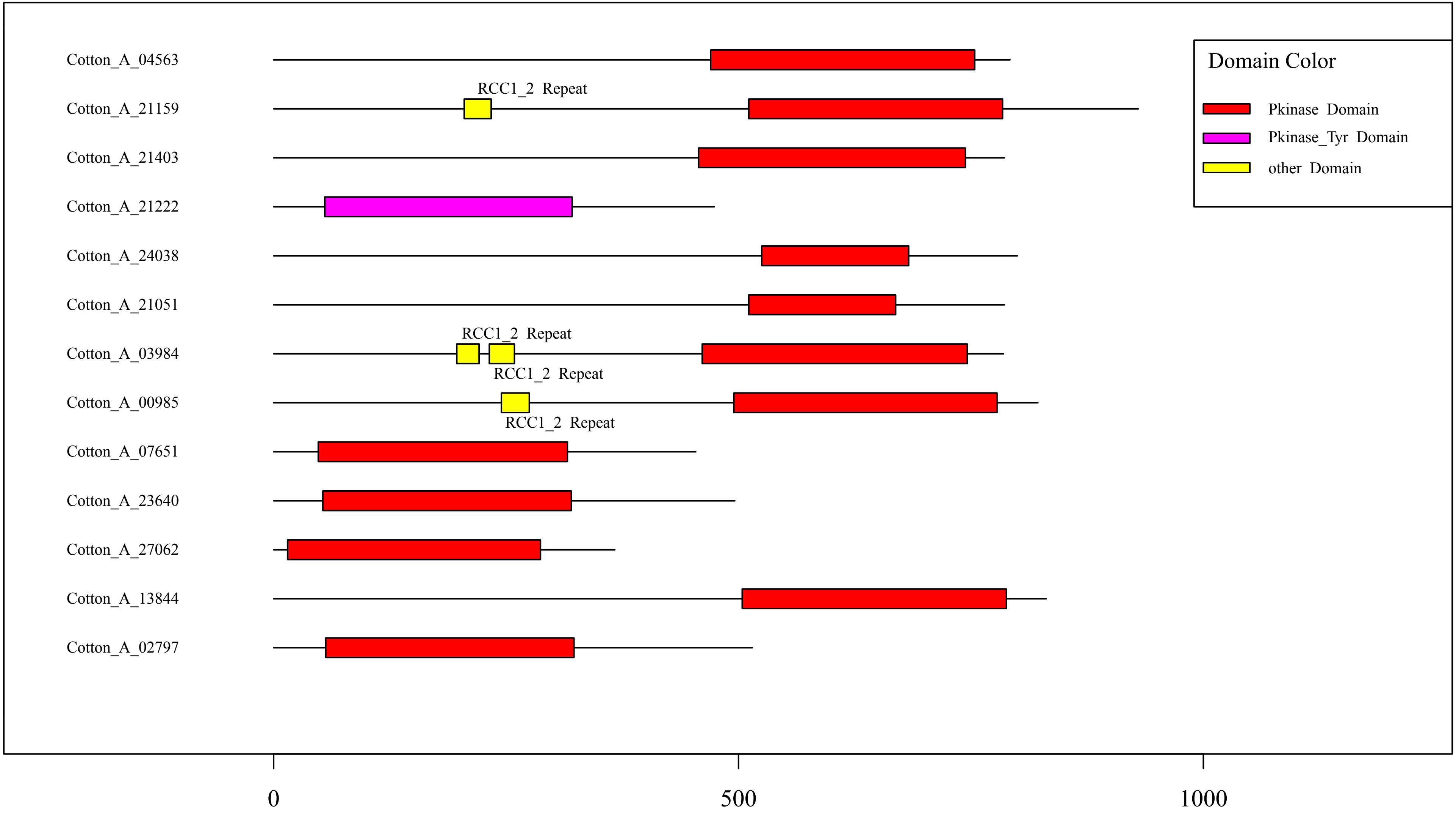

G.ar RLK−Pelle_CrRLK1L−1 domain diagram (part 1)

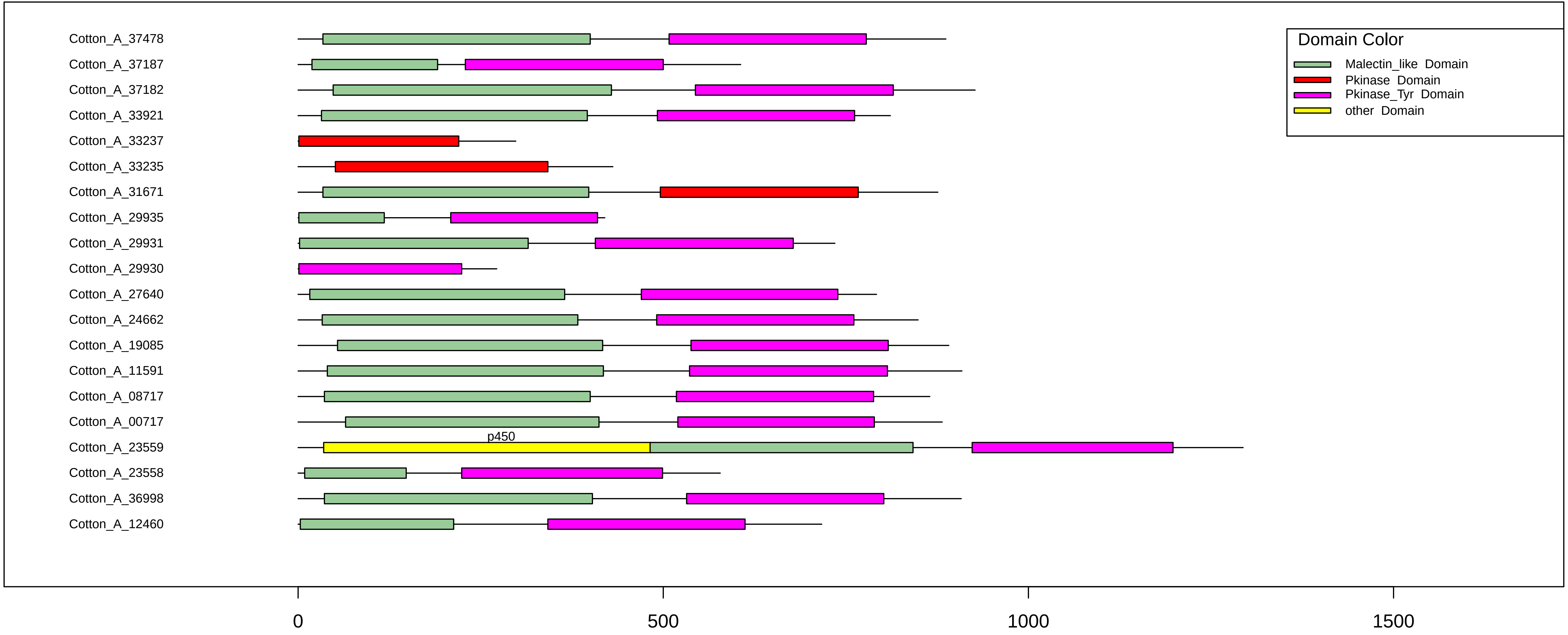

G.ar RLK−Pelle_CrRLK1L−1 domain diagram (part 2)

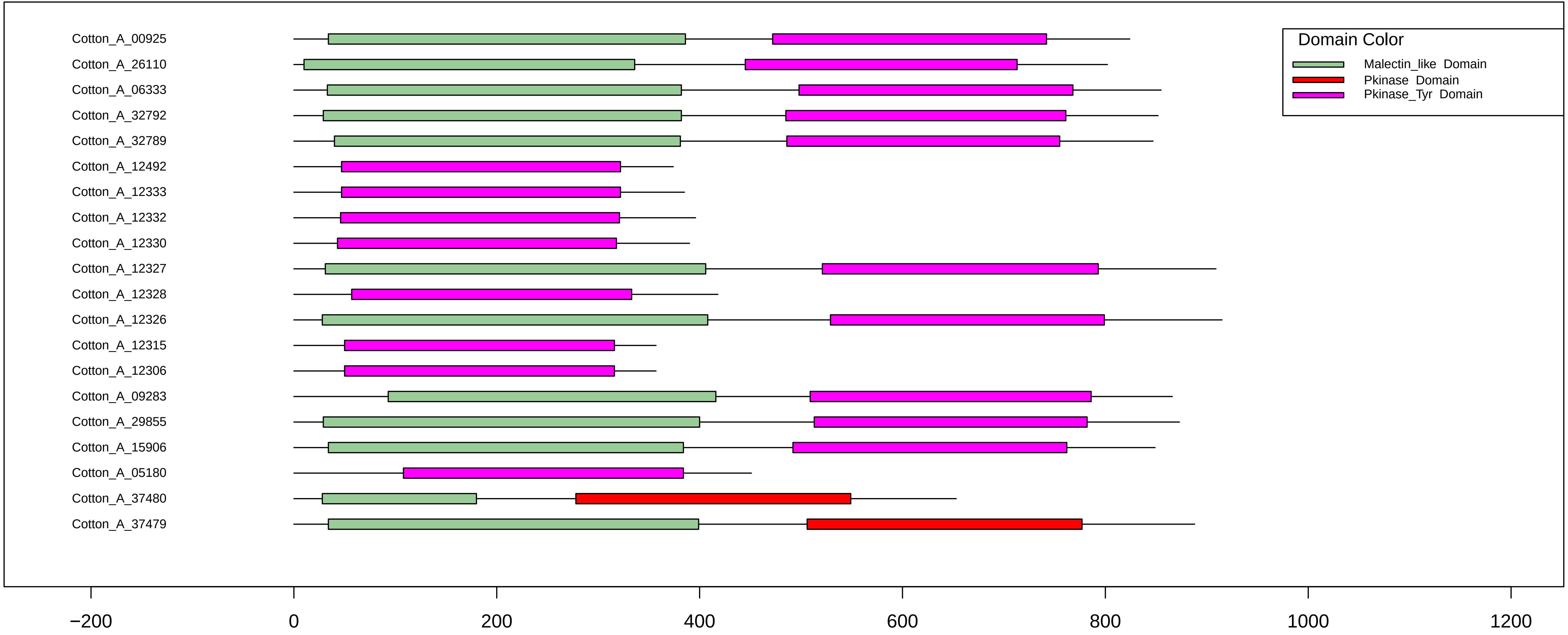

G.ar RLK−Pelle_CrRLK1L−1 domain diagram (part 3)

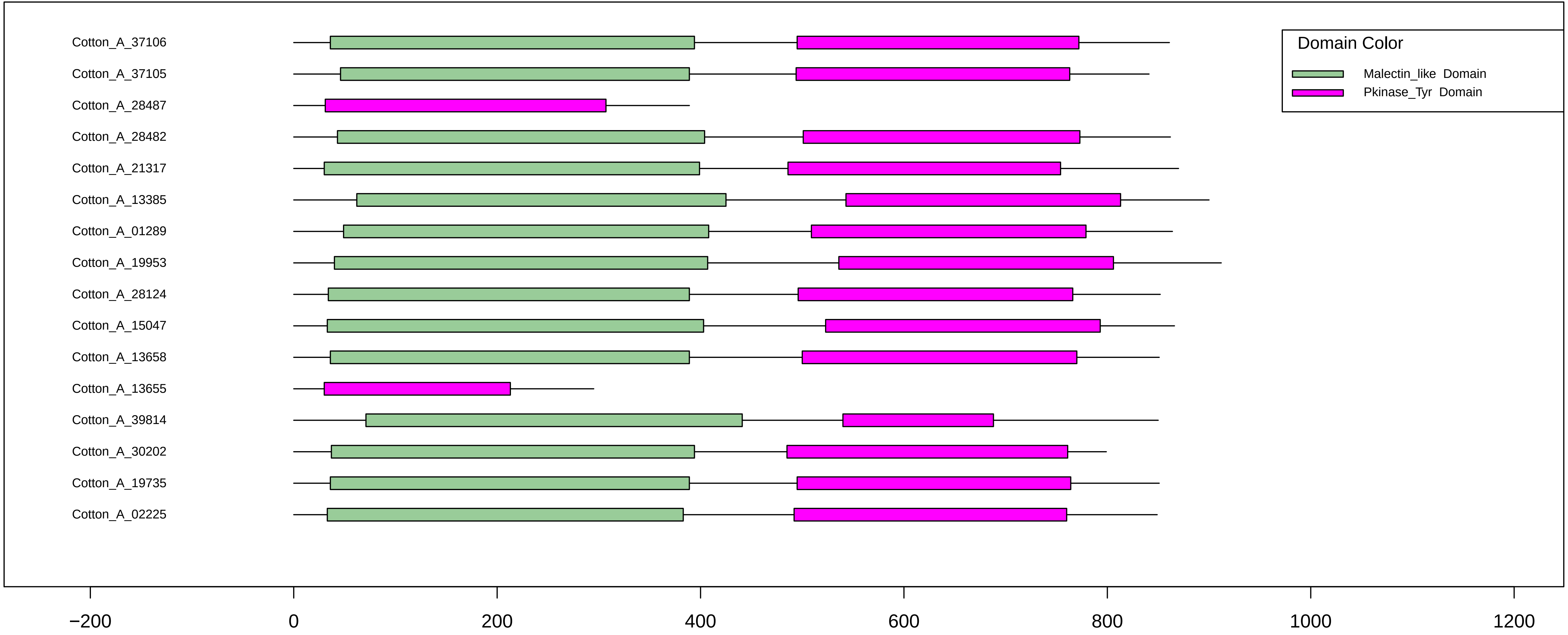

G.ar RLK−Pelle_DLSV domain diagram (part 1)

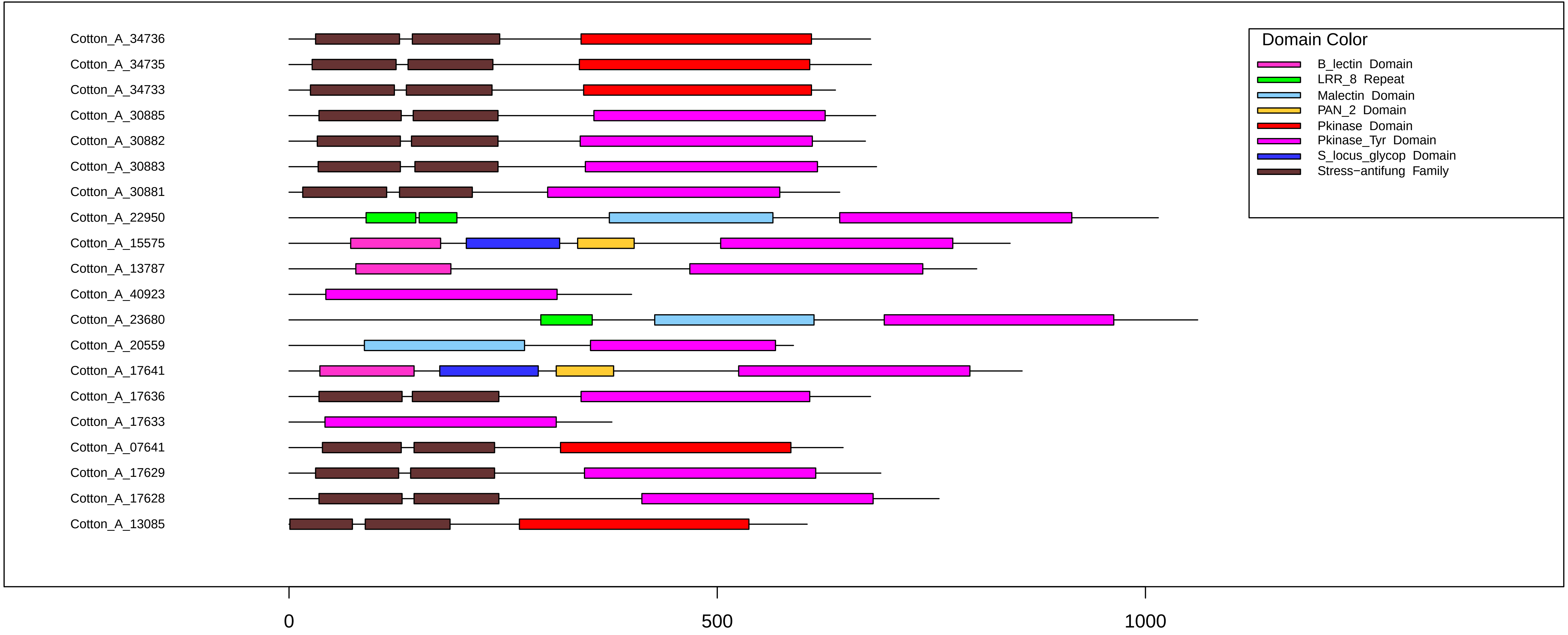

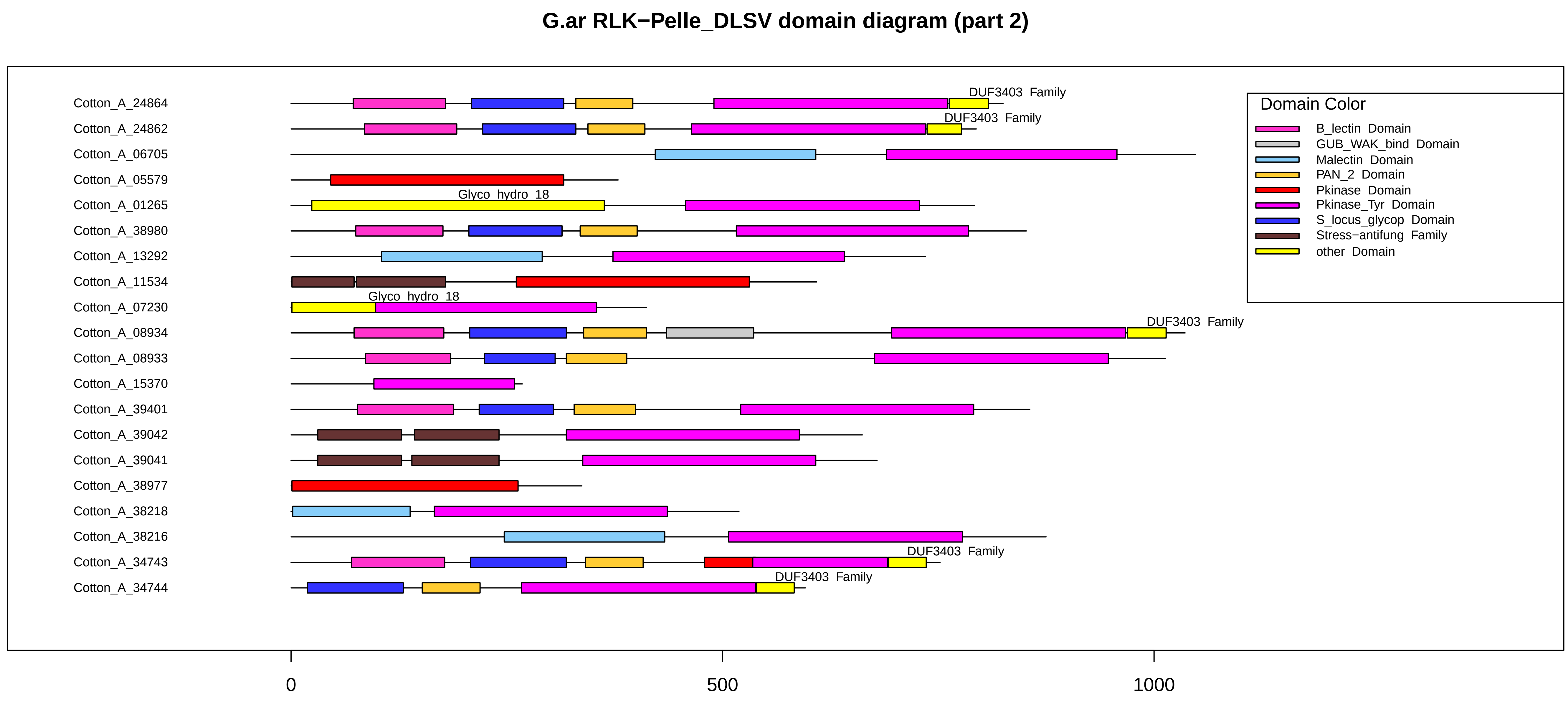
G.ar RLK−Pelle_DLSV domain diagram (part 2)
Cotton_A_24864
Cotton_A_24862
Cotton_A_06705
Cotton_A_05579
Cotton_A_01265
Cotton_A_38980
Cotton_A_13292
Cotton_A_11534
Cotton_A_07230
Cotton_A_08934
Cotton_A_08933
Cotton_A_15370
Cotton_A_39401
Cotton_A_39042
Cotton_A_39041
Cotton_A_38977
Cotton_A_38218
Cotton_A_38216
Cotton_A_34743
Cotton_A_34744
DUF3403 Family
Glyco_hydro_18
Domain Color
B_lectin Domain
GUB_WAK_bind Domain
Malectin Domain
PAN_2 Domain
Pkinase Domain
Pkinase_Tyr Domain
S_locus_glycop Domain
Stress−antifung Family
other Domain
0
500
1000

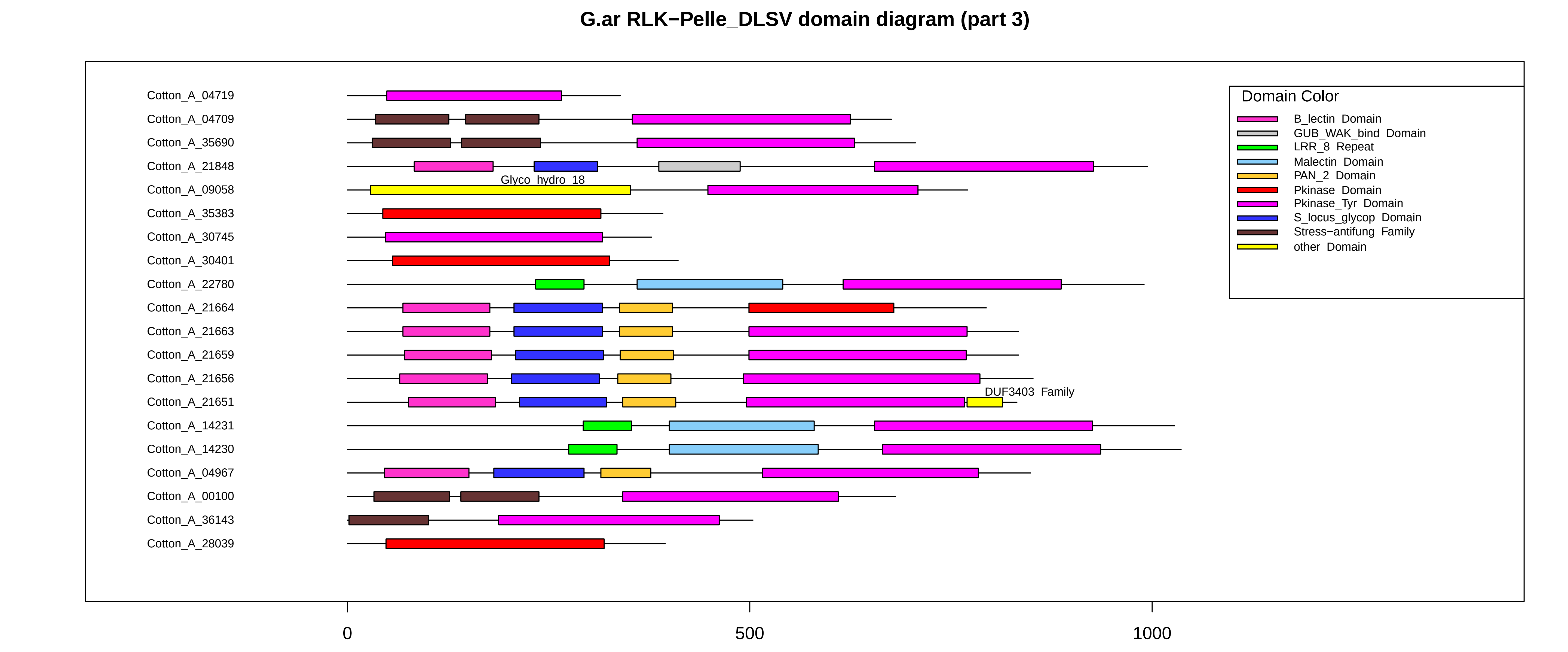
G.ar RLK−Pelle_DLSV domain diagram (part 3)
Cotton_A_04719
Cotton_A_04709
Cotton_A_35690
Cotton_A_21848
Cotton_A_09058
Cotton_A_35383
Cotton_A_30745
Cotton_A_30401
Cotton_A_22780
Cotton_A_21664
Cotton_A_21663
Cotton_A_21659
Cotton_A_21656
Cotton_A_21651
Cotton_A_14231
Cotton_A_14230
Cotton_A_04967
Cotton_A_00100
Cotton_A_36143
Cotton_A_28039
Glyco_hydro_18
DUF3403 Family
Domain Color
B_lectin Domain
GUB_WAK_bind Domain
LRR_8 Repeat
Malectin Domain
PAN_2 Domain
Pkinase Domain
Pkinase_Tyr Domain
S_locus_glycop Domain
Stress−antifung Family
other Domain
0
500
1000

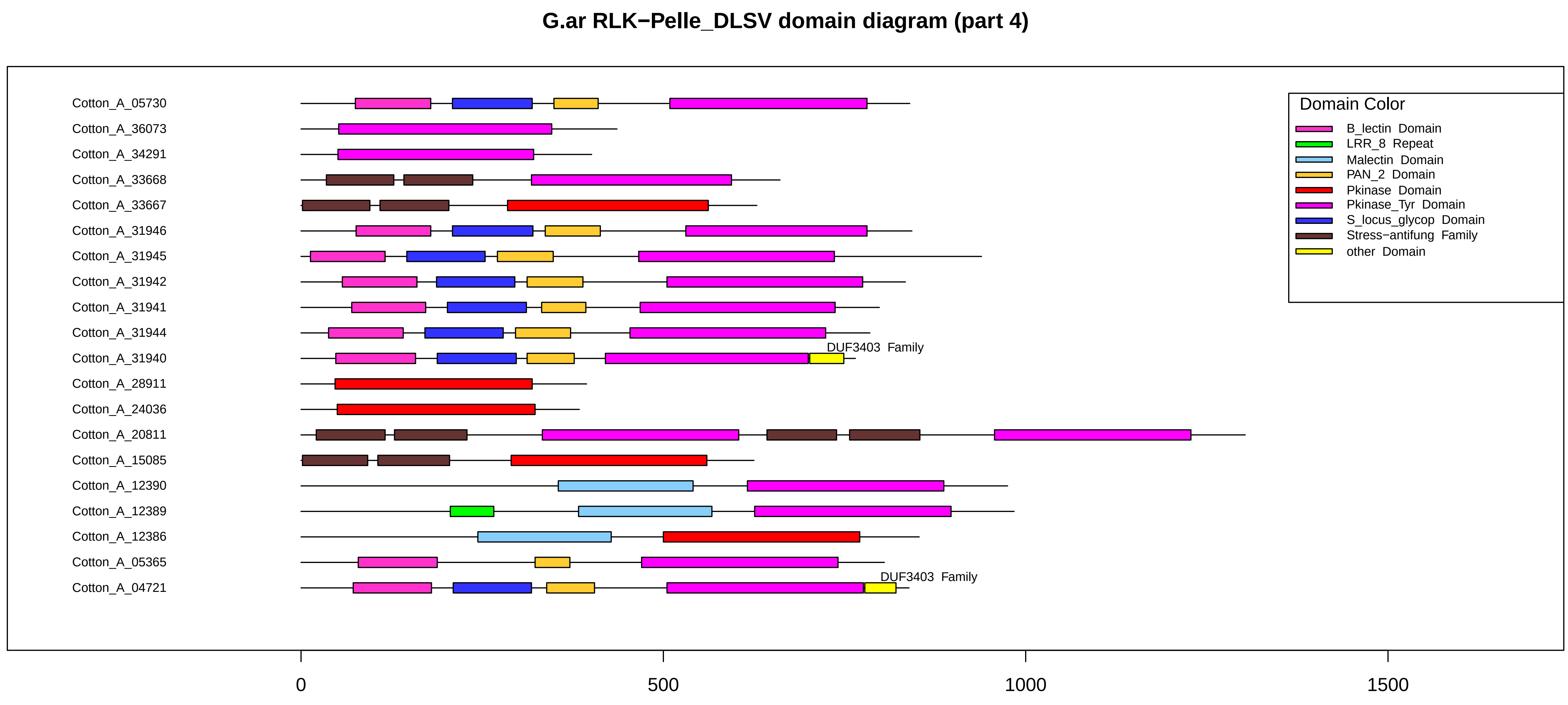
G.ar RLK−Pelle_DLSV domain diagram (part 4)
Cotton_A_05730
Cotton_A_36073
Cotton_A_34291
Cotton_A_33668
Cotton_A_33667
Cotton_A_31946
Cotton_A_31945
Cotton_A_31942
Cotton_A_31941
Cotton_A_31944
Cotton_A_31940
Cotton_A_28911
Cotton_A_24036
Cotton_A_20811
Cotton_A_15085
Cotton_A_12390
Cotton_A_12389
Cotton_A_12386
Cotton_A_05365
Cotton_A_04721
DUF3403 Family
DUF3403 Family
Domain Color
B_lectin Domain
LRR_8 Repeat
Malectin Domain
PAN_2 Domain
Pkinase Domain
Pkinase_Tyr Domain
S_locus_glycop Domain
Stress−antifung Family
other Domain
0
500
1000
1500

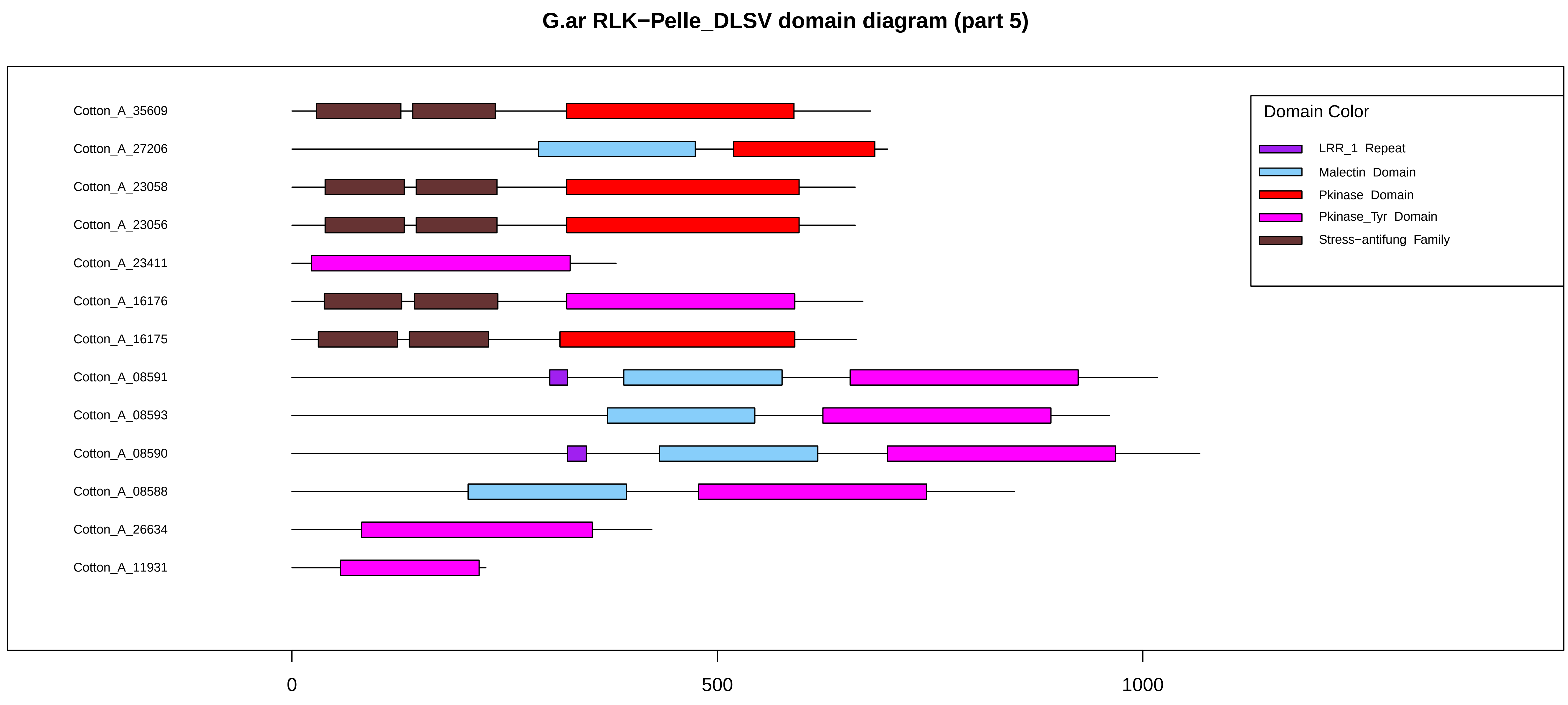
G.ar RLK−Pelle_DLSV domain diagram (part 5)
Cotton_A_35609
Cotton_A_27206
Cotton_A_23058
Cotton_A_23056
Cotton_A_23411
Cotton_A_16176
Cotton_A_16175
Cotton_A_08591
Cotton_A_08593
Cotton_A_08590
Cotton_A_08588
Cotton_A_26634
Cotton_A_11931
Domain Color
LRR_1 Repeat
Malectin Domain
Pkinase Domain
Pkinase_Tyr Domain
Stress−antifung Family
0
500
1000

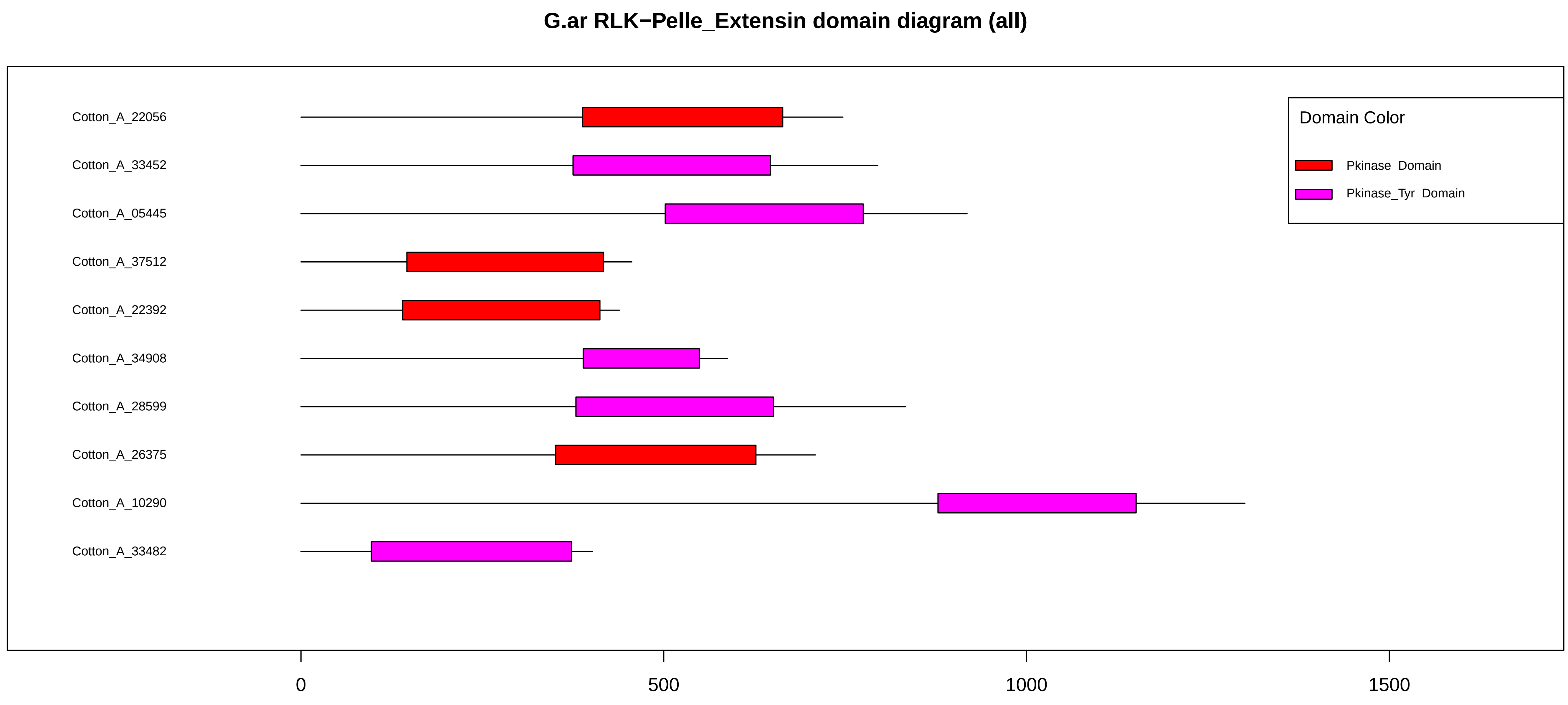
G.ar RLK−Pelle_Extensin domain diagram (all)
Cotton_A_22056
Cotton_A_33452
Cotton_A_05445
Cotton_A_37512
Cotton_A_22392
Cotton_A_34908
Cotton_A_28599
Cotton_A_26375
Cotton_A_10290
Cotton_A_33482
Domain Color
Pkinase Domain
Pkinase_Tyr Domain
0
500
1000
1500

G.ar RLK−Pelle_L−LEC domain diagram (part 1)

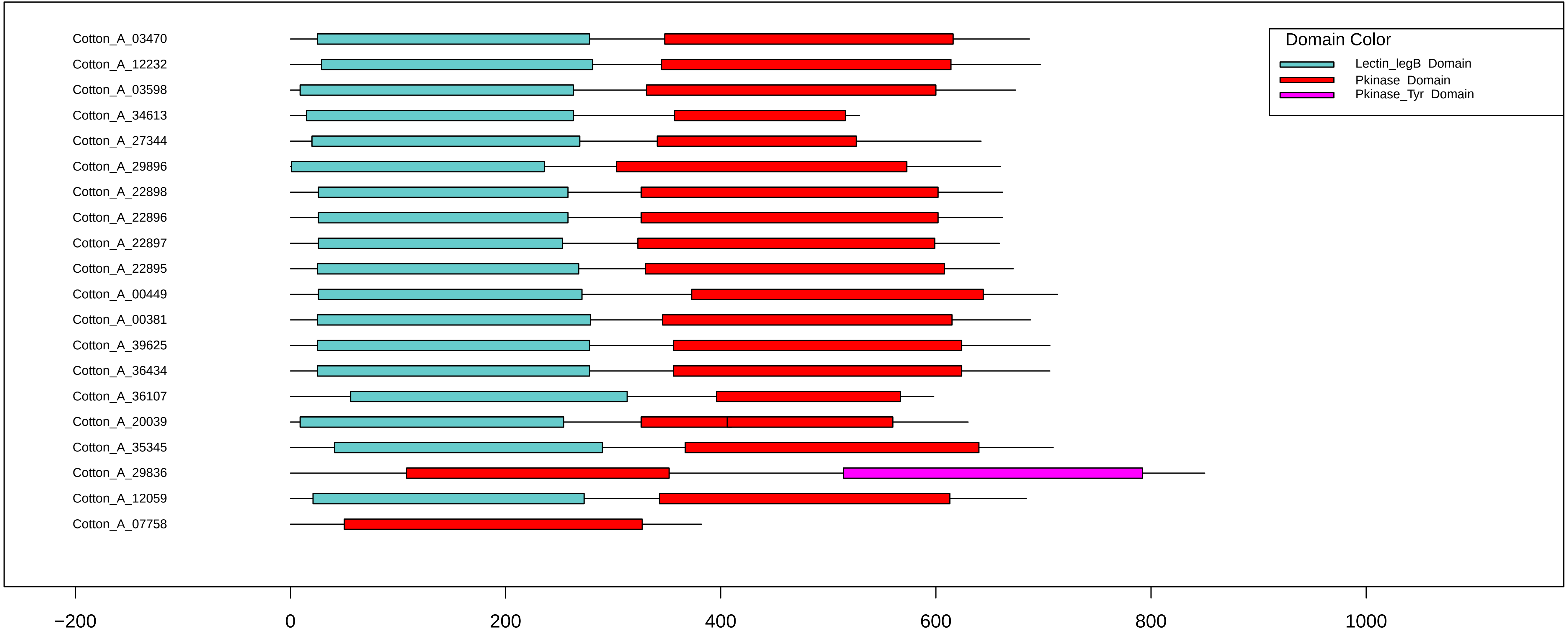

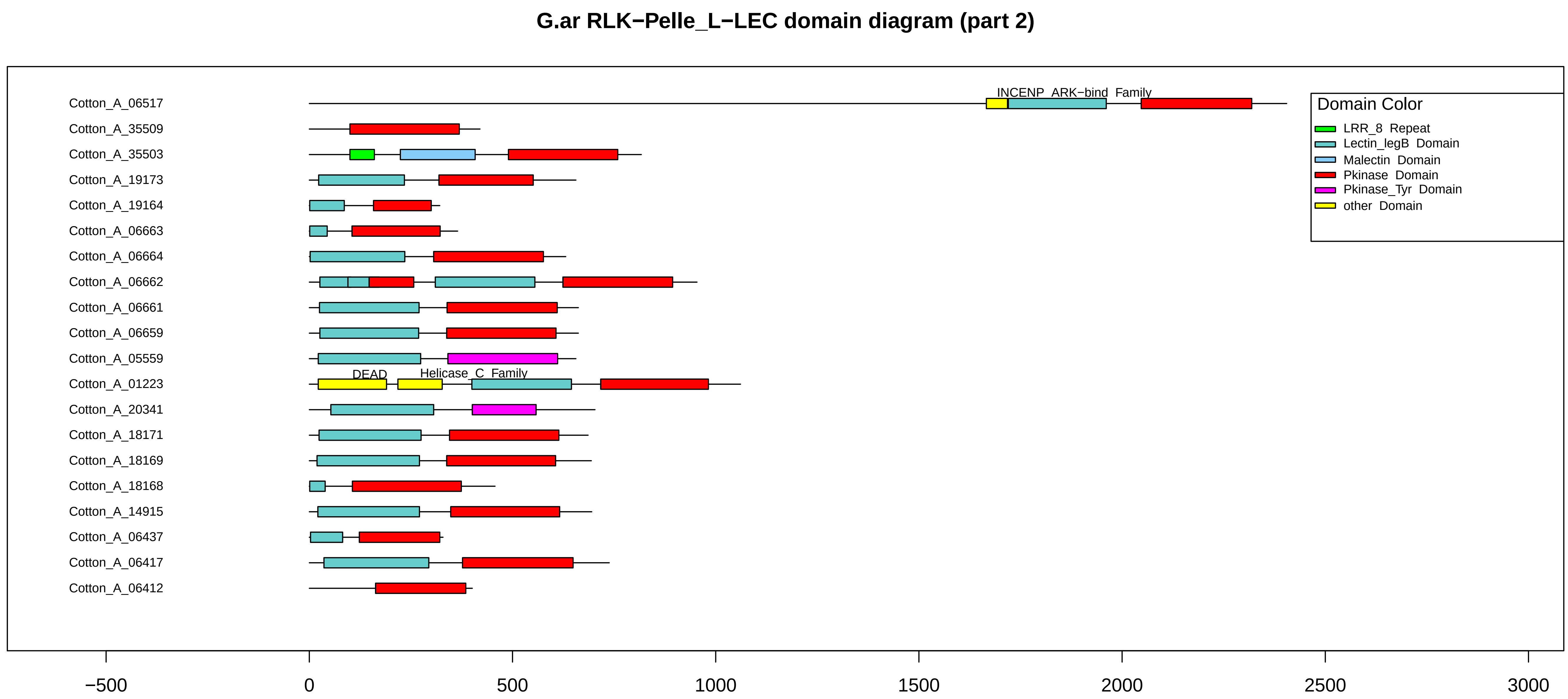
G.ar RLK−Pelle_L−LEC domain diagram (part 2)
Cotton_A_06517
Cotton_A_35509
Cotton_A_35503
Cotton_A_19173
Cotton_A_19164
Cotton_A_06663
Cotton_A_06664
Cotton_A_06662
Cotton_A_06661
Cotton_A_06659
Cotton_A_05559
Cotton_A_01223
Cotton_A_20341
Cotton_A_18171
Cotton_A_18169
Cotton_A_18168
Cotton_A_14915
Cotton_A_06437
Cotton_A_06417
Cotton_A_06412
INCENP_ARK−bind Family
DEAD
Helicase_C Family
Domain Color
LRR_8 Repeat
Lectin_legB Domain
Malectin Domain
Pkinase Domain
Pkinase_Tyr Domain
other Domain
−500
0
500
1000
1500
2000
2500
3000

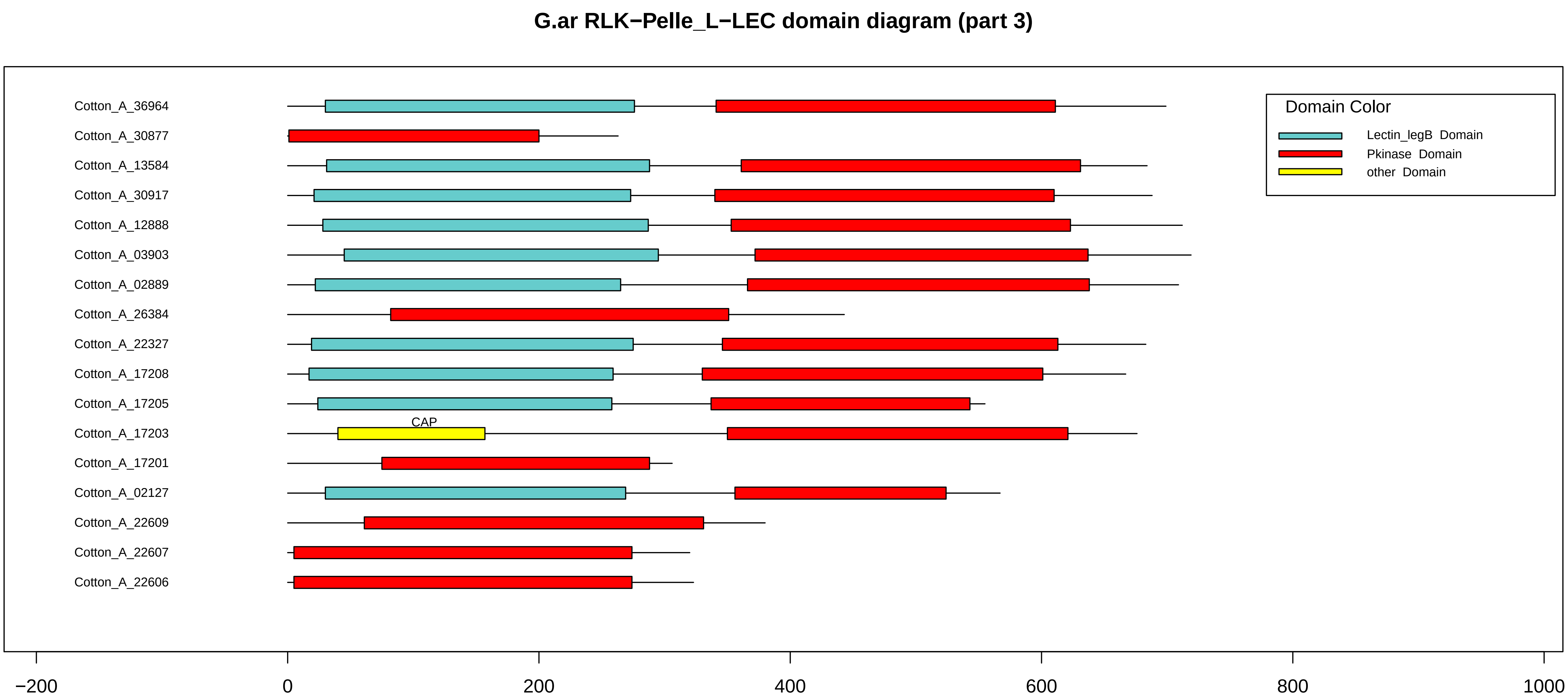
G.ar RLK−Pelle_L−LEC domain diagram (part 3)
Cotton_A_36964
Cotton_A_30877
Cotton_A_13584
Cotton_A_30917
Cotton_A_12888
Cotton_A_03903
Cotton_A_02889
Cotton_A_26384
Cotton_A_22327
Cotton_A_17208
Cotton_A_17205
Cotton_A_17203
CAP
Cotton_A_17201
Cotton_A_02127
Cotton_A_22609
Cotton_A_22607
Cotton_A_22606
Domain Color
Lectin_legB Domain
Pkinase Domain
other Domain
−200
0
200
400
600
800
1000

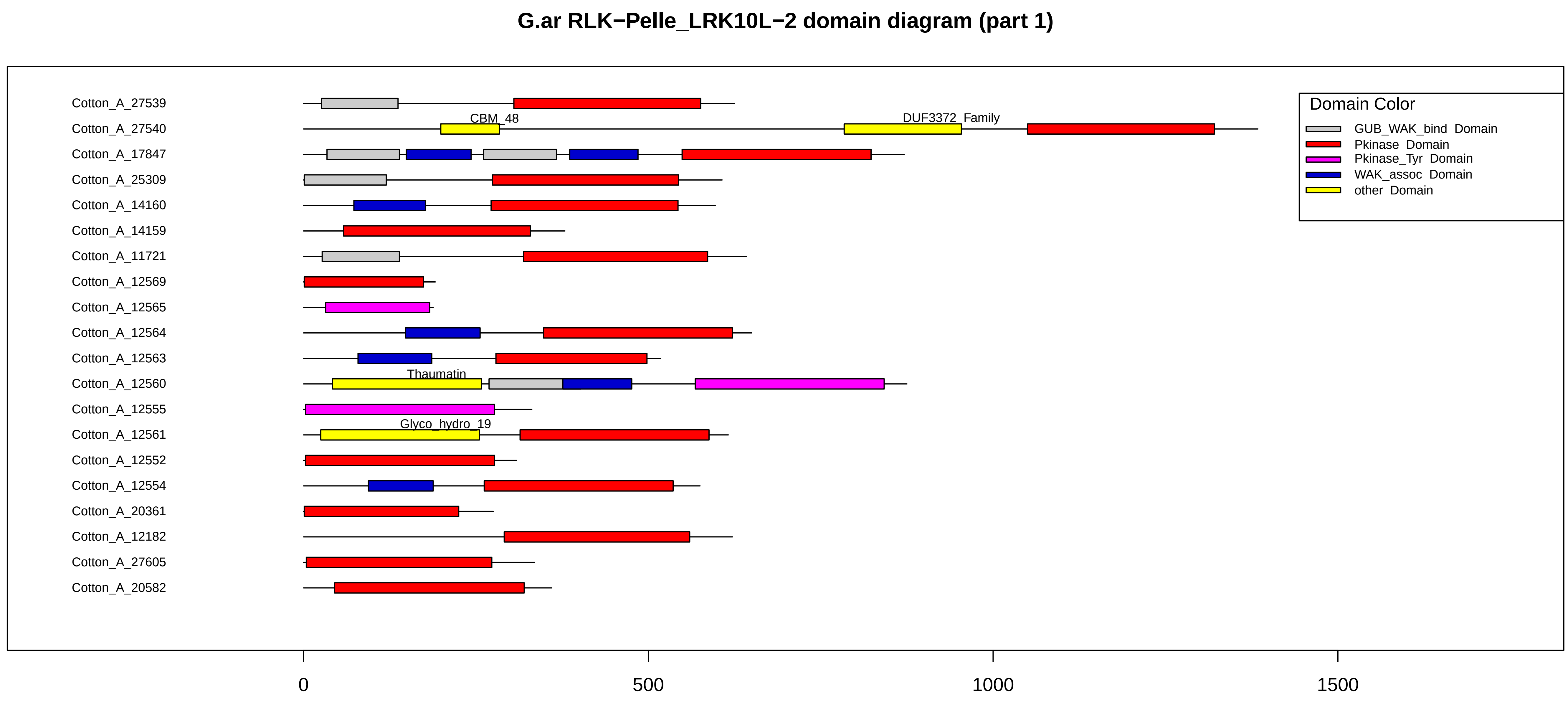
G.ar RLK−Pelle_LRK10L−2 domain diagram (part 1)
Cotton_A_27539
Cotton_A_27540
Cotton_A_17847
Cotton_A_25309
Cotton_A_14160
Cotton_A_14159
Cotton_A_11721
Cotton_A_12569
Cotton_A_12565
Cotton_A_12564
Cotton_A_12563
Cotton_A_12560
Cotton_A_12555
Cotton_A_12561
Cotton_A_12552
Cotton_A_12554
Cotton_A_20361
Cotton_A_12182
Cotton_A_27605
Cotton_A_20582
CBM_48
DUF3372_Family
Thaumatin
Glyco_hydro_19
Domain Color
GUB_WAK_bind Domain
Pkinase Domain
Pkinase_Tyr Domain
WAK_assoc Domain
other Domain
0
500
1000
1500

G.ar RLK−Pelle_LRK10L−2 domain diagram (part 2)

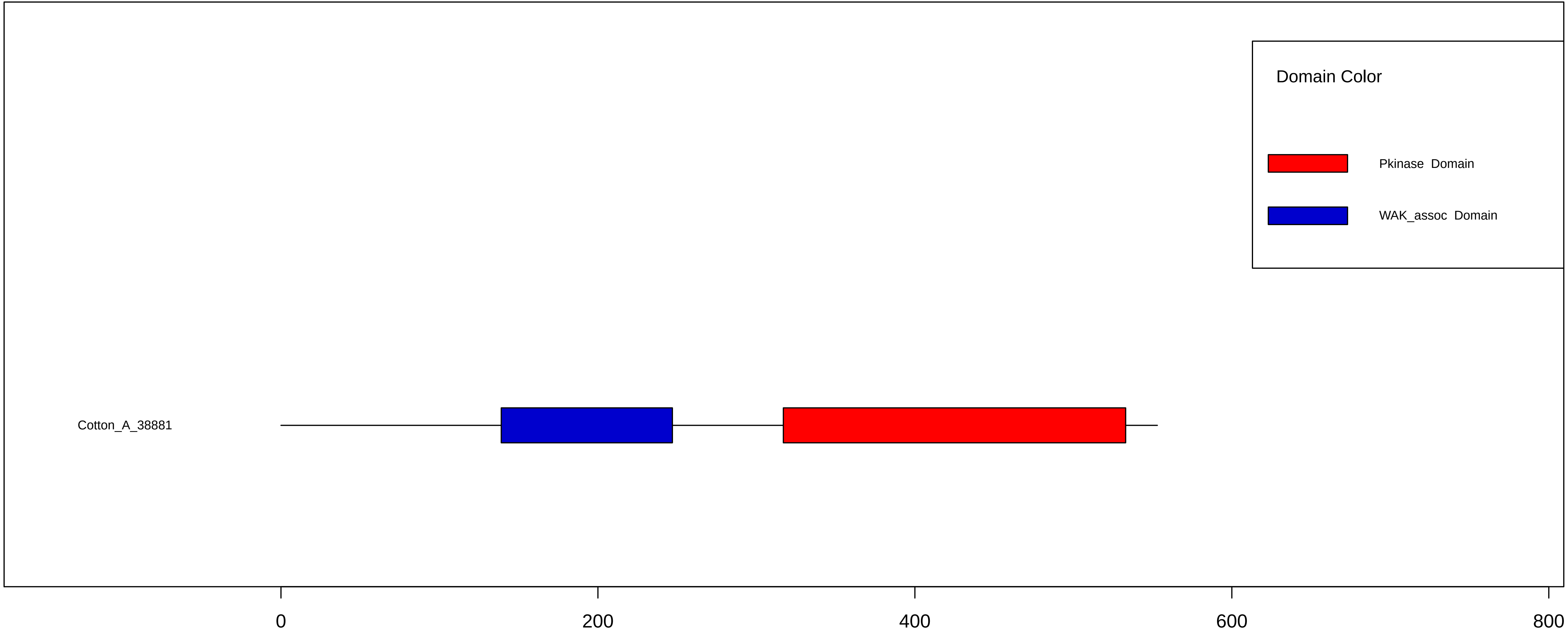

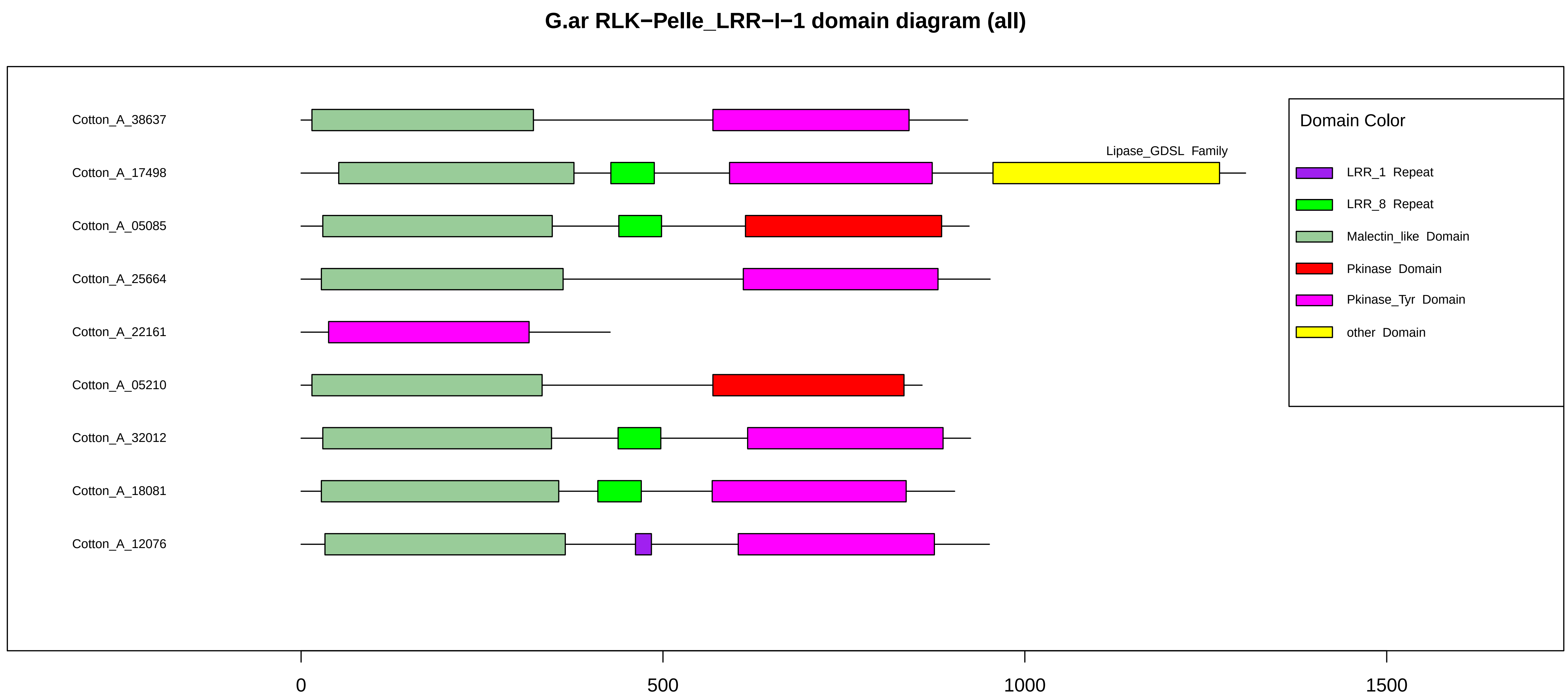
G.ar RLK−Pelle_LRR−I−1 domain diagram (all)
Cotton_A_38637
Cotton_A_17498
Cotton_A_05085
Cotton_A_25664
Cotton_A_22161
Cotton_A_05210
Cotton_A_32012
Cotton_A_18081
Cotton_A_12076
Lipase_GDSL Family
Domain Color
LRR_1 Repeat
LRR_8 Repeat
Malectin_like Domain
Pkinase Domain
Pkinase_Tyr Domain
other Domain
0
500
1000
1500

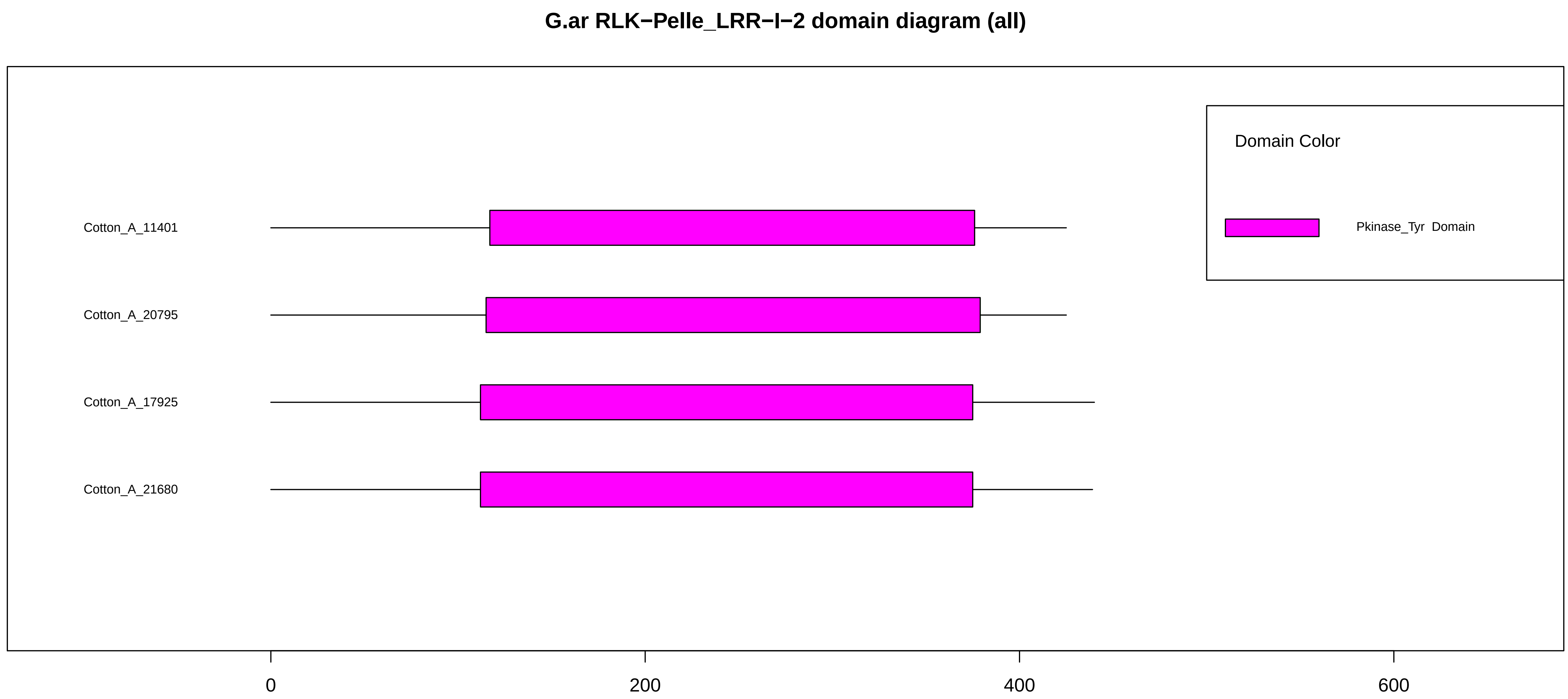
G.ar RLK−Pelle_LRR−I−2 domain diagram (all)
Domain Color
Pkinase_Tyr Domain
Cotton_A_11401
Cotton_A_20795
Cotton_A_17925
Cotton_A_21680
0
200
400
600

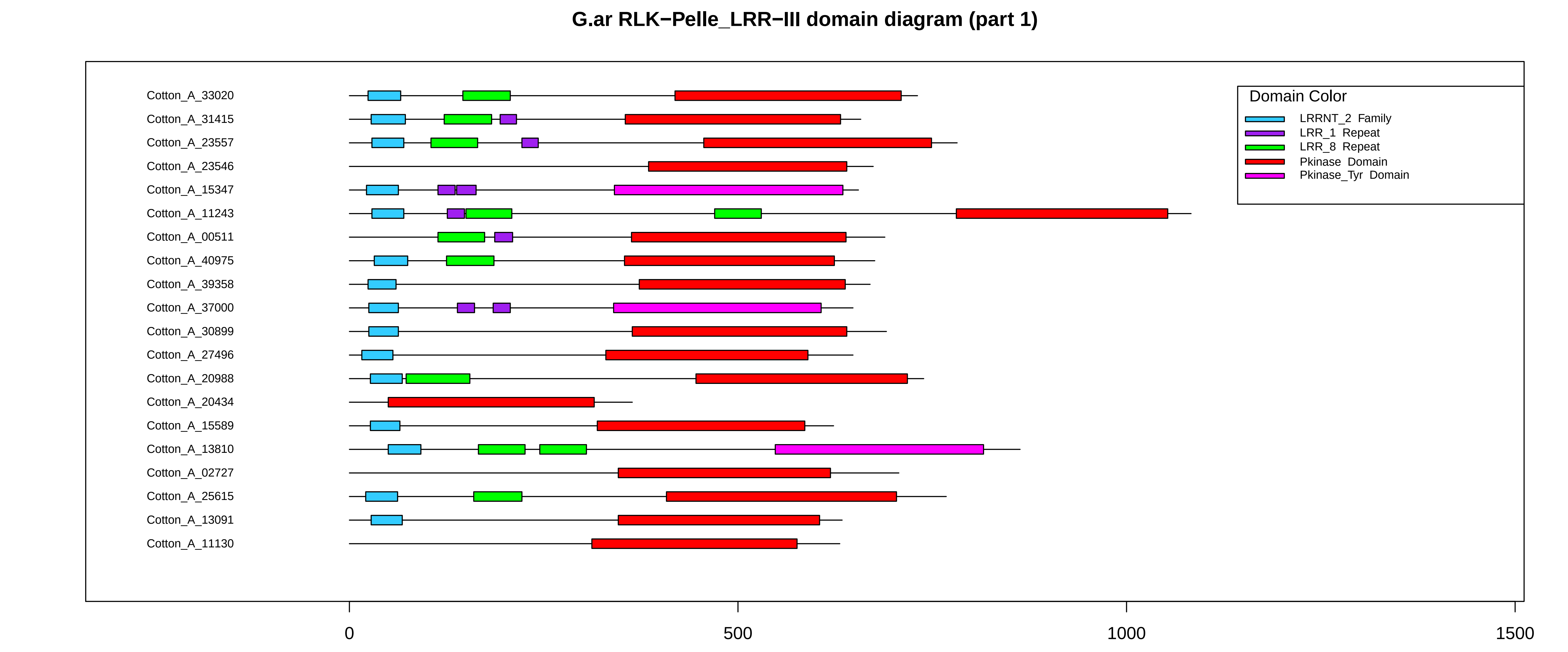
G.ar RLK−Pelle_LRR−III domain diagram (part 1)
Cotton_A_33020
Cotton_A_31415
Cotton_A_23557
Cotton_A_23546
Cotton_A_15347
Cotton_A_11243
Cotton_A_00511
Cotton_A_40975
Cotton_A_39358
Cotton_A_37000
Cotton_A_30899
Cotton_A_27496
Cotton_A_20988
Cotton_A_20434
Cotton_A_15589
Cotton_A_13810
Cotton_A_02727
Cotton_A_25615
Cotton_A_13091
Cotton_A_11130
Domain Color
LRRNT_2 Family
LRR_1 Repeat
LRR_8 Repeat
Pkinase Domain
Pkinase_Tyr Domain
0
500
1000
1500

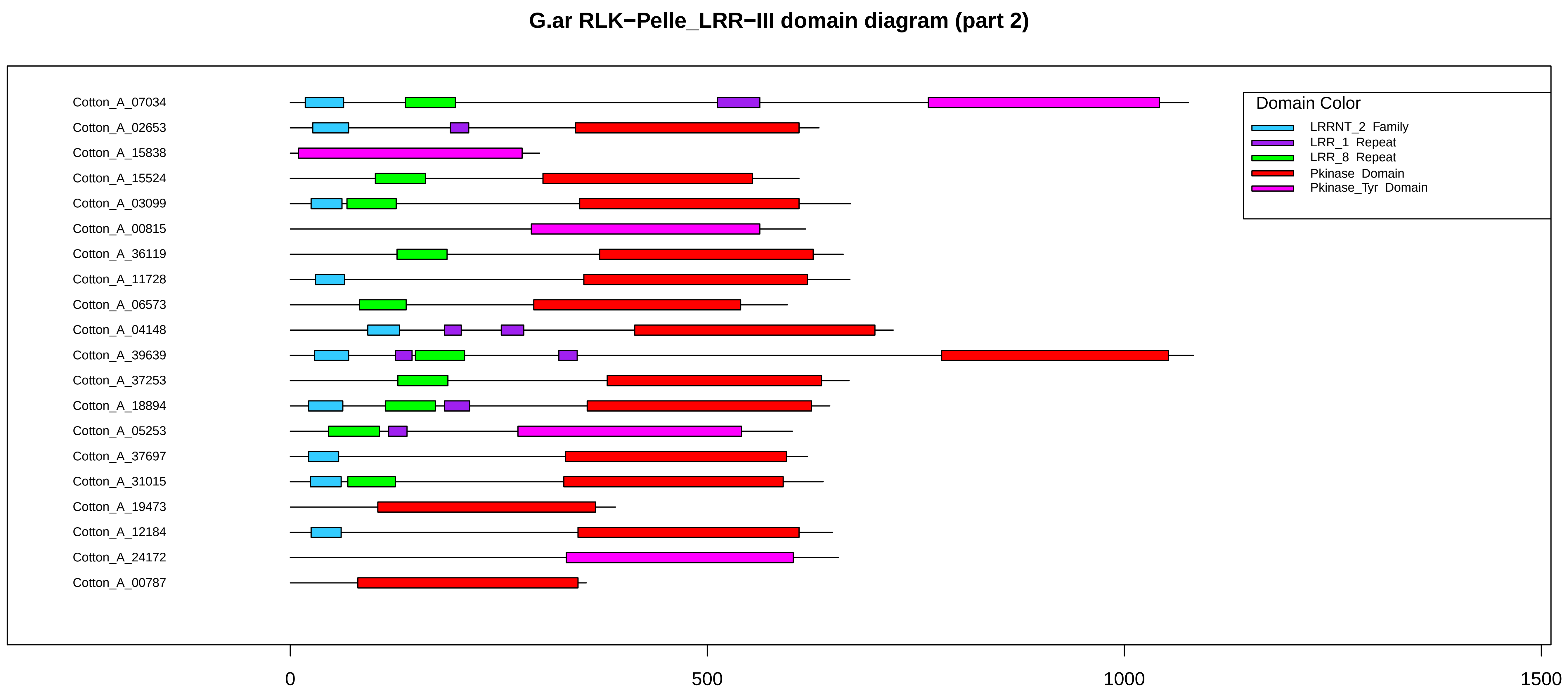
G.ar RLK−Pelle_LRR−III domain diagram (part 2)
Cotton_A_07034
Cotton_A_02653
Cotton_A_15838
Cotton_A_15524
Cotton_A_03099
Cotton_A_00815
Cotton_A_36119
Cotton_A_11728
Cotton_A_06573
Cotton_A_04148
Cotton_A_39639
Cotton_A_37253
Cotton_A_18894
Cotton_A_05253
Cotton_A_37697
Cotton_A_31015
Cotton_A_19473
Cotton_A_12184
Cotton_A_24172
Cotton_A_00787
Domain Color
LRRNT_2 Family
LRR_1 Repeat
LRR_8 Repeat
Pkinase Domain
Pkinase_Tyr Domain
0
500
1000
1500

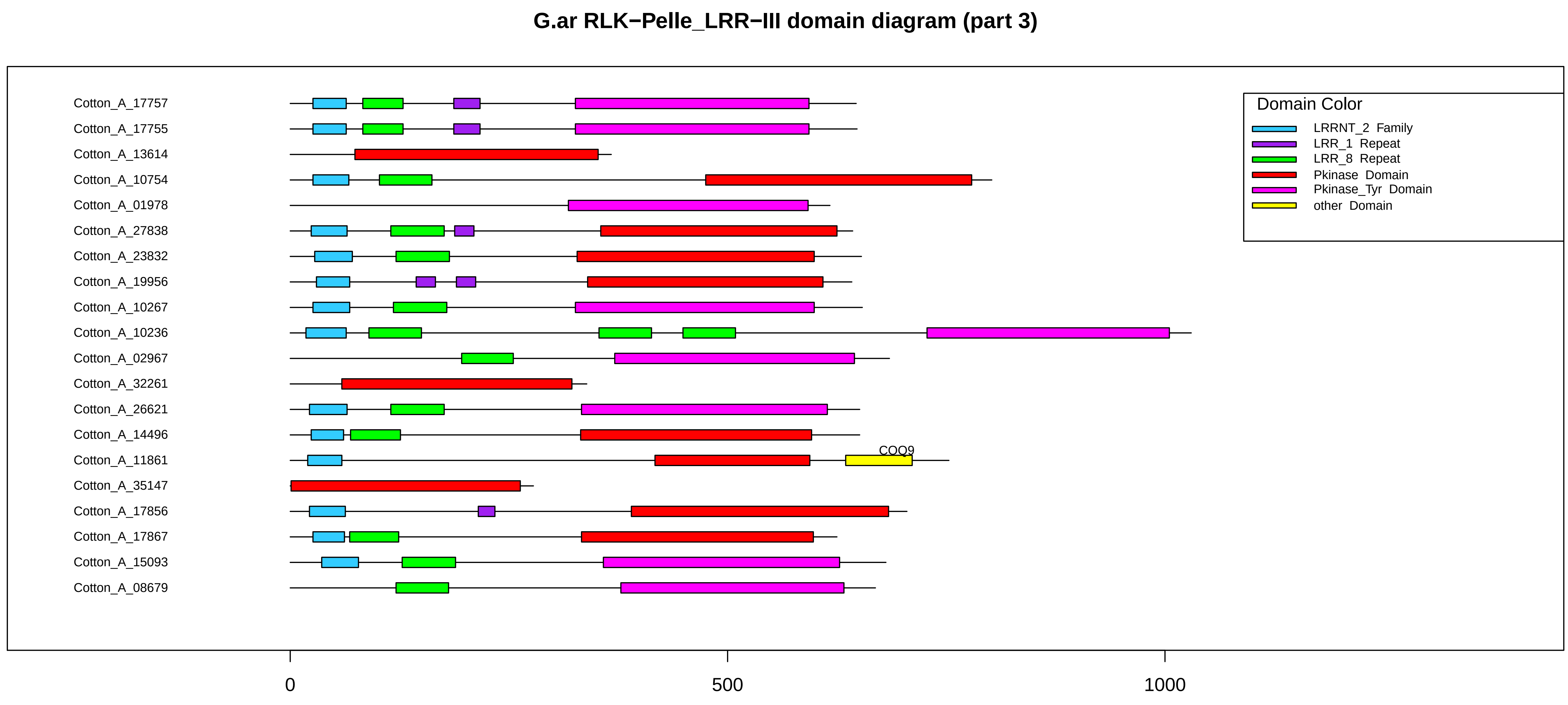
G.ar RLK−Pelle_LRR−III domain diagram (part 3)
Cotton_A_17757
Cotton_A_17755
Cotton_A_13614
Cotton_A_10754
Cotton_A_01978
Cotton_A_27838
Cotton_A_23832
Cotton_A_19956
Cotton_A_10267
Cotton_A_10236
Cotton_A_02967
Cotton_A_32261
Cotton_A_26621
Cotton_A_14496
Cotton_A_11861
Cotton_A_35147
Cotton_A_17856
Cotton_A_17867
Cotton_A_15093
Cotton_A_08679
COQ9
Domain Color
LRRNT_2 Family
LRR_1 Repeat
LRR_8 Repeat
Pkinase Domain
Pkinase_Tyr Domain
other Domain
0
500
1000

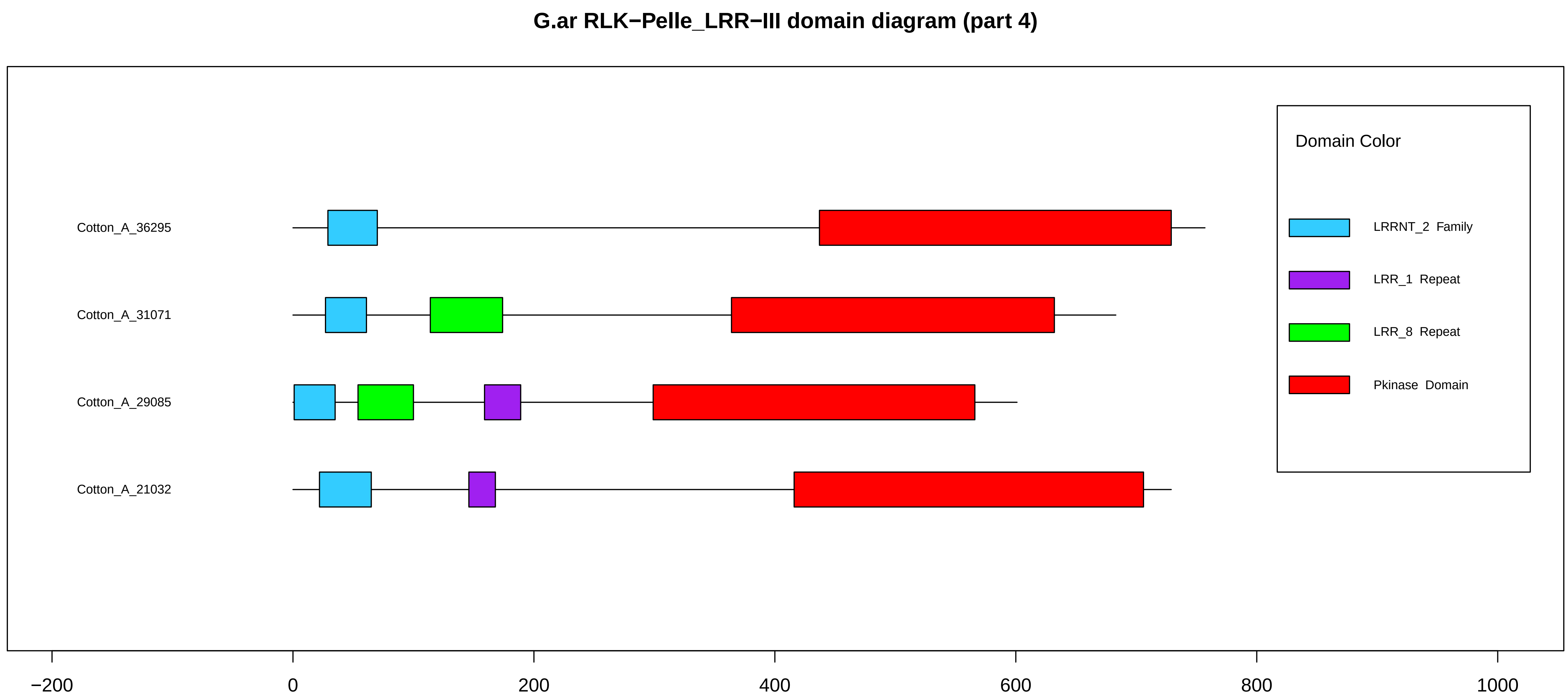
G.ar RLK−Pelle_LRR−III domain diagram (part 4)
Cotton_A_36295
Cotton_A_31071
Cotton_A_29085
Cotton_A_21032
Domain Color
LRRNT_2 Family
LRR_1 Repeat
LRR_8 Repeat
Pkinase Domain
−200
0
200
400
600
800
1000

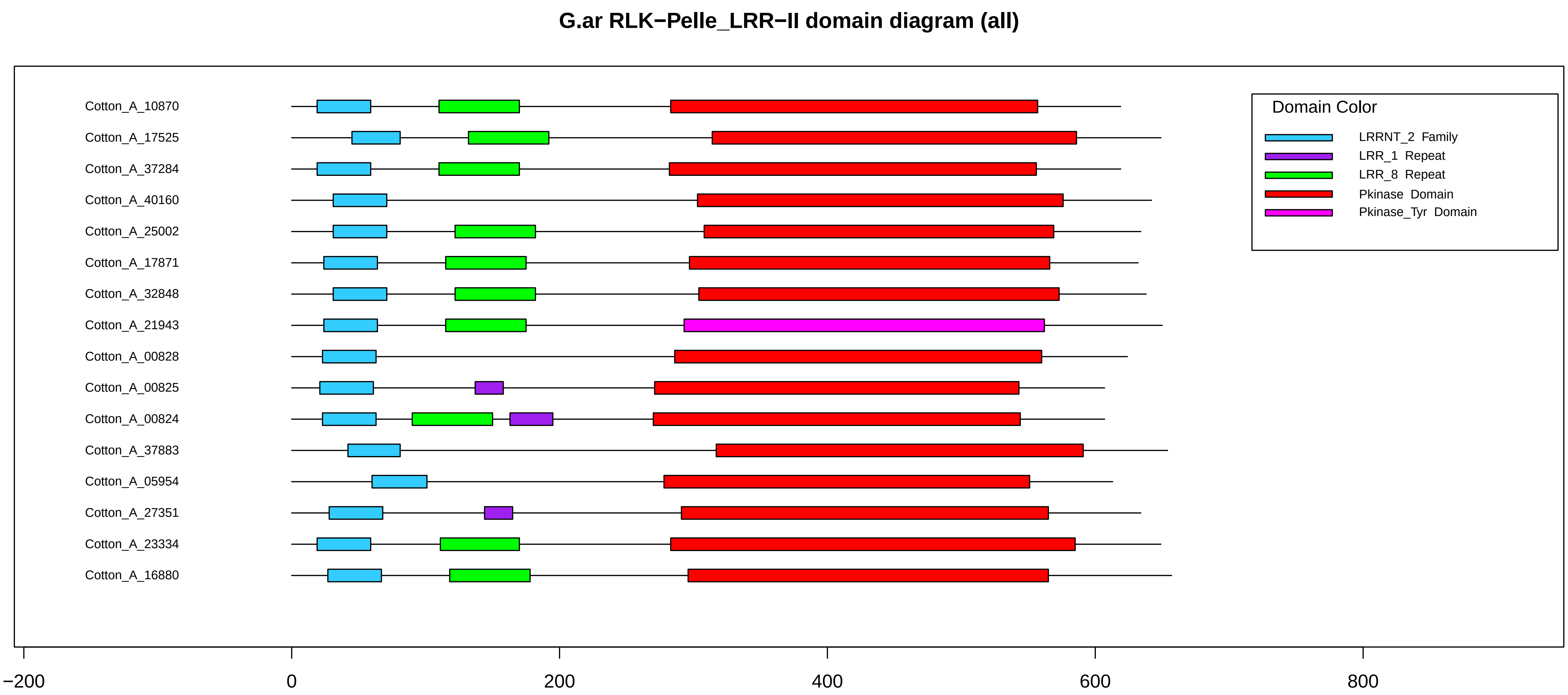
G.ar RLK−Pelle_LRR−II domain diagram (all)
Cotton_A_10870
Cotton_A_17525
Cotton_A_37284
Cotton_A_40160
Cotton_A_25002
Cotton_A_17871
Cotton_A_32848
Cotton_A_21943
Cotton_A_00828
Cotton_A_00825
Cotton_A_00824
Cotton_A_37883
Cotton_A_05954
Cotton_A_27351
Cotton_A_23334
Cotton_A_16880
Domain Color
LRRNT_2 Family
LRR_1 Repeat
LRR_8 Repeat
Pkinase Domain
Pkinase_Tyr Domain
−200
0
200
400
600
800

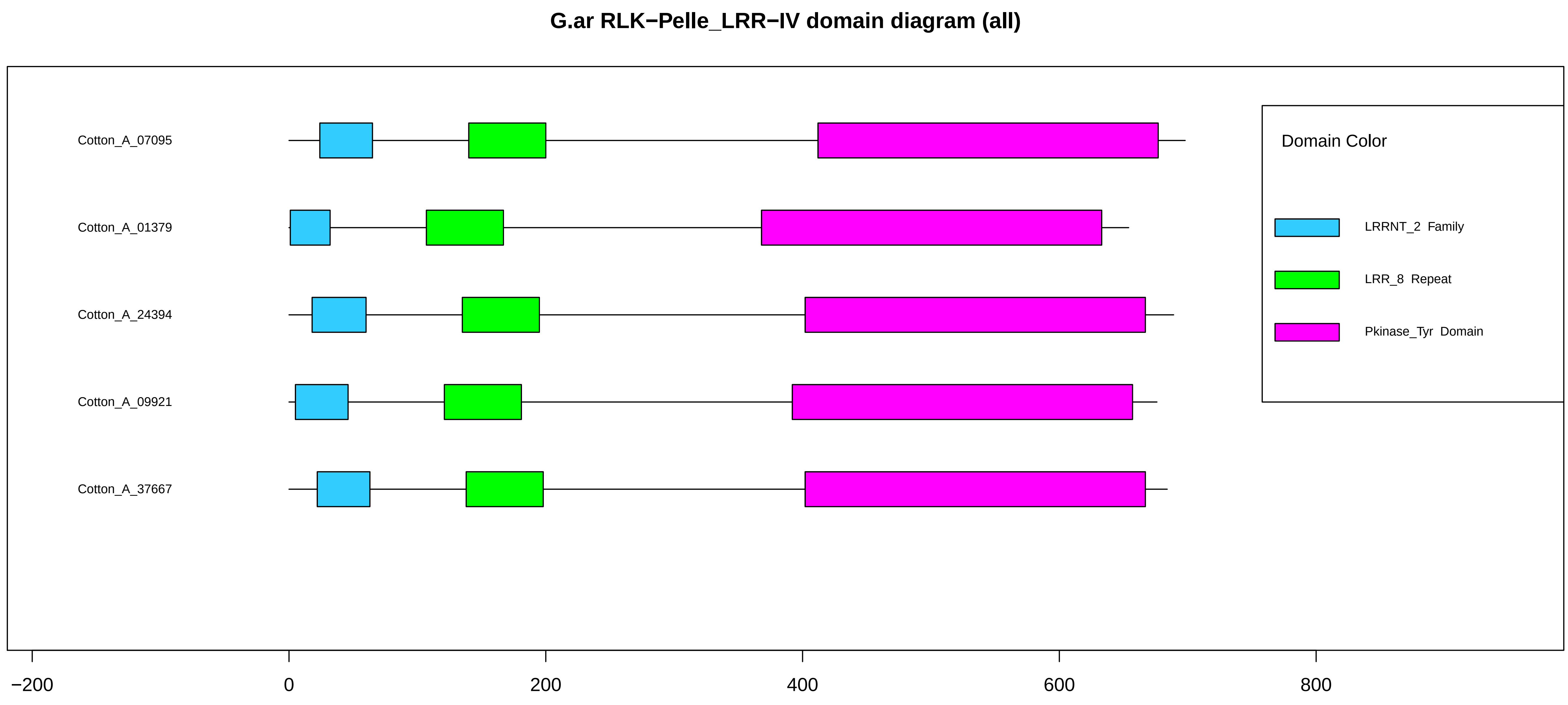
G.ar RLK−Pelle_LRR−IV domain diagram (all)
Cotton_A_07095
Cotton_A_01379
Cotton_A_24394
Cotton_A_09921
Cotton_A_37667
Domain Color
LRRNT_2 Family
LRR_8 Repeat
Pkinase_Tyr Domain
−200
0
200
400
600
800

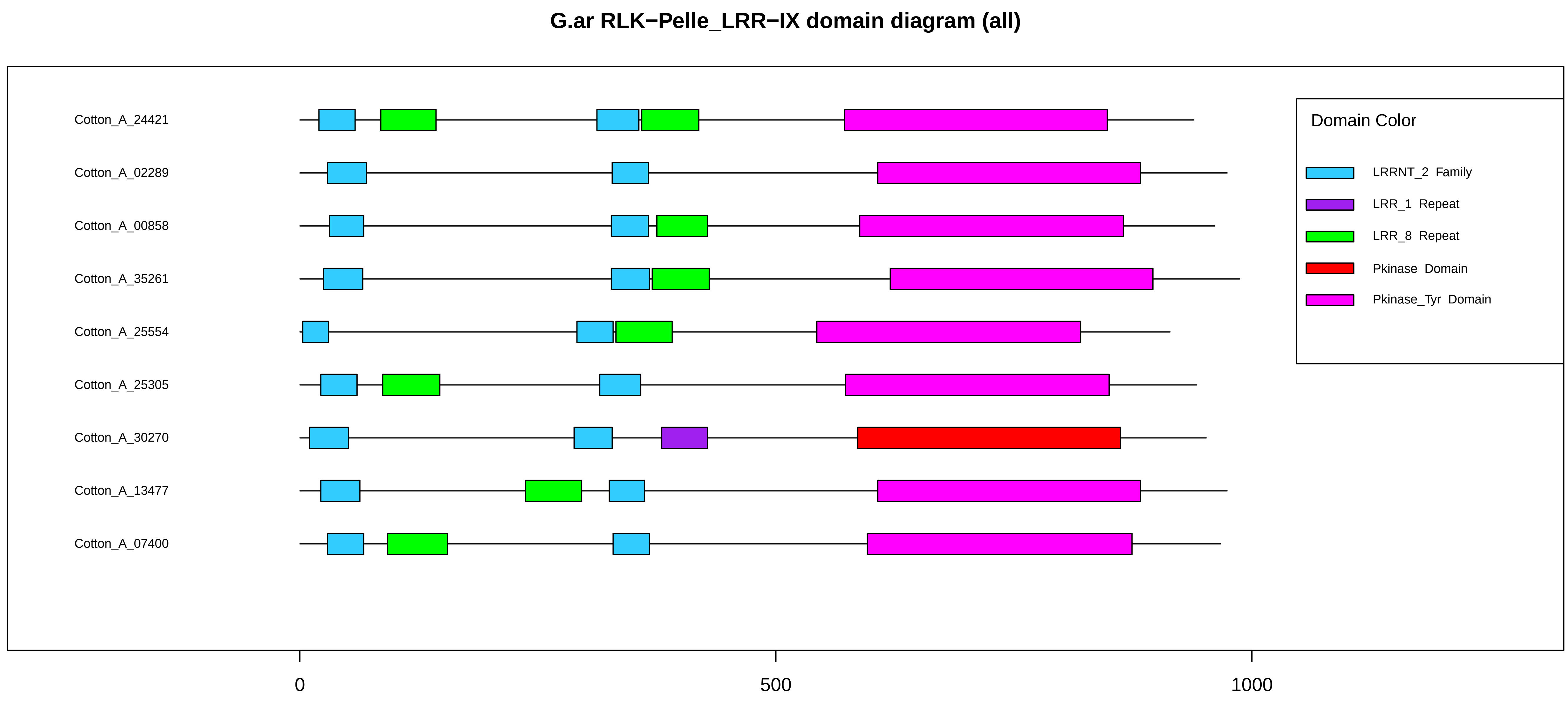

G.ar RLK−Pelle_LRR−IX domain diagram (all)
Cotton_A_24421
Cotton_A_02289
Cotton_A_00858
Cotton_A_35261
Cotton_A_25554
Cotton_A_25305
Cotton_A_30270
Cotton_A_13477
Cotton_A_07400
Domain Color
LRRNT_2 Family
LRR_1 Repeat
LRR_8 Repeat
Pkinase Domain
Pkinase_Tyr Domain
0
500
1000

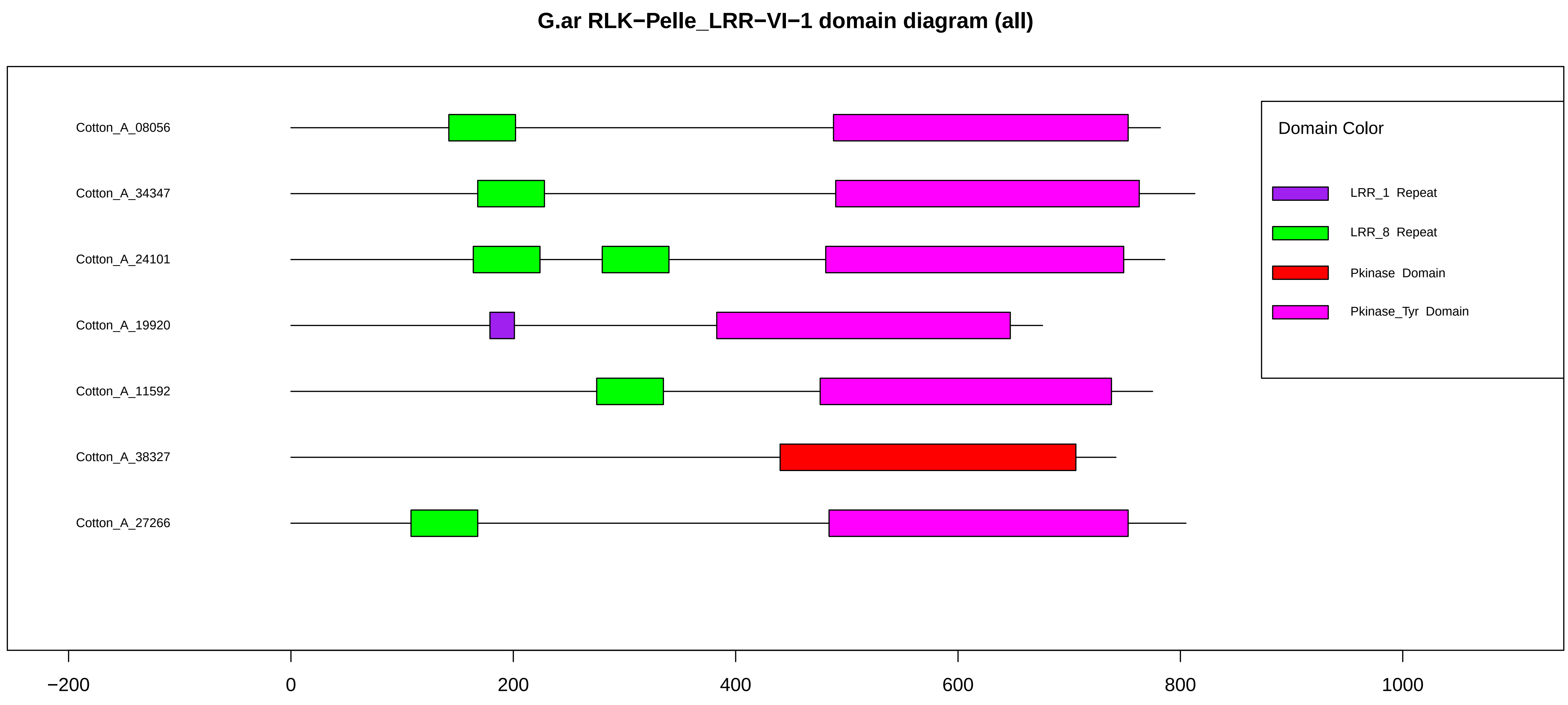

G.ar RLK−Pelle_LRR−VI−1 domain diagram (all)
Cotton_A_08056
Cotton_A_34347
Cotton_A_24101
Cotton_A_19920
Cotton_A_11592
Cotton_A_38327
Cotton_A_27266
Domain Color
LRR_1 Repeat
LRR_8 Repeat
Pkinase Domain
Pkinase_Tyr Domain
−200
0
200
400
600
800
1000

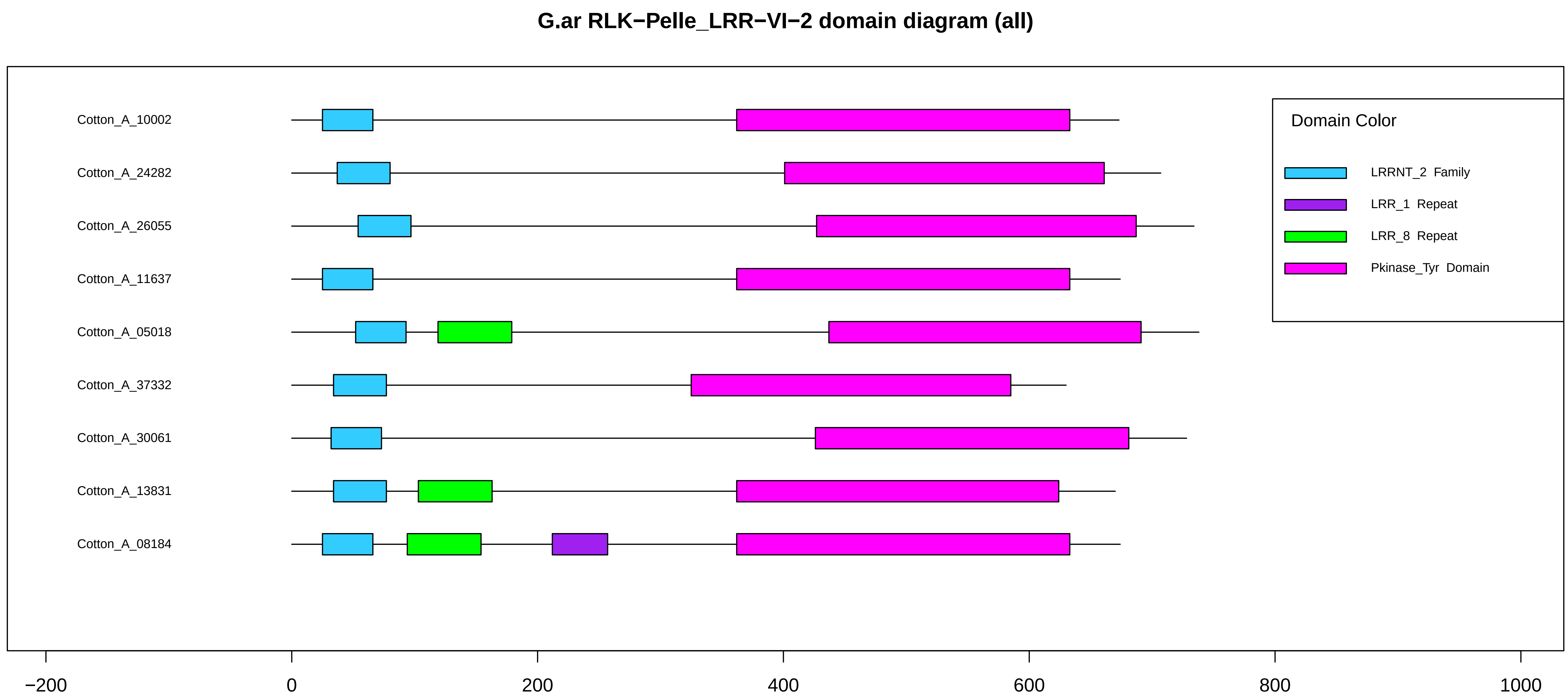

G.ar RLK−Pelle_LRR−VI−2 domain diagram (all)
Cotton_A_10002
Cotton_A_24282
Cotton_A_26055
Cotton_A_11637
Cotton_A_05018
Cotton_A_37332
Cotton_A_30061
Cotton_A_13831
Cotton_A_08184
Domain Color
LRRNT_2 Family
LRR_1 Repeat
LRR_8 Repeat
Pkinase_Tyr Domain
−200
0
200
400
600
800
1000

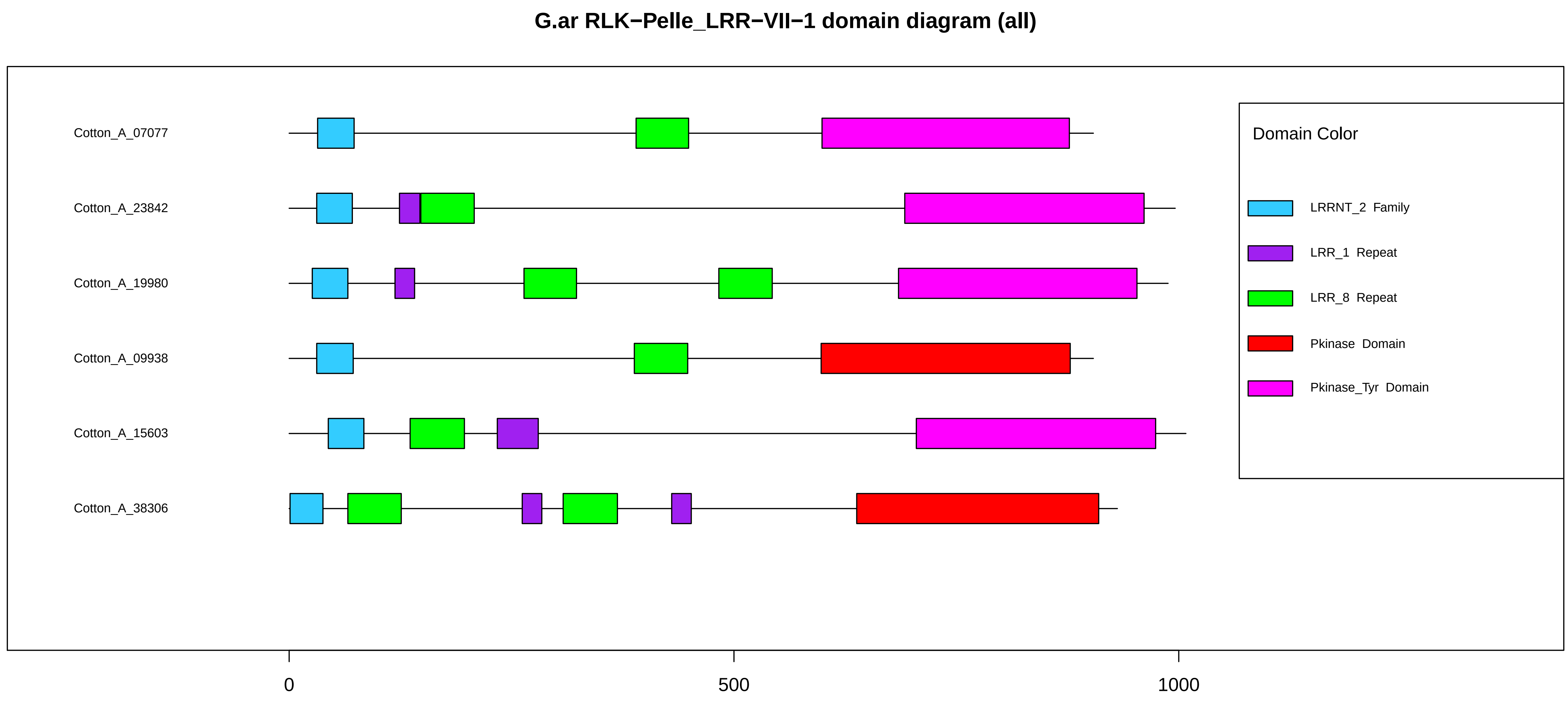
G.ar RLK−Pelle_LRR−VII−1 domain diagram (all)
Cotton_A_07077
Cotton_A_23842
Cotton_A_19980
Cotton_A_09938
Cotton_A_15603
Cotton_A_38306
Domain Color
LRRNT_2 Family
LRR_1 Repeat
LRR_8 Repeat
Pkinase Domain
Pkinase_Tyr Domain
0
500
1000

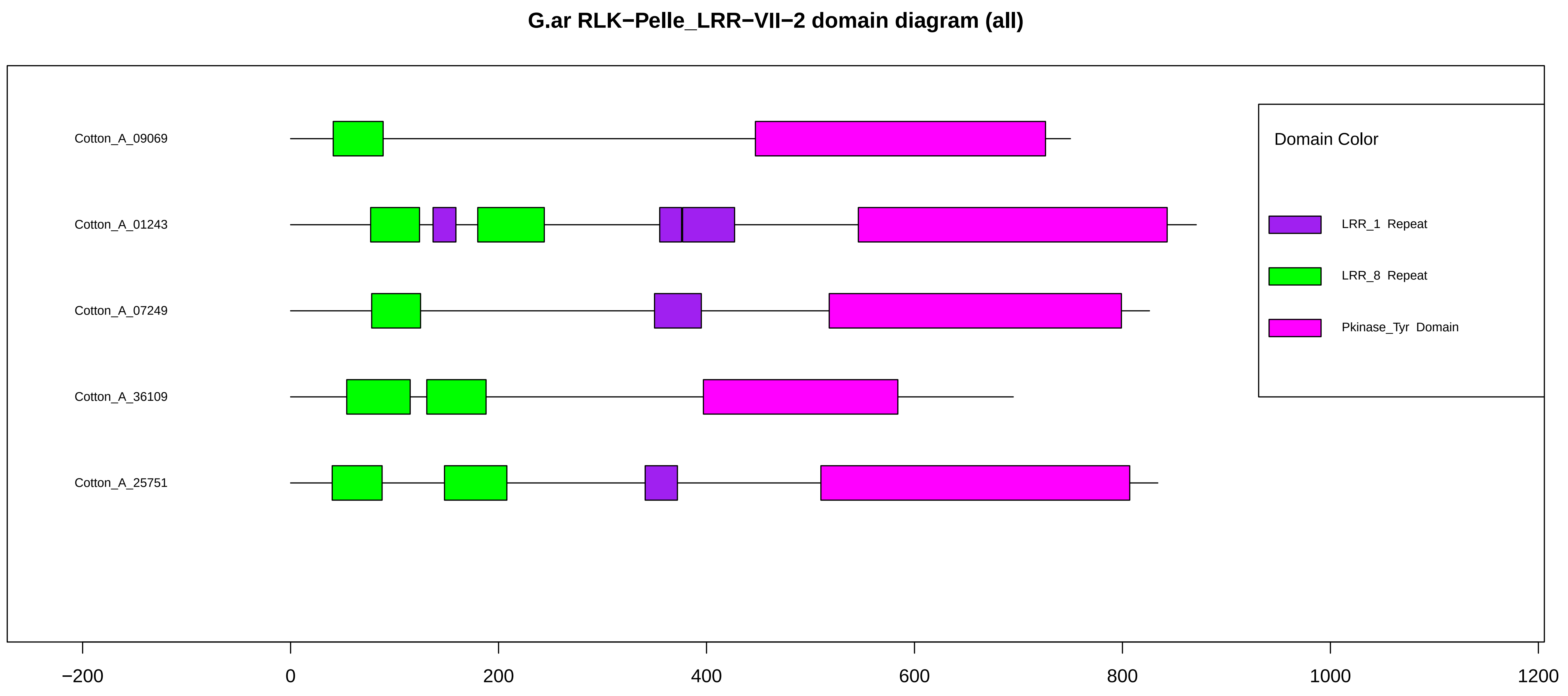
G.ar RLK−Pelle_LRR−VII−2 domain diagram (all)
Cotton_A_09069
Cotton_A_01243
Cotton_A_07249
Cotton_A_36109
Cotton_A_25751
Domain Color
LRR_1 Repeat
LRR_8 Repeat
Pkinase_Tyr Domain
−200
0
200
400
600
800
1000
1200

G.ar RLK−Pelle_LRR−VII−3 domain diagram (all)

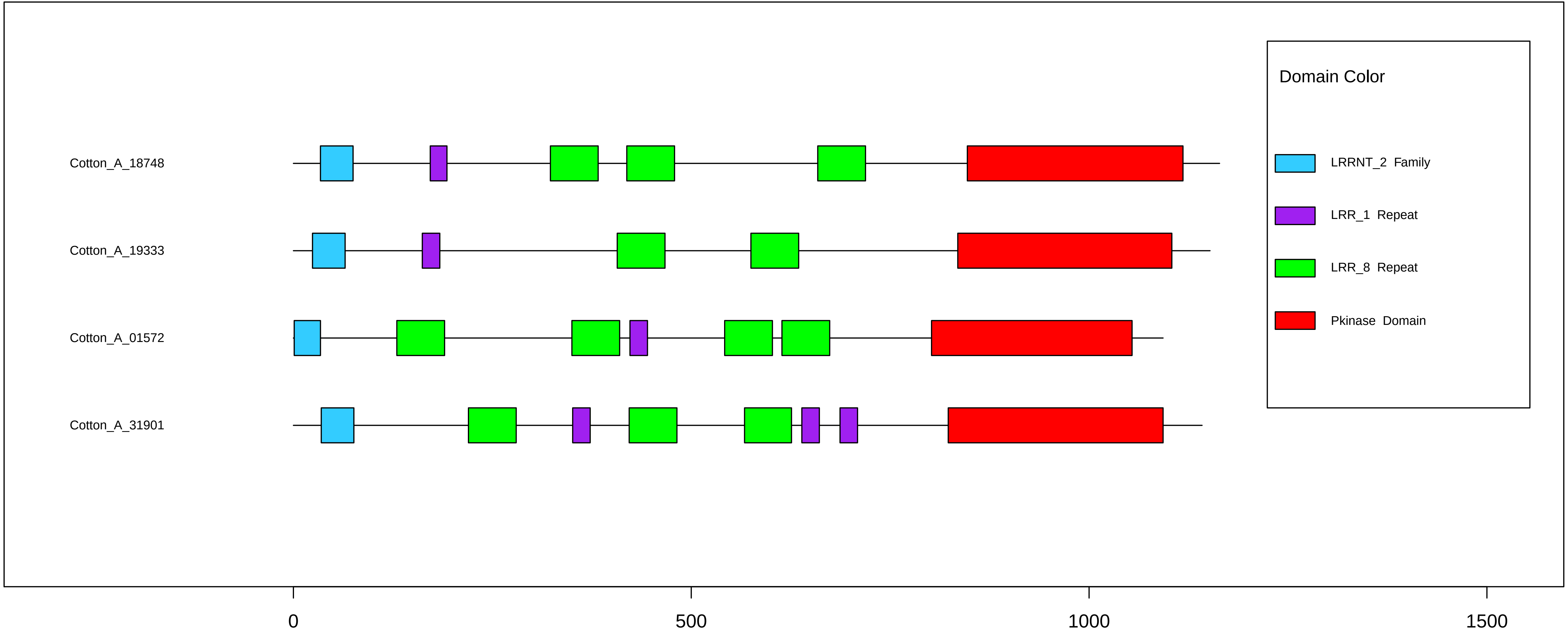

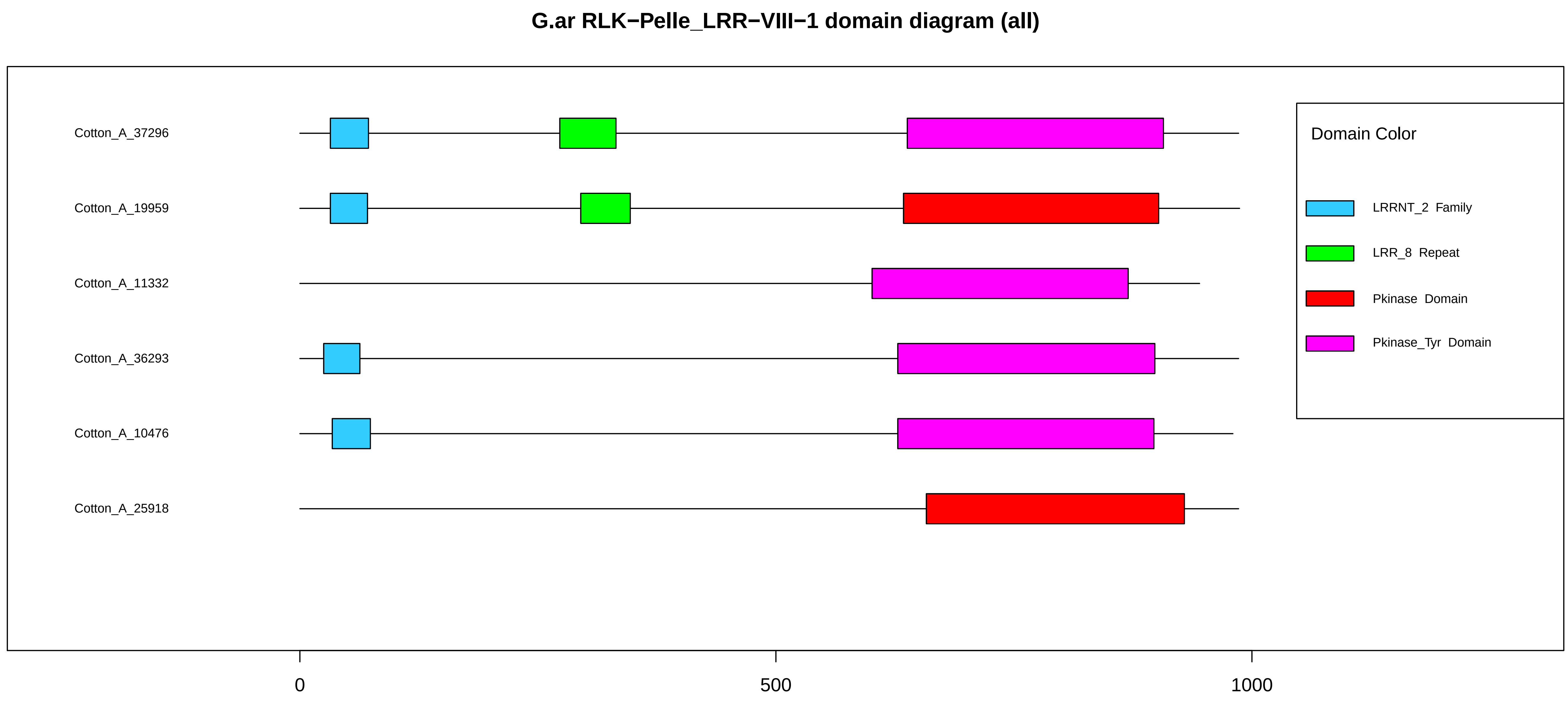
G.ar RLK−Pelle_LRR−VIII−1 domain diagram (all)
Cotton_A_37296
Cotton_A_19959
Cotton_A_11332
Cotton_A_36293
Cotton_A_10476
Cotton_A_25918
Domain Color
LRRNT_2 Family
LRR_8 Repeat
Pkinase Domain
Pkinase_Tyr Domain
0
500
1000

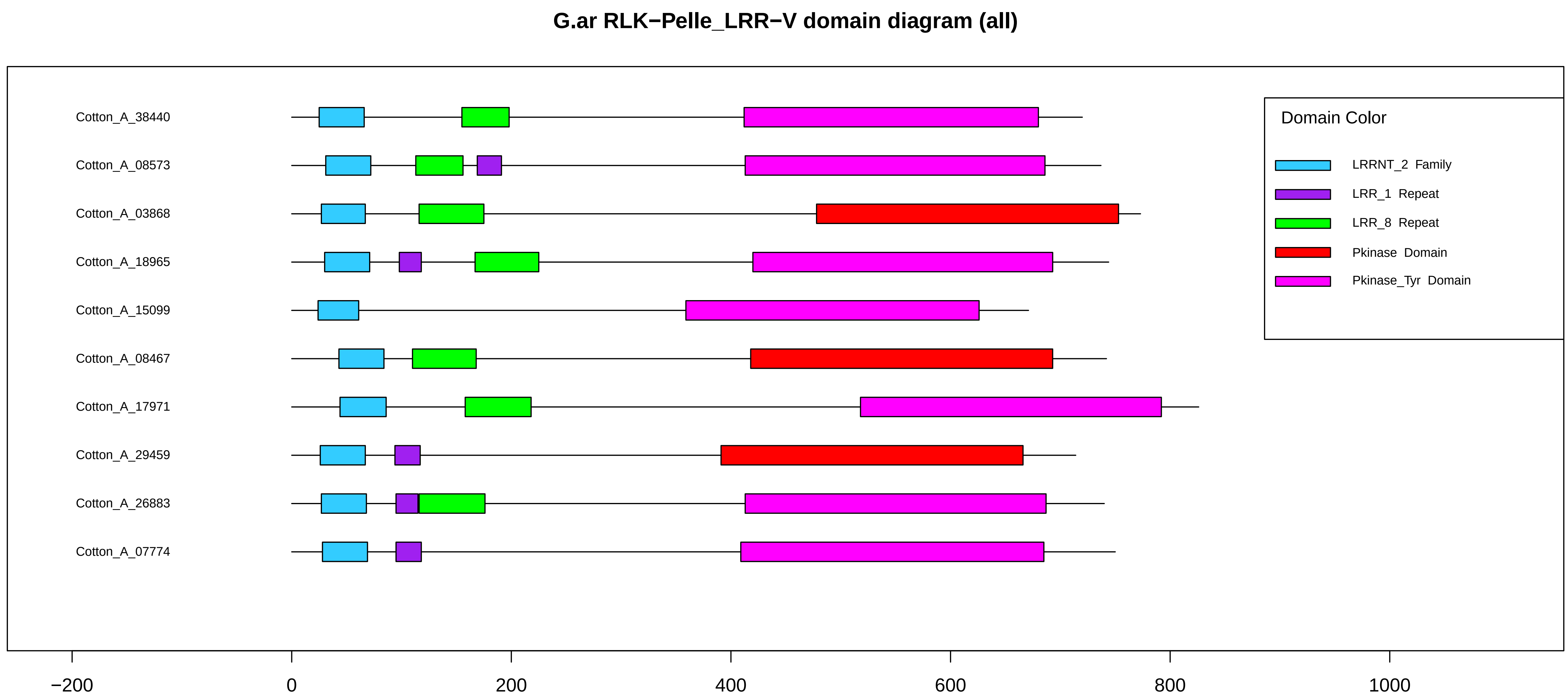

G.ar RLK−Pelle_LRR−V domain diagram (all)
Cotton_A_38440
Cotton_A_08573
Cotton_A_03868
Cotton_A_18965
Cotton_A_15099
Cotton_A_08467
Cotton_A_17971
Cotton_A_29459
Cotton_A_26883
Cotton_A_07774
Domain Color
LRRNT_2 Family
LRR_1 Repeat
LRR_8 Repeat
Pkinase Domain
Pkinase_Tyr Domain
−200
0
200
400
600
800
1000

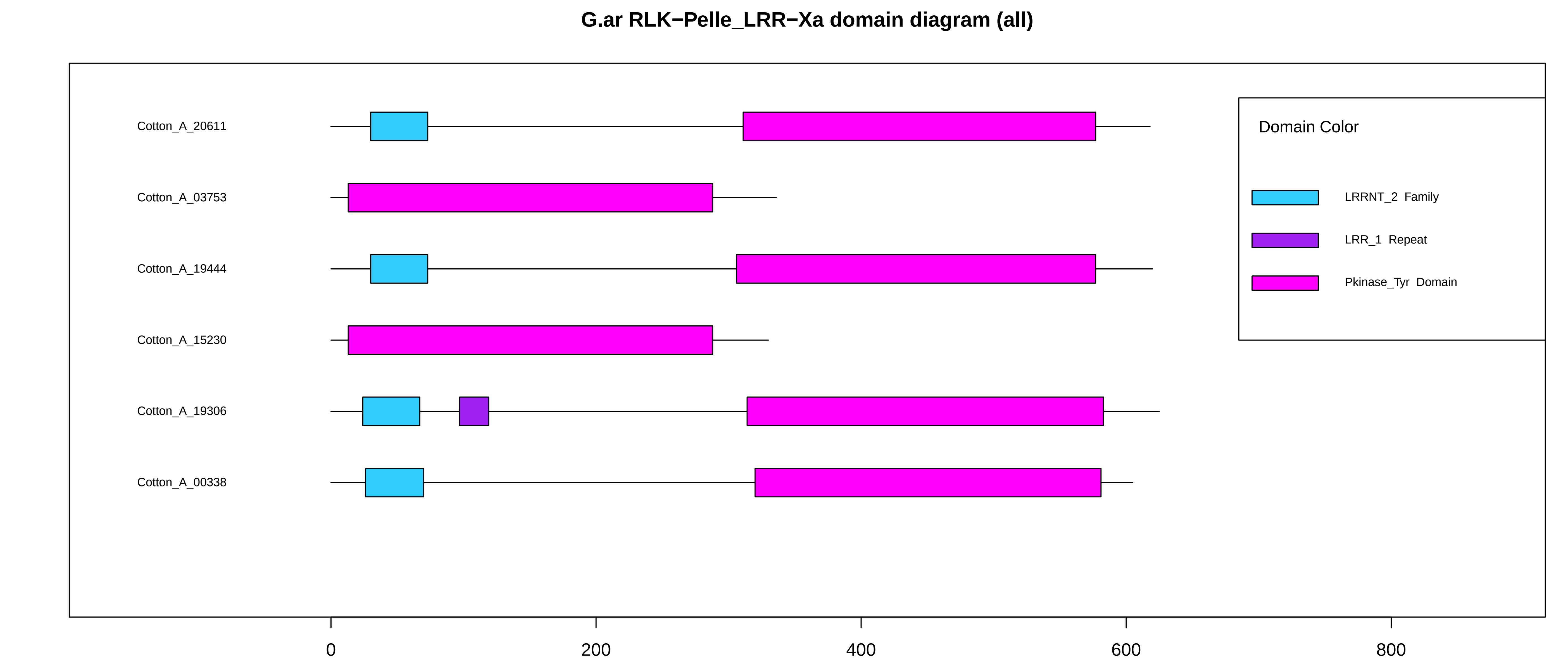

G.ar RLK−Pelle_LRR−Xa domain diagram (all)
Cotton_A_20611
Cotton_A_03753
Cotton_A_19444
Cotton_A_15230
Cotton_A_19306
Cotton_A_00338
Domain Color
LRRNT_2 Family
LRR_1 Repeat
Pkinase_Tyr Domain
0
200
400
600
800

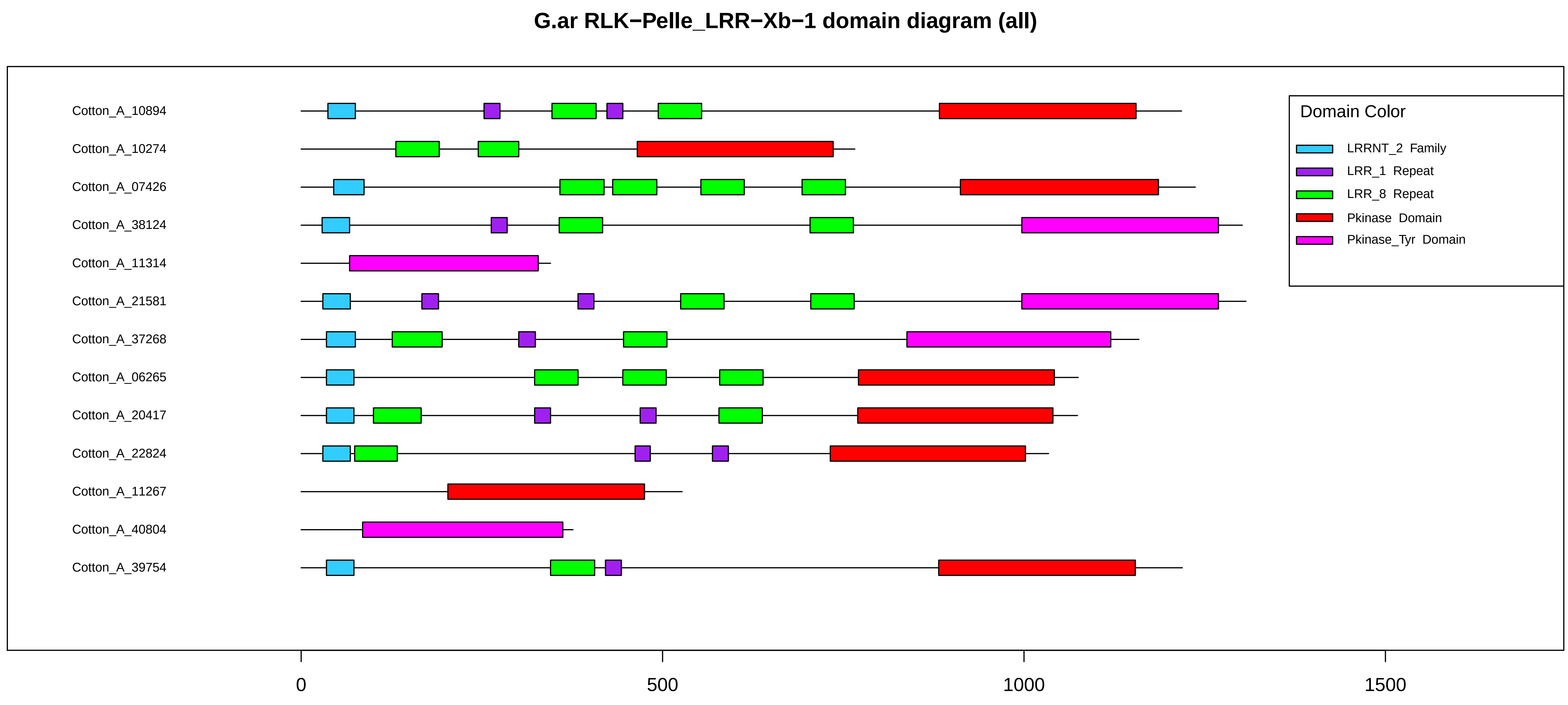

G.ar RLK−Pelle_LRR−Xb−1 domain diagram (all)
Cotton_A_10894
Cotton_A_10274
Cotton_A_07426
Cotton_A_38124
Cotton_A_11314
Cotton_A_21581
Cotton_A_37268
Cotton_A_06265
Cotton_A_20417
Cotton_A_22824
Cotton_A_11267
Cotton_A_40804
Cotton_A_39754
Domain Color
LRRNT_2 Family
LRR_1 Repeat
LRR_8 Repeat
Pkinase Domain
Pkinase_Tyr Domain
0
500
1000
1500

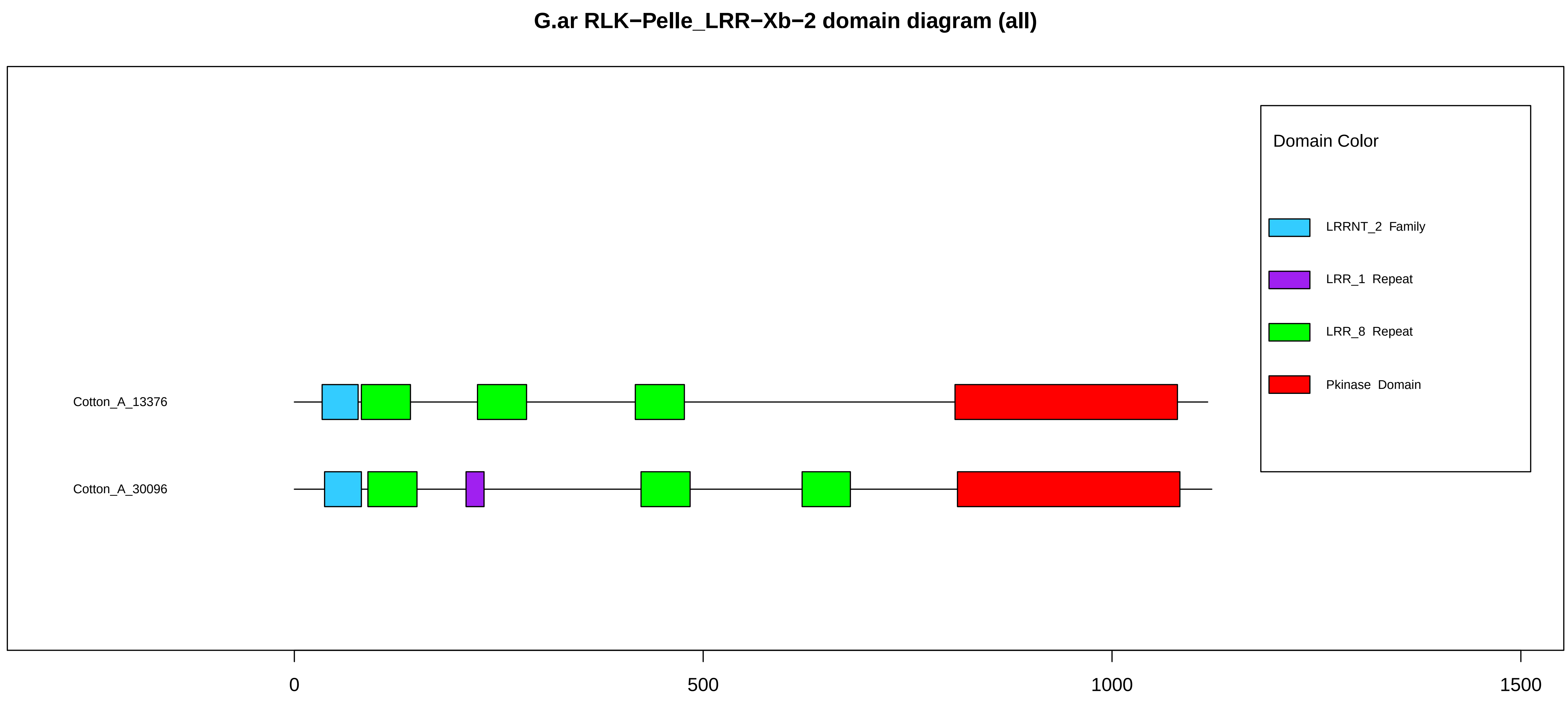

G.ar RLK−Pelle_LRR−Xb−2 domain diagram (all)
Domain Color
LRRNT_2 Family
LRR_1 Repeat
LRR_8 Repeat
Pkinase Domain
Cotton_A_13376
Cotton_A_30096
0
500
1000
1500

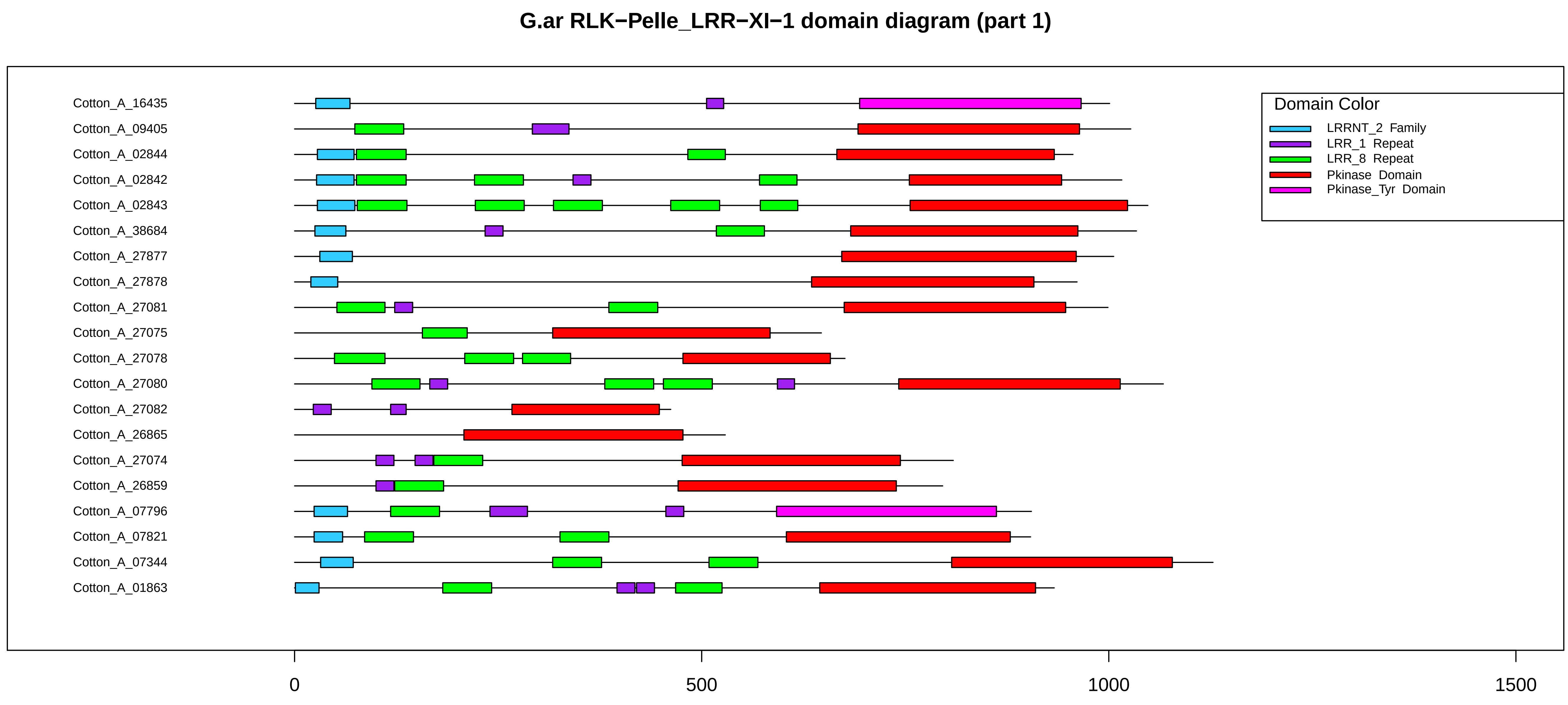
G.ar RLK−Pelle_LRR−XI−1 domain diagram (part 1)
Cotton_A_16435
Cotton_A_09405
Cotton_A_02844
Cotton_A_02842
Cotton_A_02843
Cotton_A_38684
Cotton_A_27877
Cotton_A_27878
Cotton_A_27081
Cotton_A_27075
Cotton_A_27078
Cotton_A_27080
Cotton_A_27082
Cotton_A_26865
Cotton_A_27074
Cotton_A_26859
Cotton_A_07796
Cotton_A_07821
Cotton_A_07344
Cotton_A_01863
Domain Color
LRRNT_2 Family
LRR_1 Repeat
LRR_8 Repeat
Pkinase Domain
Pkinase_Tyr Domain
0
500
1000
1500

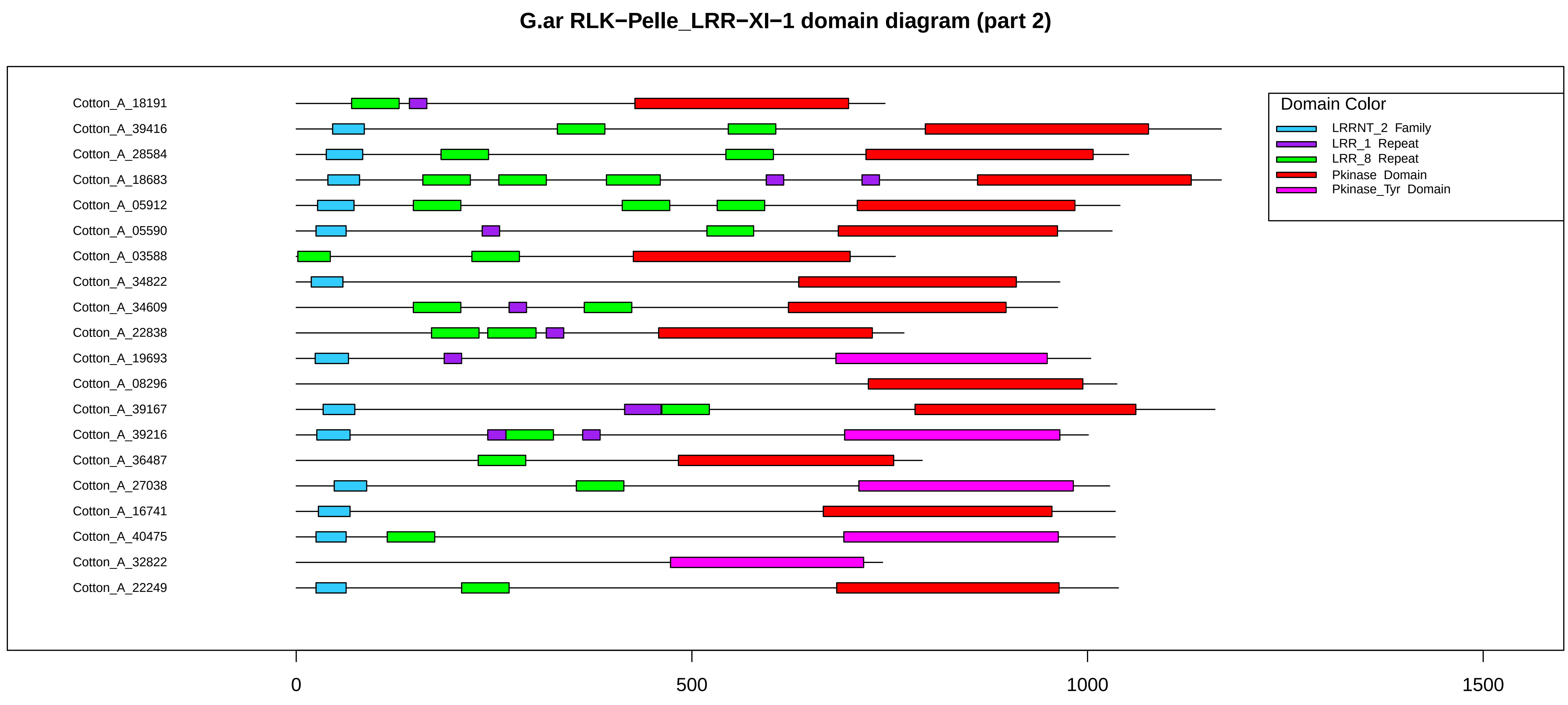
G.ar RLK−Pelle_LRR−XI−1 domain diagram (part 2)
Cotton_A_18191
Cotton_A_39416
Cotton_A_28584
Cotton_A_18683
Cotton_A_05912
Cotton_A_05590
Cotton_A_03588
Cotton_A_34822
Cotton_A_34609
Cotton_A_22838
Cotton_A_19693
Cotton_A_08296
Cotton_A_39167
Cotton_A_39216
Cotton_A_36487
Cotton_A_27038
Cotton_A_16741
Cotton_A_40475
Cotton_A_32822
Cotton_A_22249
Domain Color
LRRNT_2 Family
LRR_1 Repeat
LRR_8 Repeat
Pkinase Domain
Pkinase_Tyr Domain
0
500
1000
1500

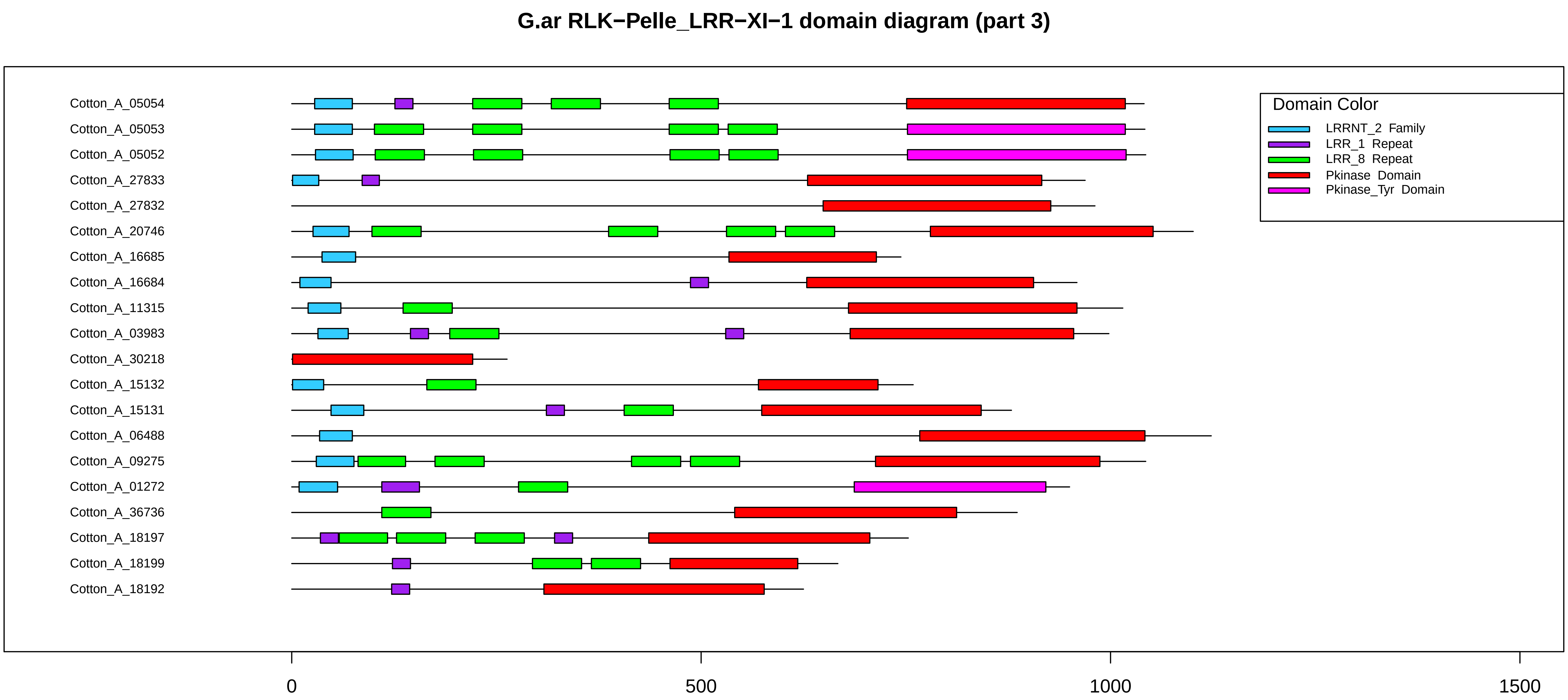
G.ar RLK−Pelle_LRR−XI−1 domain diagram (part 3)
Cotton_A_05054
Cotton_A_05053
Cotton_A_05052
Cotton_A_27833
Cotton_A_27832
Cotton_A_20746
Cotton_A_16685
Cotton_A_16684
Cotton_A_11315
Cotton_A_03983
Cotton_A_30218
Cotton_A_15132
Cotton_A_15131
Cotton_A_06488
Cotton_A_09275
Cotton_A_01272
Cotton_A_36736
Cotton_A_18197
Cotton_A_18199
Cotton_A_18192
Domain Color
LRRNT_2 Family
LRR_1 Repeat
LRR_8 Repeat
Pkinase Domain
Pkinase_Tyr Domain
0
500
1000
1500

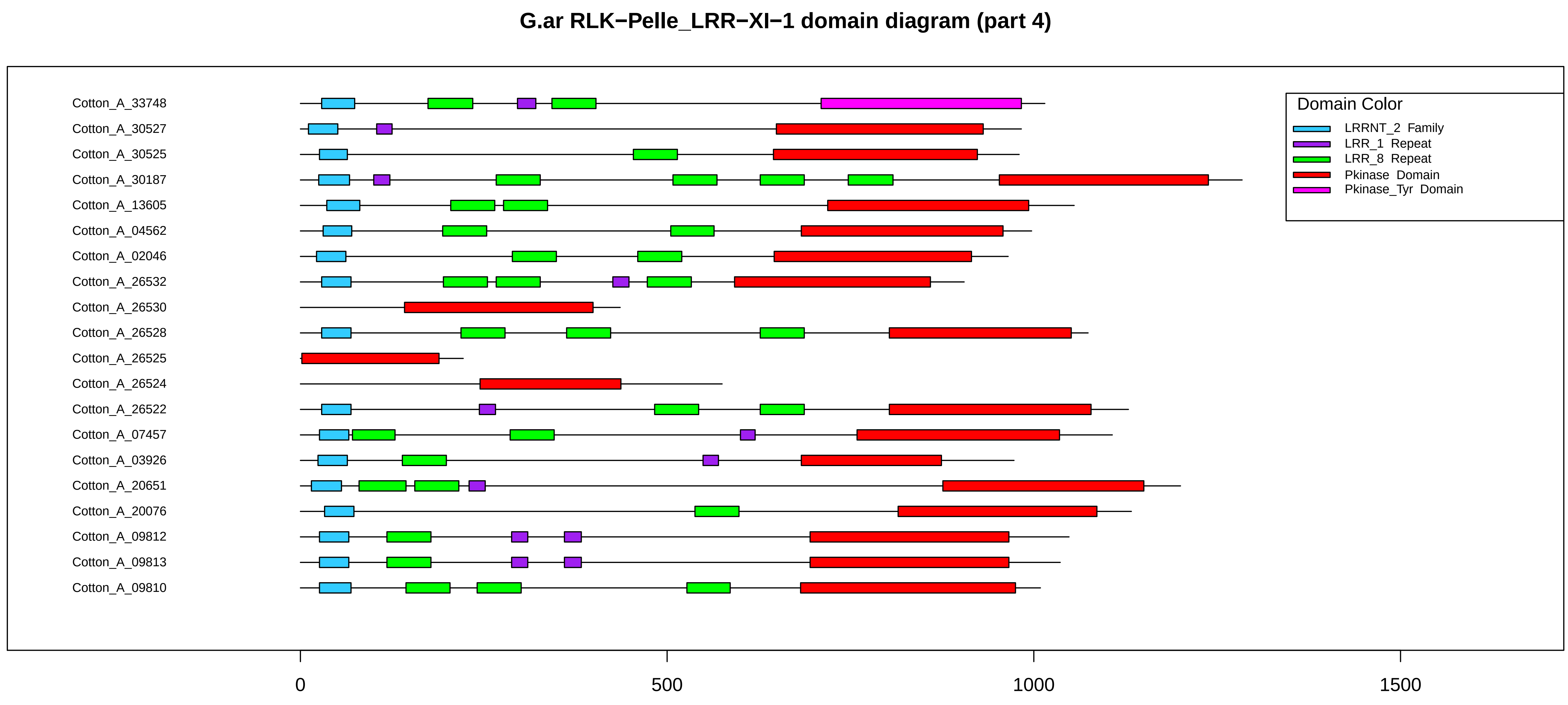

G.ar RLK−Pelle_LRR−XI−1 domain diagram (part 4)
Cotton_A_33748
Cotton_A_30527
Cotton_A_30525
Cotton_A_30187
Cotton_A_13605
Cotton_A_04562
Cotton_A_02046
Cotton_A_26532
Cotton_A_26530
Cotton_A_26528
Cotton_A_26525
Cotton_A_26524
Cotton_A_26522
Cotton_A_07457
Cotton_A_03926
Cotton_A_20651
Cotton_A_20076
Cotton_A_09812
Cotton_A_09813
Cotton_A_09810
Domain Color
LRRNT_2 Family
LRR_1 Repeat
LRR_8 Repeat
Pkinase Domain
Pkinase_Tyr Domain
0
500
1000
1500

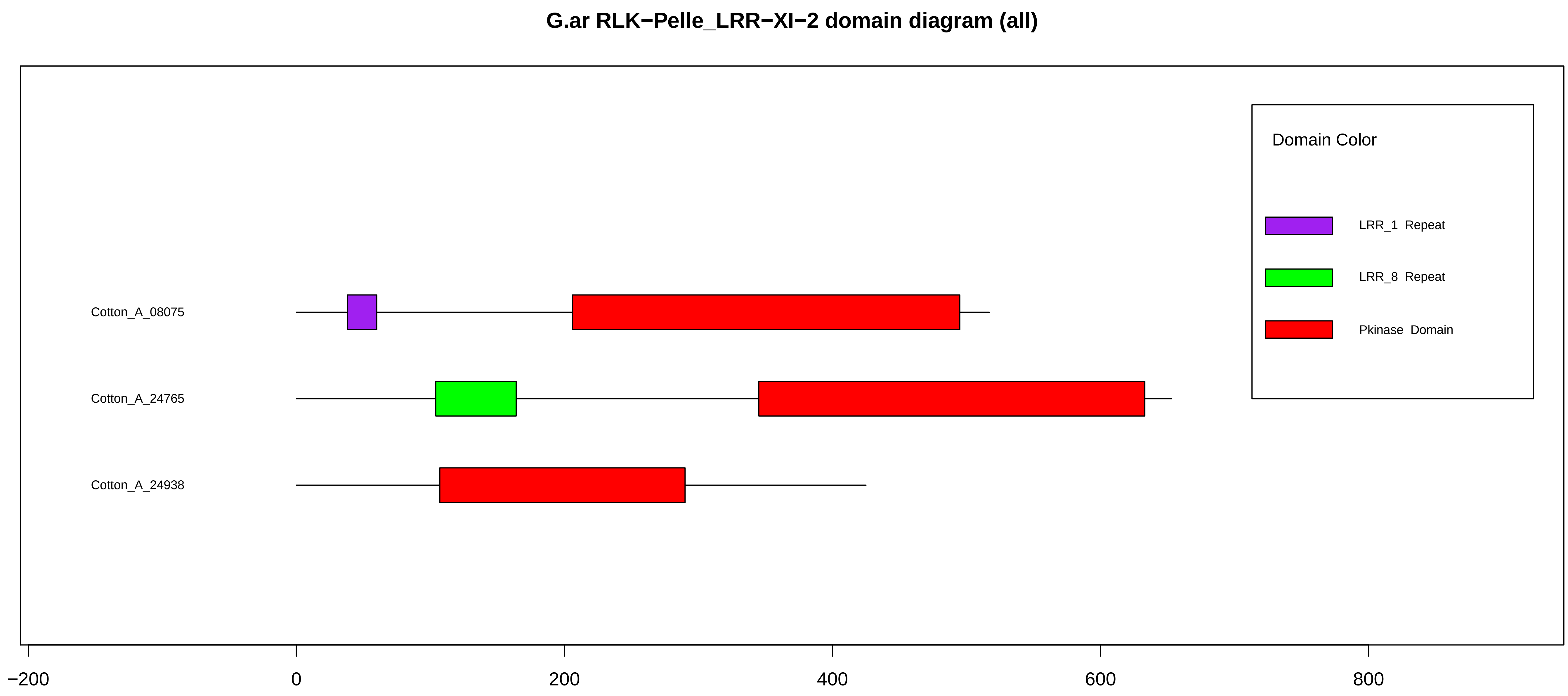
G.ar RLK−Pelle_LRR−XI−2 domain diagram (all)
Domain Color
LRR_1 Repeat
LRR_8 Repeat
Pkinase Domain
Cotton_A_08075
Cotton_A_24765
Cotton_A_24938
−200
0
200
400
600
800

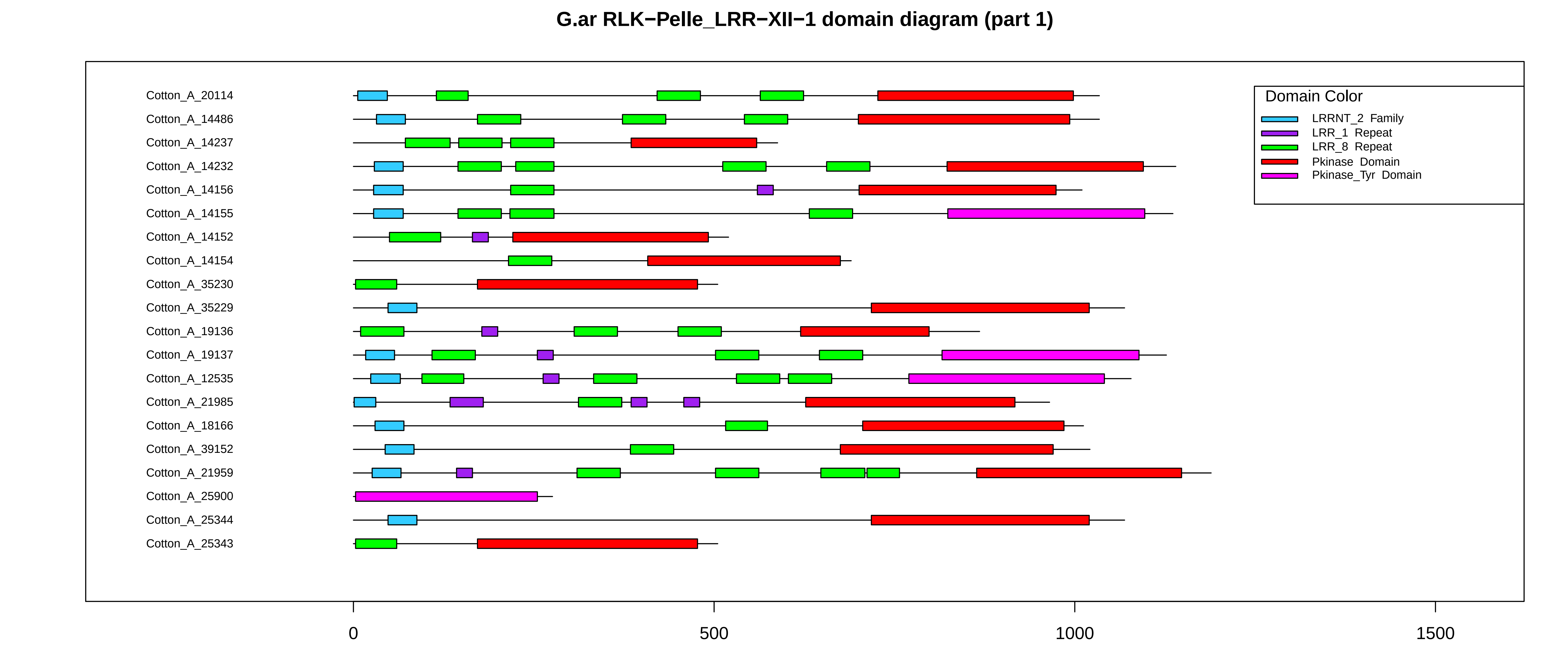
G.ar RLK−Pelle_LRR−XII−1 domain diagram (part 1)
Cotton_A_20114
Cotton_A_14486
Cotton_A_14237
Cotton_A_14232
Cotton_A_14156
Cotton_A_14155
Cotton_A_14152
Cotton_A_14154
Cotton_A_35230
Cotton_A_35229
Cotton_A_19136
Cotton_A_19137
Cotton_A_12535
Cotton_A_21985
Cotton_A_18166
Cotton_A_39152
Cotton_A_21959
Cotton_A_25900
Cotton_A_25344
Cotton_A_25343
Domain Color
LRRNT_2 Family
LRR_1 Repeat
LRR_8 Repeat
Pkinase Domain
Pkinase_Tyr Domain
0
500
1000
1500

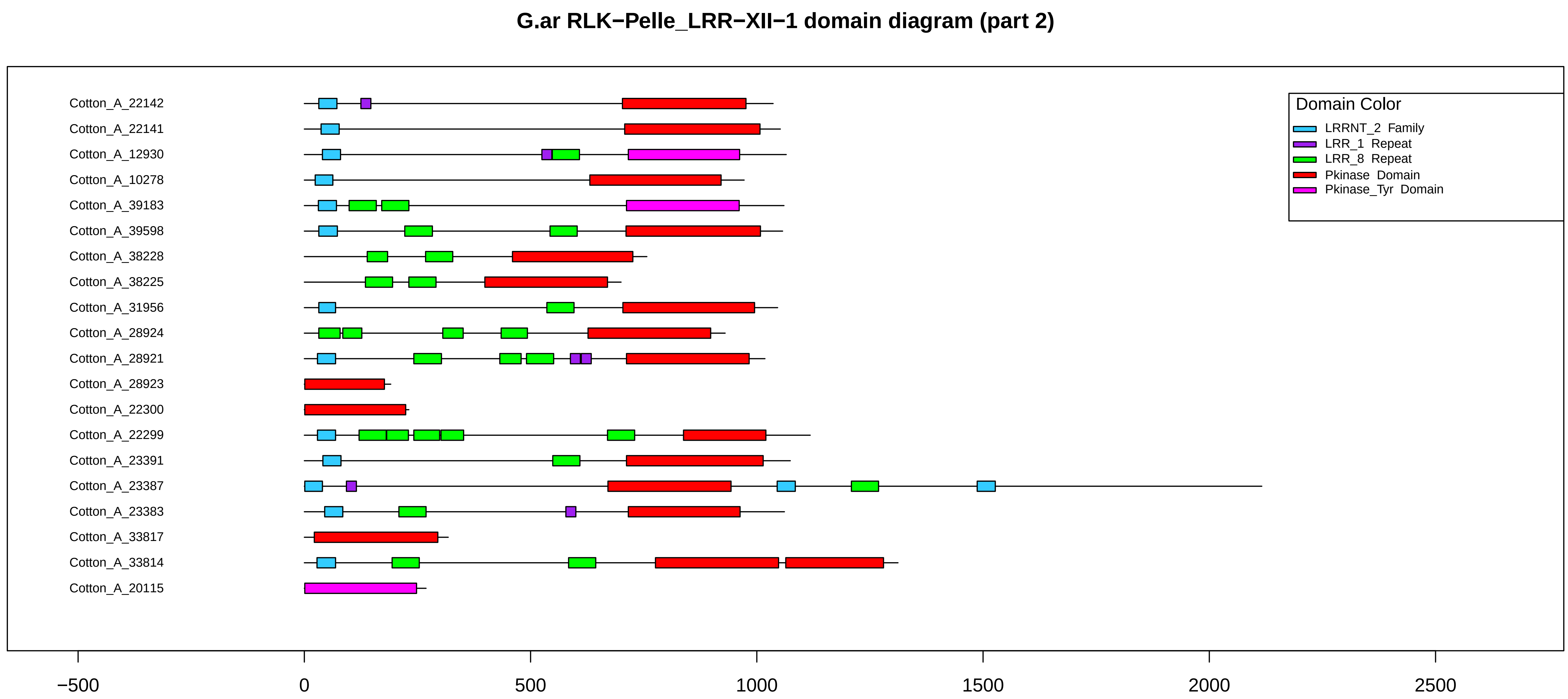

G.ar RLK−Pelle_LRR−XII−1 domain diagram (part 2)
Cotton_A_22142
Cotton_A_22141
Cotton_A_12930
Cotton_A_10278
Cotton_A_39183
Cotton_A_39598
Cotton_A_38228
Cotton_A_38225
Cotton_A_31956
Cotton_A_28924
Cotton_A_28921
Cotton_A_28923
Cotton_A_22300
Cotton_A_22299
Cotton_A_23391
Cotton_A_23387
Cotton_A_23383
Cotton_A_33817
Cotton_A_33814
Cotton_A_20115
Domain Color
LRRNT_2 Family
LRR_1 Repeat
LRR_8 Repeat
Pkinase Domain
Pkinase_Tyr Domain
−500
0
500
1000
1500
2000
2500

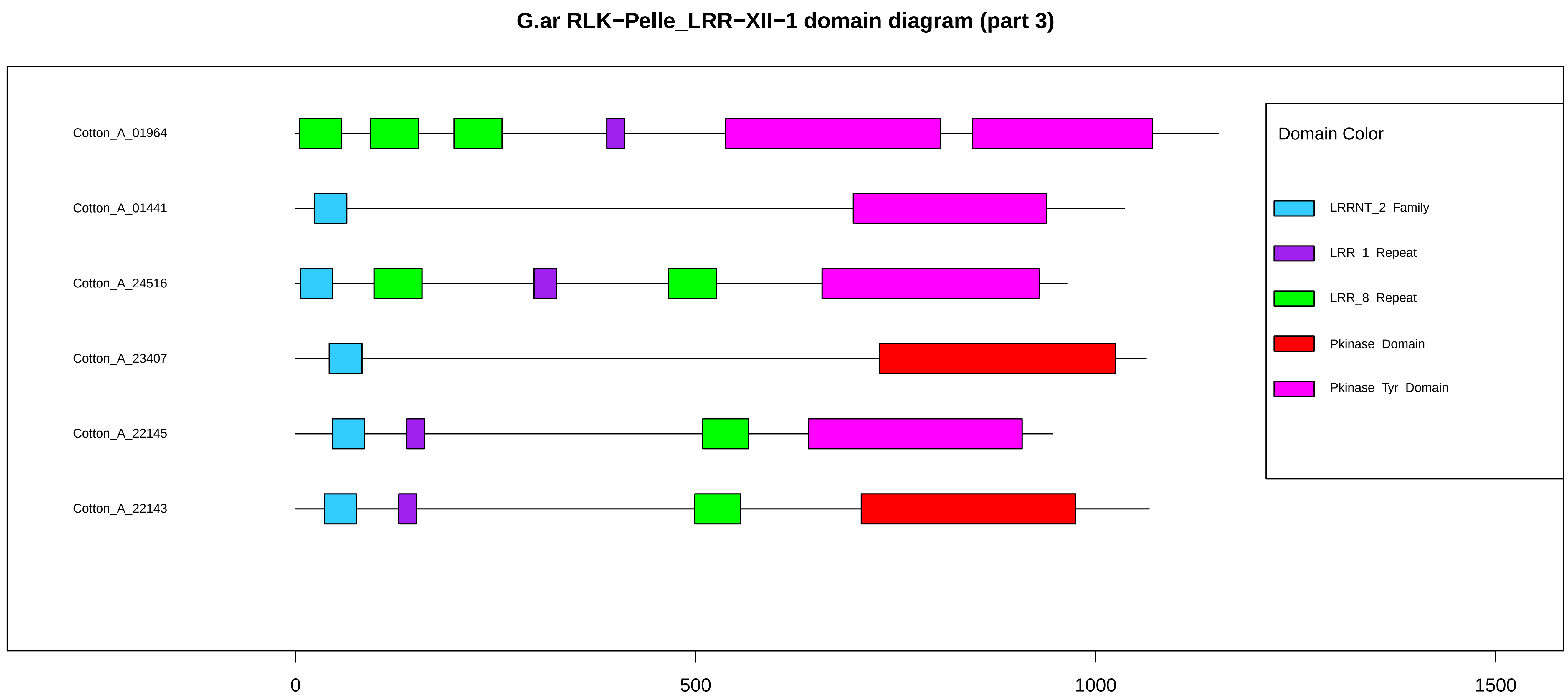

G.ar RLK−Pelle_LRR−XII−1 domain diagram (part 3)
Cotton_A_01964
Cotton_A_01441
Cotton_A_24516
Cotton_A_23407
Cotton_A_22145
Cotton_A_22143
Domain Color
LRRNT_2 Family
LRR_1 Repeat
LRR_8 Repeat
Pkinase Domain
Pkinase_Tyr Domain
0
500
1000
1500

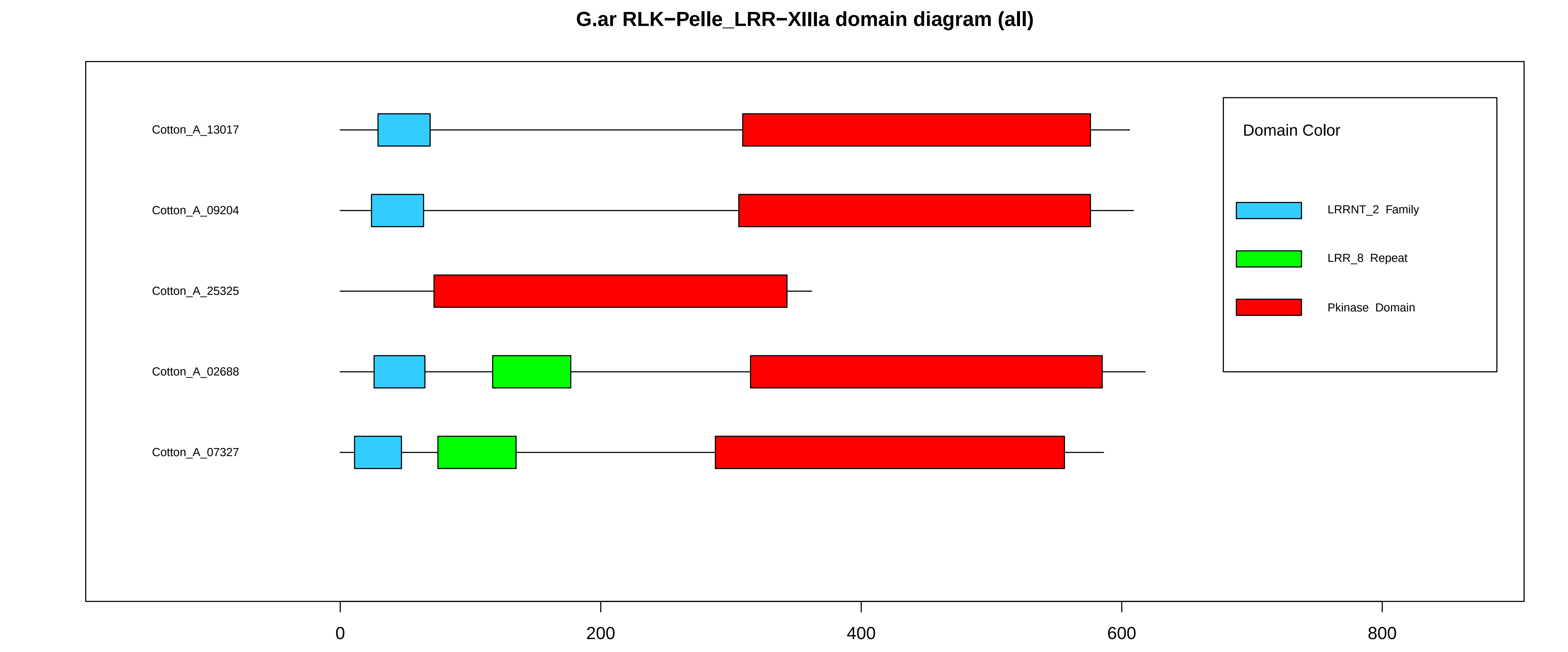
G.ar RLK−Pelle_LRR−XIIIa domain diagram (all)
Cotton_A_13017
Cotton_A_09204
Cotton_A_25325
Cotton_A_02688
Cotton_A_07327
Domain Color
LRRNT_2 Family
LRR_8 Repeat
Pkinase Domain
0
200
400
600
800

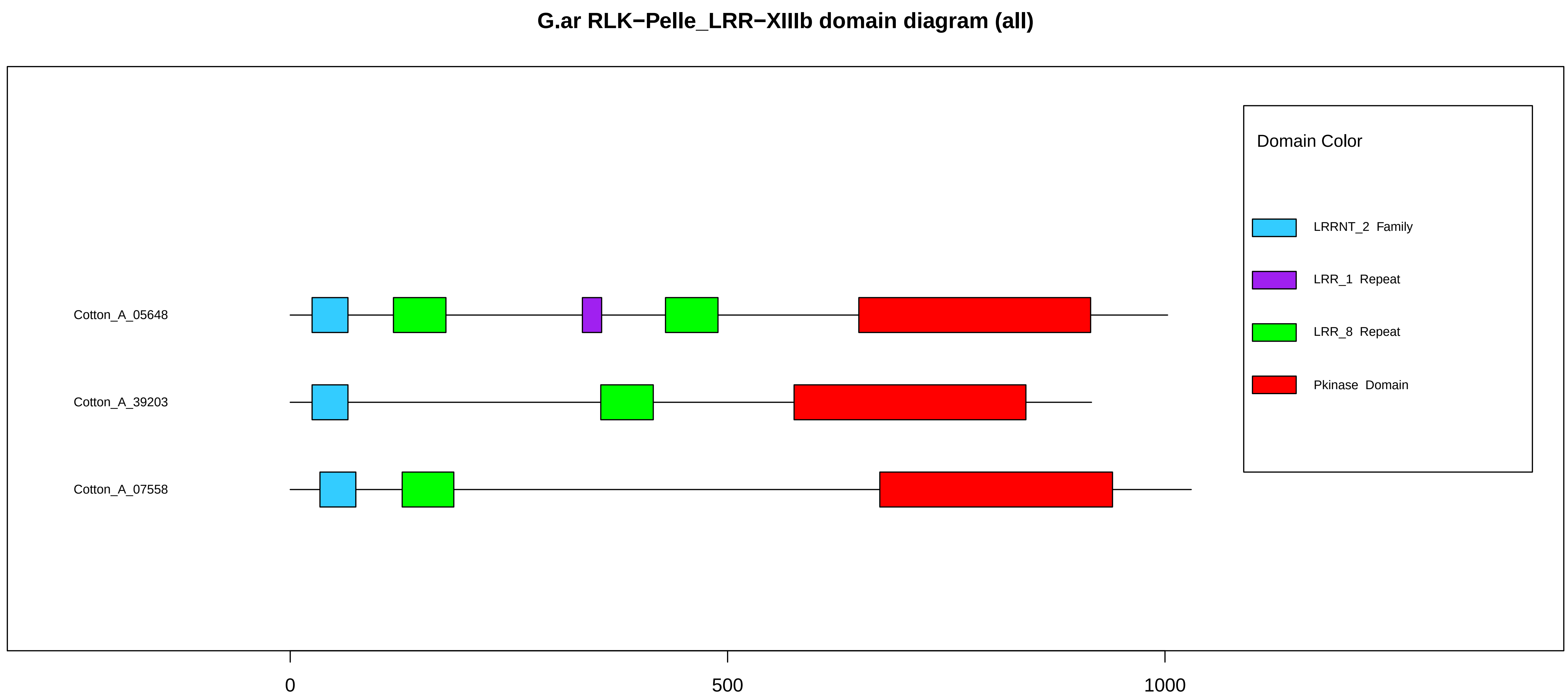
G.ar RLK−Pelle_LRR−XIIIb domain diagram (all)
Domain Color
LRRNT_2 Family
LRR_1 Repeat
LRR_8 Repeat
Pkinase Domain
Cotton_A_05648
Cotton_A_39203
Cotton_A_07558
0
500
1000

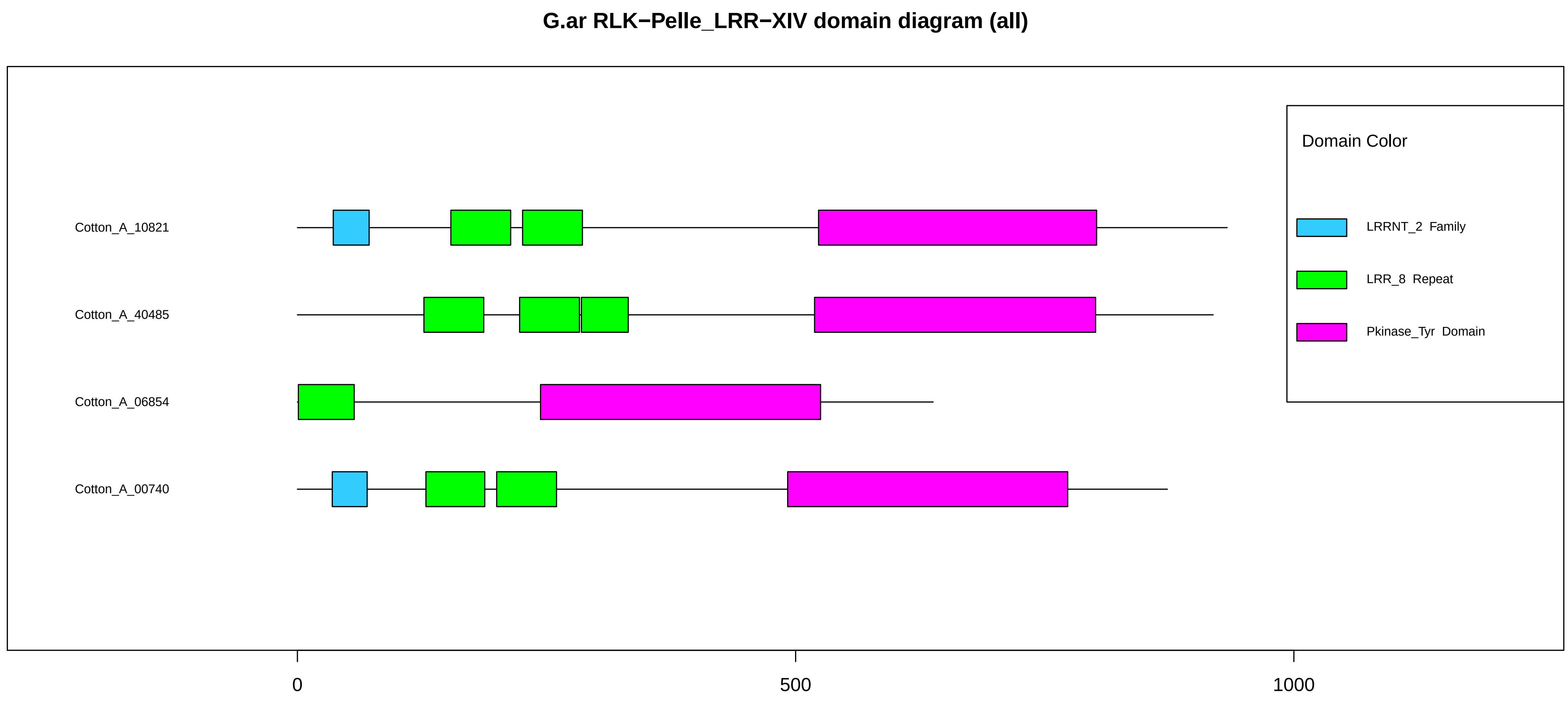

G.ar RLK−Pelle_LRR−XIV domain diagram (all)
Cotton_A_10821
Cotton_A_40485
Cotton_A_06854
Cotton_A_00740
Domain Color
LRRNT_2 Family
LRR_8 Repeat
Pkinase_Tyr Domain
0
500
1000

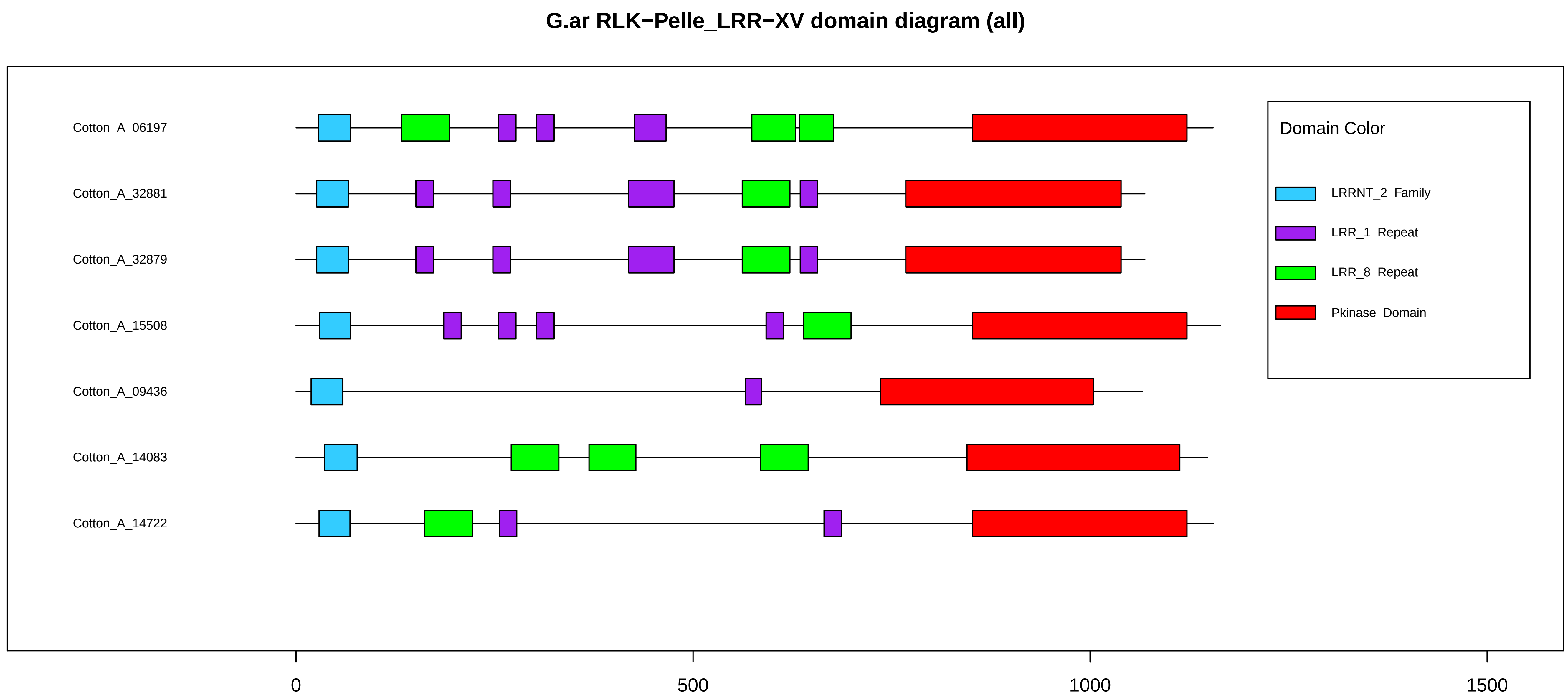
G.ar RLK−Pelle_LRR−XV domain diagram (all)
Cotton_A_06197
Cotton_A_32881
Cotton_A_32879
Cotton_A_15508
Cotton_A_09436
Cotton_A_14083
Cotton_A_14722
Domain Color
LRRNT_2 Family
LRR_1 Repeat
LRR_8 Repeat
Pkinase Domain
0
500
1000
1500

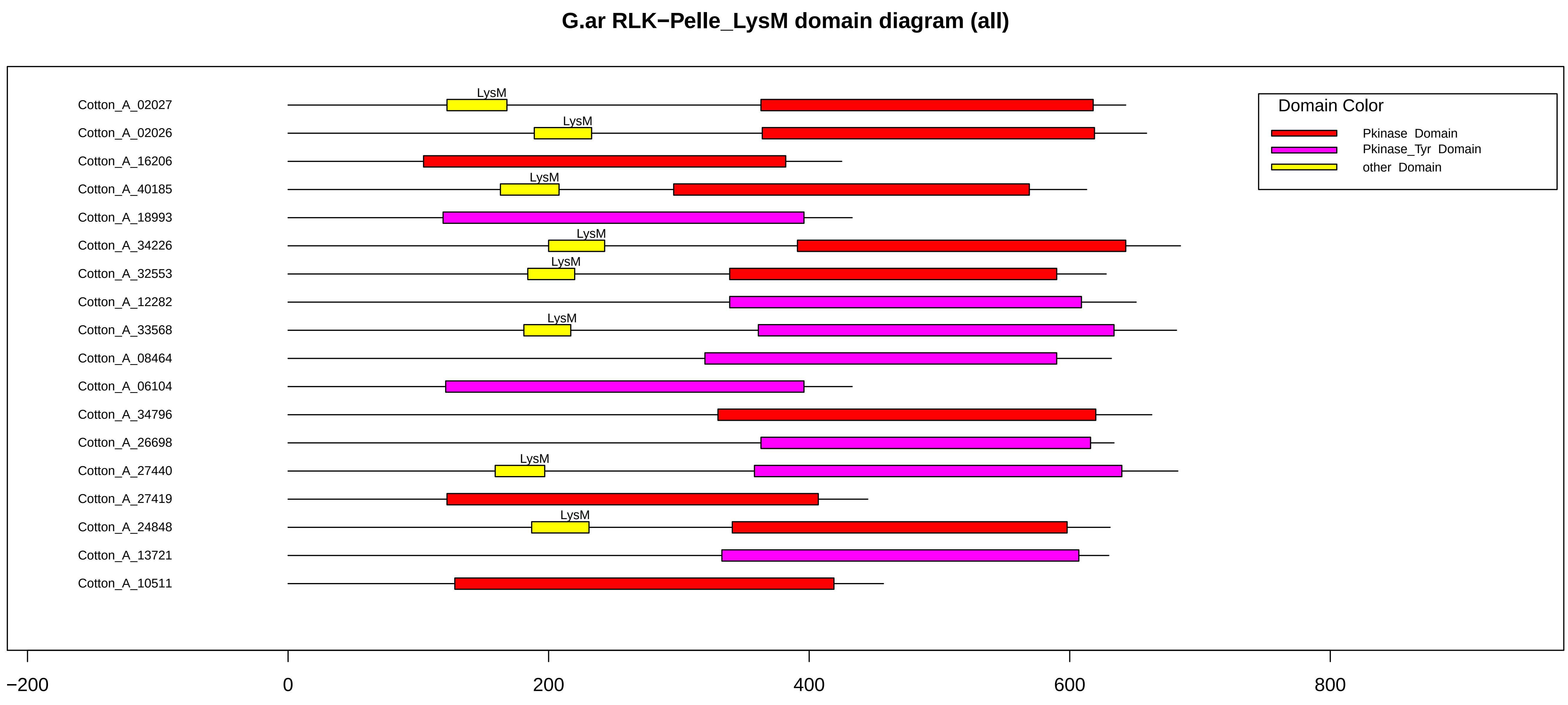

G.ar RLK−Pelle_LysM domain diagram (all)
Cotton_A_02027
Cotton_A_02026
Cotton_A_16206
Cotton_A_40185
Cotton_A_18993
Cotton_A_34226
Cotton_A_32553
Cotton_A_12282
Cotton_A_33568
Cotton_A_08464
Cotton_A_06104
Cotton_A_34796
Cotton_A_26698
Cotton_A_27440
Cotton_A_27419
Cotton_A_24848
Cotton_A_13721
Cotton_A_10511
LysM
Domain Color
Pkinase Domain
Pkinase_Tyr Domain
other Domain
−200
0
200
400
600
800

G.ar RLK−Pelle_PERK−1 domain diagram (all)

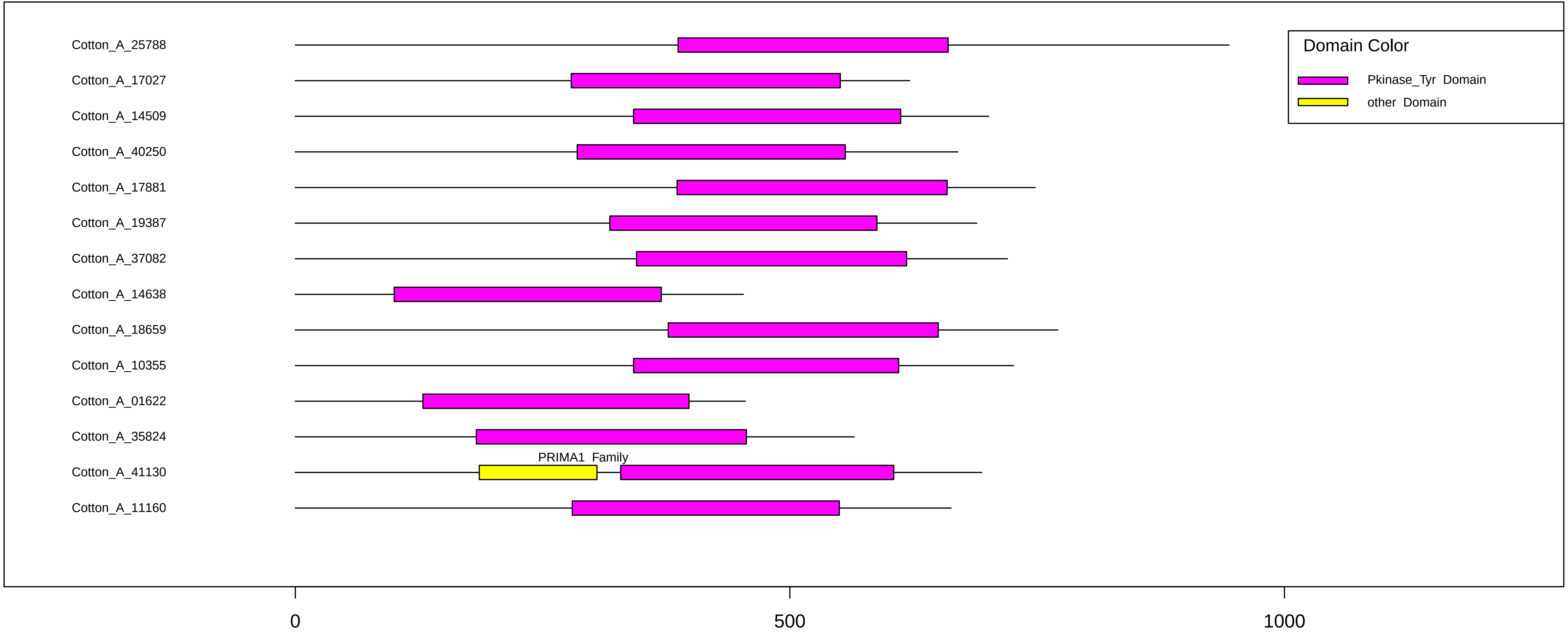

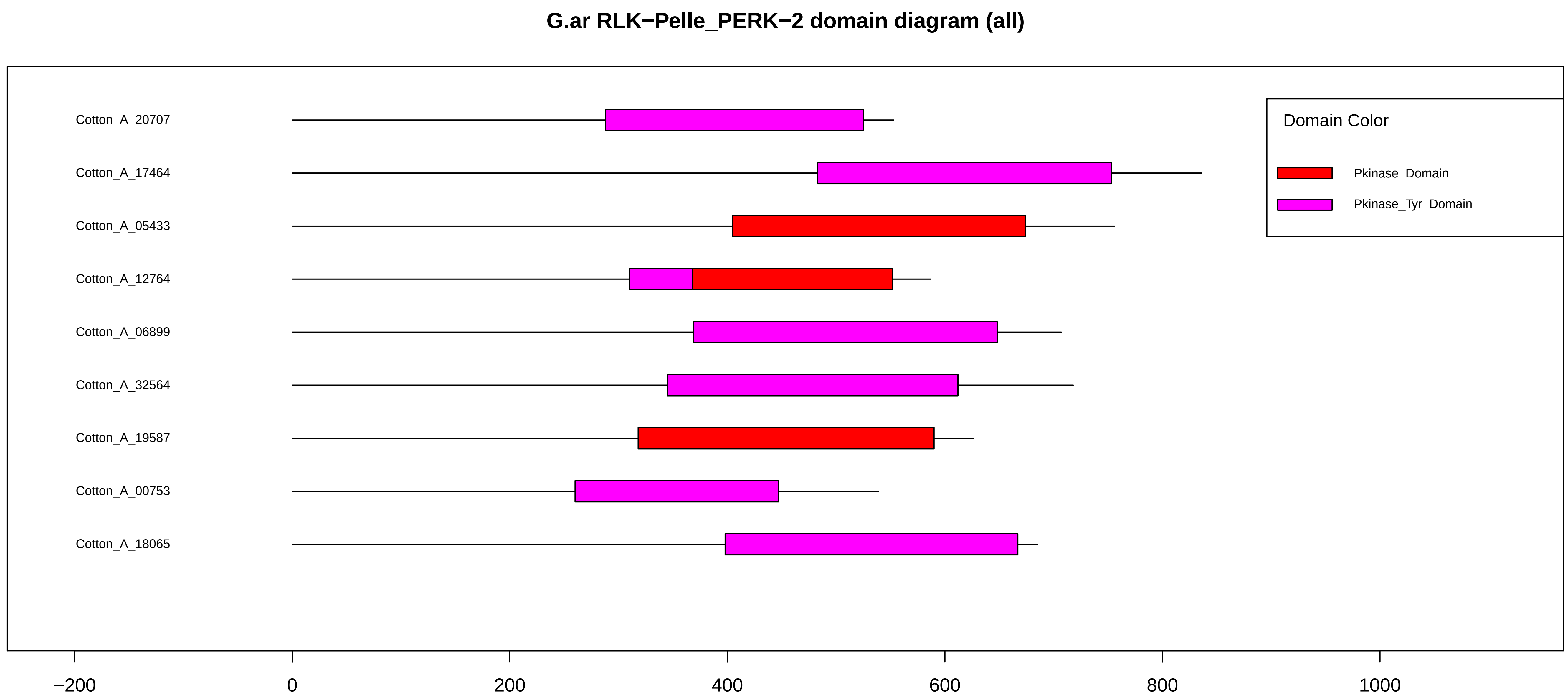
G.ar RLK−Pelle_PERK−2 domain diagram (all)
Cotton_A_20707
Cotton_A_17464
Cotton_A_05433
Cotton_A_12764
Cotton_A_06899
Cotton_A_32564
Cotton_A_19587
Cotton_A_00753
Cotton_A_18065
Domain Color
Pkinase Domain
Pkinase_Tyr Domain
−200
0
200
400
600
800
1000

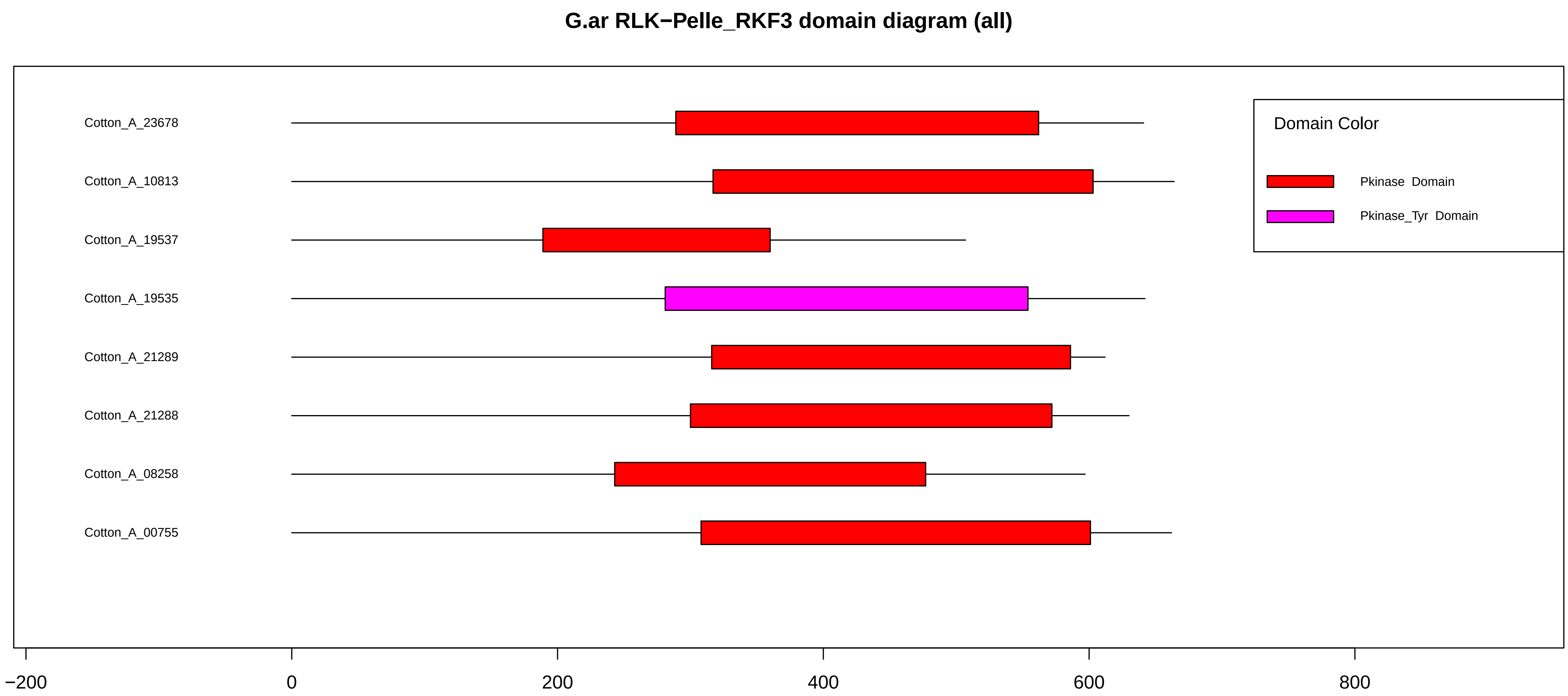
G.ar RLK−Pelle_RKF3 domain diagram (all)
Cotton_A_23678
Cotton_A_10813
Cotton_A_19537
Cotton_A_19535
Cotton_A_21289
Cotton_A_21288
Cotton_A_08258
Cotton_A_00755
Domain Color
Pkinase Domain
Pkinase_Tyr Domain
−200
0
200
400
600
800

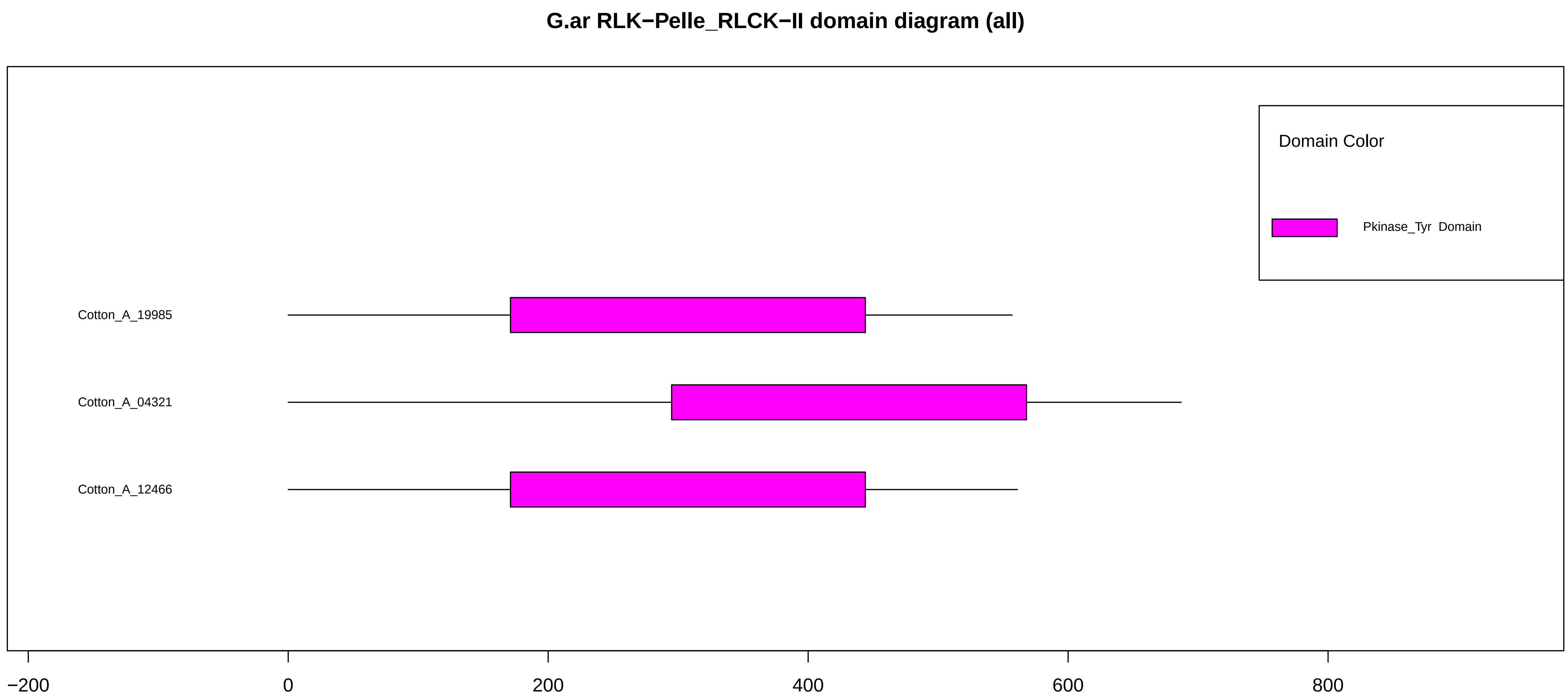
G.ar RLK−Pelle_RLCK−II domain diagram (all)
Domain Color
Pkinase_Tyr Domain
Cotton_A_19985
Cotton_A_04321
Cotton_A_12466
−200
0
200
400
600
800

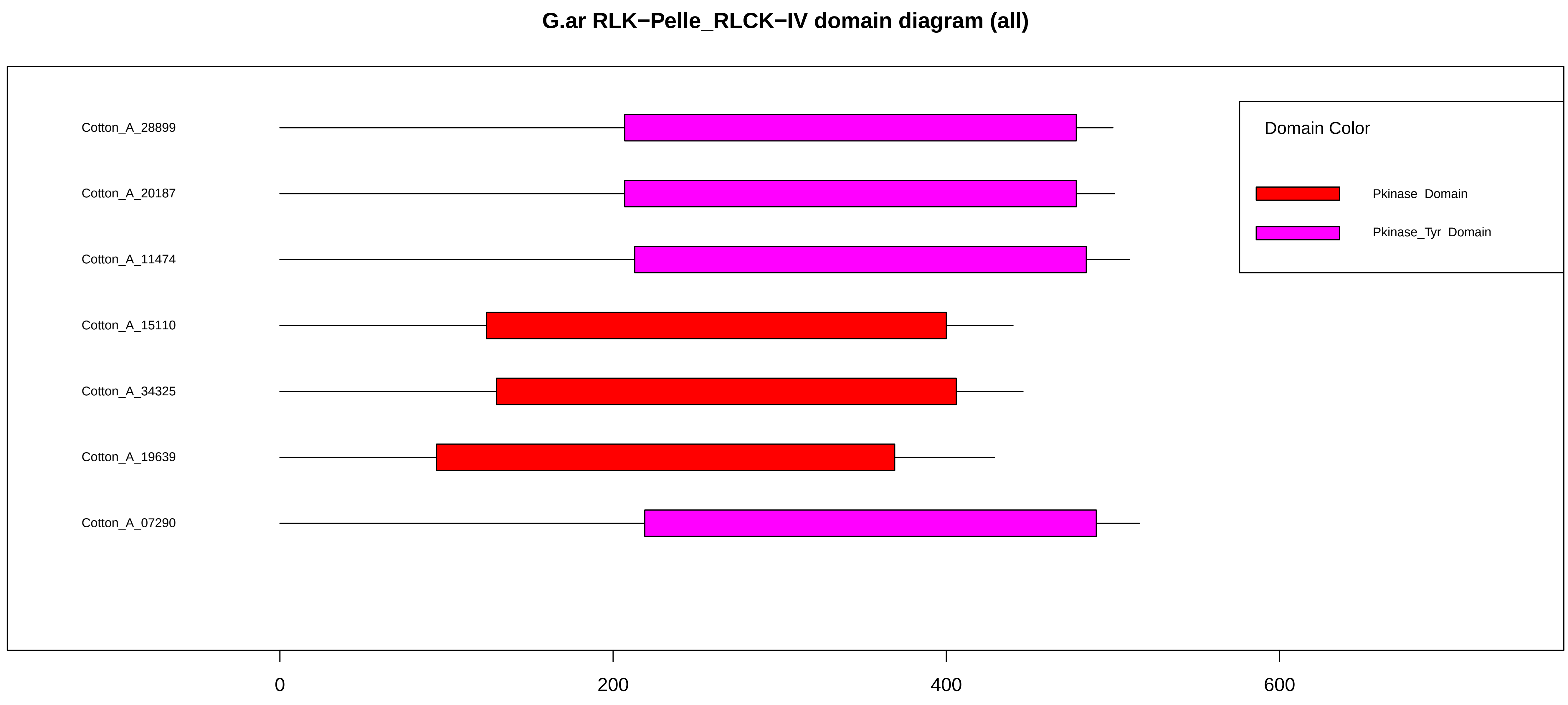
G.ar RLK−Pelle_RLCK−IV domain diagram (all)
Cotton_A_28899
Cotton_A_20187
Cotton_A_11474
Cotton_A_15110
Cotton_A_34325
Cotton_A_19639
Cotton_A_07290
Domain Color
Pkinase Domain
Pkinase_Tyr Domain
0
200
400
600

G.ar RLK−Pelle_RLCK−IXa domain diagram (all)

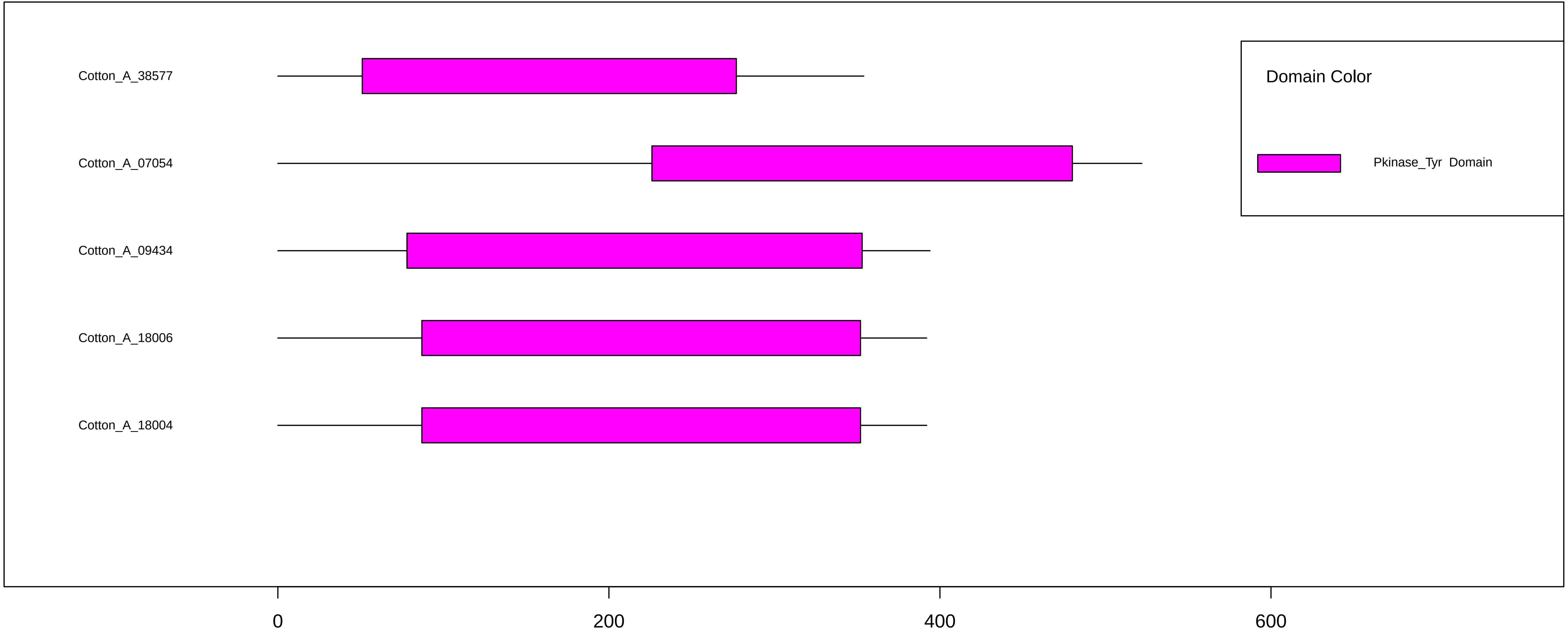

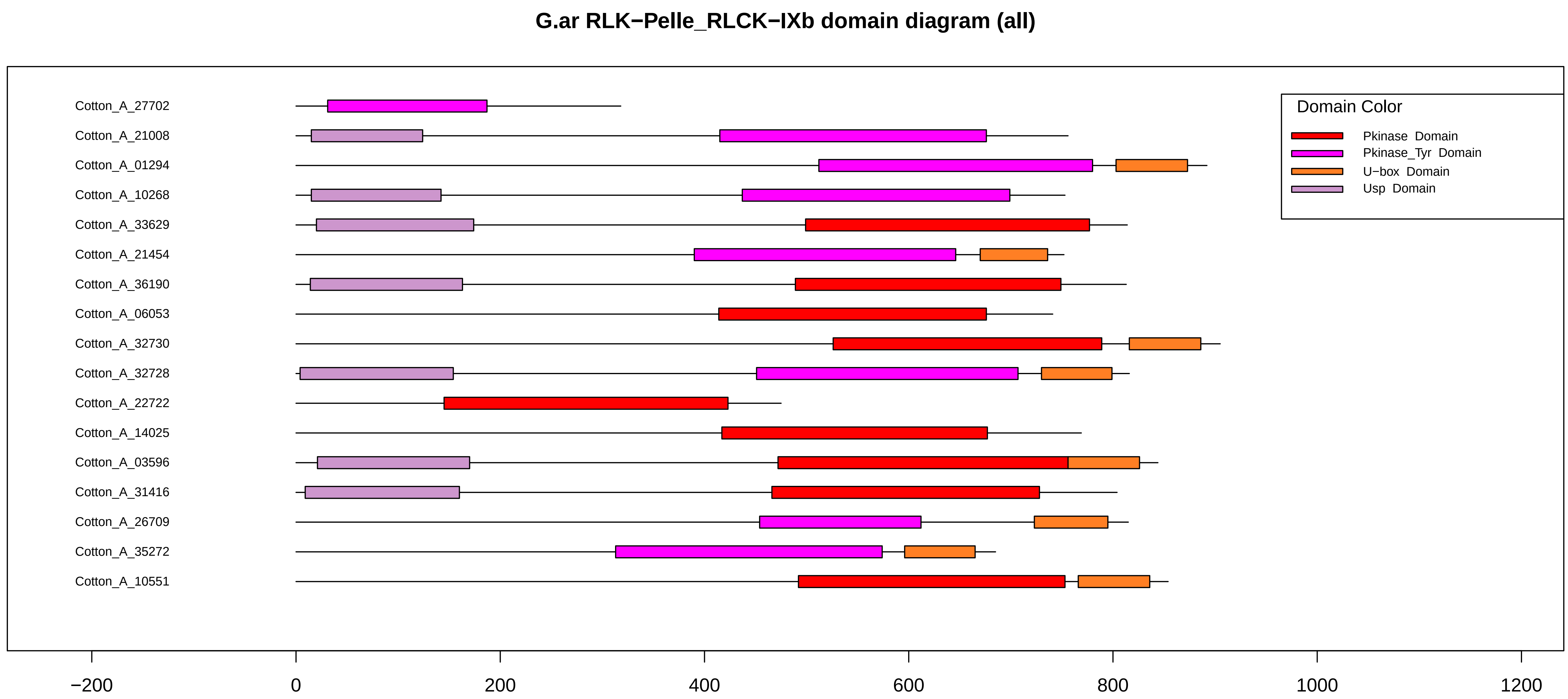
G.ar RLK−Pelle_RLCK−IXb domain diagram (all)
Cotton_A_27702
Cotton_A_21008
Cotton_A_01294
Cotton_A_10268
Cotton_A_33629
Cotton_A_21454
Cotton_A_36190
Cotton_A_06053
Cotton_A_32730
Cotton_A_32728
Cotton_A_22722
Cotton_A_14025
Cotton_A_03596
Cotton_A_31416
Cotton_A_26709
Cotton_A_35272
Cotton_A_10551
Domain Color
Pkinase Domain
Pkinase_Tyr Domain
U−box Domain
Usp Domain
−200
0
200
400
600
800
1000
1200

G.ar RLK−Pelle_RLCK−Os domain diagram (all)

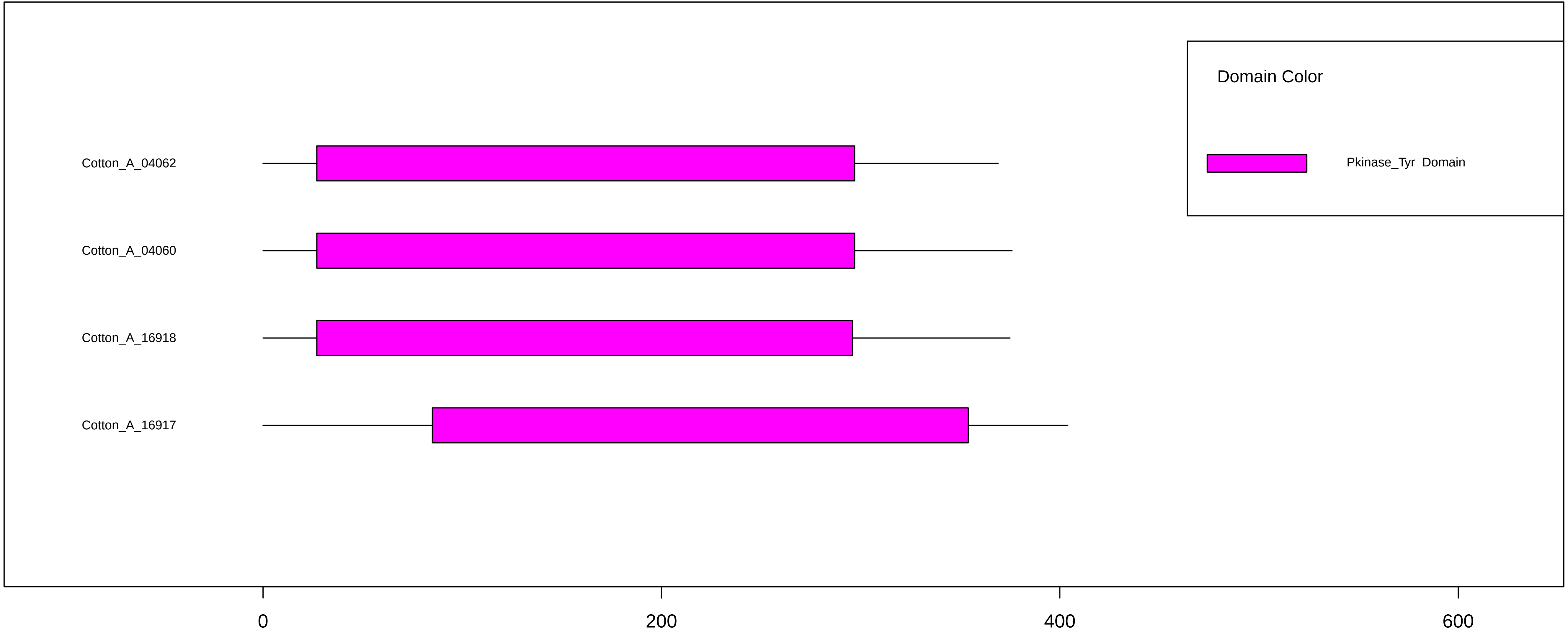

G.ar RLK−Pelle_RLCK−VIIa−1 domain diagram (all)

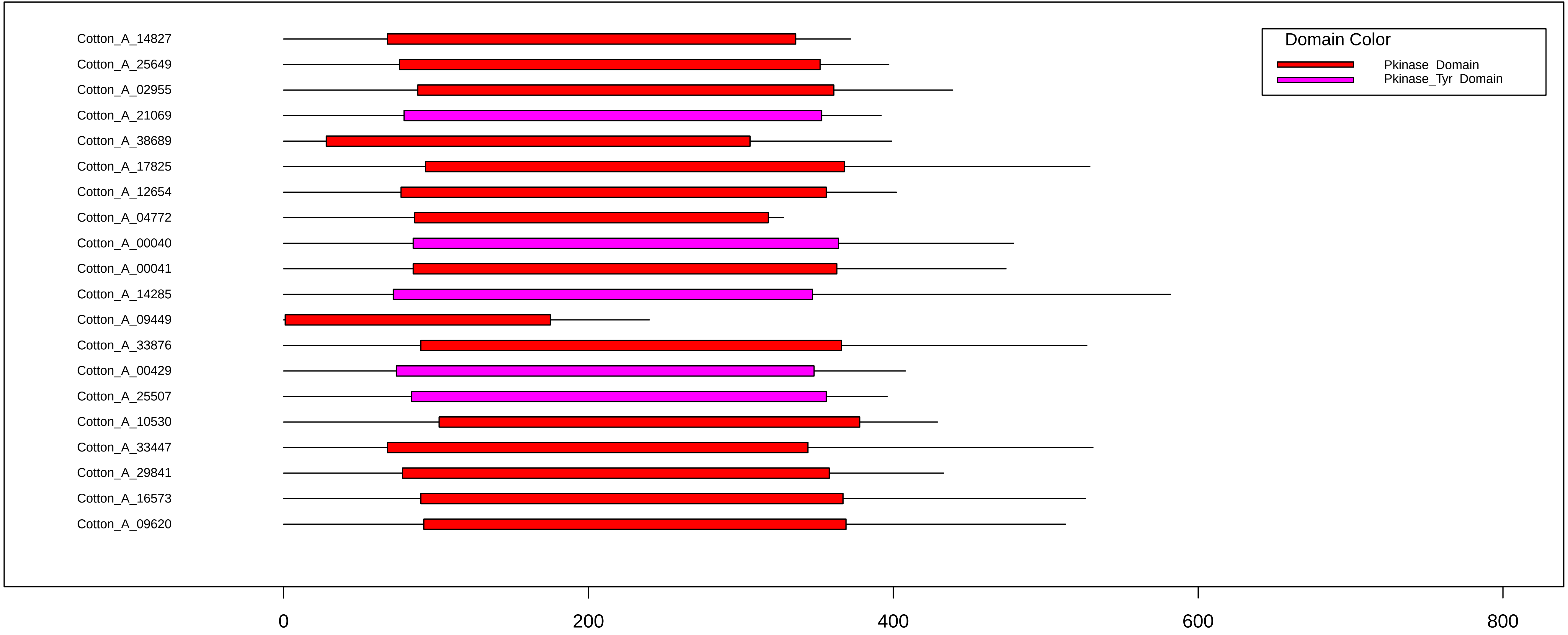

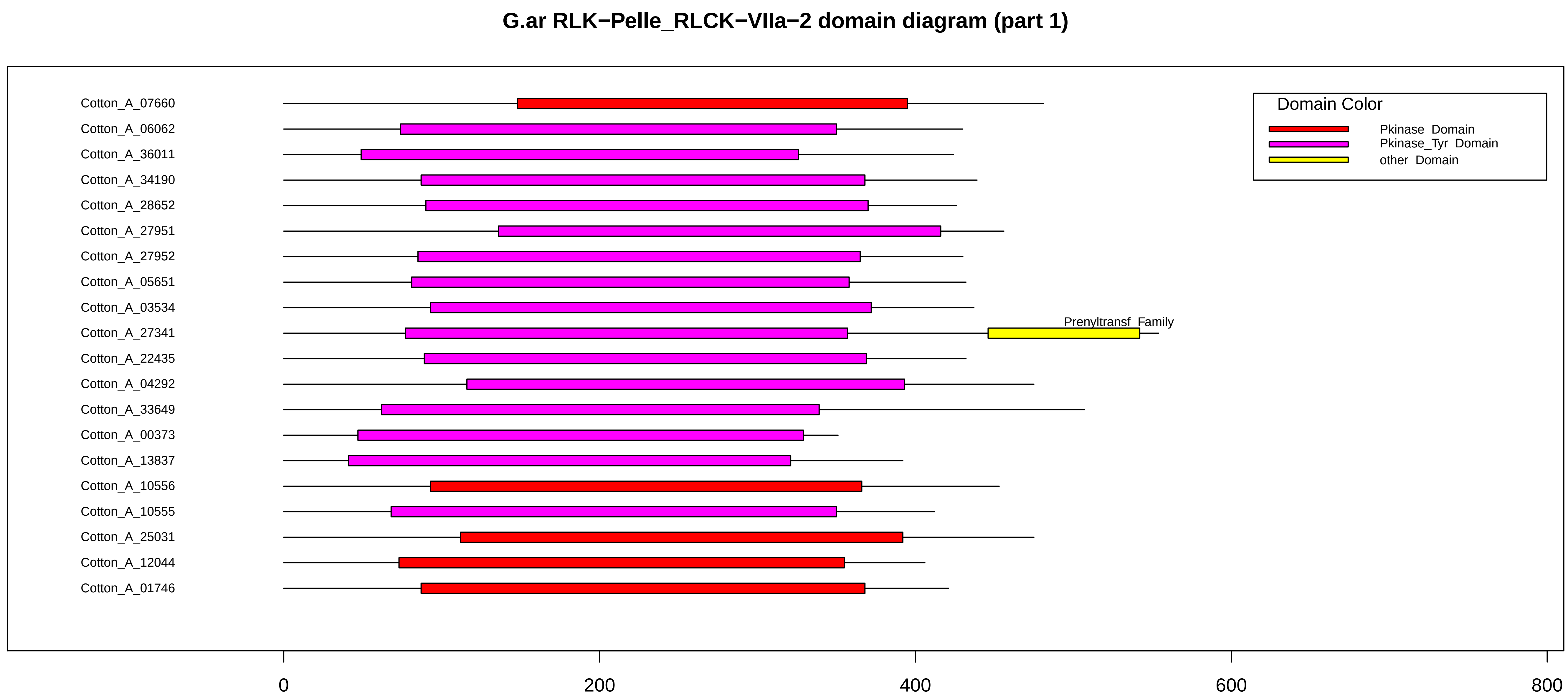
G.ar RLK−Pelle_RLCK−VIIa−2 domain diagram (part 1)
Cotton_A_07660
Cotton_A_06062
Cotton_A_36011
Cotton_A_34190
Cotton_A_28652
Cotton_A_27951
Cotton_A_27952
Cotton_A_05651
Cotton_A_03534
Cotton_A_27341
Cotton_A_22435
Cotton_A_04292
Cotton_A_33649
Cotton_A_00373
Cotton_A_13837
Cotton_A_10556
Cotton_A_10555
Cotton_A_25031
Cotton_A_12044
Cotton_A_01746
Prenyltransf Family
Domain Color
Pkinase Domain
Pkinase_Tyr Domain
other Domain
0
200
400
600
800

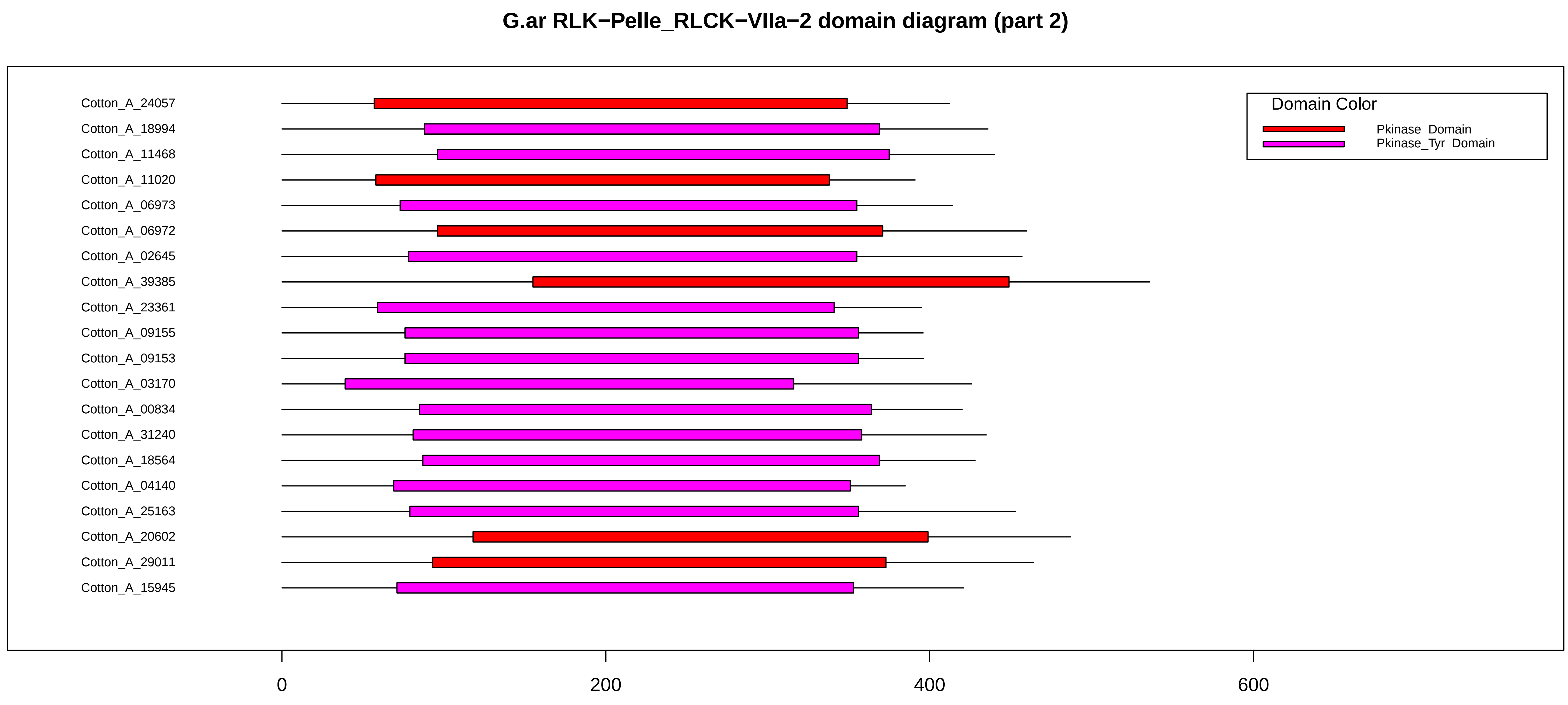
G.ar RLK−Pelle_RLCK−VIIa−2 domain diagram (part 2)
Cotton_A_24057
Cotton_A_18994
Cotton_A_11468
Cotton_A_11020
Cotton_A_06973
Cotton_A_06972
Cotton_A_02645
Cotton_A_39385
Cotton_A_23361
Cotton_A_09155
Cotton_A_09153
Cotton_A_03170
Cotton_A_00834
Cotton_A_31240
Cotton_A_18564
Cotton_A_04140
Cotton_A_25163
Cotton_A_20602
Cotton_A_29011
Cotton_A_15945
Domain Color
Pkinase Domain
Pkinase_Tyr Domain
0
200
400
600

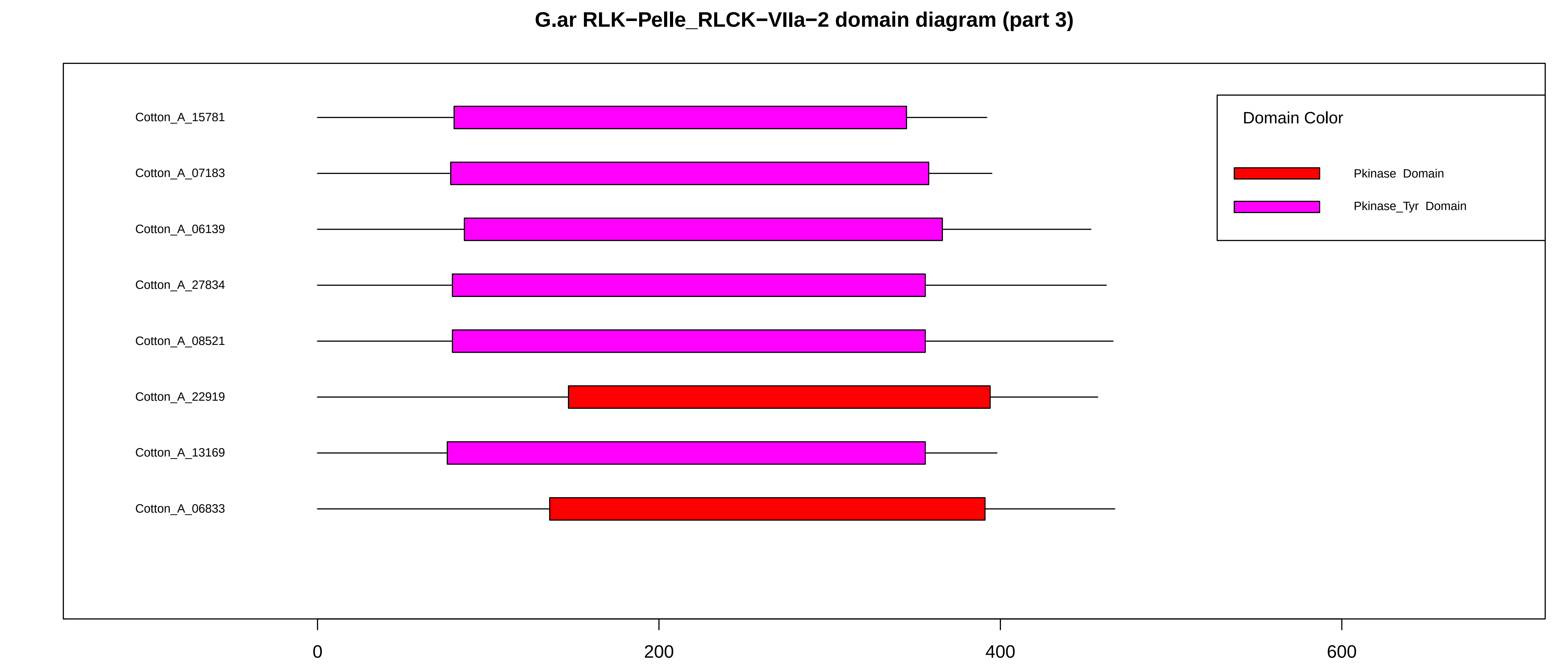
G.ar RLK−Pelle_RLCK−VIIa−2 domain diagram (part 3)
Cotton_A_15781
Cotton_A_07183
Cotton_A_06139
Cotton_A_27834
Cotton_A_08521
Cotton_A_22919
Cotton_A_13169
Cotton_A_06833
Domain Color
Pkinase Domain
Pkinase_Tyr Domain
0
200
400
600

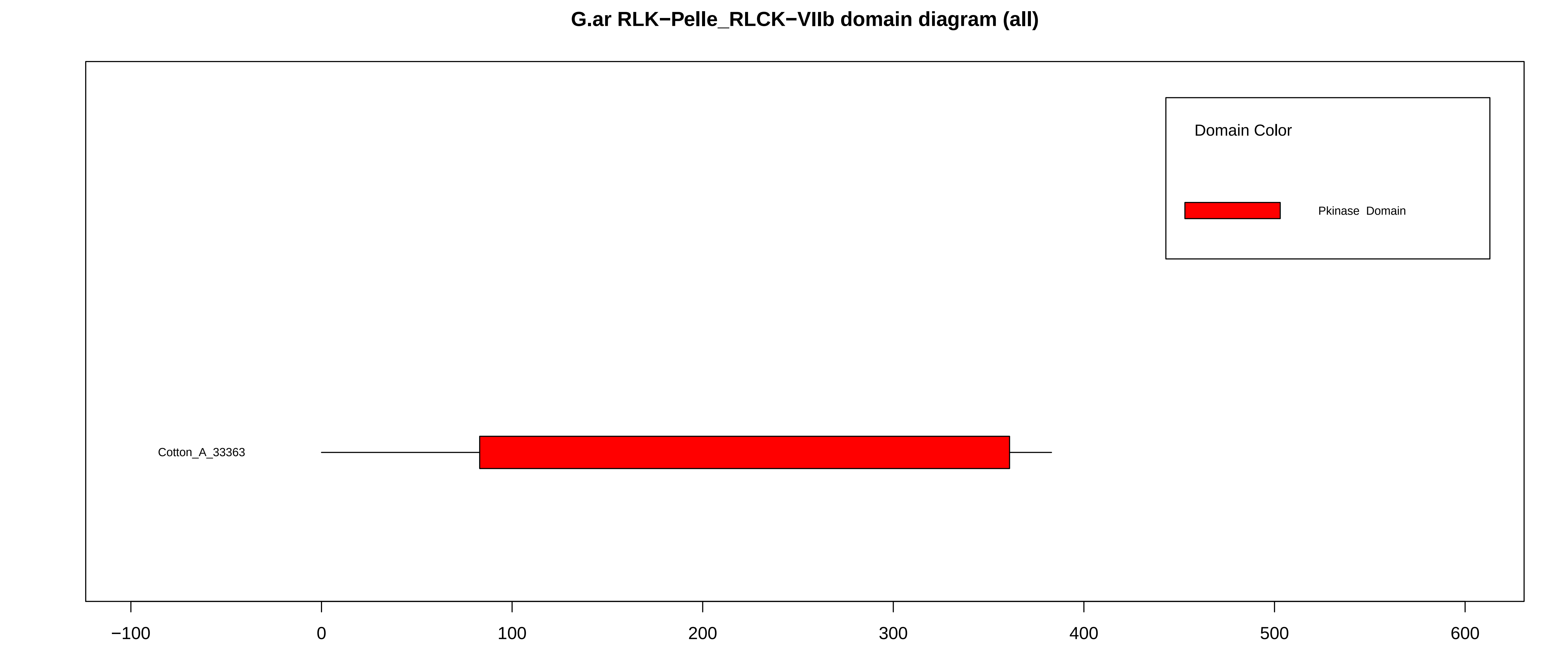
G.ar RLK−Pelle_RLCK−VIIb domain diagram (all)
Domain Color
Pkinase Domain
Cotton_A_33363
−100
0
100
200
300
400
500
600

G.ar RLK−Pelle_RLCK−VIII domain diagram (all)

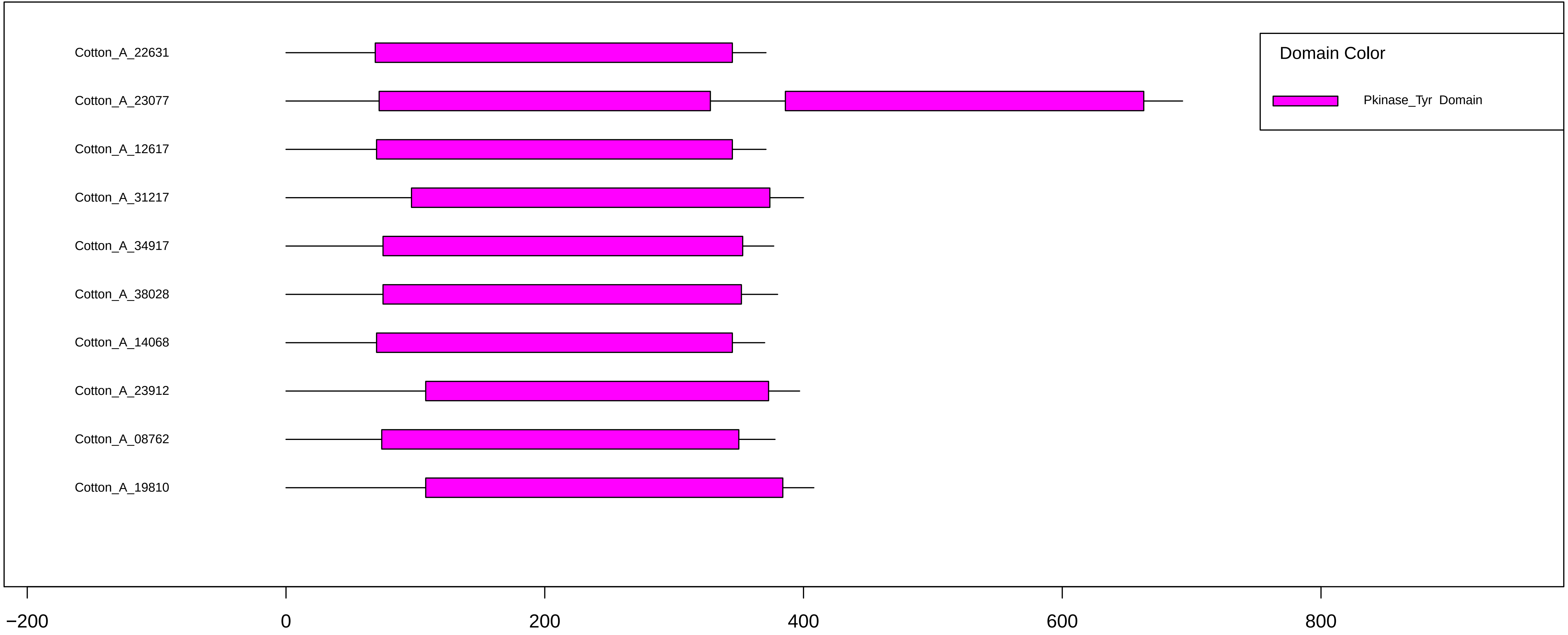

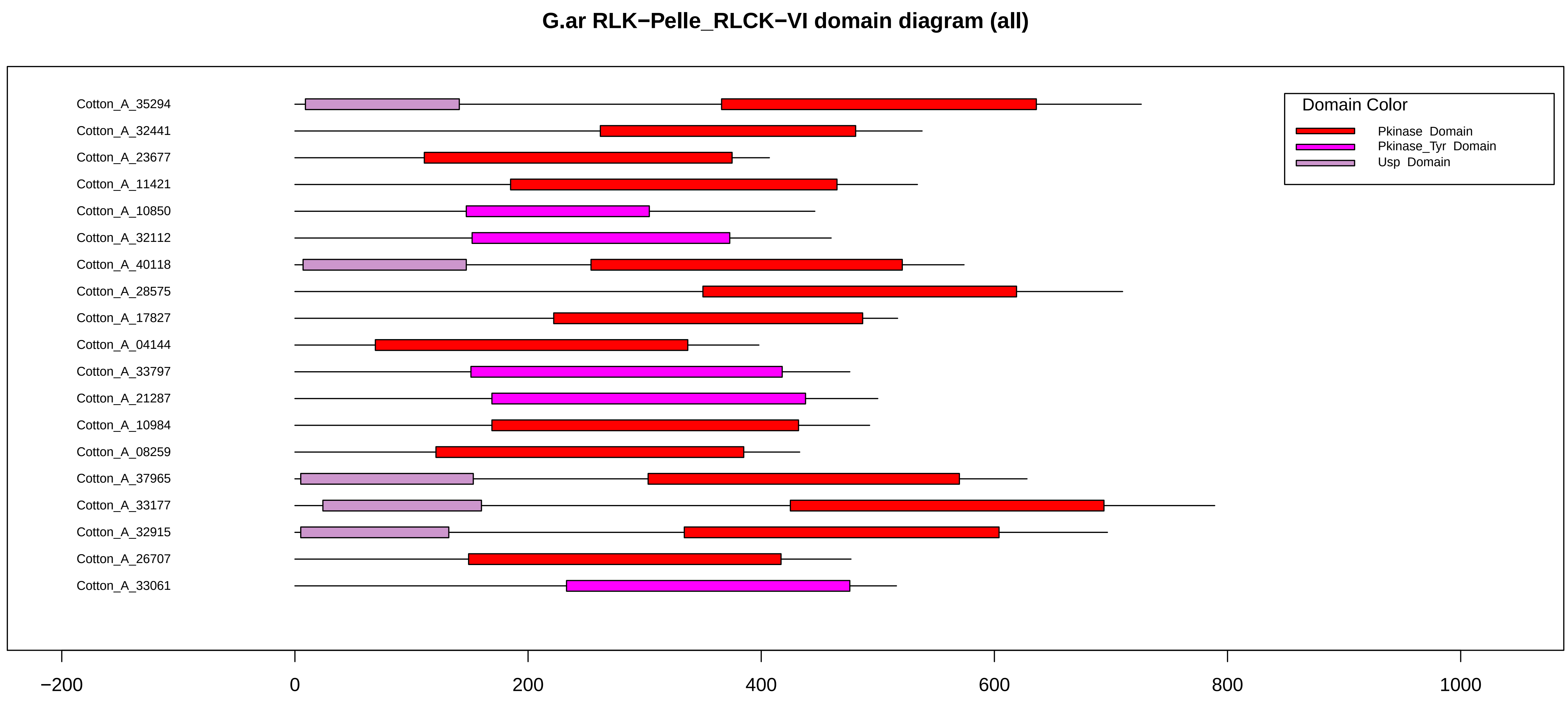
G.ar RLK−Pelle_RLCK−VI domain diagram (all)
Cotton_A_35294
Cotton_A_32441
Cotton_A_23677
Cotton_A_11421
Cotton_A_10850
Cotton_A_32112
Cotton_A_40118
Cotton_A_28575
Cotton_A_17827
Cotton_A_04144
Cotton_A_33797
Cotton_A_21287
Cotton_A_10984
Cotton_A_08259
Cotton_A_37965
Cotton_A_33177
Cotton_A_32915
Cotton_A_26707
Cotton_A_33061
Domain Color
Pkinase Domain
Pkinase_Tyr Domain
Usp Domain
−200
0
200
400
600
800
1000

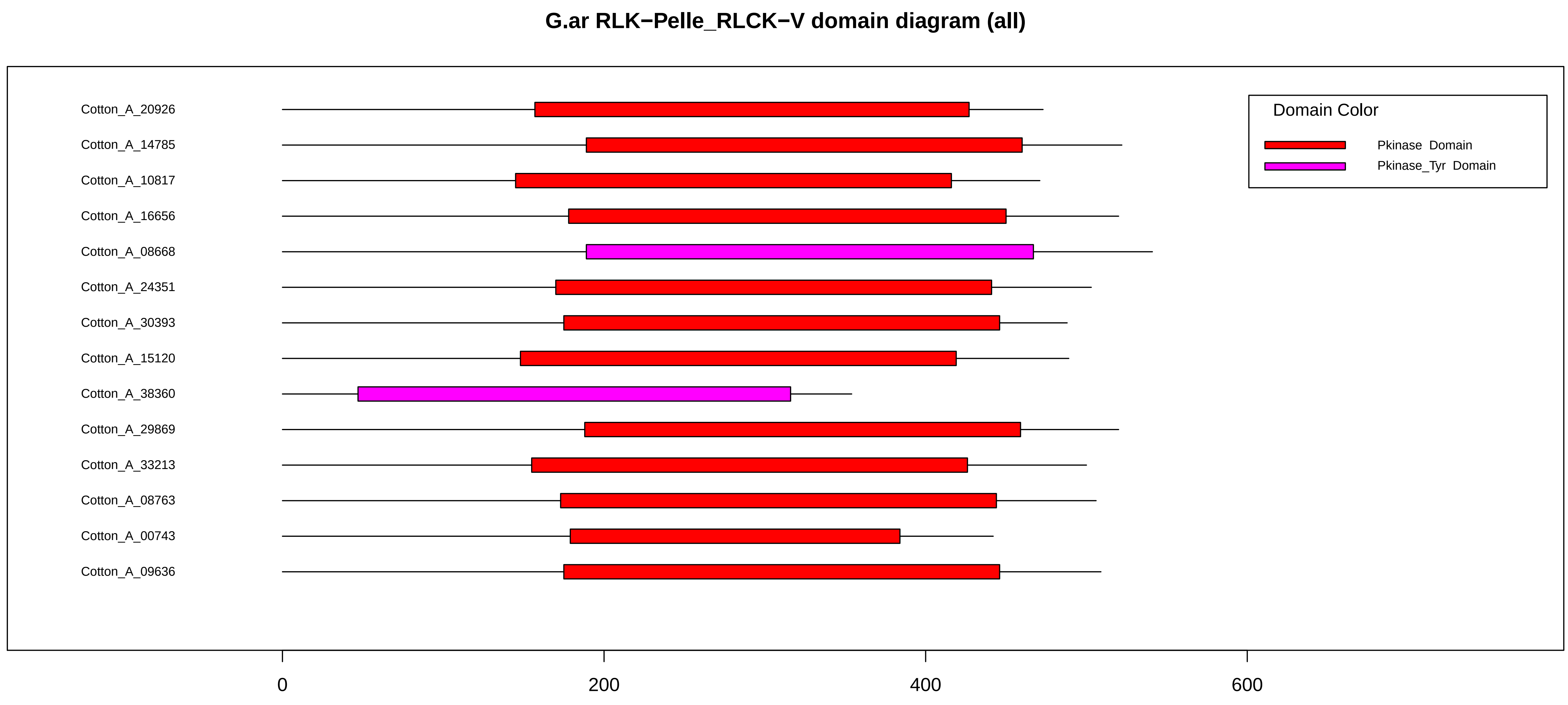

G.ar RLK−Pelle_RLCK−V domain diagram (all)
Cotton_A_20926
Cotton_A_14785
Cotton_A_10817
Cotton_A_16656
Cotton_A_08668
Cotton_A_24351
Cotton_A_30393
Cotton_A_15120
Cotton_A_38360
Cotton_A_29869
Cotton_A_33213
Cotton_A_08763
Cotton_A_00743
Cotton_A_09636
Domain Color
Pkinase Domain
Pkinase_Tyr Domain
0
200
400
600

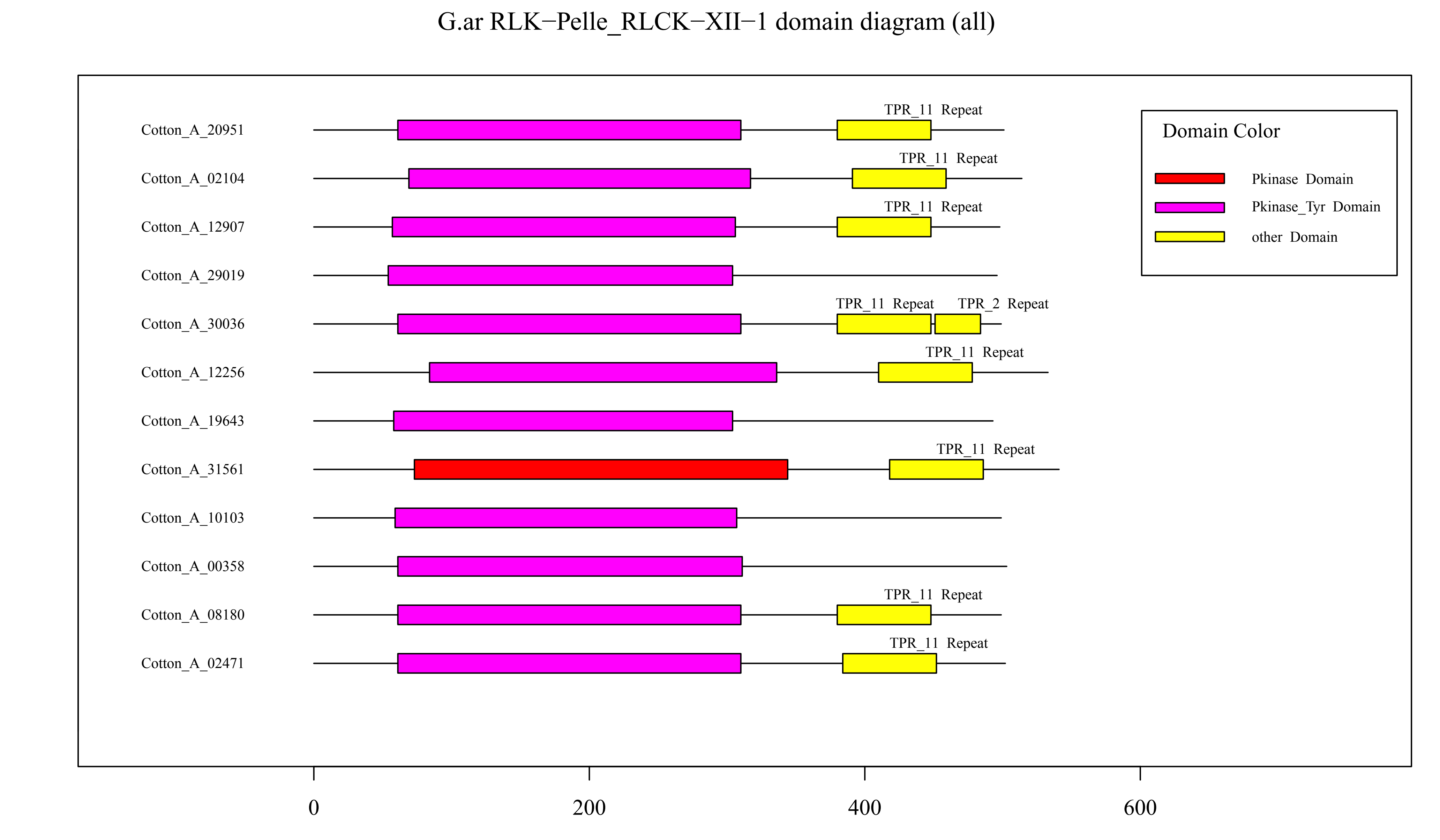

G.ar RLK−Pelle_RLCK−XII−1 domain diagram (all)
Cotton_A_20951
Cotton_A_02104
Cotton_A_12907
Cotton_A_29019
Cotton_A_30036
Cotton_A_12256
Cotton_A_19643
Cotton_A_31561
Cotton_A_10103
Cotton_A_00358
Cotton_A_08180
Cotton_A_02471
TPR_11 Repeat
TPR_2 Repeat
Domain Color
Pkinase Domain
Pkinase_Tyr Domain
other Domain
0
200
400
600

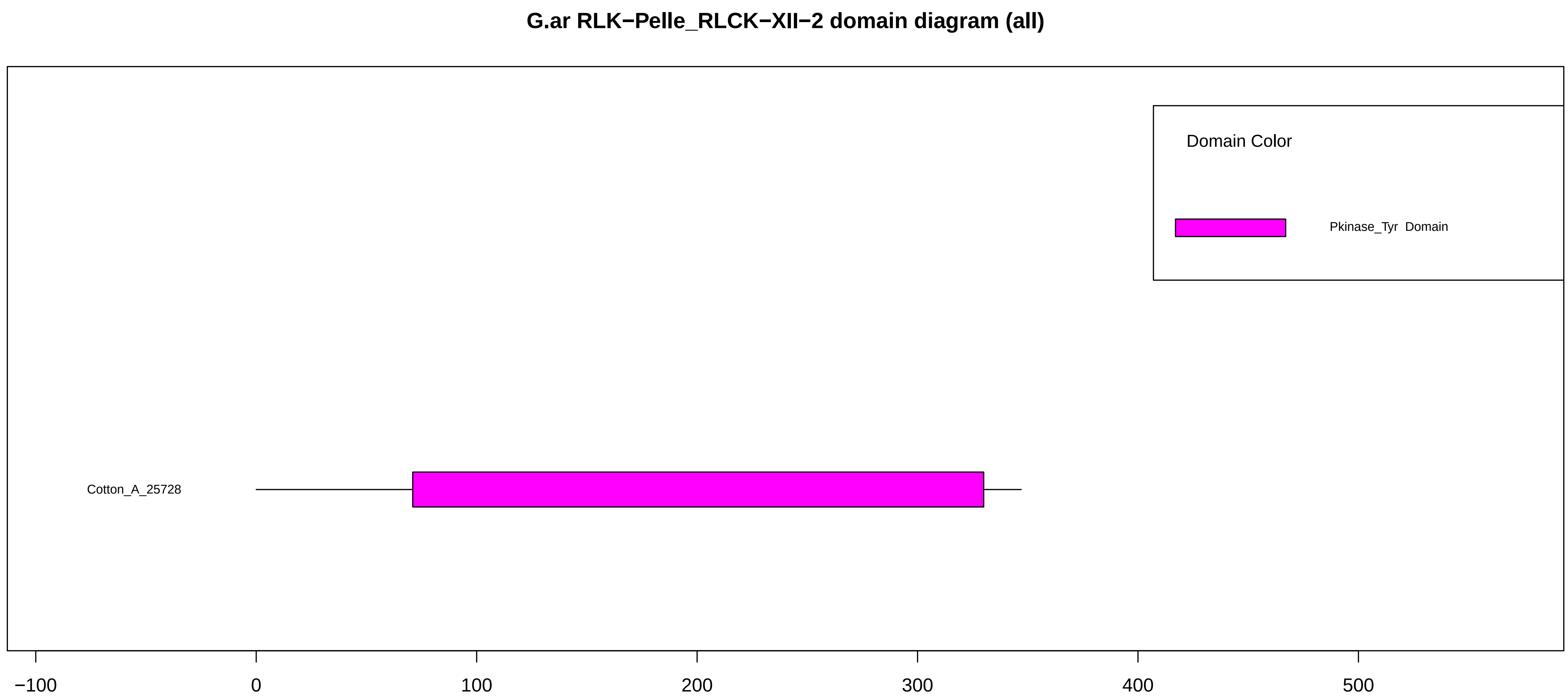
G.ar RLK−Pelle_RLCK−XII−2 domain diagram (all)
Domain Color
Pkinase_Tyr Domain
Cotton_A_25728
−100
0
100
200
300
400
500

G.ar RLK−Pelle_RLCK−XIII domain diagram (all)

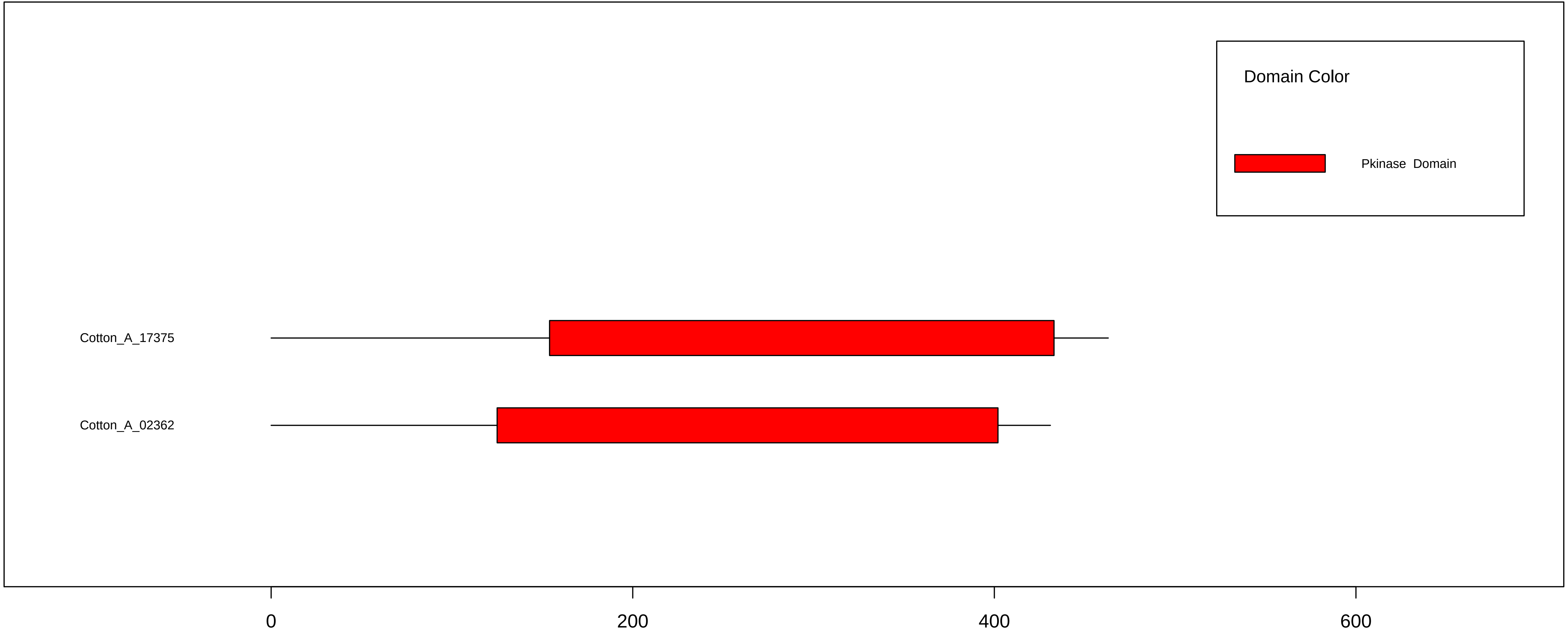

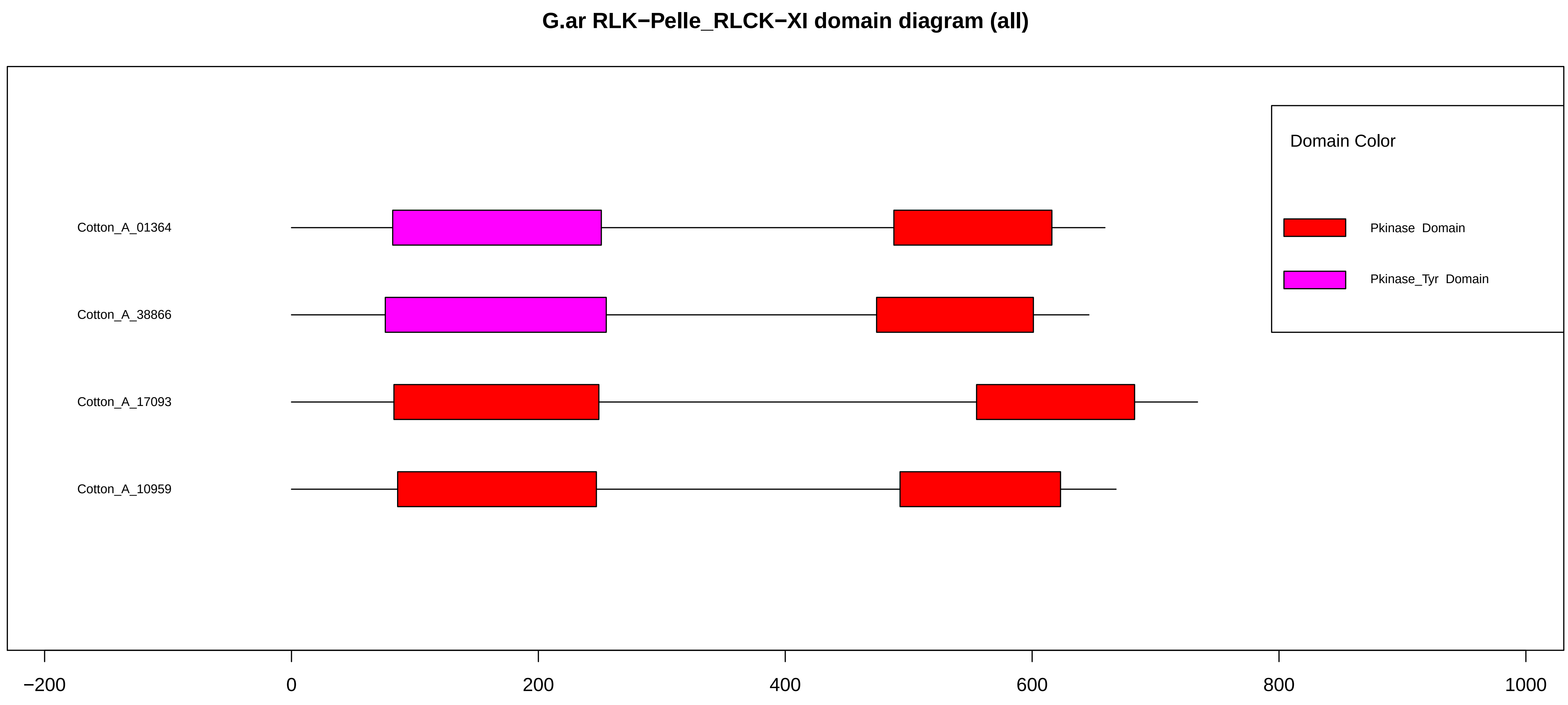
G.ar RLK−Pelle_RLCK−XI domain diagram (all)
Domain Color
Pkinase Domain
Pkinase_Tyr Domain
Cotton_A_01364
Cotton_A_38866
Cotton_A_17093
Cotton_A_10959
−200
0
200
400
600
800
1000

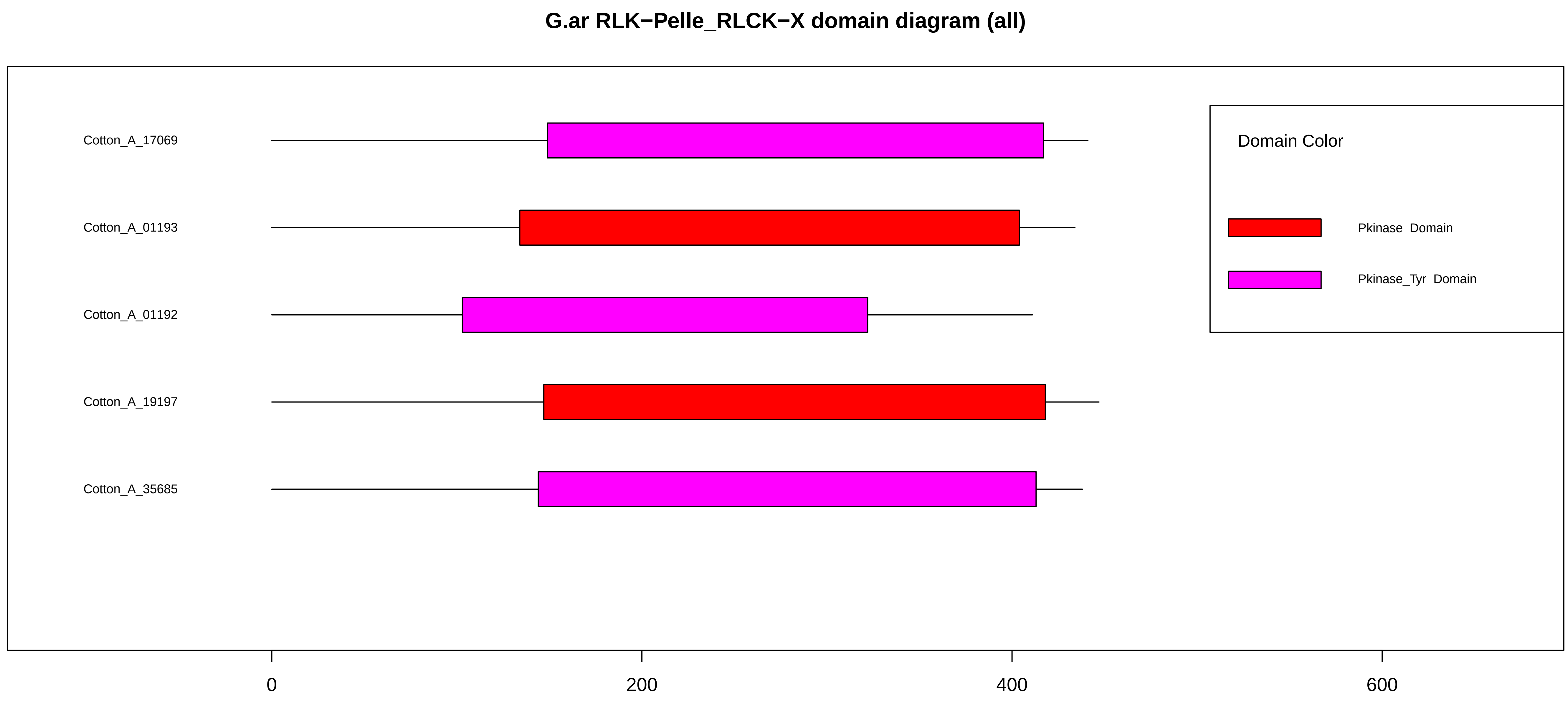

G.ar RLK−Pelle_RLCK−X domain diagram (all)
Cotton_A_17069
Cotton_A_01193
Cotton_A_01192
Cotton_A_19197
Cotton_A_35685
Domain Color
Pkinase Domain
Pkinase_Tyr Domain
0
200
400
600

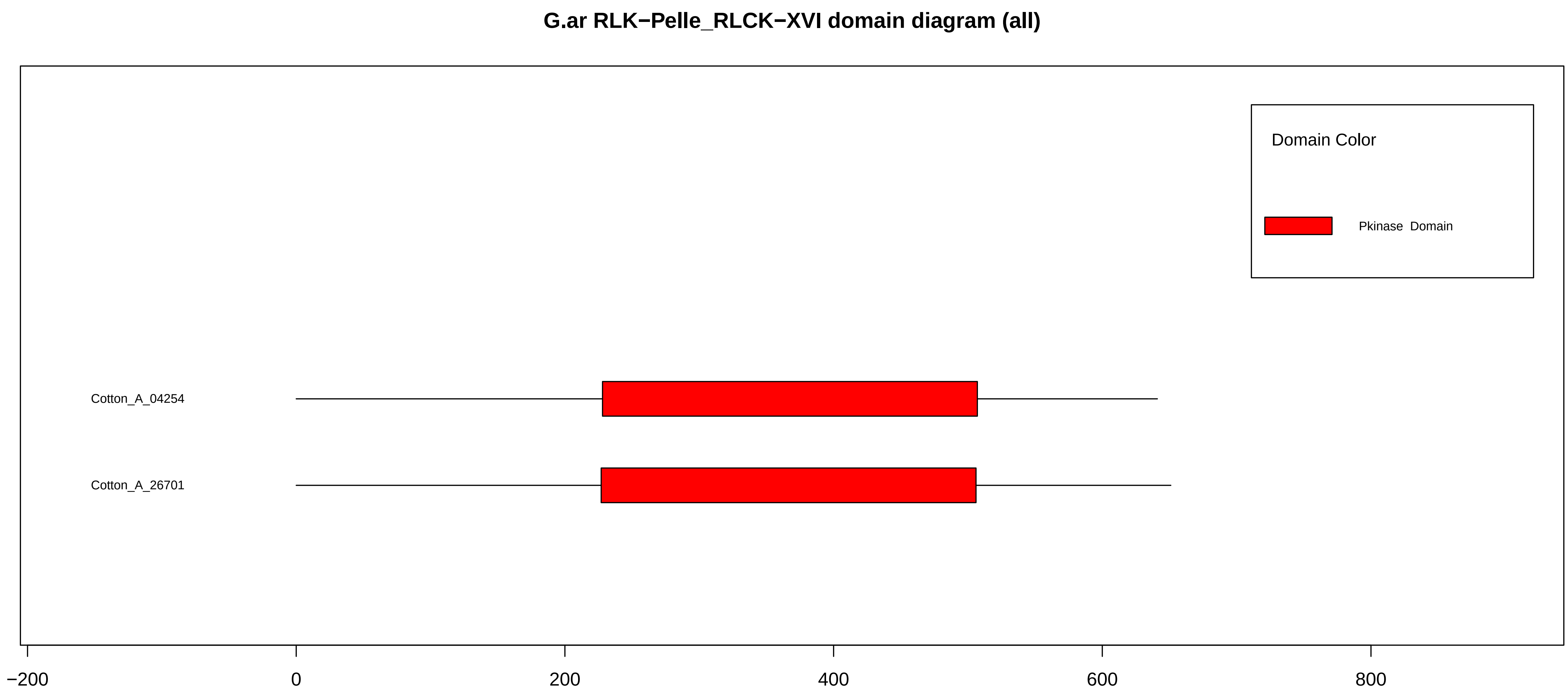
G.ar RLK−Pelle_RLCK−XVI domain diagram (all)
Domain Color
Pkinase Domain
Cotton_A_04254
Cotton_A_26701
−200
0
200
400
600
800

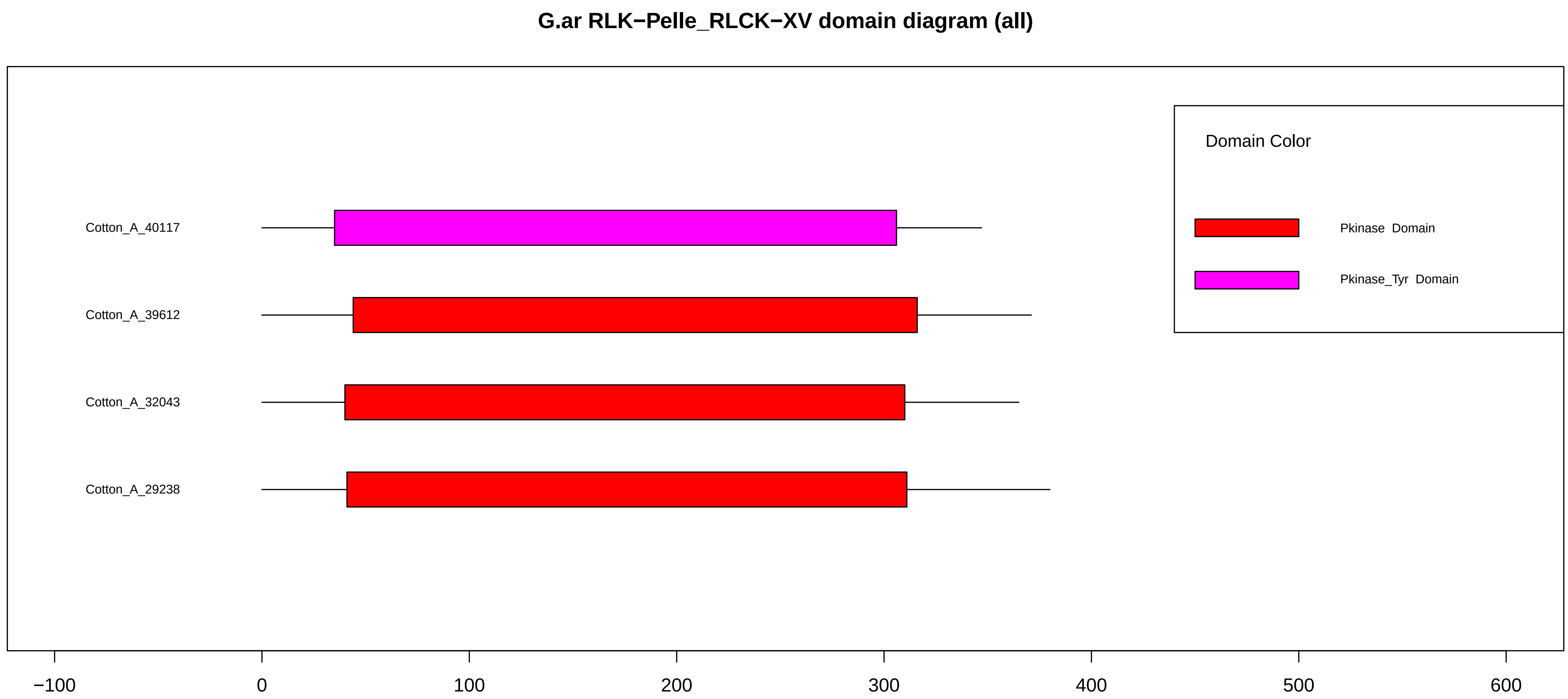
G.ar RLK−Pelle_RLCK−XV domain diagram (all)
Domain Color
Pkinase Domain
Pkinase_Tyr Domain
Cotton_A_40117
Cotton_A_39612
Cotton_A_32043
Cotton_A_29238
−100
0
100
200
300
400
500
600

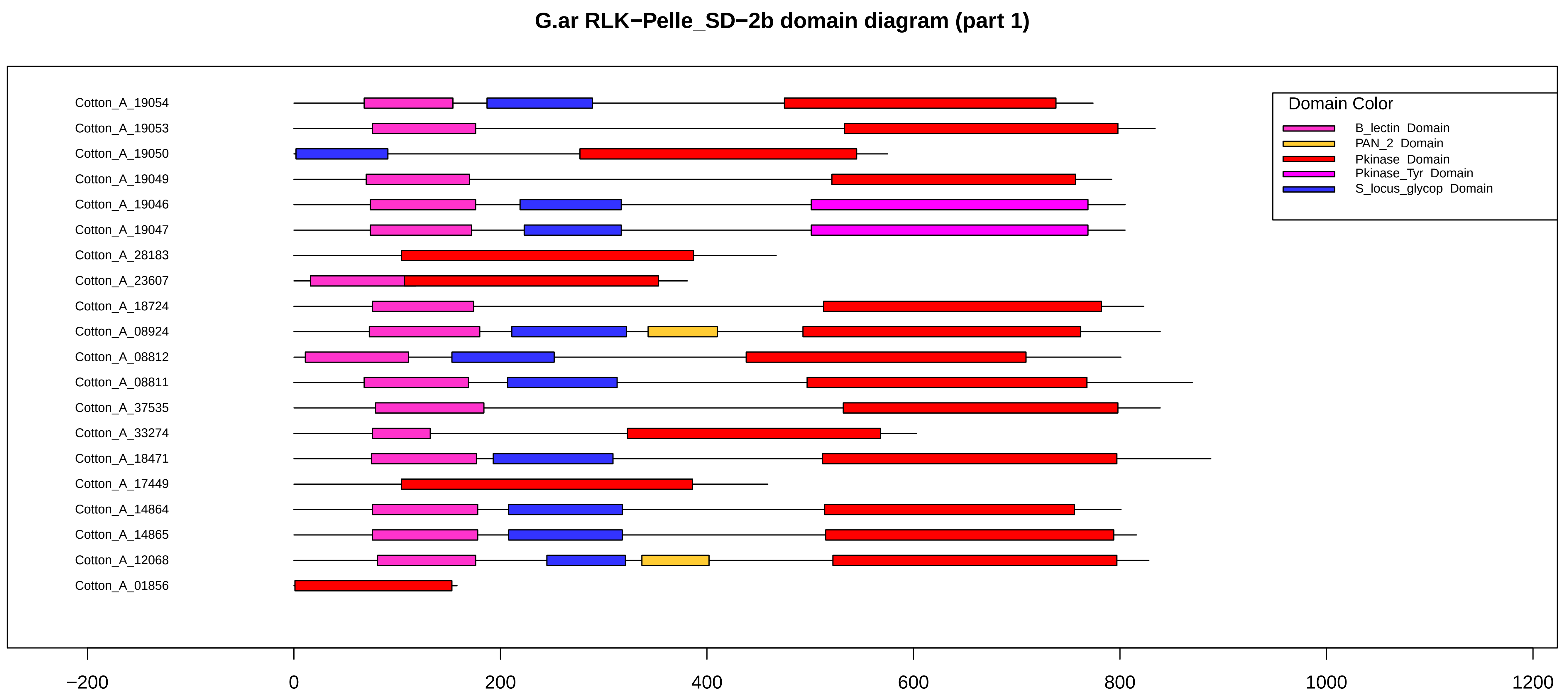
G.ar RLK−Pelle_SD−2b domain diagram (part 1)
Cotton_A_19054
Cotton_A_19053
Cotton_A_19050
Cotton_A_19049
Cotton_A_19046
Cotton_A_19047
Cotton_A_28183
Cotton_A_23607
Cotton_A_18724
Cotton_A_08924
Cotton_A_08812
Cotton_A_08811
Cotton_A_37535
Cotton_A_33274
Cotton_A_18471
Cotton_A_17449
Cotton_A_14864
Cotton_A_14865
Cotton_A_12068
Cotton_A_01856
Domain Color
B_lectin Domain
PAN_2 Domain
Pkinase Domain
Pkinase_Tyr Domain
S_locus_glycop Domain
−200
0
200
400
600
800
1000
1200

G.ar RLK−Pelle_SD−2b domain diagram (part 2)

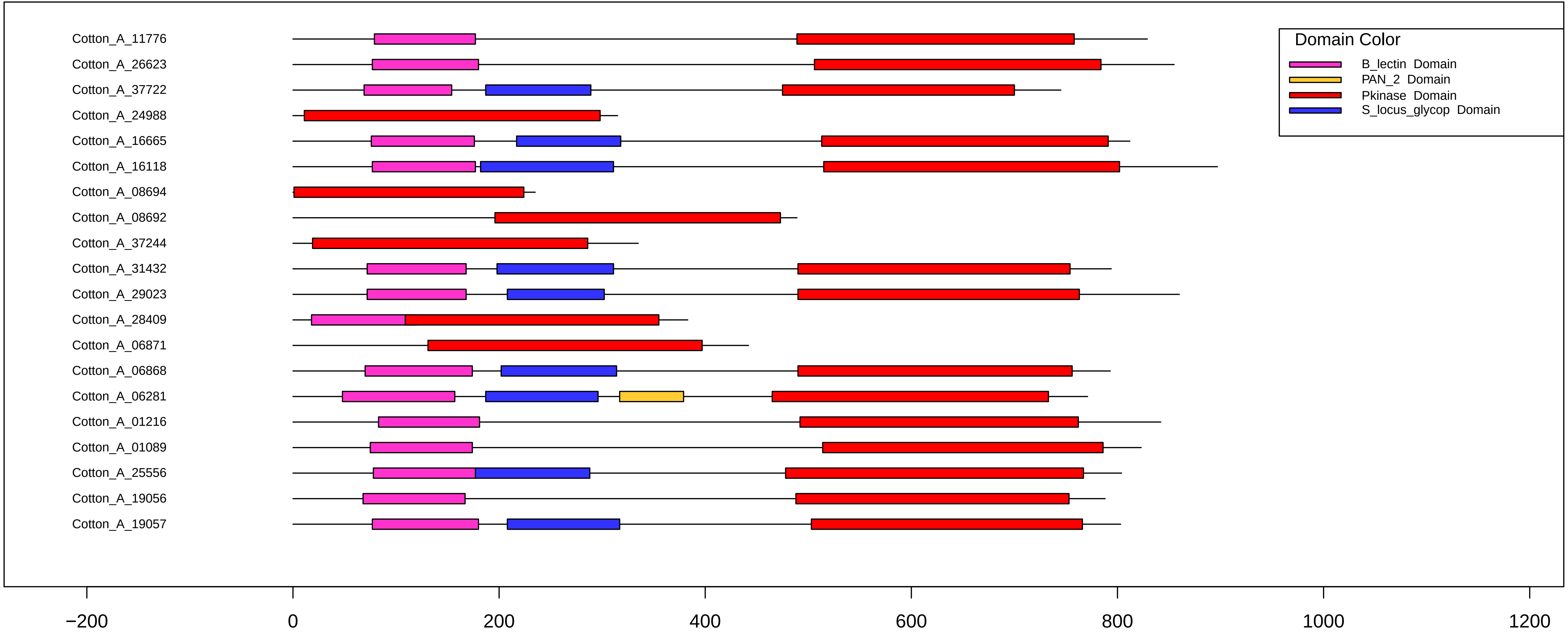

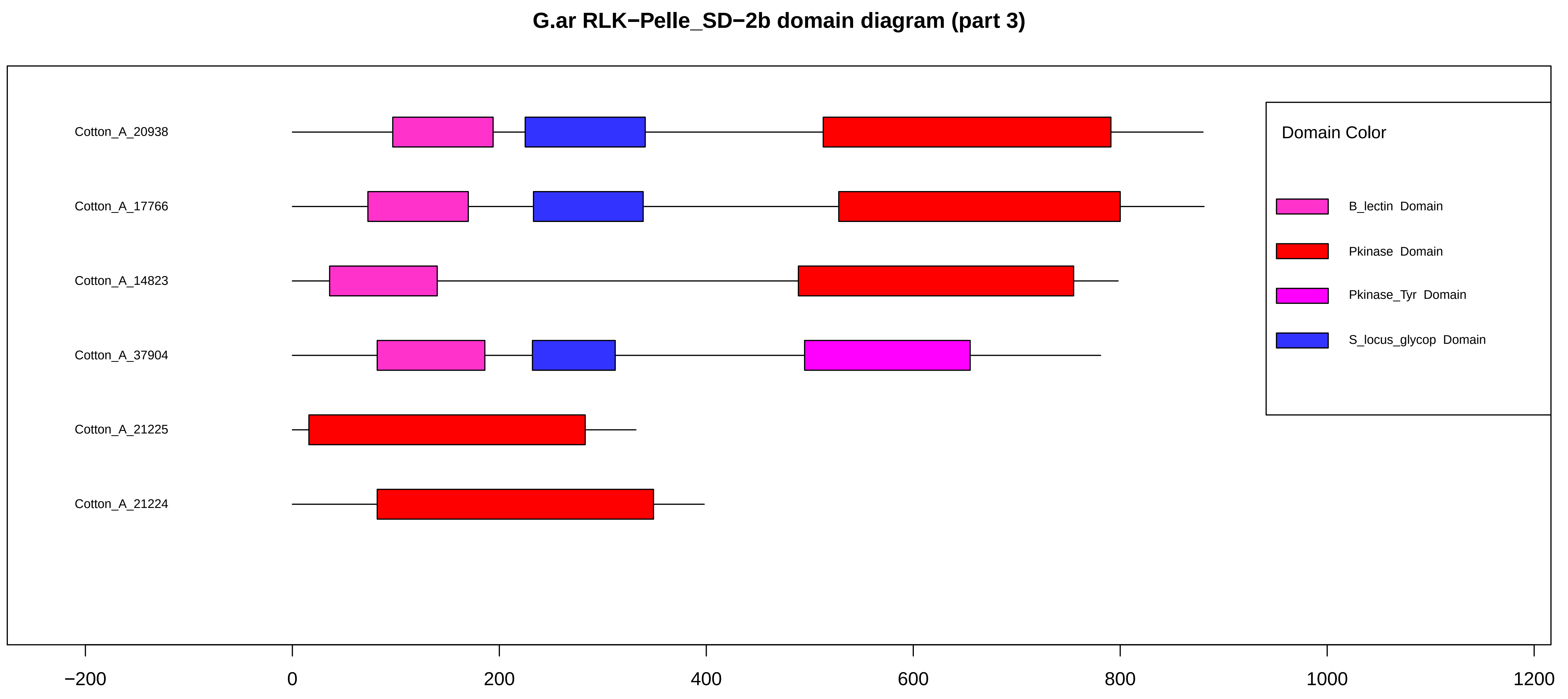

G.ar RLK−Pelle_SD−2b domain diagram (part 3)
Cotton_A_20938
Cotton_A_17766
Cotton_A_14823
Cotton_A_37904
Cotton_A_21225
Cotton_A_21224
Domain Color
B_lectin Domain
Pkinase Domain
Pkinase_Tyr Domain
S_locus_glycop Domain
−200
0
200
400
600
800
1000
1200

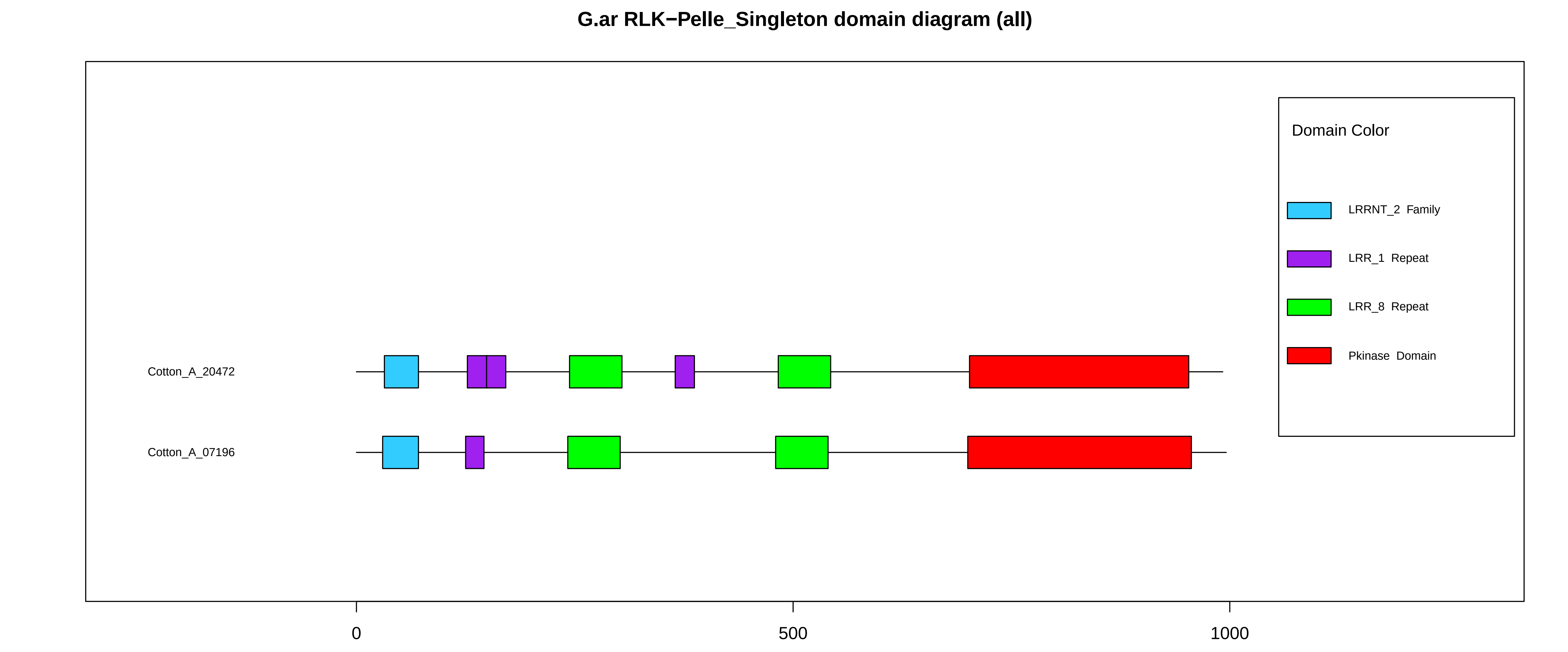
G.ar RLK−Pelle_Singleton domain diagram (all)
Domain Color
LRRNT_2 Family
LRR_1 Repeat
LRR_8 Repeat
Pkinase Domain
Cotton_A_20472
Cotton_A_07196
0
500
1000

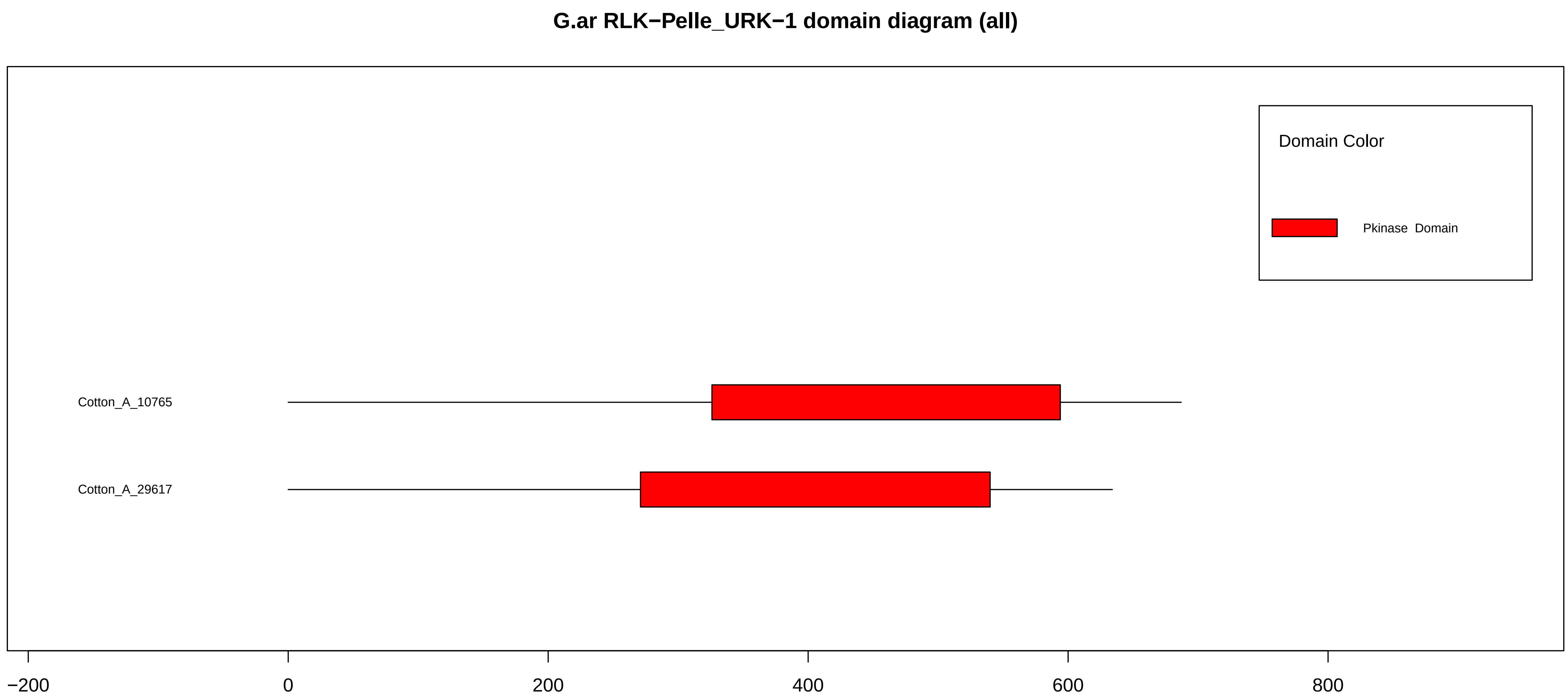
G.ar RLK−Pelle_URK−1 domain diagram (all)
Domain Color
Pkinase Domain
Cotton_A_10765
Cotton_A_29617
−200
0
200
400
600
800

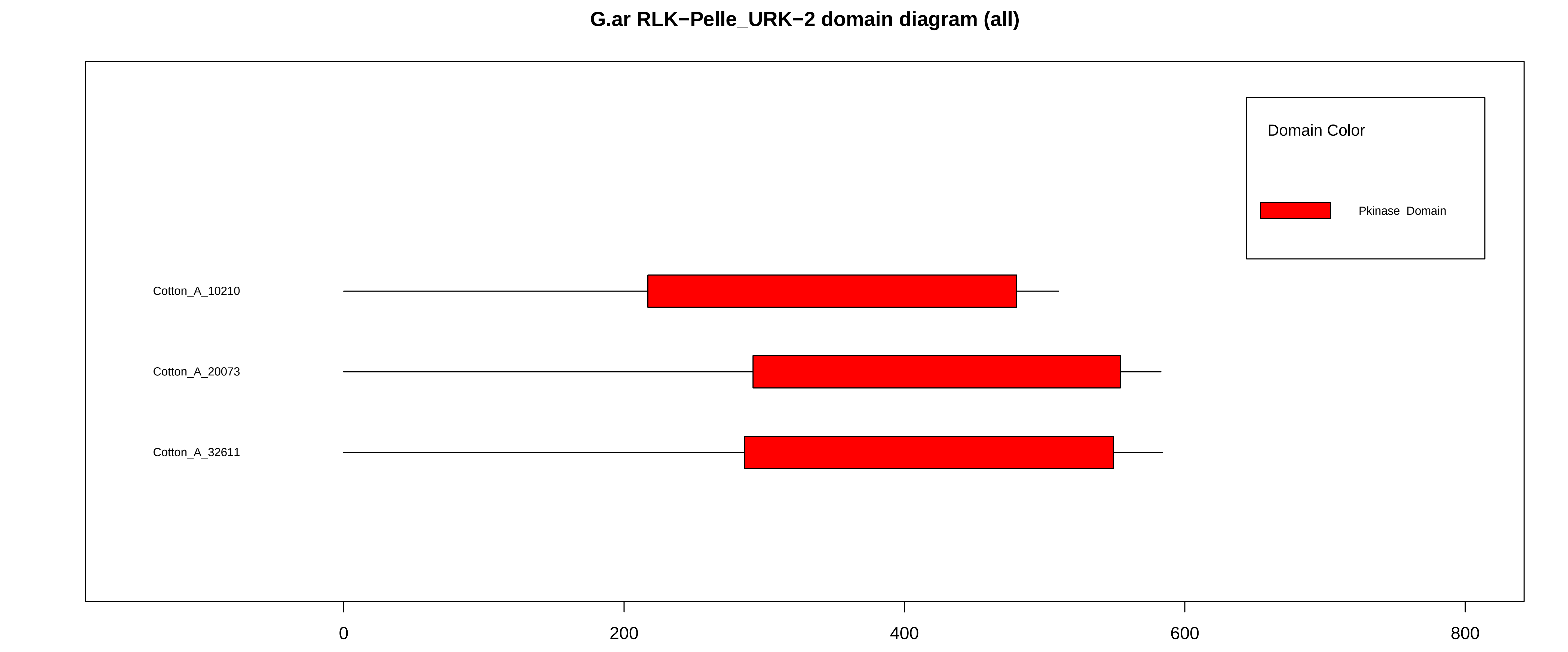

G.ar RLK−Pelle_URK−2 domain diagram (all)
Domain Color
Pkinase Domain
Cotton_A_10210
Cotton_A_20073
Cotton_A_32611
0
200
400
600
800

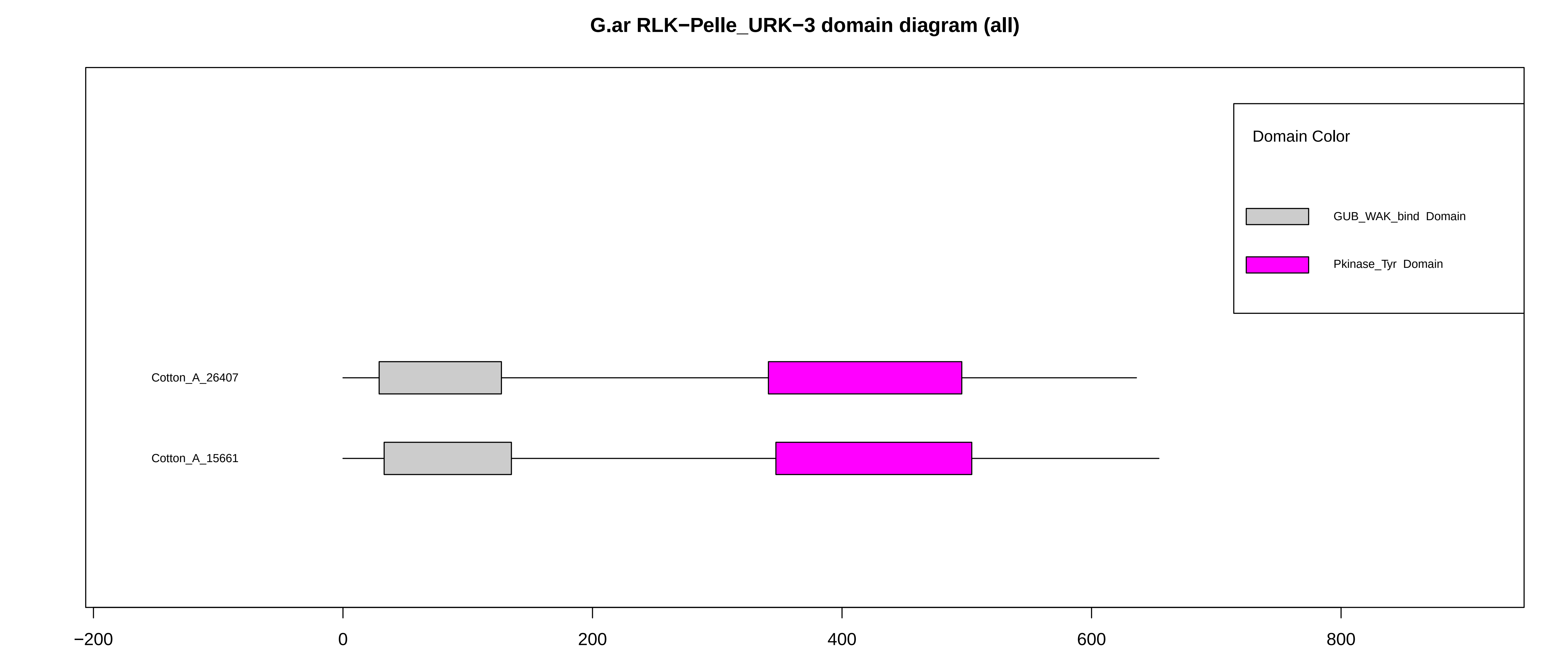
G.ar RLK−Pelle_URK−3 domain diagram (all)
Domain Color
GUB_WAK_bind Domain
Pkinase_Tyr Domain
Cotton_A_26407
Cotton_A_15661
−200
0
200
400
600
800

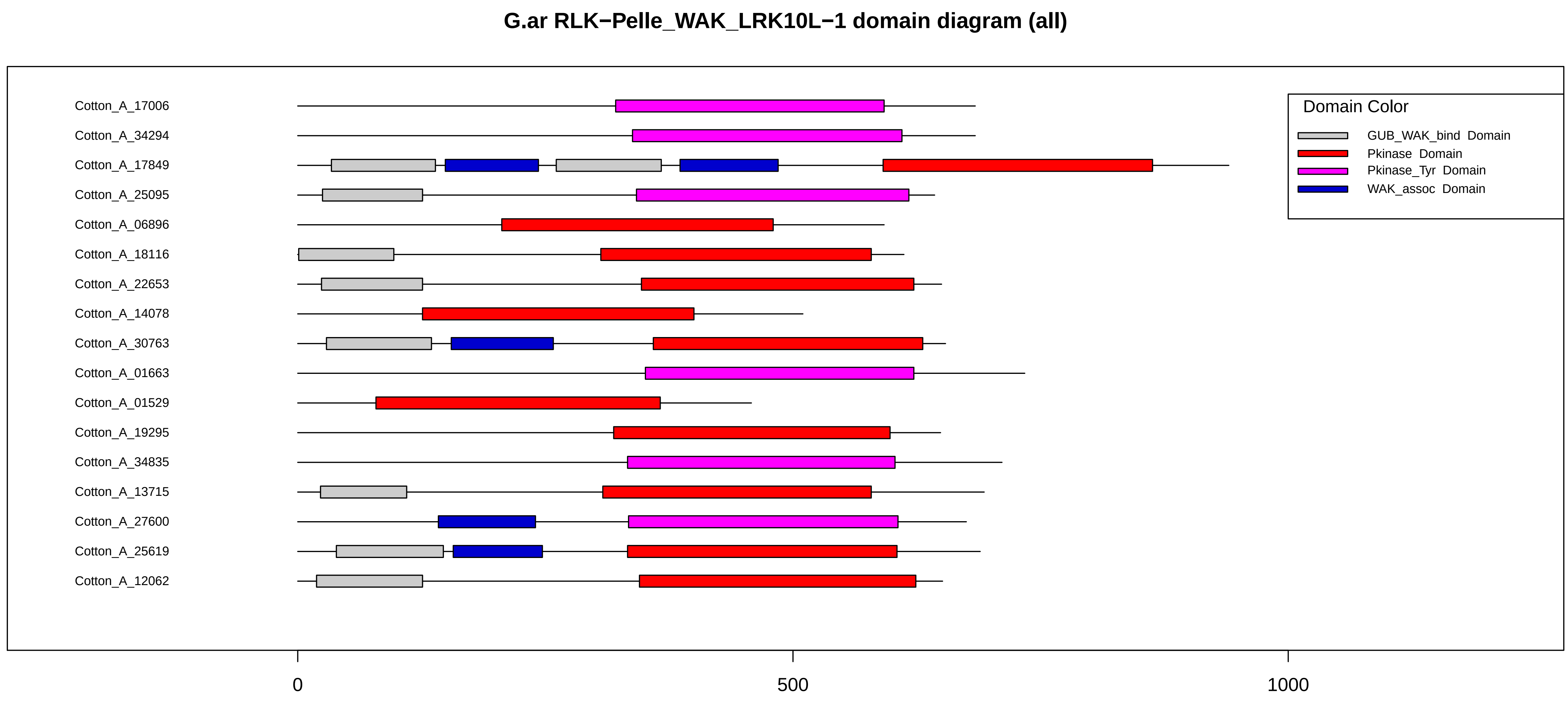
G.ar RLK−Pelle_WAK_LRK10L−1 domain diagram (all)
Cotton_A_17006
Cotton_A_34294
Cotton_A_17849
Cotton_A_25095
Cotton_A_06896
Cotton_A_18116
Cotton_A_22653
Cotton_A_14078
Cotton_A_30763
Cotton_A_01663
Cotton_A_01529
Cotton_A_19295
Cotton_A_34835
Cotton_A_13715
Cotton_A_27600
Cotton_A_25619
Cotton_A_12062
Domain Color
GUB_WAK_bind Domain
Pkinase Domain
Pkinase_Tyr Domain
WAK_assoc Domain
0
500
1000

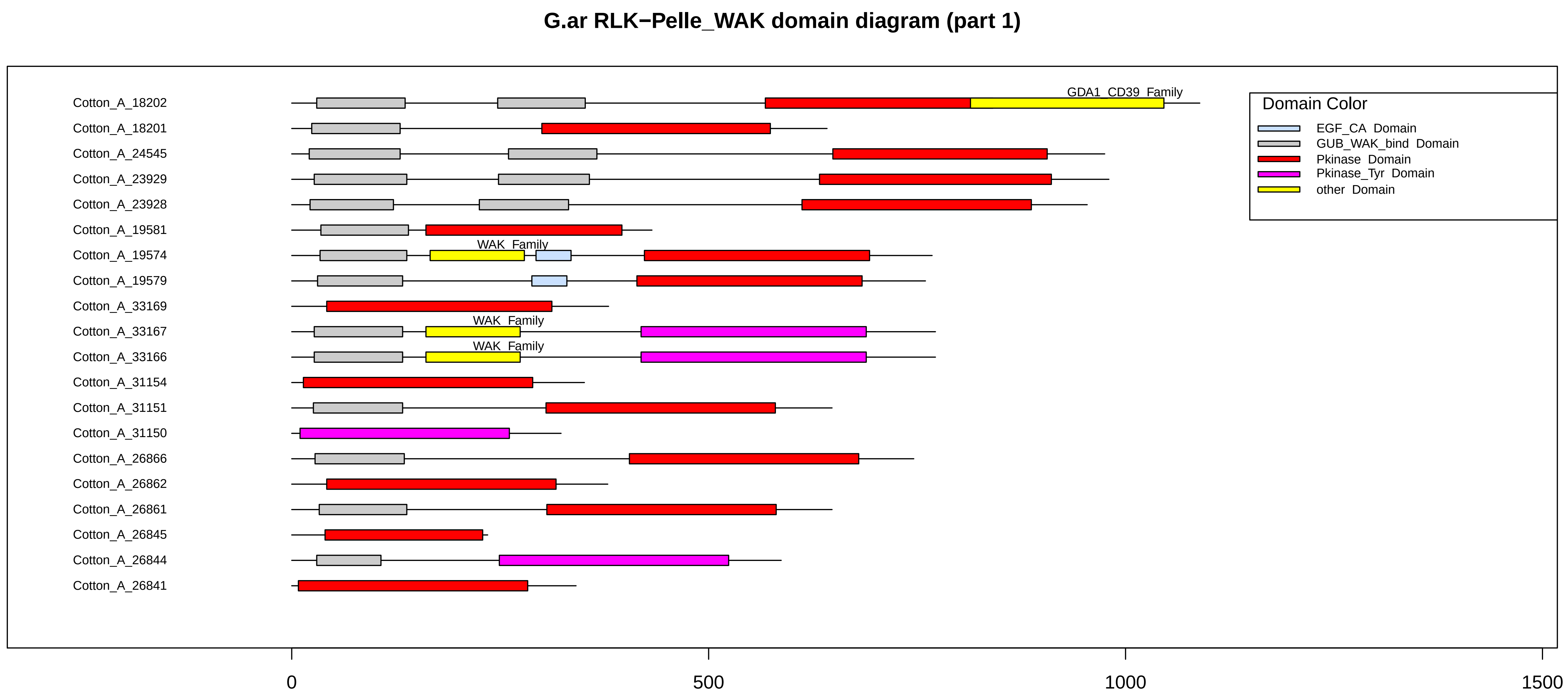
G.ar RLK−Pelle_WAK domain diagram (part 1)
Cotton_A_18202
Cotton_A_18201
Cotton_A_24545
Cotton_A_23929
Cotton_A_23928
Cotton_A_19581
Cotton_A_19574
Cotton_A_19579
Cotton_A_33169
Cotton_A_33167
Cotton_A_33166
Cotton_A_31154
Cotton_A_31151
Cotton_A_31150
Cotton_A_26866
Cotton_A_26862
Cotton_A_26861
Cotton_A_26845
Cotton_A_26844
Cotton_A_26841
GDA1_CD39_Family
WAK_Family
WAK_Family
WAK_Family
Domain Color
EGF_CA Domain
GUB_WAK_bind Domain
Pkinase Domain
Pkinase_Tyr Domain
other Domain
0
500
1000
1500

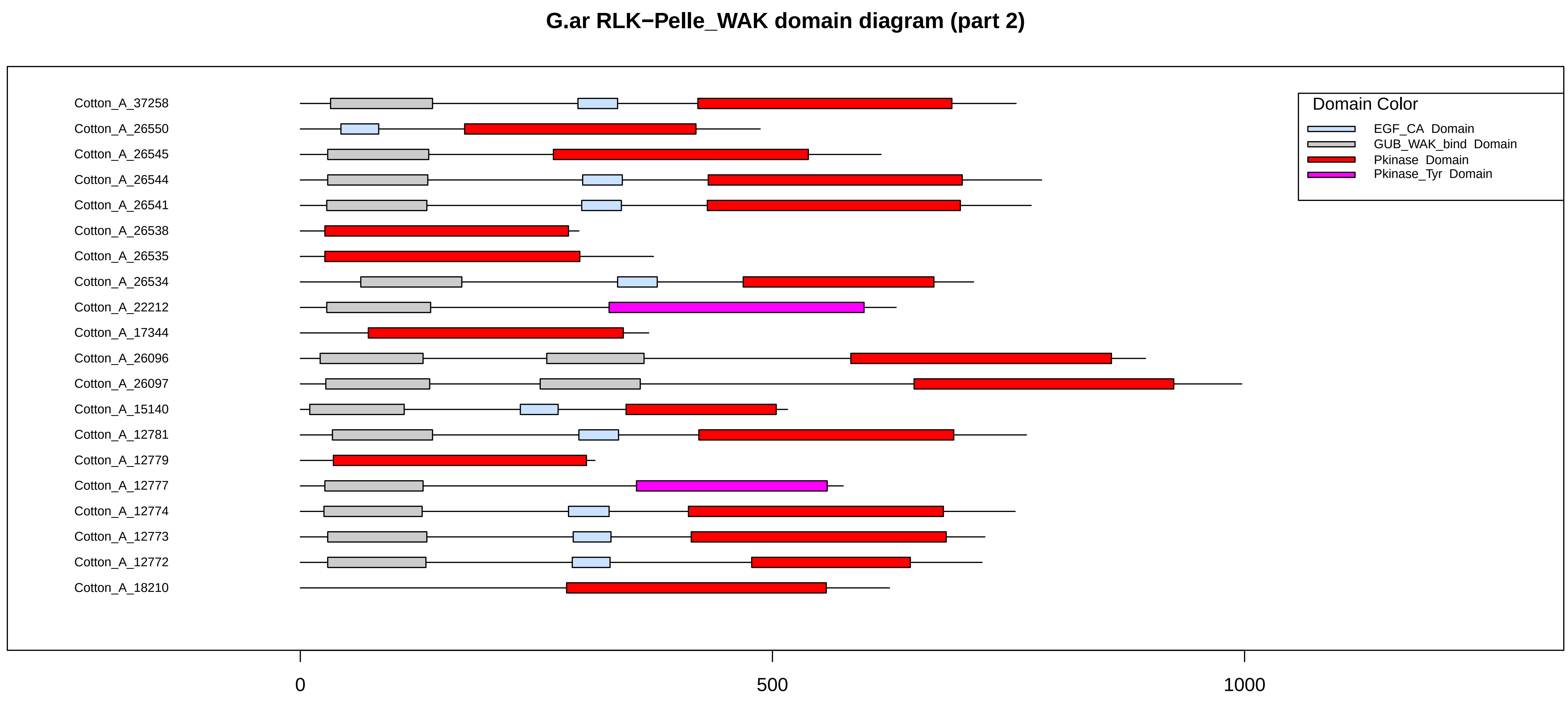

G.ar RLK−Pelle_WAK domain diagram (part 2)
Cotton_A_37258
Cotton_A_26550
Cotton_A_26545
Cotton_A_26544
Cotton_A_26541
Cotton_A_26538
Cotton_A_26535
Cotton_A_26534
Cotton_A_22212
Cotton_A_17344
Cotton_A_26096
Cotton_A_26097
Cotton_A_15140
Cotton_A_12781
Cotton_A_12779
Cotton_A_12777
Cotton_A_12774
Cotton_A_12773
Cotton_A_12772
Cotton_A_18210
Domain Color
EGF_CA Domain
GUB_WAK_bind Domain
Pkinase Domain
Pkinase_Tyr Domain
0
500
1000

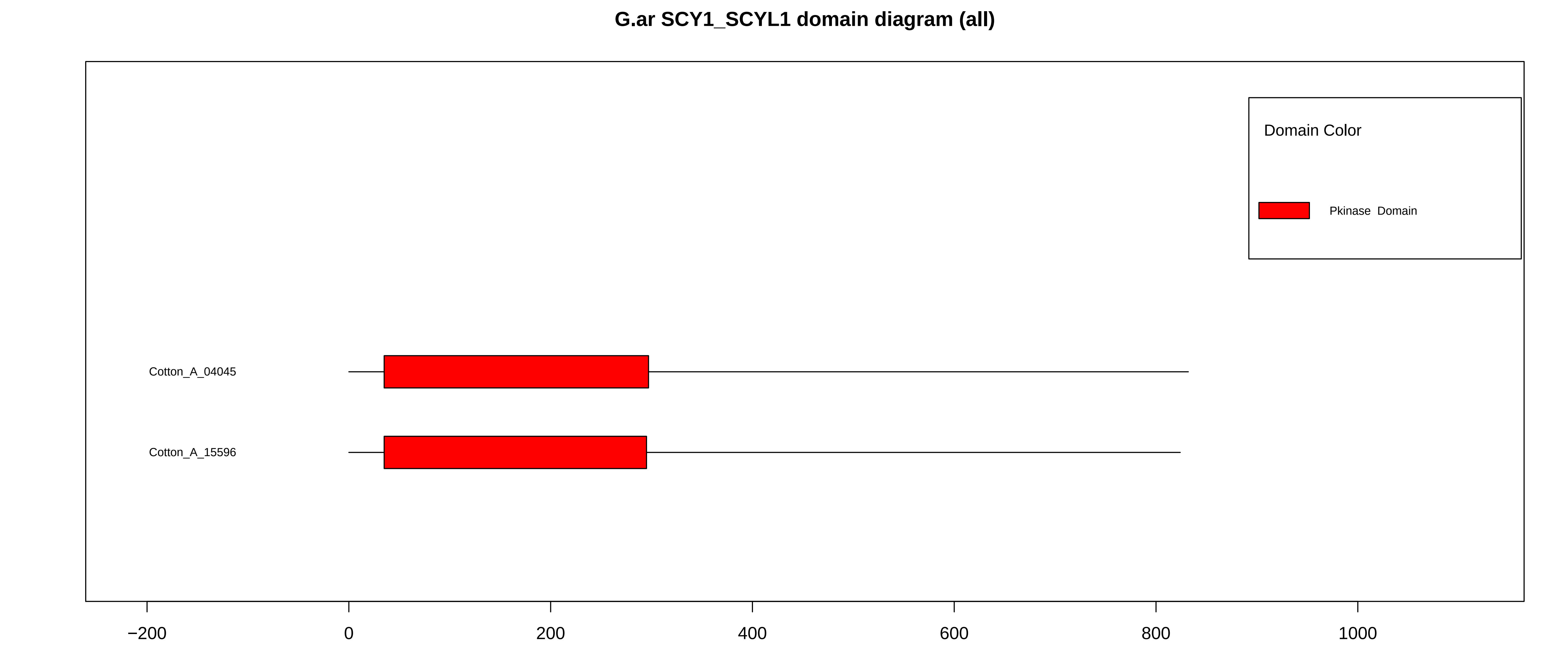
G.ar SCY1_SCYL1 domain diagram (all)
Domain Color
Pkinase Domain
Cotton_A_04045
Cotton_A_15596
−200
0
200
400
600
800
1000

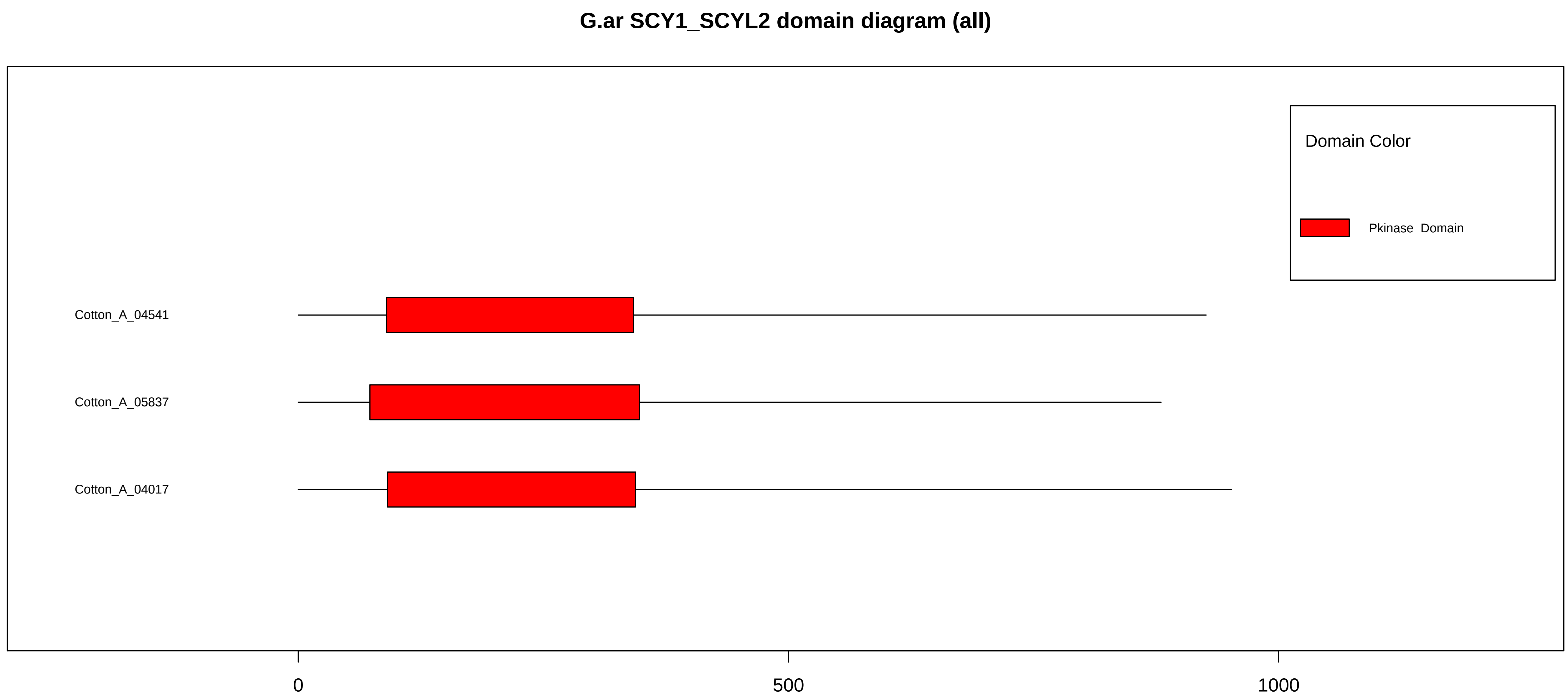
G.ar SCY1_SCYL2 domain diagram (all)
Domain Color
Pkinase Domain
Cotton_A_04541
Cotton_A_05837
Cotton_A_04017
0
500
1000

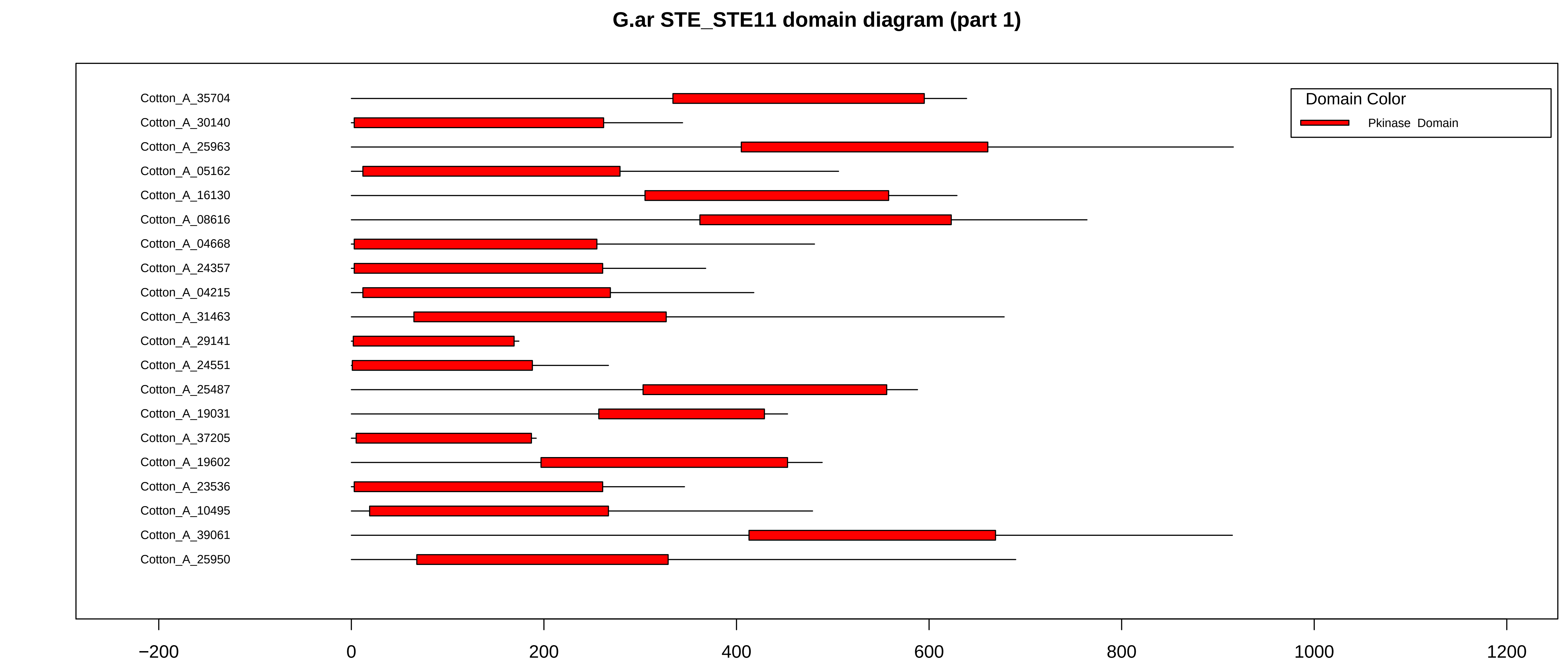
G.ar STE_STE11 domain diagram (part 1)
Cotton_A_35704
Cotton_A_30140
Cotton_A_25963
Cotton_A_05162
Cotton_A_16130
Cotton_A_08616
Cotton_A_04668
Cotton_A_24357
Cotton_A_04215
Cotton_A_31463
Cotton_A_29141
Cotton_A_24551
Cotton_A_25487
Cotton_A_19031
Cotton_A_37205
Cotton_A_19602
Cotton_A_23536
Cotton_A_10495
Cotton_A_39061
Cotton_A_25950
Domain Color
Pkinase Domain
−200
0
200
400
600
800
1000
1200

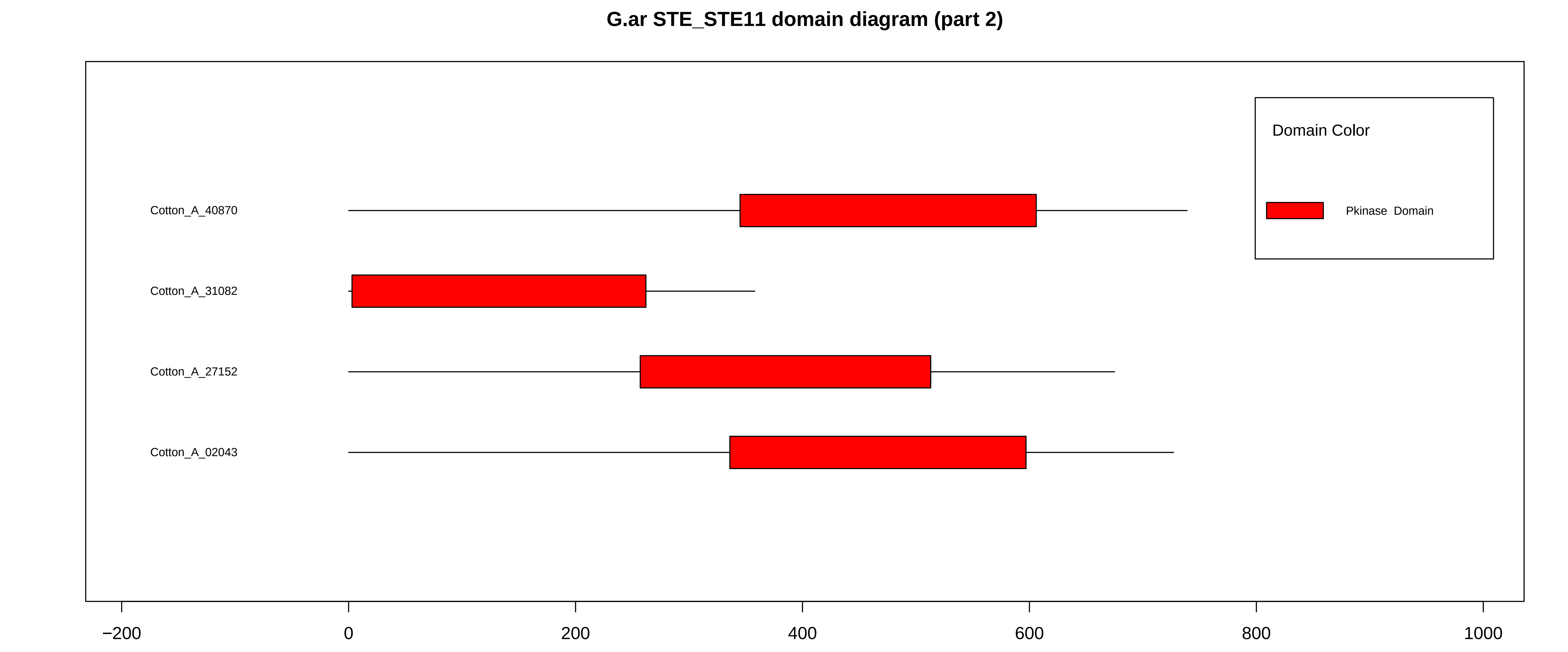
G.ar STE_STE11 domain diagram (part 2)
Domain Color
Pkinase Domain
Cotton_A_40870
Cotton_A_31082
Cotton_A_27152
Cotton_A_02043
−200
0
200
400
600
800
1000

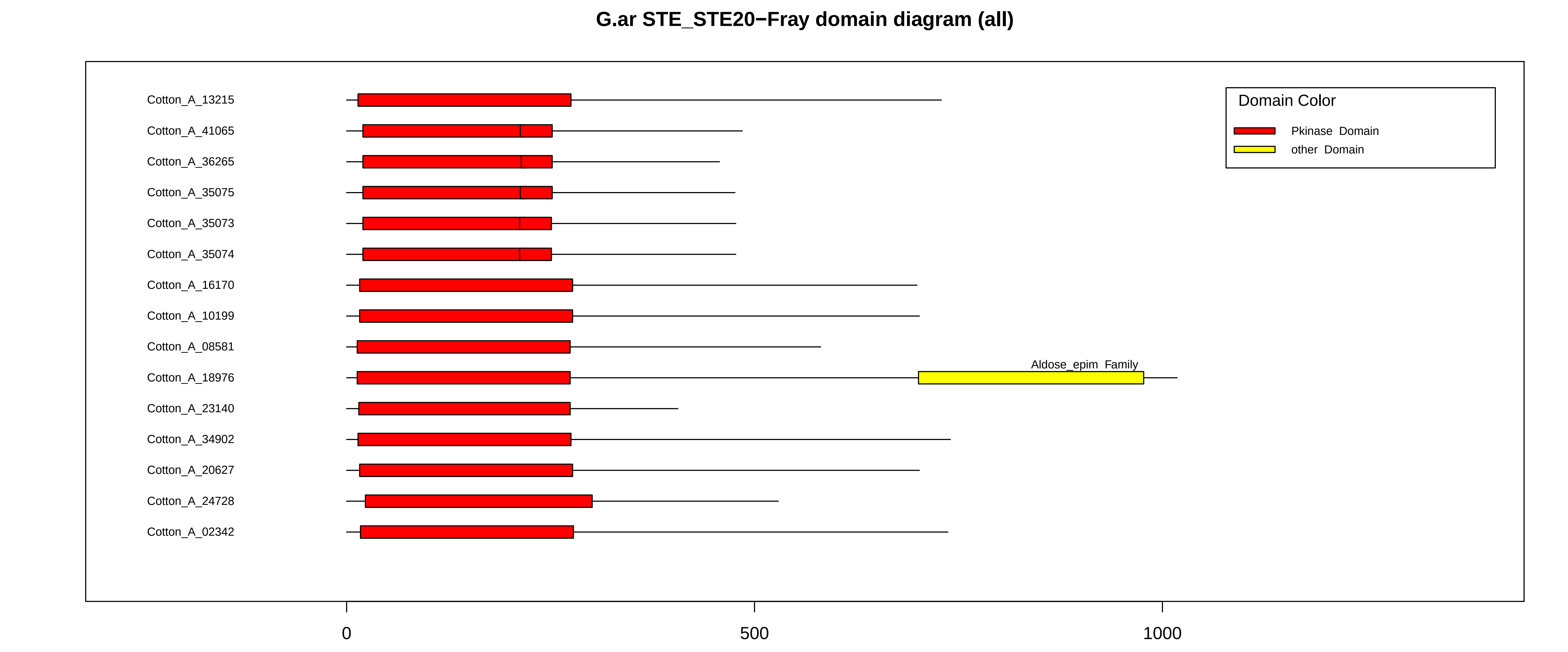
G.ar STE_STE20−Fray domain diagram (all)
Cotton_A_13215
Cotton_A_41065
Cotton_A_36265
Cotton_A_35075
Cotton_A_35073
Cotton_A_35074
Cotton_A_16170
Cotton_A_10199
Cotton_A_08581
Cotton_A_18976
Cotton_A_23140
Cotton_A_34902
Cotton_A_20627
Cotton_A_24728
Cotton_A_02342
Aldose_epim Family
Domain Color
Pkinase Domain
other Domain
0
500
1000

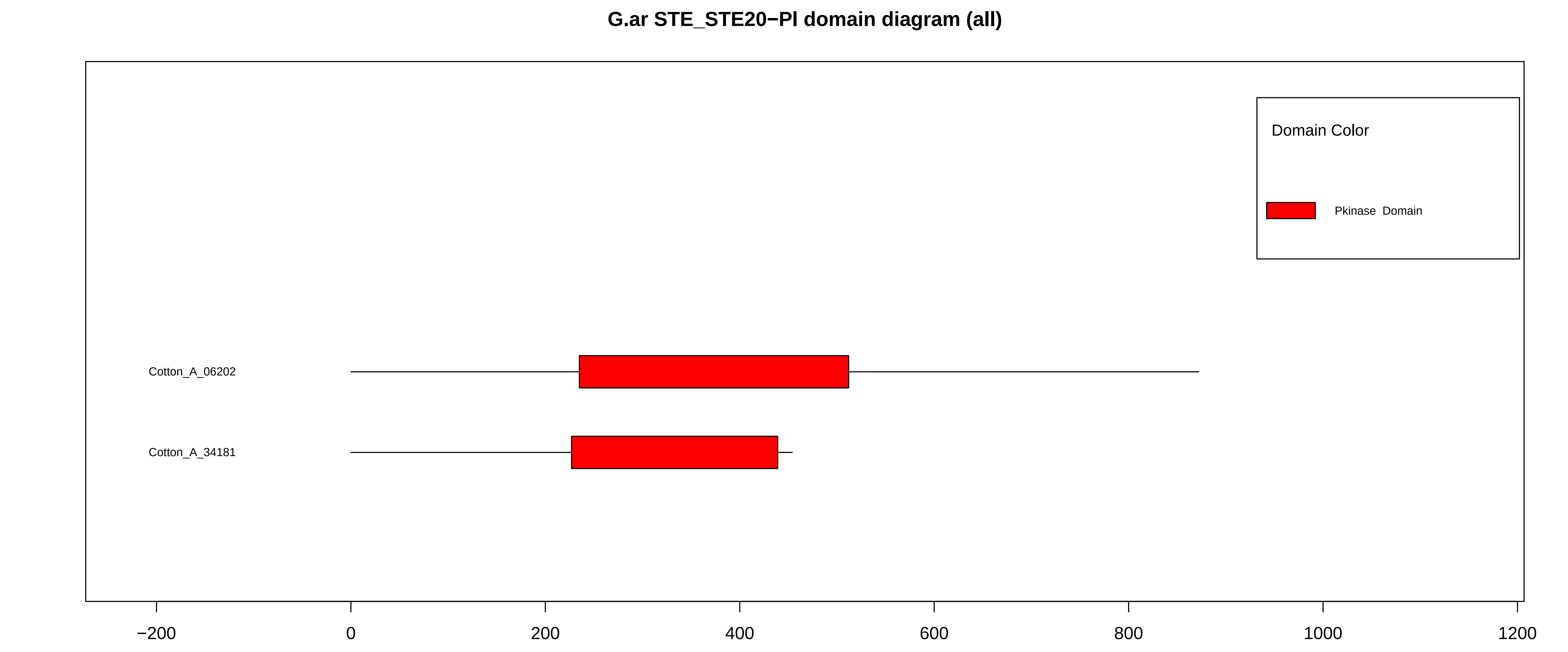
G.ar STE_STE20−Pl domain diagram (all)
Domain Color
Pkinase Domain
Cotton_A_06202
Cotton_A_34181
−200
0
200
400
600
800
1000
1200

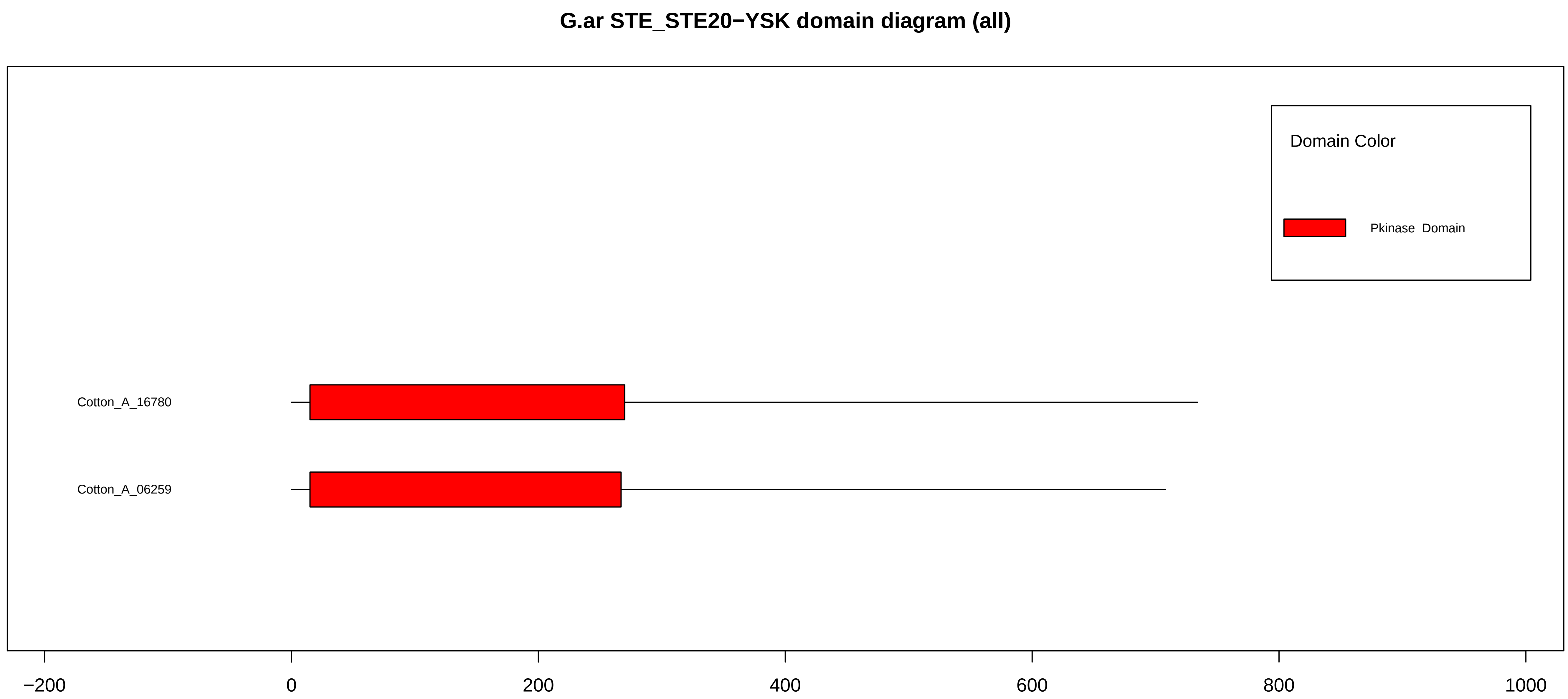
G.ar STE_STE20−YSK domain diagram (all)
Domain Color
Pkinase Domain
Cotton_A_16780
Cotton_A_06259
−200
0
200
400
600
800
1000

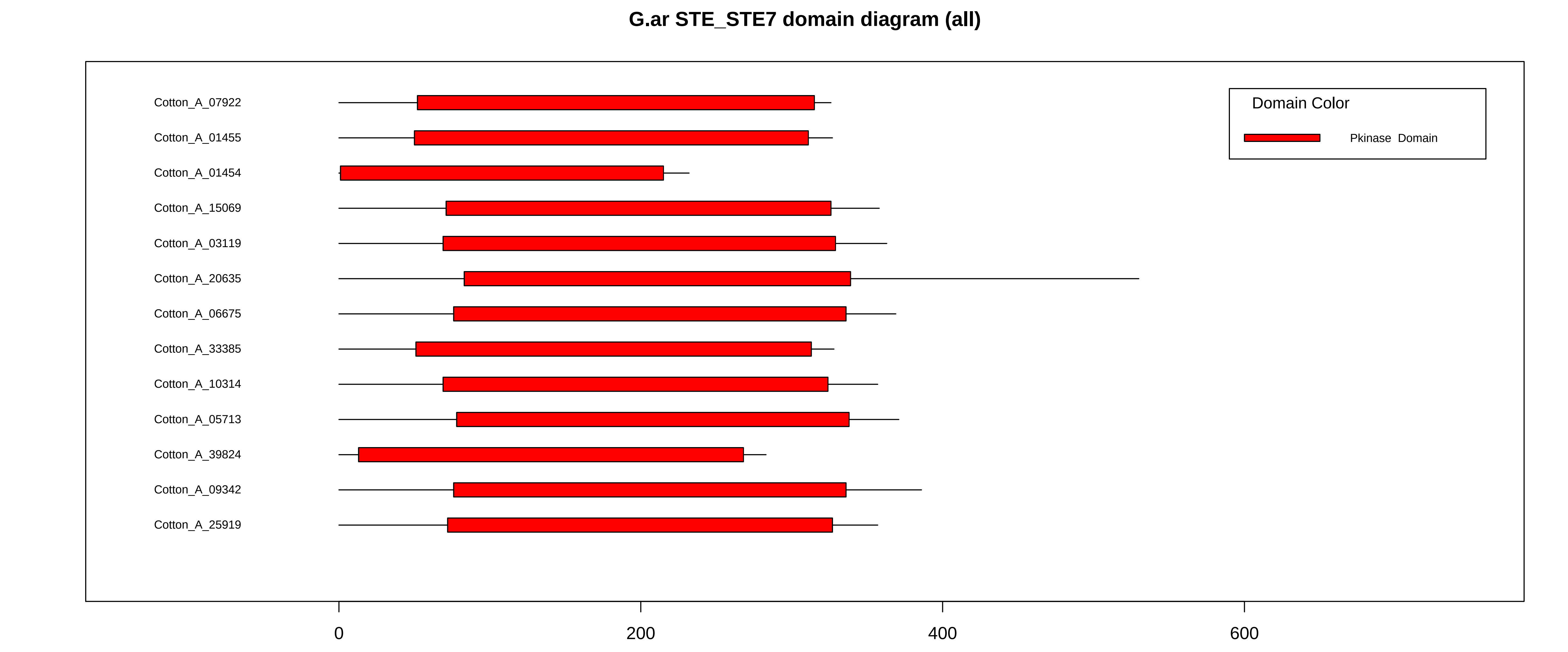
G.ar STE_STE7 domain diagram (all)
Cotton_A_07922
Cotton_A_01455
Cotton_A_01454
Cotton_A_15069
Cotton_A_03119
Cotton_A_20635
Cotton_A_06675
Cotton_A_33385
Cotton_A_10314
Cotton_A_05713
Cotton_A_39824
Cotton_A_09342
Cotton_A_25919
Domain Color
Pkinase Domain
0
200
400
600

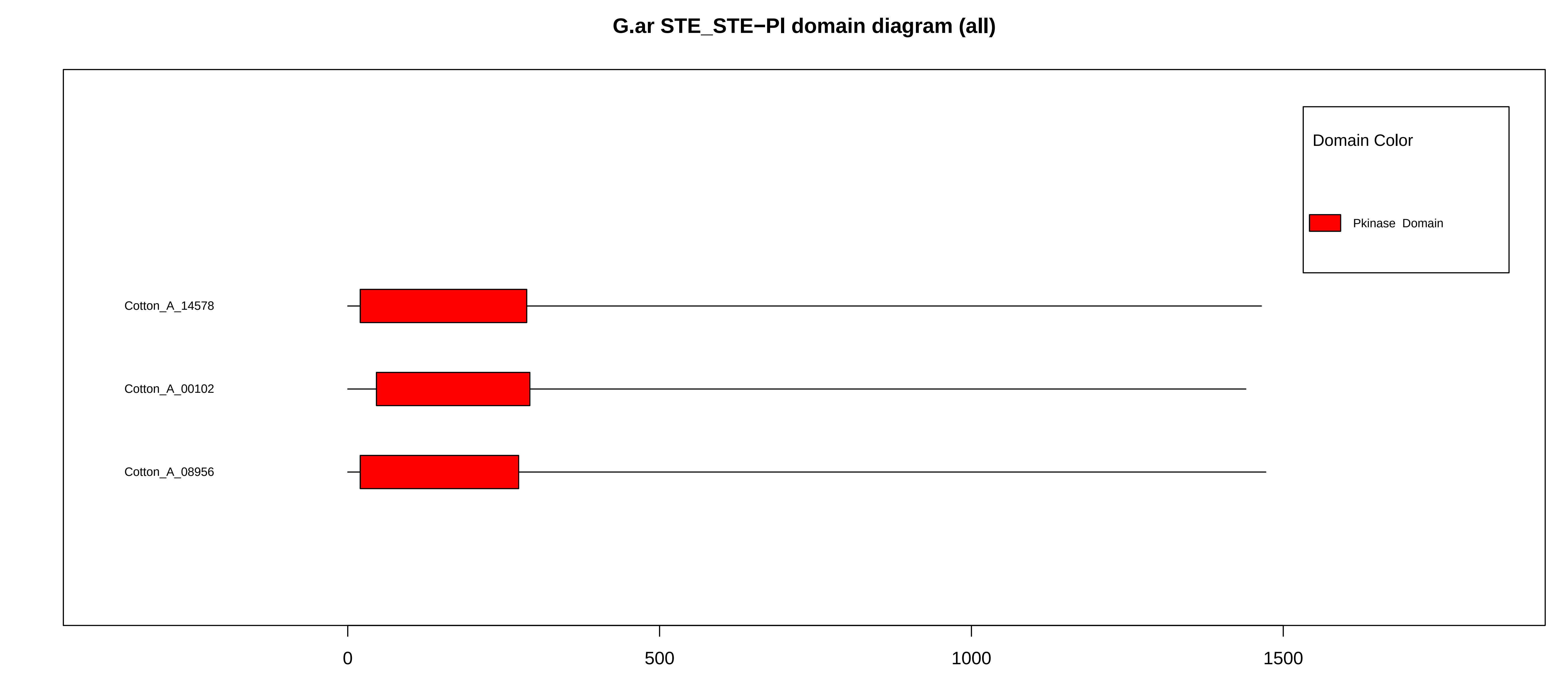
G.ar STE_STE−PI domain diagram (all)
Domain Color
Pkinase Domain
Cotton_A_14578
Cotton_A_00102
Cotton_A_08956
0
500
1000
1500

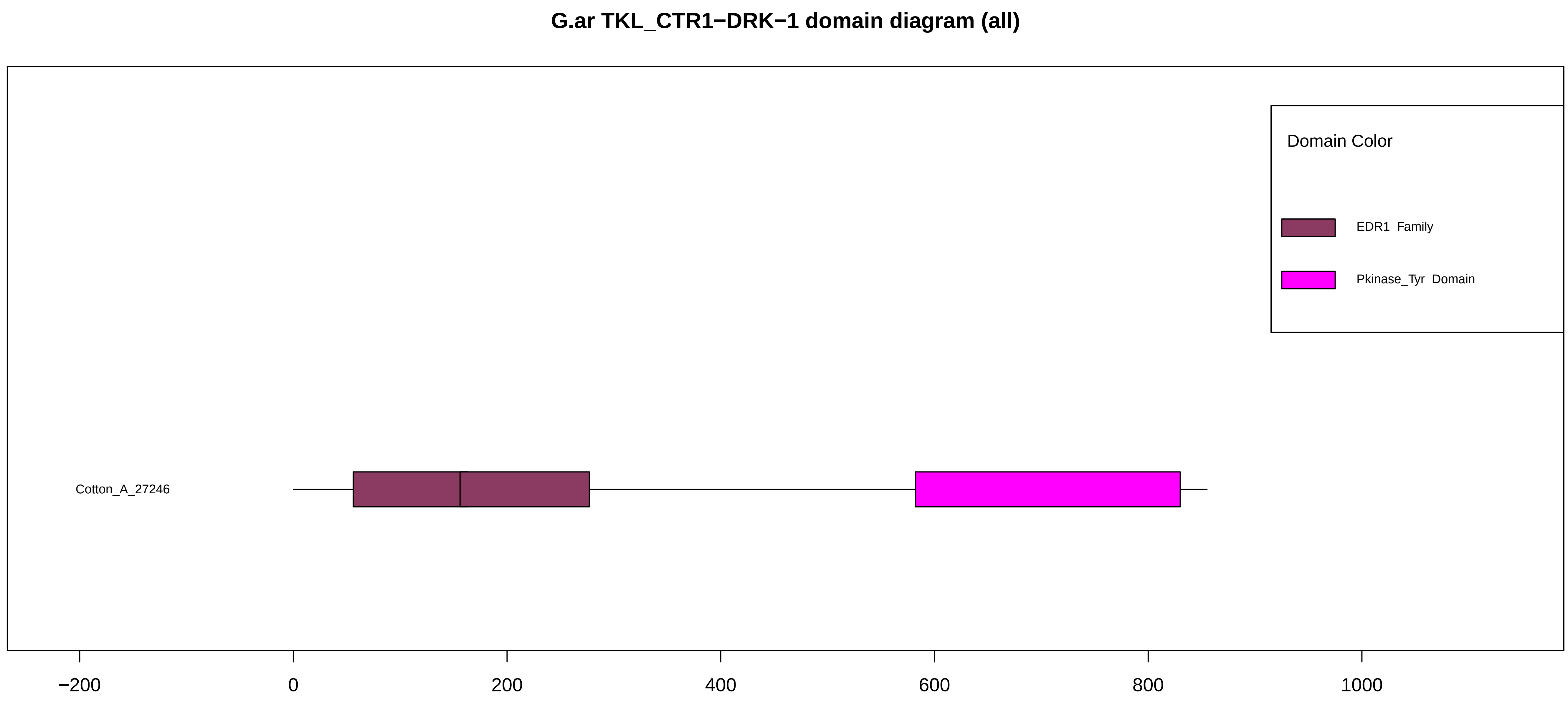
G.ar TKL_CTR1−DRK−1 domain diagram (all)
Domain Color
EDR1 Family
Pkinase_Tyr Domain
Cotton_A_27246
−200
0
200
400
600
800
1000

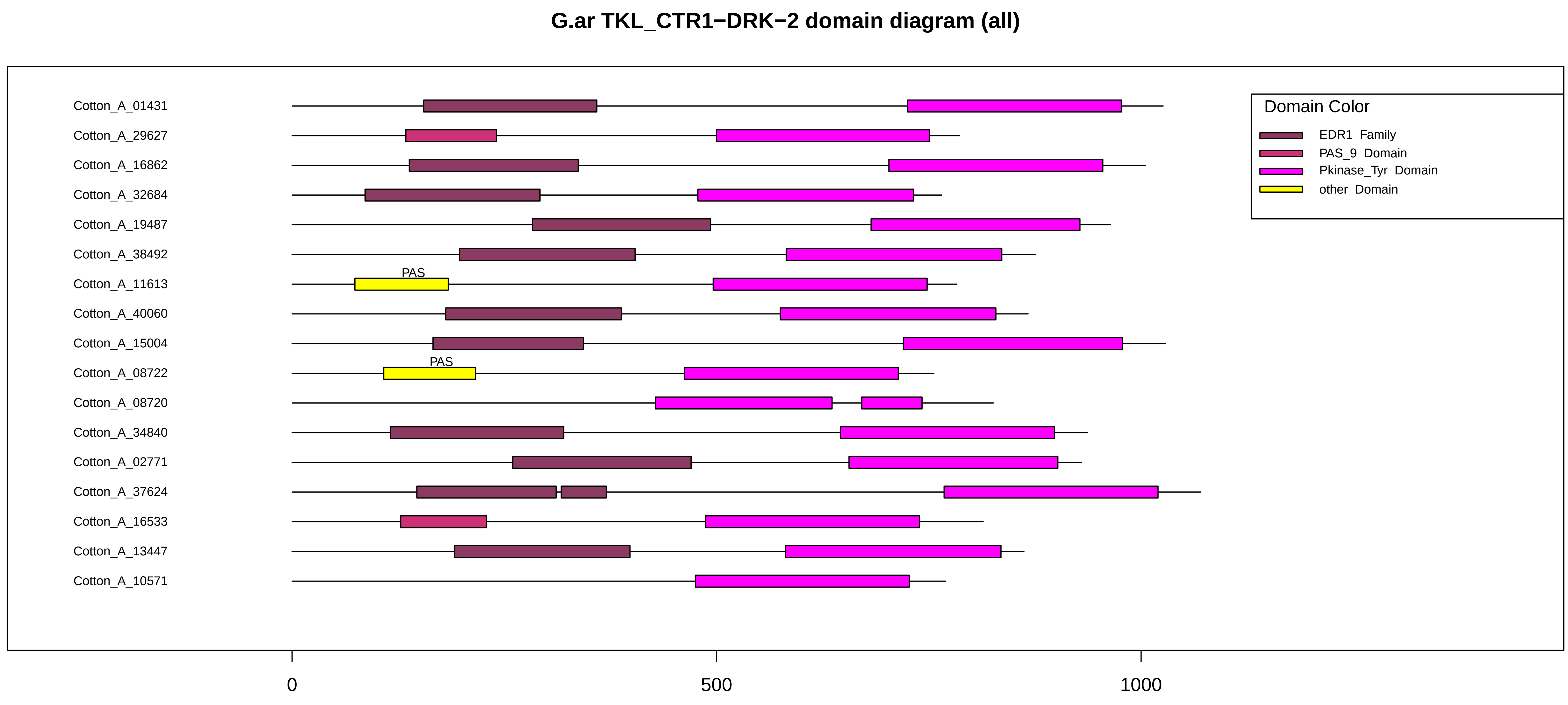
G.ar TKL_CTR1−DRK−2 domain diagram (all)
Cotton_A_01431
Cotton_A_29627
Cotton_A_16862
Cotton_A_32684
Cotton_A_19487
Cotton_A_38492
Cotton_A_11613
Cotton_A_40060
Cotton_A_15004
Cotton_A_08722
Cotton_A_08720
Cotton_A_34840
Cotton_A_02771
Cotton_A_37624
Cotton_A_16533
Cotton_A_13447
Cotton_A_10571
PAS
PAS
Domain Color
EDR1 Family
PAS_9 Domain
Pkinase_Tyr Domain
other Domain
0
500
1000

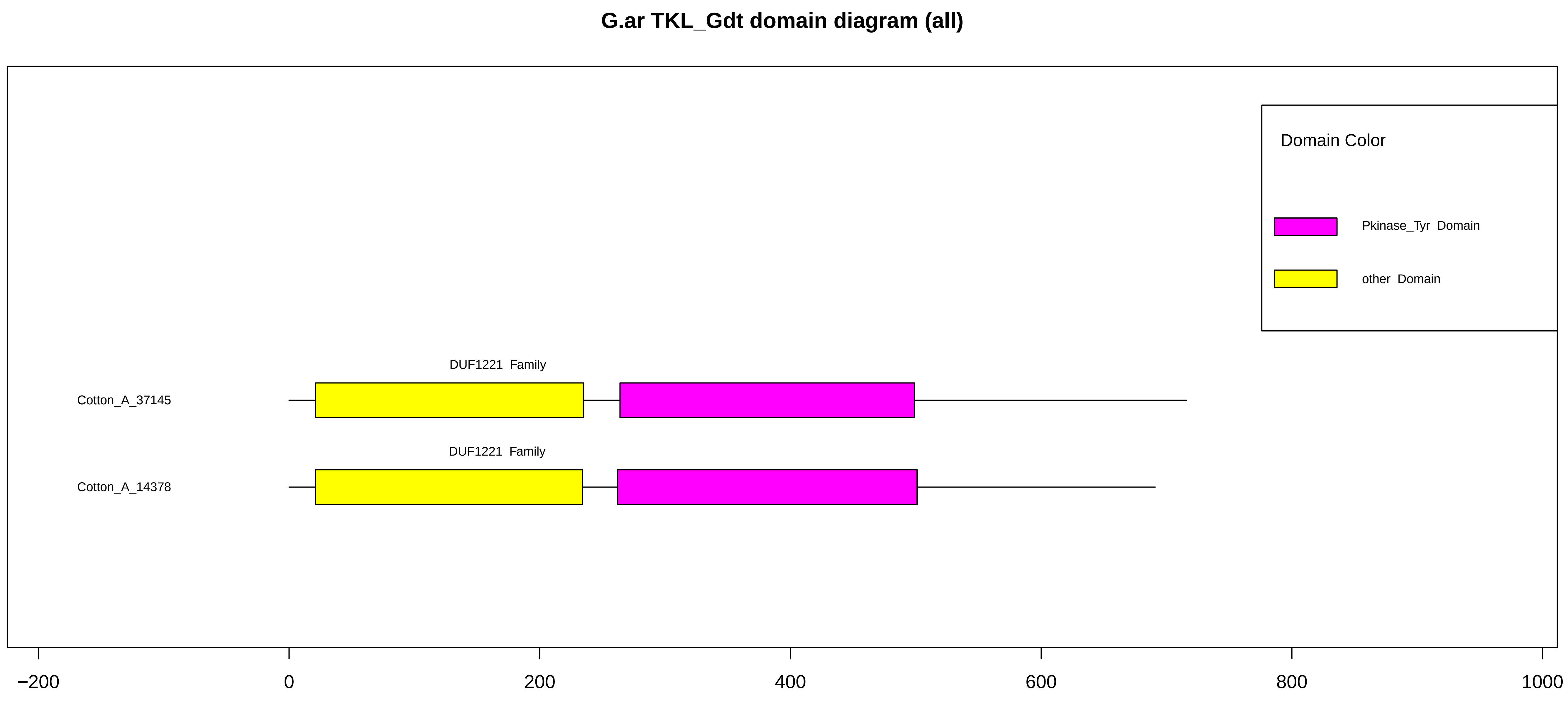
G.ar TKL_Gdt domain diagram (all)
Domain Color
Pkinase_Tyr Domain
other Domain
DUF1221 Family
Cotton_A_37145
DUF1221 Family
Cotton_A_14378
−200
0
200
400
600
800
1000

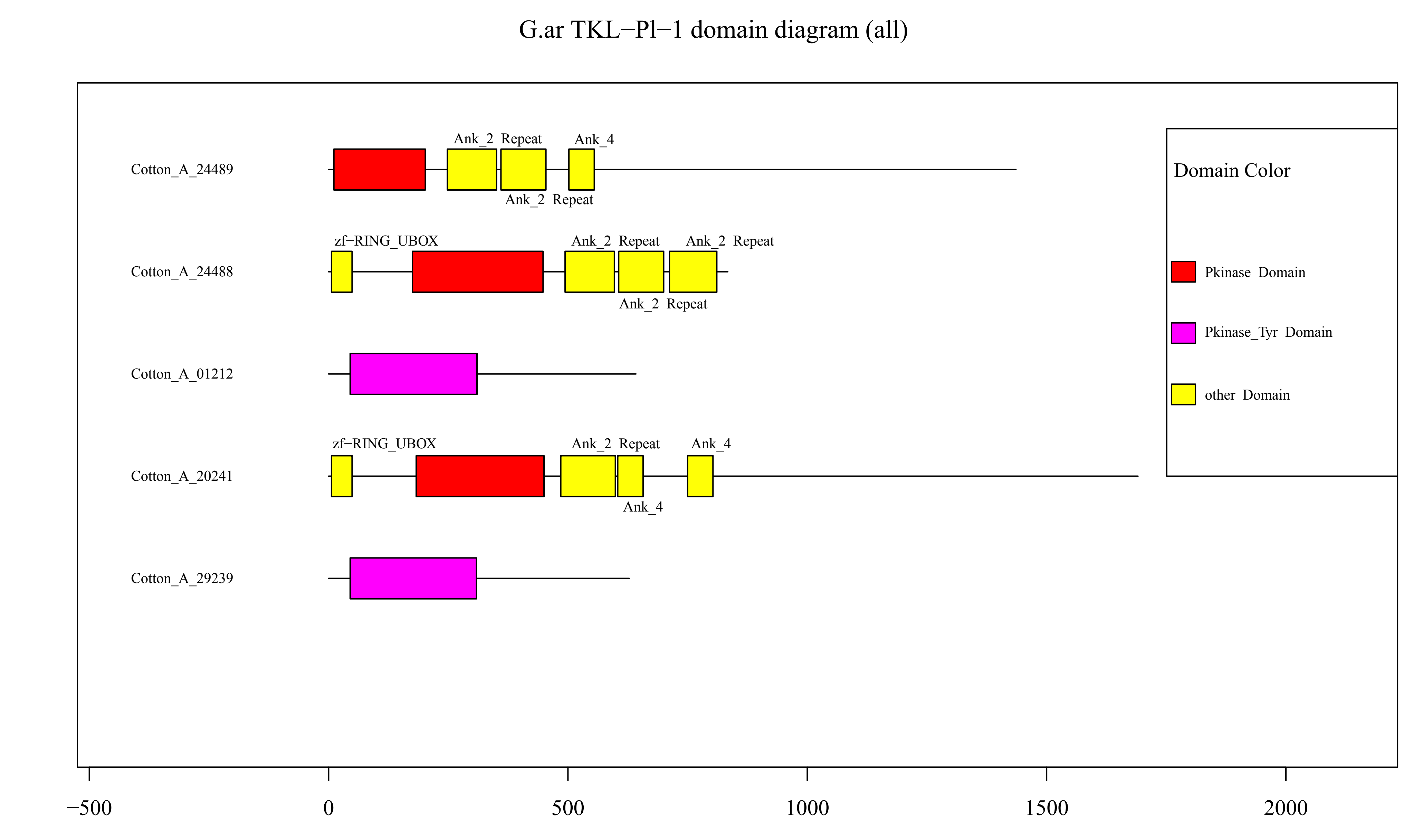
G.ar TKL−Pl−1 domain diagram (all)
Cotton_A_24489
Ank_2 Repeat
Ank_2 Repeat
Ank_4
Cotton_A_24488
zf−RING_UBOX
Ank_2 Repeat
Ank_2 Repeat
Ank_2 Repeat
Cotton_A_01212
Cotton_A_20241
zf−RING_UBOX
Ank_2 Repeat
Ank_4
Ank_4
Cotton_A_29239
Domain Color
Pkinase Domain
Pkinase_Tyr Domain
other Domain
−500
0
500
1000
1500
2000

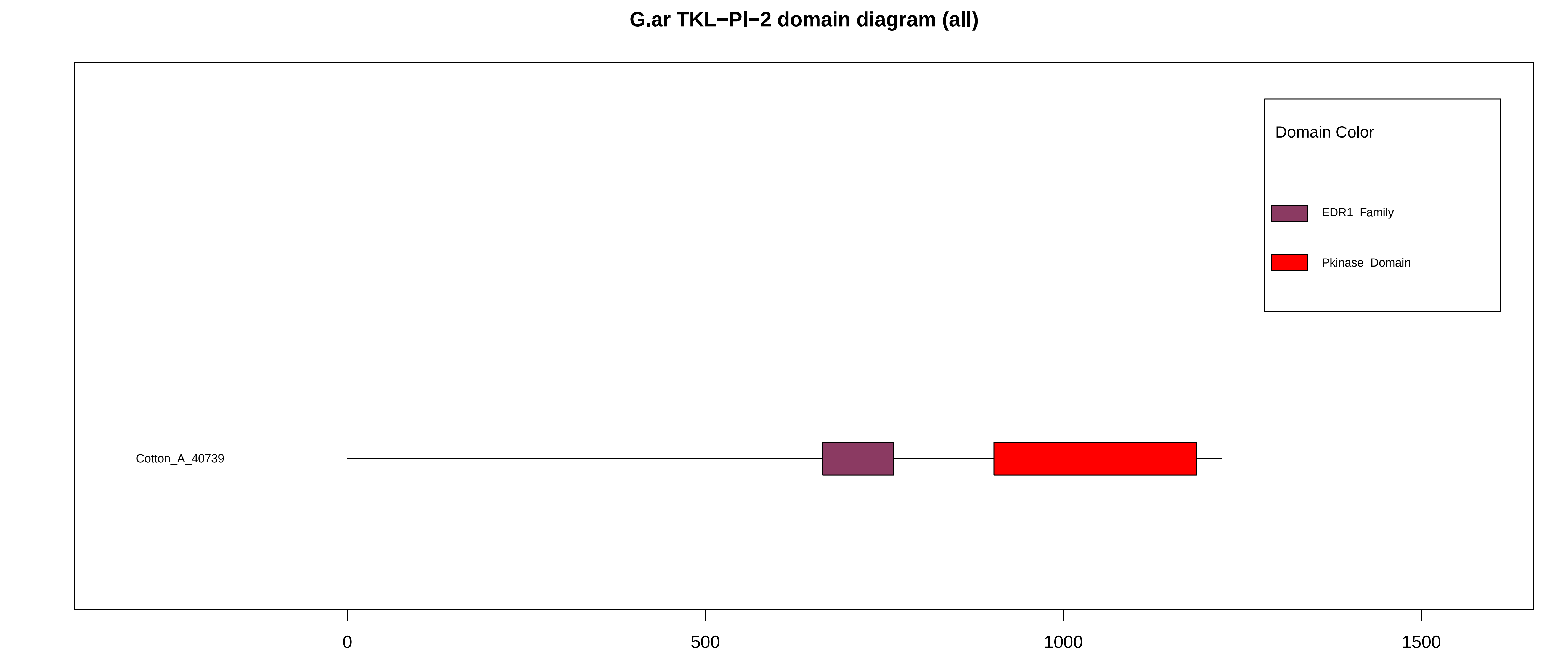
G.ar TKL−Pl−2 domain diagram (all)
Domain Color
EDR1 Family
Pkinase Domain
Cotton_A_40739
0
500
1000
1500

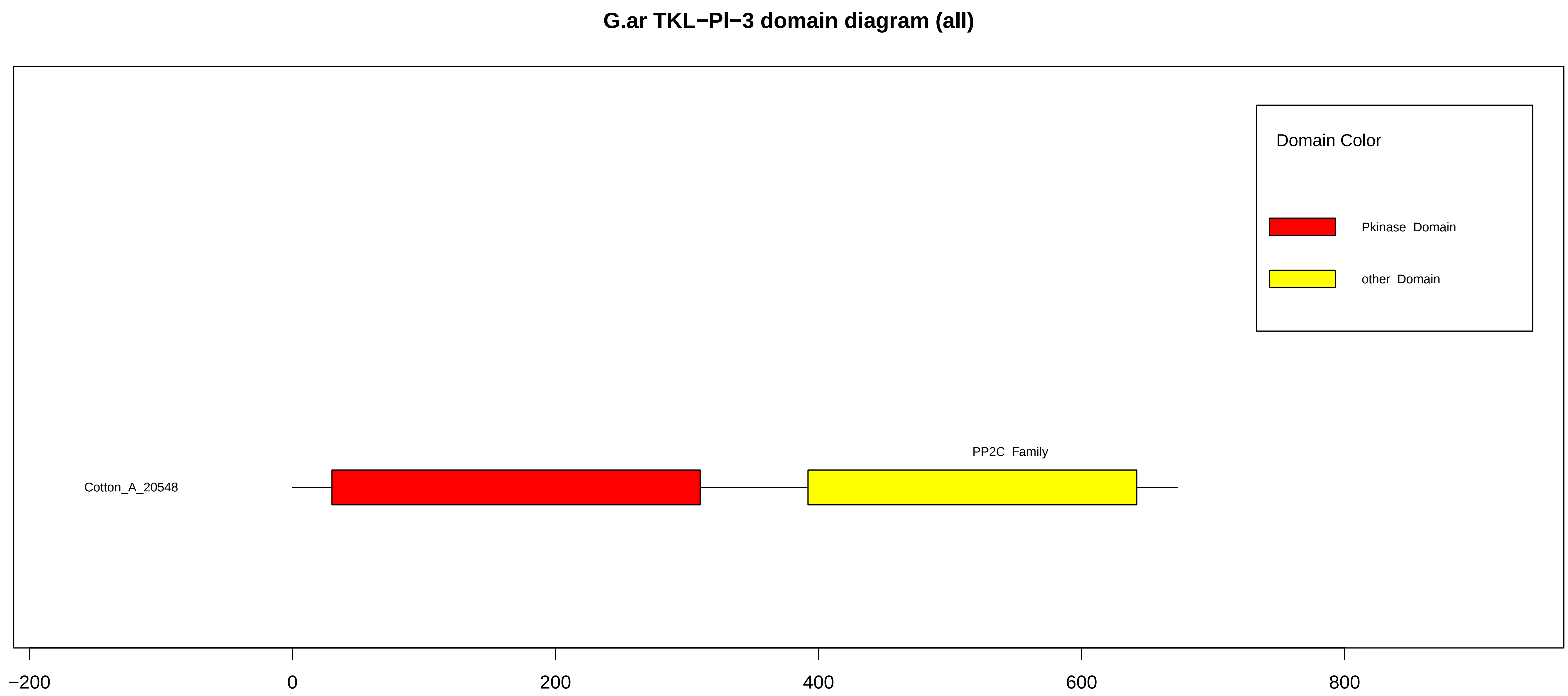
G.ar TKL−Pl−3 domain diagram (all)
Domain Color
Pkinase Domain
other Domain
PP2C Family
Cotton_A_20548
−200
0
200
400
600
800

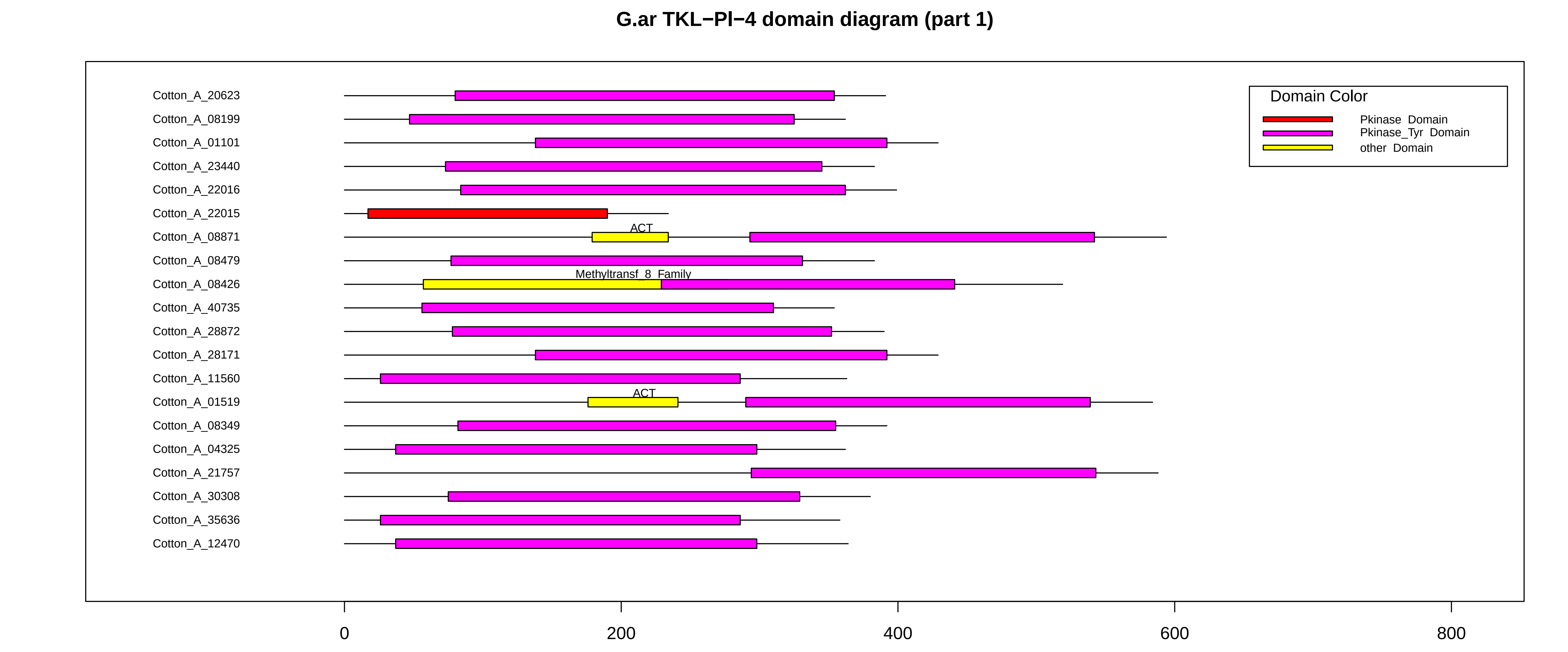

G.ar TKL−Pl−4 domain diagram (part 1)
Cotton_A_20623
Cotton_A_08199
Cotton_A_01101
Cotton_A_23440
Cotton_A_22016
Cotton_A_22015
Cotton_A_08871
Cotton_A_08479
Cotton_A_08426
Cotton_A_40735
Cotton_A_28872
Cotton_A_28171
Cotton_A_11560
Cotton_A_01519
Cotton_A_08349
Cotton_A_04325
Cotton_A_21757
Cotton_A_30308
Cotton_A_35636
Cotton_A_12470
ACT
Methyltransf_8_Family
ACT
Domain Color
Pkinase Domain
Pkinase_Tyr Domain
other Domain
0
200
400
600
800

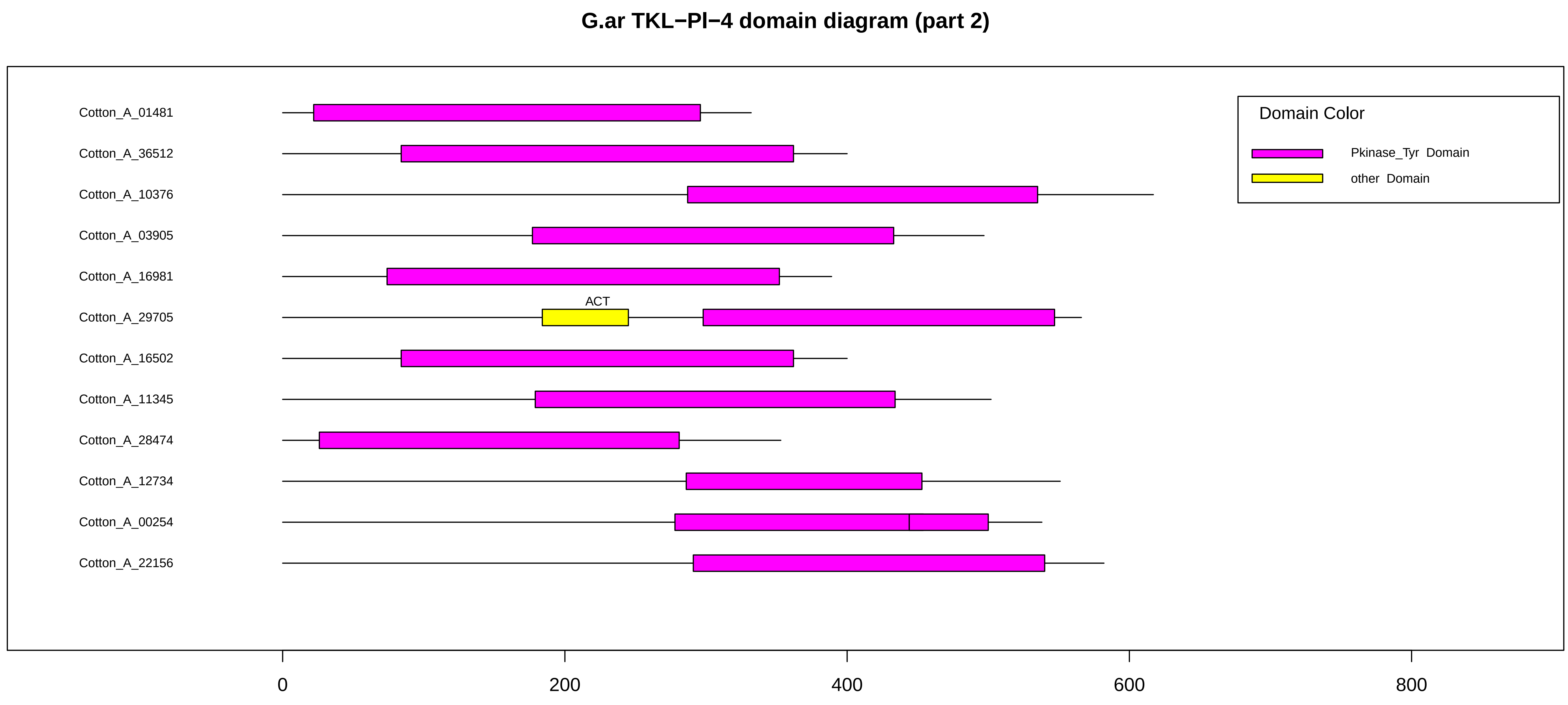
G.ar TKL−Pl−4 domain diagram (part 2)
Cotton_A_01481
Cotton_A_36512
Cotton_A_10376
Cotton_A_03905
Cotton_A_16981
Cotton_A_29705
Cotton_A_16502
Cotton_A_11345
Cotton_A_28474
Cotton_A_12734
Cotton_A_00254
Cotton_A_22156
ACT
Domain Color
Pkinase_Tyr Domain
other Domain
0
200
400
600
800

G.ar TKL−Pl−5 domain diagram (all)

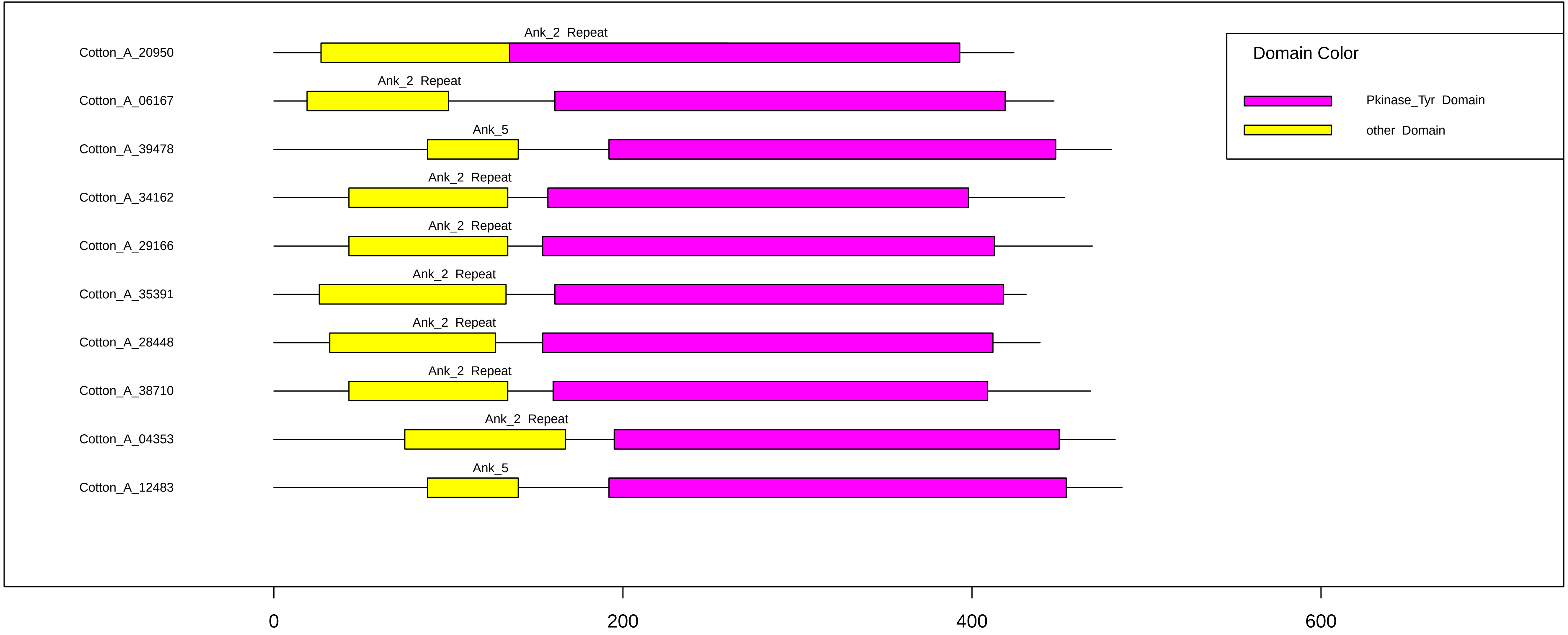

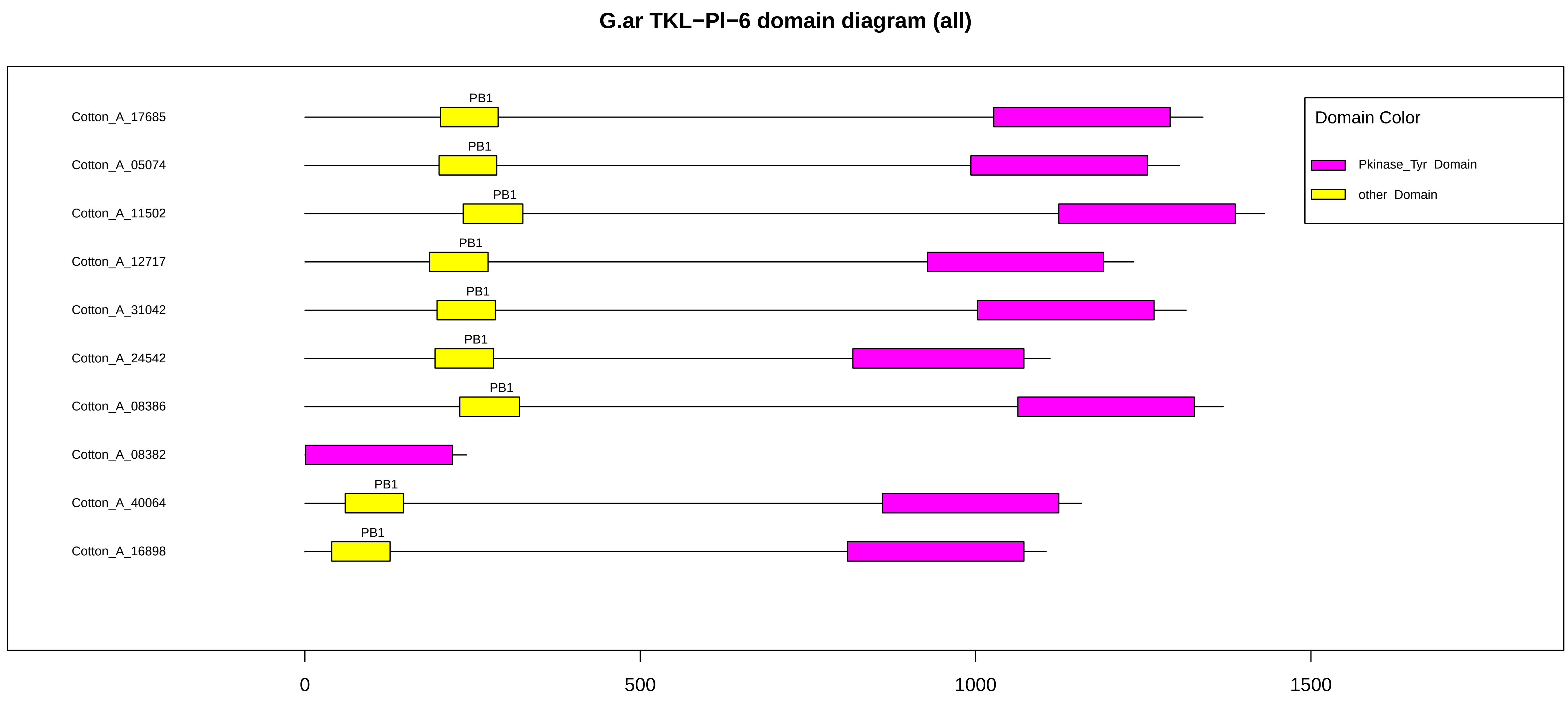
G.ar TKL−Pl−6 domain diagram (all)
Cotton_A_17685
Cotton_A_05074
Cotton_A_11502
Cotton_A_12717
Cotton_A_31042
Cotton_A_24542
Cotton_A_08386
Cotton_A_08382
Cotton_A_40064
Cotton_A_16898
PB1
PB1
PB1
PB1
PB1
PB1
PB1
PB1
PB1
Domain Color
Pkinase_Tyr Domain
other Domain
0
500
1000
1500

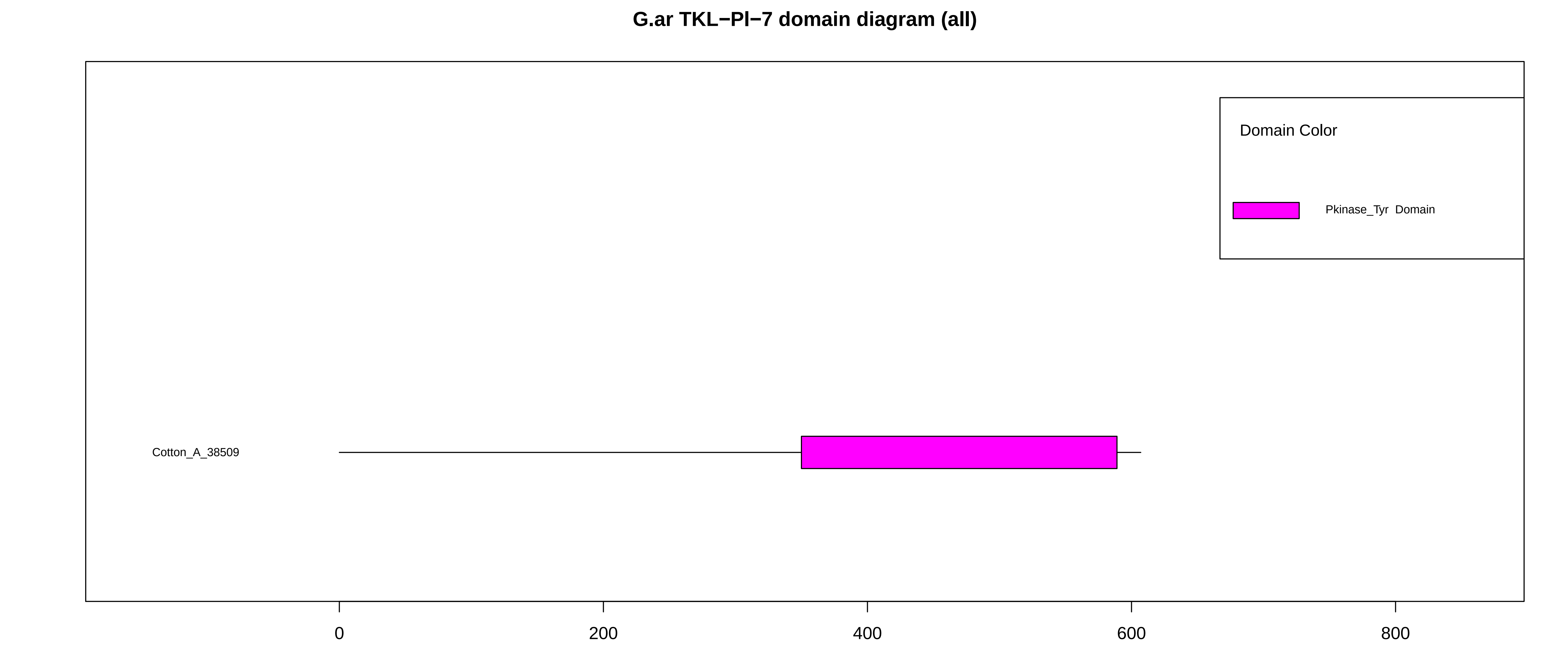
G.ar TKL−Pl−7 domain diagram (all)
Domain Color
Pkinase_Tyr Domain
Cotton_A_38509
0
200
400
600
800

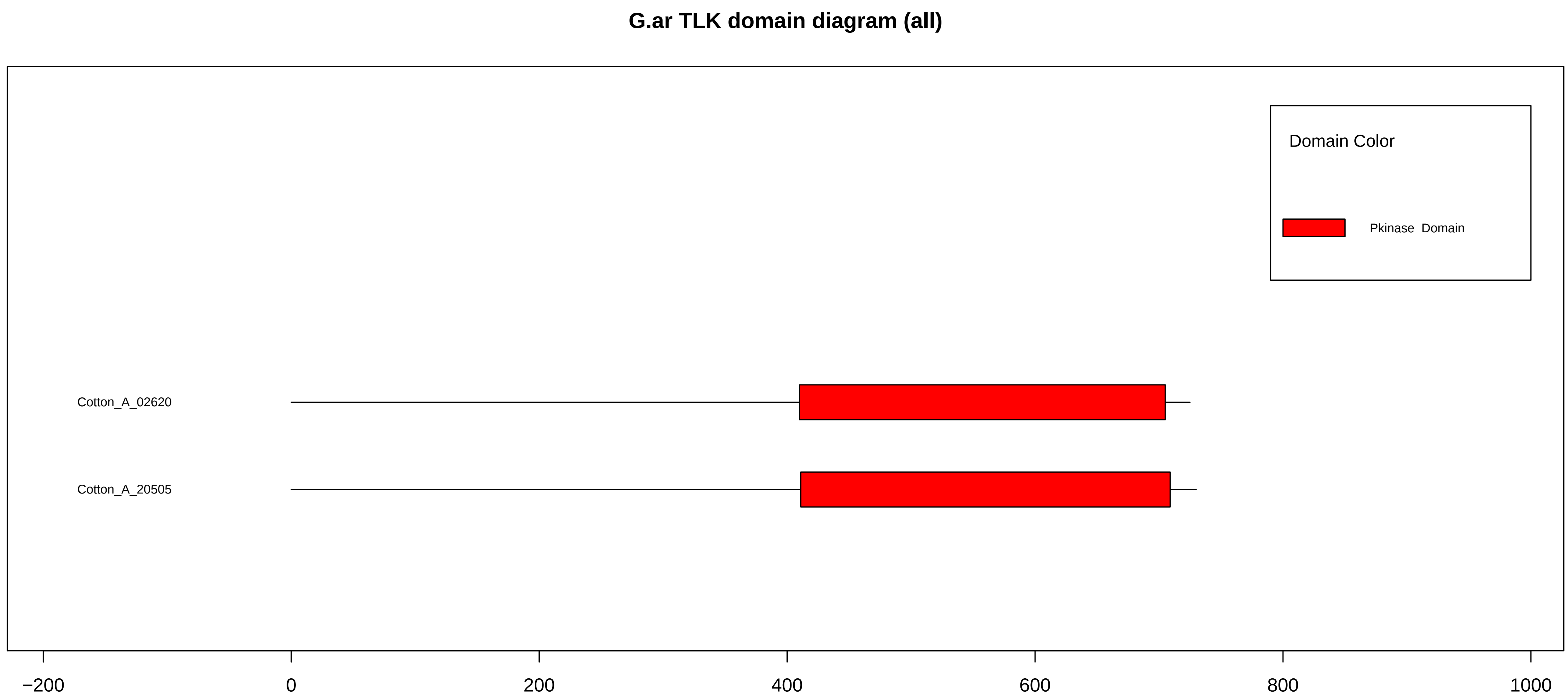
G.ar TLK domain diagram (all)
Domain Color
Pkinase Domain
Cotton_A_02620
Cotton_A_20505
−200
0
200
400
600
800
1000

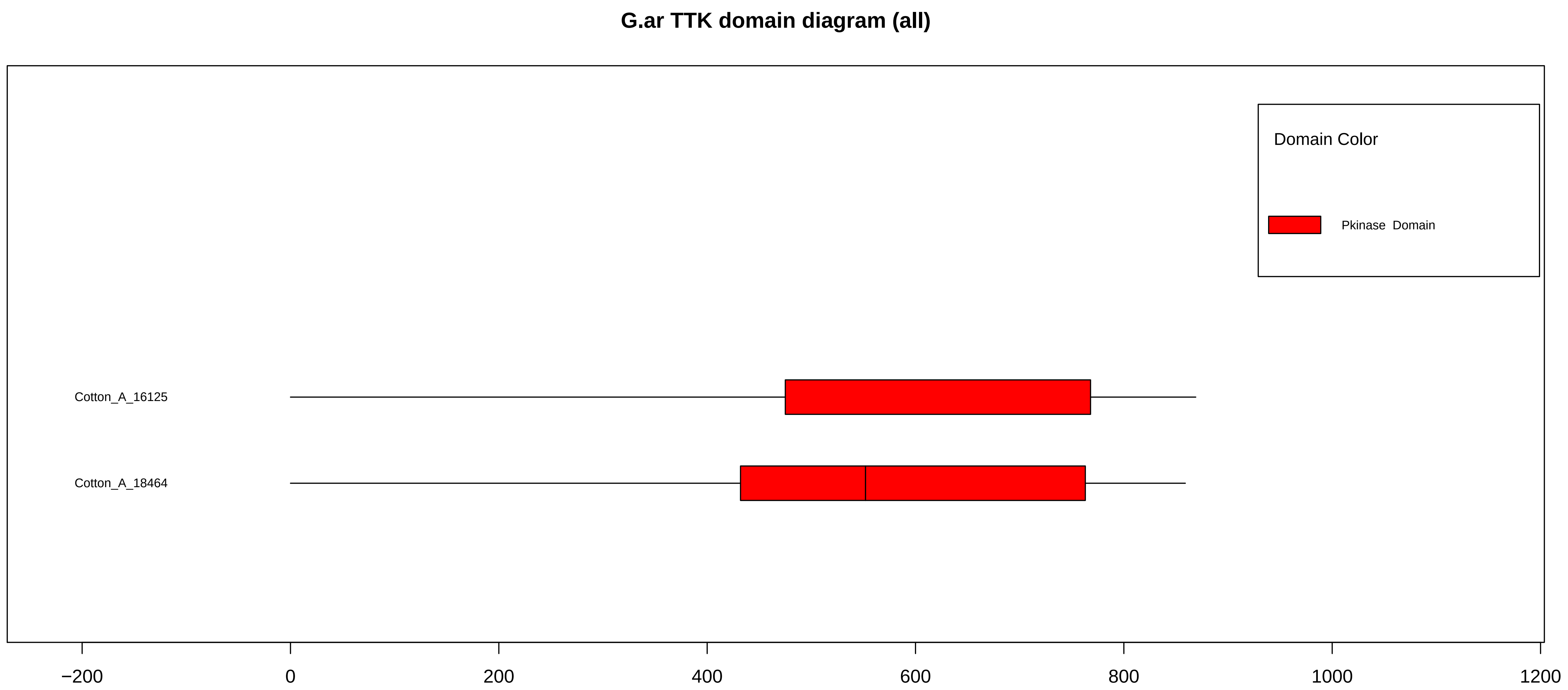
G.ar TTK domain diagram (all)
Domain Color
Pkinase Domain
Cotton_A_16125
Cotton_A_18464
−200
0
200
400
600
800
1000
1200

G.ar ULK_Fused domain diagram (all)

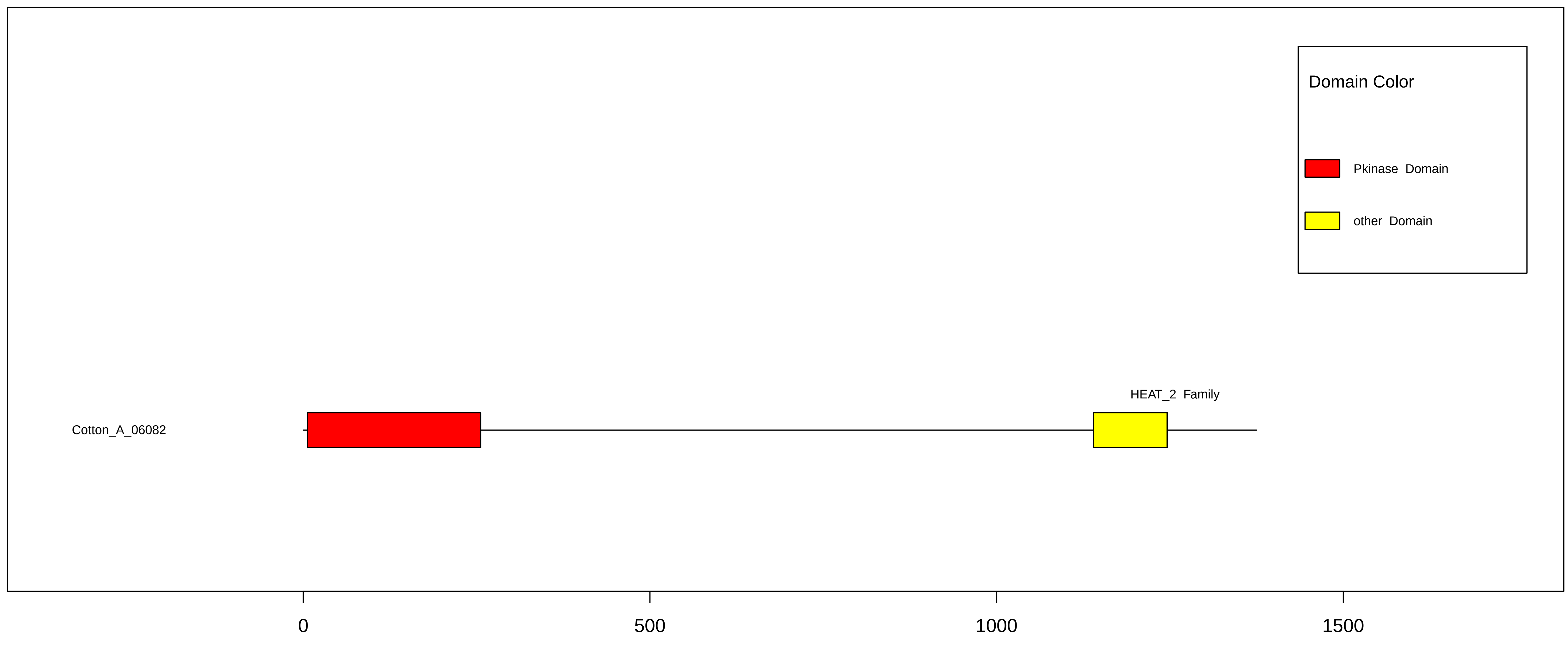

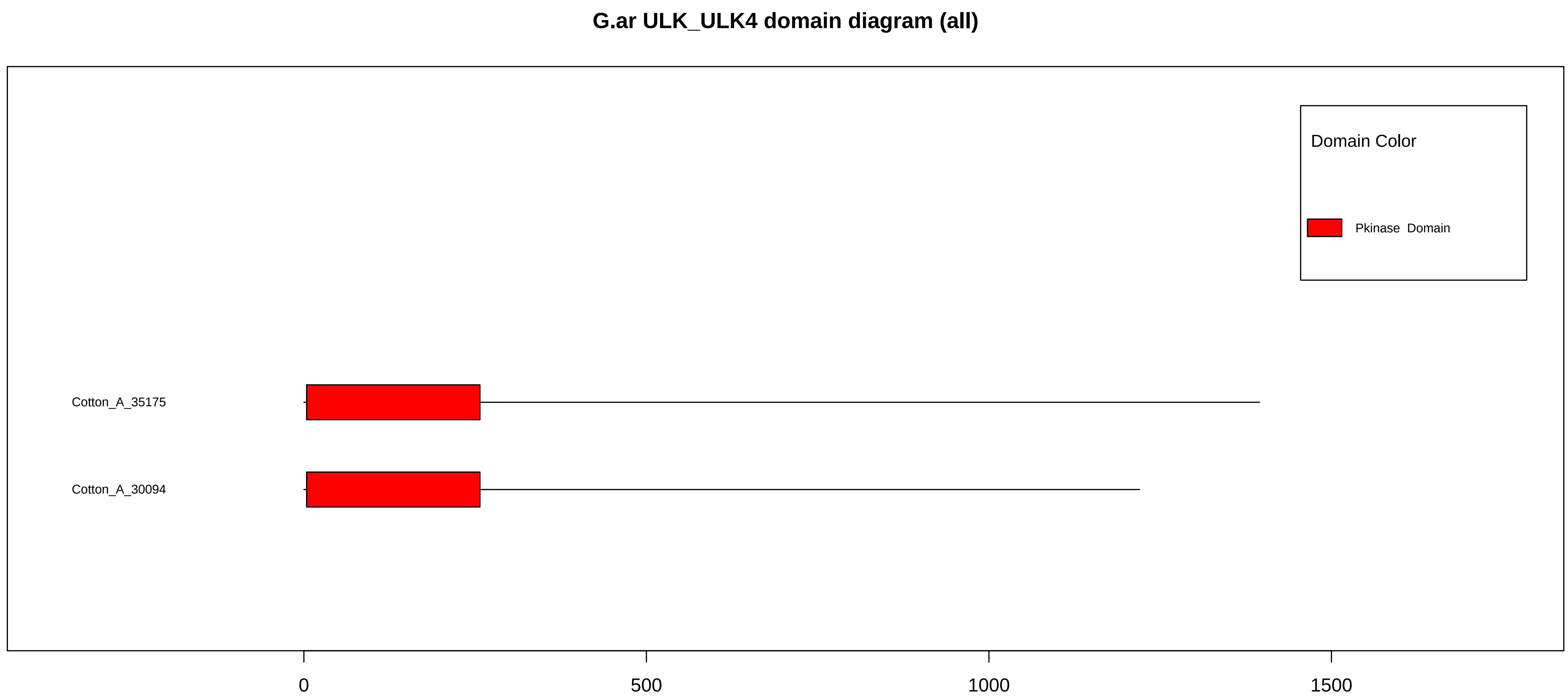
G.ar ULK_ULK4 domain diagram (all)
Domain Color
Pkinase Domain
Cotton_A_35175
Cotton_A_30094
0
500
1000
1500

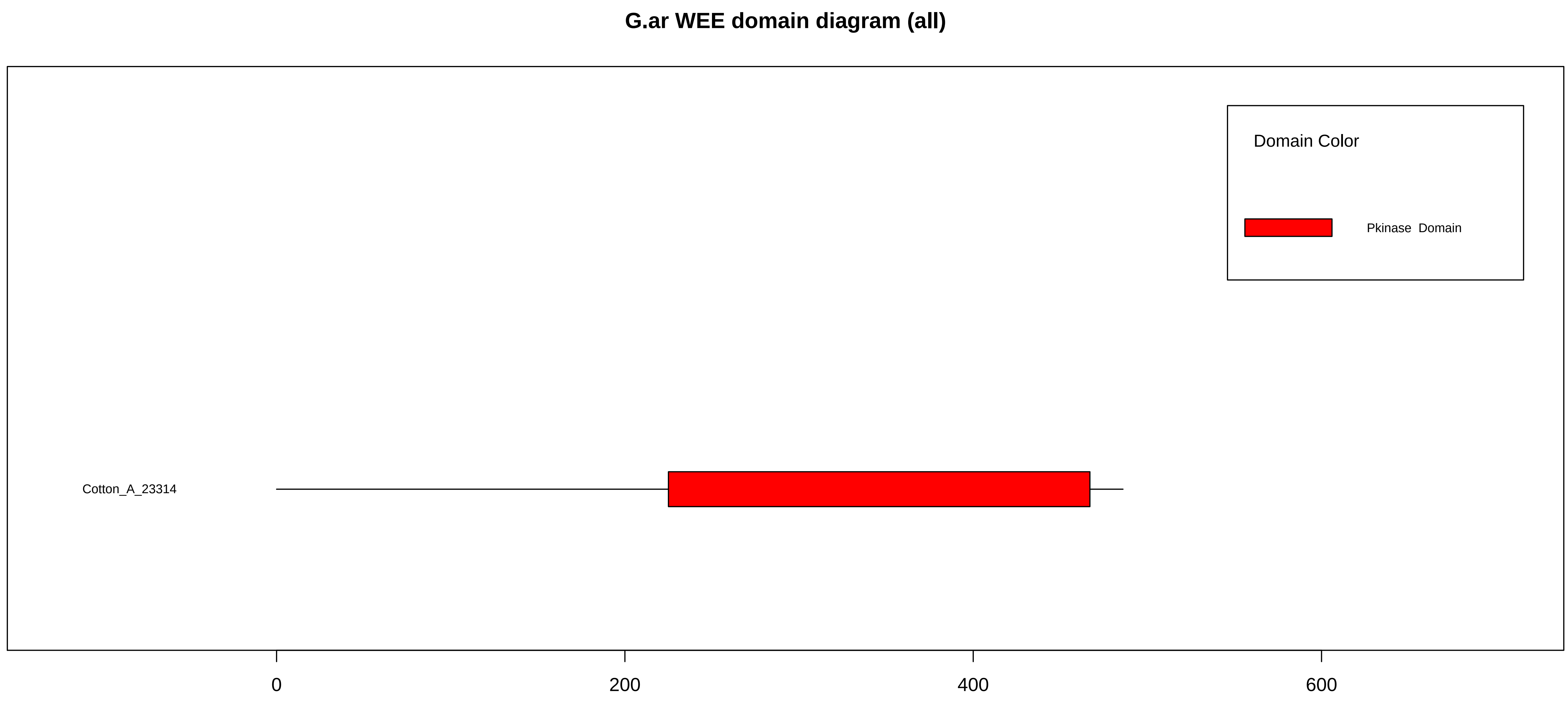
G.ar WEE domain diagram (all)
Domain Color
Pkinase Domain
Cotton_A_23314
0
200
400
600

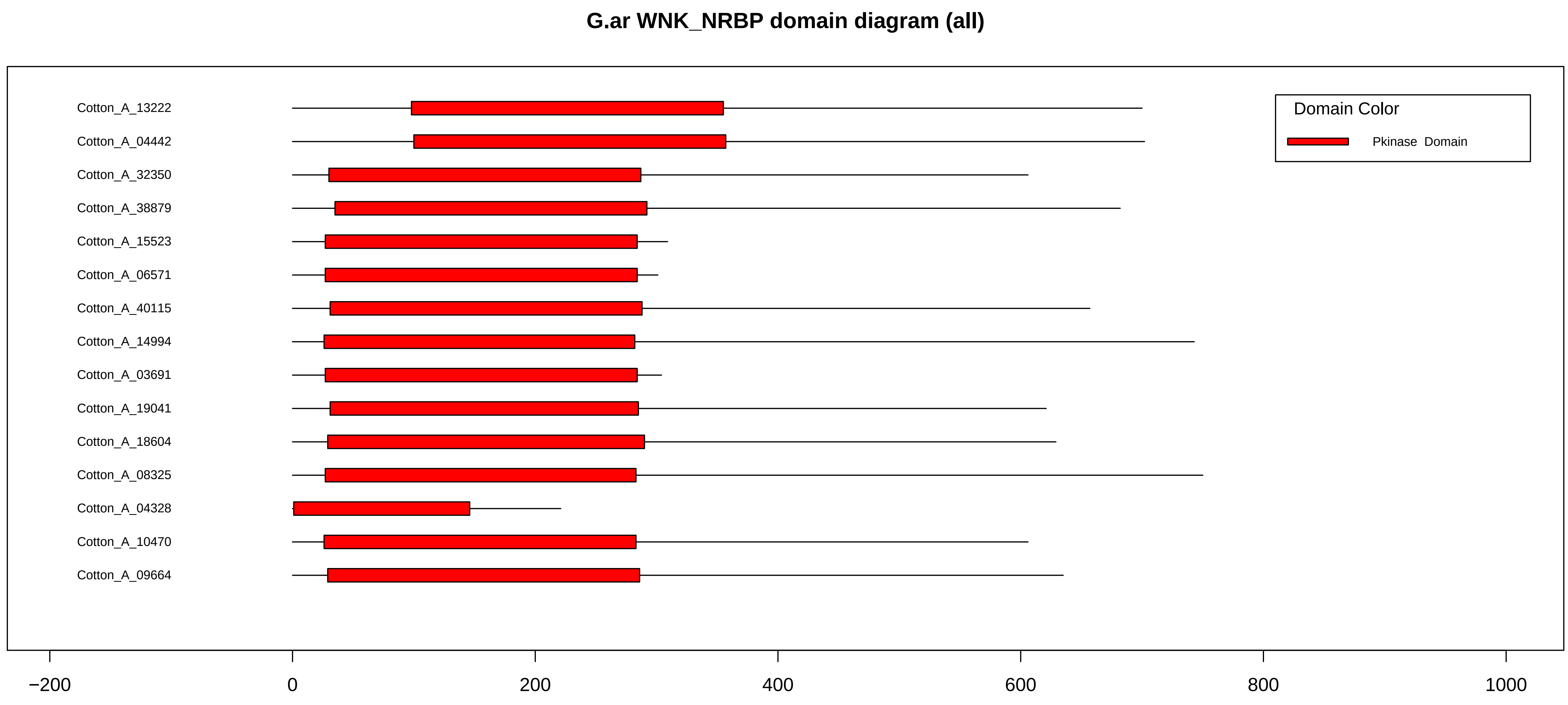

G.ar WNK_NRBP domain diagram (all)
Cotton_A_13222
Cotton_A_04442
Cotton_A_32350
Cotton_A_38879
Cotton_A_15523
Cotton_A_06571
Cotton_A_40115
Cotton_A_14994
Cotton_A_03691
Cotton_A_19041
Cotton_A_18604
Cotton_A_08325
Cotton_A_04328
Cotton_A_10470
Cotton_A_09664
Domain Color
Pkinase Domain
−200
0
200
400
600
800
1000

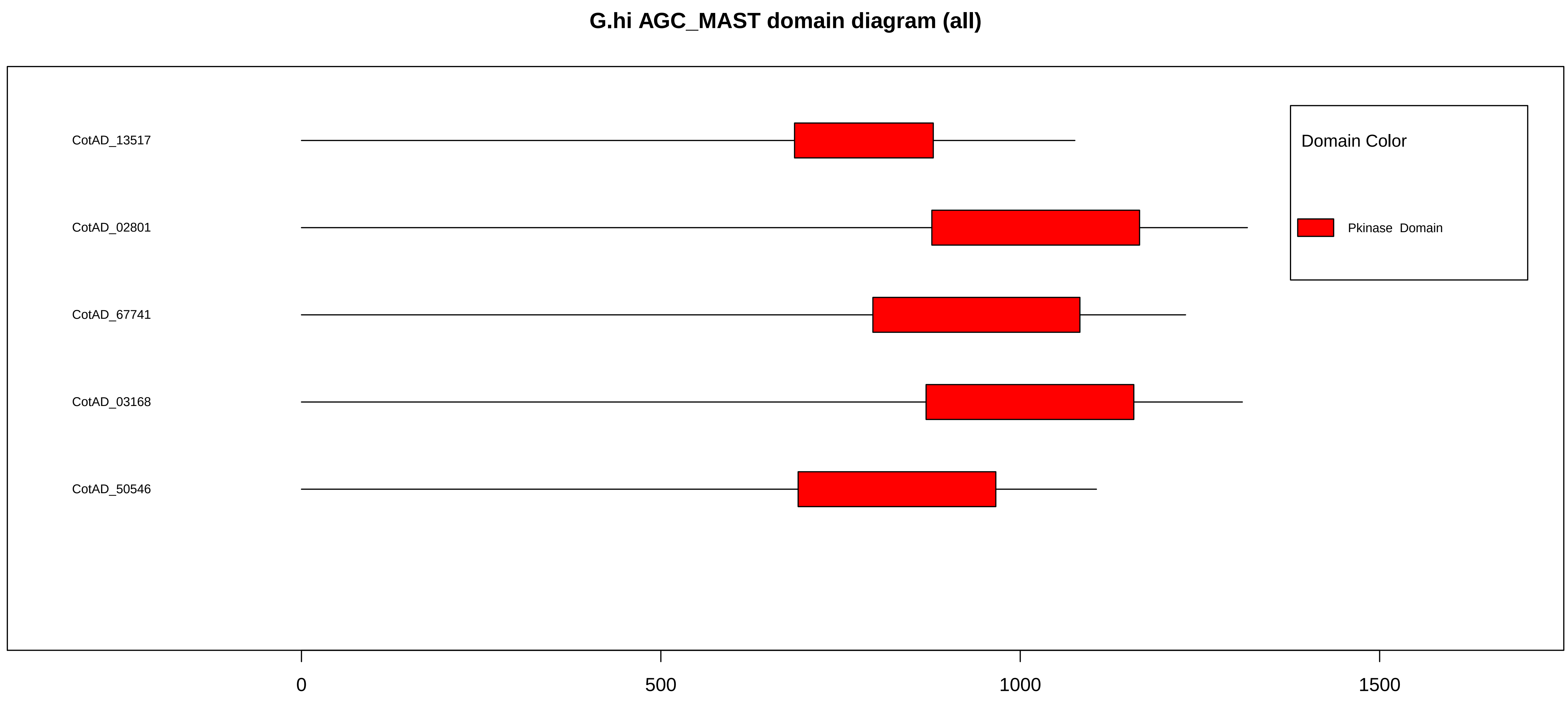
G.hi AGC_MAST domain diagram (all)
CotAD_13517
CotAD_02801
CotAD_67741
CotAD_03168
CotAD_50546
Domain Color
Pkinase Domain
0
500
1000
1500

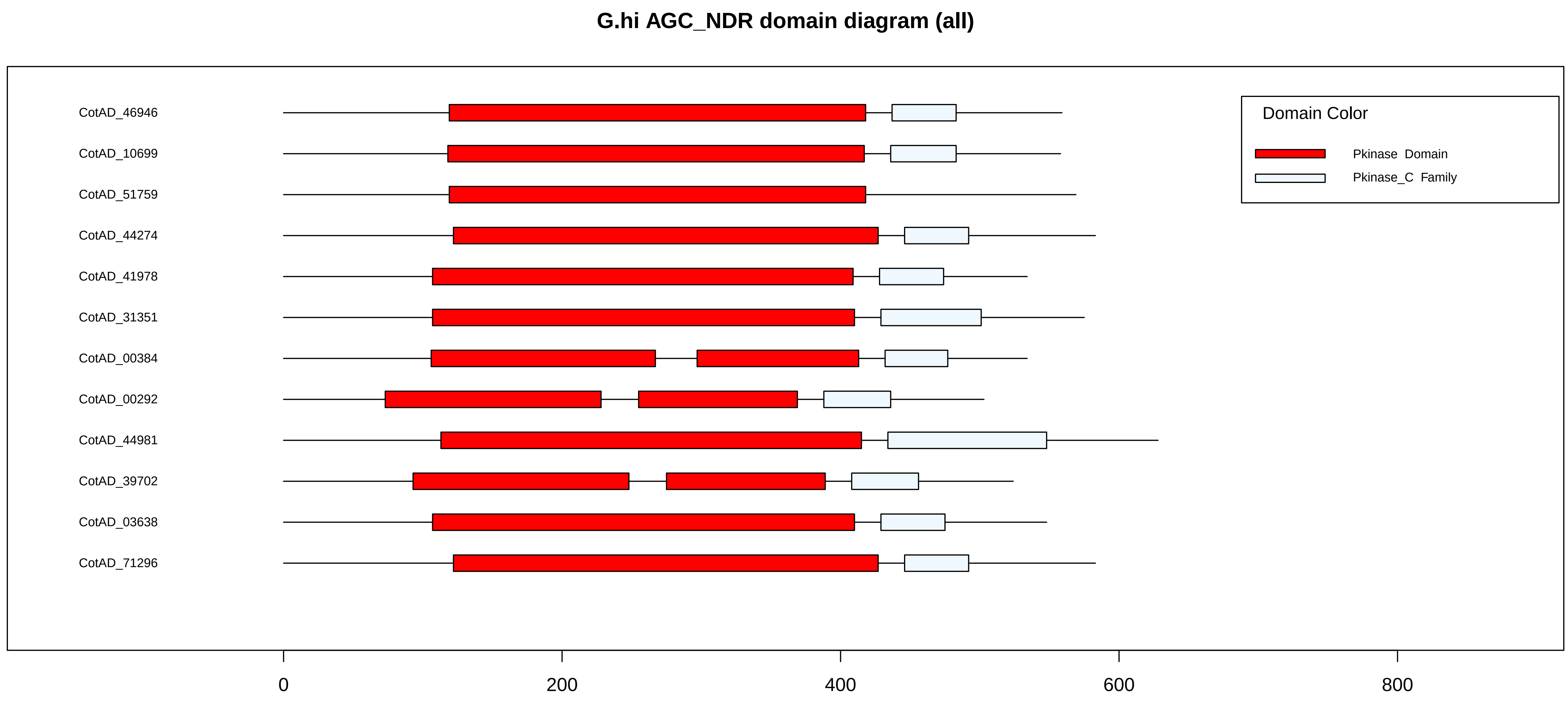
G.hi AGC_NDR domain diagram (all)
CotAD_46946
CotAD_10699
CotAD_51759
CotAD_44274
CotAD_41978
CotAD_31351
CotAD_00384
CotAD_00292
CotAD_44981
CotAD_39702
CotAD_03638
CotAD_71296
Domain Color
Pkinase Domain
Pkinase_C Family
0
200
400
600
800

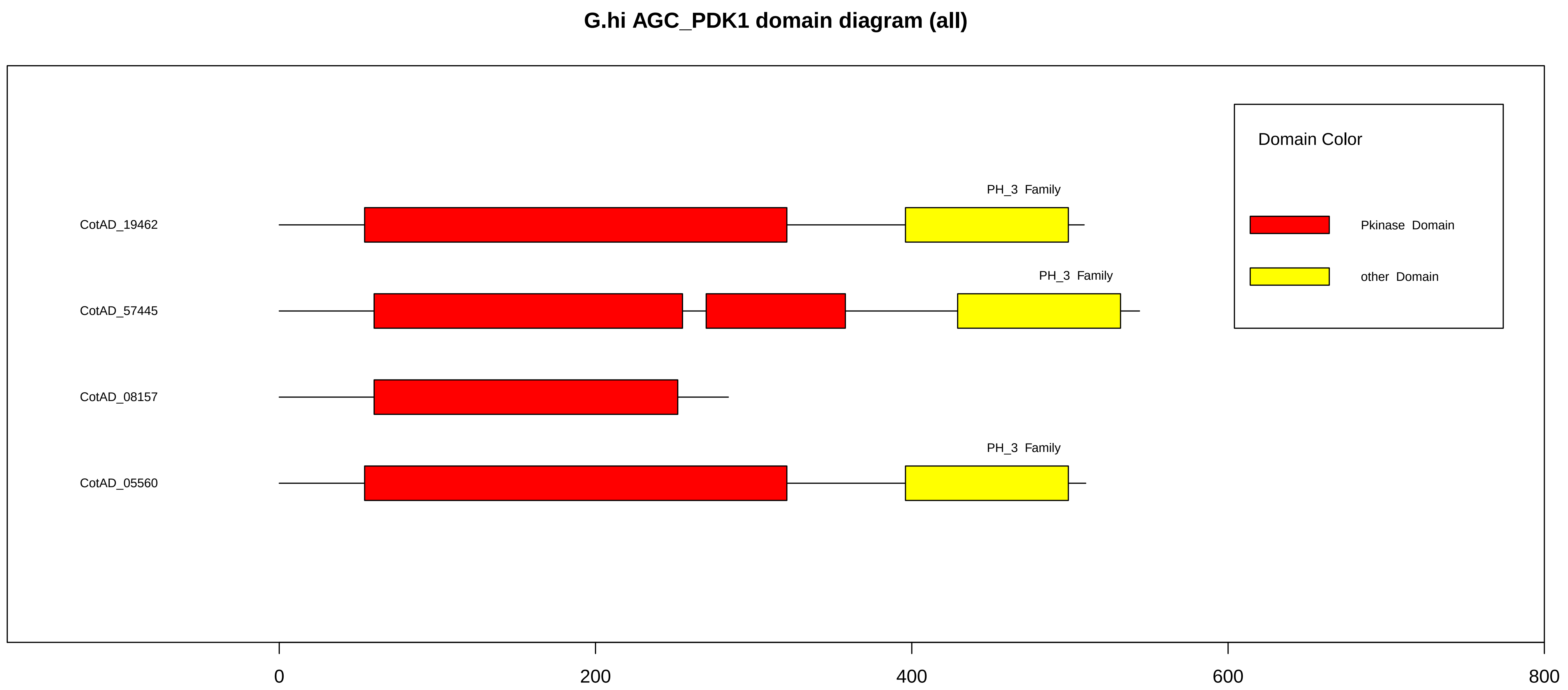
G.hi AGC_PDK1 domain diagram (all)
Domain Color
Pkinase Domain
other Domain
CotAD_19462
PH_3 Family
CotAD_57445
PH_3 Family
CotAD_08157
CotAD_05560
PH_3 Family
0
200
400
600
800

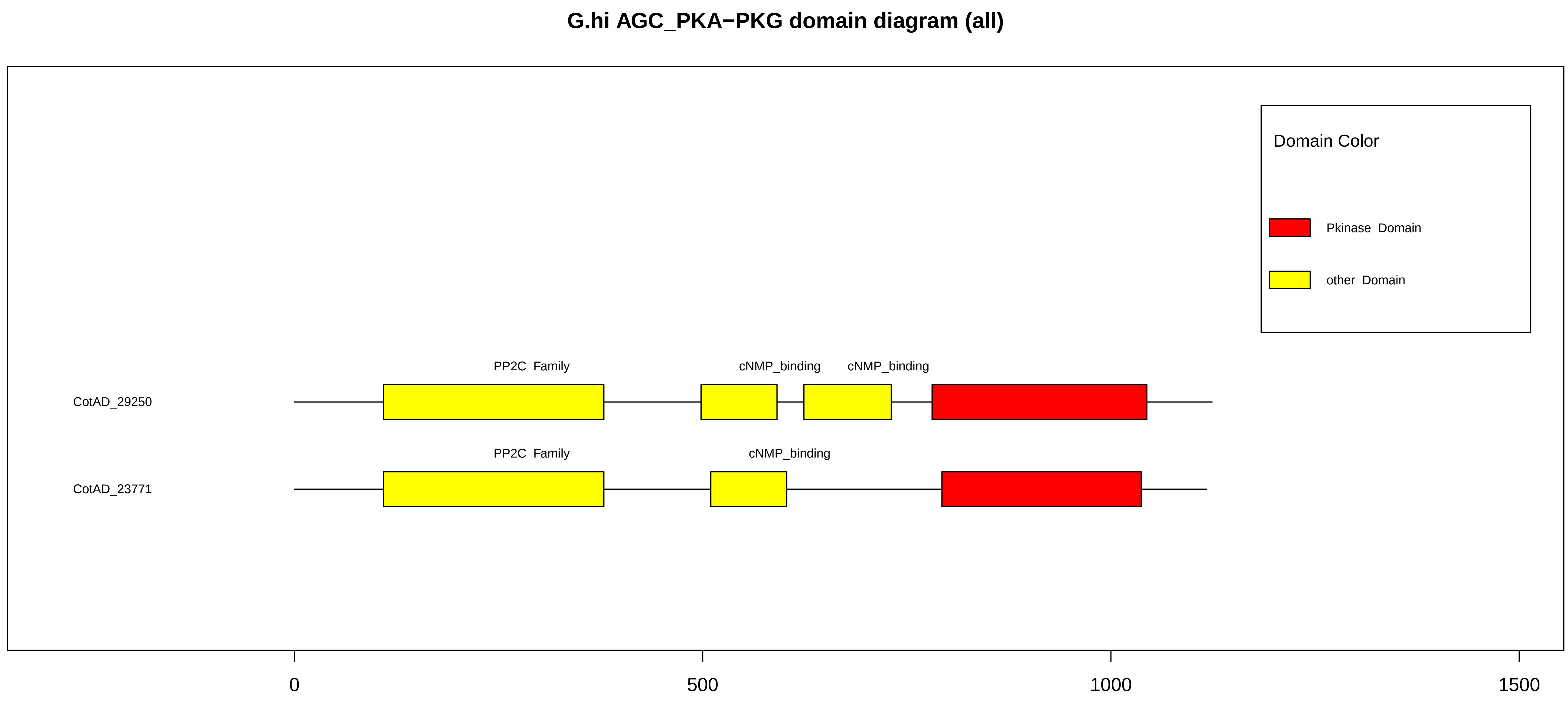
G.hi AGC_PKA−PKG domain diagram (all)
Domain Color
Pkinase Domain
other Domain
PP2C Family
cNMP_binding
cNMP_binding
CotAD_29250
PP2C Family
cNMP_binding
CotAD_23771
0
500
1000
1500

G.hi AGC_RSK−2 domain diagram (part 1)

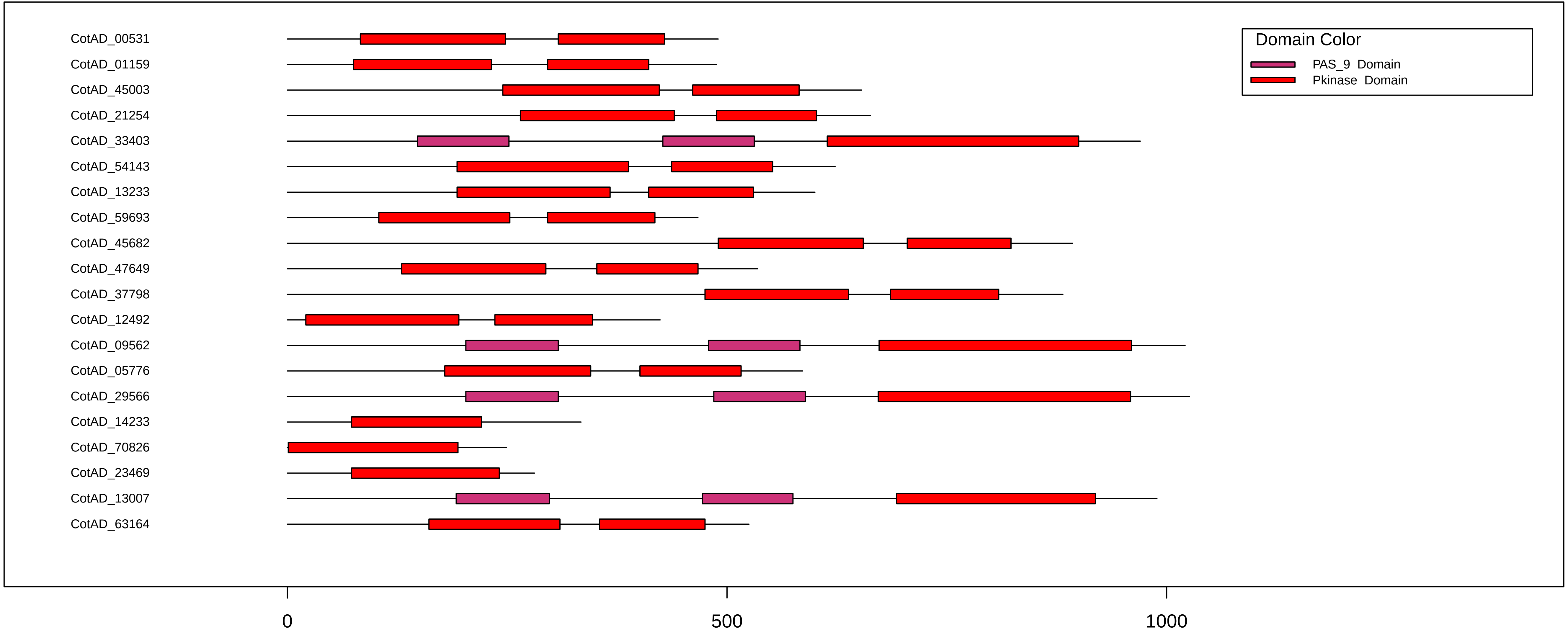

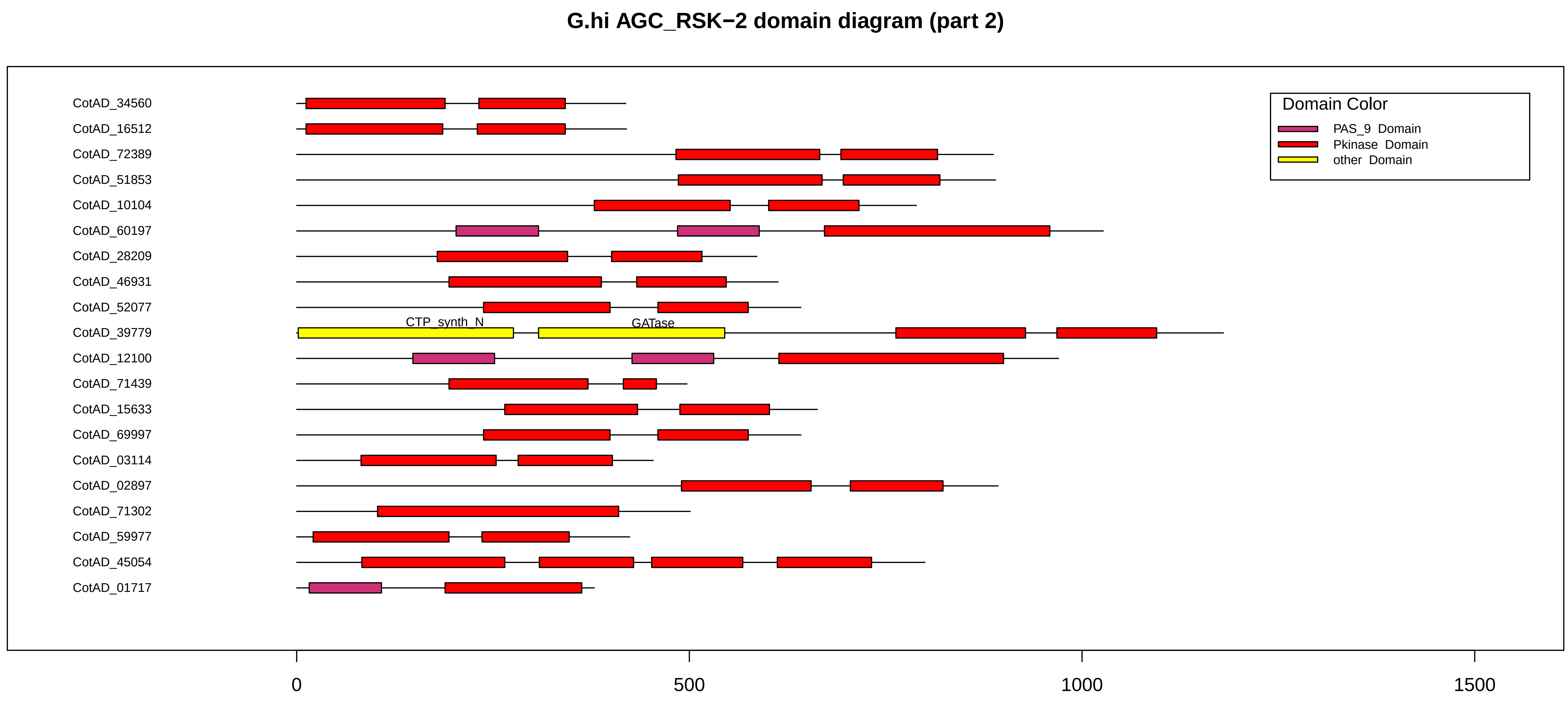
G.hi AGC_RSK−2 domain diagram (part 2)
Domain Color
PAS_9 Domain
Pkinase Domain
other Domain
CotAD_34560
CotAD_16512
CotAD_72389
CotAD_51853
CotAD_10104
CotAD_60197
CotAD_28209
CotAD_46931
CotAD_52077
CotAD_39779
CotAD_12100
CotAD_71439
CotAD_15633
CotAD_69997
CotAD_03114
CotAD_02897
CotAD_71302
CotAD_59977
CotAD_45054
CotAD_01717
CTP_synth_N
GATase
0
500
1000
1500

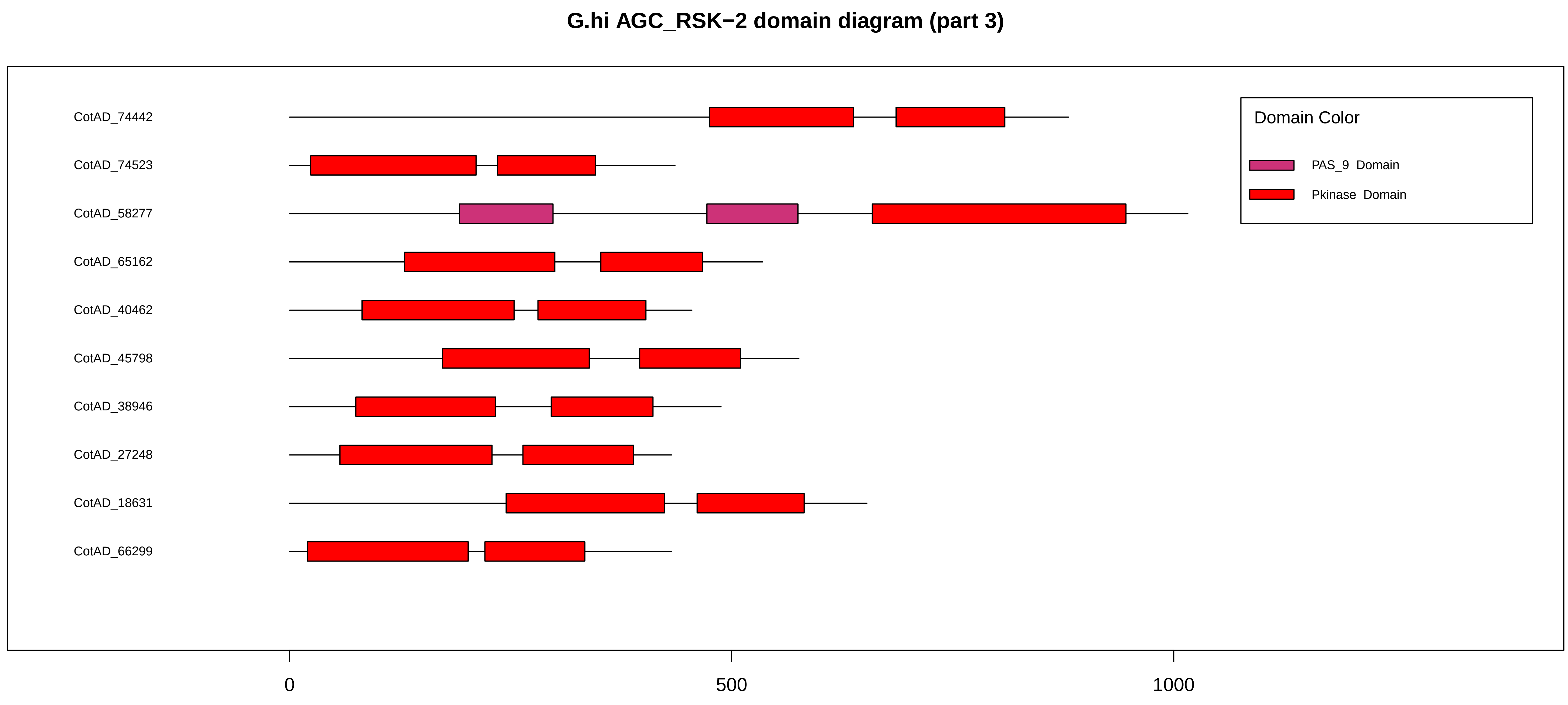
G.hi AGC_RSK−2 domain diagram (part 3)
CotAD_74442
CotAD_74523
CotAD_58277
CotAD_65162
CotAD_40462
CotAD_45798
CotAD_38946
CotAD_27248
CotAD_18631
CotAD_66299
Domain Color
PAS_9 Domain
Pkinase Domain
0
500
1000

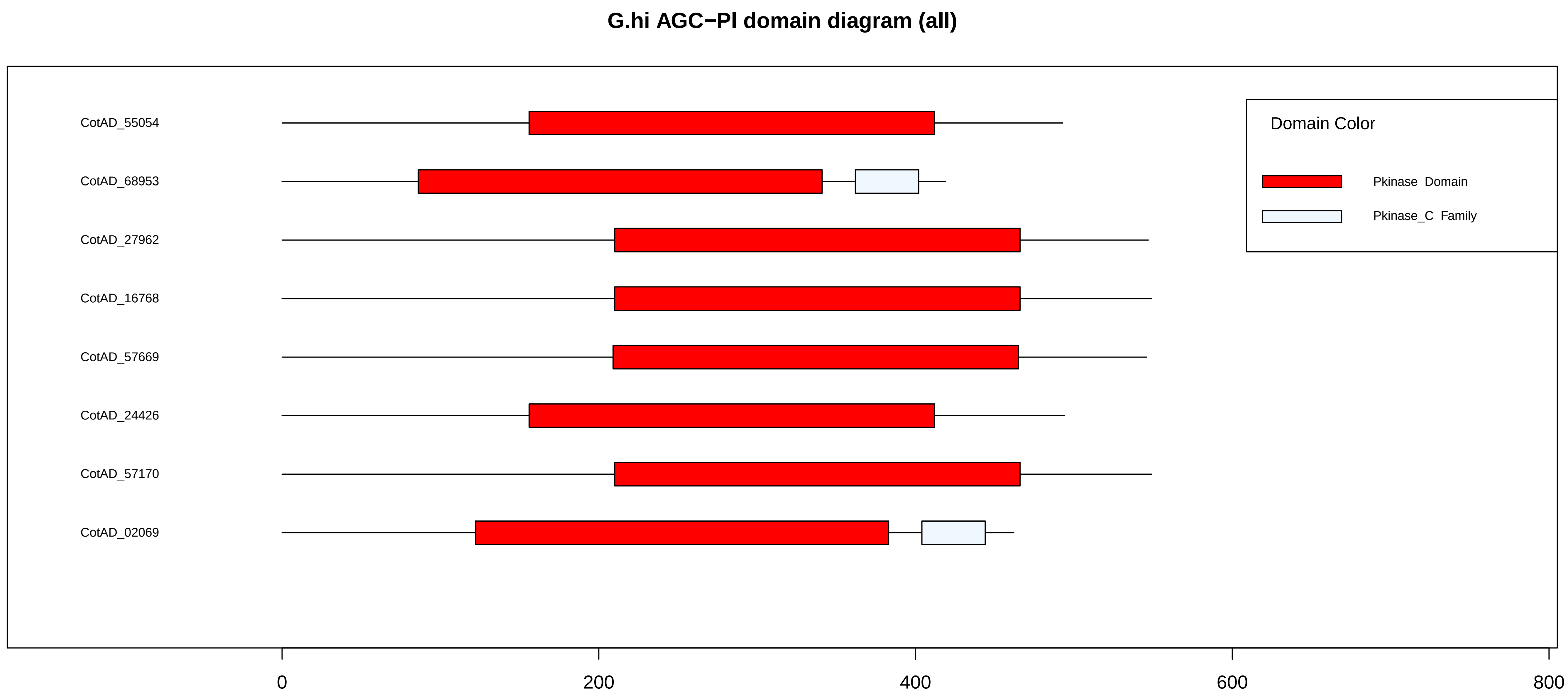
G.hi AGC−PI domain diagram (all)
CotAD_55054
CotAD_68953
CotAD_27962
CotAD_16768
CotAD_57669
CotAD_24426
CotAD_57170
CotAD_02069
Domain Color
Pkinase Domain
Pkinase_C Family
0
200
400
600
800

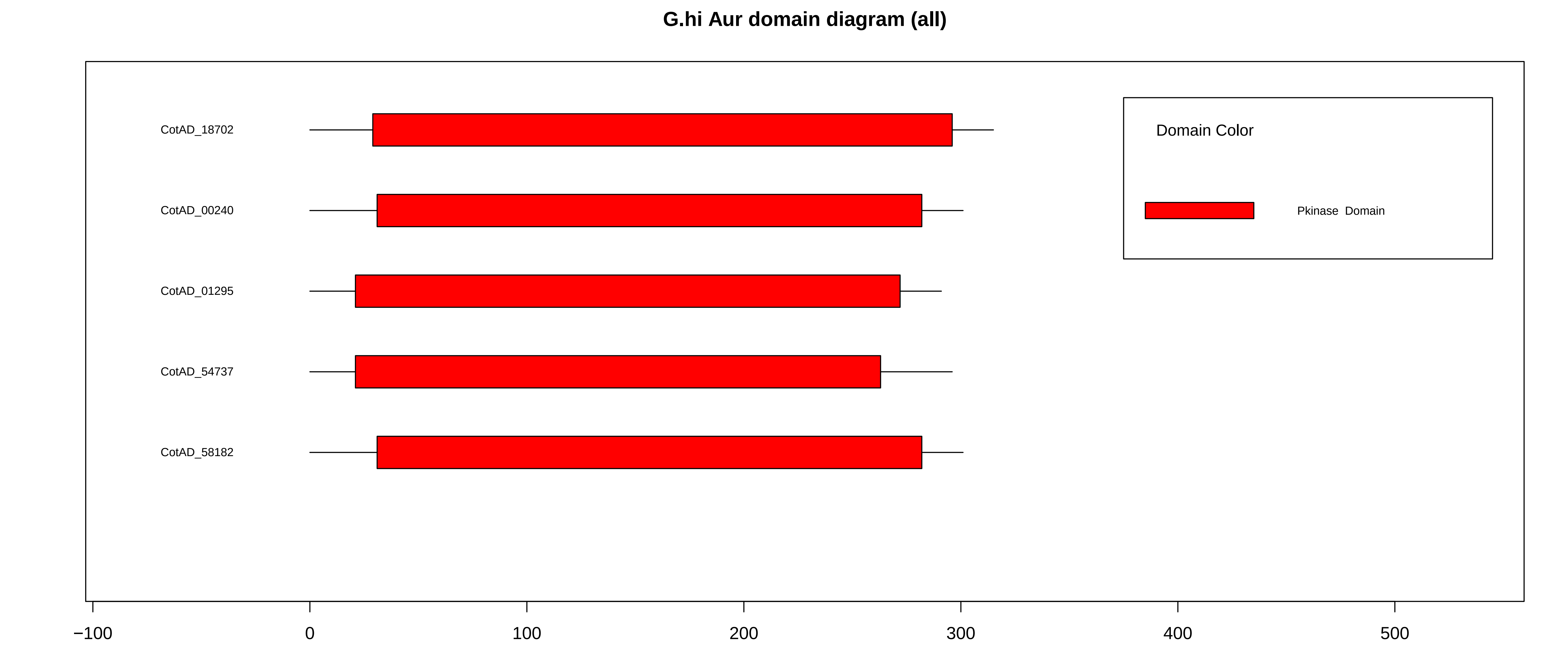
G.hi Aur domain diagram (all)
CotAD_18702
CotAD_00240
CotAD_01295
CotAD_54737
CotAD_58182
Domain Color
Pkinase Domain
−100
0
100
200
300
400
500

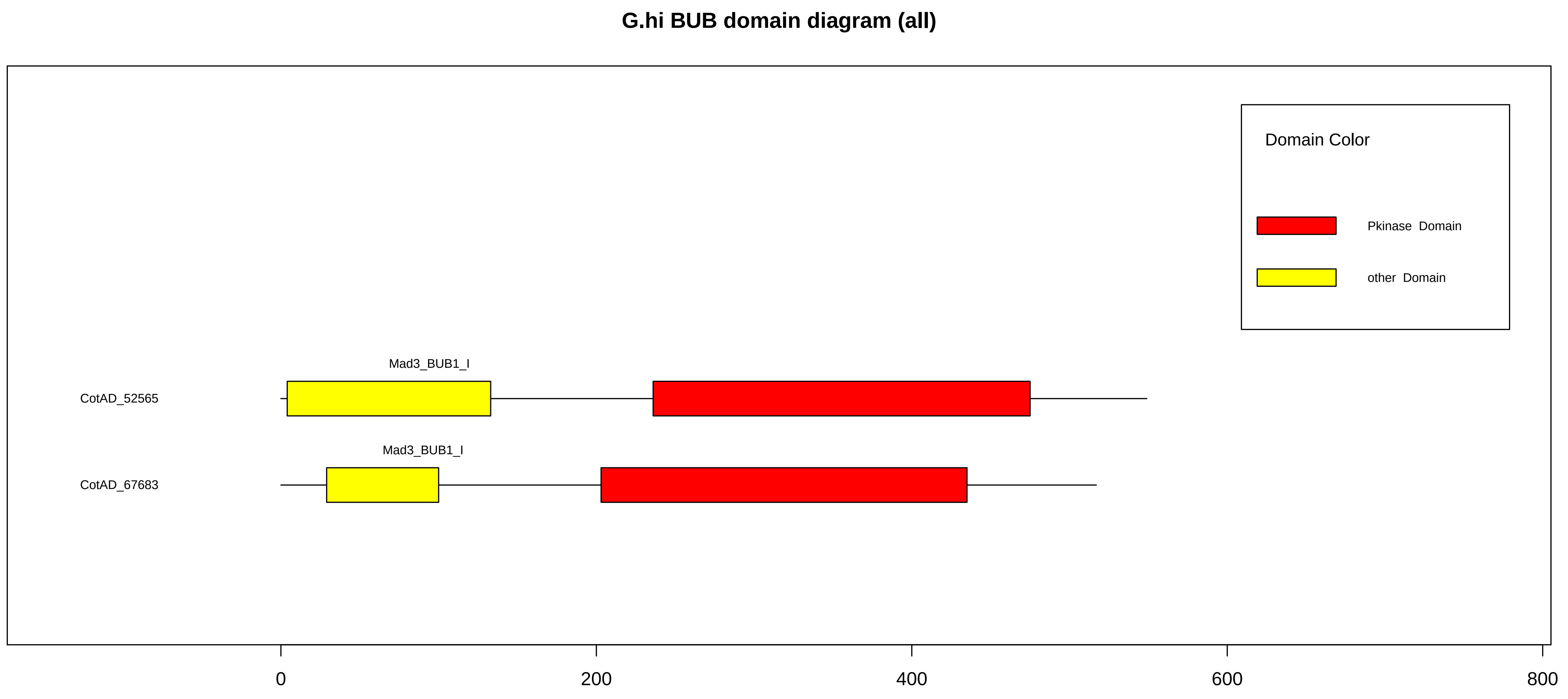
G.hi BUB domain diagram (all)
Domain Color
Pkinase Domain
other Domain
Mad3_BUB1_I
CotAD_52565
Mad3_BUB1_I
CotAD_67683
0
200
400
600
800

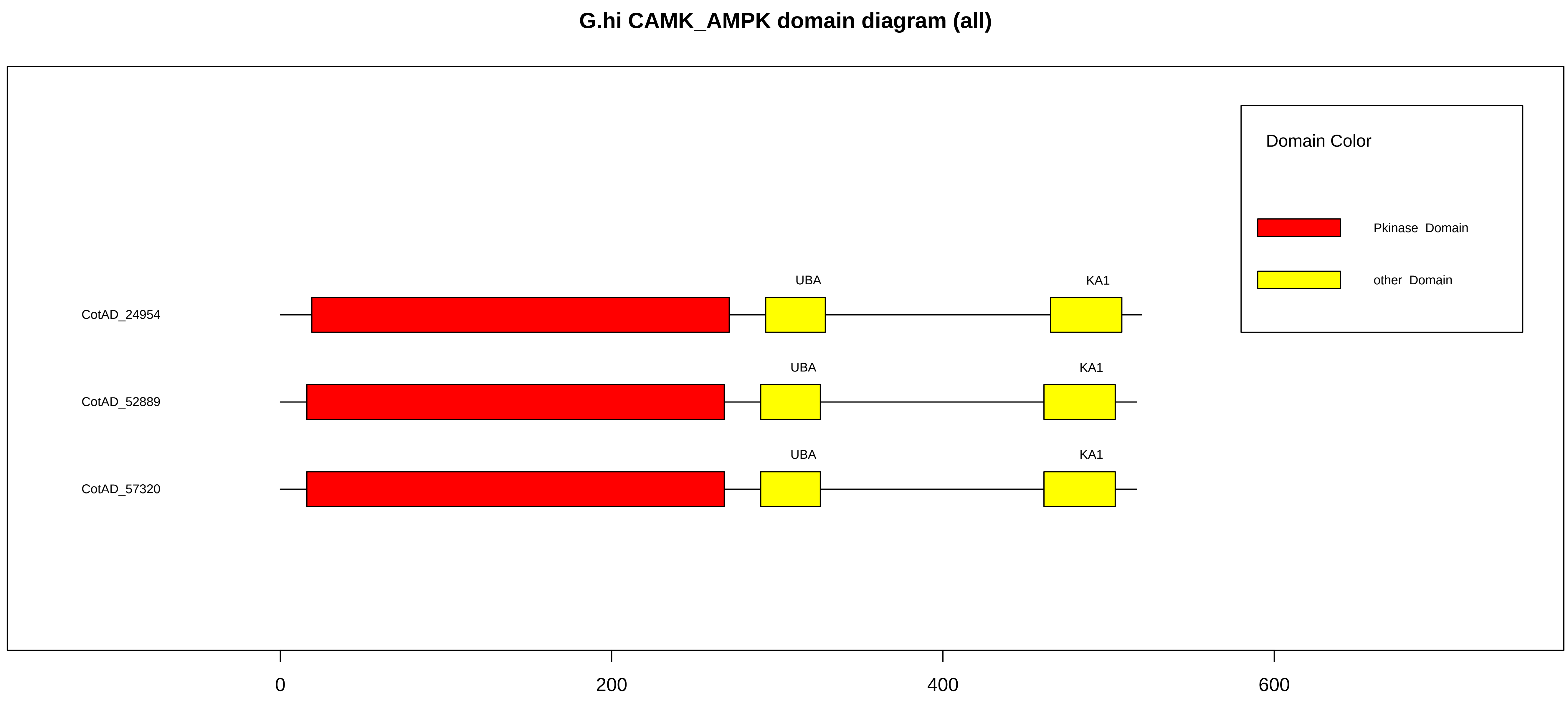

G.hi CAMK_AMPK domain diagram (all)
Domain Color
Pkinase Domain
other Domain
CotAD_24954
CotAD_52889
CotAD_57320
UBA
KA1
UBA
KA1
UBA
KA1
0
200
400
600

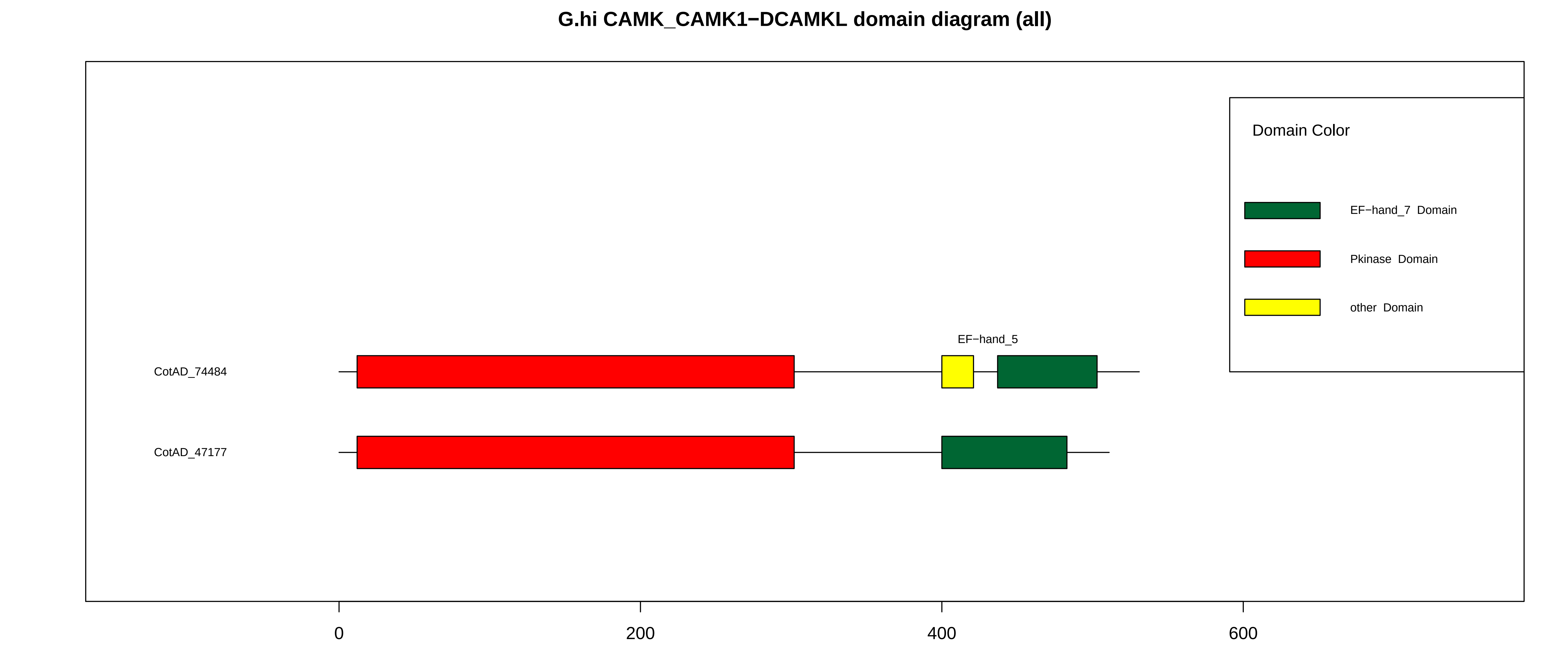

G.hi CAMK_CAMK1−DCAMKL domain diagram (all)
Domain Color
EF−hand_7 Domain
Pkinase Domain
other Domain
EF−hand_5
CotAD_74484
CotAD_47177
0
200
400
600

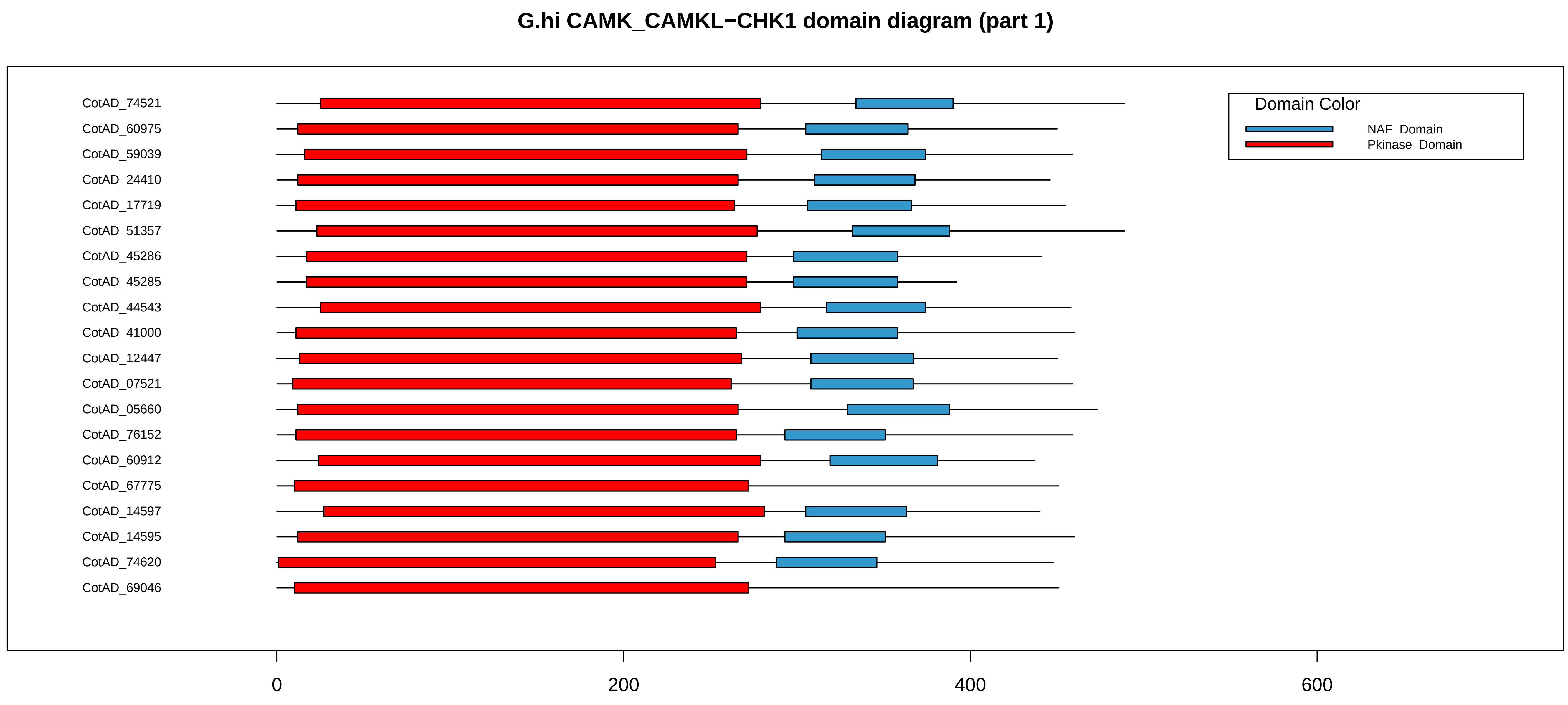
G.hi CAMK_CAMKL−CHK1 domain diagram (part 1)
CotAD_74521
CotAD_60975
CotAD_59039
CotAD_24410
CotAD_17719
CotAD_51357
CotAD_45286
CotAD_45285
CotAD_44543
CotAD_41000
CotAD_12447
CotAD_07521
CotAD_05660
CotAD_76152
CotAD_60912
CotAD_67775
CotAD_14597
CotAD_14595
CotAD_74620
CotAD_69046
Domain Color
NAF Domain
Pkinase Domain
0
200
400
600

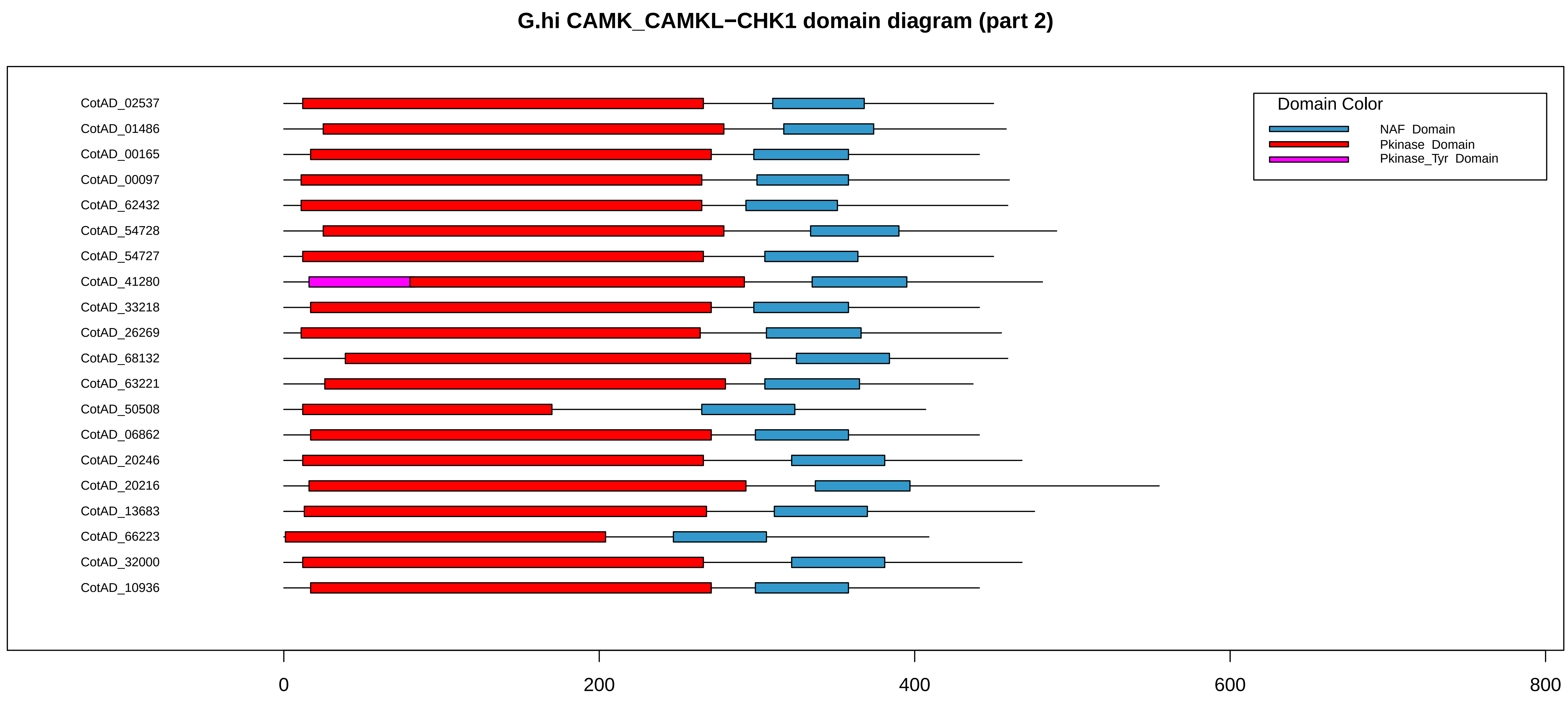

G.hi CAMK_CAMKL−CHK1 domain diagram (part 2)
CotAD_02537
CotAD_01486
CotAD_00165
CotAD_00097
CotAD_62432
CotAD_54728
CotAD_54727
CotAD_41280
CotAD_33218
CotAD_26269
CotAD_68132
CotAD_63221
CotAD_50508
CotAD_06862
CotAD_20246
CotAD_20216
CotAD_13683
CotAD_66223
CotAD_32000
CotAD_10936
Domain Color
NAF Domain
Pkinase Domain
Pkinase_Tyr Domain
0
200
400
600
800

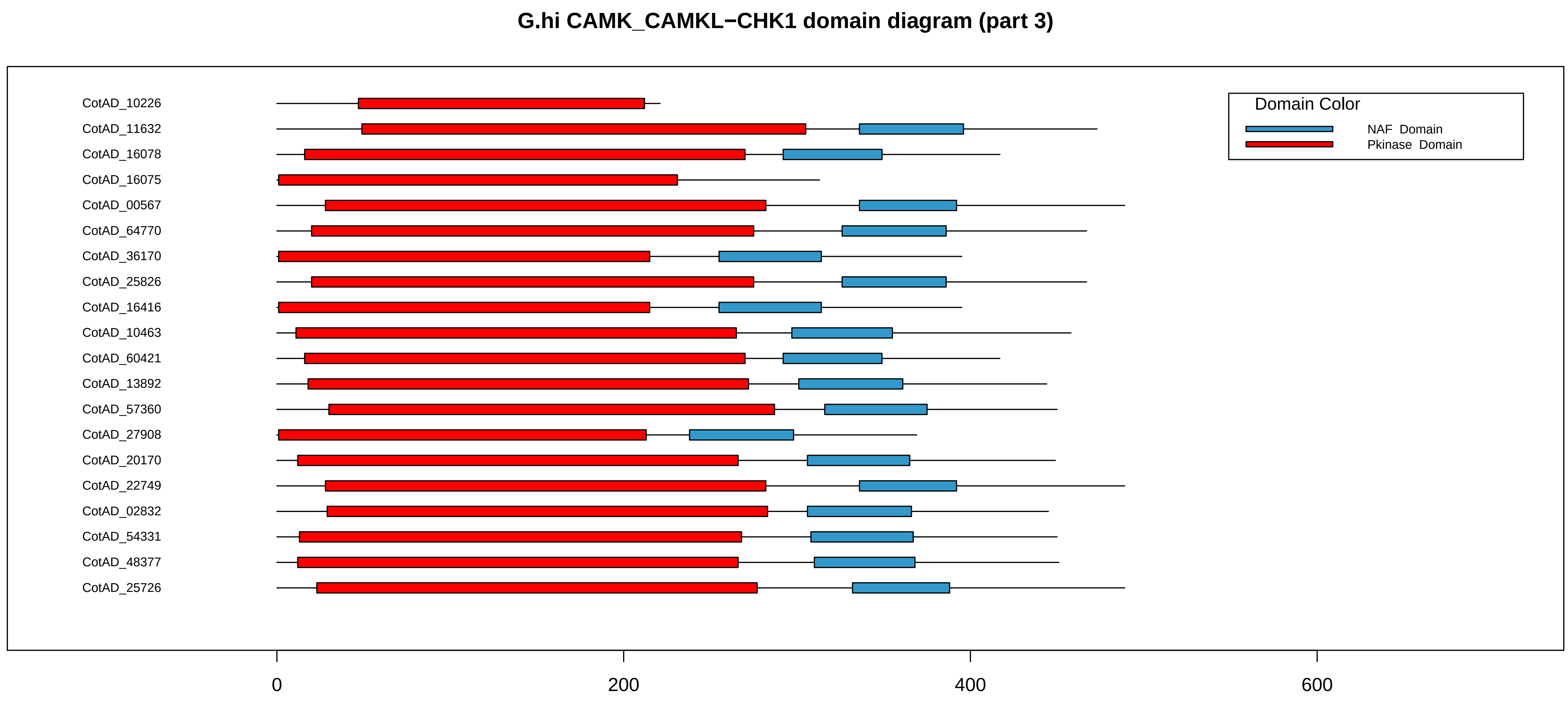

G.hi CAMK_CAMKL−CHK1 domain diagram (part 3)
CotAD_10226
CotAD_11632
CotAD_16078
CotAD_16075
CotAD_00567
CotAD_64770
CotAD_36170
CotAD_25826
CotAD_16416
CotAD_10463
CotAD_60421
CotAD_13892
CotAD_57360
CotAD_27908
CotAD_20170
CotAD_22749
CotAD_02832
CotAD_54331
CotAD_48377
CotAD_25726
Domain Color
NAF Domain
Pkinase Domain
0
200
400
600

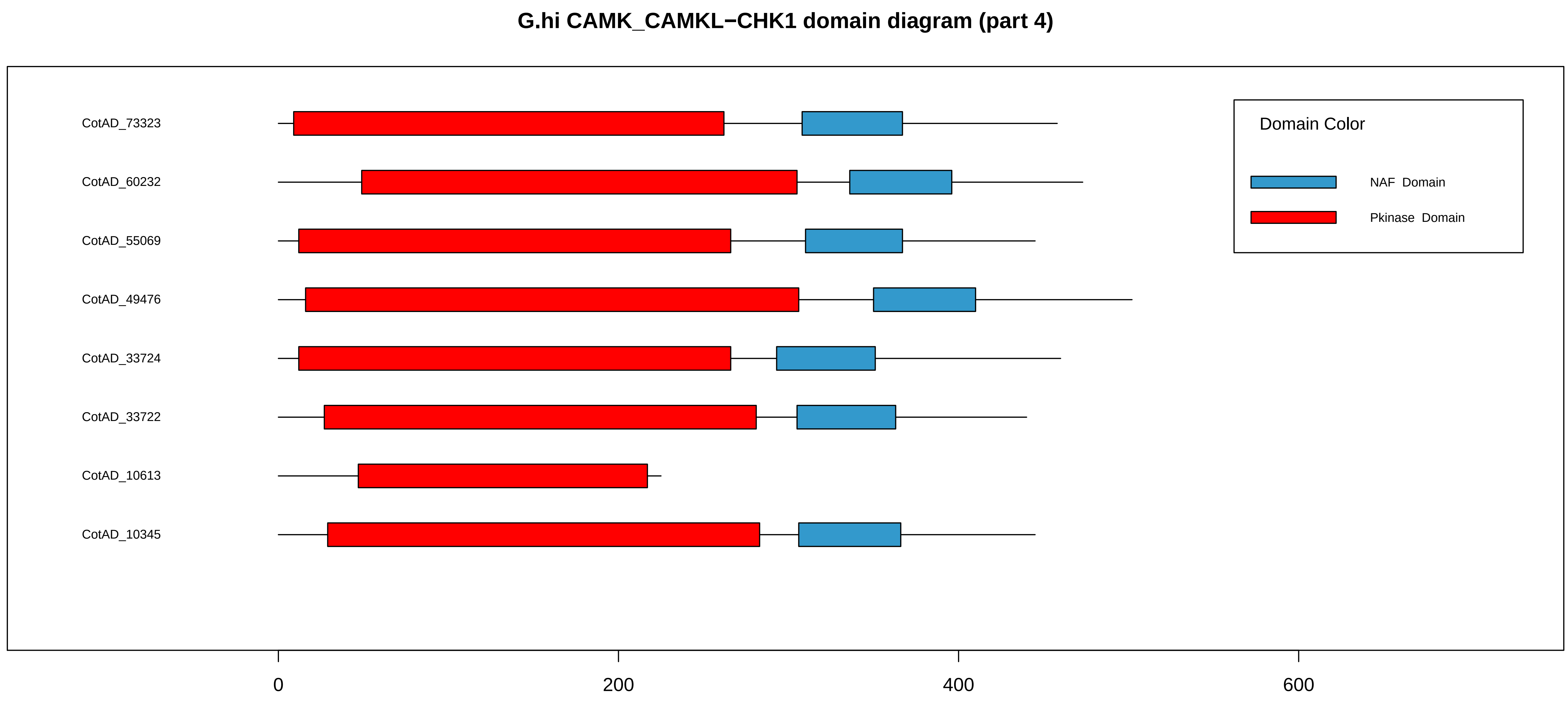
G.hi CAMK_CAMKL−CHK1 domain diagram (part 4)
CotAD_73323
CotAD_60232
CotAD_55069
CotAD_49476
CotAD_33724
CotAD_33722
CotAD_10613
CotAD_10345
Domain Color
NAF Domain
Pkinase Domain
0
200
400
600

G.hi CAMK_CAMKL−LKB domain diagram (all)

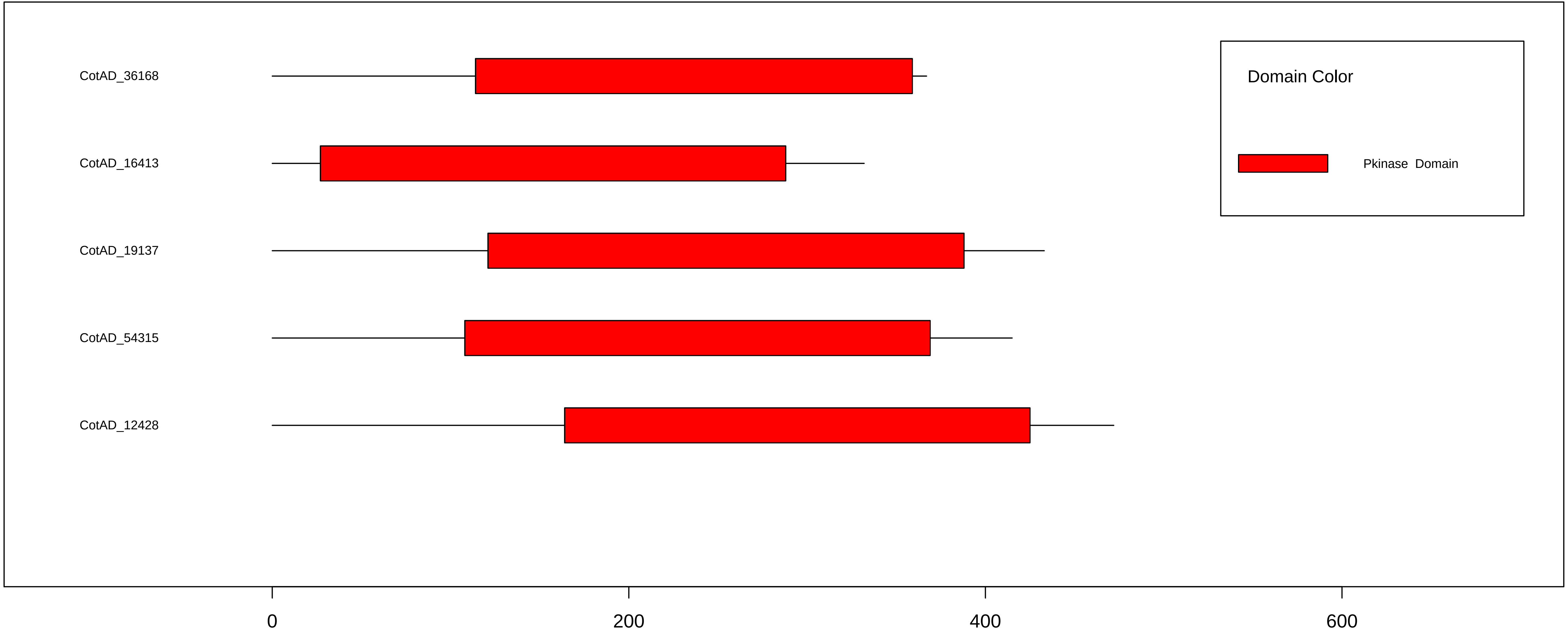

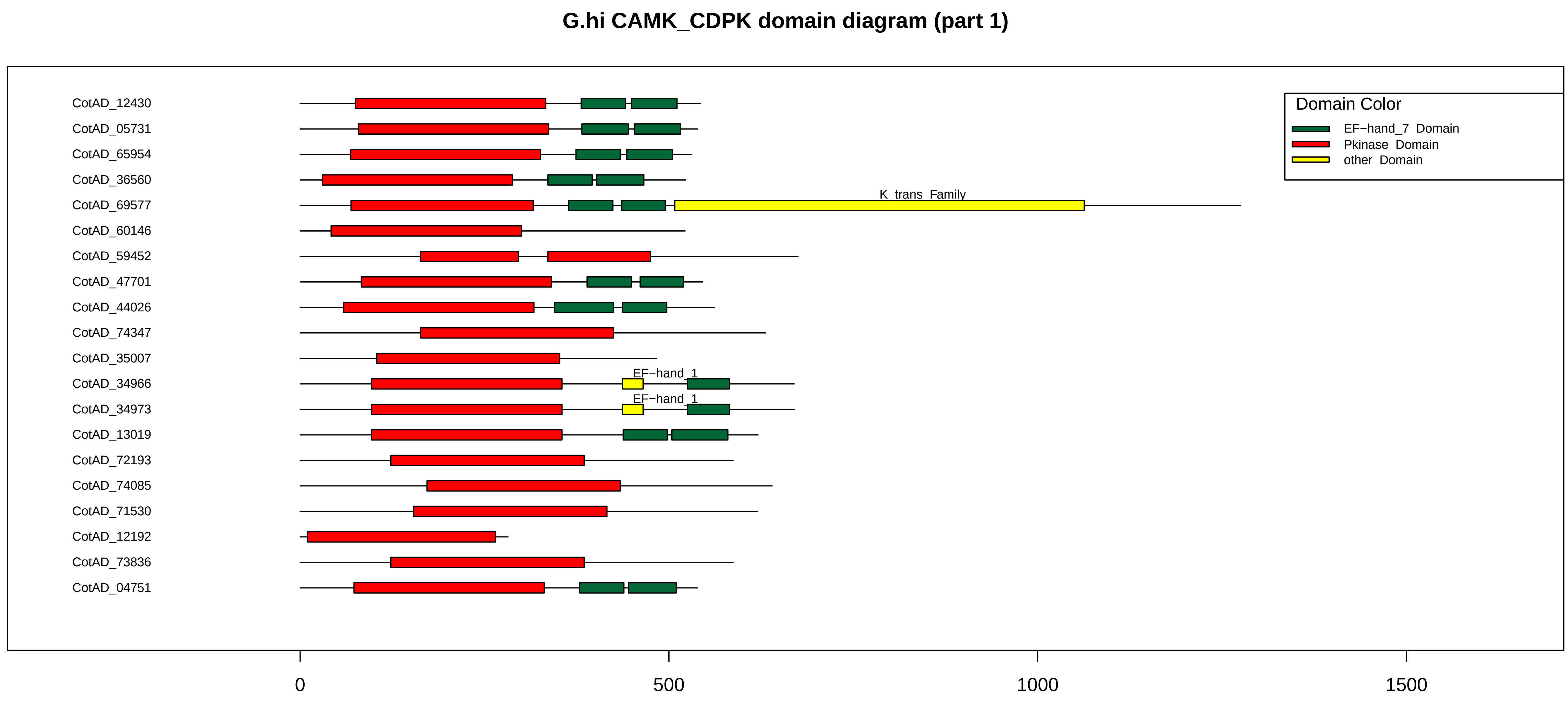
G.hi CAMK_CDPK domain diagram (part 1)
CotAD_12430
CotAD_05731
CotAD_65954
CotAD_36560
CotAD_69577
CotAD_60146
CotAD_59452
CotAD_47701
CotAD_44026
CotAD_74347
CotAD_35007
CotAD_34966
CotAD_34973
CotAD_13019
CotAD_72193
CotAD_74085
CotAD_71530
CotAD_12192
CotAD_73836
CotAD_04751
K_trans_Family
EF−hand_1
EF−hand_1
Domain Color
EF−hand_7 Domain
Pkinase Domain
other Domain
0
500
1000
1500

G.hi CAMK_CDPK domain diagram (part 2)

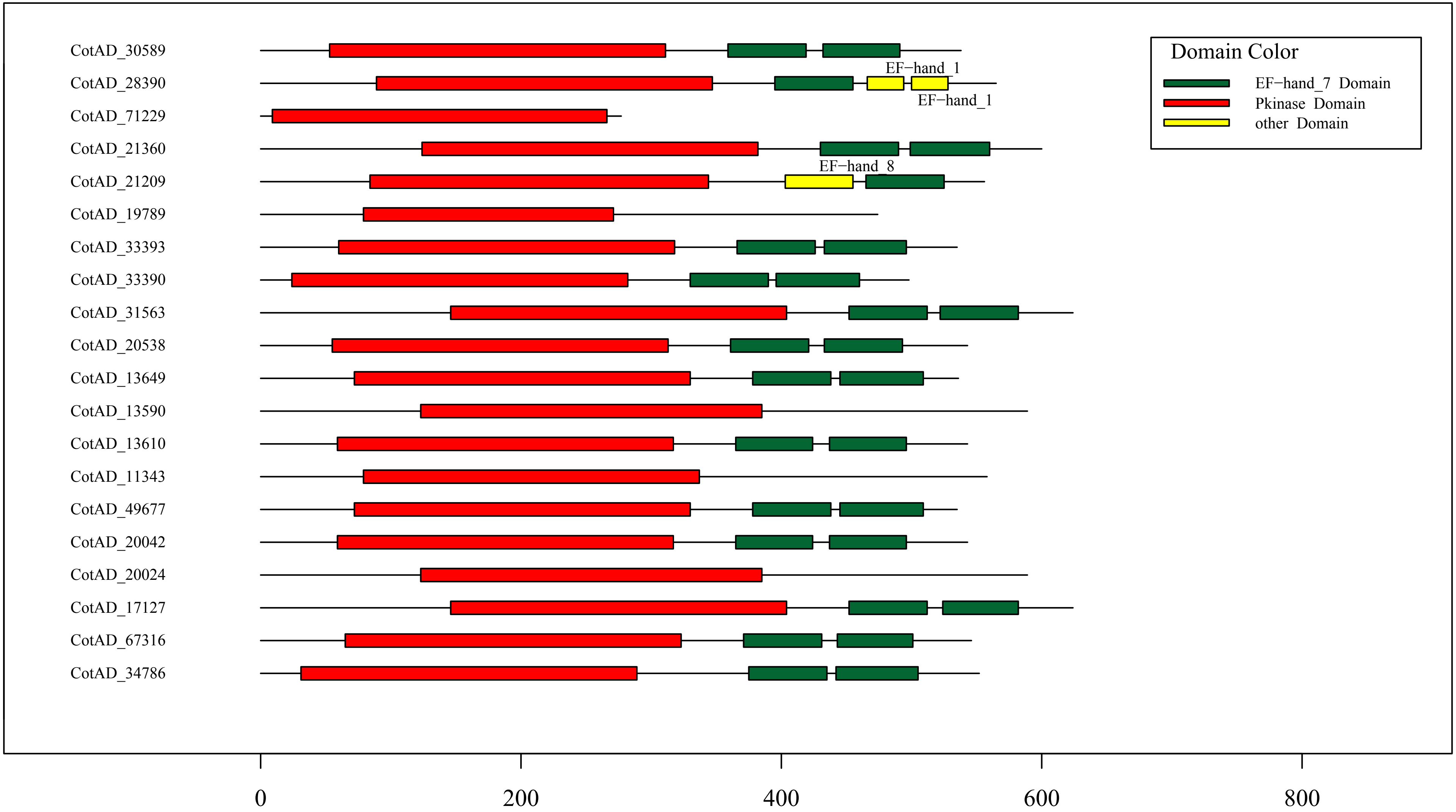

G.hi CAMK_CDPK domain diagram (part 3)

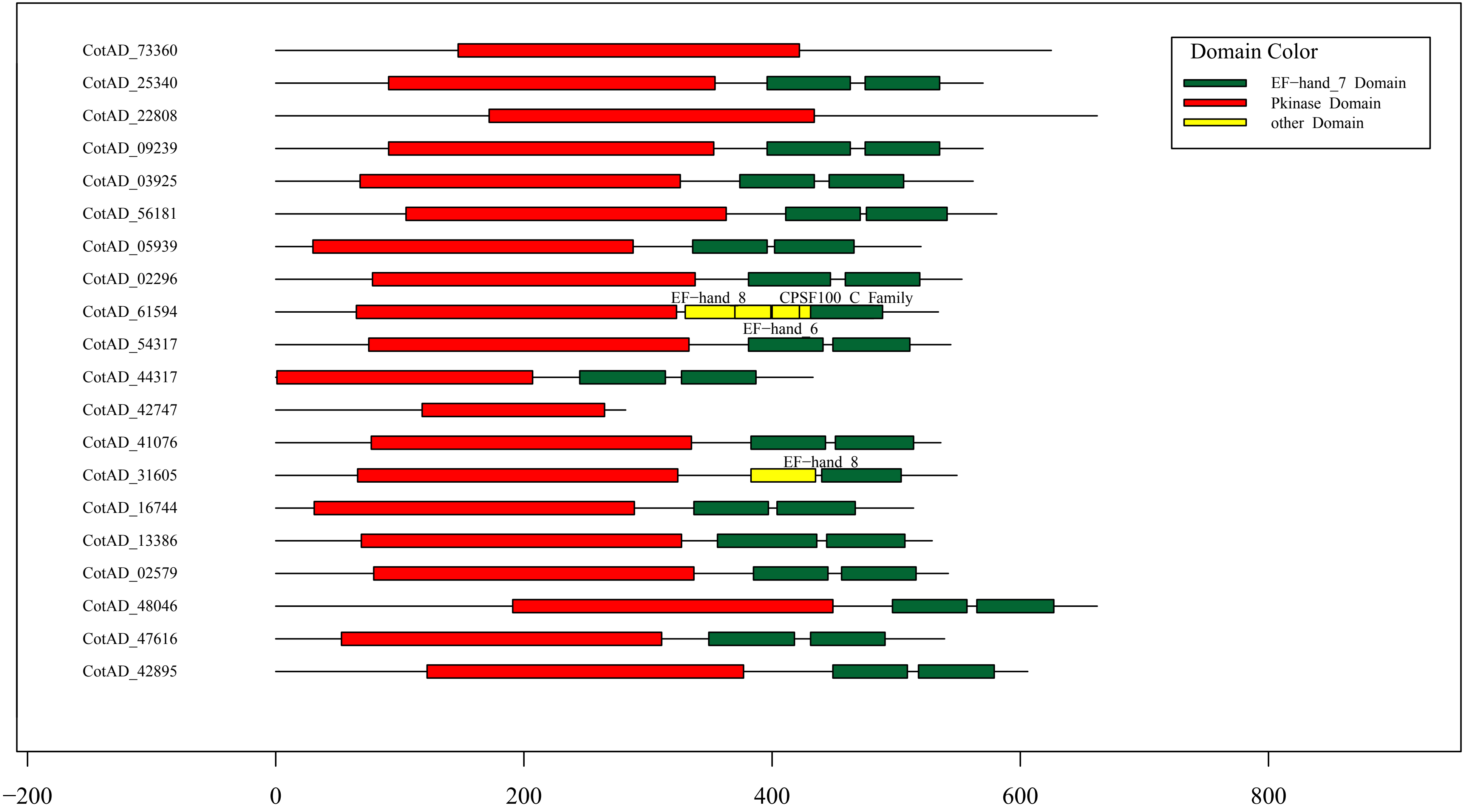

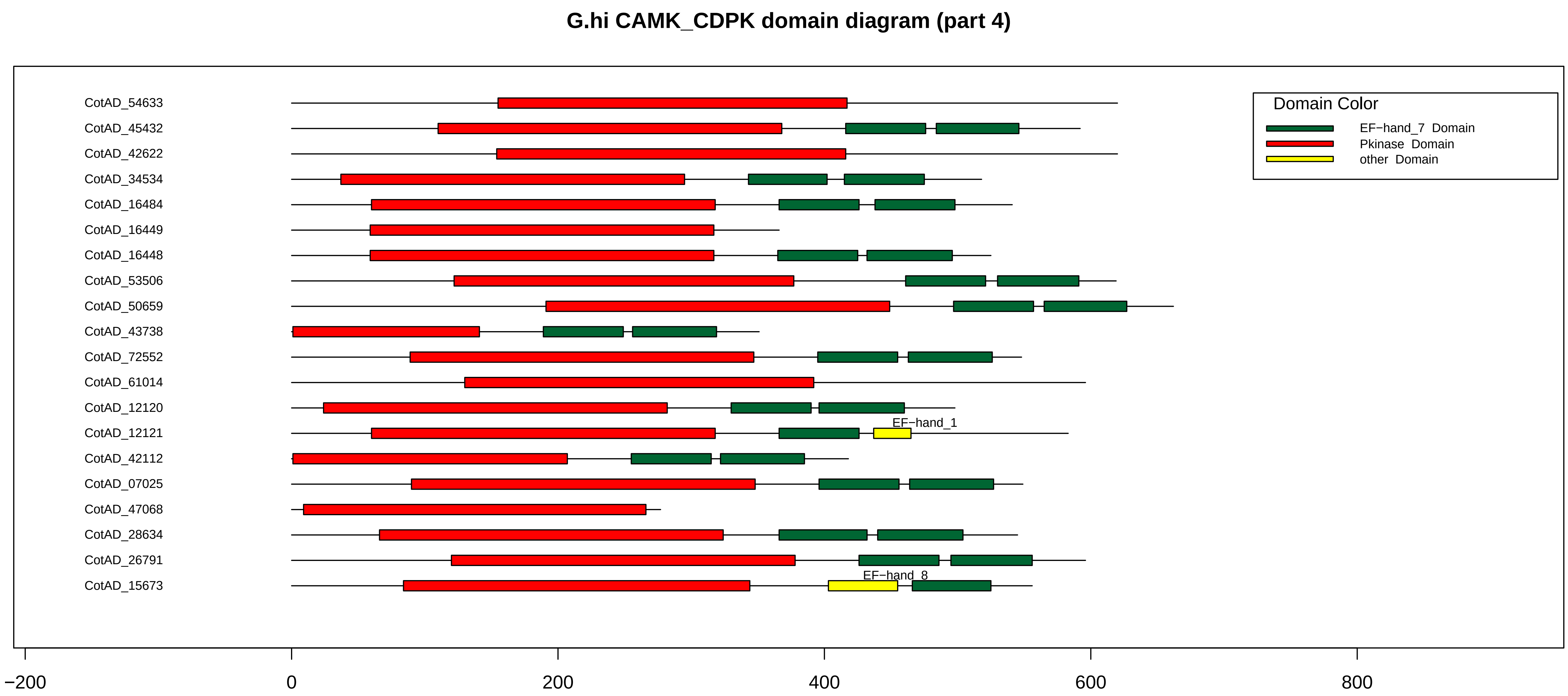

G.hi CAMK_CDPK domain diagram (part 4)
Domain Color
EF−hand_7 Domain
Pkinase Domain
other Domain
CotAD_54633
CotAD_45432
CotAD_42622
CotAD_34534
CotAD_16484
CotAD_16449
CotAD_16448
CotAD_53506
CotAD_50659
CotAD_43738
CotAD_72552
CotAD_61014
CotAD_12120
CotAD_12121
CotAD_42112
CotAD_07025
CotAD_47068
CotAD_28634
CotAD_26791
CotAD_15673
EF−hand_1
EF−hand_8
−200
0
200
400
600
800

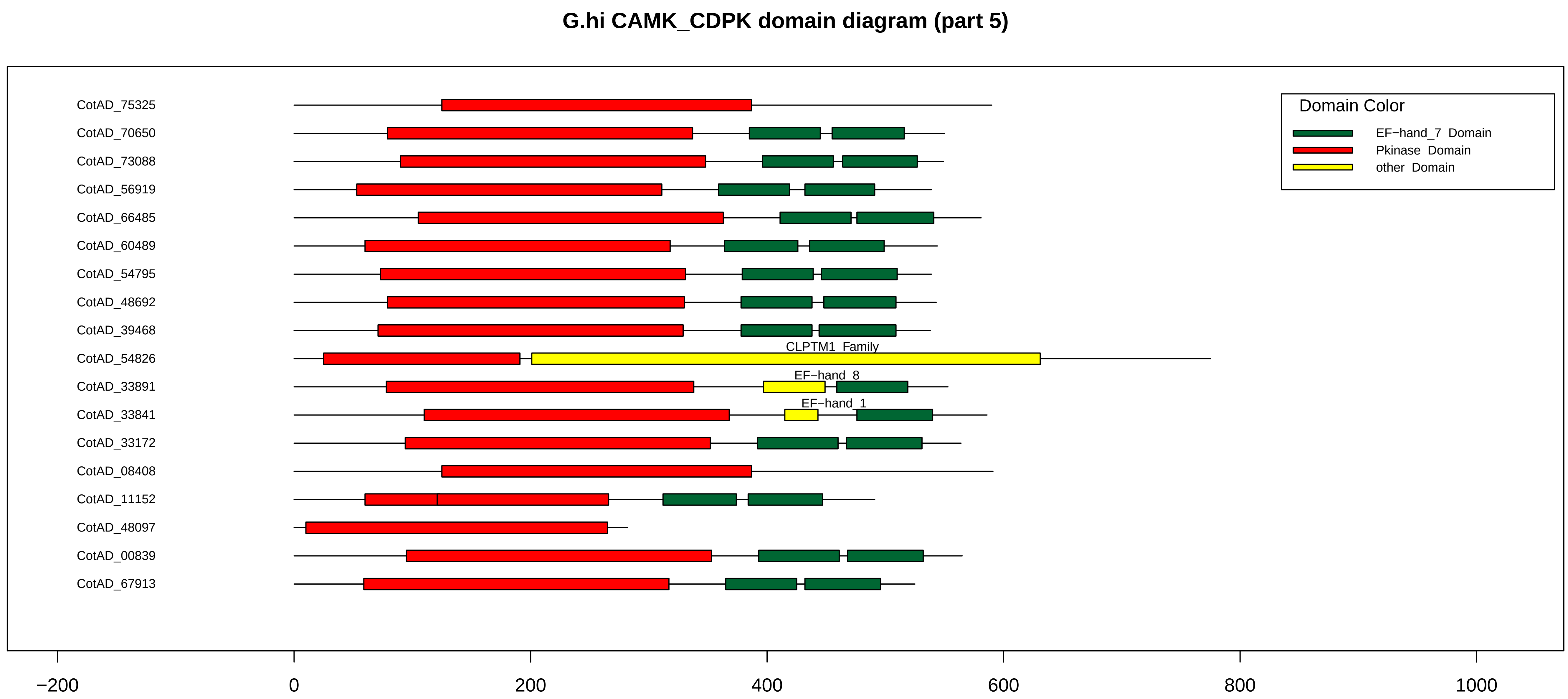
G.hi CAMK_CDPK domain diagram (part 5)
CotAD_75325
CotAD_70650
CotAD_73088
CotAD_56919
CotAD_66485
CotAD_60489
CotAD_54795
CotAD_48692
CotAD_39468
CotAD_54826
CotAD_33891
CotAD_33841
CotAD_33172
CotAD_08408
CotAD_11152
CotAD_48097
CotAD_00839
CotAD_67913
CLPTM1_Family
EF−hand_8
EF−hand_1
Domain Color
EF−hand_7 Domain
Pkinase Domain
other Domain
−200
0
200
400
600
800
1000

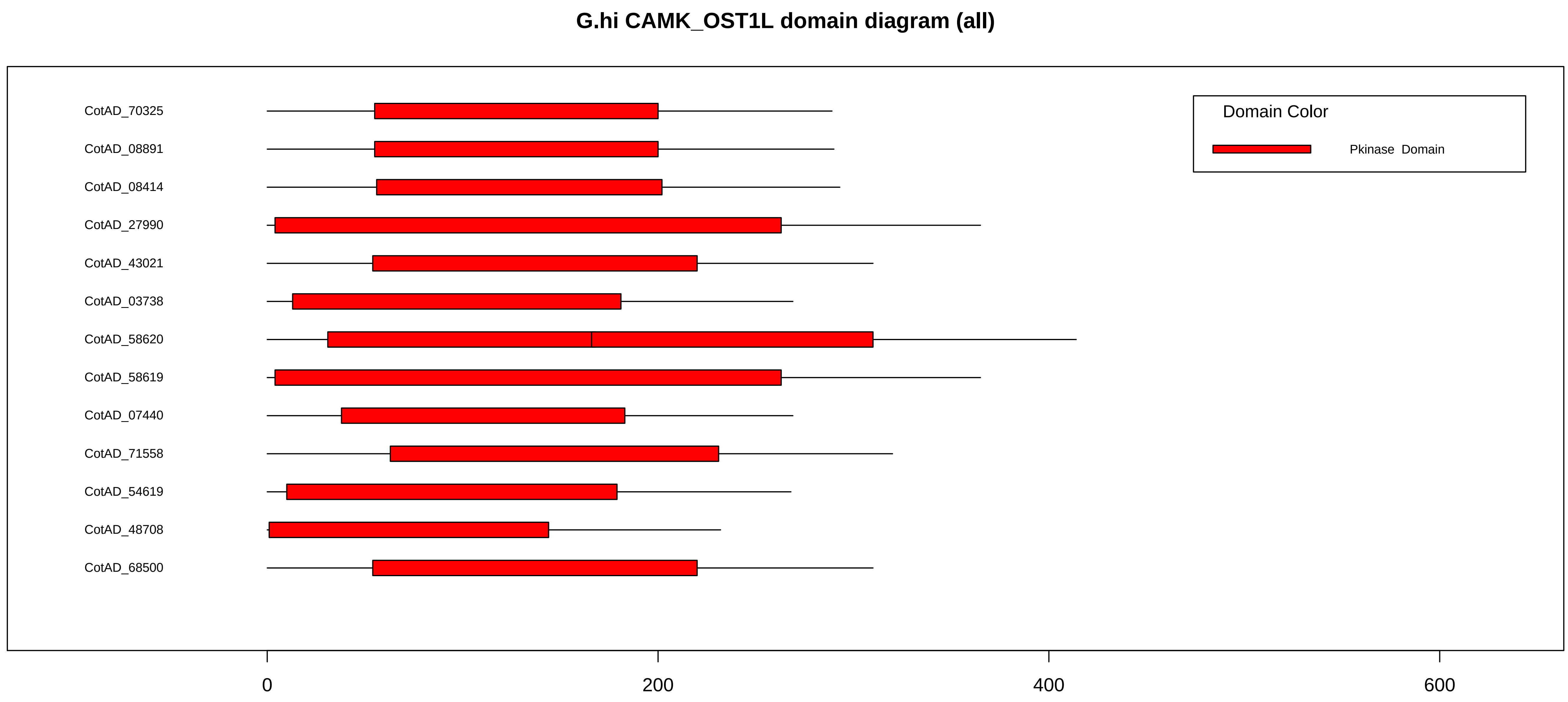
G.hi CAMK_OST1L domain diagram (all)
CotAD_70325
CotAD_08891
CotAD_08414
CotAD_27990
CotAD_43021
CotAD_03738
CotAD_58620
CotAD_58619
CotAD_07440
CotAD_71558
CotAD_54619
CotAD_48708
CotAD_68500
Domain Color
Pkinase Domain
0
200
400
600

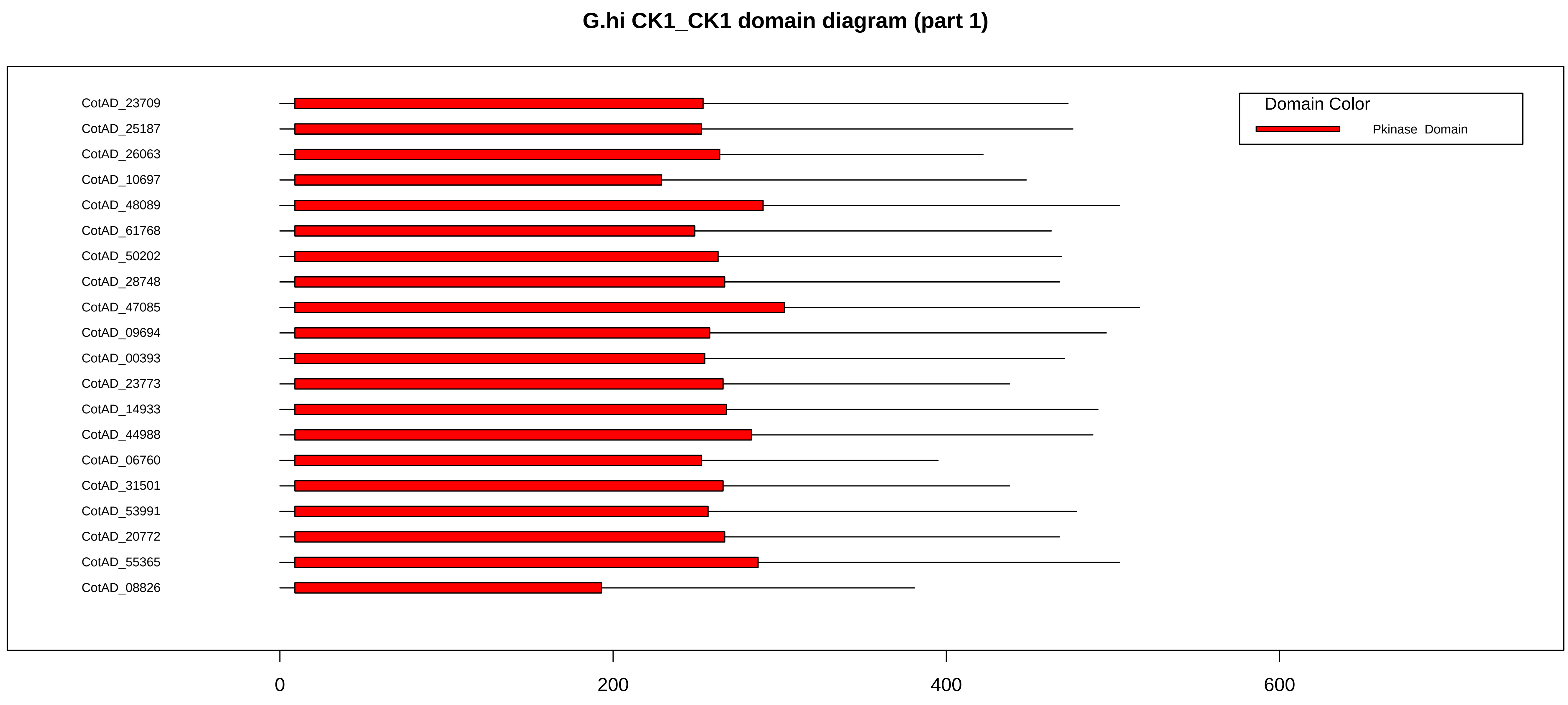

G.hi CK1_CK1 domain diagram (part 1)
CotAD_23709
CotAD_25187
CotAD_26063
CotAD_10697
CotAD_48089
CotAD_61768
CotAD_50202
CotAD_28748
CotAD_47085
CotAD_09694
CotAD_00393
CotAD_23773
CotAD_14933
CotAD_44988
CotAD_06760
CotAD_31501
CotAD_53991
CotAD_20772
CotAD_55365
CotAD_08826
Domain Color
Pkinase Domain
0
200
400
600

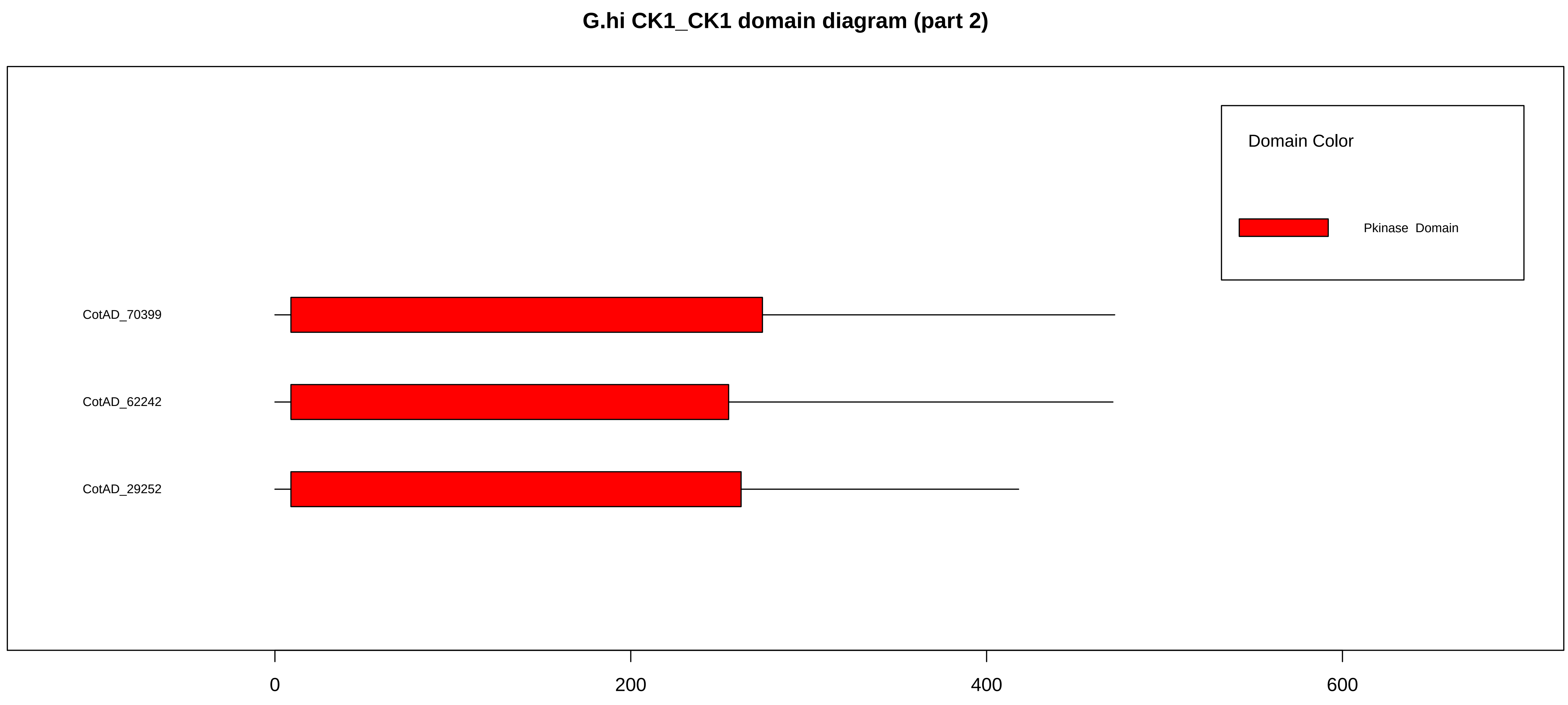
G.hi CK1_CK1 domain diagram (part 2)
Domain Color
Pkinase Domain
CotAD_70399
CotAD_62242
CotAD_29252
0
200
400
600

G.hi CK1_CK1−Pl domain diagram (part 1)

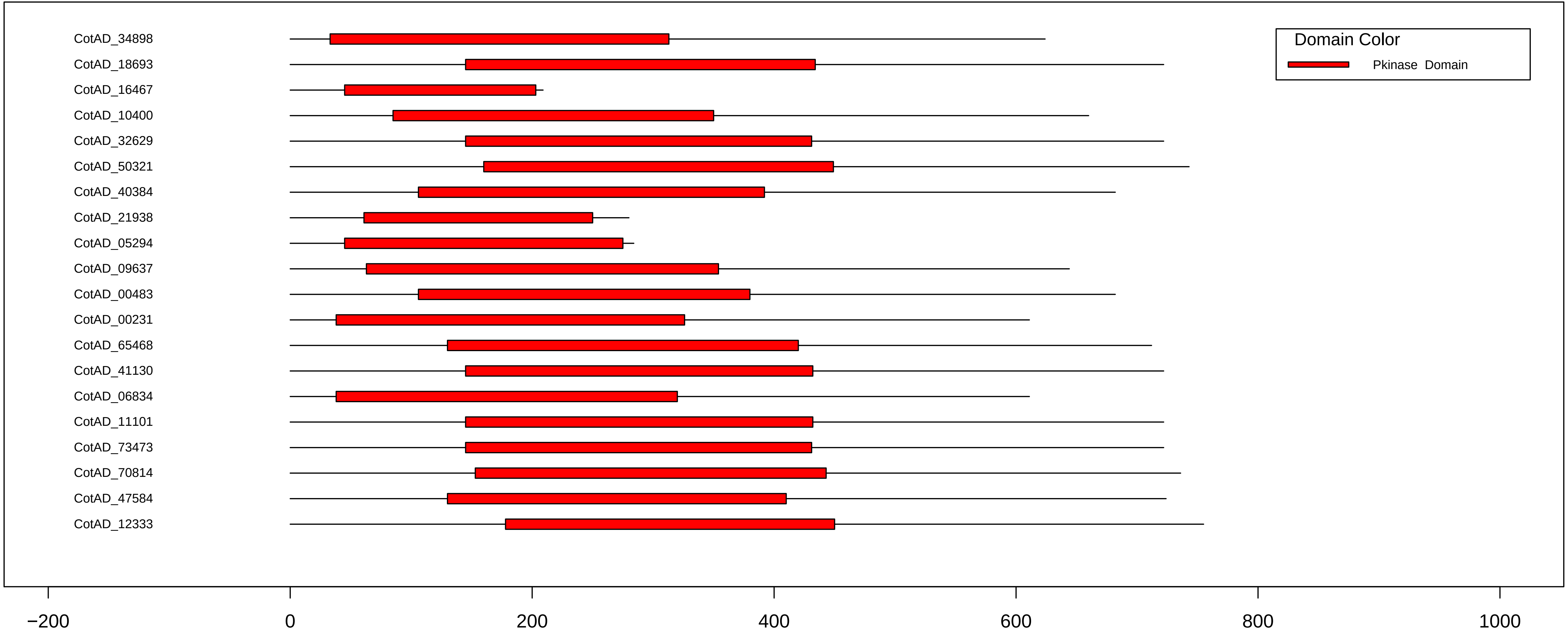

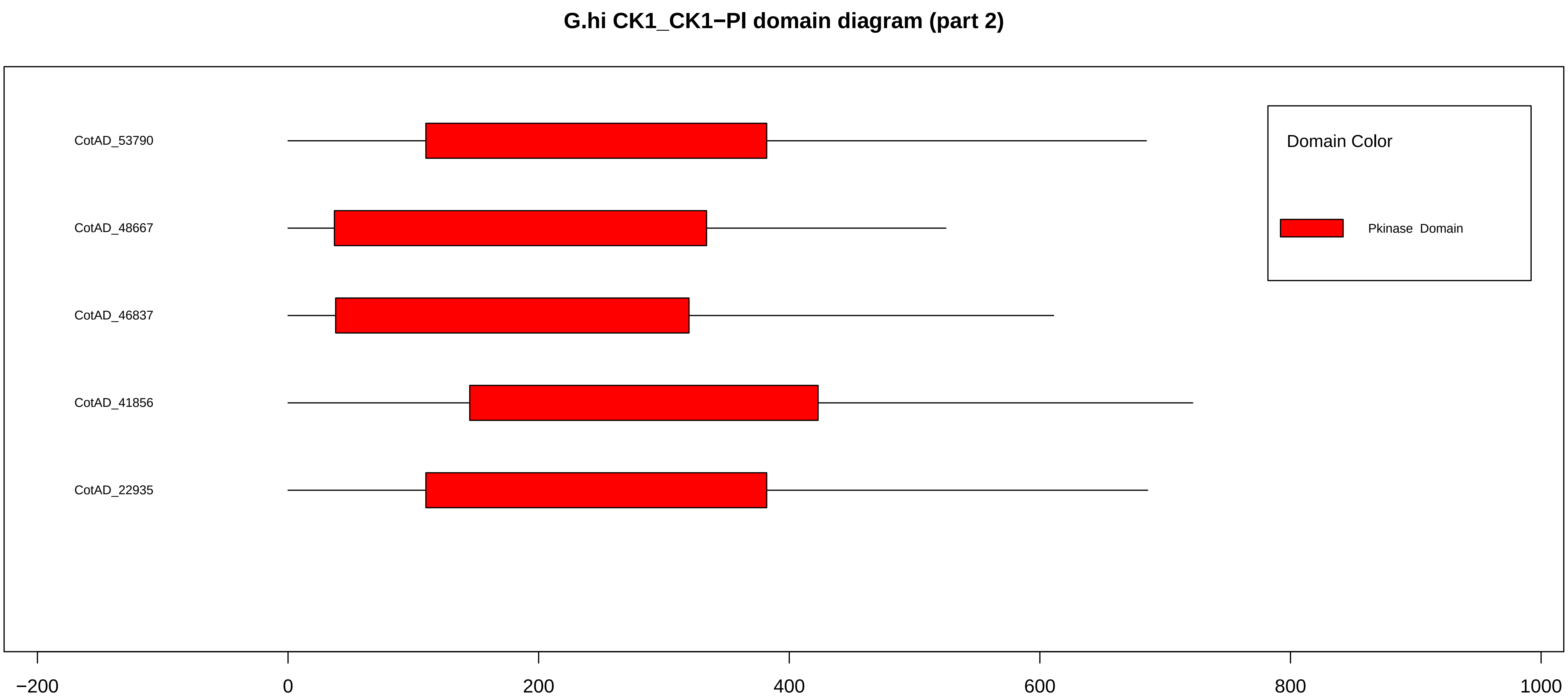
G.hi CK1_CK1−PI domain diagram (part 2)
CotAD_53790
CotAD_48667
CotAD_46837
CotAD_41856
CotAD_22935
Domain Color
Pkinase Domain
−200
0
200
400
600
800
1000

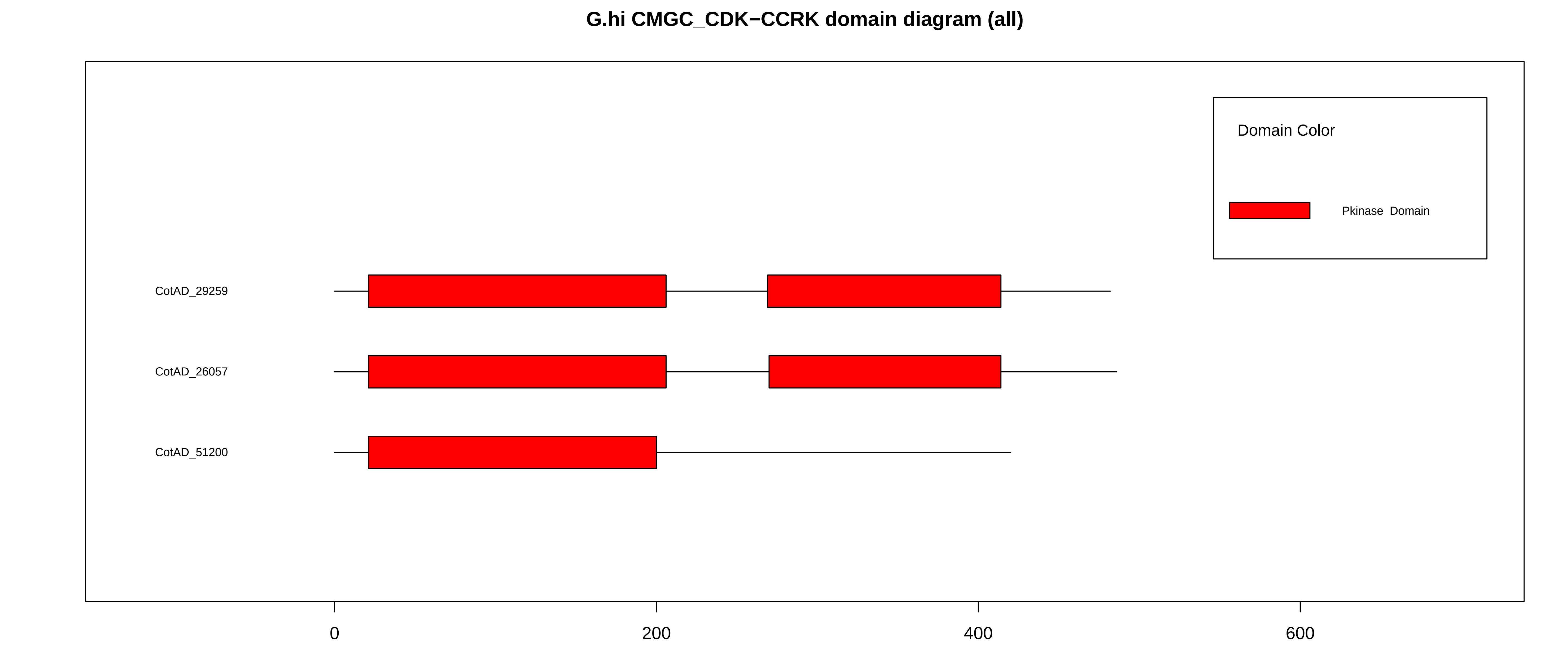
G.hi CMGC_CDK−CCRK domain diagram (all)
Domain Color
Pkinase Domain
CotAD_29259
CotAD_26057
CotAD_51200
0
200
400
600

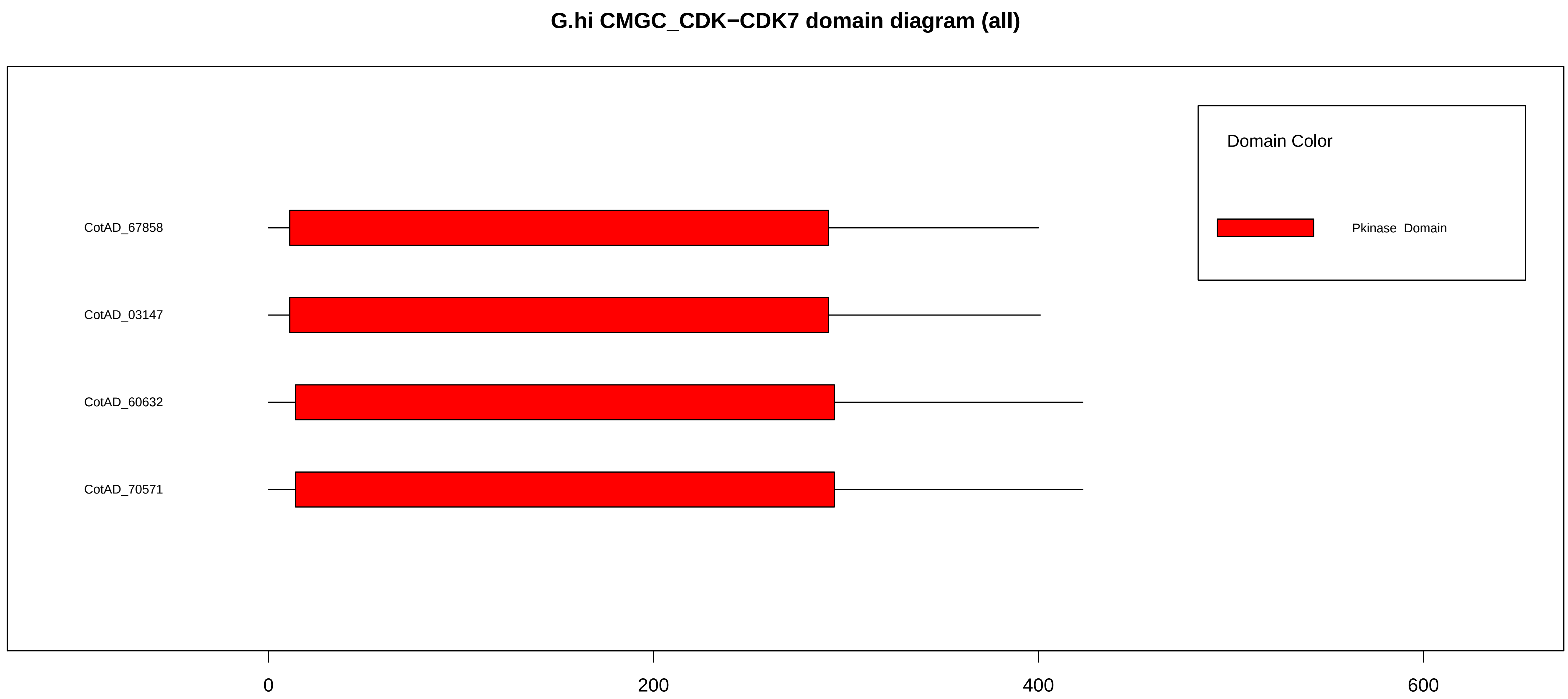
G.hi CMGC_CDK−CDK7 domain diagram (all)
CotAD_67858
CotAD_03147
CotAD_60632
CotAD_70571
Domain Color
Pkinase Domain
0
200
400
600

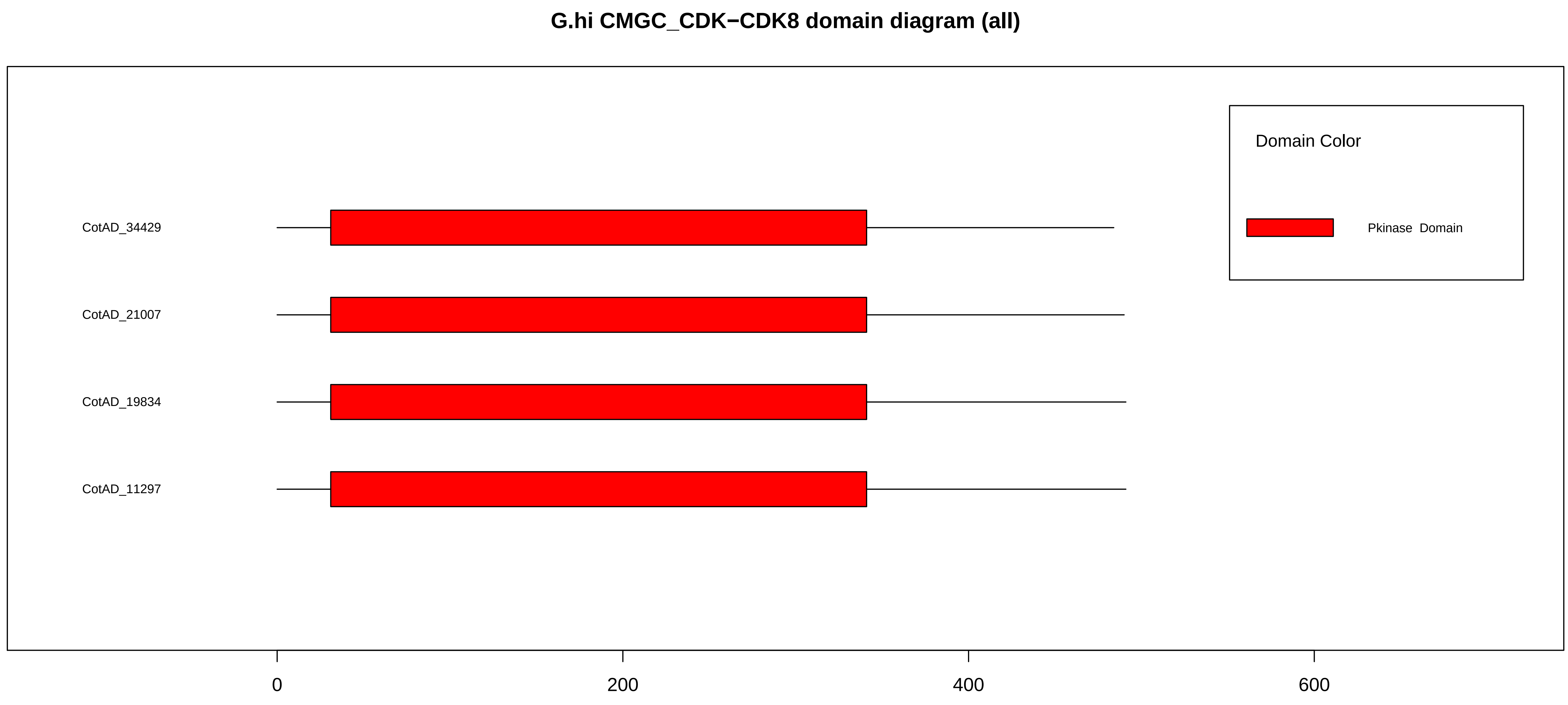
G.hi CMGC_CDK−CDK8 domain diagram (all)
Domain Color
Pkinase Domain
CotAD_34429
CotAD_21007
CotAD_19834
CotAD_11297
0
200
400
600

G.hi CMGC_CDK−CRK7−CDK9 domain diagram (part 1)

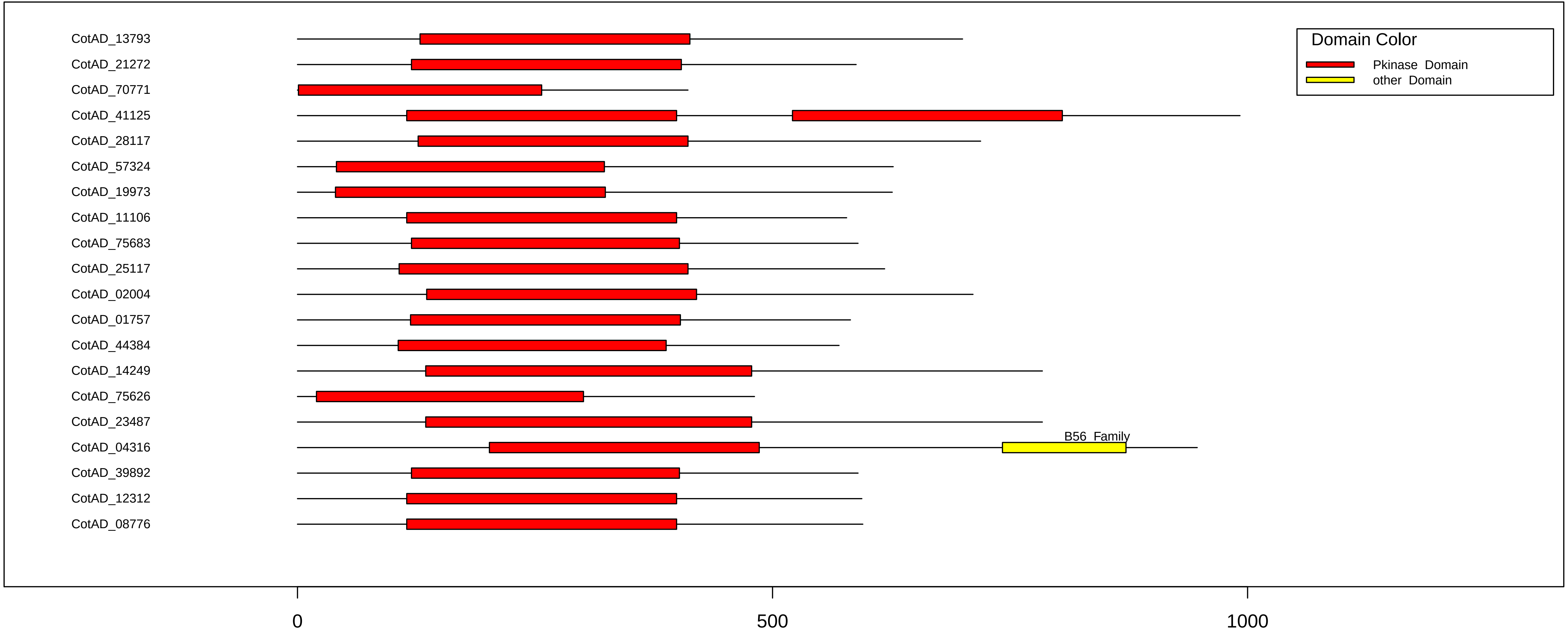

G.hi CMGC_CDK−CRK7−CDK9 domain diagram (part 2)

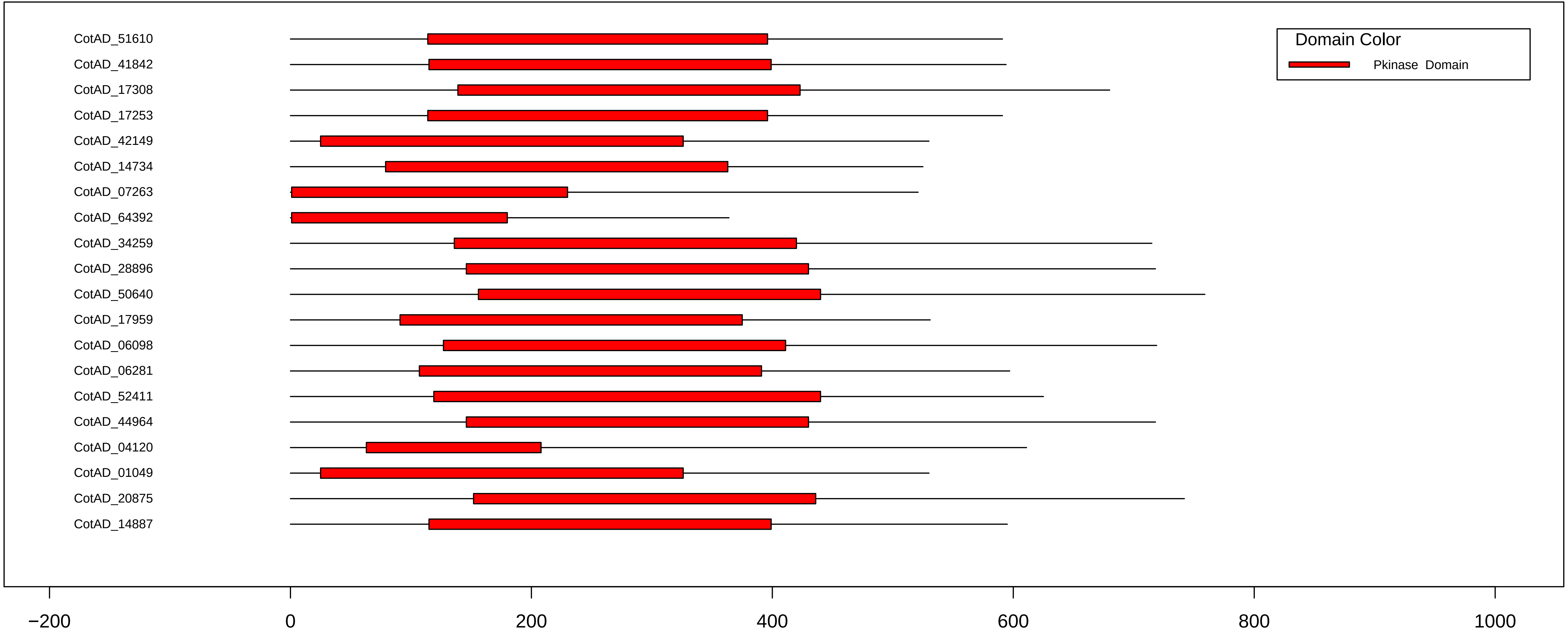

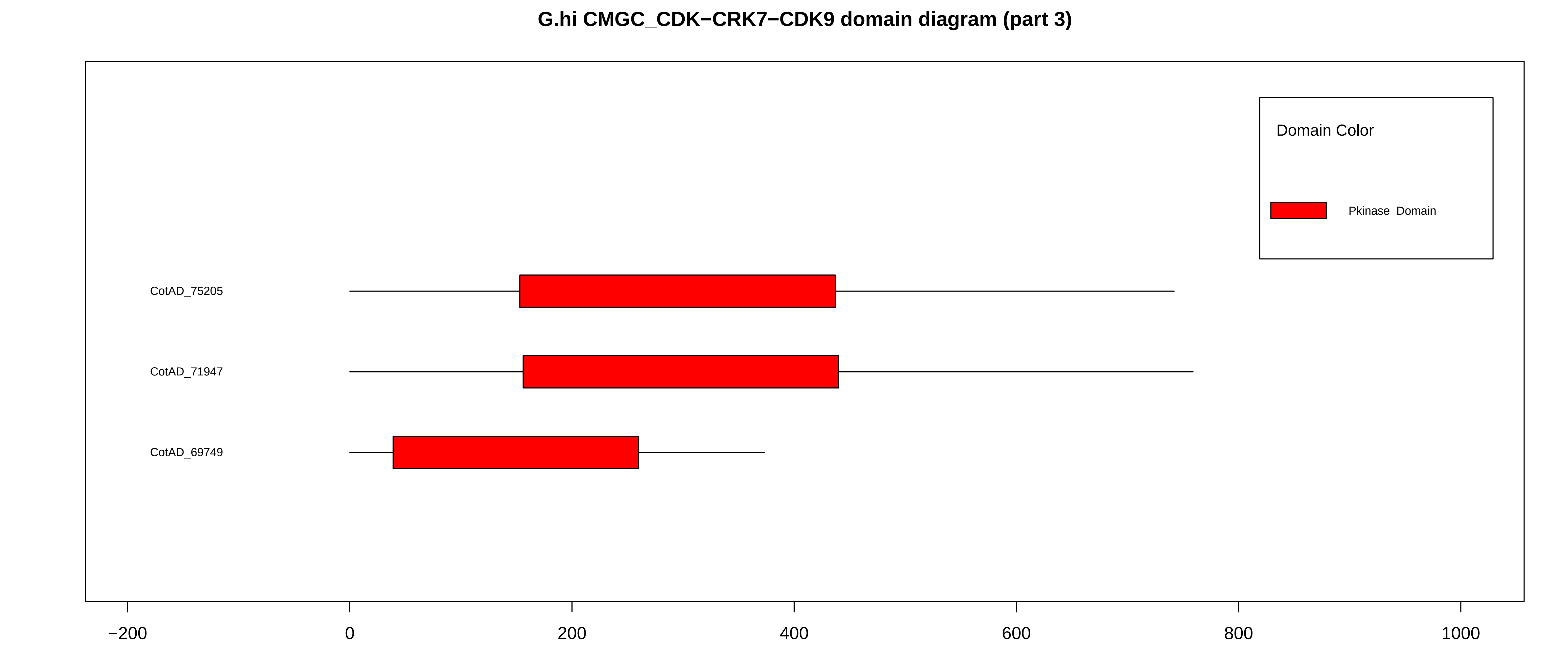
G.hi CMGC_CDK−CRK7−CDK9 domain diagram (part 3)
Domain Color
Pkinase Domain
CotAD_75205
CotAD_71947
CotAD_69749
−200
0
200
400
600
800
1000

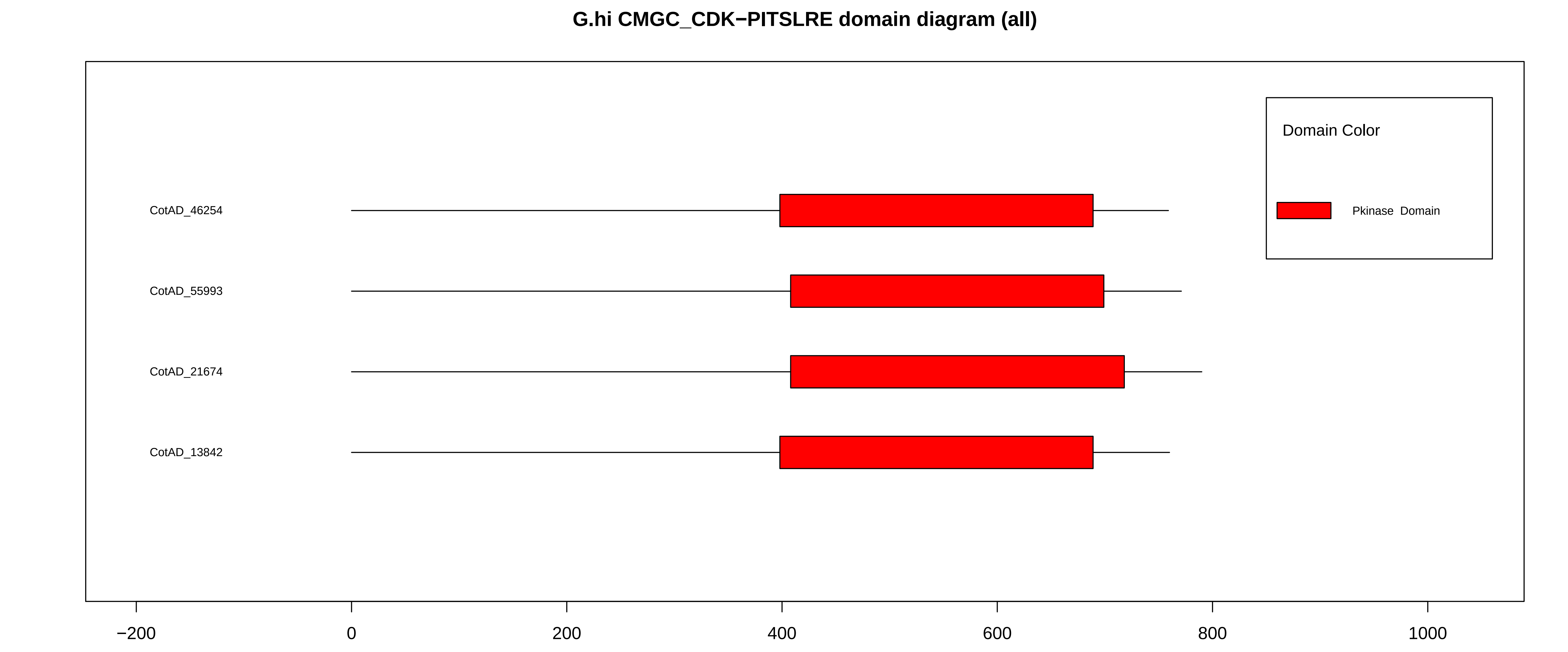
G.hi CMGC_CDK−PITSLRE domain diagram (all)
Domain Color
Pkinase Domain
CotAD_46254
CotAD_55993
CotAD_21674
CotAD_13842
−200
0
200
400
600
800
1000

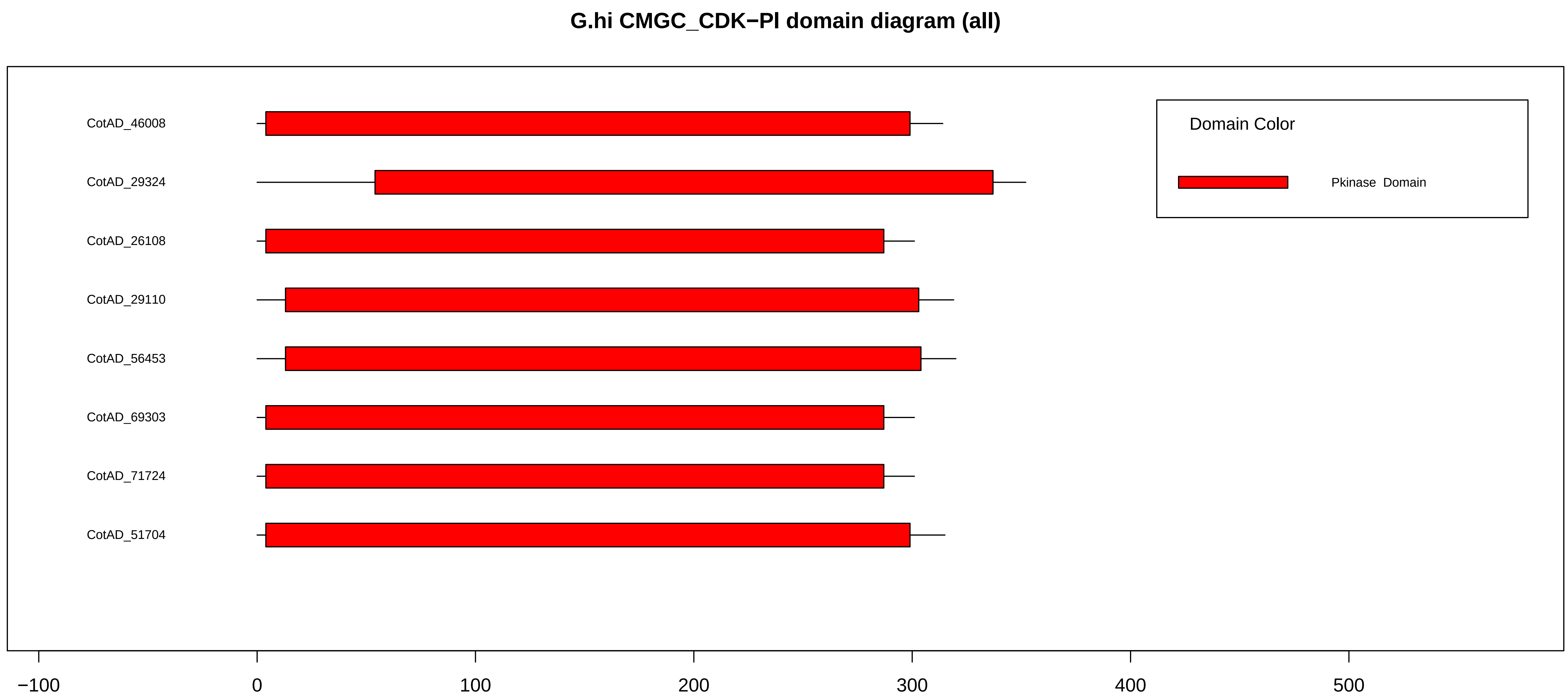
G.hi CMGC_CDK−PI domain diagram (all)
CotAD_46008
CotAD_29324
CotAD_26108
CotAD_29110
CotAD_56453
CotAD_69303
CotAD_71724
CotAD_51704
Domain Color
Pkinase Domain
−100
0
100
200
300
400
500

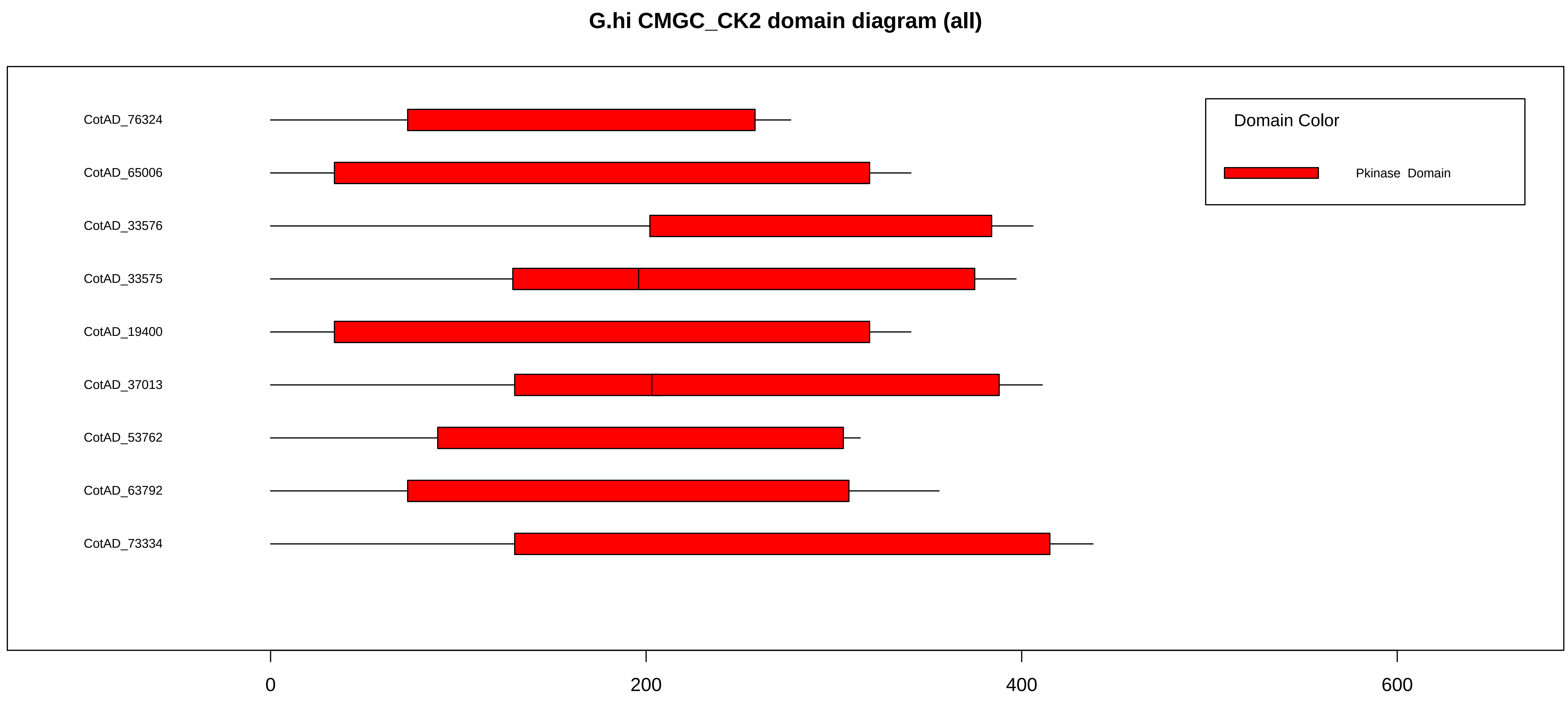

G.hi CMGC_CK2 domain diagram (all)
CotAD_76324
CotAD_65006
CotAD_33576
CotAD_33575
CotAD_19400
CotAD_37013
CotAD_53762
CotAD_63792
CotAD_73334
Domain Color
Pkinase Domain
0
200
400
600

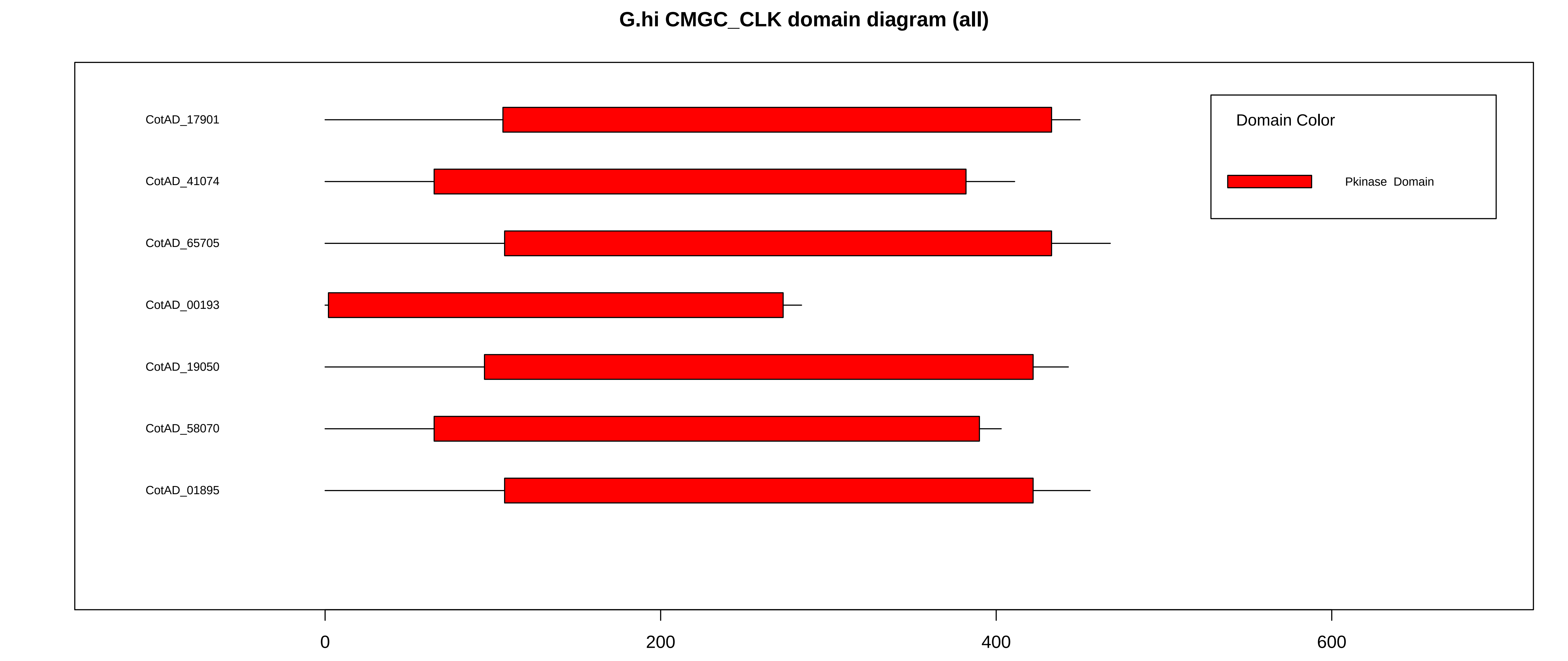
G.hi CMGC_CLK domain diagram (all)
CotAD_17901
CotAD_41074
CotAD_65705
CotAD_00193
CotAD_19050
CotAD_58070
CotAD_01895
Domain Color
Pkinase Domain
0
200
400
600

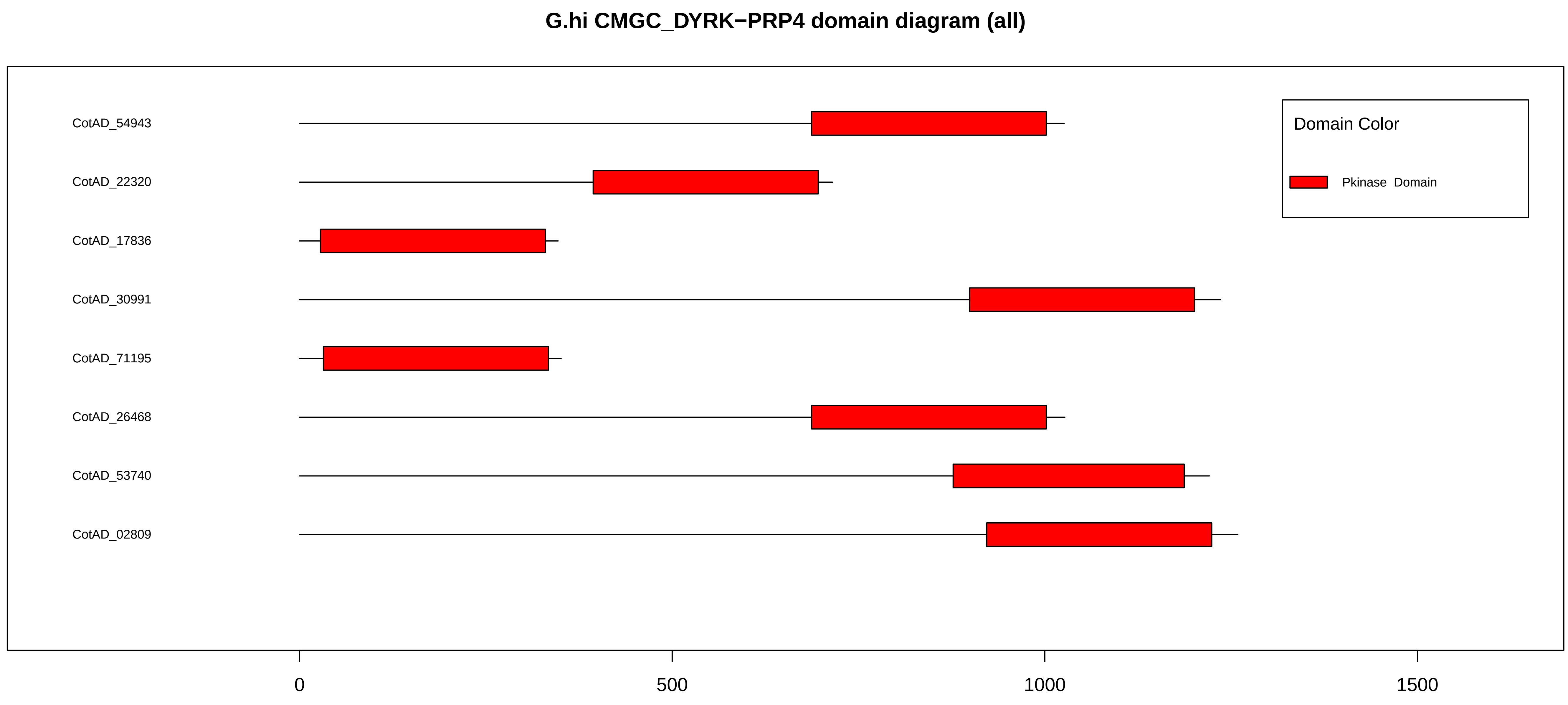
G.hi CMGC_DYRK−PRP4 domain diagram (all)
CotAD_54943
CotAD_22320
CotAD_17836
CotAD_30991
CotAD_71195
CotAD_26468
CotAD_53740
CotAD_02809
Domain Color
Pkinase Domain
0
500
1000
1500

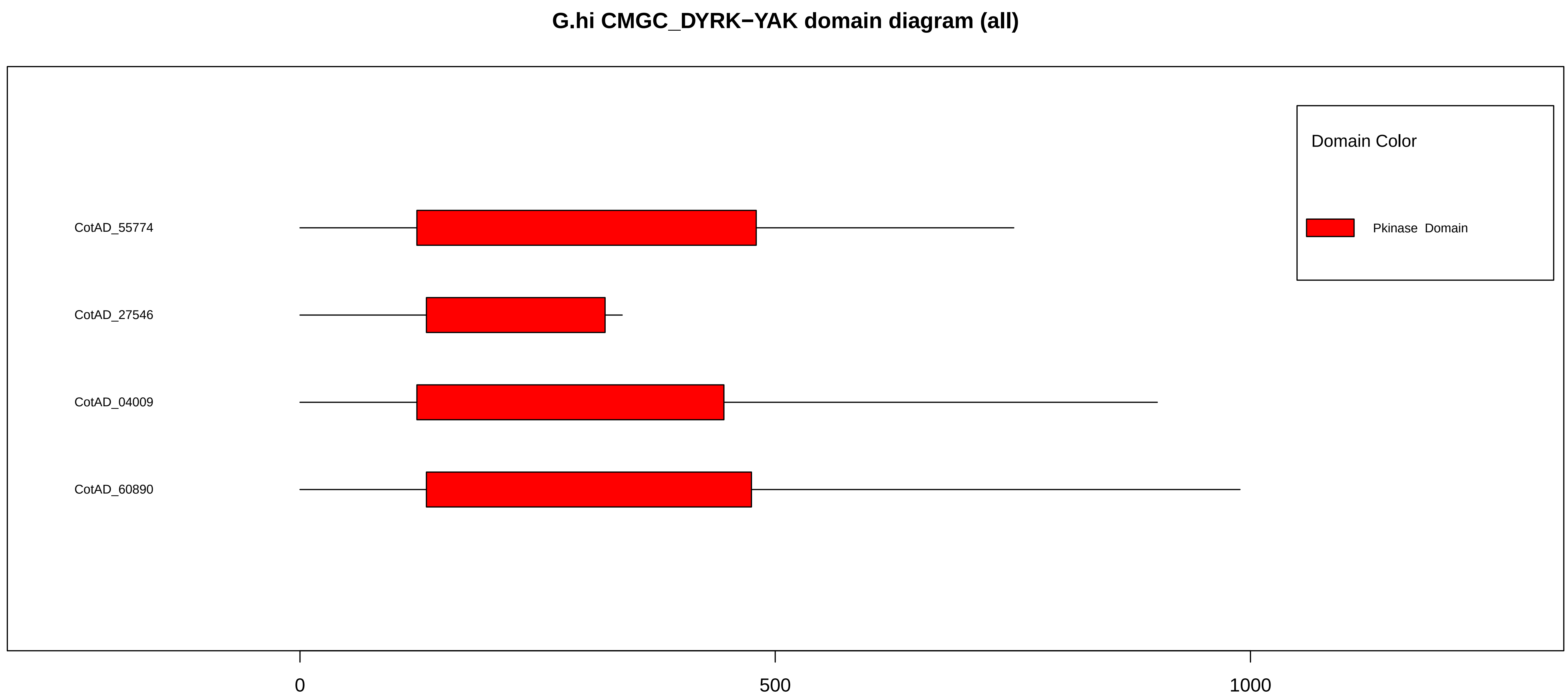
G.hi CMGC_DYRK−YAK domain diagram (all)
CotAD_55774
CotAD_27546
CotAD_04009
CotAD_60890
Domain Color
Pkinase Domain
0
500
1000

G.hi CMGC_GSKL domain diagram (all)

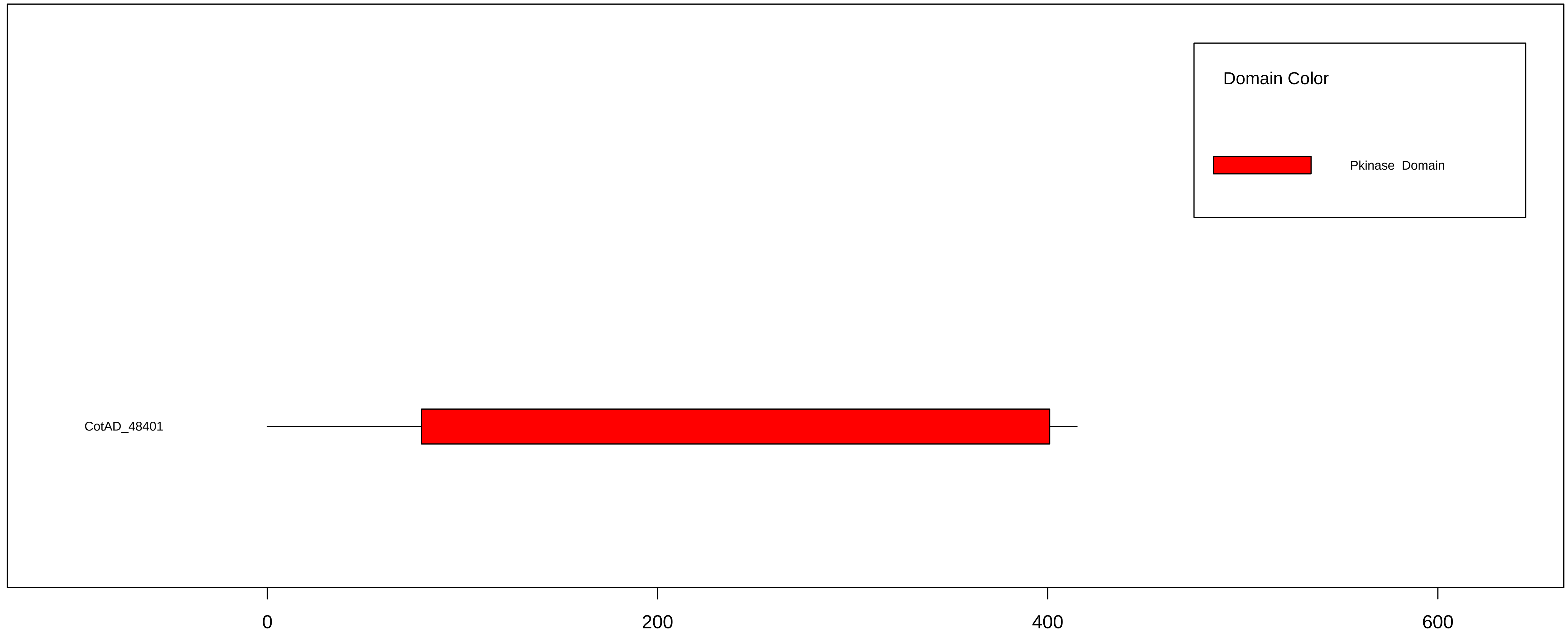

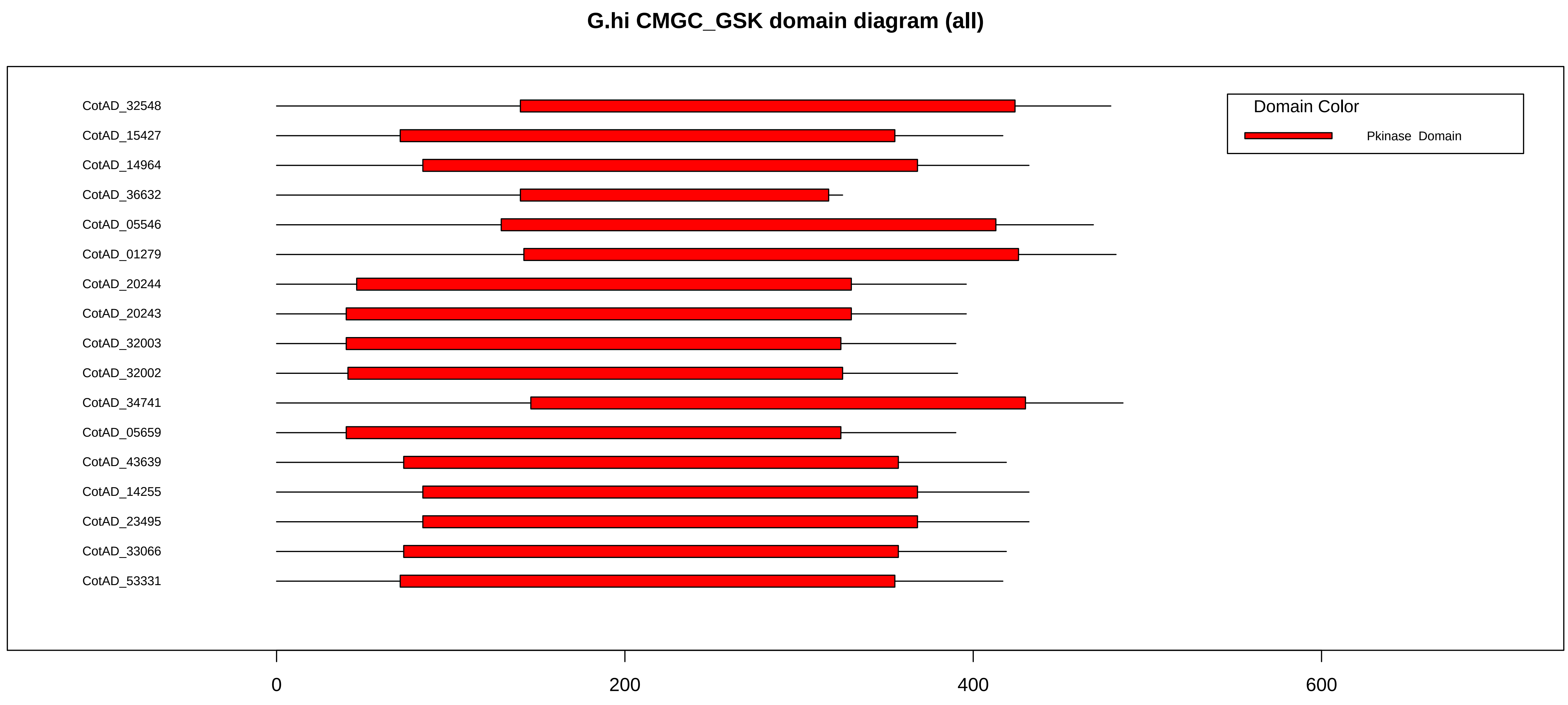

G.hi CMGC_GSK domain diagram (all)
CotAD_32548
CotAD_15427
CotAD_14964
CotAD_36632
CotAD_05546
CotAD_01279
CotAD_20244
CotAD_20243
CotAD_32003
CotAD_32002
CotAD_34741
CotAD_05659
CotAD_43639
CotAD_14255
CotAD_23495
CotAD_33066
CotAD_53331
Domain Color
Pkinase Domain
0
200
400
600

G.hi CMGC_MAPK domain diagram (part 1)

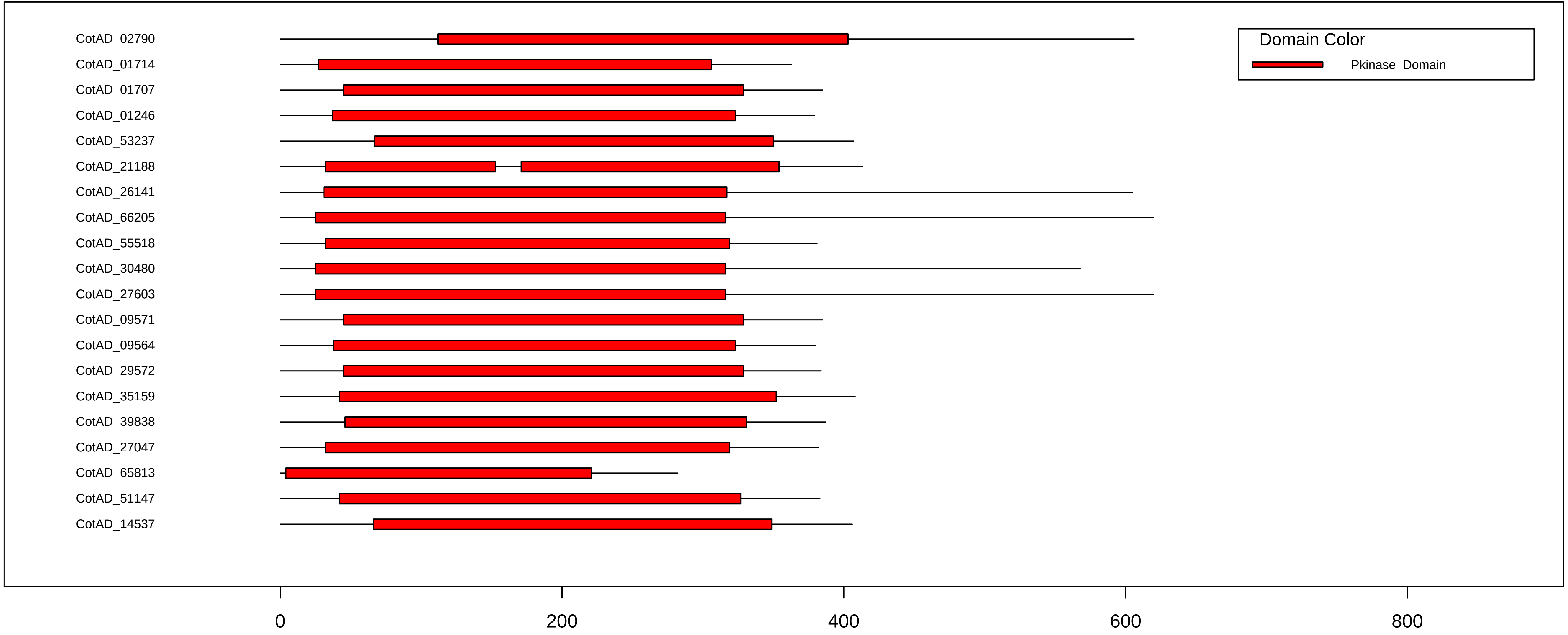

G.hi CMGC_MAPK domain diagram (part 2)

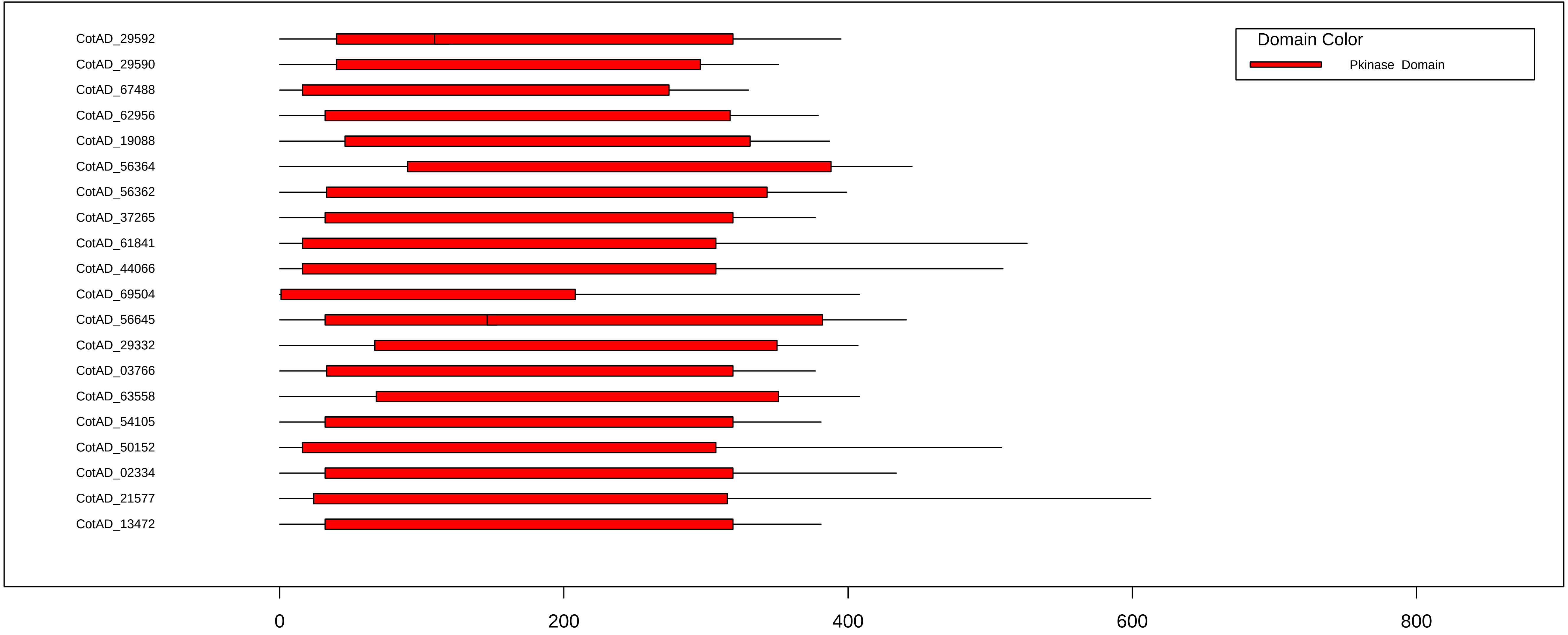

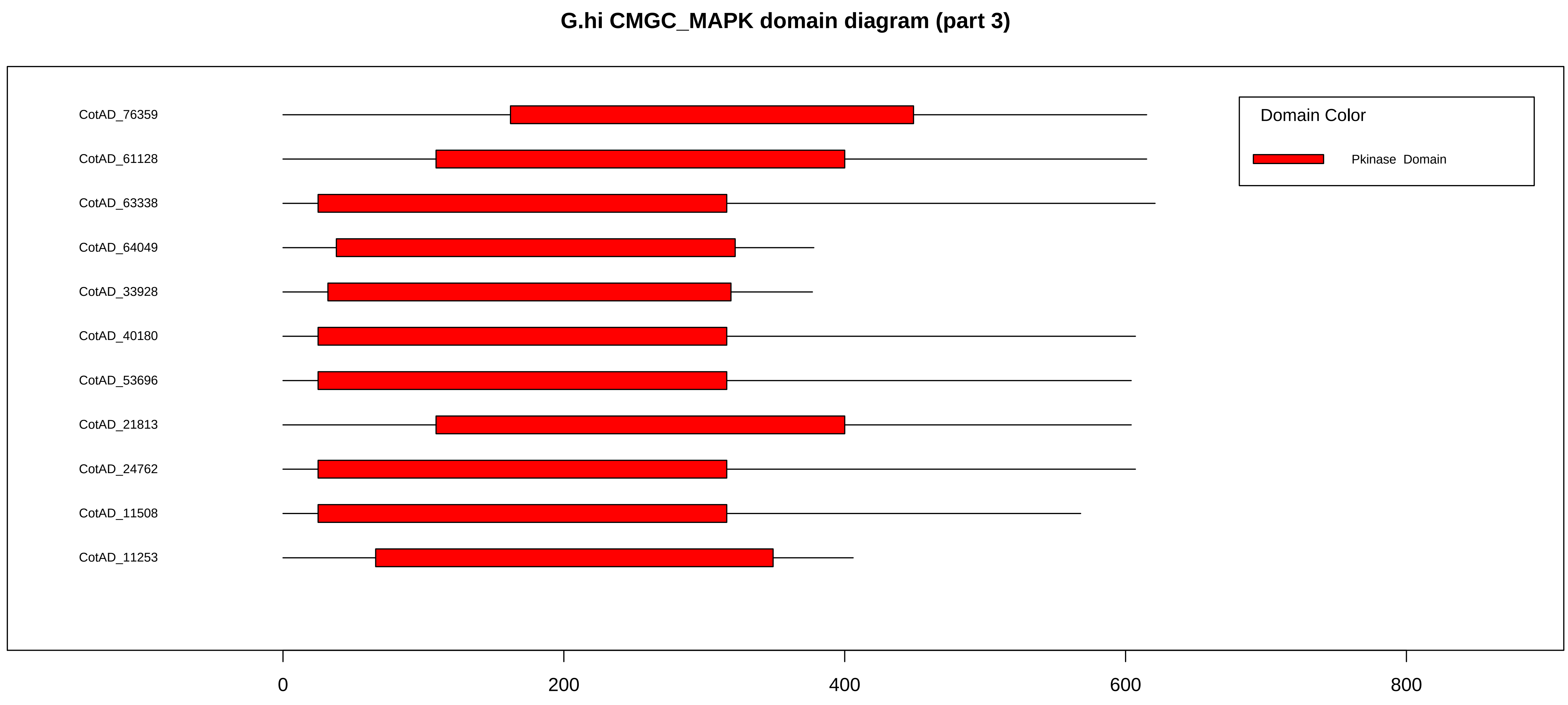
G.hi CMGC_MAPK domain diagram (part 3)
CotAD_76359
CotAD_61128
CotAD_63338
CotAD_64049
CotAD_33928
CotAD_40180
CotAD_53696
CotAD_21813
CotAD_24762
CotAD_11508
CotAD_11253
Domain Color
Pkinase Domain
0
200
400
600
800

G.hi CMGC_Pl−Tthe domain diagram (all)

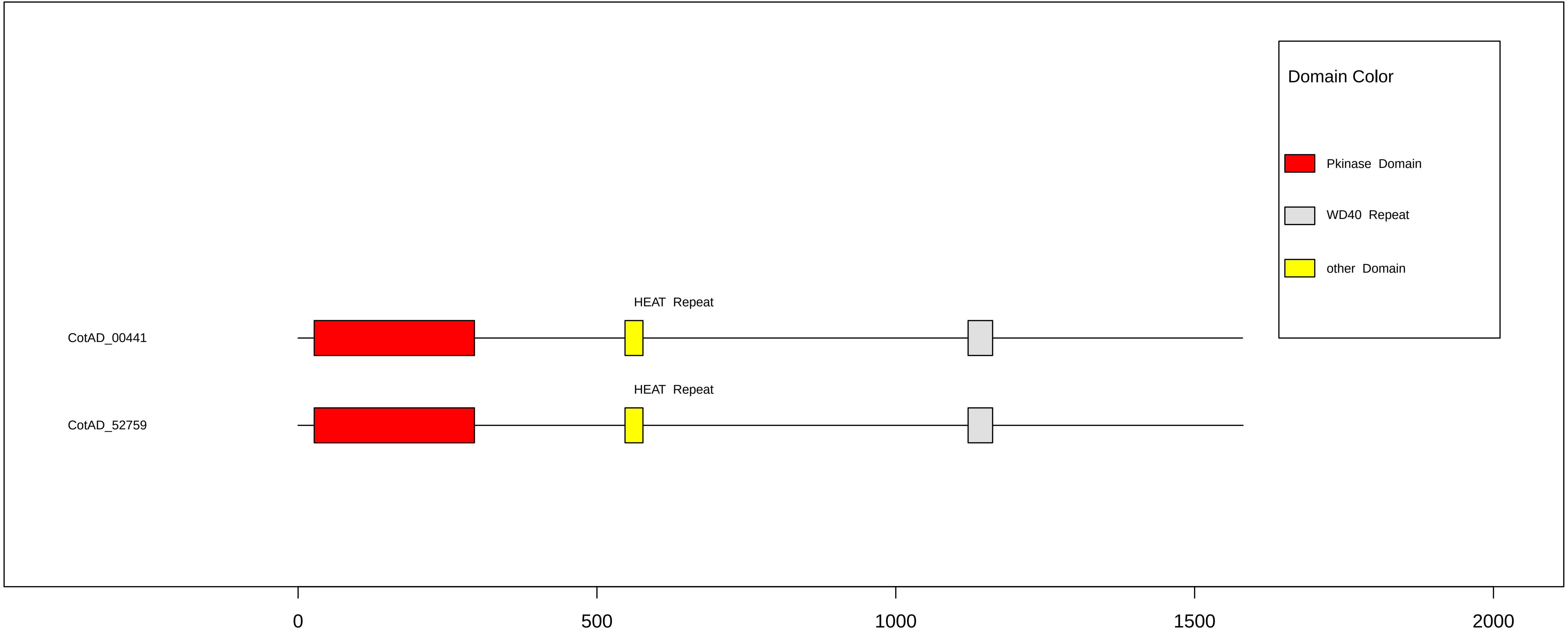

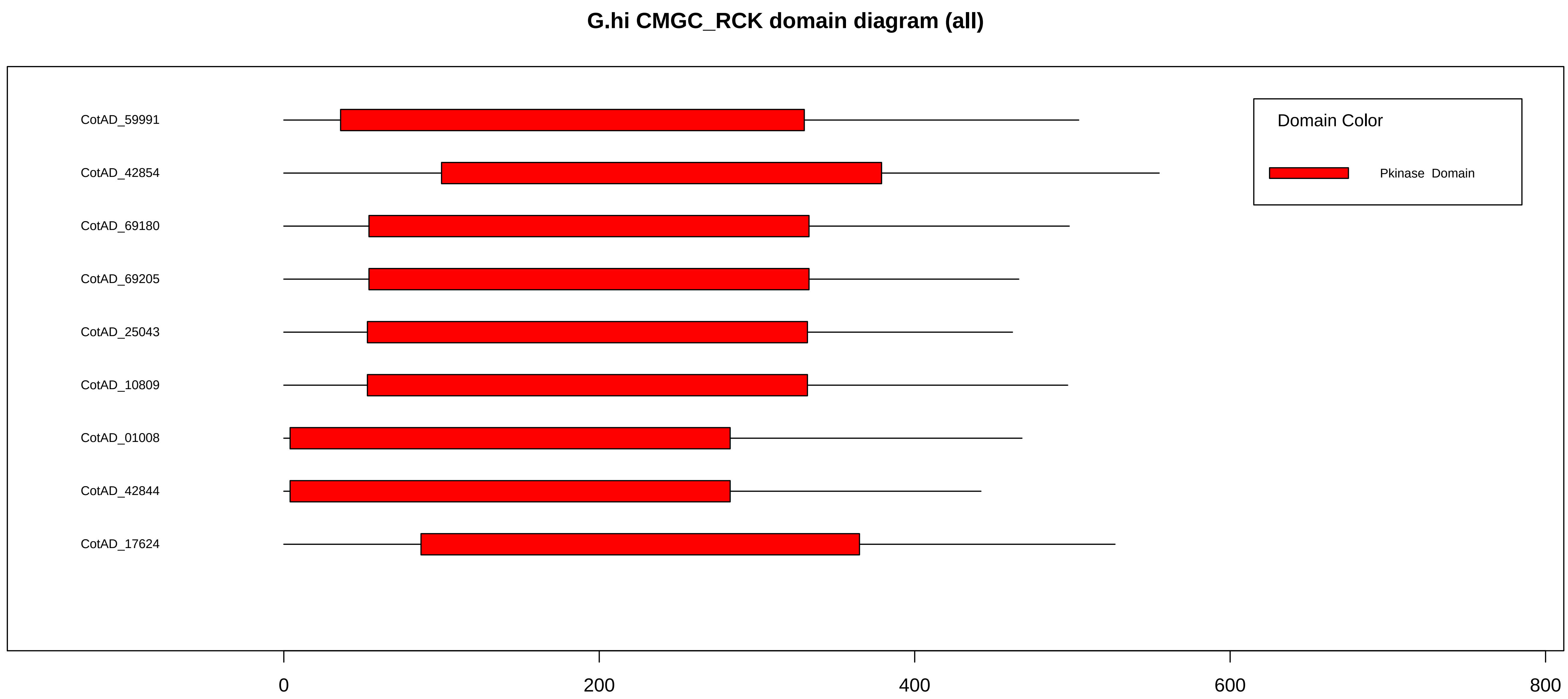
G.hi CMGC_RCK domain diagram (all)
CotAD_59991
CotAD_42854
CotAD_69180
CotAD_69205
CotAD_25043
CotAD_10809
CotAD_01008
CotAD_42844
CotAD_17624
Domain Color
Pkinase Domain
0
200
400
600
800

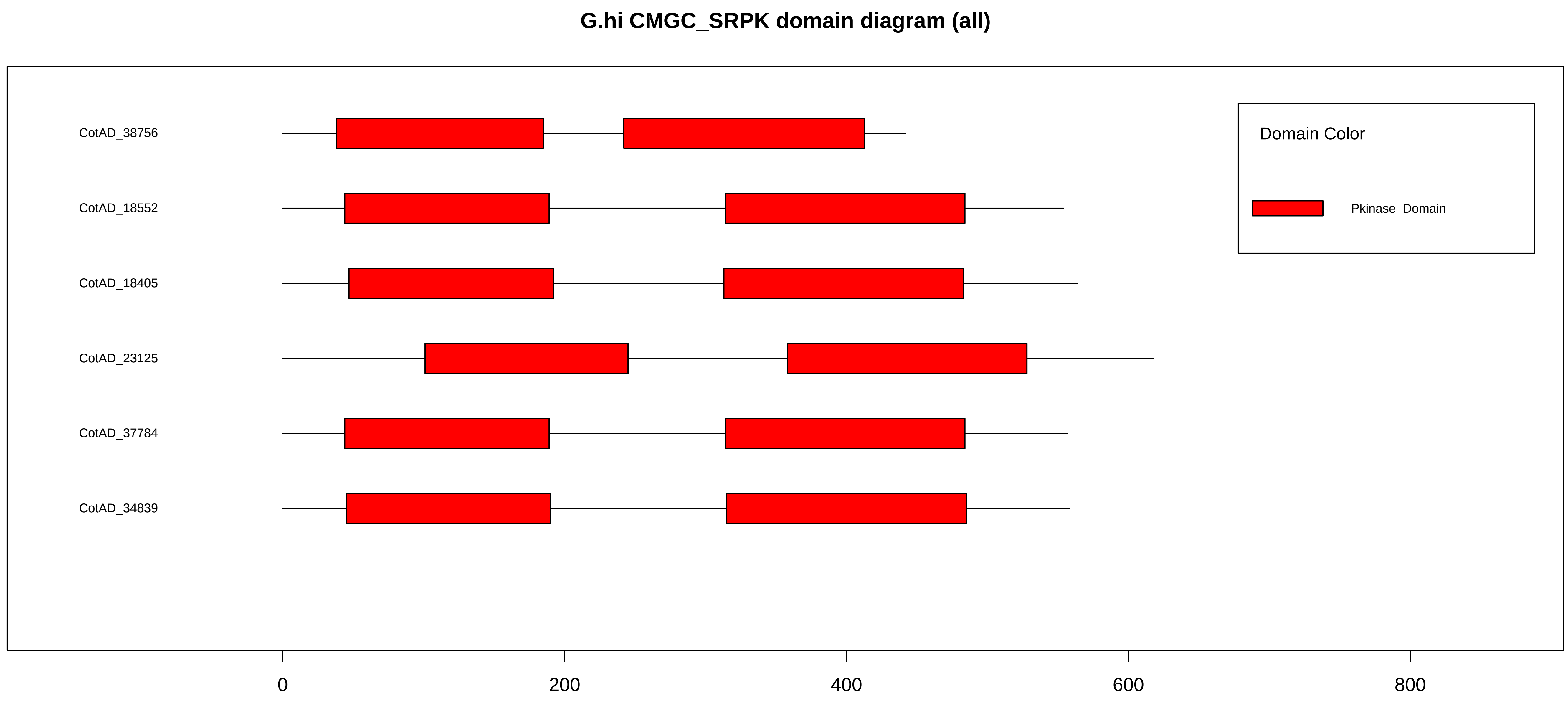

G.hi CMGC_SRPK domain diagram (all)
CotAD_38756
CotAD_18552
CotAD_18405
CotAD_23125
CotAD_37784
CotAD_34839
Domain Color
Pkinase Domain
0
200
400
600
800

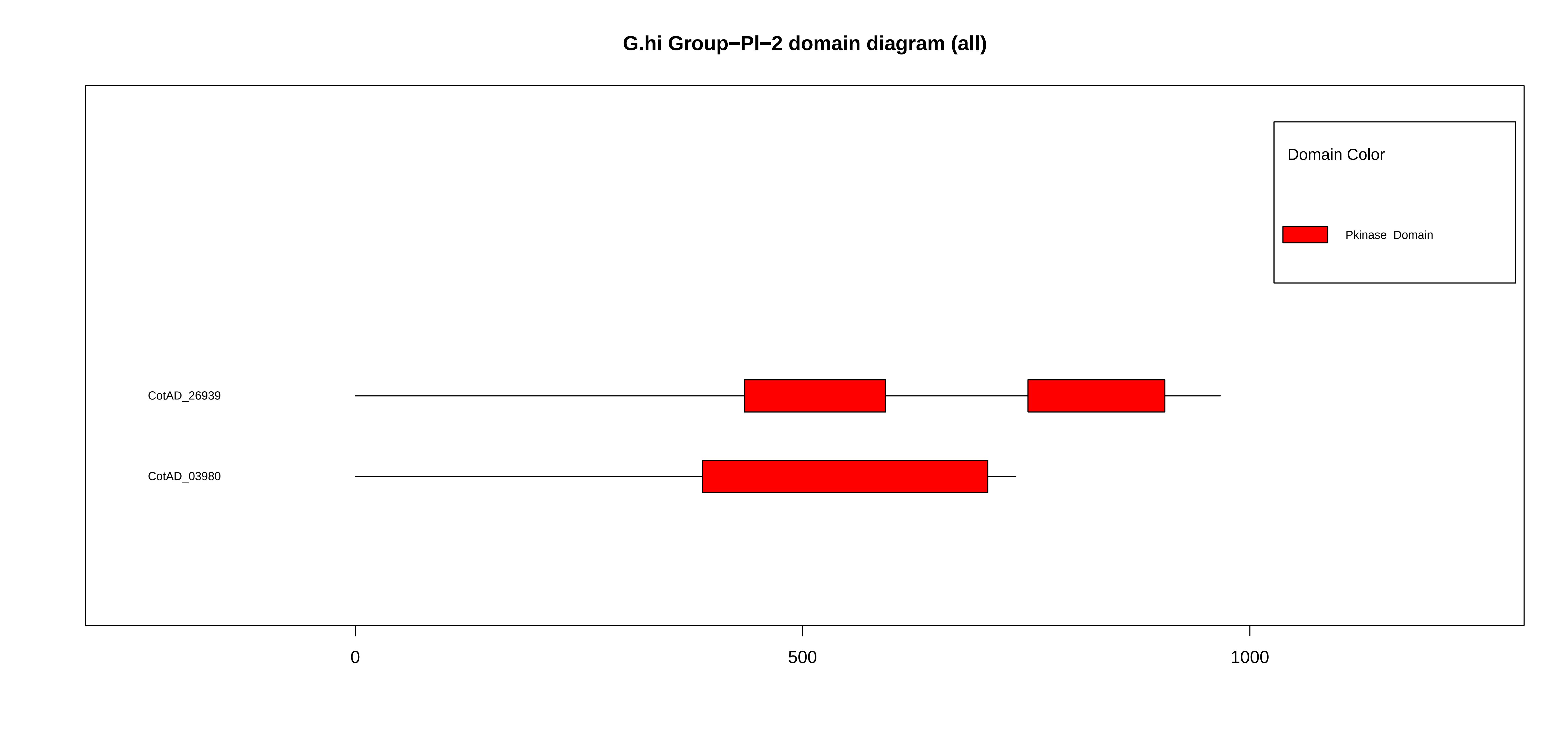
G.hi Group−PI−2 domain diagram (all)
Domain Color
Pkinase Domain
CotAD_26939
CotAD_03980
0
500
1000

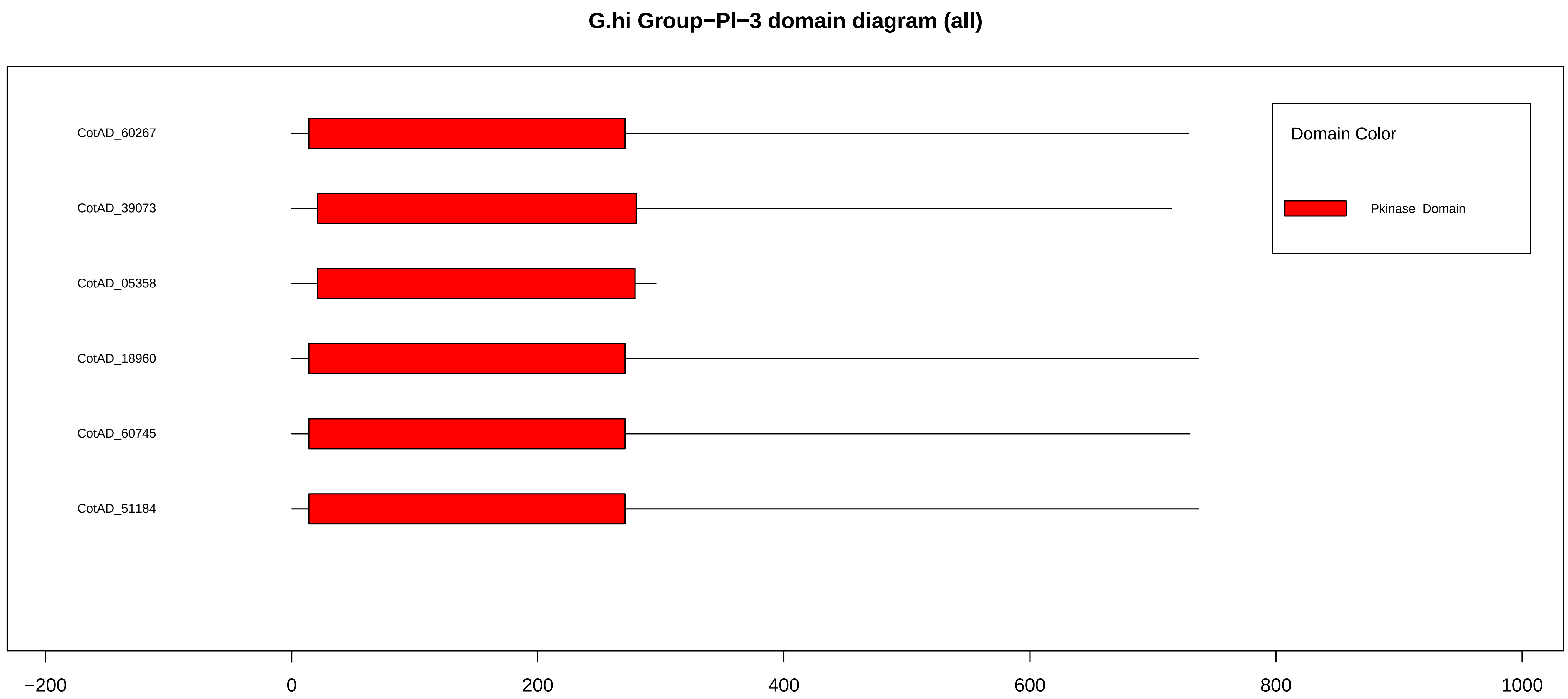
G.hi Group−PI−3 domain diagram (all)
CotAD_60267
CotAD_39073
CotAD_05358
CotAD_18960
CotAD_60745
CotAD_51184
Domain Color
Pkinase Domain
−200
0
200
400
600
800
1000

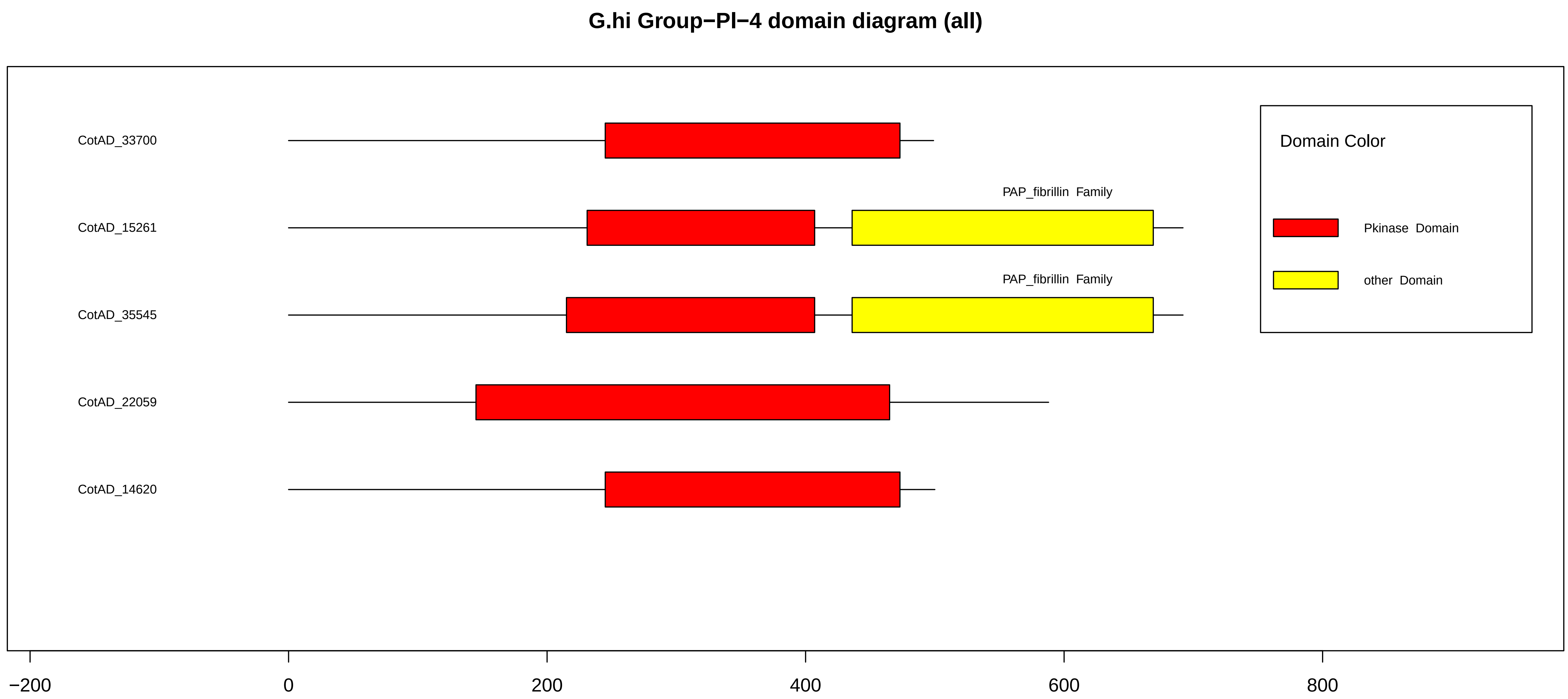
G.hi Group−PI−4 domain diagram (all)
CotAD_33700
CotAD_15261
CotAD_35545
CotAD_22059
CotAD_14620
PAP_fibrillin Family
PAP_fibrillin Family
Domain Color
Pkinase Domain
other Domain
−200
0
200
400
600
800

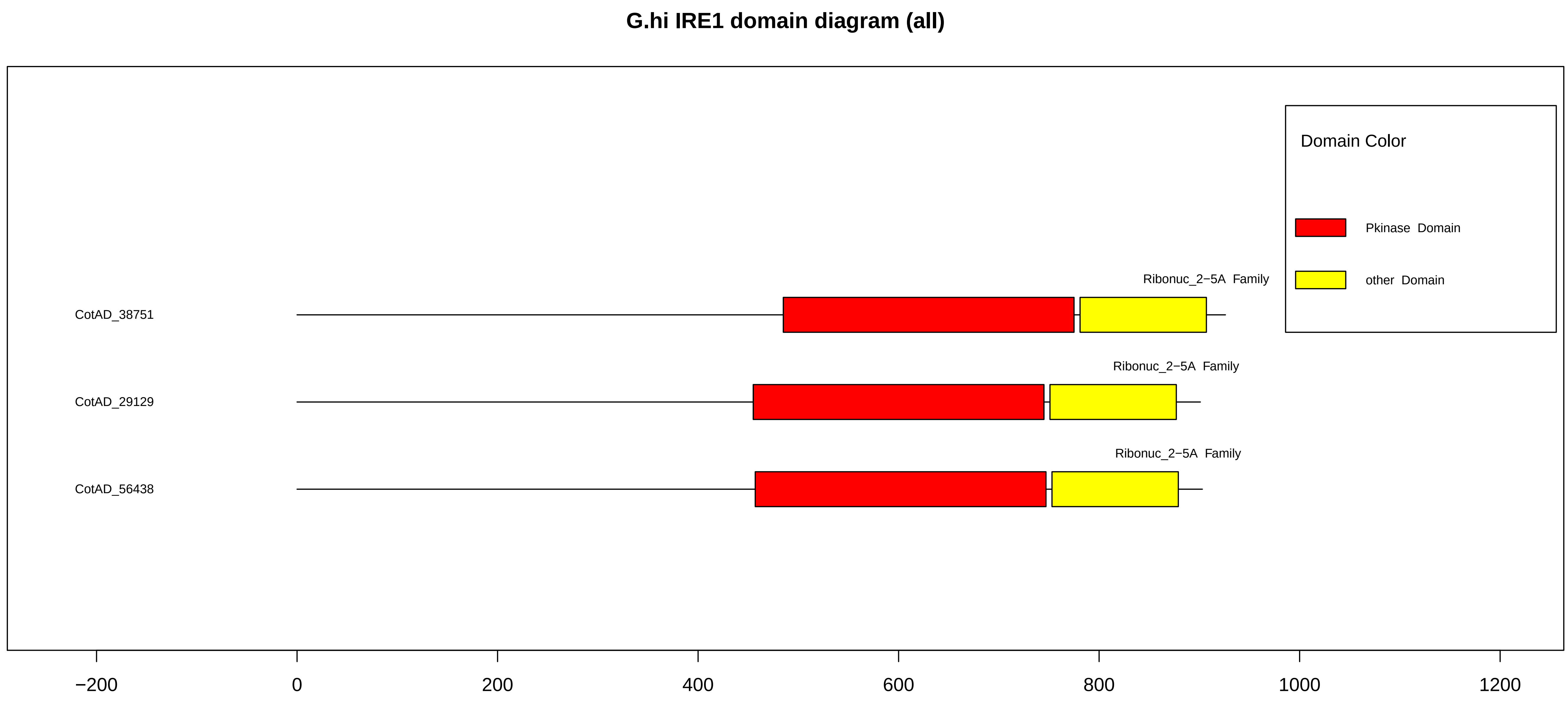
G.hi IRE1 domain diagram (all)
Domain Color
Pkinase Domain
other Domain
Ribonuc_2−5A Family
CotAD_38751
Ribonuc_2−5A Family
CotAD_29129
Ribonuc_2−5A Family
CotAD_56438
−200
0
200
400
600
800
1000
1200

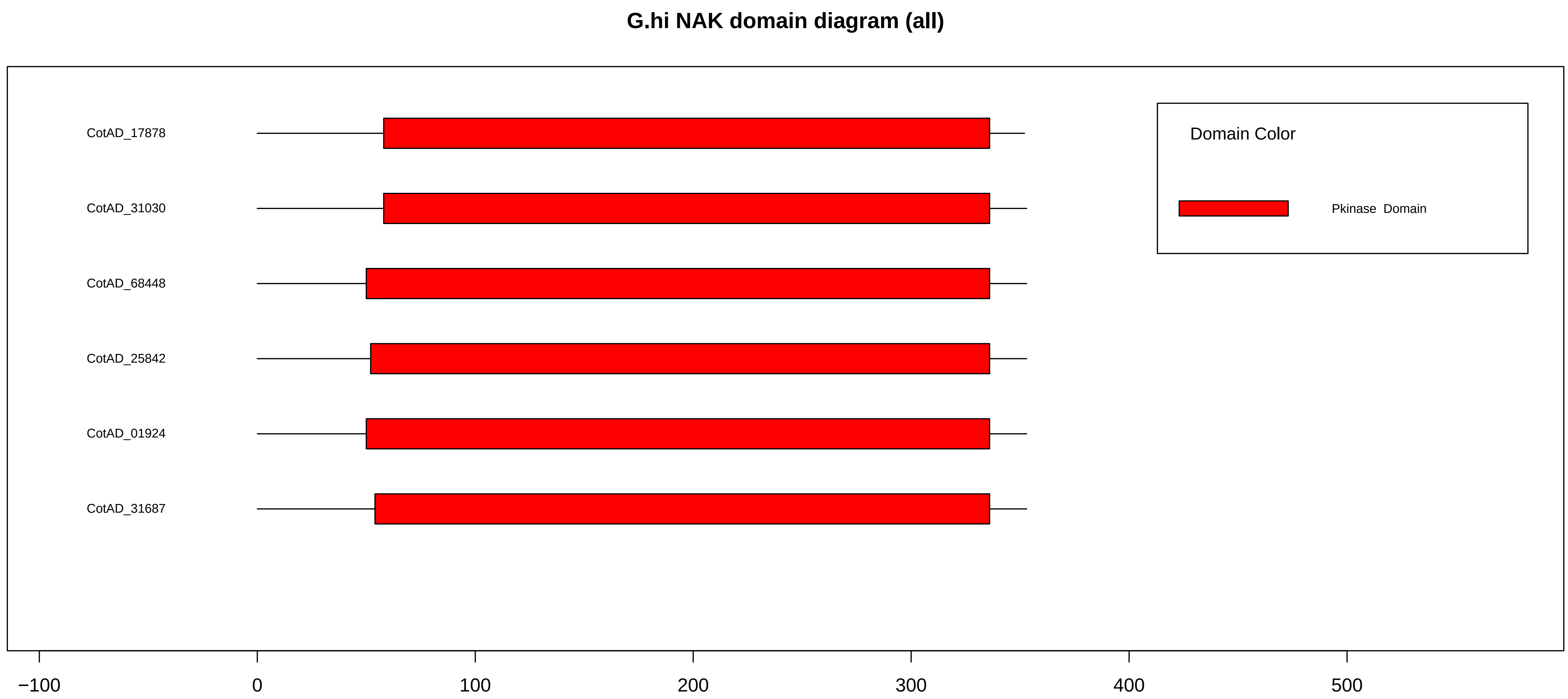
G.hi NAK domain diagram (all)
CotAD_17878
CotAD_31030
CotAD_68448
CotAD_25842
CotAD_01924
CotAD_31687
Domain Color
Pkinase Domain
−100
0
100
200
300
400
500

G.hi NEK domain diagram (all)

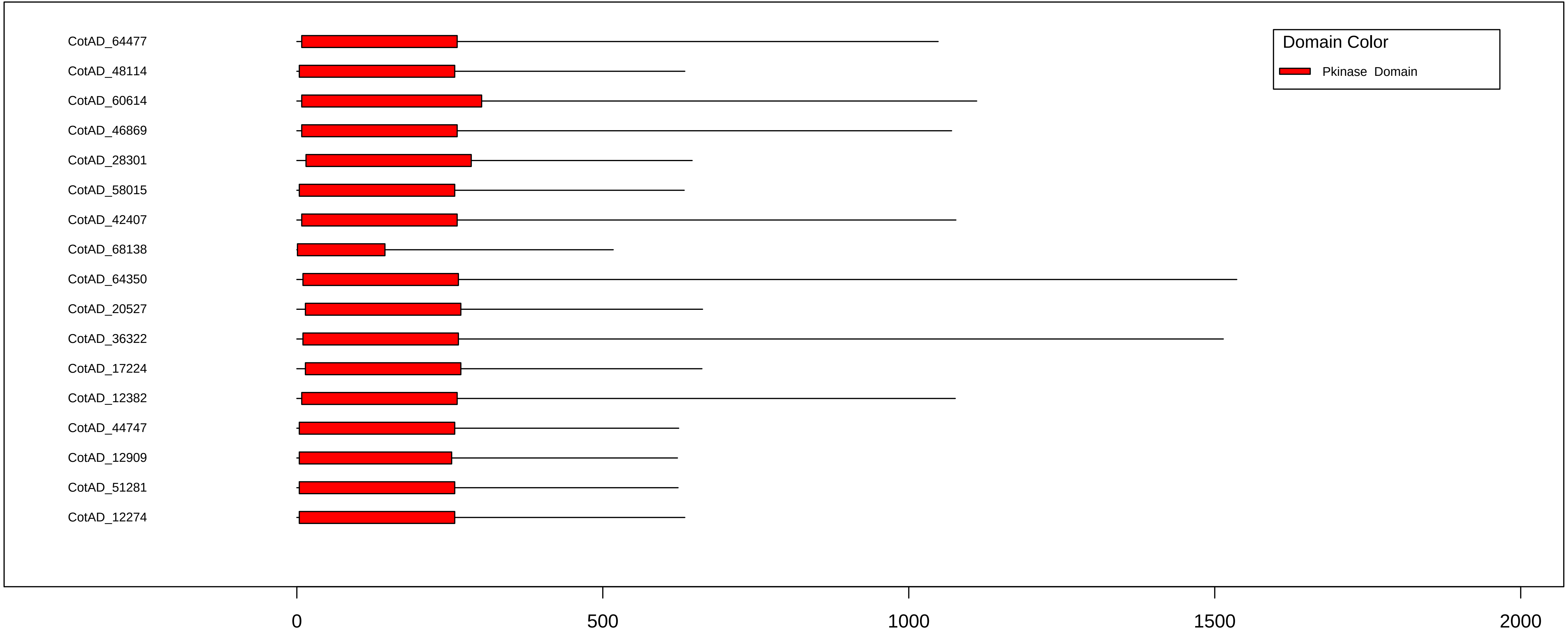

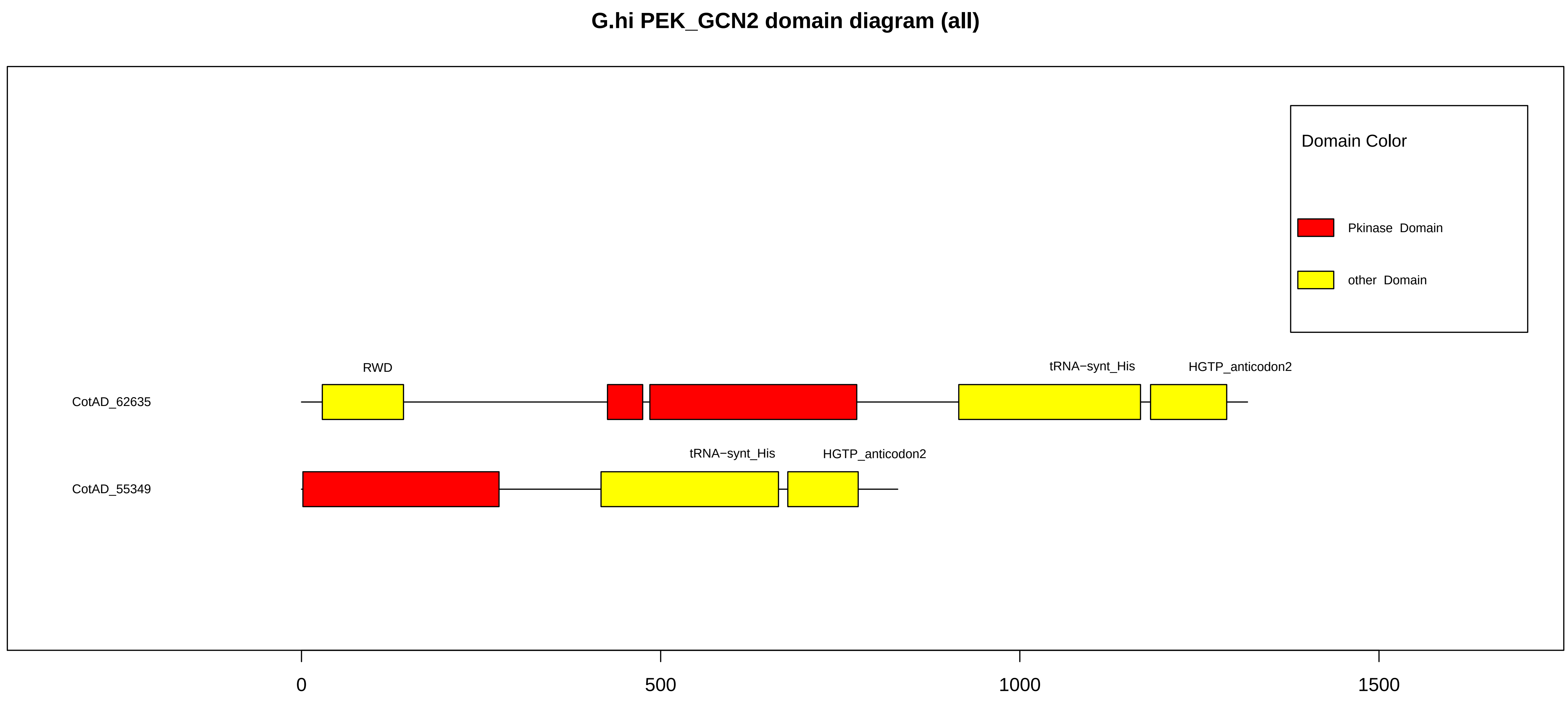
G.hi PEK_GCN2 domain diagram (all)
Domain Color
Pkinase Domain
other Domain
RWD
tRNA−synt_His
HGTP_anticodon2
CotAD_62635
tRNA−synt_His
HGTP_anticodon2
CotAD_55349
0
500
1000
1500

G.hi PEK_PEK domain diagram (all)

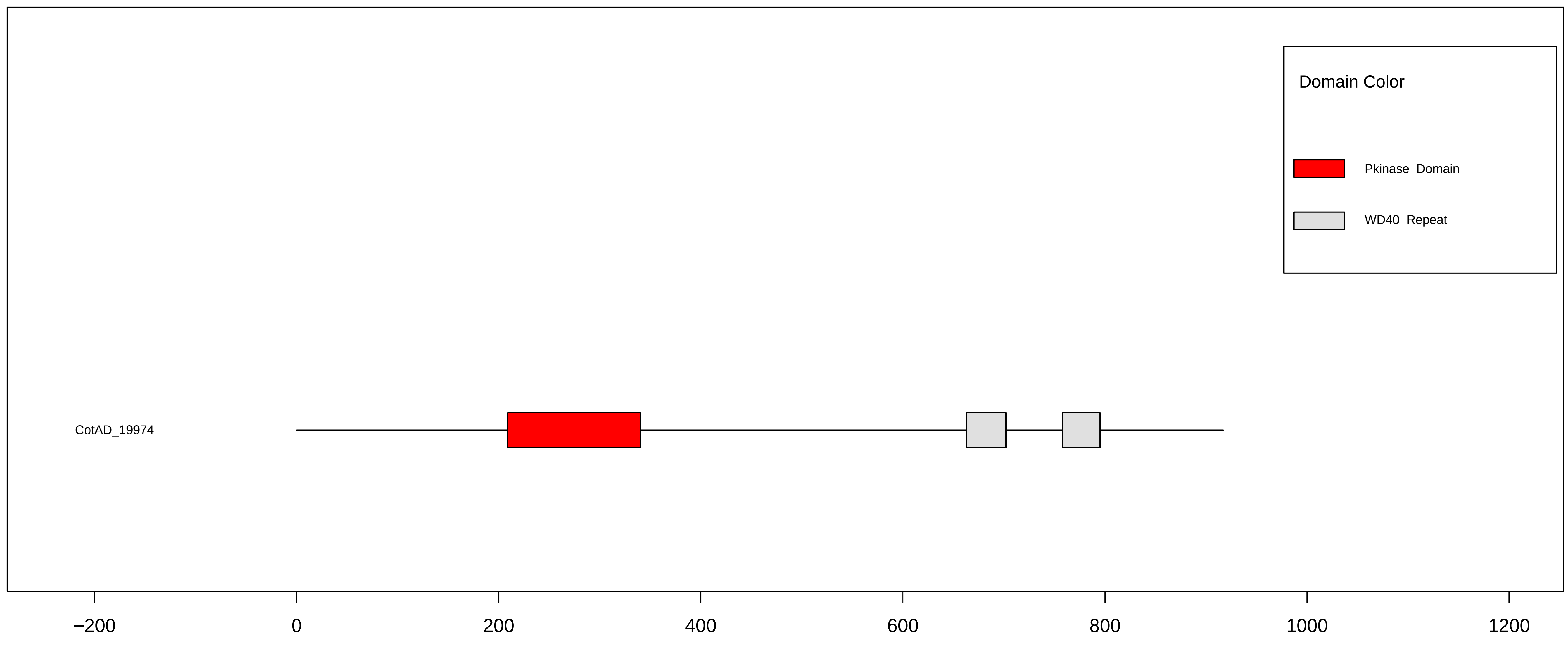

G.hi RLK−Pelle_C−LEC domain diagram (all)

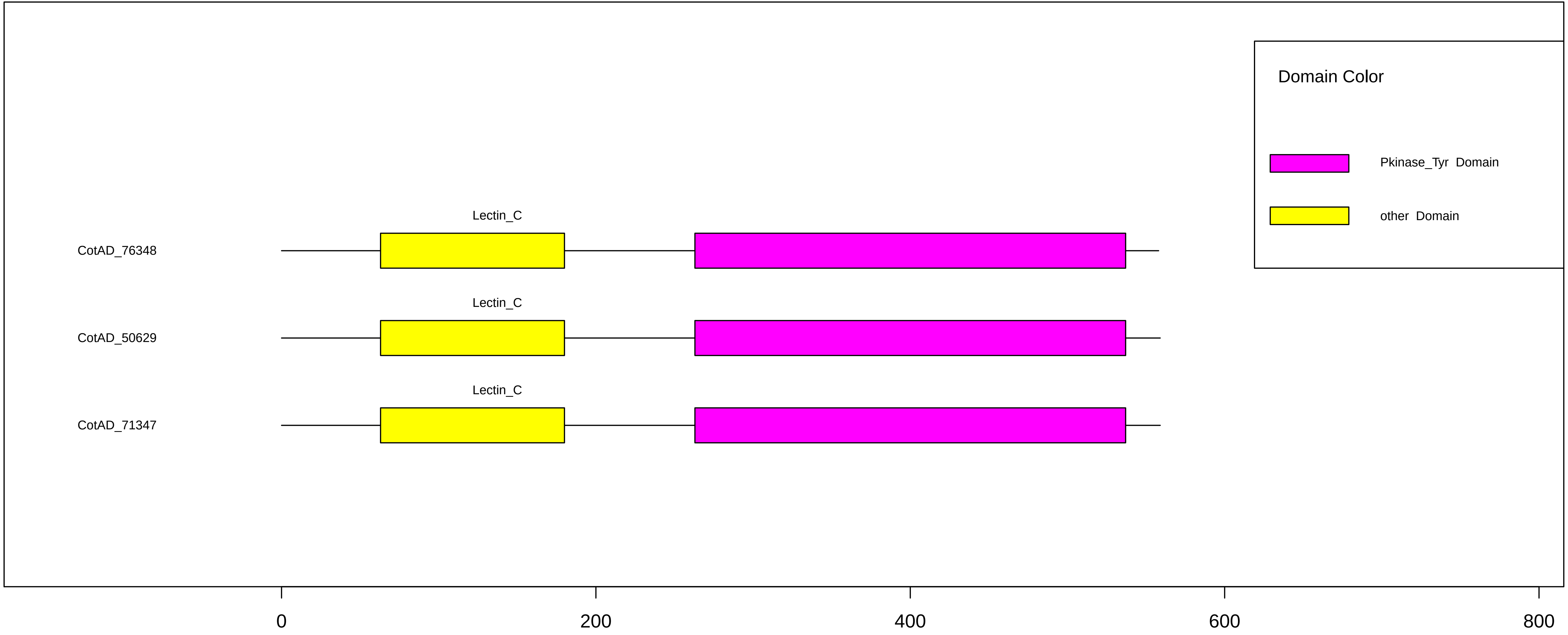

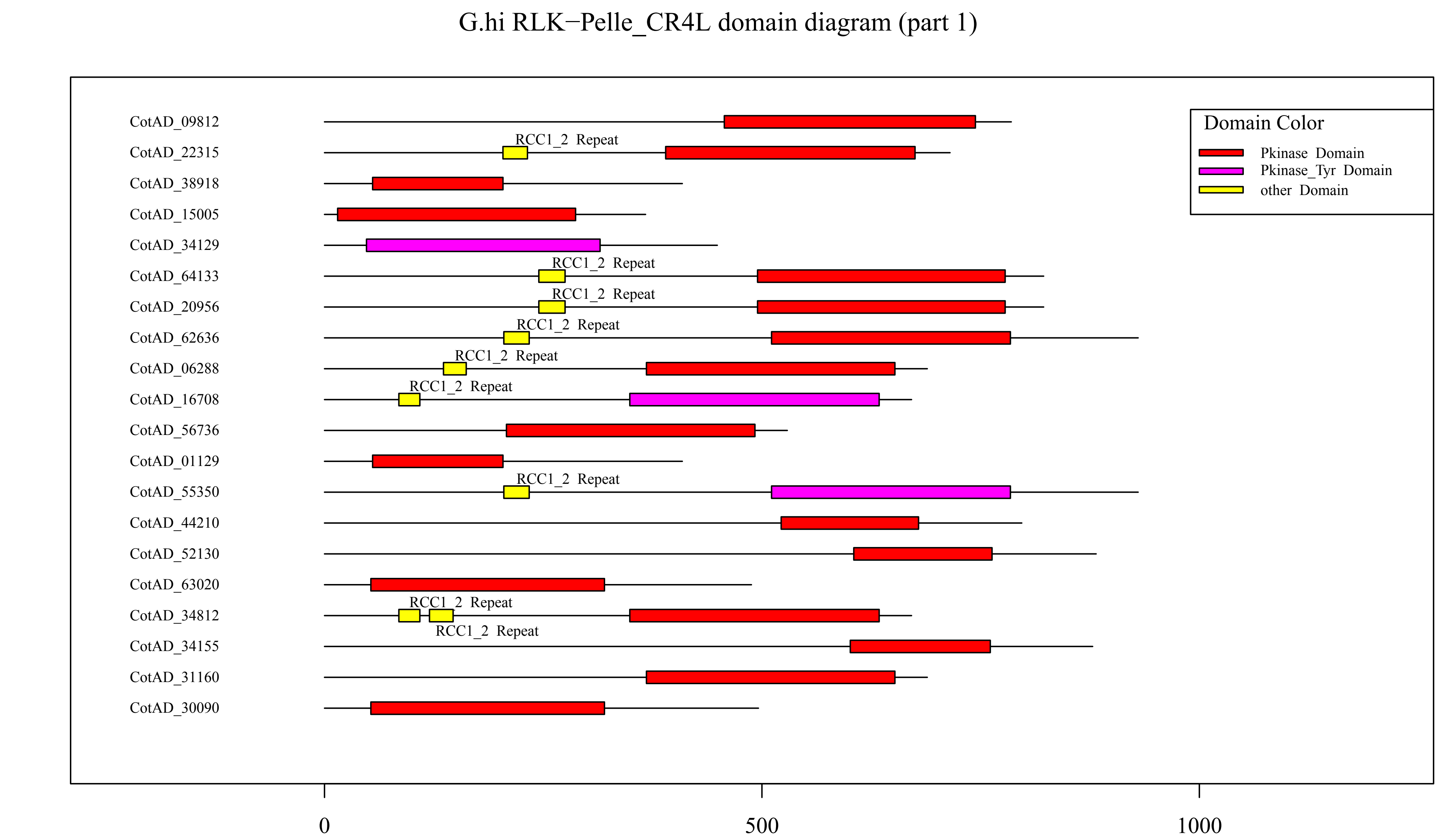
G.hi RLK−Pelle_CR4L domain diagram (part 1)
CotAD_09812
CotAD_22315
CotAD_38918
CotAD_15005
CotAD_34129
CotAD_64133
CotAD_20956
CotAD_62636
CotAD_06288
CotAD_16708
CotAD_56736
CotAD_01129
CotAD_55350
CotAD_44210
CotAD_52130
CotAD_63020
CotAD_34812
CotAD_34155
CotAD_31160
CotAD_30090
RCC1_2 Repeat
Domain Color
Pkinase Domain
Pkinase_Tyr Domain
other Domain
0
500
1000

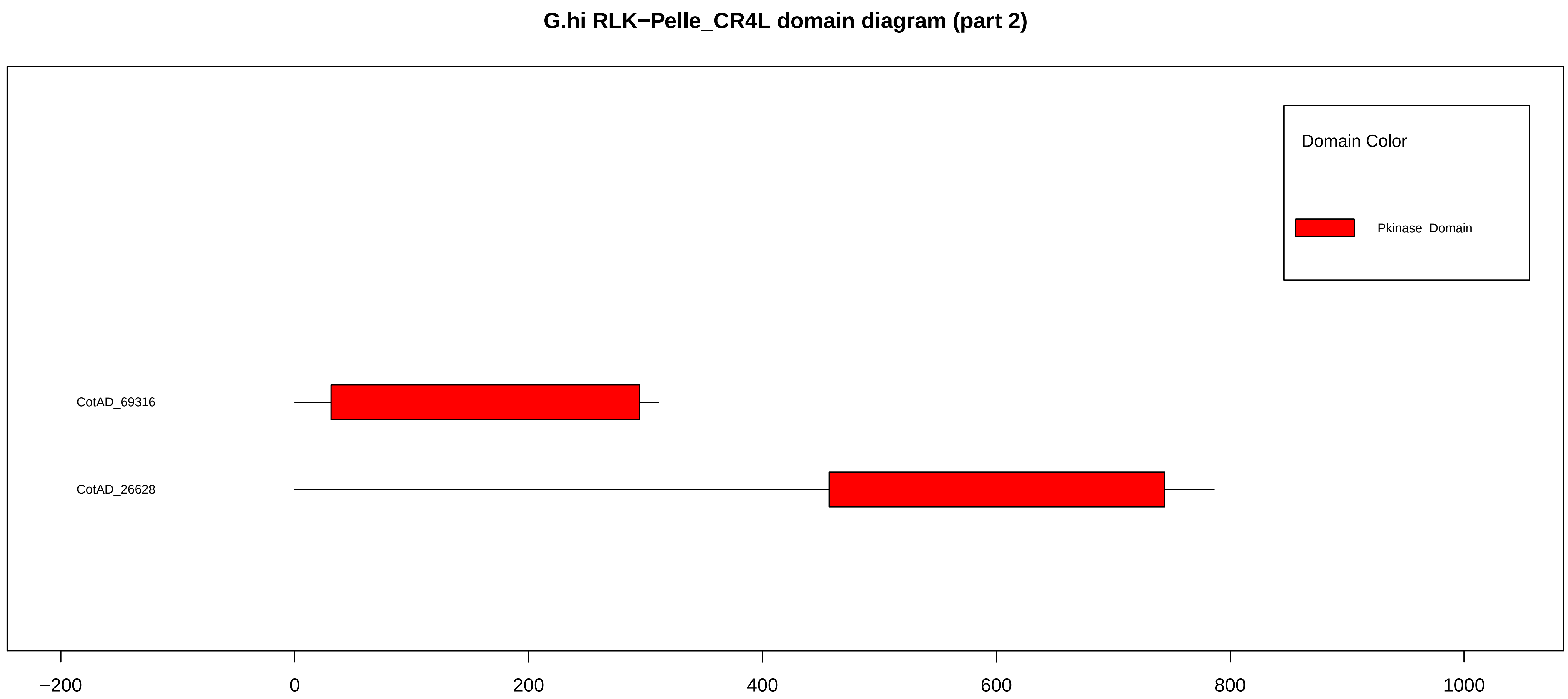
G.hi RLK−Pelle_CR4L domain diagram (part 2)
Domain Color
Pkinase Domain
CotAD_69316
CotAD_26628
−200
0
200
400
600
800
1000

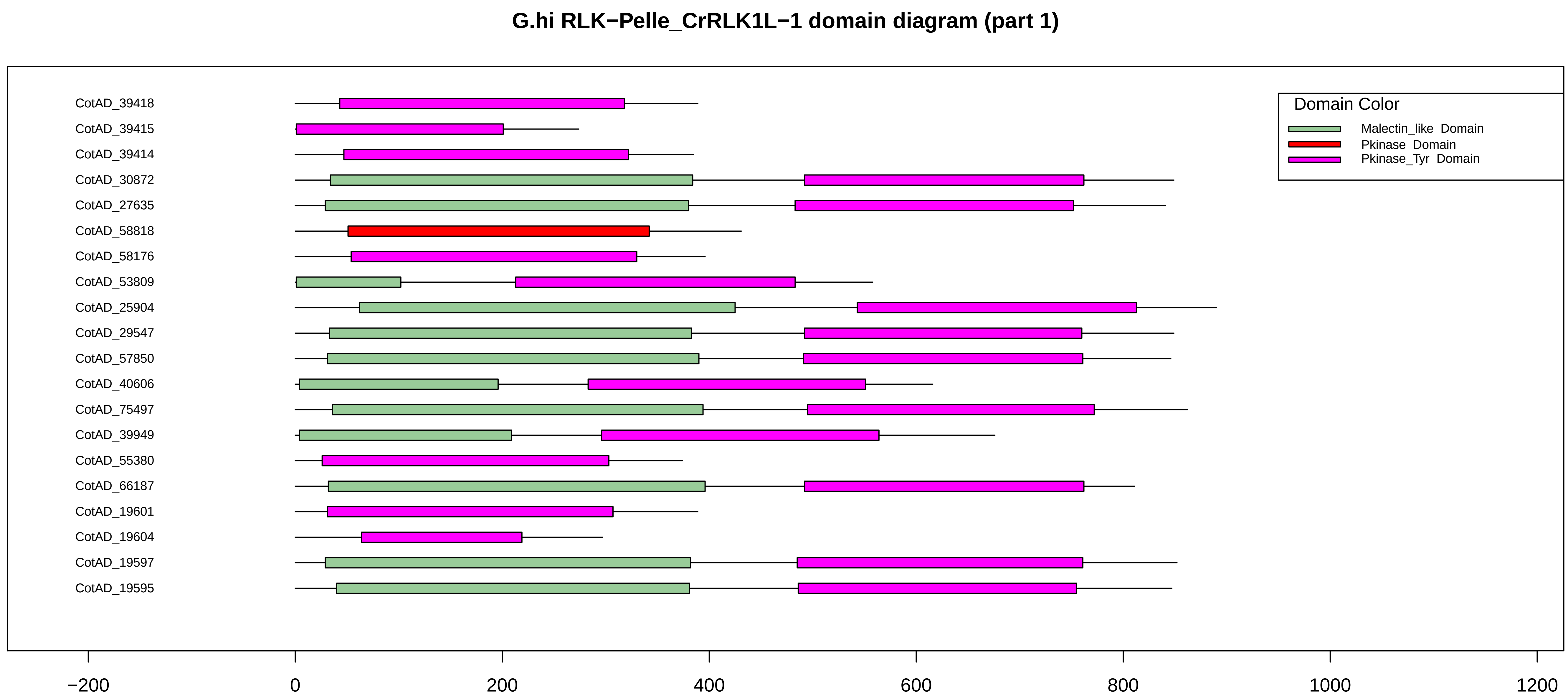
G.hi RLK−Pelle_CrRLK1L−1 domain diagram (part 1)
CotAD_39418
CotAD_39415
CotAD_39414
CotAD_30872
CotAD_27635
CotAD_58818
CotAD_58176
CotAD_53809
CotAD_25904
CotAD_29547
CotAD_57850
CotAD_40606
CotAD_75497
CotAD_39949
CotAD_55380
CotAD_66187
CotAD_19601
CotAD_19604
CotAD_19597
CotAD_19595
Domain Color
Malectin_like Domain
Pkinase Domain
Pkinase_Tyr Domain
−200
0
200
400
600
800
1000
1200

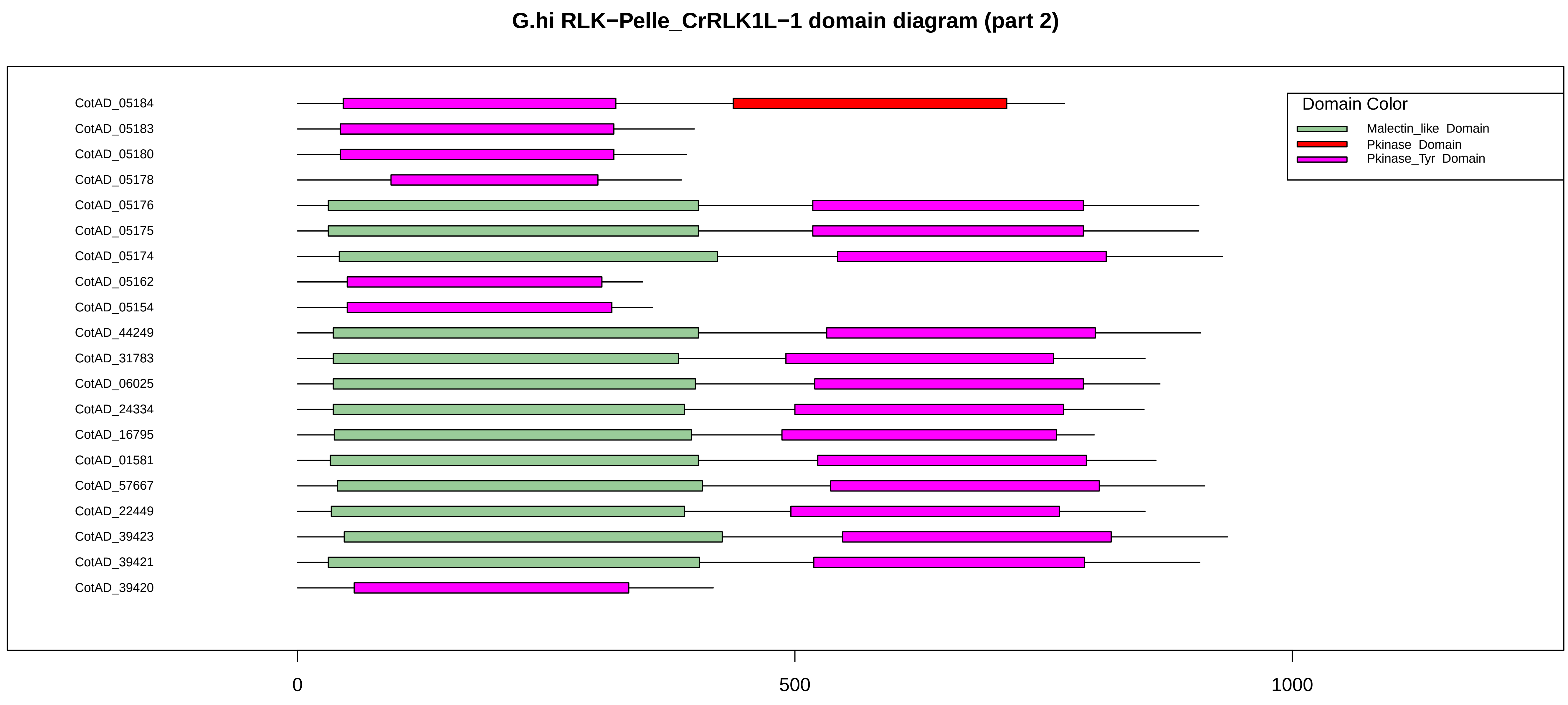
G.hi RLK−Pelle_CrRLK1L−1 domain diagram (part 2)
CotAD_05184
CotAD_05183
CotAD_05180
CotAD_05178
CotAD_05176
CotAD_05175
CotAD_05174
CotAD_05162
CotAD_05154
CotAD_44249
CotAD_31783
CotAD_06025
CotAD_24334
CotAD_16795
CotAD_01581
CotAD_57667
CotAD_22449
CotAD_39423
CotAD_39421
CotAD_39420
Domain Color
Malectin_like Domain
Pkinase Domain
Pkinase_Tyr Domain
0
500
1000

G.hi RLK−Pelle_CrRLK1L−1 domain diagram (part 3)

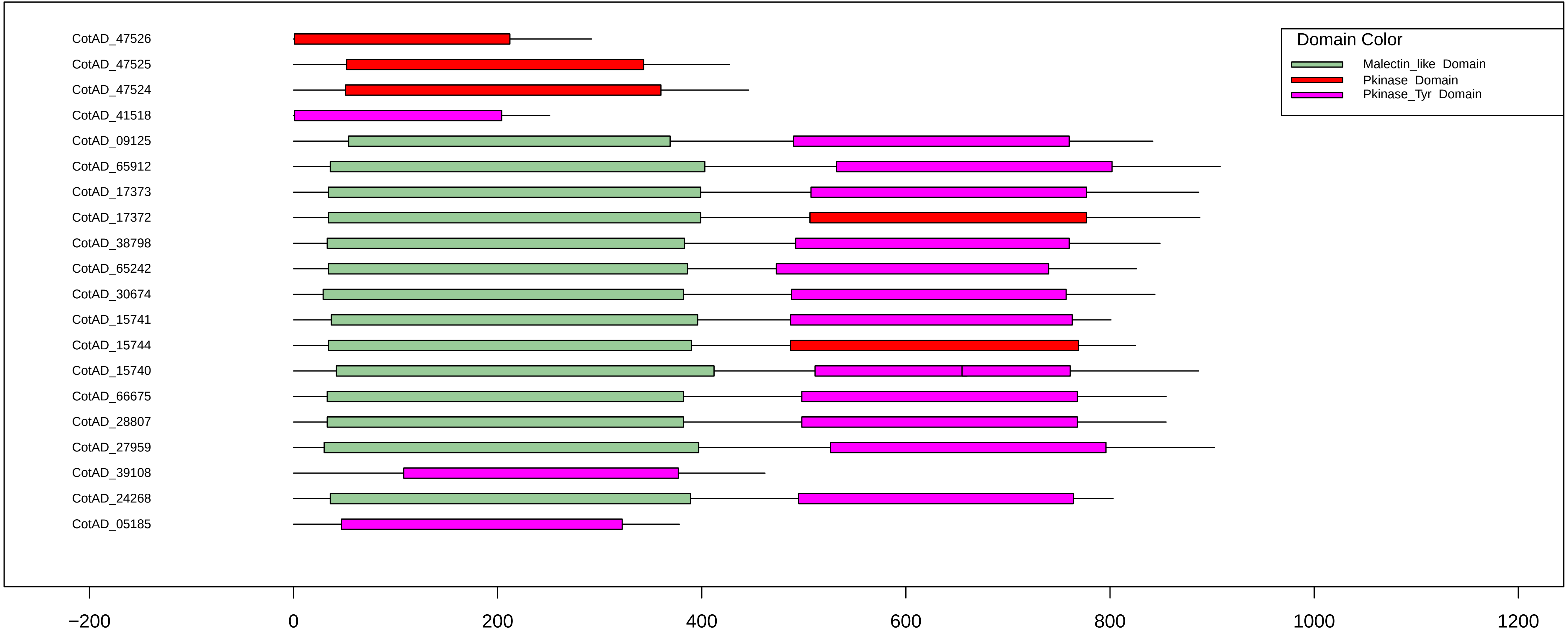

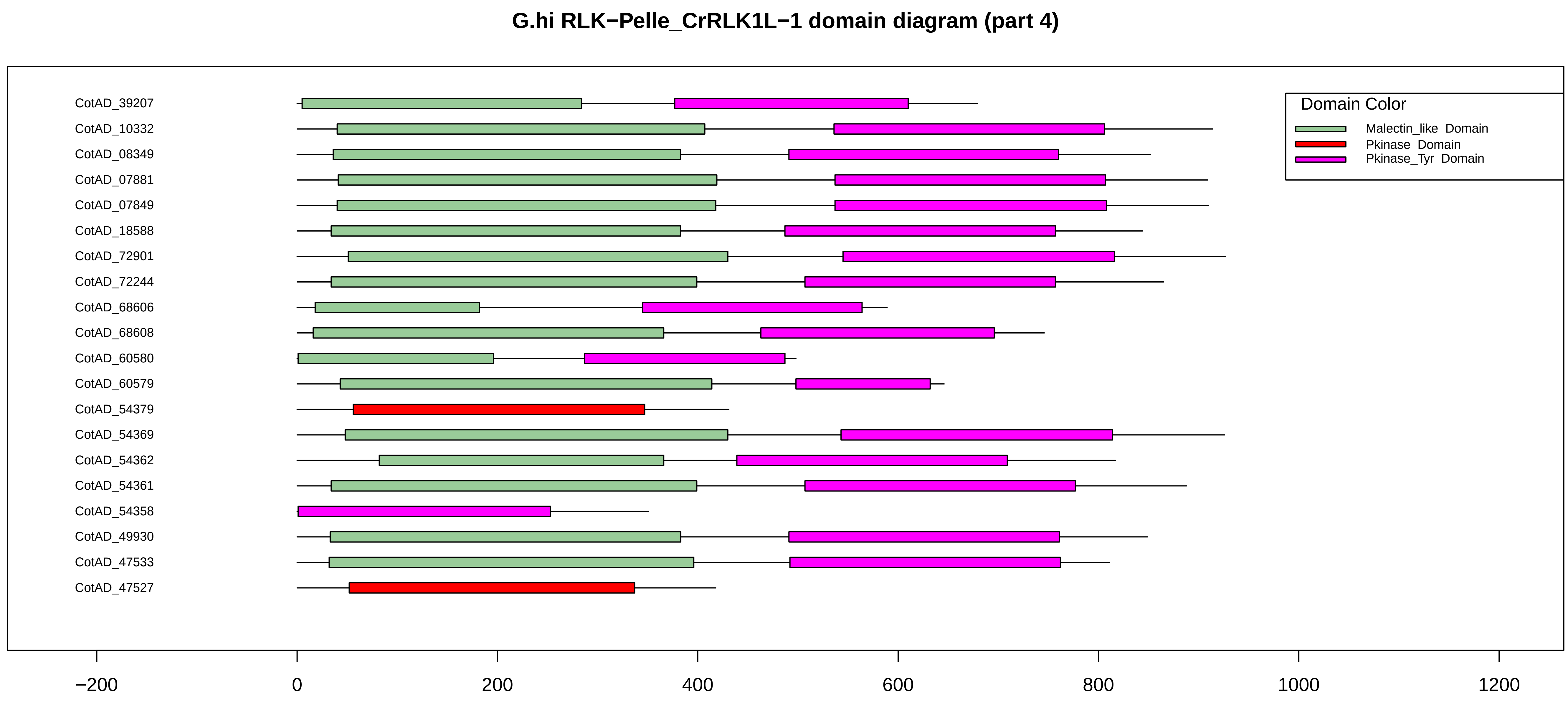
G.hi RLK−Pelle_CrRLK1L−1 domain diagram (part 4)
CotAD_39207
CotAD_10332
CotAD_08349
CotAD_07881
CotAD_07849
CotAD_18588
CotAD_72901
CotAD_72244
CotAD_68606
CotAD_68608
CotAD_60580
CotAD_60579
CotAD_54379
CotAD_54369
CotAD_54362
CotAD_54361
CotAD_54358
CotAD_49930
CotAD_47533
CotAD_47527
Domain Color
Malectin_like Domain
Pkinase Domain
Pkinase_Tyr Domain
−200
0
200
400
600
800
1000
1200

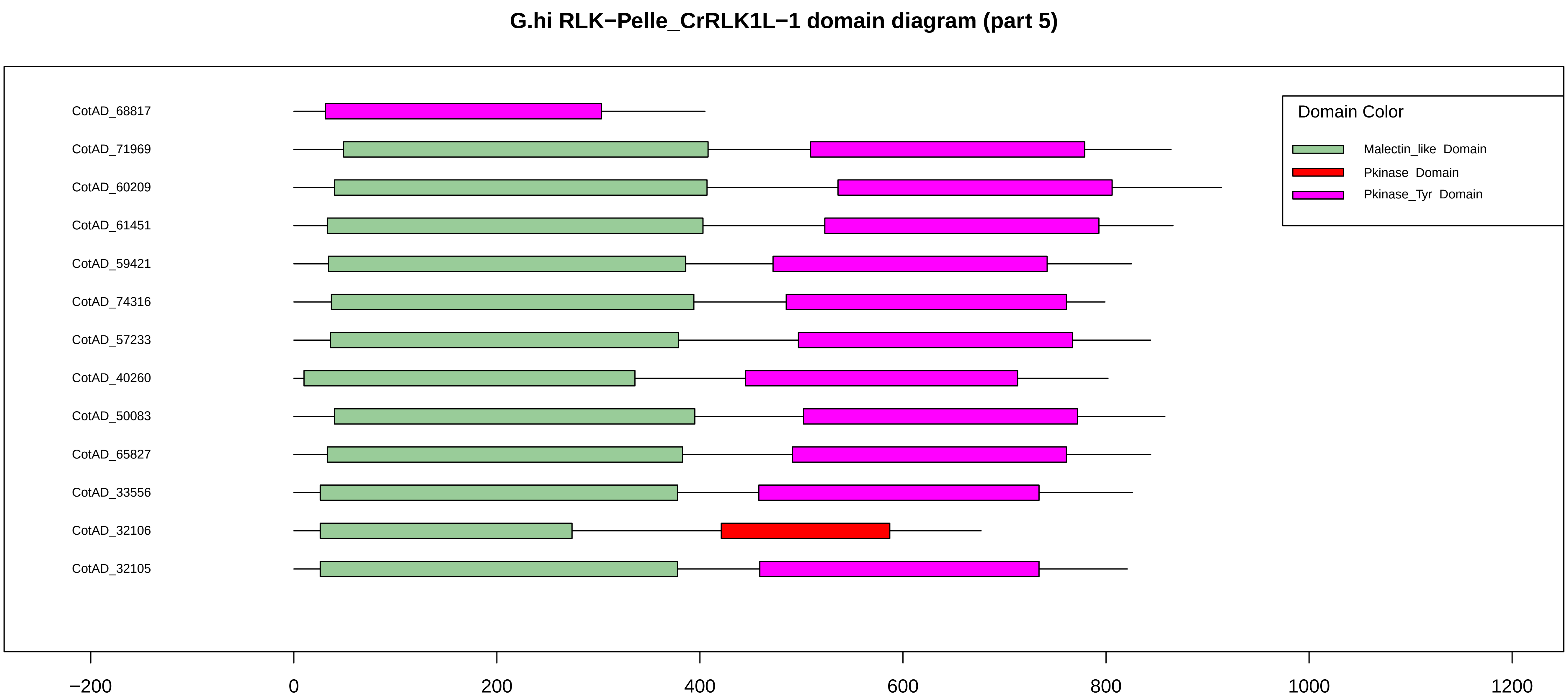
G.hi RLK−Pelle_CrRLK1L−1 domain diagram (part 5)
CotAD_68817
CotAD_71969
CotAD_60209
CotAD_61451
CotAD_59421
CotAD_74316
CotAD_57233
CotAD_40260
CotAD_50083
CotAD_65827
CotAD_33556
CotAD_32106
CotAD_32105
Domain Color
Malectin_like Domain
Pkinase Domain
Pkinase_Tyr Domain
−200
0
200
400
600
800
1000
1200

G.hi RLK−Pelle_DLSV domain diagram (part 1)

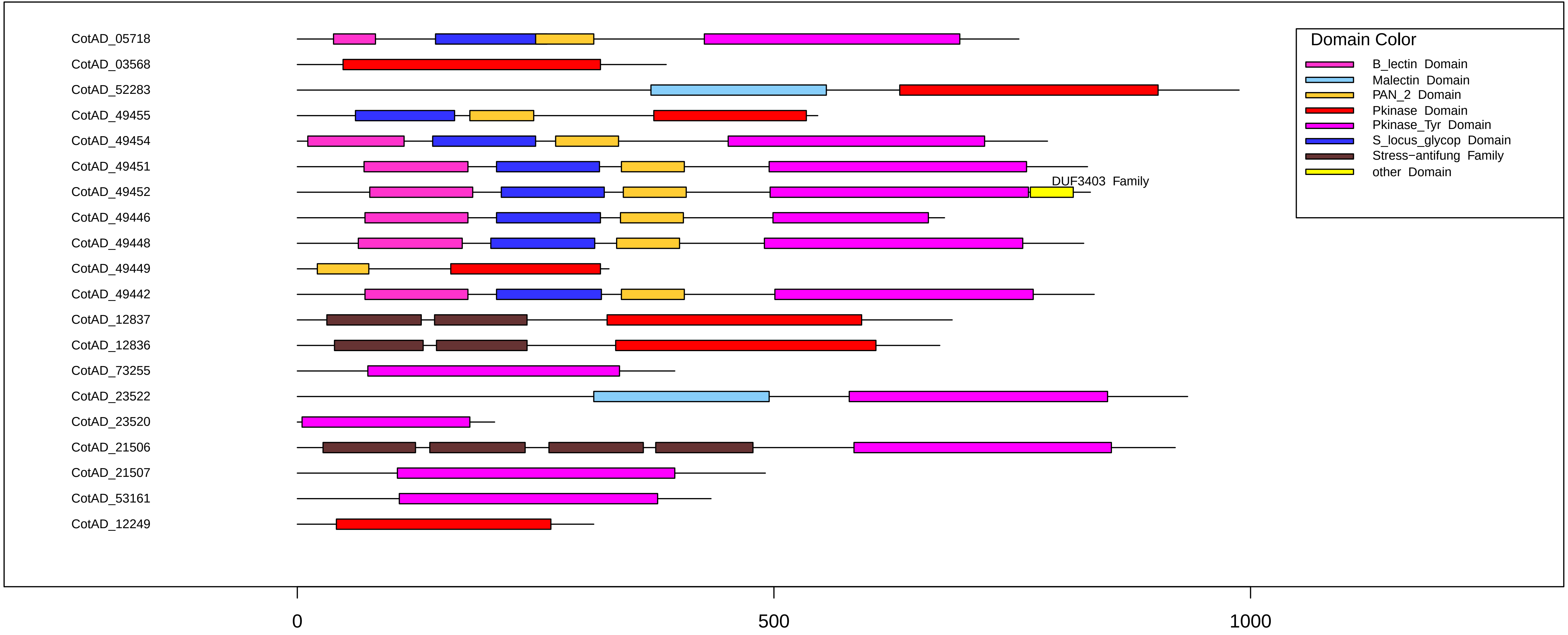

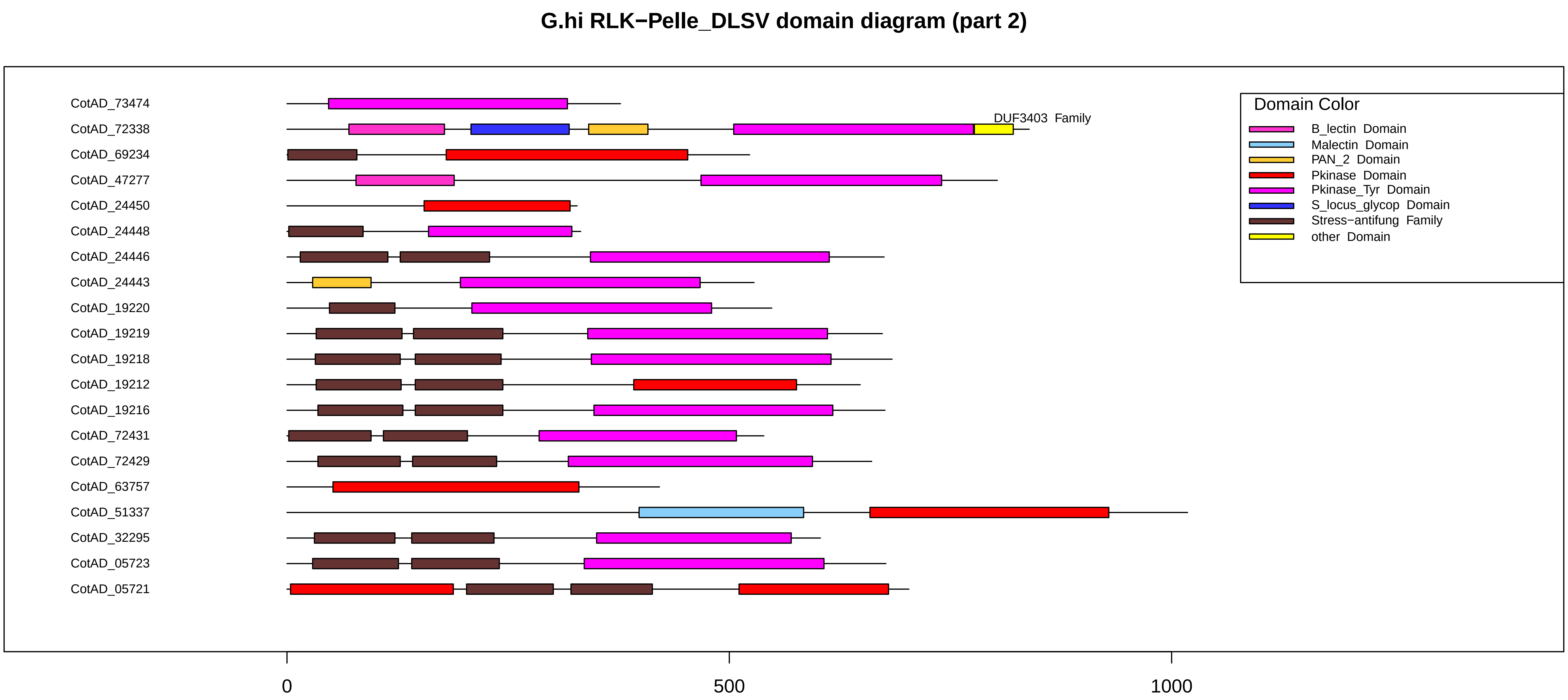

G.hi RLK−Pelle_DLSV domain diagram (part 2)
CotAD_73474
CotAD_72338
CotAD_69234
CotAD_47277
CotAD_24450
CotAD_24448
CotAD_24446
CotAD_24443
CotAD_19220
CotAD_19219
CotAD_19218
CotAD_19212
CotAD_19216
CotAD_72431
CotAD_72429
CotAD_63757
CotAD_51337
CotAD_32295
CotAD_05723
CotAD_05721
DUF3403 Family
Domain Color
B_lectin Domain
Malectin Domain
PAN_2 Domain
Pkinase Domain
Pkinase_Tyr Domain
S_locus_glycop Domain
Stress−antifung Family
other Domain
0
500
1000

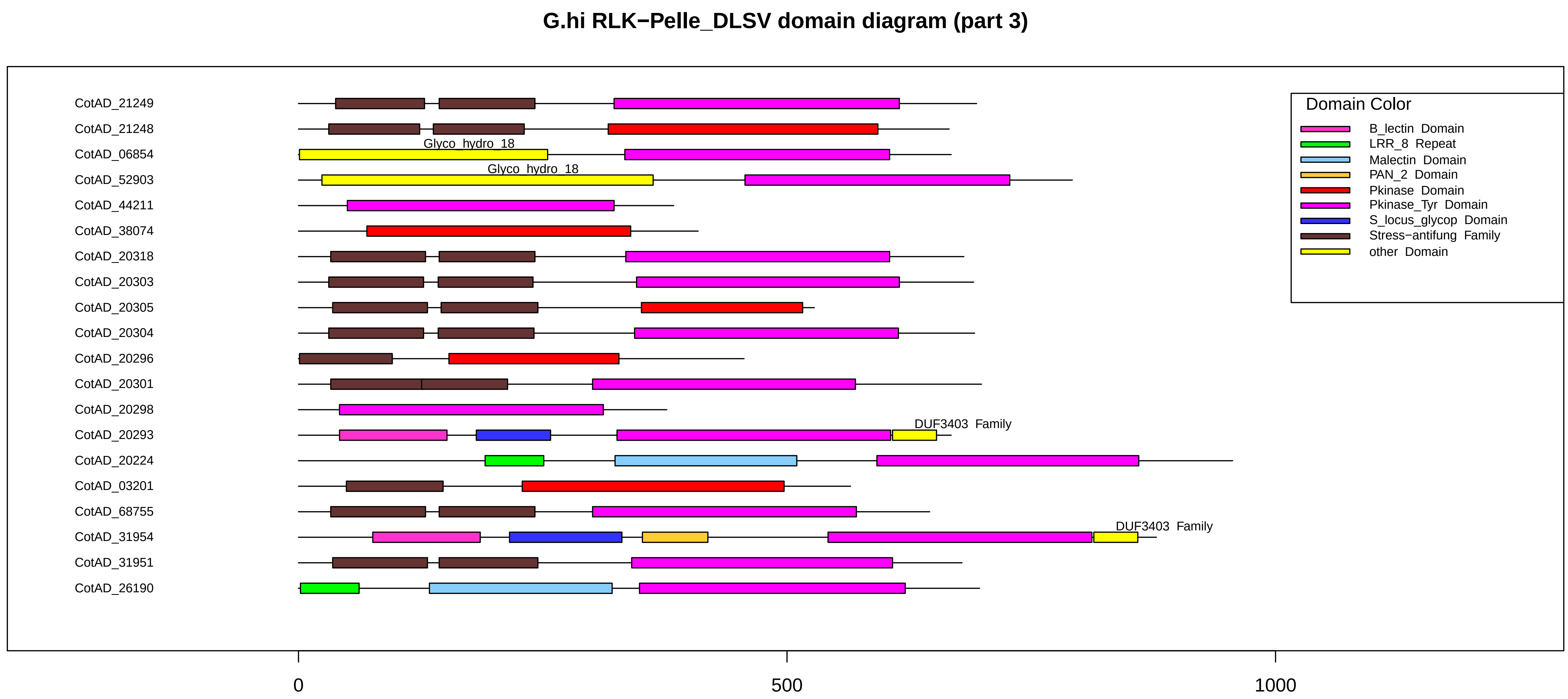
G.hi RLK−Pelle_DLSV domain diagram (part 3)
CotAD_21249
CotAD_21248
CotAD_06854
Glyco_hydro_18
CotAD_52903
Glyco_hydro_18
CotAD_44211
CotAD_38074
CotAD_20318
CotAD_20303
CotAD_20305
CotAD_20304
CotAD_20296
CotAD_20301
CotAD_20298
CotAD_20293
DUF3403 Family
CotAD_20224
CotAD_03201
CotAD_68755
CotAD_31954
DUF3403 Family
CotAD_31951
CotAD_26190
Domain Color
B_lectin Domain
LRR_8 Repeat
Malectin Domain
PAN_2 Domain
Pkinase Domain
Pkinase_Tyr Domain
S_locus_glycop Domain
Stress−antifung Family
other Domain
0
500
1000

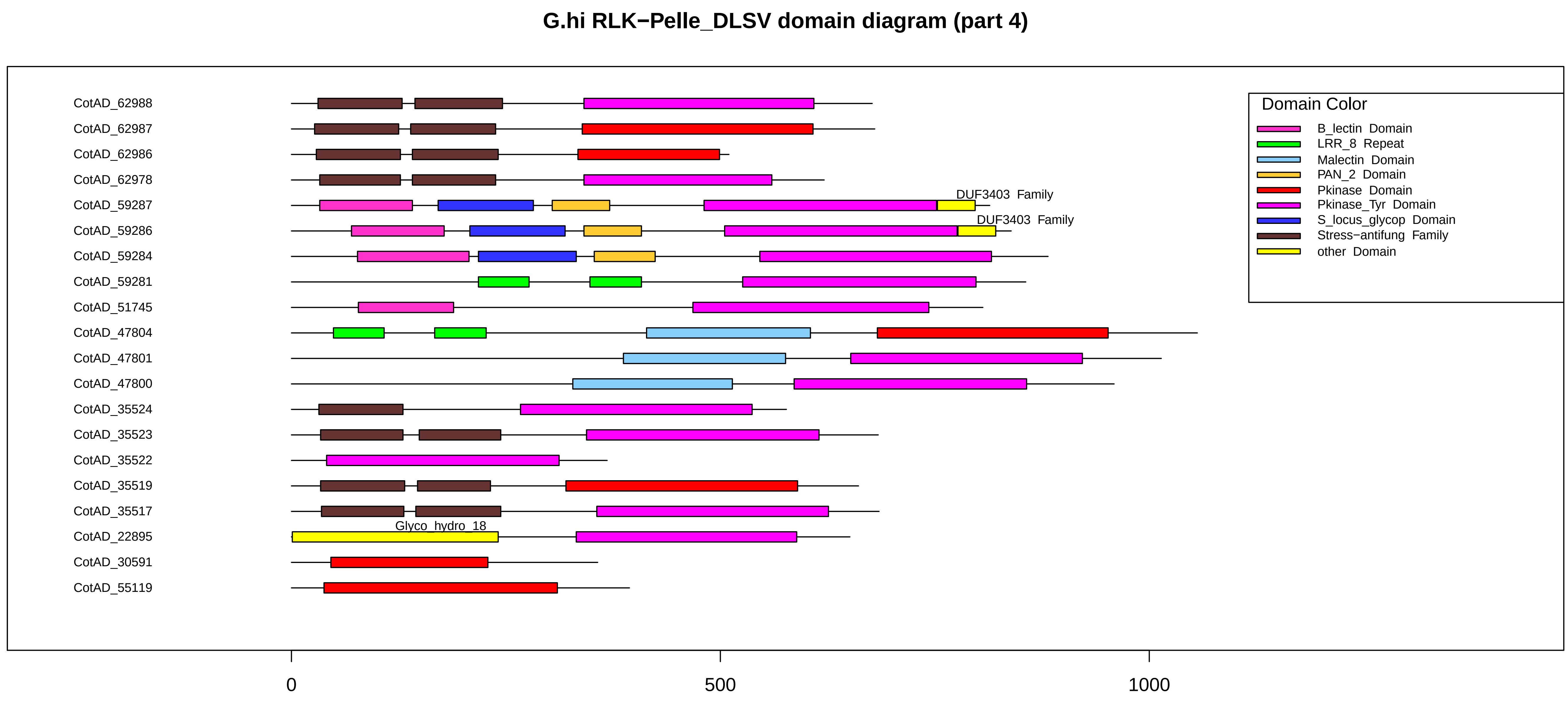
G.hi RLK−Pelle_DLSV domain diagram (part 4)
CotAD_62988
CotAD_62987
CotAD_62986
CotAD_62978
CotAD_59287
CotAD_59286
CotAD_59284
CotAD_59281
CotAD_51745
CotAD_47804
CotAD_47801
CotAD_47800
CotAD_35524
CotAD_35523
CotAD_35522
CotAD_35519
CotAD_35517
CotAD_22895
CotAD_30591
CotAD_55119
DUF3403 Family
DUF3403 Family
Glyco_hydro_18
Domain Color
B_lectin Domain
LRR_8 Repeat
Malectin Domain
PAN_2 Domain
Pkinase Domain
Pkinase_Tyr Domain
S_locus_glycop Domain
Stress−antifung Family
other Domain
0
500
1000

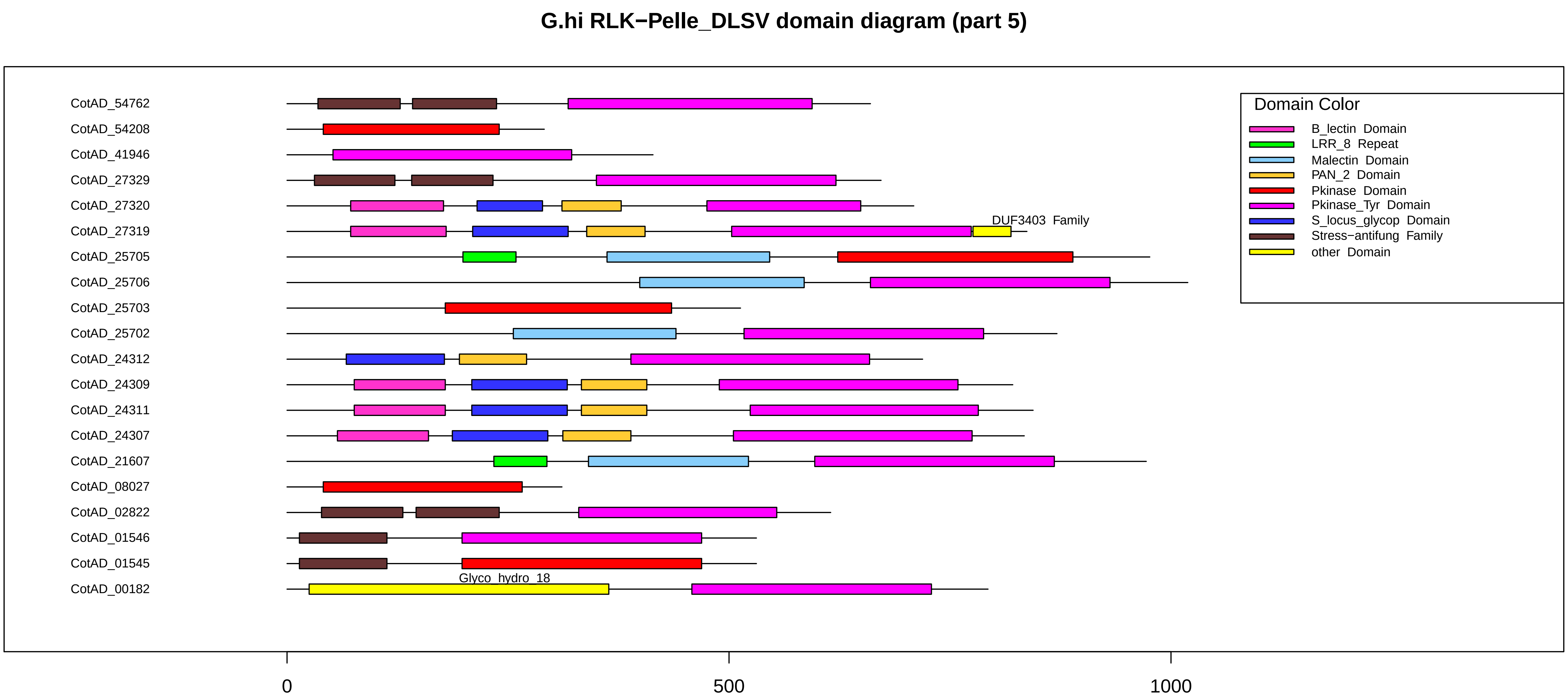
G.hi RLK−Pelle_DLSV domain diagram (part 5)
CotAD_54762
CotAD_54208
CotAD_41946
CotAD_27329
CotAD_27320
CotAD_27319
CotAD_25705
CotAD_25706
CotAD_25703
CotAD_25702
CotAD_24312
CotAD_24309
CotAD_24311
CotAD_24307
CotAD_21607
CotAD_08027
CotAD_02822
CotAD_01546
CotAD_01545
CotAD_00182
DUF3403 Family
Glyco_hydro_18
0
500
1000
Domain Color
B_lectin Domain
LRR_8 Repeat
Malectin Domain
PAN_2 Domain
Pkinase Domain
Pkinase_Tyr Domain
S_locus_glycop Domain
Stress−antifung Family
other Domain

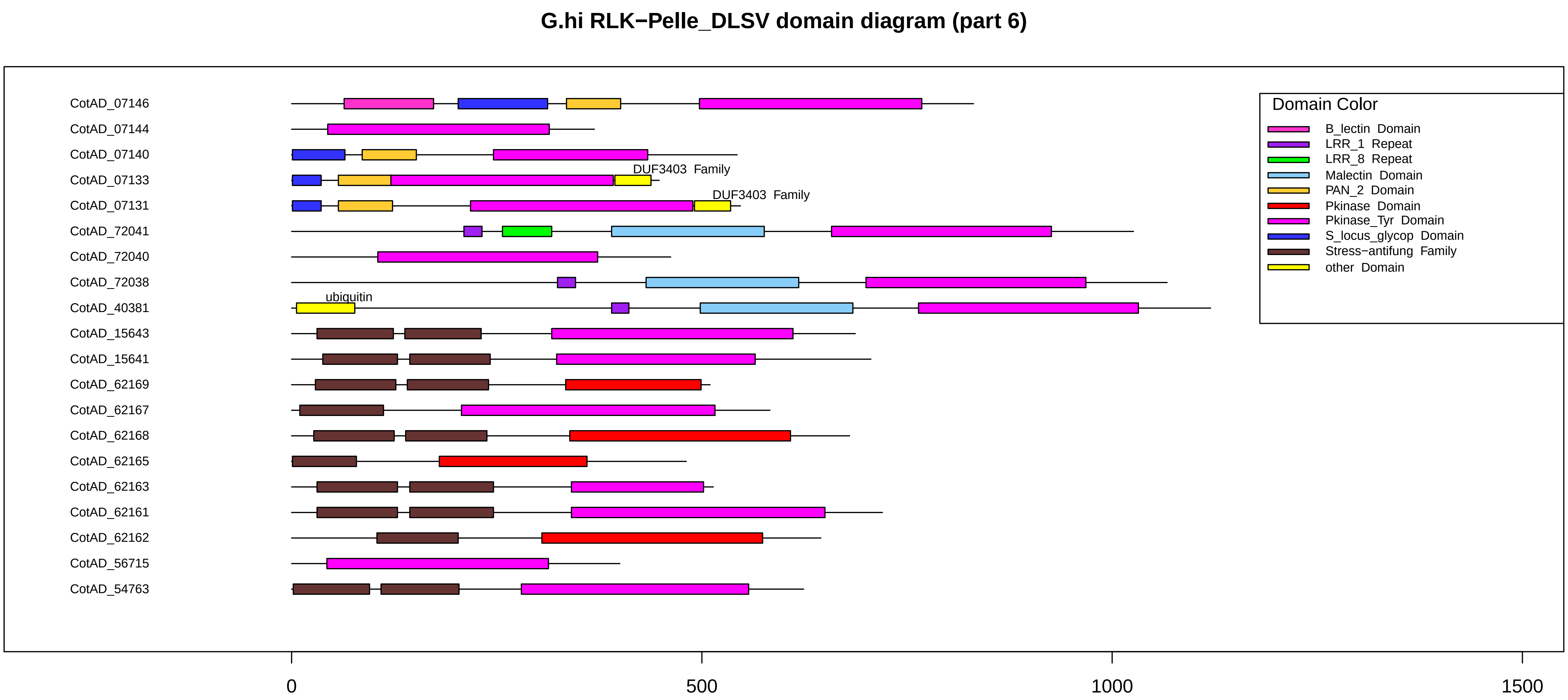
G.hi RLK−Pelle_DLSV domain diagram (part 6)
CotAD_07146
CotAD_07144
CotAD_07140
CotAD_07133
CotAD_07131
CotAD_72041
CotAD_72040
CotAD_72038
CotAD_40381
CotAD_15643
CotAD_15641
CotAD_62169
CotAD_62167
CotAD_62168
CotAD_62165
CotAD_62163
CotAD_62161
CotAD_62162
CotAD_56715
CotAD_54763
DUF3403 Family
DUF3403 Family
ubiquitin
Domain Color
B_lectin Domain
LRR_1 Repeat
LRR_8 Repeat
Malectin Domain
PAN_2 Domain
Pkinase Domain
Pkinase_Tyr Domain
S_locus_glycop Domain
Stress−antifung Family
other Domain
0
500
1000
1500

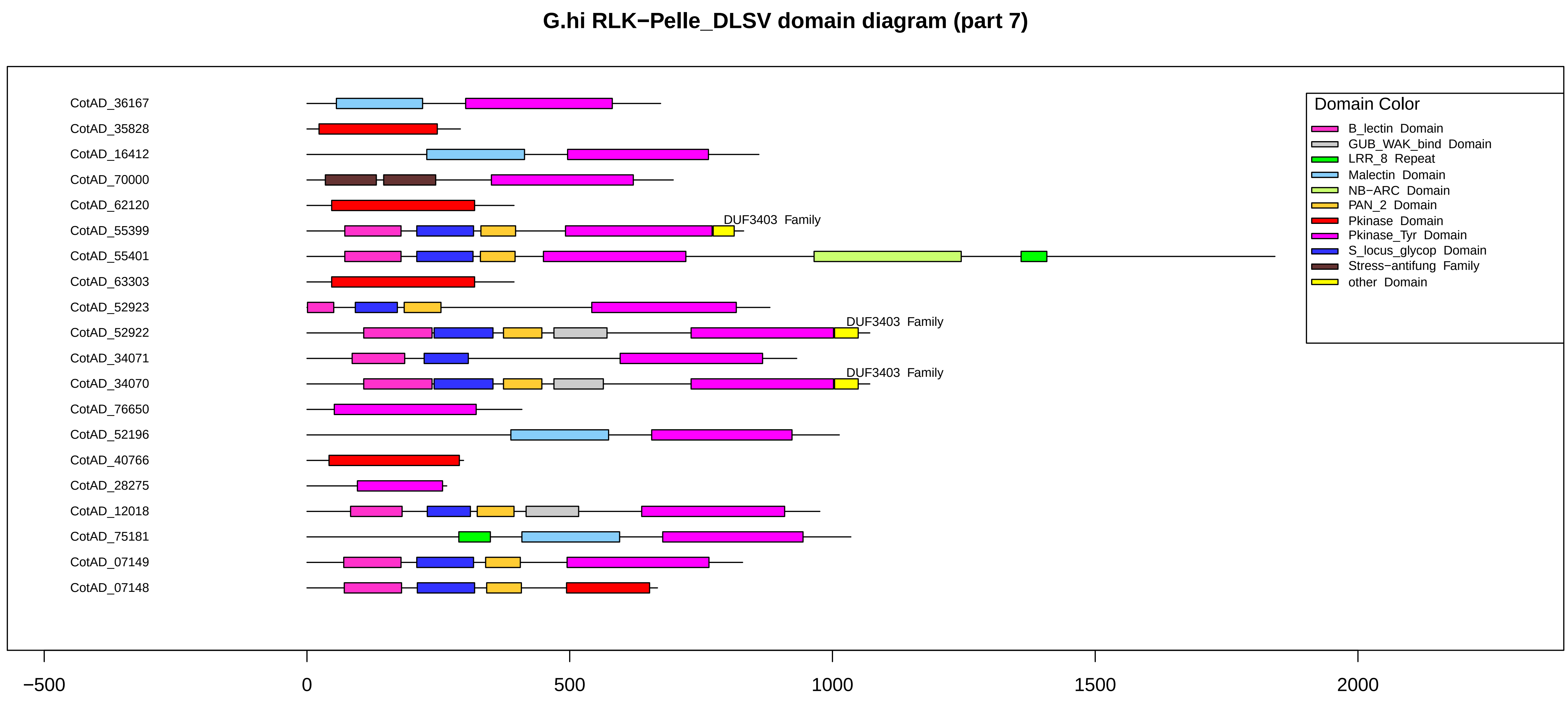
G.hi RLK−Pelle_DLSV domain diagram (part 7)
CotAD_36167
CotAD_35828
CotAD_16412
CotAD_70000
CotAD_62120
CotAD_55399
CotAD_55401
CotAD_63303
CotAD_52923
CotAD_52922
CotAD_34071
CotAD_34070
CotAD_76650
CotAD_52196
CotAD_40766
CotAD_28275
CotAD_12018
CotAD_75181
CotAD_07149
CotAD_07148
DUF3403 Family
DUF3403 Family
DUF3403 Family
Domain Color
B_lectin Domain
GUB_WAK_bind Domain
LRR_8 Repeat
Malectin Domain
NB−ARC Domain
PAN_2 Domain
Pkinase Domain
Pkinase_Tyr Domain
S_locus_glycop Domain
Stress−antifung Family
other Domain
−500
0
500
1000
1500
2000

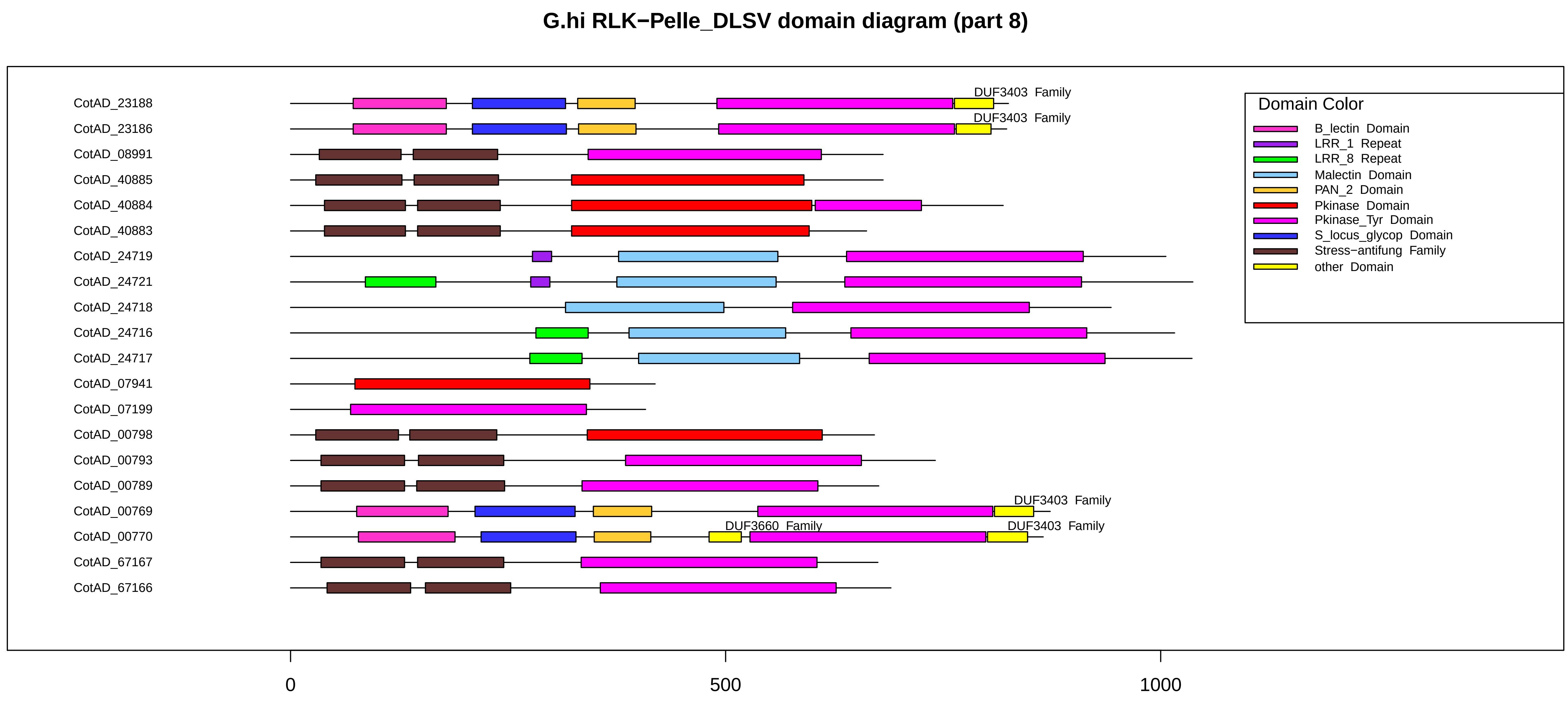

G.hi RLK−Pelle_DLSV domain diagram (part 8)
CotAD_23188
CotAD_23186
CotAD_08991
CotAD_40885
CotAD_40884
CotAD_40883
CotAD_24719
CotAD_24721
CotAD_24718
CotAD_24716
CotAD_24717
CotAD_07941
CotAD_07199
CotAD_00798
CotAD_00793
CotAD_00789
CotAD_00769
CotAD_00770
CotAD_67167
CotAD_67166
DUF3403 Family
DUF3660 Family
Domain Color
B_lectin Domain
LRR_1 Repeat
LRR_8 Repeat
Malectin Domain
PAN_2 Domain
Pkinase Domain
Pkinase_Tyr Domain
S_locus_glycop Domain
Stress−antifung Family
other Domain
0
500
1000

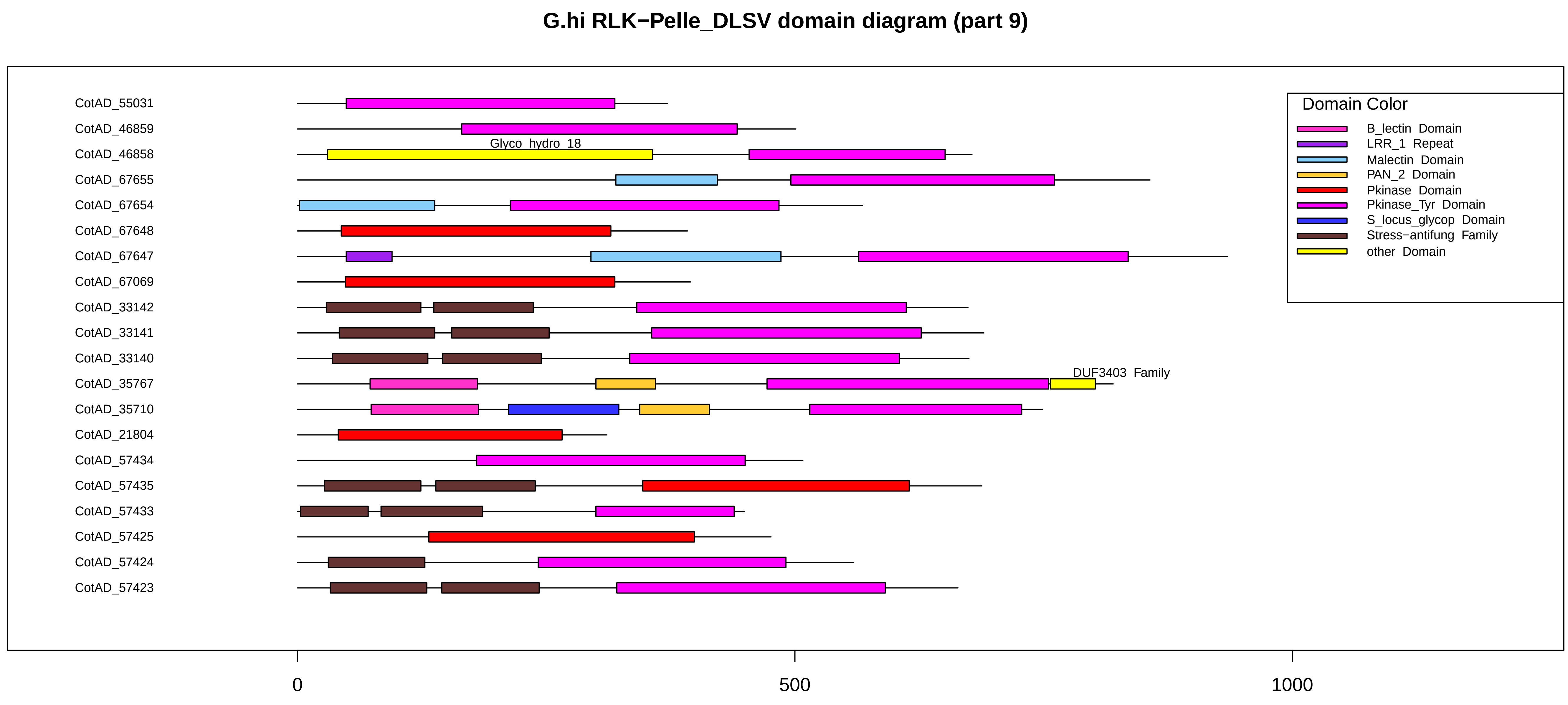

G.hi RLK−Pelle_DLSV domain diagram (part 9)
CotAD_55031
CotAD_46859
CotAD_46858
CotAD_67655
CotAD_67654
CotAD_67648
CotAD_67647
CotAD_67069
CotAD_33142
CotAD_33141
CotAD_33140
CotAD_35767
CotAD_35710
CotAD_21804
CotAD_57434
CotAD_57435
CotAD_57433
CotAD_57425
CotAD_57424
CotAD_57423
Glyco_hydro_18
DUF3403 Family
Domain Color
B_lectin Domain
LRR_1 Repeat
Malectin Domain
PAN_2 Domain
Pkinase Domain
Pkinase_Tyr Domain
S_locus_glycop Domain
Stress−antifung Family
other Domain
0
500
1000

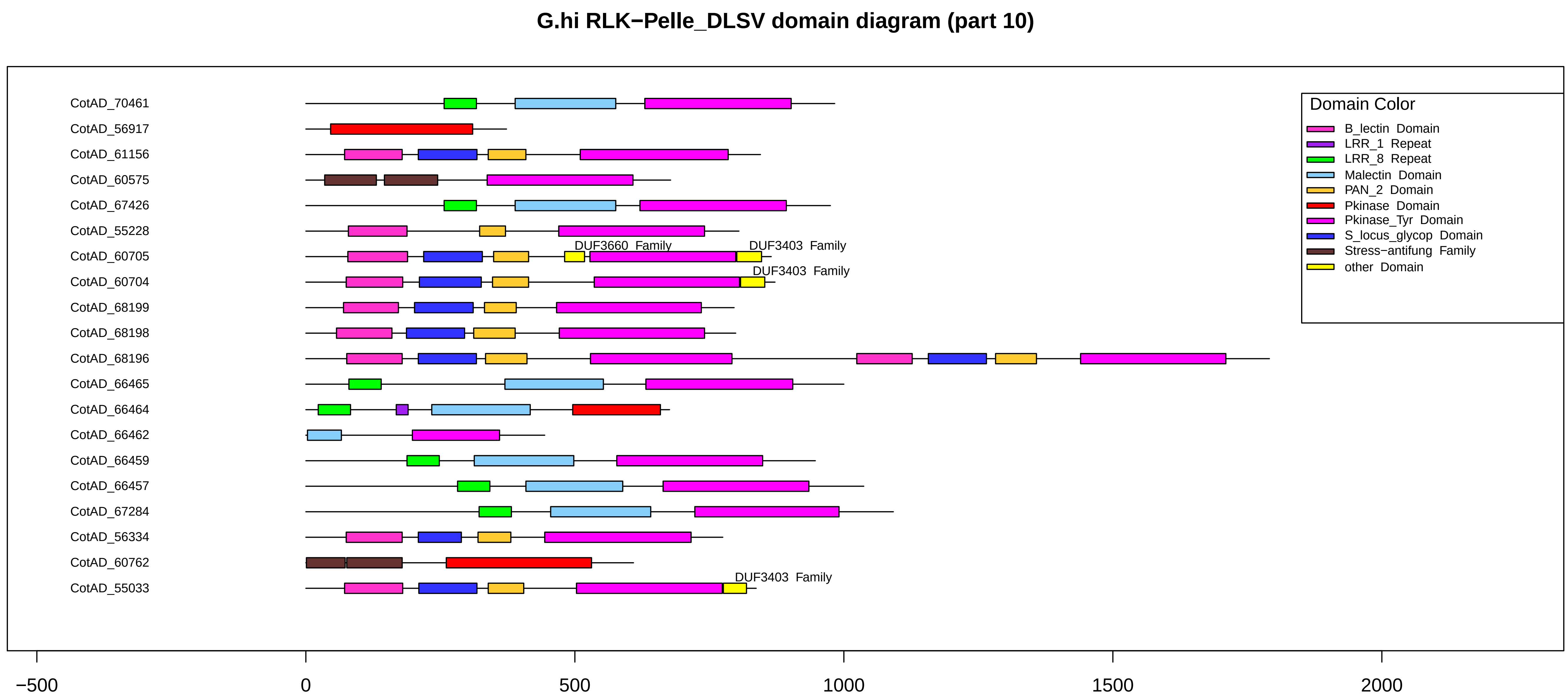
G.hi RLK−Pelle_DLSV domain diagram (part 10)
CotAD_70461
CotAD_56917
CotAD_61156
CotAD_60575
CotAD_67426
CotAD_55228
CotAD_60705
CotAD_60704
CotAD_68199
CotAD_68198
CotAD_68196
CotAD_66465
CotAD_66464
CotAD_66462
CotAD_66459
CotAD_66457
CotAD_67284
CotAD_56334
CotAD_60762
CotAD_55033
DUF3660 Family
DUF3403 Family
DUF3403 Family
DUF3403 Family
Domain Color
B_lectin Domain
LRR_1 Repeat
LRR_8 Repeat
Malectin Domain
PAN_2 Domain
Pkinase Domain
Pkinase_Tyr Domain
S_locus_glycop Domain
Stress−antifung Family
other Domain
−500
0
500
1000
1500
2000

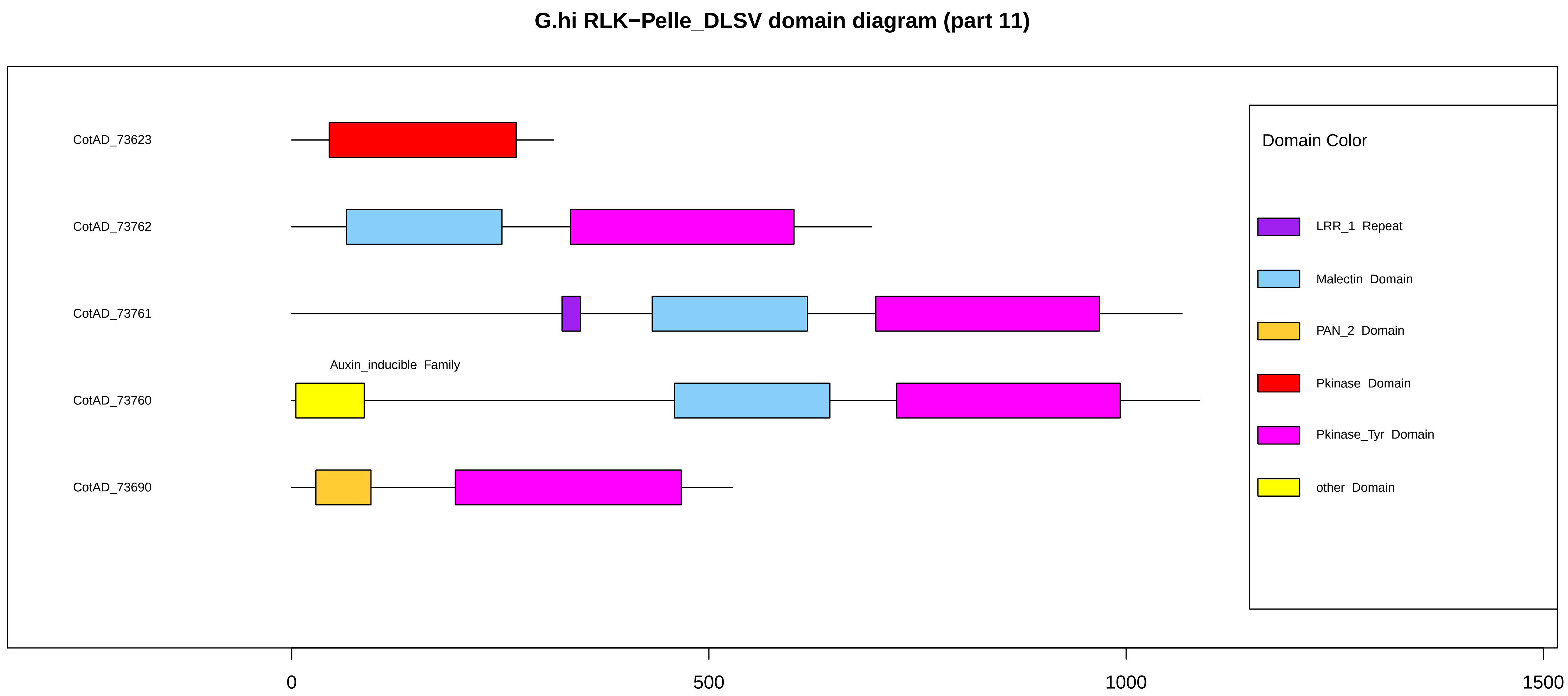
G.hi RLK−Pelle_DLSV domain diagram (part 11)
CotAD_73623
CotAD_73762
CotAD_73761
Auxin_inducible Family
CotAD_73760
CotAD_73690
Domain Color
LRR_1 Repeat
Malectin Domain
PAN_2 Domain
Pkinase Domain
Pkinase_Tyr Domain
other Domain
0
500
1000
1500

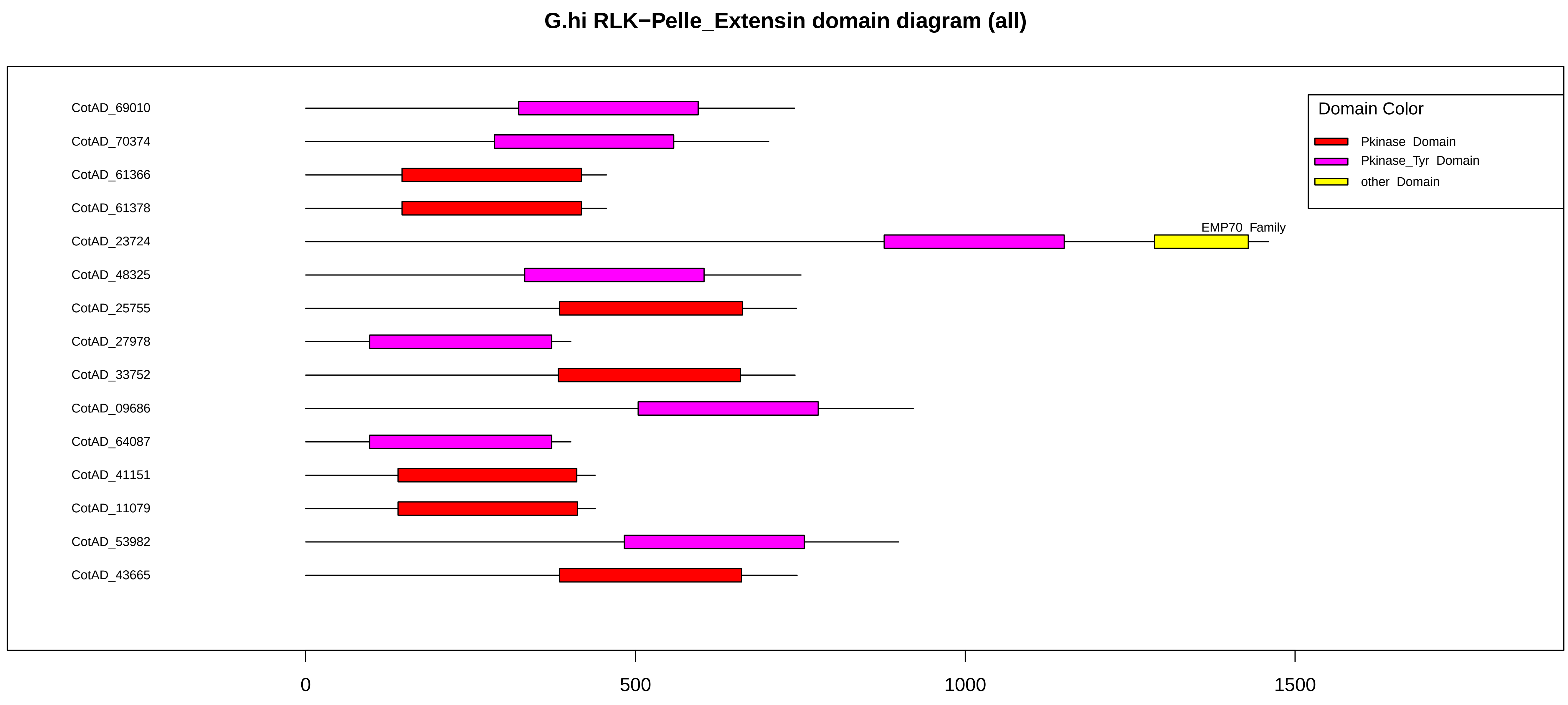
G.hi RLK−Pelle_Extensin domain diagram (all)
CotAD_69010
CotAD_70374
CotAD_61366
CotAD_61378
CotAD_23724
CotAD_48325
CotAD_25755
CotAD_27978
CotAD_33752
CotAD_09686
CotAD_64087
CotAD_41151
CotAD_11079
CotAD_53982
CotAD_43665
EMP70 Family
Domain Color
Pkinase Domain
Pkinase_Tyr Domain
other Domain
0
500
1000
1500

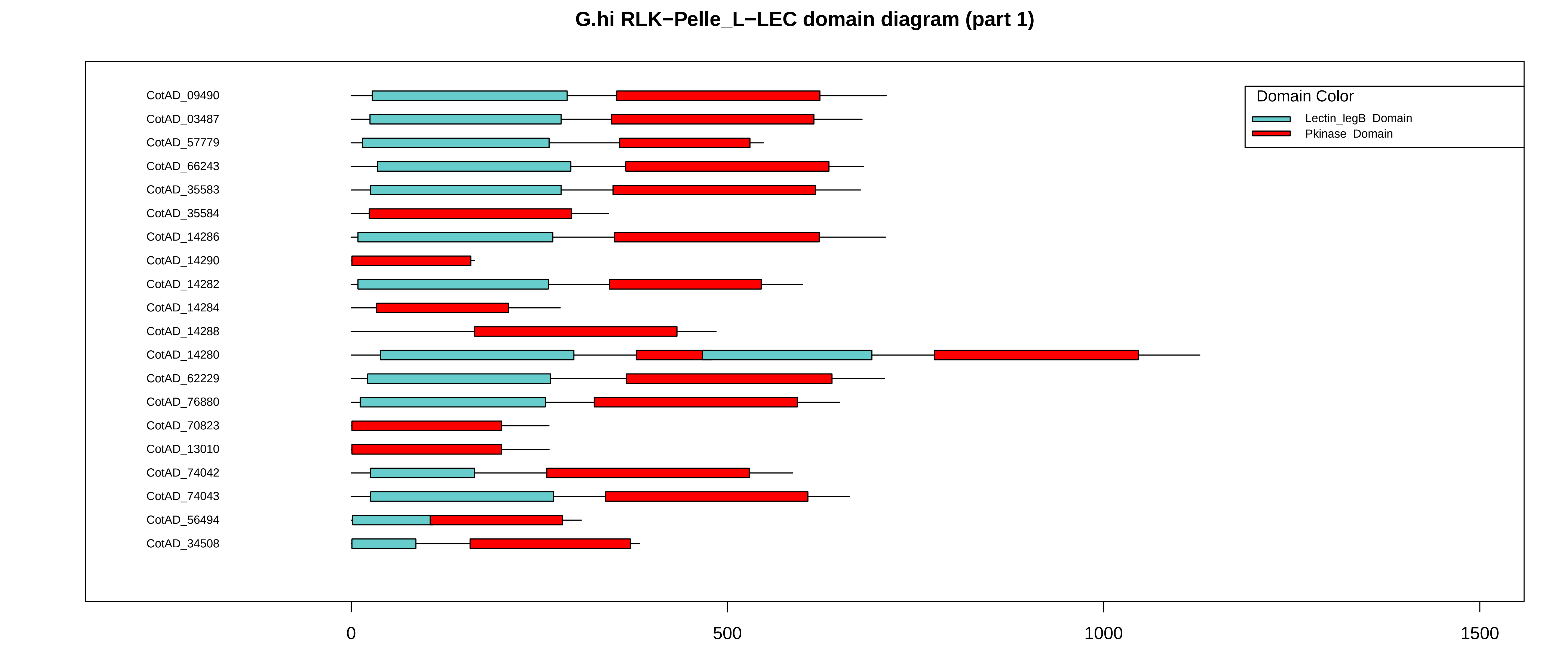

G.hi RLK−Pelle_L−LEC domain diagram (part 1)
CotAD_09490
CotAD_03487
CotAD_57779
CotAD_66243
CotAD_35583
CotAD_35584
CotAD_14286
CotAD_14290
CotAD_14282
CotAD_14284
CotAD_14288
CotAD_14280
CotAD_62229
CotAD_76880
CotAD_70823
CotAD_13010
CotAD_74042
CotAD_74043
CotAD_56494
CotAD_34508
Domain Color
Lectin_legB Domain
Pkinase Domain
0
500
1000
1500

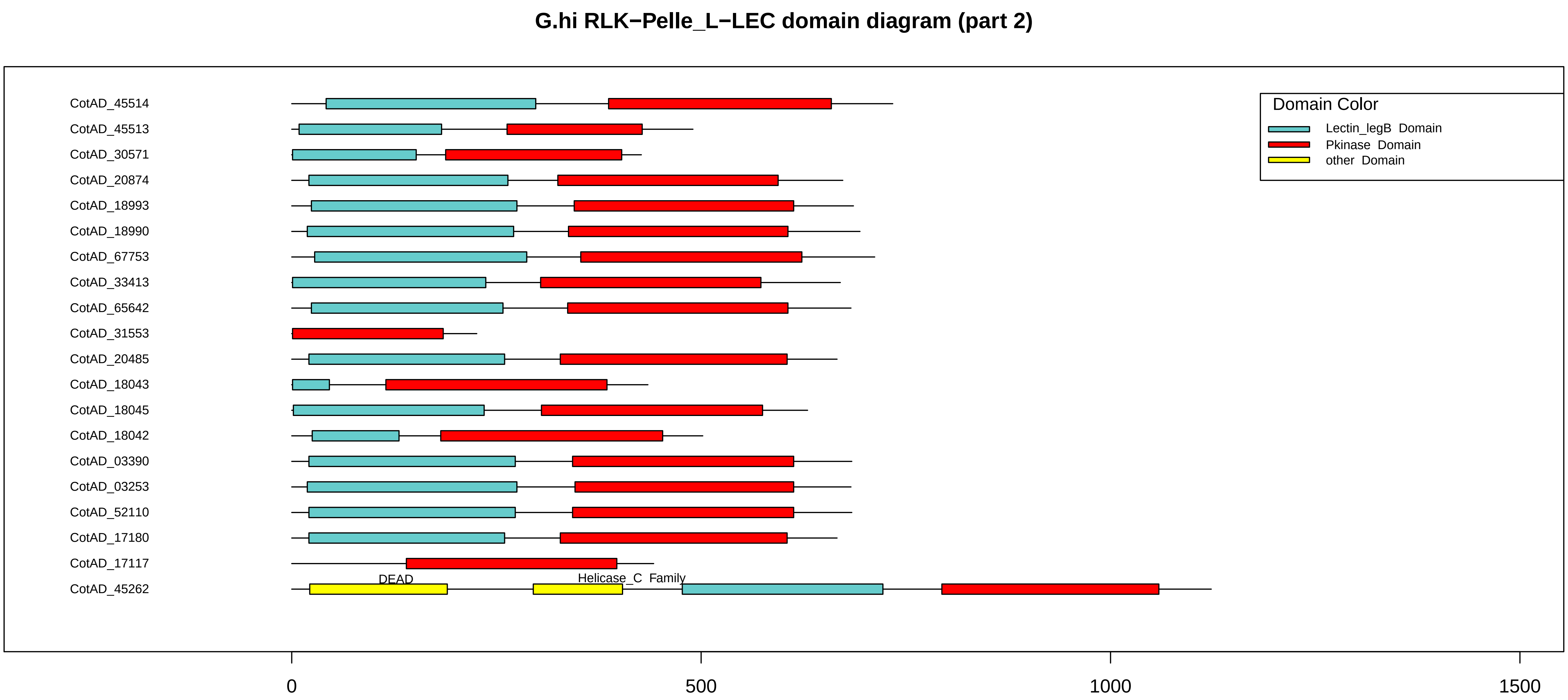
G.hi RLK−Pelle_L−LEC domain diagram (part 2)
CotAD_45514
CotAD_45513
CotAD_30571
CotAD_20874
CotAD_18993
CotAD_18990
CotAD_67753
CotAD_33413
CotAD_65642
CotAD_31553
CotAD_20485
CotAD_18043
CotAD_18045
CotAD_18042
CotAD_03390
CotAD_03253
CotAD_52110
CotAD_17180
CotAD_17117
CotAD_45262
DEAD
Helicase_C Family
Domain Color
Lectin_legB Domain
Pkinase Domain
other Domain
0
500
1000
1500

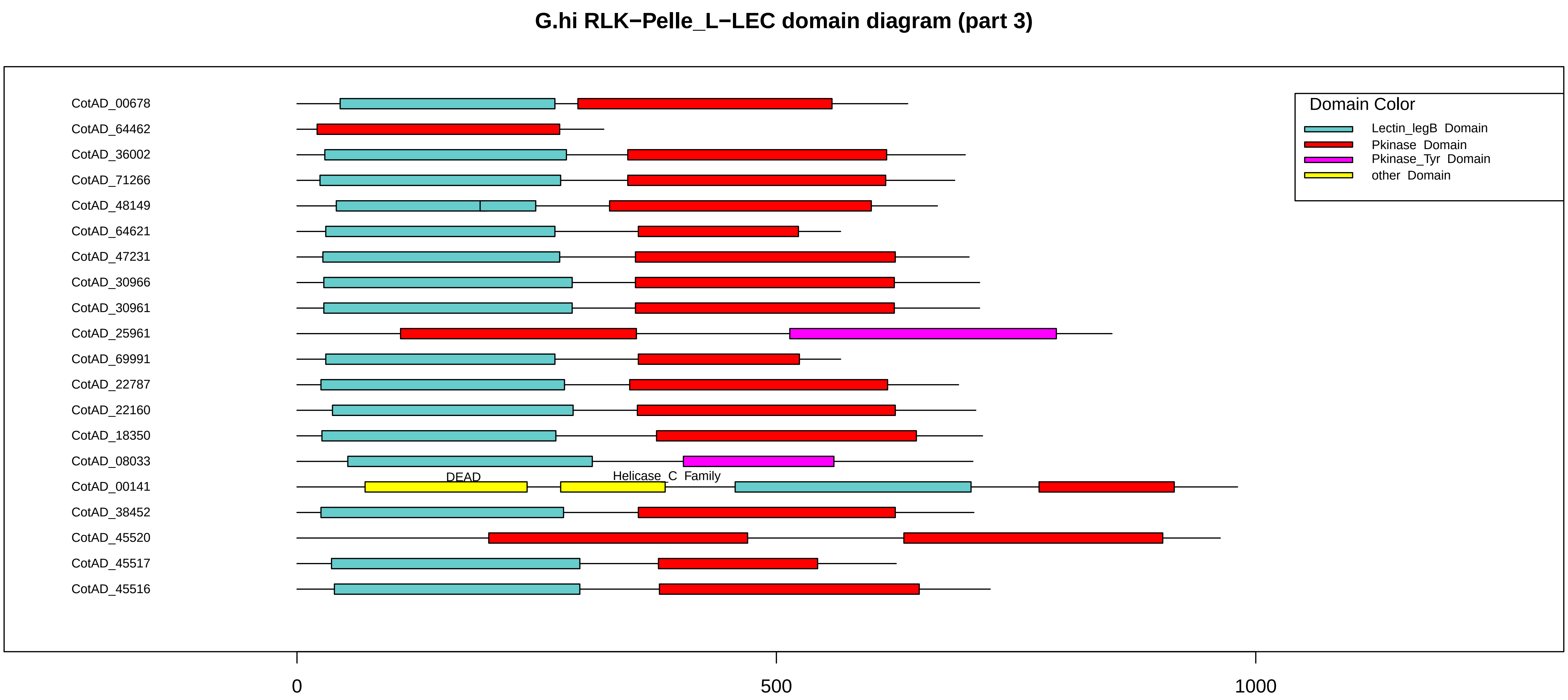
G.hi RLK−Pelle_L−LEC domain diagram (part 3)
CotAD_00678
CotAD_64462
CotAD_36002
CotAD_71266
CotAD_48149
CotAD_64621
CotAD_47231
CotAD_30966
CotAD_30961
CotAD_25961
CotAD_69991
CotAD_22787
CotAD_22160
CotAD_18350
CotAD_08033
CotAD_00141
CotAD_38452
CotAD_45520
CotAD_45517
CotAD_45516
DEAD
Helicase_C Family
Domain Color
Lectin_legB Domain
Pkinase Domain
Pkinase_Tyr Domain
other Domain
0
500
1000

G.hi RLK−Pelle_L−LEC domain diagram (part 4)

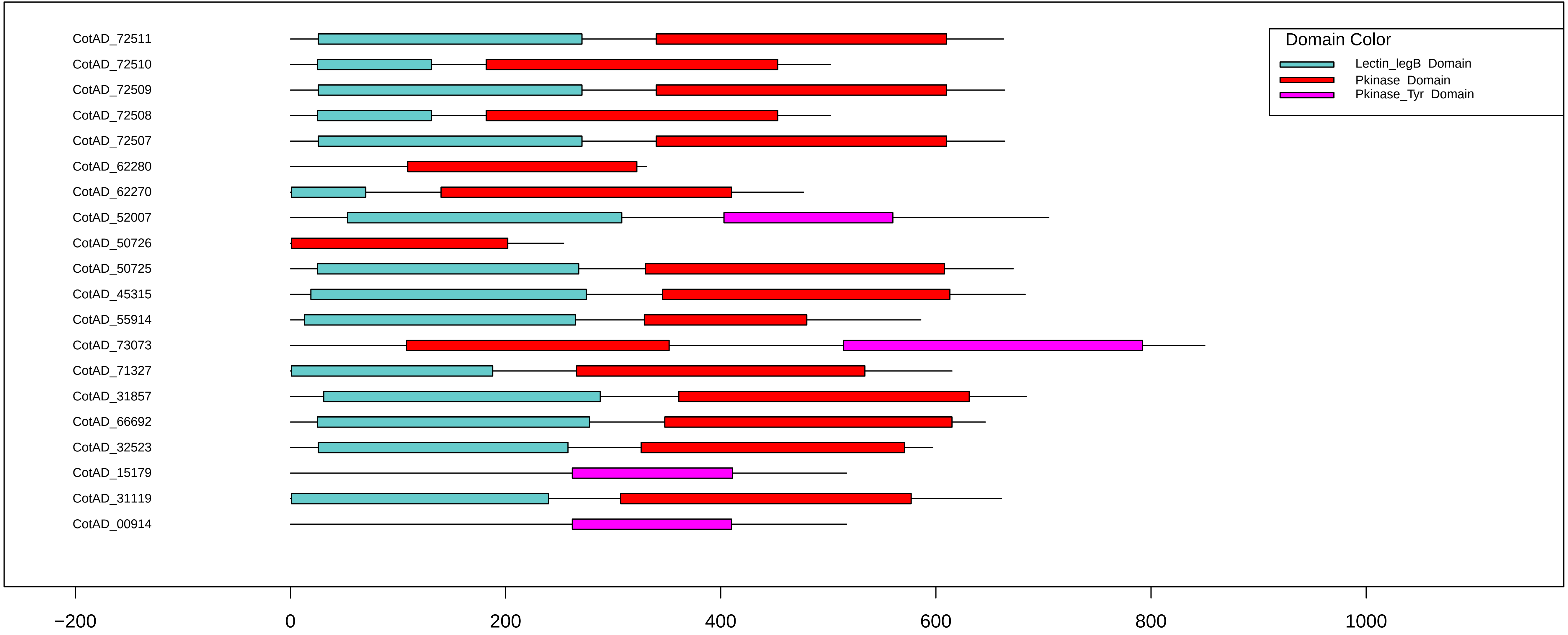

G.hi RLK−Pelle_L−LEC domain diagram (part 5)

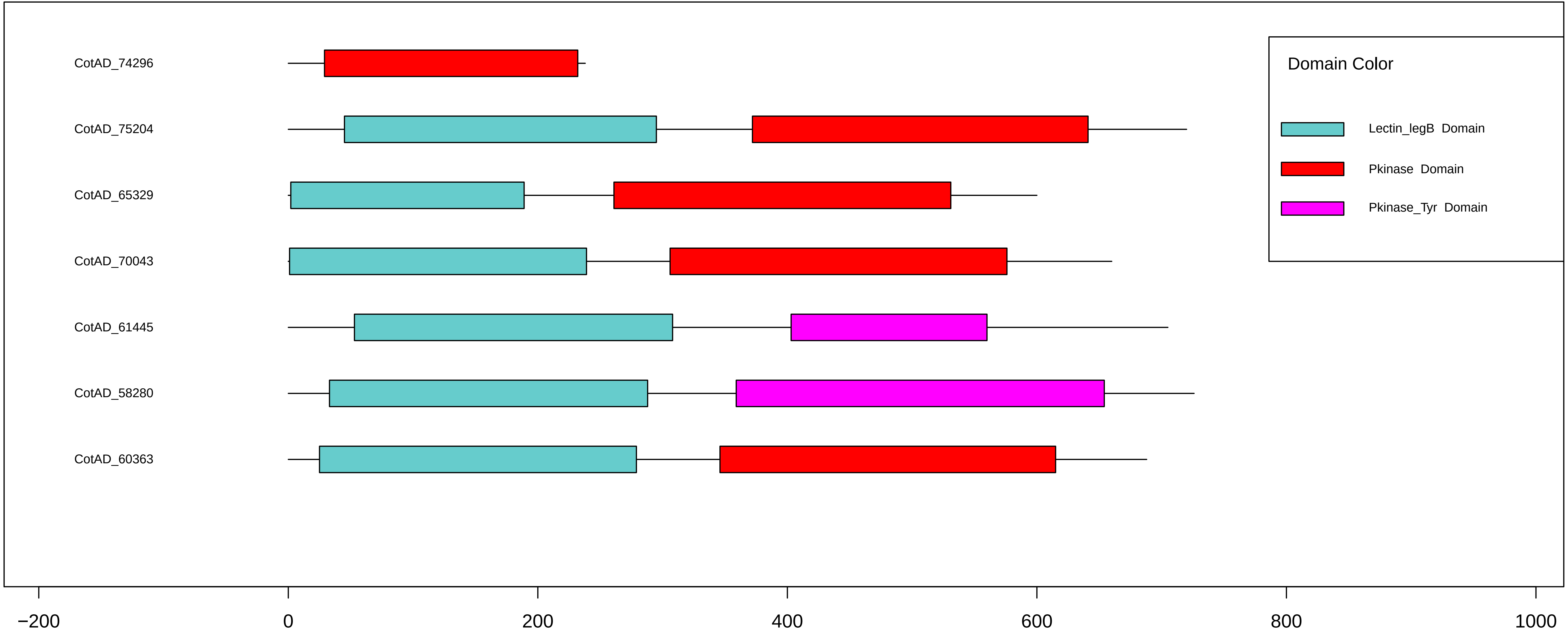

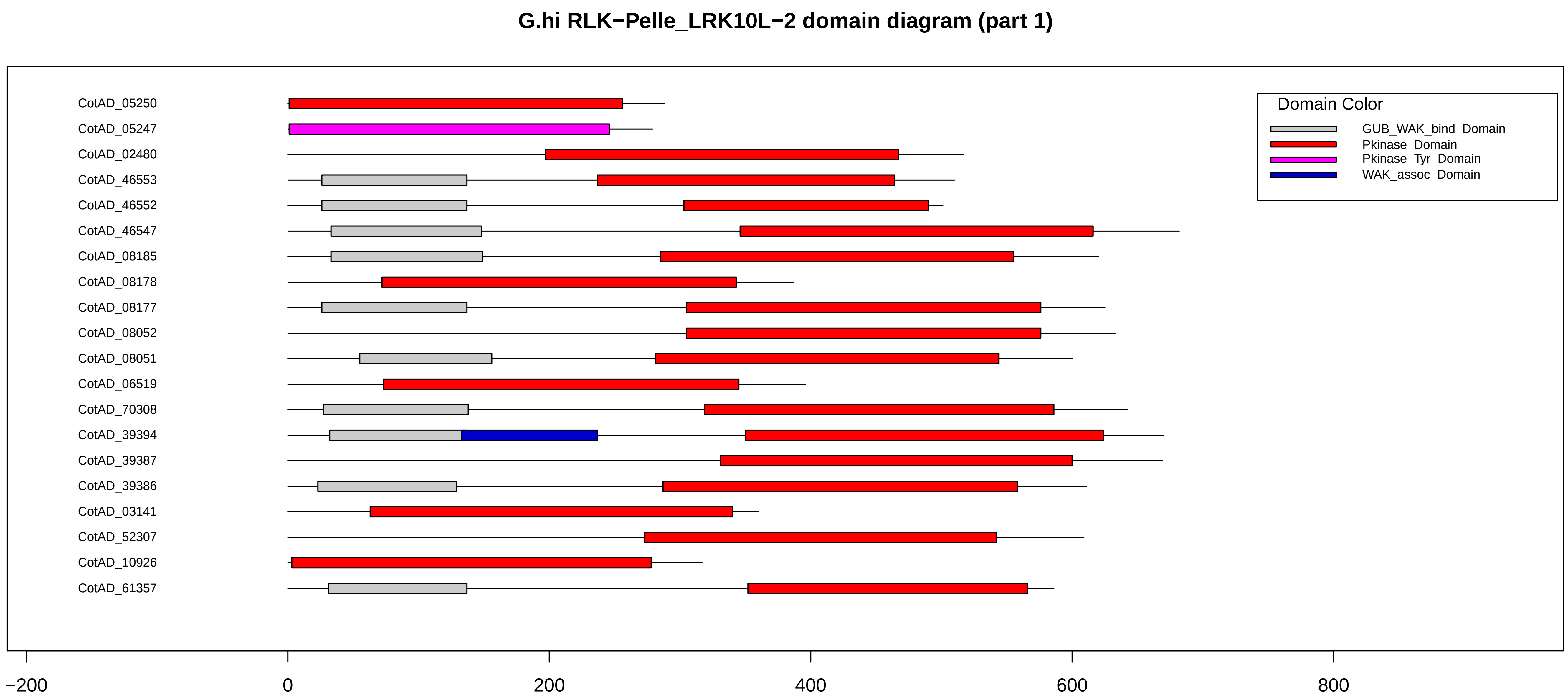

G.hi RLK−Pelle_LRK10L−2 domain diagram (part 1)
CotAD_05250
CotAD_05247
CotAD_02480
CotAD_46553
CotAD_46552
CotAD_46547
CotAD_08185
CotAD_08178
CotAD_08177
CotAD_08052
CotAD_08051
CotAD_06519
CotAD_70308
CotAD_39394
CotAD_39387
CotAD_39386
CotAD_03141
CotAD_52307
CotAD_10926
CotAD_61357
Domain Color
GUB_WAK_bind Domain
Pkinase Domain
Pkinase_Tyr Domain
WAK_assoc Domain
−200
0
200
400
600
800

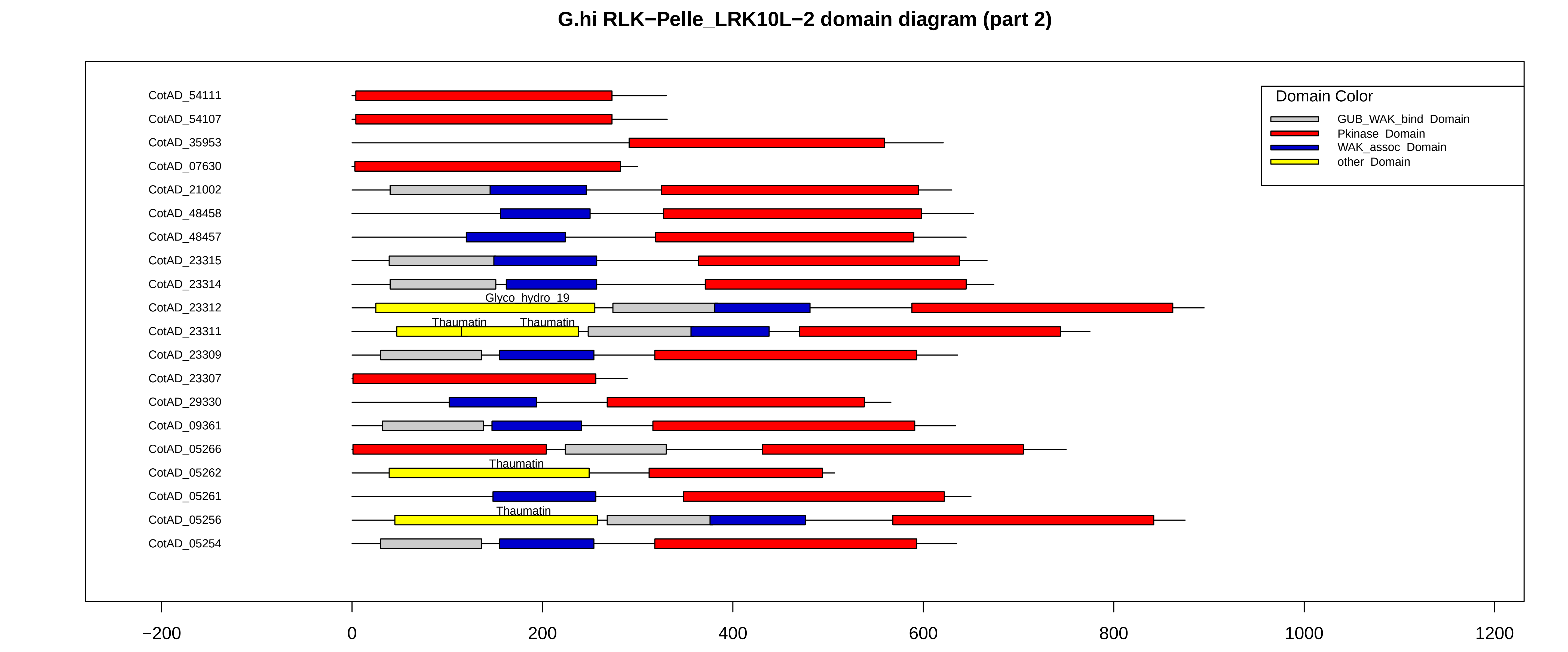
G.hi RLK−Pelle_LRK10L−2 domain diagram (part 2)
CotAD_54111
CotAD_54107
CotAD_35953
CotAD_07630
CotAD_21002
CotAD_48458
CotAD_48457
CotAD_23315
CotAD_23314
CotAD_23312
CotAD_23311
CotAD_23309
CotAD_23307
CotAD_29330
CotAD_09361
CotAD_05266
CotAD_05262
CotAD_05261
CotAD_05256
CotAD_05254
Glyco_hydro_19
Thaumatin
Thaumatin
Thaumatin
Thaumatin
Domain Color
GUB_WAK_bind Domain
Pkinase Domain
WAK_assoc Domain
other Domain
−200
0
200
400
600
800
1000
1200

G.hi RLK−Pelle_LRK10L−2 domain diagram (part 3)

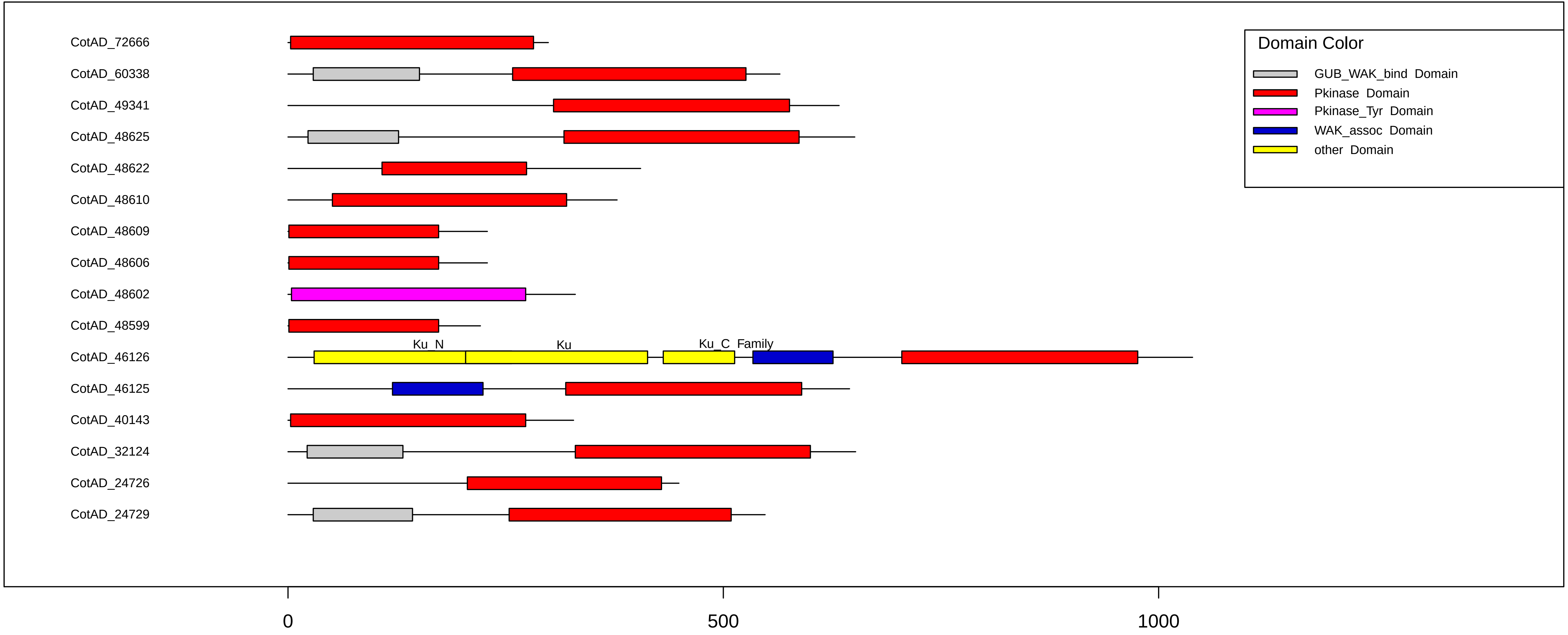

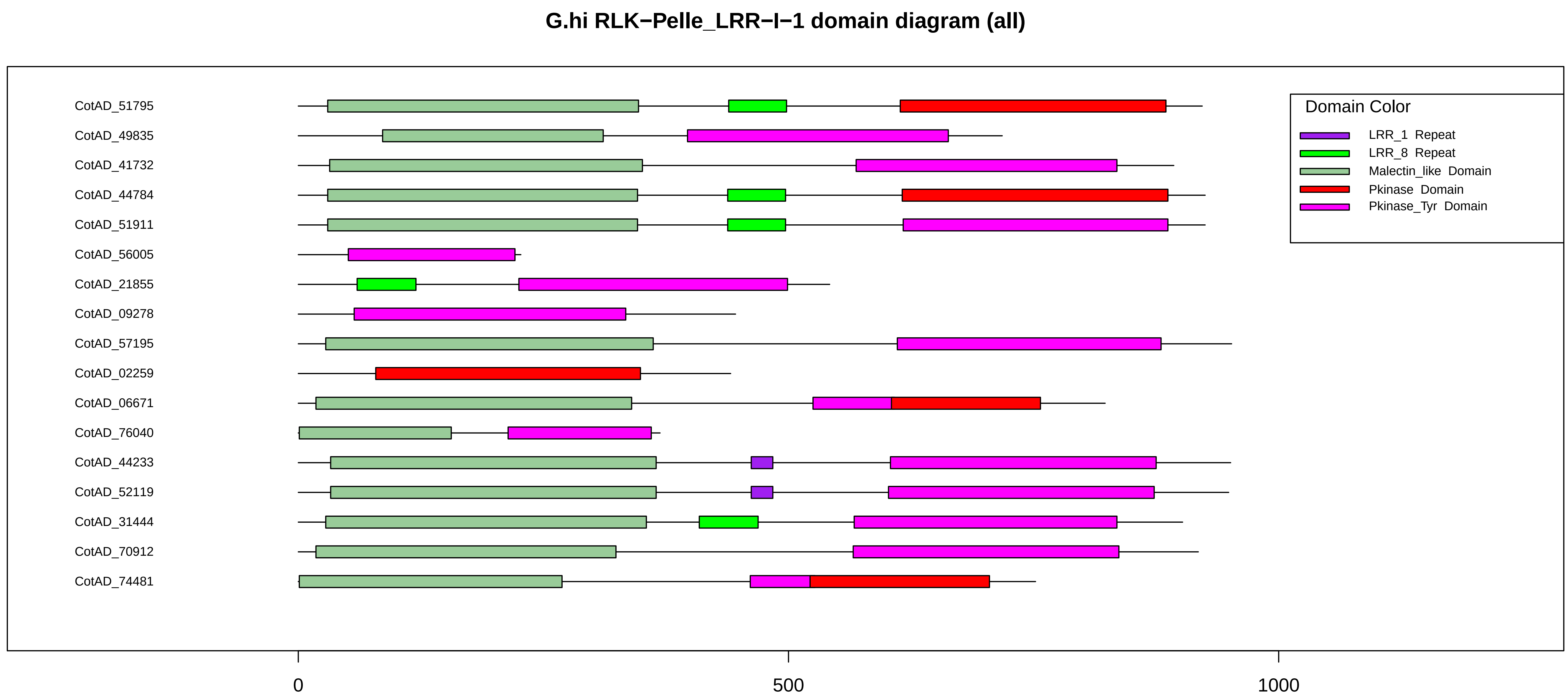

G.hi RLK−Pelle_LRR−I−1 domain diagram (all)
CotAD_51795
CotAD_49835
CotAD_41732
CotAD_44784
CotAD_51911
CotAD_56005
CotAD_21855
CotAD_09278
CotAD_57195
CotAD_02259
CotAD_06671
CotAD_76040
CotAD_44233
CotAD_52119
CotAD_31444
CotAD_70912
CotAD_74481
Domain Color
LRR_1 Repeat
LRR_8 Repeat
Malectin_like Domain
Pkinase Domain
Pkinase_Tyr Domain
0
500
1000

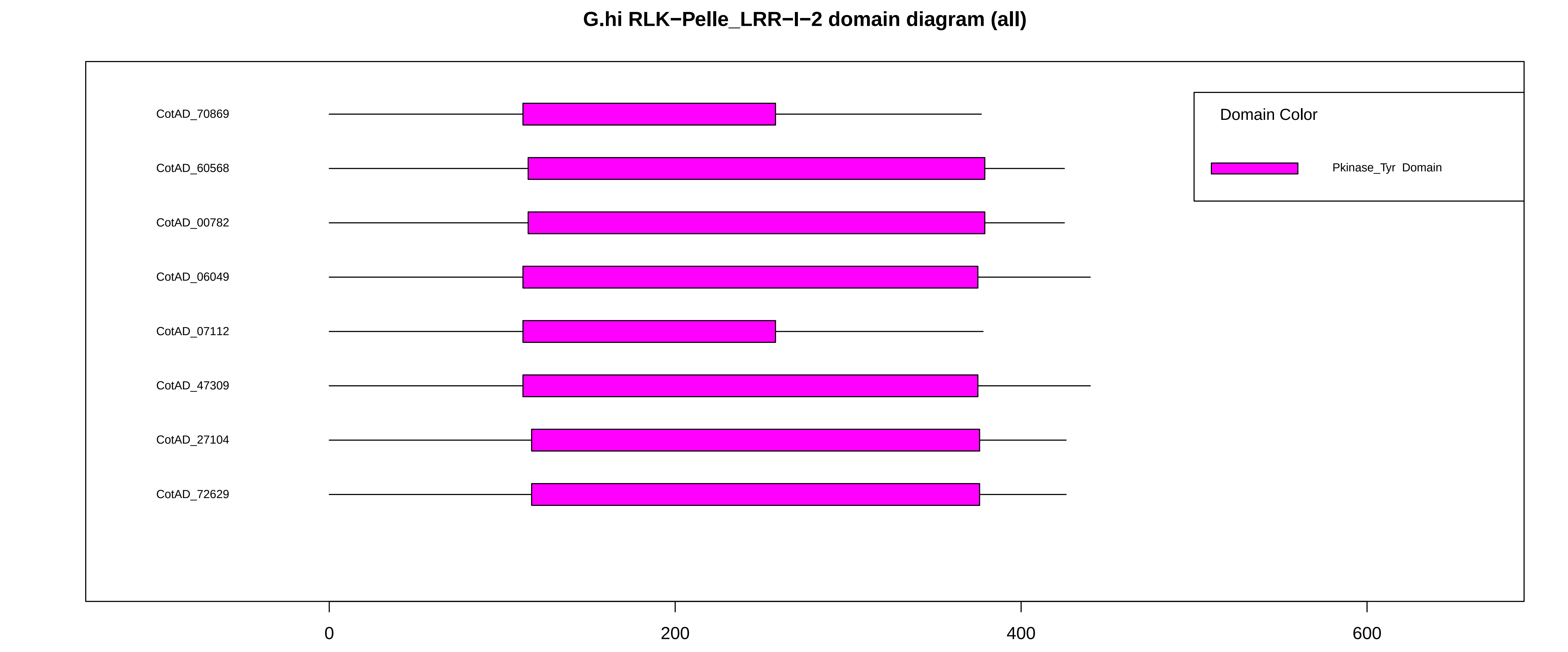
G.hi RLK−Pelle_LRR−I−2 domain diagram (all)
CotAD_70869
CotAD_60568
CotAD_00782
CotAD_06049
CotAD_07112
CotAD_47309
CotAD_27104
CotAD_72629
Domain Color
Pkinase_Tyr Domain
0
200
400
600

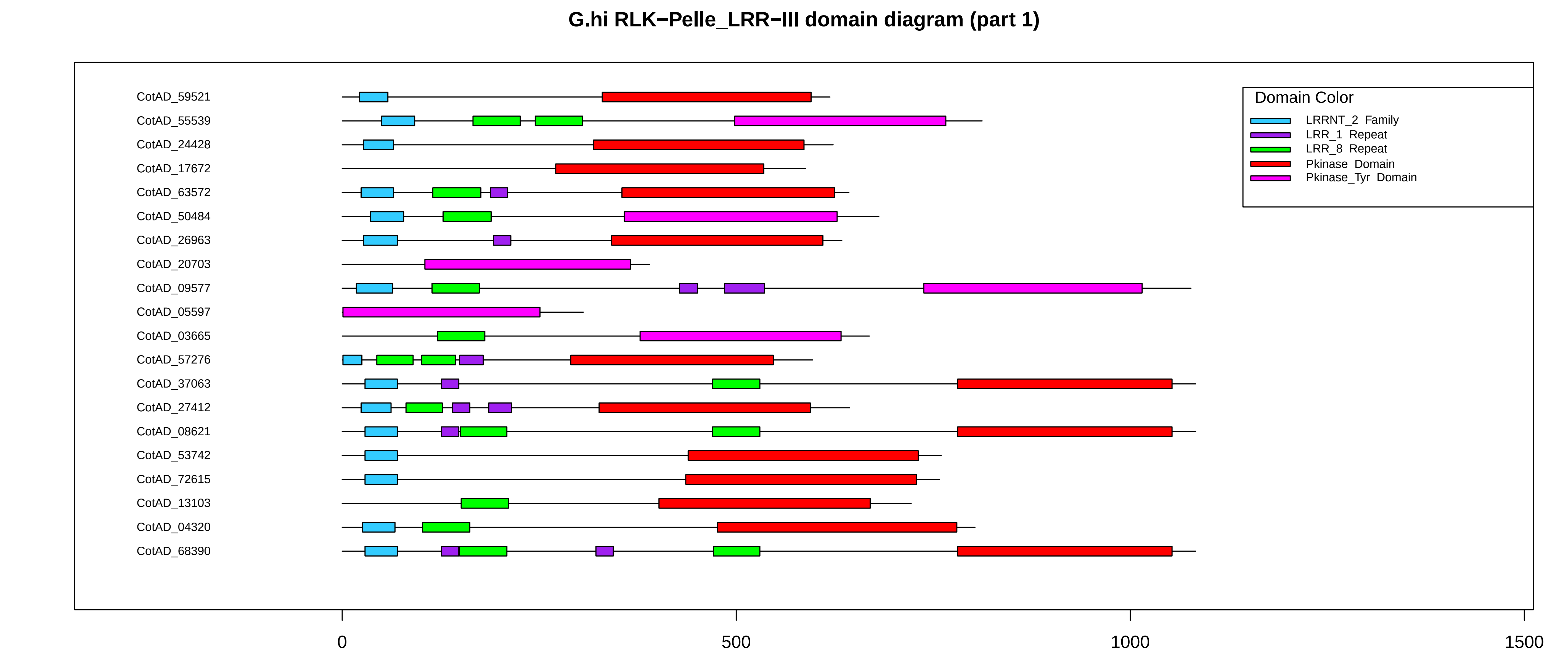
G.hi RLK−Pelle_LRR−III domain diagram (part 1)
CotAD_59521
CotAD_55539
CotAD_24428
CotAD_17672
CotAD_63572
CotAD_50484
CotAD_26963
CotAD_20703
CotAD_09577
CotAD_05597
CotAD_03665
CotAD_57276
CotAD_37063
CotAD_27412
CotAD_08621
CotAD_53742
CotAD_72615
CotAD_13103
CotAD_04320
CotAD_68390
Domain Color
LRRNT_2 Family
LRR_1 Repeat
LRR_8 Repeat
Pkinase Domain
Pkinase_Tyr Domain
0
500
1000
1500

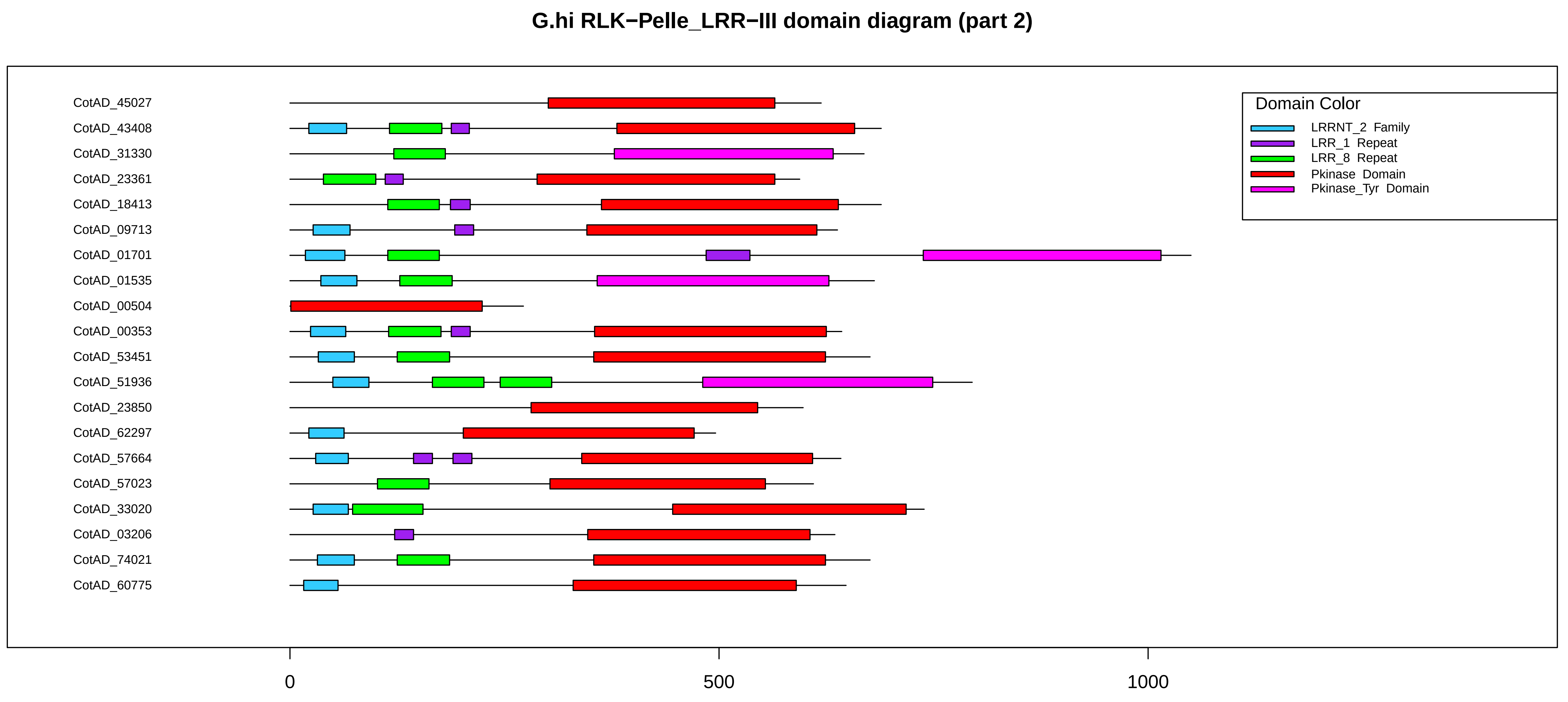
G.hi RLK−Pelle_LRR−III domain diagram (part 2)
CotAD_45027
CotAD_43408
CotAD_31330
CotAD_23361
CotAD_18413
CotAD_09713
CotAD_01701
CotAD_01535
CotAD_00504
CotAD_00353
CotAD_53451
CotAD_51936
CotAD_23850
CotAD_62297
CotAD_57664
CotAD_57023
CotAD_33020
CotAD_03206
CotAD_74021
CotAD_60775
Domain Color
LRRNT_2 Family
LRR_1 Repeat
LRR_8 Repeat
Pkinase Domain
Pkinase_Tyr Domain
0
500
1000

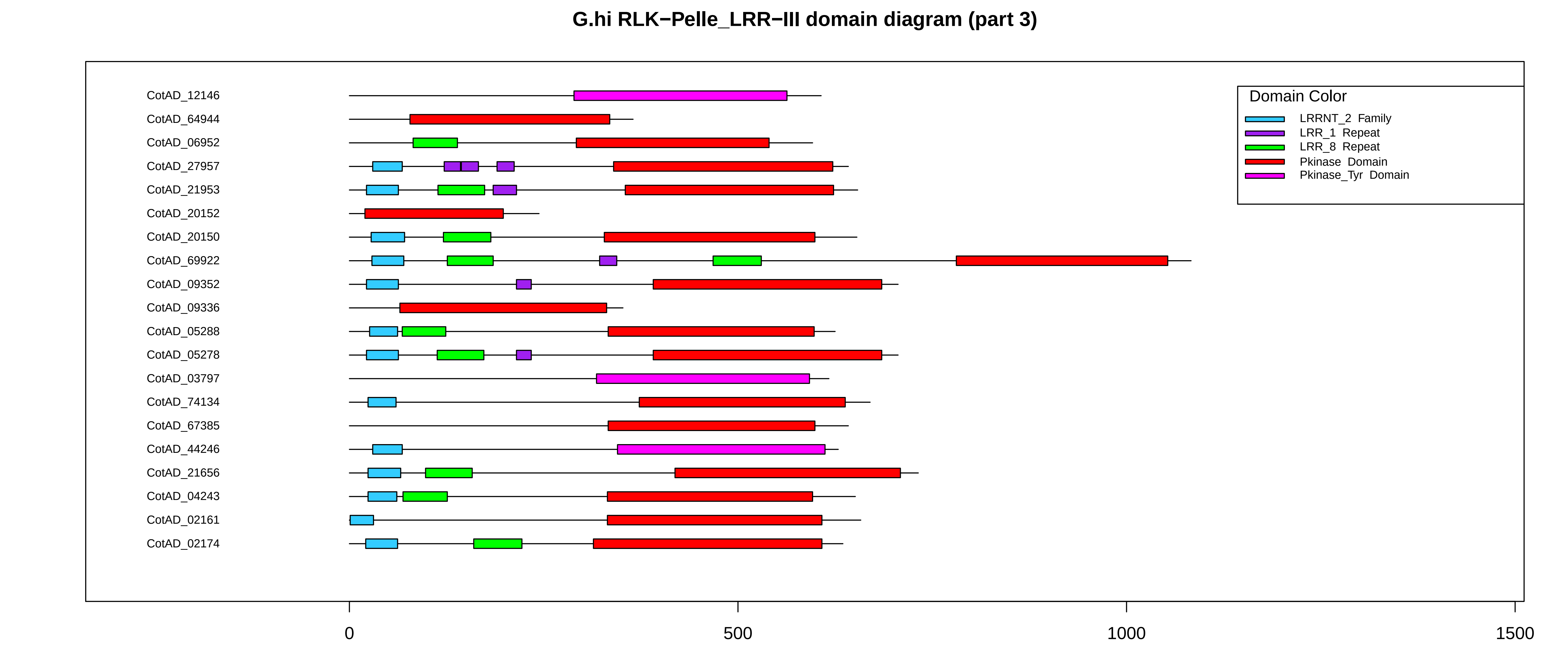
G.hi RLK−Pelle_LRR−III domain diagram (part 3)
CotAD_12146
CotAD_64944
CotAD_06952
CotAD_27957
CotAD_21953
CotAD_20152
CotAD_20150
CotAD_69922
CotAD_09352
CotAD_09336
CotAD_05288
CotAD_05278
CotAD_03797
CotAD_74134
CotAD_67385
CotAD_44246
CotAD_21656
CotAD_04243
CotAD_02161
CotAD_02174
Domain Color
LRRNT_2 Family
LRR_1 Repeat
LRR_8 Repeat
Pkinase Domain
Pkinase_Tyr Domain
0
500
1000
1500

G.hi RLK−Pelle_LRR−III domain diagram (part 4)

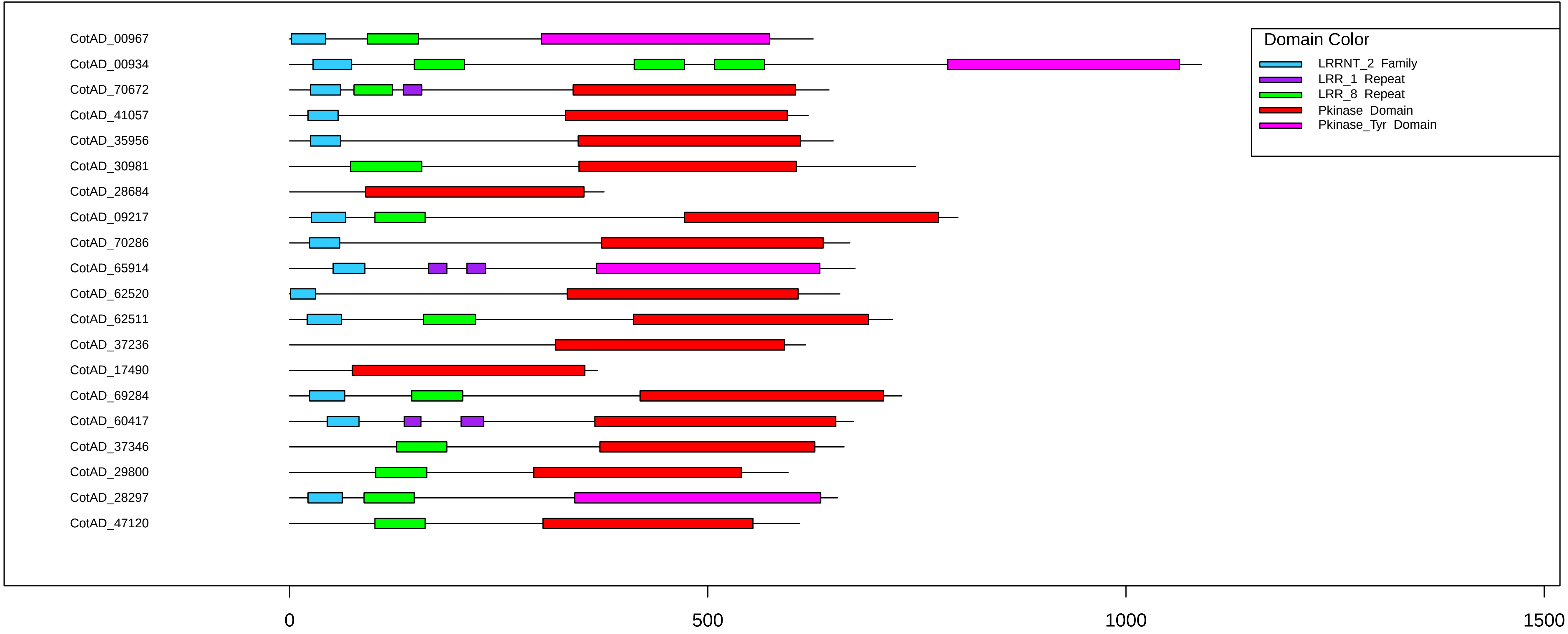

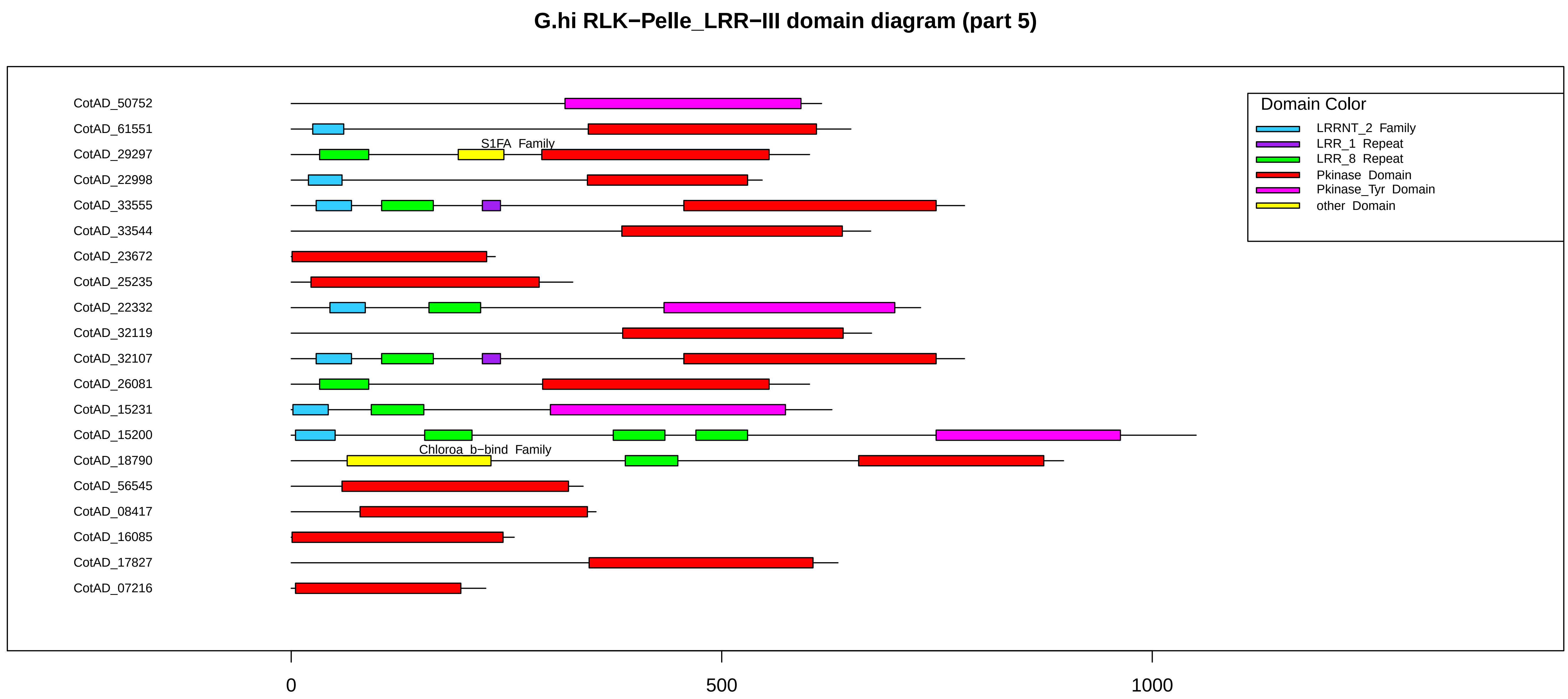

G.hi RLK−Pelle_LRR−III domain diagram (part 5)
CotAD_50752
CotAD_61551
CotAD_29297
CotAD_22998
CotAD_33555
CotAD_33544
CotAD_23672
CotAD_25235
CotAD_22332
CotAD_32119
CotAD_32107
CotAD_26081
CotAD_15231
CotAD_15200
CotAD_18790
CotAD_56545
CotAD_08417
CotAD_16085
CotAD_17827
CotAD_07216
S1FA Family
Chloroa_b−bind Family
Domain Color
LRRNT_2 Family
LRR_1 Repeat
LRR_8 Repeat
Pkinase Domain
Pkinase_Tyr Domain
other Domain
0
500
1000

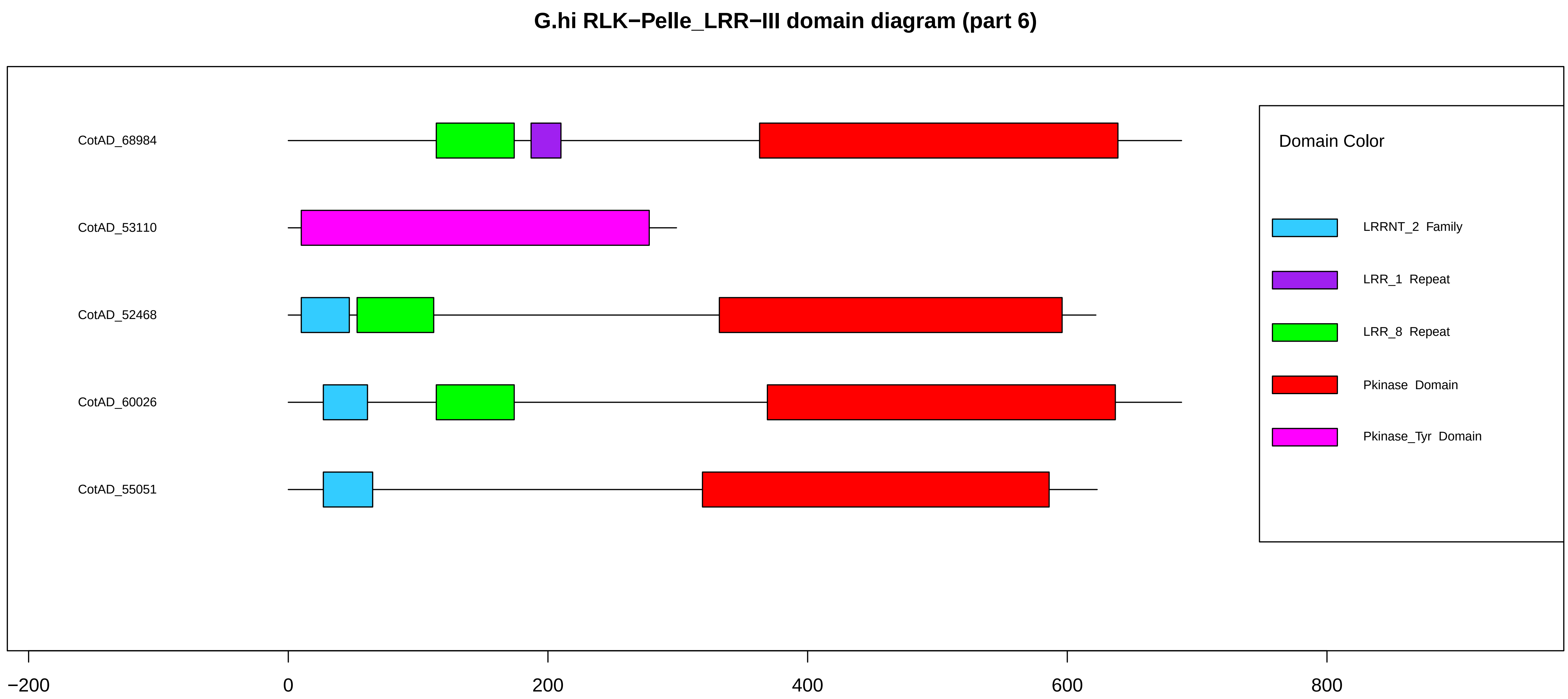

G.hi RLK−Pelle_LRR−III domain diagram (part 6)
CotAD_68984
CotAD_53110
CotAD_52468
CotAD_60026
CotAD_55051
Domain Color
LRRNT_2 Family
LRR_1 Repeat
LRR_8 Repeat
Pkinase Domain
Pkinase_Tyr Domain
−200
0
200
400
600
800

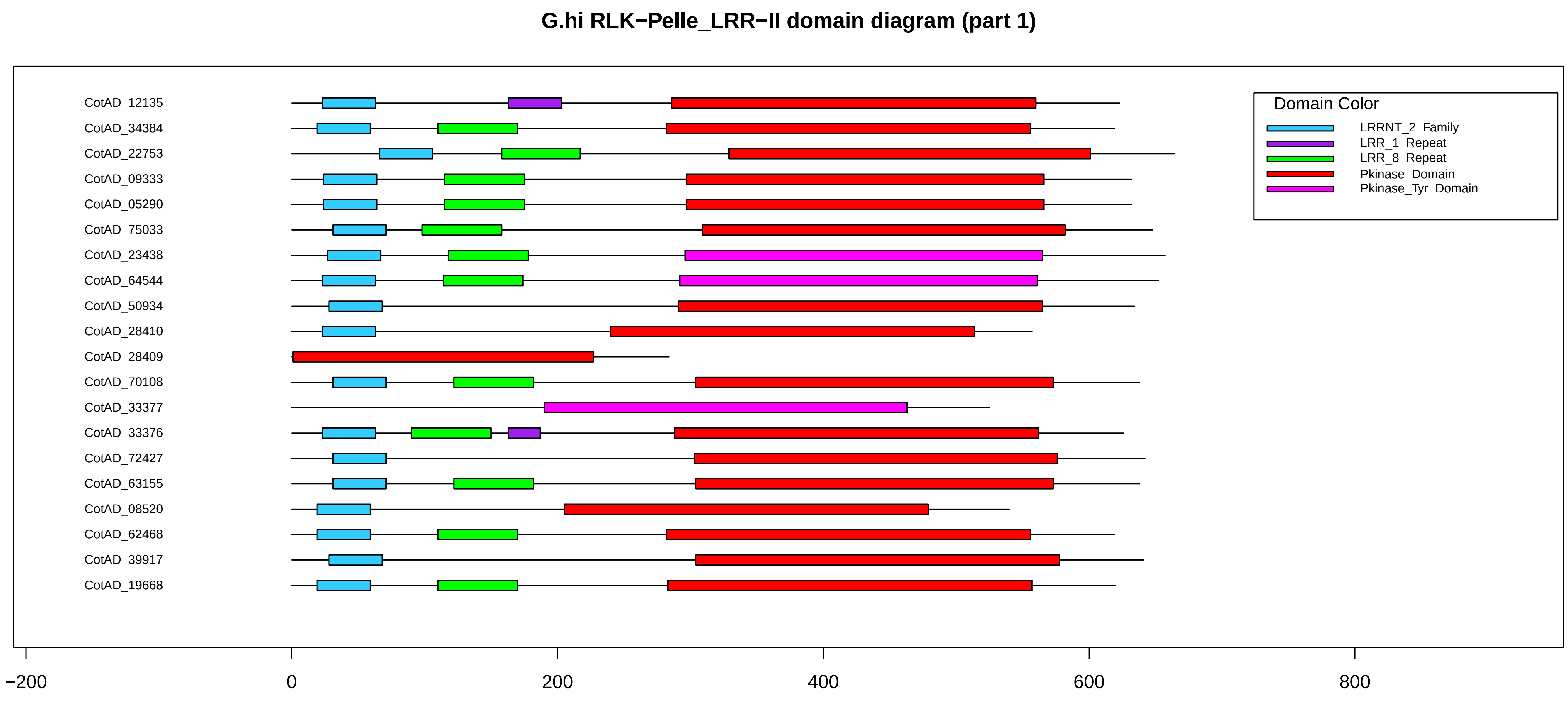
G.hi RLK−Pelle_LRR−II domain diagram (part 1)
CotAD_12135
CotAD_34384
CotAD_22753
CotAD_09333
CotAD_05290
CotAD_75033
CotAD_23438
CotAD_64544
CotAD_50934
CotAD_28410
CotAD_28409
CotAD_70108
CotAD_33377
CotAD_33376
CotAD_72427
CotAD_63155
CotAD_08520
CotAD_62468
CotAD_39917
CotAD_19668
Domain Color
LRRNT_2 Family
LRR_1 Repeat
LRR_8 Repeat
Pkinase Domain
Pkinase_Tyr Domain
−200
0
200
400
600
800

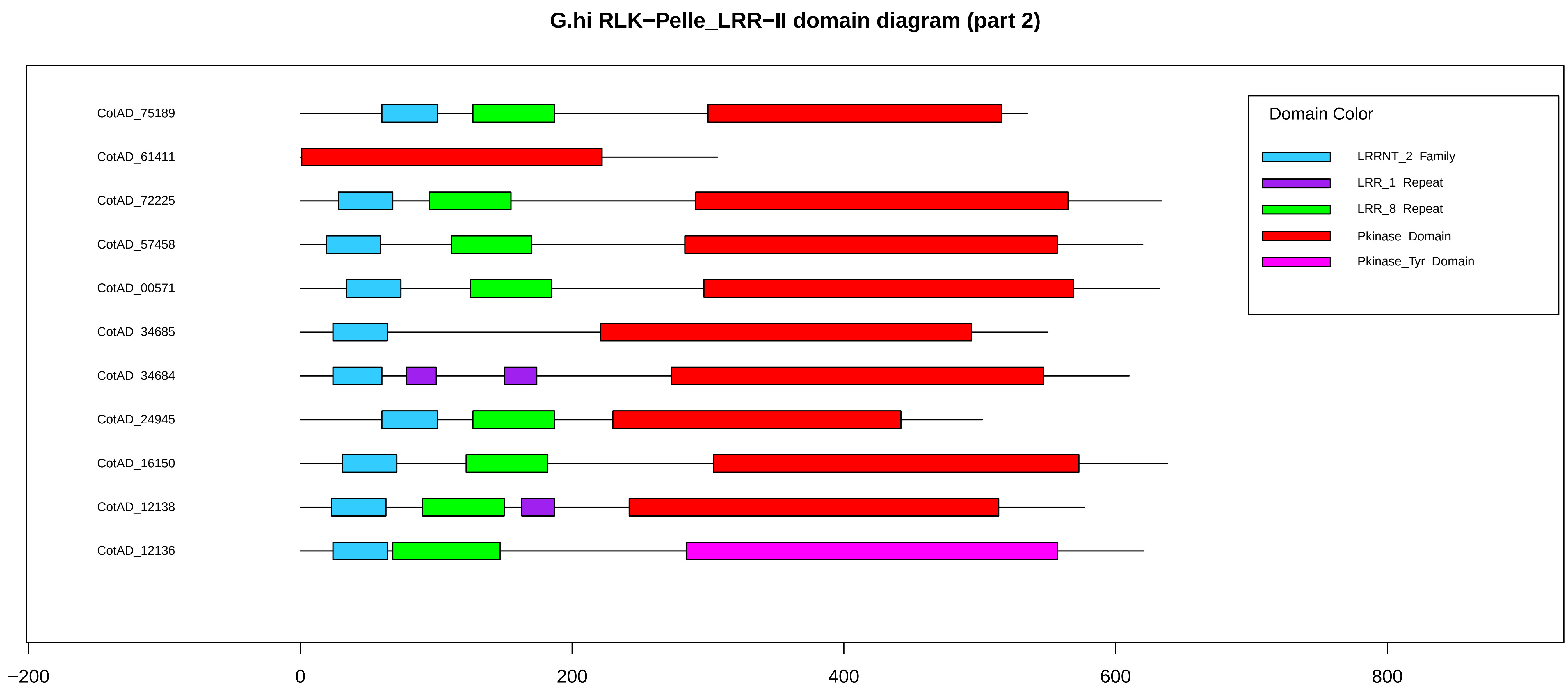

G.hi RLK−Pelle_LRR−II domain diagram (part 2)
CotAD_75189
CotAD_61411
CotAD_72225
CotAD_57458
CotAD_00571
CotAD_34685
CotAD_34684
CotAD_24945
CotAD_16150
CotAD_12138
CotAD_12136
Domain Color
LRRNT_2 Family
LRR_1 Repeat
LRR_8 Repeat
Pkinase Domain
Pkinase_Tyr Domain
−200
0
200
400
600
800

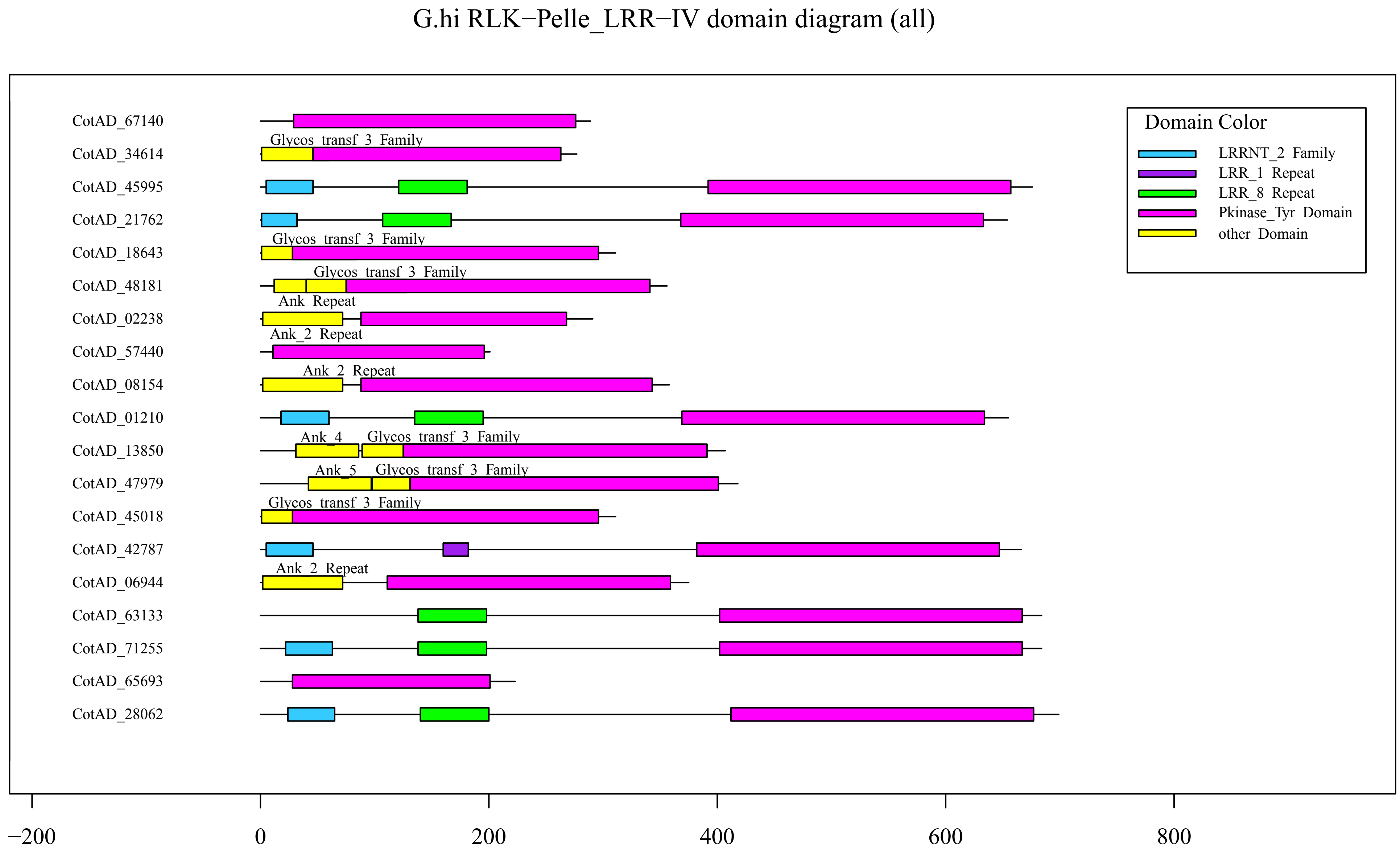
G.hi RLK−Pelle_LRR−IV domain diagram (all)
Domain Color
LRRNT_2 Family
LRR_1 Repeat
LRR_8 Repeat
Pkinase_Tyr Domain
other Domain
CotAD_67140
CotAD_34614
Glycos_transf_3 Family
CotAD_45995
CotAD_21762
CotAD_18643
Glycos_transf_3 Family
CotAD_48181
Glycos_transf_3 Family
Ank_Repeat
CotAD_02238
Ank_2 Repeat
CotAD_57440
CotAD_08154
Ank_2 Repeat
CotAD_01210
CotAD_13850
Ank_4
Glycos_transf_3 Family
CotAD_47979
Ank_5
Glycos_transf_3 Family
CotAD_45018
Glycos_transf_3 Family
CotAD_42787
CotAD_06944
Ank_2 Repeat
CotAD_63133
CotAD_71255
CotAD_65693
CotAD_28062
−200
0
200
400
600
800

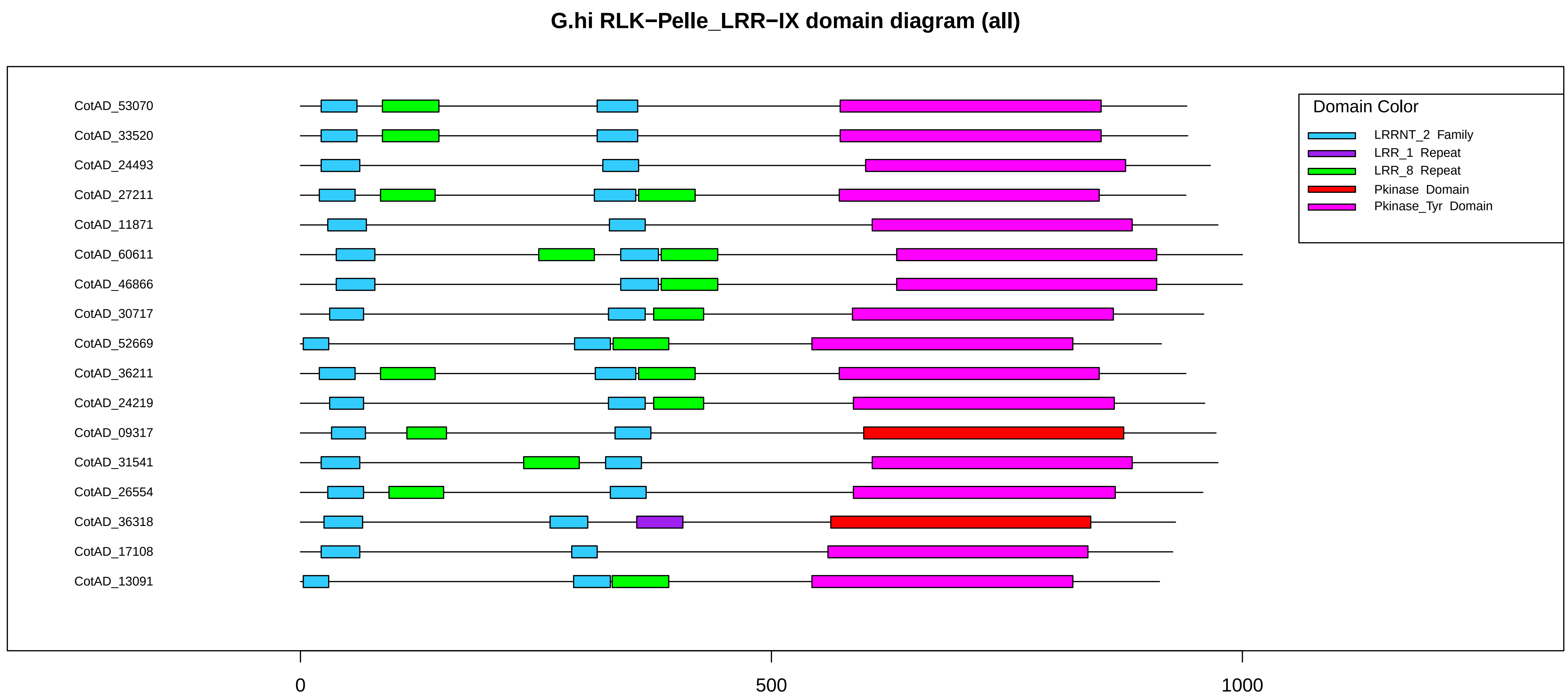

G.hi RLK−Pelle_LRR−IX domain diagram (all)
CotAD_53070
CotAD_33520
CotAD_24493
CotAD_27211
CotAD_11871
CotAD_60611
CotAD_46866
CotAD_30717
CotAD_52669
CotAD_36211
CotAD_24219
CotAD_09317
CotAD_31541
CotAD_26554
CotAD_36318
CotAD_17108
CotAD_13091
Domain Color
LRRNT_2 Family
LRR_1 Repeat
LRR_8 Repeat
Pkinase Domain
Pkinase_Tyr Domain
0
500
1000

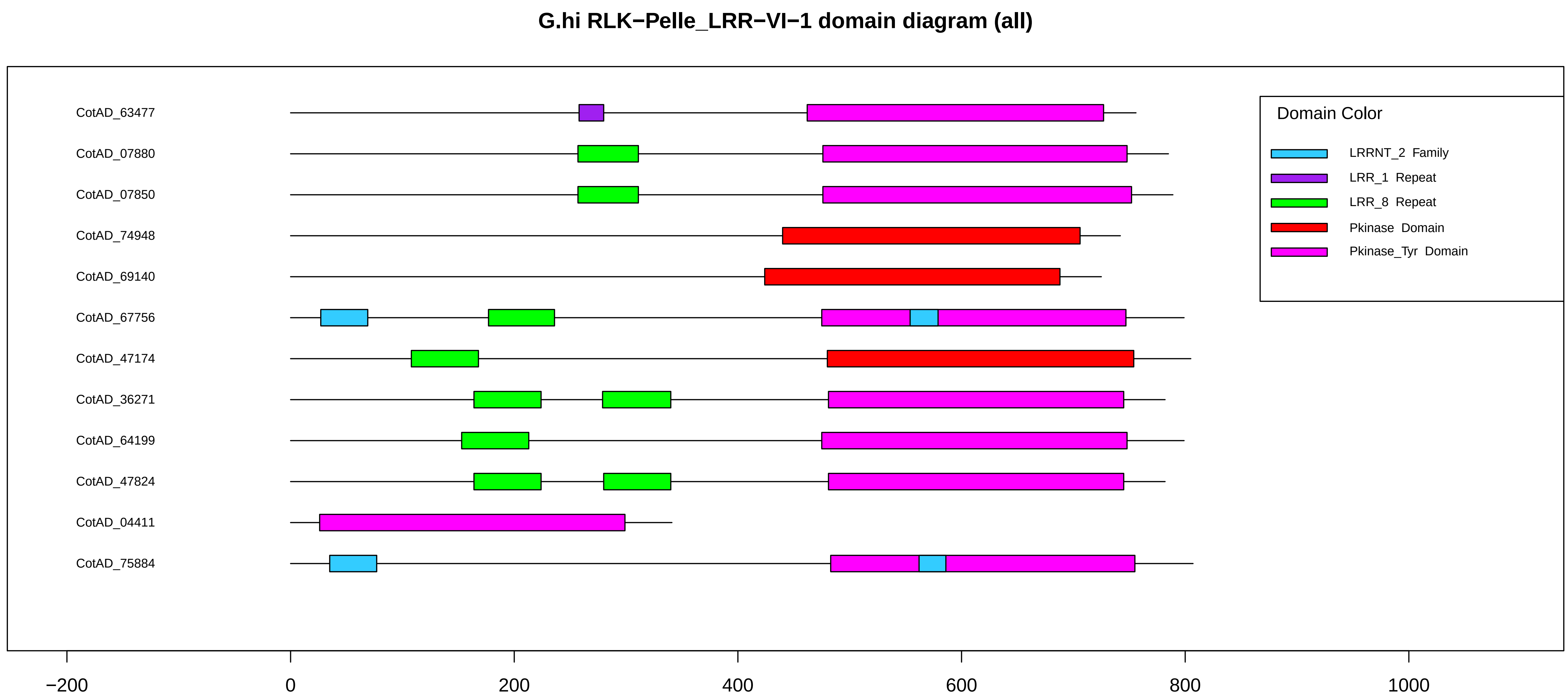
G.hi RLK−Pelle_LRR−VI−1 domain diagram (all)
CotAD_63477
CotAD_07880
CotAD_07850
CotAD_74948
CotAD_69140
CotAD_67756
CotAD_47174
CotAD_36271
CotAD_64199
CotAD_47824
CotAD_04411
CotAD_75884
Domain Color
LRRNT_2 Family
LRR_1 Repeat
LRR_8 Repeat
Pkinase Domain
Pkinase_Tyr Domain
−200
0
200
400
600
800
1000

G.hi RLK−Pelle_LRR−VI−2 domain diagram (all)

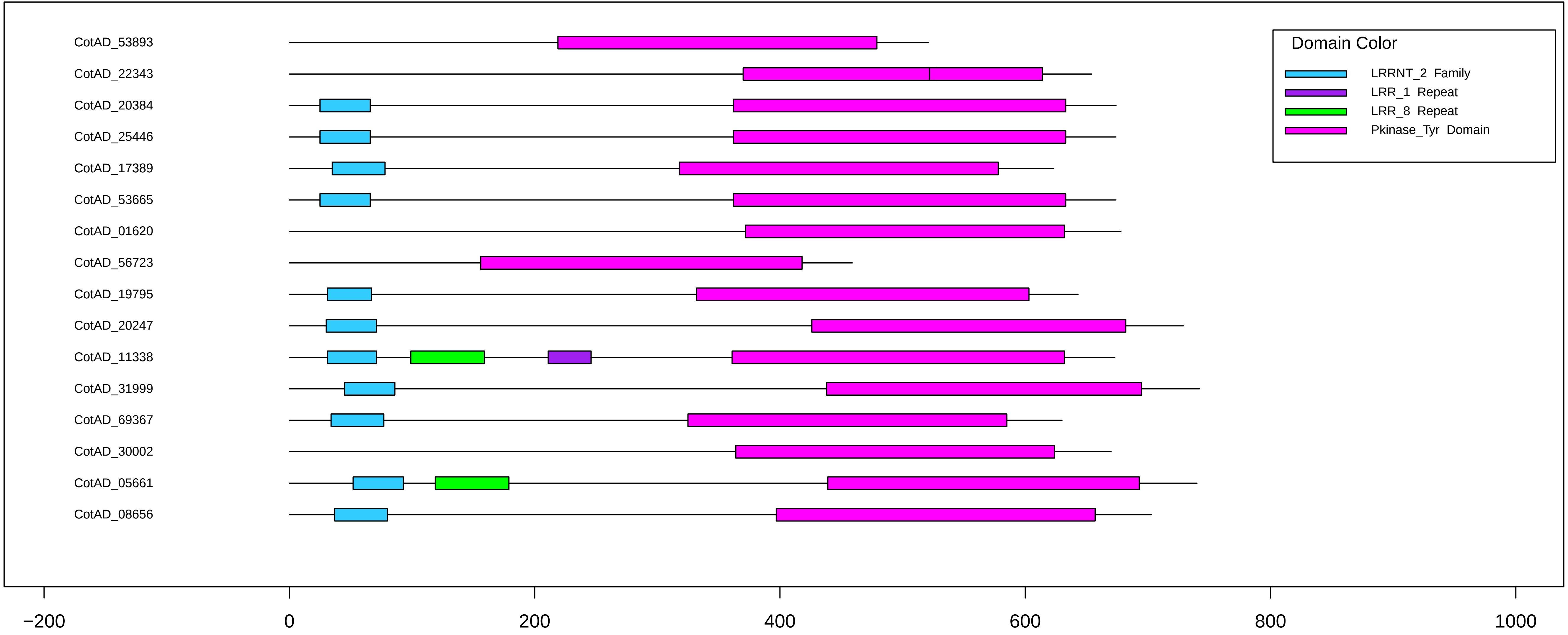

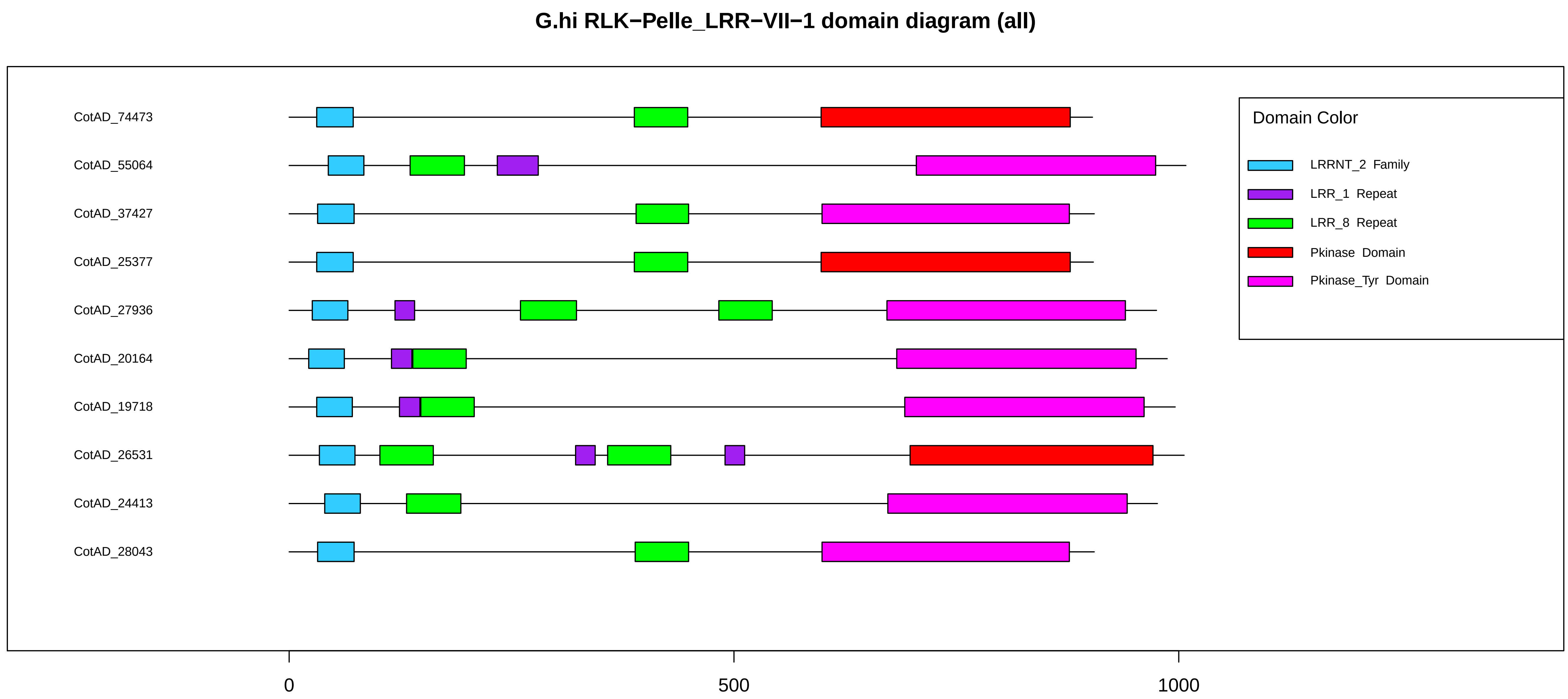
G.hi RLK−Pelle_LRR−VII−1 domain diagram (all)
CotAD_74473
CotAD_55064
CotAD_37427
CotAD_25377
CotAD_27936
CotAD_20164
CotAD_19718
CotAD_26531
CotAD_24413
CotAD_28043
Domain Color
LRRNT_2 Family
LRR_1 Repeat
LRR_8 Repeat
Pkinase Domain
Pkinase_Tyr Domain
0
500
1000

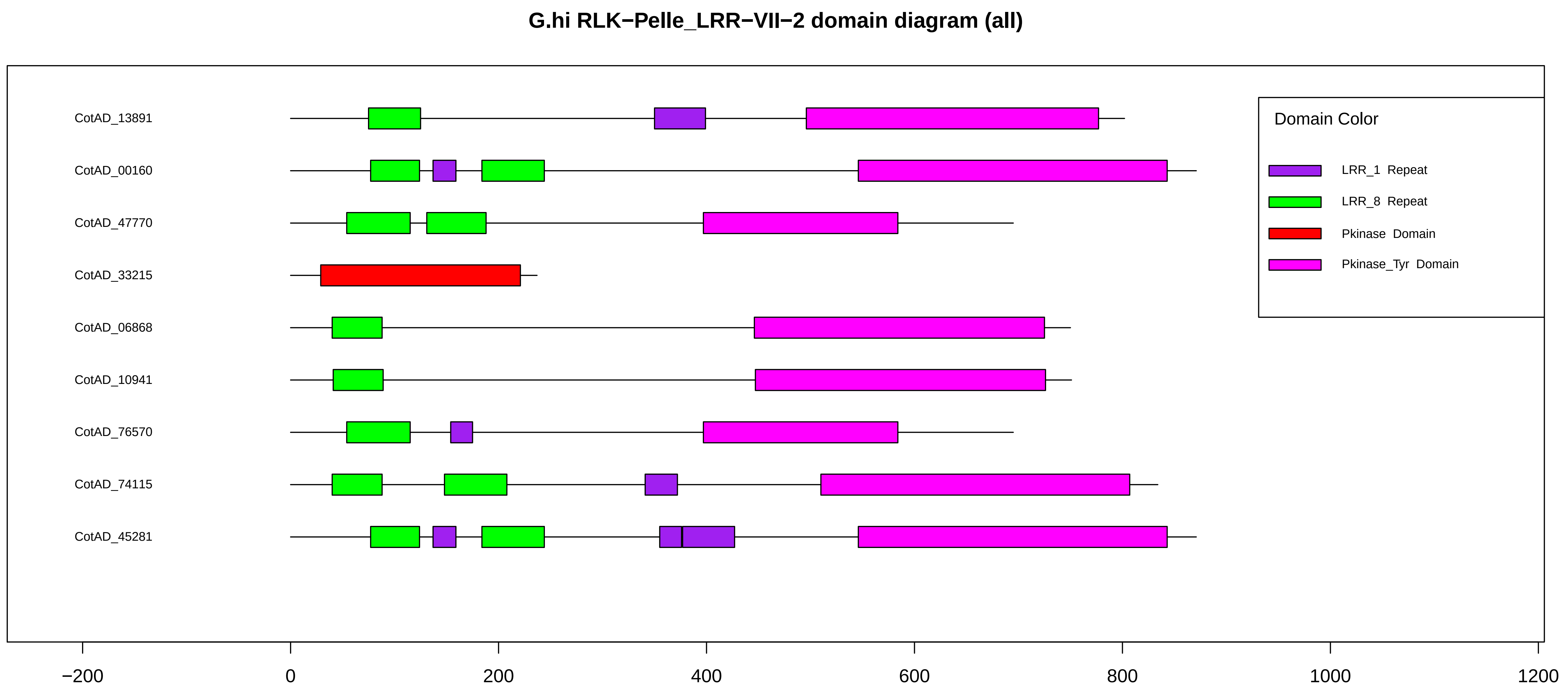
G.hi RLK−Pelle_LRR−VII−2 domain diagram (all)
CotAD_13891
CotAD_00160
CotAD_47770
CotAD_33215
CotAD_06868
CotAD_10941
CotAD_76570
CotAD_74115
CotAD_45281
Domain Color
LRR_1 Repeat
LRR_8 Repeat
Pkinase Domain
Pkinase_Tyr Domain
−200
0
200
400
600
800
1000
1200

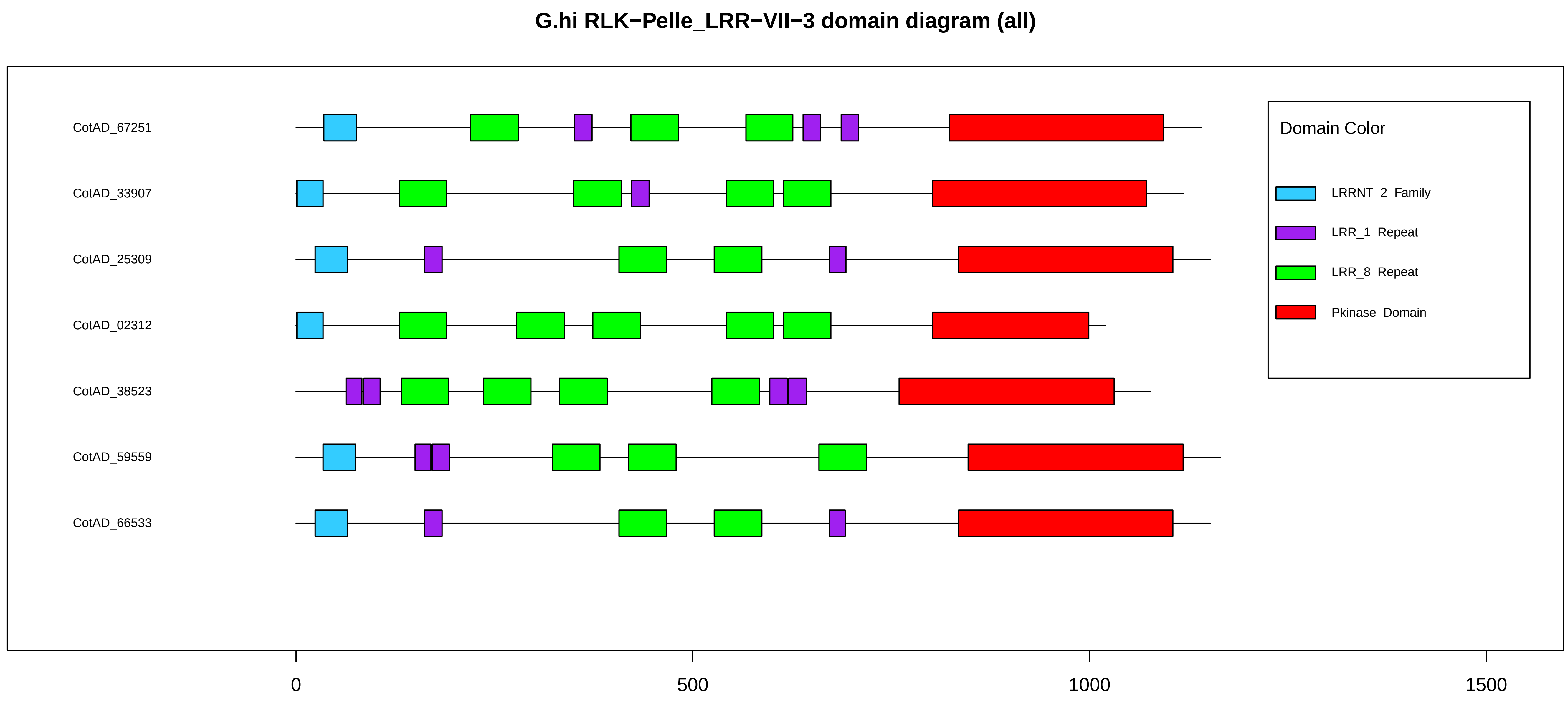

G.hi RLK−Pelle_LRR−VII−3 domain diagram (all)
CotAD_67251
CotAD_33907
CotAD_25309
CotAD_02312
CotAD_38523
CotAD_59559
CotAD_66533
Domain Color
LRRNT_2 Family
LRR_1 Repeat
LRR_8 Repeat
Pkinase Domain
0
500
1000
1500

G.hi RLK−Pelle_LRR−VIII−1 domain diagram (all)

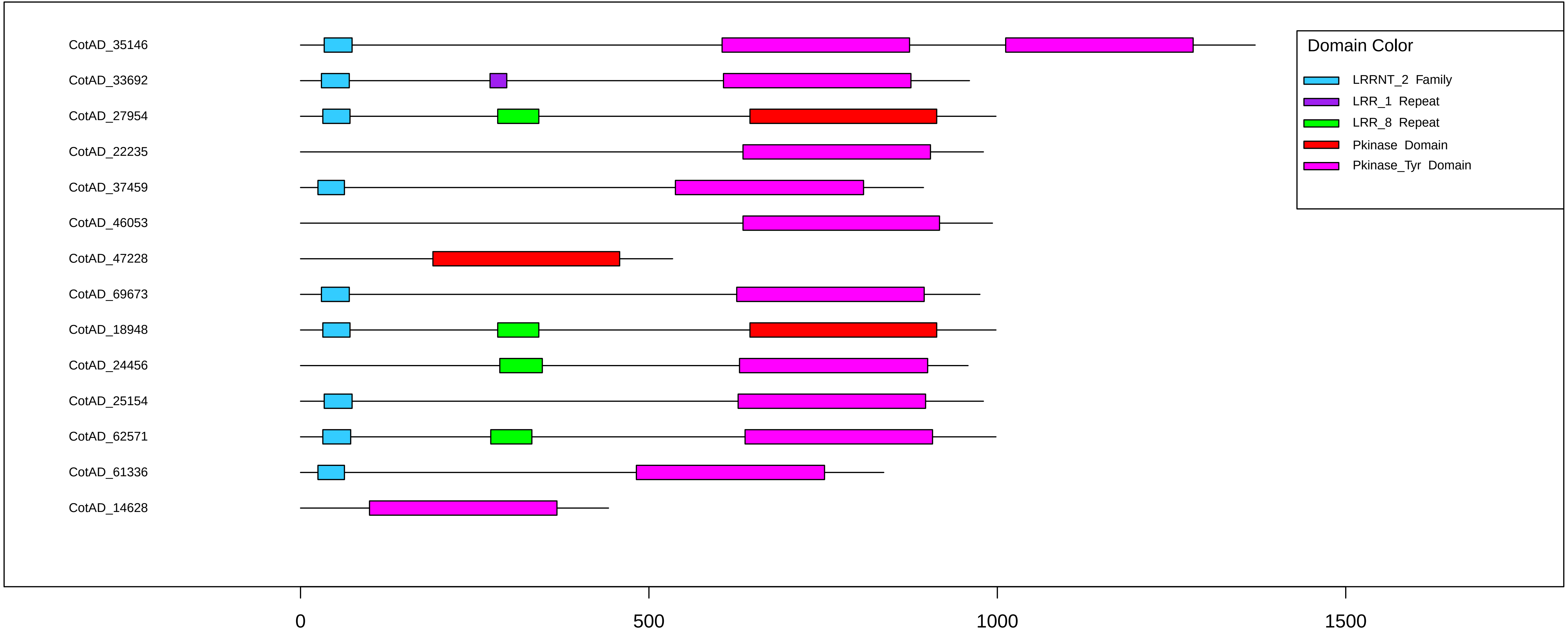

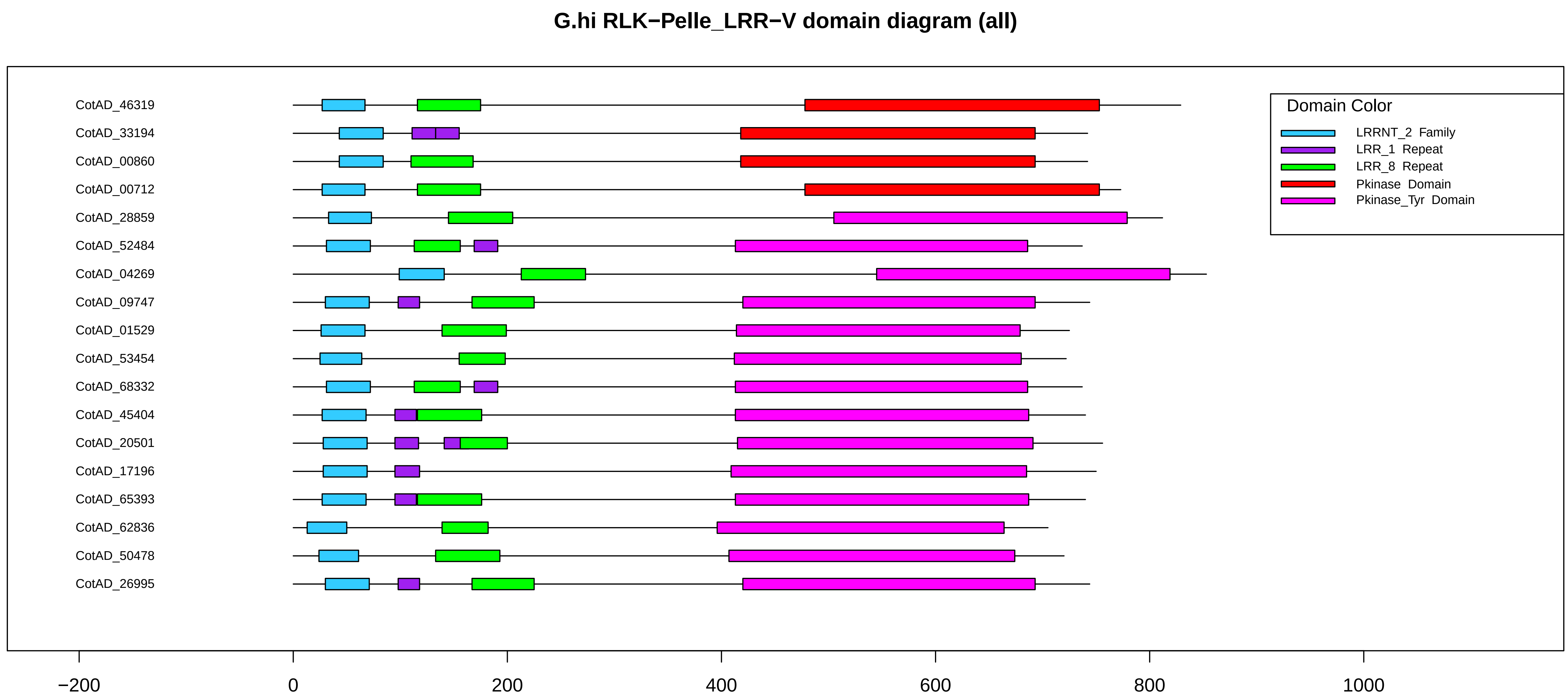
G.hi RLK−Pelle_LRR−V domain diagram (all)
CotAD_46319
CotAD_33194
CotAD_00860
CotAD_00712
CotAD_28859
CotAD_52484
CotAD_04269
CotAD_09747
CotAD_01529
CotAD_53454
CotAD_68332
CotAD_45404
CotAD_20501
CotAD_17196
CotAD_65393
CotAD_62836
CotAD_50478
CotAD_26995
Domain Color
LRRNT_2 Family
LRR_1 Repeat
LRR_8 Repeat
Pkinase Domain
Pkinase_Tyr Domain
−200
0
200
400
600
800
1000

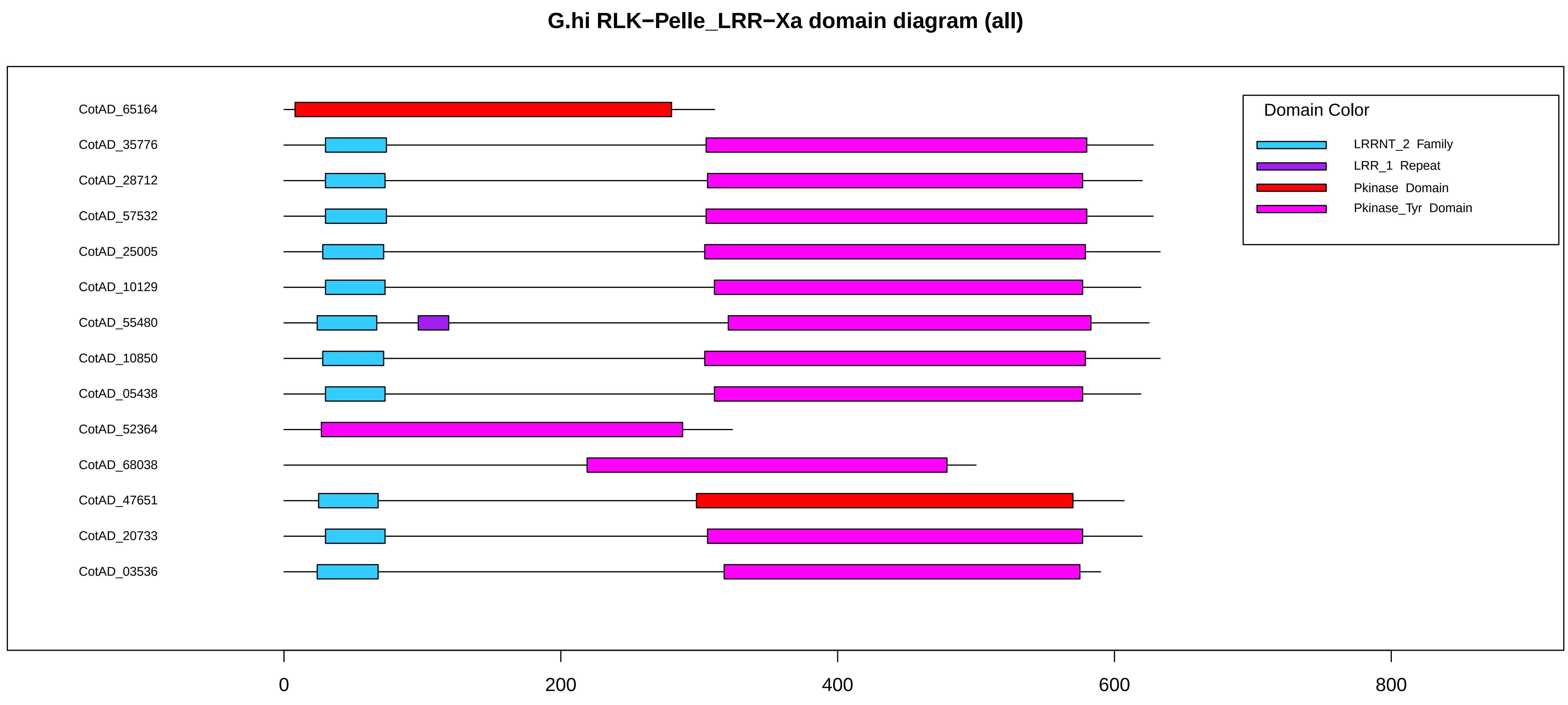
G.hi RLK−Pelle_LRR−Xa domain diagram (all)
CotAD_65164
CotAD_35776
CotAD_28712
CotAD_57532
CotAD_25005
CotAD_10129
CotAD_55480
CotAD_10850
CotAD_05438
CotAD_52364
CotAD_68038
CotAD_47651
CotAD_20733
CotAD_03536
Domain Color
LRRNT_2 Family
LRR_1 Repeat
Pkinase Domain
Pkinase_Tyr Domain
0
200
400
600
800

G.hi RLK−Pelle_LRR−Xb−1 domain diagram (all)

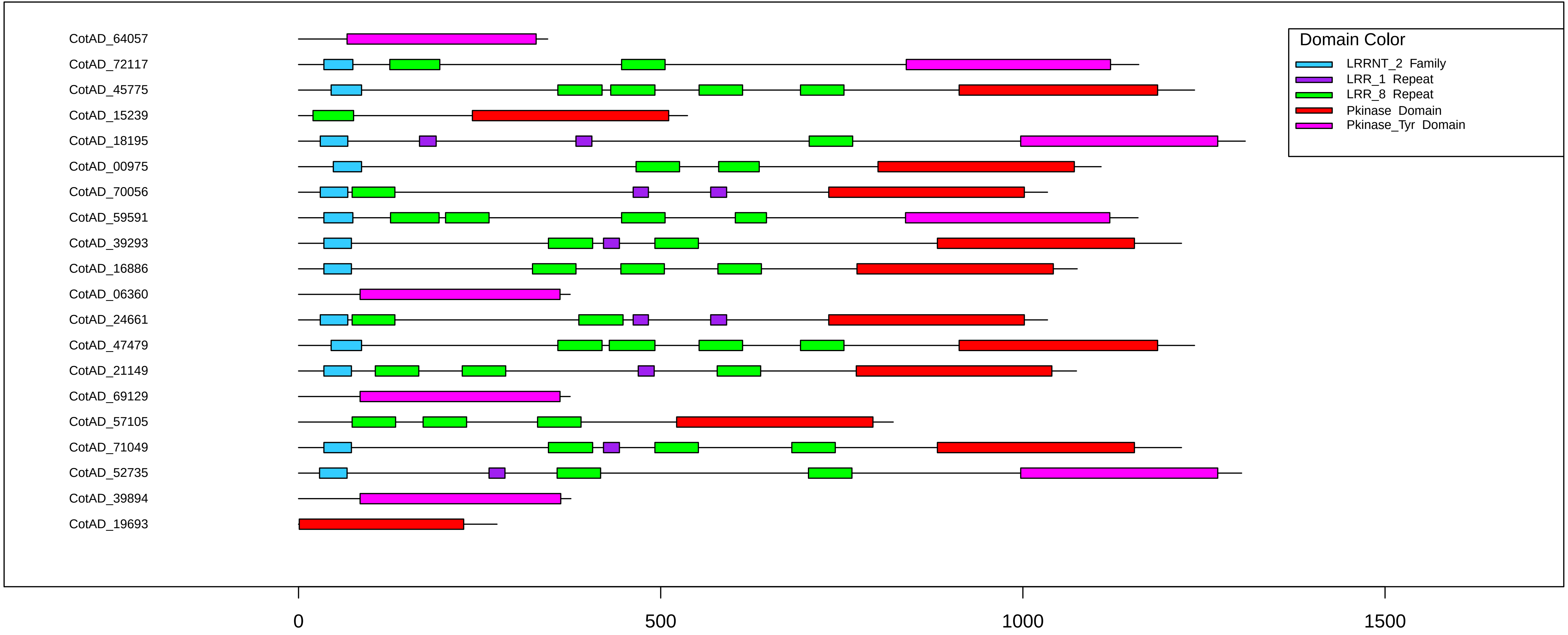

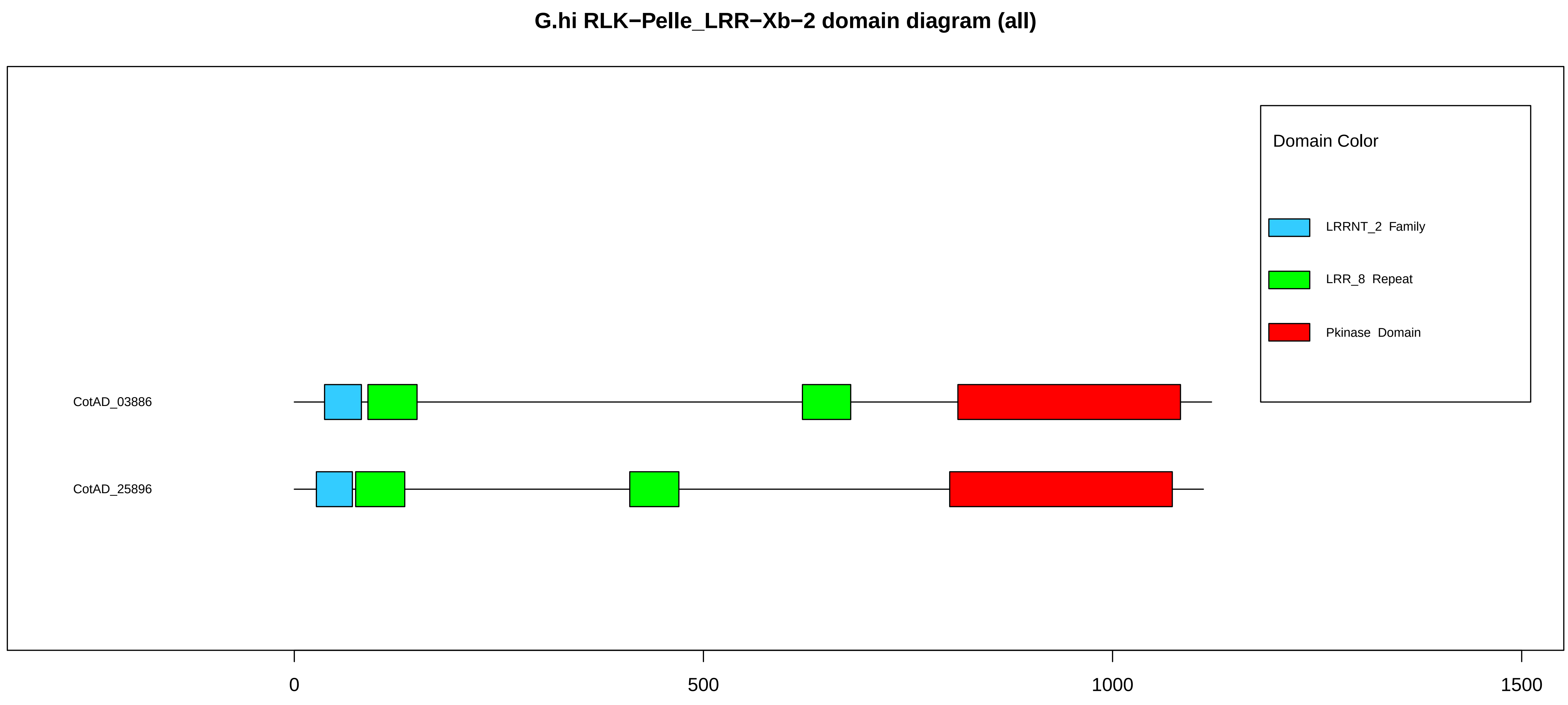
G.hi RLK−Pelle_LRR−Xb−2 domain diagram (all)
Domain Color
LRRNT_2 Family
LRR_8 Repeat
Pkinase Domain
CotAD_03886
CotAD_25896
0
500
1000
1500

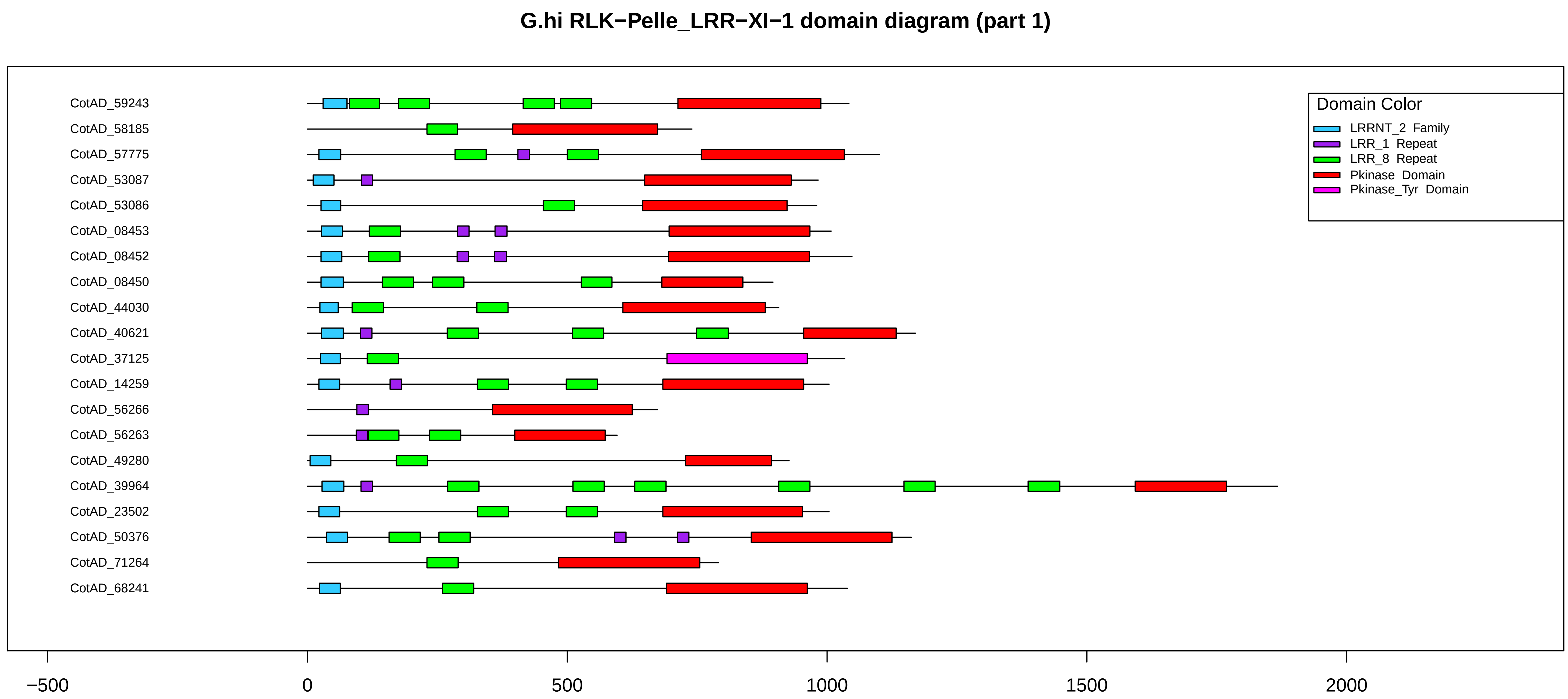
G.hi RLK−Pelle_LRR−XI−1 domain diagram (part 1)
CotAD_59243
CotAD_58185
CotAD_57775
CotAD_53087
CotAD_53086
CotAD_08453
CotAD_08452
CotAD_08450
CotAD_44030
CotAD_40621
CotAD_37125
CotAD_14259
CotAD_56266
CotAD_56263
CotAD_49280
CotAD_39964
CotAD_23502
CotAD_50376
CotAD_71264
CotAD_68241
Domain Color
LRRNT_2 Family
LRR_1 Repeat
LRR_8 Repeat
Pkinase Domain
Pkinase_Tyr Domain
−500
0
500
1000
1500
2000

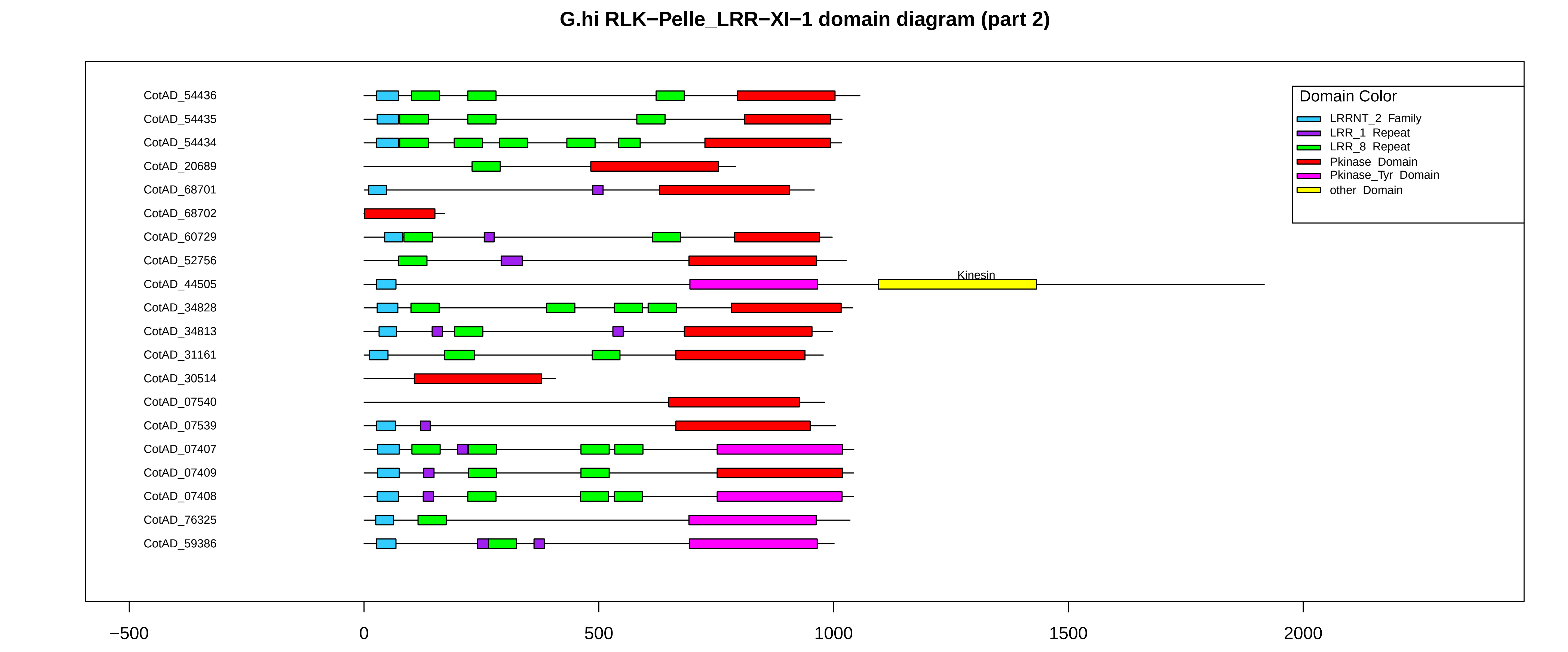
G.hi RLK−Pelle_LRR−XI−1 domain diagram (part 2)
CotAD_54436
CotAD_54435
CotAD_54434
CotAD_20689
CotAD_68701
CotAD_68702
CotAD_60729
CotAD_52756
CotAD_44505
CotAD_34828
CotAD_34813
CotAD_31161
CotAD_30514
CotAD_07540
CotAD_07539
CotAD_07407
CotAD_07409
CotAD_07408
CotAD_76325
CotAD_59386
Kinesin
Domain Color
LRRNT_2 Family
LRR_1 Repeat
LRR_8 Repeat
Pkinase Domain
Pkinase_Tyr Domain
other Domain
−500
0
500
1000
1500
2000

G.hi RLK−Pelle_LRR−XI−1 domain diagram (part 3)

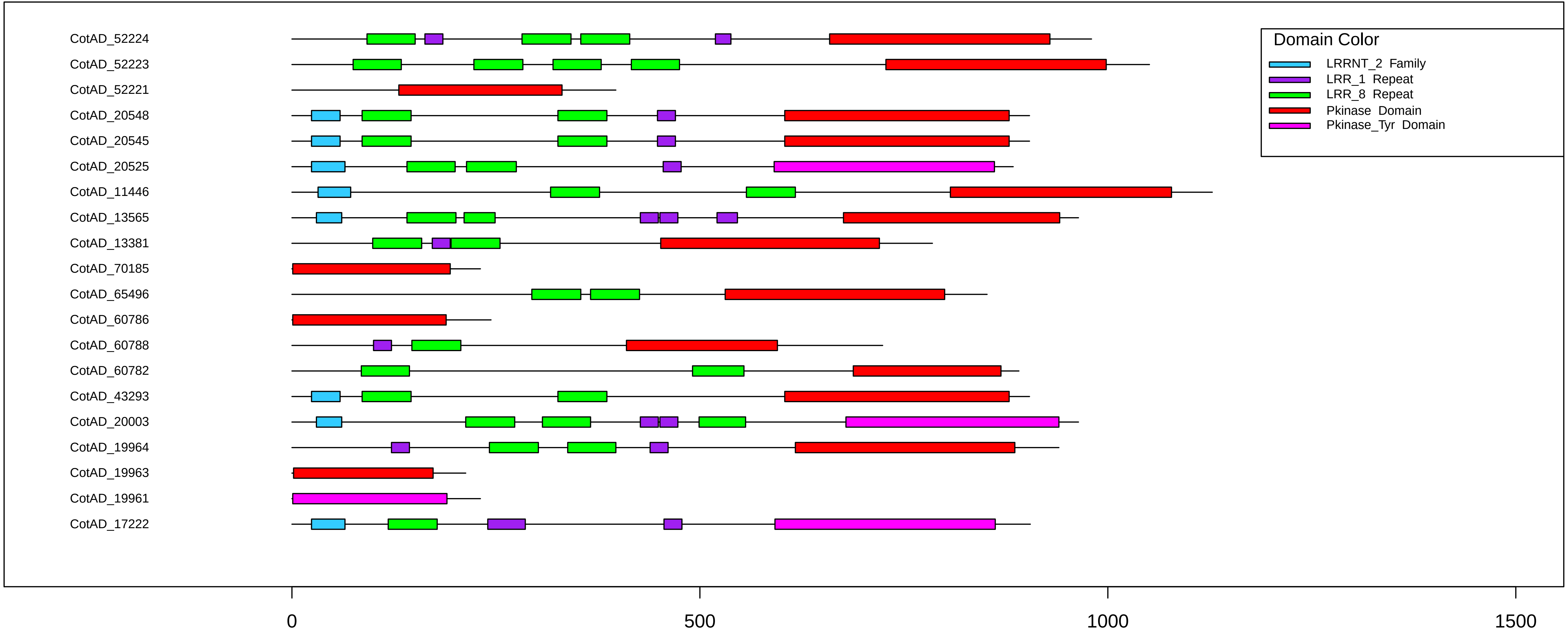

G.hi RLK−Pelle_LRR−XI−1 domain diagram (part 4)

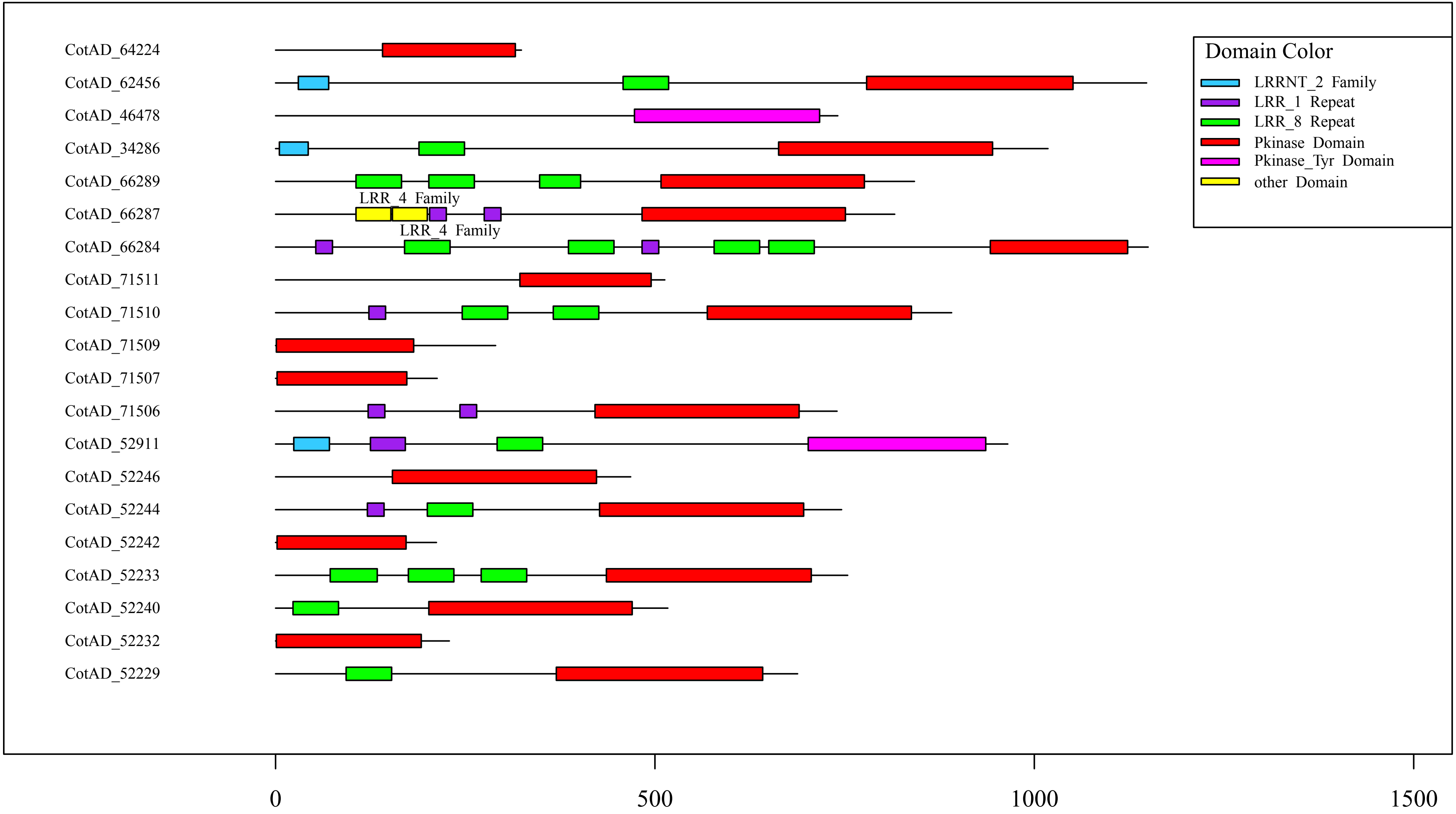

G.hi RLK−Pelle_LRR−XI−1 domain diagram (part 5)

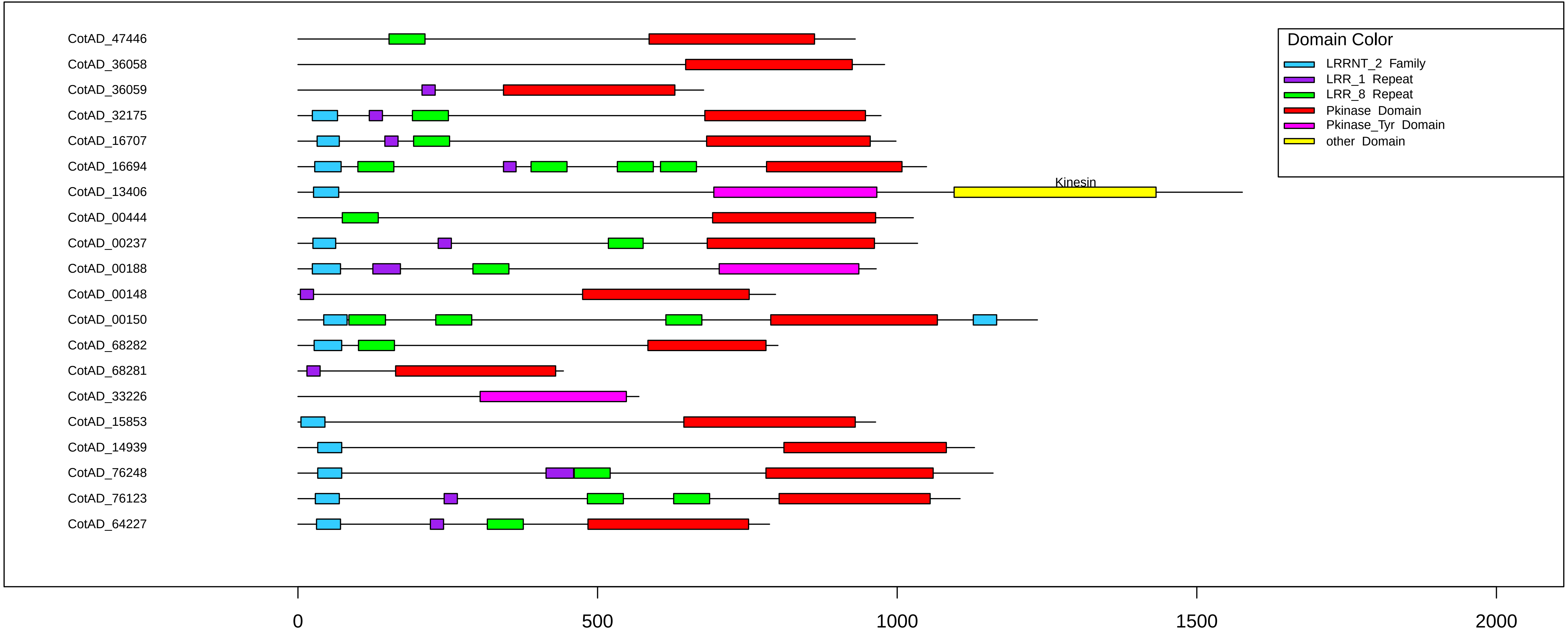

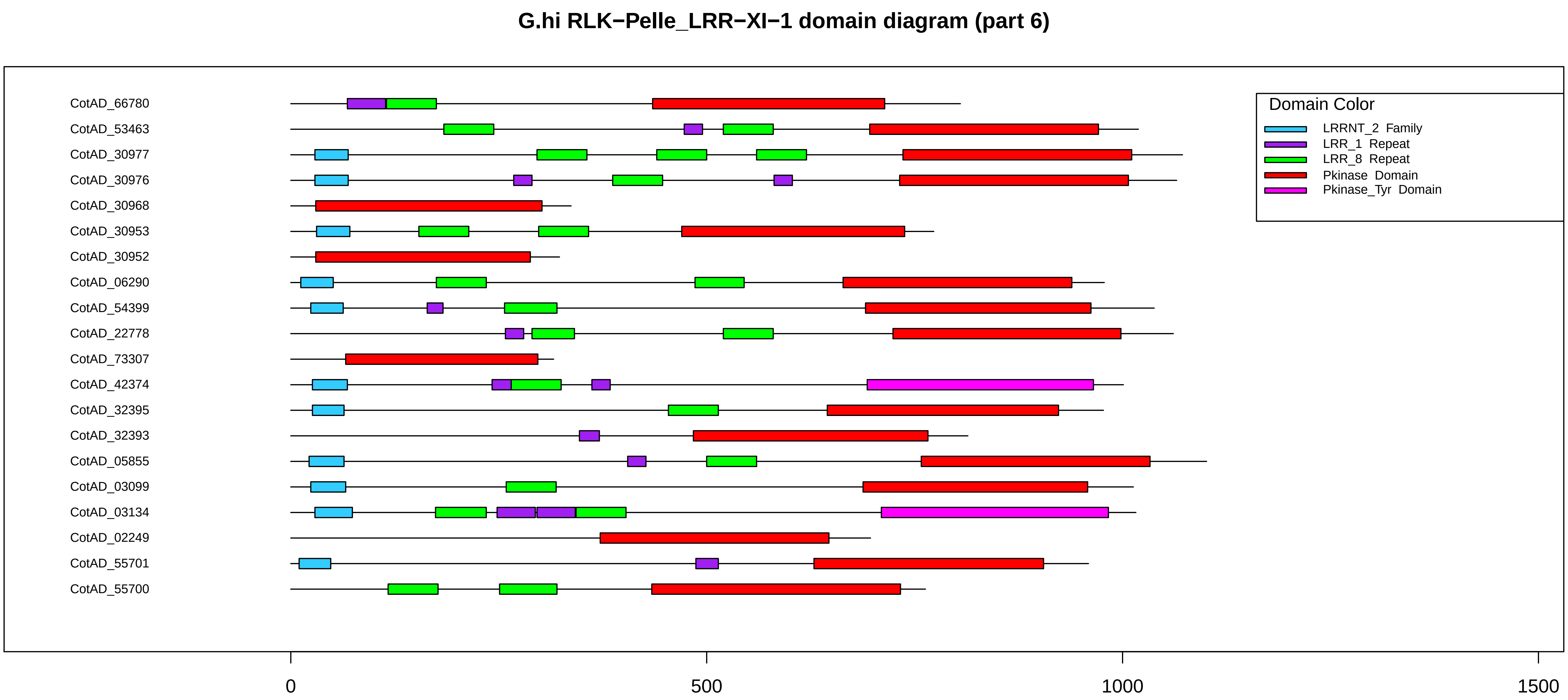

G.hi RLK−Pelle_LRR−XI−1 domain diagram (part 6)
CotAD_66780
CotAD_53463
CotAD_30977
CotAD_30976
CotAD_30968
CotAD_30953
CotAD_30952
CotAD_06290
CotAD_54399
CotAD_22778
CotAD_73307
CotAD_42374
CotAD_32395
CotAD_32393
CotAD_05855
CotAD_03099
CotAD_03134
CotAD_02249
CotAD_55701
CotAD_55700
Domain Color
LRRNT_2 Family
LRR_1 Repeat
LRR_8 Repeat
Pkinase Domain
Pkinase_Tyr Domain
0
500
1000
1500

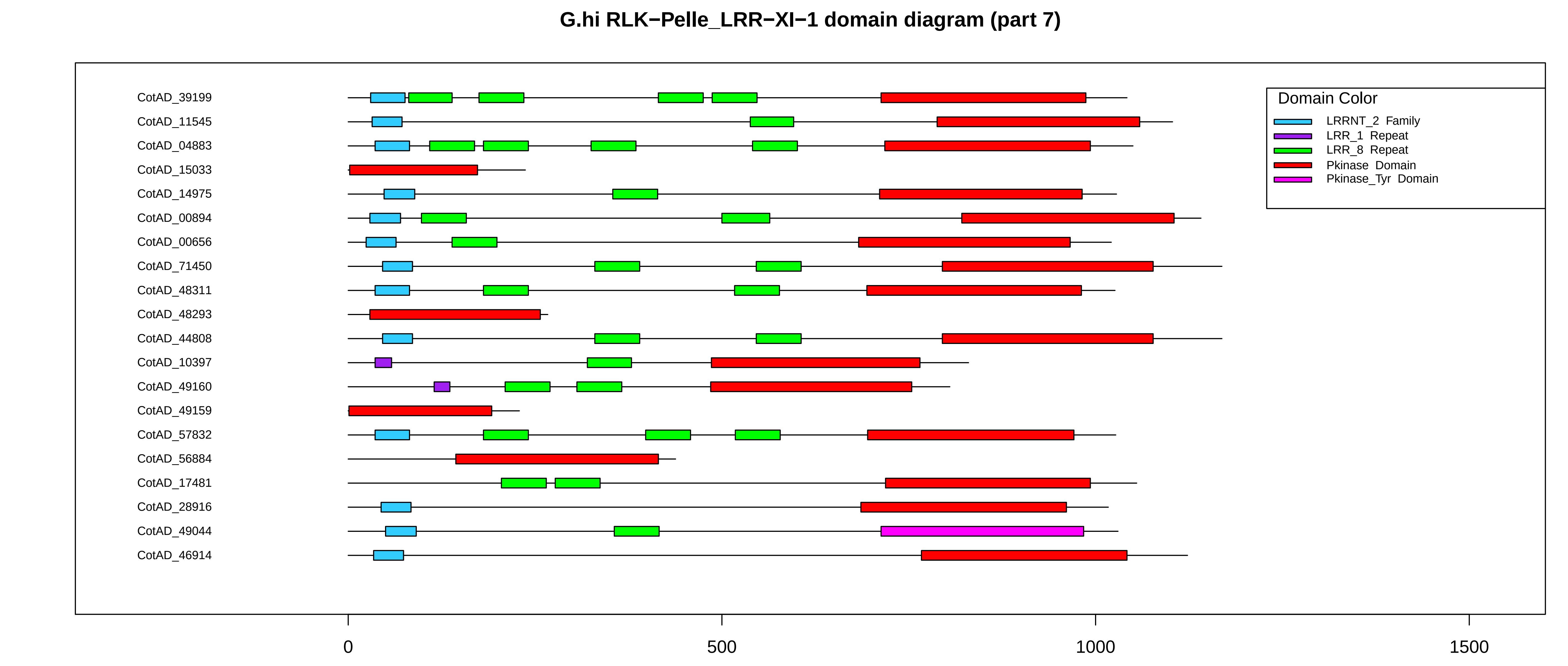
G.hi RLK−Pelle_LRR−XI−1 domain diagram (part 7)
CotAD_39199
CotAD_11545
CotAD_04883
CotAD_15033
CotAD_14975
CotAD_00894
CotAD_00656
CotAD_71450
CotAD_48311
CotAD_48293
CotAD_44808
CotAD_10397
CotAD_49160
CotAD_49159
CotAD_57832
CotAD_56884
CotAD_17481
CotAD_28916
CotAD_49044
CotAD_46914
Domain Color
LRRNT_2 Family
LRR_1 Repeat
LRR_8 Repeat
Pkinase Domain
Pkinase_Tyr Domain
0
500
1000
1500

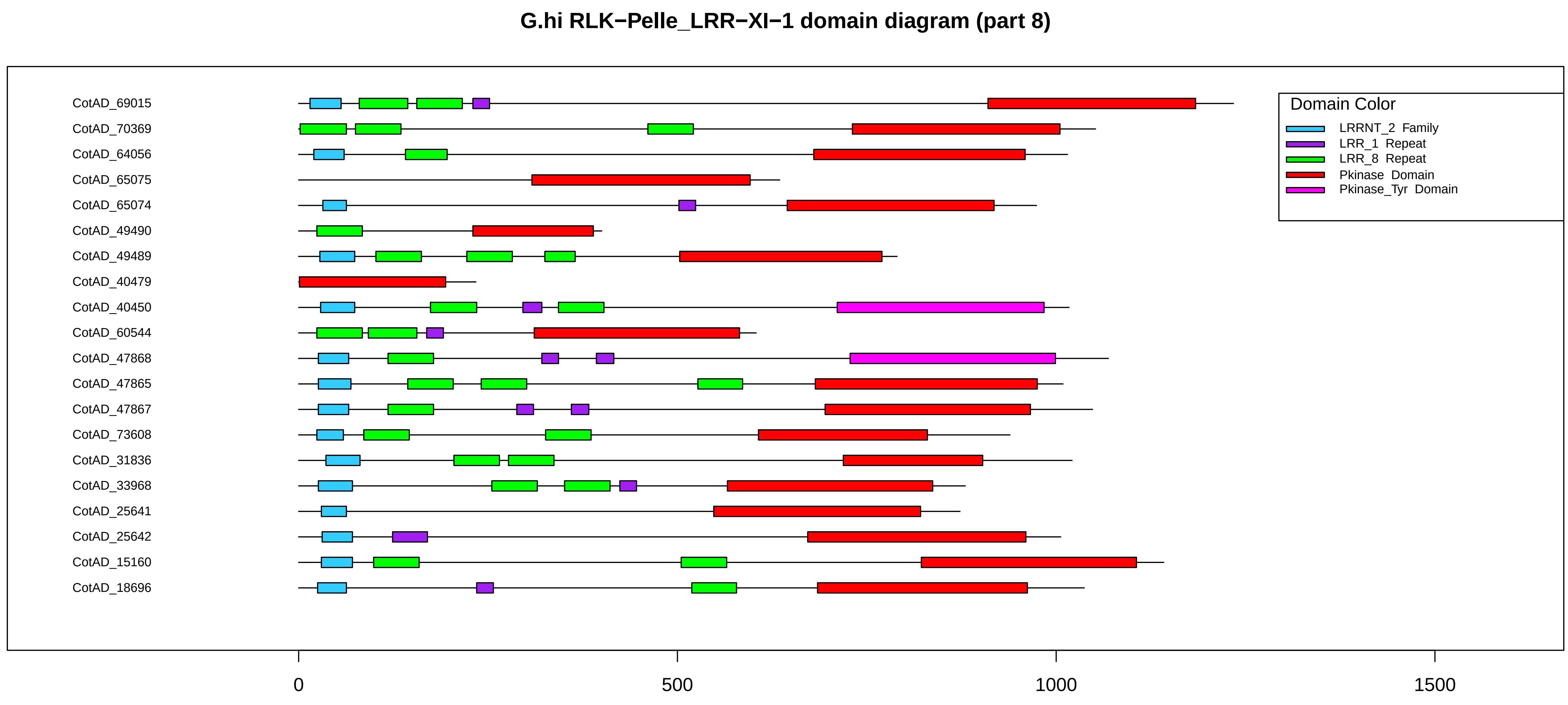
G.hi RLK−Pelle_LRR−XI−1 domain diagram (part 8)
CotAD_69015
CotAD_70369
CotAD_64056
CotAD_65075
CotAD_65074
CotAD_49490
CotAD_49489
CotAD_40479
CotAD_40450
CotAD_60544
CotAD_47868
CotAD_47865
CotAD_47867
CotAD_73608
CotAD_31836
CotAD_33968
CotAD_25641
CotAD_25642
CotAD_15160
CotAD_18696
Domain Color
LRRNT_2 Family
LRR_1 Repeat
LRR_8 Repeat
Pkinase Domain
Pkinase_Tyr Domain
0
500
1000
1500

G.hi RLK−Pelle_LRR−XI−1 domain diagram (part 9)

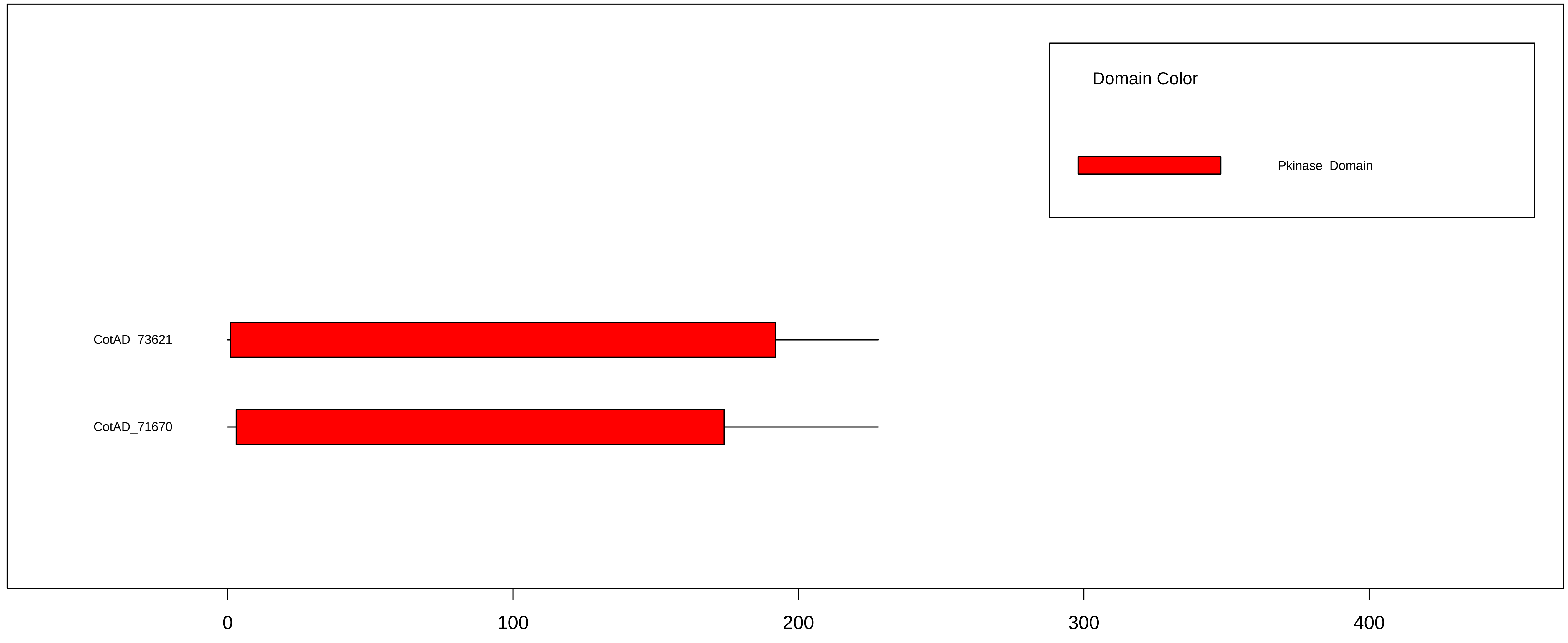

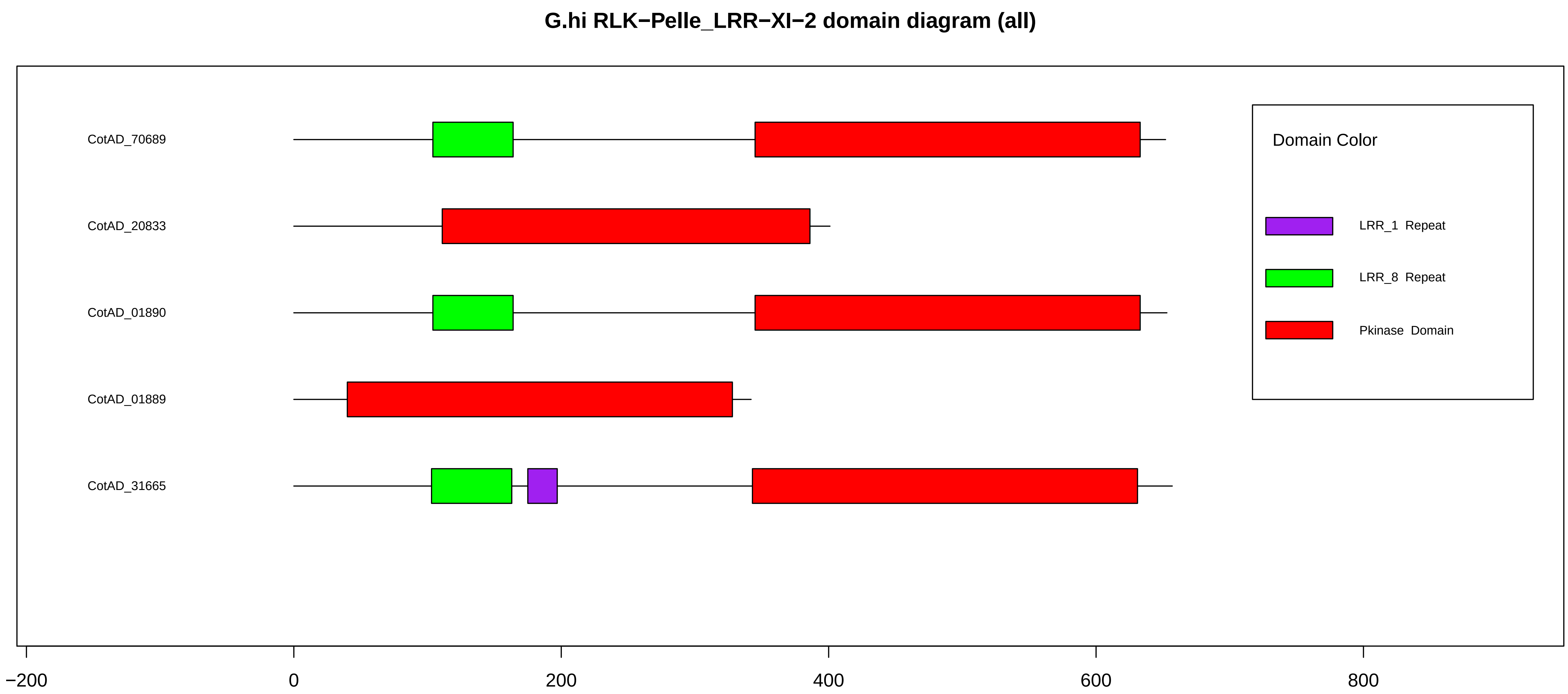

G.hi RLK−Pelle_LRR−XI−2 domain diagram (all)
CotAD_70689
CotAD_20833
CotAD_01890
CotAD_01889
CotAD_31665
Domain Color
LRR_1 Repeat
LRR_8 Repeat
Pkinase Domain
−200
0
200
400
600
800

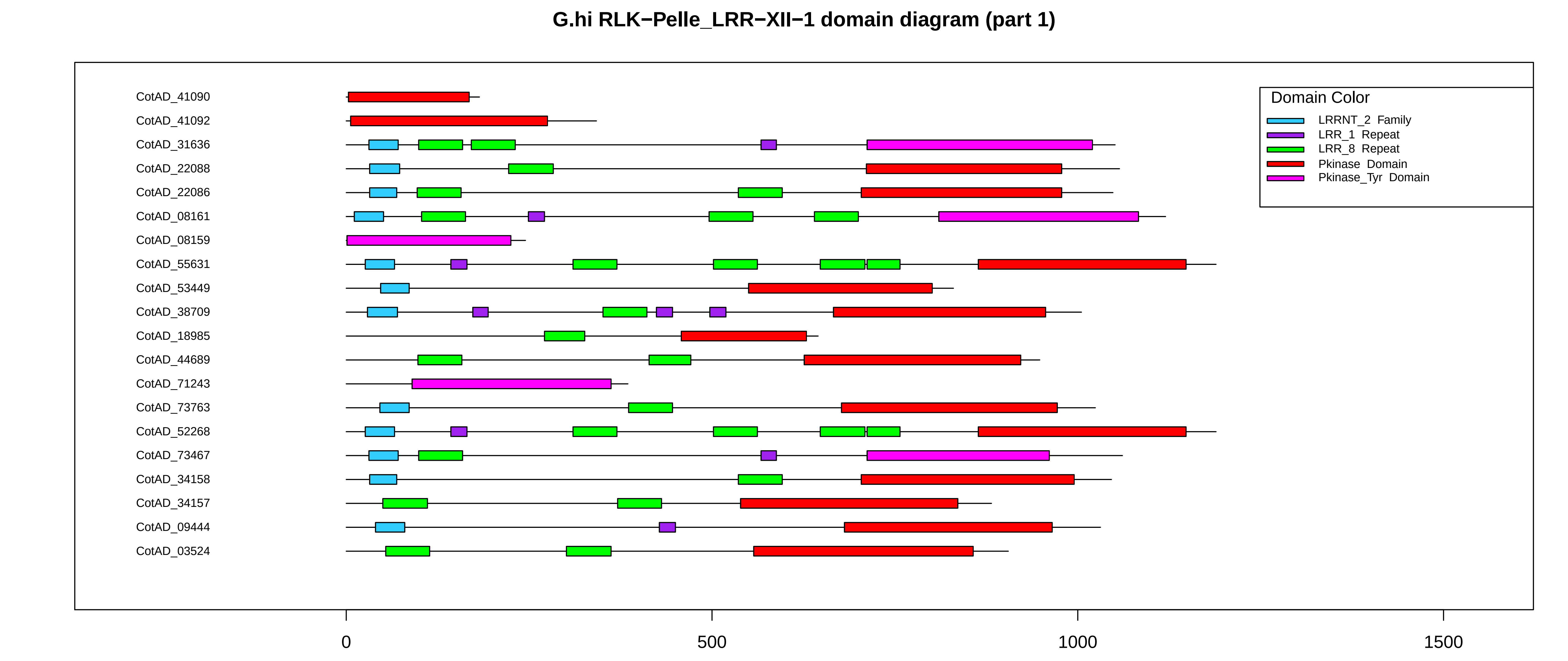

G.hi RLK−Pelle_LRR−XII−1 domain diagram (part 1)
CotAD_41090
CotAD_41092
CotAD_31636
CotAD_22088
CotAD_22086
CotAD_08161
CotAD_08159
CotAD_55631
CotAD_53449
CotAD_38709
CotAD_18985
CotAD_44689
CotAD_71243
CotAD_73763
CotAD_52268
CotAD_73467
CotAD_34158
CotAD_34157
CotAD_09444
CotAD_03524
Domain Color
LRRNT_2 Family
LRR_1 Repeat
LRR_8 Repeat
Pkinase Domain
Pkinase_Tyr Domain
0
500
1000
1500

G.hi RLK−Pelle_LRR−XII−1 domain diagram (part 2)

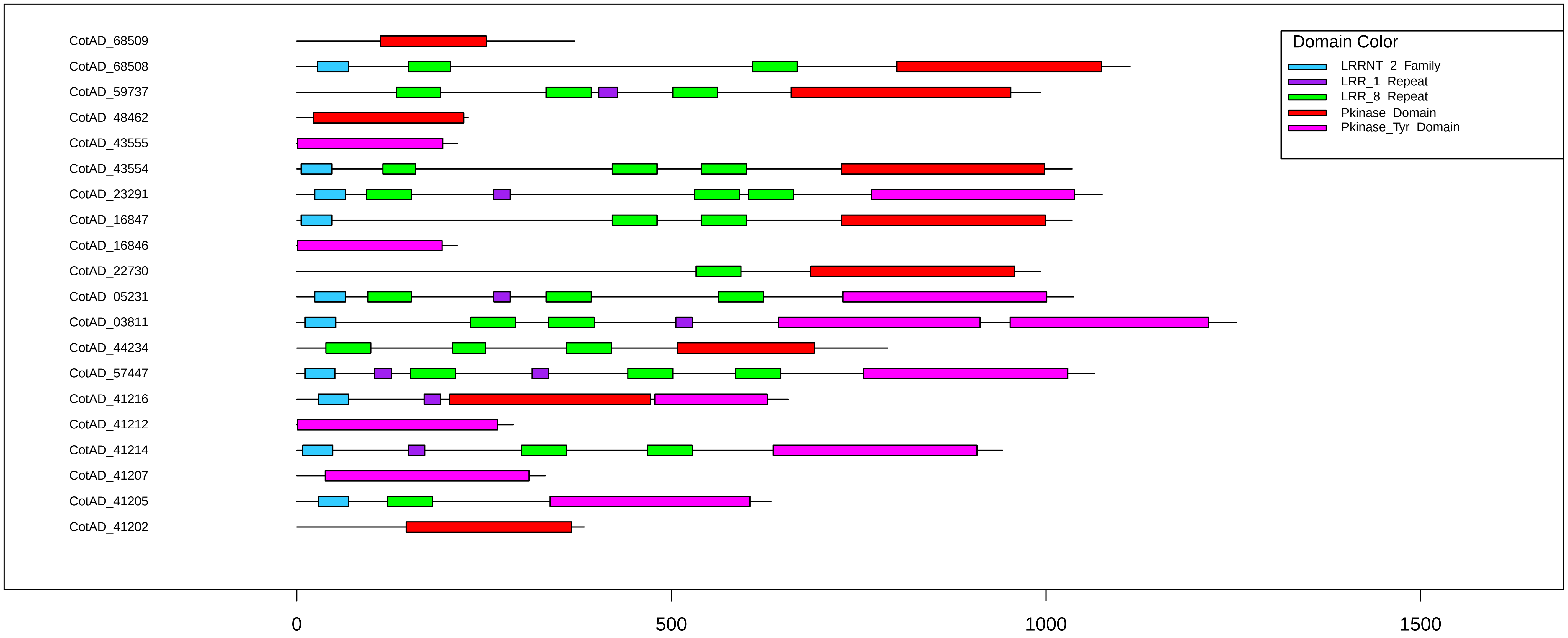

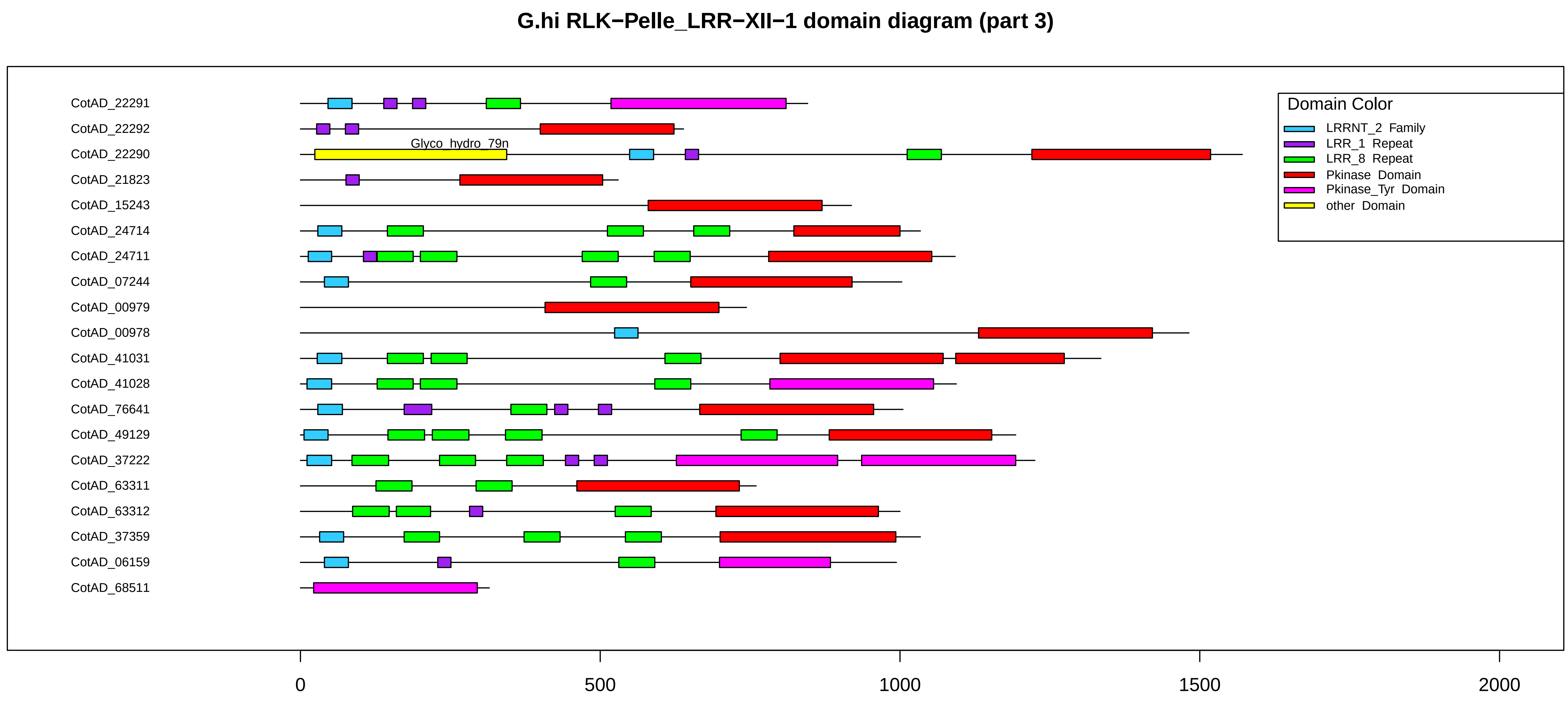
G.hi RLK−Pelle_LRR−XII−1 domain diagram (part 3)
CotAD_22291
CotAD_22292
CotAD_22290
CotAD_21823
CotAD_15243
CotAD_24714
CotAD_24711
CotAD_07244
CotAD_00979
CotAD_00978
CotAD_41031
CotAD_41028
CotAD_76641
CotAD_49129
CotAD_37222
CotAD_63311
CotAD_63312
CotAD_37359
CotAD_06159
CotAD_68511
Glyco_hydro_79n
Domain Color
LRRNT_2 Family
LRR_1 Repeat
LRR_8 Repeat
Pkinase Domain
Pkinase_Tyr Domain
other Domain
0
500
1000
1500
2000

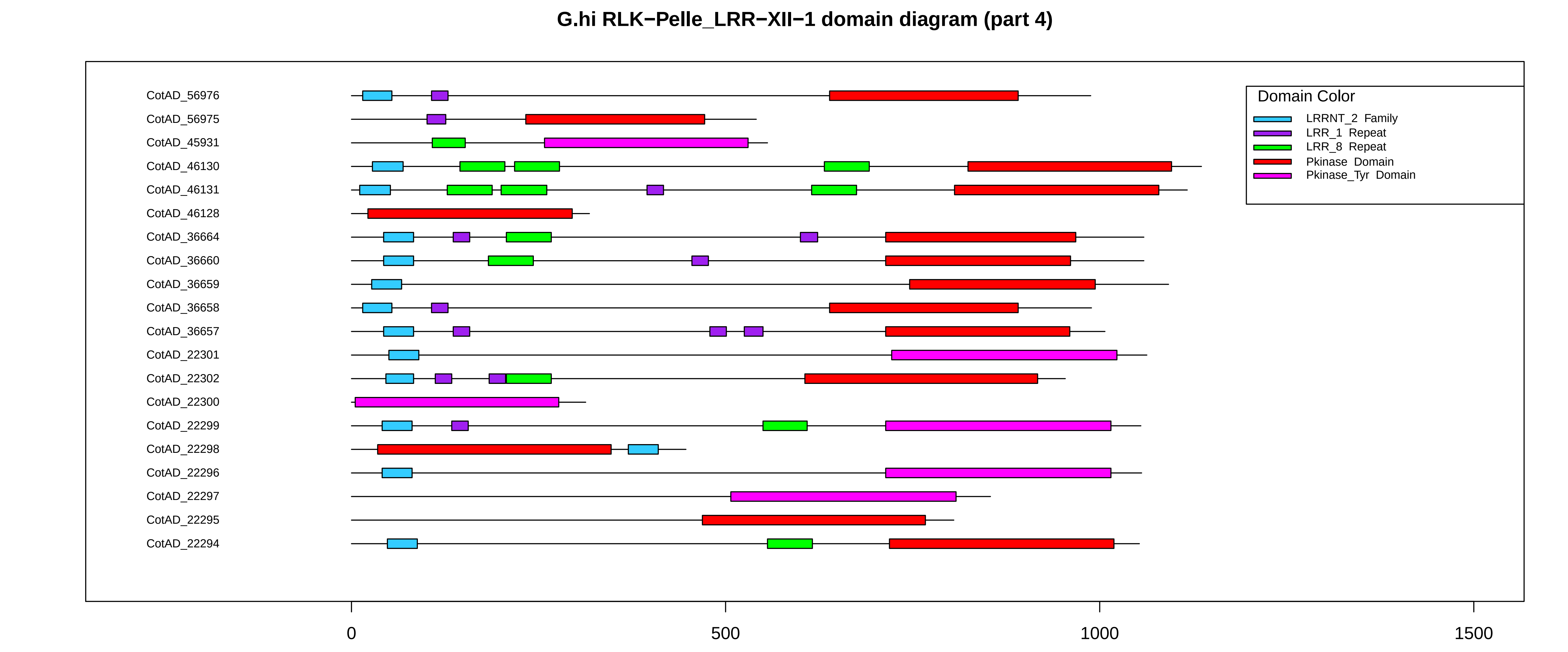
G.hi RLK−Pelle_LRR−XII−1 domain diagram (part 4)
CotAD_56976
CotAD_56975
CotAD_45931
CotAD_46130
CotAD_46131
CotAD_46128
CotAD_36664
CotAD_36660
CotAD_36659
CotAD_36658
CotAD_36657
CotAD_22301
CotAD_22302
CotAD_22300
CotAD_22299
CotAD_22298
CotAD_22296
CotAD_22297
CotAD_22295
CotAD_22294
Domain Color
LRRNT_2 Family
LRR_1 Repeat
LRR_8 Repeat
Pkinase Domain
Pkinase_Tyr Domain
0
500
1000
1500

G.hi RLK−Pelle_LRR−XII−1 domain diagram (part 5)

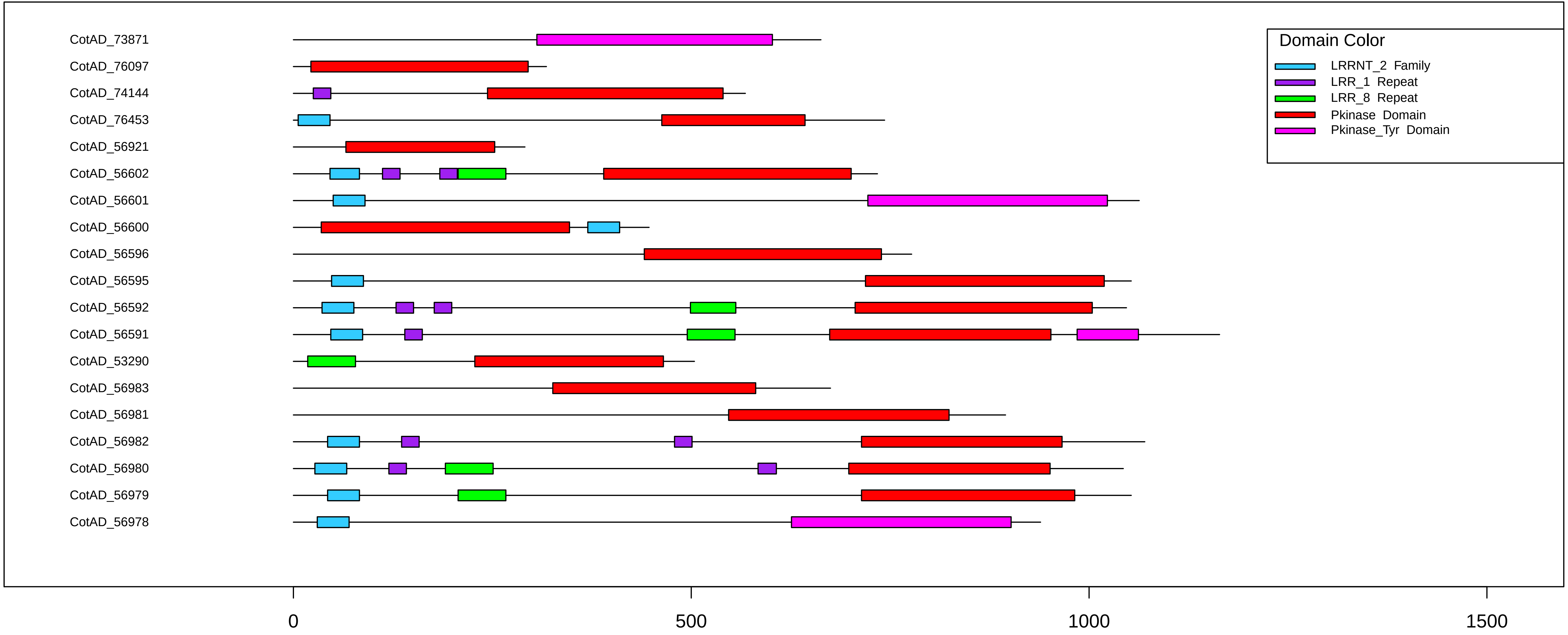

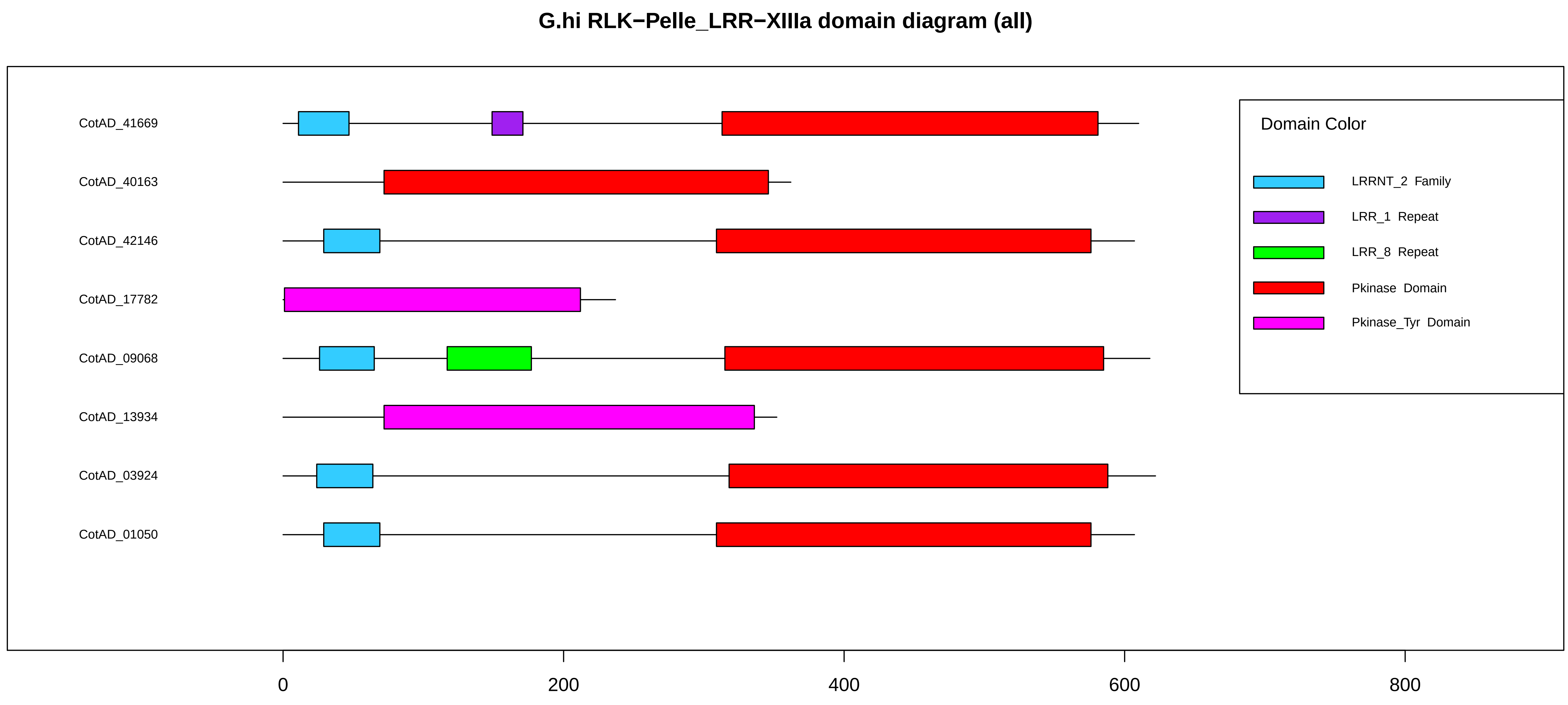
G.hi RLK−Pelle_LRR−XIIIa domain diagram (all)
CotAD_41669
CotAD_40163
CotAD_42146
CotAD_17782
CotAD_09068
CotAD_13934
CotAD_03924
CotAD_01050
Domain Color
LRRNT_2 Family
LRR_1 Repeat
LRR_8 Repeat
Pkinase Domain
Pkinase_Tyr Domain
0
200
400
600
800

G.hi RLK−Pelle_LRR−XIIIb domain diagram (all)

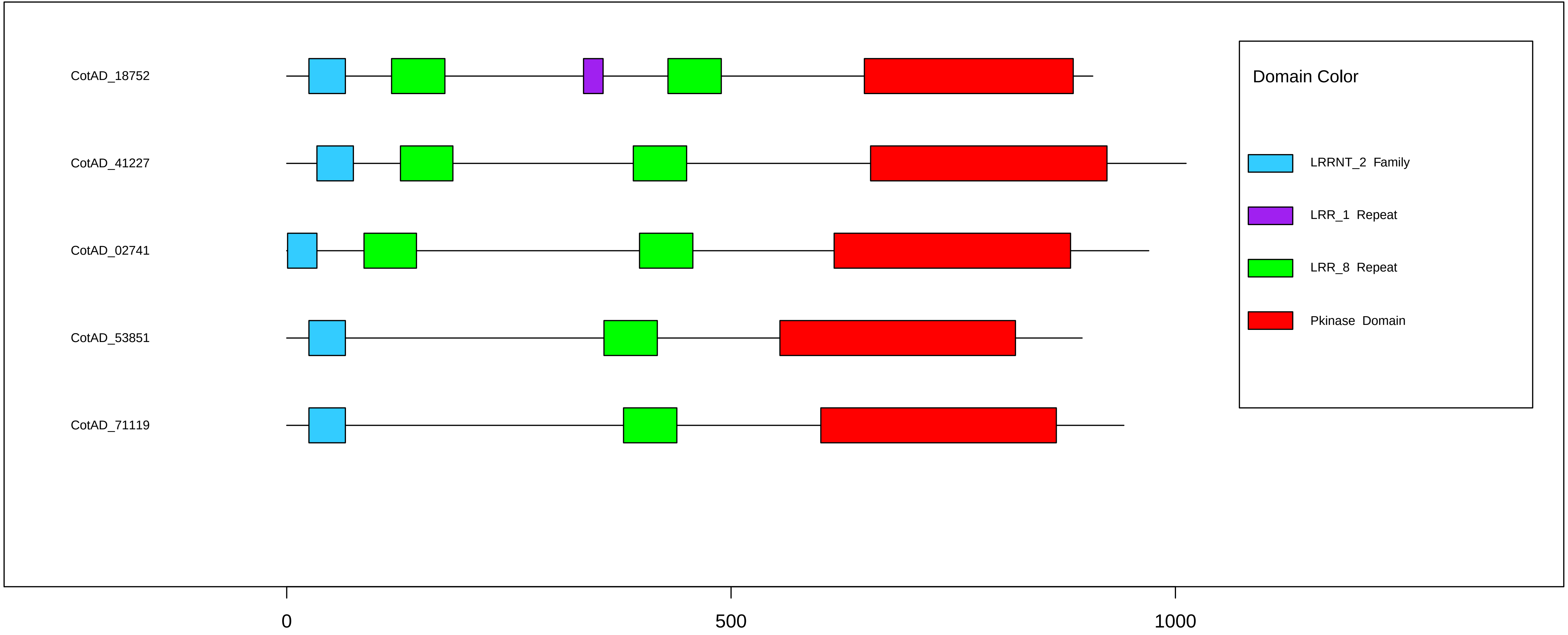

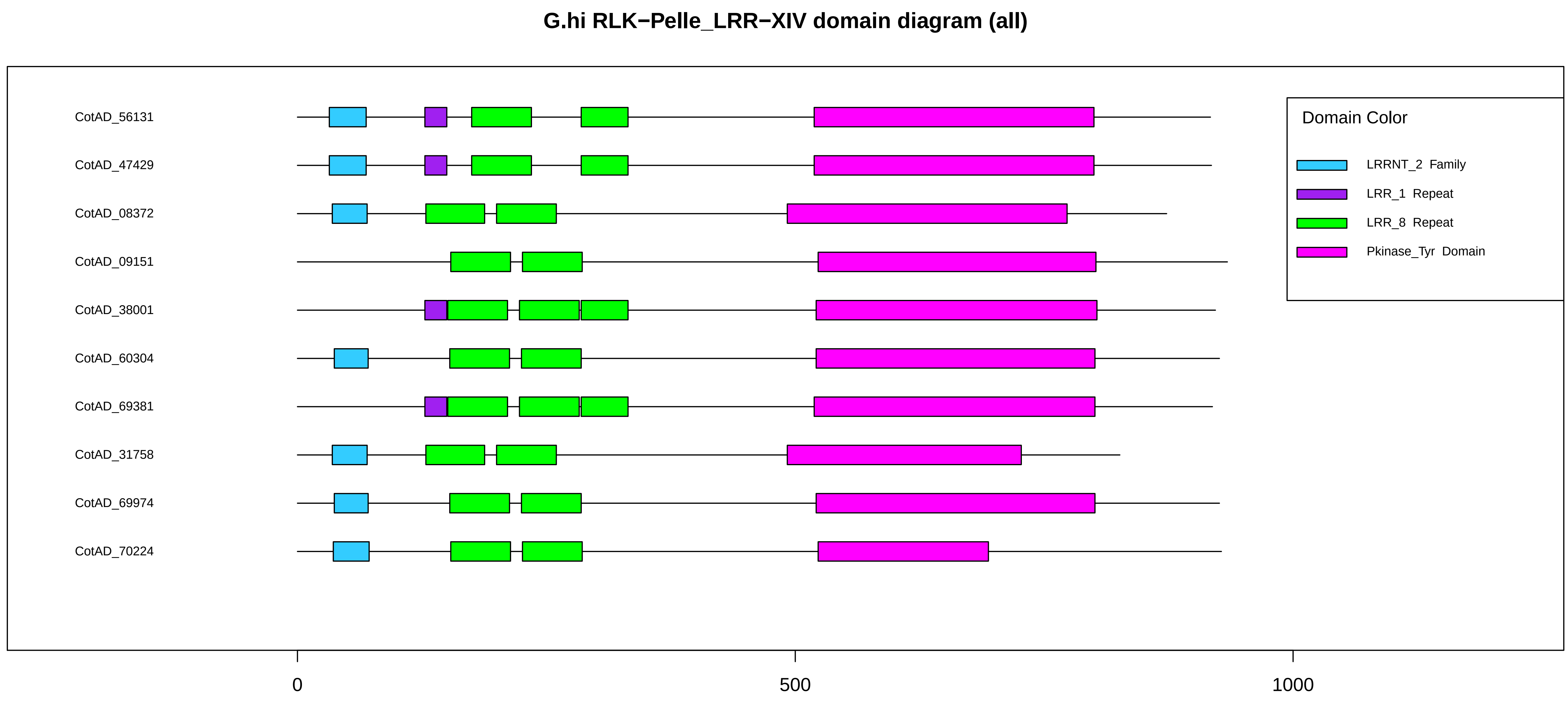
G.hi RLK−Pelle_LRR−XIV domain diagram (all)
CotAD_56131
CotAD_47429
CotAD_08372
CotAD_09151
CotAD_38001
CotAD_60304
CotAD_69381
CotAD_31758
CotAD_69974
CotAD_70224
Domain Color
LRRNT_2 Family
LRR_1 Repeat
LRR_8 Repeat
Pkinase_Tyr Domain
0
500
1000

G.hi RLK−Pelle_LRR−XV domain diagram (all)

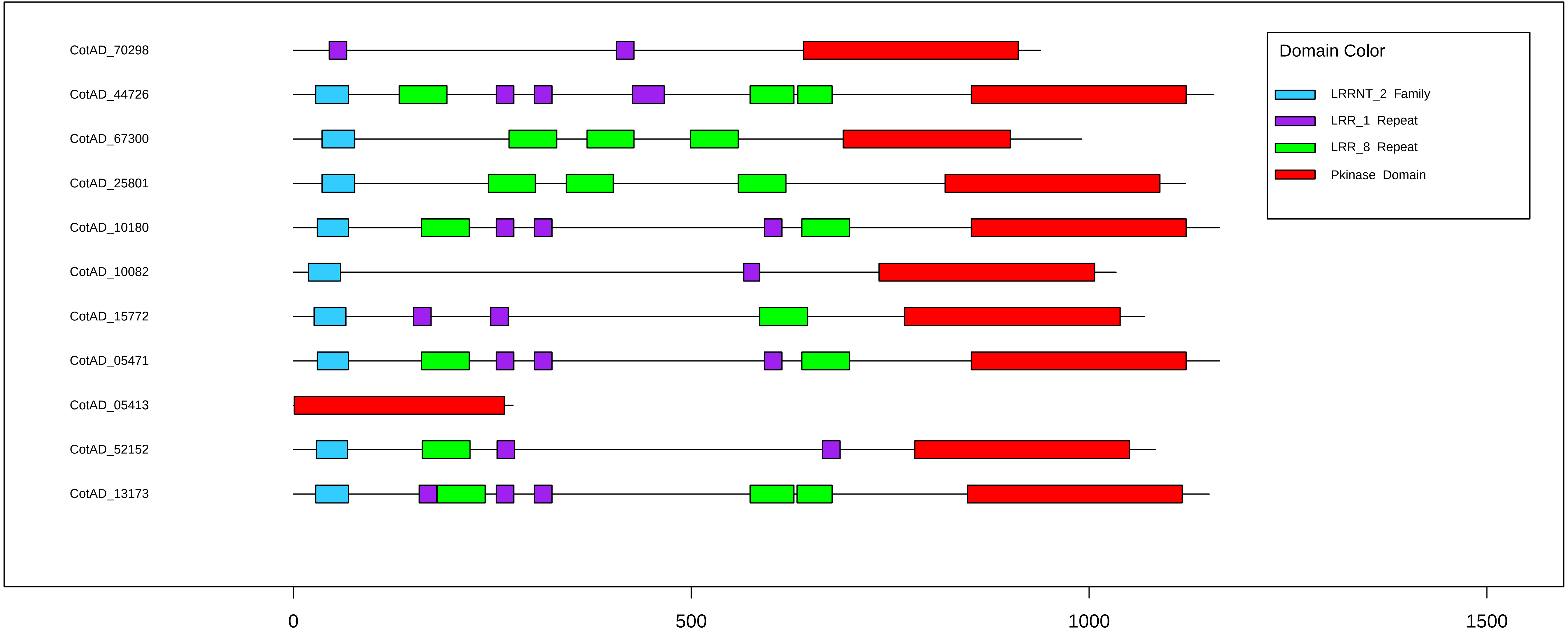

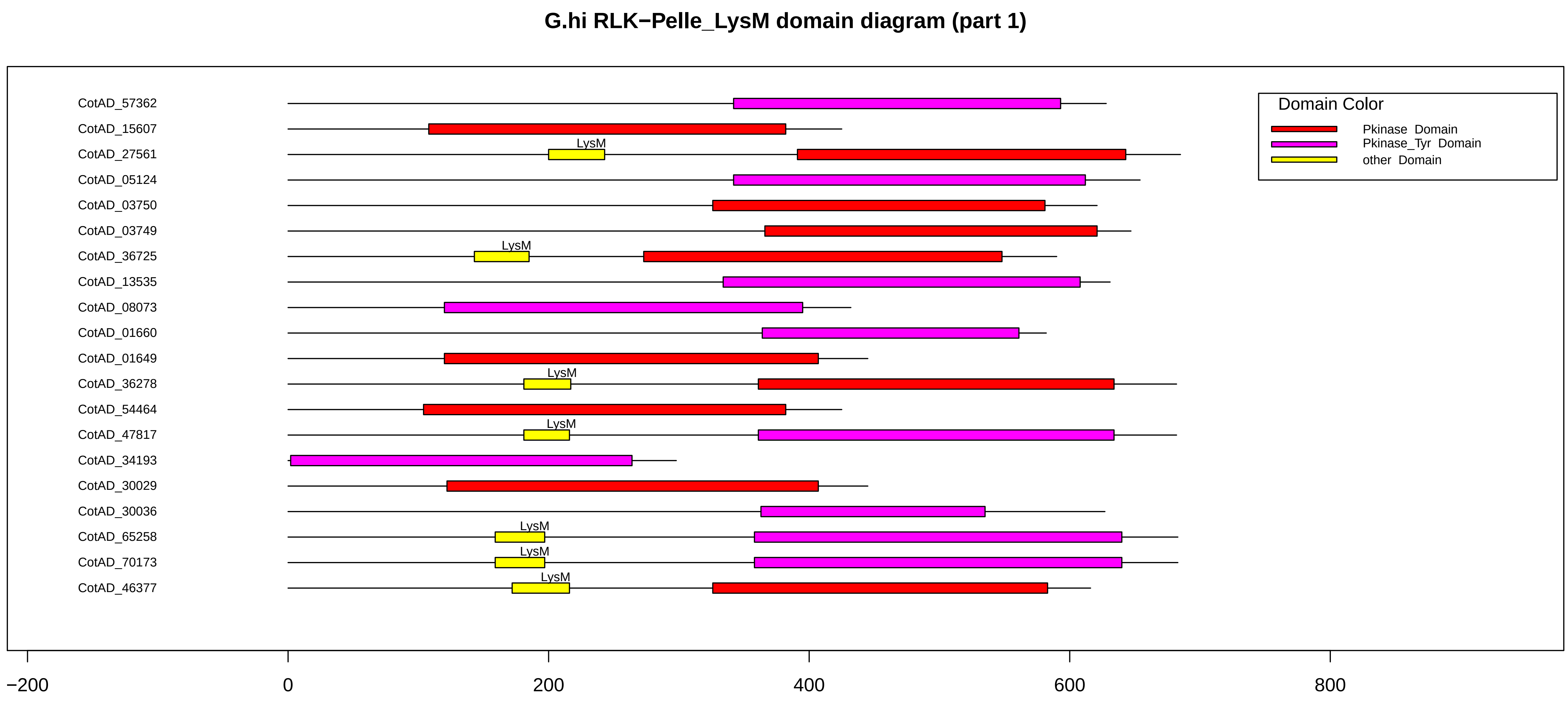

G.hi RLK−Pelle_LysM domain diagram (part 1)
Domain Color
Pkinase Domain
Pkinase_Tyr Domain
other Domain
CotAD_57362
CotAD_15607
CotAD_27561
CotAD_05124
CotAD_03750
CotAD_03749
CotAD_36725
CotAD_13535
CotAD_08073
CotAD_01660
CotAD_01649
CotAD_36278
CotAD_54464
CotAD_47817
CotAD_34193
CotAD_30029
CotAD_30036
CotAD_65258
CotAD_70173
CotAD_46377
LysM
−200
0
200
400
600
800

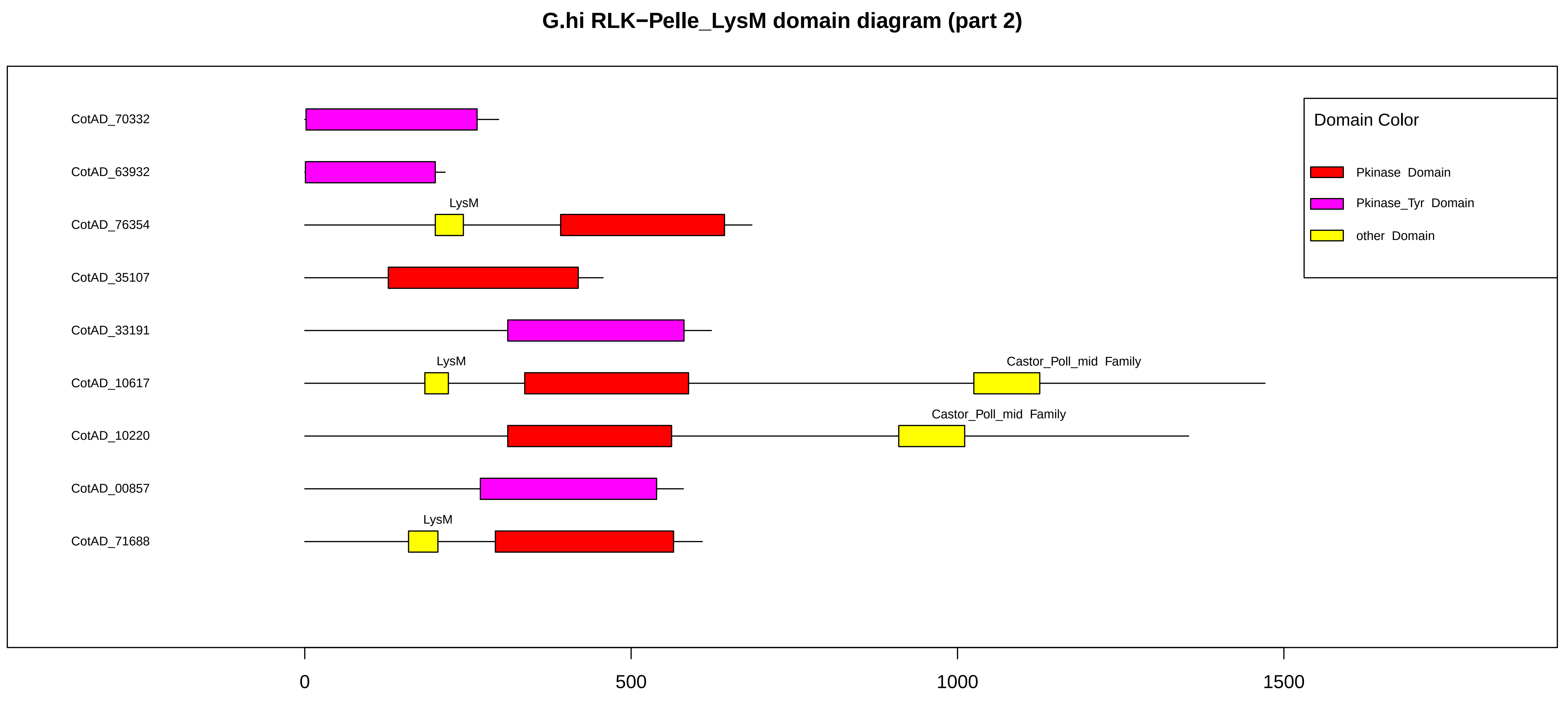
G.hi RLK−Pelle_LysM domain diagram (part 2)
CotAD_70332
CotAD_63932
CotAD_76354
LysM
CotAD_35107
CotAD_33191
CotAD_10617
LysM
Castor_Poll_mid Family
CotAD_10220
Castor_Poll_mid Family
CotAD_00857
CotAD_71688
LysM
Domain Color
Pkinase Domain
Pkinase_Tyr Domain
other Domain
0
500
1000
1500

G.hi RLK−Pelle_PERK−1 domain diagram (part 1)

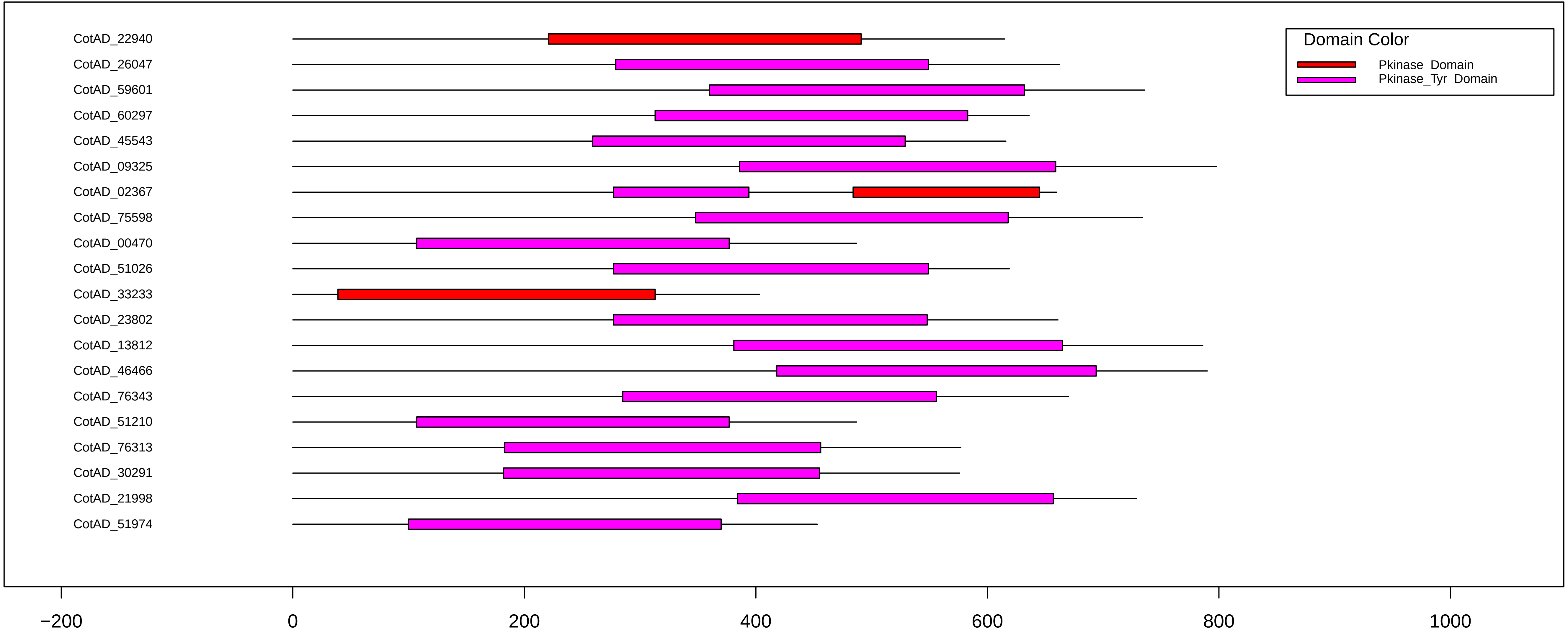

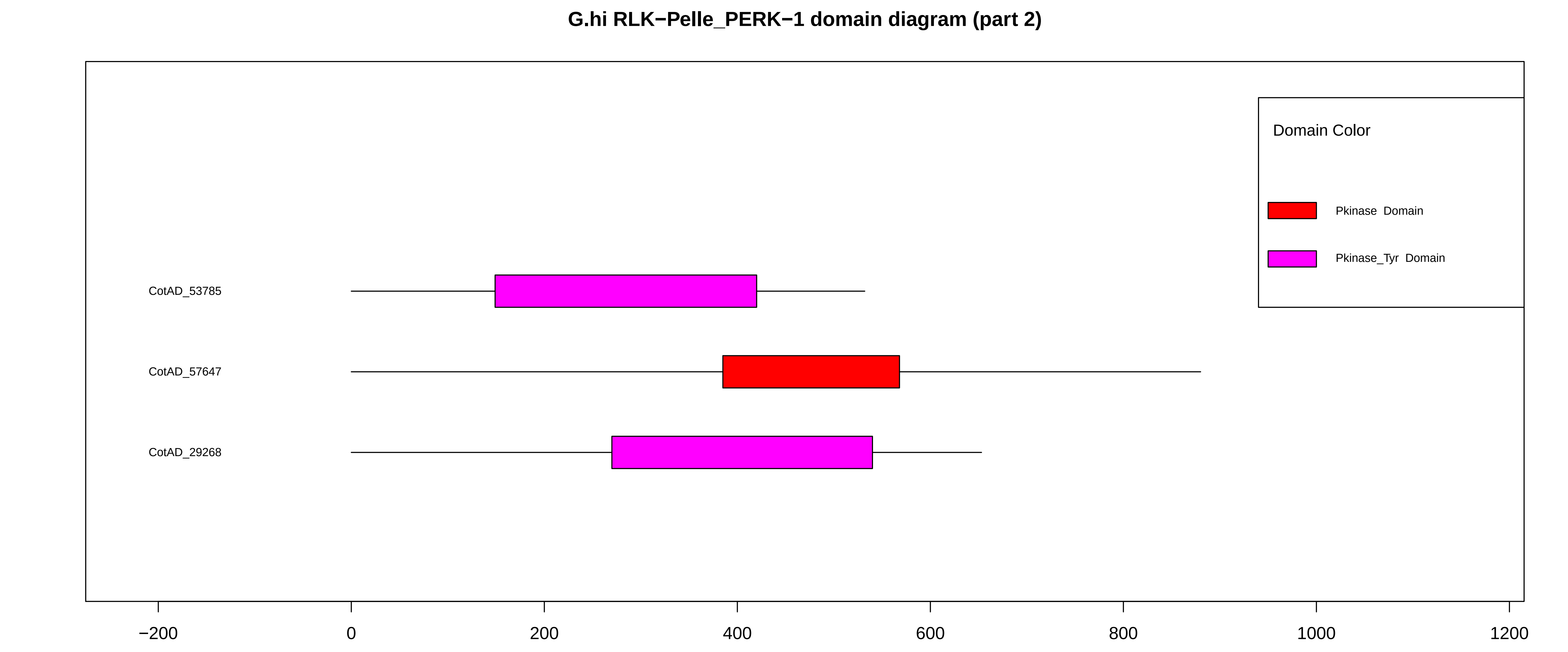
G.hi RLK−Pelle_PERK−1 domain diagram (part 2)
Domain Color
Pkinase Domain
Pkinase_Tyr Domain
CotAD_53785
CotAD_57647
CotAD_29268
−200
0
200
400
600
800
1000
1200

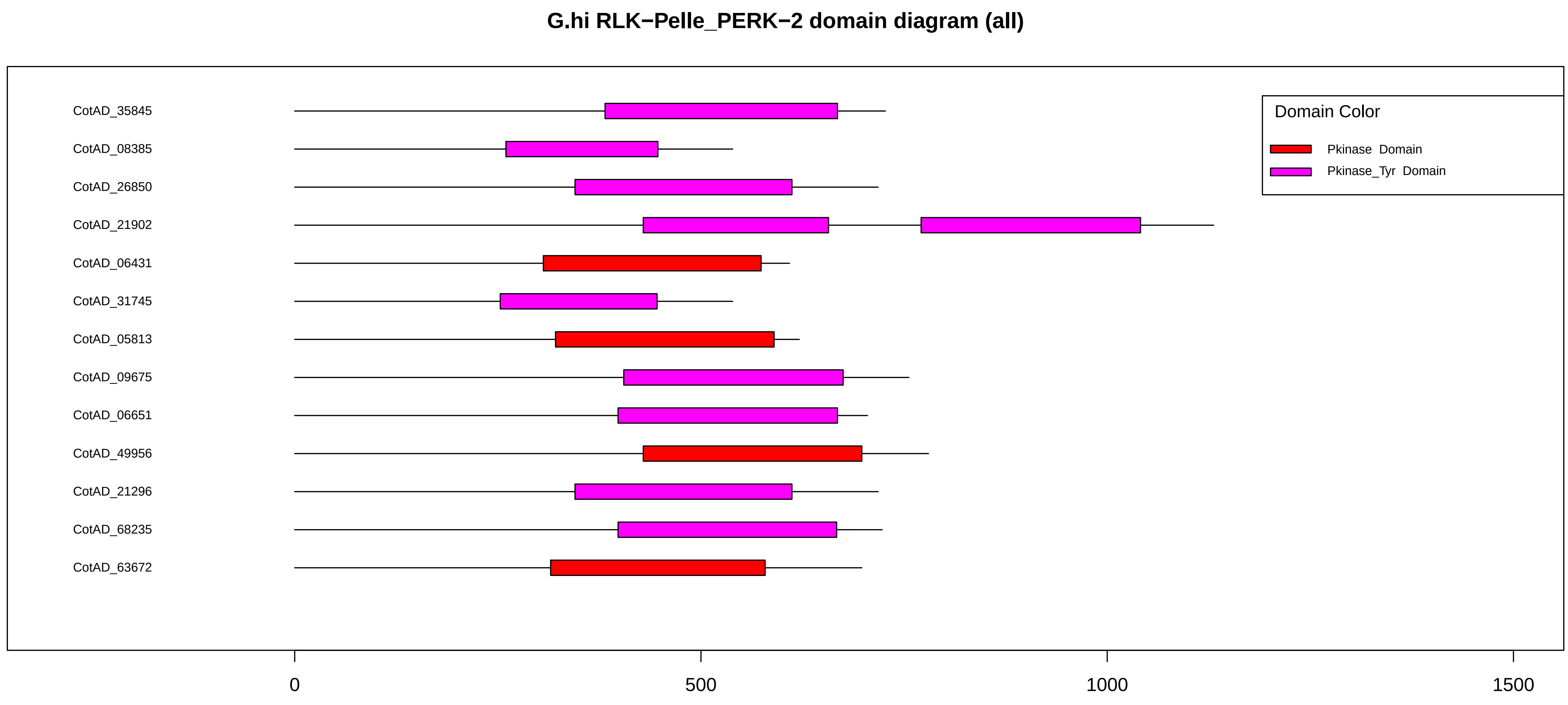
G.hi RLK−Pelle_PERK−2 domain diagram (all)
CotAD_35845
CotAD_08385
CotAD_26850
CotAD_21902
CotAD_06431
CotAD_31745
CotAD_05813
CotAD_09675
CotAD_06651
CotAD_49956
CotAD_21296
CotAD_68235
CotAD_63672
Domain Color
Pkinase Domain
Pkinase_Tyr Domain
0
500
1000
1500

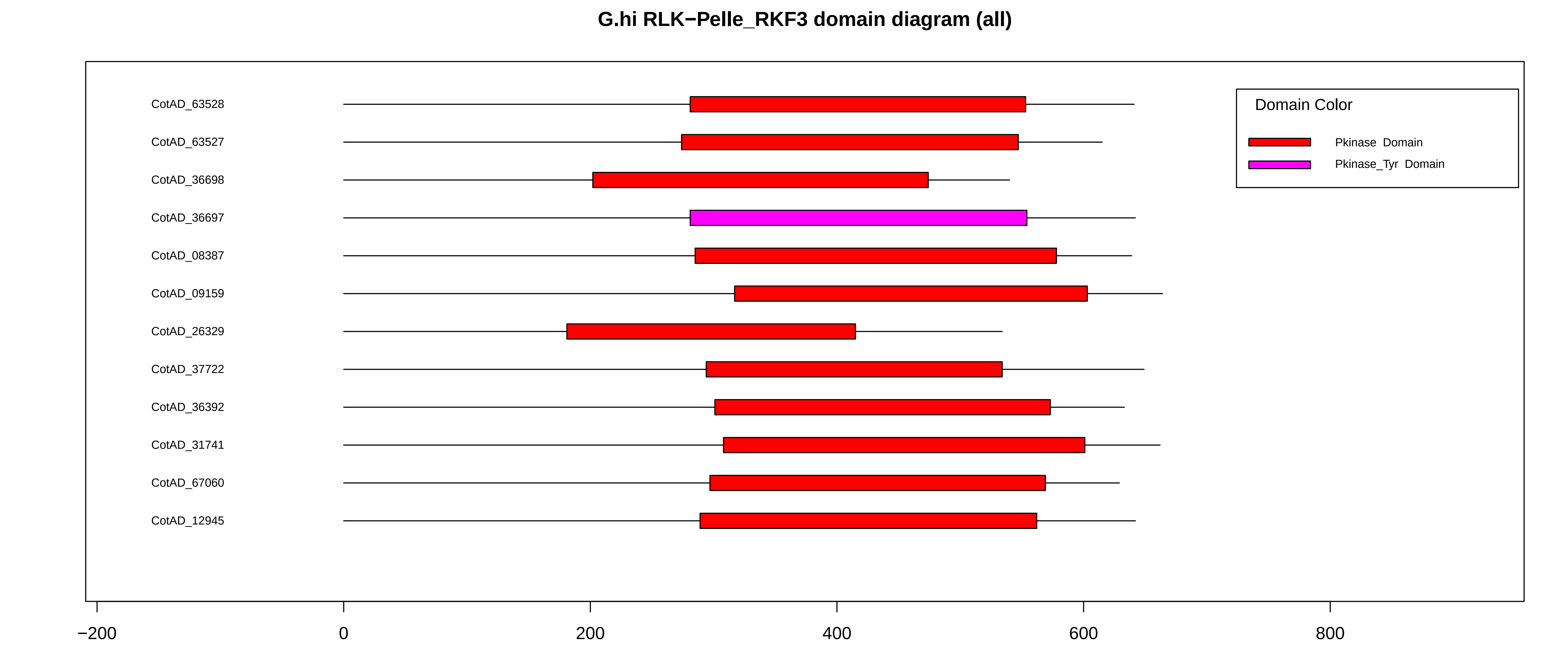
G.hi RLK−Pelle_RKF3 domain diagram (all)
CotAD_63528
CotAD_63527
CotAD_36698
CotAD_36697
CotAD_08387
CotAD_09159
CotAD_26329
CotAD_37722
CotAD_36392
CotAD_31741
CotAD_67060
CotAD_12945
Domain Color
Pkinase Domain
Pkinase_Tyr Domain
−200
0
200
400
600
800

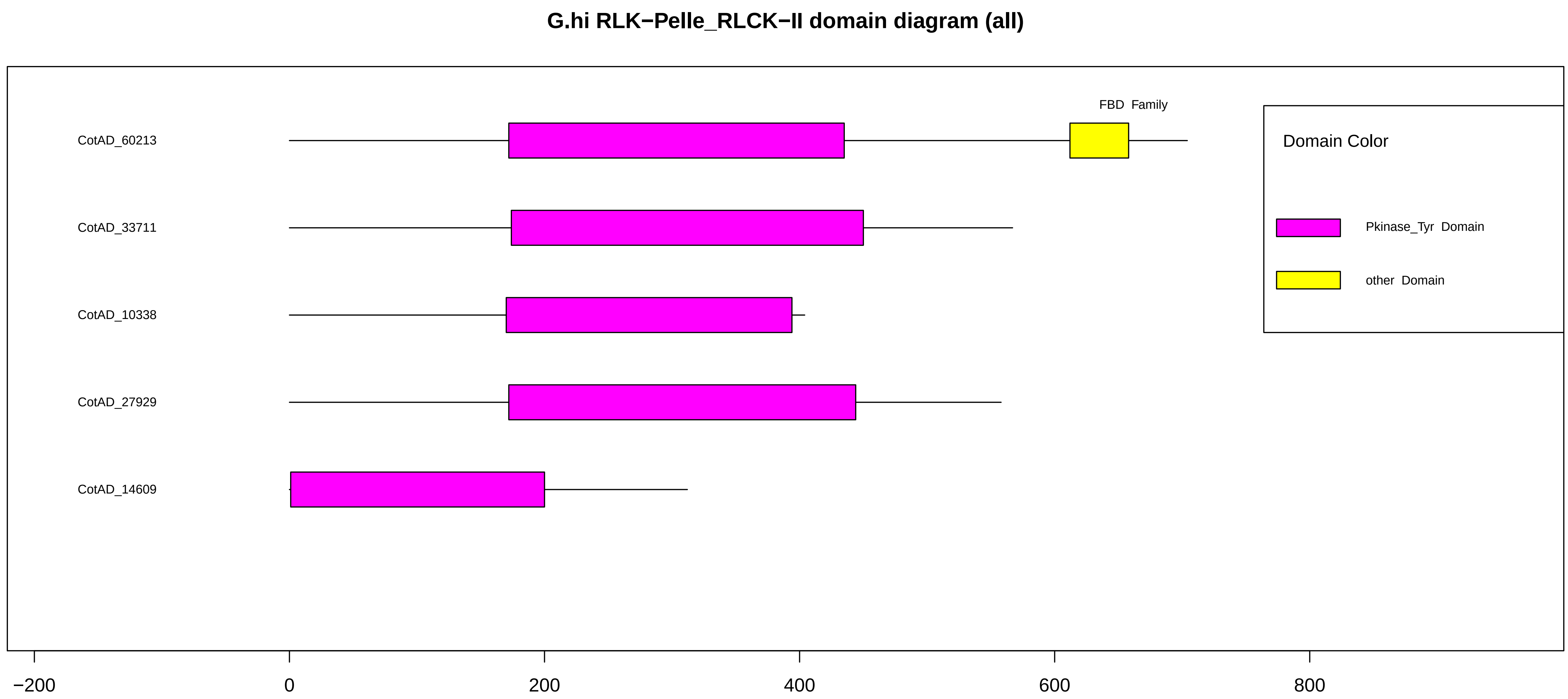
G.hi RLK−Pelle_RLCK−II domain diagram (all)
FBD Family
CotAD_60213
CotAD_33711
CotAD_10338
CotAD_27929
CotAD_14609
Domain Color
Pkinase_Tyr Domain
other Domain
−200
0
200
400
600
800

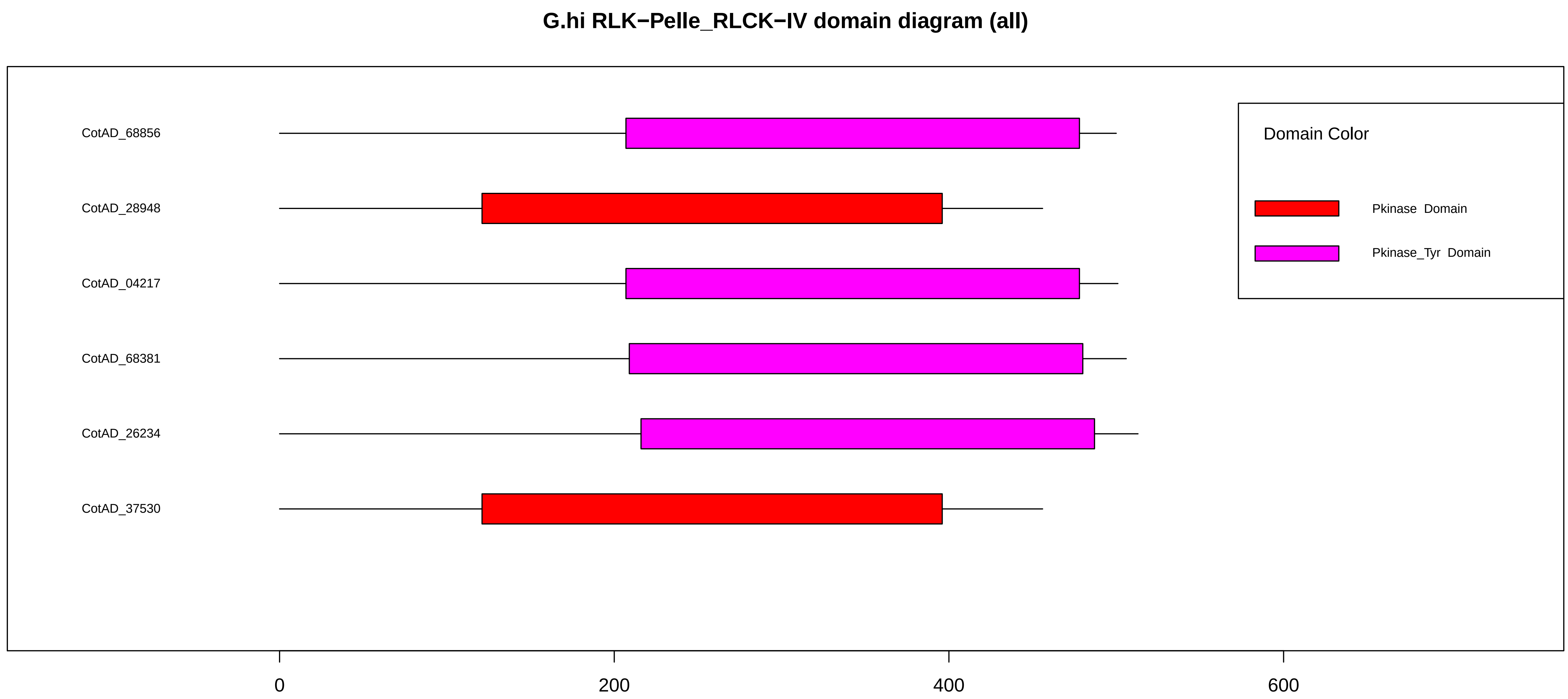
G.hi RLK−Pelle_RLCK−IV domain diagram (all)
CotAD_68856
CotAD_28948
CotAD_04217
CotAD_68381
CotAD_26234
CotAD_37530
Domain Color
Pkinase Domain
Pkinase_Tyr Domain
0
200
400
600

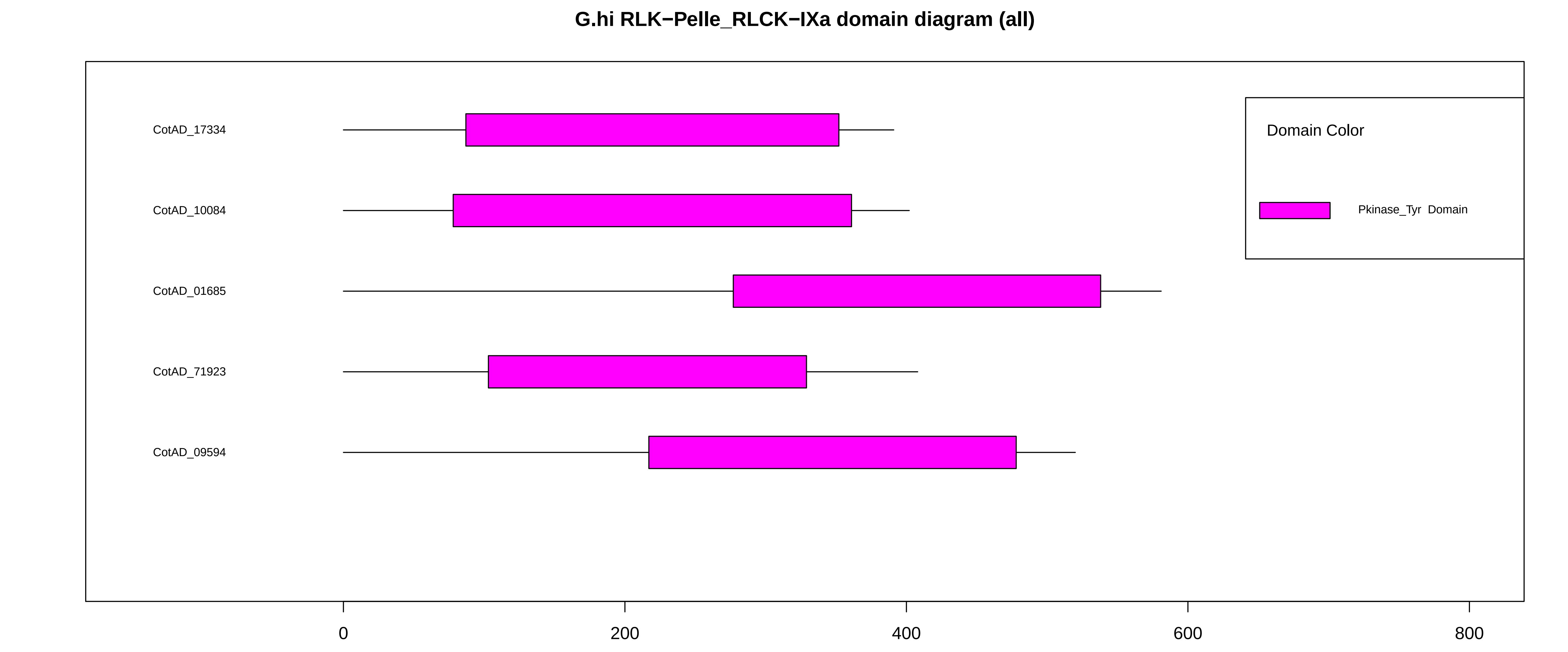
G.hi RLK−Pelle_RLCK−IXa domain diagram (all)
CotAD_17334
CotAD_10084
CotAD_01685
CotAD_71923
CotAD_09594
Domain Color
Pkinase_Tyr Domain
0
200
400
600
800

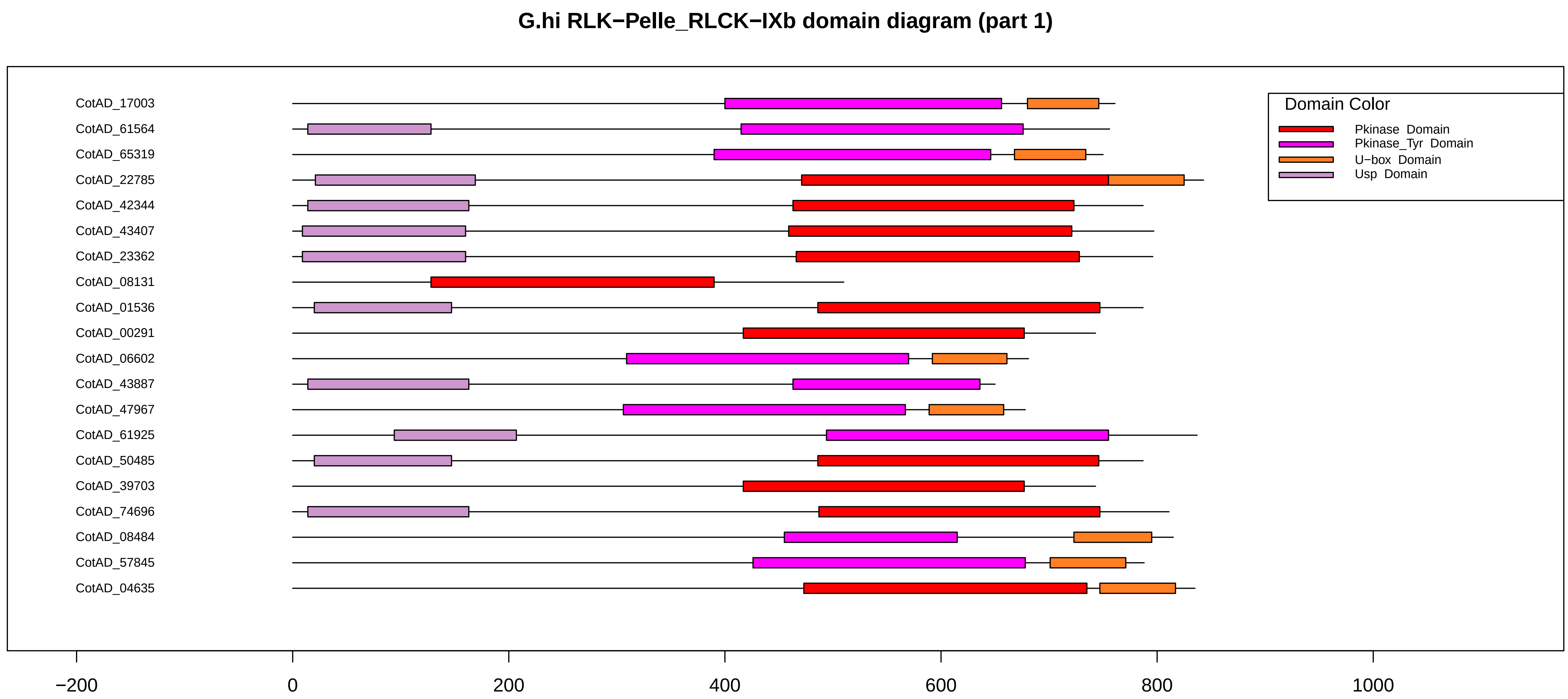
G.hi RLK−Pelle_RLCK−IXb domain diagram (part 1)
CotAD_17003
CotAD_61564
CotAD_65319
CotAD_22785
CotAD_42344
CotAD_43407
CotAD_23362
CotAD_08131
CotAD_01536
CotAD_00291
CotAD_06602
CotAD_43887
CotAD_47967
CotAD_61925
CotAD_50485
CotAD_39703
CotAD_74696
CotAD_08484
CotAD_57845
CotAD_04635
Domain Color
Pkinase Domain
Pkinase_Tyr Domain
U−box Domain
Usp Domain
−200
0
200
400
600
800
1000

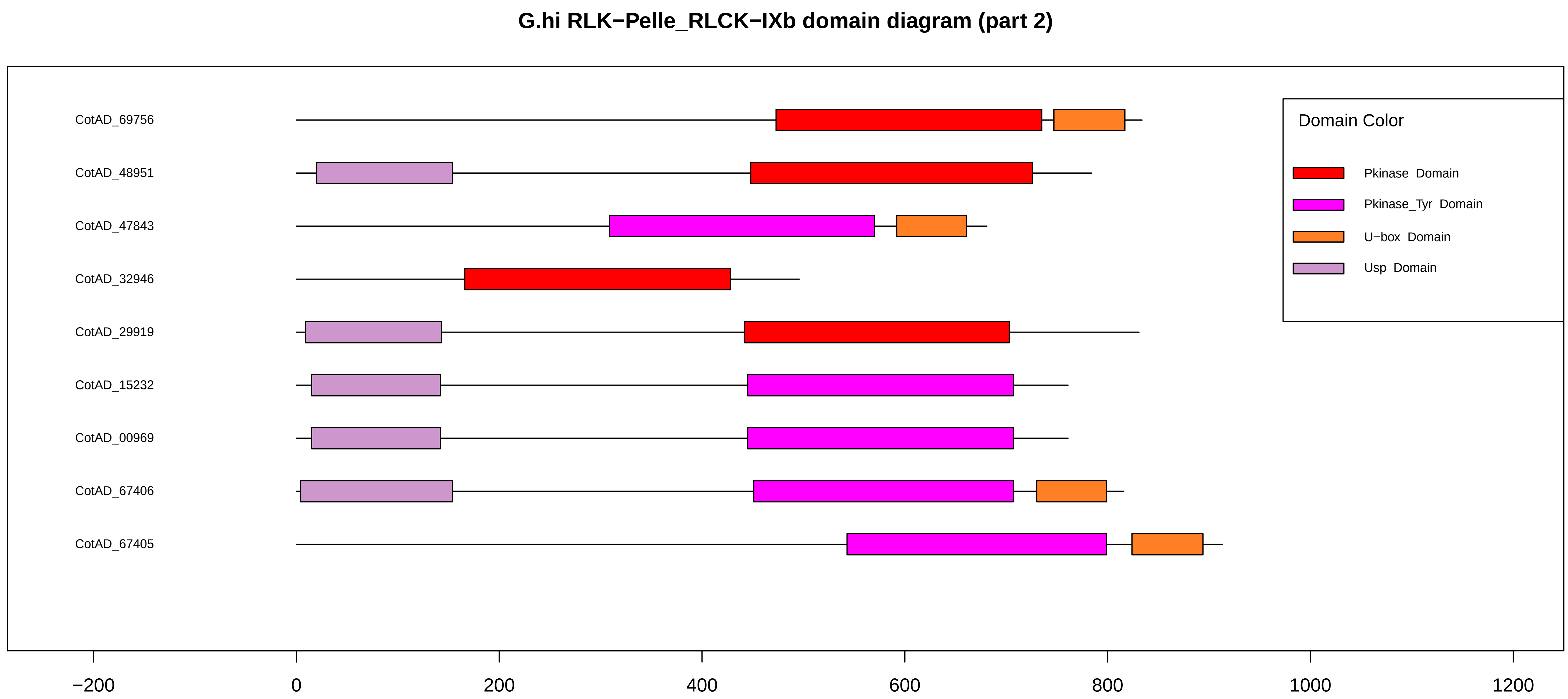
G.hi RLK−Pelle_RLCK−IXb domain diagram (part 2)
CotAD_69756
CotAD_48951
CotAD_47843
CotAD_32946
CotAD_29919
CotAD_15232
CotAD_00969
CotAD_67406
CotAD_67405
Domain Color
Pkinase Domain
Pkinase_Tyr Domain
U−box Domain
Usp Domain
−200
0
200
400
600
800
1000
1200

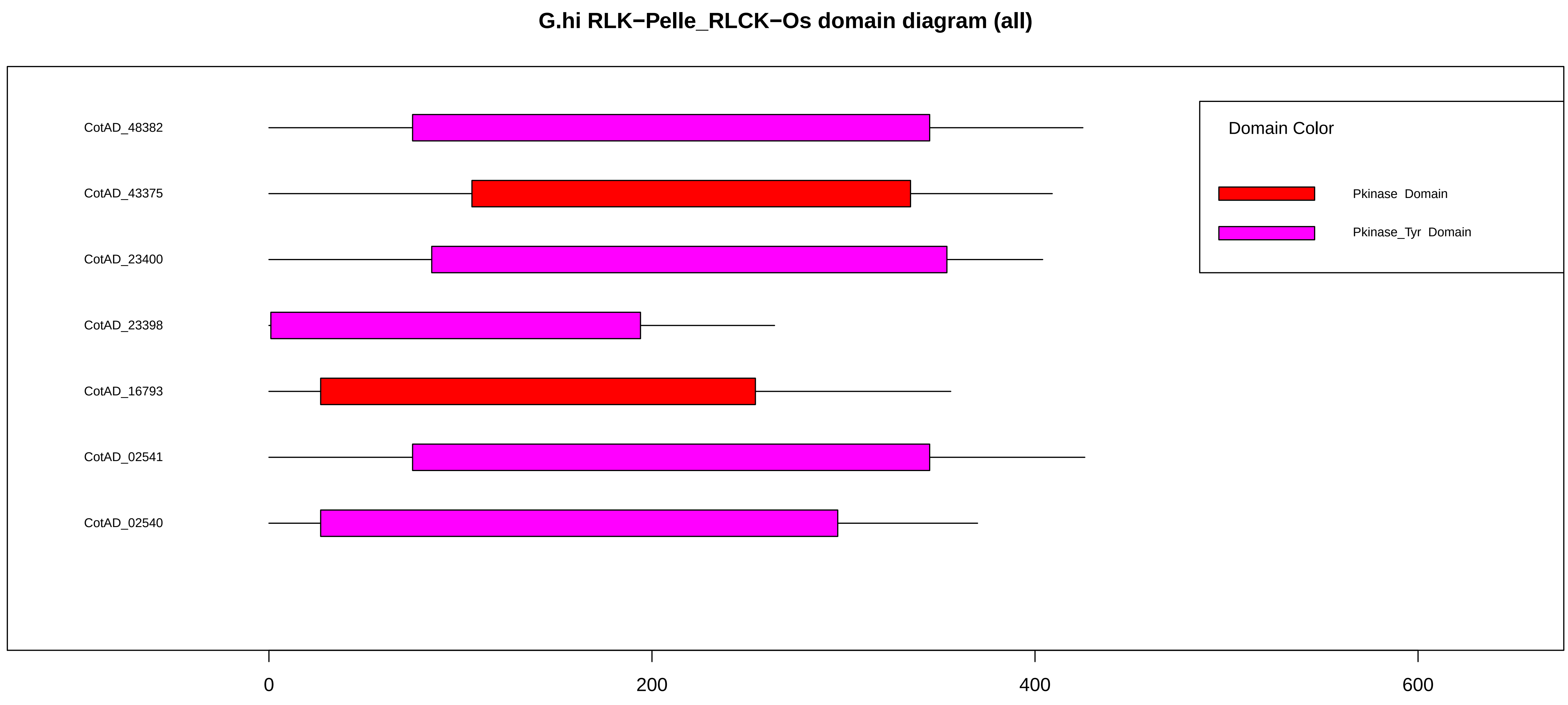
G.hi RLK−Pelle_RLCK−Os domain diagram (all)
CotAD_48382
CotAD_43375
CotAD_23400
CotAD_23398
CotAD_16793
CotAD_02541
CotAD_02540
Domain Color
Pkinase Domain
Pkinase_Tyr Domain
0
200
400
600

G.hi RLK−Pelle_RLCK−VIIa−1 domain diagram (part 1)

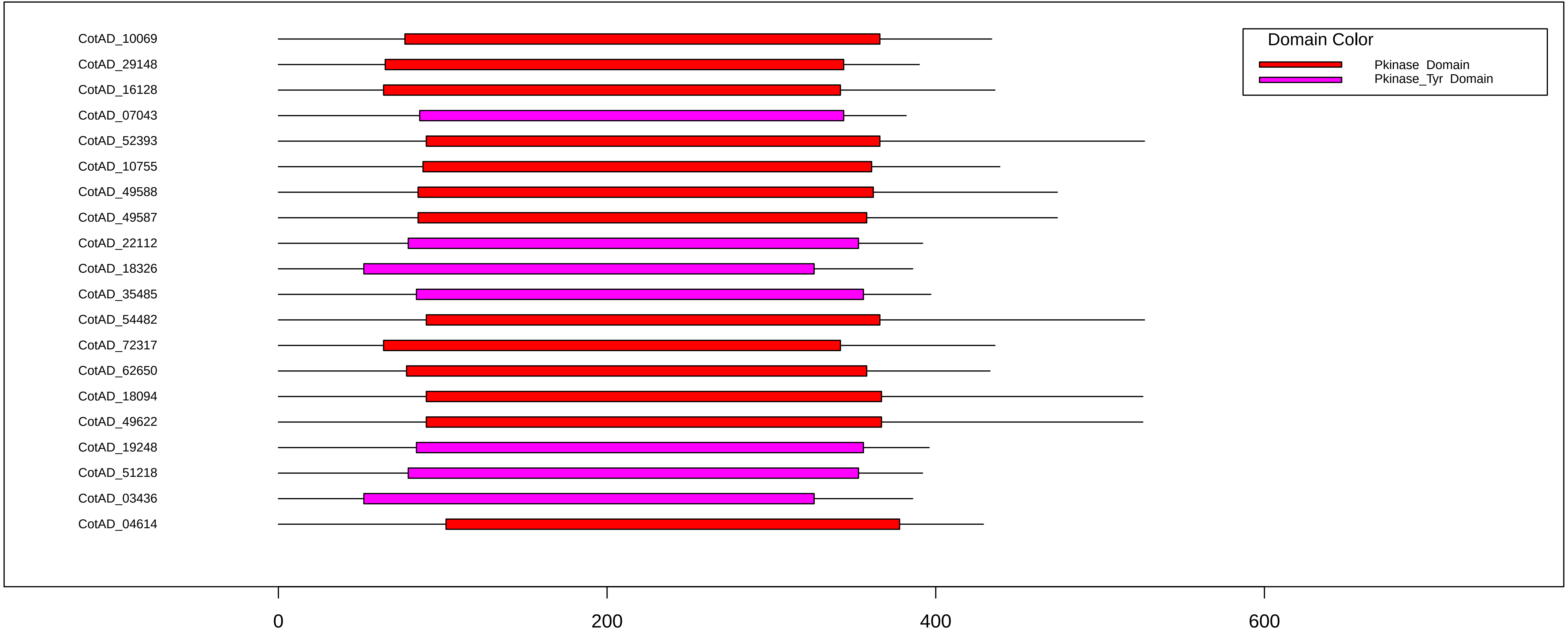

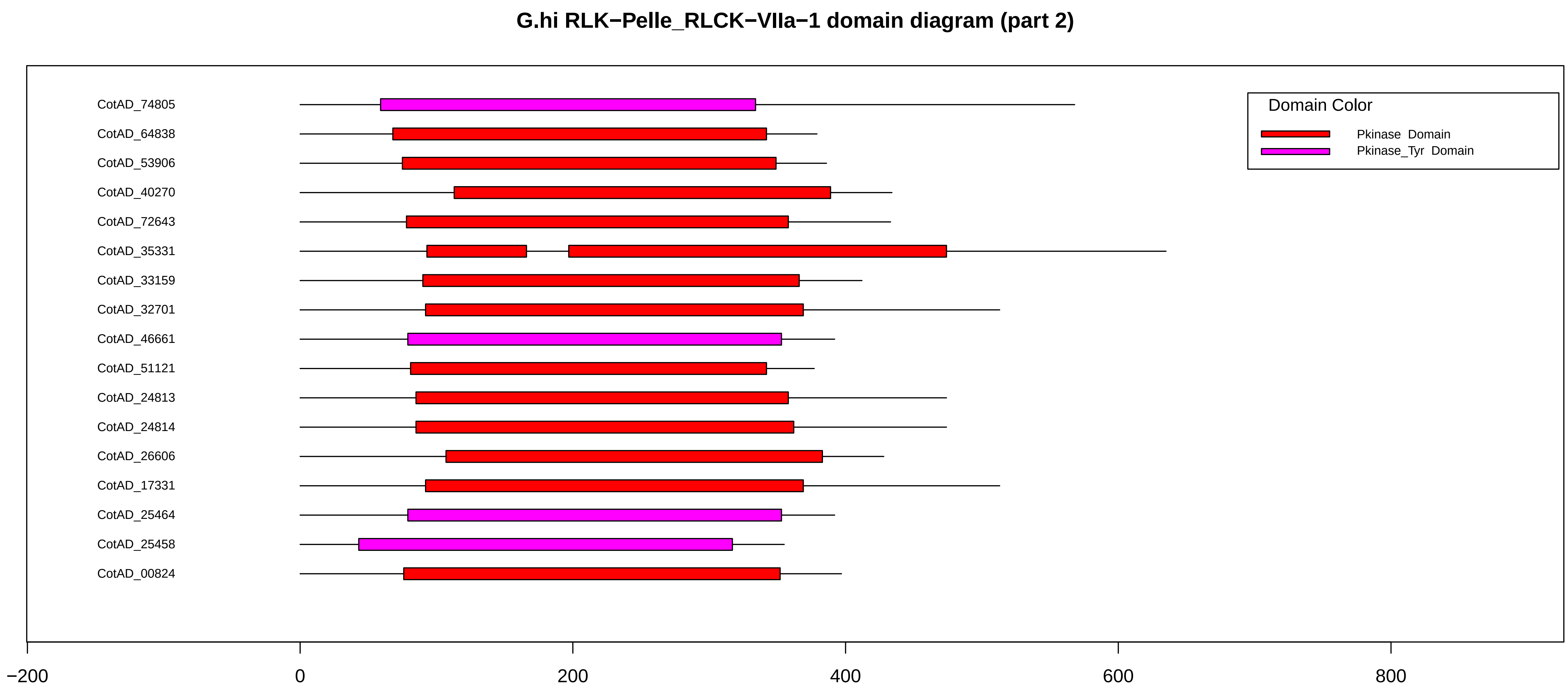
G.hi RLK−Pelle_RLCK−VIIa−1 domain diagram (part 2)
CotAD_74805
CotAD_64838
CotAD_53906
CotAD_40270
CotAD_72643
CotAD_35331
CotAD_33159
CotAD_32701
CotAD_46661
CotAD_51121
CotAD_24813
CotAD_24814
CotAD_26606
CotAD_17331
CotAD_25464
CotAD_25458
CotAD_00824
Domain Color
Pkinase Domain
Pkinase_Tyr Domain
−200
0
200
400
600
800

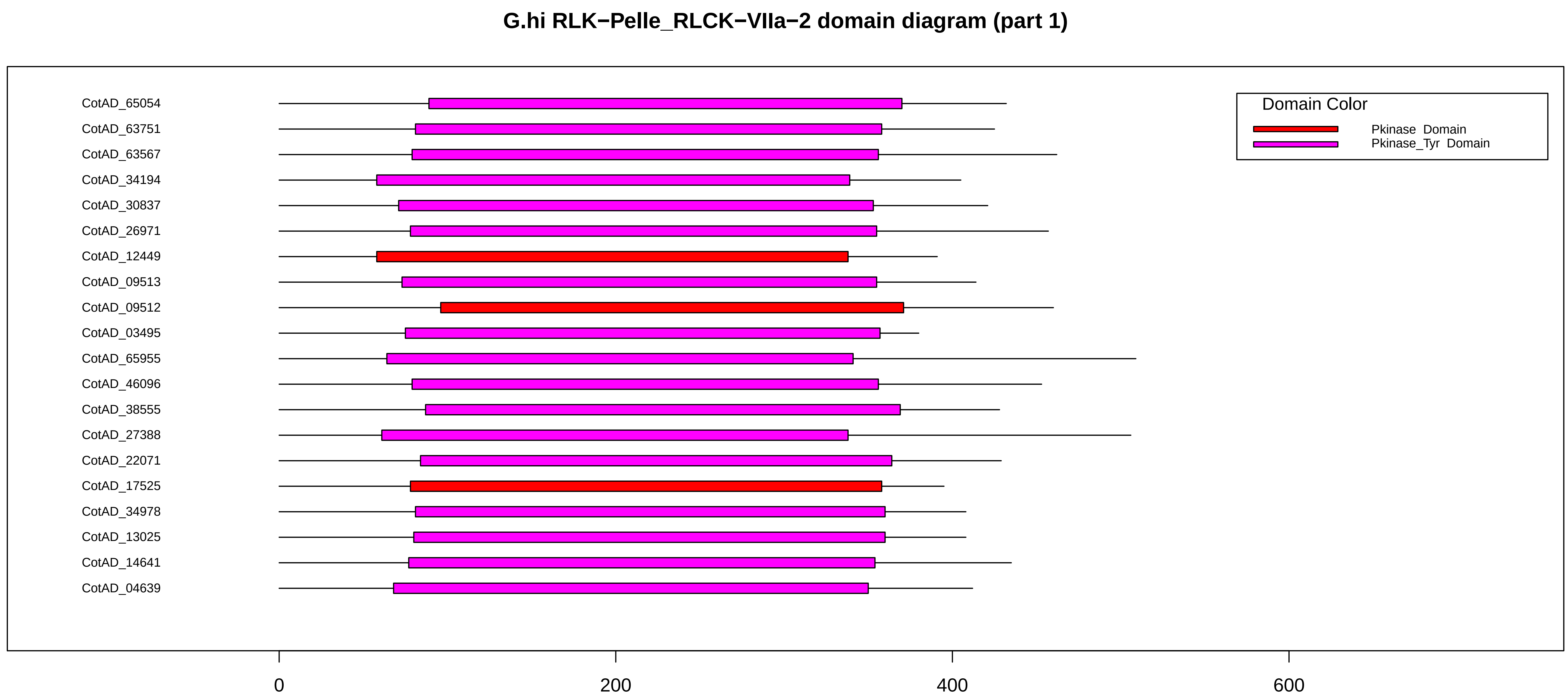

G.hi RLK−Pelle_RLCK−VIIa−2 domain diagram (part 1)
CotAD_65054
CotAD_63751
CotAD_63567
CotAD_34194
CotAD_30837
CotAD_26971
CotAD_12449
CotAD_09513
CotAD_09512
CotAD_03495
CotAD_65955
CotAD_46096
CotAD_38555
CotAD_27388
CotAD_22071
CotAD_17525
CotAD_34978
CotAD_13025
CotAD_14641
CotAD_04639
Domain Color
Pkinase Domain
Pkinase_Tyr Domain
0
200
400
600

G.hi RLK−Pelle_RLCK−VIIa−2 domain diagram (part 2)

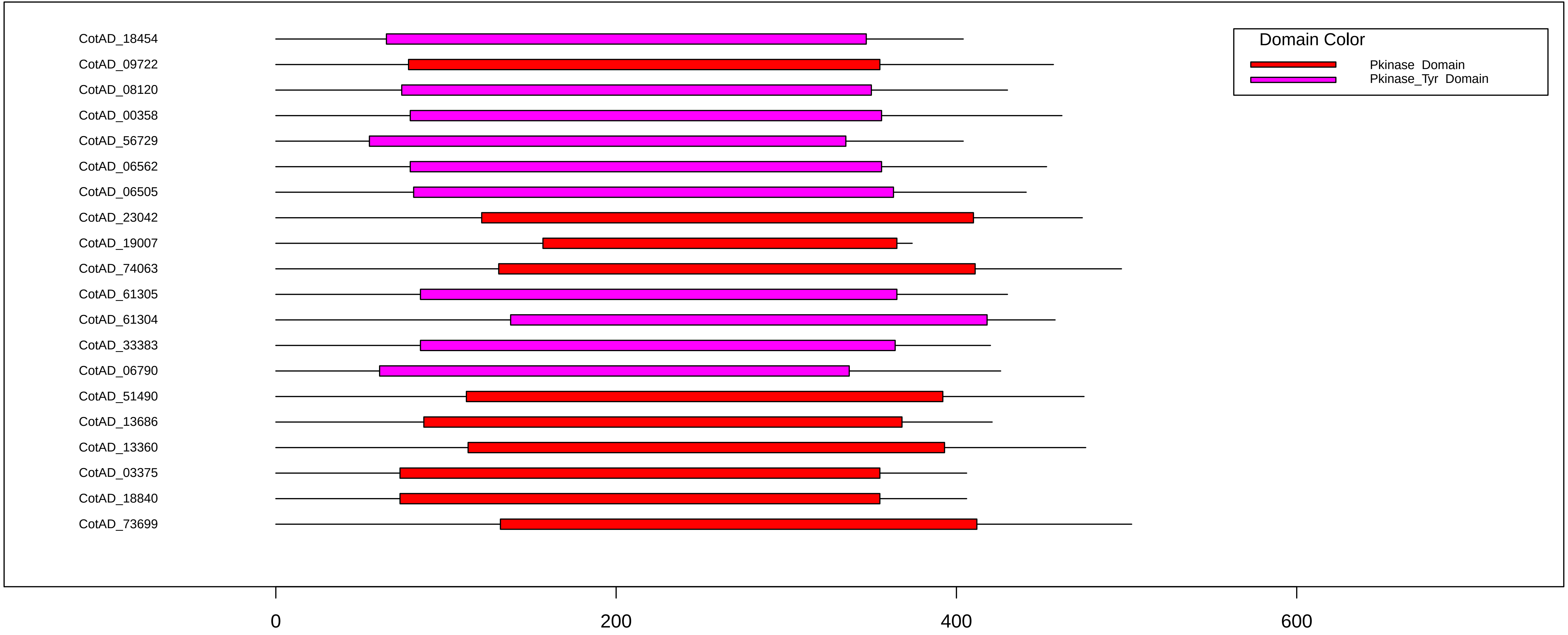

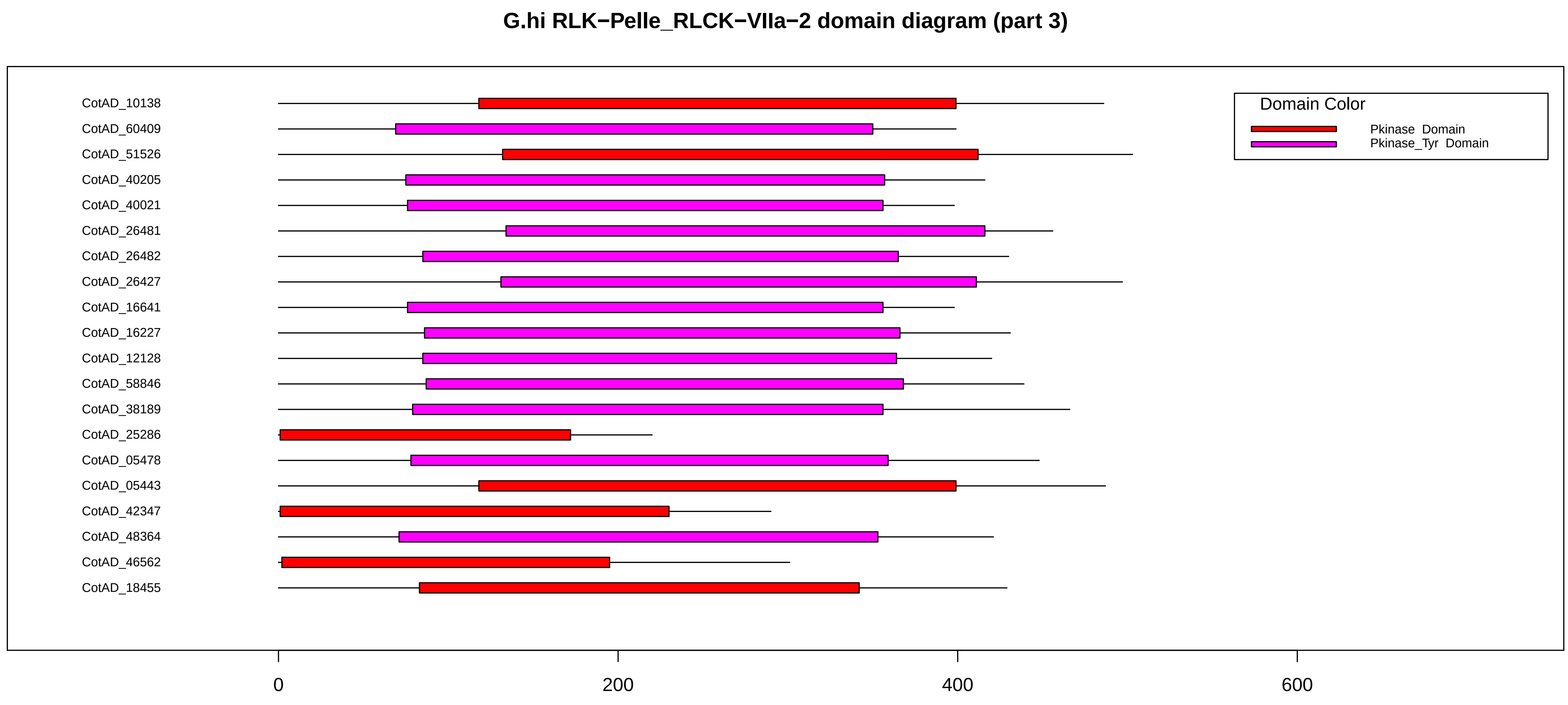
G.hi RLK−Pelle_RLCK−VIIa−2 domain diagram (part 3)
CotAD_10138
CotAD_60409
CotAD_51526
CotAD_40205
CotAD_40021
CotAD_26481
CotAD_26482
CotAD_26427
CotAD_16641
CotAD_16227
CotAD_12128
CotAD_58846
CotAD_38189
CotAD_25286
CotAD_05478
CotAD_05443
CotAD_42347
CotAD_48364
CotAD_46562
CotAD_18455
Domain Color
Pkinase Domain
Pkinase_Tyr Domain
0
200
400
600

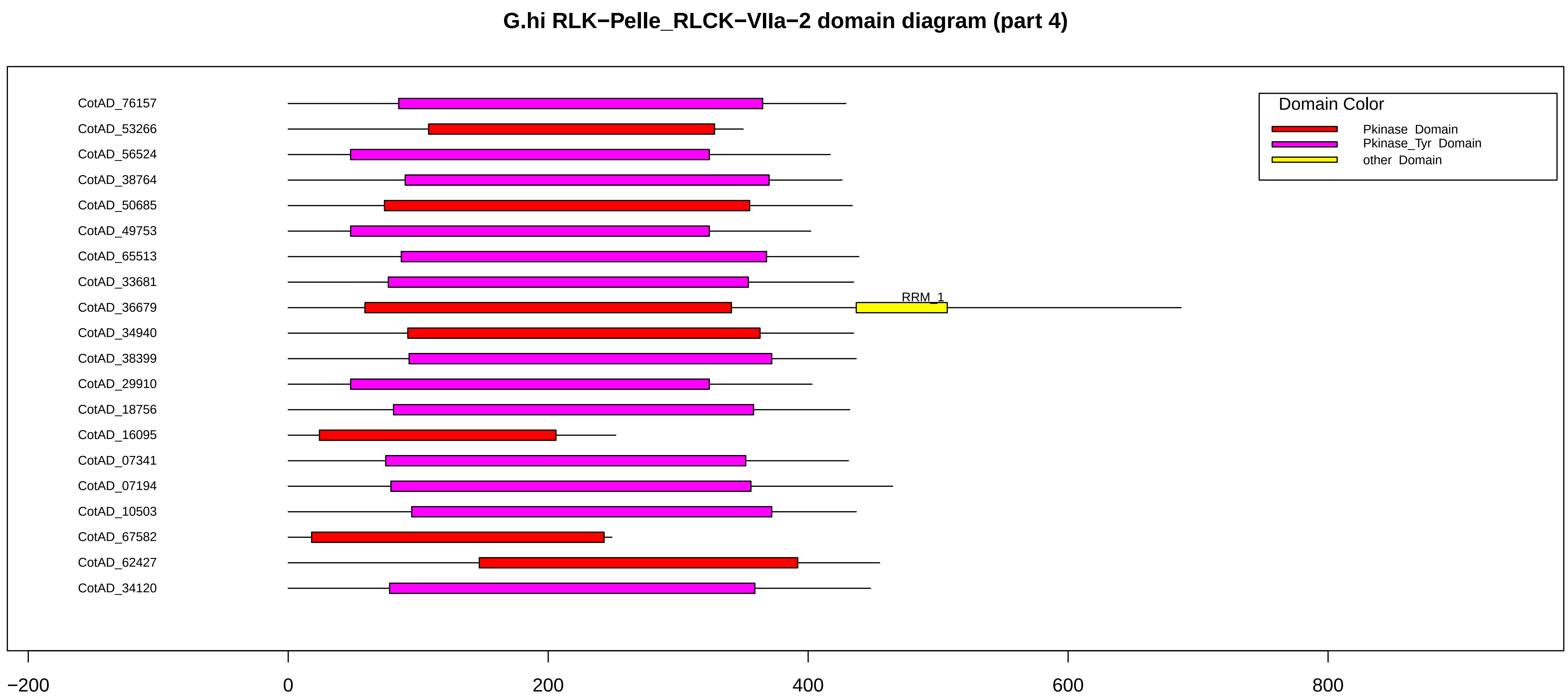

G.hi RLK−Pelle_RLCK−VIIa−2 domain diagram (part 4)
CotAD_76157
CotAD_53266
CotAD_56524
CotAD_38764
CotAD_50685
CotAD_49753
CotAD_65513
CotAD_33681
CotAD_36679
CotAD_34940
CotAD_38399
CotAD_29910
CotAD_18756
CotAD_16095
CotAD_07341
CotAD_07194
CotAD_10503
CotAD_67582
CotAD_62427
CotAD_34120
RRM_1
Domain Color
Pkinase Domain
Pkinase_Tyr Domain
other Domain
−200
0
200
400
600
800

G.hi RLK−Pelle_RLCK−VIIa−2 domain diagram (part 5)

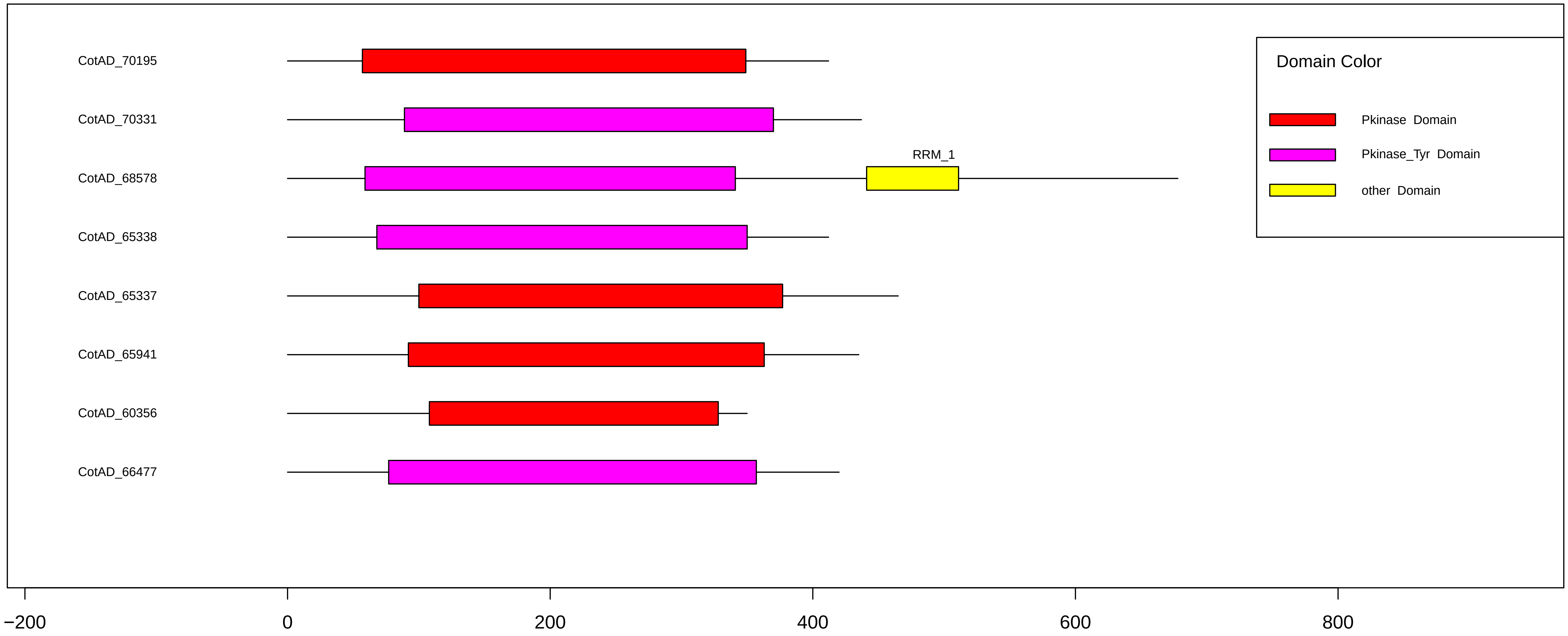

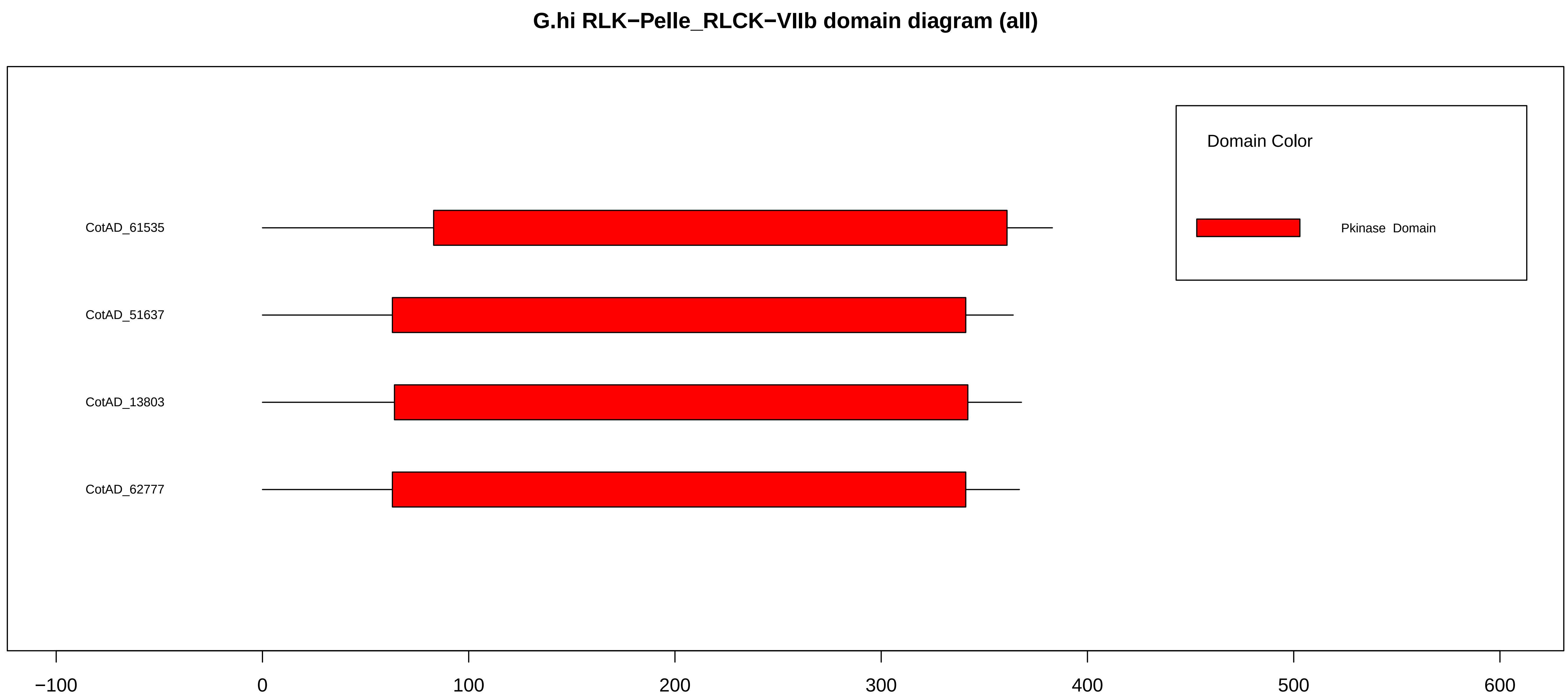
G.hi RLK−Pelle_RLCK−VIIb domain diagram (all)
CotAD_61535
CotAD_51637
CotAD_13803
CotAD_62777
Domain Color
Pkinase Domain
−100
0
100
200
300
400
500
600

G.hi RLK−Pelle_RLCK−VIII domain diagram (all)

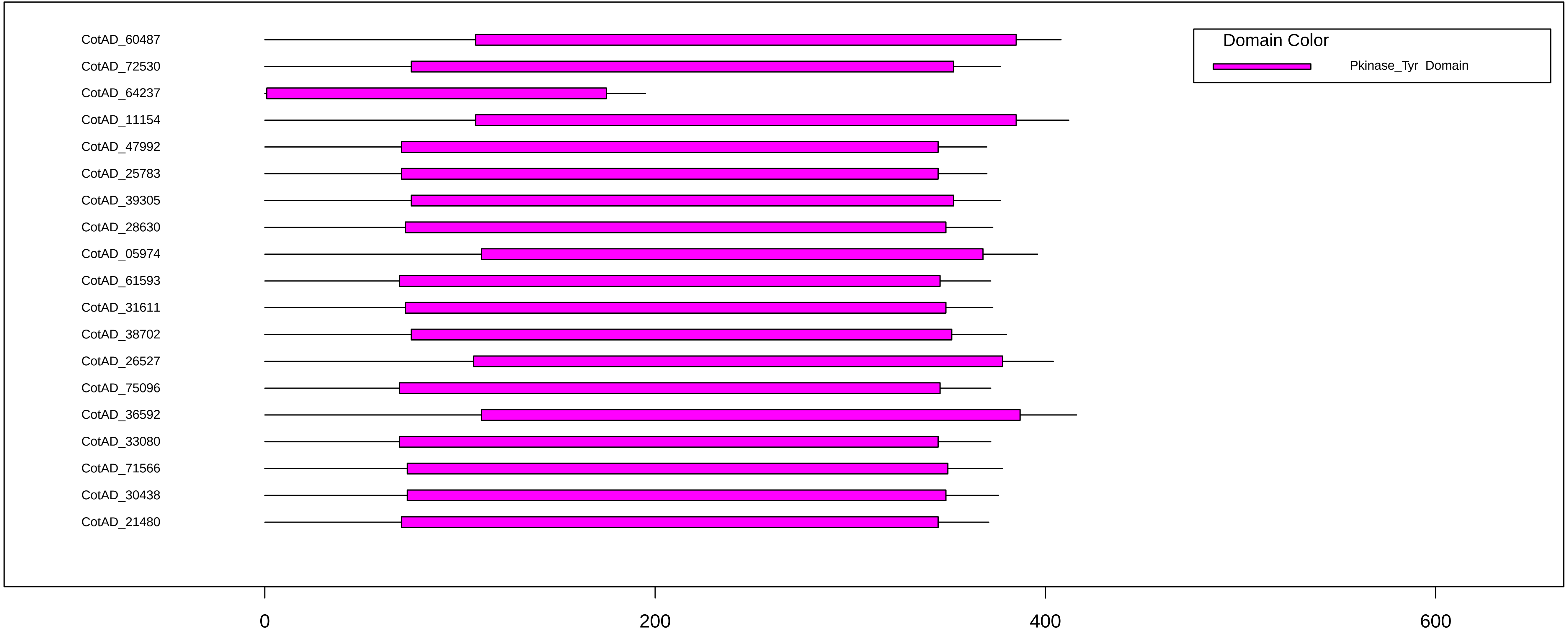

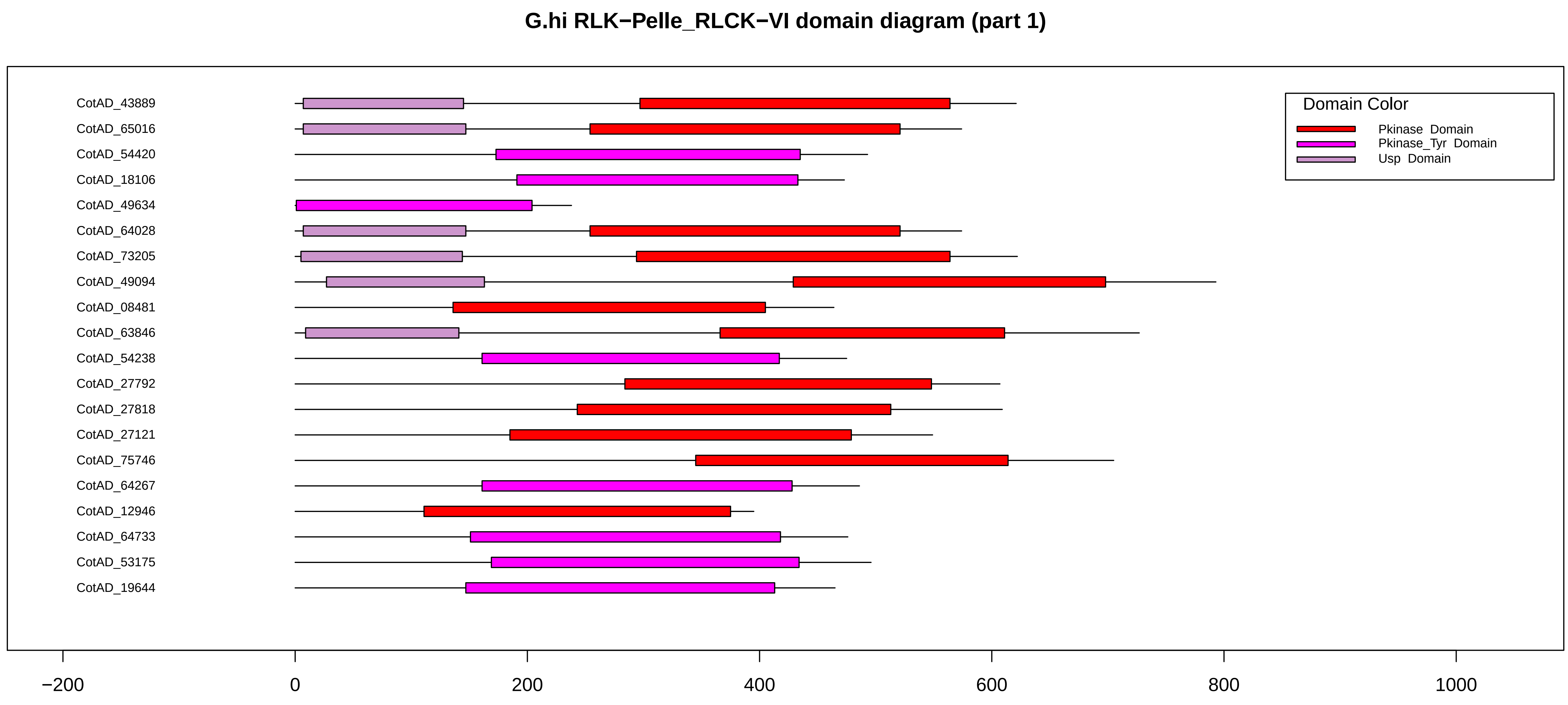
G.hi RLK−Pelle_RLCK−VI domain diagram (part 1)
Domain Color
Pkinase Domain
Pkinase_Tyr Domain
Usp Domain
CotAD_43889
CotAD_65016
CotAD_54420
CotAD_18106
CotAD_49634
CotAD_64028
CotAD_73205
CotAD_49094
CotAD_08481
CotAD_63846
CotAD_54238
CotAD_27792
CotAD_27818
CotAD_27121
CotAD_75746
CotAD_64267
CotAD_12946
CotAD_64733
CotAD_53175
CotAD_19644
−200
0
200
400
600
800
1000

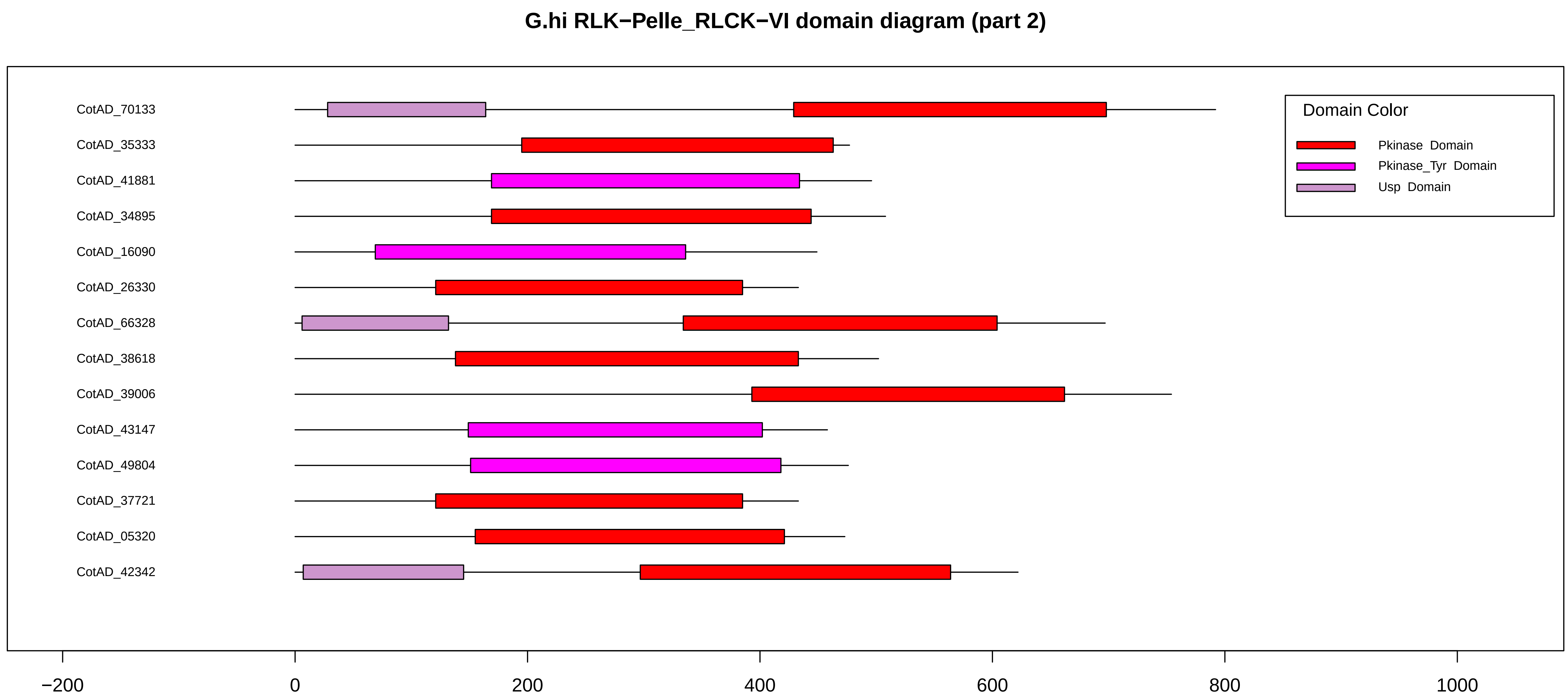
G.hi RLK−Pelle_RLCK−VI domain diagram (part 2)
CotAD_70133
CotAD_35333
CotAD_41881
CotAD_34895
CotAD_16090
CotAD_26330
CotAD_66328
CotAD_38618
CotAD_39006
CotAD_43147
CotAD_49804
CotAD_37721
CotAD_05320
CotAD_42342
Domain Color
Pkinase Domain
Pkinase_Tyr Domain
Usp Domain
−200
0
200
400
600
800
1000

G.hi RLK−Pelle_RLCK−V domain diagram (part 1)

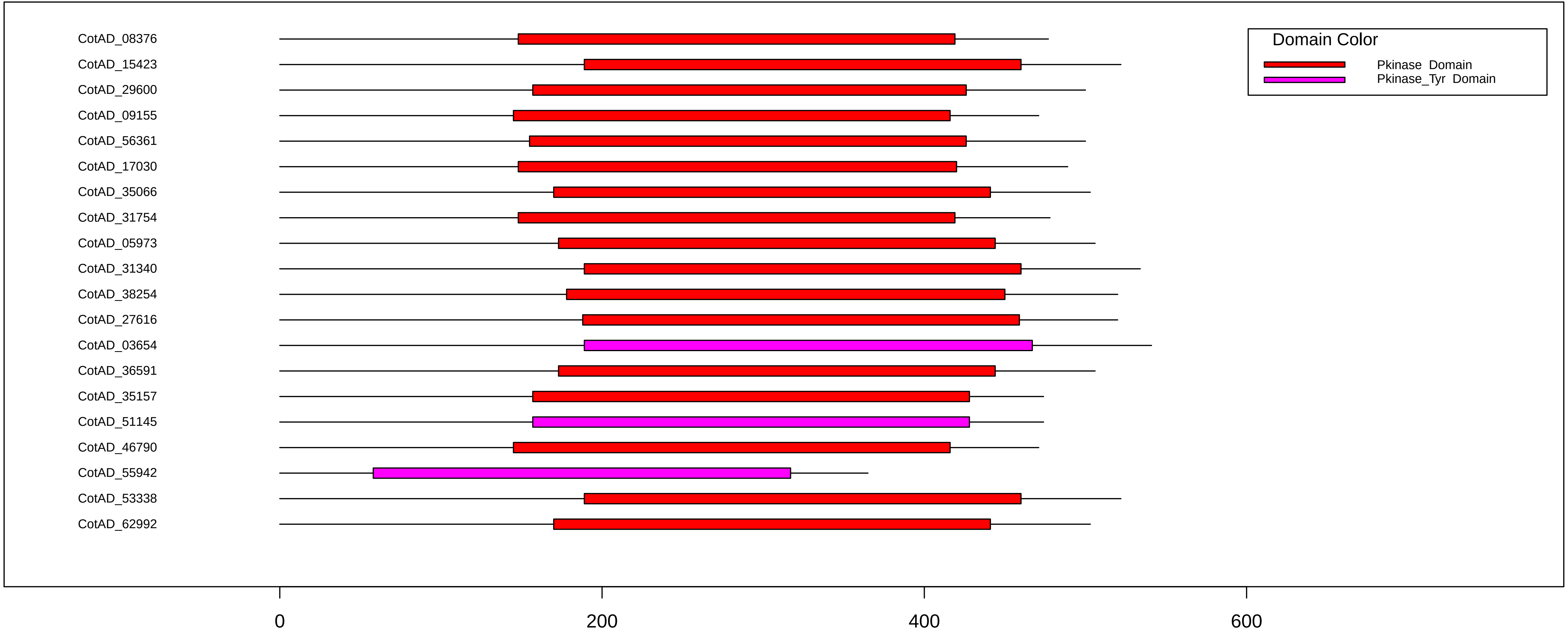

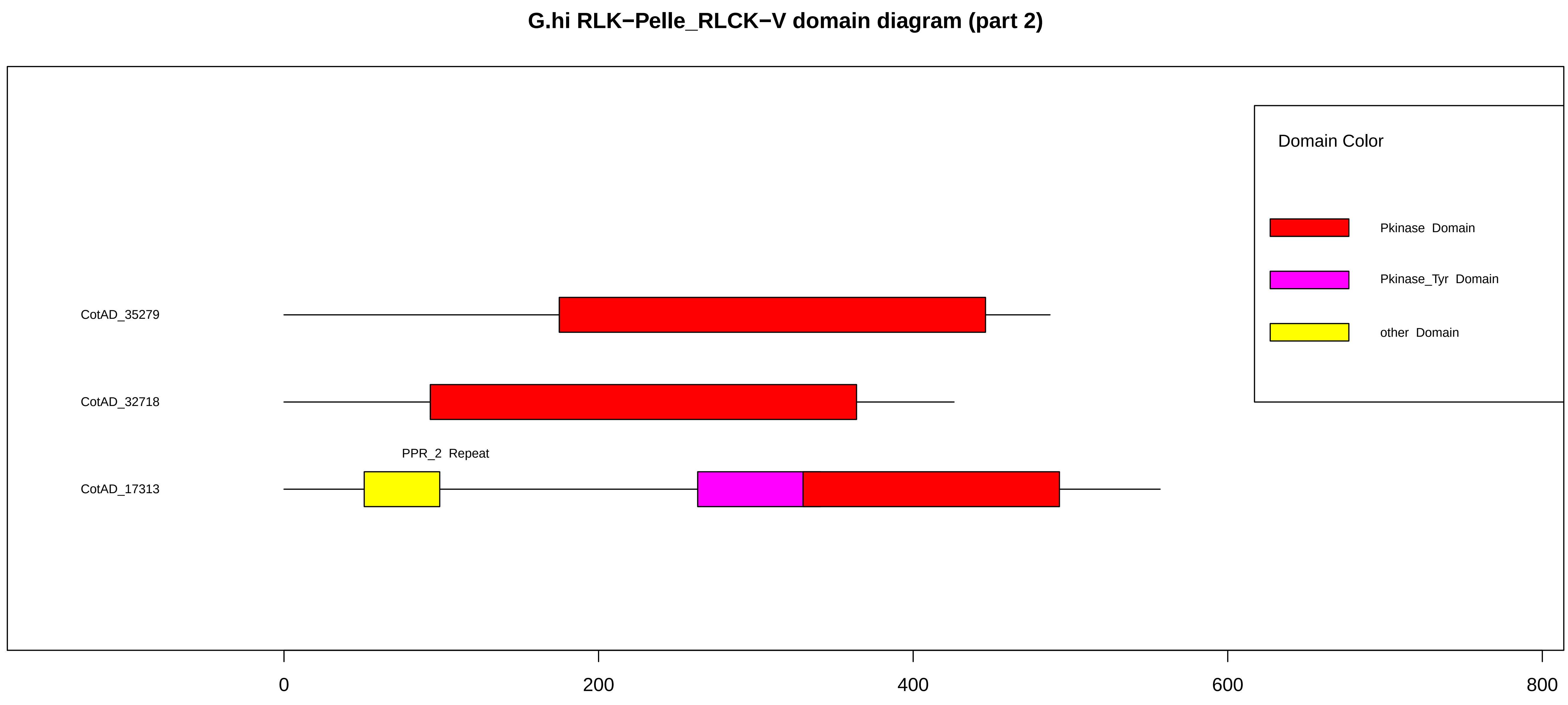
G.hi RLK−Pelle_RLCK−V domain diagram (part 2)
Domain Color
Pkinase Domain
Pkinase_Tyr Domain
other Domain
CotAD_35279
CotAD_32718
PPR_2 Repeat
CotAD_17313
0
200
400
600
800

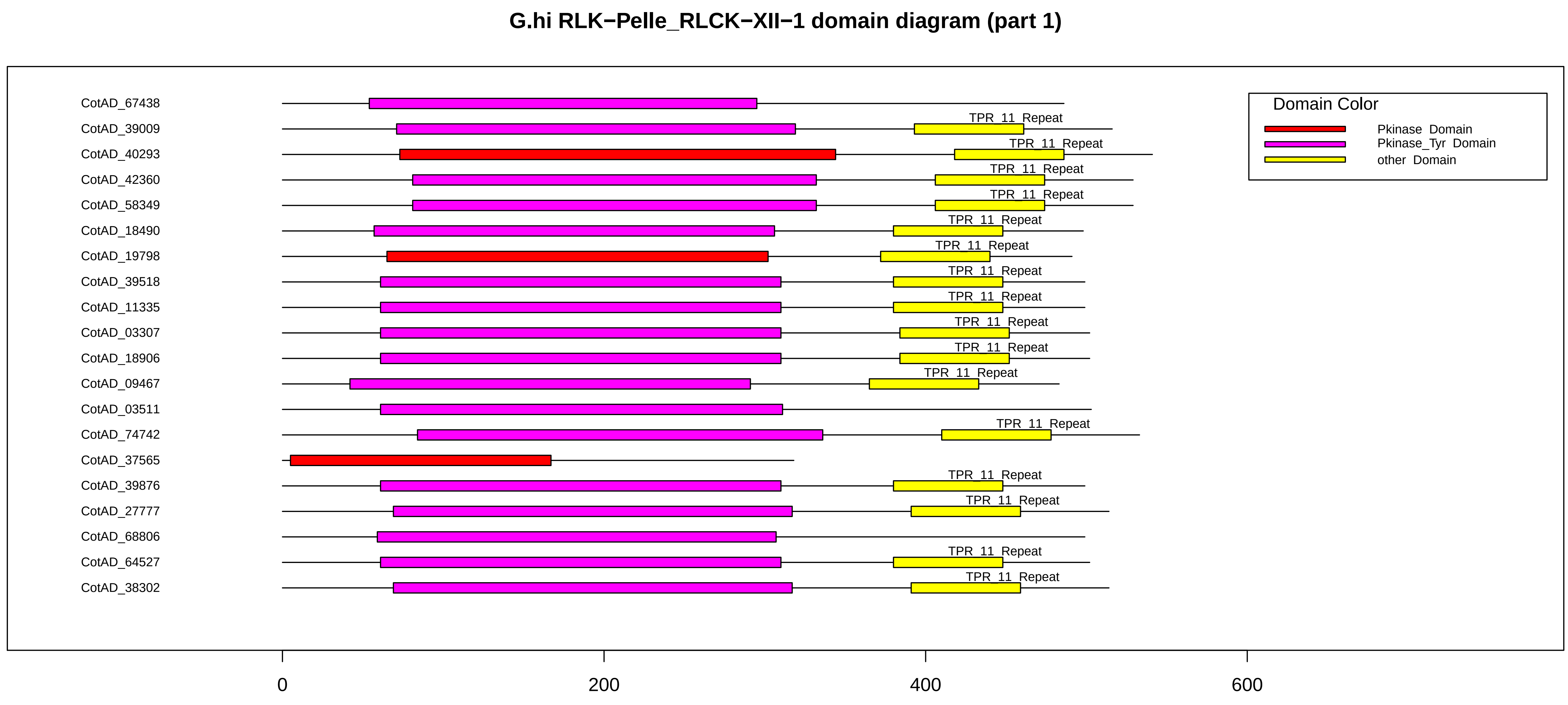
G.hi RLK−Pelle_RLCK−XII−1 domain diagram (part 1)
Domain Color
Pkinase_Domain
Pkinase_Tyr_Domain
other_Domain
CotAD_67438
CotAD_39009
CotAD_40293
CotAD_42360
CotAD_58349
CotAD_18490
CotAD_19798
CotAD_39518
CotAD_11335
CotAD_03307
CotAD_18906
CotAD_09467
CotAD_03511
CotAD_74742
CotAD_37565
CotAD_39876
CotAD_27777
CotAD_68806
CotAD_64527
CotAD_38302
TPR_11_Repeat
0
200
400
600

G.hi RLK−Pelle_RLCK−XII−1 domain diagram (part 2)

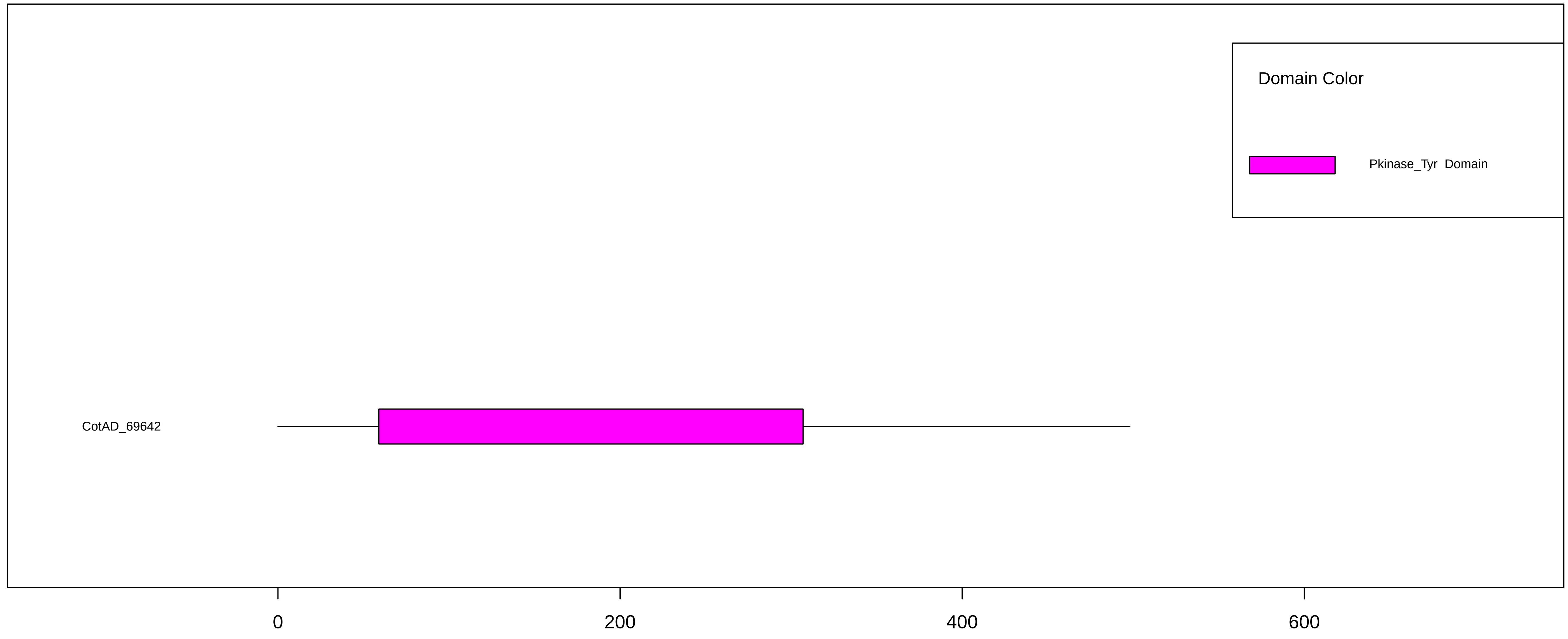

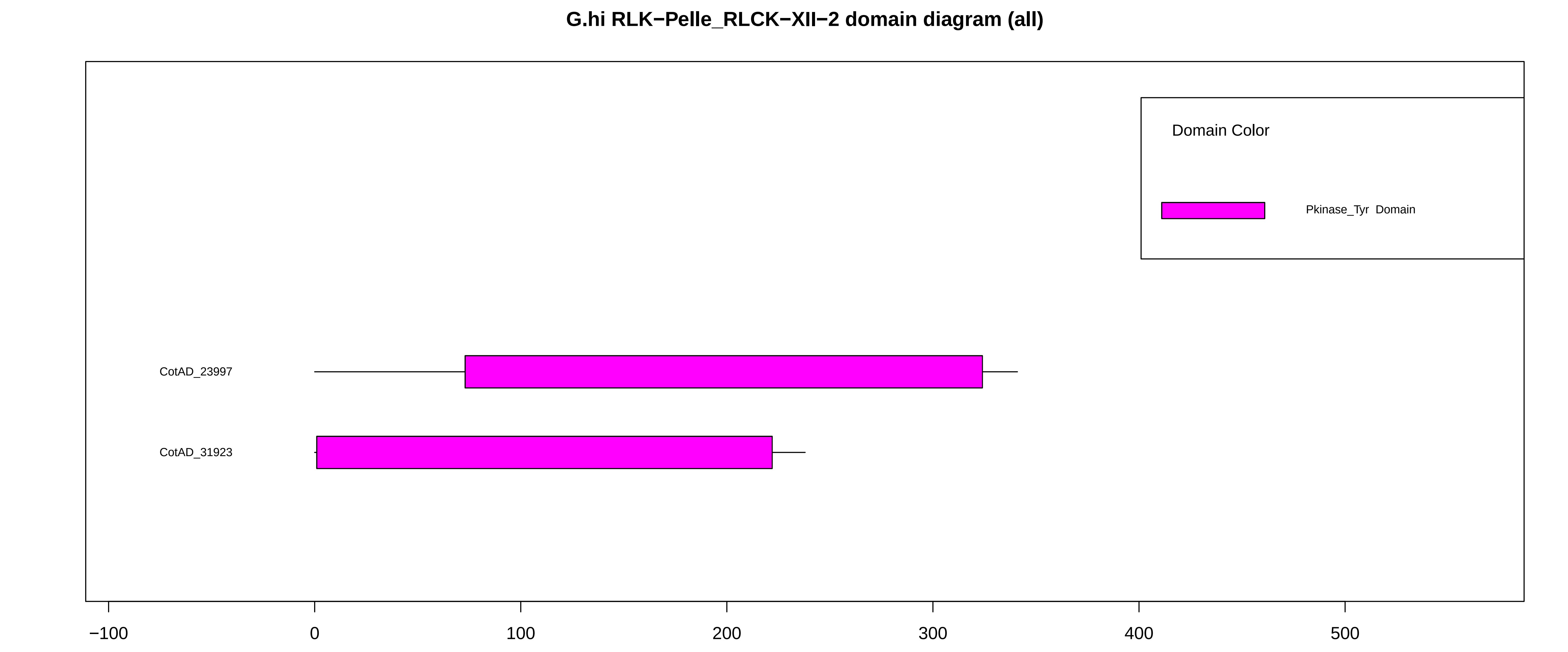

G.hi RLK−Pelle_RLCK−XII−2 domain diagram (all)
Domain Color
Pkinase_Tyr Domain
CotAD_23997
CotAD_31923
−100
0
100
200
300
400
500

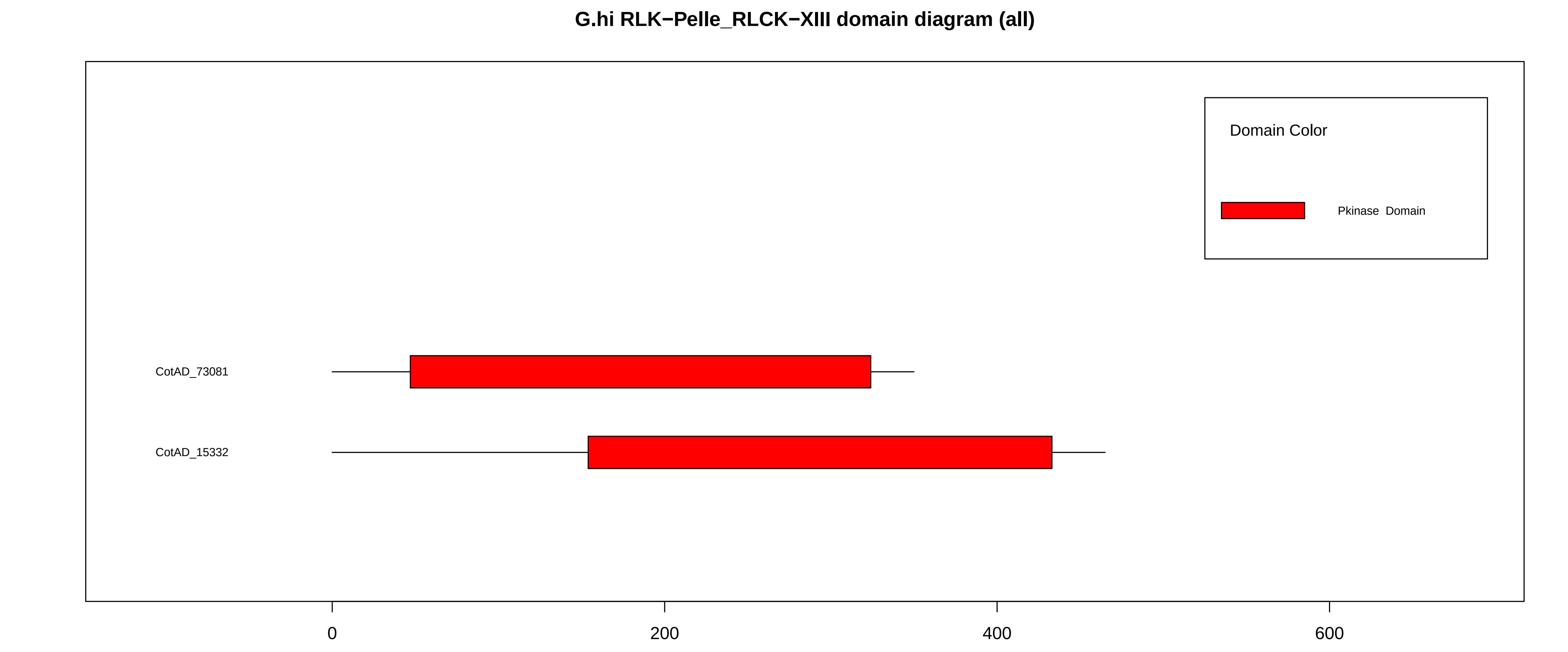
G.hi RLK−Pelle_RLCK−XIII domain diagram (all)
Domain Color
Pkinase Domain
CotAD_73081
CotAD_15332
0
200
400
600

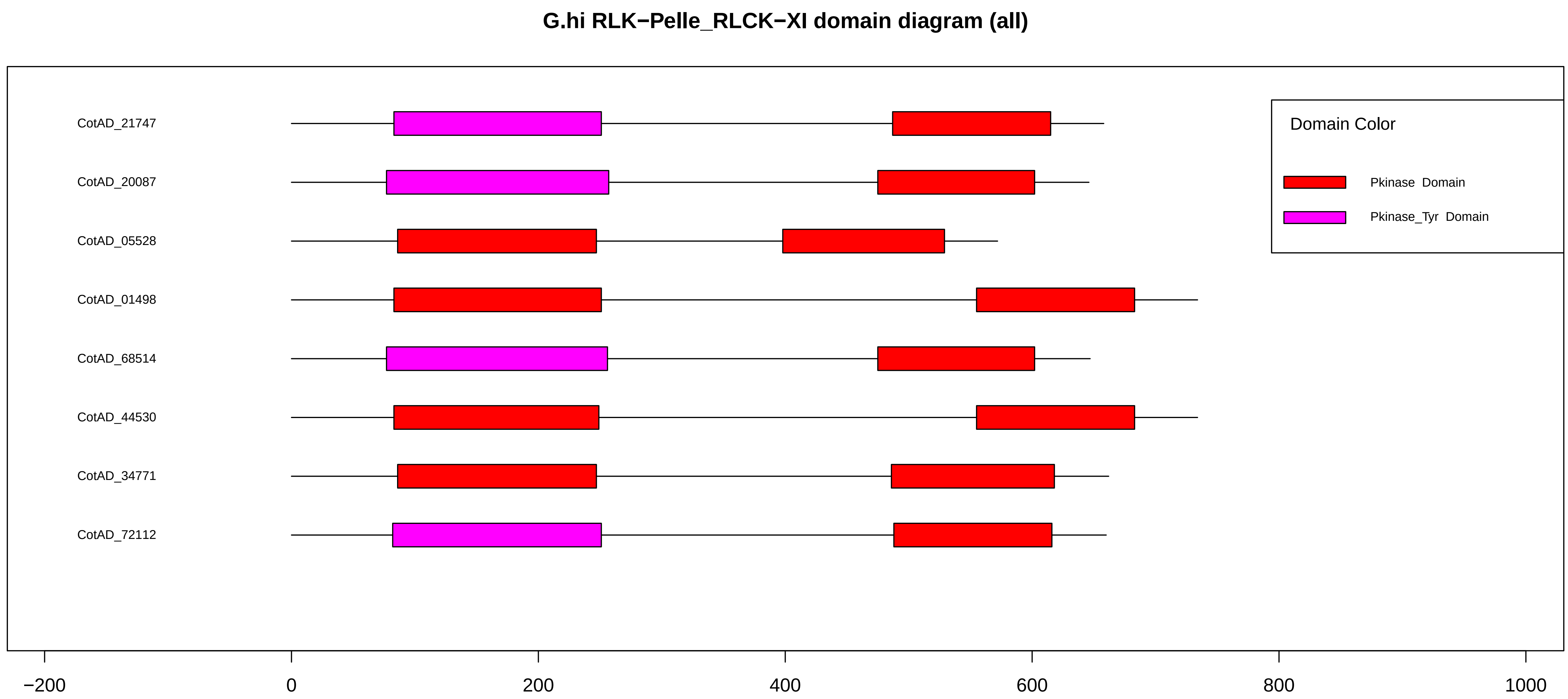

G.hi RLK−Pelle_RLCK−XI domain diagram (all)
CotAD_21747
CotAD_20087
CotAD_05528
CotAD_01498
CotAD_68514
CotAD_44530
CotAD_34771
CotAD_72112
Domain Color
Pkinase Domain
Pkinase_Tyr Domain
−200
0
200
400
600
800
1000

G.hi RLK−Pelle_RLCK−X domain diagram (all)

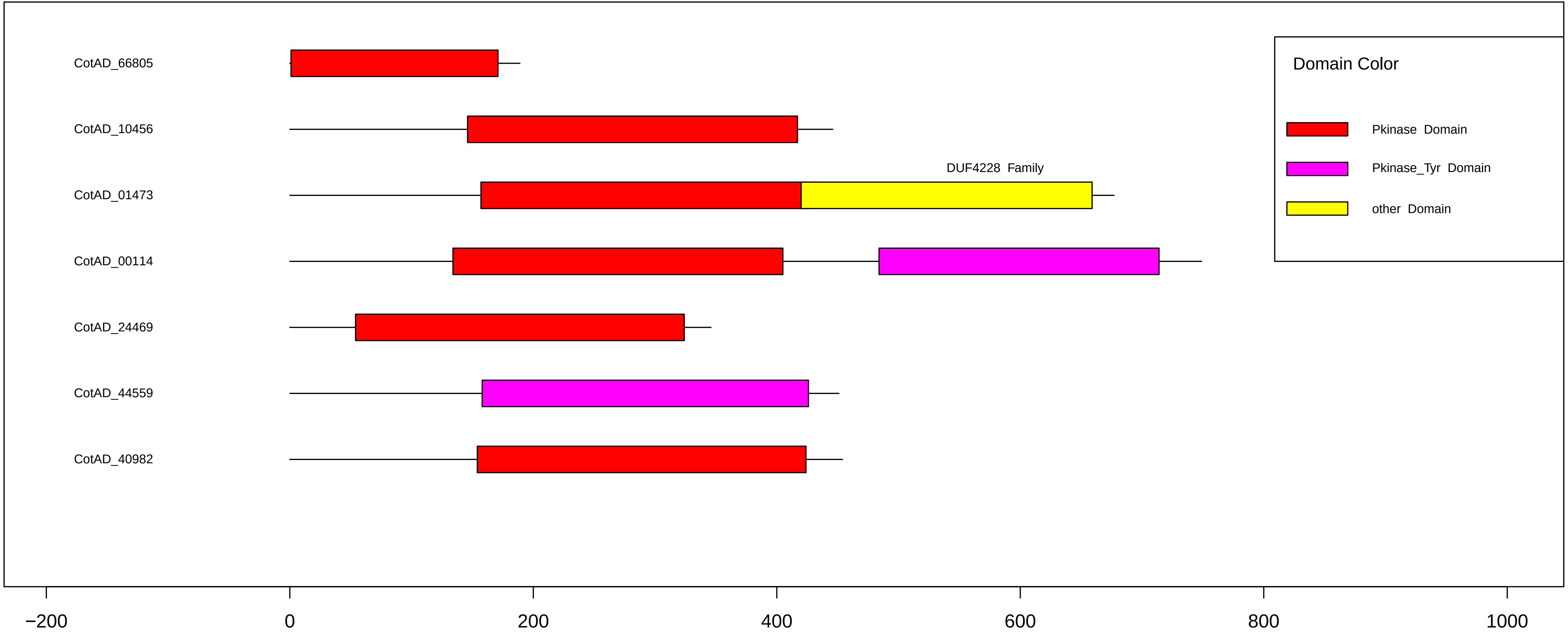

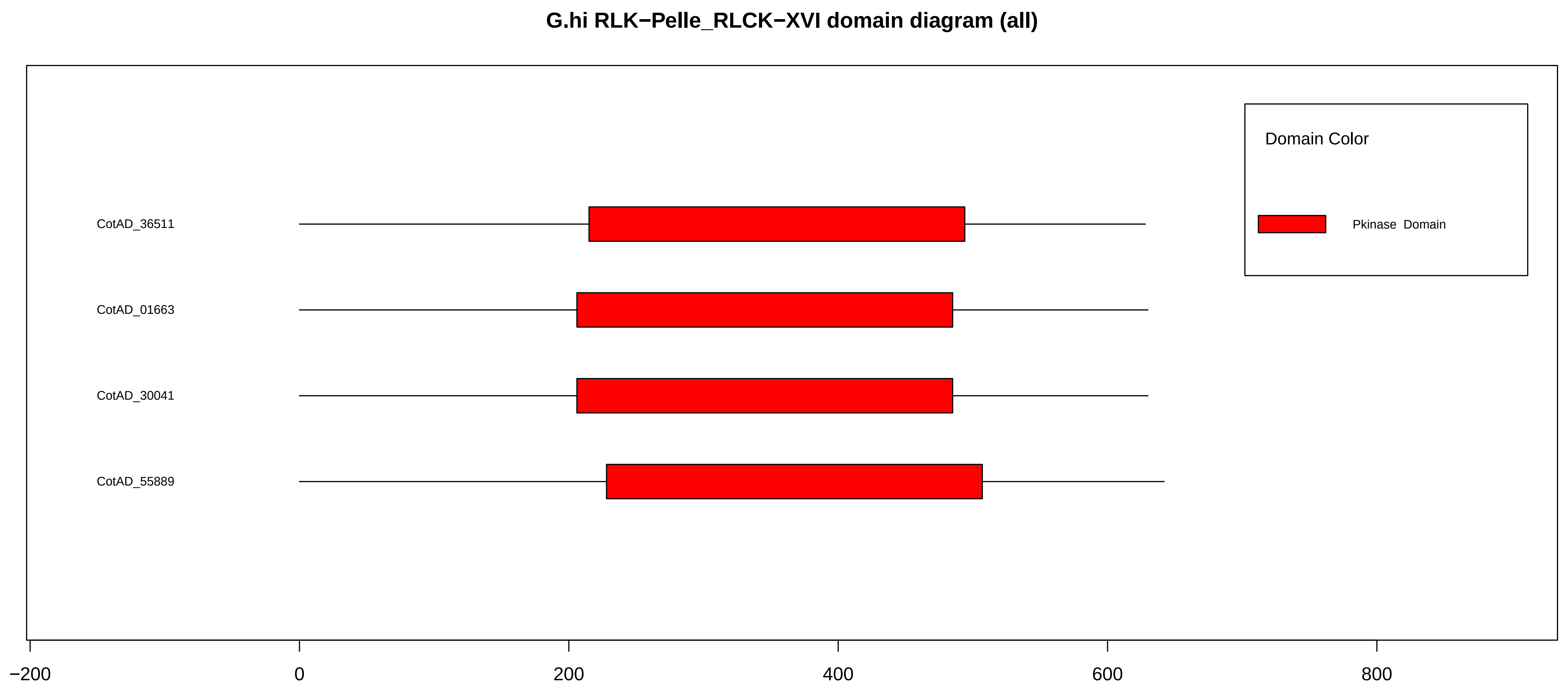

G.hi RLK−Pelle_RLCK−XVI domain diagram (all)
Domain Color
Pkinase Domain
CotAD_36511
CotAD_01663
CotAD_30041
CotAD_55889
−200
0
200
400
600
800

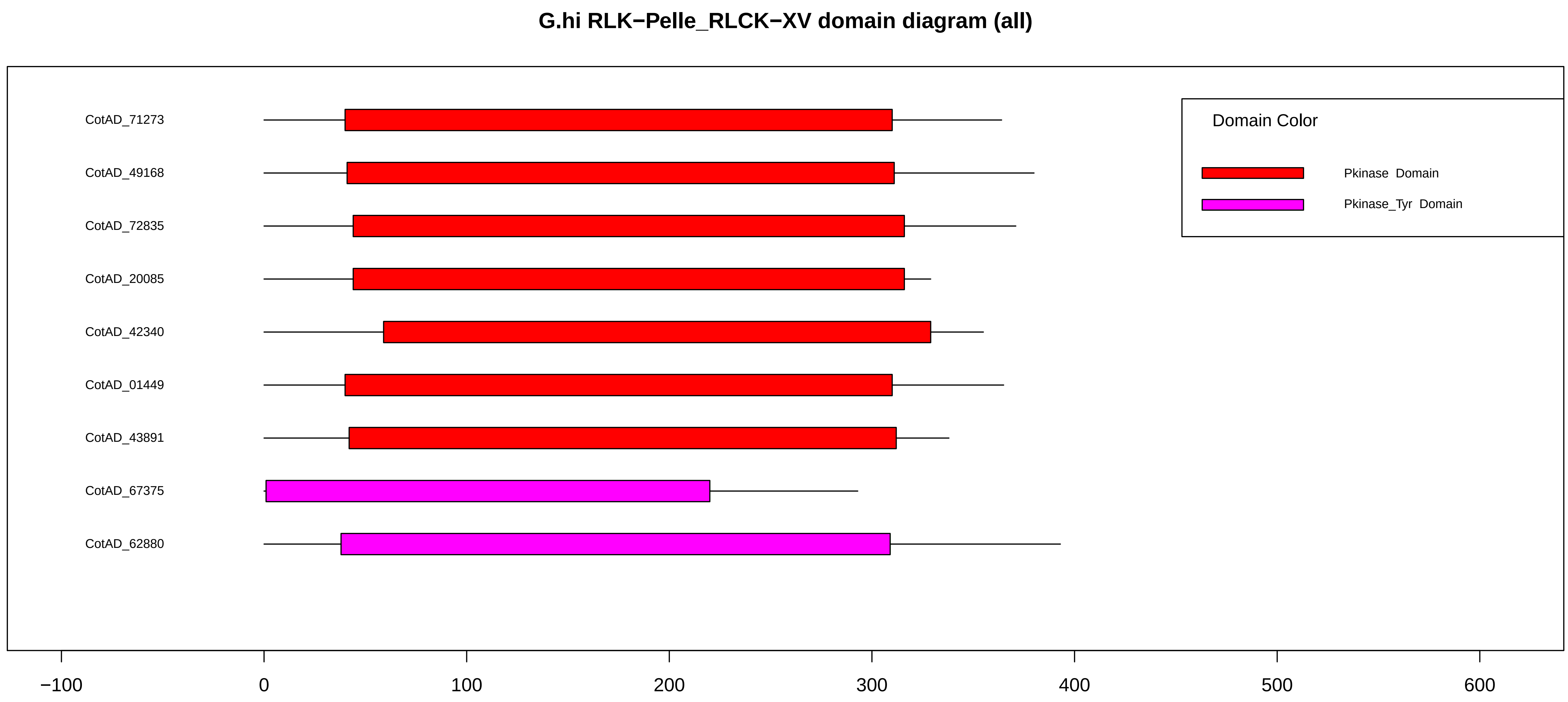
G.hi RLK−Pelle_RLCK−XV domain diagram (all)
CotAD_71273
CotAD_49168
CotAD_72835
CotAD_20085
CotAD_42340
CotAD_01449
CotAD_43891
CotAD_67375
CotAD_62880
Domain Color
Pkinase Domain
Pkinase_Tyr Domain
−100
0
100
200
300
400
500
600

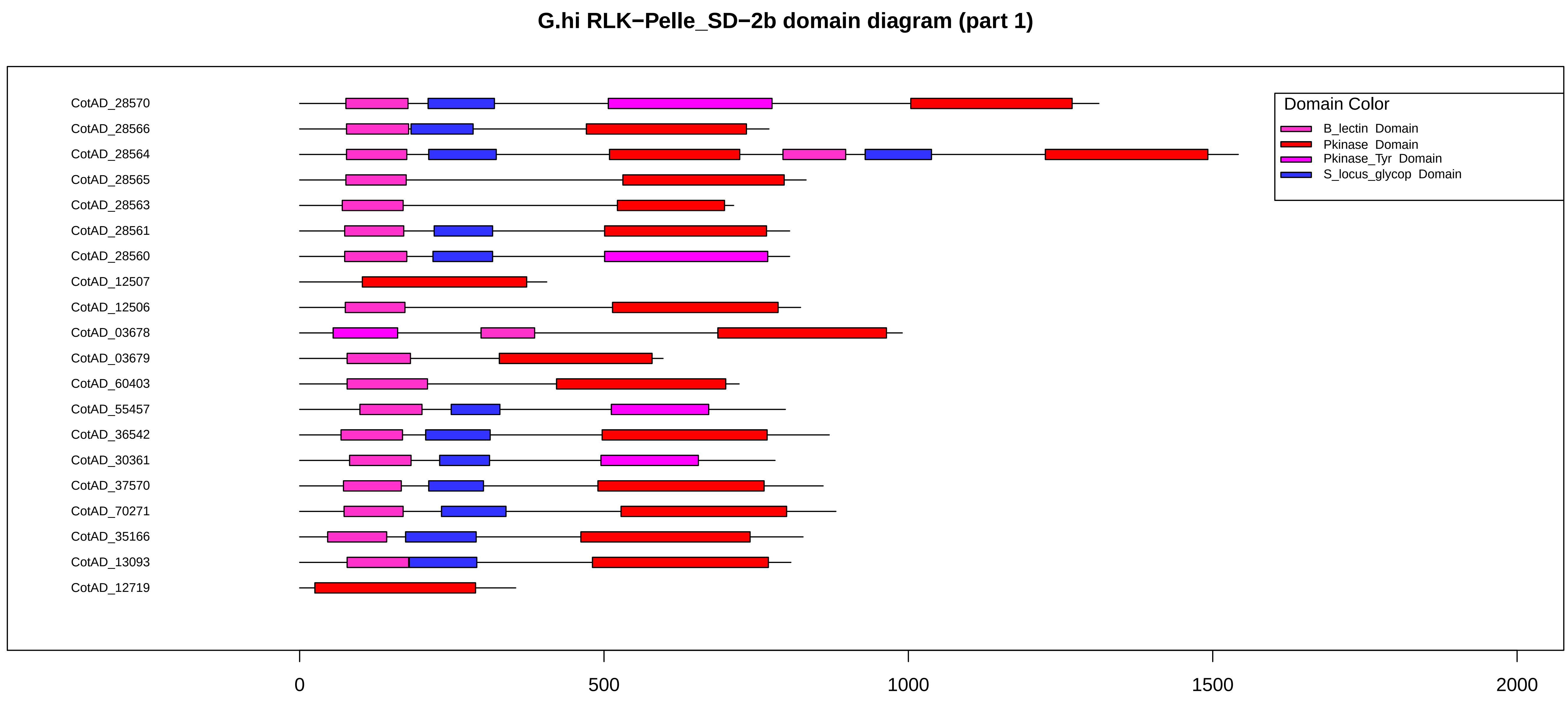

G.hi RLK−Pelle_SD−2b domain diagram (part 1)
CotAD_28570
CotAD_28566
CotAD_28564
CotAD_28565
CotAD_28563
CotAD_28561
CotAD_28560
CotAD_12507
CotAD_12506
CotAD_03678
CotAD_03679
CotAD_60403
CotAD_55457
CotAD_36542
CotAD_30361
CotAD_37570
CotAD_70271
CotAD_35166
CotAD_13093
CotAD_12719
Domain Color
B_lectin Domain
Pkinase Domain
Pkinase_Tyr Domain
S_locus_glycop Domain
0
500
1000
1500
2000

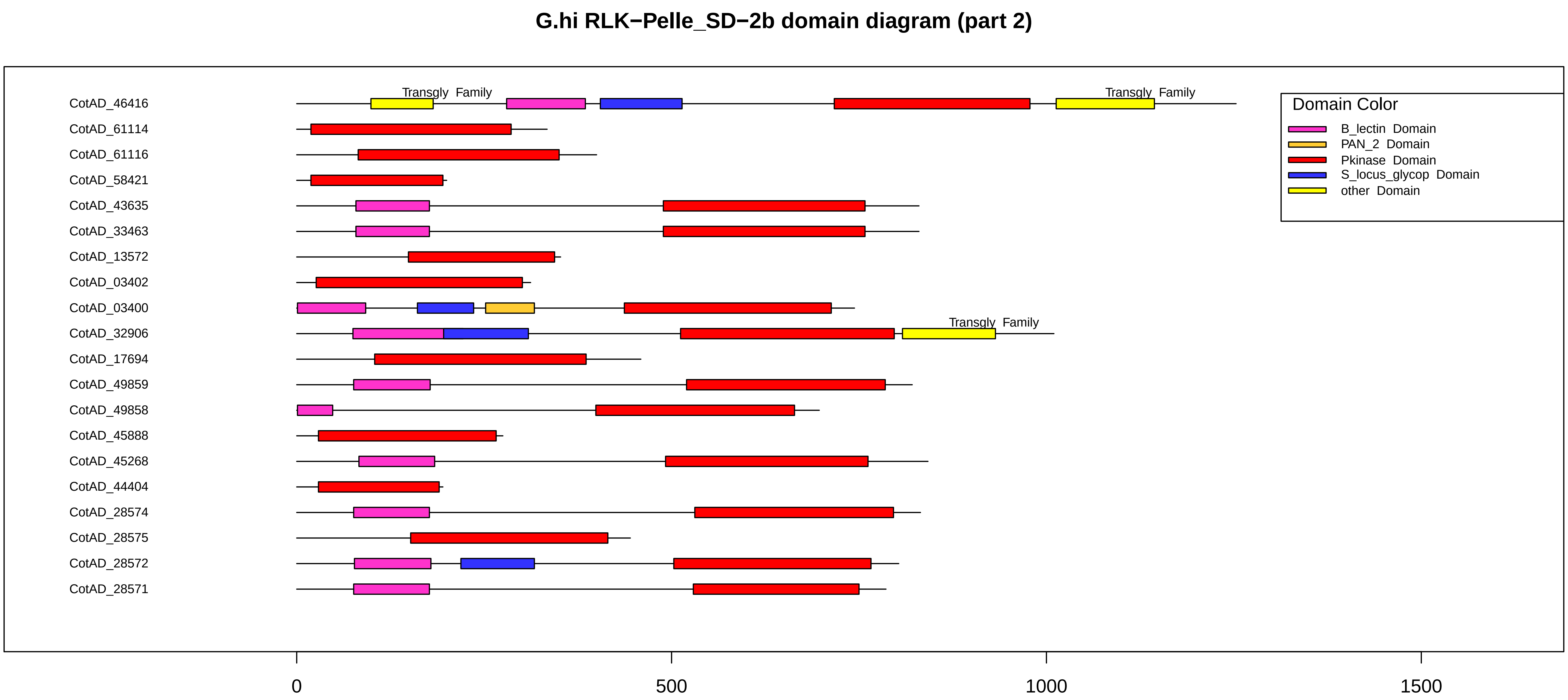
G.hi RLK−Pelle_SD−2b domain diagram (part 2)
CotAD_46416
CotAD_61114
CotAD_61116
CotAD_58421
CotAD_43635
CotAD_33463
CotAD_13572
CotAD_03402
CotAD_03400
CotAD_32906
CotAD_17694
CotAD_49859
CotAD_49858
CotAD_45888
CotAD_45268
CotAD_44404
CotAD_28574
CotAD_28575
CotAD_28572
CotAD_28571
Transgly Family
Transgly Family
Transgly Family
Domain Color
B_lectin Domain
PAN_2 Domain
Pkinase Domain
S_locus_glycop Domain
other Domain
0
500
1000
1500

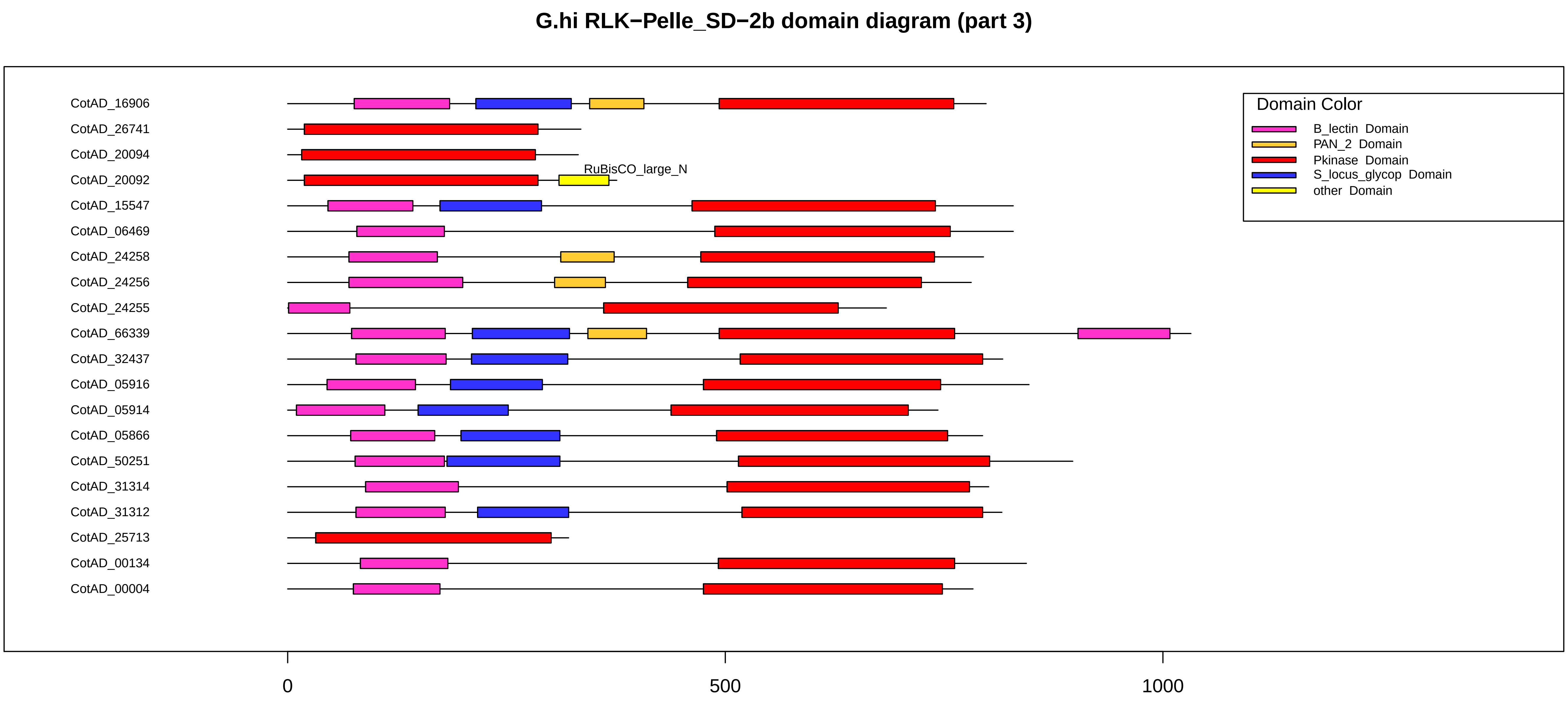
G.hi RLK−Pelle_SD−2b domain diagram (part 3)
CotAD_16906
CotAD_26741
CotAD_20094
CotAD_20092
RuBisCO_large_N
CotAD_15547
CotAD_06469
CotAD_24258
CotAD_24256
CotAD_24255
CotAD_66339
CotAD_32437
CotAD_05916
CotAD_05914
CotAD_05866
CotAD_50251
CotAD_31314
CotAD_31312
CotAD_25713
CotAD_00134
CotAD_00004
Domain Color
B_lectin Domain
PAN_2 Domain
Pkinase Domain
S_locus_glycop Domain
other Domain
0
500
1000

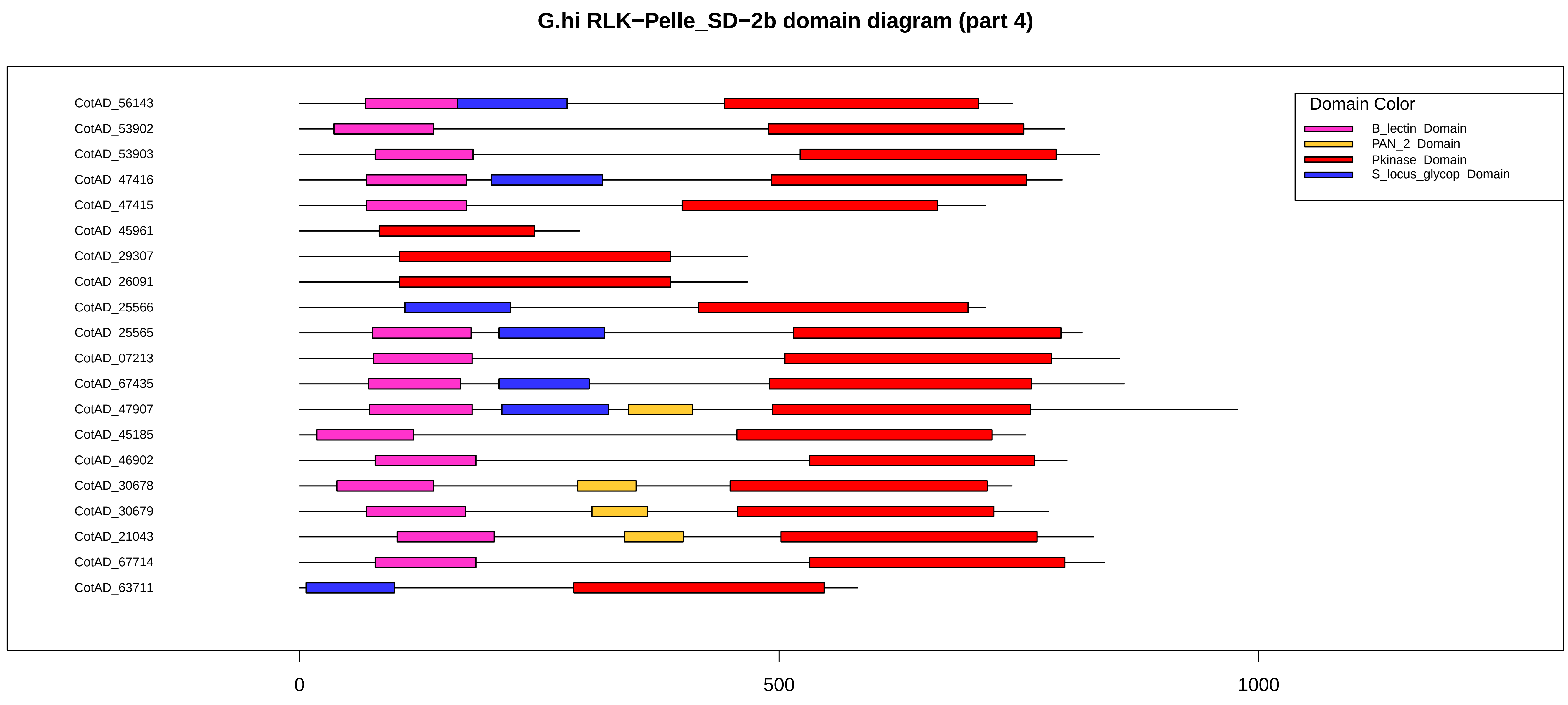
G.hi RLK−Pelle_SD−2b domain diagram (part 4)
CotAD_56143
CotAD_53902
CotAD_53903
CotAD_47416
CotAD_47415
CotAD_45961
CotAD_29307
CotAD_26091
CotAD_25566
CotAD_25565
CotAD_07213
CotAD_67435
CotAD_47907
CotAD_45185
CotAD_46902
CotAD_30678
CotAD_30679
CotAD_21043
CotAD_67714
CotAD_63711
Domain Color
B_lectin Domain
PAN_2 Domain
Pkinase Domain
S_locus_glycop Domain
0
500
1000

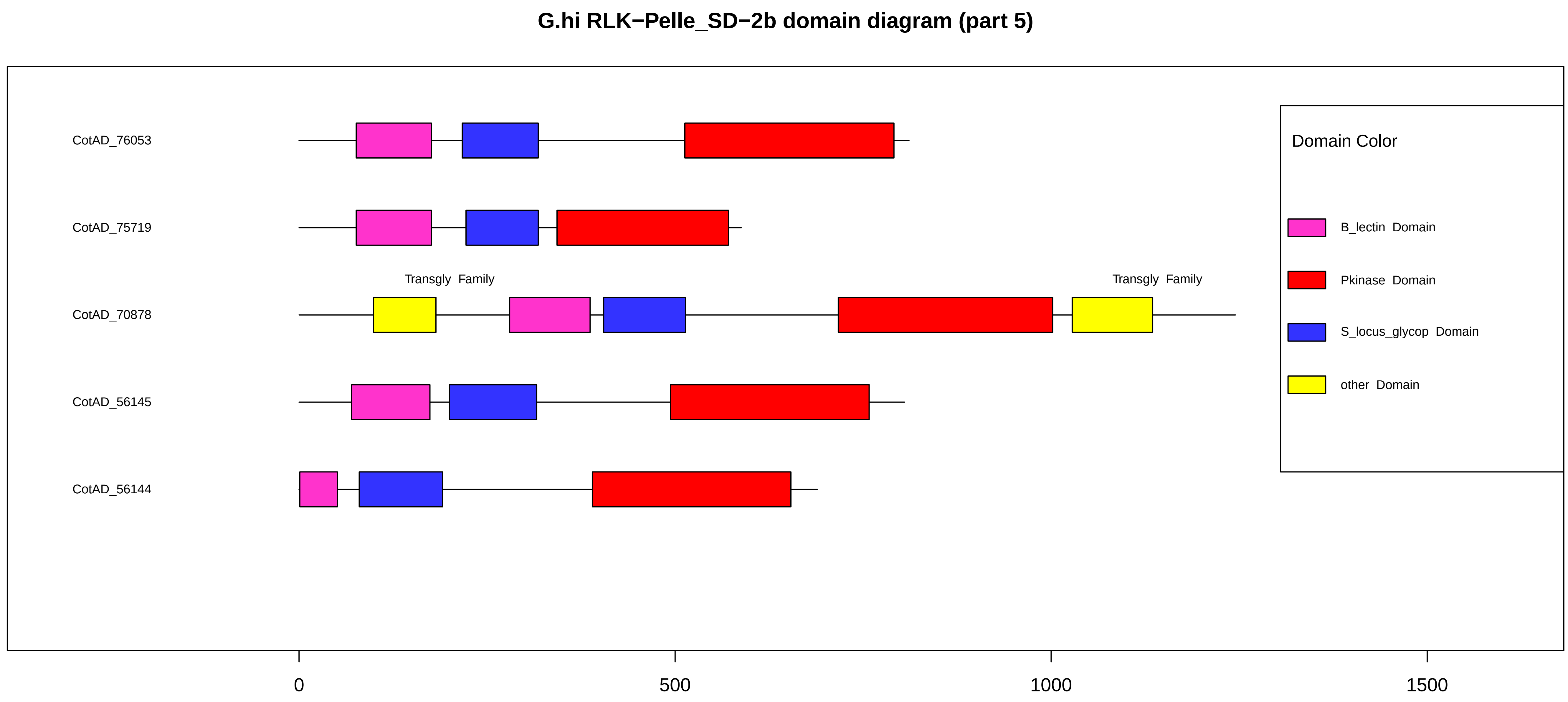
G.hi RLK−Pelle_SD−2b domain diagram (part 5)
CotAD_76053
CotAD_75719
CotAD_70878
CotAD_56145
CotAD_56144
Transgly Family
Transgly Family
Domain Color
B_lectin Domain
Pkinase Domain
S_locus_glycop Domain
other Domain
0
500
1000
1500

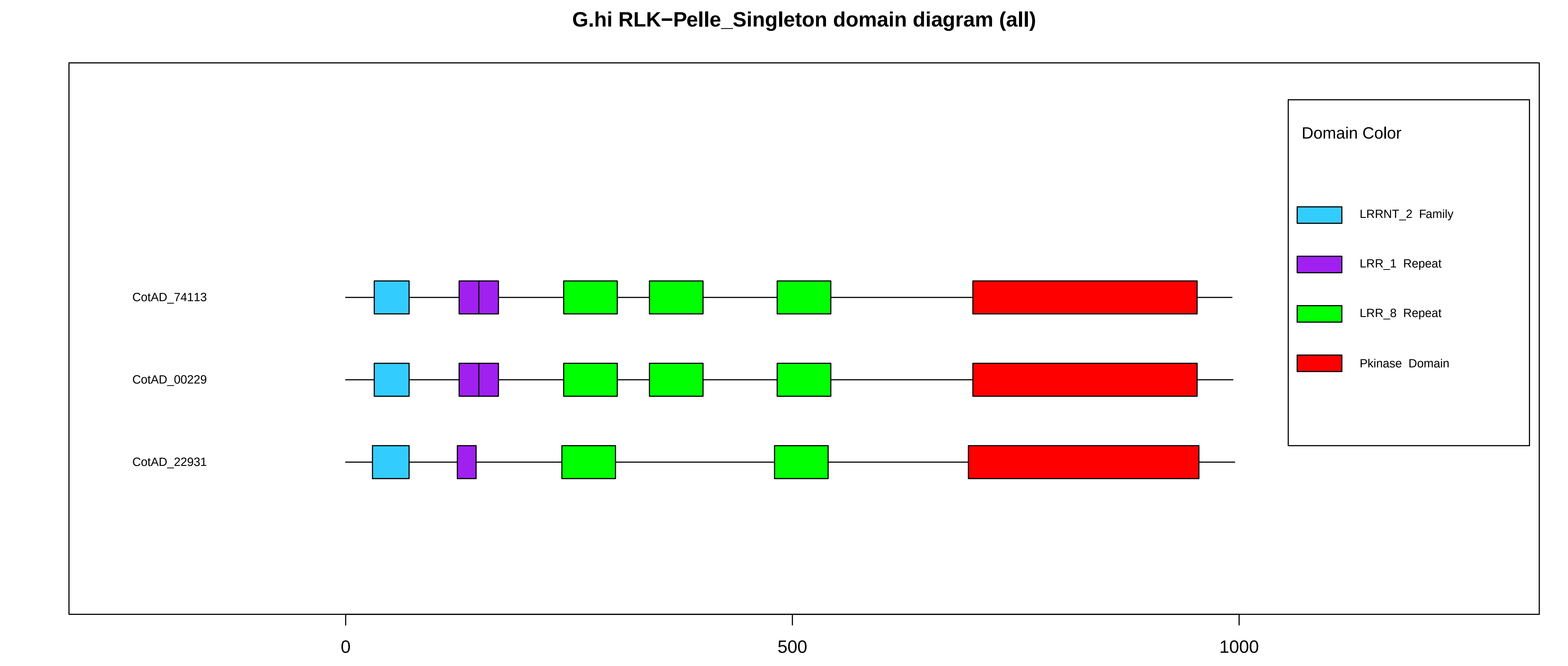
G.hi RLK−Pelle_Singleton domain diagram (all)
CotAD_74113
CotAD_00229
CotAD_22931
Domain Color
LRRNT_2 Family
LRR_1 Repeat
LRR_8 Repeat
Pkinase Domain
0
500
1000

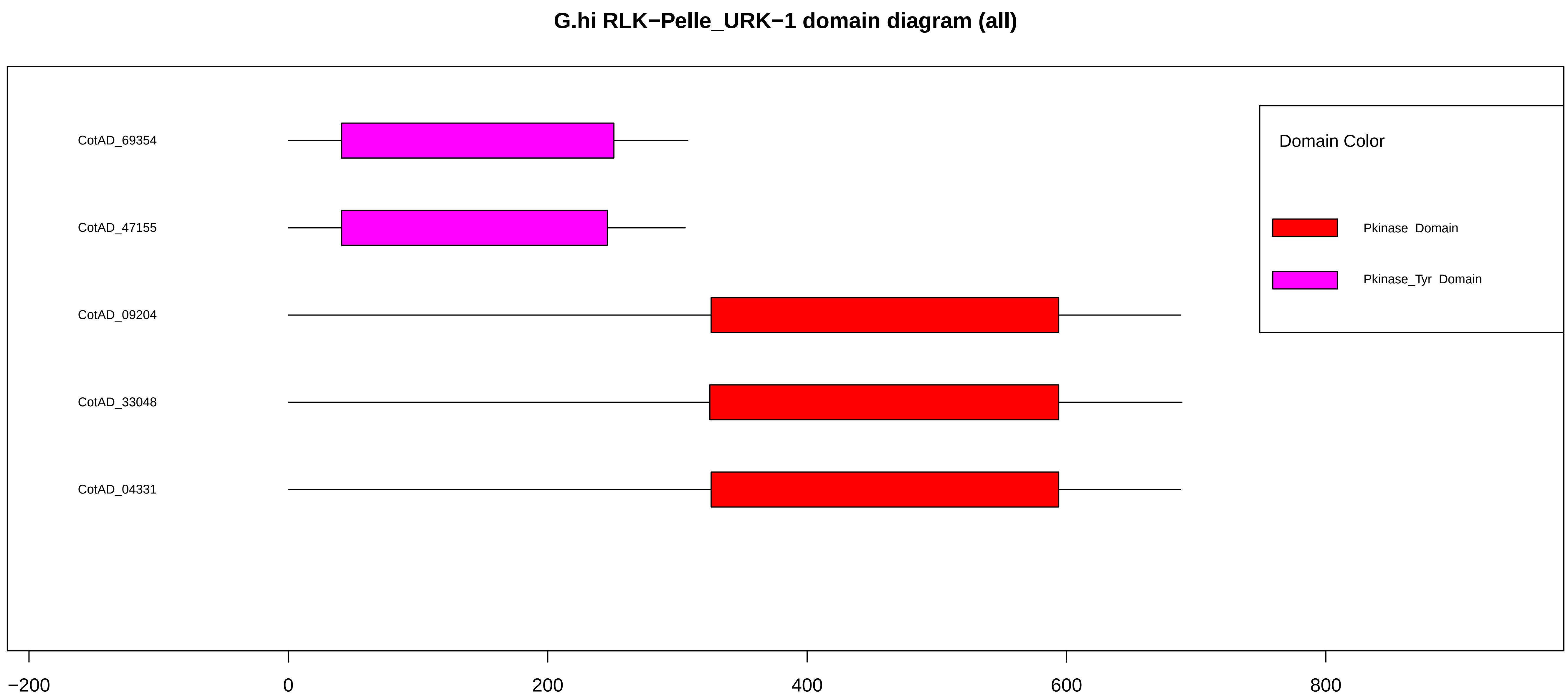

G.hi RLK−Pelle_URK−1 domain diagram (all)
CotAD_69354
CotAD_47155
CotAD_09204
CotAD_33048
CotAD_04331
Domain Color
Pkinase Domain
Pkinase_Tyr Domain
−200
0
200
400
600
800

G.hi RLK−Pelle_URK−2 domain diagram (all)

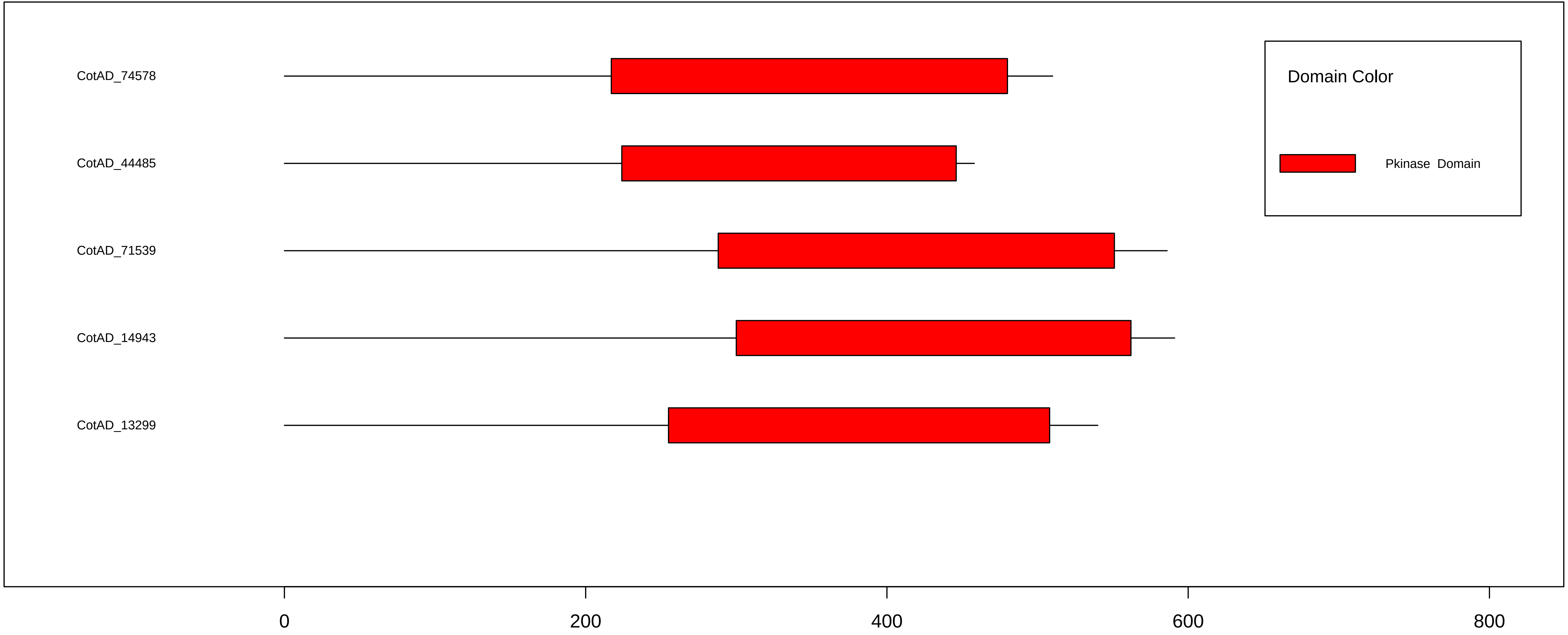

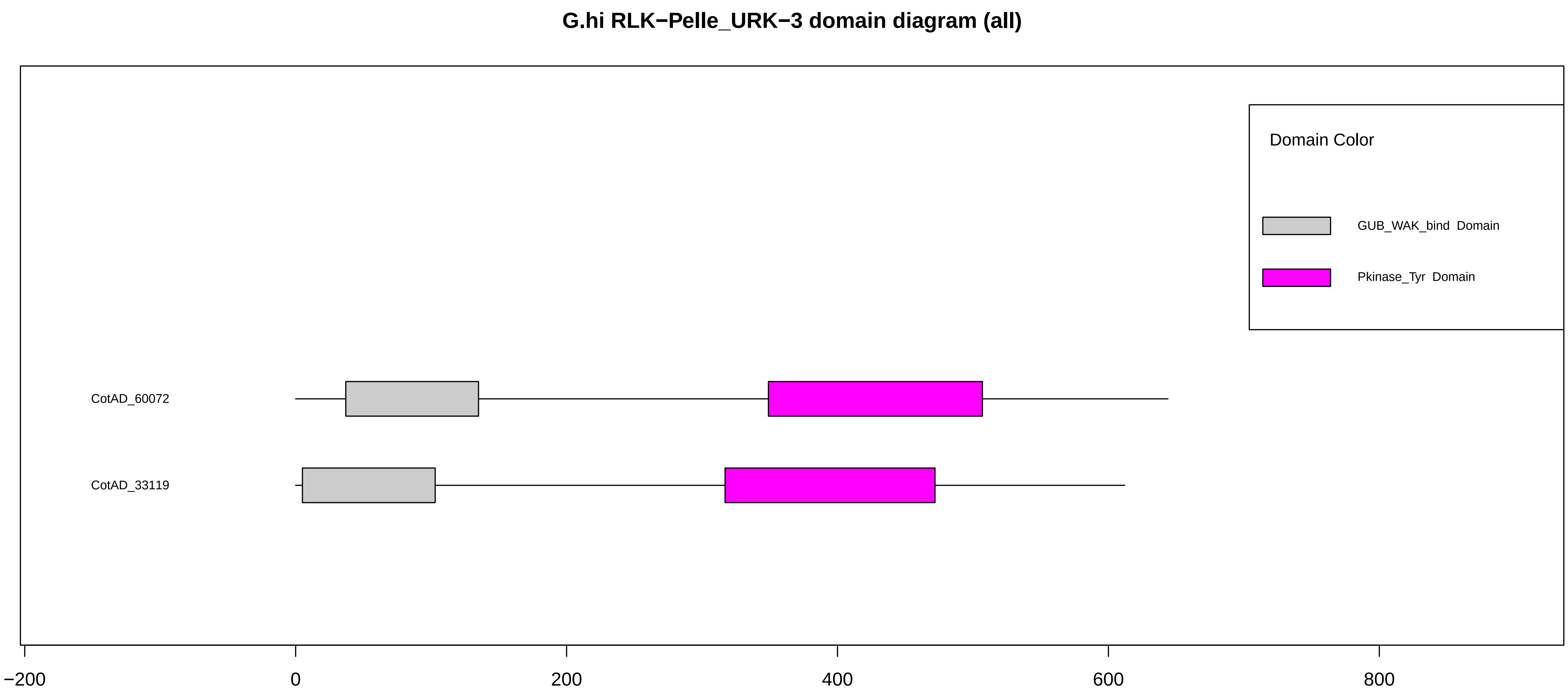
G.hi RLK−Pelle_URK−3 domain diagram (all)
Domain Color
GUB_WAK_bind Domain
Pkinase_Tyr Domain
CotAD_60072
CotAD_33119
−200
0
200
400
600
800

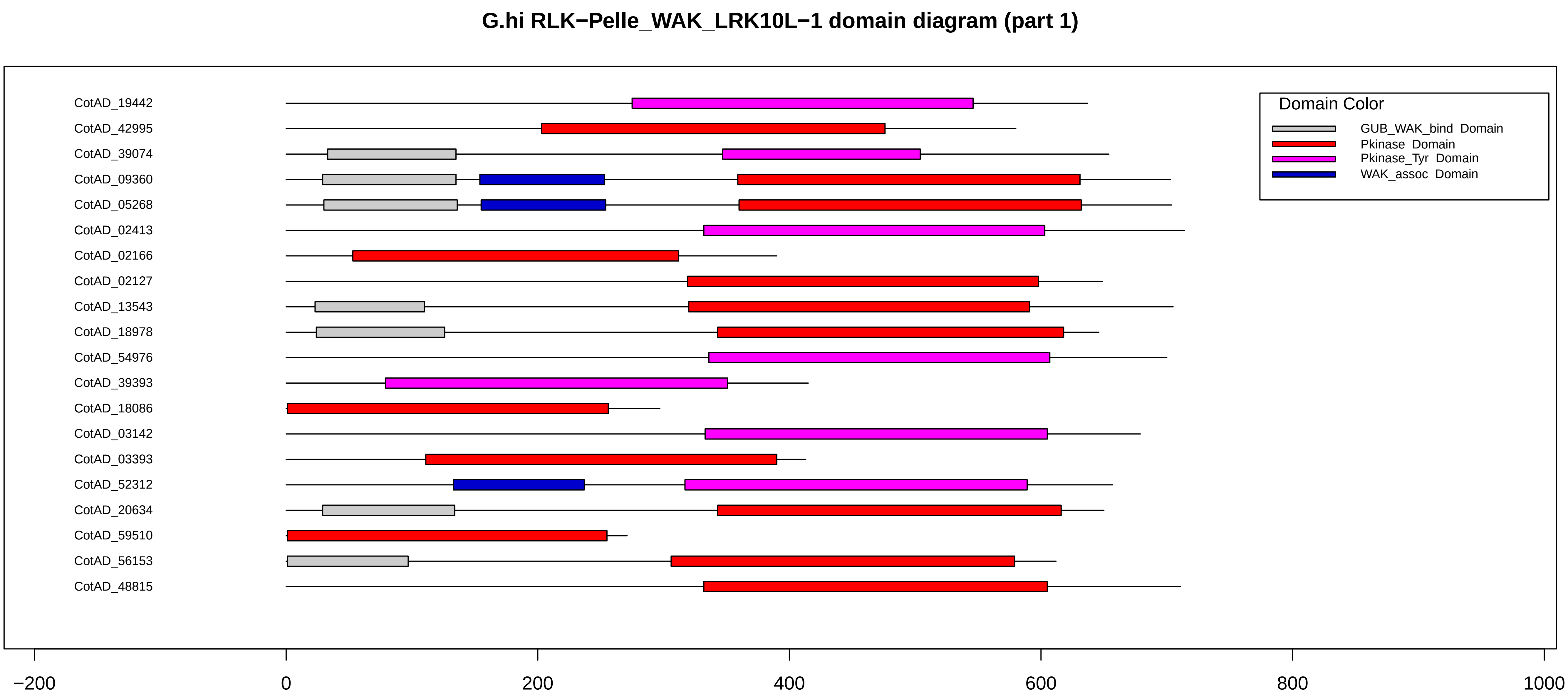
G.hi RLK−Pelle_WAK_LRK10L−1 domain diagram (part 1)
CotAD_19442
CotAD_42995
CotAD_39074
CotAD_09360
CotAD_05268
CotAD_02413
CotAD_02166
CotAD_02127
CotAD_13543
CotAD_18978
CotAD_54976
CotAD_39393
CotAD_18086
CotAD_03142
CotAD_03393
CotAD_52312
CotAD_20634
CotAD_59510
CotAD_56153
CotAD_48815
Domain Color
GUB_WAK_bind Domain
Pkinase Domain
Pkinase_Tyr Domain
WAK_assoc Domain
−200
0
200
400
600
800
1000

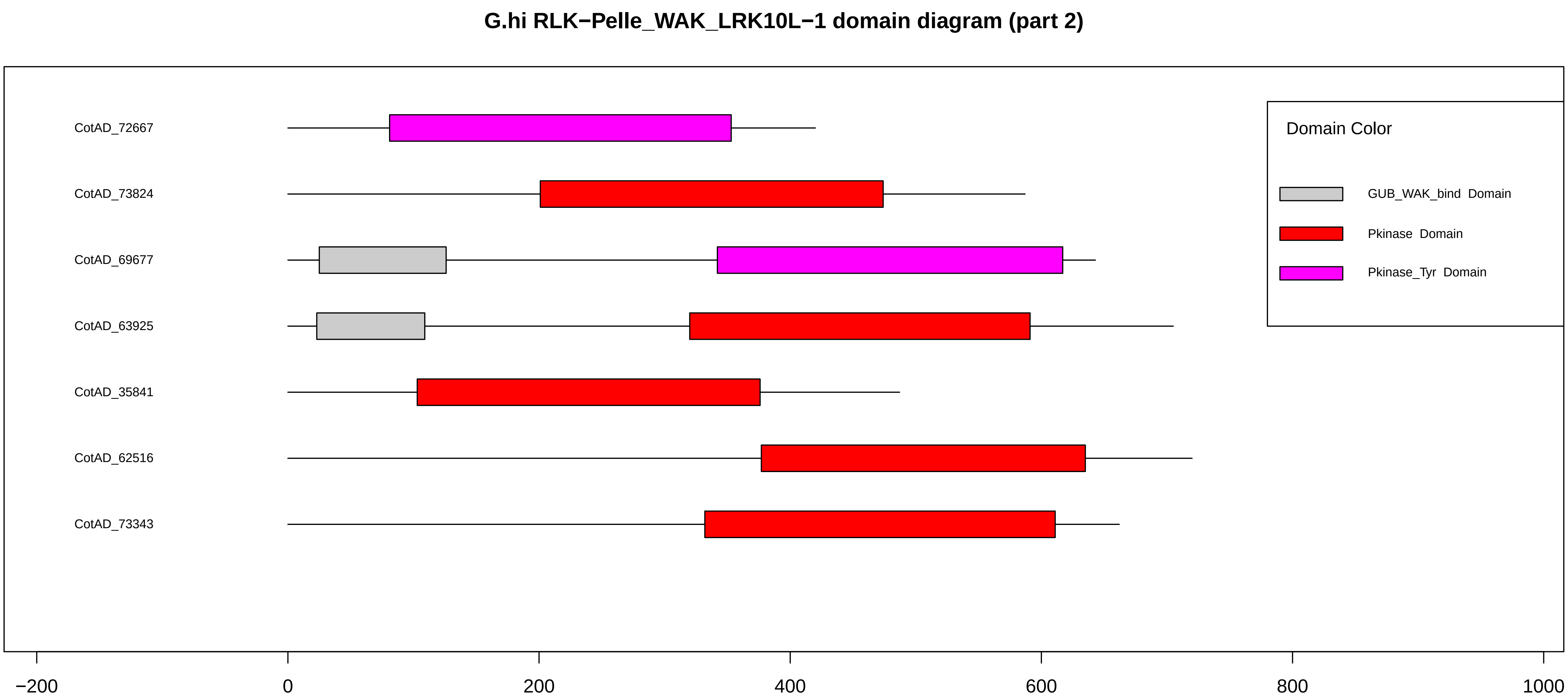

G.hi RLK−Pelle_WAK_LRK10L−1 domain diagram (part 2)
CotAD_72667
CotAD_73824
CotAD_69677
CotAD_63925
CotAD_35841
CotAD_62516
CotAD_73343
Domain Color
GUB_WAK_bind Domain
Pkinase Domain
Pkinase_Tyr Domain
−200
0
200
400
600
800
1000

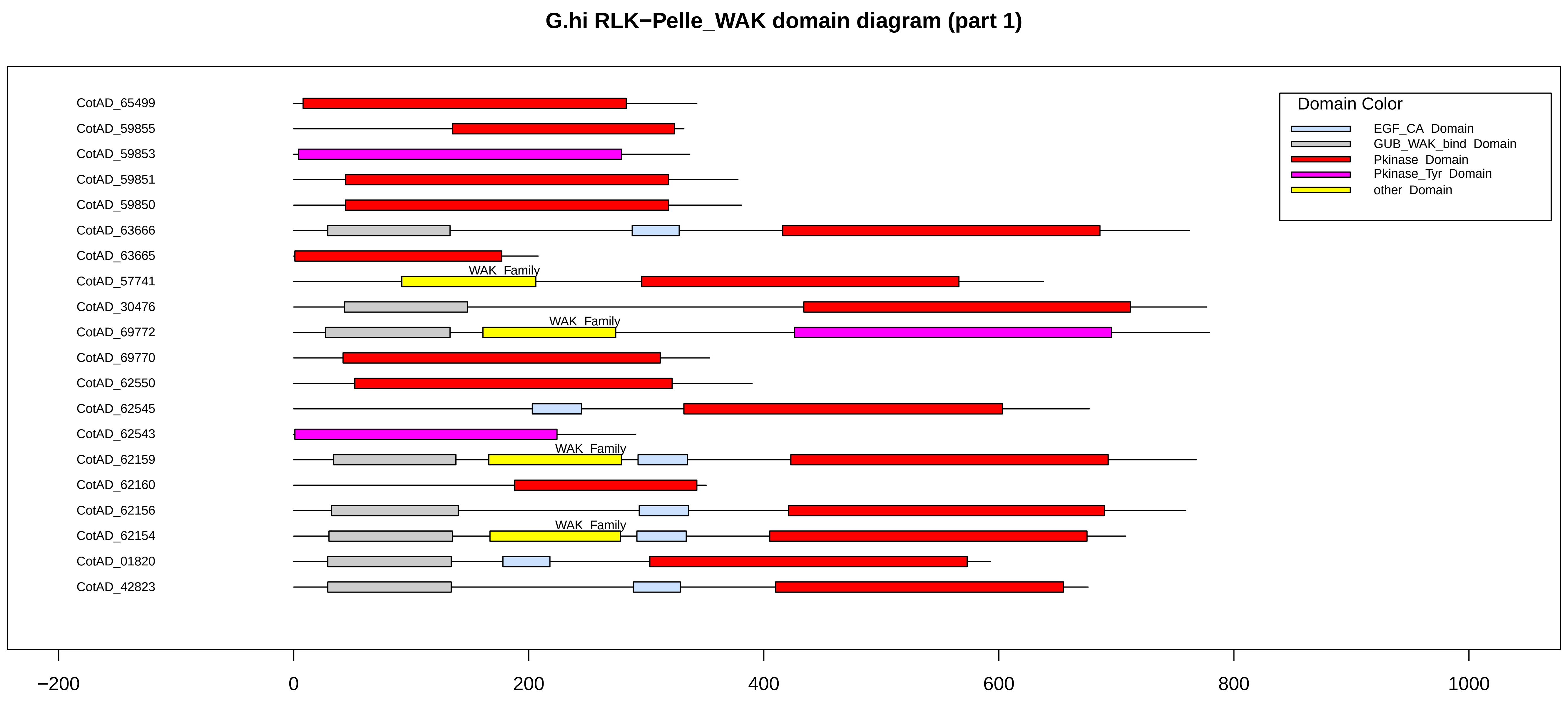
G.hi RLK−Pelle_WAK domain diagram (part 1)
CotAD_65499
CotAD_59855
CotAD_59853
CotAD_59851
CotAD_59850
CotAD_63666
CotAD_63665
CotAD_57741
CotAD_30476
CotAD_69772
CotAD_69770
CotAD_62550
CotAD_62545
CotAD_62543
CotAD_62159
CotAD_62160
CotAD_62156
CotAD_62154
CotAD_01820
CotAD_42823
WAK_Family
Domain Color
EGF_CA Domain
GUB_WAK_bind Domain
Pkinase Domain
Pkinase_Tyr Domain
other Domain
−200
0
200
400
600
800
1000

G.hi RLK−Pelle_WAK domain diagram (part 2)

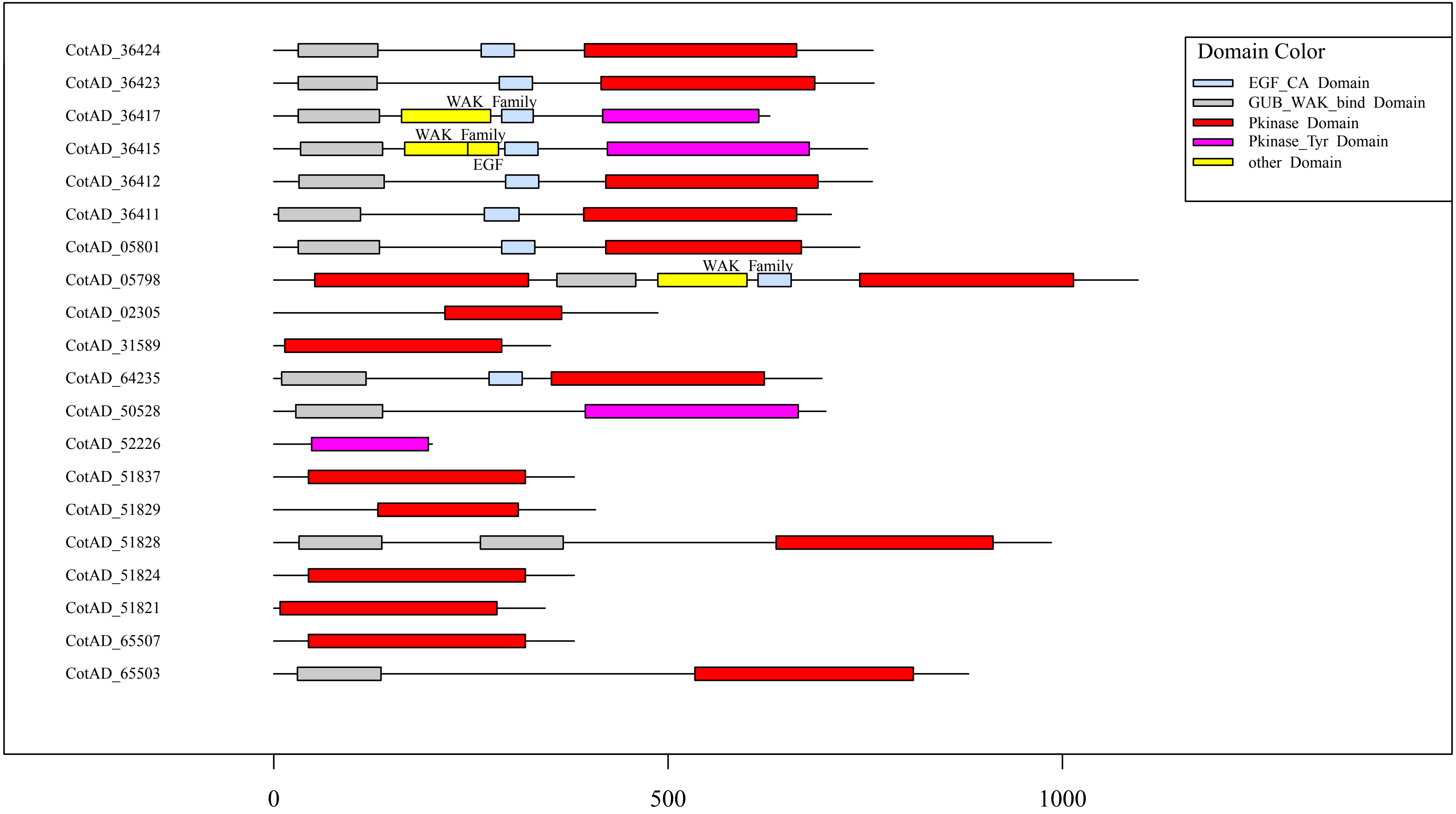

G.hi RLK−Pelle_WAK domain diagram (part 3)

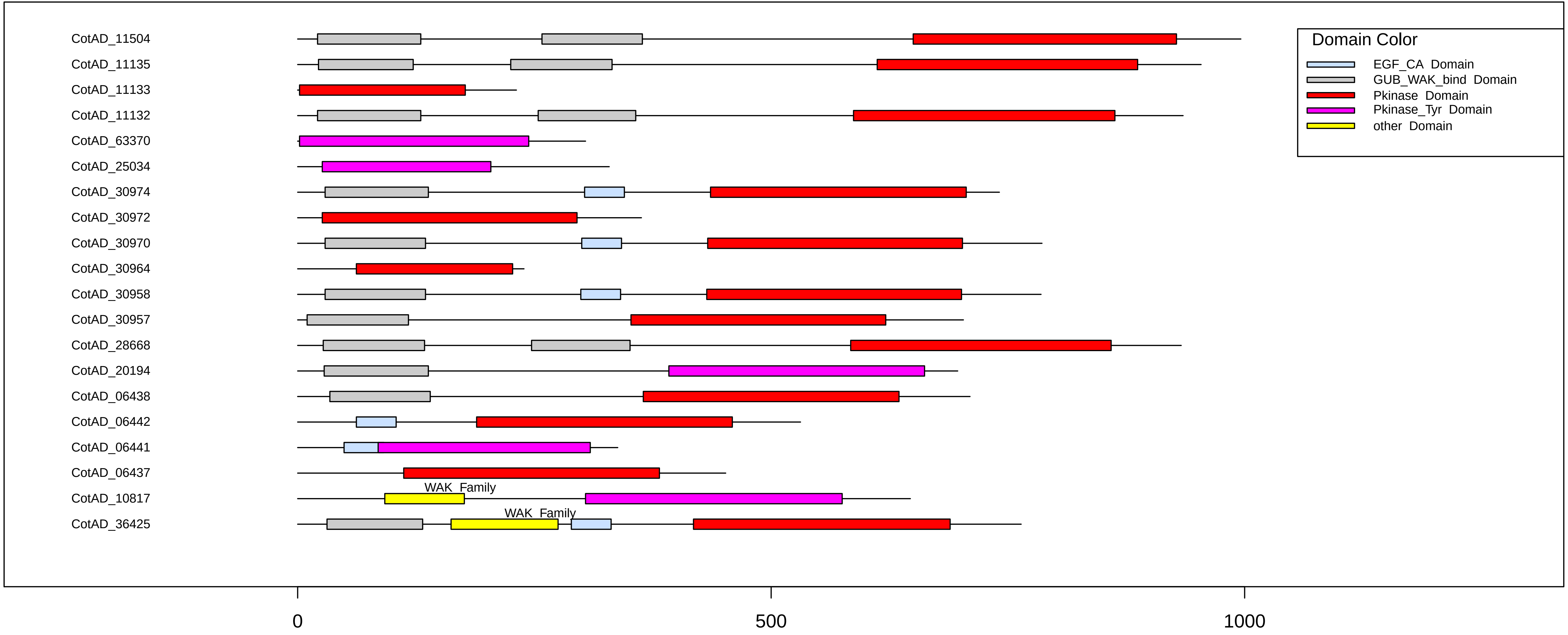

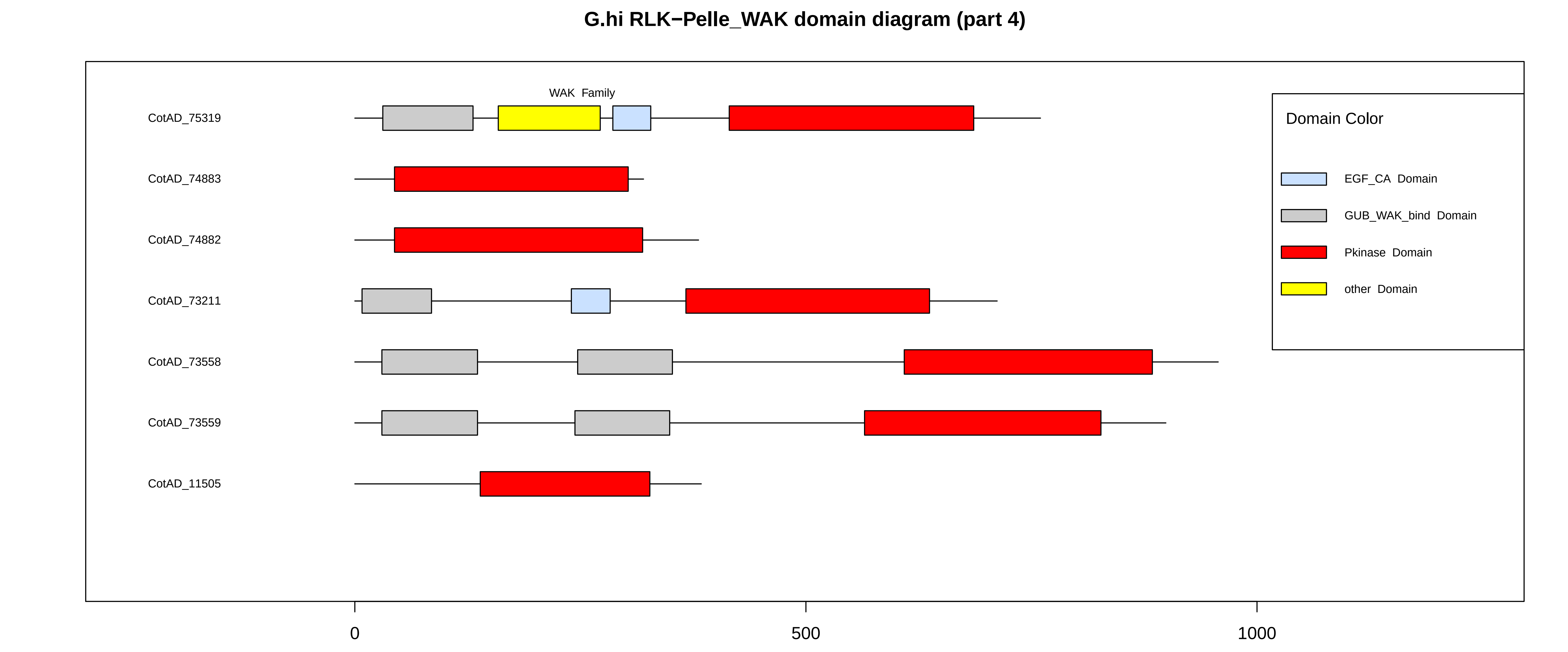
G.hi RLK−Pelle_WAK domain diagram (part 4)
WAK Family
CotAD_75319
CotAD_74883
CotAD_74882
CotAD_73211
CotAD_73558
CotAD_73559
CotAD_11505
Domain Color
EGF_CA Domain
GUB_WAK_bind Domain
Pkinase Domain
other Domain
0
500
1000

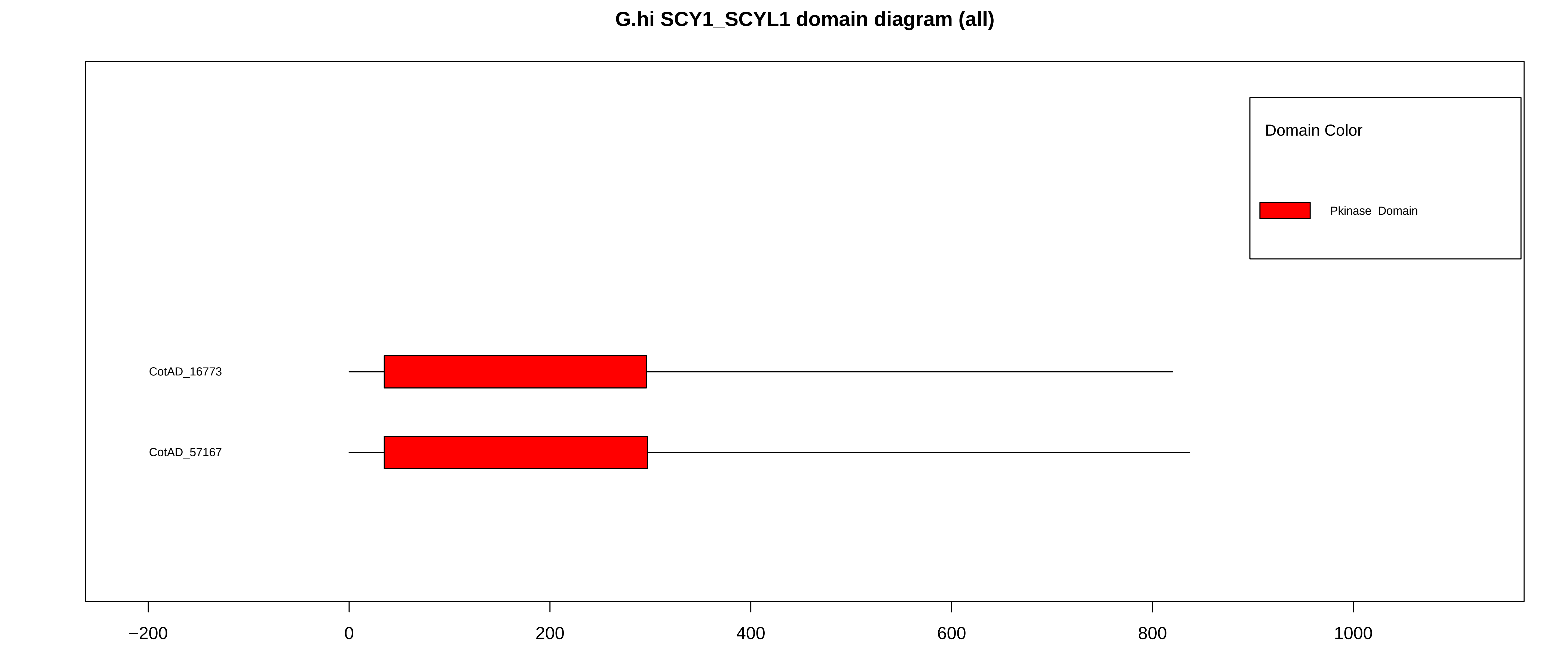
G.hi SCY1_SCYL1 domain diagram (all)
Domain Color
Pkinase Domain
CotAD_16773
CotAD_57167
−200
0
200
400
600
800
1000

G.hi SCY1_SCYL2 domain diagram (all)

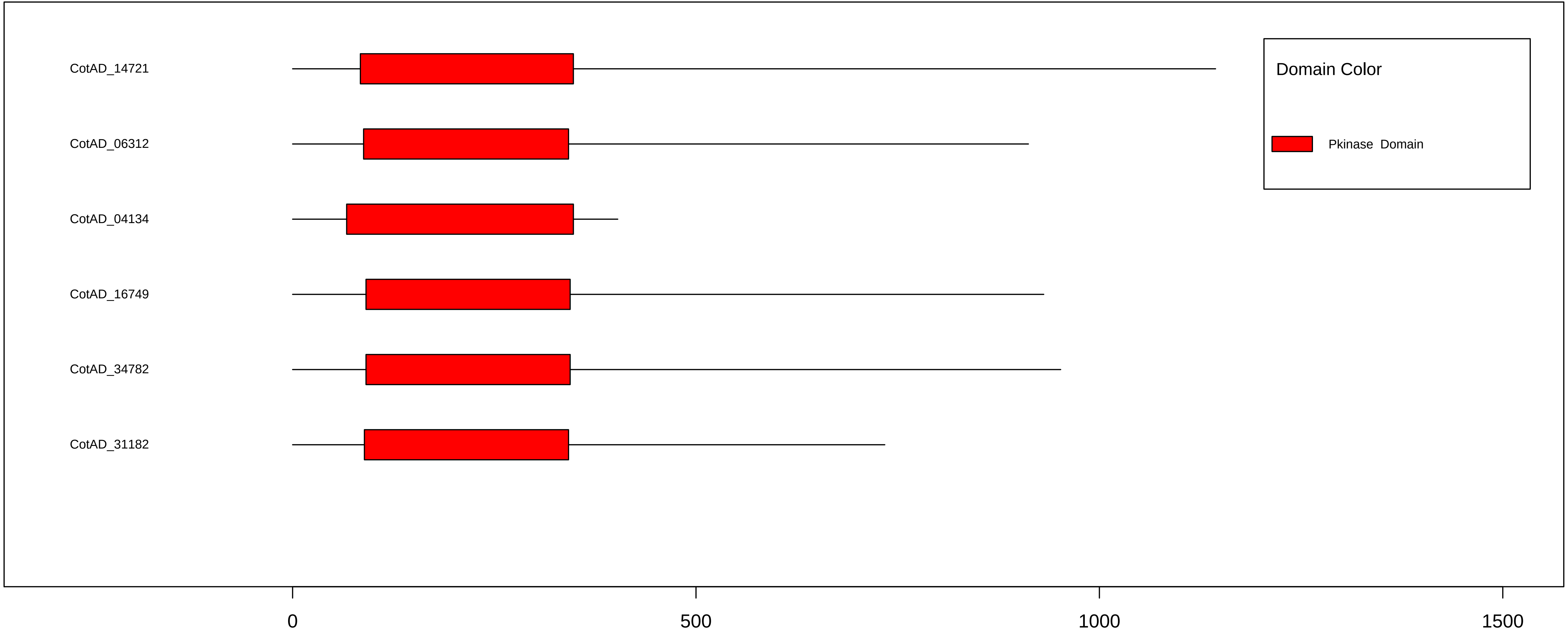

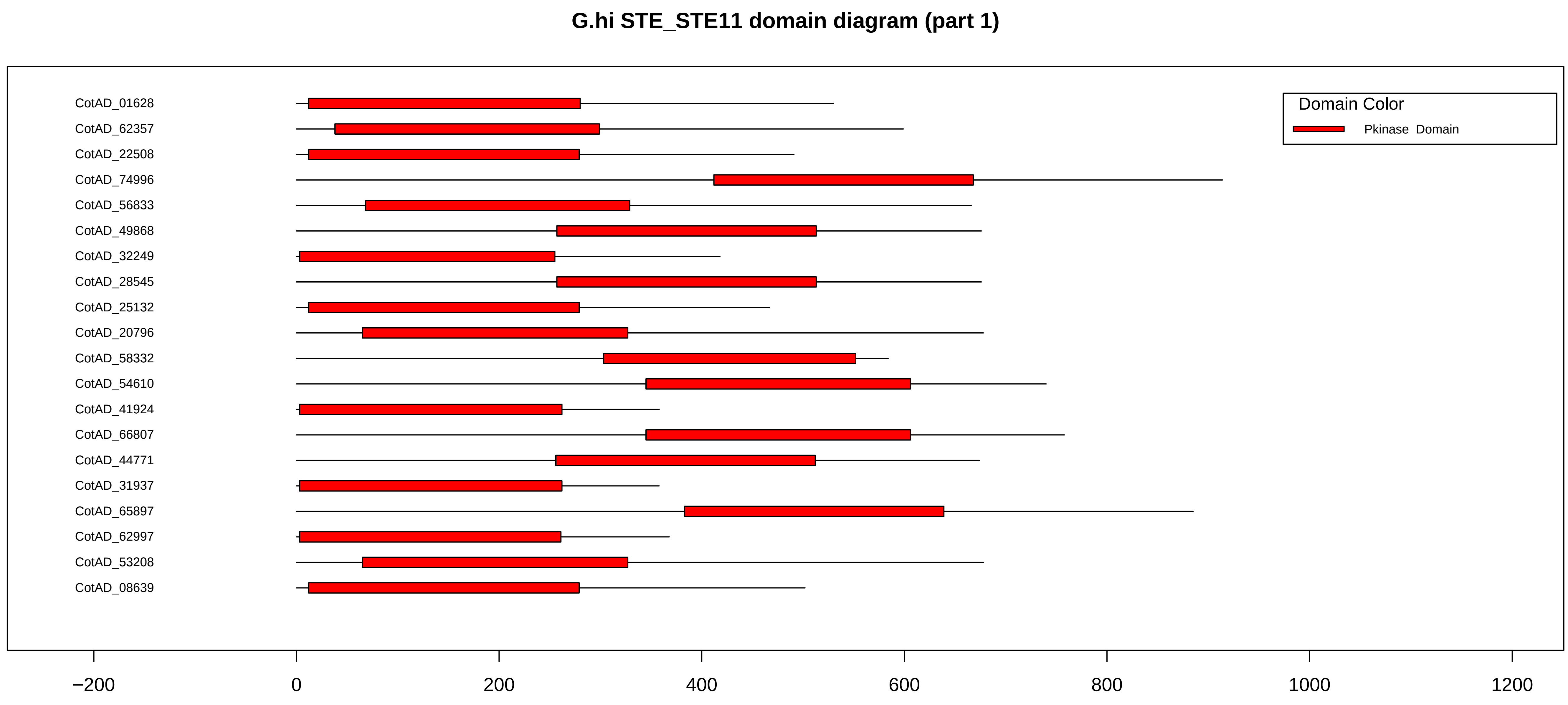
G.hi STE_STE11 domain diagram (part 1)
CotAD_01628
CotAD_62357
CotAD_22508
CotAD_74996
CotAD_56833
CotAD_49868
CotAD_32249
CotAD_28545
CotAD_25132
CotAD_20796
CotAD_58332
CotAD_54610
CotAD_41924
CotAD_66807
CotAD_44771
CotAD_31937
CotAD_65897
CotAD_62997
CotAD_53208
CotAD_08639
Domain Color
Pkinase Domain
−200
0
200
400
600
800
1000
1200

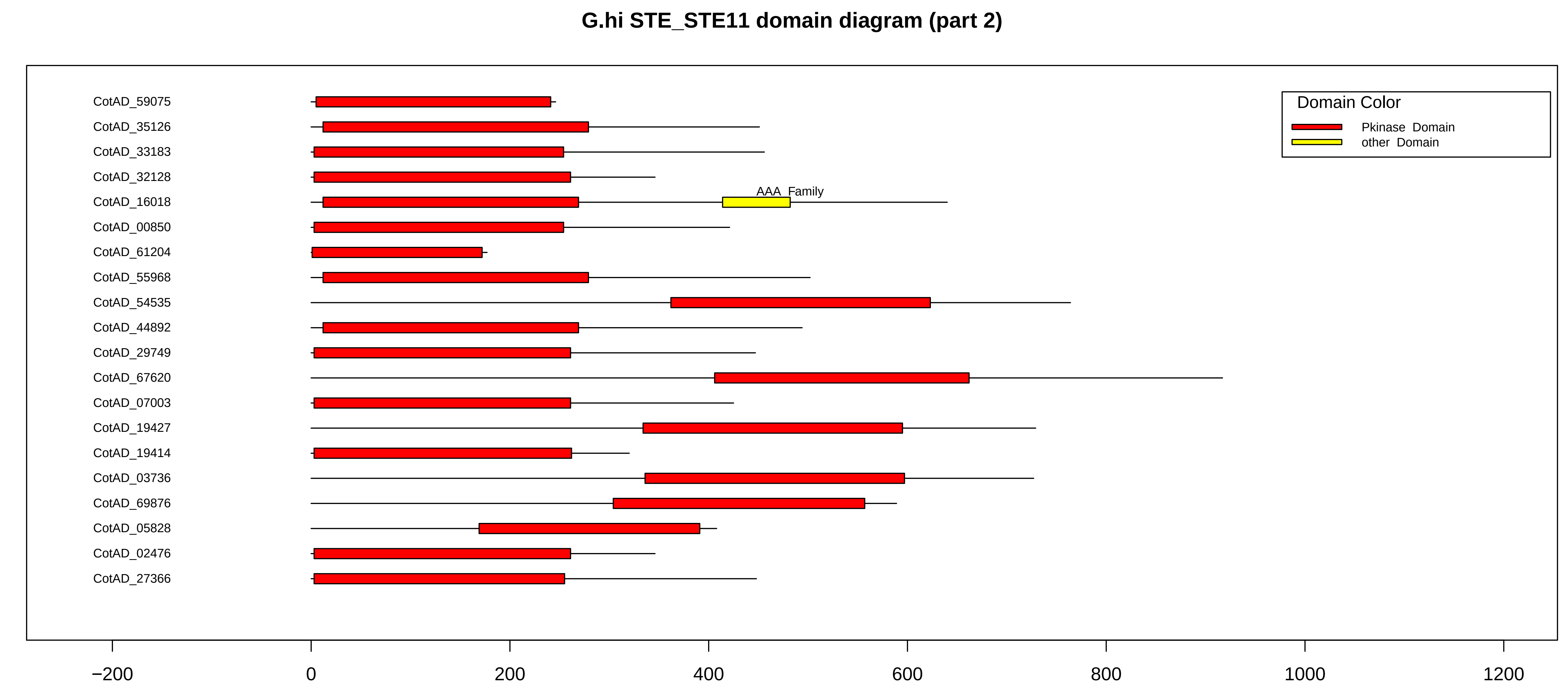
G.hi STE_STE11 domain diagram (part 2)
CotAD_59075
CotAD_35126
CotAD_33183
CotAD_32128
CotAD_16018
CotAD_00850
CotAD_61204
CotAD_55968
CotAD_54535
CotAD_44892
CotAD_29749
CotAD_67620
CotAD_07003
CotAD_19427
CotAD_19414
CotAD_03736
CotAD_69876
CotAD_05828
CotAD_02476
CotAD_27366
AAA_Family
Domain Color
Pkinase Domain
other Domain
−200
0
200
400
600
800
1000
1200

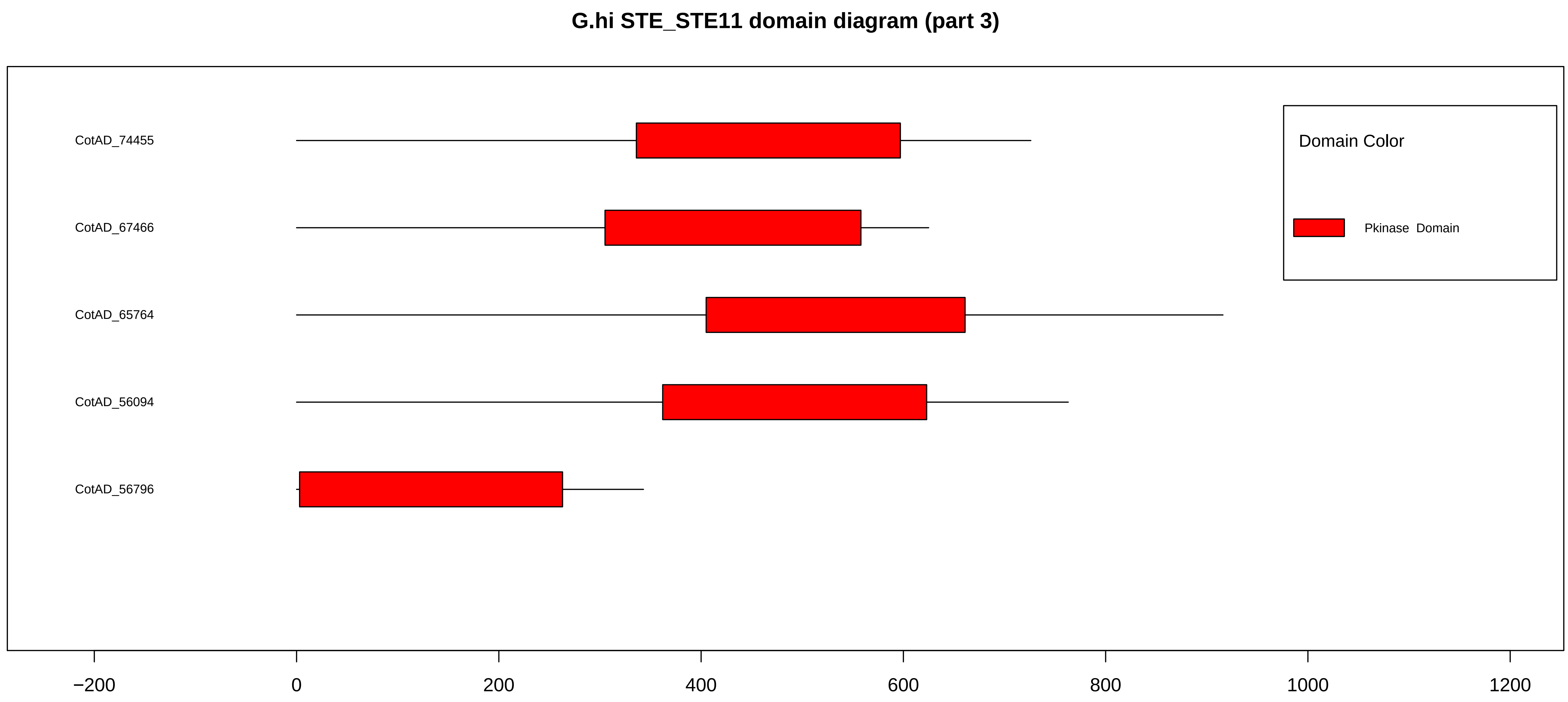
G.hi STE_STE11 domain diagram (part 3)
CotAD_74455
CotAD_67466
CotAD_65764
CotAD_56094
CotAD_56796
Domain Color
Pkinase Domain
−200
0
200
400
600
800
1000
1200

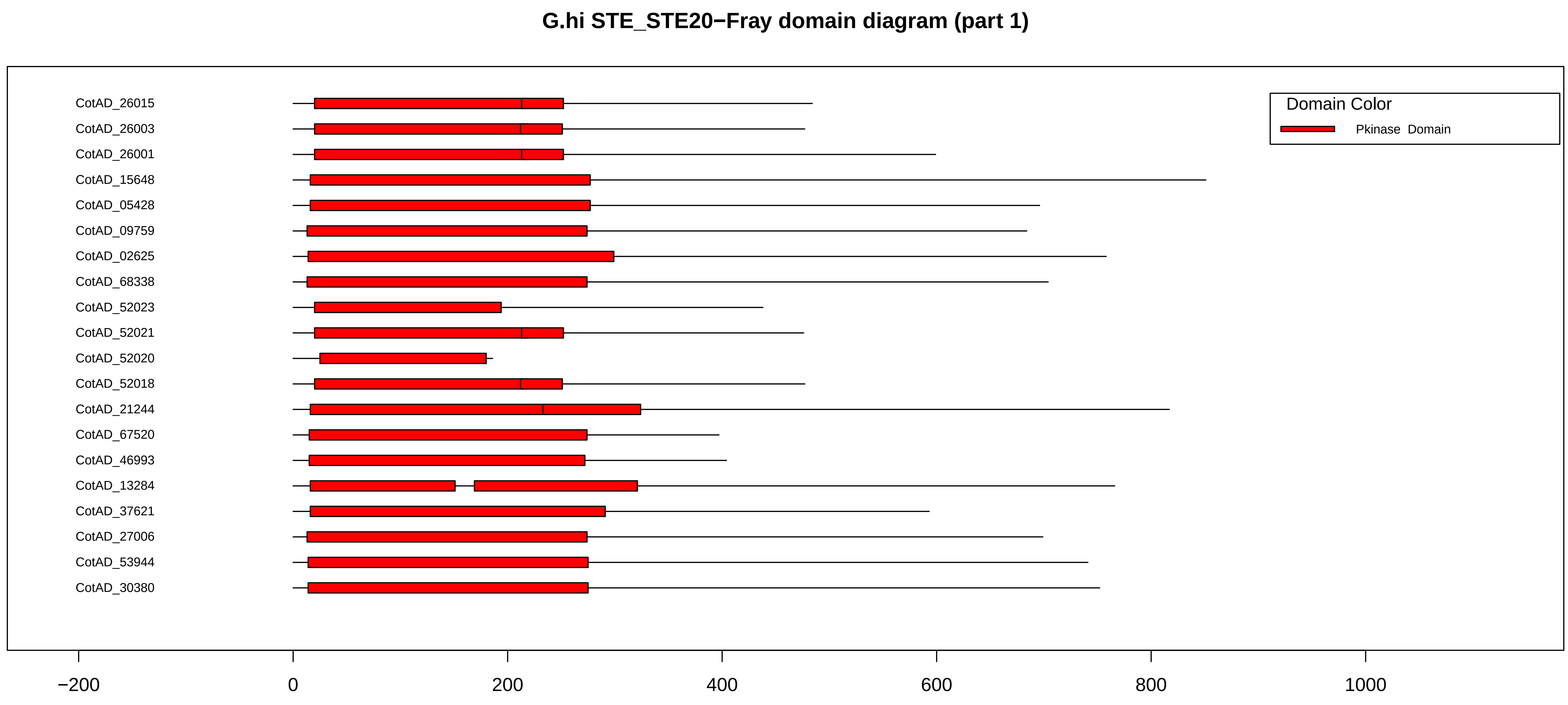
G.hi STE_STE20−Fray domain diagram (part 1)
CotAD_26015
CotAD_26003
CotAD_26001
CotAD_15648
CotAD_05428
CotAD_09759
CotAD_02625
CotAD_68338
CotAD_52023
CotAD_52021
CotAD_52020
CotAD_52018
CotAD_21244
CotAD_67520
CotAD_46993
CotAD_13284
CotAD_37621
CotAD_27006
CotAD_53944
CotAD_30380
Domain Color
Pkinase Domain
−200
0
200
400
600
800
1000

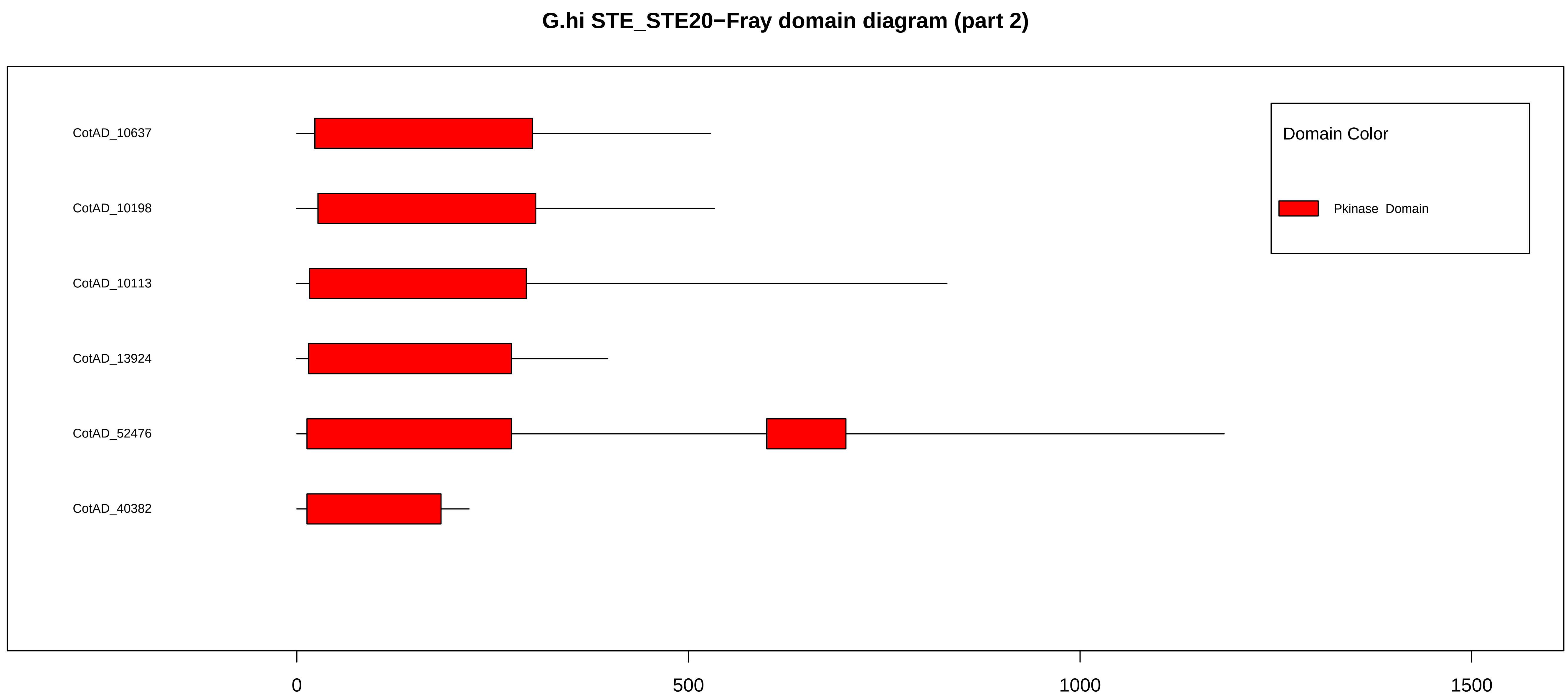
G.hi STE_STE20−Fray domain diagram (part 2)
CotAD_10637
CotAD_10198
CotAD_10113
CotAD_13924
CotAD_52476
CotAD_40382
Domain Color
Pkinase Domain
0
500
1000
1500

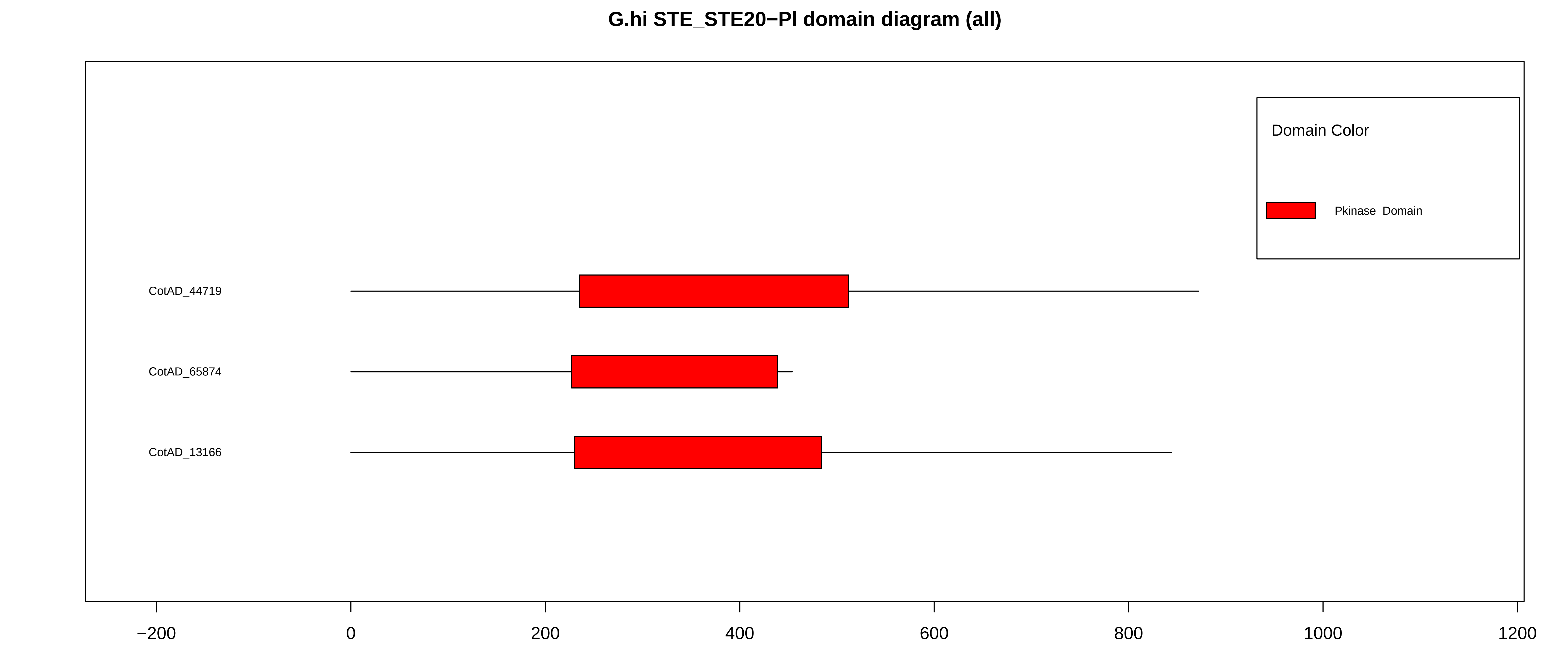
G.hi STE_STE20−PI domain diagram (all)
Domain Color
Pkinase Domain
CotAD_44719
CotAD_65874
CotAD_13166
−200
0
200
400
600
800
1000
1200

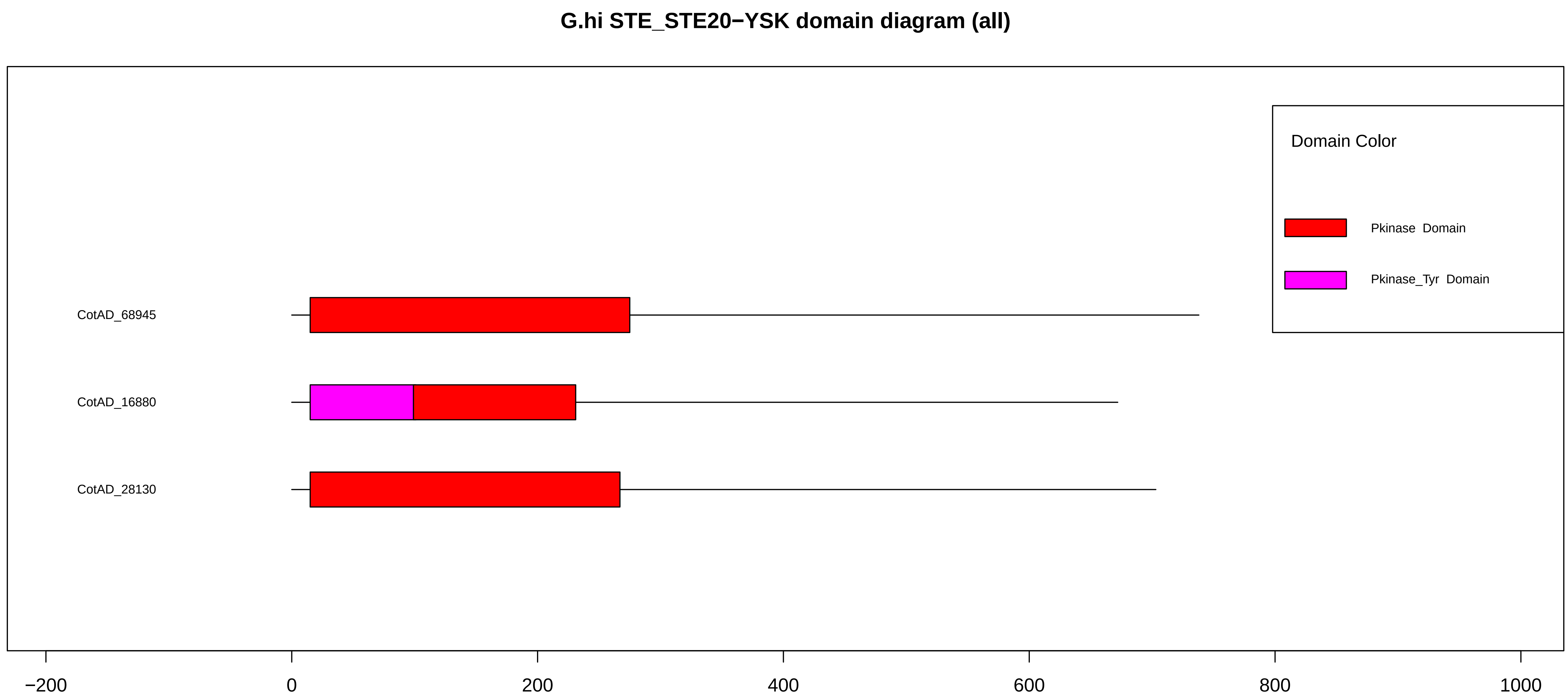
G.hi STE_STE20−YSK domain diagram (all)
Domain Color
Pkinase Domain
Pkinase_Tyr Domain
CotAD_68945
CotAD_16880
CotAD_28130
−200
0
200
400
600
800
1000

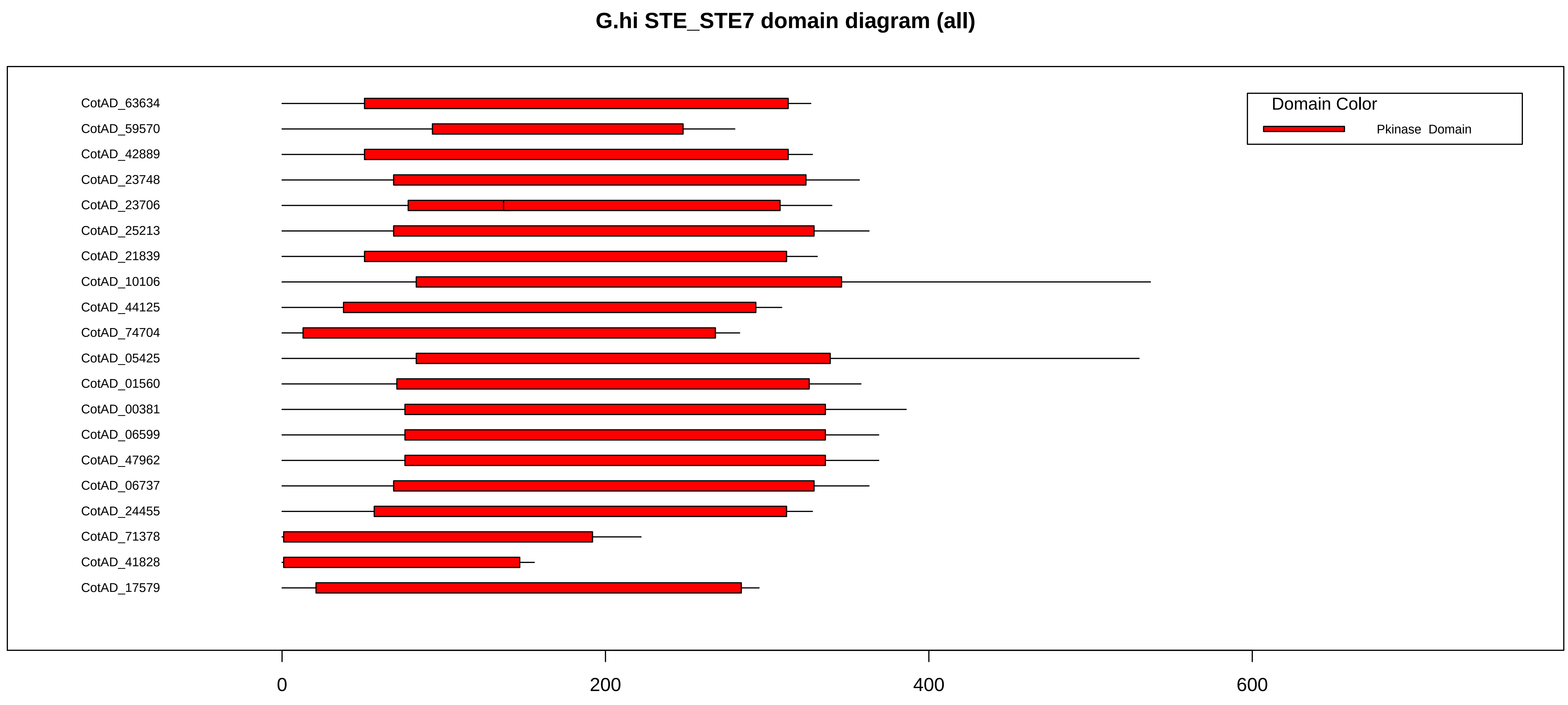

G.hi STE_STE7 domain diagram (all)
Domain Color
Pkinase Domain
CotAD_63634
CotAD_59570
CotAD_42889
CotAD_23748
CotAD_23706
CotAD_25213
CotAD_21839
CotAD_10106
CotAD_44125
CotAD_74704
CotAD_05425
CotAD_01560
CotAD_00381
CotAD_06599
CotAD_47962
CotAD_06737
CotAD_24455
CotAD_71378
CotAD_41828
CotAD_17579
0
200
400
600

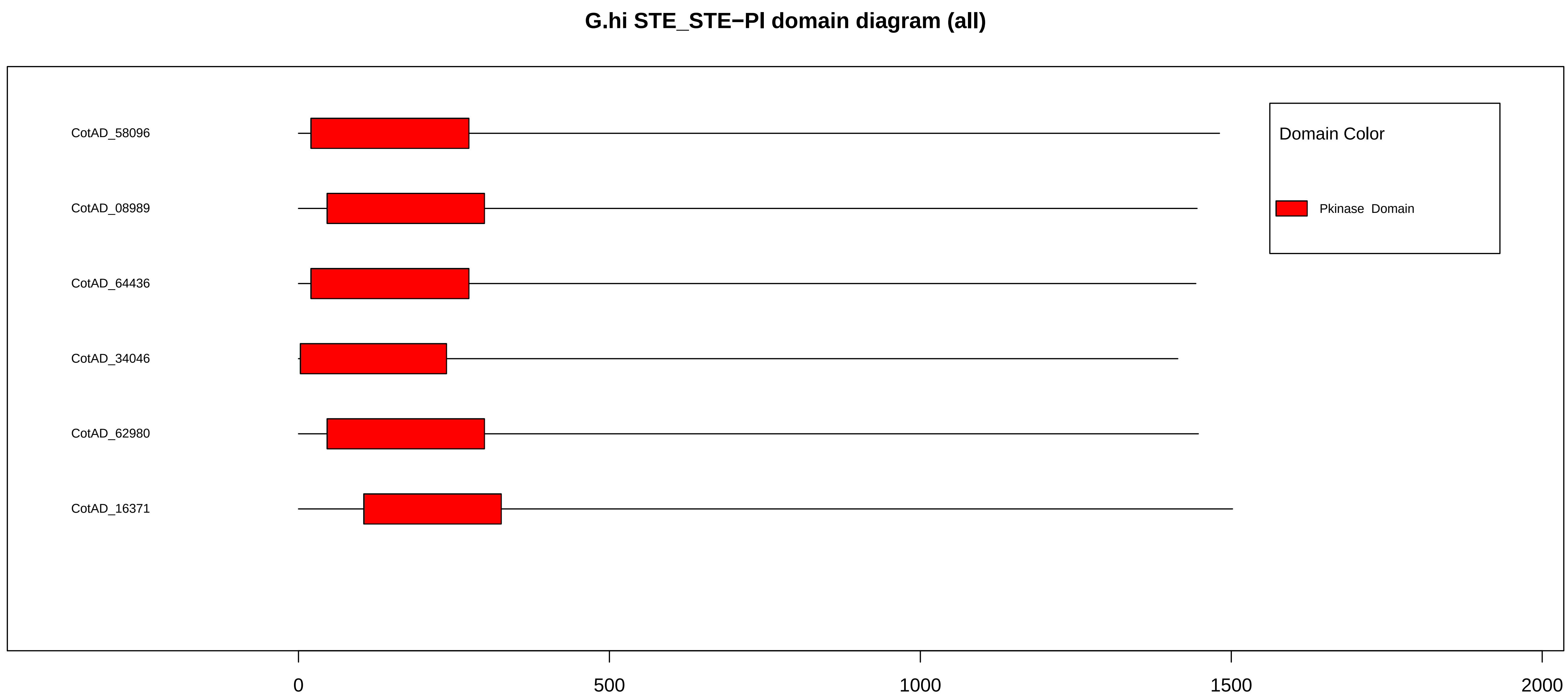

G.hi STE_STE−Pl domain diagram (all)
CotAD_58096
CotAD_08989
CotAD_64436
CotAD_34046
CotAD_62980
CotAD_16371
Domain Color
Pkinase Domain
0
500
1000
1500
2000

G.hi TKL_CTR1−DRK−1 domain diagram (all)

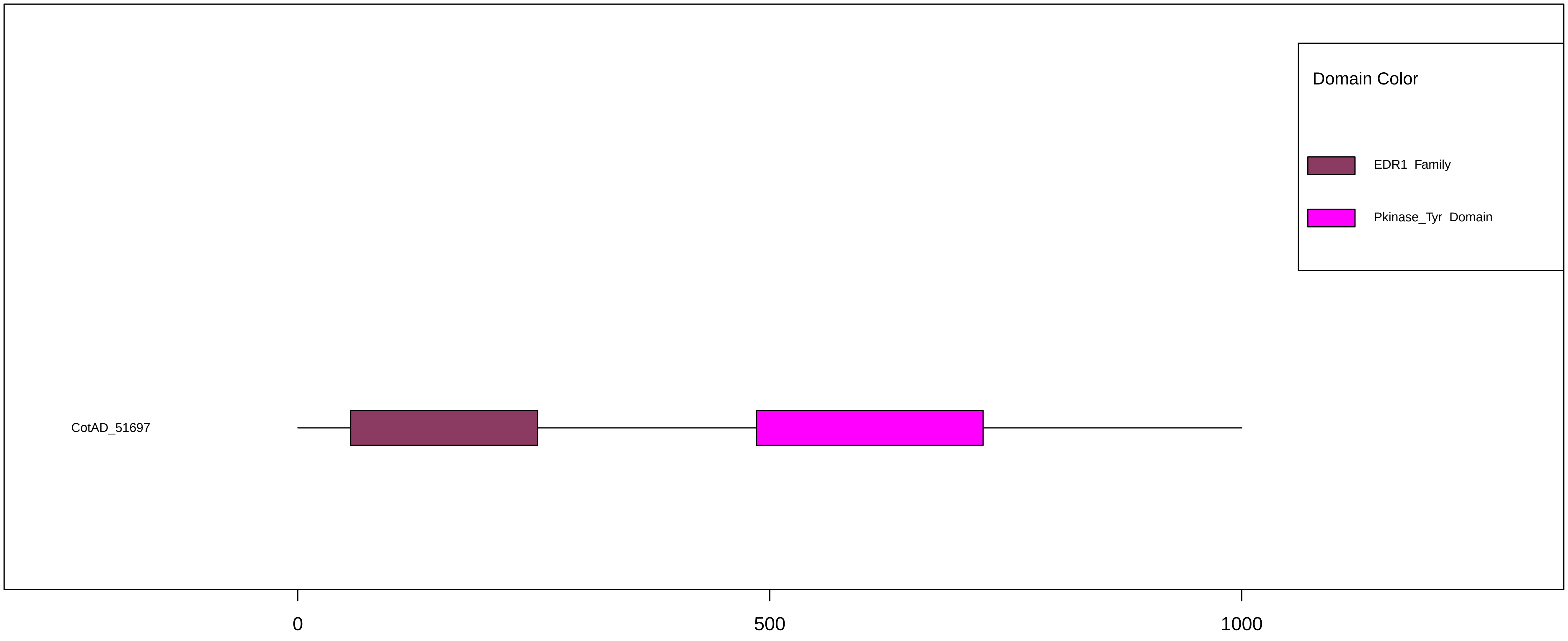

G.hi TKL_CTR1−DRK−2 domain diagram (part 1)

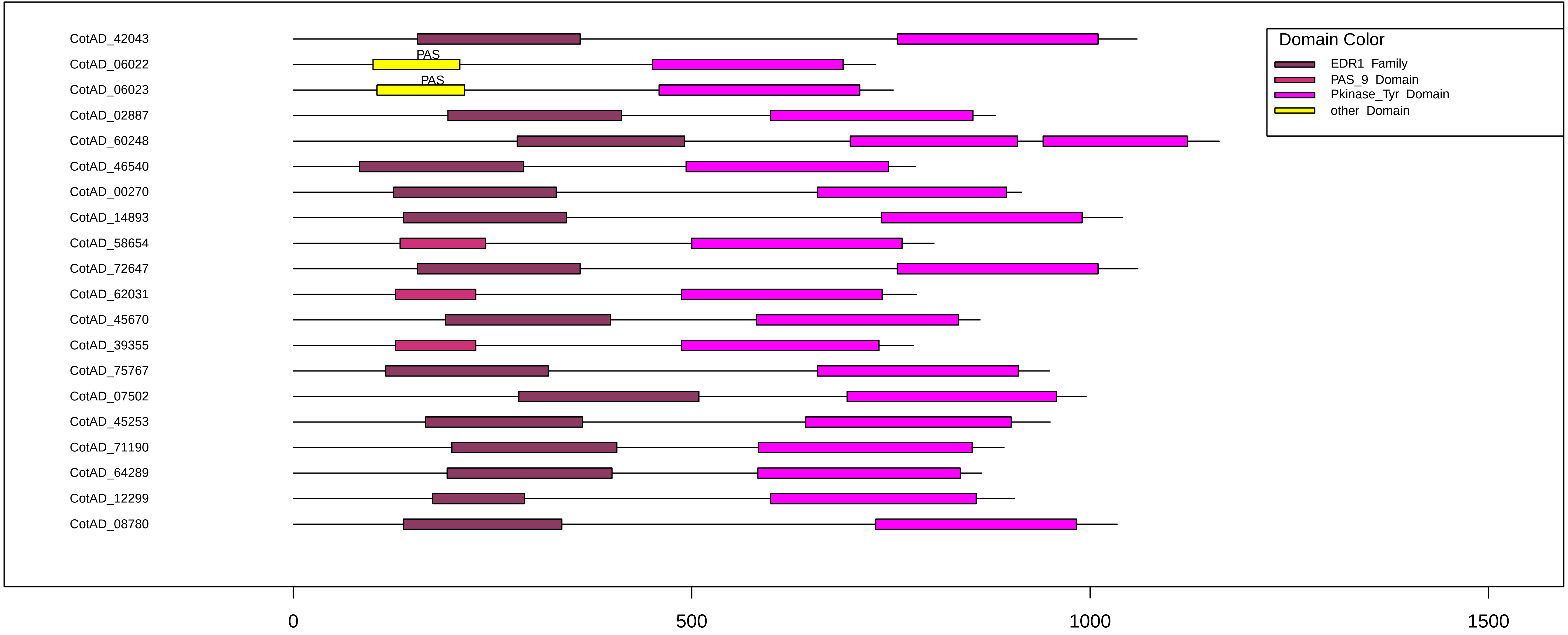

G.hi TKL_CTR1−DRK−2 domain diagram (part 2)

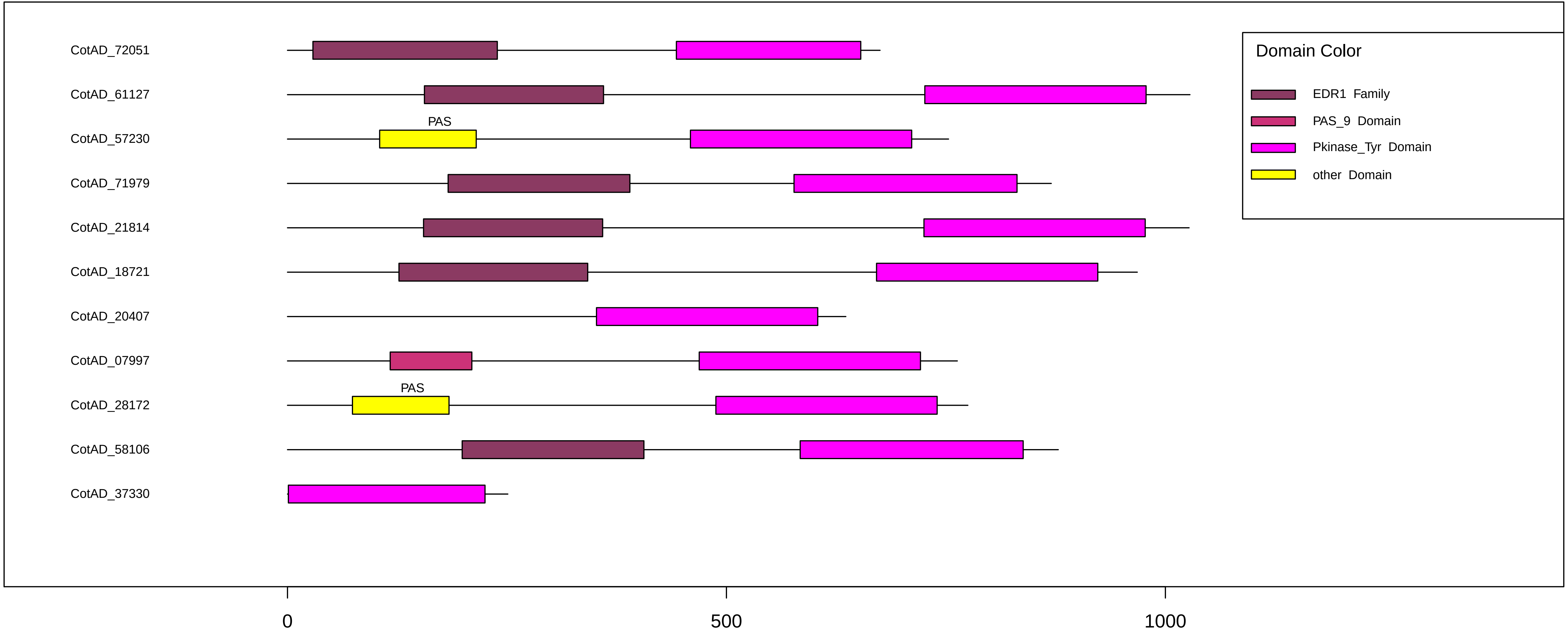

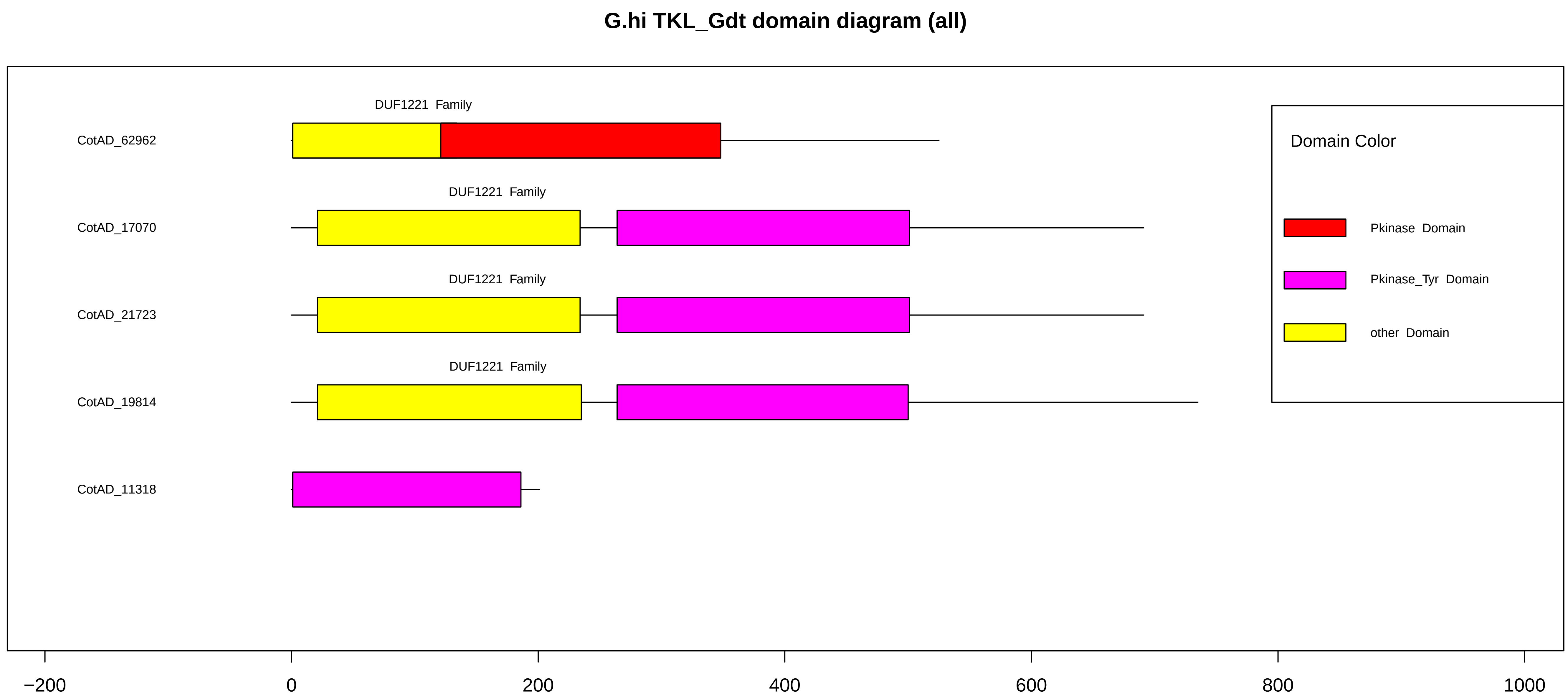
G.hi TKL_Gdt domain diagram (all)
DUF1221 Family
CotAD_62962
DUF1221 Family
CotAD_17070
DUF1221 Family
CotAD_21723
DUF1221 Family
CotAD_19814
CotAD_11318
Domain Color
Pkinase Domain
Pkinase_Tyr Domain
other Domain
−200
0
200
400
600
800
1000

G.hi TKL−Pl−1 domain diagram (part 1)

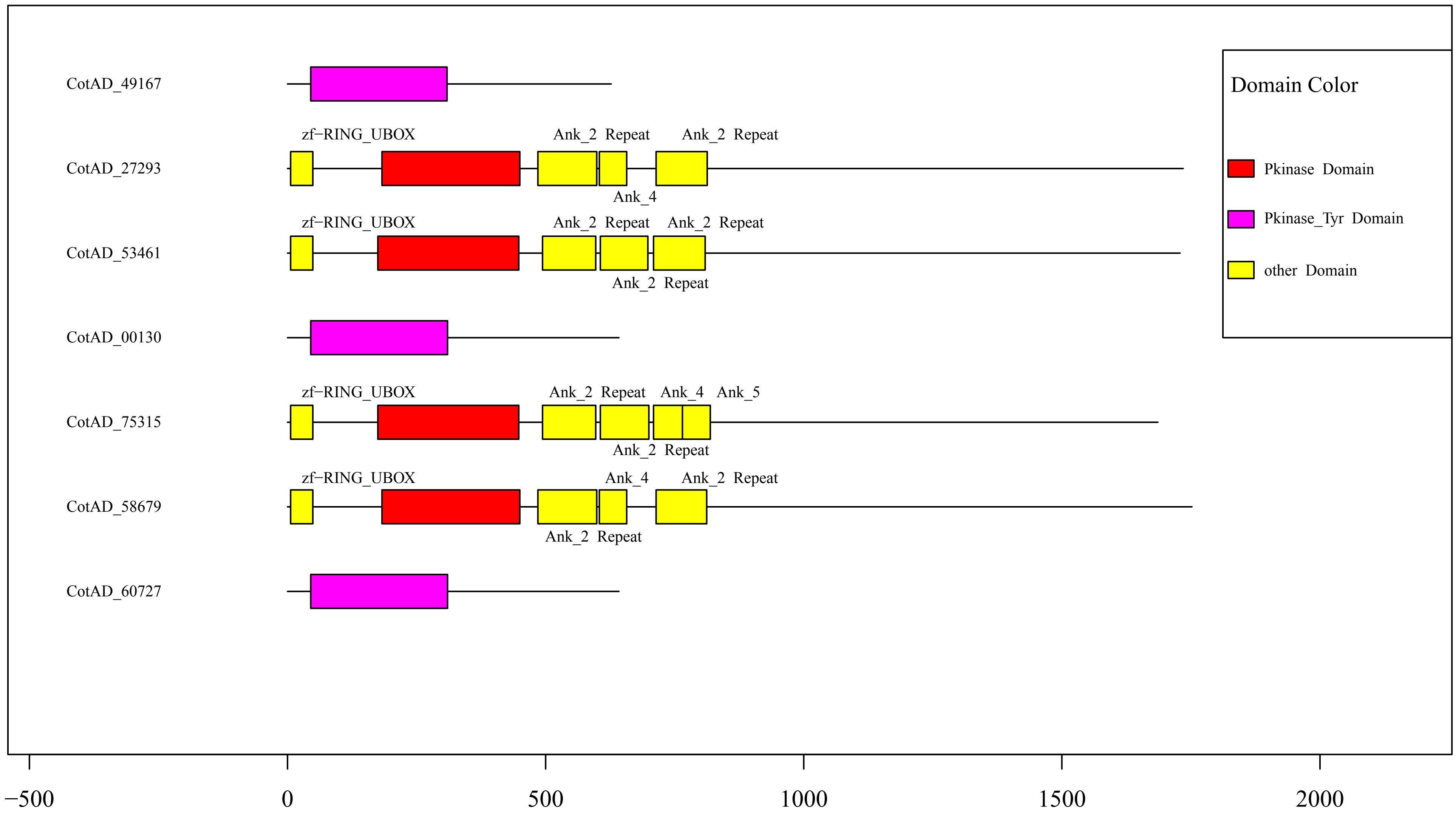

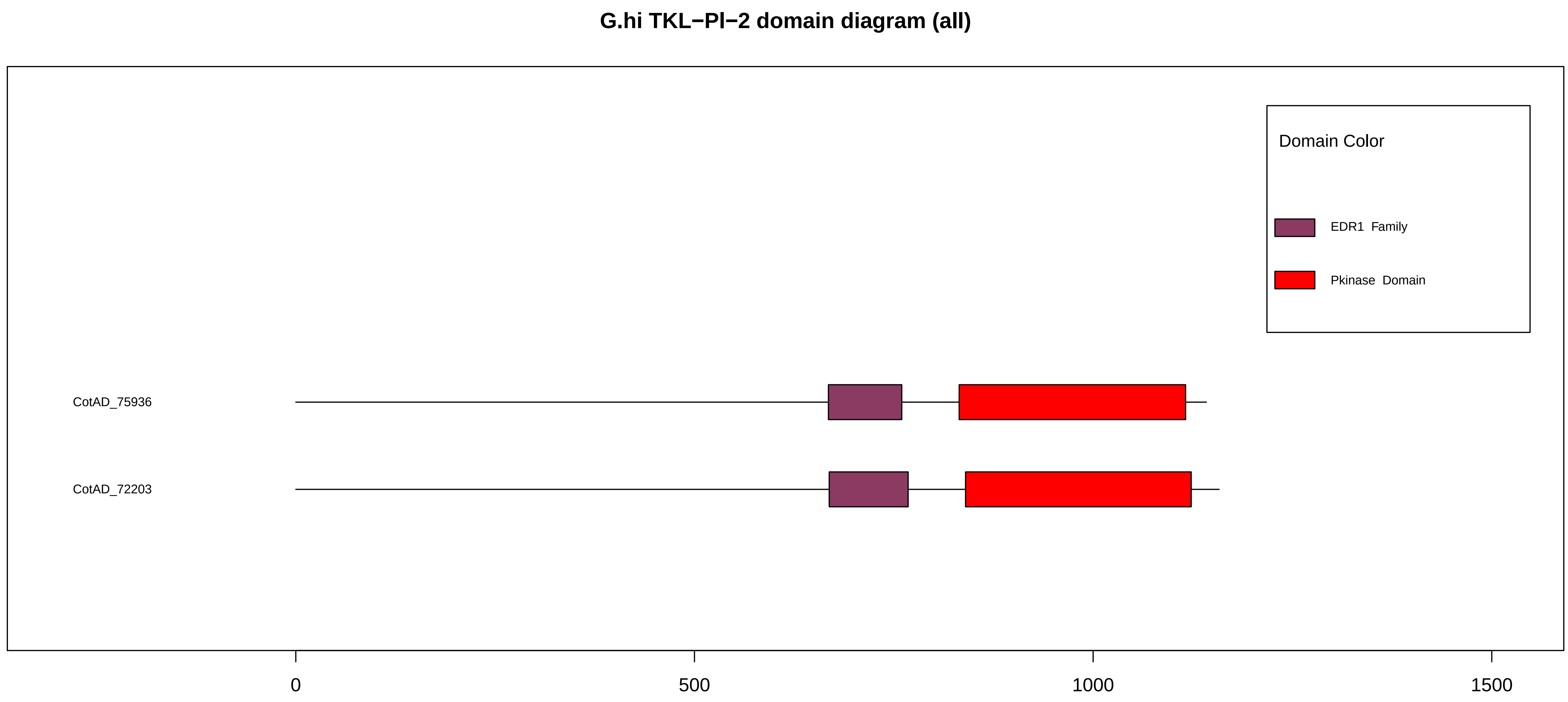
G.hi TKL−Pl−2 domain diagram (all)
Domain Color
EDR1 Family
Pkinase Domain
CotAD_75936
CotAD_72203
0
500
1000
1500

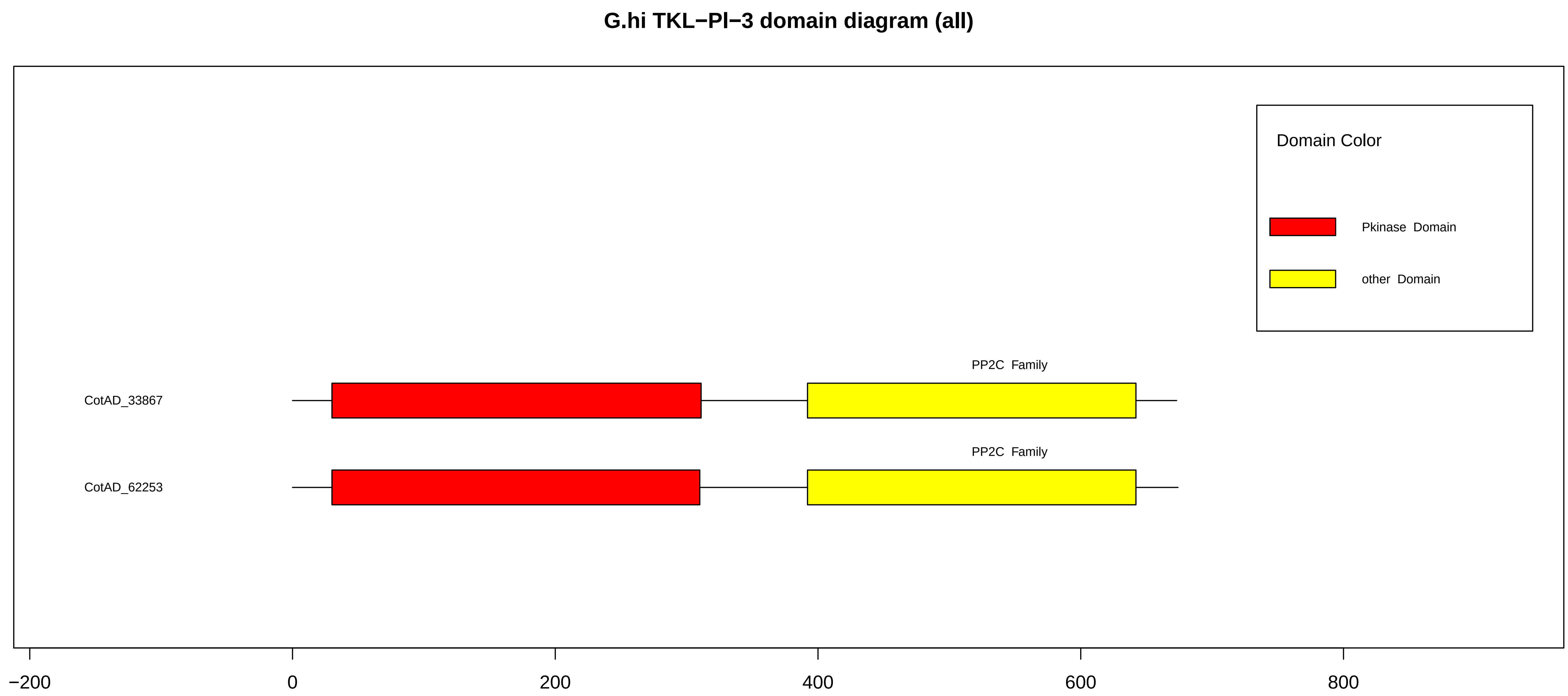
G.hi TKL−Pl−3 domain diagram (all)
Domain Color
Pkinase Domain
other Domain
PP2C Family
CotAD_33867
PP2C Family
CotAD_62253
−200
0
200
400
600
800

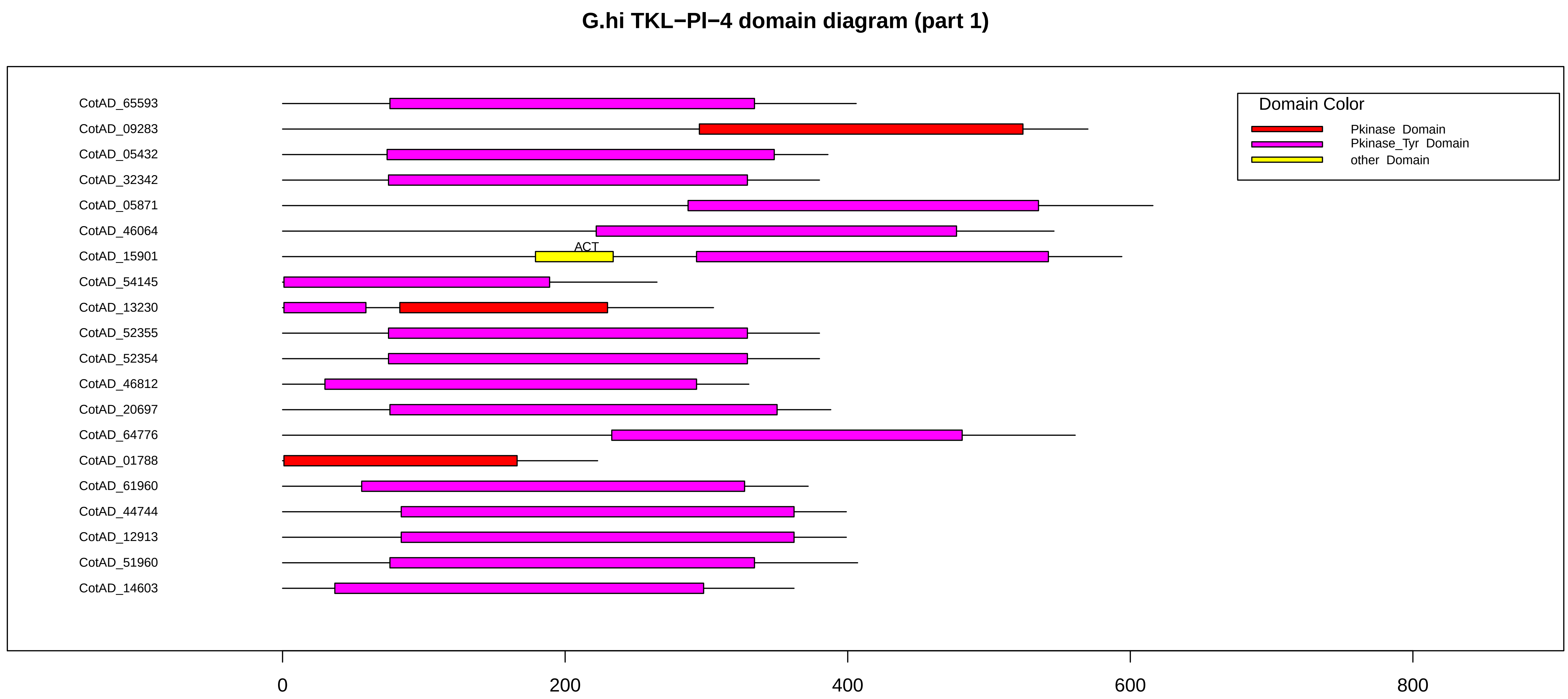
G.hi TKL−Pl−4 domain diagram (part 1)
CotAD_65593
CotAD_09283
CotAD_05432
CotAD_32342
CotAD_05871
CotAD_46064
CotAD_15901
ACT
CotAD_54145
CotAD_13230
CotAD_52355
CotAD_52354
CotAD_46812
CotAD_20697
CotAD_64776
CotAD_01788
CotAD_61960
CotAD_44744
CotAD_12913
CotAD_51960
CotAD_14603
Domain Color
Pkinase Domain
Pkinase_Tyr Domain
other Domain
0
200
400
600
800

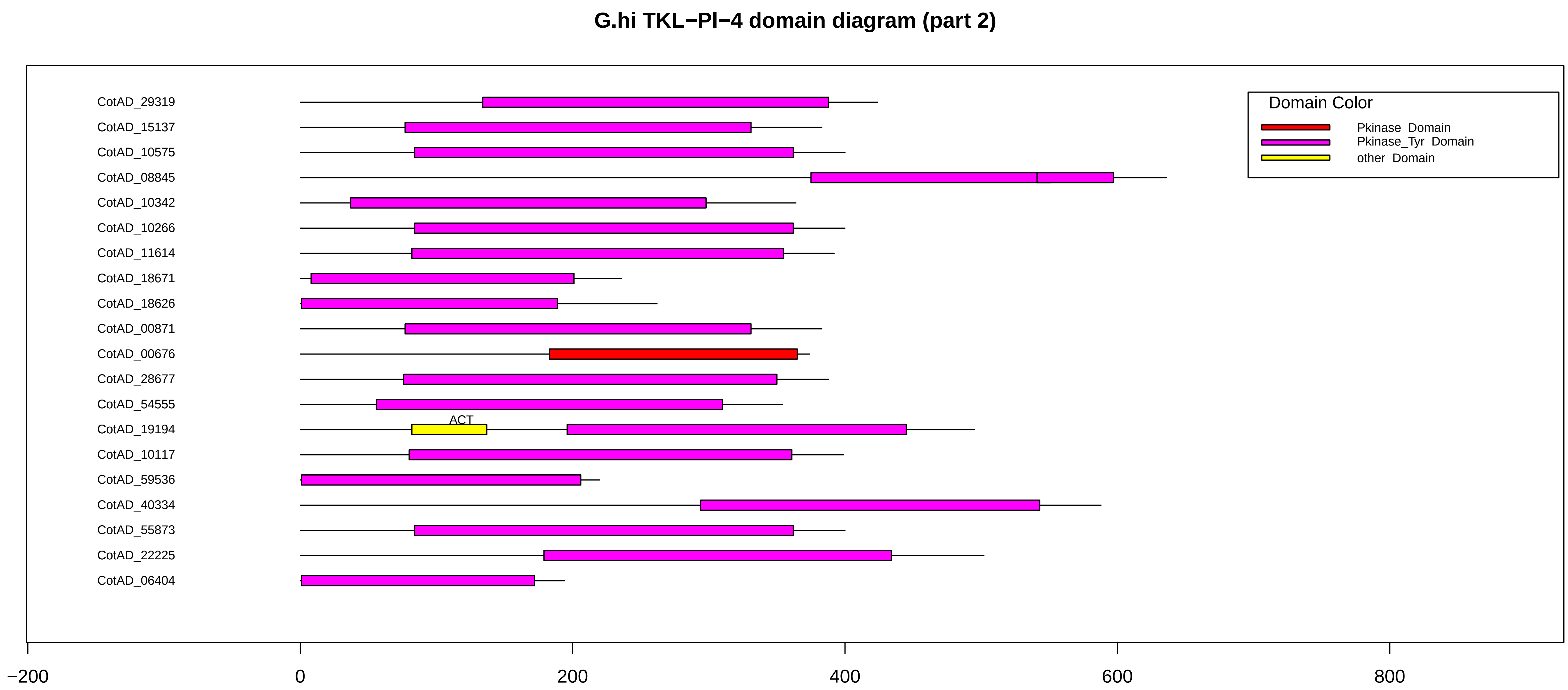
G.hi TKL−Pl−4 domain diagram (part 2)
Domain Color
Pkinase Domain
Pkinase_Tyr Domain
other Domain
CotAD_29319
CotAD_15137
CotAD_10575
CotAD_08845
CotAD_10342
CotAD_10266
CotAD_11614
CotAD_18671
CotAD_18626
CotAD_00871
CotAD_00676
CotAD_28677
CotAD_54555
CotAD_19194
ACT
CotAD_10117
CotAD_59536
CotAD_40334
CotAD_55873
CotAD_22225
CotAD_06404
−200
0
200
400
600
800

G.hi TKL−Pl−4 domain diagram (part 3)

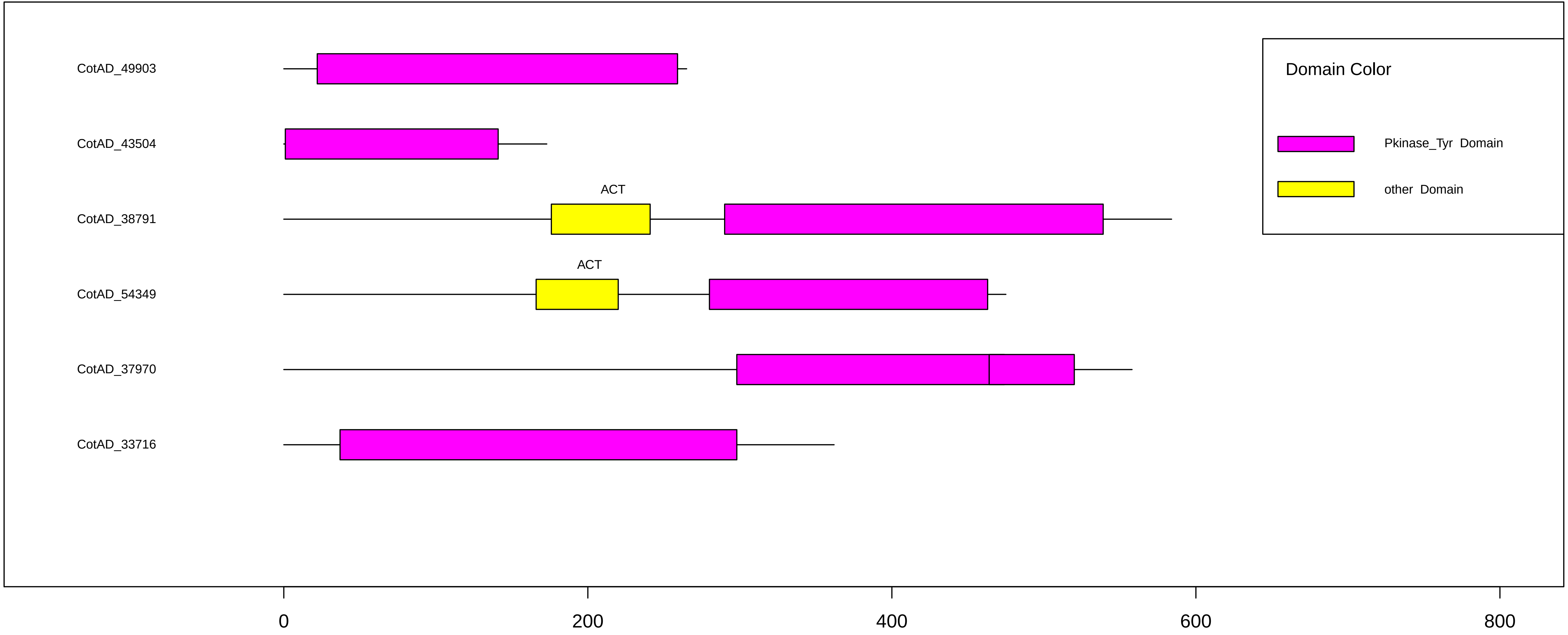

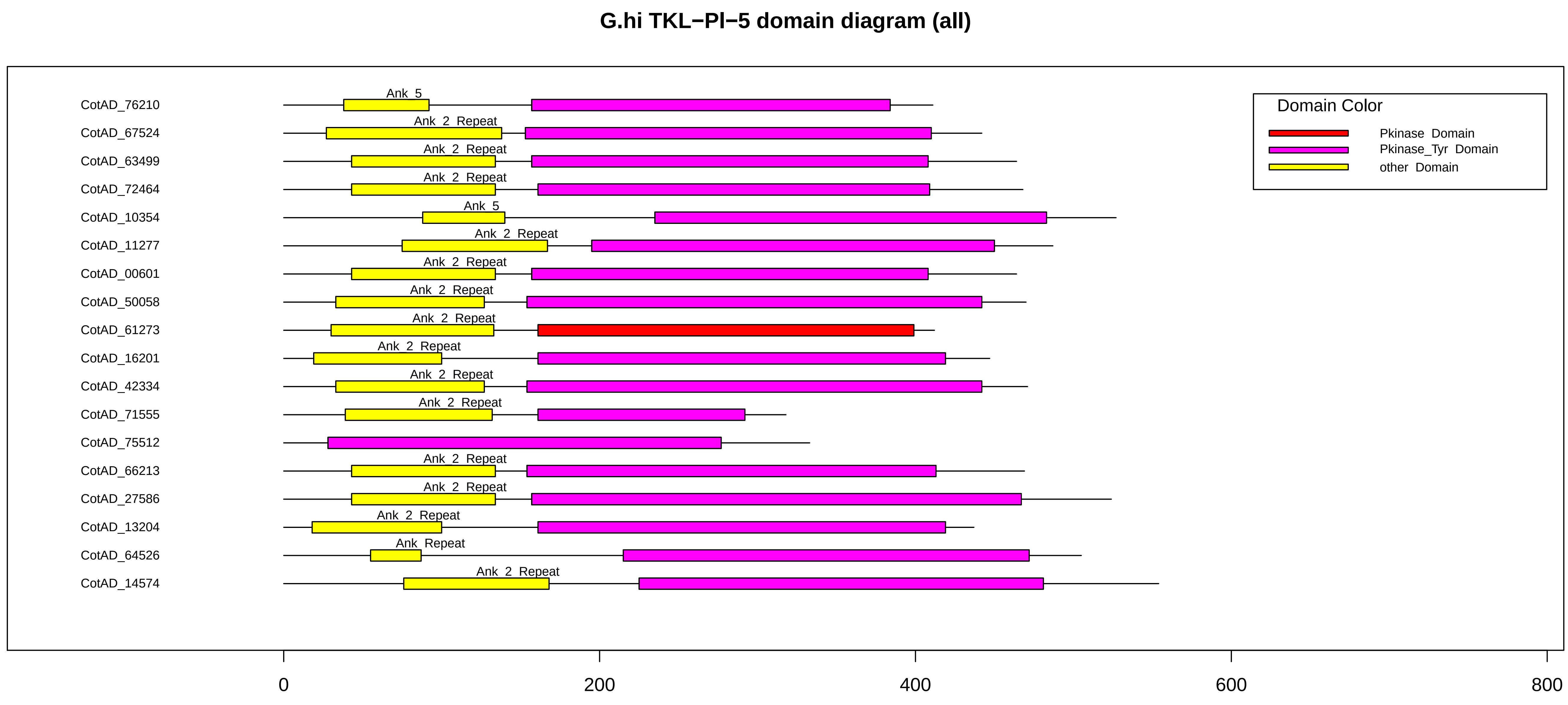
G.hi TKL−Pl−5 domain diagram (all)
CotAD_76210
CotAD_67524
CotAD_63499
CotAD_72464
CotAD_10354
CotAD_11277
CotAD_00601
CotAD_50058
CotAD_61273
CotAD_16201
CotAD_42334
CotAD_71555
CotAD_75512
CotAD_66213
CotAD_27586
CotAD_13204
CotAD_64526
CotAD_14574
Ank_5
Ank_2_Repeat
Ank_Repeat
Domain Color
Pkinase_Domain
Pkinase_Tyr_Domain
other_Domain
0
200
400
600
800

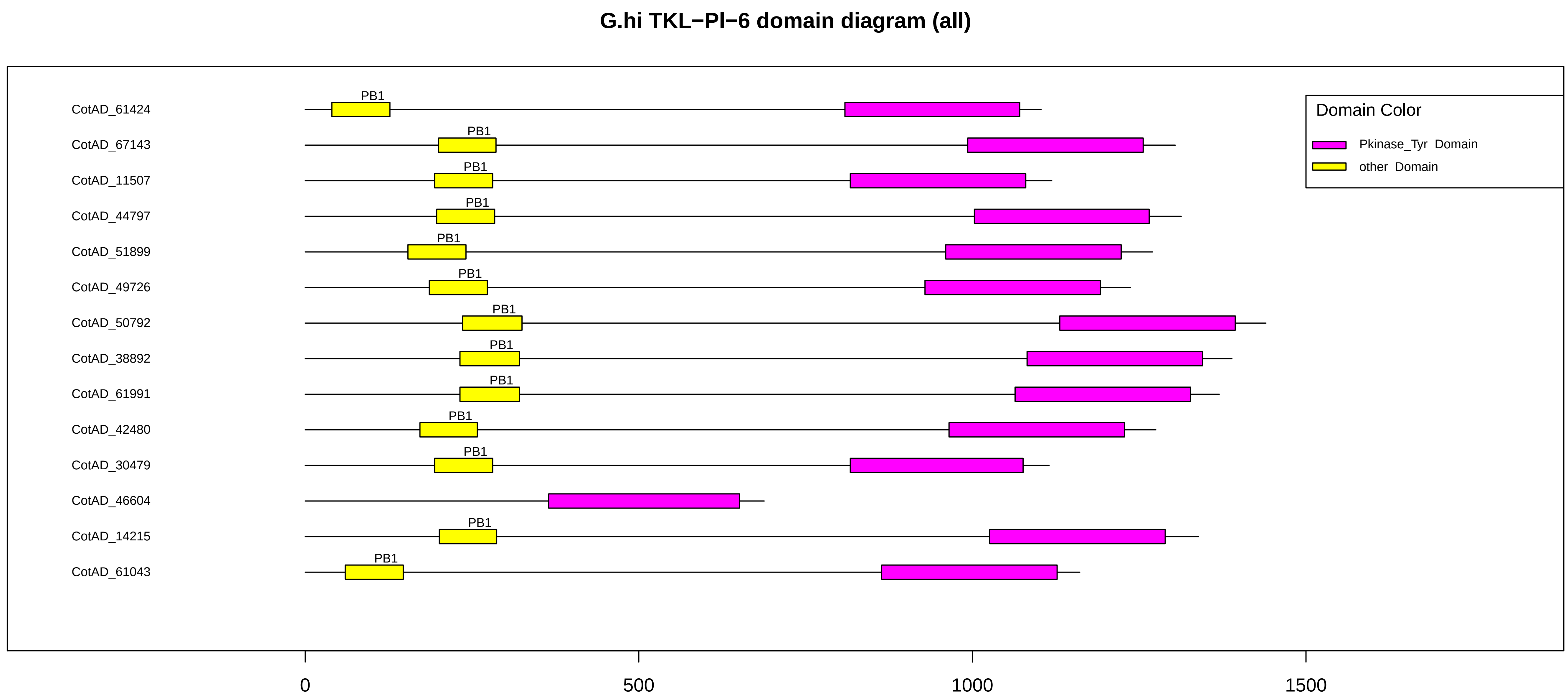

G.hi TKL−Pl−6 domain diagram (all)
CotAD_61424
CotAD_67143
CotAD_11507
CotAD_44797
CotAD_51899
CotAD_49726
CotAD_50792
CotAD_38892
CotAD_61991
CotAD_42480
CotAD_30479
CotAD_46604
CotAD_14215
CotAD_61043
PB1
Domain Color
Pkinase_Tyr Domain
other Domain
0
500
1000
1500

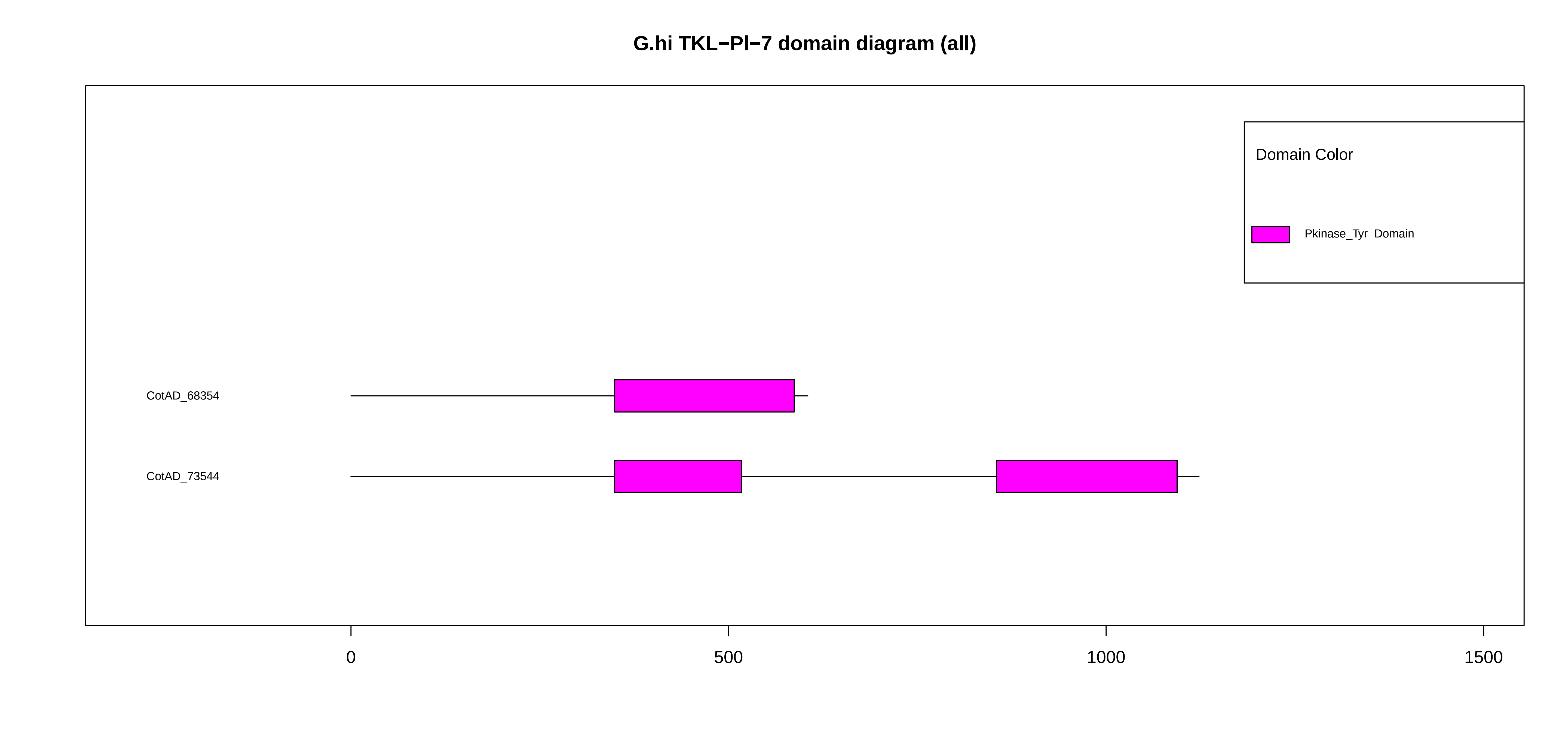

G.hi TKL−Pl−7 domain diagram (all)
Domain Color
Pkinase_Tyr Domain
CotAD_68354
CotAD_73544
0
500
1000
1500

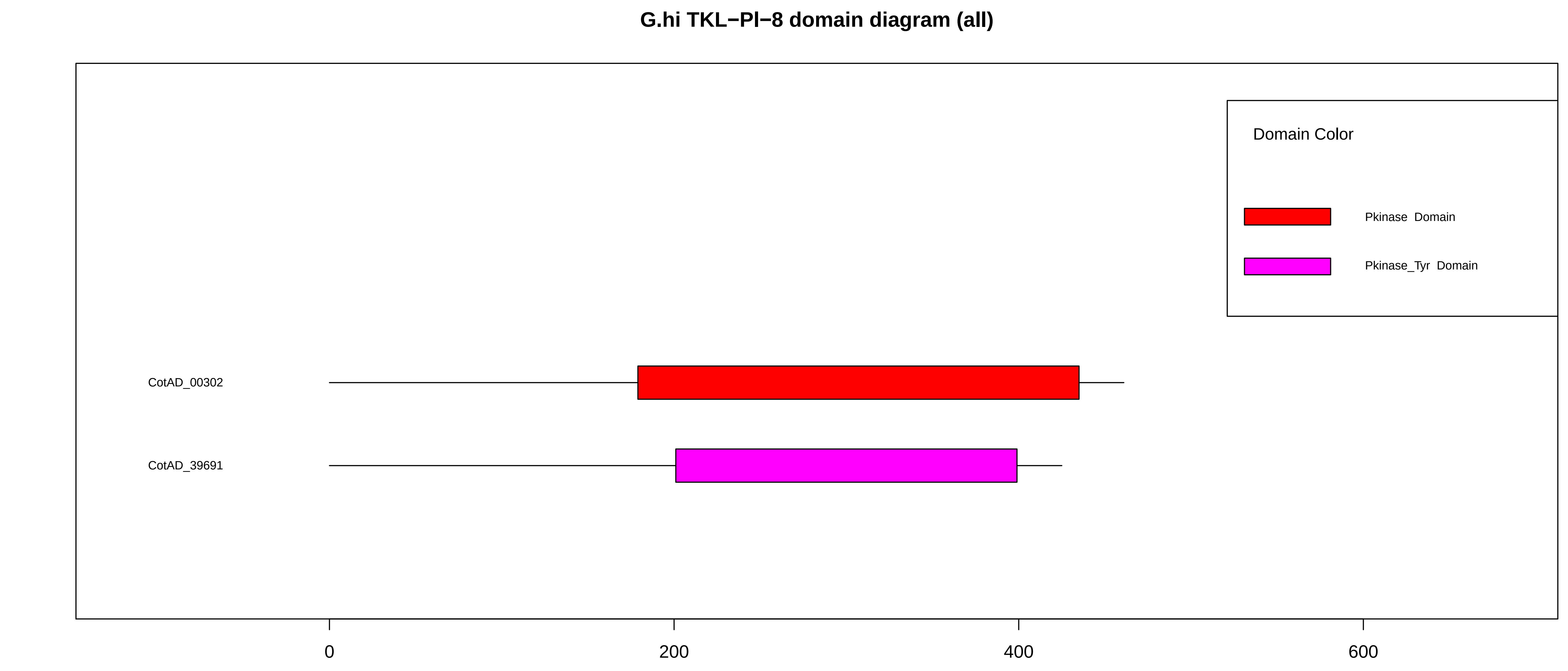
G.hi TKL−Pl−8 domain diagram (all)
Domain Color
Pkinase Domain
Pkinase_Tyr Domain
CotAD_00302
CotAD_39691
0
200
400
600

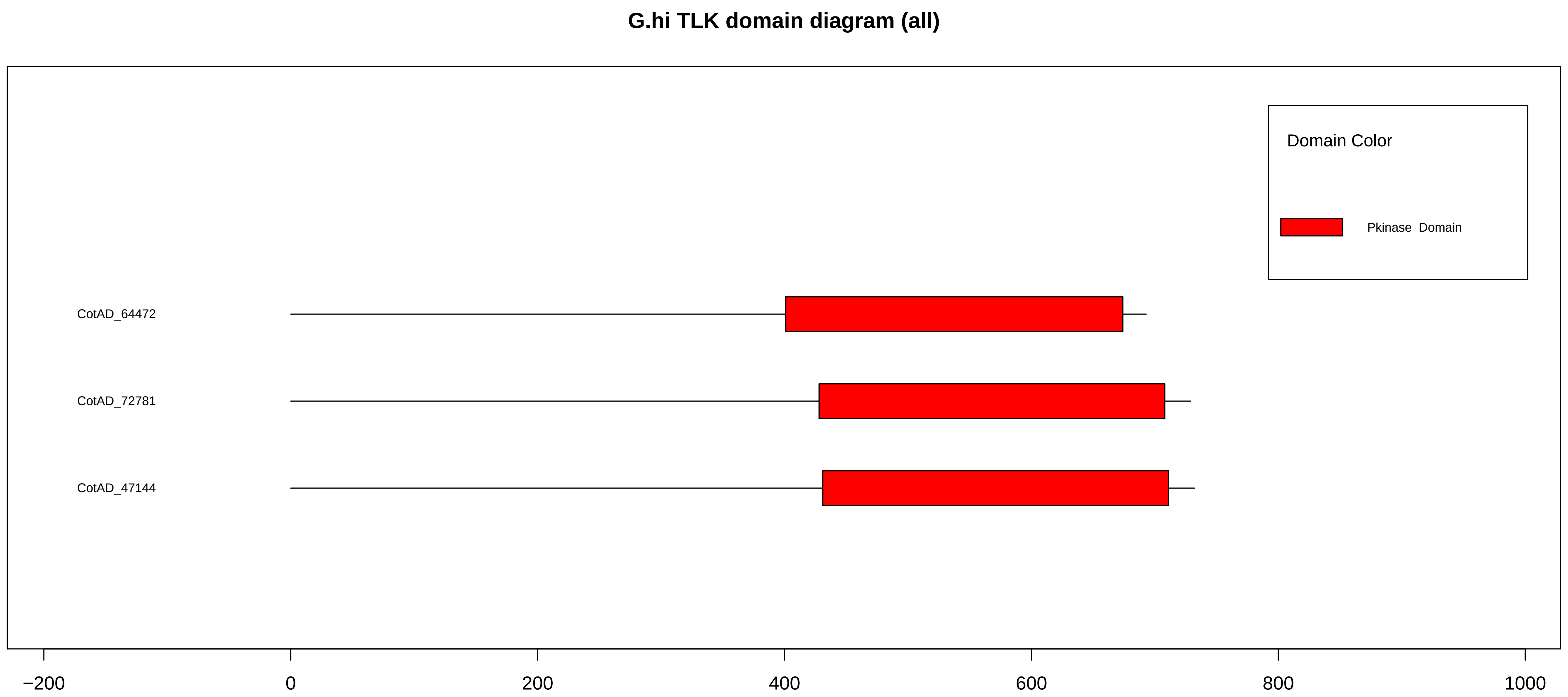

G.hi TLK domain diagram (all)
Domain Color
Pkinase Domain
CotAD_64472
CotAD_72781
CotAD_47144
−200
0
200
400
600
800
1000

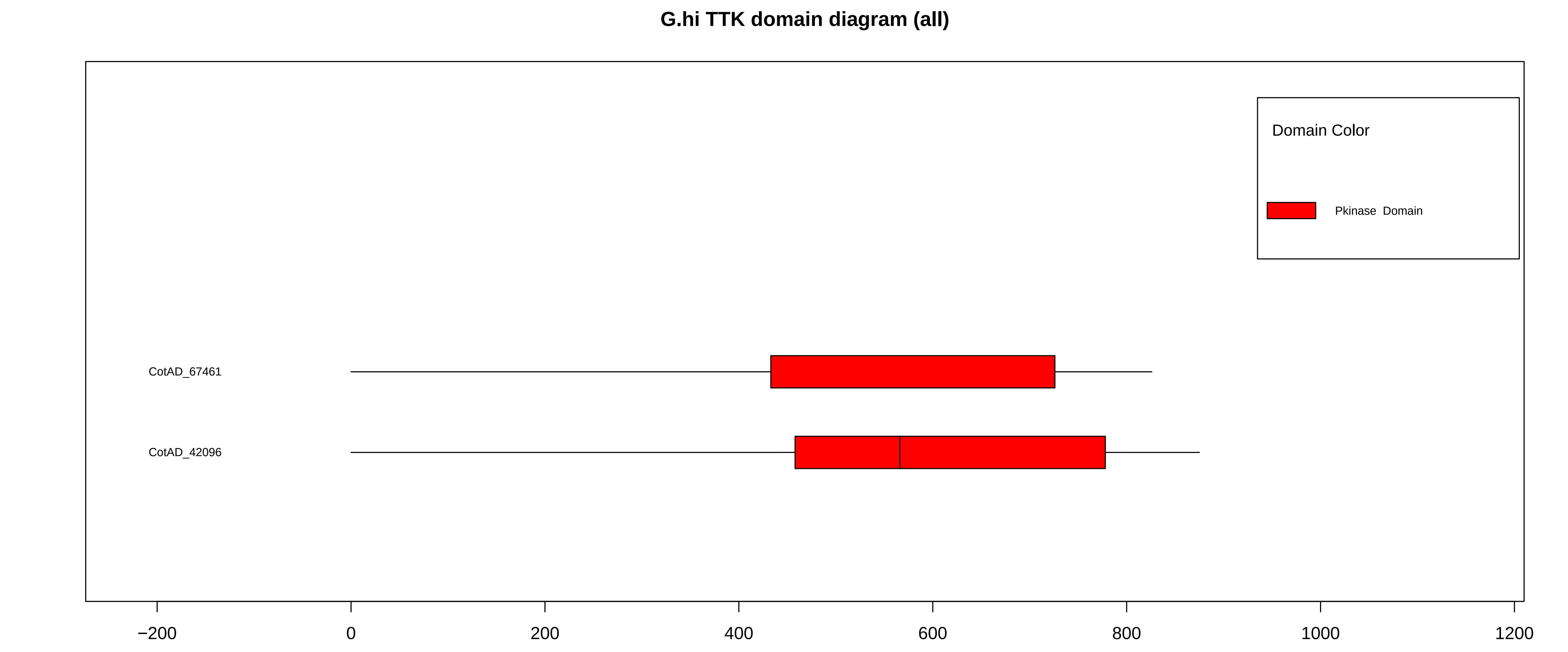
G.hi TTK domain diagram (all)
Domain Color
Pkinase Domain
CotAD_67461
CotAD_42096
−200
0
200
400
600
800
1000
1200

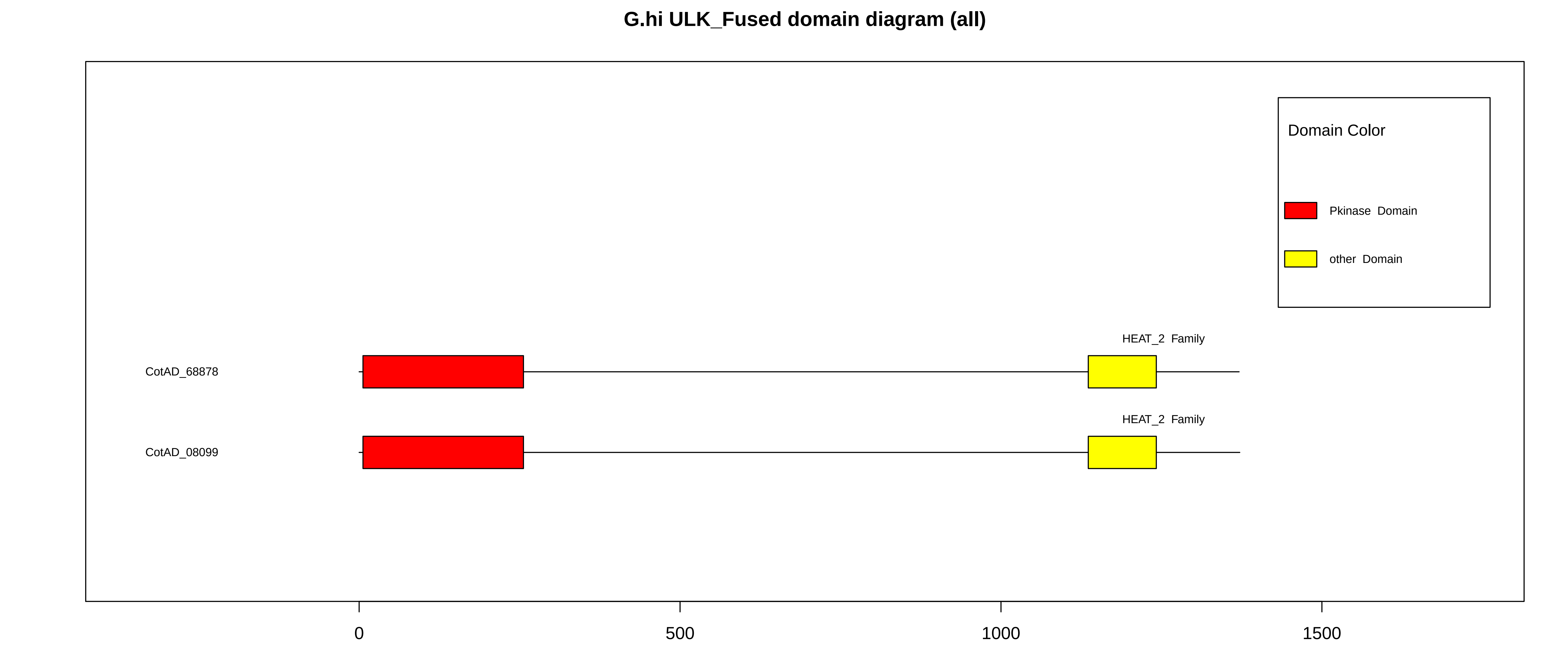

G.hi ULK_Fused domain diagram (all)
Domain Color
Pkinase Domain
other Domain
HEAT_2 Family
CotAD_68878
HEAT_2 Family
CotAD_08099
0
500
1000
1500

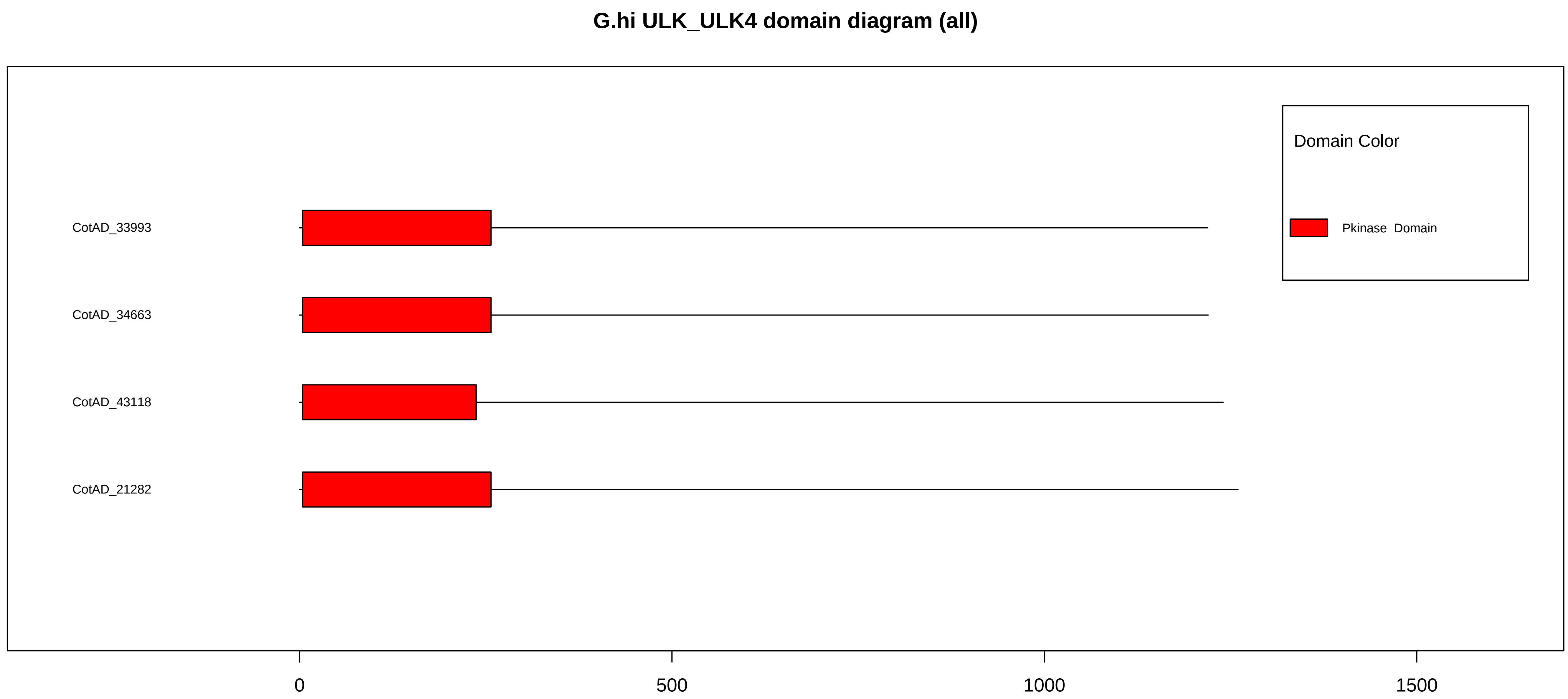
G.hi ULK_ULK4 domain diagram (all)
Domain Color
Pkinase Domain
CotAD_33993
CotAD_34663
CotAD_43118
CotAD_21282
0
500
1000
1500

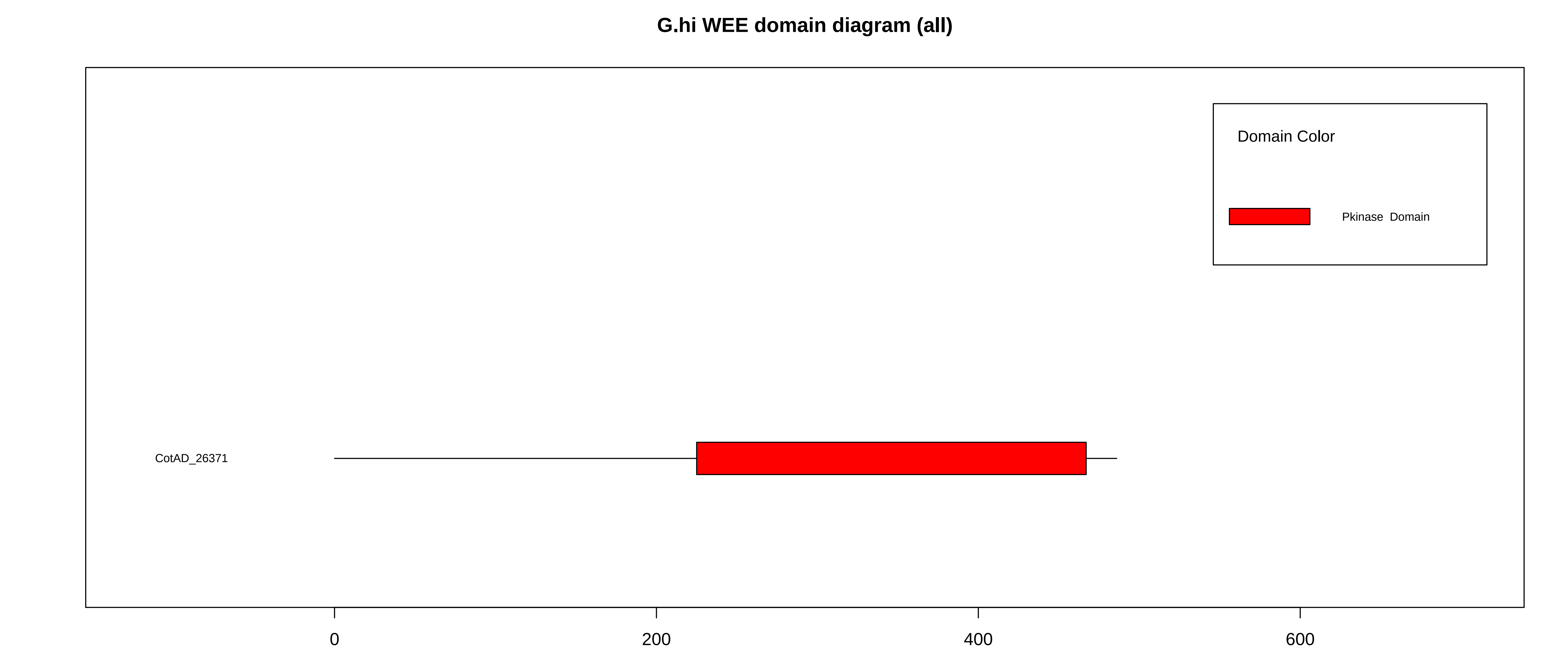
G.hi WEE domain diagram (all)
Domain Color
Pkinase Domain
CotAD_26371
0
200
400
600

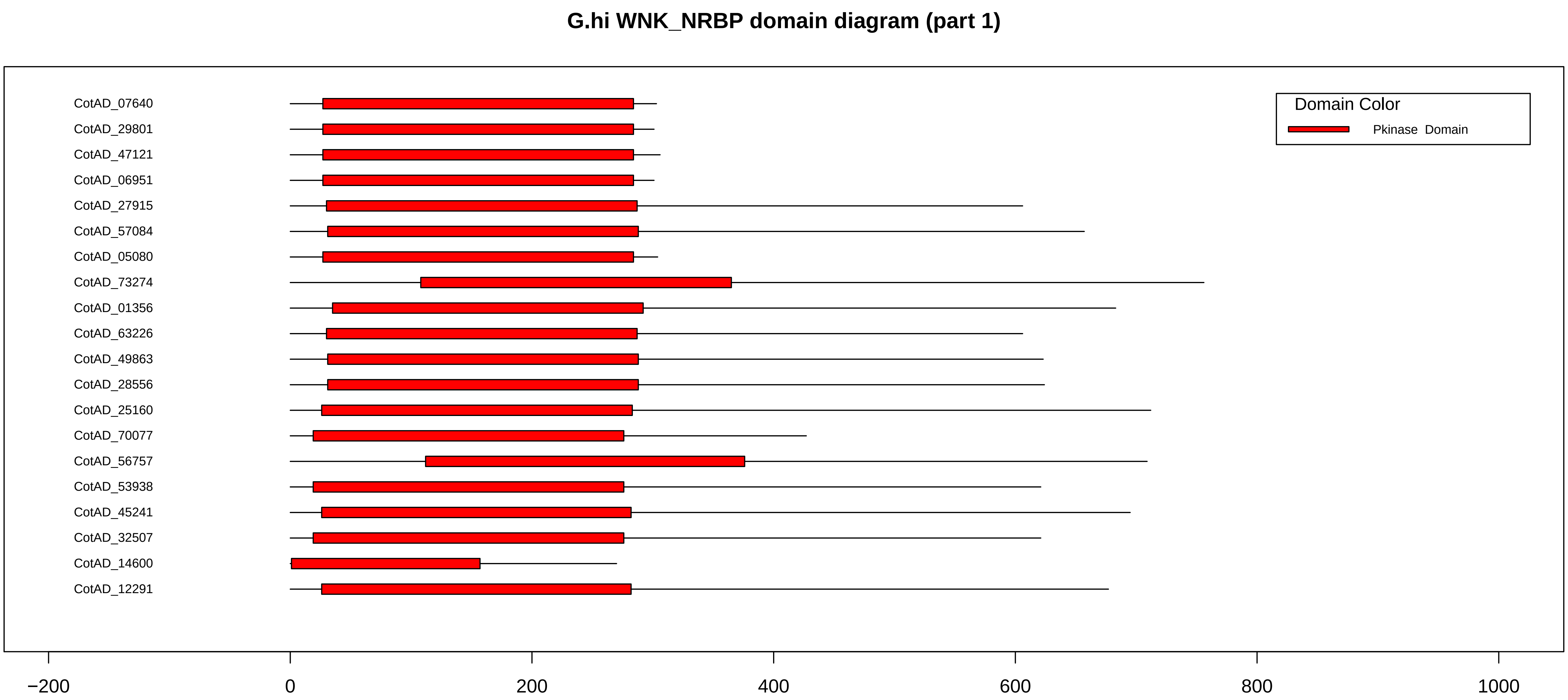
G.hi WNK_NRBP domain diagram (part 1)
CotAD_07640
CotAD_29801
CotAD_47121
CotAD_06951
CotAD_27915
CotAD_57084
CotAD_05080
CotAD_73274
CotAD_01356
CotAD_63226
CotAD_49863
CotAD_28556
CotAD_25160
CotAD_70077
CotAD_56757
CotAD_53938
CotAD_45241
CotAD_32507
CotAD_14600
CotAD_12291
Domain Color
Pkinase Domain
−200
0
200
400
600
800
1000

G.hi WNK_NRBP domain diagram (part 2)

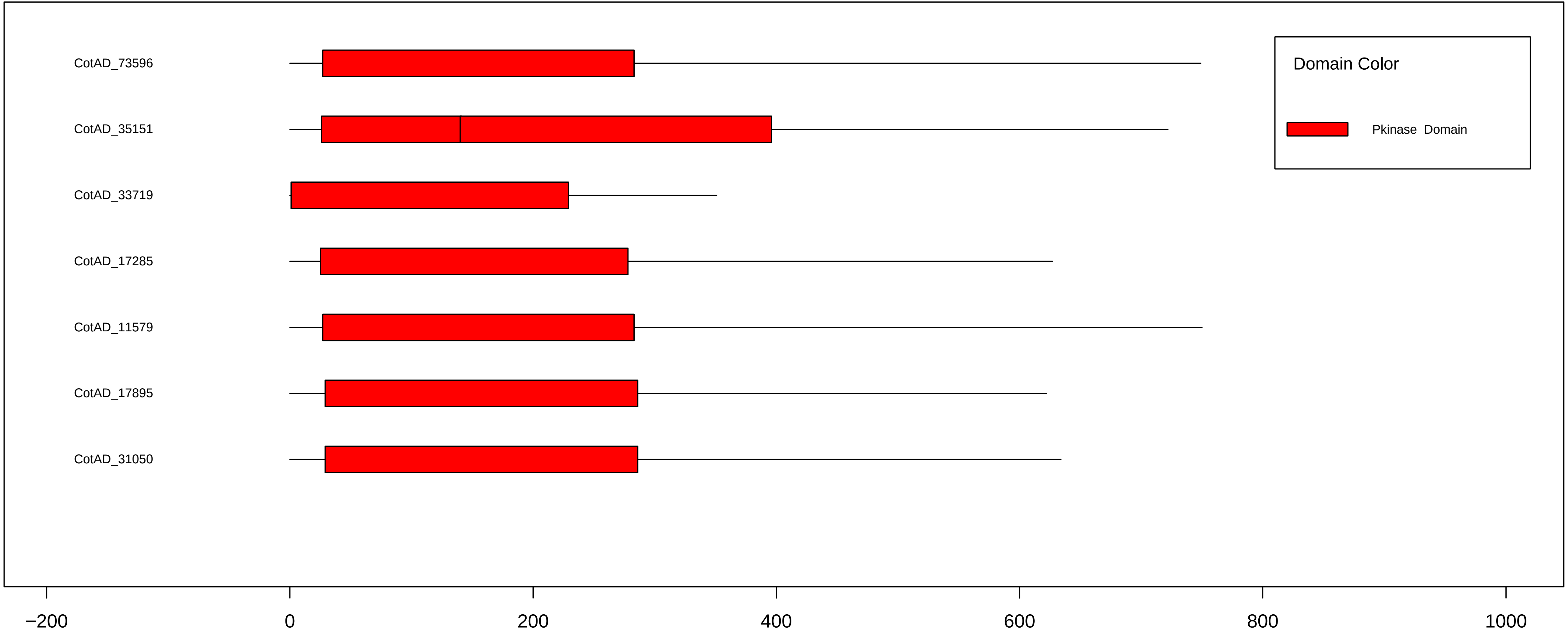

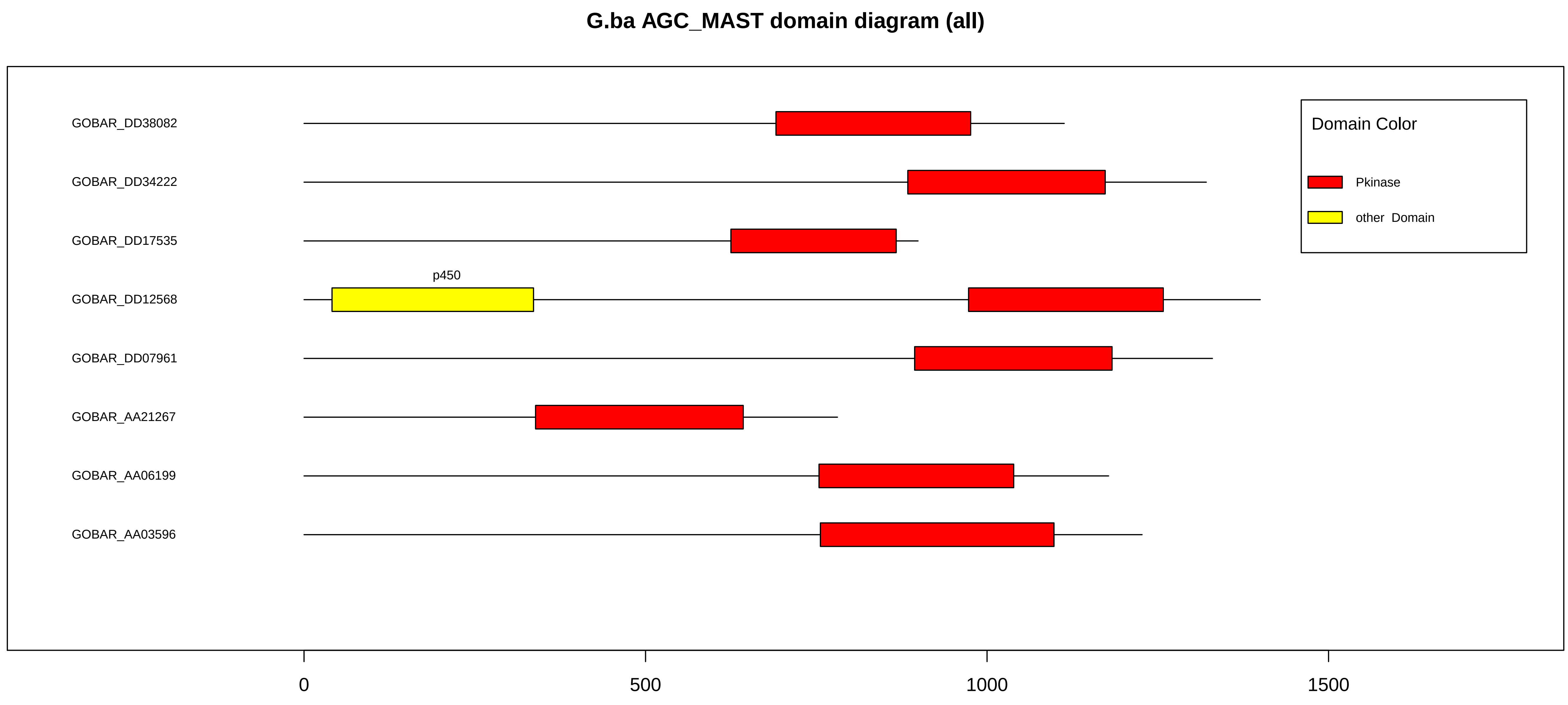
G.ba AGC_MAST domain diagram (all)
GOBAR_DD38082
GOBAR_DD34222
GOBAR_DD17535
GOBAR_DD12568
GOBAR_DD07961
GOBAR_AA21267
GOBAR_AA06199
GOBAR_AA03596
p450
Domain Color
Pkinase
other Domain
0
500
1000
1500

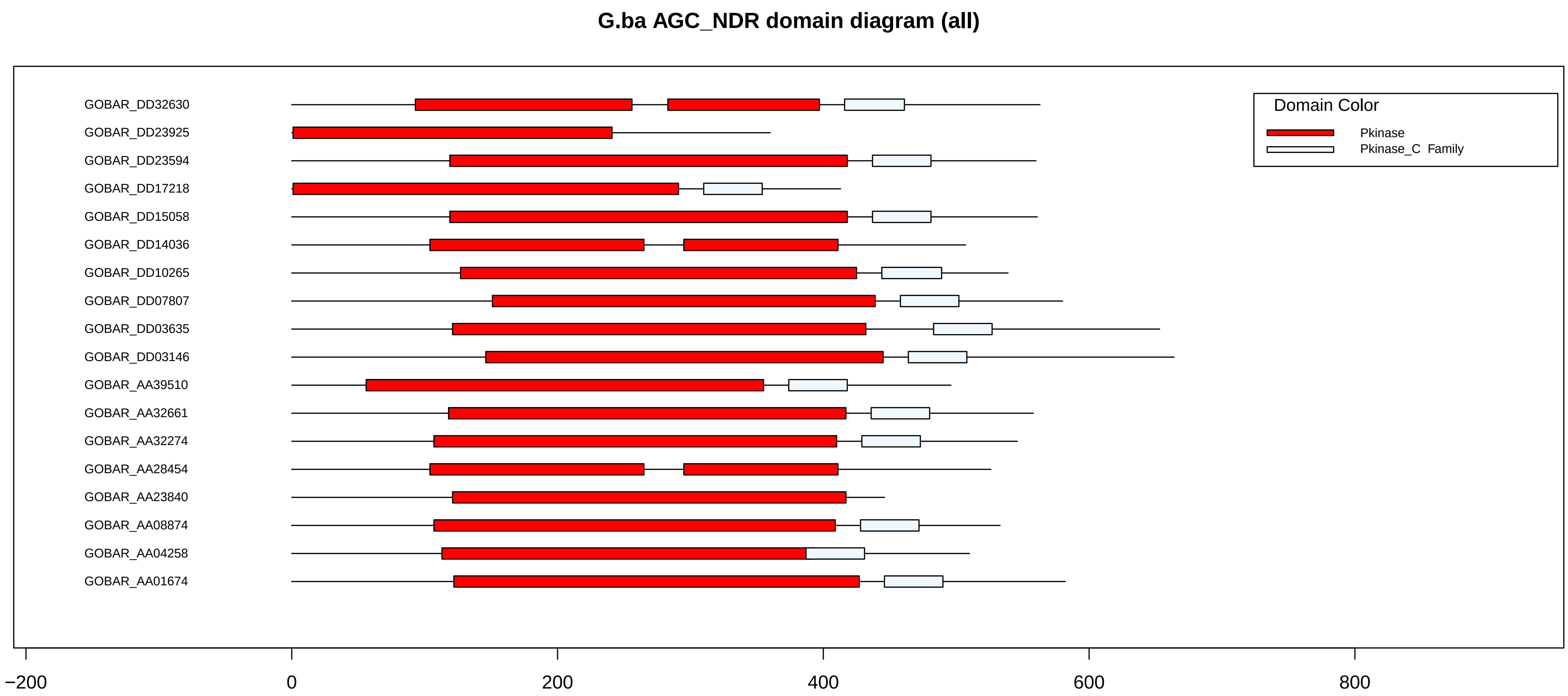
G.ba AGC_NDR domain diagram (all)
GOBAR_DD32630
GOBAR_DD23925
GOBAR_DD23594
GOBAR_DD17218
GOBAR_DD15058
GOBAR_DD14036
GOBAR_DD10265
GOBAR_DD07807
GOBAR_DD03635
GOBAR_DD03146
GOBAR_AA39510
GOBAR_AA32661
GOBAR_AA32274
GOBAR_AA28454
GOBAR_AA23840
GOBAR_AA08874
GOBAR_AA04258
GOBAR_AA01674
Domain Color
Pkinase
Pkinase_C Family
−200
0
200
400
600
800

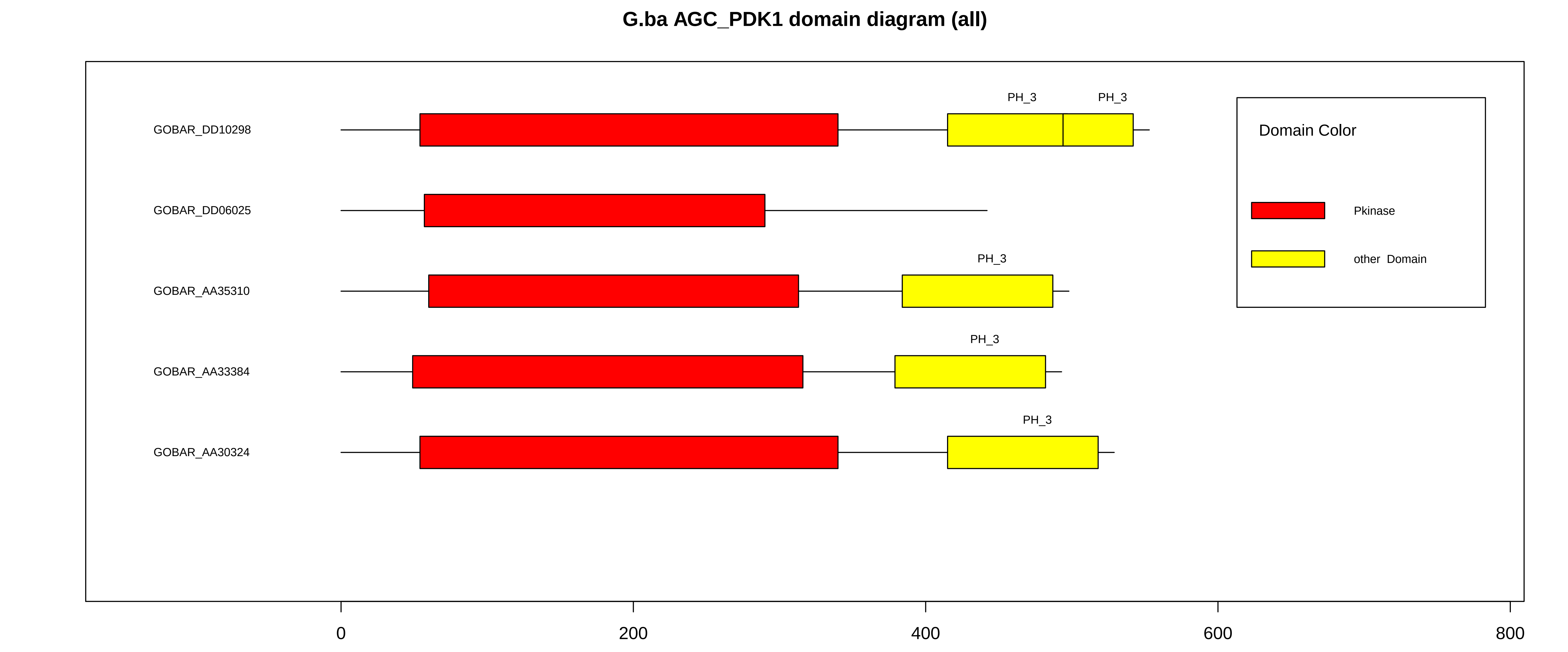
G.ba AGC_PDK1 domain diagram (all)
GOBAR_DD10298
GOBAR_DD06025
GOBAR_AA35310
GOBAR_AA33384
GOBAR_AA30324
PH_3
PH_3
PH_3
PH_3
PH_3
Domain Color
Pkinase
other Domain
0
200
400
600
800

G.ba AGC_PKA−PKG domain diagram (all)

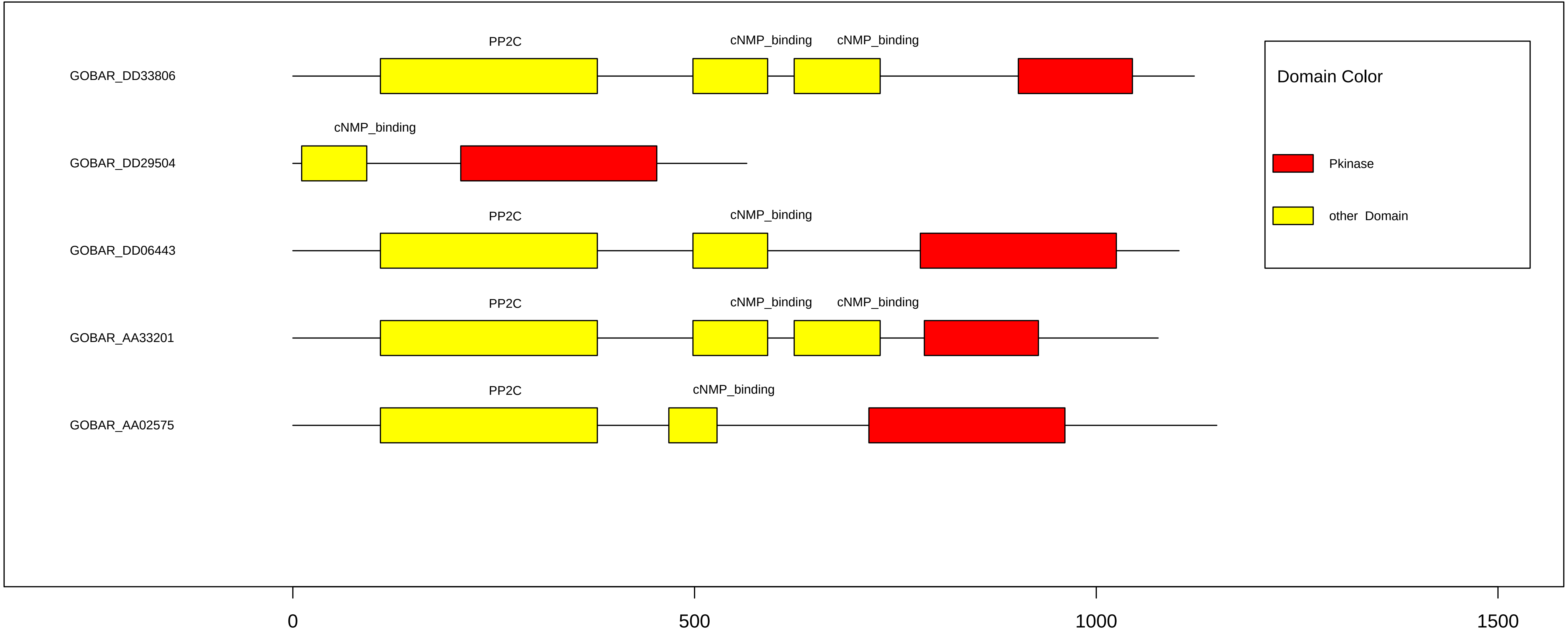

G.ba AGC_RSK−2 domain diagram (part 1)

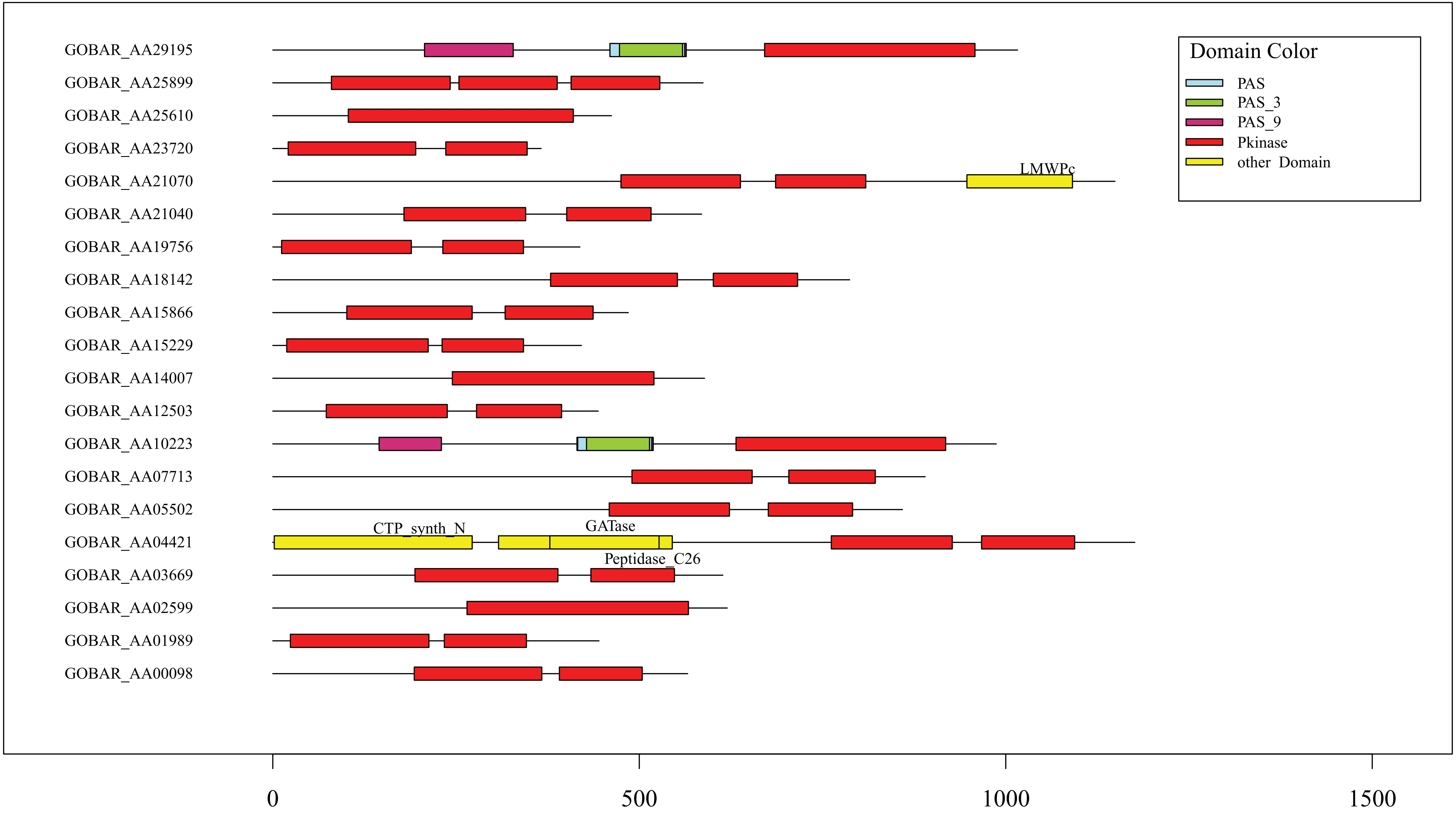

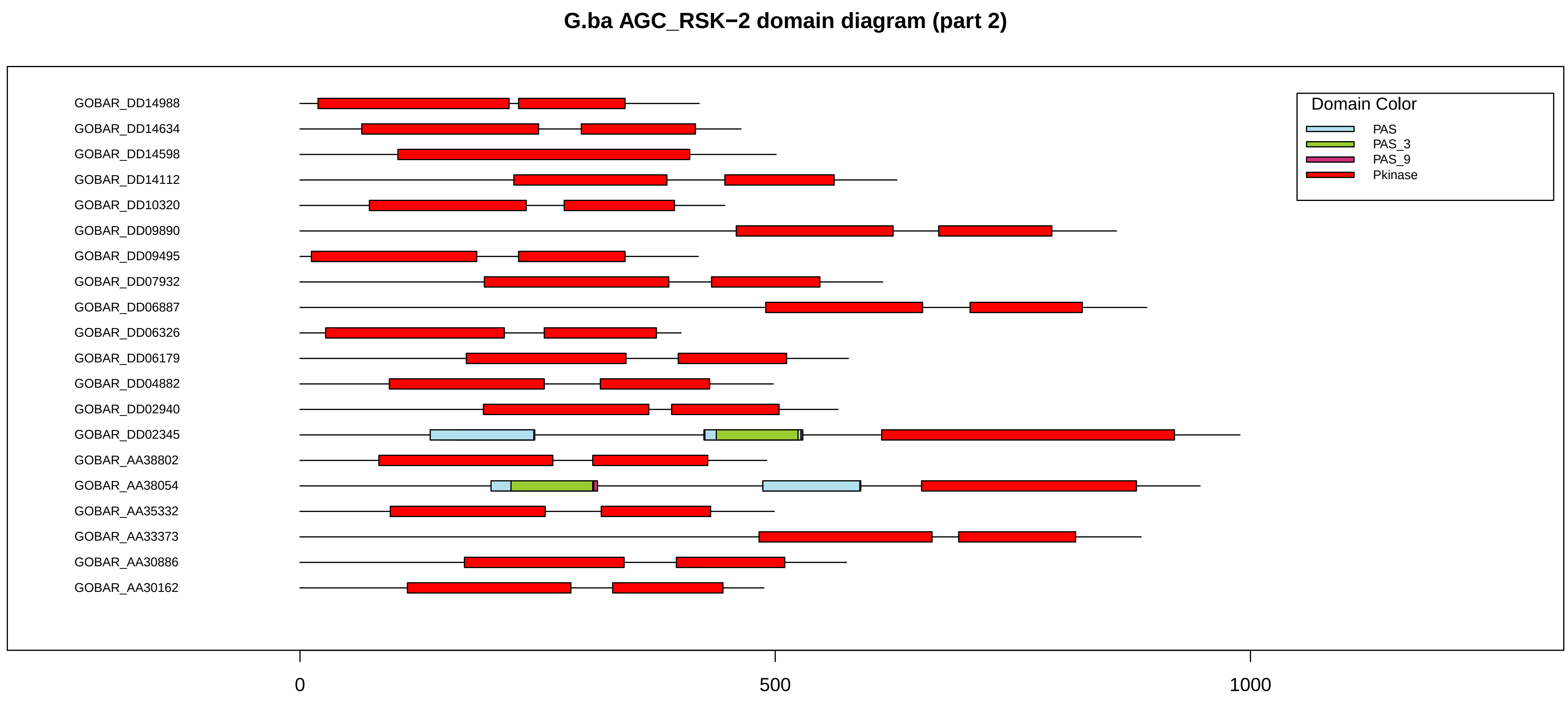
G.ba AGC_RSK−2 domain diagram (part 2)
GOBAR_DD14988
GOBAR_DD14634
GOBAR_DD14598
GOBAR_DD14112
GOBAR_DD10320
GOBAR_DD09890
GOBAR_DD09495
GOBAR_DD07932
GOBAR_DD06887
GOBAR_DD06326
GOBAR_DD06179
GOBAR_DD04882
GOBAR_DD02940
GOBAR_DD02345
GOBAR_AA38802
GOBAR_AA38054
GOBAR_AA35332
GOBAR_AA33373
GOBAR_AA30886
GOBAR_AA30162
Domain Color
PAS
PAS_3
PAS_9
Pkinase
0
500
1000

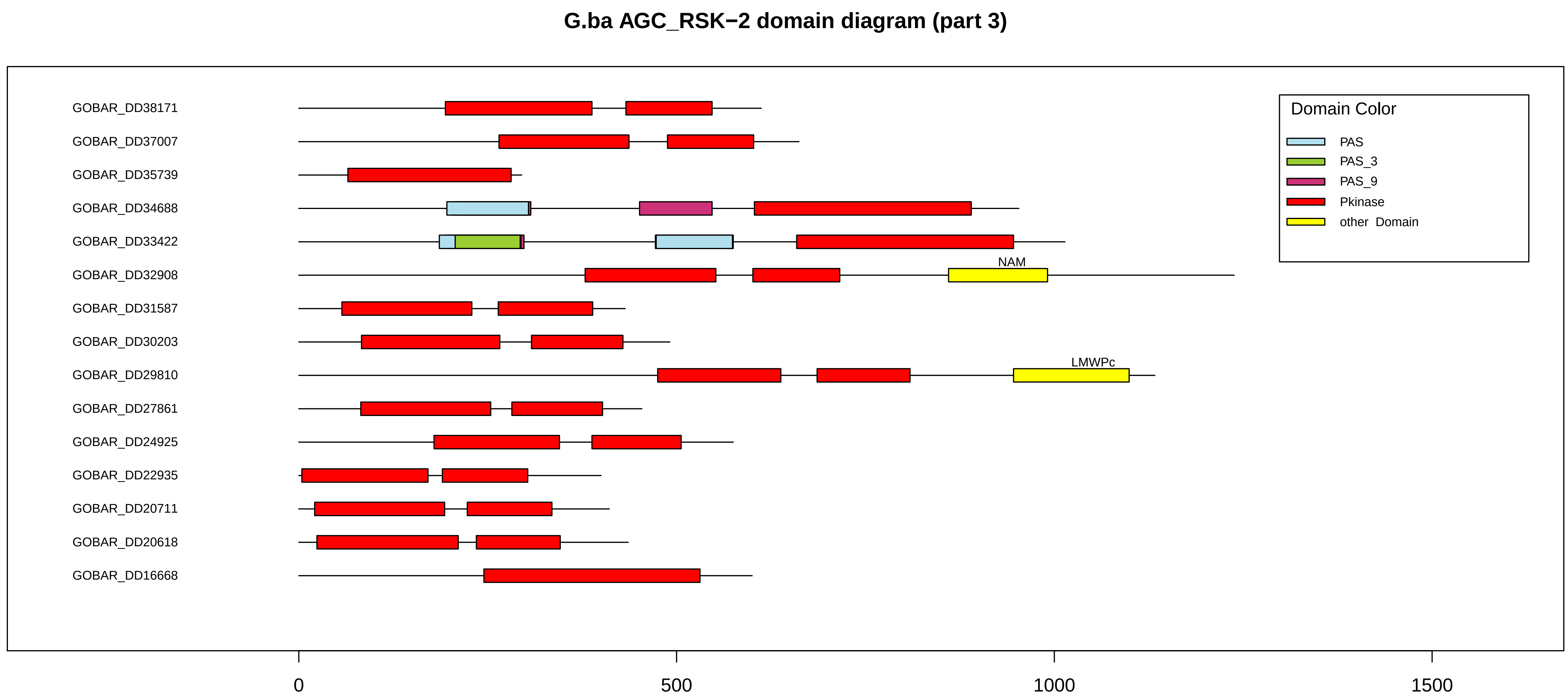

G.ba AGC_RSK−2 domain diagram (part 3)
GOBAR_DD38171
GOBAR_DD37007
GOBAR_DD35739
GOBAR_DD34688
GOBAR_DD33422
GOBAR_DD32908
GOBAR_DD31587
GOBAR_DD30203
GOBAR_DD29810
GOBAR_DD27861
GOBAR_DD24925
GOBAR_DD22935
GOBAR_DD20711
GOBAR_DD20618
GOBAR_DD16668
NAM
LMWPc
Domain Color
PAS
PAS_3
PAS_9
Pkinase
other Domain
0
500
1000
1500

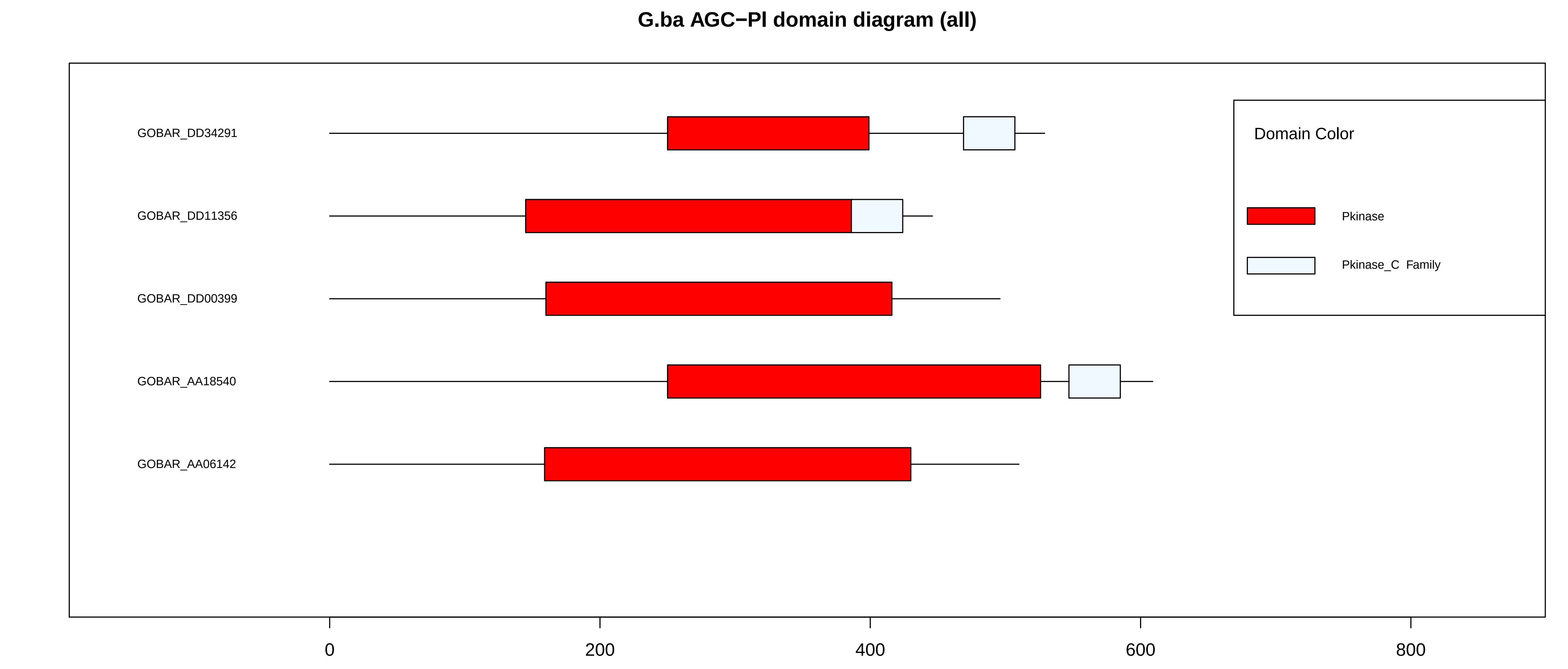

G.ba AGC−PI domain diagram (all)
GOBAR_DD34291
GOBAR_DD11356
GOBAR_DD00399
GOBAR_AA18540
GOBAR_AA06142
Domain Color
Pkinase
Pkinase_C Family
0
200
400
600
800

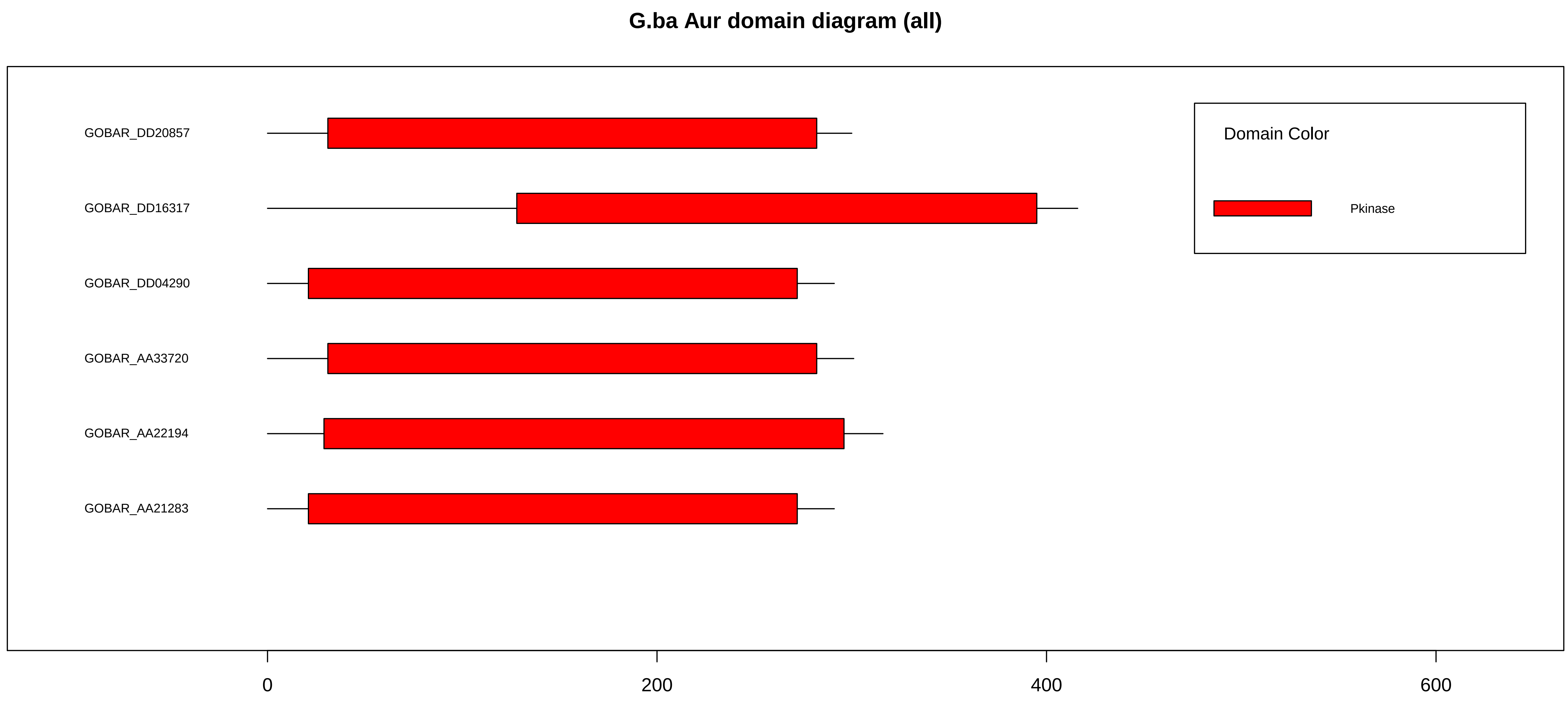
G.ba Aur domain diagram (all)
GOBAR_DD20857
GOBAR_DD16317
GOBAR_DD04290
GOBAR_AA33720
GOBAR_AA22194
GOBAR_AA21283
Domain Color
Pkinase
0
200
400
600

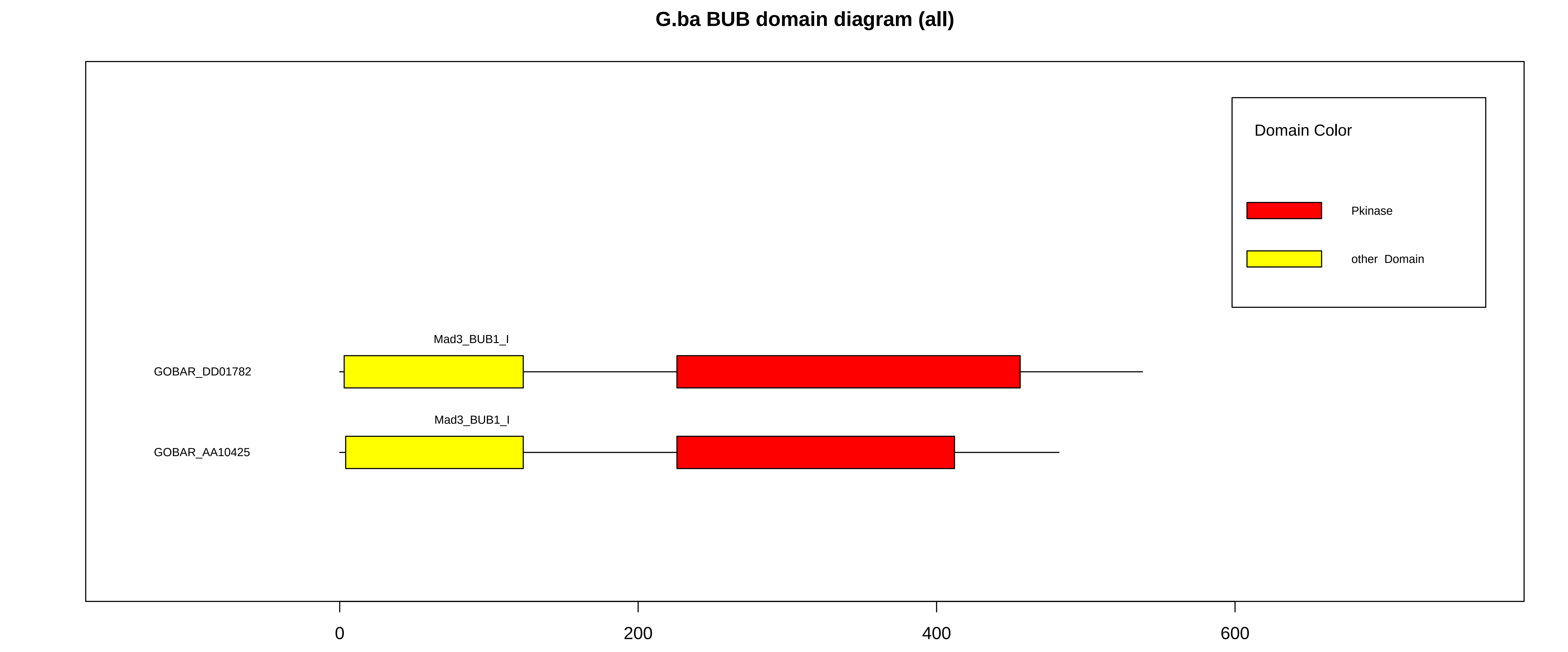
G.ba BUB domain diagram (all)
Domain Color
Pkinase
other Domain
Mad3_BUB1_I
GOBAR_DD01782
Mad3_BUB1_I
GOBAR_AA10425
0
200
400
600

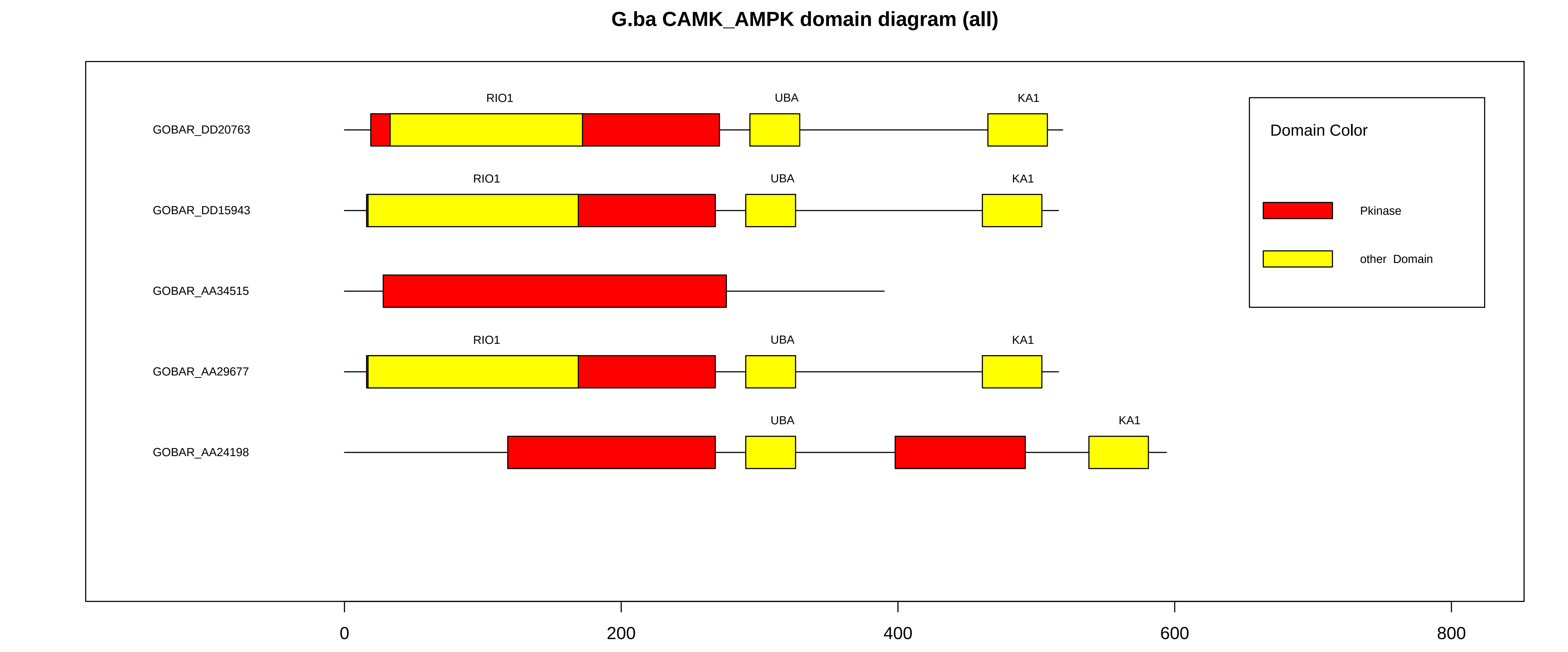
G.ba CAMK_AMPK domain diagram (all)
GOBAR_DD20763
RIO1
UBA
KA1
GOBAR_DD15943
RIO1
UBA
KA1
GOBAR_AA34515
GOBAR_AA29677
RIO1
UBA
KA1
GOBAR_AA24198
UBA
KA1
Domain Color
Pkinase
other Domain
0
200
400
600
800

G.ba CAMK_CAMK1−DCAMKL domain diagram (all)

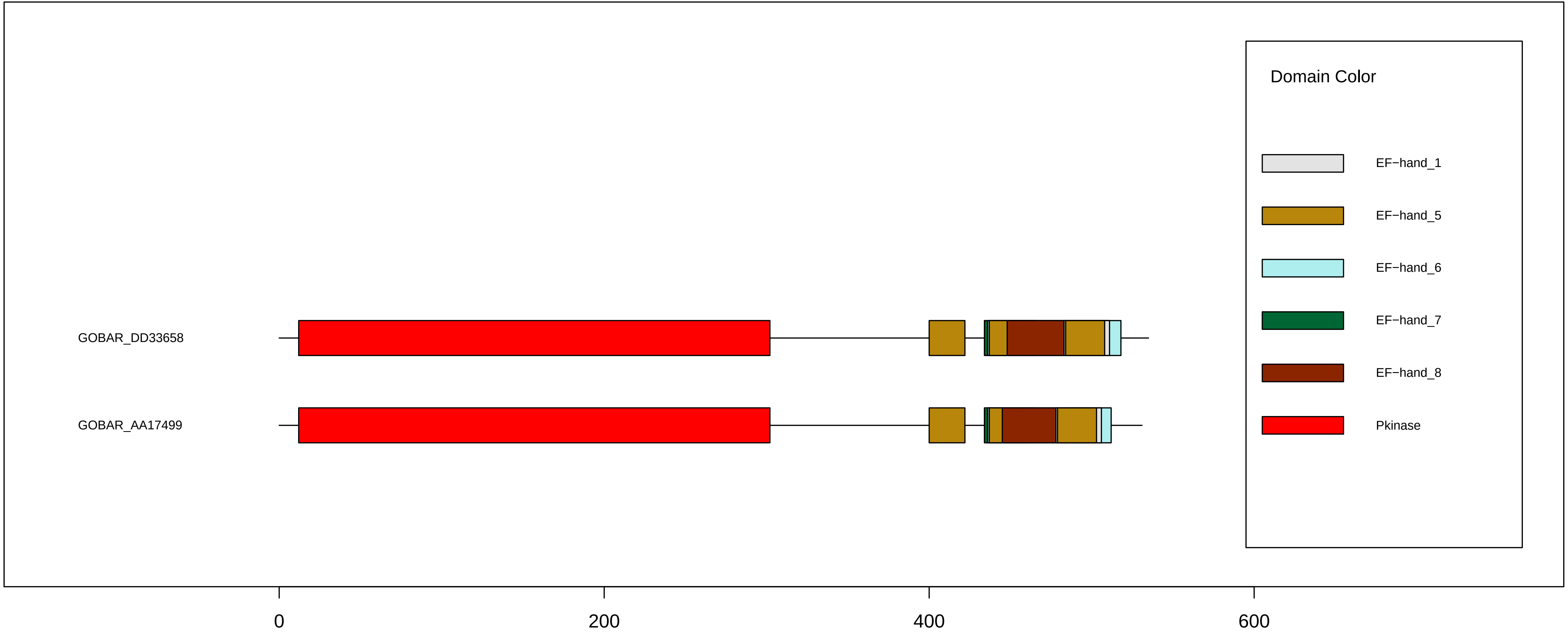

G.ba CAMK_CAMKL−CHK1 domain diagram (part 1)

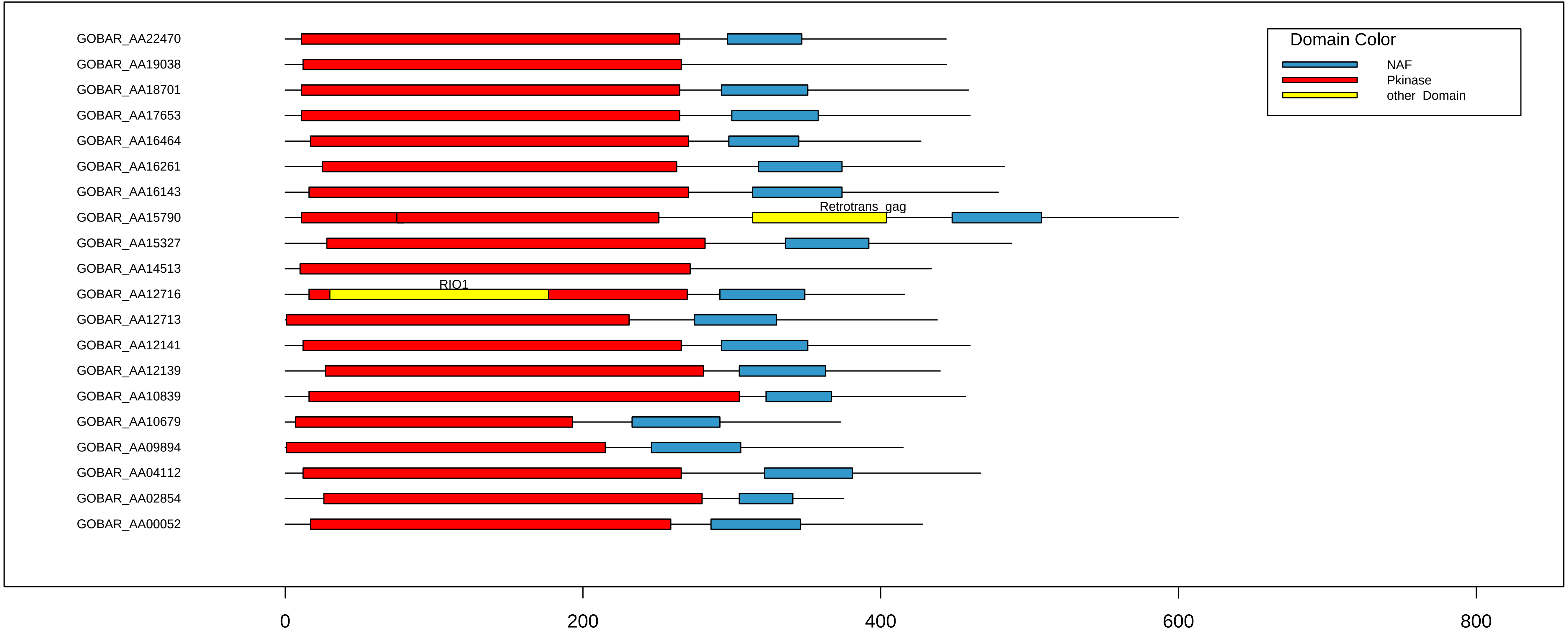

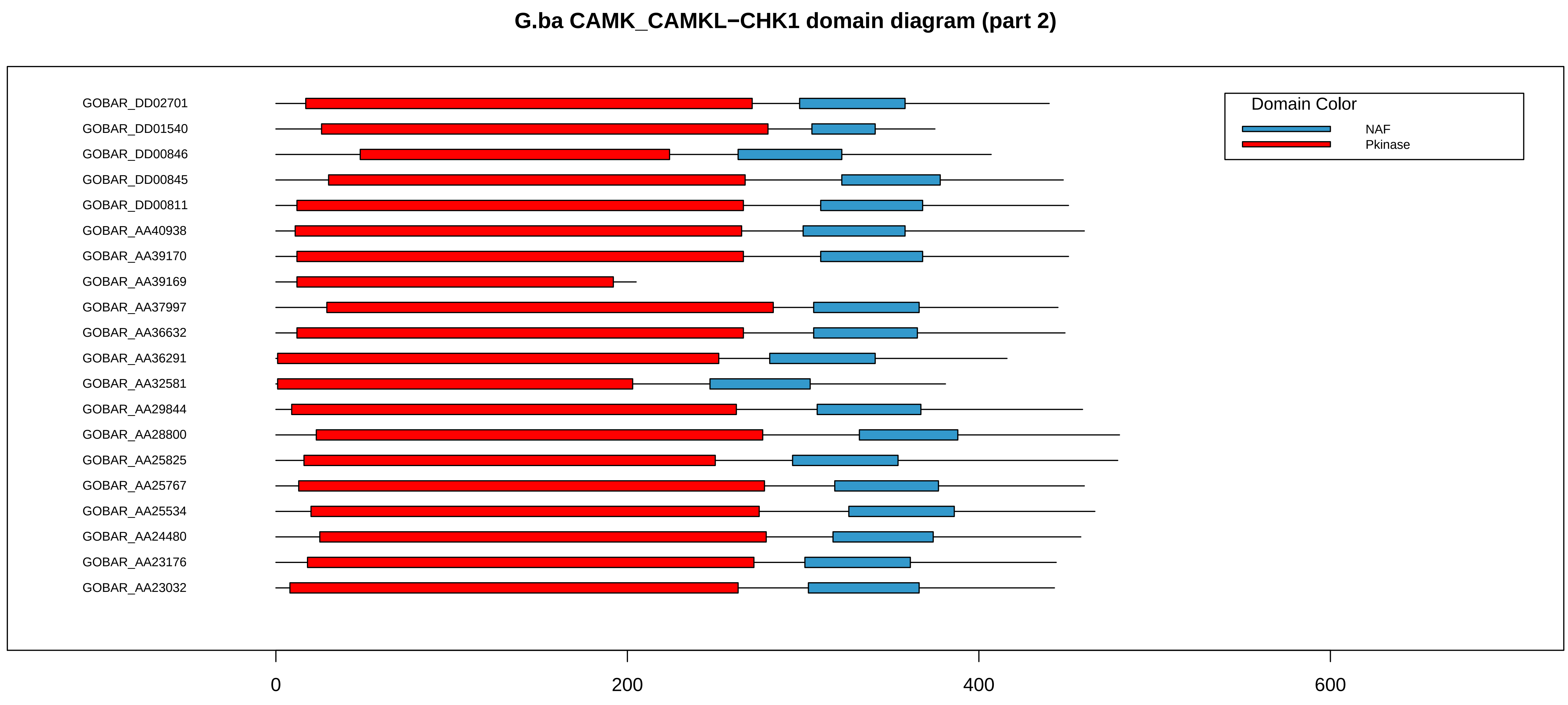
G.ba CAMK_CAMKL−CHK1 domain diagram (part 2)
GOBAR_DD02701
GOBAR_DD01540
GOBAR_DD00846
GOBAR_DD00845
GOBAR_DD00811
GOBAR_AA40938
GOBAR_AA39170
GOBAR_AA39169
GOBAR_AA37997
GOBAR_AA36632
GOBAR_AA36291
GOBAR_AA32581
GOBAR_AA29844
GOBAR_AA28800
GOBAR_AA25825
GOBAR_AA25767
GOBAR_AA25534
GOBAR_AA24480
GOBAR_AA23176
GOBAR_AA23032
Domain Color
NAF
Pkinase
0
200
400
600

G.ba CAMK_CAMKL−CHK1 domain diagram (part 3)

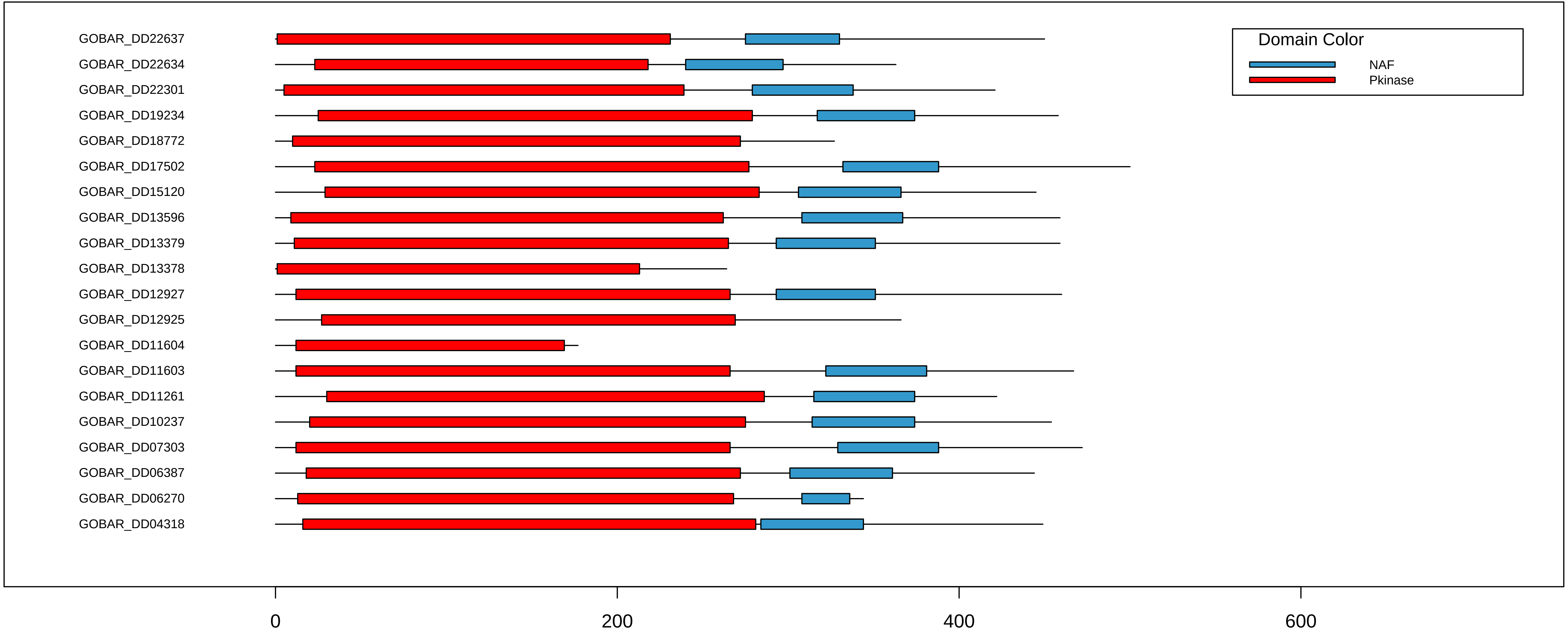

G.ba CAMK_CAMKL−CHK1 domain diagram (part 4)

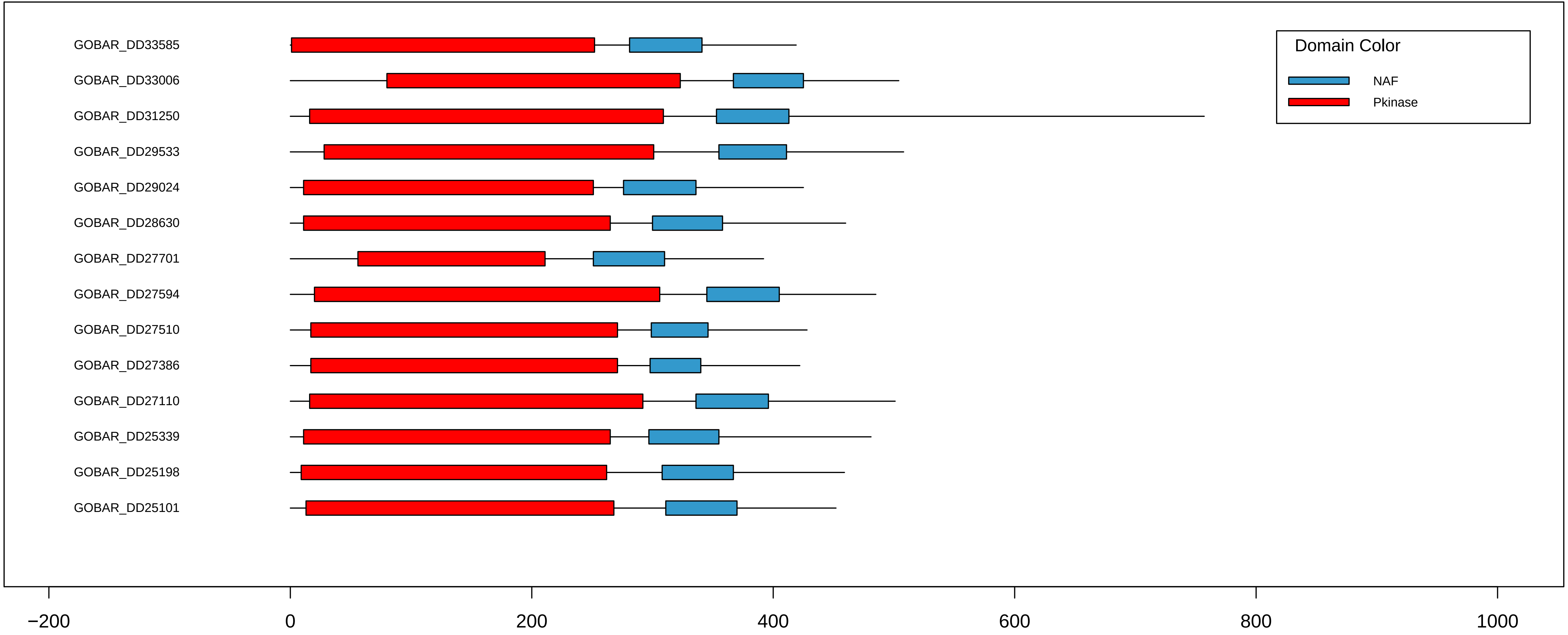

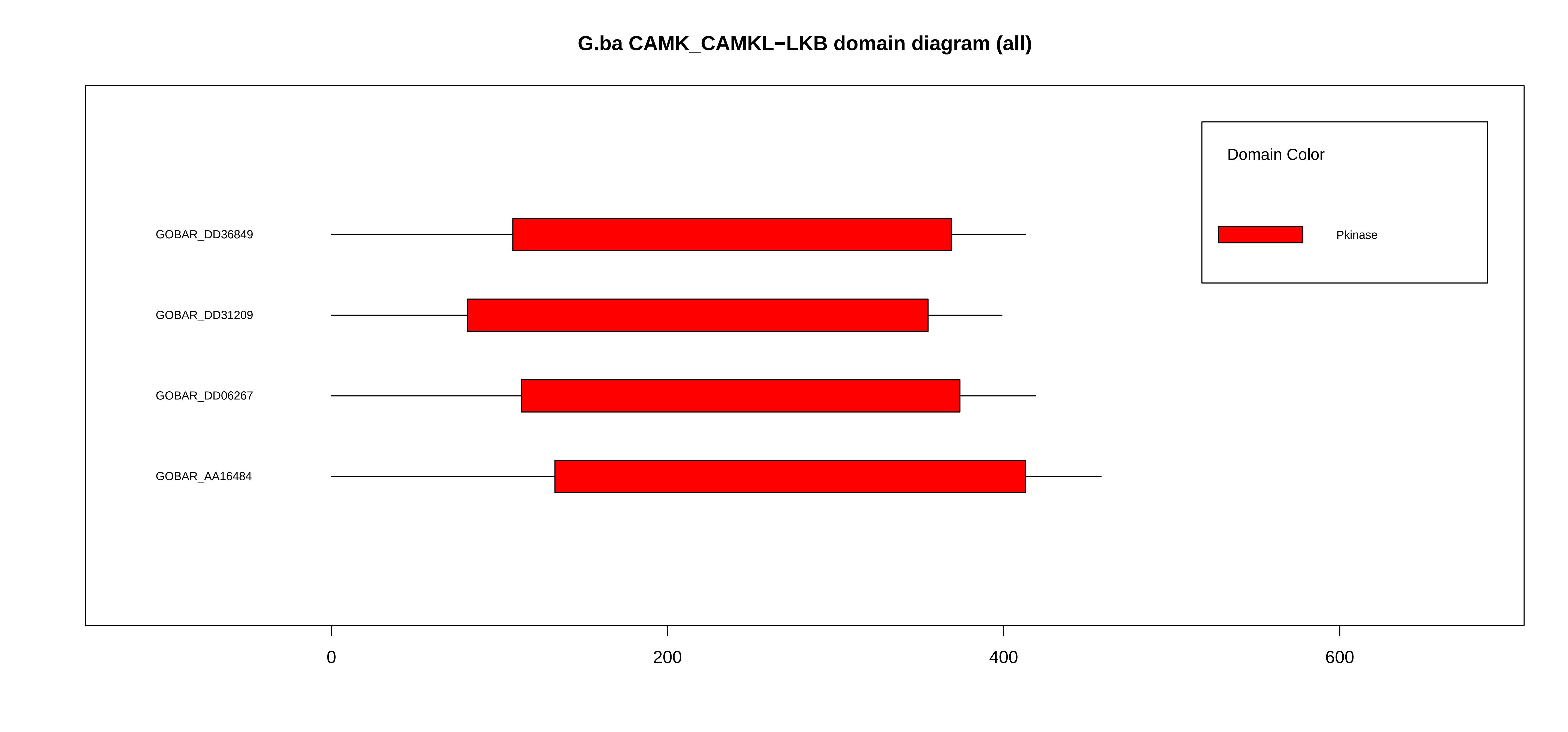
G.ba CAMK_CAMKL−LKB domain diagram (all)
Domain Color
Pkinase
GOBAR_DD36849
GOBAR_DD31209
GOBAR_DD06267
GOBAR_AA16484
0
200
400
600

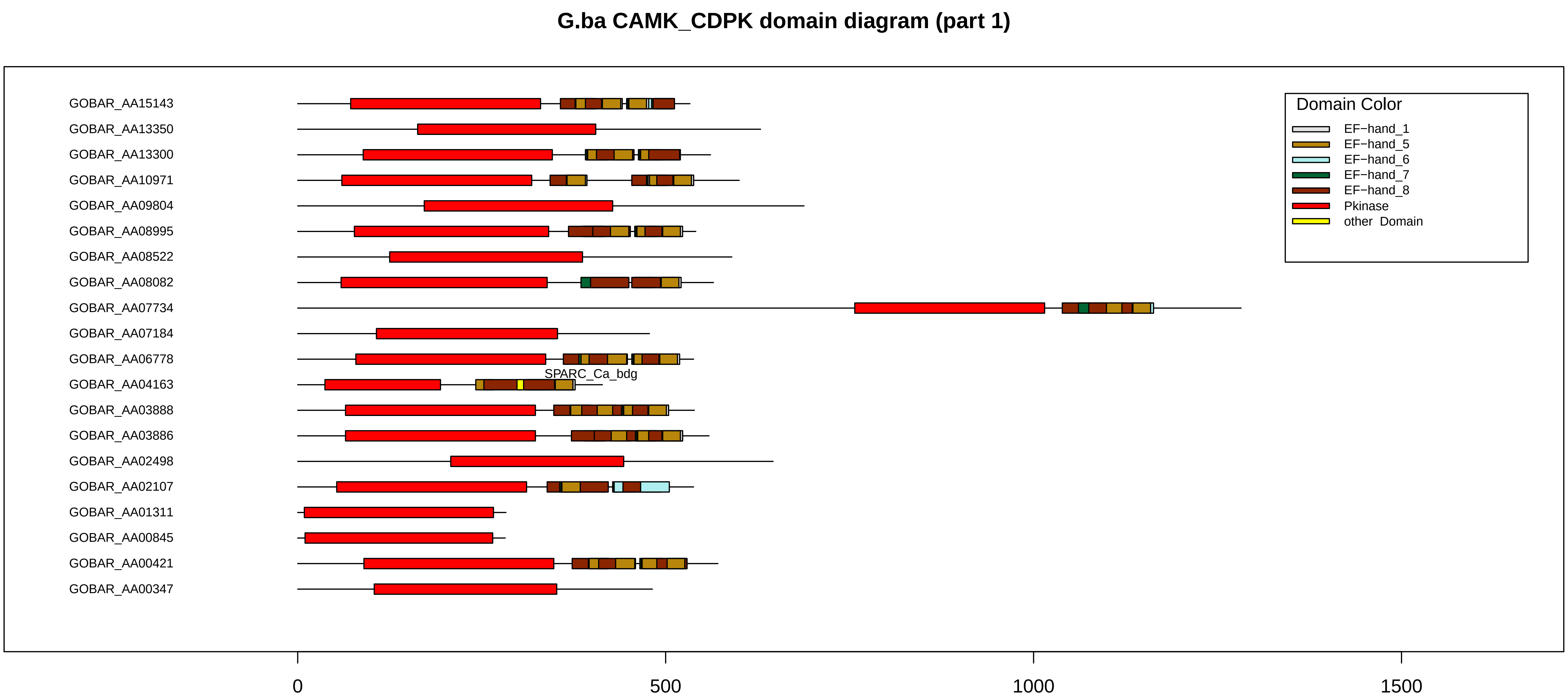

G.ba CAMK_CDPK domain diagram (part 1)
GOBAR_AA15143
GOBAR_AA13350
GOBAR_AA13300
GOBAR_AA10971
GOBAR_AA09804
GOBAR_AA08995
GOBAR_AA08522
GOBAR_AA08082
GOBAR_AA07734
GOBAR_AA07184
GOBAR_AA06778
GOBAR_AA04163
SPARC_Ca_bdg
GOBAR_AA03888
GOBAR_AA03886
GOBAR_AA02498
GOBAR_AA02107
GOBAR_AA01311
GOBAR_AA00845
GOBAR_AA00421
GOBAR_AA00347
Domain Color
EF−hand_1
EF−hand_5
EF−hand_6
EF−hand_7
EF−hand_8
Pkinase
other Domain
0
500
1000
1500

G.ba CAMK_CDPK domain diagram (part 2)

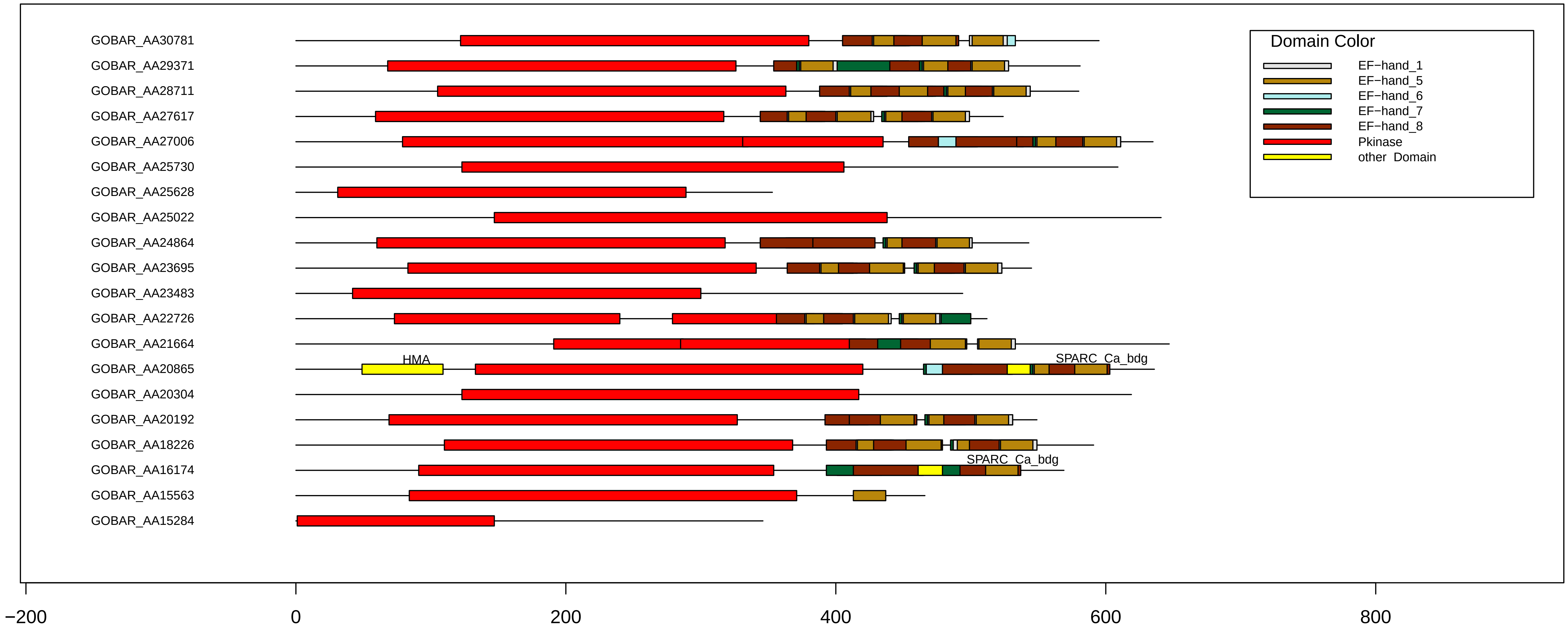

G.ba CAMK_CDPK domain diagram (part 3)

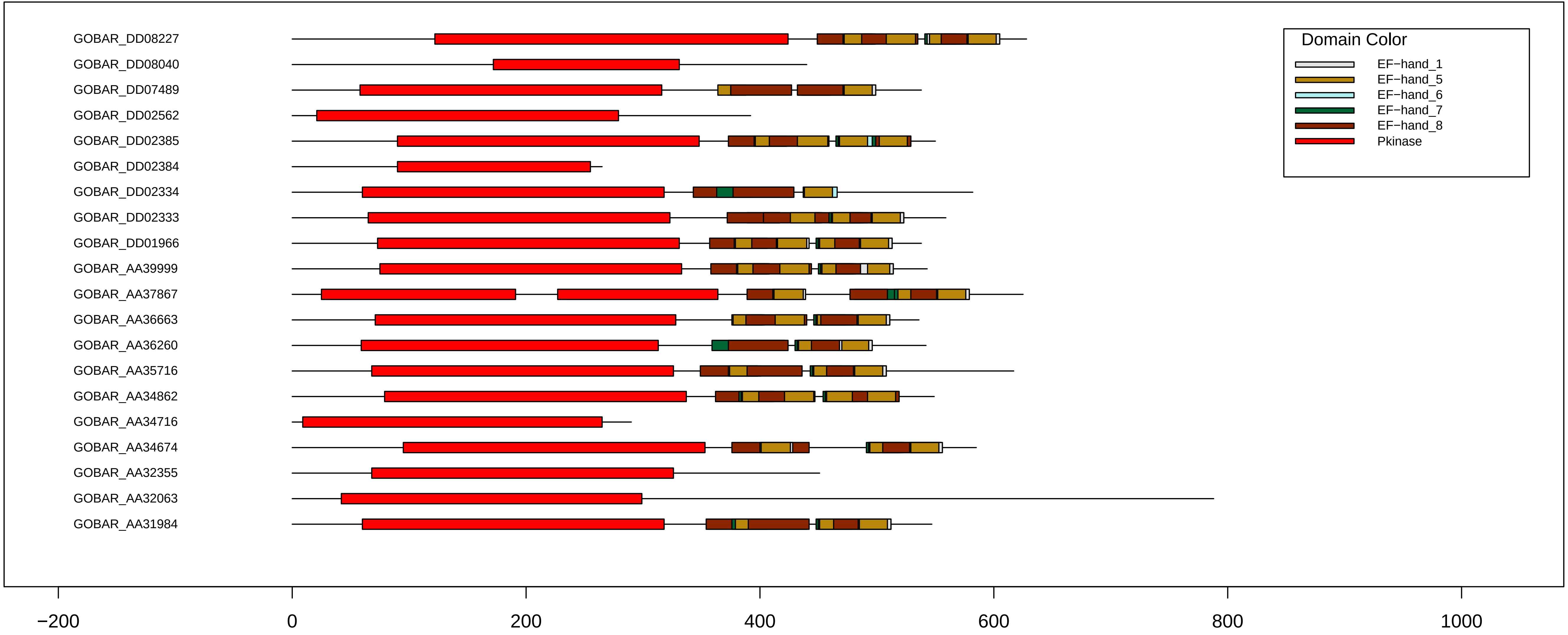

G.ba CAMK_CDPK domain diagram (part 4)

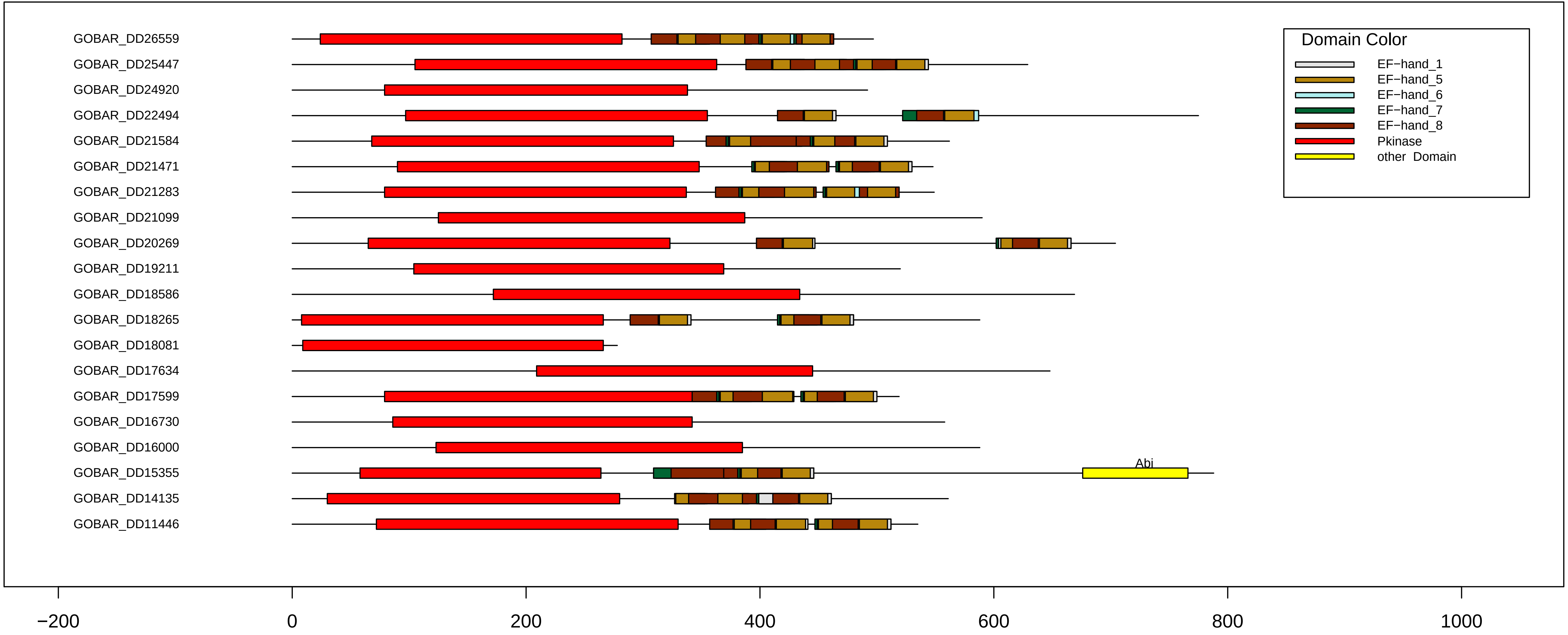

G.ba CAMK_CDPK domain diagram (part 5)

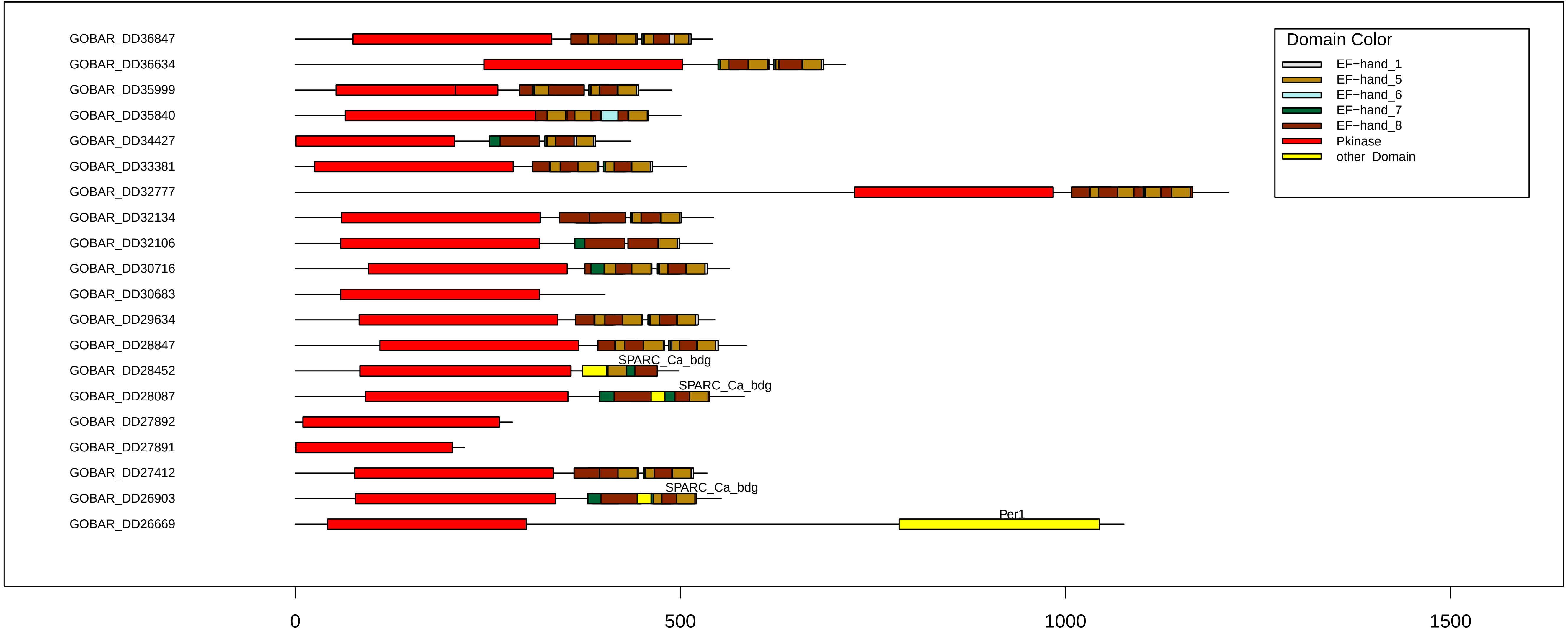

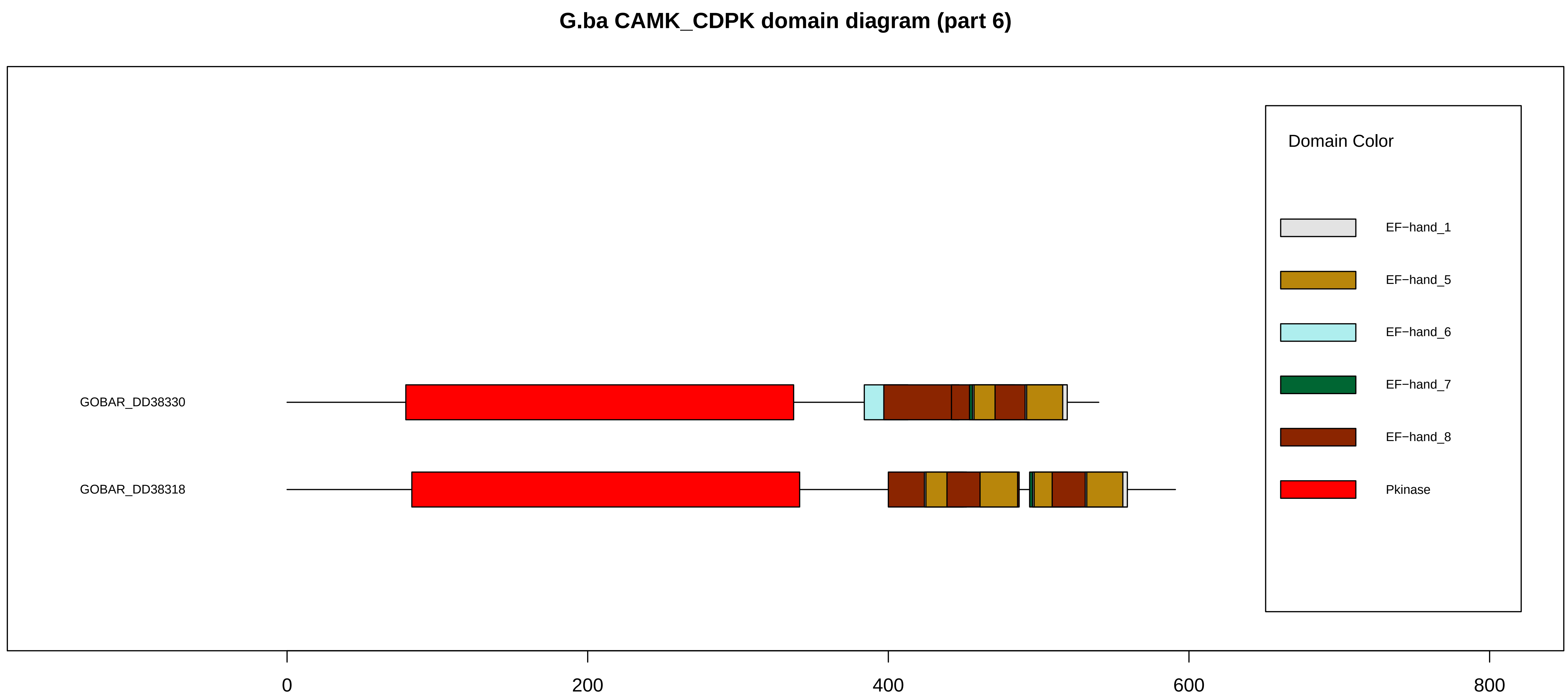
G.ba CAMK_CDPK domain diagram (part 6)
GOBAR_DD38330
GOBAR_DD38318
Domain Color
EF−hand_1
EF−hand_5
EF−hand_6
EF−hand_7
EF−hand_8
Pkinase
0
200
400
600
800

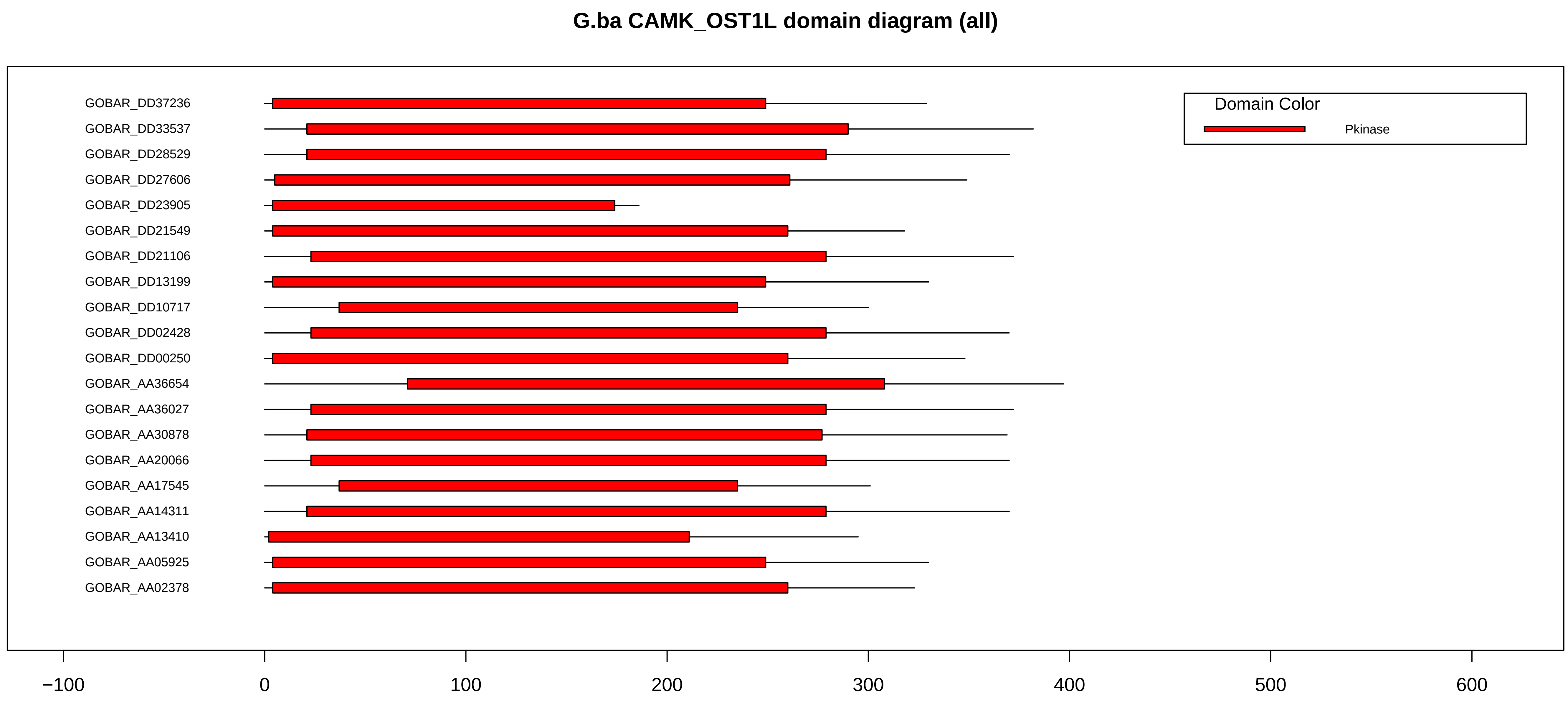

G.ba CAMK_OST1L domain diagram (all)
GOBAR_DD37236
GOBAR_DD33537
GOBAR_DD28529
GOBAR_DD27606
GOBAR_DD23905
GOBAR_DD21549
GOBAR_DD21106
GOBAR_DD13199
GOBAR_DD10717
GOBAR_DD02428
GOBAR_DD00250
GOBAR_AA36654
GOBAR_AA36027
GOBAR_AA30878
GOBAR_AA20066
GOBAR_AA17545
GOBAR_AA14311
GOBAR_AA13410
GOBAR_AA05925
GOBAR_AA02378
Domain Color
Pkinase
−100
0
100
200
300
400
500
600

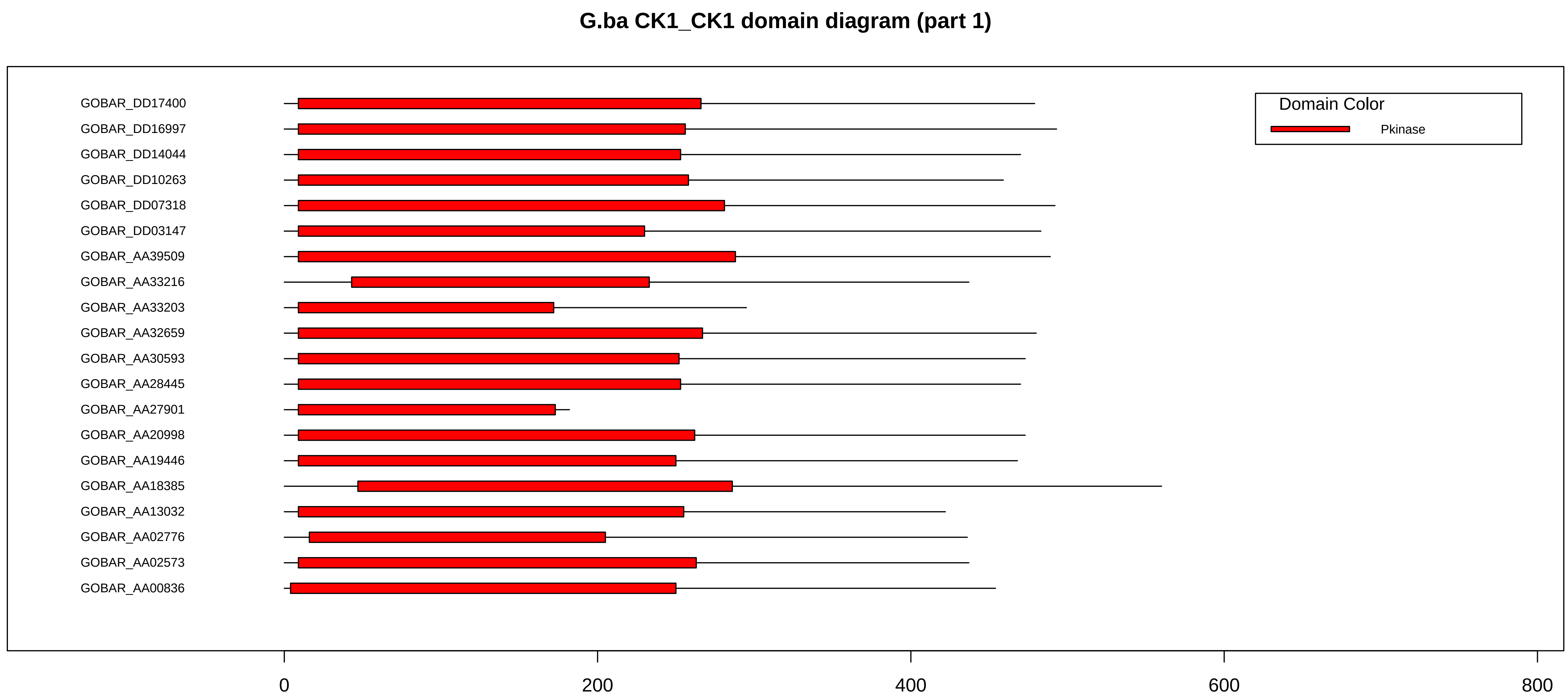

G.ba CK1_CK1 domain diagram (part 1)
GOBAR_DD17400
GOBAR_DD16997
GOBAR_DD14044
GOBAR_DD10263
GOBAR_DD07318
GOBAR_DD03147
GOBAR_AA39509
GOBAR_AA33216
GOBAR_AA33203
GOBAR_AA32659
GOBAR_AA30593
GOBAR_AA28445
GOBAR_AA27901
GOBAR_AA20998
GOBAR_AA19446
GOBAR_AA18385
GOBAR_AA13032
GOBAR_AA02776
GOBAR_AA02573
GOBAR_AA00836
Domain Color
Pkinase
0
200
400
600
800

G.ba CK1_CK1 domain diagram (part 2)

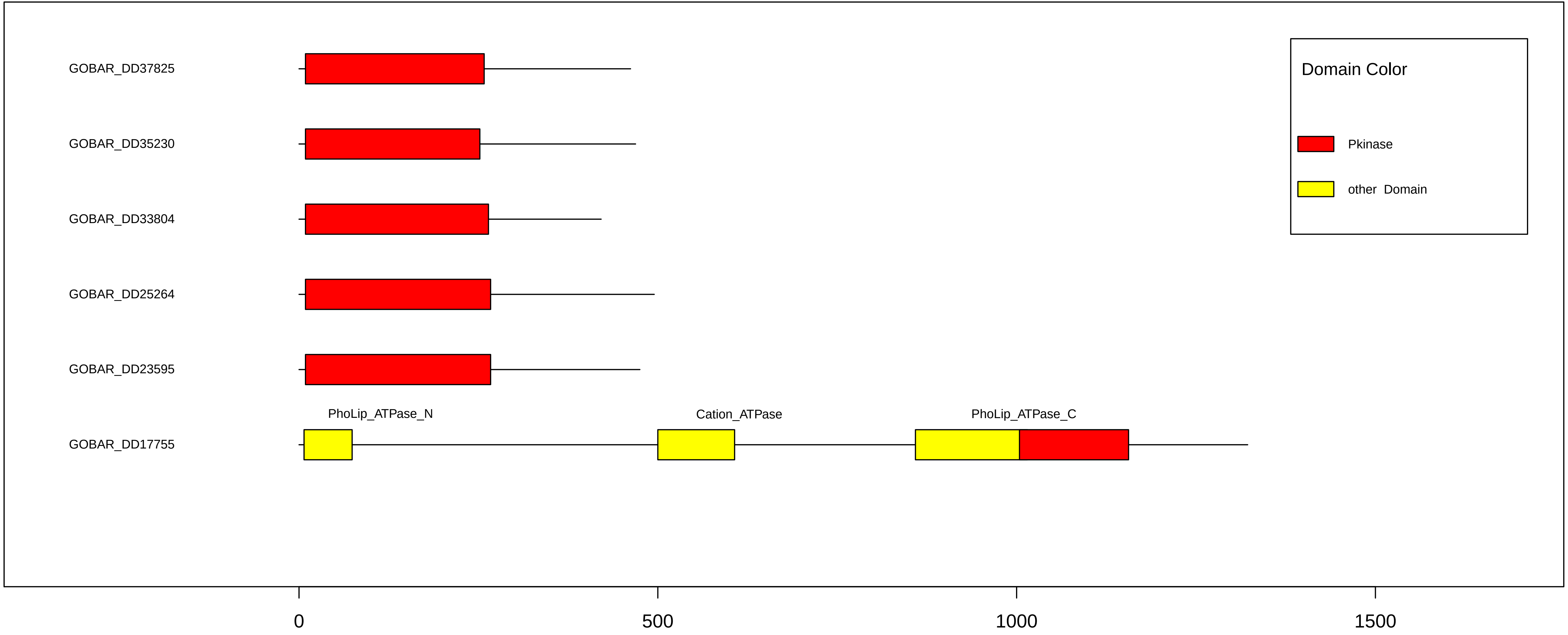

G.ba CK1_CK1−PI domain diagram (all)

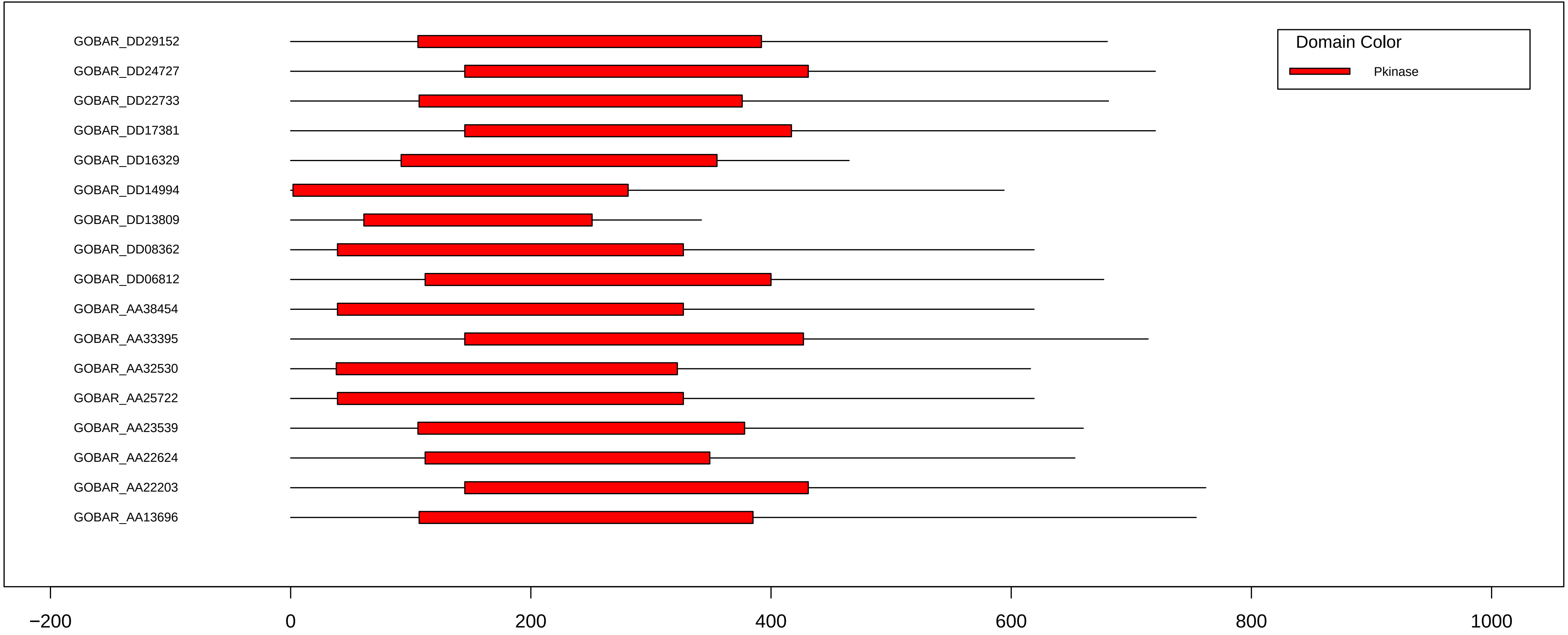

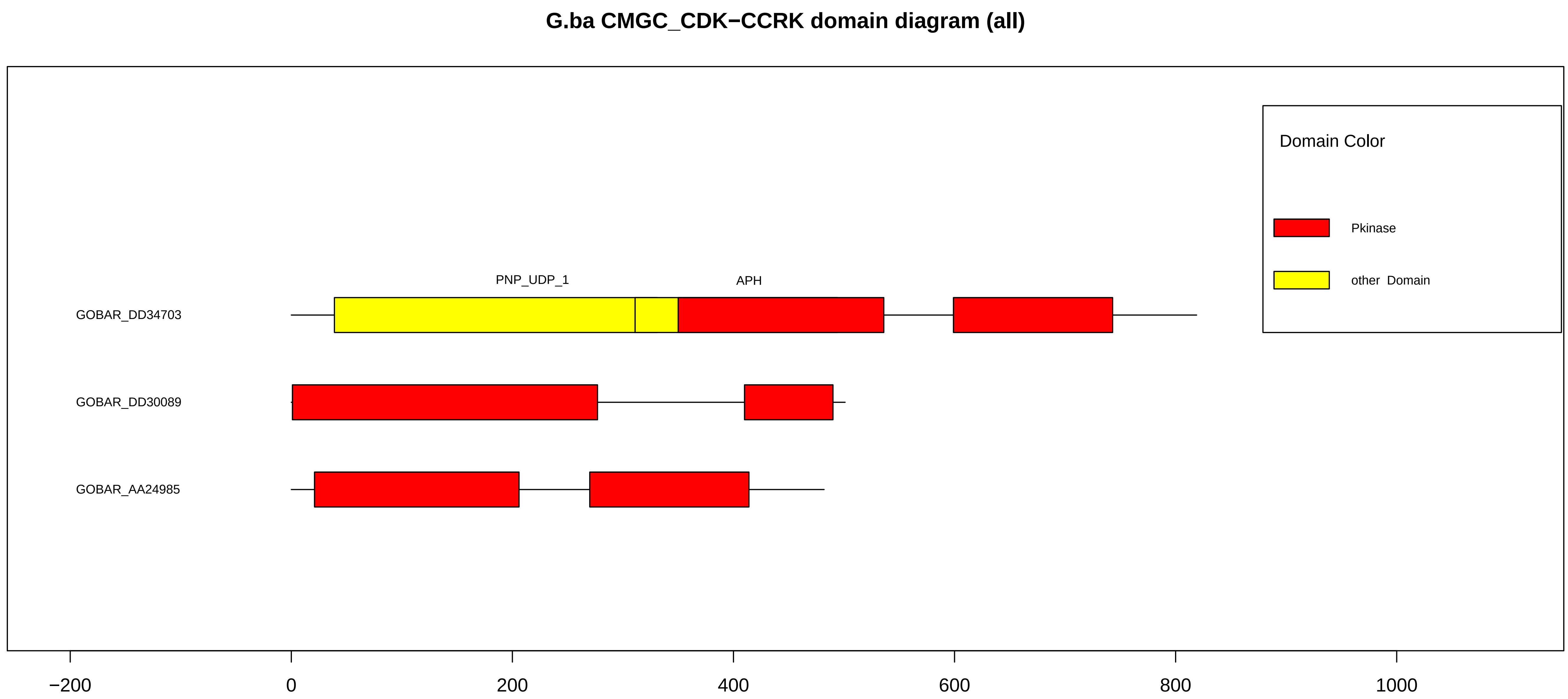
G.ba CMGC_CDK−CCRK domain diagram (all)
Domain Color
Pkinase
other Domain
PNP_UDP_1
APH
GOBAR_DD34703
GOBAR_DD30089
GOBAR_AA24985
−200
0
200
400
600
800
1000

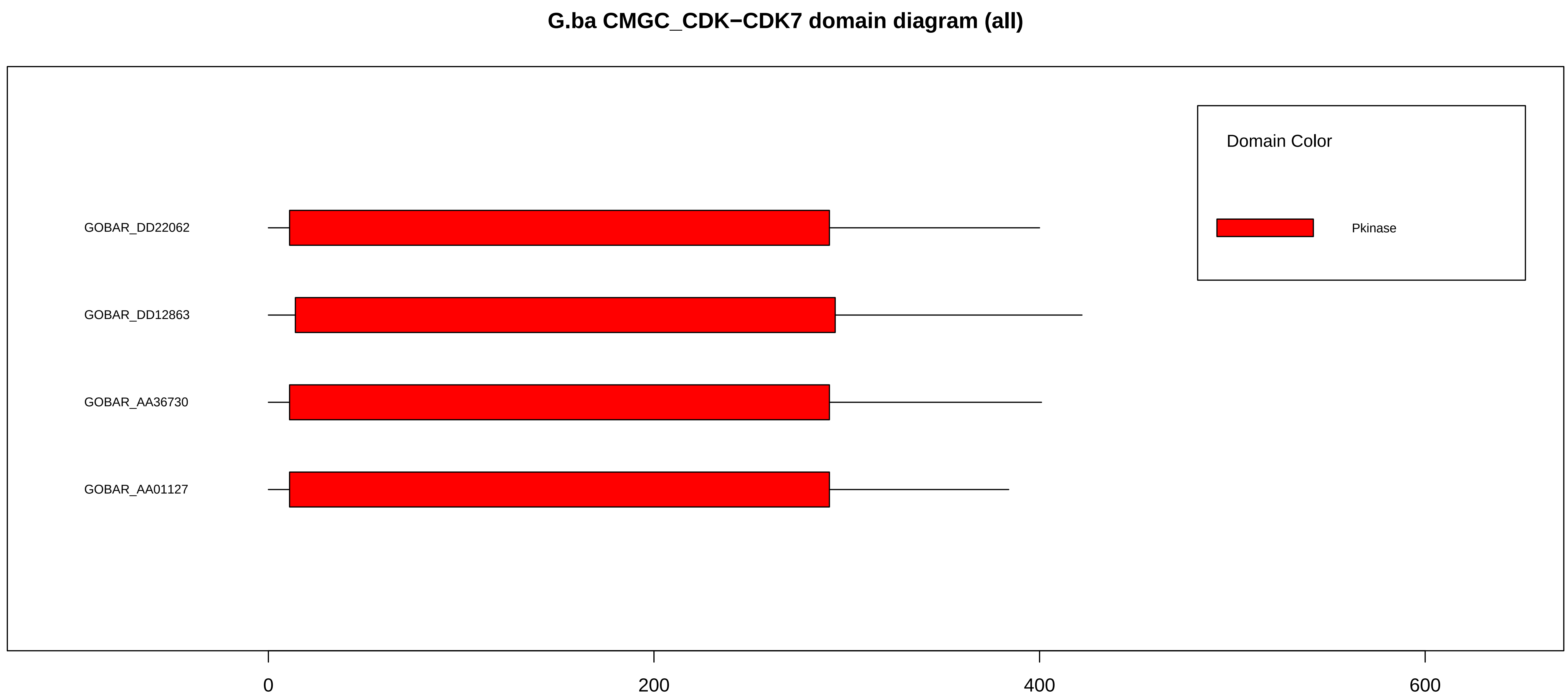

G.ba CMGC_CDK−CDK7 domain diagram (all)
Domain Color
Pkinase
GOBAR_DD22062
GOBAR_DD12863
GOBAR_AA36730
GOBAR_AA01127
0
200
400
600

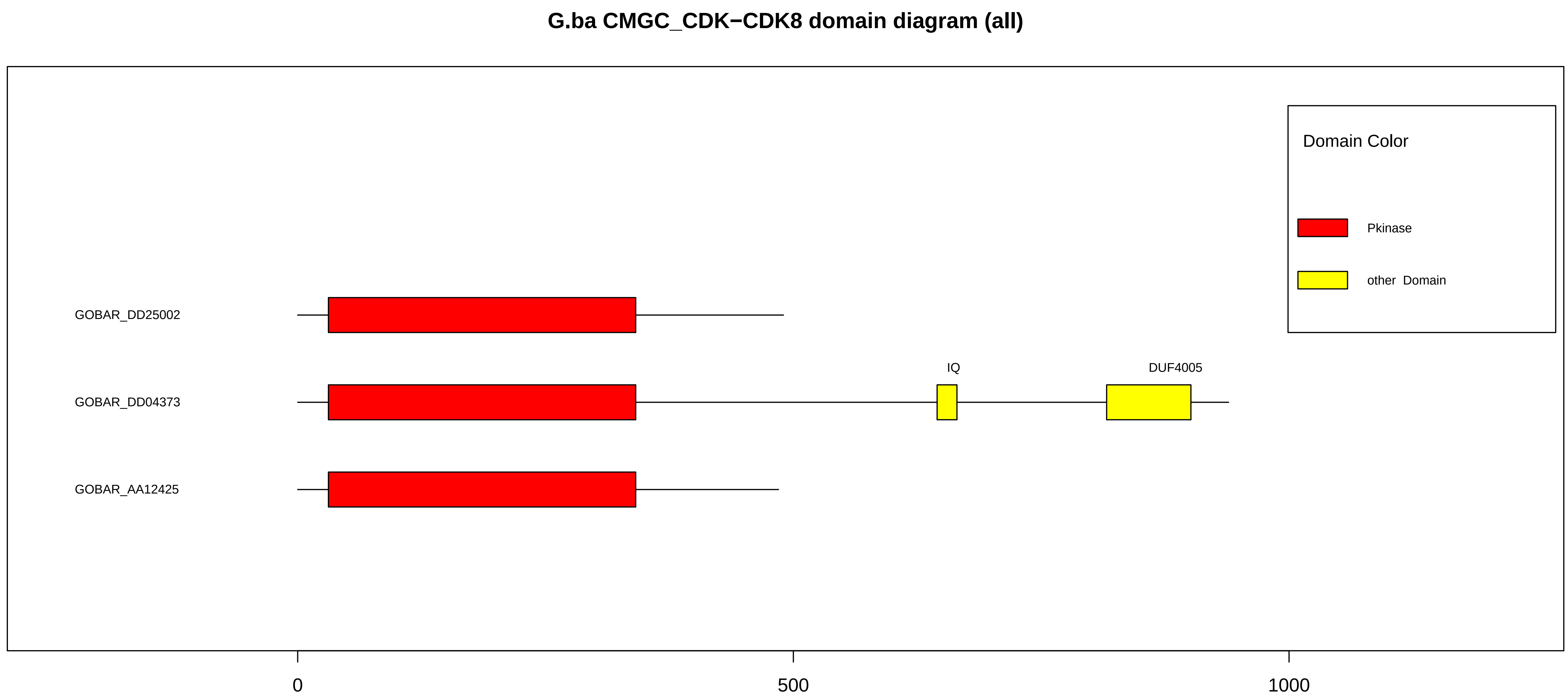
G.ba CMGC_CDK−CDK8 domain diagram (all)
Domain Color
Pkinase
other Domain
GOBAR_DD25002
IQ
DUF4005
GOBAR_DD04373
GOBAR_AA12425
0
500
1000

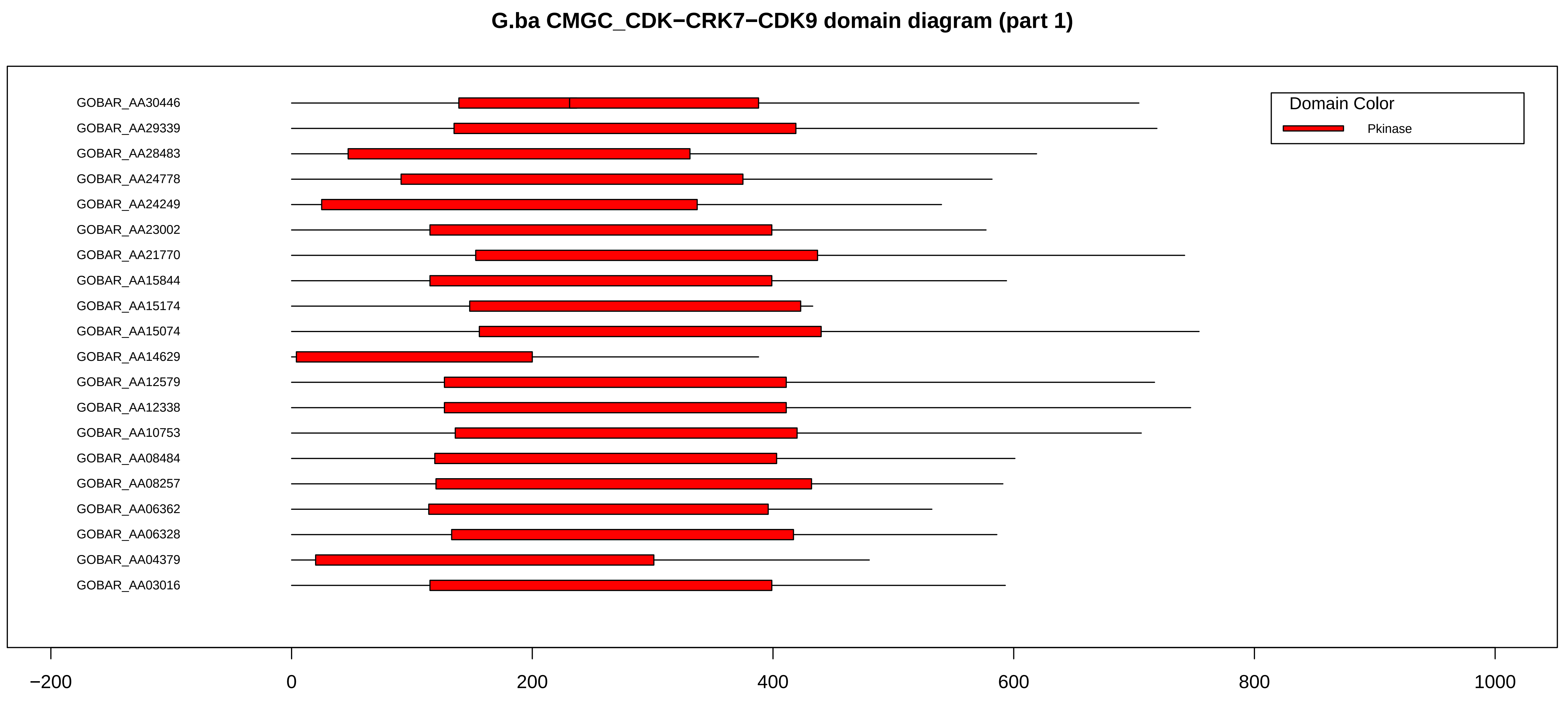

G.ba CMGC_CDK−CRK7−CDK9 domain diagram (part 1)
GOBAR_AA30446
GOBAR_AA29339
GOBAR_AA28483
GOBAR_AA24778
GOBAR_AA24249
GOBAR_AA23002
GOBAR_AA21770
GOBAR_AA15844
GOBAR_AA15174
GOBAR_AA15074
GOBAR_AA14629
GOBAR_AA12579
GOBAR_AA12338
GOBAR_AA10753
GOBAR_AA08484
GOBAR_AA08257
GOBAR_AA06362
GOBAR_AA06328
GOBAR_AA04379
GOBAR_AA03016
Domain Color
Pkinase
−200
0
200
400
600
800
1000

G.ba CMGC_CDK−CRK7−CDK9 domain diagram (part 2)

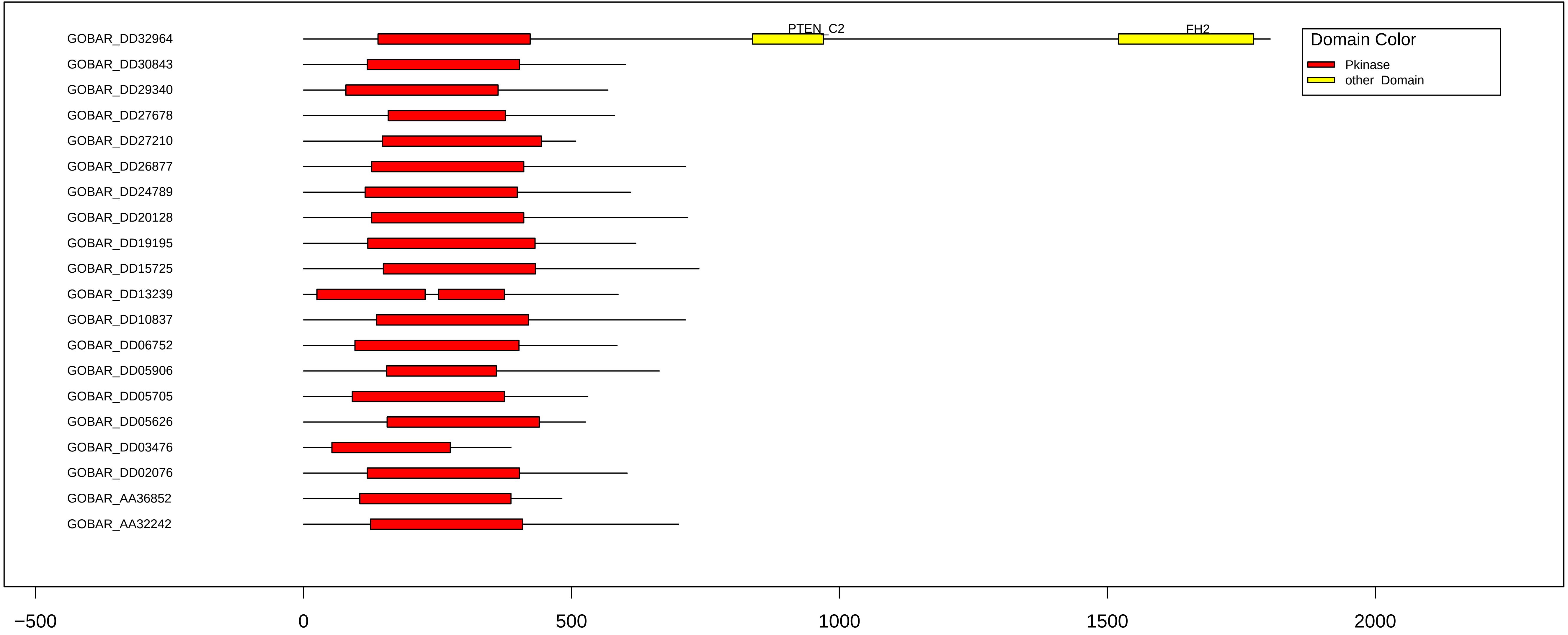

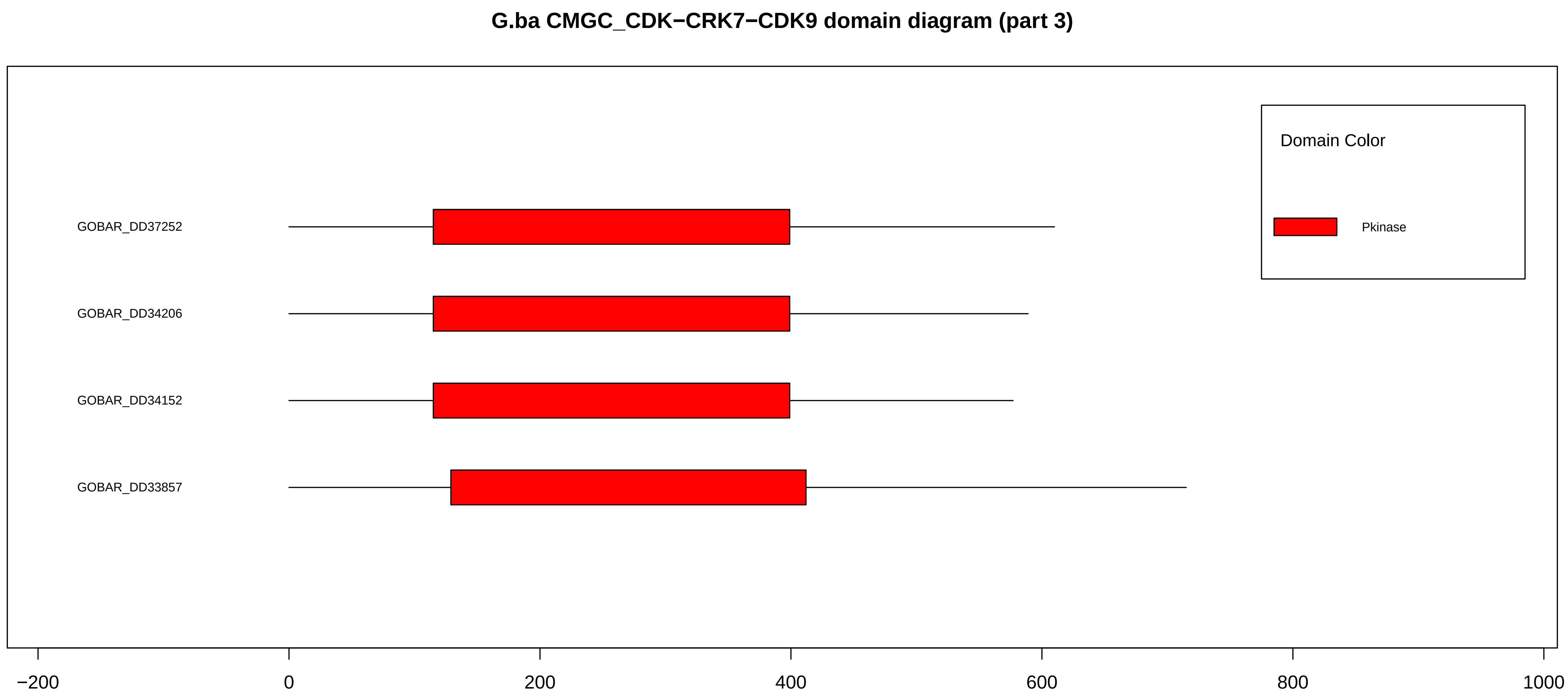
G.ba CMGC_CDK−CRK7−CDK9 domain diagram (part 3)
Domain Color
Pkinase
GOBAR_DD37252
GOBAR_DD34206
GOBAR_DD34152
GOBAR_DD33857
−200
0
200
400
600
800
1000

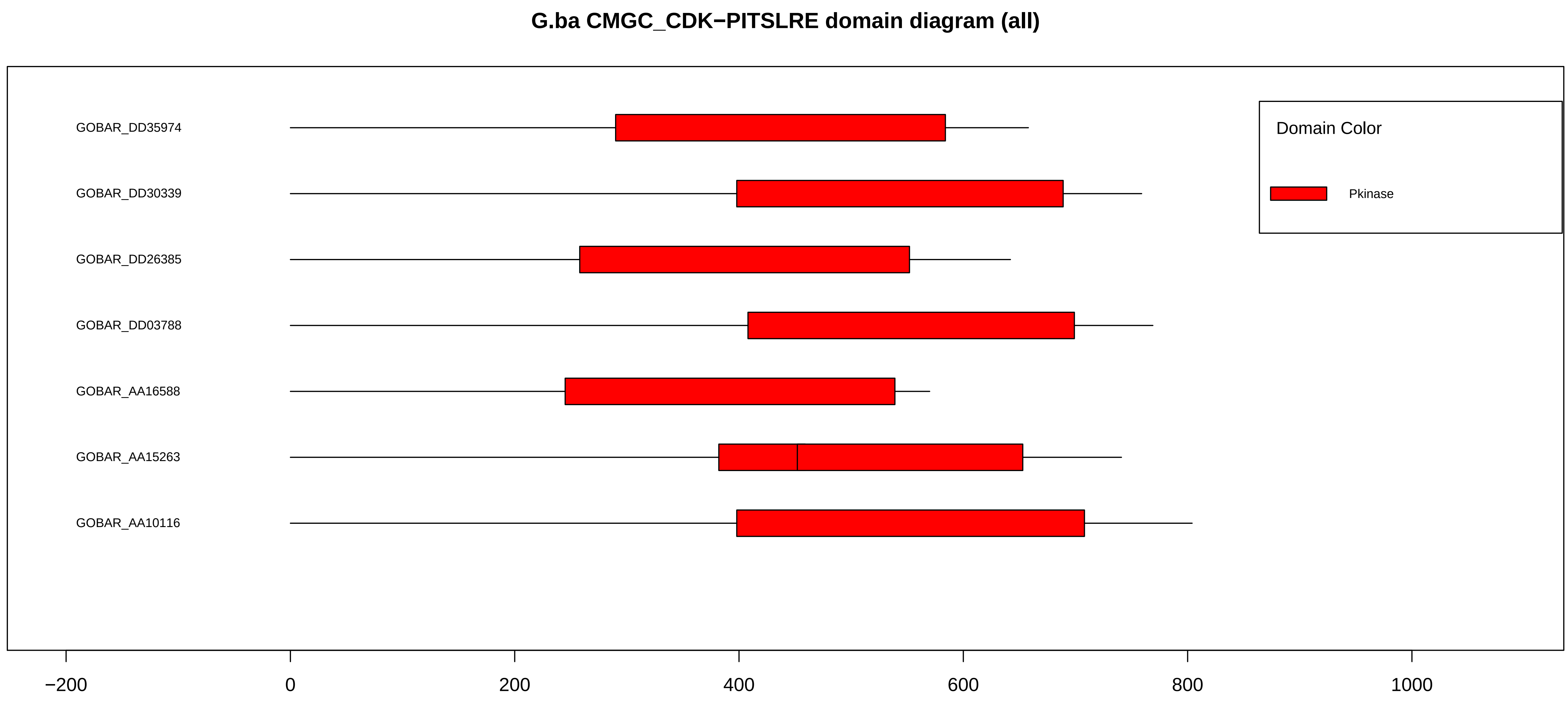

G.ba CMGC_CDK−PITSLRE domain diagram (all)
GOBAR_DD35974
GOBAR_DD30339
GOBAR_DD26385
GOBAR_DD03788
GOBAR_AA16588
GOBAR_AA15263
GOBAR_AA10116
Domain Color
Pkinase
−200
0
200
400
600
800
1000

G.ba CMGC_CDK−Pl domain diagram (all)

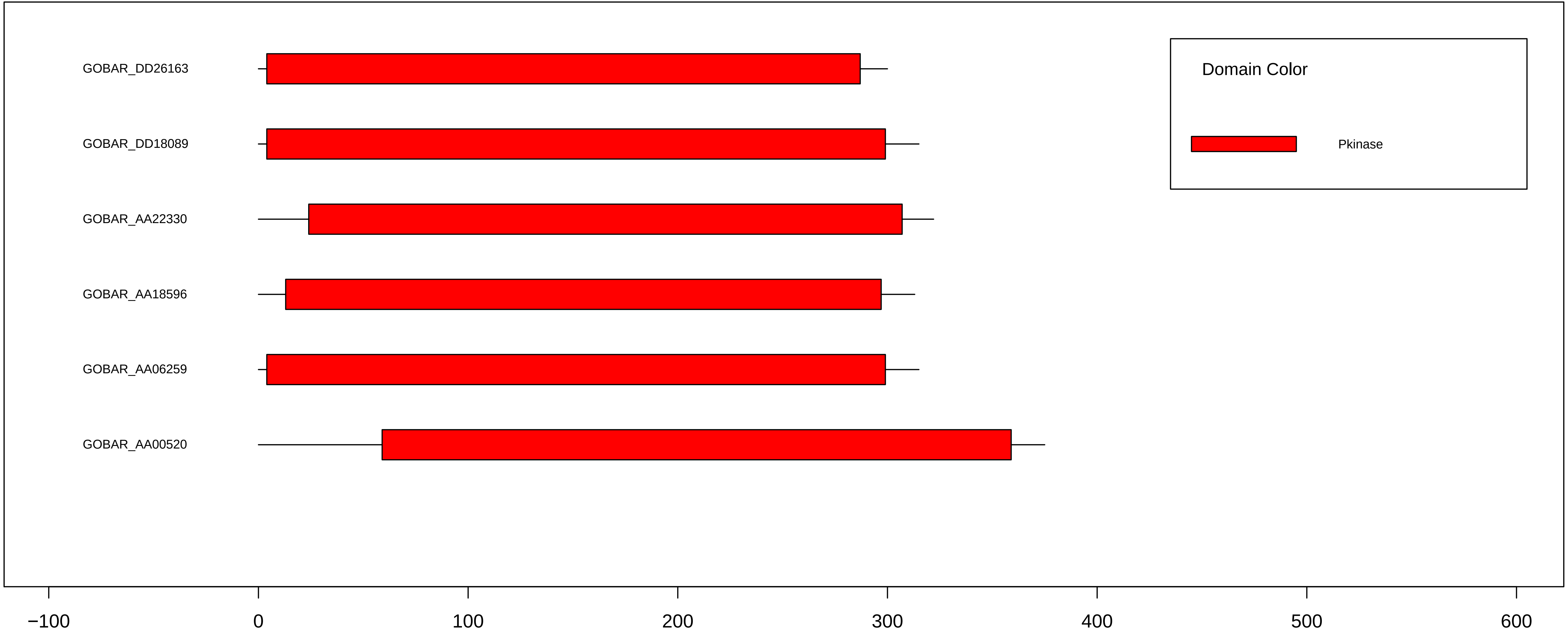

G.ba CMGC_CK2 domain diagram (all)

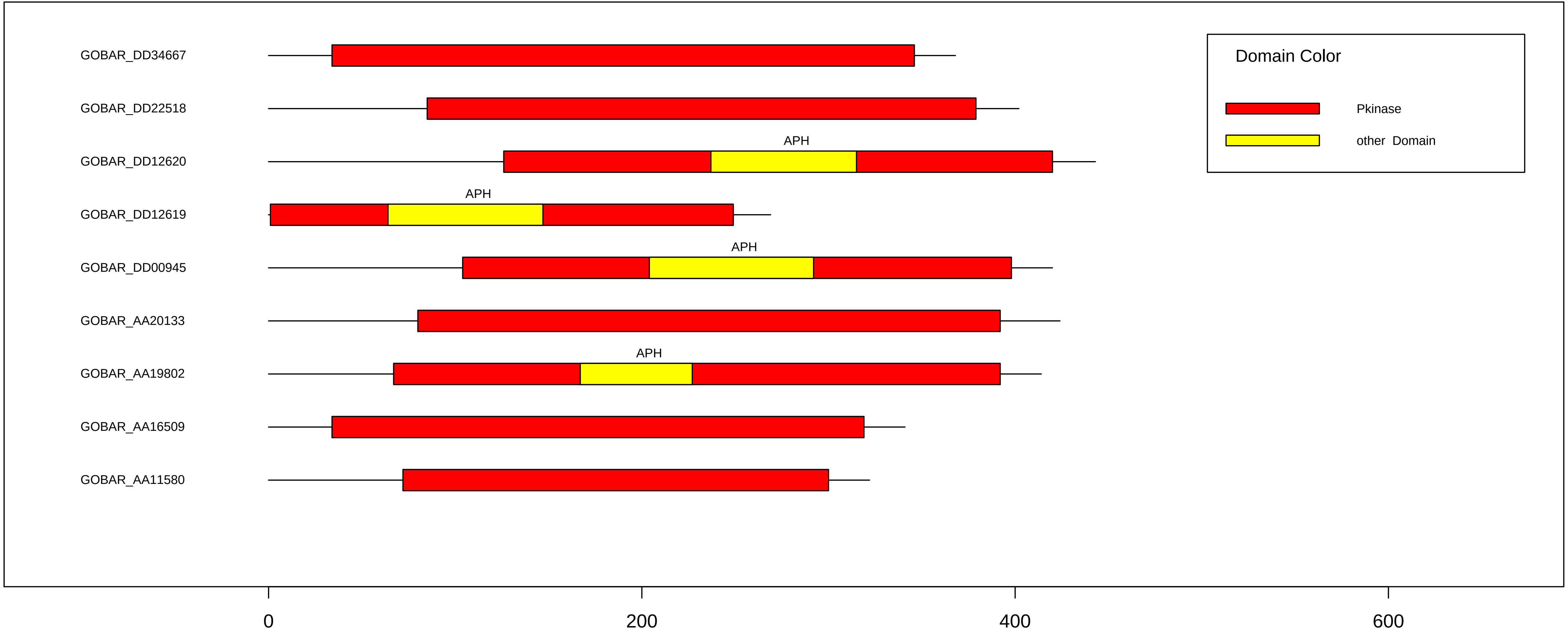

G.ba CMGC_CLK domain diagram (all)

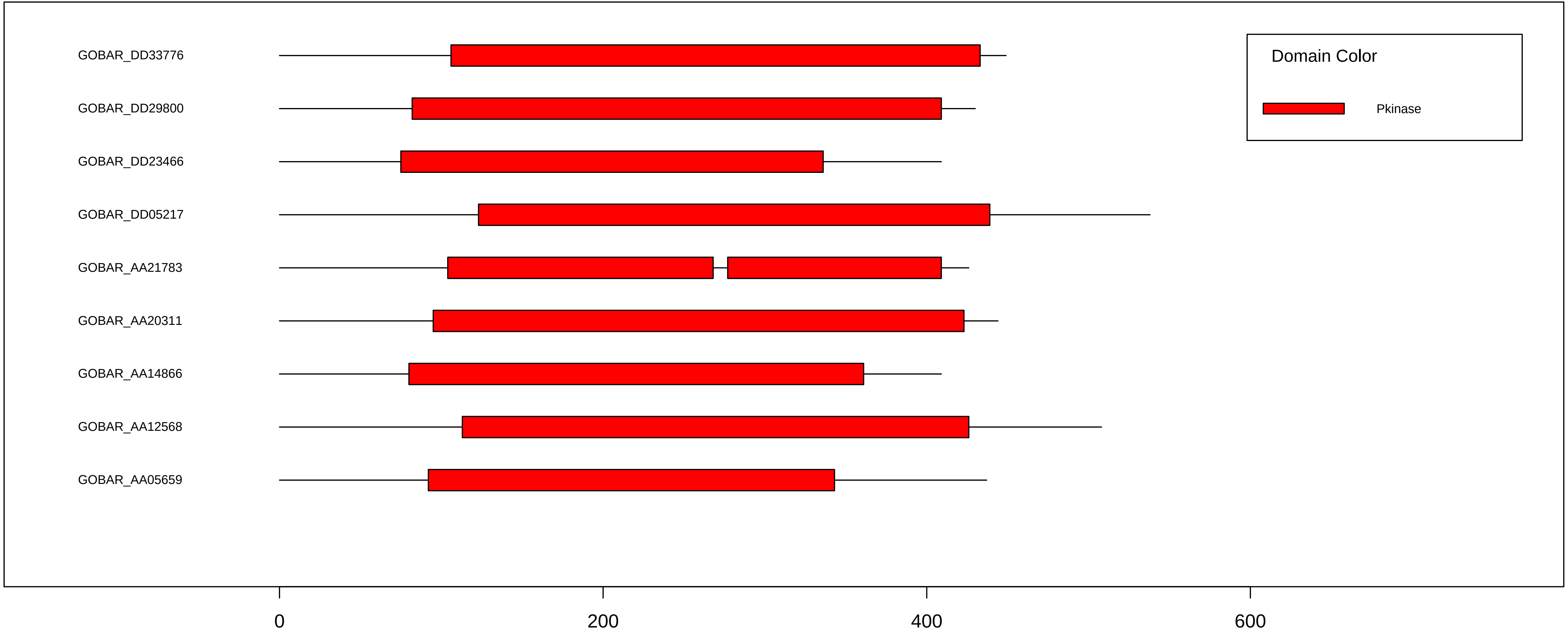

G.ba CMGC_DYRK−PRP4 domain diagram (all)

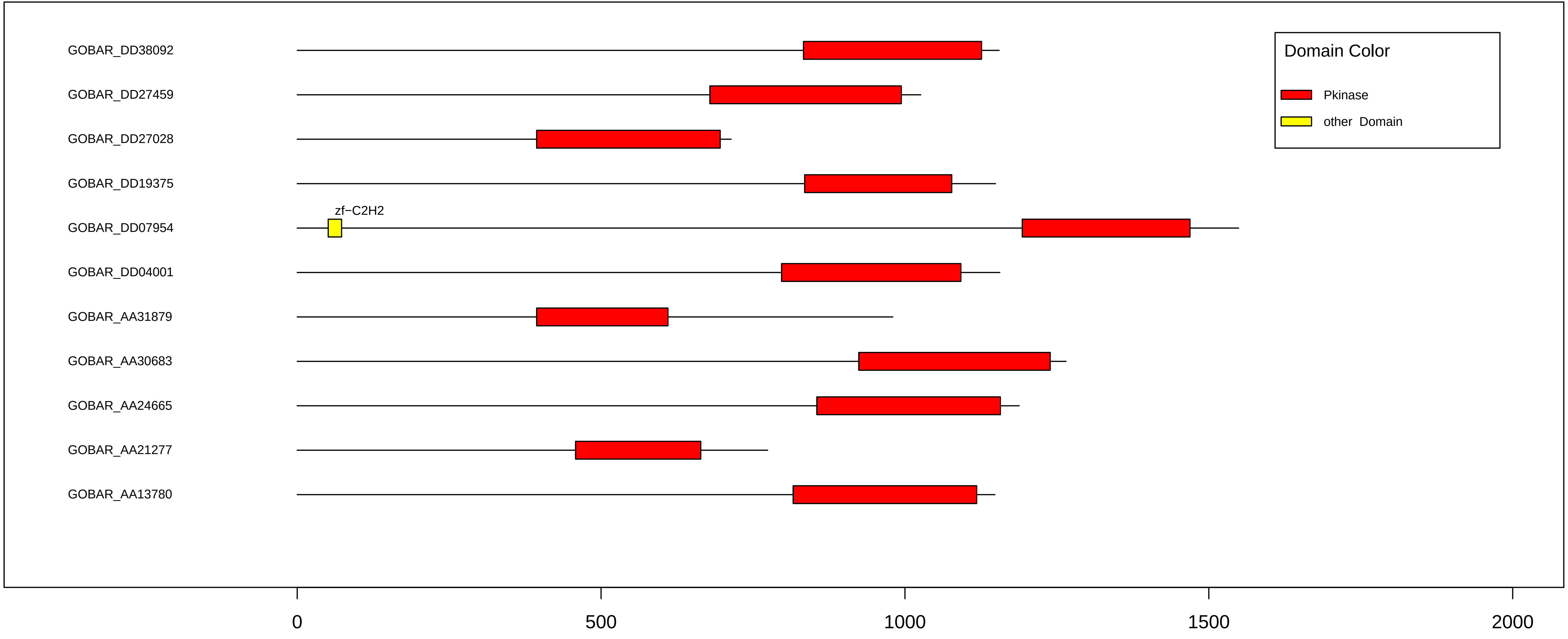

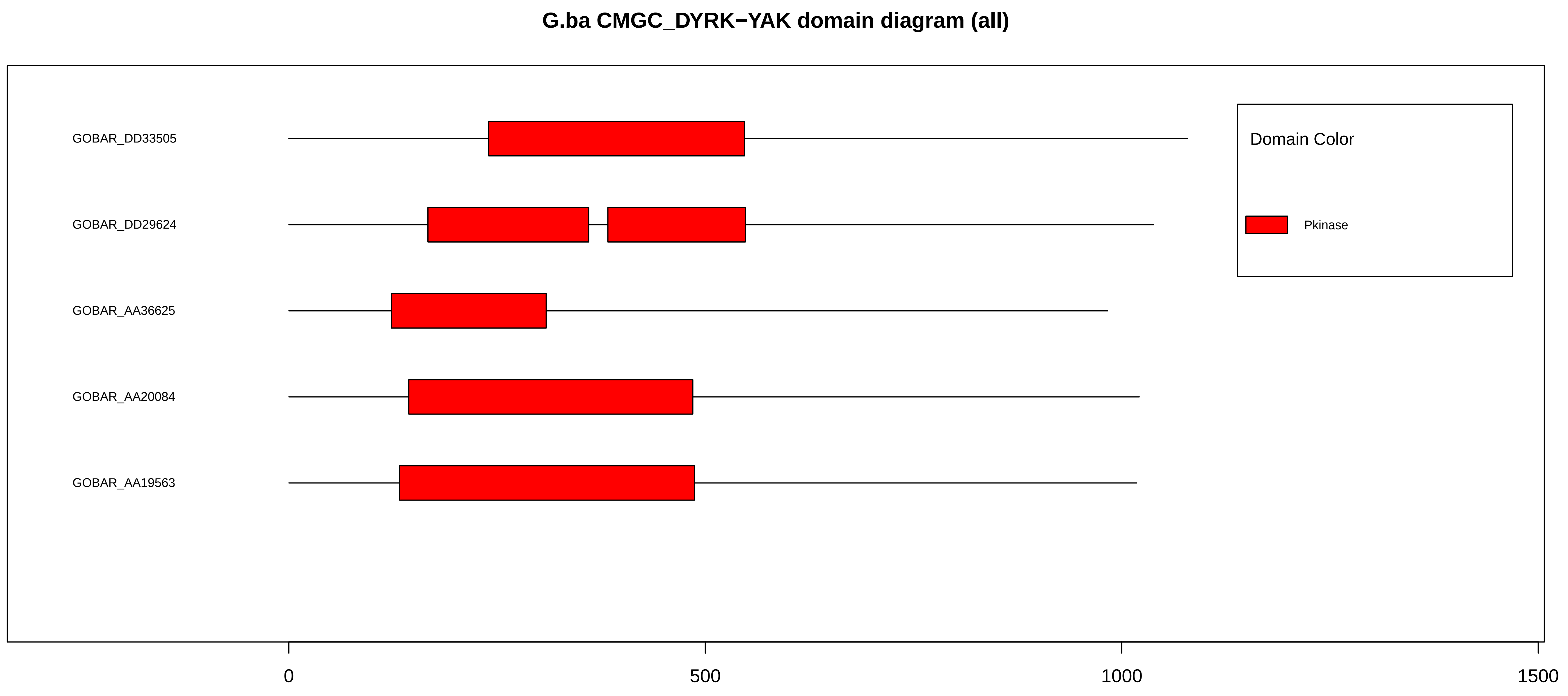
G.ba CMGC_DYRK−YAK domain diagram (all)
GOBAR_DD33505
GOBAR_DD29624
GOBAR_AA36625
GOBAR_AA20084
GOBAR_AA19563
Domain Color
Pkinase
0
500
1000
1500

G.ba CMGC_GSK domain diagram (all)

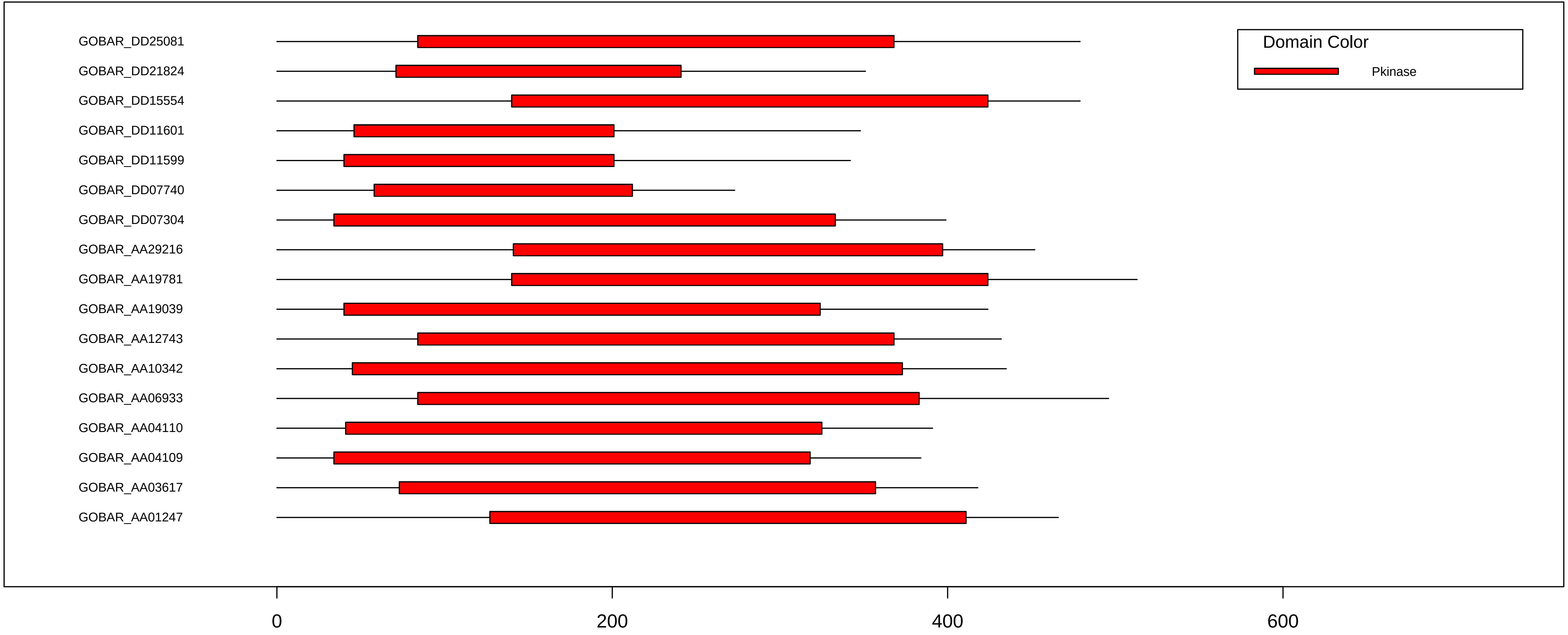

G.ba CMGC_MAPK domain diagram (part 1)

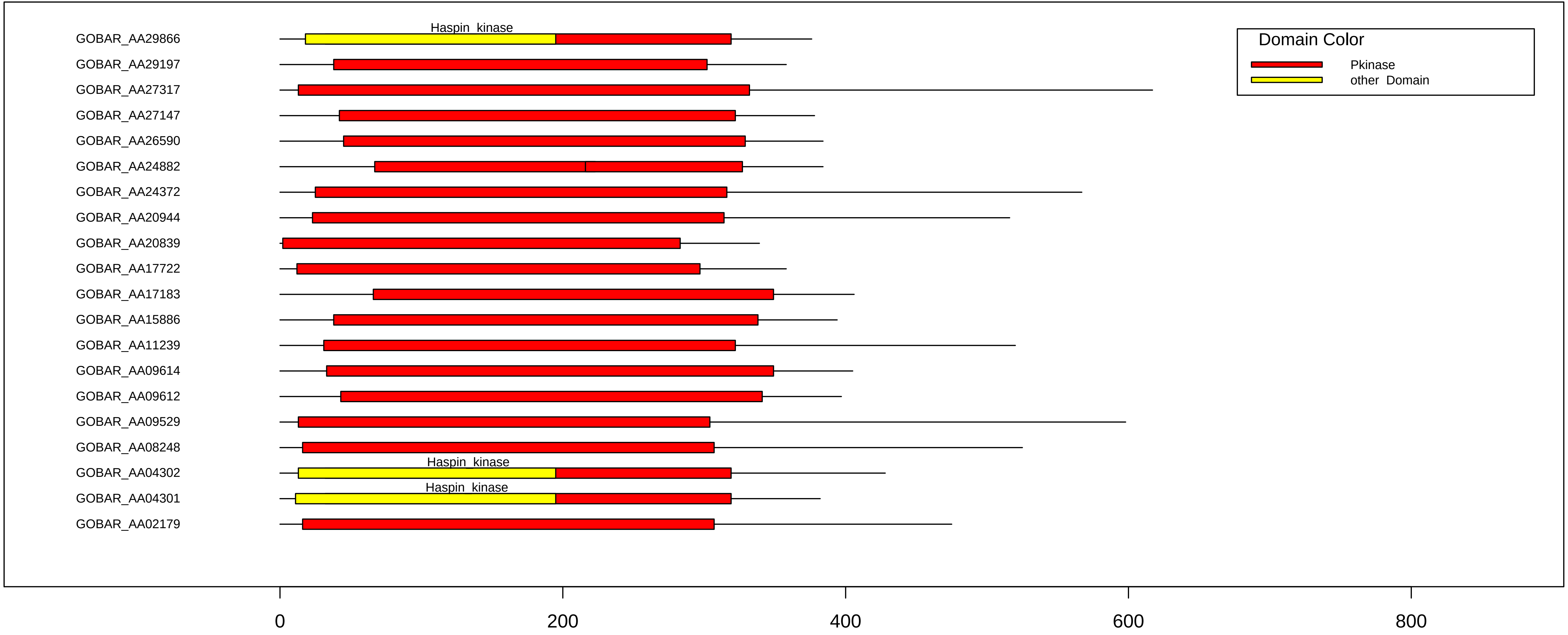

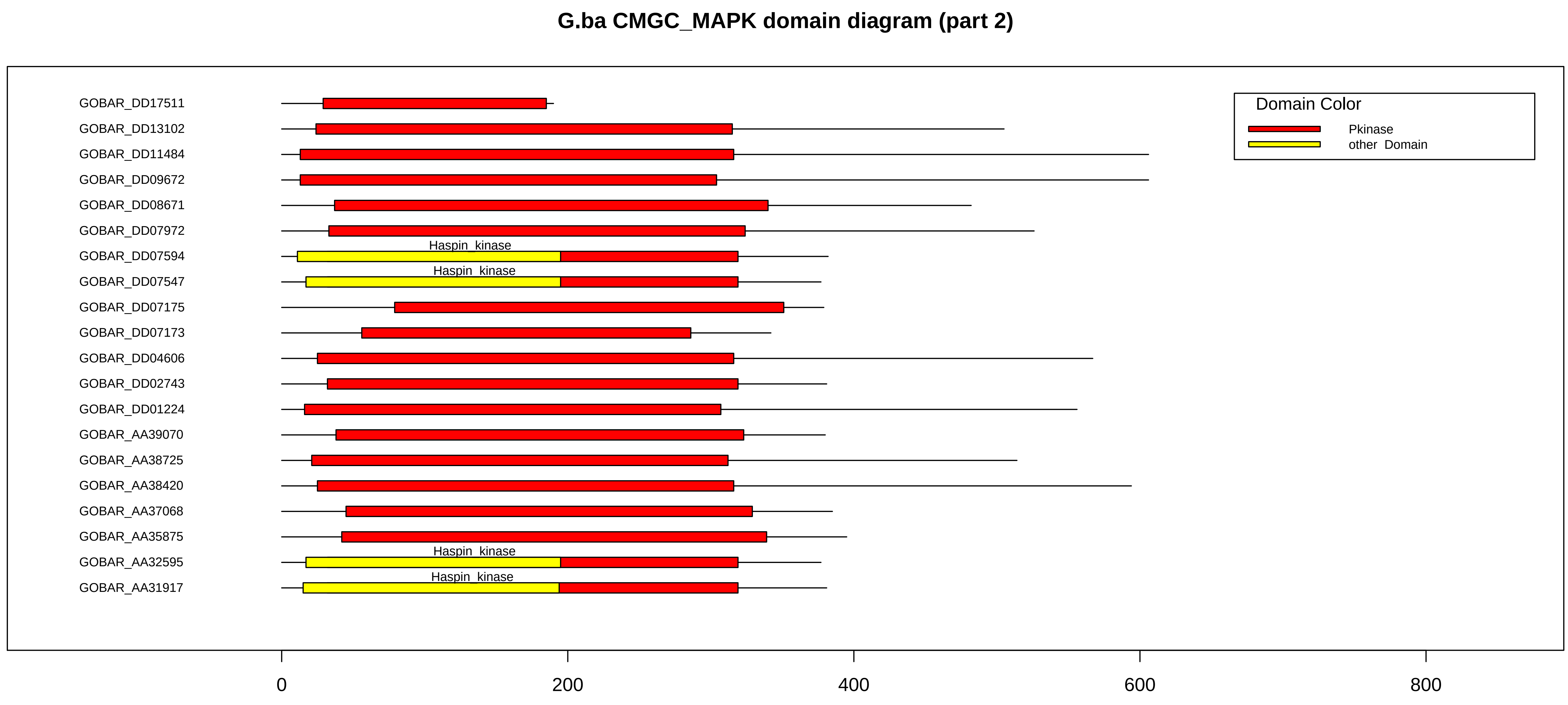
G.ba CMGC_MAPK domain diagram (part 2)
Domain Color
Pkinase
other Domain
GOBAR_DD17511
GOBAR_DD13102
GOBAR_DD11484
GOBAR_DD09672
GOBAR_DD08671
GOBAR_DD07972
GOBAR_DD07594
Haspin_kinase
GOBAR_DD07547
Haspin_kinase
GOBAR_DD07175
GOBAR_DD07173
GOBAR_DD04606
GOBAR_DD02743
GOBAR_DD01224
GOBAR_AA39070
GOBAR_AA38725
GOBAR_AA38420
GOBAR_AA37068
GOBAR_AA35875
GOBAR_AA32595
Haspin_kinase
GOBAR_AA31917
Haspin_kinase
0
200
400
600
800

G.ba CMGC_MAPK domain diagram (part 3)

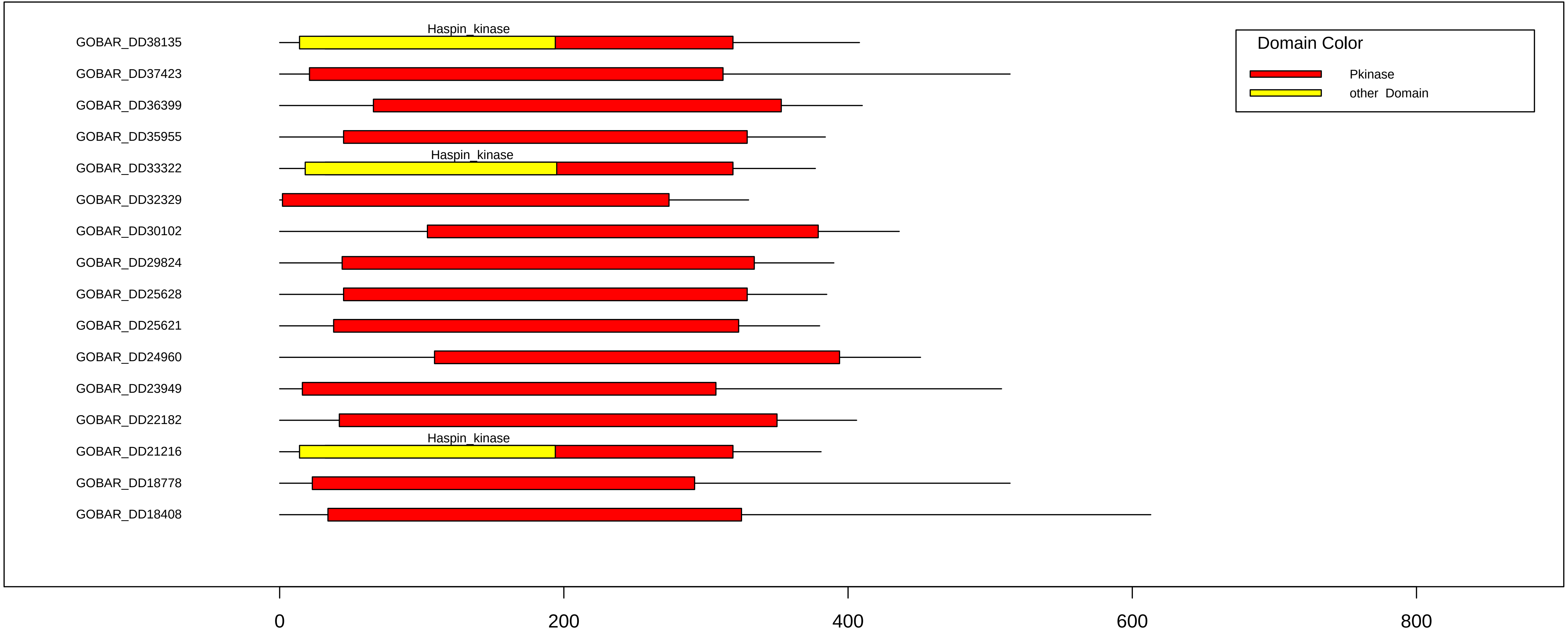

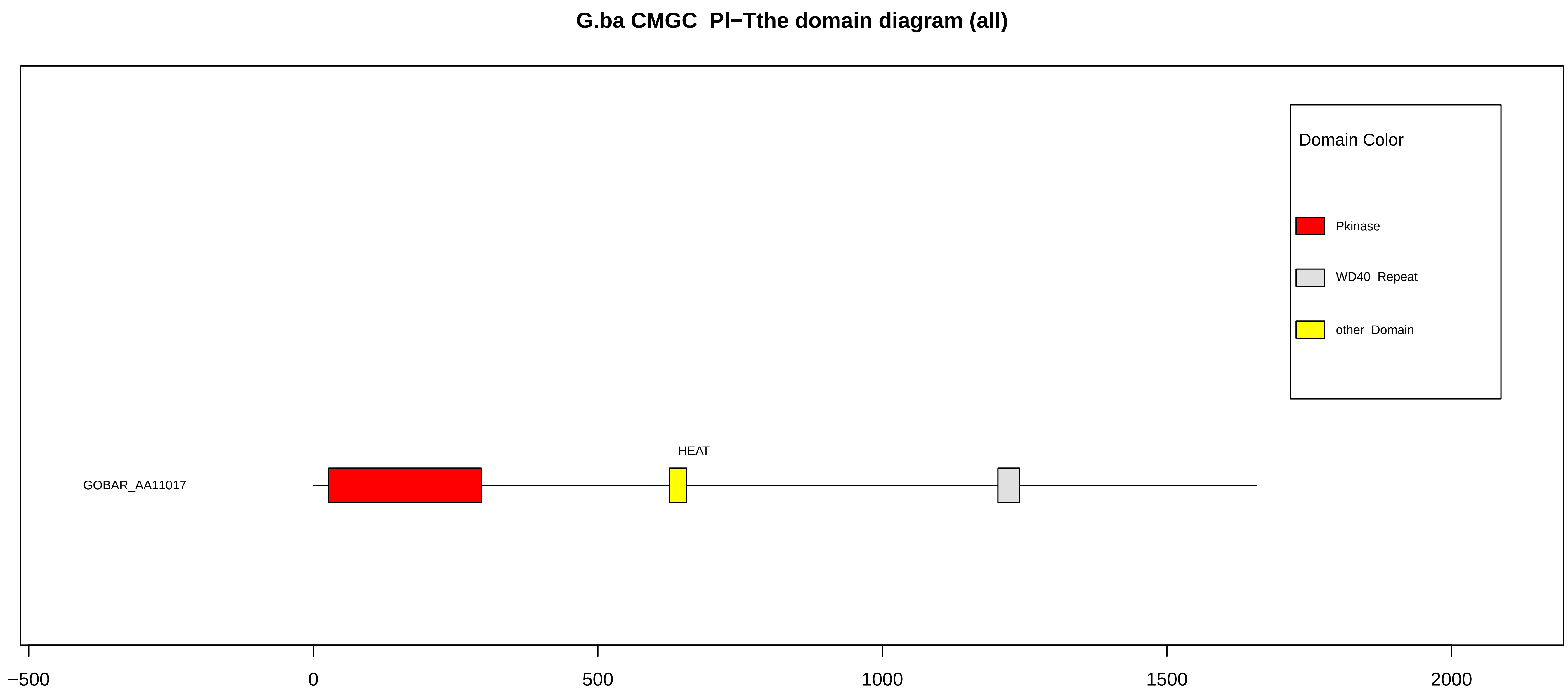
G.ba CMGC_Pl−Tthe domain diagram (all)
Domain Color
Pkinase
WD40 Repeat
other Domain
HEAT
GOBAR_AA11017
−500
0
500
1000
1500
2000

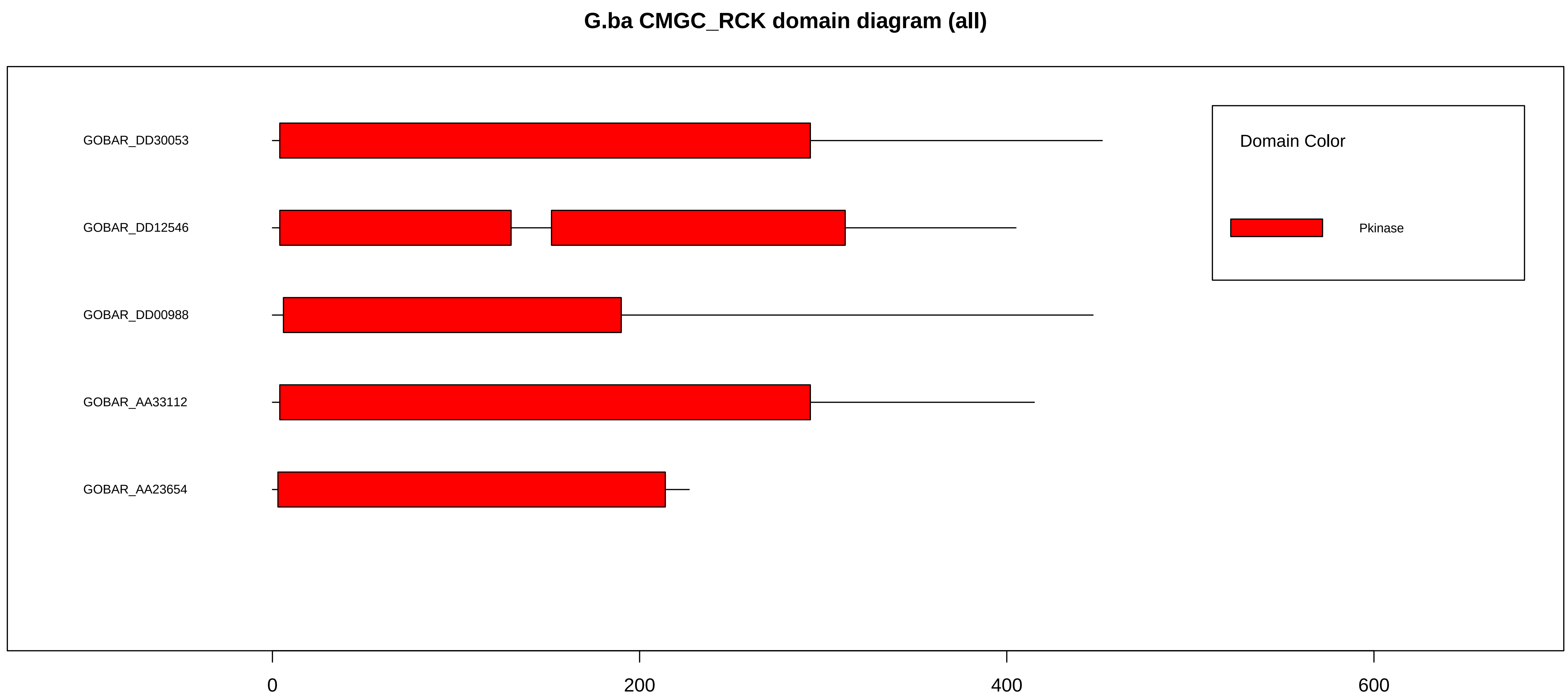
G.ba CMGC_RCK domain diagram (all)
GOBAR_DD30053
GOBAR_DD12546
GOBAR_DD00988
GOBAR_AA33112
GOBAR_AA23654
Domain Color
Pkinase
0
200
400
600

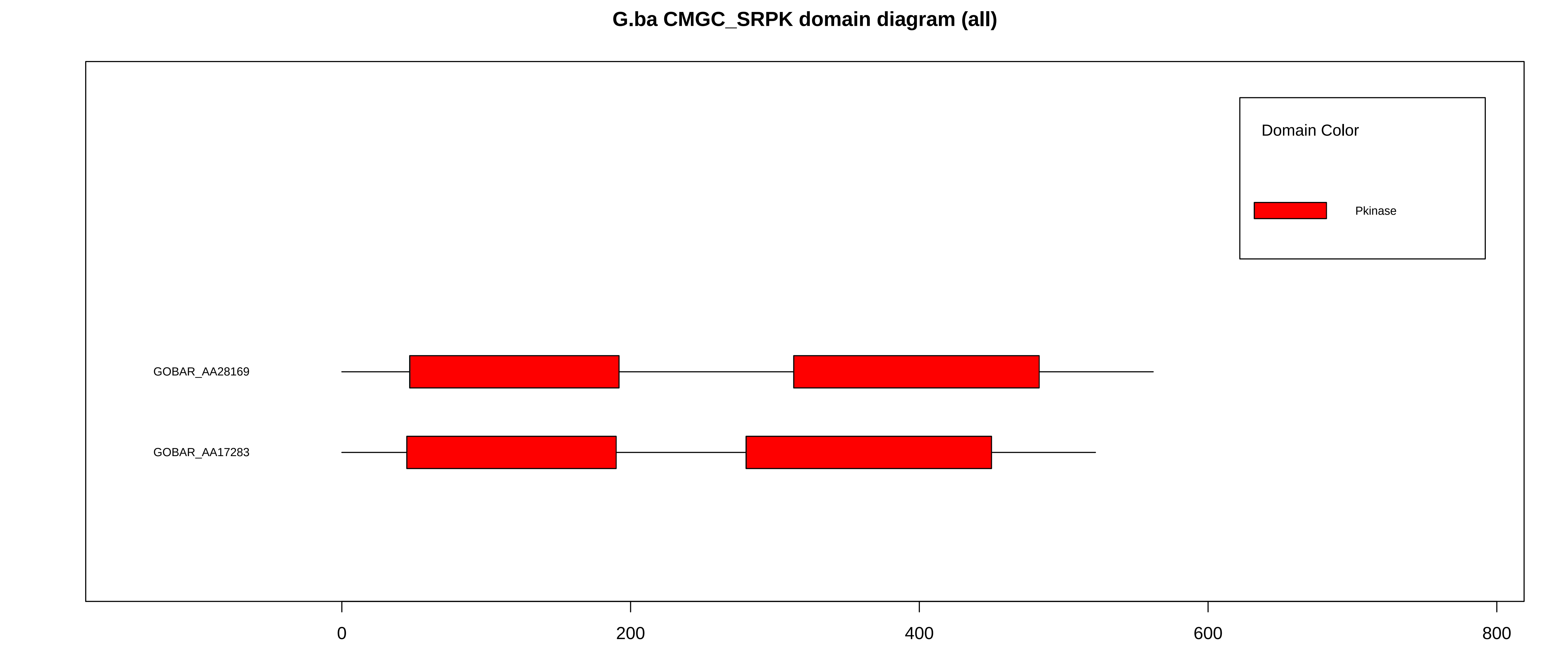
G.ba CMGC_SRPK domain diagram (all)
Domain Color
Pkinase
GOBAR_AA28169
GOBAR_AA17283
0
200
400
600
800

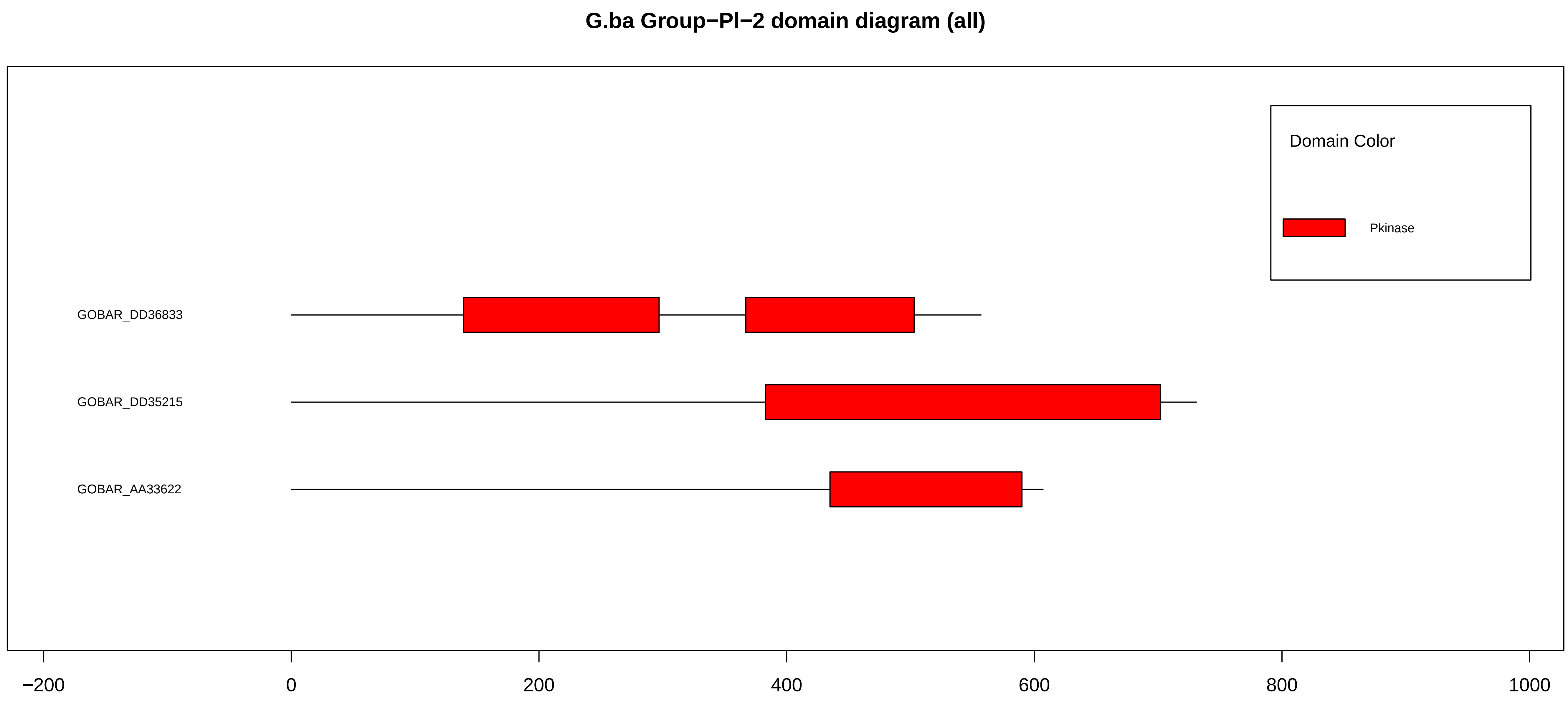
G.ba Group−PI−2 domain diagram (all)
Domain Color
Pkinase
GOBAR_DD36833
GOBAR_DD35215
GOBAR_AA33622
−200
0
200
400
600
800
1000

G.ba Group−PI−3 domain diagram (all)

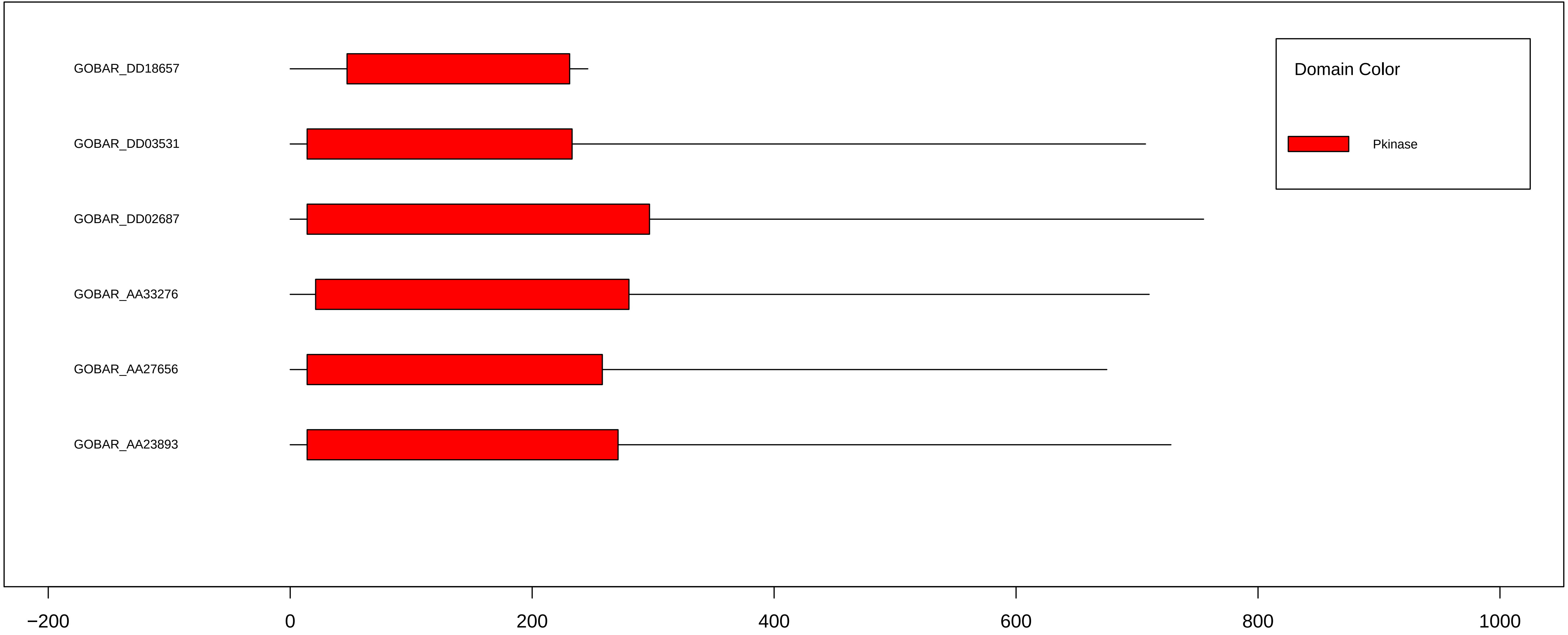

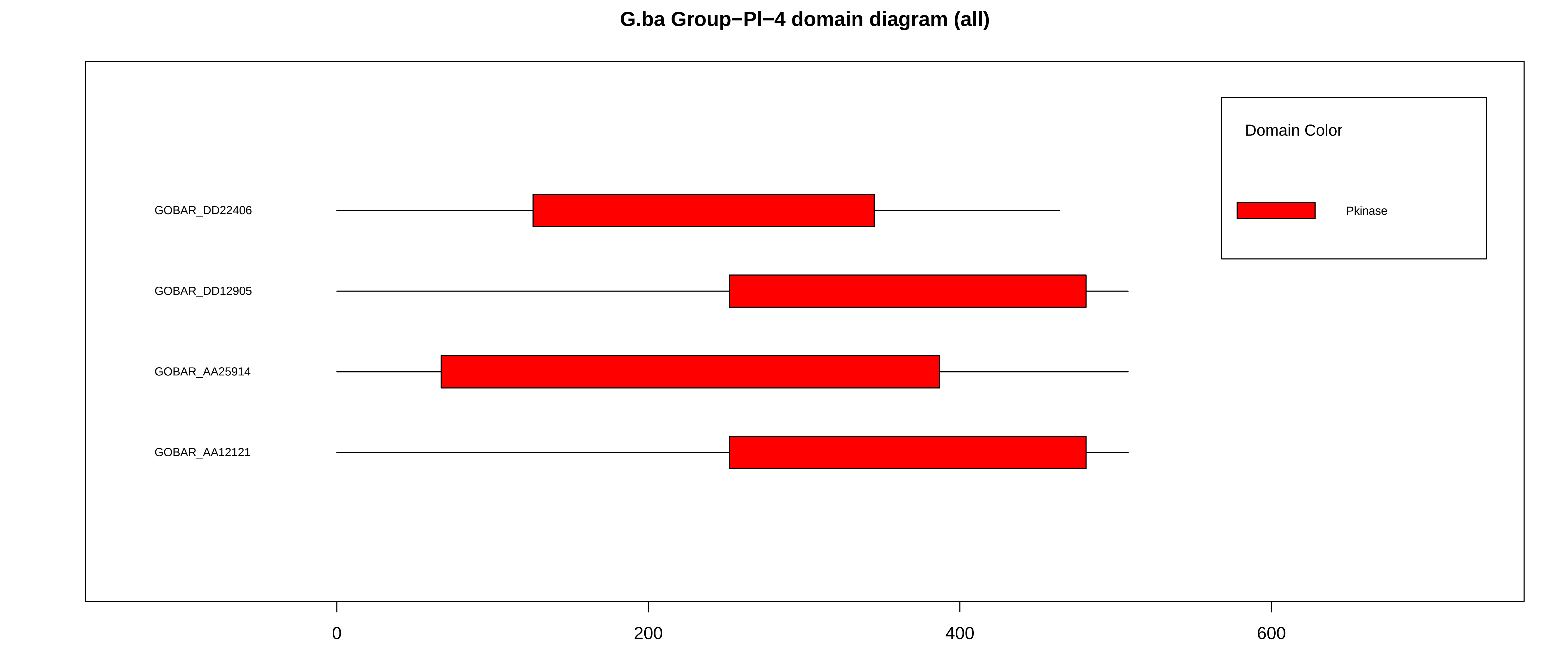
G.ba Group−PI−4 domain diagram (all)
Domain Color
Pkinase
GOBAR_DD22406
GOBAR_DD12905
GOBAR_AA25914
GOBAR_AA12121
0
200
400
600

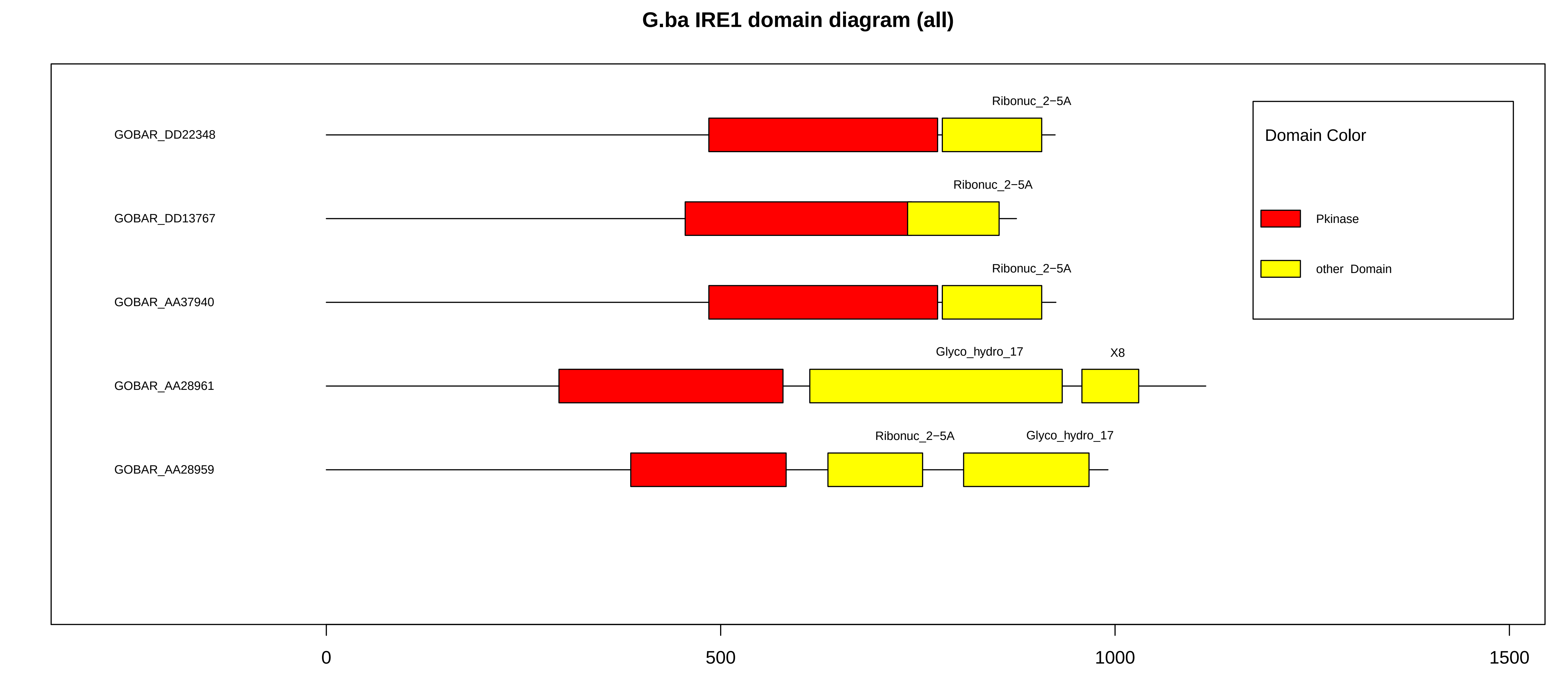

G.ba IRE1 domain diagram (all)
GOBAR_DD22348
Ribonuc_2−5A
GOBAR_DD13767
Ribonuc_2−5A
GOBAR_AA37940
Ribonuc_2−5A
GOBAR_AA28961
Glyco_hydro_17
X8
GOBAR_AA28959
Ribonuc_2−5A
Glyco_hydro_17
Domain Color
Pkinase
other Domain
0
500
1000
1500

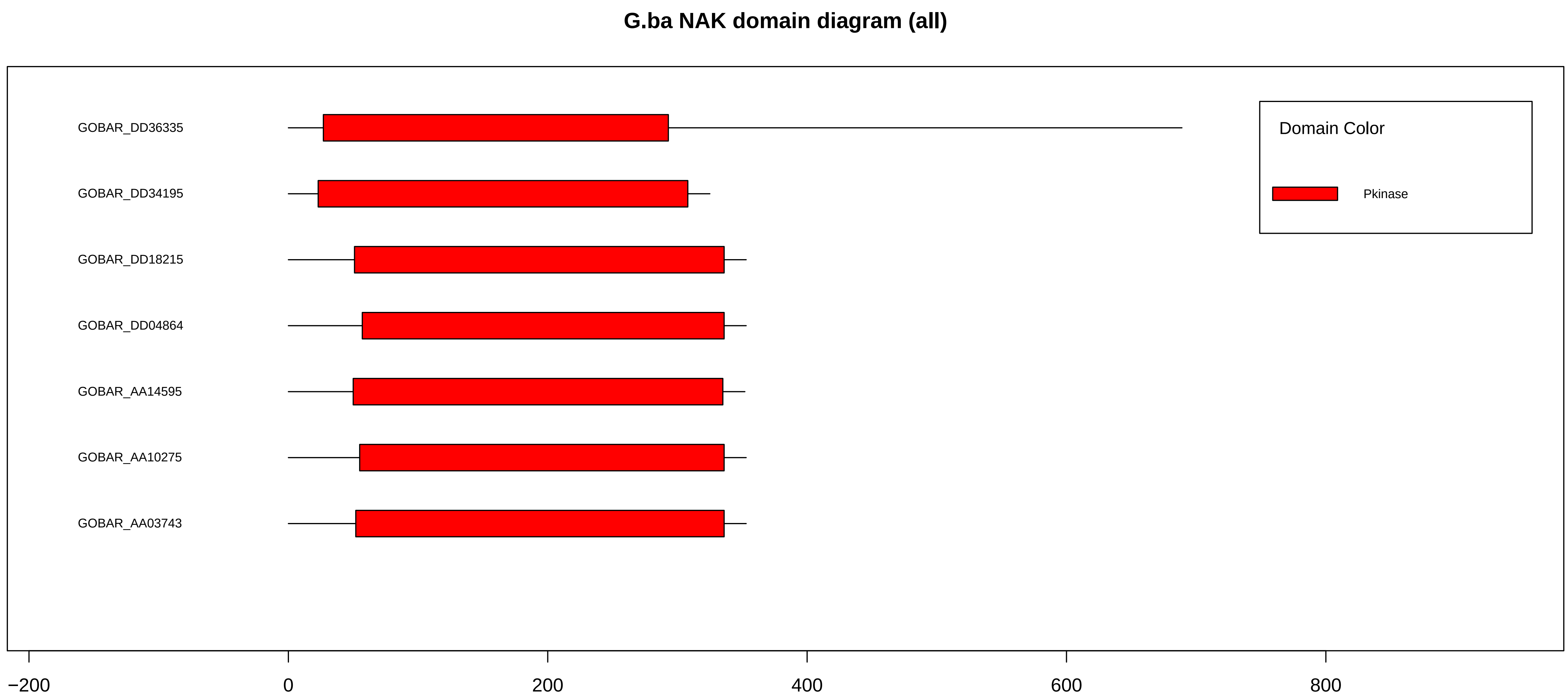
G.ba NAK domain diagram (all)
GOBAR_DD36335
GOBAR_DD34195
GOBAR_DD18215
GOBAR_DD04864
GOBAR_AA14595
GOBAR_AA10275
GOBAR_AA03743
Domain Color
Pkinase
−200
0
200
400
600
800

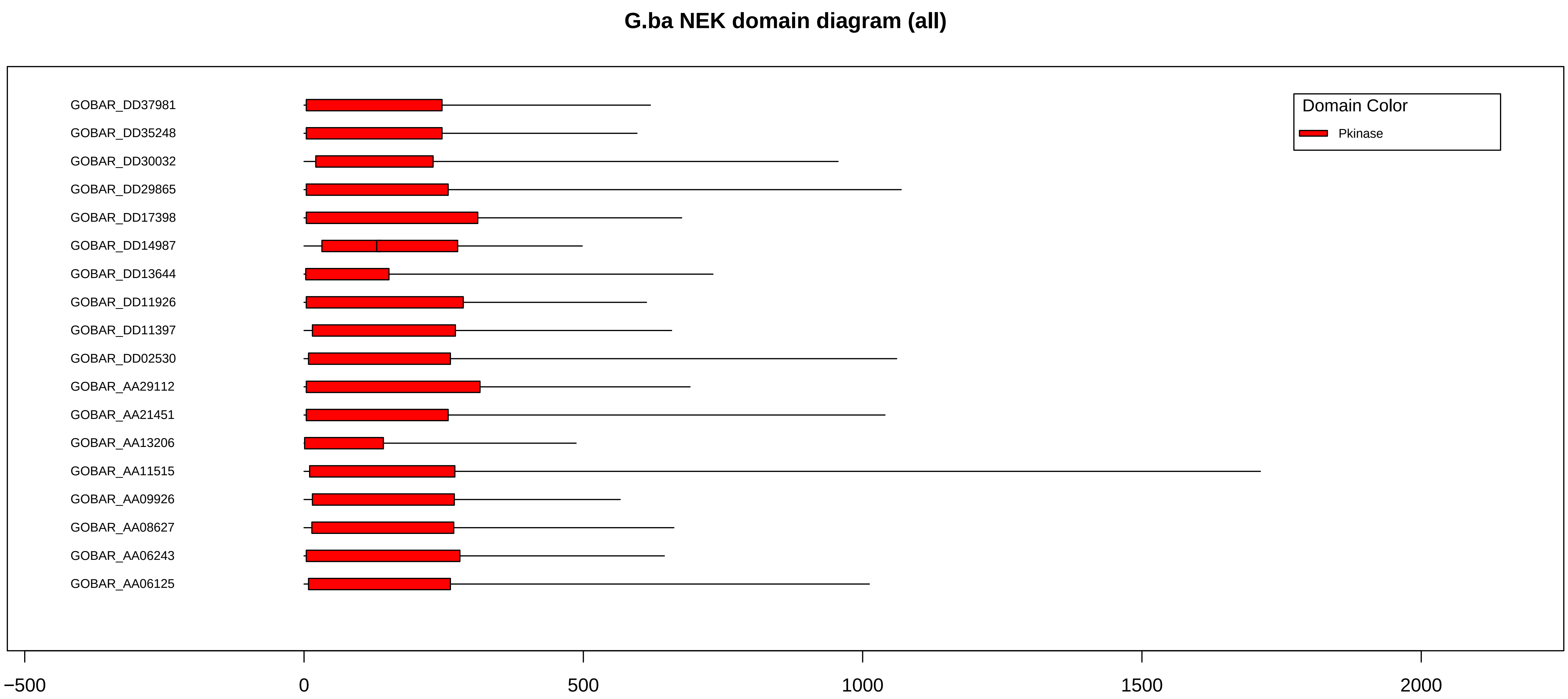

G.ba NEK domain diagram (all)
GOBAR_DD37981
GOBAR_DD35248
GOBAR_DD30032
GOBAR_DD29865
GOBAR_DD17398
GOBAR_DD14987
GOBAR_DD13644
GOBAR_DD11926
GOBAR_DD11397
GOBAR_DD02530
GOBAR_AA29112
GOBAR_AA21451
GOBAR_AA13206
GOBAR_AA11515
GOBAR_AA09926
GOBAR_AA08627
GOBAR_AA06243
GOBAR_AA06125
Domain Color
Pkinase
−500
0
500
1000
1500
2000

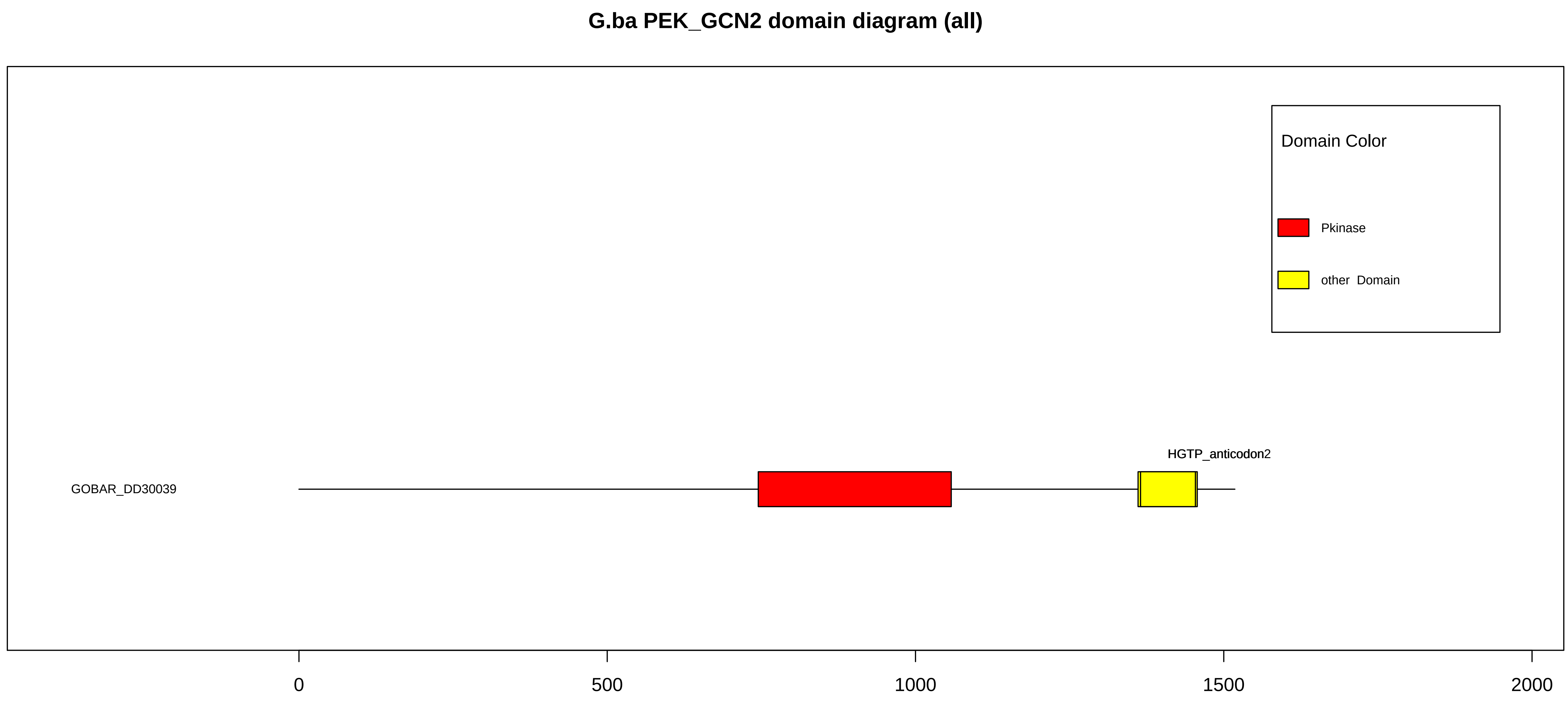
G.ba PEK_GCN2 domain diagram (all)
Domain Color
Pkinase
other Domain
HGTP_anticodon2
GOBAR_DD30039
0
500
1000
1500
2000

G.ba PEK_PEK domain diagram (all)

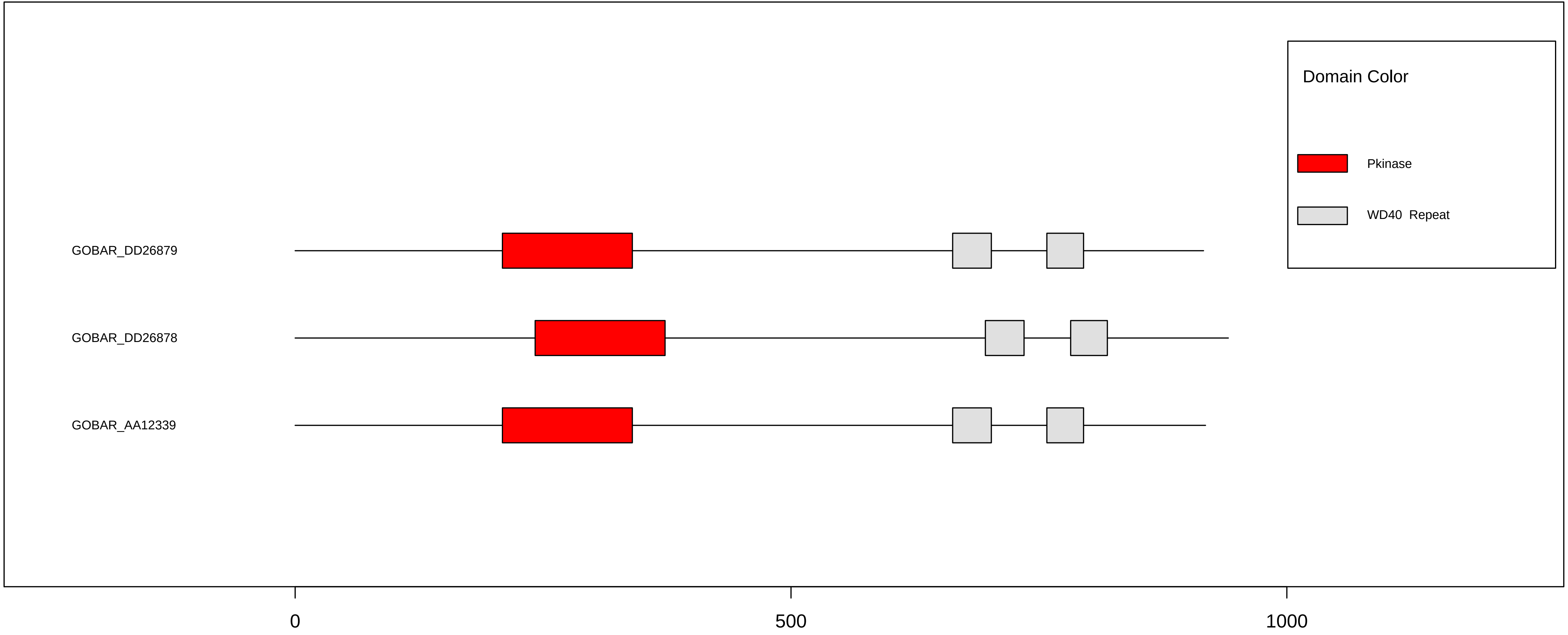

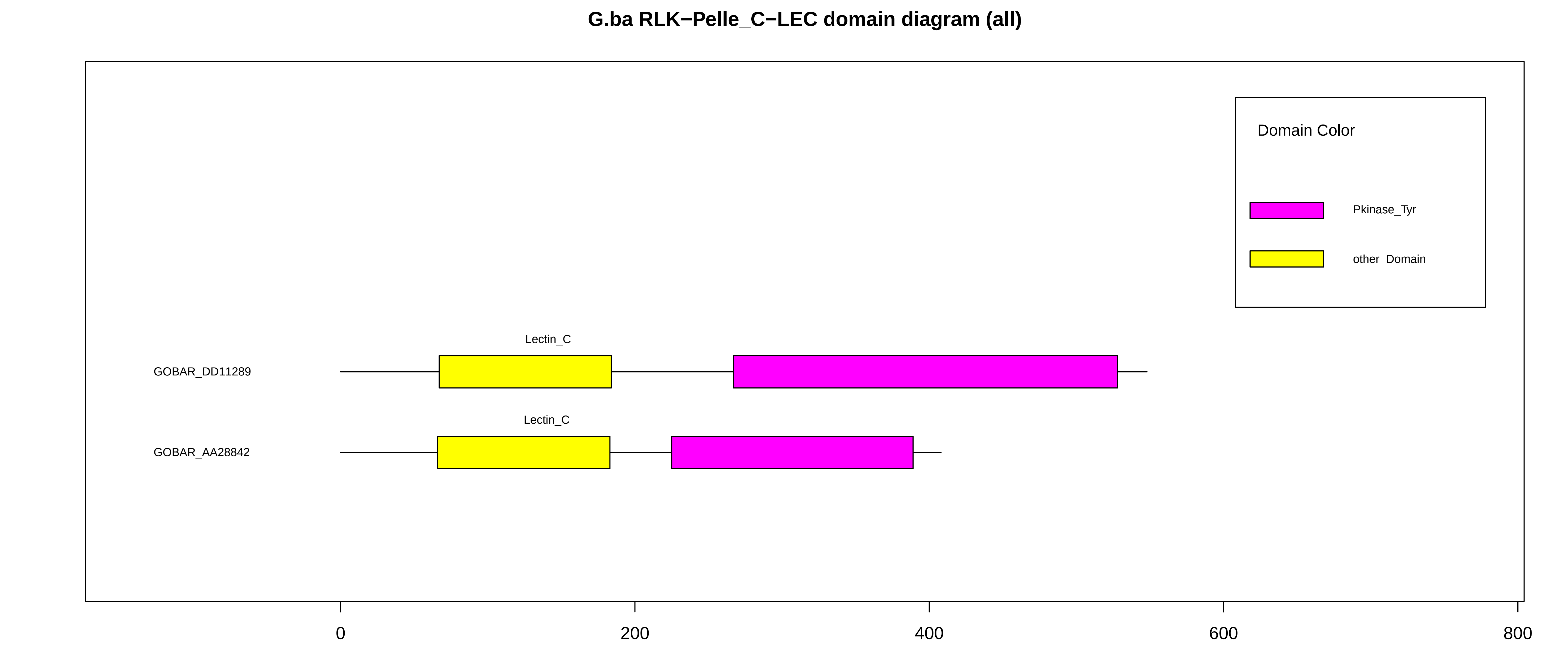

G.ba RLK−Pelle_C−LEC domain diagram (all)
Domain Color
Pkinase_Tyr
other Domain
Lectin_C
GOBAR_DD11289
Lectin_C
GOBAR_AA28842
0
200
400
600
800

G.ba RLK−Pelle_CR4L domain diagram (part 1)

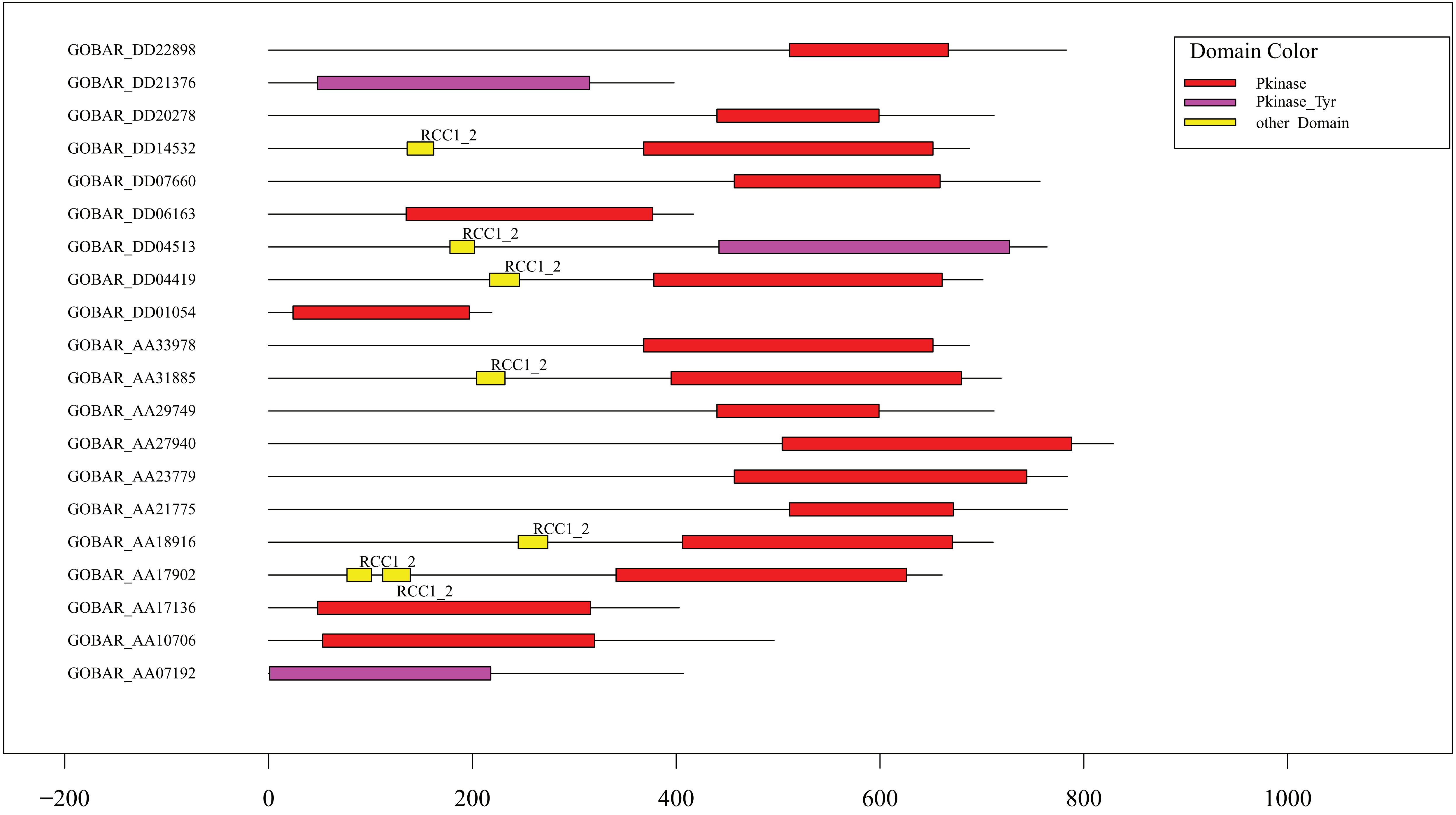

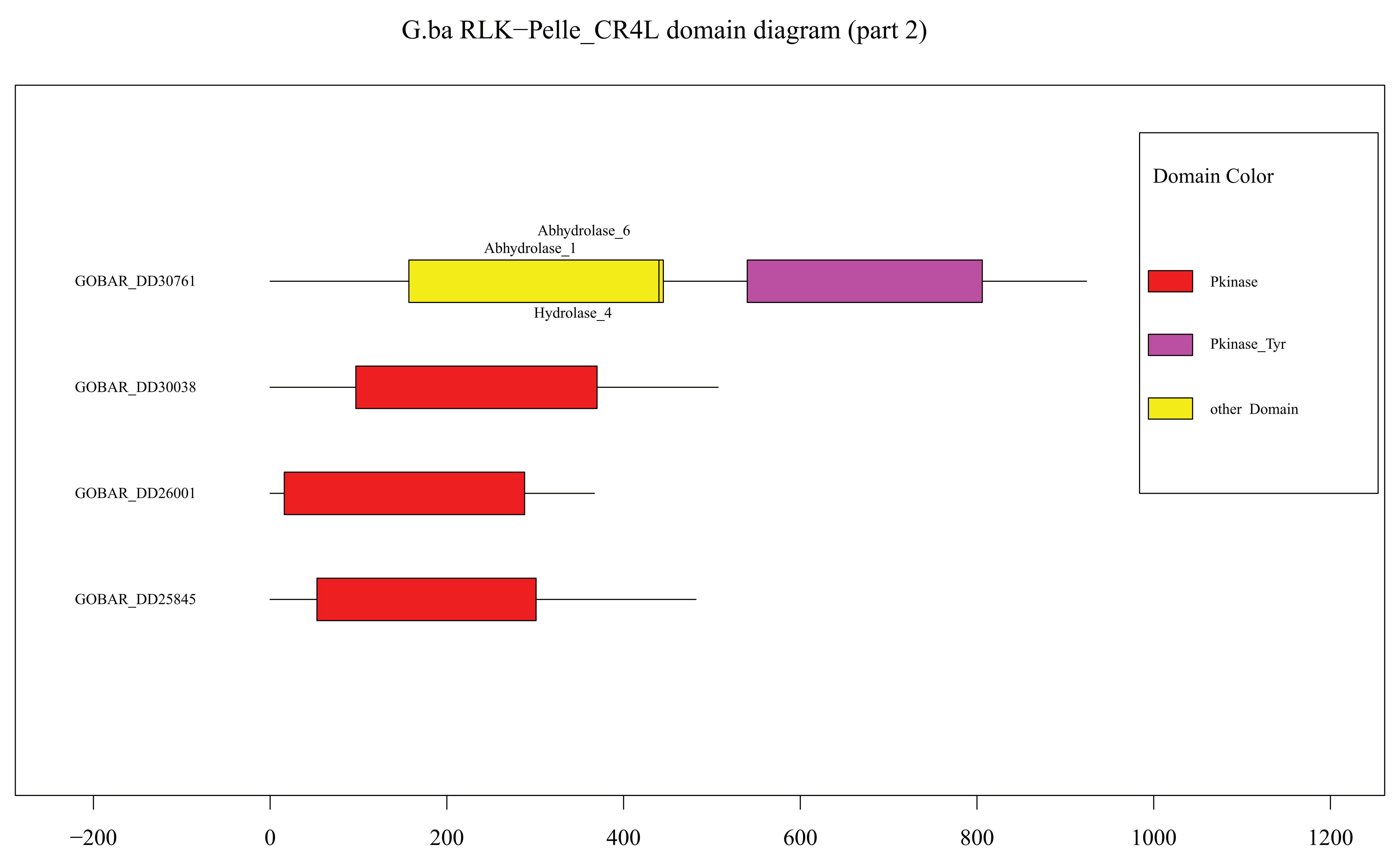
G.ba RLK−Pelle_CR4L domain diagram (part 2)
Abhydrolase_6
Abhydrolase_1
GOBAR_DD30761
Hydrolase_4
GOBAR_DD30038
GOBAR_DD26001
GOBAR_DD25845
Domain Color
Pkinase
Pkinase_Tyr
other Domain
−200
0
200
400
600
800
1000
1200

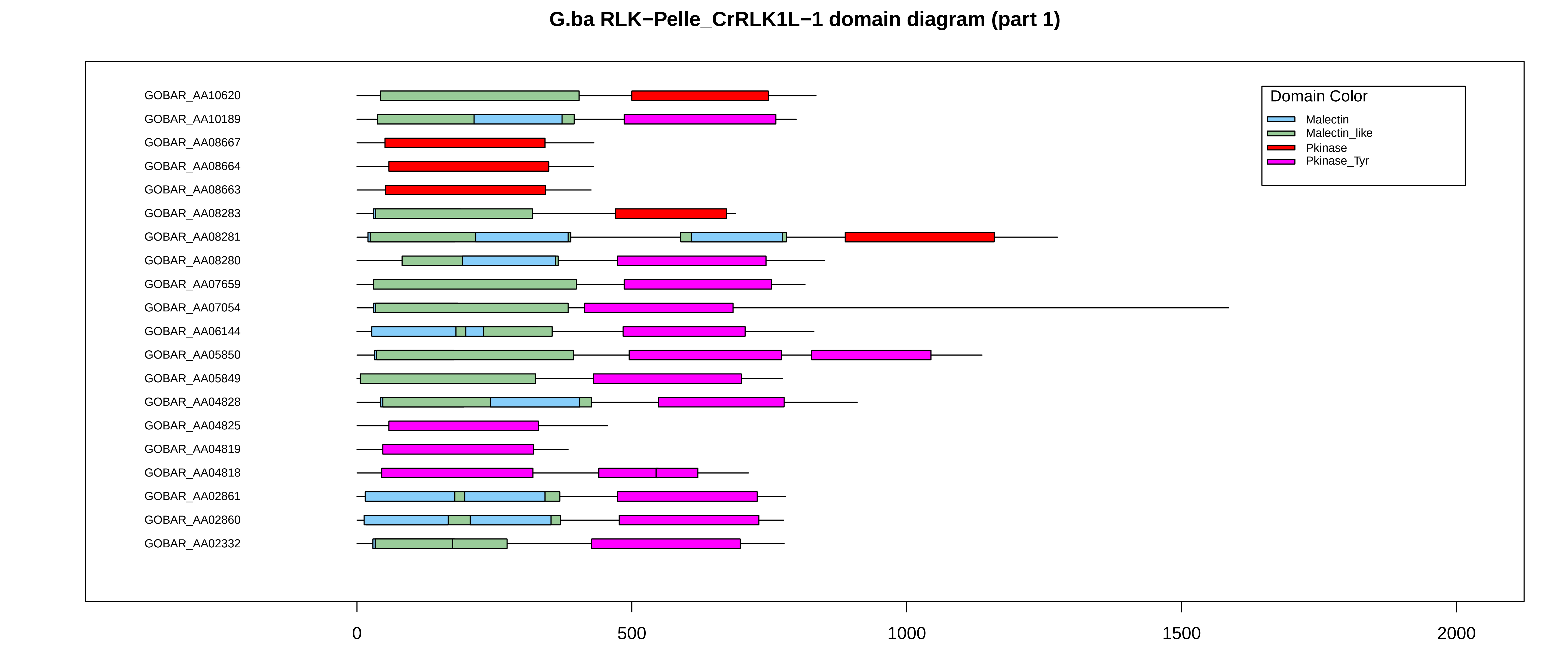
G.ba RLK−Pelle_CrRLK1L−1 domain diagram (part 1)
GOBAR_AA10620
GOBAR_AA10189
GOBAR_AA08667
GOBAR_AA08664
GOBAR_AA08663
GOBAR_AA08283
GOBAR_AA08281
GOBAR_AA08280
GOBAR_AA07659
GOBAR_AA07054
GOBAR_AA06144
GOBAR_AA05850
GOBAR_AA05849
GOBAR_AA04828
GOBAR_AA04825
GOBAR_AA04819
GOBAR_AA04818
GOBAR_AA02861
GOBAR_AA02860
GOBAR_AA02332
Domain Color
Malectin
Malectin_like
Pkinase
Pkinase_Tyr
0
500
1000
1500
2000

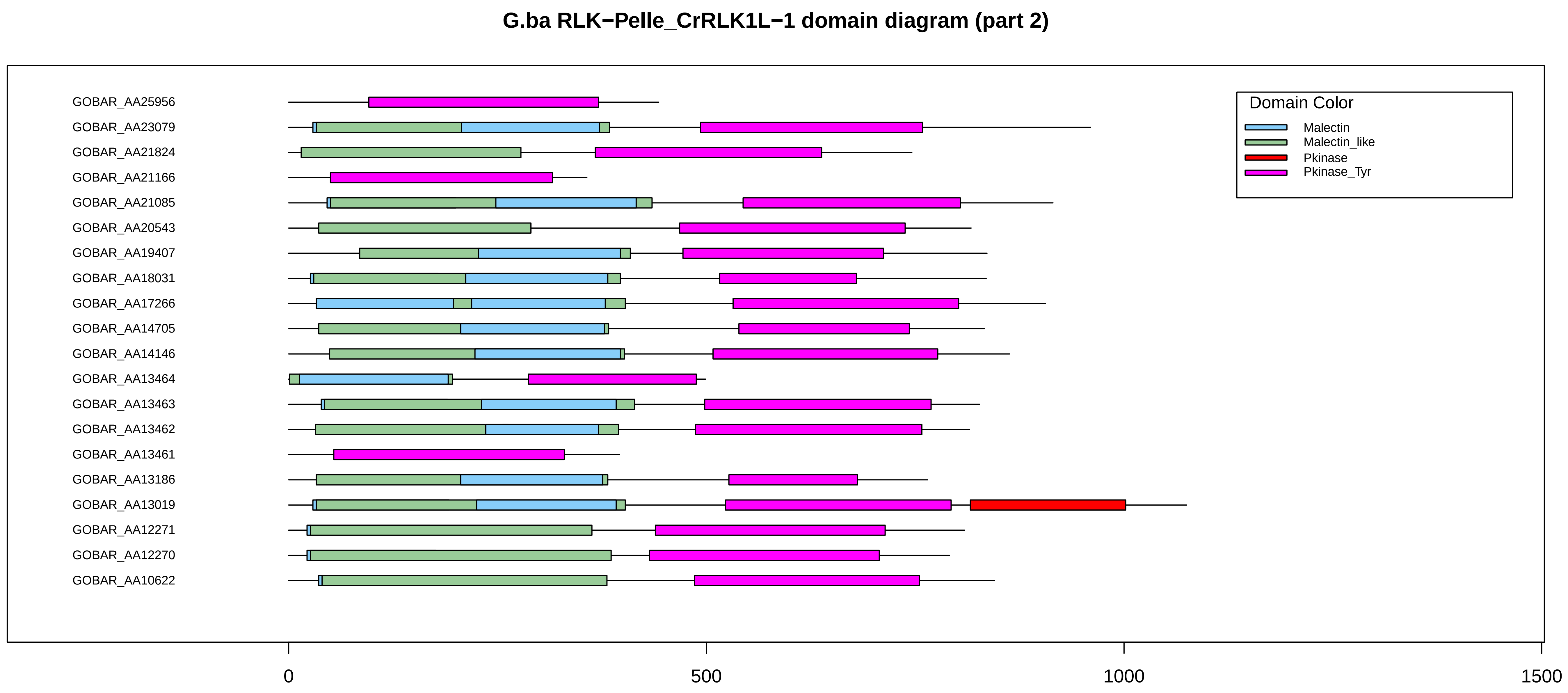

G.ba RLK−Pelle_CrRLK1L−1 domain diagram (part 2)
GOBAR_AA25956
GOBAR_AA23079
GOBAR_AA21824
GOBAR_AA21166
GOBAR_AA21085
GOBAR_AA20543
GOBAR_AA19407
GOBAR_AA18031
GOBAR_AA17266
GOBAR_AA14705
GOBAR_AA14146
GOBAR_AA13464
GOBAR_AA13463
GOBAR_AA13462
GOBAR_AA13461
GOBAR_AA13186
GOBAR_AA13019
GOBAR_AA12271
GOBAR_AA12270
GOBAR_AA10622
Domain Color
Malectin
Malectin_like
Pkinase
Pkinase_Tyr
0
500
1000
1500

G.ba RLK−Pelle_CrRLK1L−1 domain diagram (part 3)

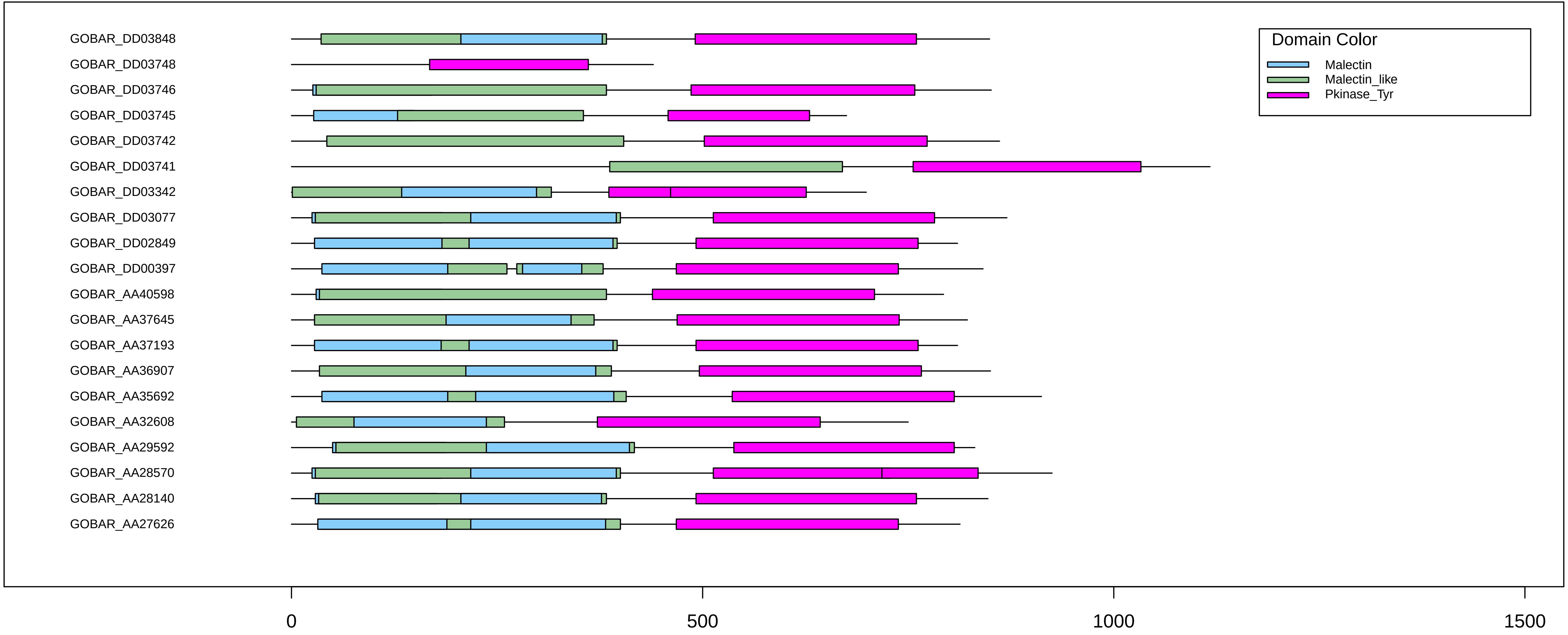

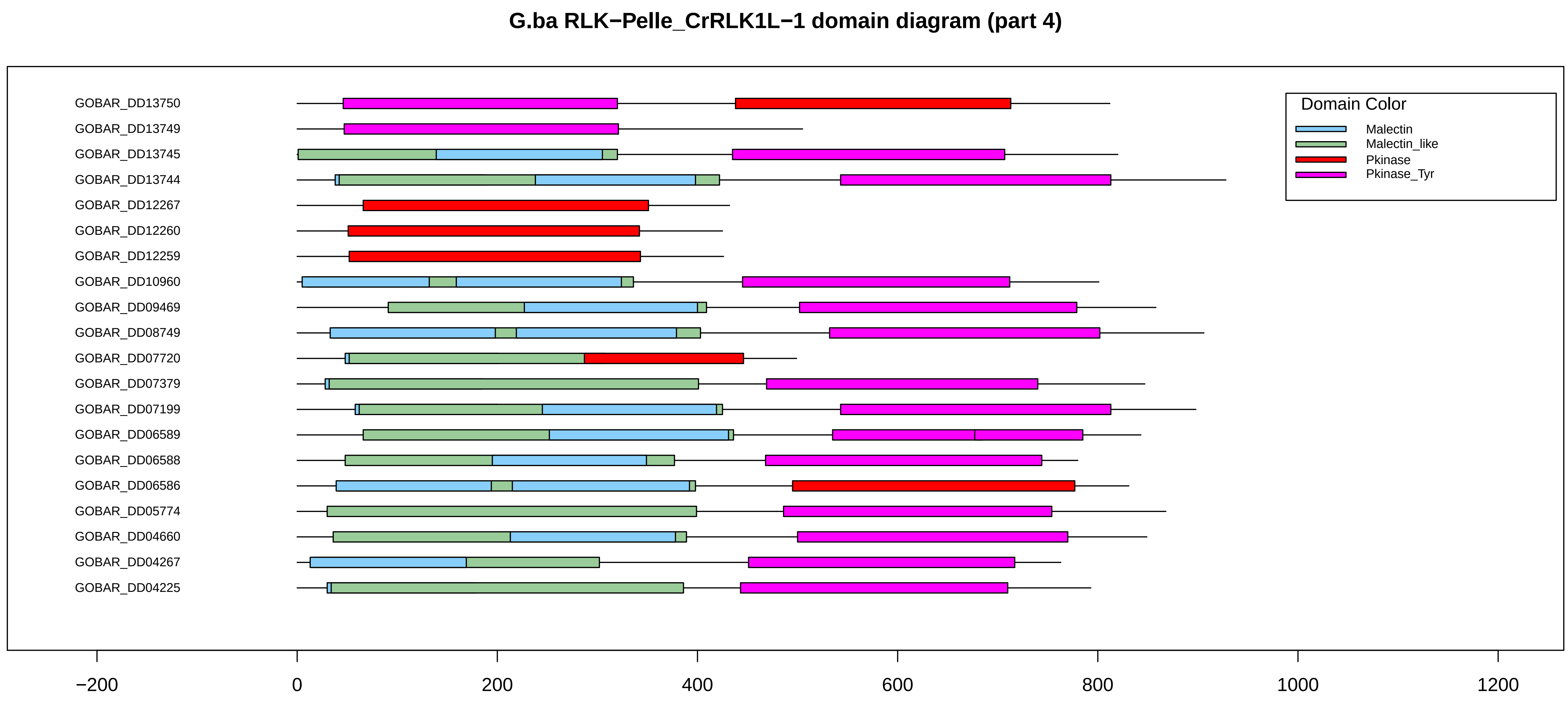
G.ba RLK−Pelle_CrRLK1L−1 domain diagram (part 4)
GOBAR_DD13750
GOBAR_DD13749
GOBAR_DD13745
GOBAR_DD13744
GOBAR_DD12267
GOBAR_DD12260
GOBAR_DD12259
GOBAR_DD10960
GOBAR_DD09469
GOBAR_DD08749
GOBAR_DD07720
GOBAR_DD07379
GOBAR_DD07199
GOBAR_DD06589
GOBAR_DD06588
GOBAR_DD06586
GOBAR_DD05774
GOBAR_DD04660
GOBAR_DD04267
GOBAR_DD04225
Domain Color
Malectin
Malectin_like
Pkinase
Pkinase_Tyr
−200
0
200
400
600
800
1000
1200

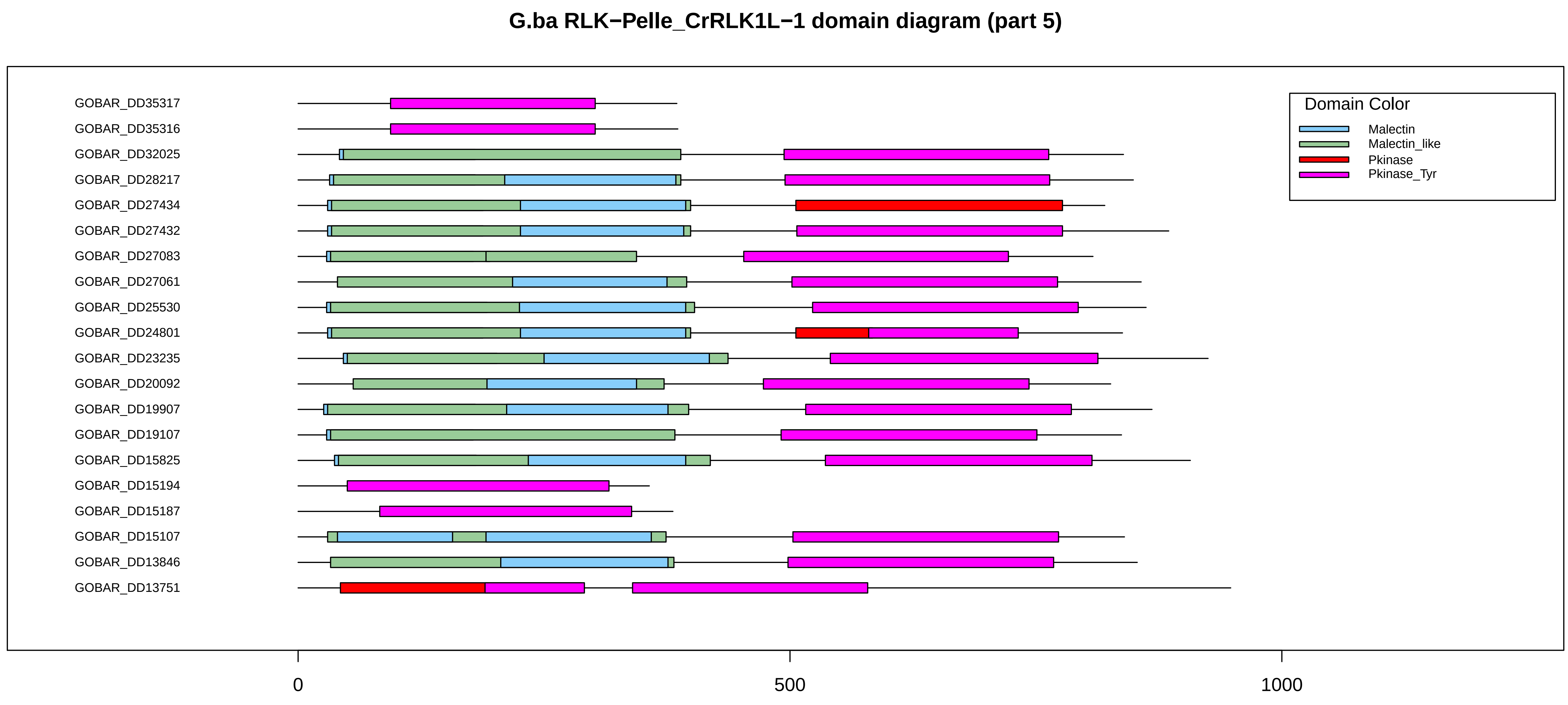
G.ba RLK−Pelle_CrRLK1L−1 domain diagram (part 5)
GOBAR_DD35317
GOBAR_DD35316
GOBAR_DD32025
GOBAR_DD28217
GOBAR_DD27434
GOBAR_DD27432
GOBAR_DD27083
GOBAR_DD27061
GOBAR_DD25530
GOBAR_DD24801
GOBAR_DD23235
GOBAR_DD20092
GOBAR_DD19907
GOBAR_DD19107
GOBAR_DD15825
GOBAR_DD15194
GOBAR_DD15187
GOBAR_DD15107
GOBAR_DD13846
GOBAR_DD13751
Domain Color
Malectin
Malectin_like
Pkinase
Pkinase_Tyr
0
500
1000

G.ba RLK−Pelle_CrRLK1L−1 domain diagram (part 6)

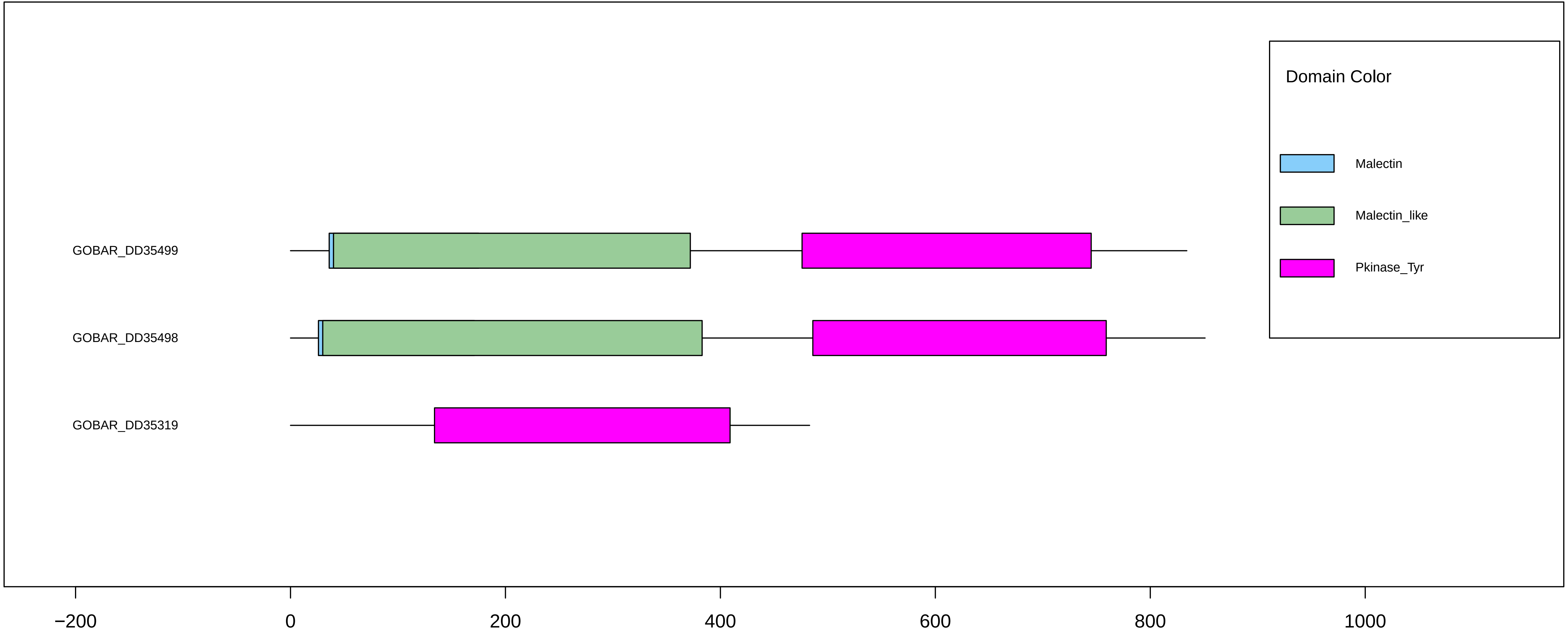

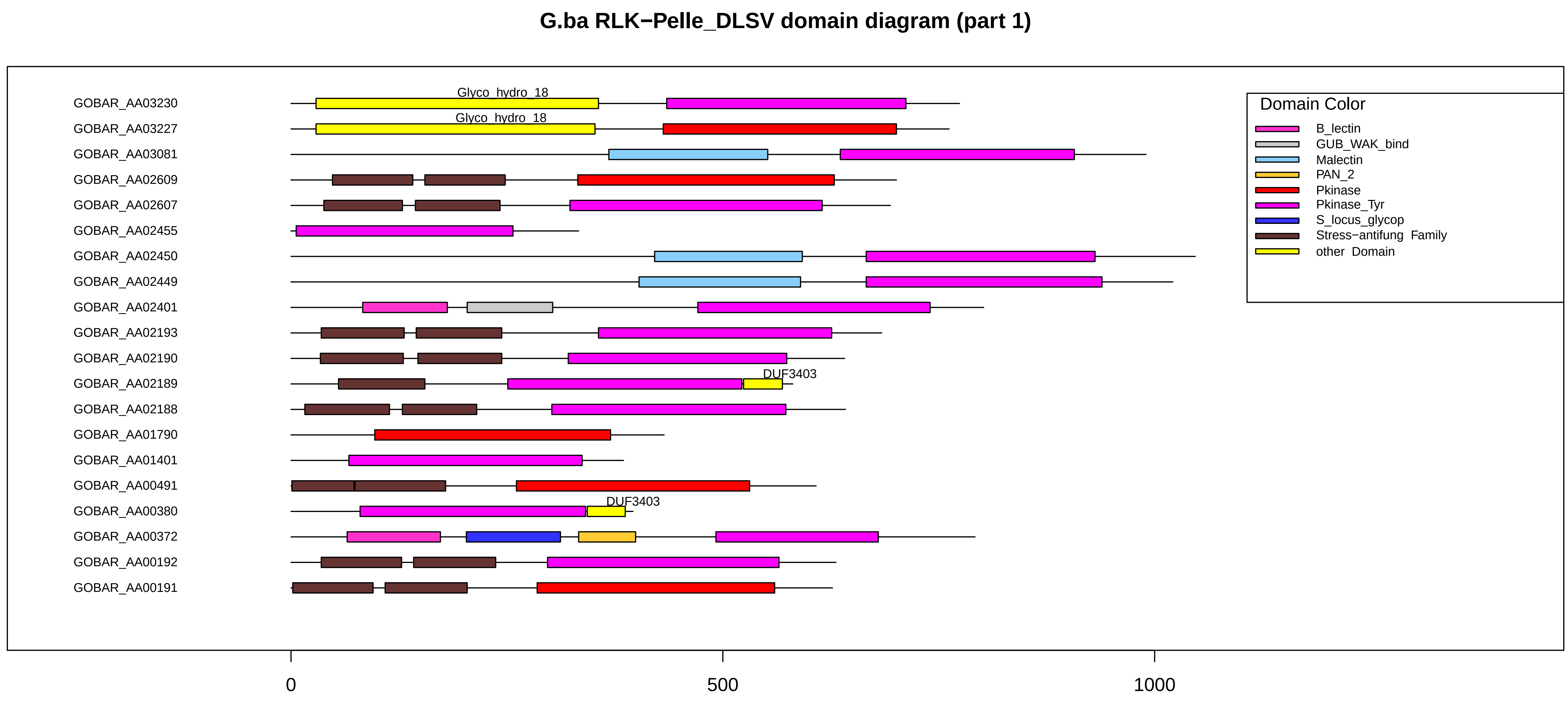
G.ba RLK−Pelle_DLSV domain diagram (part 1)
GOBAR_AA03230
GOBAR_AA03227
GOBAR_AA03081
GOBAR_AA02609
GOBAR_AA02607
GOBAR_AA02455
GOBAR_AA02450
GOBAR_AA02449
GOBAR_AA02401
GOBAR_AA02193
GOBAR_AA02190
GOBAR_AA02189
GOBAR_AA02188
GOBAR_AA01790
GOBAR_AA01401
GOBAR_AA00491
GOBAR_AA00380
GOBAR_AA00372
GOBAR_AA00192
GOBAR_AA00191
Glyco_hydro_18
Glyco_hydro_18
DUF3403
DUF3403
Domain Color
B_lectin
GUB_WAK_bind
Malectin
PAN_2
Pkinase
Pkinase_Tyr
S_locus_glycop
Stress−antifung Family
other Domain
0
500
1000

G.ba RLK−Pelle_DLSV domain diagram (part 2)

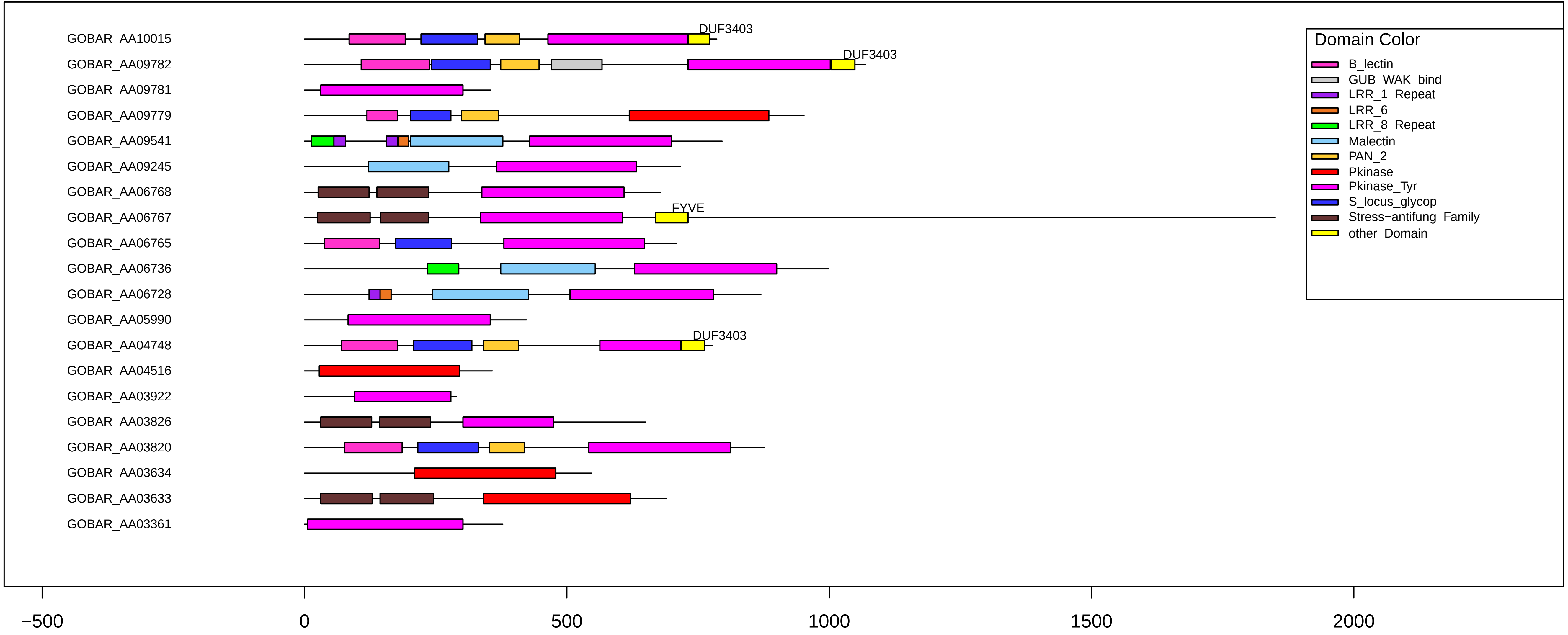

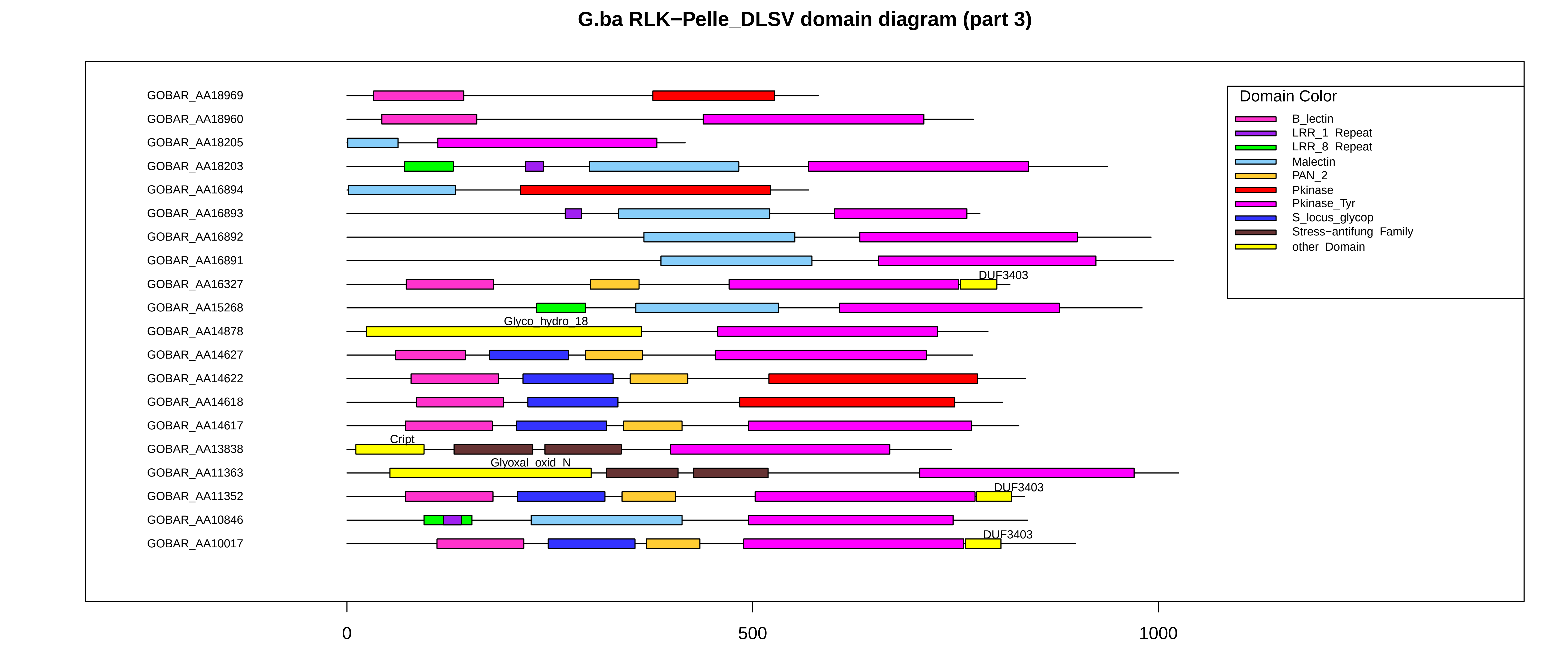
G.ba RLK−Pelle_DLSV domain diagram (part 3)
GOBAR_AA18969
GOBAR_AA18960
GOBAR_AA18205
GOBAR_AA18203
GOBAR_AA16894
GOBAR_AA16893
GOBAR_AA16892
GOBAR_AA16891
GOBAR_AA16327
GOBAR_AA15268
GOBAR_AA14878
GOBAR_AA14627
GOBAR_AA14622
GOBAR_AA14618
GOBAR_AA14617
GOBAR_AA13838
GOBAR_AA11363
GOBAR_AA11352
GOBAR_AA10846
GOBAR_AA10017
DUF3403
Glyco_hydro_18
Cript
Glyoxal_oxid_N
DUF3403
DUF3403
Domain Color
B_lectin
LRR_1 Repeat
LRR_8 Repeat
Malectin
PAN_2
Pkinase
Pkinase_Tyr
S_locus_glycop
Stress−antifung Family
other Domain
0
500
1000

G.ba RLK−Pelle_DLSV domain diagram (part 4)

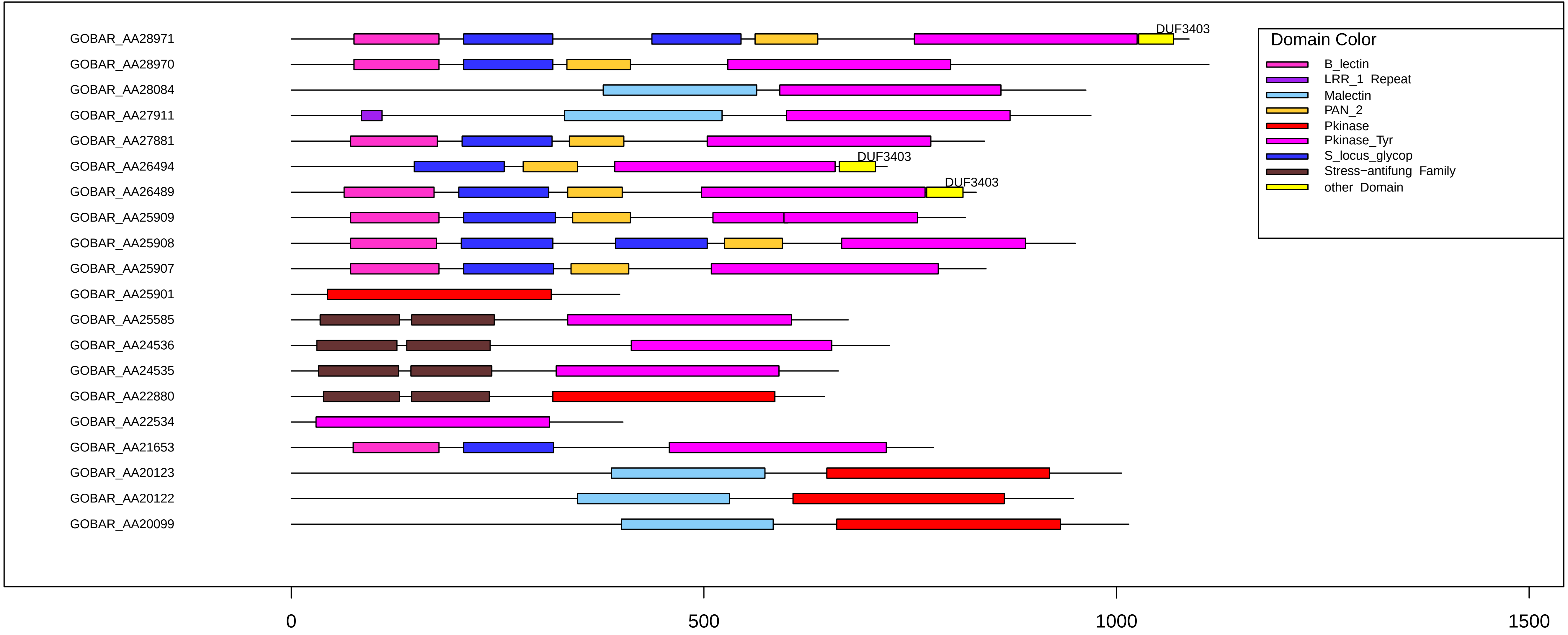

G.ba RLK−Pelle_DLSV domain diagram (part 5)

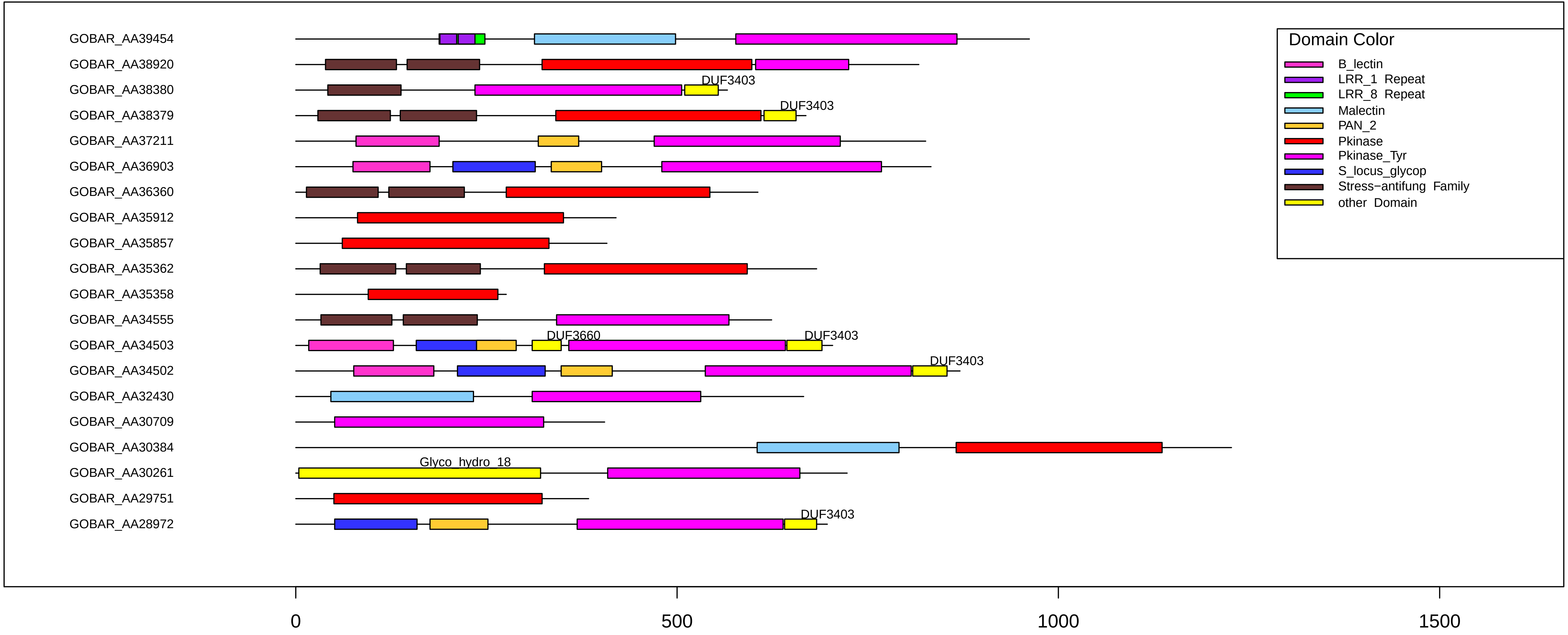

G.ba RLK−Pelle_DLSV domain diagram (part 6)

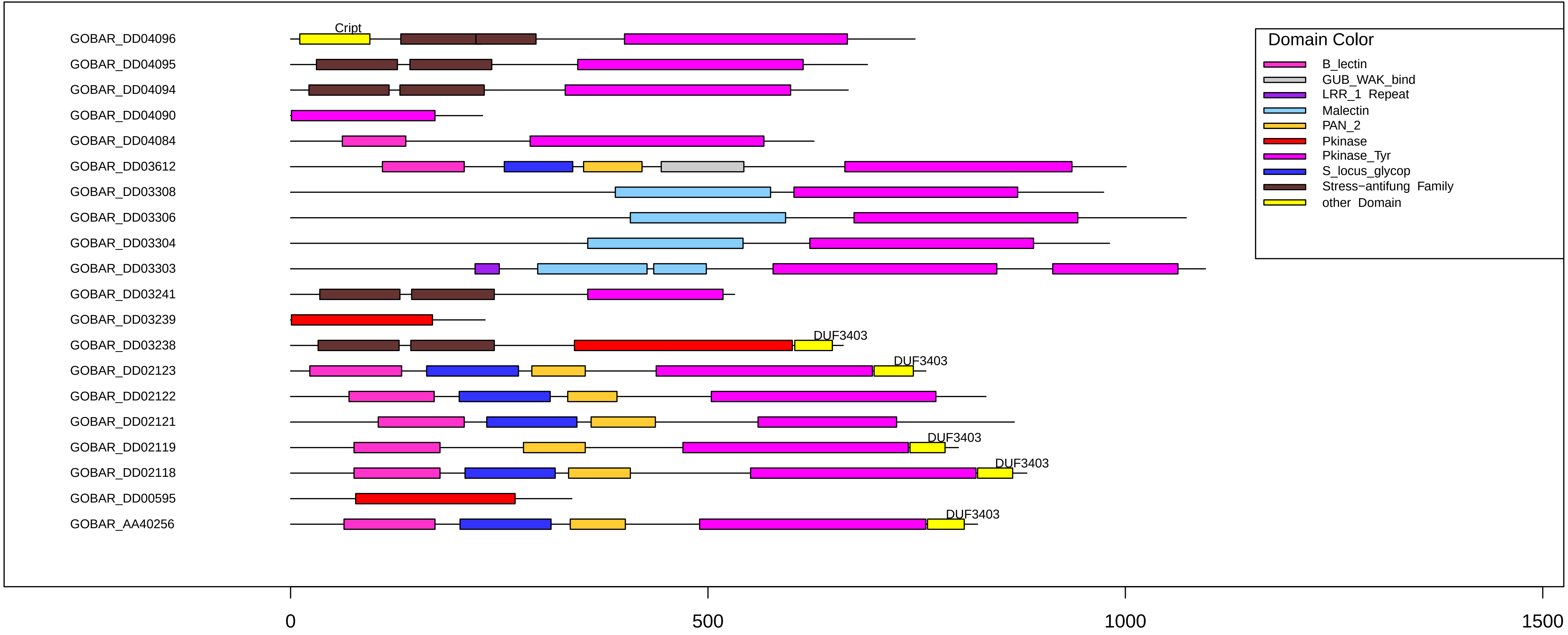

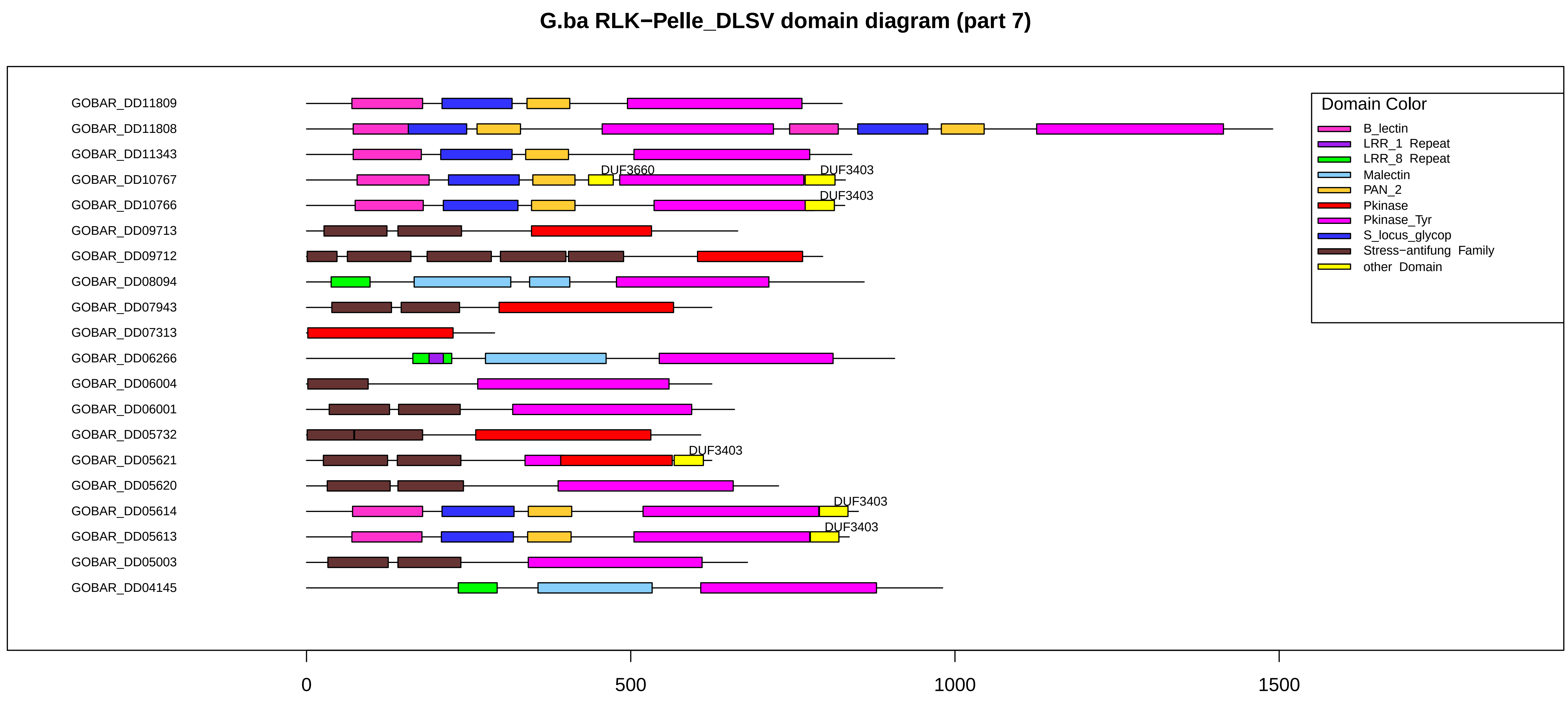

G.ba RLK−Pelle_DLSV domain diagram (part 7)
GOBAR_DD11809
GOBAR_DD11808
GOBAR_DD11343
GOBAR_DD10767
GOBAR_DD10766
GOBAR_DD09713
GOBAR_DD09712
GOBAR_DD08094
GOBAR_DD07943
GOBAR_DD07313
GOBAR_DD06266
GOBAR_DD06004
GOBAR_DD06001
GOBAR_DD05732
GOBAR_DD05621
GOBAR_DD05620
GOBAR_DD05614
GOBAR_DD05613
GOBAR_DD05003
GOBAR_DD04145
DUF3660
DUF3403
Domain Color
B_lectin
LRR_1 Repeat
LRR_8 Repeat
Malectin
PAN_2
Pkinase
Pkinase_Tyr
S_locus_glycop
Stress−antifung Family
other Domain
0
500
1000
1500

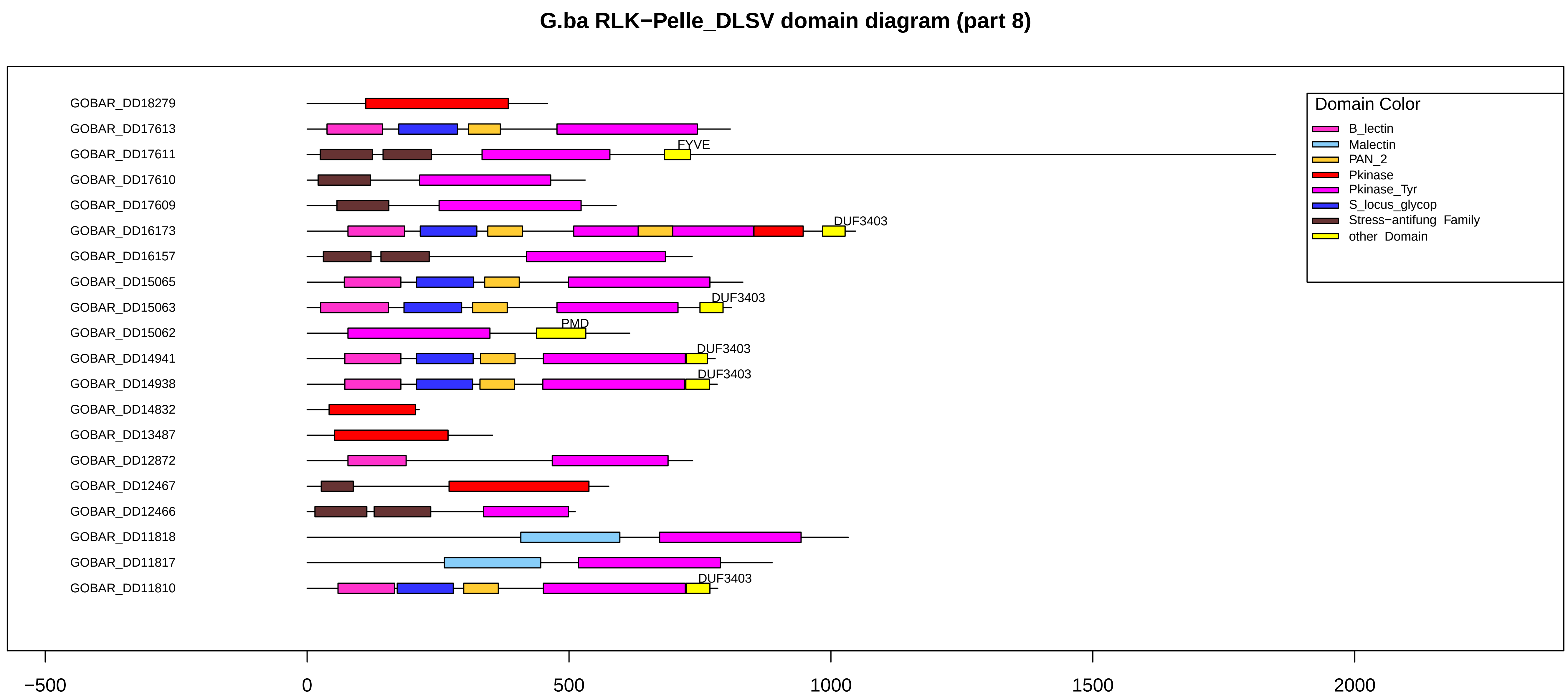
G.ba RLK−Pelle_DLSV domain diagram (part 8)
GOBAR_DD18279
GOBAR_DD17613
GOBAR_DD17611
GOBAR_DD17610
GOBAR_DD17609
GOBAR_DD16173
GOBAR_DD16157
GOBAR_DD15065
GOBAR_DD15063
GOBAR_DD15062
GOBAR_DD14941
GOBAR_DD14938
GOBAR_DD14832
GOBAR_DD13487
GOBAR_DD12872
GOBAR_DD12467
GOBAR_DD12466
GOBAR_DD11818
GOBAR_DD11817
GOBAR_DD11810
FYVE
DUF3403
DUF3403
PMD
DUF3403
DUF3403
DUF3403
Domain Color
B_lectin
Malectin
PAN_2
Pkinase
Pkinase_Tyr
S_locus_glycop
Stress−antifung Family
other Domain
−500
0
500
1000
1500
2000

G.ba RLK−Pelle_DLSV domain diagram (part 9)

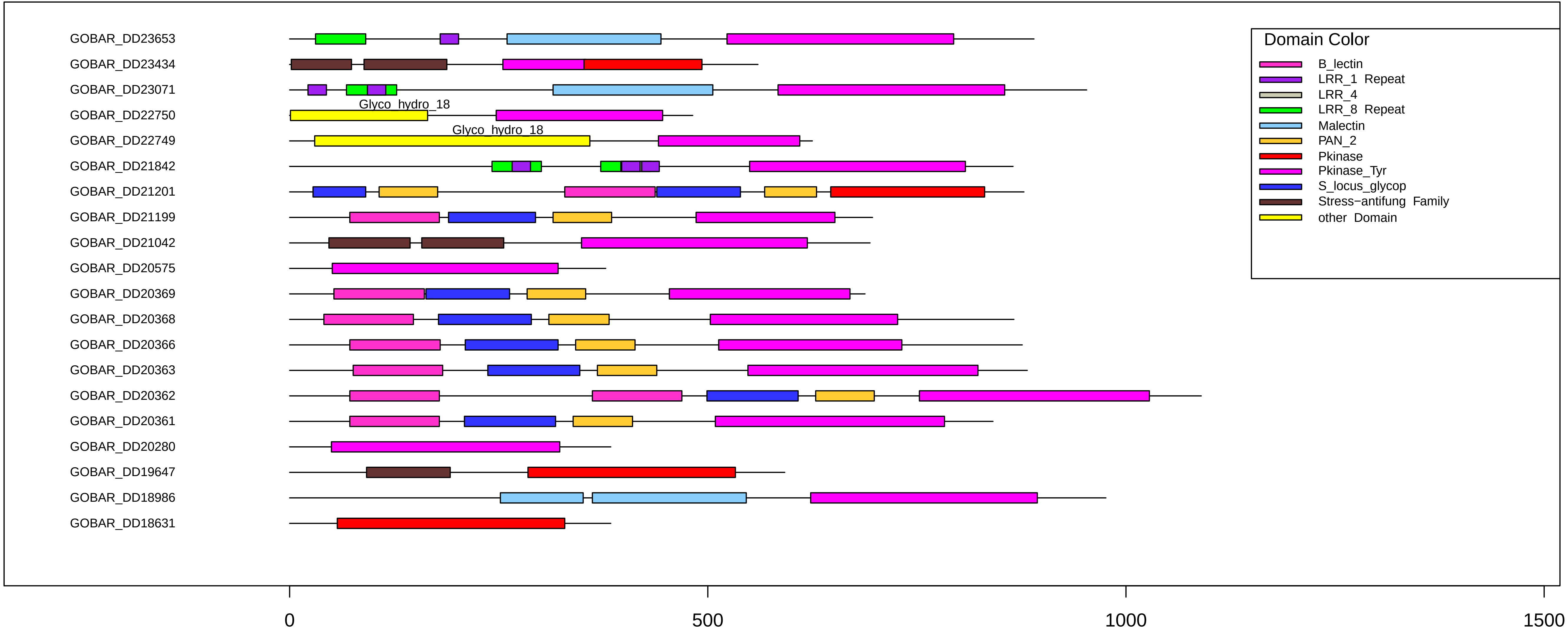

G.ba RLK−Pelle_DLSV domain diagram (part 10)

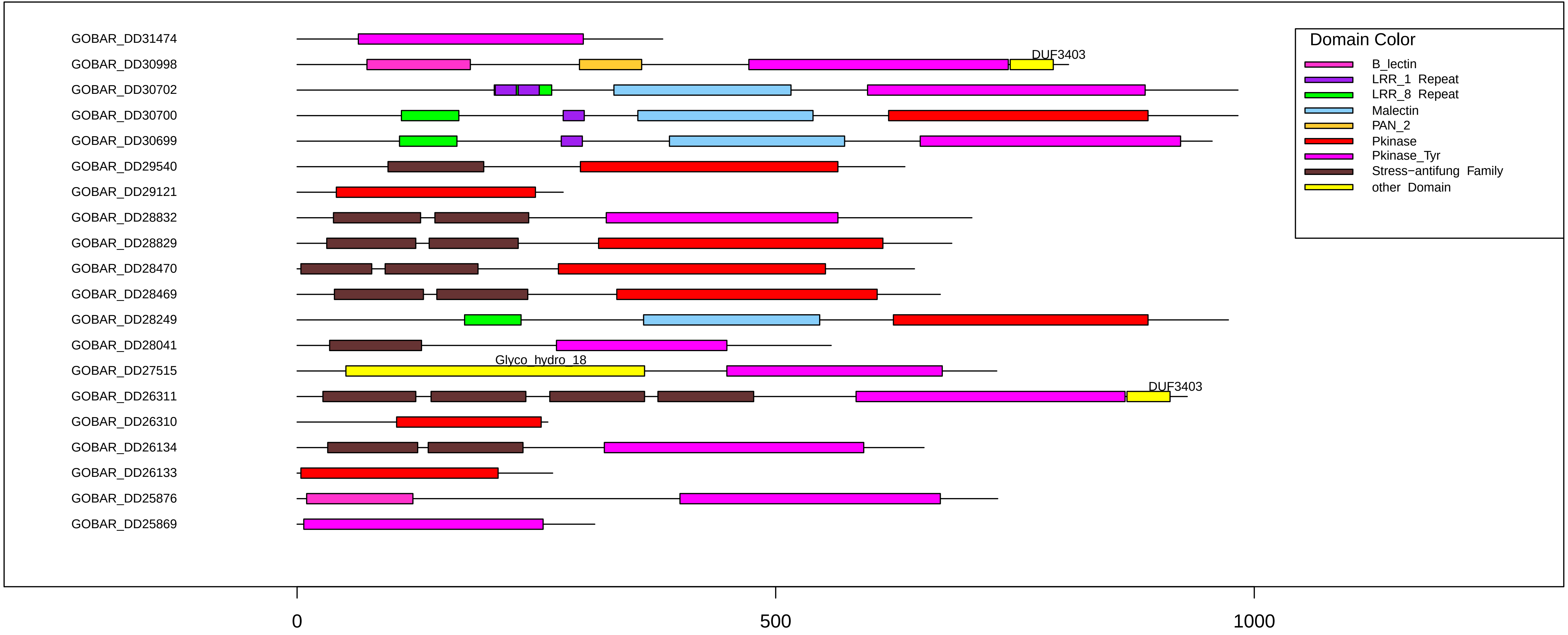

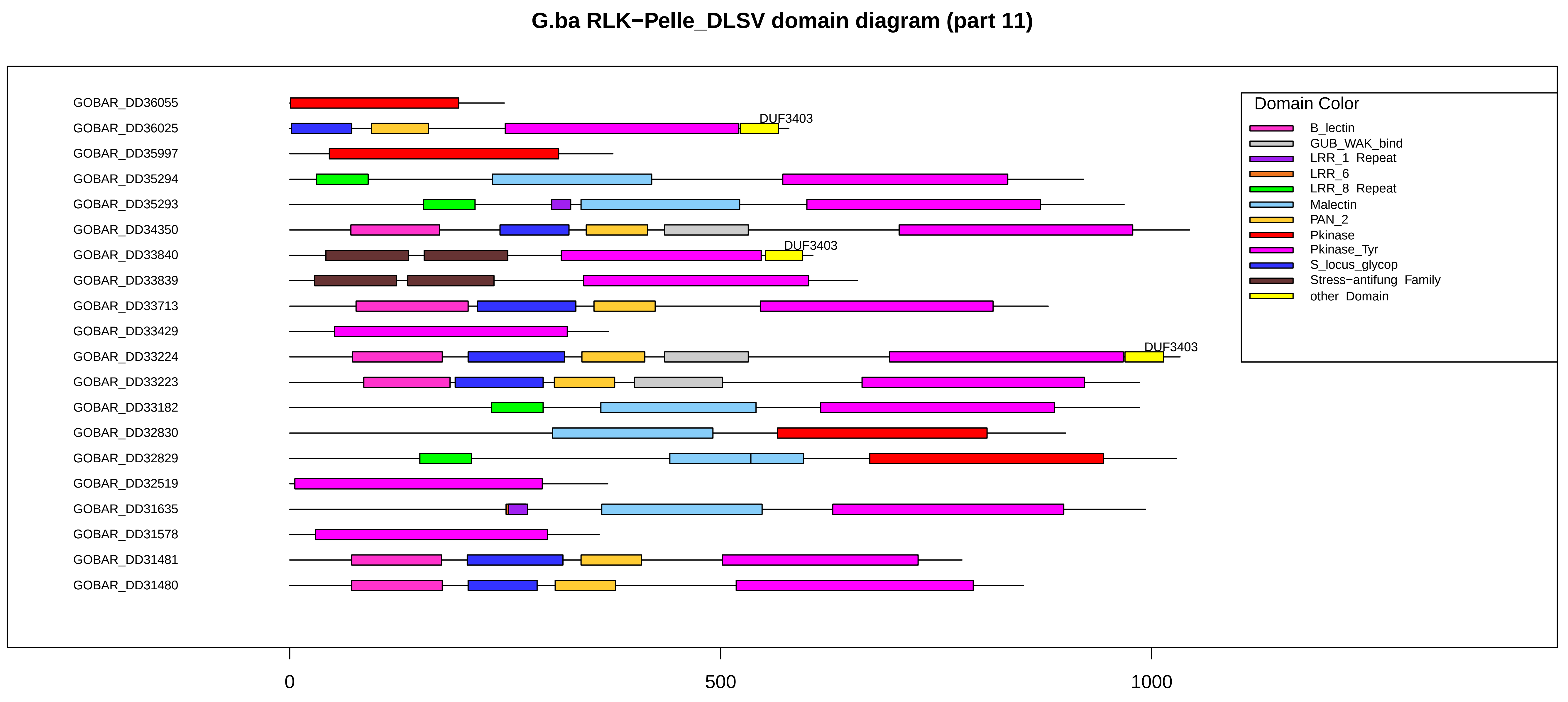
G.ba RLK−Pelle_DLSV domain diagram (part 11)
GOBAR_DD36055
GOBAR_DD36025
GOBAR_DD35997
GOBAR_DD35294
GOBAR_DD35293
GOBAR_DD34350
GOBAR_DD33840
GOBAR_DD33839
GOBAR_DD33713
GOBAR_DD33429
GOBAR_DD33224
GOBAR_DD33223
GOBAR_DD33182
GOBAR_DD32830
GOBAR_DD32829
GOBAR_DD32519
GOBAR_DD31635
GOBAR_DD31578
GOBAR_DD31481
GOBAR_DD31480
DUF3403
DUF3403
DUF3403
Domain Color
B_lectin
GUB_WAK_bind
LRR_1 Repeat
LRR_6
LRR_8 Repeat
Malectin
PAN_2
Pkinase
Pkinase_Tyr
S_locus_glycop
Stress−antifung Family
other Domain
0
500
1000

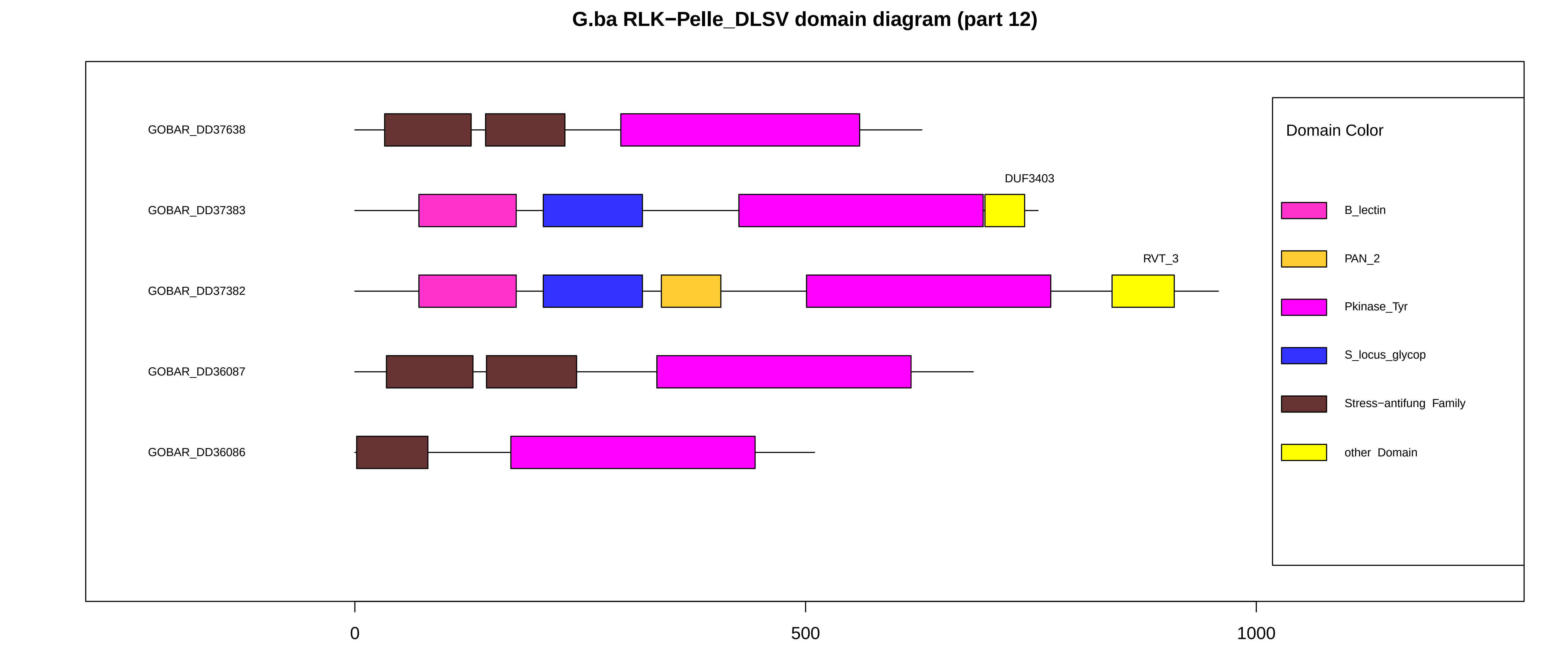
G.ba RLK−Pelle_DLSV domain diagram (part 12)
GOBAR_DD37638
GOBAR_DD37383
GOBAR_DD37382
GOBAR_DD36087
GOBAR_DD36086
DUF3403
RVT_3
Domain Color
B_lectin
PAN_2
Pkinase_Tyr
S_locus_glycop
Stress−antifung Family
other Domain
0
500
1000

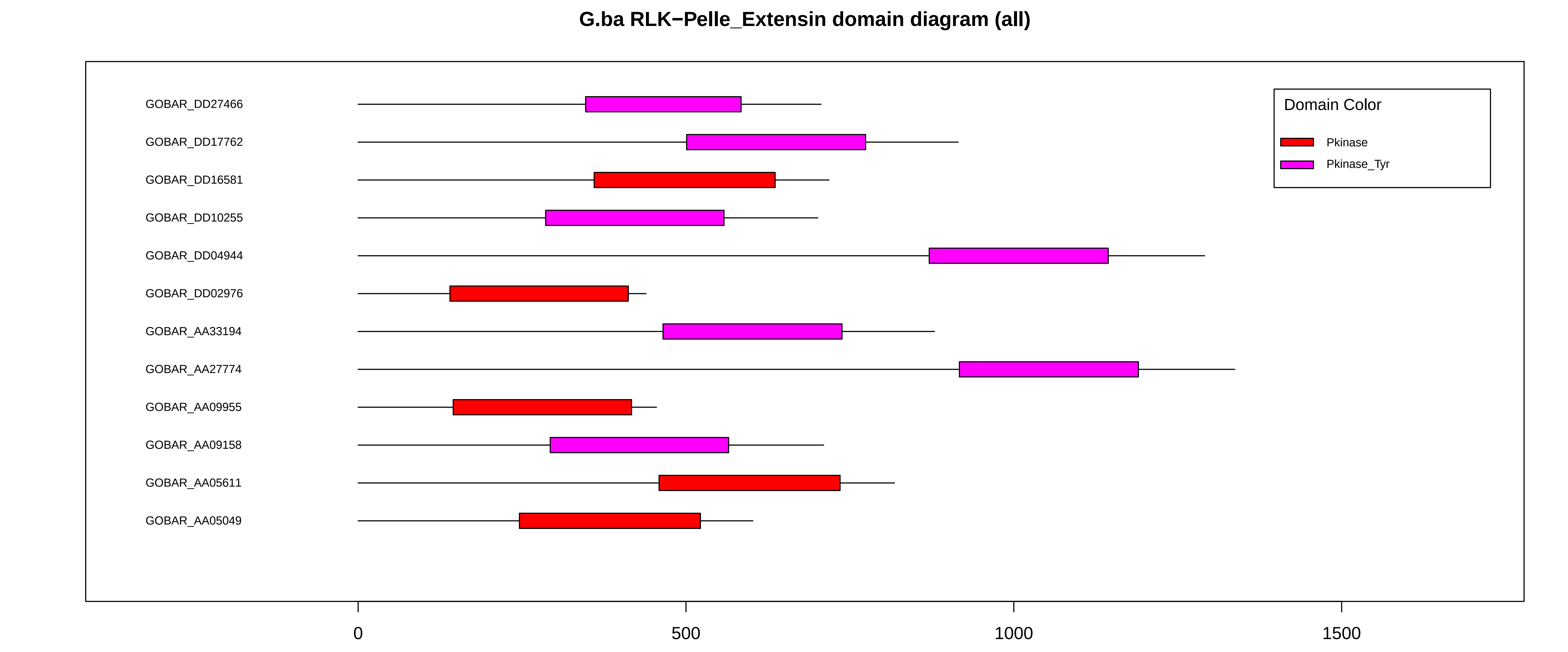

G.ba RLK−Pelle_Extensin domain diagram (all)
GOBAR_DD27466
GOBAR_DD17762
GOBAR_DD16581
GOBAR_DD10255
GOBAR_DD04944
GOBAR_DD02976
GOBAR_AA33194
GOBAR_AA27774
GOBAR_AA09955
GOBAR_AA09158
GOBAR_AA05611
GOBAR_AA05049
Domain Color
Pkinase
Pkinase_Tyr
0
500
1000
1500

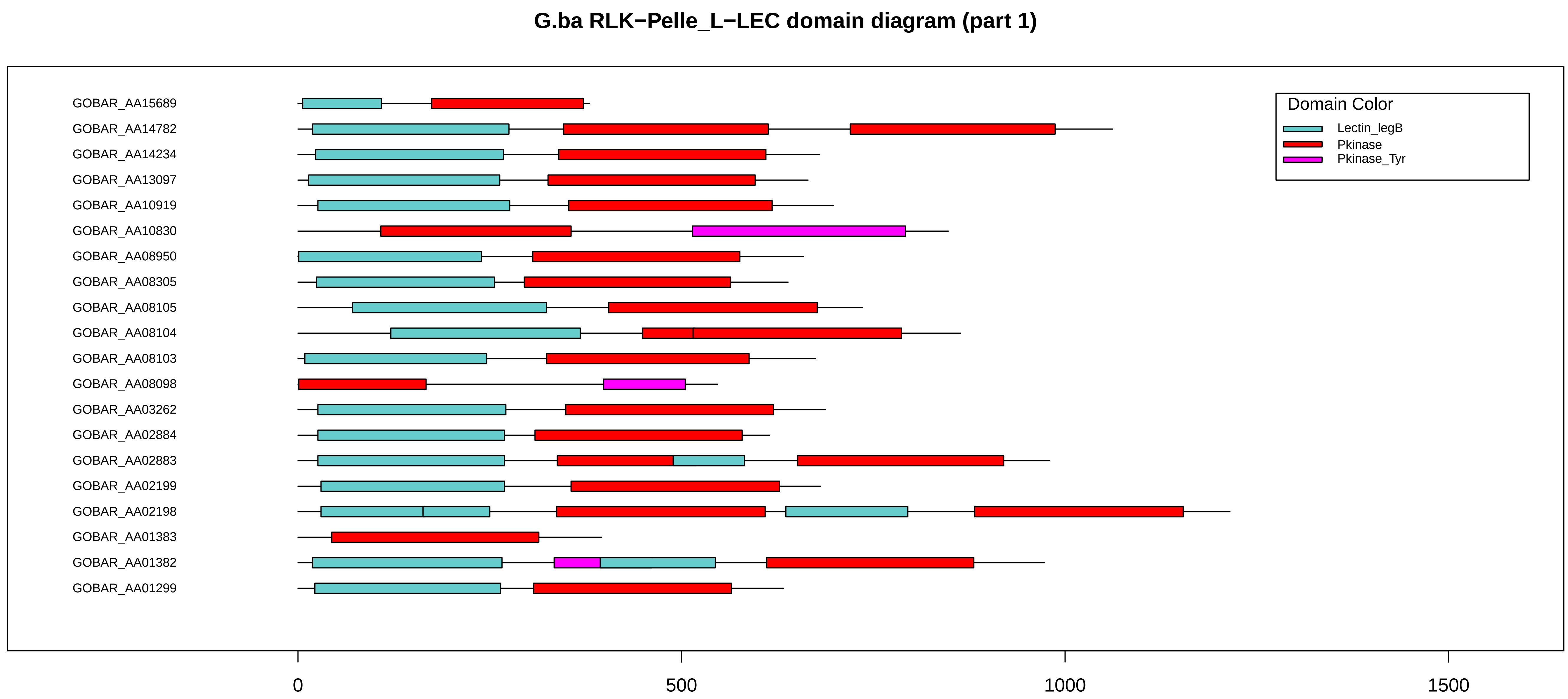

G.ba RLK−Pelle_L−LEC domain diagram (part 1)
GOBAR_AA15689
GOBAR_AA14782
GOBAR_AA14234
GOBAR_AA13097
GOBAR_AA10919
GOBAR_AA10830
GOBAR_AA08950
GOBAR_AA08305
GOBAR_AA08105
GOBAR_AA08104
GOBAR_AA08103
GOBAR_AA08098
GOBAR_AA03262
GOBAR_AA02884
GOBAR_AA02883
GOBAR_AA02199
GOBAR_AA02198
GOBAR_AA01383
GOBAR_AA01382
GOBAR_AA01299
Domain Color
Lectin_legB
Pkinase
Pkinase_Tyr
0
500
1000
1500

G.ba RLK−Pelle_L−LEC domain diagram (part 2)

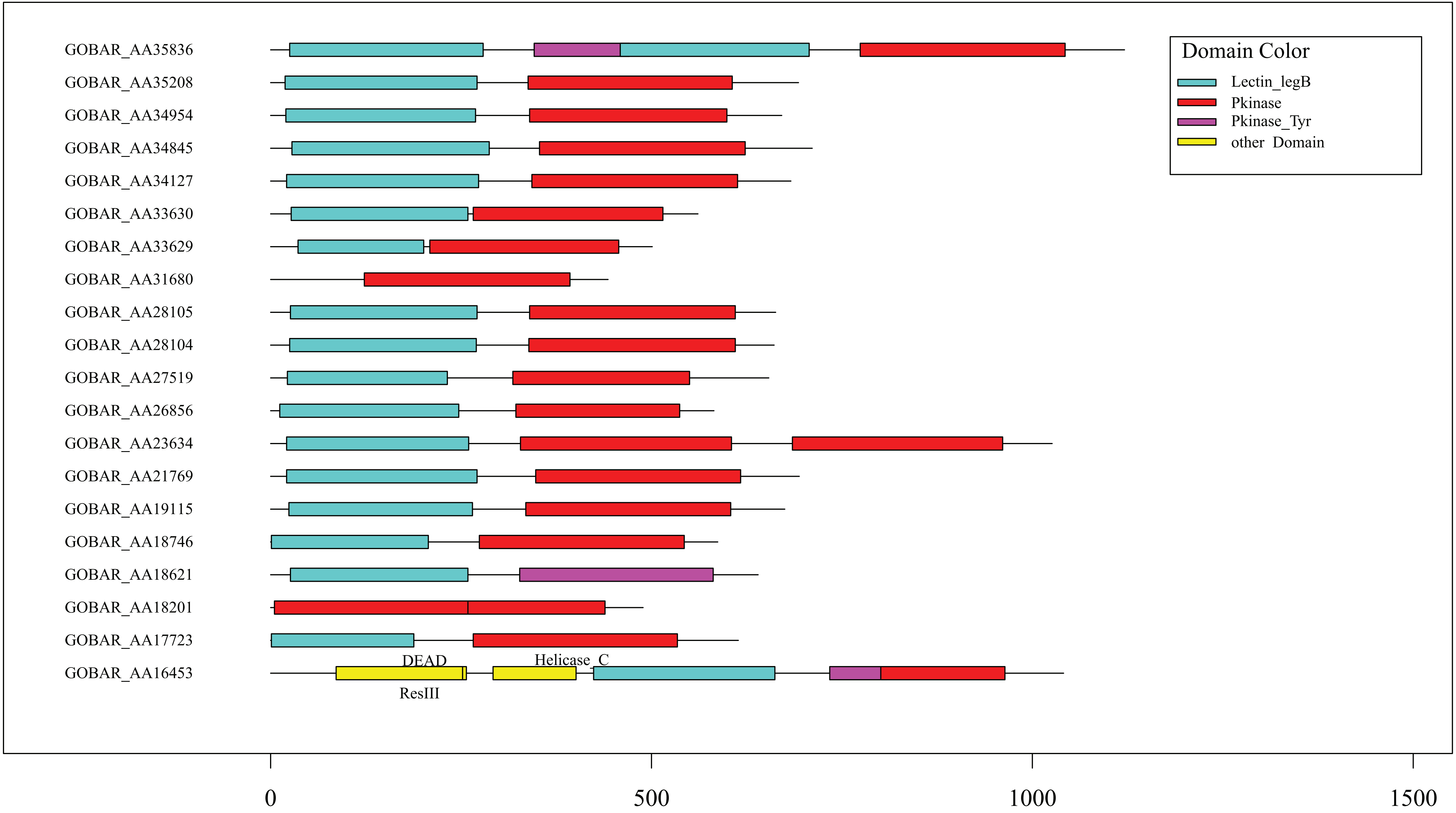

G.ba RLK−Pelle_L−LEC domain diagram (part 3)

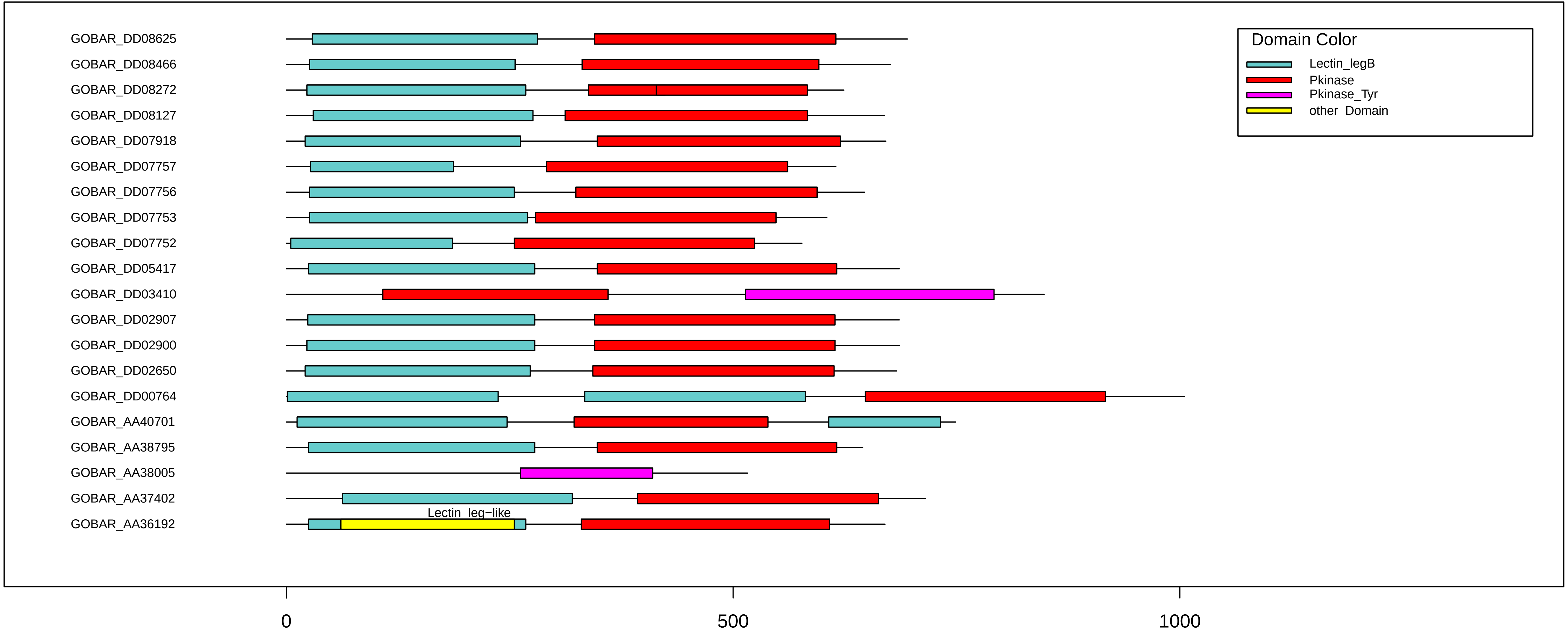

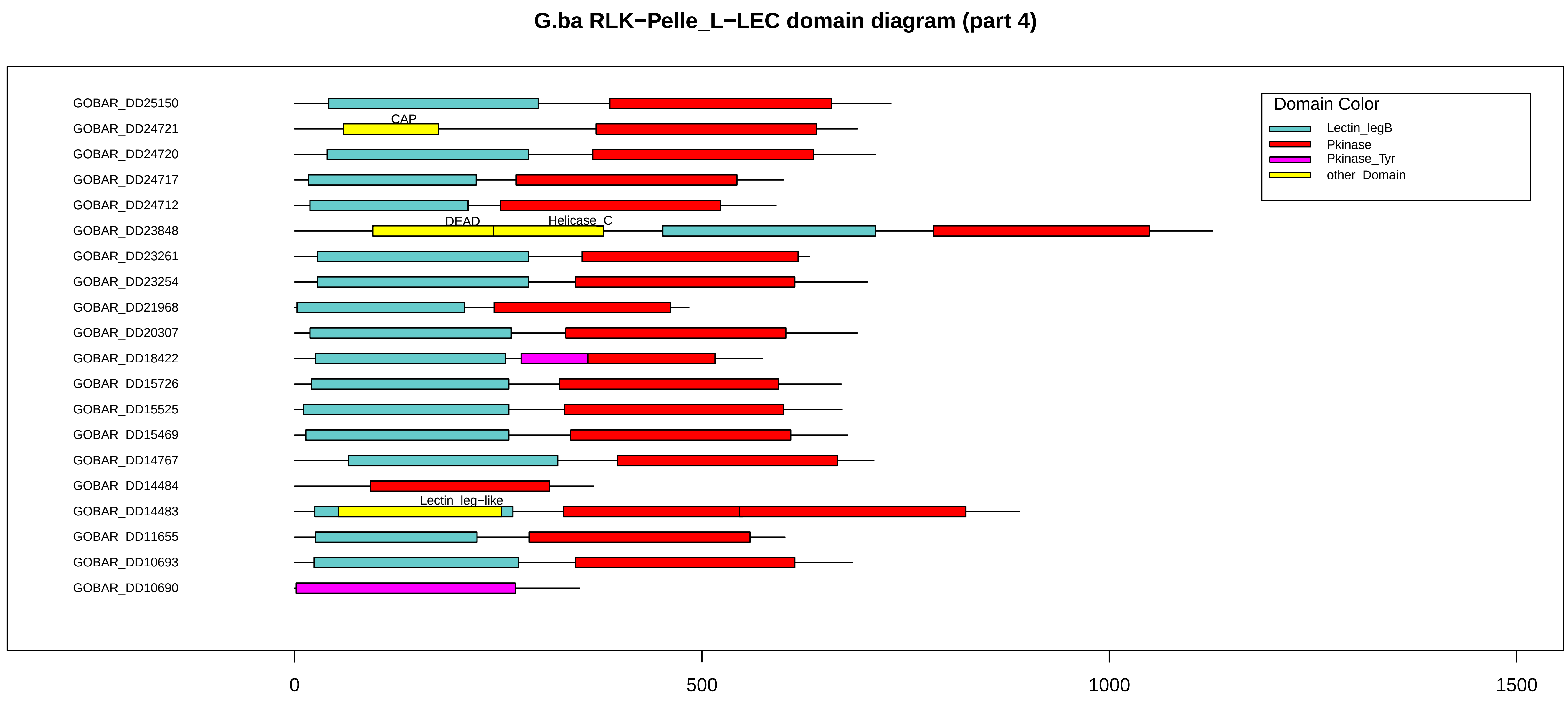

G.ba RLK−Pelle_L−LEC domain diagram (part 4)
GOBAR_DD25150
GOBAR_DD24721
GOBAR_DD24720
GOBAR_DD24717
GOBAR_DD24712
GOBAR_DD23848
GOBAR_DD23261
GOBAR_DD23254
GOBAR_DD21968
GOBAR_DD20307
GOBAR_DD18422
GOBAR_DD15726
GOBAR_DD15525
GOBAR_DD15469
GOBAR_DD14767
GOBAR_DD14484
GOBAR_DD14483
GOBAR_DD11655
GOBAR_DD10693
GOBAR_DD10690
CAP
DEAD
Helicase_C
Lectin_leg−like
Domain Color
Lectin_legB
Pkinase
Pkinase_Tyr
other Domain
0
500
1000
1500

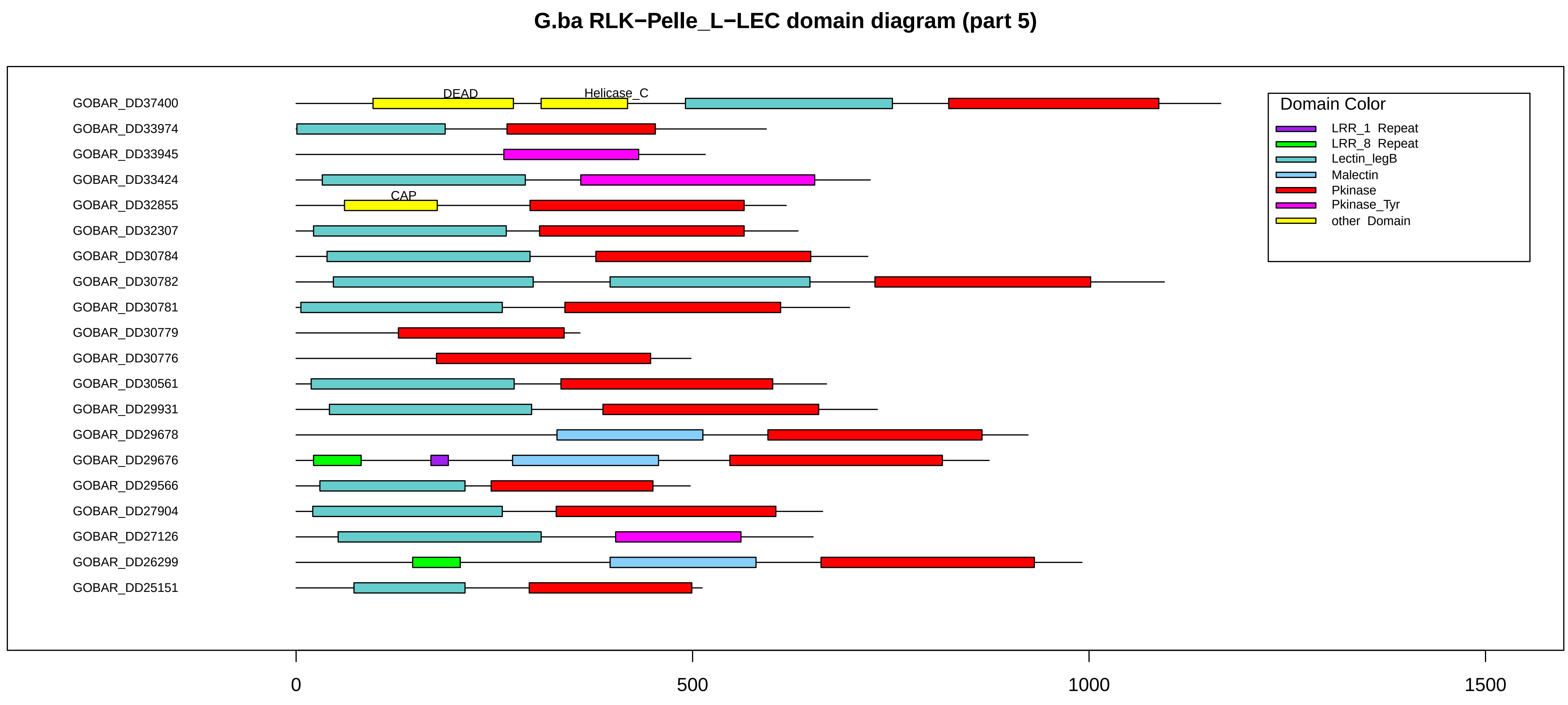

G.ba RLK−Pelle_L−LEC domain diagram (part 5)
GOBAR_DD37400
GOBAR_DD33974
GOBAR_DD33945
GOBAR_DD33424
GOBAR_DD32855
GOBAR_DD32307
GOBAR_DD30784
GOBAR_DD30782
GOBAR_DD30781
GOBAR_DD30779
GOBAR_DD30776
GOBAR_DD30561
GOBAR_DD29931
GOBAR_DD29678
GOBAR_DD29676
GOBAR_DD29566
GOBAR_DD27904
GOBAR_DD27126
GOBAR_DD26299
GOBAR_DD25151
DEAD
Helicase_C
CAP
Domain Color
LRR_1 Repeat
LRR_8 Repeat
Lectin_legB
Malectin
Pkinase
Pkinase_Tyr
other Domain
0
500
1000
1500

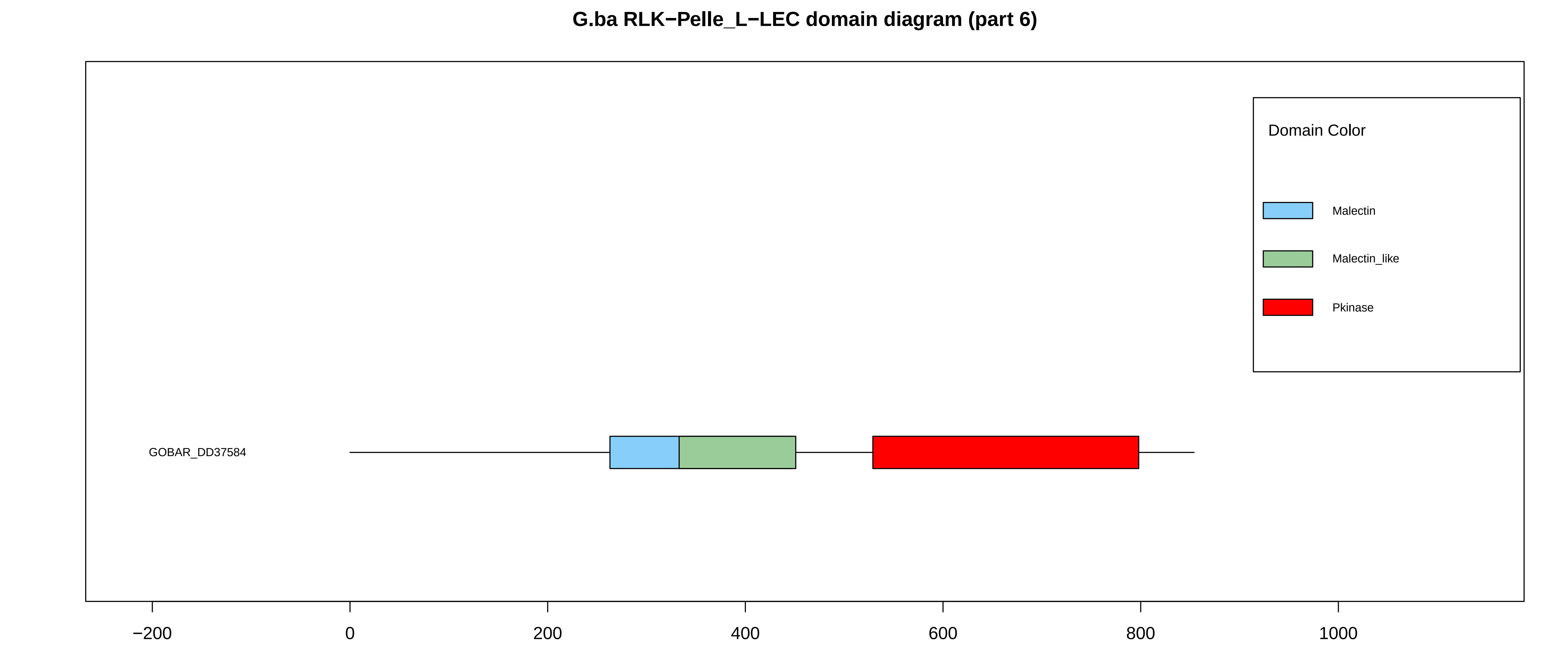
G.ba RLK−Pelle_L−LEC domain diagram (part 6)
Domain Color
Malectin
Malectin_like
Pkinase
GOBAR_DD37584
−200
0
200
400
600
800
1000

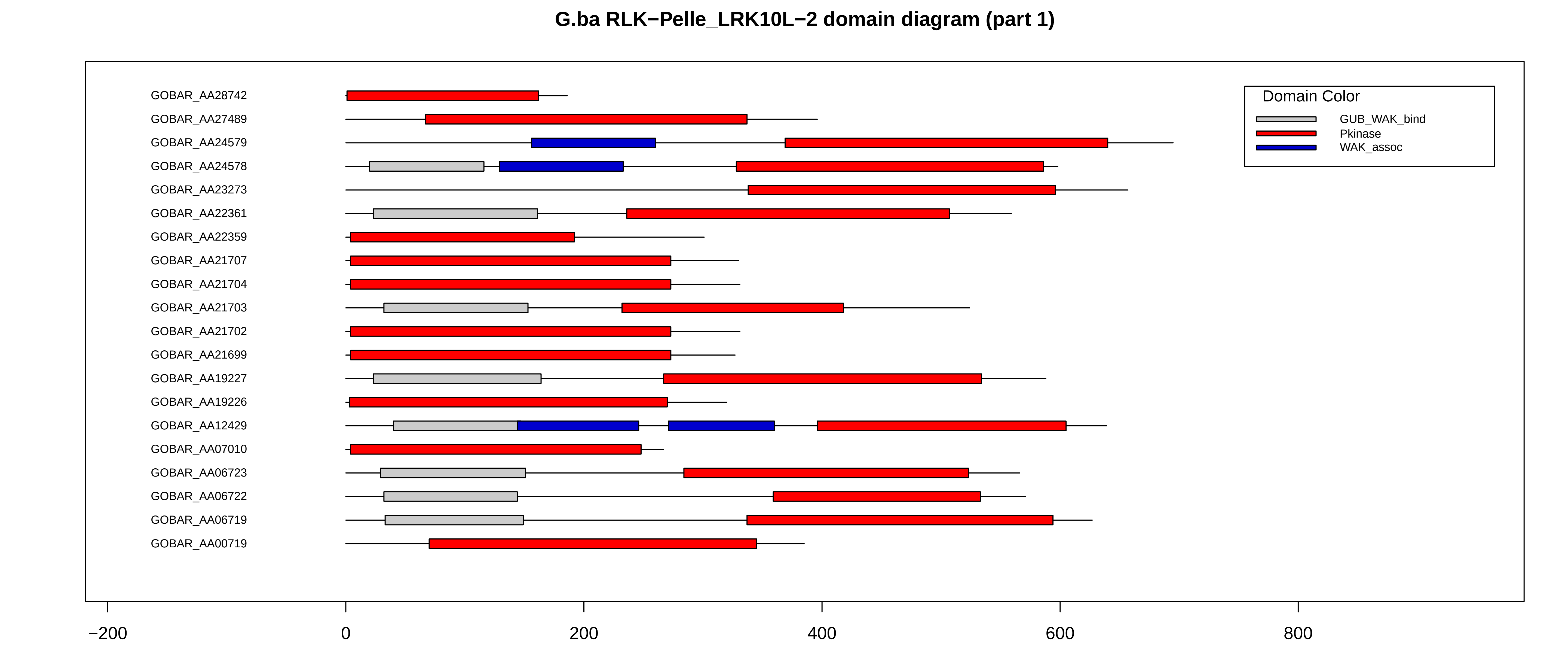
G.ba RLK−Pelle_LRK10L−2 domain diagram (part 1)
GOBAR_AA28742
GOBAR_AA27489
GOBAR_AA24579
GOBAR_AA24578
GOBAR_AA23273
GOBAR_AA22361
GOBAR_AA22359
GOBAR_AA21707
GOBAR_AA21704
GOBAR_AA21703
GOBAR_AA21702
GOBAR_AA21699
GOBAR_AA19227
GOBAR_AA19226
GOBAR_AA12429
GOBAR_AA07010
GOBAR_AA06723
GOBAR_AA06722
GOBAR_AA06719
GOBAR_AA00719
Domain Color
GUB_WAK_bind
Pkinase
WAK_assoc
−200
0
200
400
600
800

G.ba RLK−Pelle_LRK10L−2 domain diagram (part 2)

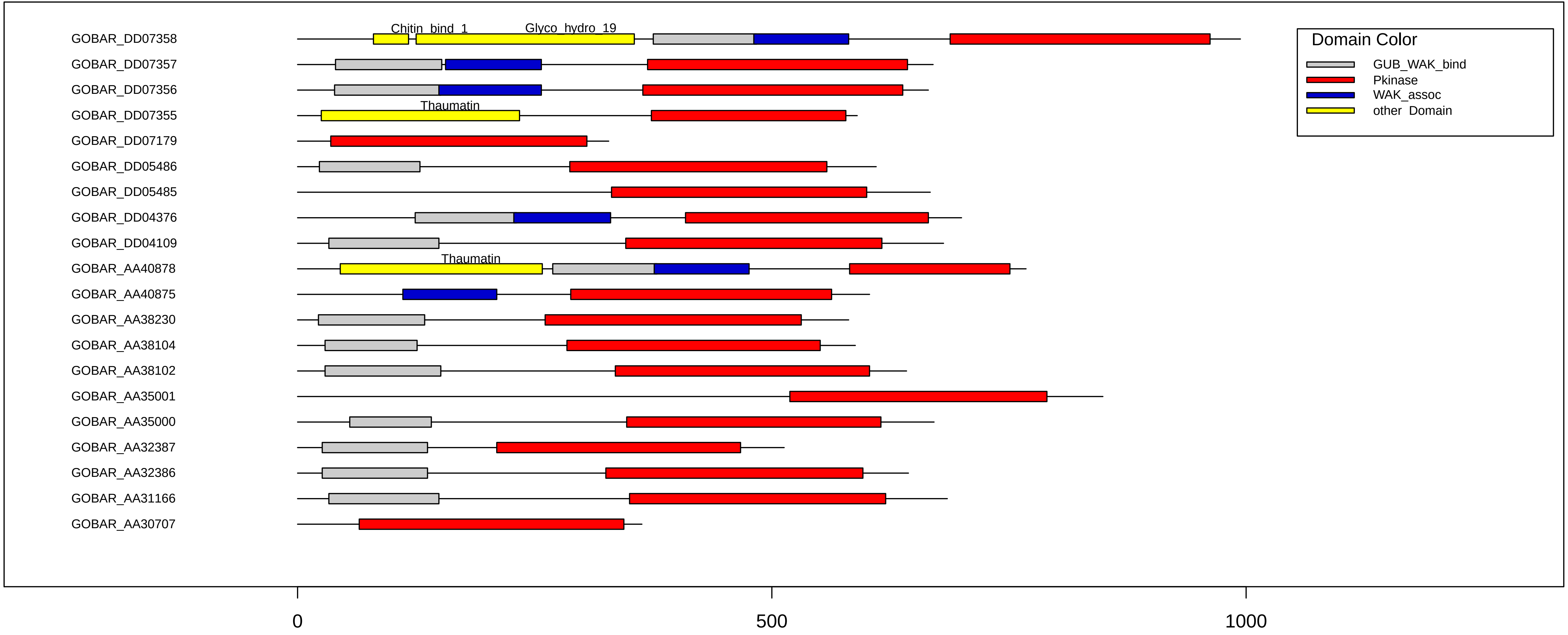

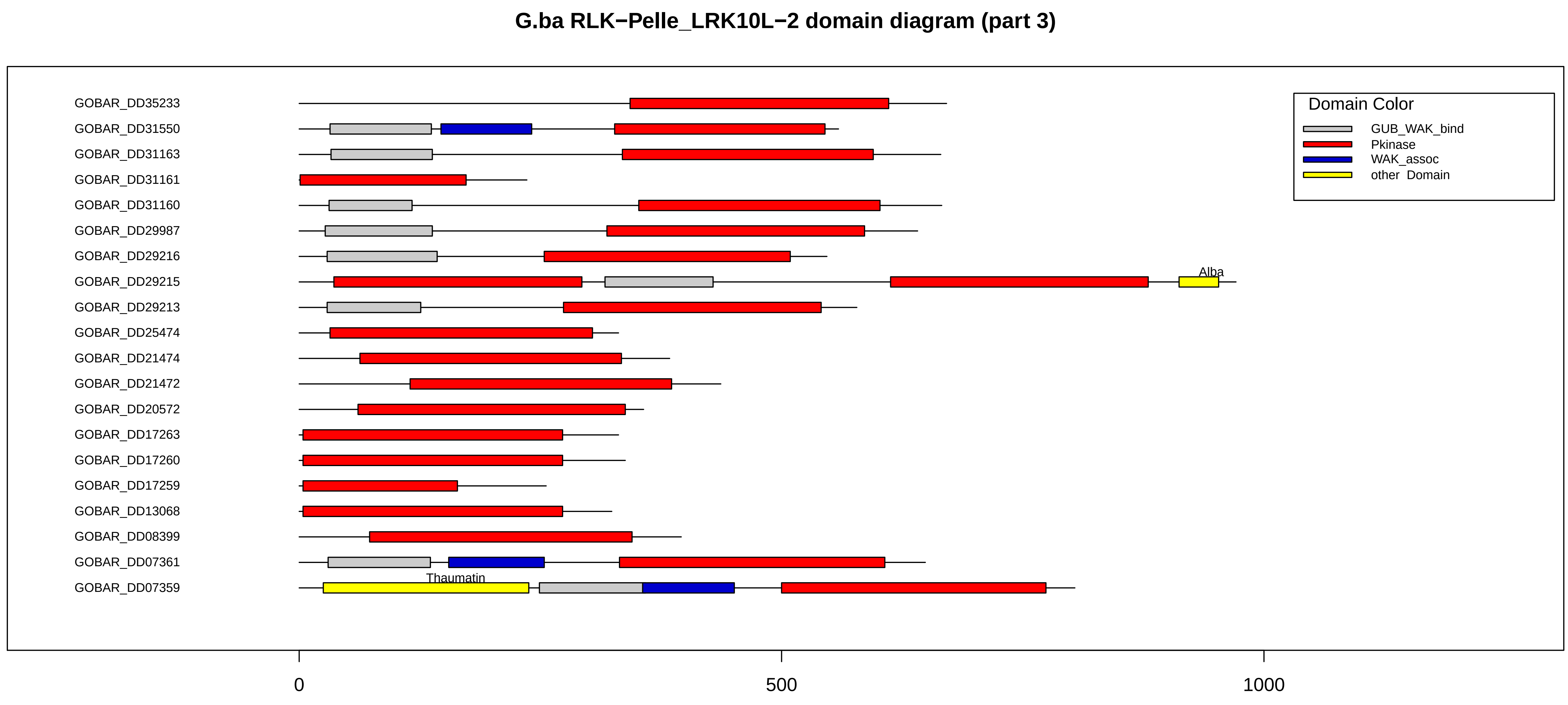
G.ba RLK−Pelle_LRK10L−2 domain diagram (part 3)
GOBAR_DD35233
GOBAR_DD31550
GOBAR_DD31163
GOBAR_DD31161
GOBAR_DD31160
GOBAR_DD29987
GOBAR_DD29216
GOBAR_DD29215
GOBAR_DD29213
GOBAR_DD25474
GOBAR_DD21474
GOBAR_DD21472
GOBAR_DD20572
GOBAR_DD17263
GOBAR_DD17260
GOBAR_DD17259
GOBAR_DD13068
GOBAR_DD08399
GOBAR_DD07361
GOBAR_DD07359
Alba
Thaumatin
Domain Color
GUB_WAK_bind
Pkinase
WAK_assoc
other Domain
0
500
1000

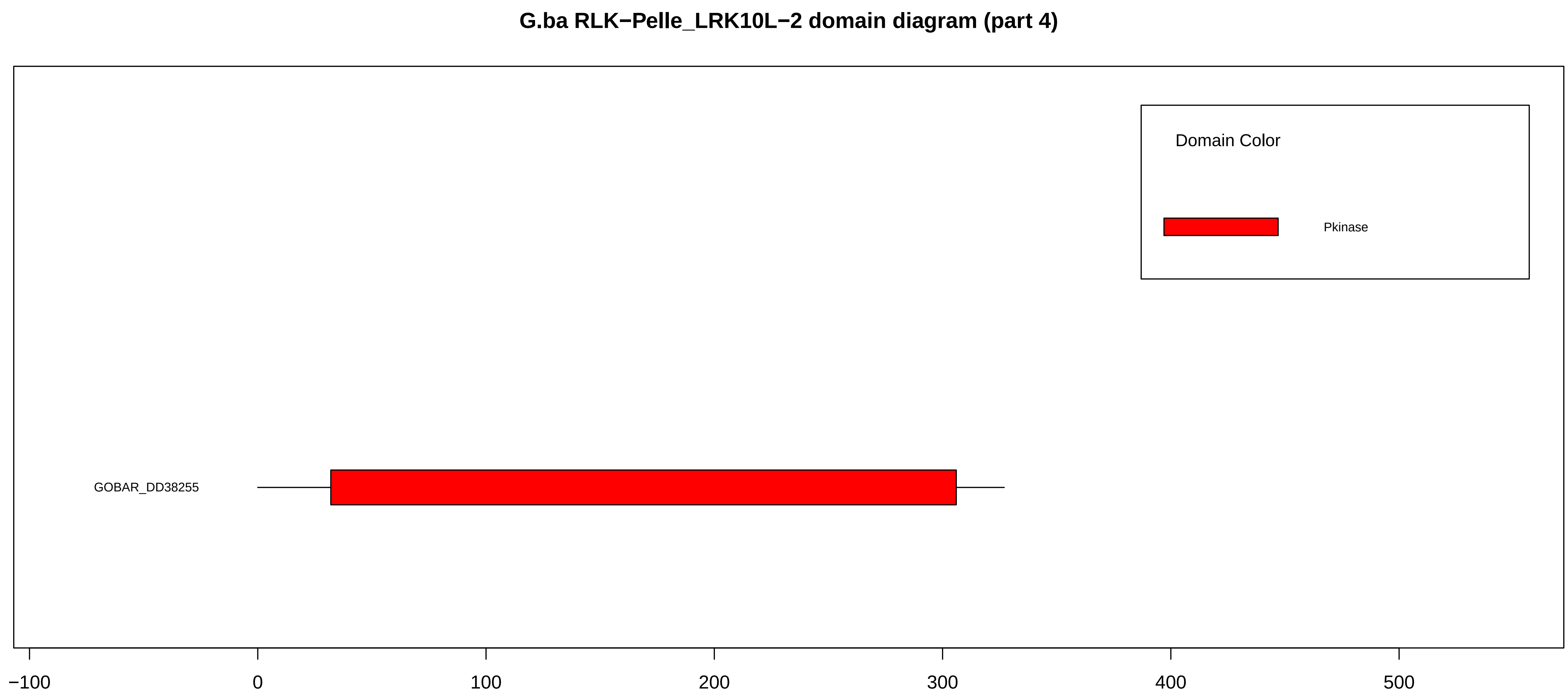
G.ba RLK−Pelle_LRK10L−2 domain diagram (part 4)
Domain Color
Pkinase
GOBAR_DD38255
−100
0
100
200
300
400
500

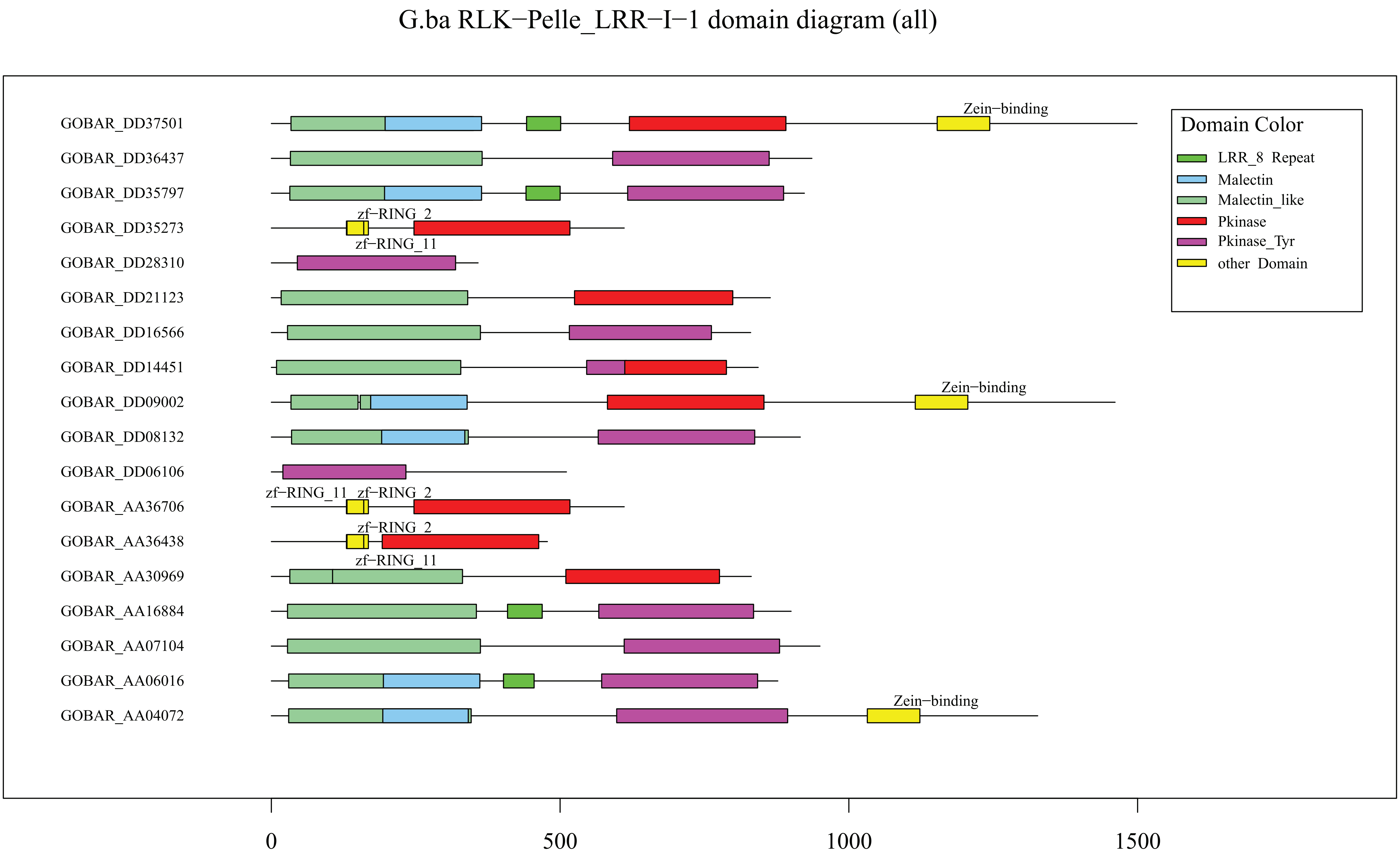
G.ba RLK−Pelle_LRR−I−1 domain diagram (all)
GOBAR_DD37501
GOBAR_DD36437
GOBAR_DD35797
GOBAR_DD35273
GOBAR_DD28310
GOBAR_DD21123
GOBAR_DD16566
GOBAR_DD14451
GOBAR_DD09002
GOBAR_DD08132
GOBAR_DD06106
GOBAR_AA36706
GOBAR_AA36438
GOBAR_AA30969
GOBAR_AA16884
GOBAR_AA07104
GOBAR_AA06016
GOBAR_AA04072
Zein−binding
zf−RING_2
zf−RING_11
Domain Color
LRR_8 Repeat
Malectin
Malectin_like
Pkinase
Pkinase_Tyr
other Domain
0
500
1000
1500

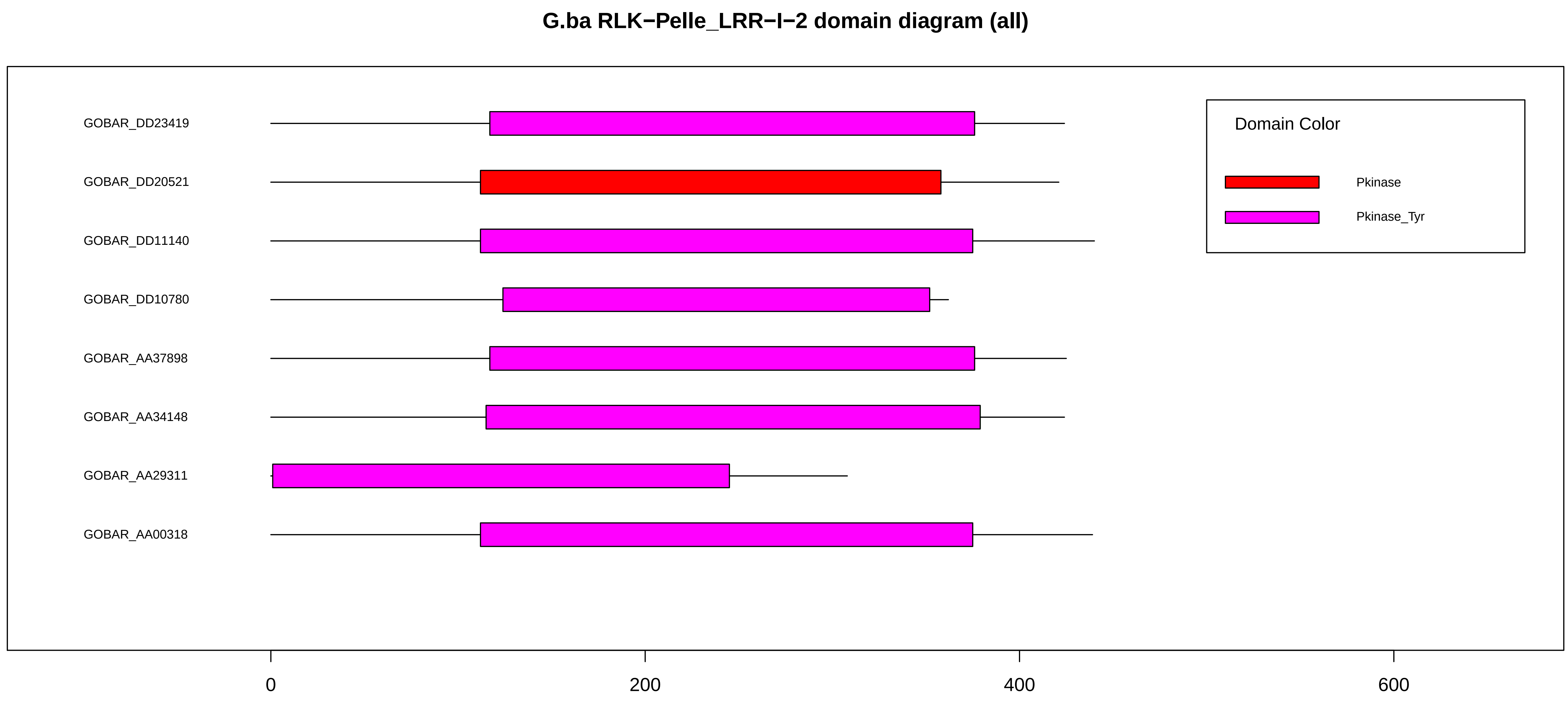

G.ba RLK−Pelle_LRR−I−2 domain diagram (all)
GOBAR_DD23419
GOBAR_DD20521
GOBAR_DD11140
GOBAR_DD10780
GOBAR_AA37898
GOBAR_AA34148
GOBAR_AA29311
GOBAR_AA00318
Domain Color
Pkinase
Pkinase_Tyr
0
200
400
600

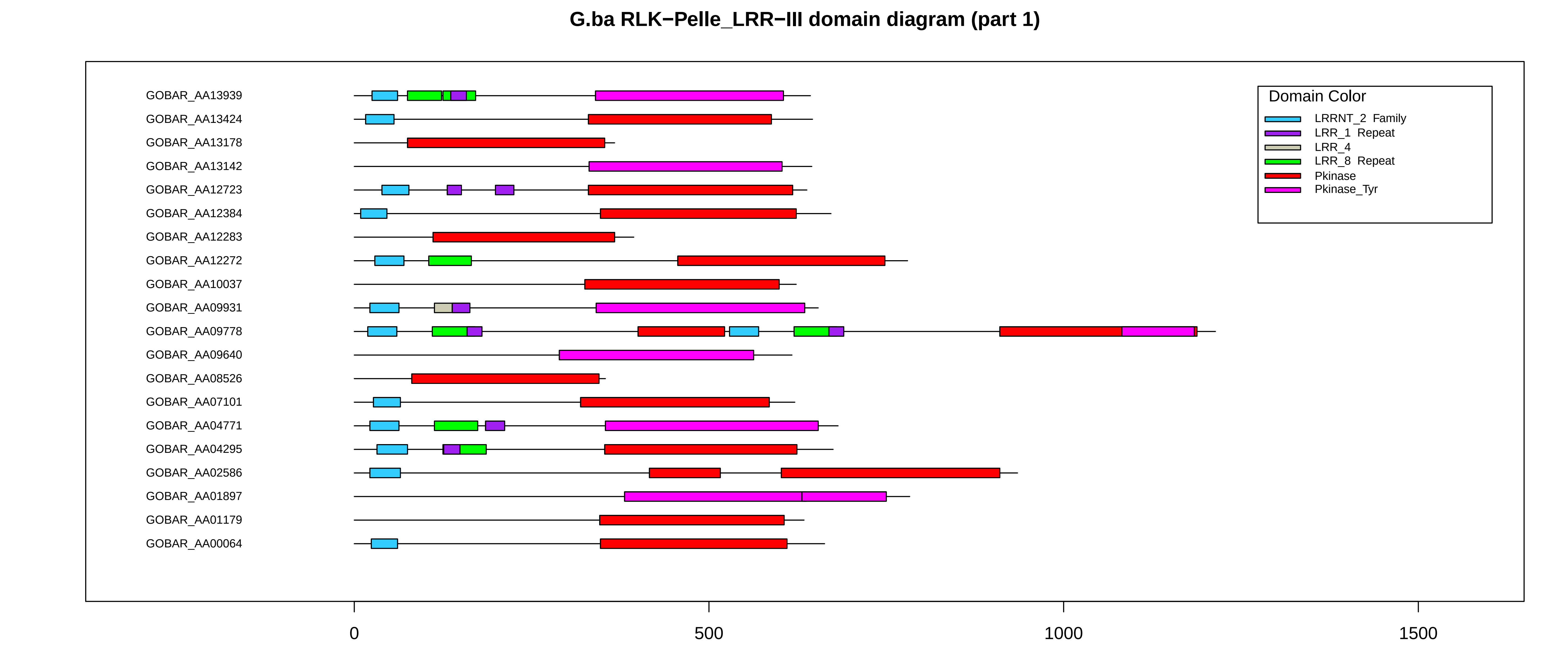
G.ba RLK−Pelle_LRR−III domain diagram (part 1)
GOBAR_AA13939
GOBAR_AA13424
GOBAR_AA13178
GOBAR_AA13142
GOBAR_AA12723
GOBAR_AA12384
GOBAR_AA12283
GOBAR_AA12272
GOBAR_AA10037
GOBAR_AA09931
GOBAR_AA09778
GOBAR_AA09640
GOBAR_AA08526
GOBAR_AA07101
GOBAR_AA04771
GOBAR_AA04295
GOBAR_AA02586
GOBAR_AA01897
GOBAR_AA01179
GOBAR_AA00064
Domain Color
LRRNT_2 Family
LRR_1 Repeat
LRR_4
LRR_8 Repeat
Pkinase
Pkinase_Tyr
0
500
1000
1500

G.ba RLK−Pelle_LRR−III domain diagram (part 2)

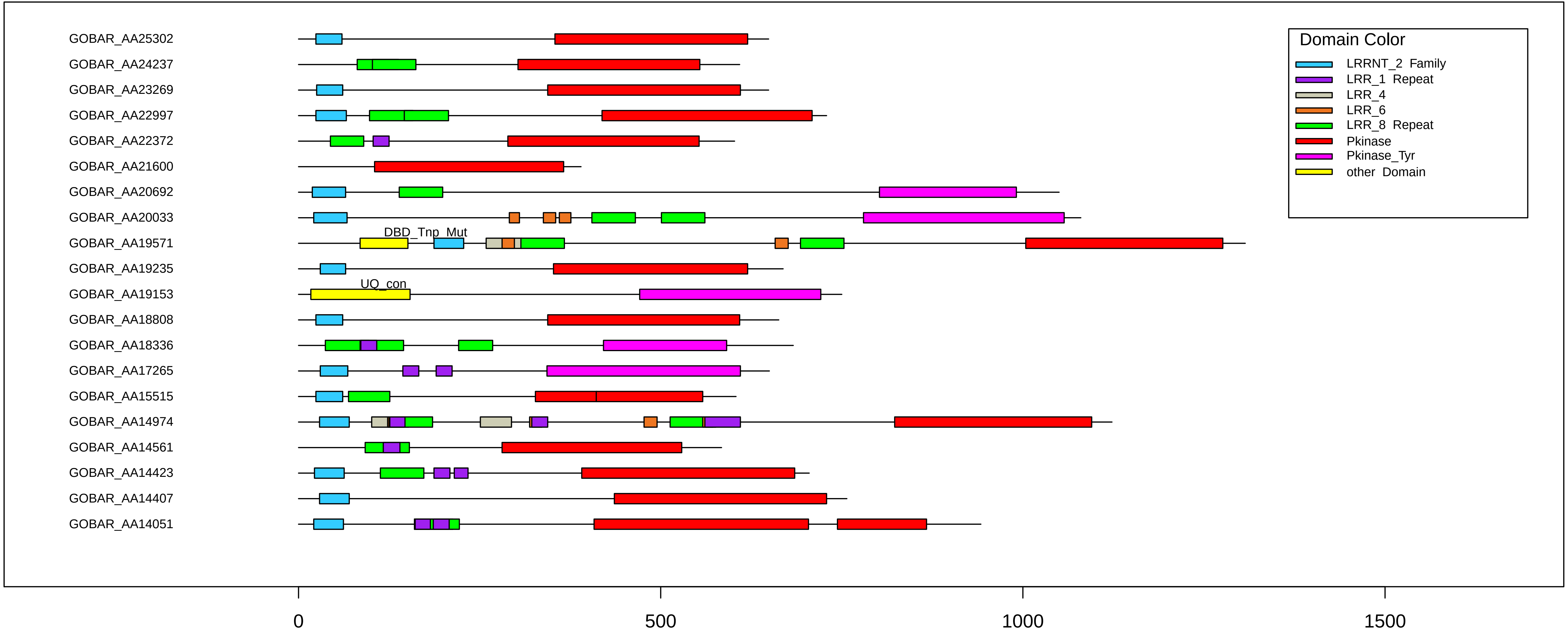

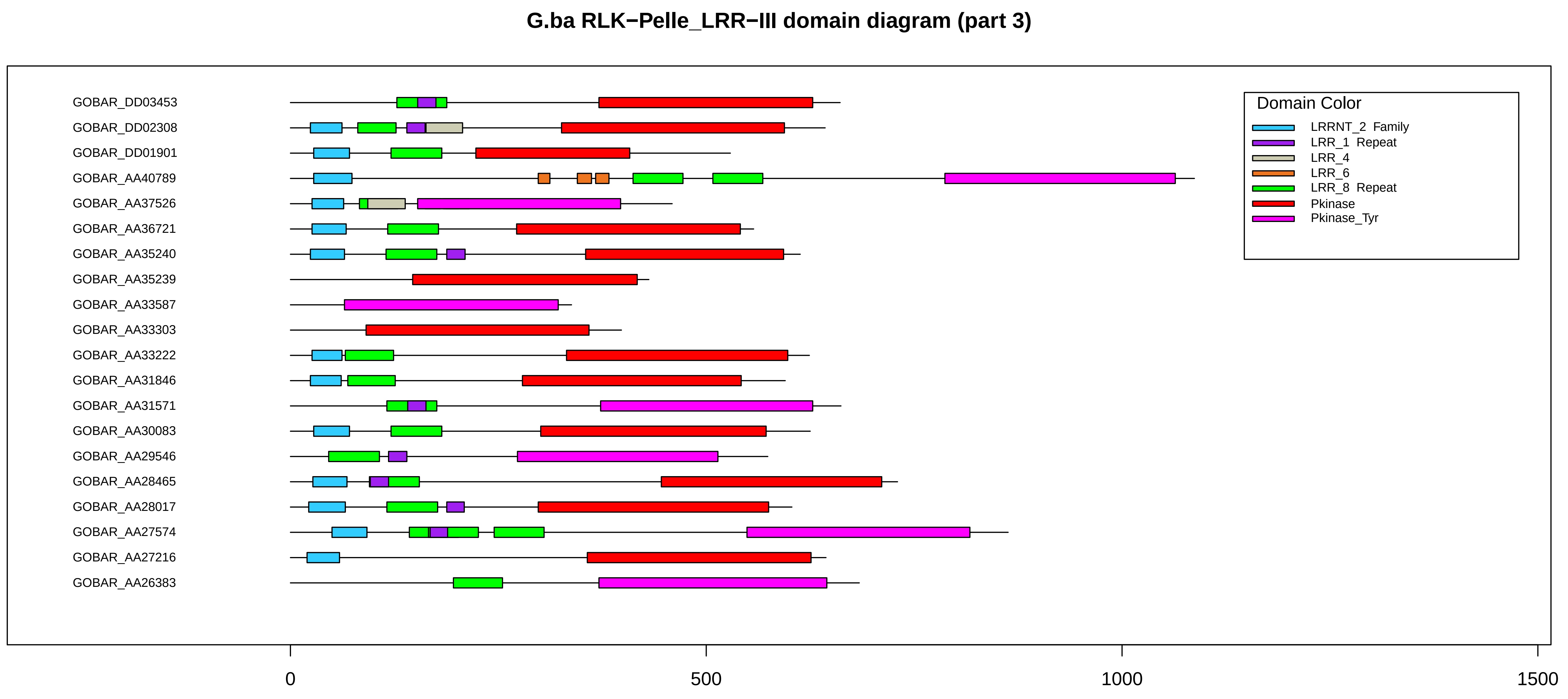
G.ba RLK−Pelle_LRR−III domain diagram (part 3)
GOBAR_DD03453
GOBAR_DD02308
GOBAR_DD01901
GOBAR_AA40789
GOBAR_AA37526
GOBAR_AA36721
GOBAR_AA35240
GOBAR_AA35239
GOBAR_AA33587
GOBAR_AA33303
GOBAR_AA33222
GOBAR_AA31846
GOBAR_AA31571
GOBAR_AA30083
GOBAR_AA29546
GOBAR_AA28465
GOBAR_AA28017
GOBAR_AA27574
GOBAR_AA27216
GOBAR_AA26383
Domain Color
LRRNT_2 Family
LRR_1 Repeat
LRR_4
LRR_6
LRR_8 Repeat
Pkinase
Pkinase_Tyr
0
500
1000
1500

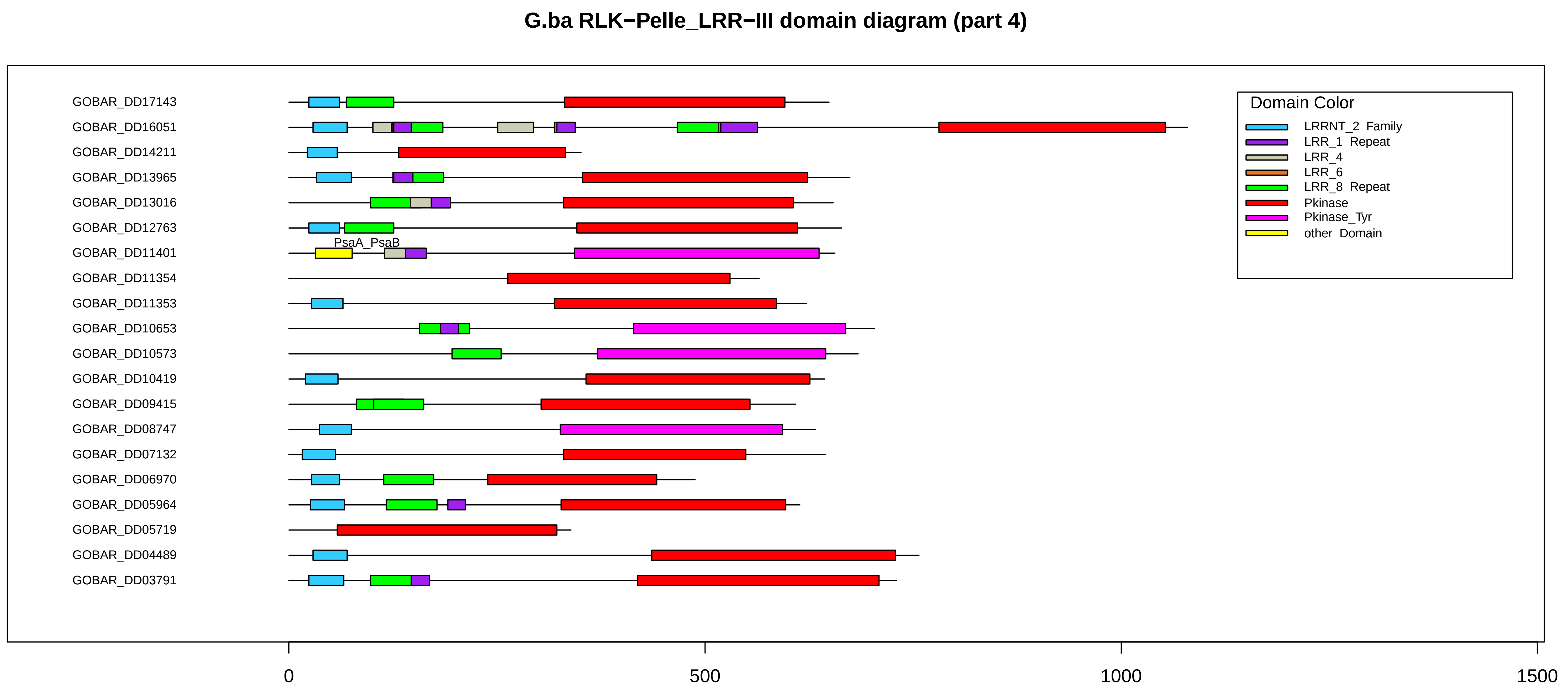
G.ba RLK−Pelle_LRR−III domain diagram (part 4)
GOBAR_DD17143
GOBAR_DD16051
GOBAR_DD14211
GOBAR_DD13965
GOBAR_DD13016
GOBAR_DD12763
GOBAR_DD11401
GOBAR_DD11354
GOBAR_DD11353
GOBAR_DD10653
GOBAR_DD10573
GOBAR_DD10419
GOBAR_DD09415
GOBAR_DD08747
GOBAR_DD07132
GOBAR_DD06970
GOBAR_DD05964
GOBAR_DD05719
GOBAR_DD04489
GOBAR_DD03791
PsaA_PsaB
Domain Color
LRRNT_2 Family
LRR_1 Repeat
LRR_4
LRR_6
LRR_8 Repeat
Pkinase
Pkinase_Tyr
other Domain
0
500
1000
1500

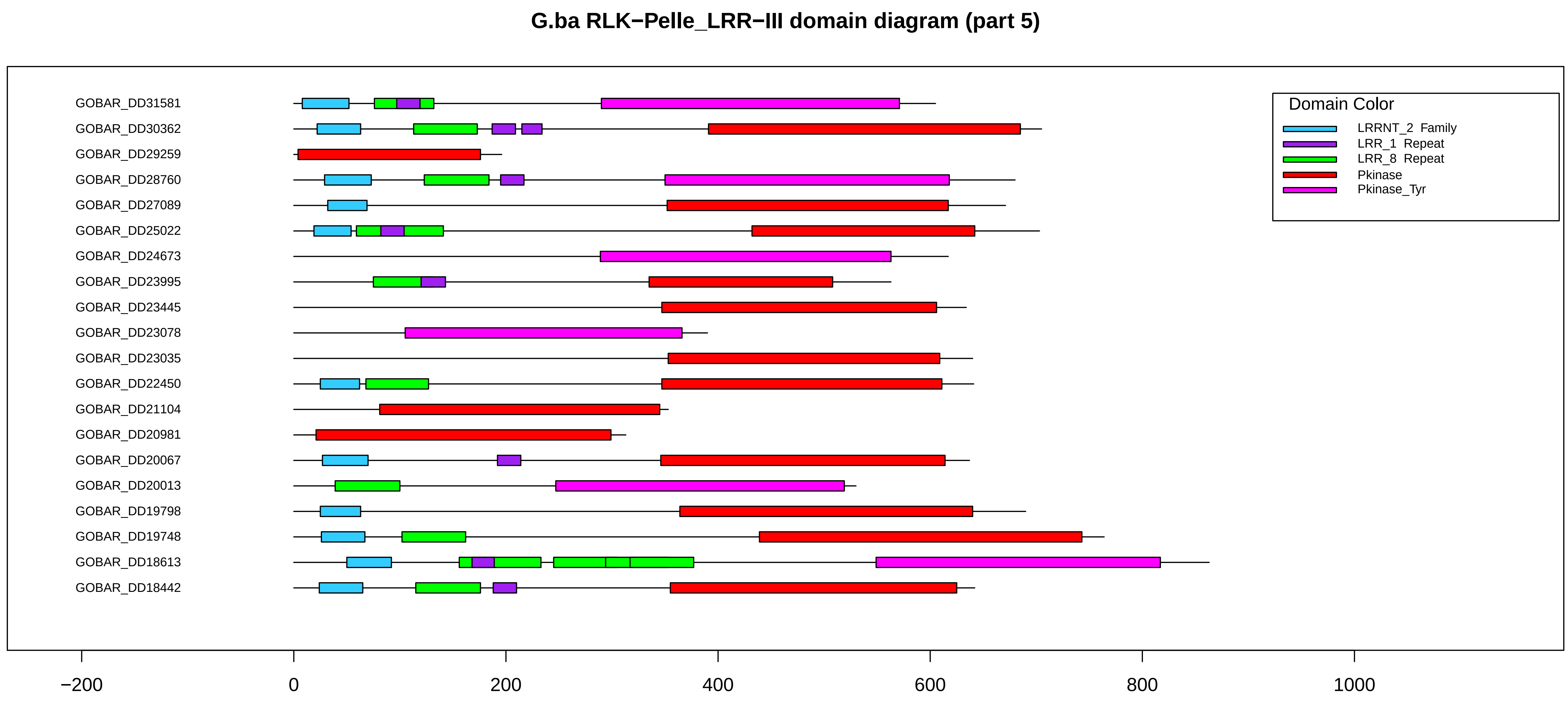

G.ba RLK−Pelle_LRR−III domain diagram (part 5)
GOBAR_DD31581
GOBAR_DD30362
GOBAR_DD29259
GOBAR_DD28760
GOBAR_DD27089
GOBAR_DD25022
GOBAR_DD24673
GOBAR_DD23995
GOBAR_DD23445
GOBAR_DD23078
GOBAR_DD23035
GOBAR_DD22450
GOBAR_DD21104
GOBAR_DD20981
GOBAR_DD20067
GOBAR_DD20013
GOBAR_DD19798
GOBAR_DD19748
GOBAR_DD18613
GOBAR_DD18442
Domain Color
LRRNT_2 Family
LRR_1 Repeat
LRR_8 Repeat
Pkinase
Pkinase_Tyr
−200
0
200
400
600
800
1000

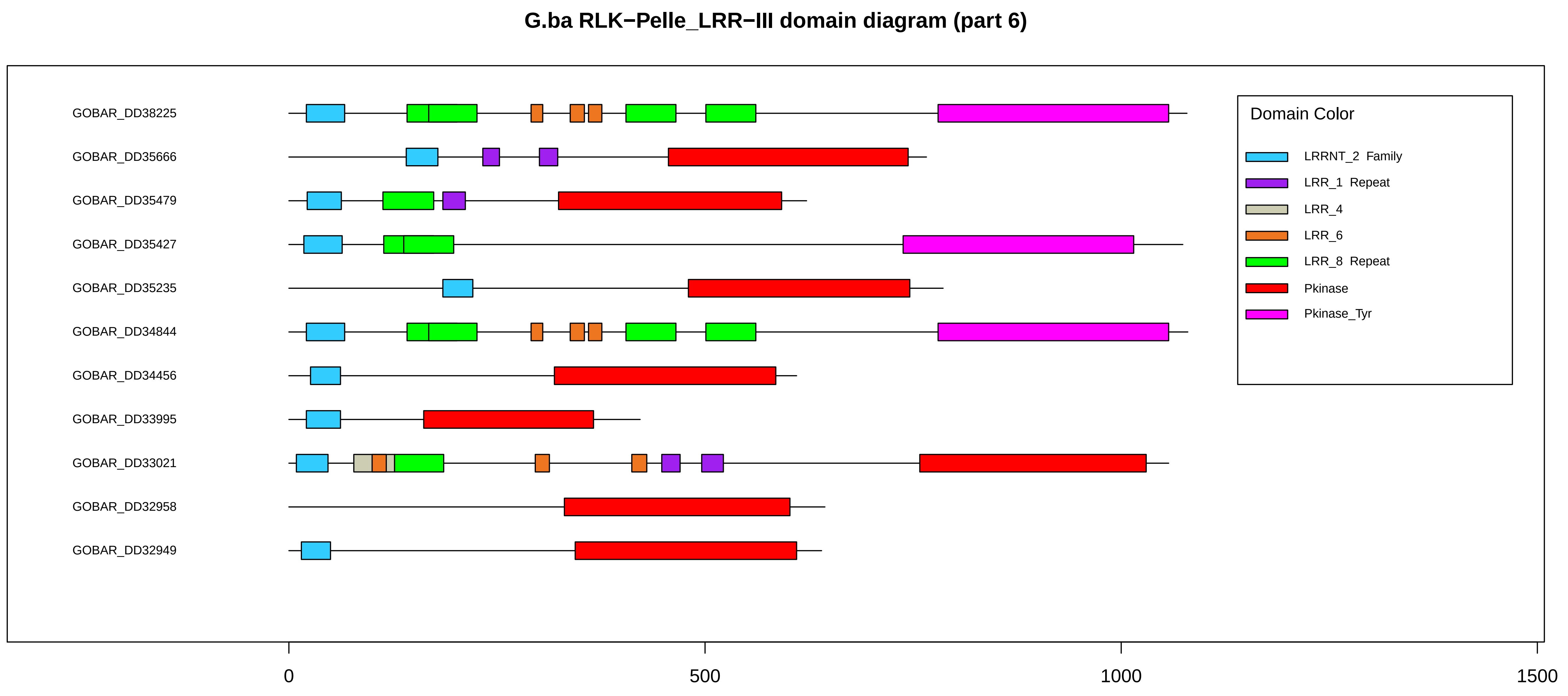
G.ba RLK−Pelle_LRR−III domain diagram (part 6)
GOBAR_DD38225
GOBAR_DD35666
GOBAR_DD35479
GOBAR_DD35427
GOBAR_DD35235
GOBAR_DD34844
GOBAR_DD34456
GOBAR_DD33995
GOBAR_DD33021
GOBAR_DD32958
GOBAR_DD32949
Domain Color
LRRNT_2 Family
LRR_1 Repeat
LRR_4
LRR_6
LRR_8 Repeat
Pkinase
Pkinase_Tyr
0
500
1000
1500

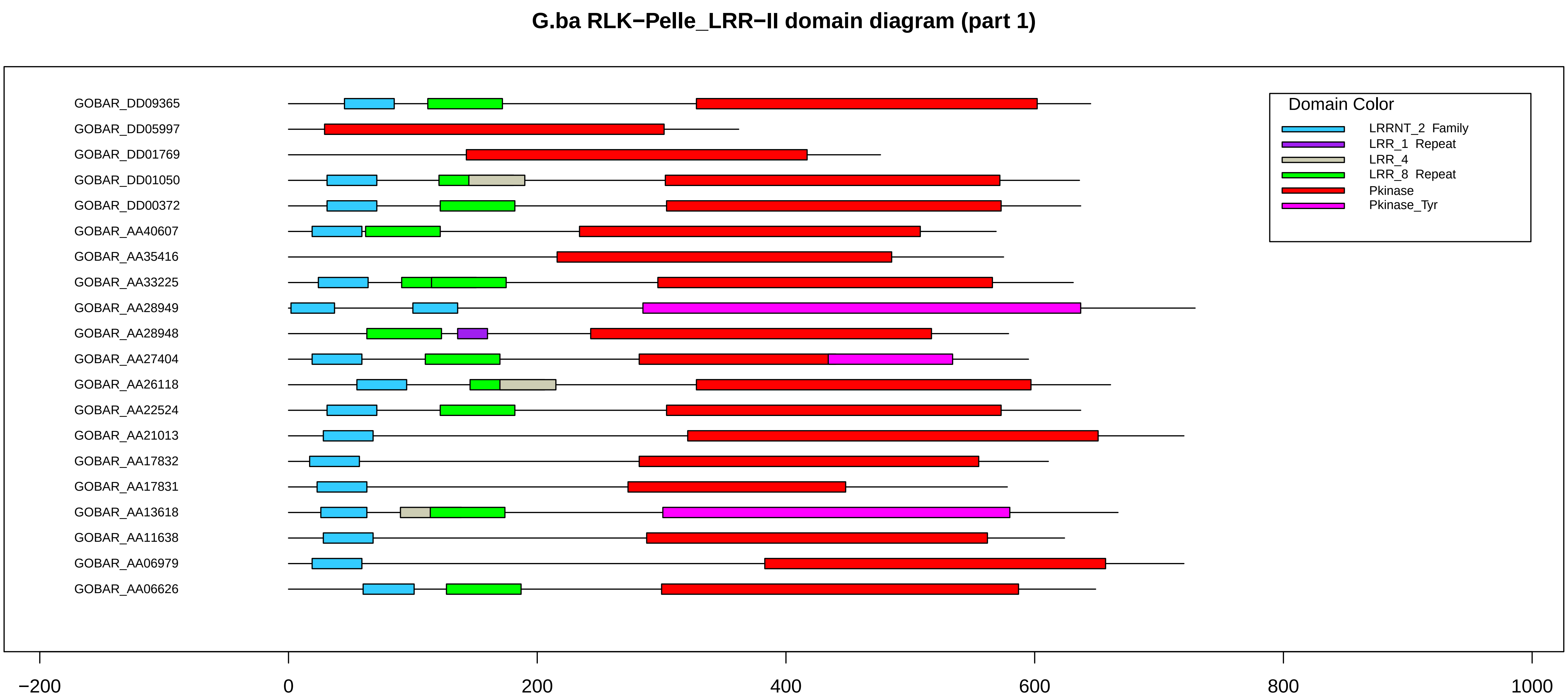
G.ba RLK−Pelle_LRR−II domain diagram (part 1)
GOBAR_DD09365
GOBAR_DD05997
GOBAR_DD01769
GOBAR_DD01050
GOBAR_DD00372
GOBAR_AA40607
GOBAR_AA35416
GOBAR_AA33225
GOBAR_AA28949
GOBAR_AA28948
GOBAR_AA27404
GOBAR_AA26118
GOBAR_AA22524
GOBAR_AA21013
GOBAR_AA17832
GOBAR_AA17831
GOBAR_AA13618
GOBAR_AA11638
GOBAR_AA06979
GOBAR_AA06626
Domain Color
LRRNT_2 Family
LRR_1 Repeat
LRR_4
LRR_8 Repeat
Pkinase
Pkinase_Tyr
−200
0
200
400
600
800
1000

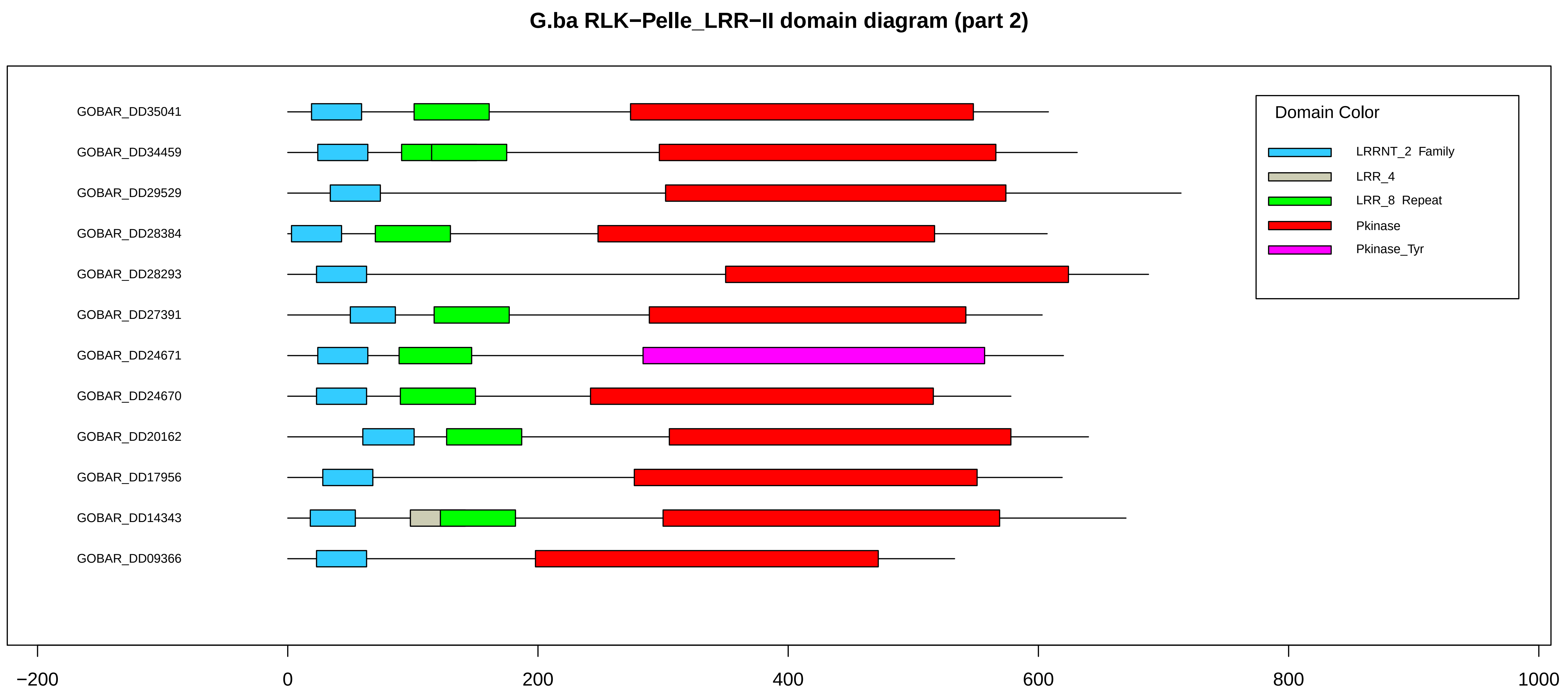
G.ba RLK−Pelle_LRR−II domain diagram (part 2)
GOBAR_DD35041
GOBAR_DD34459
GOBAR_DD29529
GOBAR_DD28384
GOBAR_DD28293
GOBAR_DD27391
GOBAR_DD24671
GOBAR_DD24670
GOBAR_DD20162
GOBAR_DD17956
GOBAR_DD14343
GOBAR_DD09366
Domain Color
LRRNT_2 Family
LRR_4
LRR_8 Repeat
Pkinase
Pkinase_Tyr
−200
0
200
400
600
800
1000

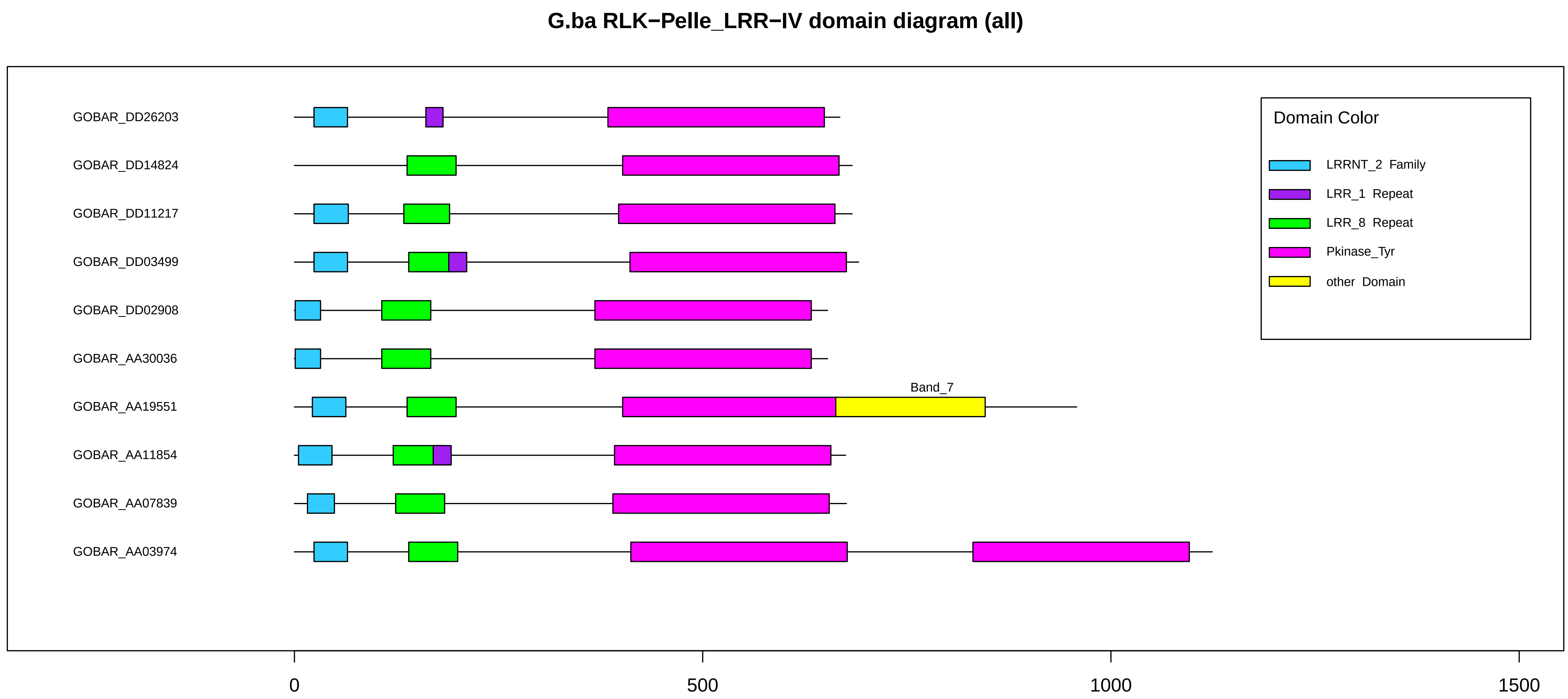
G.ba RLK−Pelle_LRR−IV domain diagram (all)
GOBAR_DD26203
GOBAR_DD14824
GOBAR_DD11217
GOBAR_DD03499
GOBAR_DD02908
GOBAR_AA30036
GOBAR_AA19551
GOBAR_AA11854
GOBAR_AA07839
GOBAR_AA03974
Band_7
Domain Color
LRRNT_2 Family
LRR_1 Repeat
LRR_8 Repeat
Pkinase_Tyr
other Domain
0
500
1000
1500

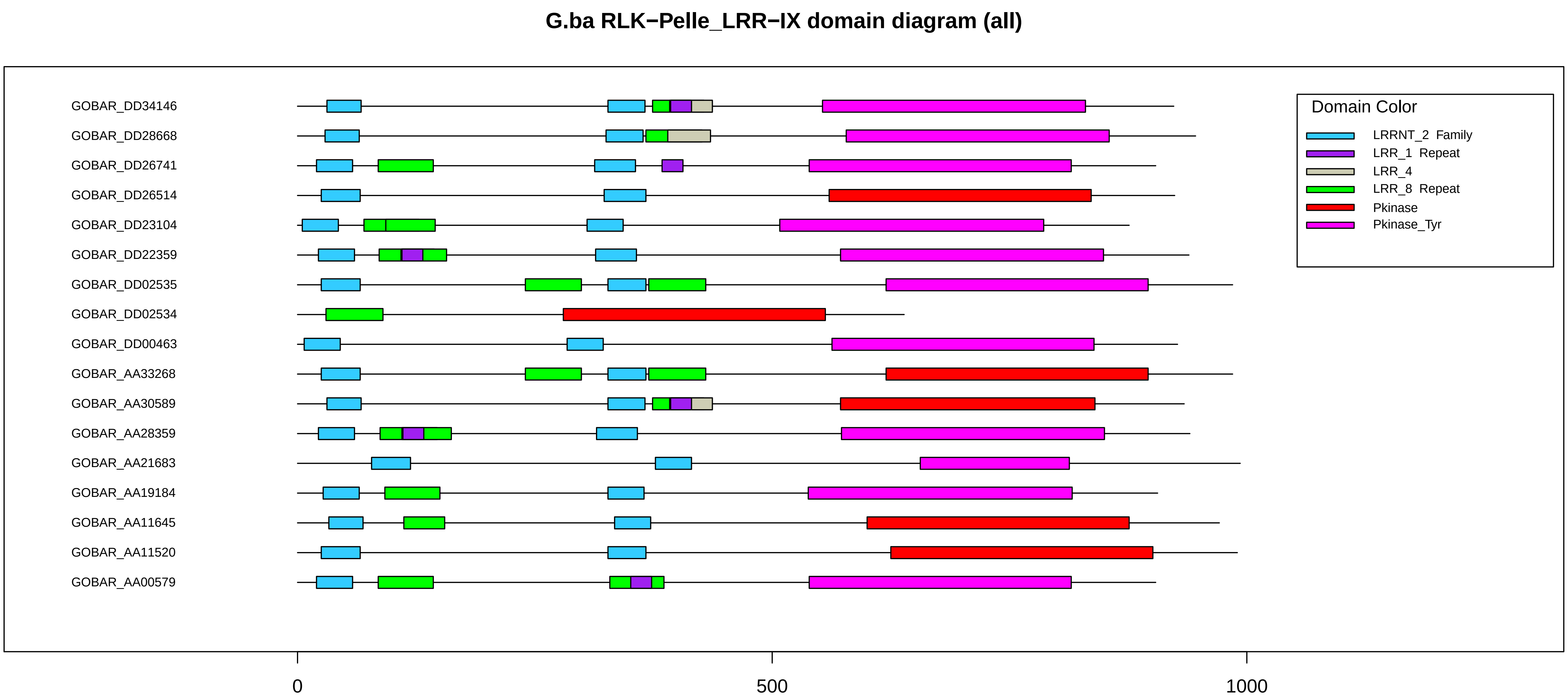
G.ba RLK−Pelle_LRR−IX domain diagram (all)
GOBAR_DD34146
GOBAR_DD28668
GOBAR_DD26741
GOBAR_DD26514
GOBAR_DD23104
GOBAR_DD22359
GOBAR_DD02535
GOBAR_DD02534
GOBAR_DD00463
GOBAR_AA33268
GOBAR_AA30589
GOBAR_AA28359
GOBAR_AA21683
GOBAR_AA19184
GOBAR_AA11645
GOBAR_AA11520
GOBAR_AA00579
Domain Color
LRRNT_2 Family
LRR_1 Repeat
LRR_4
LRR_8 Repeat
Pkinase
Pkinase_Tyr
0
500
1000

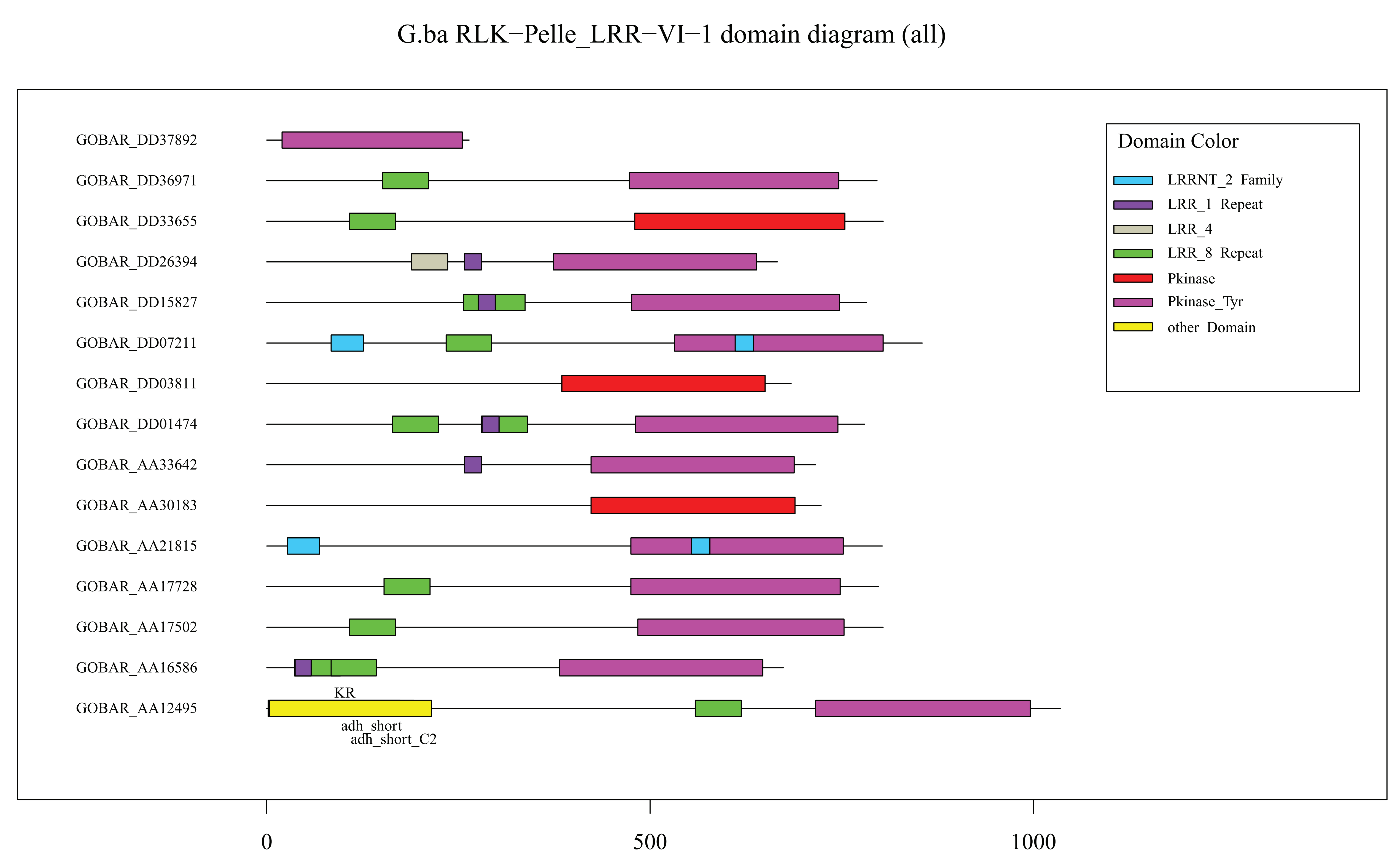

G.ba RLK−Pelle_LRR−VI−1 domain diagram (all)
GOBAR_DD37892
GOBAR_DD36971
GOBAR_DD33655
GOBAR_DD26394
GOBAR_DD15827
GOBAR_DD07211
GOBAR_DD03811
GOBAR_DD01474
GOBAR_AA33642
GOBAR_AA30183
GOBAR_AA21815
GOBAR_AA17728
GOBAR_AA17502
GOBAR_AA16586
GOBAR_AA12495
KR
adh_short
adh_short_C2
Domain Color
LRRNT_2 Family
LRR_1 Repeat
LRR_4
LRR_8 Repeat
Pkinase
Pkinase_Tyr
other Domain
0
500
1000

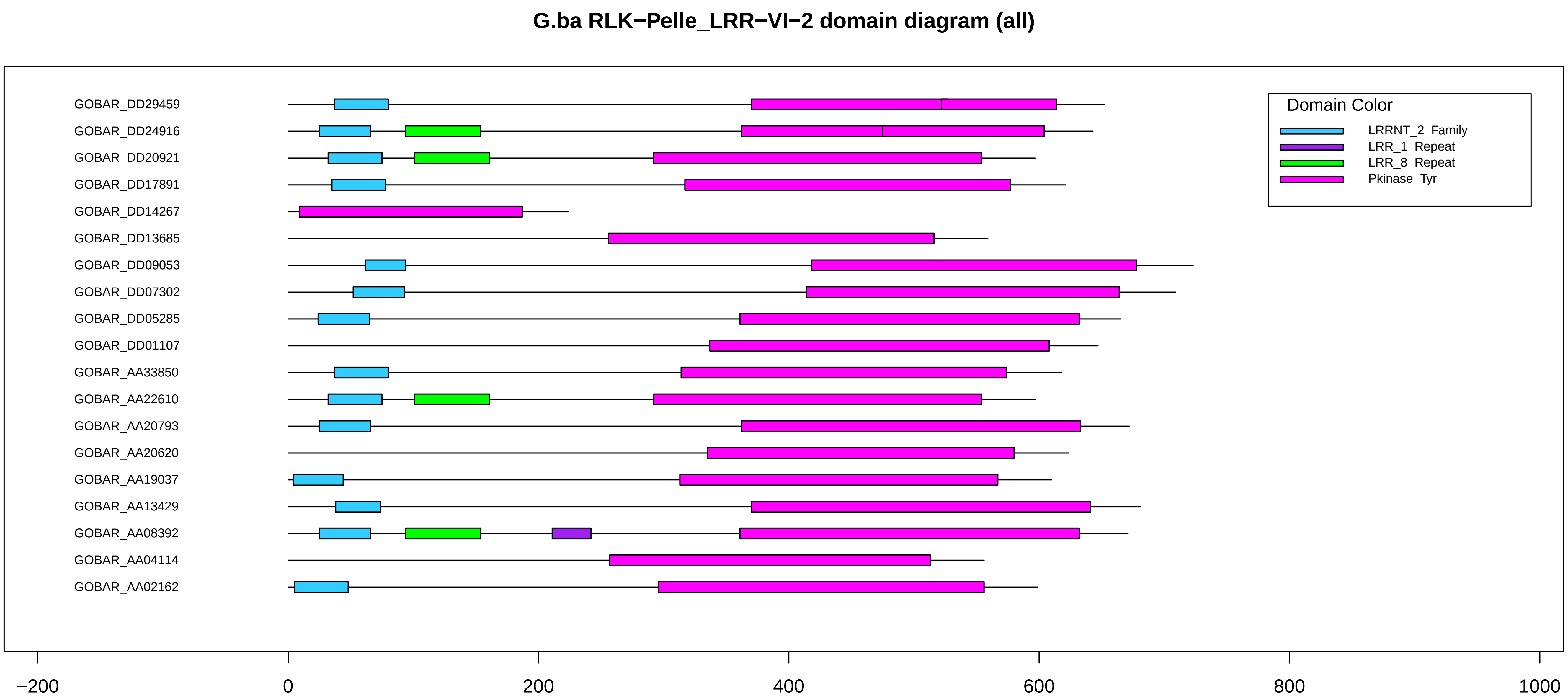

G.ba RLK−Pelle_LRR−VI−2 domain diagram (all)
GOBAR_DD29459
GOBAR_DD24916
GOBAR_DD20921
GOBAR_DD17891
GOBAR_DD14267
GOBAR_DD13685
GOBAR_DD09053
GOBAR_DD07302
GOBAR_DD05285
GOBAR_DD01107
GOBAR_AA33850
GOBAR_AA22610
GOBAR_AA20793
GOBAR_AA20620
GOBAR_AA19037
GOBAR_AA13429
GOBAR_AA08392
GOBAR_AA04114
GOBAR_AA02162
Domain Color
LRRNT_2 Family
LRR_1 Repeat
LRR_8 Repeat
Pkinase_Tyr
−200
0
200
400
600
800
1000

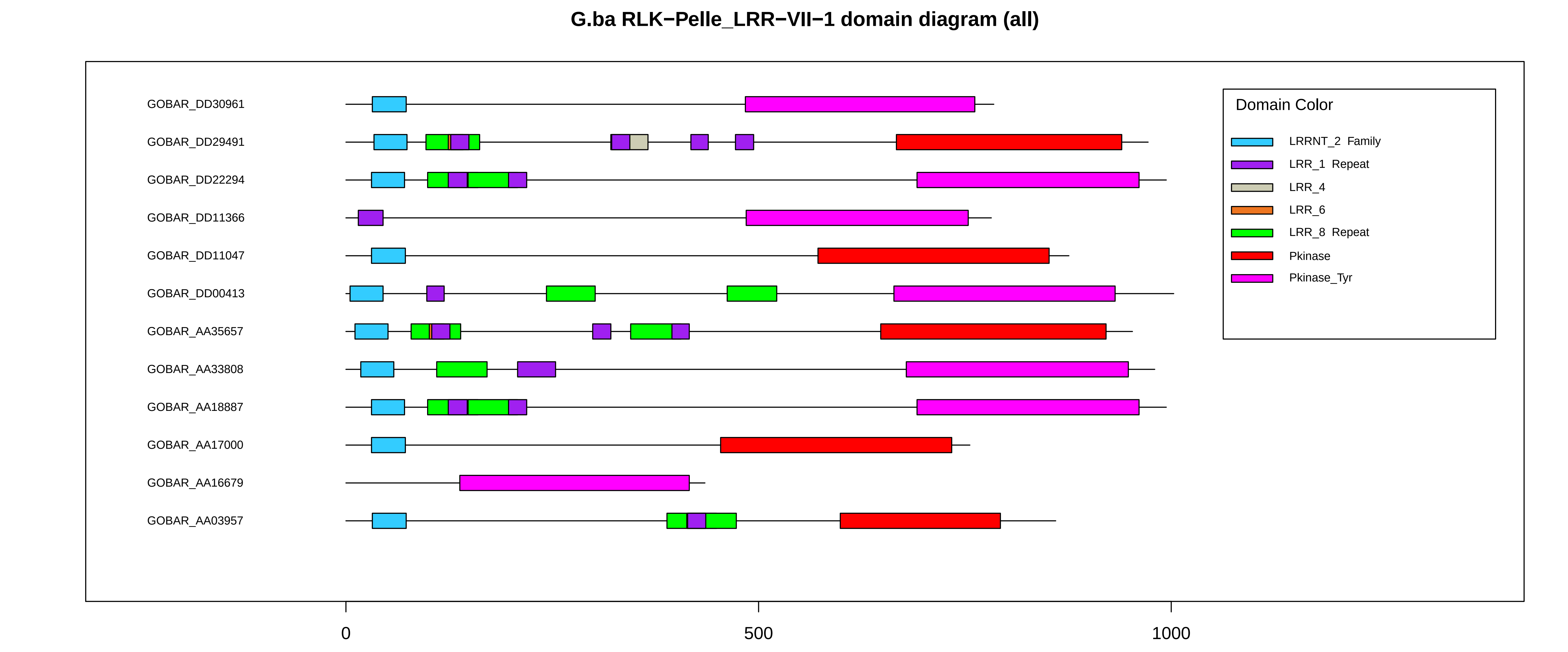

G.ba RLK−Pelle_LRR−VII−1 domain diagram (all)
GOBAR_DD30961
GOBAR_DD29491
GOBAR_DD22294
GOBAR_DD11366
GOBAR_DD11047
GOBAR_DD00413
GOBAR_AA35657
GOBAR_AA33808
GOBAR_AA18887
GOBAR_AA17000
GOBAR_AA16679
GOBAR_AA03957
0
500
1000
Domain Color
LRRNT_2 Family
LRR_1 Repeat
LRR_4
LRR_6
LRR_8 Repeat
Pkinase
Pkinase_Tyr

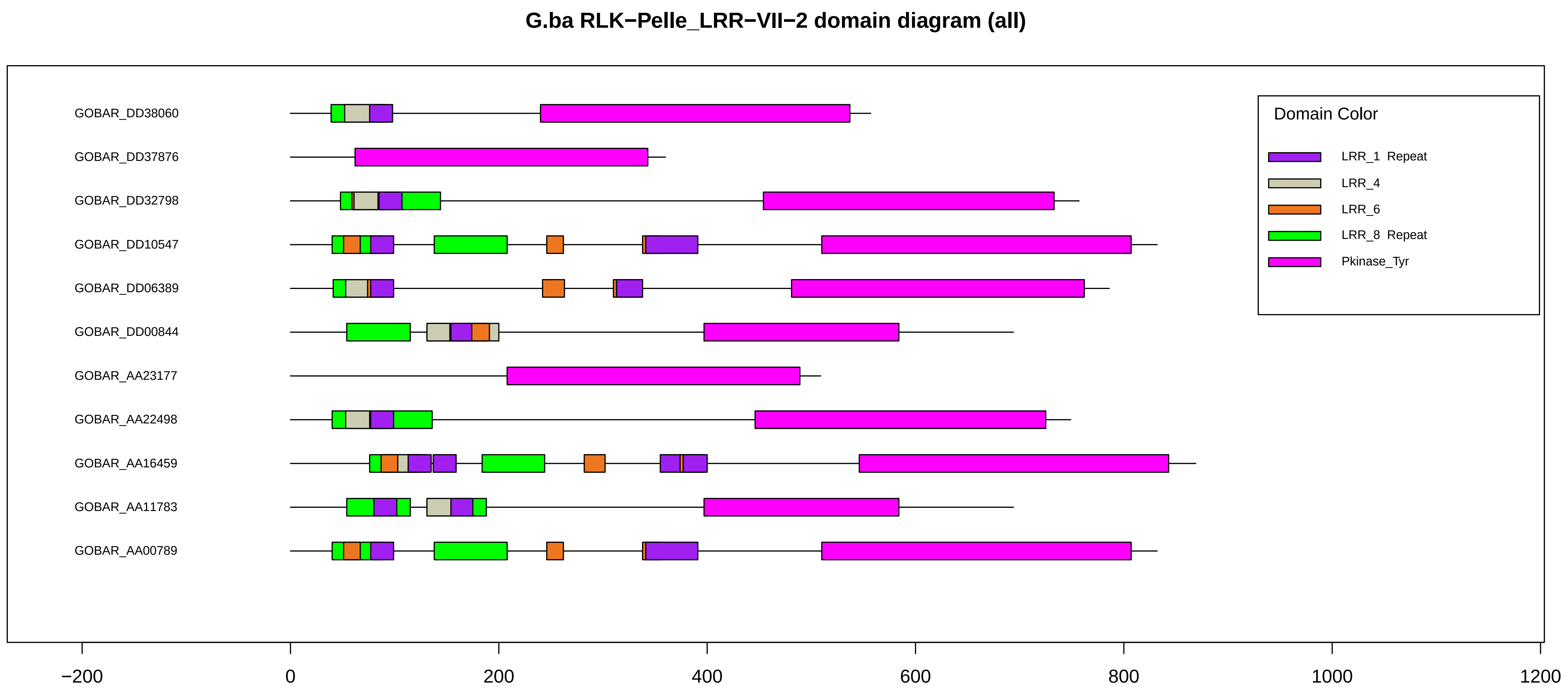
G.ba RLK−Pelle_LRR−VII−2 domain diagram (all)
GOBAR_DD38060
GOBAR_DD37876
GOBAR_DD32798
GOBAR_DD10547
GOBAR_DD06389
GOBAR_DD00844
GOBAR_AA23177
GOBAR_AA22498
GOBAR_AA16459
GOBAR_AA11783
GOBAR_AA00789
Domain Color
LRR_1 Repeat
LRR_4
LRR_6
LRR_8 Repeat
Pkinase_Tyr
−200
0
200
400
600
800
1000
1200

G.ba RLK−Pelle_LRR−VII−3 domain diagram (all)

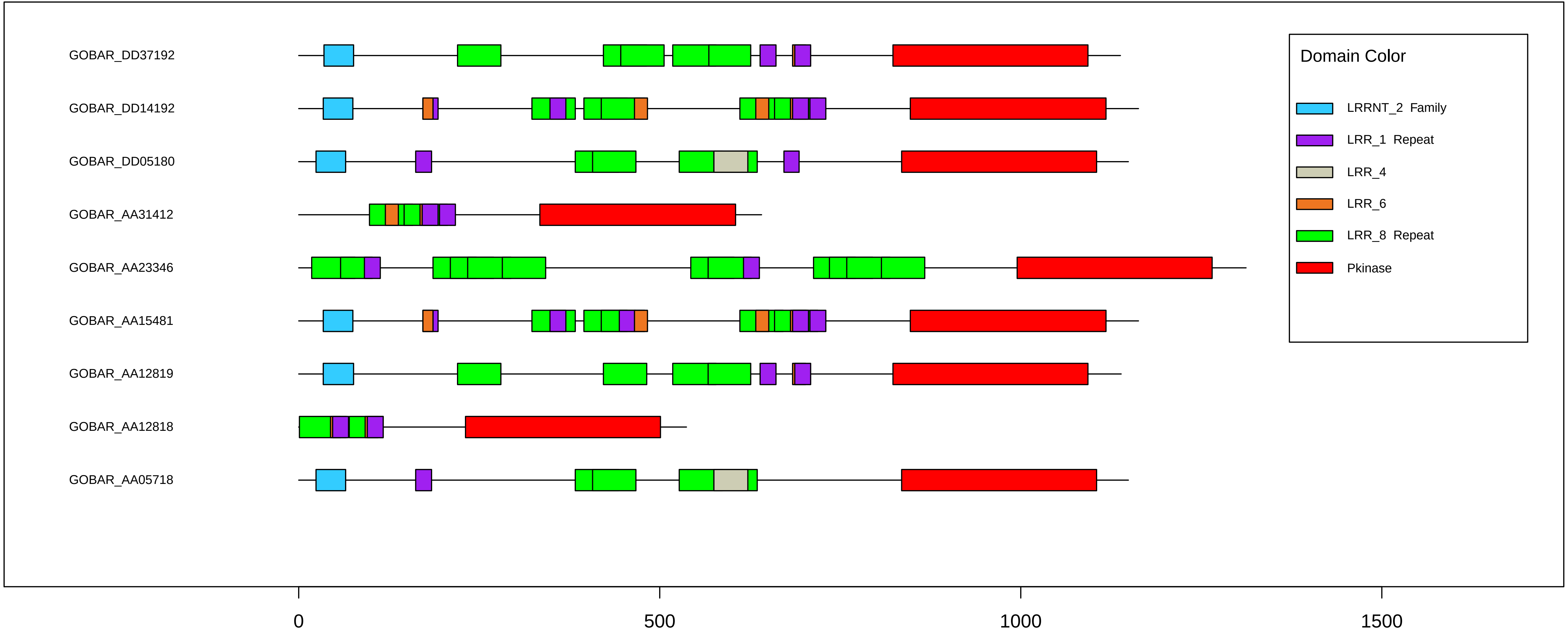

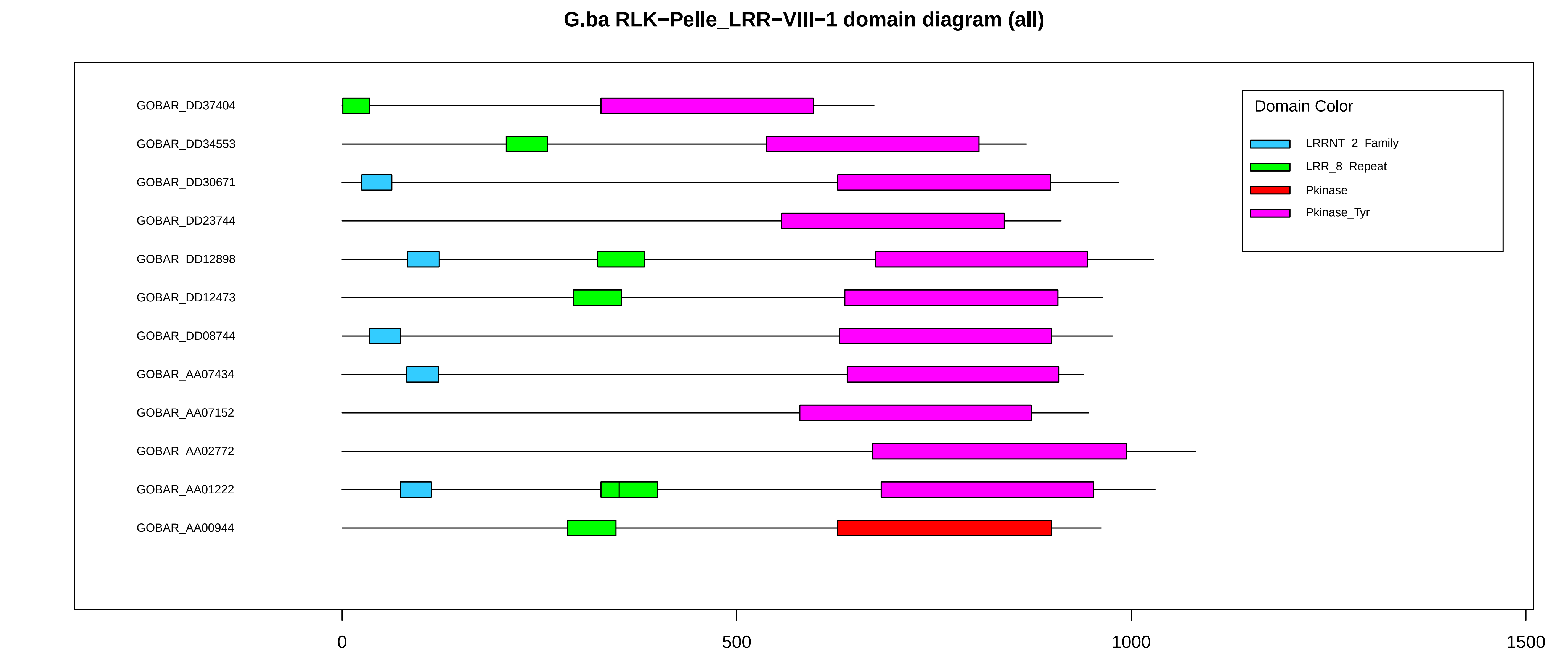
G.ba RLK−Pelle_LRR−VIII−1 domain diagram (all)
GOBAR_DD37404
GOBAR_DD34553
GOBAR_DD30671
GOBAR_DD23744
GOBAR_DD12898
GOBAR_DD12473
GOBAR_DD08744
GOBAR_AA07434
GOBAR_AA07152
GOBAR_AA02772
GOBAR_AA01222
GOBAR_AA00944
Domain Color
LRRNT_2 Family
LRR_8 Repeat
Pkinase
Pkinase_Tyr
0
500
1000
1500

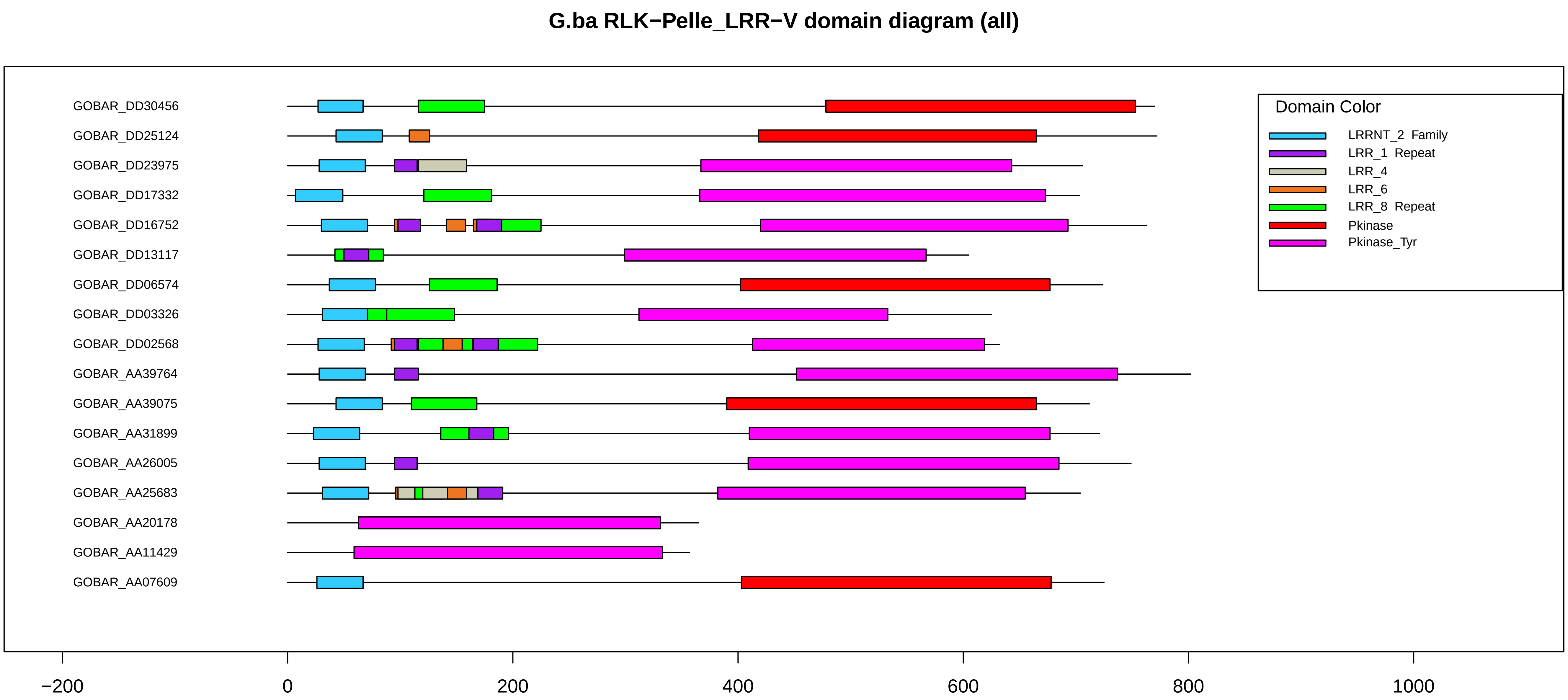

G.ba RLK−Pelle_LRR−V domain diagram (all)
GOBAR_DD30456
GOBAR_DD25124
GOBAR_DD23975
GOBAR_DD17332
GOBAR_DD16752
GOBAR_DD13117
GOBAR_DD06574
GOBAR_DD03326
GOBAR_DD02568
GOBAR_AA39764
GOBAR_AA39075
GOBAR_AA31899
GOBAR_AA26005
GOBAR_AA25683
GOBAR_AA20178
GOBAR_AA11429
GOBAR_AA07609
Domain Color
LRRNT_2 Family
LRR_1 Repeat
LRR_4
LRR_6
LRR_8 Repeat
Pkinase
Pkinase_Tyr
−200
0
200
400
600
800
1000

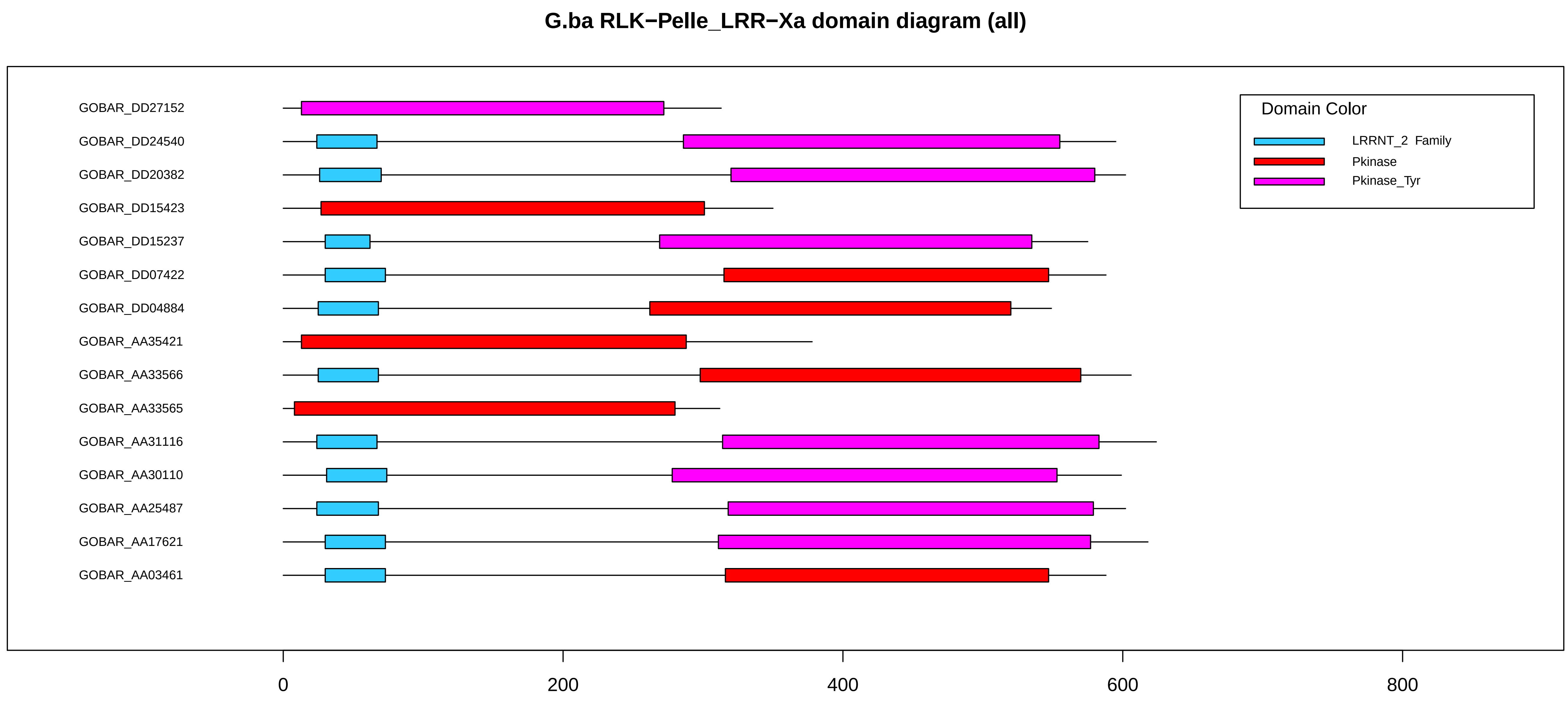
G.ba RLK−Pelle_LRR−Xa domain diagram (all)
GOBAR_DD27152
GOBAR_DD24540
GOBAR_DD20382
GOBAR_DD15423
GOBAR_DD15237
GOBAR_DD07422
GOBAR_DD04884
GOBAR_AA35421
GOBAR_AA33566
GOBAR_AA33565
GOBAR_AA31116
GOBAR_AA30110
GOBAR_AA25487
GOBAR_AA17621
GOBAR_AA03461
Domain Color
LRRNT_2 Family
Pkinase
Pkinase_Tyr
0
200
400
600
800

G.ba RLK−Pelle_LRR−Xb−1 domain diagram (part 1)

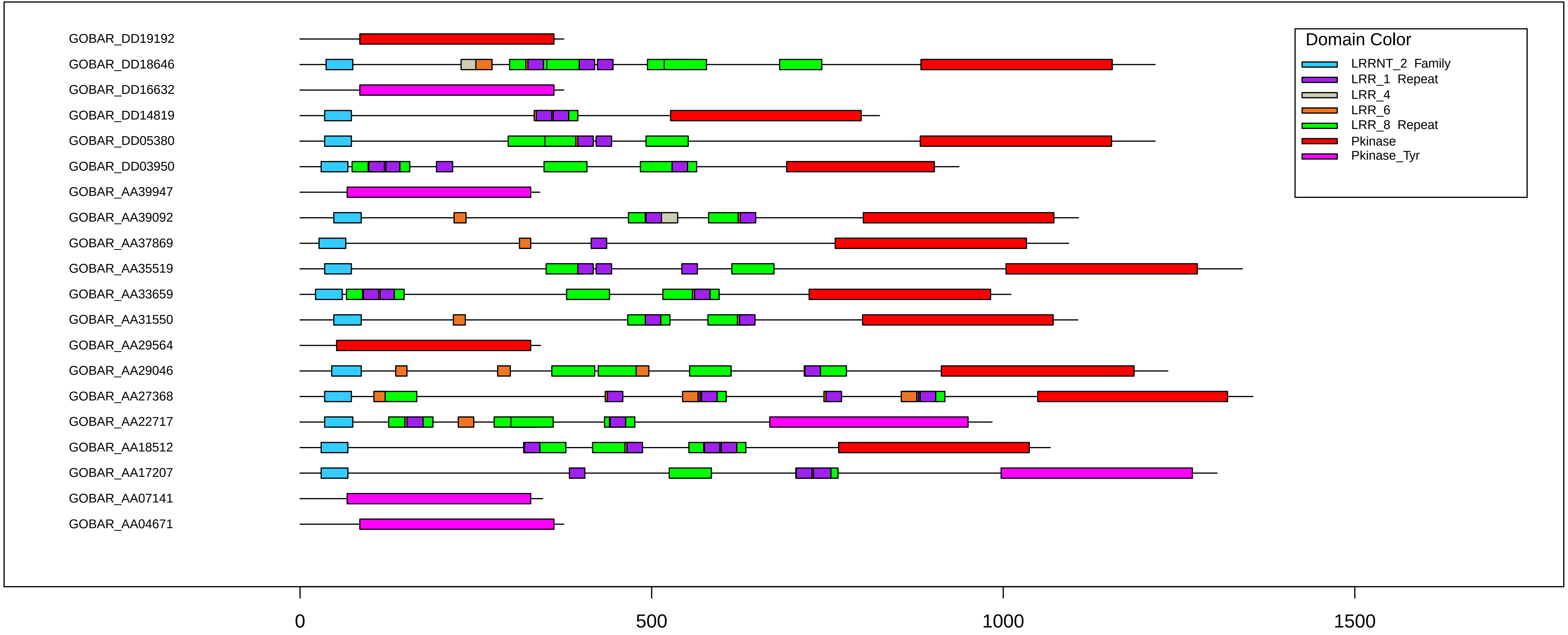

G.ba RLK−Pelle_LRR−Xb−1 domain diagram (part 2)

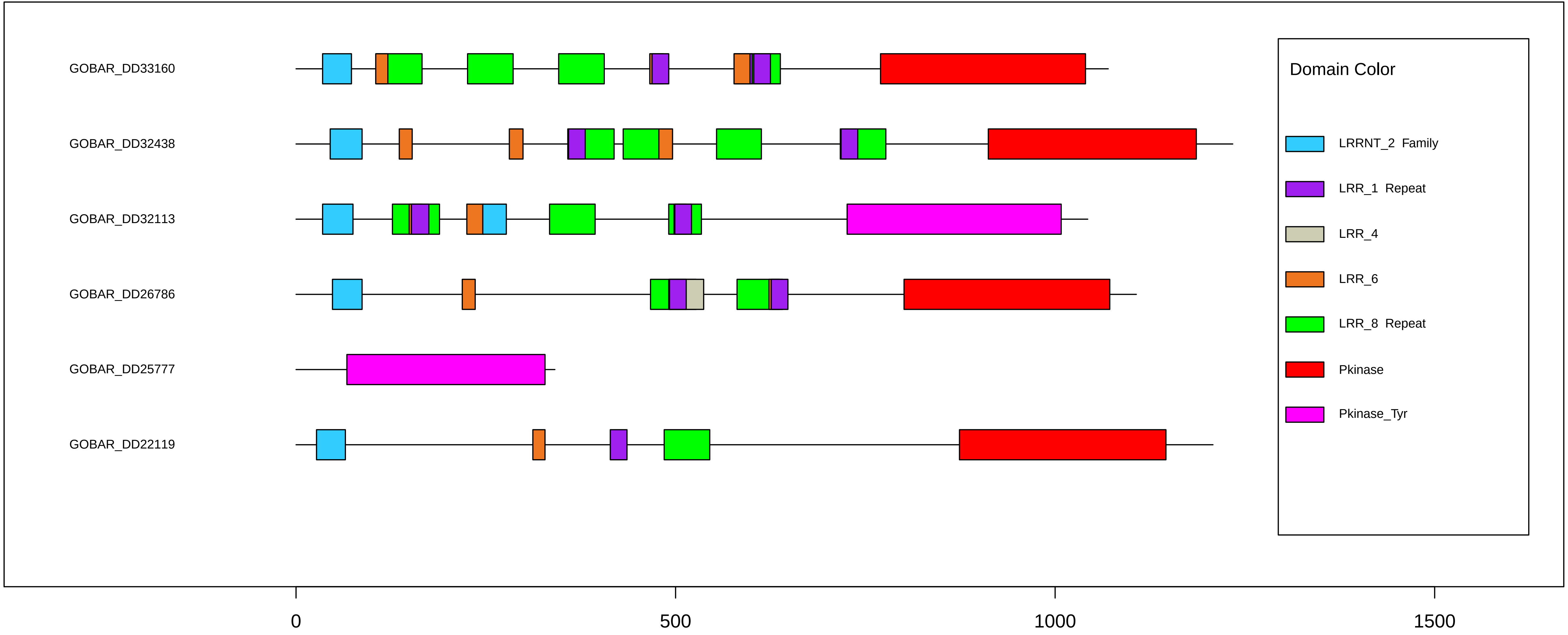

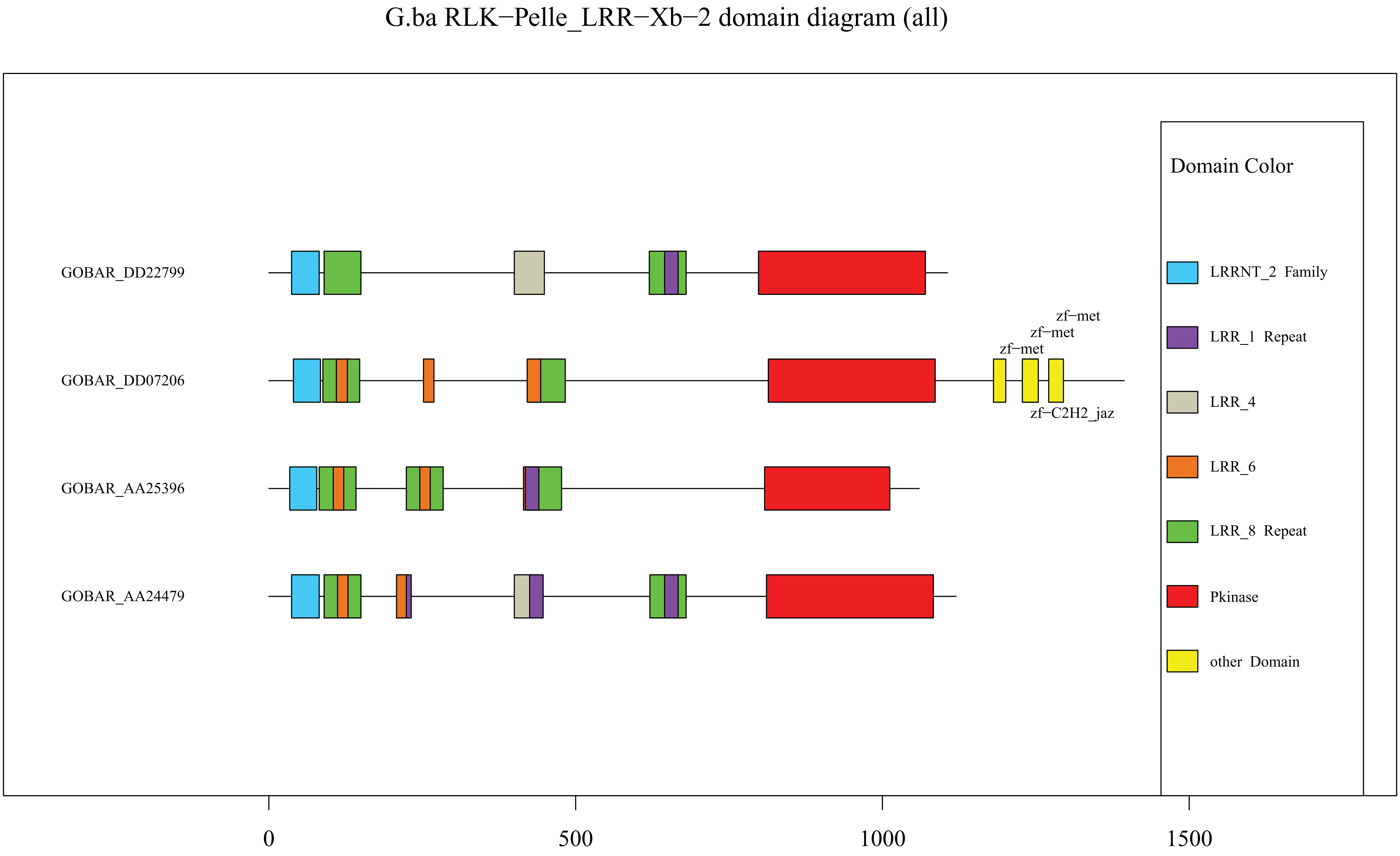
G.ba RLK−Pelle_LRR−Xb−2 domain diagram (all)
GOBAR_DD22799
GOBAR_DD07206
GOBAR_AA25396
GOBAR_AA24479
zf−met
zf−met
zf−met
zf−C2H2_jaz
Domain Color
LRRNT_2 Family
LRR_1 Repeat
LRR_4
LRR_6
LRR_8 Repeat
Pkinase
other Domain
0
500
1000
1500

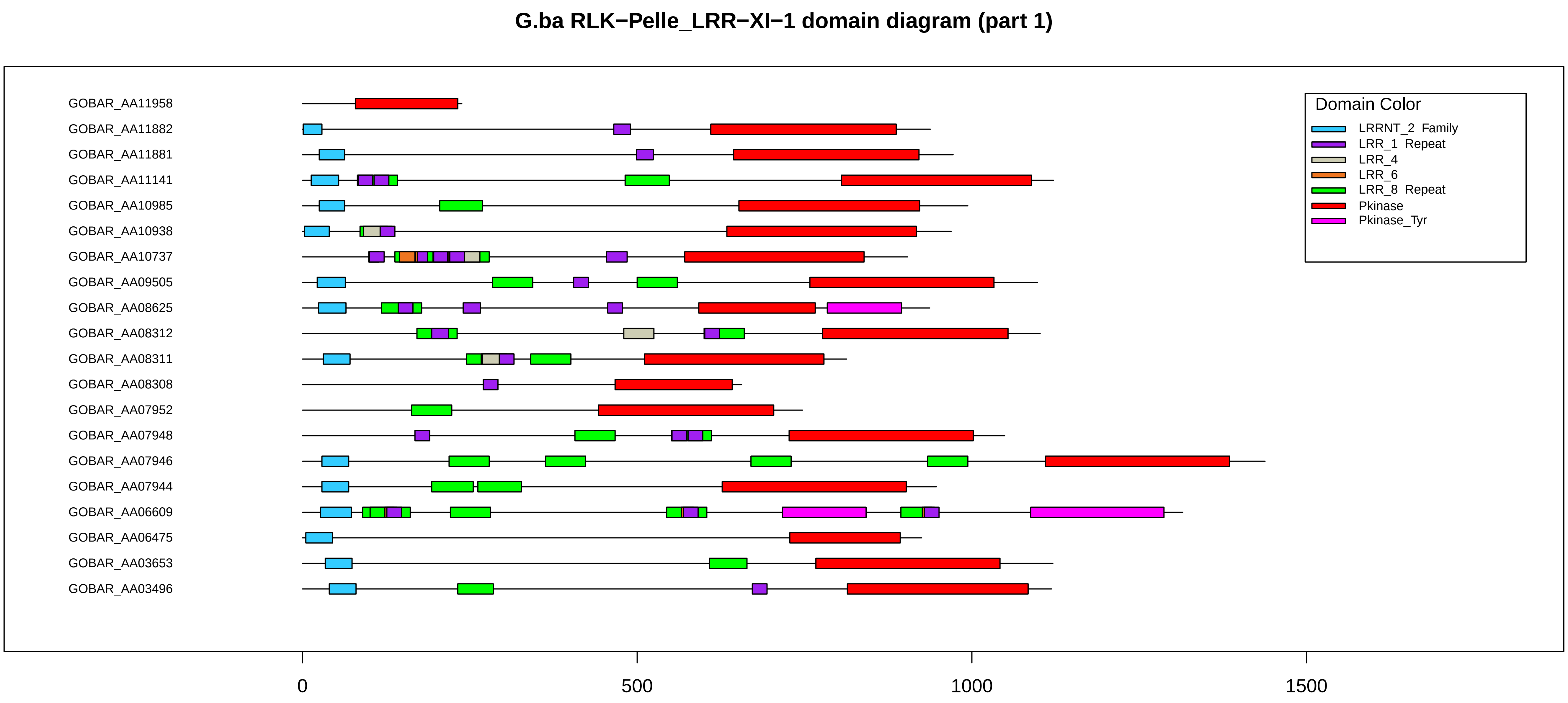

G.ba RLK−Pelle_LRR−XI−1 domain diagram (part 1)
GOBAR_AA11958
GOBAR_AA11882
GOBAR_AA11881
GOBAR_AA11141
GOBAR_AA10985
GOBAR_AA10938
GOBAR_AA10737
GOBAR_AA09505
GOBAR_AA08625
GOBAR_AA08312
GOBAR_AA08311
GOBAR_AA08308
GOBAR_AA07952
GOBAR_AA07948
GOBAR_AA07946
GOBAR_AA07944
GOBAR_AA06609
GOBAR_AA06475
GOBAR_AA03653
GOBAR_AA03496
Domain Color
LRRNT_2 Family
LRR_1 Repeat
LRR_4
LRR_6
LRR_8 Repeat
Pkinase
Pkinase_Tyr
0
500
1000
1500

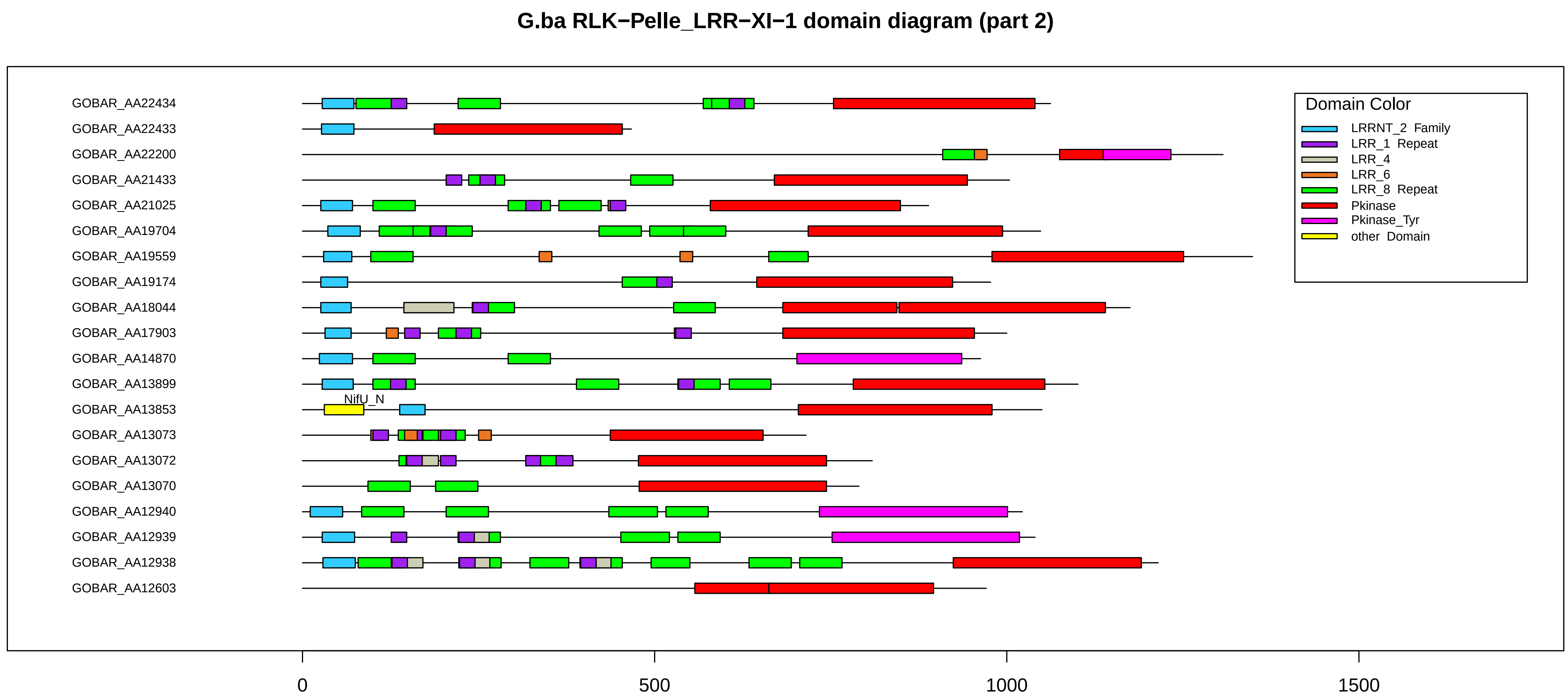
G.ba RLK−Pelle_LRR−XI−1 domain diagram (part 2)
GOBAR_AA22434
GOBAR_AA22433
GOBAR_AA22200
GOBAR_AA21433
GOBAR_AA21025
GOBAR_AA19704
GOBAR_AA19559
GOBAR_AA19174
GOBAR_AA18044
GOBAR_AA17903
GOBAR_AA14870
GOBAR_AA13899
GOBAR_AA13853
NifU_N
GOBAR_AA13073
GOBAR_AA13072
GOBAR_AA13070
GOBAR_AA12940
GOBAR_AA12939
GOBAR_AA12938
GOBAR_AA12603
Domain Color
LRRNT_2 Family
LRR_1 Repeat
LRR_4
LRR_6
LRR_8 Repeat
Pkinase
Pkinase_Tyr
other Domain
0
500
1000
1500

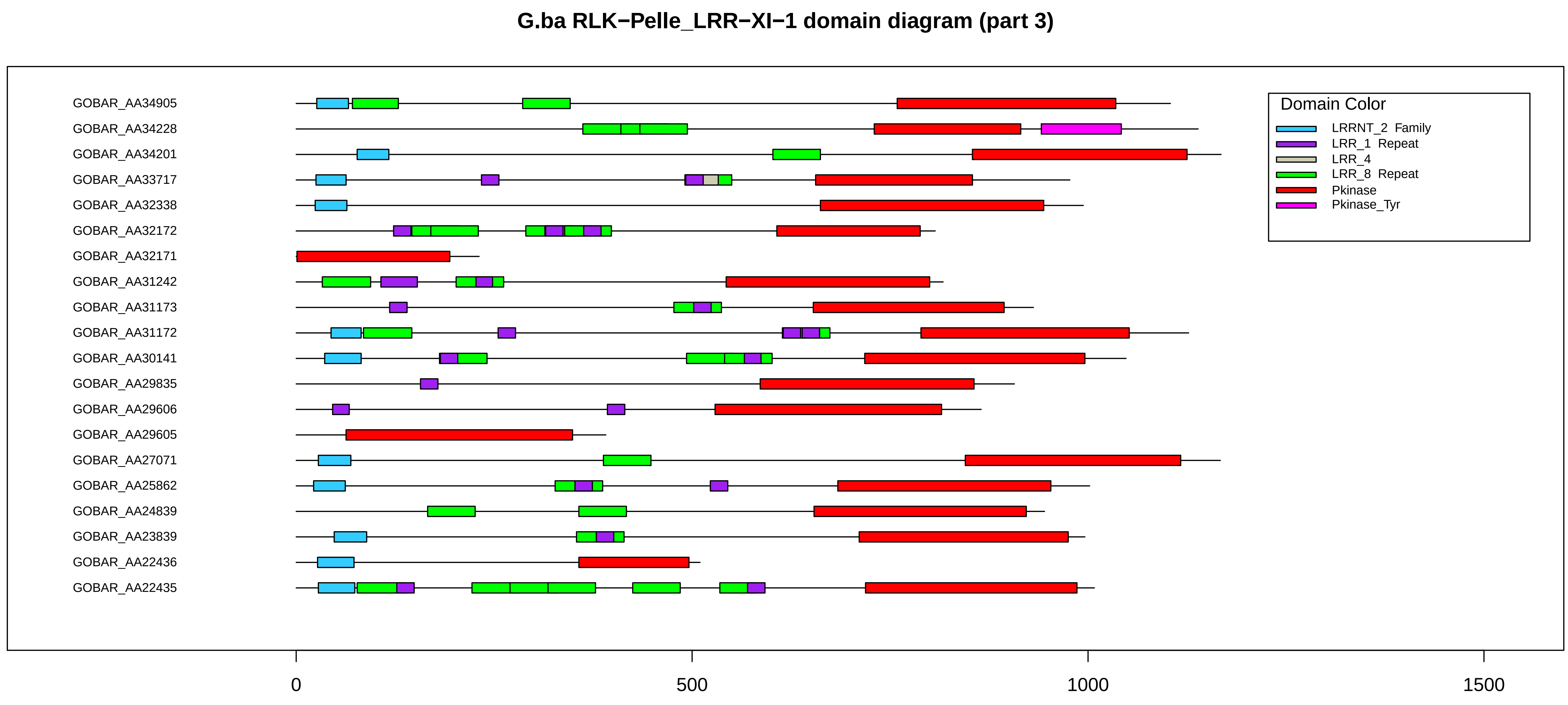
G.ba RLK−Pelle_LRR−XI−1 domain diagram (part 3)
GOBAR_AA34905
GOBAR_AA34228
GOBAR_AA34201
GOBAR_AA33717
GOBAR_AA32338
GOBAR_AA32172
GOBAR_AA32171
GOBAR_AA31242
GOBAR_AA31173
GOBAR_AA31172
GOBAR_AA30141
GOBAR_AA29835
GOBAR_AA29606
GOBAR_AA29605
GOBAR_AA27071
GOBAR_AA25862
GOBAR_AA24839
GOBAR_AA23839
GOBAR_AA22436
GOBAR_AA22435
Domain Color
LRRNT_2 Family
LRR_1 Repeat
LRR_4
LRR_8 Repeat
Pkinase
Pkinase_Tyr
0
500
1000
1500

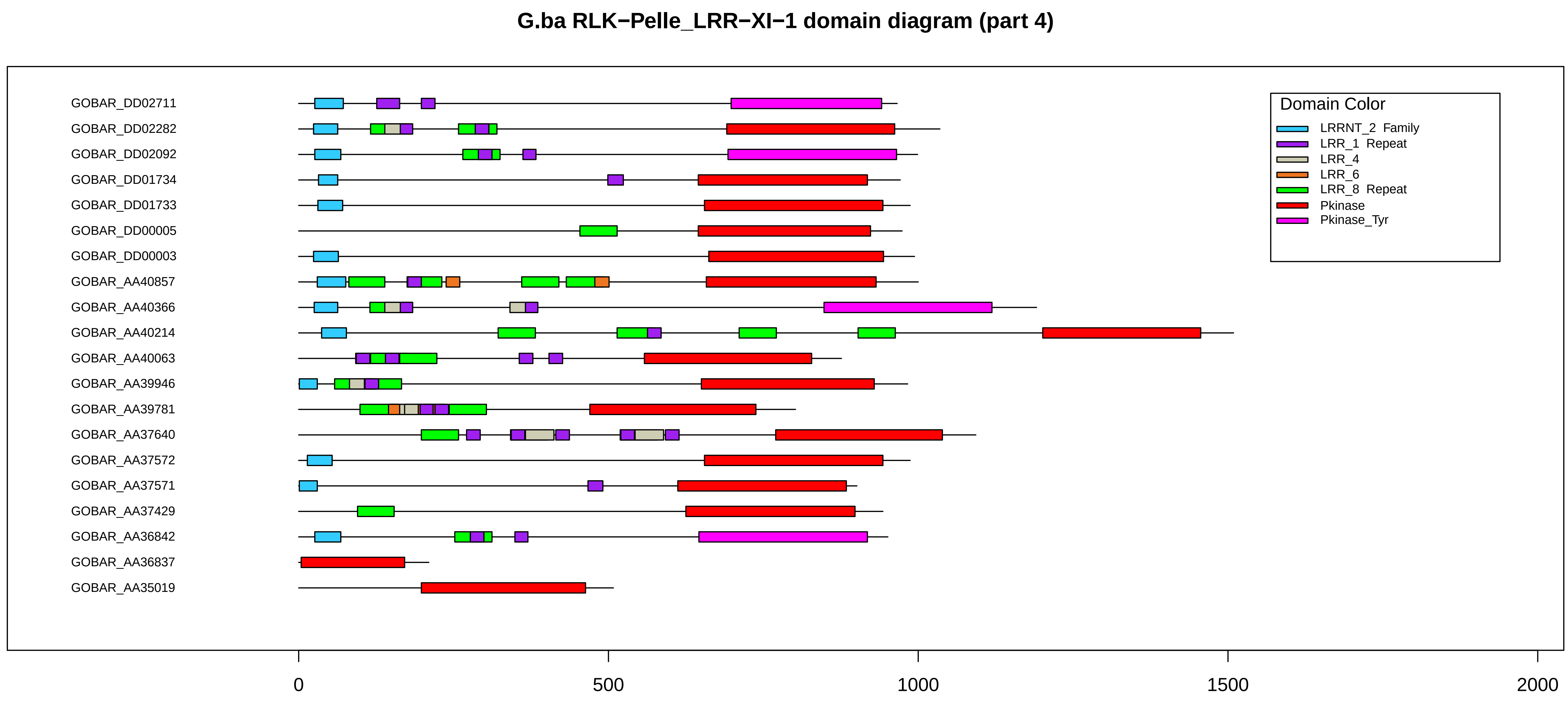
G.ba RLK−Pelle_LRR−XI−1 domain diagram (part 4)
GOBAR_DD02711
GOBAR_DD02282
GOBAR_DD02092
GOBAR_DD01734
GOBAR_DD01733
GOBAR_DD00005
GOBAR_DD00003
GOBAR_AA40857
GOBAR_AA40366
GOBAR_AA40214
GOBAR_AA40063
GOBAR_AA39946
GOBAR_AA39781
GOBAR_AA37640
GOBAR_AA37572
GOBAR_AA37571
GOBAR_AA37429
GOBAR_AA36842
GOBAR_AA36837
GOBAR_AA35019
Domain Color
LRRNT_2 Family
LRR_1 Repeat
LRR_4
LRR_6
LRR_8 Repeat
Pkinase
Pkinase_Tyr
0
500
1000
1500
2000

G.ba RLK−Pelle_LRR−XI−1 domain diagram (part 5)

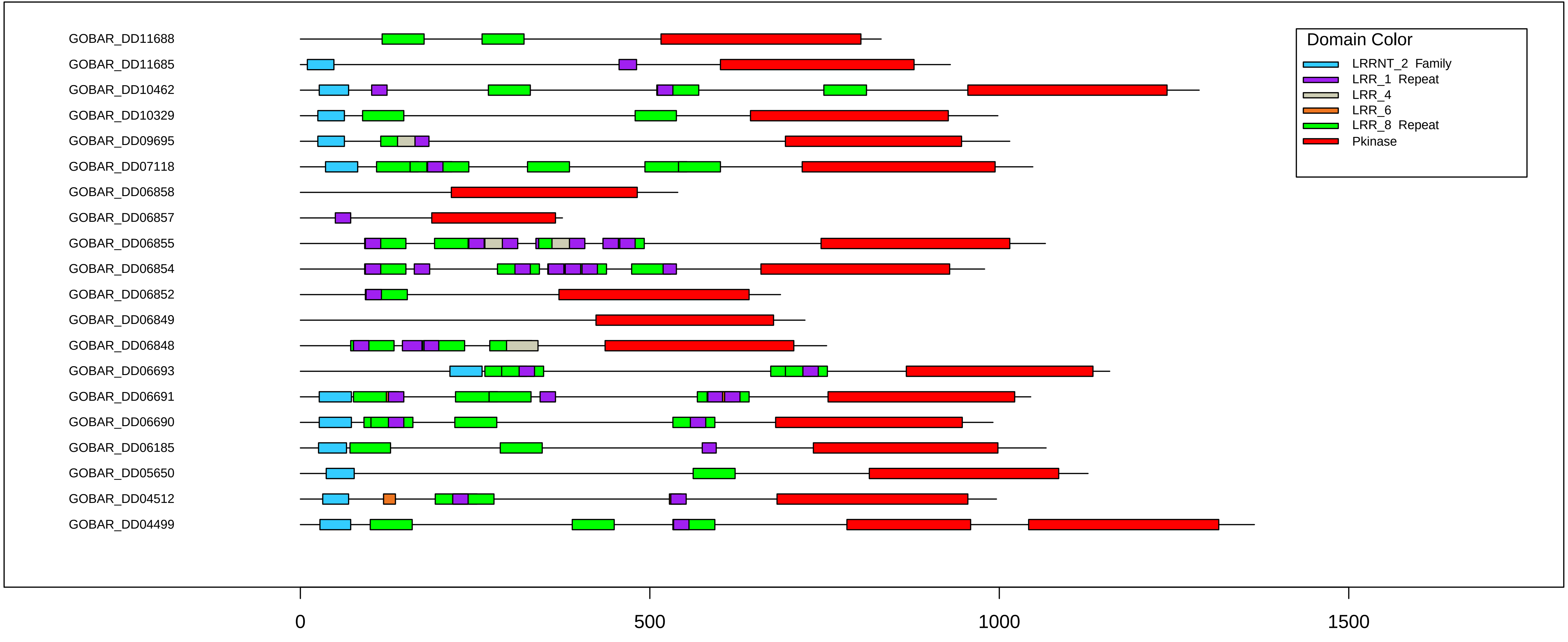

G.ba RLK−Pelle_LRR−XI−1 domain diagram (part 6)

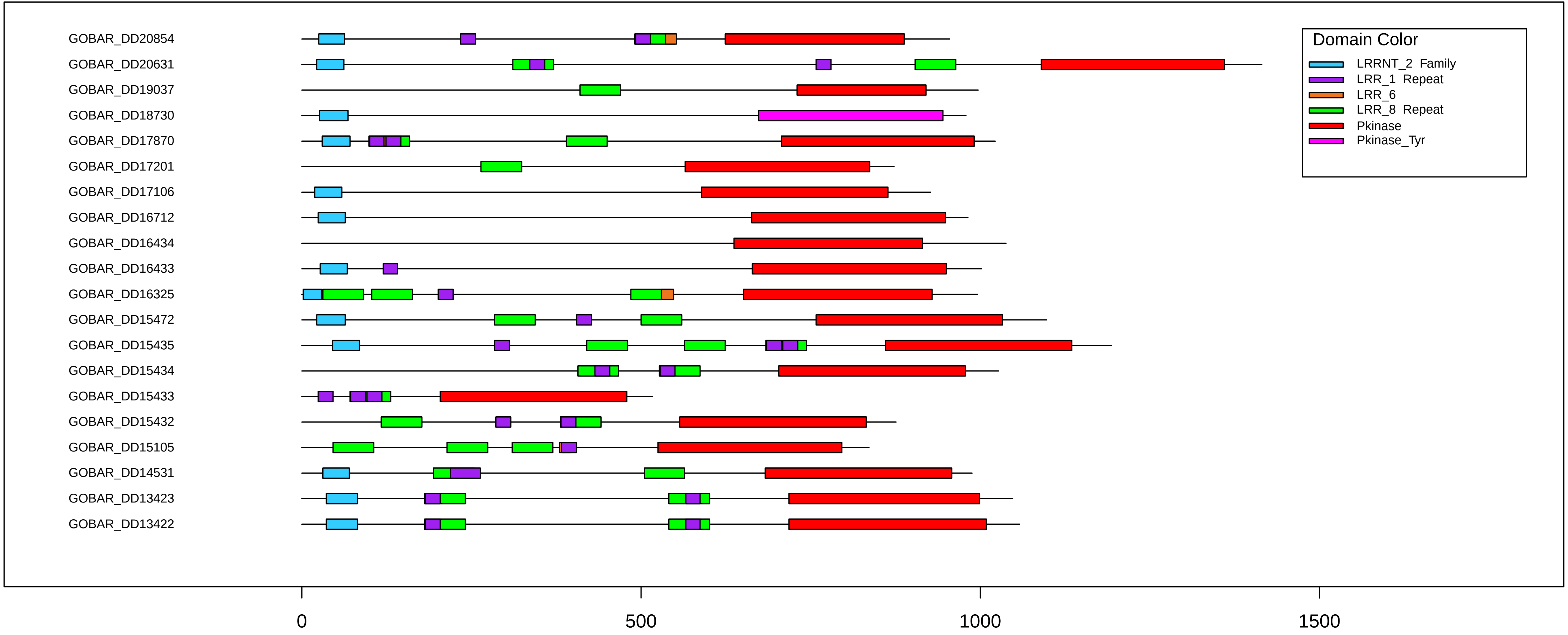

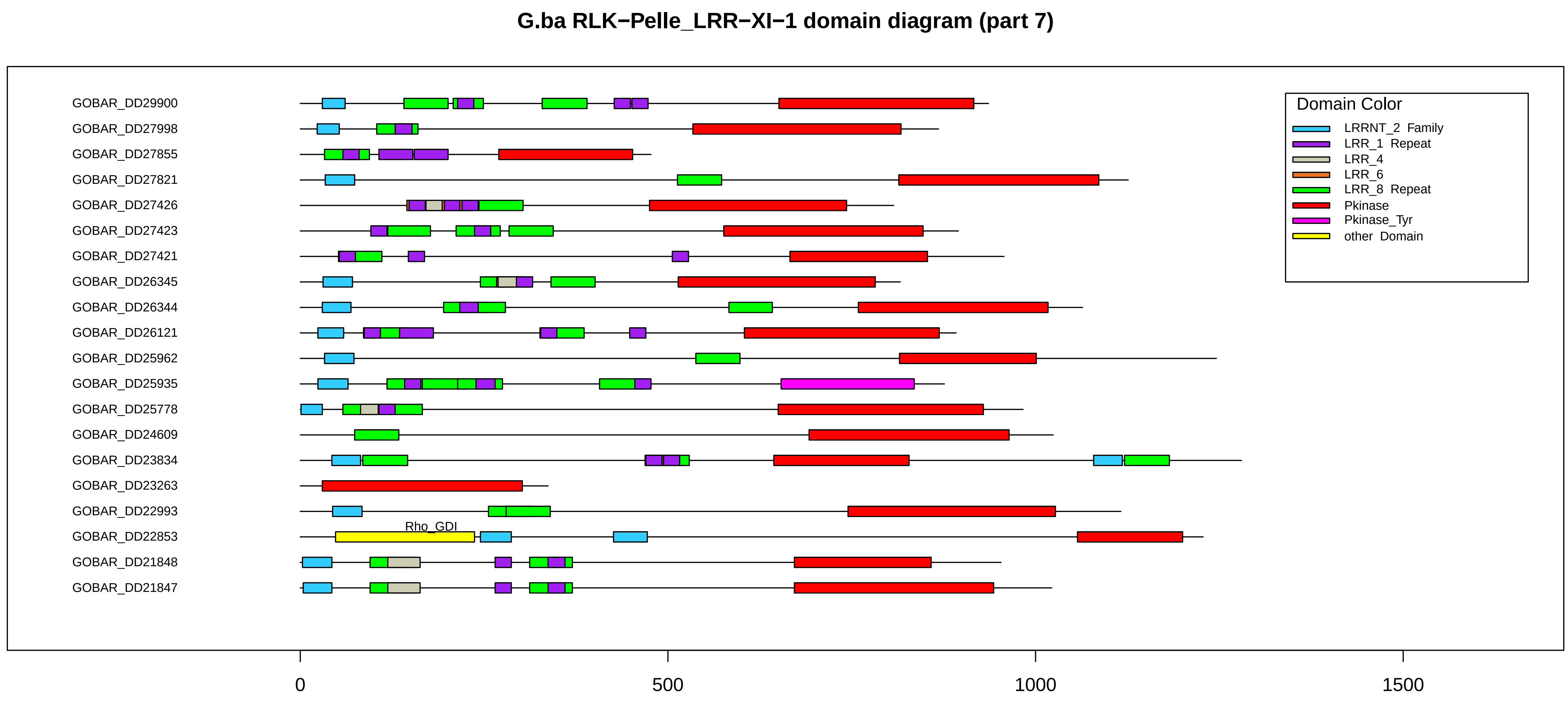
G.ba RLK−Pelle_LRR−XI−1 domain diagram (part 7)
GOBAR_DD29900
GOBAR_DD27998
GOBAR_DD27855
GOBAR_DD27821
GOBAR_DD27426
GOBAR_DD27423
GOBAR_DD27421
GOBAR_DD26345
GOBAR_DD26344
GOBAR_DD26121
GOBAR_DD25962
GOBAR_DD25935
GOBAR_DD25778
GOBAR_DD24609
GOBAR_DD23834
GOBAR_DD23263
GOBAR_DD22993
GOBAR_DD22853
GOBAR_DD21848
GOBAR_DD21847
Rho_GDI
Domain Color
LRRNT_2 Family
LRR_1 Repeat
LRR_4
LRR_6
LRR_8 Repeat
Pkinase
Pkinase_Tyr
other Domain
0
500
1000
1500

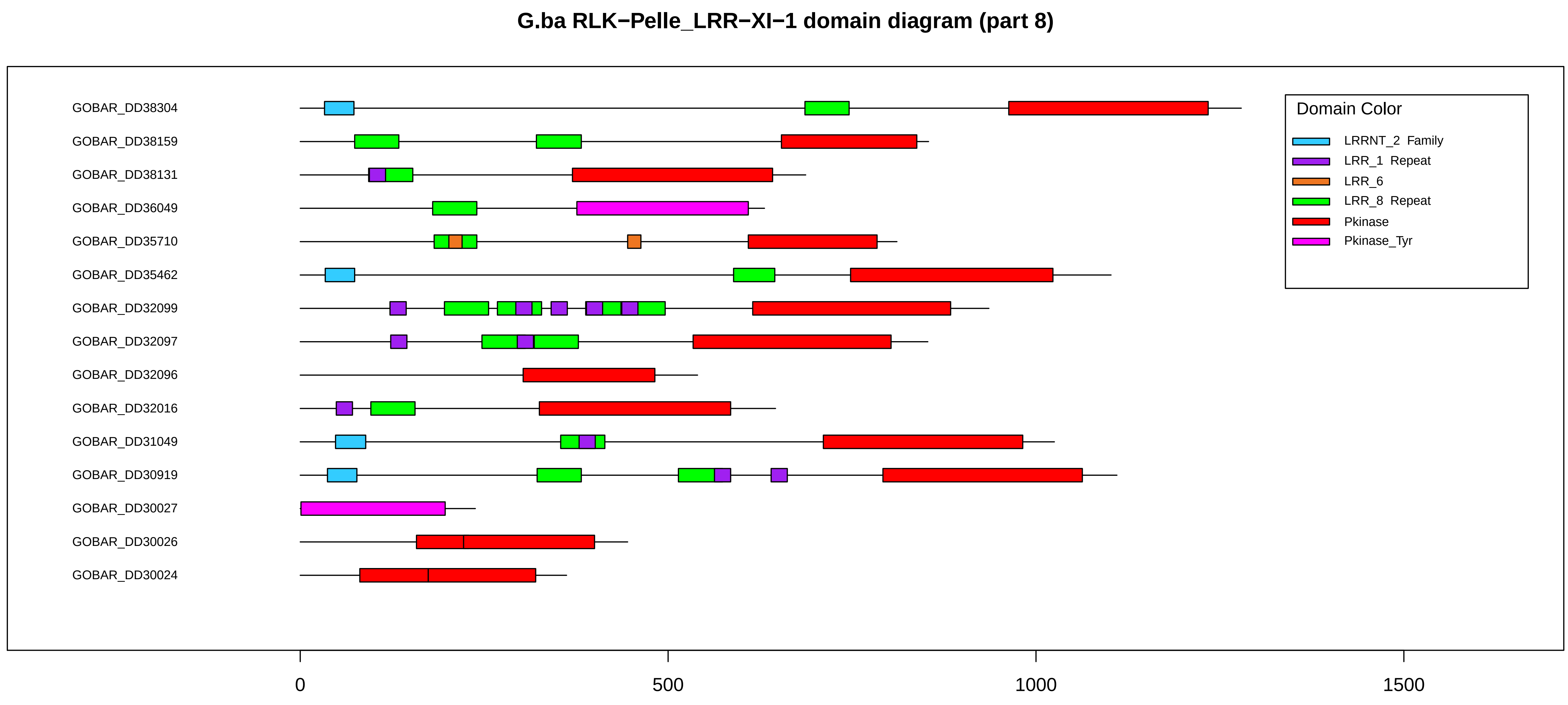
G.ba RLK−Pelle_LRR−XI−1 domain diagram (part 8)
GOBAR_DD38304
GOBAR_DD38159
GOBAR_DD38131
GOBAR_DD36049
GOBAR_DD35710
GOBAR_DD35462
GOBAR_DD32099
GOBAR_DD32097
GOBAR_DD32096
GOBAR_DD32016
GOBAR_DD31049
GOBAR_DD30919
GOBAR_DD30027
GOBAR_DD30026
GOBAR_DD30024
Domain Color
LRRNT_2 Family
LRR_1 Repeat
LRR_6
LRR_8 Repeat
Pkinase
Pkinase_Tyr
0
500
1000
1500

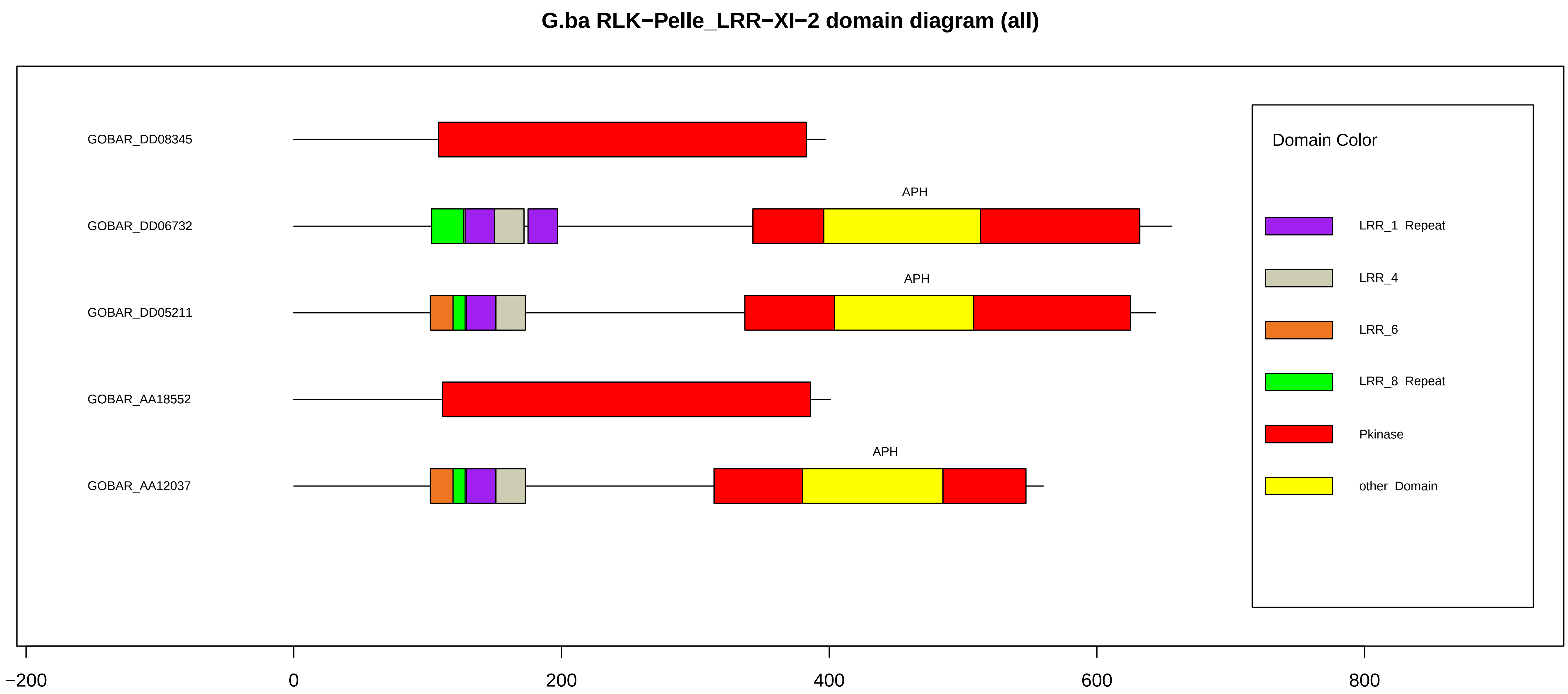
G.ba RLK−Pelle_LRR−XI−2 domain diagram (all)
GOBAR_DD08345
GOBAR_DD06732
GOBAR_DD05211
GOBAR_AA18552
GOBAR_AA12037
APH
APH
APH
Domain Color
LRR_1 Repeat
LRR_4
LRR_6
LRR_8 Repeat
Pkinase
other Domain
−200
0
200
400
600
800

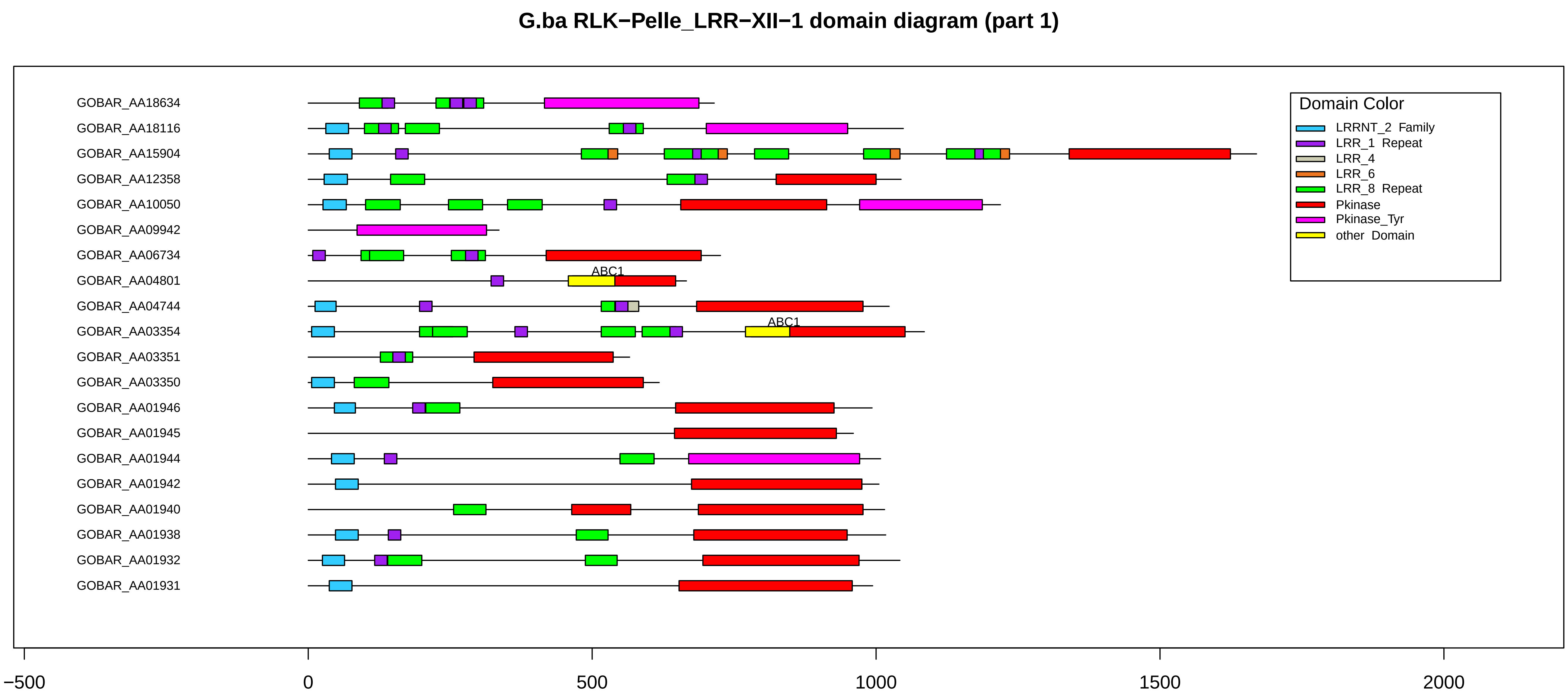
G.ba RLK−Pelle_LRR−XII−1 domain diagram (part 1)
GOBAR_AA18634
GOBAR_AA18116
GOBAR_AA15904
GOBAR_AA12358
GOBAR_AA10050
GOBAR_AA09942
GOBAR_AA06734
GOBAR_AA04801
GOBAR_AA04744
GOBAR_AA03354
GOBAR_AA03351
GOBAR_AA03350
GOBAR_AA01946
GOBAR_AA01945
GOBAR_AA01944
GOBAR_AA01942
GOBAR_AA01940
GOBAR_AA01938
GOBAR_AA01932
GOBAR_AA01931
ABC1
ABC1
Domain Color
LRRNT_2 Family
LRR_1 Repeat
LRR_4
LRR_6
LRR_8 Repeat
Pkinase
Pkinase_Tyr
other Domain
−500
0
500
1000
1500
2000

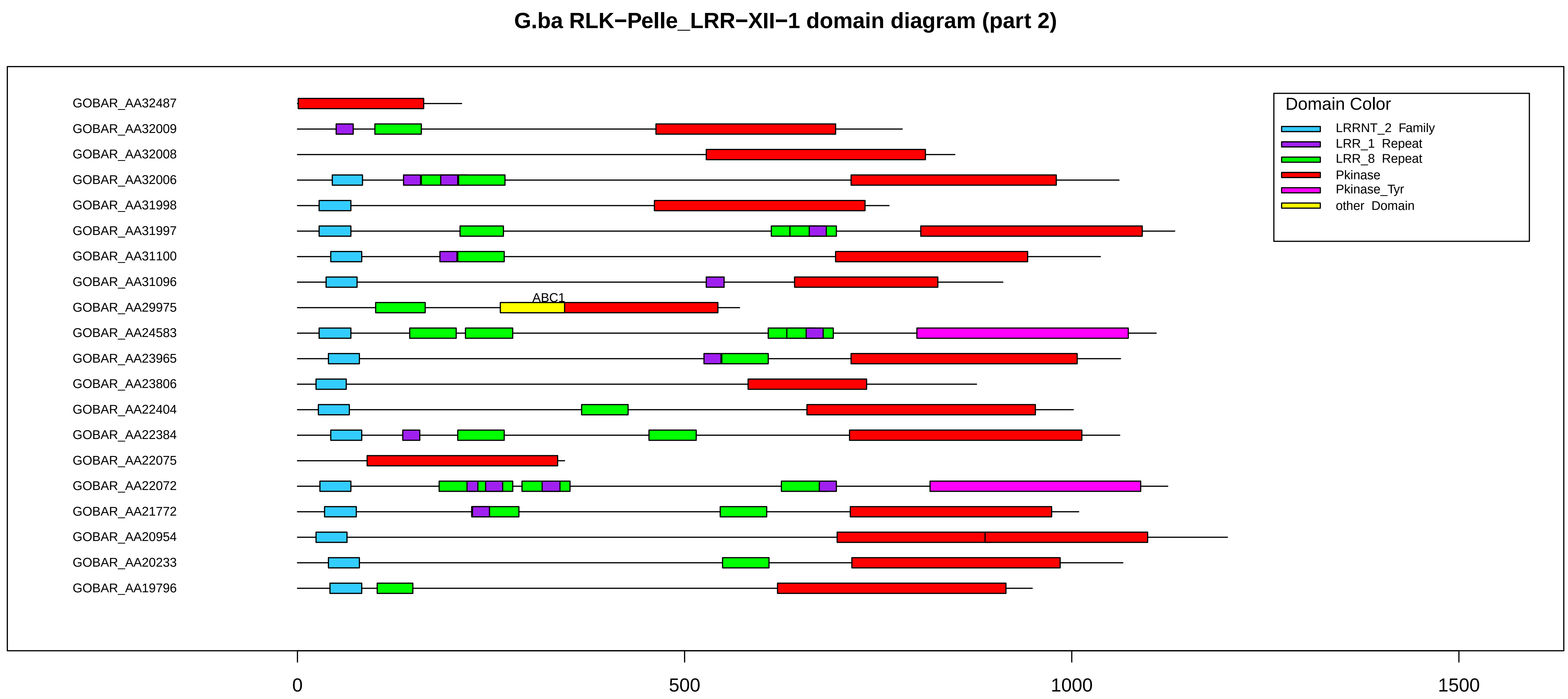
G.ba RLK−Pelle_LRR−XII−1 domain diagram (part 2)
GOBAR_AA32487
GOBAR_AA32009
GOBAR_AA32008
GOBAR_AA32006
GOBAR_AA31998
GOBAR_AA31997
GOBAR_AA31100
GOBAR_AA31096
GOBAR_AA29975
ABC1
GOBAR_AA24583
GOBAR_AA23965
GOBAR_AA23806
GOBAR_AA22404
GOBAR_AA22384
GOBAR_AA22075
GOBAR_AA22072
GOBAR_AA21772
GOBAR_AA20954
GOBAR_AA20233
GOBAR_AA19796
Domain Color
LRRNT_2 Family
LRR_1 Repeat
LRR_8 Repeat
Pkinase
Pkinase_Tyr
other Domain
0
500
1000
1500

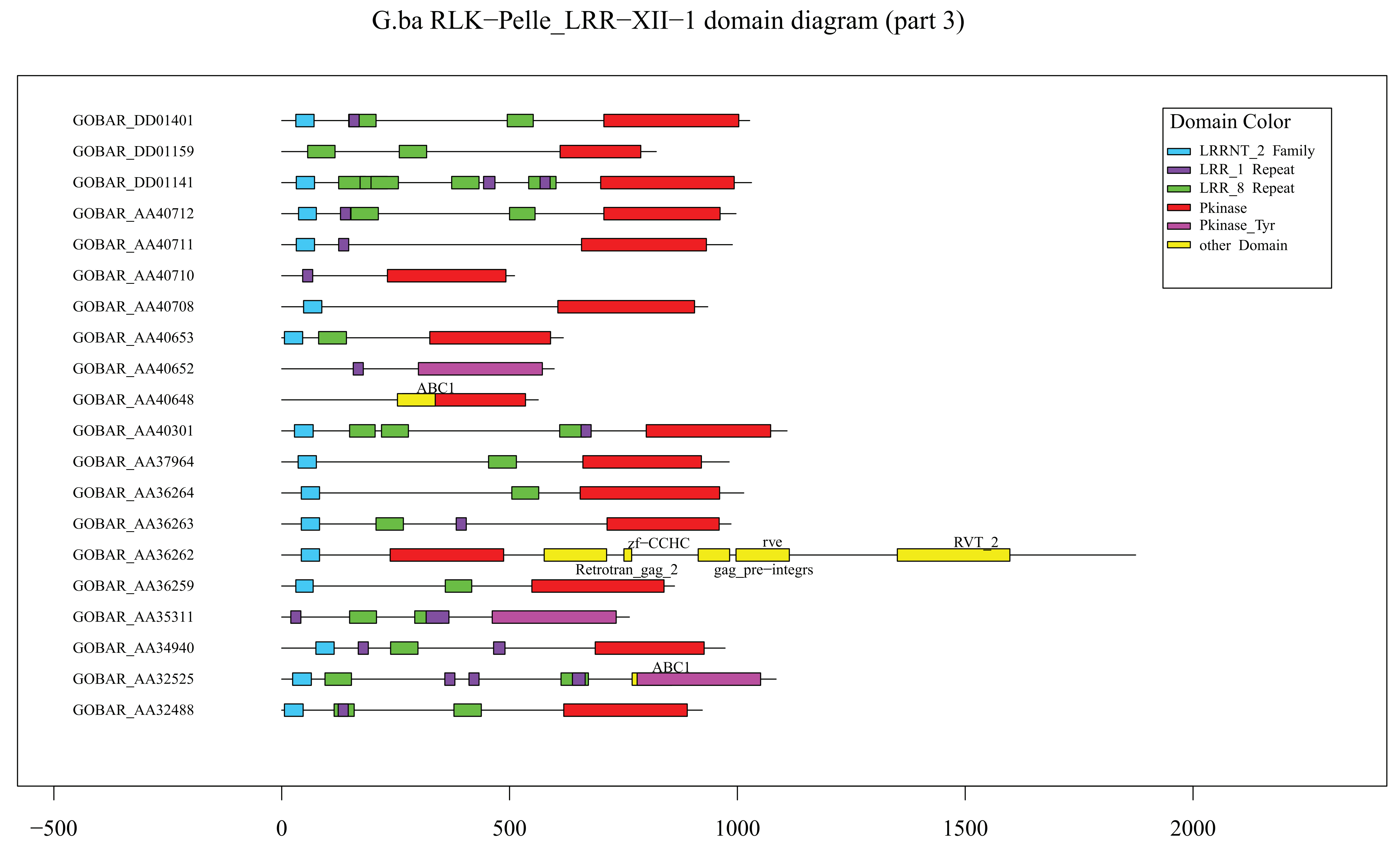
G.ba RLK−Pelle_LRR−XII−1 domain diagram (part 3)
GOBAR_DD01401
GOBAR_DD01159
GOBAR_DD01141
GOBAR_AA40712
GOBAR_AA40711
GOBAR_AA40710
GOBAR_AA40708
GOBAR_AA40653
GOBAR_AA40652
GOBAR_AA40648
GOBAR_AA40301
GOBAR_AA37964
GOBAR_AA36264
GOBAR_AA36263
GOBAR_AA36262
GOBAR_AA36259
GOBAR_AA35311
GOBAR_AA34940
GOBAR_AA32525
GOBAR_AA32488
ABC1
zf−CCHC
rve
RVT_2
Retrotran_gag_2
gag_pre−integrs
ABC1
Domain Color
LRRNT_2 Family
LRR_1 Repeat
LRR_8 Repeat
Pkinase
Pkinase_Tyr
other Domain
−500
0
500
1000
1500
2000

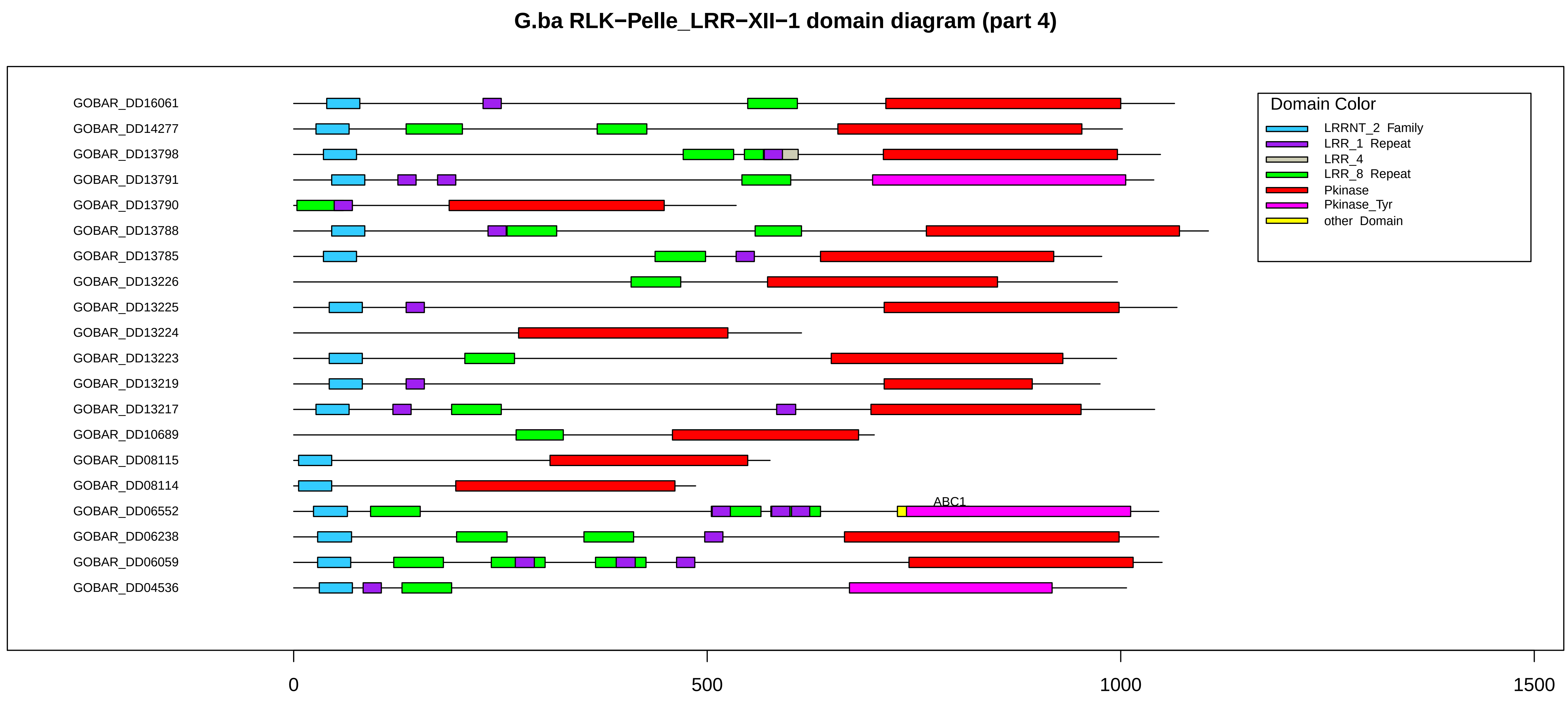
G.ba RLK−Pelle_LRR−XII−1 domain diagram (part 4)
GOBAR_DD16061
GOBAR_DD14277
GOBAR_DD13798
GOBAR_DD13791
GOBAR_DD13790
GOBAR_DD13788
GOBAR_DD13785
GOBAR_DD13226
GOBAR_DD13225
GOBAR_DD13224
GOBAR_DD13223
GOBAR_DD13219
GOBAR_DD13217
GOBAR_DD10689
GOBAR_DD08115
GOBAR_DD08114
GOBAR_DD06552
GOBAR_DD06238
GOBAR_DD06059
GOBAR_DD04536
ABC1
Domain Color
LRRNT_2 Family
LRR_1 Repeat
LRR_4
LRR_8 Repeat
Pkinase
Pkinase_Tyr
other Domain
0
500
1000
1500

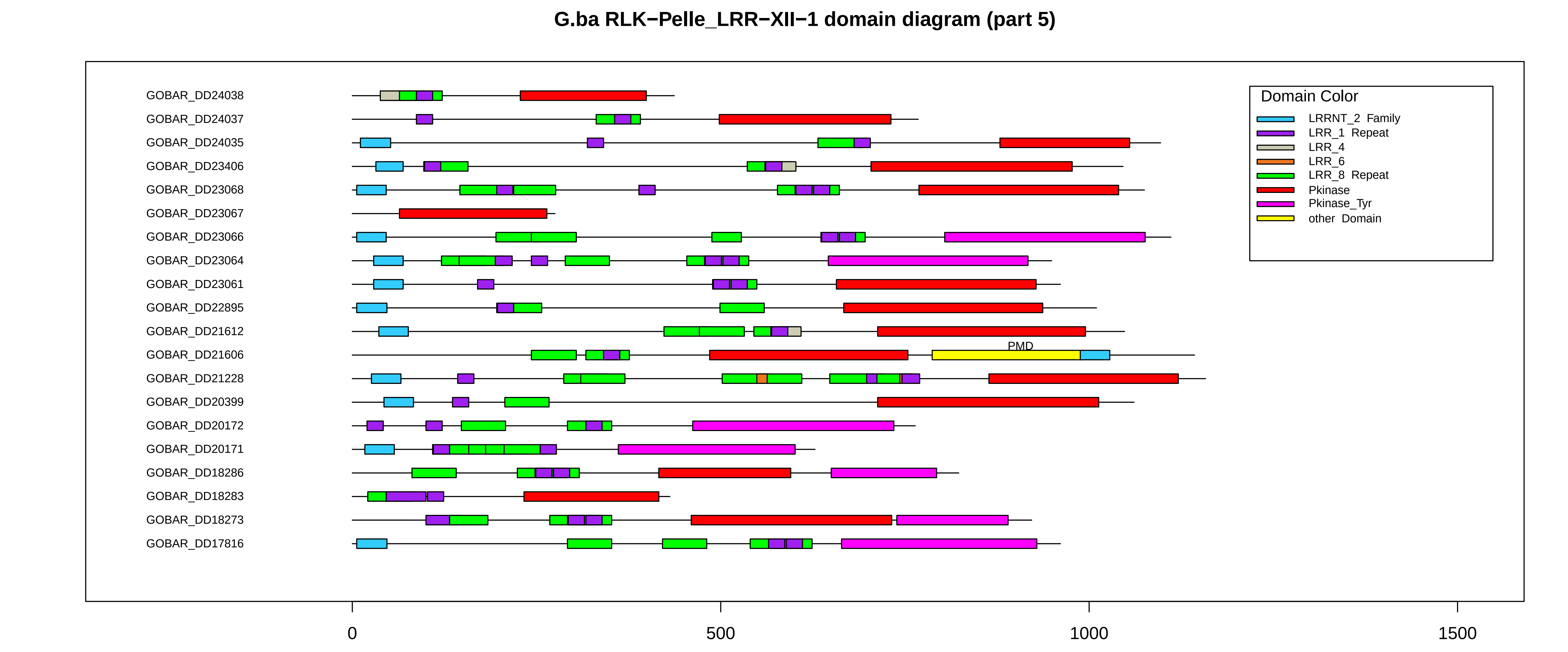
G.ba RLK−Pelle_LRR−XII−1 domain diagram (part 5)
GOBAR_DD24038
GOBAR_DD24037
GOBAR_DD24035
GOBAR_DD23406
GOBAR_DD23068
GOBAR_DD23067
GOBAR_DD23066
GOBAR_DD23064
GOBAR_DD23061
GOBAR_DD22895
GOBAR_DD21612
GOBAR_DD21606
GOBAR_DD21228
GOBAR_DD20399
GOBAR_DD20172
GOBAR_DD20171
GOBAR_DD18286
GOBAR_DD18283
GOBAR_DD18273
GOBAR_DD17816
PMD
Domain Color
LRRNT_2 Family
LRR_1 Repeat
LRR_4
LRR_6
LRR_8 Repeat
Pkinase
Pkinase_Tyr
other Domain
0
500
1000
1500

G.ba RLK−Pelle_LRR−XII−1 domain diagram (part 6)

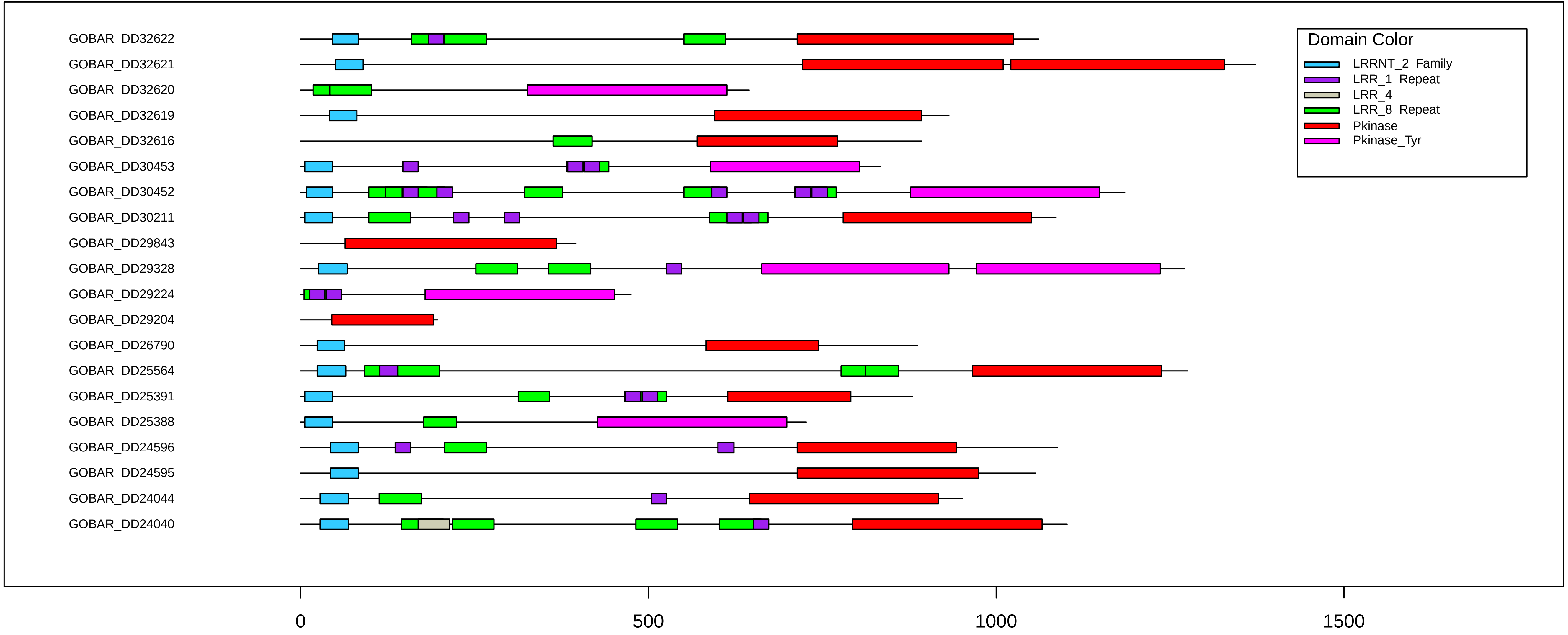

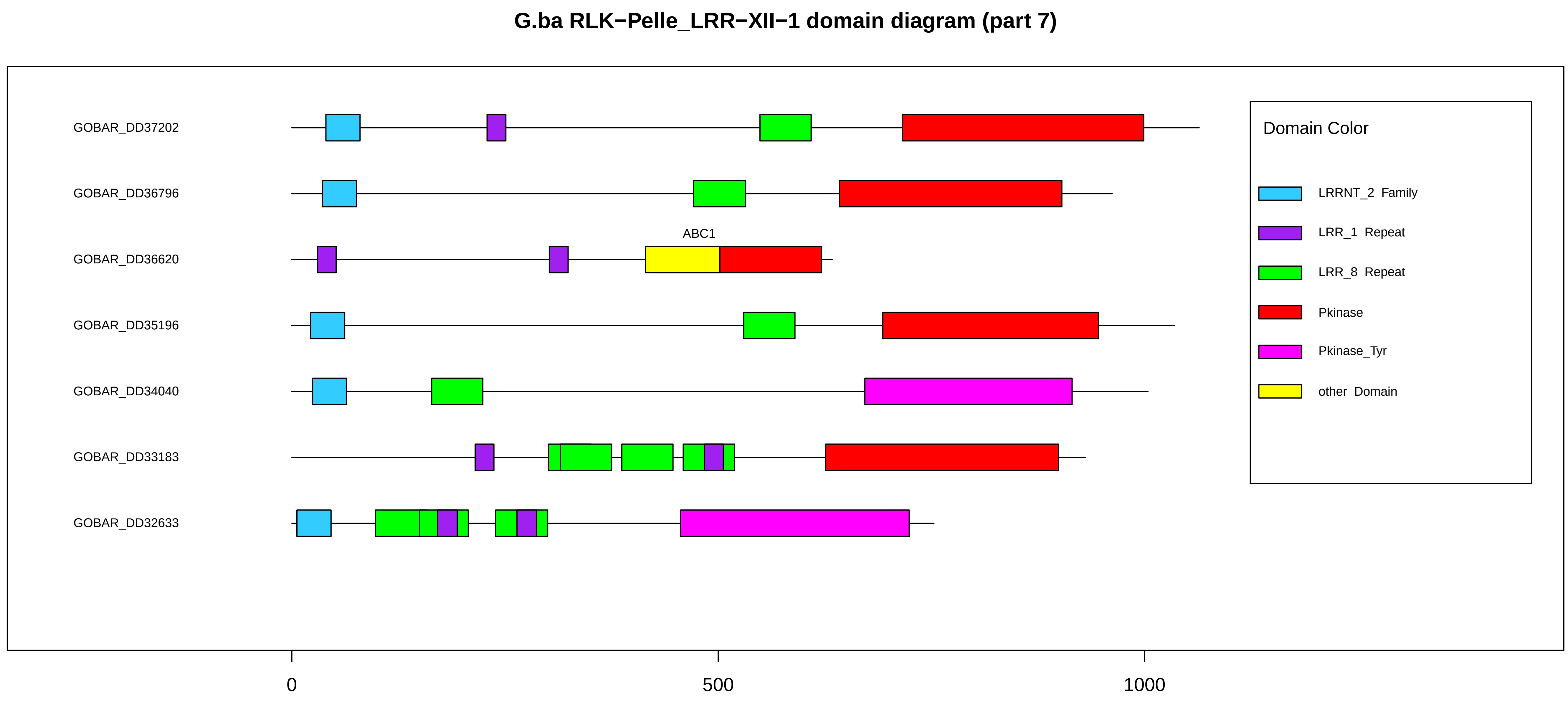

G.ba RLK−Pelle_LRR−XII−1 domain diagram (part 7)
GOBAR_DD37202
GOBAR_DD36796
GOBAR_DD36620
GOBAR_DD35196
GOBAR_DD34040
GOBAR_DD33183
GOBAR_DD32633
ABC1
Domain Color
LRRNT_2 Family
LRR_1 Repeat
LRR_8 Repeat
Pkinase
Pkinase_Tyr
other Domain
0
500
1000

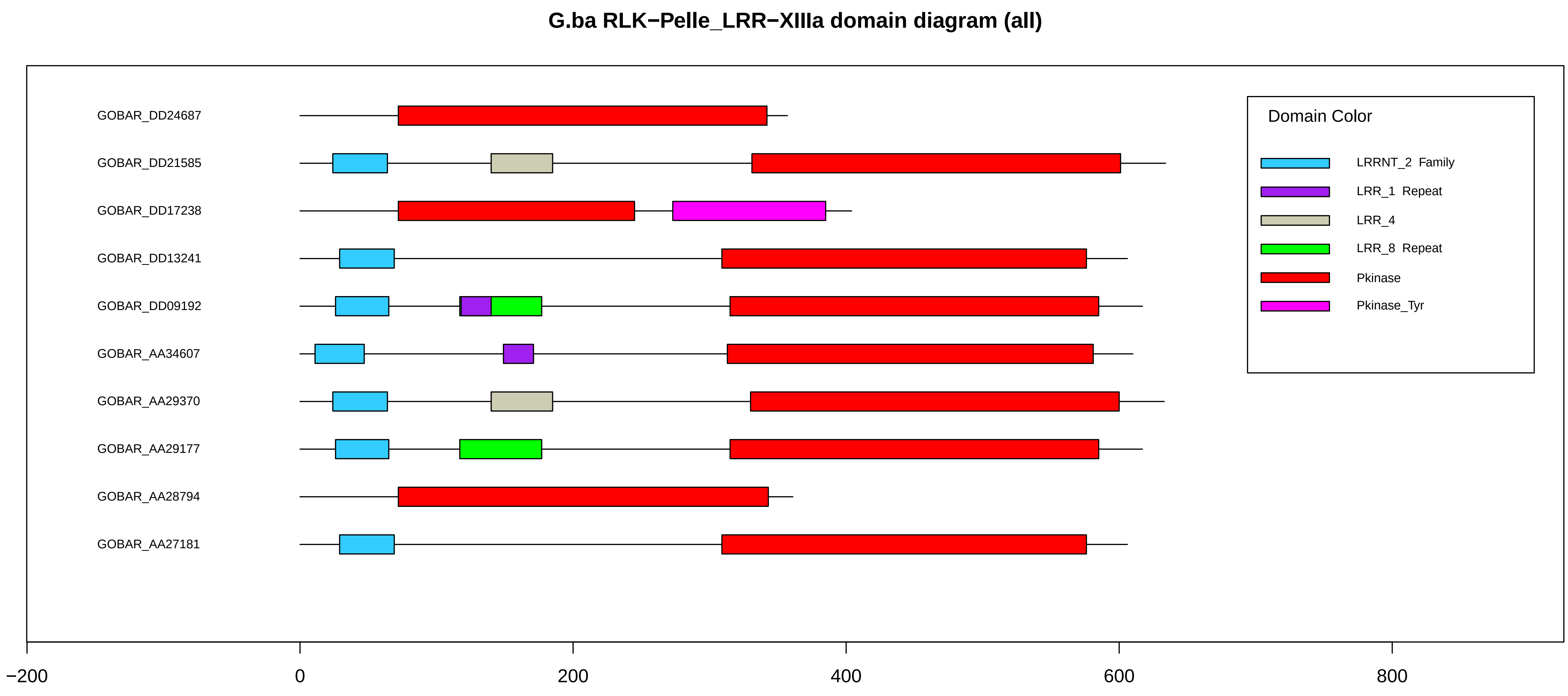
G.ba RLK−Pelle_LRR−XIIIa domain diagram (all)
GOBAR_DD24687
GOBAR_DD21585
GOBAR_DD17238
GOBAR_DD13241
GOBAR_DD09192
GOBAR_AA34607
GOBAR_AA29370
GOBAR_AA29177
GOBAR_AA28794
GOBAR_AA27181
Domain Color
LRRNT_2 Family
LRR_1 Repeat
LRR_4
LRR_8 Repeat
Pkinase
Pkinase_Tyr
−200
0
200
400
600
800

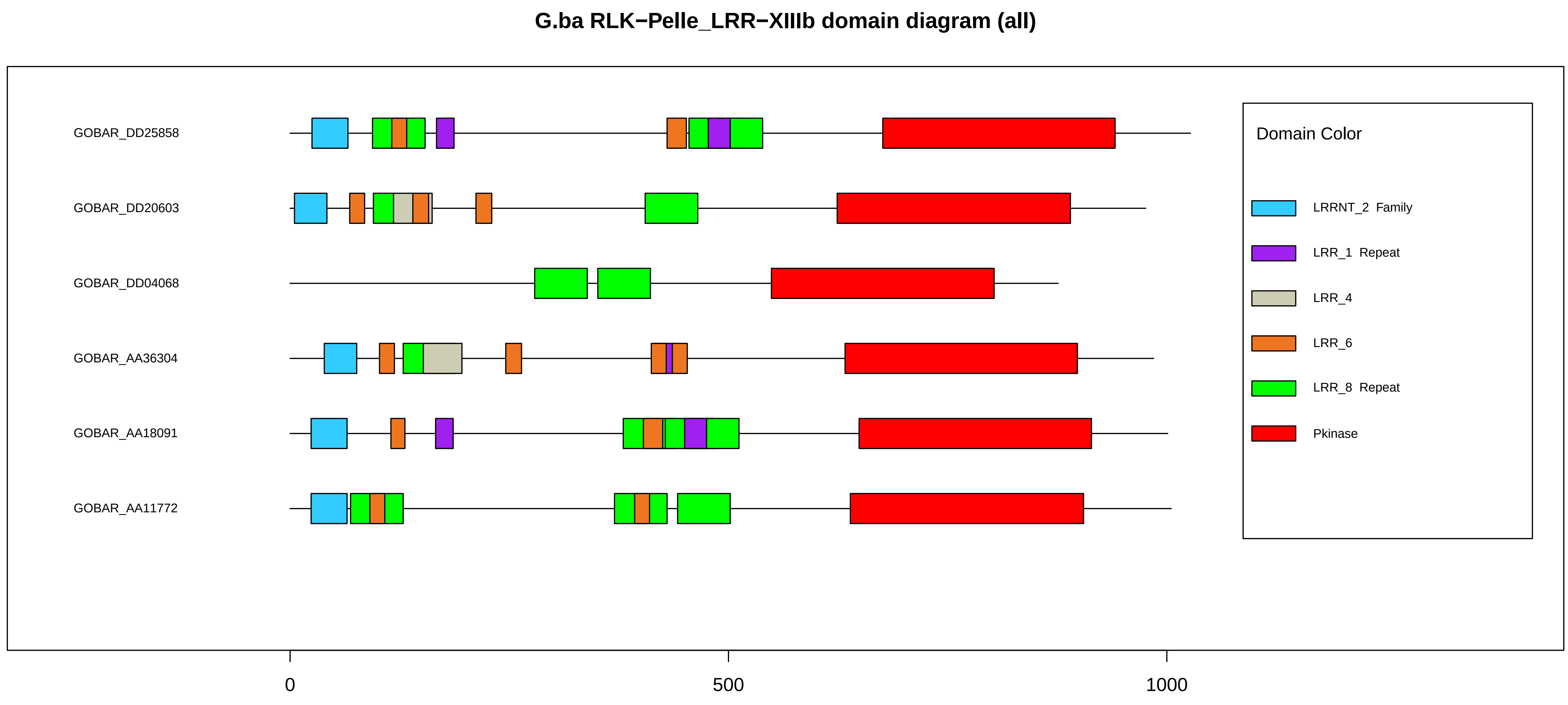
G.ba RLK−Pelle_LRR−XIIIb domain diagram (all)
GOBAR_DD25858
GOBAR_DD20603
GOBAR_DD04068
GOBAR_AA36304
GOBAR_AA18091
GOBAR_AA11772
Domain Color
LRRNT_2 Family
LRR_1 Repeat
LRR_4
LRR_6
LRR_8 Repeat
Pkinase
0
500
1000

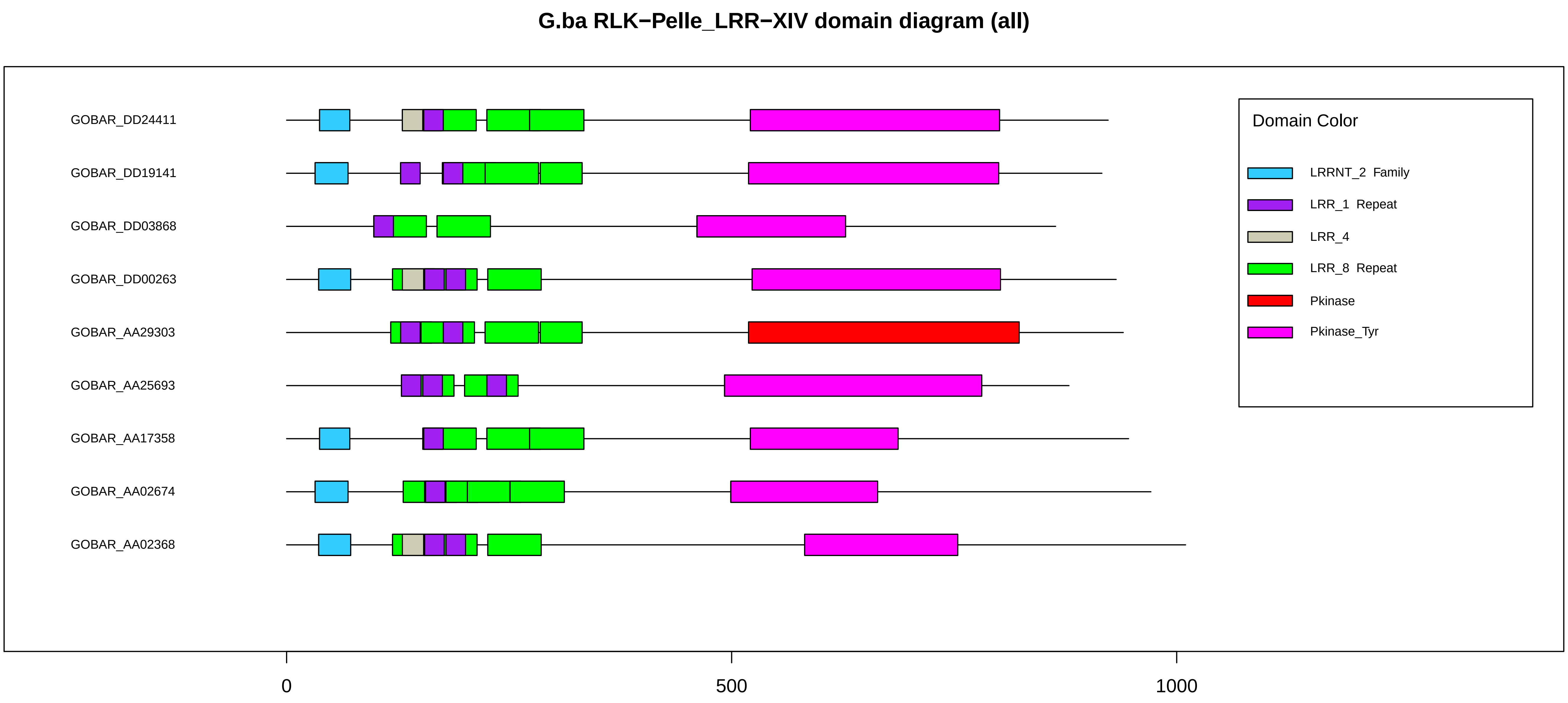
G.ba RLK−Pelle_LRR−XIV domain diagram (all)
GOBAR_DD24411
GOBAR_DD19141
GOBAR_DD03868
GOBAR_DD00263
GOBAR_AA29303
GOBAR_AA25693
GOBAR_AA17358
GOBAR_AA02674
GOBAR_AA02368
Domain Color
LRRNT_2 Family
LRR_1 Repeat
LRR_4
LRR_8 Repeat
Pkinase
Pkinase_Tyr
0
500
1000

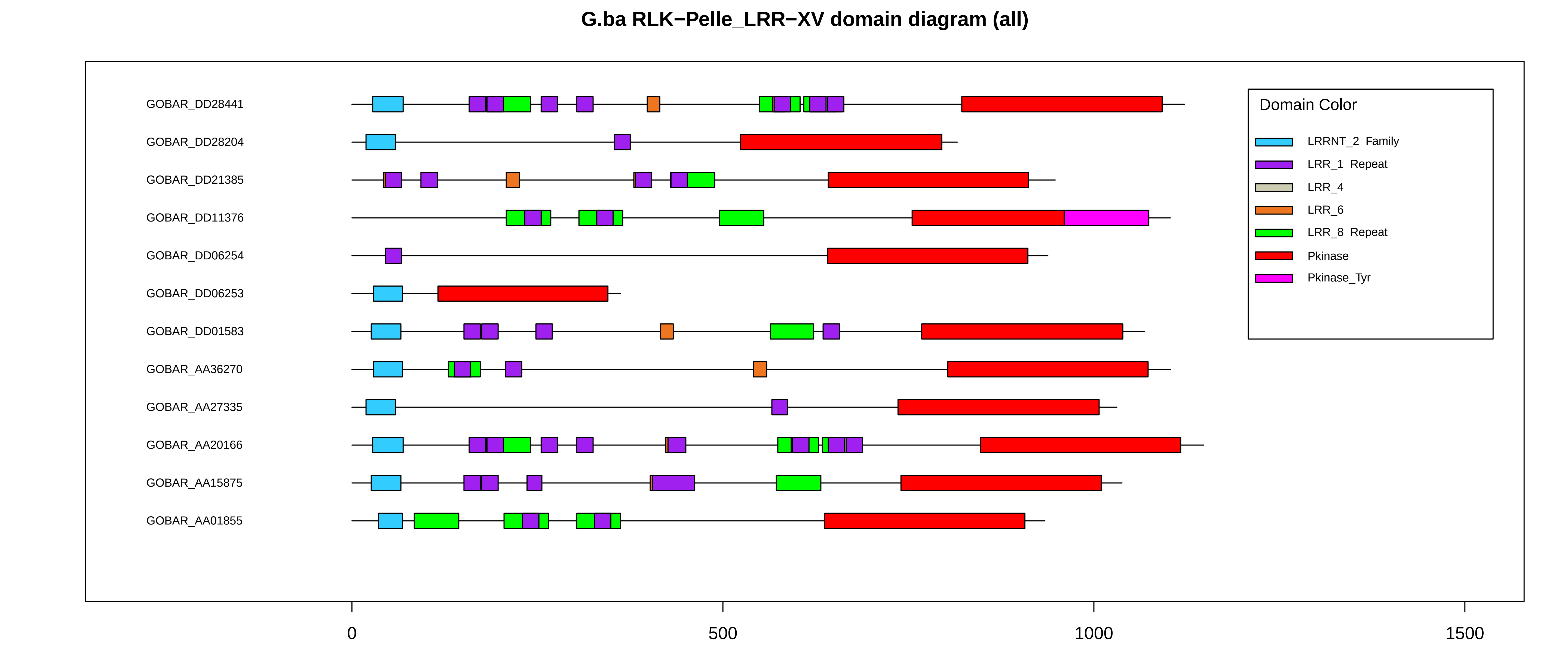
G.ba RLK−Pelle_LRR−XV domain diagram (all)
GOBAR_DD28441
GOBAR_DD28204
GOBAR_DD21385
GOBAR_DD11376
GOBAR_DD06254
GOBAR_DD06253
GOBAR_DD01583
GOBAR_AA36270
GOBAR_AA27335
GOBAR_AA20166
GOBAR_AA15875
GOBAR_AA01855
Domain Color
LRRNT_2 Family
LRR_1 Repeat
LRR_4
LRR_6
LRR_8 Repeat
Pkinase
Pkinase_Tyr
0
500
1000
1500

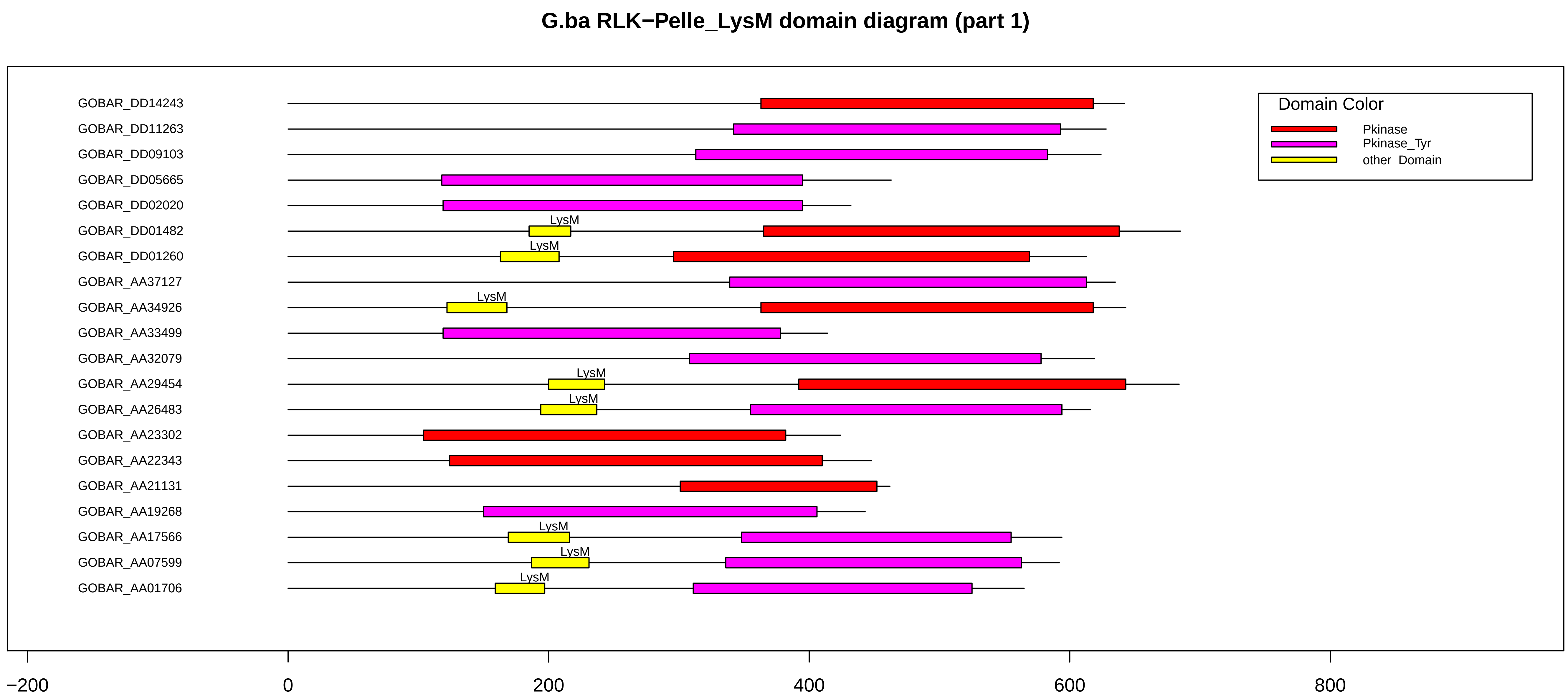
G.ba RLK−Pelle_LysM domain diagram (part 1)
Domain Color
Pkinase
Pkinase_Tyr
other Domain
GOBAR_DD14243
GOBAR_DD11263
GOBAR_DD09103
GOBAR_DD05665
GOBAR_DD02020
GOBAR_DD01482
GOBAR_DD01260
GOBAR_AA37127
GOBAR_AA34926
GOBAR_AA33499
GOBAR_AA32079
GOBAR_AA29454
GOBAR_AA26483
GOBAR_AA23302
GOBAR_AA22343
GOBAR_AA21131
GOBAR_AA19268
GOBAR_AA17566
GOBAR_AA07599
GOBAR_AA01706
LysM
−200
0
200
400
600
800

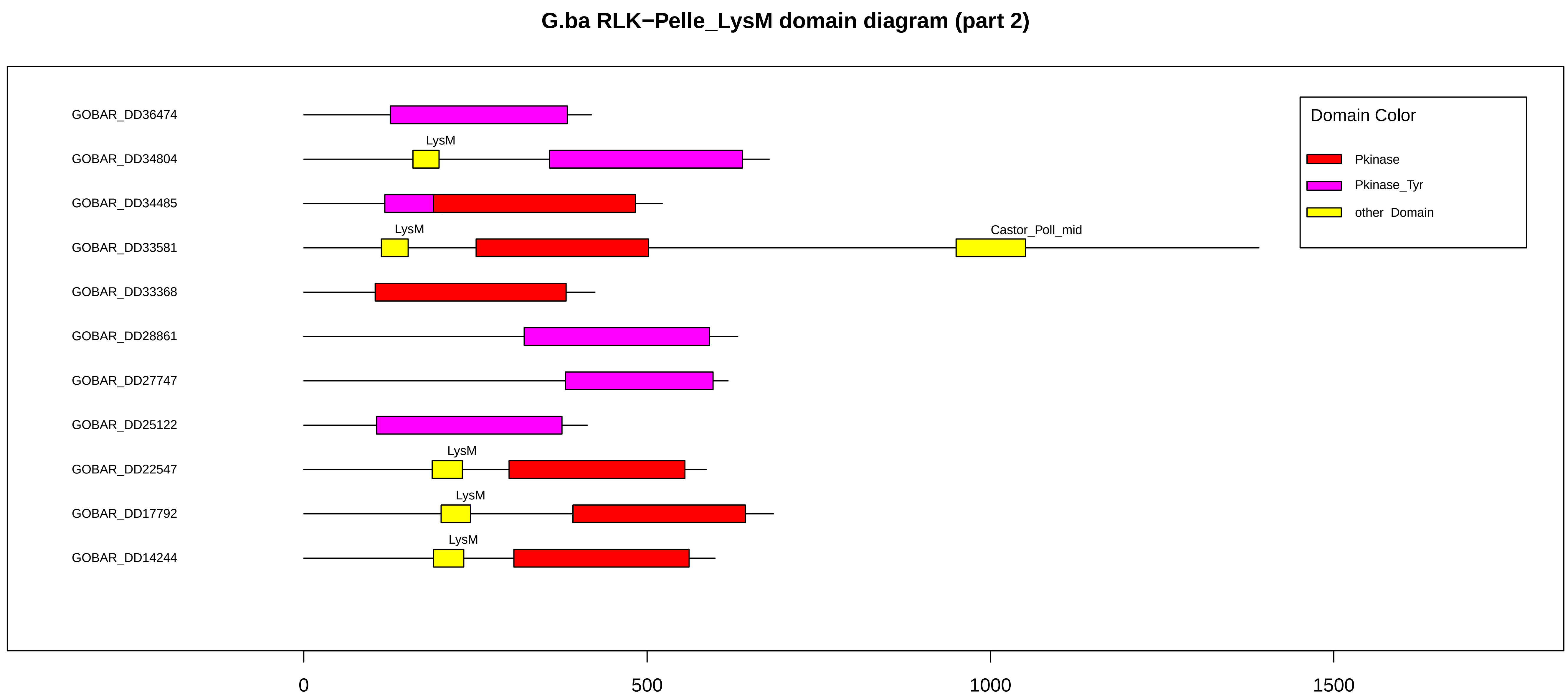
G.ba RLK−Pelle_LysM domain diagram (part 2)
GOBAR_DD36474
GOBAR_DD34804
GOBAR_DD34485
GOBAR_DD33581
GOBAR_DD33368
GOBAR_DD28861
GOBAR_DD27747
GOBAR_DD25122
GOBAR_DD22547
GOBAR_DD17792
GOBAR_DD14244
LysM
LysM
Castor_Poll_mid
LysM
LysM
LysM
Domain Color
Pkinase
Pkinase_Tyr
other Domain
0
500
1000
1500

G.ba RLK−Pelle_PERK−1 domain diagram (part 1)

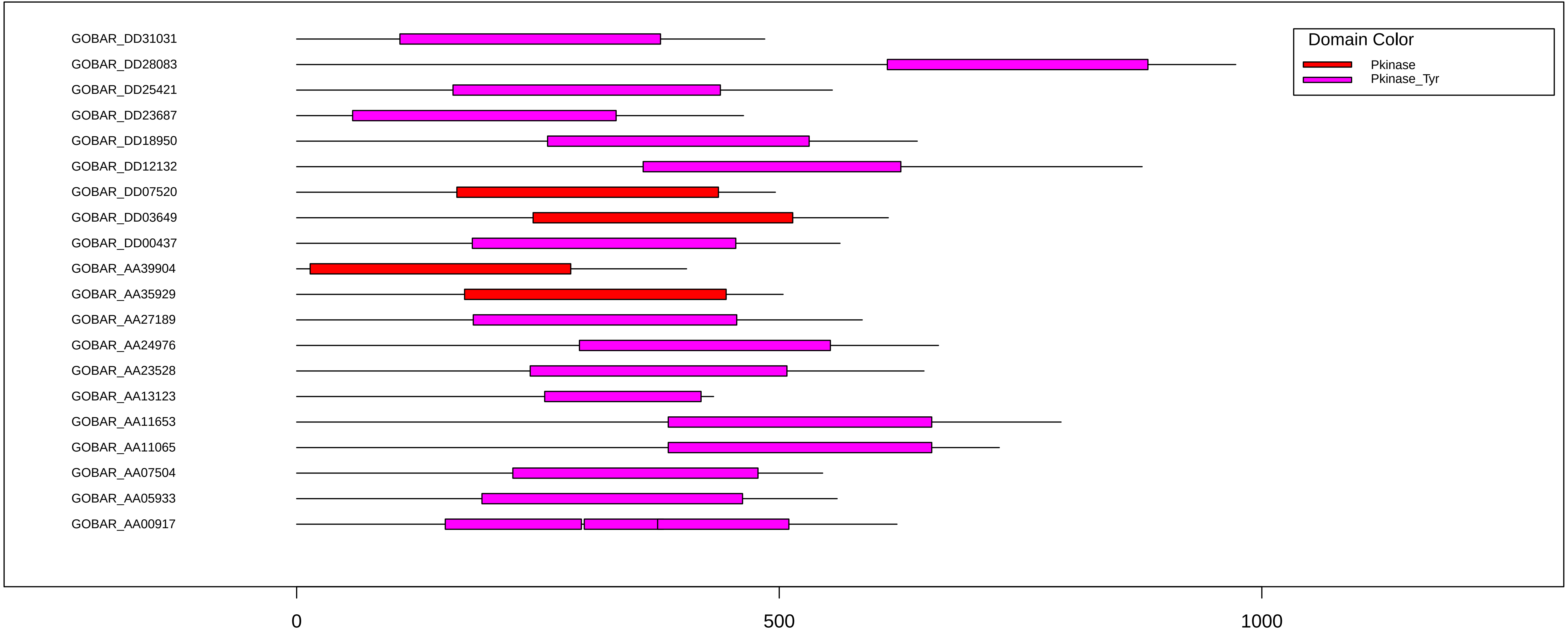

G.ba RLK−Pelle_PERK−1 domain diagram (part 2)

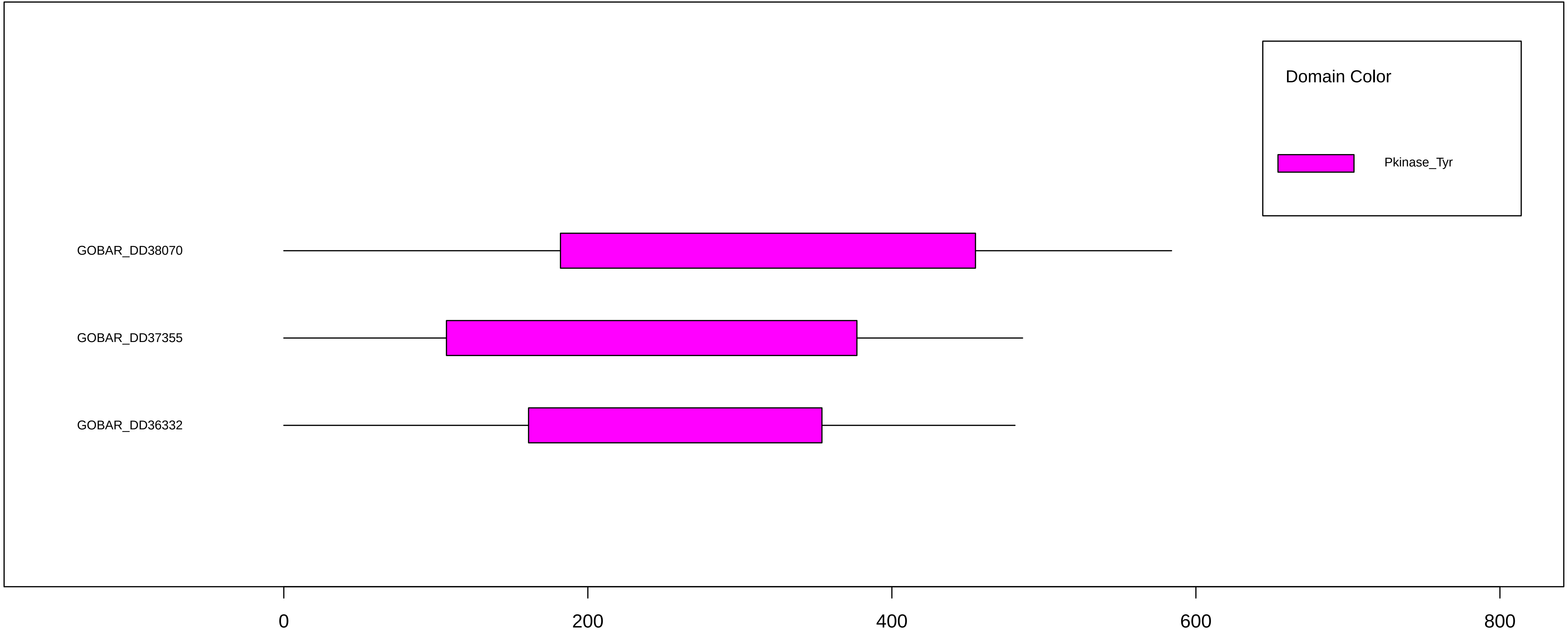

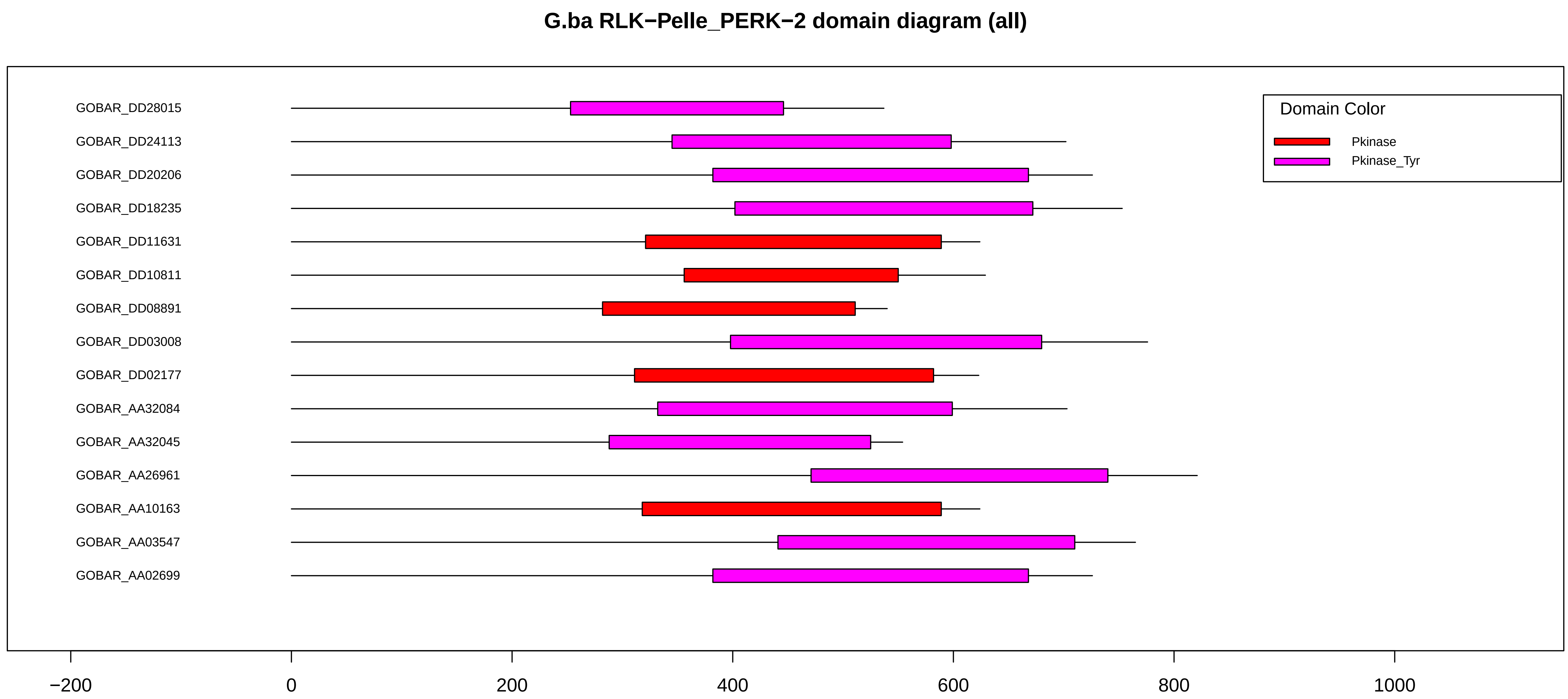
G.ba RLK−Pelle_PERK−2 domain diagram (all)
GOBAR_DD28015
GOBAR_DD24113
GOBAR_DD20206
GOBAR_DD18235
GOBAR_DD11631
GOBAR_DD10811
GOBAR_DD08891
GOBAR_DD03008
GOBAR_DD02177
GOBAR_AA32084
GOBAR_AA32045
GOBAR_AA26961
GOBAR_AA10163
GOBAR_AA03547
GOBAR_AA02699
Domain Color
Pkinase
Pkinase_Tyr
−200
0
200
400
600
800
1000

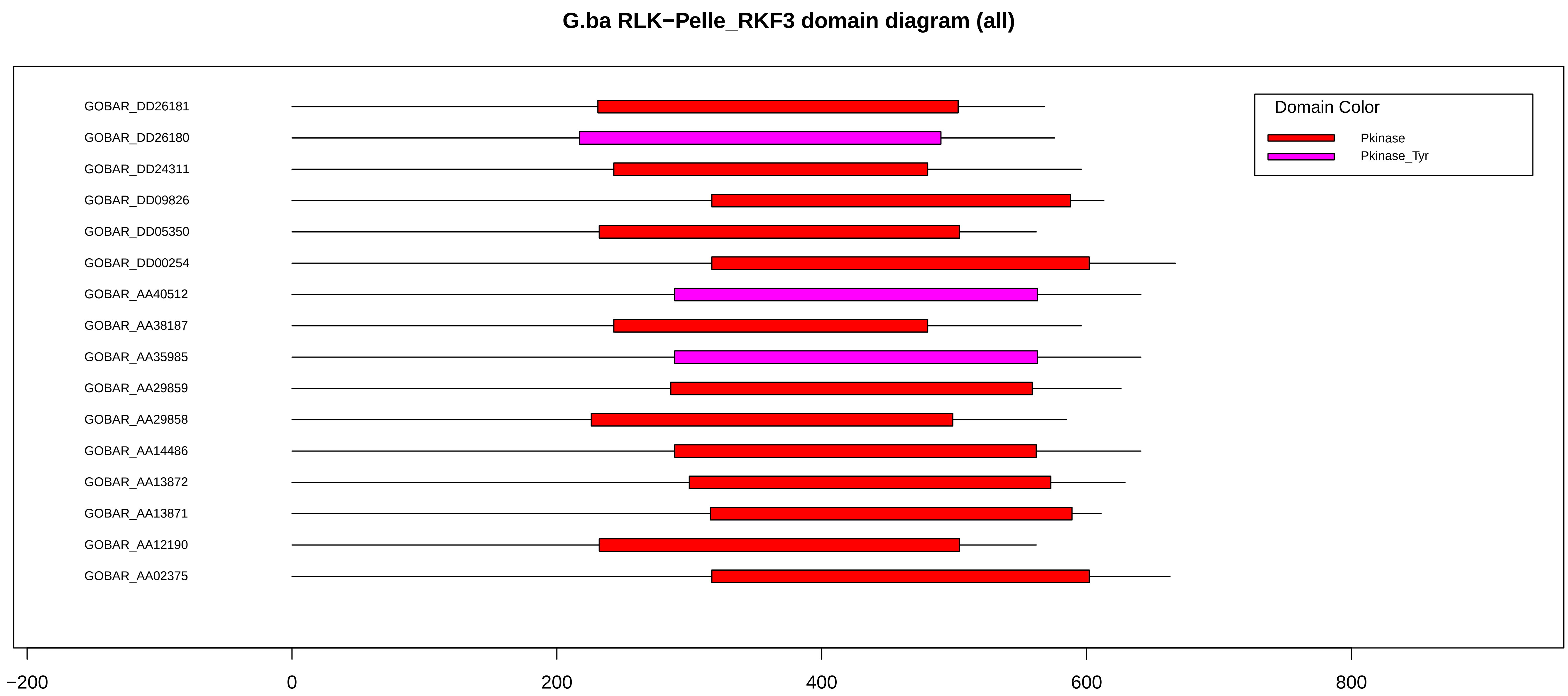
G.ba RLK−Pelle_RKF3 domain diagram (all)
GOBAR_DD26181
GOBAR_DD26180
GOBAR_DD24311
GOBAR_DD09826
GOBAR_DD05350
GOBAR_DD00254
GOBAR_AA40512
GOBAR_AA38187
GOBAR_AA35985
GOBAR_AA29859
GOBAR_AA29858
GOBAR_AA14486
GOBAR_AA13872
GOBAR_AA13871
GOBAR_AA12190
GOBAR_AA02375
Domain Color
Pkinase
Pkinase_Tyr
−200
0
200
400
600
800

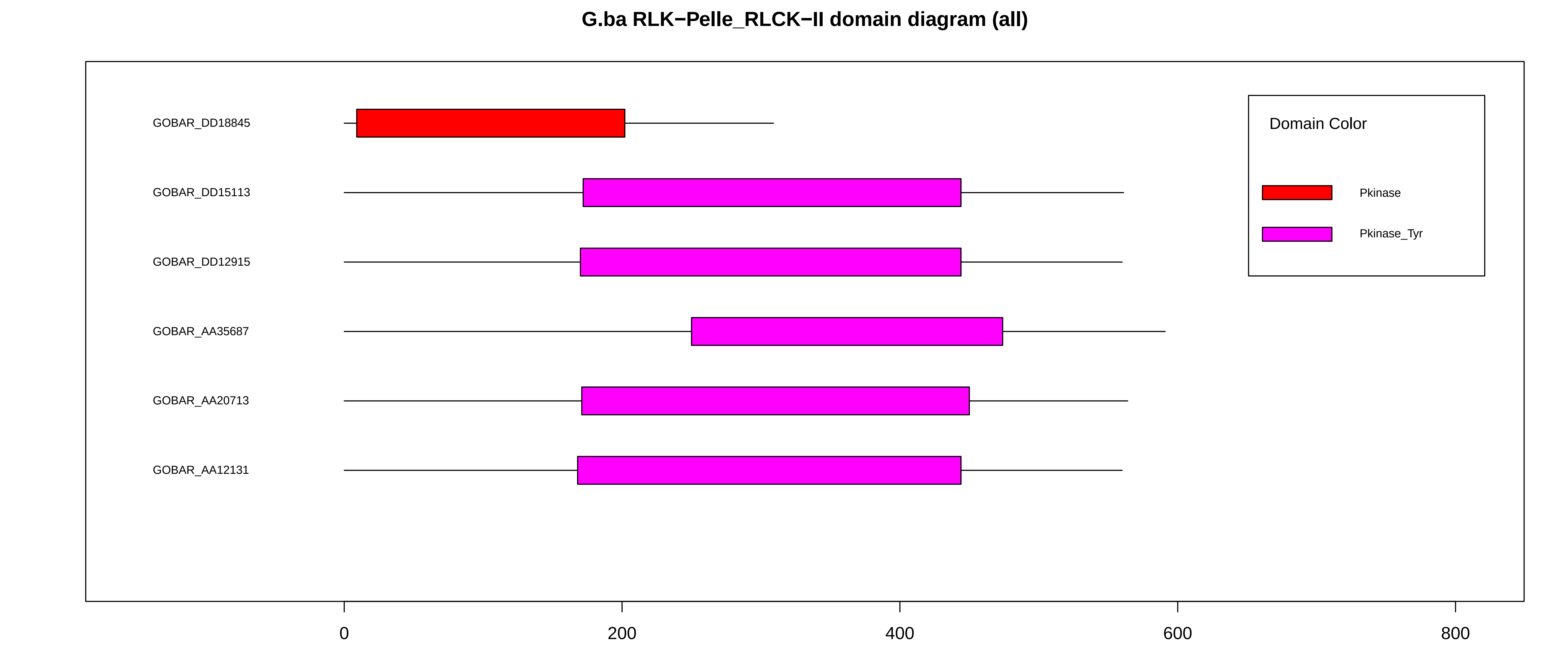

G.ba RLK−Pelle_RLCK−II domain diagram (all)
GOBAR_DD18845
GOBAR_DD15113
GOBAR_DD12915
GOBAR_AA35687
GOBAR_AA20713
GOBAR_AA12131
Domain Color
Pkinase
Pkinase_Tyr
0
200
400
600
800

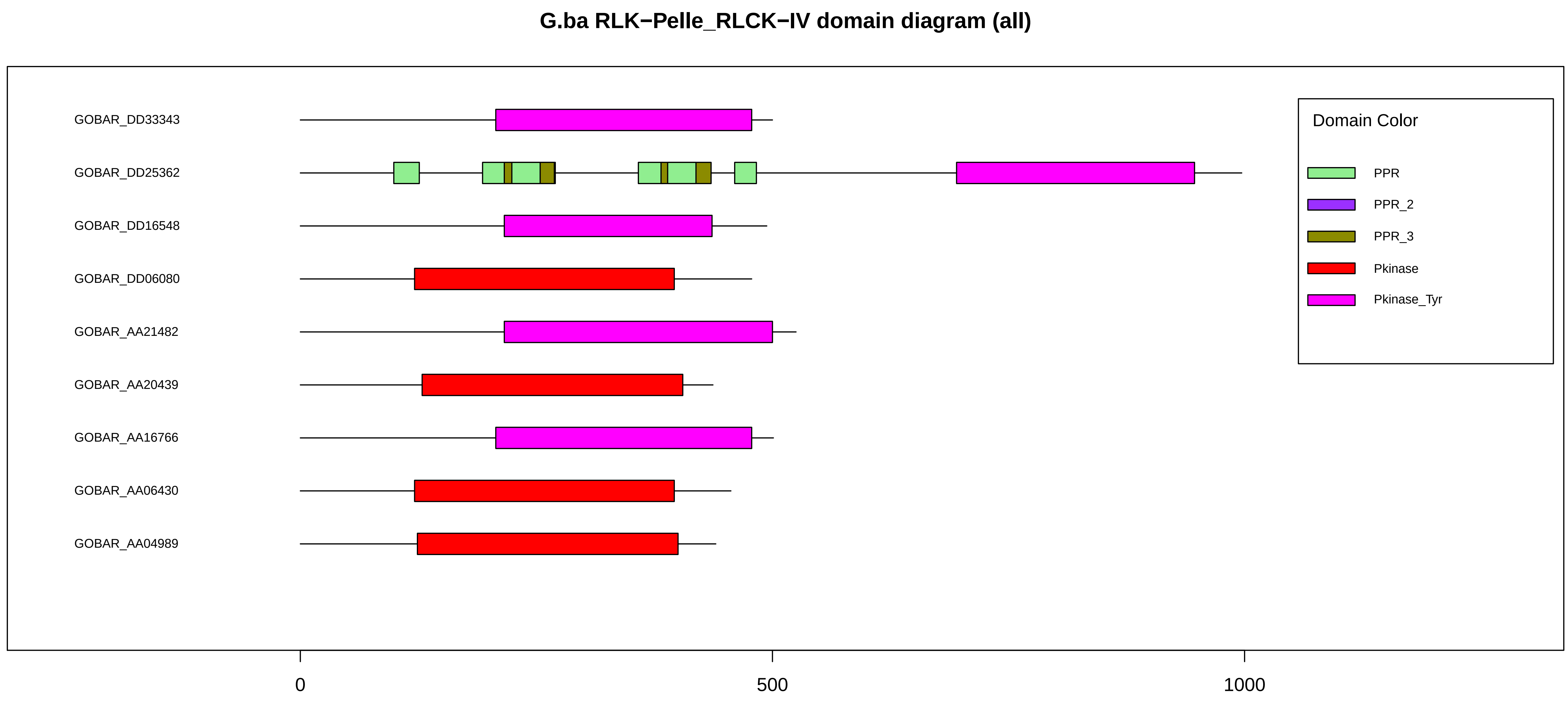

G.ba RLK−Pelle_RLCK−IV domain diagram (all)
GOBAR_DD33343
GOBAR_DD25362
GOBAR_DD16548
GOBAR_DD06080
GOBAR_AA21482
GOBAR_AA20439
GOBAR_AA16766
GOBAR_AA06430
GOBAR_AA04989
Domain Color
PPR
PPR_2
PPR_3
Pkinase
Pkinase_Tyr
0
500
1000

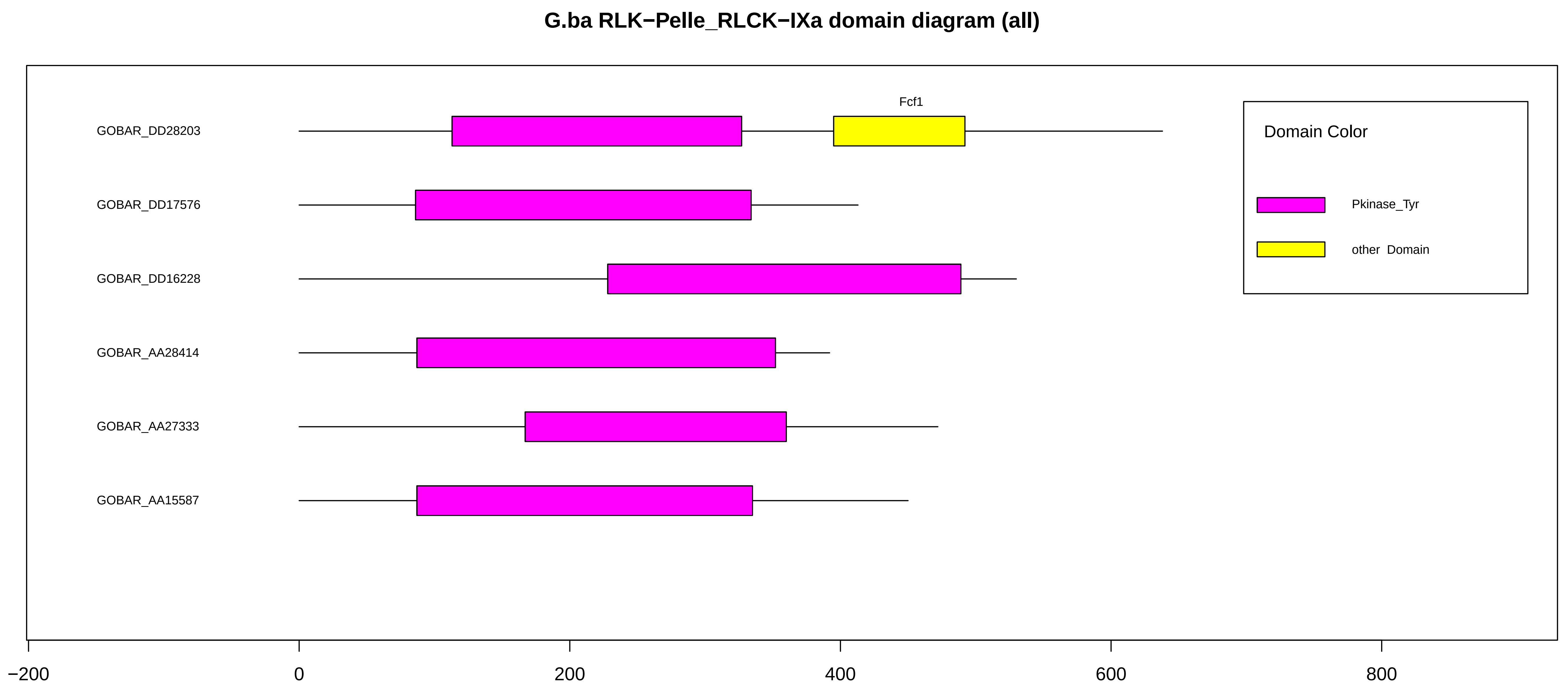

G.ba RLK−Pelle_RLCK−IXa domain diagram (all)
Fcf1
GOBAR_DD28203
GOBAR_DD17576
GOBAR_DD16228
GOBAR_AA28414
GOBAR_AA27333
GOBAR_AA15587
Domain Color
Pkinase_Tyr
other Domain
−200
0
200
400
600
800

G.ba RLK−Pelle_RLCK−IXb domain diagram (part 1)

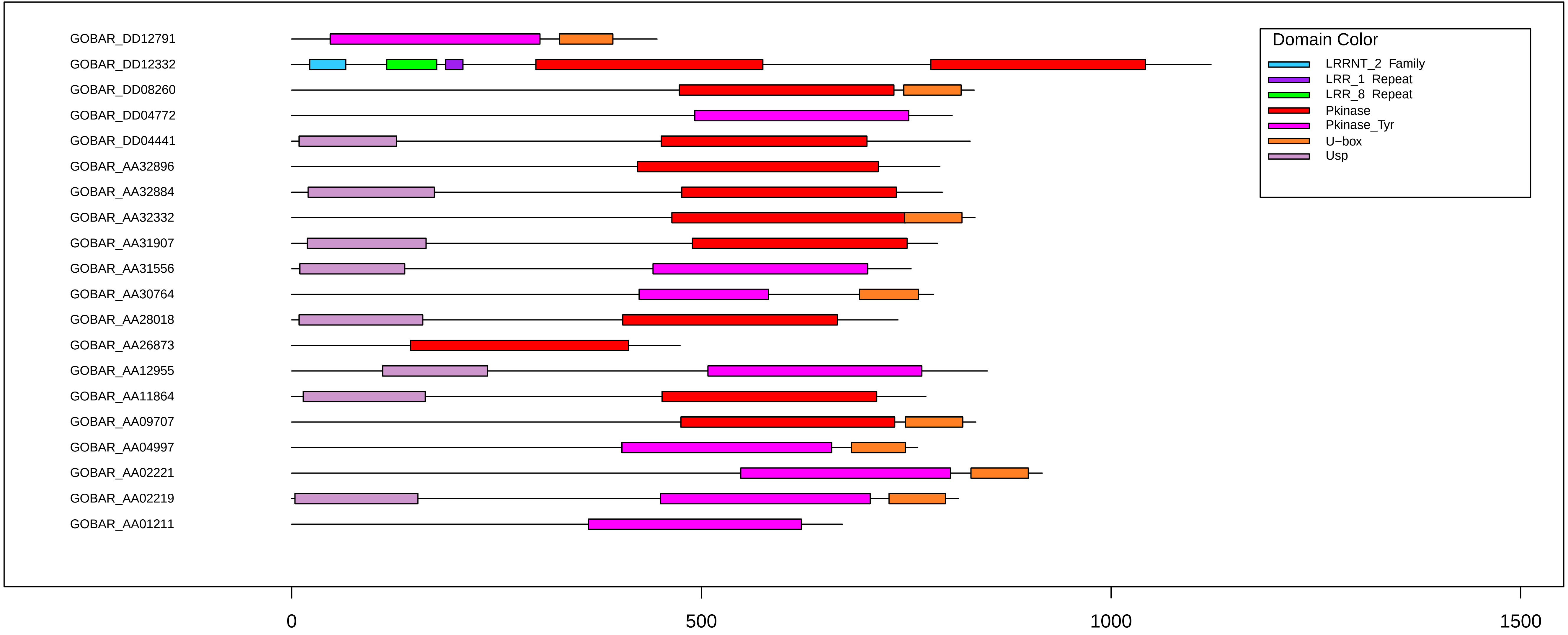

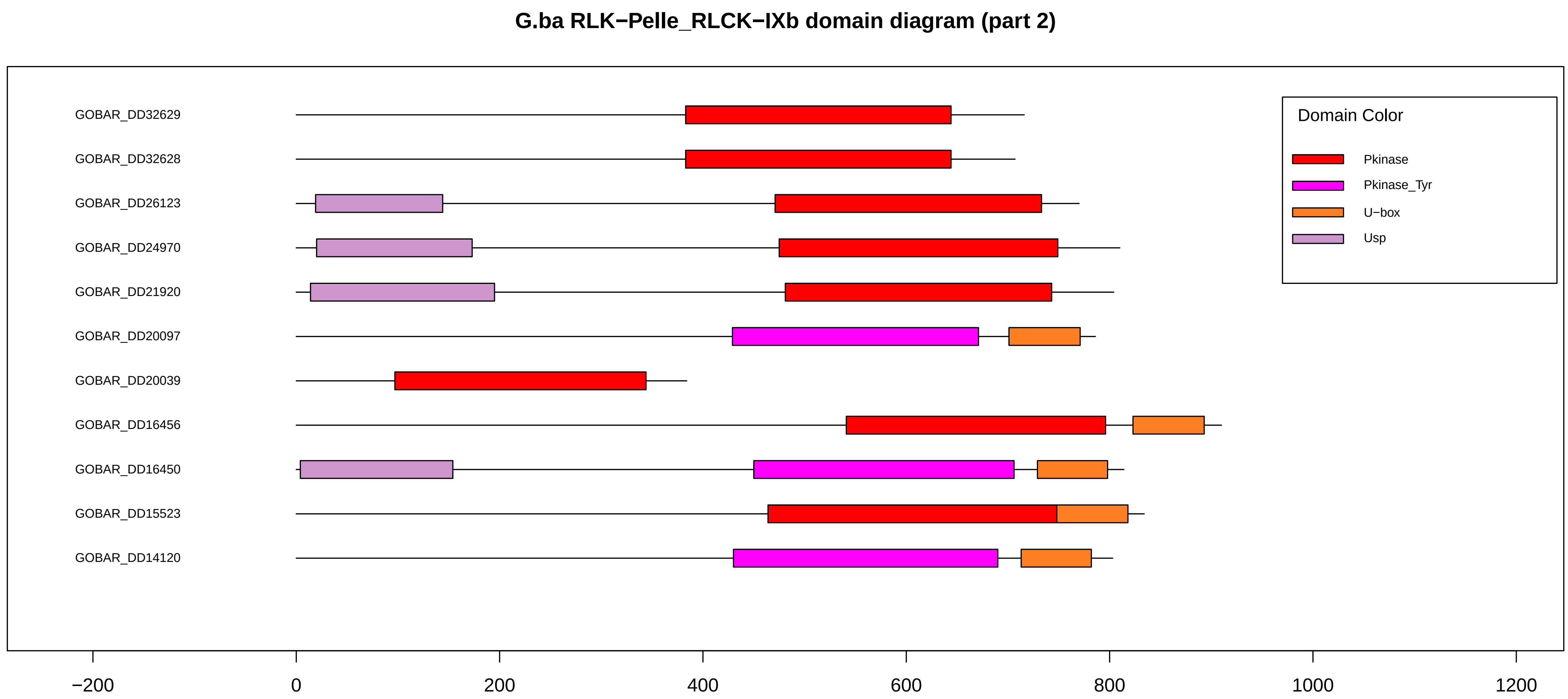
G.ba RLK−Pelle_RLCK−IXb domain diagram (part 2)
GOBAR_DD32629
GOBAR_DD32628
GOBAR_DD26123
GOBAR_DD24970
GOBAR_DD21920
GOBAR_DD20097
GOBAR_DD20039
GOBAR_DD16456
GOBAR_DD16450
GOBAR_DD15523
GOBAR_DD14120
Domain Color
Pkinase
Pkinase_Tyr
U−box
Usp
−200
0
200
400
600
800
1000
1200

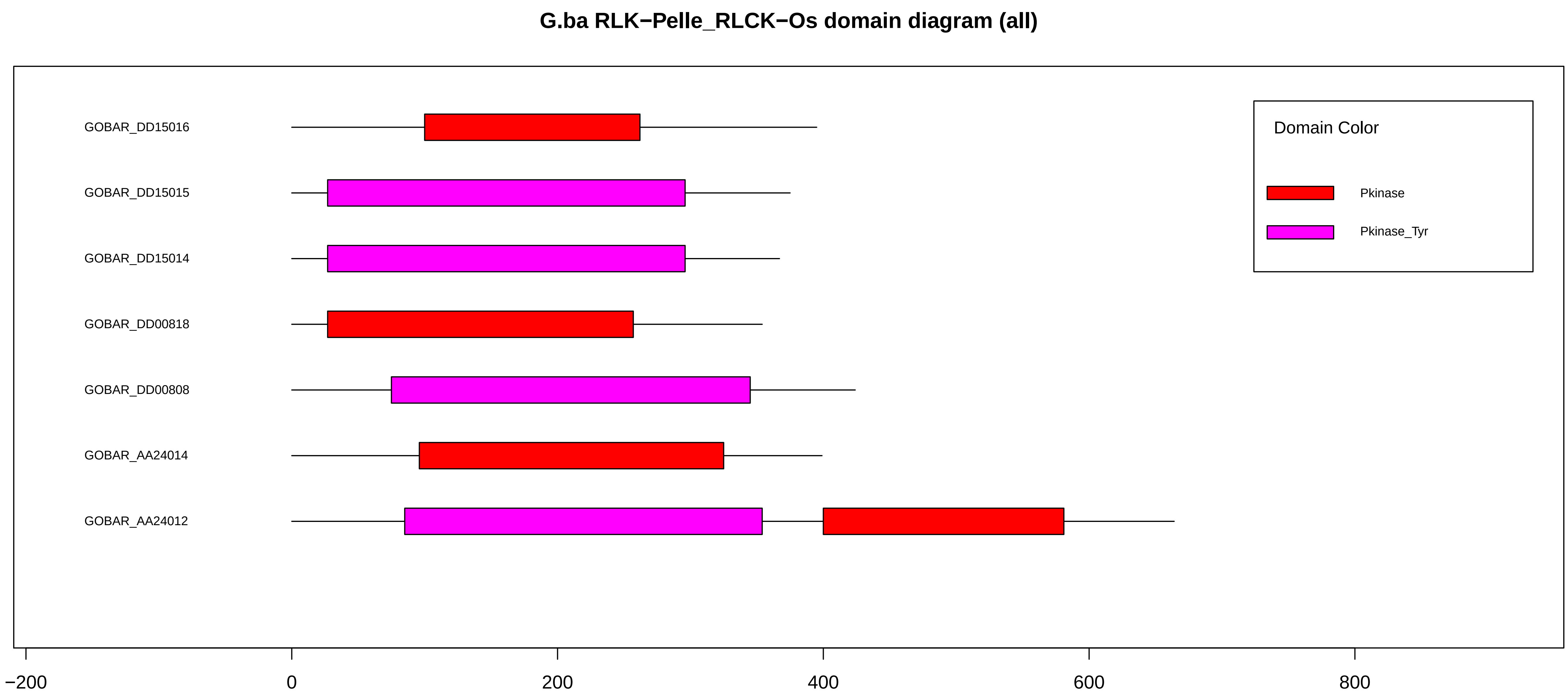
G.ba RLK−Pelle_RLCK−Os domain diagram (all)
GOBAR_DD15016
GOBAR_DD15015
GOBAR_DD15014
GOBAR_DD00818
GOBAR_DD00808
GOBAR_AA24014
GOBAR_AA24012
Domain Color
Pkinase
Pkinase_Tyr
−200
0
200
400
600
800

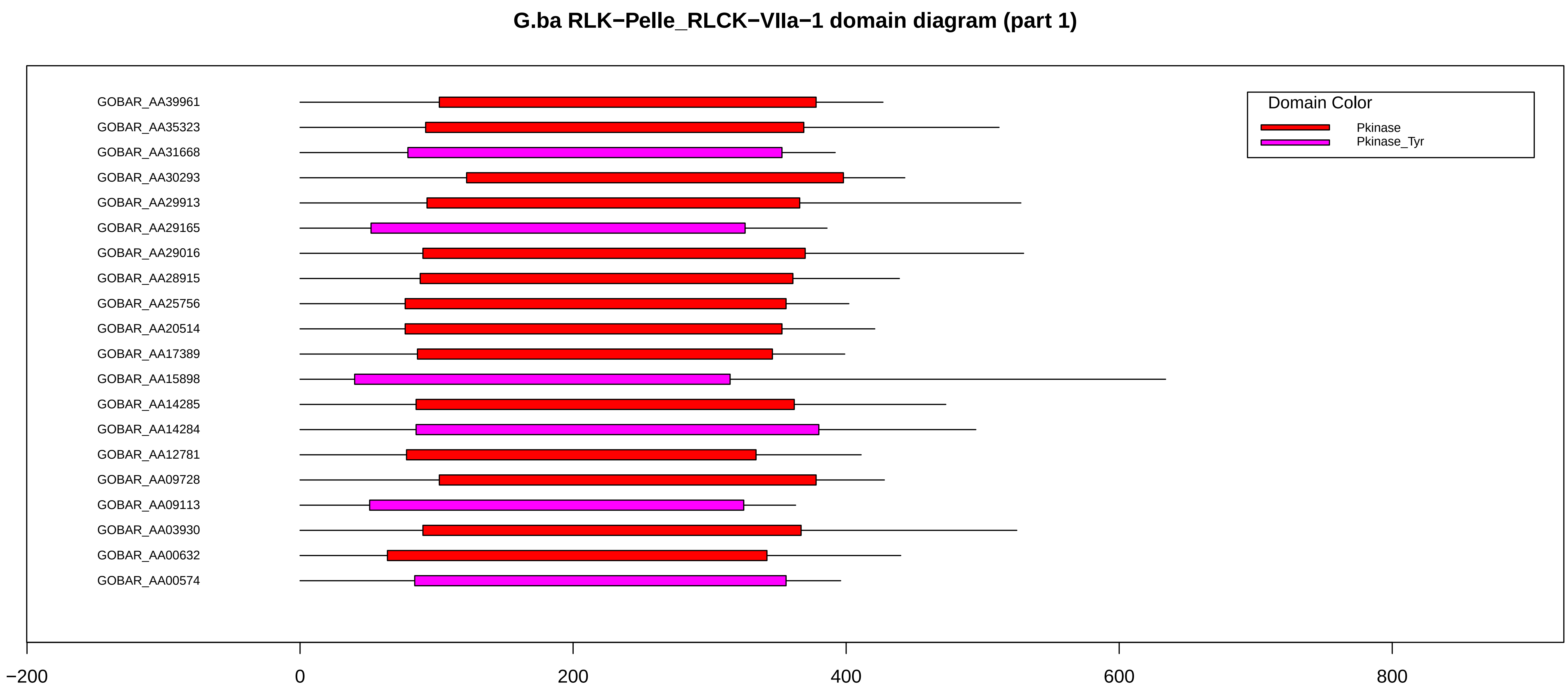
G.ba RLK−Pelle_RLCK−VIIa−1 domain diagram (part 1)
GOBAR_AA39961
GOBAR_AA35323
GOBAR_AA31668
GOBAR_AA30293
GOBAR_AA29913
GOBAR_AA29165
GOBAR_AA29016
GOBAR_AA28915
GOBAR_AA25756
GOBAR_AA20514
GOBAR_AA17389
GOBAR_AA15898
GOBAR_AA14285
GOBAR_AA14284
GOBAR_AA12781
GOBAR_AA09728
GOBAR_AA09113
GOBAR_AA03930
GOBAR_AA00632
GOBAR_AA00574
Domain Color
Pkinase
Pkinase_Tyr
−200
0
200
400
600
800

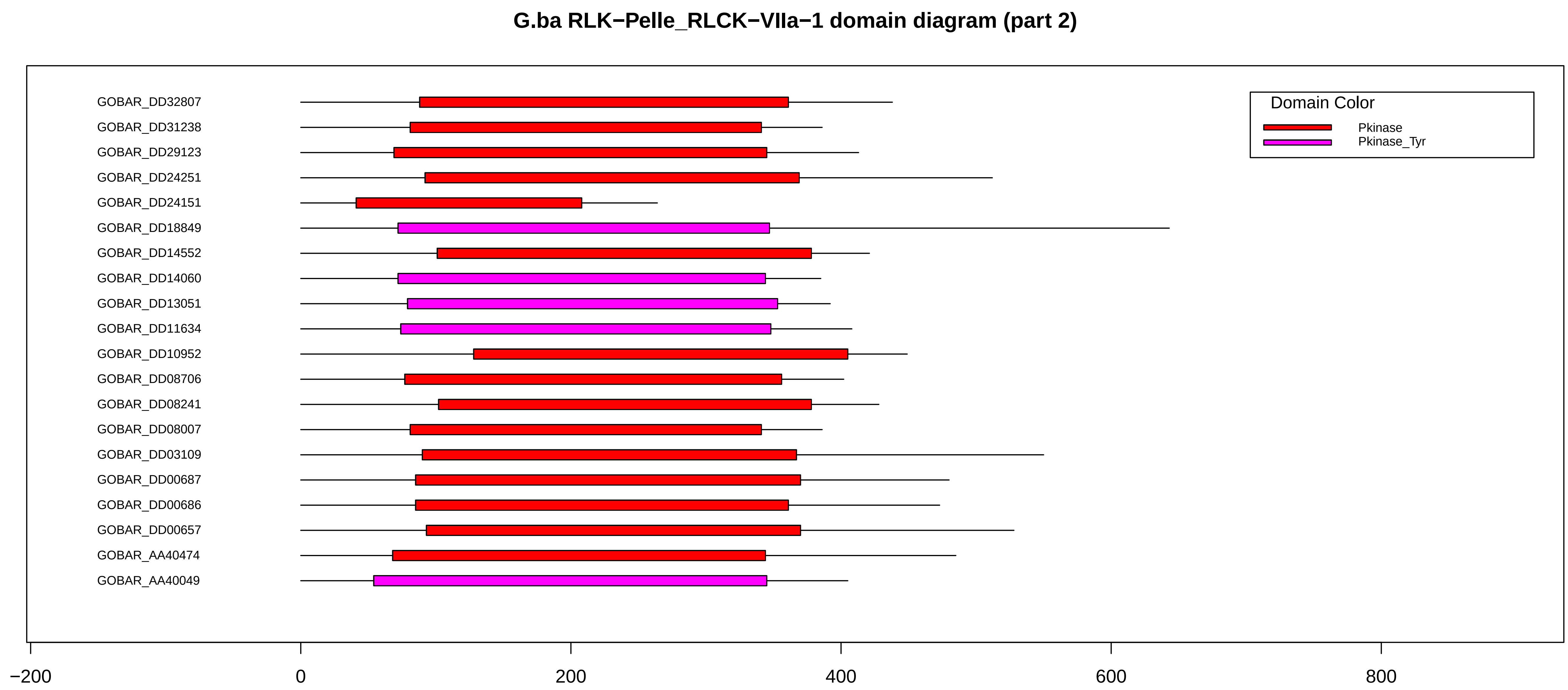
G.ba RLK−Pelle_RLCK−VIIa−1 domain diagram (part 2)
GOBAR_DD32807
GOBAR_DD31238
GOBAR_DD29123
GOBAR_DD24251
GOBAR_DD24151
GOBAR_DD18849
GOBAR_DD14552
GOBAR_DD14060
GOBAR_DD13051
GOBAR_DD11634
GOBAR_DD10952
GOBAR_DD08706
GOBAR_DD08241
GOBAR_DD08007
GOBAR_DD03109
GOBAR_DD00687
GOBAR_DD00686
GOBAR_DD00657
GOBAR_AA40474
GOBAR_AA40049
Domain Color
Pkinase
Pkinase_Tyr
−200
0
200
400
600
800

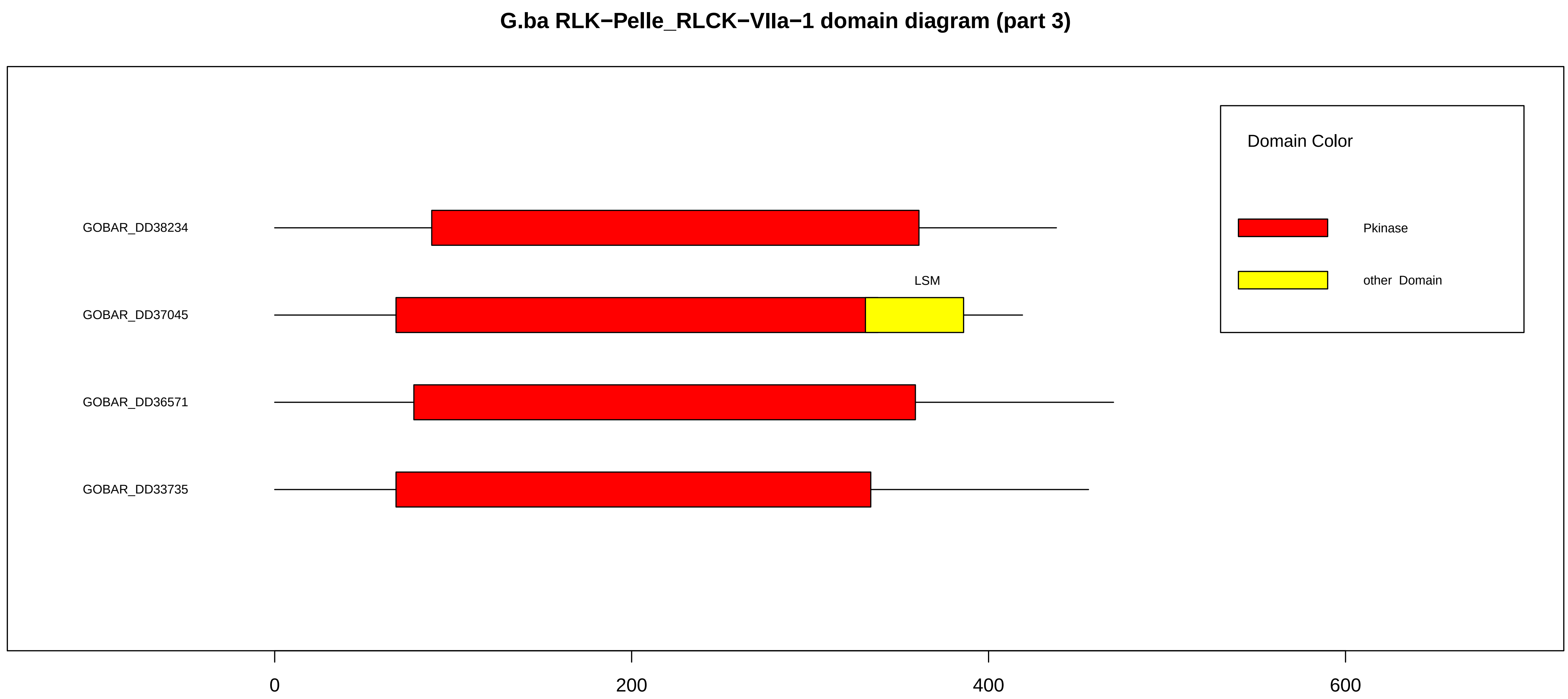
G.ba RLK−Pelle_RLCK−VIIa−1 domain diagram (part 3)
GOBAR_DD38234
GOBAR_DD37045
GOBAR_DD36571
GOBAR_DD33735
LSM
Domain Color
Pkinase
other Domain
0
200
400
600

G.ba RLK−Pelle_RLCK−VIIa−2 domain diagram (part 1)

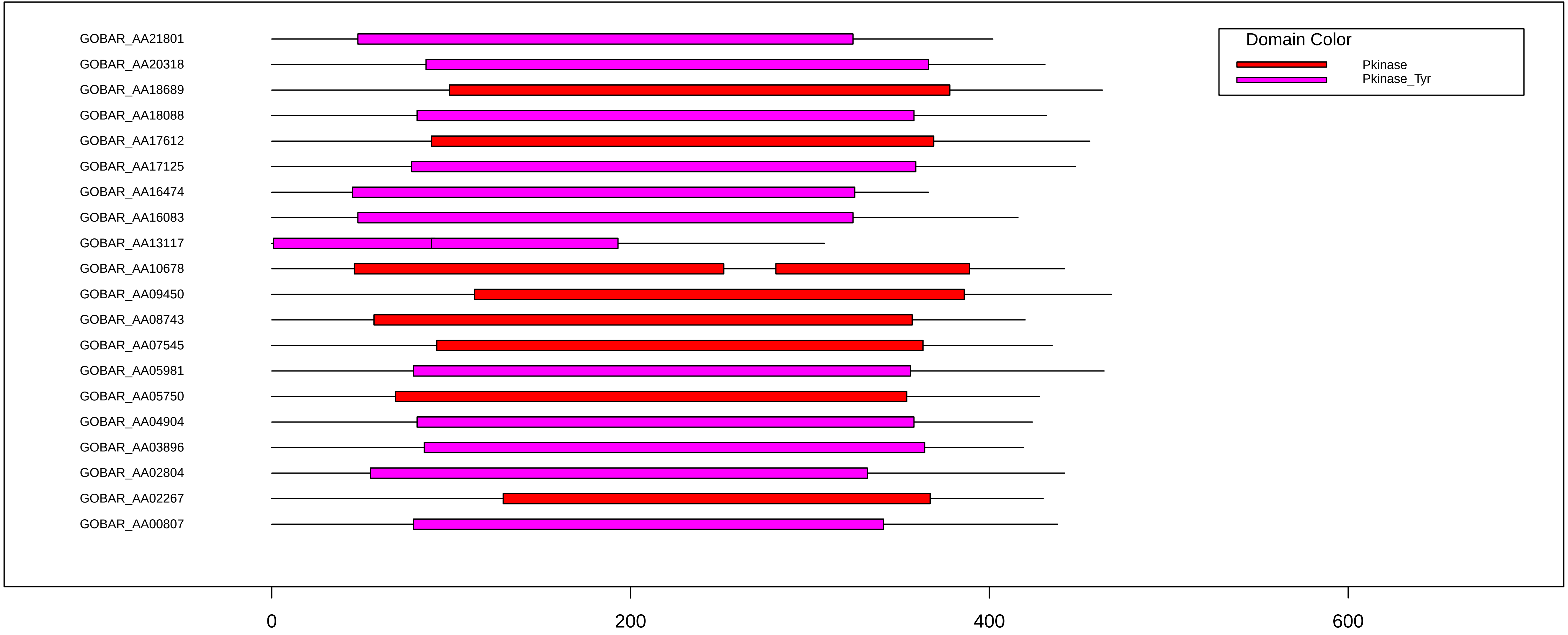

G.ba RLK−Pelle_RLCK−VIIa−2 domain diagram (part 2)

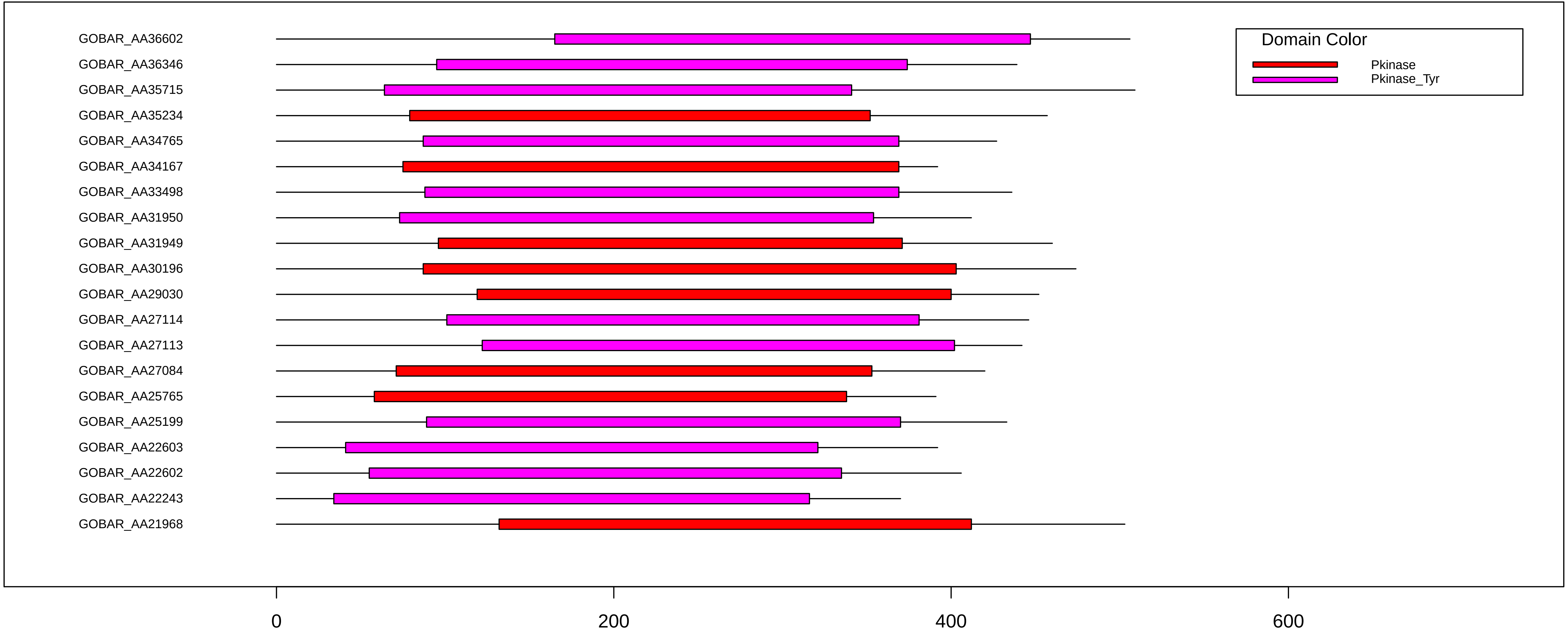

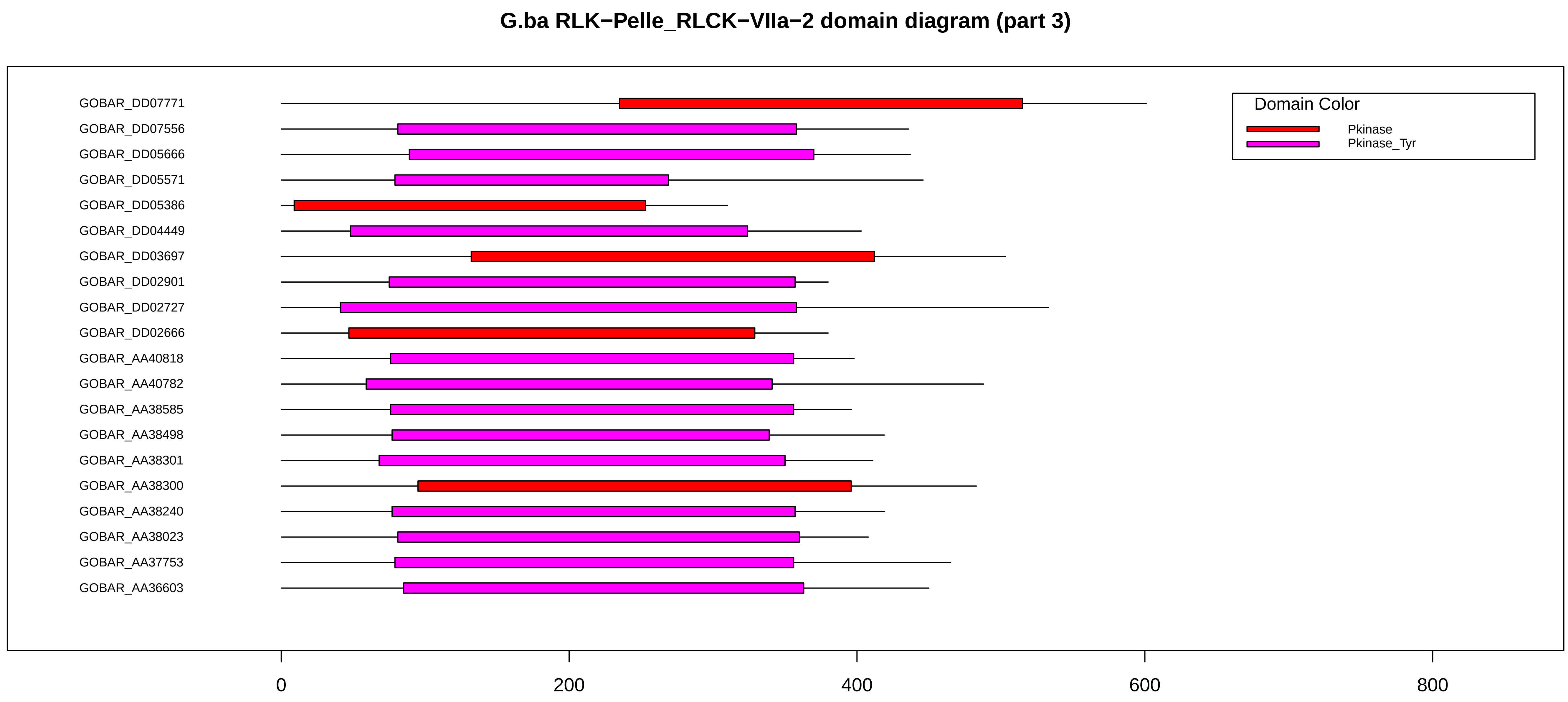
G.ba RLK−Pelle_RLCK−VIIa−2 domain diagram (part 3)
GOBAR_DD07771
GOBAR_DD07556
GOBAR_DD05666
GOBAR_DD05571
GOBAR_DD05386
GOBAR_DD04449
GOBAR_DD03697
GOBAR_DD02901
GOBAR_DD02727
GOBAR_DD02666
GOBAR_AA40818
GOBAR_AA40782
GOBAR_AA38585
GOBAR_AA38498
GOBAR_AA38301
GOBAR_AA38300
GOBAR_AA38240
GOBAR_AA38023
GOBAR_AA37753
GOBAR_AA36603
Domain Color
Pkinase
Pkinase_Tyr
0
200
400
600
800

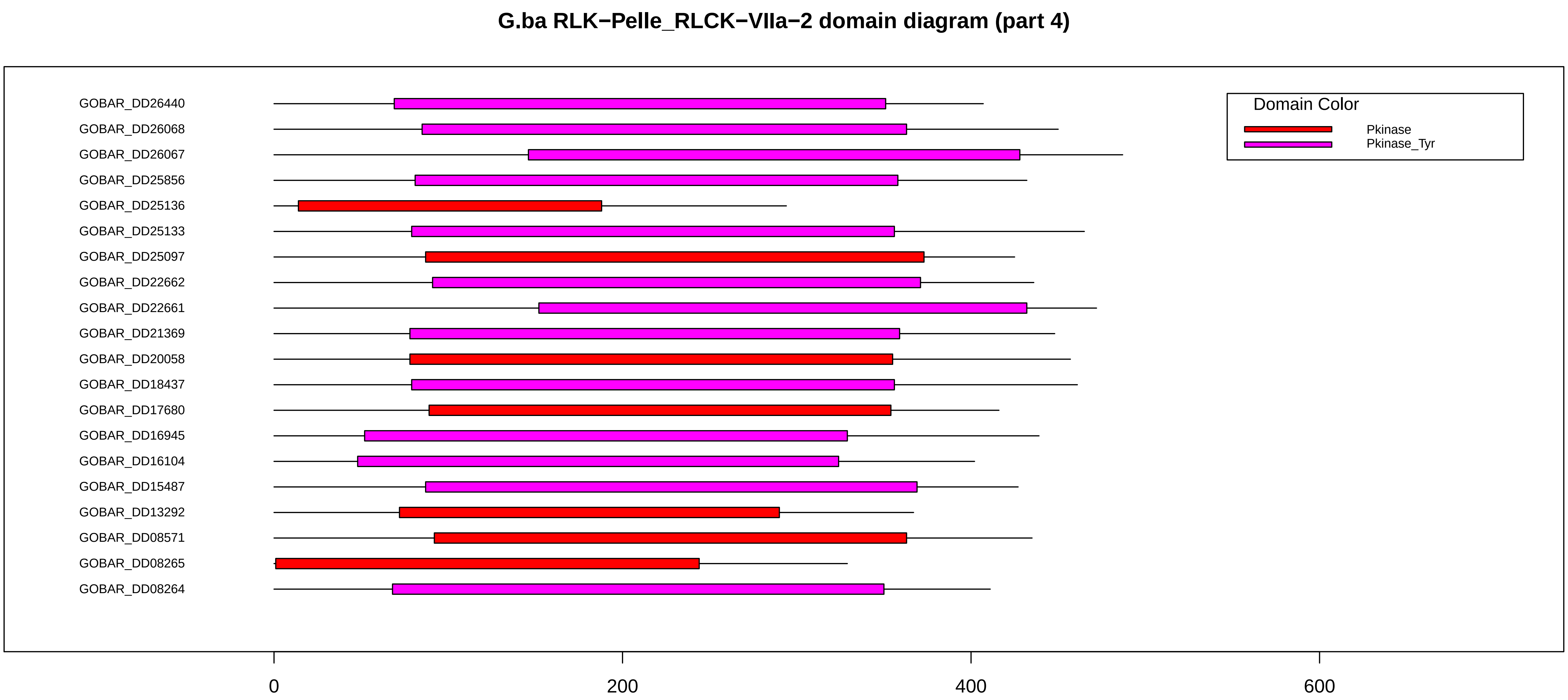

G.ba RLK−Pelle_RLCK−VIIa−2 domain diagram (part 4)
GOBAR_DD26440
GOBAR_DD26068
GOBAR_DD26067
GOBAR_DD25856
GOBAR_DD25136
GOBAR_DD25133
GOBAR_DD25097
GOBAR_DD22662
GOBAR_DD22661
GOBAR_DD21369
GOBAR_DD20058
GOBAR_DD18437
GOBAR_DD17680
GOBAR_DD16945
GOBAR_DD16104
GOBAR_DD15487
GOBAR_DD13292
GOBAR_DD08571
GOBAR_DD08265
GOBAR_DD08264
Domain Color
Pkinase
Pkinase_Tyr
0
200
400
600

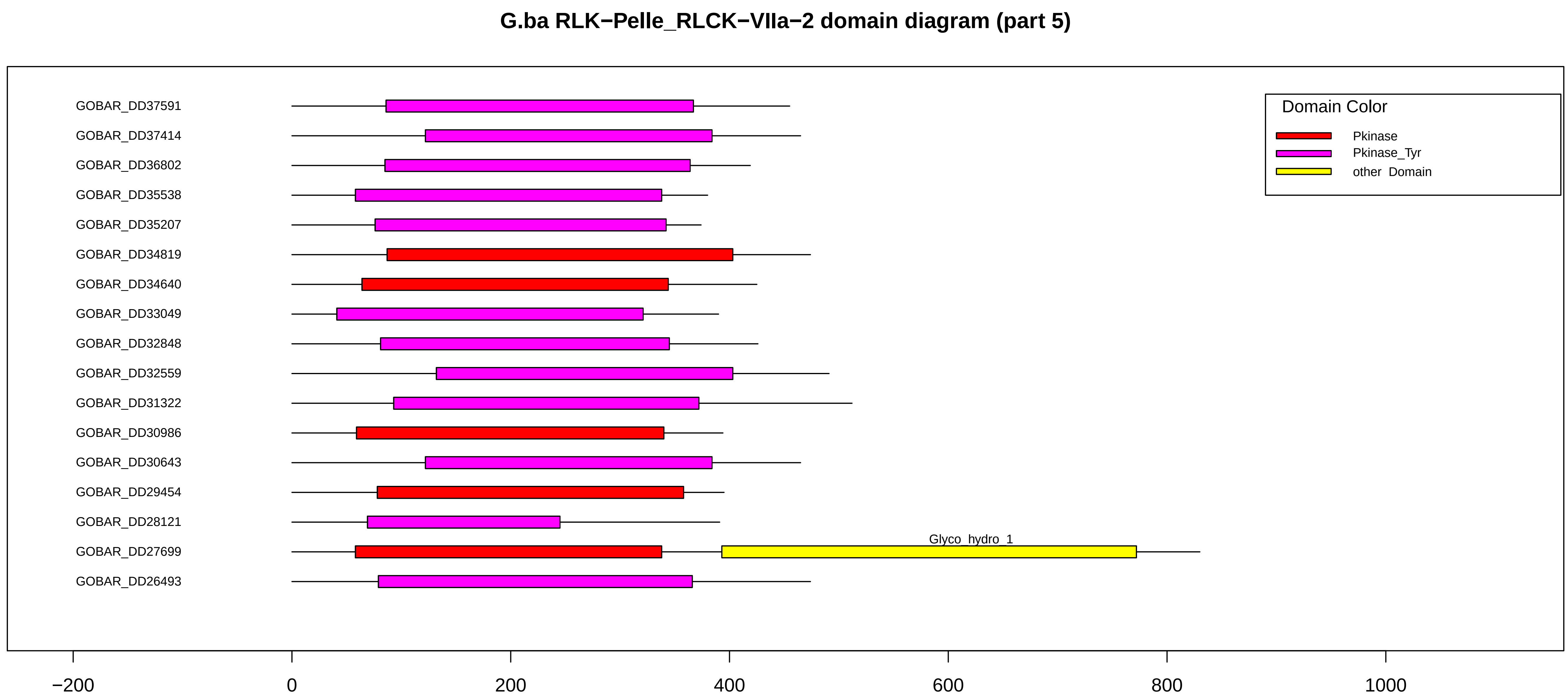
G.ba RLK−Pelle_RLCK−VIIa−2 domain diagram (part 5)
GOBAR_DD37591
GOBAR_DD37414
GOBAR_DD36802
GOBAR_DD35538
GOBAR_DD35207
GOBAR_DD34819
GOBAR_DD34640
GOBAR_DD33049
GOBAR_DD32848
GOBAR_DD32559
GOBAR_DD31322
GOBAR_DD30986
GOBAR_DD30643
GOBAR_DD29454
GOBAR_DD28121
GOBAR_DD27699
GOBAR_DD26493
Glyco_hydro_1
Domain Color
Pkinase
Pkinase_Tyr
other Domain
−200
0
200
400
600
800
1000

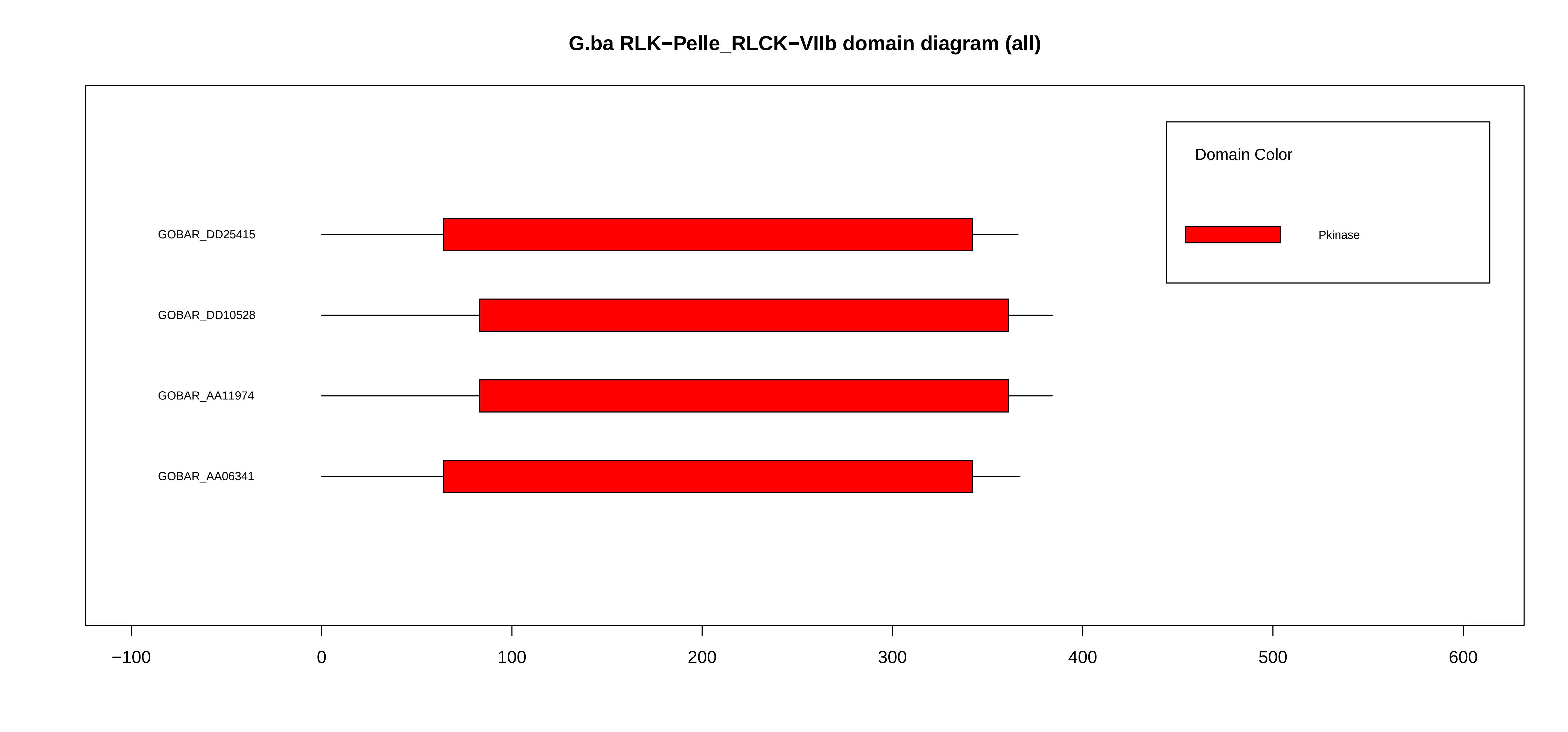
G.ba RLK−Pelle_RLCK−VIIb domain diagram (all)
Domain Color
Pkinase
GOBAR_DD25415
GOBAR_DD10528
GOBAR_AA11974
GOBAR_AA06341
−100
0
100
200
300
400
500
600

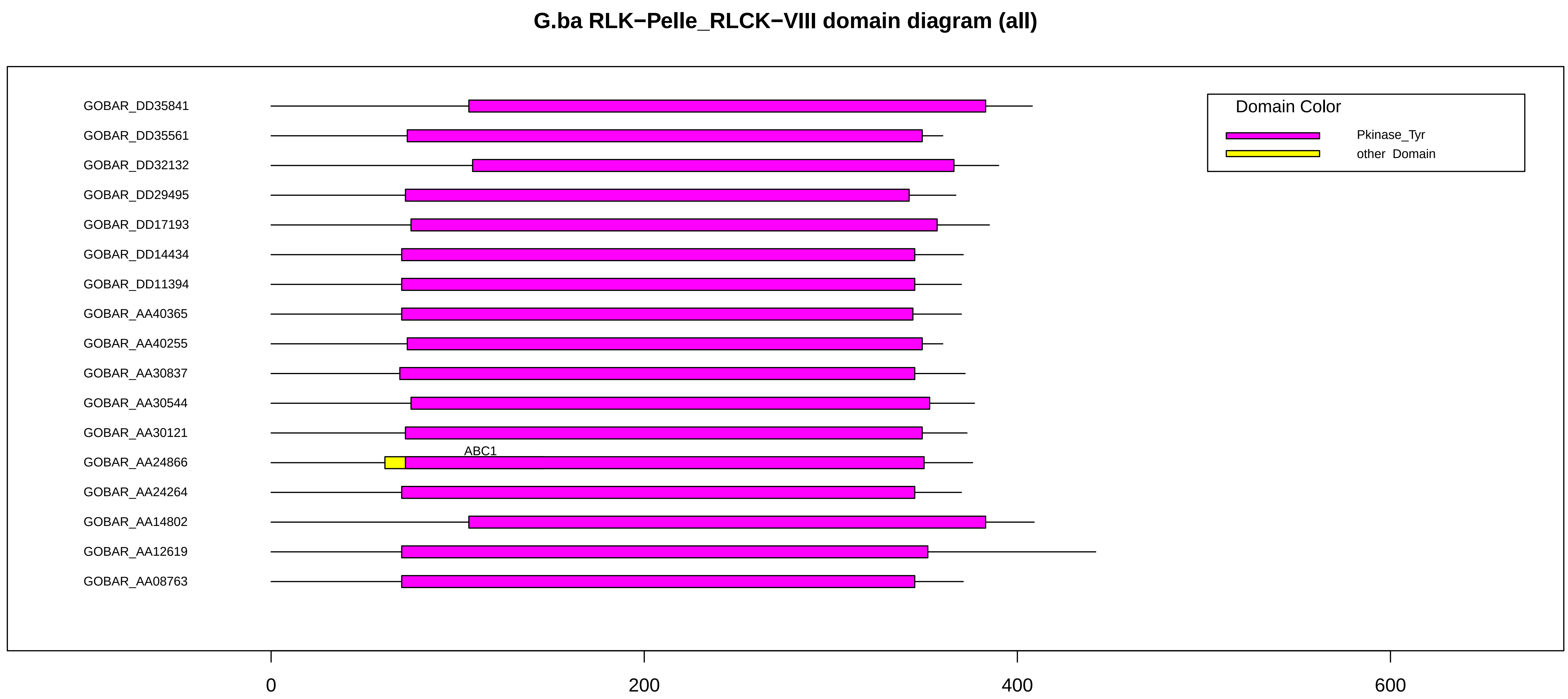
G.ba RLK−Pelle_RLCK−VIII domain diagram (all)
GOBAR_DD35841
GOBAR_DD35561
GOBAR_DD32132
GOBAR_DD29495
GOBAR_DD17193
GOBAR_DD14434
GOBAR_DD11394
GOBAR_AA40365
GOBAR_AA40255
GOBAR_AA30837
GOBAR_AA30544
GOBAR_AA30121
GOBAR_AA24866
ABC1
GOBAR_AA24264
GOBAR_AA14802
GOBAR_AA12619
GOBAR_AA08763
Domain Color
Pkinase_Tyr
other Domain
0
200
400
600

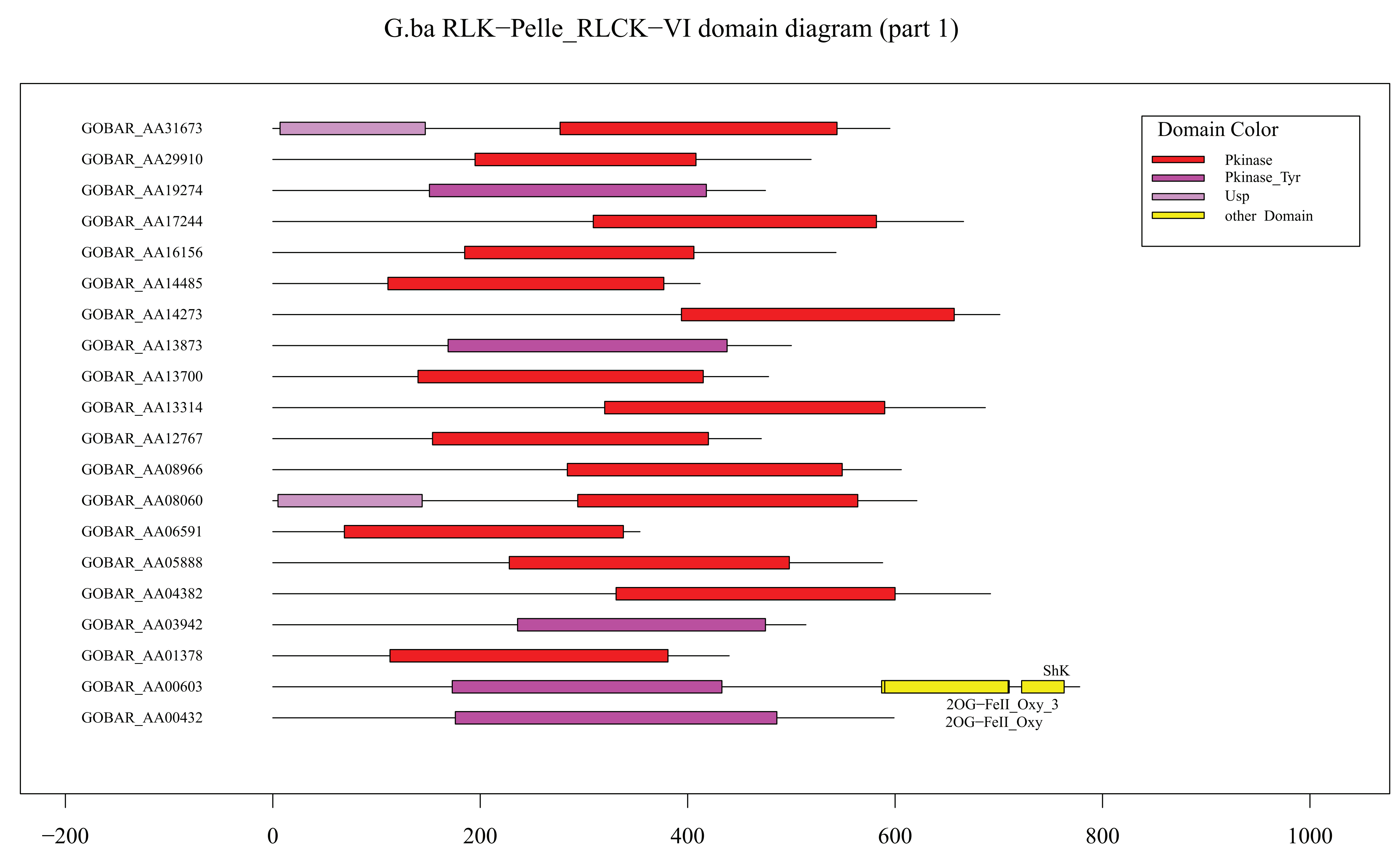

G.ba RLK−Pelle_RLCK−VI domain diagram (part 1)
GOBAR_AA31673
GOBAR_AA29910
GOBAR_AA19274
GOBAR_AA17244
GOBAR_AA16156
GOBAR_AA14485
GOBAR_AA14273
GOBAR_AA13873
GOBAR_AA13700
GOBAR_AA13314
GOBAR_AA12767
GOBAR_AA08966
GOBAR_AA08060
GOBAR_AA06591
GOBAR_AA05888
GOBAR_AA04382
GOBAR_AA03942
GOBAR_AA01378
GOBAR_AA00603
GOBAR_AA00432
ShK
2OG−FeII_Oxy_3
2OG−FeII_Oxy
Domain Color
Pkinase
Pkinase_Tyr
Usp
other Domain
−200
0
200
400
600
800
1000

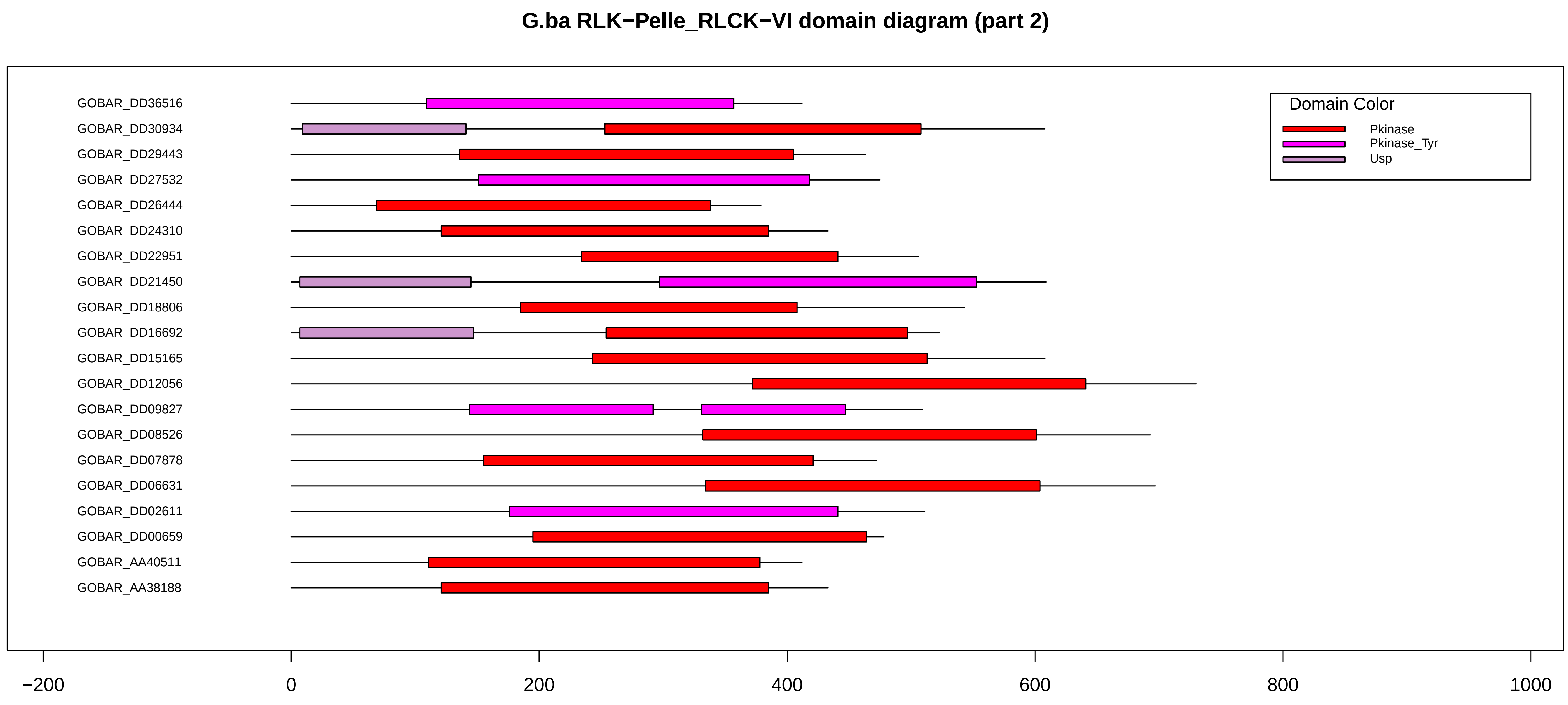

G.ba RLK−Pelle_RLCK−VI domain diagram (part 2)
GOBAR_DD36516
GOBAR_DD30934
GOBAR_DD29443
GOBAR_DD27532
GOBAR_DD26444
GOBAR_DD24310
GOBAR_DD22951
GOBAR_DD21450
GOBAR_DD18806
GOBAR_DD16692
GOBAR_DD15165
GOBAR_DD12056
GOBAR_DD09827
GOBAR_DD08526
GOBAR_DD07878
GOBAR_DD06631
GOBAR_DD02611
GOBAR_DD00659
GOBAR_AA40511
GOBAR_AA38188
Domain Color
Pkinase
Pkinase_Tyr
Usp
−200
0
200
400
600
800
1000

G.ba RLK−Pelle_RLCK−V domain diagram (part 1)

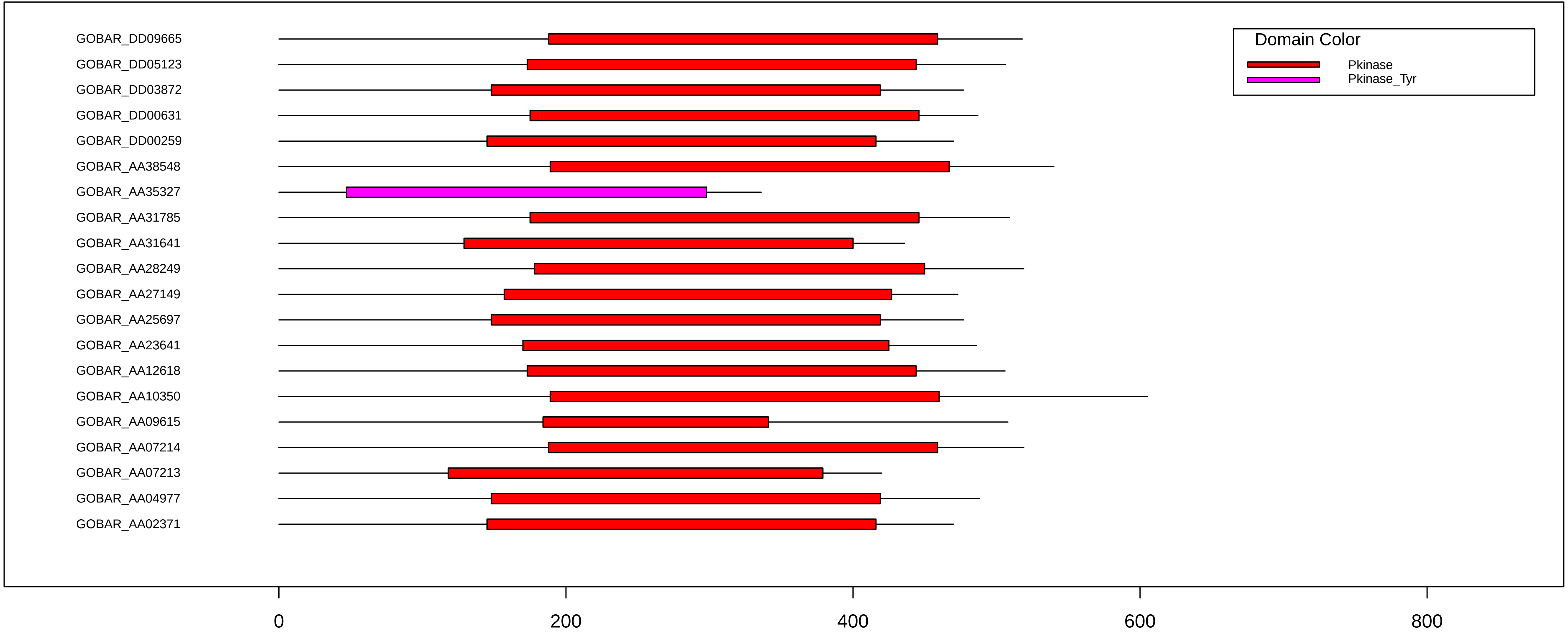

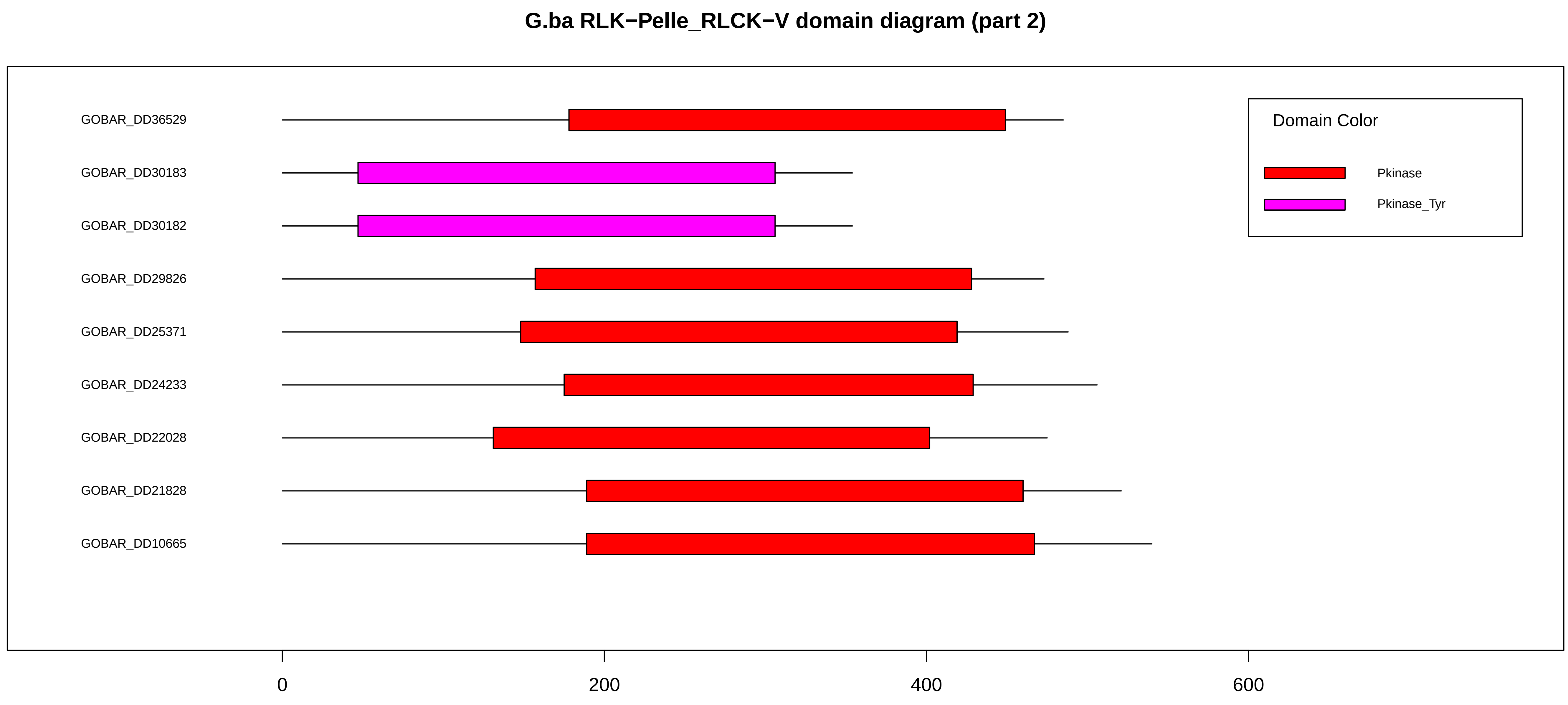
G.ba RLK−Pelle_RLCK−V domain diagram (part 2)
GOBAR_DD36529
GOBAR_DD30183
GOBAR_DD30182
GOBAR_DD29826
GOBAR_DD25371
GOBAR_DD24233
GOBAR_DD22028
GOBAR_DD21828
GOBAR_DD10665
Domain Color
Pkinase
Pkinase_Tyr
0
200
400
600

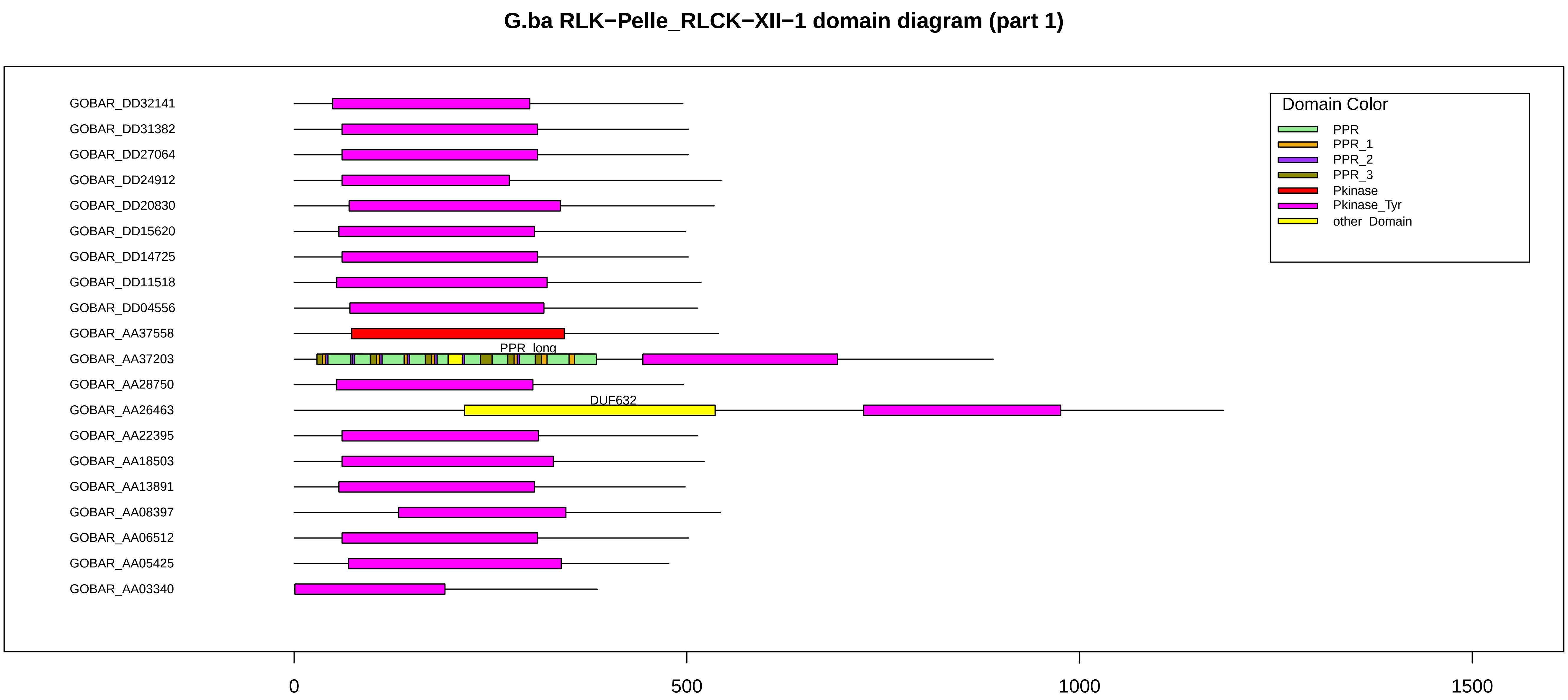
G.ba RLK−Pelle_RLCK−XII−1 domain diagram (part 1)
GOBAR_DD32141
GOBAR_DD31382
GOBAR_DD27064
GOBAR_DD24912
GOBAR_DD20830
GOBAR_DD15620
GOBAR_DD14725
GOBAR_DD11518
GOBAR_DD04556
GOBAR_AA37558
GOBAR_AA37203
GOBAR_AA28750
GOBAR_AA26463
GOBAR_AA22395
GOBAR_AA18503
GOBAR_AA13891
GOBAR_AA08397
GOBAR_AA06512
GOBAR_AA05425
GOBAR_AA03340
PPR_long
DUF632
Domain Color
PPR
PPR_1
PPR_2
PPR_3
Pkinase
Pkinase_Tyr
other Domain
0
500
1000
1500

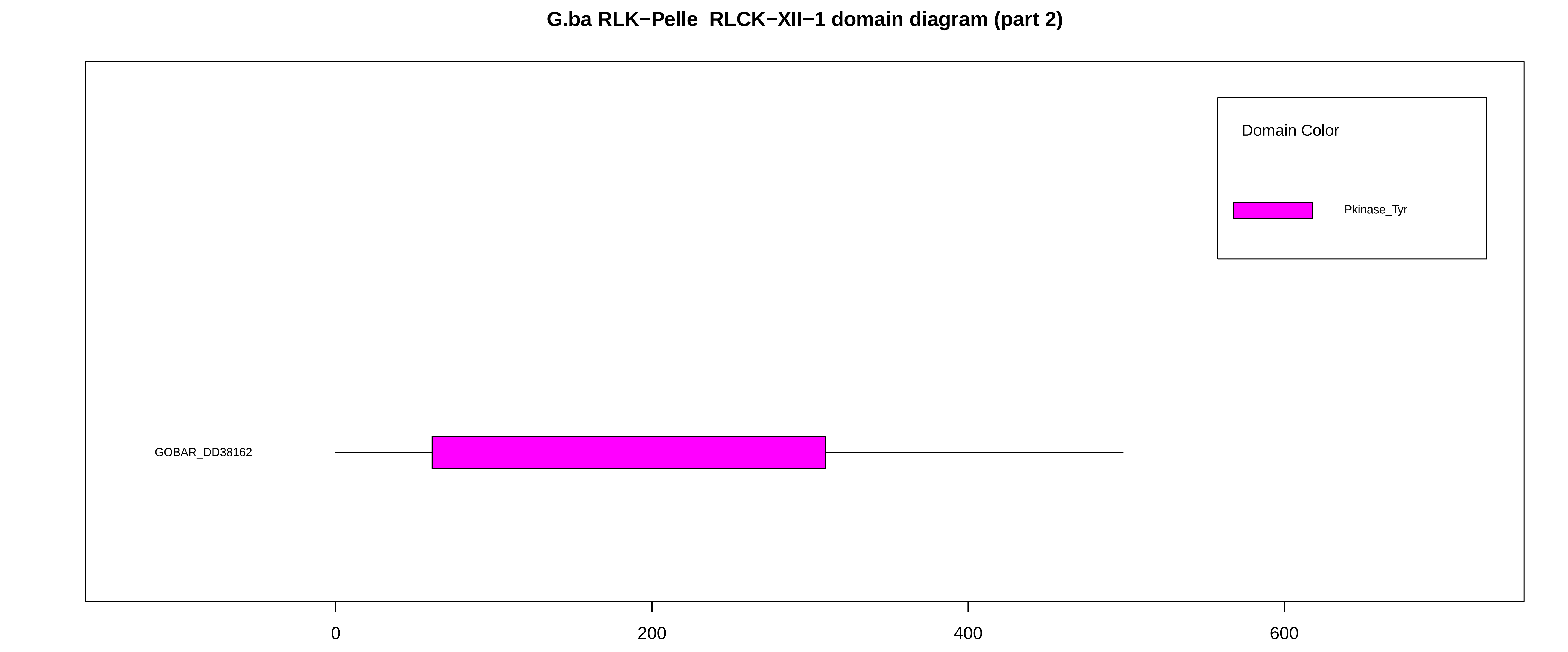
G.ba RLK−Pelle_RLCK−XII−1 domain diagram (part 2)
Domain Color
Pkinase_Tyr
GOBAR_DD38162
0
200
400
600

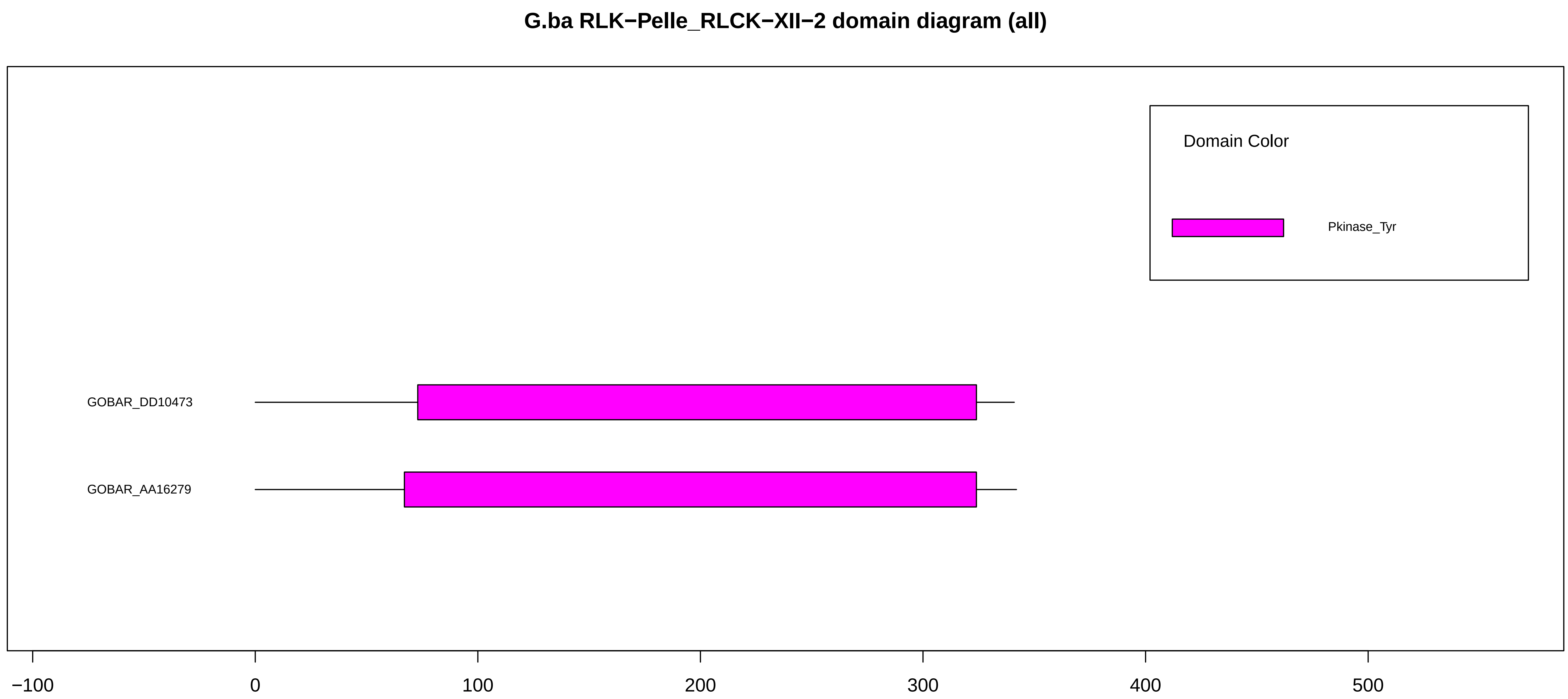

G.ba RLK−Pelle_RLCK−XII−2 domain diagram (all)
Domain Color
Pkinase_Tyr
GOBAR_DD10473
GOBAR_AA16279
−100
0
100
200
300
400
500

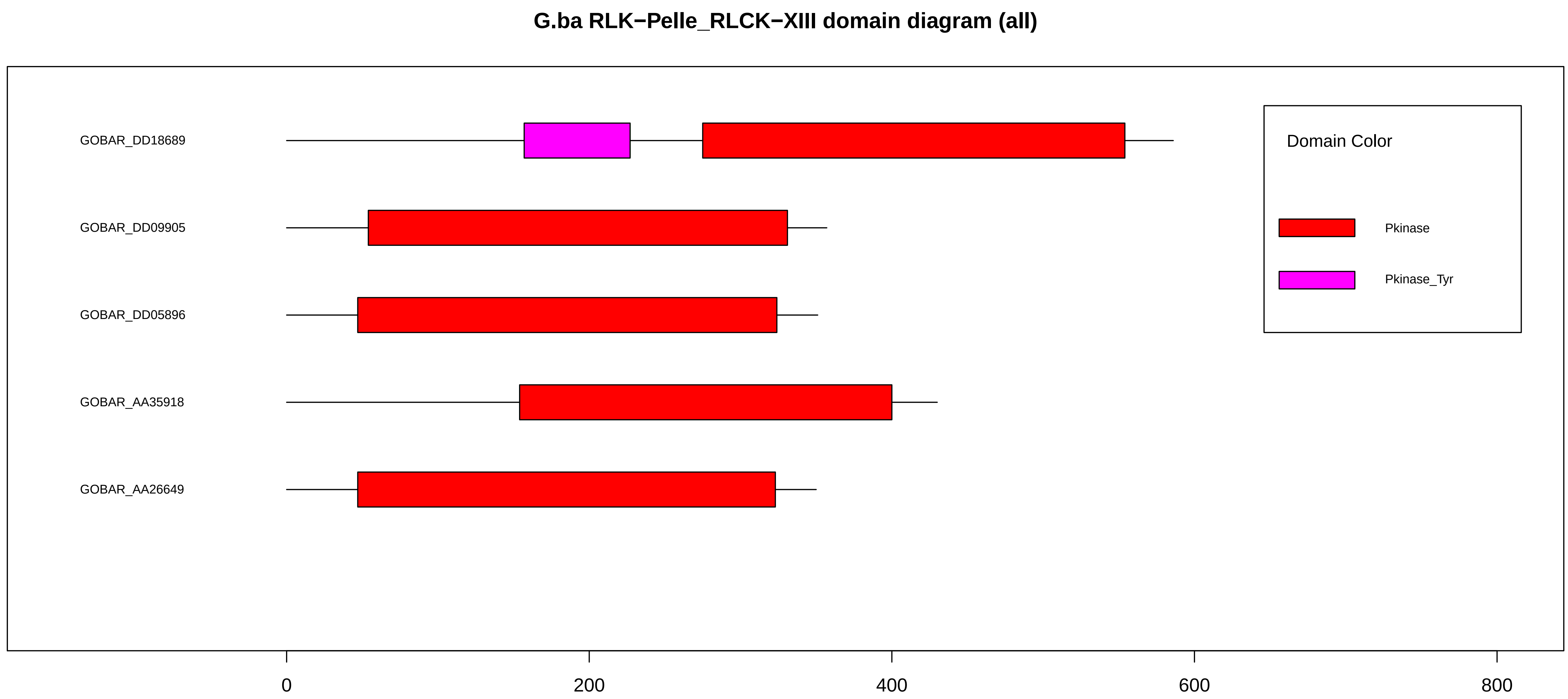
G.ba RLK−Pelle_RLCK−XIII domain diagram (all)
GOBAR_DD18689
GOBAR_DD09905
GOBAR_DD05896
GOBAR_AA35918
GOBAR_AA26649
Domain Color
Pkinase
Pkinase_Tyr
0
200
400
600
800

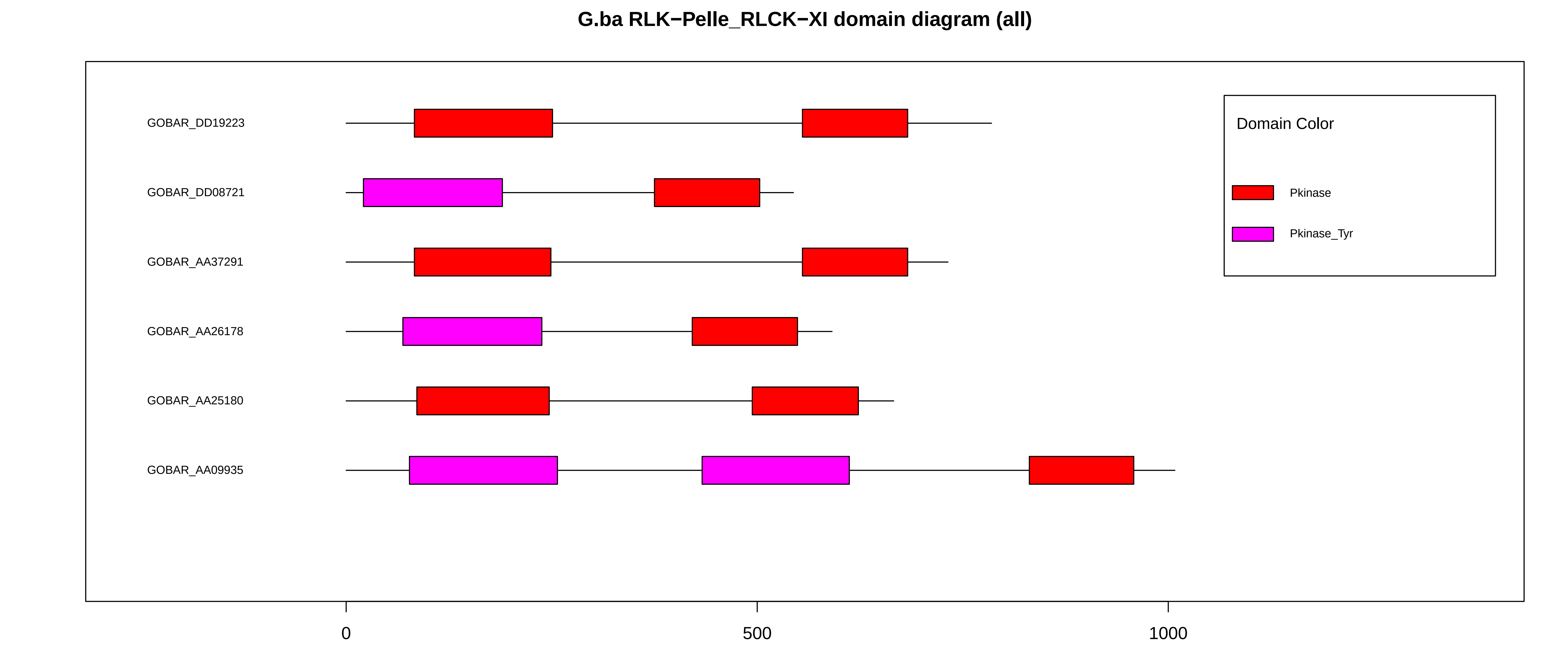
G.ba RLK−Pelle_RLCK−XI domain diagram (all)
GOBAR_DD19223
GOBAR_DD08721
GOBAR_AA37291
GOBAR_AA26178
GOBAR_AA25180
GOBAR_AA09935
Domain Color
Pkinase
Pkinase_Tyr
0
500
1000

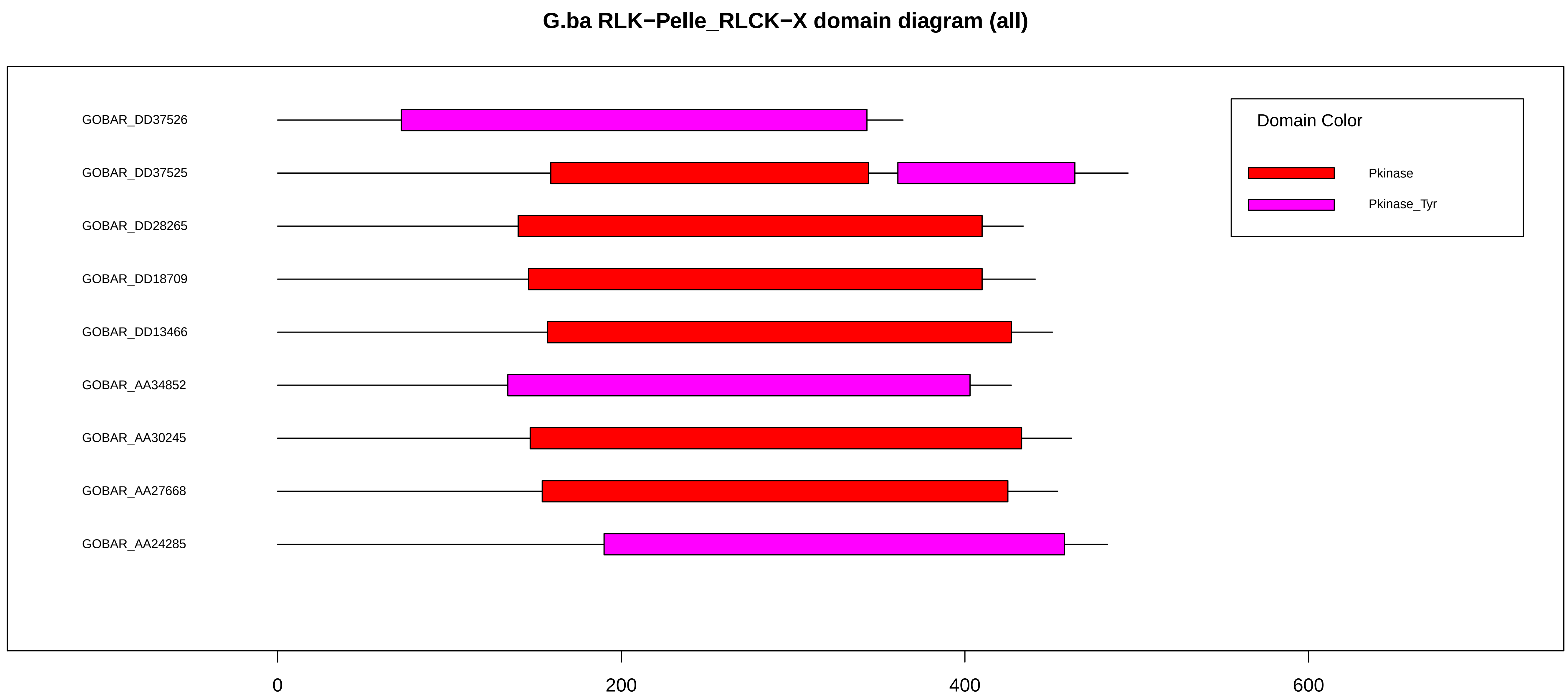

G.ba RLK−Pelle_RLCK−X domain diagram (all)
GOBAR_DD37526
GOBAR_DD37525
GOBAR_DD28265
GOBAR_DD18709
GOBAR_DD13466
GOBAR_AA34852
GOBAR_AA30245
GOBAR_AA27668
GOBAR_AA24285
Domain Color
Pkinase
Pkinase_Tyr
0
200
400
600

G.ba RLK−Pelle_RLCK−XVI domain diagram (all)

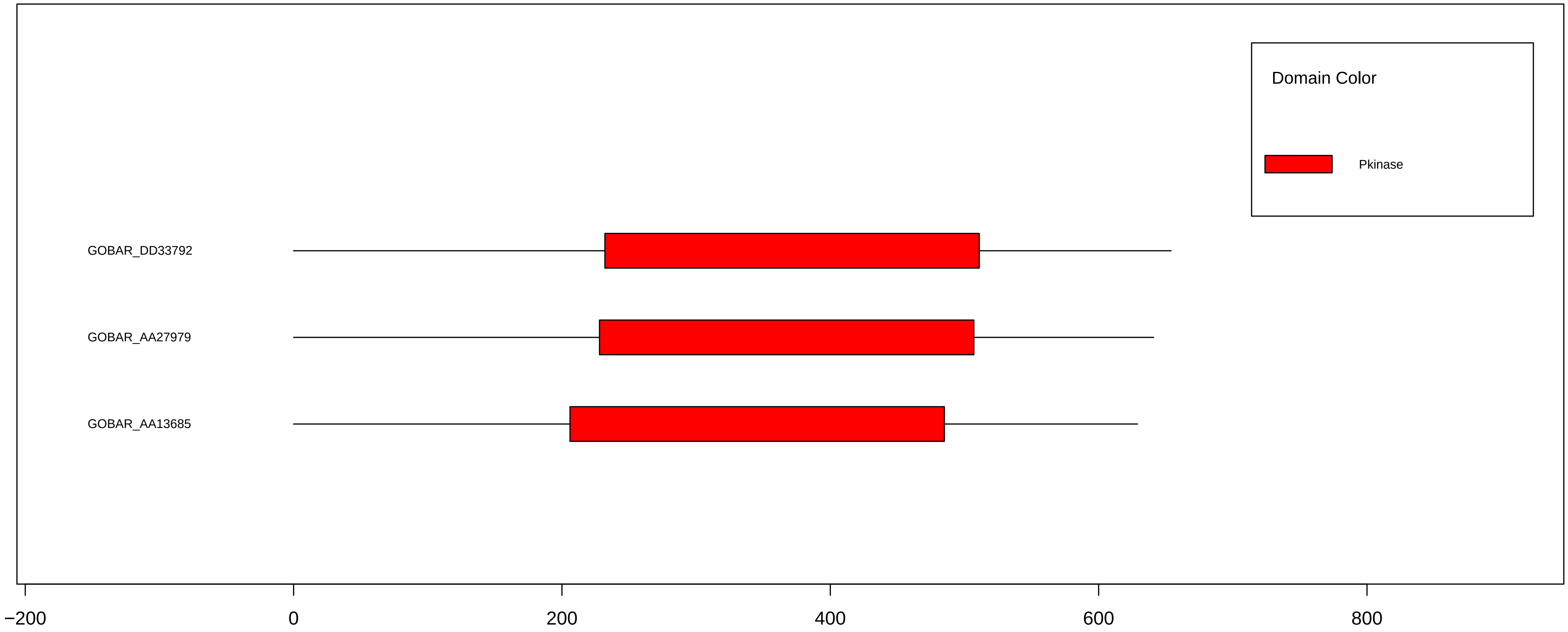

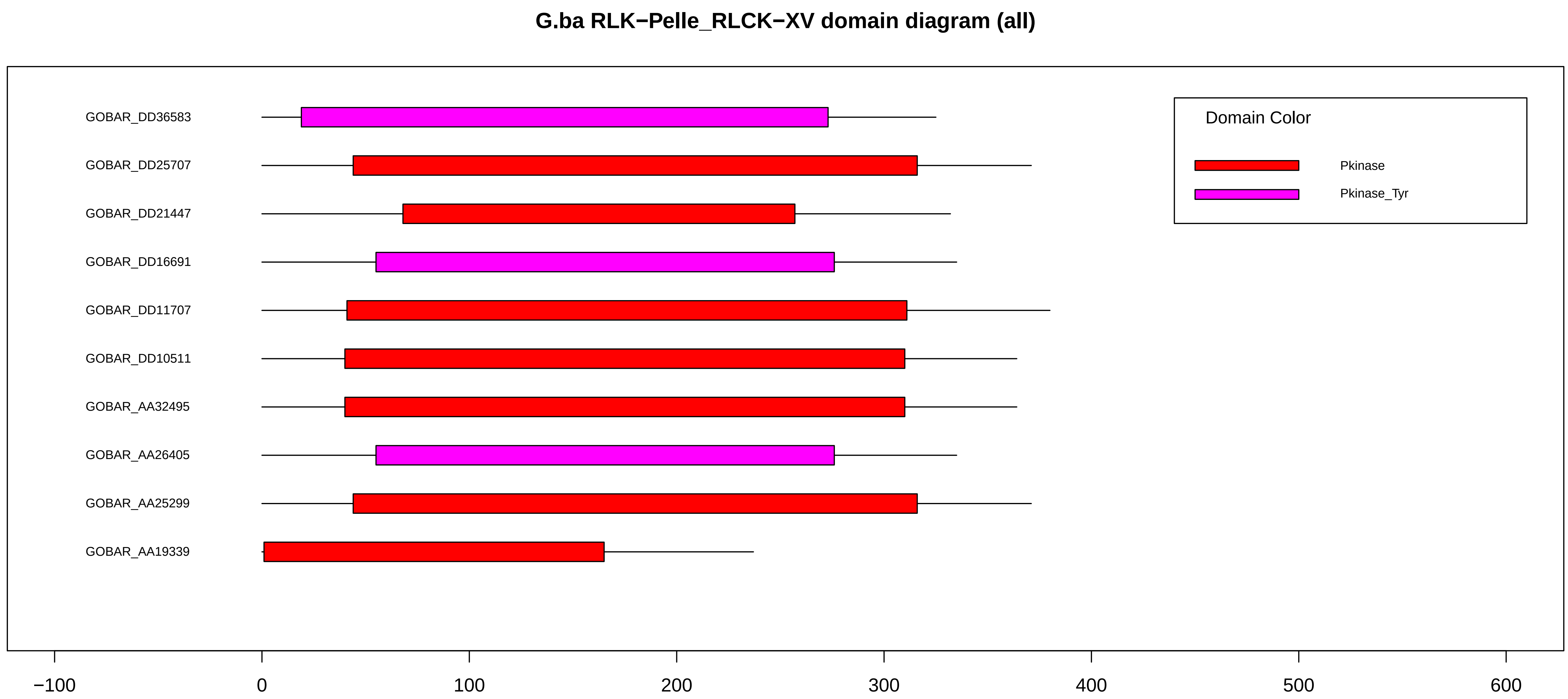
G.ba RLK−Pelle_RLCK−XV domain diagram (all)
GOBAR_DD36583
GOBAR_DD25707
GOBAR_DD21447
GOBAR_DD16691
GOBAR_DD11707
GOBAR_DD10511
GOBAR_AA32495
GOBAR_AA26405
GOBAR_AA25299
GOBAR_AA19339
Domain Color
Pkinase
Pkinase_Tyr
−100
0
100
200
300
400
500
600

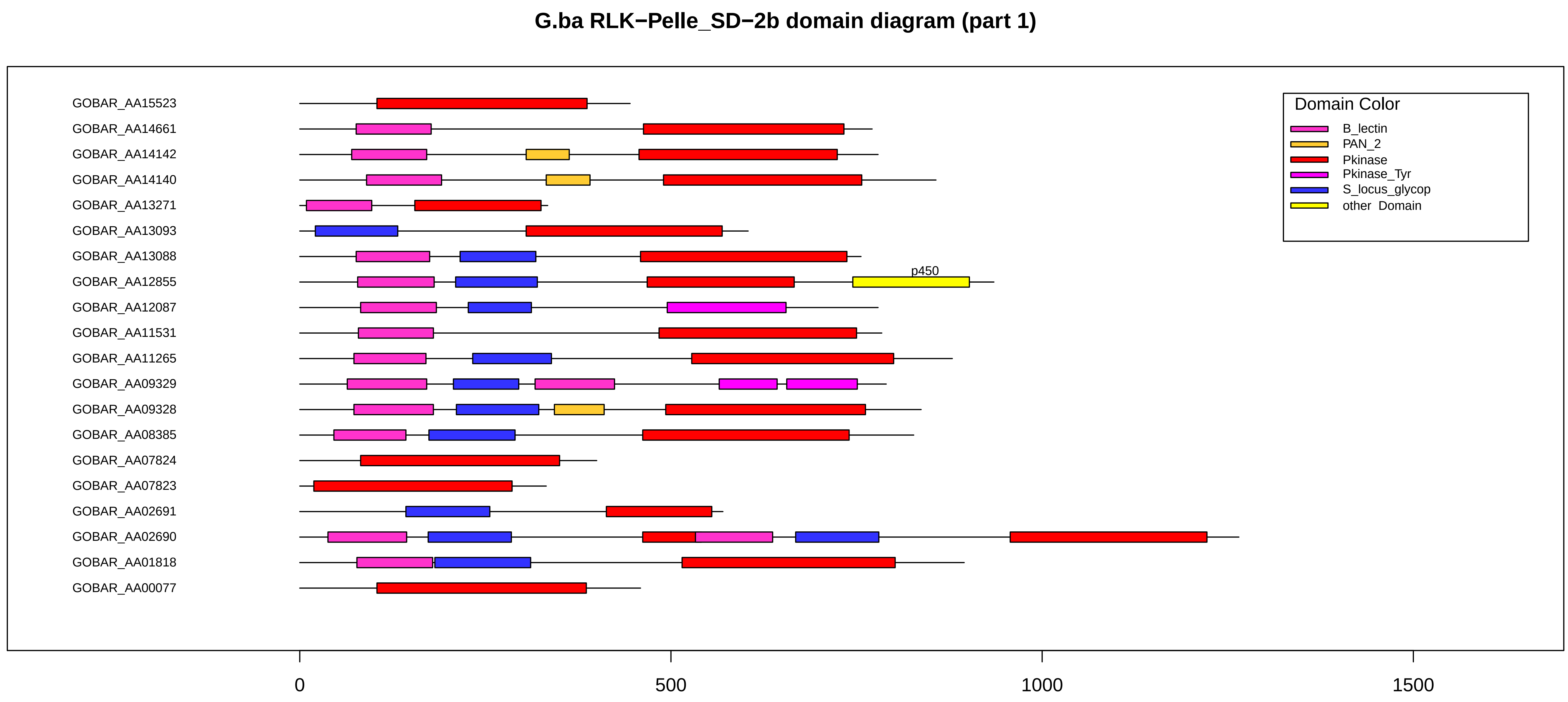
G.ba RLK−Pelle_SD−2b domain diagram (part 1)
GOBAR_AA15523
GOBAR_AA14661
GOBAR_AA14142
GOBAR_AA14140
GOBAR_AA13271
GOBAR_AA13093
GOBAR_AA13088
GOBAR_AA12855
GOBAR_AA12087
GOBAR_AA11531
GOBAR_AA11265
GOBAR_AA09329
GOBAR_AA09328
GOBAR_AA08385
GOBAR_AA07824
GOBAR_AA07823
GOBAR_AA02691
GOBAR_AA02690
GOBAR_AA01818
GOBAR_AA00077
p450
Domain Color
B_lectin
PAN_2
Pkinase
Pkinase_Tyr
S_locus_glycop
other Domain
0
500
1000
1500

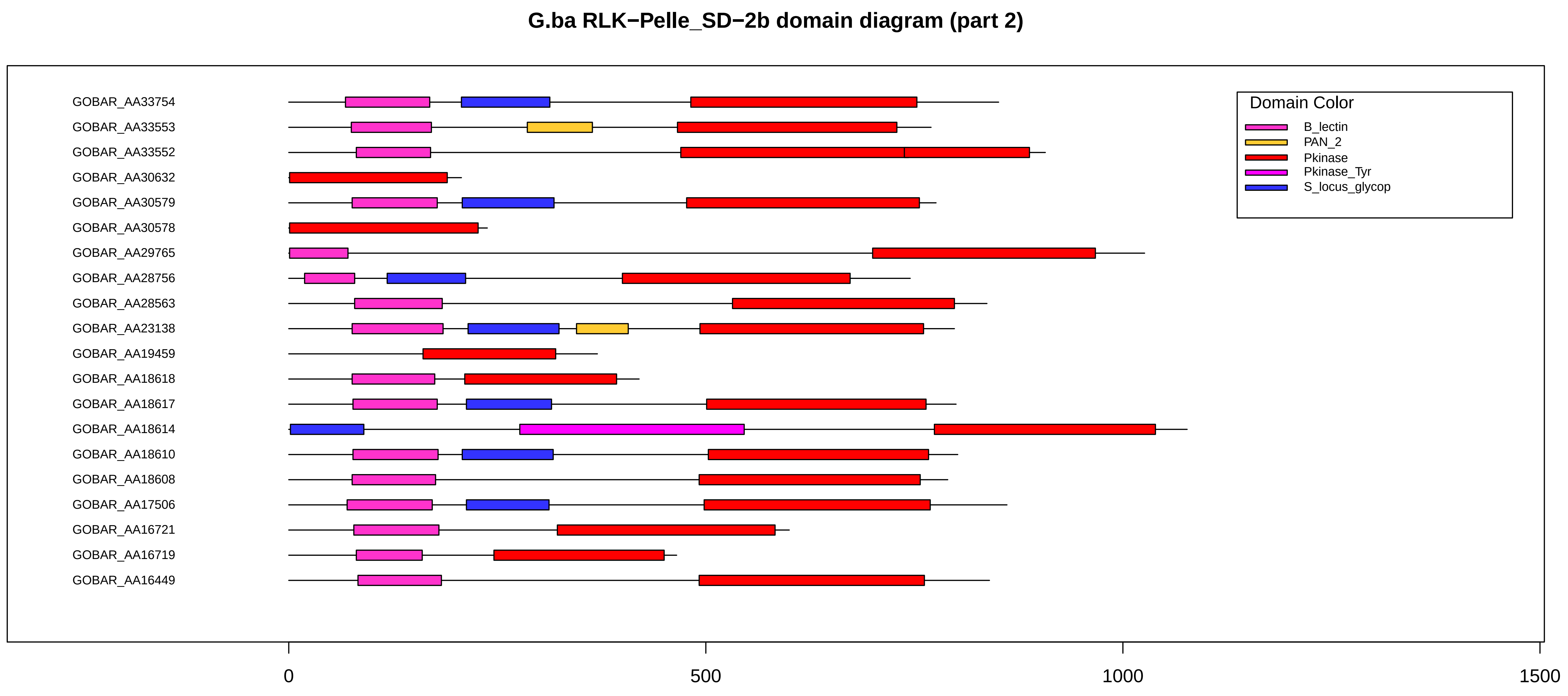
G.ba RLK−Pelle_SD−2b domain diagram (part 2)
GOBAR_AA33754
GOBAR_AA33553
GOBAR_AA33552
GOBAR_AA30632
GOBAR_AA30579
GOBAR_AA30578
GOBAR_AA29765
GOBAR_AA28756
GOBAR_AA28563
GOBAR_AA23138
GOBAR_AA19459
GOBAR_AA18618
GOBAR_AA18617
GOBAR_AA18614
GOBAR_AA18610
GOBAR_AA18608
GOBAR_AA17506
GOBAR_AA16721
GOBAR_AA16719
GOBAR_AA16449
Domain Color
B_lectin
PAN_2
Pkinase
Pkinase_Tyr
S_locus_glycop
0
500
1000
1500

G.ba RLK−Pelle_SD−2b domain diagram (part 3)

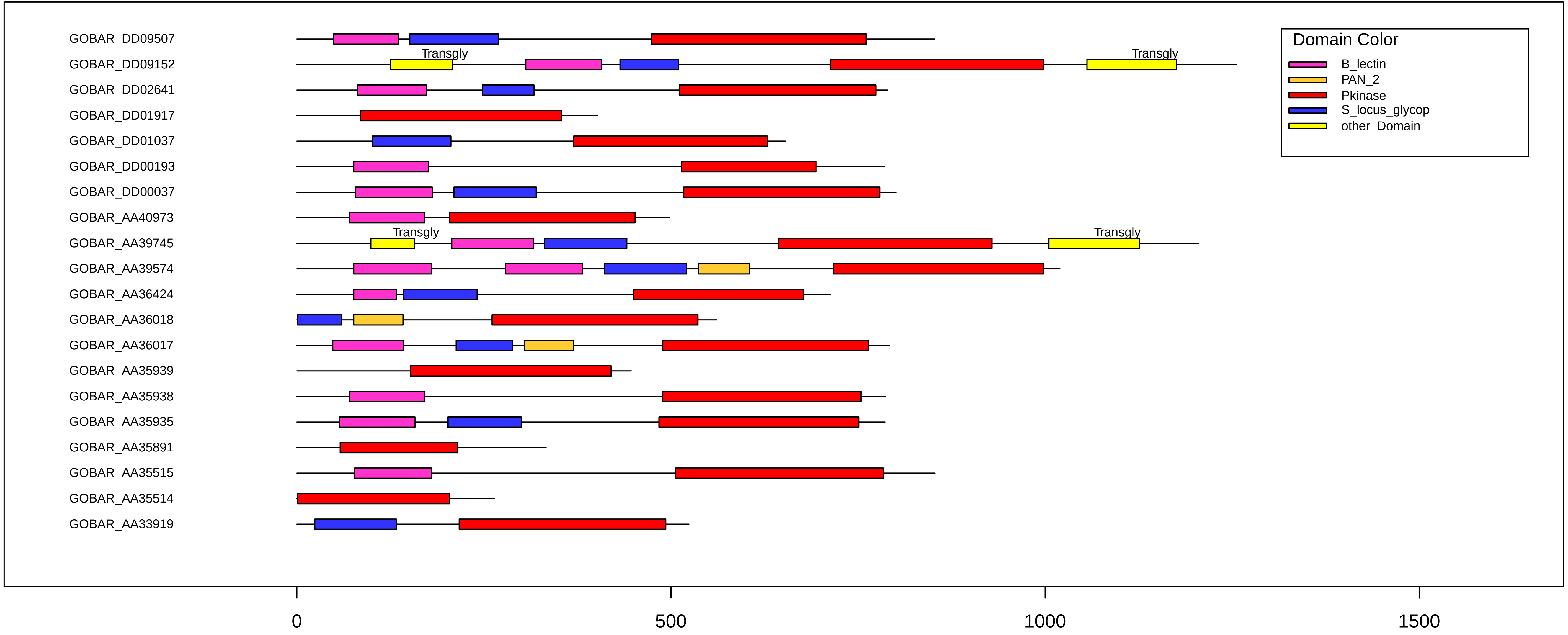

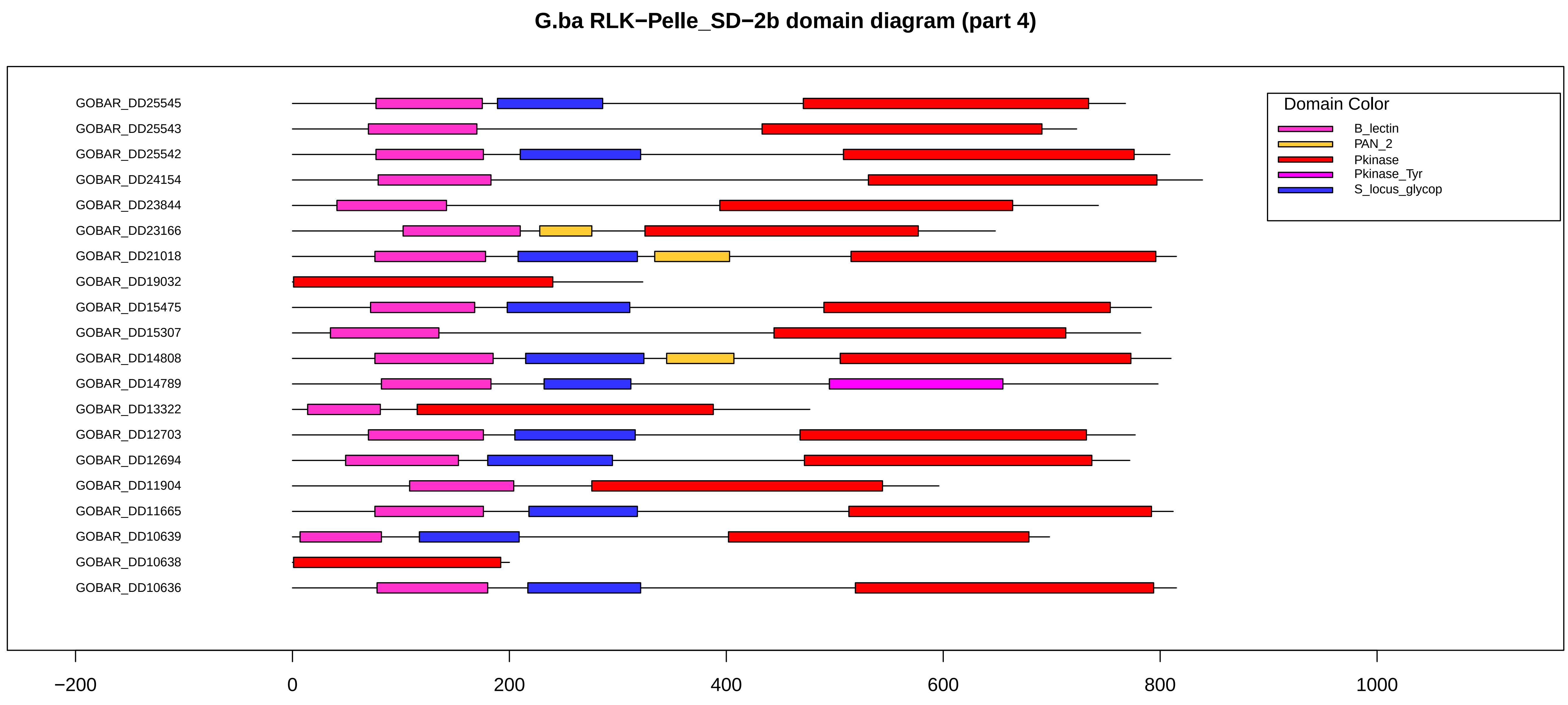
G.ba RLK−Pelle_SD−2b domain diagram (part 4)
GOBAR_DD25545
GOBAR_DD25543
GOBAR_DD25542
GOBAR_DD24154
GOBAR_DD23844
GOBAR_DD23166
GOBAR_DD21018
GOBAR_DD19032
GOBAR_DD15475
GOBAR_DD15307
GOBAR_DD14808
GOBAR_DD14789
GOBAR_DD13322
GOBAR_DD12703
GOBAR_DD12694
GOBAR_DD11904
GOBAR_DD11665
GOBAR_DD10639
GOBAR_DD10638
GOBAR_DD10636
Domain Color
B_lectin
PAN_2
Pkinase
Pkinase_Tyr
S_locus_glycop
−200
0
200
400
600
800
1000

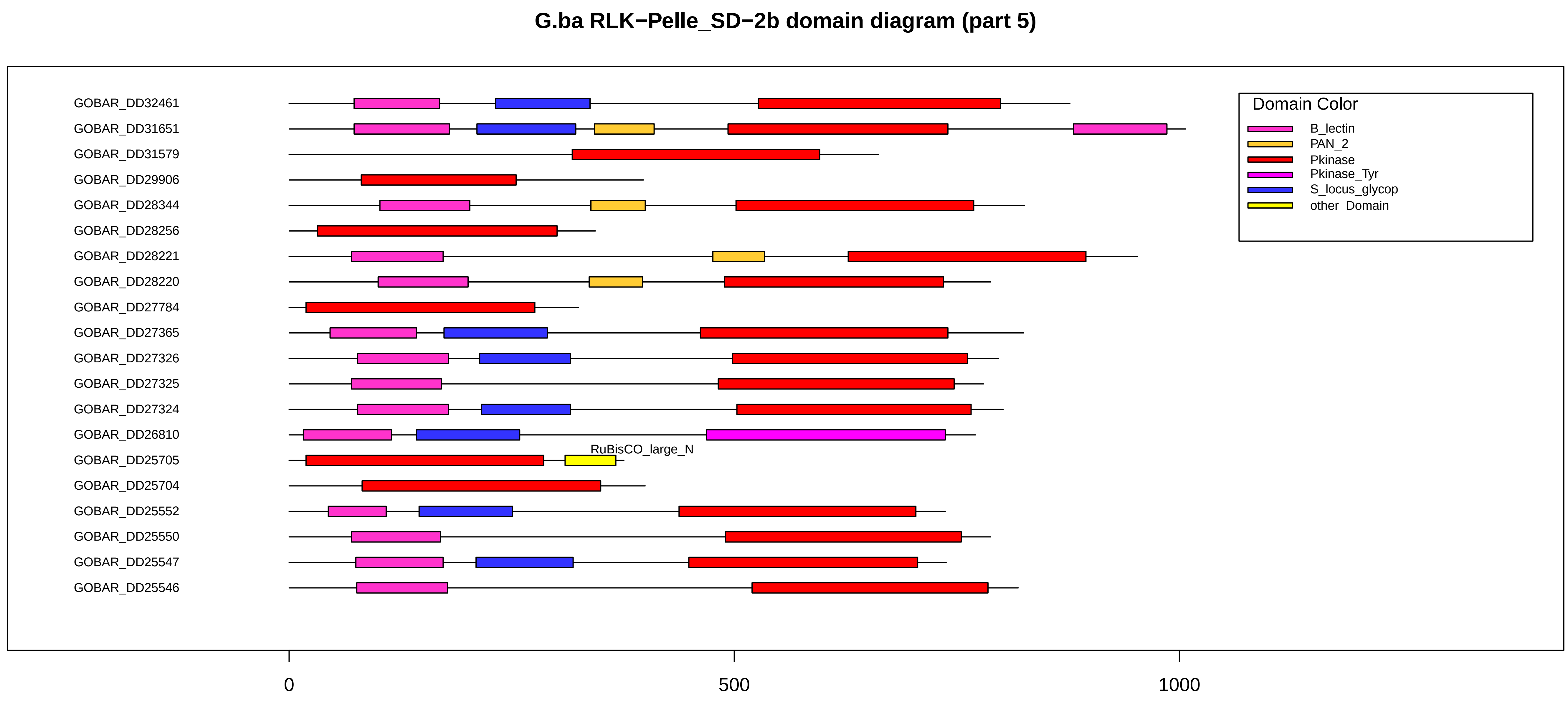

G.ba RLK−Pelle_SD−2b domain diagram (part 5)
GOBAR_DD32461
GOBAR_DD31651
GOBAR_DD31579
GOBAR_DD29906
GOBAR_DD28344
GOBAR_DD28256
GOBAR_DD28221
GOBAR_DD28220
GOBAR_DD27784
GOBAR_DD27365
GOBAR_DD27326
GOBAR_DD27325
GOBAR_DD27324
GOBAR_DD26810
GOBAR_DD25705
GOBAR_DD25704
GOBAR_DD25552
GOBAR_DD25550
GOBAR_DD25547
GOBAR_DD25546
RuBisCO_large_N
Domain Color
B_lectin
PAN_2
Pkinase
Pkinase_Tyr
S_locus_glycop
other Domain
0
500
1000

G.ba RLK−Pelle_SD−2b domain diagram (part 6)

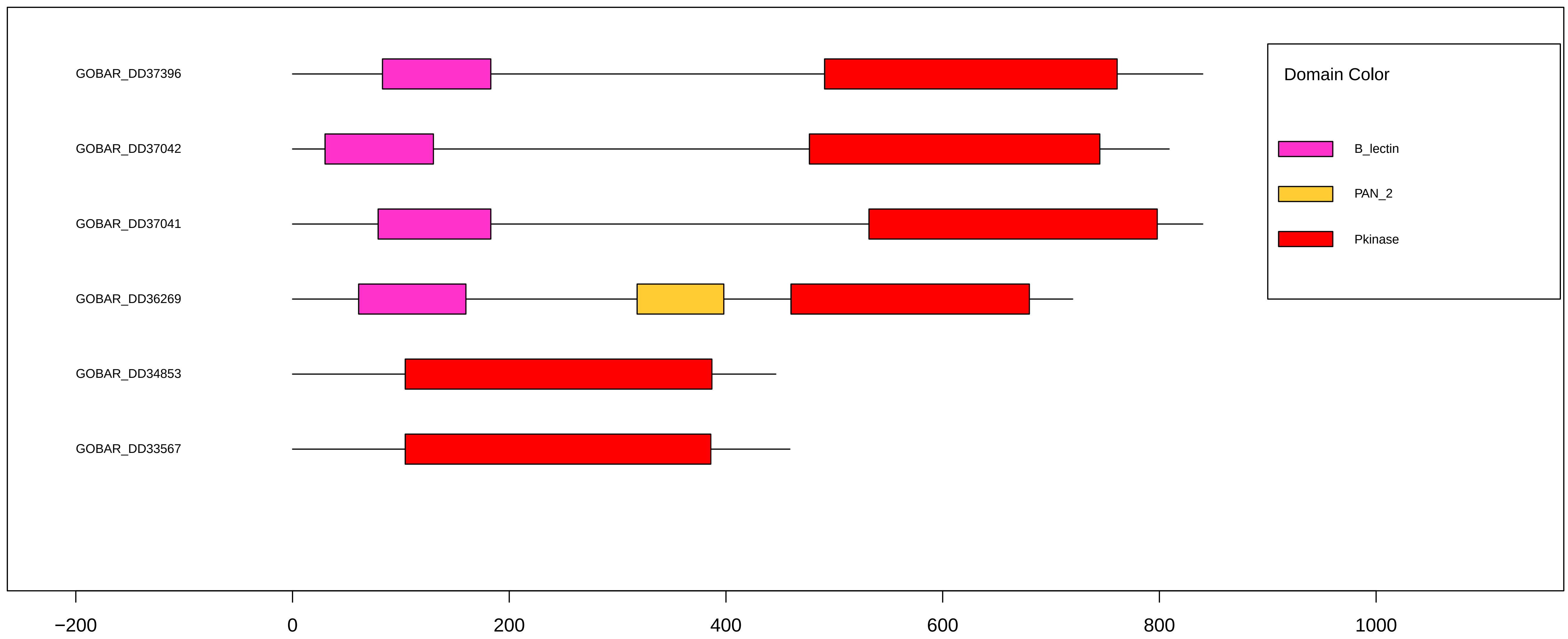

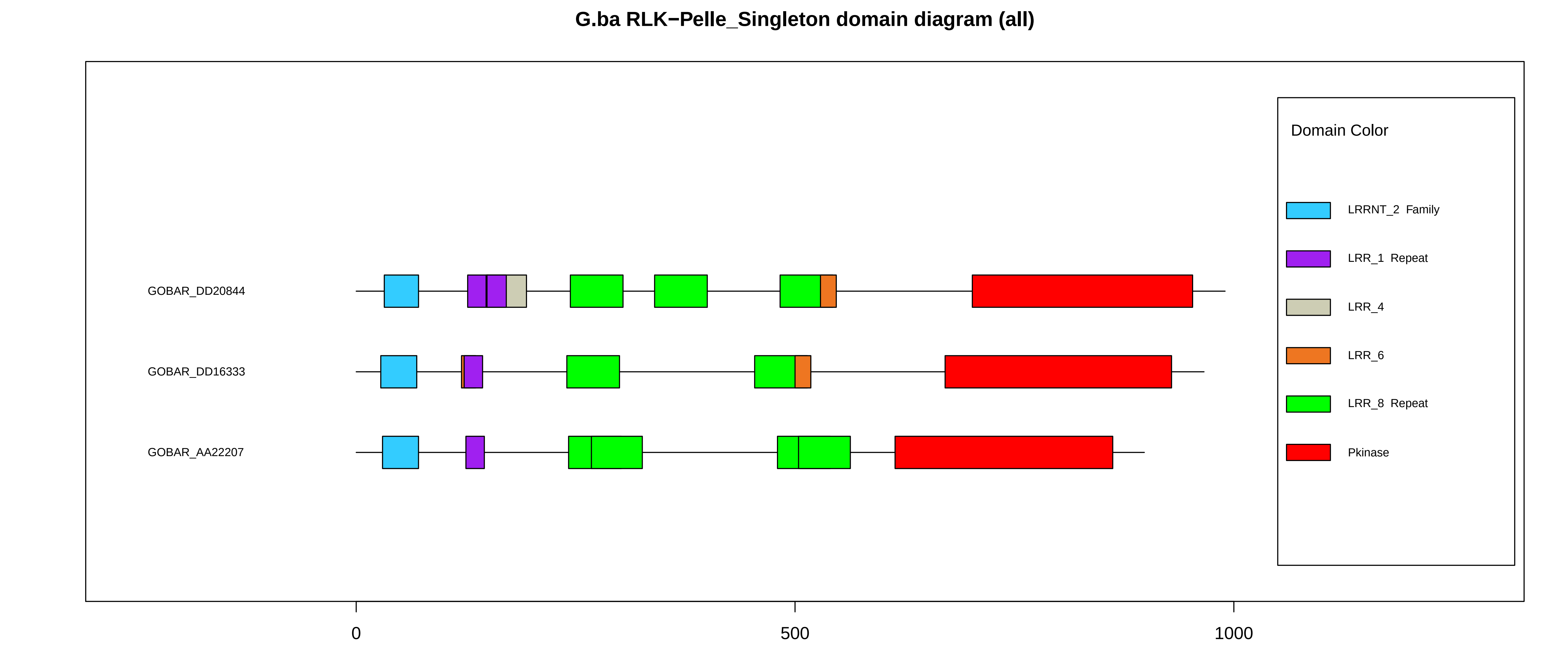
G.ba RLK−Pelle_Singleton domain diagram (all)
GOBAR_DD20844
GOBAR_DD16333
GOBAR_AA22207
0
500
1000
Domain Color
LRRNT_2 Family
LRR_1 Repeat
LRR_4
LRR_6
LRR_8 Repeat
Pkinase

G.ba RLK−Pelle_URK−1 domain diagram (all)

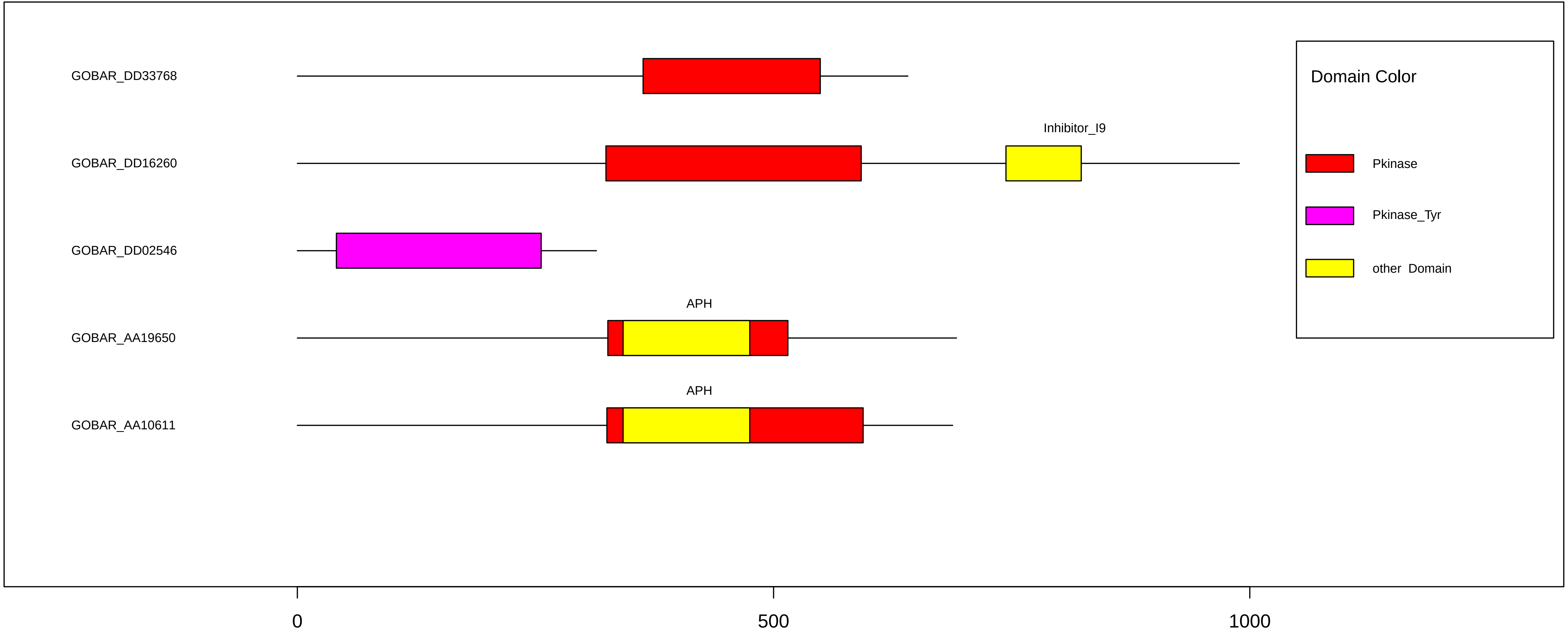

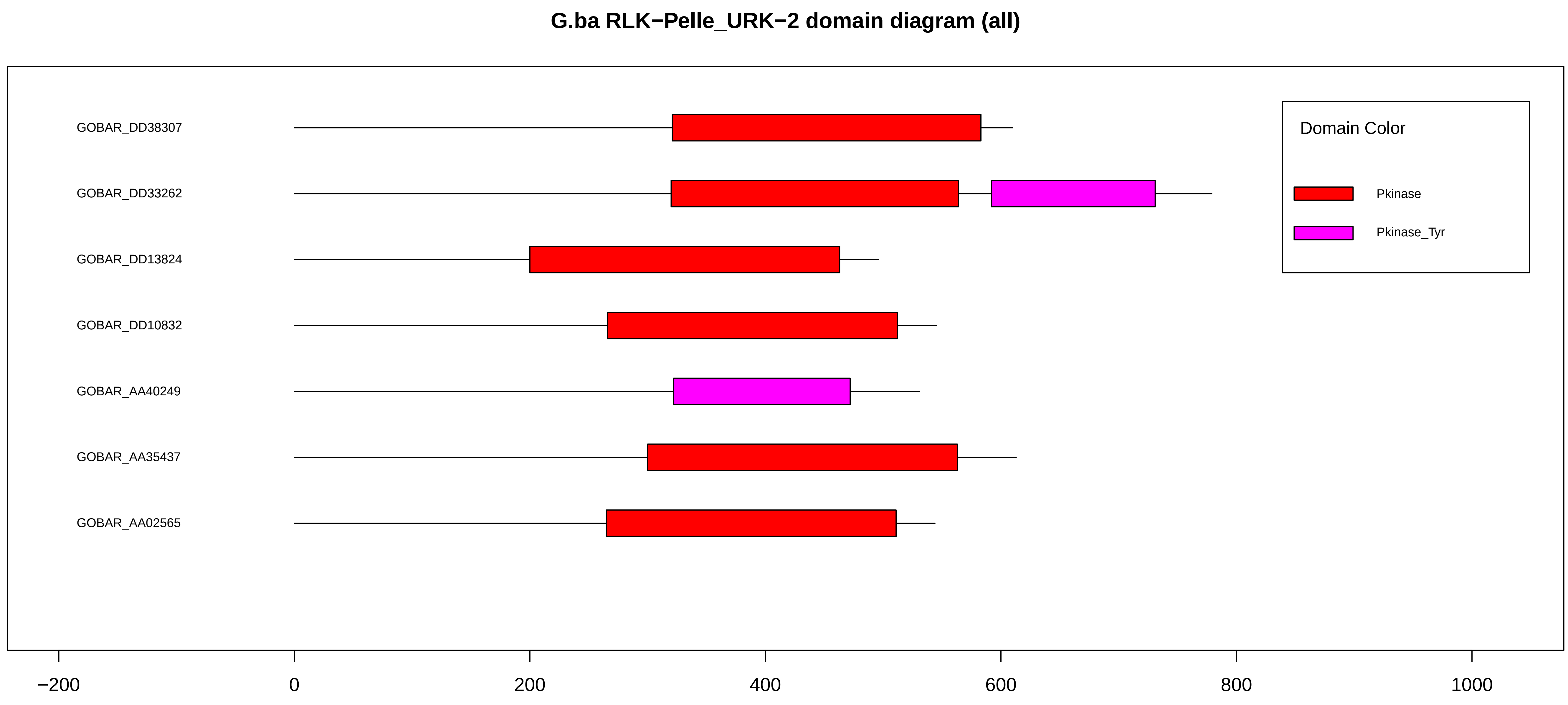

G.ba RLK−Pelle_URK−2 domain diagram (all)
GOBAR_DD38307
GOBAR_DD33262
GOBAR_DD13824
GOBAR_DD10832
GOBAR_AA40249
GOBAR_AA35437
GOBAR_AA02565
Domain Color
Pkinase
Pkinase_Tyr
−200
0
200
400
600
800
1000

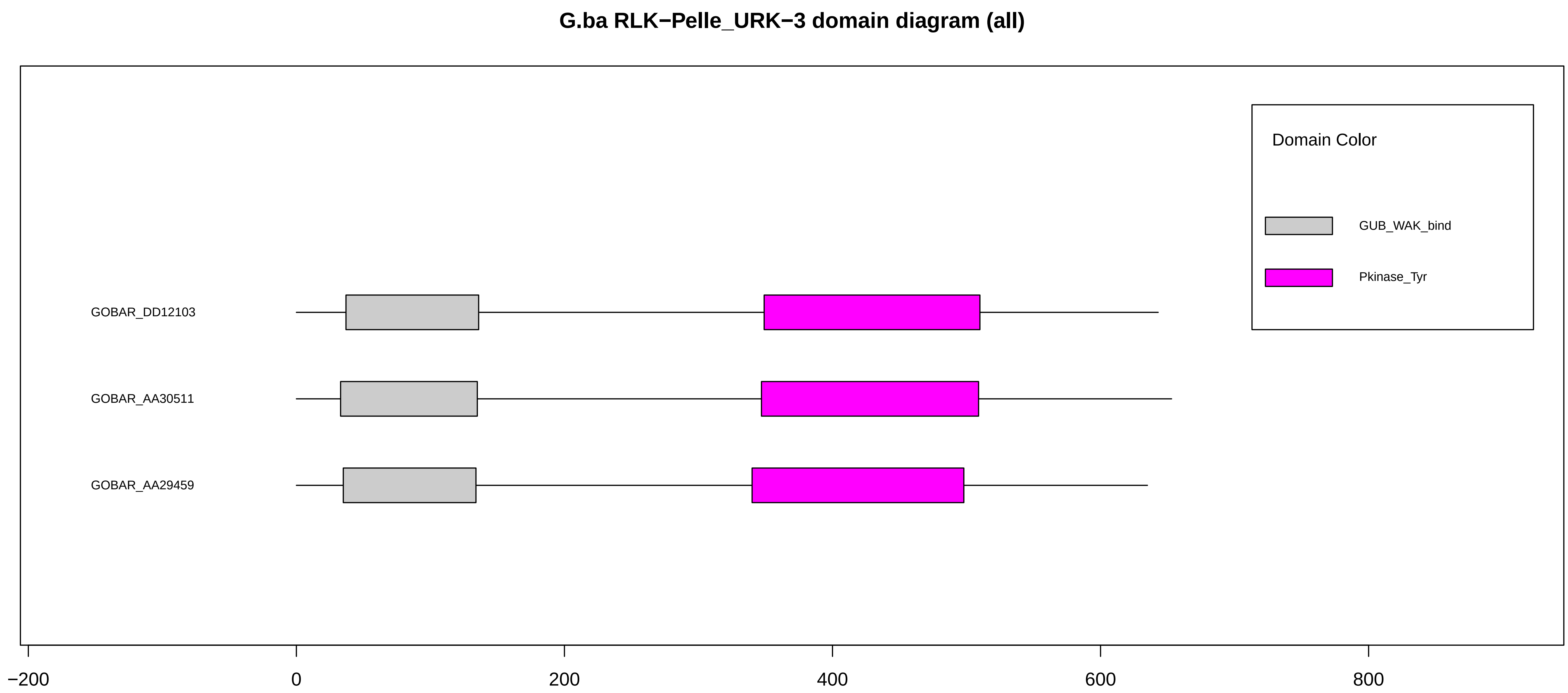
G.ba RLK−Pelle_URK−3 domain diagram (all)
Domain Color
GUB_WAK_bind
Pkinase_Tyr
GOBAR_DD12103
GOBAR_AA30511
GOBAR_AA29459
−200
0
200
400
600
800

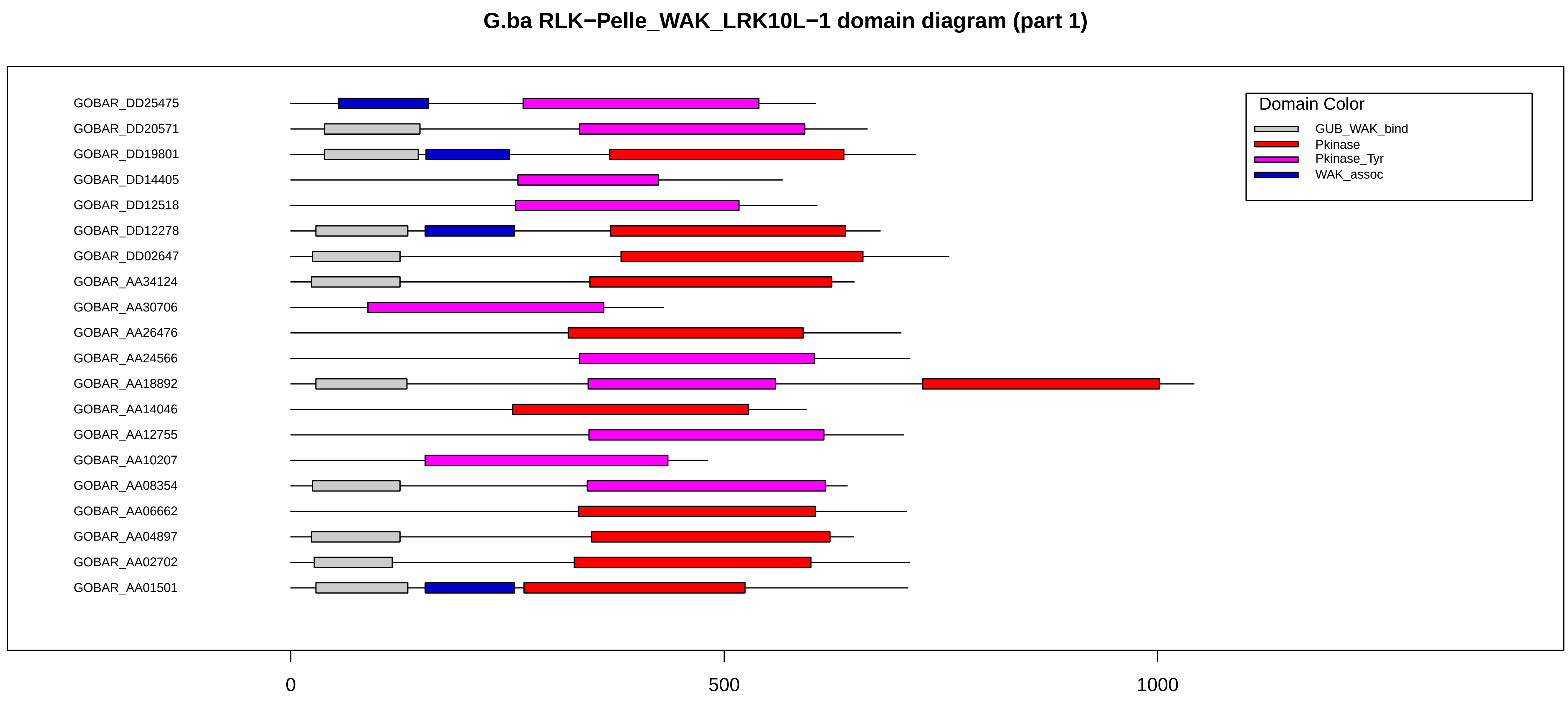
G.ba RLK−Pelle_WAK_LRK10L−1 domain diagram (part 1)
GOBAR_DD25475
GOBAR_DD20571
GOBAR_DD19801
GOBAR_DD14405
GOBAR_DD12518
GOBAR_DD12278
GOBAR_DD02647
GOBAR_AA34124
GOBAR_AA30706
GOBAR_AA26476
GOBAR_AA24566
GOBAR_AA18892
GOBAR_AA14046
GOBAR_AA12755
GOBAR_AA10207
GOBAR_AA08354
GOBAR_AA06662
GOBAR_AA04897
GOBAR_AA02702
GOBAR_AA01501
Domain Color
GUB_WAK_bind
Pkinase
Pkinase_Tyr
WAK_assoc
0
500
1000

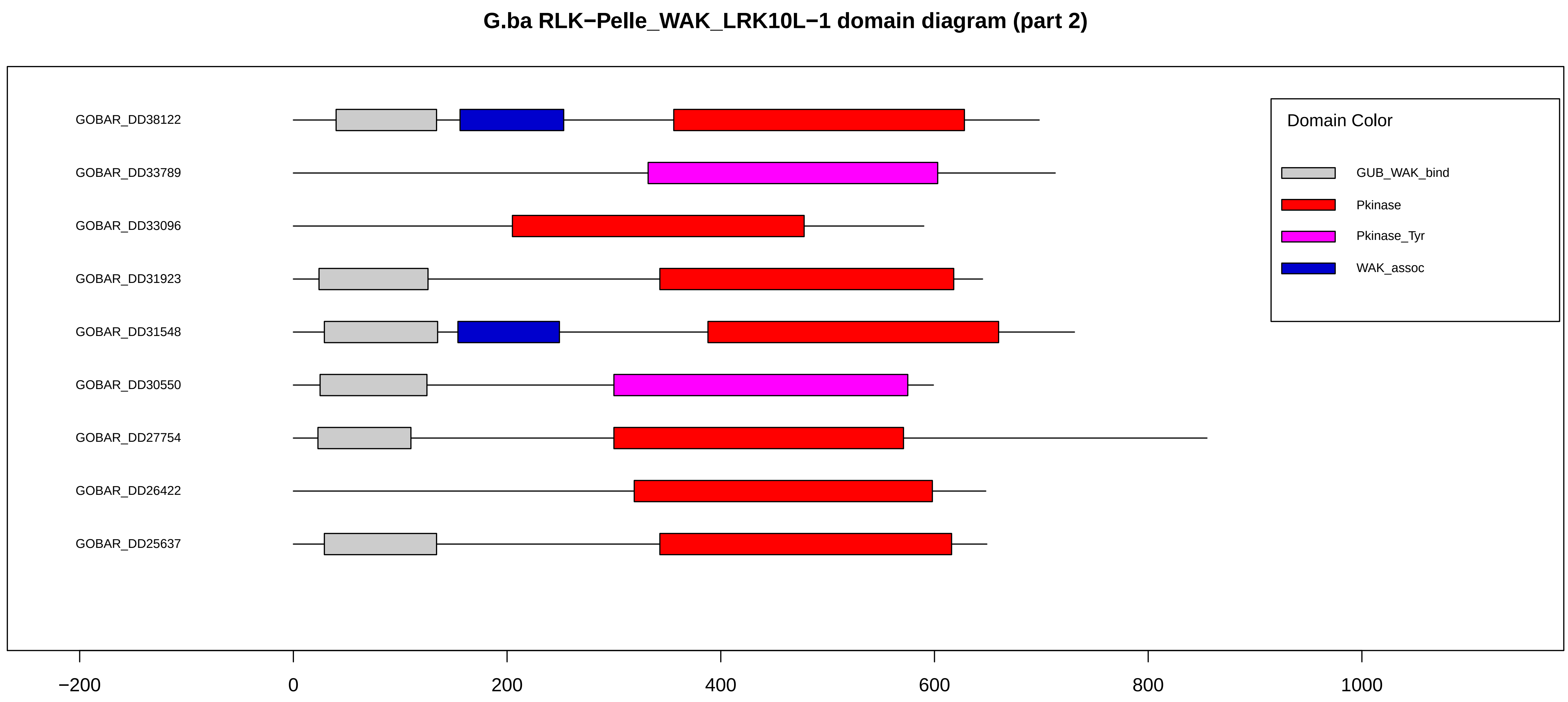
G.ba RLK−Pelle_WAK_LRK10L−1 domain diagram (part 2)
GOBAR_DD38122
GOBAR_DD33789
GOBAR_DD33096
GOBAR_DD31923
GOBAR_DD31548
GOBAR_DD30550
GOBAR_DD27754
GOBAR_DD26422
GOBAR_DD25637
Domain Color
GUB_WAK_bind
Pkinase
Pkinase_Tyr
WAK_assoc
−200
0
200
400
600
800
1000

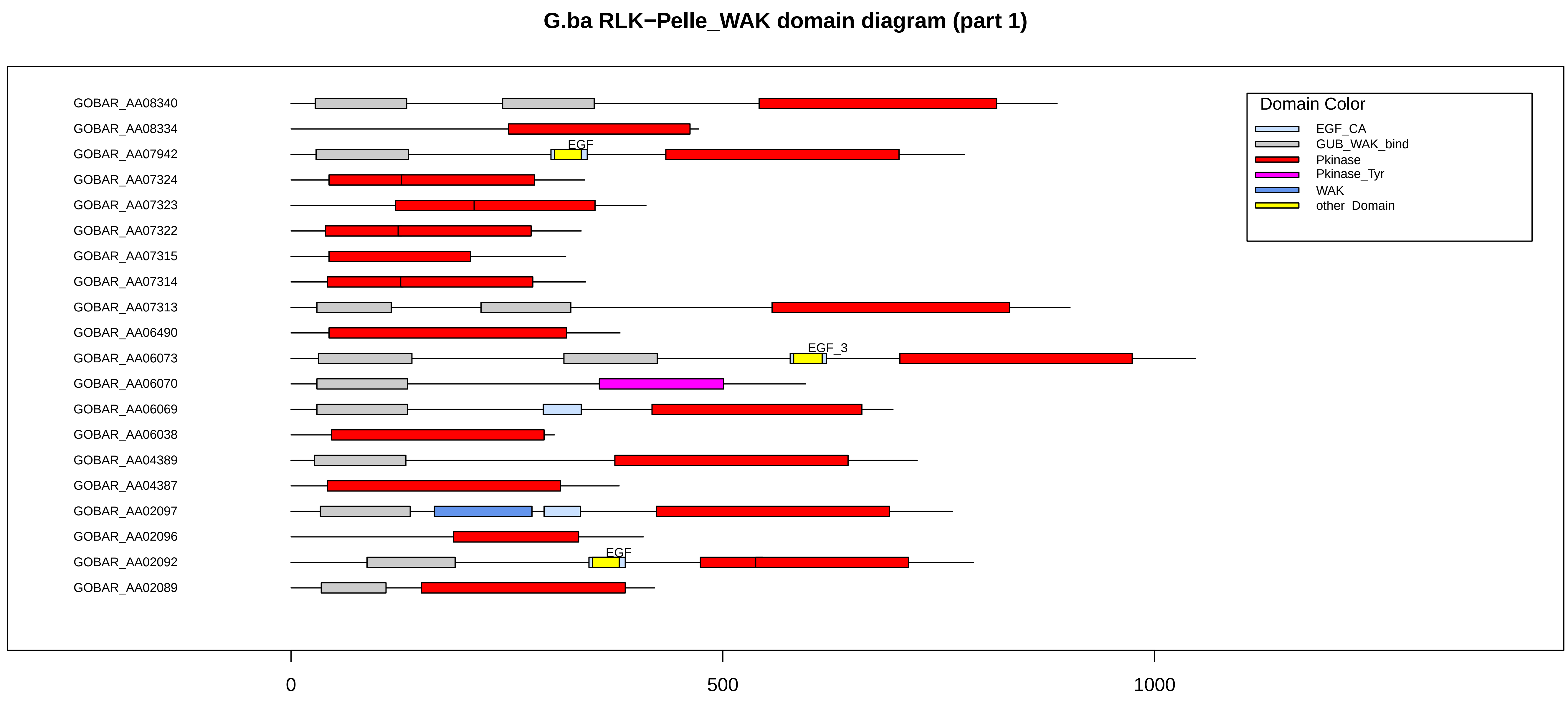
G.ba RLK−Pelle_WAK domain diagram (part 1)
GOBAR_AA08340
GOBAR_AA08334
GOBAR_AA07942
GOBAR_AA07324
GOBAR_AA07323
GOBAR_AA07322
GOBAR_AA07315
GOBAR_AA07314
GOBAR_AA07313
GOBAR_AA06490
GOBAR_AA06073
GOBAR_AA06070
GOBAR_AA06069
GOBAR_AA06038
GOBAR_AA04389
GOBAR_AA04387
GOBAR_AA02097
GOBAR_AA02096
GOBAR_AA02092
GOBAR_AA02089
EGF
EGF_3
EGF
Domain Color
EGF_CA
GUB_WAK_bind
Pkinase
Pkinase_Tyr
WAK
other Domain
0
500
1000

G.ba RLK−Pelle_WAK domain diagram (part 2)

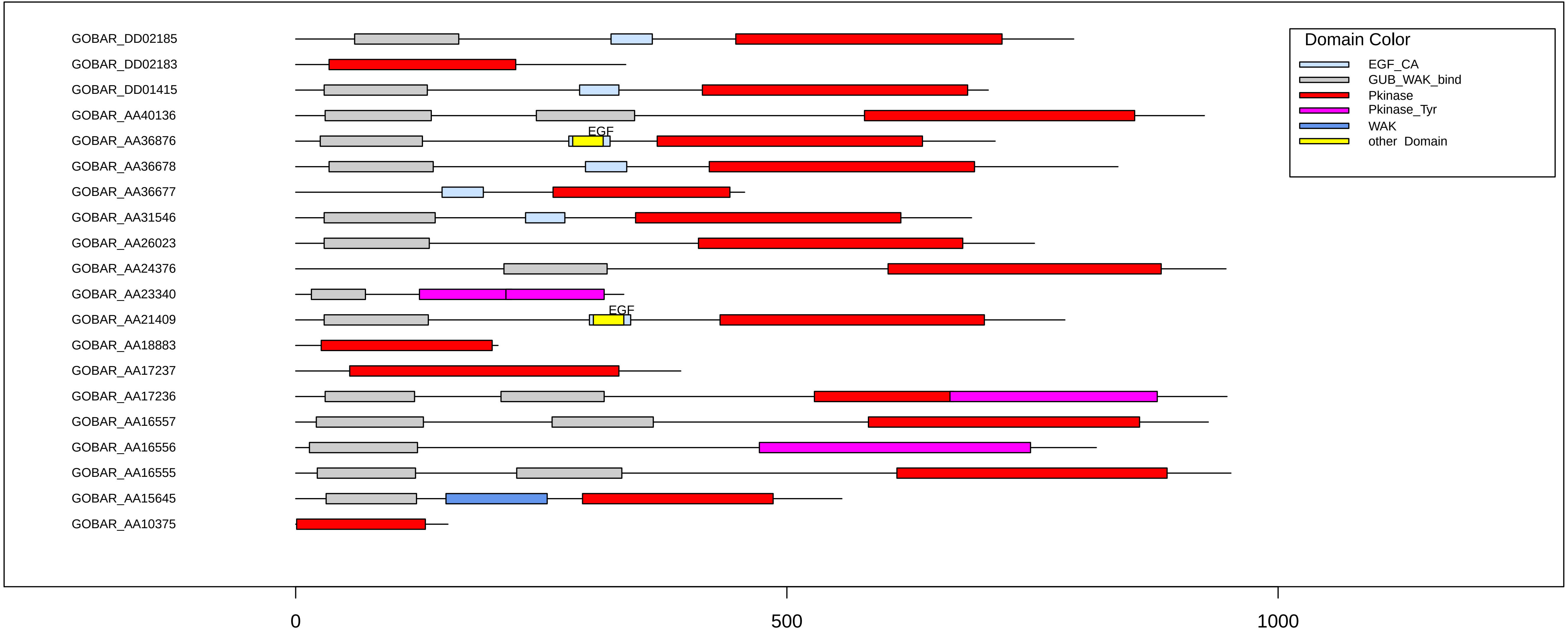

G.ba RLK−Pelle_WAK domain diagram (part 3)

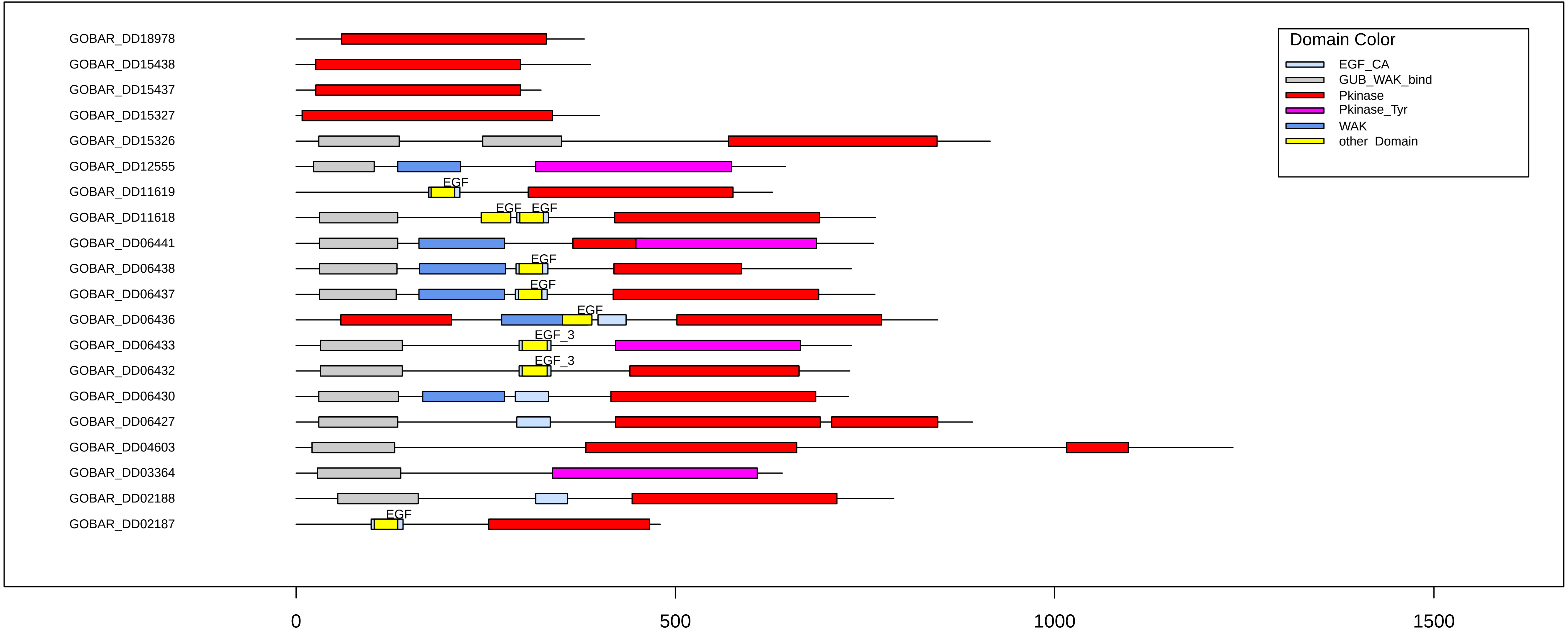

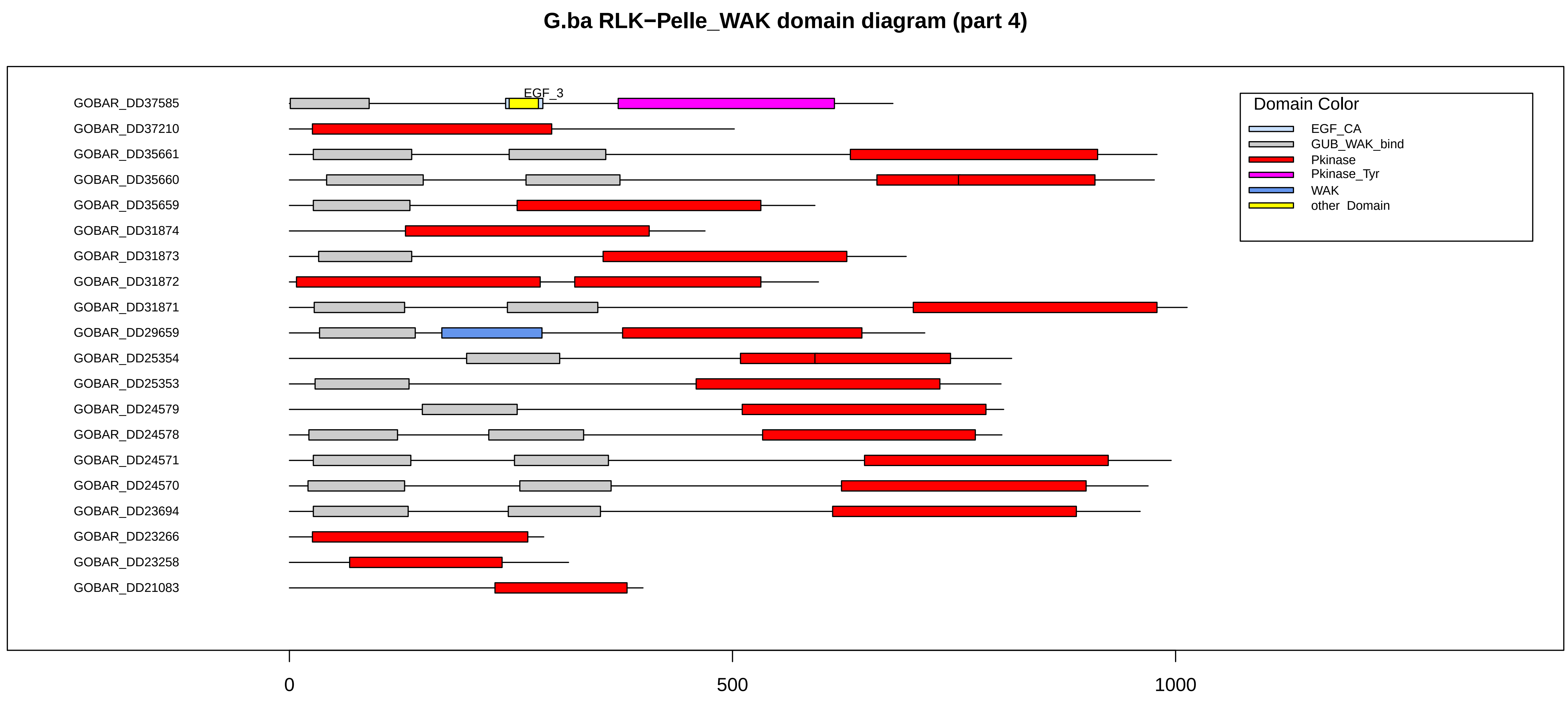

G.ba RLK−Pelle_WAK domain diagram (part 4)
GOBAR_DD37585
EGF_3
GOBAR_DD37210
GOBAR_DD35661
GOBAR_DD35660
GOBAR_DD35659
GOBAR_DD31874
GOBAR_DD31873
GOBAR_DD31872
GOBAR_DD31871
GOBAR_DD29659
GOBAR_DD25354
GOBAR_DD25353
GOBAR_DD24579
GOBAR_DD24578
GOBAR_DD24571
GOBAR_DD24570
GOBAR_DD23694
GOBAR_DD23266
GOBAR_DD23258
GOBAR_DD21083
Domain Color
EGF_CA
GUB_WAK_bind
Pkinase
Pkinase_Tyr
WAK
other Domain
0
500
1000

G.ba RLK−Pelle_WAK domain diagram (part 5)

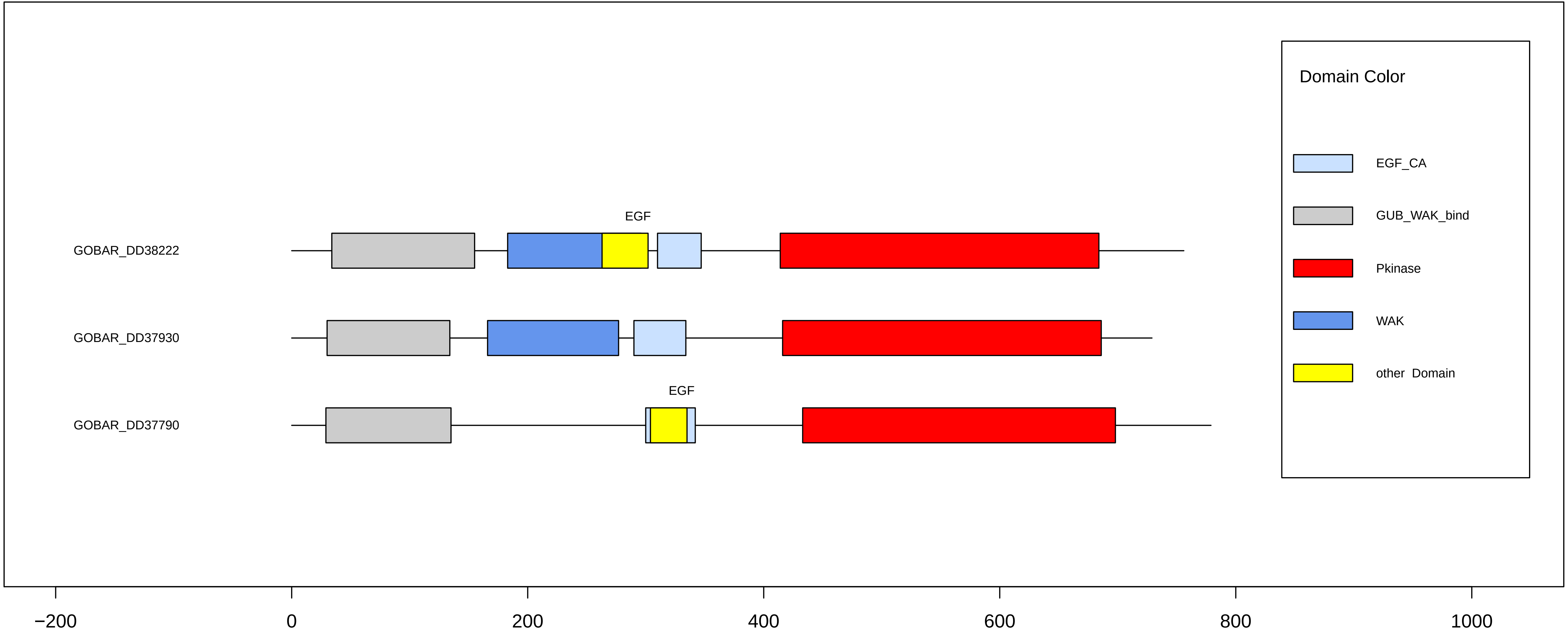

G.ba SCY1_SCYL1 domain diagram (all)

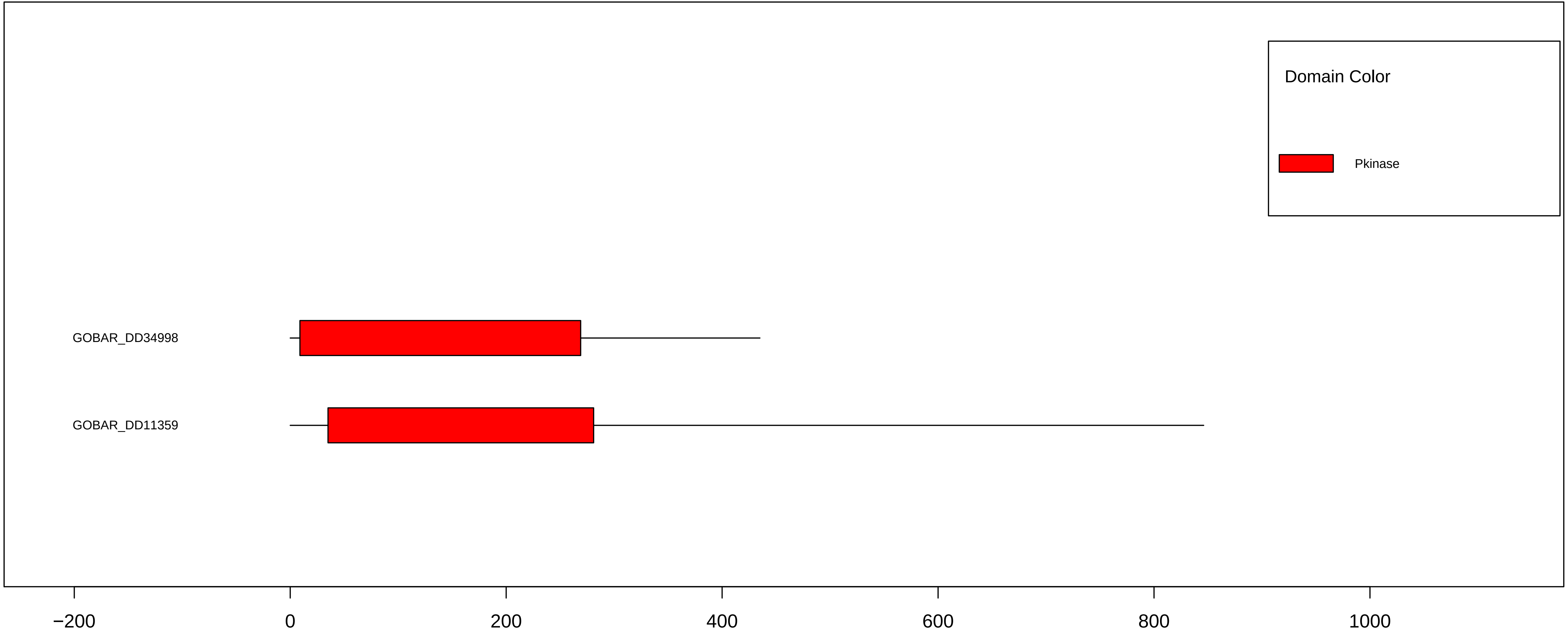

G.ba SCY1_SCYL2 domain diagram (all)

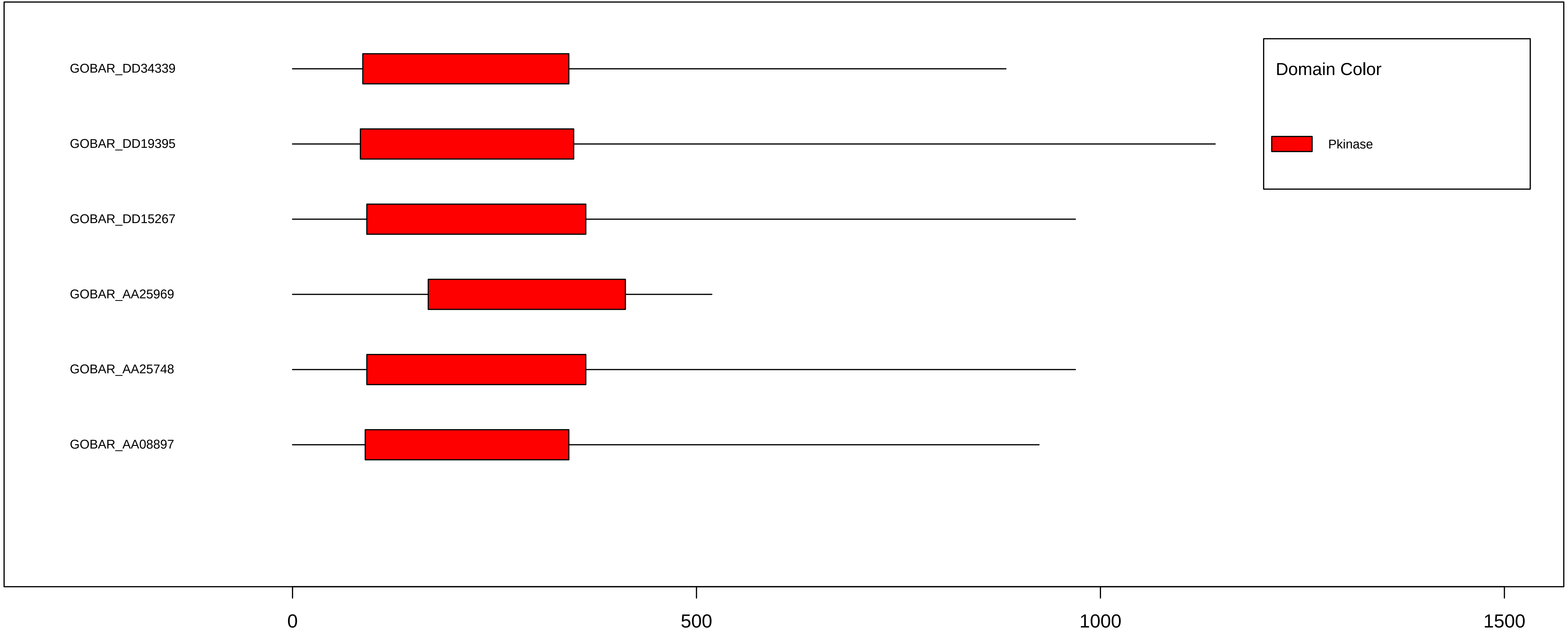

G.ba STE_STE11 domain diagram (part 1)

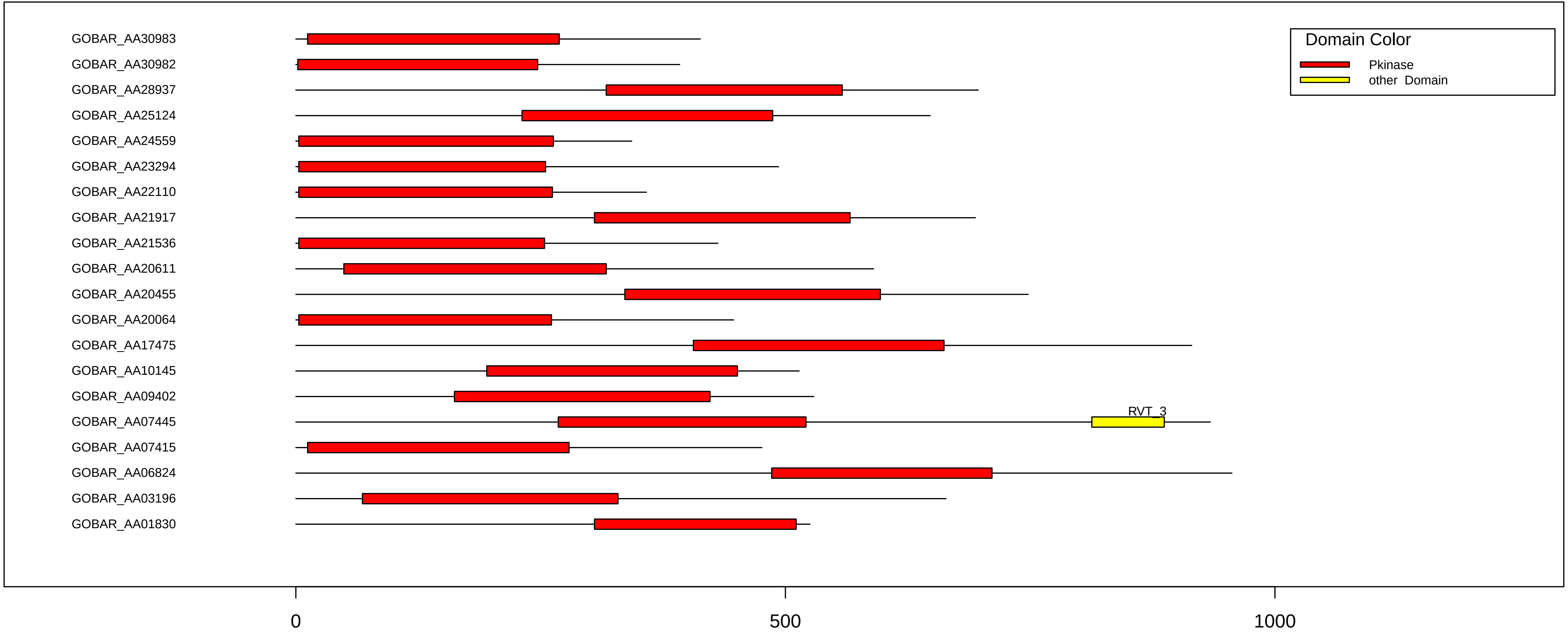

G.ba STE_STE11 domain diagram (part 2)

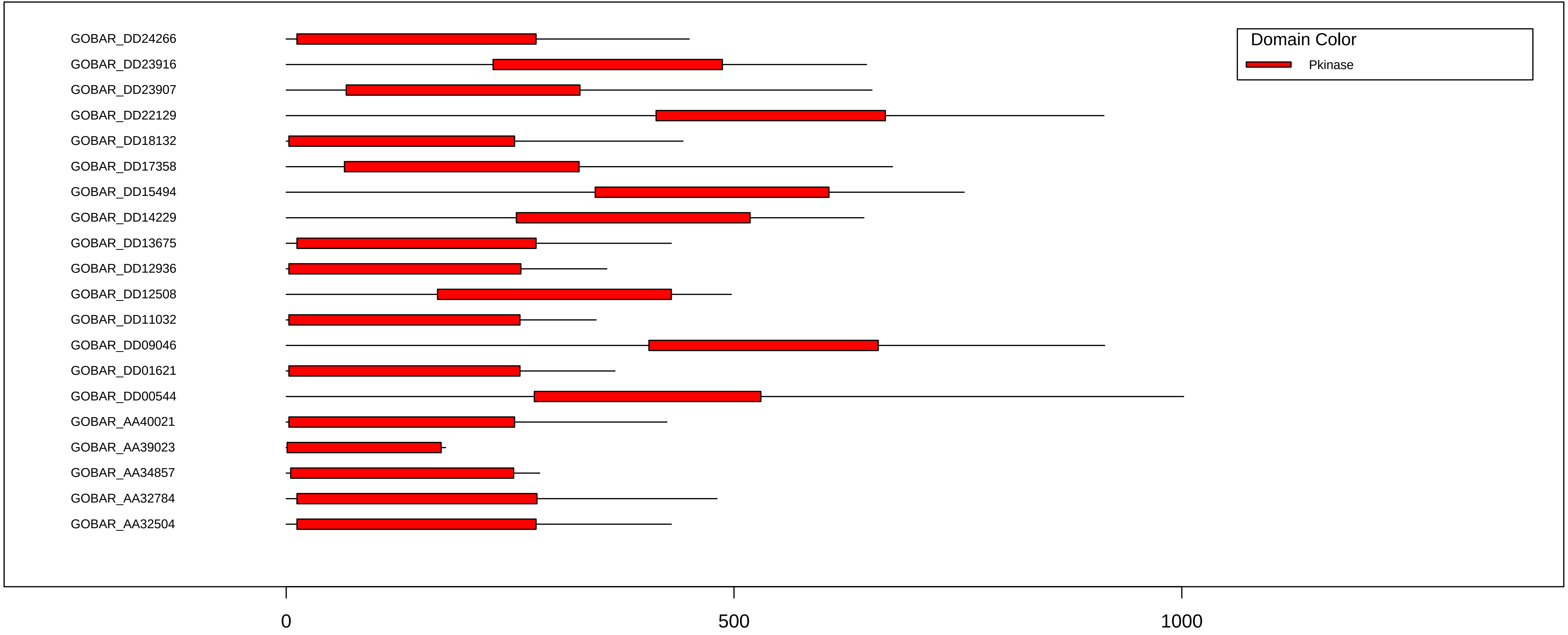

G.ba STE_STE11 domain diagram (part 3)

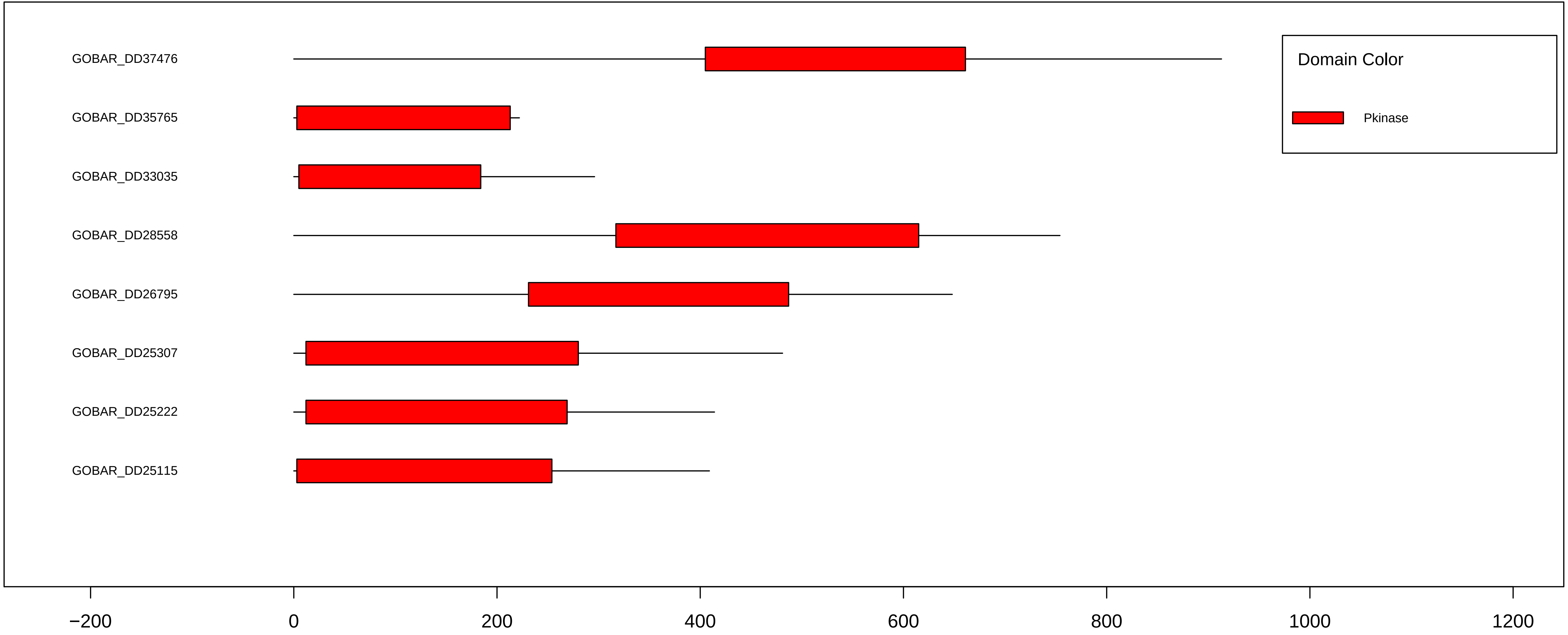

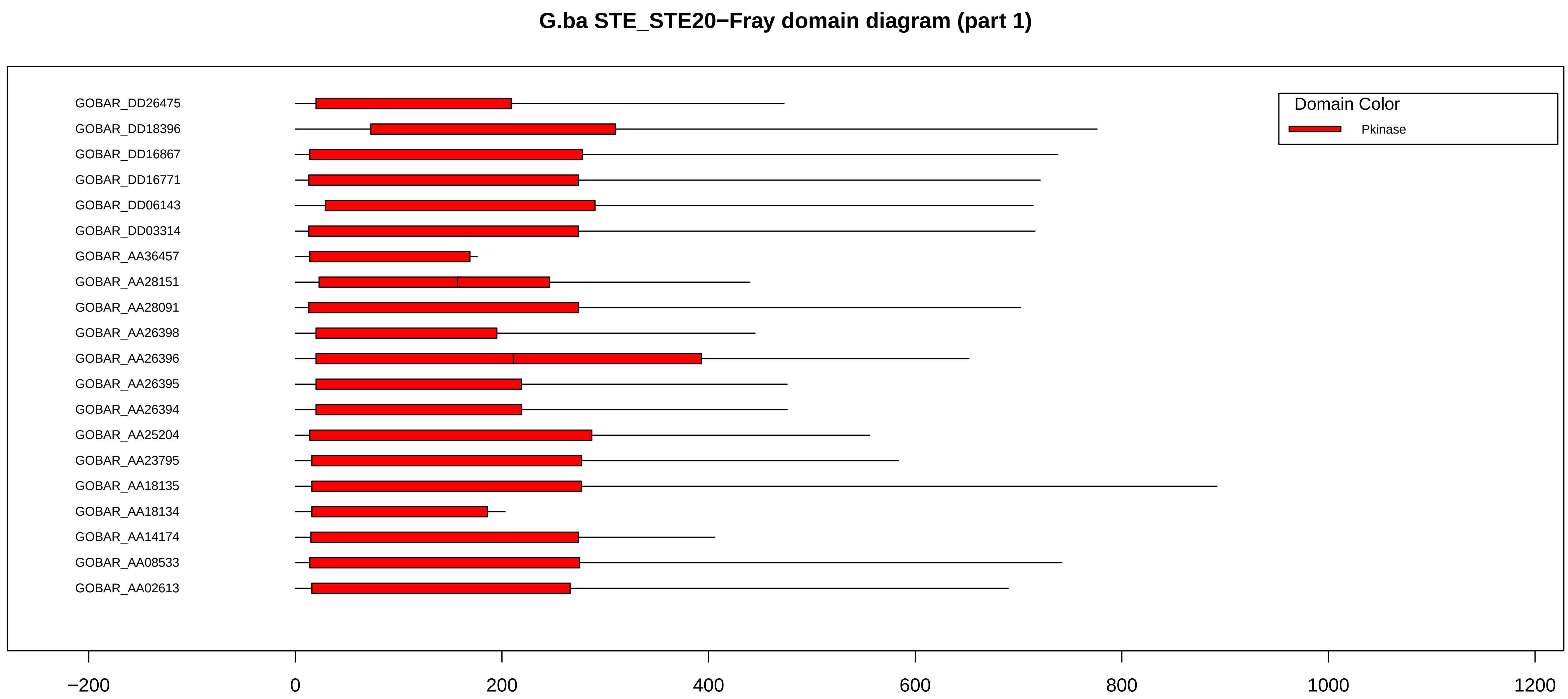
G.ba STE_STE20−Fray domain diagram (part 1)
GOBAR_DD26475
GOBAR_DD18396
GOBAR_DD16867
GOBAR_DD16771
GOBAR_DD06143
GOBAR_DD03314
GOBAR_AA36457
GOBAR_AA28151
GOBAR_AA28091
GOBAR_AA26398
GOBAR_AA26396
GOBAR_AA26395
GOBAR_AA26394
GOBAR_AA25204
GOBAR_AA23795
GOBAR_AA18135
GOBAR_AA18134
GOBAR_AA14174
GOBAR_AA08533
GOBAR_AA02613
Domain Color
Pkinase
−200
0
200
400
600
800
1000
1200

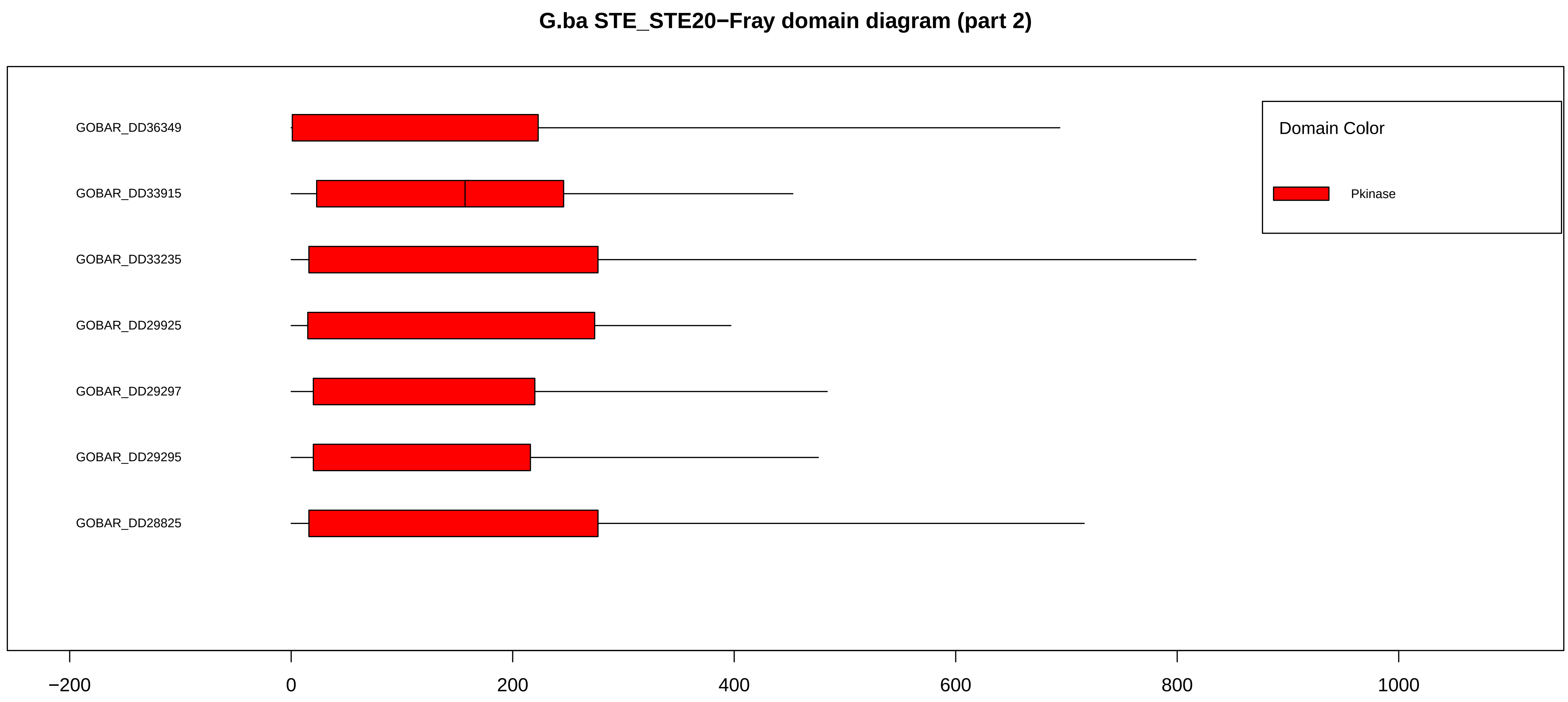

G.ba STE_STE20−Fray domain diagram (part 2)
GOBAR_DD36349
GOBAR_DD33915
GOBAR_DD33235
GOBAR_DD29925
GOBAR_DD29297
GOBAR_DD29295
GOBAR_DD28825
Domain Color
Pkinase
−200
0
200
400
600
800
1000

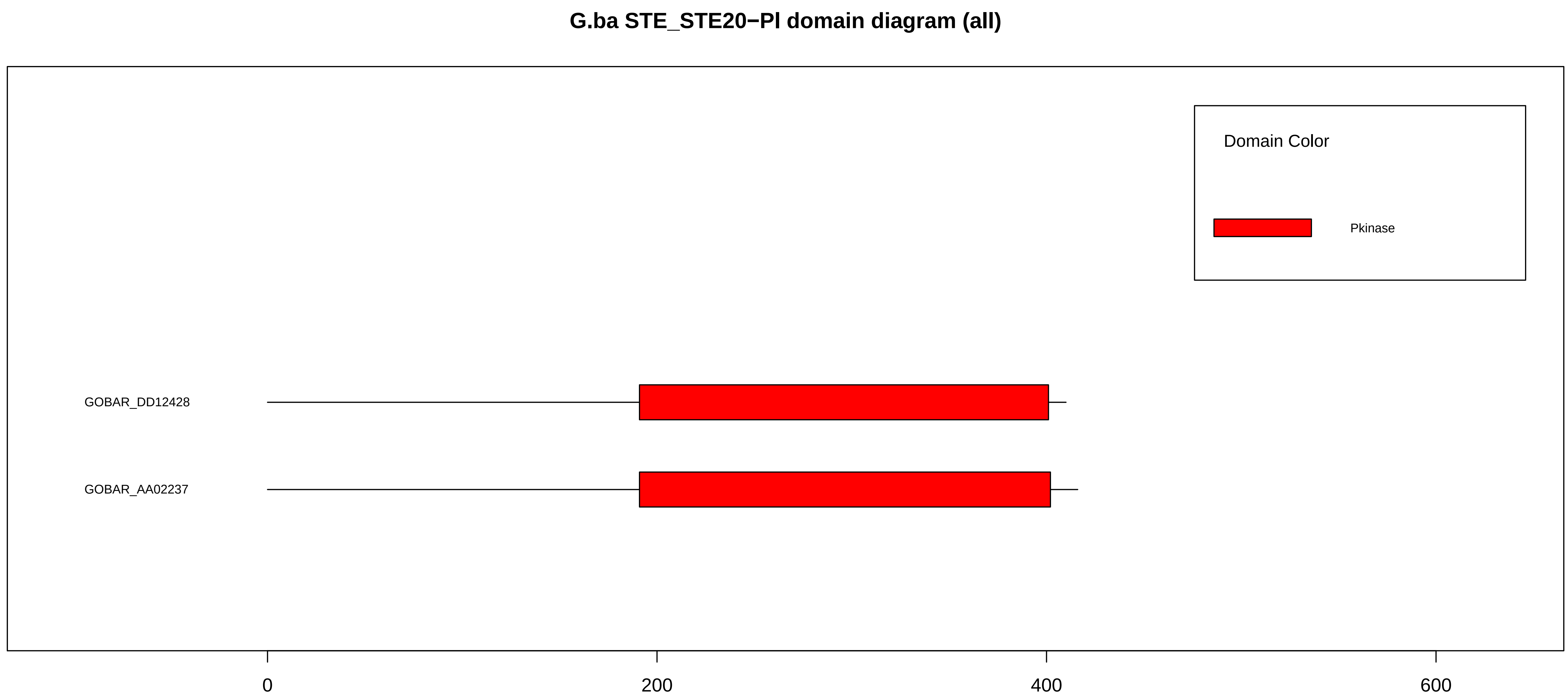
G.ba STE_STE20−Pl domain diagram (all)
Domain Color
Pkinase
GOBAR_DD12428
GOBAR_AA02237
0
200
400
600

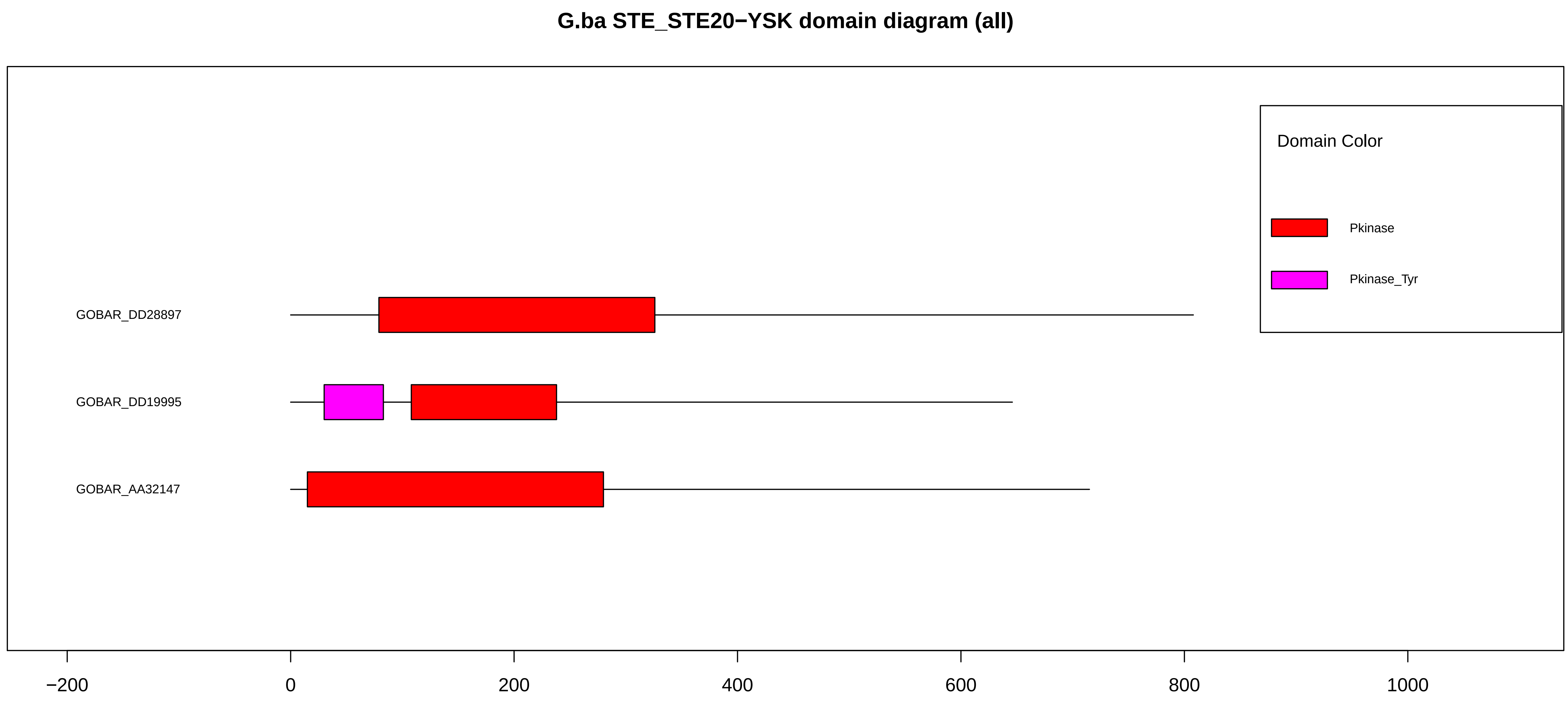
G.ba STE_STE20−YSK domain diagram (all)
Domain Color
Pkinase
Pkinase_Tyr
GOBAR_DD28897
GOBAR_DD19995
GOBAR_AA32147
−200
0
200
400
600
800
1000

G.ba STE_STE7 domain diagram (part 1)

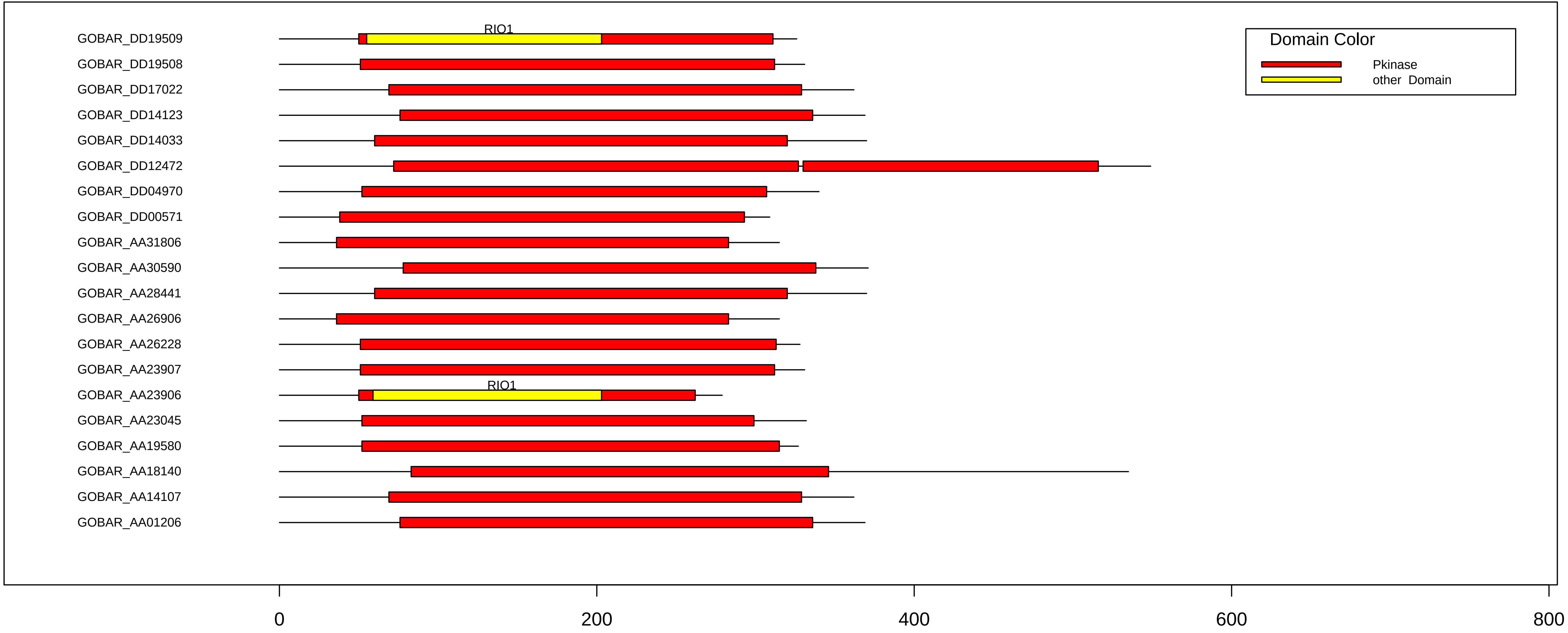

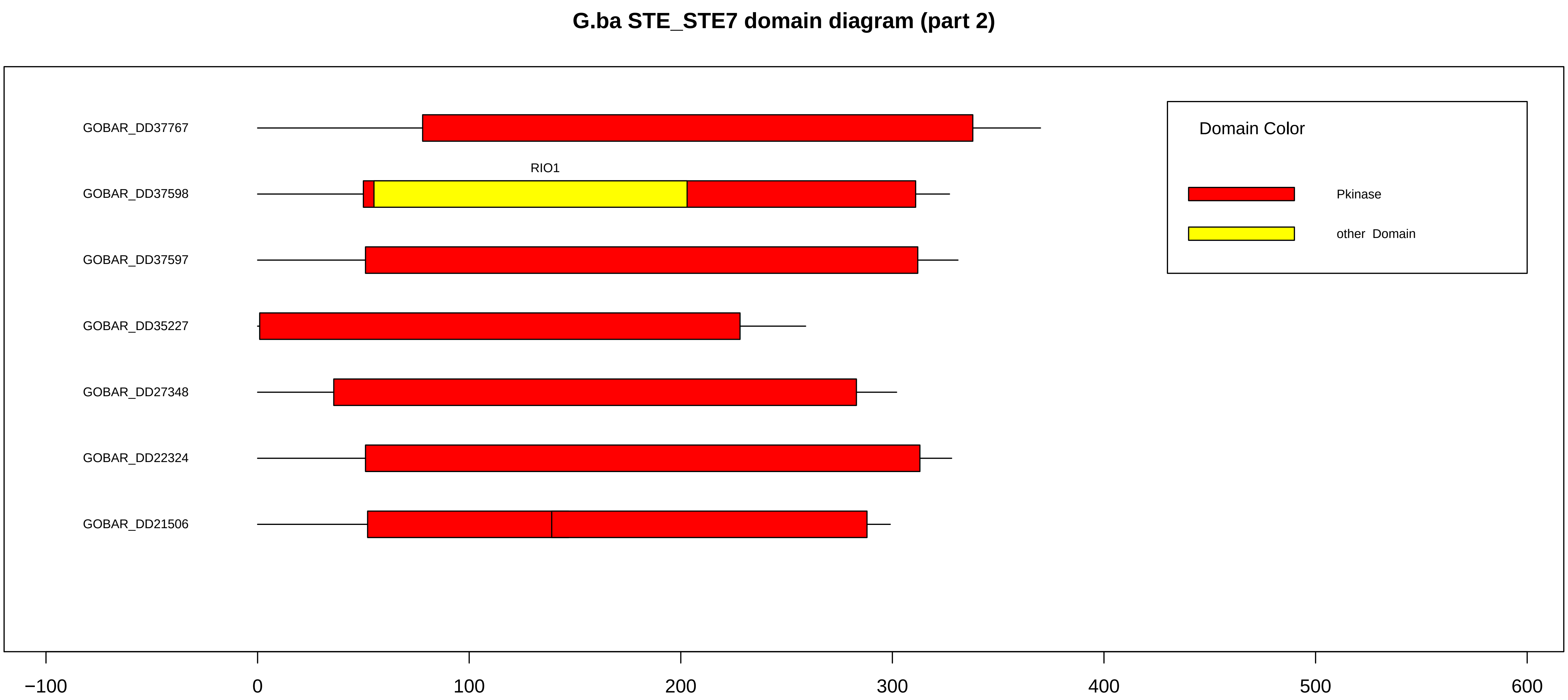

G.ba STE_STE7 domain diagram (part 2)
GOBAR_DD37767
GOBAR_DD37598
RIO1
GOBAR_DD37597
GOBAR_DD35227
GOBAR_DD27348
GOBAR_DD22324
GOBAR_DD21506
Domain Color
Pkinase
other Domain
−100
0
100
200
300
400
500
600

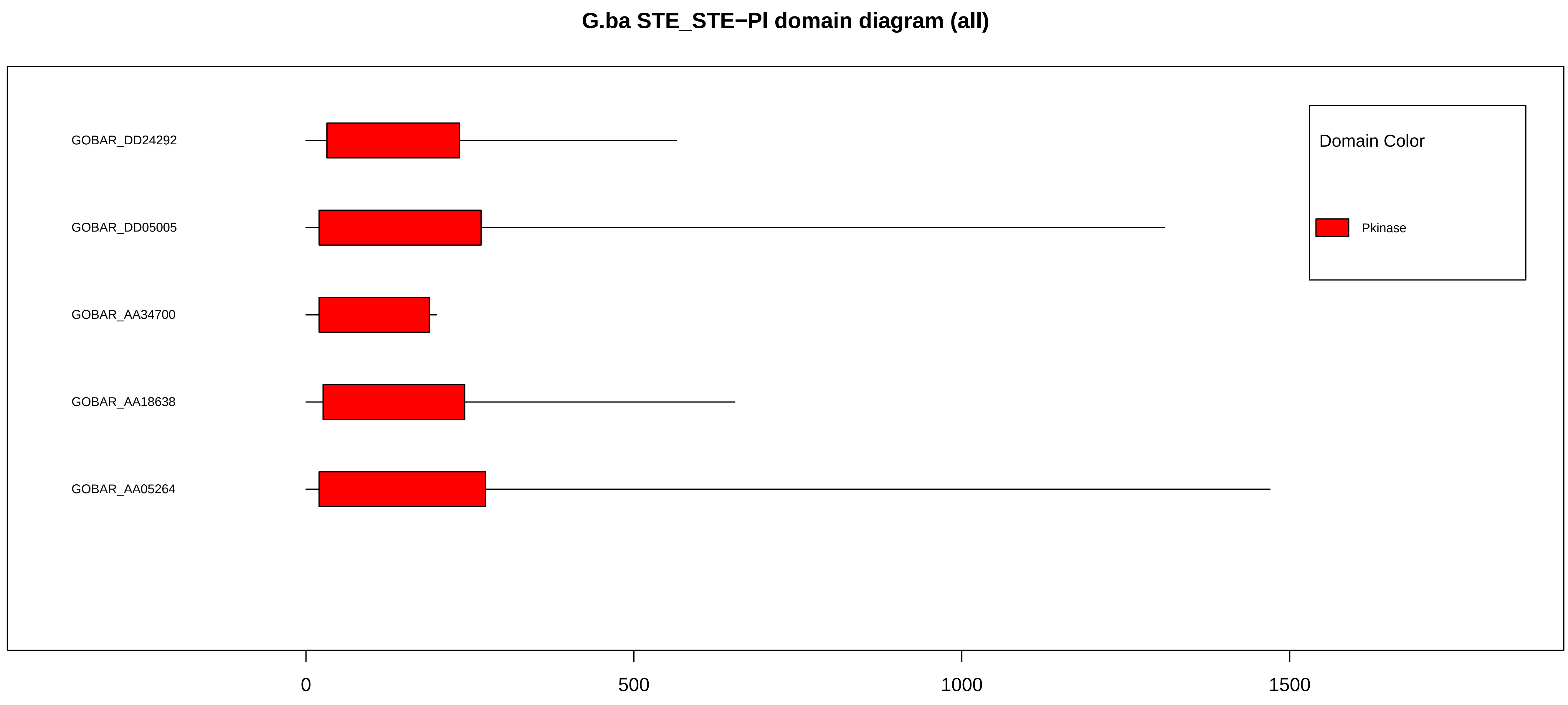
G.ba STE_STE−PI domain diagram (all)
GOBAR_DD24292
GOBAR_DD05005
GOBAR_AA34700
GOBAR_AA18638
GOBAR_AA05264
Domain Color
Pkinase
0
500
1000
1500

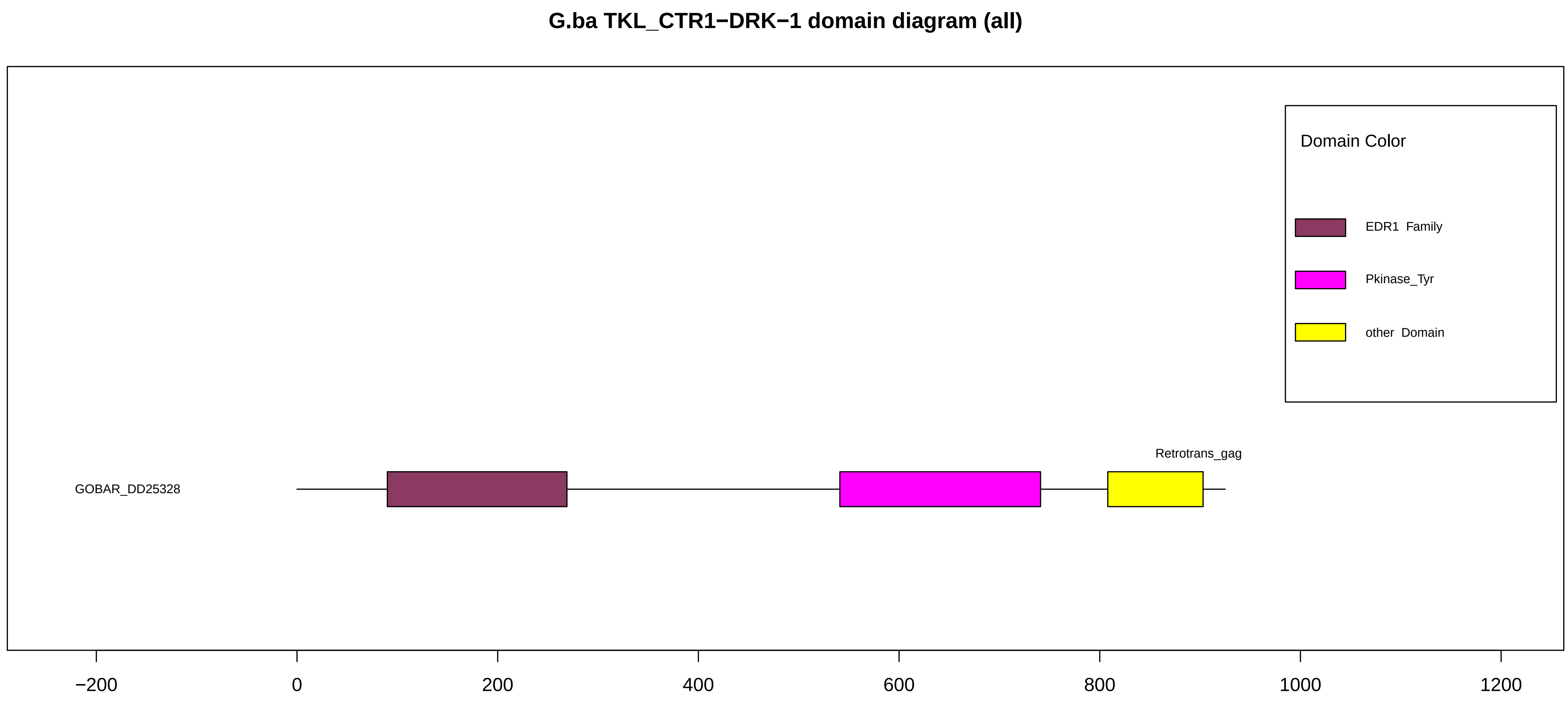
G.ba TKL_CTR1−DRK−1 domain diagram (all)
Domain Color
EDR1 Family
Pkinase_Tyr
other Domain
Retrotrans_gag
GOBAR_DD25328
−200
0
200
400
600
800
1000
1200

G.ba TKL_CTR1−DRK−2 domain diagram (part 1)

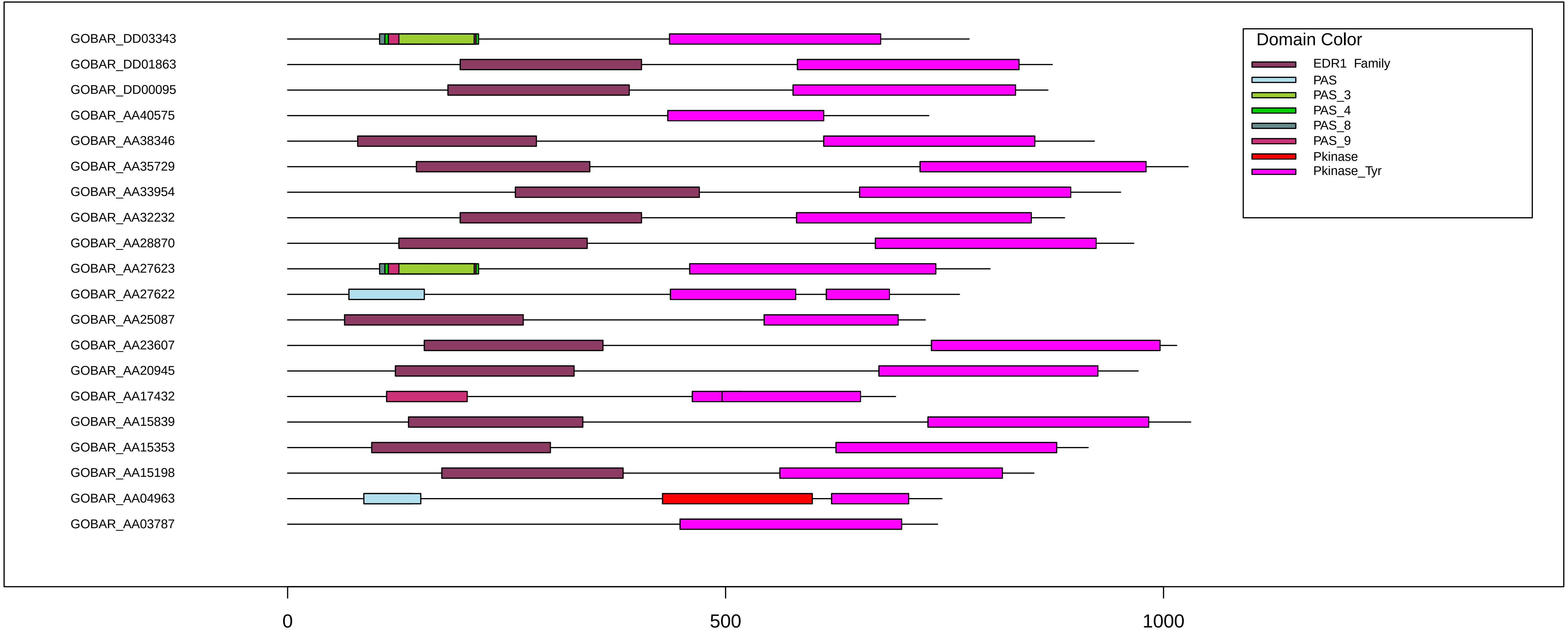

G.ba TKL_CTR1−DRK−2 domain diagram (part 2)

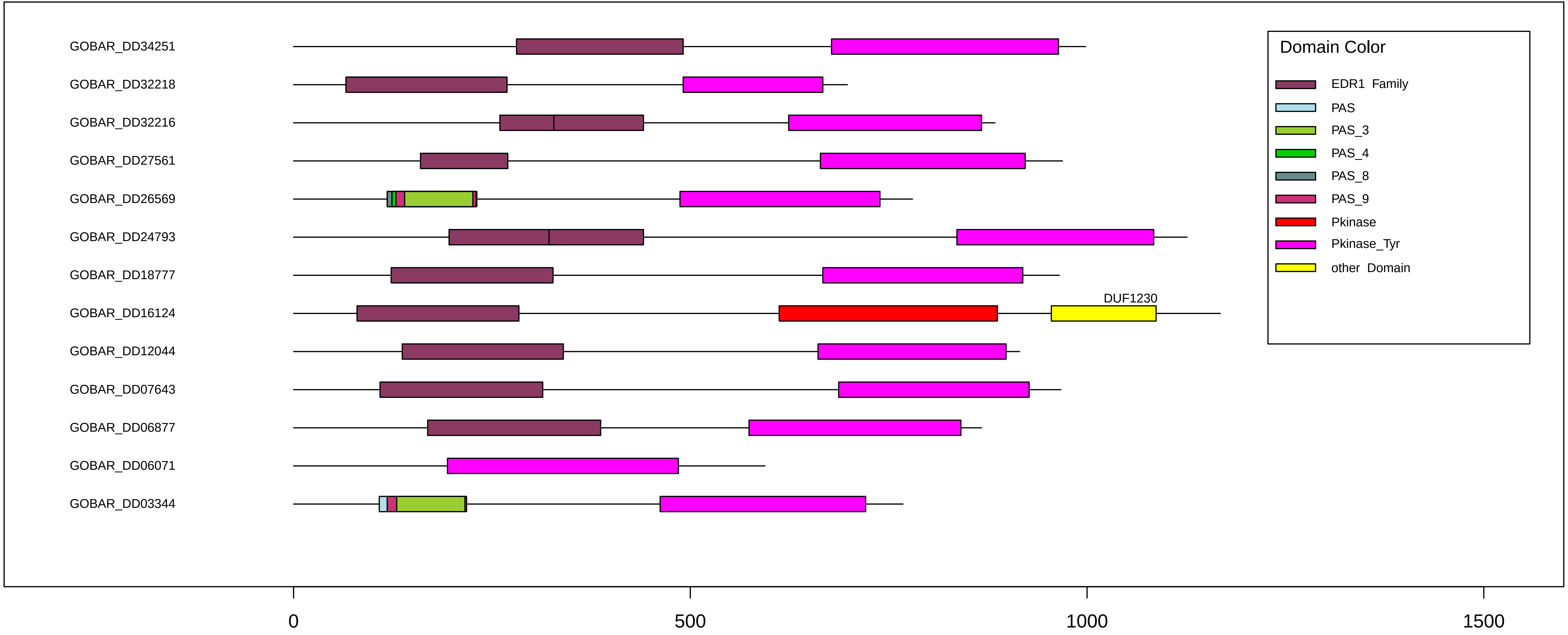

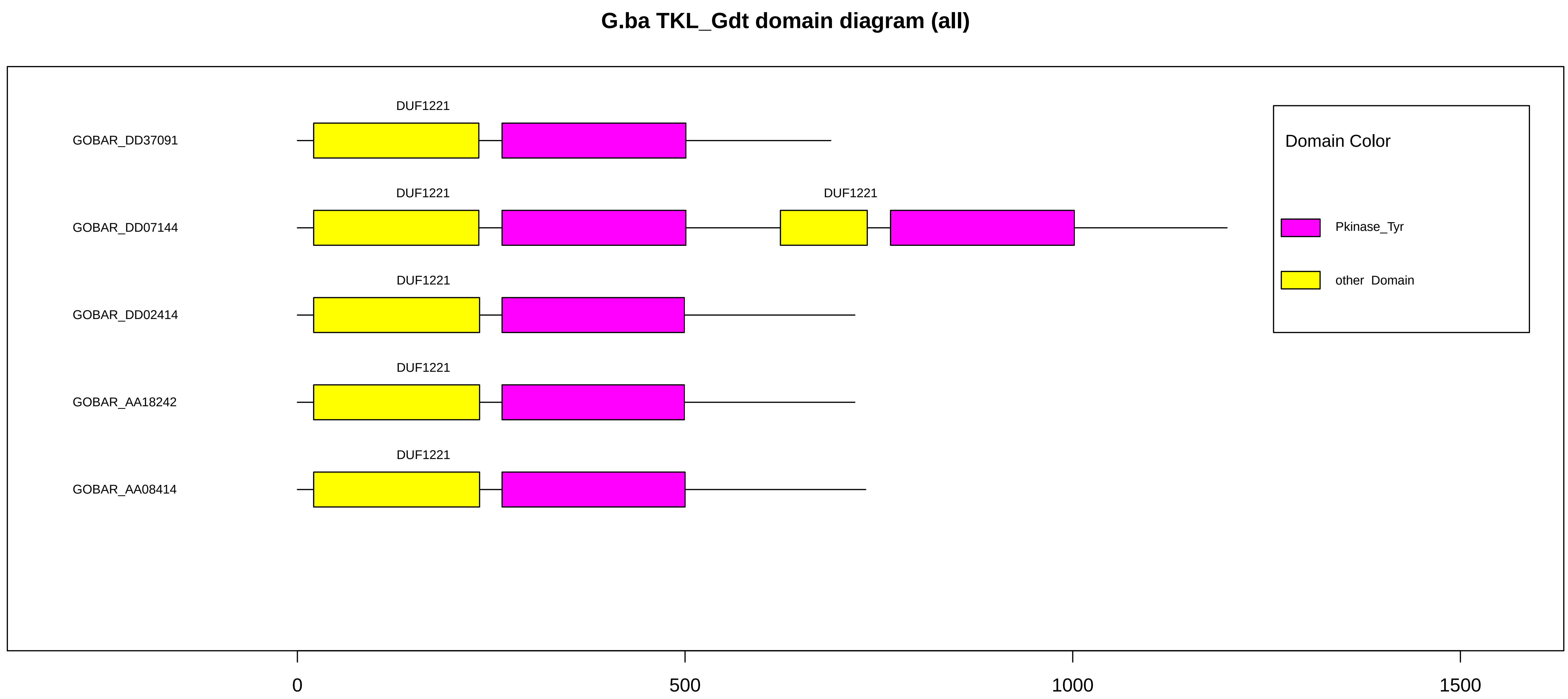
G.ba TKL_Gdt domain diagram (all)
GOBAR_DD37091
DUF1221
GOBAR_DD07144
DUF1221
DUF1221
GOBAR_DD02414
DUF1221
GOBAR_AA18242
DUF1221
GOBAR_AA08414
DUF1221
Domain Color
Pkinase_Tyr
other Domain
0
500
1000
1500

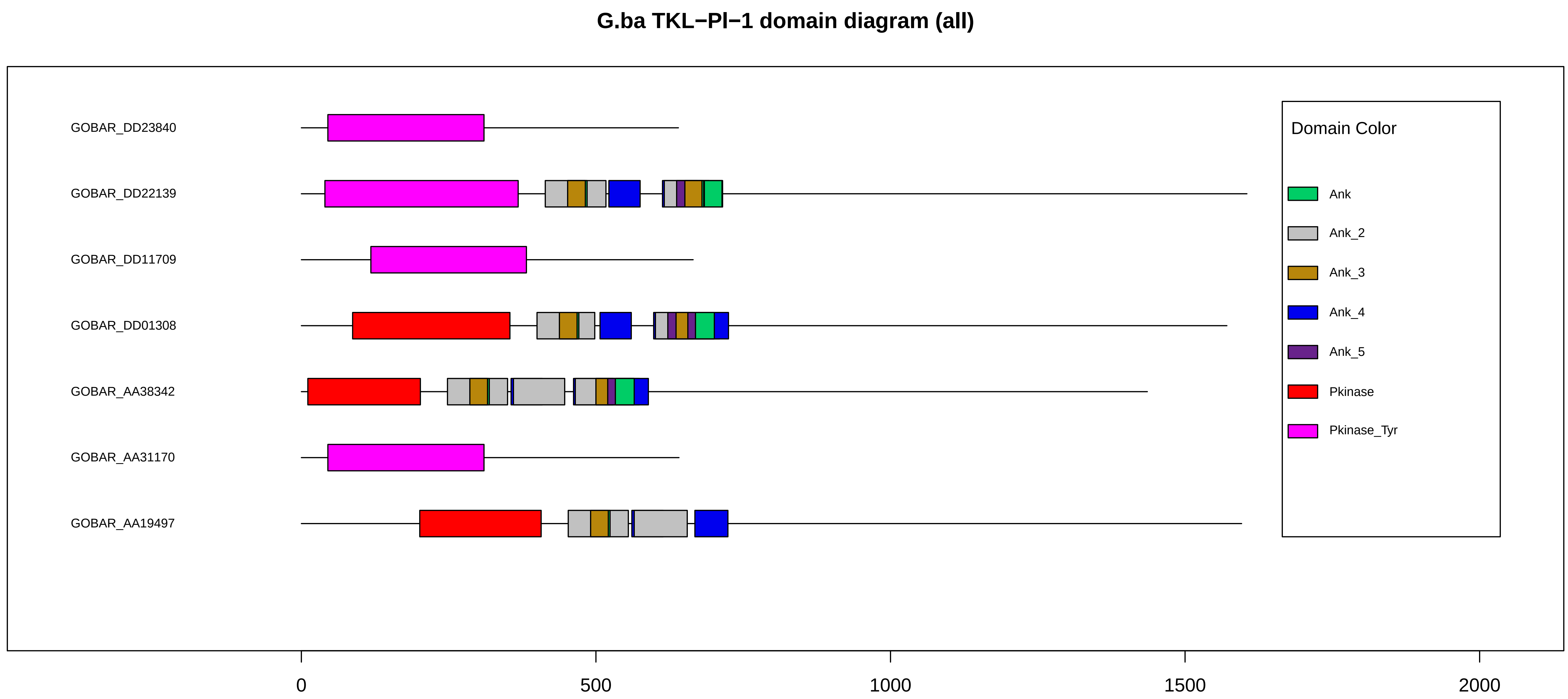
G.ba TKL−PI−1 domain diagram (all)
GOBAR_DD23840
GOBAR_DD22139
GOBAR_DD11709
GOBAR_DD01308
GOBAR_AA38342
GOBAR_AA31170
GOBAR_AA19497
Domain Color
Ank
Ank_2
Ank_3
Ank_4
Ank_5
Pkinase
Pkinase_Tyr
0
500
1000
1500
2000

G.ba TKL−Pl−2 domain diagram (all)

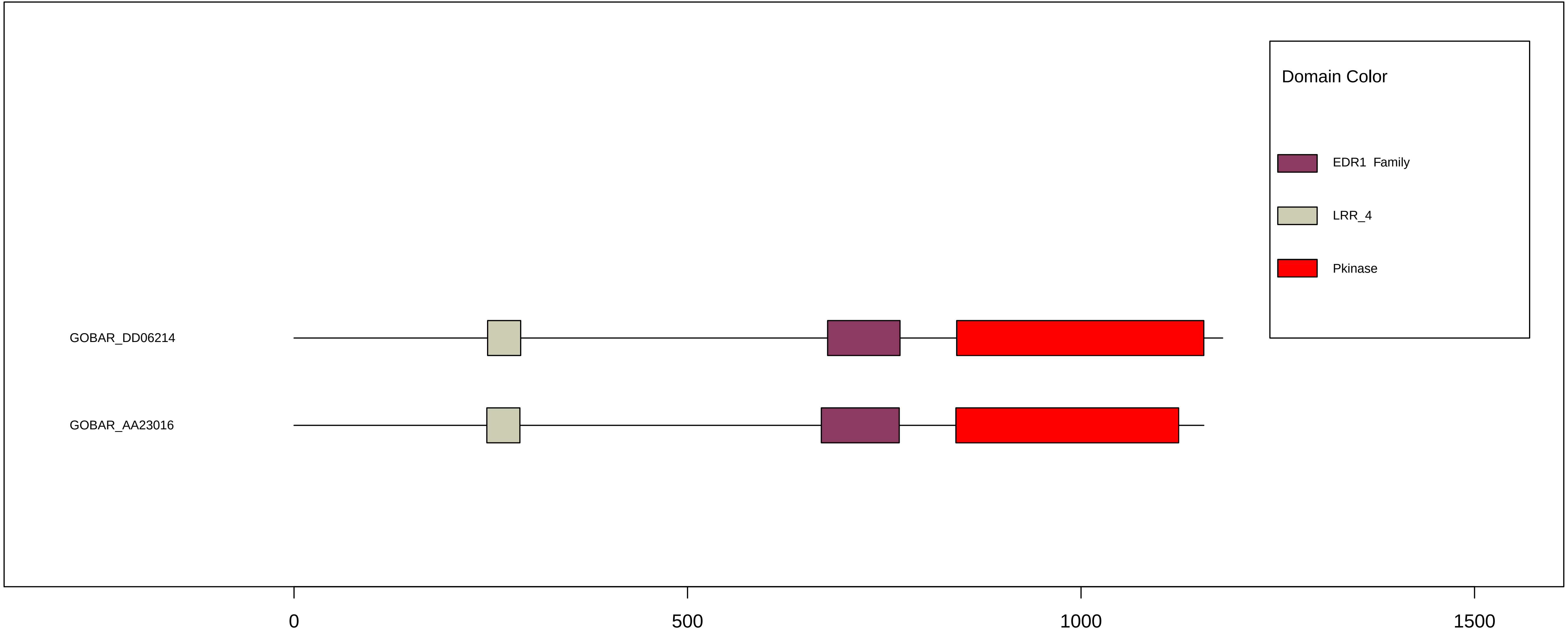

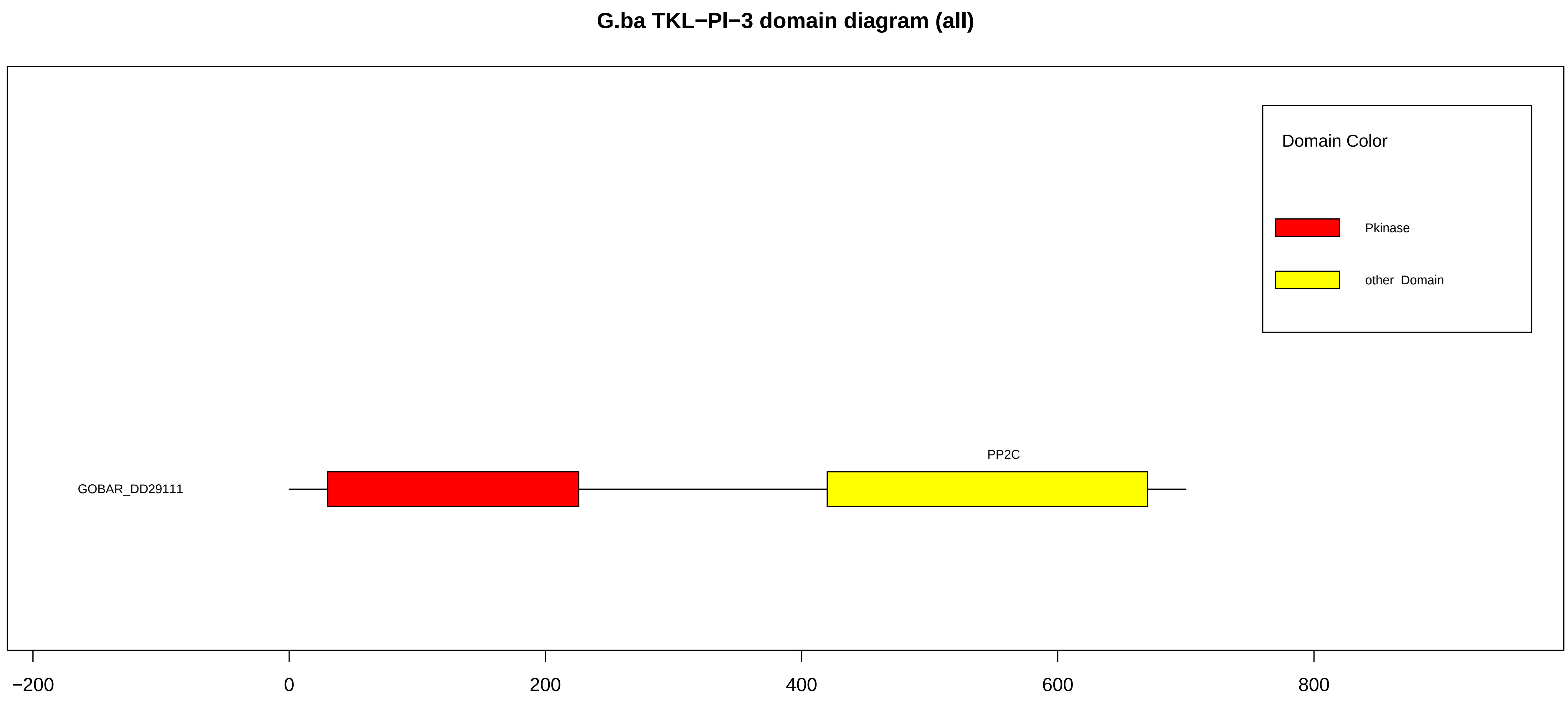
G.ba TKL−Pl−3 domain diagram (all)
Domain Color
Pkinase
other Domain
PP2C
GOBAR_DD29111
−200
0
200
400
600
800

G.ba TKL−Pl−4 domain diagram (part 1)

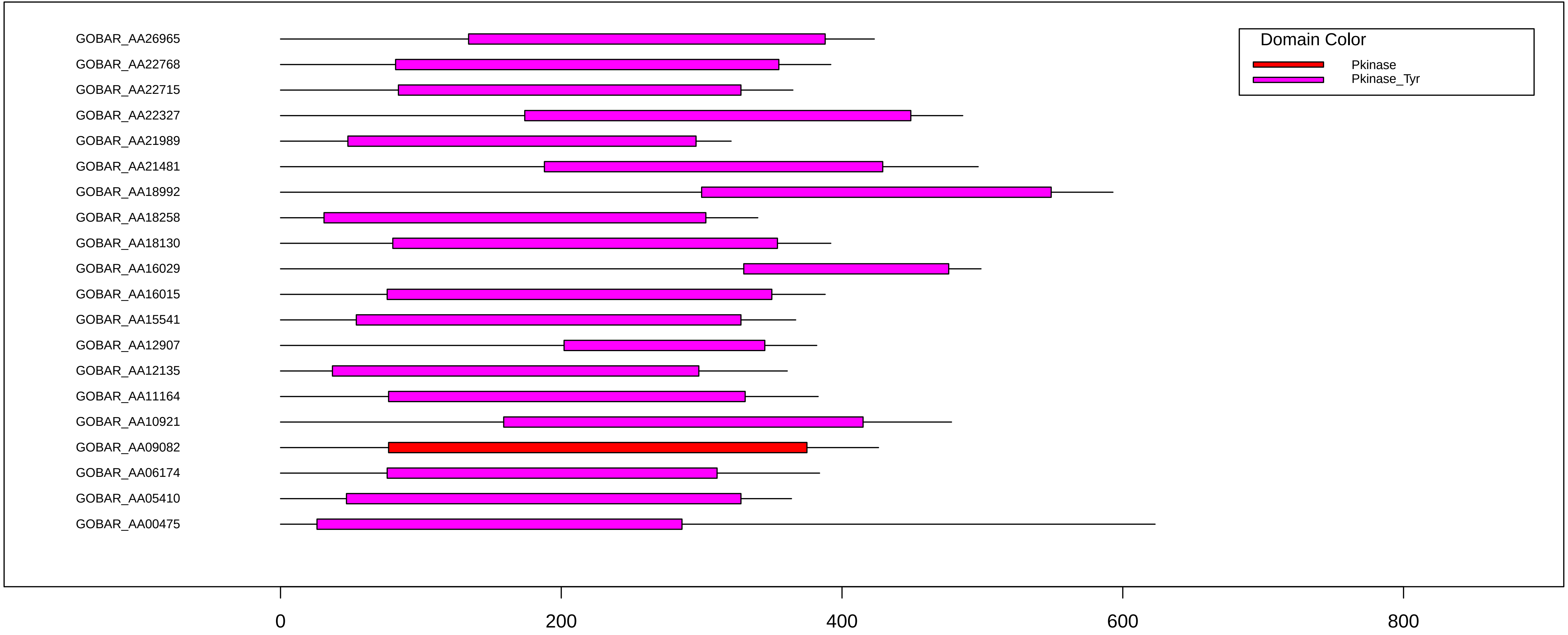

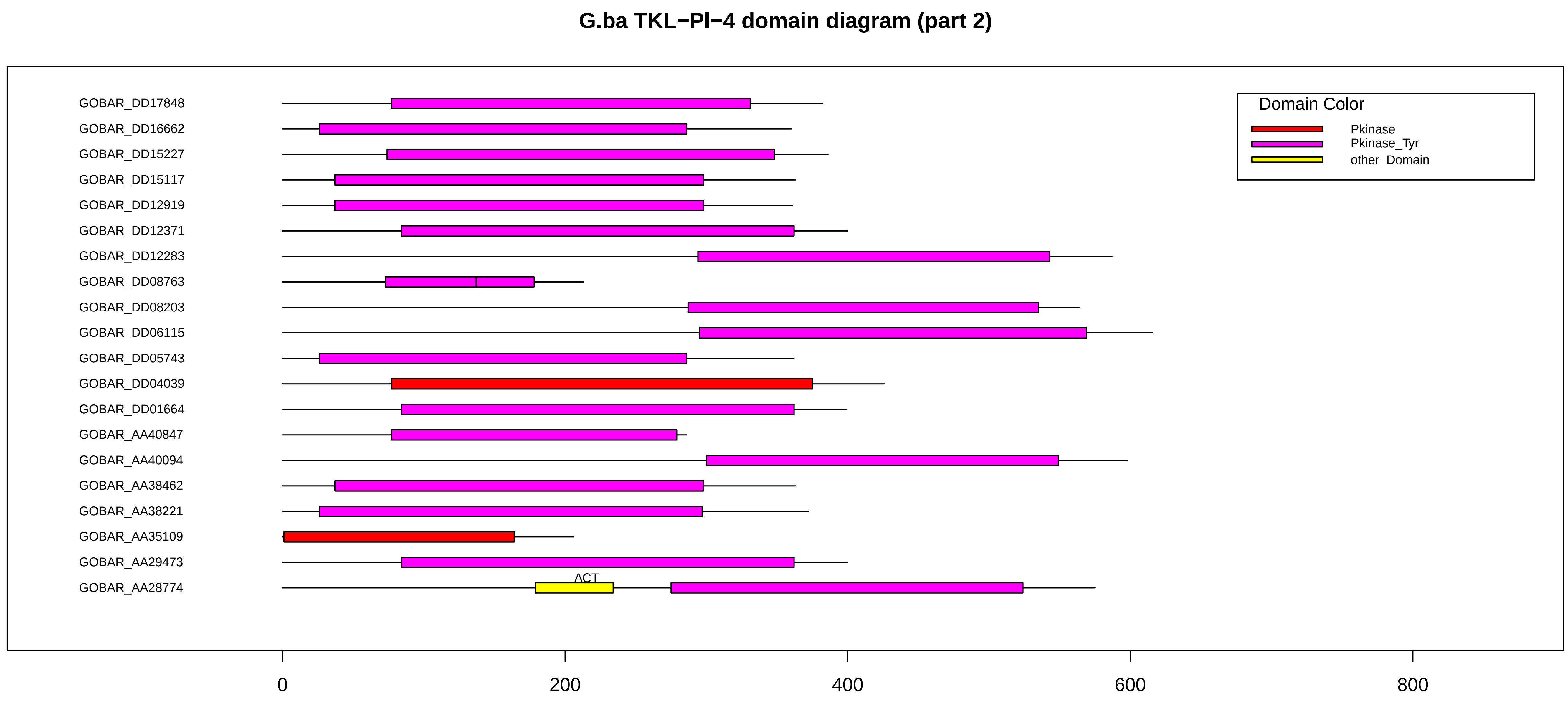
G.ba TKL−Pl−4 domain diagram (part 2)
GOBAR_DD17848
GOBAR_DD16662
GOBAR_DD15227
GOBAR_DD15117
GOBAR_DD12919
GOBAR_DD12371
GOBAR_DD12283
GOBAR_DD08763
GOBAR_DD08203
GOBAR_DD06115
GOBAR_DD05743
GOBAR_DD04039
GOBAR_DD01664
GOBAR_AA40847
GOBAR_AA40094
GOBAR_AA38462
GOBAR_AA38221
GOBAR_AA35109
GOBAR_AA29473
GOBAR_AA28774
ACT
Domain Color
Pkinase
Pkinase_Tyr
other Domain
0
200
400
600
800

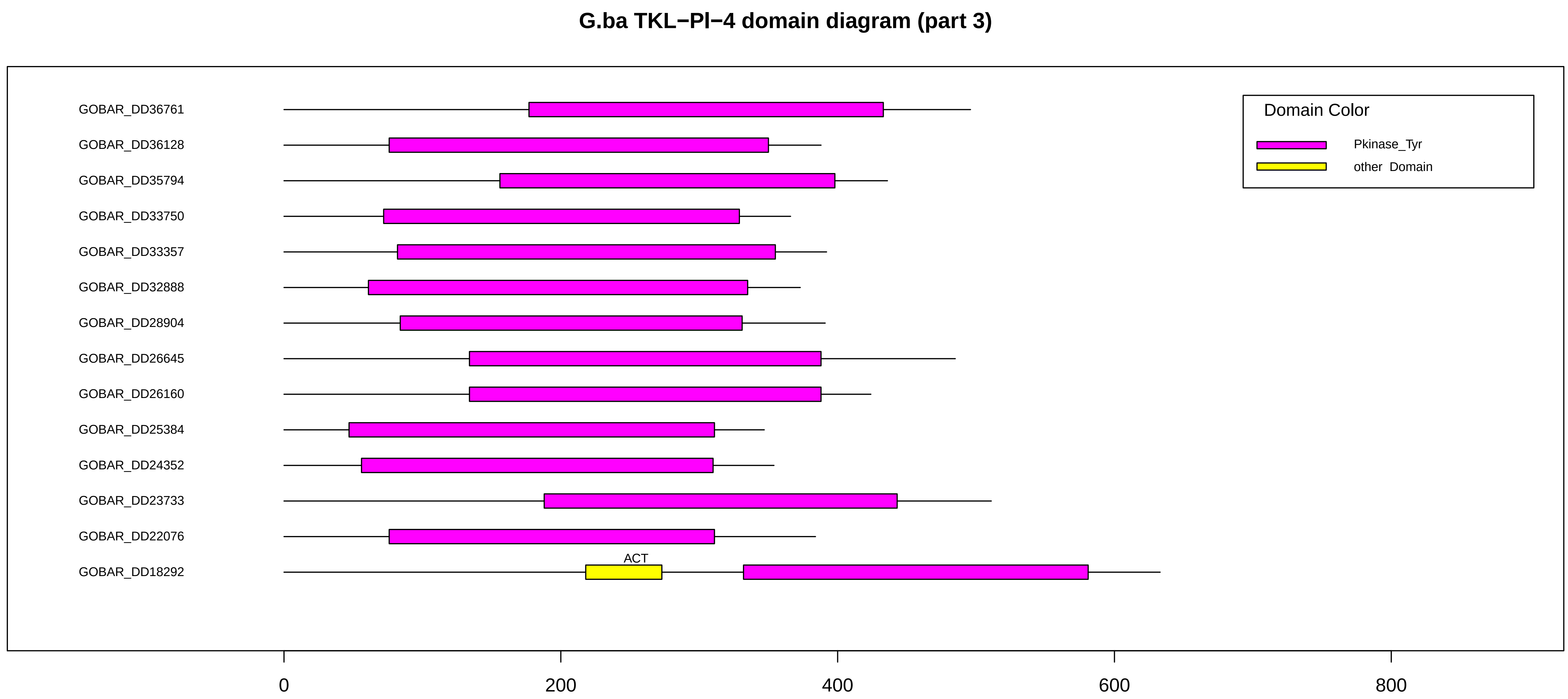
G.ba TKL−Pl−4 domain diagram (part 3)
GOBAR_DD36761
GOBAR_DD36128
GOBAR_DD35794
GOBAR_DD33750
GOBAR_DD33357
GOBAR_DD32888
GOBAR_DD28904
GOBAR_DD26645
GOBAR_DD26160
GOBAR_DD25384
GOBAR_DD24352
GOBAR_DD23733
GOBAR_DD22076
GOBAR_DD18292
ACT
Domain Color
Pkinase_Tyr
other Domain
0
200
400
600
800

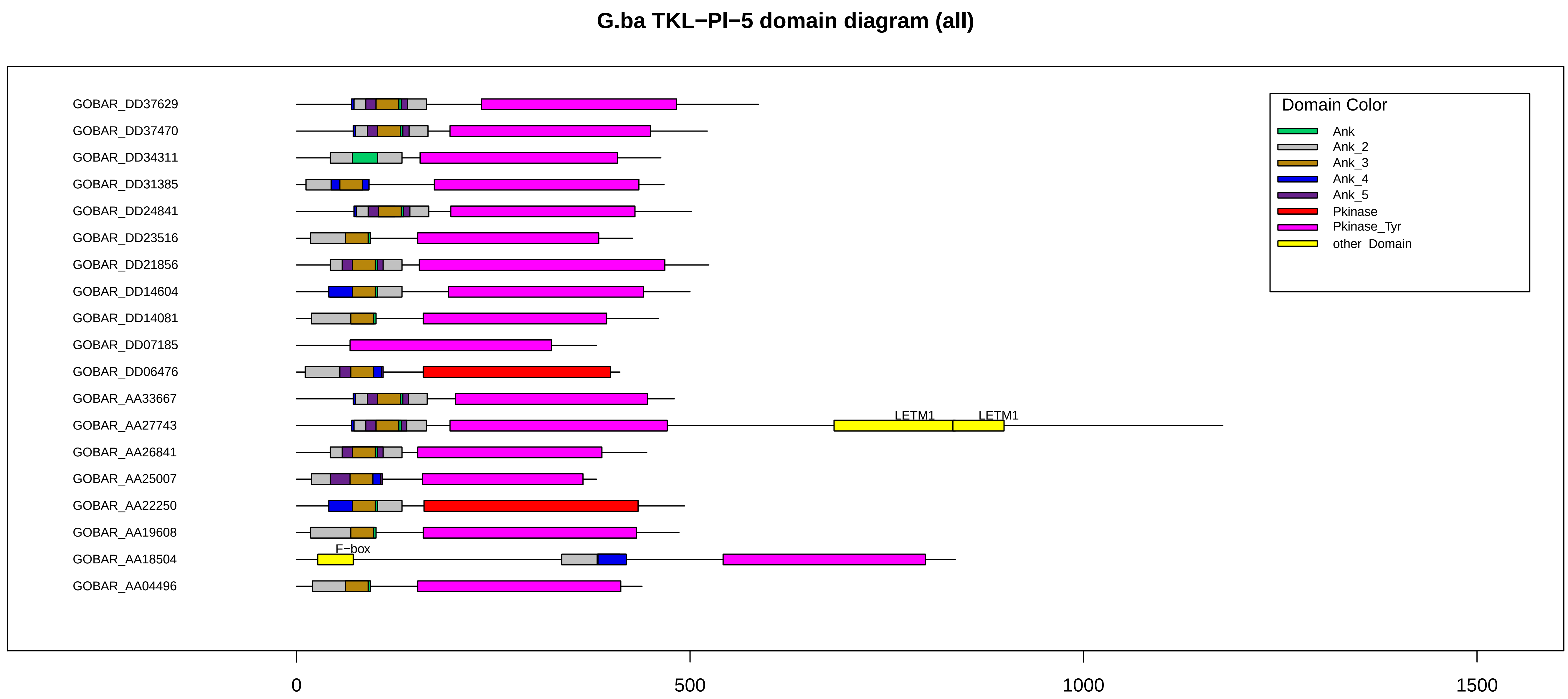

G.ba TKL−PI−5 domain diagram (all)
GOBAR_DD37629
GOBAR_DD37470
GOBAR_DD34311
GOBAR_DD31385
GOBAR_DD24841
GOBAR_DD23516
GOBAR_DD21856
GOBAR_DD14604
GOBAR_DD14081
GOBAR_DD07185
GOBAR_DD06476
GOBAR_AA33667
GOBAR_AA27743
GOBAR_AA26841
GOBAR_AA25007
GOBAR_AA22250
GOBAR_AA19608
GOBAR_AA18504
GOBAR_AA04496
LETM1
LETM1
F−box
Domain Color
Ank
Ank_2
Ank_3
Ank_4
Ank_5
Pkinase
Pkinase_Tyr
other Domain
0
500
1000
1500

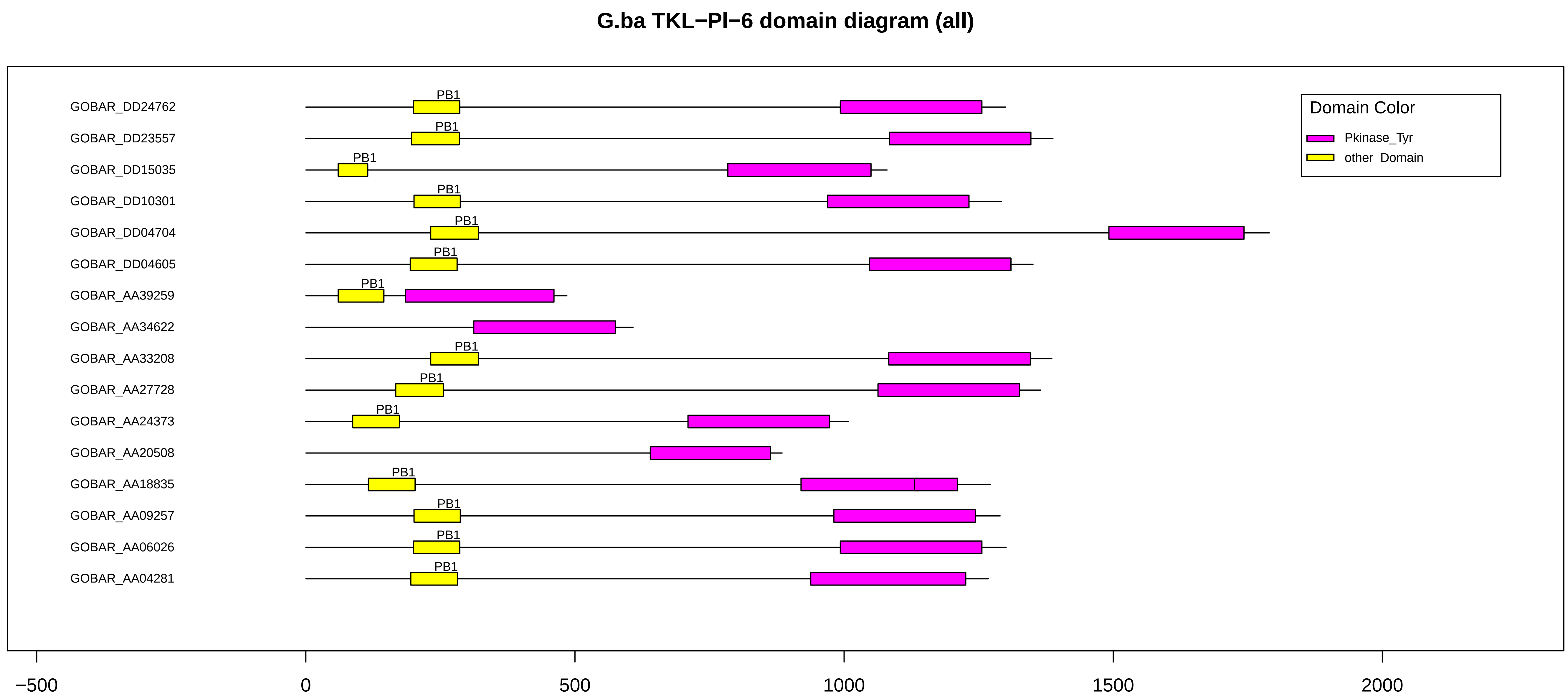
G.ba TKL−Pl−6 domain diagram (all)
GOBAR_DD24762
GOBAR_DD23557
GOBAR_DD15035
GOBAR_DD10301
GOBAR_DD04704
GOBAR_DD04605
GOBAR_AA39259
GOBAR_AA34622
GOBAR_AA33208
GOBAR_AA27728
GOBAR_AA24373
GOBAR_AA20508
GOBAR_AA18835
GOBAR_AA09257
GOBAR_AA06026
GOBAR_AA04281
PB1
Domain Color
Pkinase_Tyr
other Domain
−500
0
500
1000
1500
2000

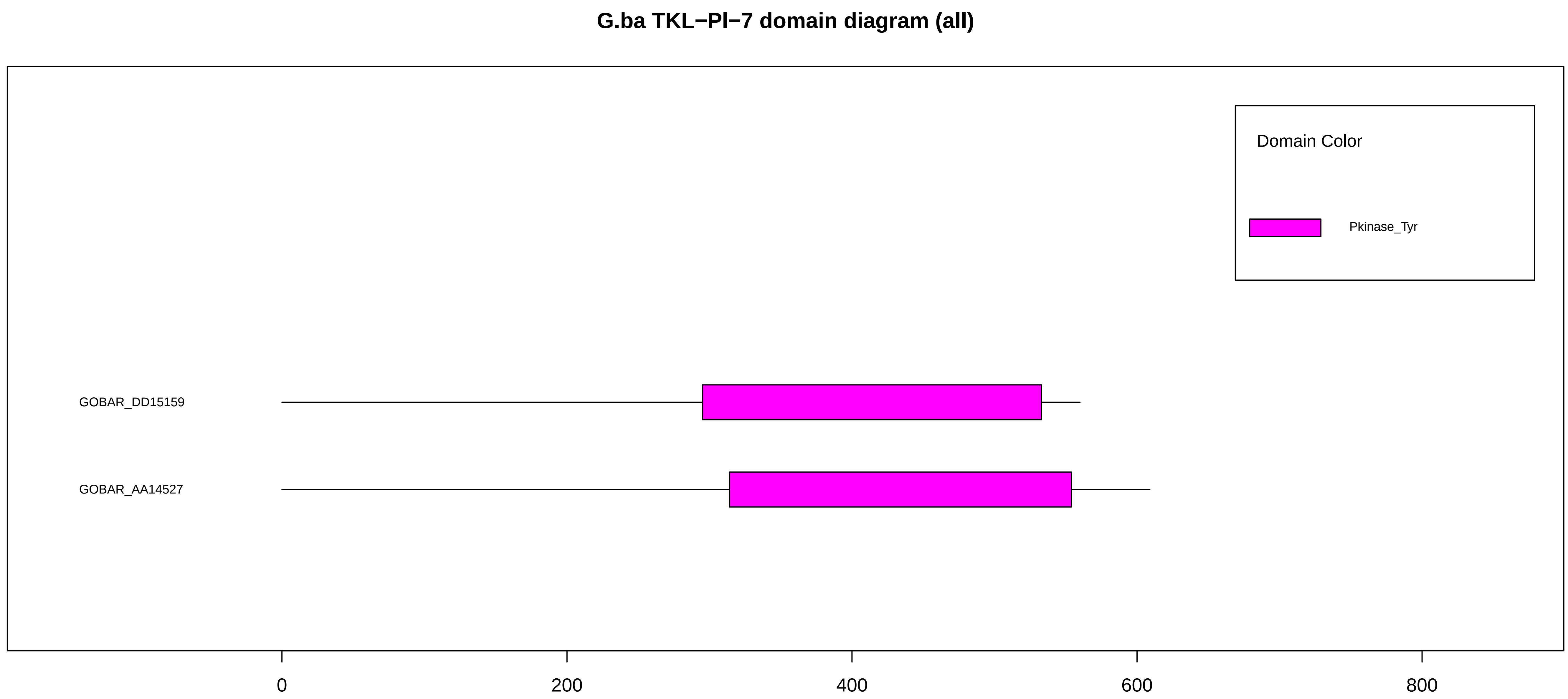
G.ba TKL−Pl−7 domain diagram (all)
Domain Color
Pkinase_Tyr
GOBAR_DD15159
GOBAR_AA14527
0
200
400
600
800

G.ba TKL−Pl−8 domain diagram (all)

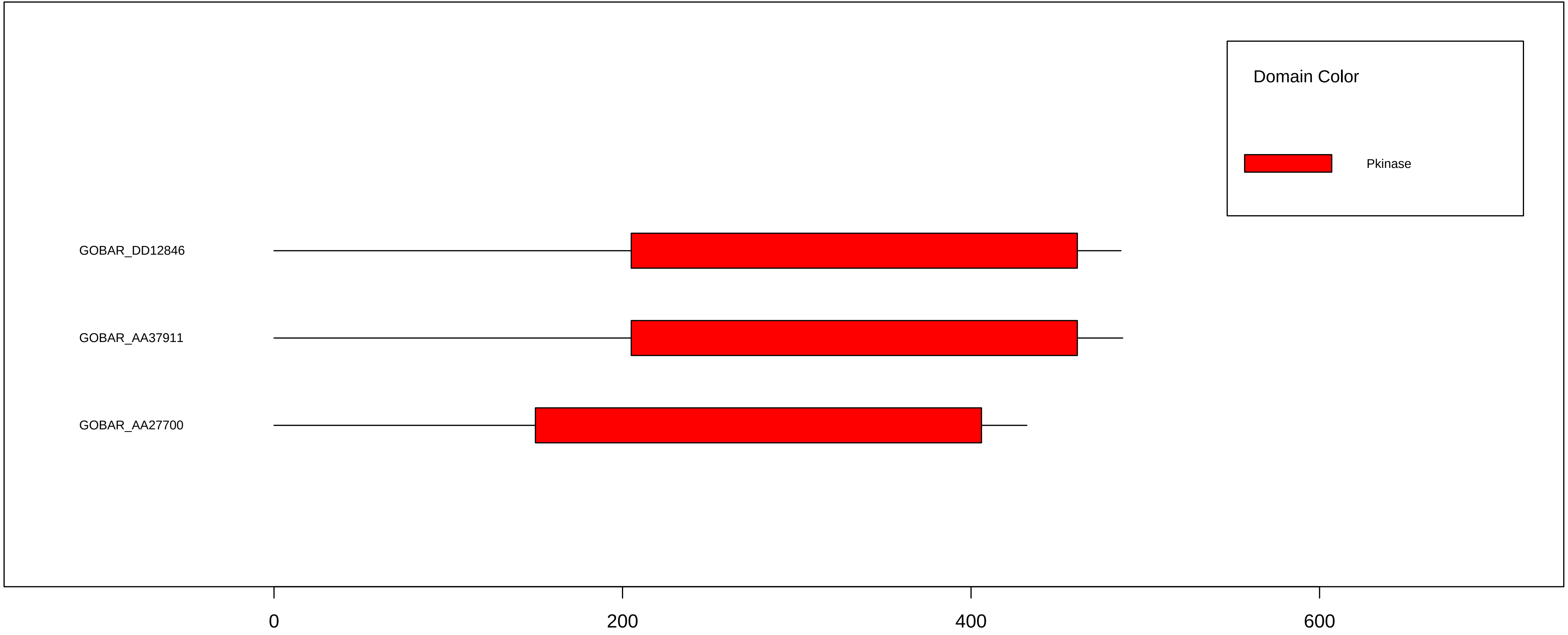

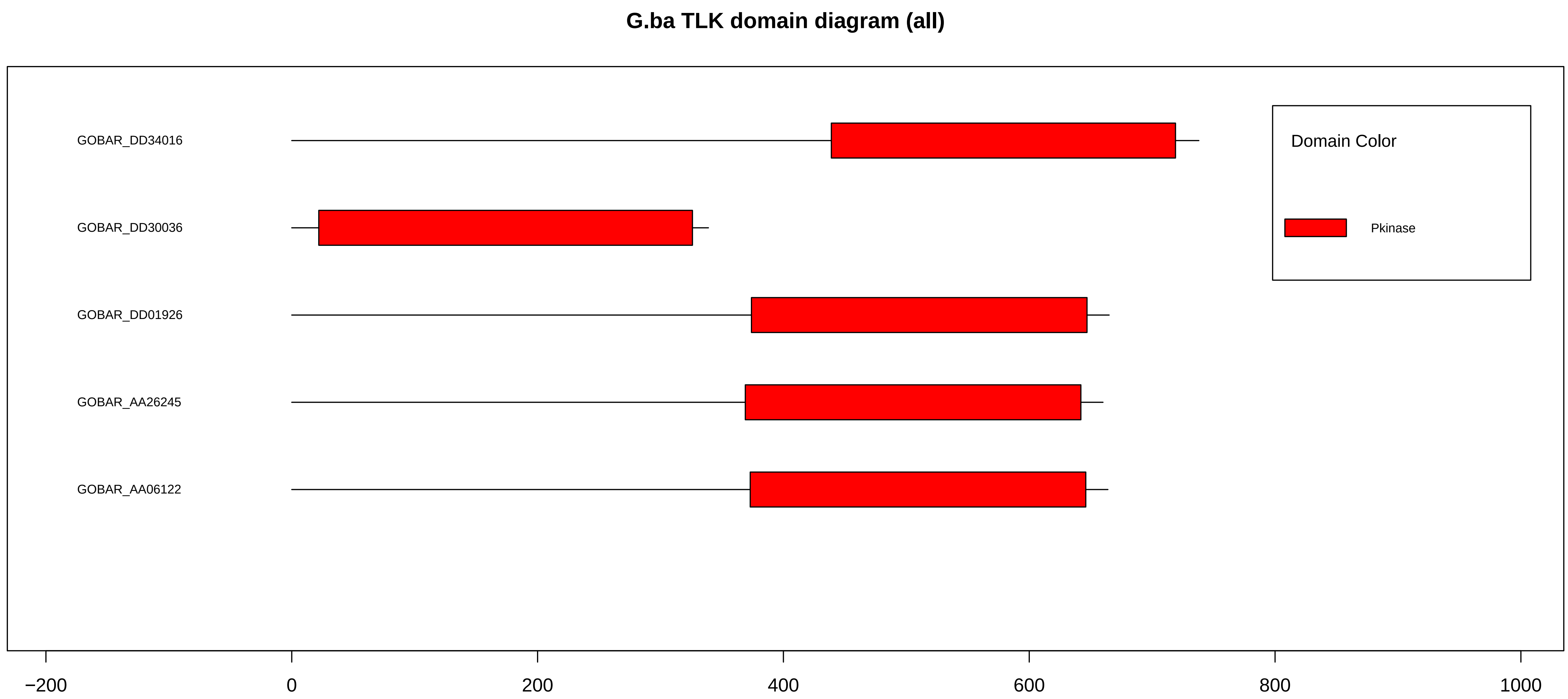
G.ba TLK domain diagram (all)
GOBAR_DD34016
GOBAR_DD30036
GOBAR_DD01926
GOBAR_AA26245
GOBAR_AA06122
Domain Color
Pkinase
−200
0
200
400
600
800
1000

G.ba TTK domain diagram (all)

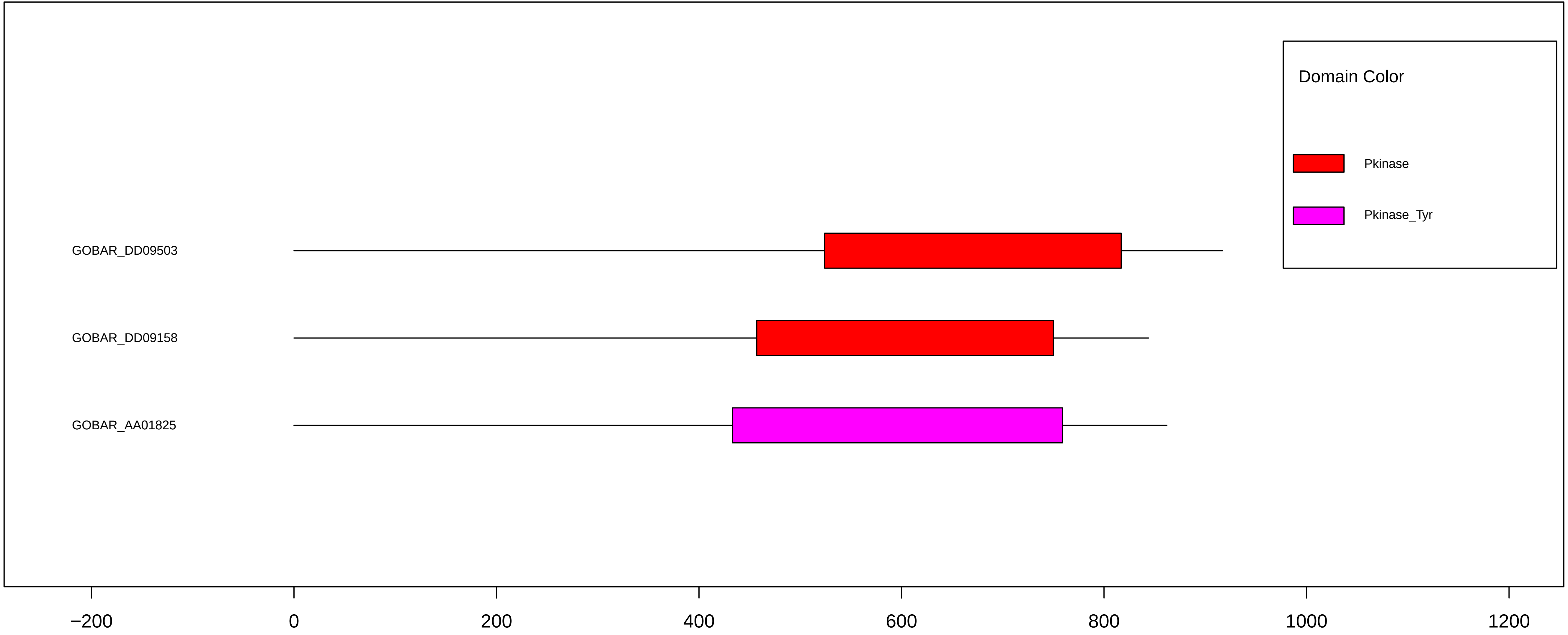

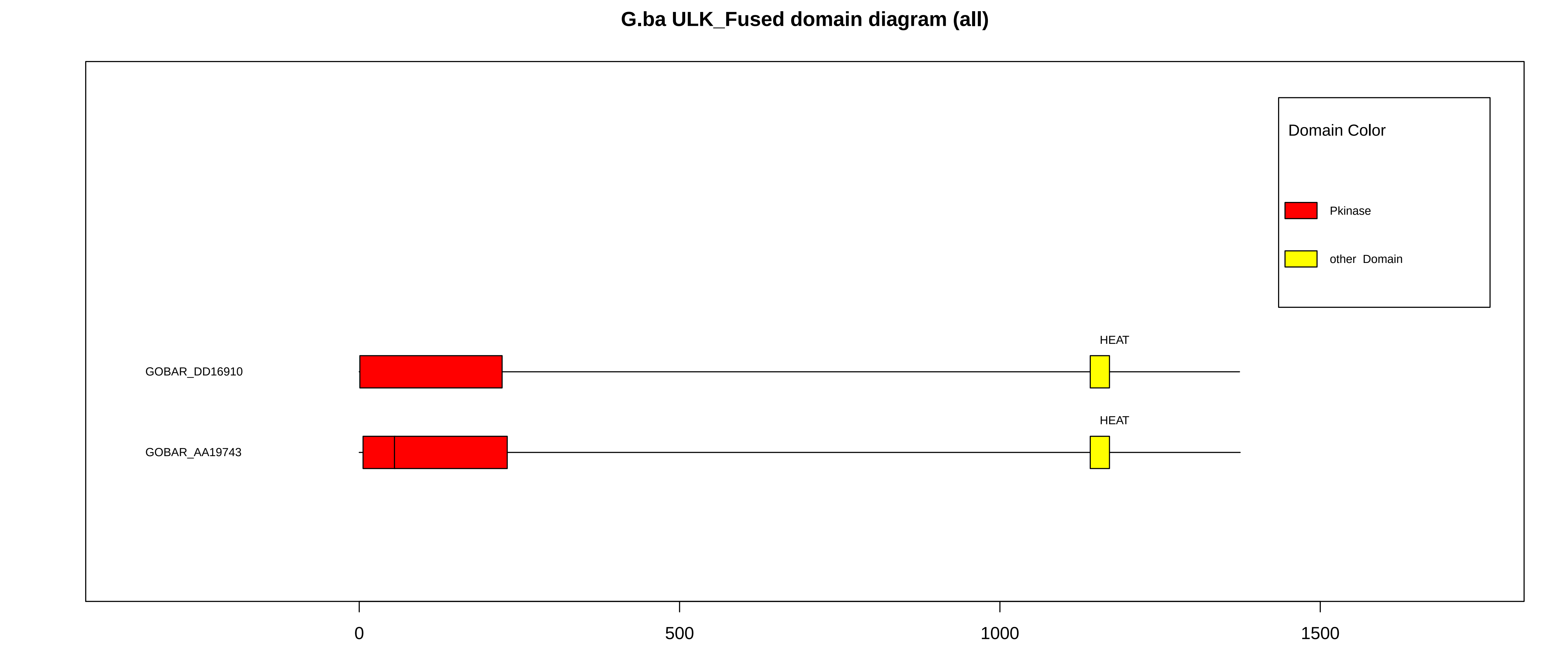

G.ba ULK_Fused domain diagram (all)
Domain Color
Pkinase
other Domain
HEAT
GOBAR_DD16910
HEAT
GOBAR_AA19743
0
500
1000
1500

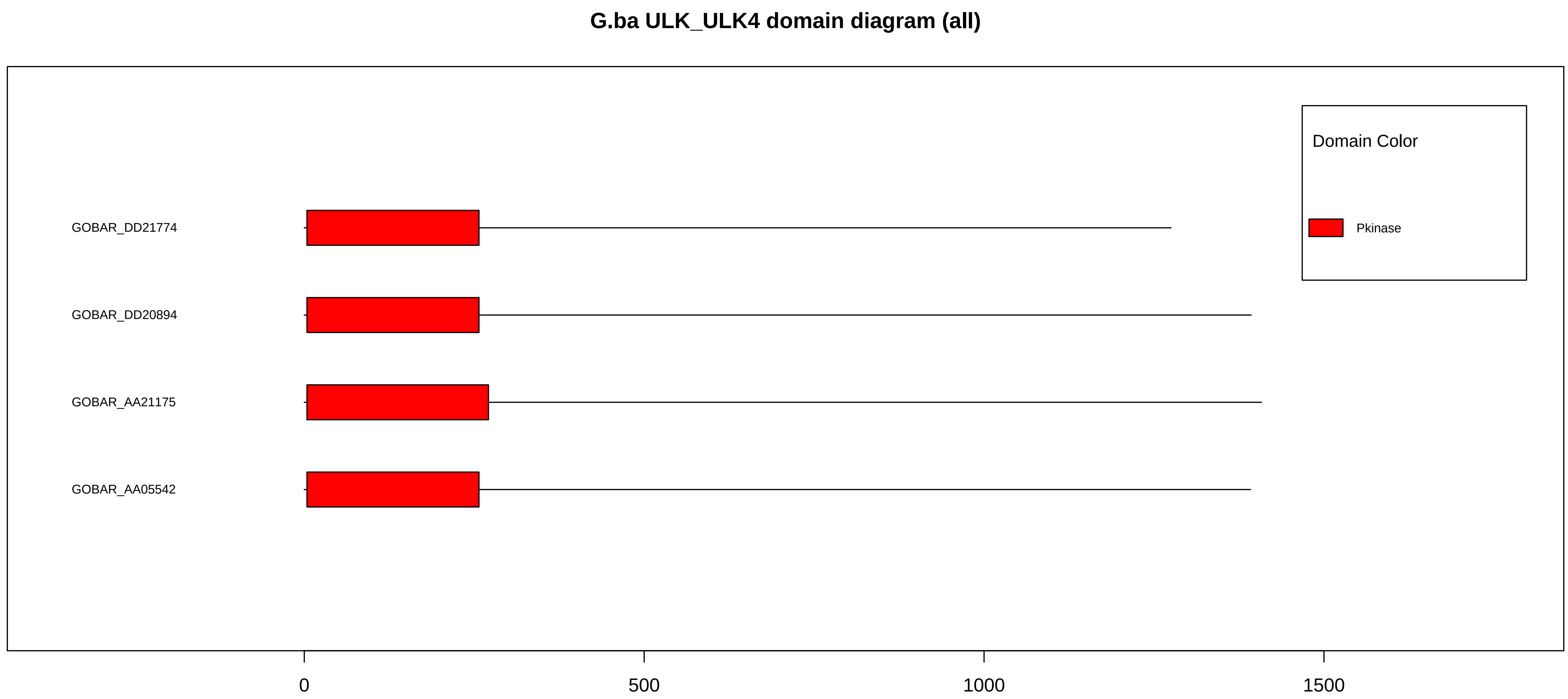
G.ba ULK_ULK4 domain diagram (all)
Domain Color
Pkinase
GOBAR_DD21774
GOBAR_DD20894
GOBAR_AA21175
GOBAR_AA05542
0
500
1000
1500

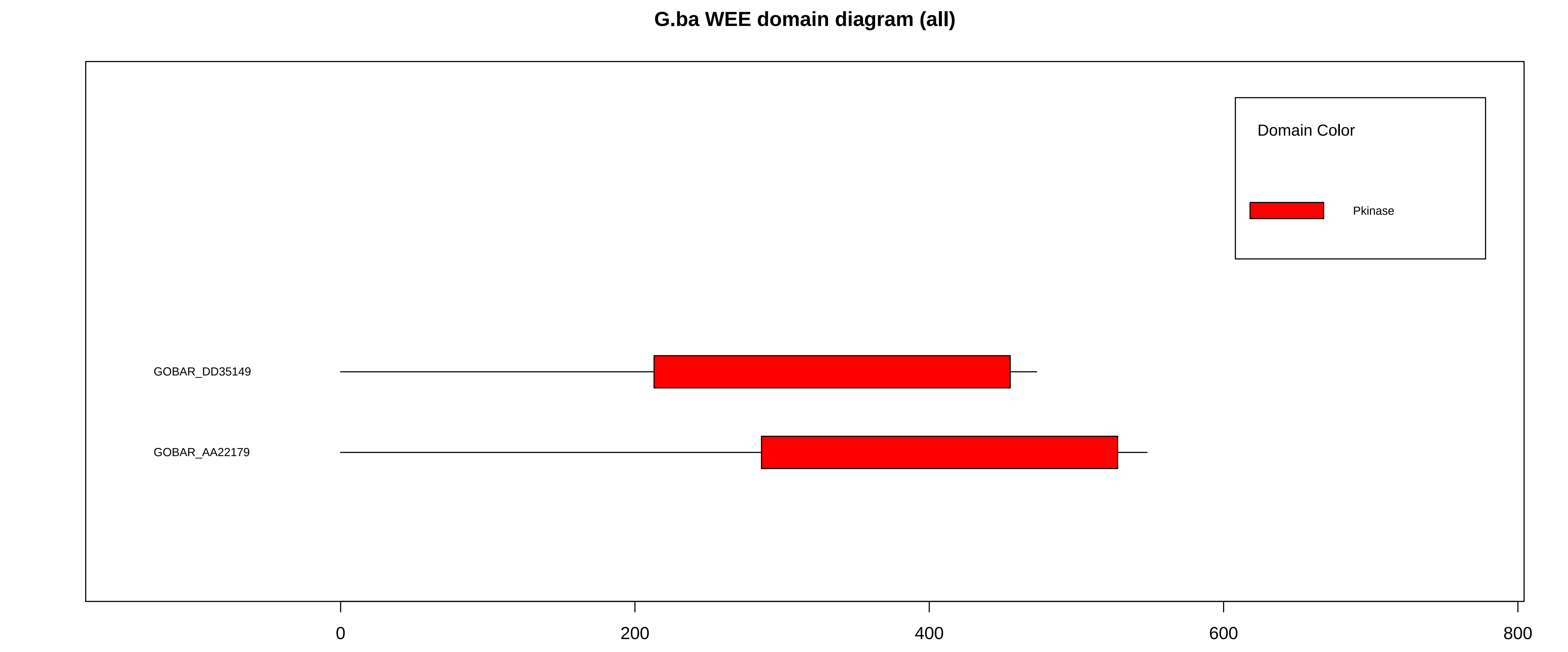
G.ba WEE domain diagram (all)
Domain Color
Pkinase
GOBAR_DD35149
GOBAR_AA22179
0
200
400
600
800

G.ba WNK_NRBP domain diagram (part 1)

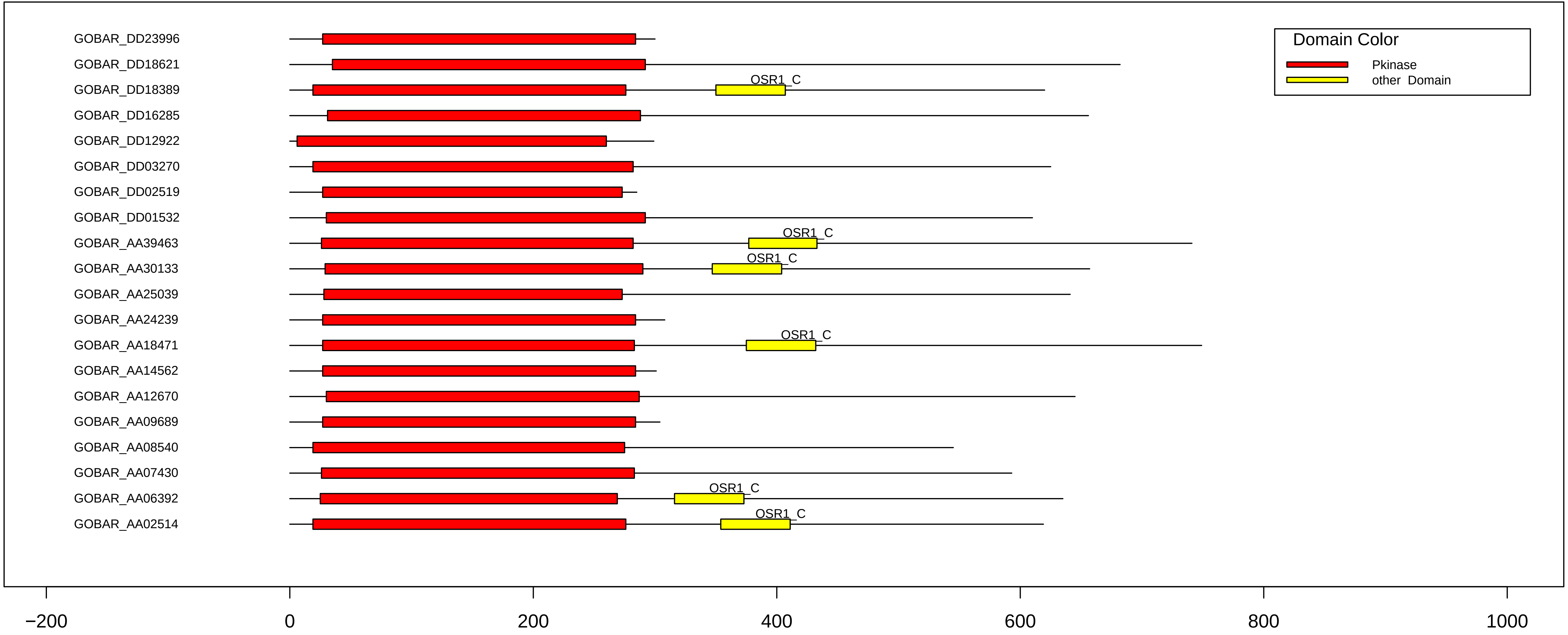

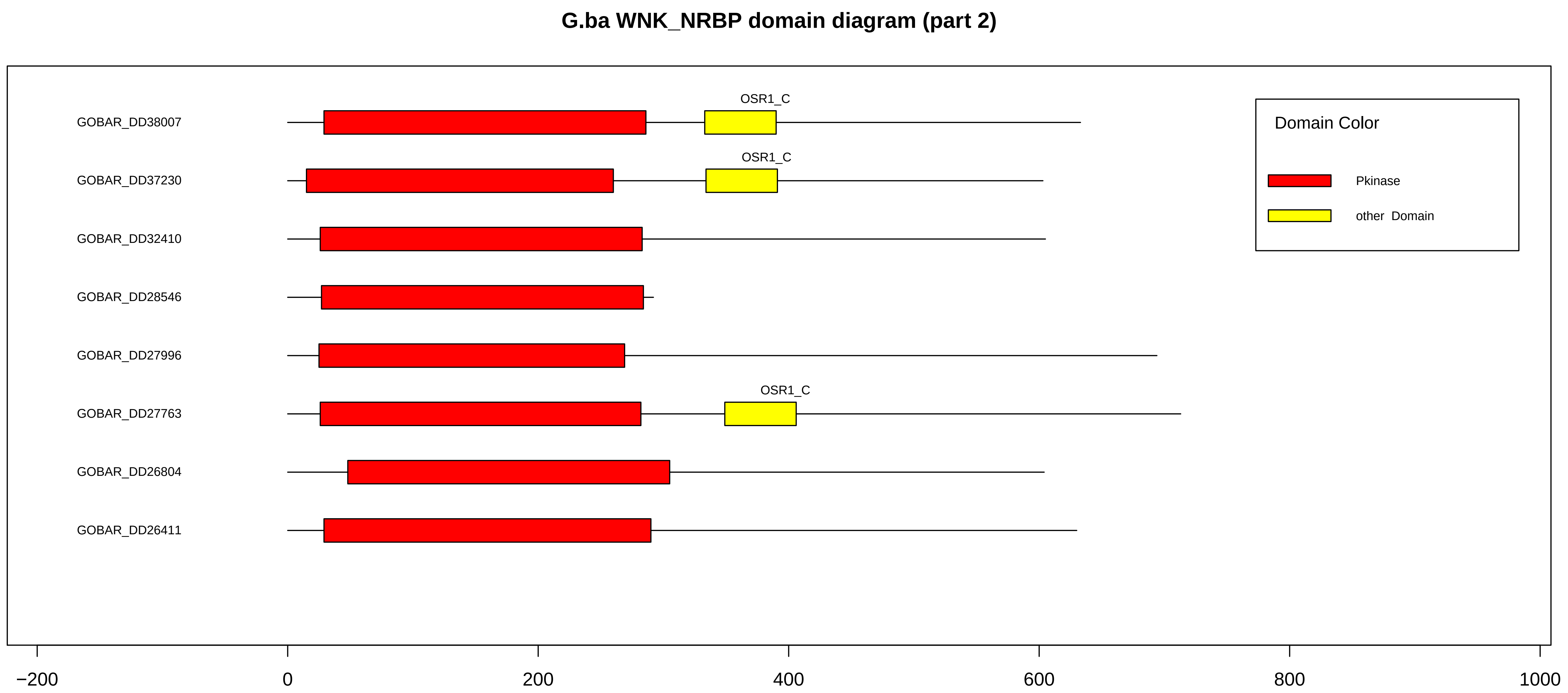
G.ba WNK_NRBP domain diagram (part 2)
GOBAR_DD38007
GOBAR_DD37230
GOBAR_DD32410
GOBAR_DD28546
GOBAR_DD27996
GOBAR_DD27763
GOBAR_DD26804
GOBAR_DD26411
OSR1_C
OSR1_C
OSR1_C
Domain Color
Pkinase
other Domain
−200
0
200
400
600
800
1000
