# Supplementary material for: Genome-wide classification, evolutionary analysis and gene expression patterns of the kinome in Gossypium
Source: PLoS One. 2018 May 16;13(5):e0197392. doi: 10.1371/journal.pone.0197392 (PMC5955557; doi:10.1371/journal.pone.0197392)
Supplement: S3 Fig — The descriptions of domain and exon phases are the same as in Fig 2. The lengths of the boxes and lines are scaled based on the length of genes. (PDF) [file pone.0197392.s003.pdf]

G.hi AGC_MAST exon−intron and domain diagram (all)

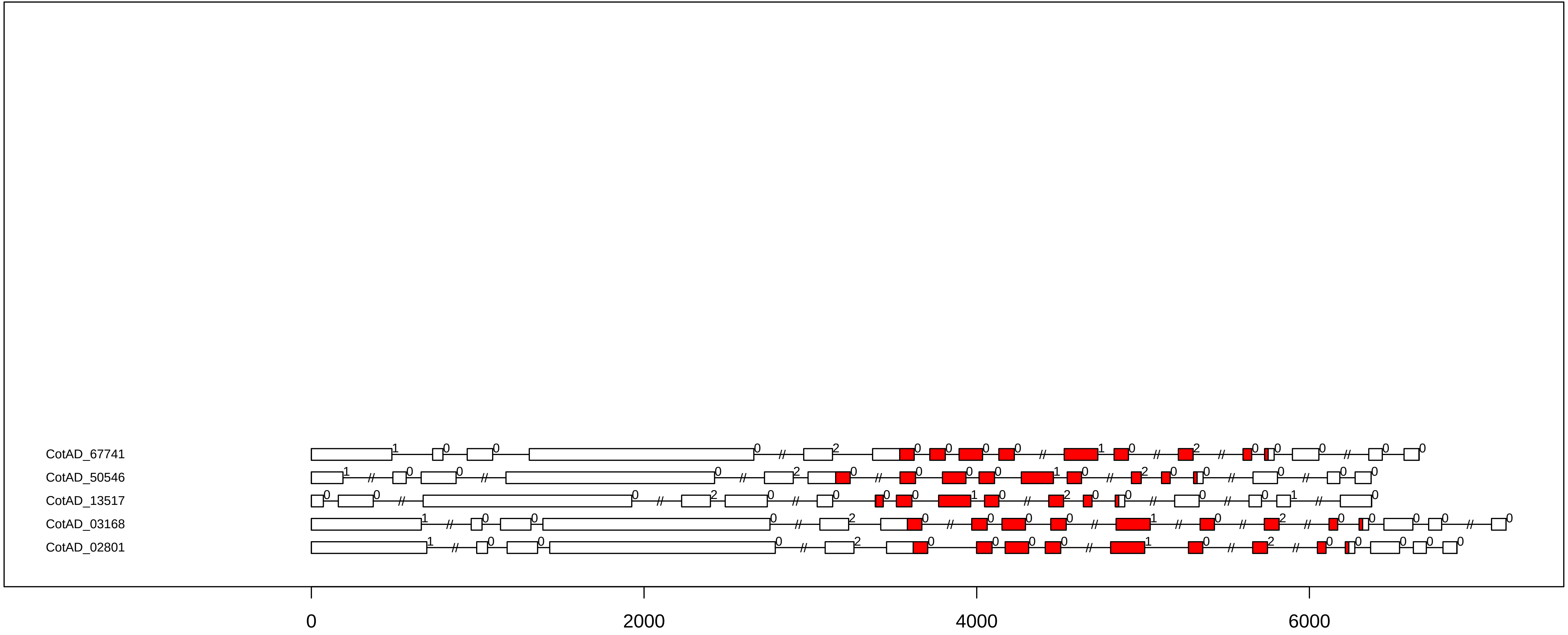

G.hi AGC_NDR exon−intron and domain diagram (all)

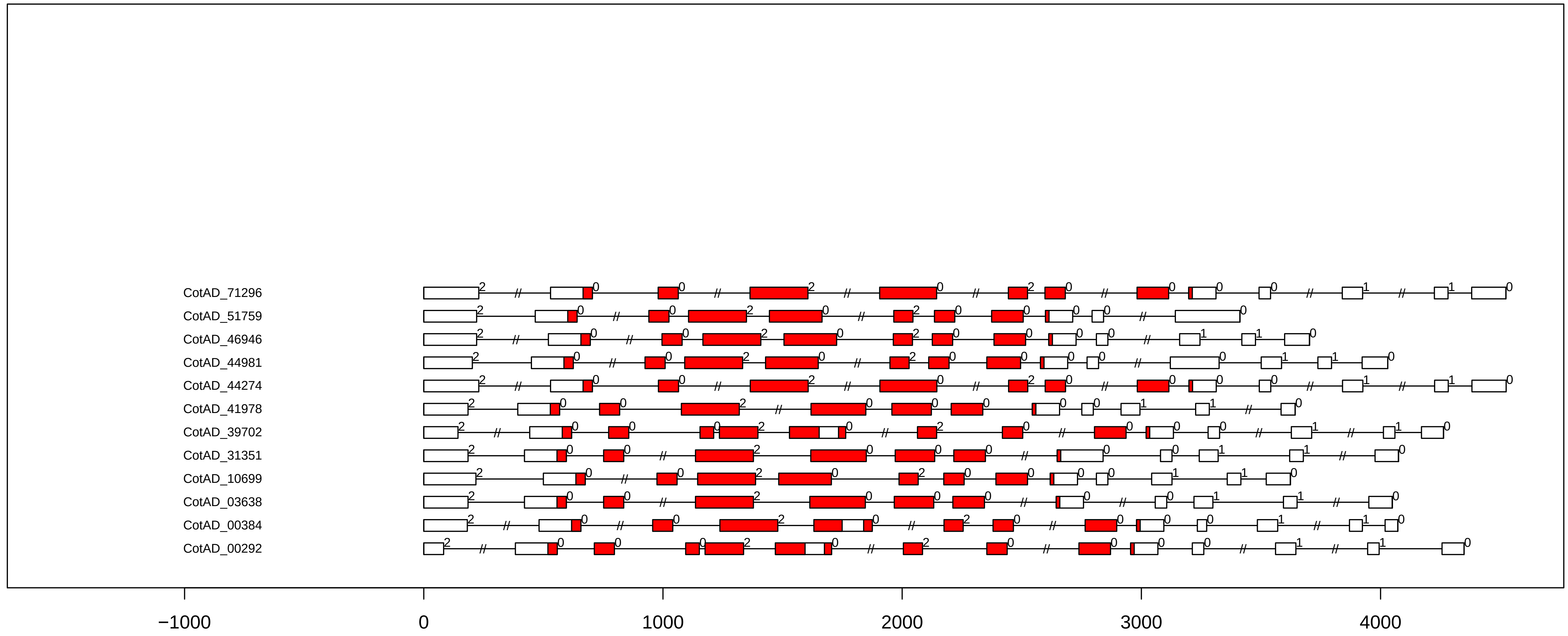

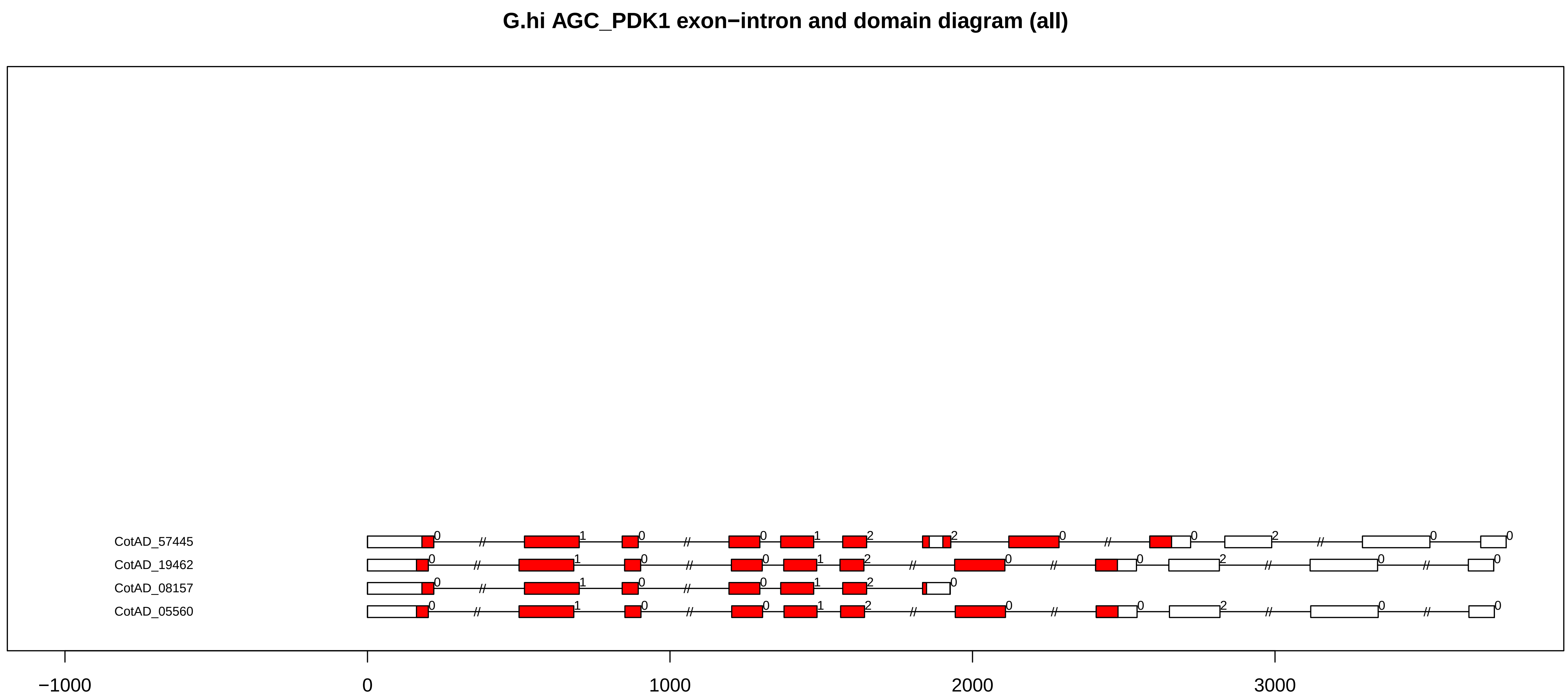
G.hi AGC_PDK1 exon−intron and domain diagram (all)
CotAD_57445
CotAD_19462
CotAD_08157
CotAD_05560
−1000
0
1000
2000
3000

G.hi AGC_PKA−PKG exon−intron and domain diagram (all)

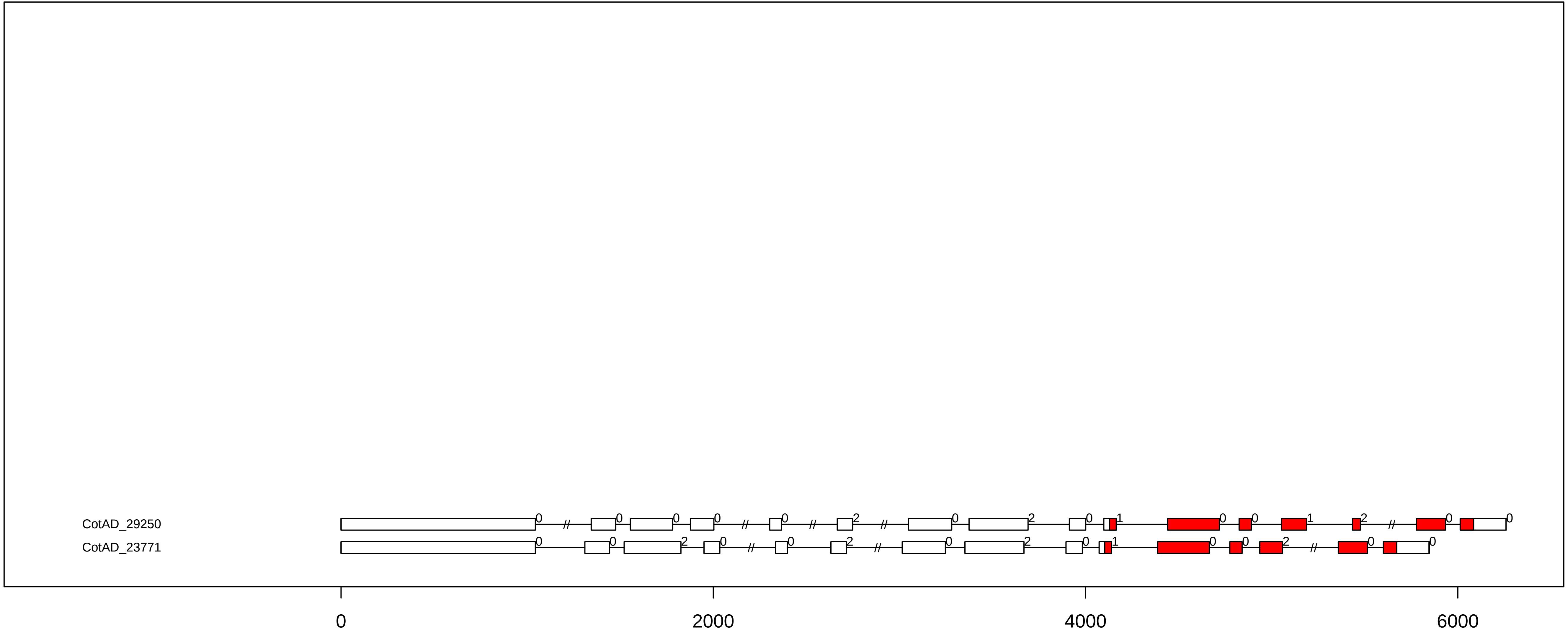

G.hi AGC_RSK−2 exon−intron and domain diagram (part 1)

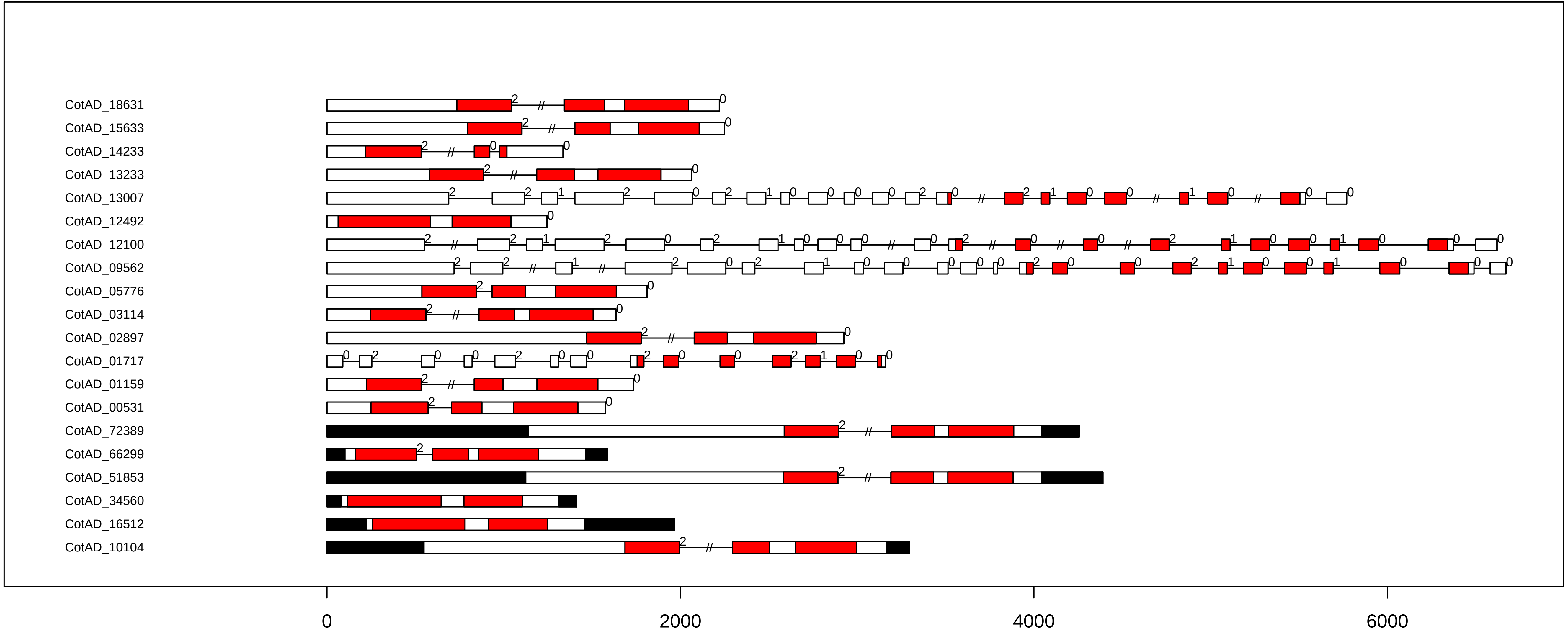

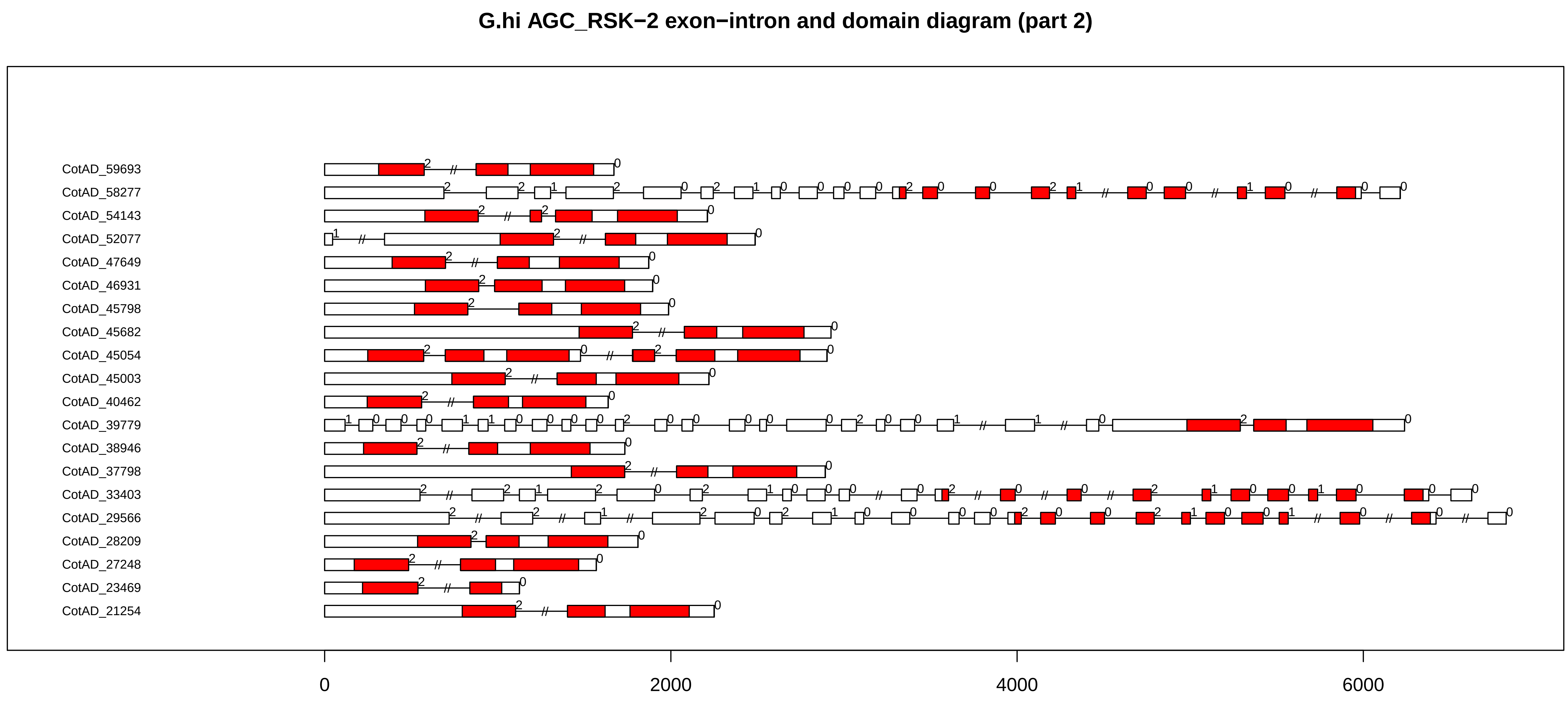
G.hi AGC_RSK−2 exon−intron and domain diagram (part 2)
CotAD_59693
CotAD_58277
CotAD_54143
CotAD_52077
CotAD_47649
CotAD_46931
CotAD_45798
CotAD_45682
CotAD_45054
CotAD_45003
CotAD_40462
CotAD_39779
CotAD_38946
CotAD_37798
CotAD_33403
CotAD_29566
CotAD_28209
CotAD_27248
CotAD_23469
CotAD_21254
0
2000
4000
6000

G.hi AGC_RSK−2 exon−intron and domain diagram (part 3)

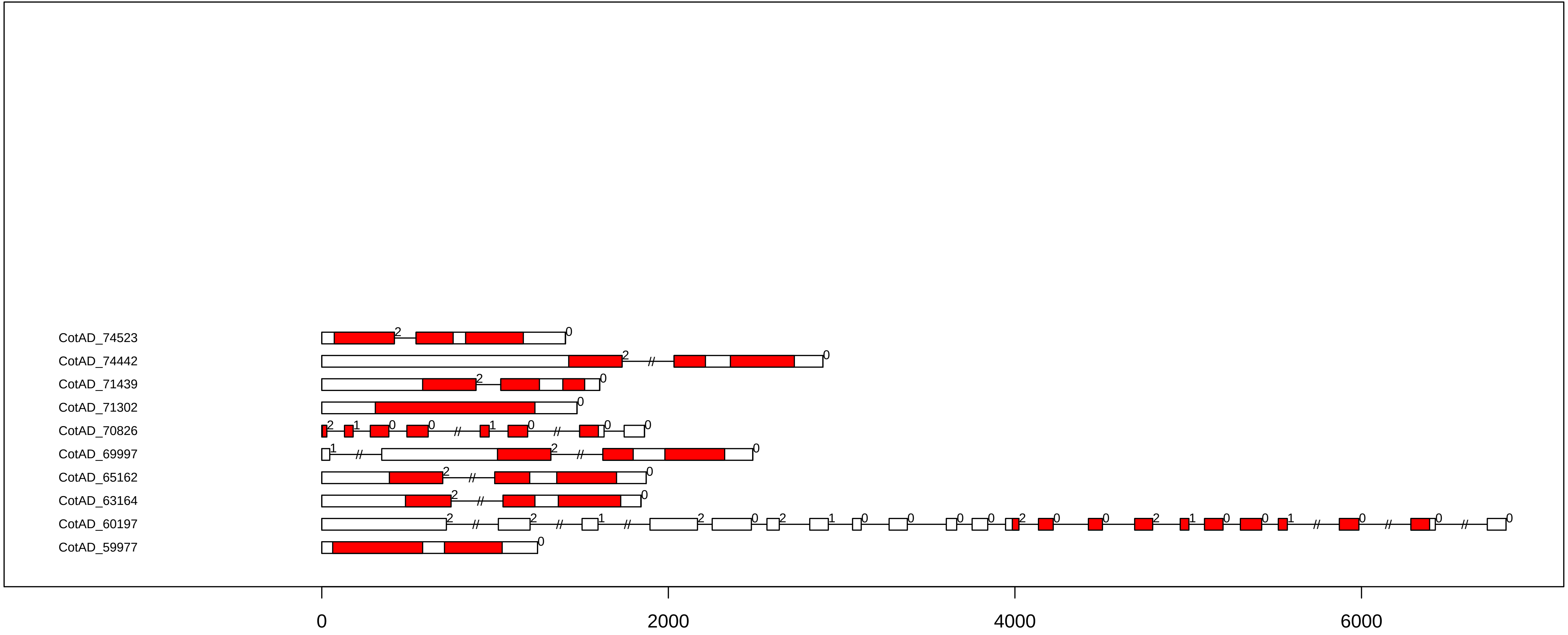

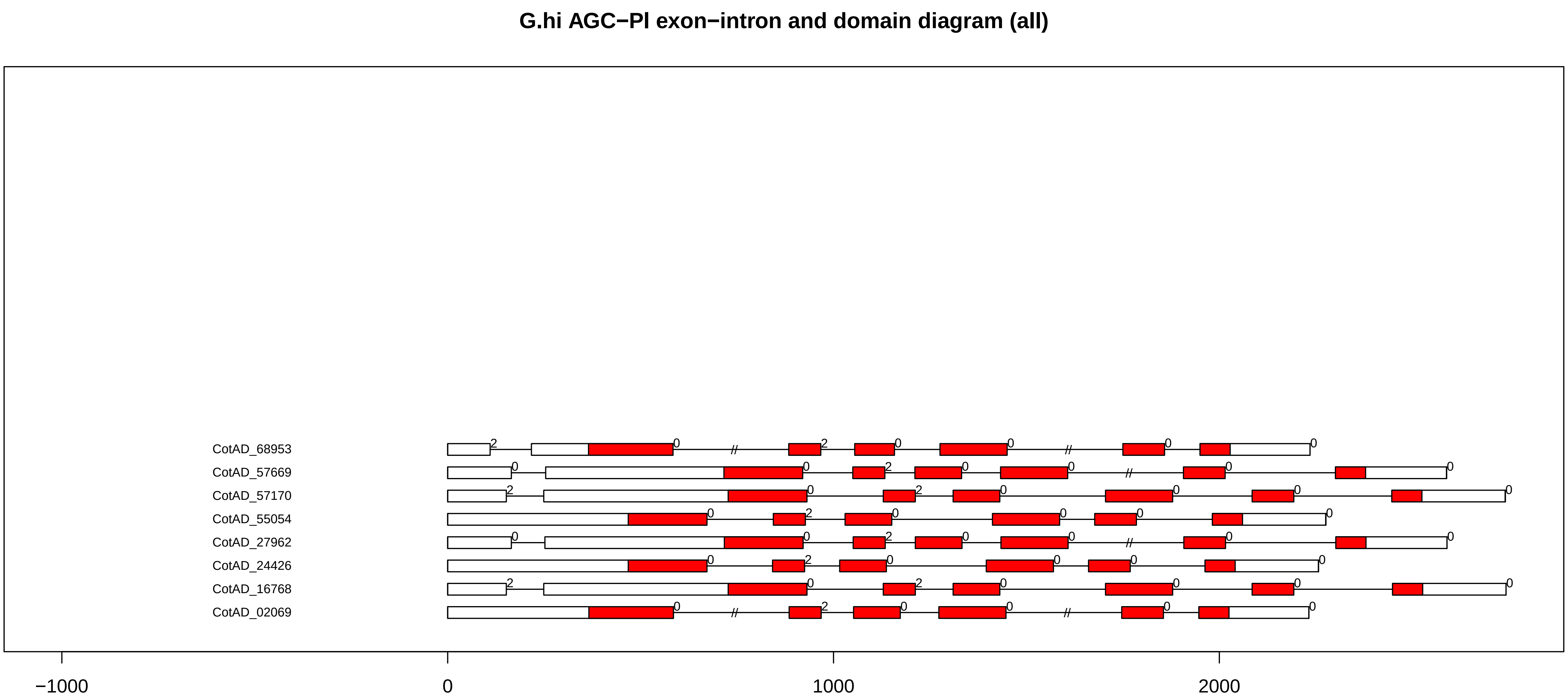
G.hi AGC−PI exon−intron and domain diagram (all)
CotAD_68953
CotAD_57669
CotAD_57170
CotAD_55054
CotAD_27962
CotAD_24426
CotAD_16768
CotAD_02069
−1000
0
1000
2000

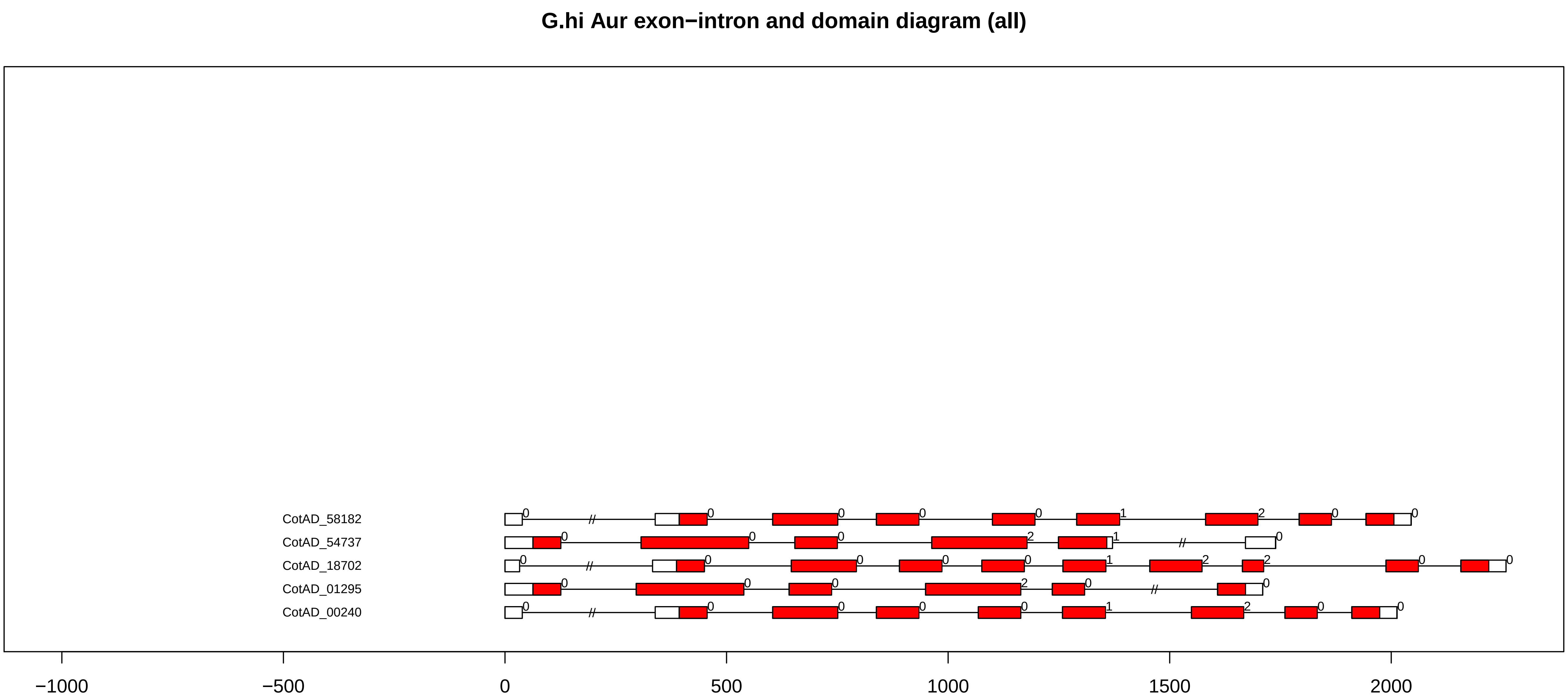
G.hi Aur exon−intron and domain diagram (all)
CotAD_58182
CotAD_54737
CotAD_18702
CotAD_01295
CotAD_00240
−1000
−500
0
500
1000
1500
2000

G.hi BUB exon−intron and domain diagram (all)

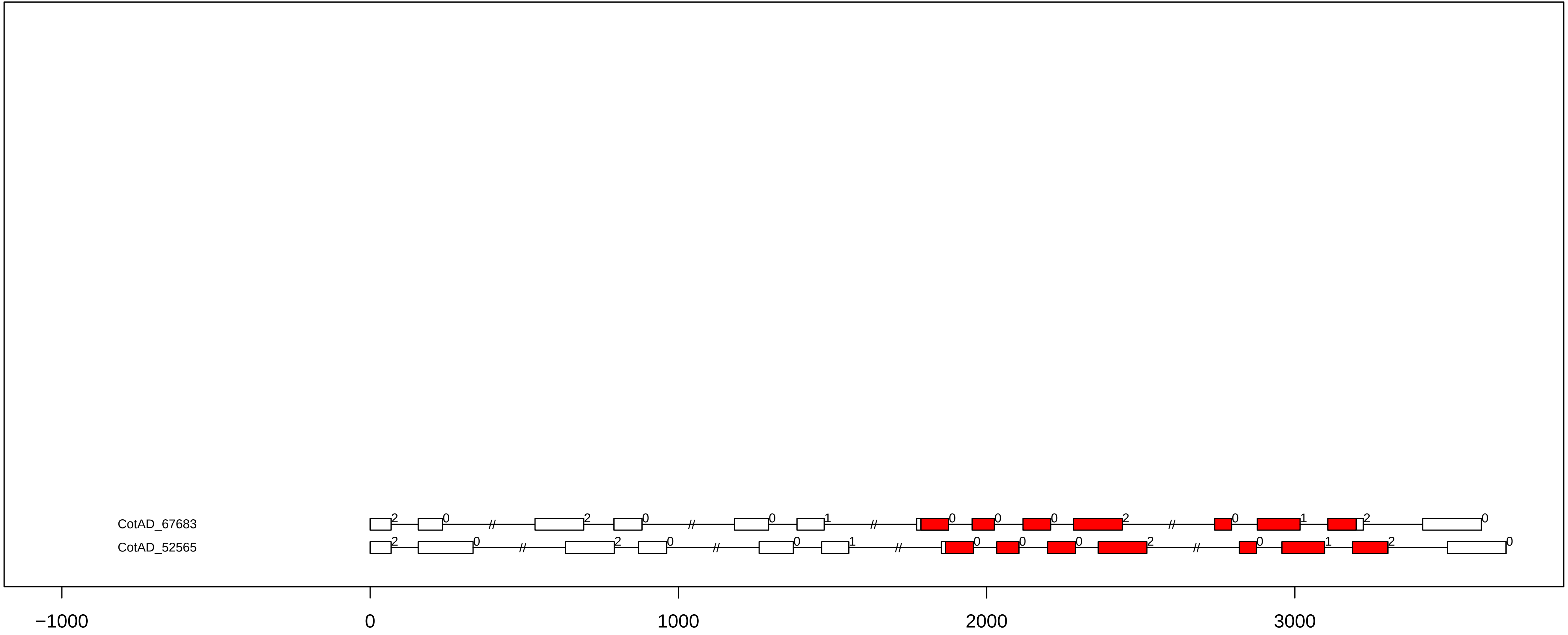

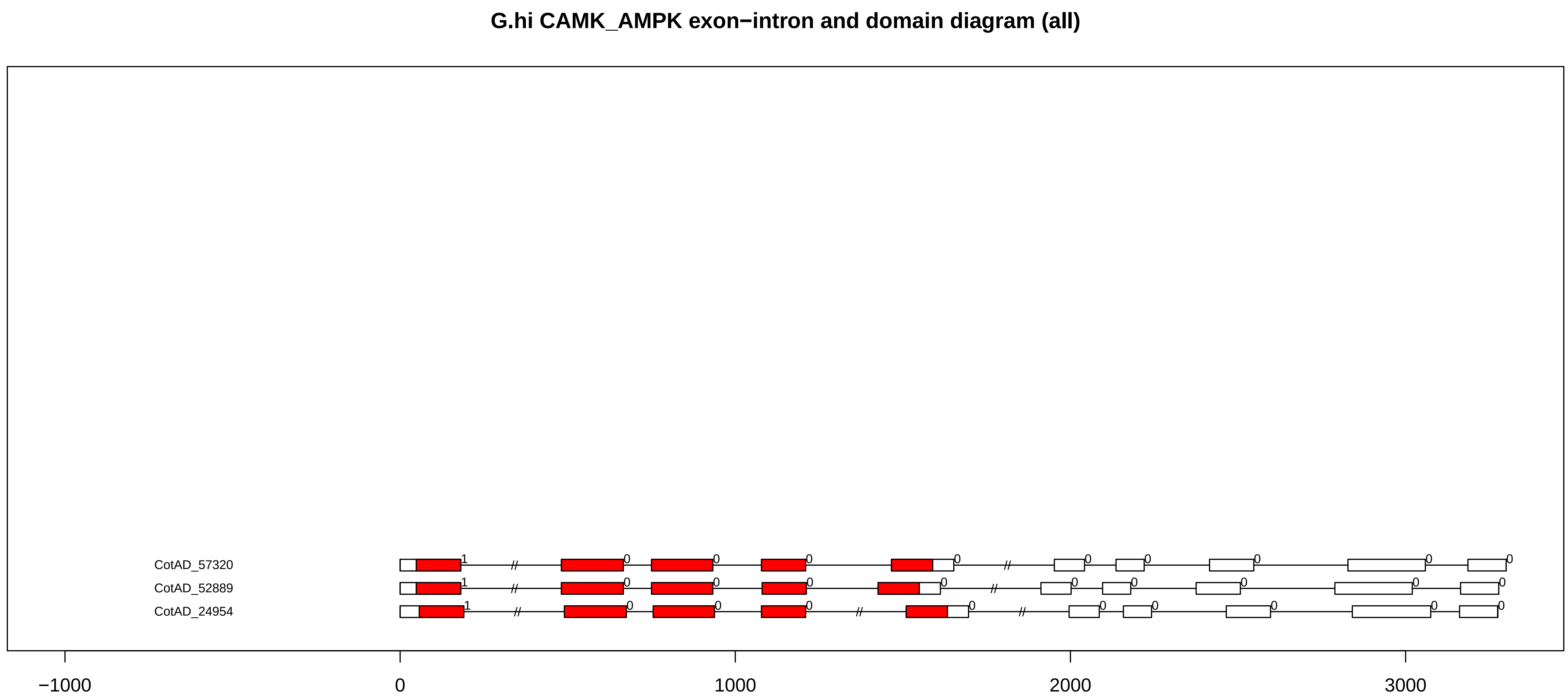
G.hi CAMK_AMPK exon−intron and domain diagram (all)
CotAD_57320
CotAD_52889
CotAD_24954
−1000
0
1000
2000
3000

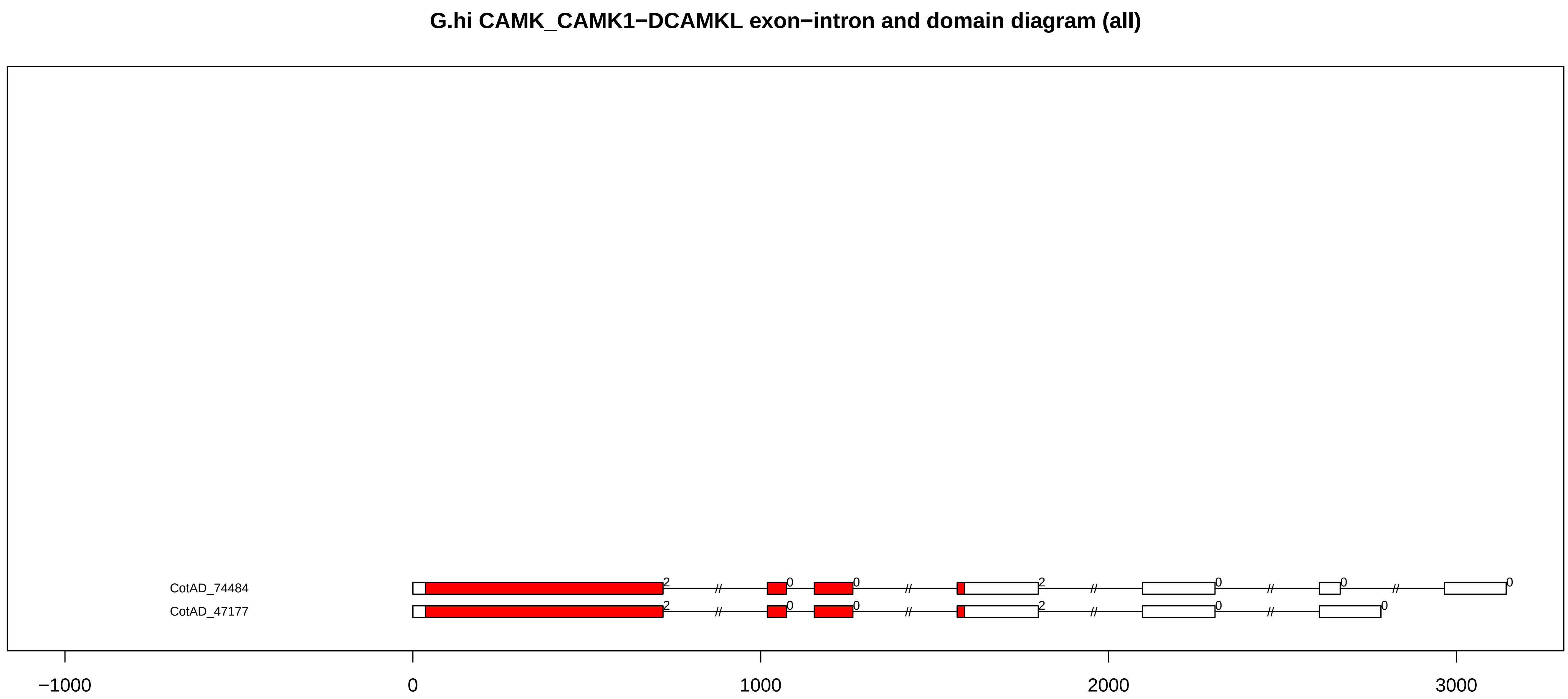
G.hi CAMK_CAMK1−DCAMKL exon−intron and domain diagram (all)
CotAD_74484
CotAD_47177
−1000
0
1000
2000
3000

G.hi CAMK_CAMKL−CHK1 exon−intron and domain diagram (part 1)

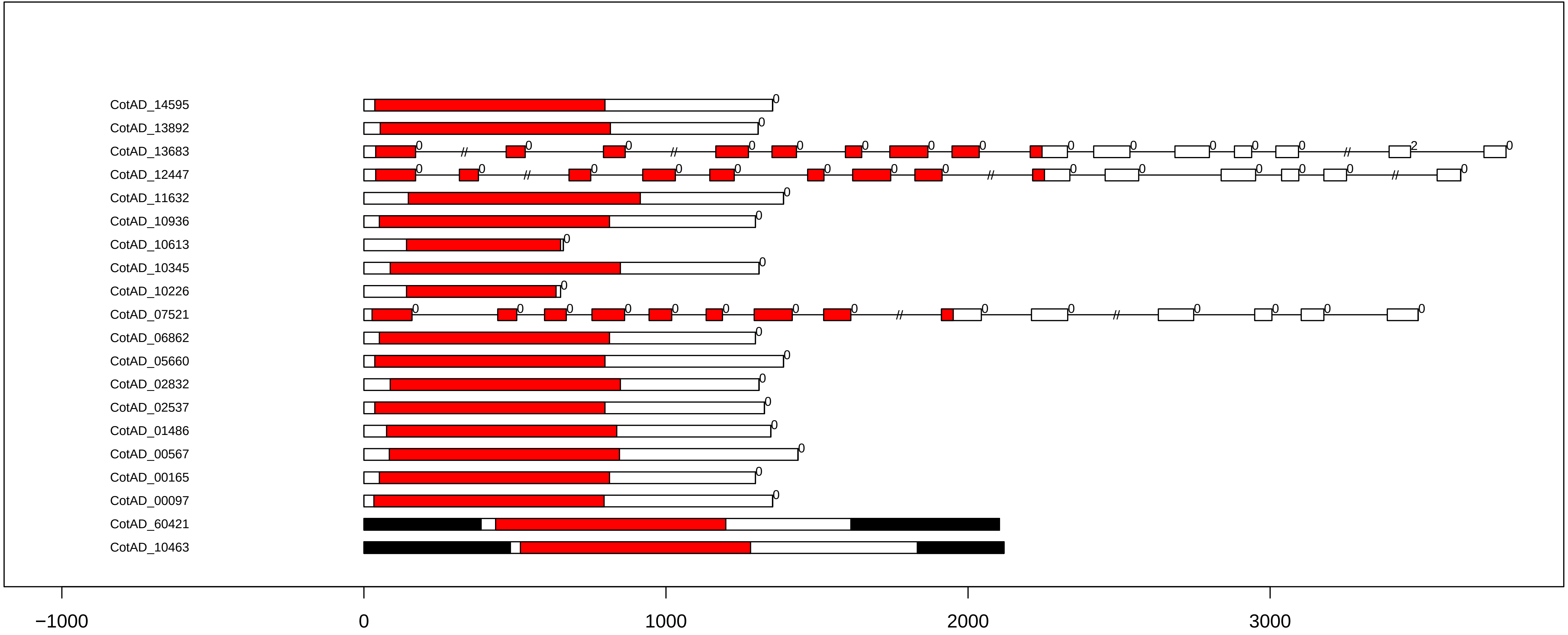

G.hi CAMK_CAMKL−CHK1 exon−intron and domain diagram (part 2)

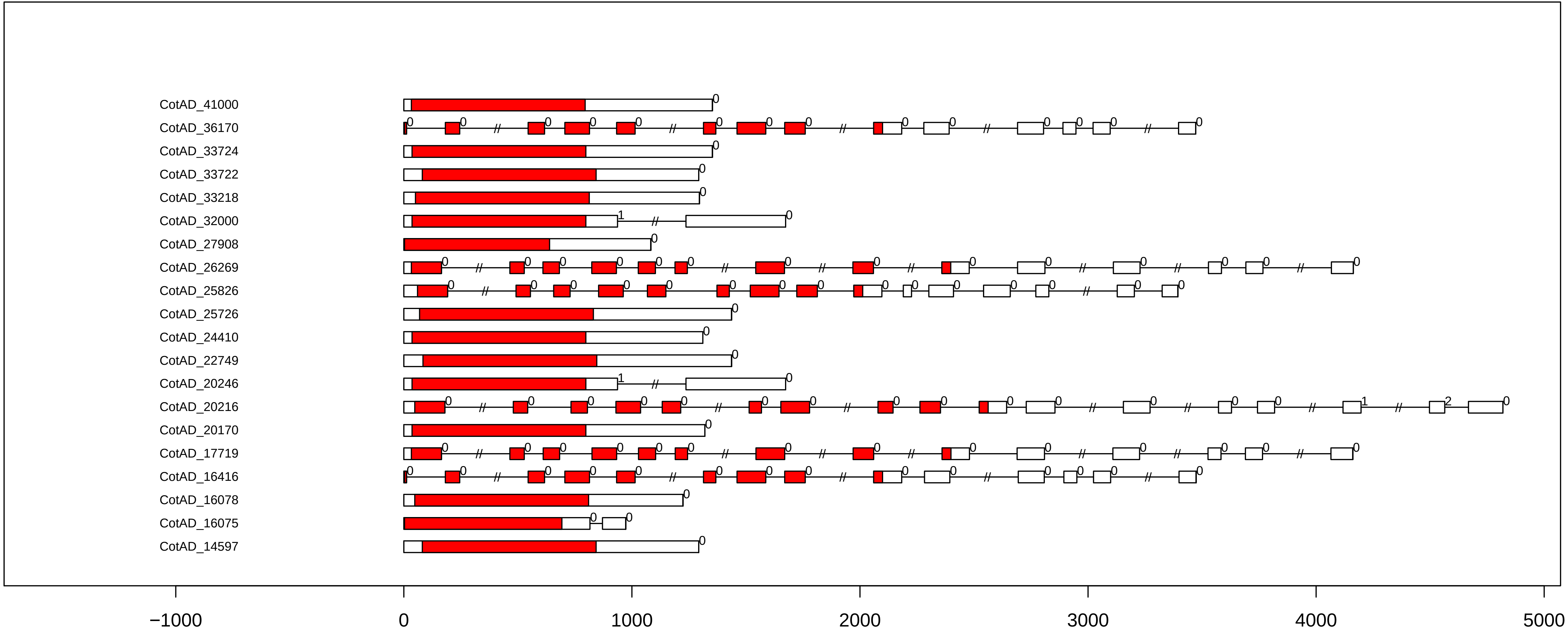

G.hi CAMK_CAMKL−CHK1 exon−intron and domain diagram (part 3)

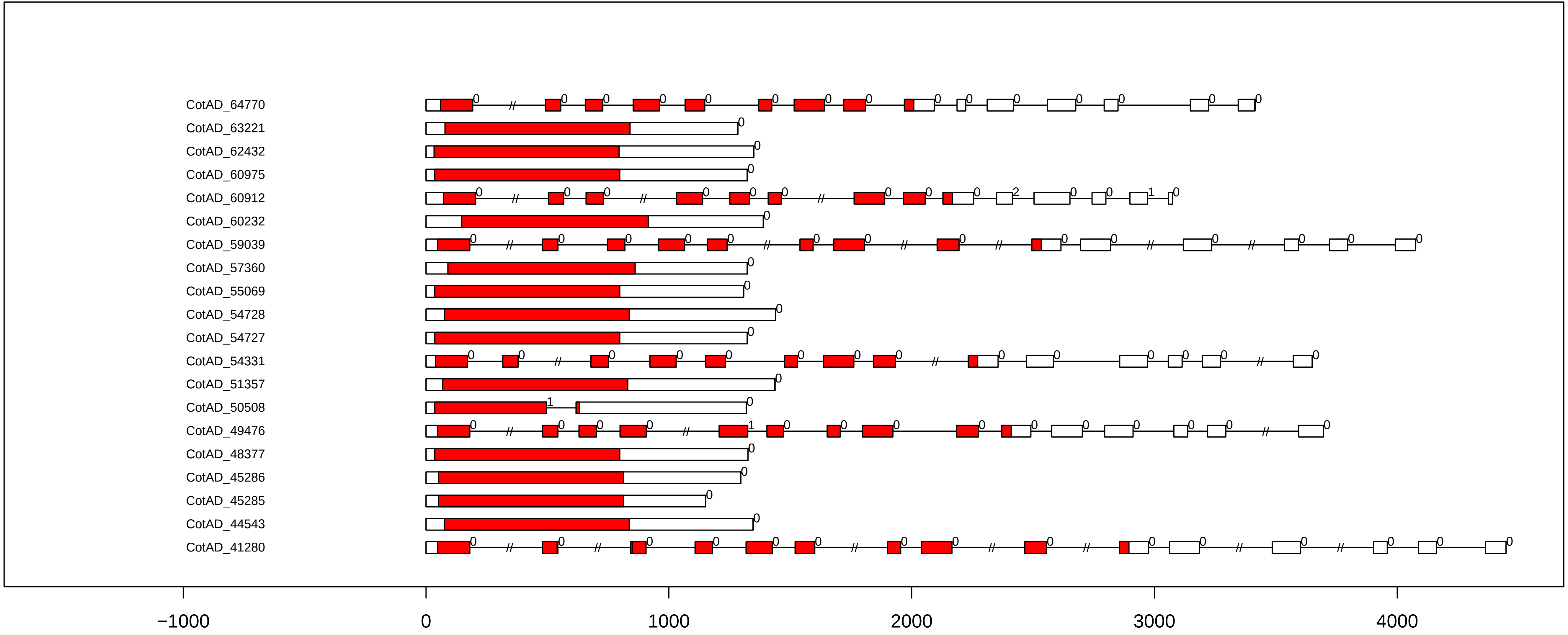

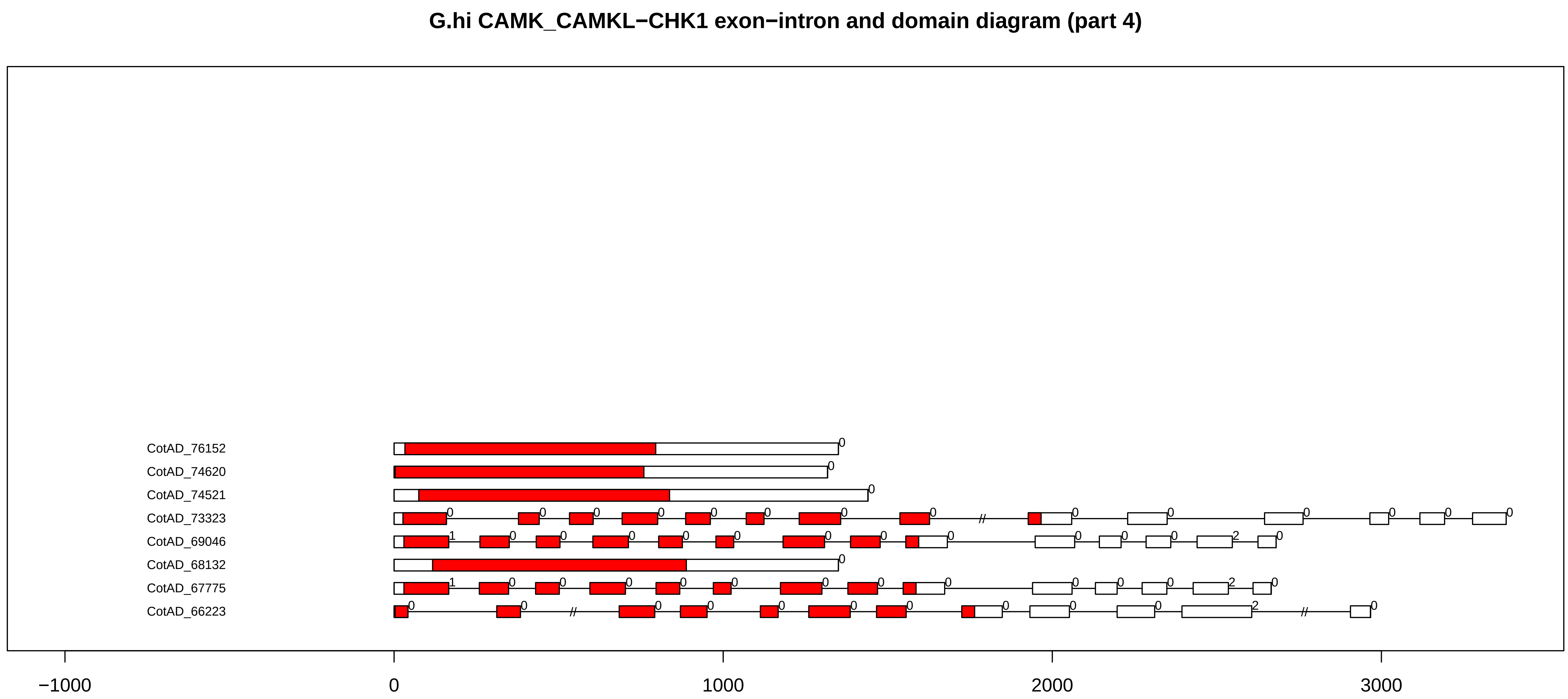
G.hi CAMK_CAMKL−CHK1 exon−intron and domain diagram (part 4)
CotAD_76152
CotAD_74620
CotAD_74521
CotAD_73323
CotAD_69046
CotAD_68132
CotAD_67775
CotAD_66223
−1000
0
1000
2000
3000

G.hi CAMK_CAMKL−LKB exon−intron and domain diagram (all)

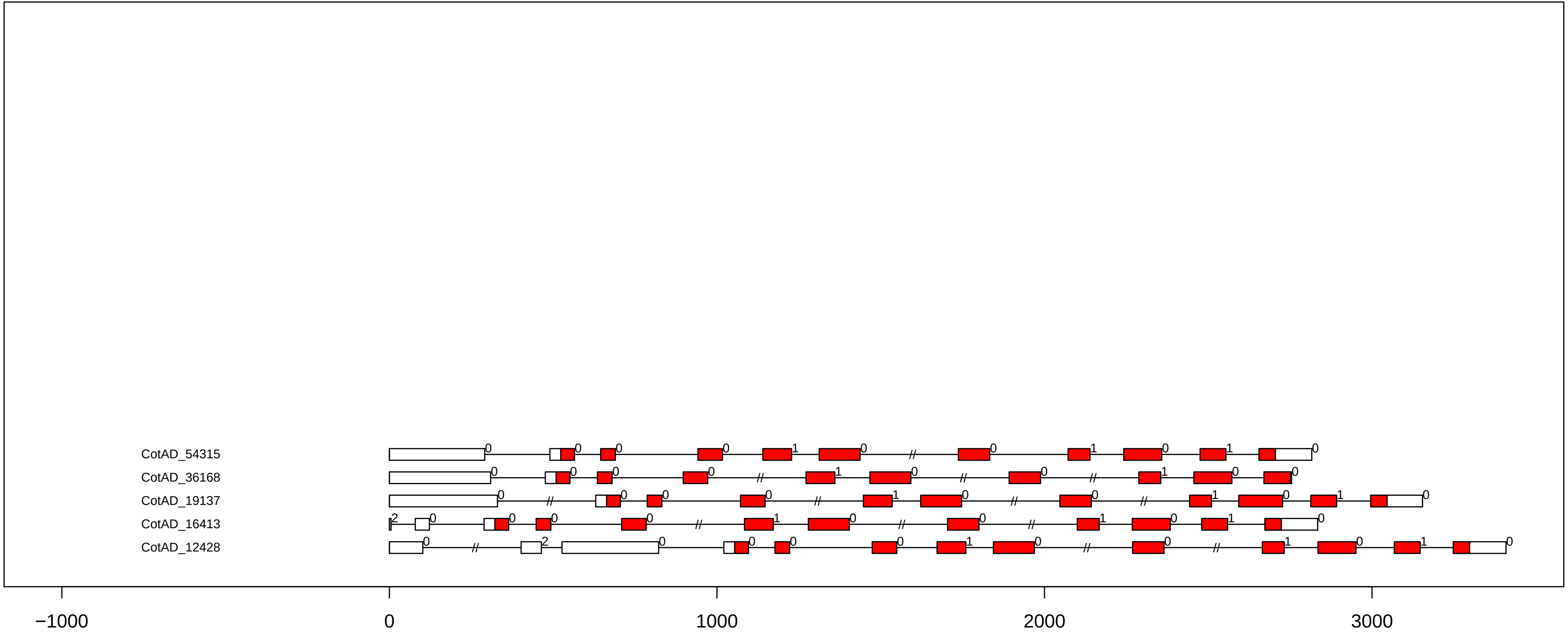

G.hi CAMK_CDPK exon−intron and domain diagram (part 1)

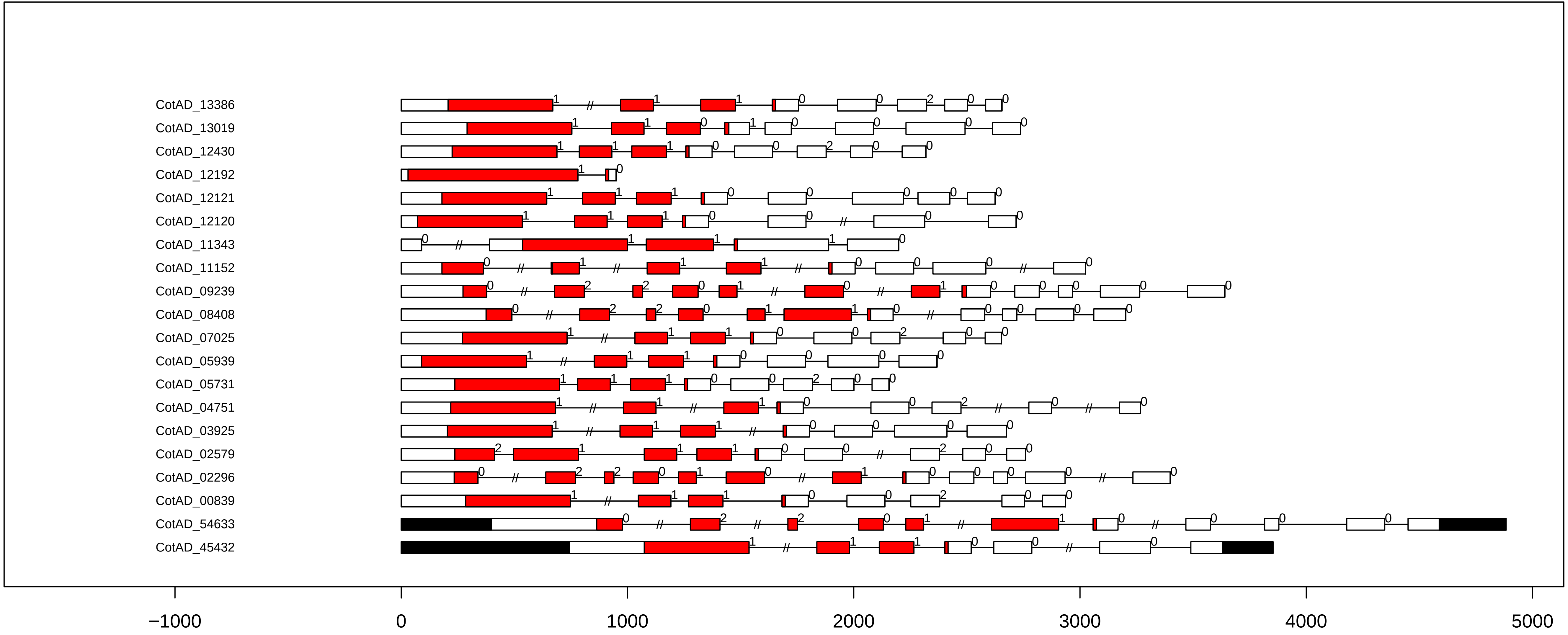

G.hi CAMK_CDPK exon−intron and domain diagram (part 2)

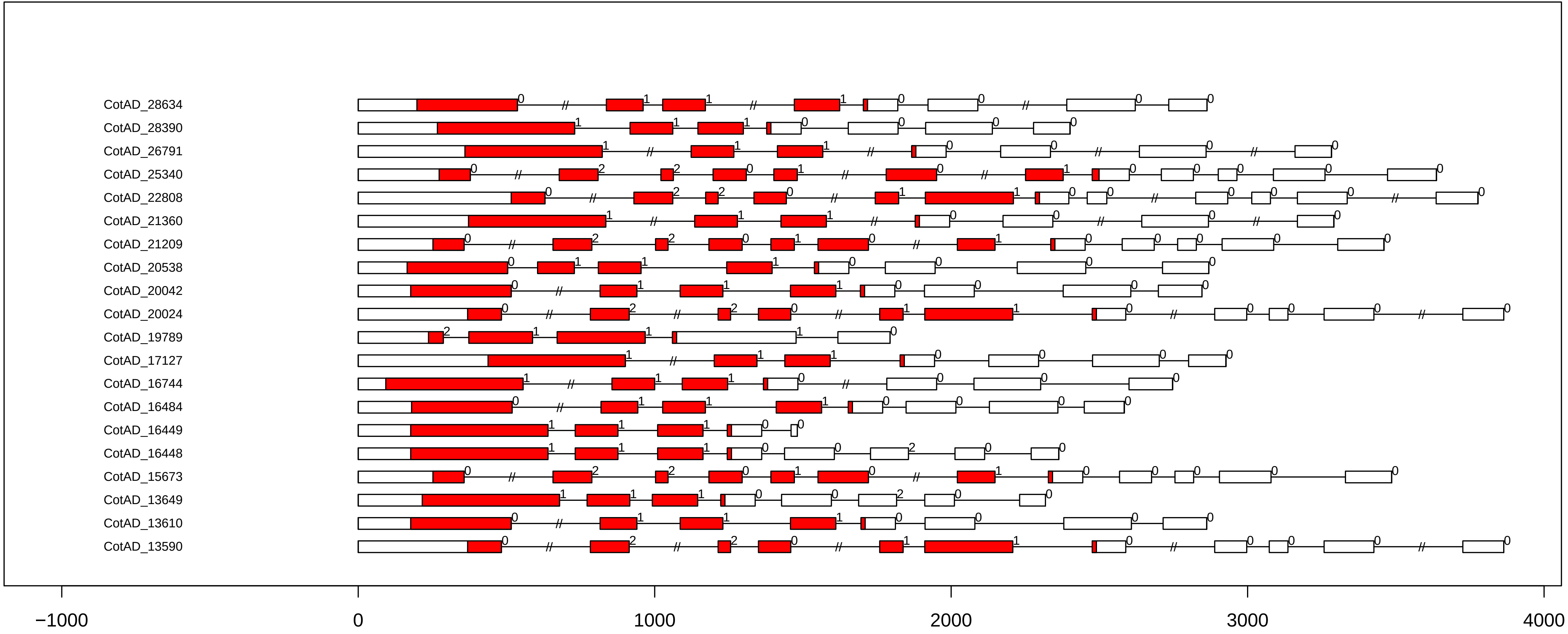

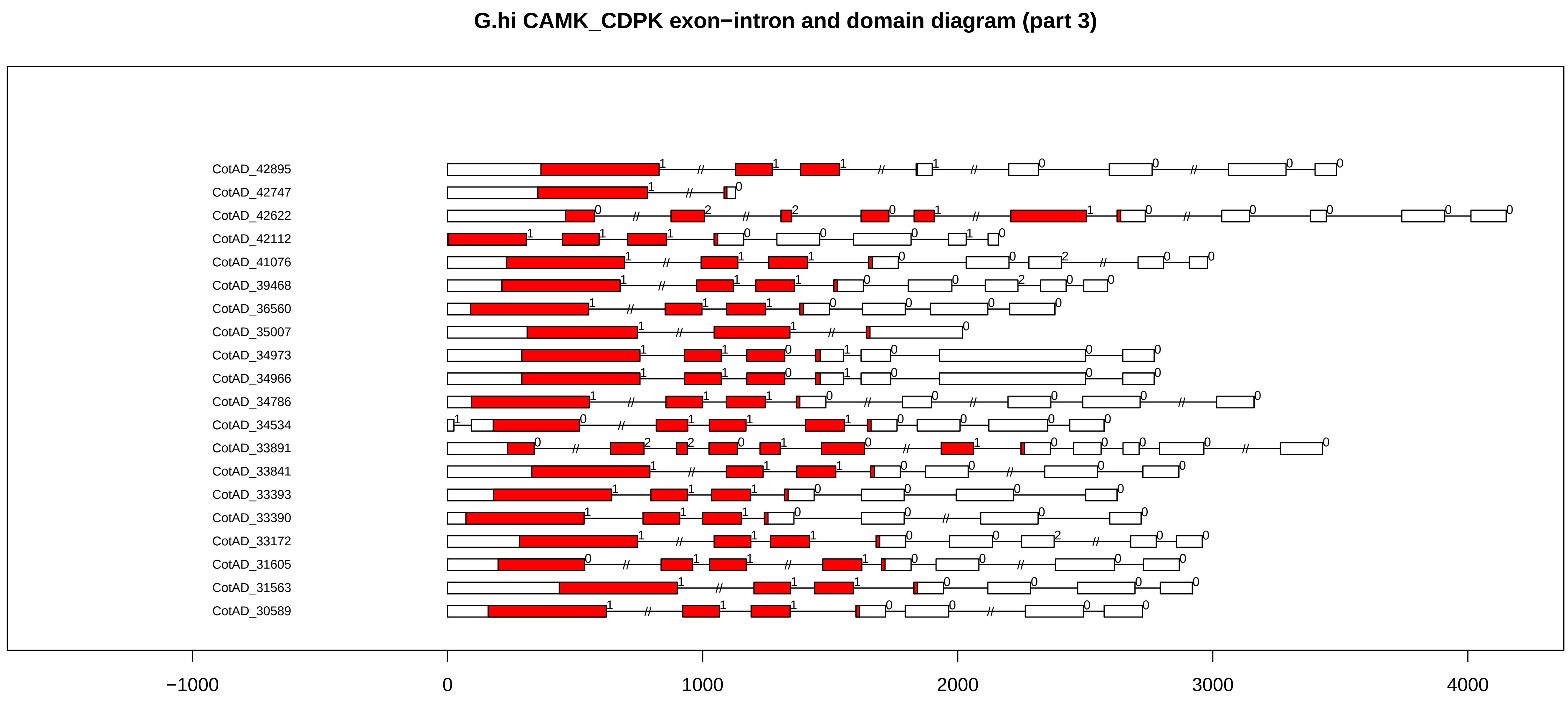
G.hi CAMK_CDPK exon−intron and domain diagram (part 3)
CotAD_42895
CotAD_42747
CotAD_42622
CotAD_42112
CotAD_41076
CotAD_39468
CotAD_36560
CotAD_35007
CotAD_34973
CotAD_34966
CotAD_34786
CotAD_34534
CotAD_33891
CotAD_33841
CotAD_33393
CotAD_33390
CotAD_33172
CotAD_31605
CotAD_31563
CotAD_30589
−1000
0
1000
2000
3000
4000

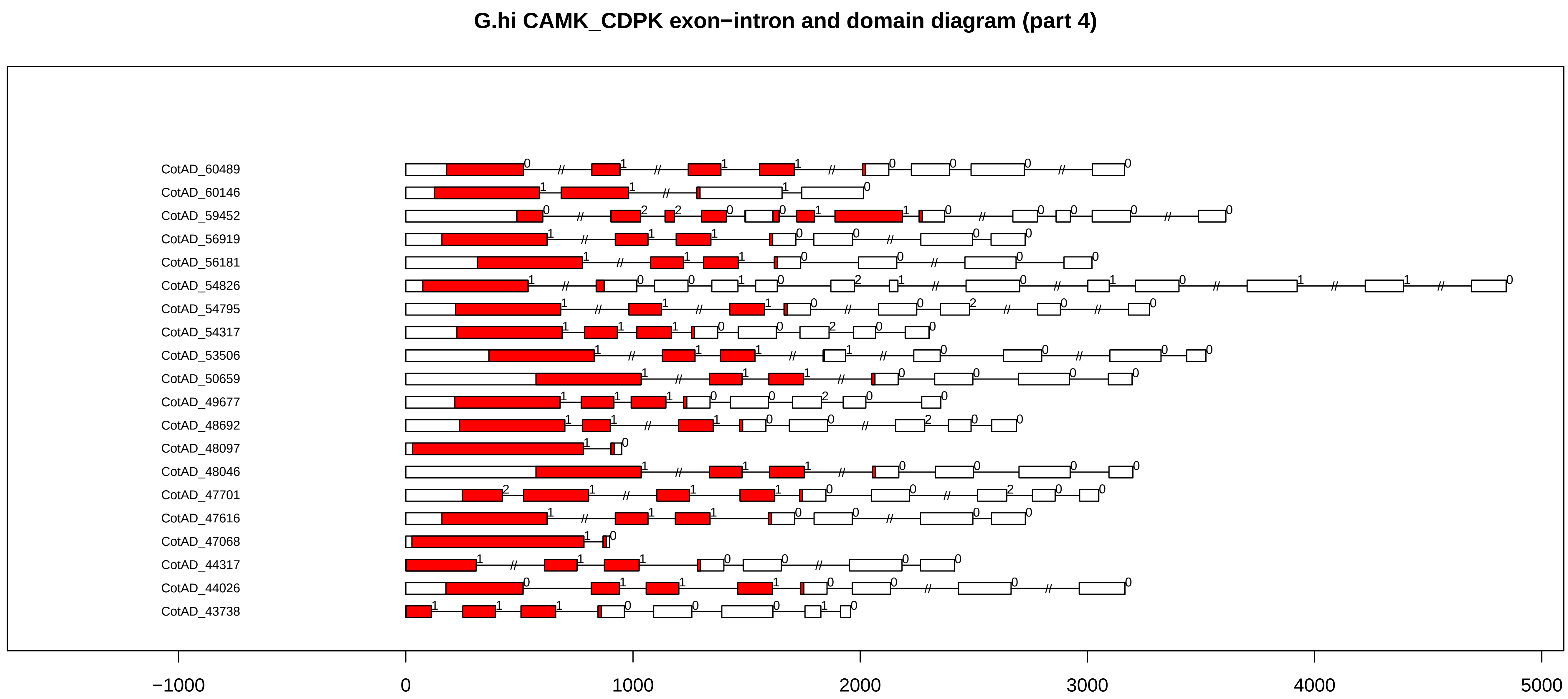

G.hi CAMK_CDPK exon−intron and domain diagram (part 4)
CotAD_60489
CotAD_60146
CotAD_59452
CotAD_56919
CotAD_56181
CotAD_54826
CotAD_54795
CotAD_54317
CotAD_53506
CotAD_50659
CotAD_49677
CotAD_48692
CotAD_48097
CotAD_48046
CotAD_47701
CotAD_47616
CotAD_47068
CotAD_44317
CotAD_44026
CotAD_43738
−1000
0
1000
2000
3000
4000
5000

G.hi CAMK_CDPK exon−intron and domain diagram (part 5)

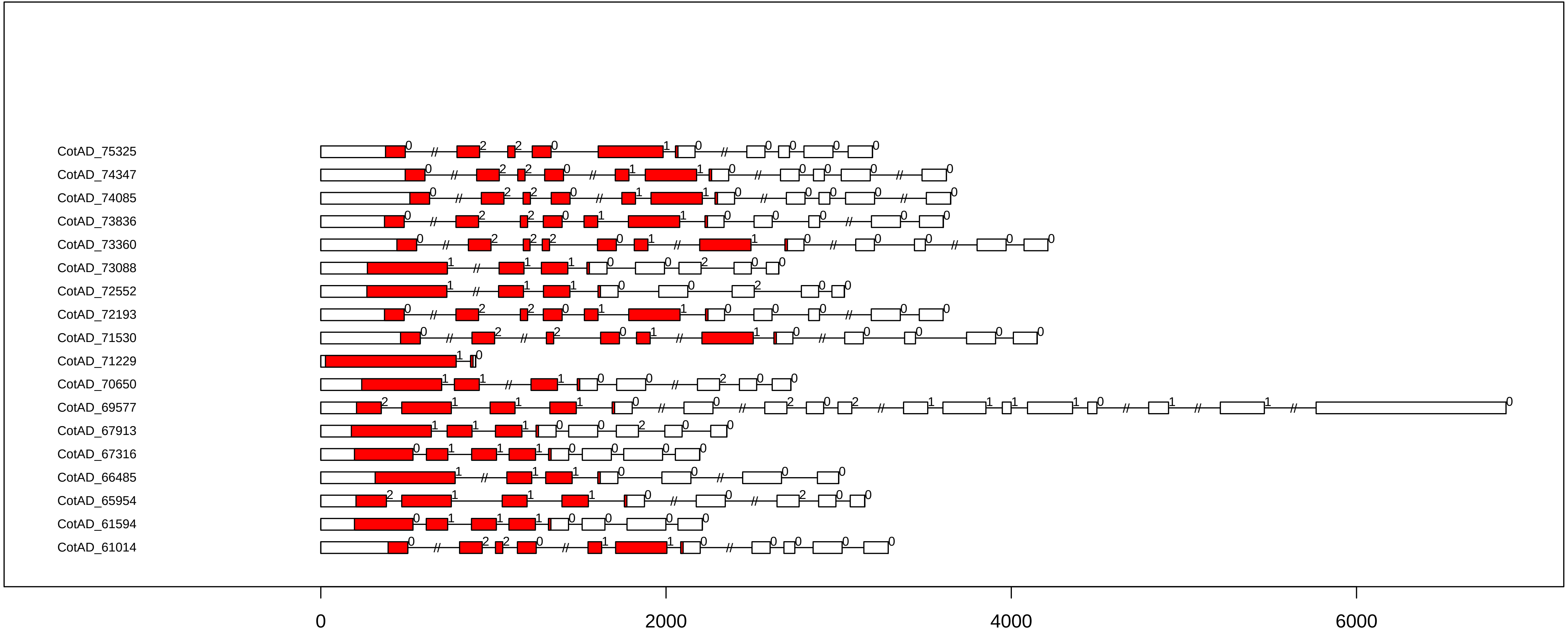

G.hi CAMK_OST1L exon−intron and domain diagram (all)

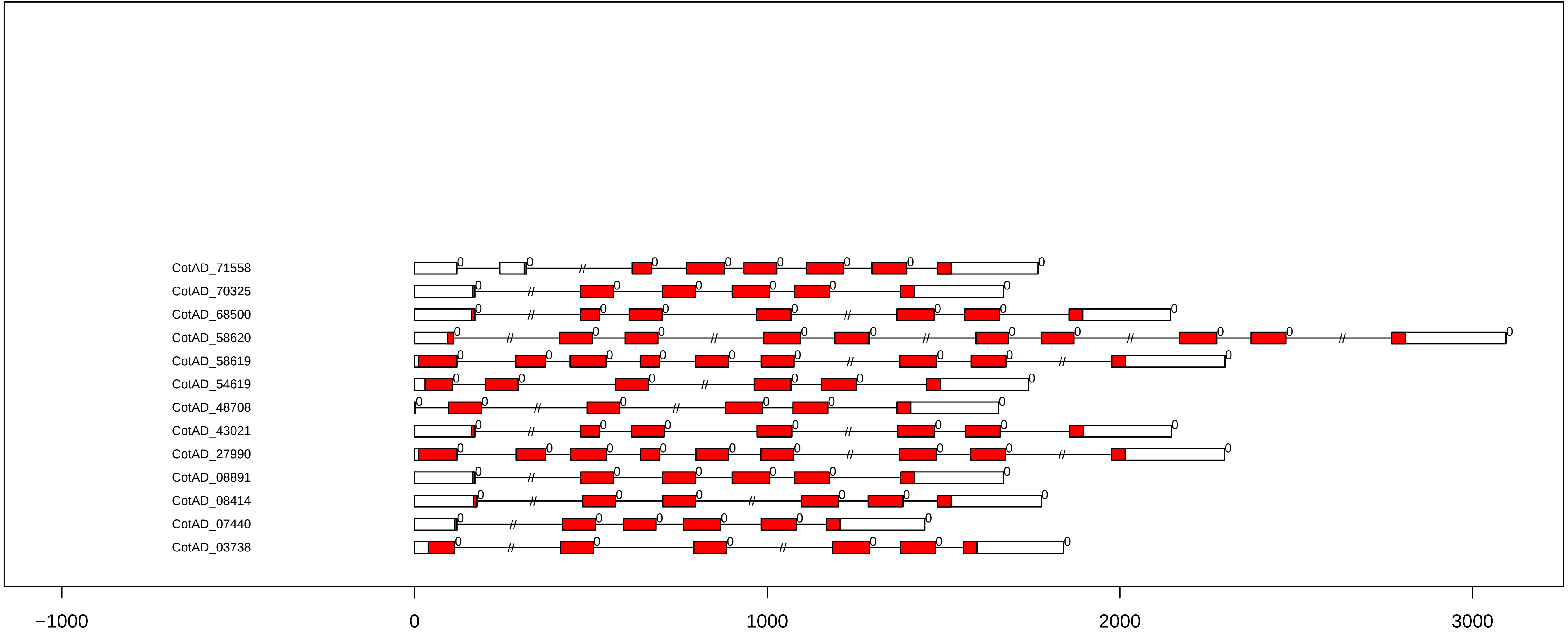

G.hi CK1_CK1 exon−intron and domain diagram (part 1)

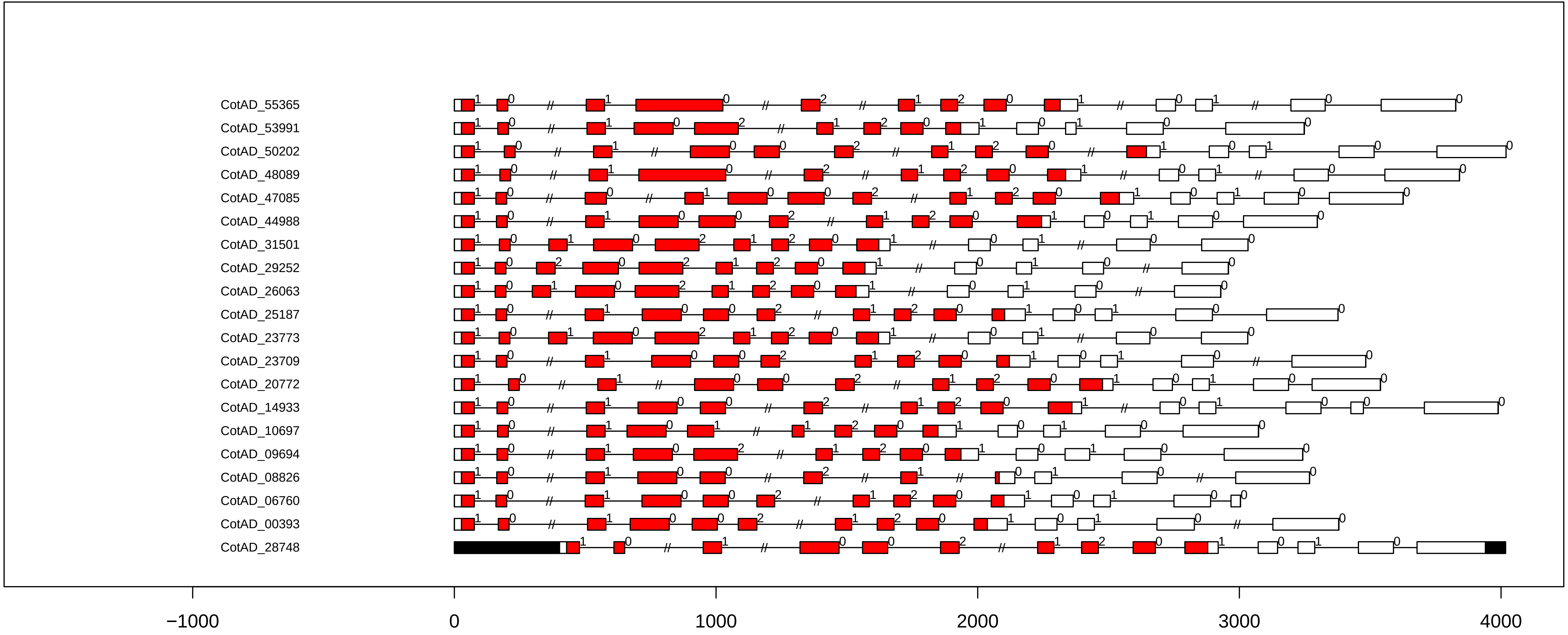

G.hi CK1_CK1 exon−intron and domain diagram (part 2)

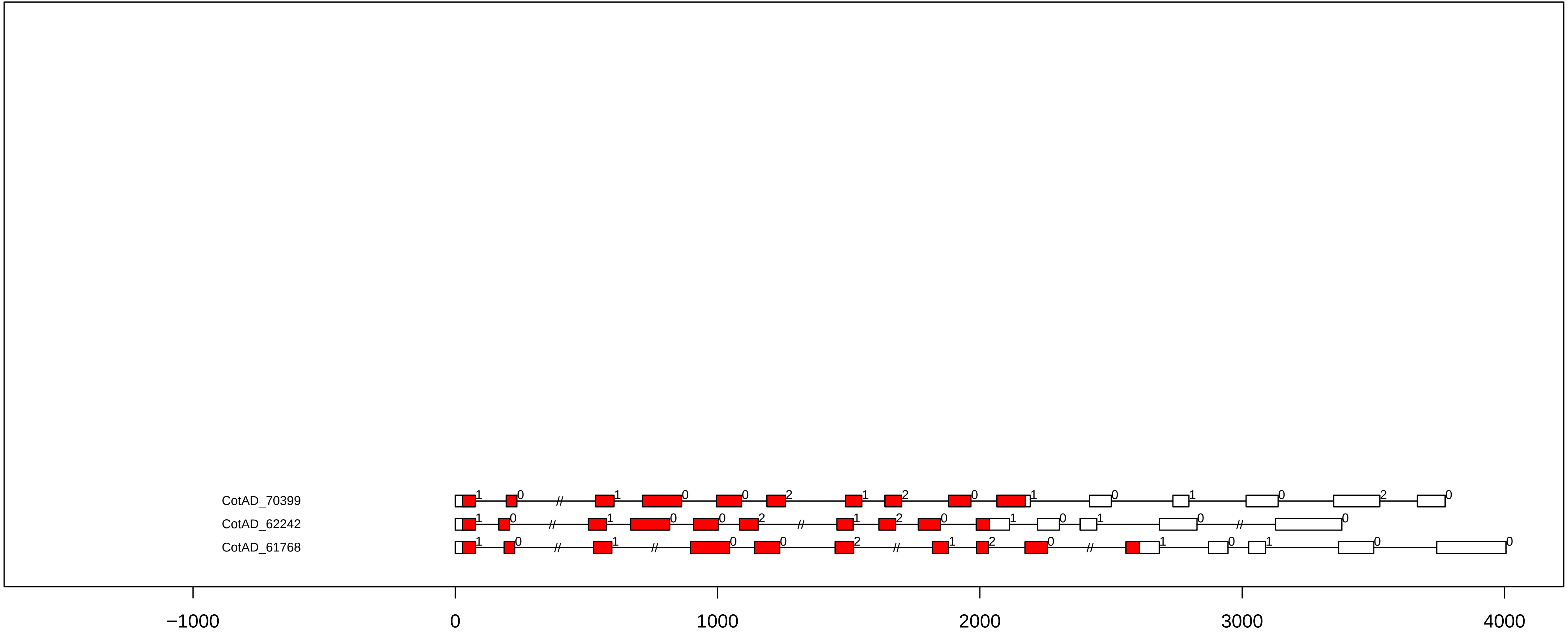

G.hi CK1_CK1−PI exon−intron and domain diagram (part 1)

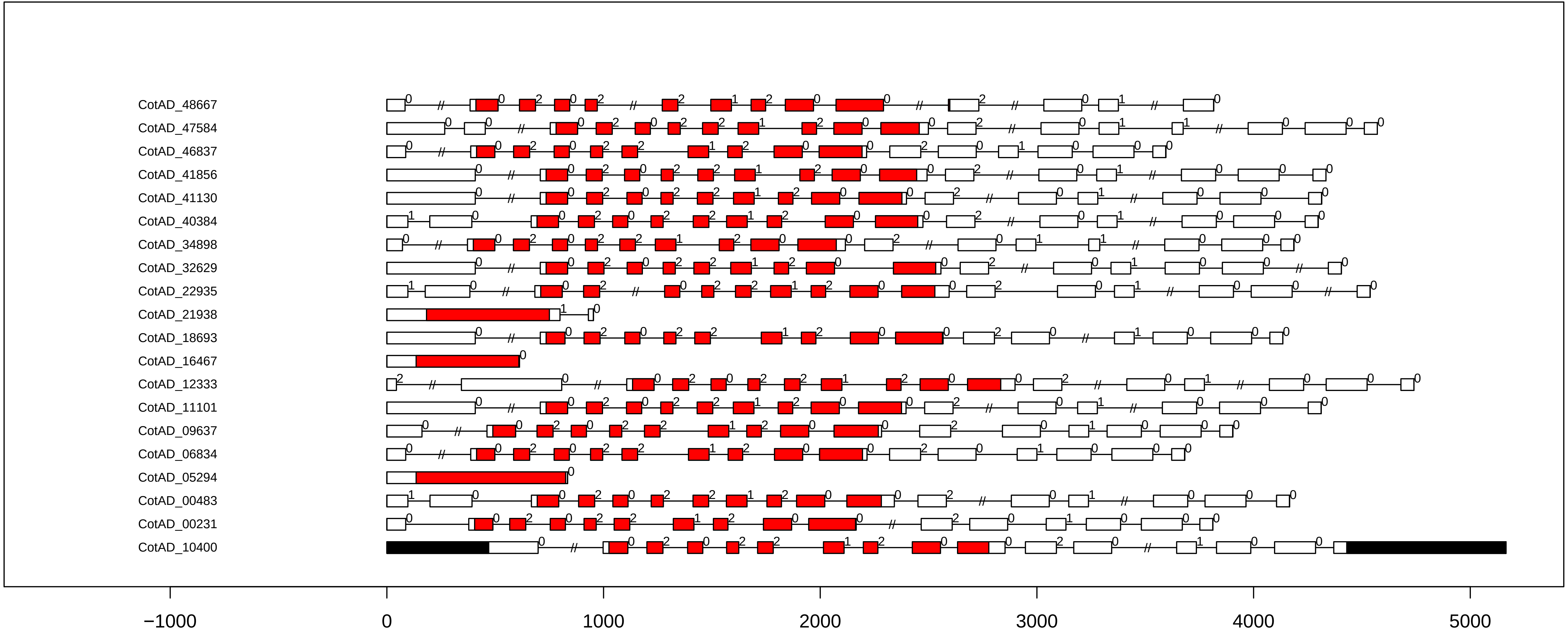

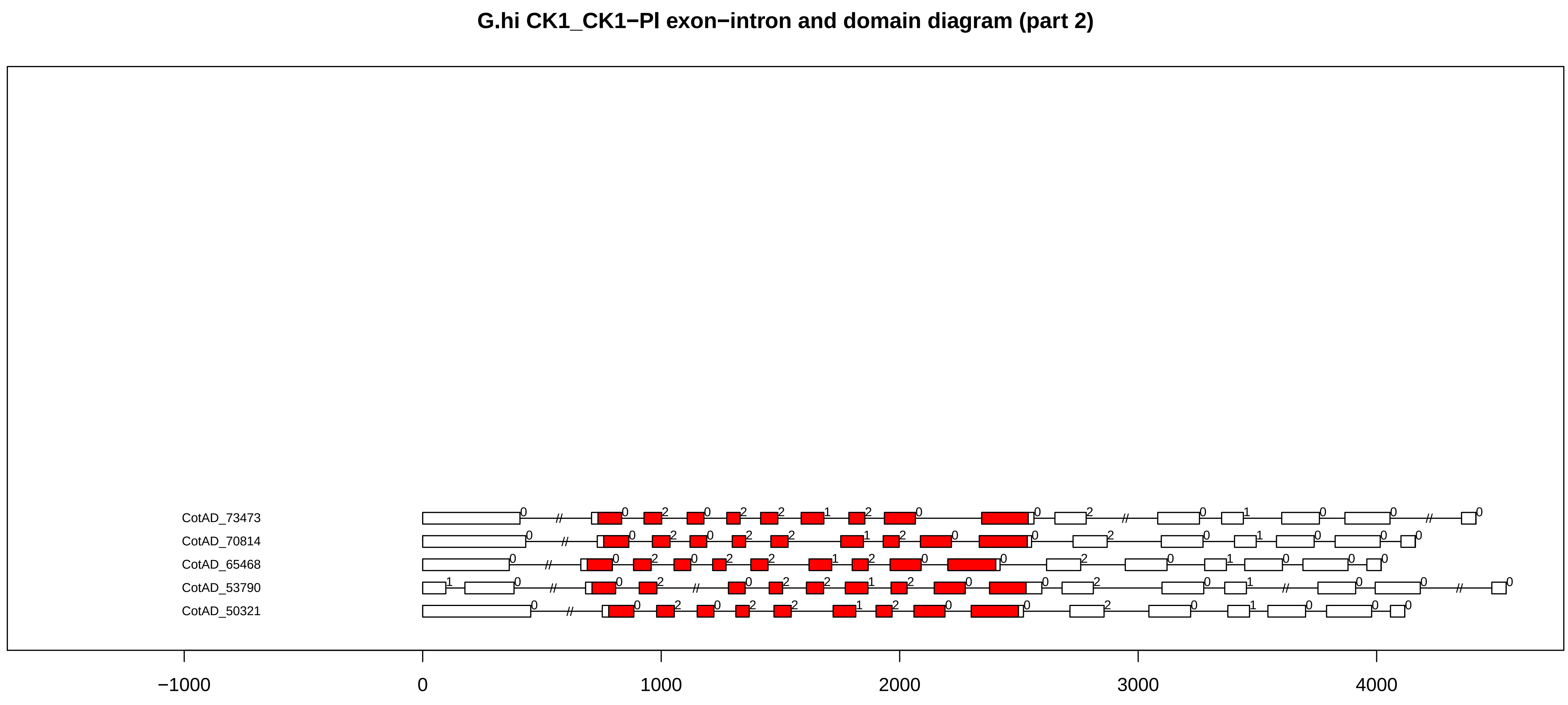

G.hi CK1_CK1−PI exon−intron and domain diagram (part 2)
CotAD_73473
CotAD_70814
CotAD_65468
CotAD_53790
CotAD_50321
−1000
0
1000
2000
3000
4000

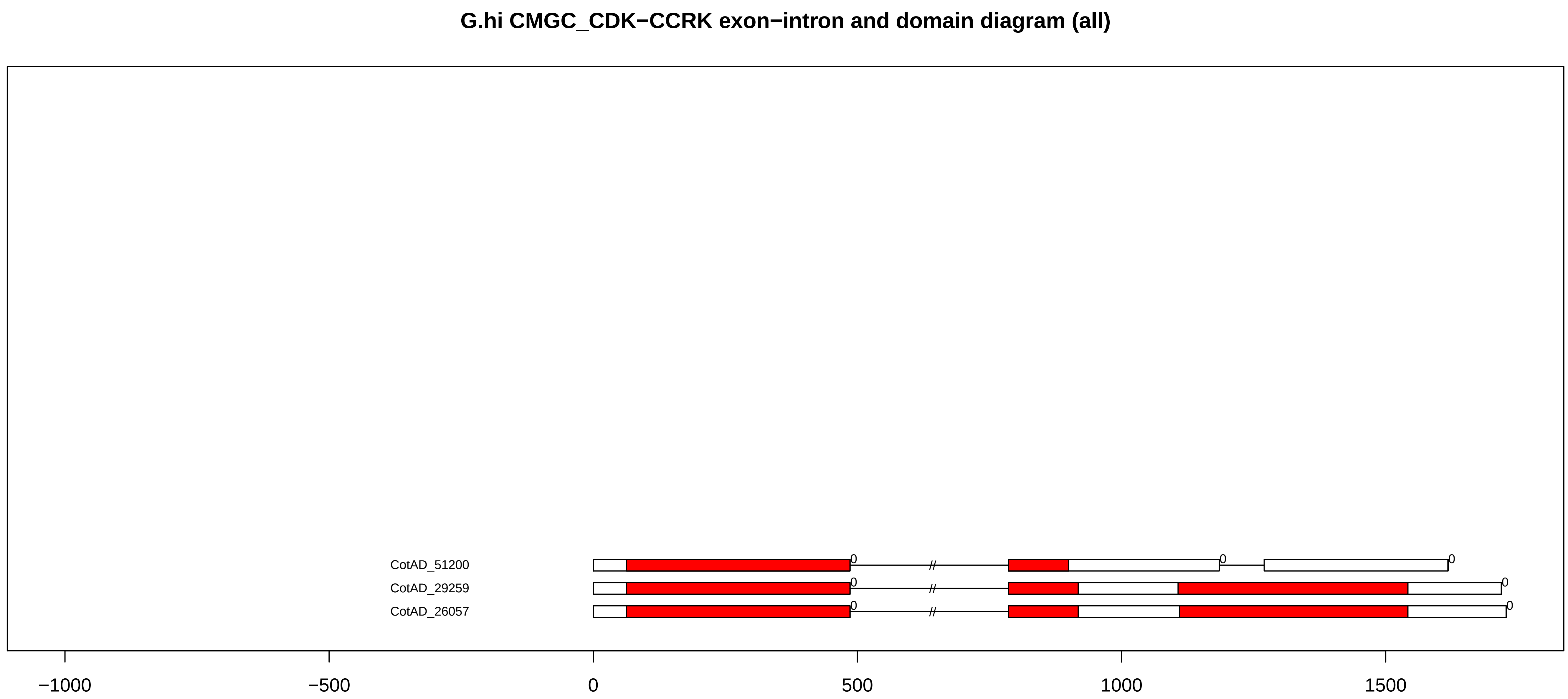
G.hi CMGC_CDK−CCRK exon−intron and domain diagram (all)
CotAD_51200
CotAD_29259
CotAD_26057
−1000
−500
0
500
1000
1500

G.hi CMGC_CDK−CDK7 exon−intron and domain diagram (all)

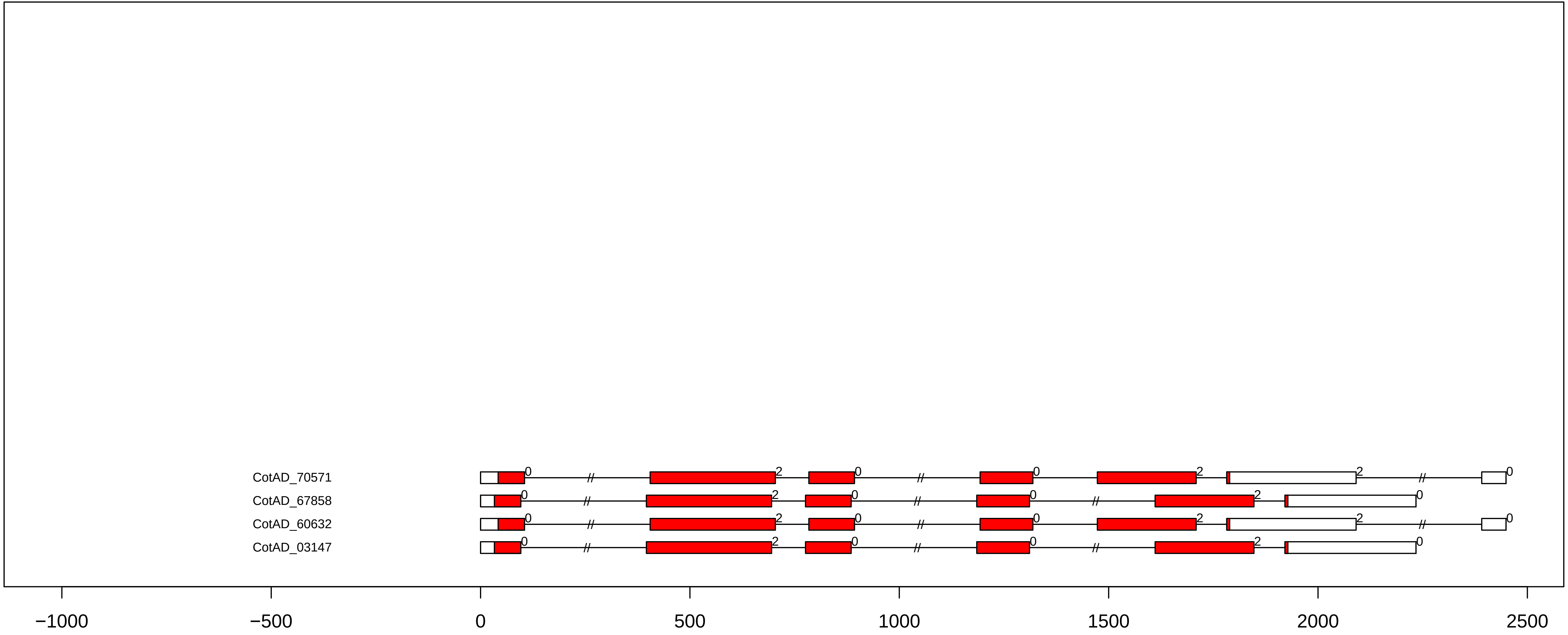

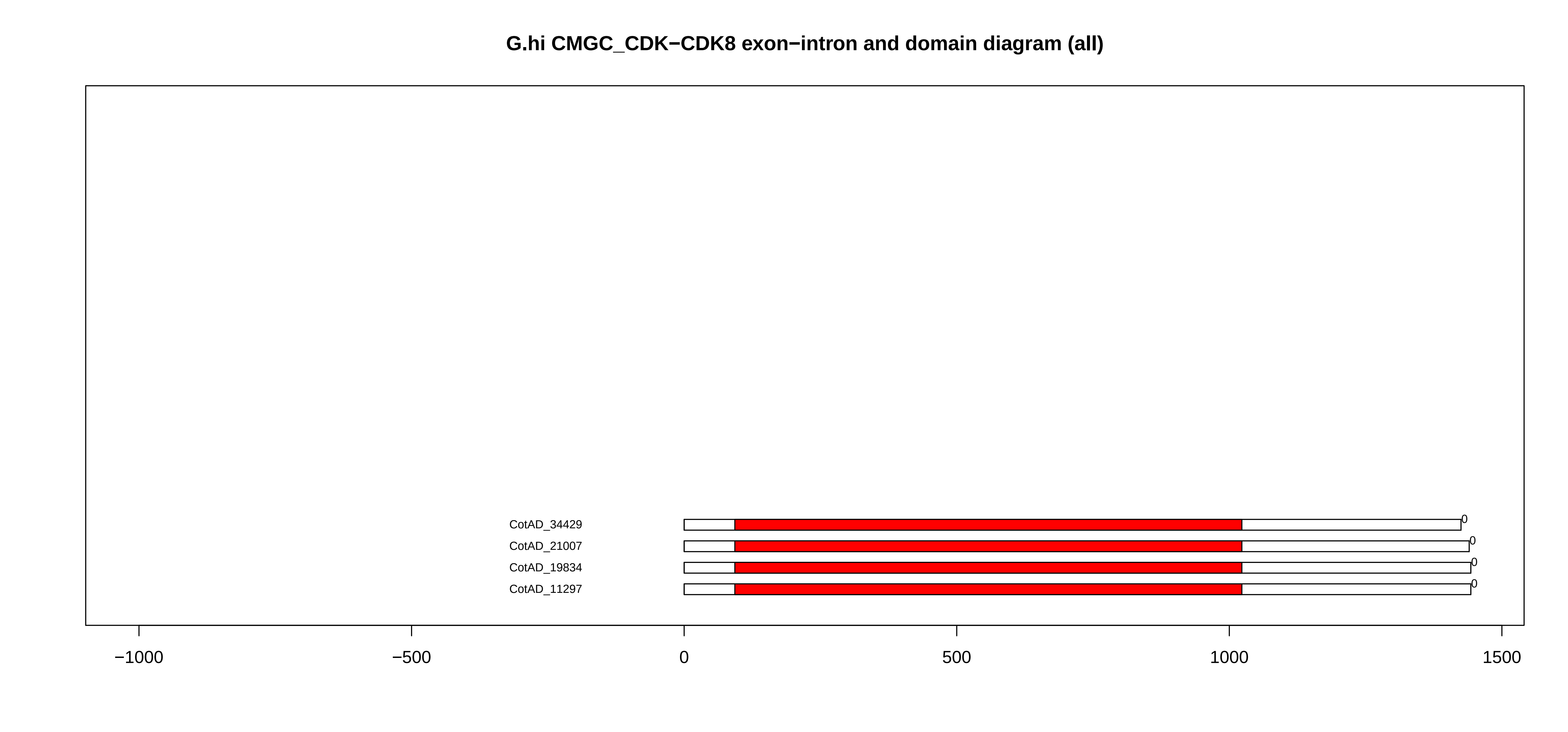

G.hi CMGC_CDK−CRK7−CDK9 exon−intron and domain diagram (part 1)

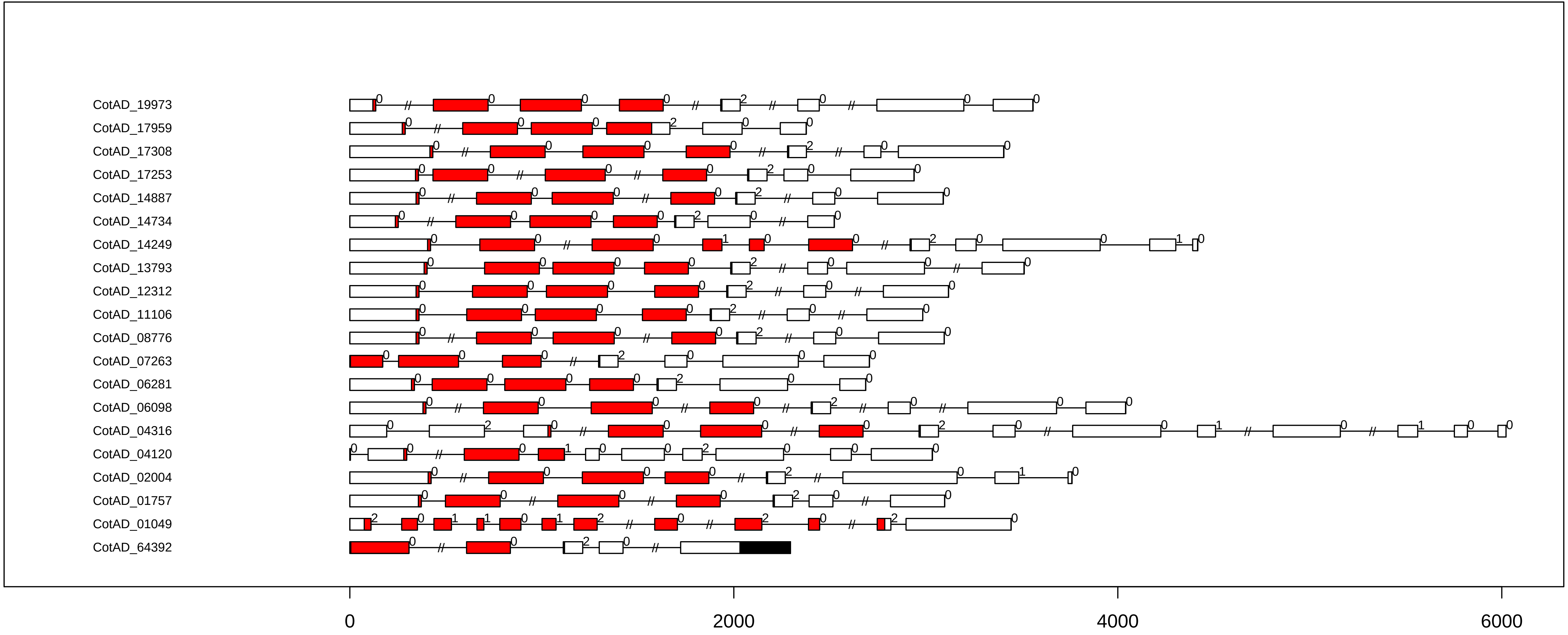

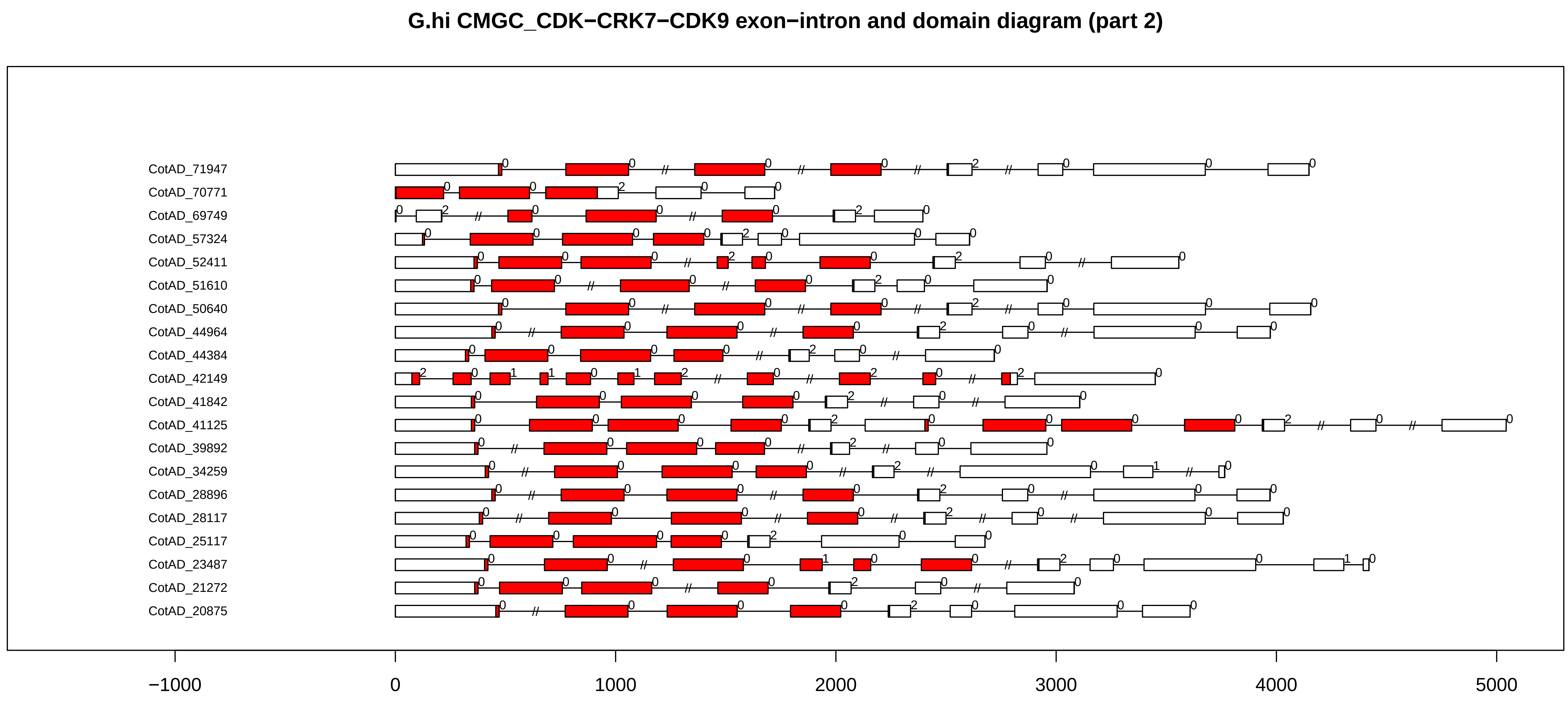
G.hi CMGC_CDK−CRK7−CDK9 exon−intron and domain diagram (part 2)
CotAD_71947
CotAD_70771
CotAD_69749
CotAD_57324
CotAD_52411
CotAD_51610
CotAD_50640
CotAD_44964
CotAD_44384
CotAD_42149
CotAD_41842
CotAD_41125
CotAD_39892
CotAD_34259
CotAD_28896
CotAD_28117
CotAD_25117
CotAD_23487
CotAD_21272
CotAD_20875
−1000
0
1000
2000
3000
4000
5000

G.hi CMGC_CDK−CRK7−CDK9 exon−intron and domain diagram (part 3)

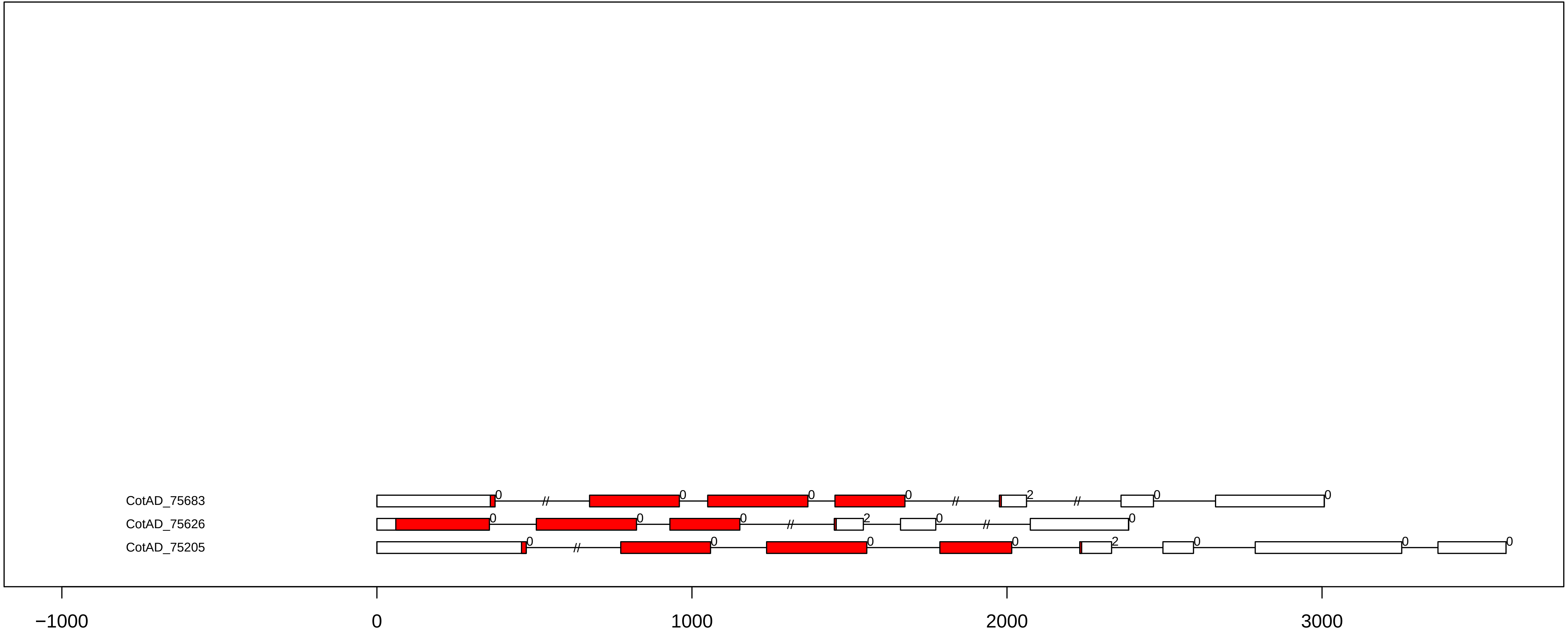

G.hi CMGC_CDK−PITSLRE exon−intron and domain diagram (all)

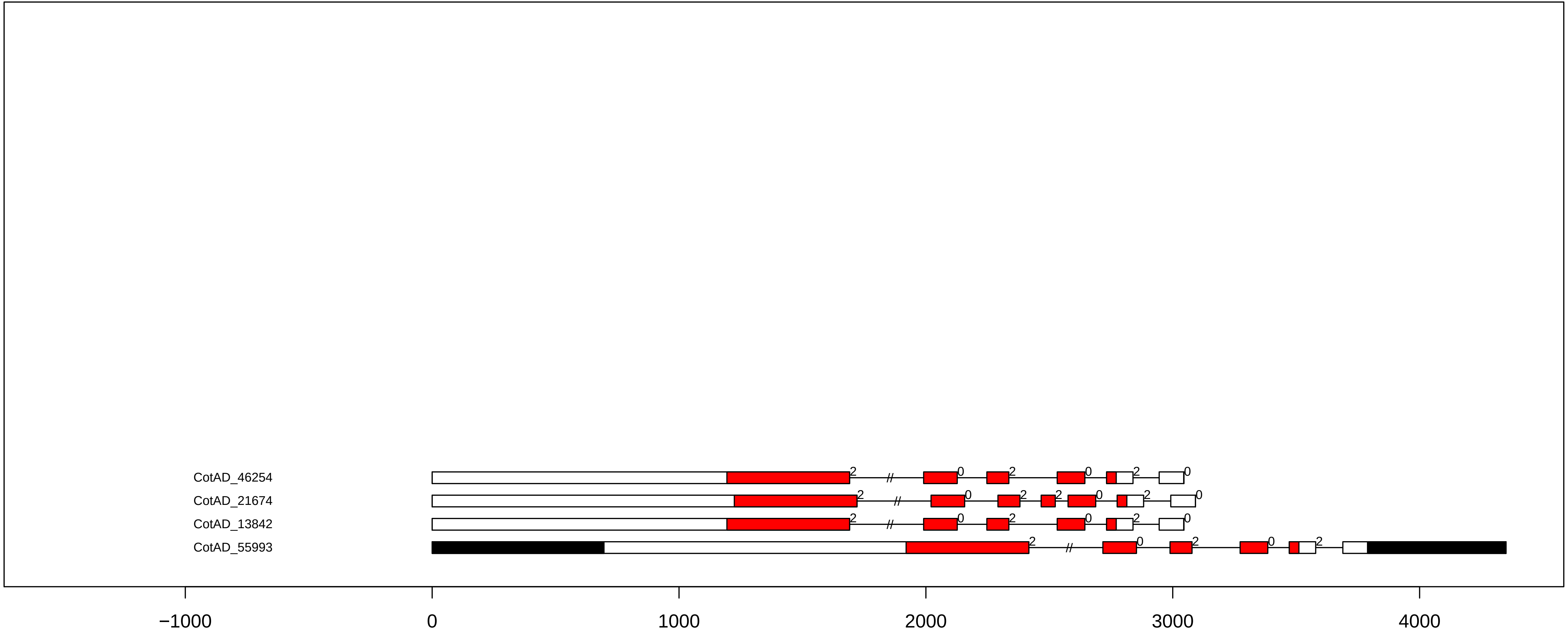

G.hi CMGC_CDK−PI exon−intron and domain diagram (all)

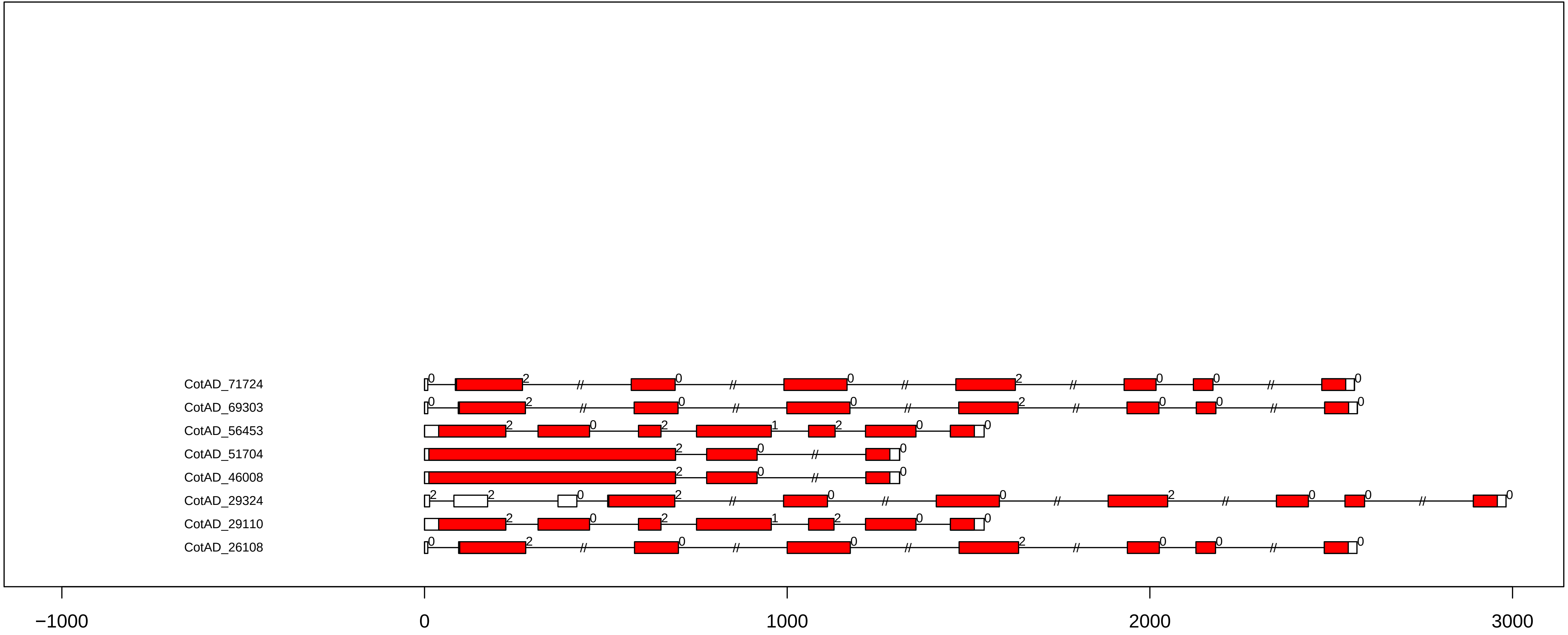

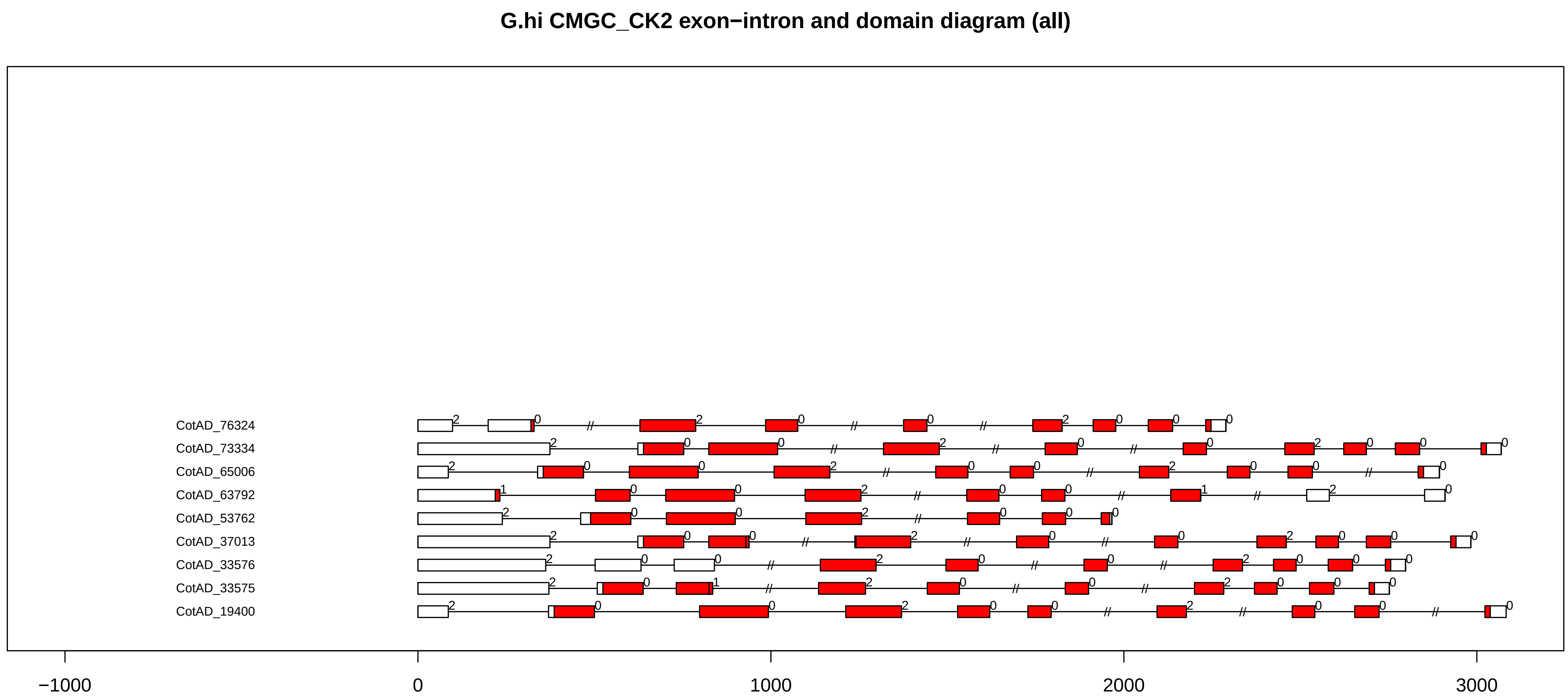

G.hi CMGC_CK2 exon−intron and domain diagram (all)
CotAD_76324
CotAD_73334
CotAD_65006
CotAD_63792
CotAD_53762
CotAD_37013
CotAD_33576
CotAD_33575
CotAD_19400
−1000
0
1000
2000
3000

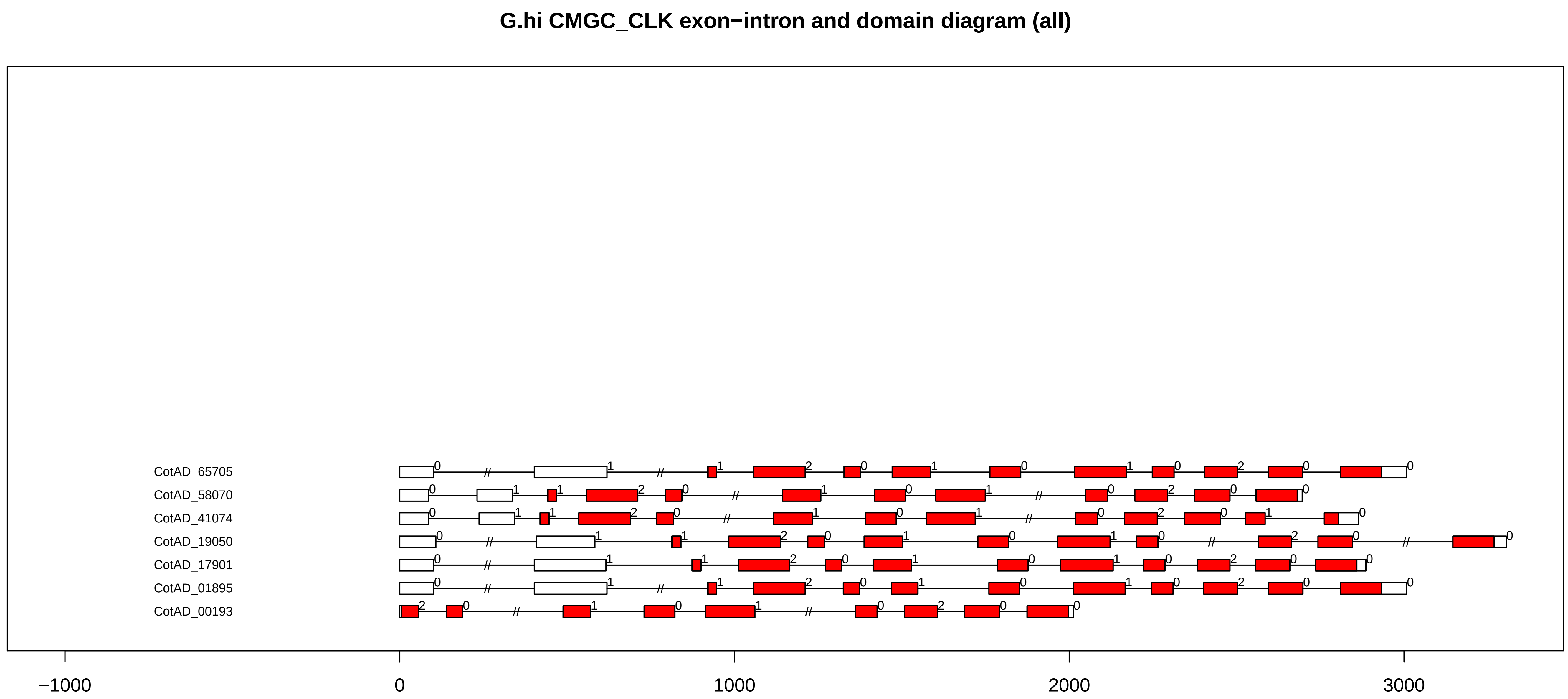

G.hi CMGC_CLK exon−intron and domain diagram (all)
CotAD_65705
CotAD_58070
CotAD_41074
CotAD_19050
CotAD_17901
CotAD_01895
CotAD_00193
−1000
0
1000
2000
3000

G.hi CMGC_DYRK−PRP4 exon−intron and domain diagram (all)

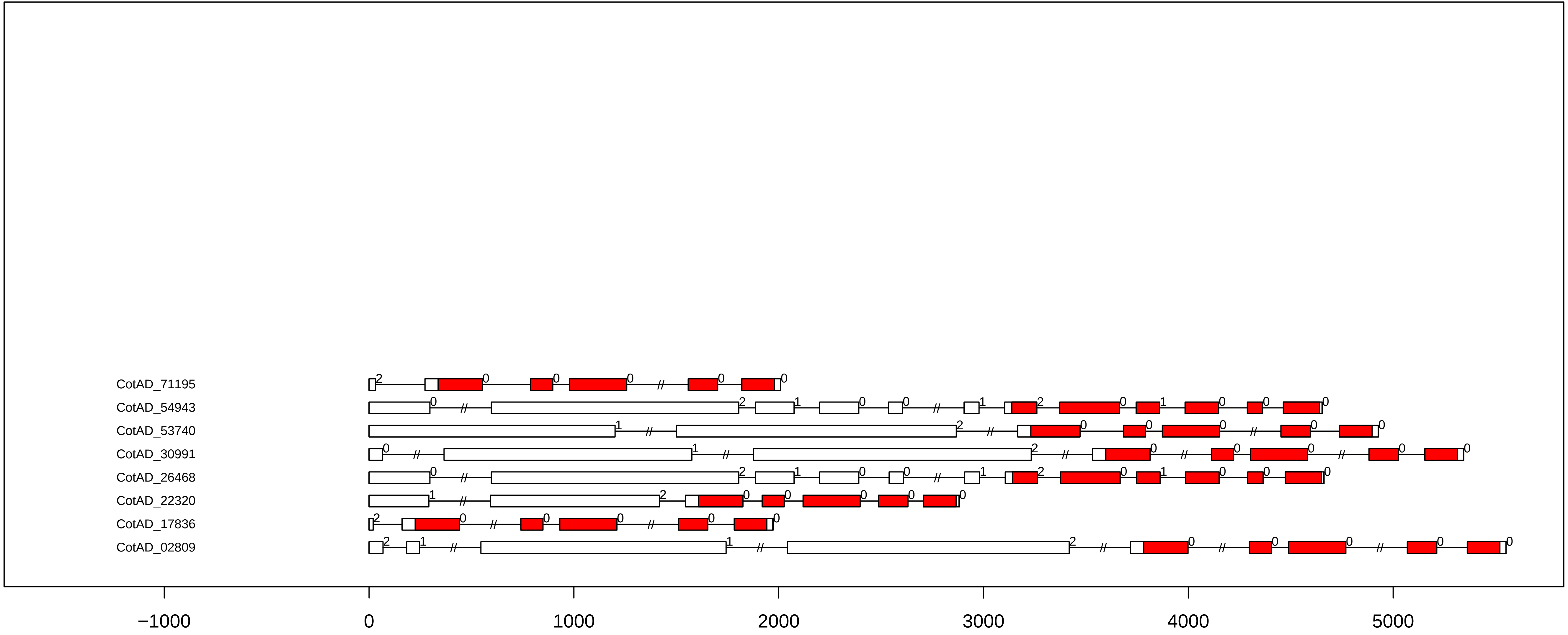

G.hi CMGC_DYRK−YAK exon−intron and domain diagram (all)

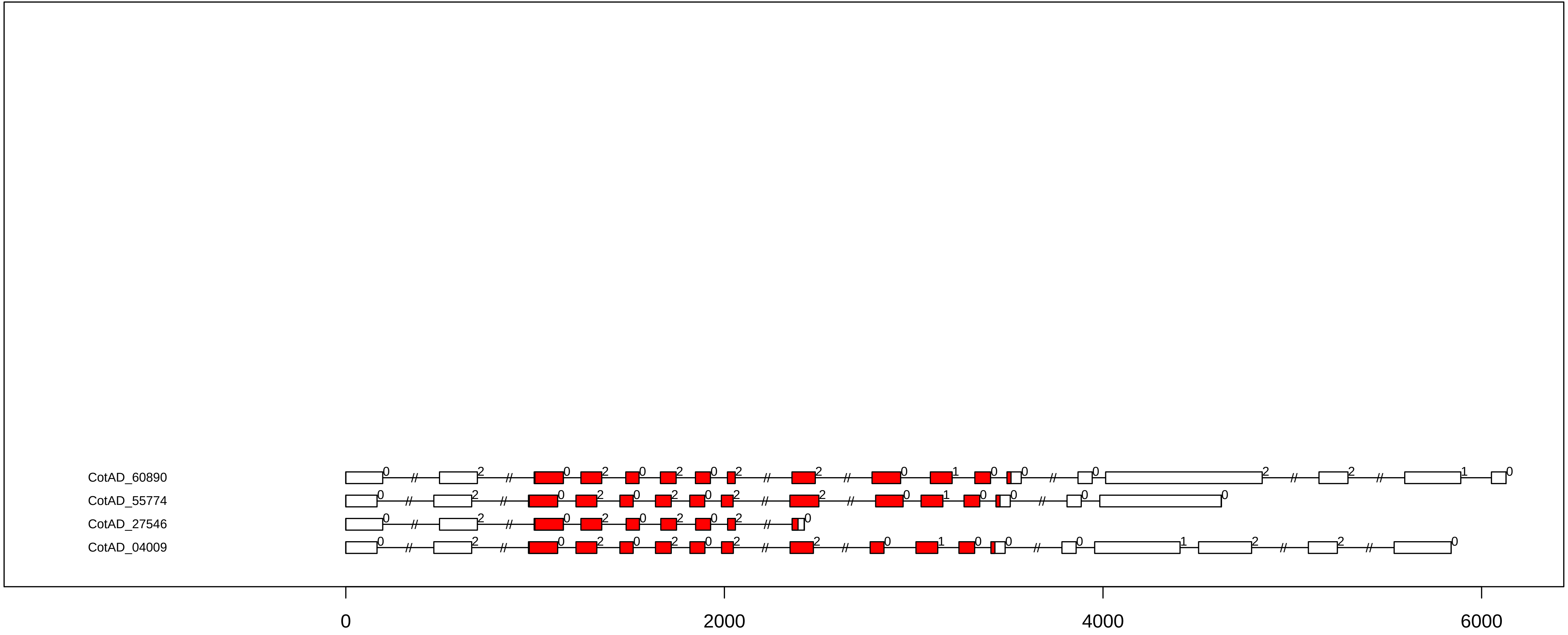

G.hi CMGC_GSKL exon−intron and domain diagram (all)

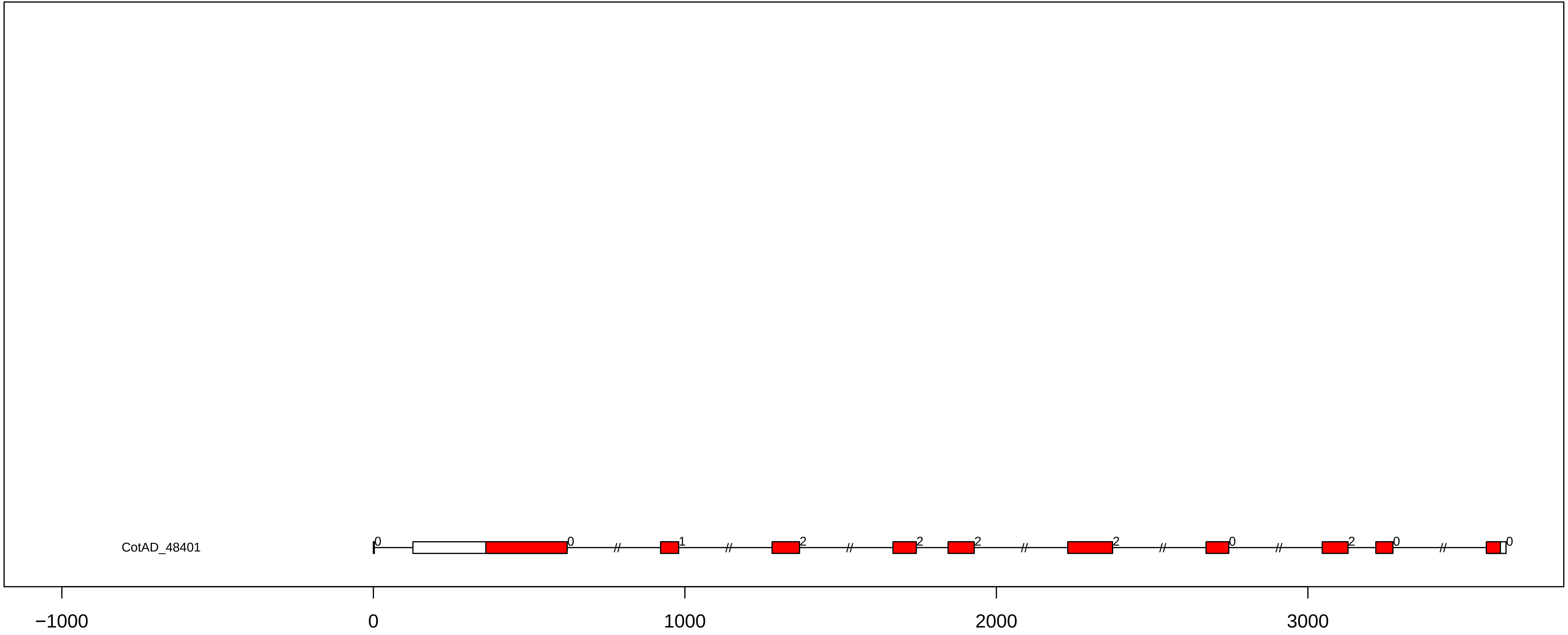

G.hi CMGC_GSK exon−intron and domain diagram (all)

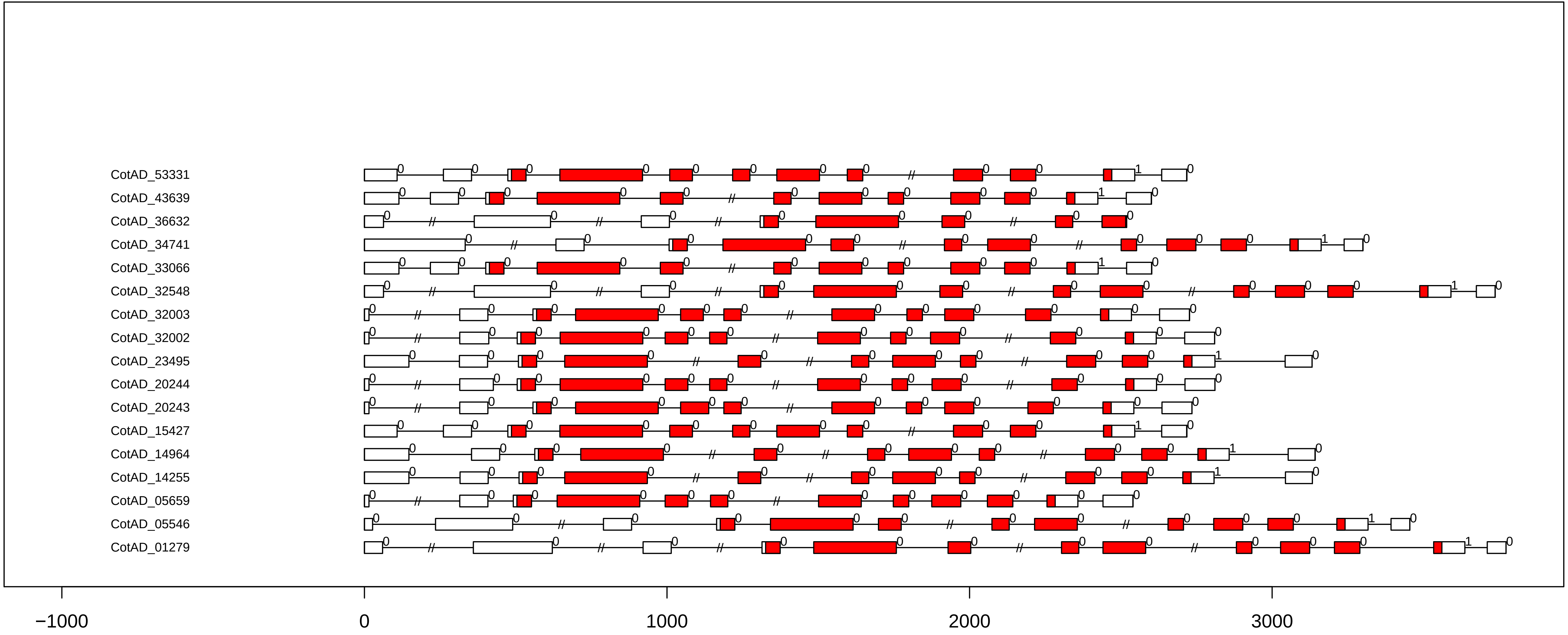

G.hi CMGC_MAPK exon−intron and domain diagram (part 1)

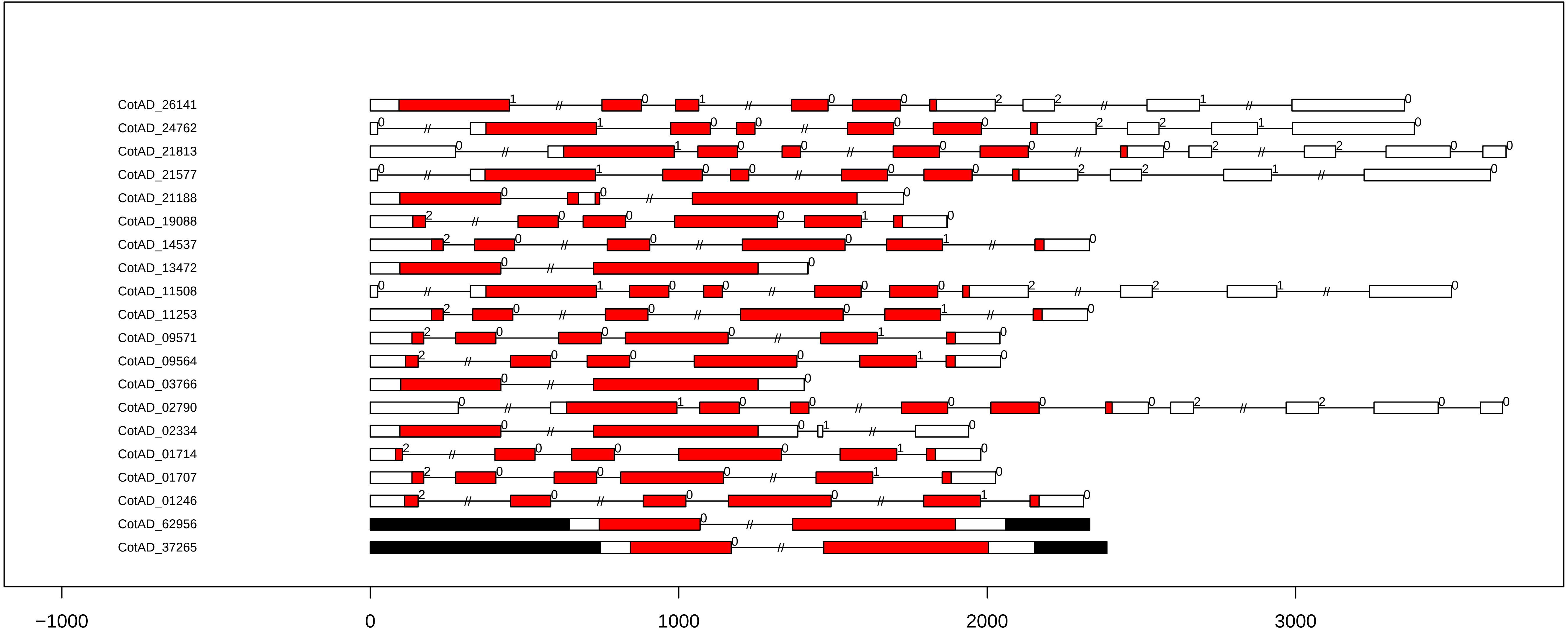

G.hi CMGC_MAPK exon−intron and domain diagram (part 2)

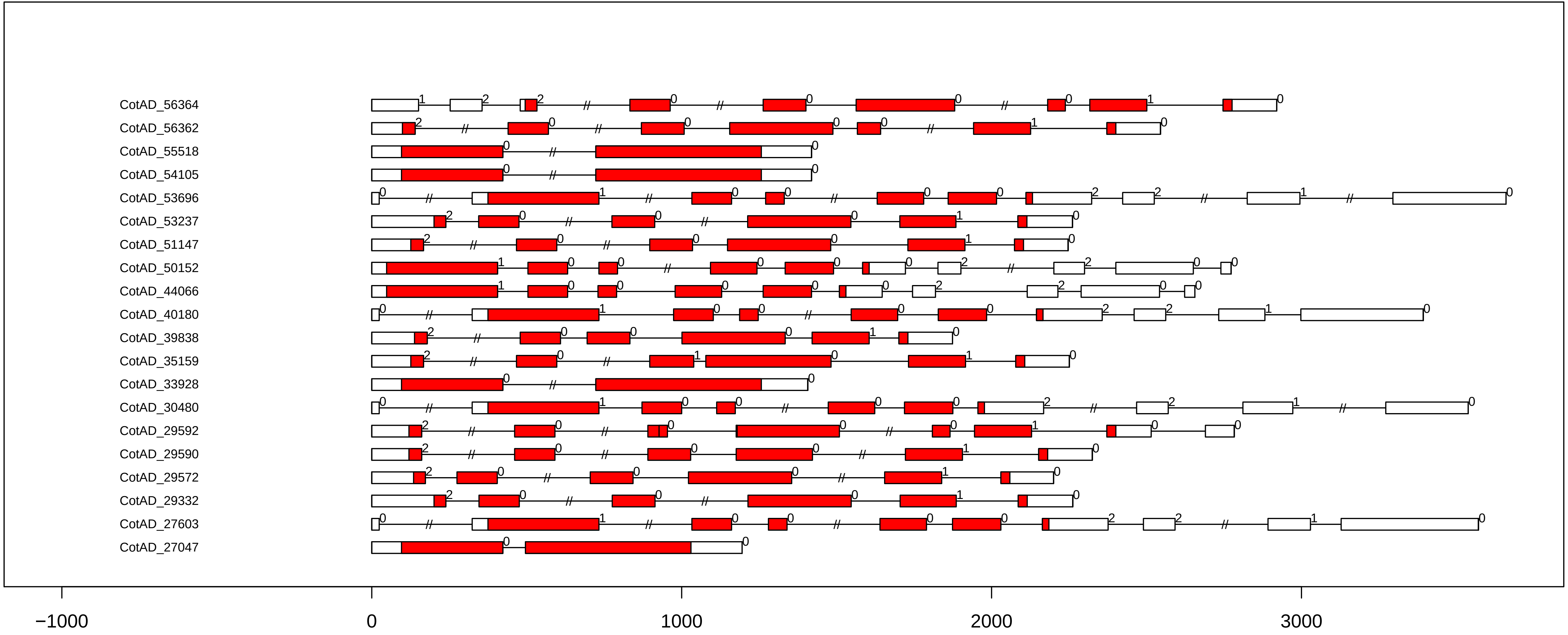

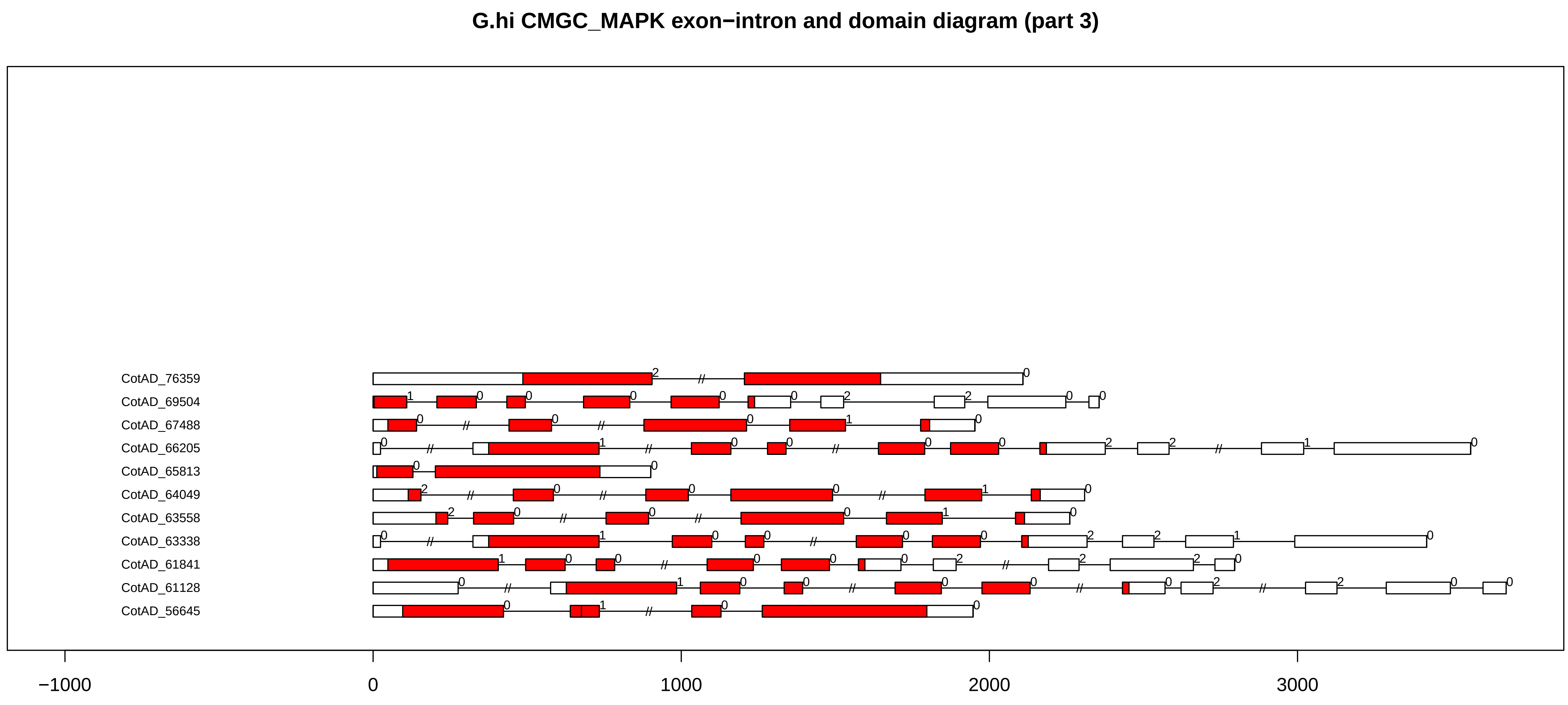
G.hi CMGC_MAPK exon−intron and domain diagram (part 3)
CotAD_76359
CotAD_69504
CotAD_67488
CotAD_66205
CotAD_65813
CotAD_64049
CotAD_63558
CotAD_63338
CotAD_61841
CotAD_61128
CotAD_56645
−1000
0
1000
2000
3000

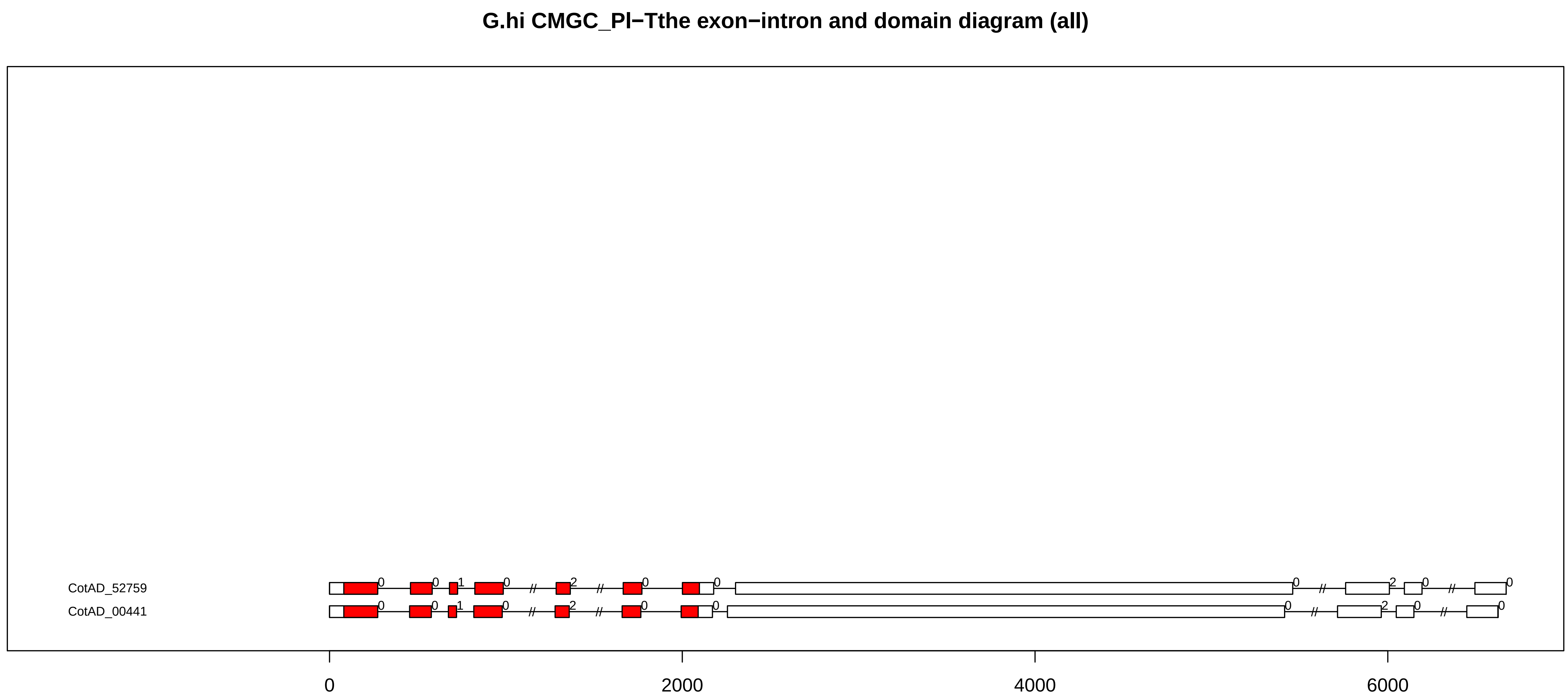

G.hi CMGC_Pl−Tthe exon−intron and domain diagram (all)
CotAD_52759
CotAD_00441
0
2000
4000
6000

G.hi CMGC_RCK exon−intron and domain diagram (all)

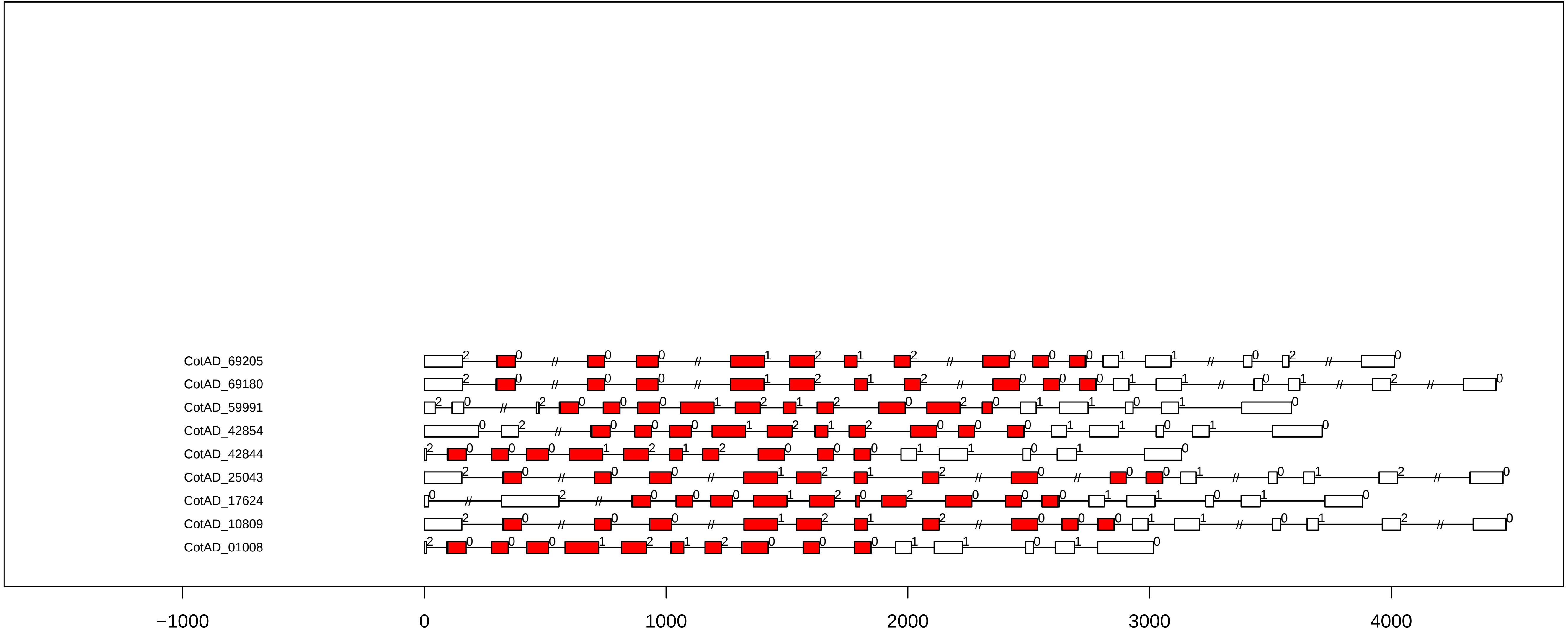

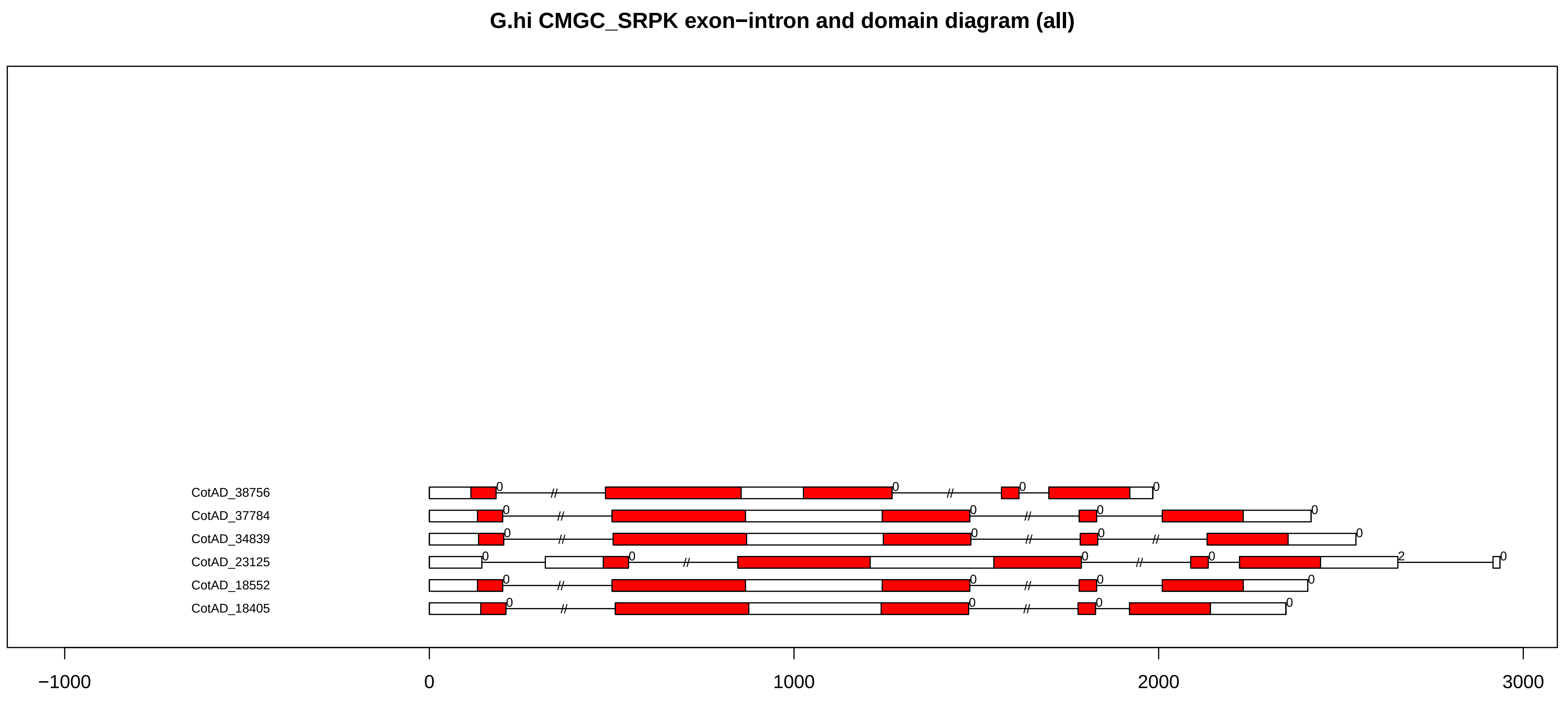
G.hi CMGC_SRPK exon−intron and domain diagram (all)
CotAD_38756
CotAD_37784
CotAD_34839
CotAD_23125
CotAD_18552
CotAD_18405
−1000
0
1000
2000
3000

G.hi Group−PI−2 exon−intron and domain diagram (all)

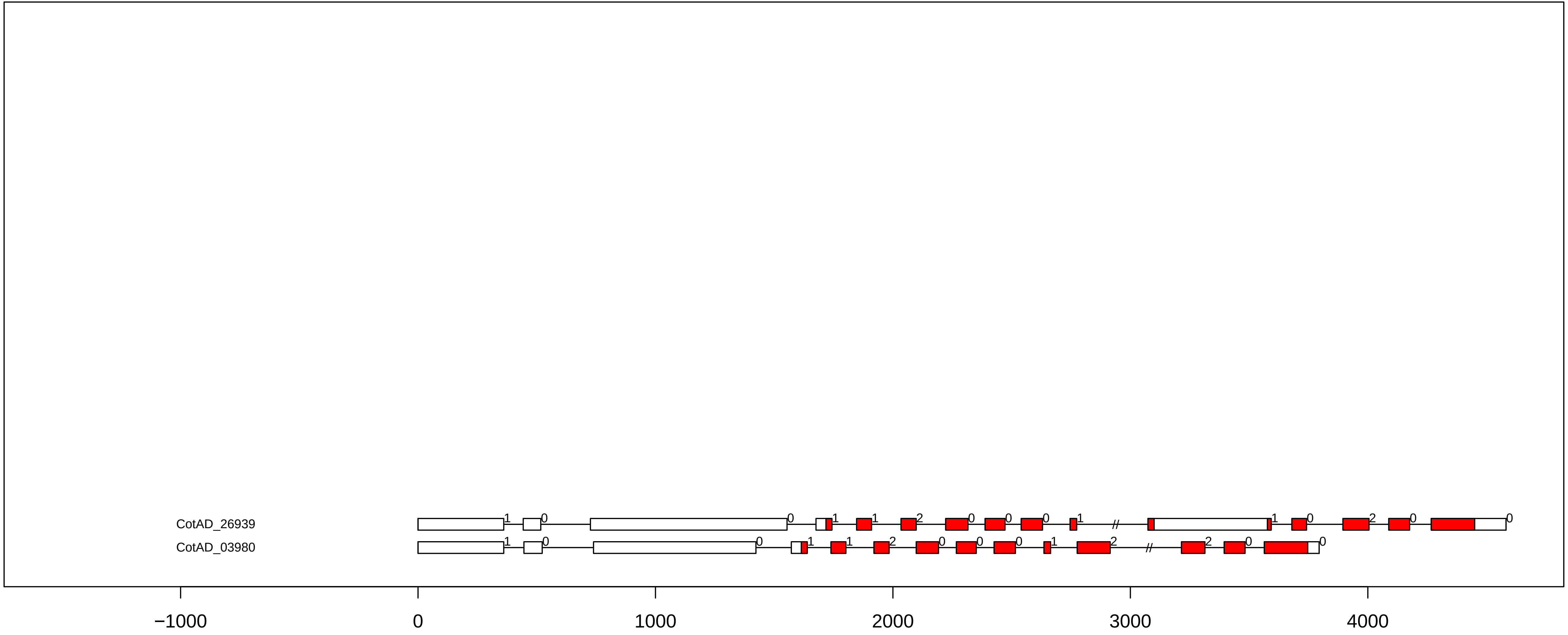

G.hi Group−PI−3 exon−intron and domain diagram (all)

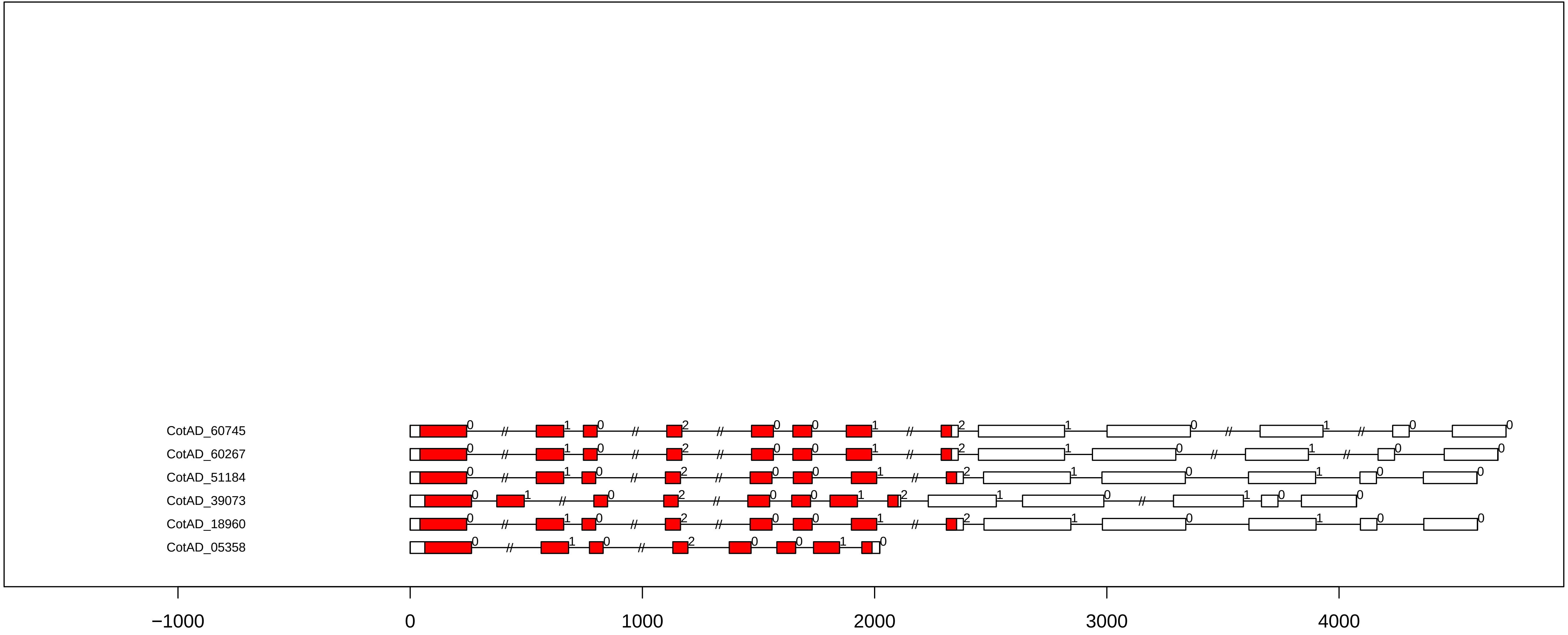

G.hi Group−PI−4 exon−intron and domain diagram (all)

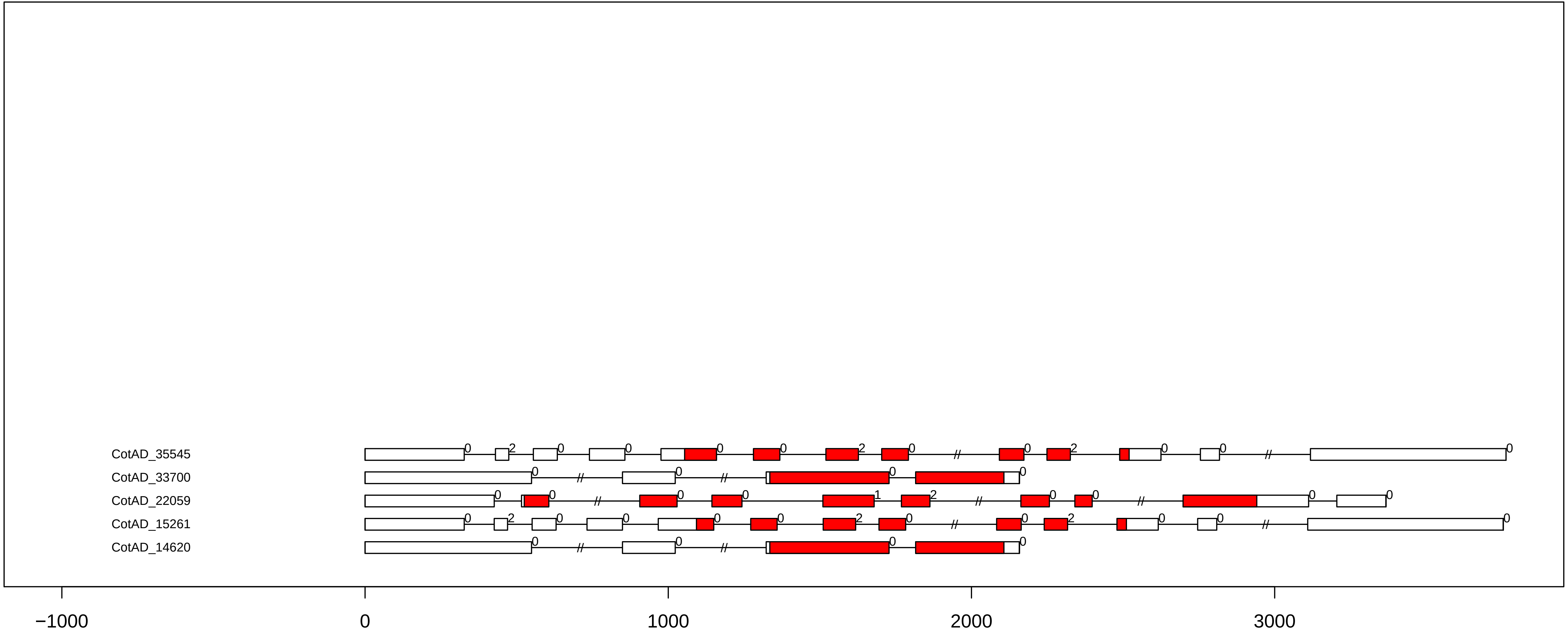

G.hi IRE1 exon−intron and domain diagram (all)

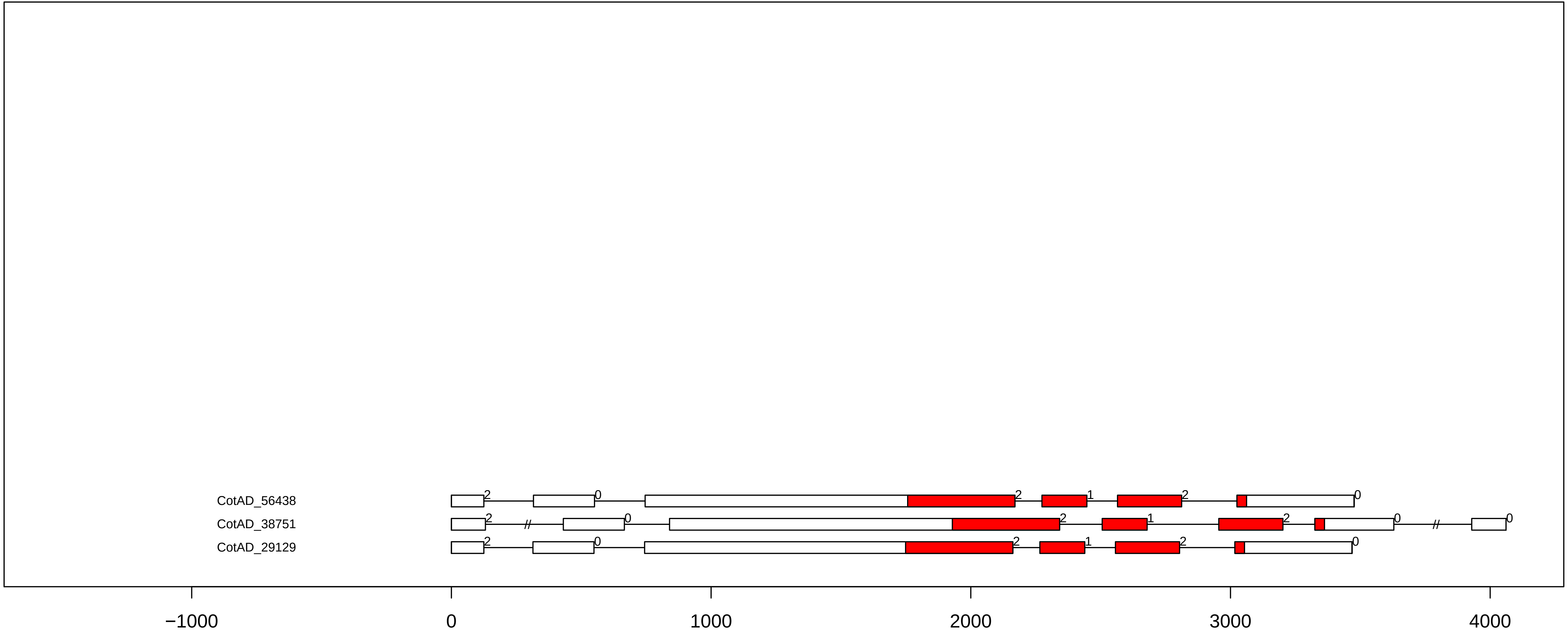

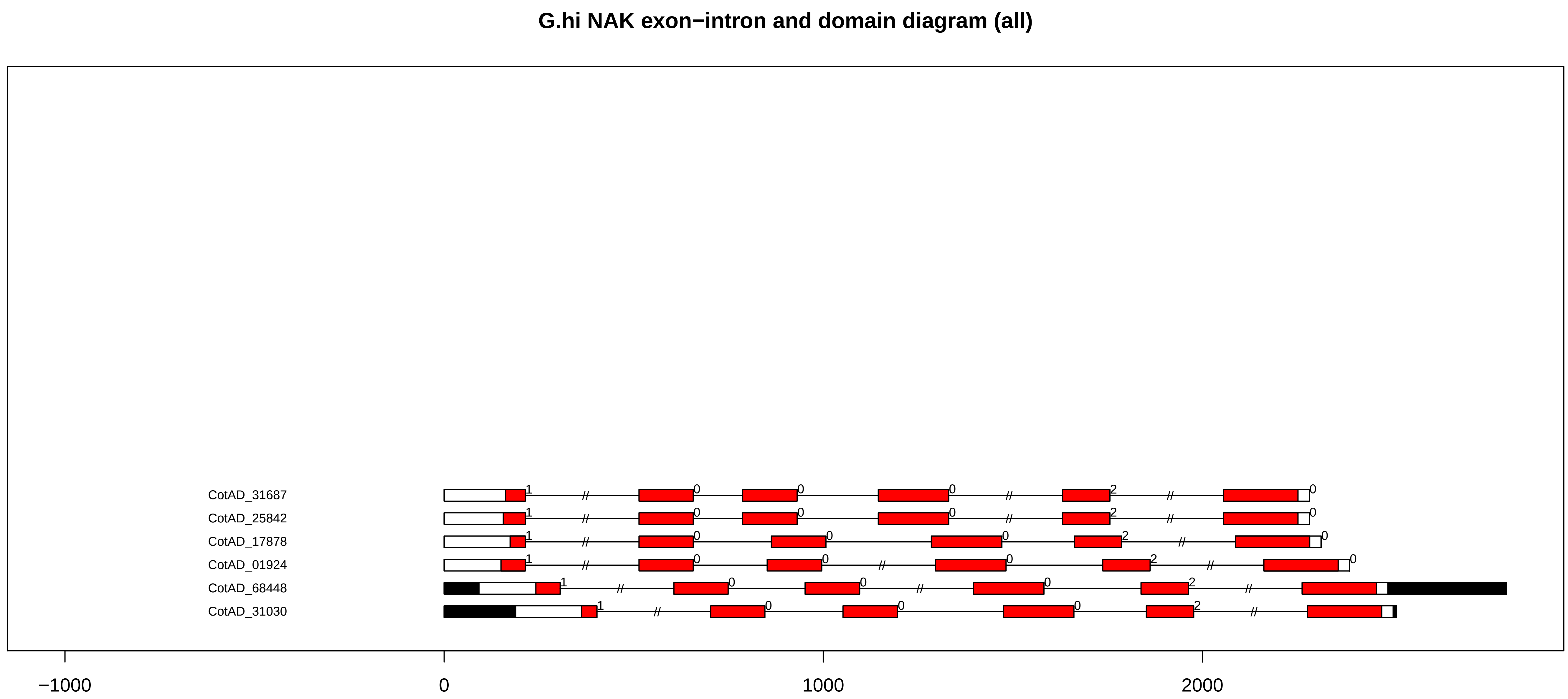

G.hi NAK exon−intron and domain diagram (all)
CotAD_31687
CotAD_25842
CotAD_17878
CotAD_01924
CotAD_68448
CotAD_31030
−1000
0
1000
2000

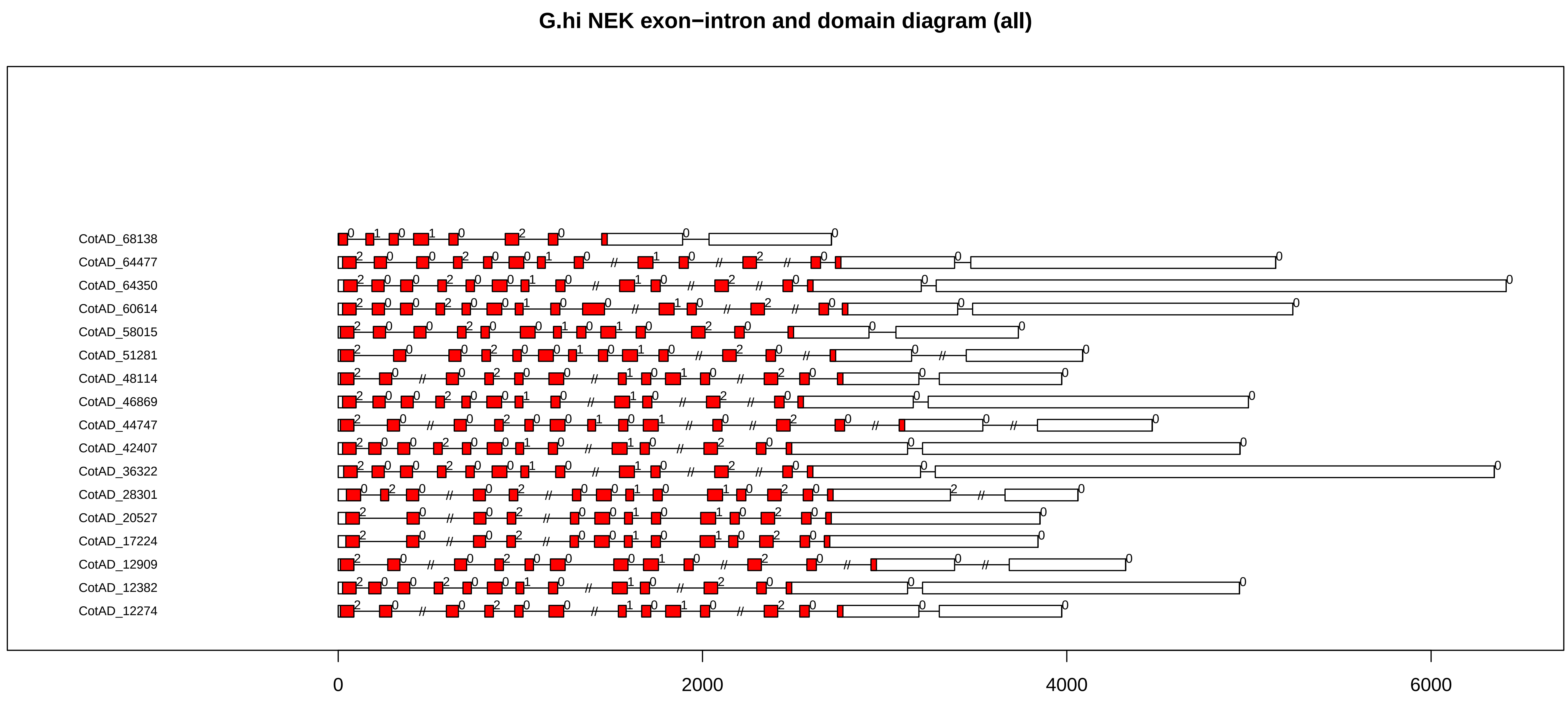

G.hi NEK exon−intron and domain diagram (all)
CotAD_68138
CotAD_64477
CotAD_64350
CotAD_60614
CotAD_58015
CotAD_51281
CotAD_48114
CotAD_46869
CotAD_44747
CotAD_42407
CotAD_36322
CotAD_28301
CotAD_20527
CotAD_17224
CotAD_12909
CotAD_12382
CotAD_12274
0
2000
4000
6000

G.hi PEK_GCN2 exon−intron and domain diagram (all)

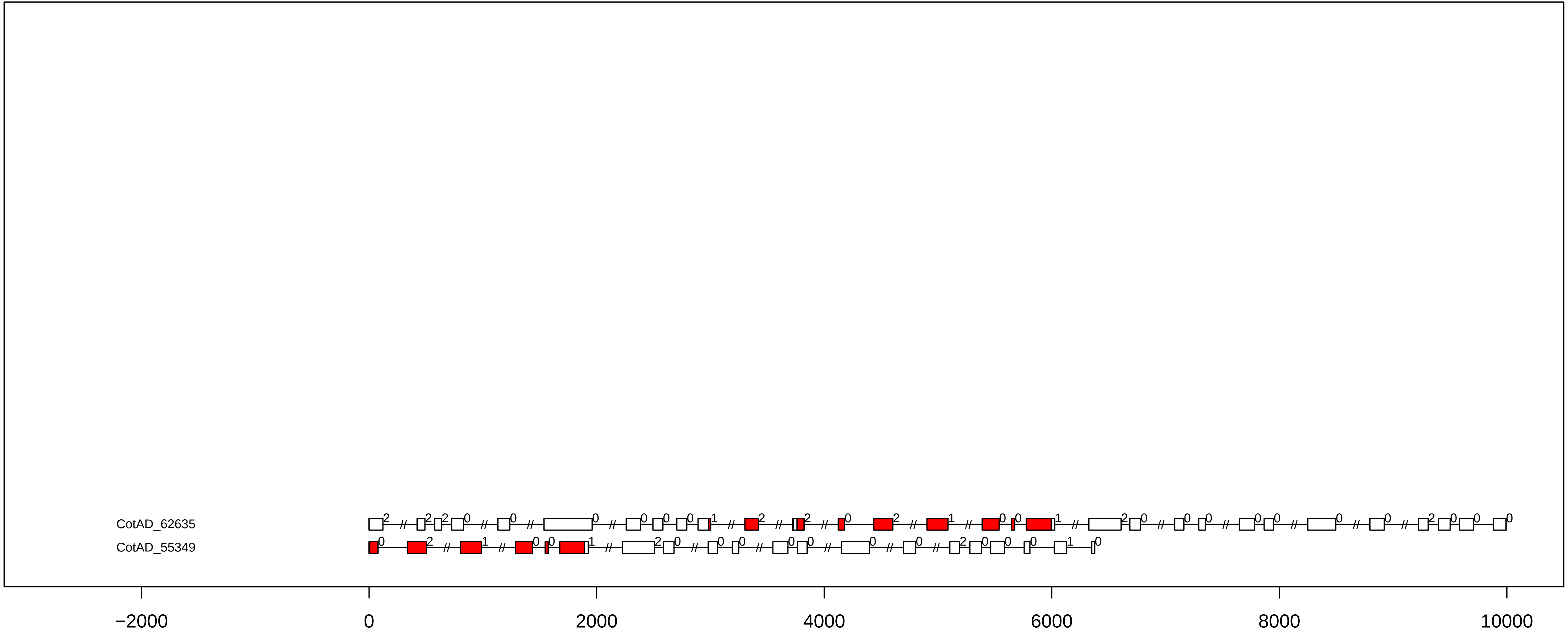

G.hi PEK_PEK exon−intron and domain diagram (all)

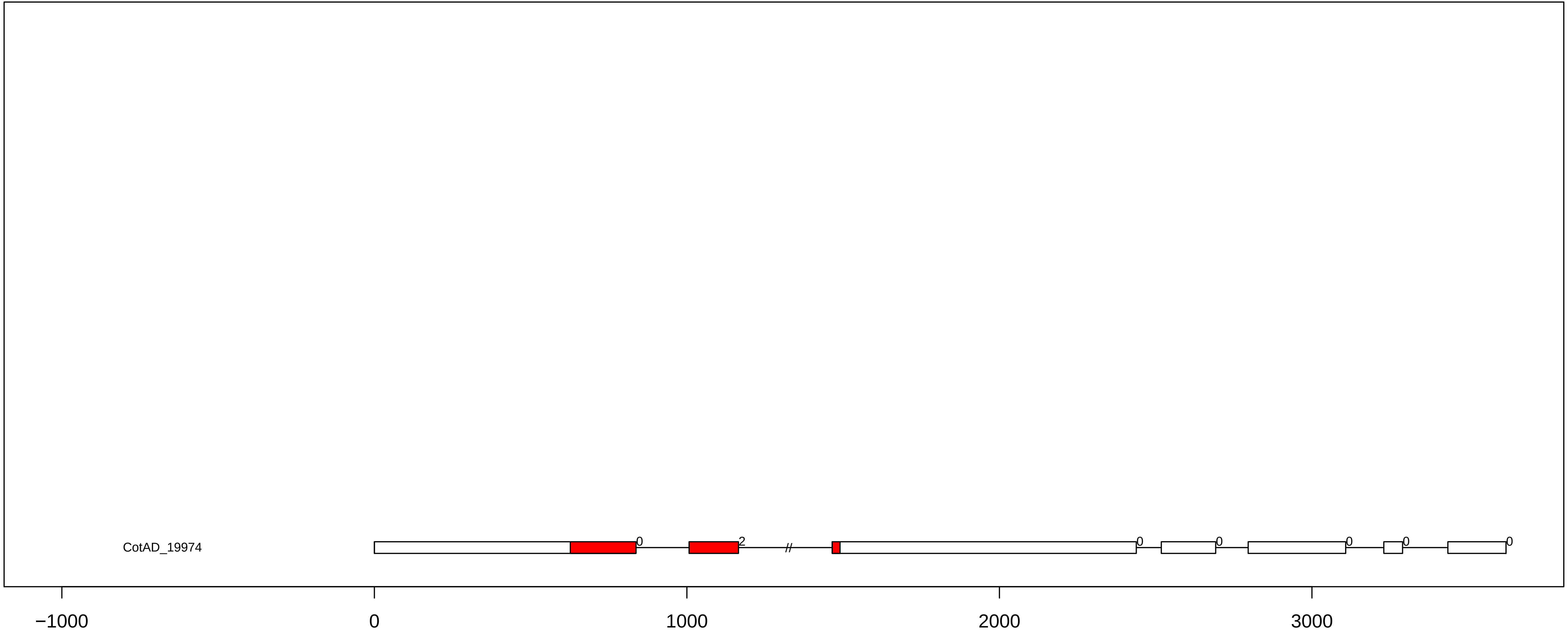

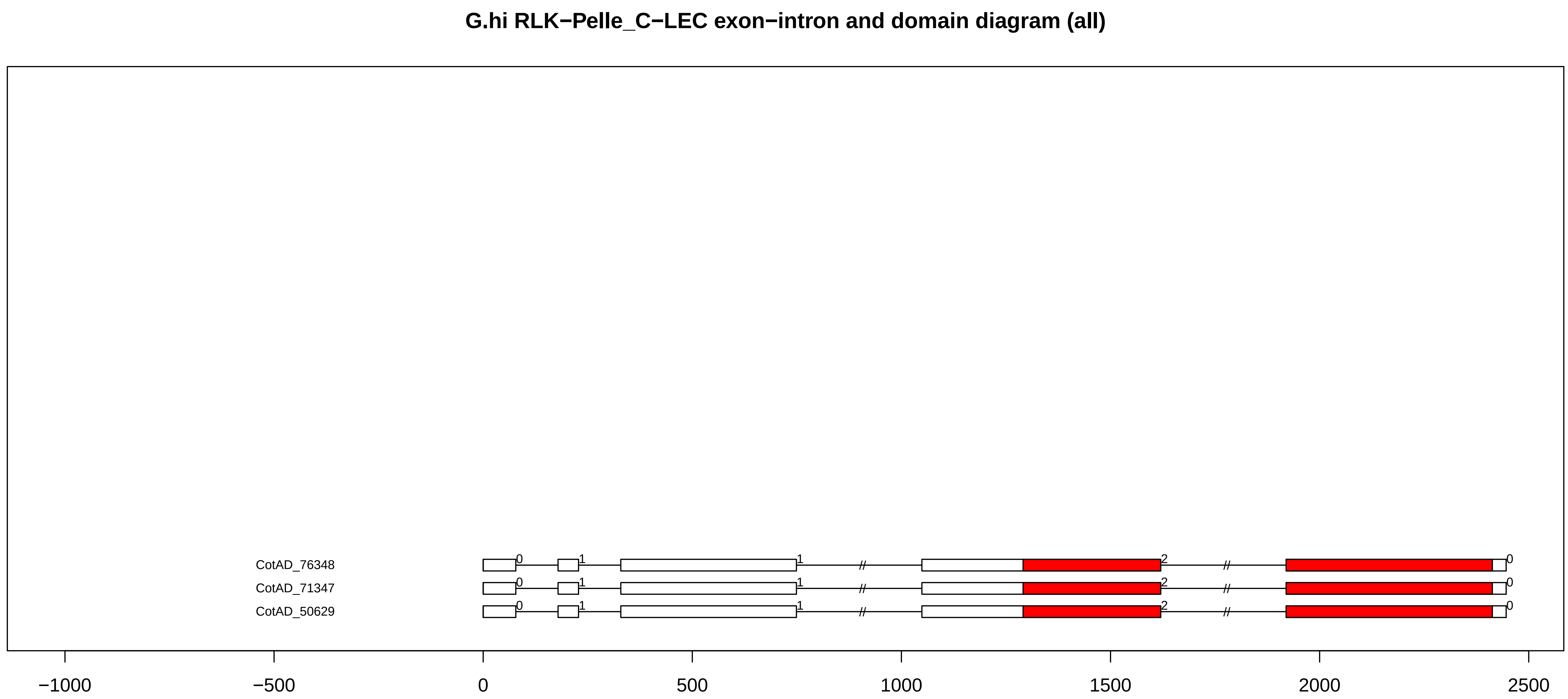

G.hi RLK−Pelle_C−LEC exon−intron and domain diagram (all)
CotAD_76348
CotAD_71347
CotAD_50629
0
1
1
2
0
−1000
−500
0
500
1000
1500
2000
2500

G.hi RLK−Pelle_CR4L exon−intron and domain diagram (part 1)

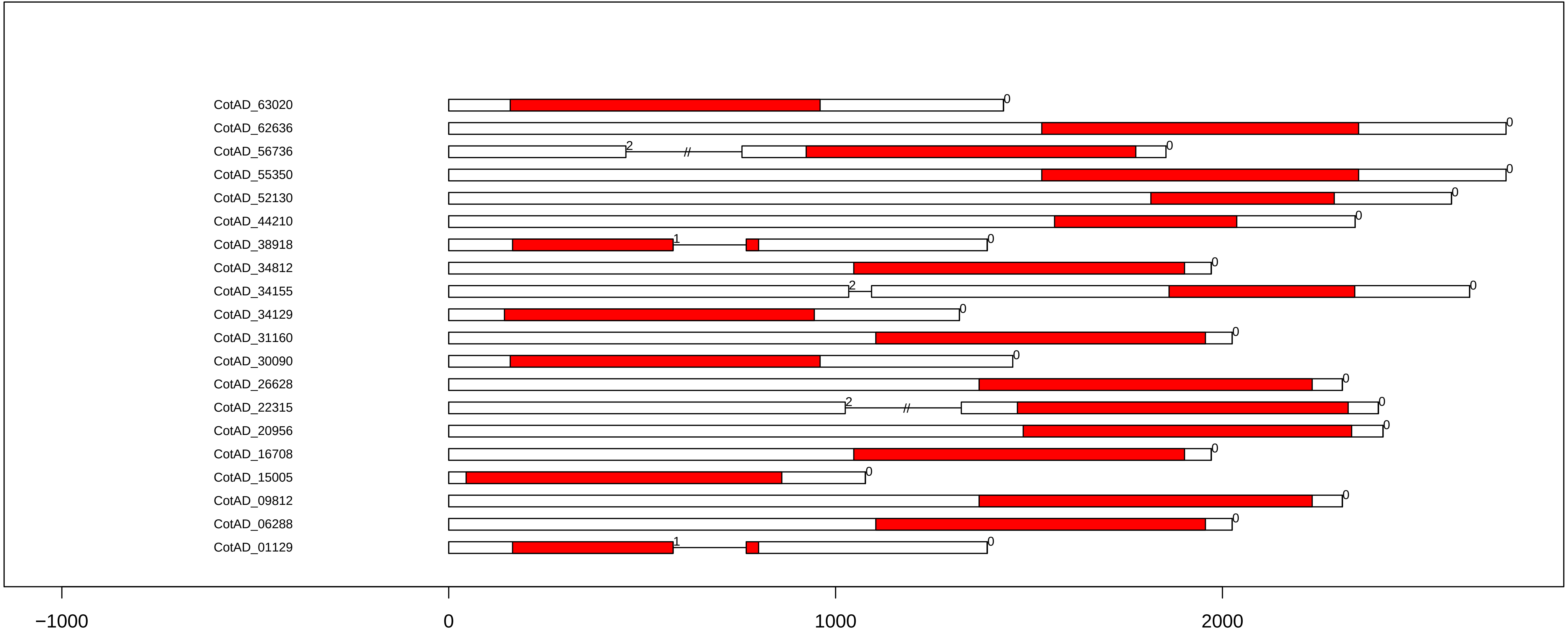

G.hi RLK−Pelle_CR4L exon−intron and domain diagram (part 2)

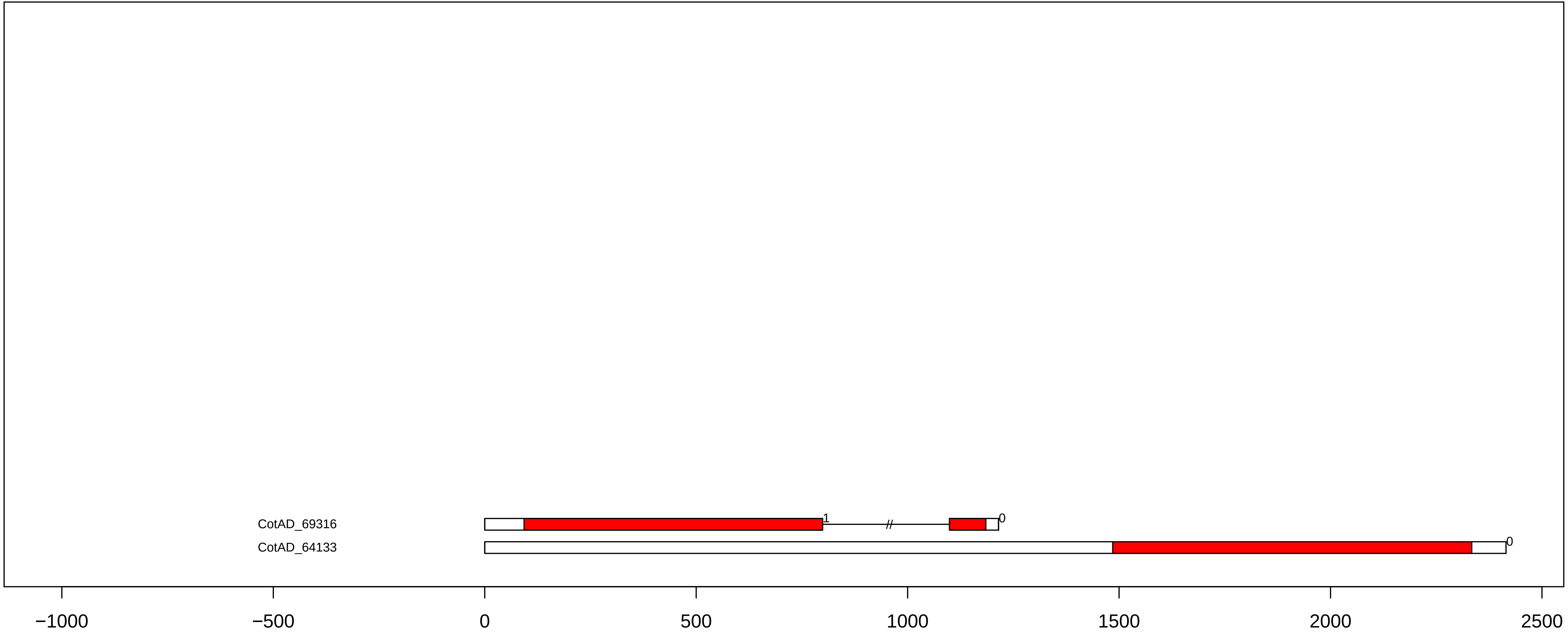

G.hi RLK−Pelle_CrRLK1L−1 exon−intron and domain diagram (part 1)

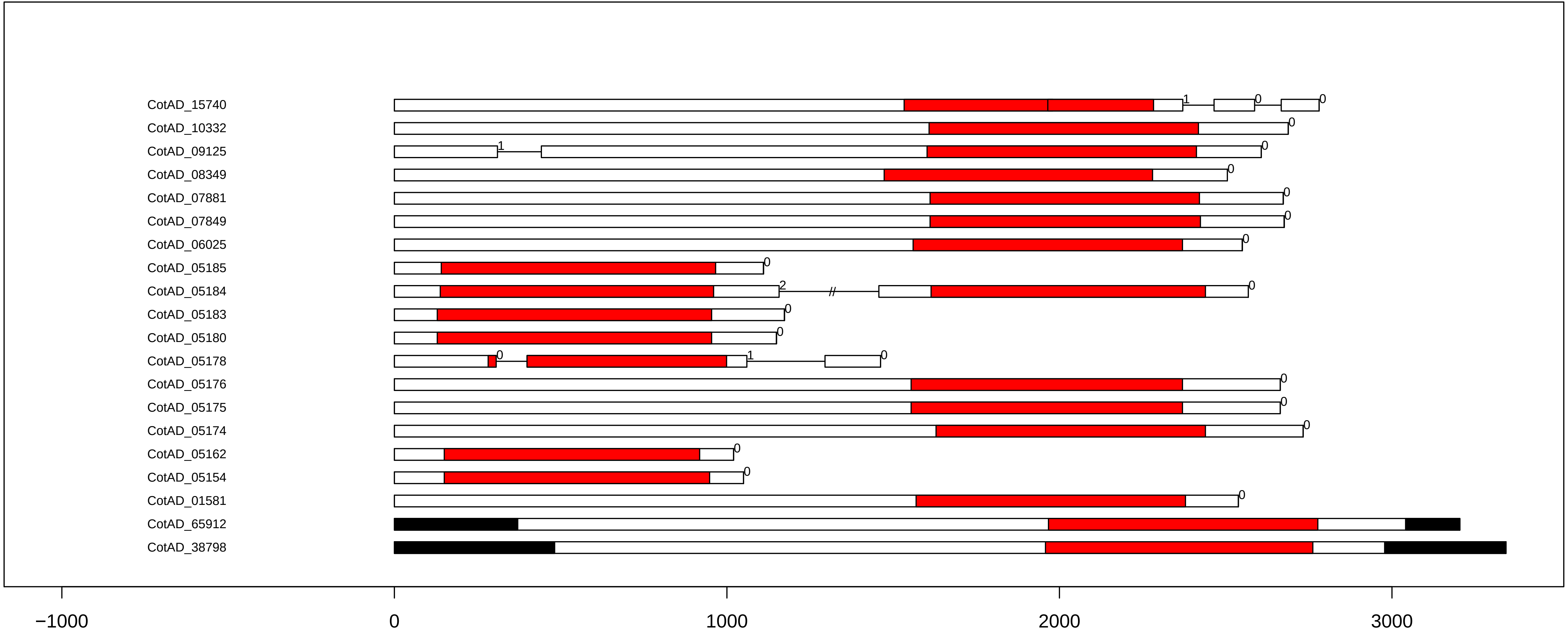

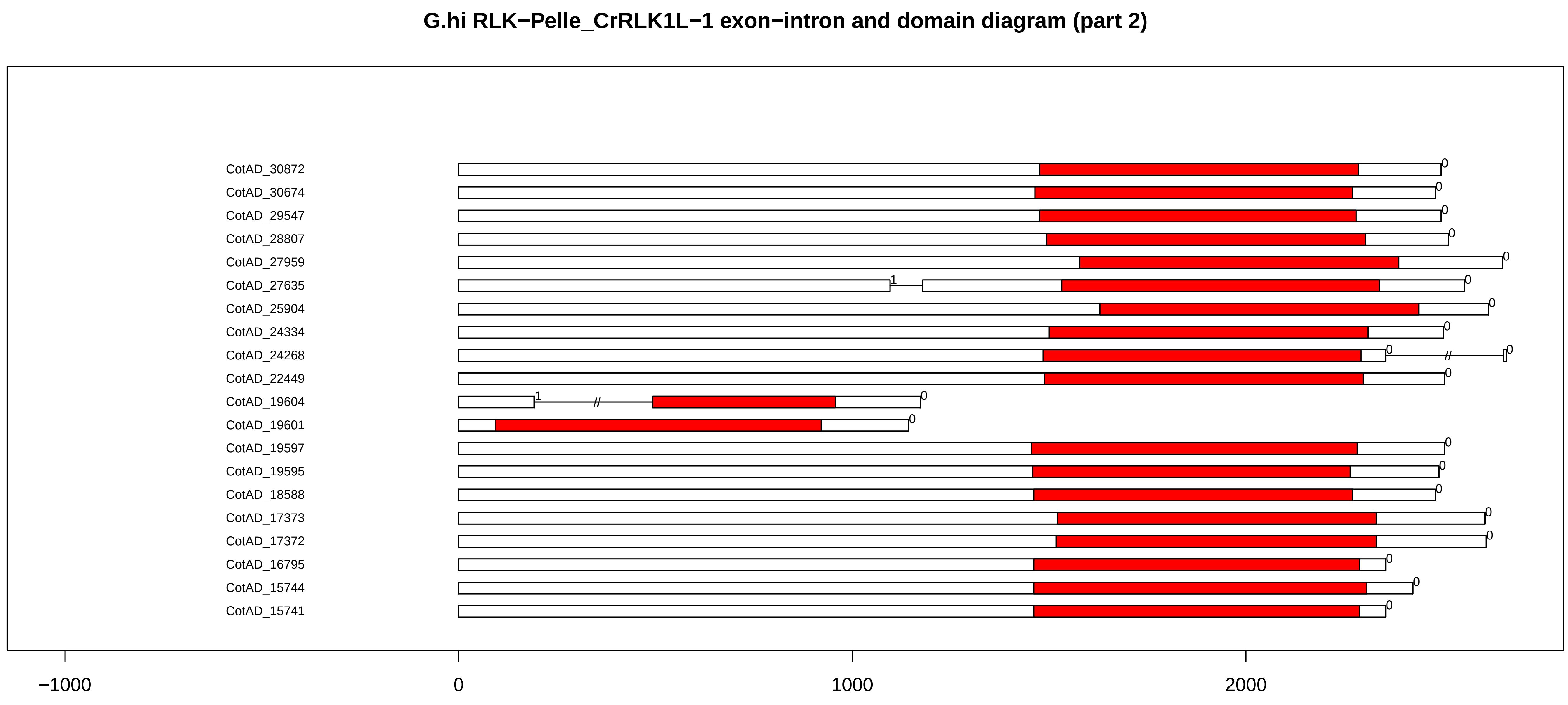
G.hi RLK−Pelle_CrRLK1L−1 exon−intron and domain diagram (part 2)
CotAD_30872
CotAD_30674
CotAD_29547
CotAD_28807
CotAD_27959
CotAD_27635
CotAD_25904
CotAD_24334
CotAD_24268
CotAD_22449
CotAD_19604
CotAD_19601
CotAD_19597
CotAD_19595
CotAD_18588
CotAD_17373
CotAD_17372
CotAD_16795
CotAD_15744
CotAD_15741
−1000
0
1000
2000

G.hi RLK−Pelle_CrRLK1L−1 exon−intron and domain diagram (part 3)

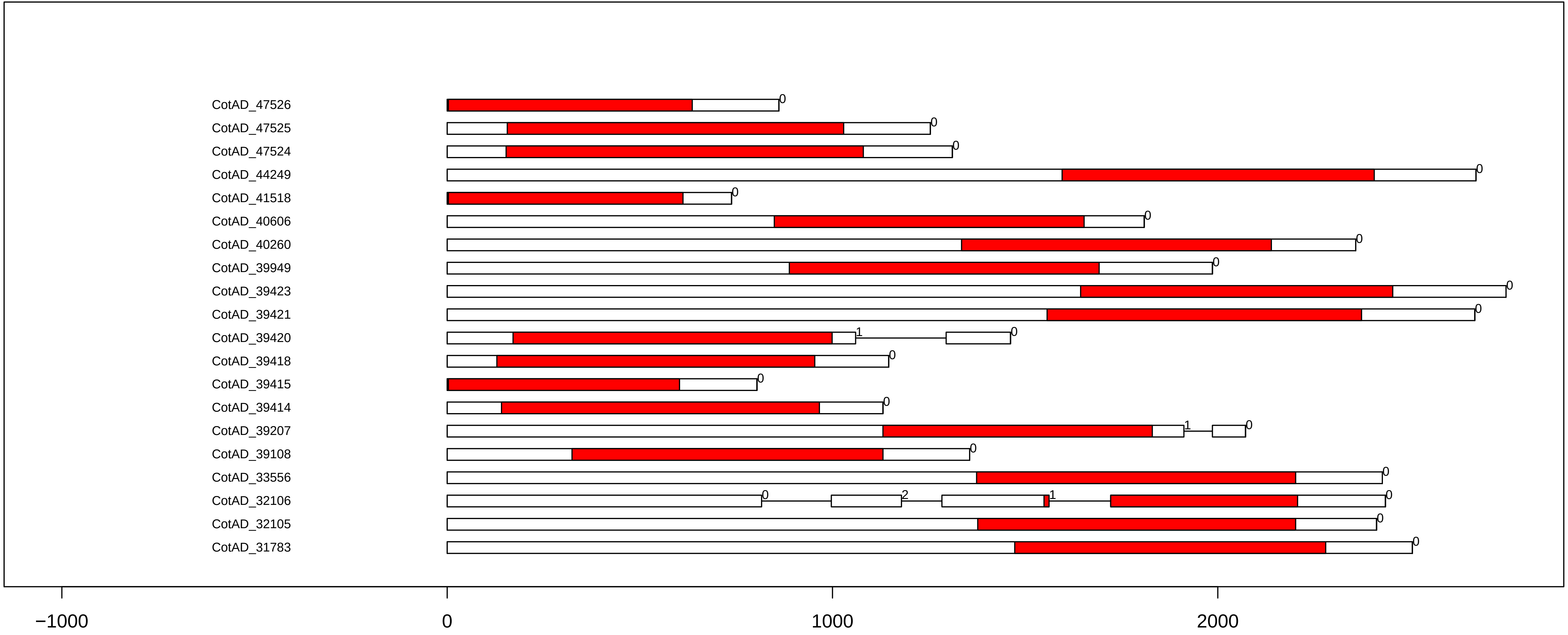

G.hi RLK−Pelle_CrRLK1L−1 exon−intron and domain diagram (part 4)

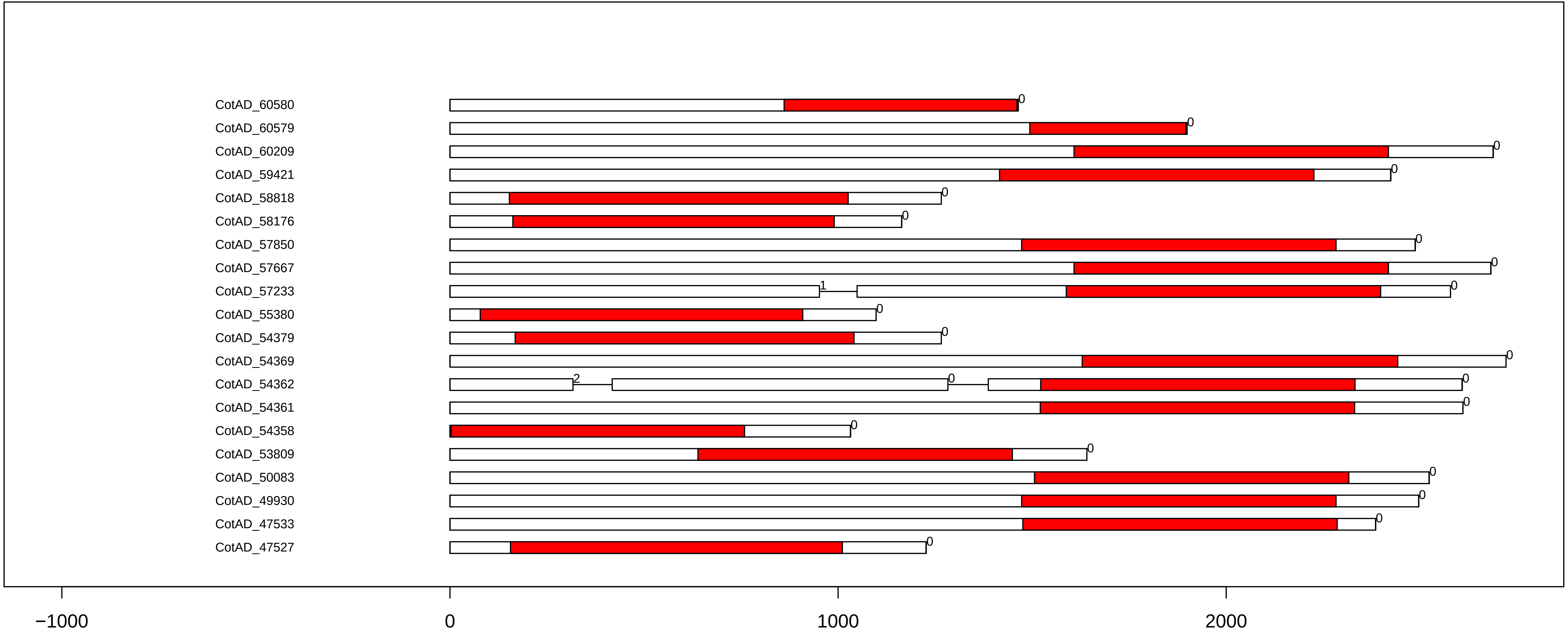

G.hi RLK−Pelle_CrRLK1L−1 exon−intron and domain diagram (part 5)

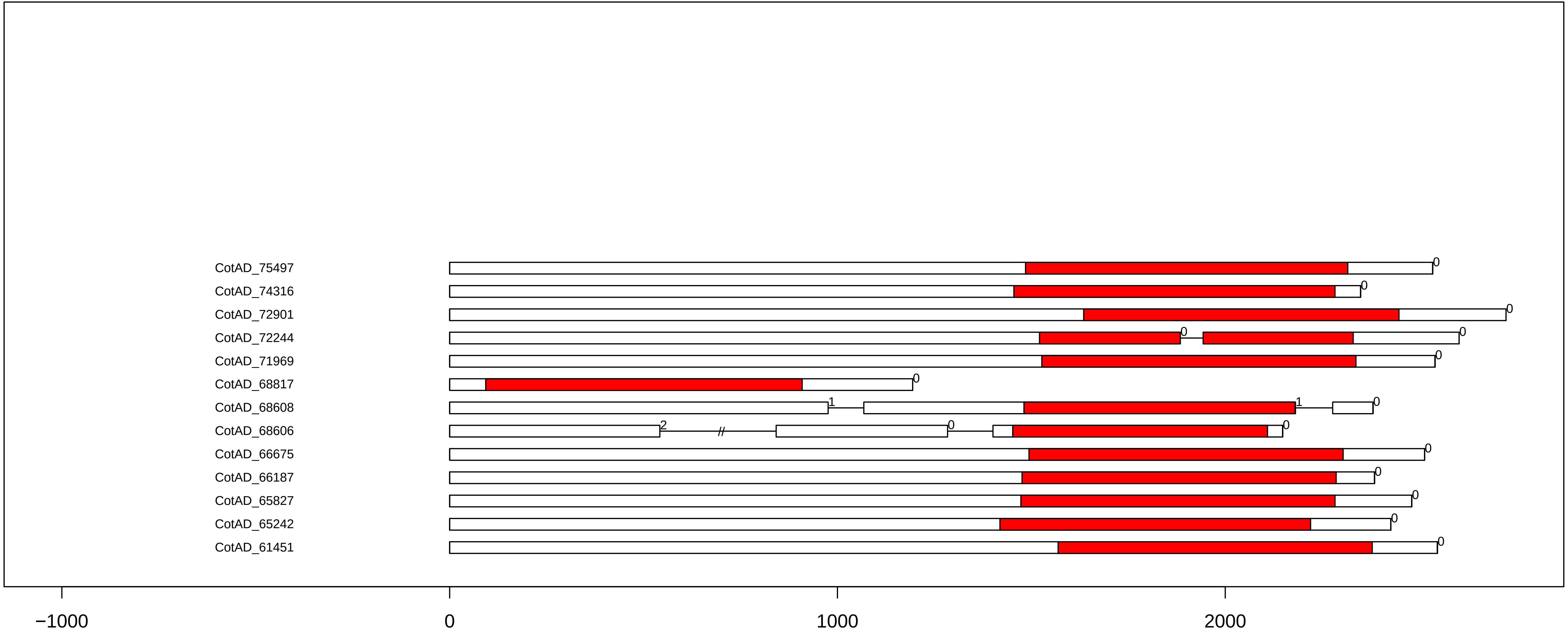

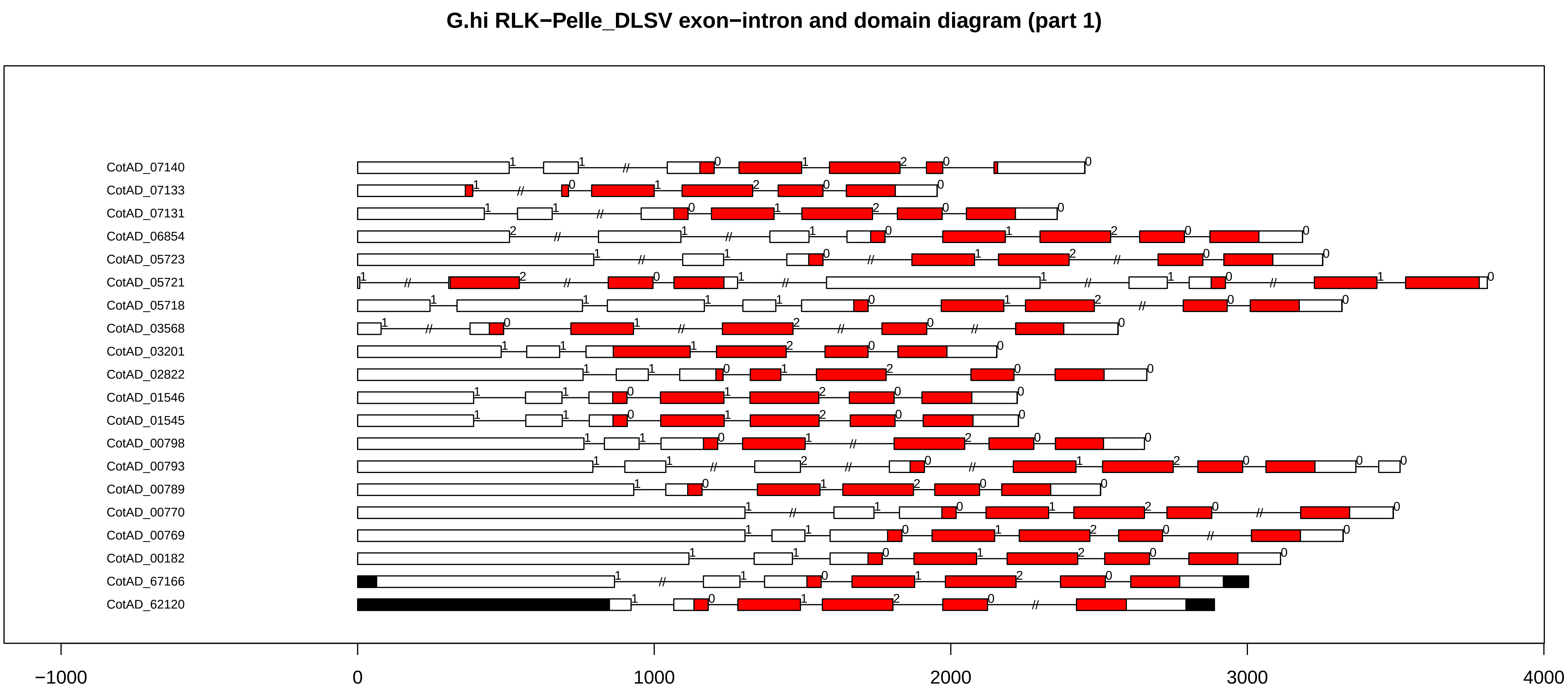
G.hi RLK−Pelle_DLSV exon−intron and domain diagram (part 1)
CotAD_07140
CotAD_07133
CotAD_07131
CotAD_06854
CotAD_05723
CotAD_05721
CotAD_05718
CotAD_03568
CotAD_03201
CotAD_02822
CotAD_01546
CotAD_01545
CotAD_00798
CotAD_00793
CotAD_00789
CotAD_00770
CotAD_00769
CotAD_00182
CotAD_67166
CotAD_62120
−1000
0
1000
2000
3000
4000

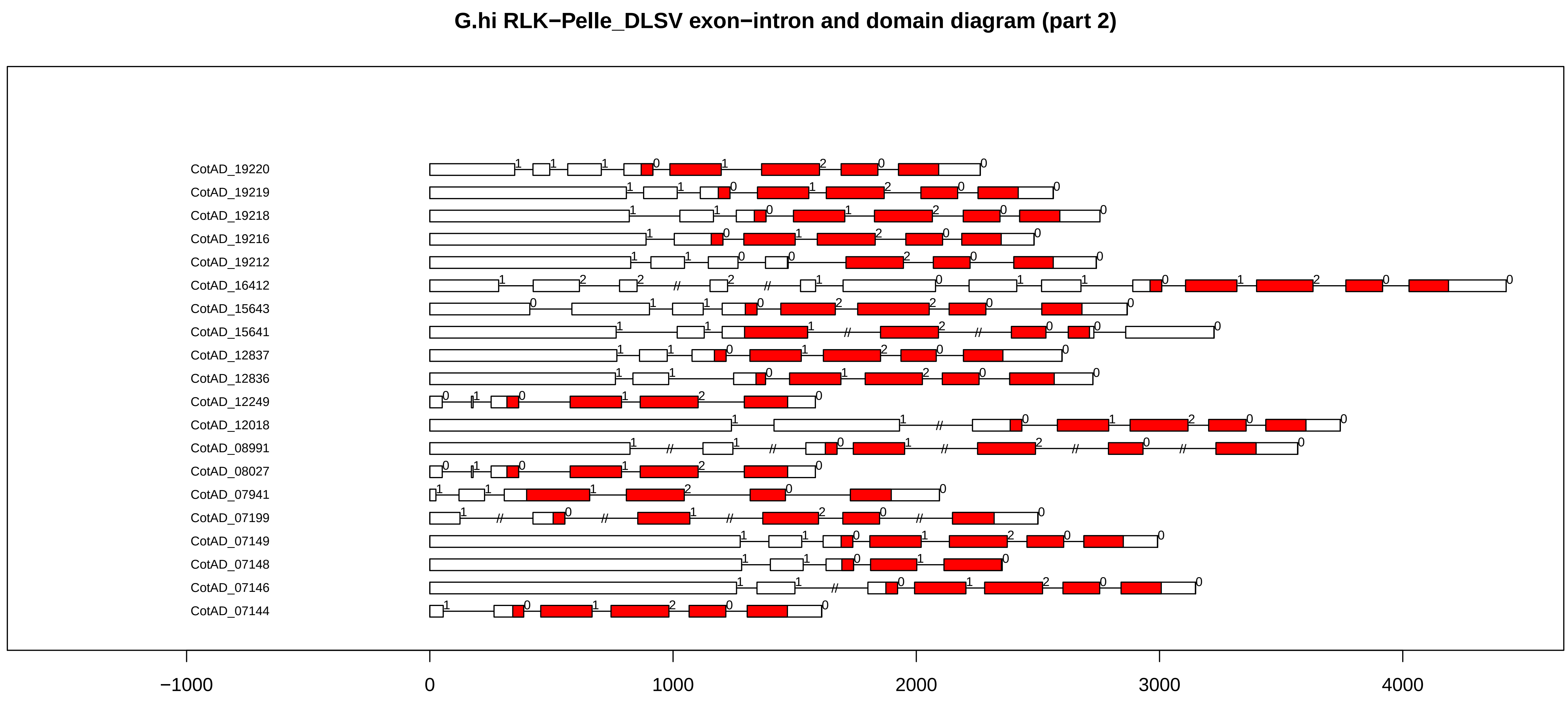
G.hi RLK−Pelle_DLSV exon−intron and domain diagram (part 2)
CotAD_19220
CotAD_19219
CotAD_19218
CotAD_19216
CotAD_19212
CotAD_16412
CotAD_15643
CotAD_15641
CotAD_12837
CotAD_12836
CotAD_12249
CotAD_12018
CotAD_08991
CotAD_08027
CotAD_07941
CotAD_07199
CotAD_07149
CotAD_07148
CotAD_07146
CotAD_07144
−1000
0
1000
2000
3000
4000

G.hi RLK−Pelle_DLSV exon−intron and domain diagram (part 3)

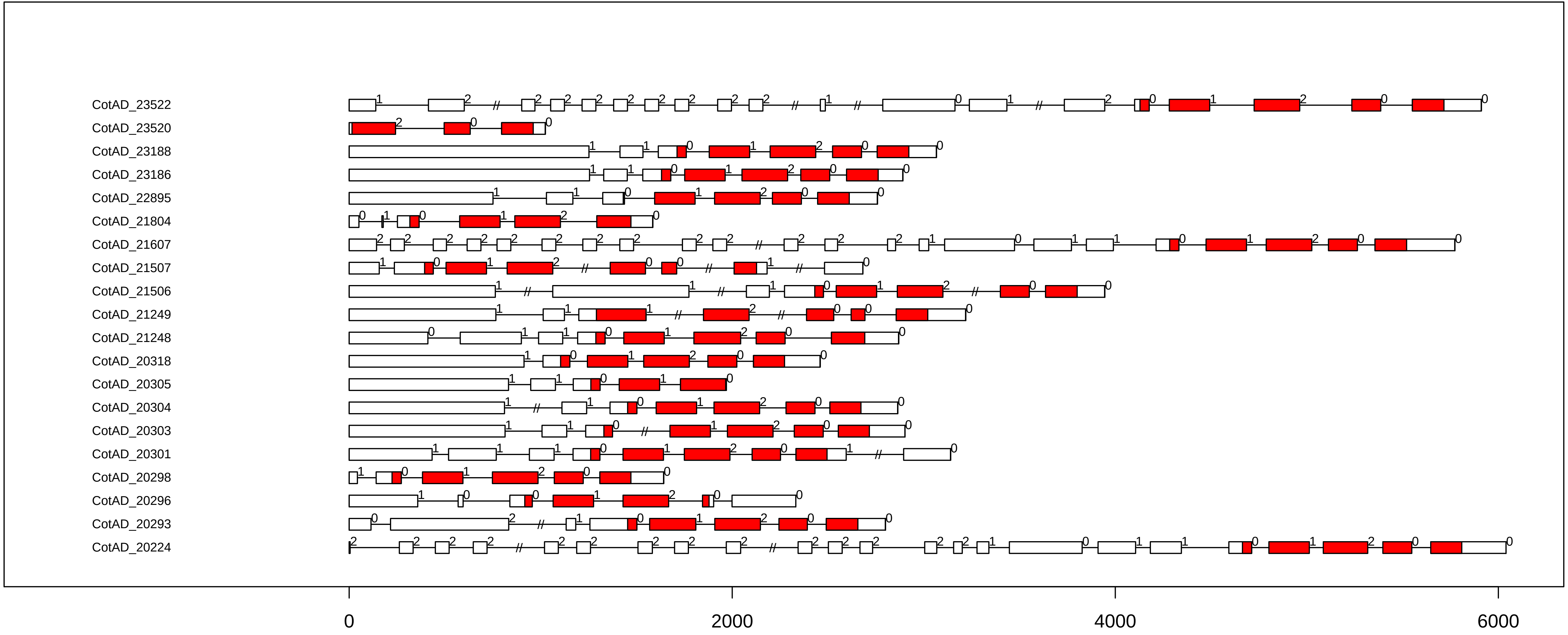

G.hi RLK−Pelle_DLSV exon−intron and domain diagram (part 4)

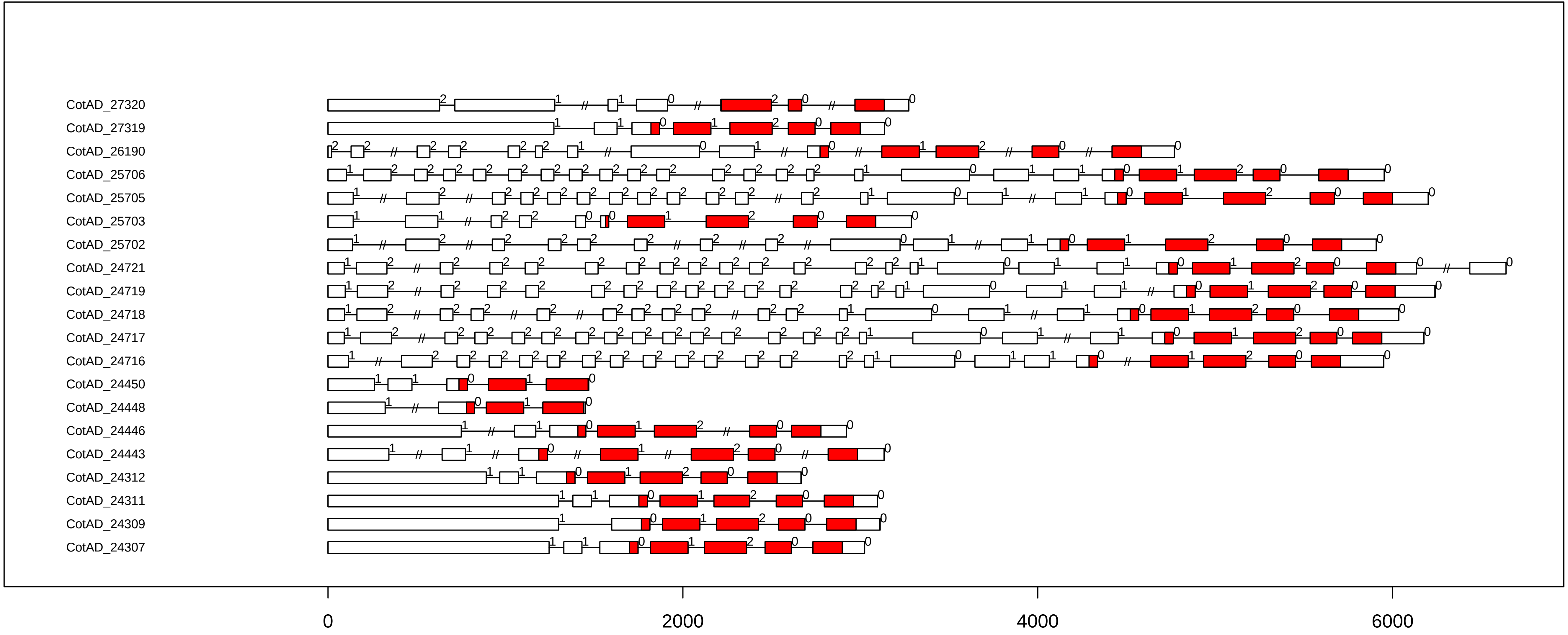

G.hi RLK−Pelle_DLSV exon−intron and domain diagram (part 5)

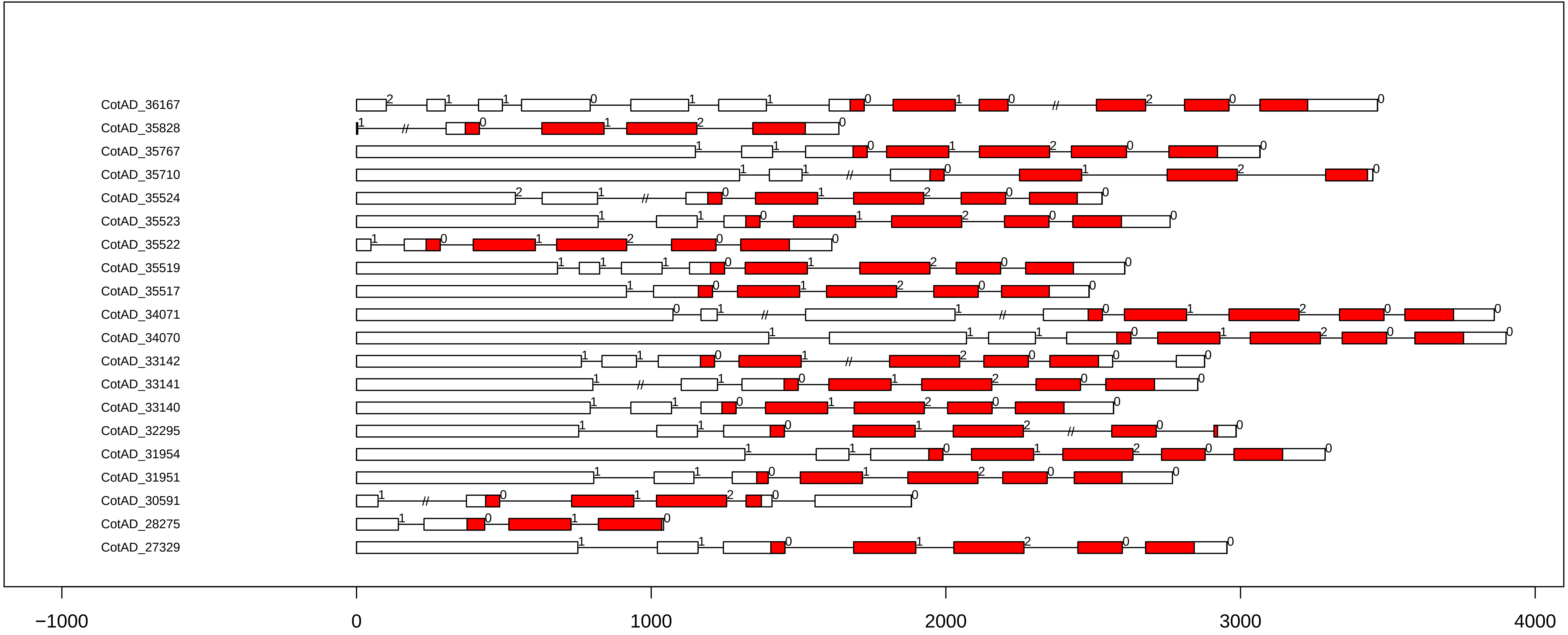

G.hi RLK−Pelle_DLSV exon−intron and domain diagram (part 6)

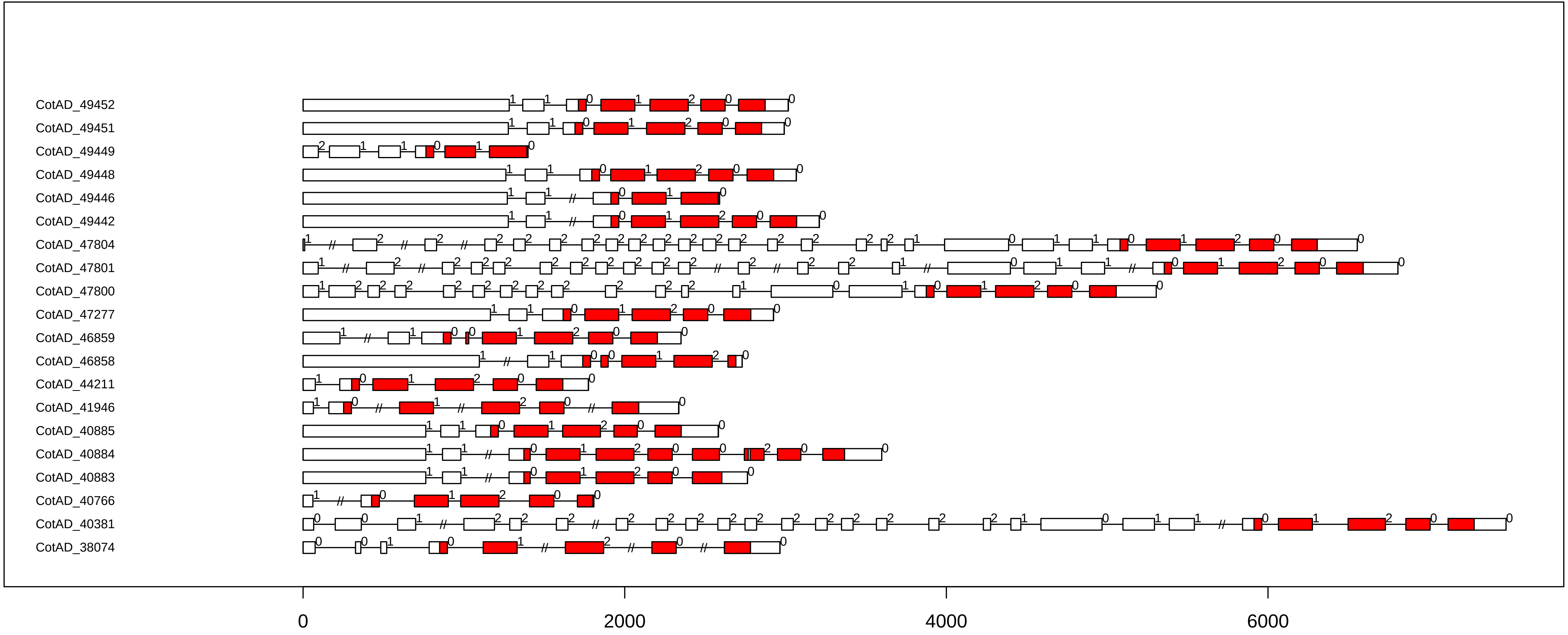

G.hi RLK−Pelle_DLSV exon−intron and domain diagram (part 7)

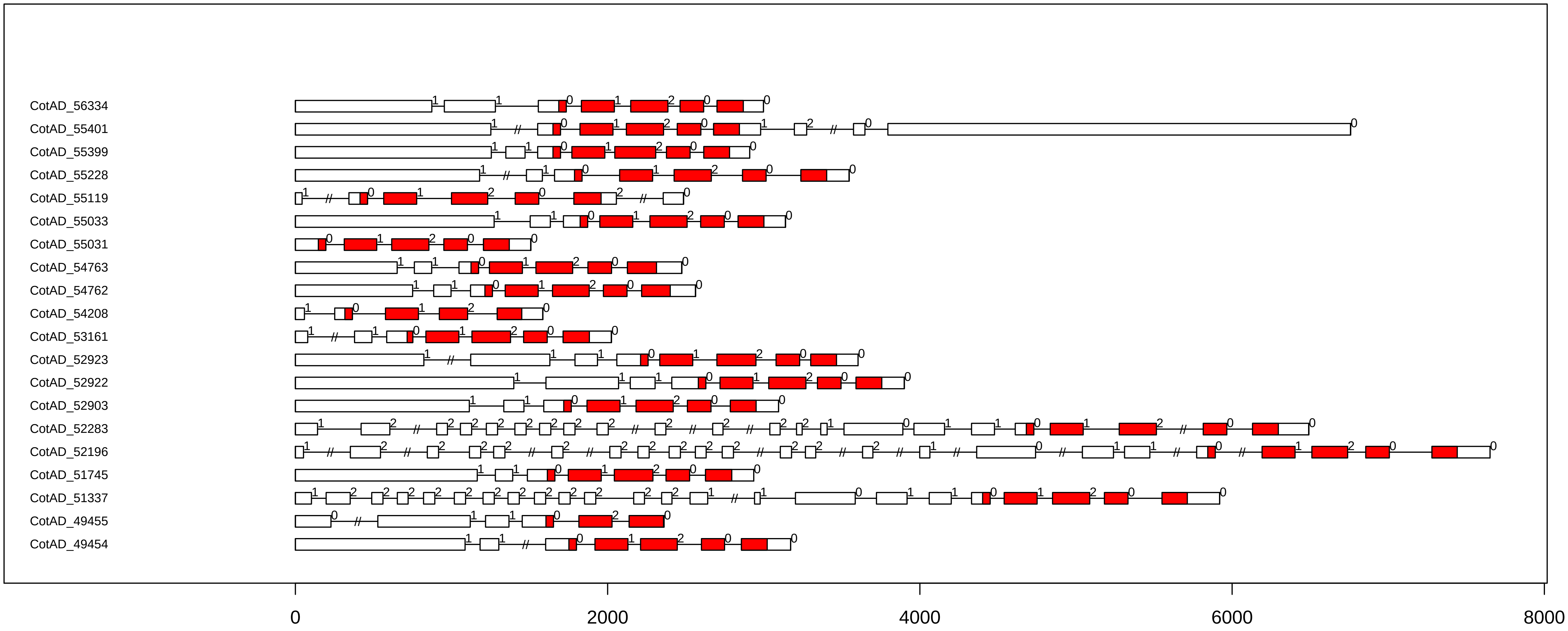

G.hi RLK−Pelle_DLSV exon−intron and domain diagram (part 8)

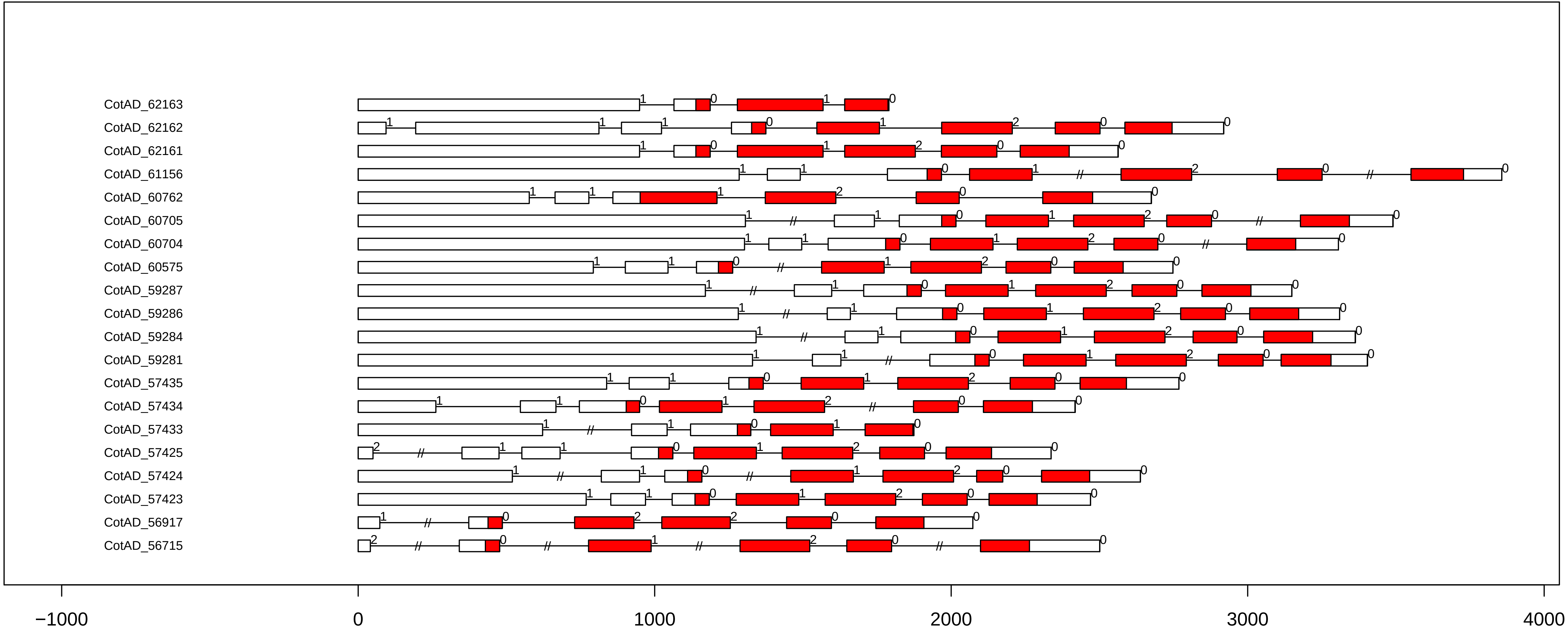

G.hi RLK−Pelle_DLSV exon−intron and domain diagram (part 9)

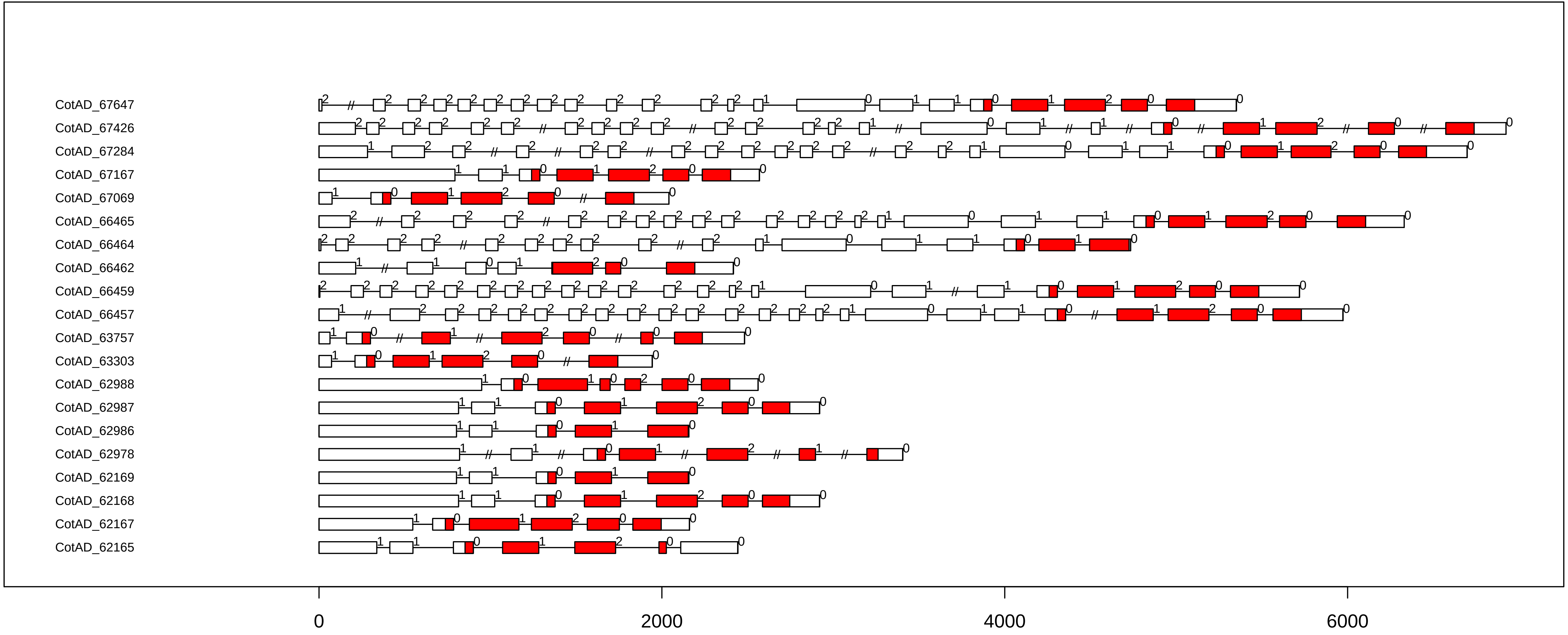

G.hi RLK−Pelle_DLSV exon−intron and domain diagram (part 10)

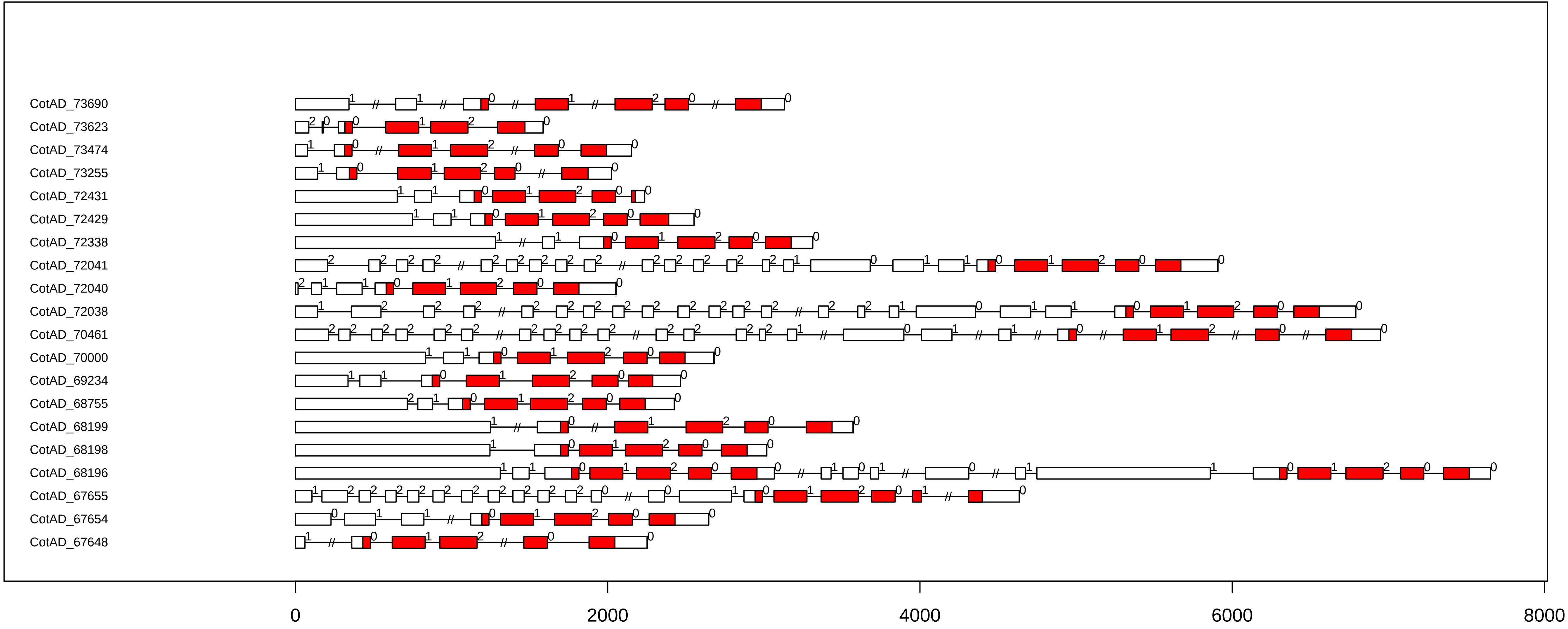

G.hi RLK−Pelle_DLSV exon−intron and domain diagram (part 11)

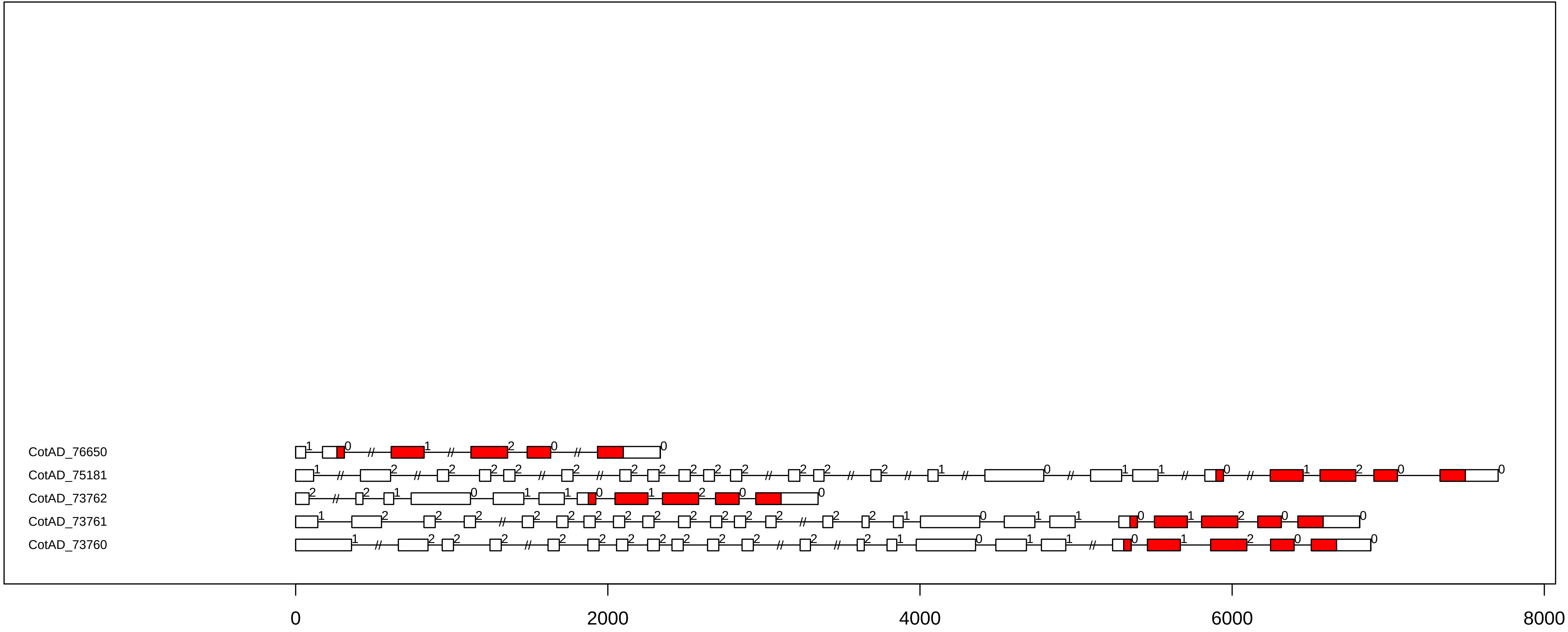

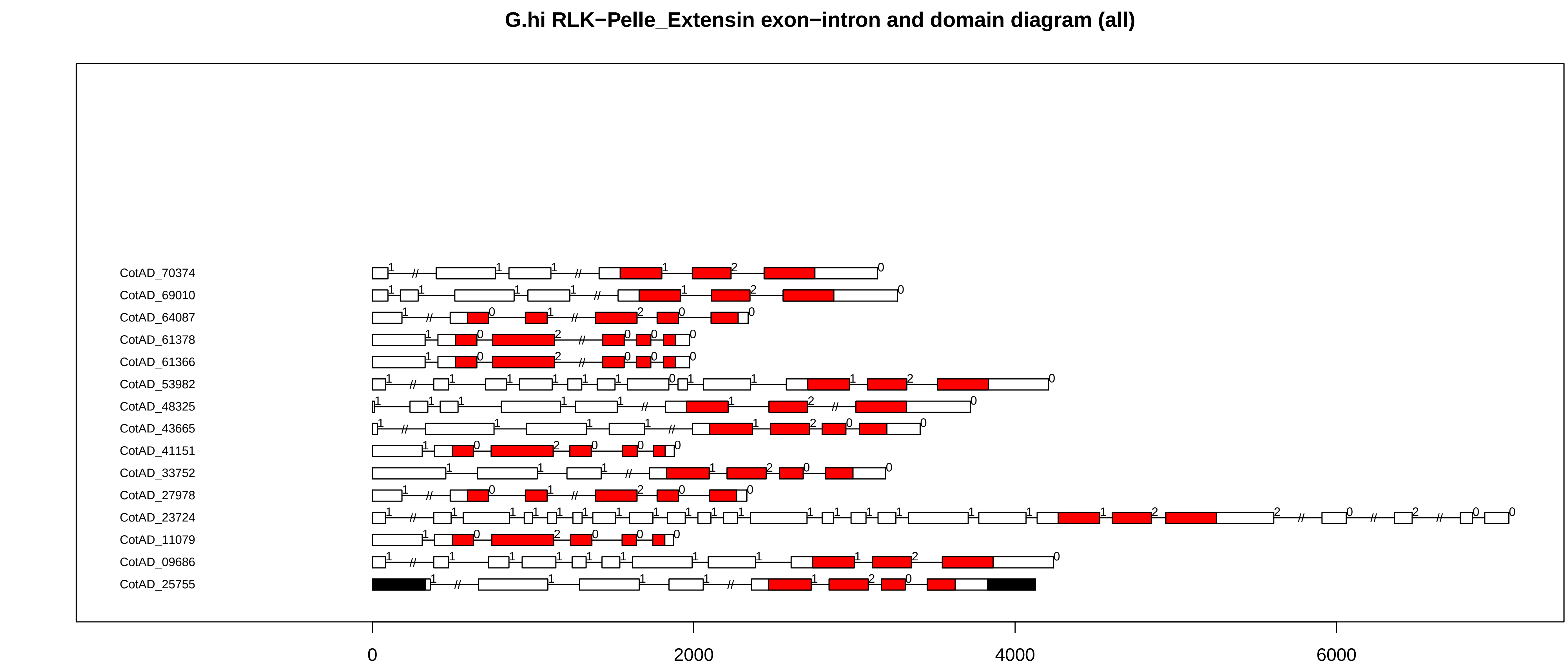
G.hi RLK−Pelle_Extensin exon−intron and domain diagram (all)
CotAD_70374
CotAD_69010
CotAD_64087
CotAD_61378
CotAD_61366
CotAD_53982
CotAD_48325
CotAD_43665
CotAD_41151
CotAD_33752
CotAD_27978
CotAD_23724
CotAD_11079
CotAD_09686
CotAD_25755
0
2000
4000
6000

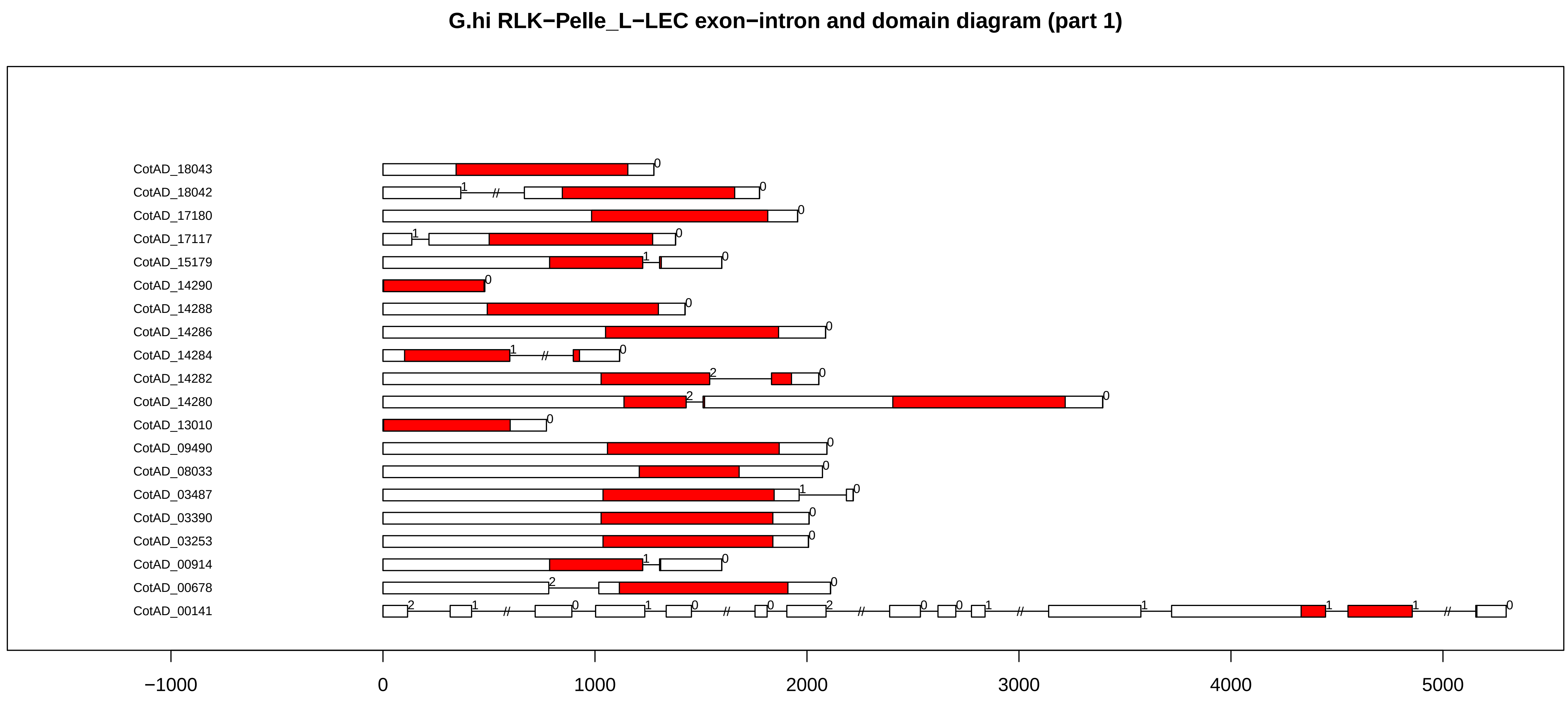
G.hi RLK−Pelle_L−LEC exon−intron and domain diagram (part 1)
CotAD_18043
CotAD_18042
CotAD_17180
CotAD_17117
CotAD_15179
CotAD_14290
CotAD_14288
CotAD_14286
CotAD_14284
CotAD_14282
CotAD_14280
CotAD_13010
CotAD_09490
CotAD_08033
CotAD_03487
CotAD_03390
CotAD_03253
CotAD_00914
CotAD_00678
CotAD_00141
−1000
0
1000
2000
3000
4000
5000

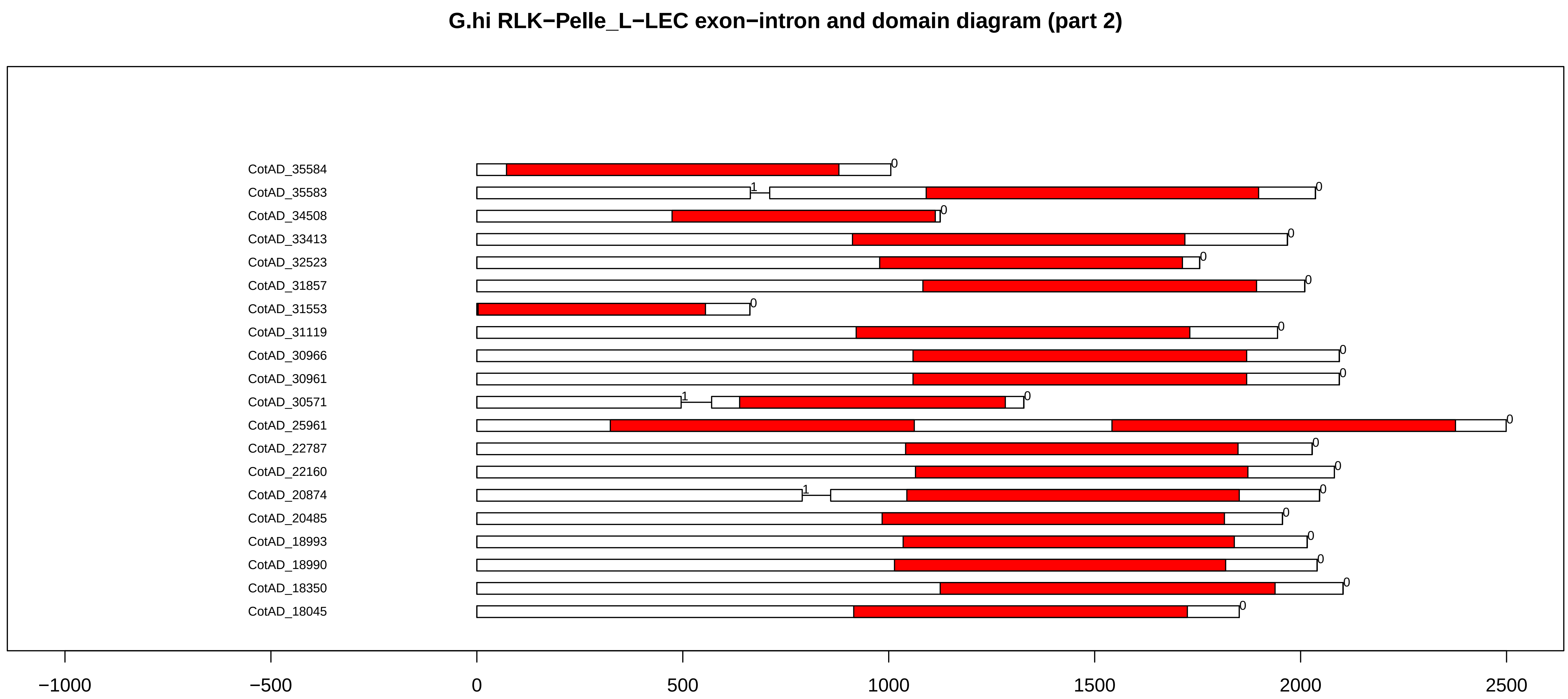
G.hi RLK−Pelle_L−LEC exon−intron and domain diagram (part 2)
CotAD_35584
CotAD_35583
CotAD_34508
CotAD_33413
CotAD_32523
CotAD_31857
CotAD_31553
CotAD_31119
CotAD_30966
CotAD_30961
CotAD_30571
CotAD_25961
CotAD_22787
CotAD_22160
CotAD_20874
CotAD_20485
CotAD_18993
CotAD_18990
CotAD_18350
CotAD_18045
−1000
−500
0
500
1000
1500
2000
2500

G.hi RLK−Pelle_L−LEC exon−intron and domain diagram (part 3)

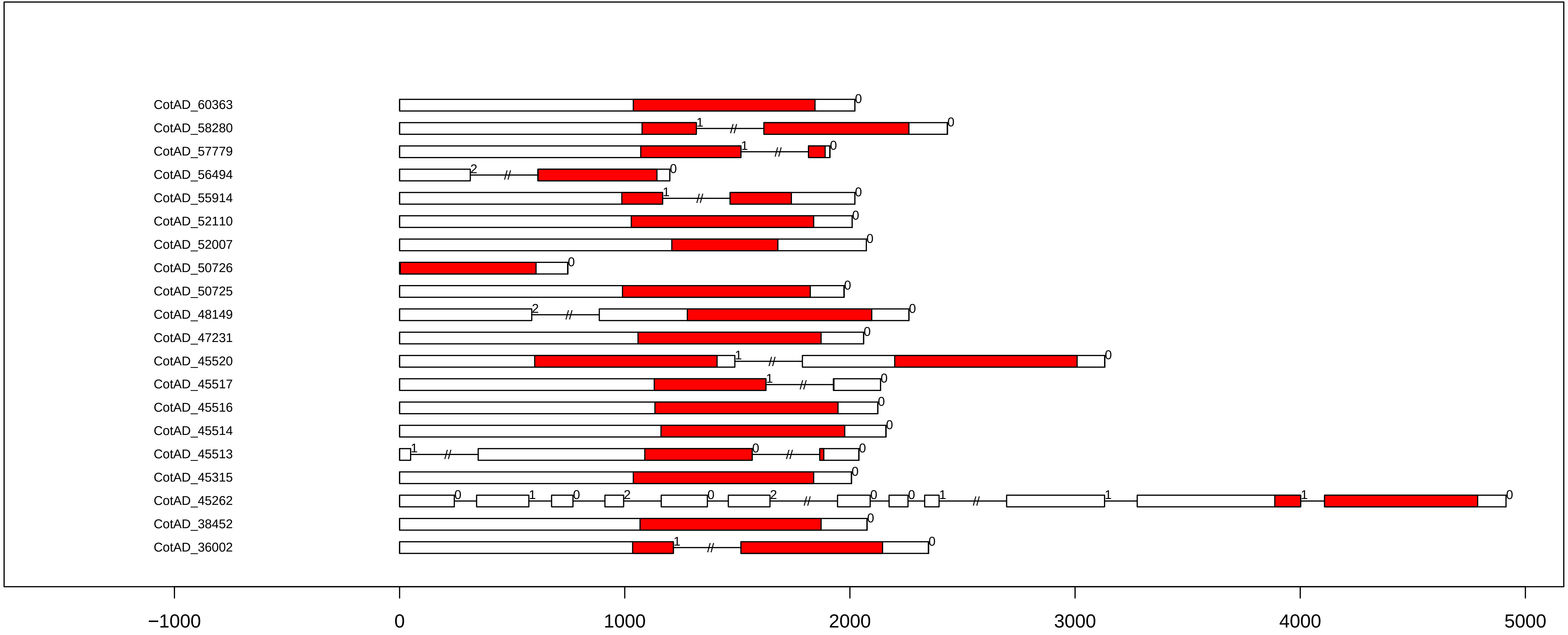

G.hi RLK−Pelle_L−LEC exon−intron and domain diagram (part 4)

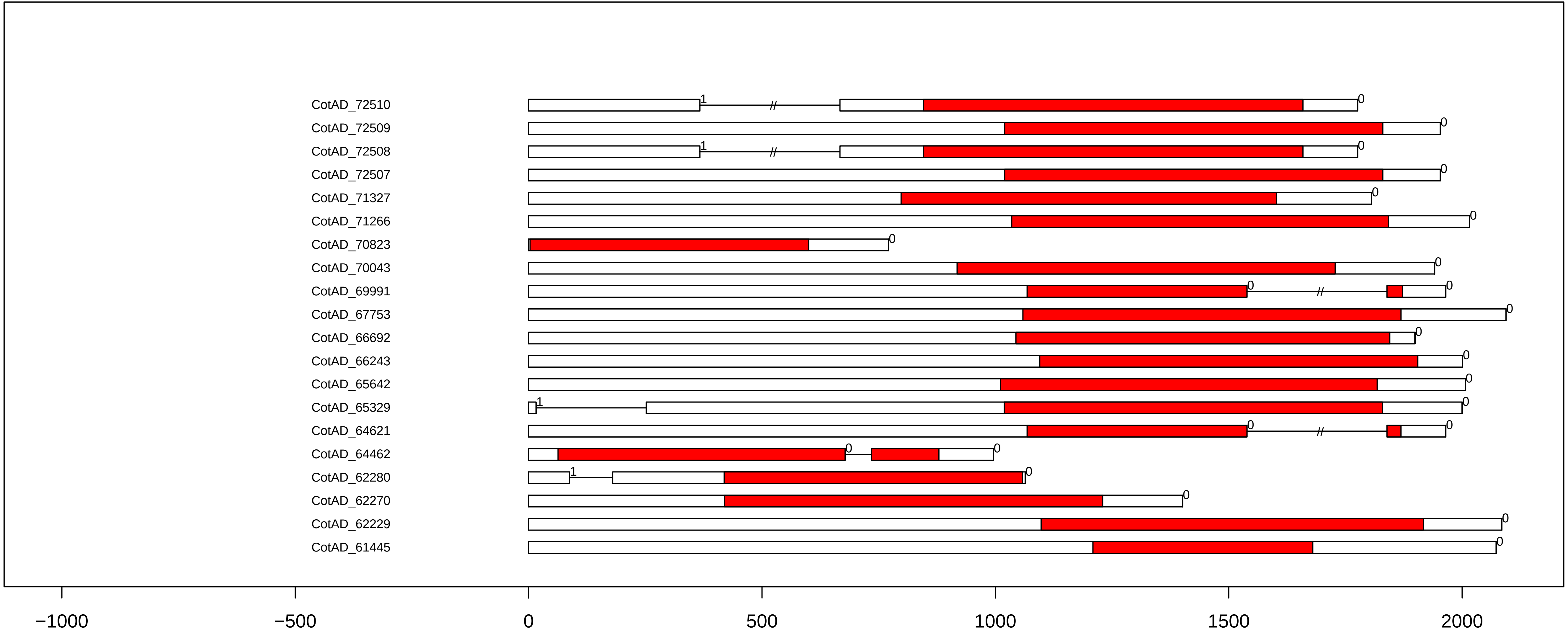

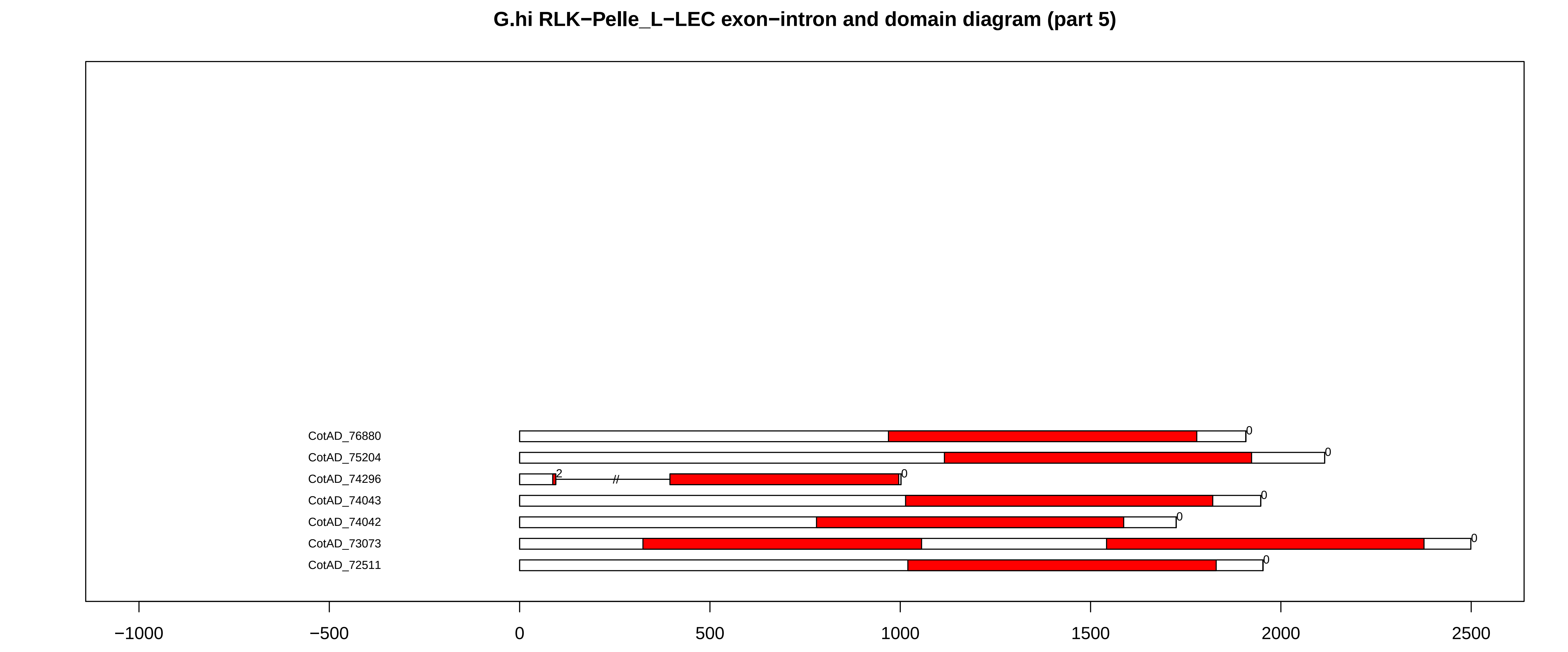

G.hi RLK−Pelle_L−LEC exon−intron and domain diagram (part 5)
CotAD_76880
CotAD_75204
CotAD_74296
CotAD_74043
CotAD_74042
CotAD_73073
CotAD_72511
0
0
2
0
0
0
0
0
−1000
−500
0
500
1000
1500
2000
2500

G.hi RLK−Pelle_LRK10L−2 exon−intron and domain diagram (part 1)

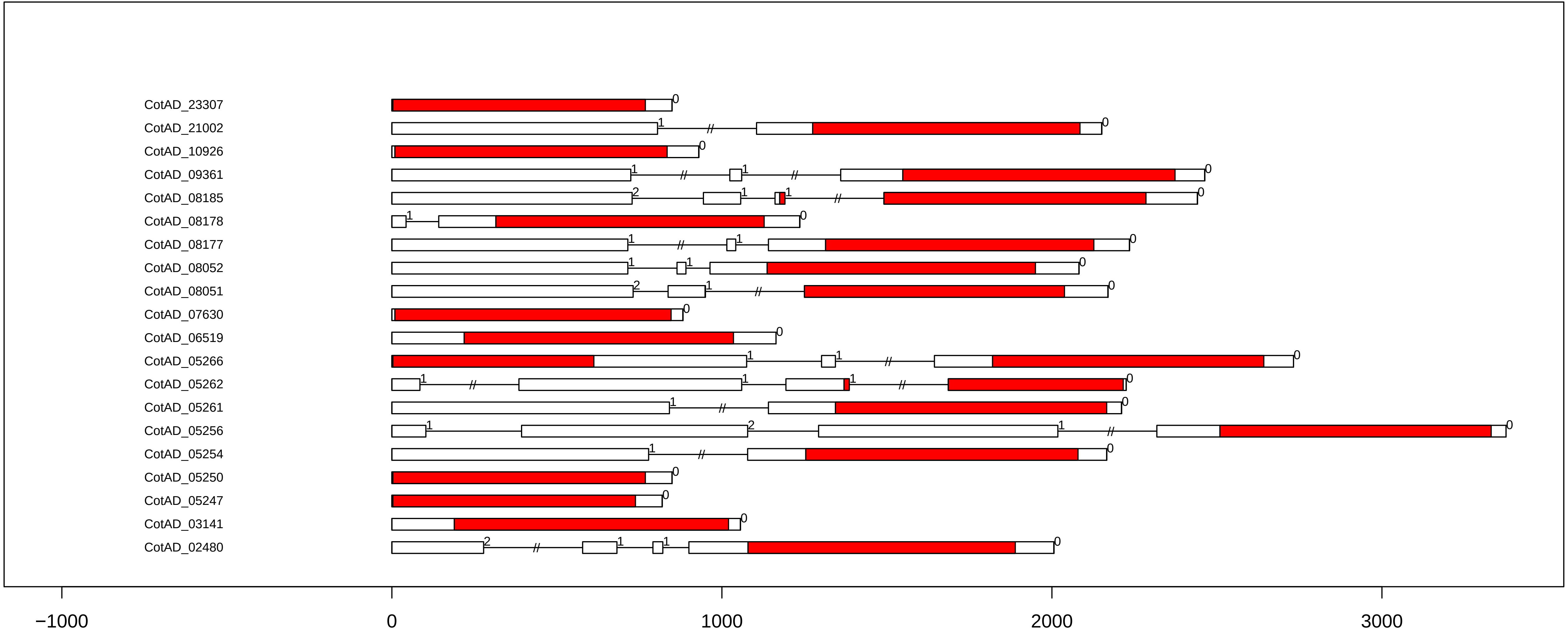

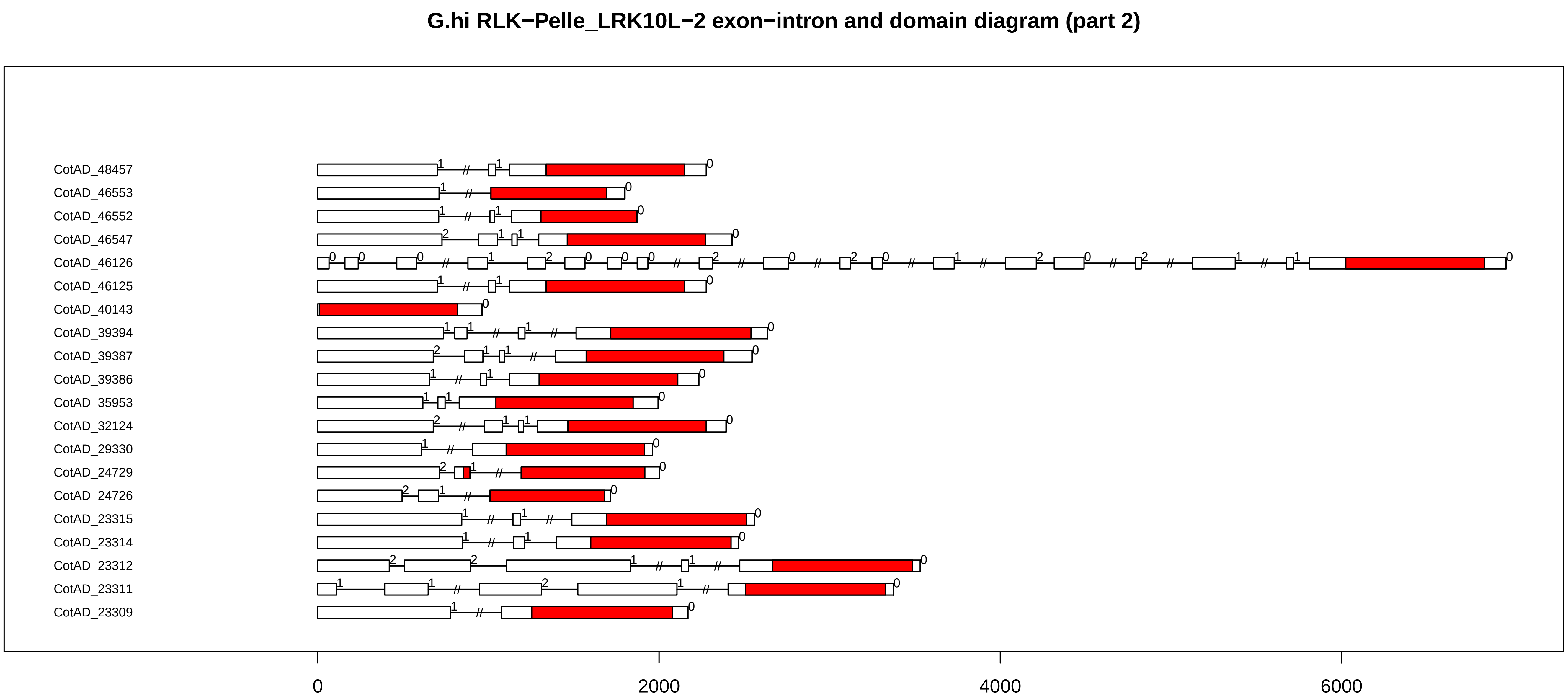
G.hi RLK−Pelle_LRK10L−2 exon−intron and domain diagram (part 2)
CotAD_48457
CotAD_46553
CotAD_46552
CotAD_46547
CotAD_46126
CotAD_46125
CotAD_40143
CotAD_39394
CotAD_39387
CotAD_39386
CotAD_35953
CotAD_32124
CotAD_29330
CotAD_24729
CotAD_24726
CotAD_23315
CotAD_23314
CotAD_23312
CotAD_23311
CotAD_23309
0
2000
4000
6000

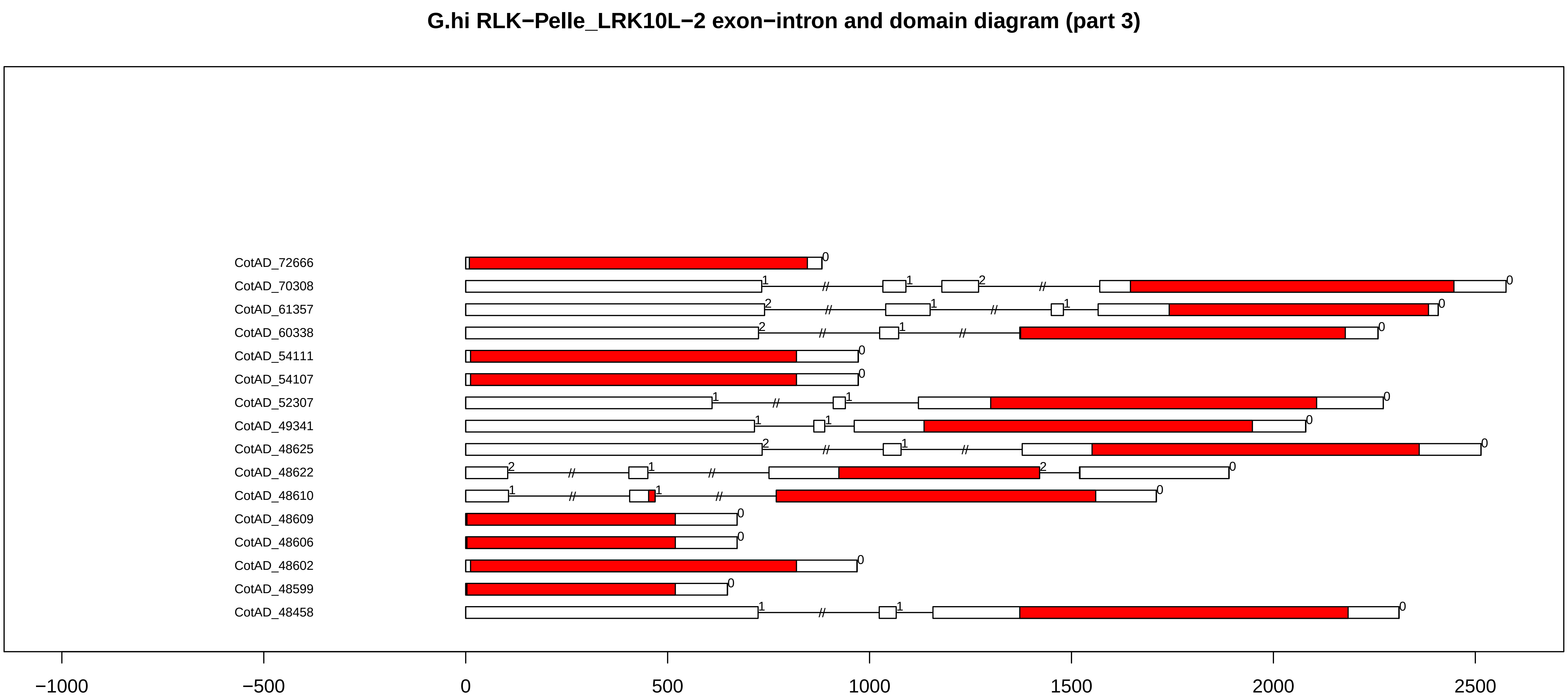
G.hi RLK−Pelle_LRK10L−2 exon−intron and domain diagram (part 3)
CotAD_72666
CotAD_70308
CotAD_61357
CotAD_60338
CotAD_54111
CotAD_54107
CotAD_52307
CotAD_49341
CotAD_48625
CotAD_48622
CotAD_48610
CotAD_48609
CotAD_48606
CotAD_48602
CotAD_48599
CotAD_48458
−1000
−500
0
500
1000
1500
2000
2500

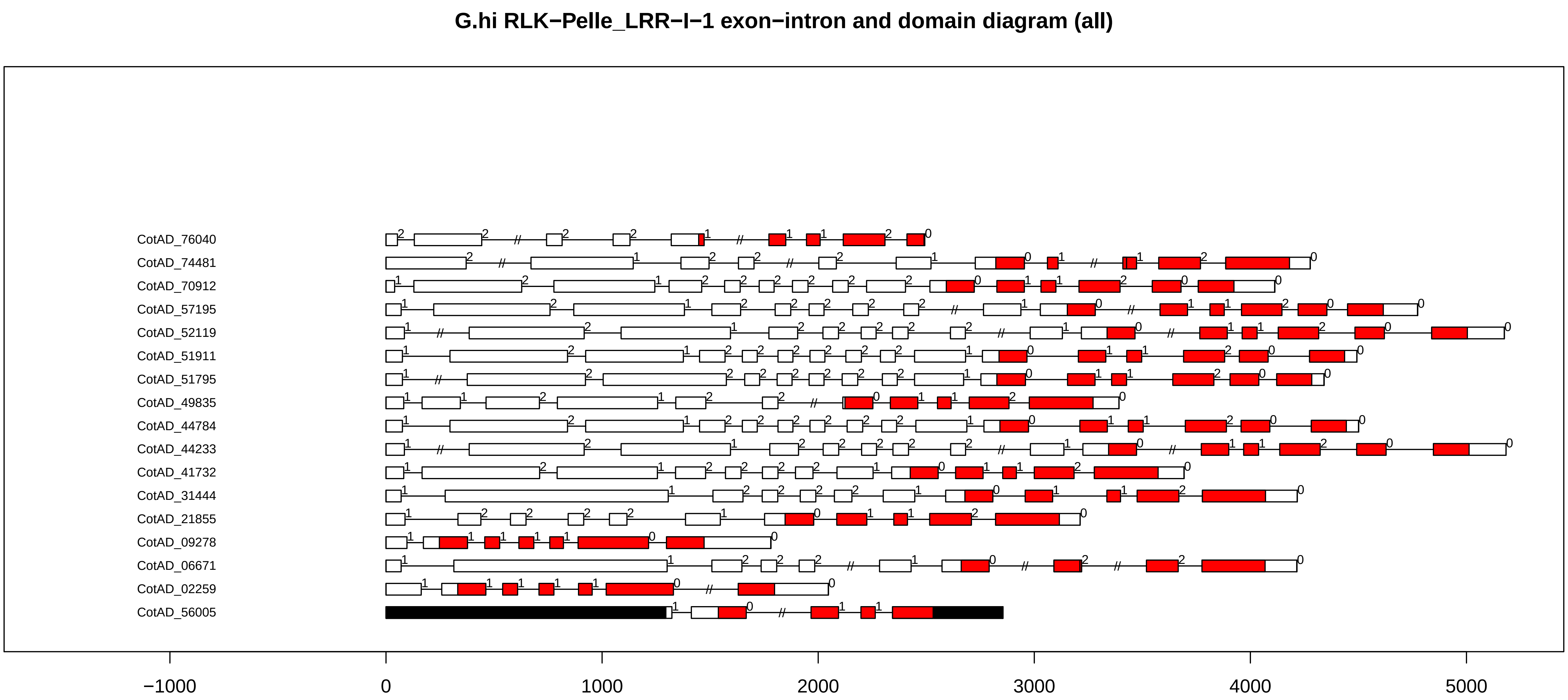
G.hi RLK−Pelle_LRR−I−1 exon−intron and domain diagram (all)
CotAD_76040
CotAD_74481
CotAD_70912
CotAD_57195
CotAD_52119
CotAD_51911
CotAD_51795
CotAD_49835
CotAD_44784
CotAD_44233
CotAD_41732
CotAD_31444
CotAD_21855
CotAD_09278
CotAD_06671
CotAD_02259
CotAD_56005
−1000
0
1000
2000
3000
4000
5000

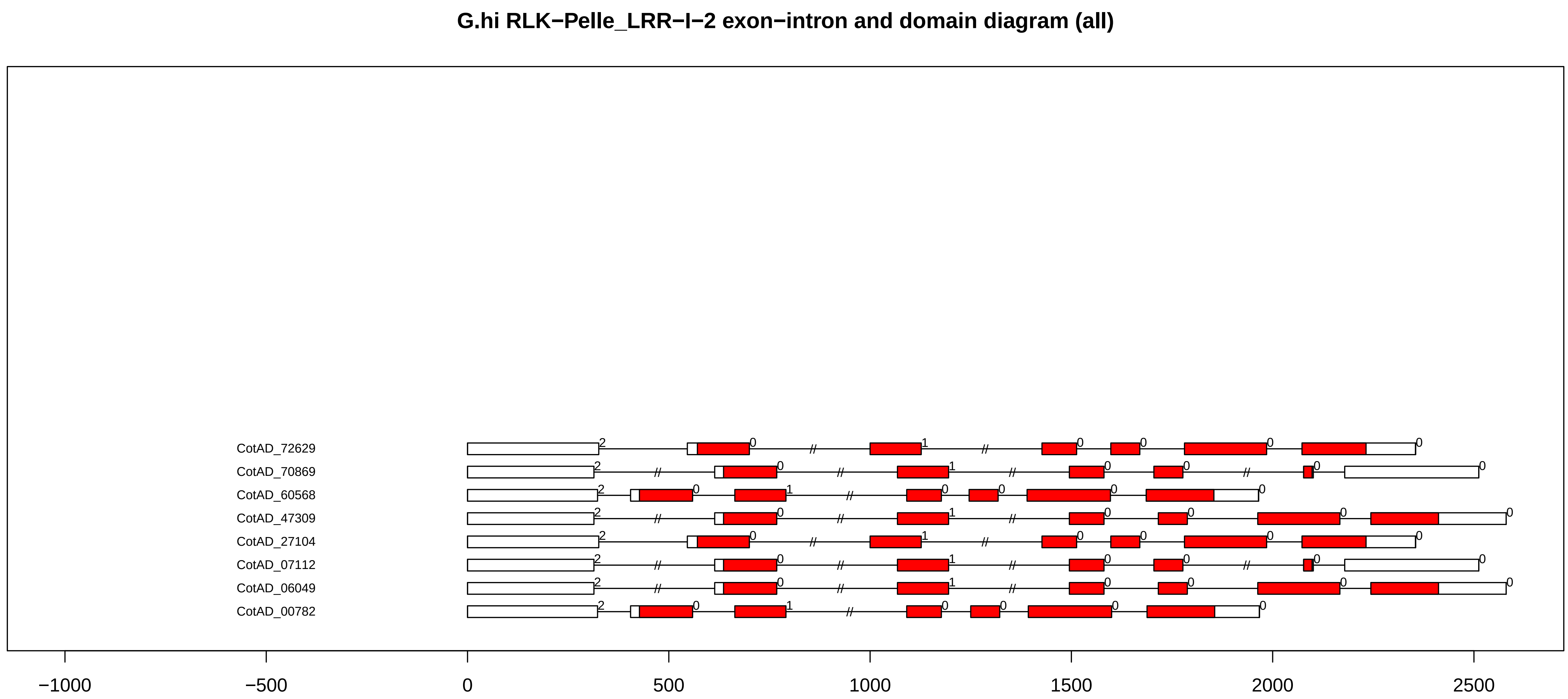
G.hi RLK−Pelle_LRR−I−2 exon−intron and domain diagram (all)
CotAD_72629
CotAD_70869
CotAD_60568
CotAD_47309
CotAD_27104
CotAD_07112
CotAD_06049
CotAD_00782
−1000
−500
0
500
1000
1500
2000
2500

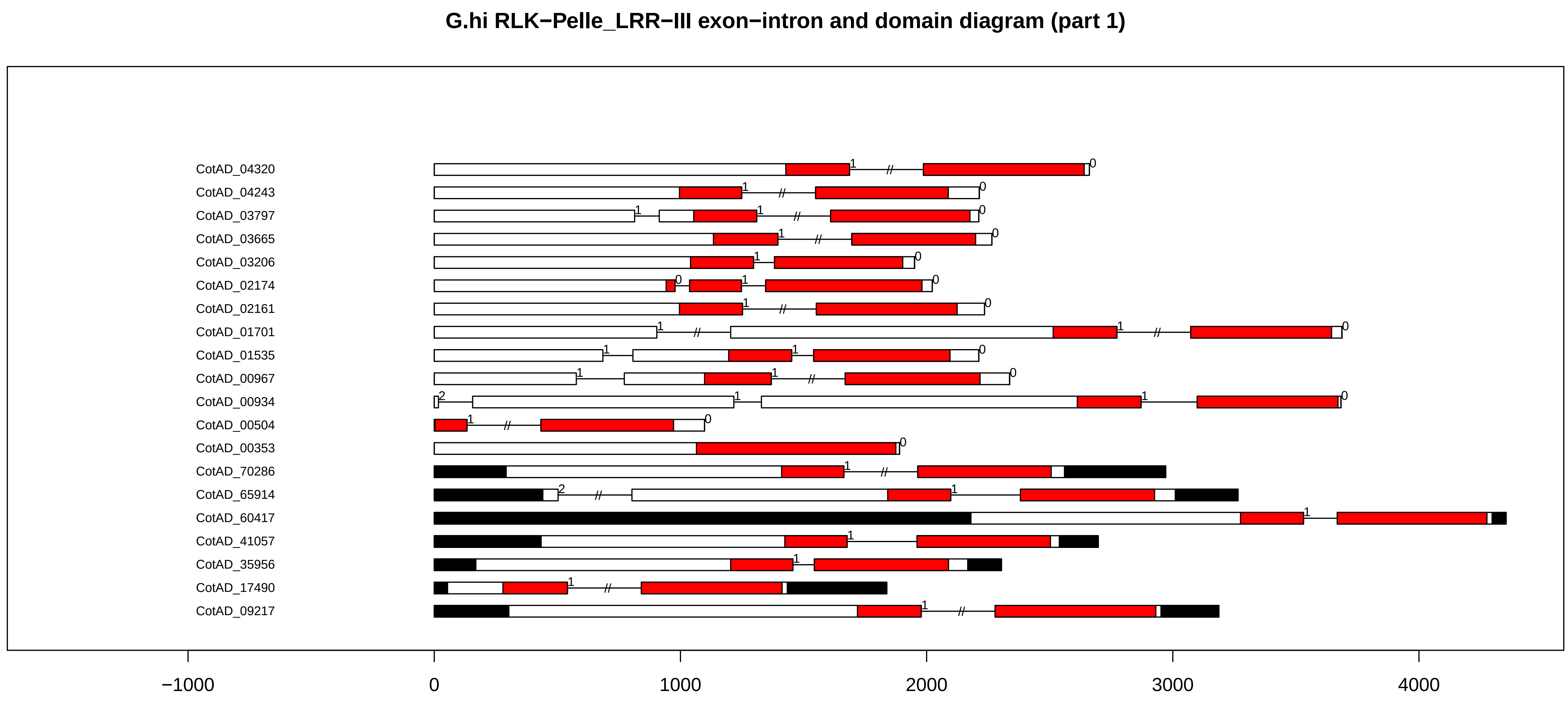
G.hi RLK−Pelle_LRR−III exon−intron and domain diagram (part 1)
CotAD_04320
CotAD_04243
CotAD_03797
CotAD_03665
CotAD_03206
CotAD_02174
CotAD_02161
CotAD_01701
CotAD_01535
CotAD_00967
CotAD_00934
CotAD_00504
CotAD_00353
CotAD_70286
CotAD_65914
CotAD_60417
CotAD_41057
CotAD_35956
CotAD_17490
CotAD_09217
−1000
0
1000
2000
3000
4000

G.hi RLK−Pelle_LRR−III exon−intron and domain diagram (part 2)

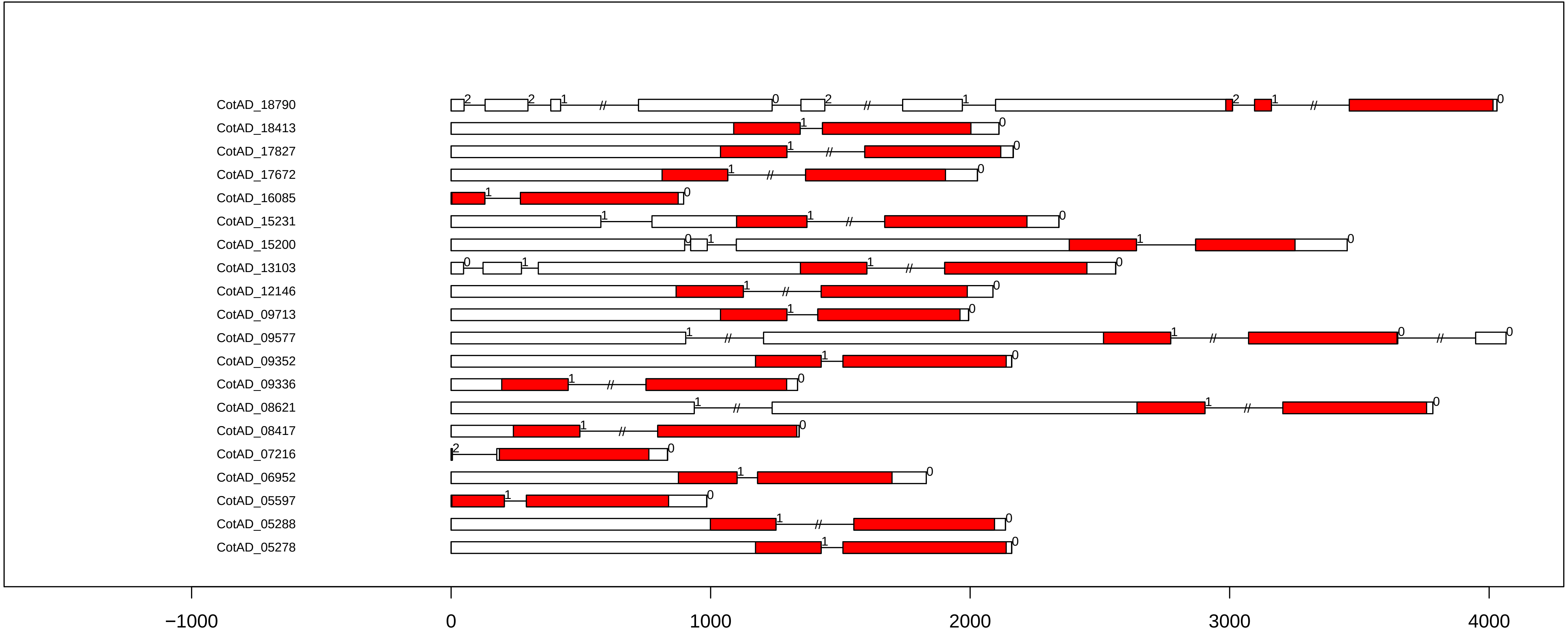

G.hi RLK−Pelle_LRR−III exon−intron and domain diagram (part 3)

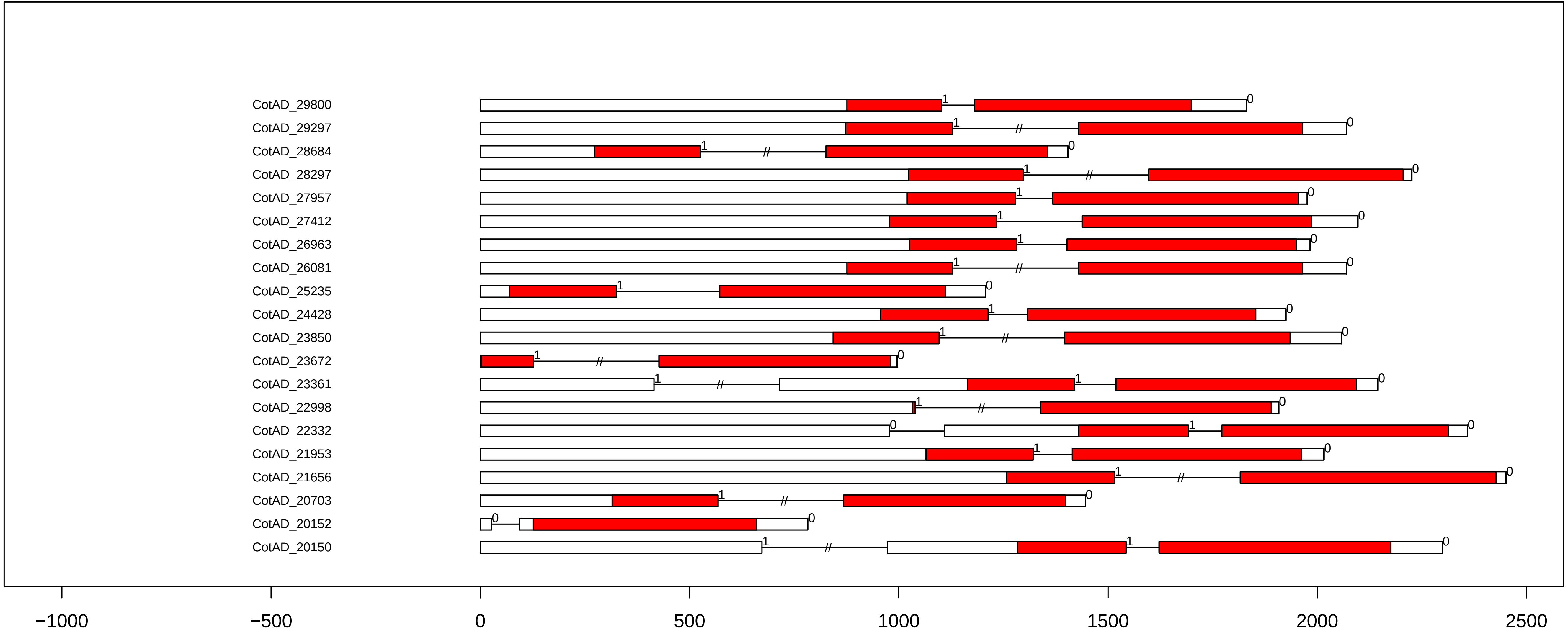

G.hi RLK−Pelle_LRR−III exon−intron and domain diagram (part 4)

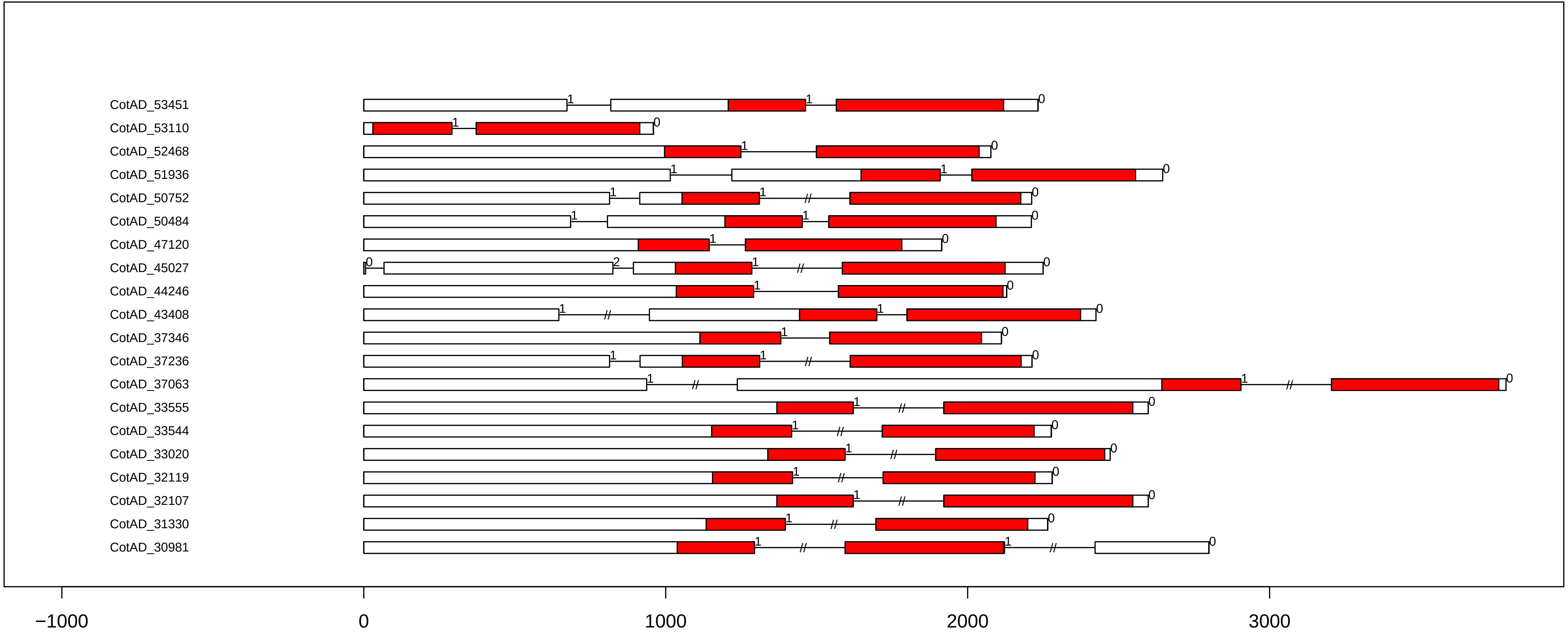

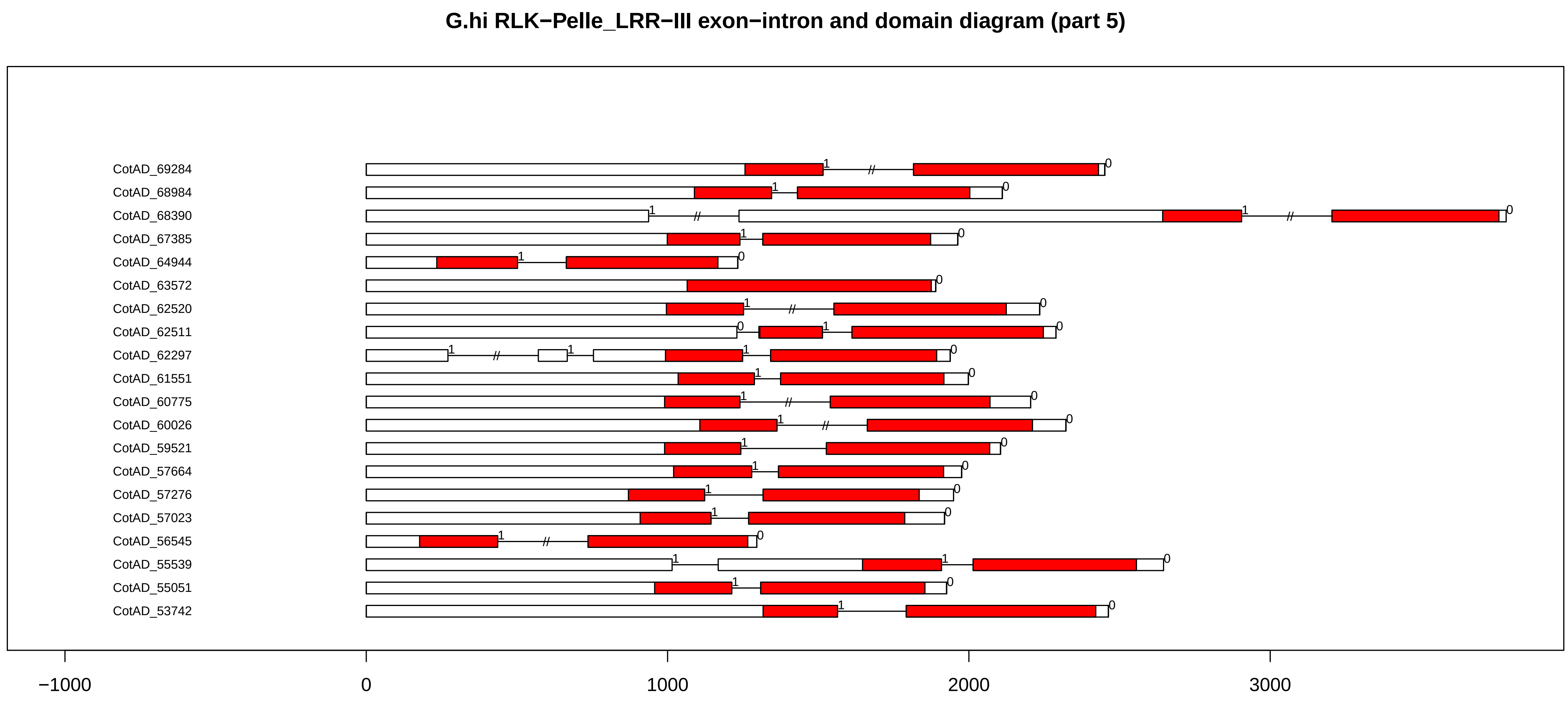
G.hi RLK−Pelle_LRR−III exon−intron and domain diagram (part 5)
CotAD_69284
CotAD_68984
CotAD_68390
CotAD_67385
CotAD_64944
CotAD_63572
CotAD_62520
CotAD_62511
CotAD_62297
CotAD_61551
CotAD_60775
CotAD_60026
CotAD_59521
CotAD_57664
CotAD_57276
CotAD_57023
CotAD_56545
CotAD_55539
CotAD_55051
CotAD_53742
−1000
0
1000
2000
3000

G.hi RLK−Pelle_LRR−III exon−intron and domain diagram (part 6)

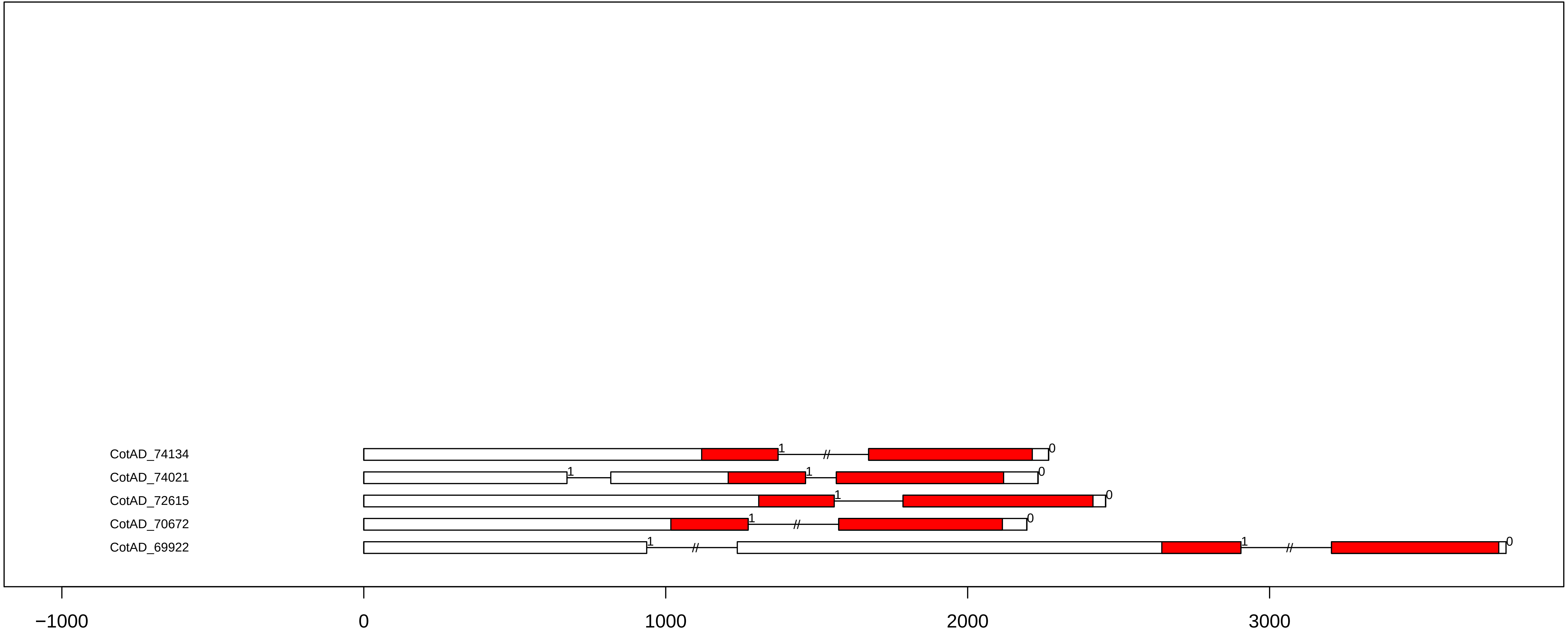

G.hi RLK−Pelle_LRR−II exon−intron and domain diagram (part 1)

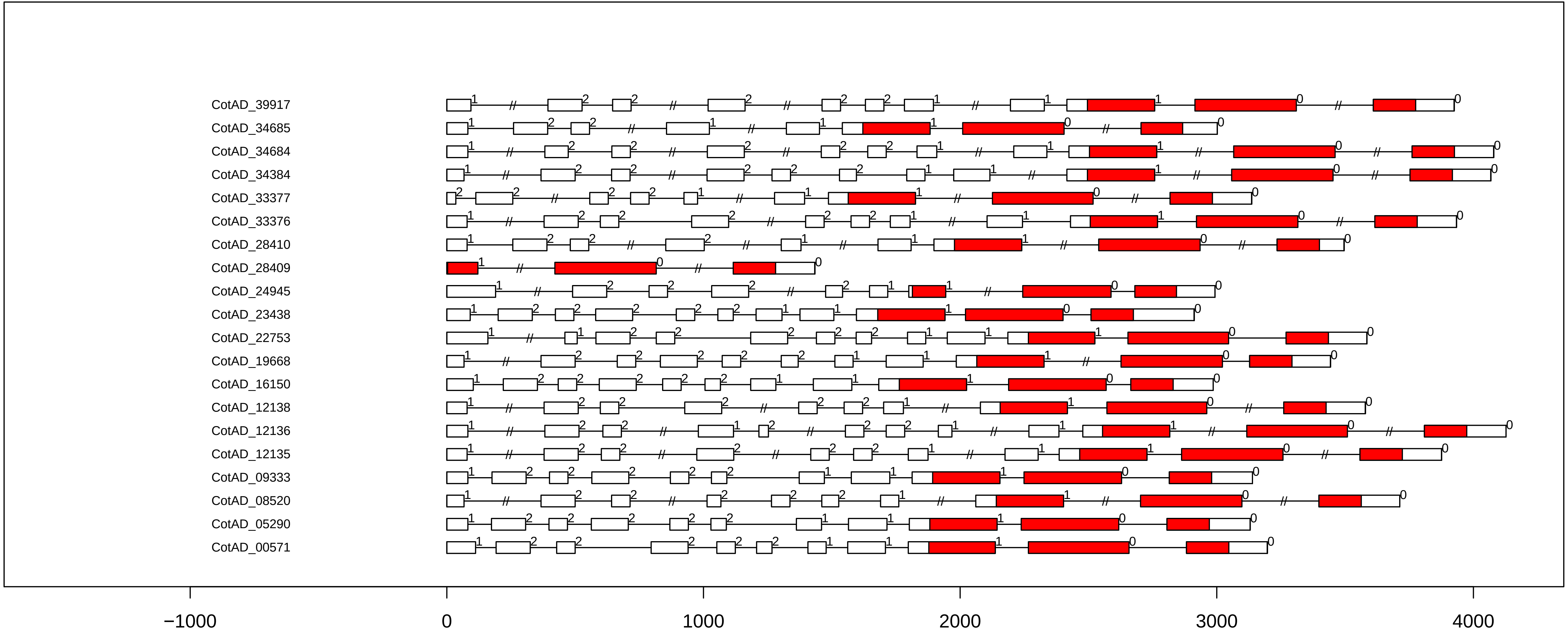

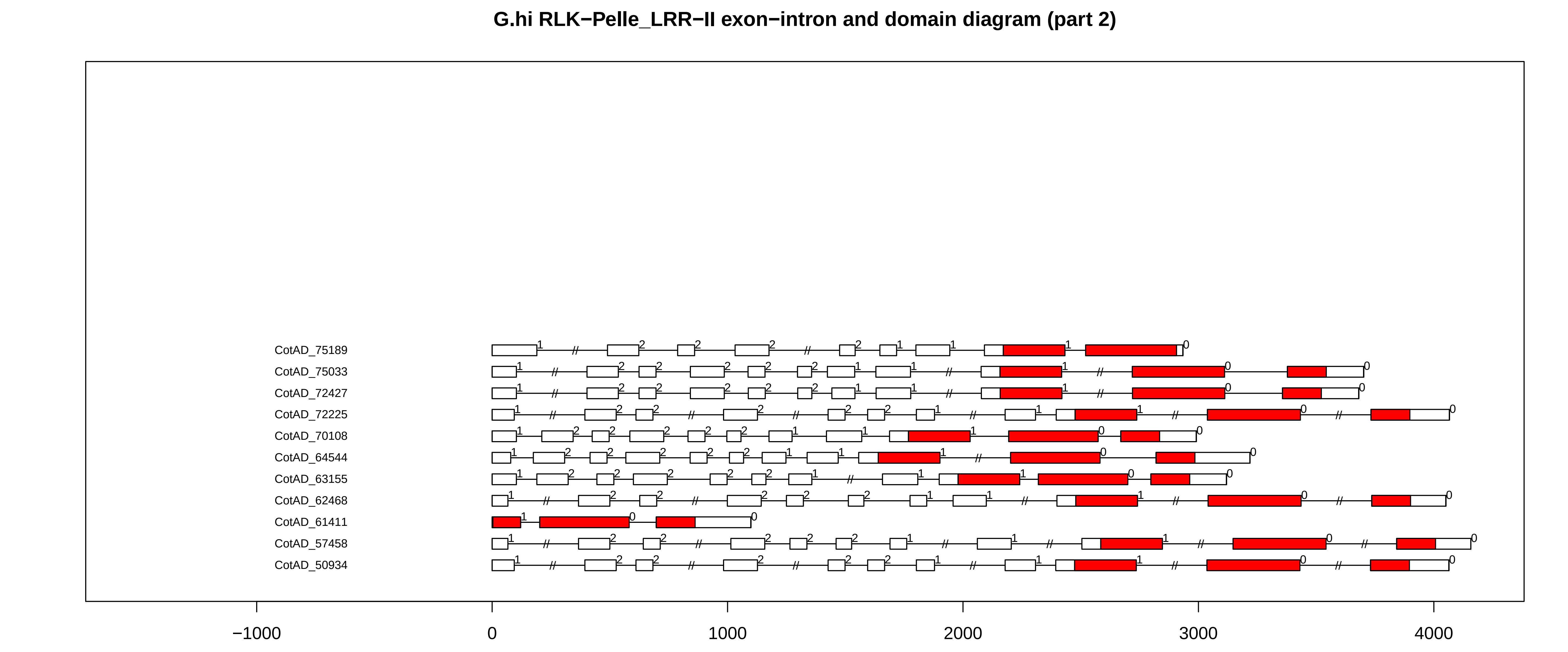

G.hi RLK−Pelle_LRR−II exon−intron and domain diagram (part 2)
CotAD_75189
CotAD_75033
CotAD_72427
CotAD_72225
CotAD_70108
CotAD_64544
CotAD_63155
CotAD_62468
CotAD_61411
CotAD_57458
CotAD_50934
−1000
0
1000
2000
3000
4000

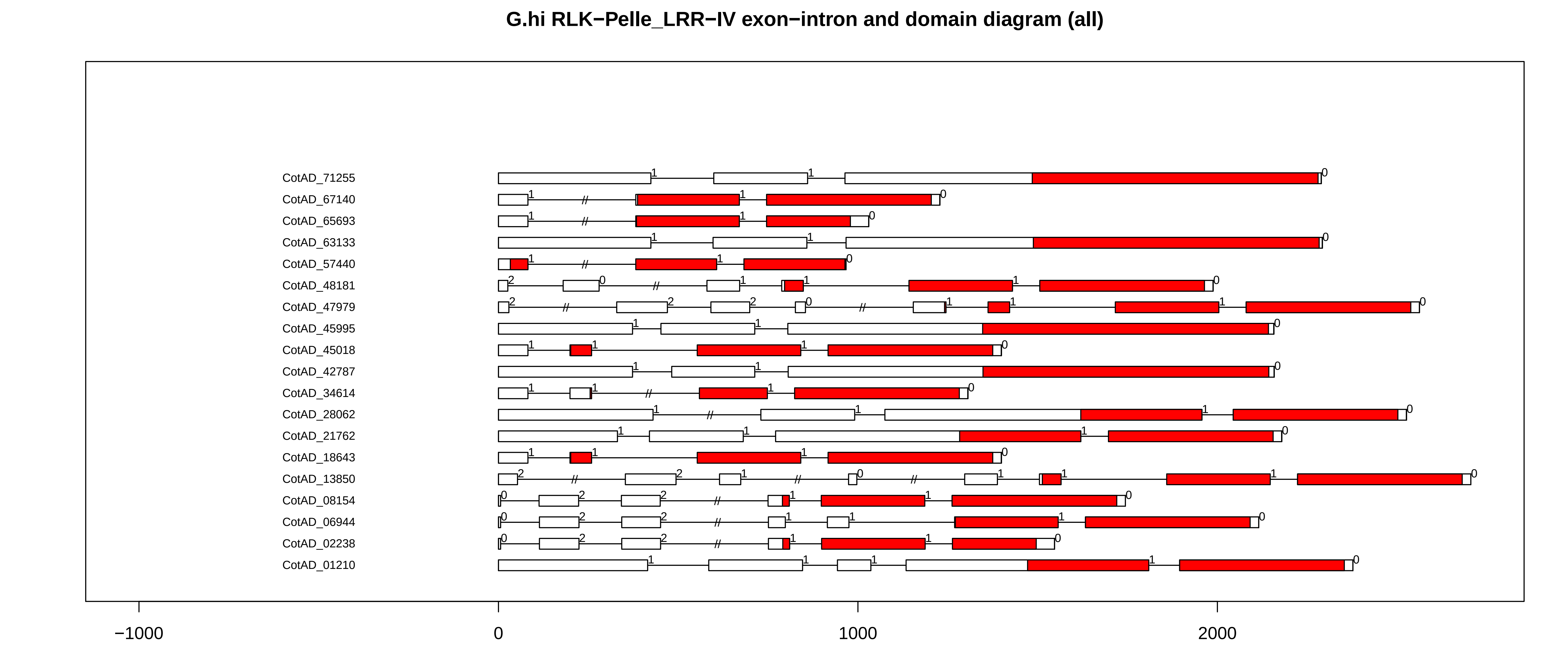

G.hi RLK−Pelle_LRR−IV exon−intron and domain diagram (all)
CotAD_71255
CotAD_67140
CotAD_65693
CotAD_63133
CotAD_57440
CotAD_48181
CotAD_47979
CotAD_45995
CotAD_45018
CotAD_42787
CotAD_34614
CotAD_28062
CotAD_21762
CotAD_18643
CotAD_13850
CotAD_08154
CotAD_06944
CotAD_02238
CotAD_01210
−1000
0
1000
2000

G.hi RLK−Pelle_LRR−IX exon−intron and domain diagram (all)

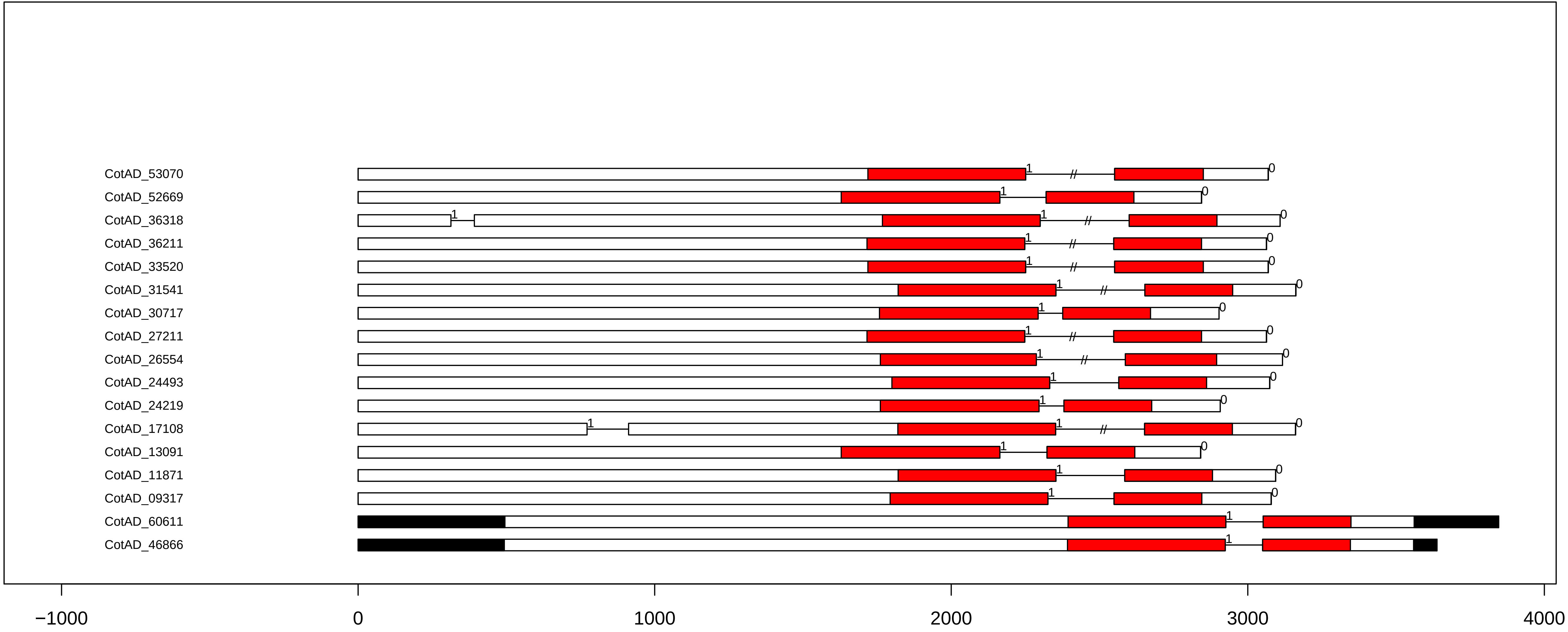

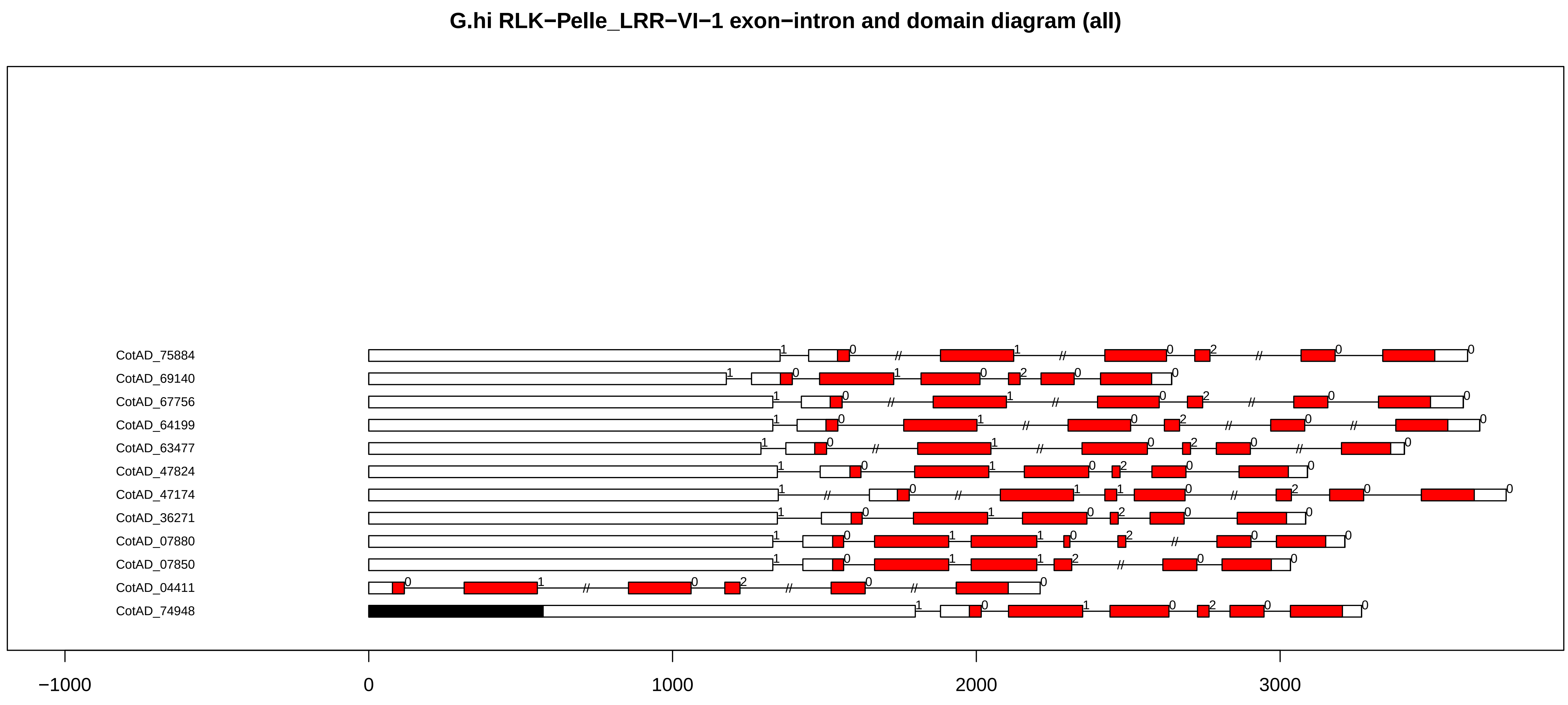
G.hi RLK−Pelle_LRR−VI−1 exon−intron and domain diagram (all)
CotAD_75884
CotAD_69140
CotAD_67756
CotAD_64199
CotAD_63477
CotAD_47824
CotAD_47174
CotAD_36271
CotAD_07880
CotAD_07850
CotAD_04411
CotAD_74948
−1000
0
1000
2000
3000

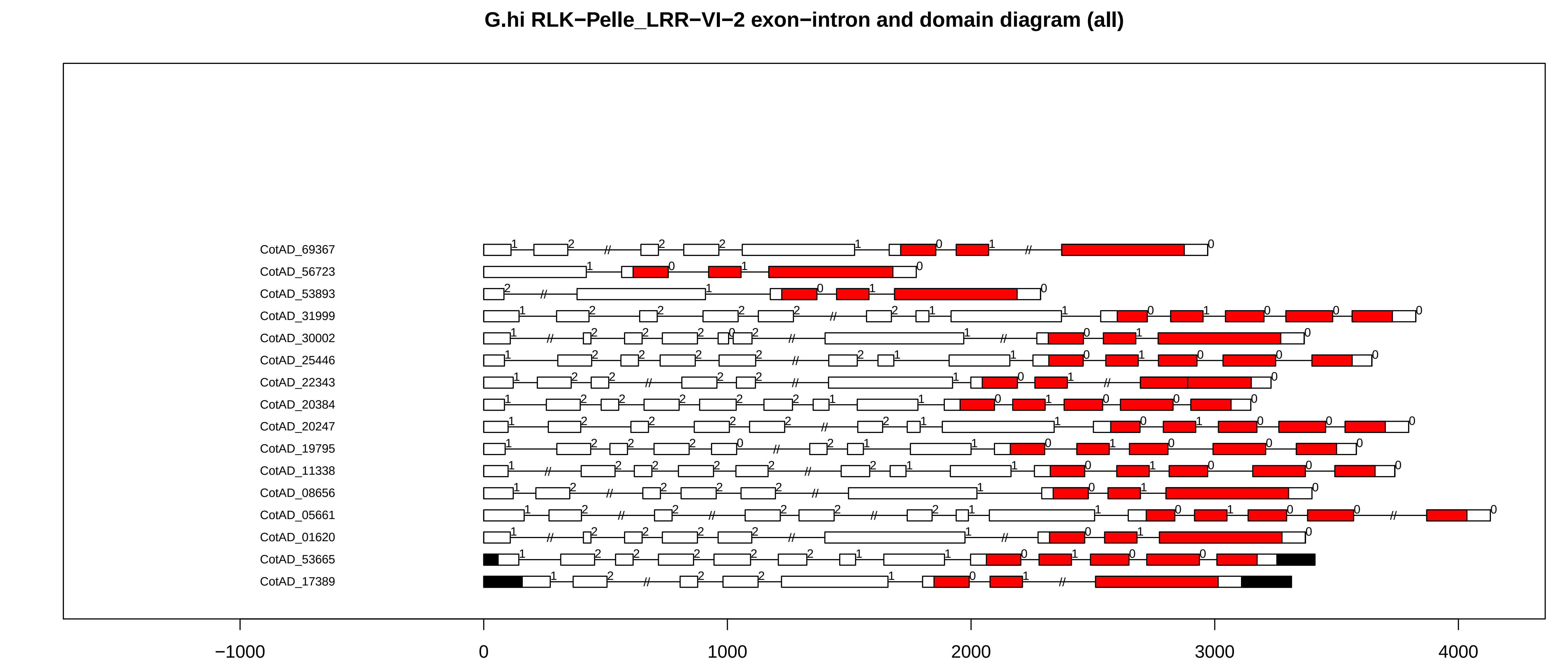

G.hi RLK−Pelle_LRR−VI−2 exon−intron and domain diagram (all)
CotAD_69367
CotAD_56723
CotAD_53893
CotAD_31999
CotAD_30002
CotAD_25446
CotAD_22343
CotAD_20384
CotAD_20247
CotAD_19795
CotAD_11338
CotAD_08656
CotAD_05661
CotAD_01620
CotAD_53665
CotAD_17389
−1000
0
1000
2000
3000
4000

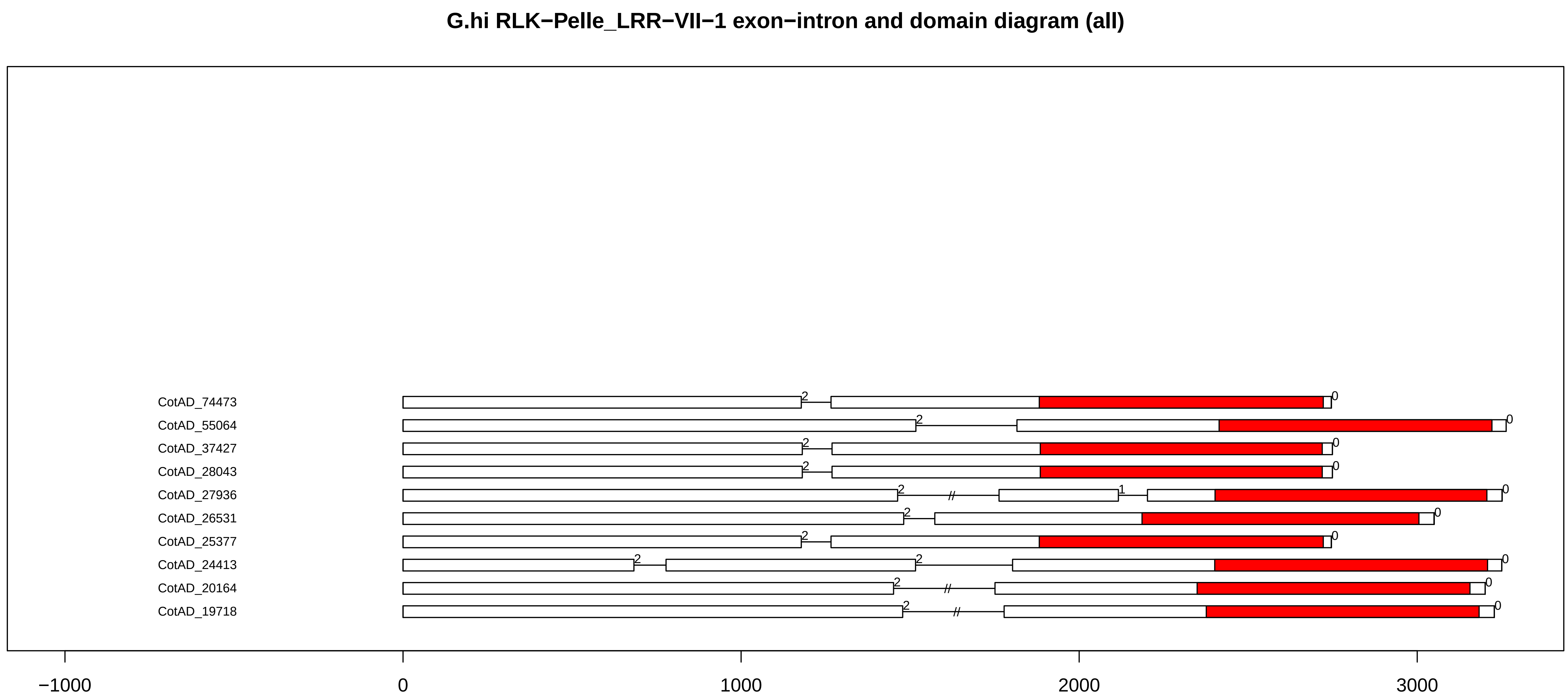
G.hi RLK−Pelle_LRR−VII−1 exon−intron and domain diagram (all)
CotAD_74473
CotAD_55064
CotAD_37427
CotAD_28043
CotAD_27936
CotAD_26531
CotAD_25377
CotAD_24413
CotAD_20164
CotAD_19718
−1000
0
1000
2000
3000

G.hi RLK−Pelle_LRR−VII−2 exon−intron and domain diagram (all)

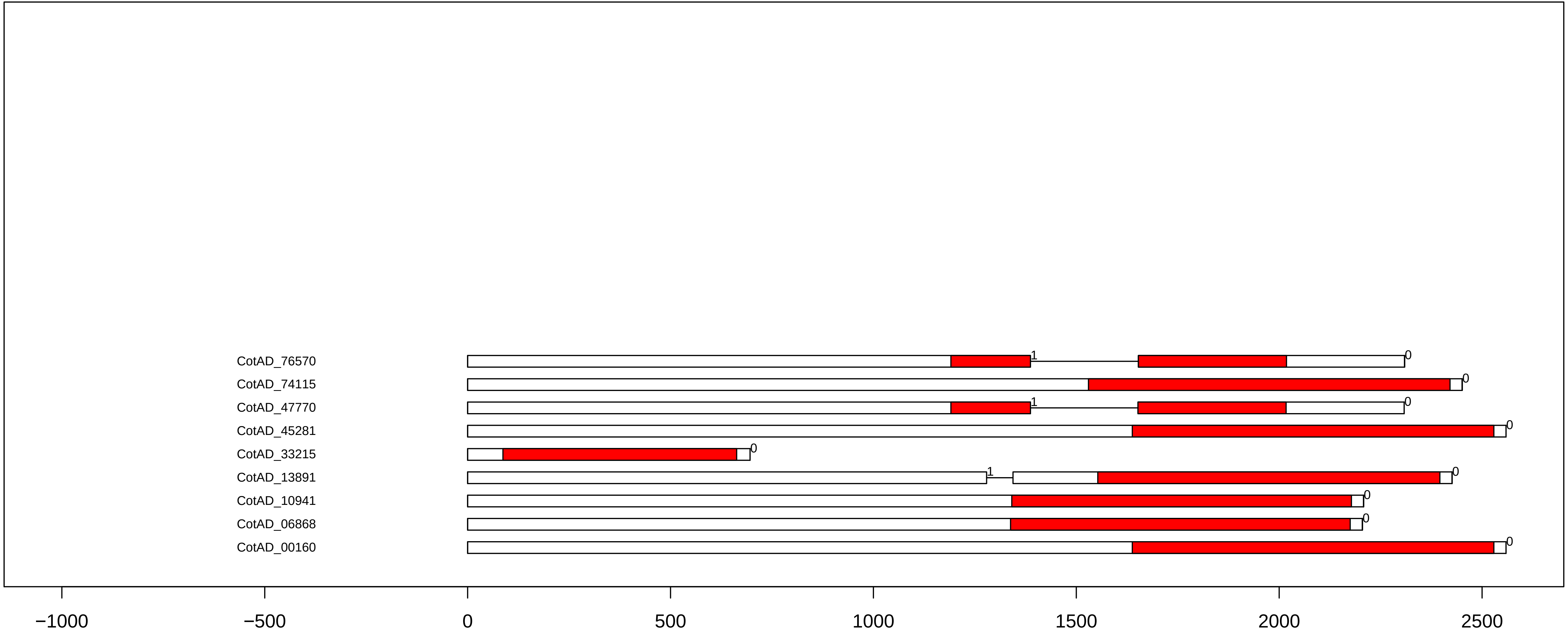

G.hi RLK−Pelle_LRR−VII−3 exon−intron and domain diagram (all)

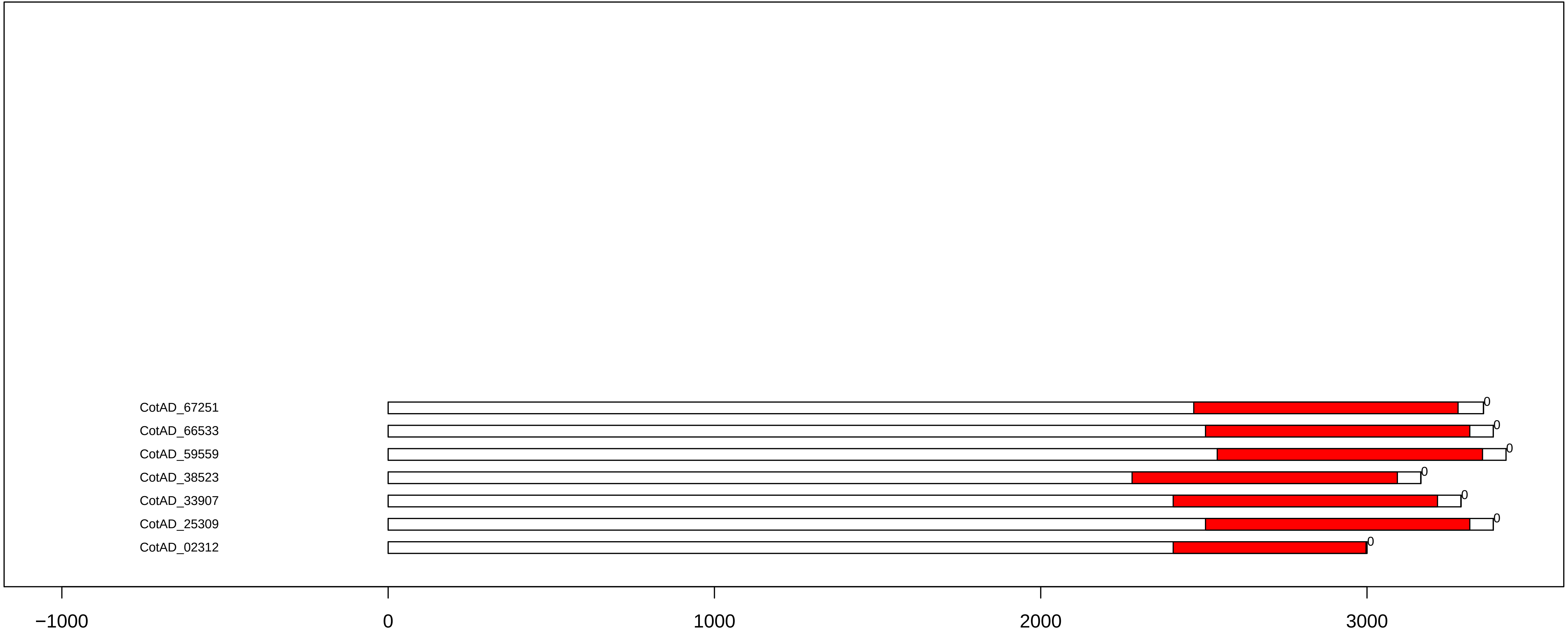

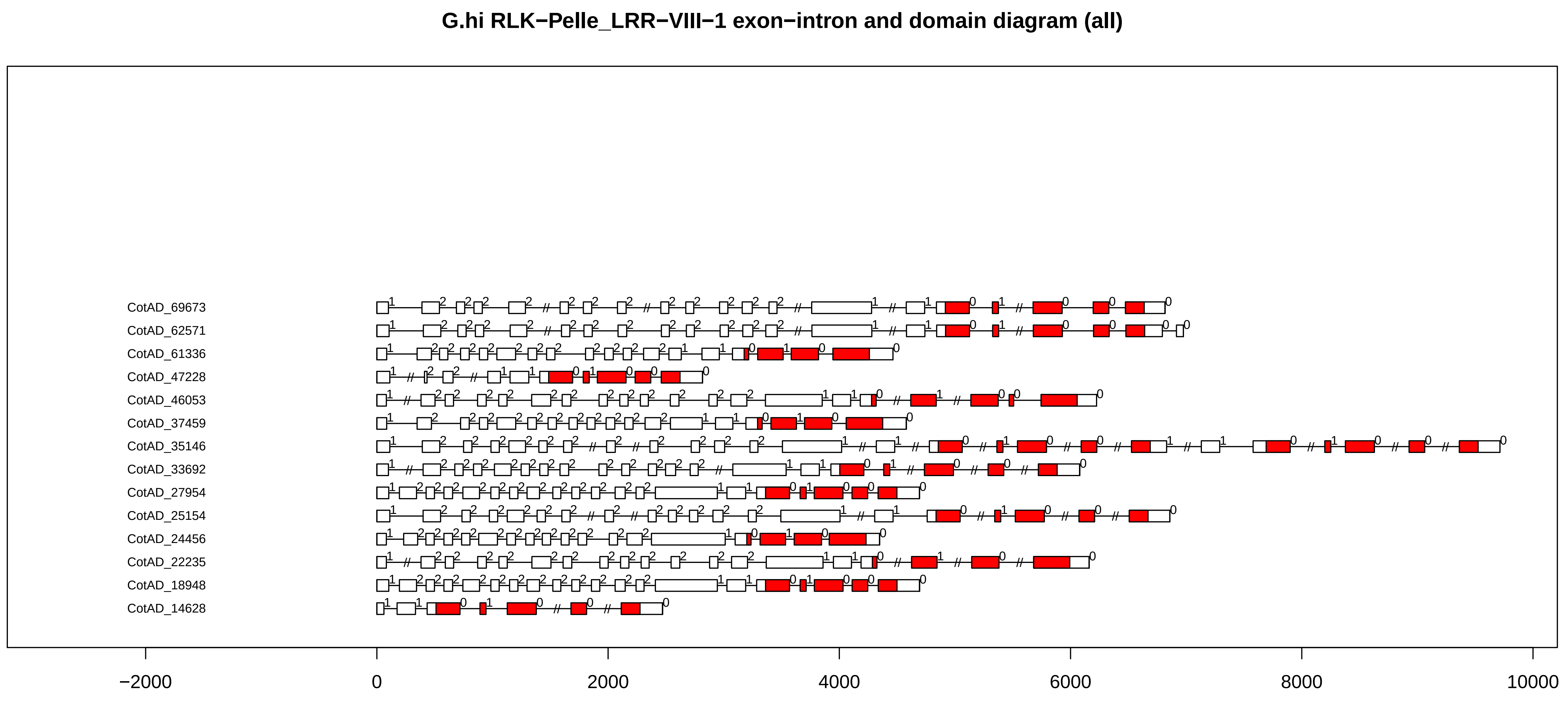
G.hi RLK−Pelle_LRR−VIII−1 exon−intron and domain diagram (all)
CotAD_69673
CotAD_62571
CotAD_61336
CotAD_47228
CotAD_46053
CotAD_37459
CotAD_35146
CotAD_33692
CotAD_27954
CotAD_25154
CotAD_24456
CotAD_22235
CotAD_18948
CotAD_14628
−2000
0
2000
4000
6000
8000
10000

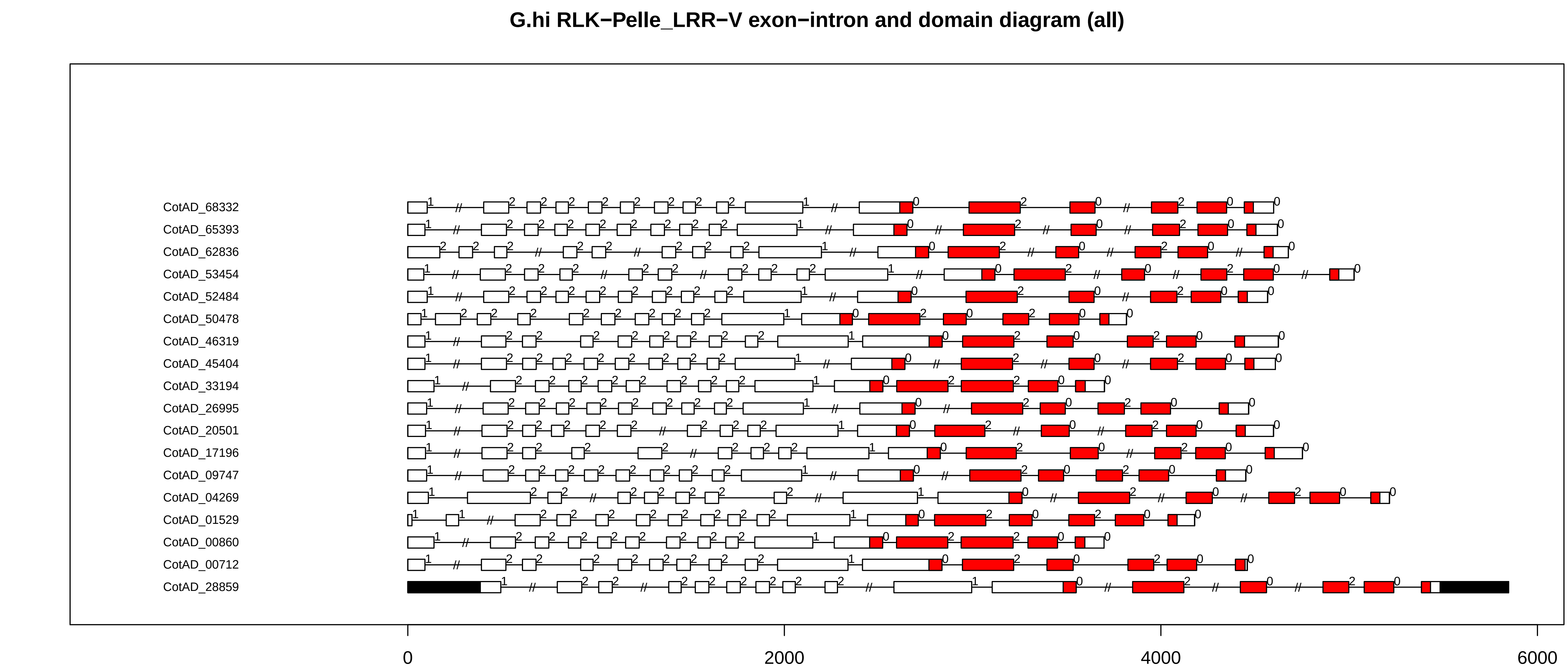

G.hi RLK−Pelle_LRR−V exon−intron and domain diagram (all)
CotAD_68332
CotAD_65393
CotAD_62836
CotAD_53454
CotAD_52484
CotAD_50478
CotAD_46319
CotAD_45404
CotAD_33194
CotAD_26995
CotAD_20501
CotAD_17196
CotAD_09747
CotAD_04269
CotAD_01529
CotAD_00860
CotAD_00712
CotAD_28859
0
2000
4000
6000

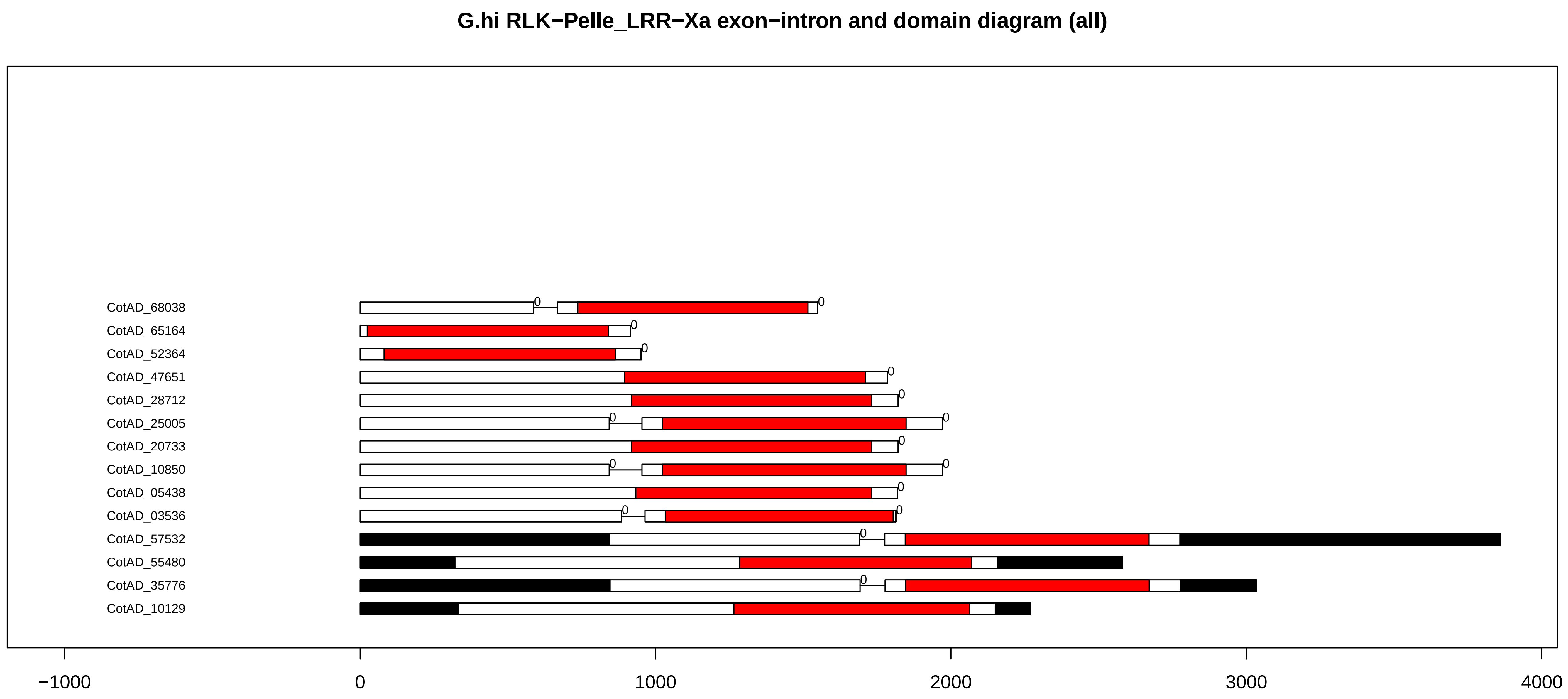

G.hi RLK−Pelle_LRR−Xa exon−intron and domain diagram (all)
CotAD_68038
CotAD_65164
CotAD_52364
CotAD_47651
CotAD_28712
CotAD_25005
CotAD_20733
CotAD_10850
CotAD_05438
CotAD_03536
CotAD_57532
CotAD_55480
CotAD_35776
CotAD_10129
−1000
0
1000
2000
3000
4000

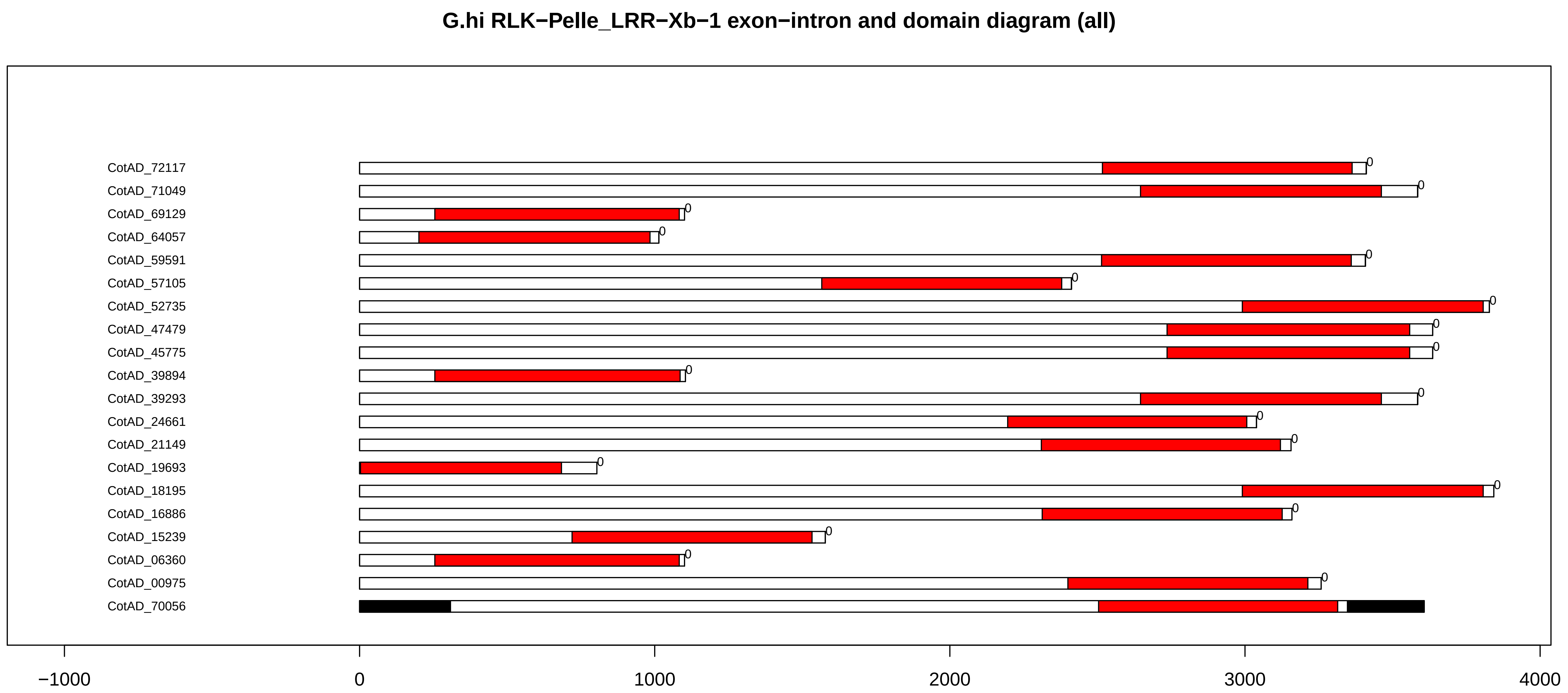
G.hi RLK−Pelle_LRR−Xb−1 exon−intron and domain diagram (all)
CotAD_72117
CotAD_71049
CotAD_69129
CotAD_64057
CotAD_59591
CotAD_57105
CotAD_52735
CotAD_47479
CotAD_45775
CotAD_39894
CotAD_39293
CotAD_24661
CotAD_21149
CotAD_19693
CotAD_18195
CotAD_16886
CotAD_15239
CotAD_06360
CotAD_00975
CotAD_70056
−1000
0
1000
2000
3000
4000

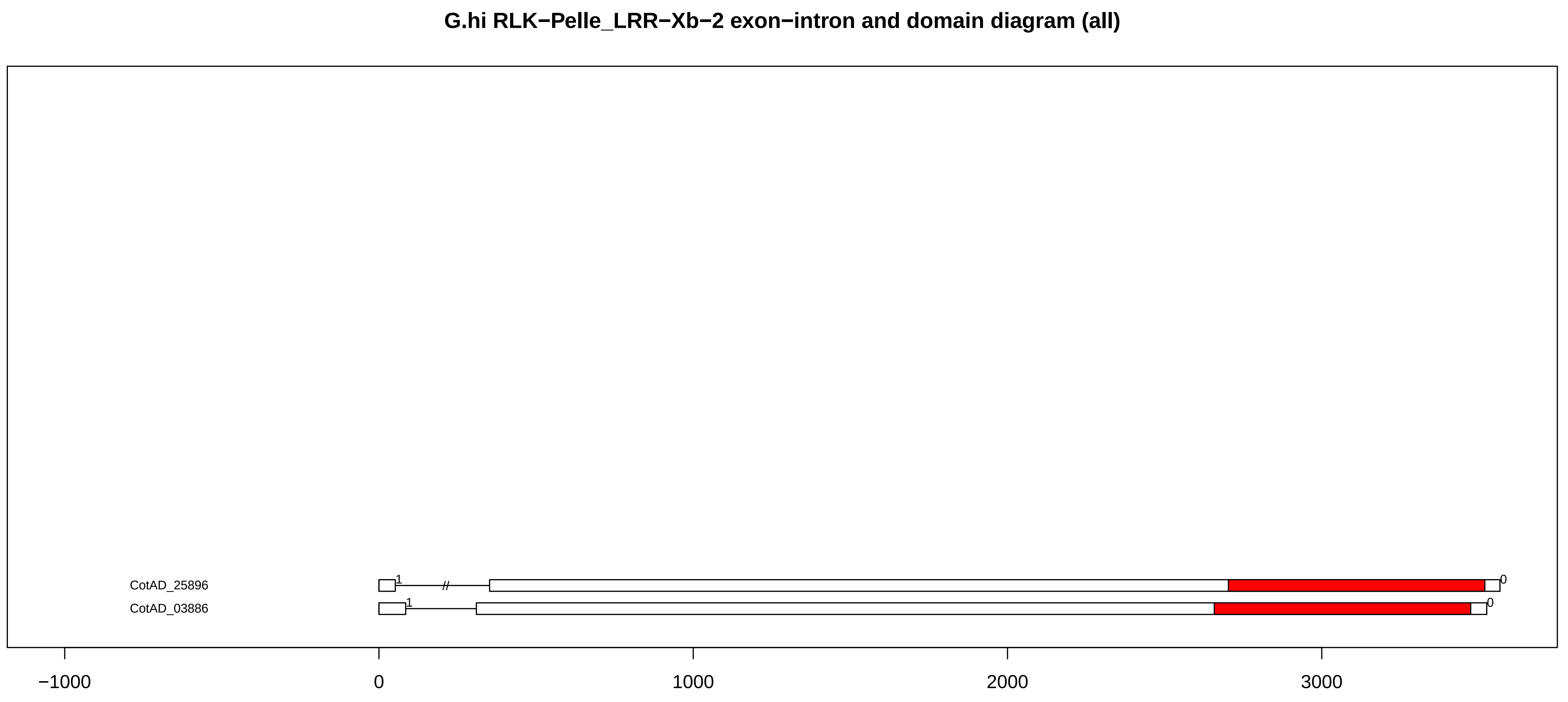
G.hi RLK−Pelle_LRR−Xb−2 exon−intron and domain diagram (all)
CotAD_25896
CotAD_03886
1
1
0
0
−1000
0
1000
2000
3000

G.hi RLK−Pelle_LRR−XI−1 exon−intron and domain diagram (part 1)

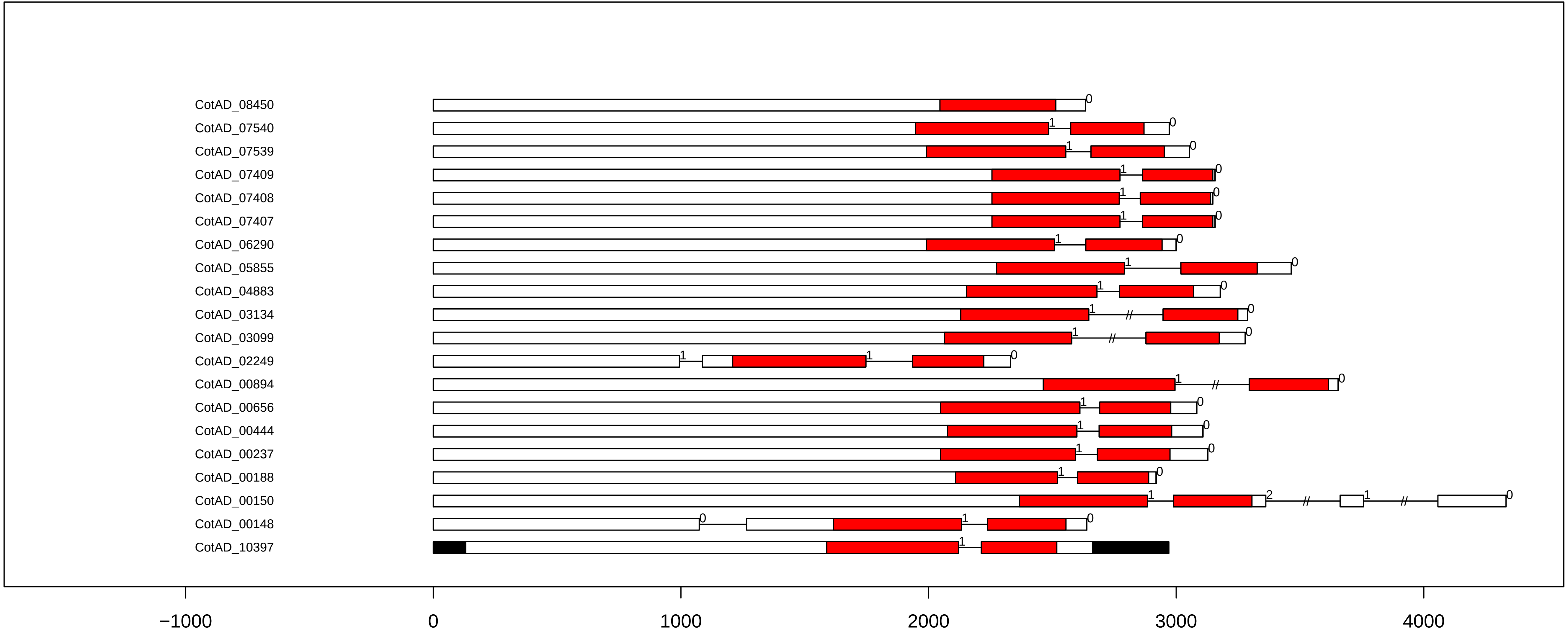

G.hi RLK−Pelle_LRR−XI−1 exon−intron and domain diagram (part 2)

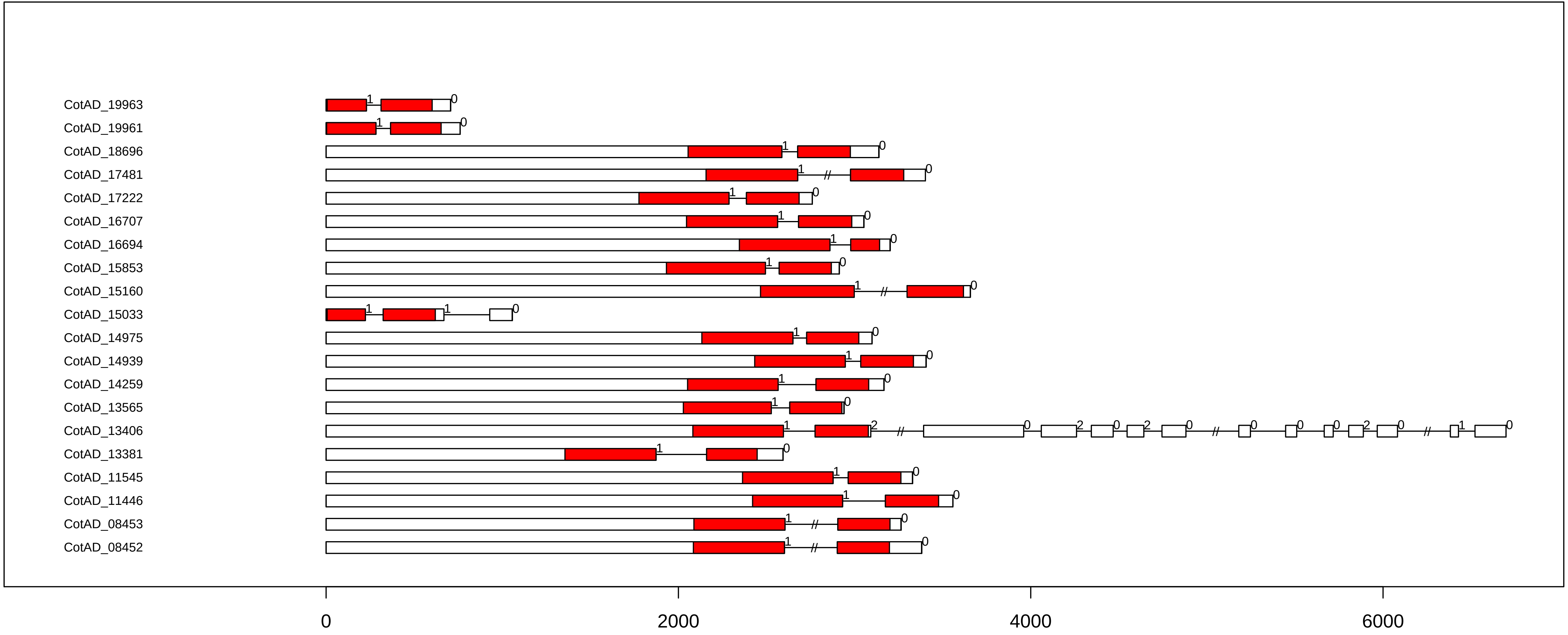

G.hi RLK−Pelle_LRR−XI−1 exon−intron and domain diagram (part 3)

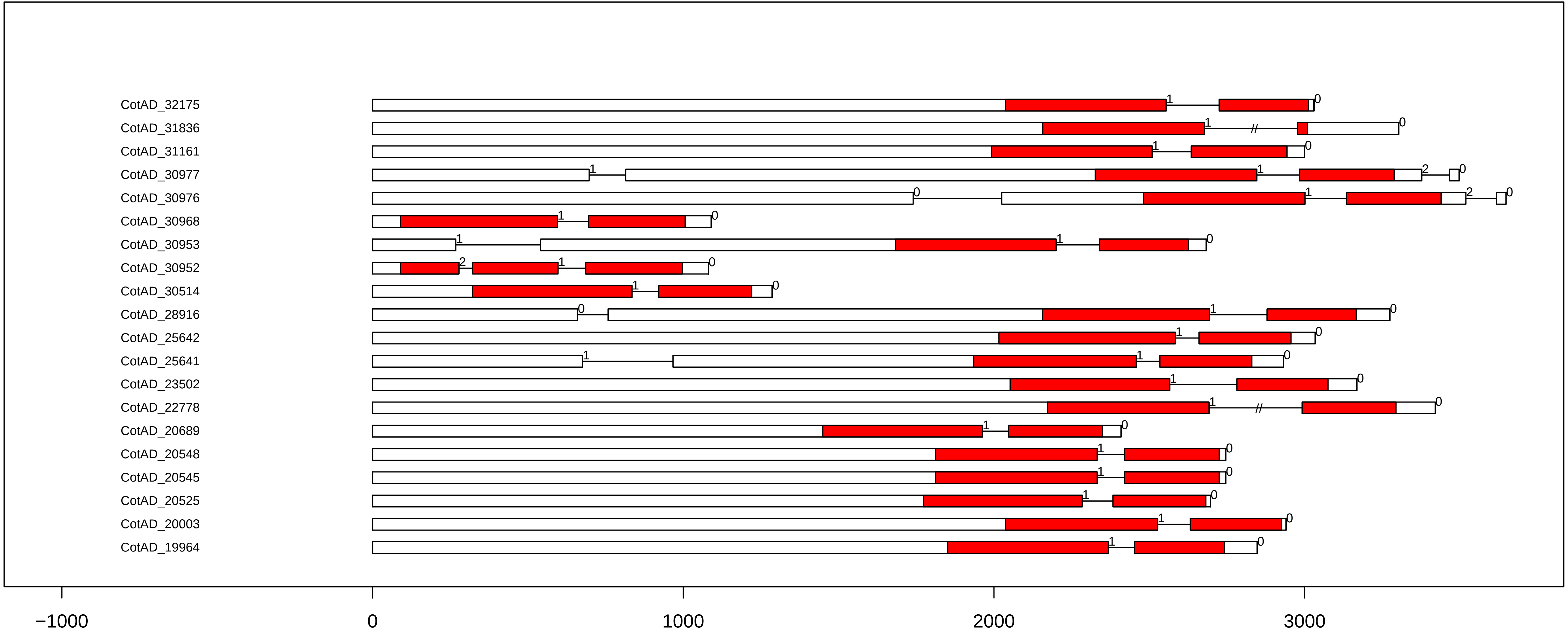

G.hi RLK−Pelle_LRR−XI−1 exon−intron and domain diagram (part 4)

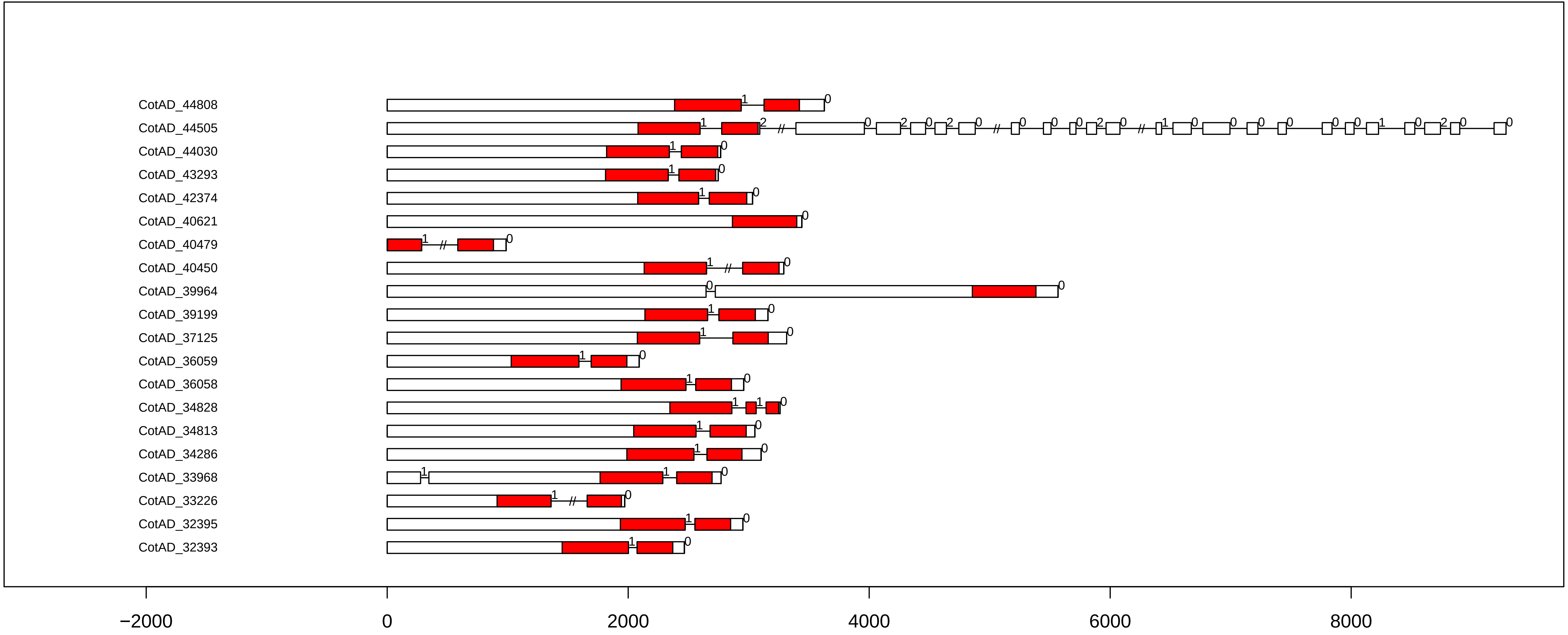

G.hi RLK−Pelle_LRR−XI−1 exon−intron and domain diagram (part 5)

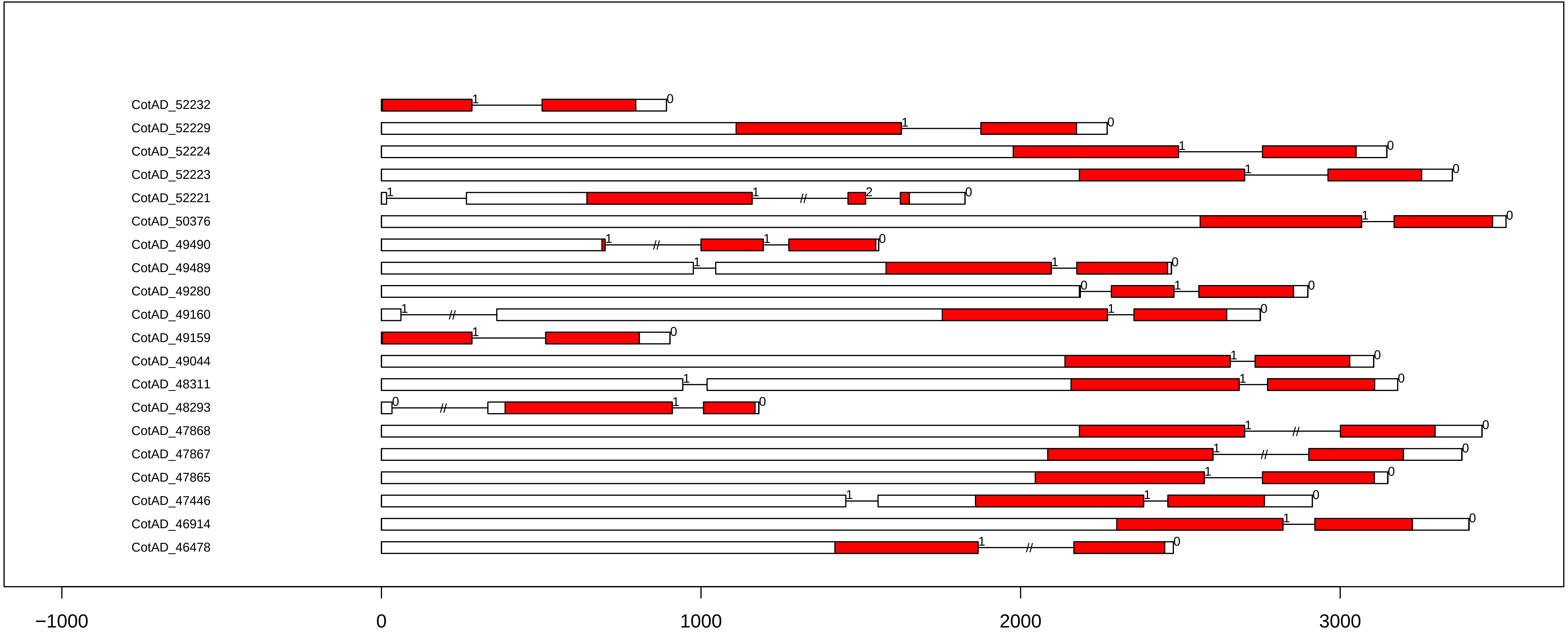

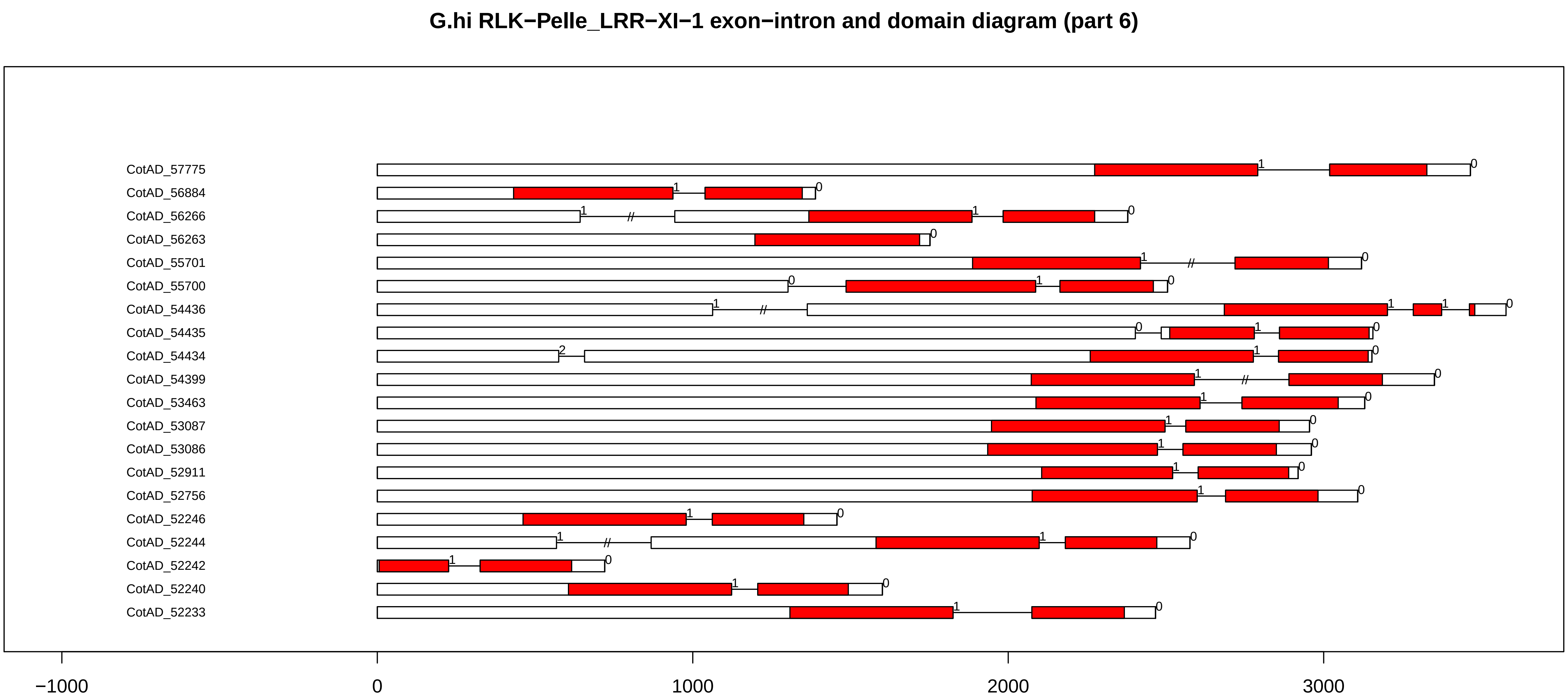
G.hi RLK−Pelle_LRR−XI−1 exon−intron and domain diagram (part 6)
CotAD_57775
CotAD_56884
CotAD_56266
CotAD_56263
CotAD_55701
CotAD_55700
CotAD_54436
CotAD_54435
CotAD_54434
CotAD_54399
CotAD_53463
CotAD_53087
CotAD_53086
CotAD_52911
CotAD_52756
CotAD_52246
CotAD_52244
CotAD_52242
CotAD_52240
CotAD_52233
−1000
0
1000
2000
3000

G.hi RLK−Pelle_LRR−XI−1 exon−intron and domain diagram (part 7)

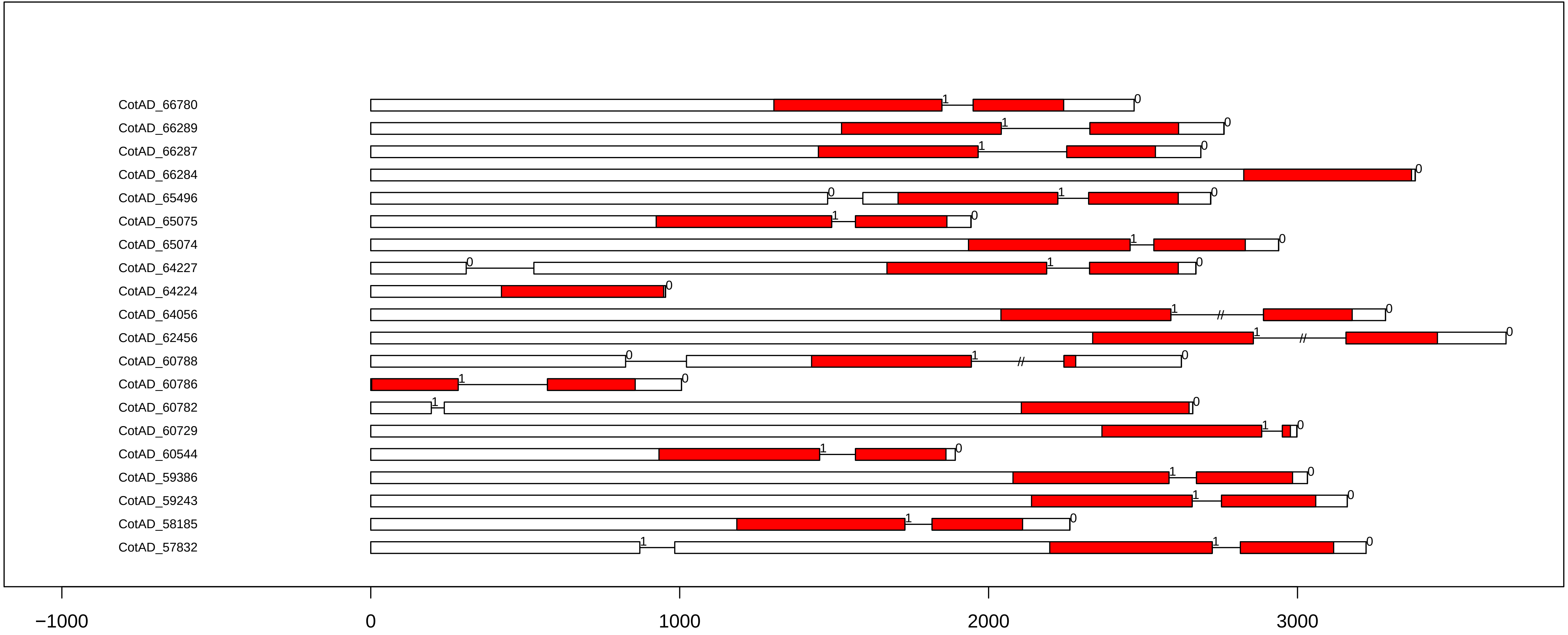

G.hi RLK−Pelle_LRR−XI−1 exon−intron and domain diagram (part 8)

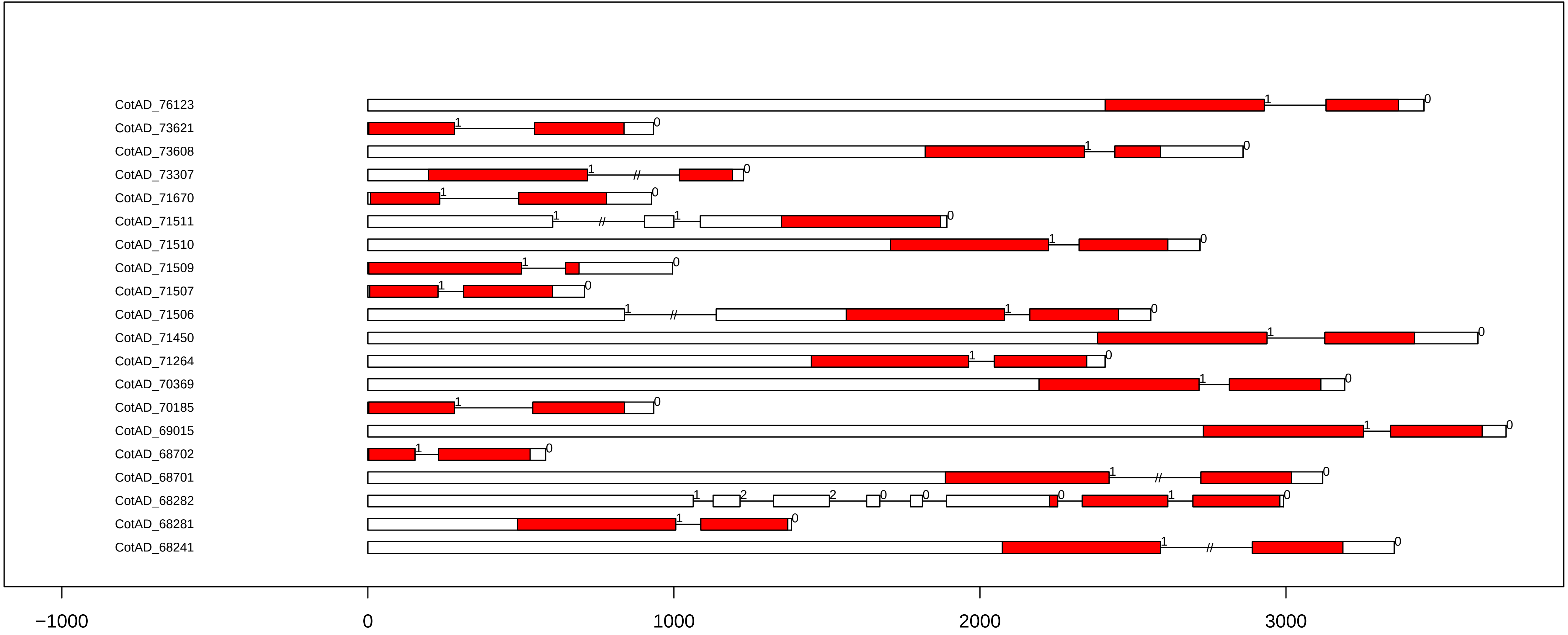

G.hi RLK−Pelle_LRR−XI−1 exon−intron and domain diagram (part 9)

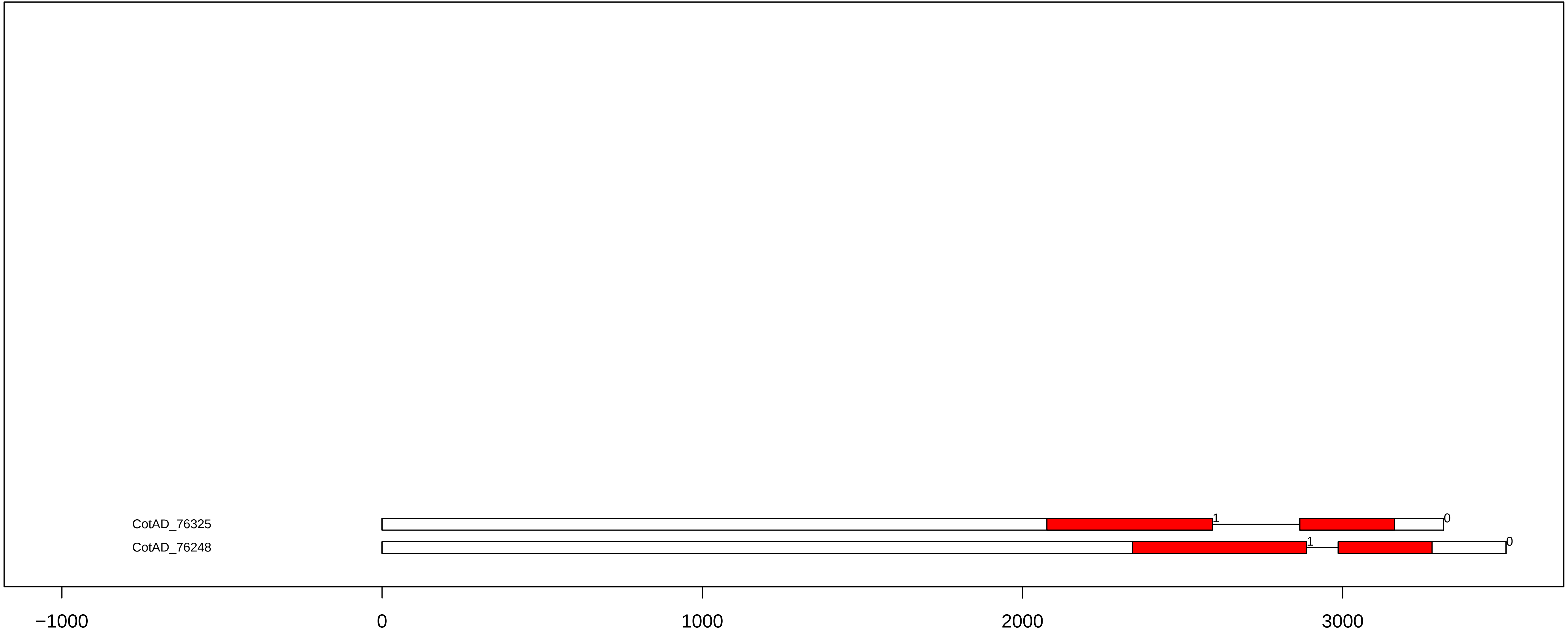

G.hi RLK−Pelle_LRR−XI−2 exon−intron and domain diagram (all)

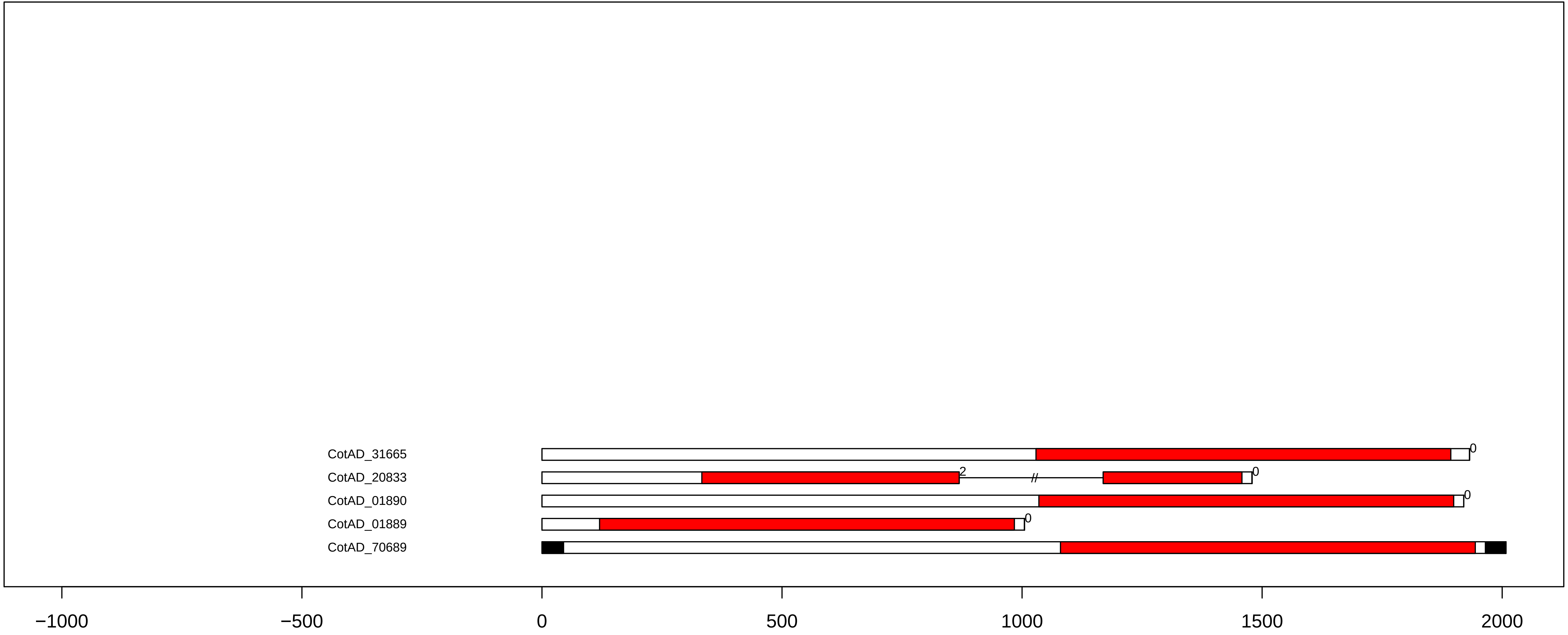

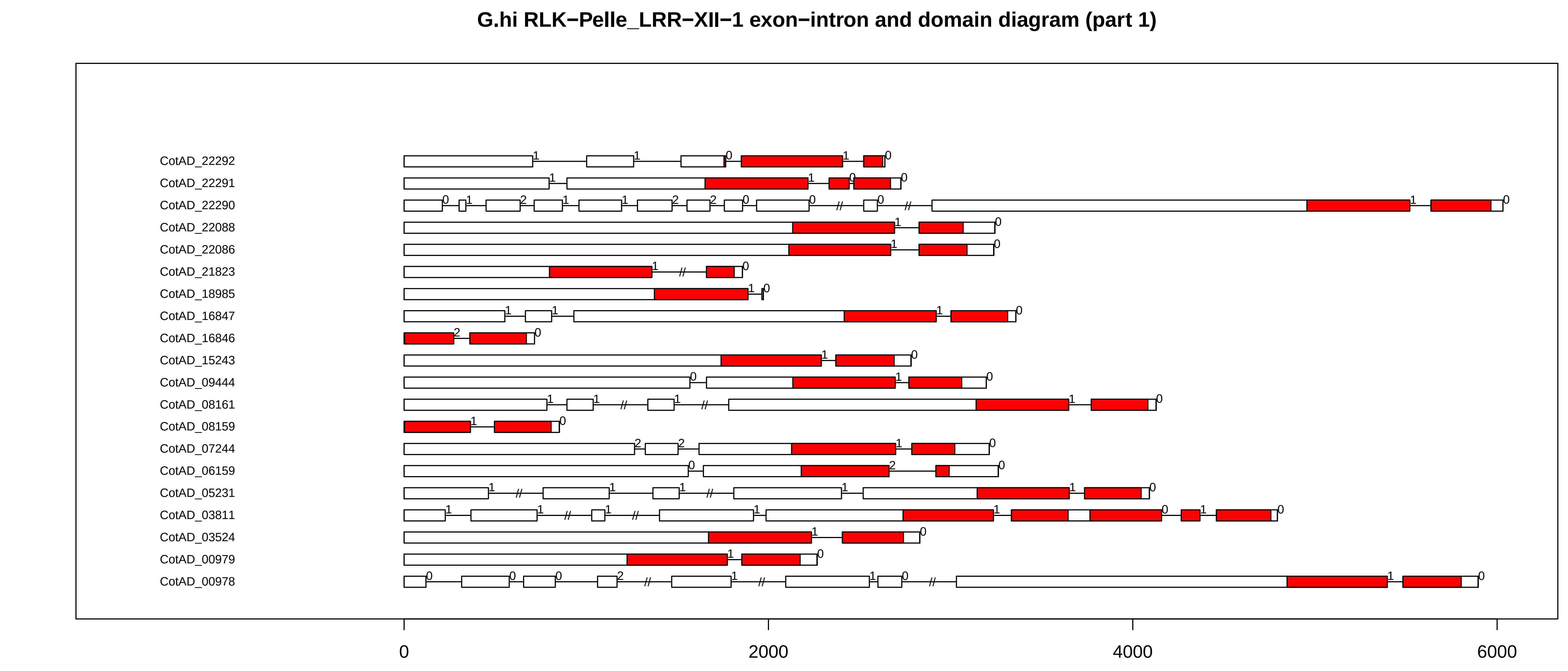

G.hi RLK−Pelle_LRR−XII−1 exon−intron and domain diagram (part 1)
CotAD_22292
CotAD_22291
CotAD_22290
CotAD_22088
CotAD_22086
CotAD_21823
CotAD_18985
CotAD_16847
CotAD_16846
CotAD_15243
CotAD_09444
CotAD_08161
CotAD_08159
CotAD_07244
CotAD_06159
CotAD_05231
CotAD_03811
CotAD_03524
CotAD_00979
CotAD_00978
0
2000
4000
6000

G.hi RLK−Pelle_LRR−XII−1 exon−intron and domain diagram (part 2)

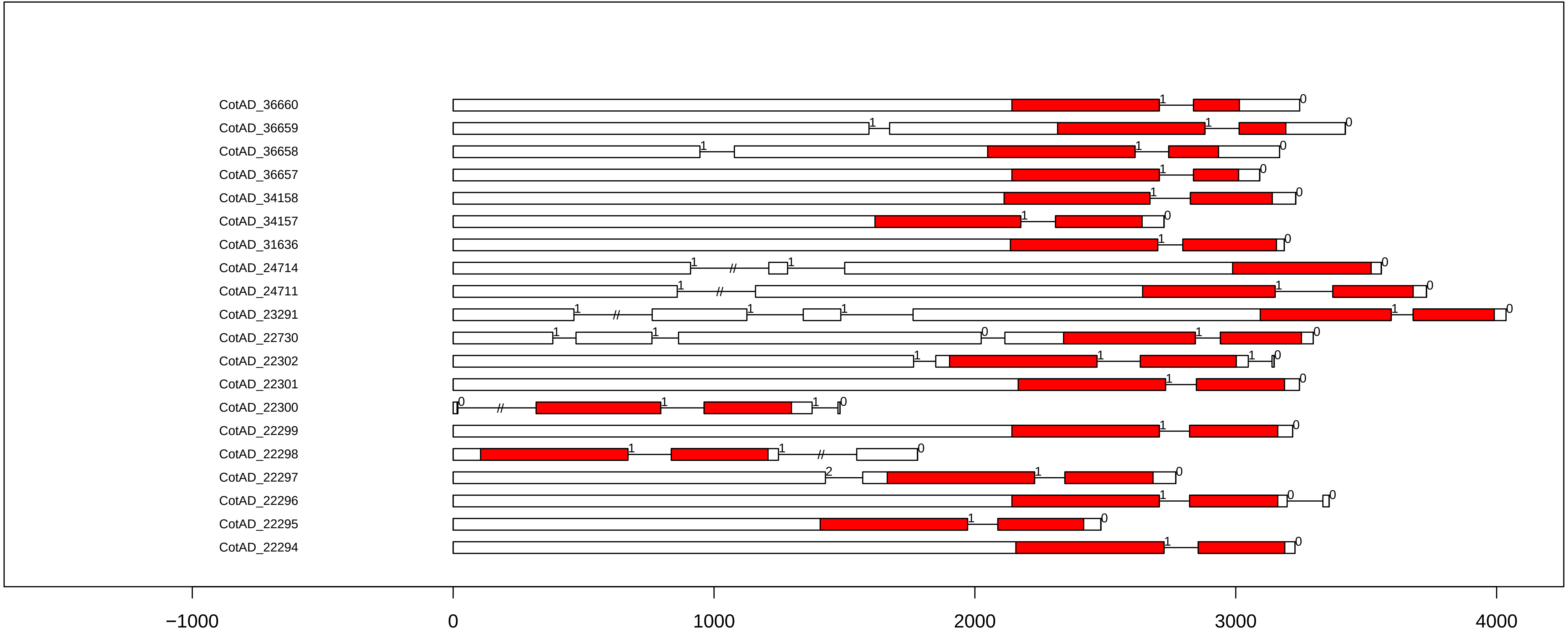

G.hi RLK−Pelle_LRR−XII−1 exon−intron and domain diagram (part 3)

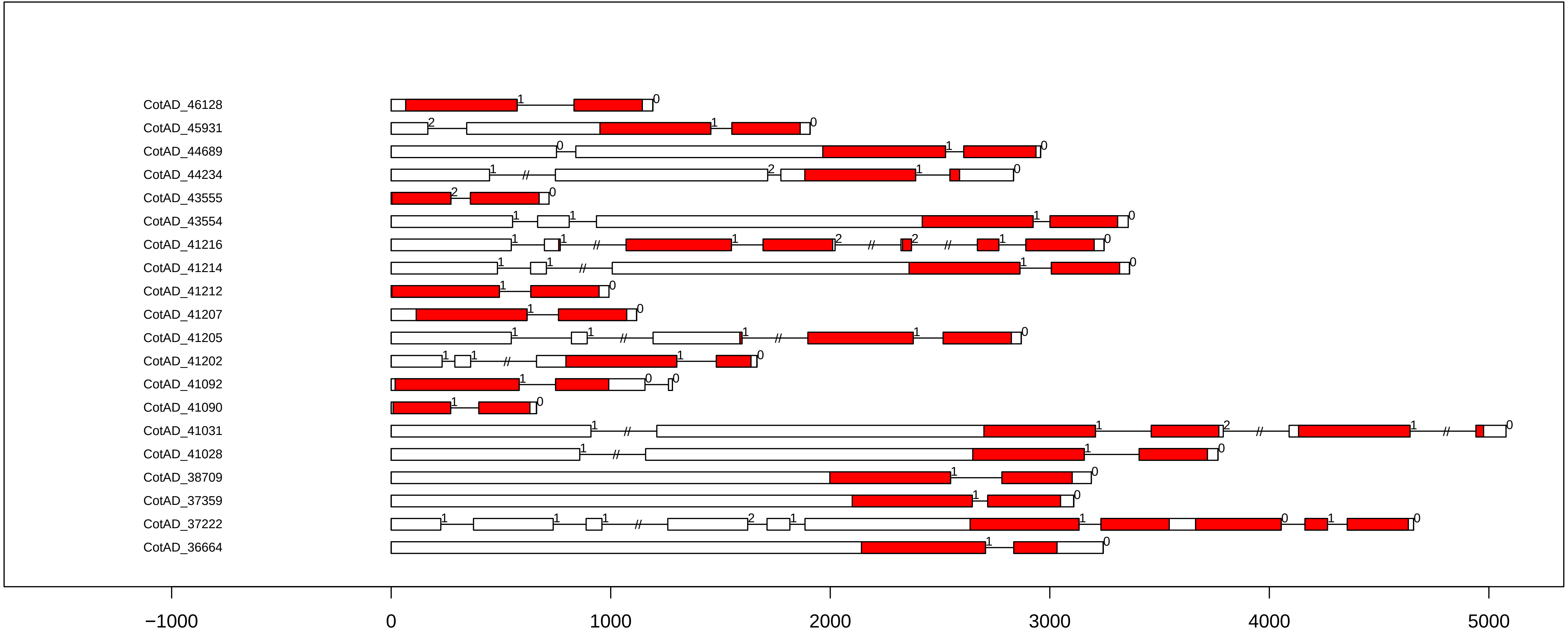

G.hi RLK−Pelle_LRR−XII−1 exon−intron and domain diagram (part 4)

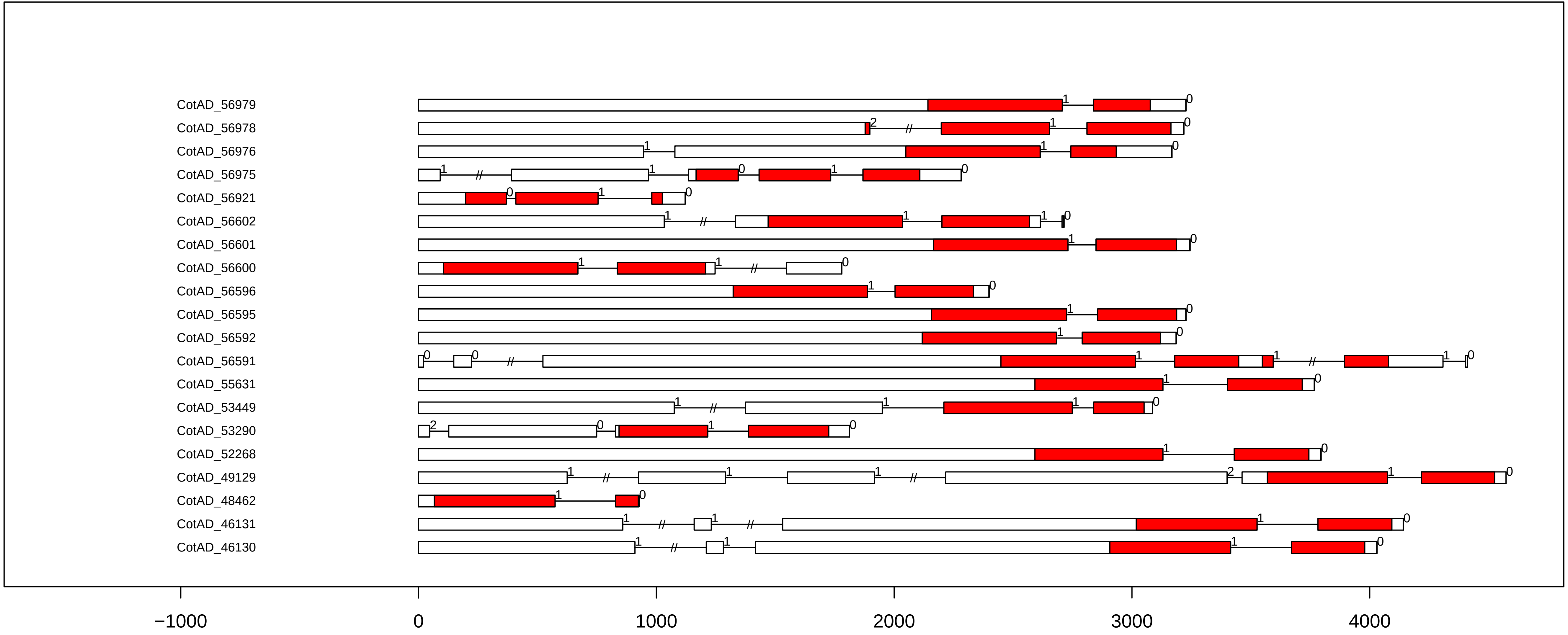

G.hi RLK−Pelle_LRR−XII−1 exon−intron and domain diagram (part 5)

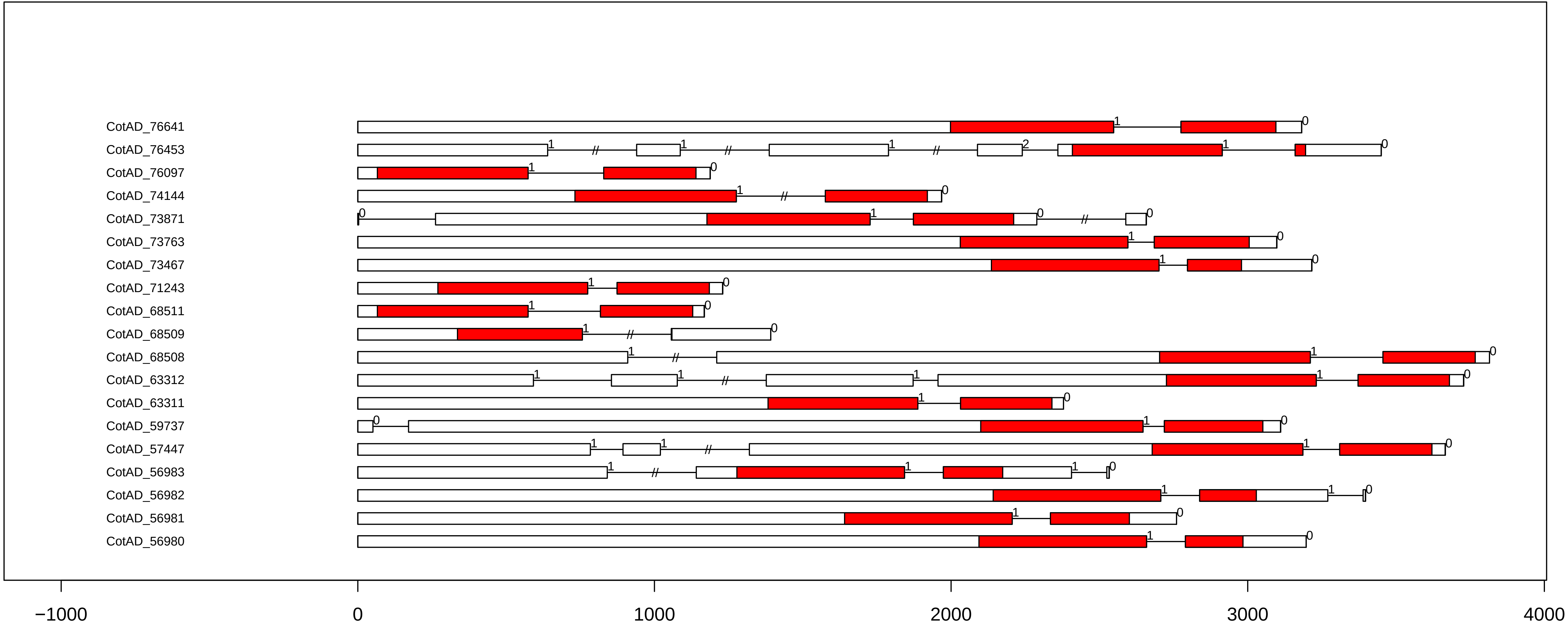

G.hi RLK−Pelle_LRR−XIIIa exon−intron and domain diagram (all)

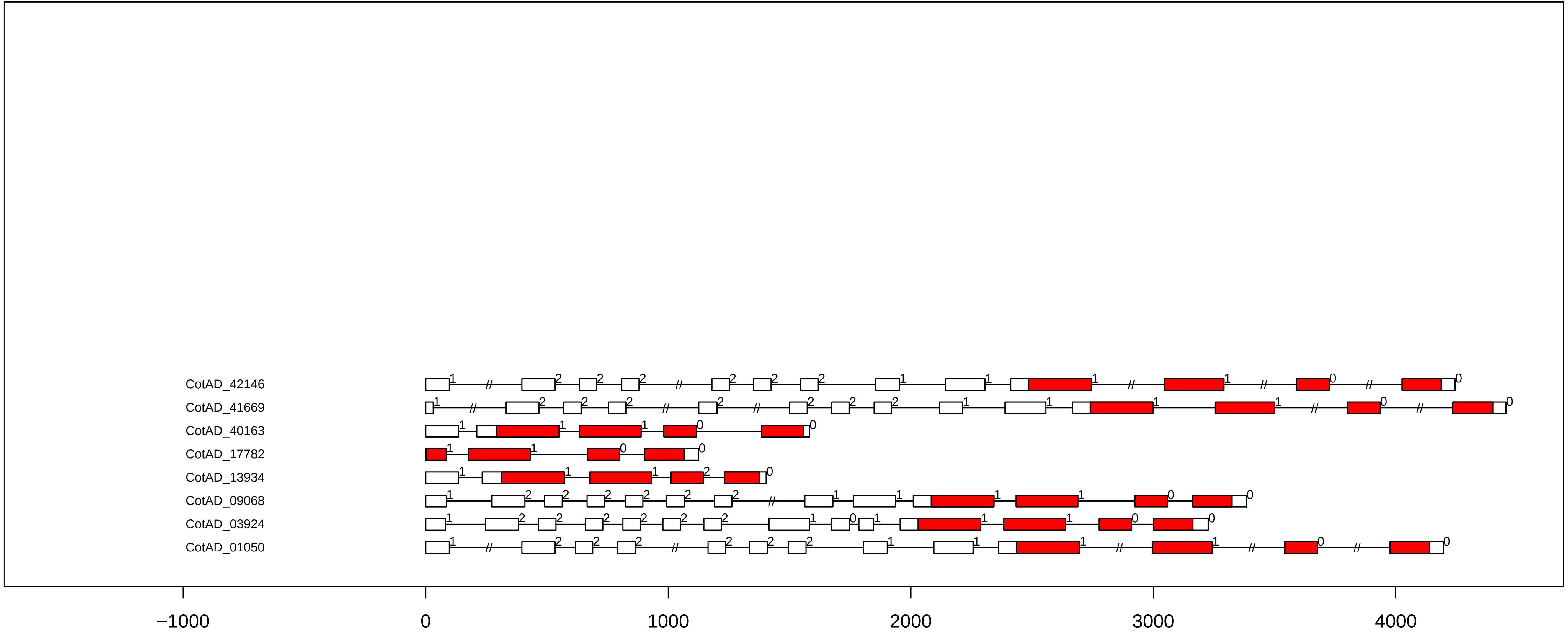

G.hi RLK−Pelle_LRR−XIIIb exon−intron and domain diagram (all)

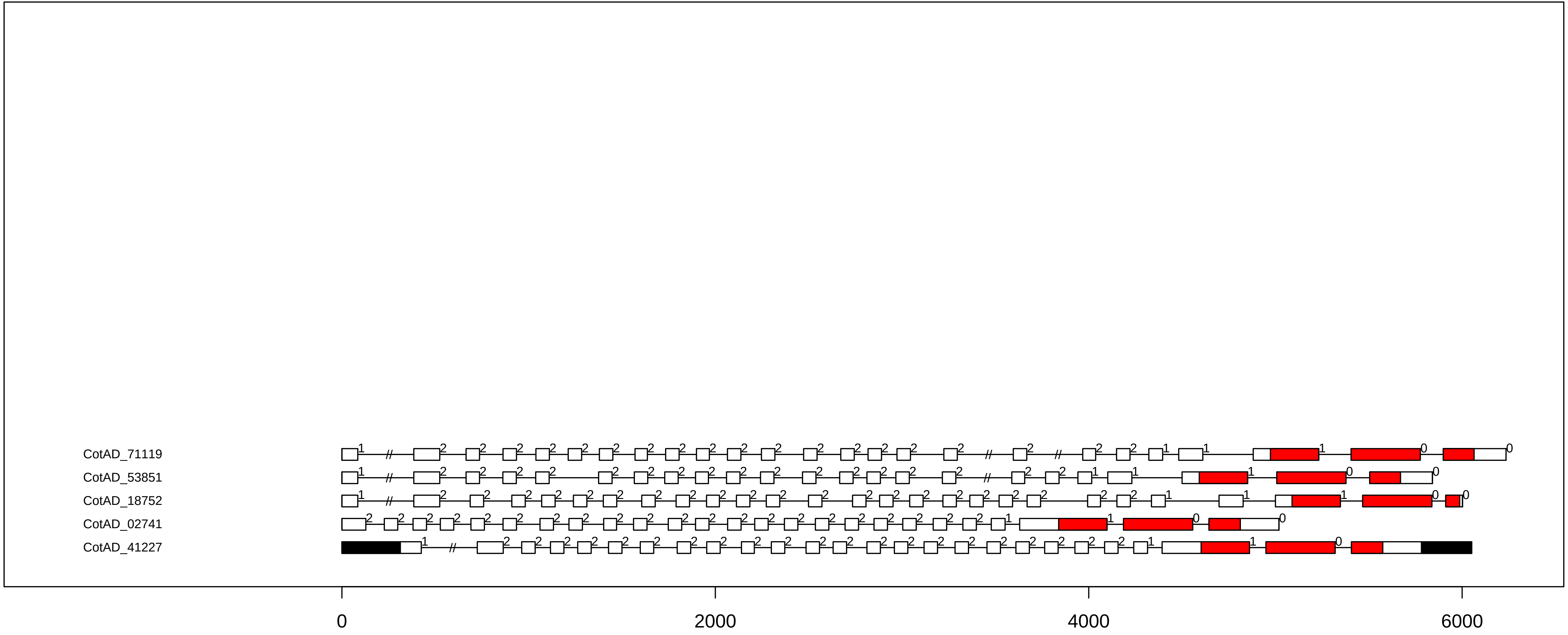

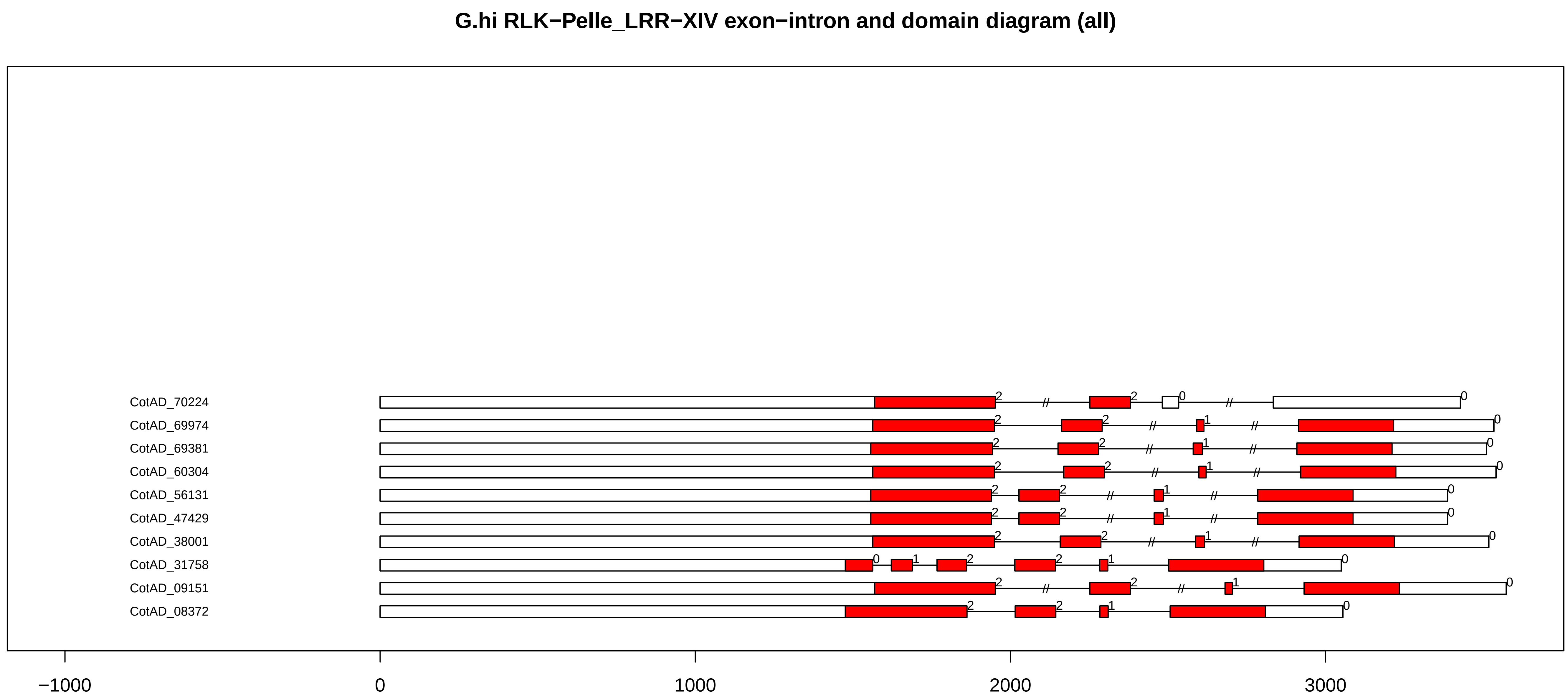

G.hi RLK−Pelle_LRR−XIV exon−intron and domain diagram (all)
CotAD_70224
CotAD_69974
CotAD_69381
CotAD_60304
CotAD_56131
CotAD_47429
CotAD_38001
CotAD_31758
CotAD_09151
CotAD_08372
−1000
0
1000
2000
3000

G.hi RLK−Pelle_LRR−XV exon−intron and domain diagram (all)

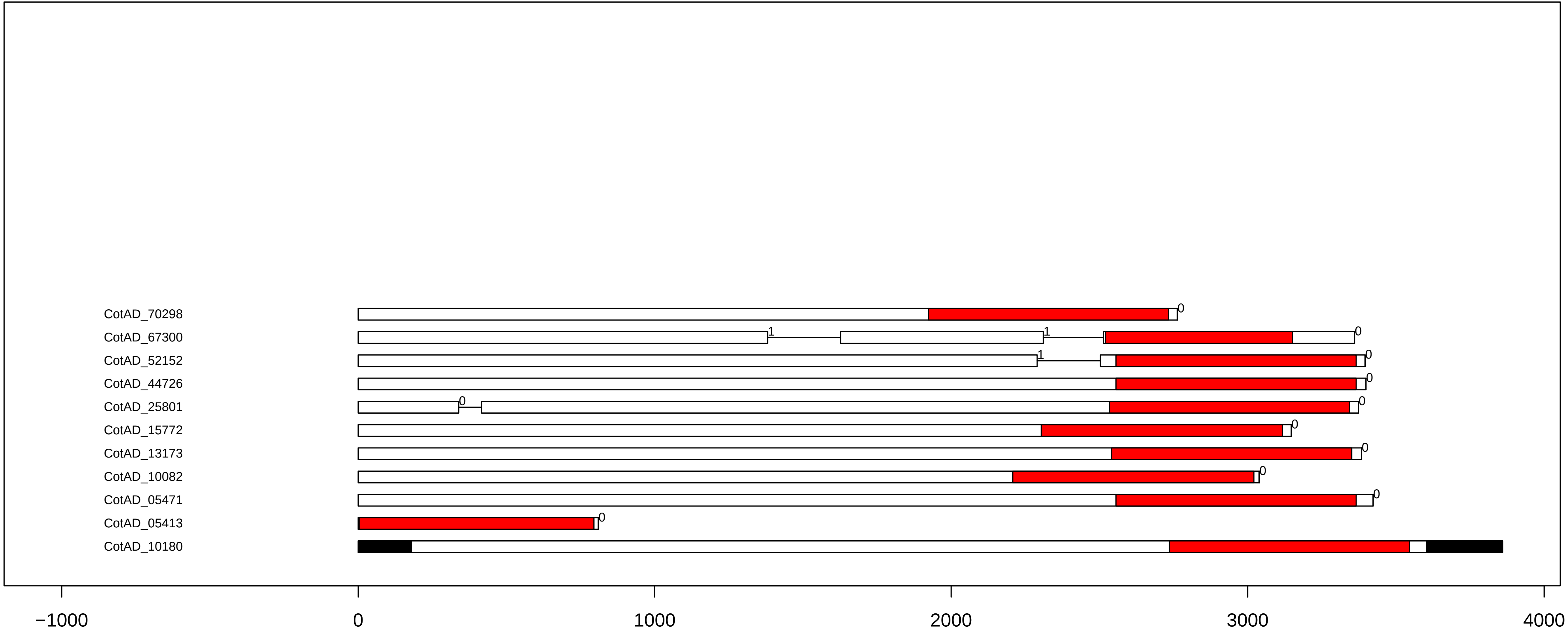

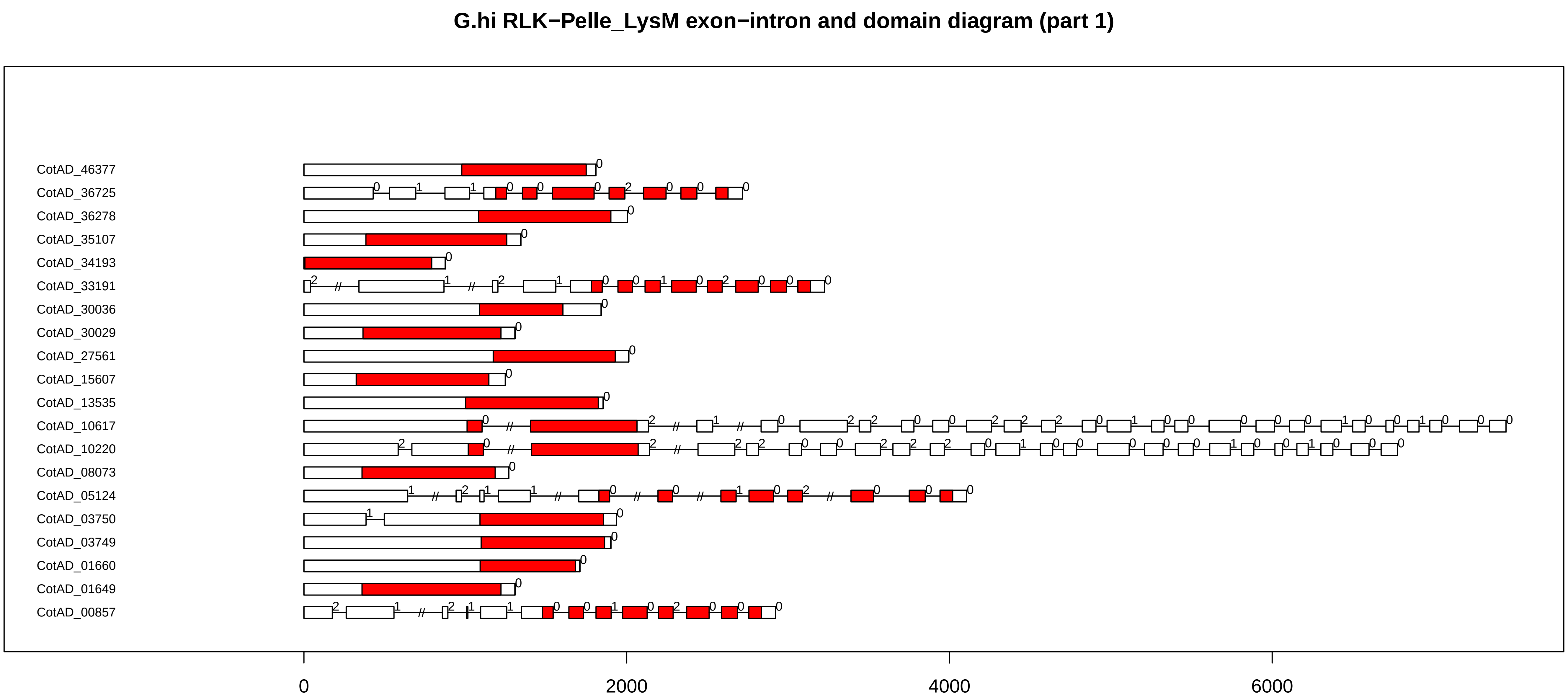
G.hi RLK−Pelle_LysM exon−intron and domain diagram (part 1)
CotAD_46377
CotAD_36725
CotAD_36278
CotAD_35107
CotAD_34193
CotAD_33191
CotAD_30036
CotAD_30029
CotAD_27561
CotAD_15607
CotAD_13535
CotAD_10617
CotAD_10220
CotAD_08073
CotAD_05124
CotAD_03750
CotAD_03749
CotAD_01660
CotAD_01649
CotAD_00857
0
2000
4000
6000

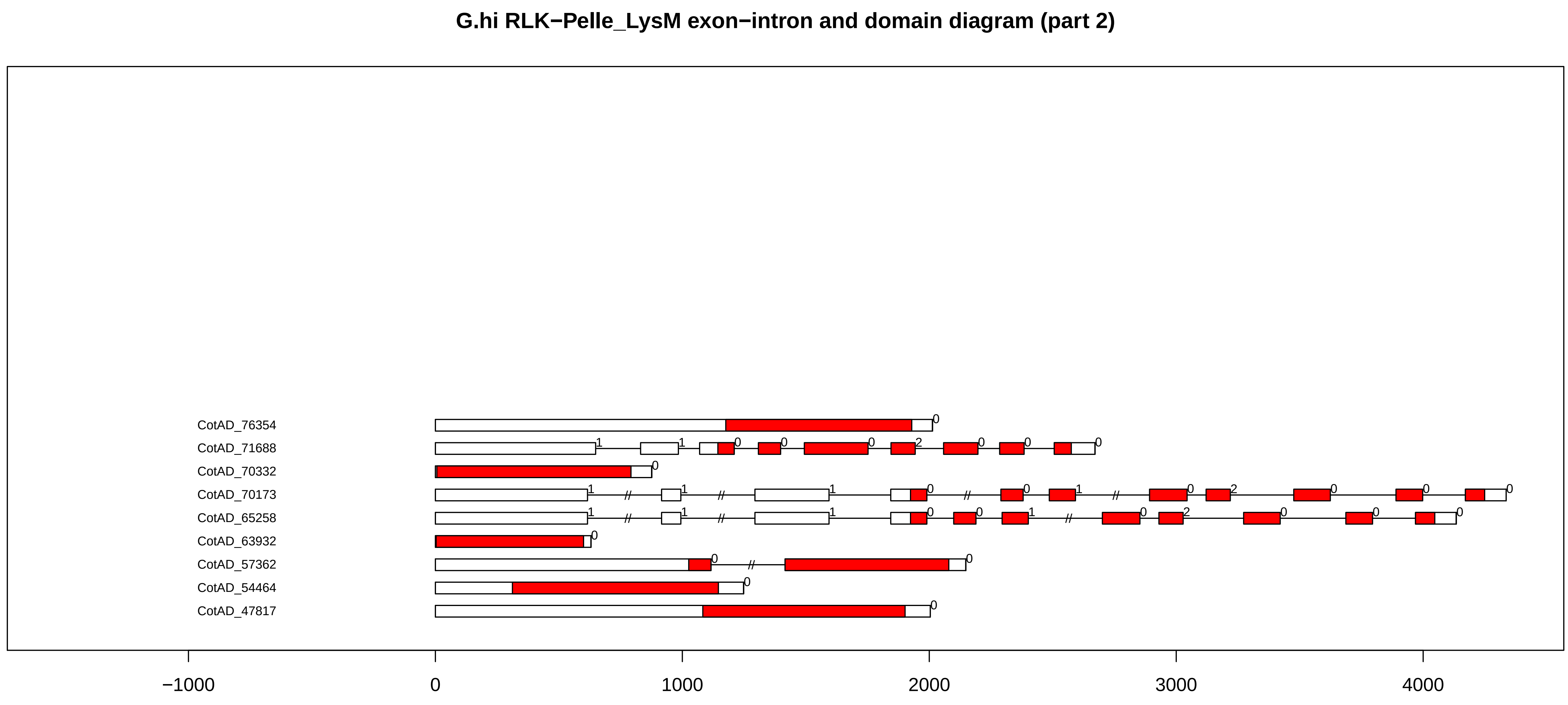
G.hi RLK−Pelle_LysM exon−intron and domain diagram (part 2)
CotAD_76354
CotAD_71688
CotAD_70332
CotAD_70173
CotAD_65258
CotAD_63932
CotAD_57362
CotAD_54464
CotAD_47817
−1000
0
1000
2000
3000
4000

G.hi RLK−Pelle_PERK−1 exon−intron and domain diagram (part 1)

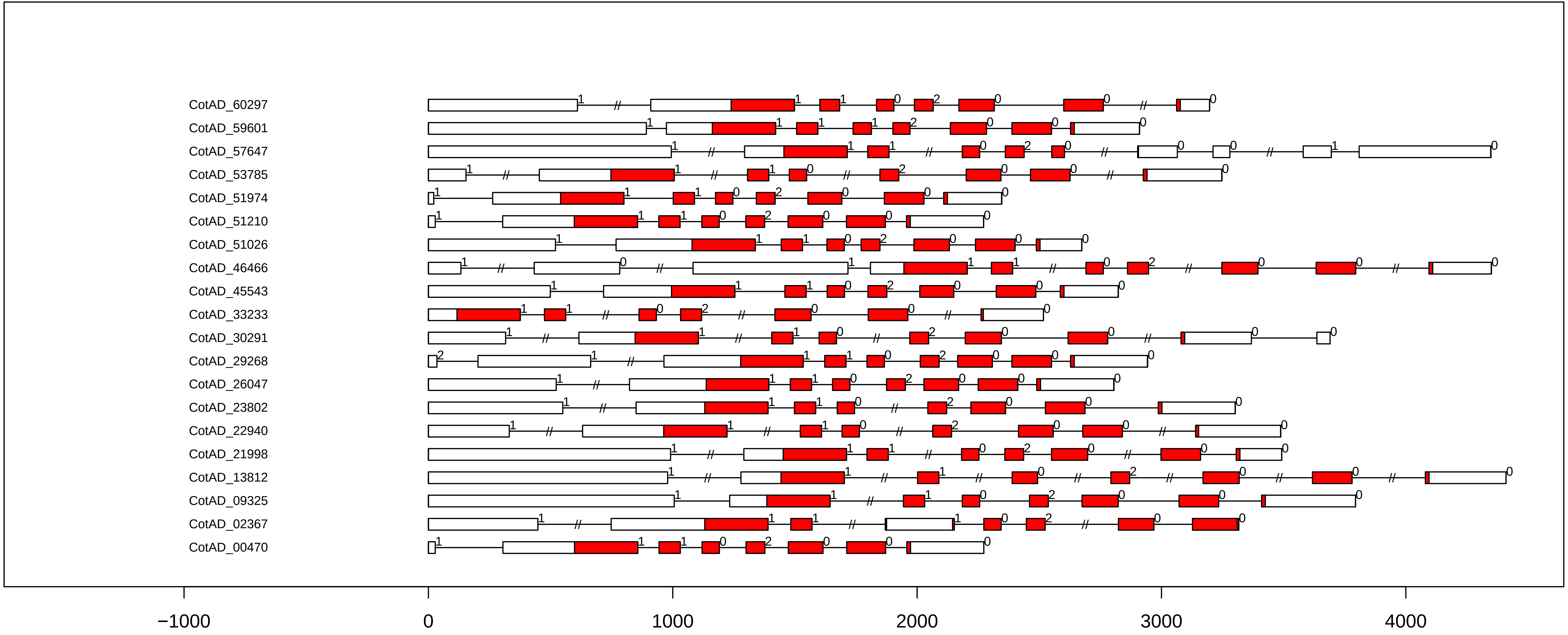

G.hi RLK−Pelle_PERK−1 exon−intron and domain diagram (part 2)

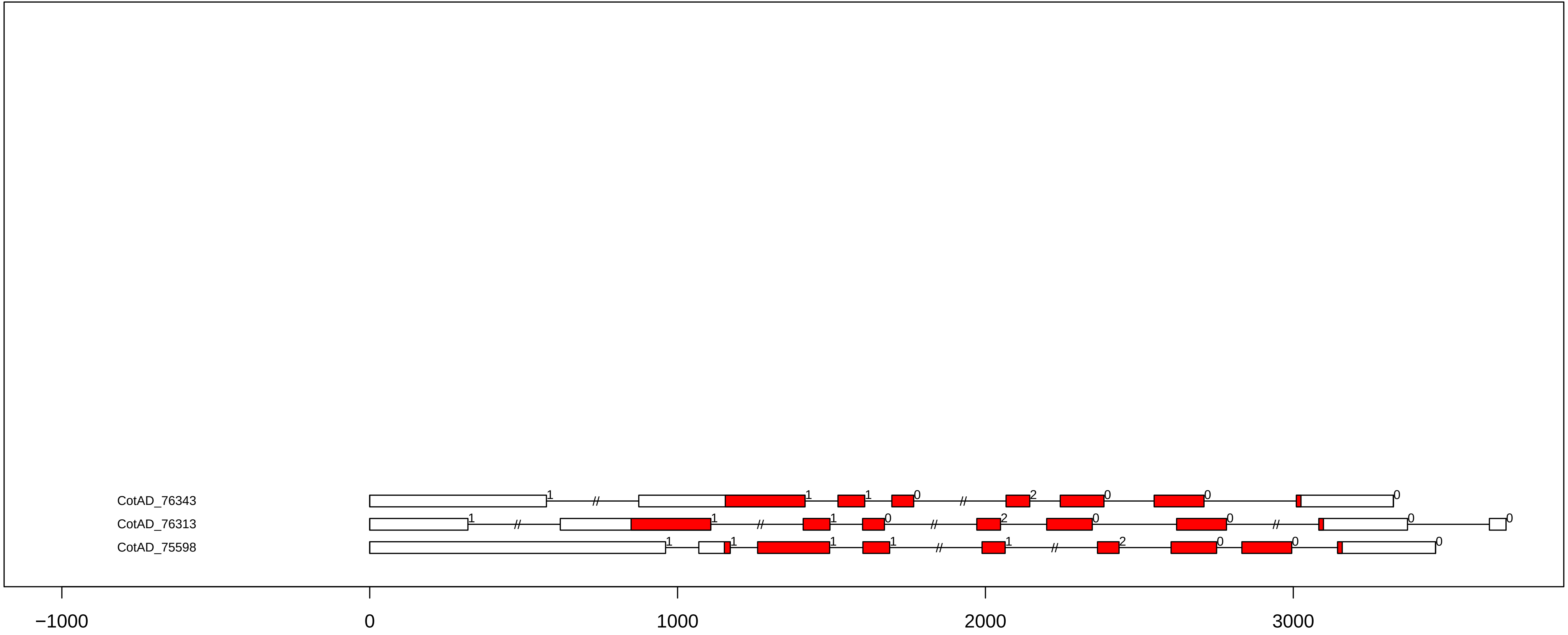

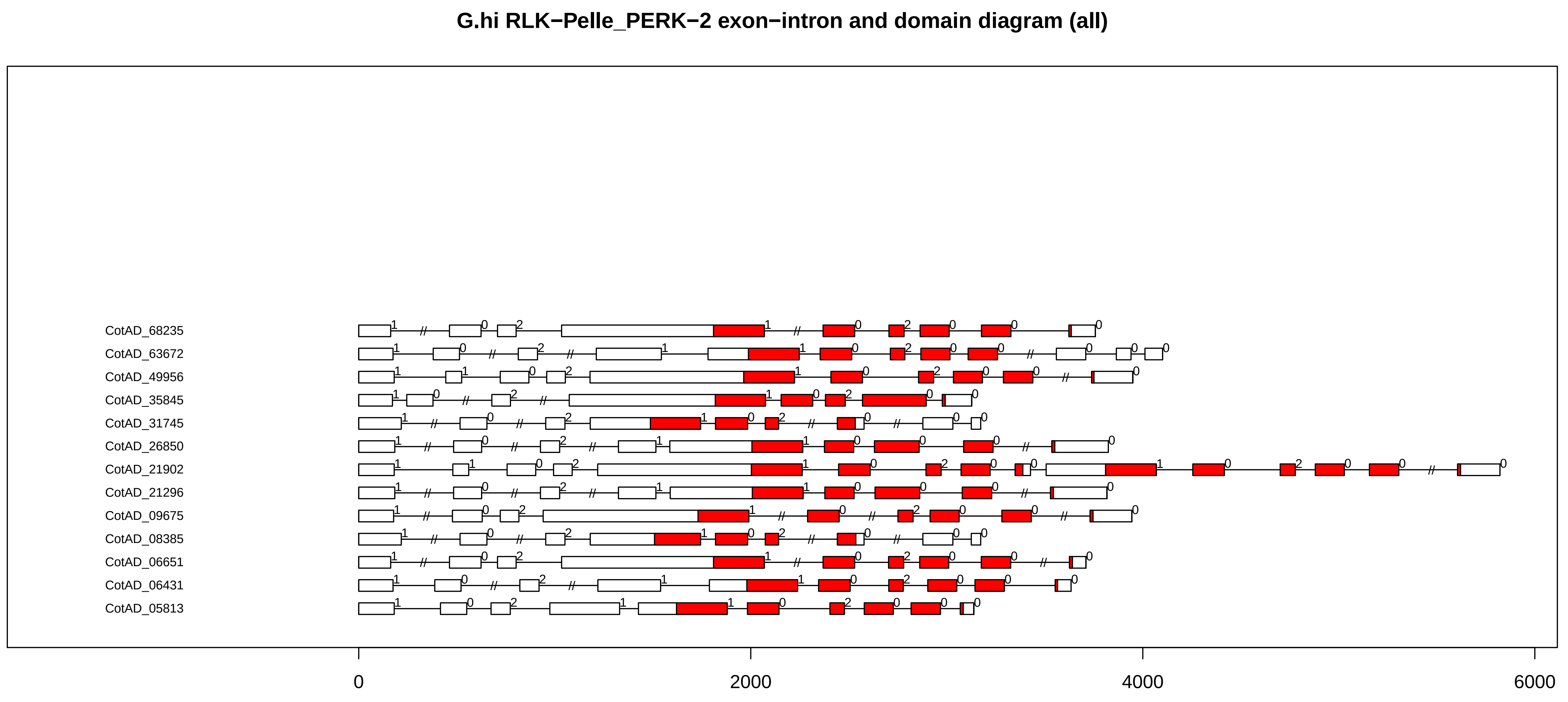
G.hi RLK−Pelle_PERK−2 exon−intron and domain diagram (all)
CotAD_68235
CotAD_63672
CotAD_49956
CotAD_35845
CotAD_31745
CotAD_26850
CotAD_21902
CotAD_21296
CotAD_09675
CotAD_08385
CotAD_06651
CotAD_06431
CotAD_05813
0
2000
4000
6000

G.hi RLK−Pelle_RKF3 exon−intron and domain diagram (all)

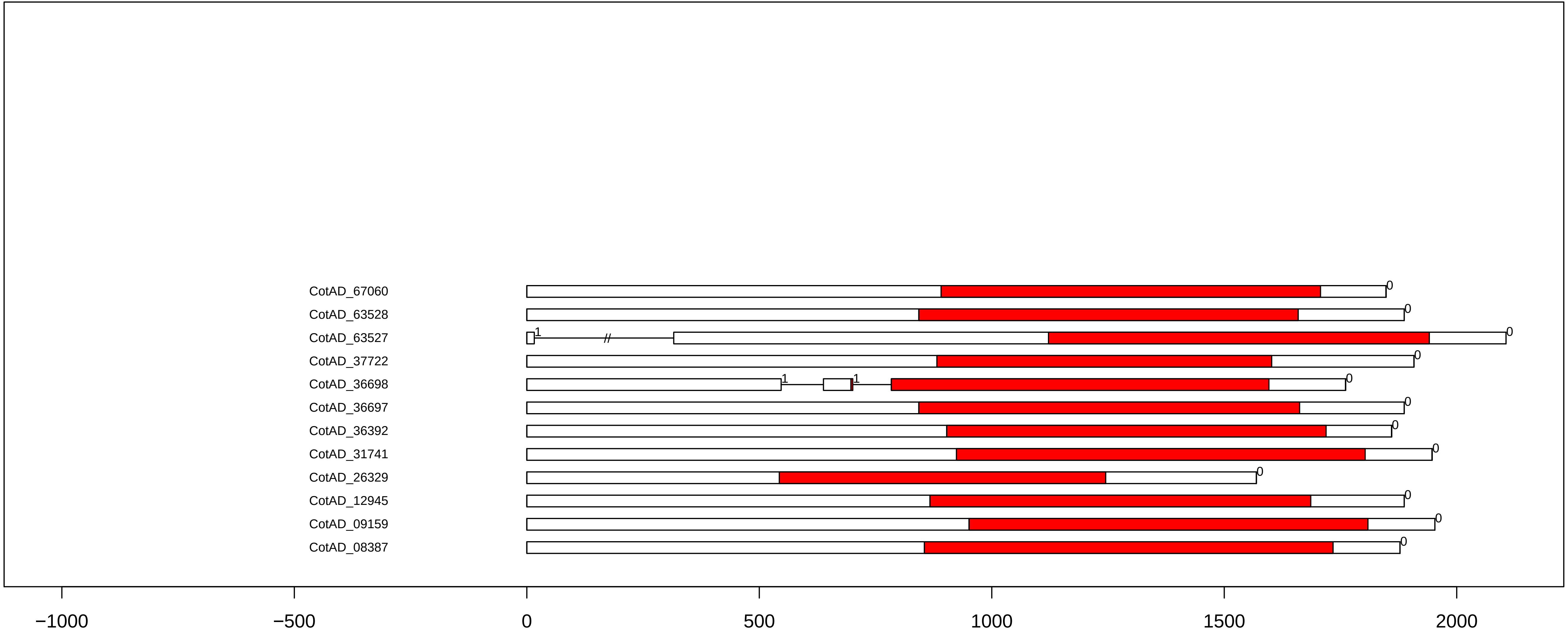

G.hi RLK−Pelle_RLCK−II exon−intron and domain diagram (all)

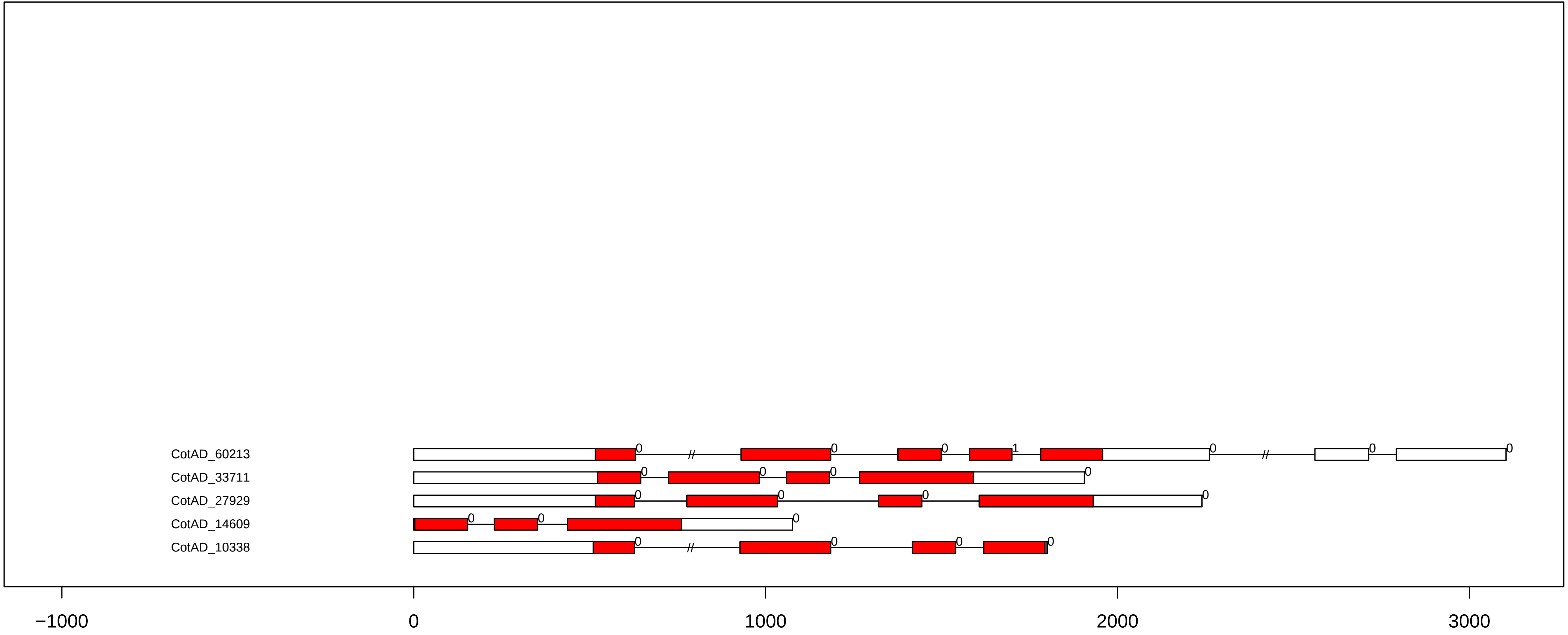

G.hi RLK−Pelle_RLCK−IV exon−intron and domain diagram (all)

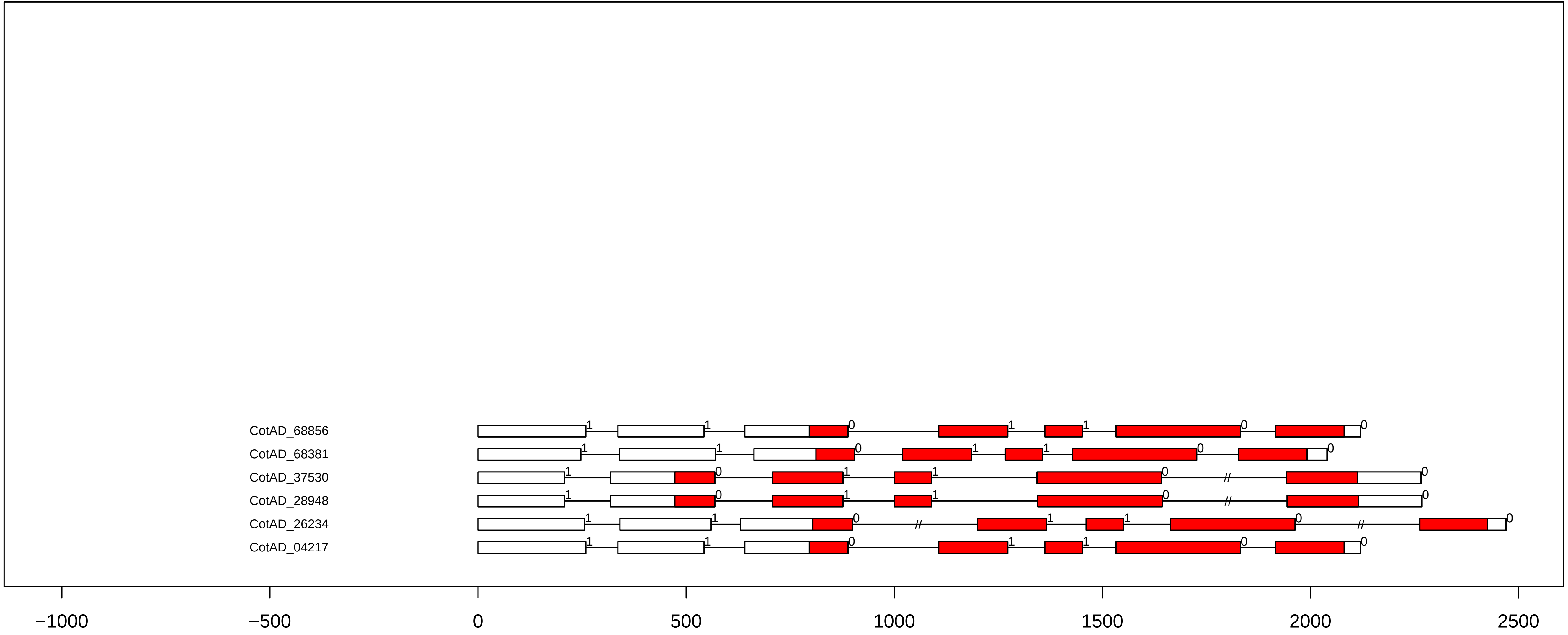

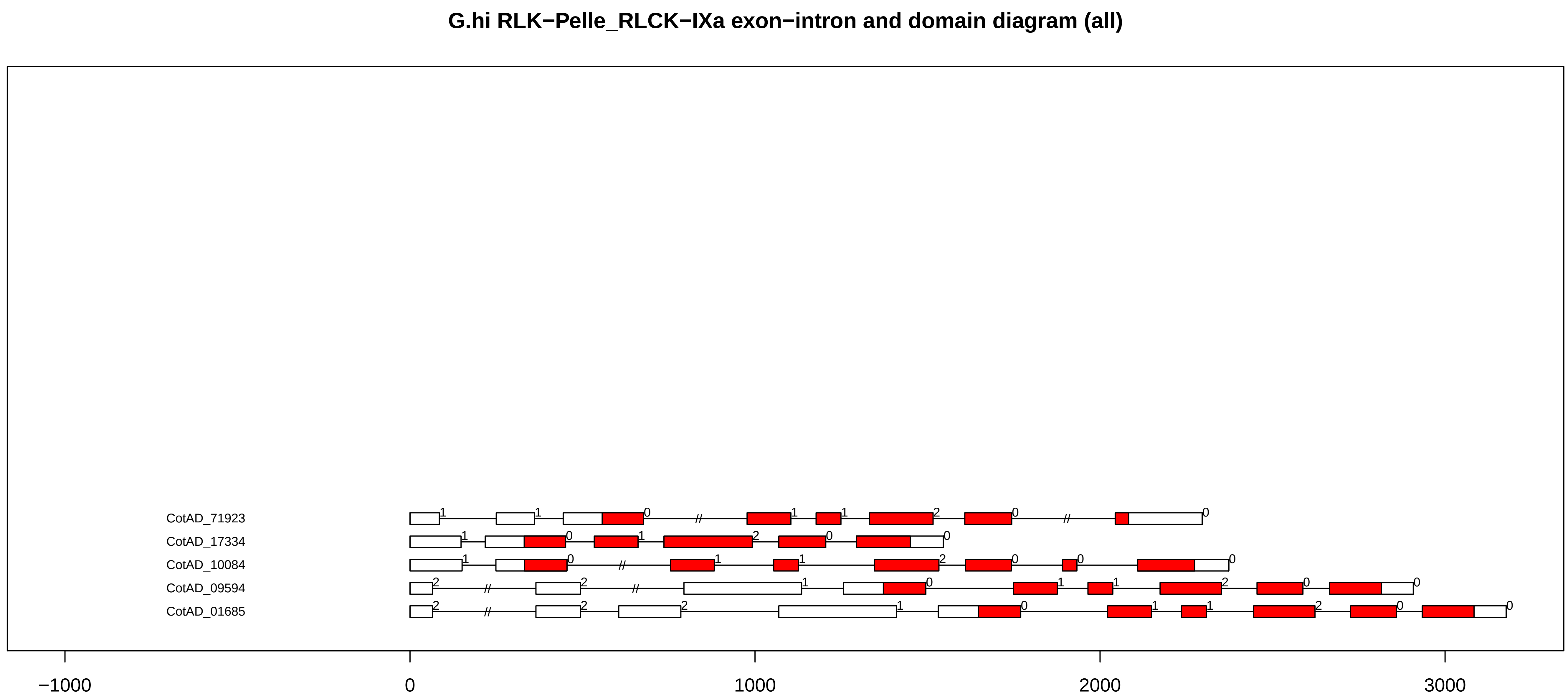

G.hi RLK−Pelle_RLCK−IXa exon−intron and domain diagram (all)
CotAD_71923
CotAD_17334
CotAD_10084
CotAD_09594
CotAD_01685
−1000
0
1000
2000
3000

G.hi RLK−Pelle_RLCK−IXb exon−intron and domain diagram (part 1)

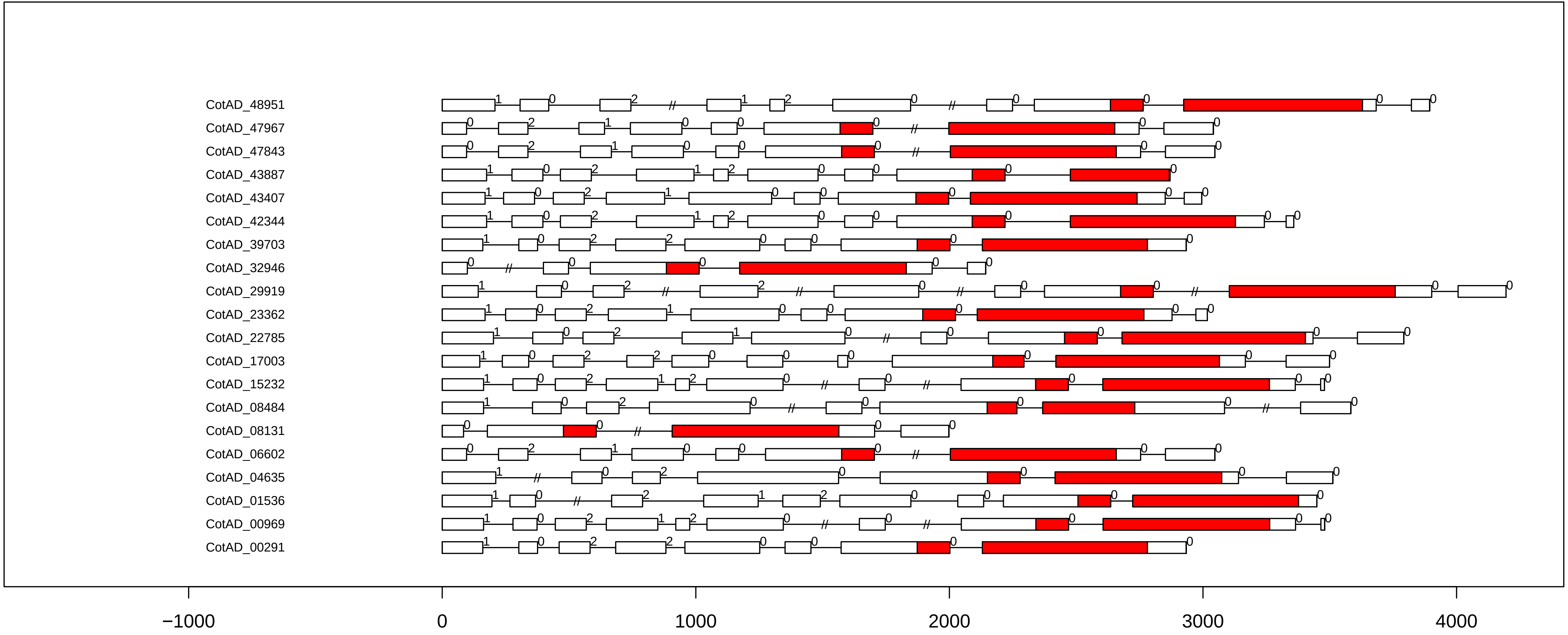

G.hi RLK−Pelle_RLCK−IXb exon−intron and domain diagram (part 2)

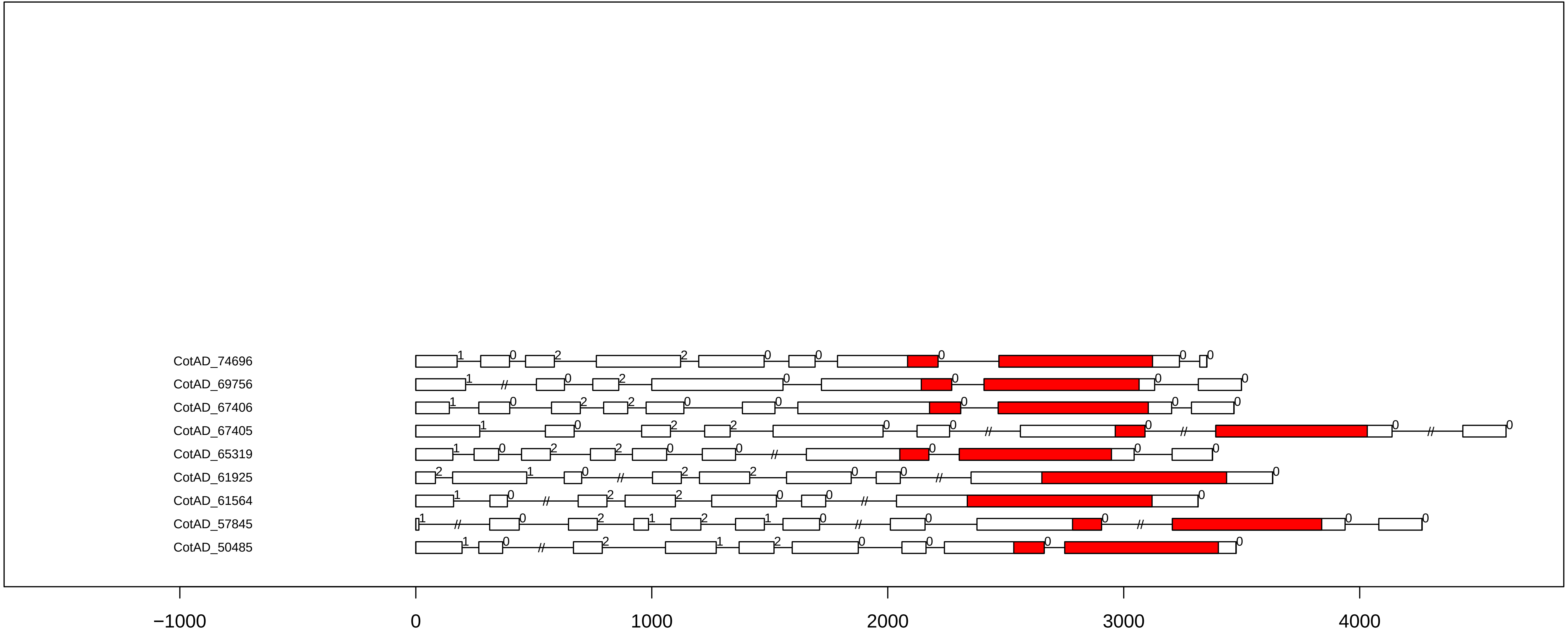

G.hi RLK−Pelle_RLCK−Os exon−intron and domain diagram (all)

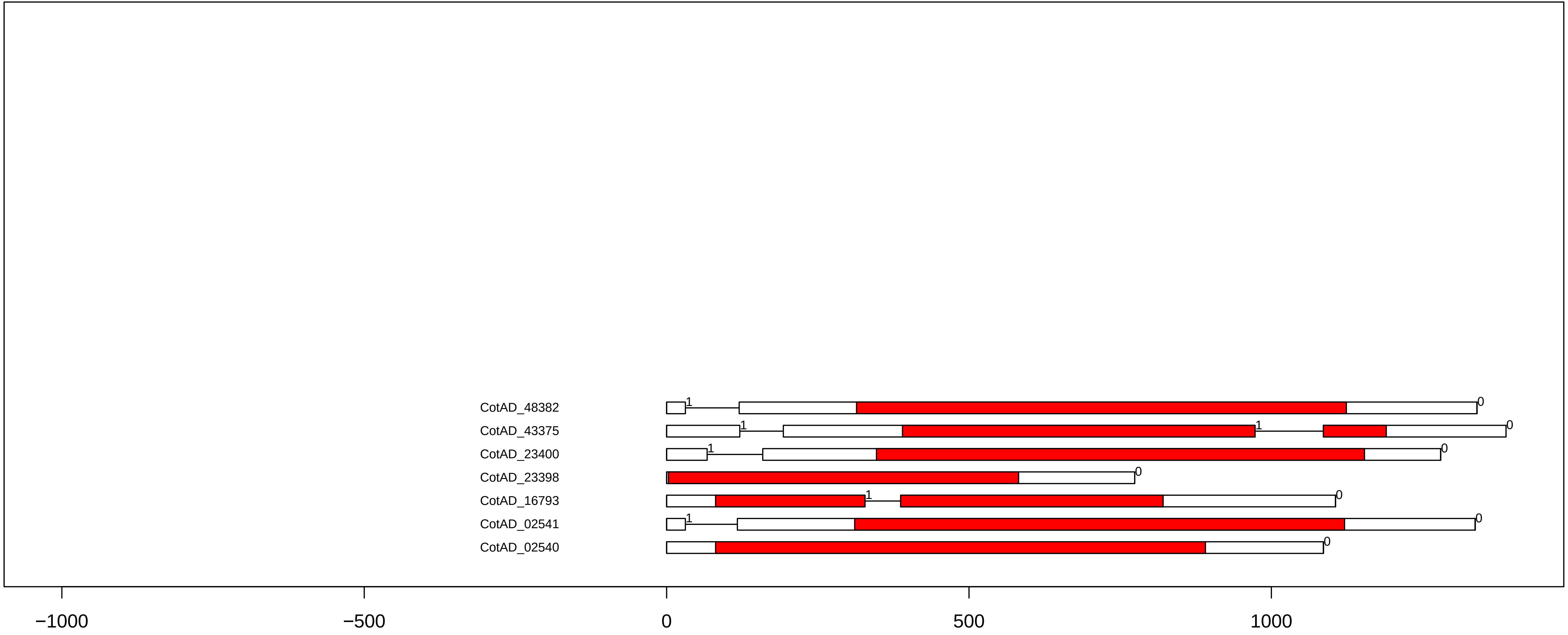

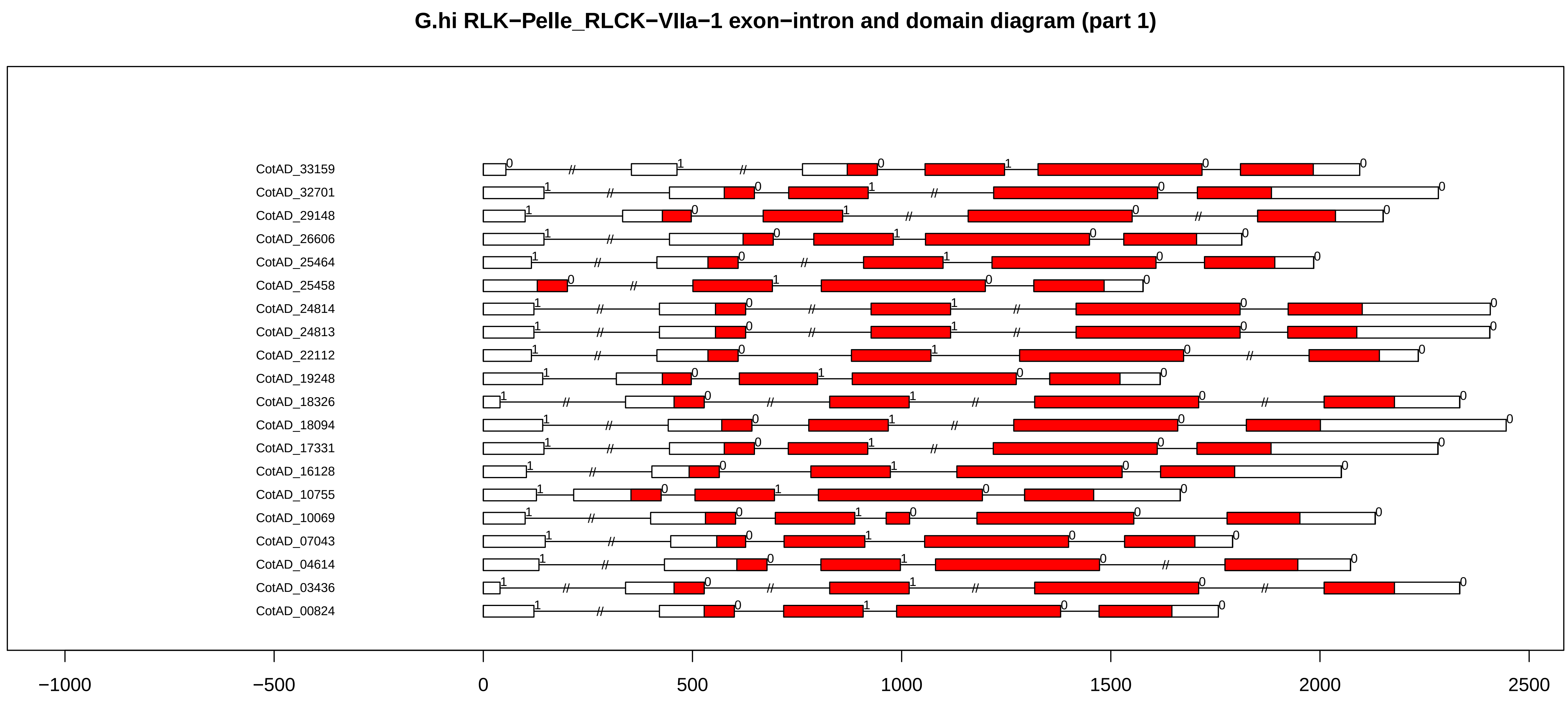
G.hi RLK−Pelle_RLCK−VIIa−1 exon−intron and domain diagram (part 1)
CotAD_33159
CotAD_32701
CotAD_29148
CotAD_26606
CotAD_25464
CotAD_25458
CotAD_24814
CotAD_24813
CotAD_22112
CotAD_19248
CotAD_18326
CotAD_18094
CotAD_17331
CotAD_16128
CotAD_10755
CotAD_10069
CotAD_07043
CotAD_04614
CotAD_03436
CotAD_00824
−1000
−500
0
500
1000
1500
2000
2500

G.hi RLK−Pelle_RLCK−VIIa−1 exon−intron and domain diagram (part 2)

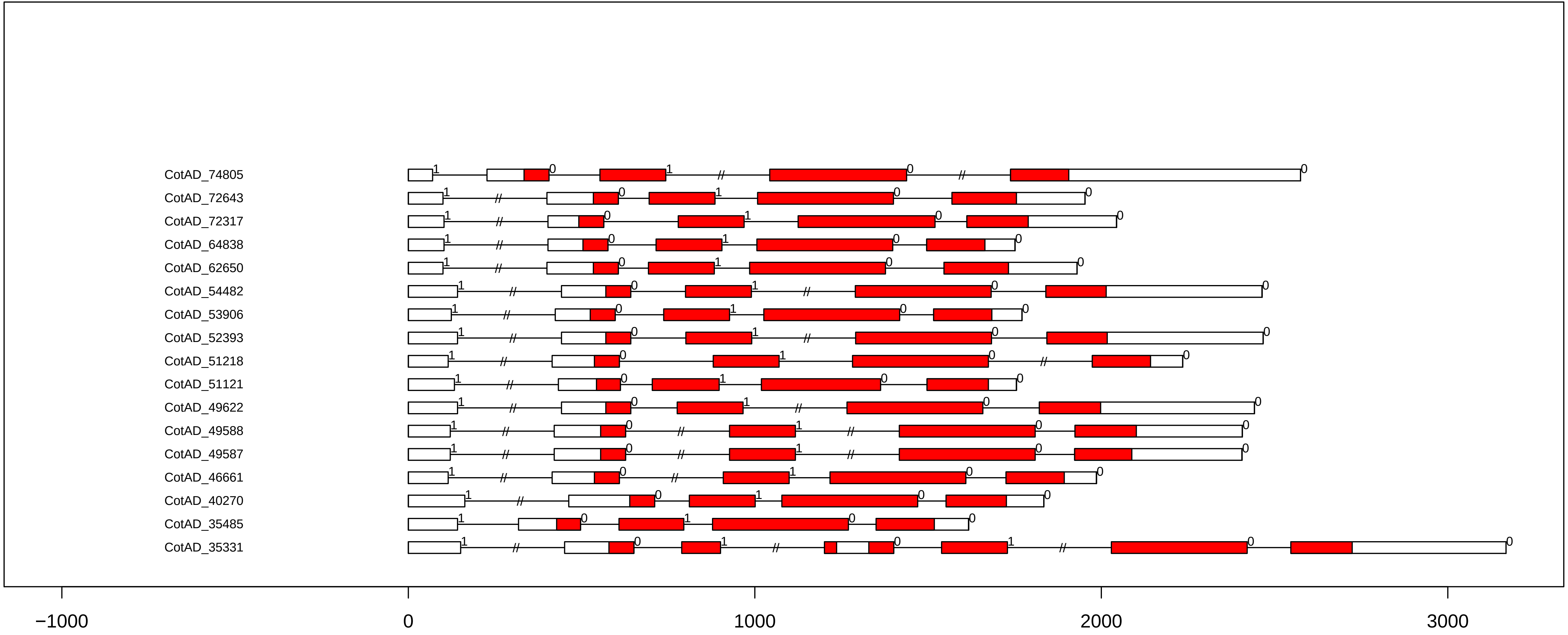

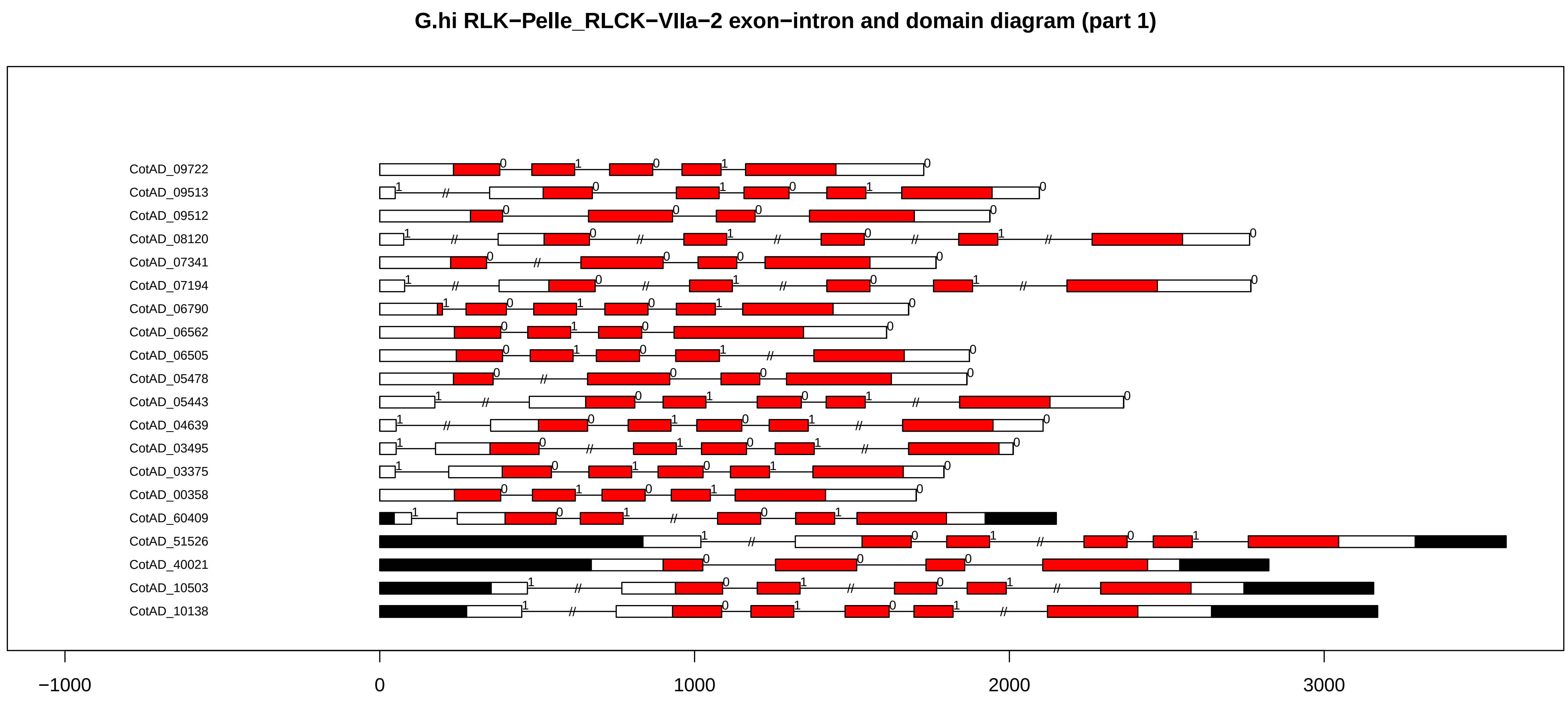
G.hi RLK−Pelle_RLCK−VIIa−2 exon−intron and domain diagram (part 1)
CotAD_09722
CotAD_09513
CotAD_09512
CotAD_08120
CotAD_07341
CotAD_07194
CotAD_06790
CotAD_06562
CotAD_06505
CotAD_05478
CotAD_05443
CotAD_04639
CotAD_03495
CotAD_03375
CotAD_00358
CotAD_60409
CotAD_51526
CotAD_40021
CotAD_10503
CotAD_10138
−1000
0
1000
2000
3000

G.hi RLK−Pelle_RLCK−VIIa−2 exon−intron and domain diagram (part 2)

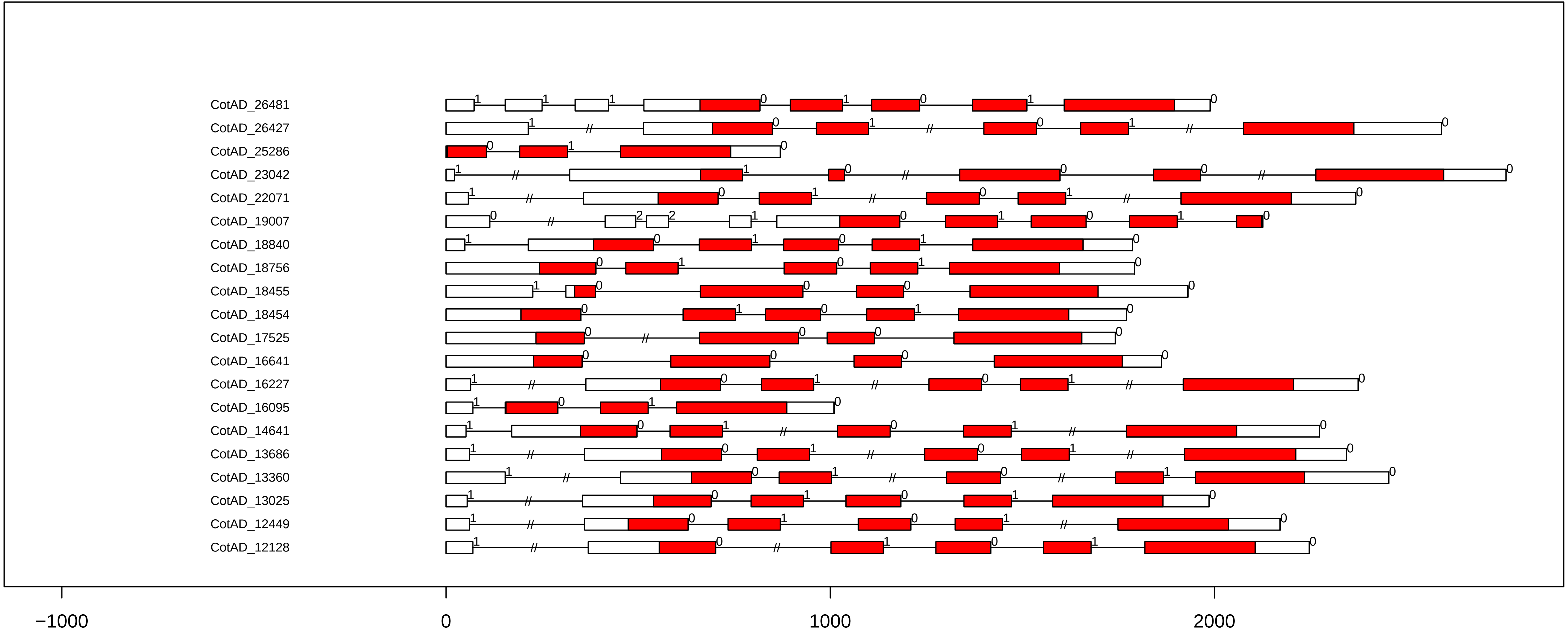

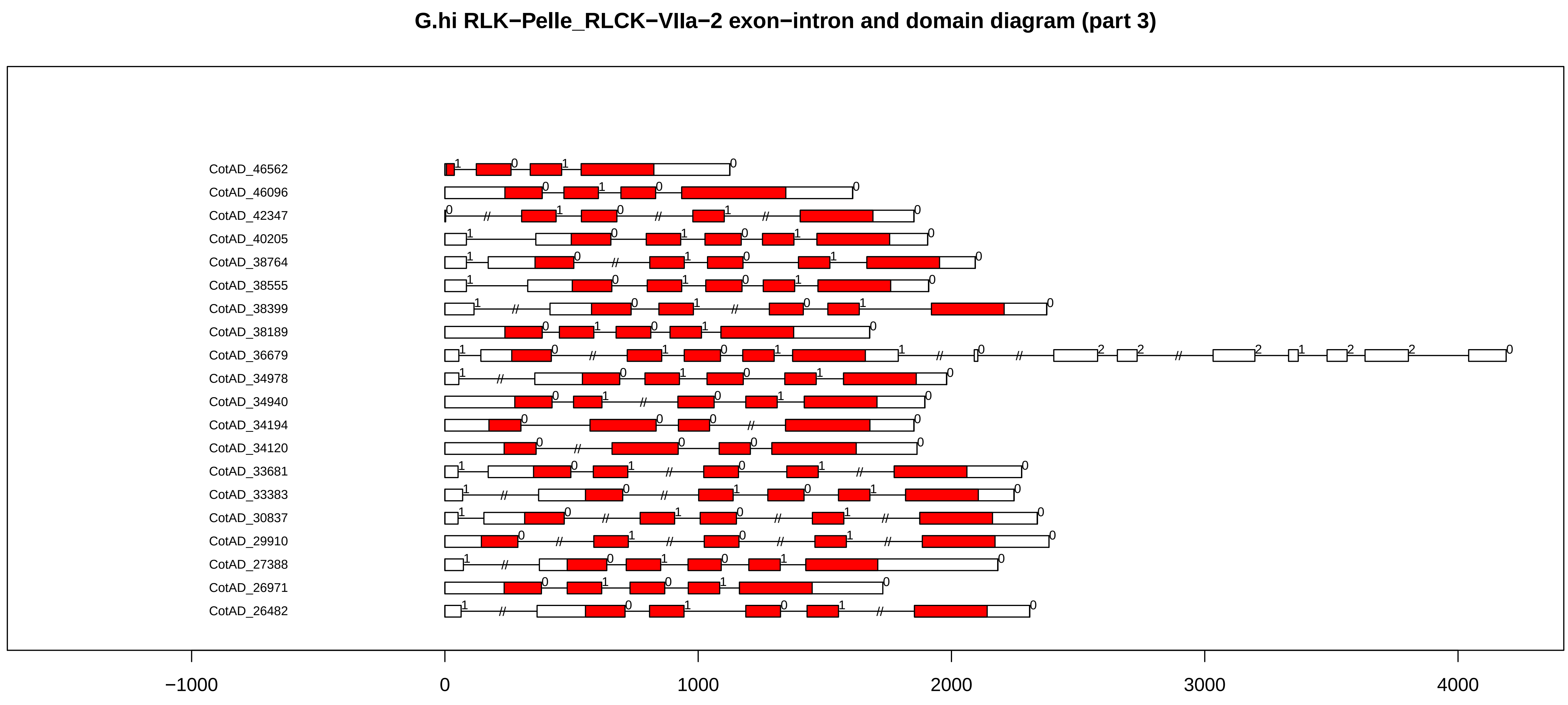
G.hi RLK−Pelle_RLCK−VIIa−2 exon−intron and domain diagram (part 3)
CotAD_46562
CotAD_46096
CotAD_42347
CotAD_40205
CotAD_38764
CotAD_38555
CotAD_38399
CotAD_38189
CotAD_36679
CotAD_34978
CotAD_34940
CotAD_34194
CotAD_34120
CotAD_33681
CotAD_33383
CotAD_30837
CotAD_29910
CotAD_27388
CotAD_26971
CotAD_26482
−1000
0
1000
2000
3000
4000

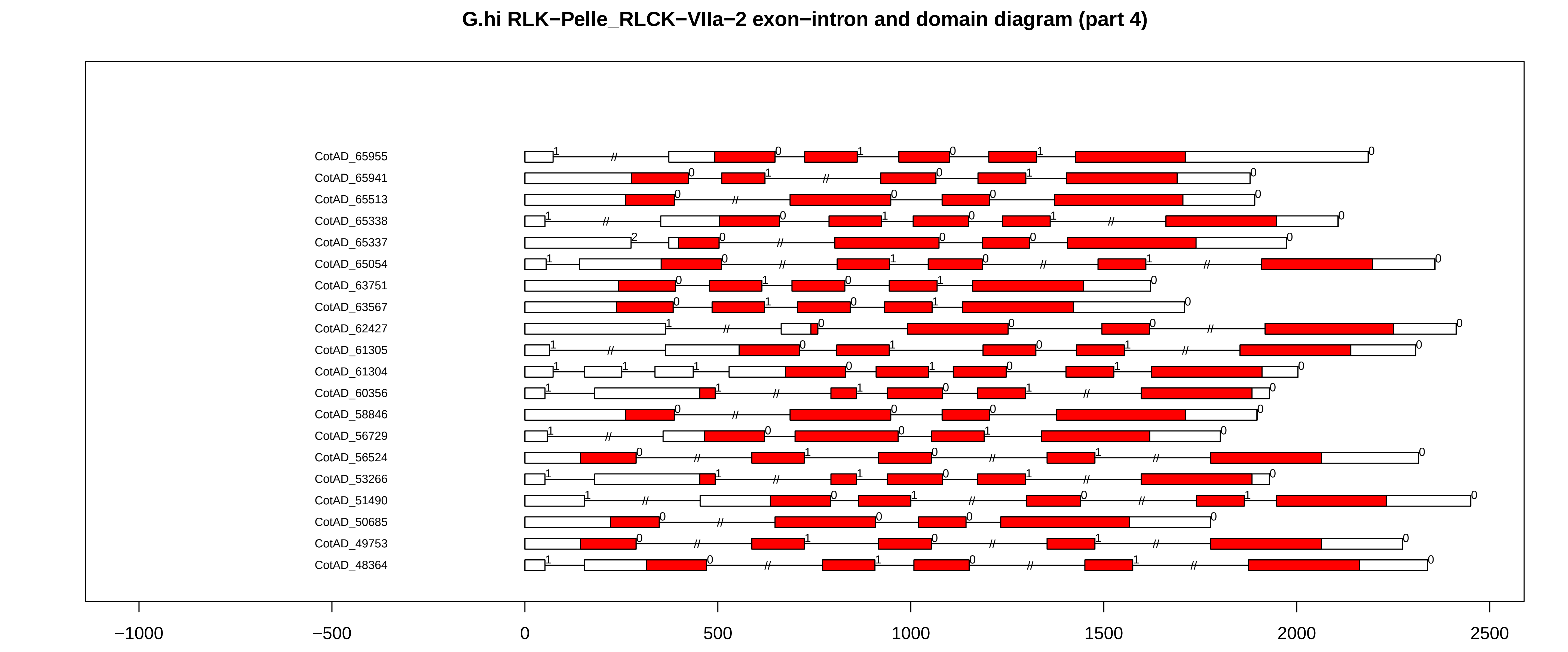
G.hi RLK−Pelle_RLCK−VIIa−2 exon−intron and domain diagram (part 4)
CotAD_65955
CotAD_65941
CotAD_65513
CotAD_65338
CotAD_65337
CotAD_65054
CotAD_63751
CotAD_63567
CotAD_62427
CotAD_61305
CotAD_61304
CotAD_60356
CotAD_58846
CotAD_56729
CotAD_56524
CotAD_53266
CotAD_51490
CotAD_50685
CotAD_49753
CotAD_48364
−1000
−500
0
500
1000
1500
2000
2500

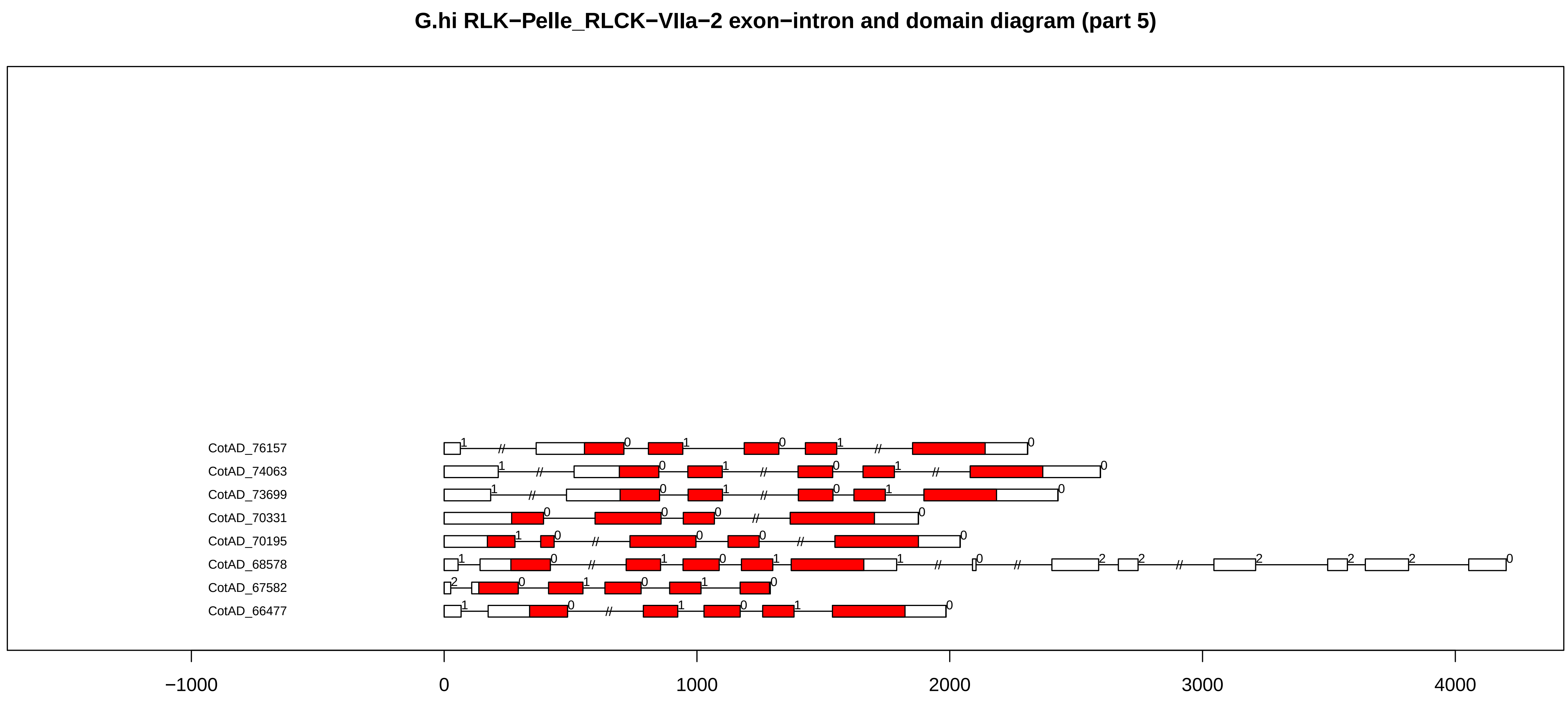

G.hi RLK−Pelle_RLCK−VIIa−2 exon−intron and domain diagram (part 5)
CotAD_76157
CotAD_74063
CotAD_73699
CotAD_70331
CotAD_70195
CotAD_68578
CotAD_67582
CotAD_66477
−1000
0
1000
2000
3000
4000

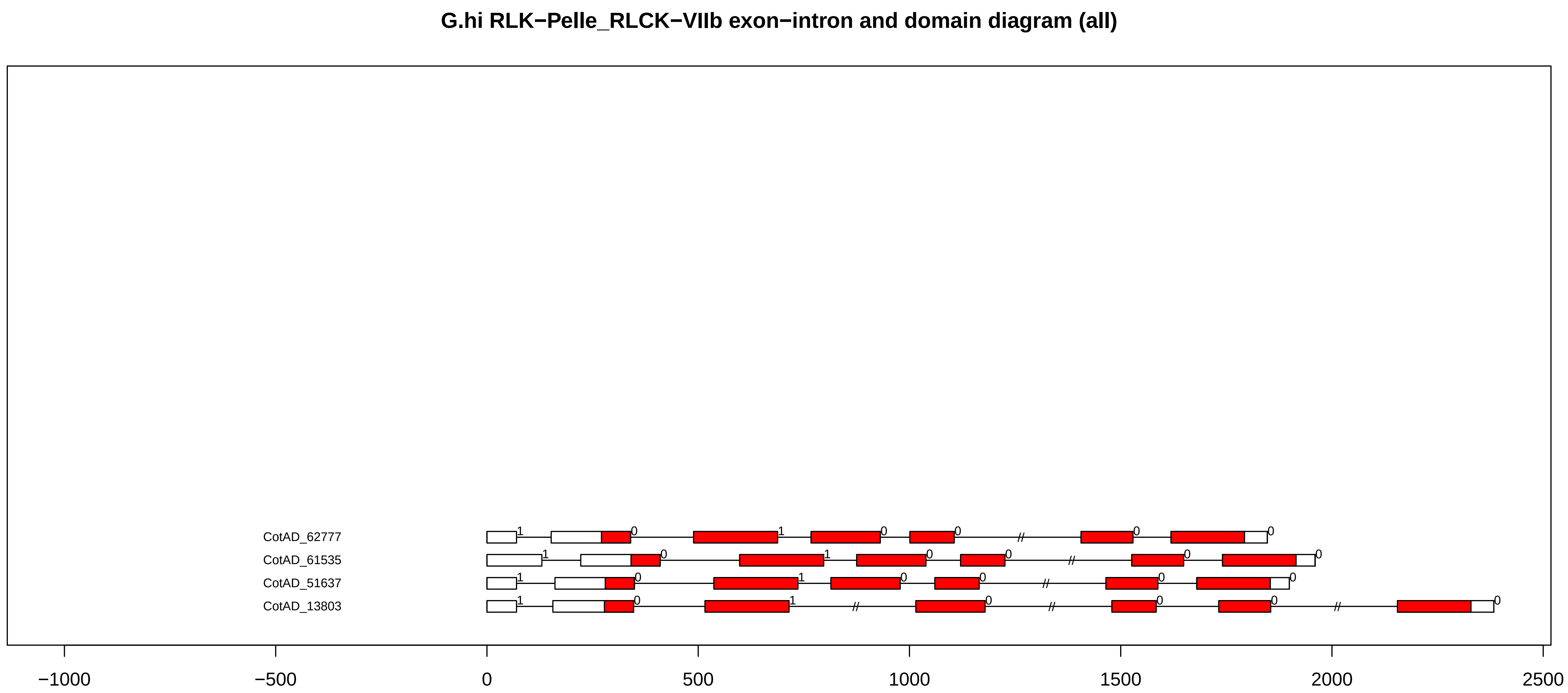
G.hi RLK−Pelle_RLCK−VIIb exon−intron and domain diagram (all)
CotAD_62777
CotAD_61535
CotAD_51637
CotAD_13803
−1000
−500
0
500
1000
1500
2000
2500

G.hi RLK−Pelle_RLCK−VIII exon−intron and domain diagram (all)

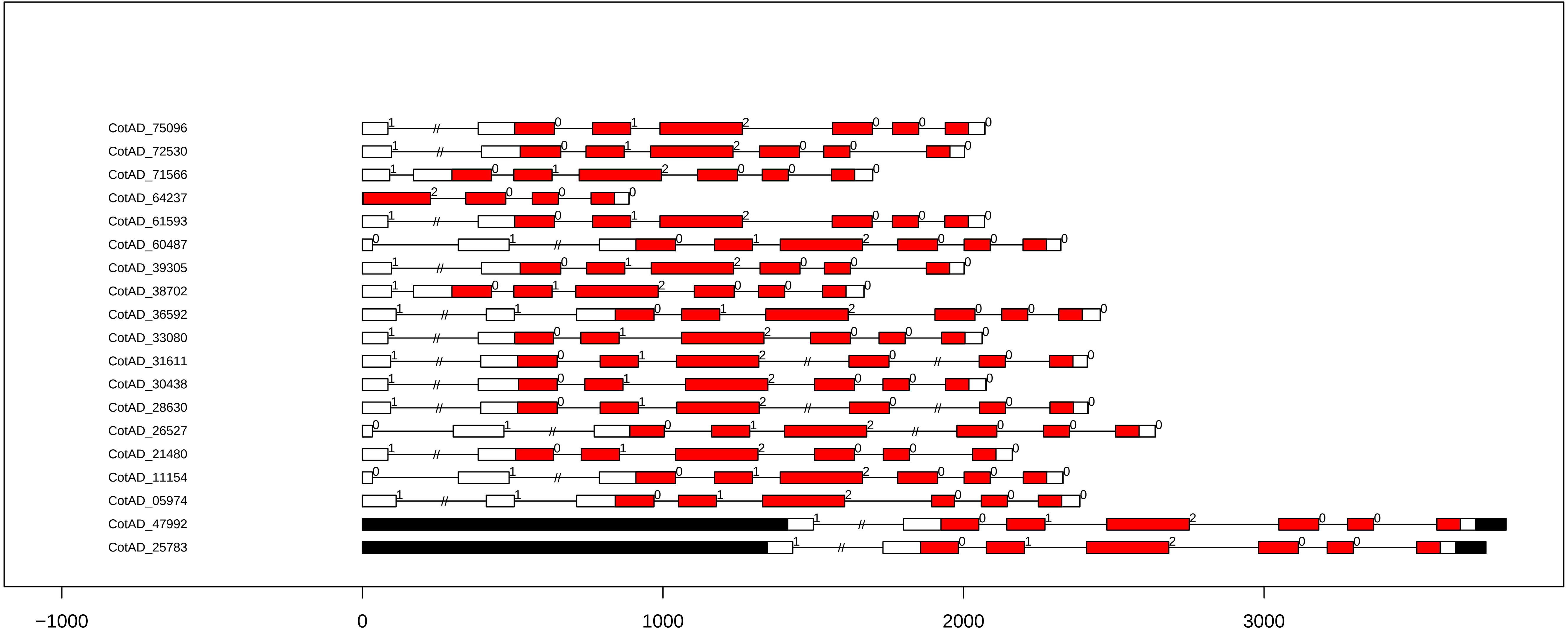

G.hi RLK−Pelle_RLCK−VI exon−intron and domain diagram (part 1)

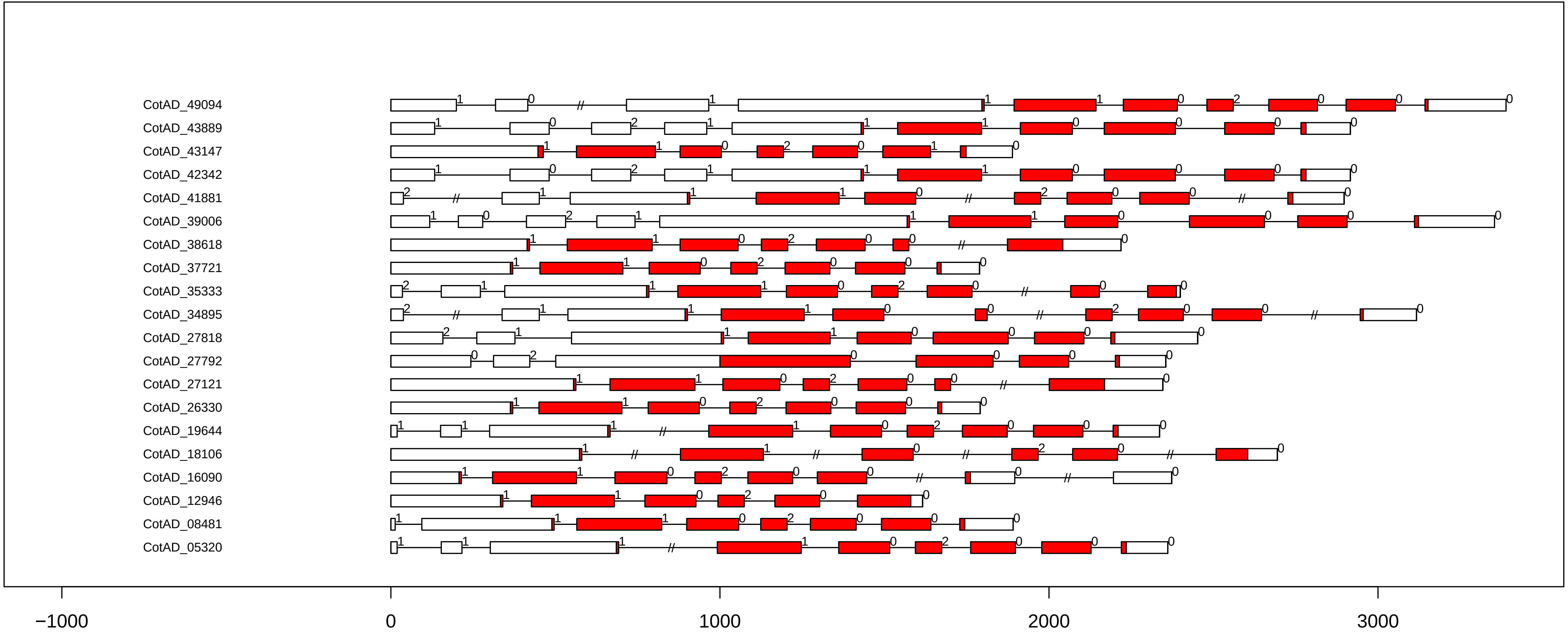

G.hi RLK−Pelle_RLCK−VI exon−intron and domain diagram (part 2)

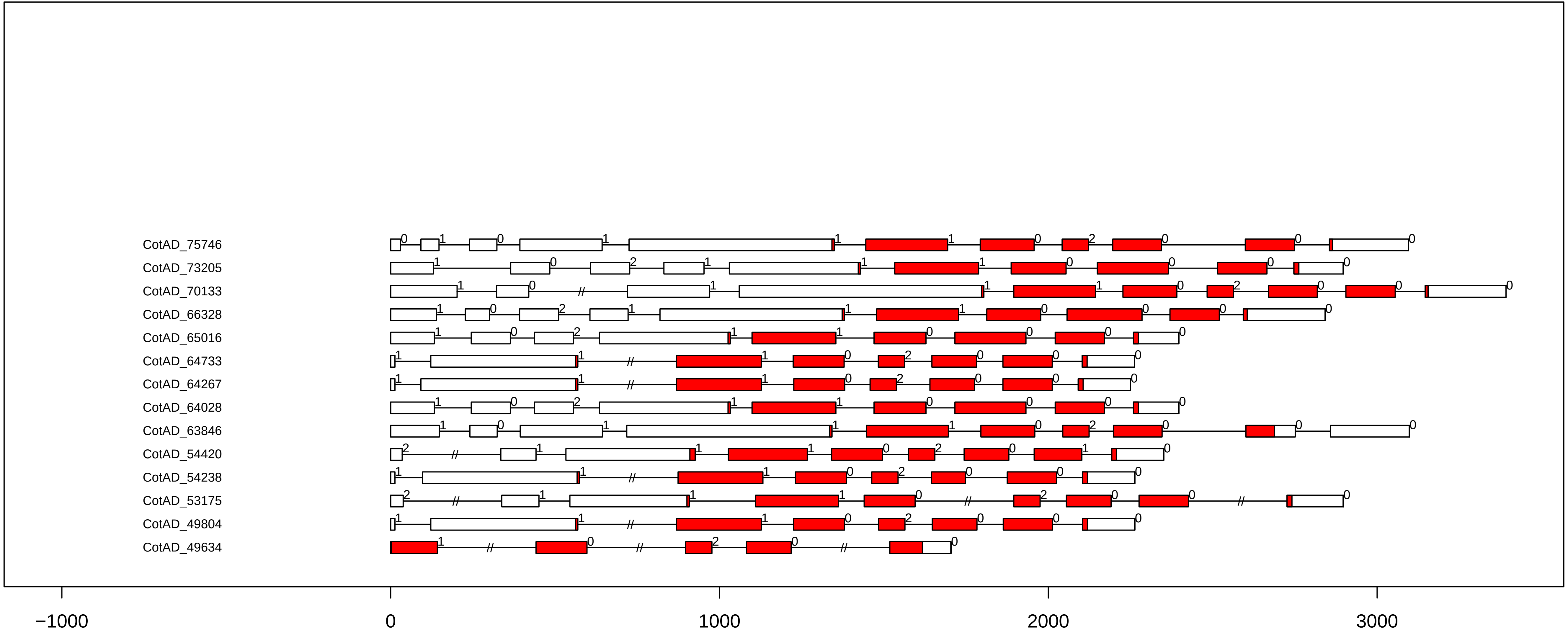

G.hi RLK−Pelle_RLCK−V exon−intron and domain diagram (part 1)

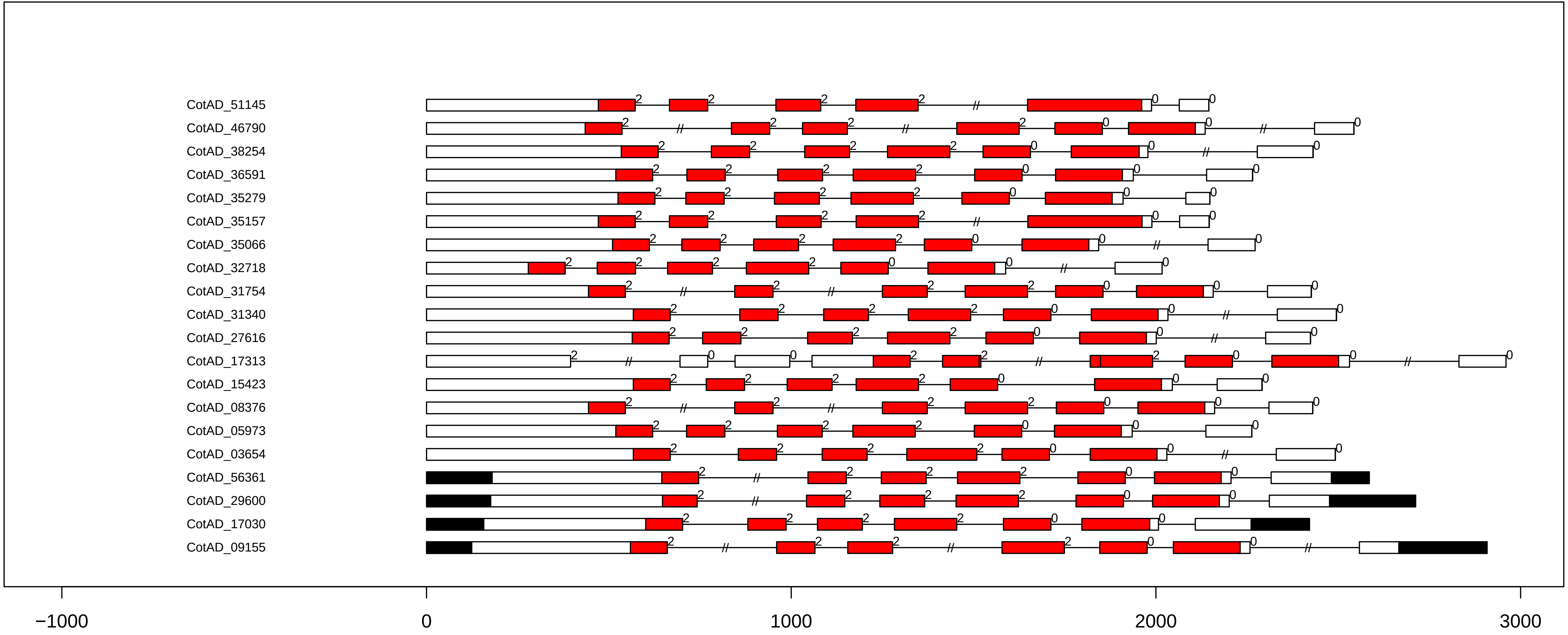

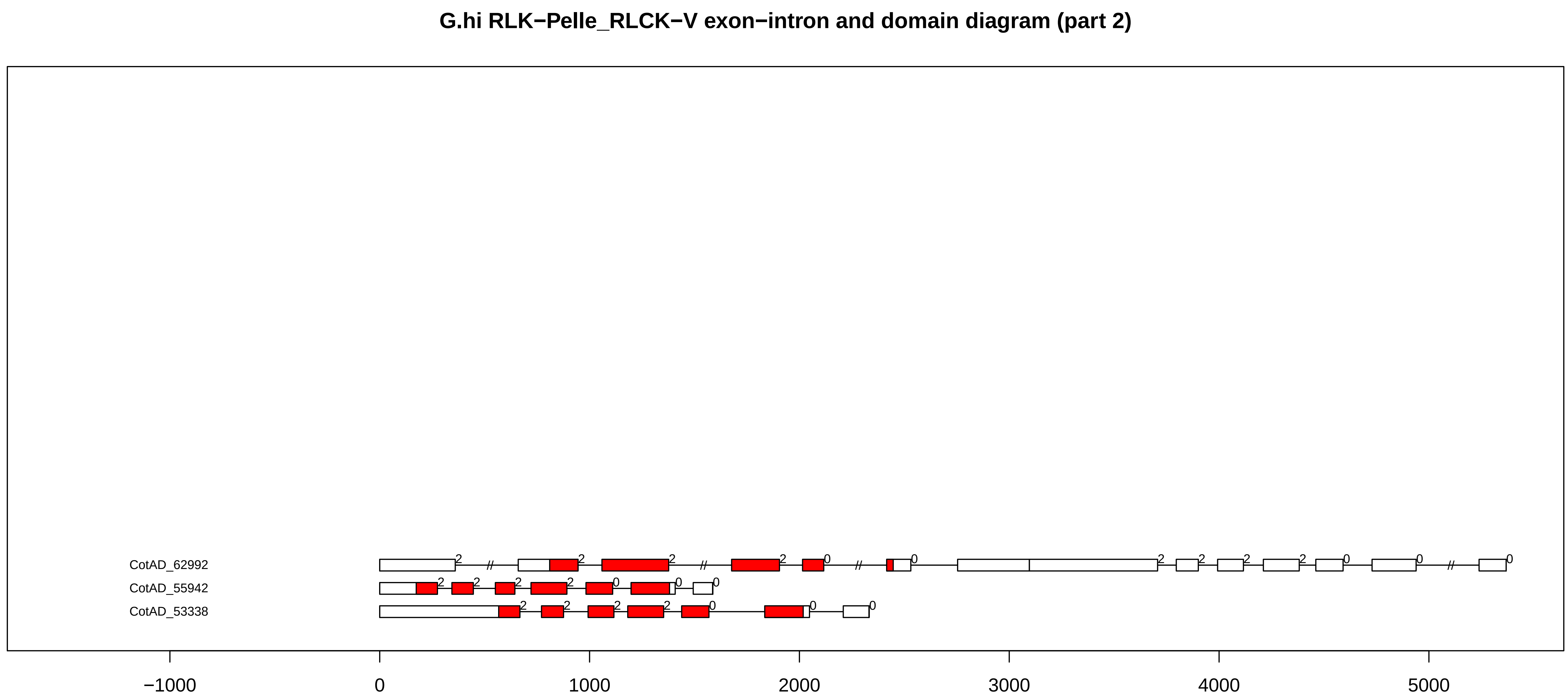

G.hi RLK−Pelle_RLCK−V exon−intron and domain diagram (part 2)
CotAD_62992
CotAD_55942
CotAD_53338
−1000
0
1000
2000
3000
4000
5000

G.hi RLK−Pelle_RLCK−XII−1 exon−intron and domain diagram (part 1)

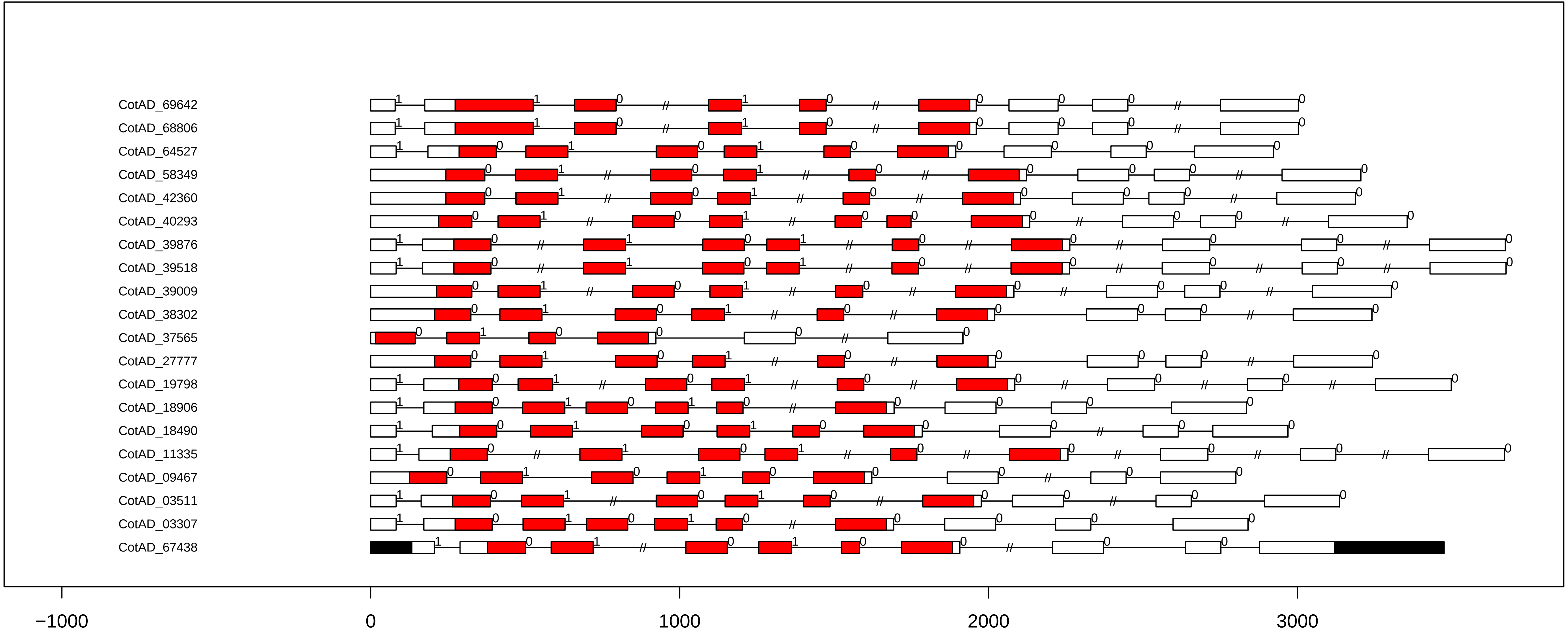

G.hi RLK−Pelle_RLCK−XII−1 exon−intron and domain diagram (part 2)

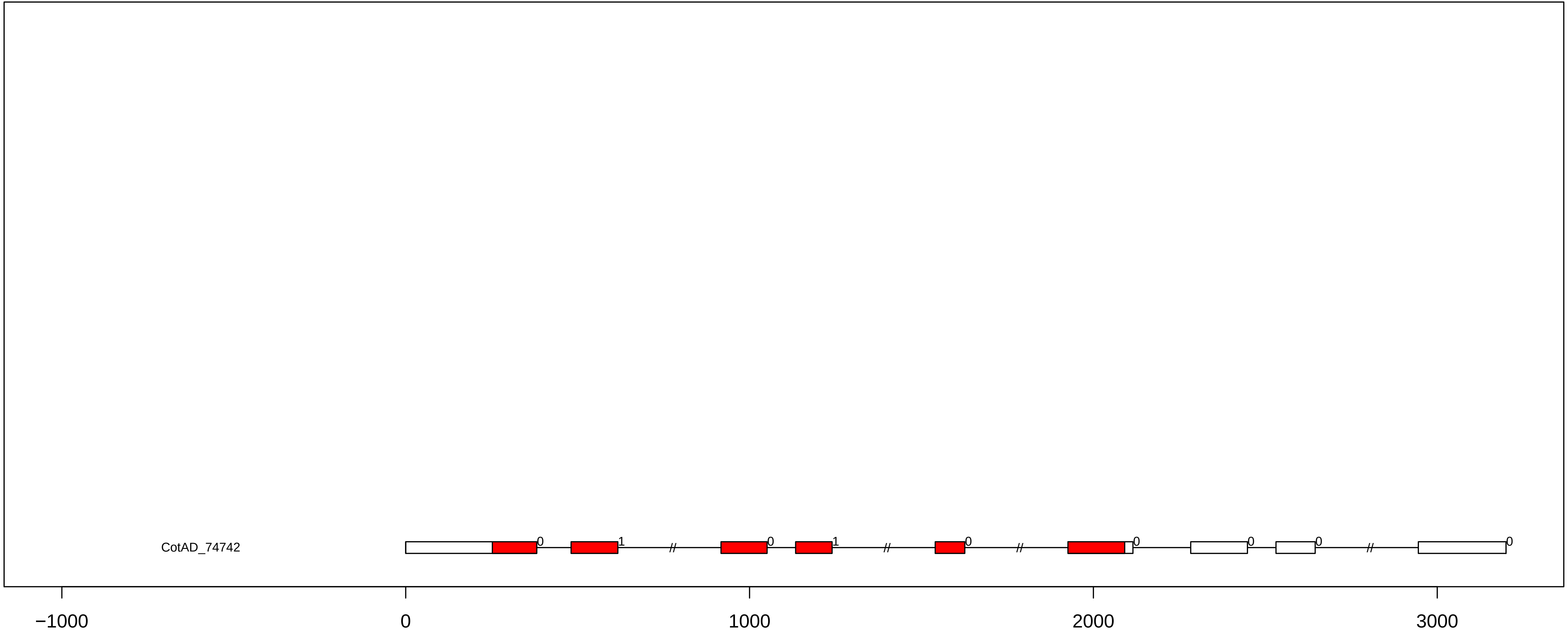

G.hi RLK−Pelle_RLCK−XII−2 exon−intron and domain diagram (all)

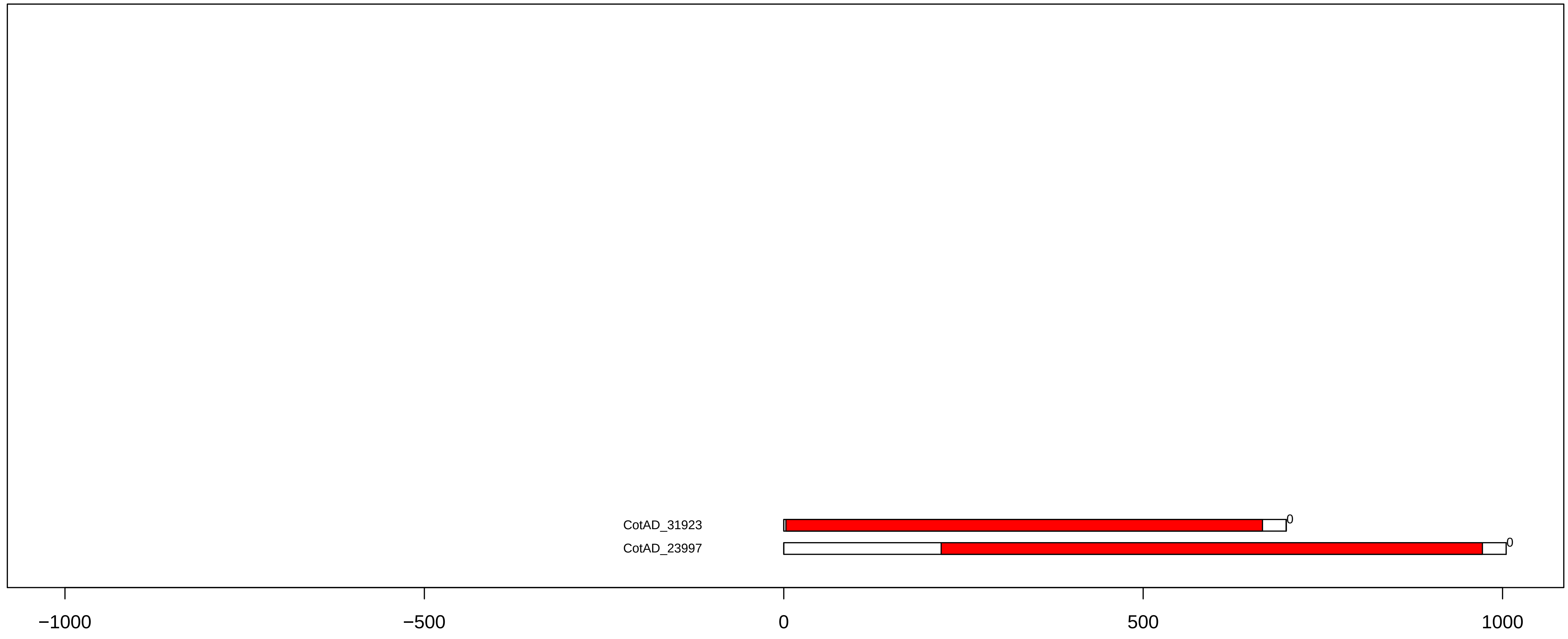

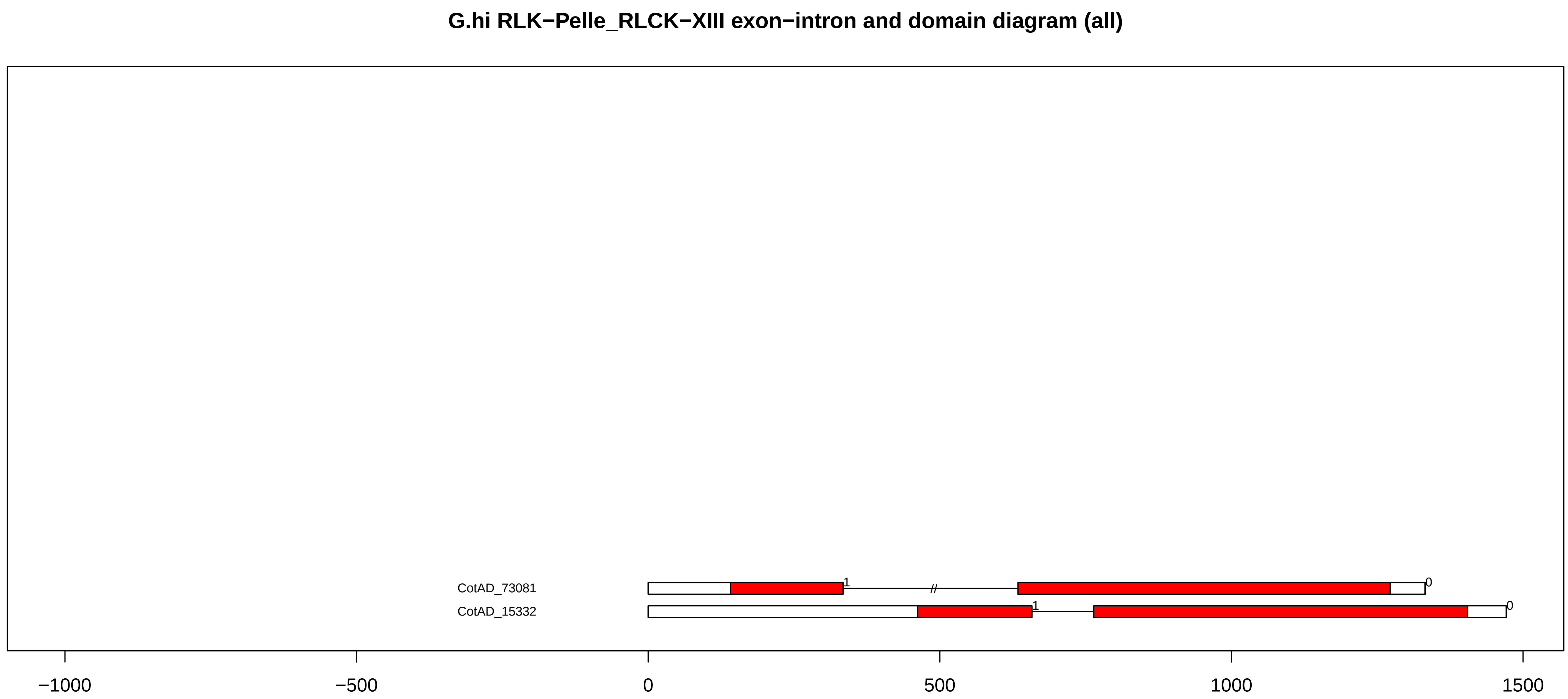

G.hi RLK−Pelle_RLCK−XIII exon−intron and domain diagram (all)
CotAD_73081
CotAD_15332
1
0
1
0
−1000
−500
0
500
1000
1500

G.hi RLK−Pelle_RLCK−XI exon−intron and domain diagram (all)

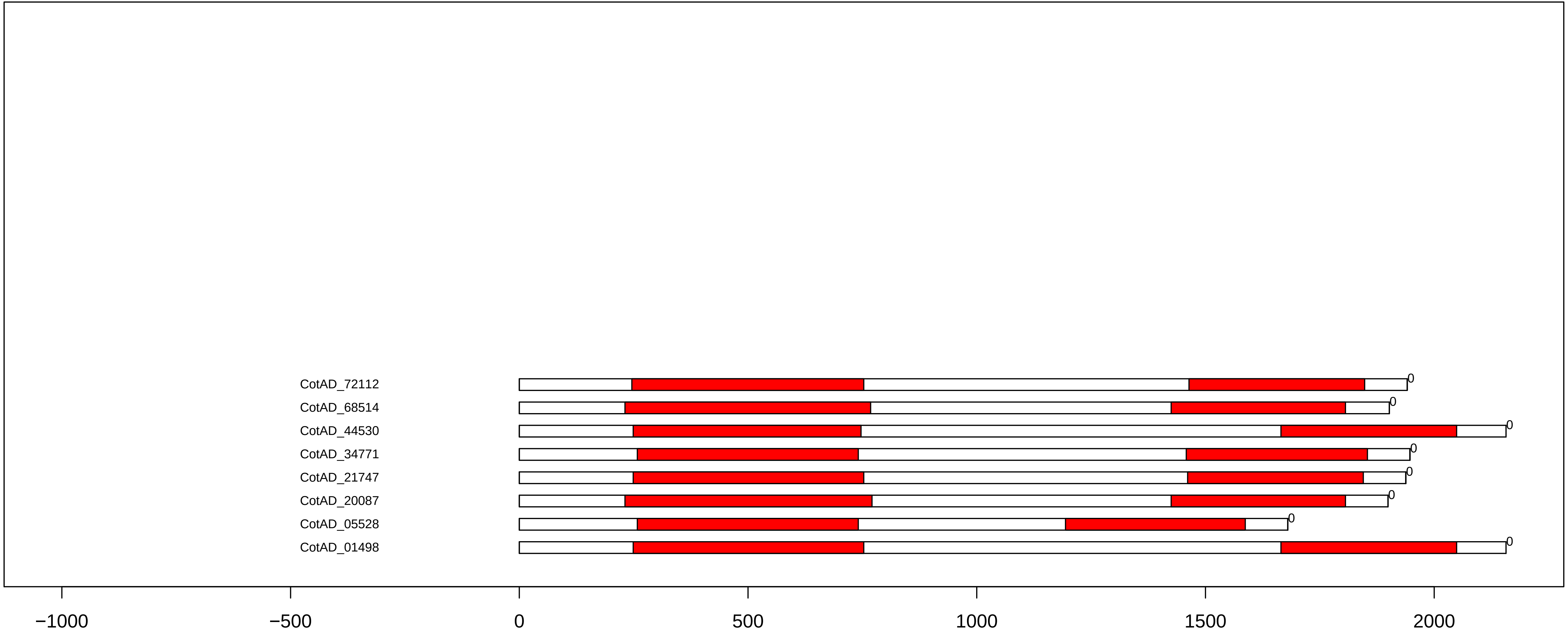

G.hi RLK−Pelle_RLCK−X exon−intron and domain diagram (all)

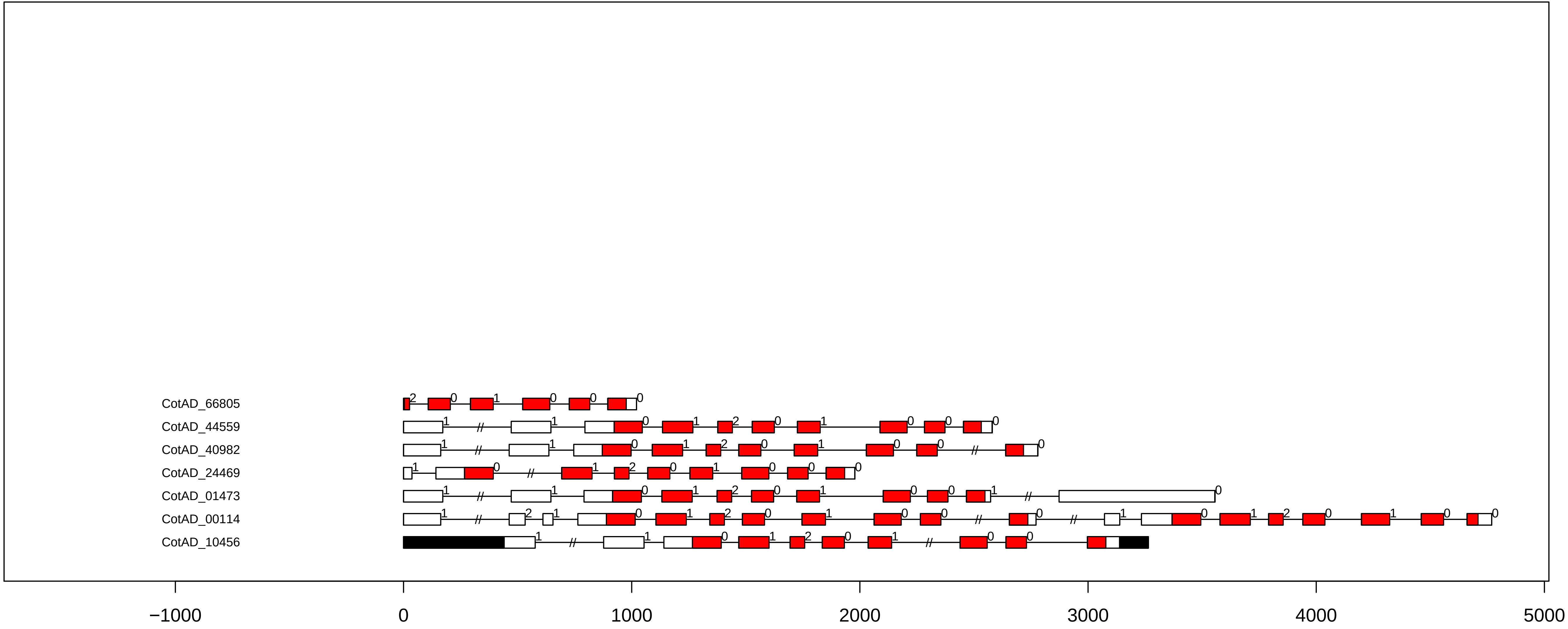

G.hi RLK−Pelle_RLCK−XVI exon−intron and domain diagram (all)

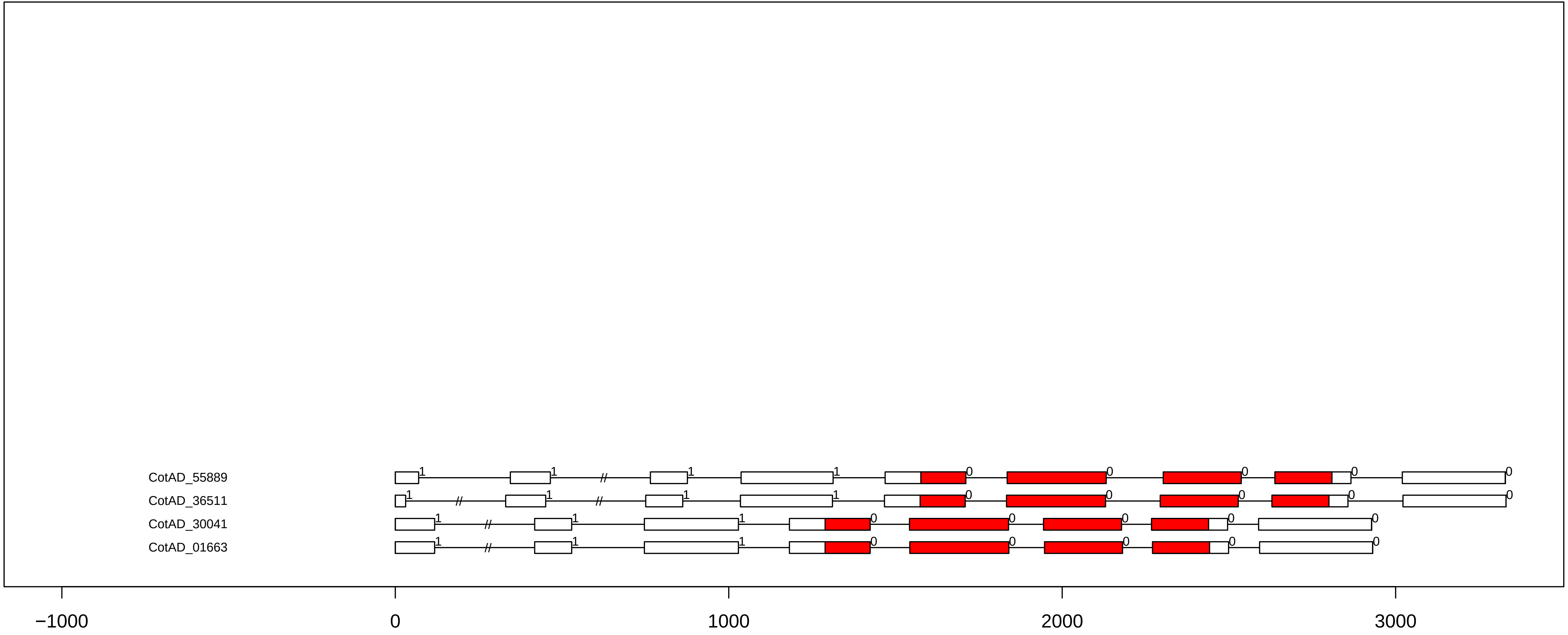

G.hi RLK−Pelle_RLCK−XV exon−intron and domain diagram (all)

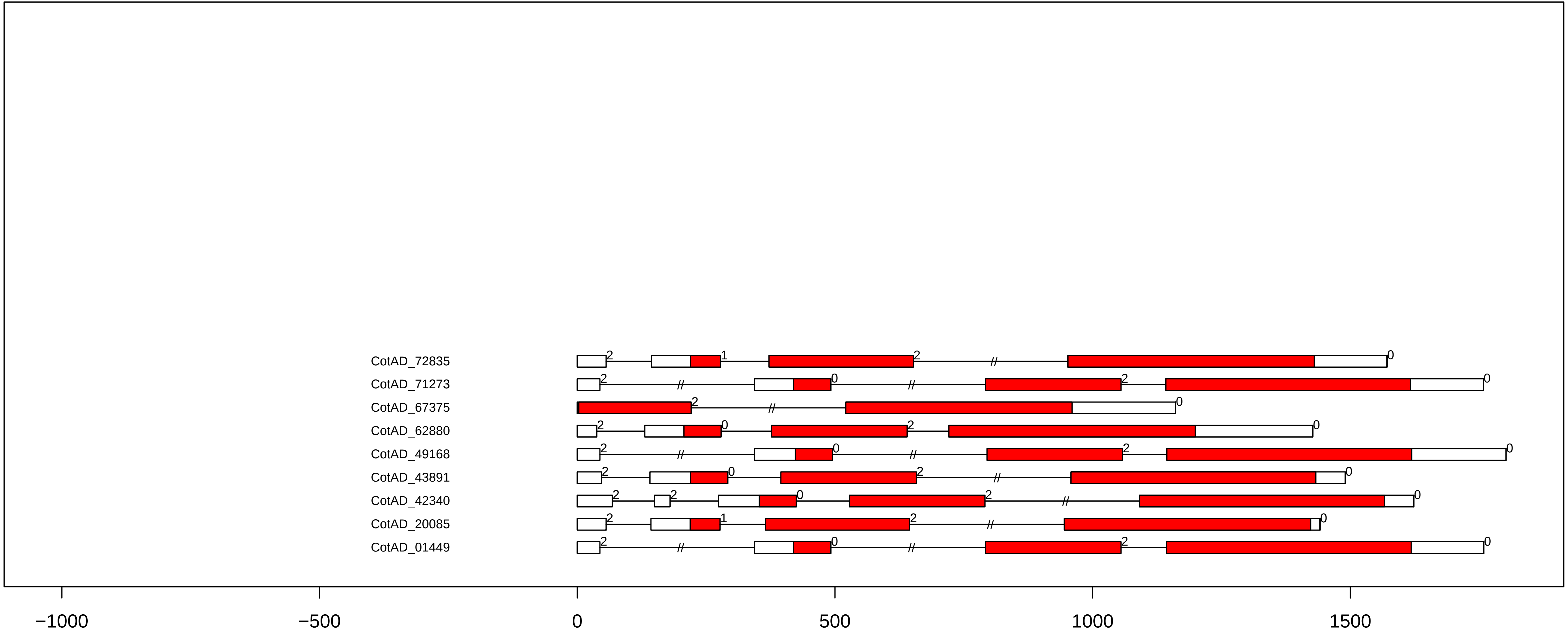

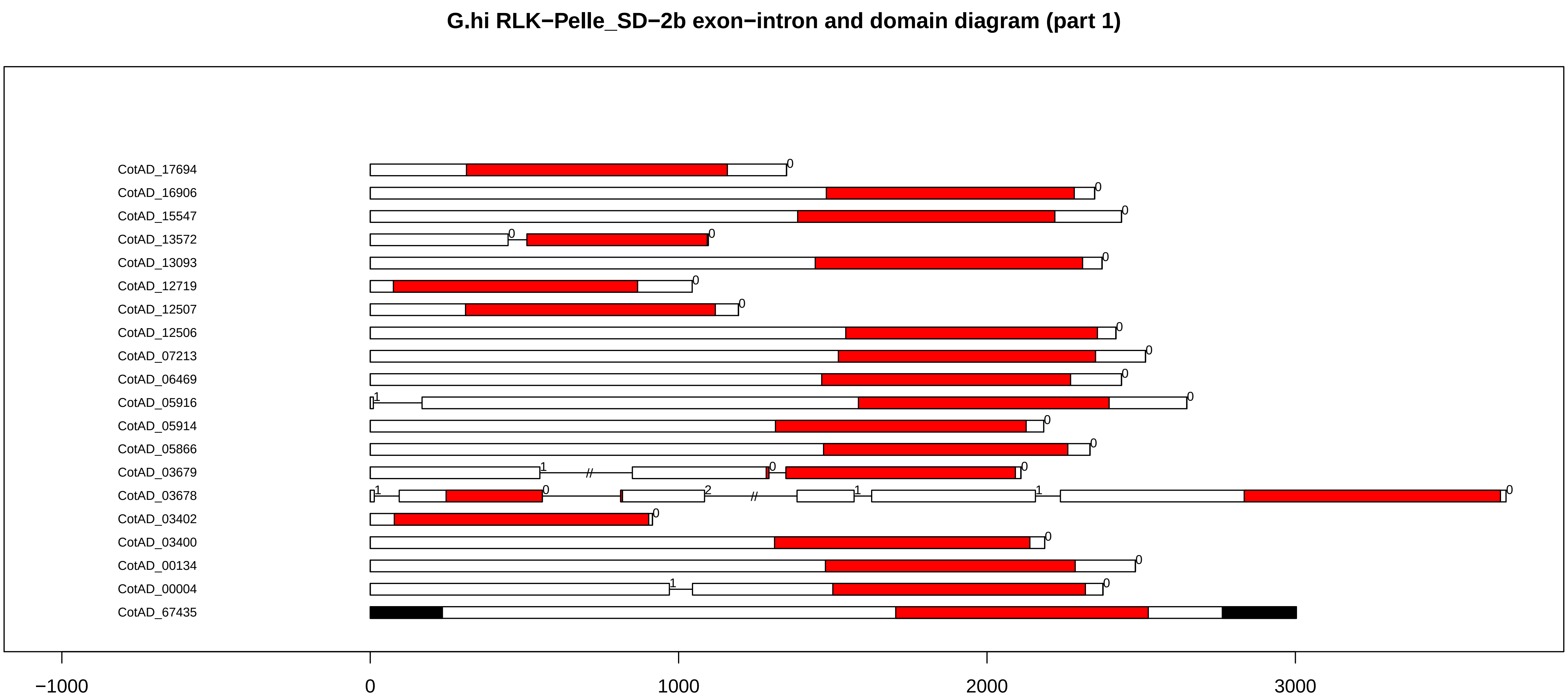

G.hi RLK−Pelle_SD−2b exon−intron and domain diagram (part 1)
CotAD_17694
CotAD_16906
CotAD_15547
CotAD_13572
CotAD_13093
CotAD_12719
CotAD_12507
CotAD_12506
CotAD_07213
CotAD_06469
CotAD_05916
CotAD_05914
CotAD_05866
CotAD_03679
CotAD_03678
CotAD_03402
CotAD_03400
CotAD_00134
CotAD_00004
CotAD_67435
−1000
0
1000
2000
3000

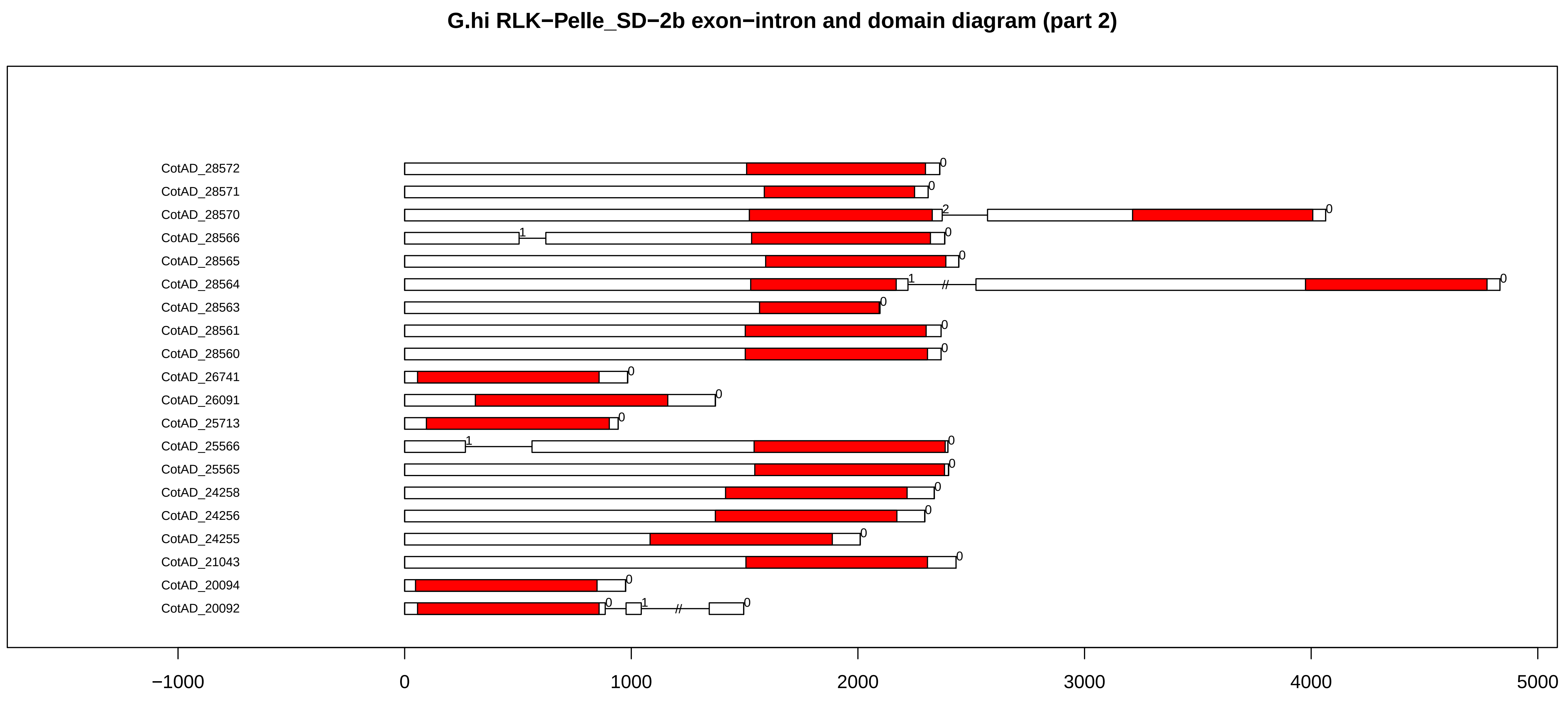
G.hi RLK−Pelle_SD−2b exon−intron and domain diagram (part 2)
CotAD_28572
CotAD_28571
CotAD_28570
CotAD_28566
CotAD_28565
CotAD_28564
CotAD_28563
CotAD_28561
CotAD_28560
CotAD_26741
CotAD_26091
CotAD_25713
CotAD_25566
CotAD_25565
CotAD_24258
CotAD_24256
CotAD_24255
CotAD_21043
CotAD_20094
CotAD_20092
−1000
0
1000
2000
3000
4000
5000

G.hi RLK−Pelle_SD−2b exon−intron and domain diagram (part 3)

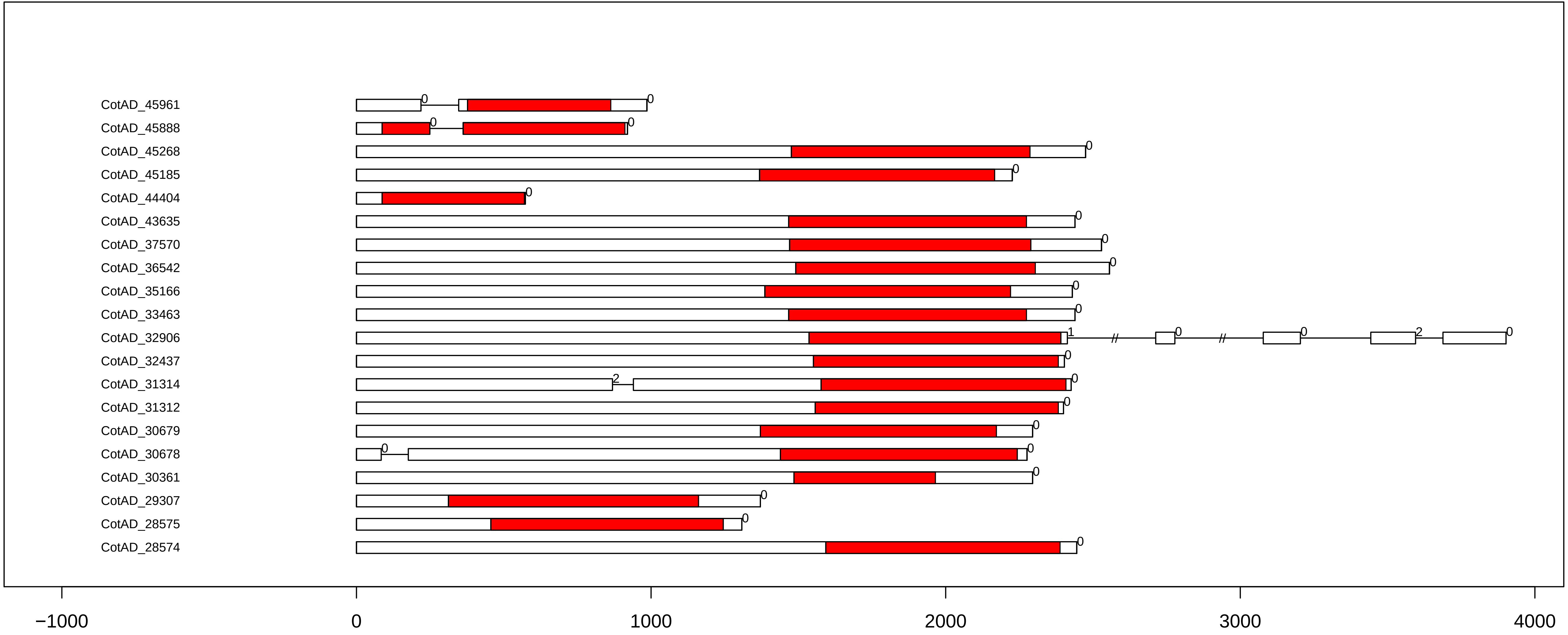

G.hi RLK−Pelle_SD−2b exon−intron and domain diagram (part 4)

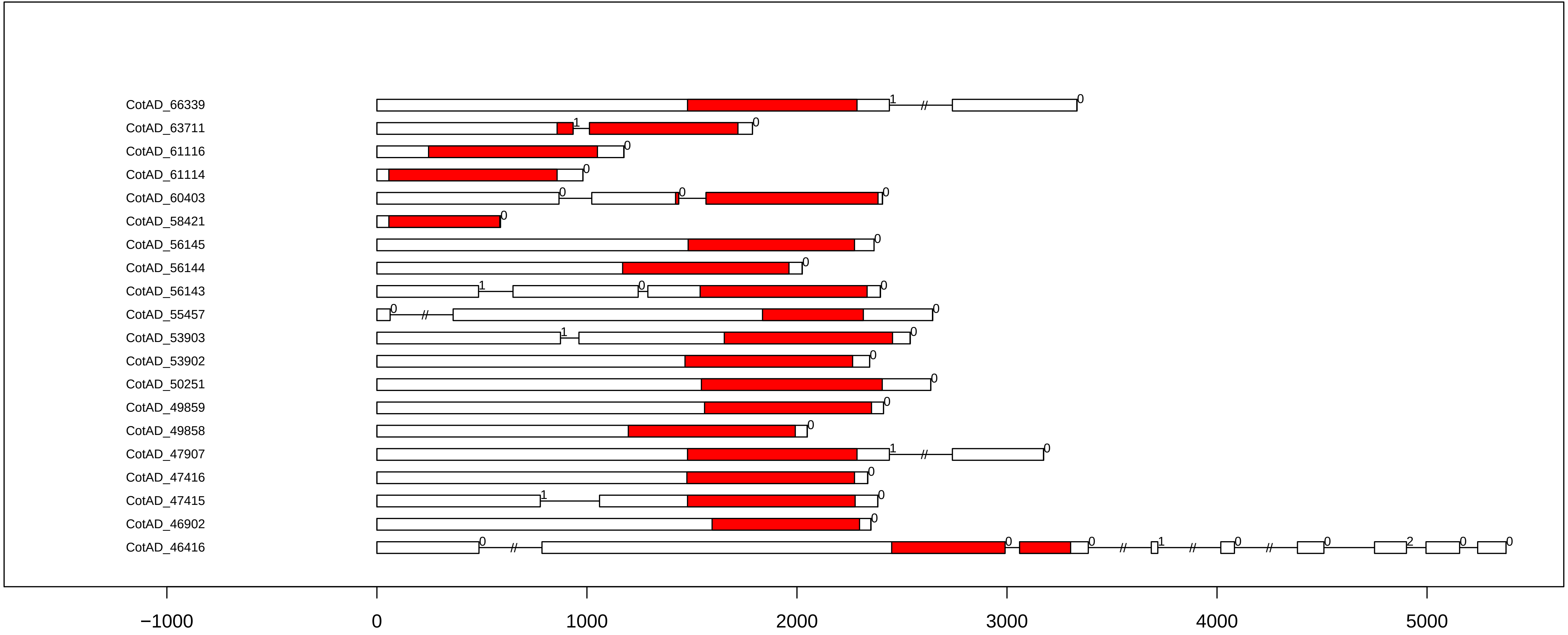

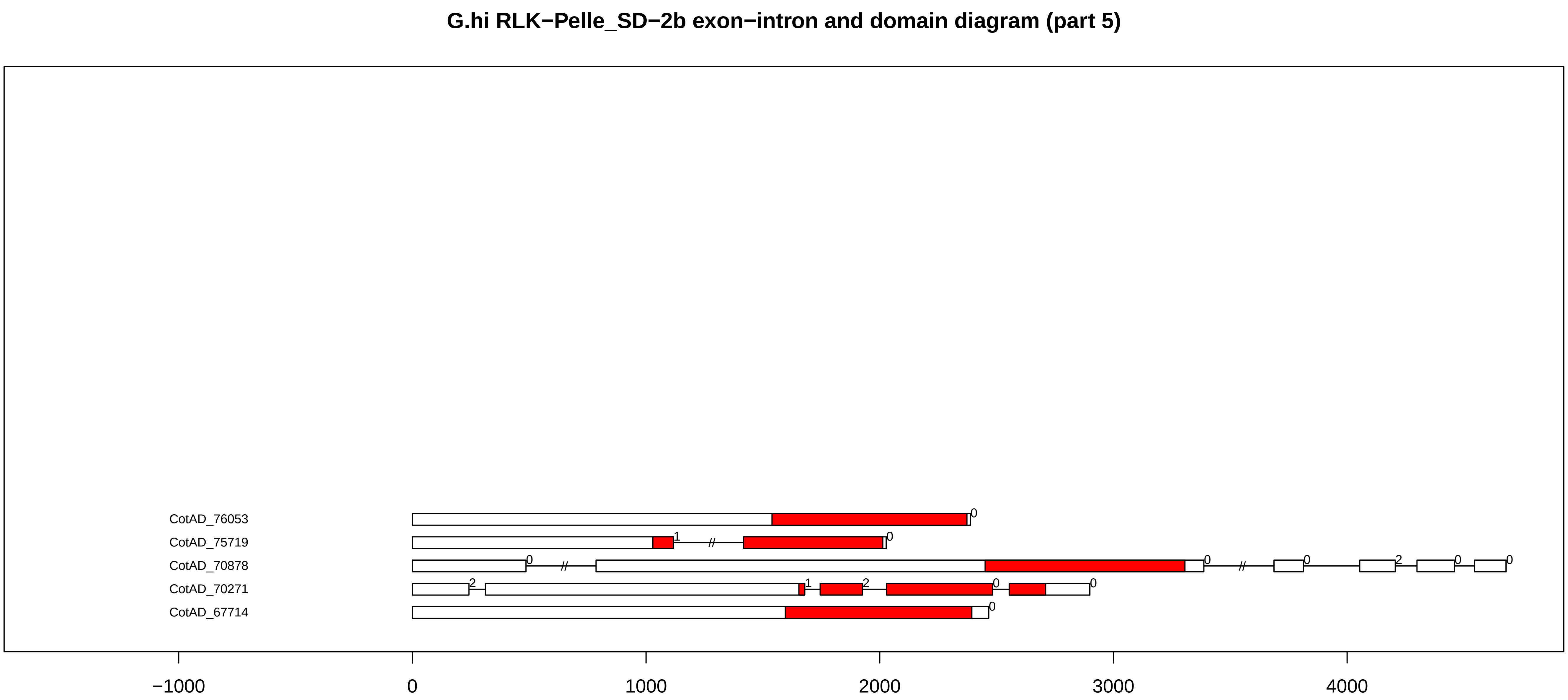
G.hi RLK−Pelle_SD−2b exon−intron and domain diagram (part 5)
CotAD_76053
CotAD_75719
CotAD_70878
CotAD_70271
CotAD_67714
−1000
0
1000
2000
3000
4000

G.hi RLK−Pelle_Singleton exon−intron and domain diagram (all)

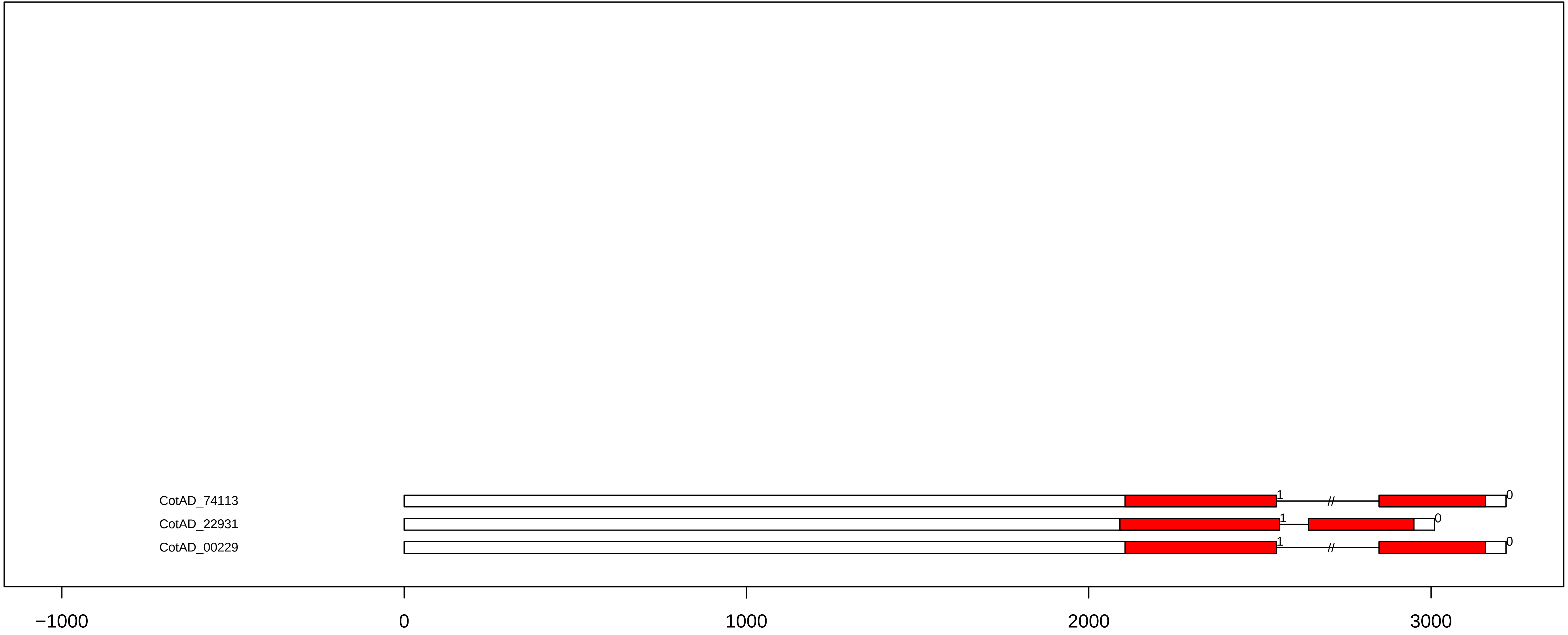

G.hi RLK−Pelle_URK−1 exon−intron and domain diagram (all)

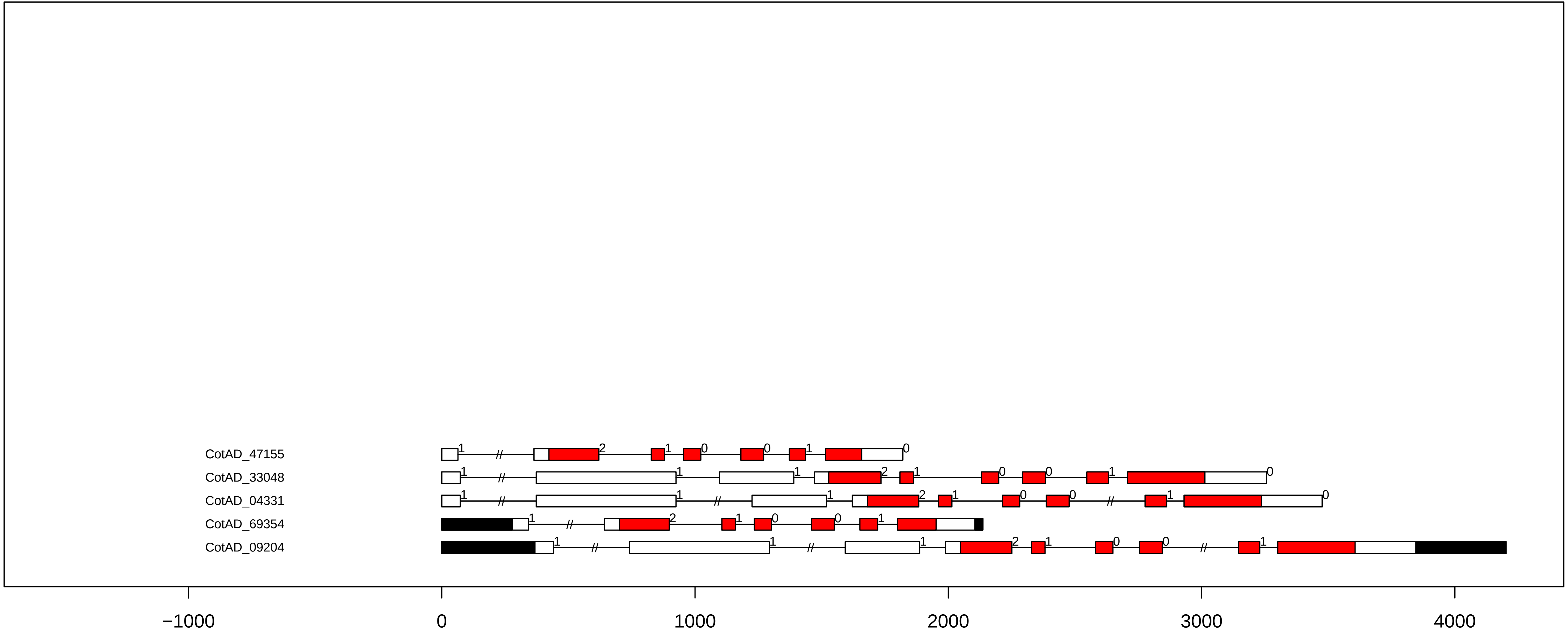

G.hi RLK−Pelle_URK−2 exon−intron and domain diagram (all)

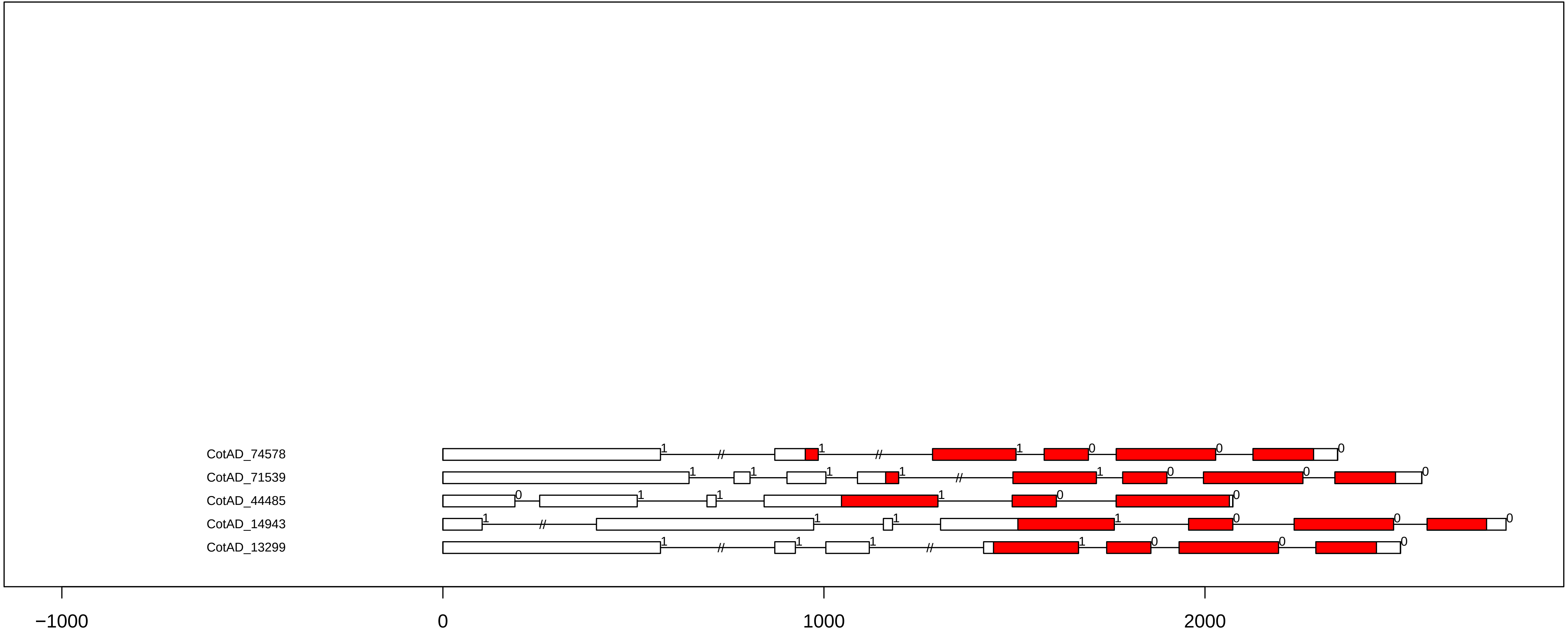

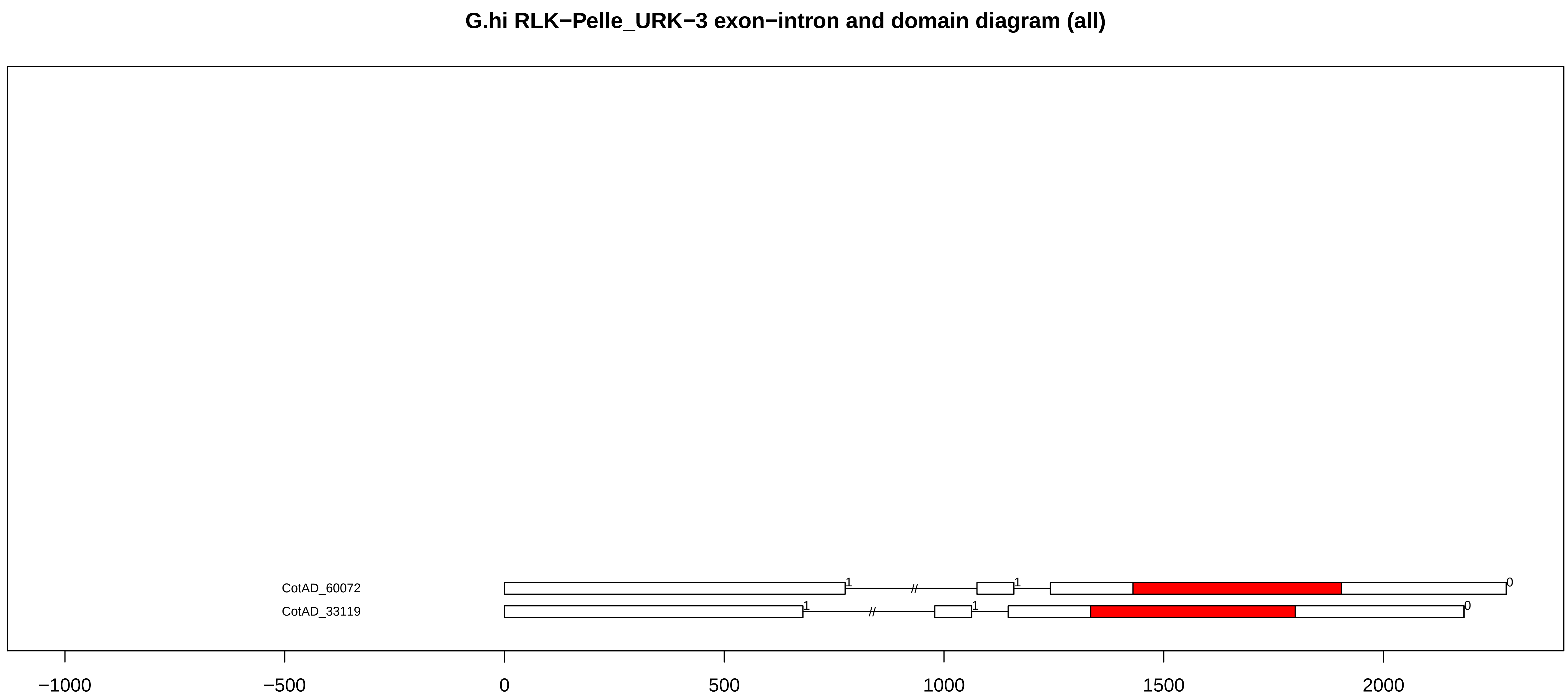

G.hi RLK−Pelle_URK−3 exon−intron and domain diagram (all)
CotAD_60072
CotAD_33119
1
1
0
1
1
0
−1000
−500
0
500
1000
1500
2000

G.hi RLK−Pelle_WAK_LRK10L−1 exon−intron and domain diagram (part 1)

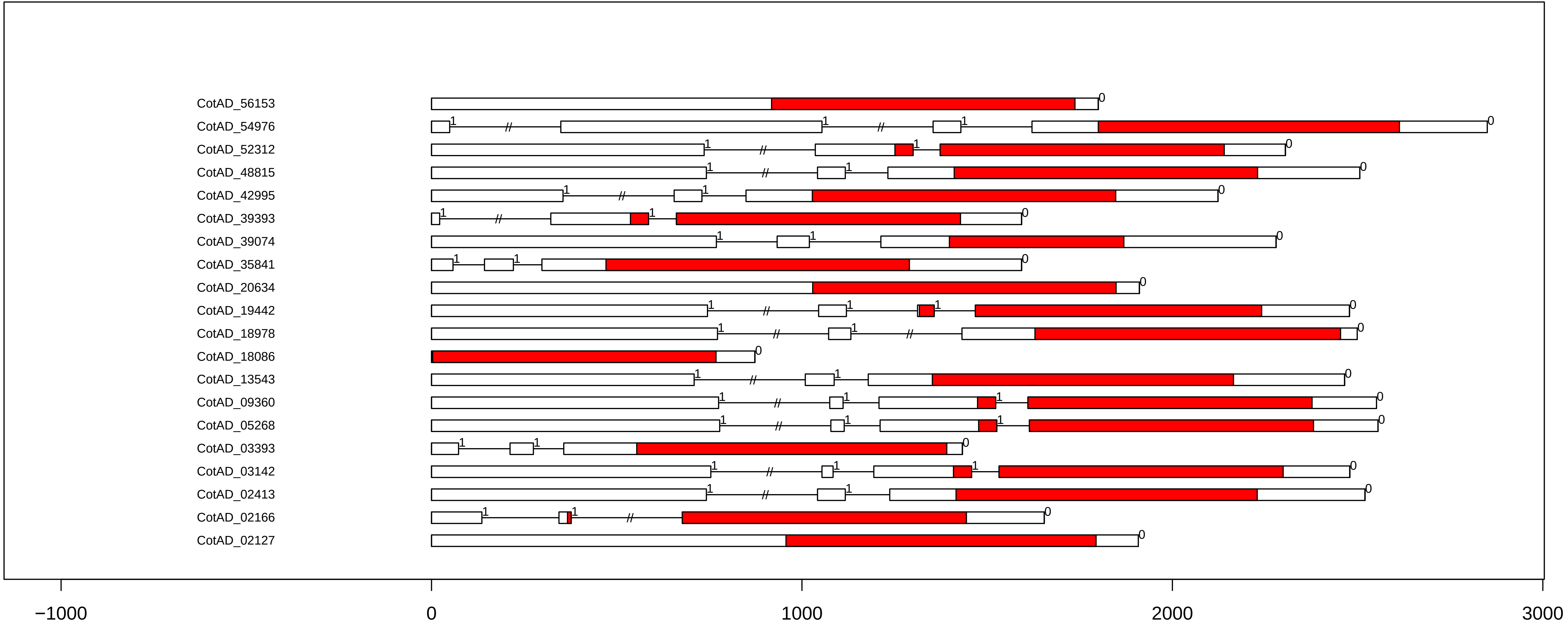

G.hi RLK−Pelle_WAK_LRK10L−1 exon−intron and domain diagram (part 2)

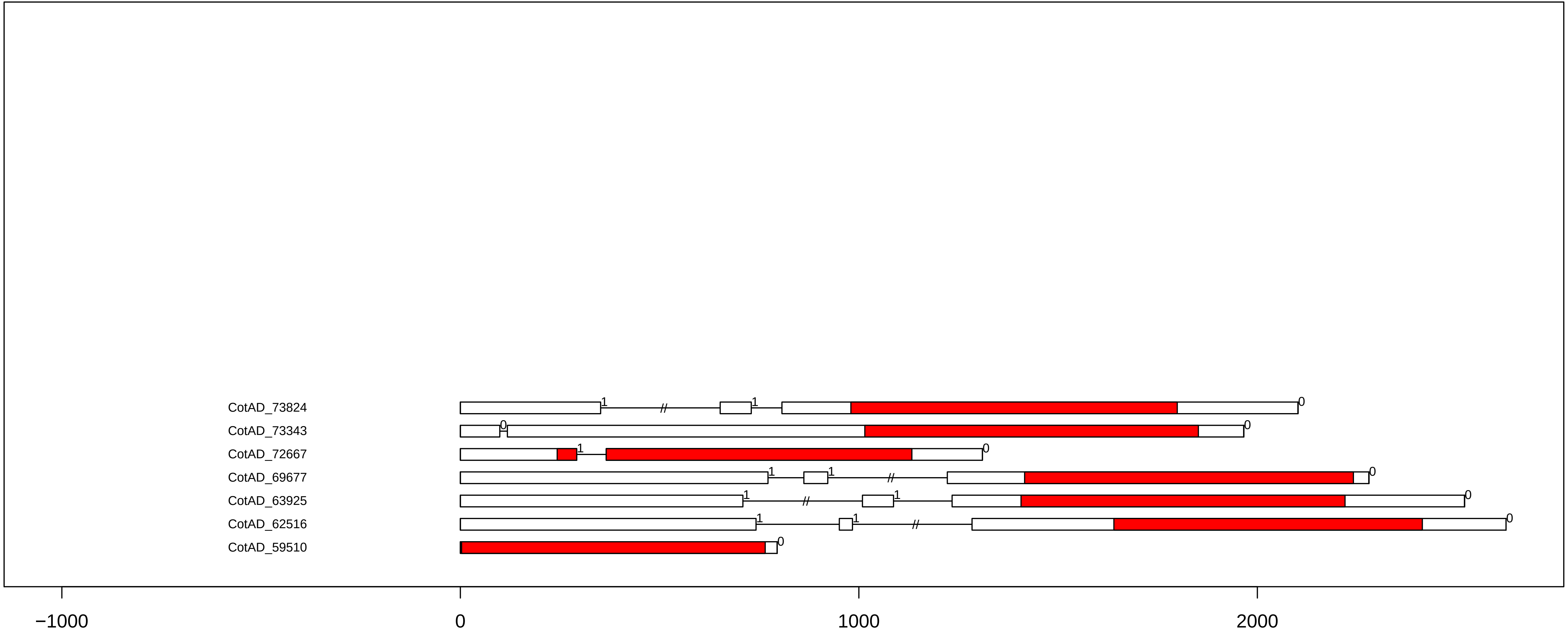

G.hi RLK−Pelle_WAK exon−intron and domain diagram (part 1)

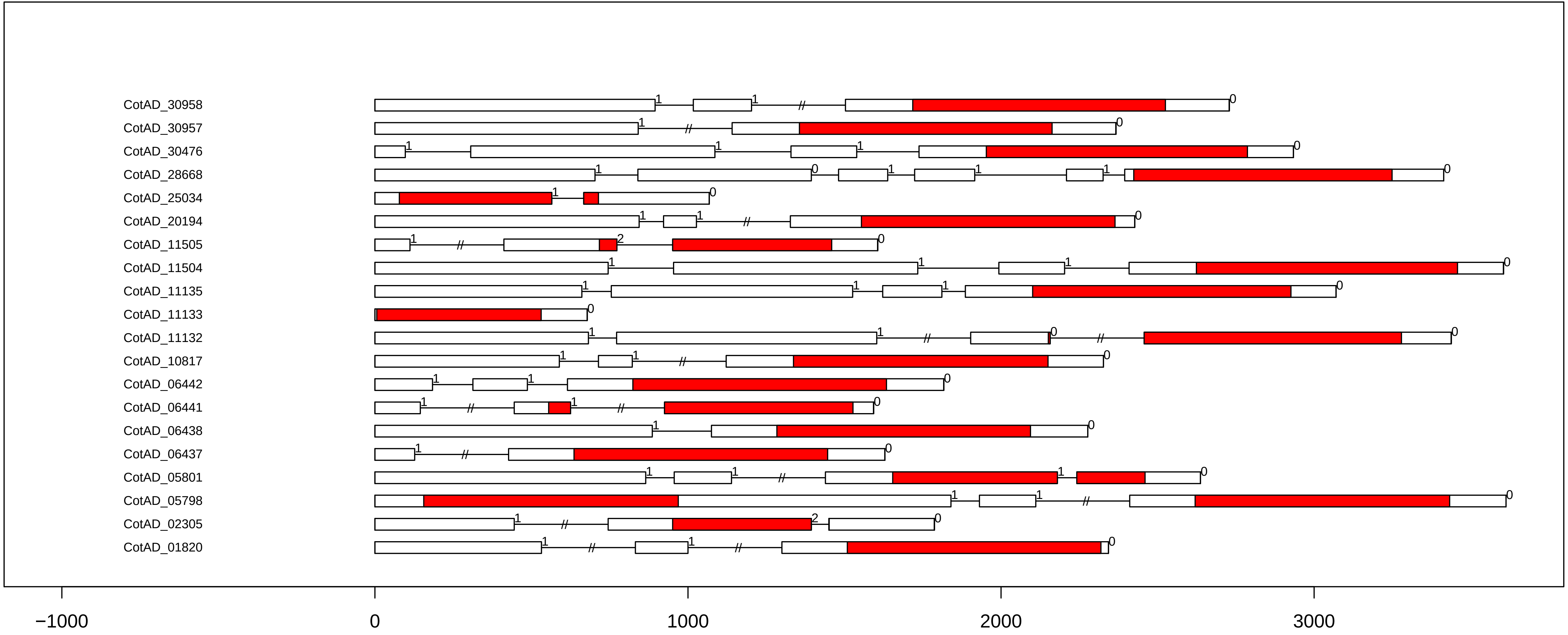

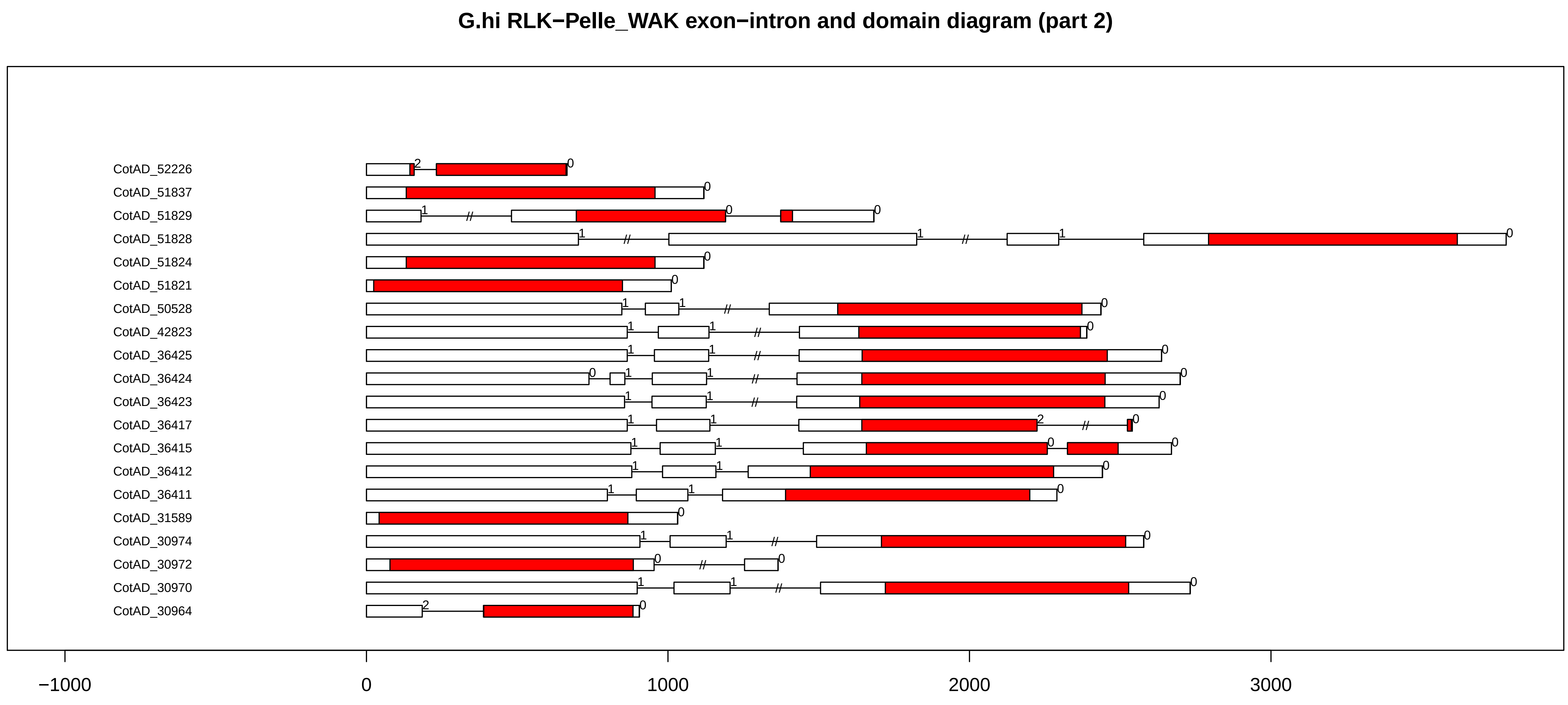

G.hi RLK−Pelle_WAK exon−intron and domain diagram (part 2)
CotAD_52226
CotAD_51837
CotAD_51829
CotAD_51828
CotAD_51824
CotAD_51821
CotAD_50528
CotAD_42823
CotAD_36425
CotAD_36424
CotAD_36423
CotAD_36417
CotAD_36415
CotAD_36412
CotAD_36411
CotAD_31589
CotAD_30974
CotAD_30972
CotAD_30970
CotAD_30964
−1000
0
1000
2000
3000

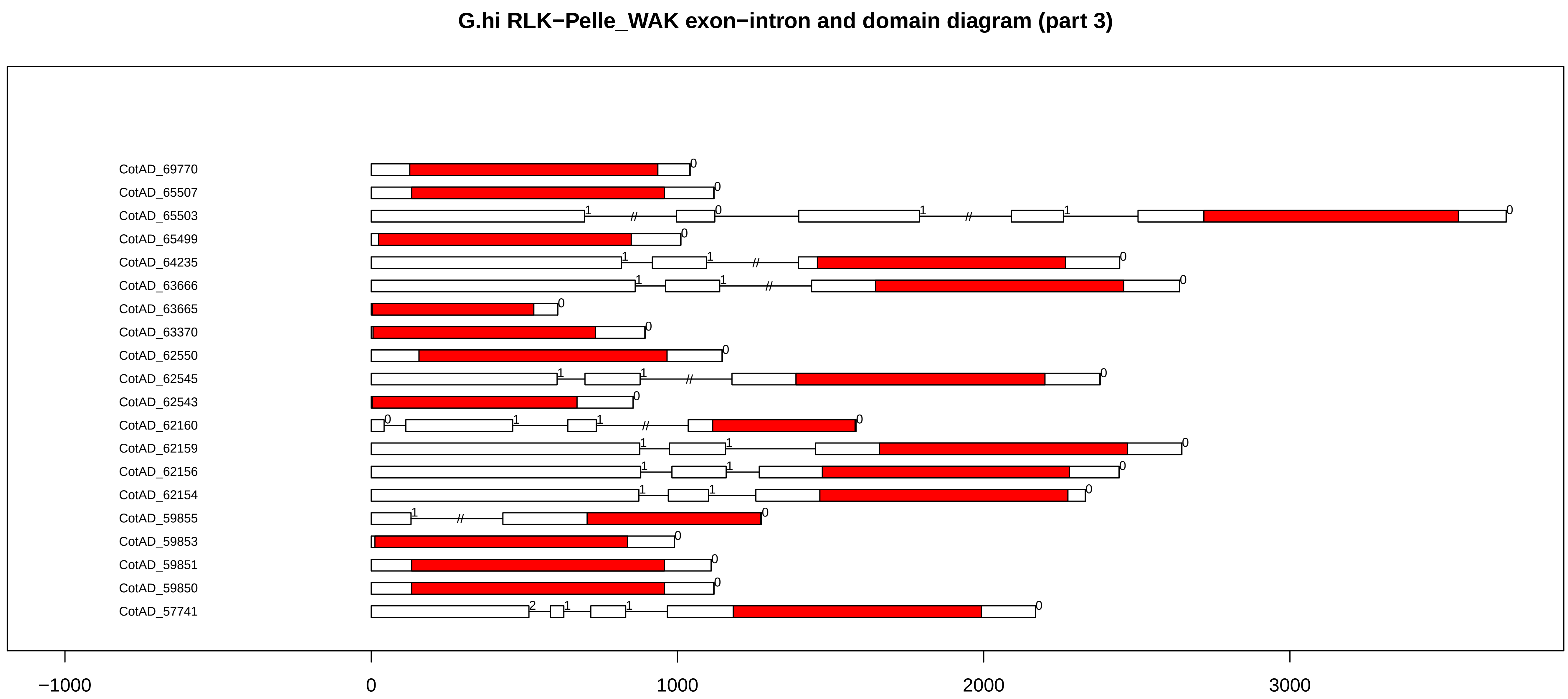
G.hi RLK−Pelle_WAK exon−intron and domain diagram (part 3)
CotAD_69770
CotAD_65507
CotAD_65503
CotAD_65499
CotAD_64235
CotAD_63666
CotAD_63665
CotAD_63370
CotAD_62550
CotAD_62545
CotAD_62543
CotAD_62160
CotAD_62159
CotAD_62156
CotAD_62154
CotAD_59855
CotAD_59853
CotAD_59851
CotAD_59850
CotAD_57741
−1000
0
1000
2000
3000

G.hi RLK−Pelle_WAK exon−intron and domain diagram (part 4)

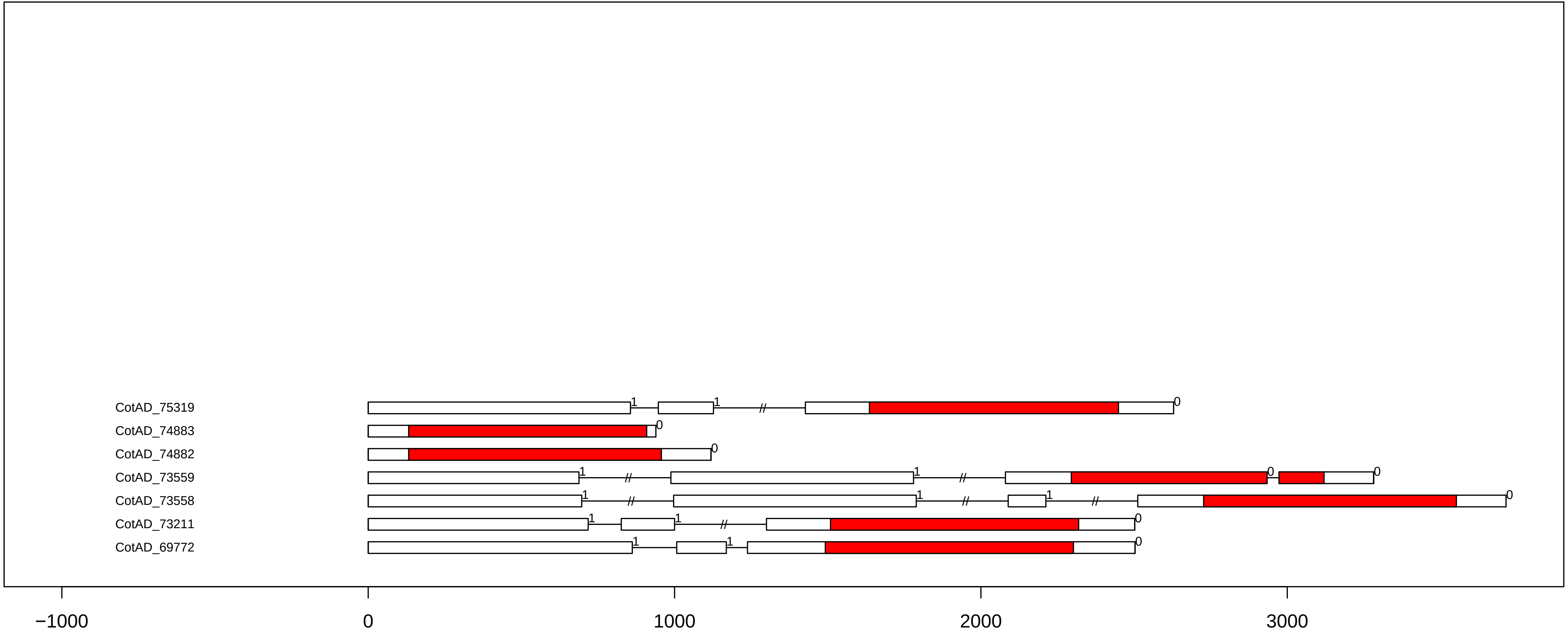

G.hi SCY1_SCYL1 exon−intron and domain diagram (all)

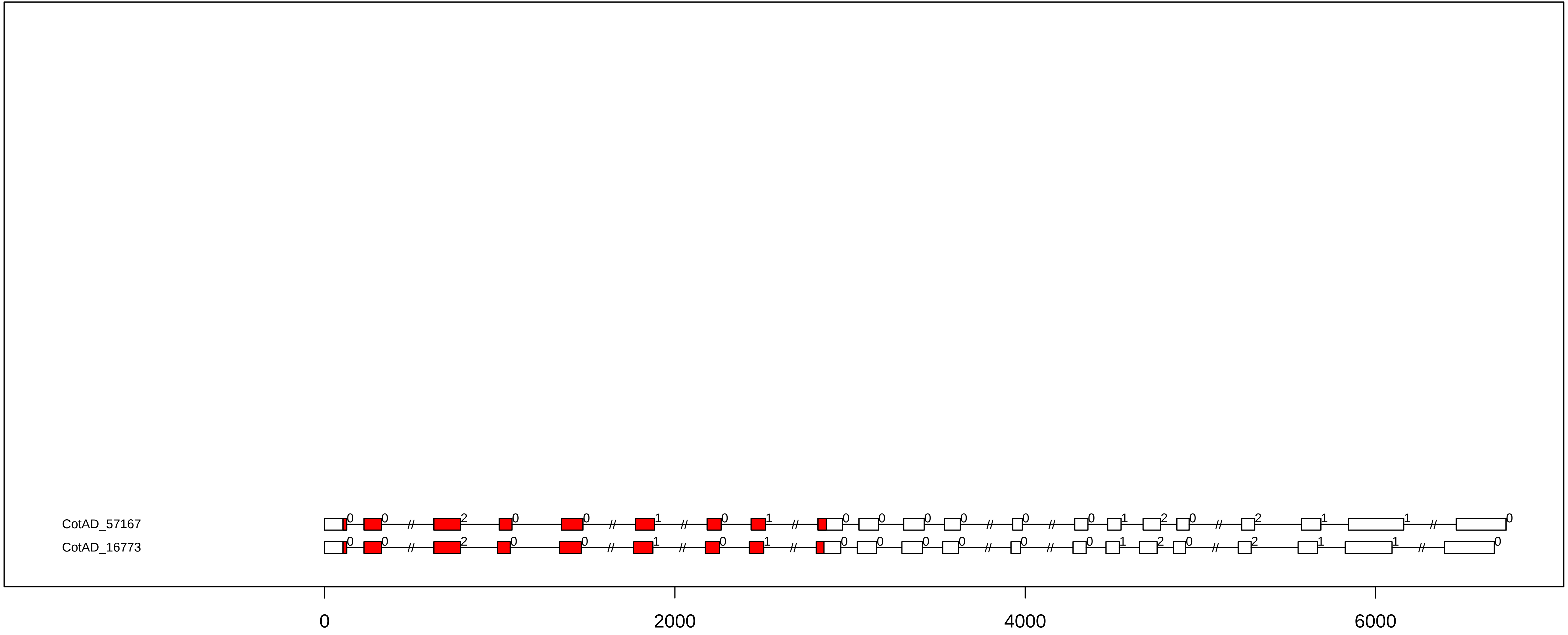

G.hi SCY1_SCYL2 exon−intron and domain diagram (all)

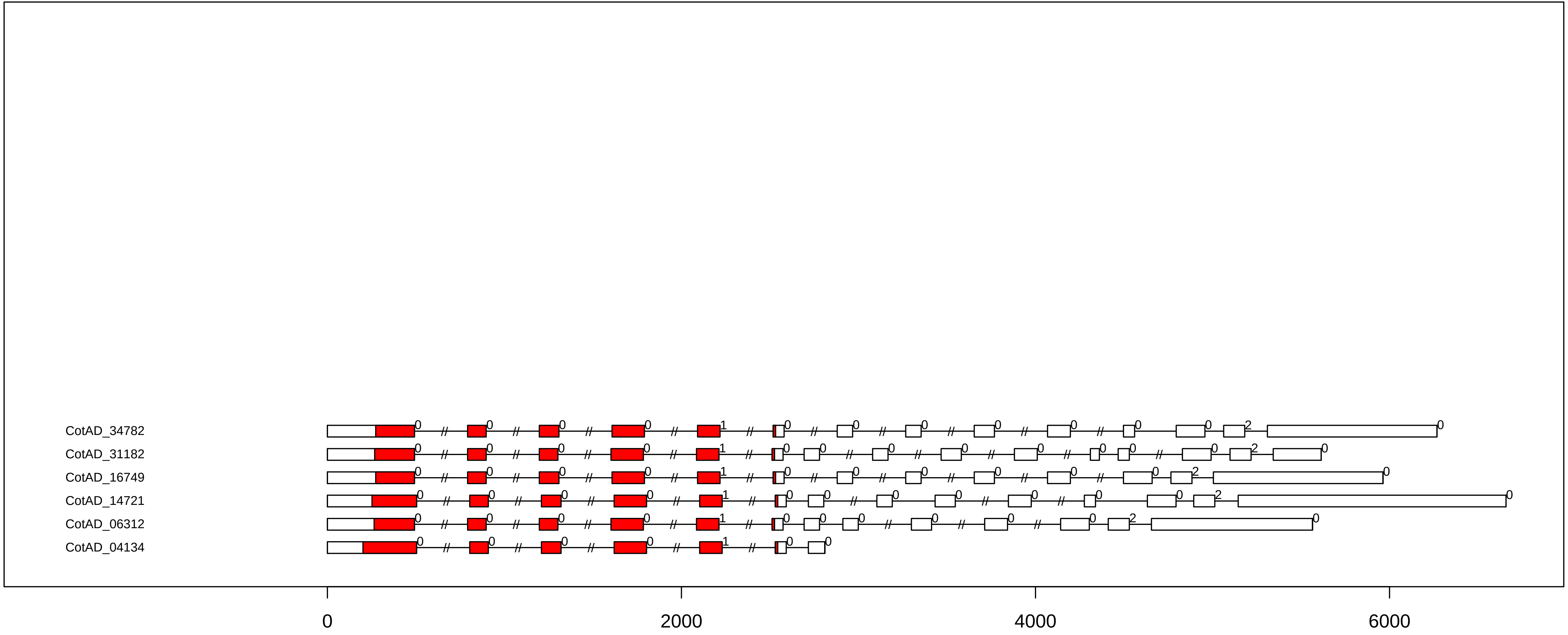

G.hi STE_STE11 exon−intron and domain diagram (part 1)

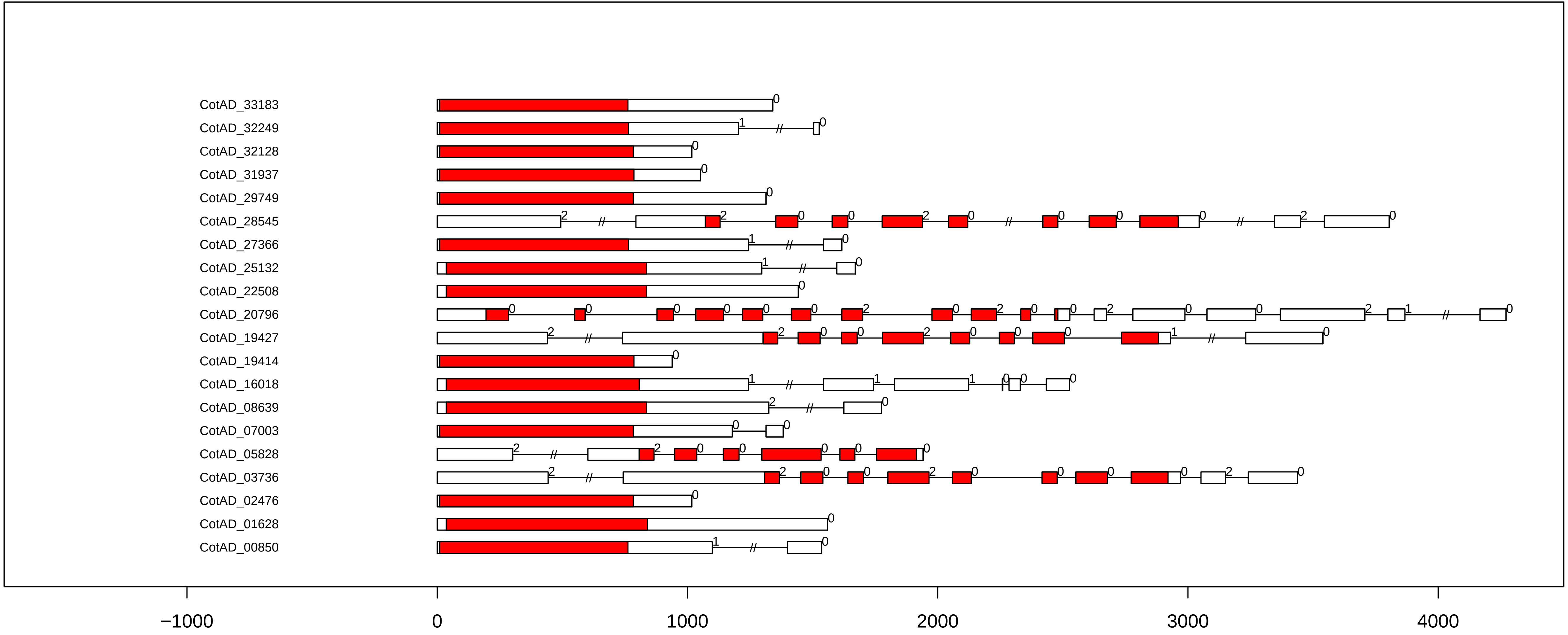

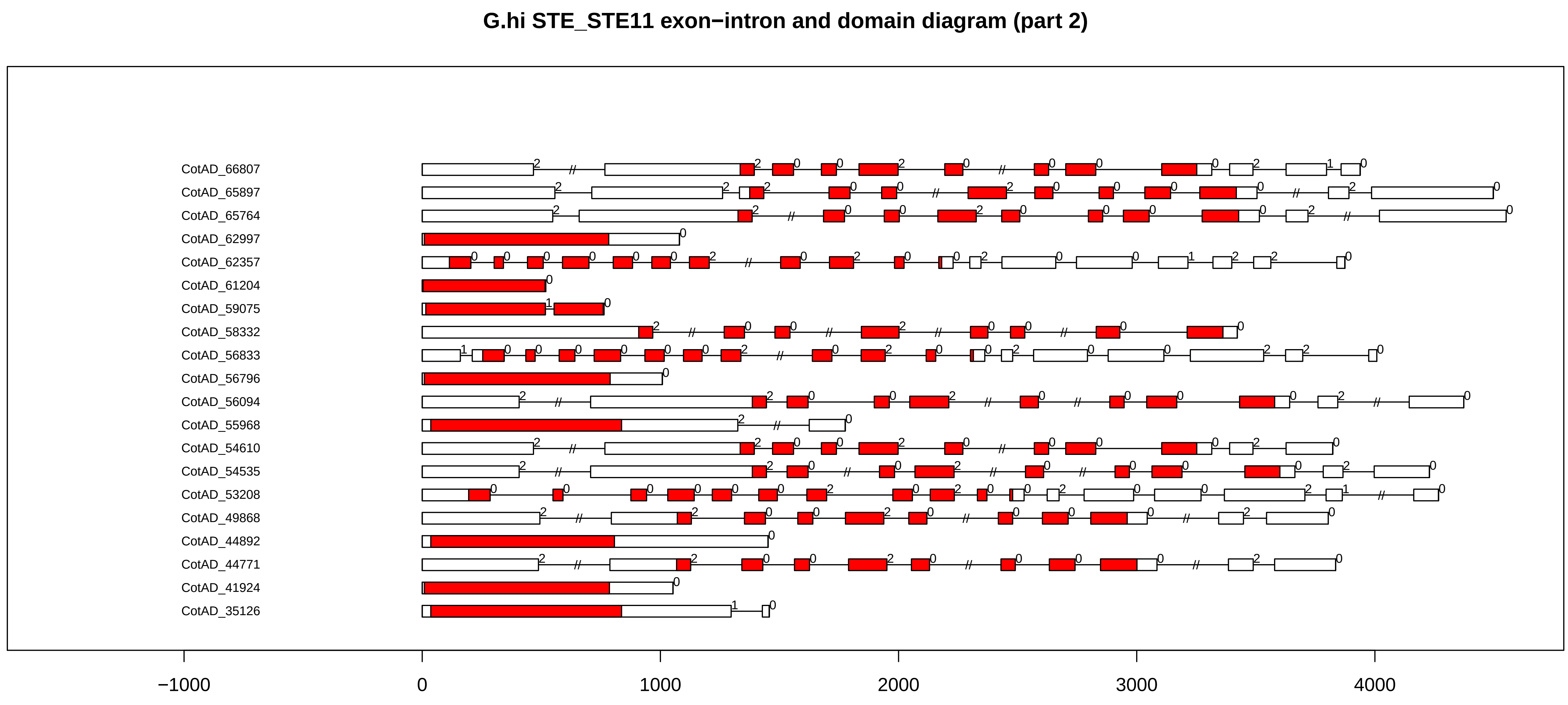

G.hi STE_STE11 exon−intron and domain diagram (part 2)
CotAD_66807
CotAD_65897
CotAD_65764
CotAD_62997
CotAD_62357
CotAD_61204
CotAD_59075
CotAD_58332
CotAD_56833
CotAD_56796
CotAD_56094
CotAD_55968
CotAD_54610
CotAD_54535
CotAD_53208
CotAD_49868
CotAD_44892
CotAD_44771
CotAD_41924
CotAD_35126
−1000
0
1000
2000
3000
4000

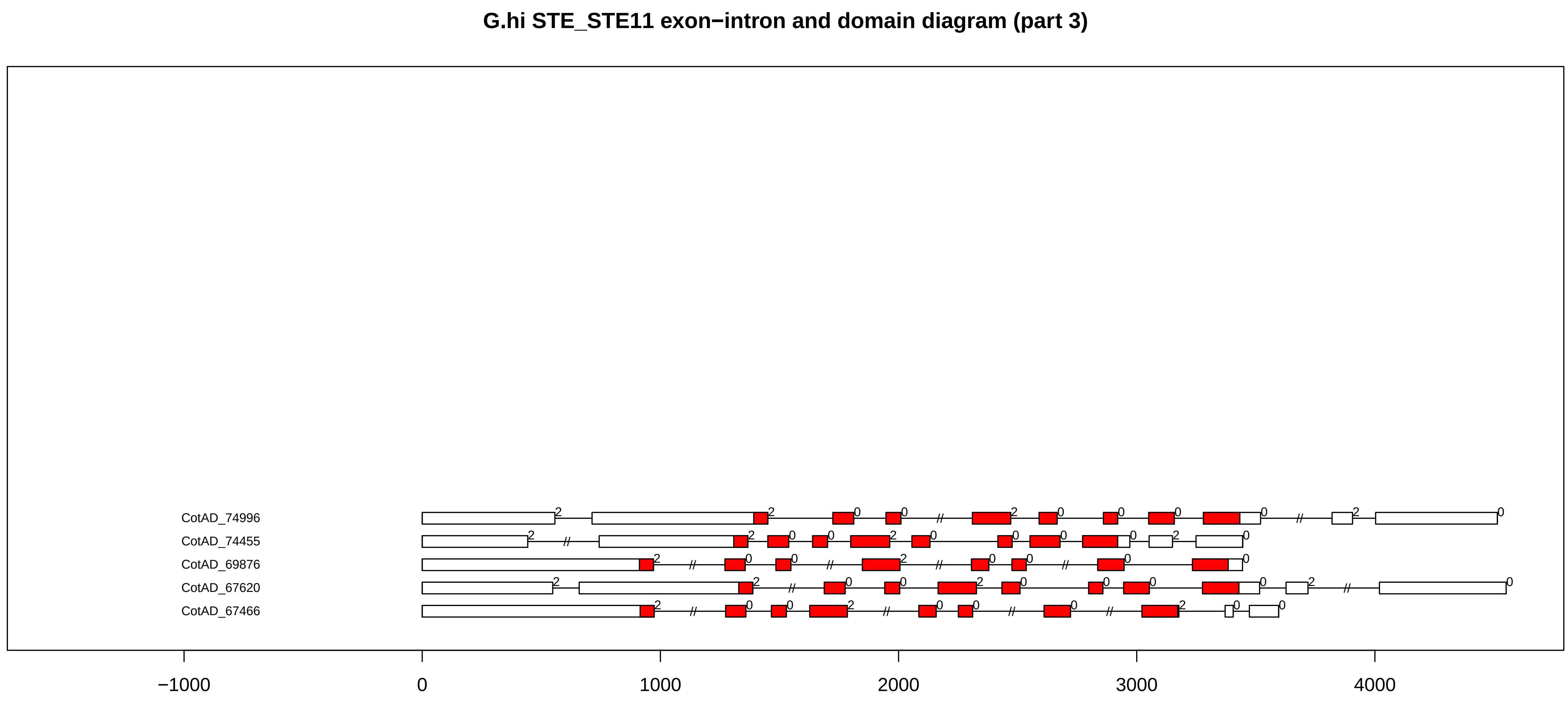
G.hi STE_STE11 exon−intron and domain diagram (part 3)
CotAD_74996
CotAD_74455
CotAD_69876
CotAD_67620
CotAD_67466
−1000
0
1000
2000
3000
4000

G.hi STE_STE20−Fray exon−intron and domain diagram (part 1)

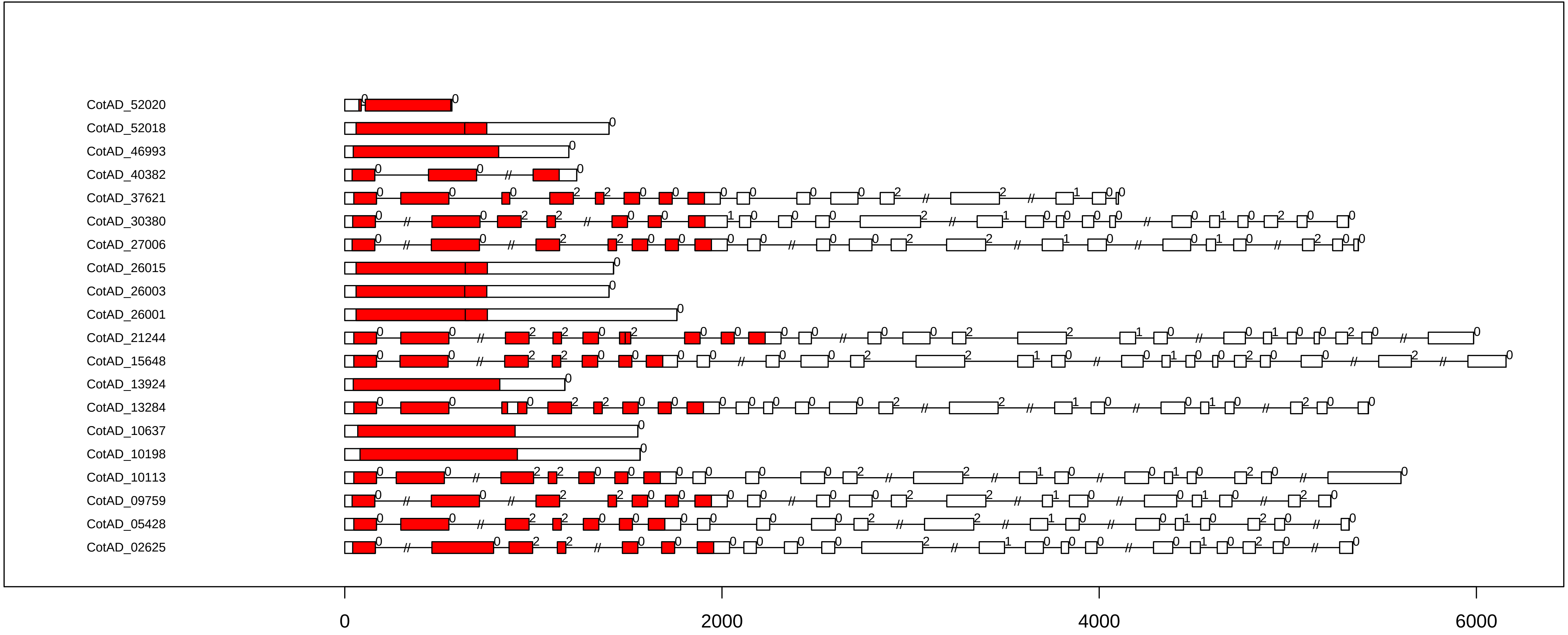

G.hi STE_STE20−Fray exon−intron and domain diagram (part 2)

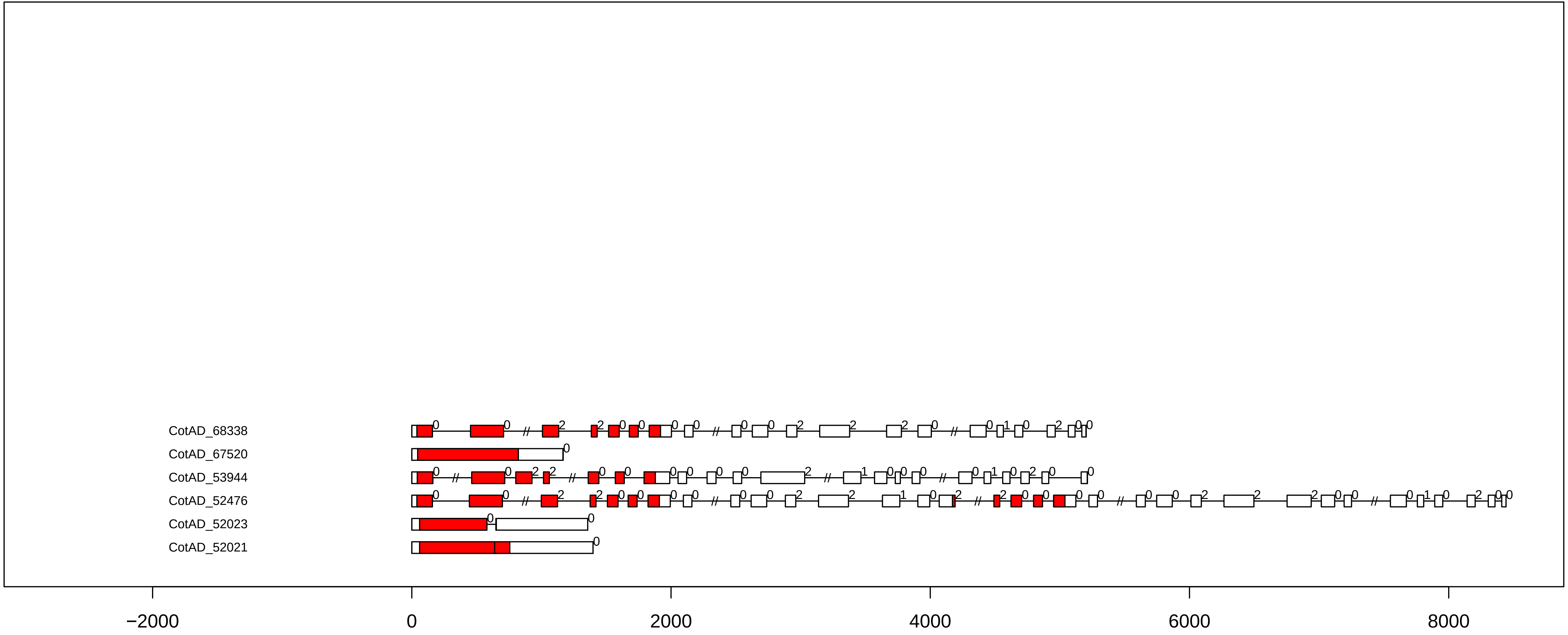

G.hi STE_STE20−PI exon−intron and domain diagram (all)

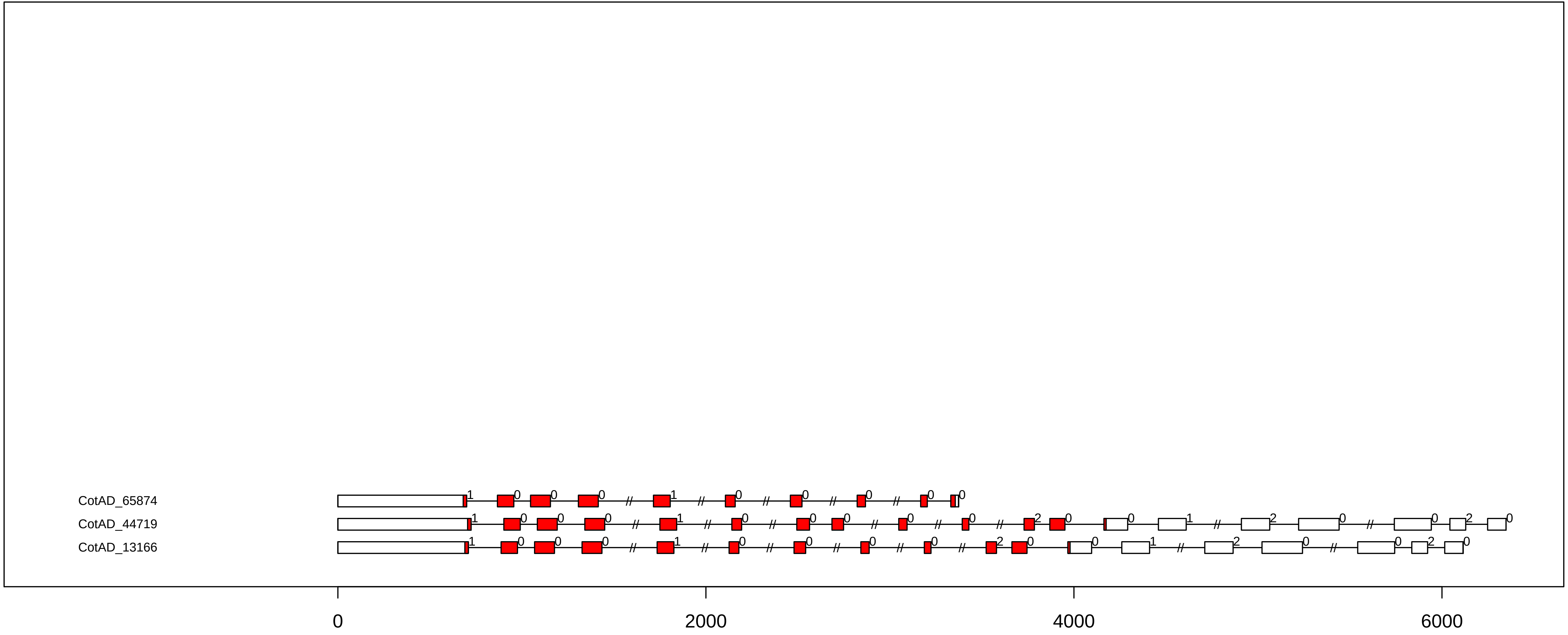

G.hi STE_STE20−YSK exon−intron and domain diagram (all)

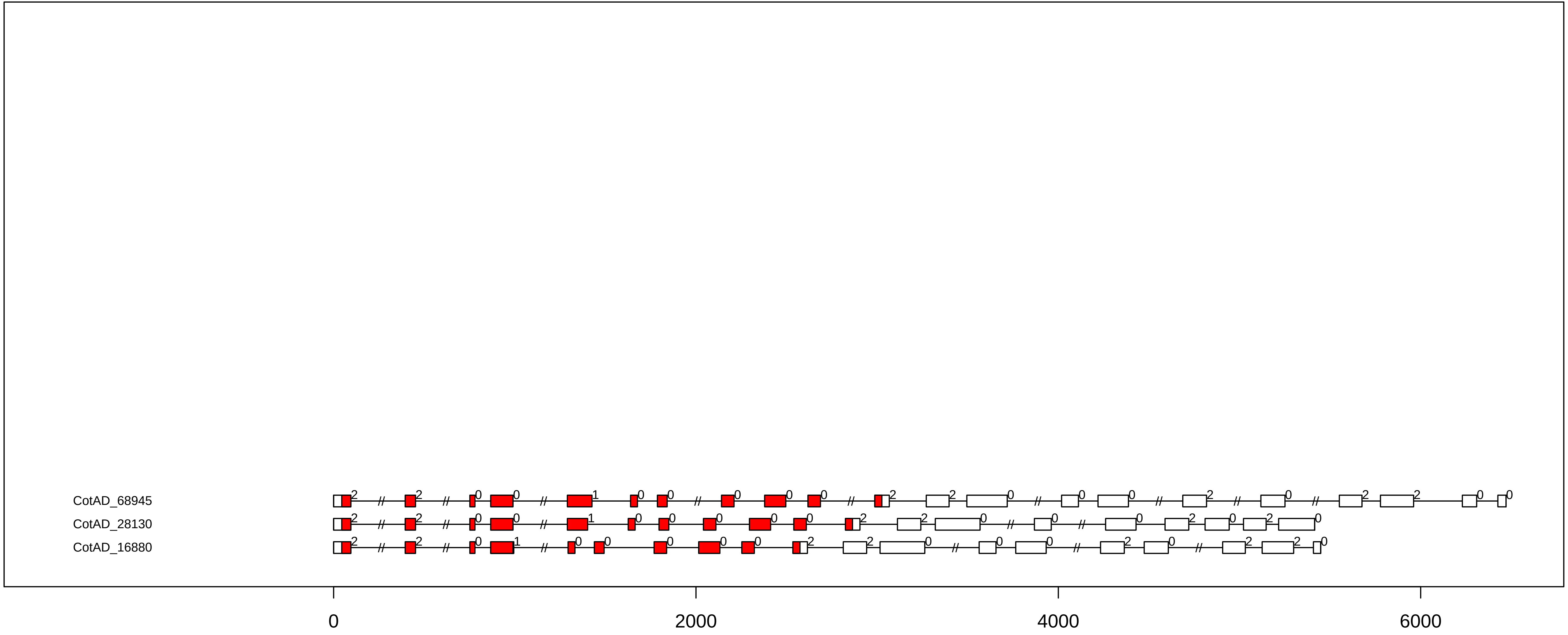

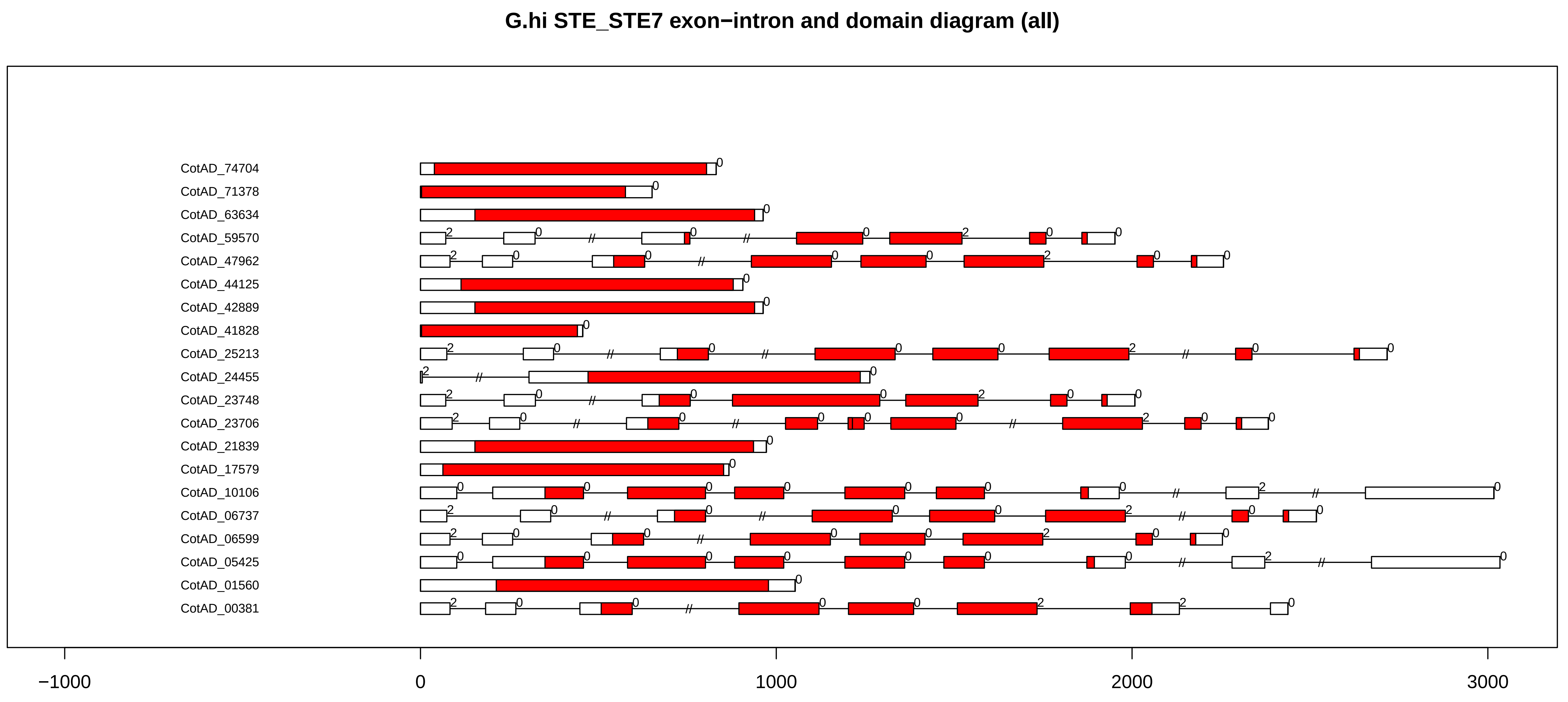

G.hi STE_STE7 exon−intron and domain diagram (all)
CotAD_74704
CotAD_71378
CotAD_63634
CotAD_59570
CotAD_47962
CotAD_44125
CotAD_42889
CotAD_41828
CotAD_25213
CotAD_24455
CotAD_23748
CotAD_23706
CotAD_21839
CotAD_17579
CotAD_10106
CotAD_06737
CotAD_06599
CotAD_05425
CotAD_01560
CotAD_00381
−1000
0
1000
2000
3000

G.hi STE_STE−Pl exon−intron and domain diagram (all)

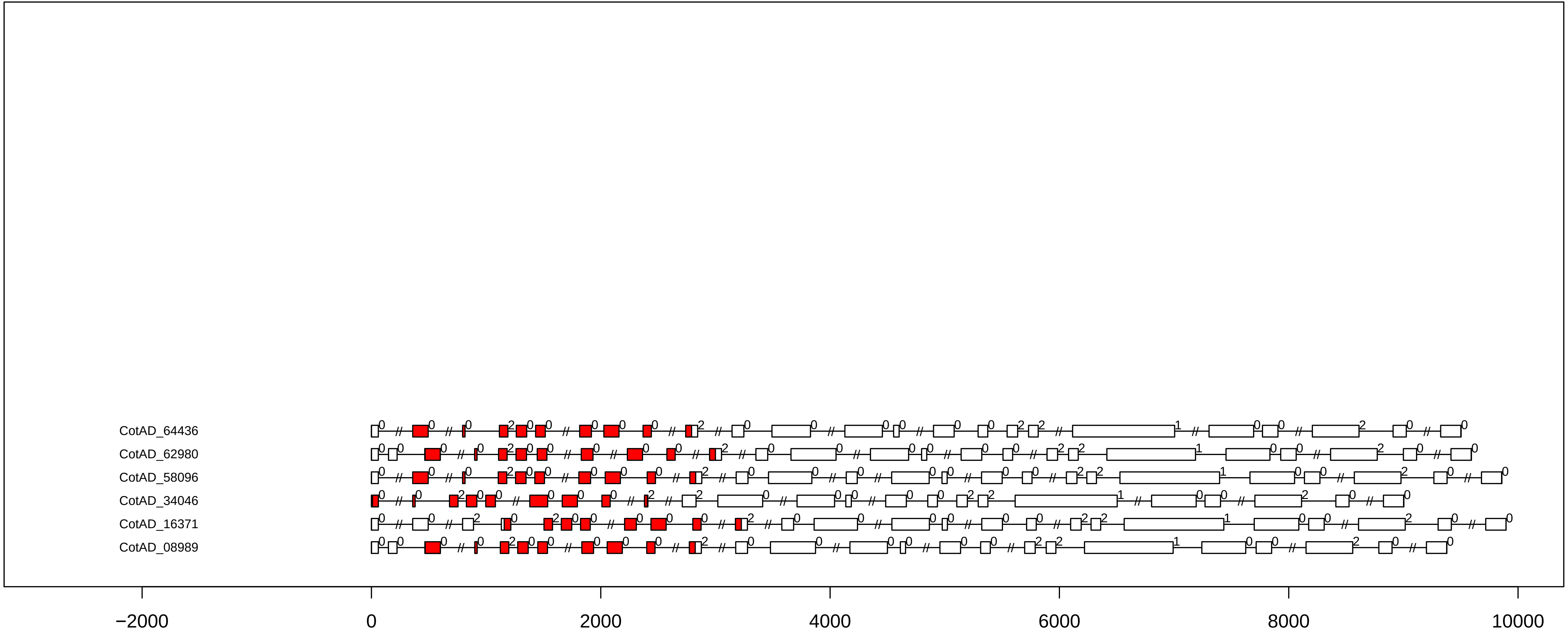

G.hi TKL_CTR1−DRK−1 exon−intron and domain diagram (all)

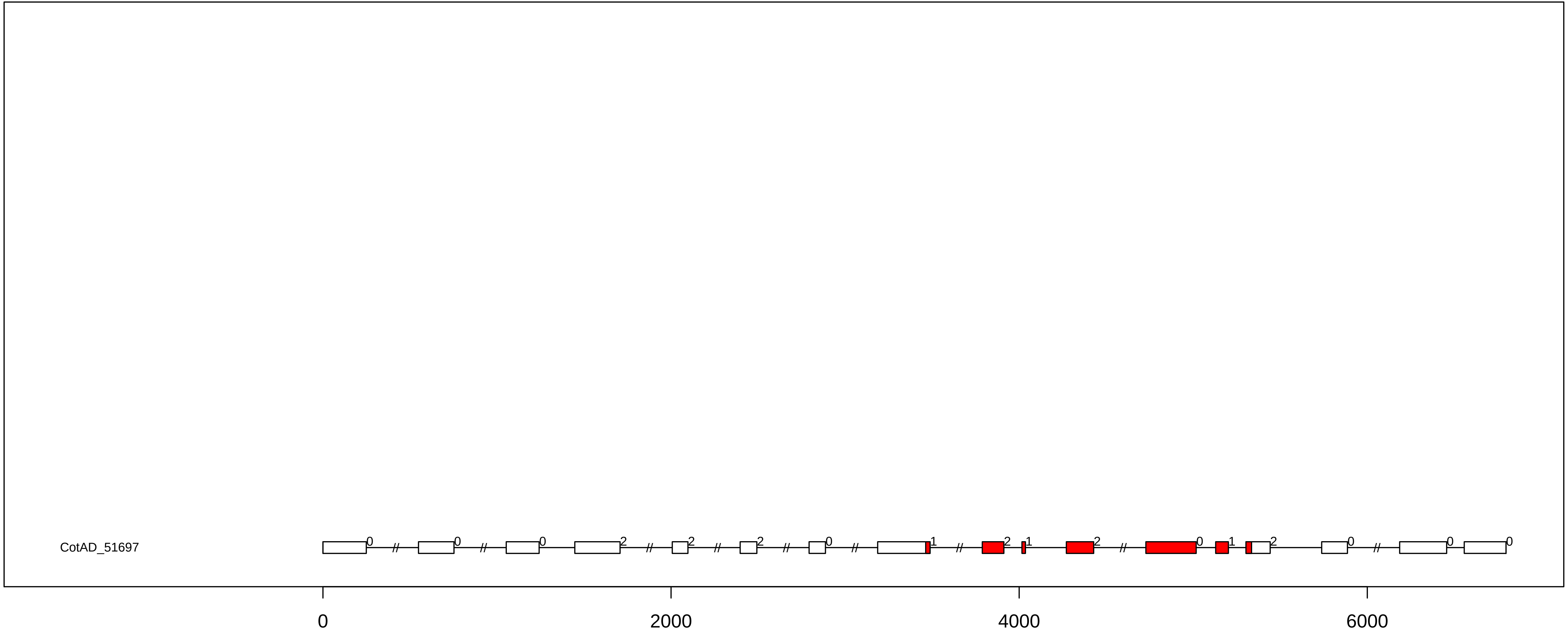

G.hi TKL_CTR1−DRK−2 exon−intron and domain diagram (part 1)

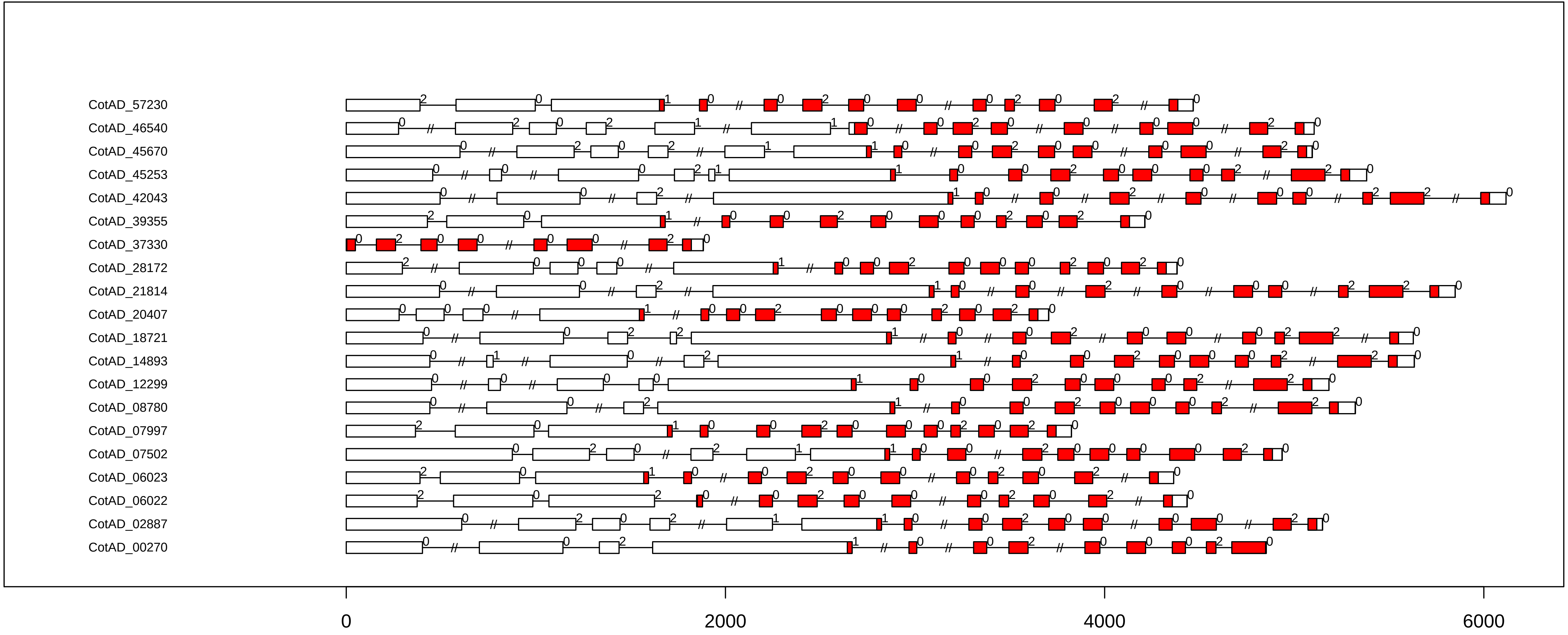

G.hi TKL_CTR1−DRK−2 exon−intron and domain diagram (part 2)

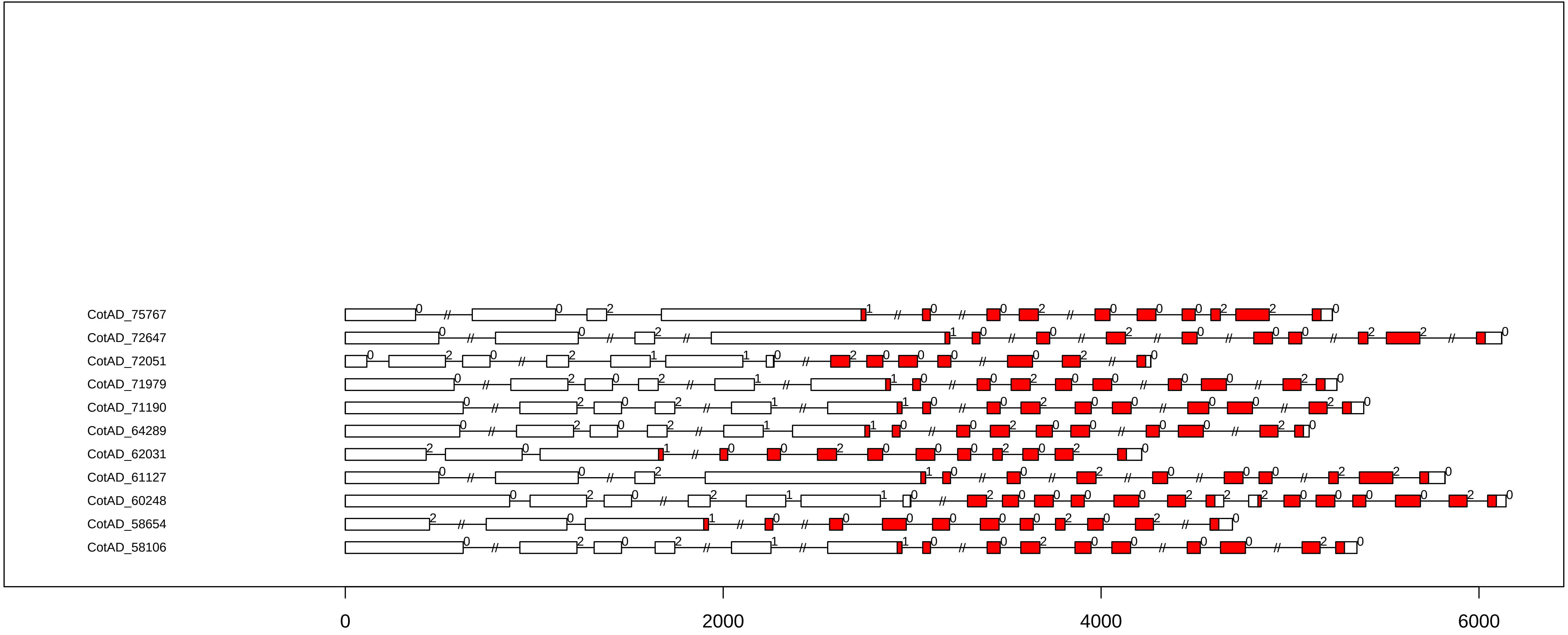

G.hi TKL_Gdt exon−intron and domain diagram (all)

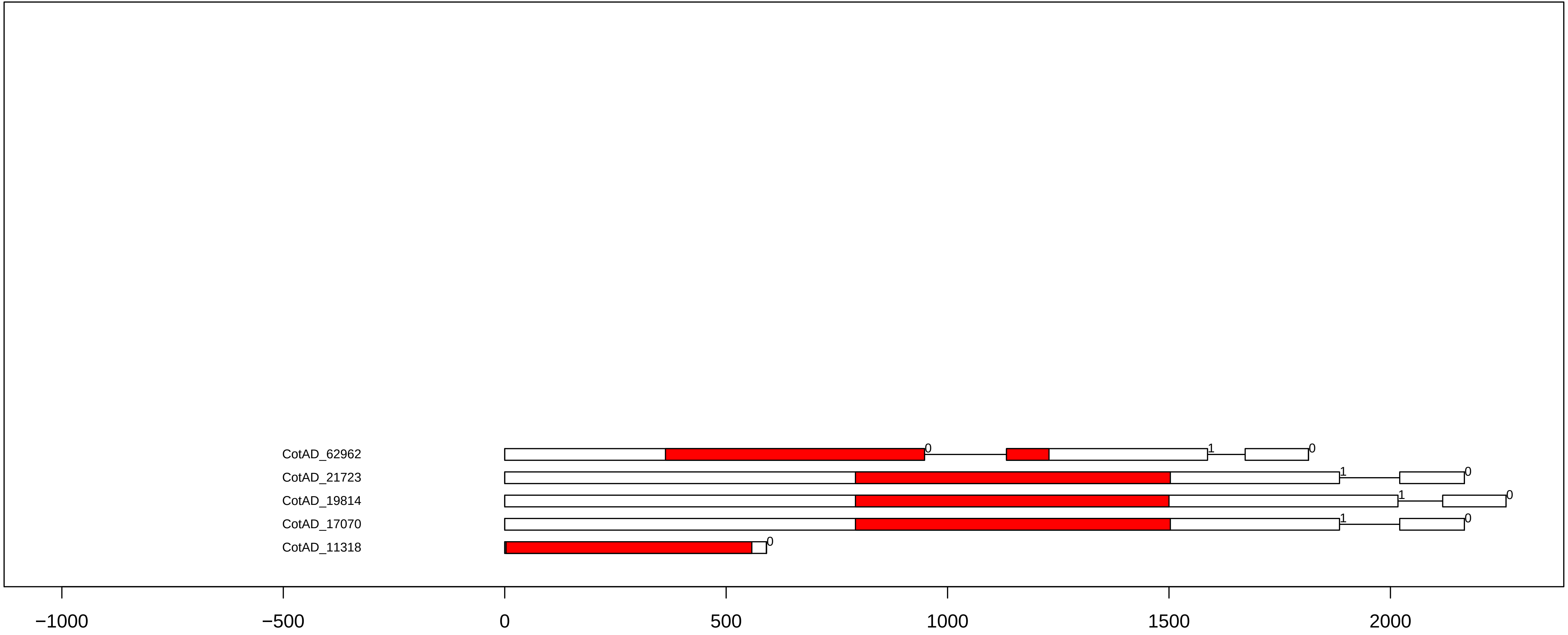

G.hi TKL−Pl−1 exon−intron and domain diagram (all)

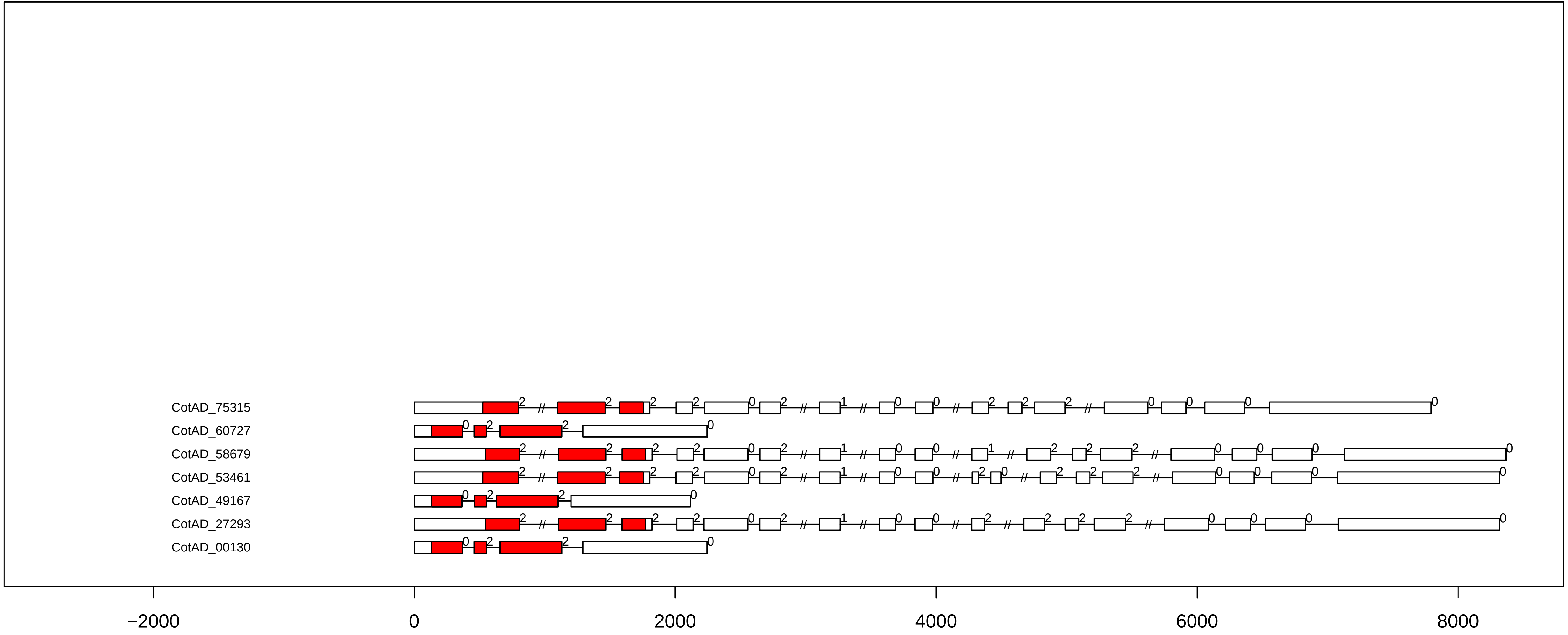

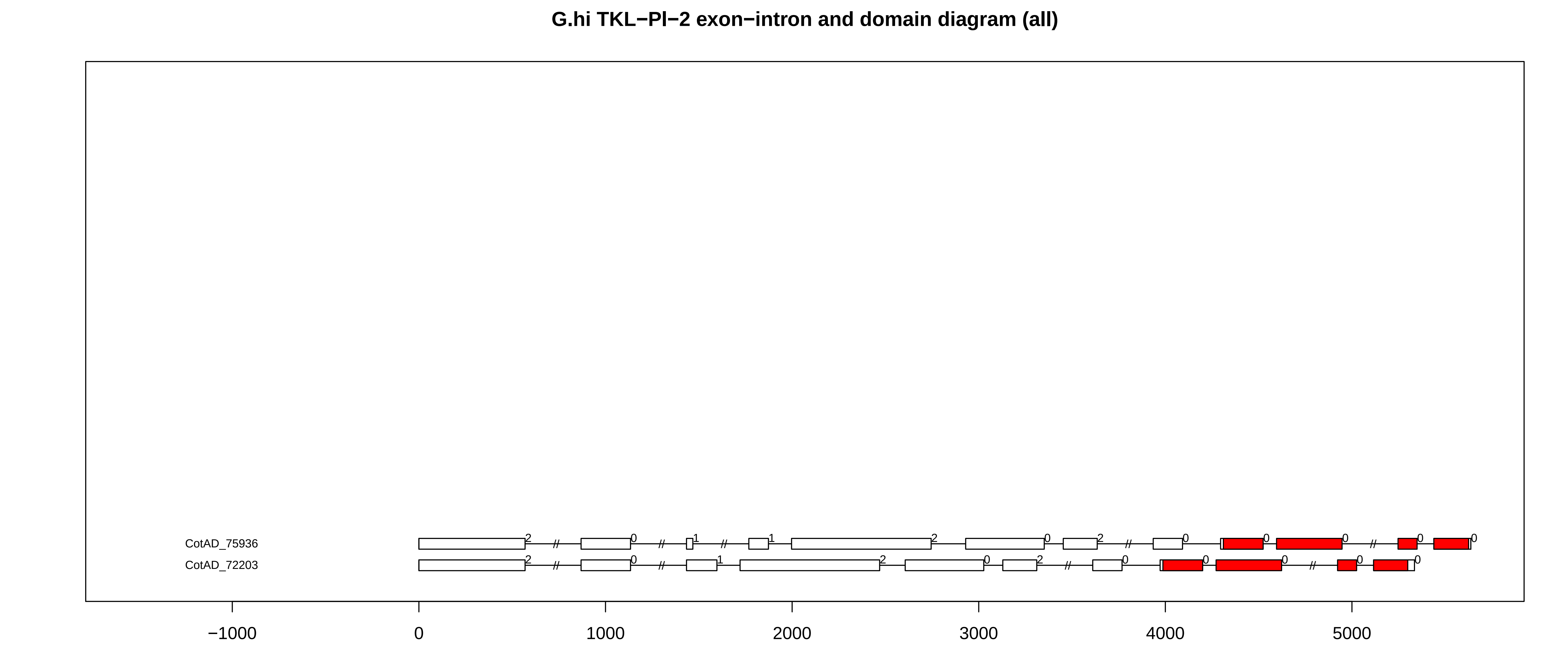

G.hi TKL−Pl−2 exon−intron and domain diagram (all)
CotAD_75936
CotAD_72203
2
0
1
−1000
0
1000
2000
3000
4000
5000

G.hi TKL−Pl−3 exon−intron and domain diagram (all)

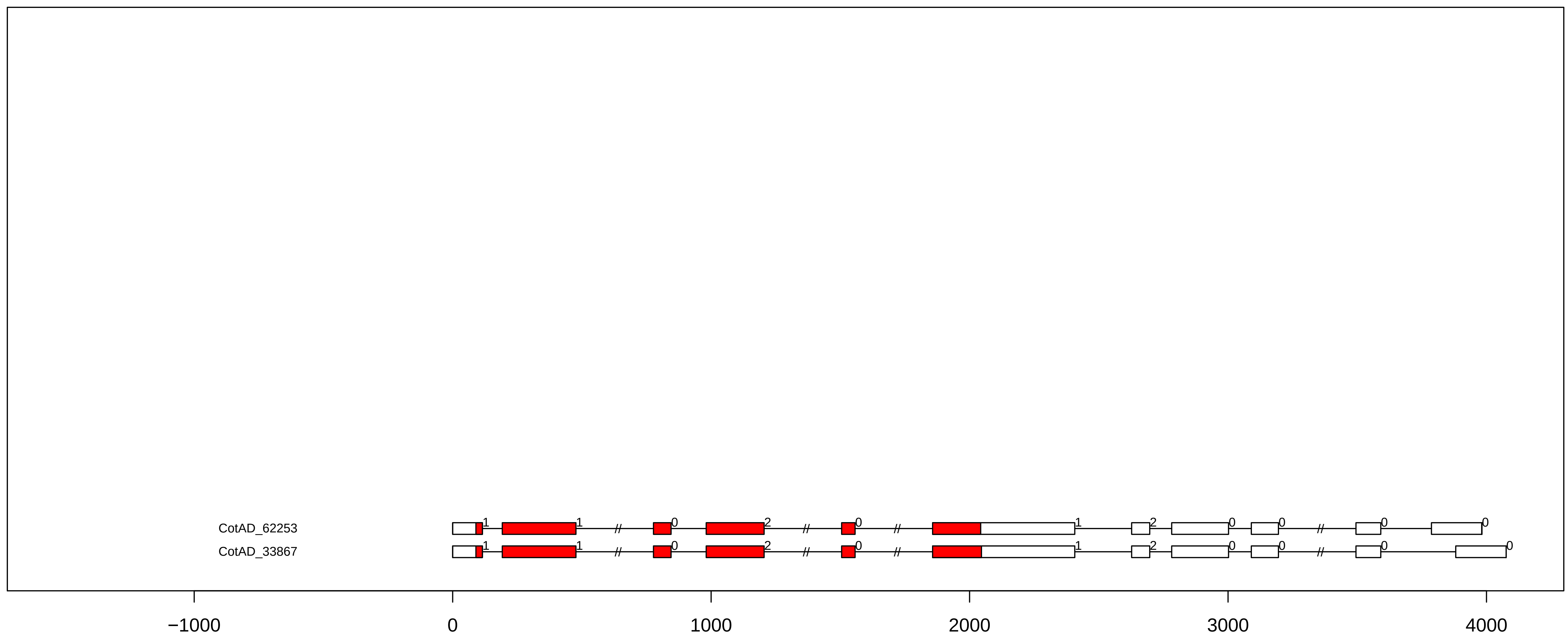

G.hi TKL−Pl−4 exon−intron and domain diagram (part 1)

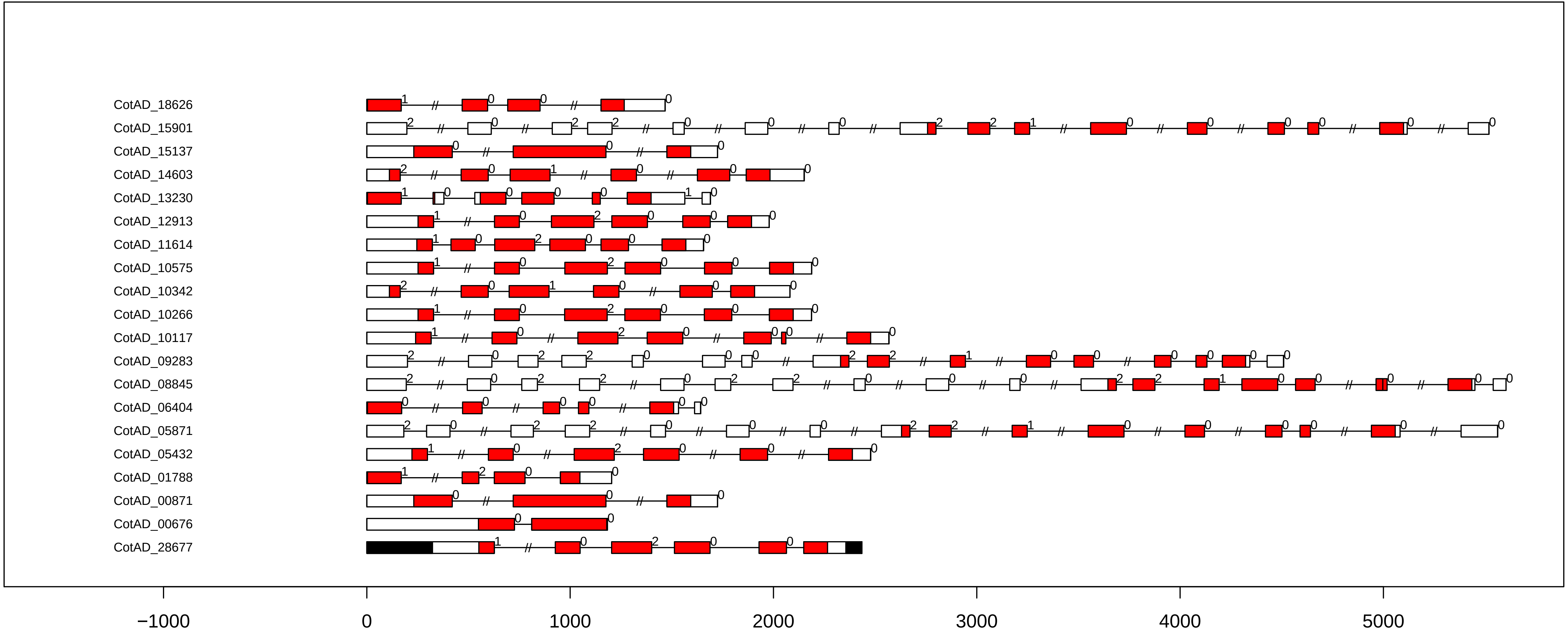

G.hi TKL−Pl−4 exon−intron and domain diagram (part 2)

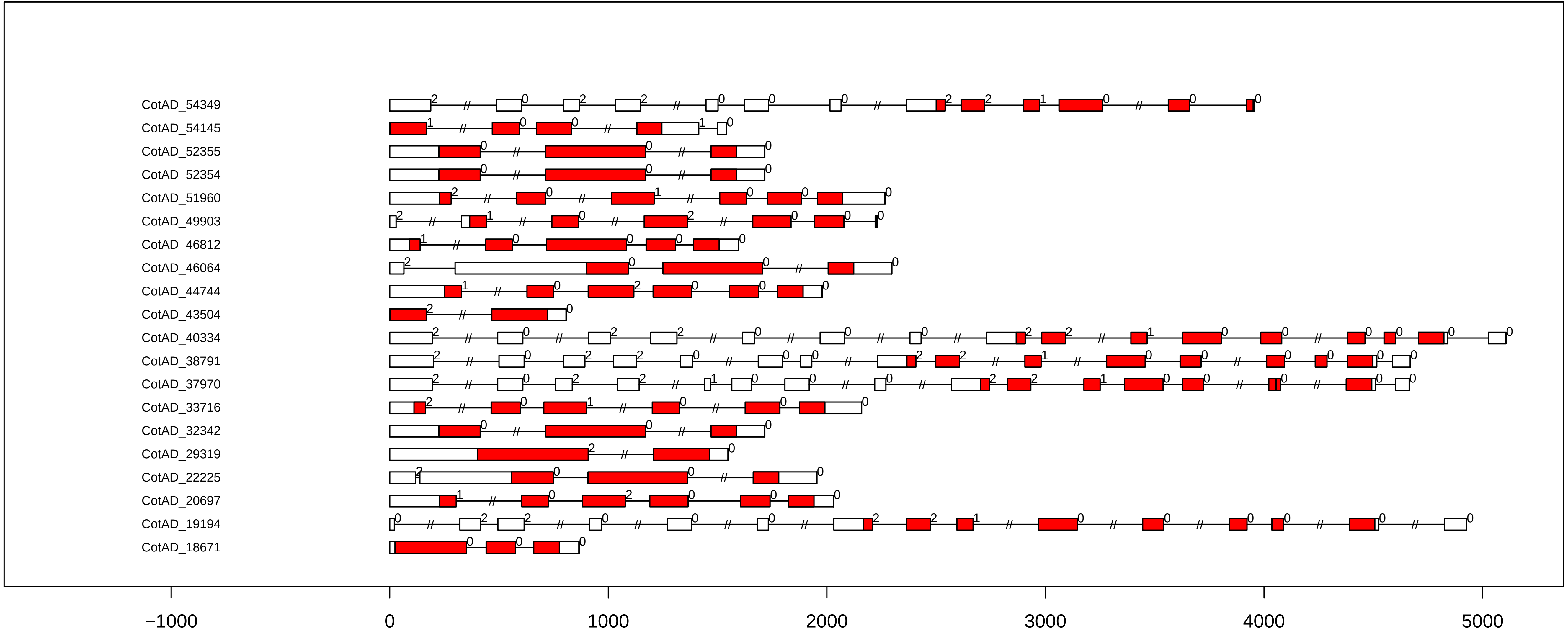

G.hi TKL−Pl−4 exon−intron and domain diagram (part 3)

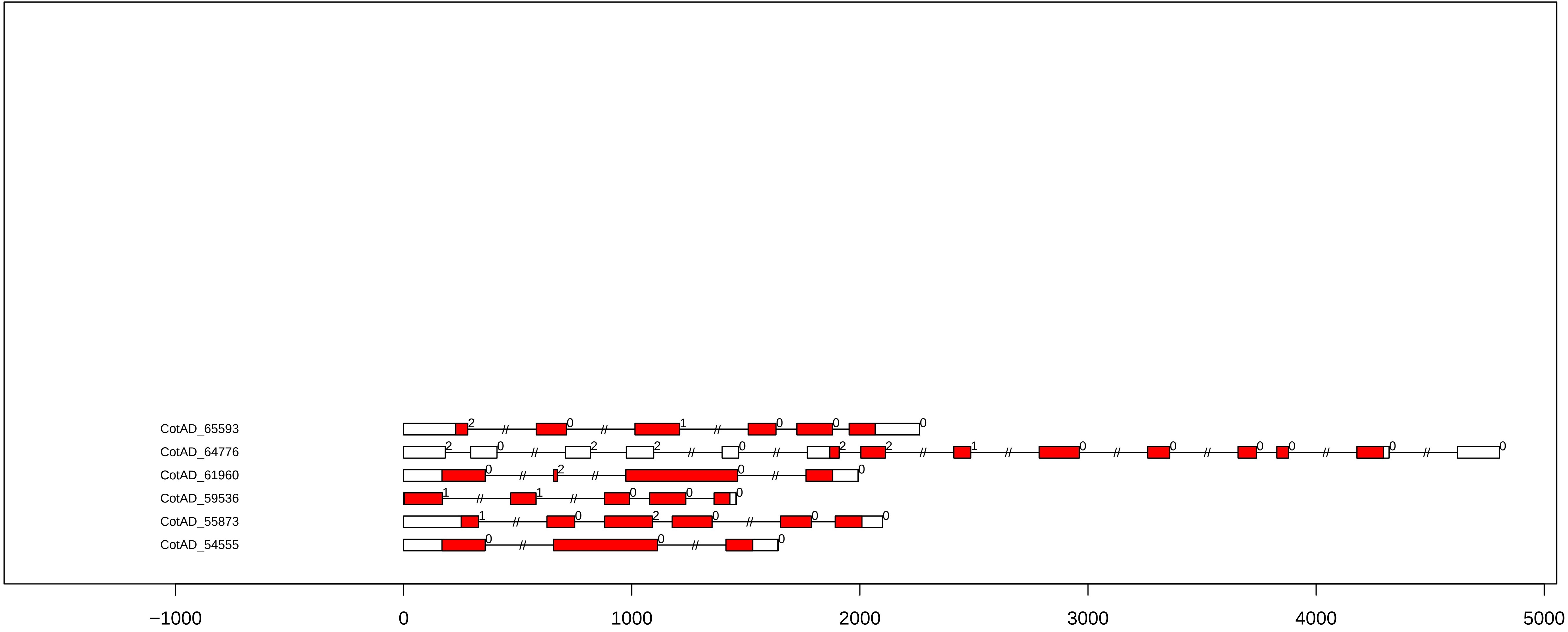

G.hi TKL−Pl−5 exon−intron and domain diagram (all)

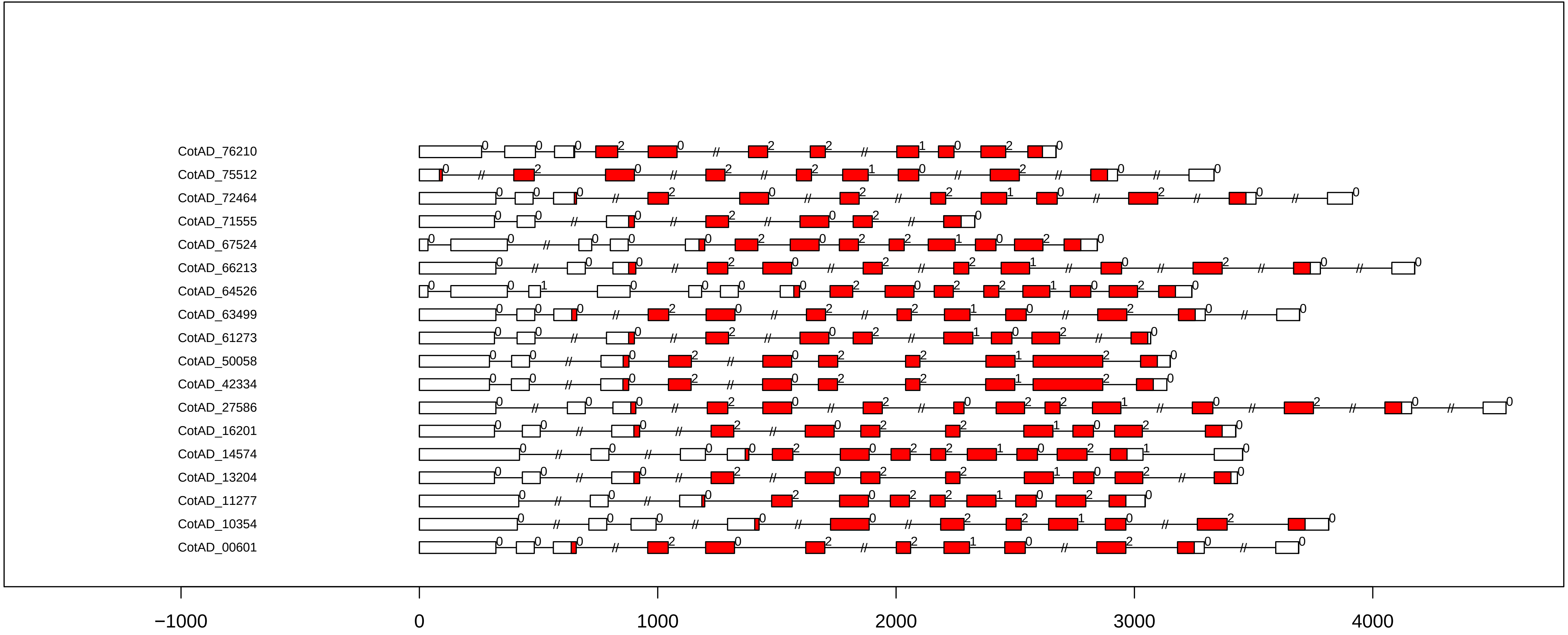

G.hi TKL−Pl−6 exon−intron and domain diagram (all)

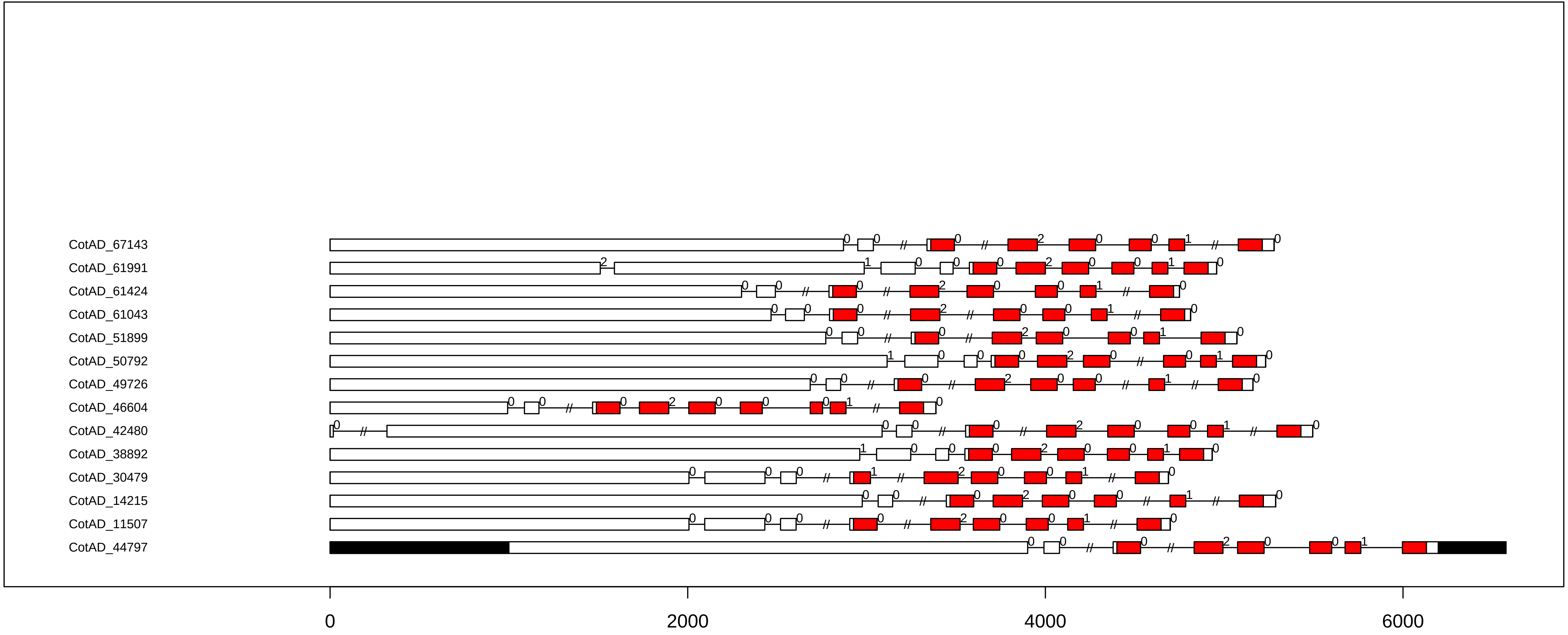

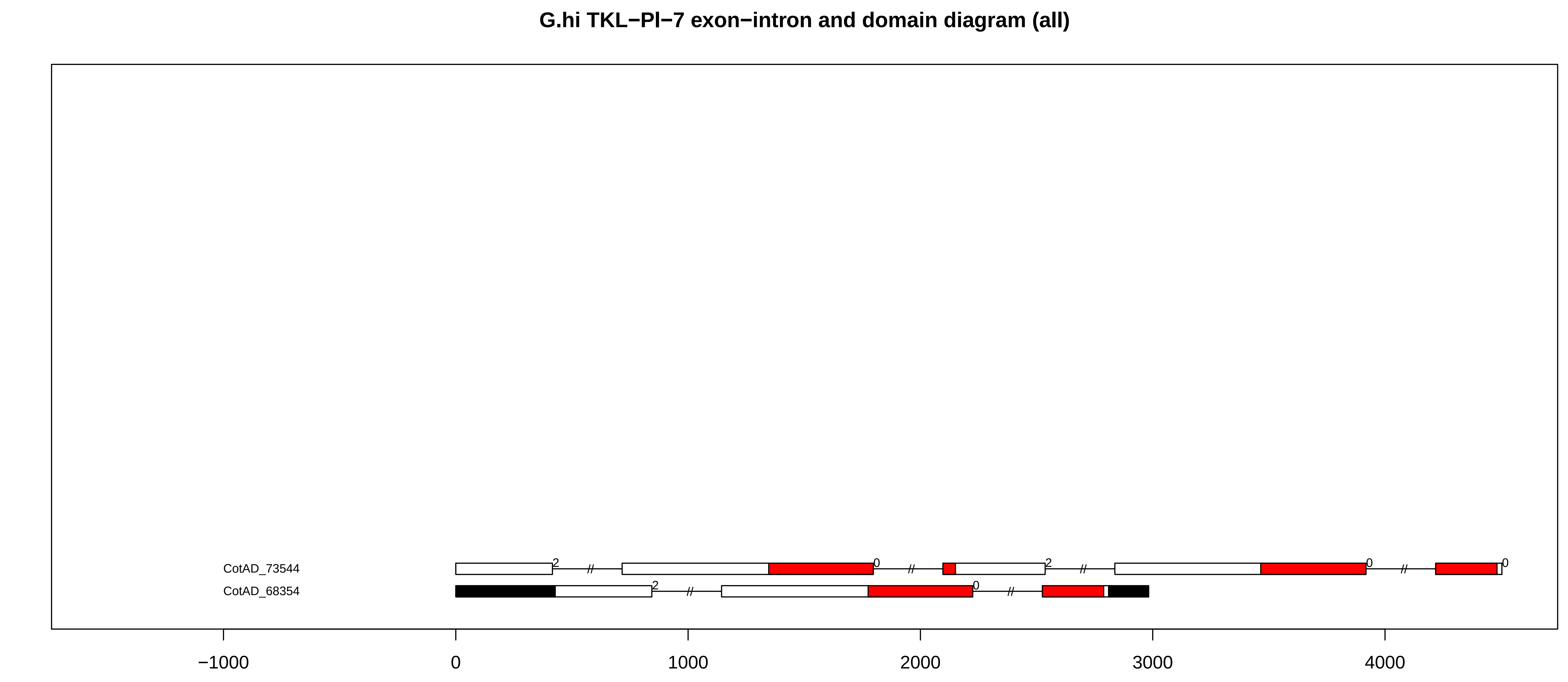

G.hi TKL−Pl−7 exon−intron and domain diagram (all)
CotAD_73544
CotAD_68354
2
0
2
0
0
2
0
−1000
0
1000
2000
3000
4000

G.hi TKL−Pl−8 exon−intron and domain diagram (all)

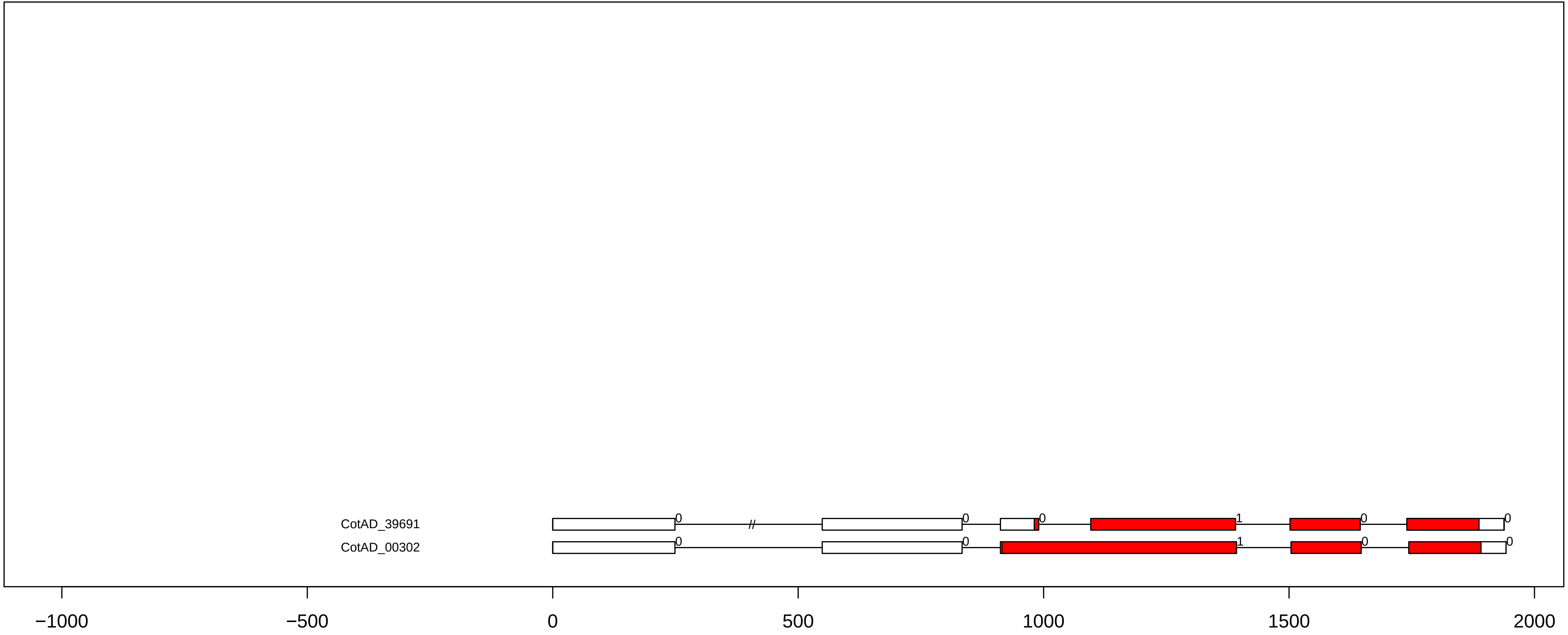

G.hi TLK exon−intron and domain diagram (all)

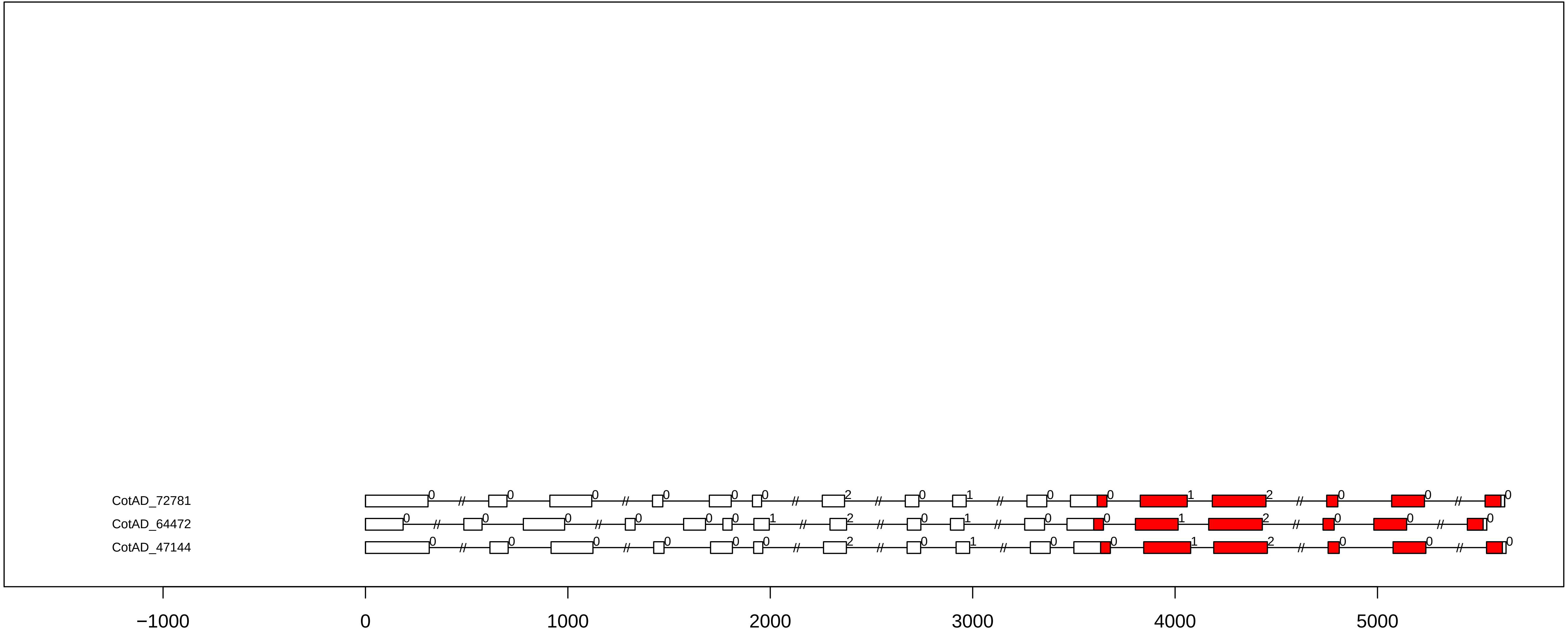

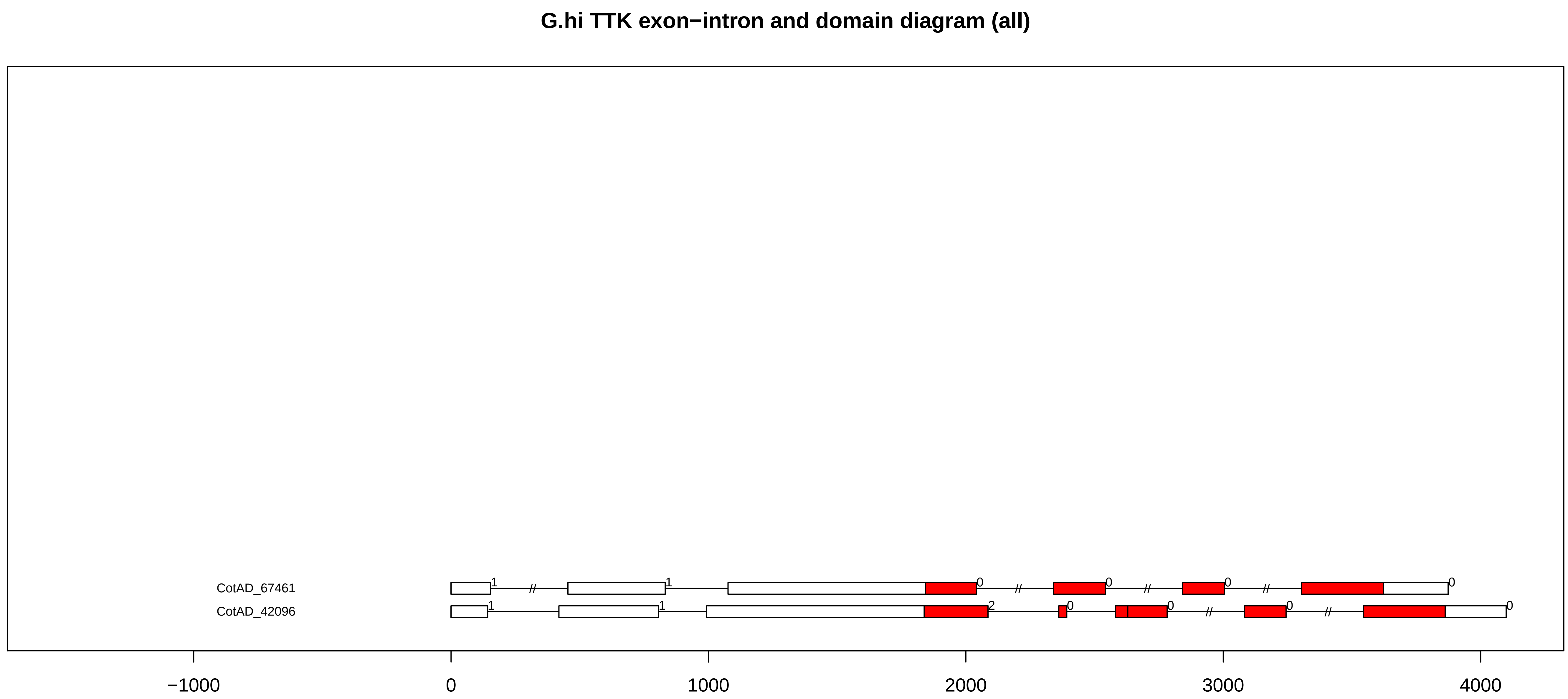
G.hi TTK exon−intron and domain diagram (all)
CotAD_67461
CotAD_42096
−1000
0
1000
2000
3000
4000

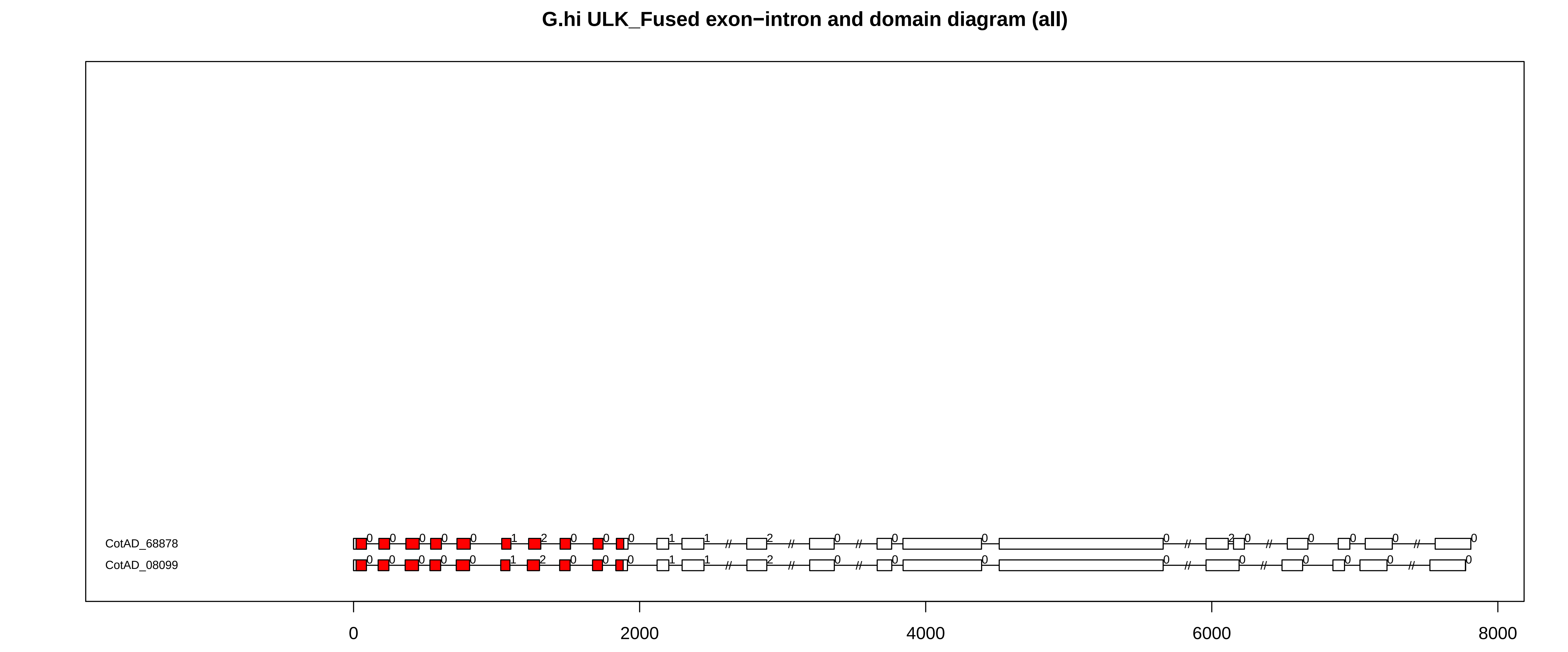
G.hi ULK_Fused exon−intron and domain diagram (all)
CotAD_68878
CotAD_08099
0
2000
4000
6000
8000

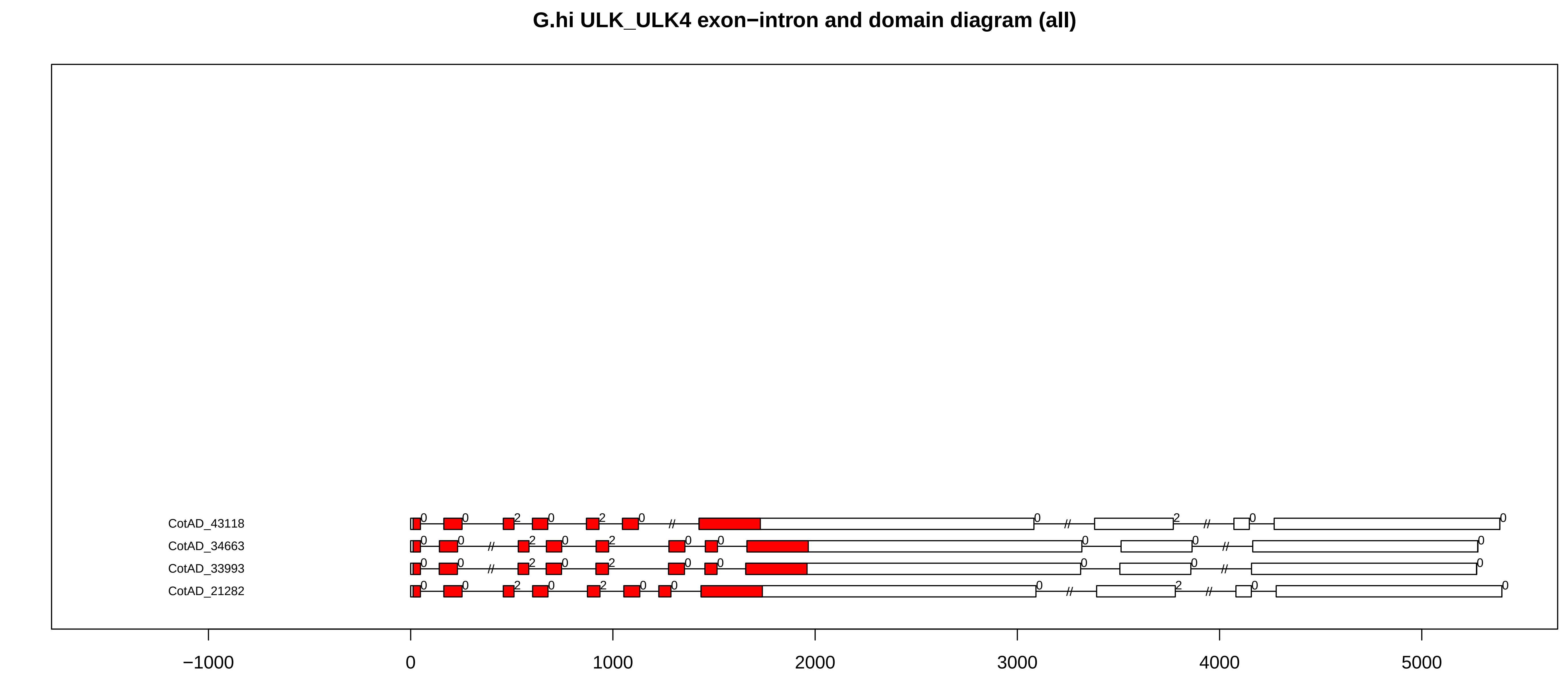
G.hi ULK_ULK4 exon−intron and domain diagram (all)
CotAD_43118
CotAD_34663
CotAD_33993
CotAD_21282
−1000
0
1000
2000
3000
4000
5000

G.hi WEE exon−intron and domain diagram (all)

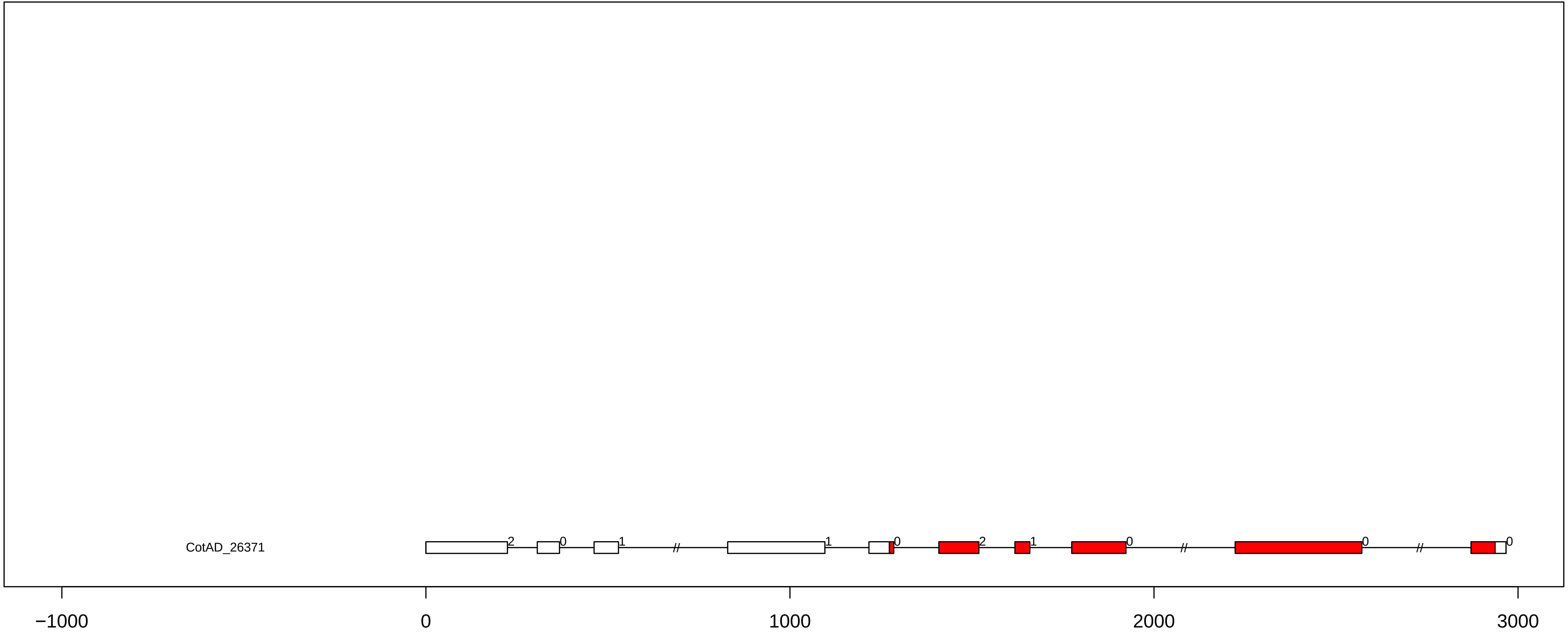

G.hi WNK_NRBP exon−intron and domain diagram (part 1)

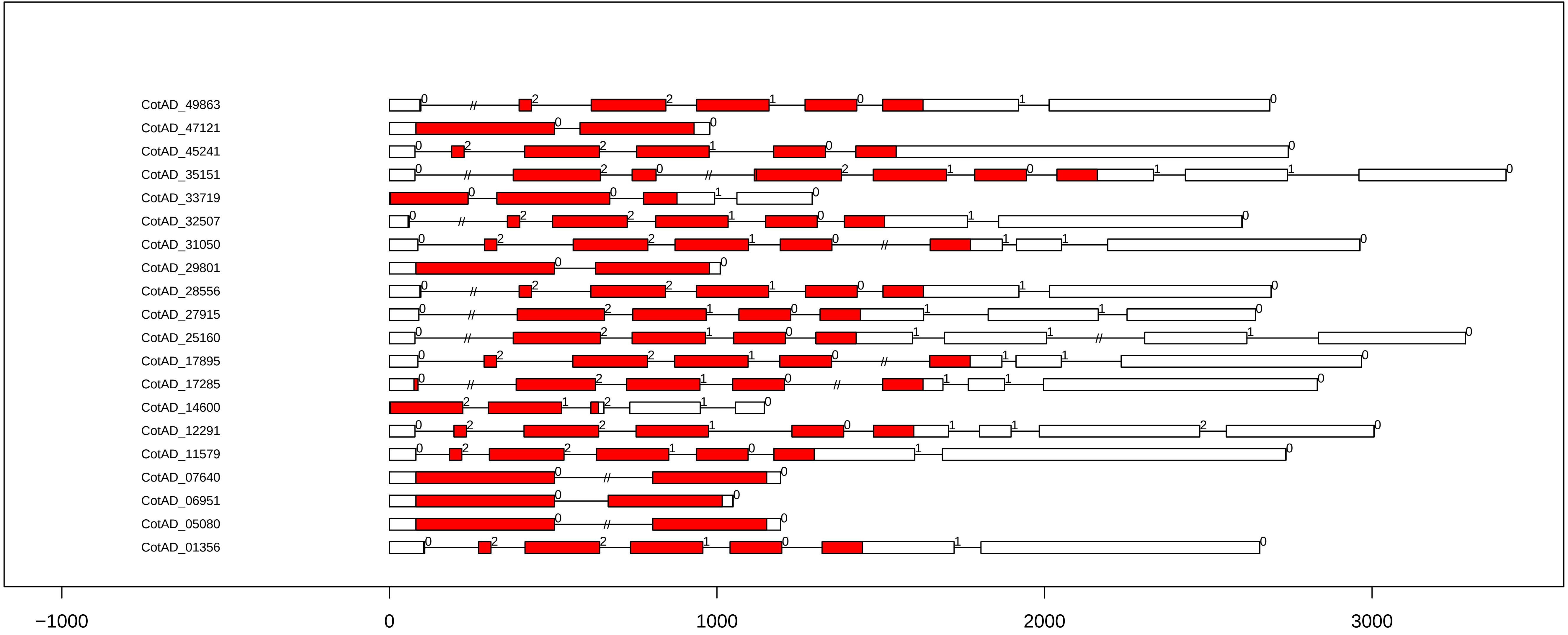

G.hi WNK_NRBP exon−intron and domain diagram (part 2)

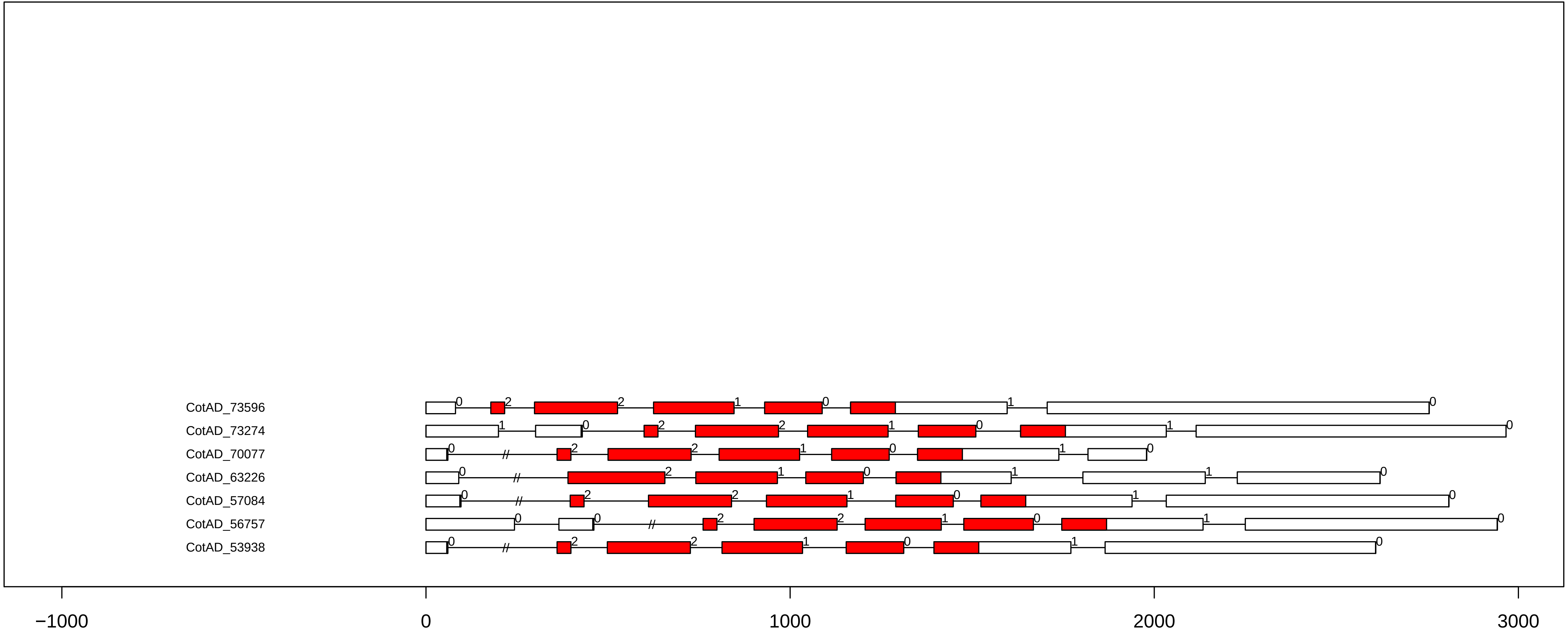

G.ba AGC_MAST exon−intron and domain diagram (all)

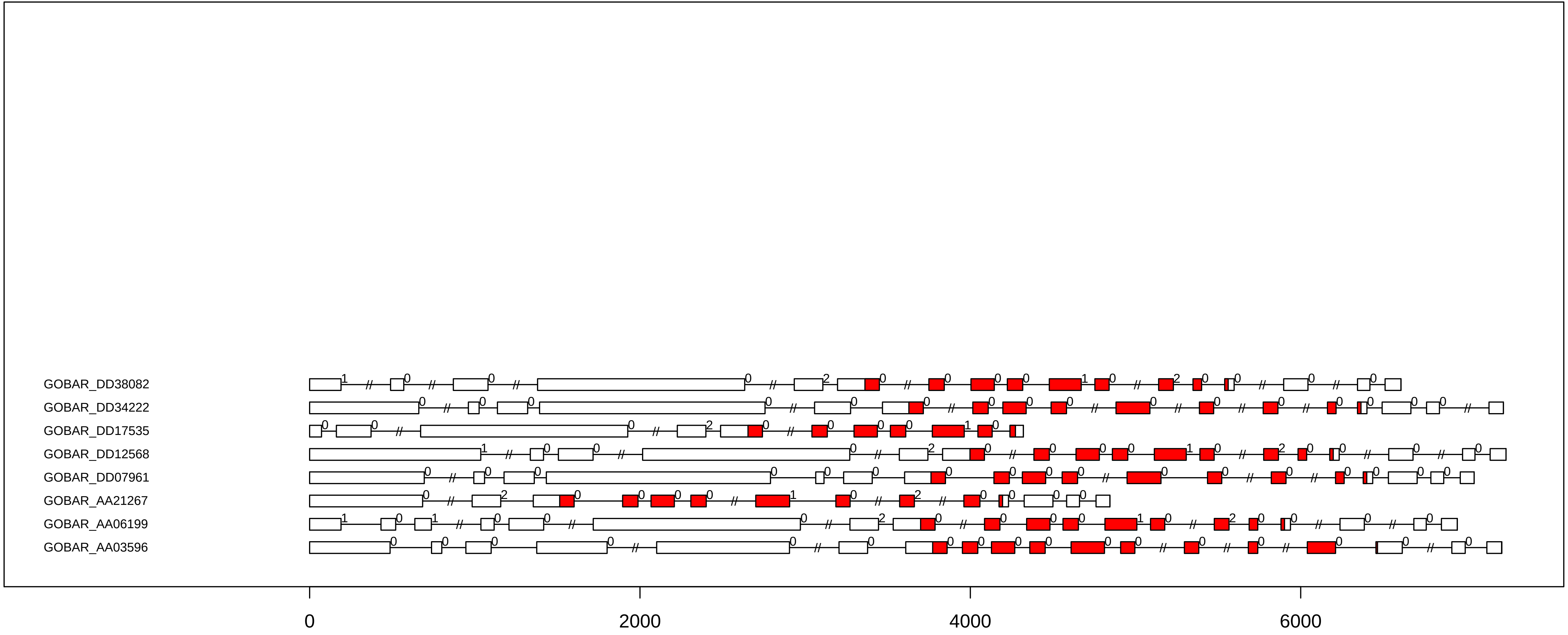

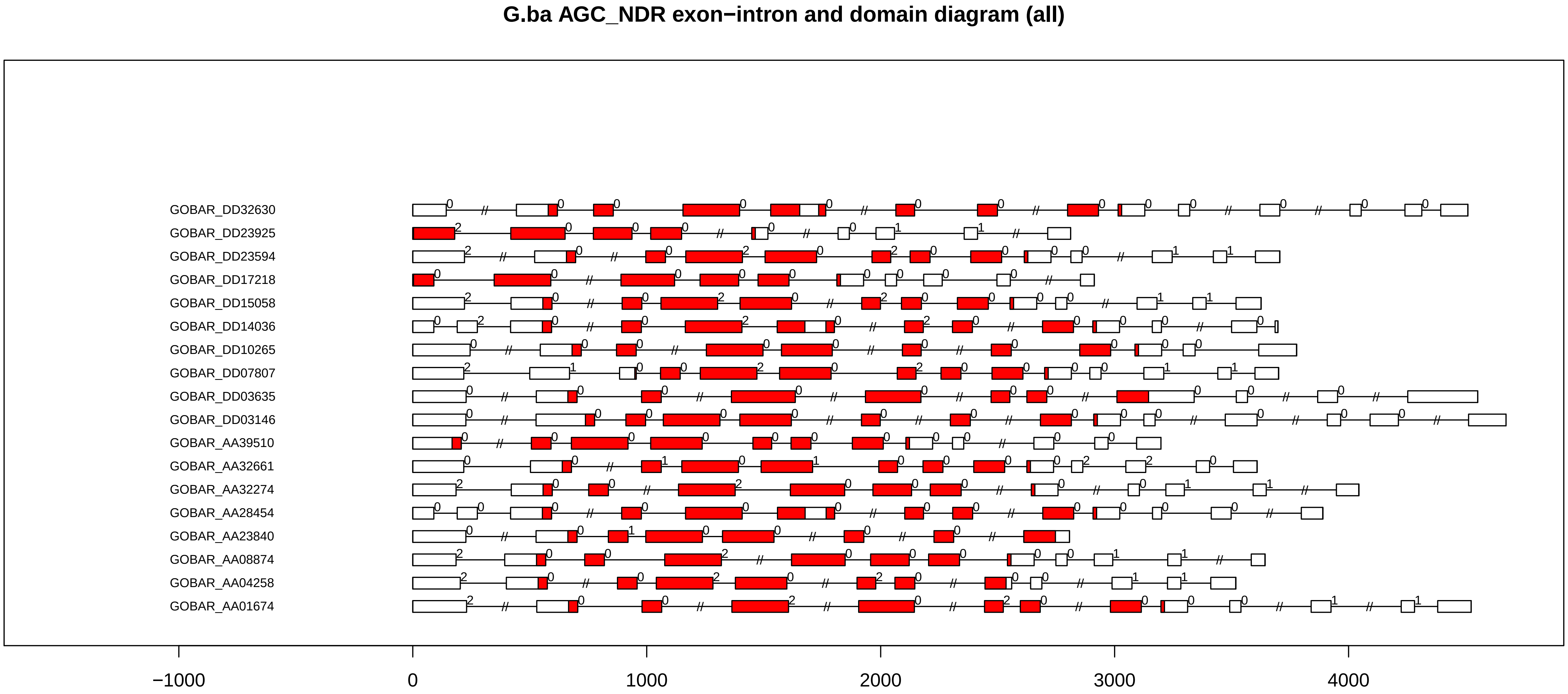

G.ba AGC_NDR exon−intron and domain diagram (all)
GOBAR_DD32630
GOBAR_DD23925
GOBAR_DD23594
GOBAR_DD17218
GOBAR_DD15058
GOBAR_DD14036
GOBAR_DD10265
GOBAR_DD07807
GOBAR_DD03635
GOBAR_DD03146
GOBAR_AA39510
GOBAR_AA32661
GOBAR_AA32274
GOBAR_AA28454
GOBAR_AA23840
GOBAR_AA08874
GOBAR_AA04258
GOBAR_AA01674
−1000
0
1000
2000
3000
4000

G.ba AGC_PDK1 exon−intron and domain diagram (all)

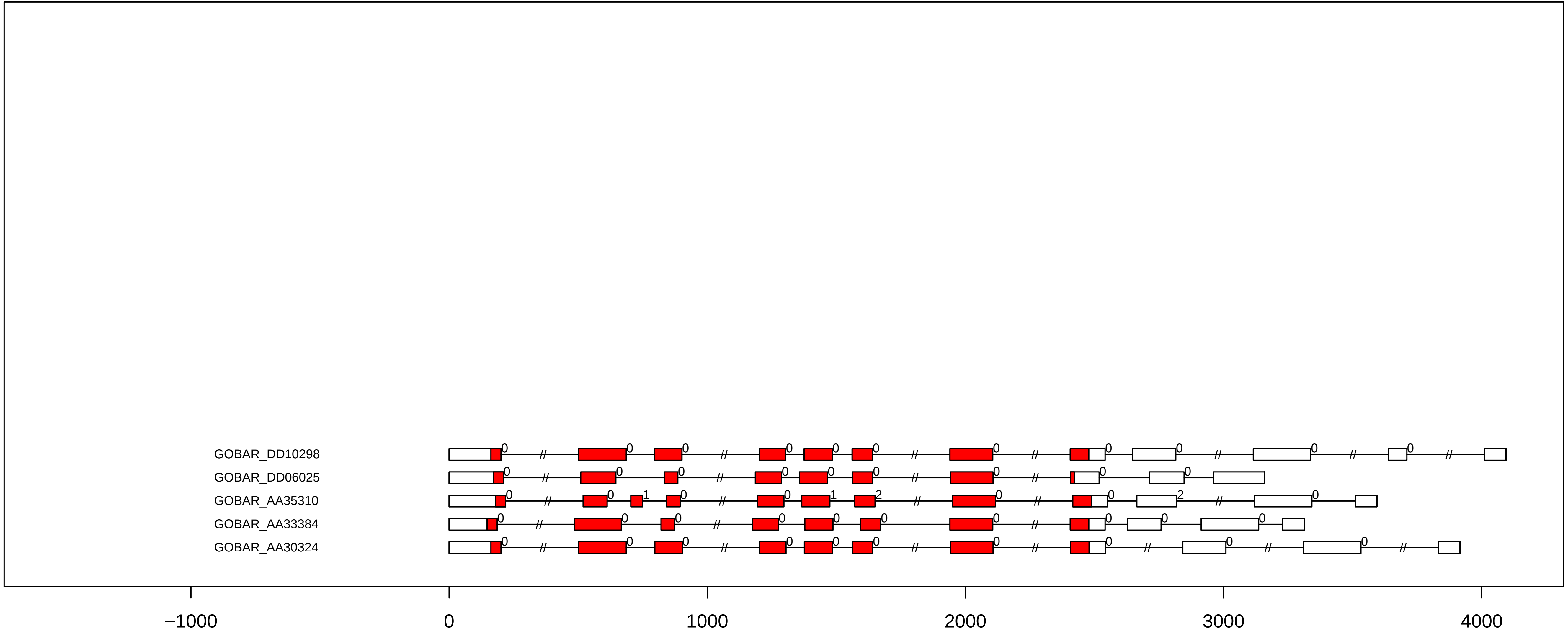

G.ba AGC_PKA−PKG exon−intron and domain diagram (all)

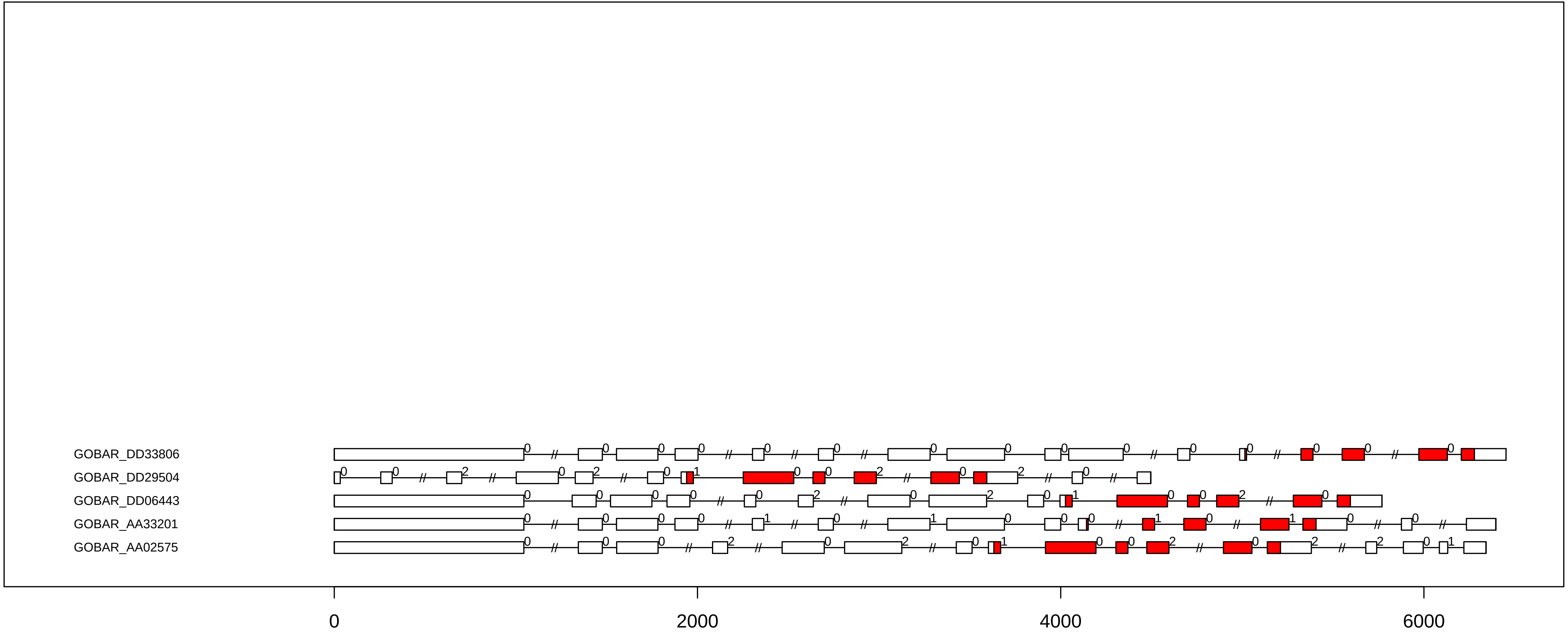

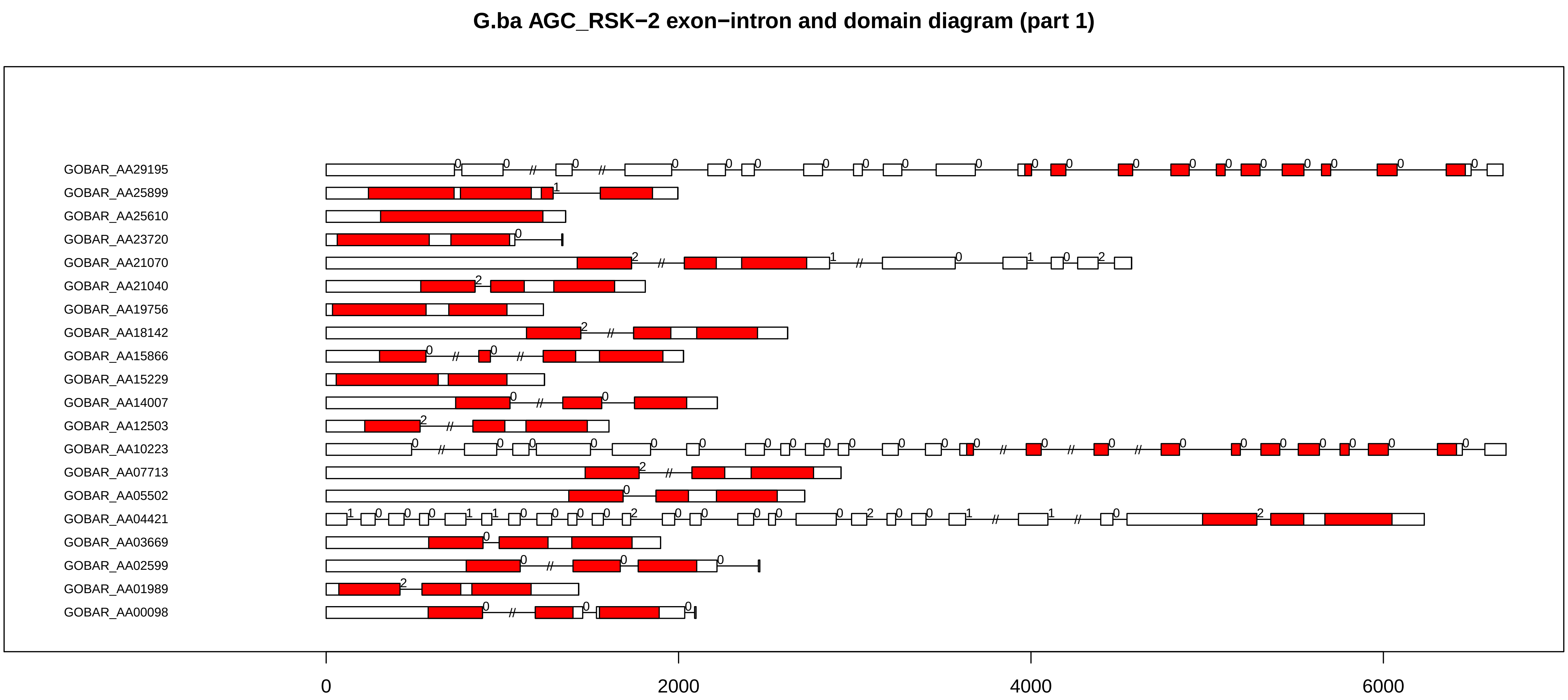
G.ba AGC_RSK−2 exon−intron and domain diagram (part 1)
GOBAR_AA29195
GOBAR_AA25899
GOBAR_AA25610
GOBAR_AA23720
GOBAR_AA21070
GOBAR_AA21040
GOBAR_AA19756
GOBAR_AA18142
GOBAR_AA15866
GOBAR_AA15229
GOBAR_AA14007
GOBAR_AA12503
GOBAR_AA10223
GOBAR_AA07713
GOBAR_AA05502
GOBAR_AA04421
GOBAR_AA03669
GOBAR_AA02599
GOBAR_AA01989
GOBAR_AA00098
0
2000
4000
6000

G.ba AGC_RSK−2 exon−intron and domain diagram (part 2)

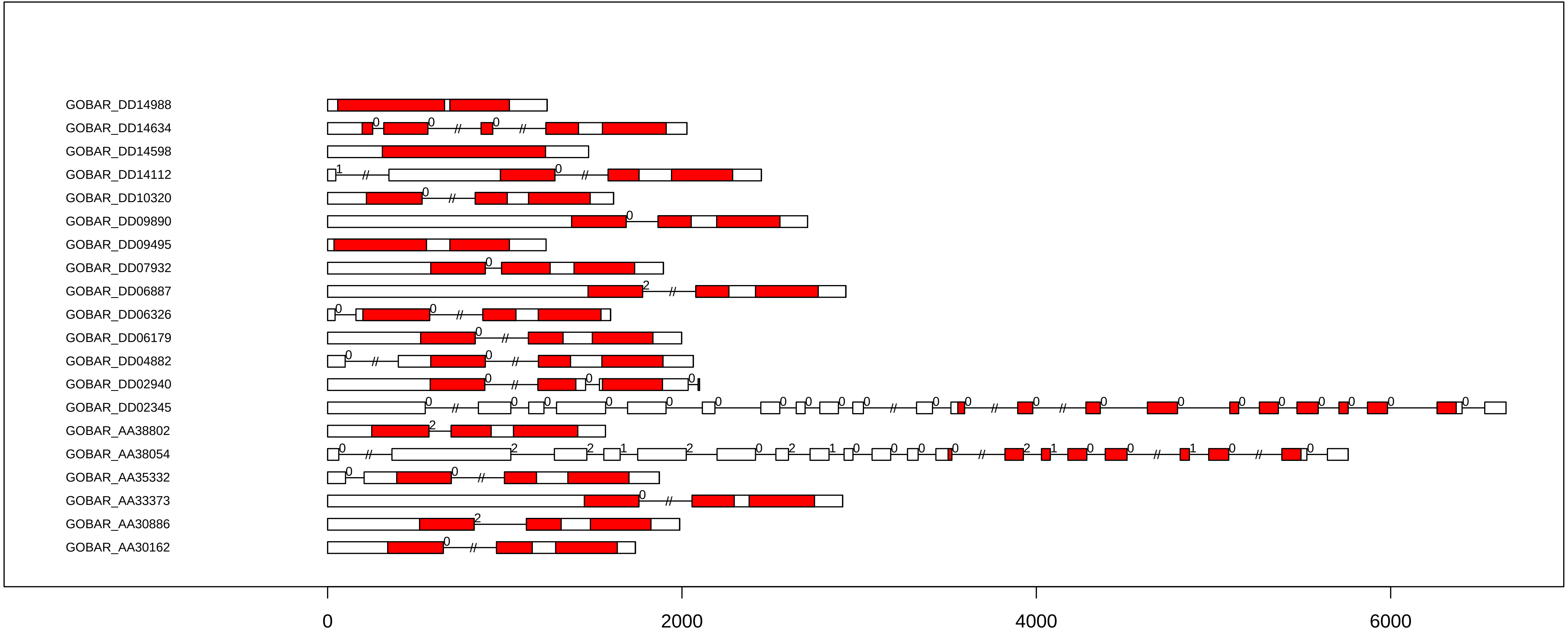

G.ba AGC_RSK−2 exon−intron and domain diagram (part 3)

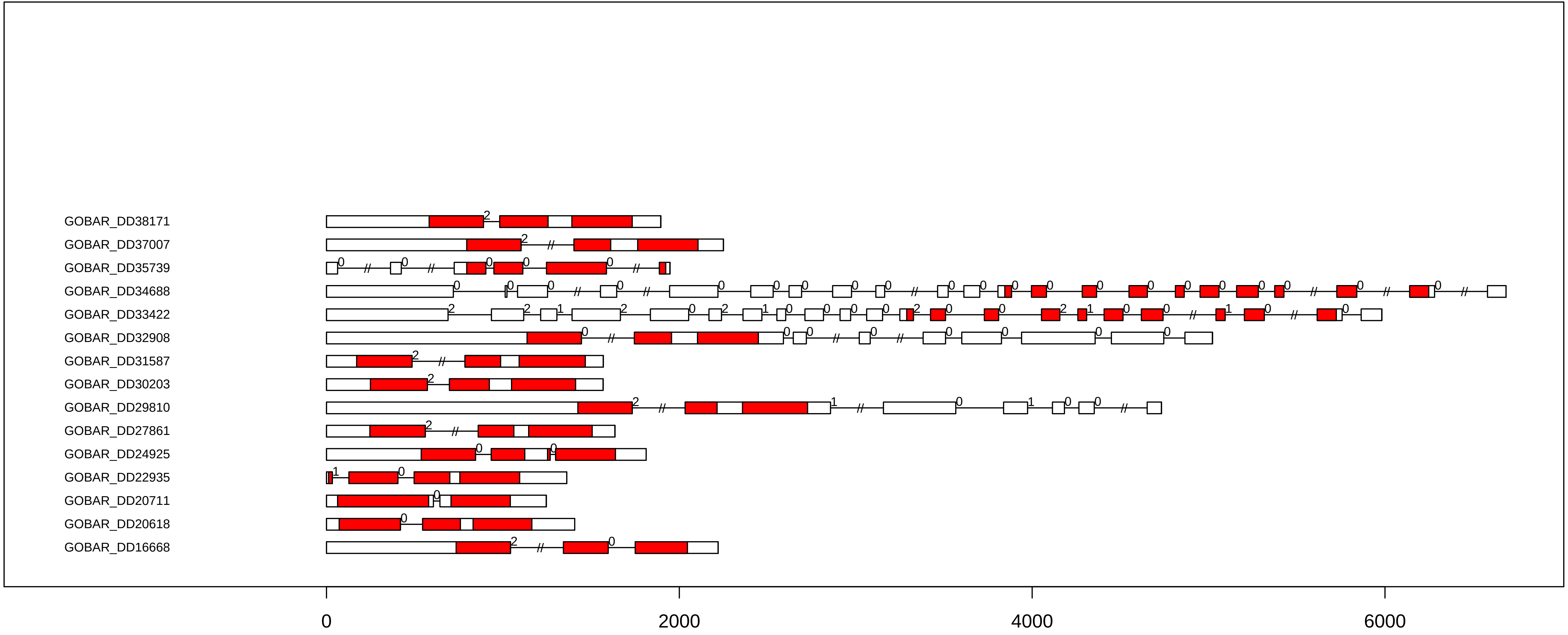

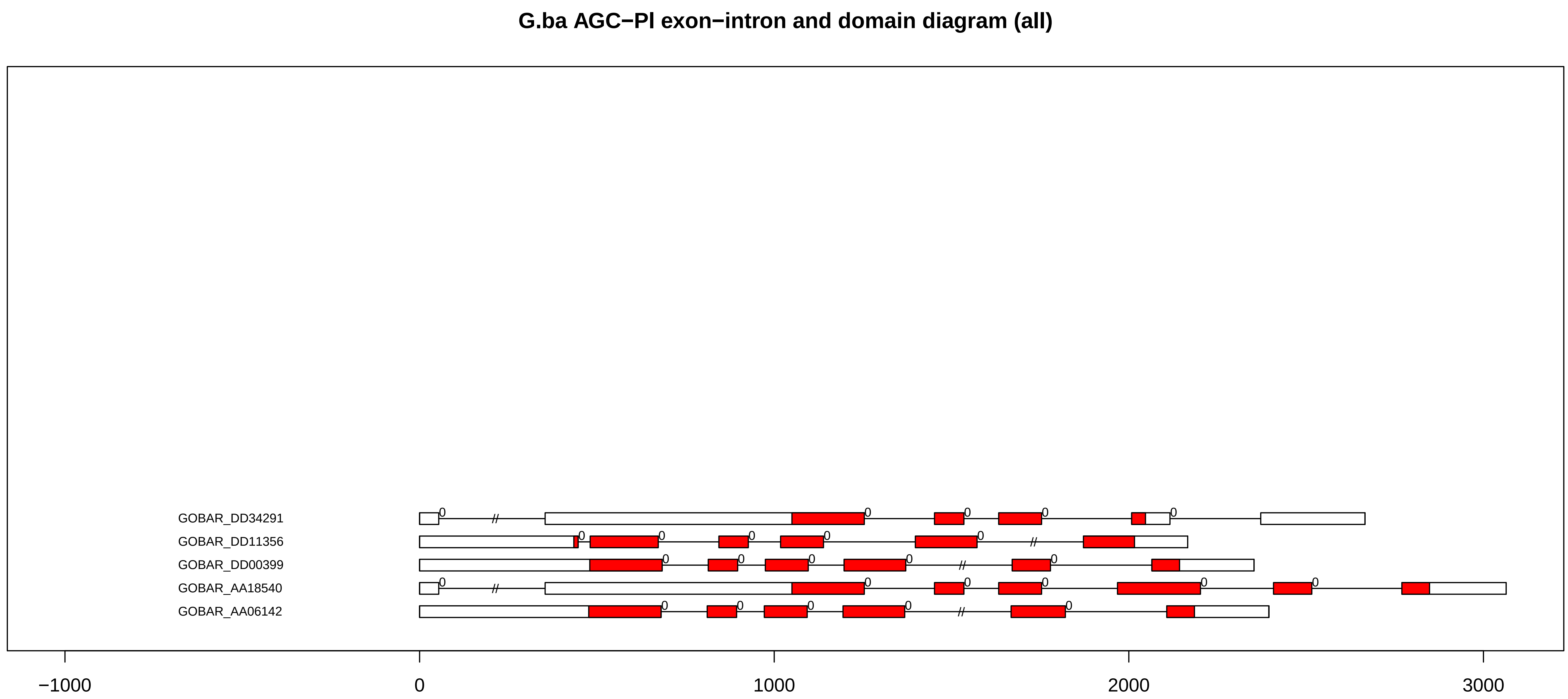
G.ba AGC−PI exon−intron and domain diagram (all)
GOBAR_DD34291
GOBAR_DD11356
GOBAR_DD00399
GOBAR_AA18540
GOBAR_AA06142
−1000
0
1000
2000
3000

G.ba Aur exon−intron and domain diagram (all)

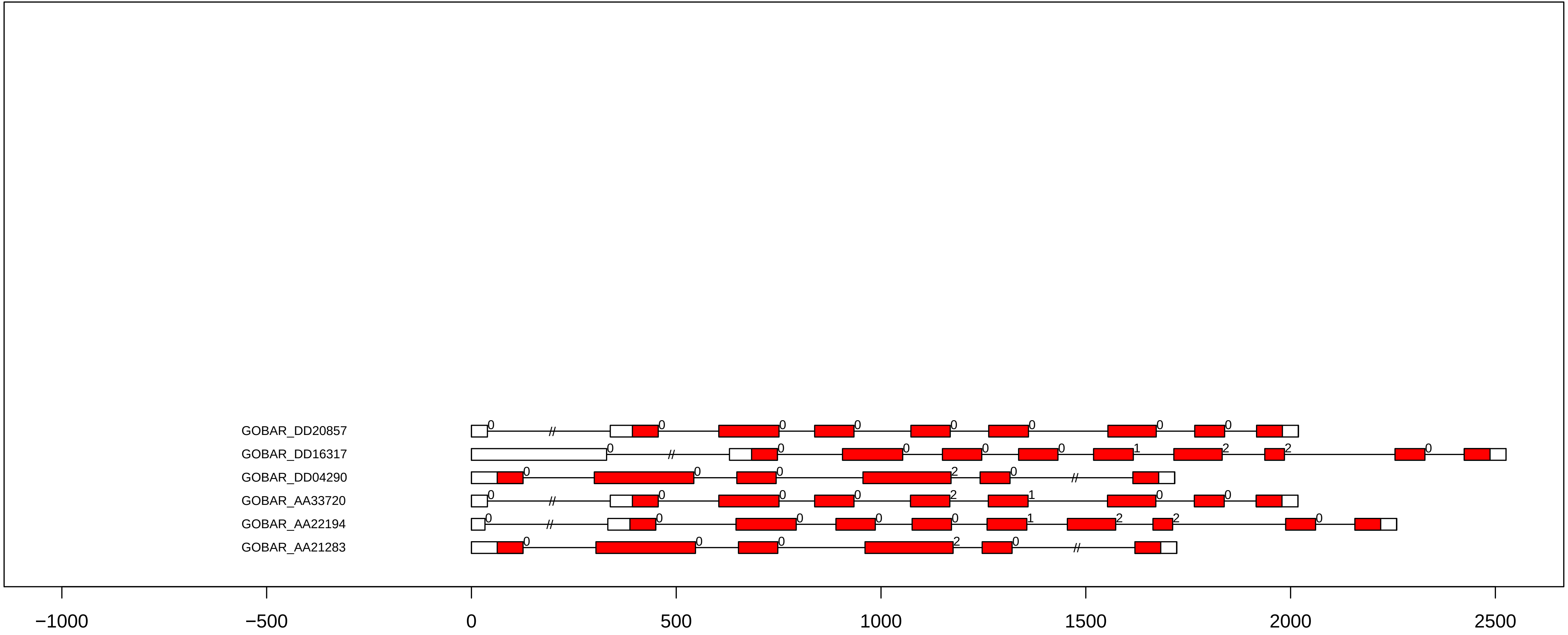

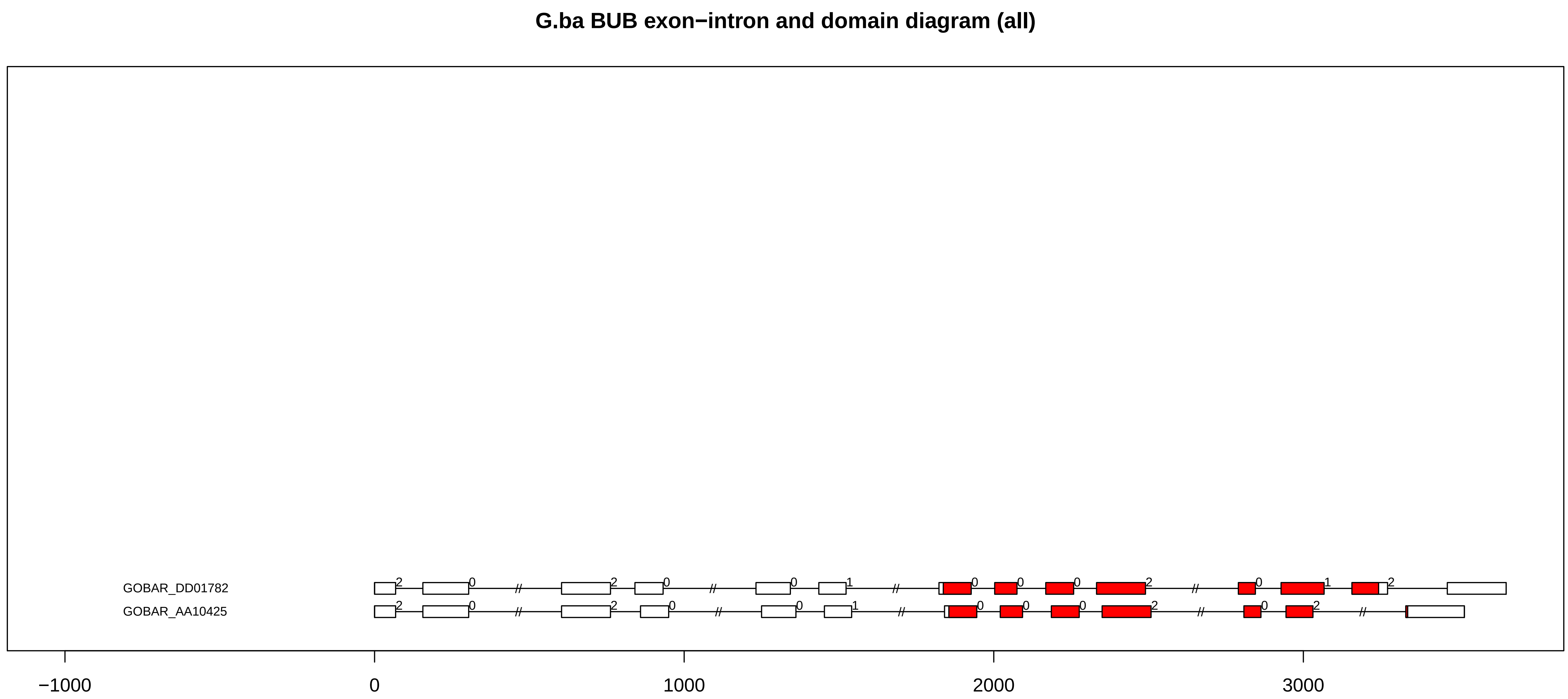

G.ba BUB exon−intron and domain diagram (all)
GOBAR_DD01782
GOBAR_AA10425
−1000
0
1000
2000
3000

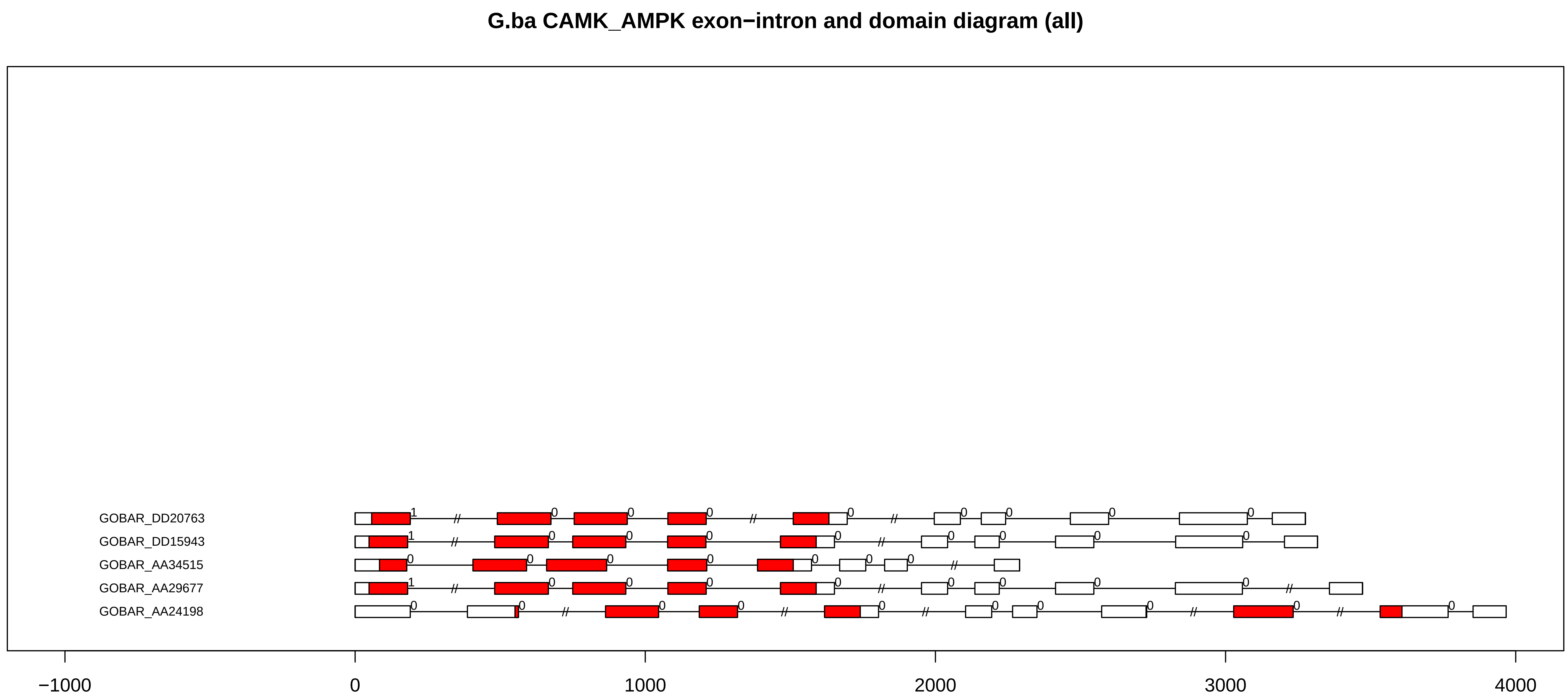

G.ba CAMK_AMPK exon−intron and domain diagram (all)
GOBAR_DD20763
GOBAR_DD15943
GOBAR_AA34515
GOBAR_AA29677
GOBAR_AA24198
−1000
0
1000
2000
3000
4000

G.ba CAMK_CAMK1−DCAMKL exon−intron and domain diagram (all)

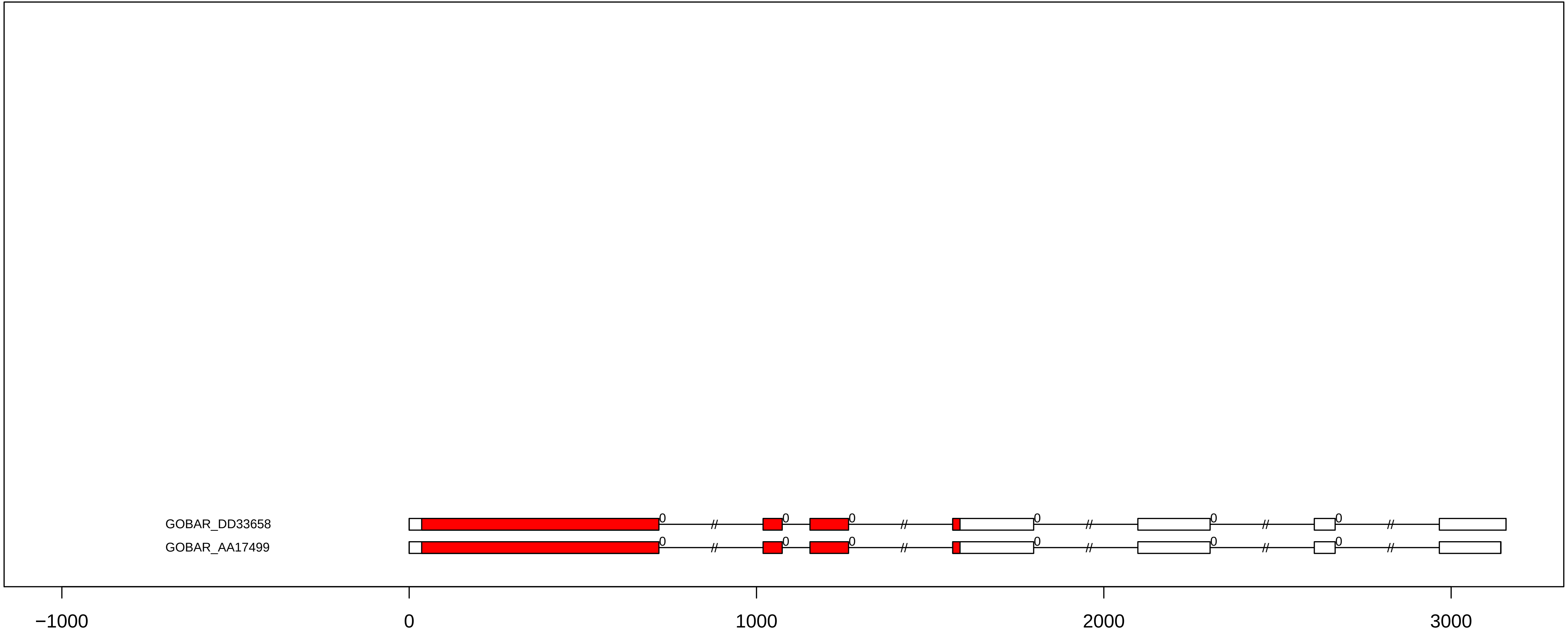

G.ba CAMK_CAMKL−CHK1 exon−intron and domain diagram (part 1)

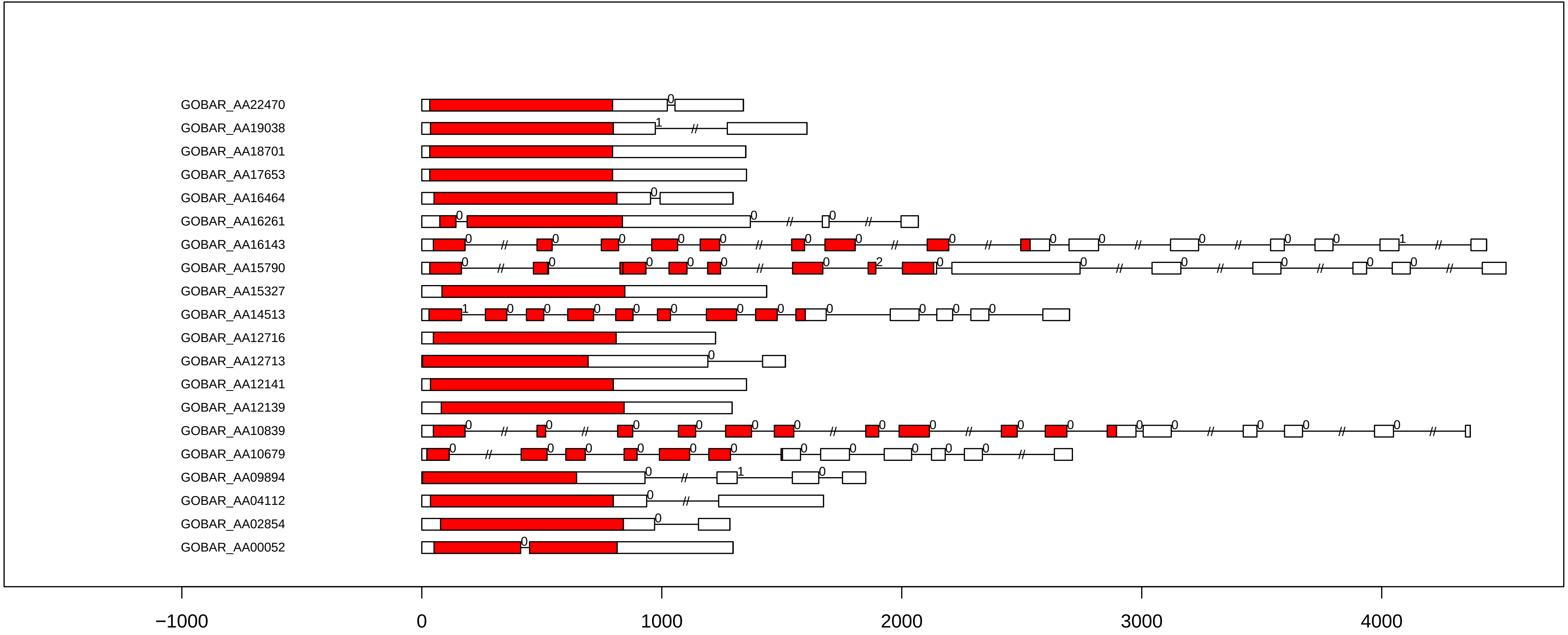

G.ba CAMK_CAMKL−CHK1 exon−intron and domain diagram (part 2)

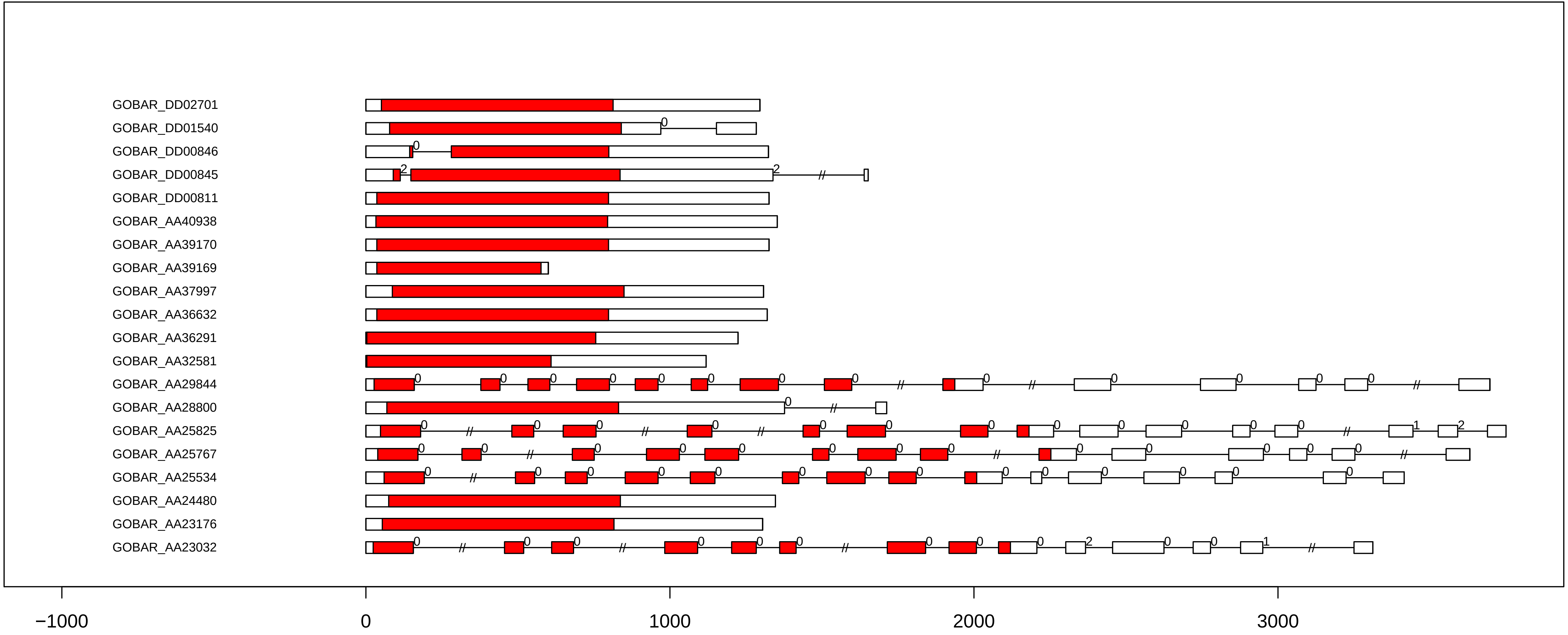

G.ba CAMK_CAMKL−CHK1 exon−intron and domain diagram (part 3)

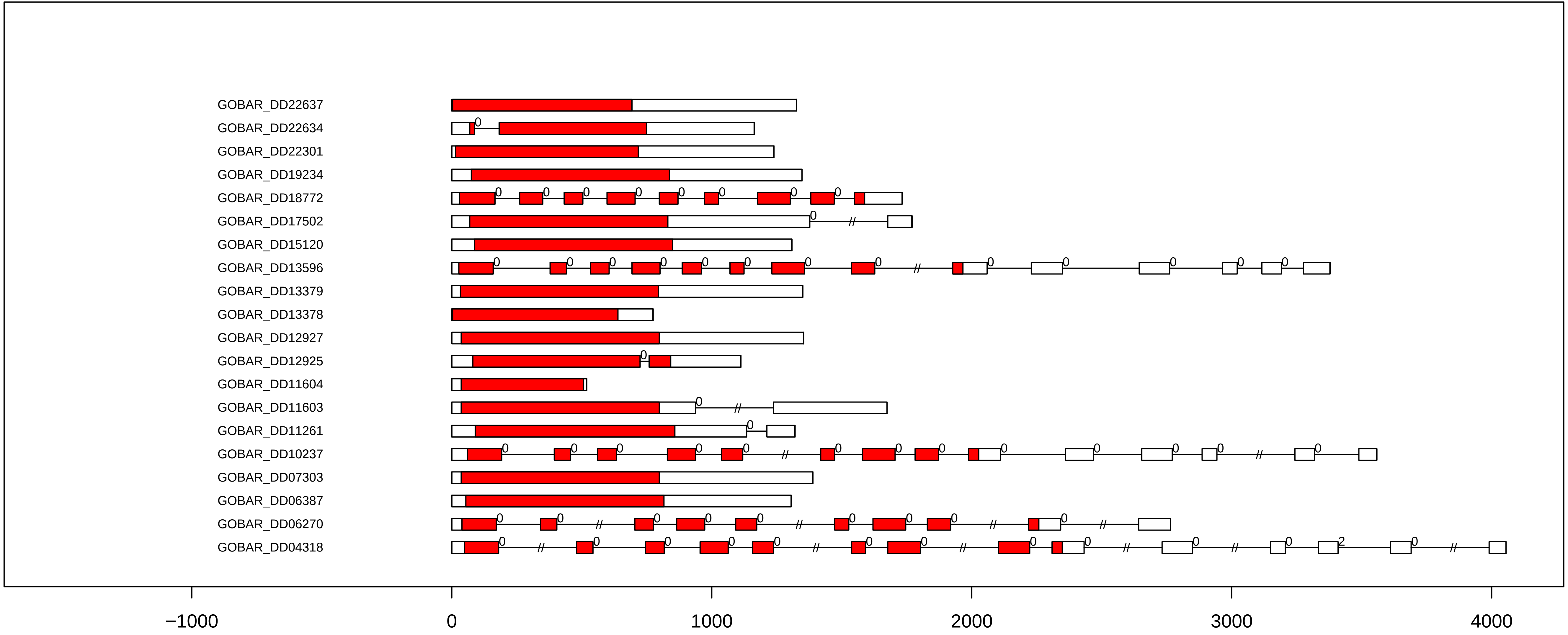

G.ba CAMK_CAMKL−CHK1 exon−intron and domain diagram (part 4)

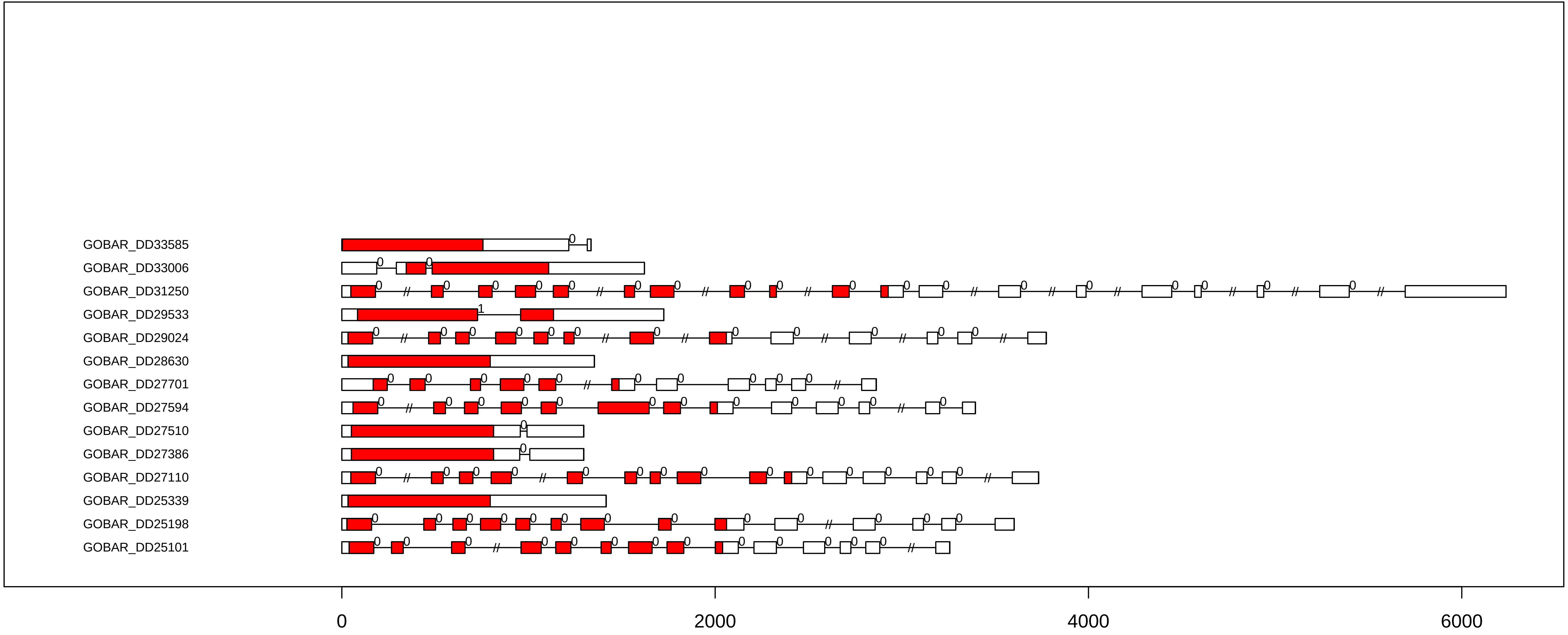

G.ba CAMK_CAMKL−LKB exon−intron and domain diagram (all)

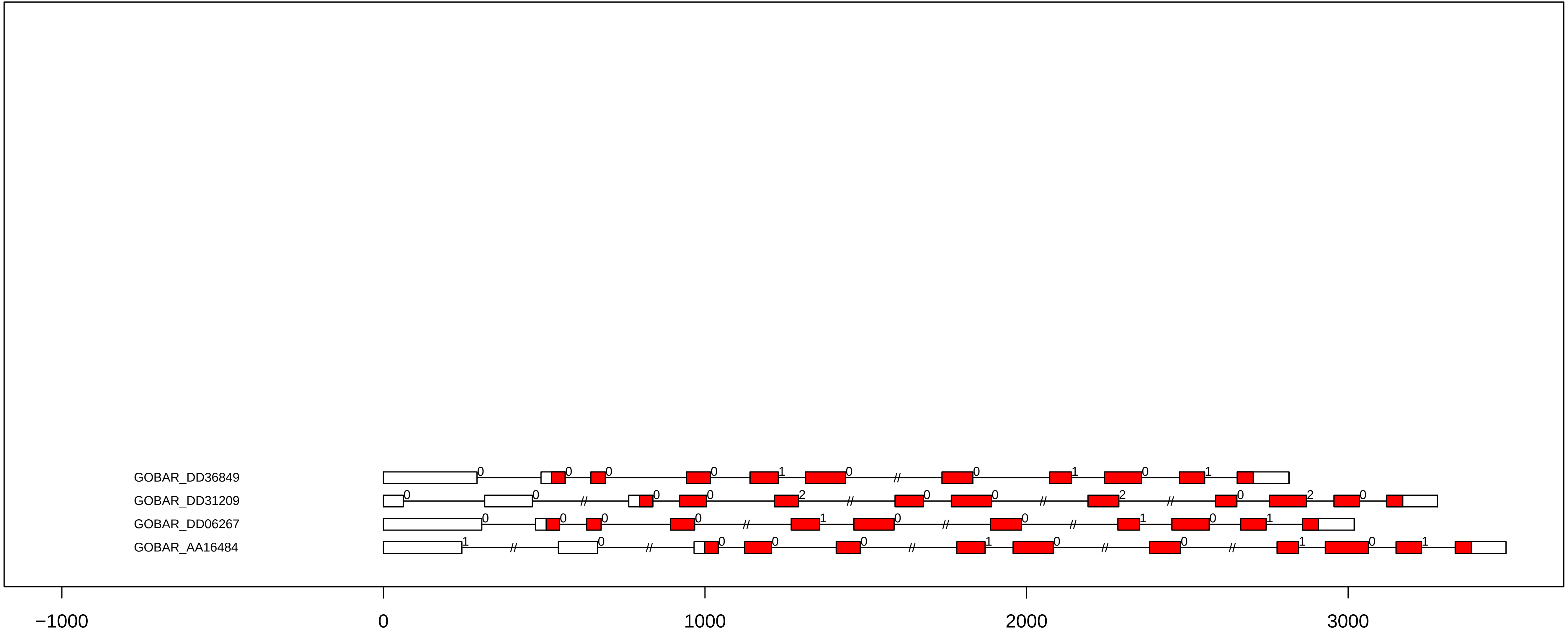

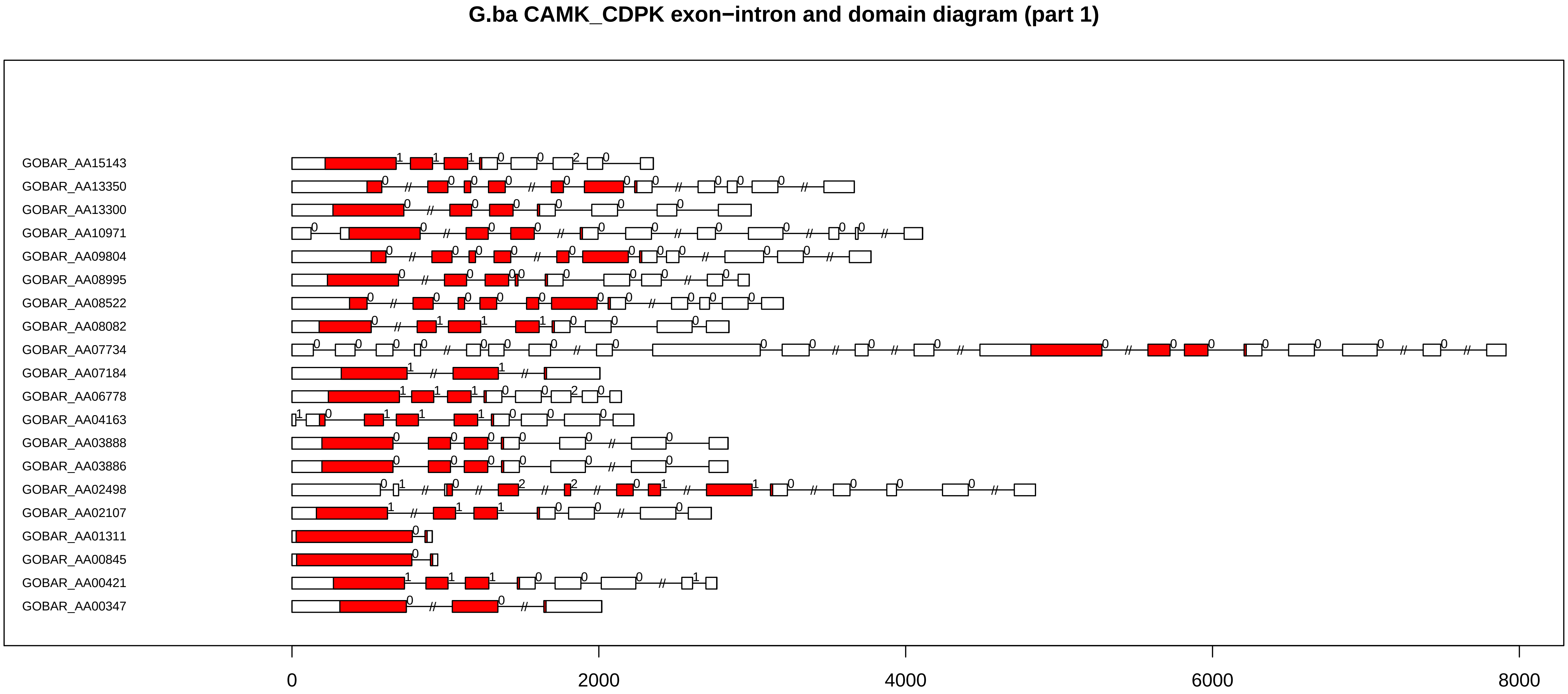
G.ba CAMK_CDPK exon−intron and domain diagram (part 1)
GOBAR_AA15143
GOBAR_AA13350
GOBAR_AA13300
GOBAR_AA10971
GOBAR_AA09804
GOBAR_AA08995
GOBAR_AA08522
GOBAR_AA08082
GOBAR_AA07734
GOBAR_AA07184
GOBAR_AA06778
GOBAR_AA04163
GOBAR_AA03888
GOBAR_AA03886
GOBAR_AA02498
GOBAR_AA02107
GOBAR_AA01311
GOBAR_AA00845
GOBAR_AA00421
GOBAR_AA00347
0
2000
4000
6000
8000

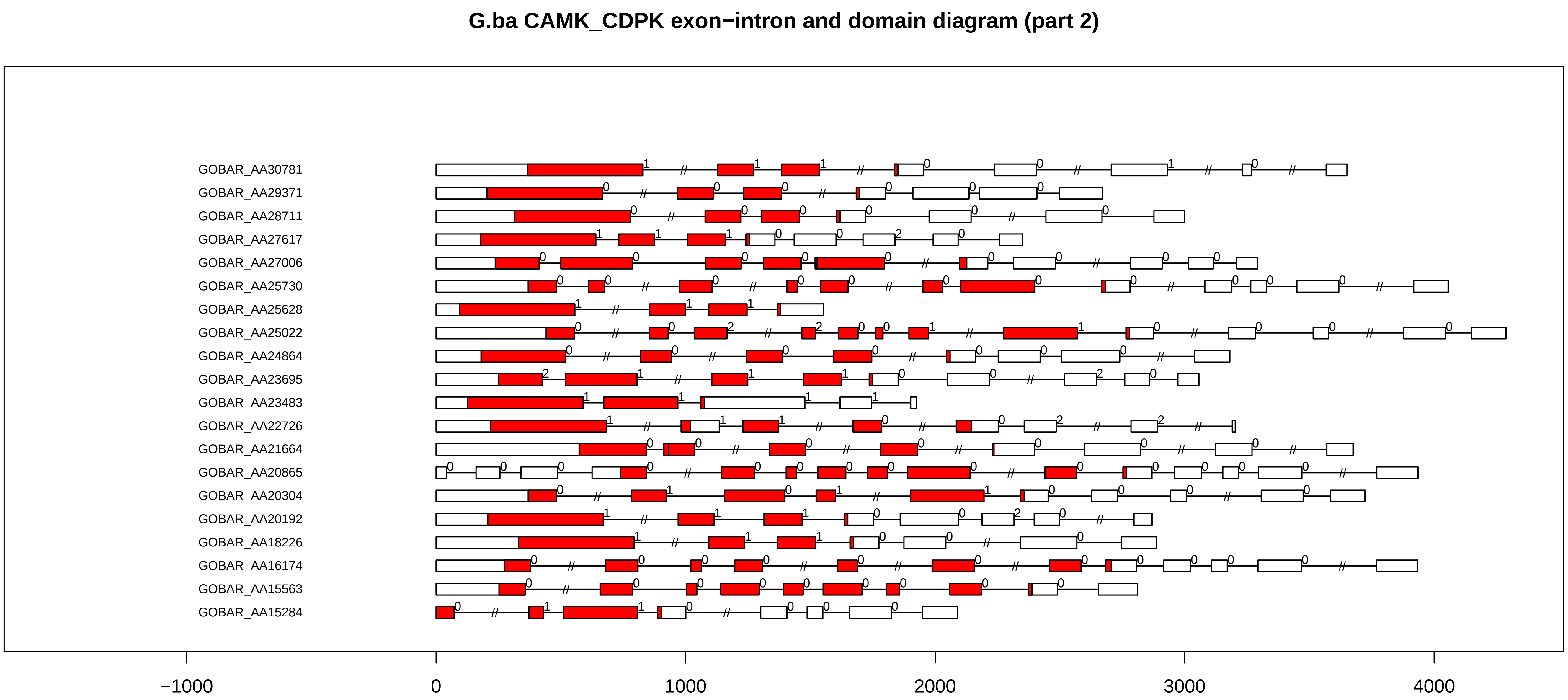

G.ba CAMK_CDPK exon−intron and domain diagram (part 2)
GOBAR_AA30781
GOBAR_AA29371
GOBAR_AA28711
GOBAR_AA27617
GOBAR_AA27006
GOBAR_AA25730
GOBAR_AA25628
GOBAR_AA25022
GOBAR_AA24864
GOBAR_AA23695
GOBAR_AA23483
GOBAR_AA22726
GOBAR_AA21664
GOBAR_AA20865
GOBAR_AA20304
GOBAR_AA20192
GOBAR_AA18226
GOBAR_AA16174
GOBAR_AA15563
GOBAR_AA15284
−1000
0
1000
2000
3000
4000

G.ba CAMK_CDPK exon−intron and domain diagram (part 3)

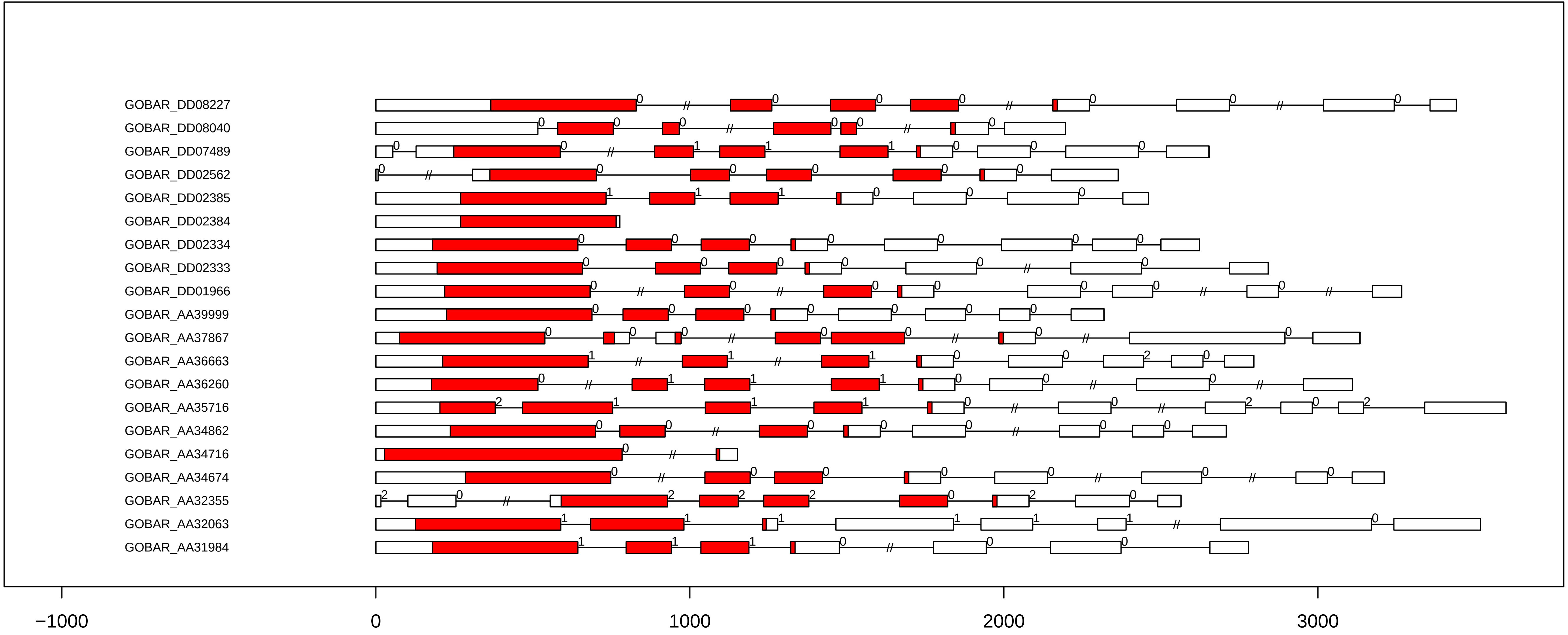

G.ba CAMK_CDPK exon−intron and domain diagram (part 4)

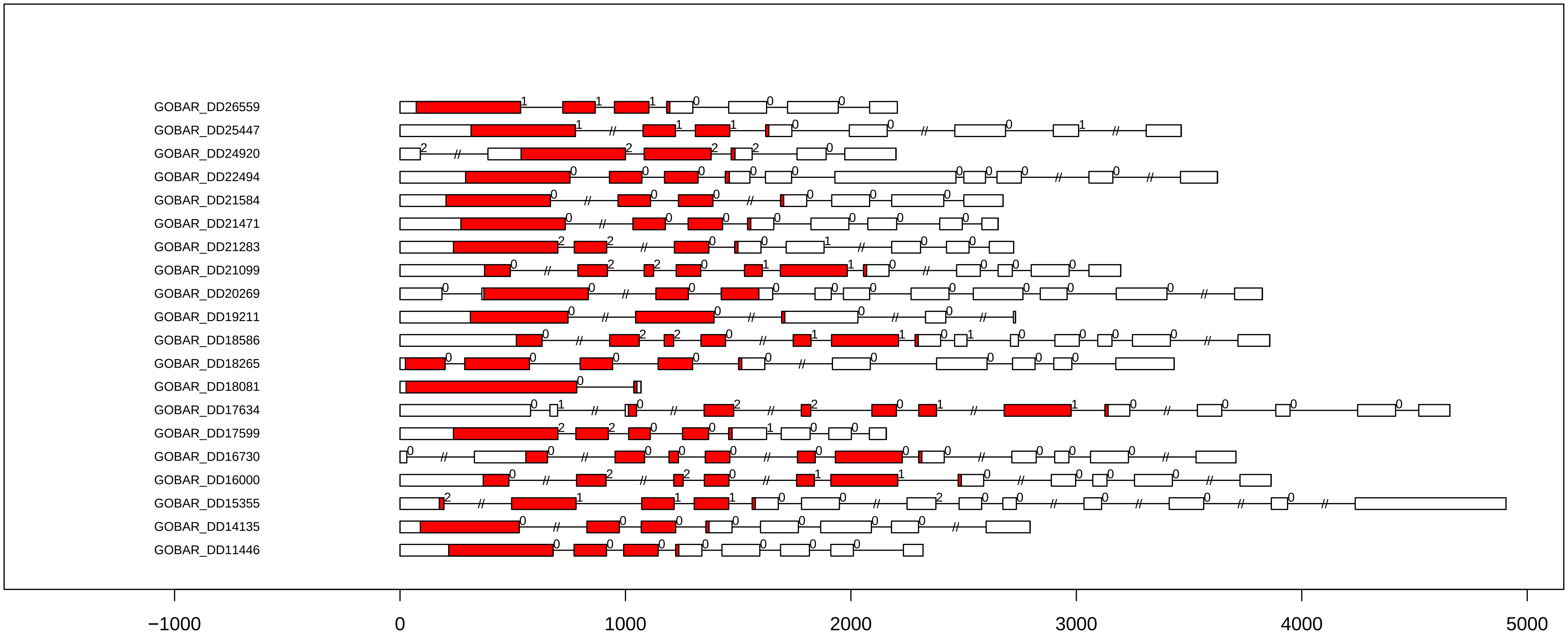

G.ba CAMK_CDPK exon−intron and domain diagram (part 5)

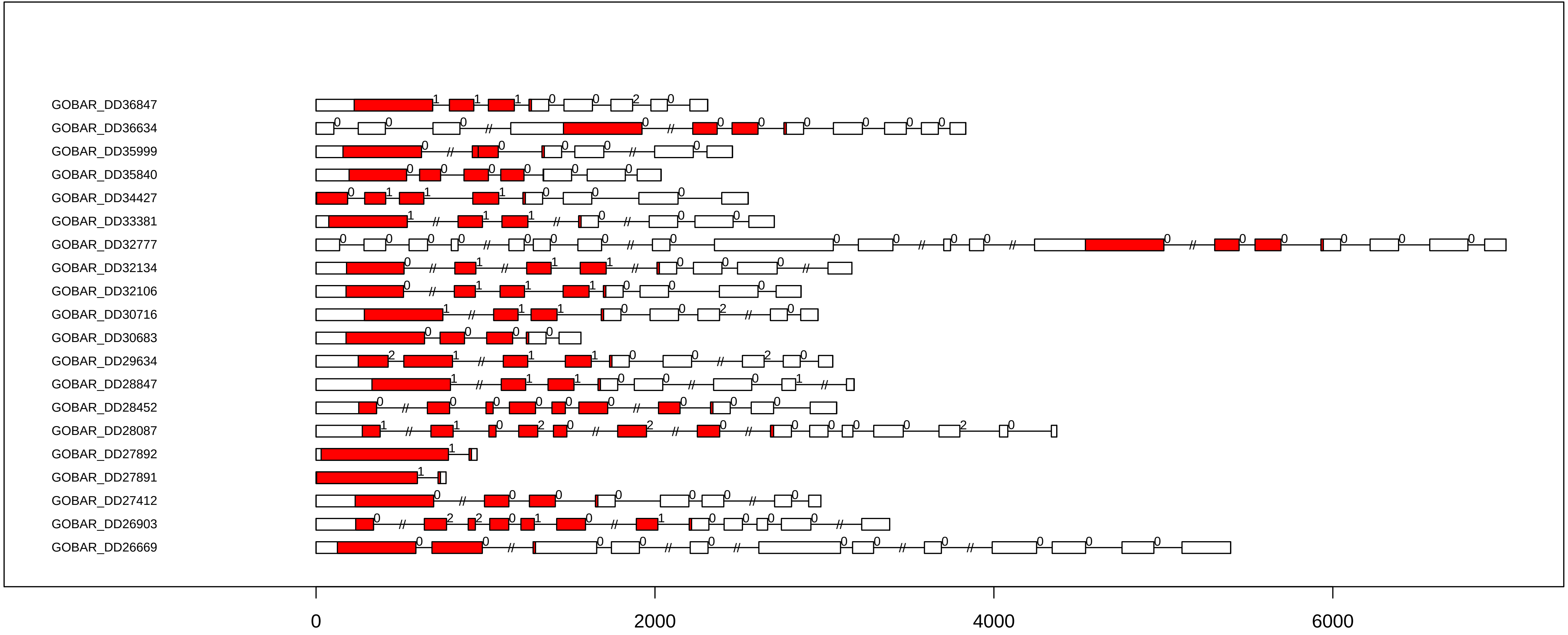

G.ba CAMK_CDPK exon−intron and domain diagram (part 6)

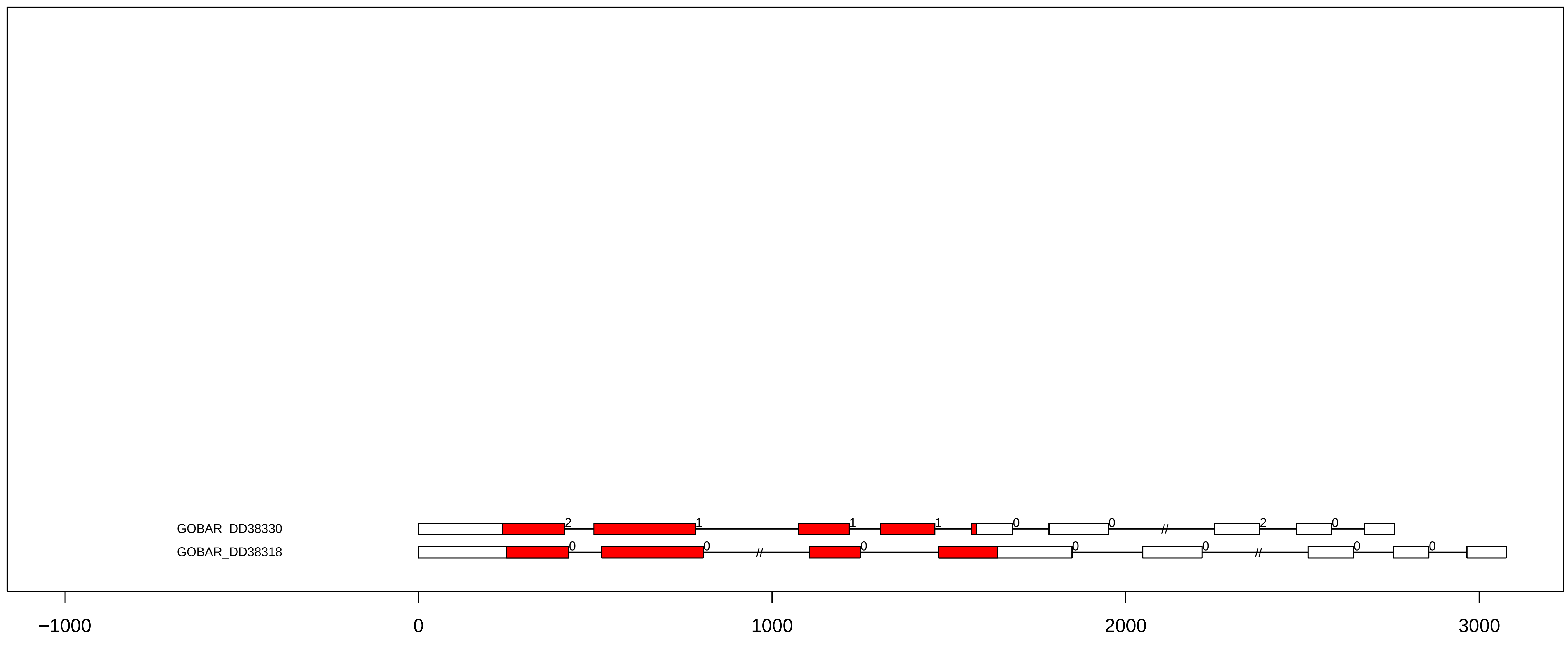

G.ba CAMK_OST1L exon−intron and domain diagram (all)

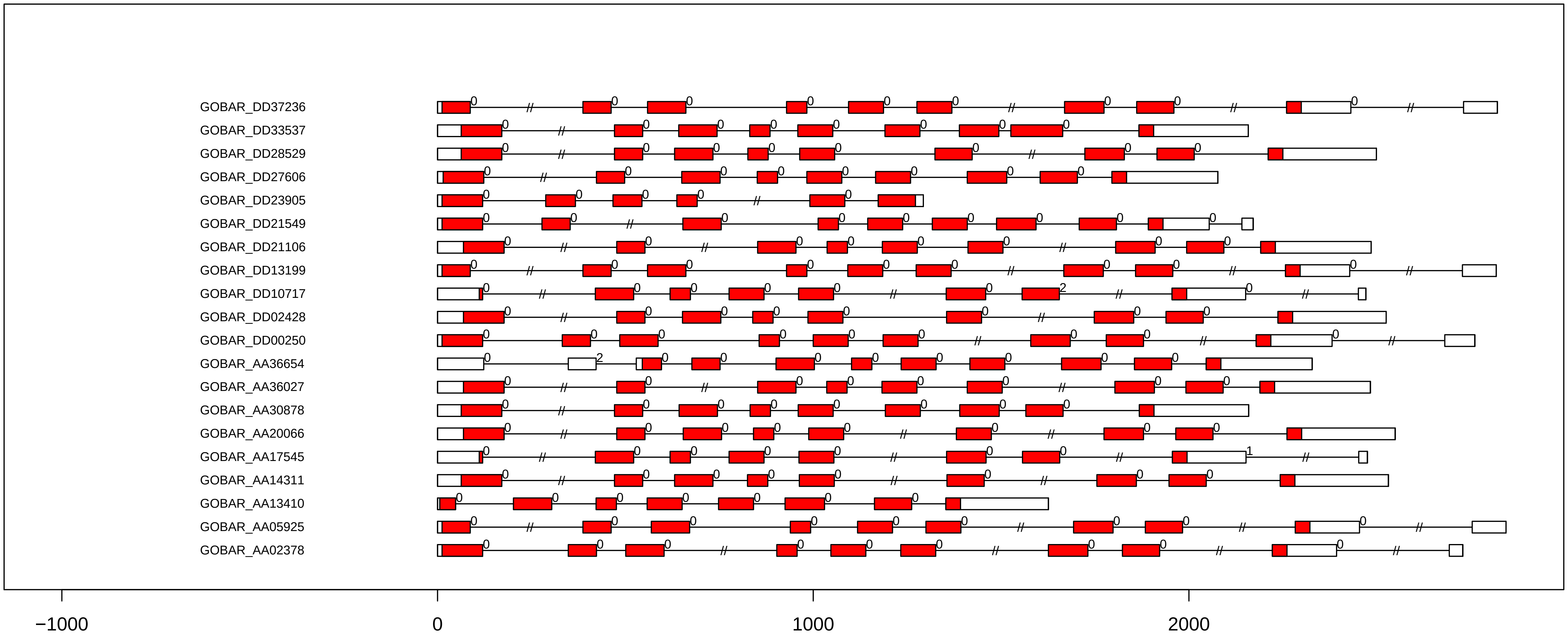

G.ba CK1_CK1 exon−intron and domain diagram (part 1)

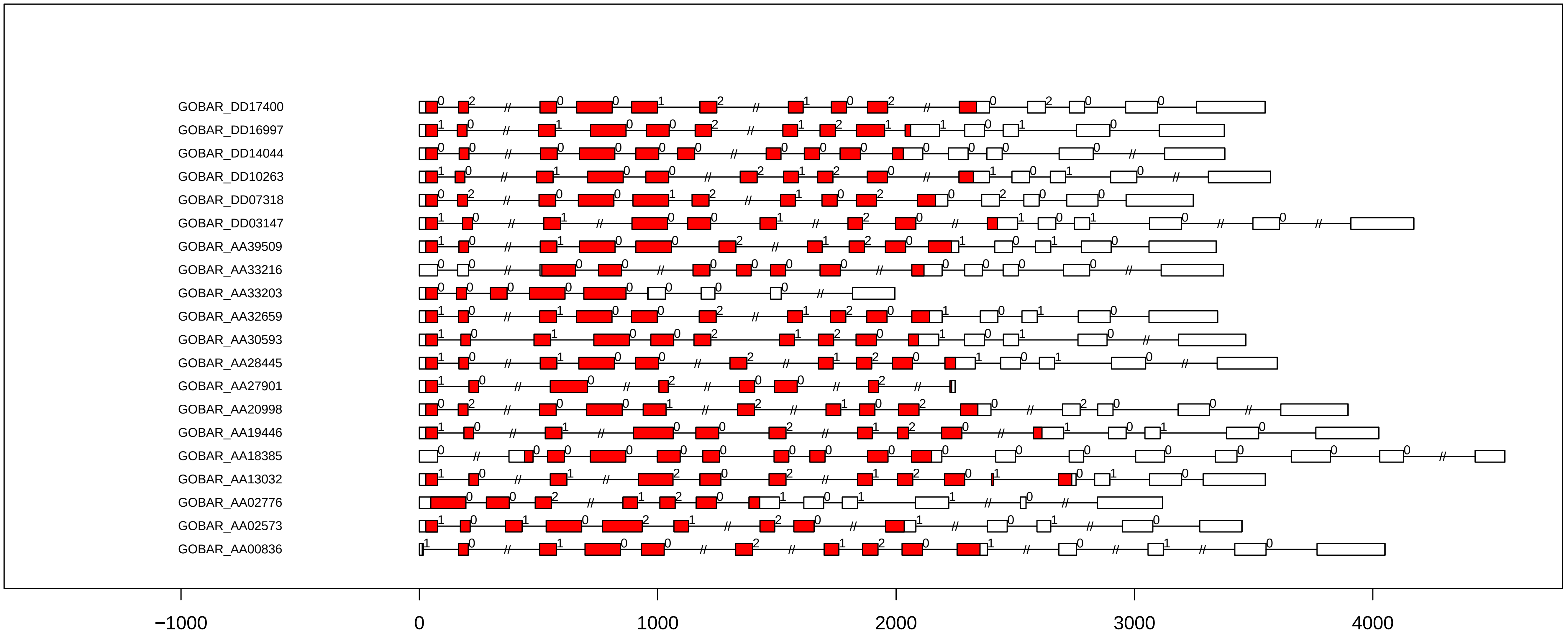

G.ba CK1_CK1 exon−intron and domain diagram (part 2)

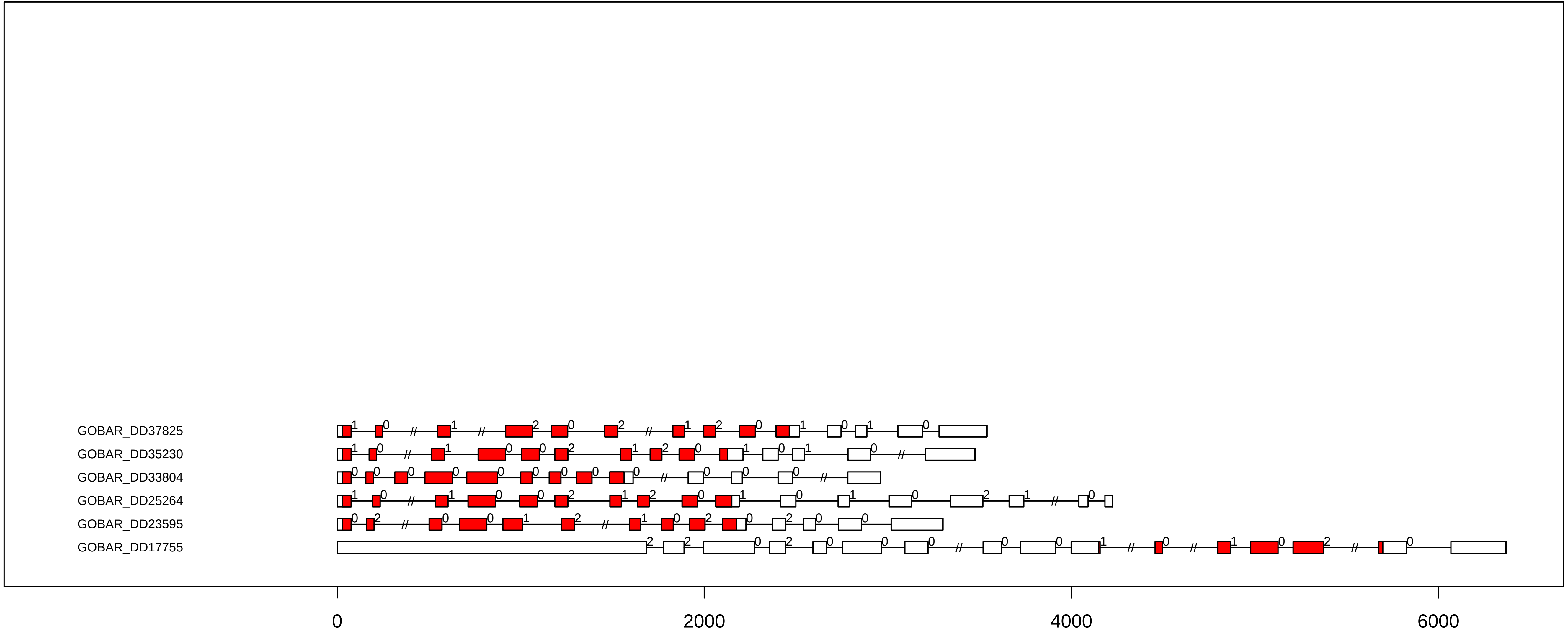

G.ba CK1_CK1−PI exon−intron and domain diagram (all)

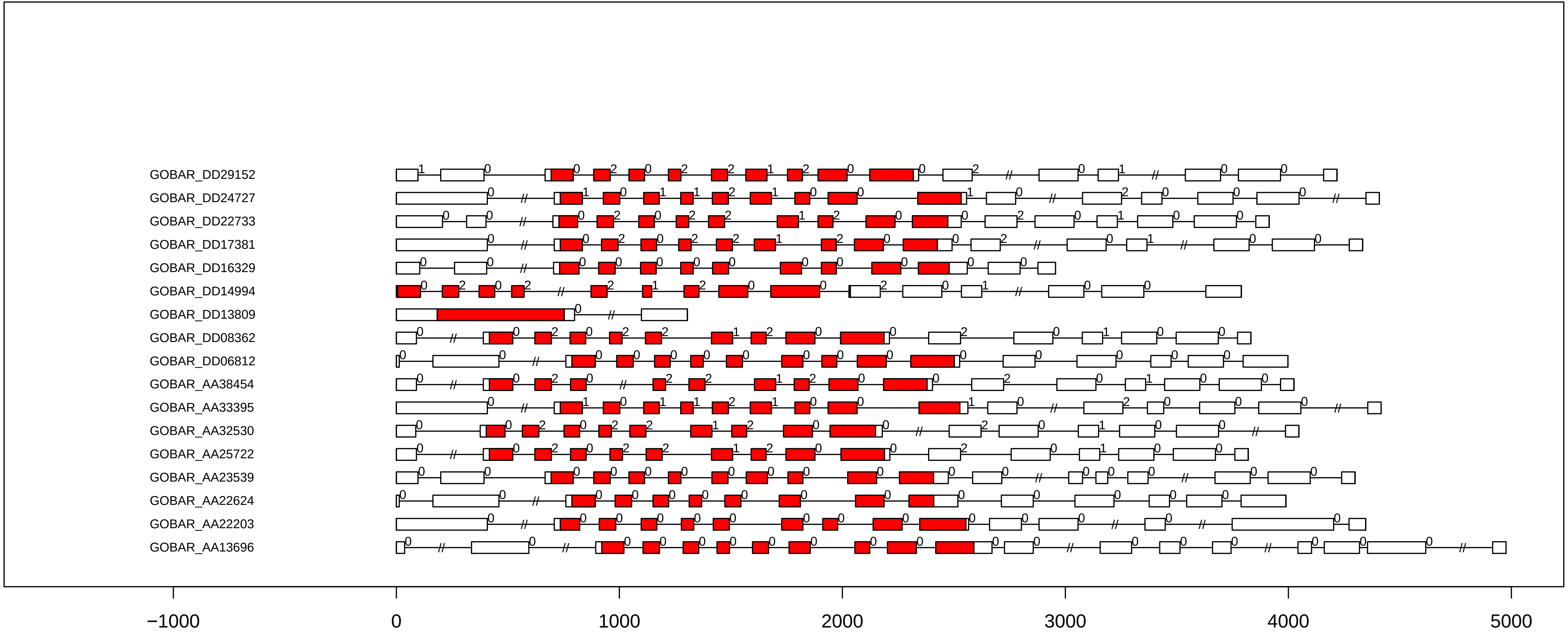

G.ba CMGC_CDK−CCRK exon−intron and domain diagram (all)

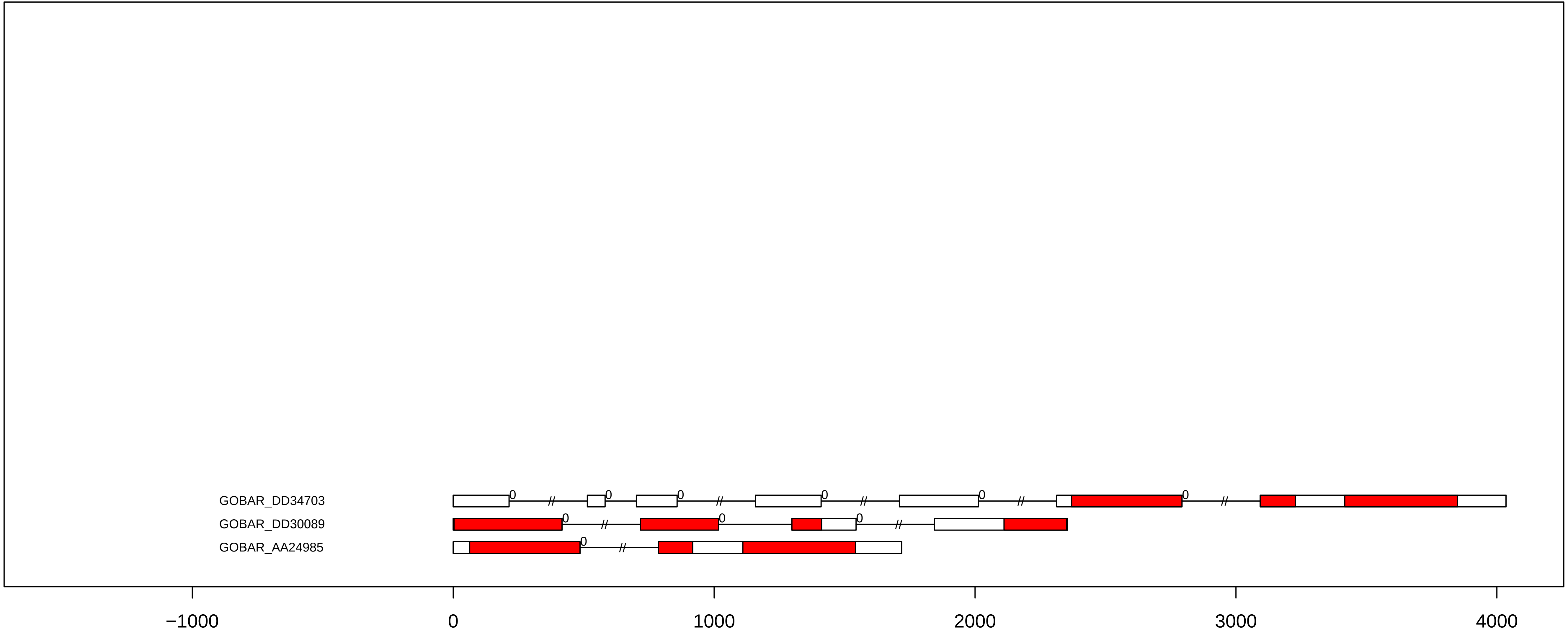

G.ba CMGC_CDK−CDK7 exon−intron and domain diagram (all)

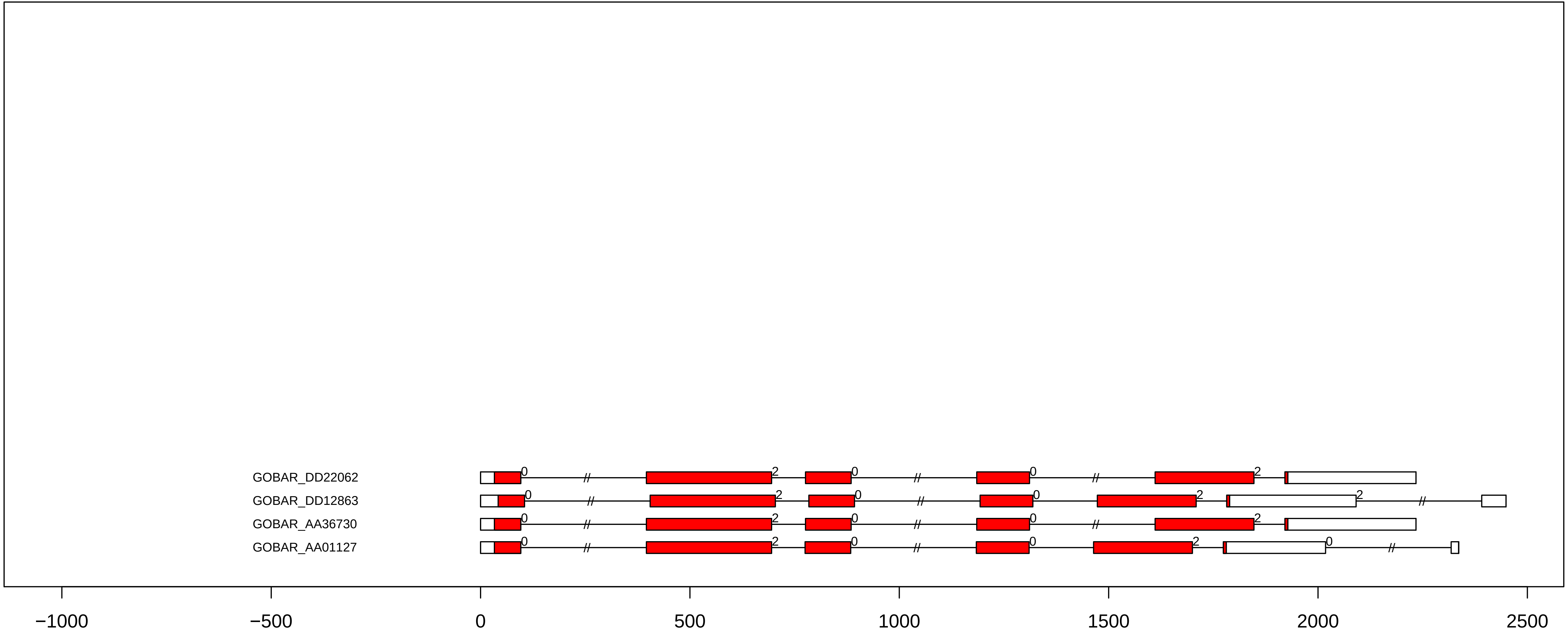

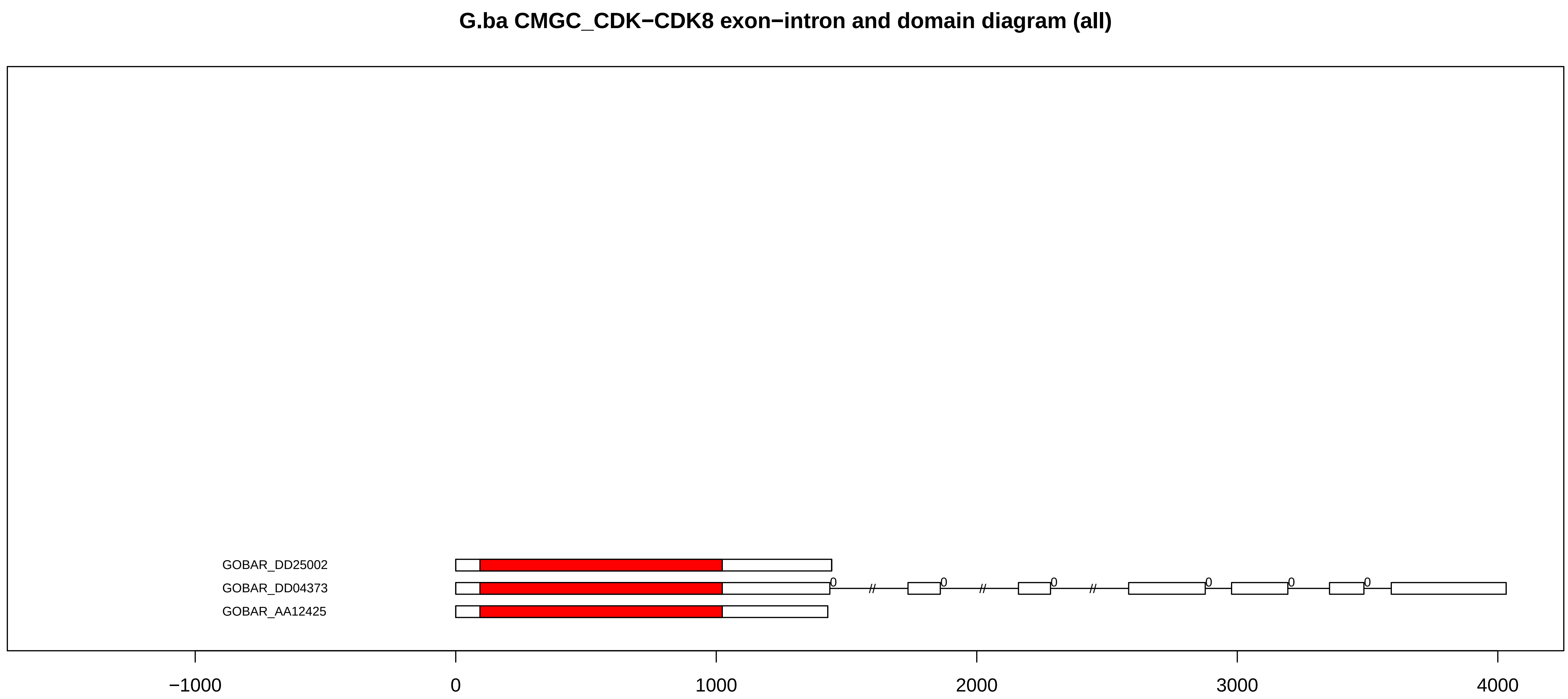
G.ba CMGC_CDK−CDK8 exon−intron and domain diagram (all)
GOBAR_DD25002
GOBAR_DD04373
GOBAR_AA12425
0
0
0
0
0
0
−1000
0
1000
2000
3000
4000

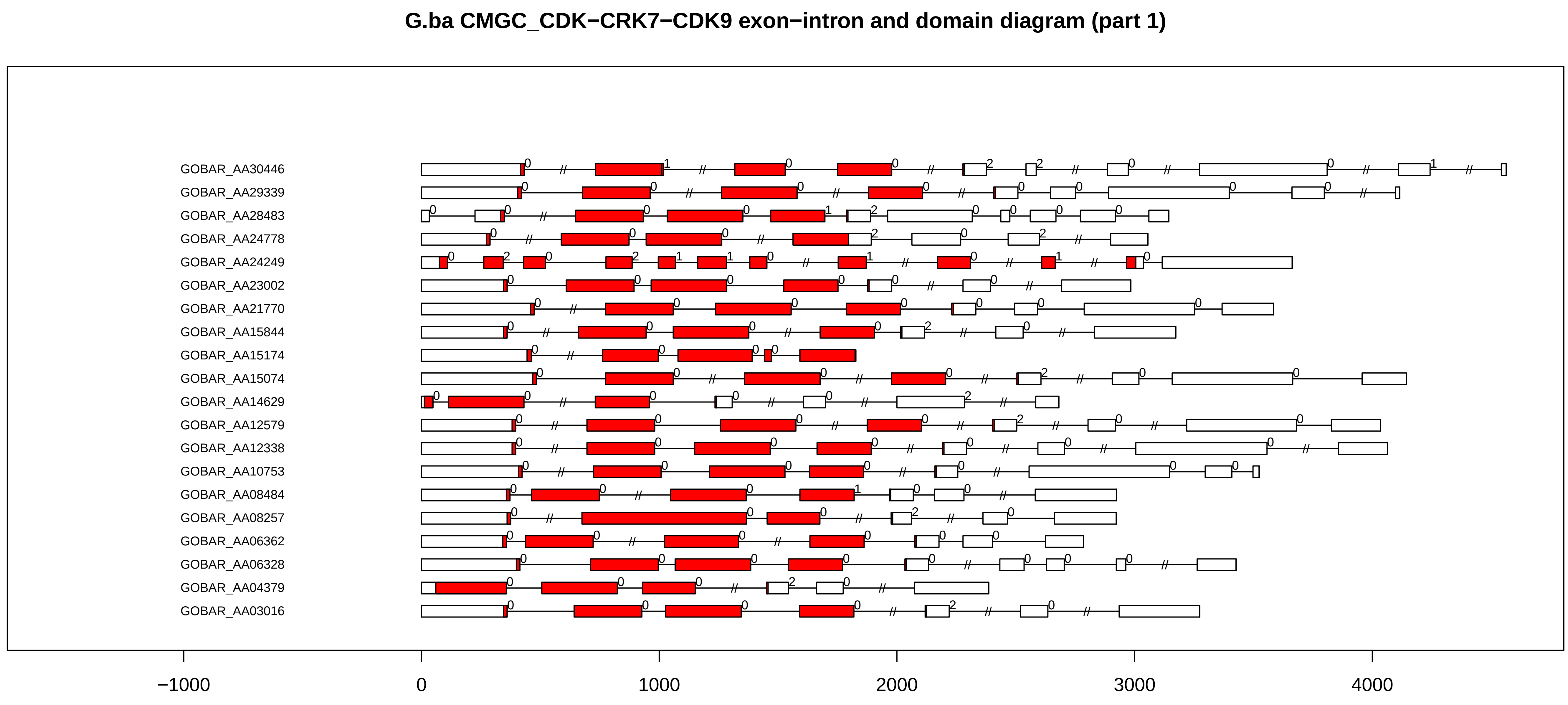
G.ba CMGC_CDK−CRK7−CDK9 exon−intron and domain diagram (part 1)
GOBAR_AA30446
GOBAR_AA29339
GOBAR_AA28483
GOBAR_AA24778
GOBAR_AA24249
GOBAR_AA23002
GOBAR_AA21770
GOBAR_AA15844
GOBAR_AA15174
GOBAR_AA15074
GOBAR_AA14629
GOBAR_AA12579
GOBAR_AA12338
GOBAR_AA10753
GOBAR_AA08484
GOBAR_AA08257
GOBAR_AA06362
GOBAR_AA06328
GOBAR_AA04379
GOBAR_AA03016
−1000
0
1000
2000
3000
4000

G.ba CMGC_CDK−CRK7−CDK9 exon−intron and domain diagram (part 2)

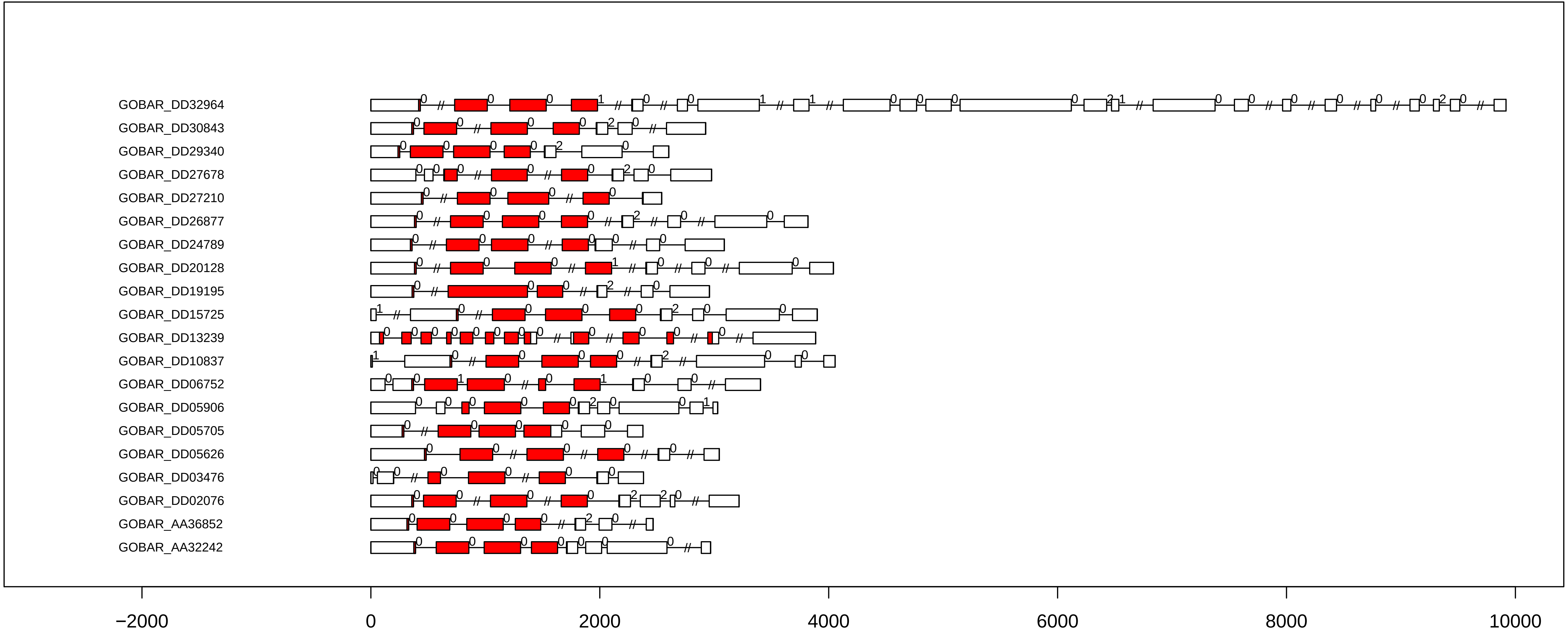

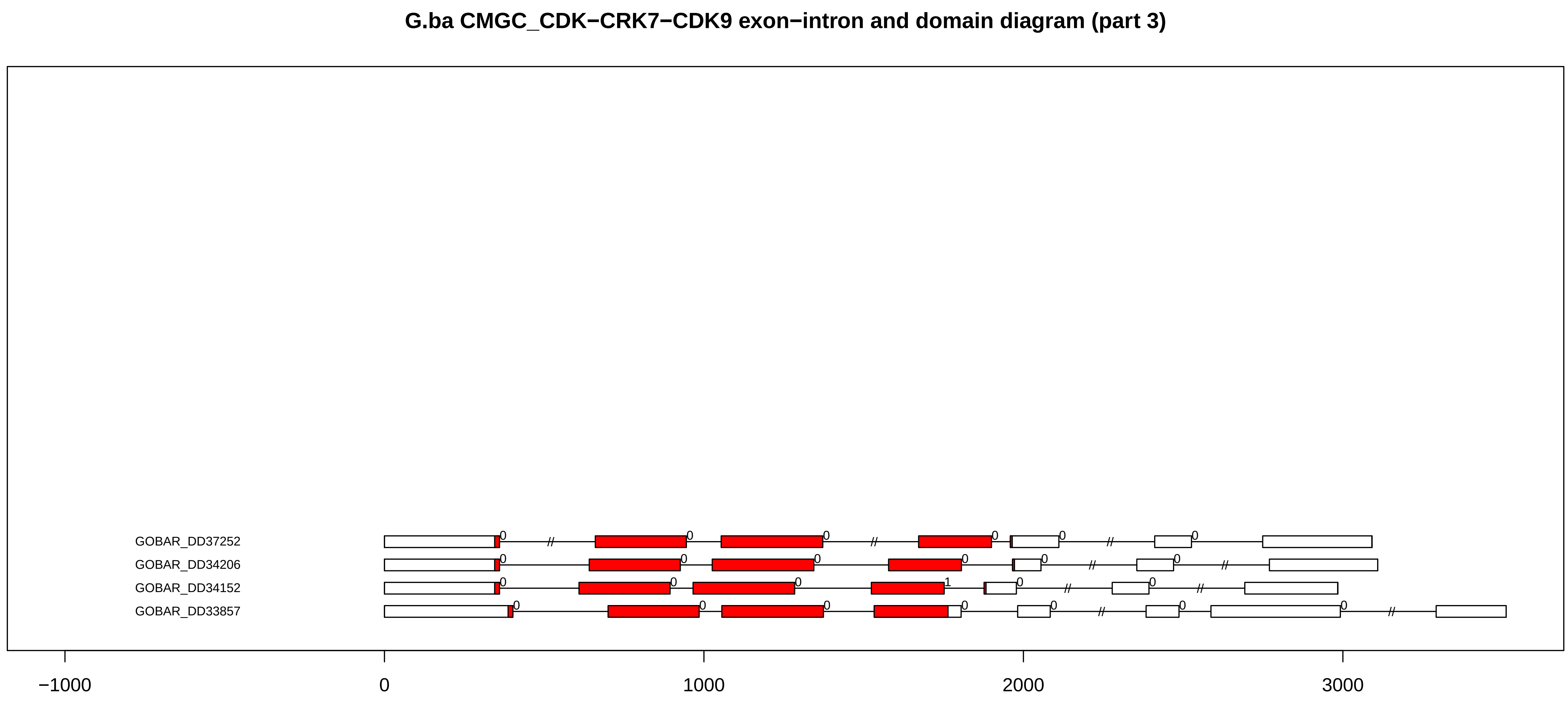

G.ba CMGC_CDK−CRK7−CDK9 exon−intron and domain diagram (part 3)
GOBAR_DD37252
GOBAR_DD34206
GOBAR_DD34152
GOBAR_DD33857
−1000
0
1000
2000
3000

G.ba CMGC_CDK−PITSLRE exon−intron and domain diagram (all)

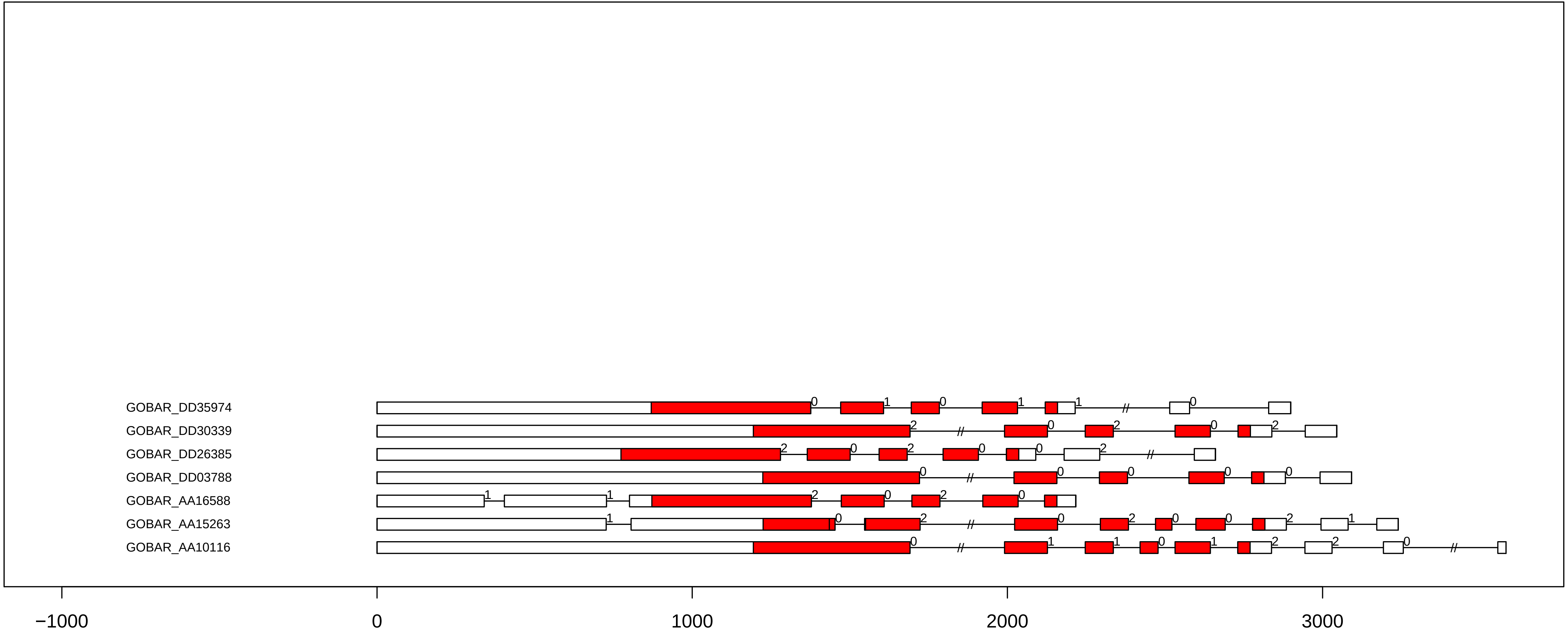

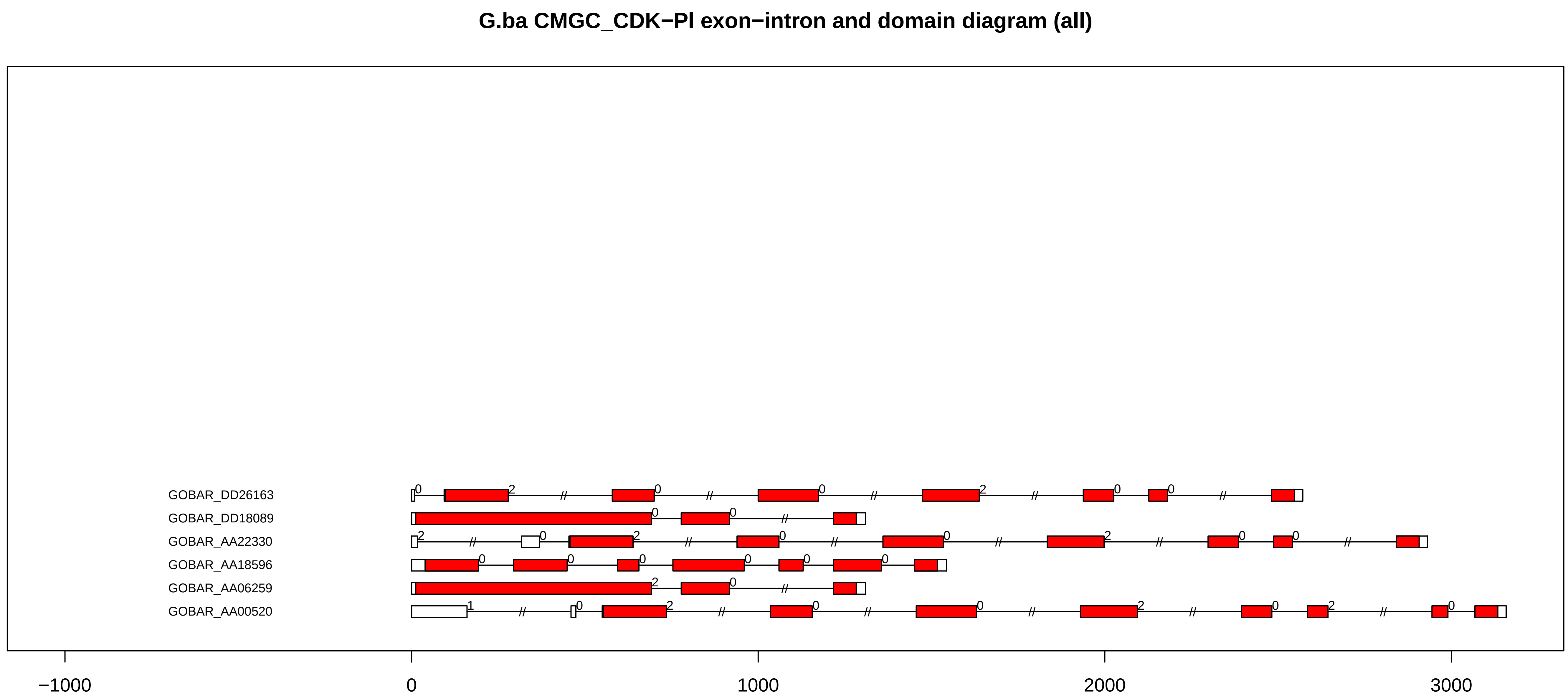
G.ba CMGC_CDK−Pl exon−intron and domain diagram (all)
GOBAR_DD26163
GOBAR_DD18089
GOBAR_AA22330
GOBAR_AA18596
GOBAR_AA06259
GOBAR_AA00520
−1000
0
1000
2000
3000

G.ba CMGC_CK2 exon−intron and domain diagram (all)

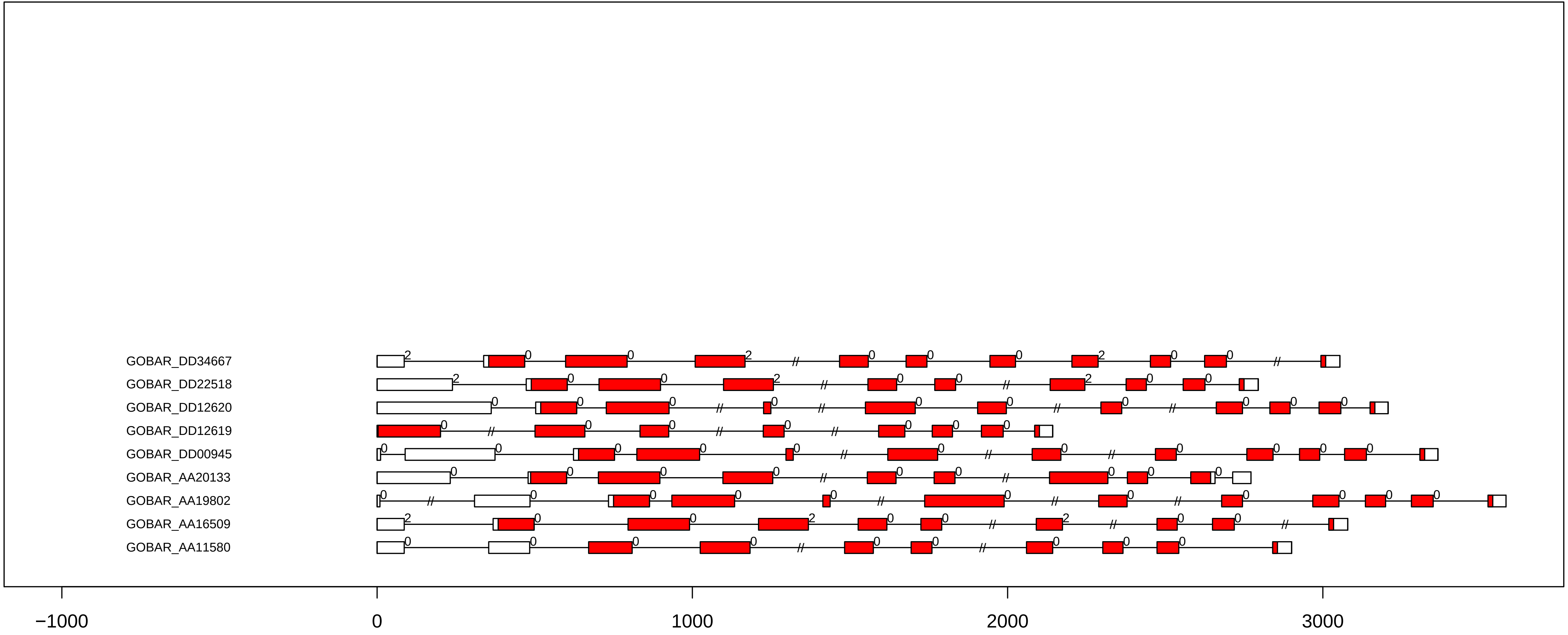

G.ba CMGC_CLK exon−intron and domain diagram (all)

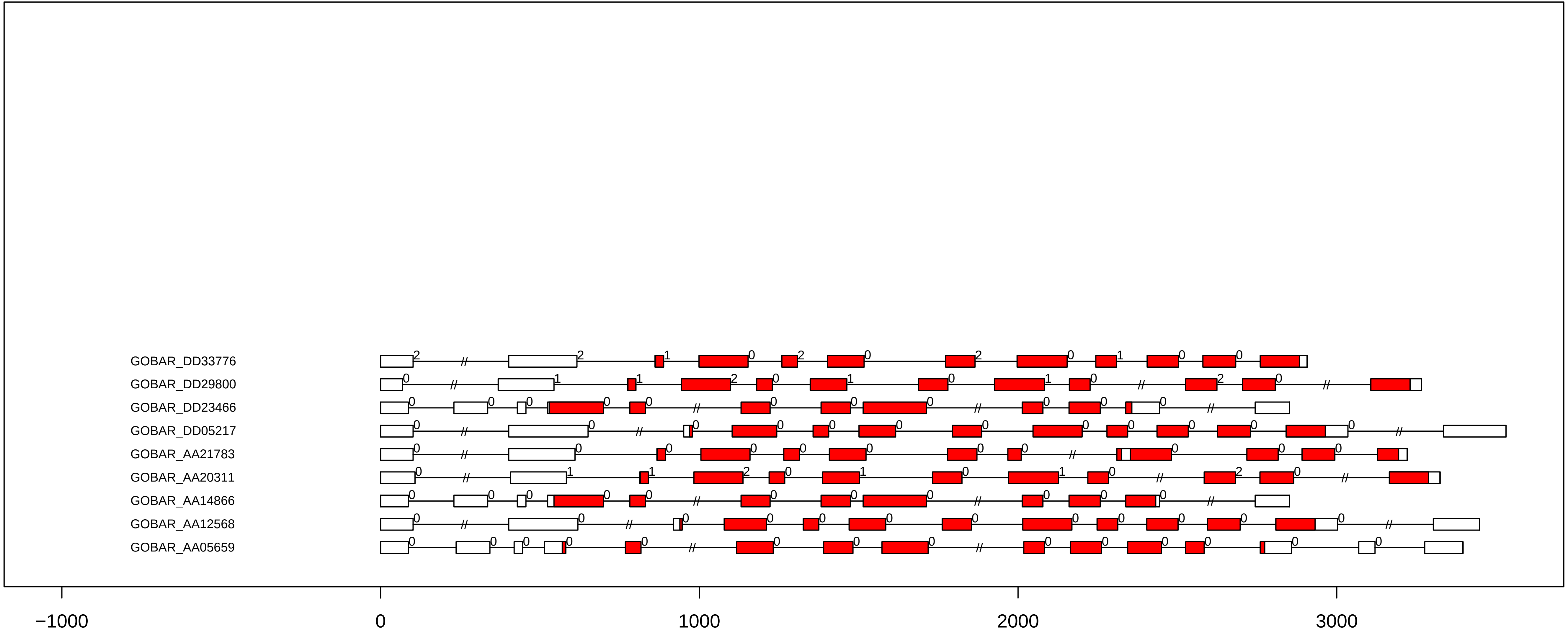

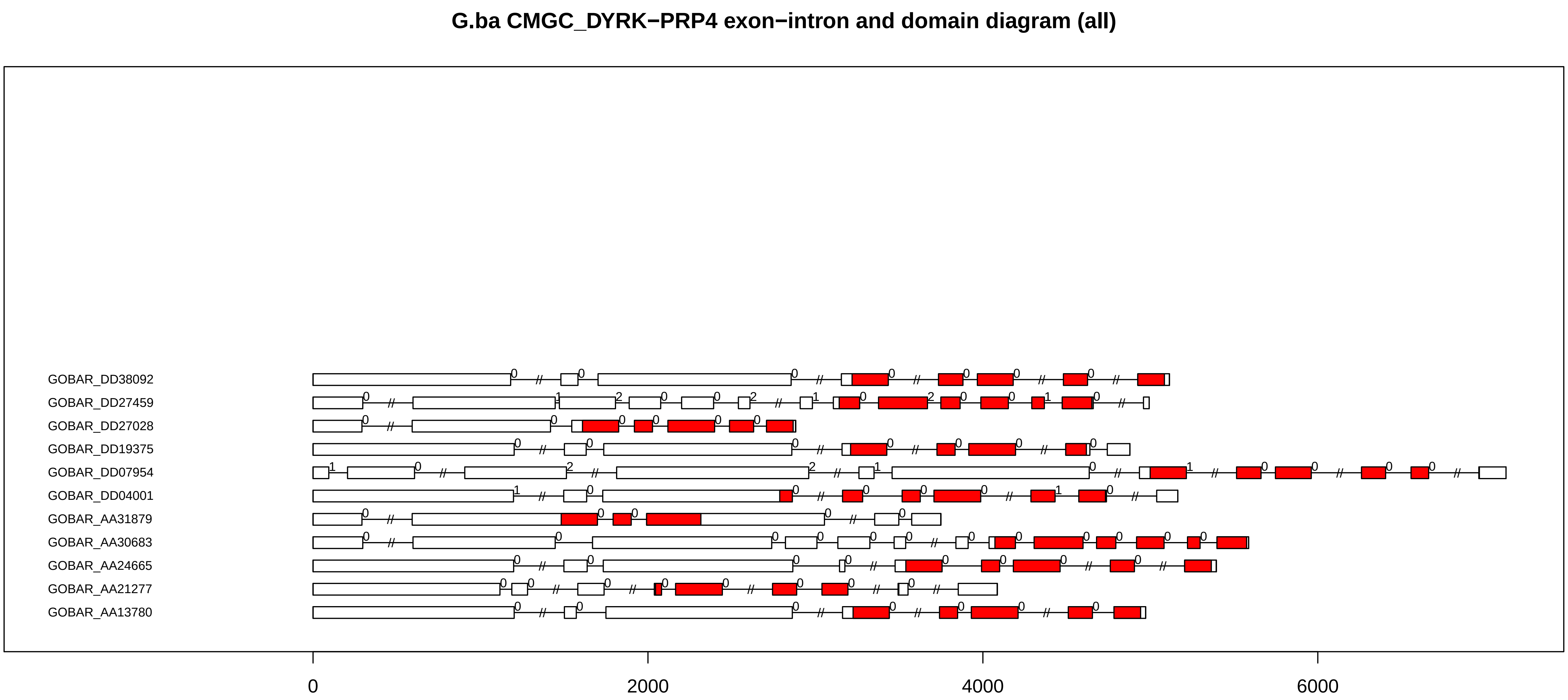
G.ba CMGC_DYRK−PRP4 exon−intron and domain diagram (all)
GOBAR_DD38092
GOBAR_DD27459
GOBAR_DD27028
GOBAR_DD19375
GOBAR_DD07954
GOBAR_DD04001
GOBAR_AA31879
GOBAR_AA30683
GOBAR_AA24665
GOBAR_AA21277
GOBAR_AA13780
0
2000
4000
6000

G.ba CMGC_DYRK−YAK exon−intron and domain diagram (all)

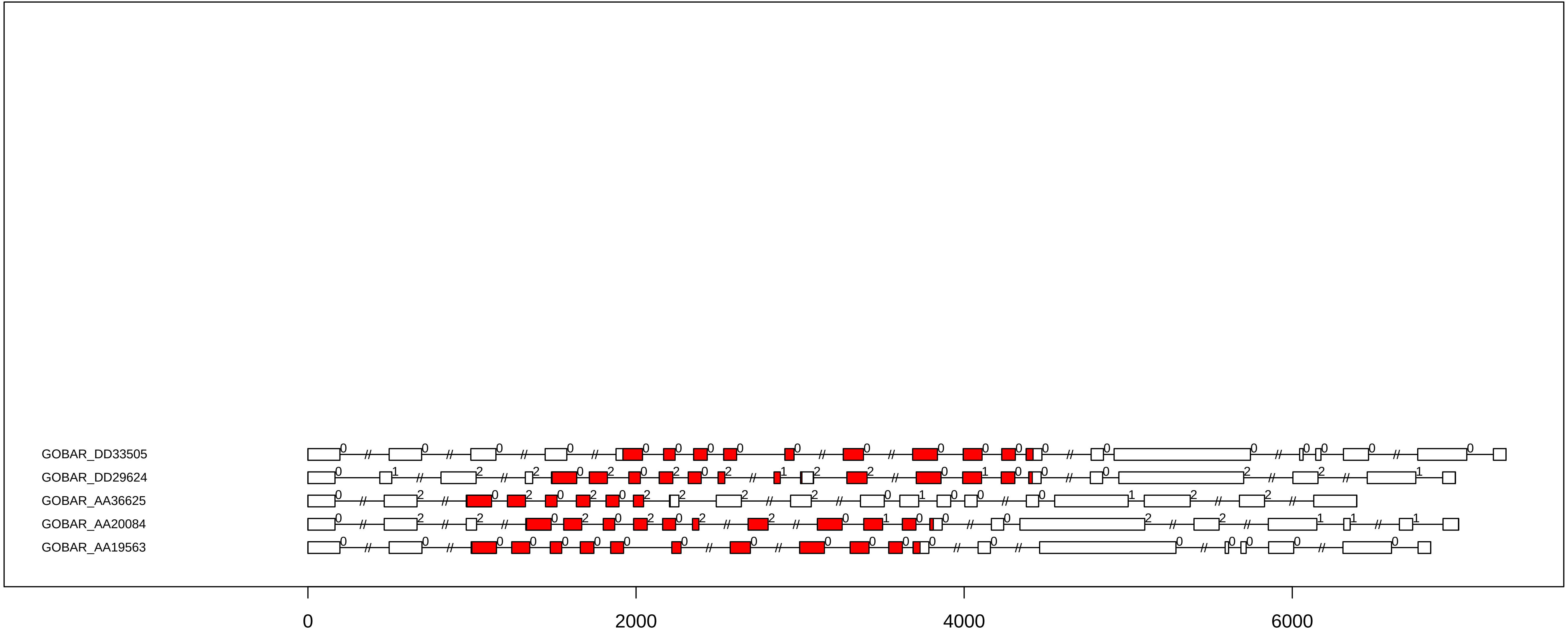

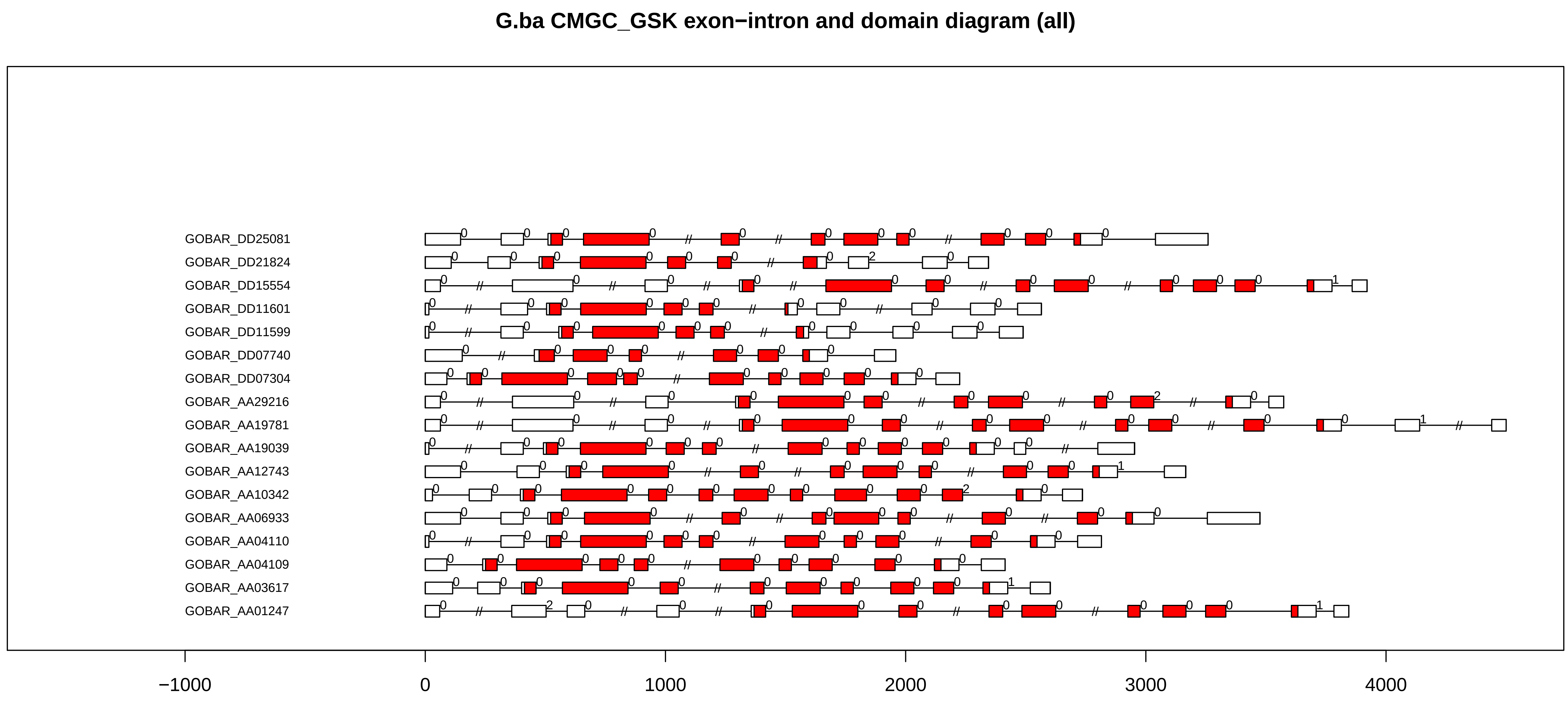

G.ba CMGC_GSK exon−intron and domain diagram (all)
GOBAR_DD25081
GOBAR_DD21824
GOBAR_DD15554
GOBAR_DD11601
GOBAR_DD11599
GOBAR_DD07740
GOBAR_DD07304
GOBAR_AA29216
GOBAR_AA19781
GOBAR_AA19039
GOBAR_AA12743
GOBAR_AA10342
GOBAR_AA06933
GOBAR_AA04110
GOBAR_AA04109
GOBAR_AA03617
GOBAR_AA01247
−1000
0
1000
2000
3000
4000

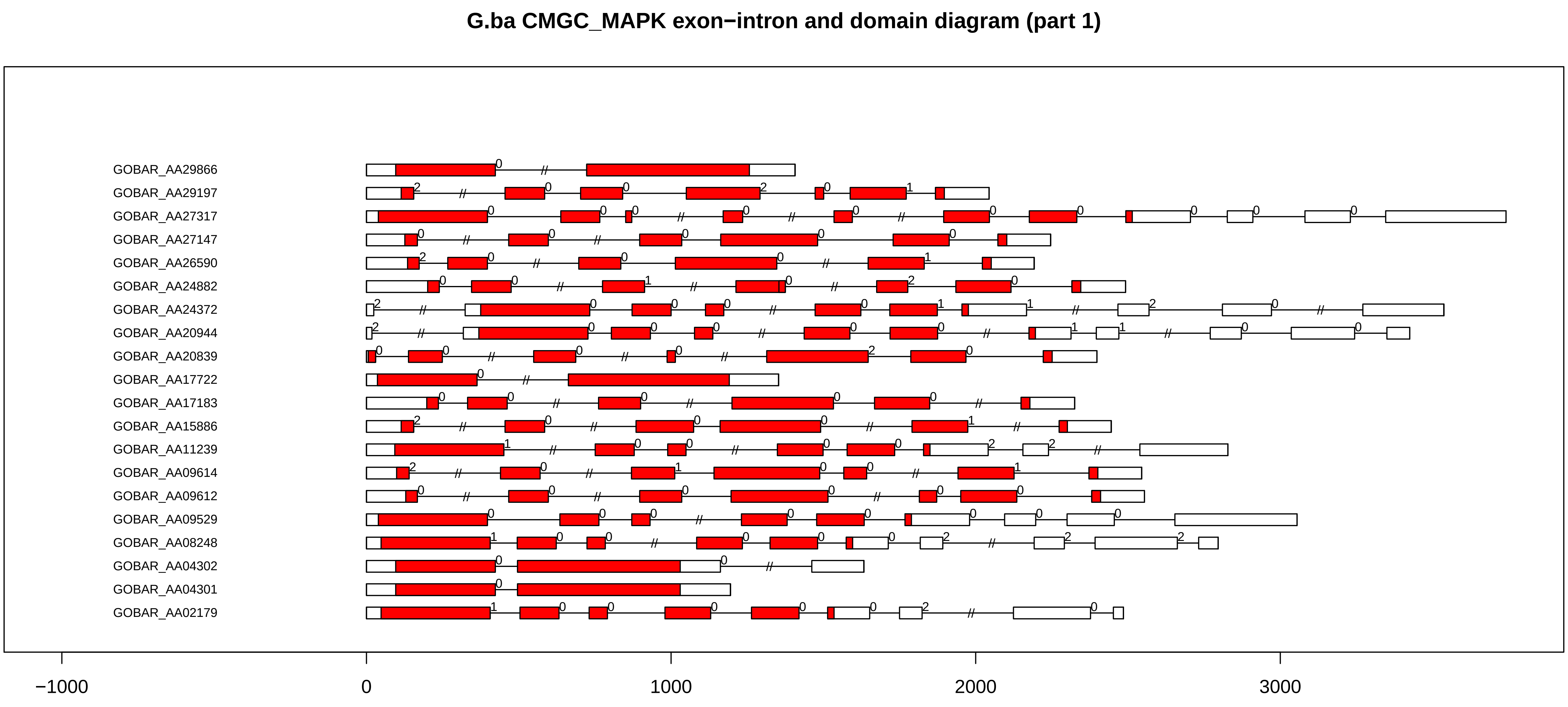
G.ba CMGC_MAPK exon−intron and domain diagram (part 1)
GOBAR_AA29866
GOBAR_AA29197
GOBAR_AA27317
GOBAR_AA27147
GOBAR_AA26590
GOBAR_AA24882
GOBAR_AA24372
GOBAR_AA20944
GOBAR_AA20839
GOBAR_AA17722
GOBAR_AA17183
GOBAR_AA15886
GOBAR_AA11239
GOBAR_AA09614
GOBAR_AA09612
GOBAR_AA09529
GOBAR_AA08248
GOBAR_AA04302
GOBAR_AA04301
GOBAR_AA02179
−1000
0
1000
2000
3000

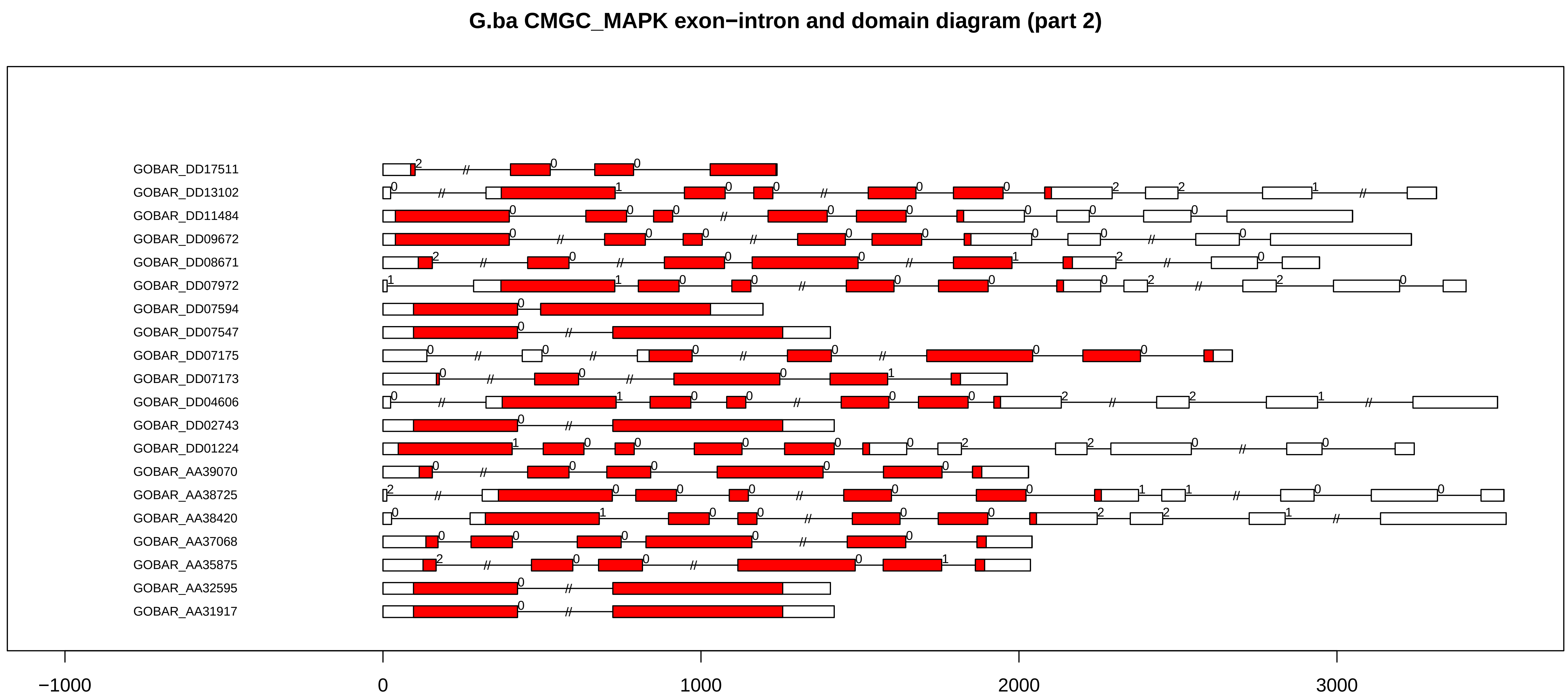
G.ba CMGC_MAPK exon−intron and domain diagram (part 2)
GOBAR_DD17511
GOBAR_DD13102
GOBAR_DD11484
GOBAR_DD09672
GOBAR_DD08671
GOBAR_DD07972
GOBAR_DD07594
GOBAR_DD07547
GOBAR_DD07175
GOBAR_DD07173
GOBAR_DD04606
GOBAR_DD02743
GOBAR_DD01224
GOBAR_AA39070
GOBAR_AA38725
GOBAR_AA38420
GOBAR_AA37068
GOBAR_AA35875
GOBAR_AA32595
GOBAR_AA31917
−1000
0
1000
2000
3000

G.ba CMGC_MAPK exon−intron and domain diagram (part 3)

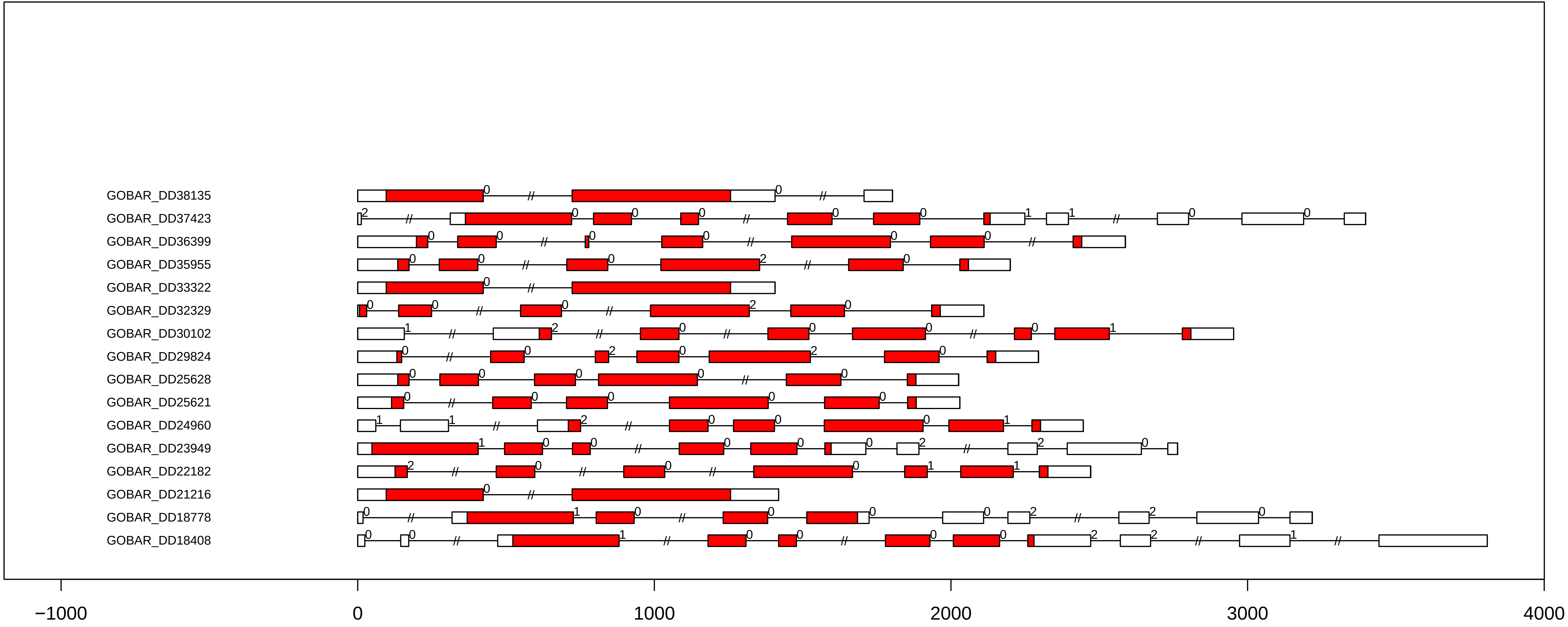

G.ba CMGC_Pl−Tthe exon−intron and domain diagram (all)

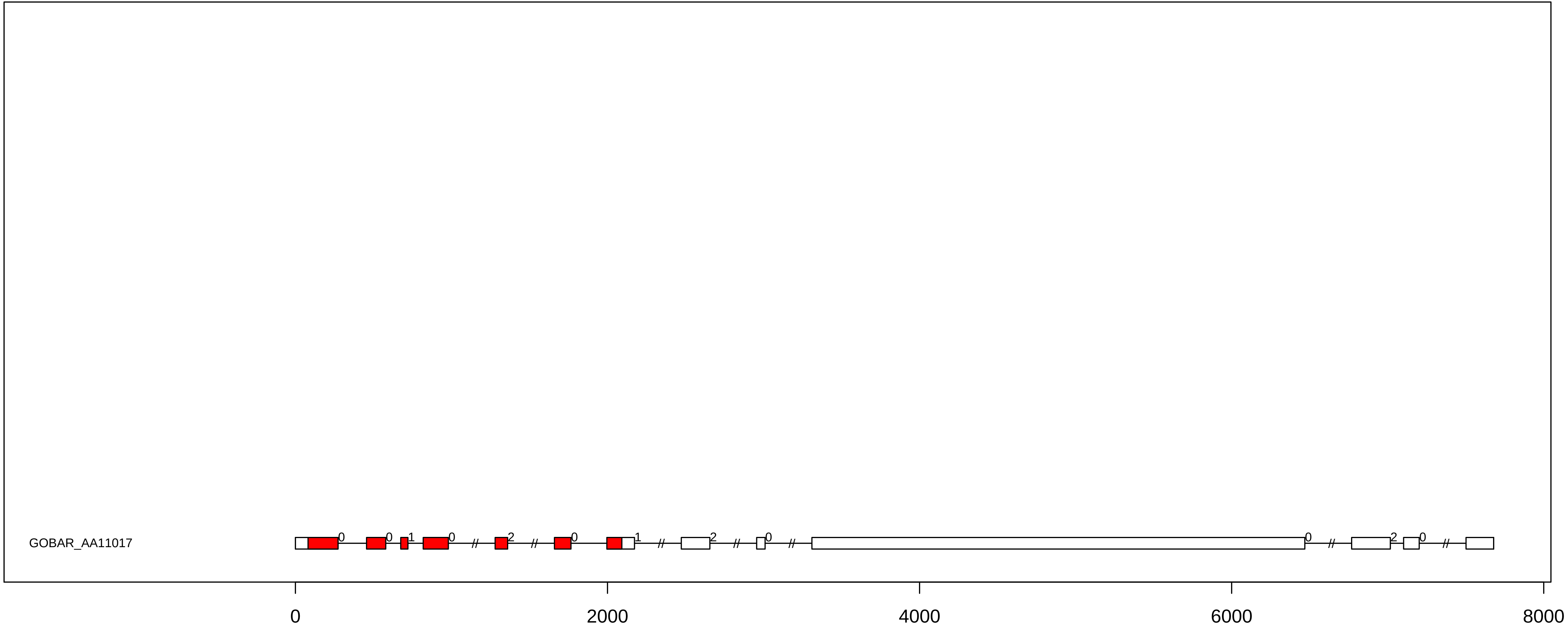

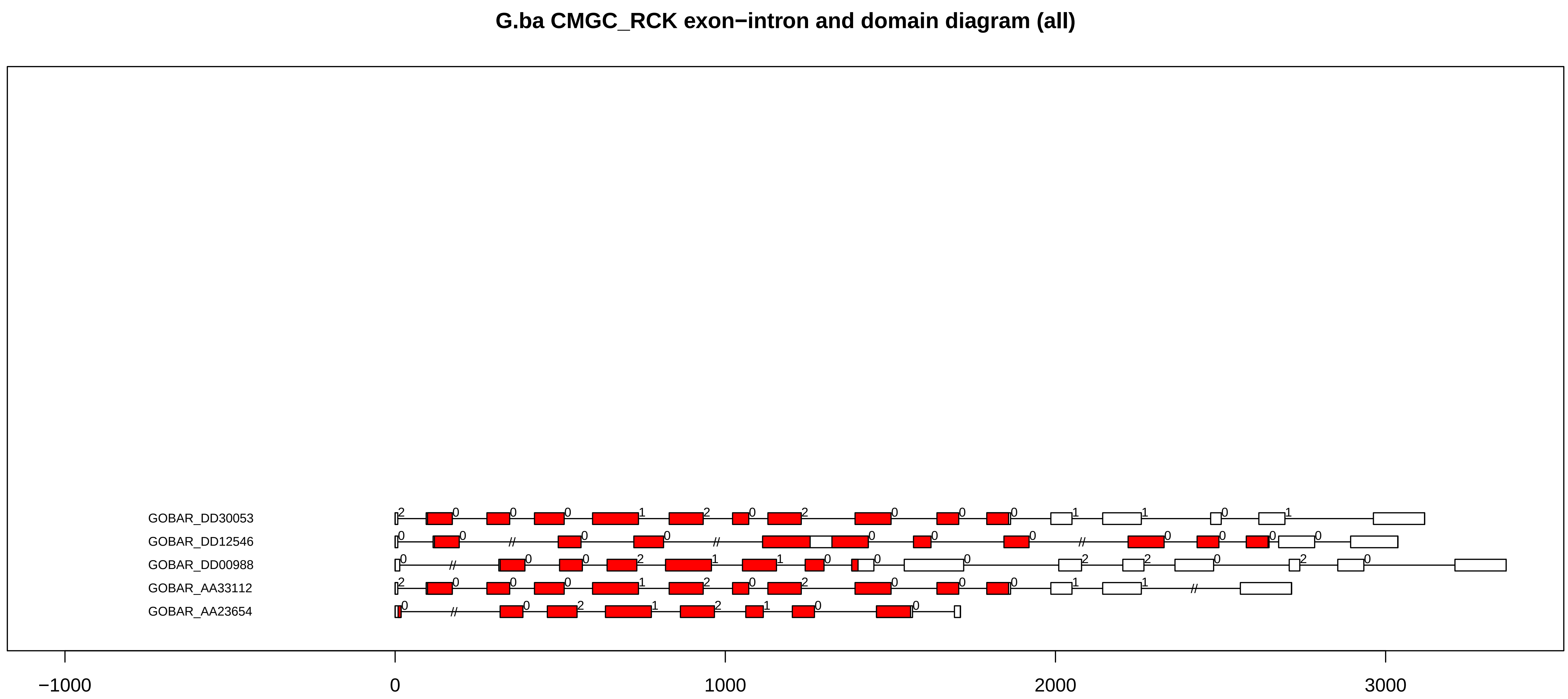
G.ba CMGC_RCK exon−intron and domain diagram (all)
GOBAR_DD30053
GOBAR_DD12546
GOBAR_DD00988
GOBAR_AA33112
GOBAR_AA23654
−1000
0
1000
2000
3000

G.ba CMGC_SRPK exon−intron and domain diagram (all)

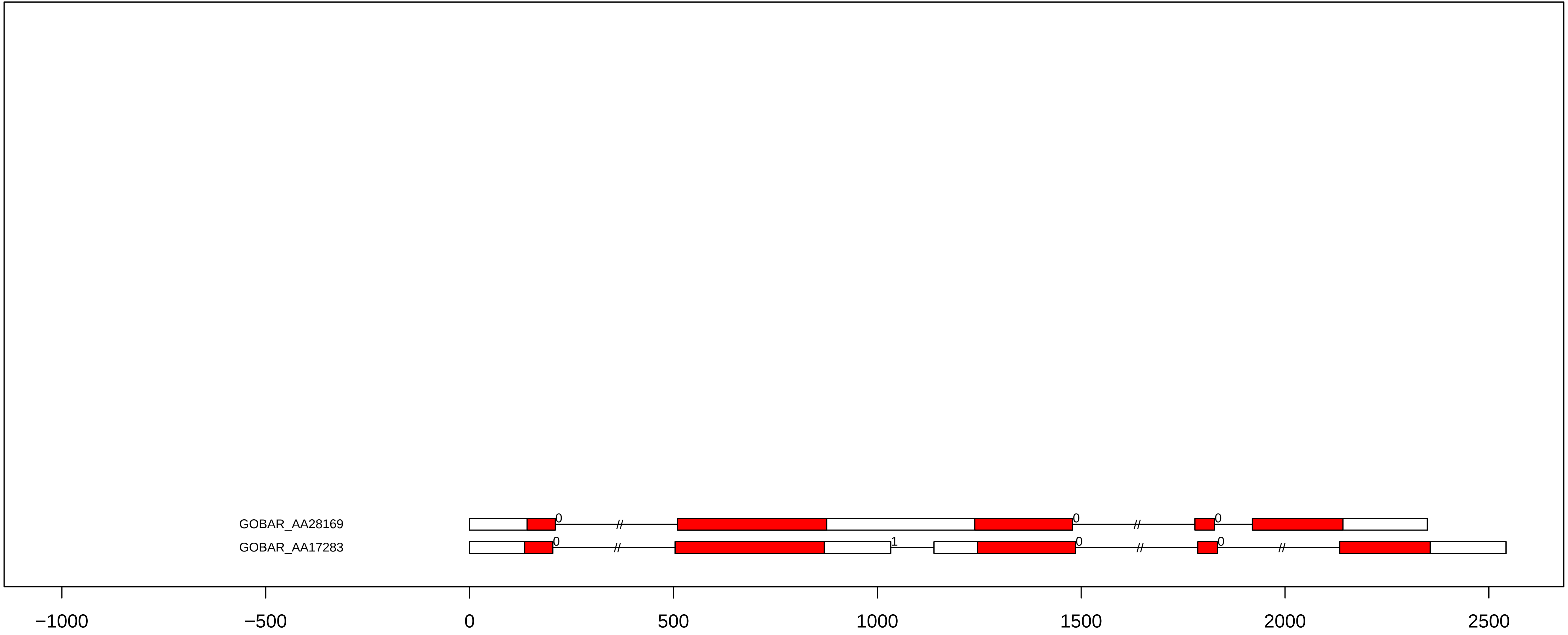

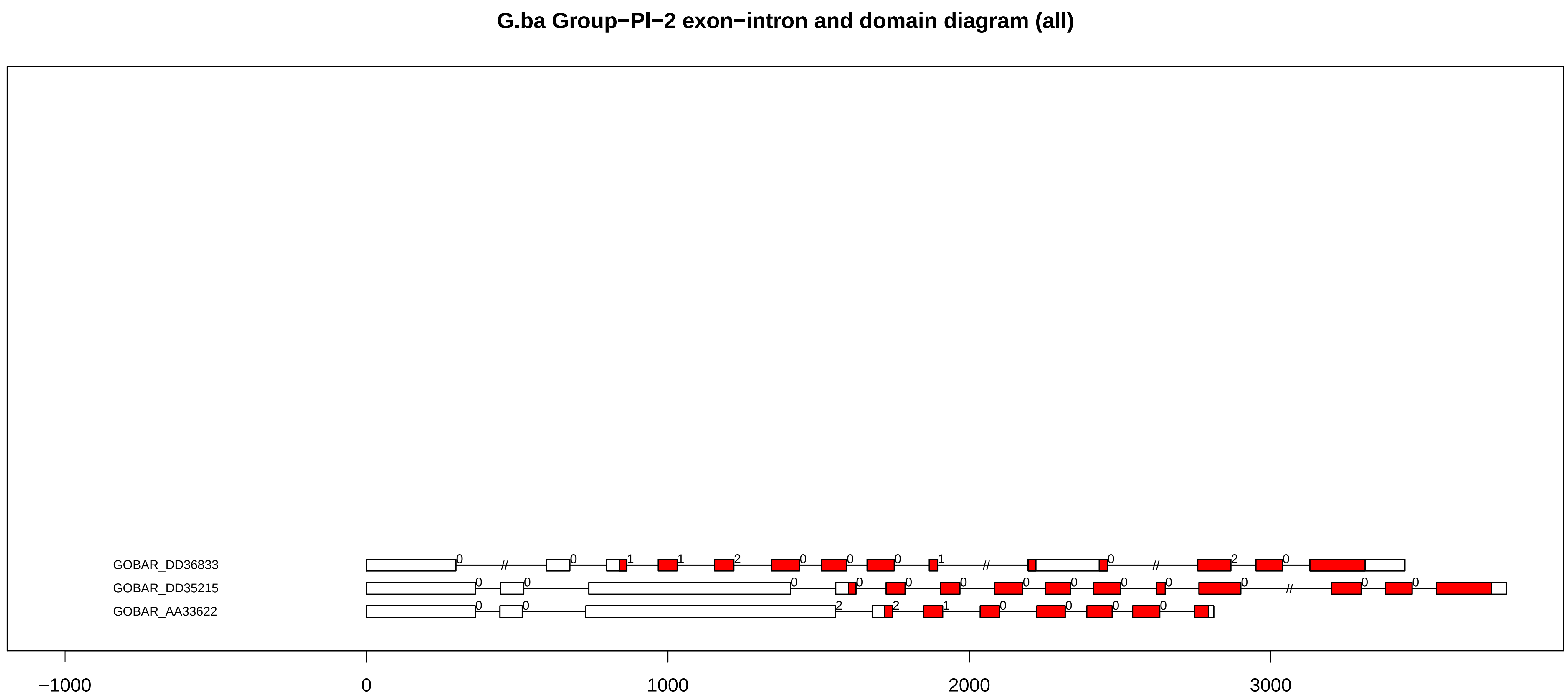
G.ba Group−PI−2 exon−intron and domain diagram (all)
GOBAR_DD36833
GOBAR_DD35215
GOBAR_AA33622
−1000
0
1000
2000
3000

G.ba Group−PI−3 exon−intron and domain diagram (all)

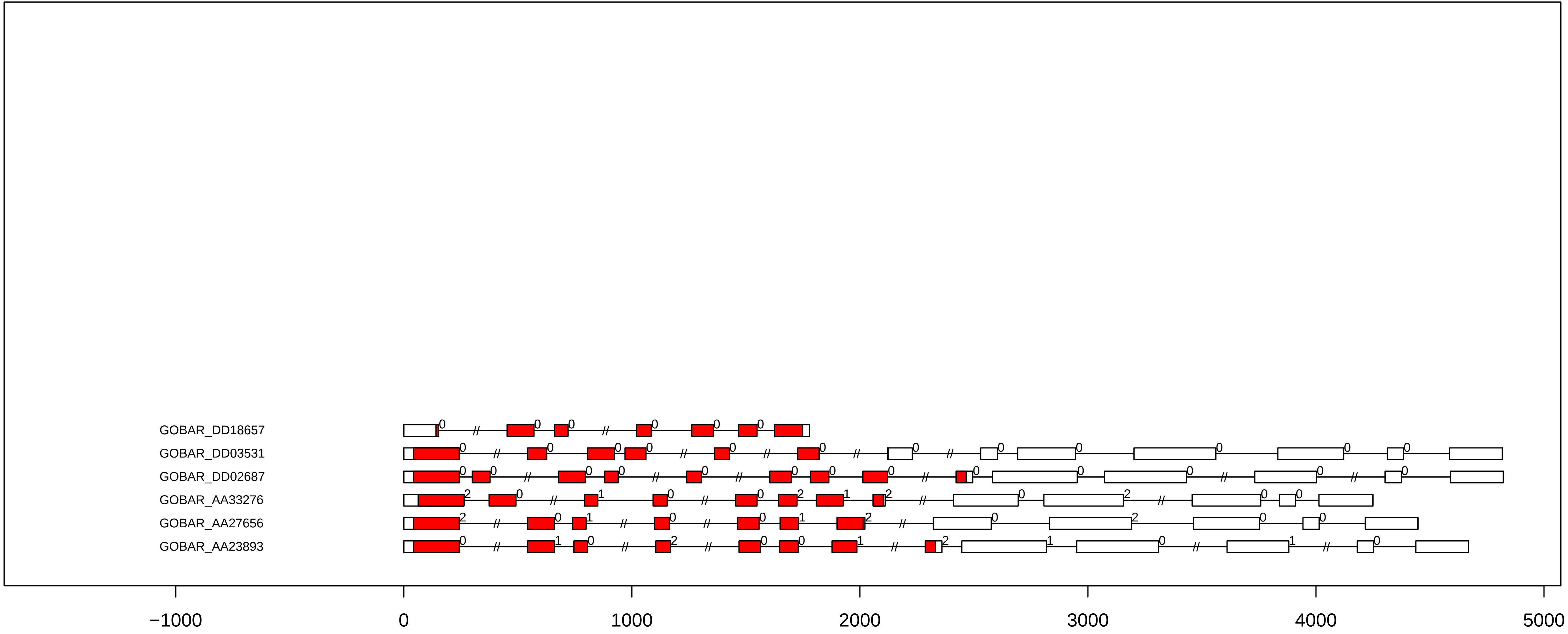

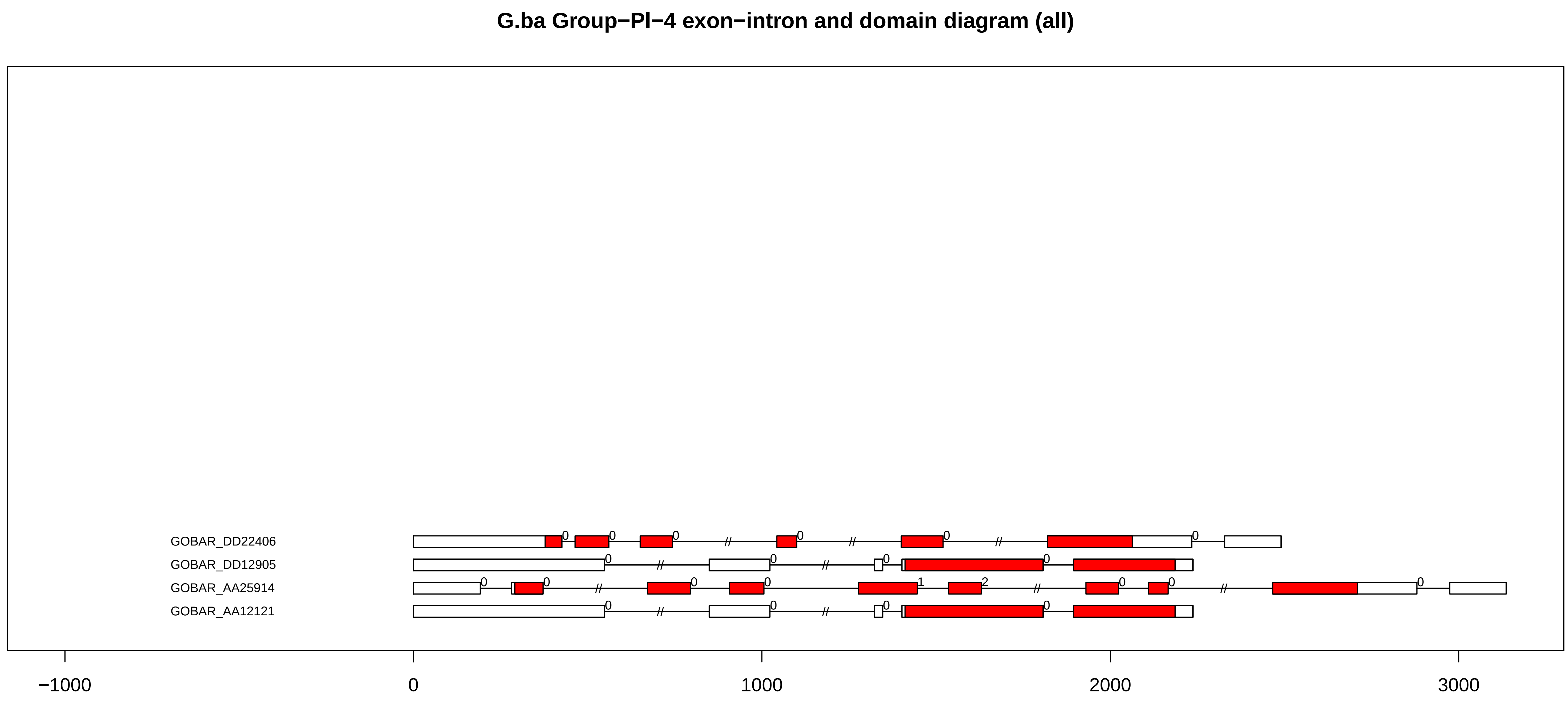
G.ba Group−PI−4 exon−intron and domain diagram (all)
GOBAR_DD22406
GOBAR_DD12905
GOBAR_AA25914
GOBAR_AA12121
−1000
0
1000
2000
3000

G.ba IRE1 exon−intron and domain diagram (all)

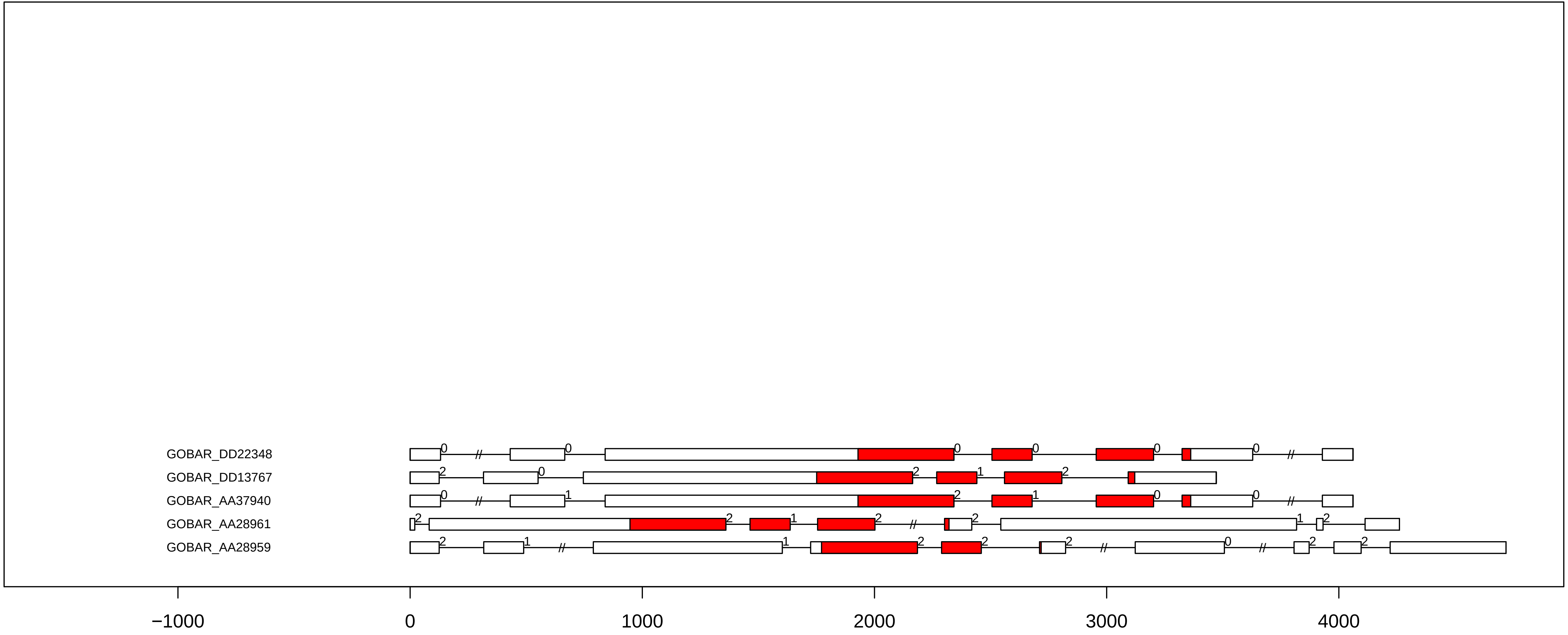

G.ba NAK exon−intron and domain diagram (all)

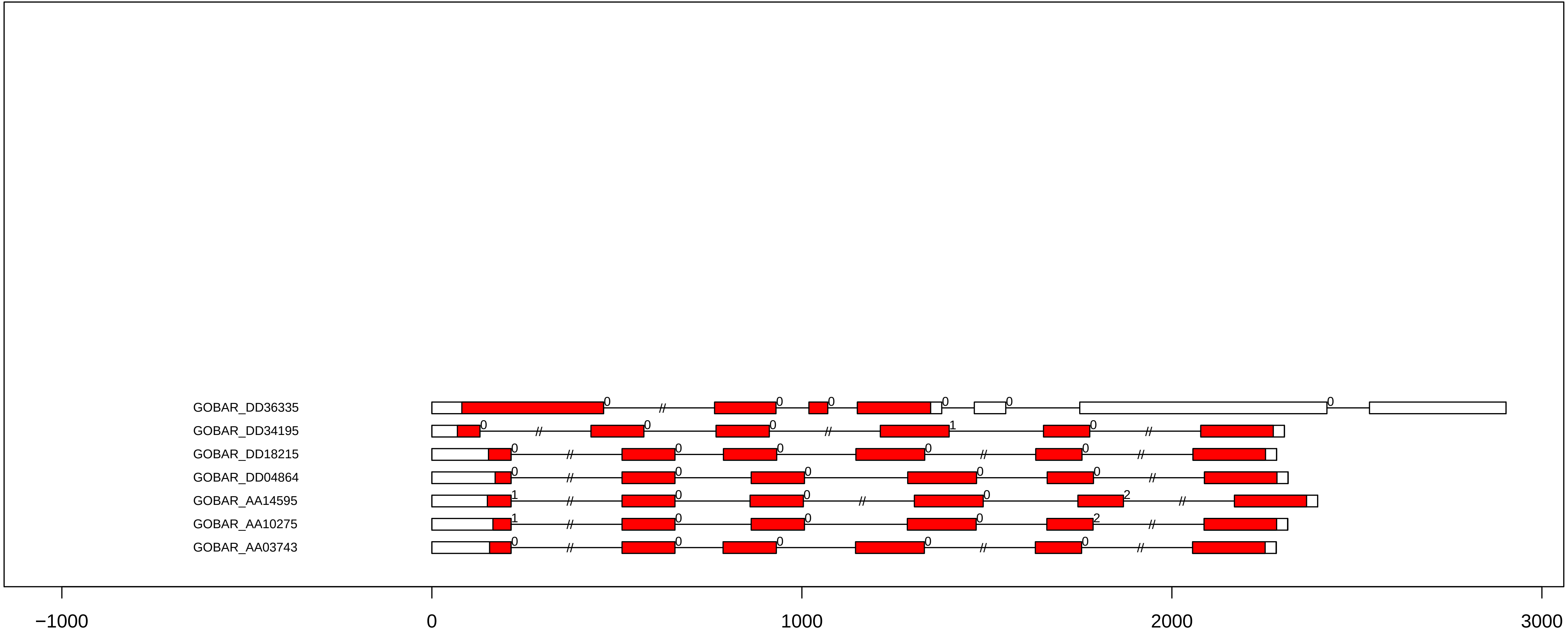

G.ba NEK exon−intron and domain diagram (all)

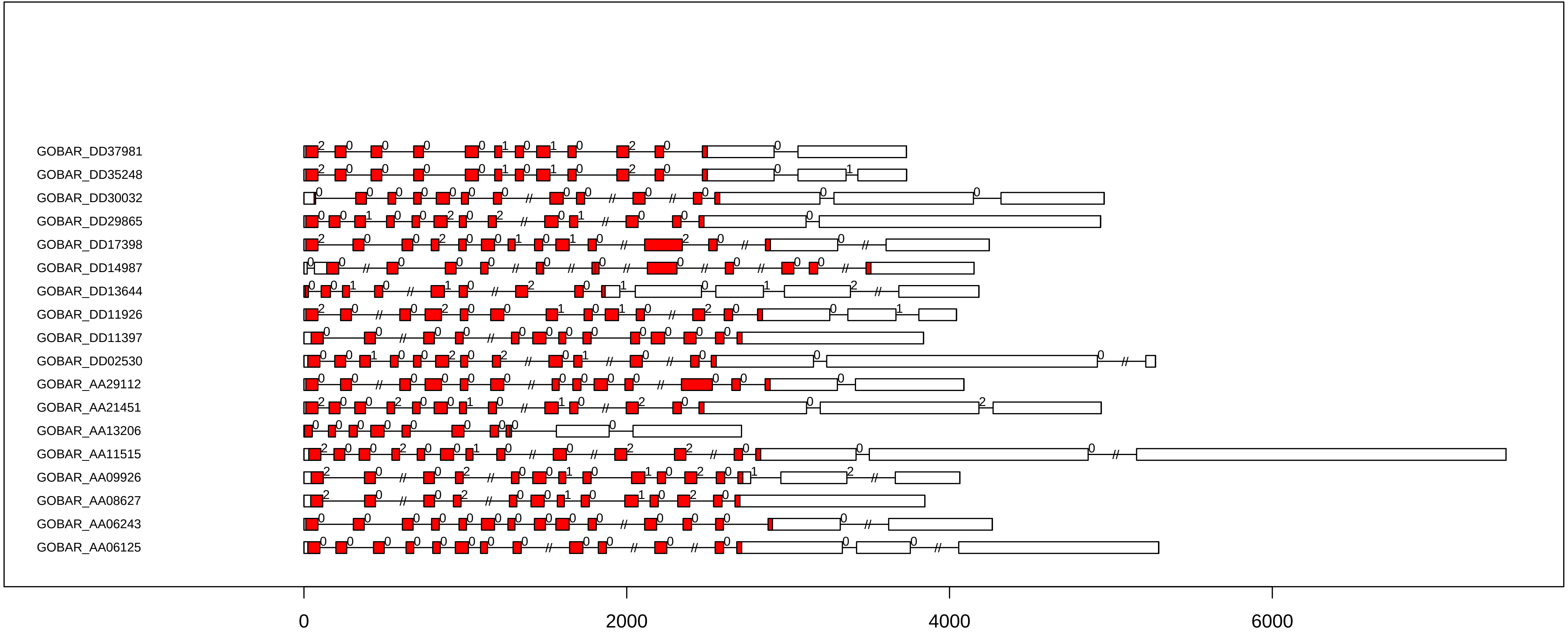

G.ba PEK_GCN2 exon−intron and domain diagram (all)

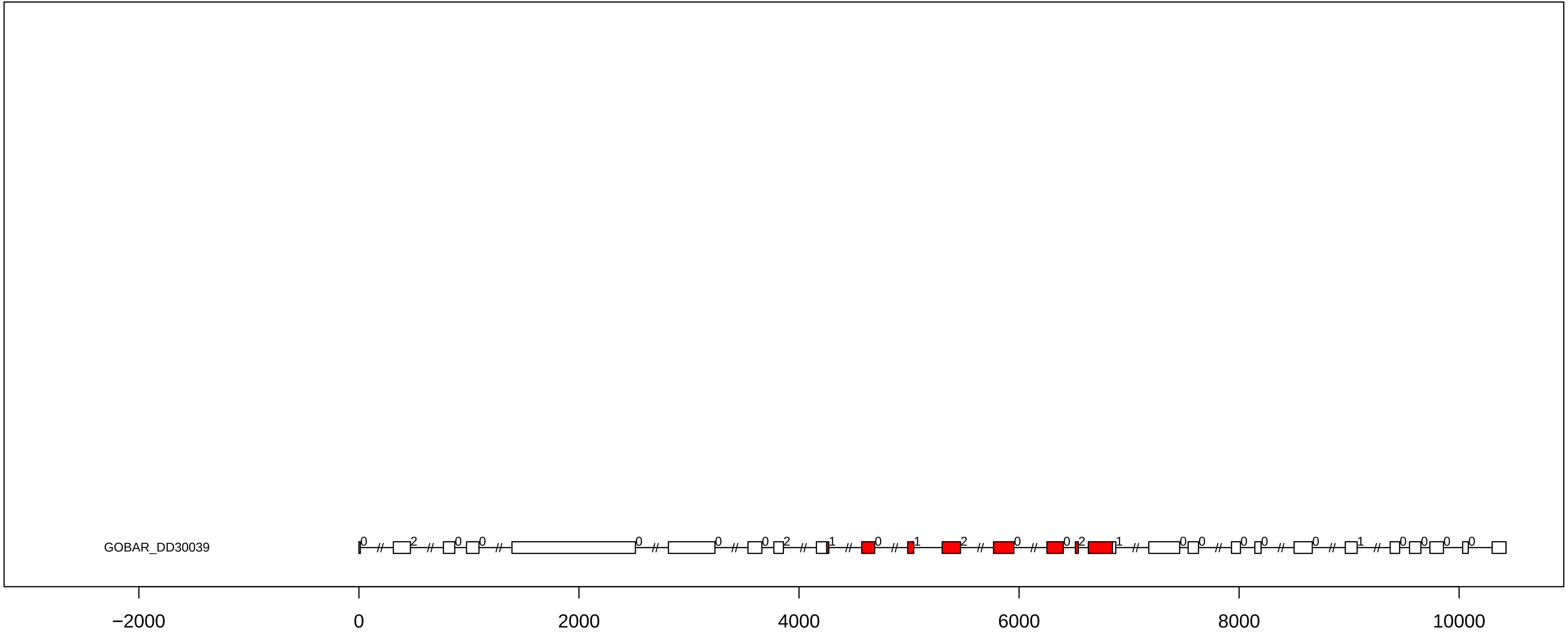

G.ba PEK_PEK exon−intron and domain diagram (all)

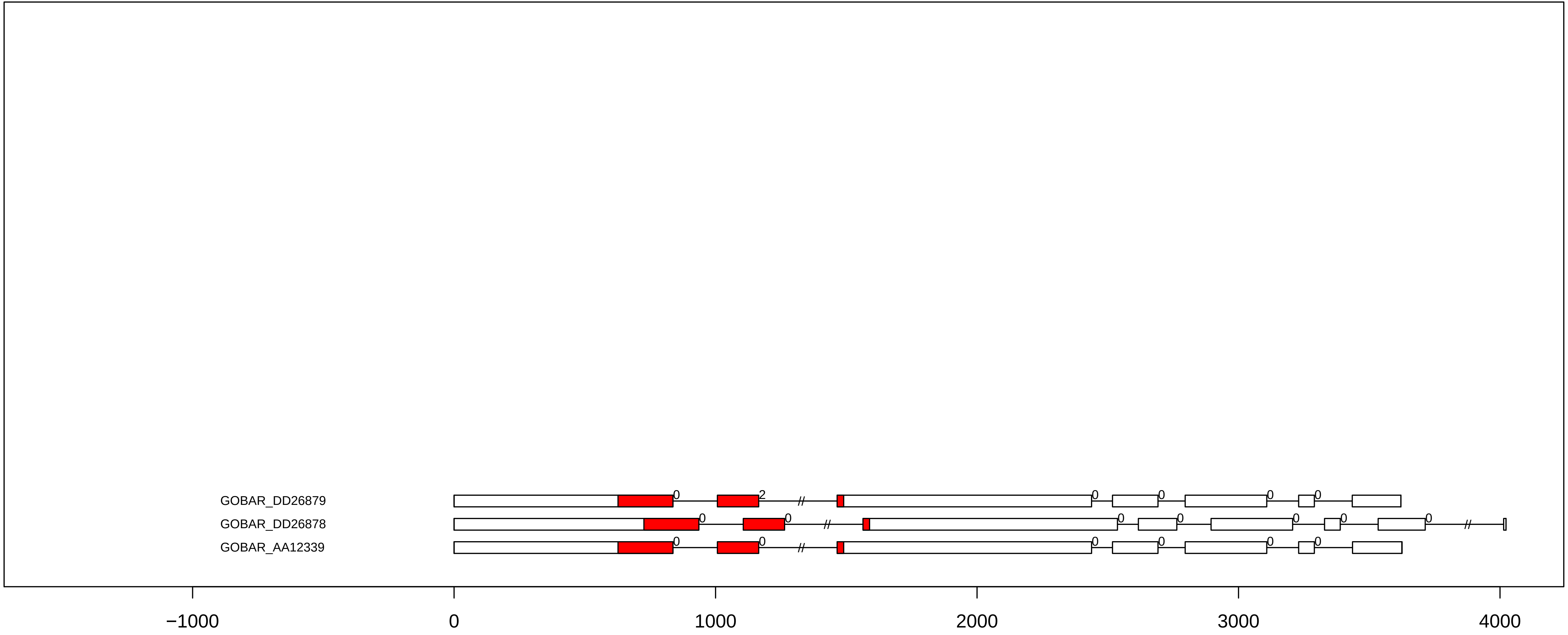

G.ba RLK−Pelle_C−LEC exon−intron and domain diagram (all)

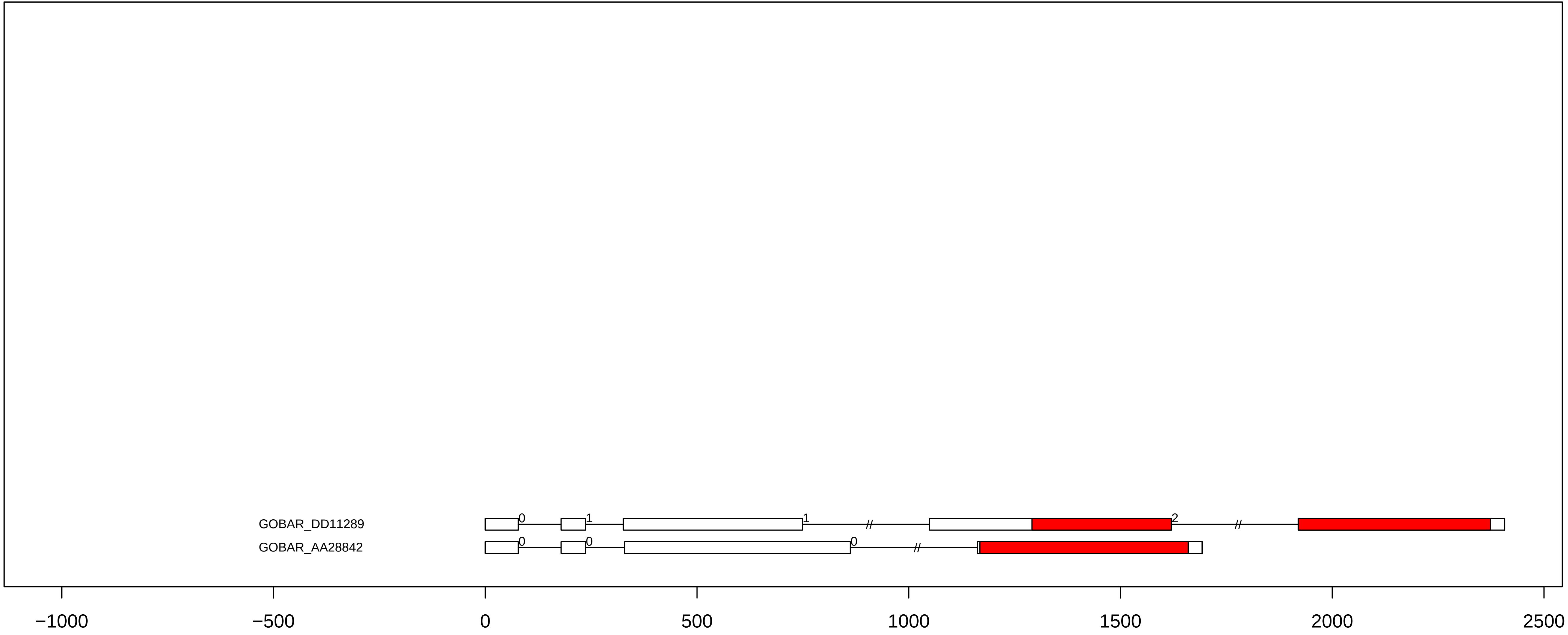

G.ba RLK−Pelle_CR4L exon−intron and domain diagram (part 1)

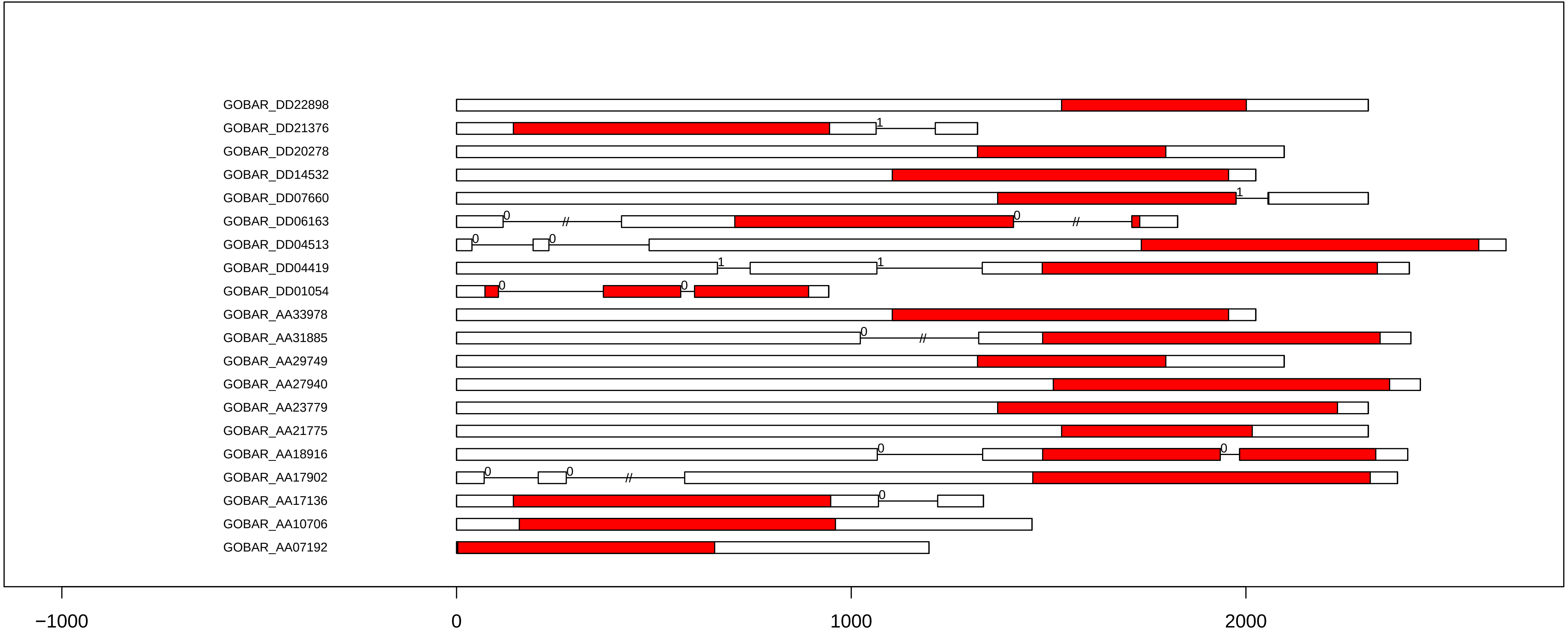

G.ba RLK−Pelle_CR4L exon−intron and domain diagram (part 2)

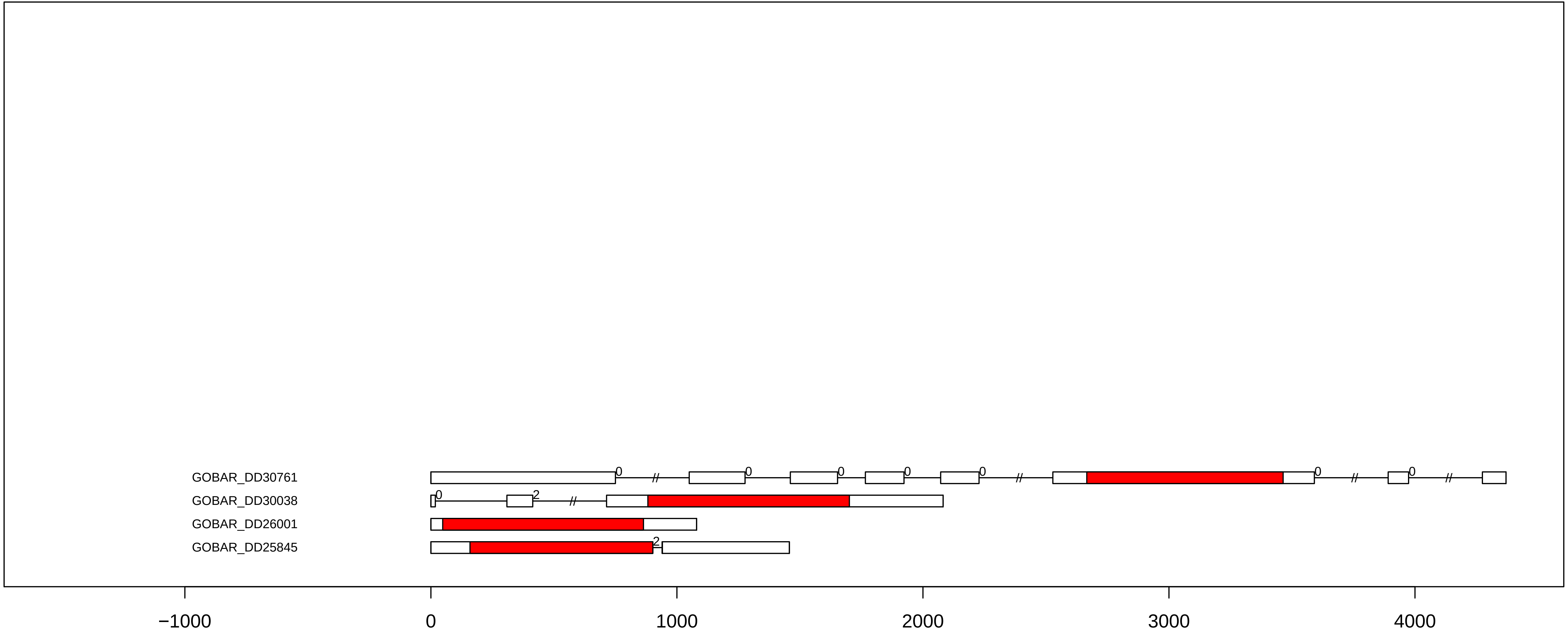

G.ba RLK−Pelle_CrRLK1L−1 exon−intron and domain diagram (part 1)

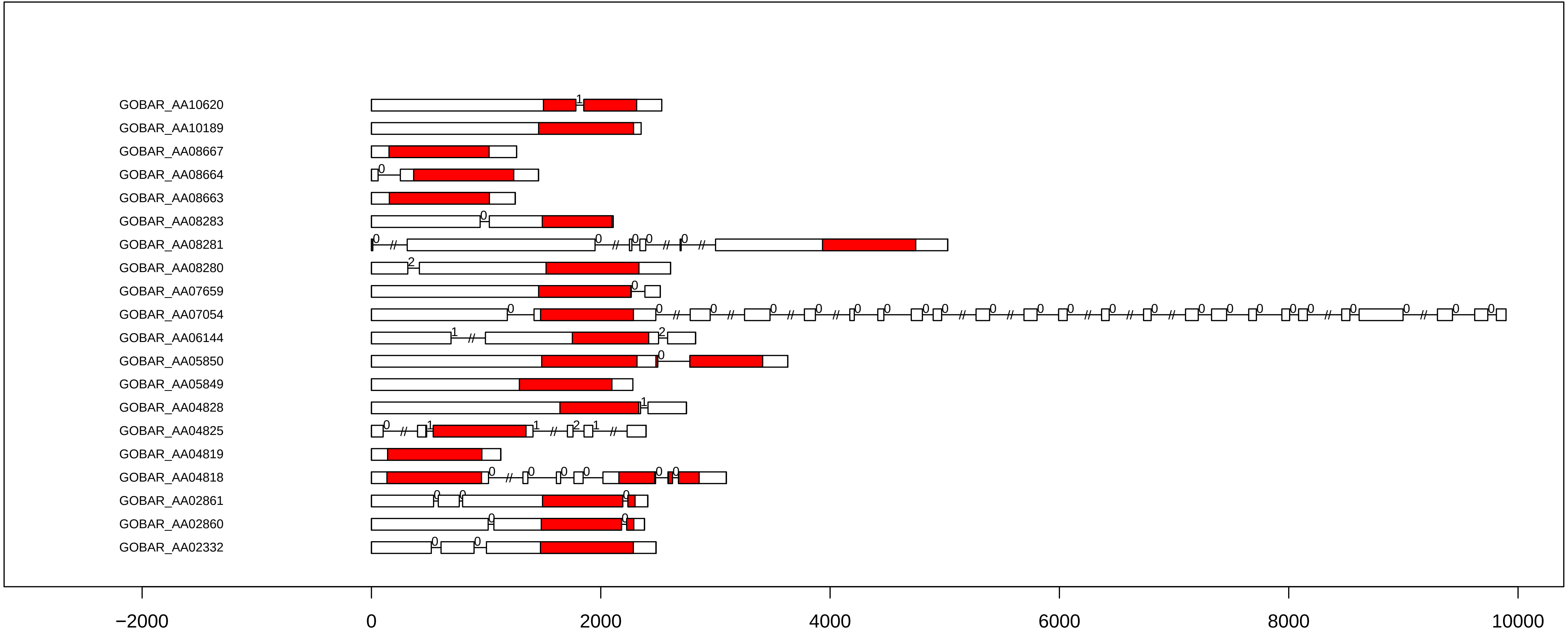

G.ba RLK−Pelle_CrRLK1L−1 exon−intron and domain diagram (part 2)

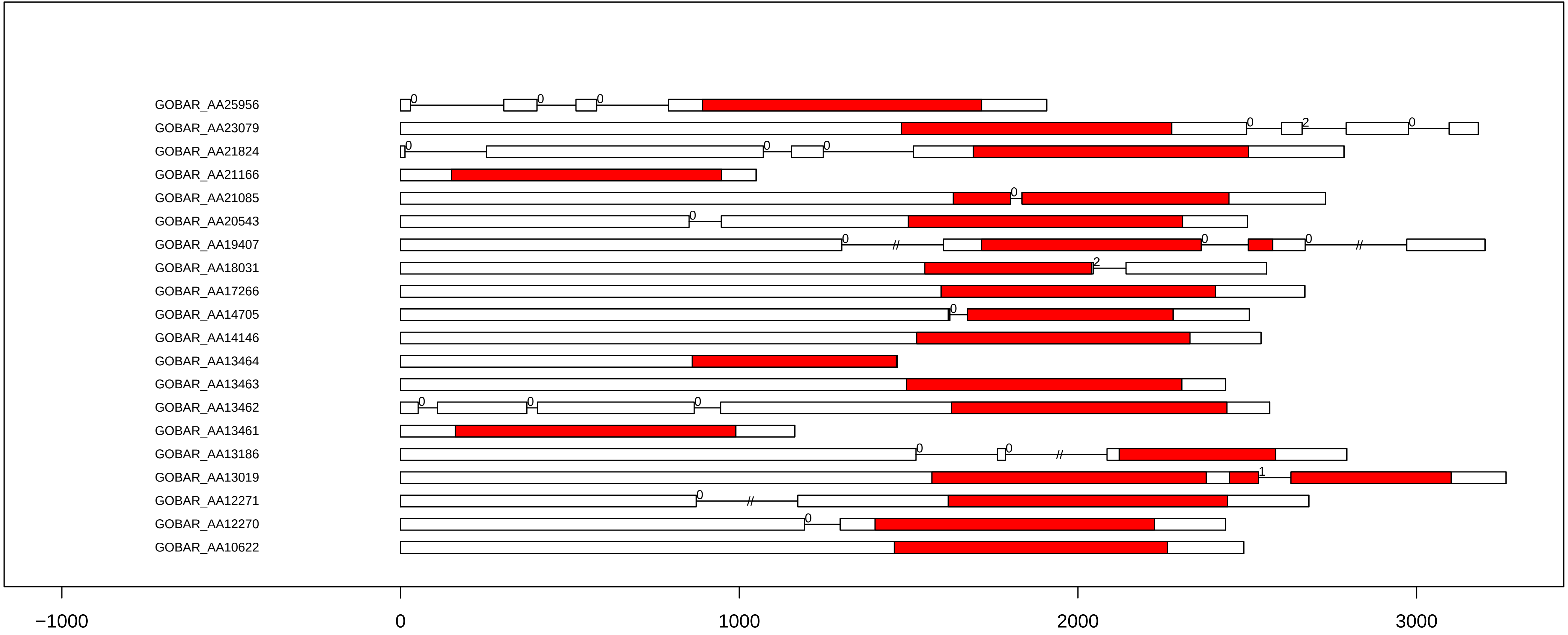

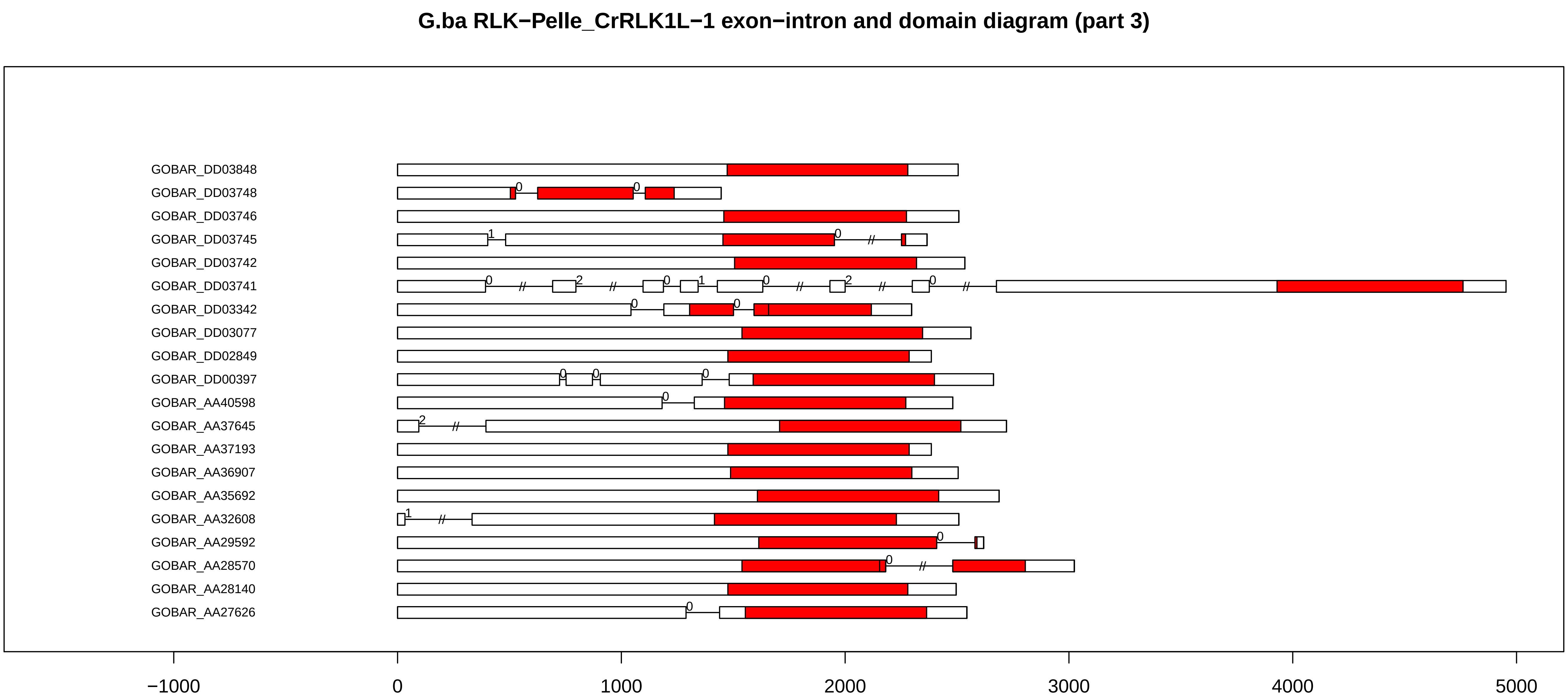
G.ba RLK−Pelle_CrRLK1L−1 exon−intron and domain diagram (part 3)
GOBAR_DD03848
GOBAR_DD03748
GOBAR_DD03746
GOBAR_DD03745
GOBAR_DD03742
GOBAR_DD03741
GOBAR_DD03342
GOBAR_DD03077
GOBAR_DD02849
GOBAR_DD00397
GOBAR_AA40598
GOBAR_AA37645
GOBAR_AA37193
GOBAR_AA36907
GOBAR_AA35692
GOBAR_AA32608
GOBAR_AA29592
GOBAR_AA28570
GOBAR_AA28140
GOBAR_AA27626
−1000
0
1000
2000
3000
4000
5000

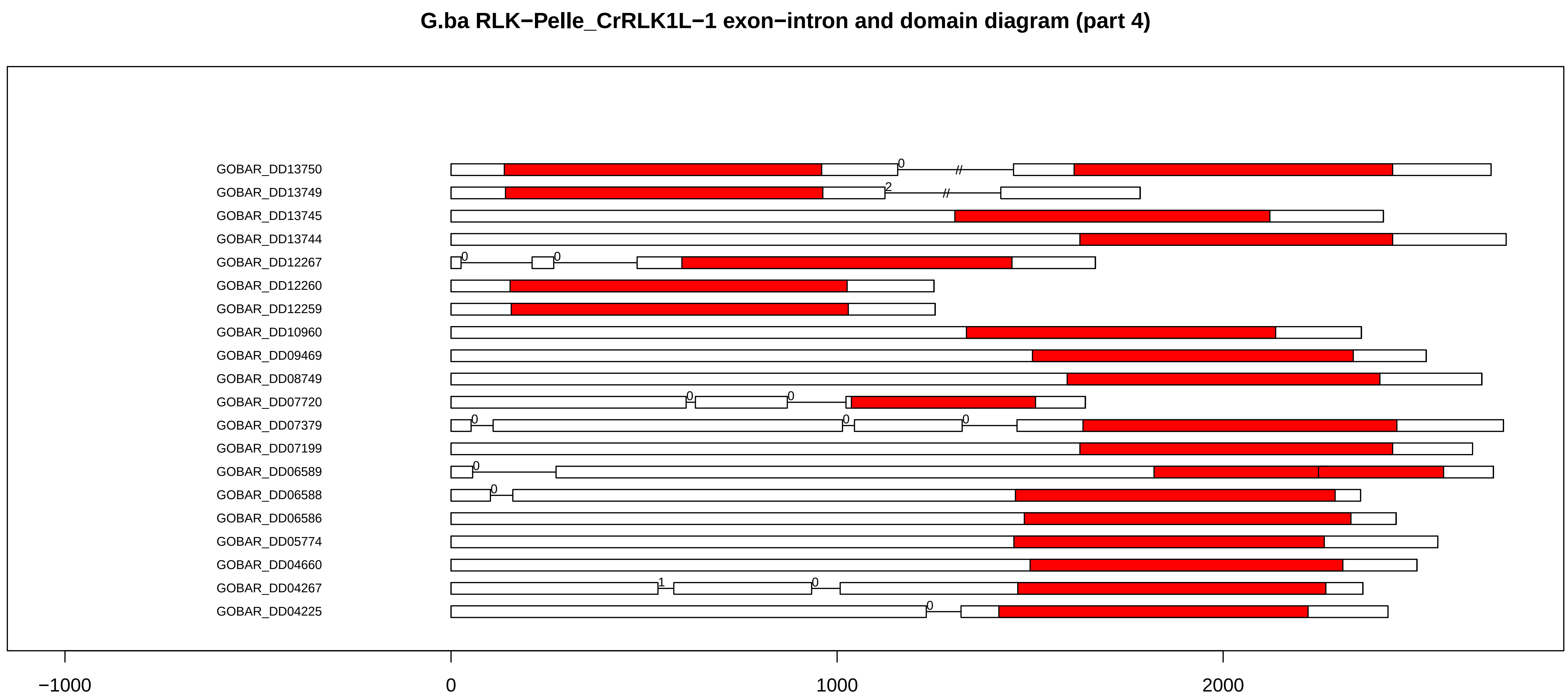
G.ba RLK−Pelle_CrRLK1L−1 exon−intron and domain diagram (part 4)
GOBAR_DD13750
GOBAR_DD13749
GOBAR_DD13745
GOBAR_DD13744
GOBAR_DD12267
GOBAR_DD12260
GOBAR_DD12259
GOBAR_DD10960
GOBAR_DD09469
GOBAR_DD08749
GOBAR_DD07720
GOBAR_DD07379
GOBAR_DD07199
GOBAR_DD06589
GOBAR_DD06588
GOBAR_DD06586
GOBAR_DD05774
GOBAR_DD04660
GOBAR_DD04267
GOBAR_DD04225
−1000
0
1000
2000

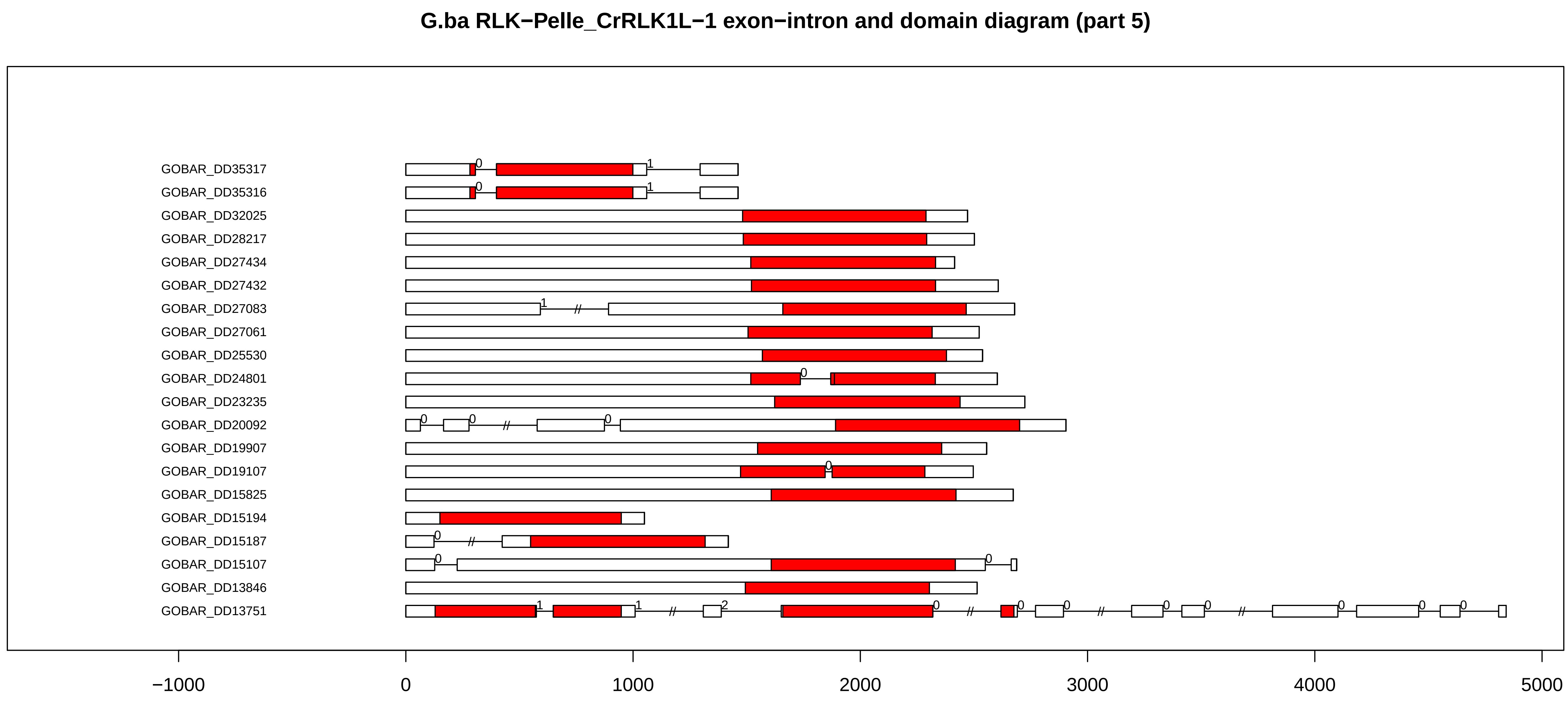
G.ba RLK−Pelle_CrRLK1L−1 exon−intron and domain diagram (part 5)
GOBAR_DD35317
GOBAR_DD35316
GOBAR_DD32025
GOBAR_DD28217
GOBAR_DD27434
GOBAR_DD27432
GOBAR_DD27083
GOBAR_DD27061
GOBAR_DD25530
GOBAR_DD24801
GOBAR_DD23235
GOBAR_DD20092
GOBAR_DD19907
GOBAR_DD19107
GOBAR_DD15825
GOBAR_DD15194
GOBAR_DD15187
GOBAR_DD15107
GOBAR_DD13846
GOBAR_DD13751
−1000
0
1000
2000
3000
4000
5000

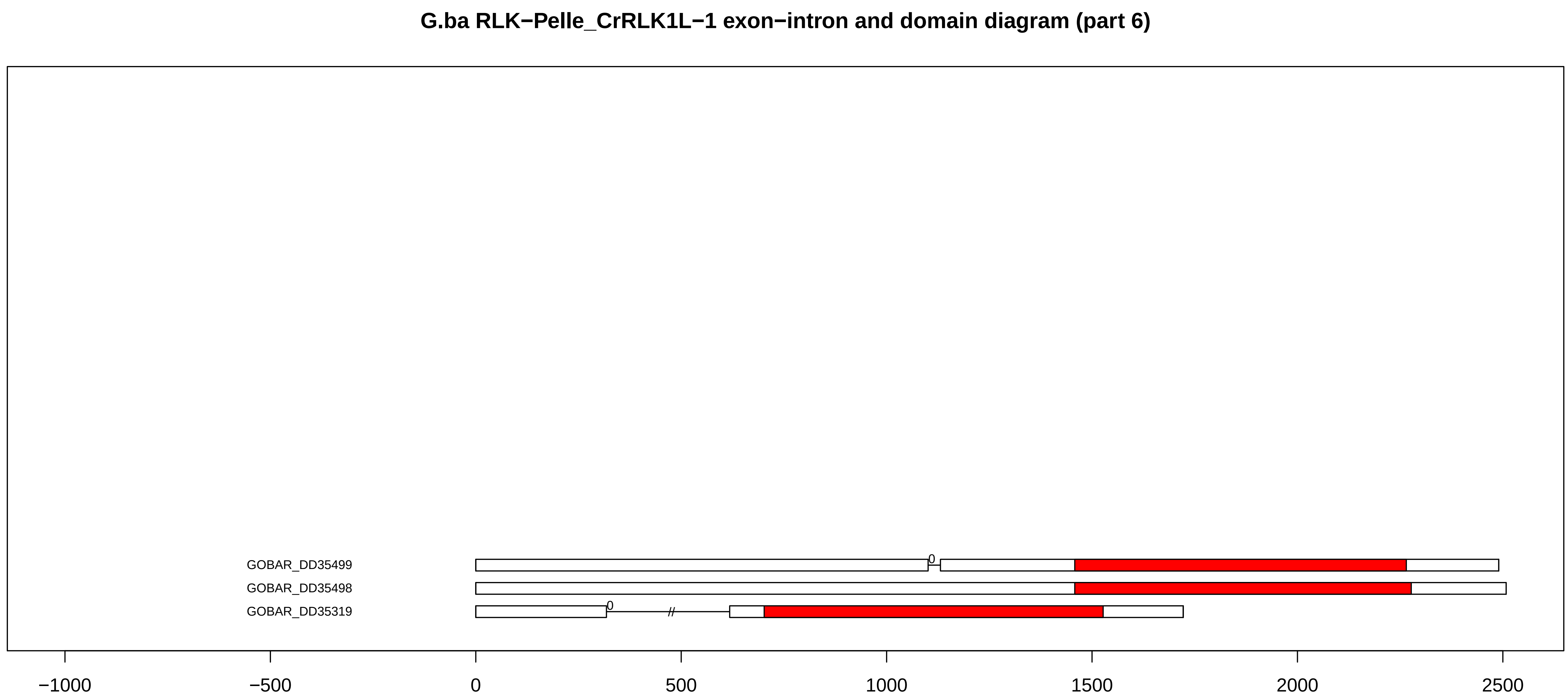
G.ba RLK−Pelle_CrRLK1L−1 exon−intron and domain diagram (part 6)
GOBAR_DD35499
GOBAR_DD35498
GOBAR_DD35319
0
0
//
−1000
−500
0
500
1000
1500
2000
2500

G.ba RLK−Pelle_DLSV exon−intron and domain diagram (part 1)

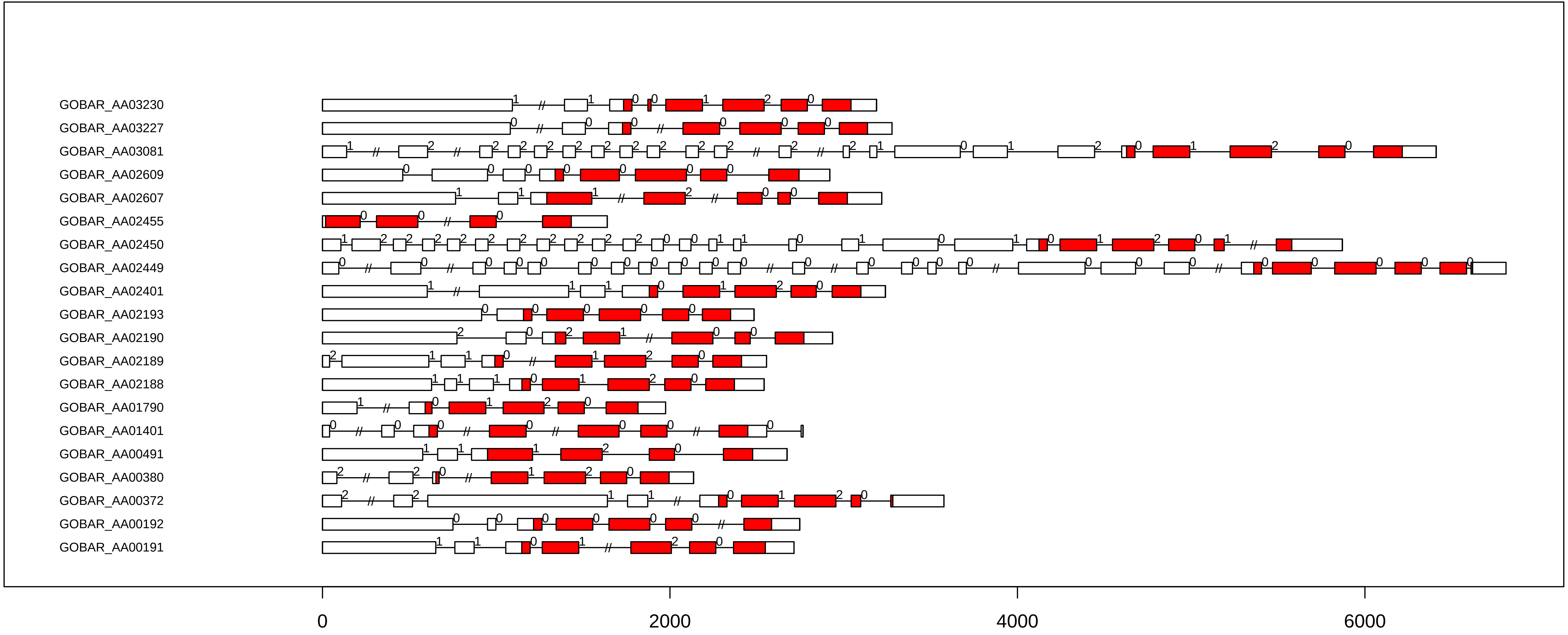

G.ba RLK−Pelle_DLSV exon−intron and domain diagram (part 2)

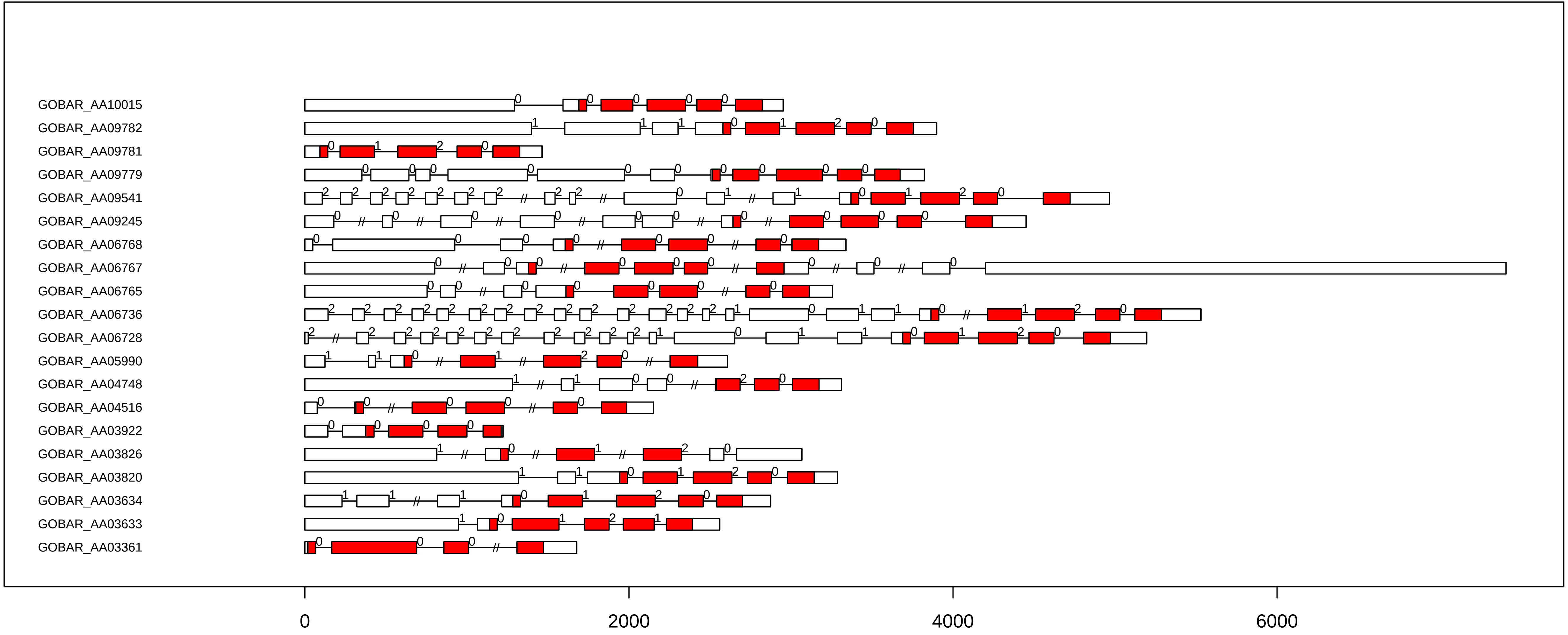

G.ba RLK−Pelle_DLSV exon−intron and domain diagram (part 3)

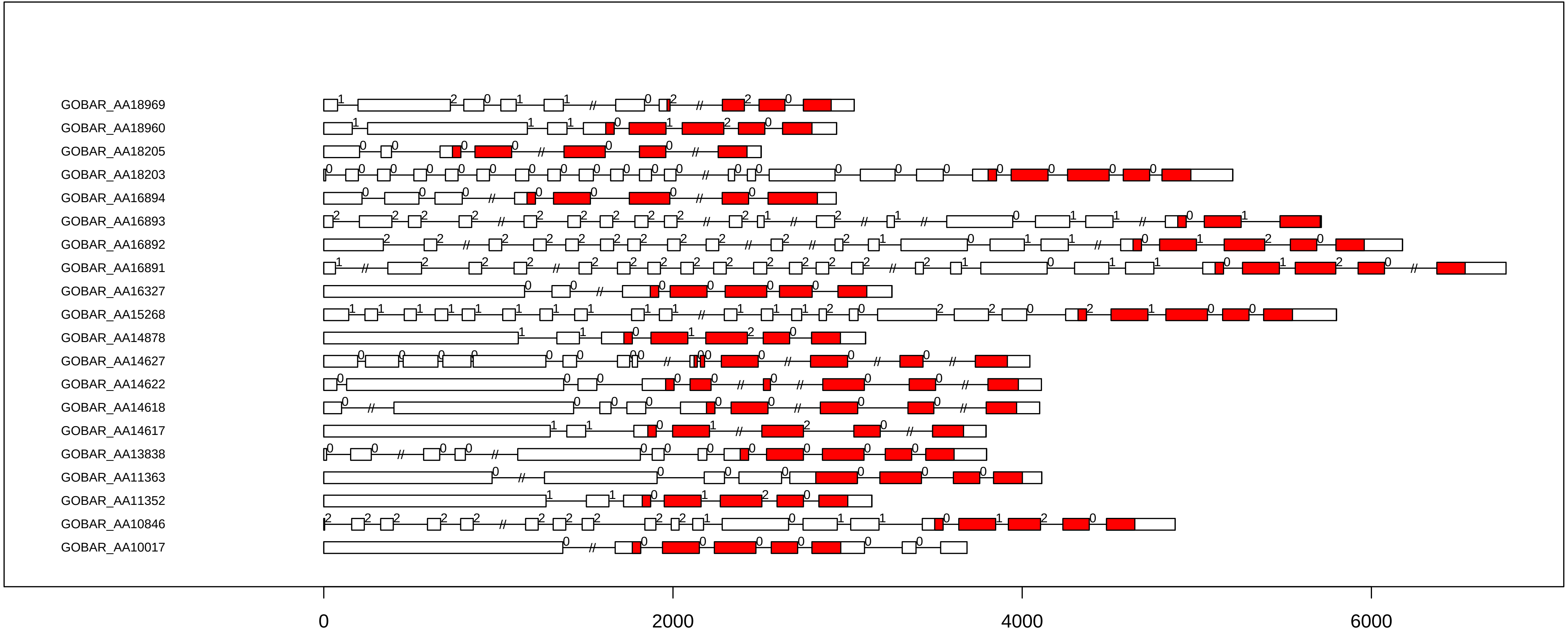

G.ba RLK−Pelle_DLSV exon−intron and domain diagram (part 4)

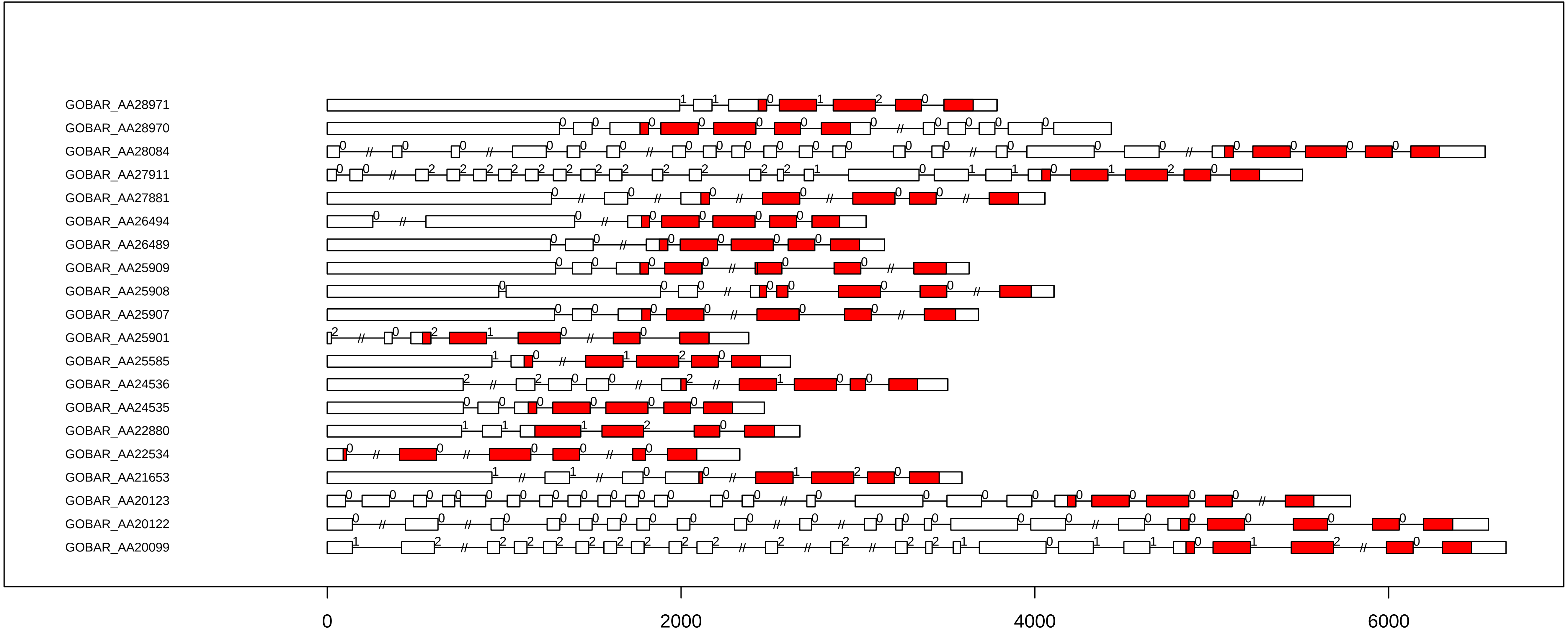

G.ba RLK−Pelle_DLSV exon−intron and domain diagram (part 5)

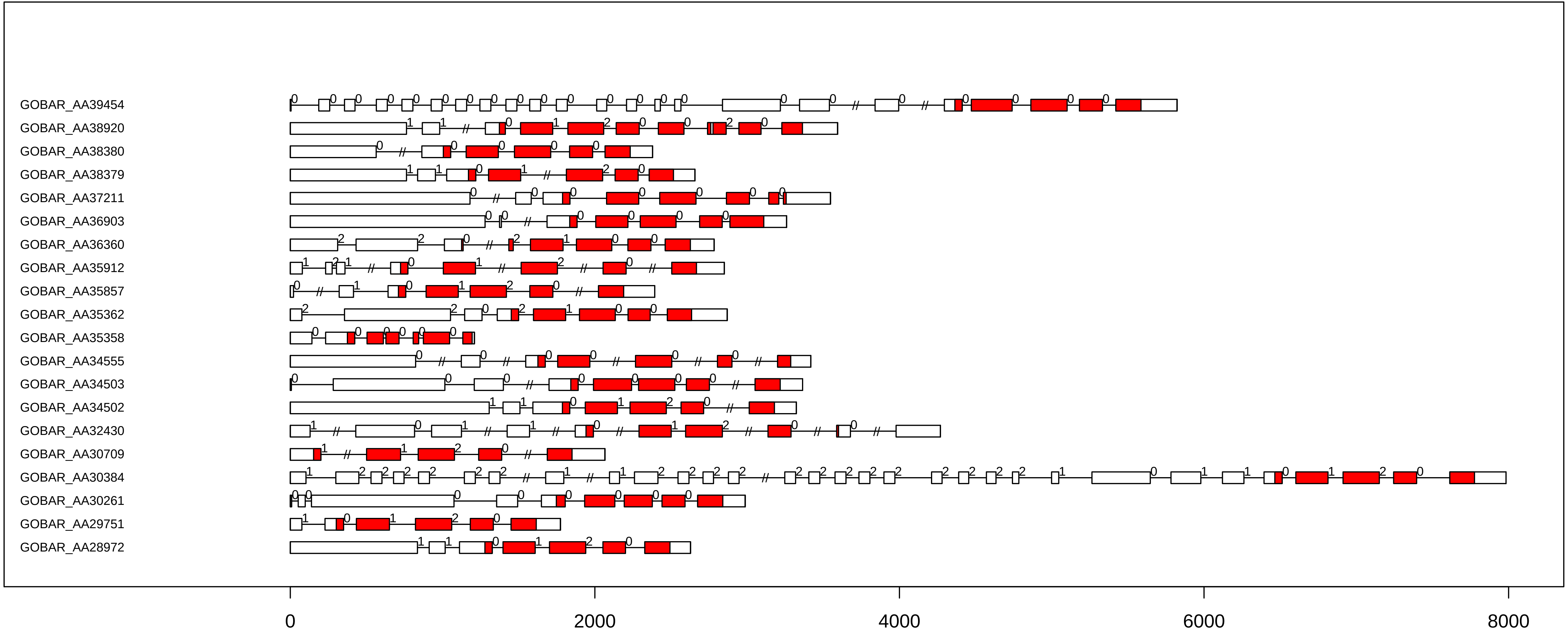

G.ba RLK−Pelle_DLSV exon−intron and domain diagram (part 6)

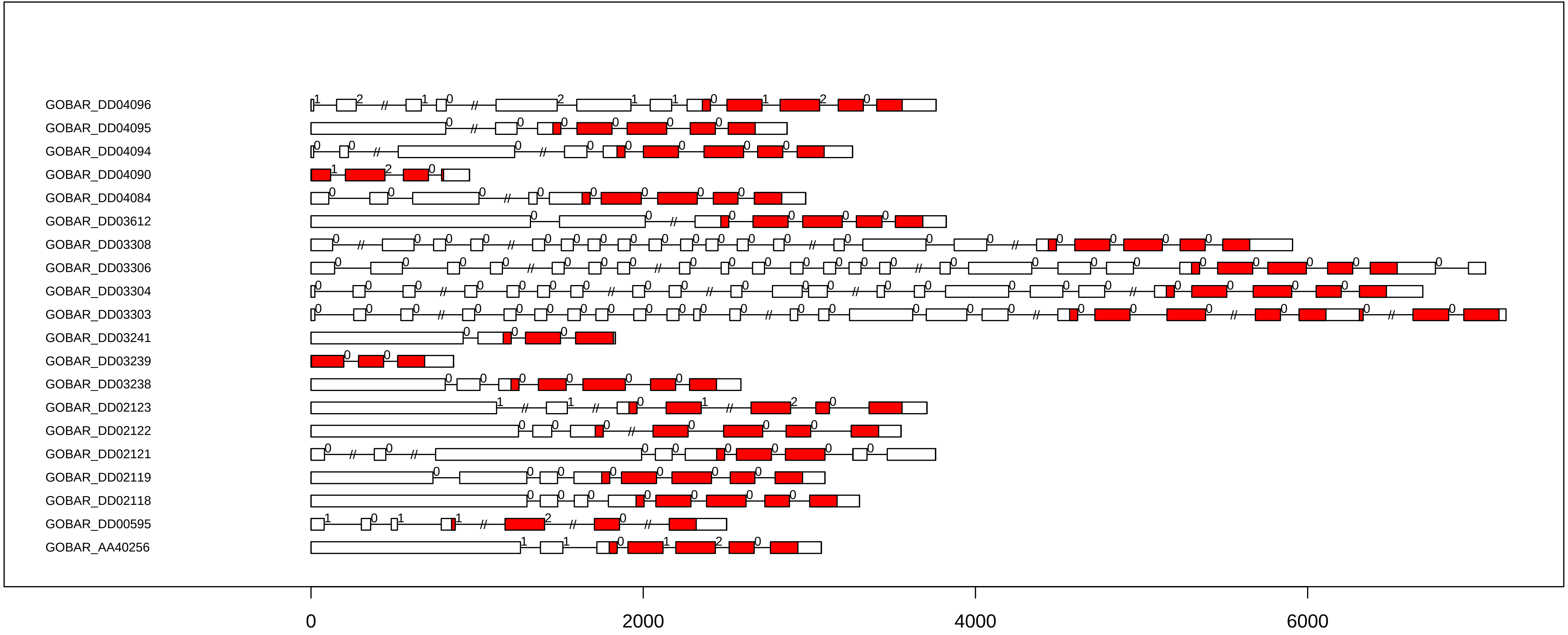

G.ba RLK−Pelle_DLSV exon−intron and domain diagram (part 7)

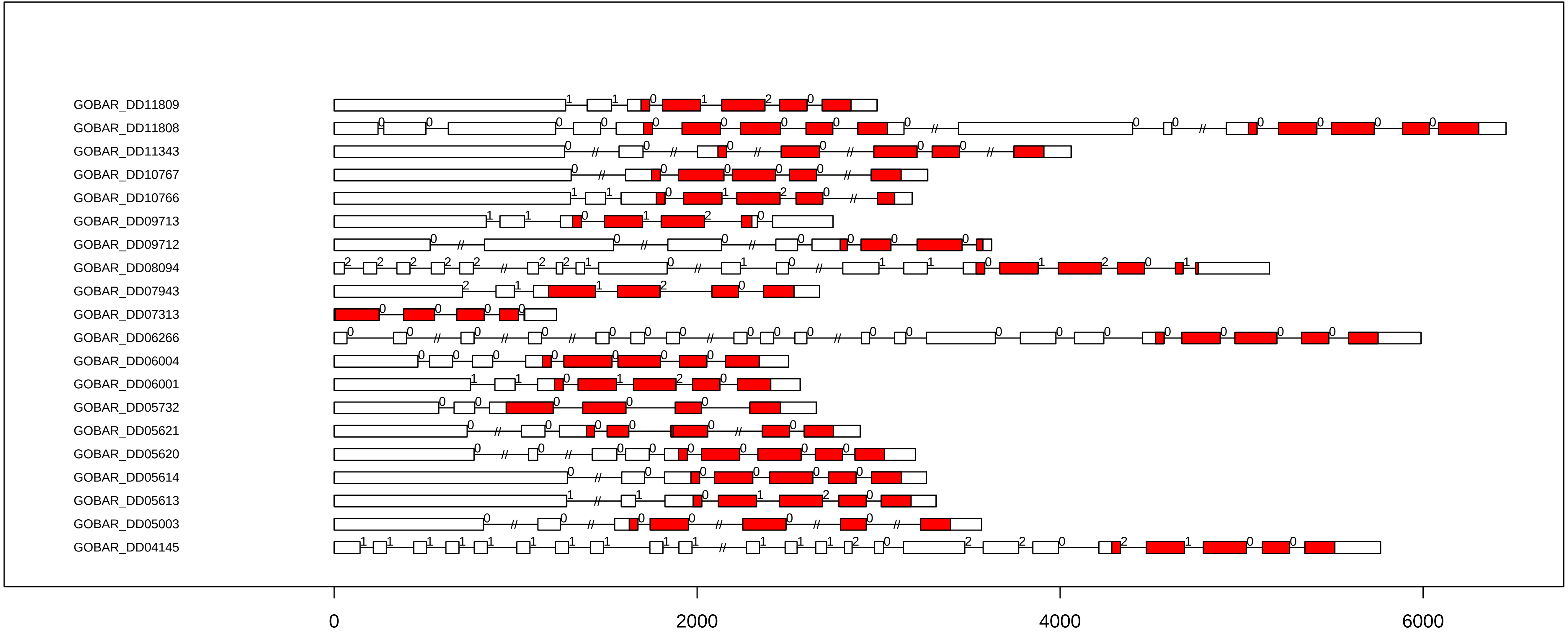

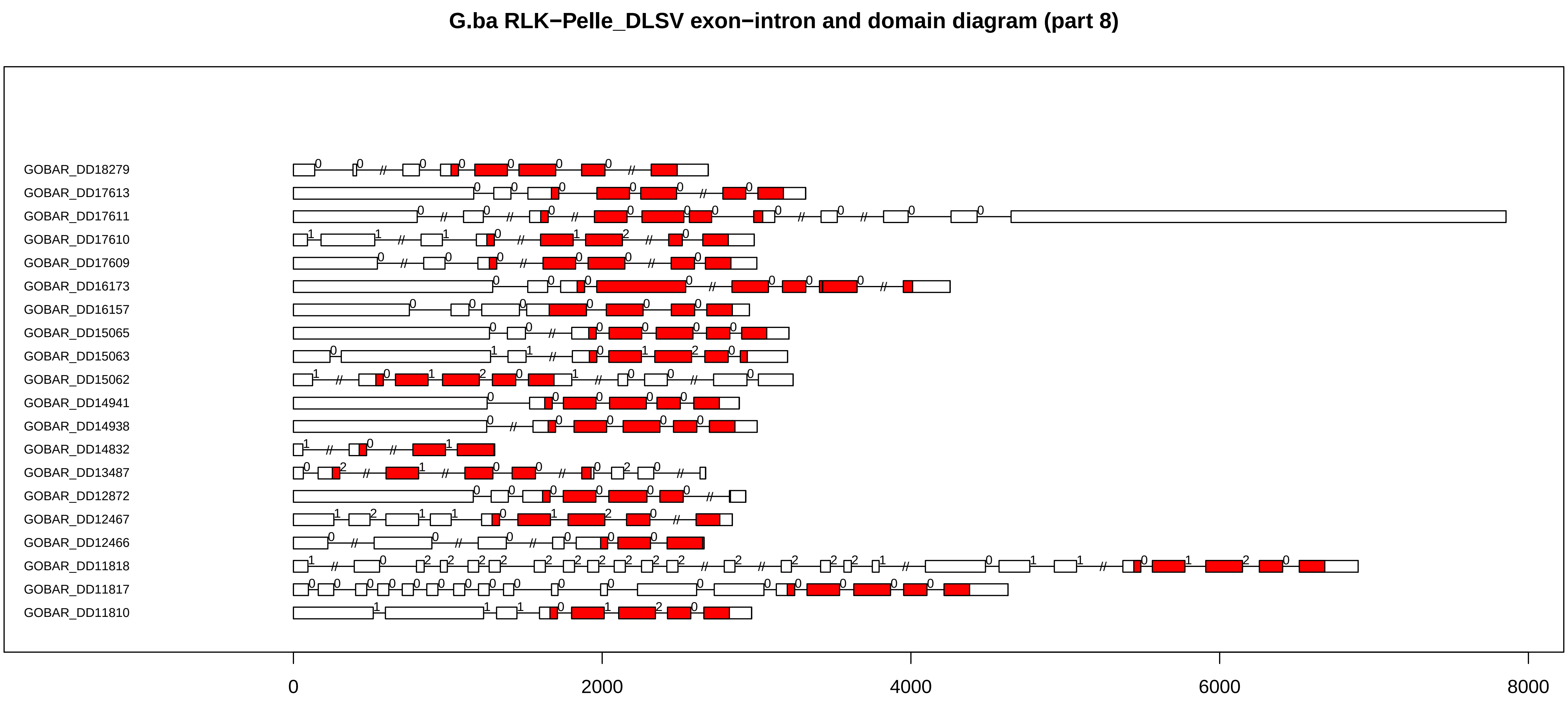
G.ba RLK−Pelle_DLSV exon−intron and domain diagram (part 8)
GOBAR_DD18279
GOBAR_DD17613
GOBAR_DD17611
GOBAR_DD17610
GOBAR_DD17609
GOBAR_DD16173
GOBAR_DD16157
GOBAR_DD15065
GOBAR_DD15063
GOBAR_DD15062
GOBAR_DD14941
GOBAR_DD14938
GOBAR_DD14832
GOBAR_DD13487
GOBAR_DD12872
GOBAR_DD12467
GOBAR_DD12466
GOBAR_DD11818
GOBAR_DD11817
GOBAR_DD11810
0
2000
4000
6000
8000

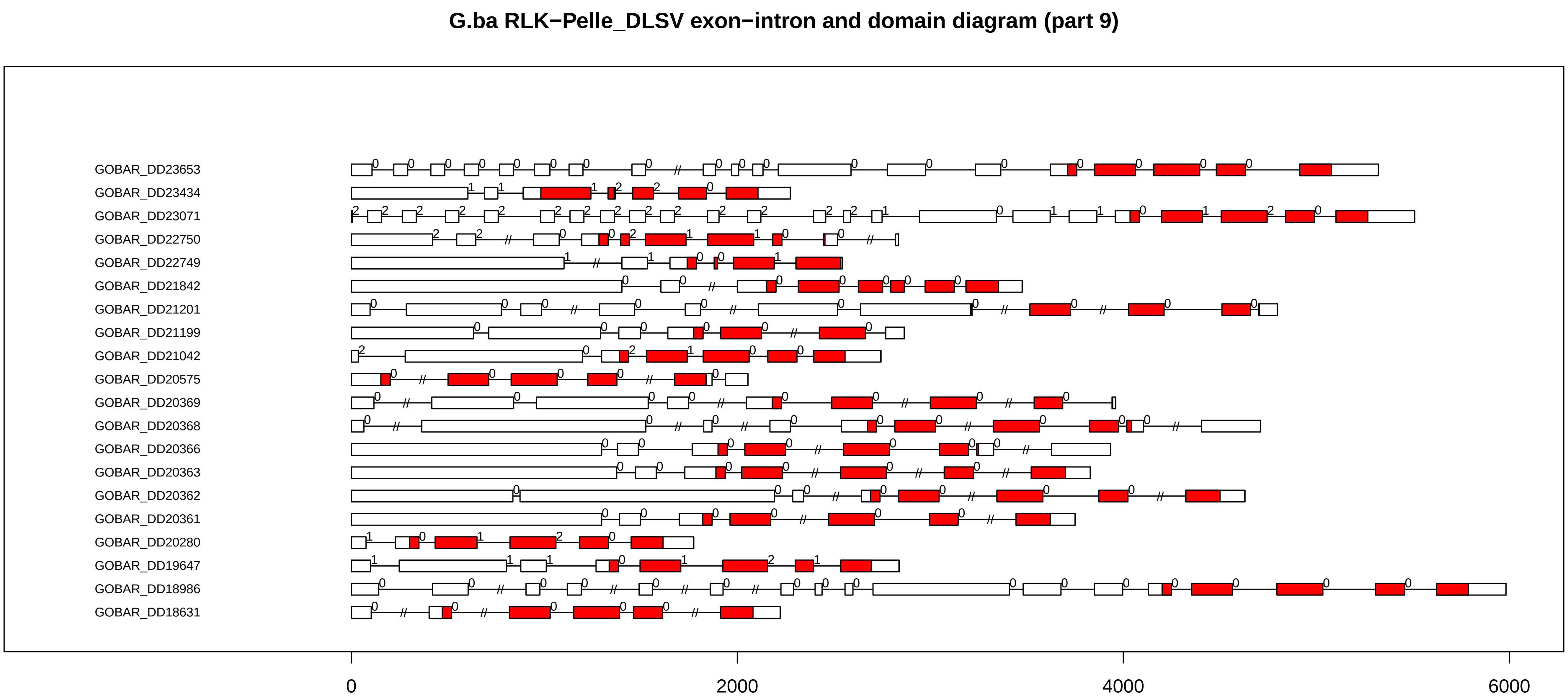
G.ba RLK−Pelle_DLSV exon−intron and domain diagram (part 9)
GOBAR_DD23653
GOBAR_DD23434
GOBAR_DD23071
GOBAR_DD22750
GOBAR_DD22749
GOBAR_DD21842
GOBAR_DD21201
GOBAR_DD21199
GOBAR_DD21042
GOBAR_DD20575
GOBAR_DD20369
GOBAR_DD20368
GOBAR_DD20366
GOBAR_DD20363
GOBAR_DD20362
GOBAR_DD20361
GOBAR_DD20280
GOBAR_DD19647
GOBAR_DD18986
GOBAR_DD18631
0
2000
4000
6000

G.ba RLK−Pelle_DLSV exon−intron and domain diagram (part 10)

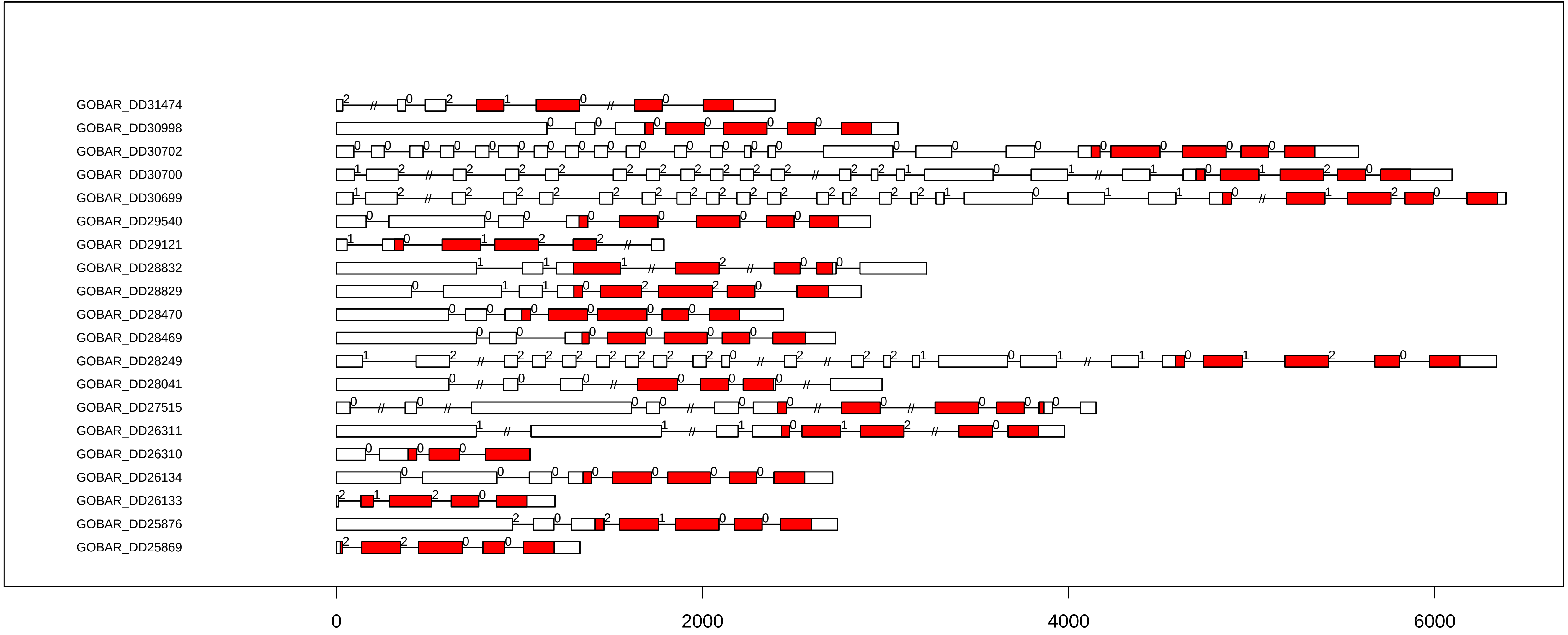

G.ba RLK−Pelle_DLSV exon−intron and domain diagram (part 11)

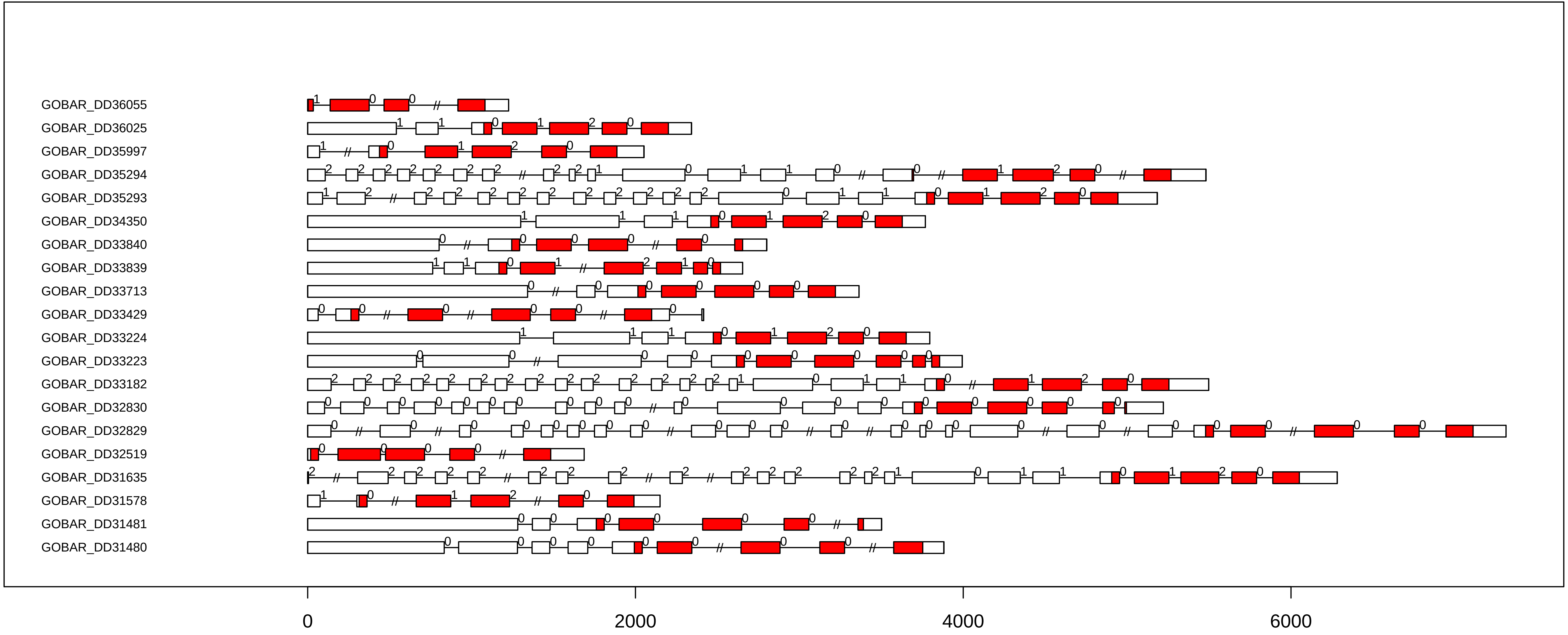

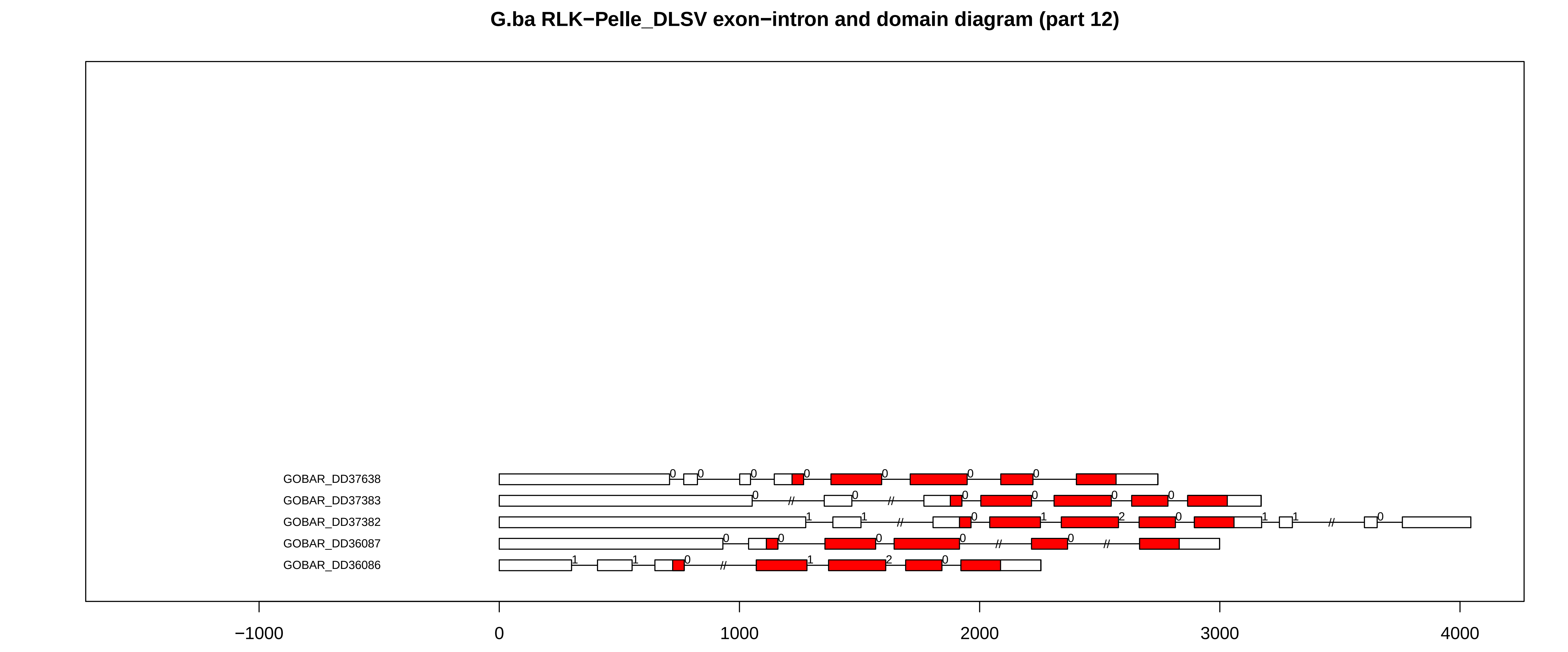

G.ba RLK−Pelle_DLSV exon−intron and domain diagram (part 12)
GOBAR_DD37638
GOBAR_DD37383
GOBAR_DD37382
GOBAR_DD36087
GOBAR_DD36086
−1000
0
1000
2000
3000
4000

G.ba RLK−Pelle_Extensin exon−intron and domain diagram (all)

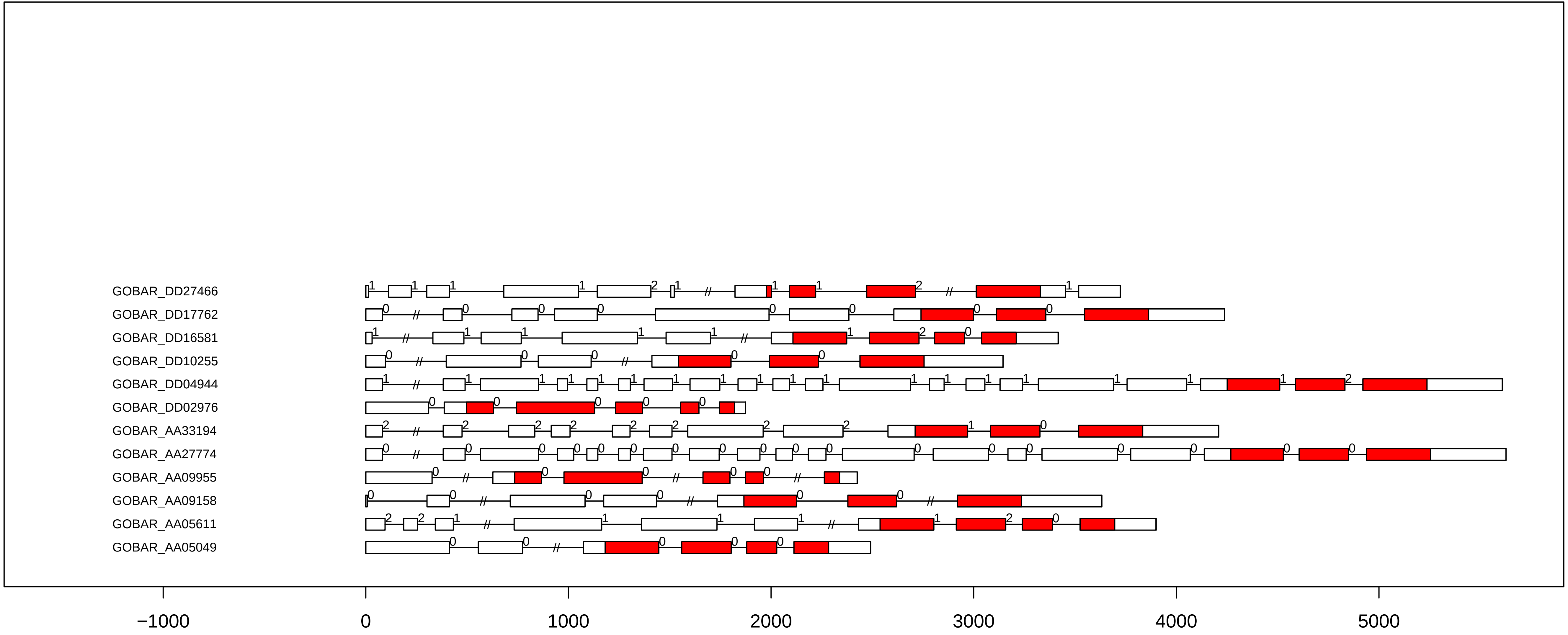

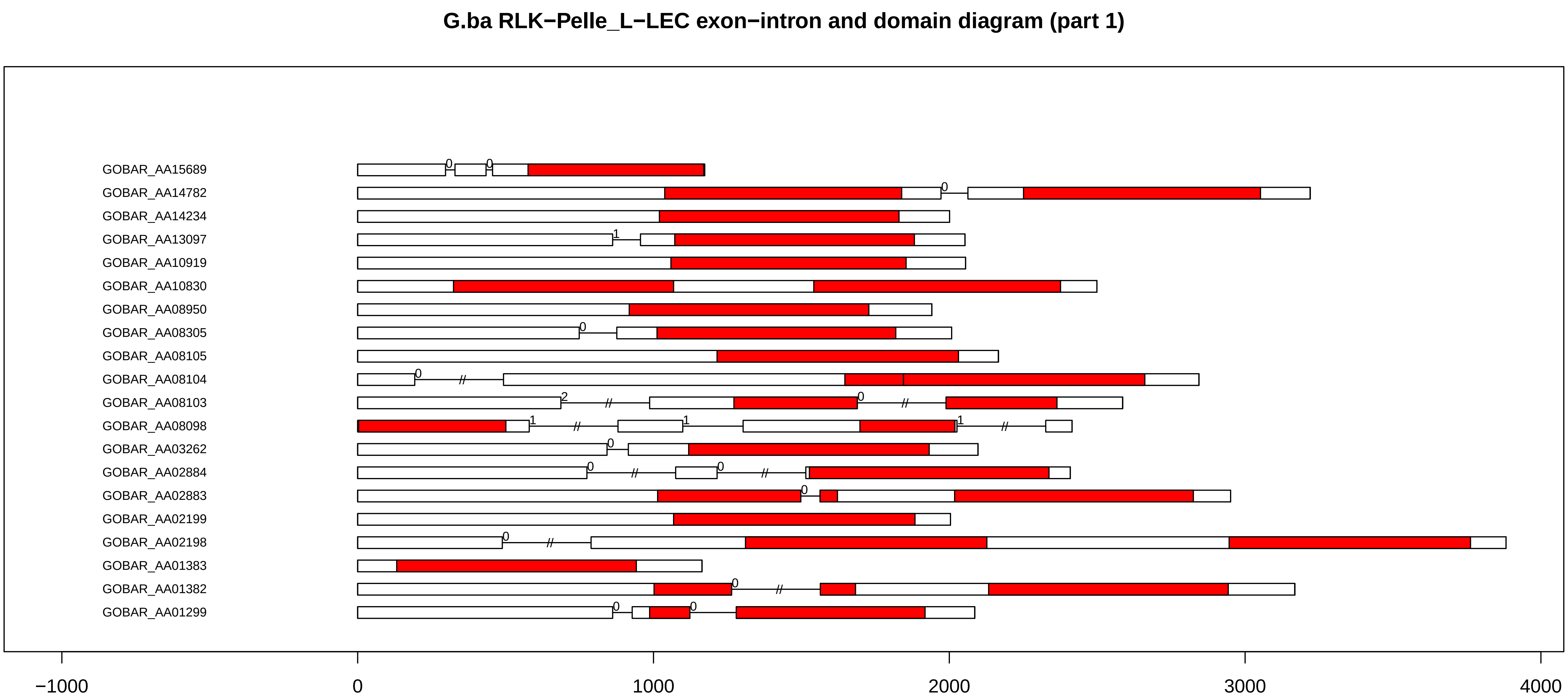
G.ba RLK−Pelle_L−LEC exon−intron and domain diagram (part 1)
GOBAR_AA15689
GOBAR_AA14782
GOBAR_AA14234
GOBAR_AA13097
GOBAR_AA10919
GOBAR_AA10830
GOBAR_AA08950
GOBAR_AA08305
GOBAR_AA08105
GOBAR_AA08104
GOBAR_AA08103
GOBAR_AA08098
GOBAR_AA03262
GOBAR_AA02884
GOBAR_AA02883
GOBAR_AA02199
GOBAR_AA02198
GOBAR_AA01383
GOBAR_AA01382
GOBAR_AA01299
−1000
0
1000
2000
3000
4000

G.ba RLK−Pelle_L−LEC exon−intron and domain diagram (part 2)

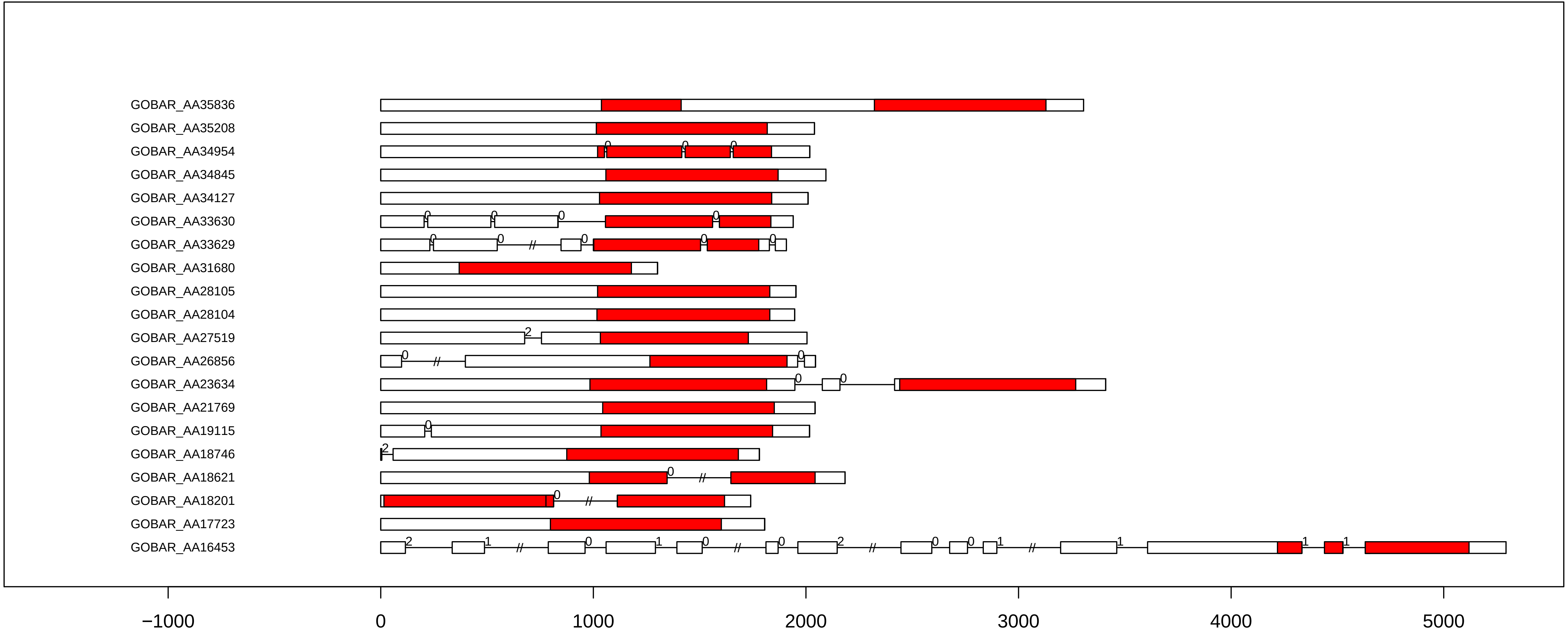

G.ba RLK−Pelle_L−LEC exon−intron and domain diagram (part 3)

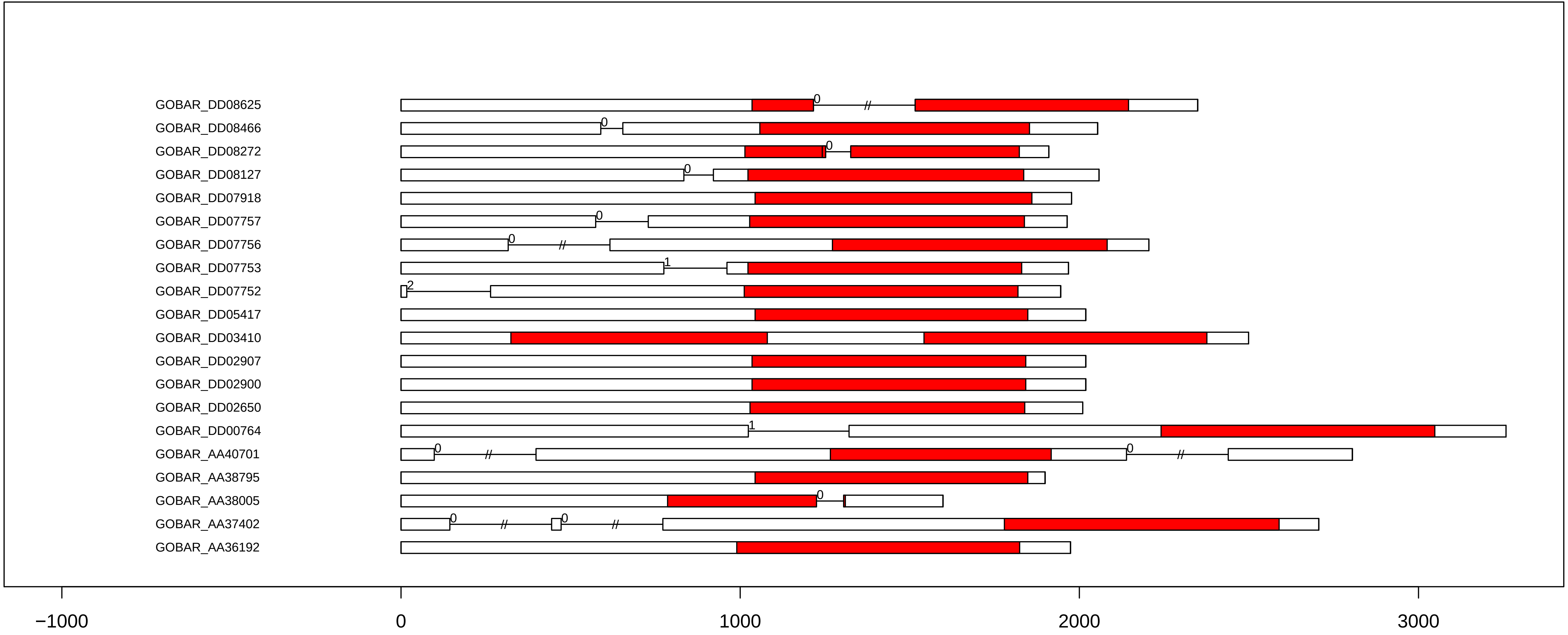

G.ba RLK−Pelle_L−LEC exon−intron and domain diagram (part 4)

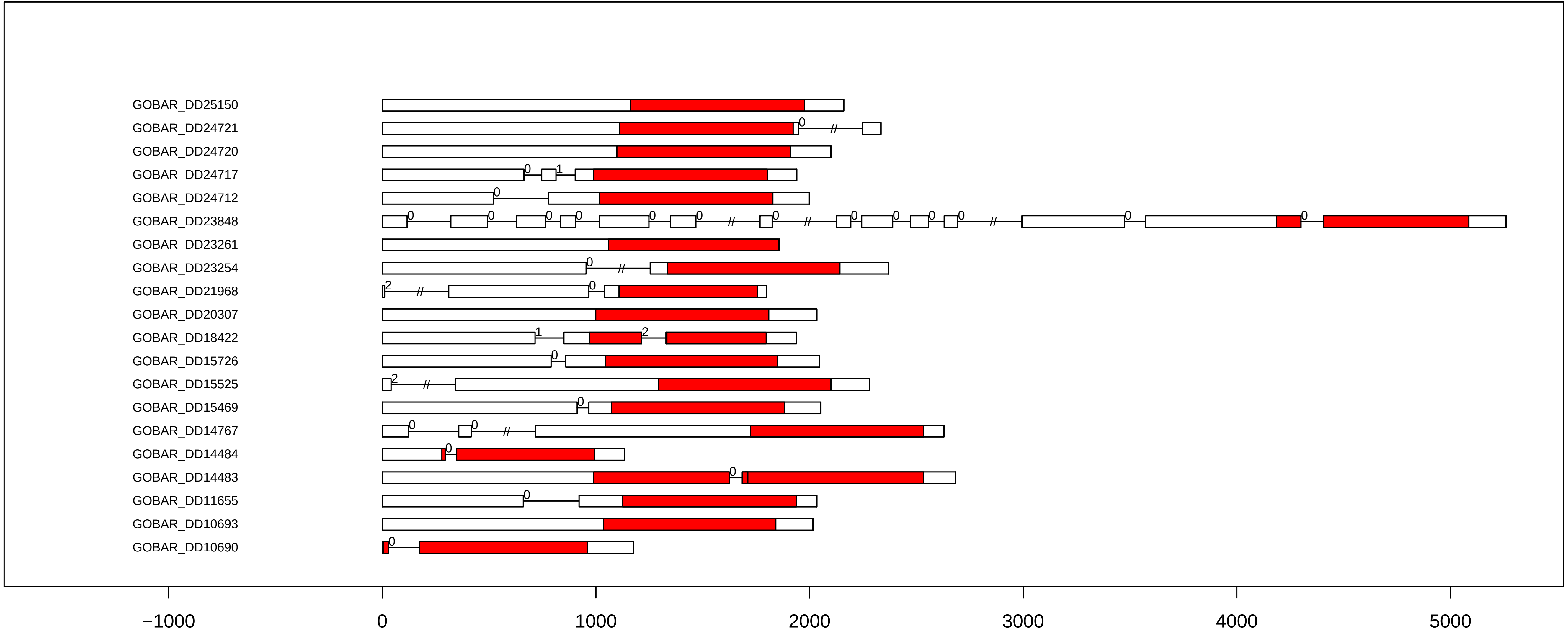

G.ba RLK−Pelle_L−LEC exon−intron and domain diagram (part 5)

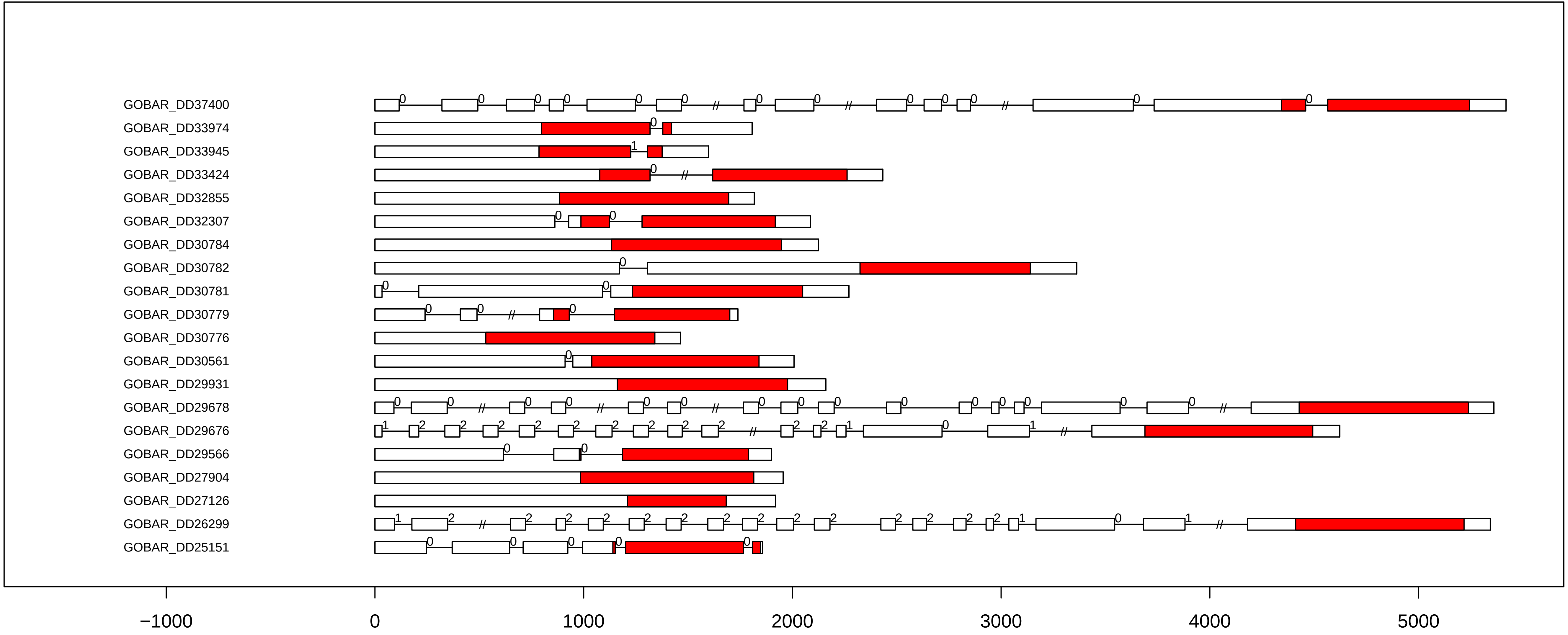

G.ba RLK−Pelle_L−LEC exon−intron and domain diagram (part 6)

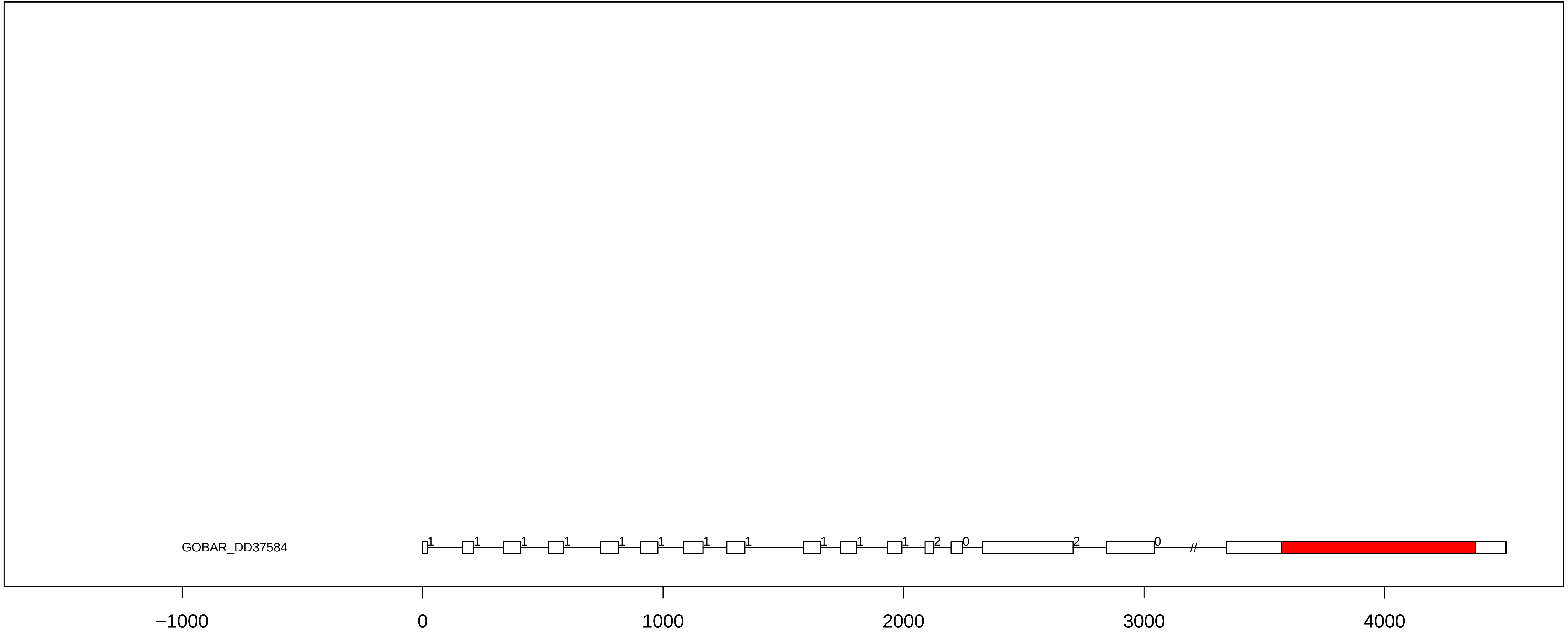

G.ba RLK−Pelle_LRK10L−2 exon−intron and domain diagram (part 1)

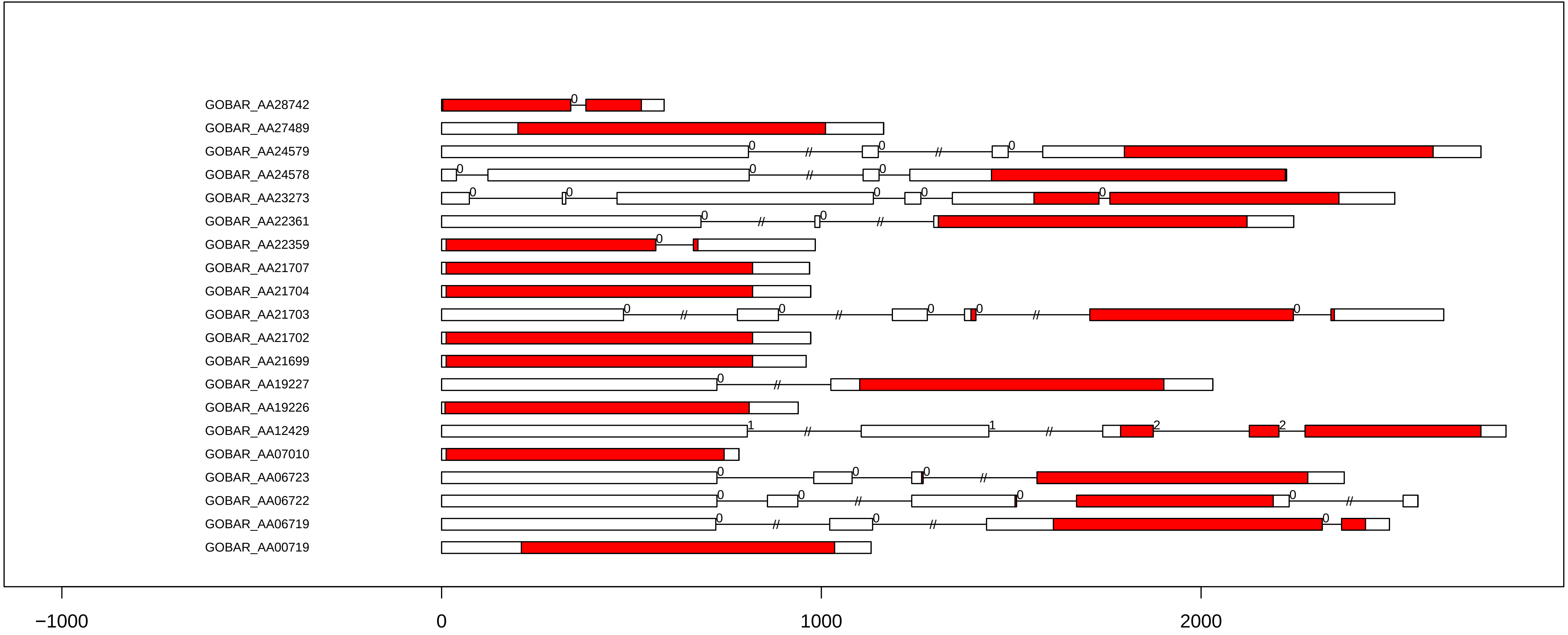

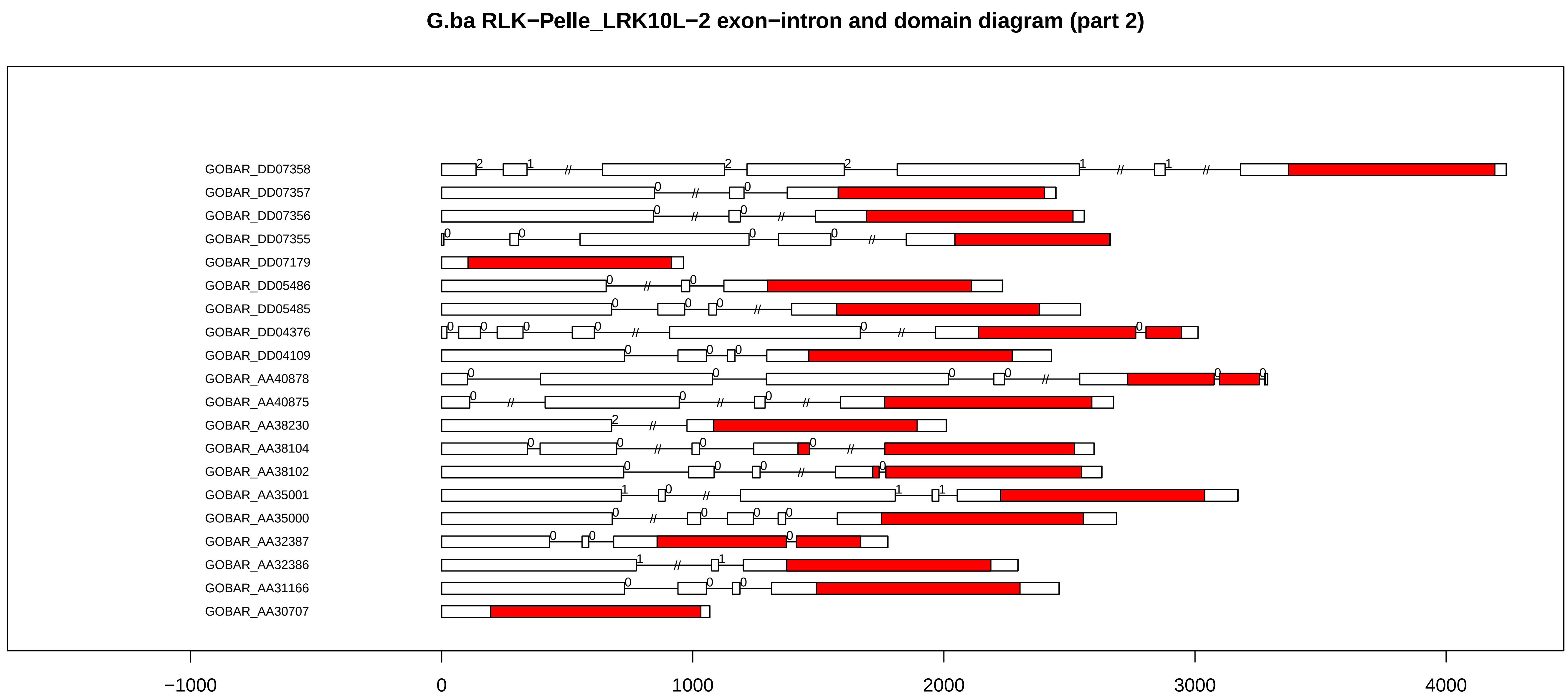
G.ba RLK−Pelle_LRK10L−2 exon−intron and domain diagram (part 2)
GOBAR_DD07358
GOBAR_DD07357
GOBAR_DD07356
GOBAR_DD07355
GOBAR_DD07179
GOBAR_DD05486
GOBAR_DD05485
GOBAR_DD04376
GOBAR_DD04109
GOBAR_AA40878
GOBAR_AA40875
GOBAR_AA38230
GOBAR_AA38104
GOBAR_AA38102
GOBAR_AA35001
GOBAR_AA35000
GOBAR_AA32387
GOBAR_AA32386
GOBAR_AA31166
GOBAR_AA30707
−1000
0
1000
2000
3000
4000

G.ba RLK−Pelle_LRK10L−2 exon−intron and domain diagram (part 3)

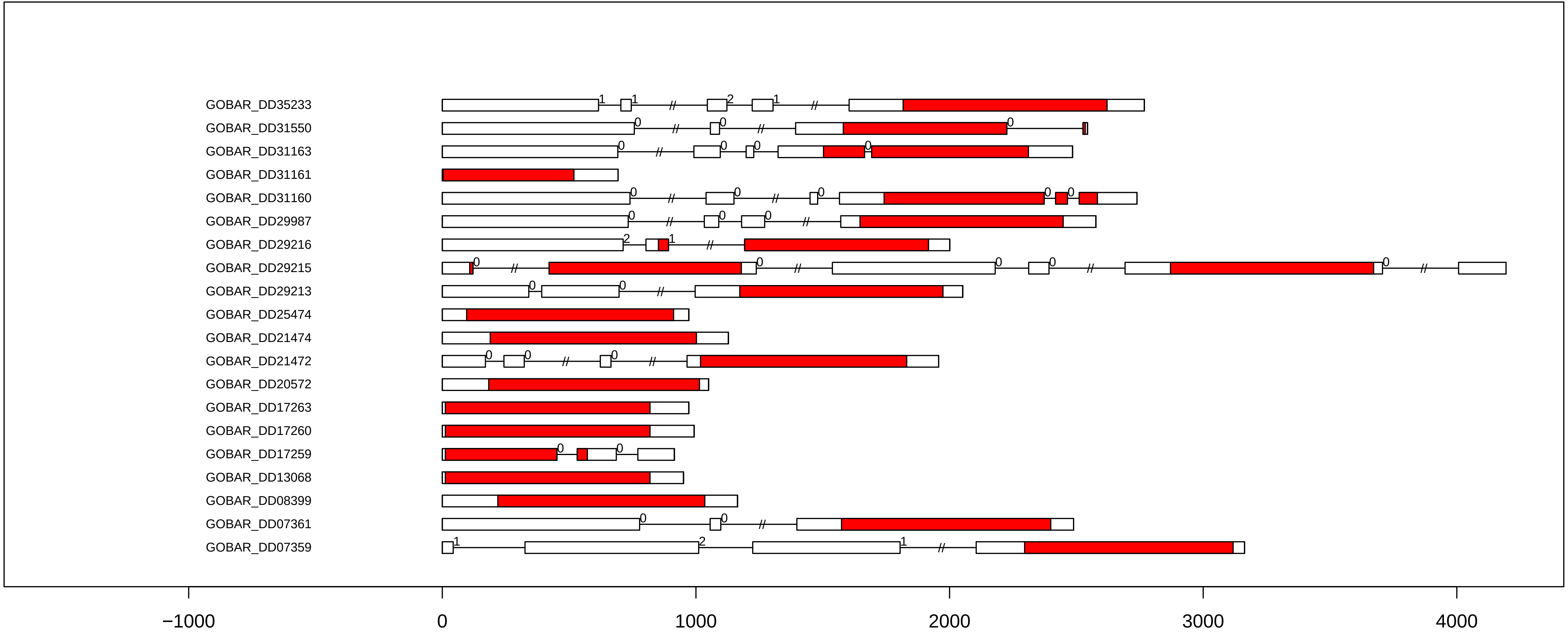

G.ba RLK−Pelle_LRK10L−2 exon−intron and domain diagram (part 4)

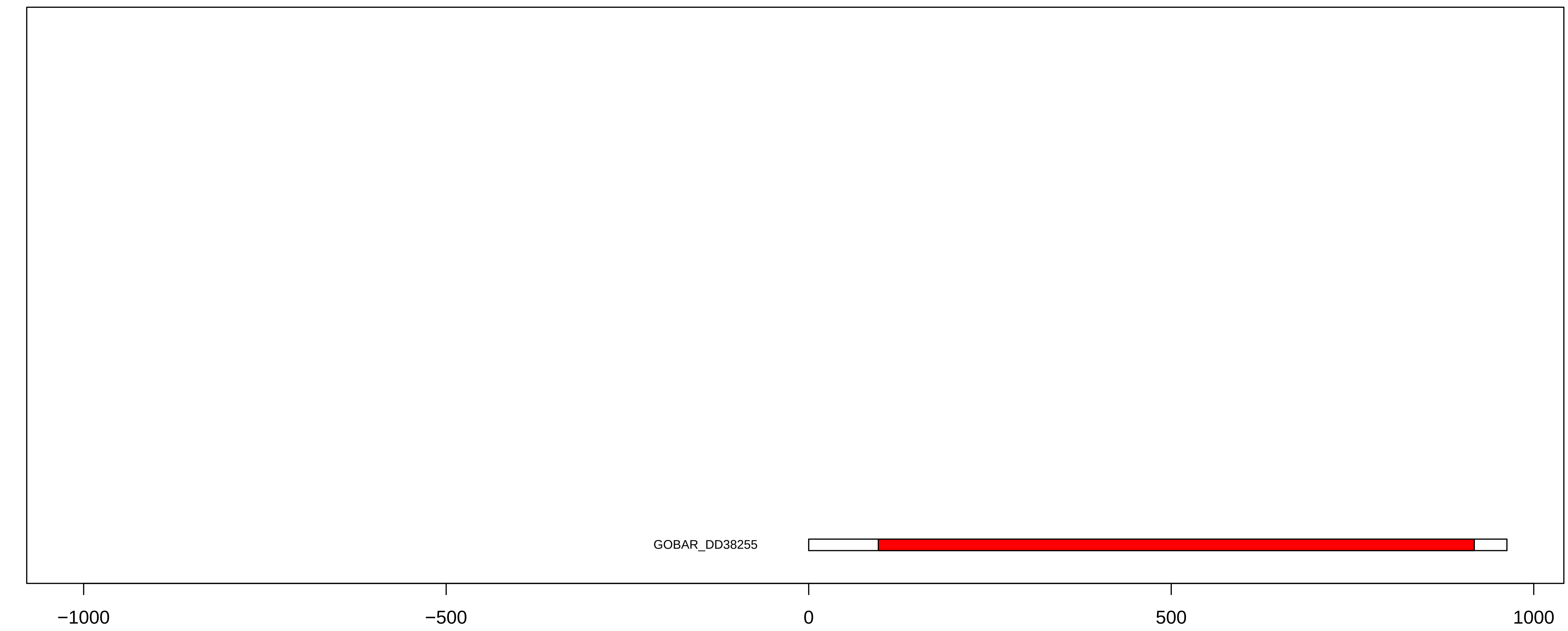

G.ba RLK−Pelle_LRR−I−1 exon−intron and domain diagram (all)

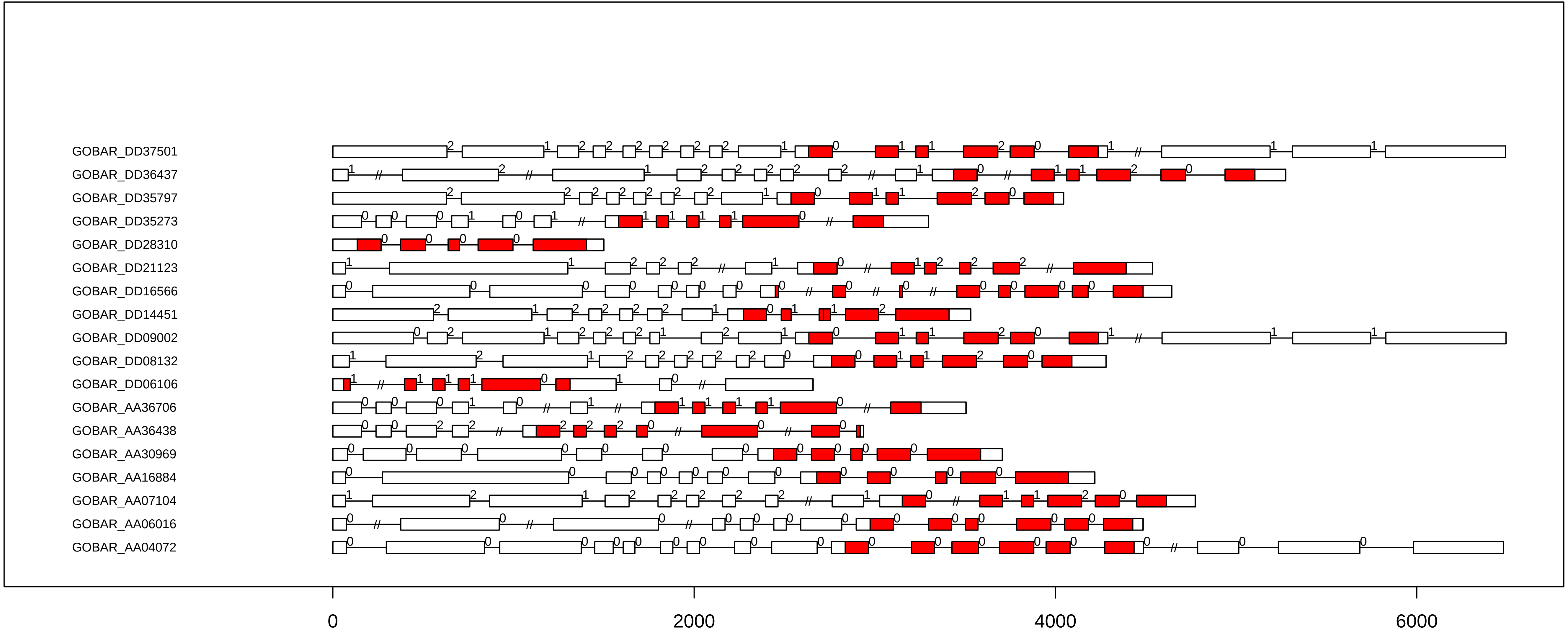

G.ba RLK−Pelle_LRR−I−2 exon−intron and domain diagram (all)

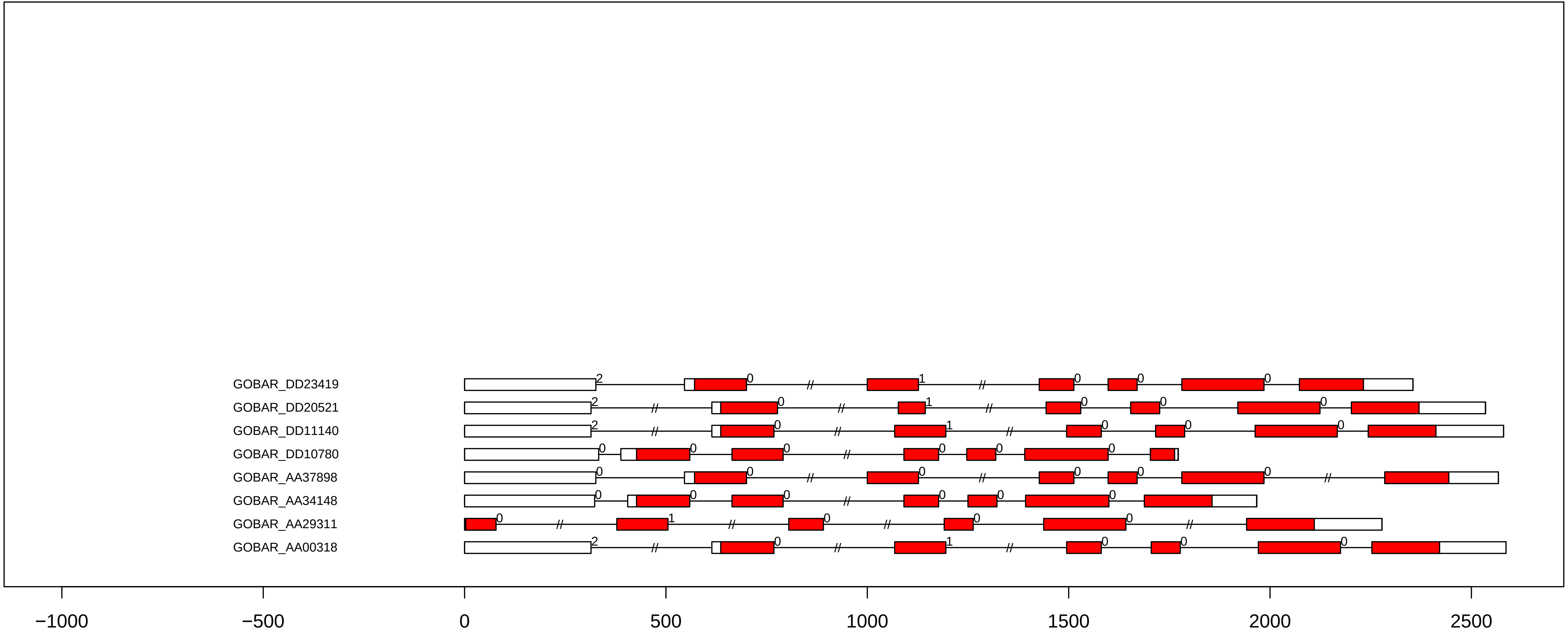

G.ba RLK−Pelle_LRR−III exon−intron and domain diagram (part 1)

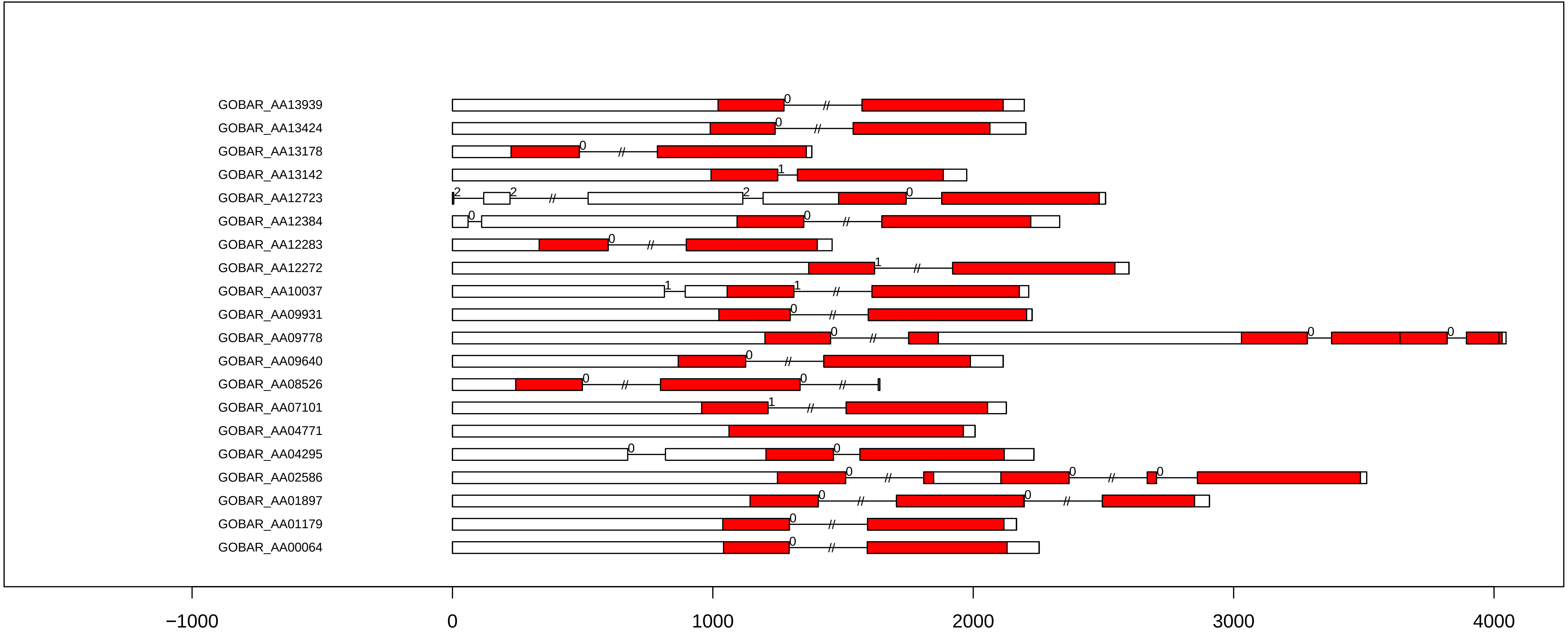

G.ba RLK−Pelle_LRR−III exon−intron and domain diagram (part 2)

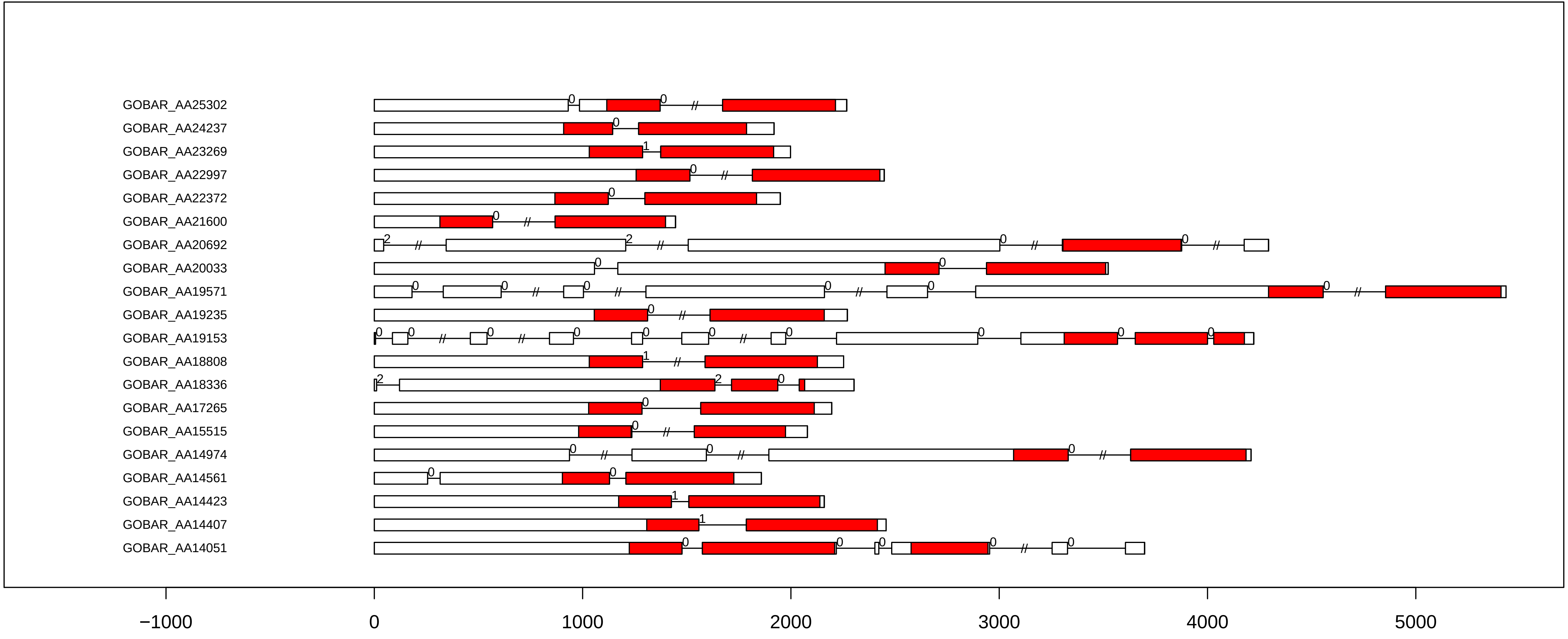

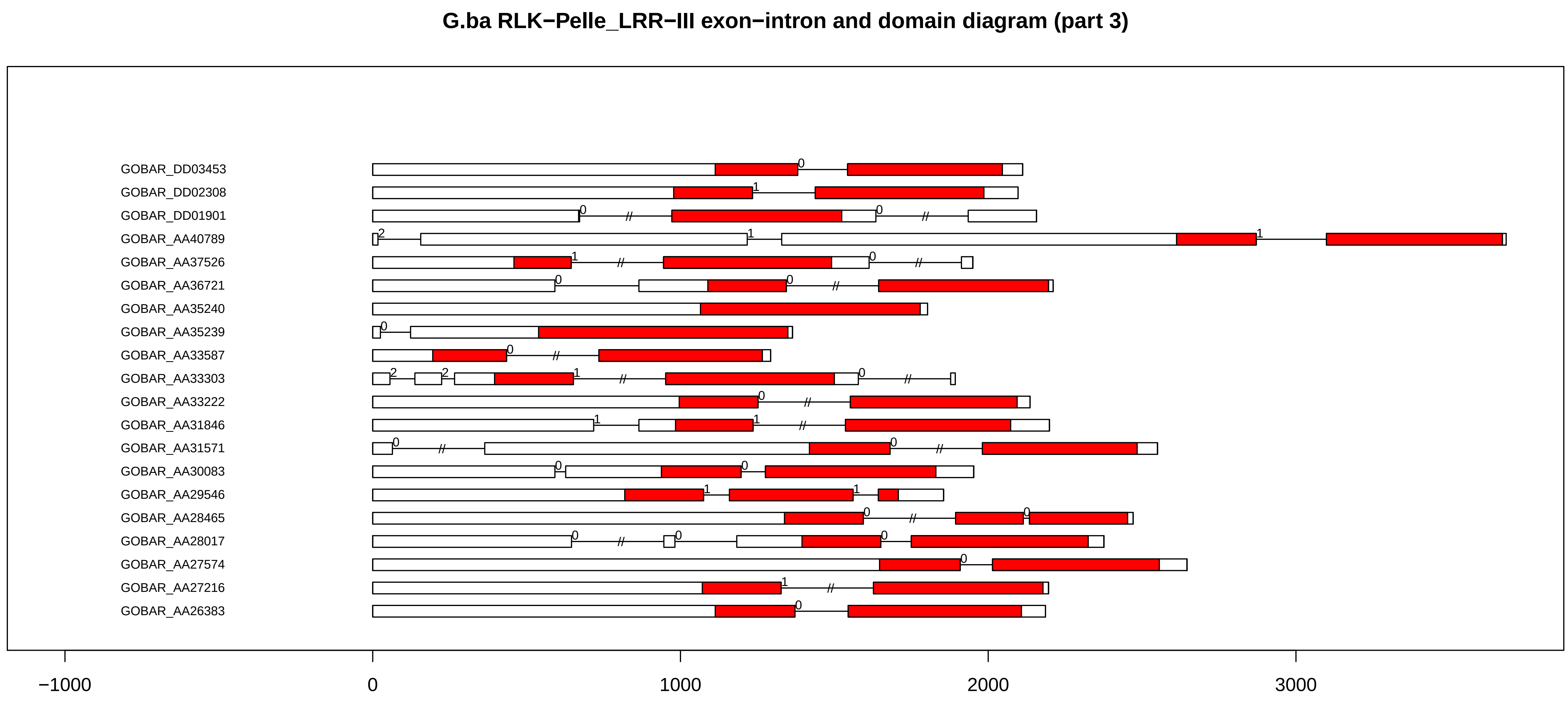
G.ba RLK−Pelle_LRR−III exon−intron and domain diagram (part 3)
GOBAR_DD03453
GOBAR_DD02308
GOBAR_DD01901
GOBAR_AA40789
GOBAR_AA37526
GOBAR_AA36721
GOBAR_AA35240
GOBAR_AA35239
GOBAR_AA33587
GOBAR_AA33303
GOBAR_AA33222
GOBAR_AA31846
GOBAR_AA31571
GOBAR_AA30083
GOBAR_AA29546
GOBAR_AA28465
GOBAR_AA28017
GOBAR_AA27574
GOBAR_AA27216
GOBAR_AA26383
−1000
0
1000
2000
3000

G.ba RLK−Pelle_LRR−III exon−intron and domain diagram (part 4)

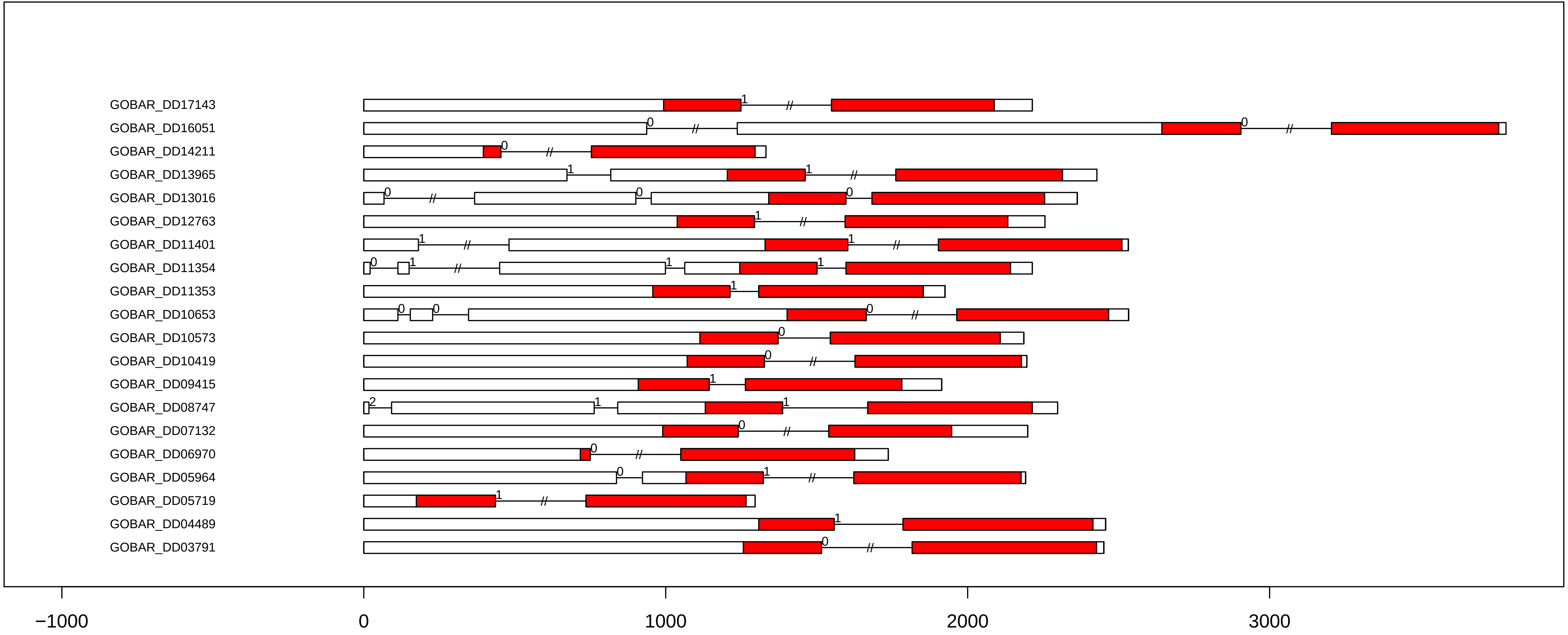

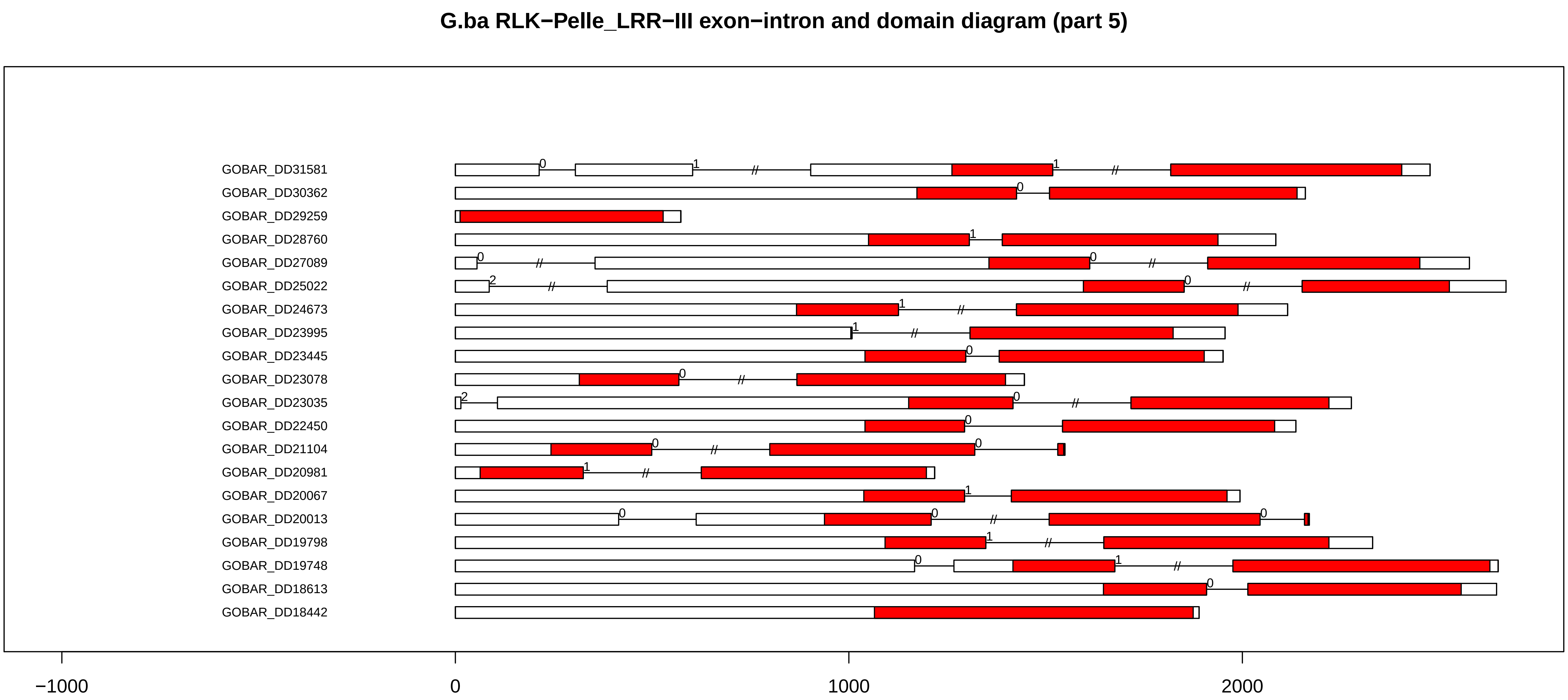

G.ba RLK−Pelle_LRR−III exon−intron and domain diagram (part 5)
GOBAR_DD31581
GOBAR_DD30362
GOBAR_DD29259
GOBAR_DD28760
GOBAR_DD27089
GOBAR_DD25022
GOBAR_DD24673
GOBAR_DD23995
GOBAR_DD23445
GOBAR_DD23078
GOBAR_DD23035
GOBAR_DD22450
GOBAR_DD21104
GOBAR_DD20981
GOBAR_DD20067
GOBAR_DD20013
GOBAR_DD19798
GOBAR_DD19748
GOBAR_DD18613
GOBAR_DD18442
−1000
0
1000
2000

G.ba RLK−Pelle_LRR−III exon−intron and domain diagram (part 6)

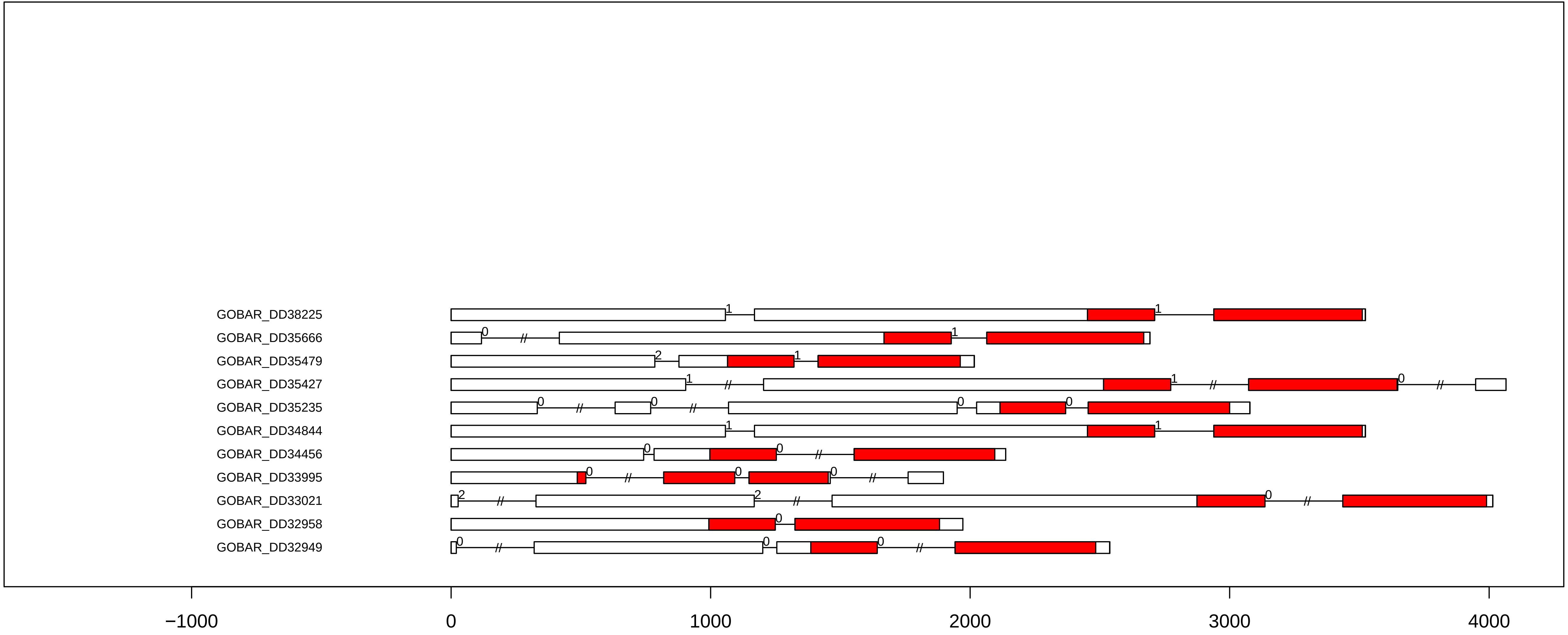

G.ba RLK−Pelle_LRR−II exon−intron and domain diagram (part 1)

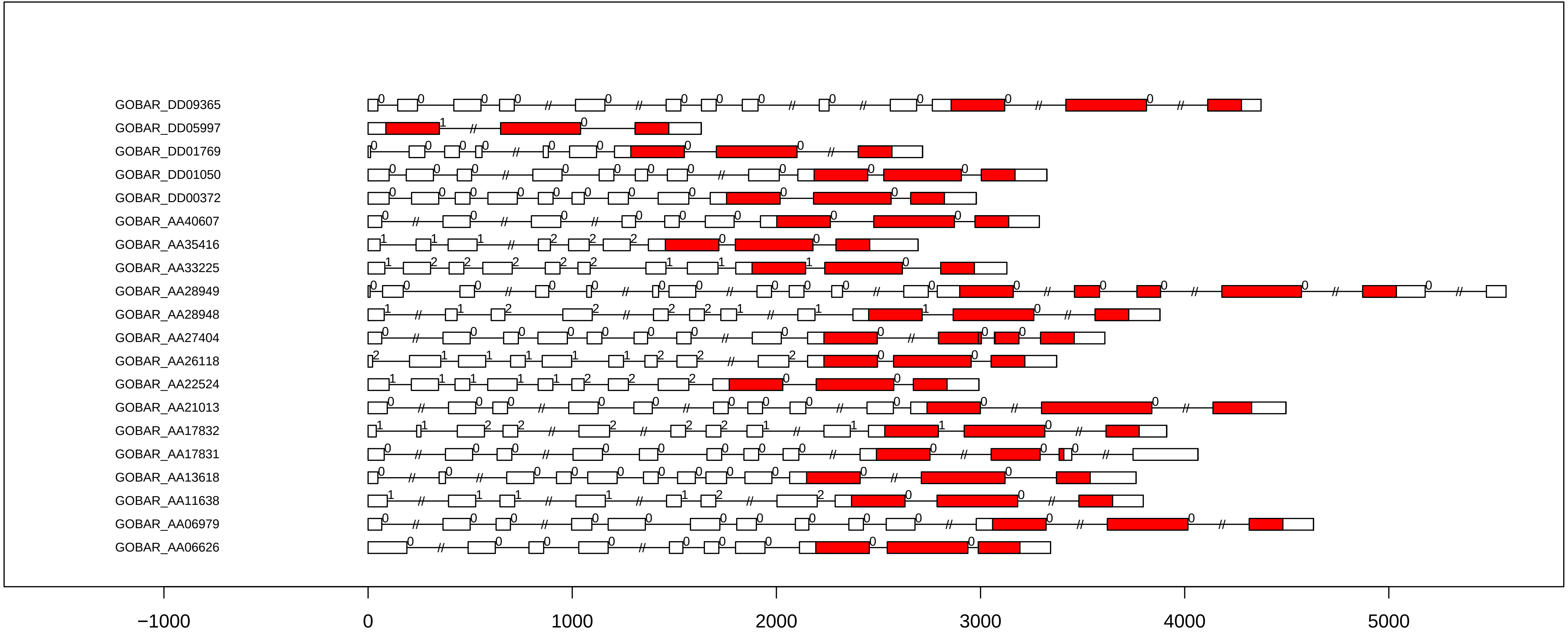

G.ba RLK−Pelle_LRR−II exon−intron and domain diagram (part 2)

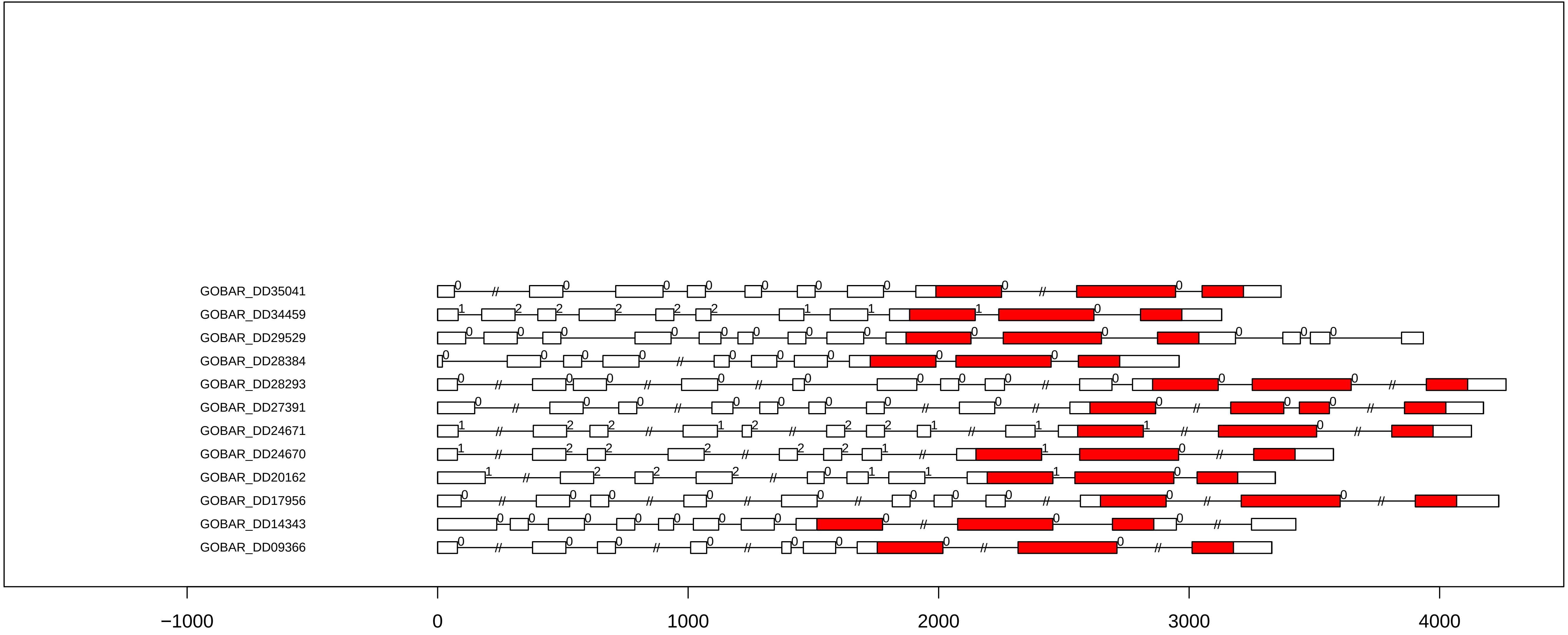

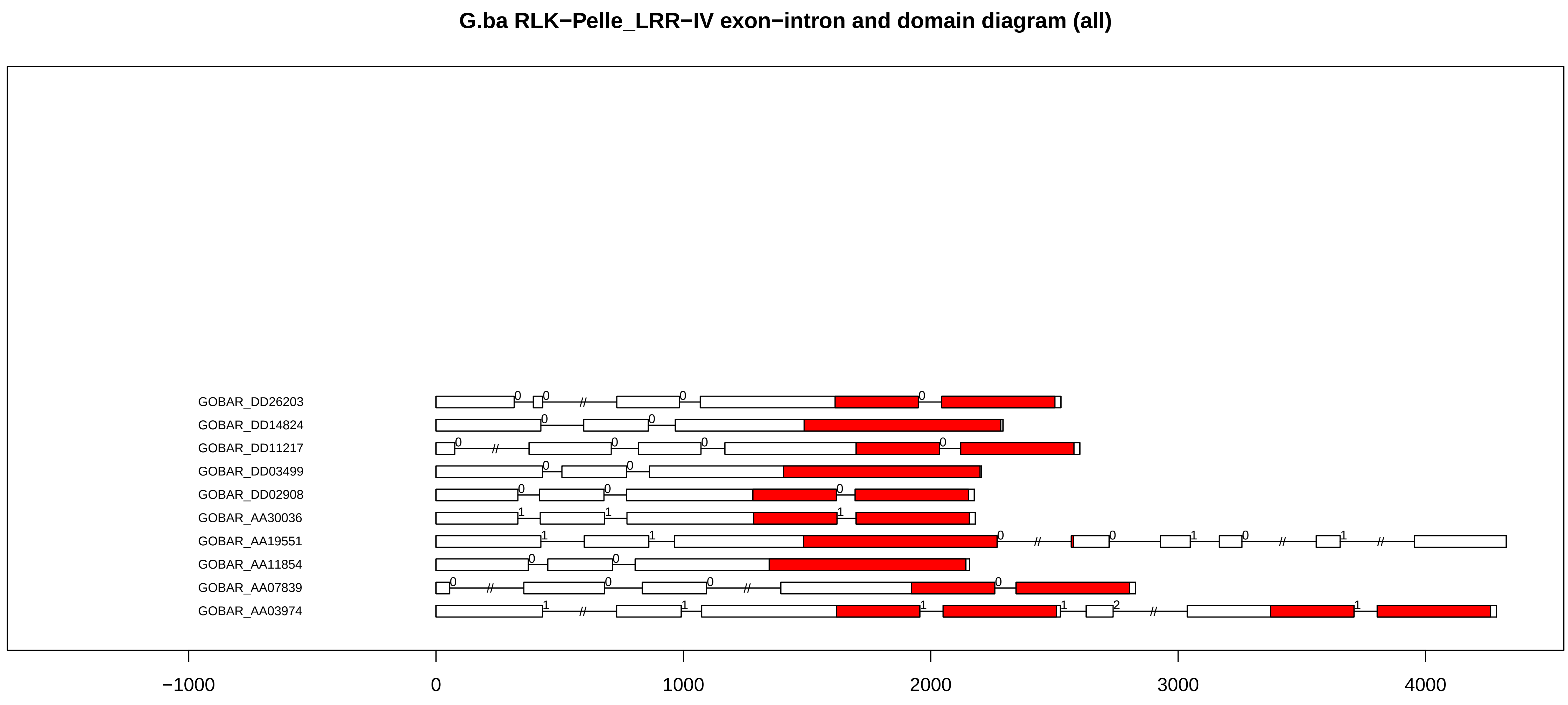
G.ba RLK−Pelle_LRR−IV exon−intron and domain diagram (all)
GOBAR_DD26203
GOBAR_DD14824
GOBAR_DD11217
GOBAR_DD03499
GOBAR_DD02908
GOBAR_AA30036
GOBAR_AA19551
GOBAR_AA11854
GOBAR_AA07839
GOBAR_AA03974
−1000
0
1000
2000
3000
4000

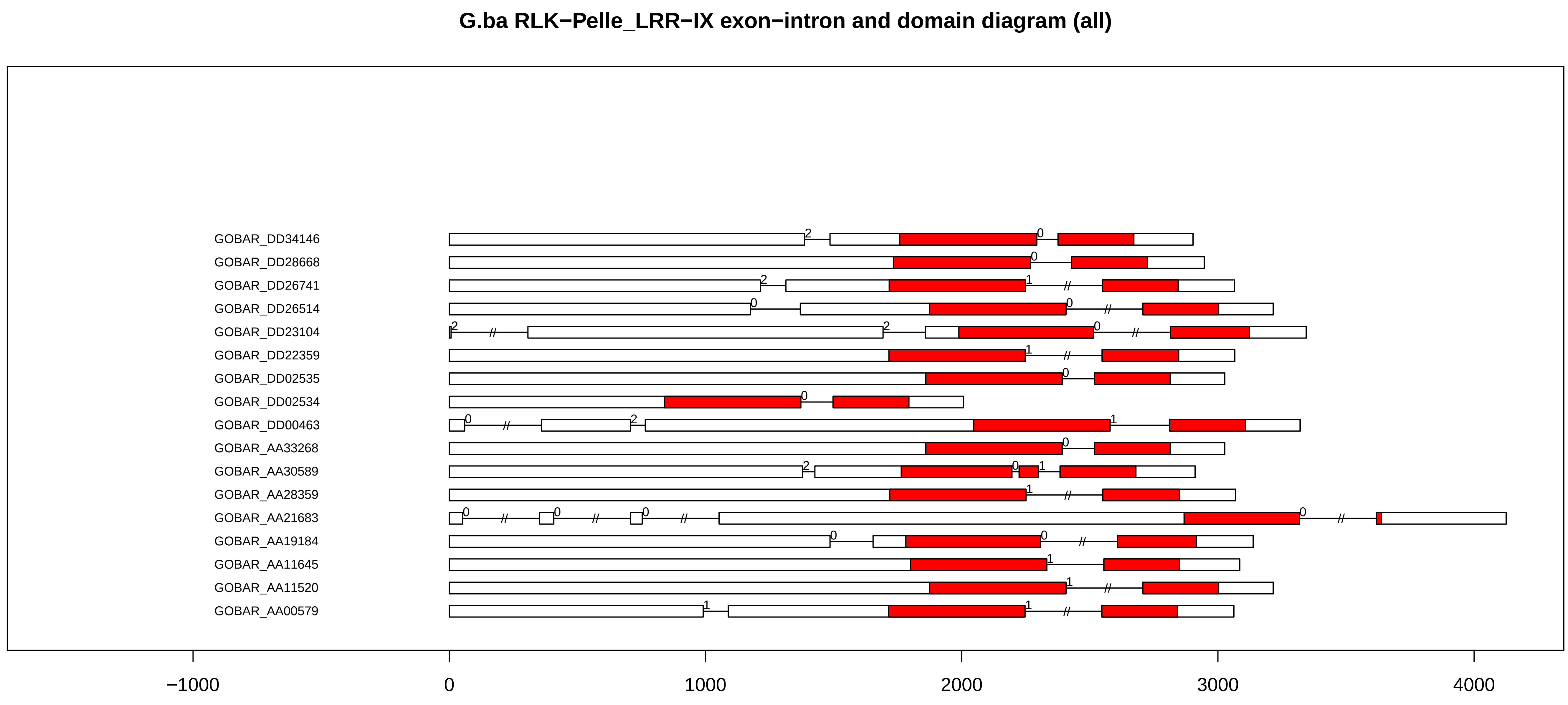
G.ba RLK−Pelle_LRR−IX exon−intron and domain diagram (all)
GOBAR_DD34146
GOBAR_DD28668
GOBAR_DD26741
GOBAR_DD26514
GOBAR_DD23104
GOBAR_DD22359
GOBAR_DD02535
GOBAR_DD02534
GOBAR_DD00463
GOBAR_AA33268
GOBAR_AA30589
GOBAR_AA28359
GOBAR_AA21683
GOBAR_AA19184
GOBAR_AA11645
GOBAR_AA11520
GOBAR_AA00579
−1000
0
1000
2000
3000
4000

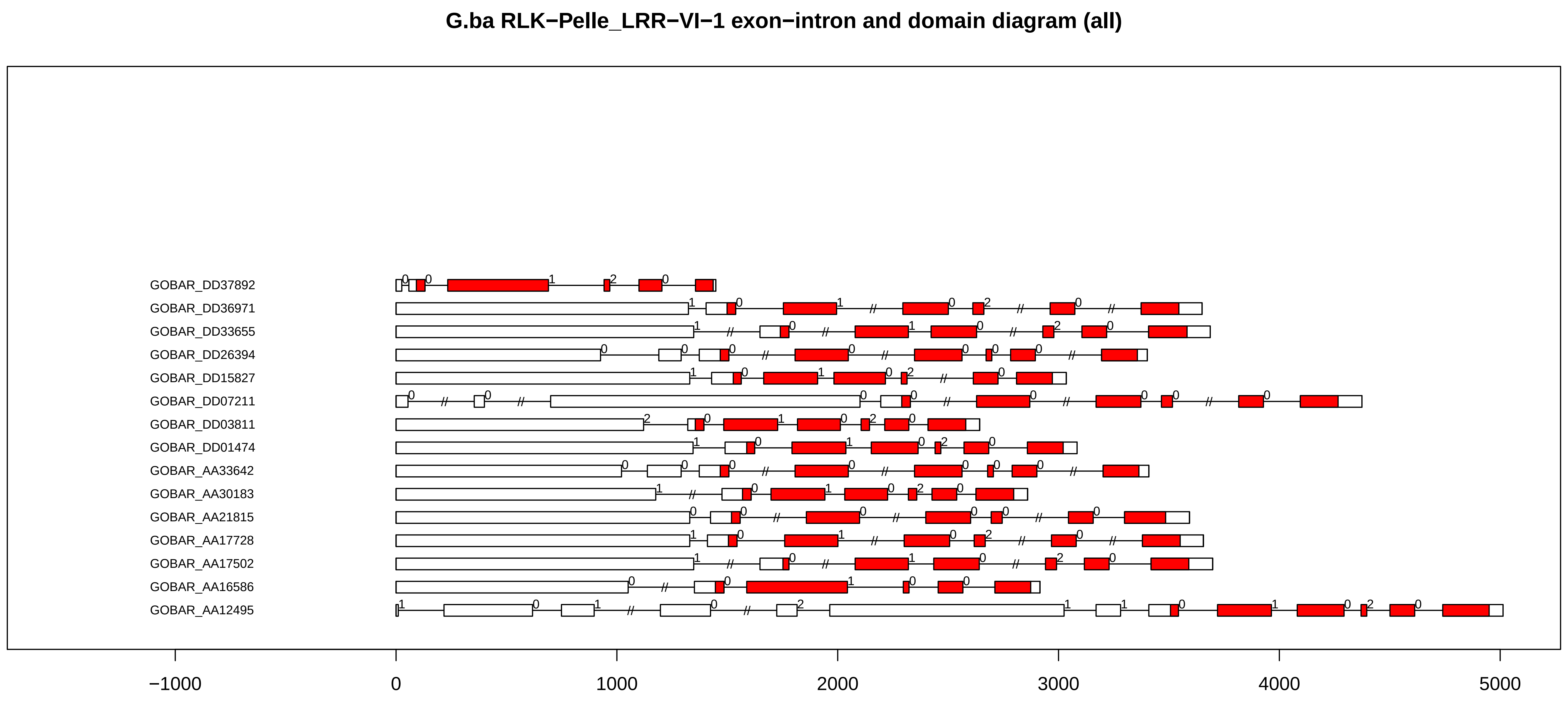
G.ba RLK−Pelle_LRR−VI−1 exon−intron and domain diagram (all)
GOBAR_DD37892
GOBAR_DD36971
GOBAR_DD33655
GOBAR_DD26394
GOBAR_DD15827
GOBAR_DD07211
GOBAR_DD03811
GOBAR_DD01474
GOBAR_AA33642
GOBAR_AA30183
GOBAR_AA21815
GOBAR_AA17728
GOBAR_AA17502
GOBAR_AA16586
GOBAR_AA12495
−1000
0
1000
2000
3000
4000
5000

G.ba RLK−Pelle_LRR−VI−2 exon−intron and domain diagram (all)

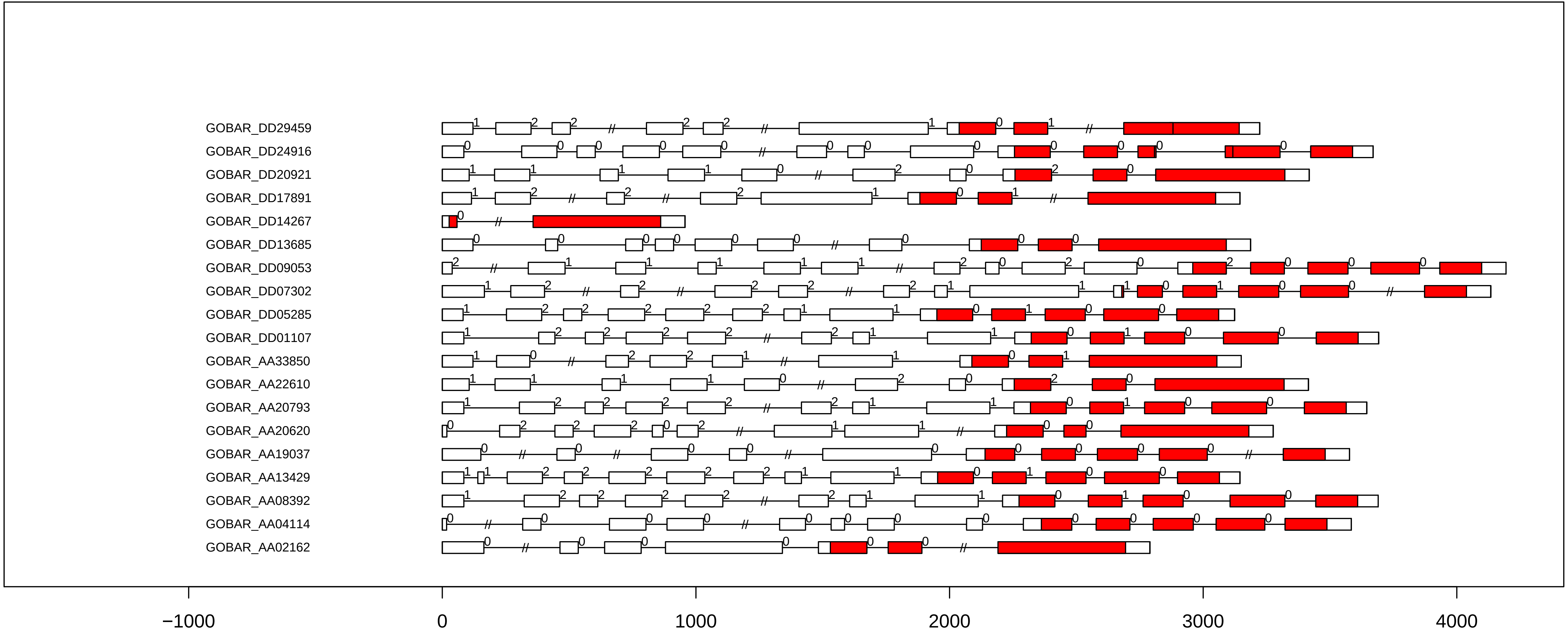

G.ba RLK−Pelle_LRR−VII−1 exon−intron and domain diagram (all)

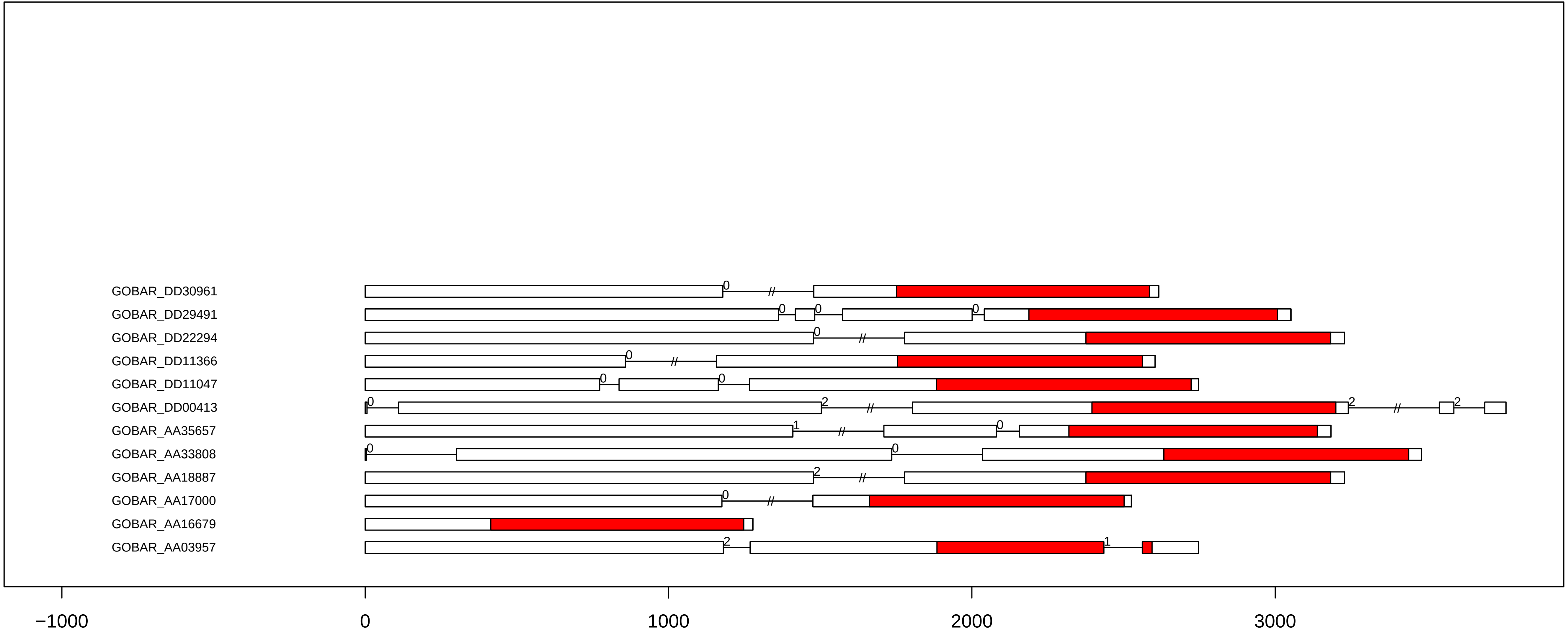

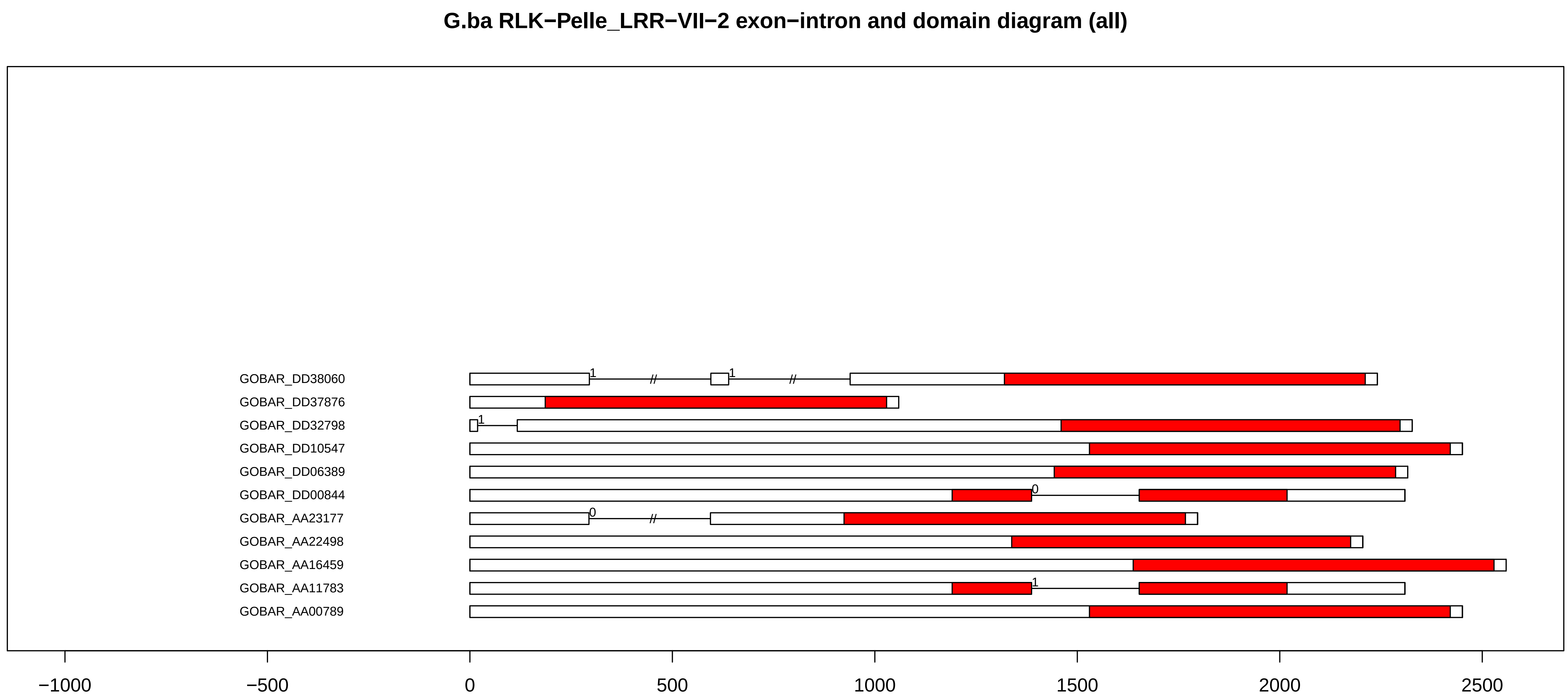
G.ba RLK−Pelle_LRR−VII−2 exon−intron and domain diagram (all)
GOBAR_DD38060
GOBAR_DD37876
GOBAR_DD32798
GOBAR_DD10547
GOBAR_DD06389
GOBAR_DD00844
GOBAR_AA23177
GOBAR_AA22498
GOBAR_AA16459
GOBAR_AA11783
GOBAR_AA00789
−1000
−500
0
500
1000
1500
2000
2500

G.ba RLK−Pelle_LRR−VII−3 exon−intron and domain diagram (all)

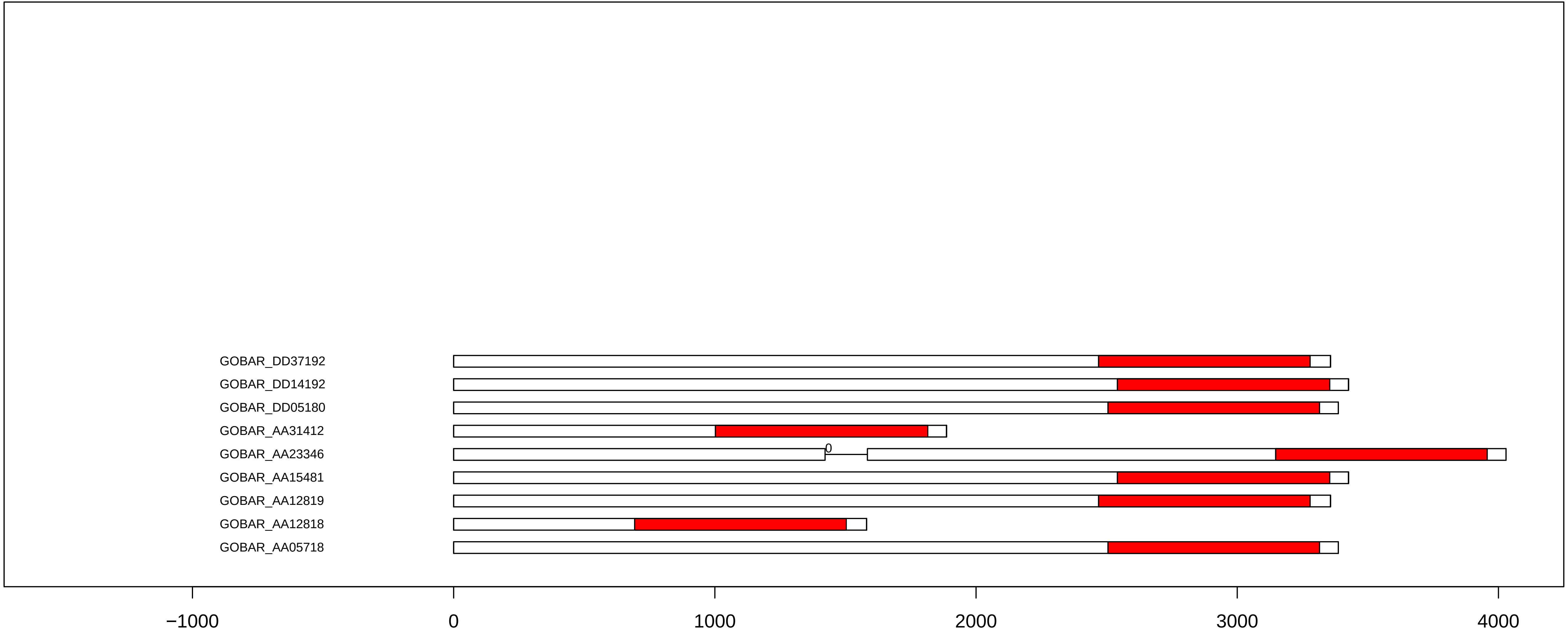

G.ba RLK−Pelle_LRR−VIII−1 exon−intron and domain diagram (all)

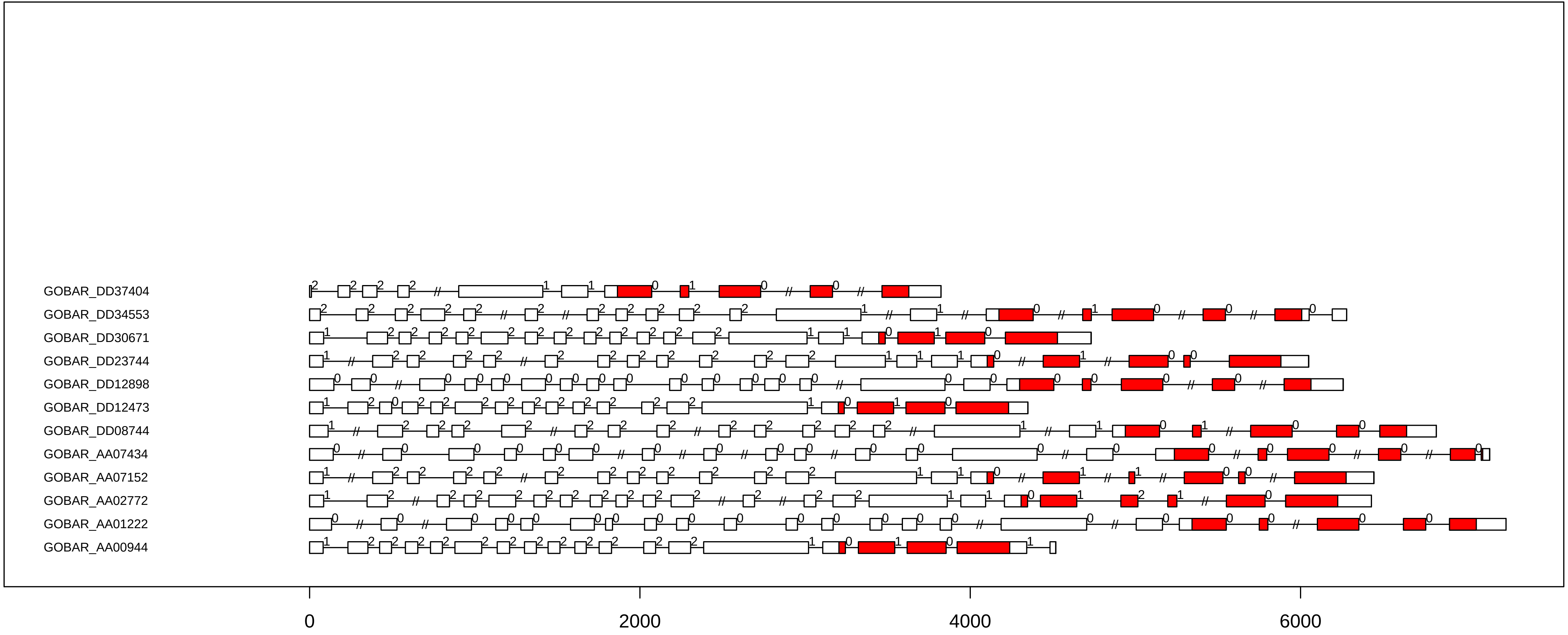

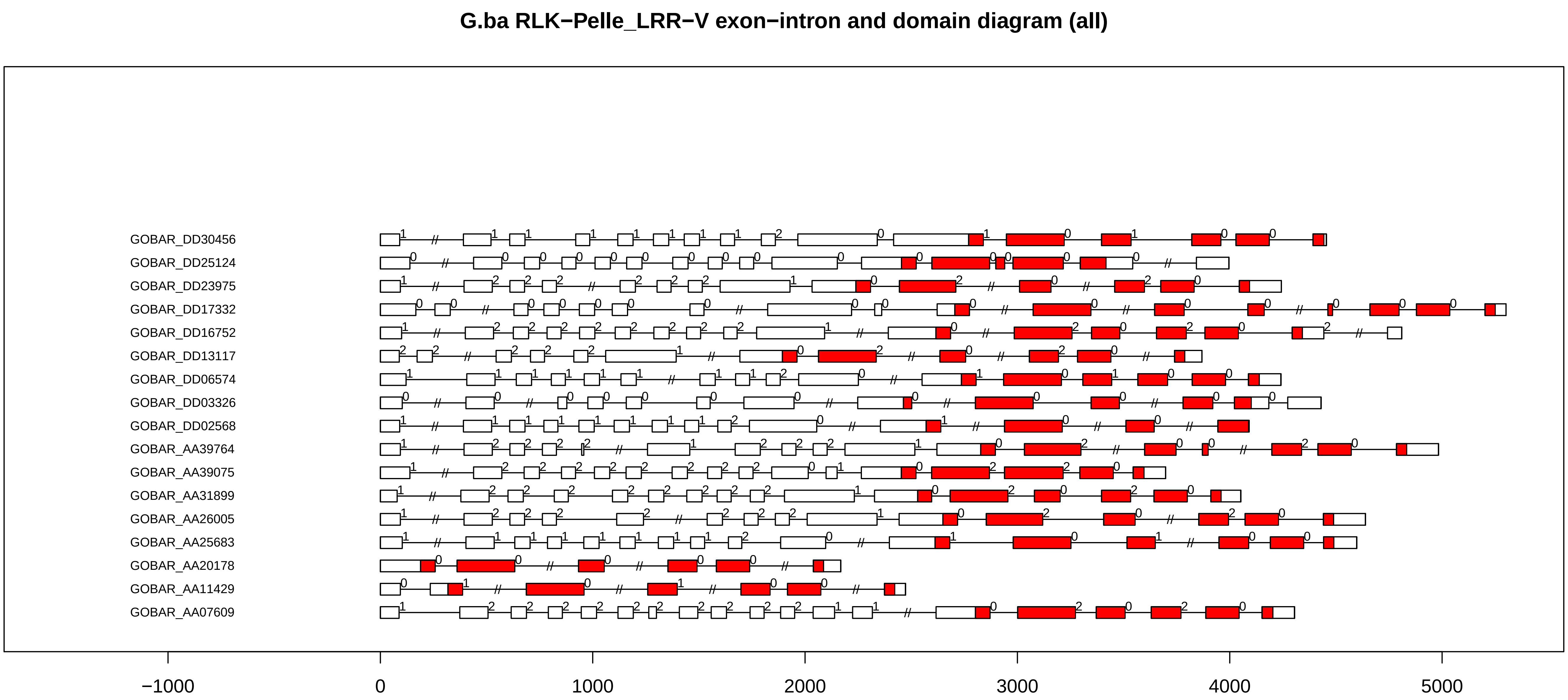
G.ba RLK−Pelle_LRR−V exon−intron and domain diagram (all)
GOBAR_DD30456
GOBAR_DD25124
GOBAR_DD23975
GOBAR_DD17332
GOBAR_DD16752
GOBAR_DD13117
GOBAR_DD06574
GOBAR_DD03326
GOBAR_DD02568
GOBAR_AA39764
GOBAR_AA39075
GOBAR_AA31899
GOBAR_AA26005
GOBAR_AA25683
GOBAR_AA20178
GOBAR_AA11429
GOBAR_AA07609
−1000
0
1000
2000
3000
4000
5000

G.ba RLK−Pelle_LRR−Xa exon−intron and domain diagram (all)

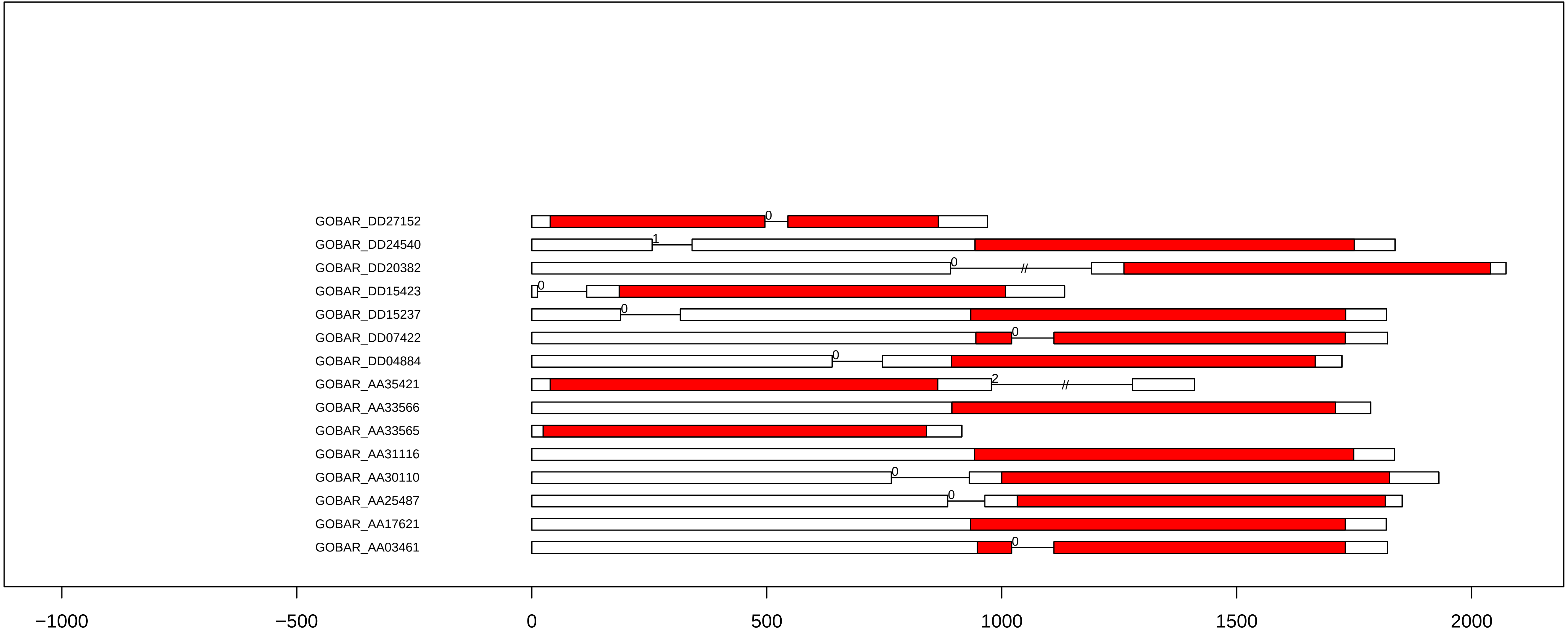

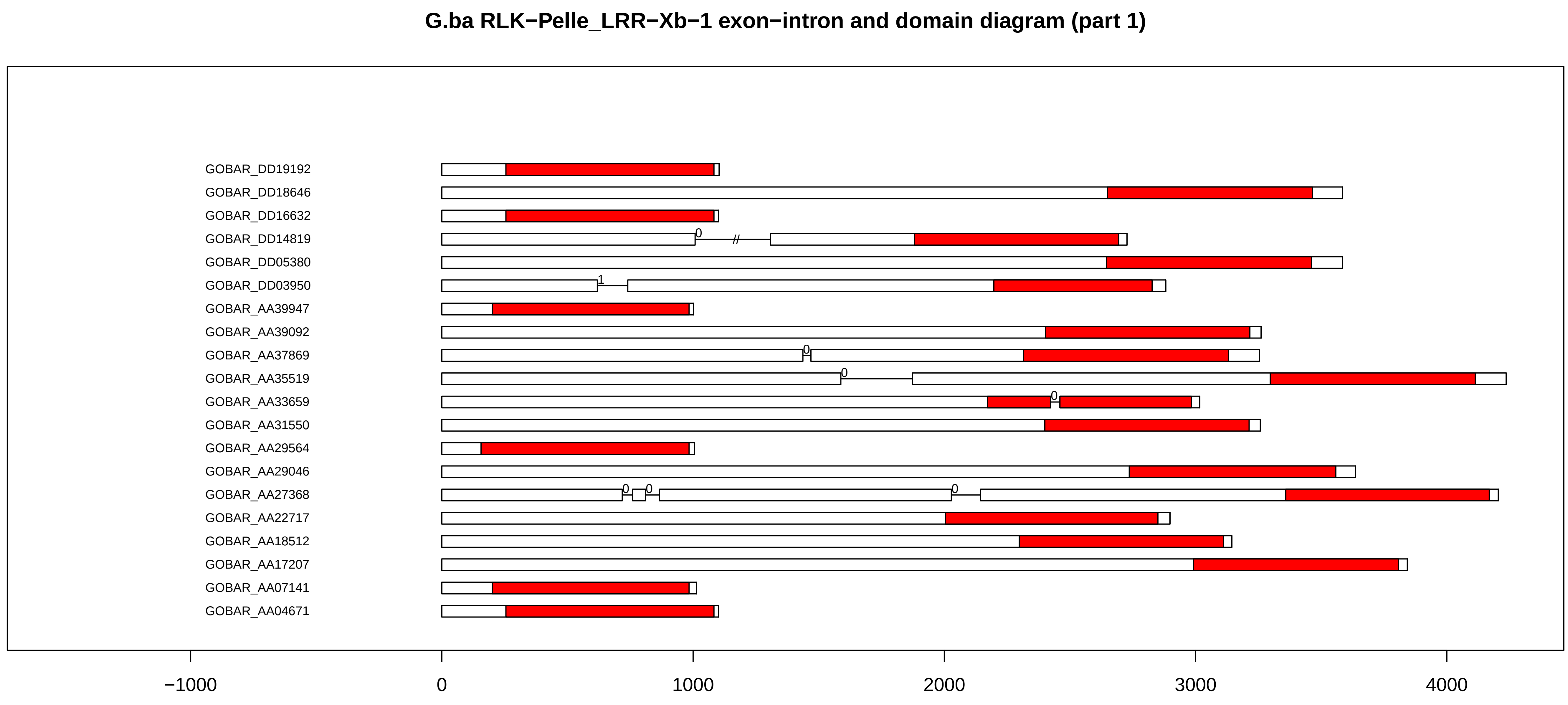

G.ba RLK−Pelle_LRR−Xb−1 exon−intron and domain diagram (part 1)
GOBAR_DD19192
GOBAR_DD18646
GOBAR_DD16632
GOBAR_DD14819
GOBAR_DD05380
GOBAR_DD03950
GOBAR_AA39947
GOBAR_AA39092
GOBAR_AA37869
GOBAR_AA35519
GOBAR_AA33659
GOBAR_AA31550
GOBAR_AA29564
GOBAR_AA29046
GOBAR_AA27368
GOBAR_AA22717
GOBAR_AA18512
GOBAR_AA17207
GOBAR_AA07141
GOBAR_AA04671
−1000
0
1000
2000
3000
4000

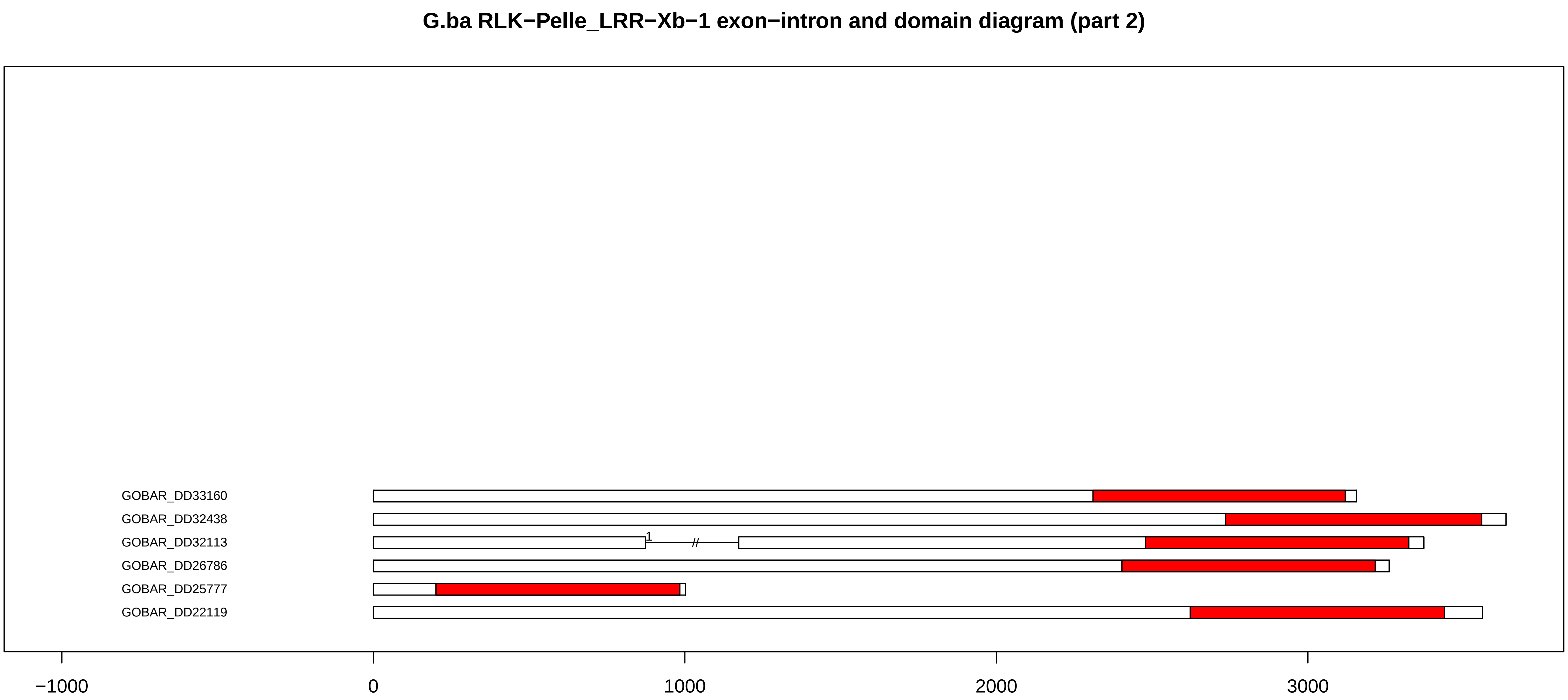
G.ba RLK−Pelle_LRR−Xb−1 exon−intron and domain diagram (part 2)
GOBAR_DD33160
GOBAR_DD32438
GOBAR_DD32113
1
GOBAR_DD26786
GOBAR_DD25777
GOBAR_DD22119
−1000
0
1000
2000
3000

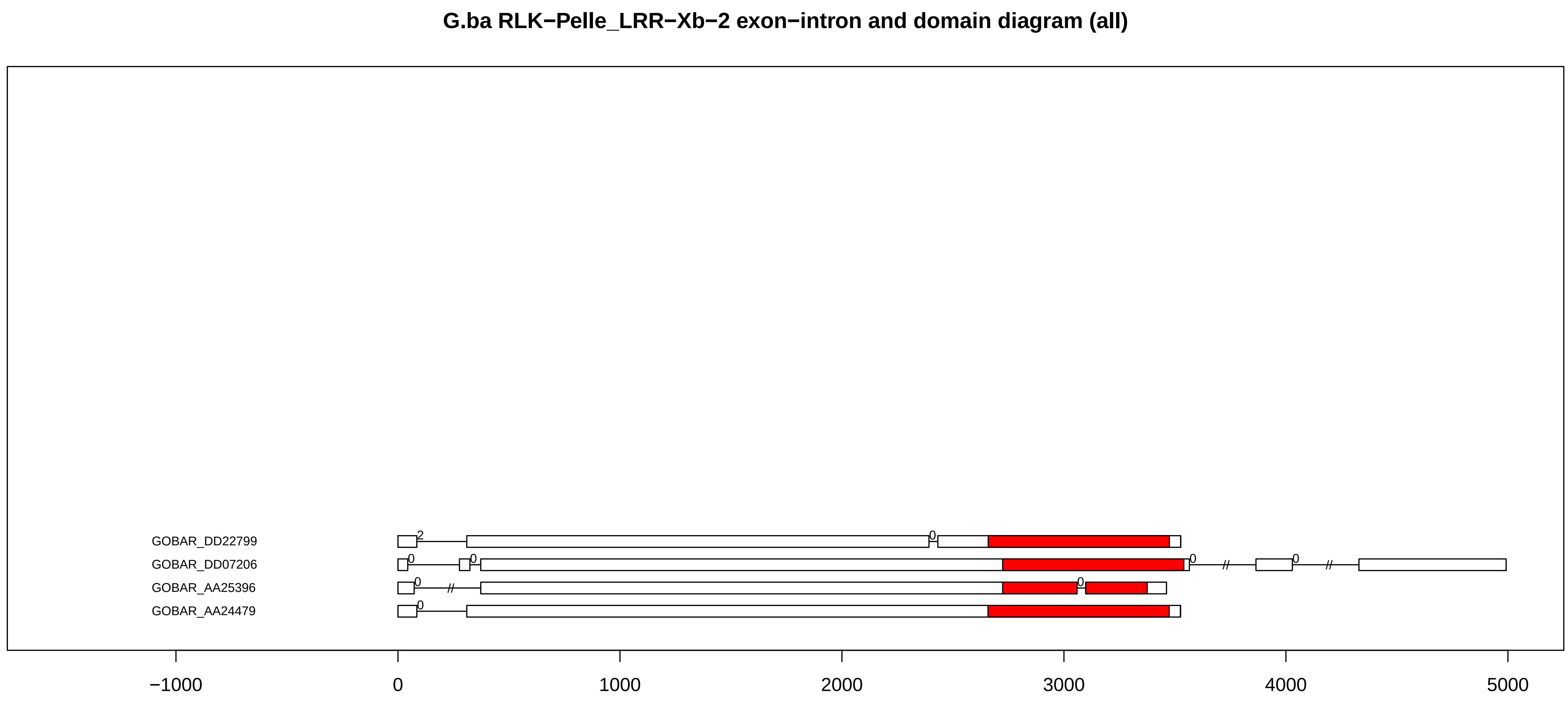
G.ba RLK−Pelle_LRR−Xb−2 exon−intron and domain diagram (all)
GOBAR_DD22799
GOBAR_DD07206
GOBAR_AA25396
GOBAR_AA24479
−1000
0
1000
2000
3000
4000
5000

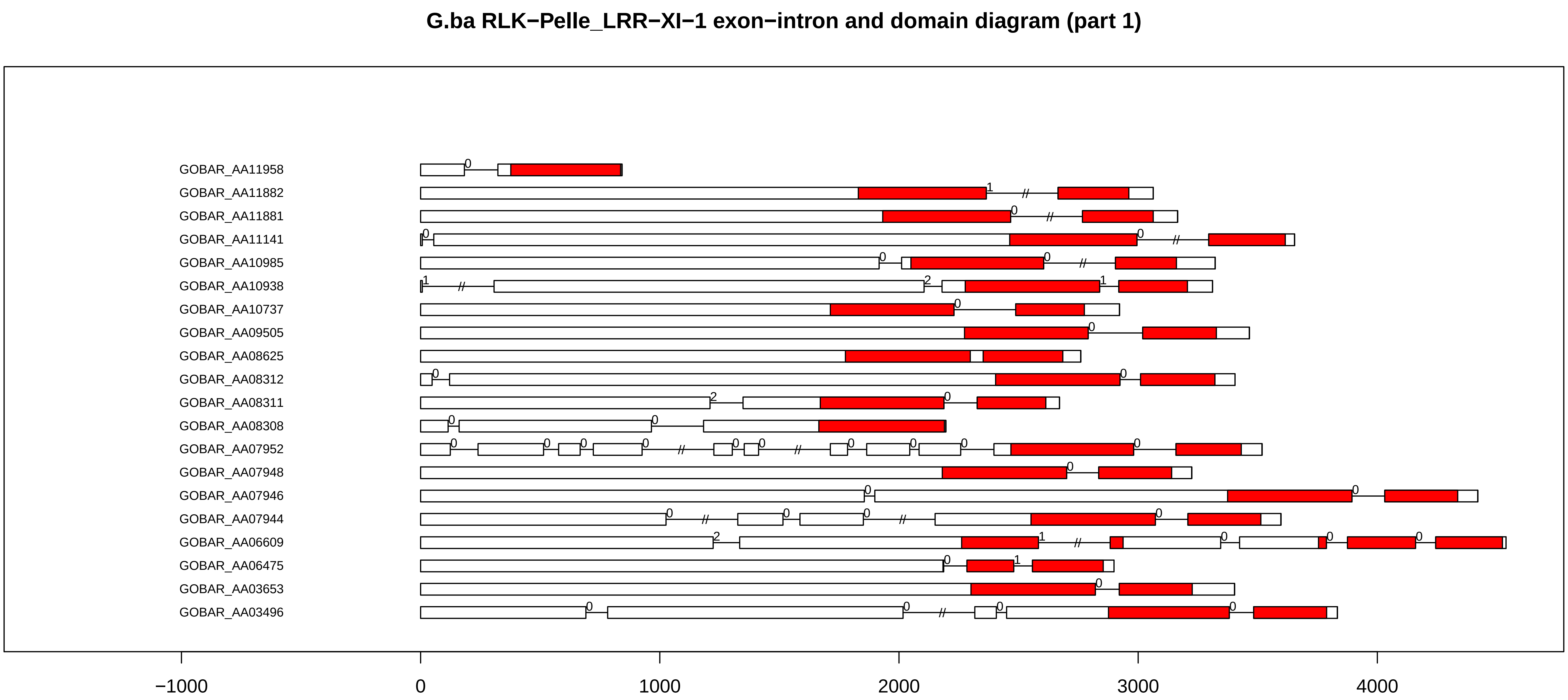

G.ba RLK−Pelle_LRR−XI−1 exon−intron and domain diagram (part 1)
GOBAR_AA11958
GOBAR_AA11882
GOBAR_AA11881
GOBAR_AA11141
GOBAR_AA10985
GOBAR_AA10938
GOBAR_AA10737
GOBAR_AA09505
GOBAR_AA08625
GOBAR_AA08312
GOBAR_AA08311
GOBAR_AA08308
GOBAR_AA07952
GOBAR_AA07948
GOBAR_AA07946
GOBAR_AA07944
GOBAR_AA06609
GOBAR_AA06475
GOBAR_AA03653
GOBAR_AA03496
−1000
0
1000
2000
3000
4000

G.ba RLK−Pelle_LRR−XI−1 exon−intron and domain diagram (part 2)

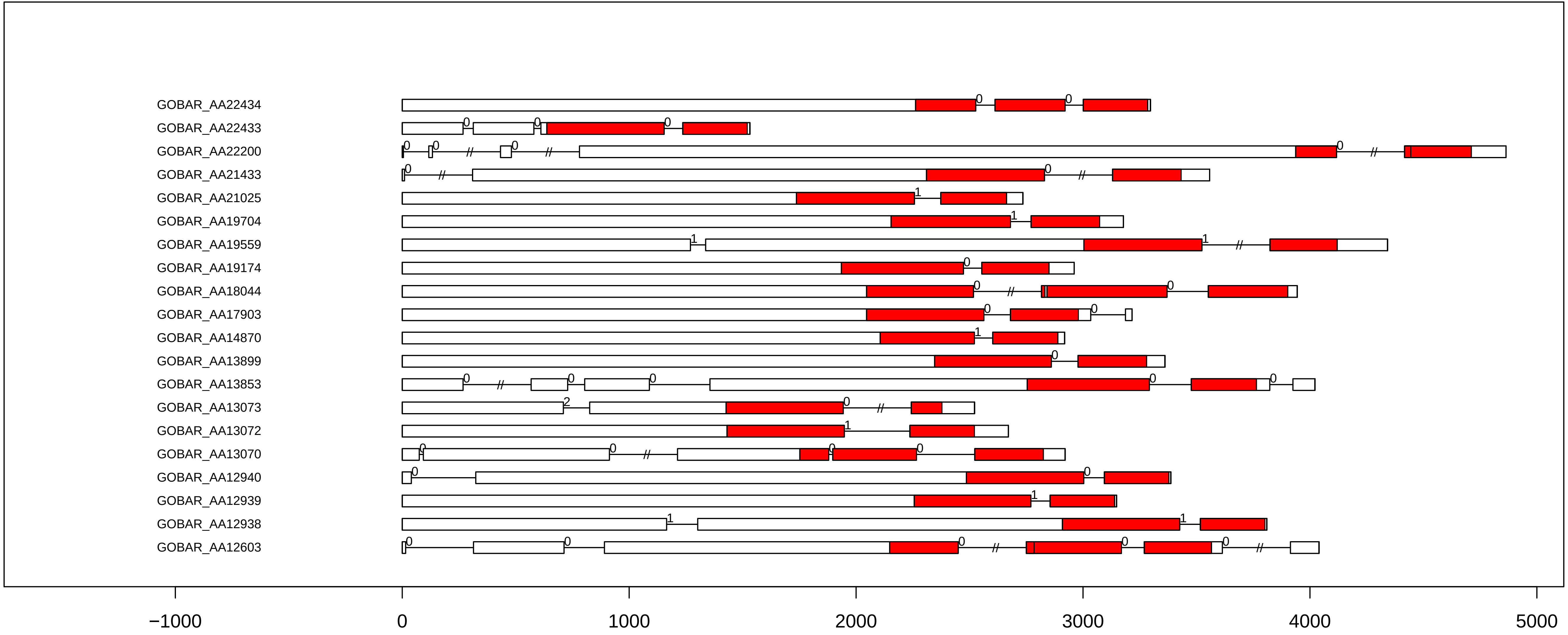

G.ba RLK−Pelle_LRR−XI−1 exon−intron and domain diagram (part 3)

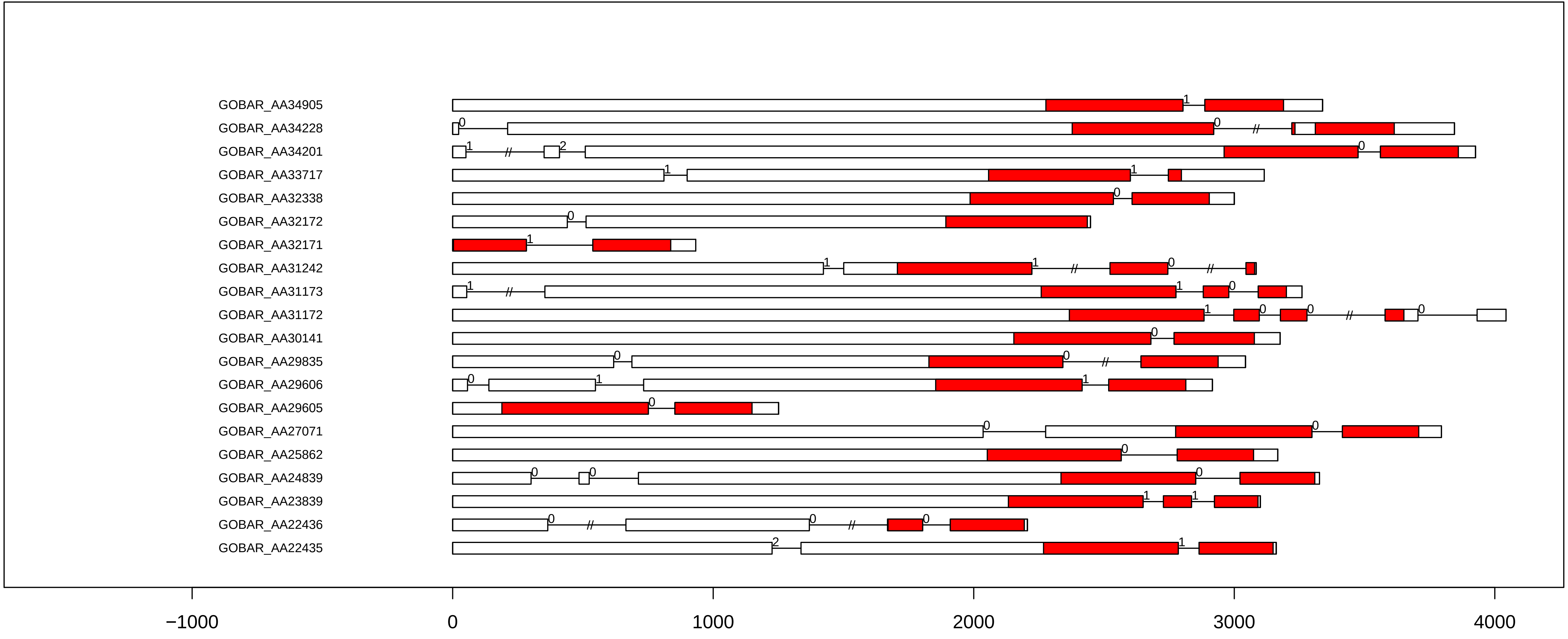

G.ba RLK−Pelle_LRR−XI−1 exon−intron and domain diagram (part 4)

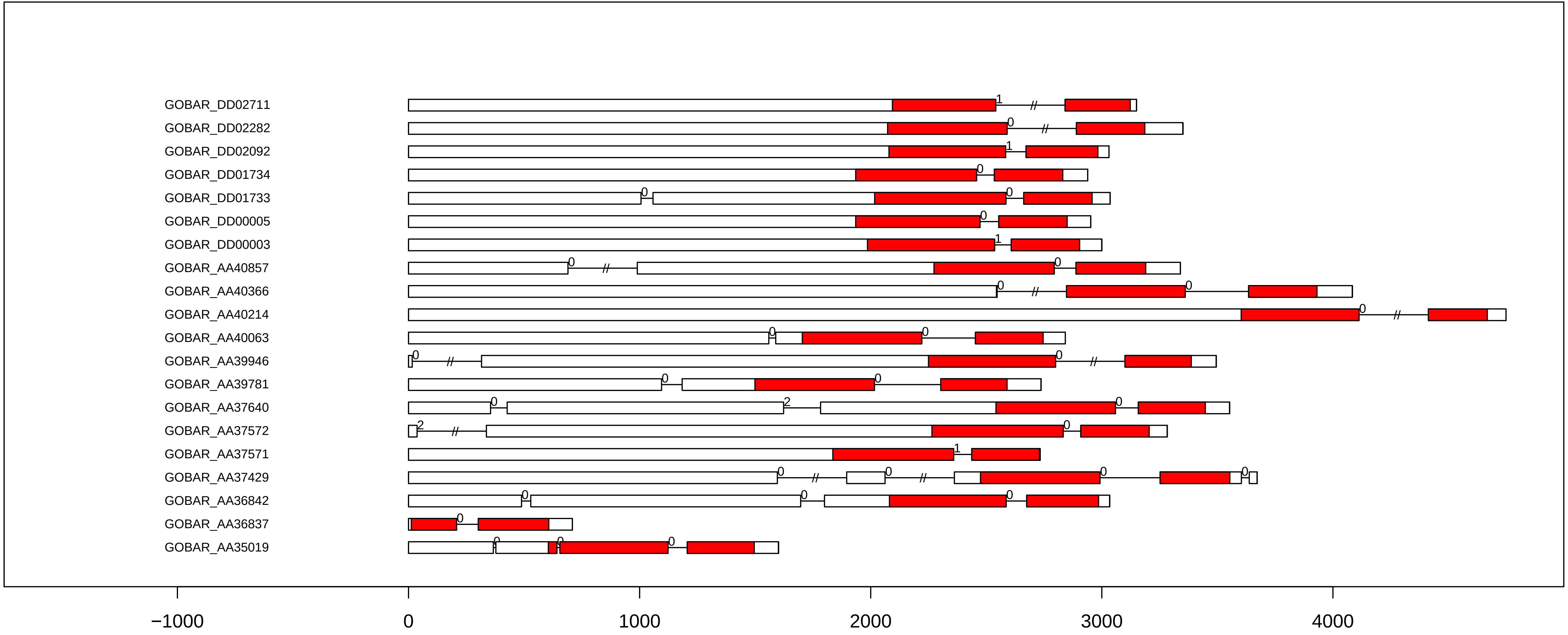

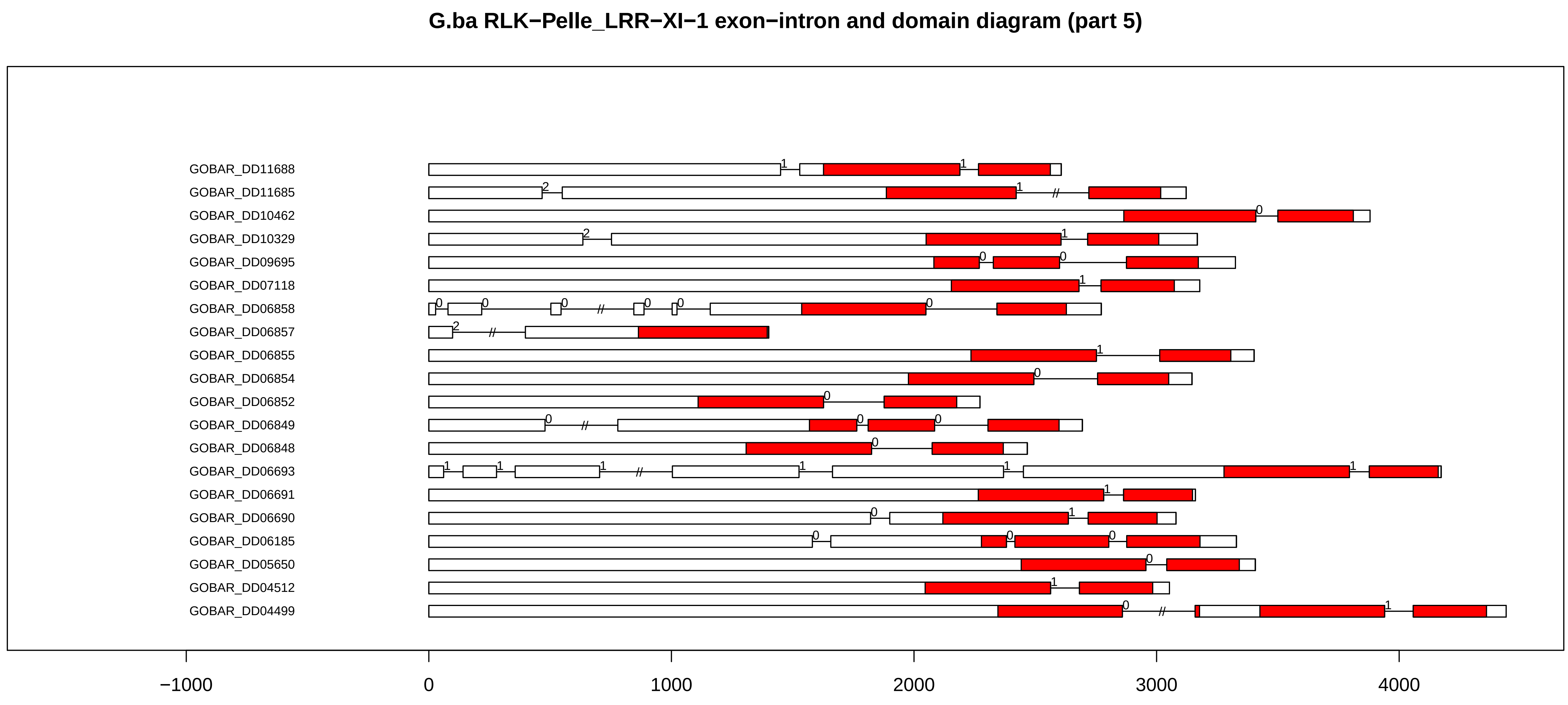
G.ba RLK−Pelle_LRR−XI−1 exon−intron and domain diagram (part 5)
GOBAR_DD11688
GOBAR_DD11685
GOBAR_DD10462
GOBAR_DD10329
GOBAR_DD09695
GOBAR_DD07118
GOBAR_DD06858
GOBAR_DD06857
GOBAR_DD06855
GOBAR_DD06854
GOBAR_DD06852
GOBAR_DD06849
GOBAR_DD06848
GOBAR_DD06693
GOBAR_DD06691
GOBAR_DD06690
GOBAR_DD06185
GOBAR_DD05650
GOBAR_DD04512
GOBAR_DD04499
−1000
0
1000
2000
3000
4000

G.ba RLK−Pelle_LRR−XI−1 exon−intron and domain diagram (part 6)

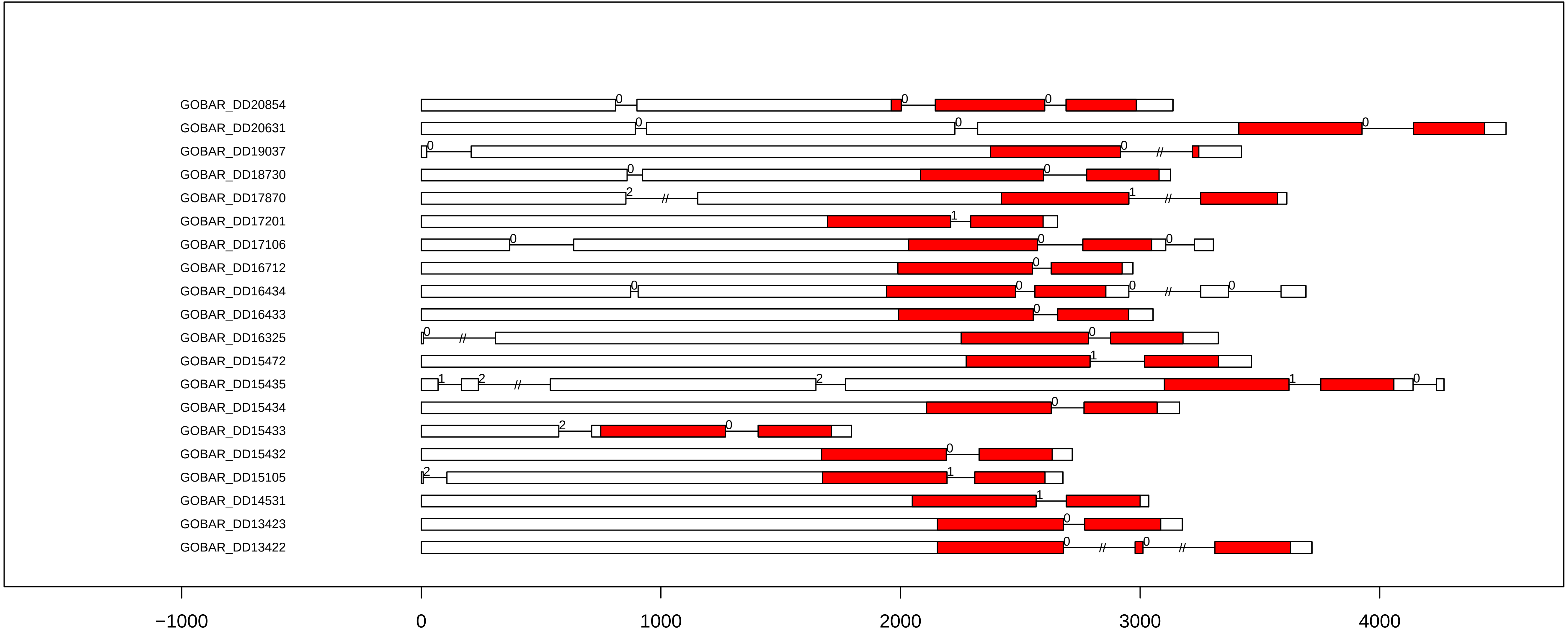

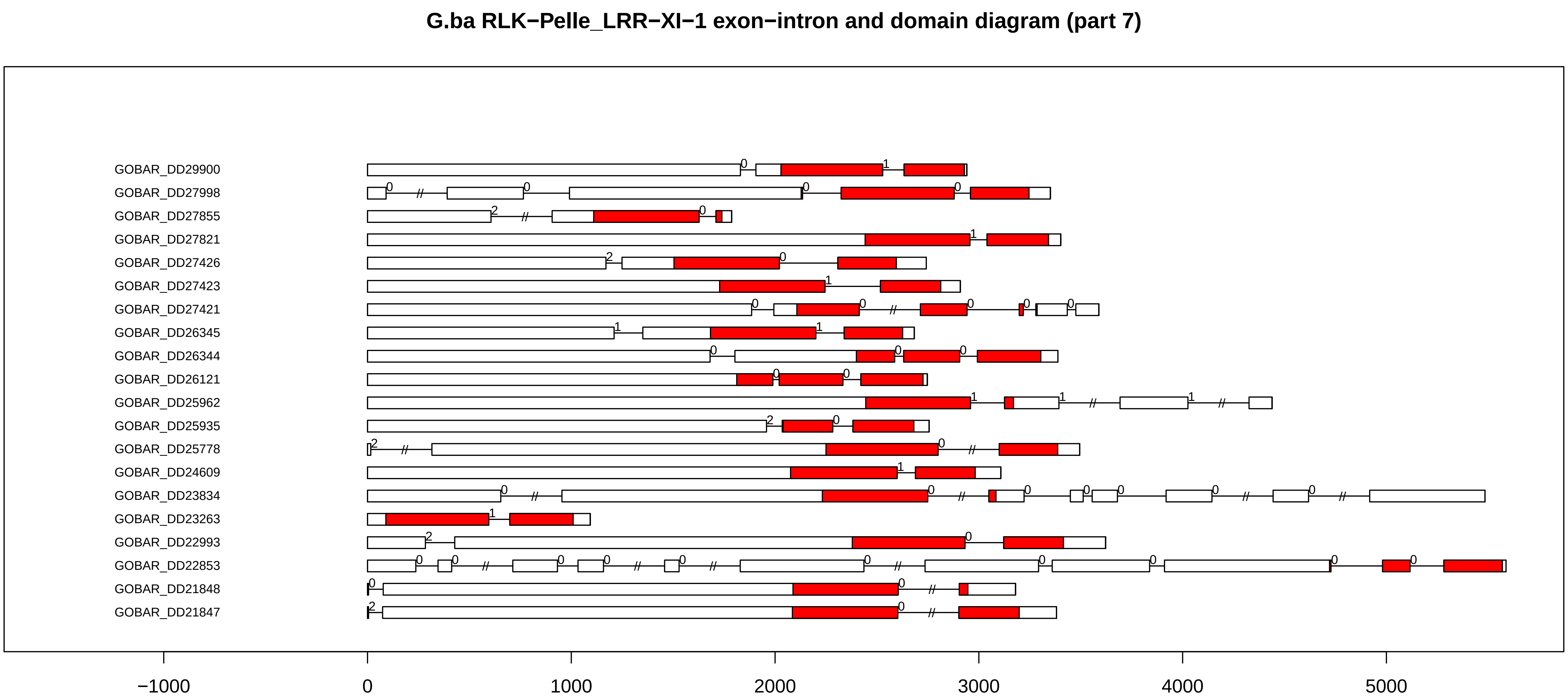

G.ba RLK−Pelle_LRR−XI−1 exon−intron and domain diagram (part 7)
GOBAR_DD29900
GOBAR_DD27998
GOBAR_DD27855
GOBAR_DD27821
GOBAR_DD27426
GOBAR_DD27423
GOBAR_DD27421
GOBAR_DD26345
GOBAR_DD26344
GOBAR_DD26121
GOBAR_DD25962
GOBAR_DD25935
GOBAR_DD25778
GOBAR_DD24609
GOBAR_DD23834
GOBAR_DD23263
GOBAR_DD22993
GOBAR_DD22853
GOBAR_DD21848
GOBAR_DD21847
−1000
0
1000
2000
3000
4000
5000

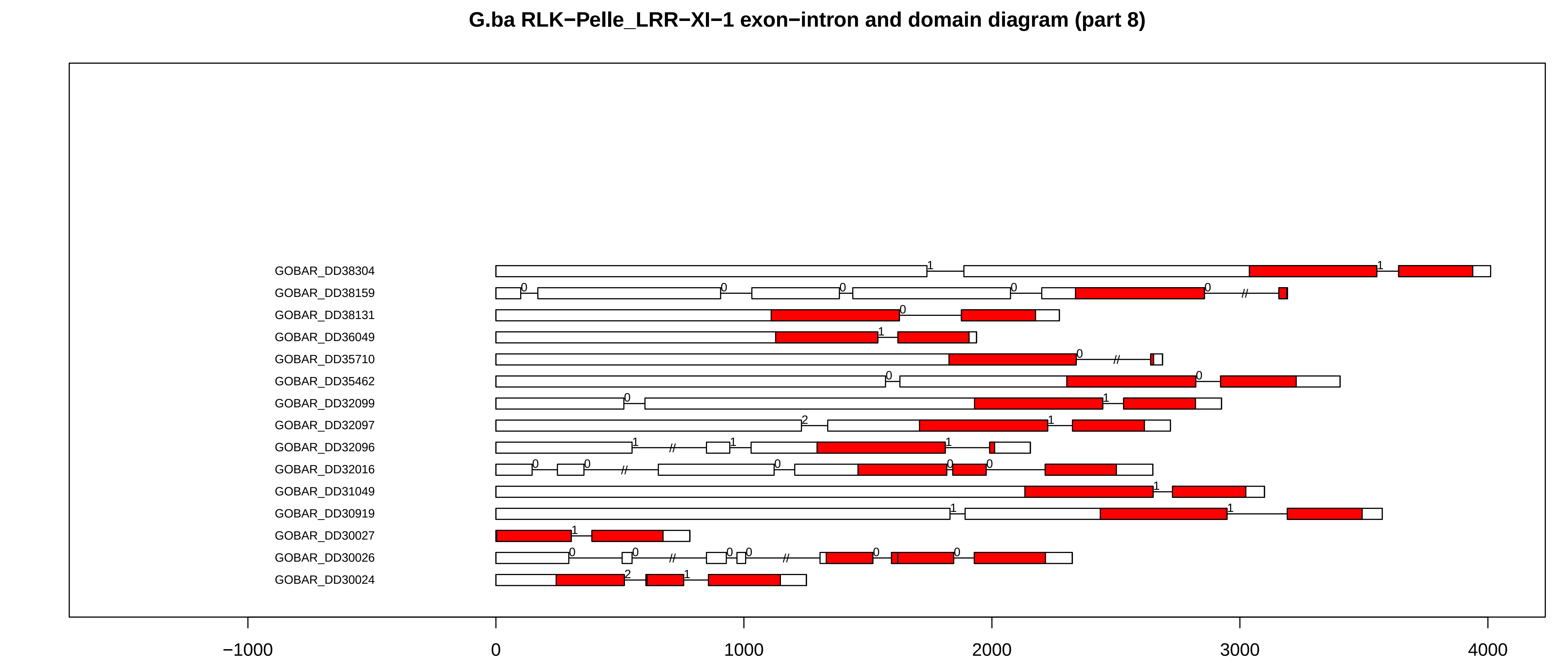
G.ba RLK−Pelle_LRR−XI−1 exon−intron and domain diagram (part 8)
GOBAR_DD38304
GOBAR_DD38159
GOBAR_DD38131
GOBAR_DD36049
GOBAR_DD35710
GOBAR_DD35462
GOBAR_DD32099
GOBAR_DD32097
GOBAR_DD32096
GOBAR_DD32016
GOBAR_DD31049
GOBAR_DD30919
GOBAR_DD30027
GOBAR_DD30026
GOBAR_DD30024
−1000
0
1000
2000
3000
4000

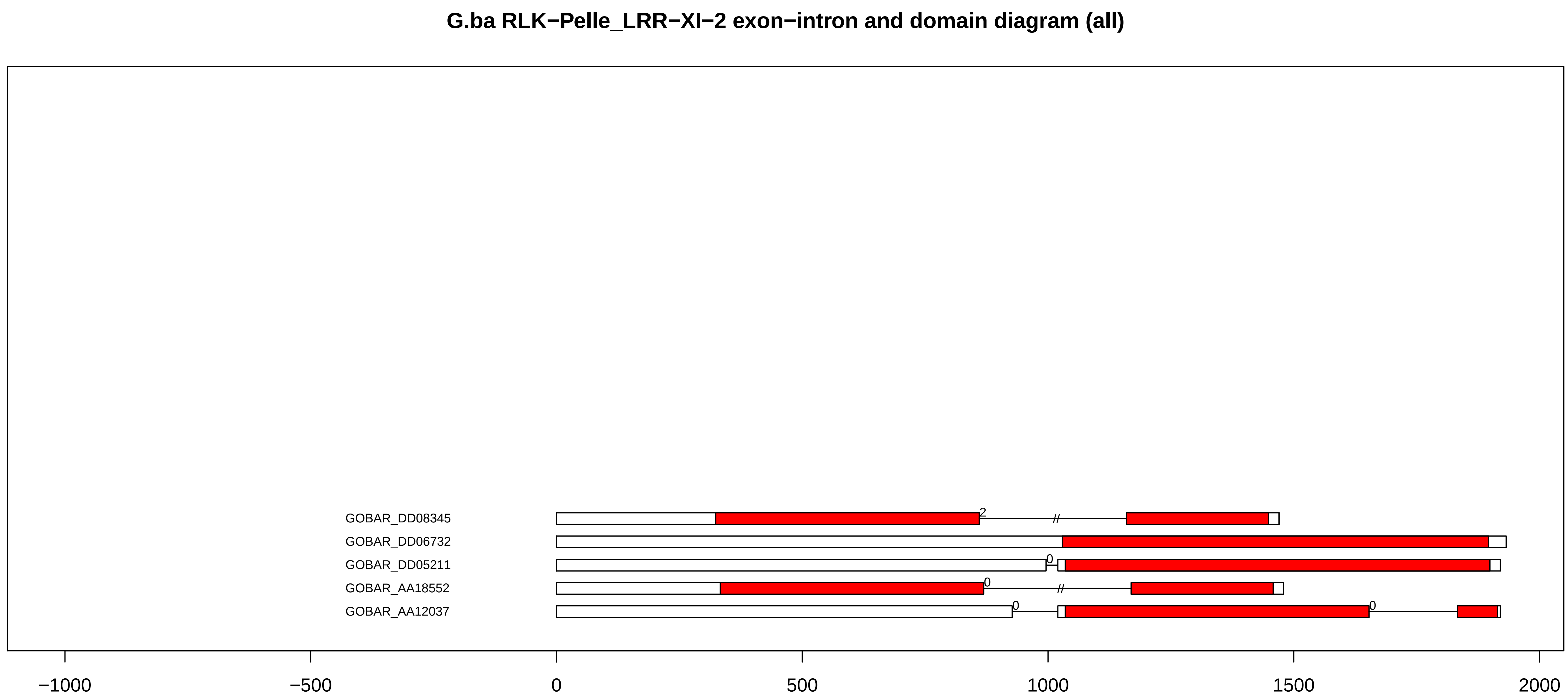

G.ba RLK−Pelle_LRR−XI−2 exon−intron and domain diagram (all)
GOBAR_DD08345
GOBAR_DD06732
GOBAR_DD05211
GOBAR_AA18552
GOBAR_AA12037
2
0
0
0
0
−1000
−500
0
500
1000
1500
2000

G.ba RLK−Pelle_LRR−XII−1 exon−intron and domain diagram (part 1)

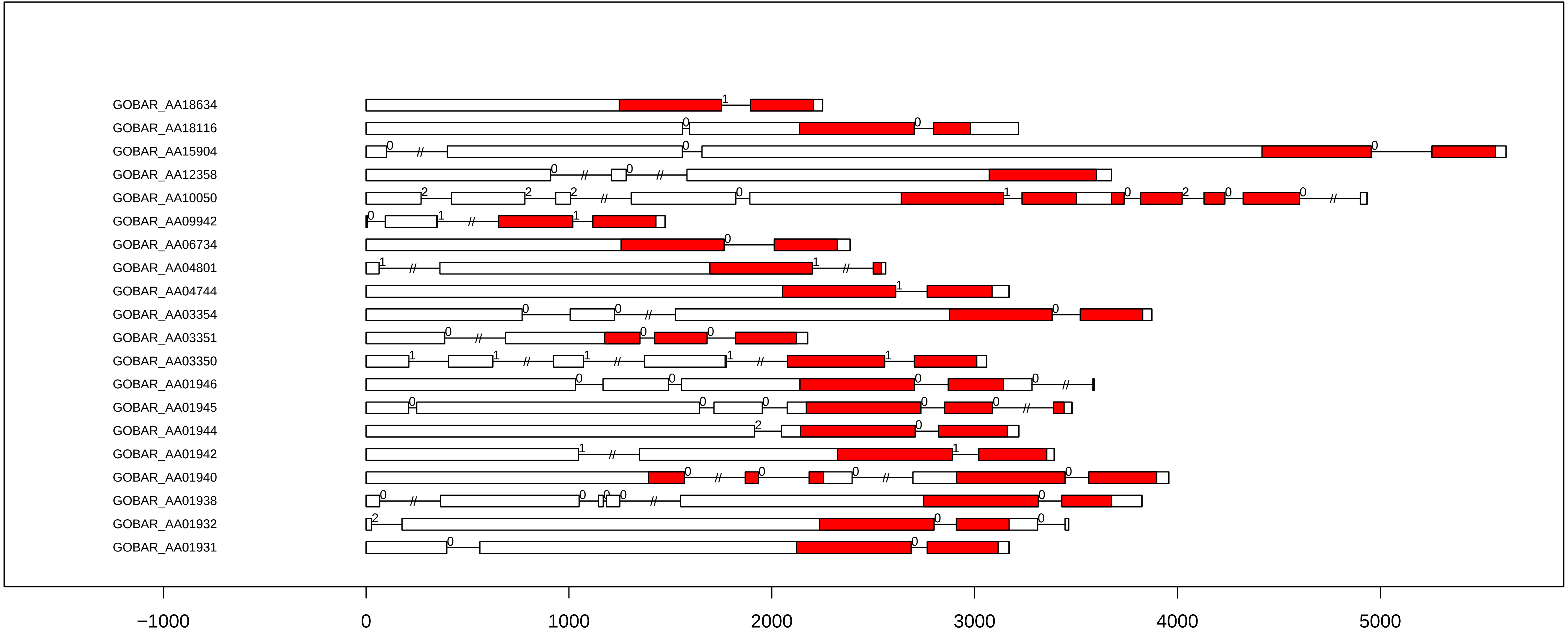

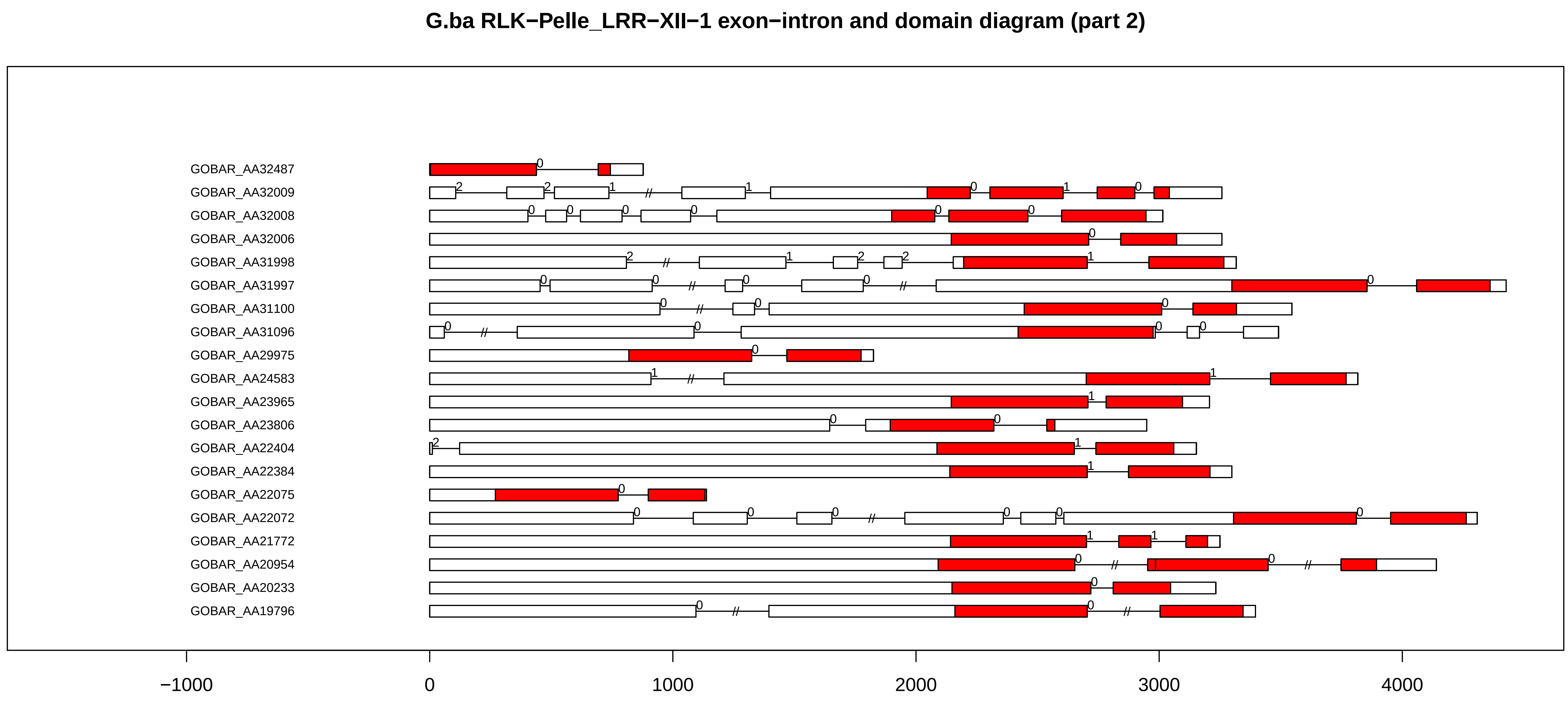
G.ba RLK−Pelle_LRR−XII−1 exon−intron and domain diagram (part 2)
GOBAR_AA32487
GOBAR_AA32009
GOBAR_AA32008
GOBAR_AA32006
GOBAR_AA31998
GOBAR_AA31997
GOBAR_AA31100
GOBAR_AA31096
GOBAR_AA29975
GOBAR_AA24583
GOBAR_AA23965
GOBAR_AA23806
GOBAR_AA22404
GOBAR_AA22384
GOBAR_AA22075
GOBAR_AA22072
GOBAR_AA21772
GOBAR_AA20954
GOBAR_AA20233
GOBAR_AA19796
−1000
0
1000
2000
3000
4000

G.ba RLK−Pelle_LRR−XII−1 exon−intron and domain diagram (part 3)

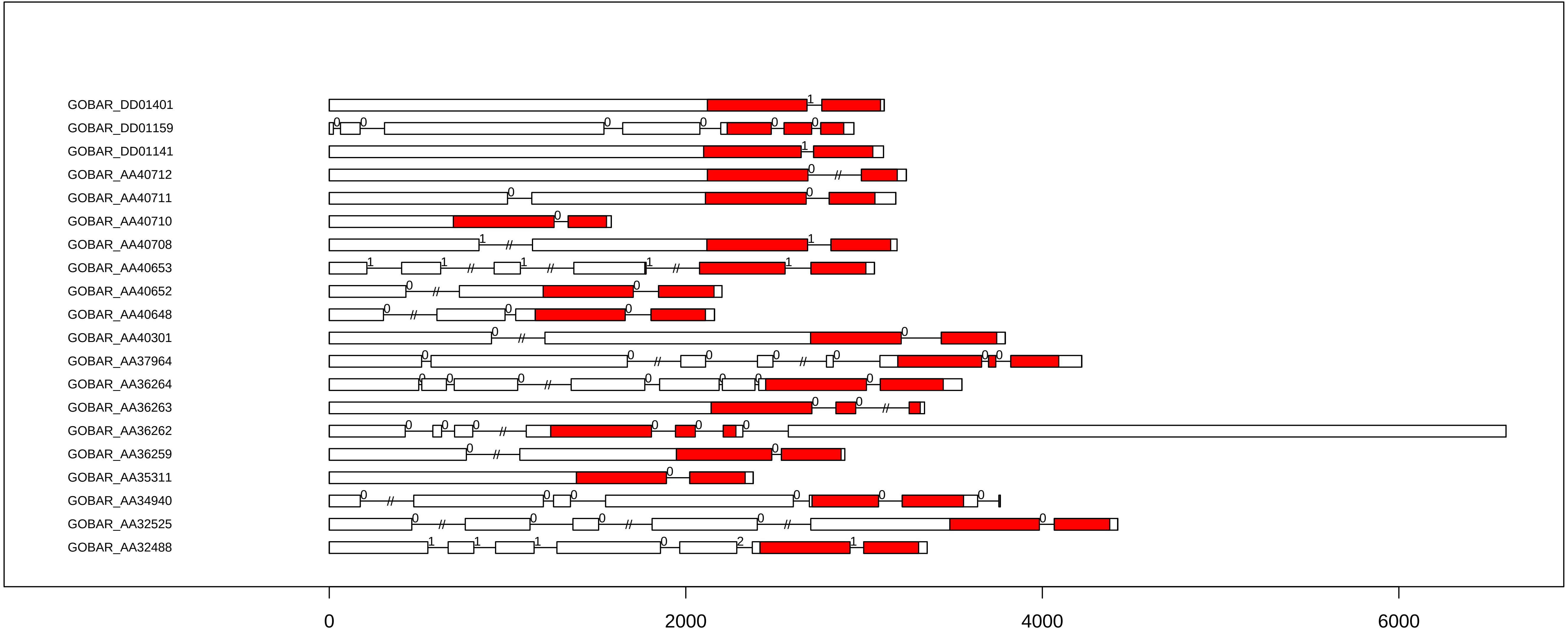

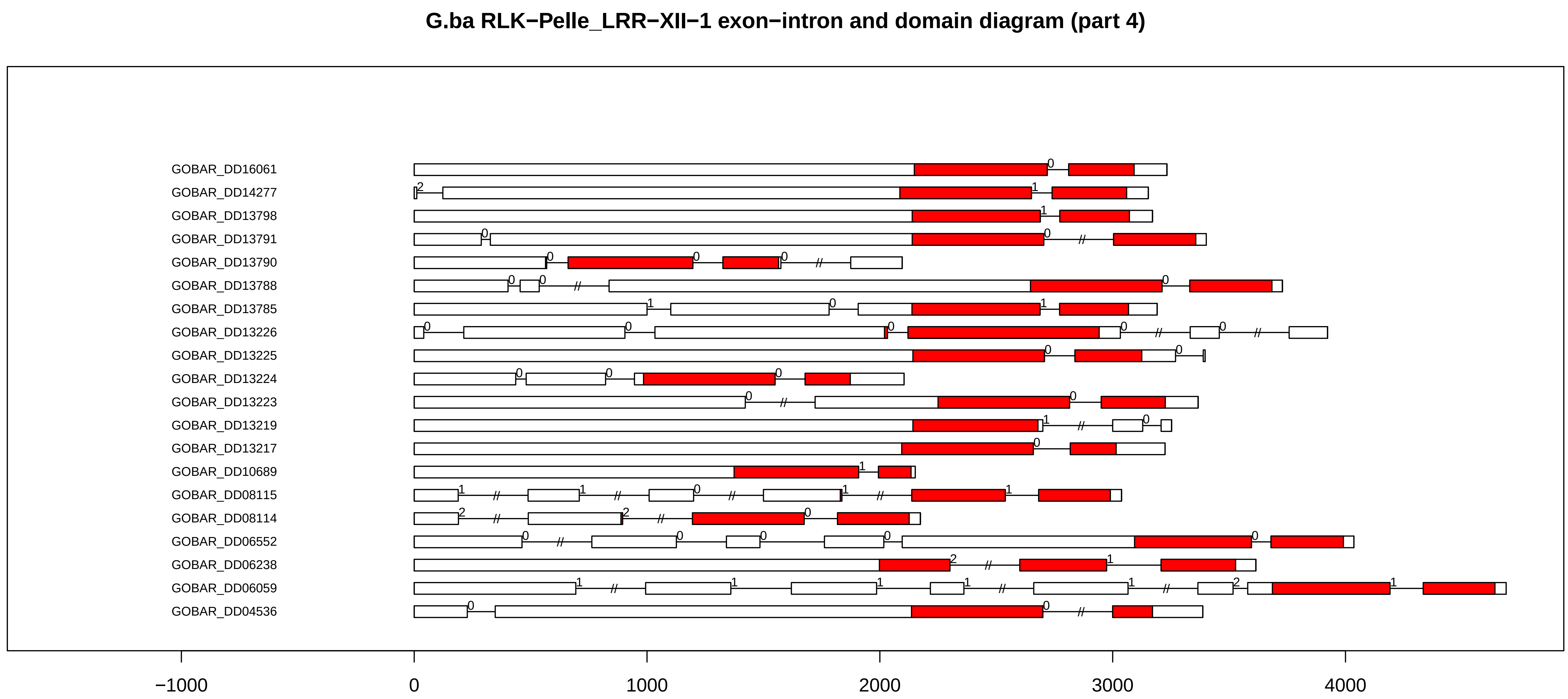
G.ba RLK−Pelle_LRR−XII−1 exon−intron and domain diagram (part 4)
GOBAR_DD16061
GOBAR_DD14277
GOBAR_DD13798
GOBAR_DD13791
GOBAR_DD13790
GOBAR_DD13788
GOBAR_DD13785
GOBAR_DD13226
GOBAR_DD13225
GOBAR_DD13224
GOBAR_DD13223
GOBAR_DD13219
GOBAR_DD13217
GOBAR_DD10689
GOBAR_DD08115
GOBAR_DD08114
GOBAR_DD06552
GOBAR_DD06238
GOBAR_DD06059
GOBAR_DD04536
−1000
0
1000
2000
3000
4000

G.ba RLK−Pelle_LRR−XII−1 exon−intron and domain diagram (part 5)

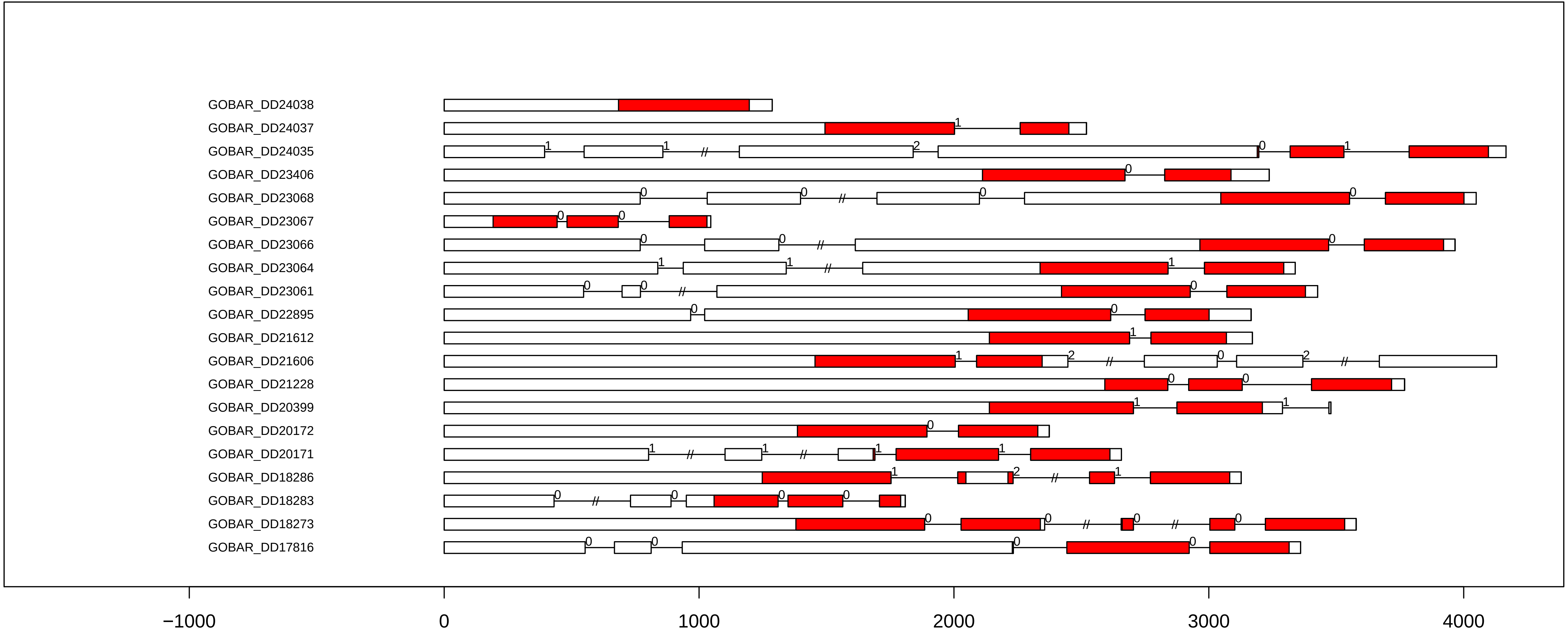

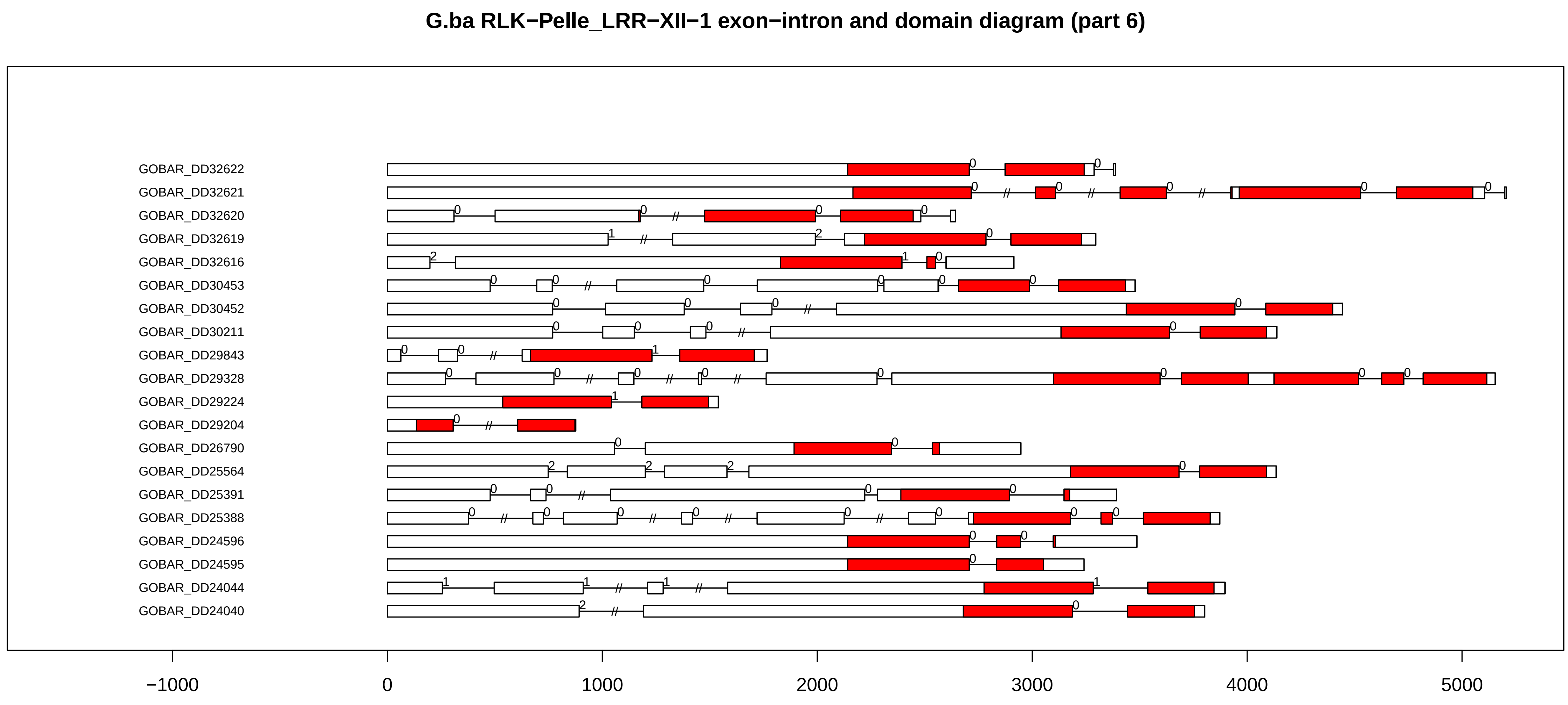
G.ba RLK−Pelle_LRR−XII−1 exon−intron and domain diagram (part 6)
GOBAR_DD32622
GOBAR_DD32621
GOBAR_DD32620
GOBAR_DD32619
GOBAR_DD32616
GOBAR_DD30453
GOBAR_DD30452
GOBAR_DD30211
GOBAR_DD29843
GOBAR_DD29328
GOBAR_DD29224
GOBAR_DD29204
GOBAR_DD26790
GOBAR_DD25564
GOBAR_DD25391
GOBAR_DD25388
GOBAR_DD24596
GOBAR_DD24595
GOBAR_DD24044
GOBAR_DD24040
−1000
0
1000
2000
3000
4000
5000

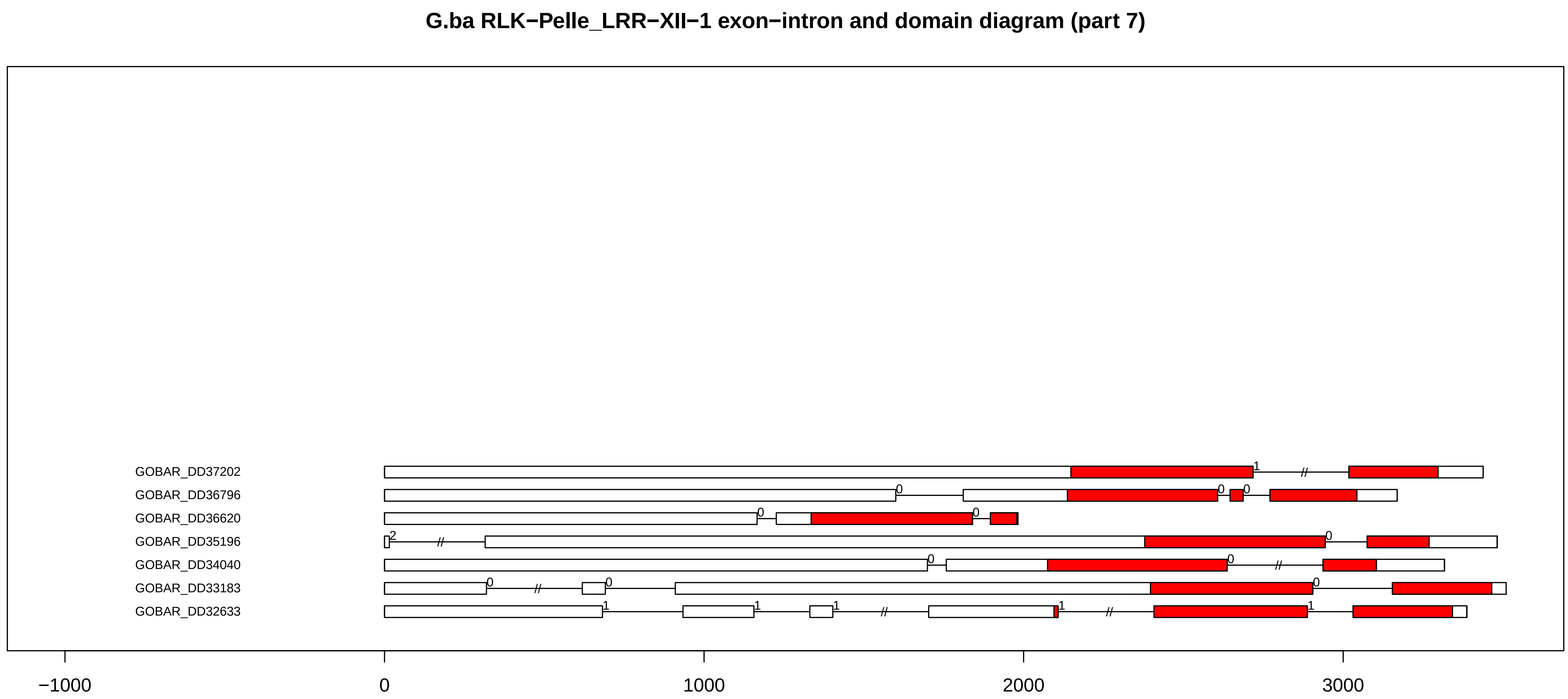
G.ba RLK−Pelle_LRR−XII−1 exon−intron and domain diagram (part 7)
GOBAR_DD37202
GOBAR_DD36796
GOBAR_DD36620
GOBAR_DD35196
GOBAR_DD34040
GOBAR_DD33183
GOBAR_DD32633
−1000
0
1000
2000
3000

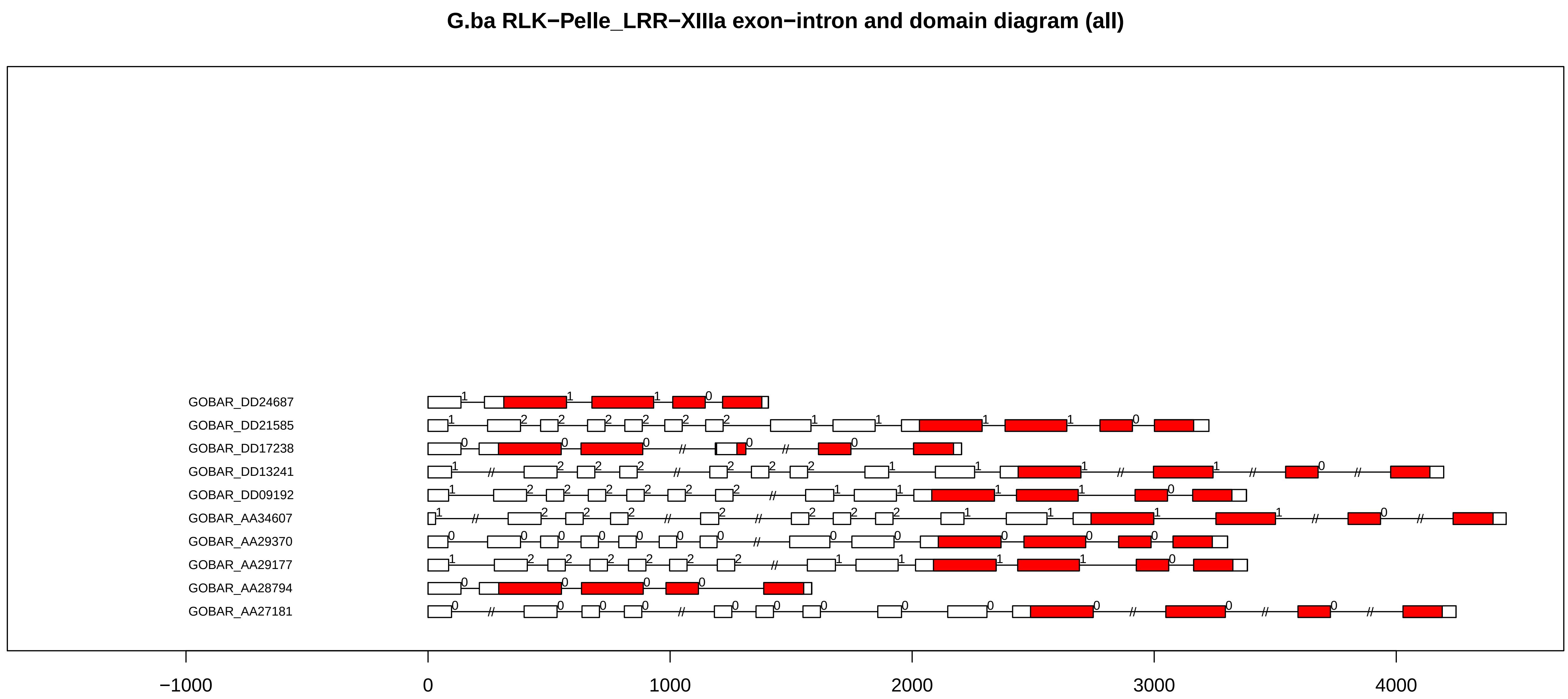
G.ba RLK−Pelle_LRR−XIIIa exon−intron and domain diagram (all)
GOBAR_DD24687
GOBAR_DD21585
GOBAR_DD17238
GOBAR_DD13241
GOBAR_DD09192
GOBAR_AA34607
GOBAR_AA29370
GOBAR_AA29177
GOBAR_AA28794
GOBAR_AA27181
−1000
0
1000
2000
3000
4000

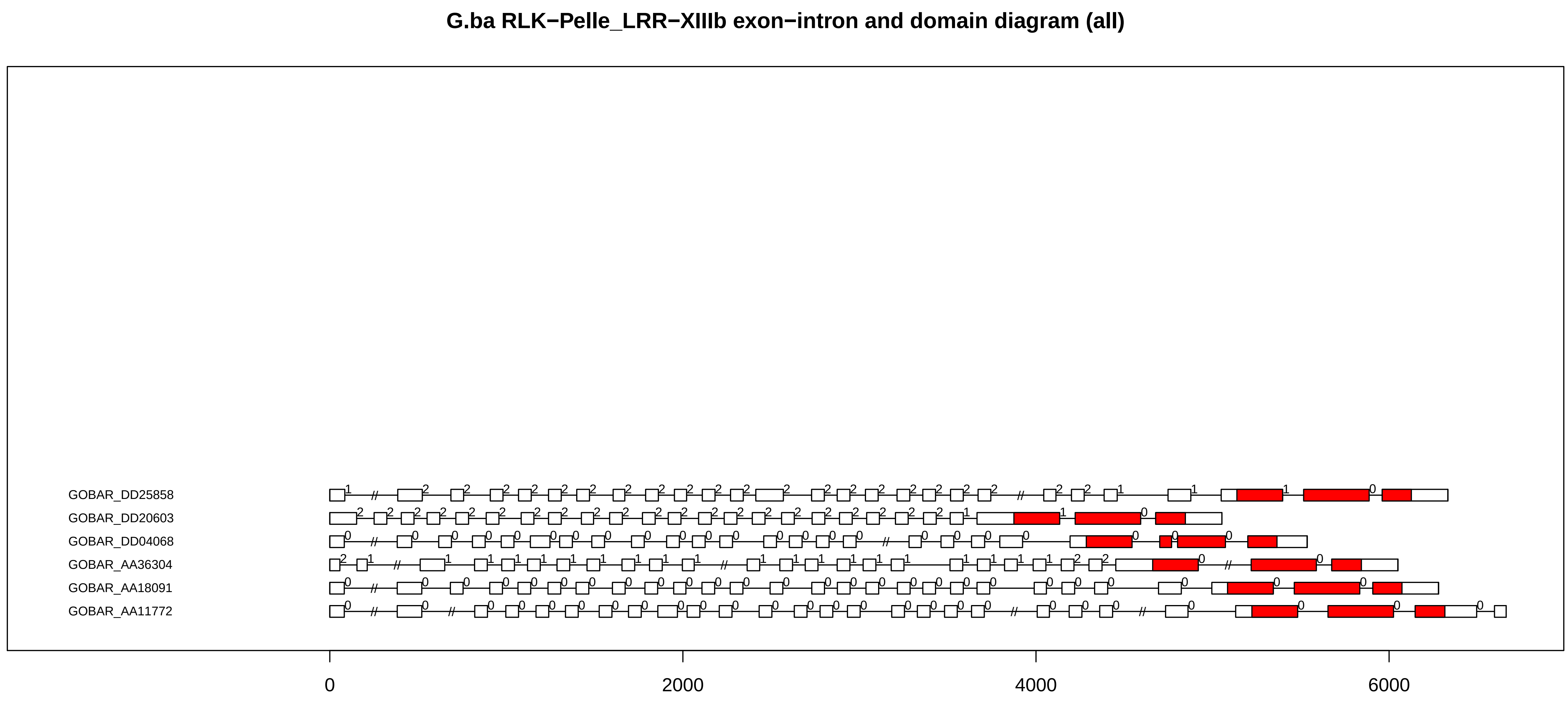
G.ba RLK−Pelle_LRR−XIIIb exon−intron and domain diagram (all)
GOBAR_DD25858
GOBAR_DD20603
GOBAR_DD04068
GOBAR_AA36304
GOBAR_AA18091
GOBAR_AA11772
0
2000
4000
6000

G.ba RLK−Pelle_LRR−XIV exon−intron and domain diagram (all)

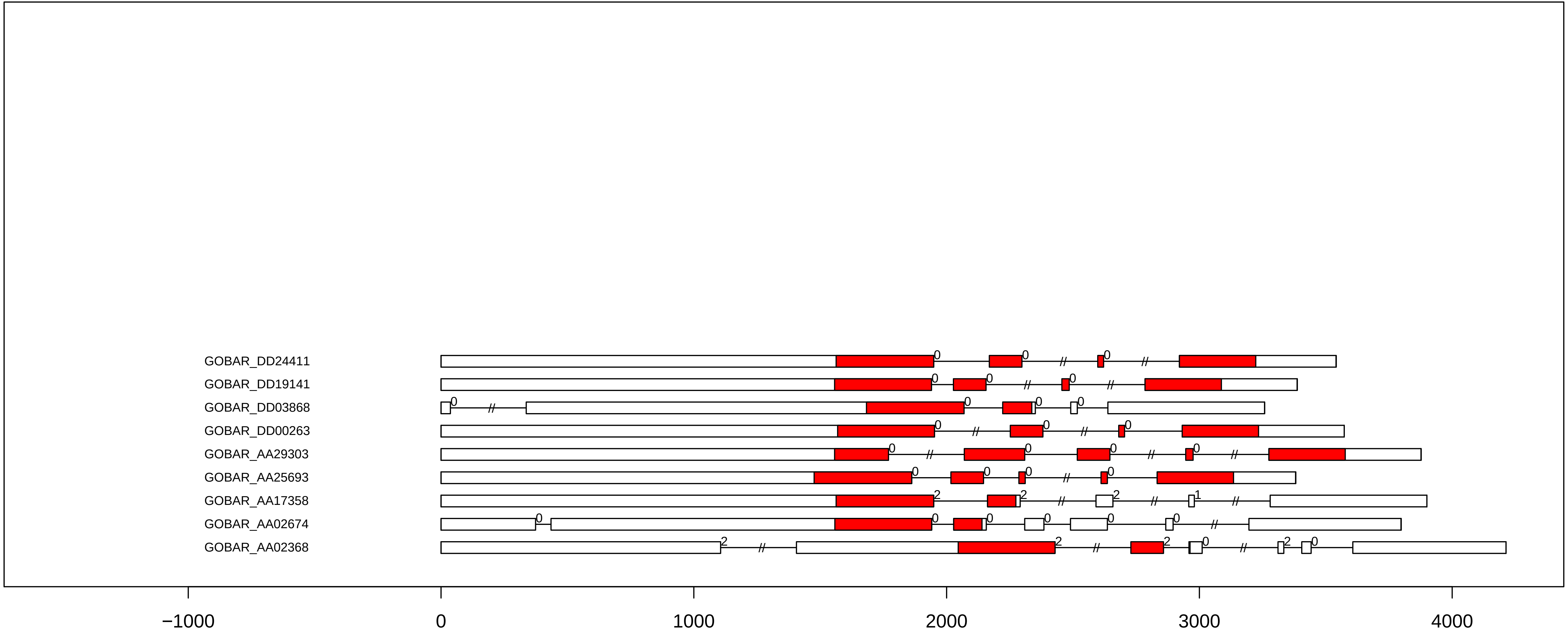

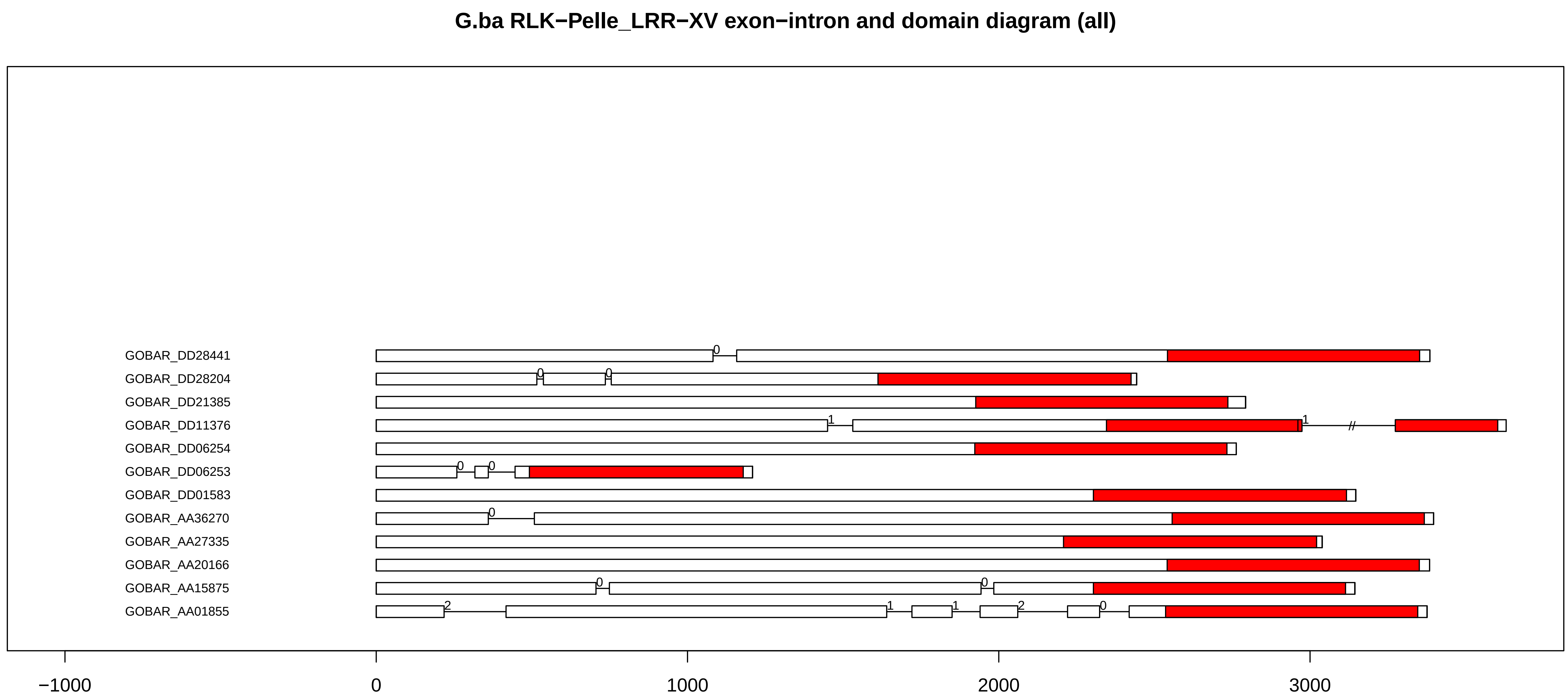

G.ba RLK−Pelle_LRR−XV exon−intron and domain diagram (all)
GOBAR_DD28441
GOBAR_DD28204
GOBAR_DD21385
GOBAR_DD11376
GOBAR_DD06254
GOBAR_DD06253
GOBAR_DD01583
GOBAR_AA36270
GOBAR_AA27335
GOBAR_AA20166
GOBAR_AA15875
GOBAR_AA01855
−1000
0
1000
2000
3000

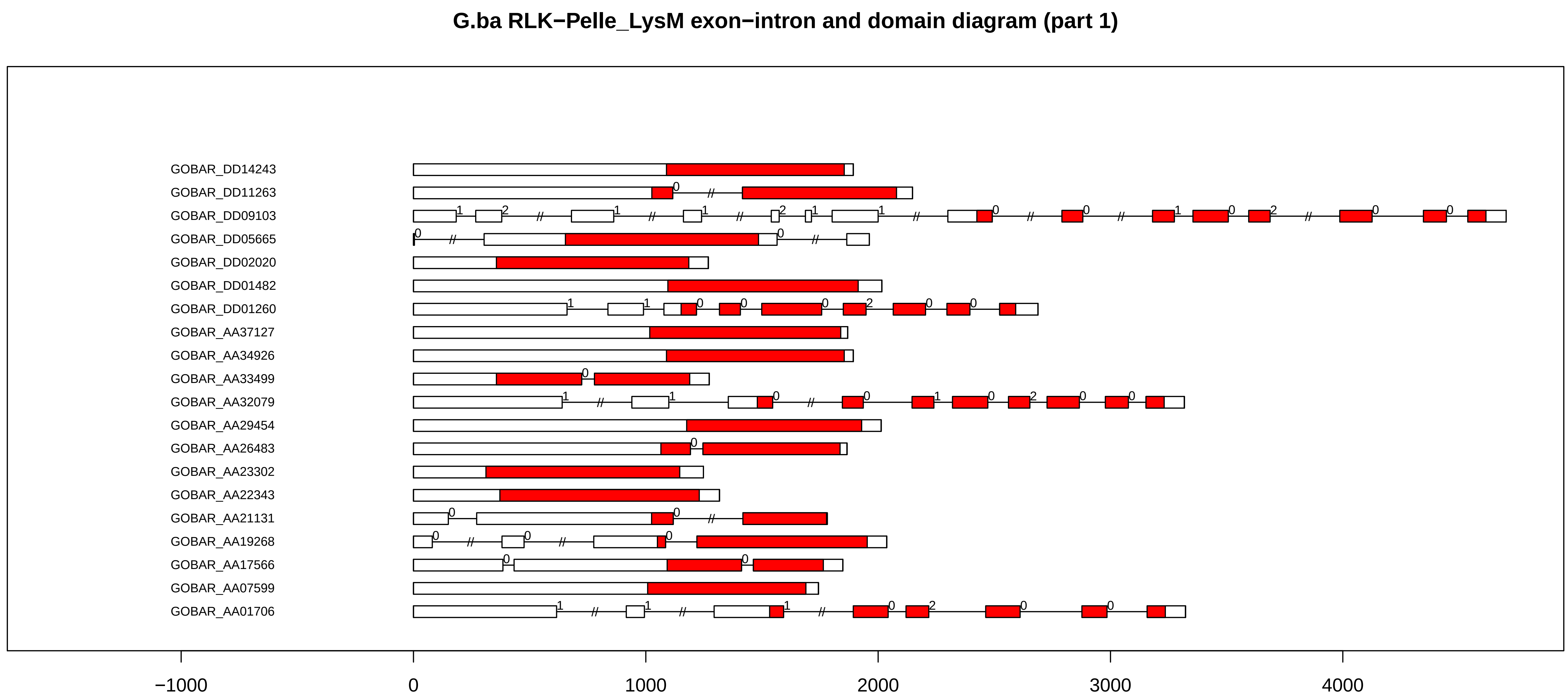
G.ba RLK−Pelle_LysM exon−intron and domain diagram (part 1)
GOBAR_DD14243
GOBAR_DD11263
GOBAR_DD09103
GOBAR_DD05665
GOBAR_DD02020
GOBAR_DD01482
GOBAR_DD01260
GOBAR_AA37127
GOBAR_AA34926
GOBAR_AA33499
GOBAR_AA32079
GOBAR_AA29454
GOBAR_AA26483
GOBAR_AA23302
GOBAR_AA22343
GOBAR_AA21131
GOBAR_AA19268
GOBAR_AA17566
GOBAR_AA07599
GOBAR_AA01706
−1000
0
1000
2000
3000
4000

G.ba RLK−Pelle_LysM exon−intron and domain diagram (part 2)

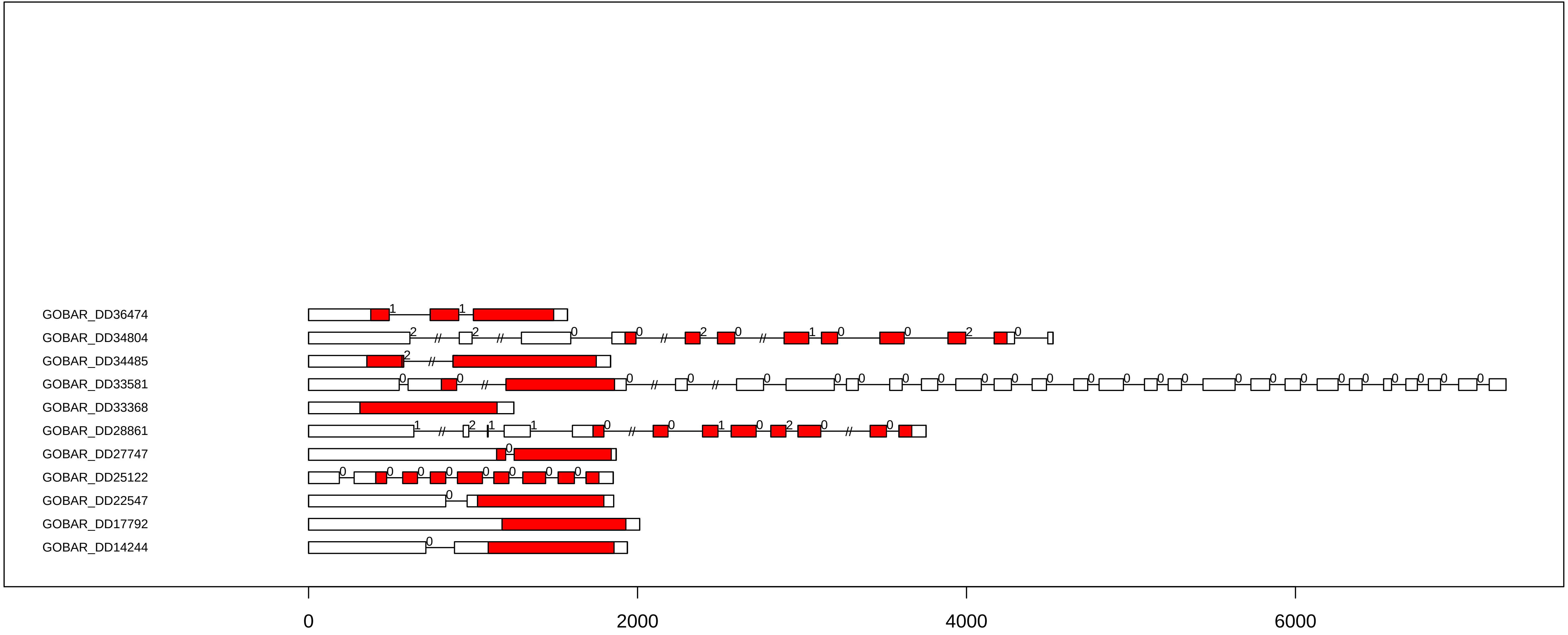

G.ba RLK−Pelle_PERK−1 exon−intron and domain diagram (part 1)

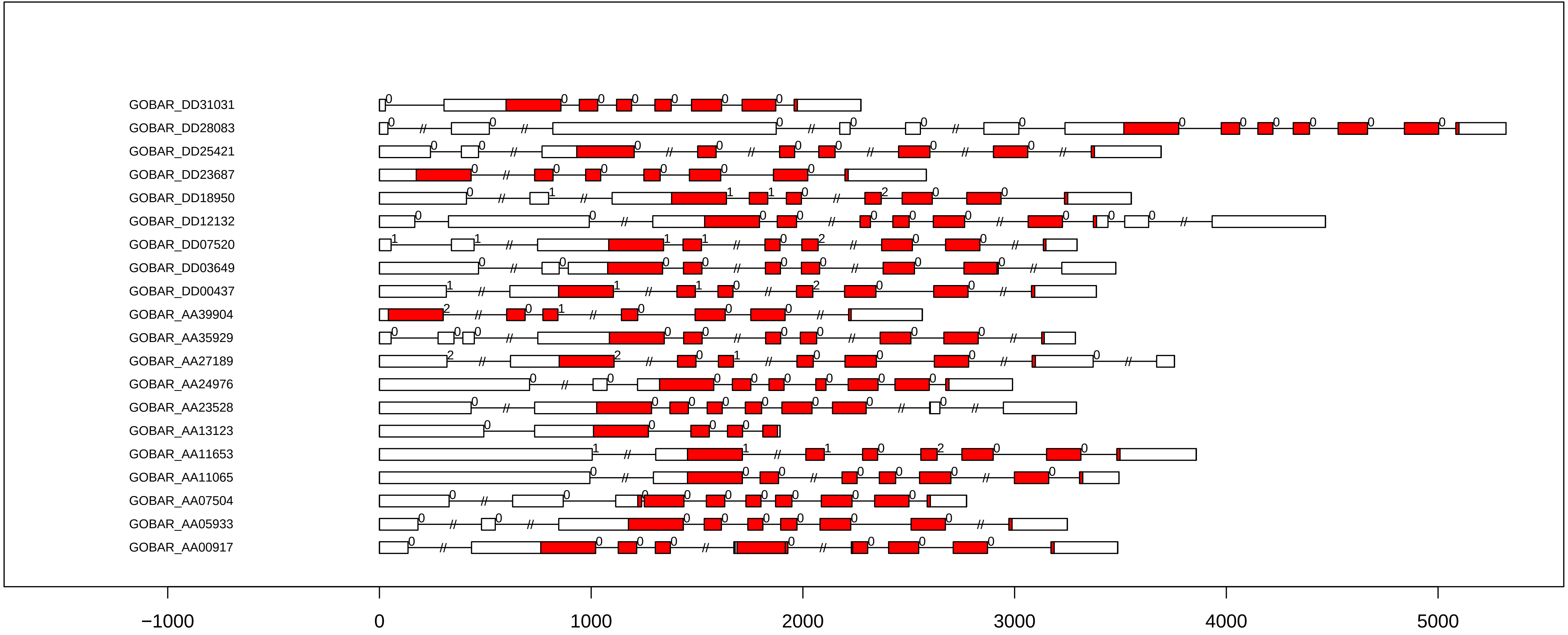

G.ba RLK−Pelle_PERK−1 exon−intron and domain diagram (part 2)

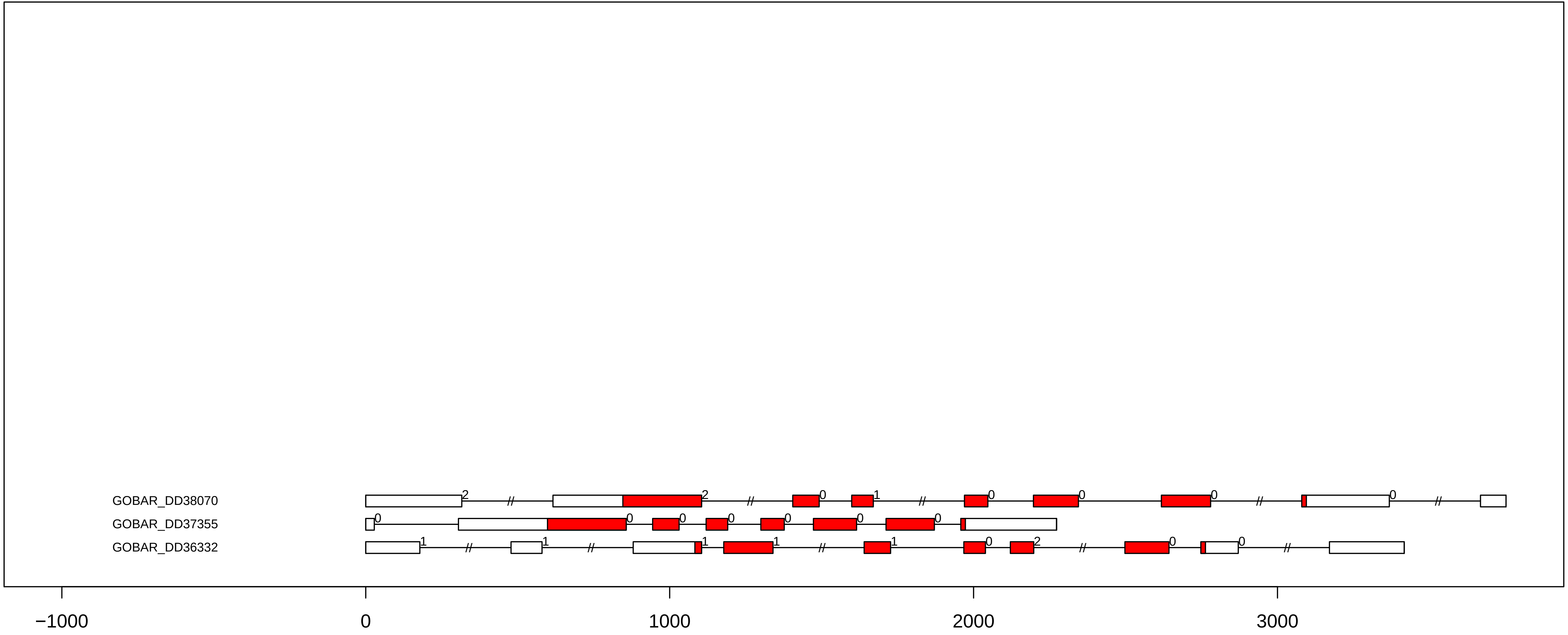

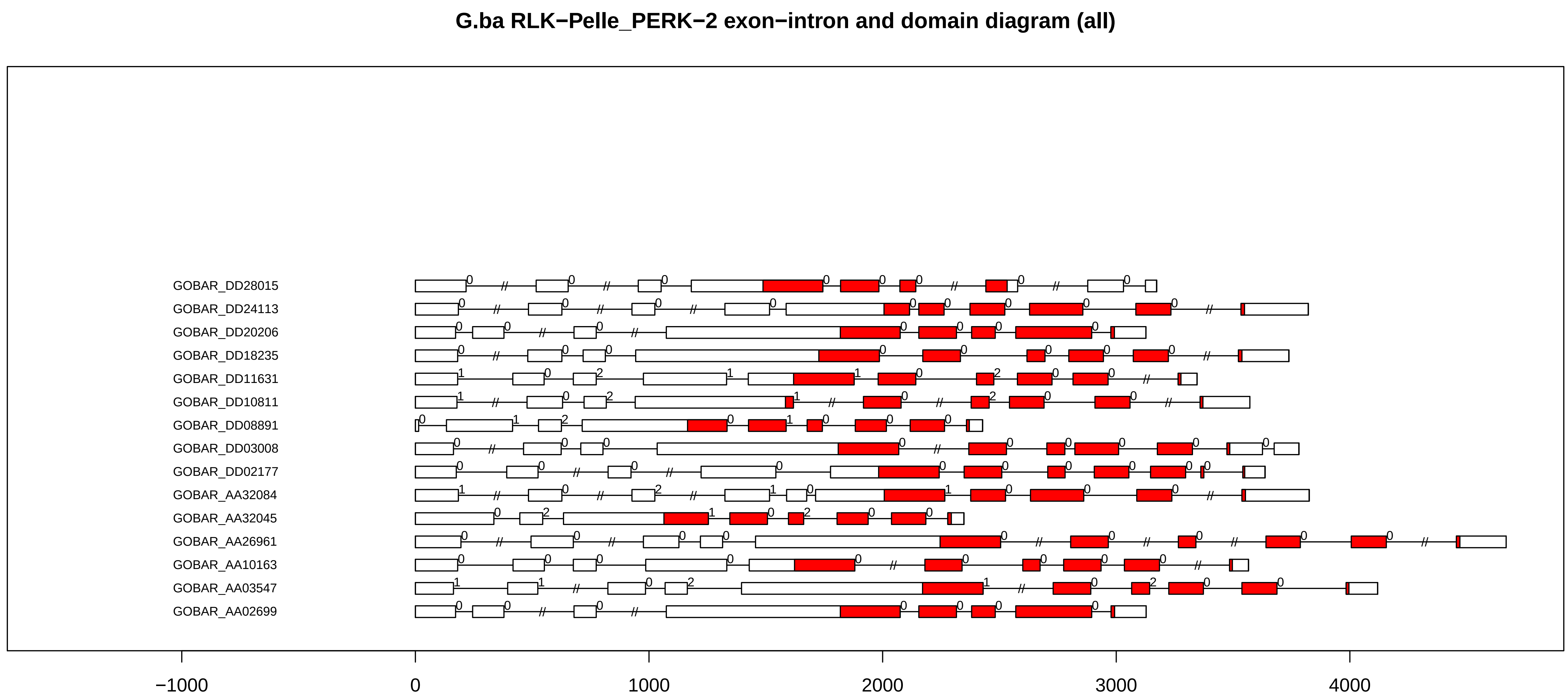
G.ba RLK−Pelle_PERK−2 exon−intron and domain diagram (all)
GOBAR_DD28015
GOBAR_DD24113
GOBAR_DD20206
GOBAR_DD18235
GOBAR_DD11631
GOBAR_DD10811
GOBAR_DD08891
GOBAR_DD03008
GOBAR_DD02177
GOBAR_AA32084
GOBAR_AA32045
GOBAR_AA26961
GOBAR_AA10163
GOBAR_AA03547
GOBAR_AA02699
−1000
0
1000
2000
3000
4000

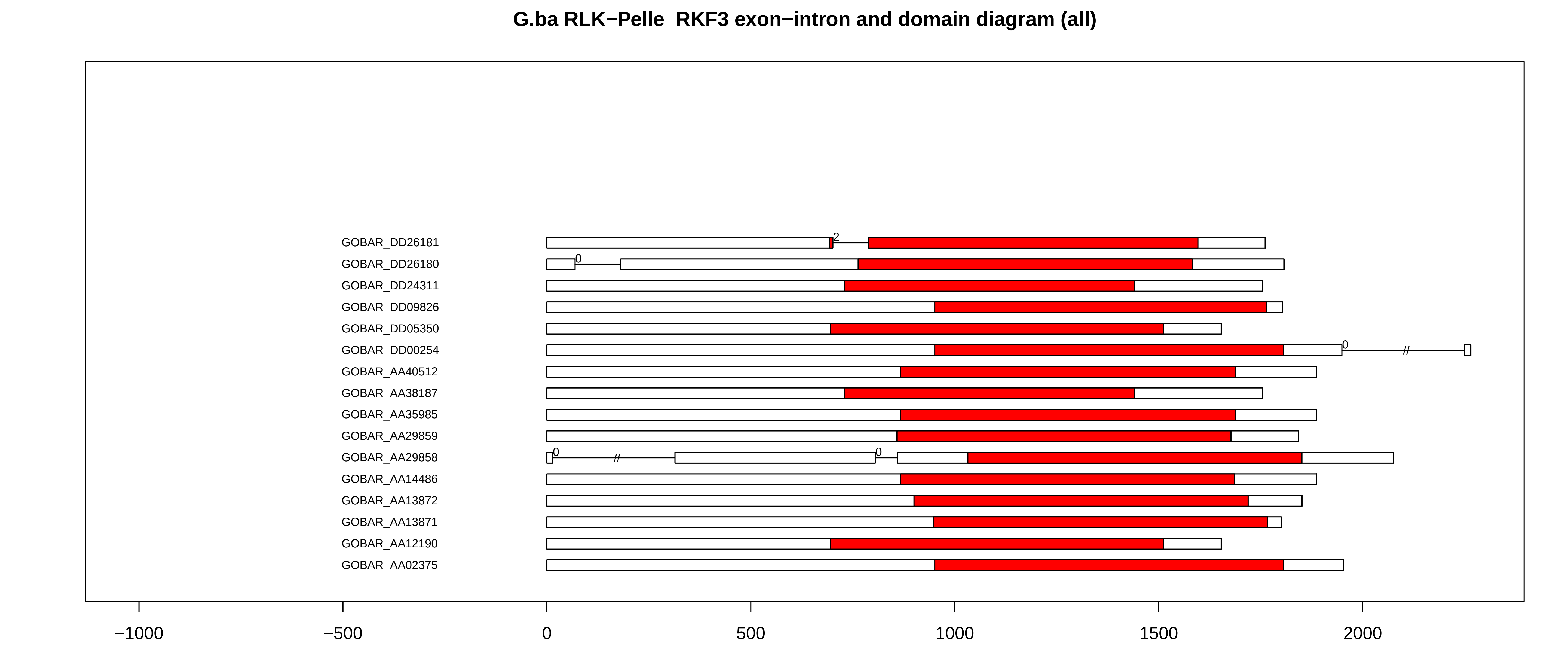

G.ba RLK−Pelle_RKF3 exon−intron and domain diagram (all)
GOBAR_DD26181
GOBAR_DD26180
GOBAR_DD24311
GOBAR_DD09826
GOBAR_DD05350
GOBAR_DD00254
GOBAR_AA40512
GOBAR_AA38187
GOBAR_AA35985
GOBAR_AA29859
GOBAR_AA29858
GOBAR_AA14486
GOBAR_AA13872
GOBAR_AA13871
GOBAR_AA12190
GOBAR_AA02375
−1000
−500
0
500
1000
1500
2000

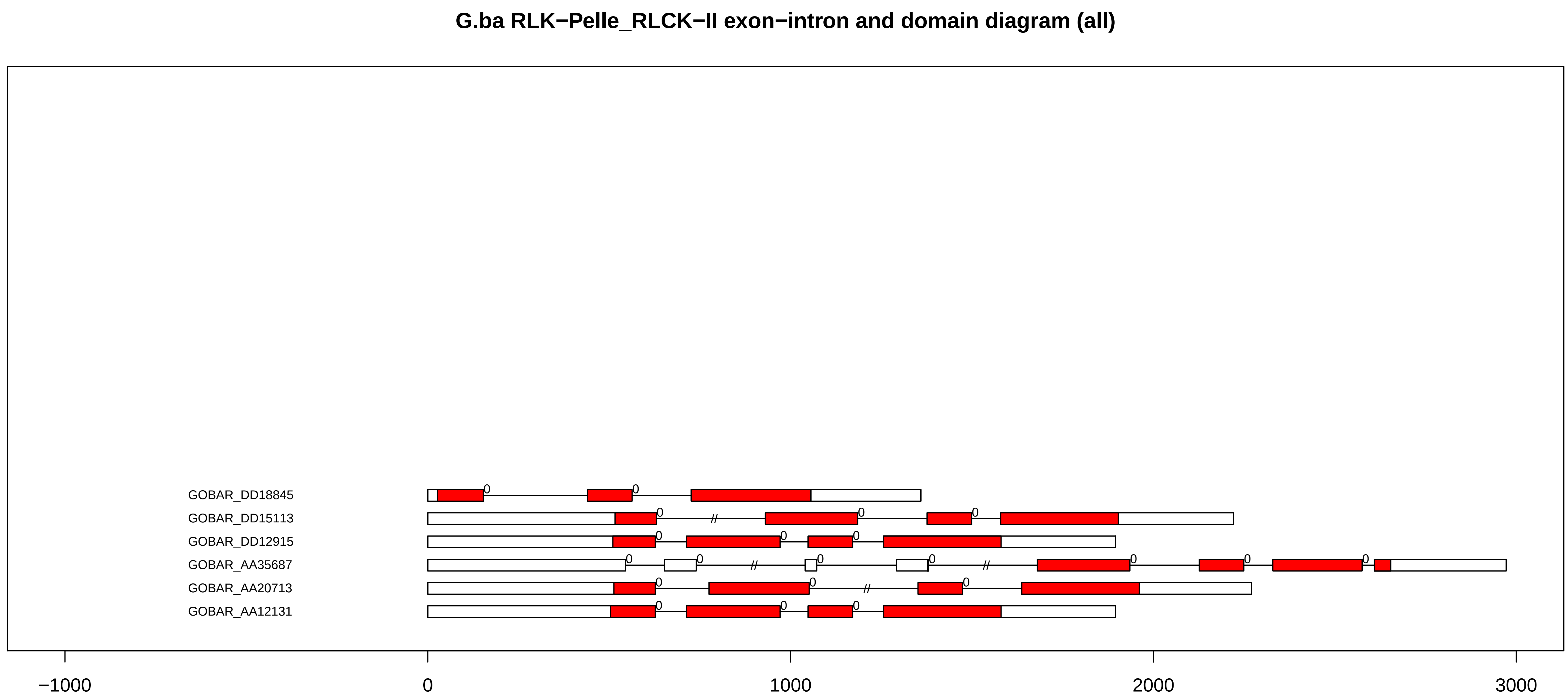

G.ba RLK−Pelle_RLCK−II exon−intron and domain diagram (all)
GOBAR_DD18845
GOBAR_DD15113
GOBAR_DD12915
GOBAR_AA35687
GOBAR_AA20713
GOBAR_AA12131
−1000
0
1000
2000
3000

G.ba RLK−Pelle_RLCK−IV exon−intron and domain diagram (all)

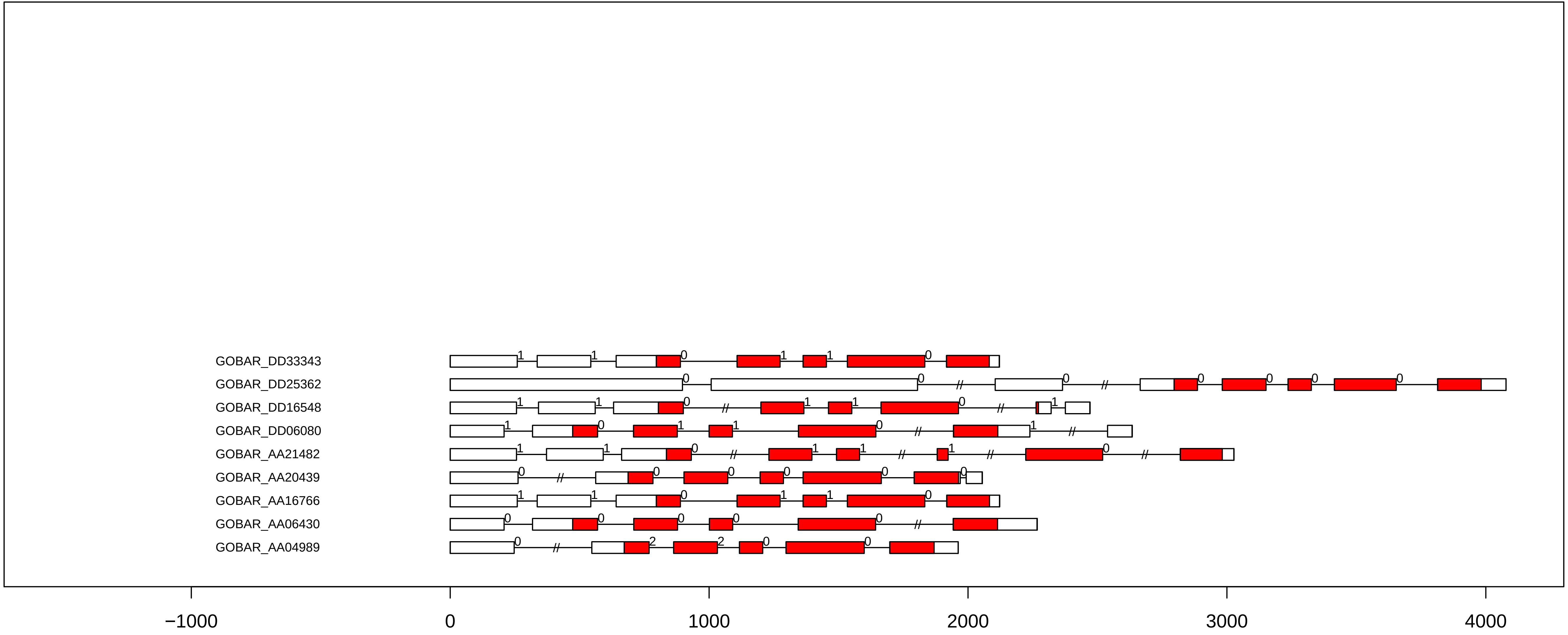

G.ba RLK−Pelle_RLCK−IXa exon−intron and domain diagram (all)

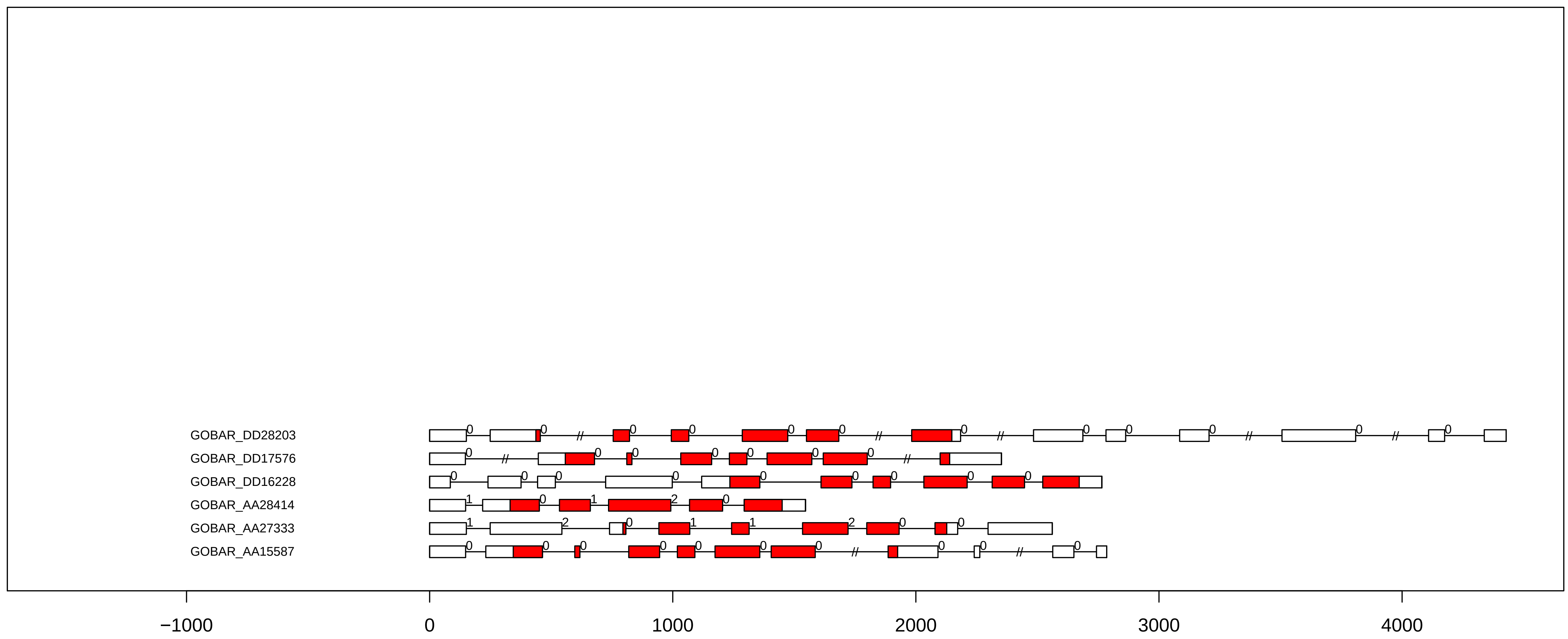

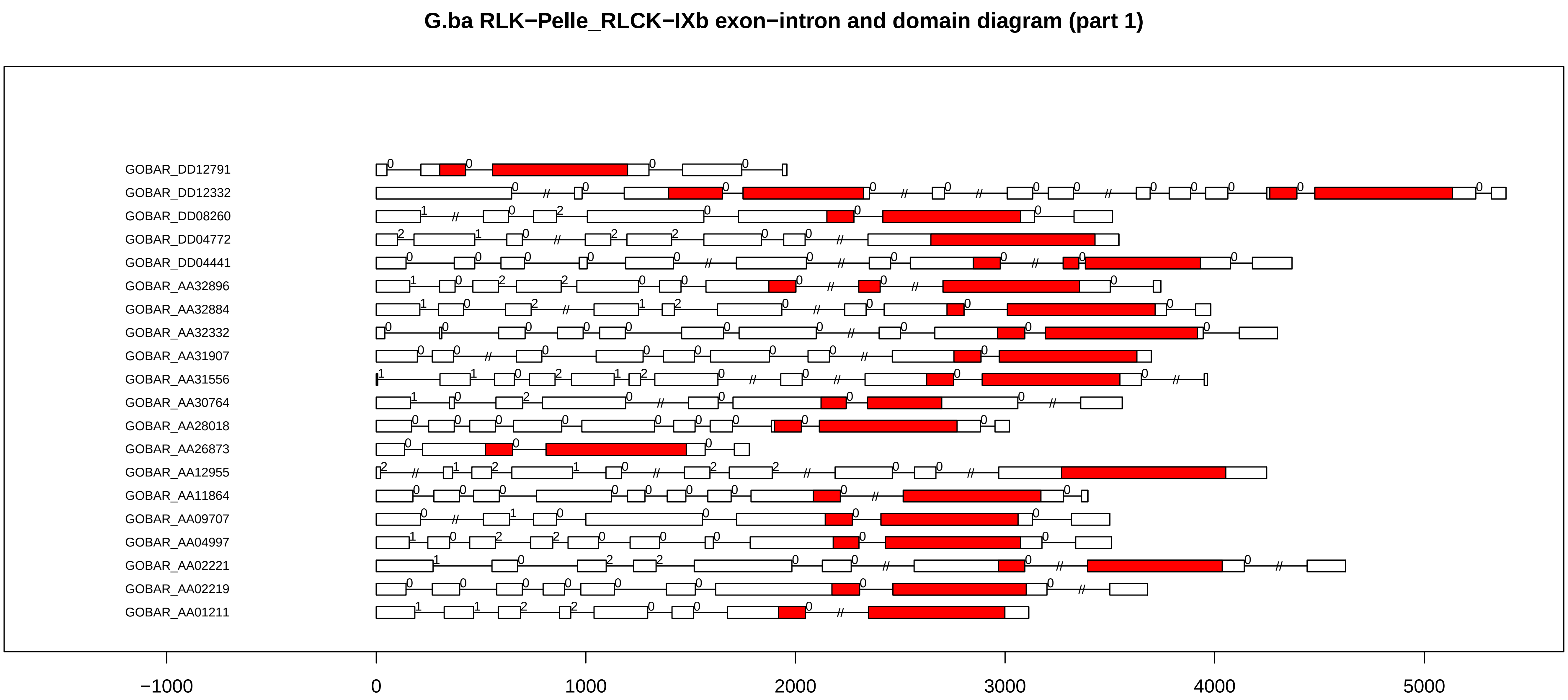
G.ba RLK−Pelle_RLCK−IXb exon−intron and domain diagram (part 1)
GOBAR_DD12791
GOBAR_DD12332
GOBAR_DD08260
GOBAR_DD04772
GOBAR_DD04441
GOBAR_AA32896
GOBAR_AA32884
GOBAR_AA32332
GOBAR_AA31907
GOBAR_AA31556
GOBAR_AA30764
GOBAR_AA28018
GOBAR_AA26873
GOBAR_AA12955
GOBAR_AA11864
GOBAR_AA09707
GOBAR_AA04997
GOBAR_AA02221
GOBAR_AA02219
GOBAR_AA01211
−1000
0
1000
2000
3000
4000
5000

G.ba RLK−Pelle_RLCK−IXb exon−intron and domain diagram (part 2)

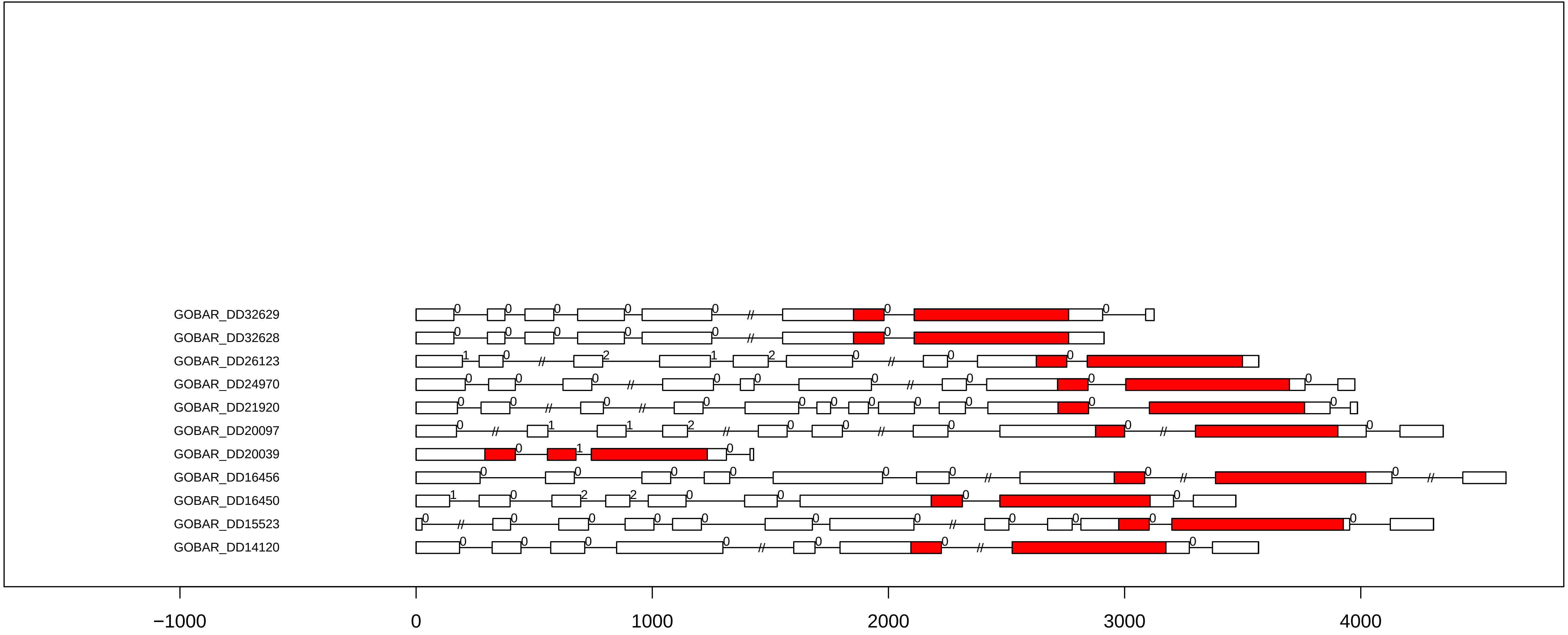

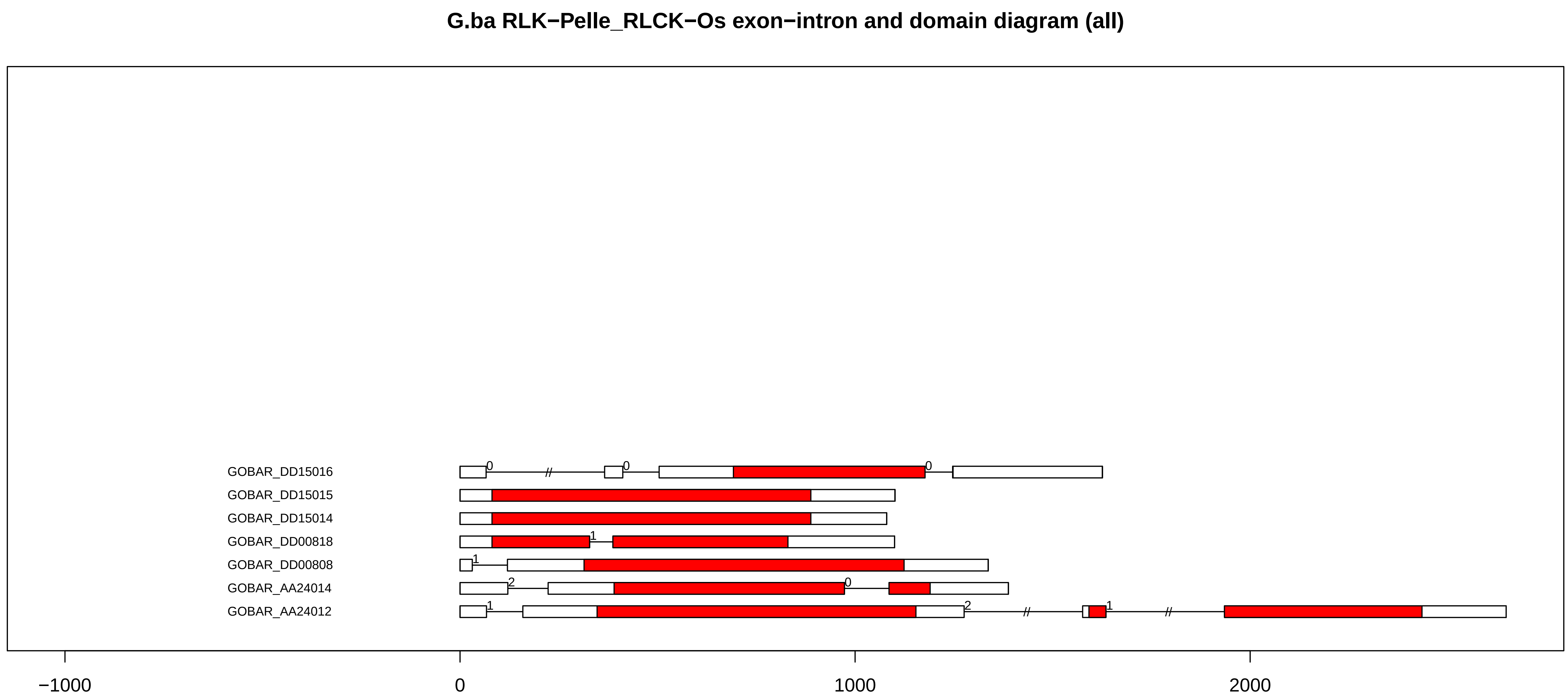
G.ba RLK−Pelle_RLCK−Os exon−intron and domain diagram (all)
GOBAR_DD15016
GOBAR_DD15015
GOBAR_DD15014
GOBAR_DD00818
GOBAR_DD00808
GOBAR_AA24014
GOBAR_AA24012
−1000
0
1000
2000

G.ba RLK−Pelle_RLCK−VIIa−1 exon−intron and domain diagram (part 1)

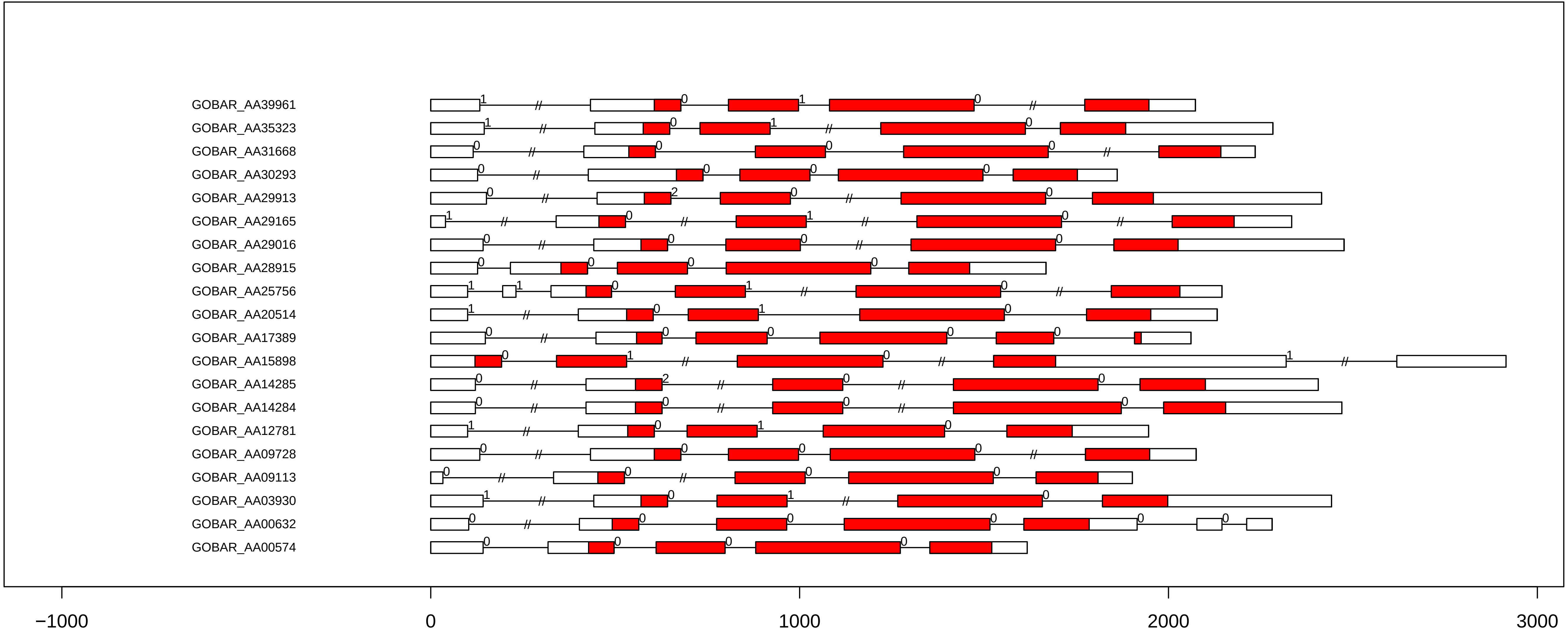

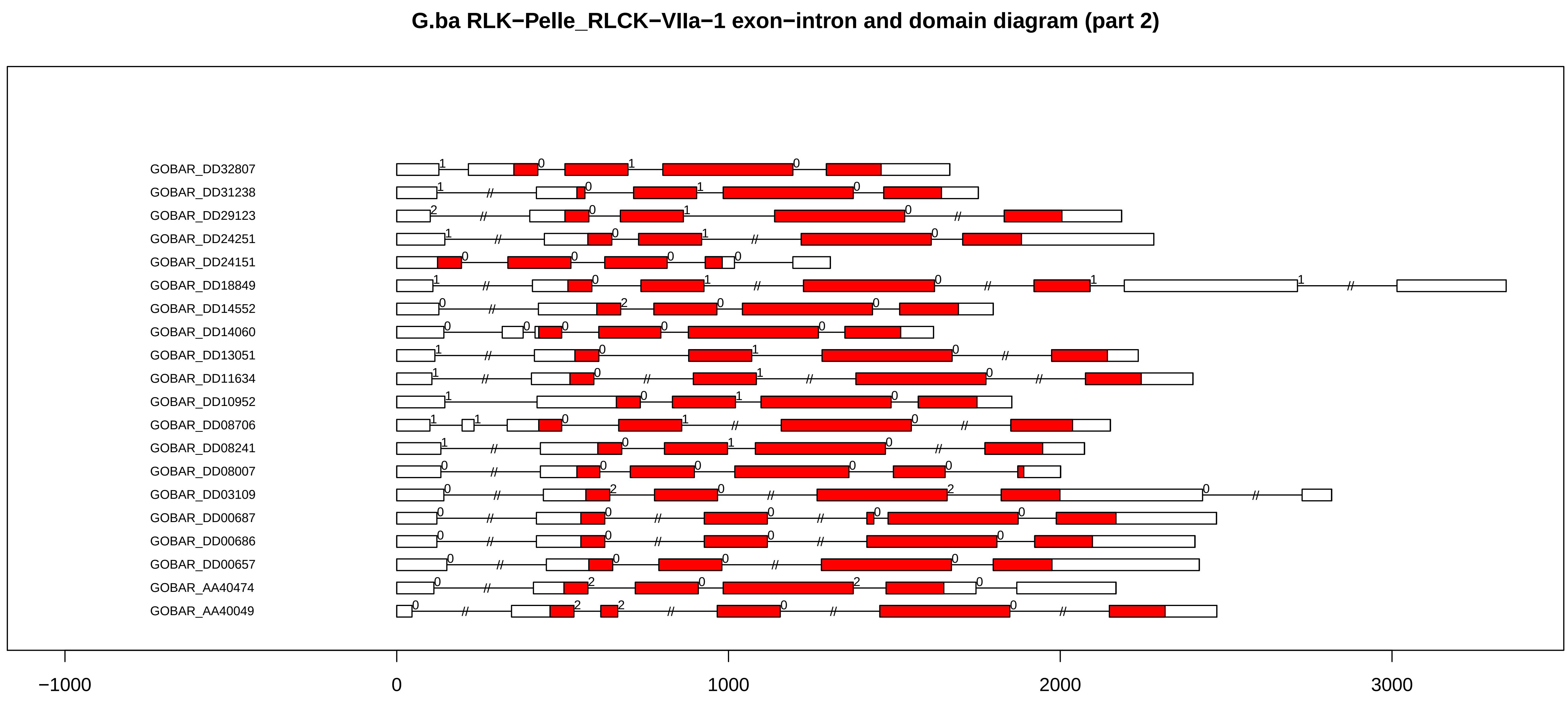
G.ba RLK−Pelle_RLCK−VIIa−1 exon−intron and domain diagram (part 2)
GOBAR_DD32807
GOBAR_DD31238
GOBAR_DD29123
GOBAR_DD24251
GOBAR_DD24151
GOBAR_DD18849
GOBAR_DD14552
GOBAR_DD14060
GOBAR_DD13051
GOBAR_DD11634
GOBAR_DD10952
GOBAR_DD08706
GOBAR_DD08241
GOBAR_DD08007
GOBAR_DD03109
GOBAR_DD00687
GOBAR_DD00686
GOBAR_DD00657
GOBAR_AA40474
GOBAR_AA40049
−1000
0
1000
2000
3000

G.ba RLK−Pelle_RLCK−VIIa−1 exon−intron and domain diagram (part 3)

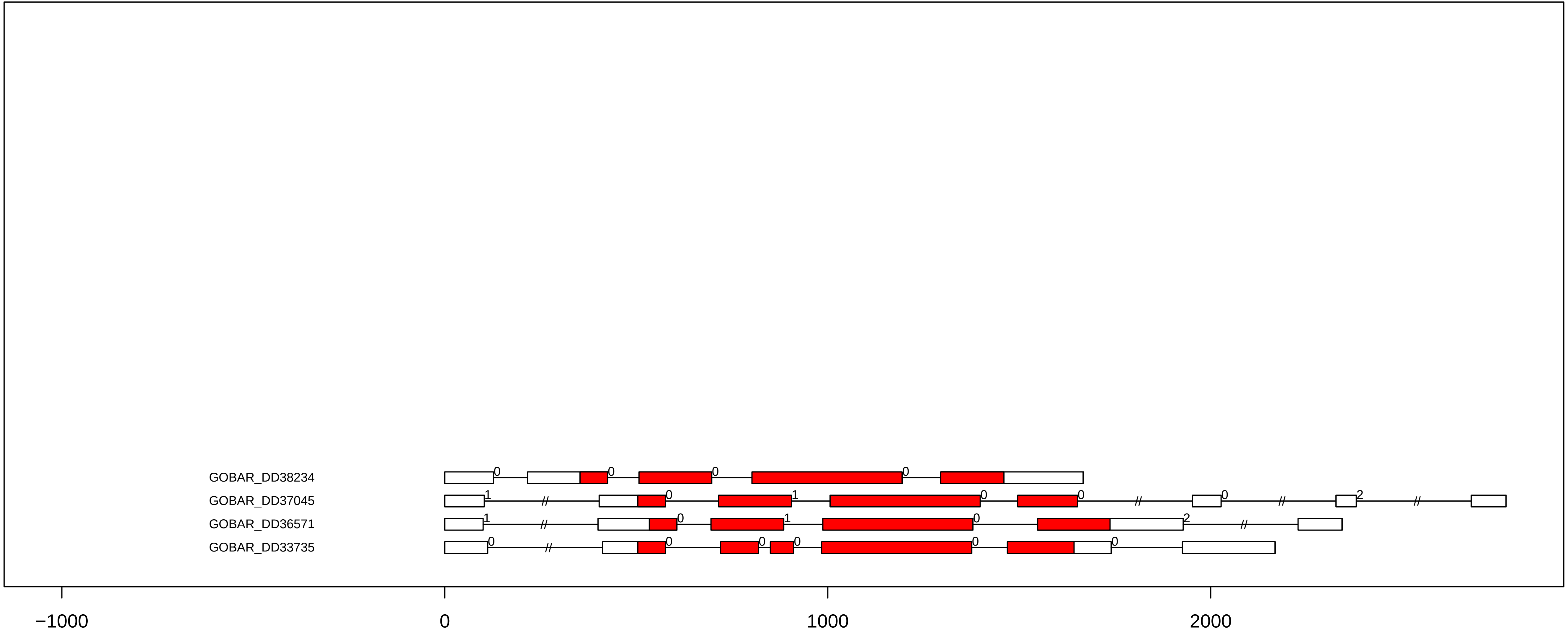

G.ba RLK−Pelle_RLCK−VIIa−2 exon−intron and domain diagram (part 1)

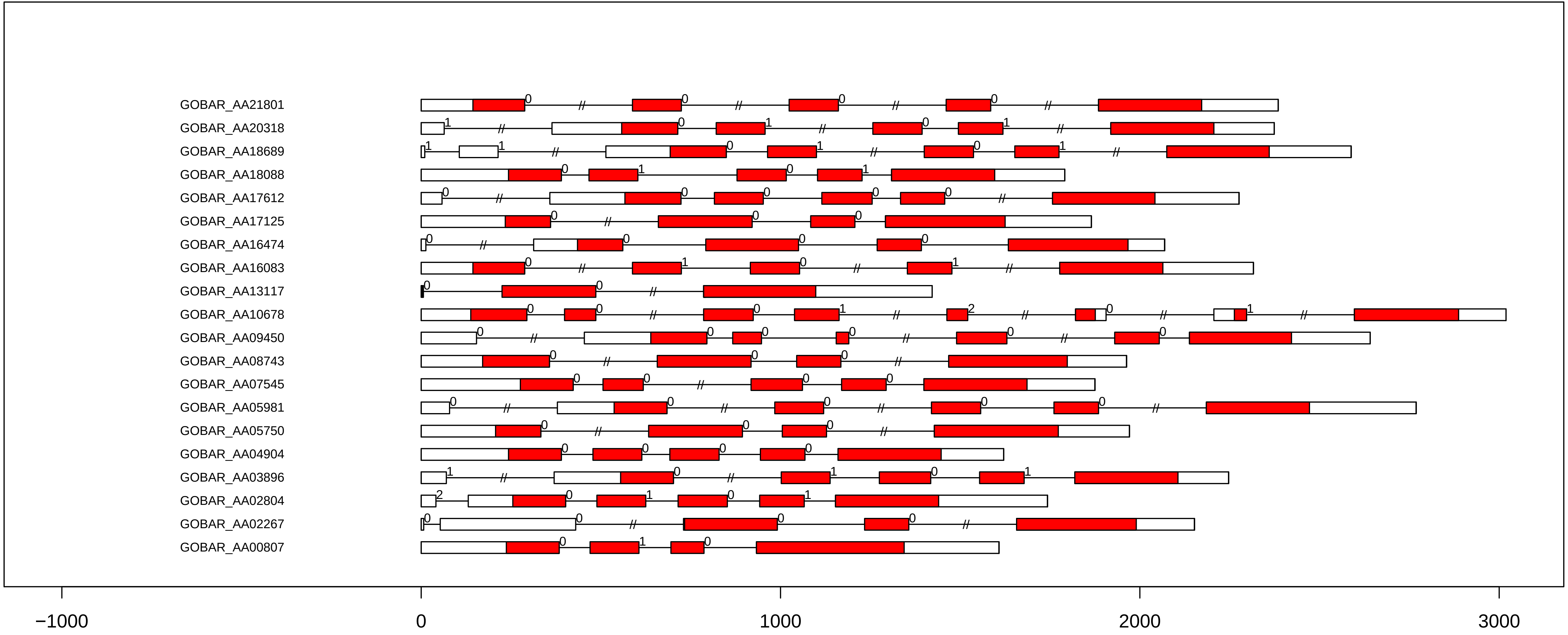

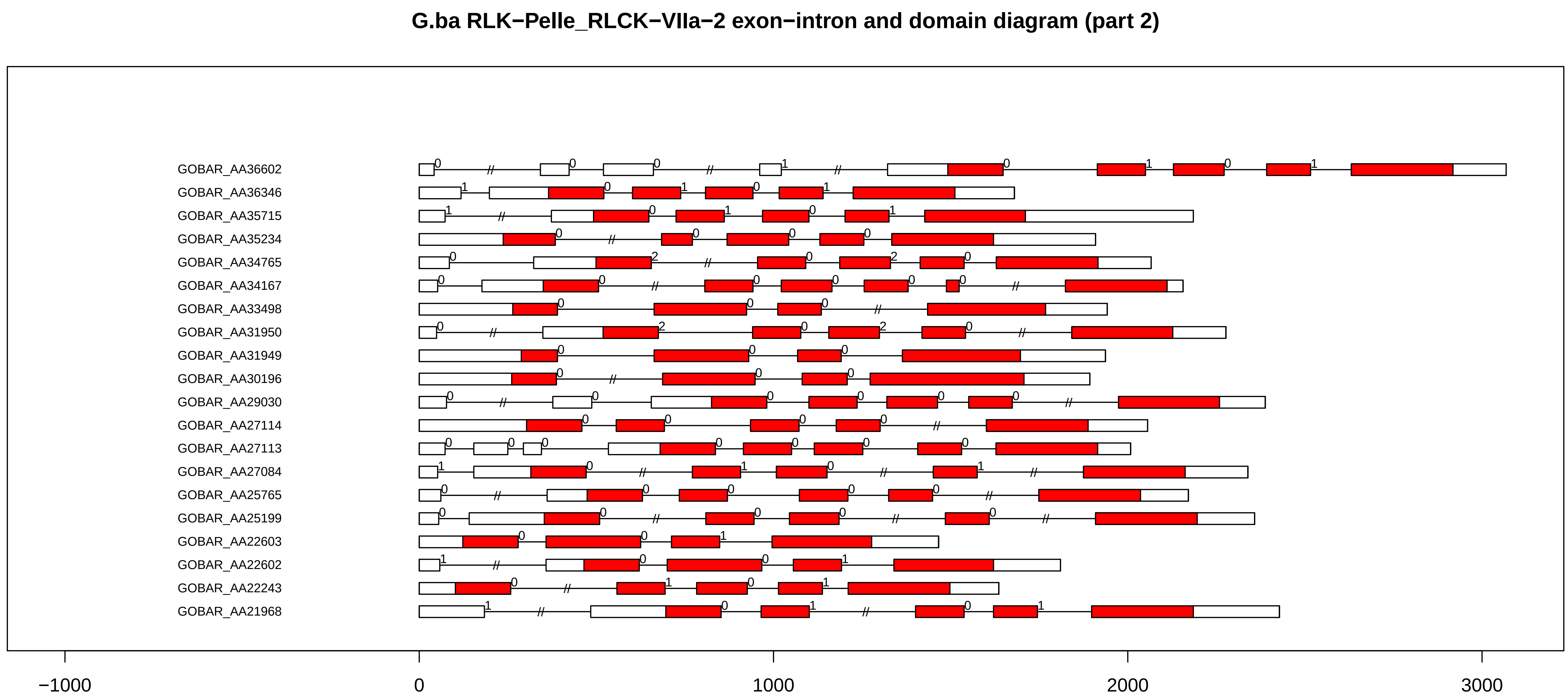

G.ba RLK−Pelle_RLCK−VIIa−2 exon−intron and domain diagram (part 2)
GOBAR_AA36602
GOBAR_AA36346
GOBAR_AA35715
GOBAR_AA35234
GOBAR_AA34765
GOBAR_AA34167
GOBAR_AA33498
GOBAR_AA31950
GOBAR_AA31949
GOBAR_AA30196
GOBAR_AA29030
GOBAR_AA27114
GOBAR_AA27113
GOBAR_AA27084
GOBAR_AA25765
GOBAR_AA25199
GOBAR_AA22603
GOBAR_AA22602
GOBAR_AA22243
GOBAR_AA21968
−1000
0
1000
2000
3000

G.ba RLK−Pelle_RLCK−VIIa−2 exon−intron and domain diagram (part 3)

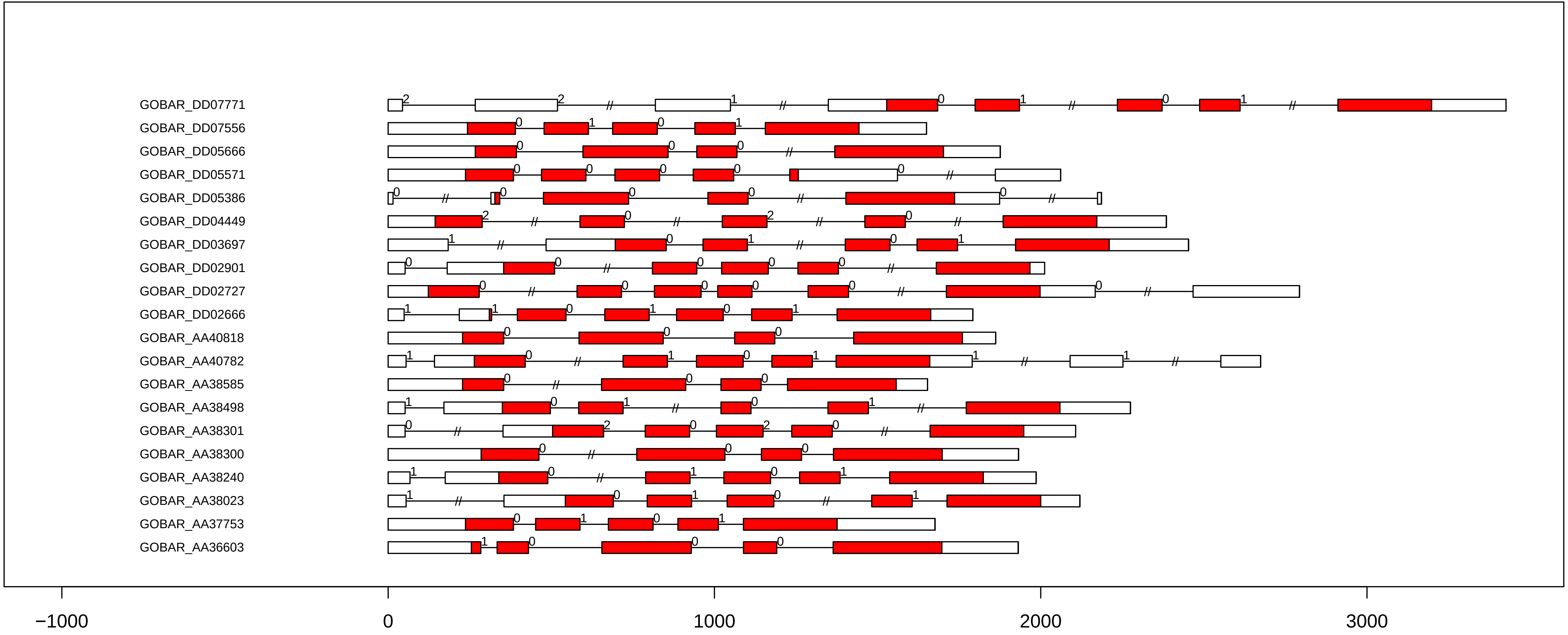

G.ba RLK−Pelle_RLCK−VIIa−2 exon−intron and domain diagram (part 4)

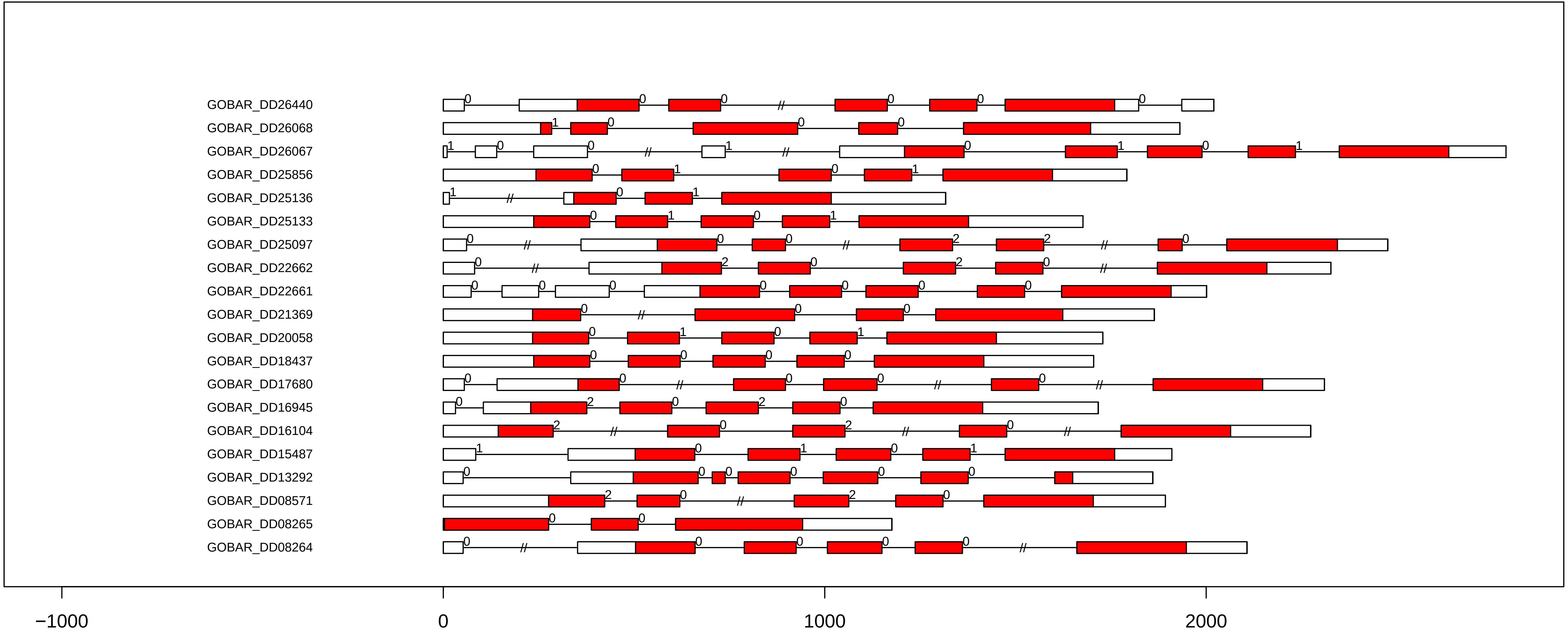

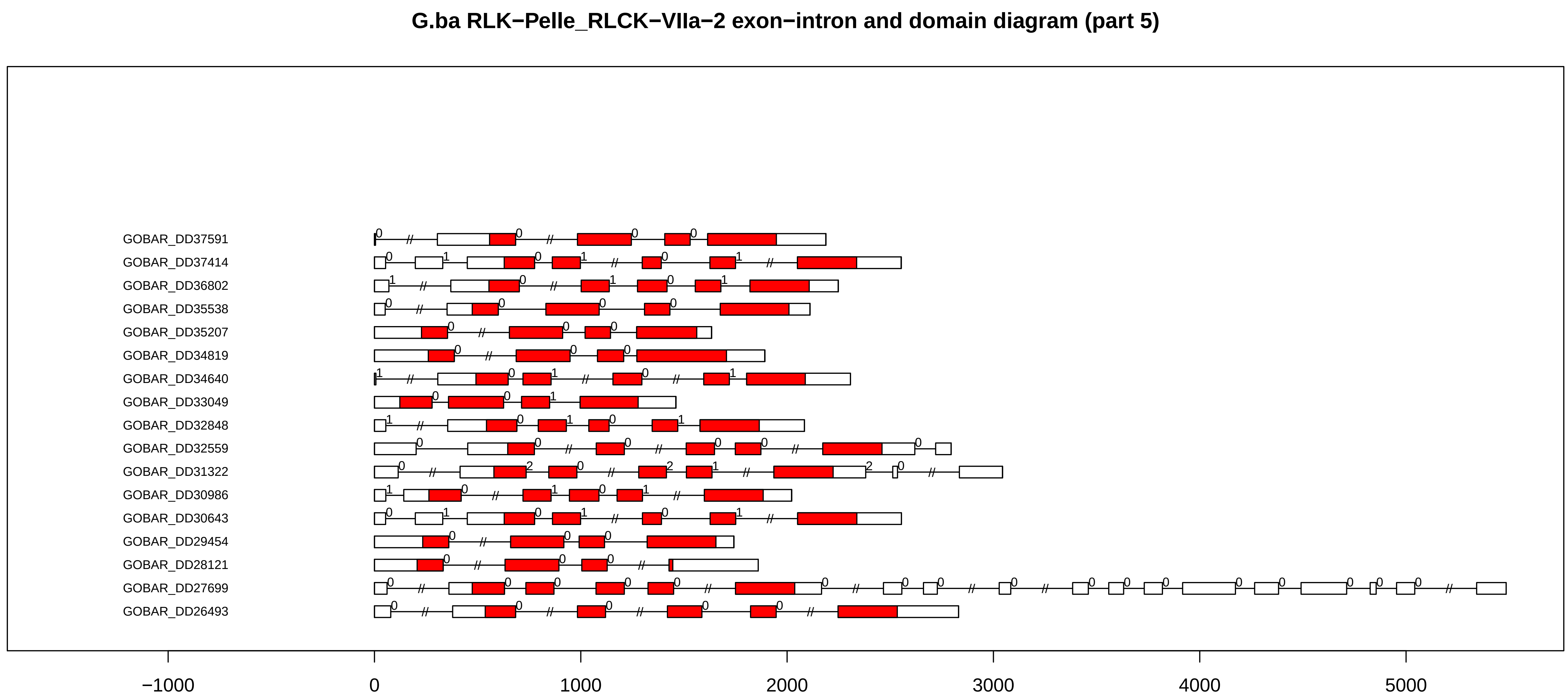
G.ba RLK−Pelle_RLCK−VIIa−2 exon−intron and domain diagram (part 5)
GOBAR_DD37591
GOBAR_DD37414
GOBAR_DD36802
GOBAR_DD35538
GOBAR_DD35207
GOBAR_DD34819
GOBAR_DD34640
GOBAR_DD33049
GOBAR_DD32848
GOBAR_DD32559
GOBAR_DD31322
GOBAR_DD30986
GOBAR_DD30643
GOBAR_DD29454
GOBAR_DD28121
GOBAR_DD27699
GOBAR_DD26493
−1000
0
1000
2000
3000
4000
5000

G.ba RLK−Pelle_RLCK−VIIb exon−intron and domain diagram (all)

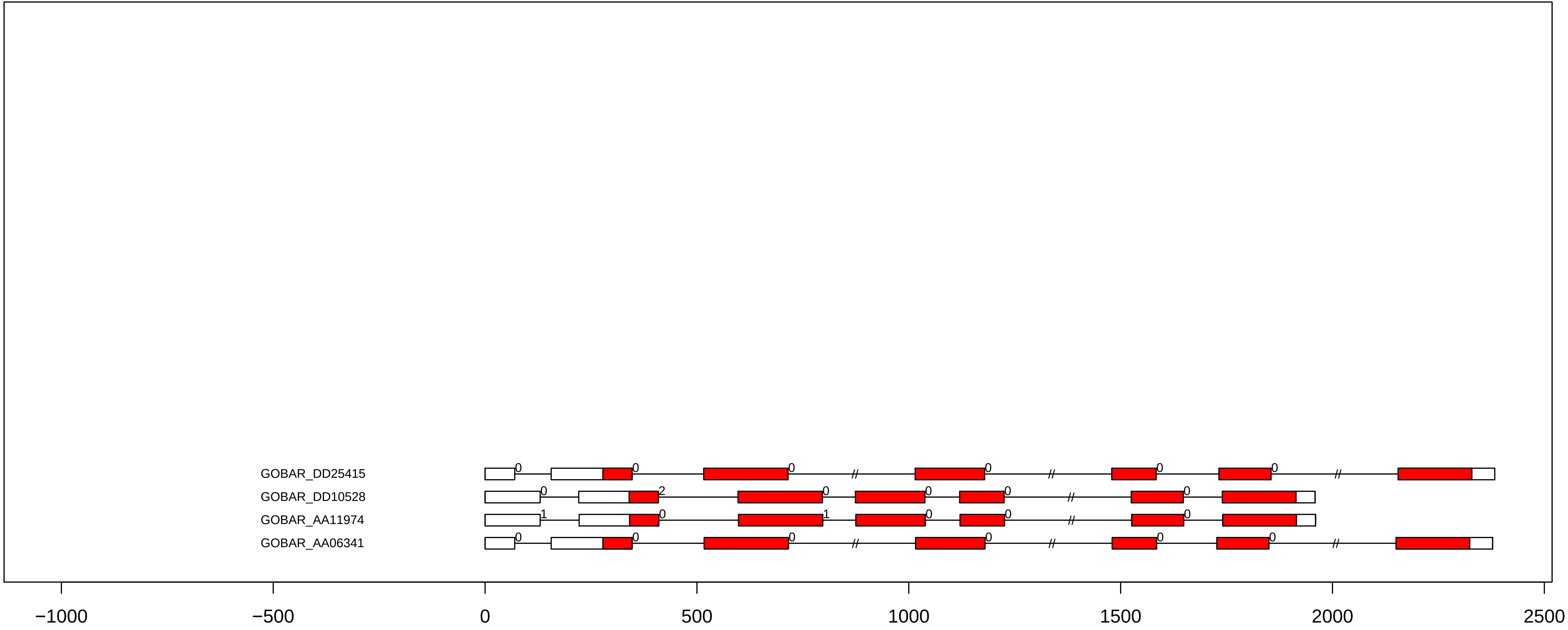

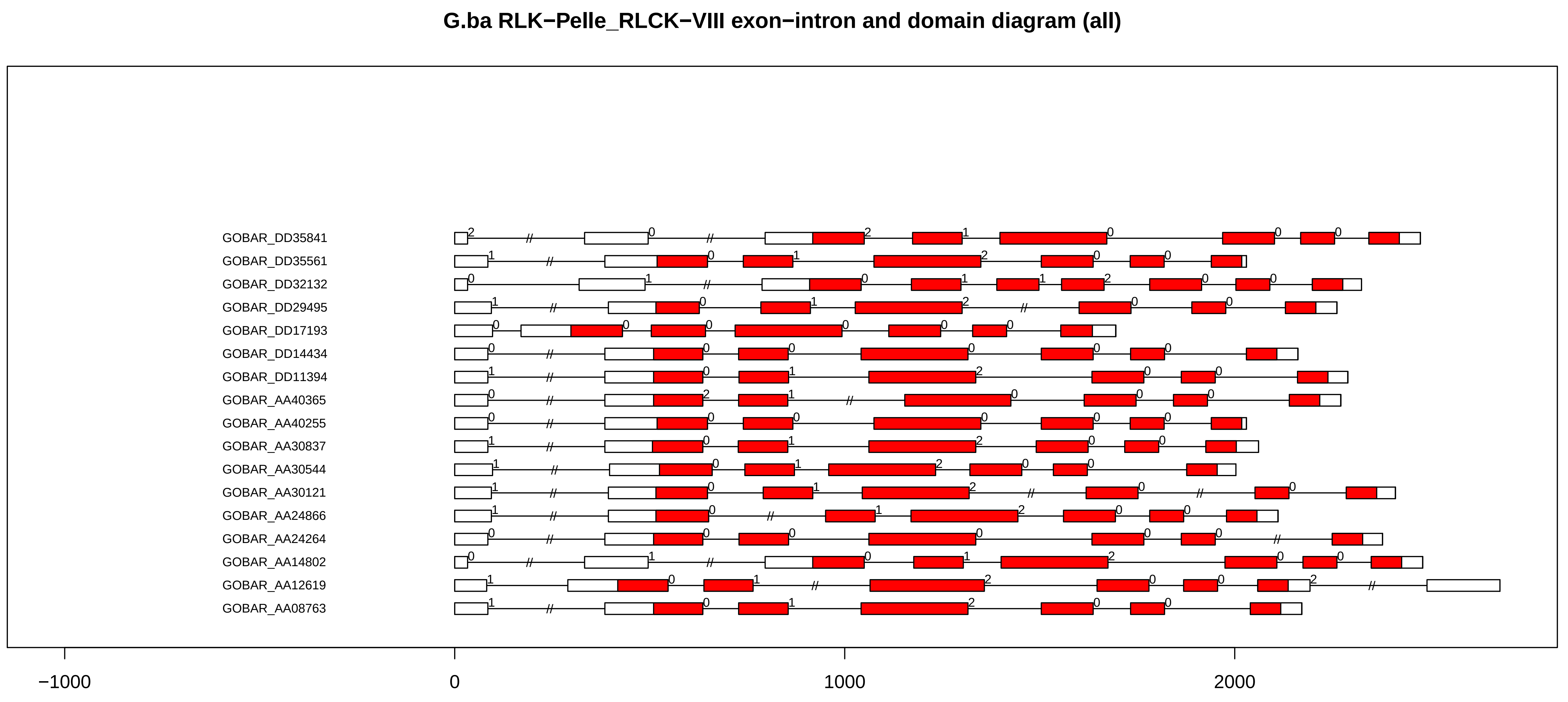
G.ba RLK−Pelle_RLCK−VIII exon−intron and domain diagram (all)
GOBAR_DD35841
GOBAR_DD35561
GOBAR_DD32132
GOBAR_DD29495
GOBAR_DD17193
GOBAR_DD14434
GOBAR_DD11394
GOBAR_AA40365
GOBAR_AA40255
GOBAR_AA30837
GOBAR_AA30544
GOBAR_AA30121
GOBAR_AA24866
GOBAR_AA24264
GOBAR_AA14802
GOBAR_AA12619
GOBAR_AA08763
−1000
0
1000
2000

G.ba RLK−Pelle_RLCK−VI exon−intron and domain diagram (part 1)

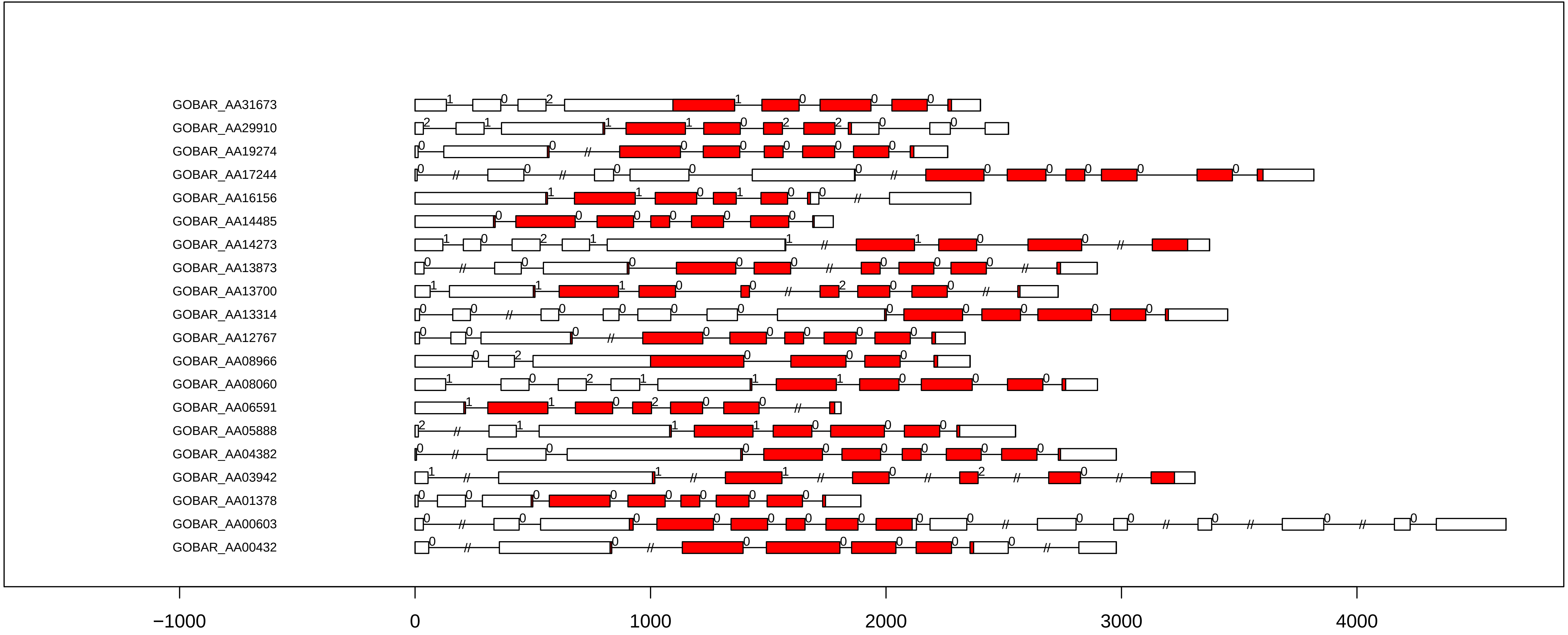

G.ba RLK−Pelle_RLCK−VI exon−intron and domain diagram (part 2)

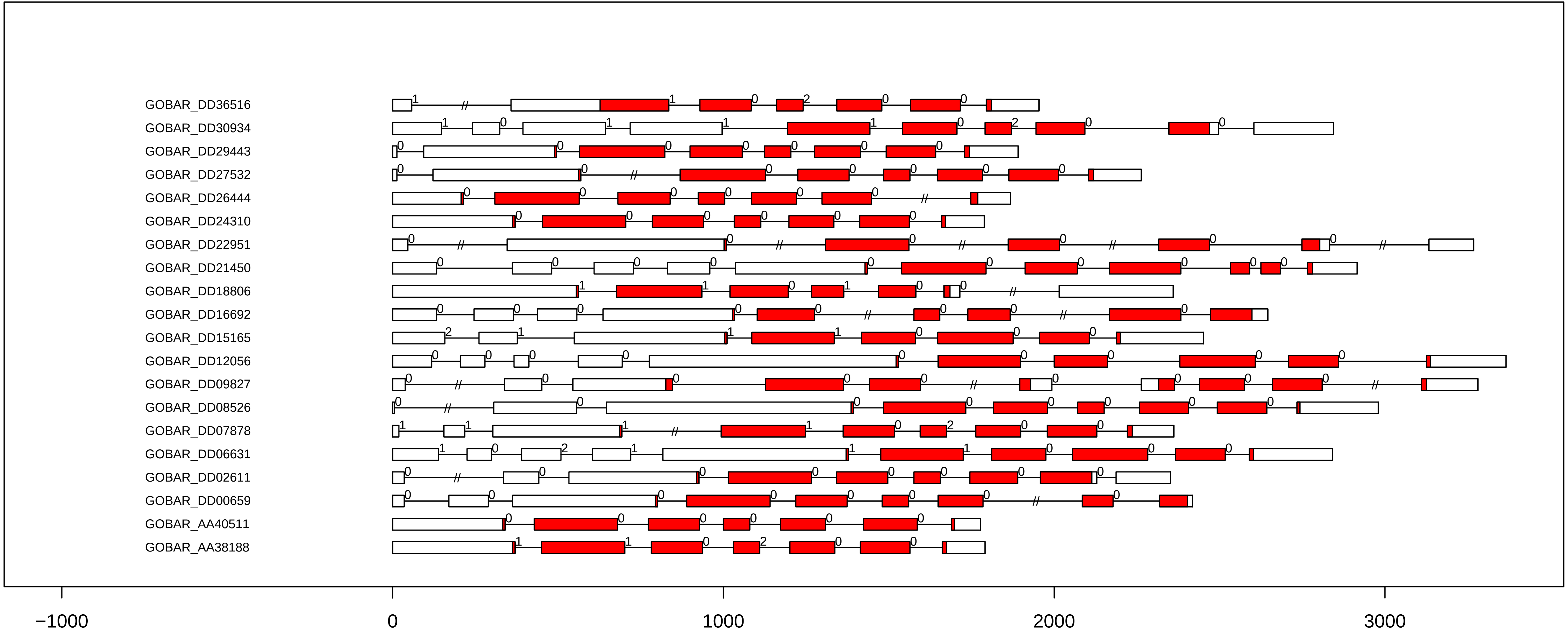

G.ba RLK−Pelle_RLCK−V exon−intron and domain diagram (part 1)

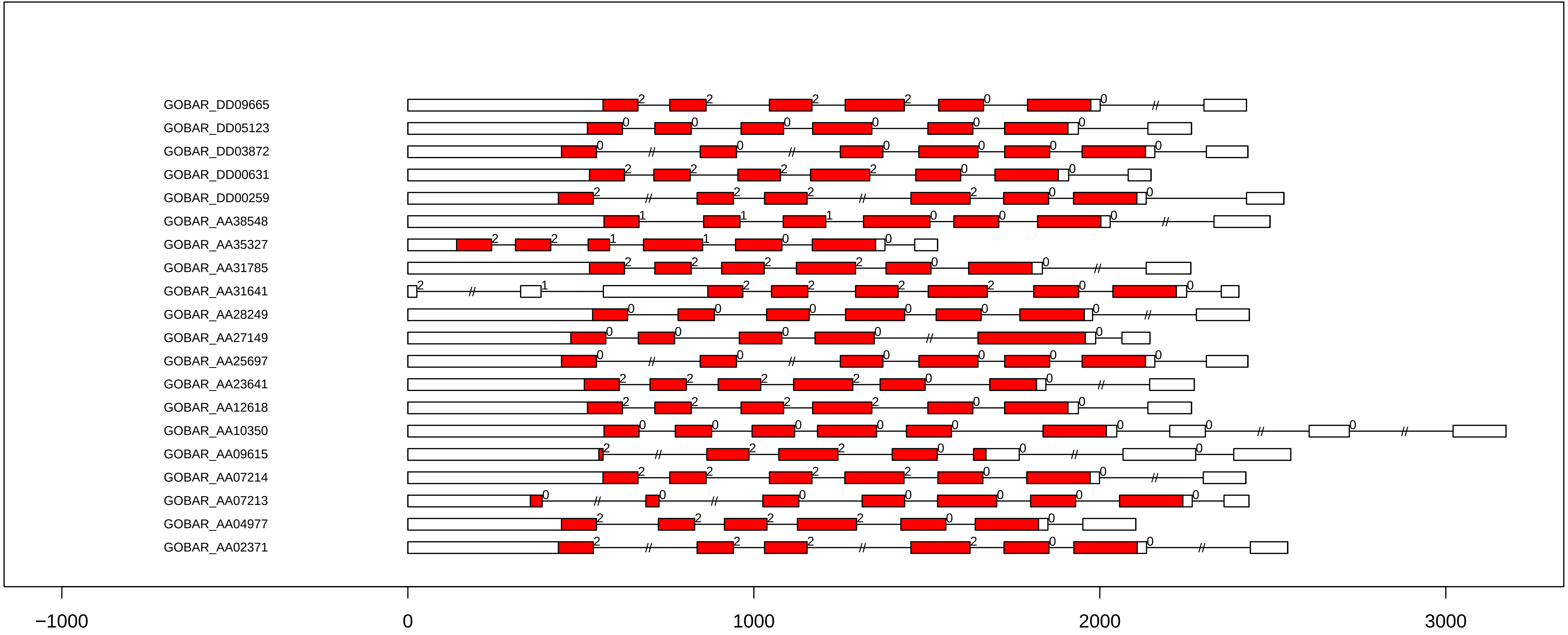

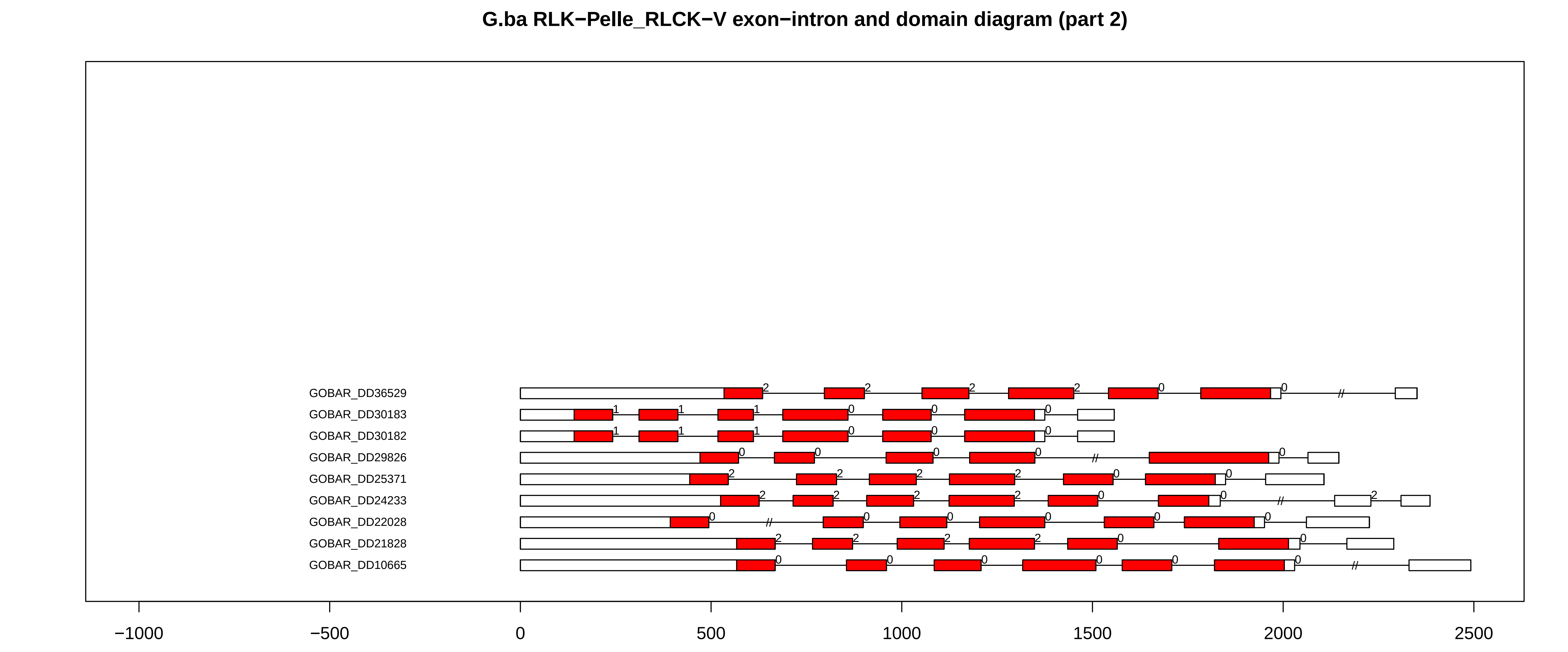
G.ba RLK−Pelle_RLCK−V exon−intron and domain diagram (part 2)
GOBAR_DD36529
GOBAR_DD30183
GOBAR_DD30182
GOBAR_DD29826
GOBAR_DD25371
GOBAR_DD24233
GOBAR_DD22028
GOBAR_DD21828
GOBAR_DD10665
−1000
−500
0
500
1000
1500
2000
2500

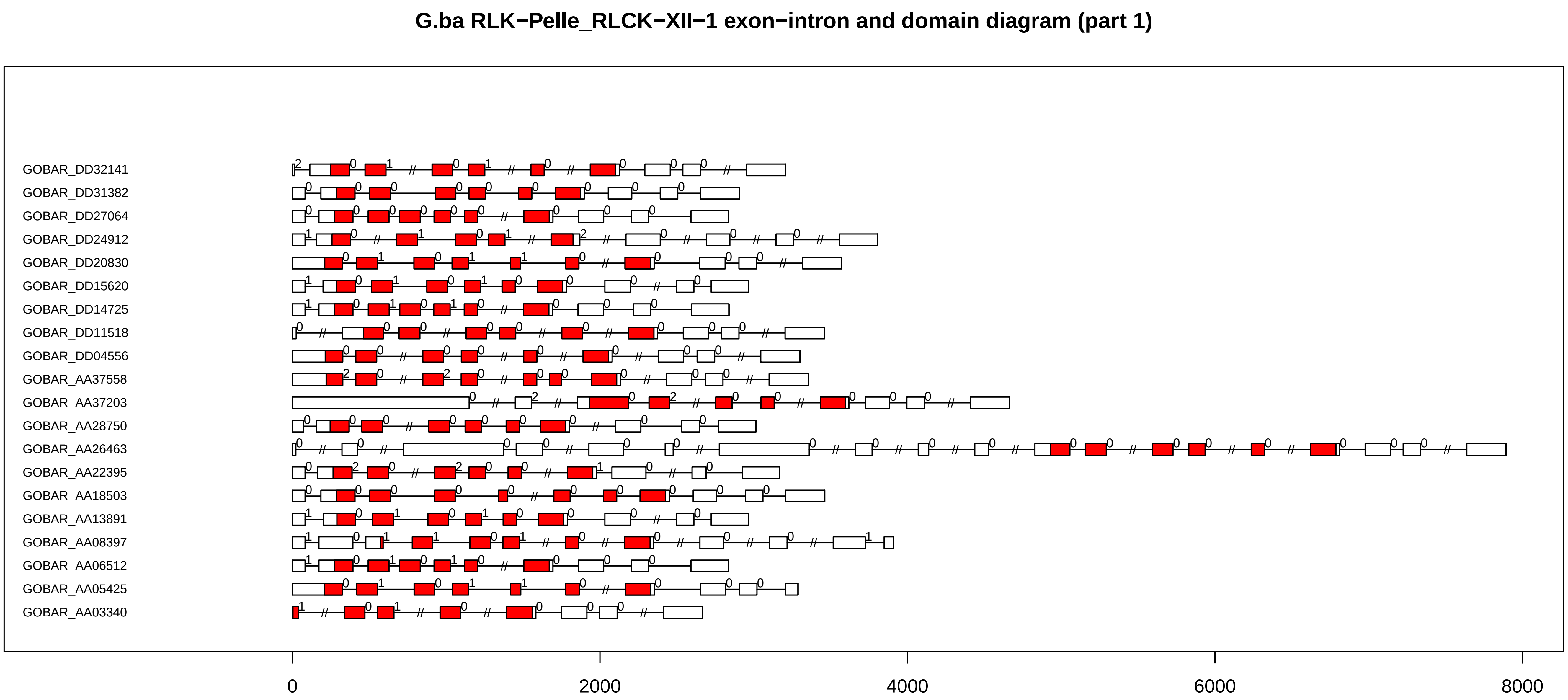

G.ba RLK−Pelle_RLCK−XII−1 exon−intron and domain diagram (part 1)
GOBAR_DD32141
GOBAR_DD31382
GOBAR_DD27064
GOBAR_DD24912
GOBAR_DD20830
GOBAR_DD15620
GOBAR_DD14725
GOBAR_DD11518
GOBAR_DD04556
GOBAR_AA37558
GOBAR_AA37203
GOBAR_AA28750
GOBAR_AA26463
GOBAR_AA22395
GOBAR_AA18503
GOBAR_AA13891
GOBAR_AA08397
GOBAR_AA06512
GOBAR_AA05425
GOBAR_AA03340
0
2000
4000
6000
8000

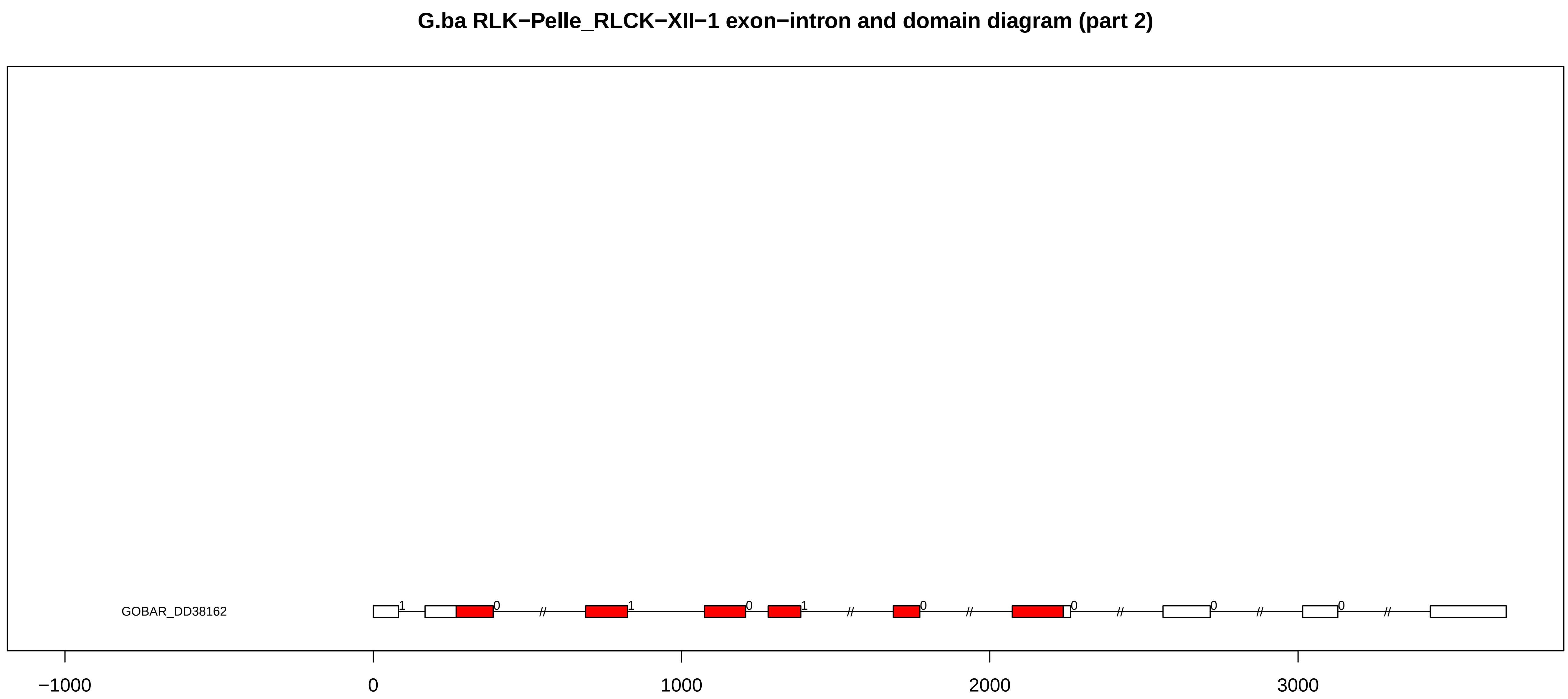
G.ba RLK−Pelle_RLCK−XII−1 exon−intron and domain diagram (part 2)
GOBAR_DD38162
1
0
1
0
1
0
0
0
0
−1000
0
1000
2000
3000

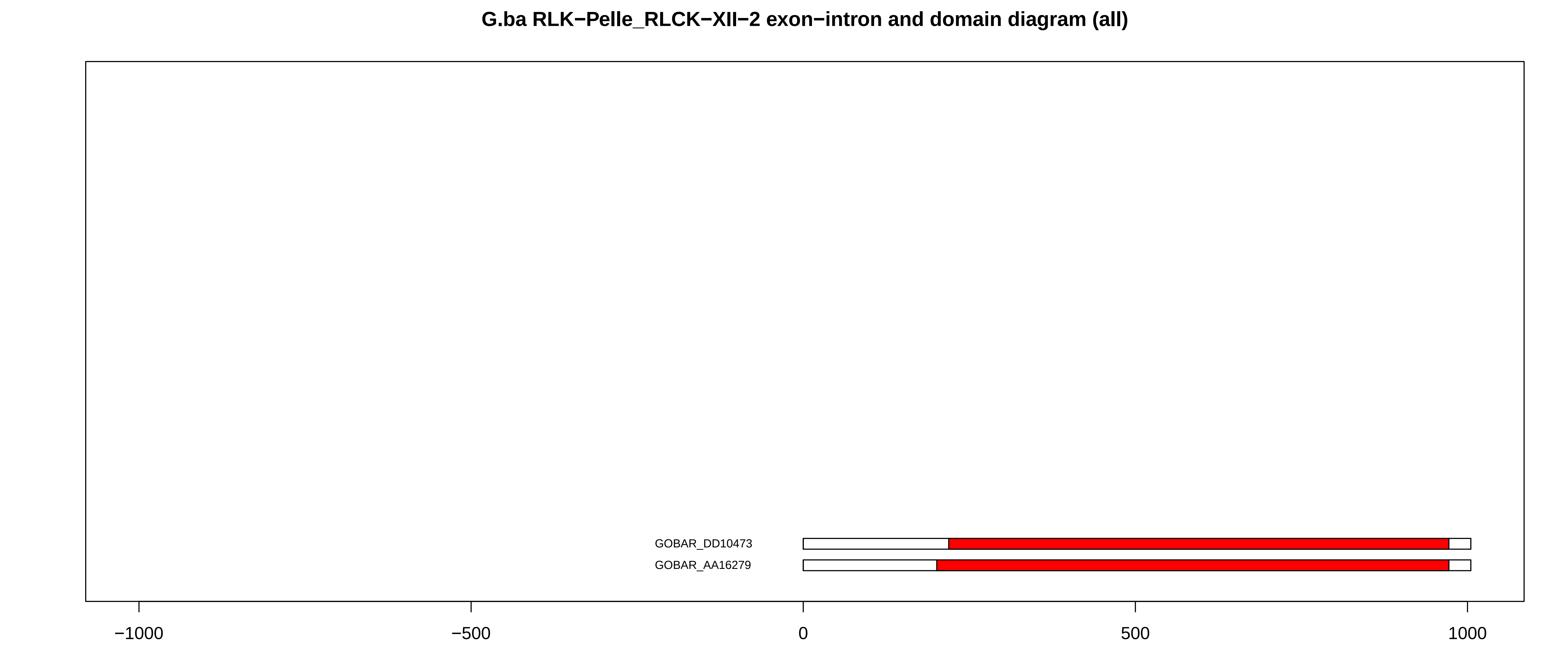

G.ba RLK−Pelle_RLCK−XII−2 exon−intron and domain diagram (all)
GOBAR_DD10473
GOBAR_AA16279
−1000
−500
0
500
1000

G.ba RLK−Pelle_RLCK−XIII exon−intron and domain diagram (all)

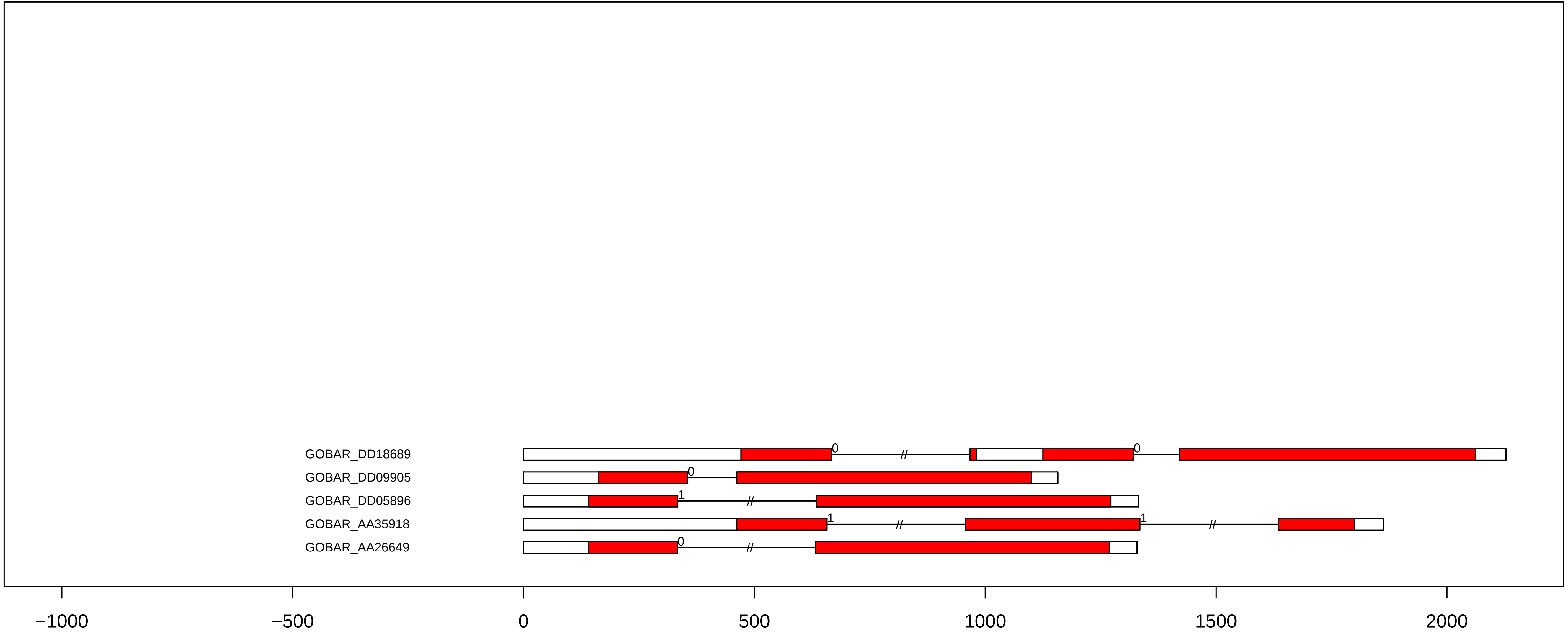

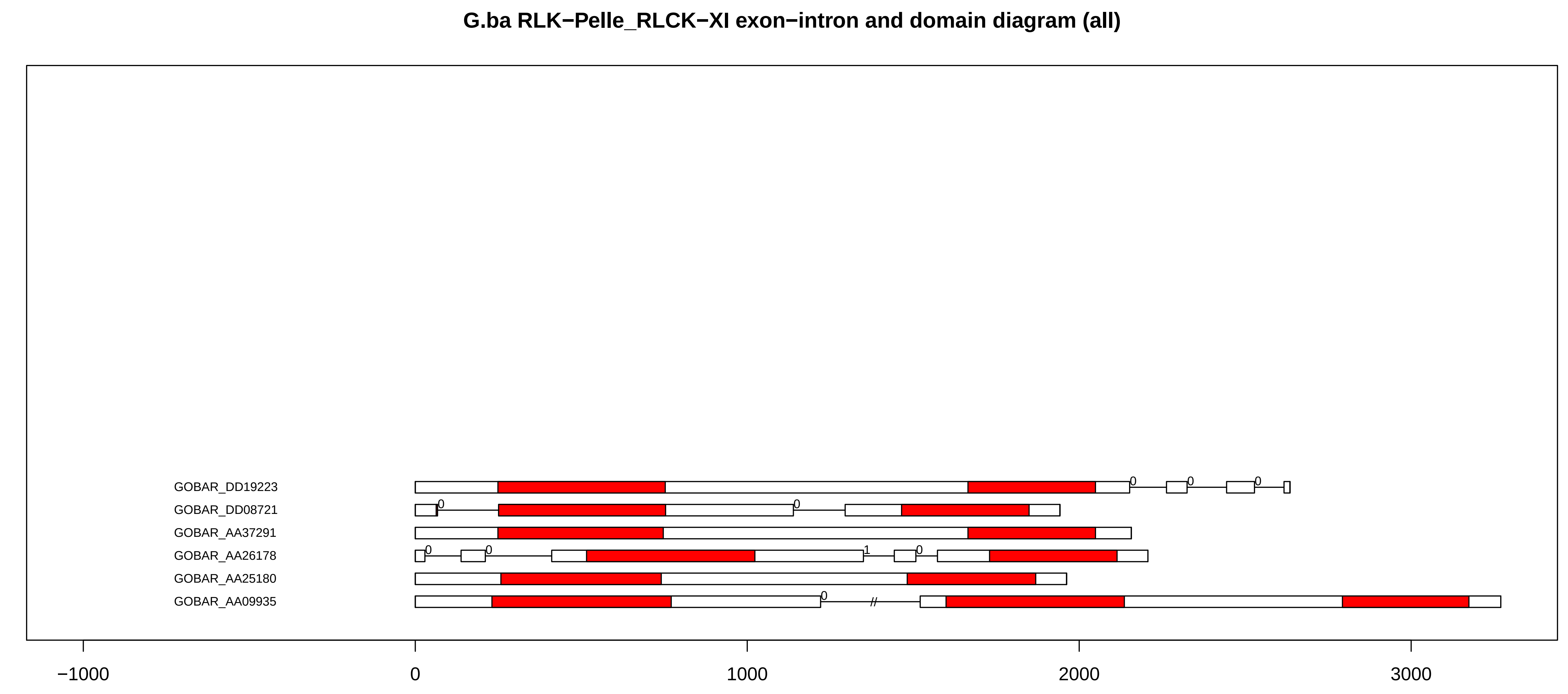

G.ba RLK−Pelle_RLCK−XI exon−intron and domain diagram (all)
GOBAR_DD19223
GOBAR_DD08721
GOBAR_AA37291
GOBAR_AA26178
GOBAR_AA25180
GOBAR_AA09935
−1000
0
1000
2000
3000

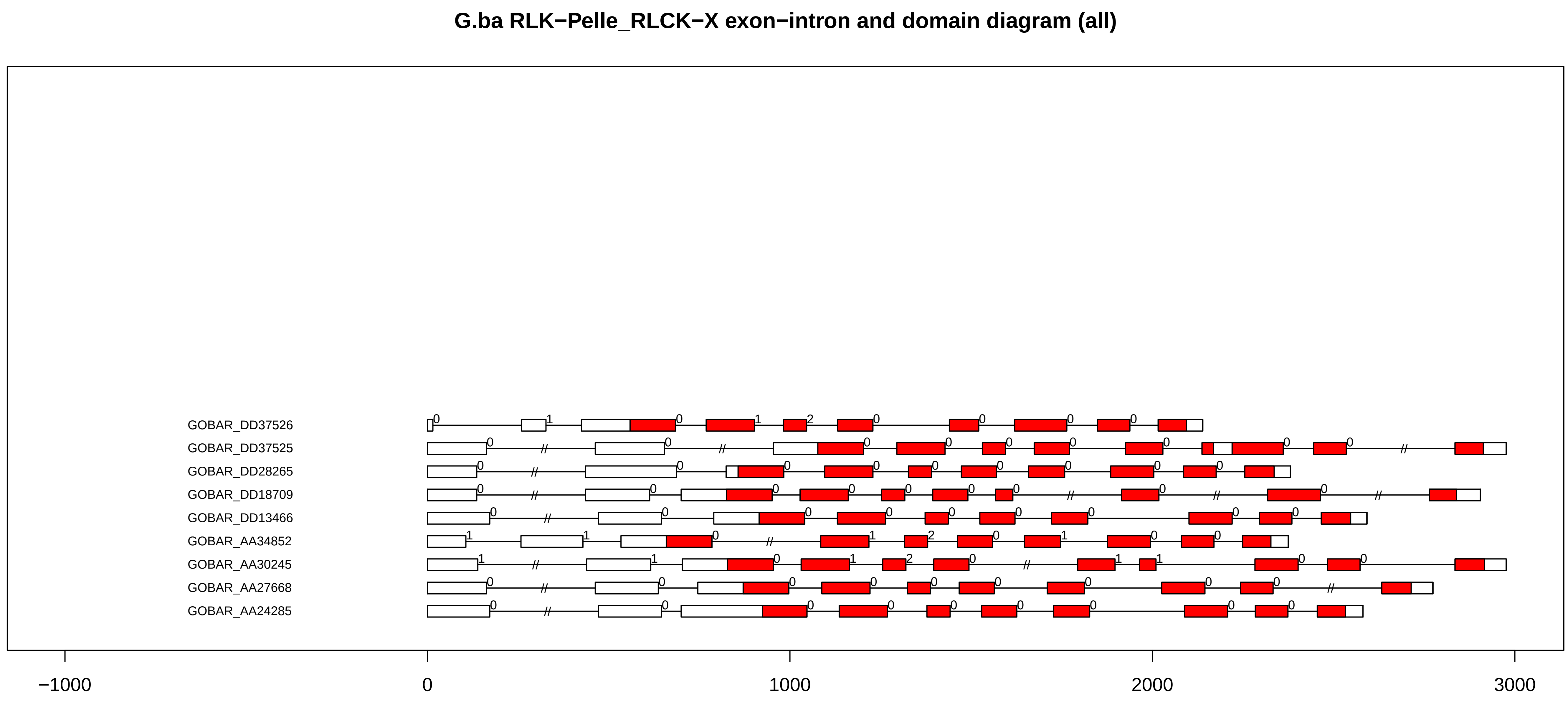
G.ba RLK−Pelle_RLCK−X exon−intron and domain diagram (all)
GOBAR_DD37526
GOBAR_DD37525
GOBAR_DD28265
GOBAR_DD18709
GOBAR_DD13466
GOBAR_AA34852
GOBAR_AA30245
GOBAR_AA27668
GOBAR_AA24285
−1000
0
1000
2000
3000

G.ba RLK−Pelle_RLCK−XVI exon−intron and domain diagram (all)

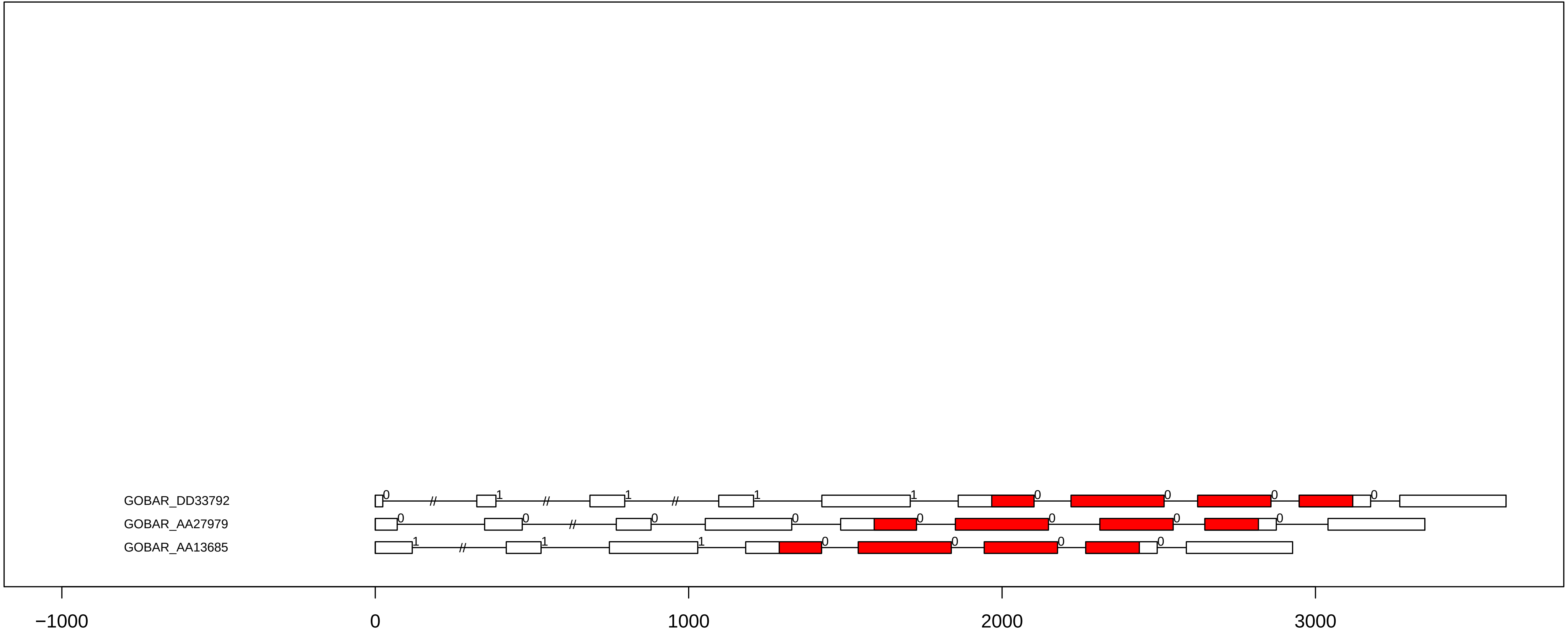

G.ba RLK−Pelle_RLCK−XV exon−intron and domain diagram (all)

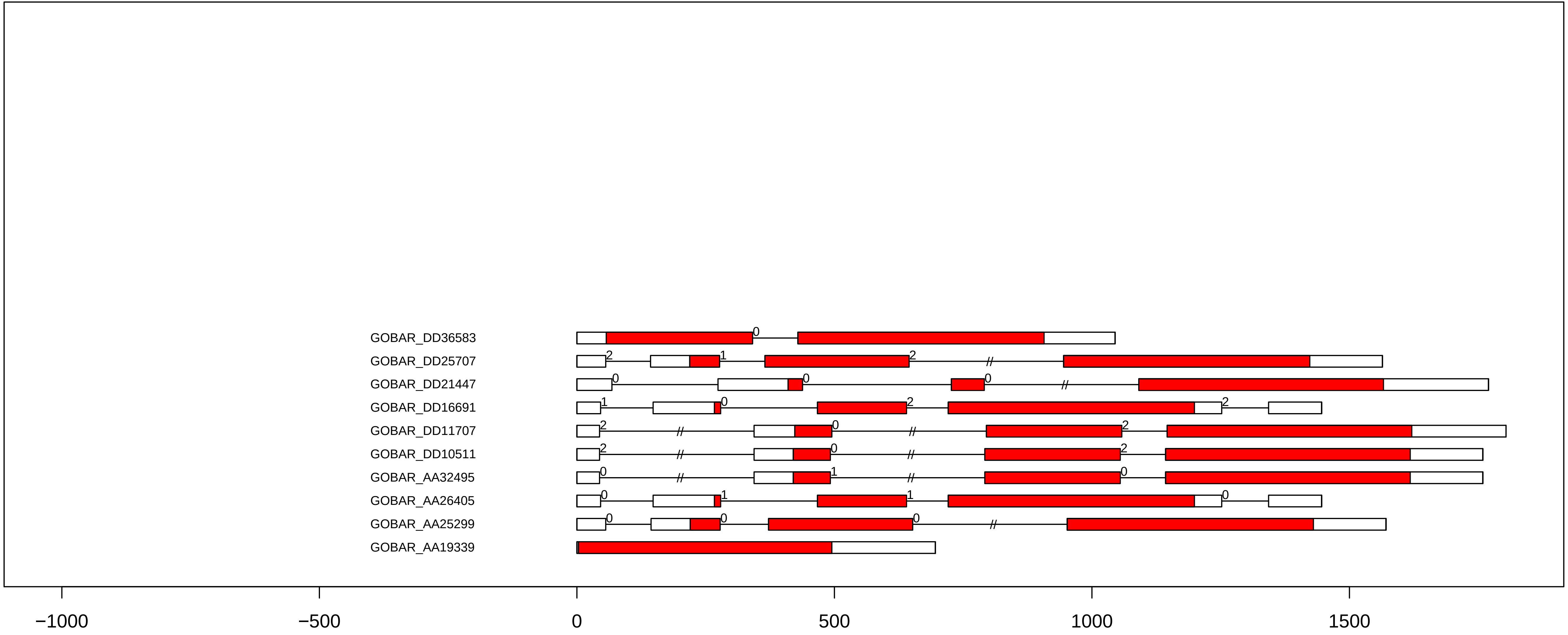

G.ba RLK−Pelle_SD−2b exon−intron and domain diagram (part 1)

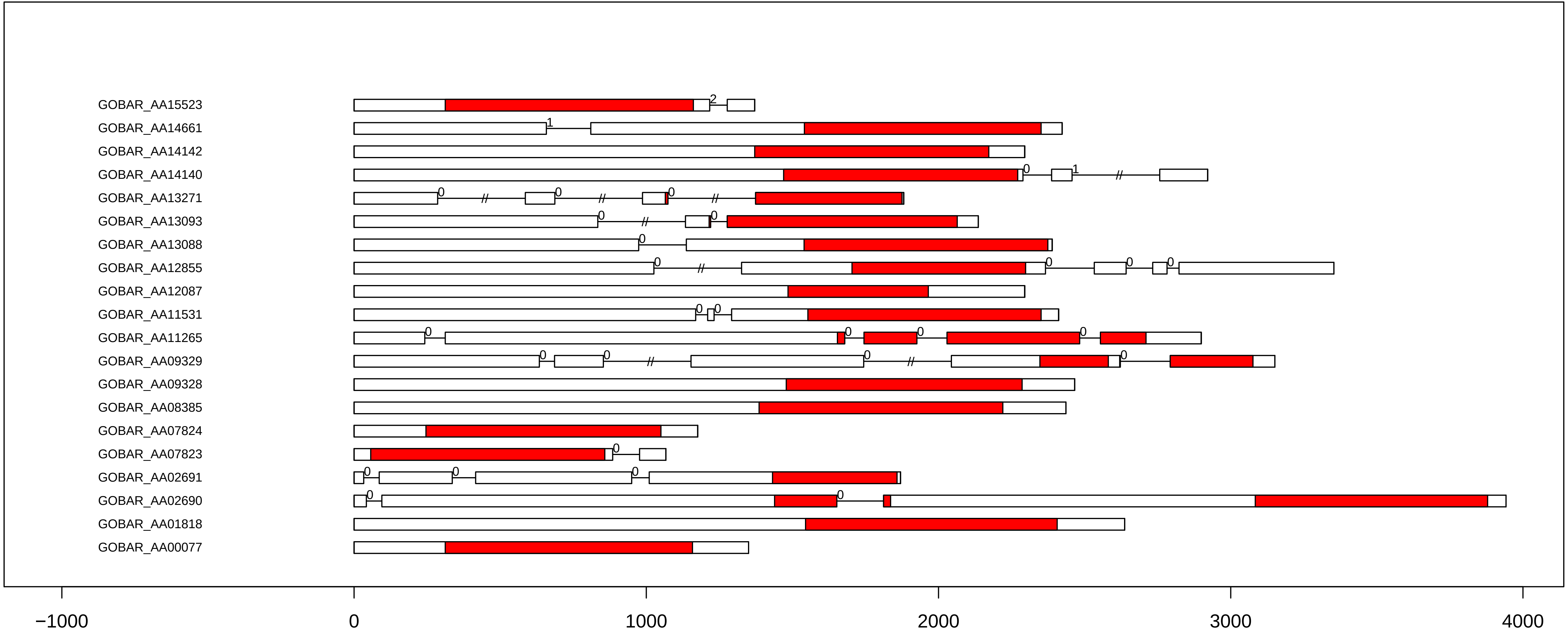

G.ba RLK−Pelle_SD−2b exon−intron and domain diagram (part 2)

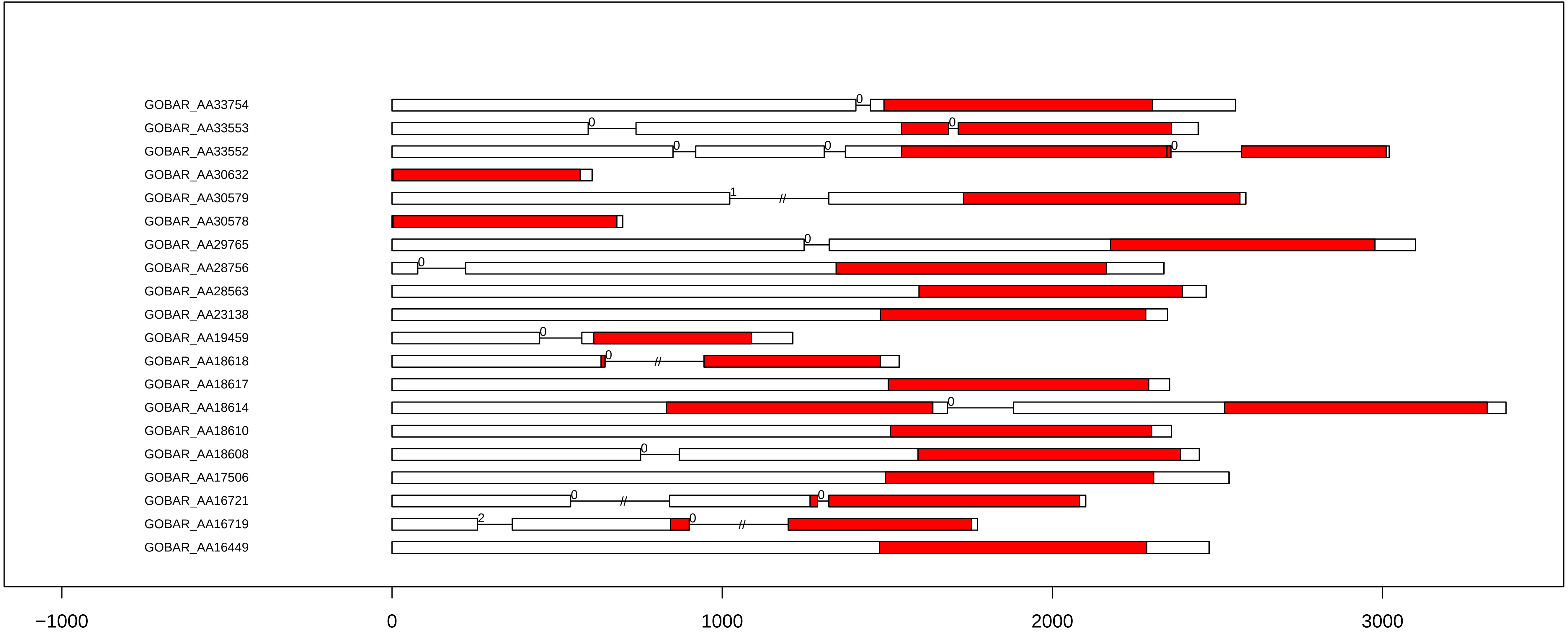

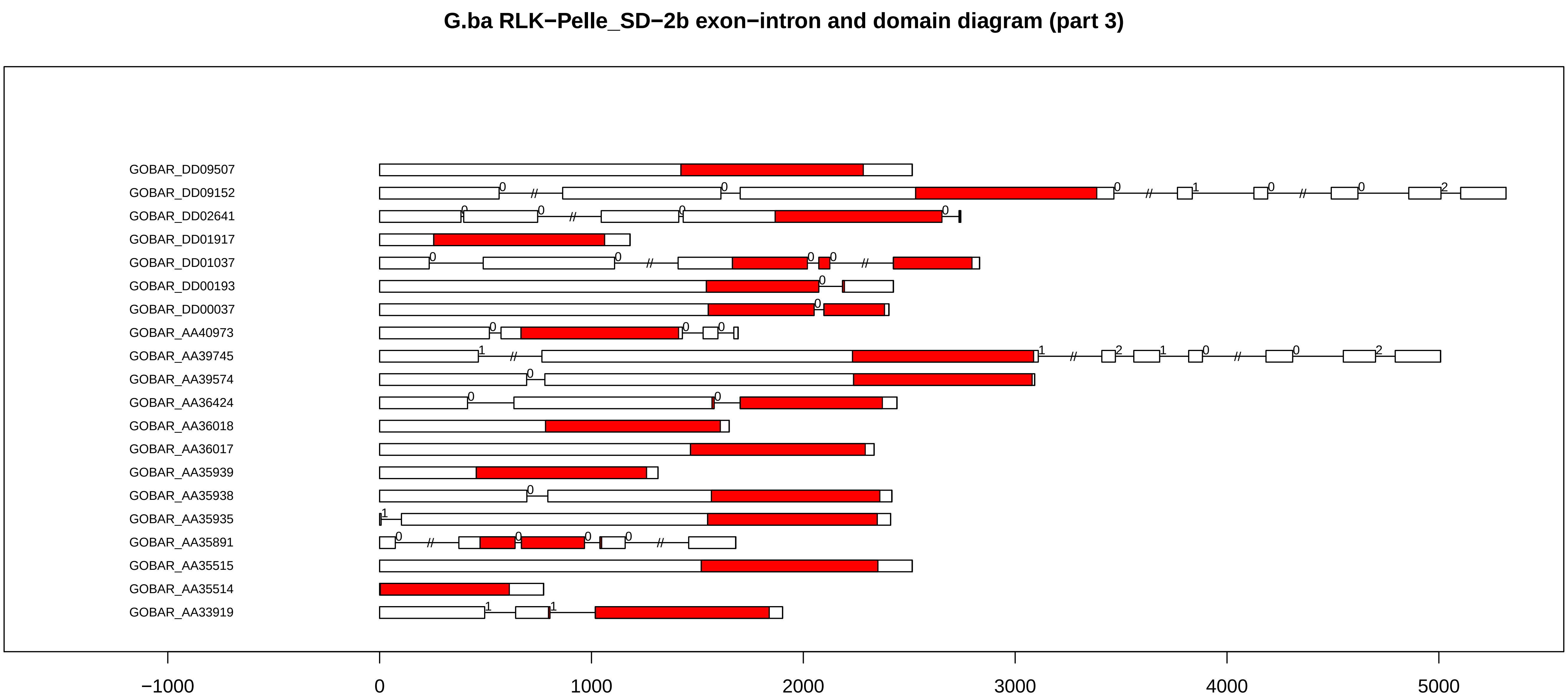
G.ba RLK−Pelle_SD−2b exon−intron and domain diagram (part 3)
GOBAR_DD09507
GOBAR_DD09152
GOBAR_DD02641
GOBAR_DD01917
GOBAR_DD01037
GOBAR_DD00193
GOBAR_DD00037
GOBAR_AA40973
GOBAR_AA39745
GOBAR_AA39574
GOBAR_AA36424
GOBAR_AA36018
GOBAR_AA36017
GOBAR_AA35939
GOBAR_AA35938
GOBAR_AA35935
GOBAR_AA35891
GOBAR_AA35515
GOBAR_AA35514
GOBAR_AA33919
−1000
0
1000
2000
3000
4000
5000

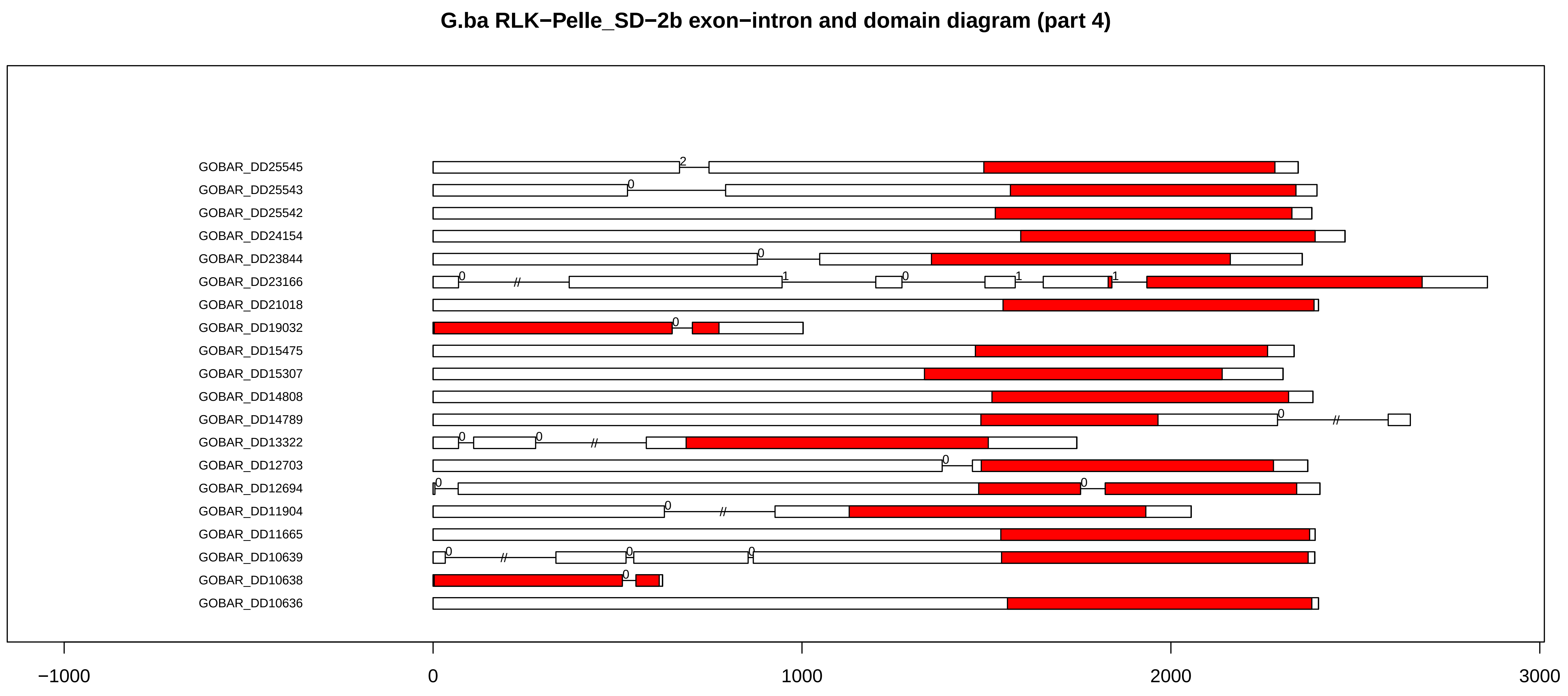

G.ba RLK−Pelle_SD−2b exon−intron and domain diagram (part 4)
GOBAR_DD25545
GOBAR_DD25543
GOBAR_DD25542
GOBAR_DD24154
GOBAR_DD23844
GOBAR_DD23166
GOBAR_DD21018
GOBAR_DD19032
GOBAR_DD15475
GOBAR_DD15307
GOBAR_DD14808
GOBAR_DD14789
GOBAR_DD13322
GOBAR_DD12703
GOBAR_DD12694
GOBAR_DD11904
GOBAR_DD11665
GOBAR_DD10639
GOBAR_DD10638
GOBAR_DD10636
−1000
0
1000
2000
3000

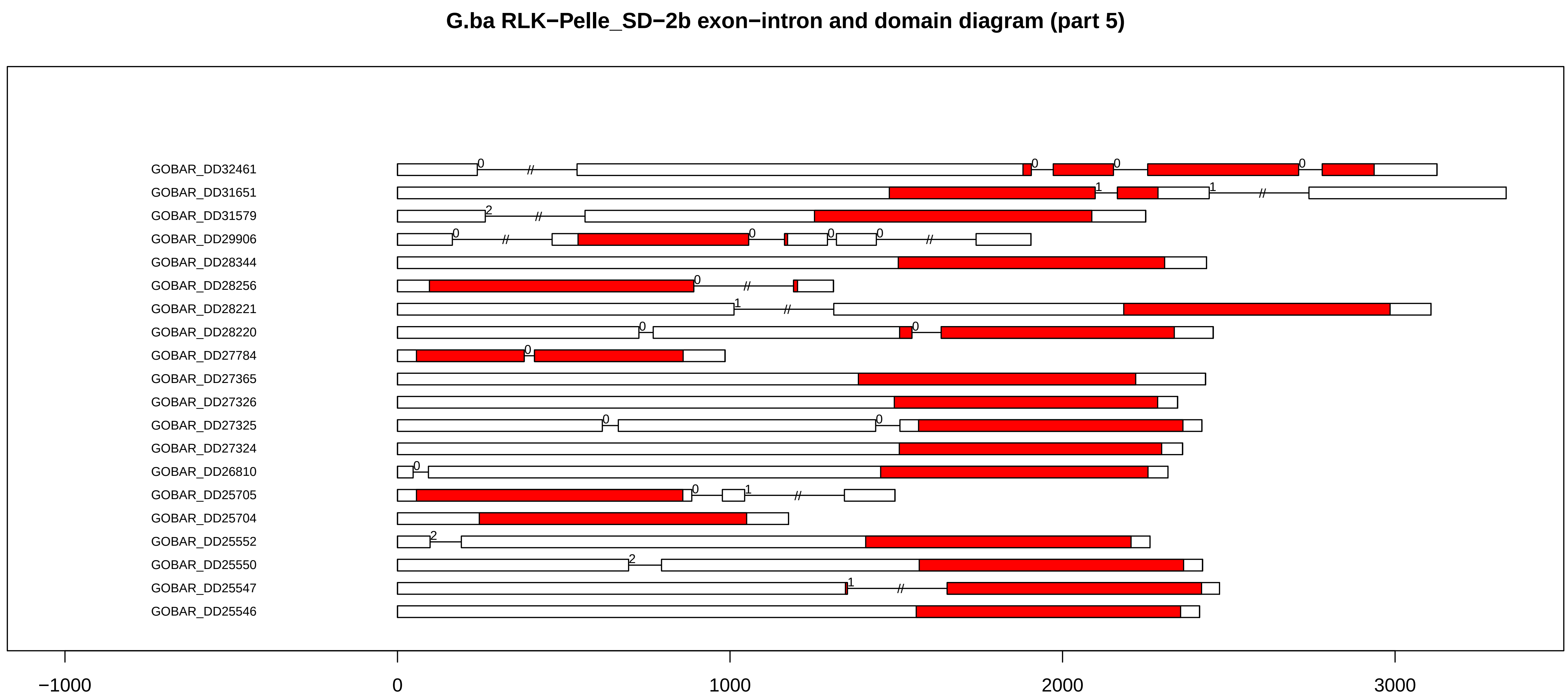
G.ba RLK−Pelle_SD−2b exon−intron and domain diagram (part 5)
GOBAR_DD32461
GOBAR_DD31651
GOBAR_DD31579
GOBAR_DD29906
GOBAR_DD28344
GOBAR_DD28256
GOBAR_DD28221
GOBAR_DD28220
GOBAR_DD27784
GOBAR_DD27365
GOBAR_DD27326
GOBAR_DD27325
GOBAR_DD27324
GOBAR_DD26810
GOBAR_DD25705
GOBAR_DD25704
GOBAR_DD25552
GOBAR_DD25550
GOBAR_DD25547
GOBAR_DD25546
−1000
0
1000
2000
3000

G.ba RLK−Pelle_SD−2b exon−intron and domain diagram (part 6)

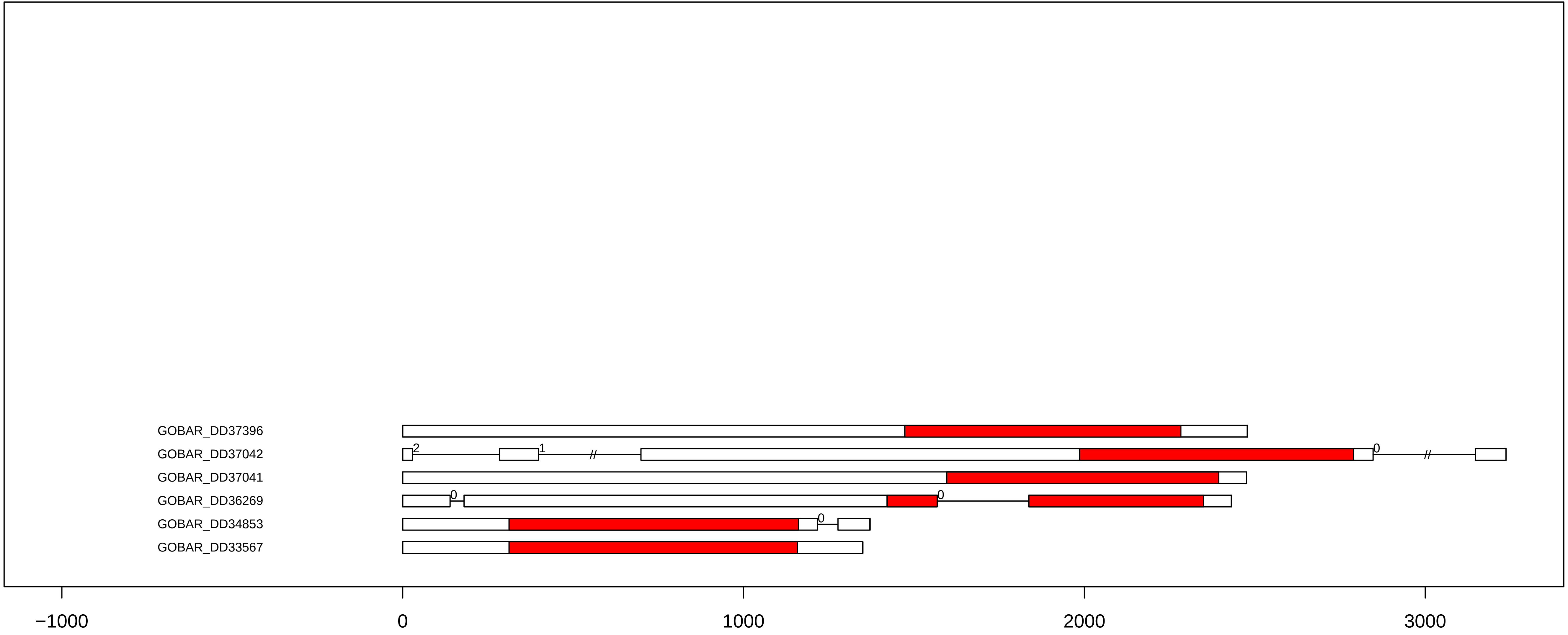

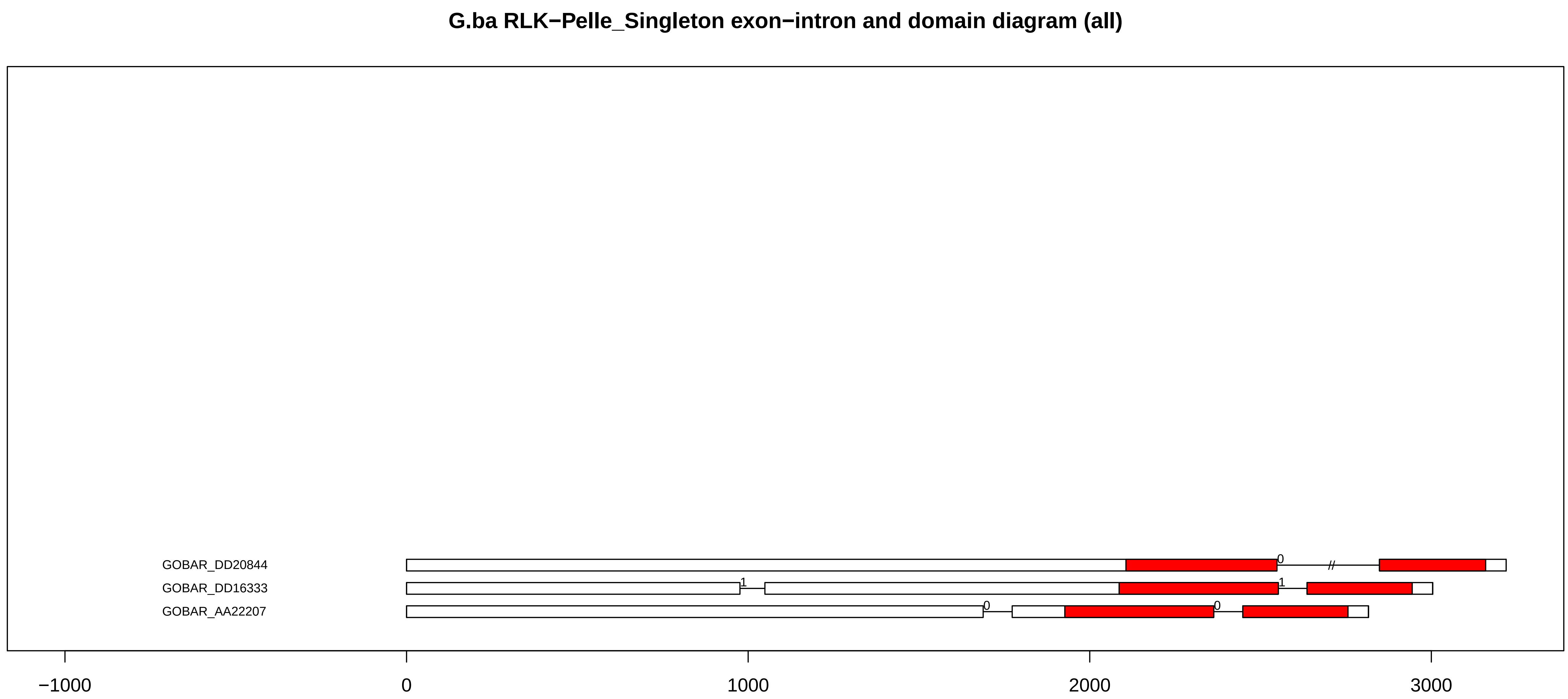
G.ba RLK−Pelle_Singleton exon−intron and domain diagram (all)
GOBAR_DD20844
GOBAR_DD16333
GOBAR_AA22207
0
1
1
0
0
−1000
0
1000
2000
3000

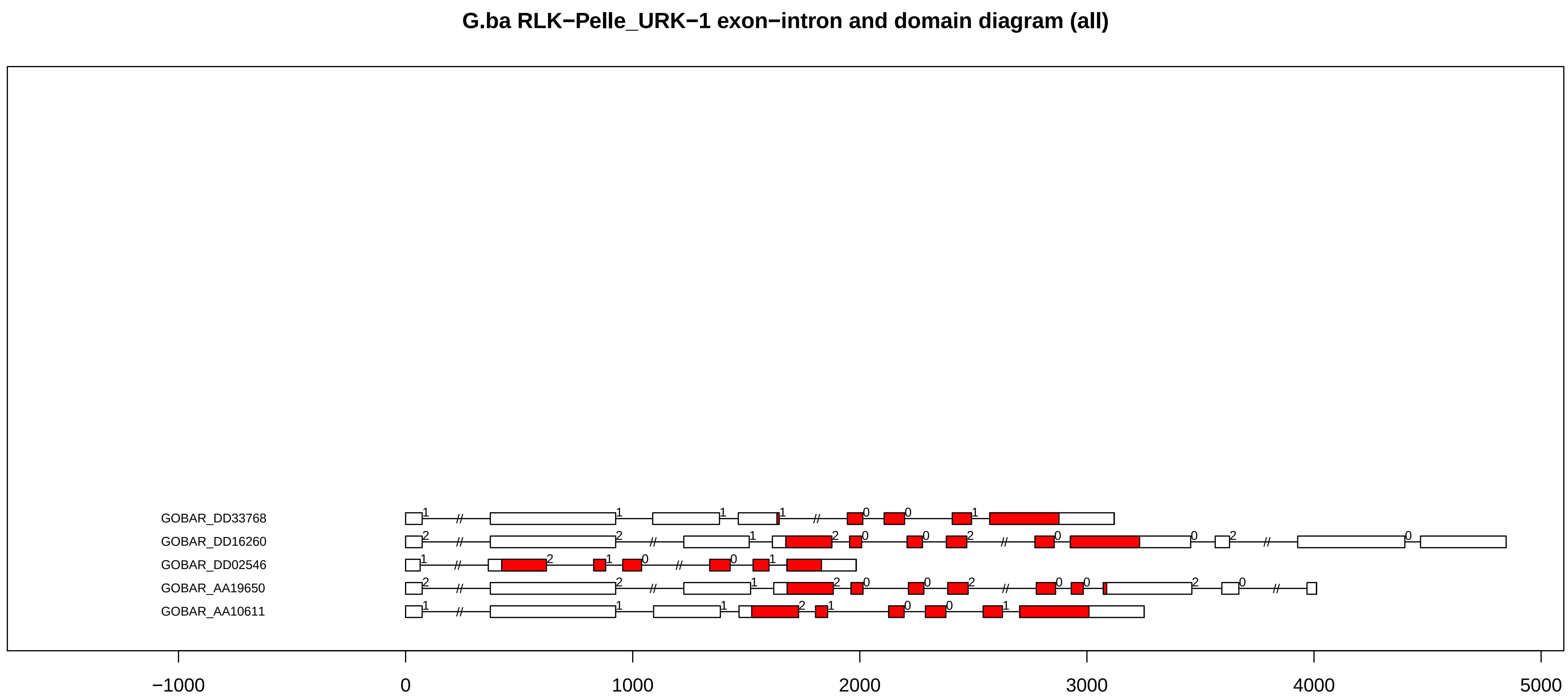

G.ba RLK−Pelle_URK−1 exon−intron and domain diagram (all)
GOBAR_DD33768
GOBAR_DD16260
GOBAR_DD02546
GOBAR_AA19650
GOBAR_AA10611
−1000
0
1000
2000
3000
4000
5000

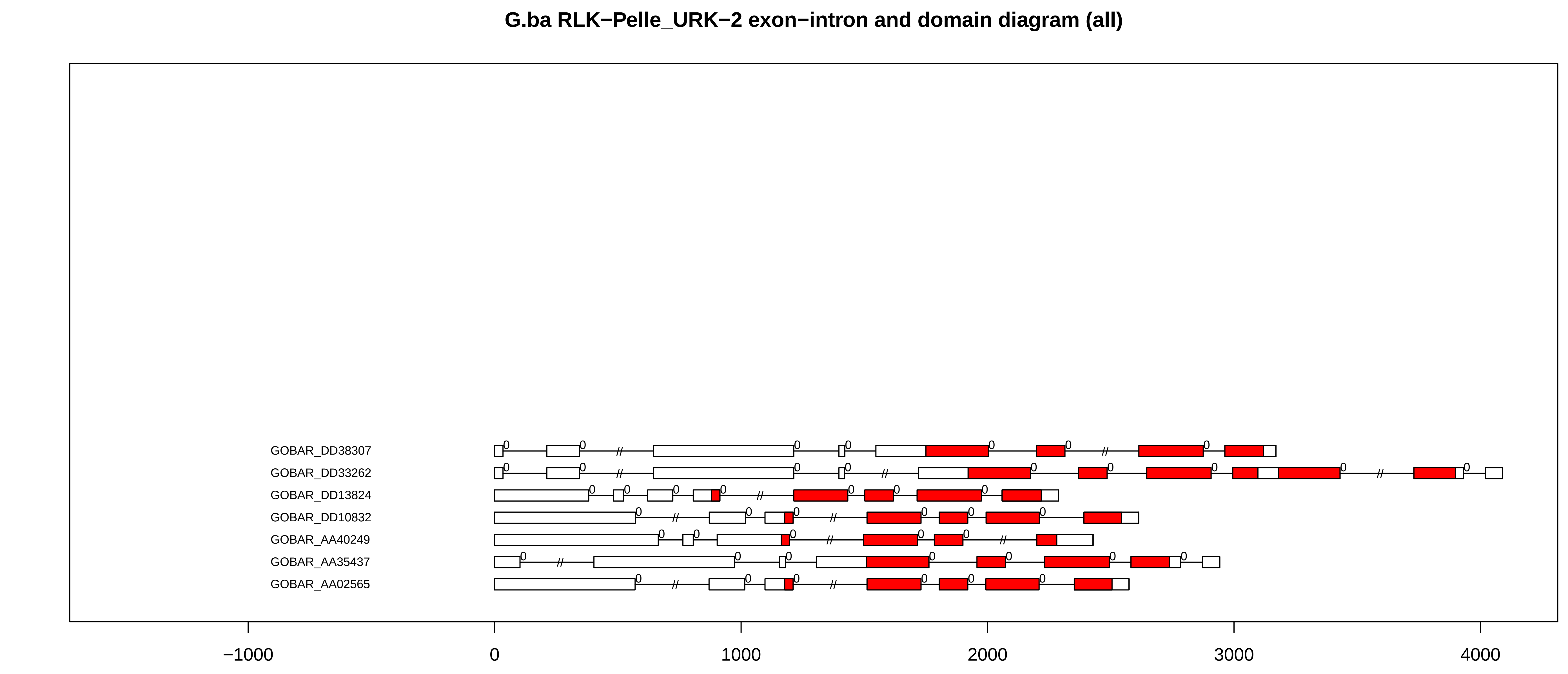
G.ba RLK−Pelle_URK−2 exon−intron and domain diagram (all)
GOBAR_DD38307
GOBAR_DD33262
GOBAR_DD13824
GOBAR_DD10832
GOBAR_AA40249
GOBAR_AA35437
GOBAR_AA02565
−1000
0
1000
2000
3000
4000

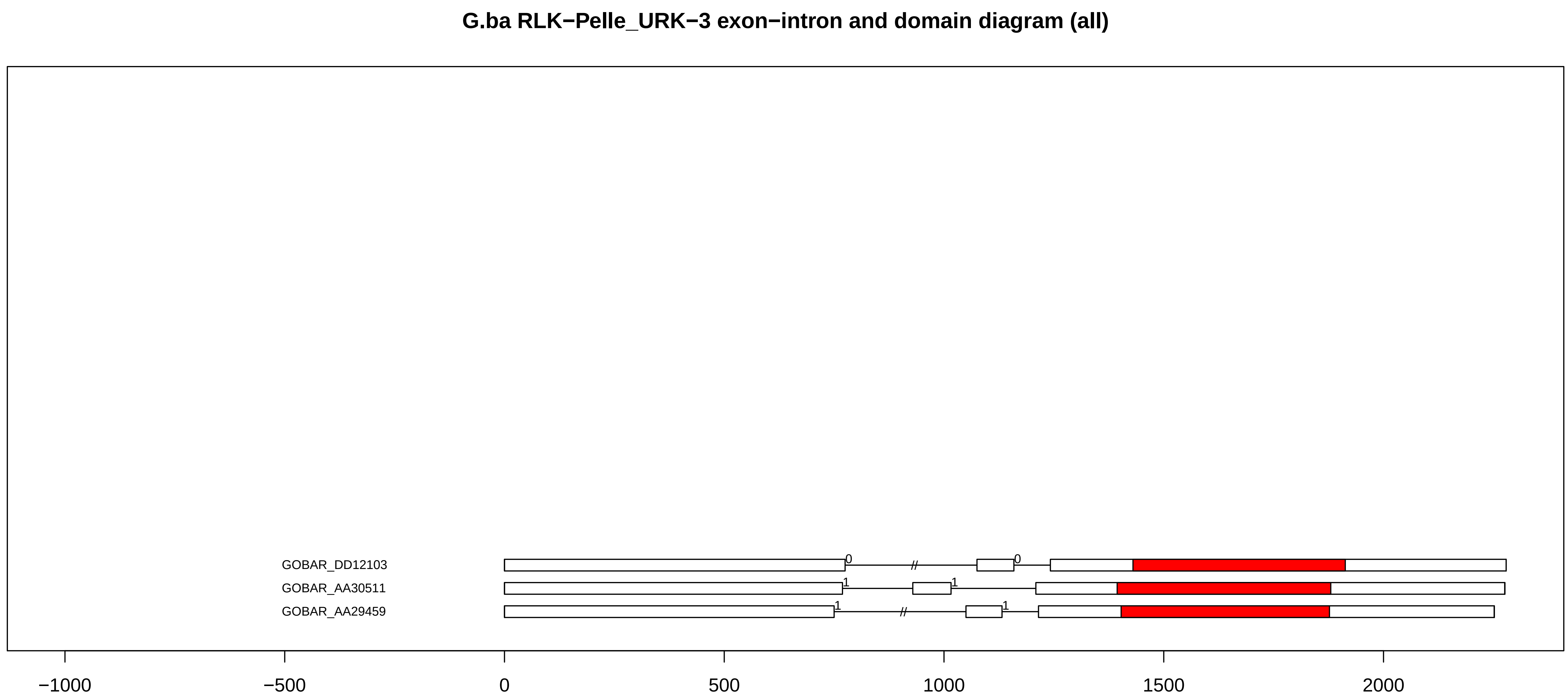

G.ba RLK−Pelle_URK−3 exon−intron and domain diagram (all)
GOBAR_DD12103
GOBAR_AA30511
GOBAR_AA29459
0
0
1
1
1
1
−1000
−500
0
500
1000
1500
2000

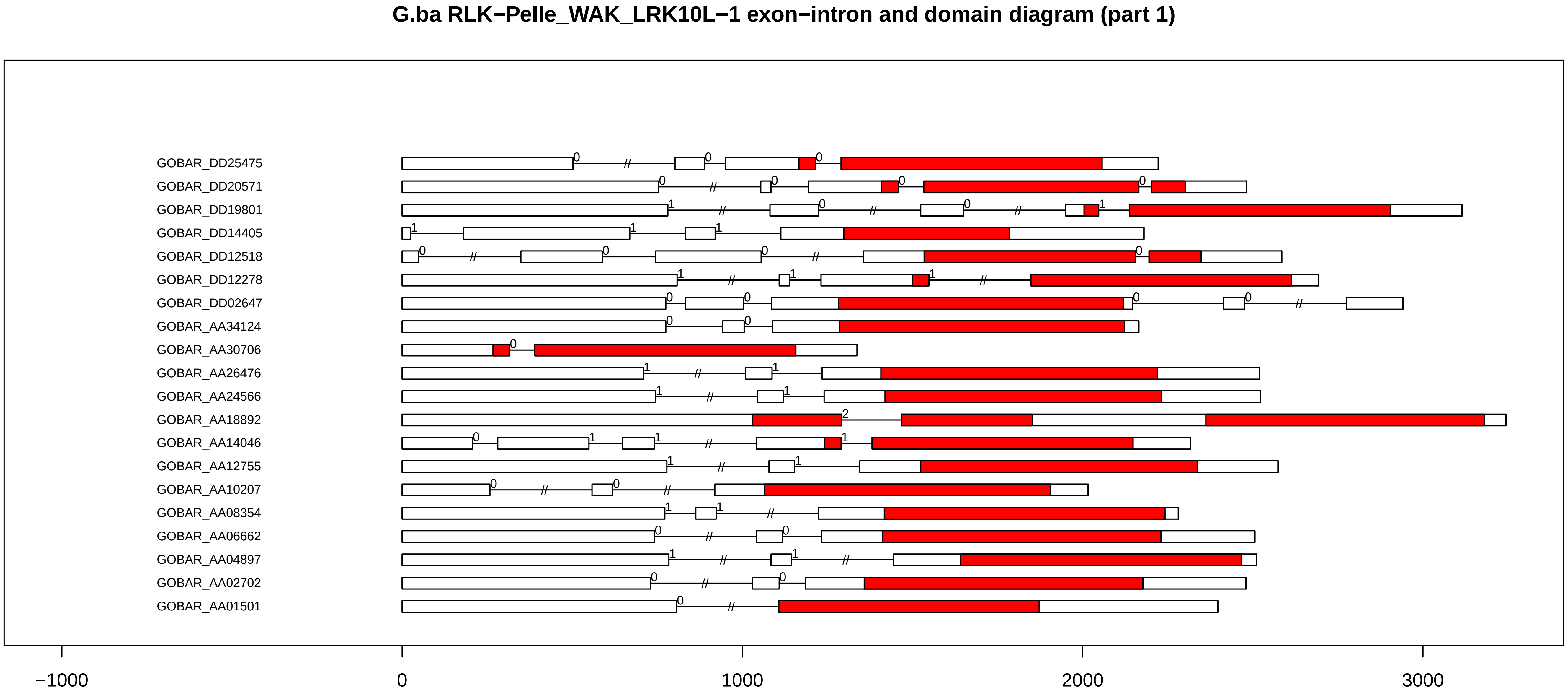
G.ba RLK−Pelle_WAK_LRK10L−1 exon−intron and domain diagram (part 1)
GOBAR_DD25475
GOBAR_DD20571
GOBAR_DD19801
GOBAR_DD14405
GOBAR_DD12518
GOBAR_DD12278
GOBAR_DD02647
GOBAR_AA34124
GOBAR_AA30706
GOBAR_AA26476
GOBAR_AA24566
GOBAR_AA18892
GOBAR_AA14046
GOBAR_AA12755
GOBAR_AA10207
GOBAR_AA08354
GOBAR_AA06662
GOBAR_AA04897
GOBAR_AA02702
GOBAR_AA01501
−1000
0
1000
2000
3000

G.ba RLK−Pelle_WAK_LRK10L−1 exon−intron and domain diagram (part 2)

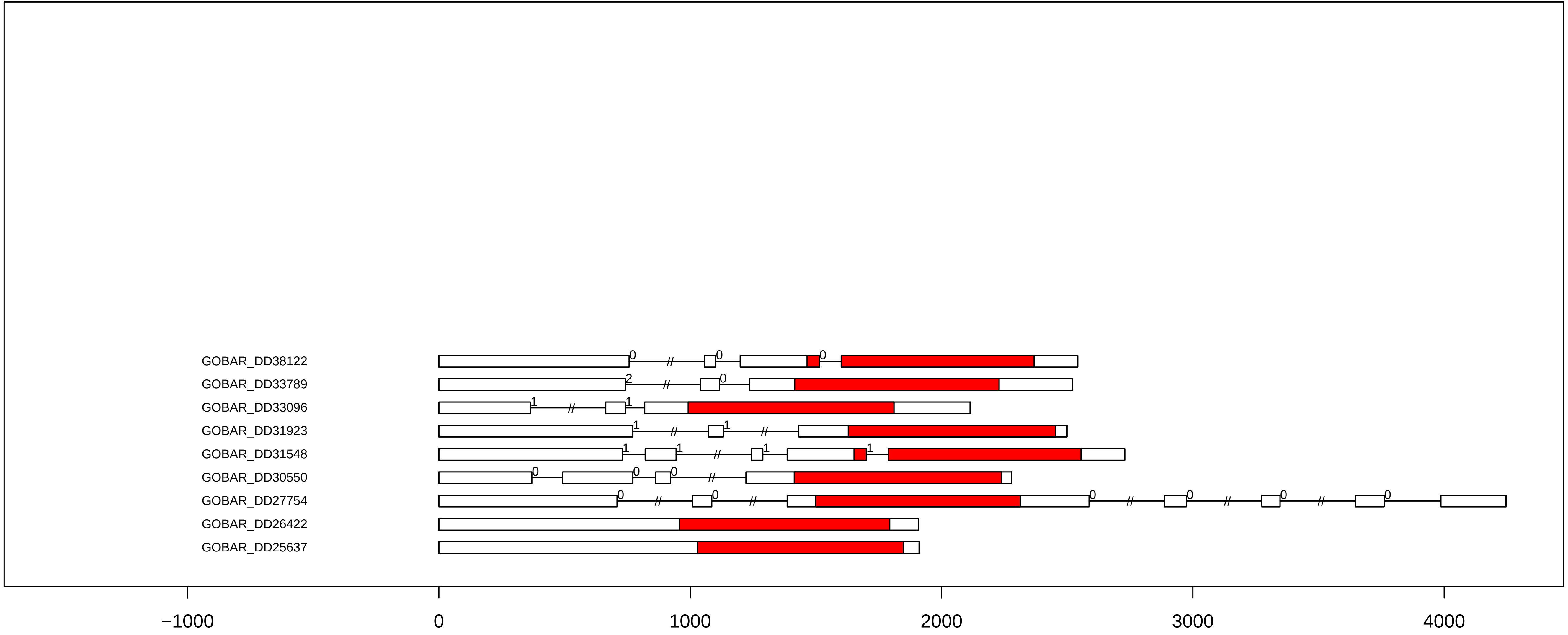

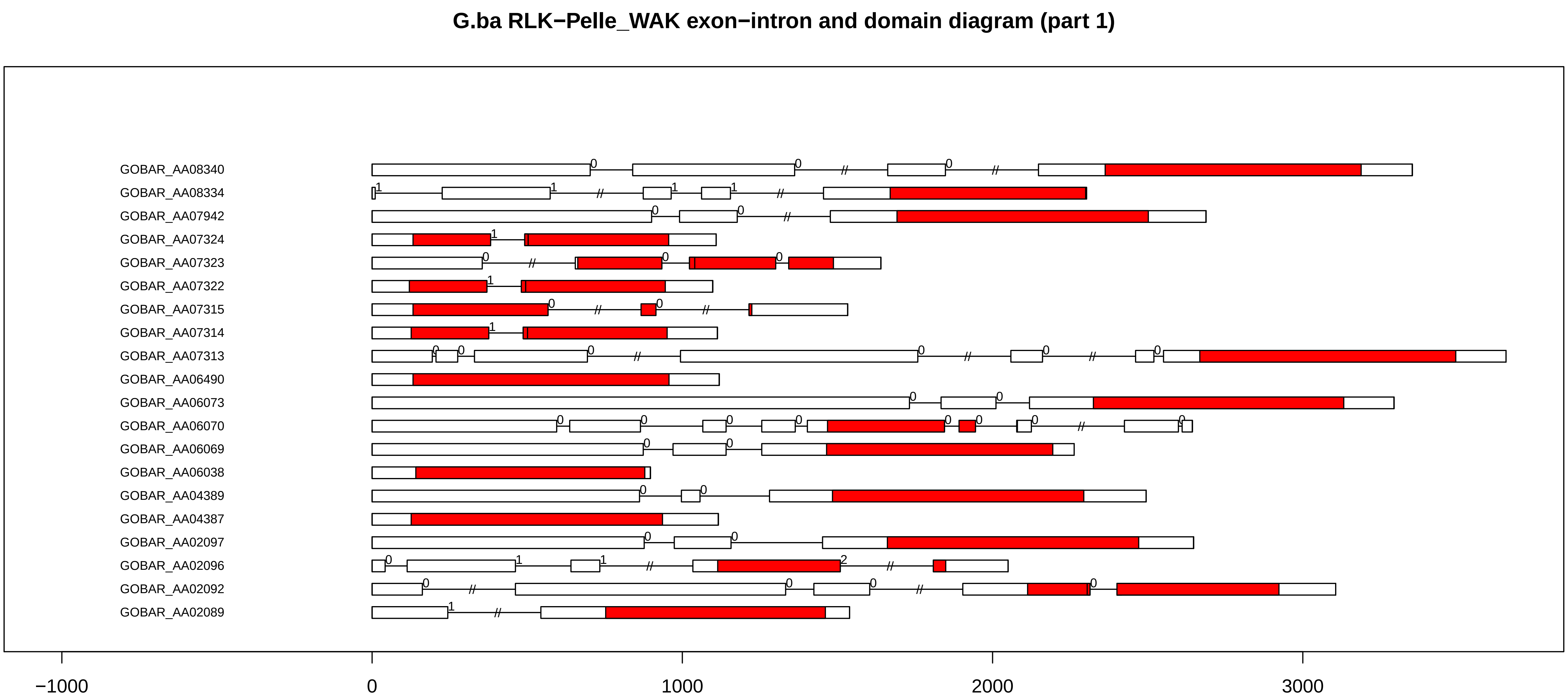
G.ba RLK−Pelle_WAK exon−intron and domain diagram (part 1)
GOBAR_AA08340
GOBAR_AA08334
GOBAR_AA07942
GOBAR_AA07324
GOBAR_AA07323
GOBAR_AA07322
GOBAR_AA07315
GOBAR_AA07314
GOBAR_AA07313
GOBAR_AA06490
GOBAR_AA06073
GOBAR_AA06070
GOBAR_AA06069
GOBAR_AA06038
GOBAR_AA04389
GOBAR_AA04387
GOBAR_AA02097
GOBAR_AA02096
GOBAR_AA02092
GOBAR_AA02089
−1000
0
1000
2000
3000

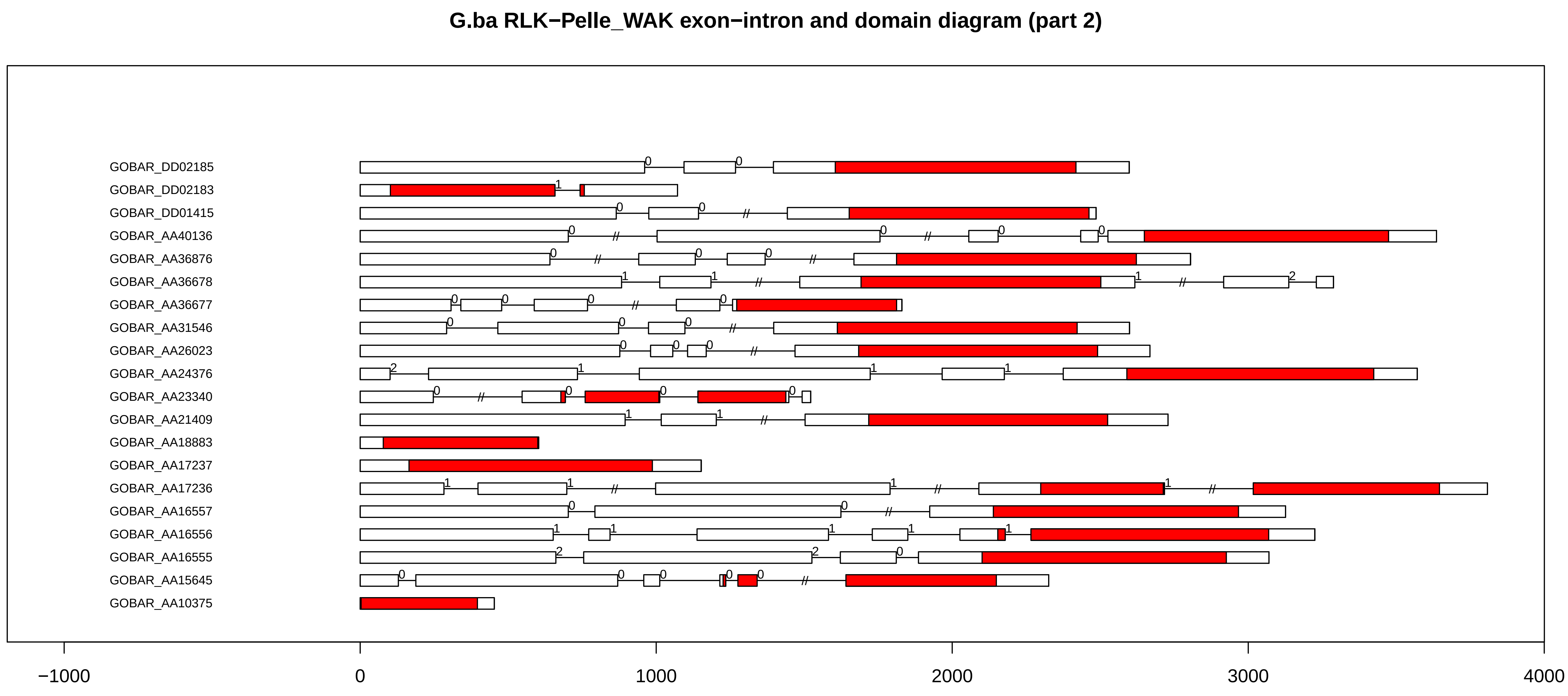
G.ba RLK−Pelle_WAK exon−intron and domain diagram (part 2)
GOBAR_DD02185
GOBAR_DD02183
GOBAR_DD01415
GOBAR_AA40136
GOBAR_AA36876
GOBAR_AA36678
GOBAR_AA36677
GOBAR_AA31546
GOBAR_AA26023
GOBAR_AA24376
GOBAR_AA23340
GOBAR_AA21409
GOBAR_AA18883
GOBAR_AA17237
GOBAR_AA17236
GOBAR_AA16557
GOBAR_AA16556
GOBAR_AA16555
GOBAR_AA15645
GOBAR_AA10375
−1000
0
1000
2000
3000
4000

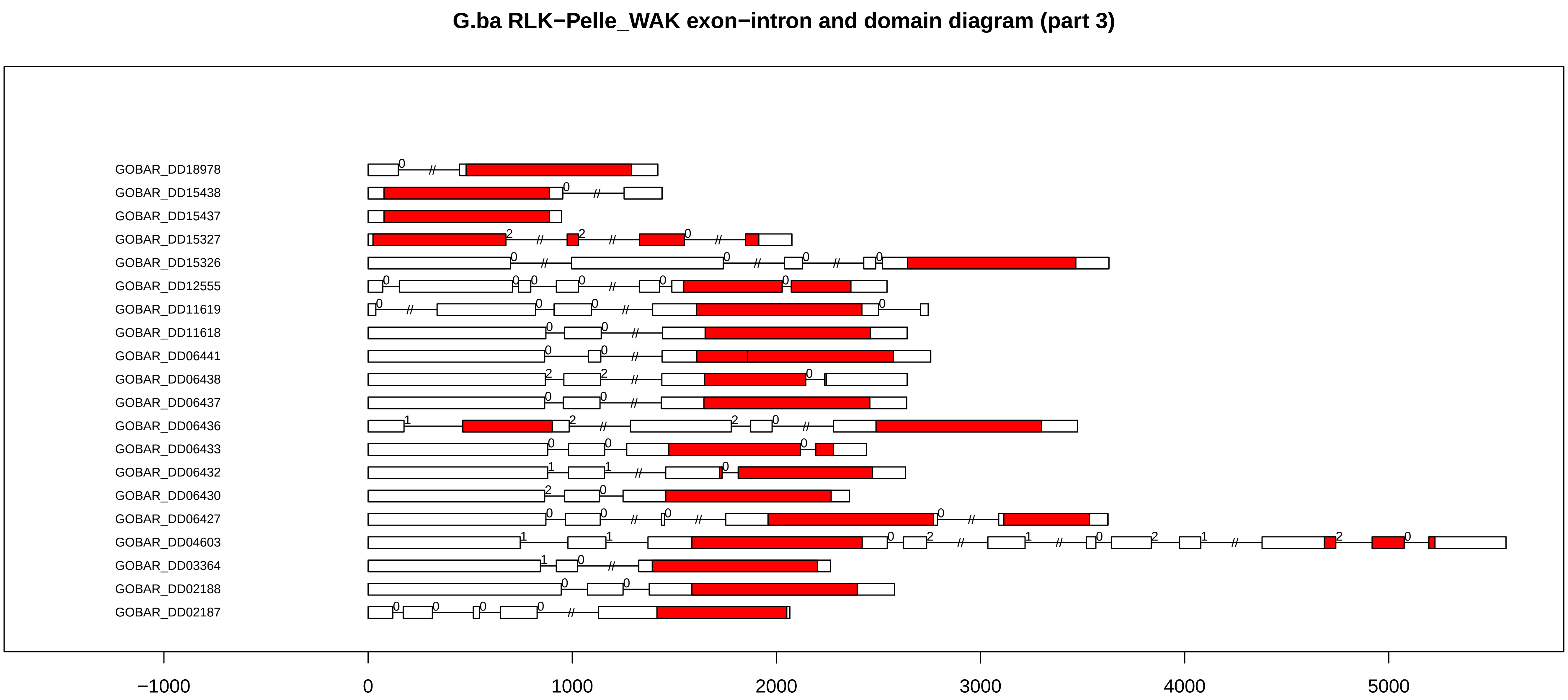
G.ba RLK−Pelle_WAK exon−intron and domain diagram (part 3)
GOBAR_DD18978
GOBAR_DD15438
GOBAR_DD15437
GOBAR_DD15327
GOBAR_DD15326
GOBAR_DD12555
GOBAR_DD11619
GOBAR_DD11618
GOBAR_DD06441
GOBAR_DD06438
GOBAR_DD06437
GOBAR_DD06436
GOBAR_DD06433
GOBAR_DD06432
GOBAR_DD06430
GOBAR_DD06427
GOBAR_DD04603
GOBAR_DD03364
GOBAR_DD02188
GOBAR_DD02187
−1000
0
1000
2000
3000
4000
5000

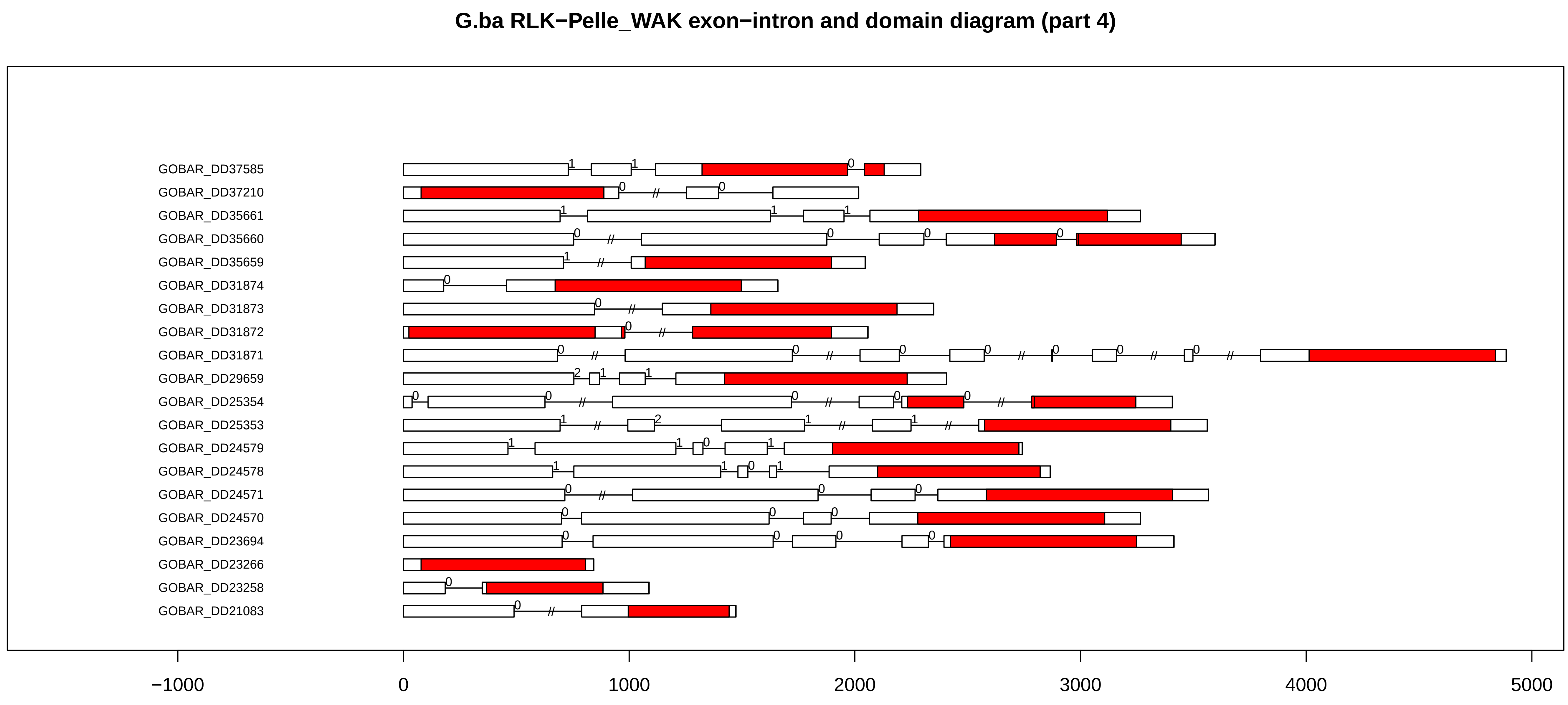

G.ba RLK−Pelle_WAK exon−intron and domain diagram (part 4)
GOBAR_DD37585
GOBAR_DD37210
GOBAR_DD35661
GOBAR_DD35660
GOBAR_DD35659
GOBAR_DD31874
GOBAR_DD31873
GOBAR_DD31872
GOBAR_DD31871
GOBAR_DD29659
GOBAR_DD25354
GOBAR_DD25353
GOBAR_DD24579
GOBAR_DD24578
GOBAR_DD24571
GOBAR_DD24570
GOBAR_DD23694
GOBAR_DD23266
GOBAR_DD23258
GOBAR_DD21083
−1000
0
1000
2000
3000
4000
5000

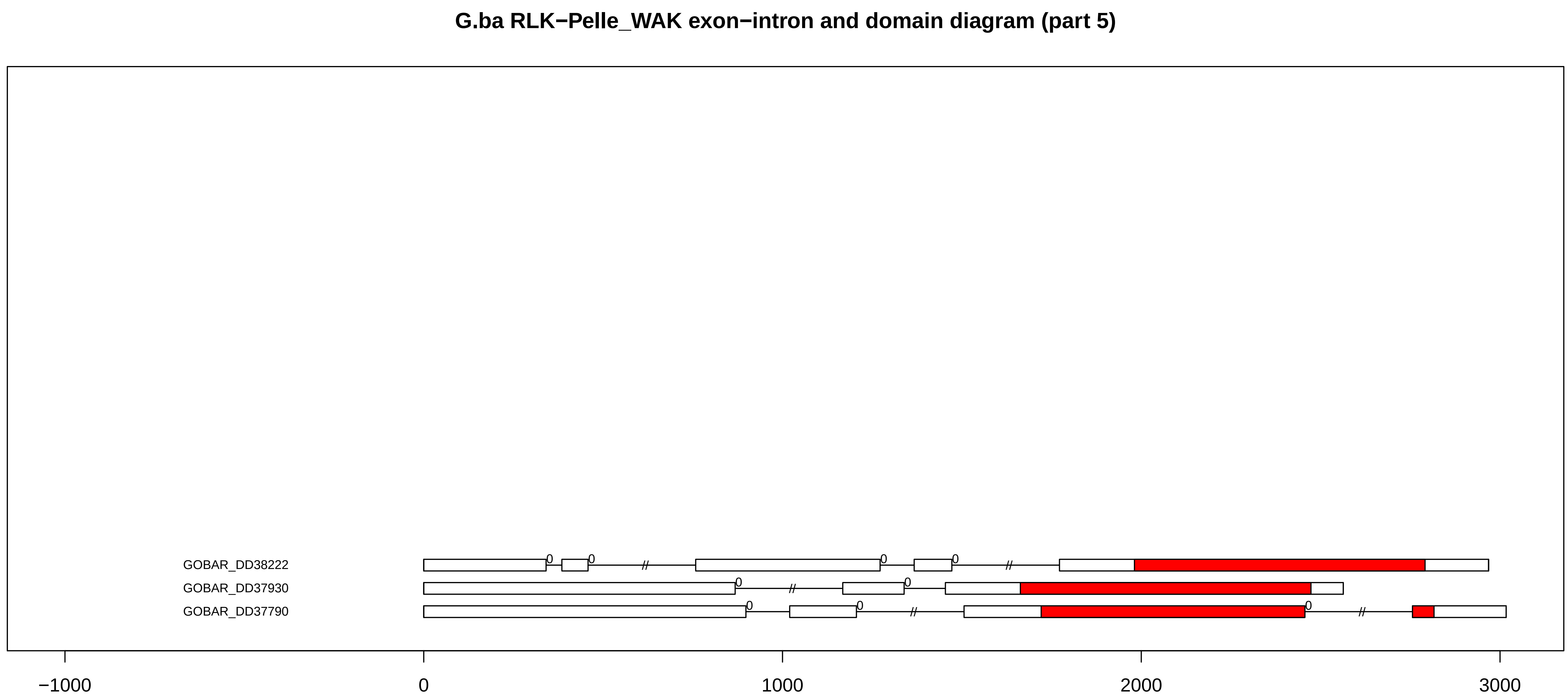
G.ba RLK−Pelle_WAK exon−intron and domain diagram (part 5)
GOBAR_DD38222
GOBAR_DD37930
GOBAR_DD37790
−1000
0
1000
2000
3000

G.ba SCY1_SCYL1 exon−intron and domain diagram (all)

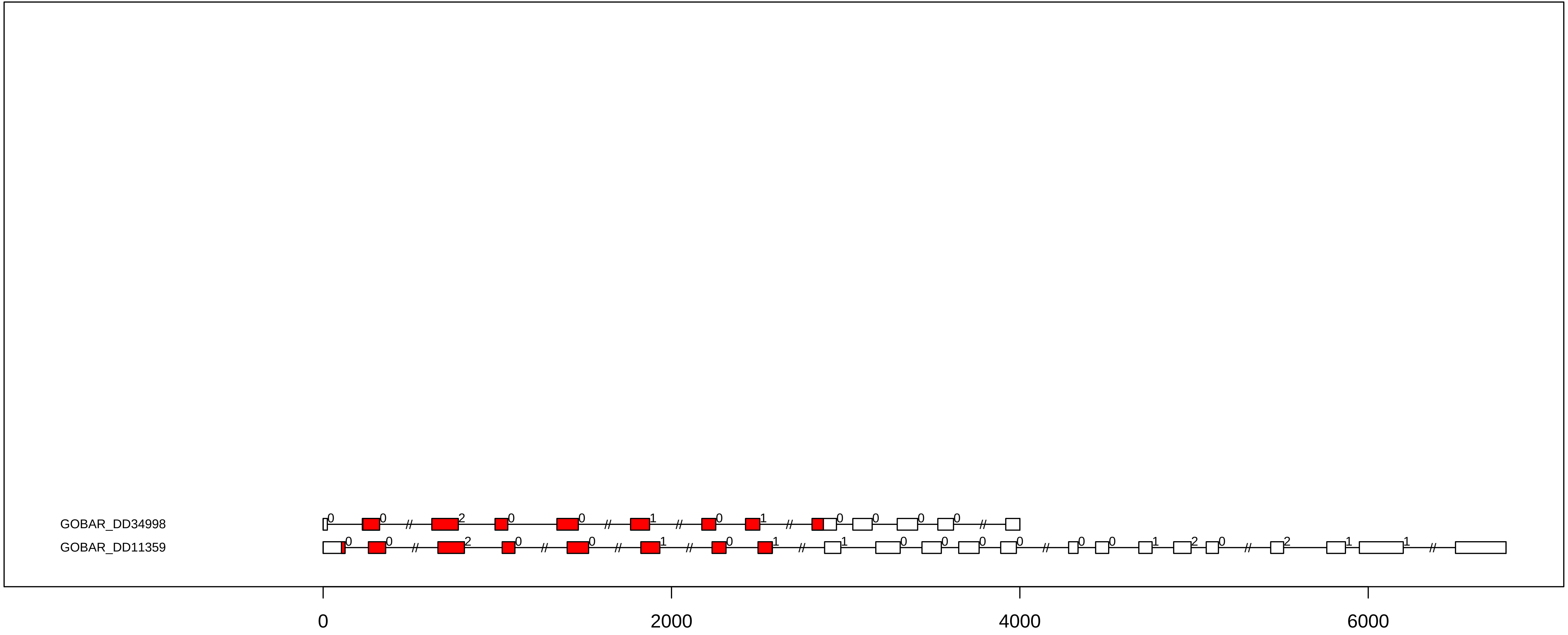

G.ba SCY1_SCYL2 exon−intron and domain diagram (all)

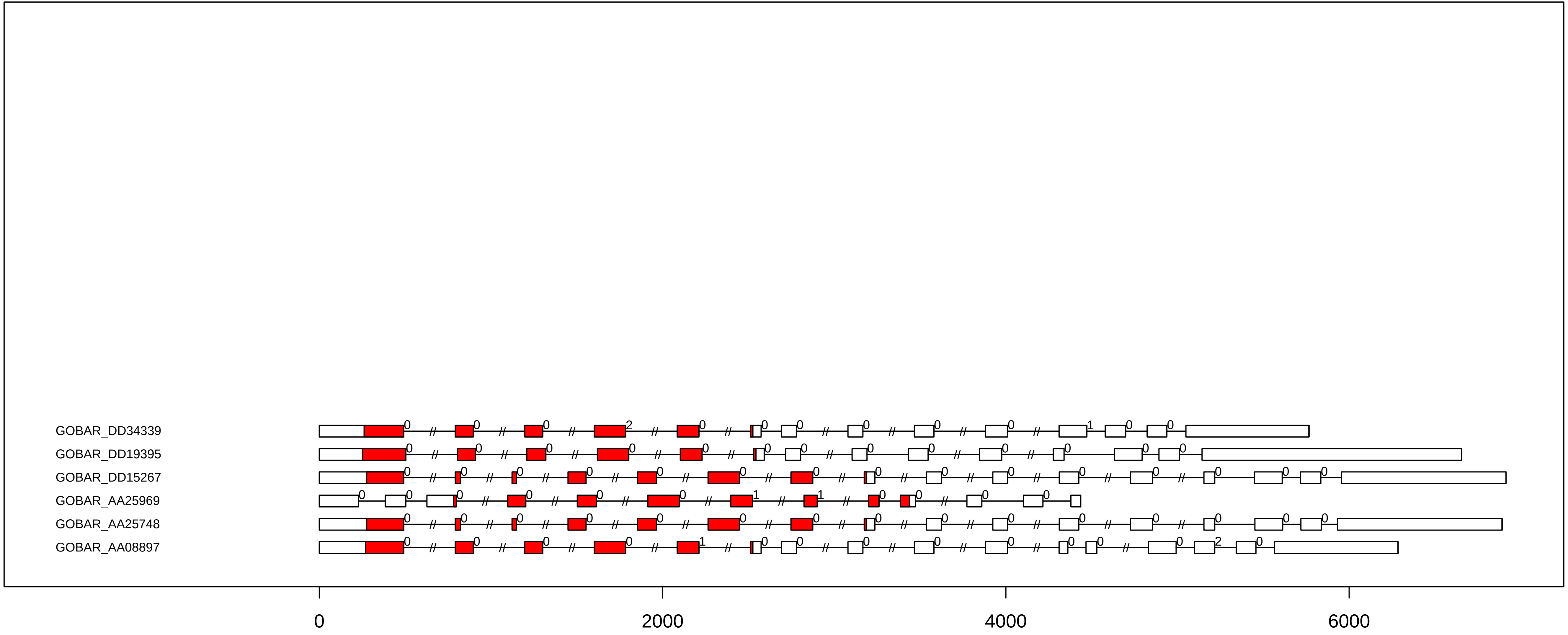

G.ba STE_STE11 exon−intron and domain diagram (part 1)

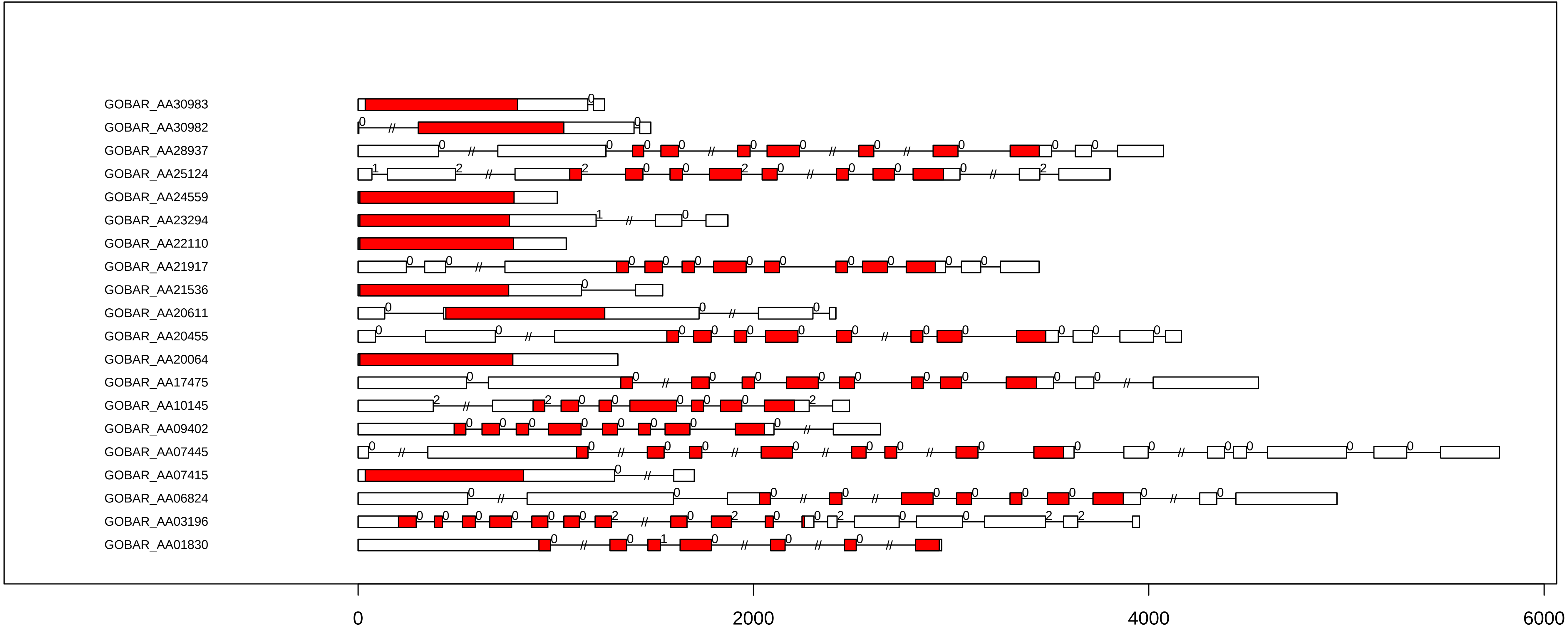

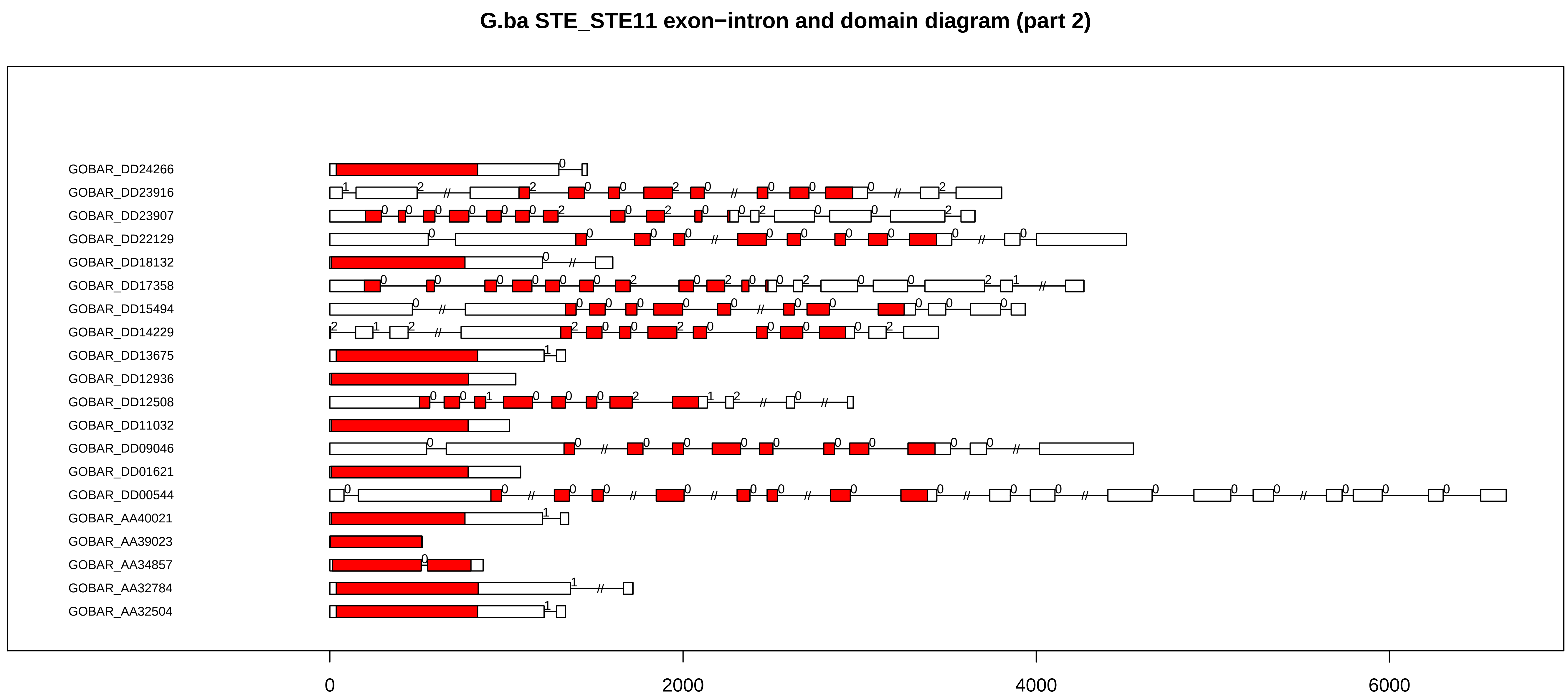
G.ba STE_STE11 exon−intron and domain diagram (part 2)
GOBAR_DD24266
GOBAR_DD23916
GOBAR_DD23907
GOBAR_DD22129
GOBAR_DD18132
GOBAR_DD17358
GOBAR_DD15494
GOBAR_DD14229
GOBAR_DD13675
GOBAR_DD12936
GOBAR_DD12508
GOBAR_DD11032
GOBAR_DD09046
GOBAR_DD01621
GOBAR_DD00544
GOBAR_AA40021
GOBAR_AA39023
GOBAR_AA34857
GOBAR_AA32784
GOBAR_AA32504
0
2000
4000
6000

G.ba STE_STE11 exon−intron and domain diagram (part 3)

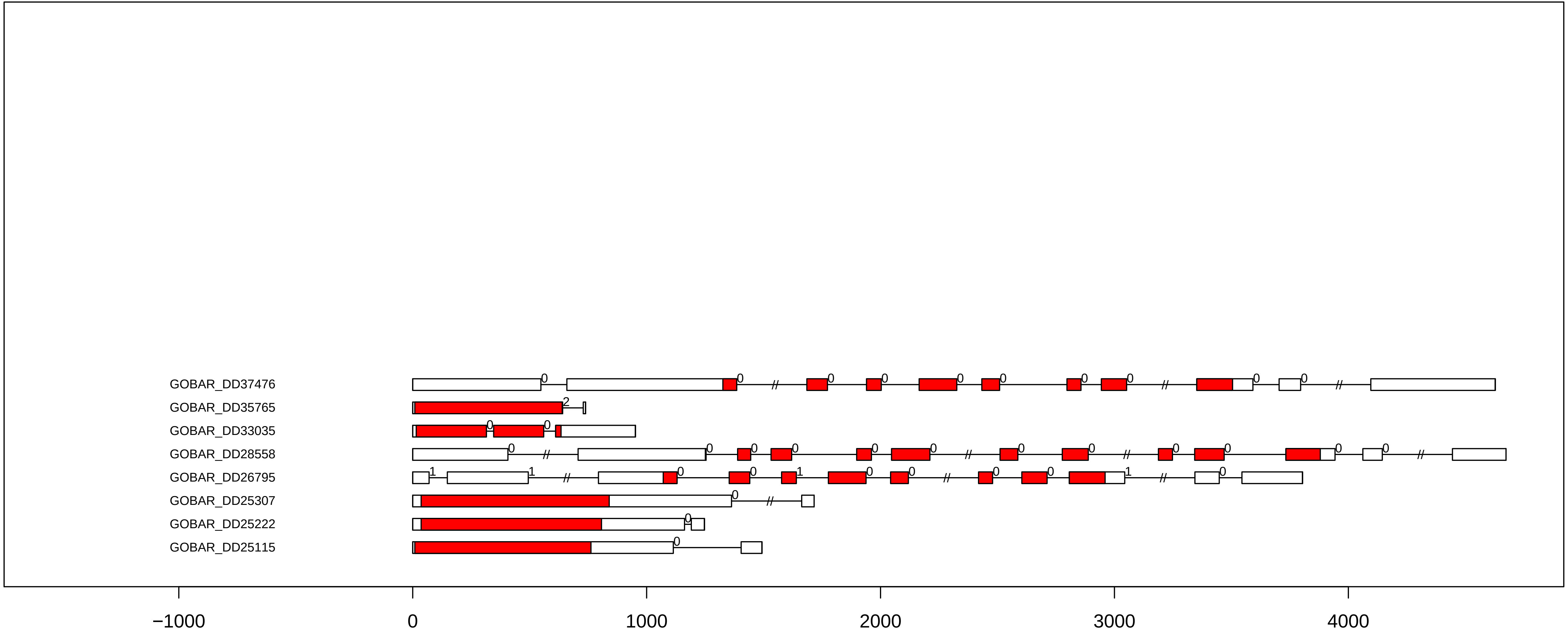

G.ba STE_STE20−Fray exon−intron and domain diagram (part 1)

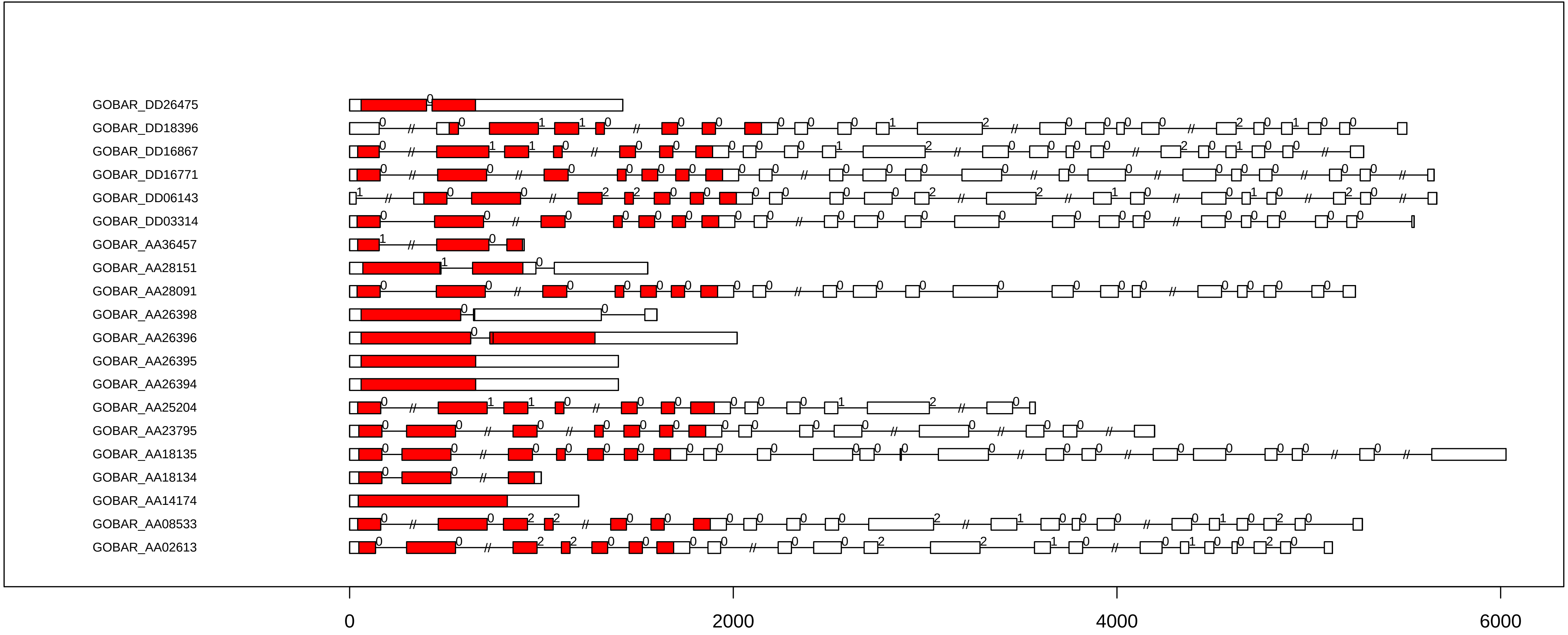

G.ba STE_STE20−Fray exon−intron and domain diagram (part 2)

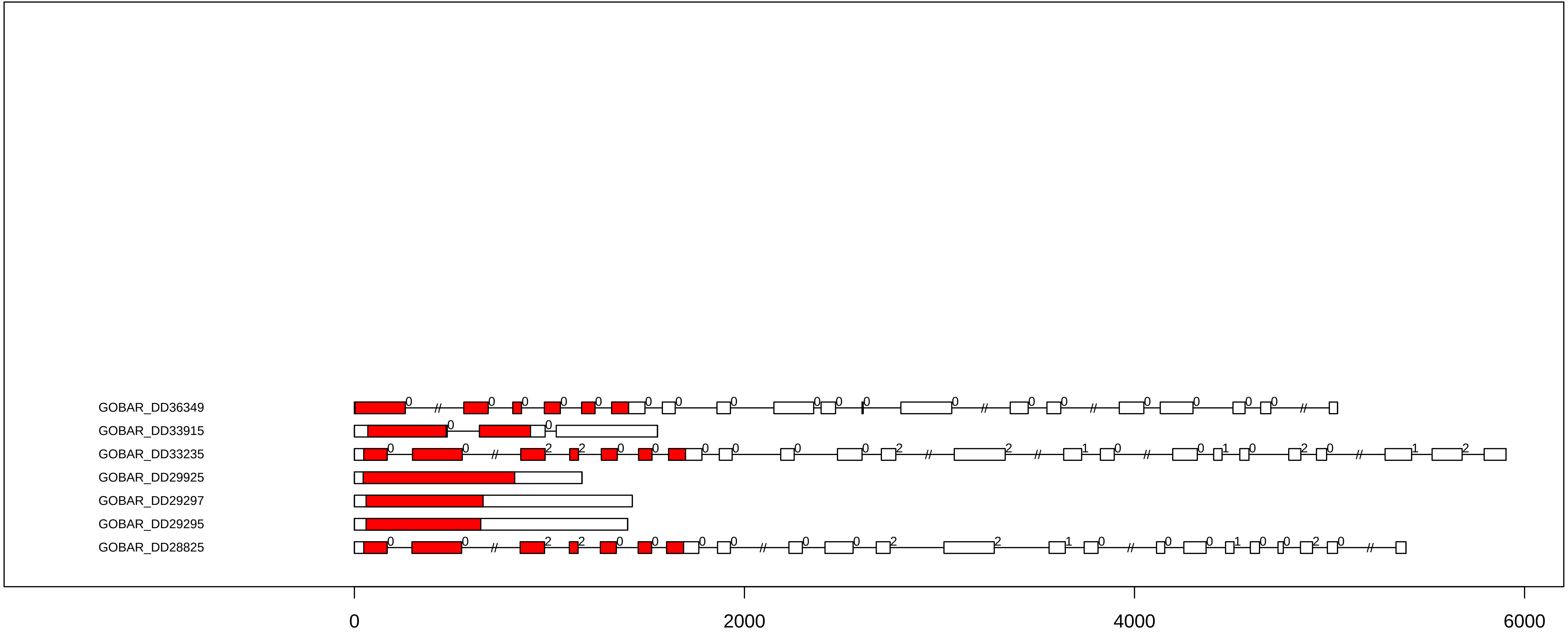

G.ba STE_STE20−PI exon−intron and domain diagram (all)

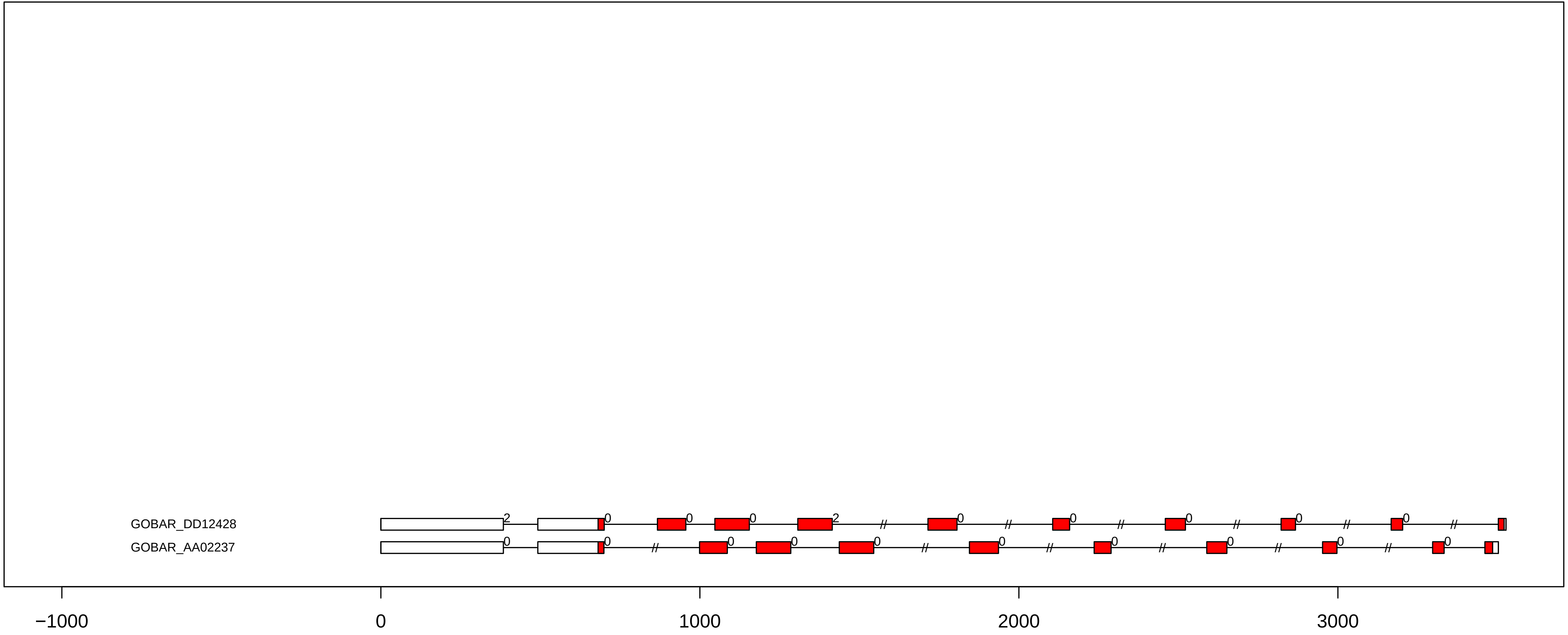

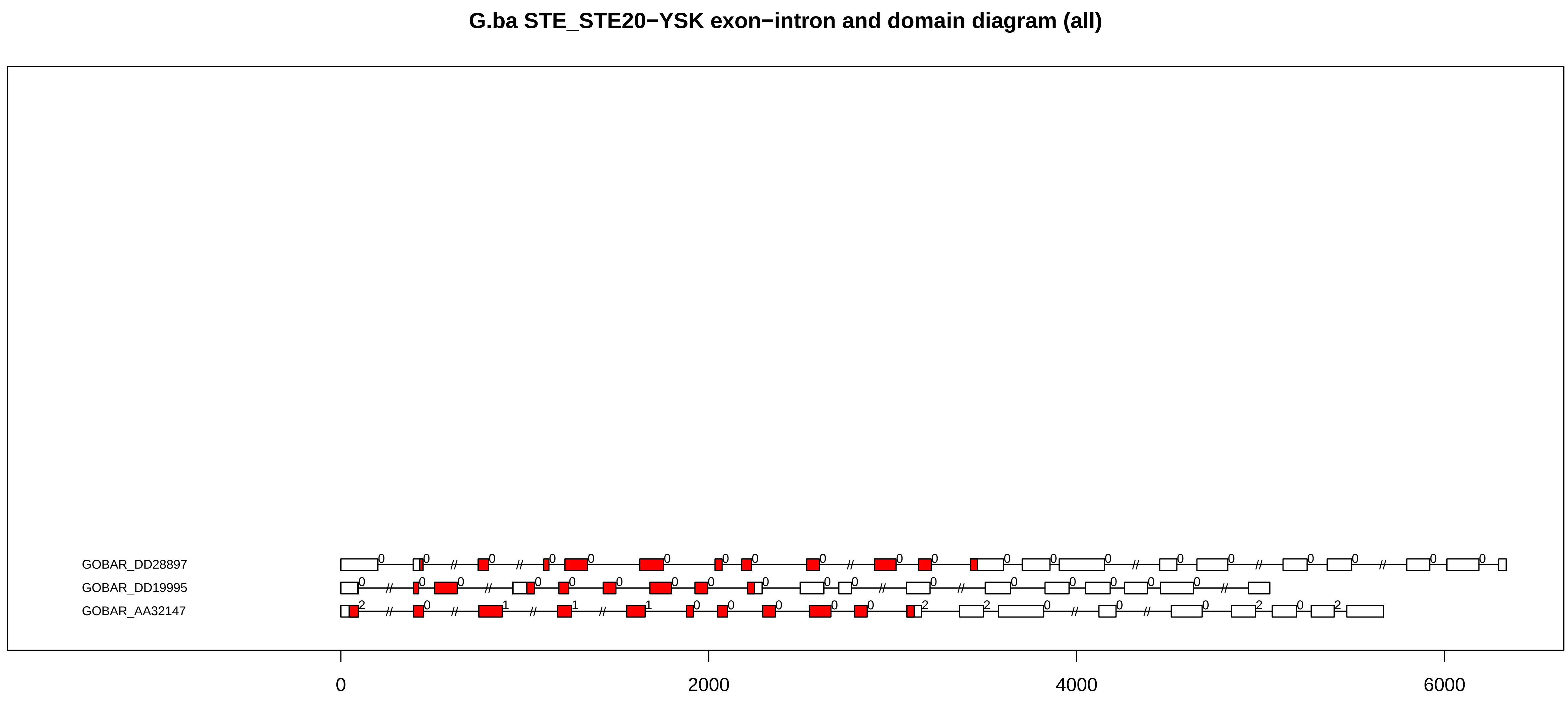

G.ba STE_STE20−YSK exon−intron and domain diagram (all)
GOBAR_DD28897
GOBAR_DD19995
GOBAR_AA32147
0
2000
4000
6000

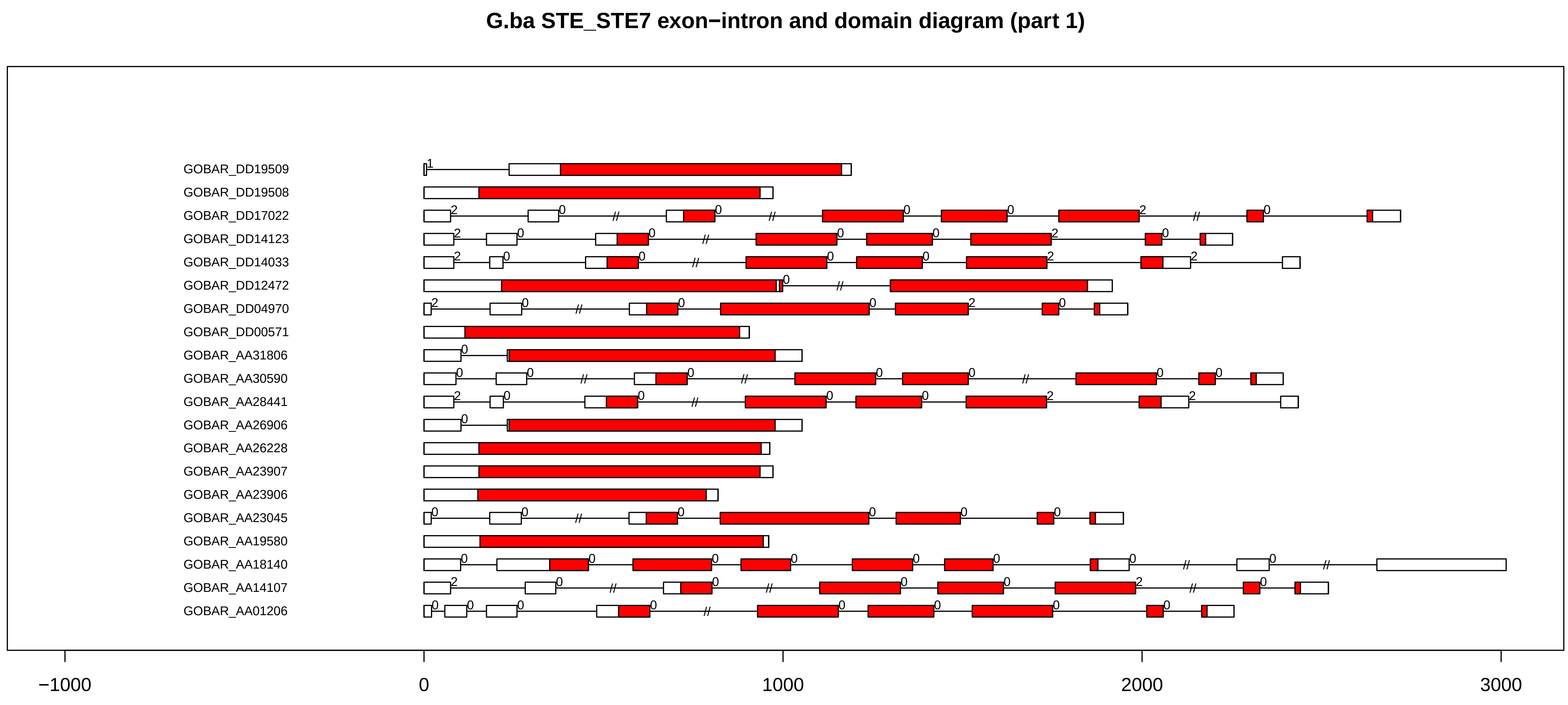

G.ba STE_STE7 exon−intron and domain diagram (part 1)
GOBAR_DD19509
GOBAR_DD19508
GOBAR_DD17022
GOBAR_DD14123
GOBAR_DD14033
GOBAR_DD12472
GOBAR_DD04970
GOBAR_DD00571
GOBAR_AA31806
GOBAR_AA30590
GOBAR_AA28441
GOBAR_AA26906
GOBAR_AA26228
GOBAR_AA23907
GOBAR_AA23906
GOBAR_AA23045
GOBAR_AA19580
GOBAR_AA18140
GOBAR_AA14107
GOBAR_AA01206
−1000
0
1000
2000
3000

G.ba STE_STE7 exon−intron and domain diagram (part 2)

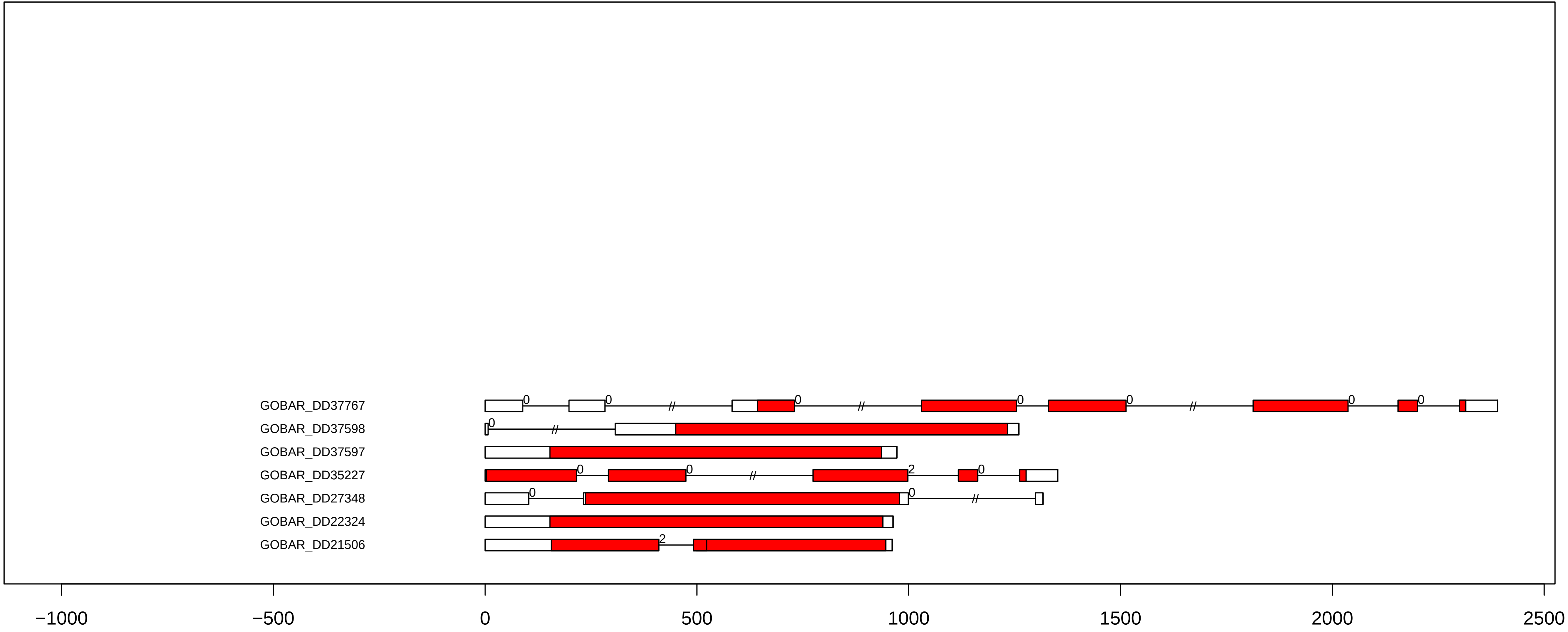

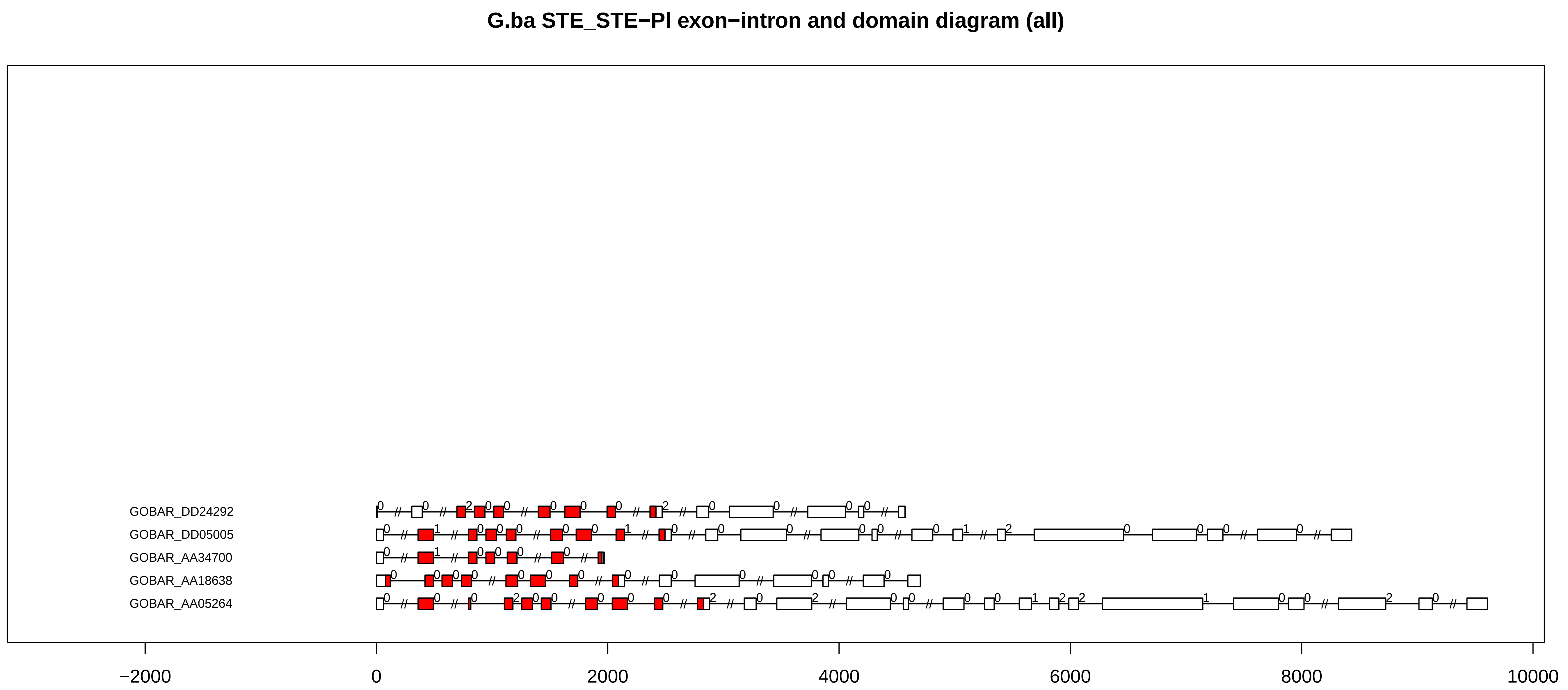
G.ba STE_STE−PI exon−intron and domain diagram (all)
GOBAR_DD24292
GOBAR_DD05005
GOBAR_AA34700
GOBAR_AA18638
GOBAR_AA05264
−2000
0
2000
4000
6000
8000
10000

G.ba TKL_CTR1−DRK−1 exon−intron and domain diagram (all)

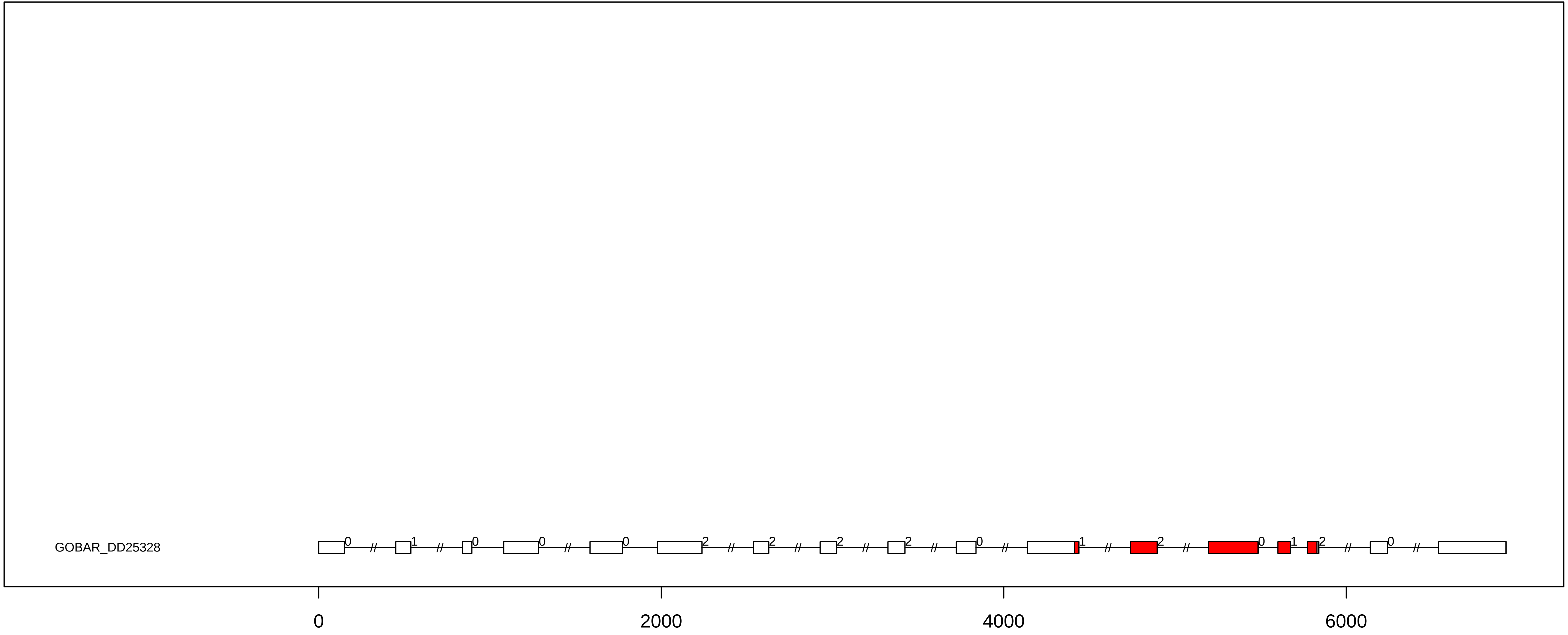

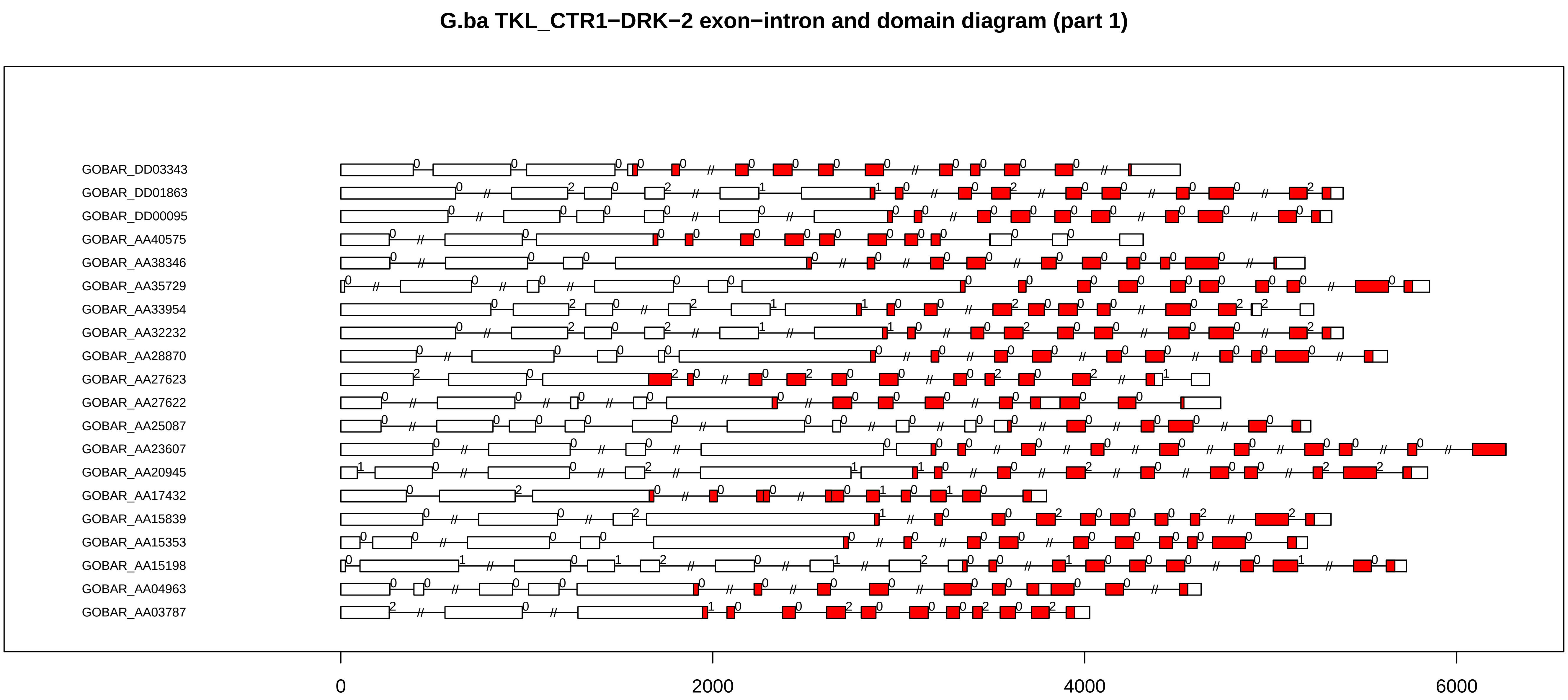
G.ba TKL_CTR1−DRK−2 exon−intron and domain diagram (part 1)
GOBAR_DD03343
GOBAR_DD01863
GOBAR_DD00095
GOBAR_AA40575
GOBAR_AA38346
GOBAR_AA35729
GOBAR_AA33954
GOBAR_AA32232
GOBAR_AA28870
GOBAR_AA27623
GOBAR_AA27622
GOBAR_AA25087
GOBAR_AA23607
GOBAR_AA20945
GOBAR_AA17432
GOBAR_AA15839
GOBAR_AA15353
GOBAR_AA15198
GOBAR_AA04963
GOBAR_AA03787
0
2000
4000
6000

G.ba TKL_CTR1−DRK−2 exon−intron and domain diagram (part 2)

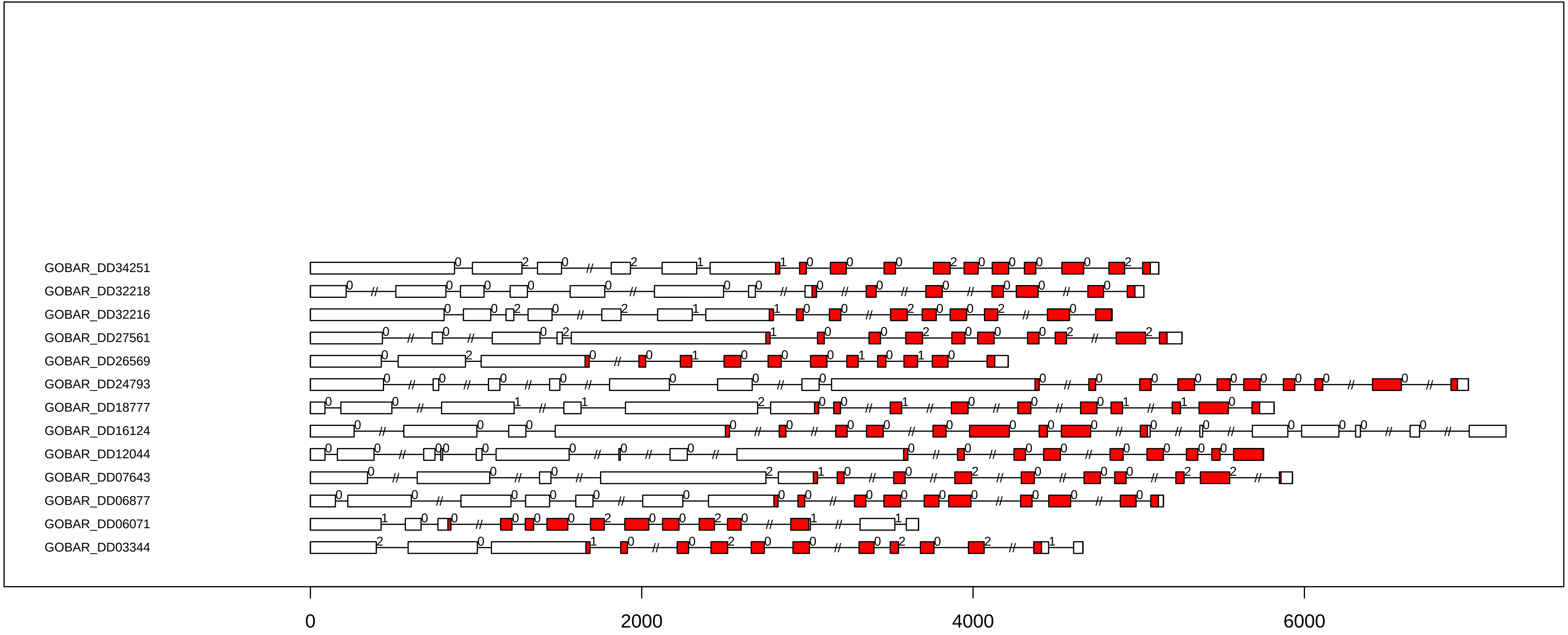

G.ba TKL_Gdt exon−intron and domain diagram (all)

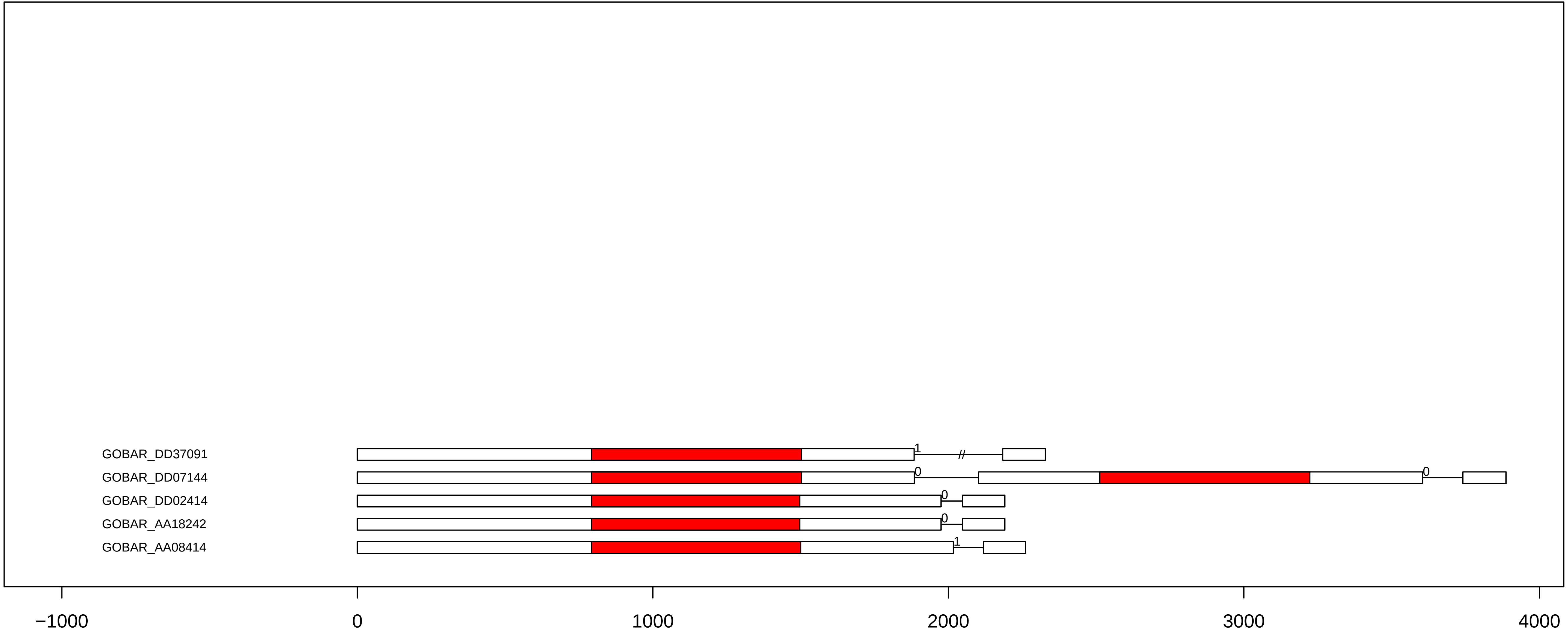

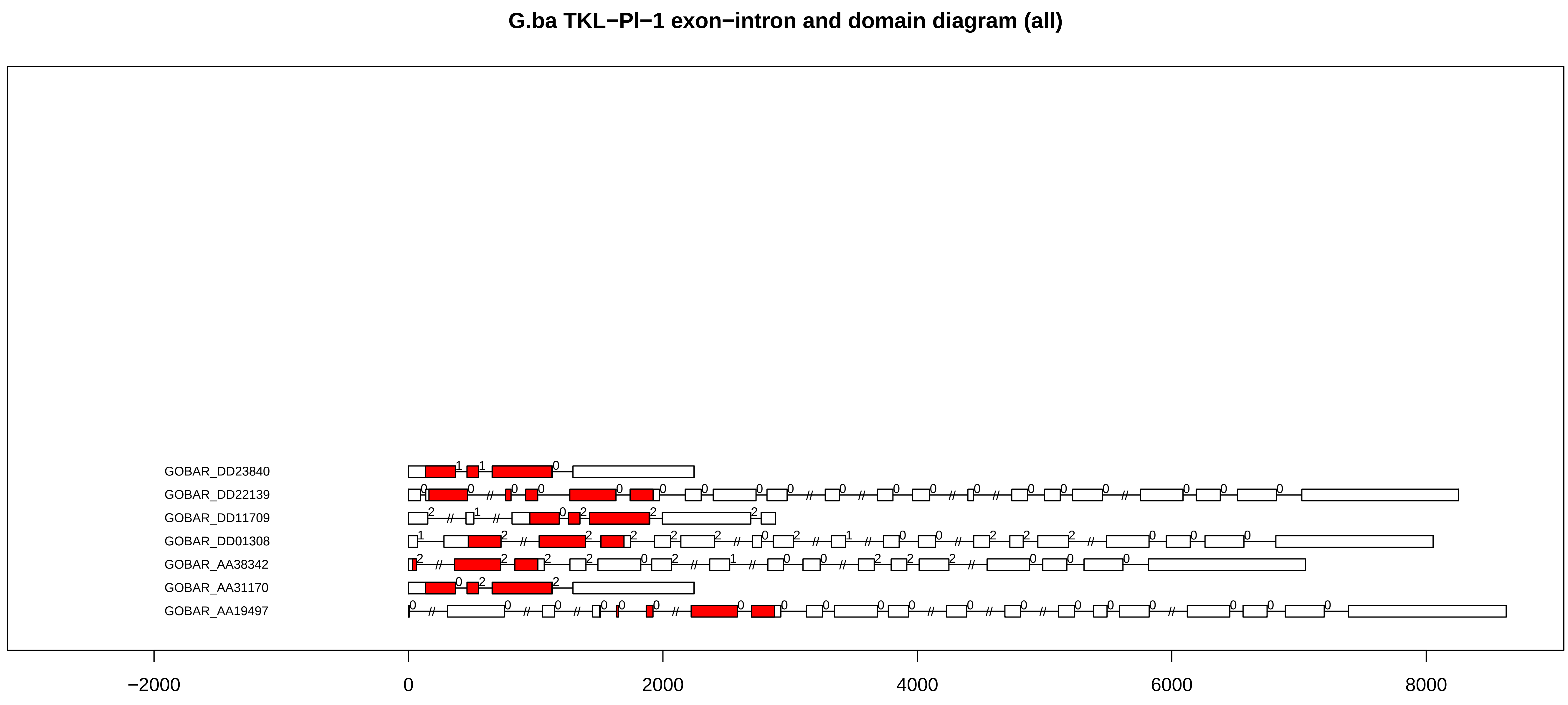
G.ba TKL−Pl−1 exon−intron and domain diagram (all)
GOBAR_DD23840
GOBAR_DD22139
GOBAR_DD11709
GOBAR_DD01308
GOBAR_AA38342
GOBAR_AA31170
GOBAR_AA19497
−2000
0
2000
4000
6000
8000

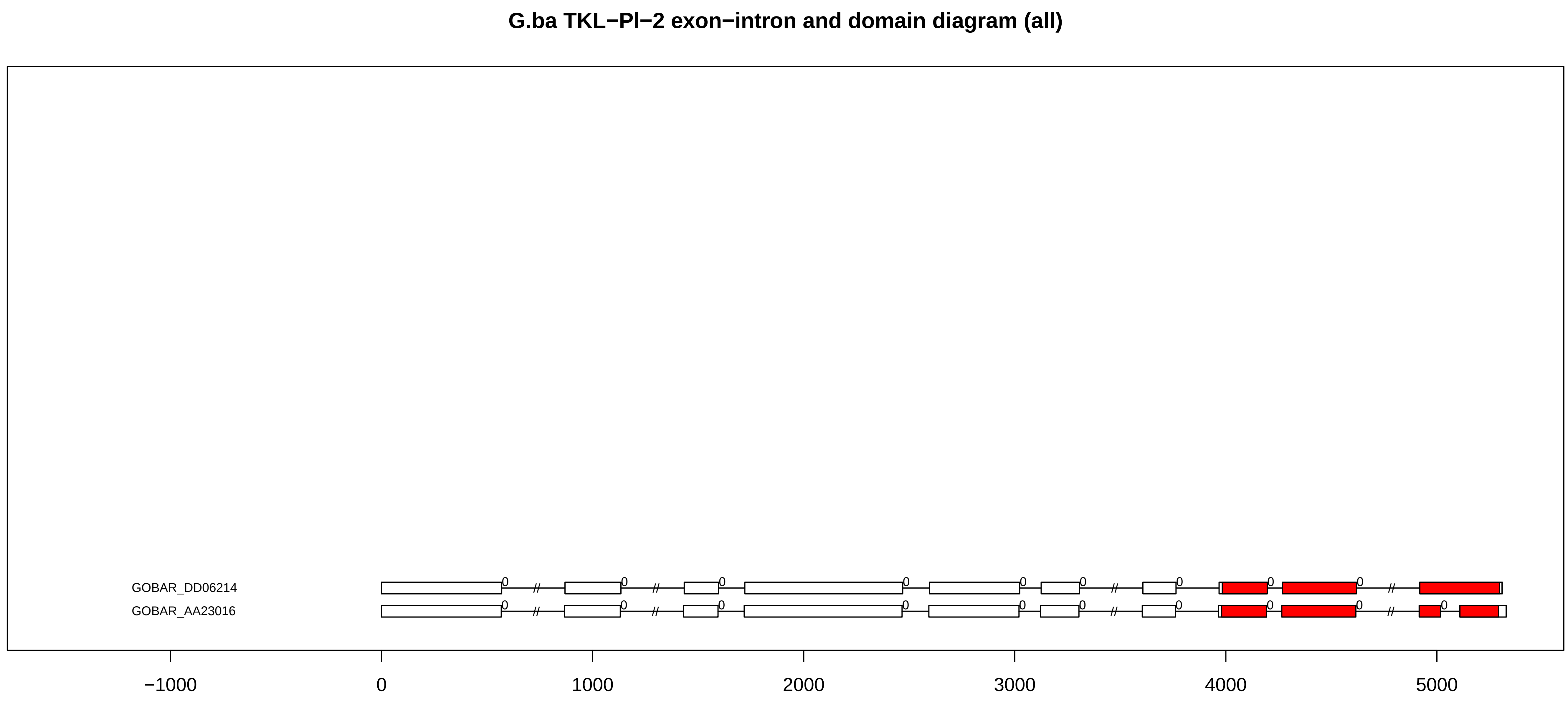
G.ba TKL−PI−2 exon−intron and domain diagram (all)
GOBAR_DD06214
GOBAR_AA23016
−1000
0
1000
2000
3000
4000
5000

G.ba TKL−Pl−3 exon−intron and domain diagram (all)

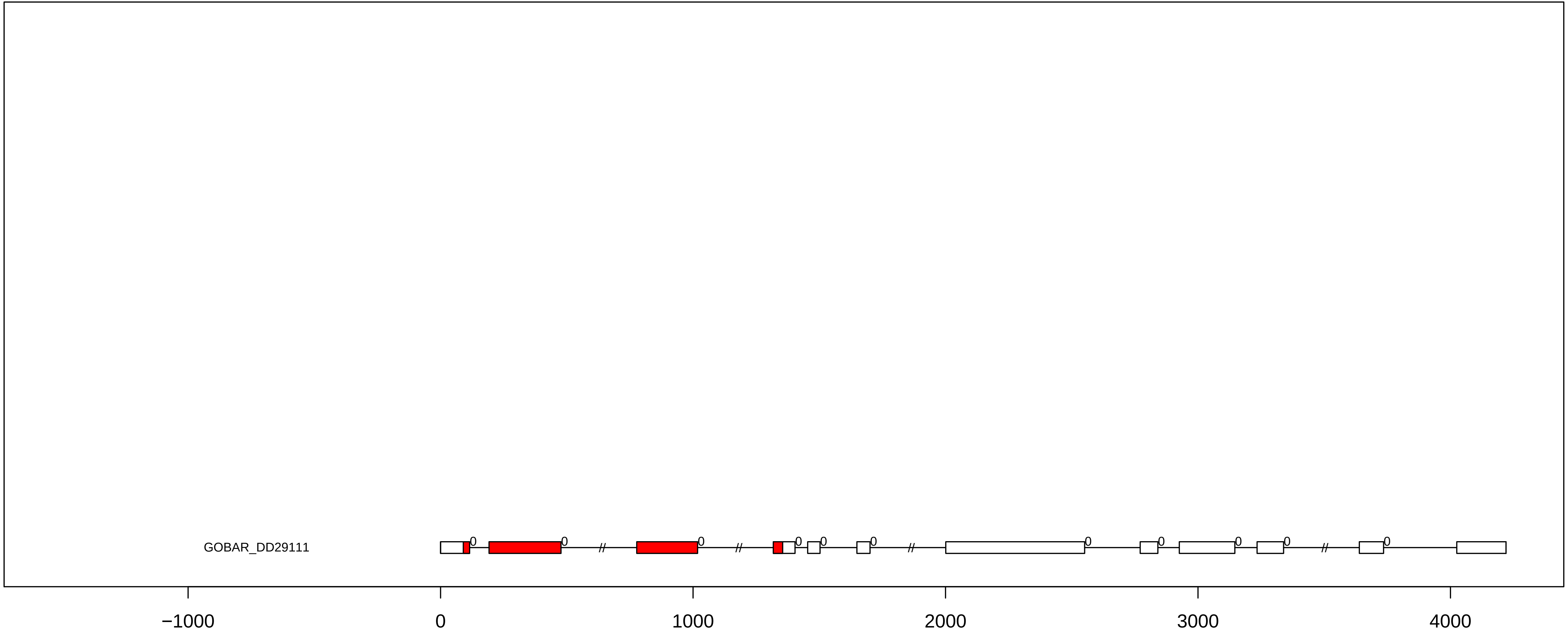

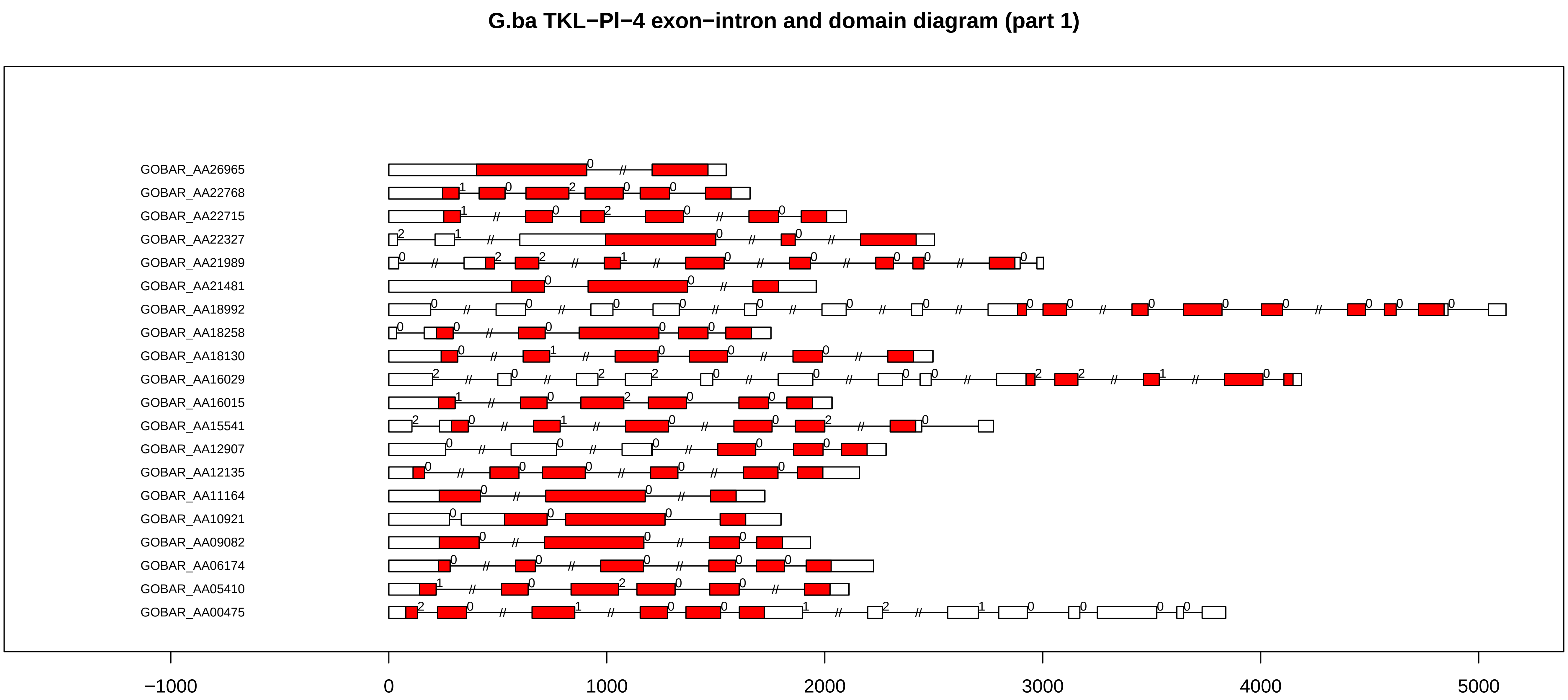
G.ba TKL−Pl−4 exon−intron and domain diagram (part 1)
GOBAR_AA26965
GOBAR_AA22768
GOBAR_AA22715
GOBAR_AA22327
GOBAR_AA21989
GOBAR_AA21481
GOBAR_AA18992
GOBAR_AA18258
GOBAR_AA18130
GOBAR_AA16029
GOBAR_AA16015
GOBAR_AA15541
GOBAR_AA12907
GOBAR_AA12135
GOBAR_AA11164
GOBAR_AA10921
GOBAR_AA09082
GOBAR_AA06174
GOBAR_AA05410
GOBAR_AA00475
−1000
0
1000
2000
3000
4000
5000

G.ba TKL−Pl−4 exon−intron and domain diagram (part 2)

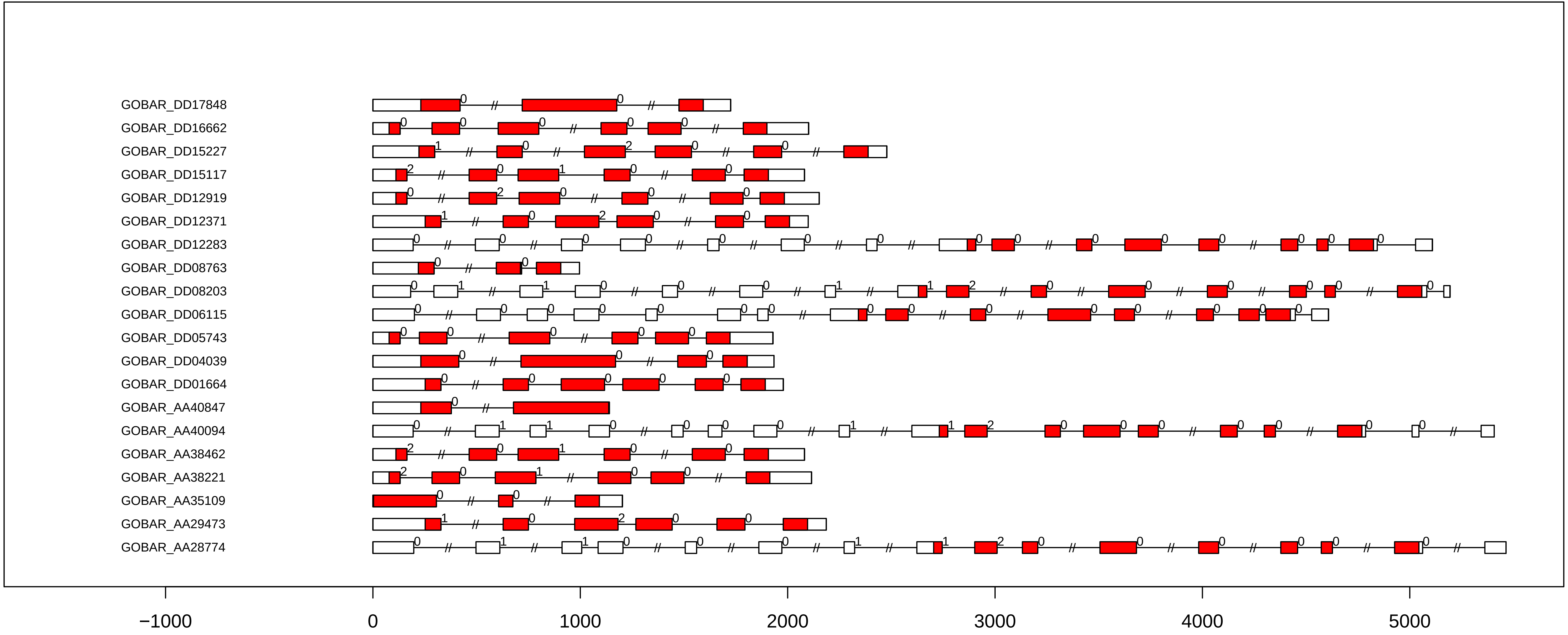

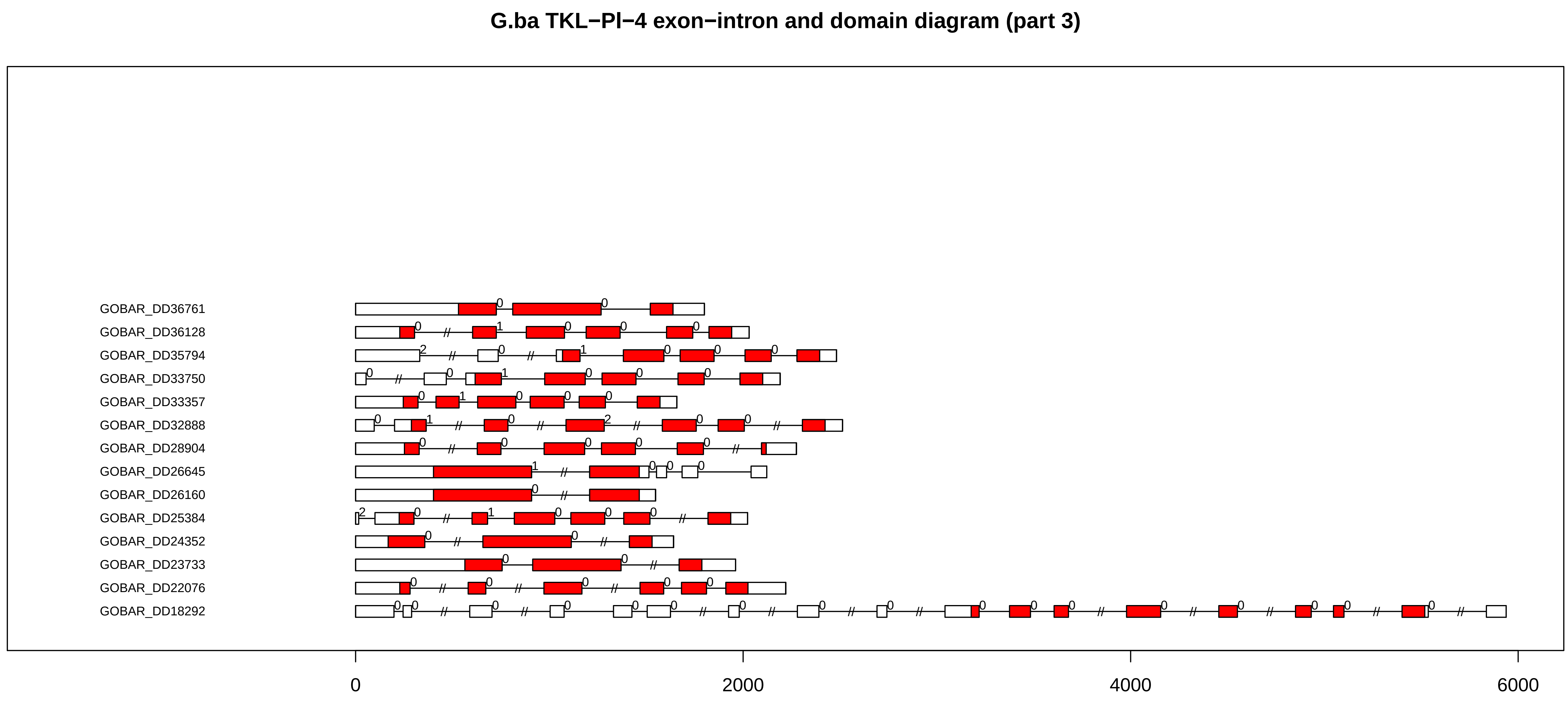

G.ba TKL−Pl−4 exon−intron and domain diagram (part 3)
GOBAR_DD36761
GOBAR_DD36128
GOBAR_DD35794
GOBAR_DD33750
GOBAR_DD33357
GOBAR_DD32888
GOBAR_DD28904
GOBAR_DD26645
GOBAR_DD26160
GOBAR_DD25384
GOBAR_DD24352
GOBAR_DD23733
GOBAR_DD22076
GOBAR_DD18292
0
2000
4000
6000

G.ba TKL−PI−5 exon−intron and domain diagram (all)

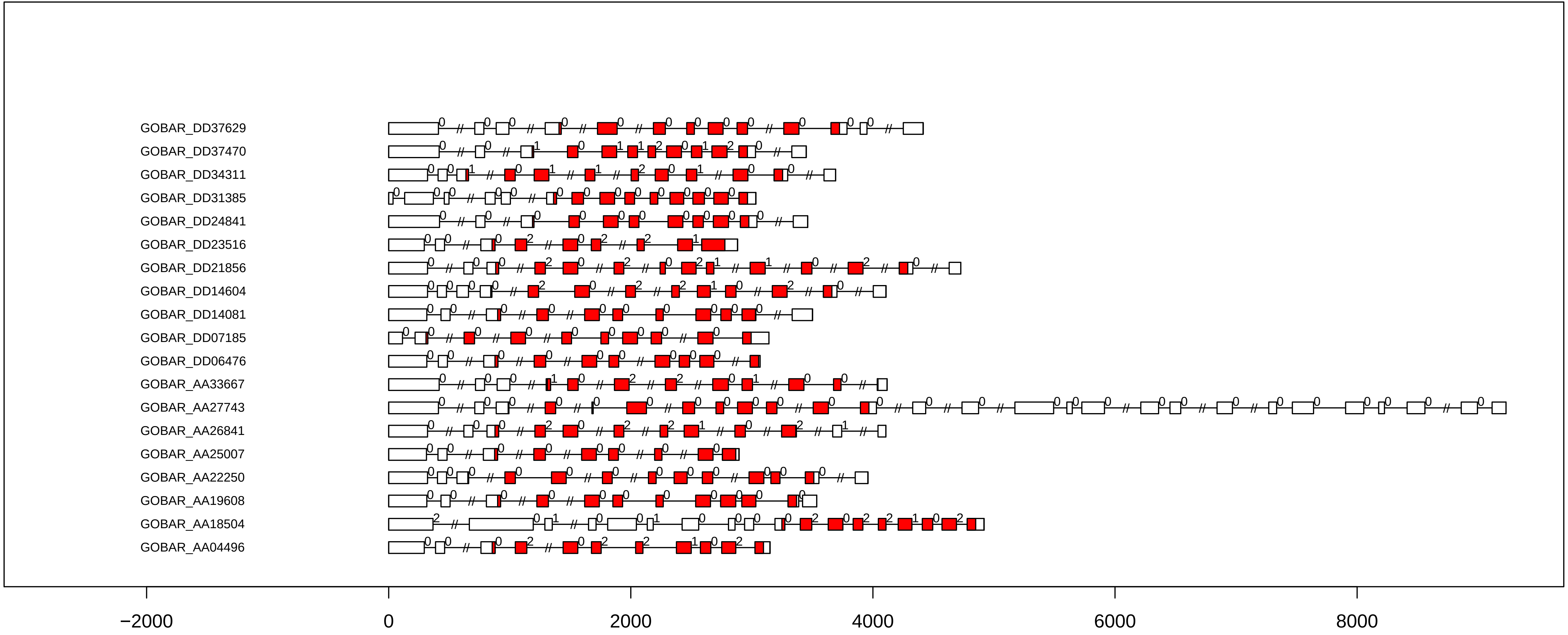

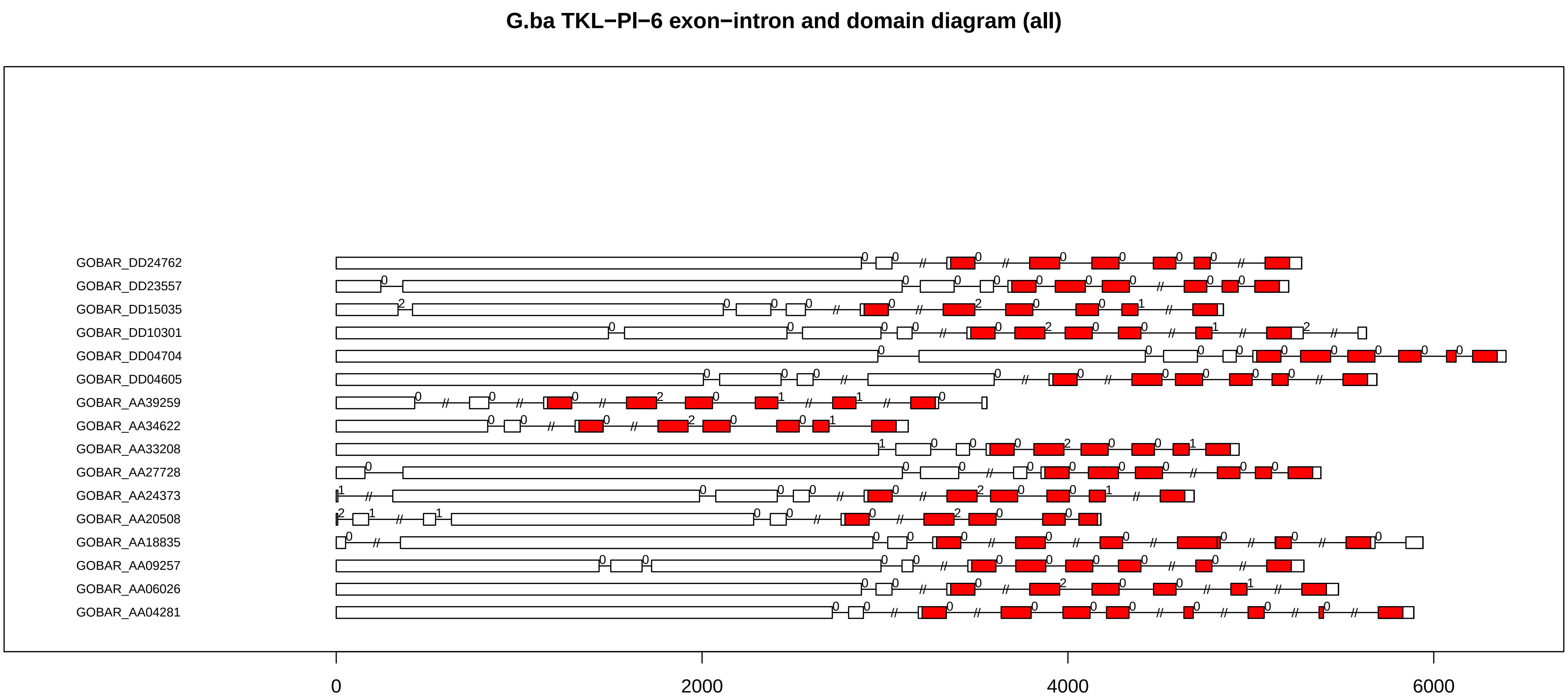

G.ba TKL−Pl−6 exon−intron and domain diagram (all)
GOBAR_DD24762
GOBAR_DD23557
GOBAR_DD15035
GOBAR_DD10301
GOBAR_DD04704
GOBAR_DD04605
GOBAR_AA39259
GOBAR_AA34622
GOBAR_AA33208
GOBAR_AA27728
GOBAR_AA24373
GOBAR_AA20508
GOBAR_AA18835
GOBAR_AA09257
GOBAR_AA06026
GOBAR_AA04281
0
2000
4000
6000

G.ba TKL−Pl−7 exon−intron and domain diagram (all)

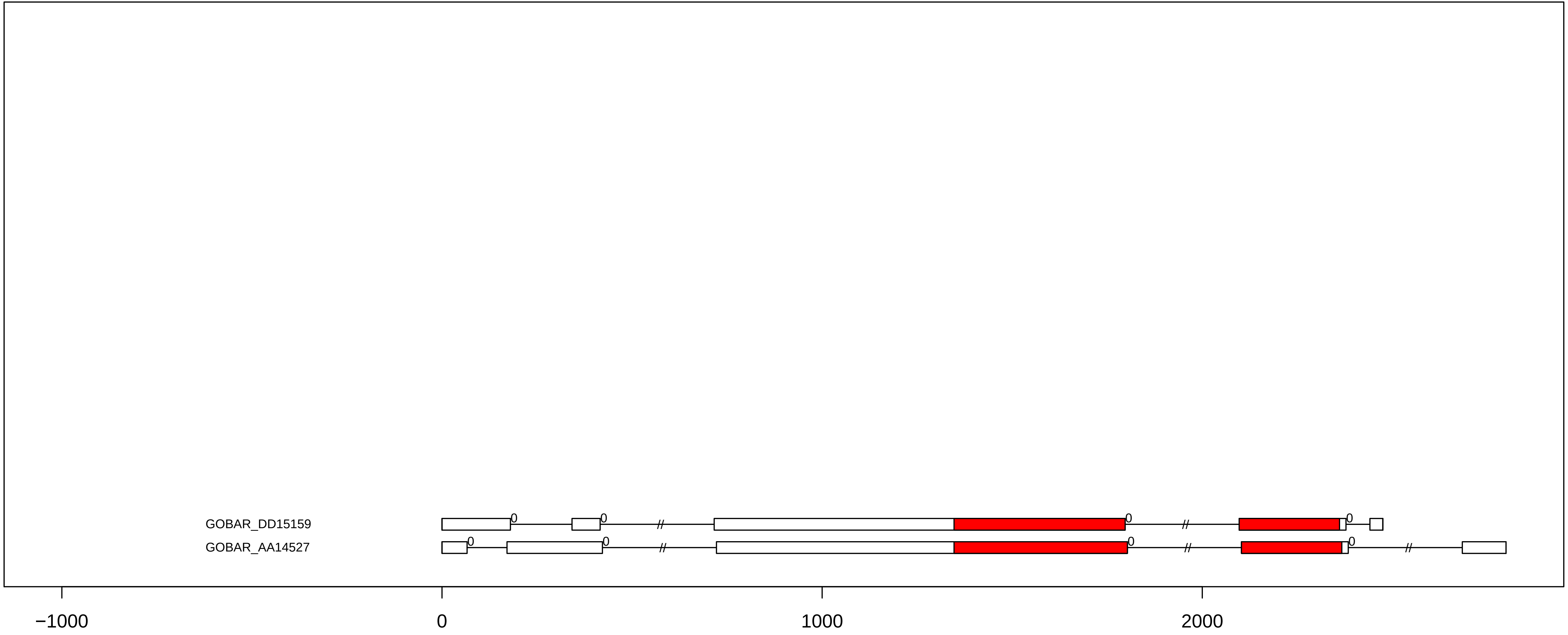

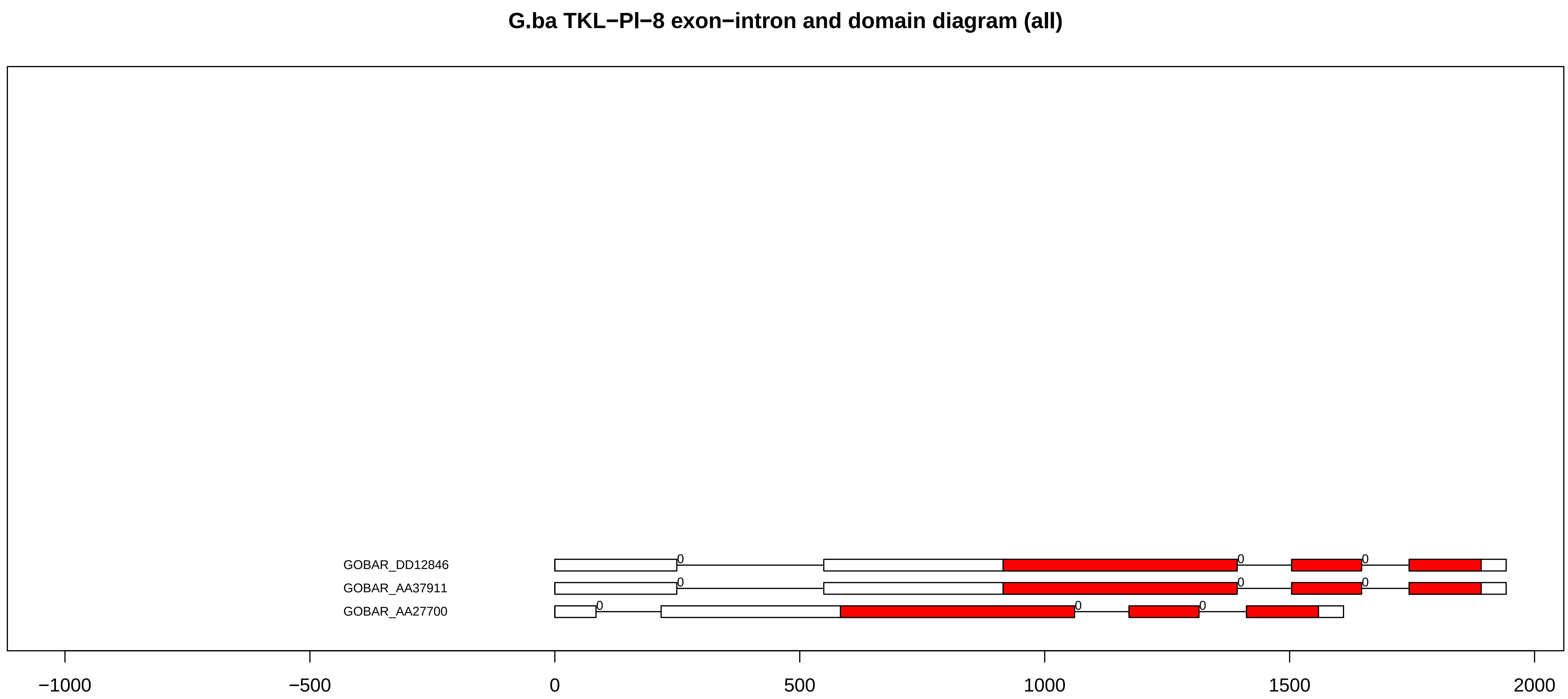
G.ba TKL−Pl−8 exon−intron and domain diagram (all)
GOBAR_DD12846
GOBAR_AA37911
GOBAR_AA27700
0
−1000
−500
0
500
1000
1500
2000

G.ba TLK exon−intron and domain diagram (all)

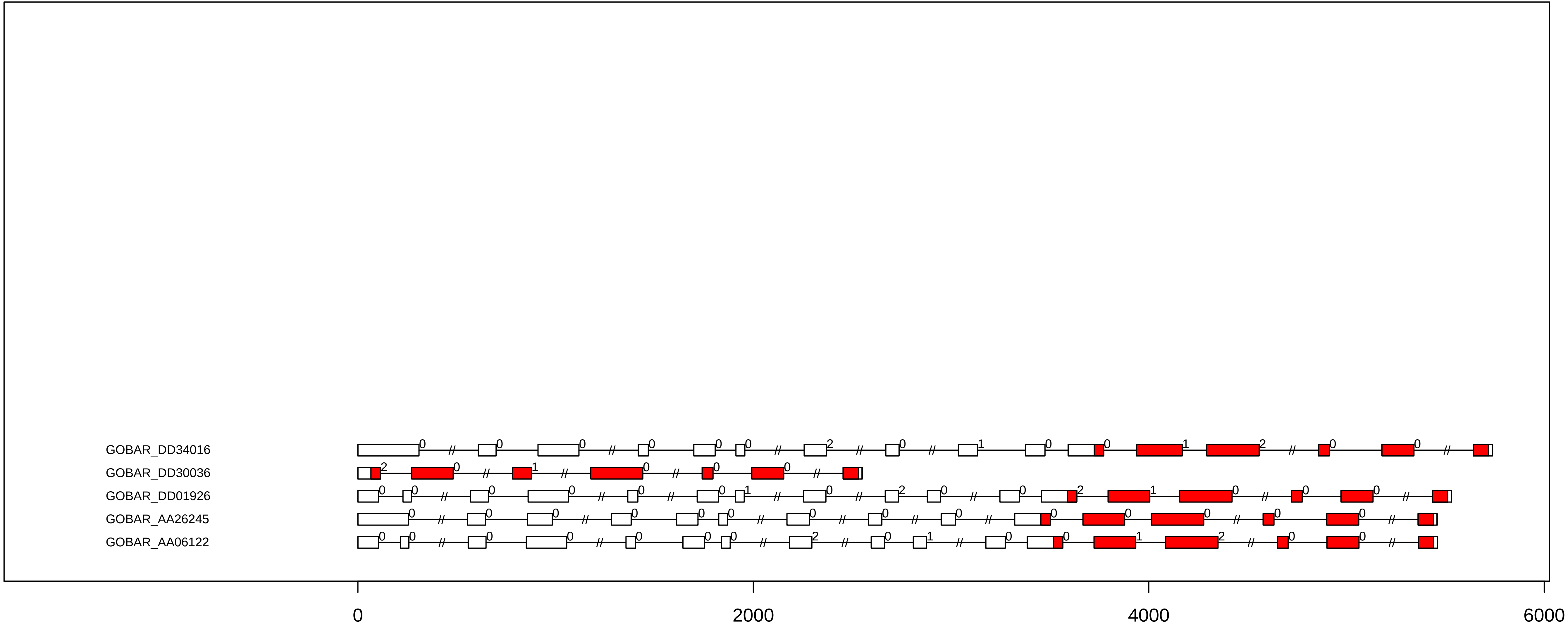

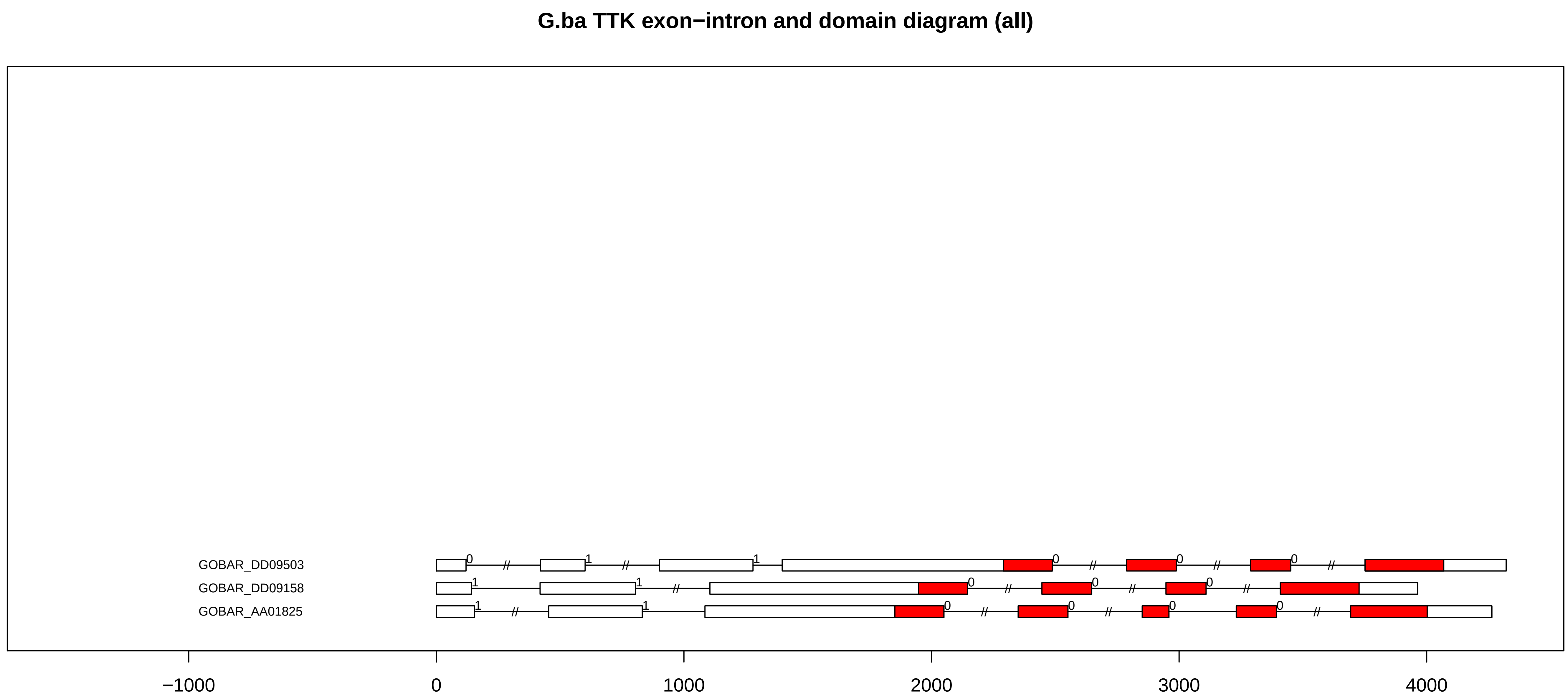
G.ba TTK exon−intron and domain diagram (all)
GOBAR_DD09503
GOBAR_DD09158
GOBAR_AA01825
−1000
0
1000
2000
3000
4000

G.ba ULK_Fused exon−intron and domain diagram (all)

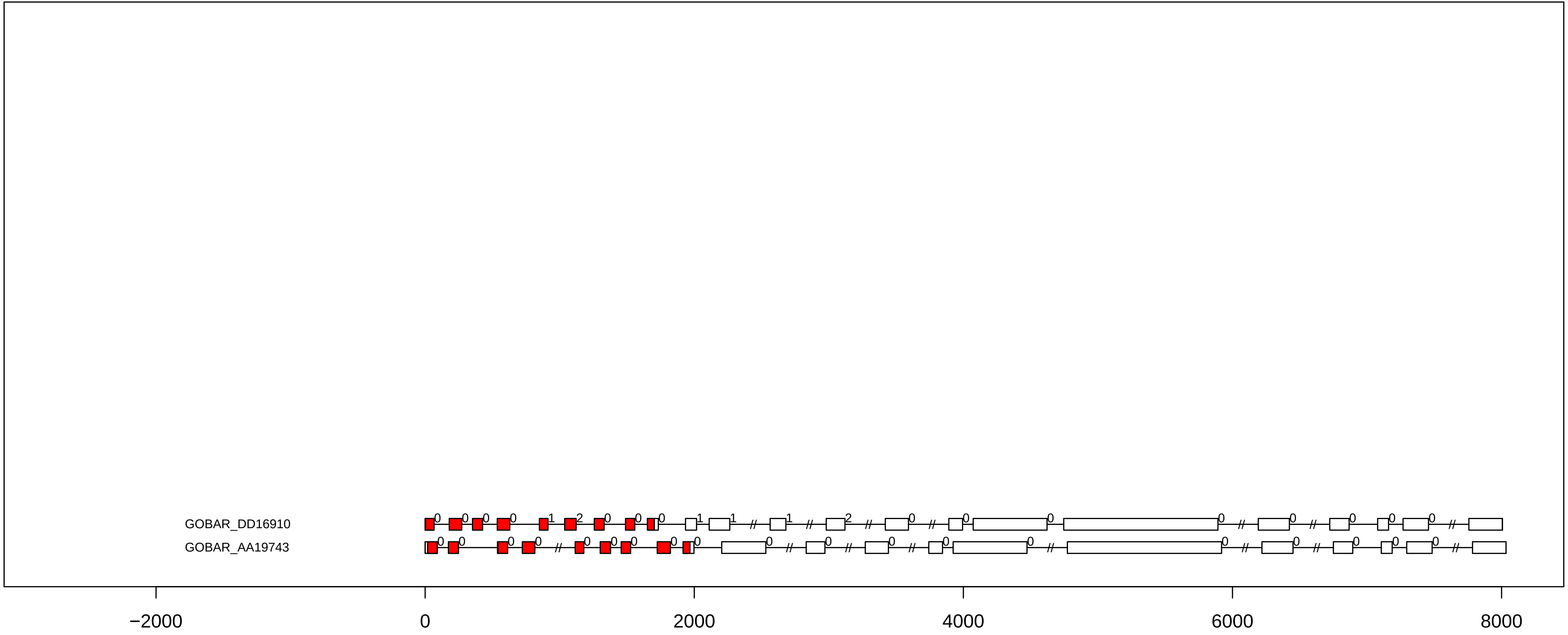

G.ba ULK_ULK4 exon−intron and domain diagram (all)

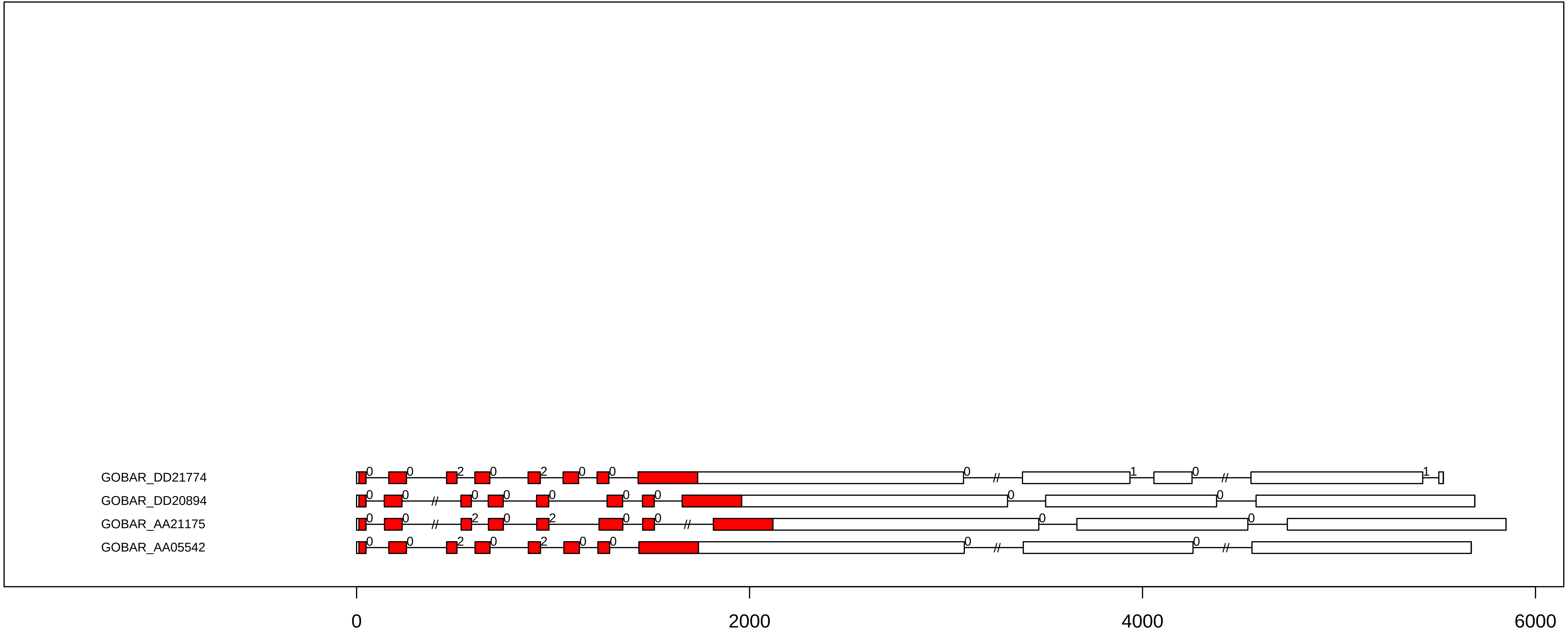

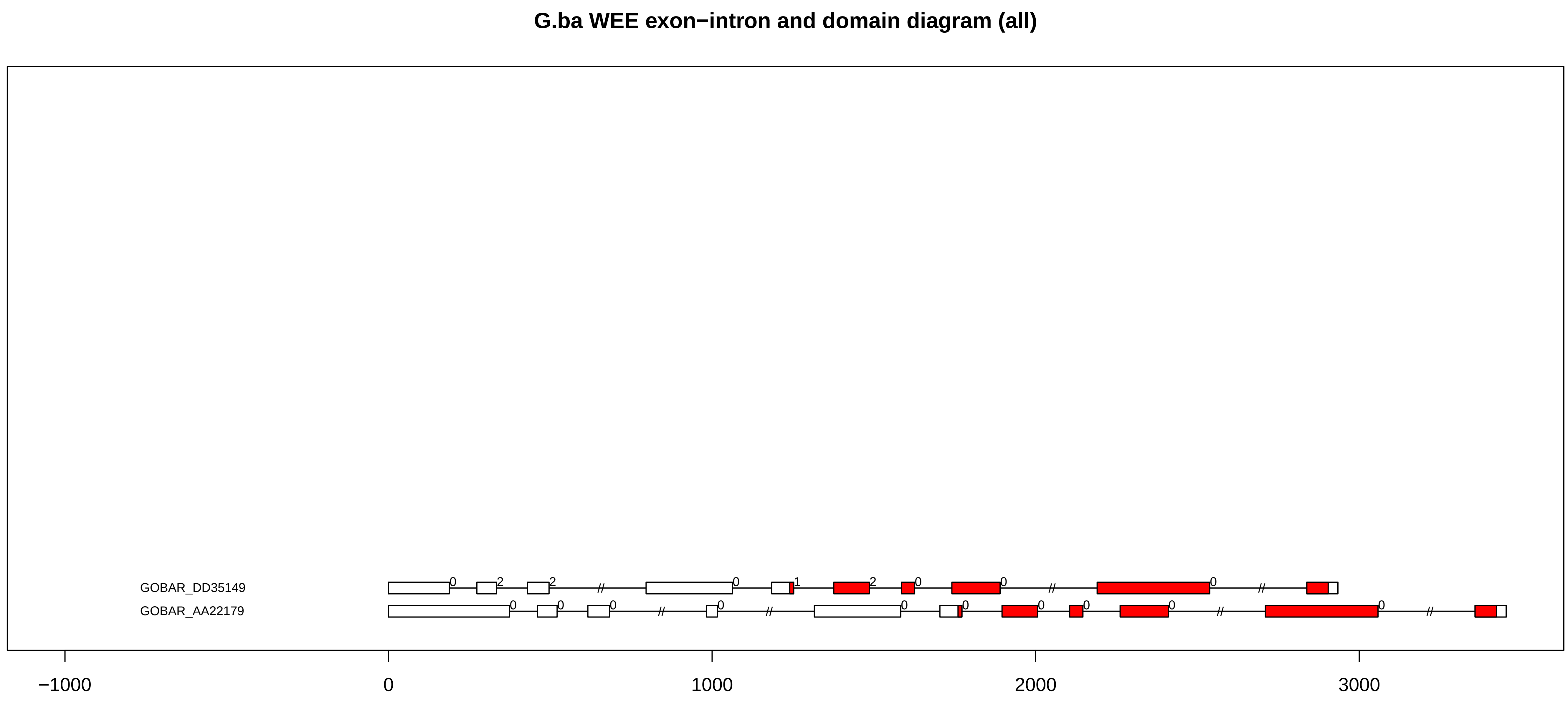

G.ba WEE exon−intron and domain diagram (all)
GOBAR_DD35149
GOBAR_AA22179
0
2
2
0
1
2
0
0
0
0
0
0
0
0
0
0
0
0
0
0
−1000
0
1000
2000
3000

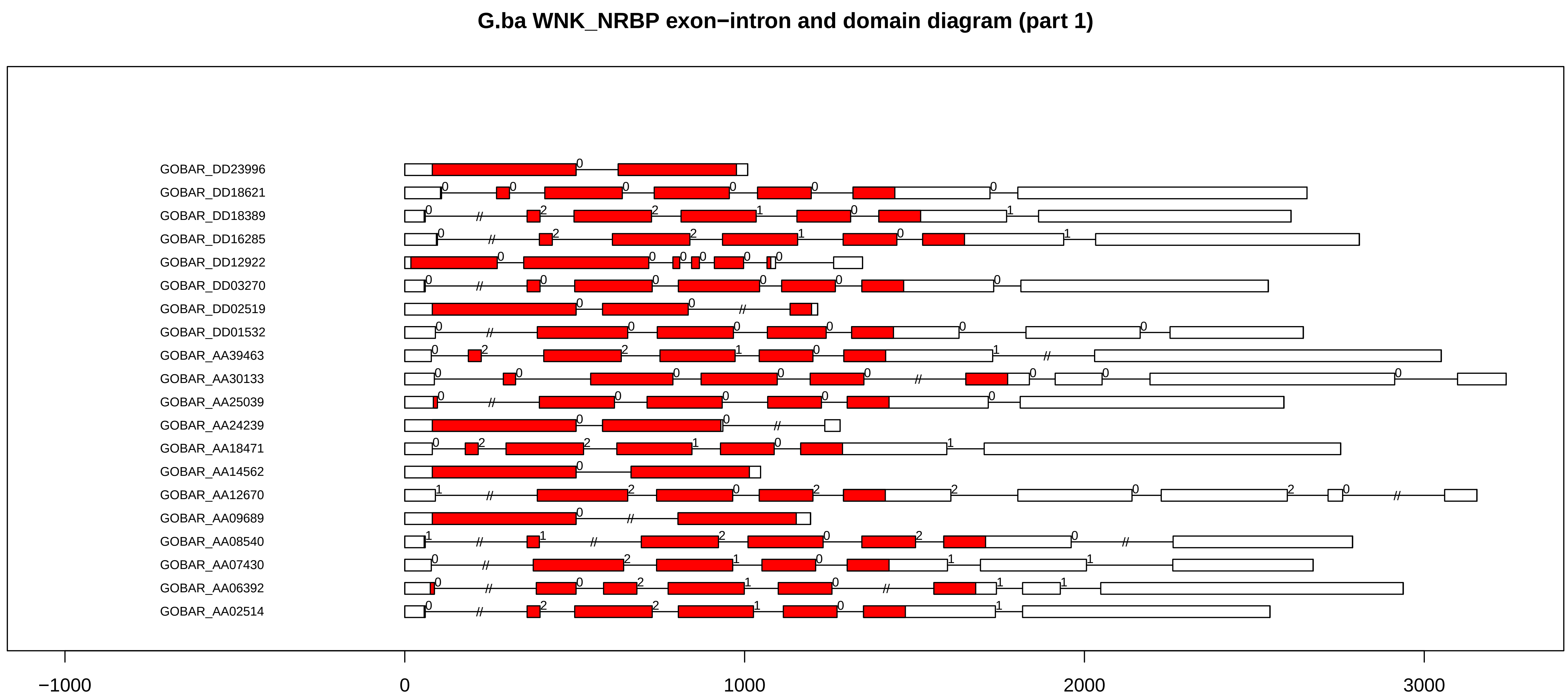
G.ba WNK_NRBP exon−intron and domain diagram (part 1)
GOBAR_DD23996
GOBAR_DD18621
GOBAR_DD18389
GOBAR_DD16285
GOBAR_DD12922
GOBAR_DD03270
GOBAR_DD02519
GOBAR_DD01532
GOBAR_AA39463
GOBAR_AA30133
GOBAR_AA25039
GOBAR_AA24239
GOBAR_AA18471
GOBAR_AA14562
GOBAR_AA12670
GOBAR_AA09689
GOBAR_AA08540
GOBAR_AA07430
GOBAR_AA06392
GOBAR_AA02514
−1000
0
1000
2000
3000

G.ba WNK_NRBP exon−intron and domain diagram (part 2)

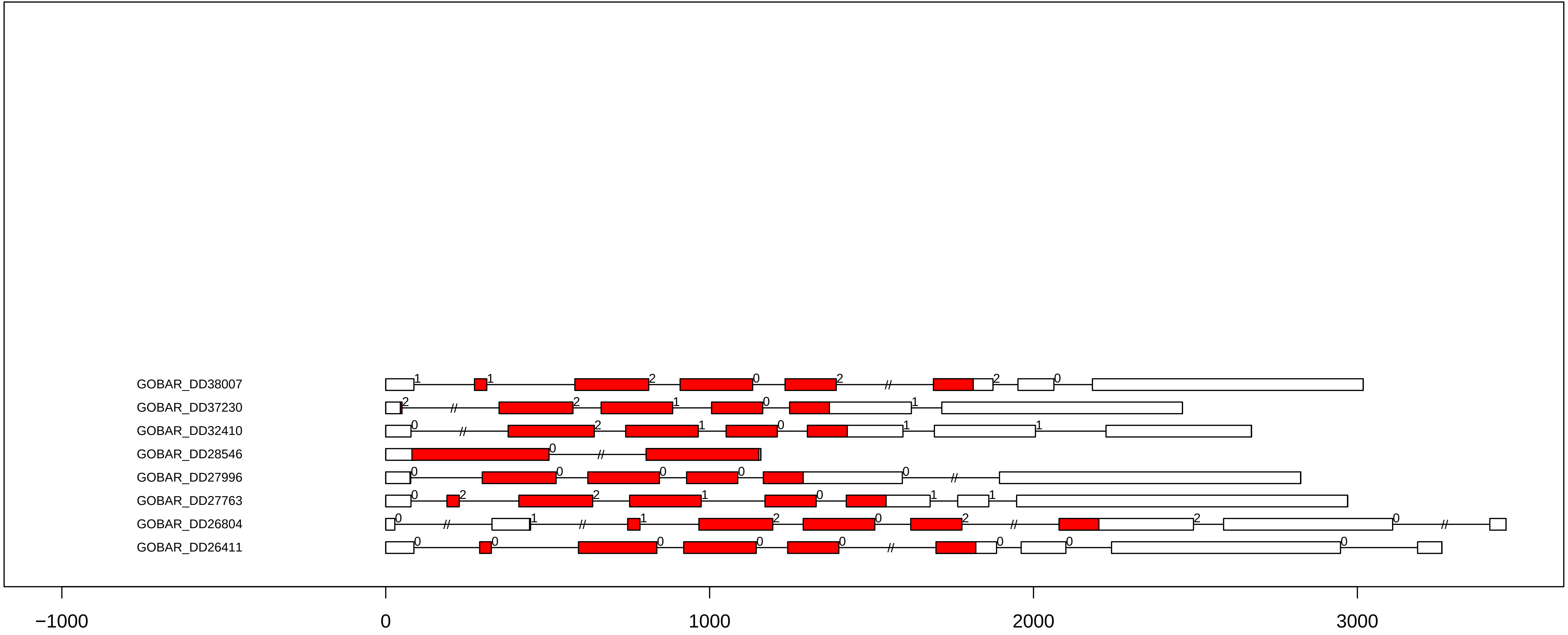

G.ar AGC_MAST exon−intron and domain diagram (all)

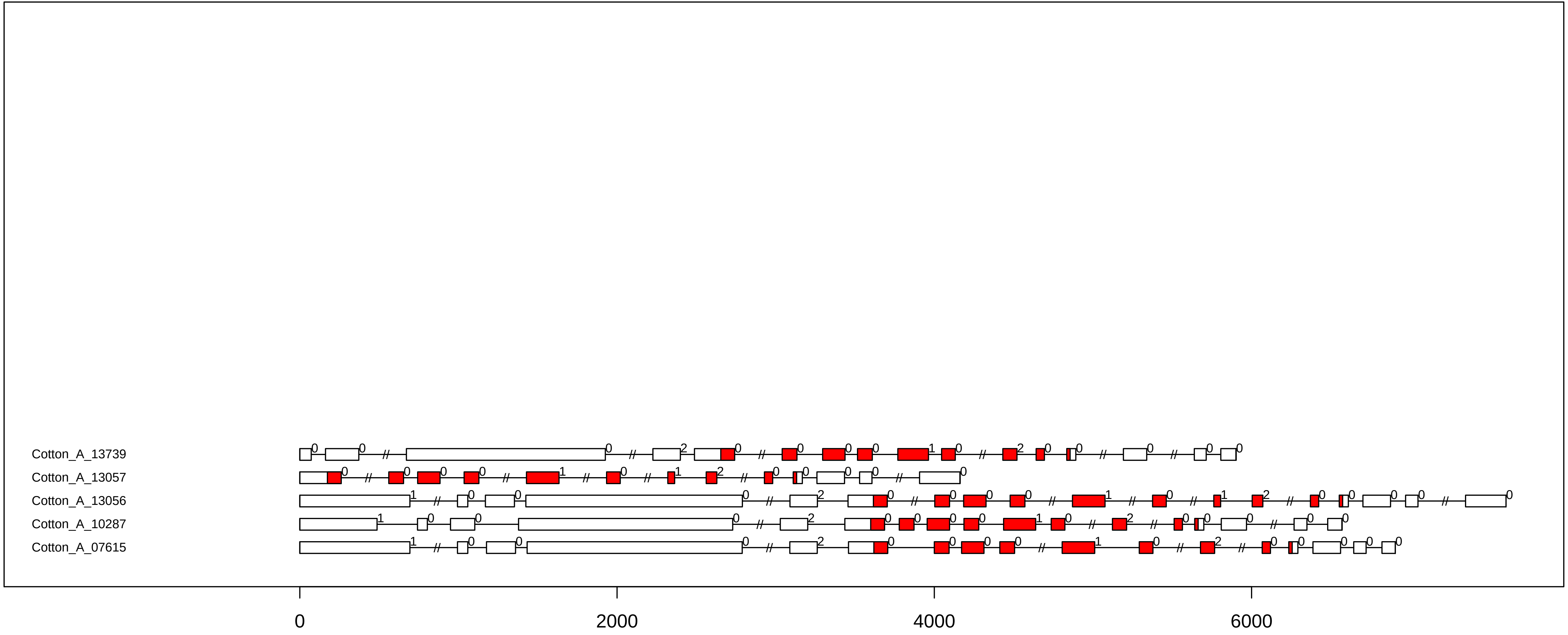

G.ar AGC_NDR exon−intron and domain diagram (all)

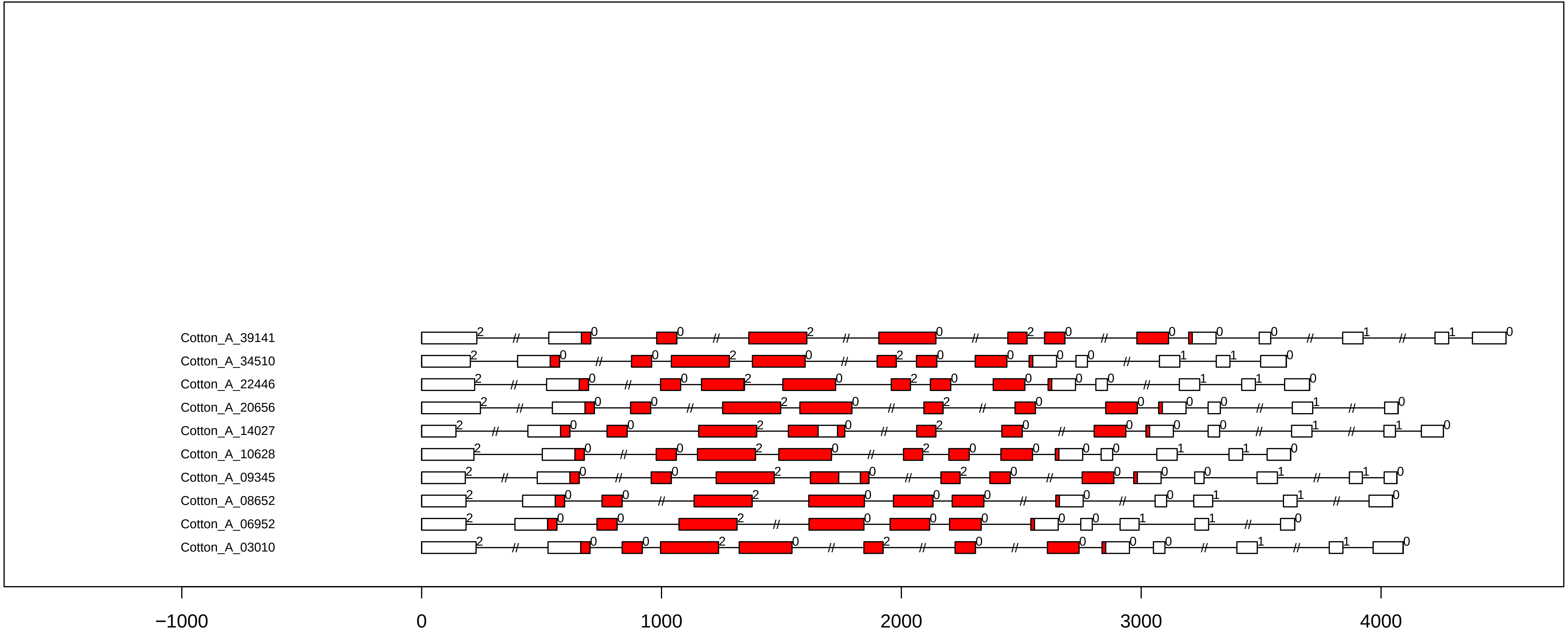

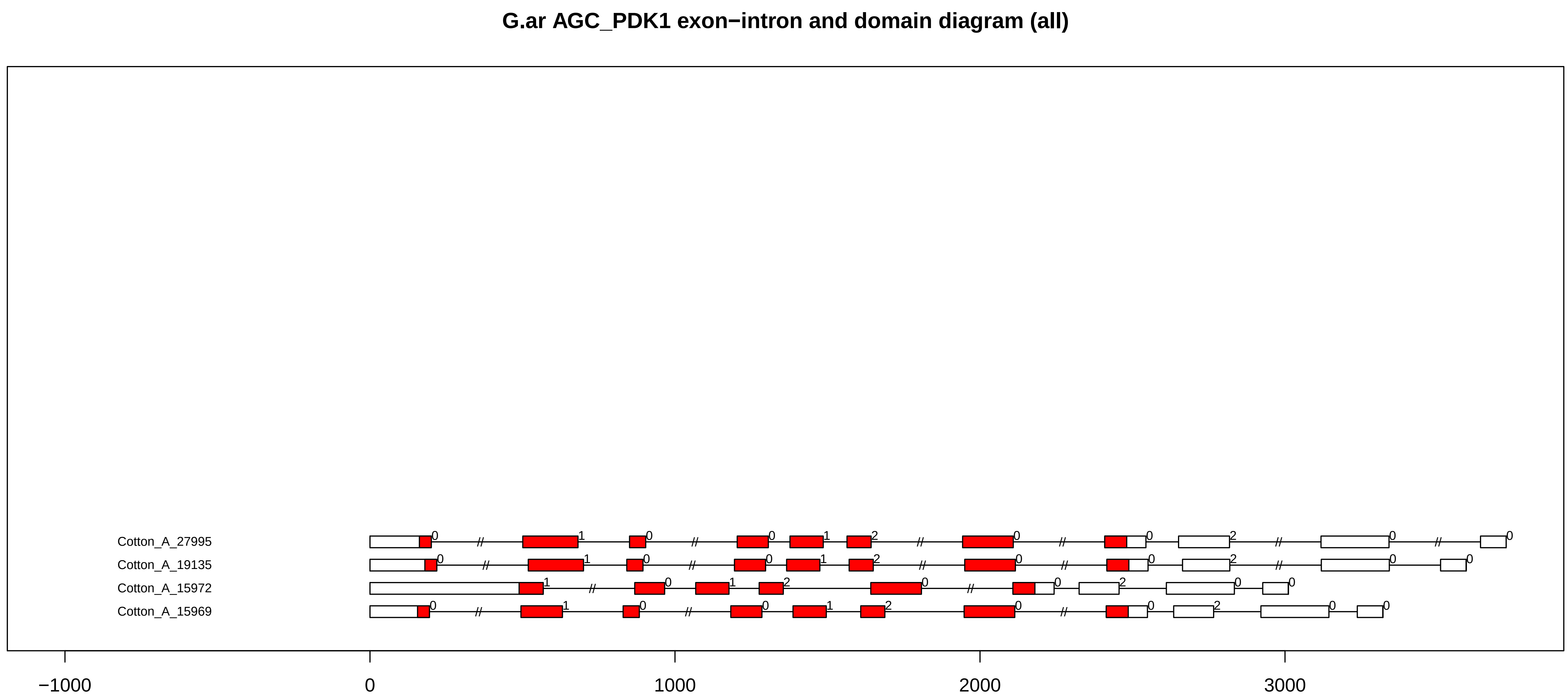
G.ar AGC_PDK1 exon−intron and domain diagram (all)
Cotton_A_27995
Cotton_A_19135
Cotton_A_15972
Cotton_A_15969
−1000
0
1000
2000
3000

G.ar AGC_PKA−PKG exon−intron and domain diagram (all)

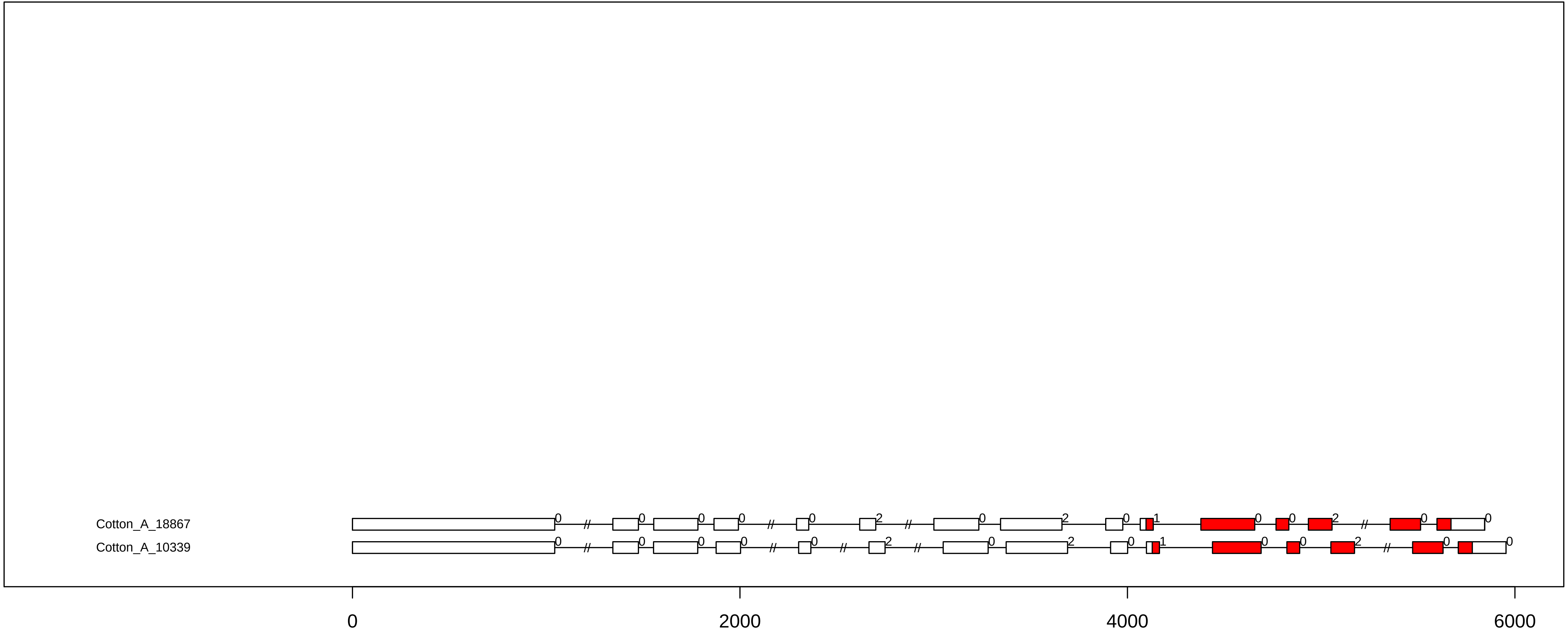

G.ar AGC_RSK−2 exon−intron and domain diagram (part 1)

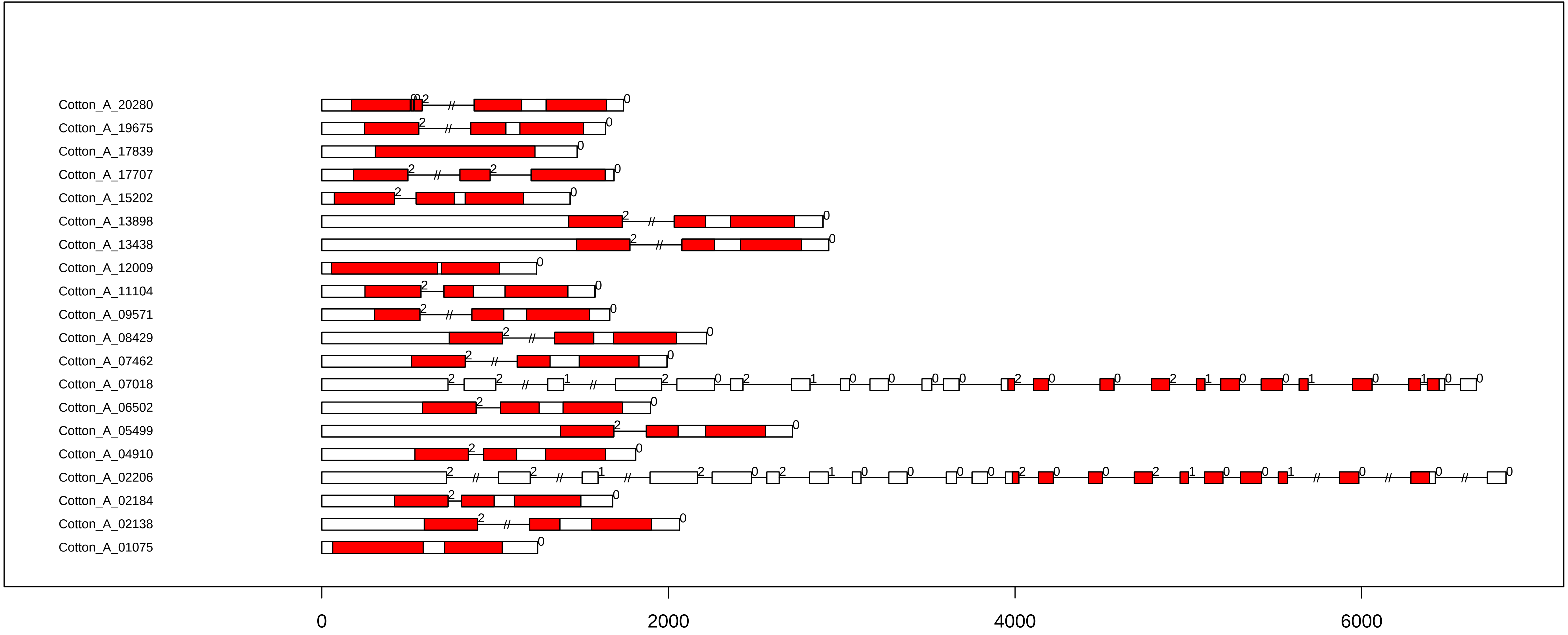

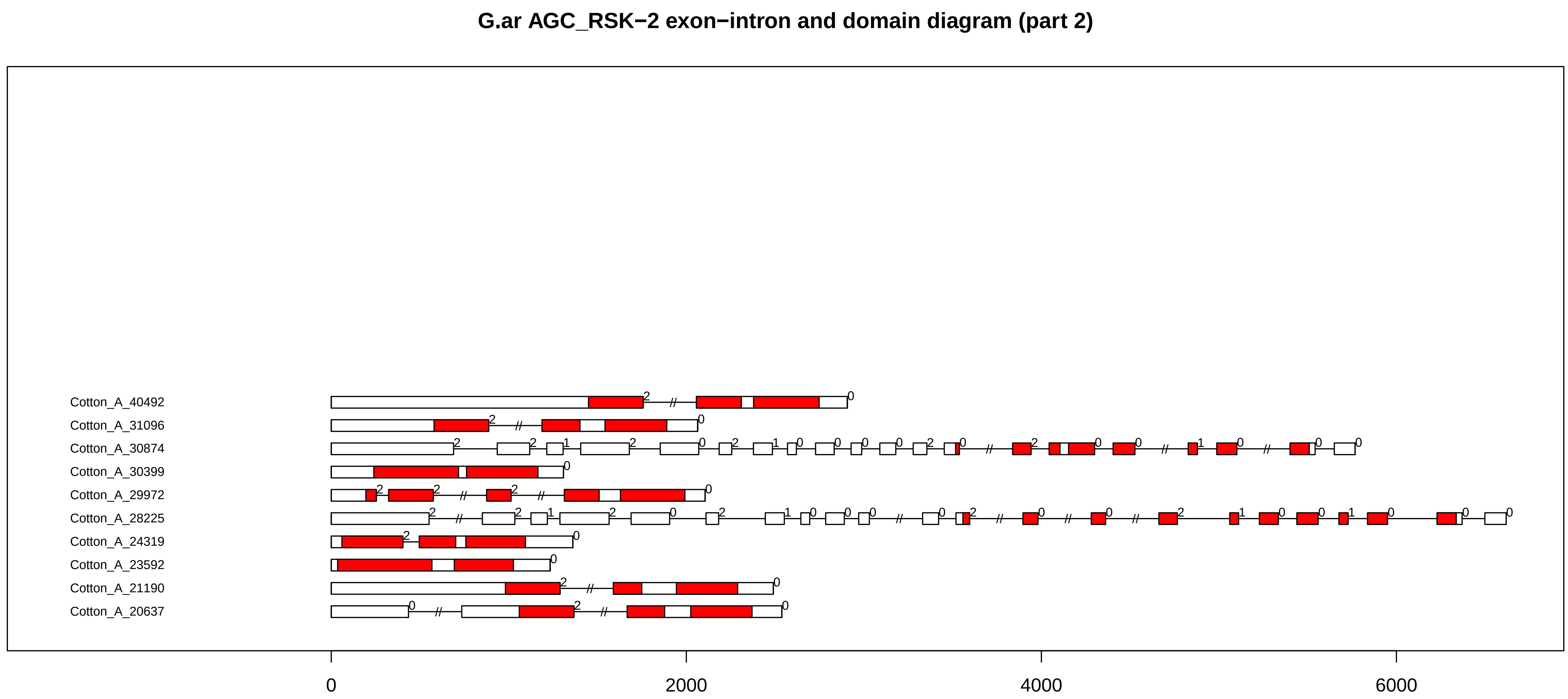

G.ar AGC_RSK−2 exon−intron and domain diagram (part 2)
Cotton_A_40492
Cotton_A_31096
Cotton_A_30874
Cotton_A_30399
Cotton_A_29972
Cotton_A_28225
Cotton_A_24319
Cotton_A_23592
Cotton_A_21190
Cotton_A_20637
0
2000
4000
6000

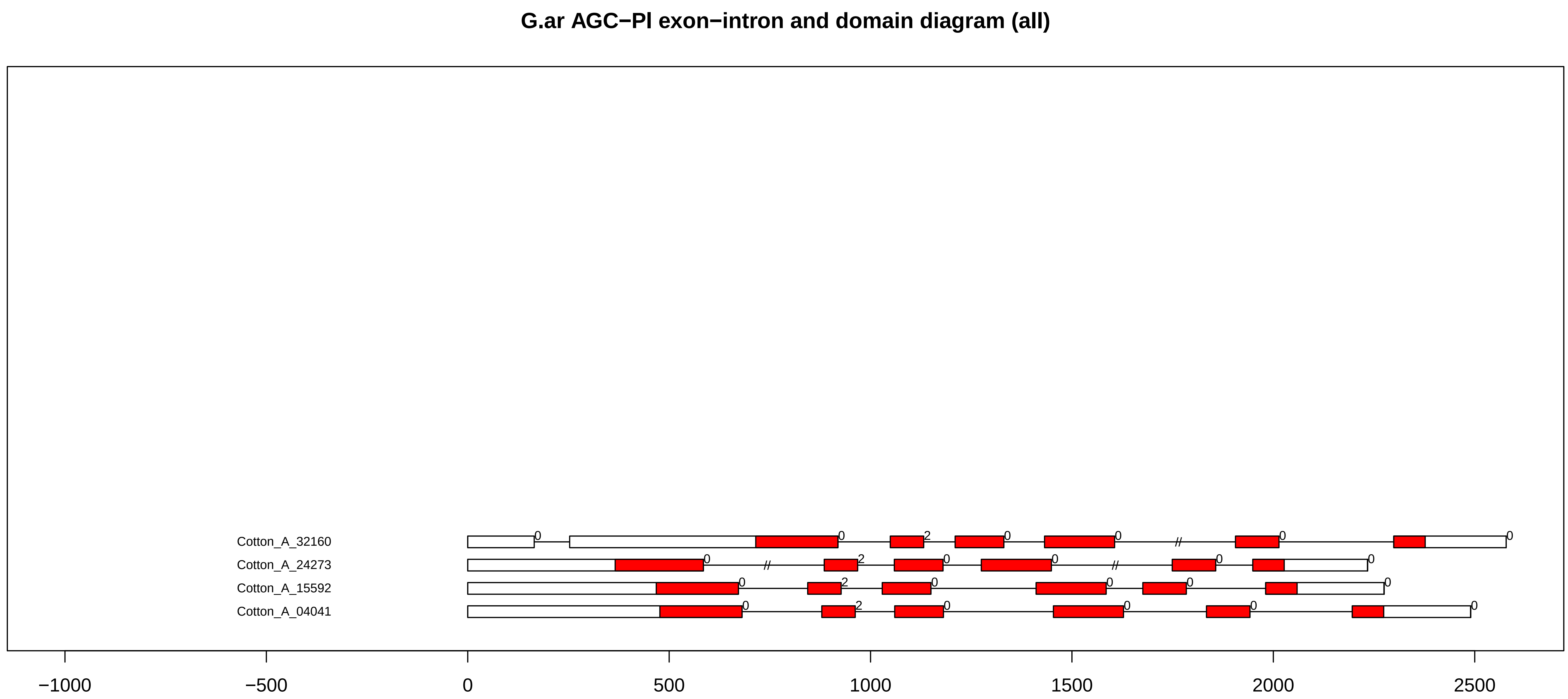
G.ar AGC−PI exon−intron and domain diagram (all)
Cotton_A_32160
Cotton_A_24273
Cotton_A_15592
Cotton_A_04041
−1000
−500
0
500
1000
1500
2000
2500

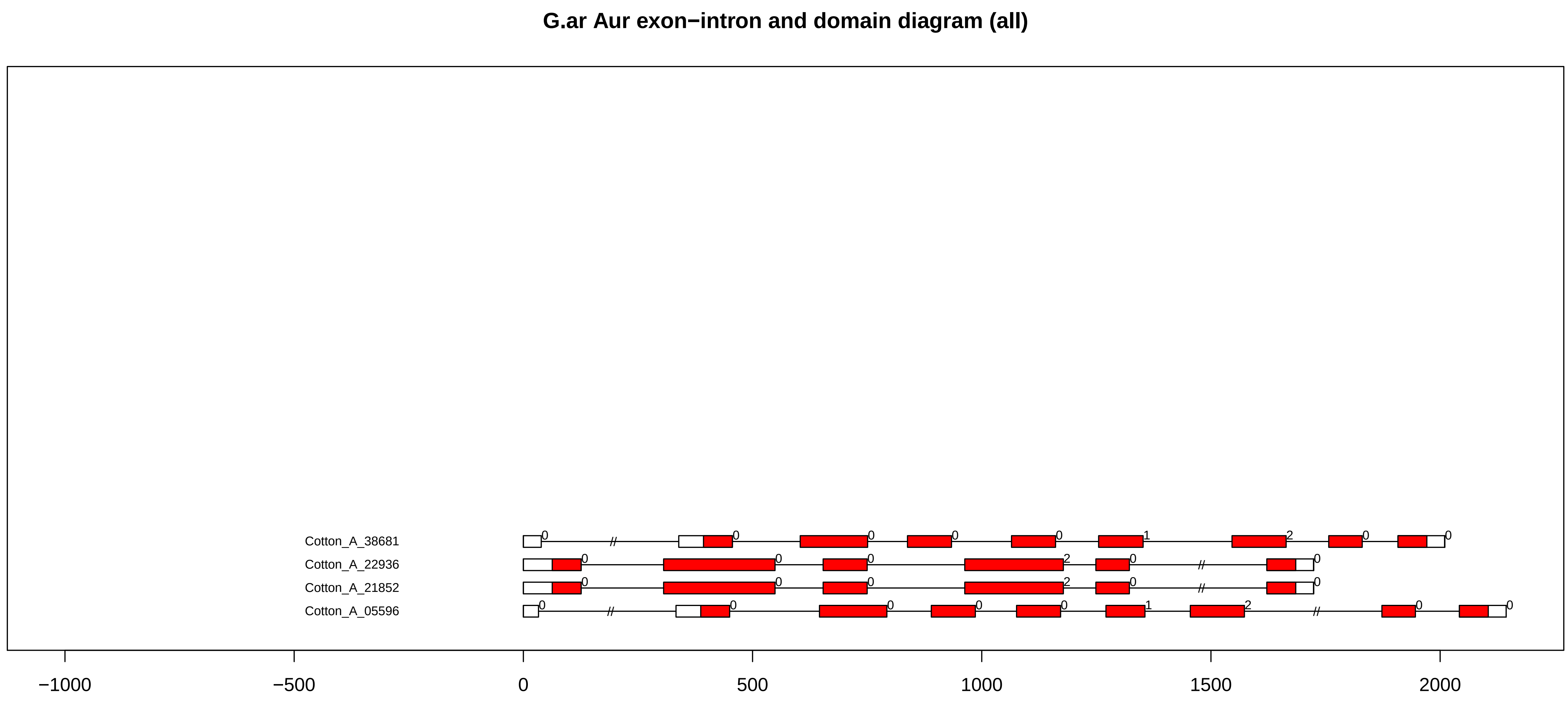
G.ar Aur exon−intron and domain diagram (all)
Cotton_A_38681
Cotton_A_22936
Cotton_A_21852
Cotton_A_05596
−1000
−500
0
500
1000
1500
2000

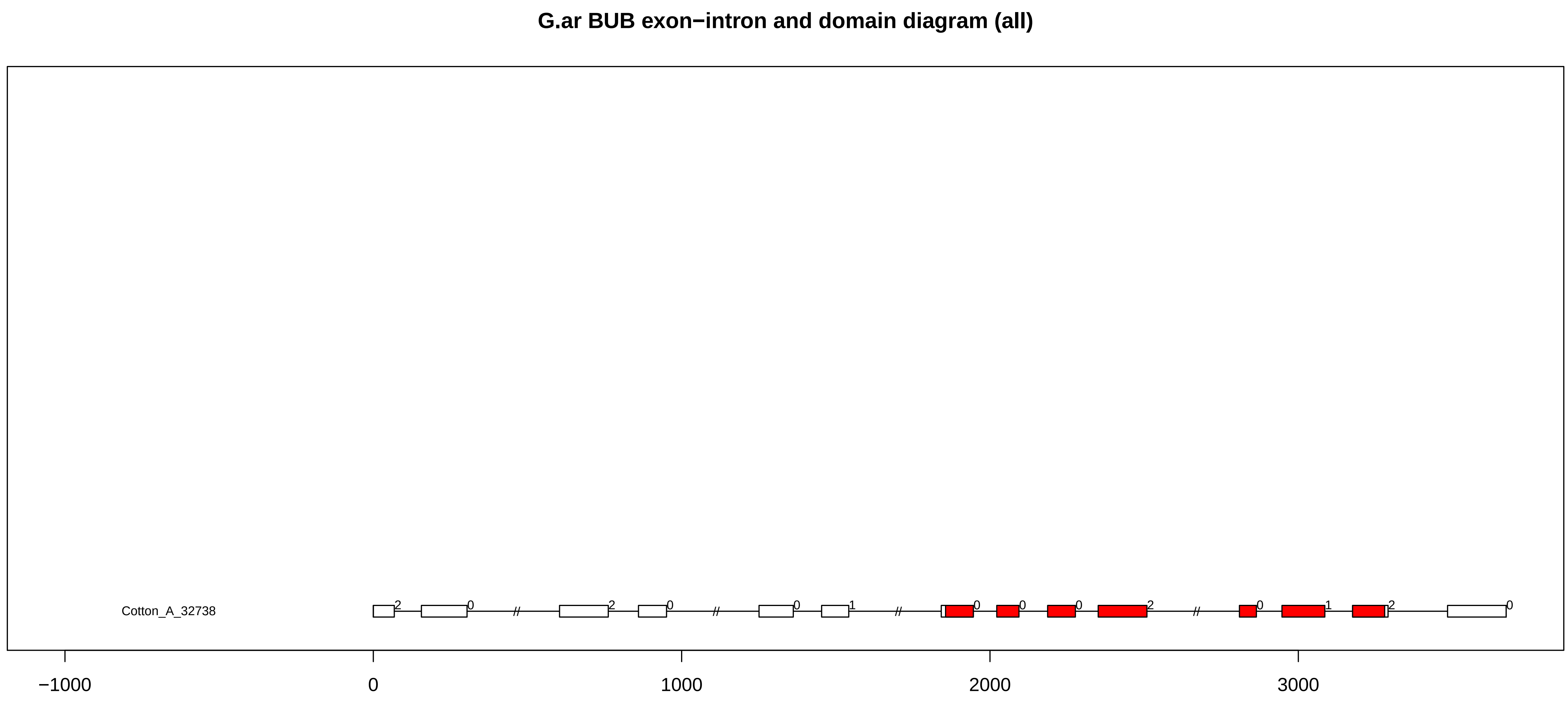
G.ar BUB exon−intron and domain diagram (all)
Cotton_A_32738
2
0
2
0
0
1
0
0
0
2
0
1
2
0
−1000
0
1000
2000
3000

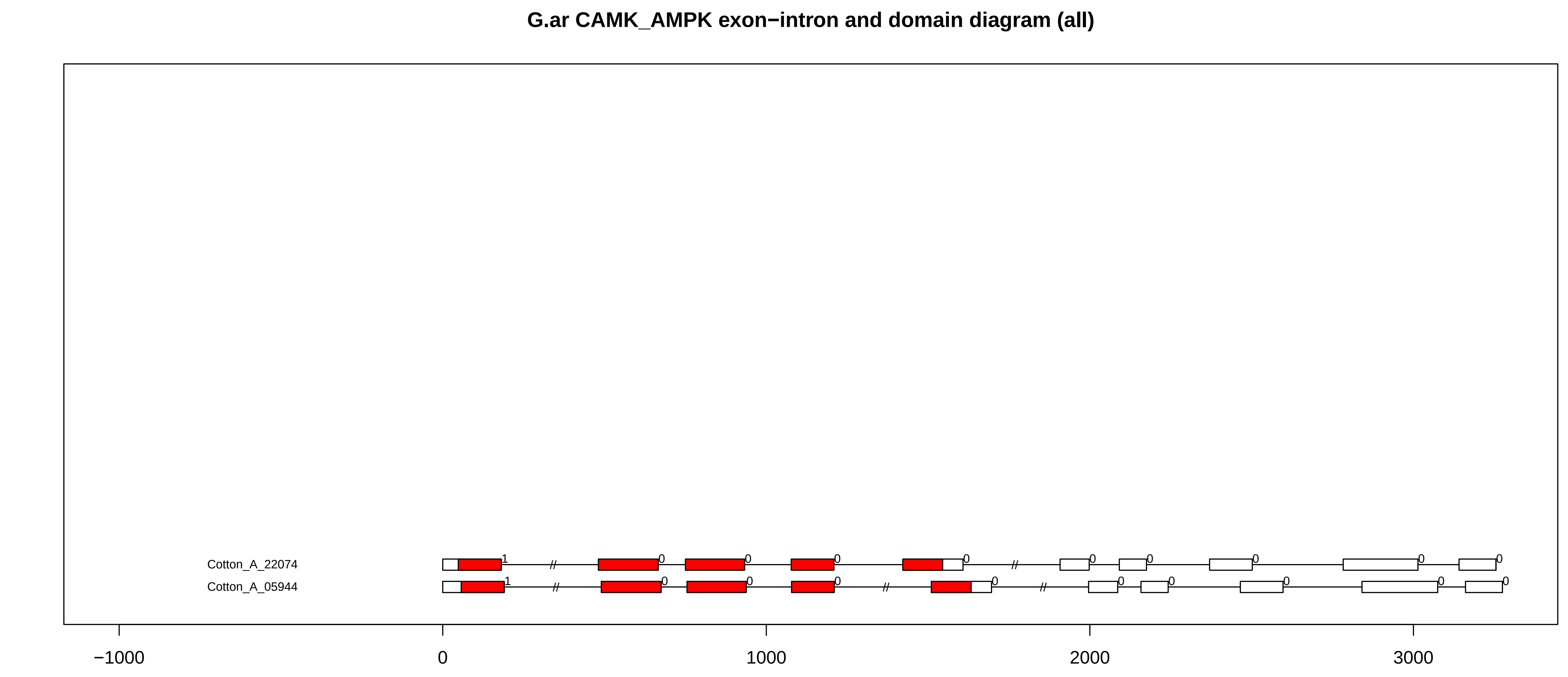
G.ar CAMK_AMPK exon−intron and domain diagram (all)
Cotton_A_22074
Cotton_A_05944
1
0
−1000
0
1000
2000
3000

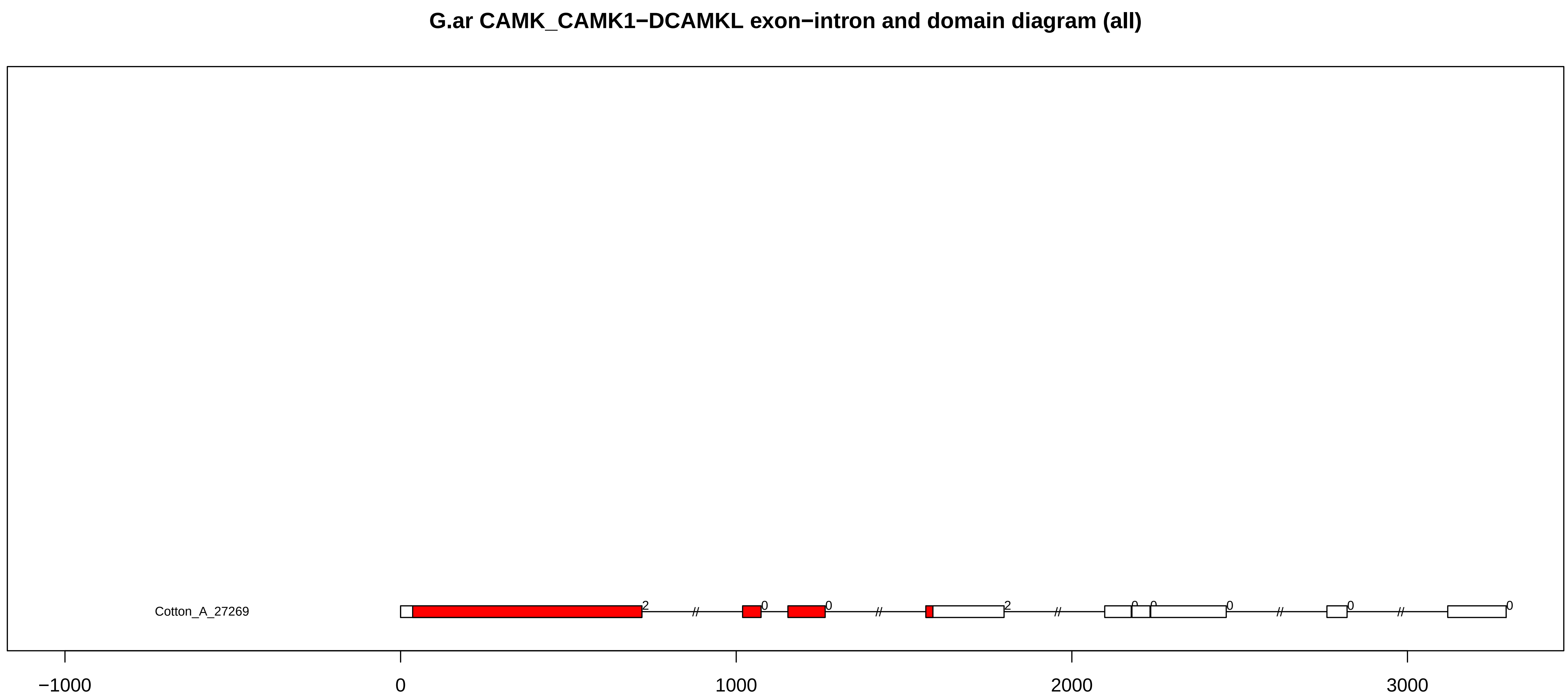
G.ar CAMK_CAMK1−DCAMKL exon−intron and domain diagram (all)
Cotton_A_27269
2
0
0
2
0
0
0
0
0
−1000
0
1000
2000
3000

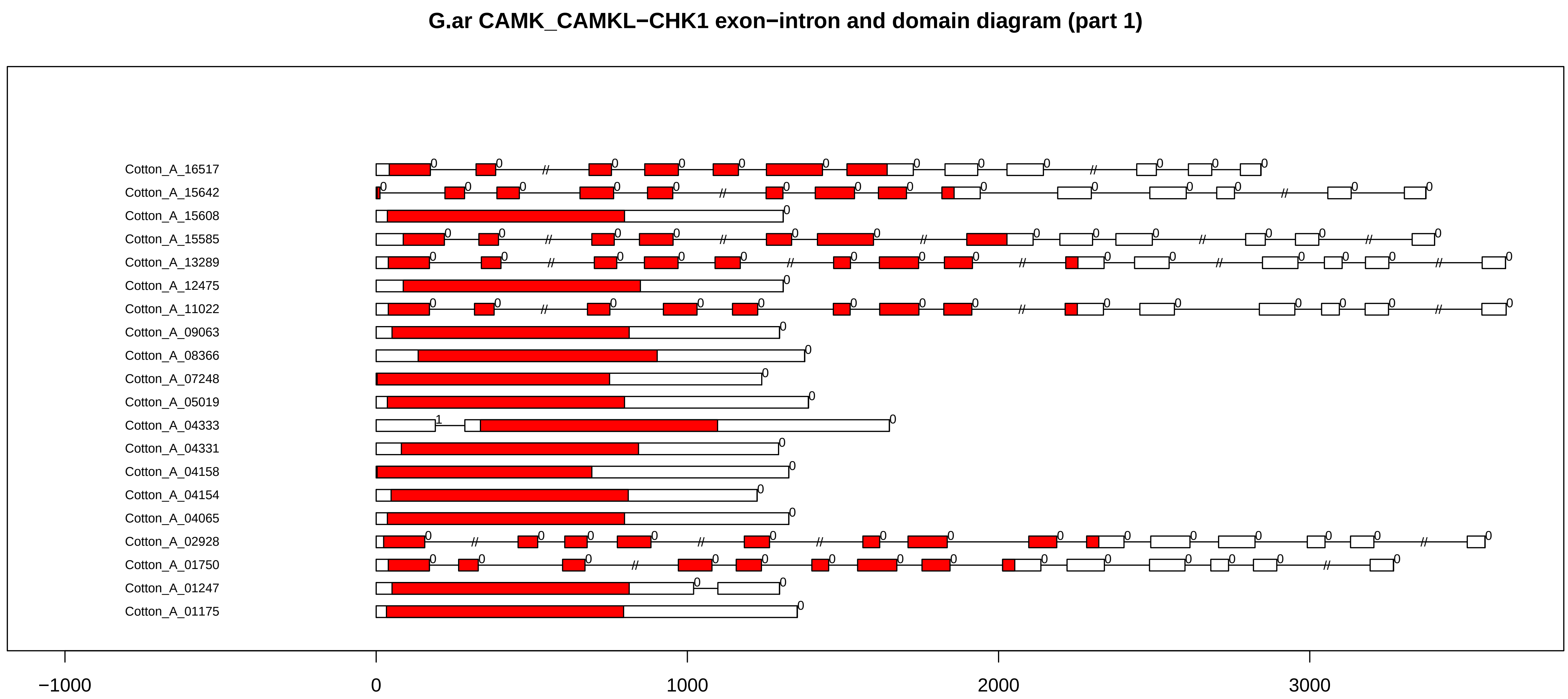
G.ar CAMK_CAMKL−CHK1 exon−intron and domain diagram (part 1)
Cotton_A_16517
Cotton_A_15642
Cotton_A_15608
Cotton_A_15585
Cotton_A_13289
Cotton_A_12475
Cotton_A_11022
Cotton_A_09063
Cotton_A_08366
Cotton_A_07248
Cotton_A_05019
Cotton_A_04333
Cotton_A_04331
Cotton_A_04158
Cotton_A_04154
Cotton_A_04065
Cotton_A_02928
Cotton_A_01750
Cotton_A_01247
Cotton_A_01175
−1000
0
1000
2000
3000

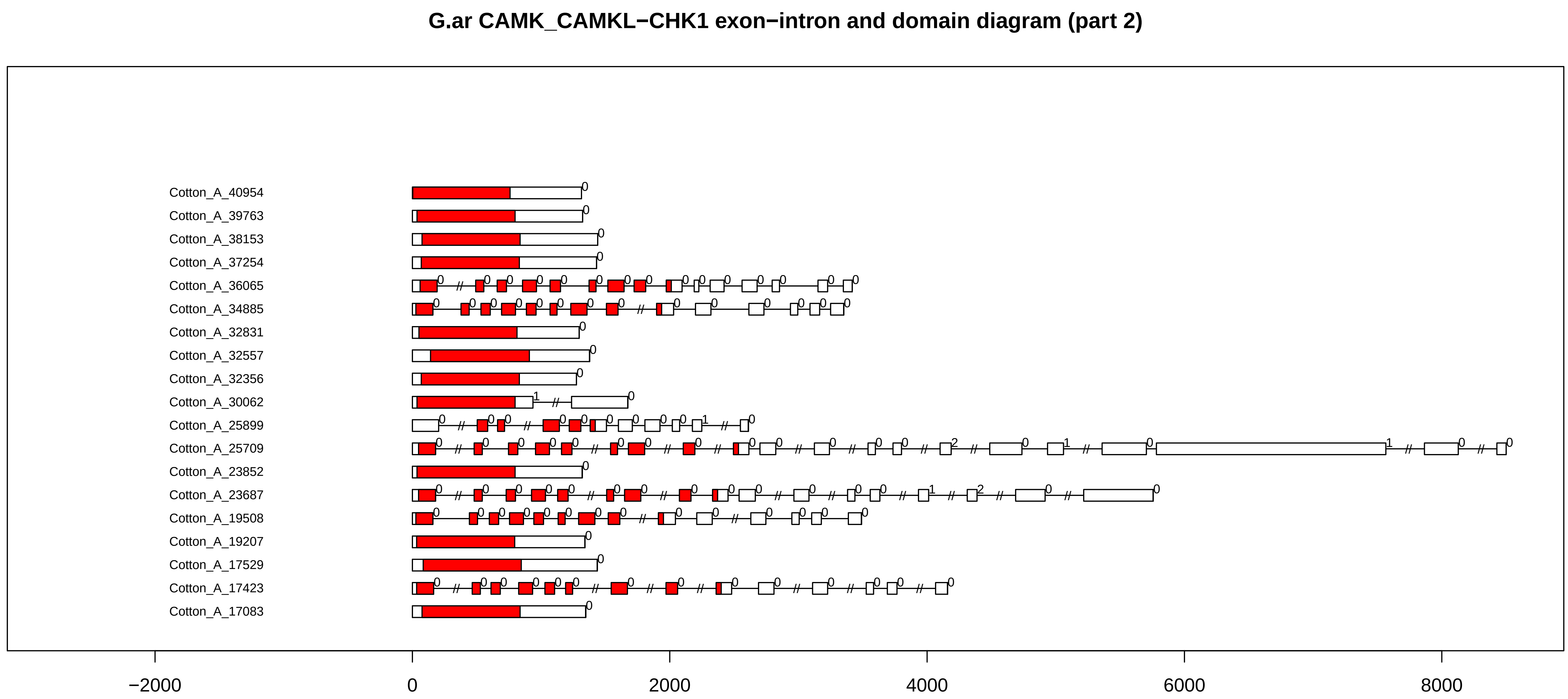
G.ar CAMK_CAMKL−CHK1 exon−intron and domain diagram (part 2)
Cotton_A_40954
Cotton_A_39763
Cotton_A_38153
Cotton_A_37254
Cotton_A_36065
Cotton_A_34885
Cotton_A_32831
Cotton_A_32557
Cotton_A_32356
Cotton_A_30062
Cotton_A_25899
Cotton_A_25709
Cotton_A_23852
Cotton_A_23687
Cotton_A_19508
Cotton_A_19207
Cotton_A_17529
Cotton_A_17423
Cotton_A_17083
−2000
0
2000
4000
6000
8000

G.ar CAMK_CAMKL−LKB exon−intron and domain diagram (all)

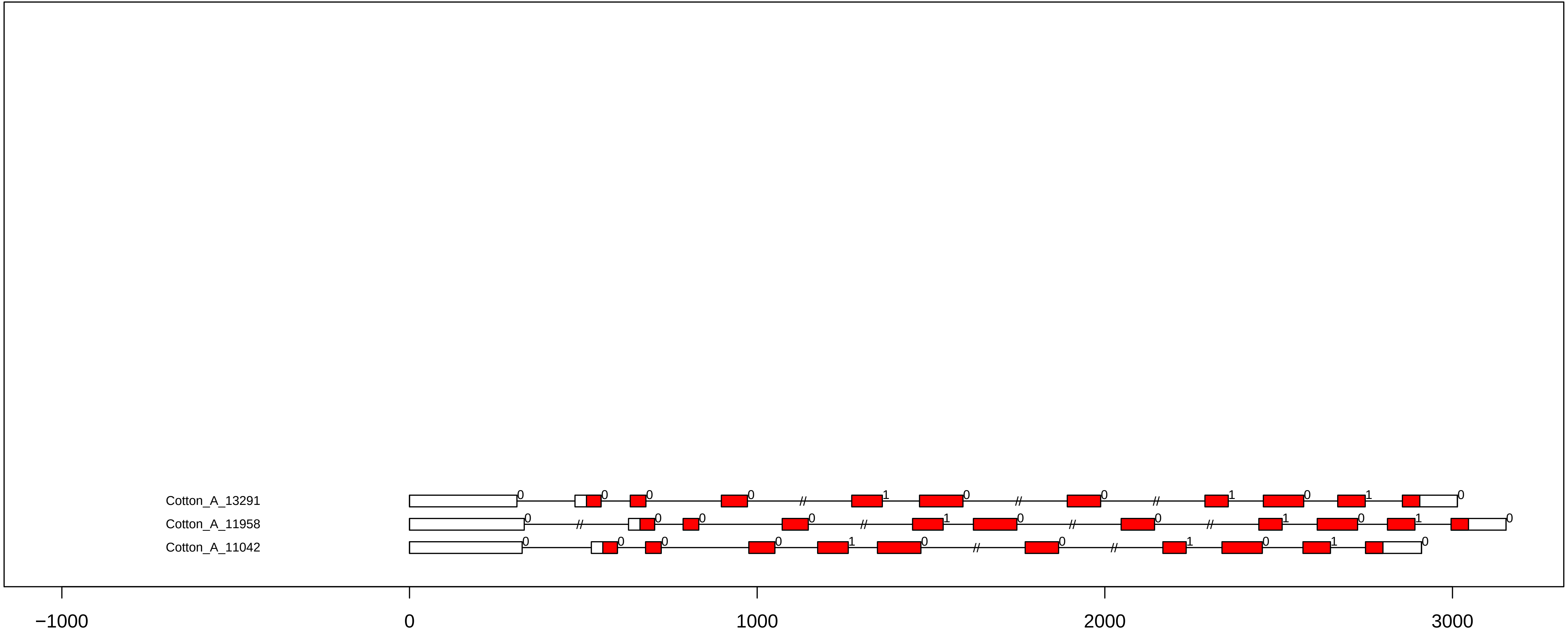

G.ar CAMK_CDPK exon−intron and domain diagram (part 1)

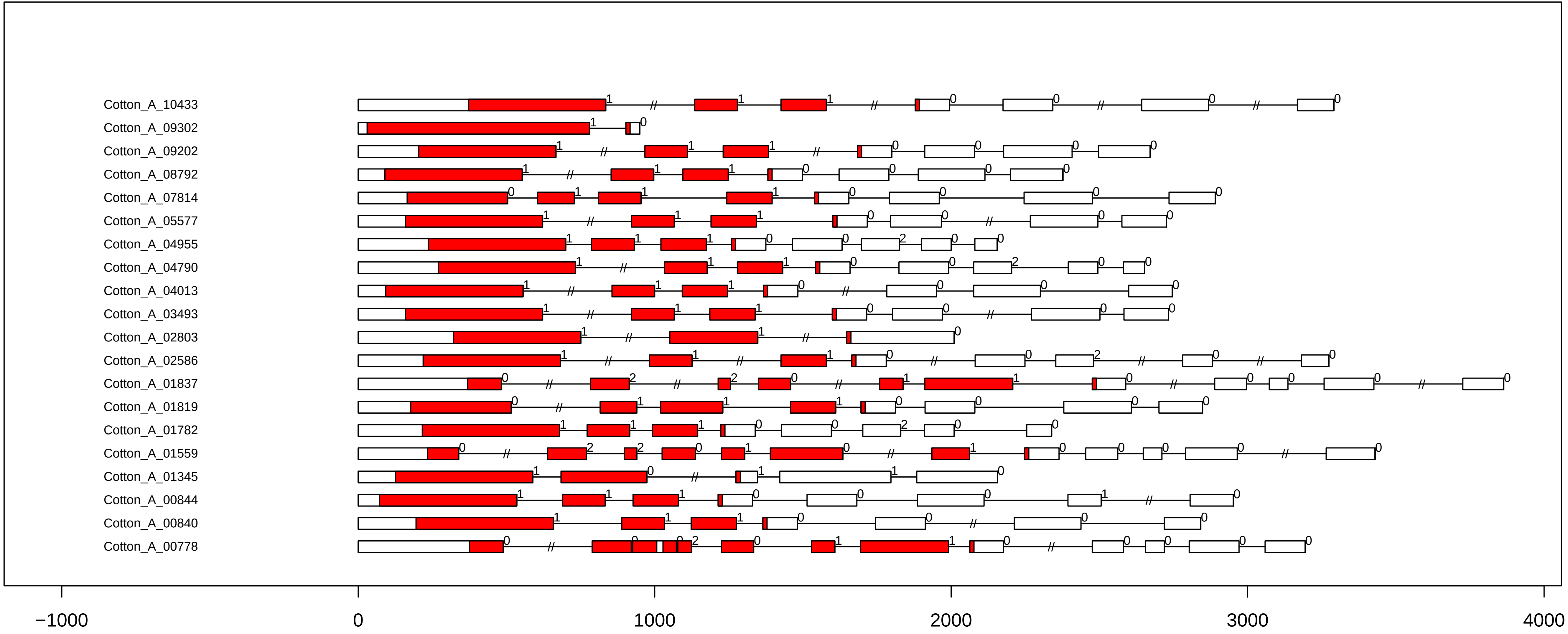

G.ar CAMK_CDPK exon−intron and domain diagram (part 2)

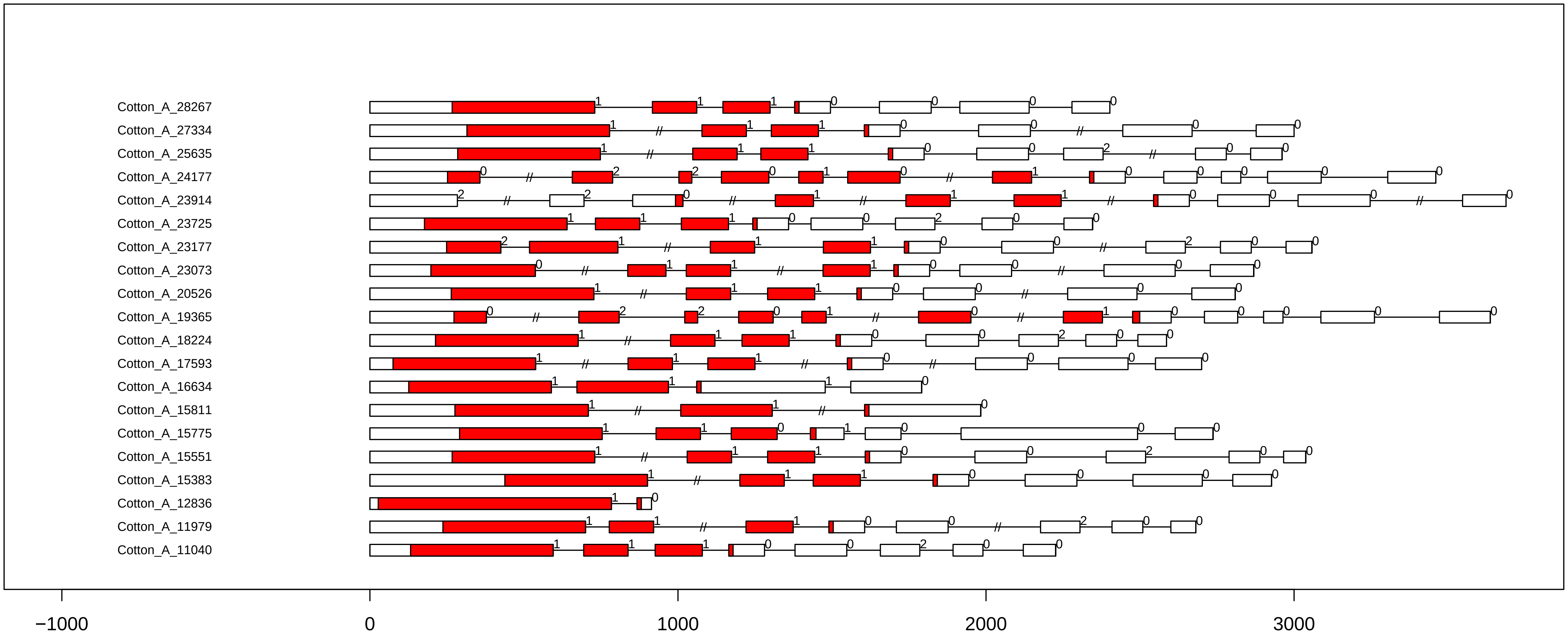

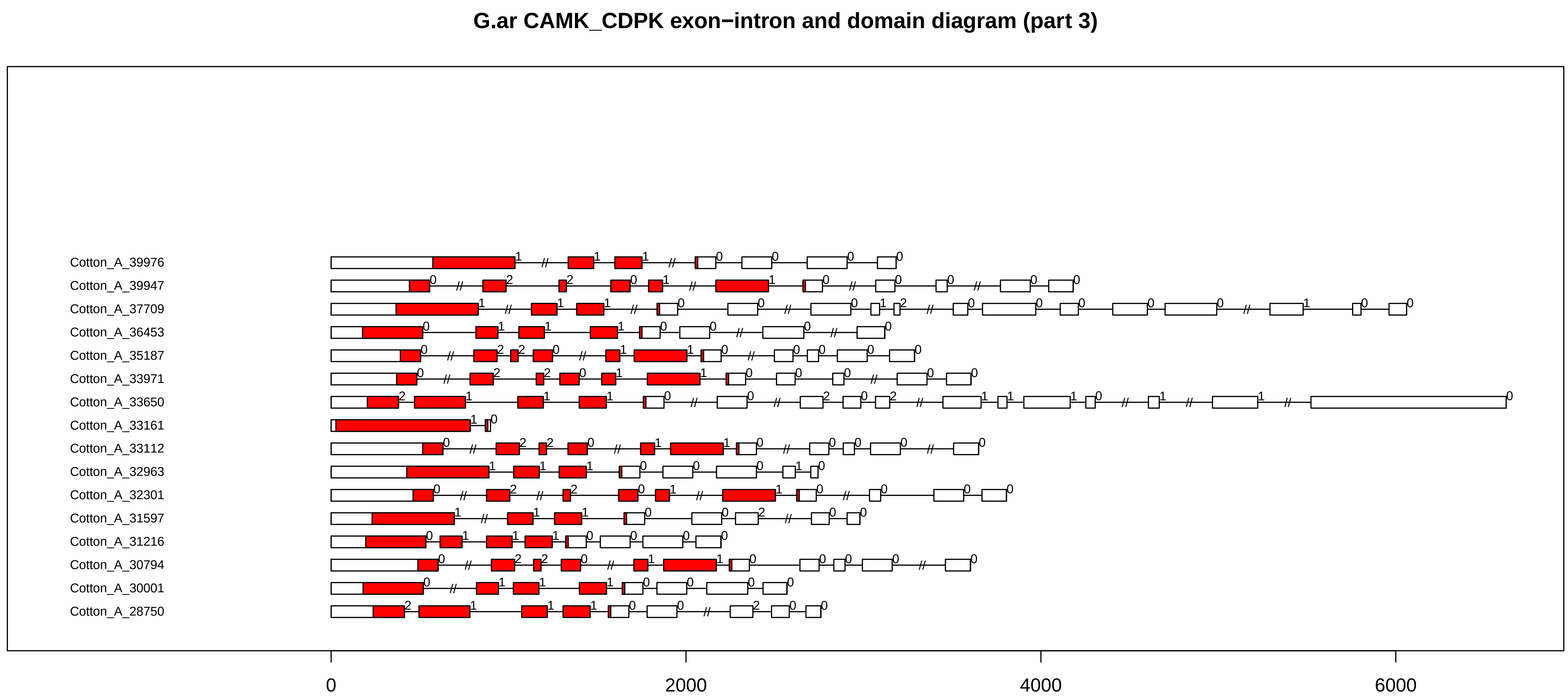
G.ar CAMK_CDPK exon−intron and domain diagram (part 3)
Cotton_A_39976
Cotton_A_39947
Cotton_A_37709
Cotton_A_36453
Cotton_A_35187
Cotton_A_33971
Cotton_A_33650
Cotton_A_33161
Cotton_A_33112
Cotton_A_32963
Cotton_A_32301
Cotton_A_31597
Cotton_A_31216
Cotton_A_30794
Cotton_A_30001
Cotton_A_28750
0
2000
4000
6000

G.ar CAMK_OST1L exon−intron and domain diagram (all)

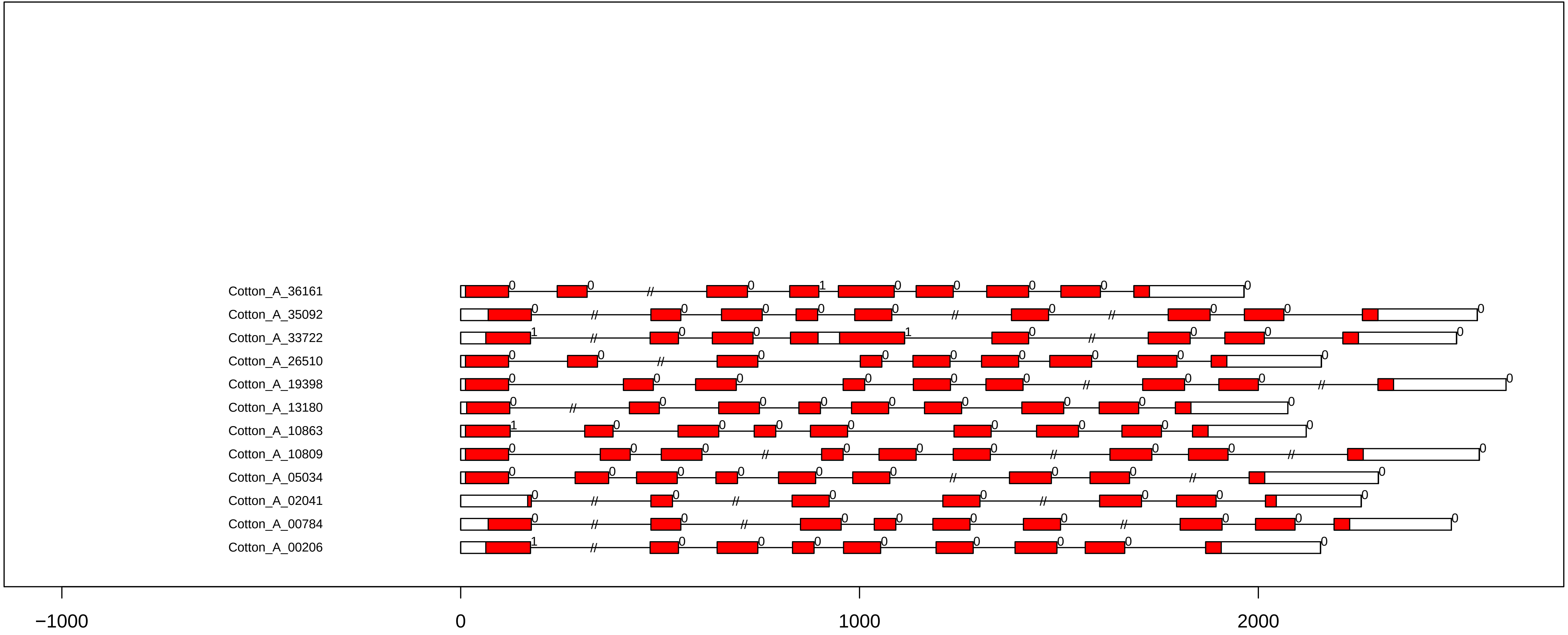

G.ar CK1_CK1 exon−intron and domain diagram (all)

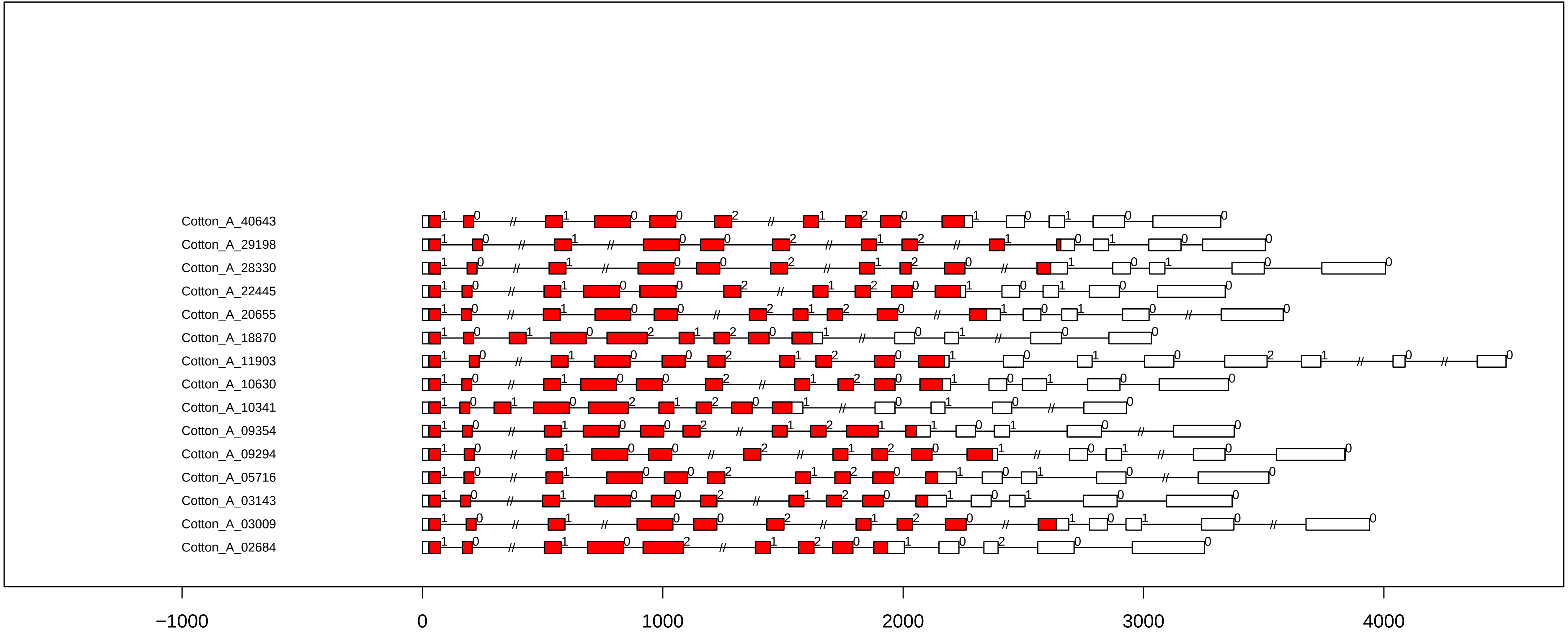

G.ar CK1_CK1−PI exon−intron and domain diagram (all)

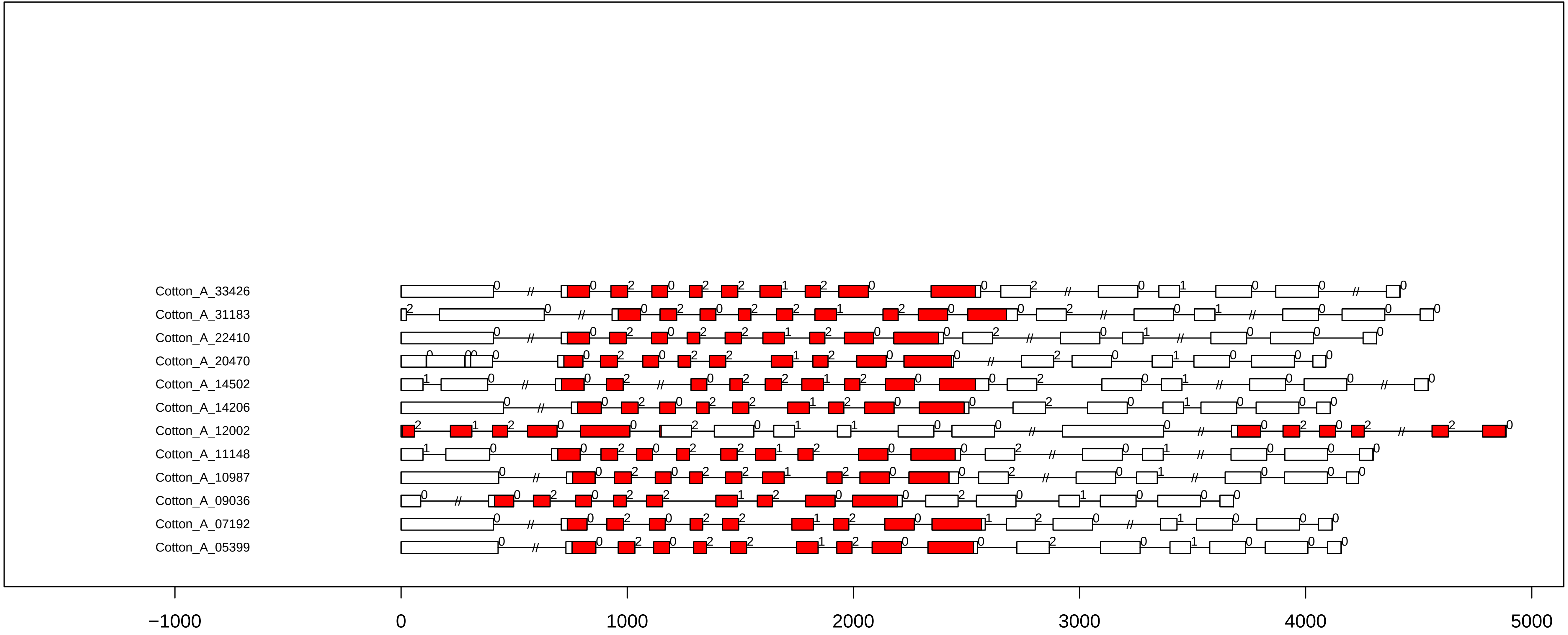

G.ar CMGC_CDK−CCRK exon−intron and domain diagram (all)

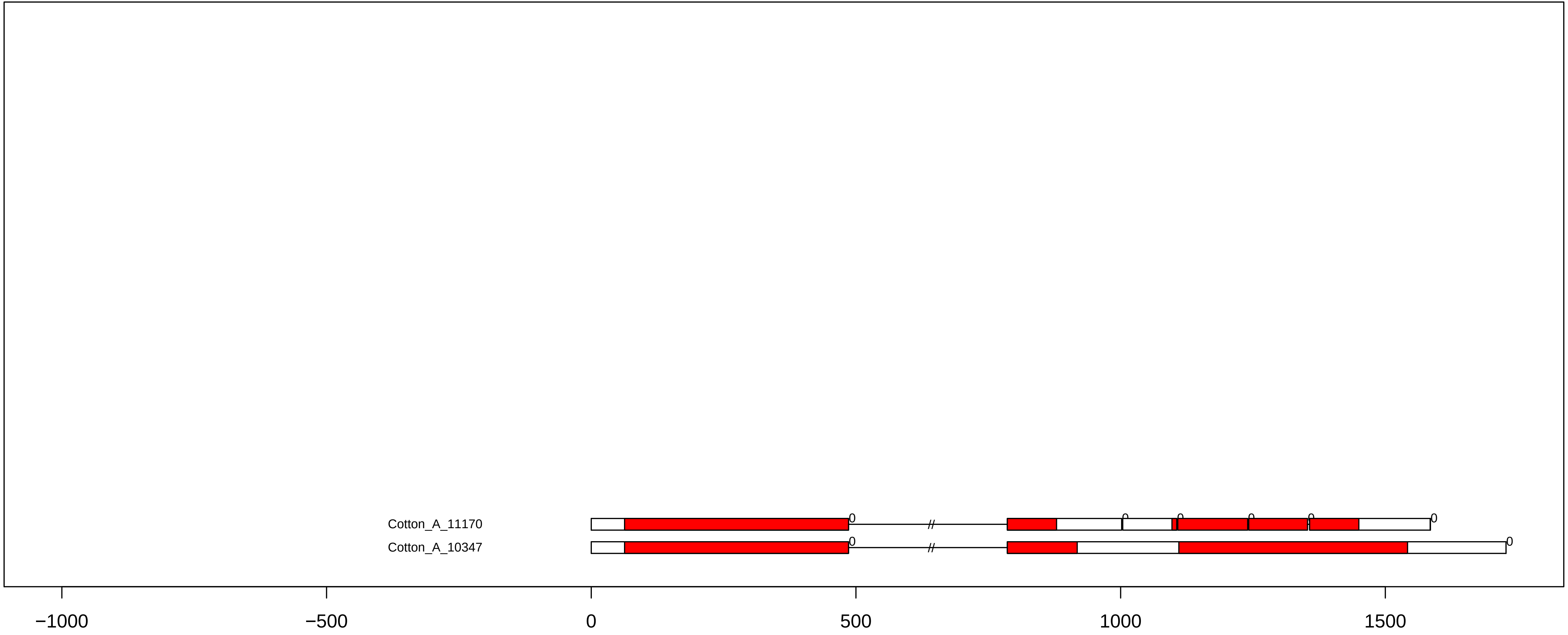

G.ar CMGC_CDK−CDK7 exon−intron and domain diagram (all)

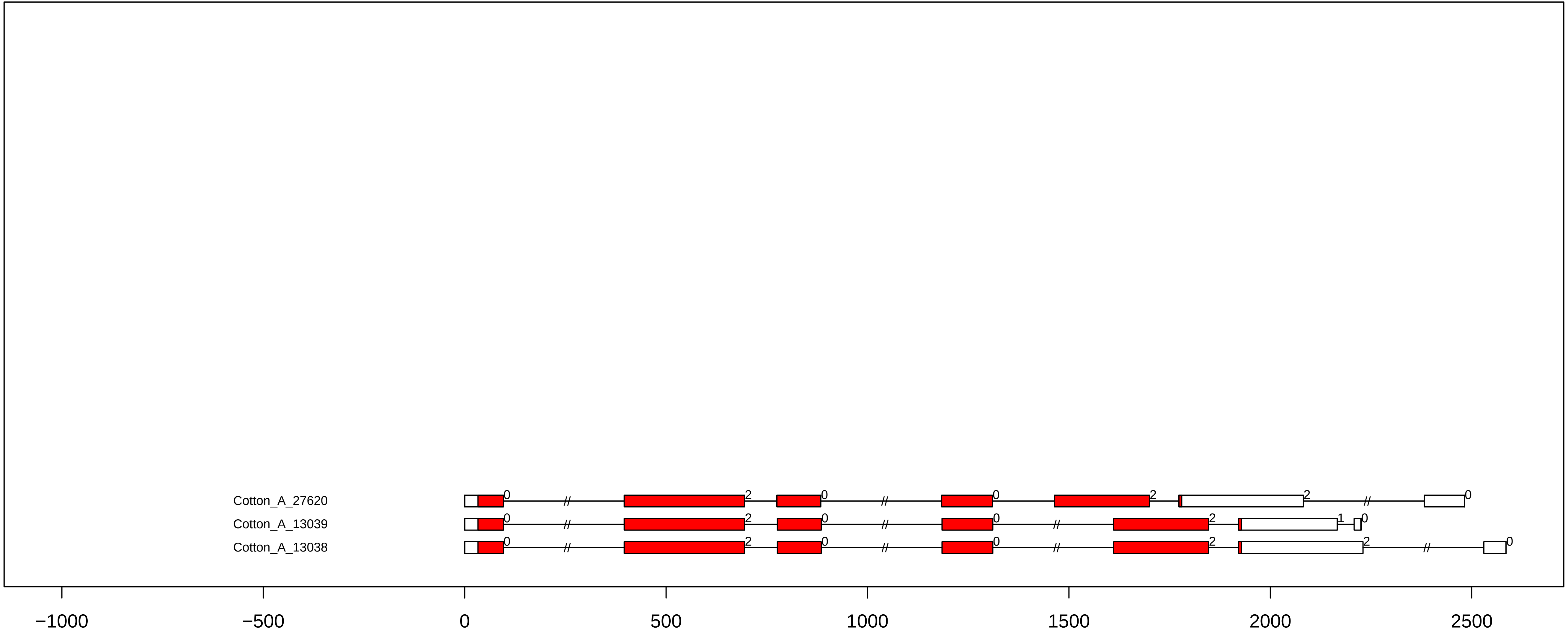

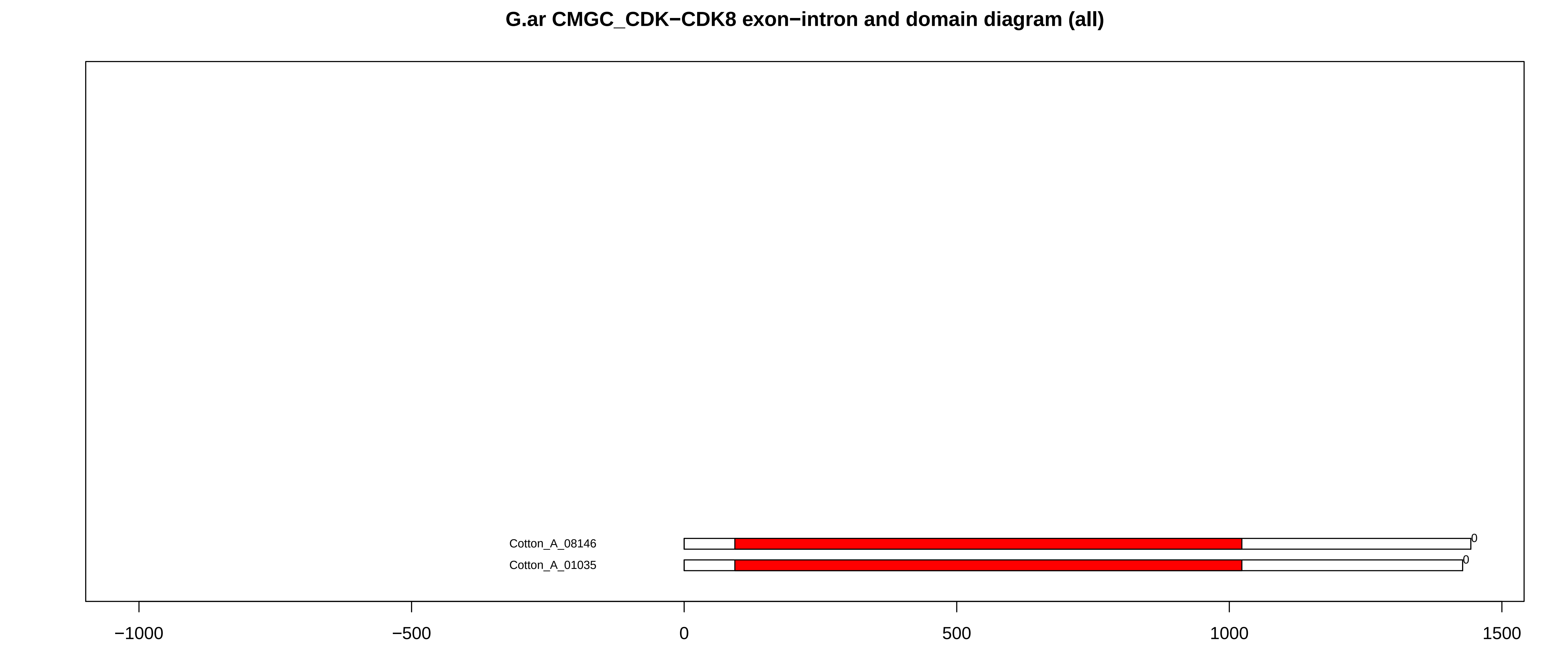
G.ar CMGC_CDK−CDK8 exon−intron and domain diagram (all)
Cotton_A_08146
Cotton_A_01035
0
0
−1000
−500
0
500
1000
1500

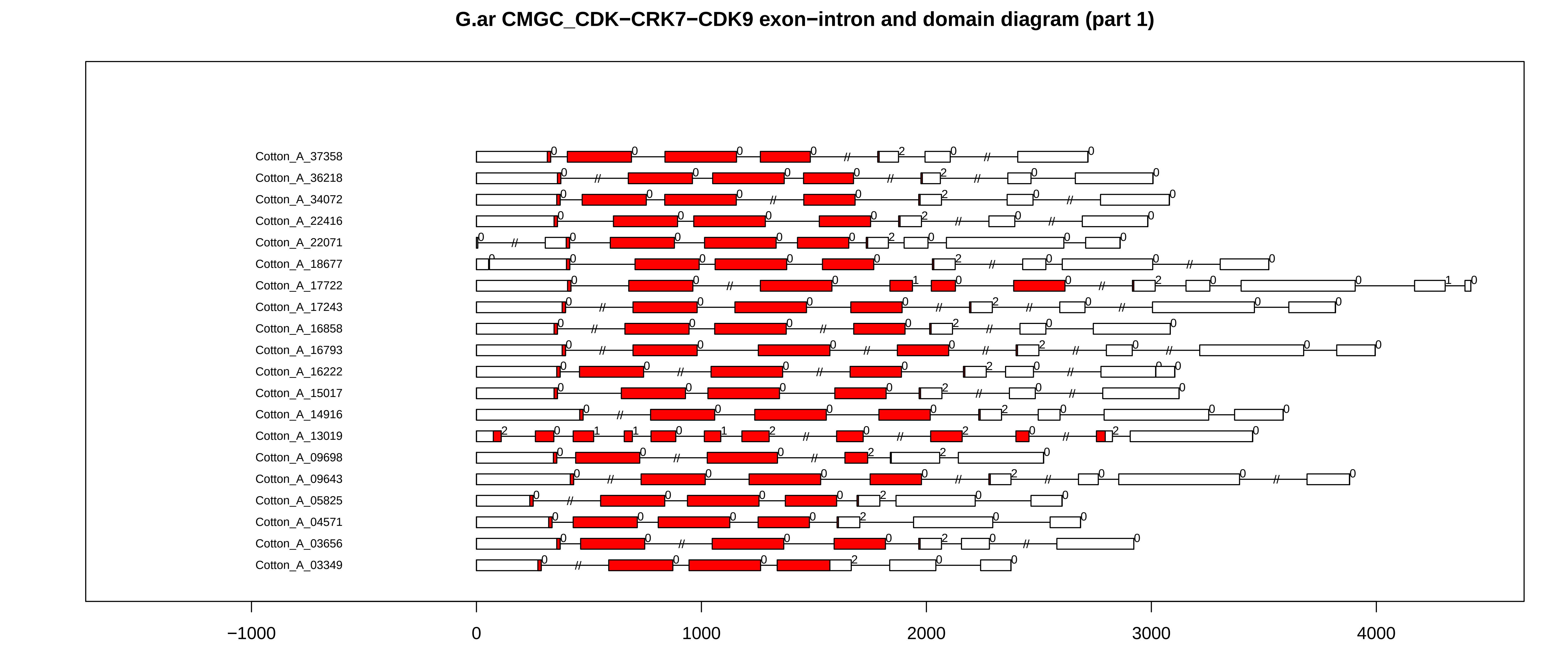

G.ar CMGC_CDK−CRK7−CDK9 exon−intron and domain diagram (part 1)
Cotton_A_37358
Cotton_A_36218
Cotton_A_34072
Cotton_A_22416
Cotton_A_22071
Cotton_A_18677
Cotton_A_17722
Cotton_A_17243
Cotton_A_16858
Cotton_A_16793
Cotton_A_16222
Cotton_A_15017
Cotton_A_14916
Cotton_A_13019
Cotton_A_09698
Cotton_A_09643
Cotton_A_05825
Cotton_A_04571
Cotton_A_03656
Cotton_A_03349
−1000
0
1000
2000
3000
4000

G.ar CMGC_CDK−CRK7−CDK9 exon−intron and domain diagram (part 2)

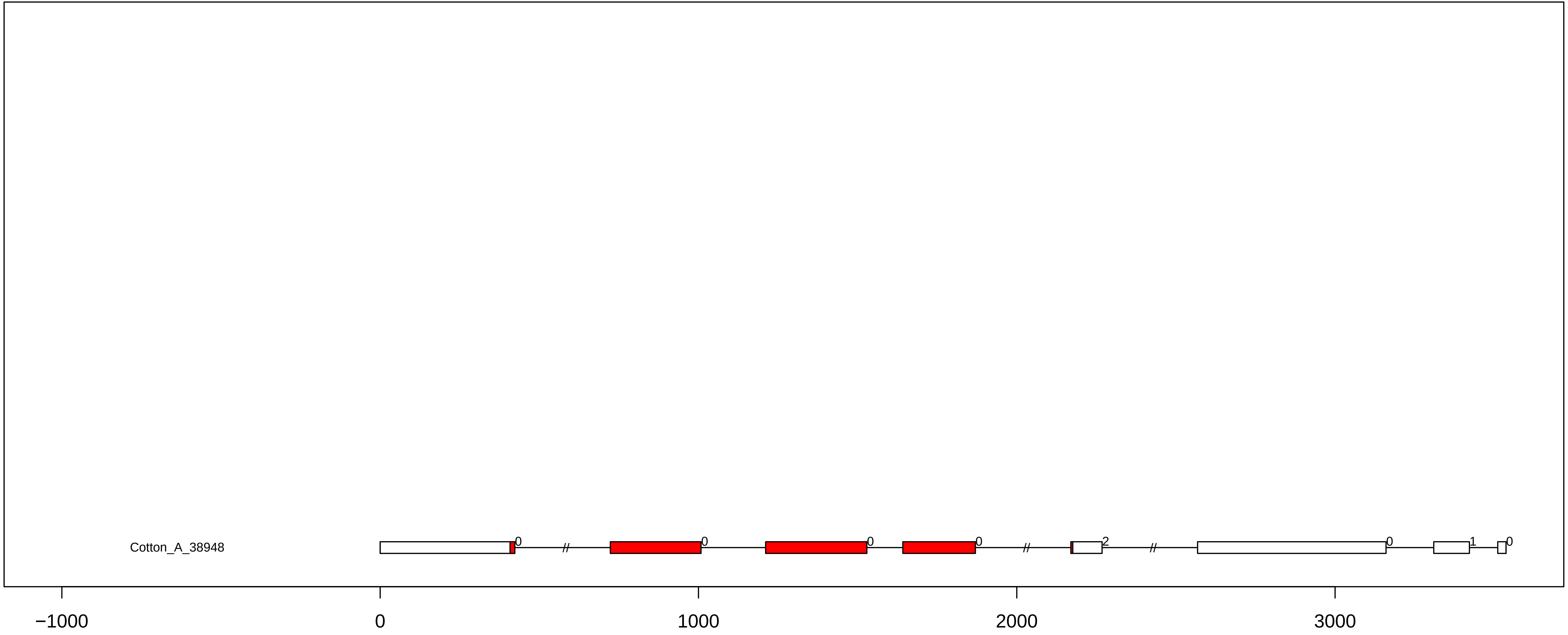

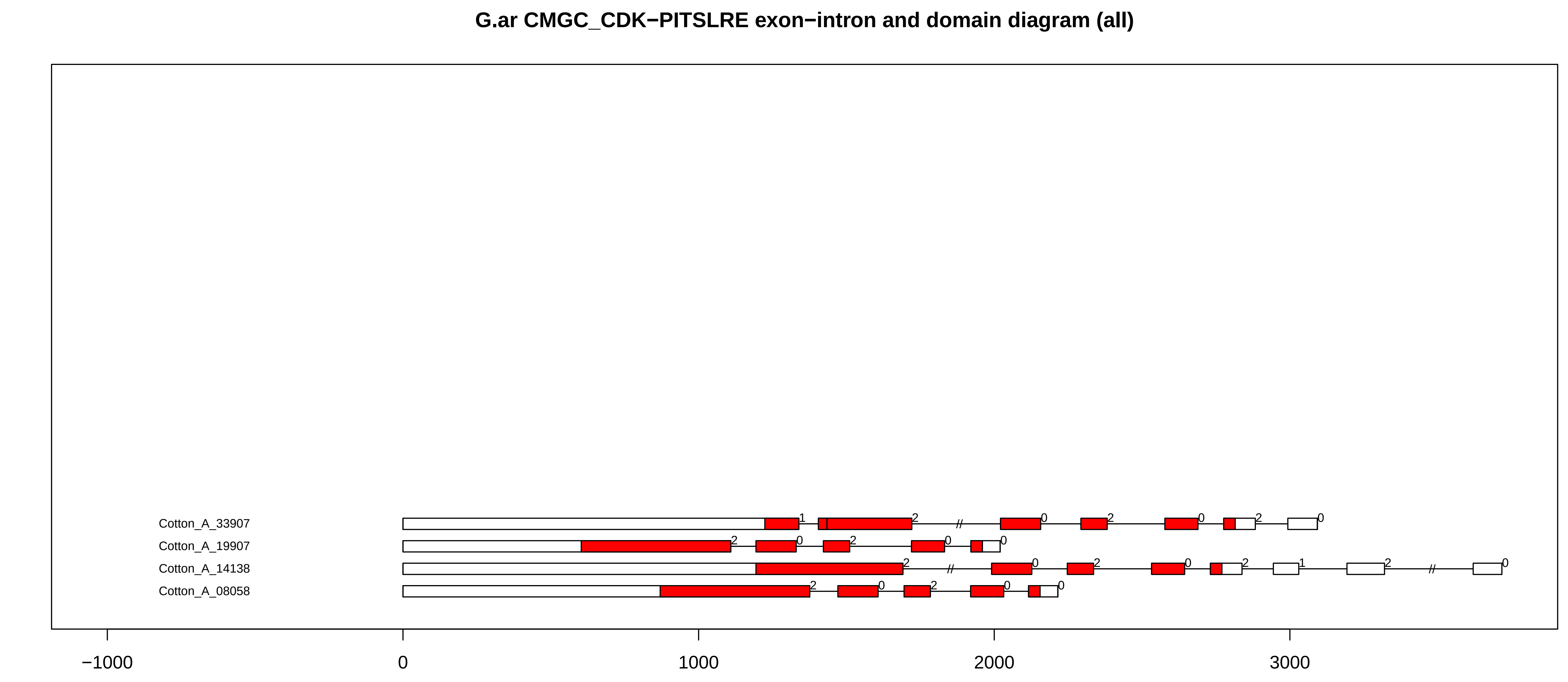
G.ar CMGC_CDK−PITSLRE exon−intron and domain diagram (all)
Cotton_A_33907
Cotton_A_19907
Cotton_A_14138
Cotton_A_08058
−1000
0
1000
2000
3000

G.ar CMGC_CDK−Pl exon−intron and domain diagram (all)

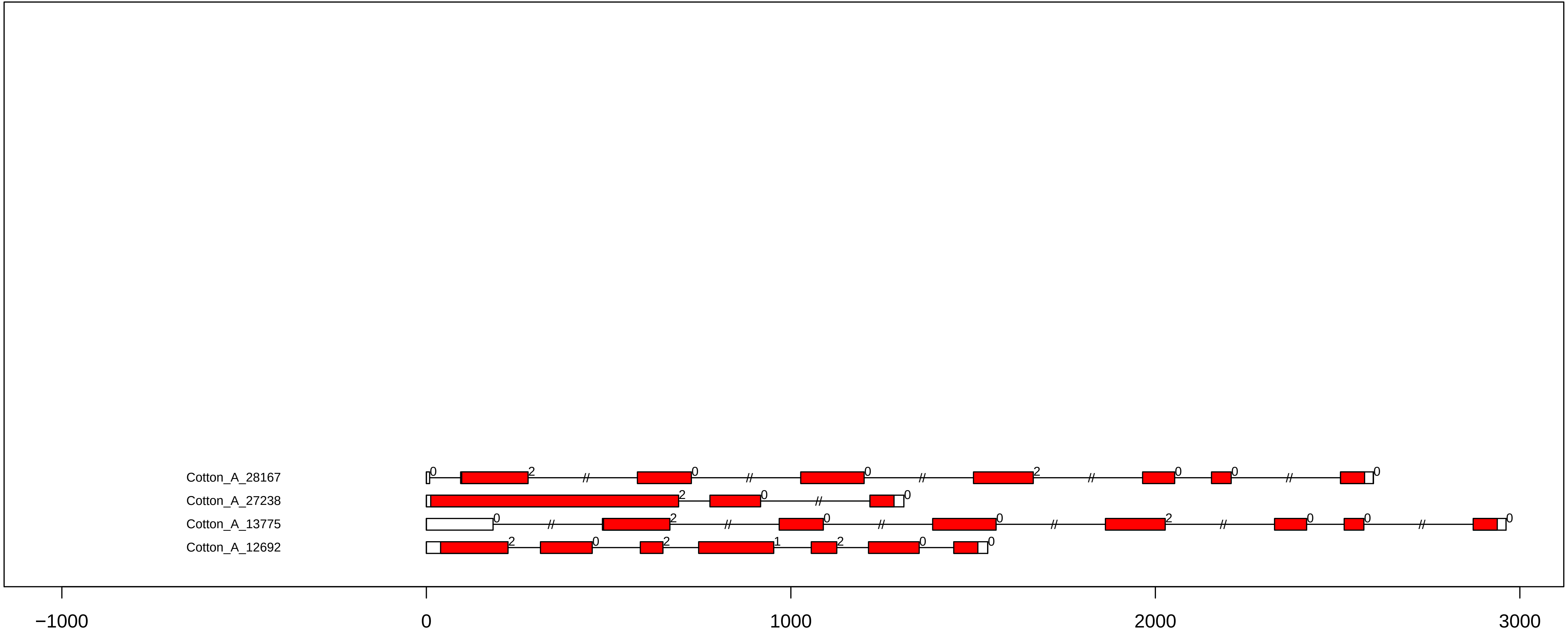

G.ar CMGC_CK2 exon−intron and domain diagram (all)

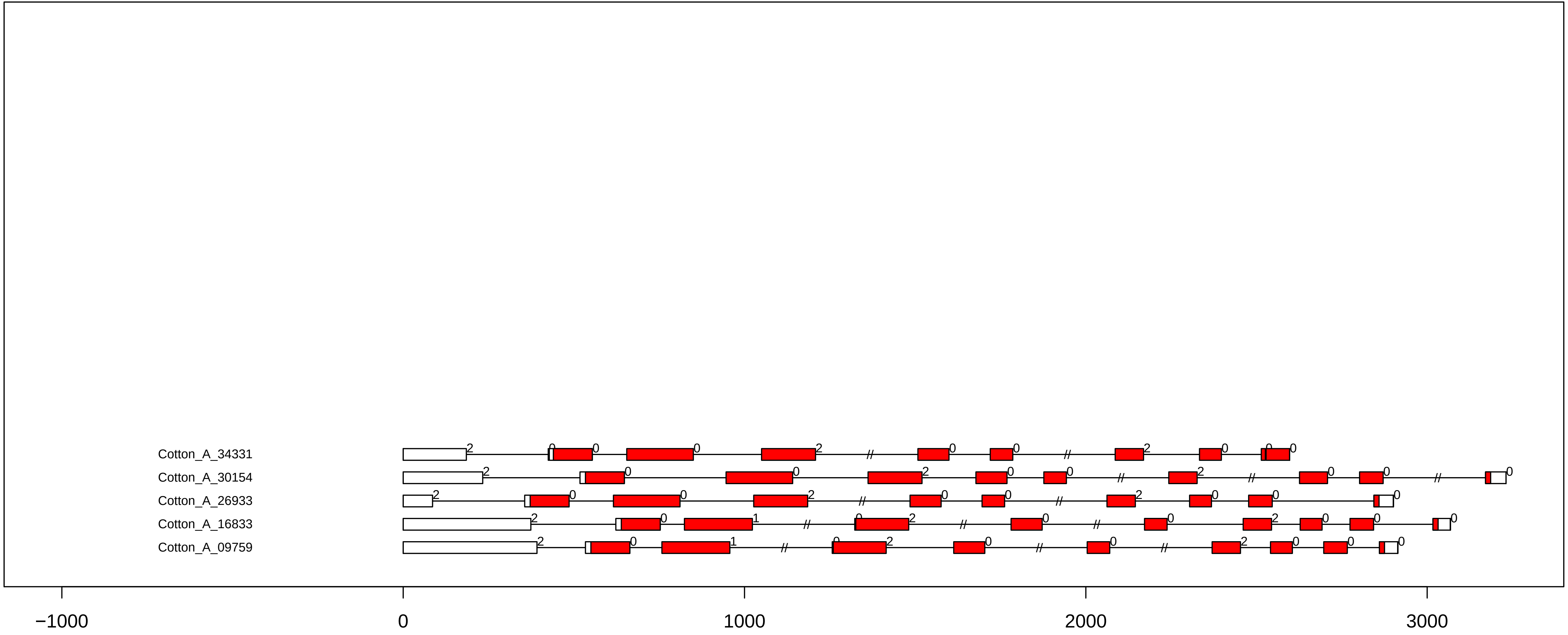

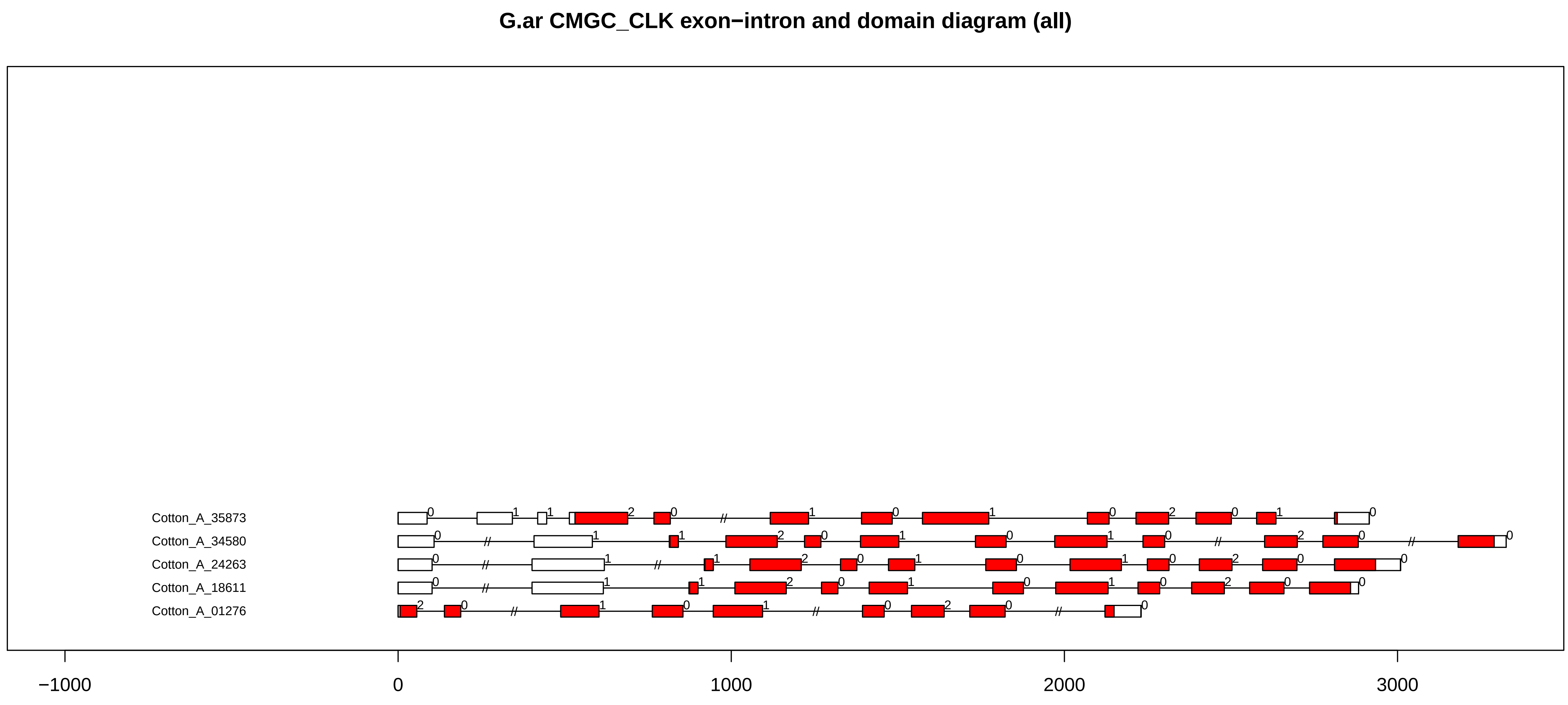
G.ar CMGC_CLK exon−intron and domain diagram (all)
Cotton_A_35873
Cotton_A_34580
Cotton_A_24263
Cotton_A_18611
Cotton_A_01276
−1000
0
1000
2000
3000

G.ar CMGC_DYRK−PRP4 exon−intron and domain diagram (all)

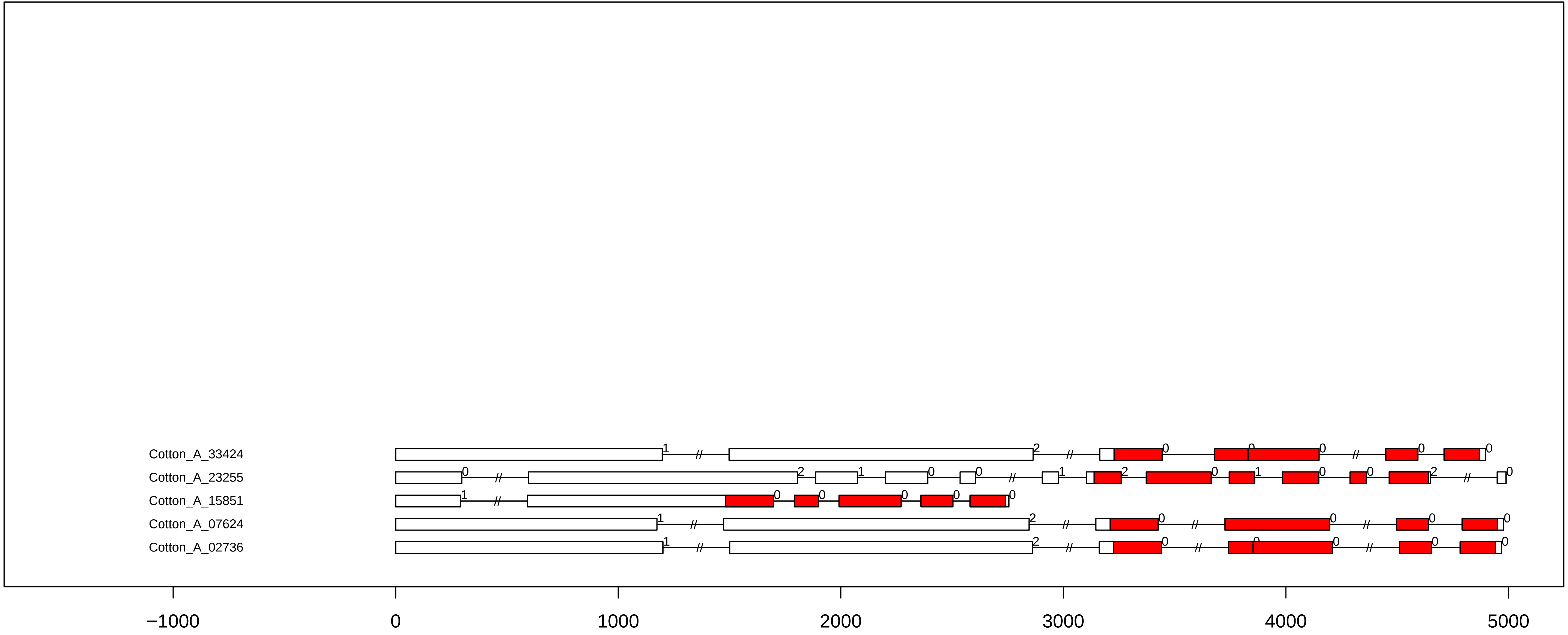

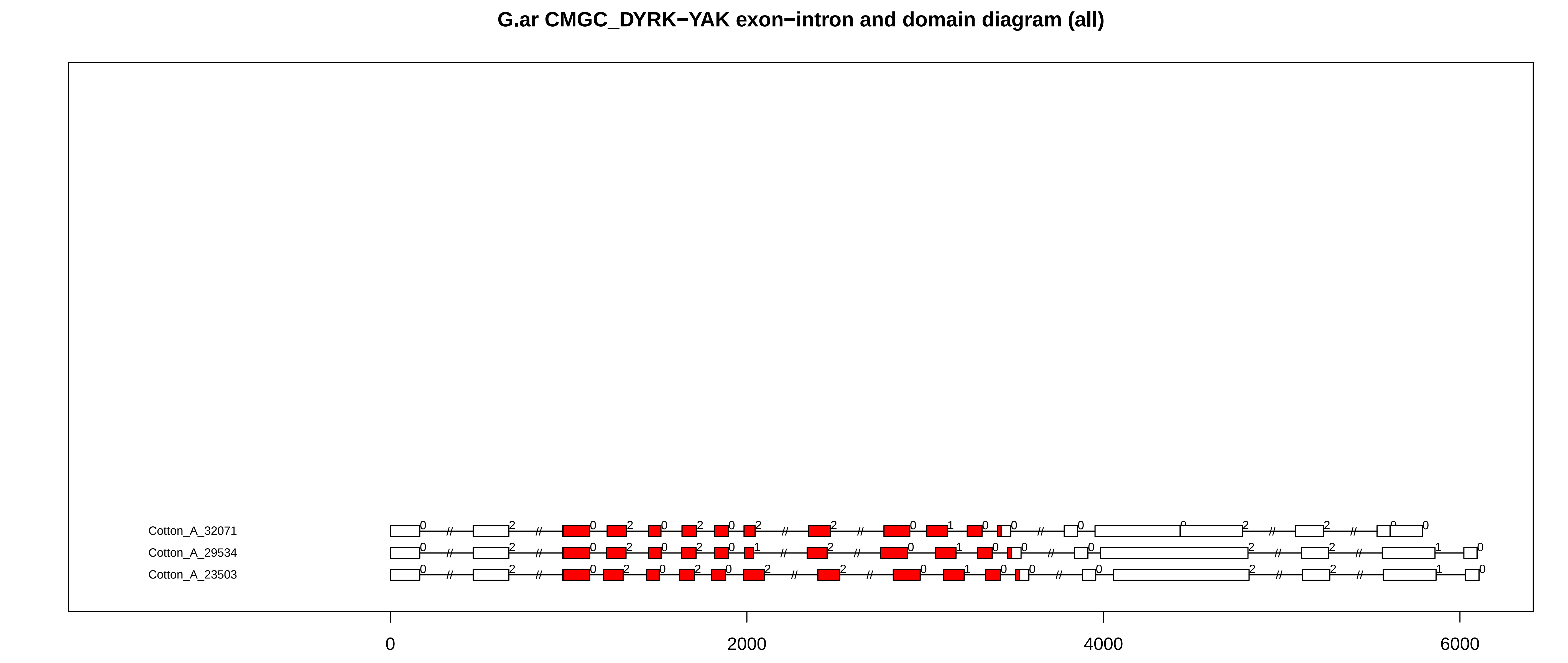
G.ar CMGC_DYRK−YAK exon−intron and domain diagram (all)
Cotton_A_32071
Cotton_A_29534
Cotton_A_23503
0
2000
4000
6000

G.ar CMGC_GSKL exon−intron and domain diagram (all)

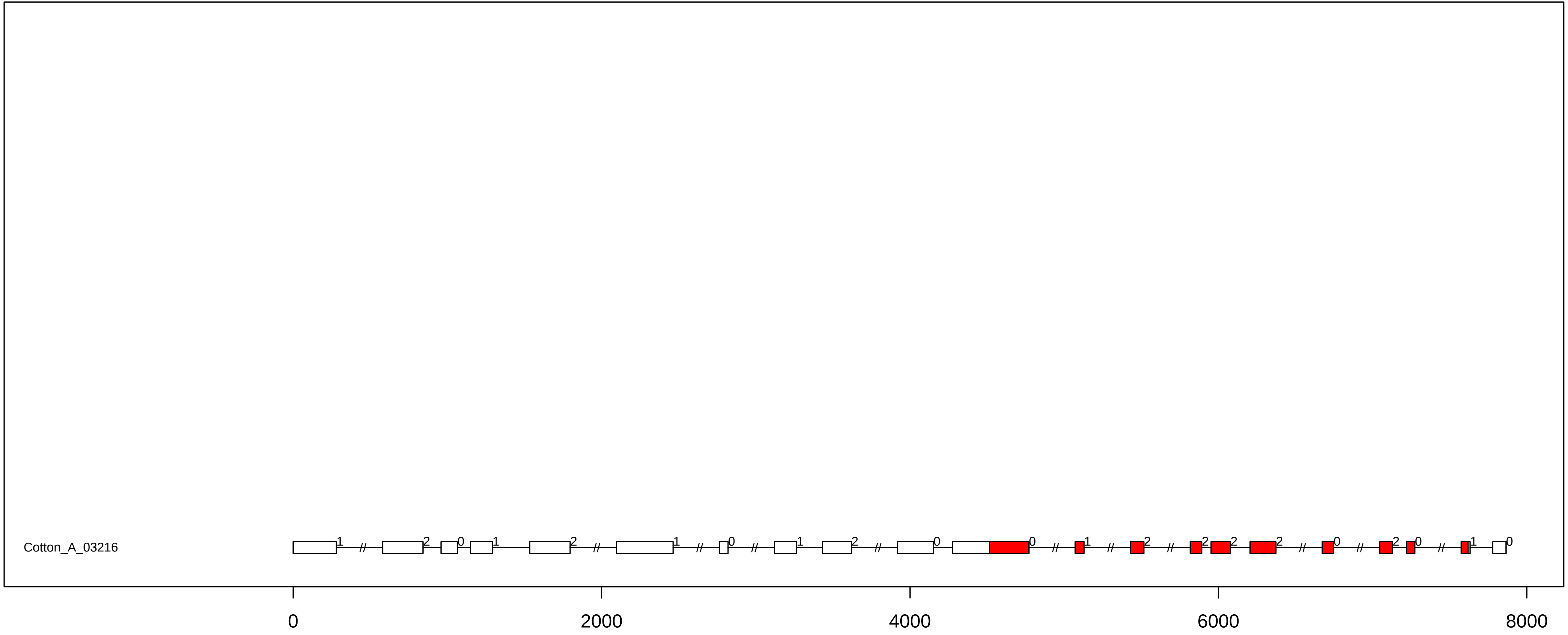

G.ar CMGC_GSK exon−intron and domain diagram (all)

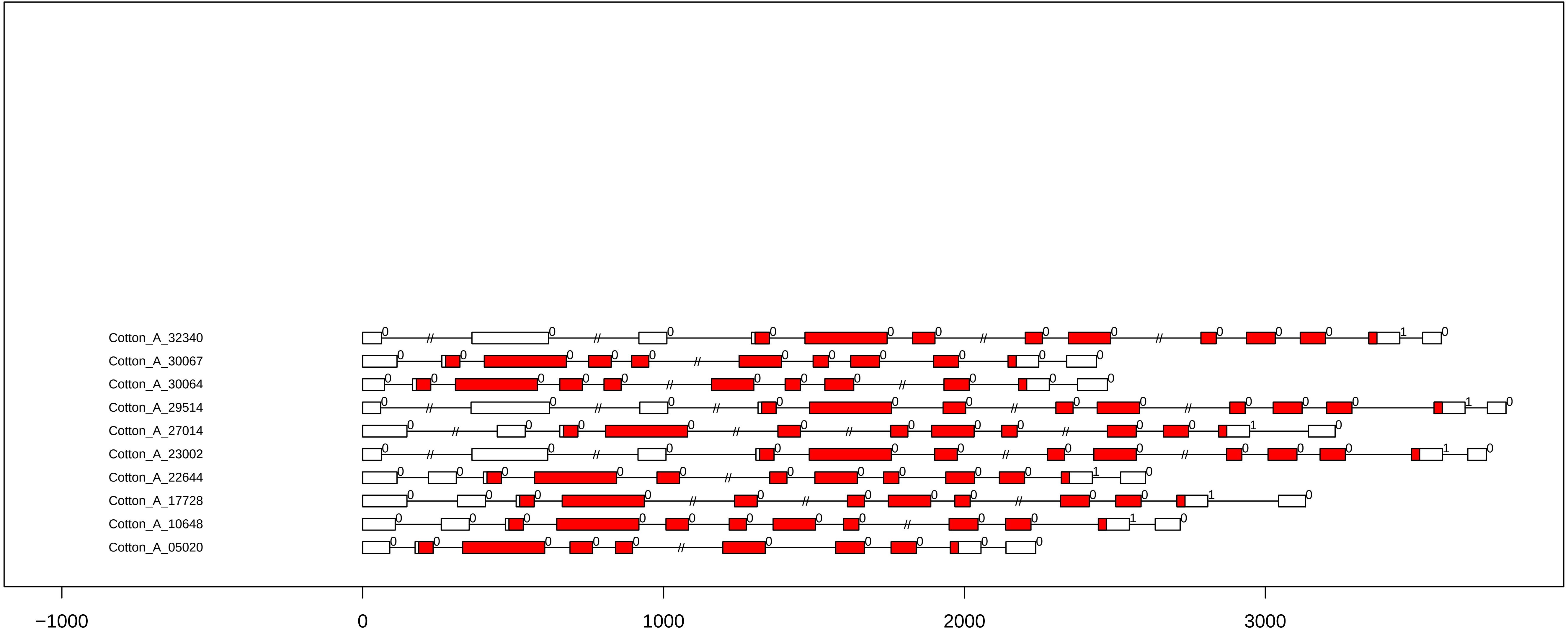

G.ar CMGC_MAPK exon−intron and domain diagram (part 1)

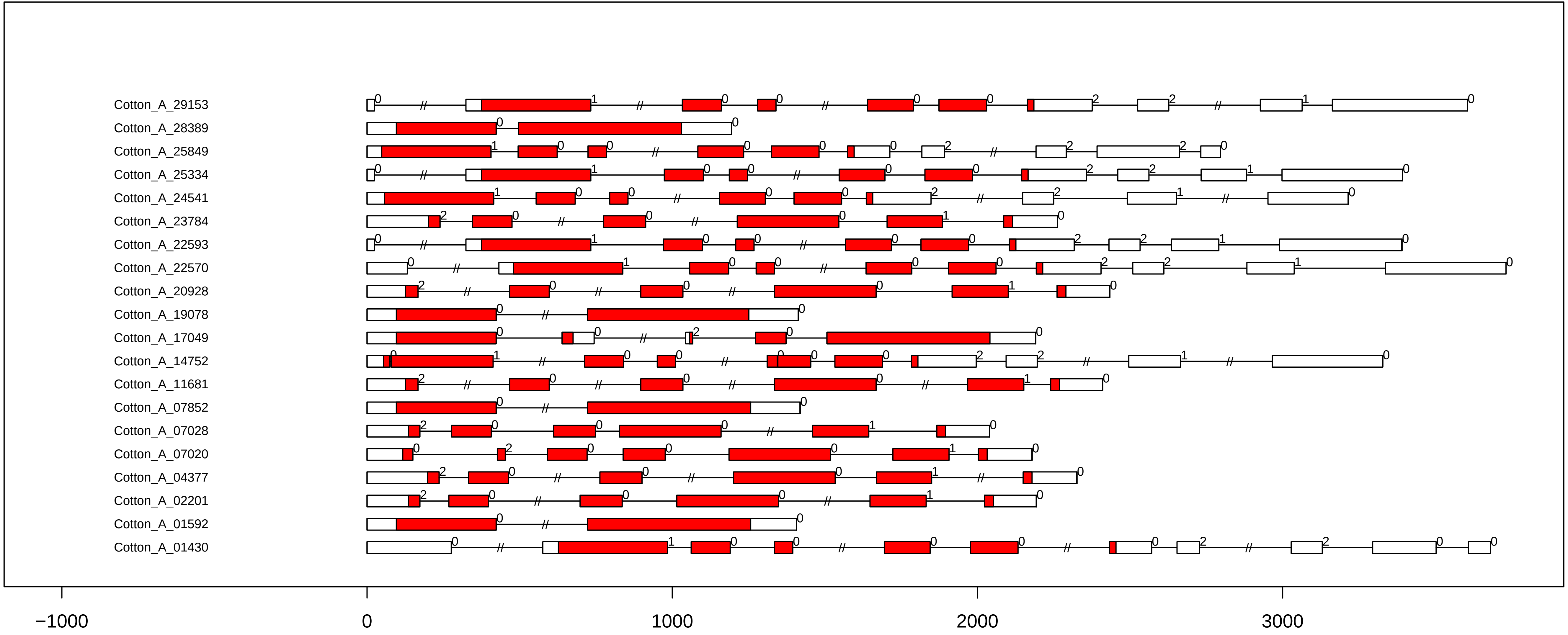

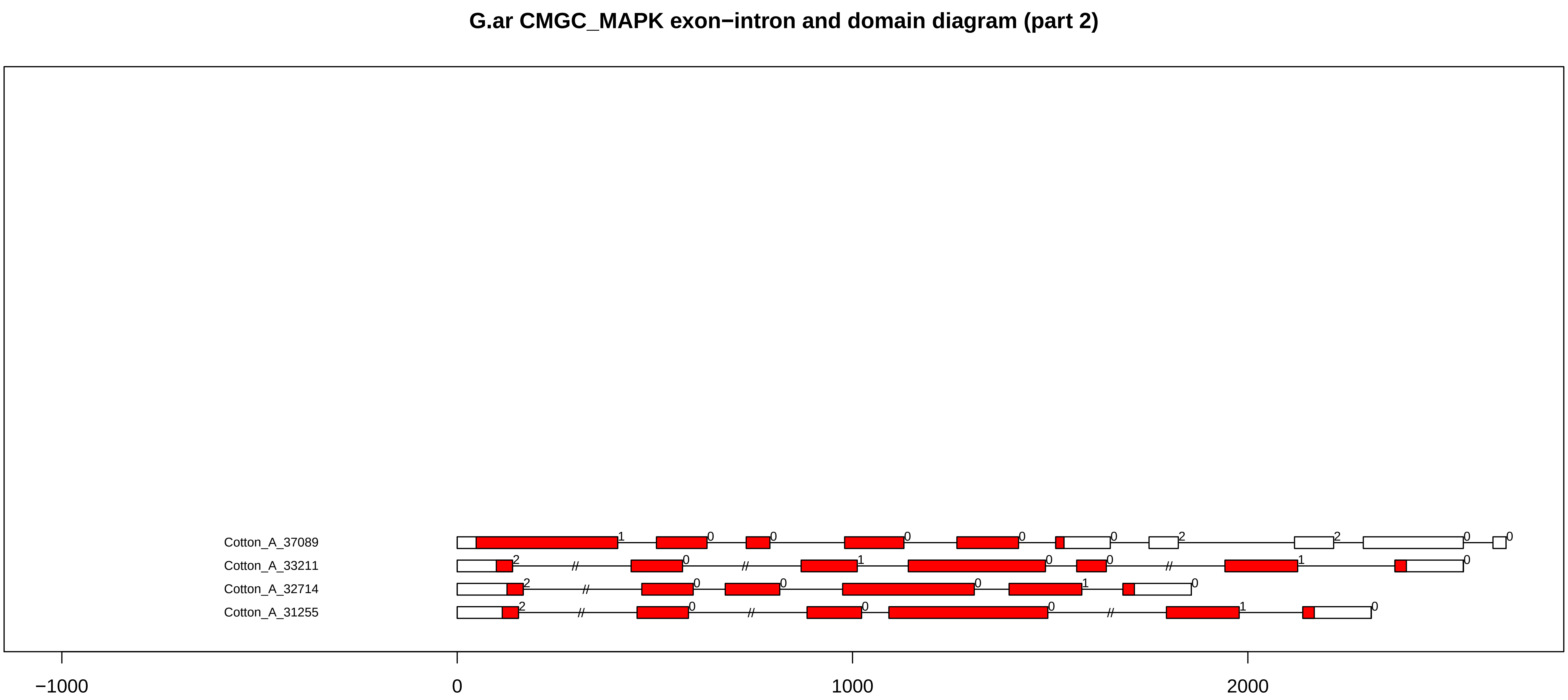
G.ar CMGC_MAPK exon−intron and domain diagram (part 2)
Cotton_A_37089
Cotton_A_33211
Cotton_A_32714
Cotton_A_31255
−1000
0
1000
2000

G.ar CMGC_PI−Tthe exon−intron and domain diagram (all)

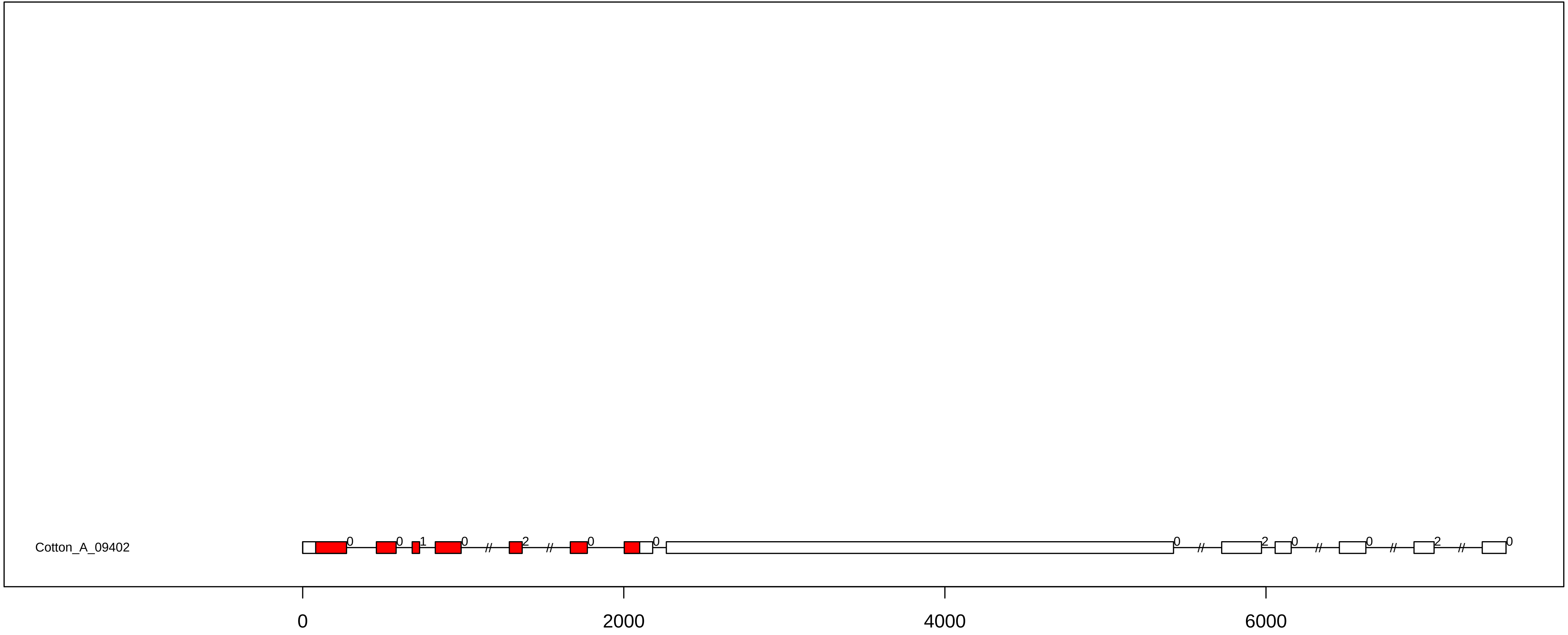

G.ar CMGC_RCK exon−intron and domain diagram (all)

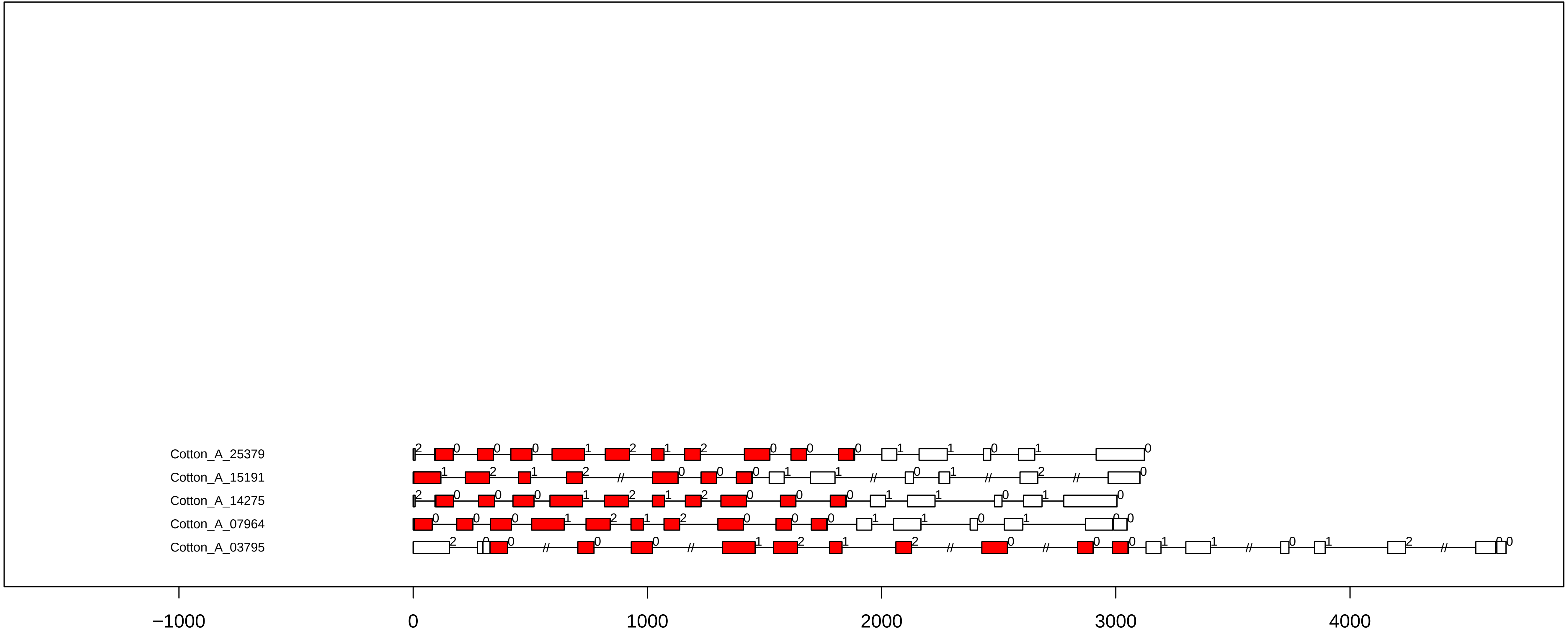

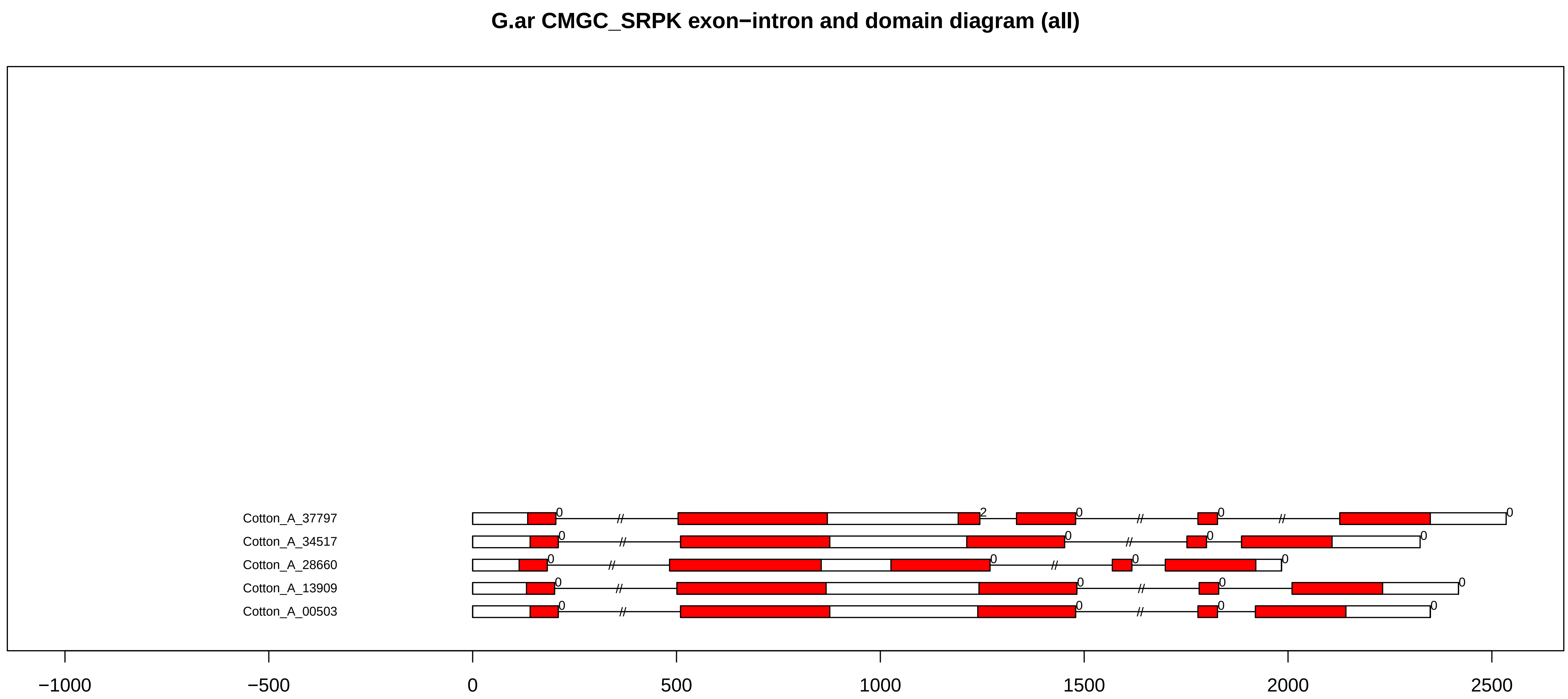
G.ar CMGC_SRPK exon−intron and domain diagram (all)
Cotton_A_37797
Cotton_A_34517
Cotton_A_28660
Cotton_A_13909
Cotton_A_00503
−1000
−500
0
500
1000
1500
2000
2500

G.ar Group−PI−2 exon−intron and domain diagram (all)

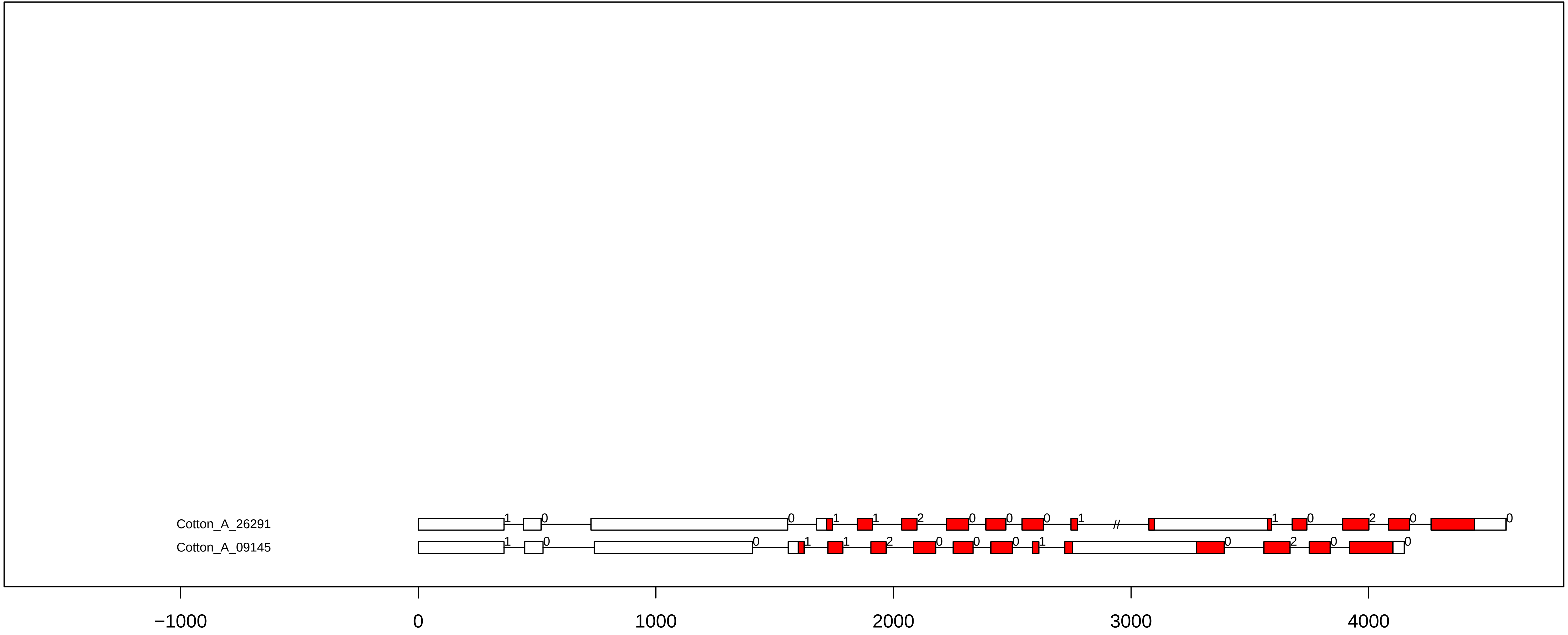

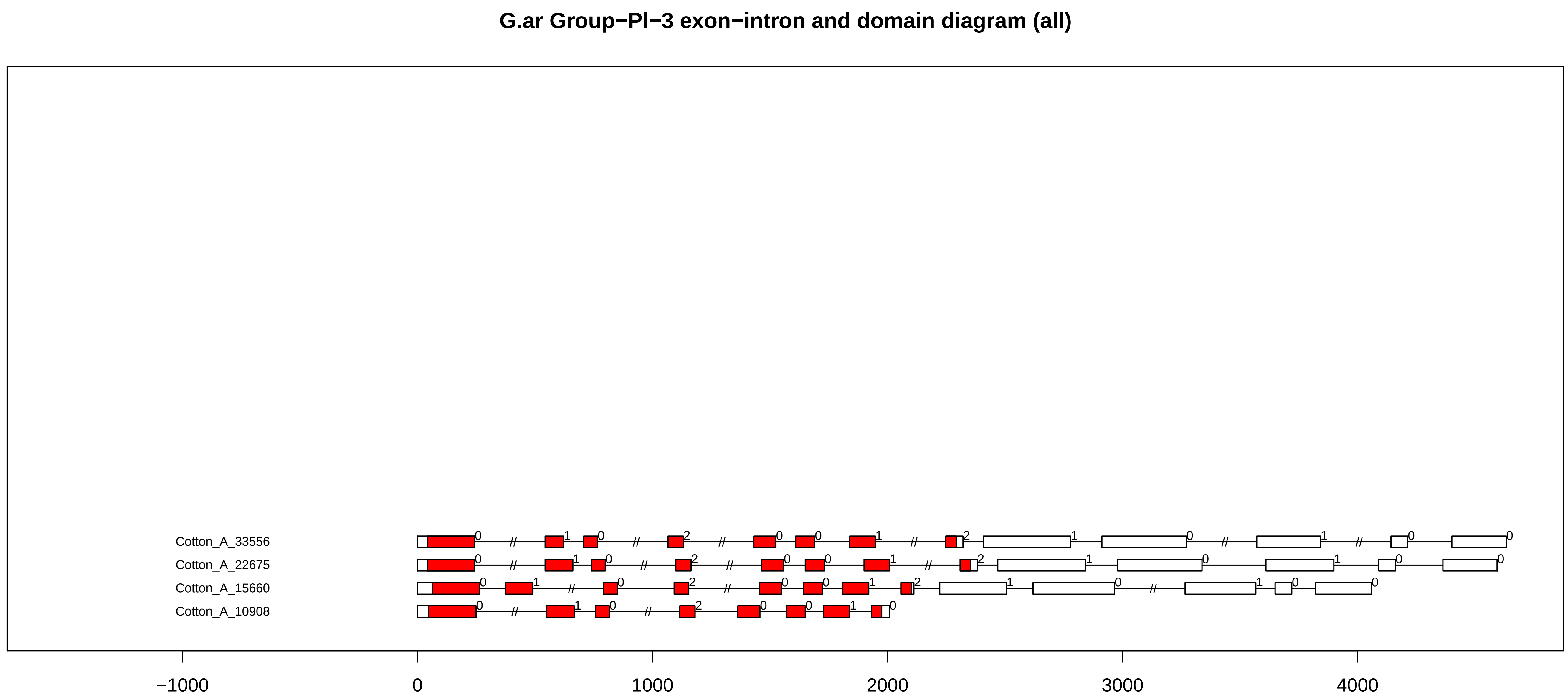
G.ar Group−PI−3 exon−intron and domain diagram (all)
Cotton_A_33556
Cotton_A_22675
Cotton_A_15660
Cotton_A_10908
−1000
0
1000
2000
3000
4000

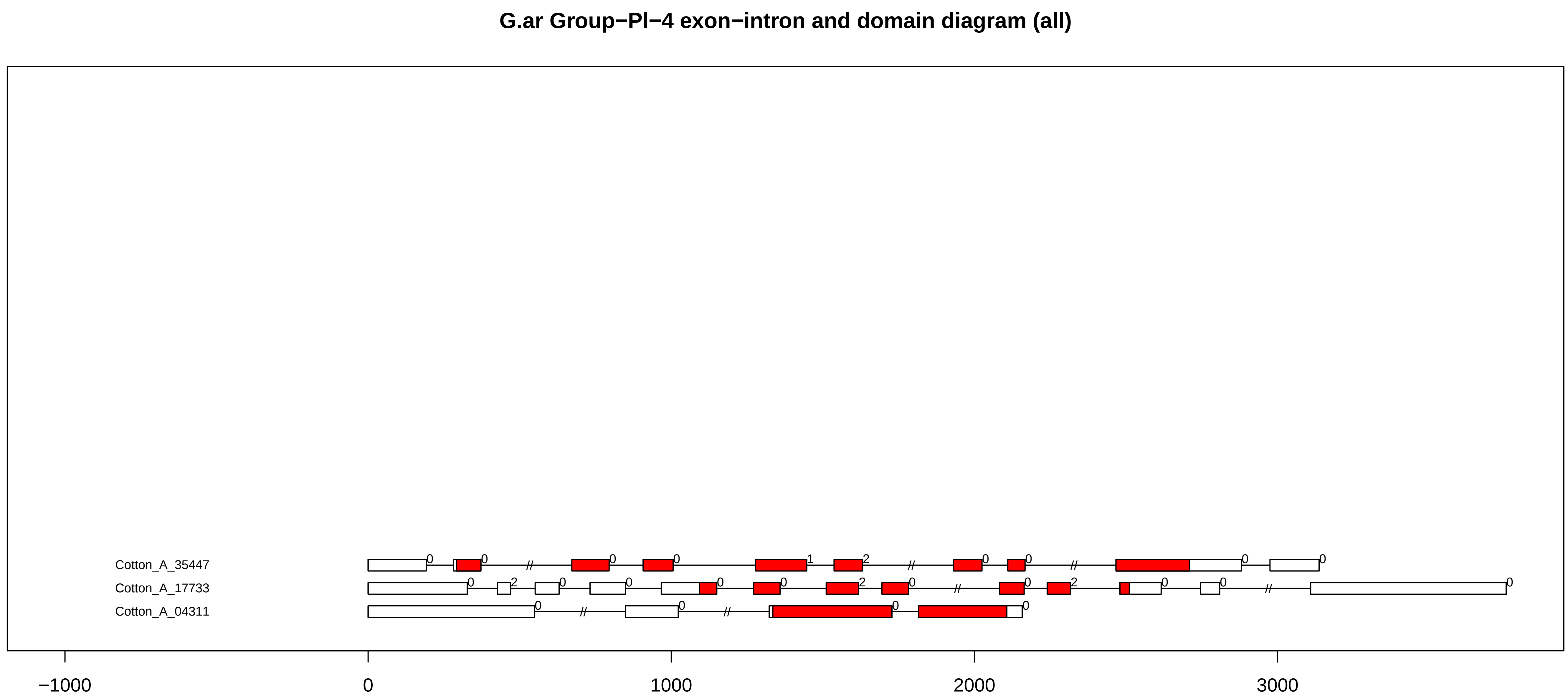
G.ar Group−PI−4 exon−intron and domain diagram (all)
Cotton_A_35447
Cotton_A_17733
Cotton_A_04311
−1000
0
1000
2000
3000

G.ar IRE1 exon−intron and domain diagram (all)

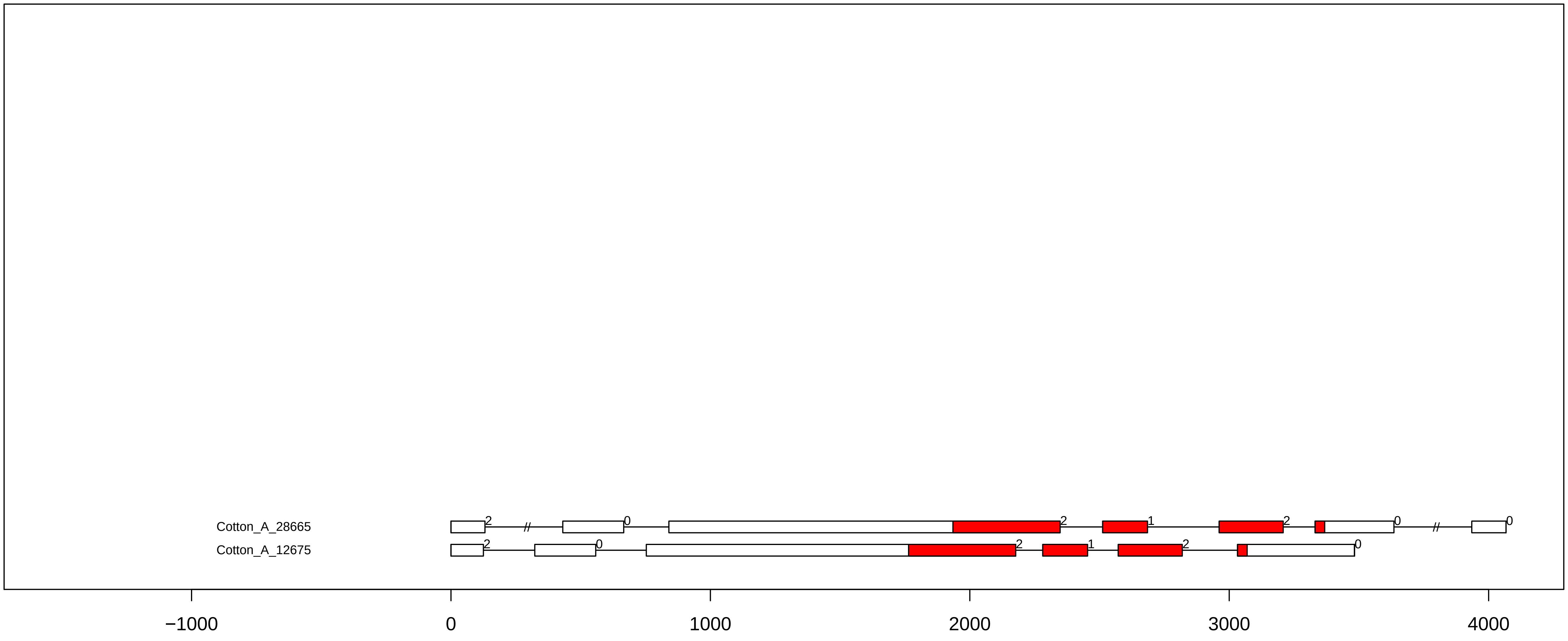

G.ar NAK exon−intron and domain diagram (all)

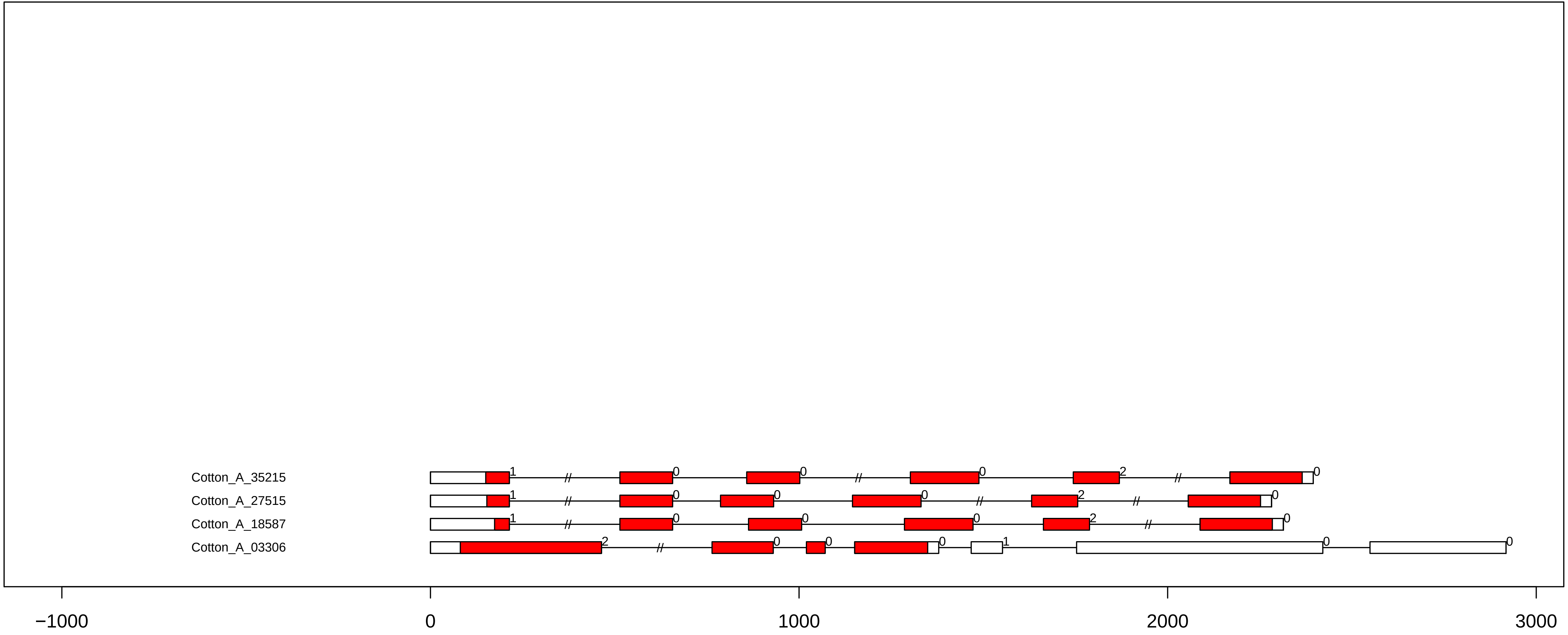

G.ar NEK exon−intron and domain diagram (all)

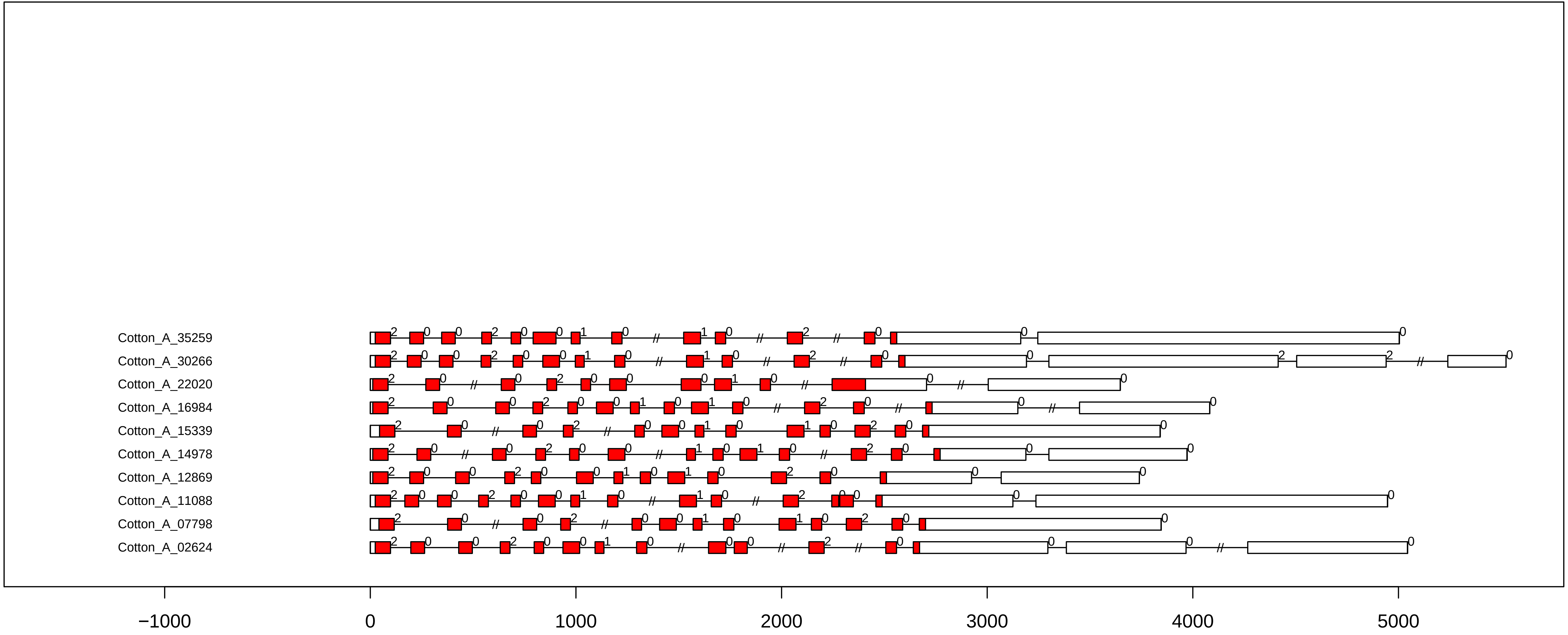

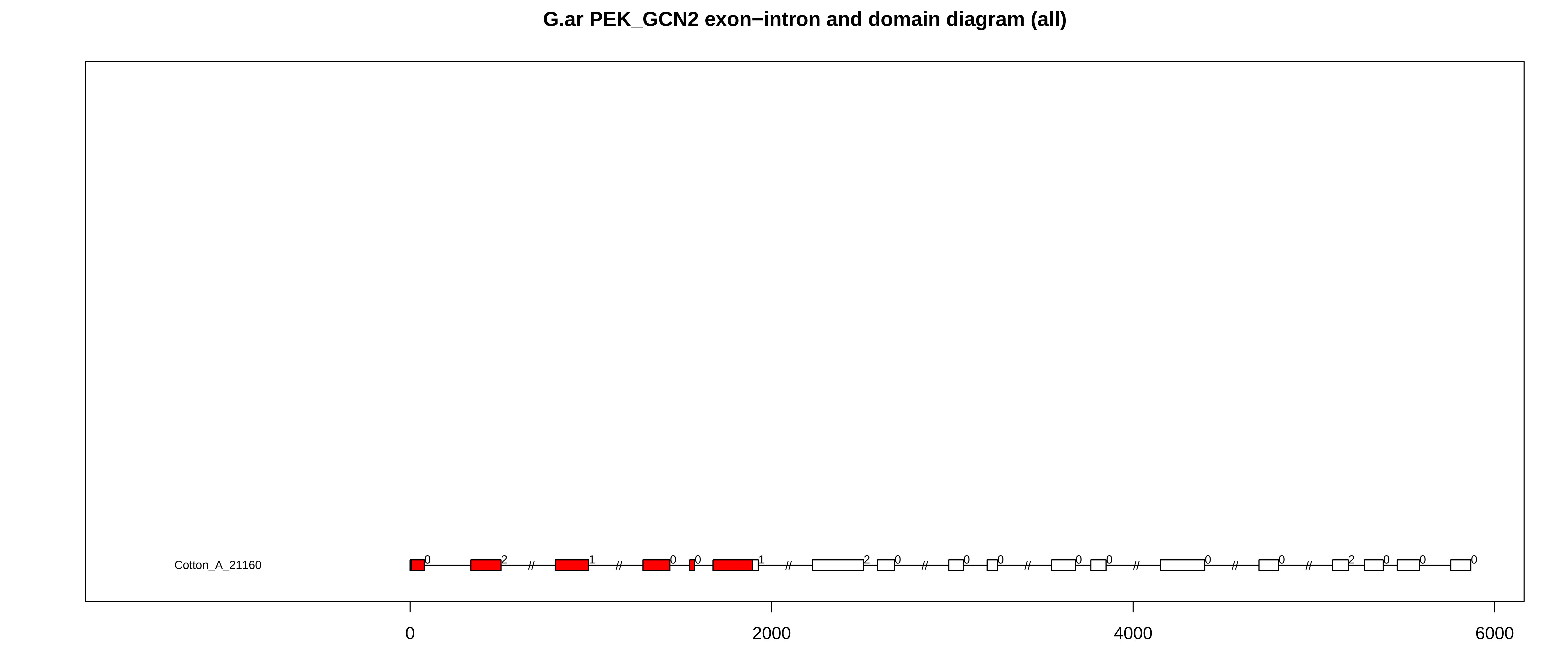
G.ar PEK_GCN2 exon−intron and domain diagram (all)
Cotton_A_21160
0
2
1
0
0
1
2
0
0
0
0
0
0
0
2
0
0
0
0
2000
4000
6000

G.ar PEK_PEK exon−intron and domain diagram (all)

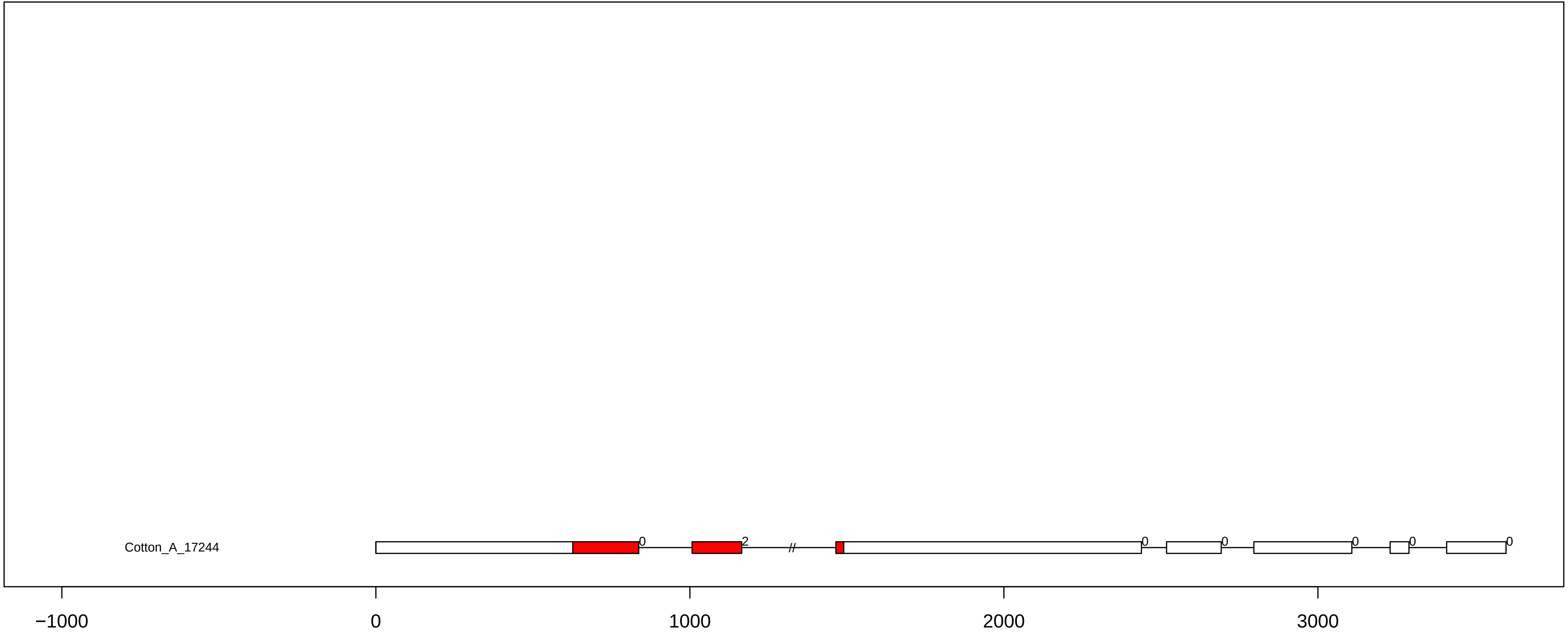

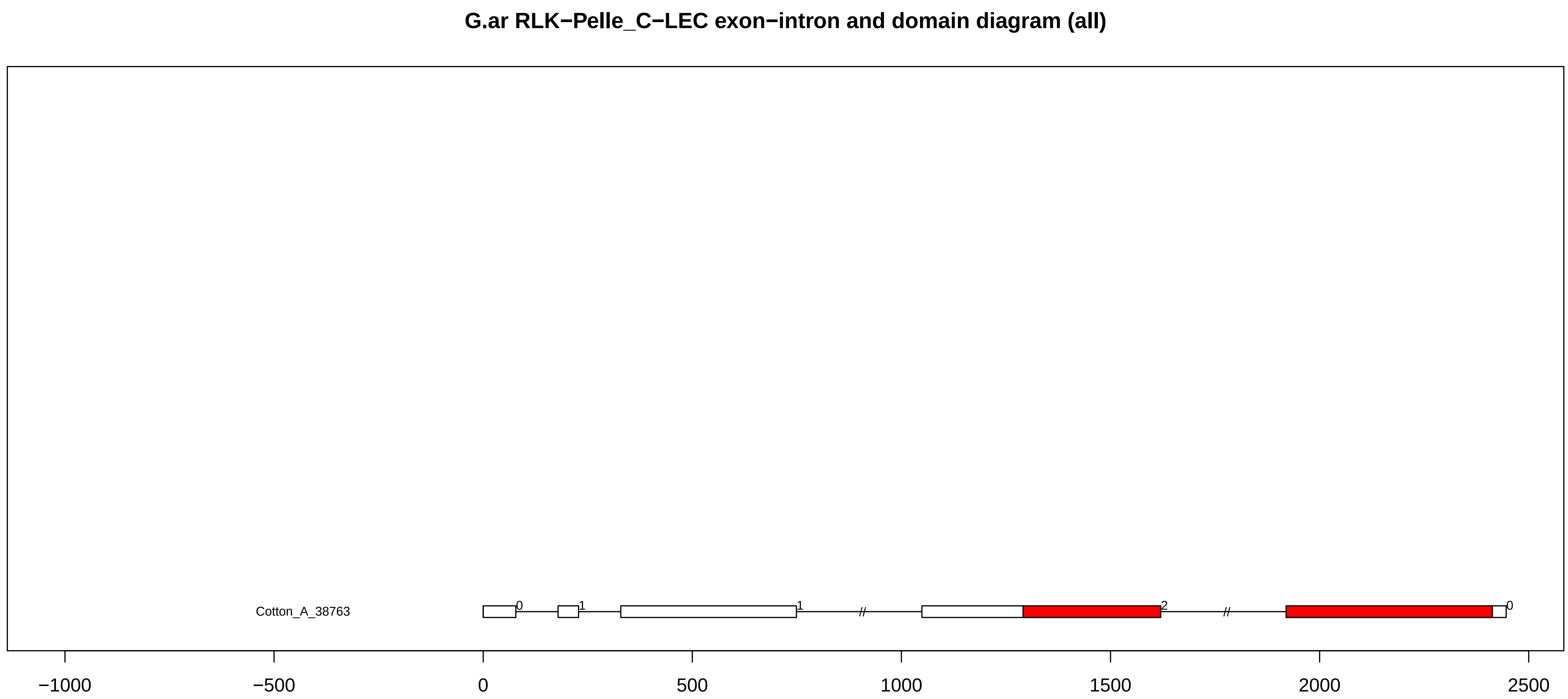
G.ar RLK−Pelle_C−LEC exon−intron and domain diagram (all)
Cotton_A_38763
0
1
1
2
0
−1000
−500
0
500
1000
1500
2000
2500

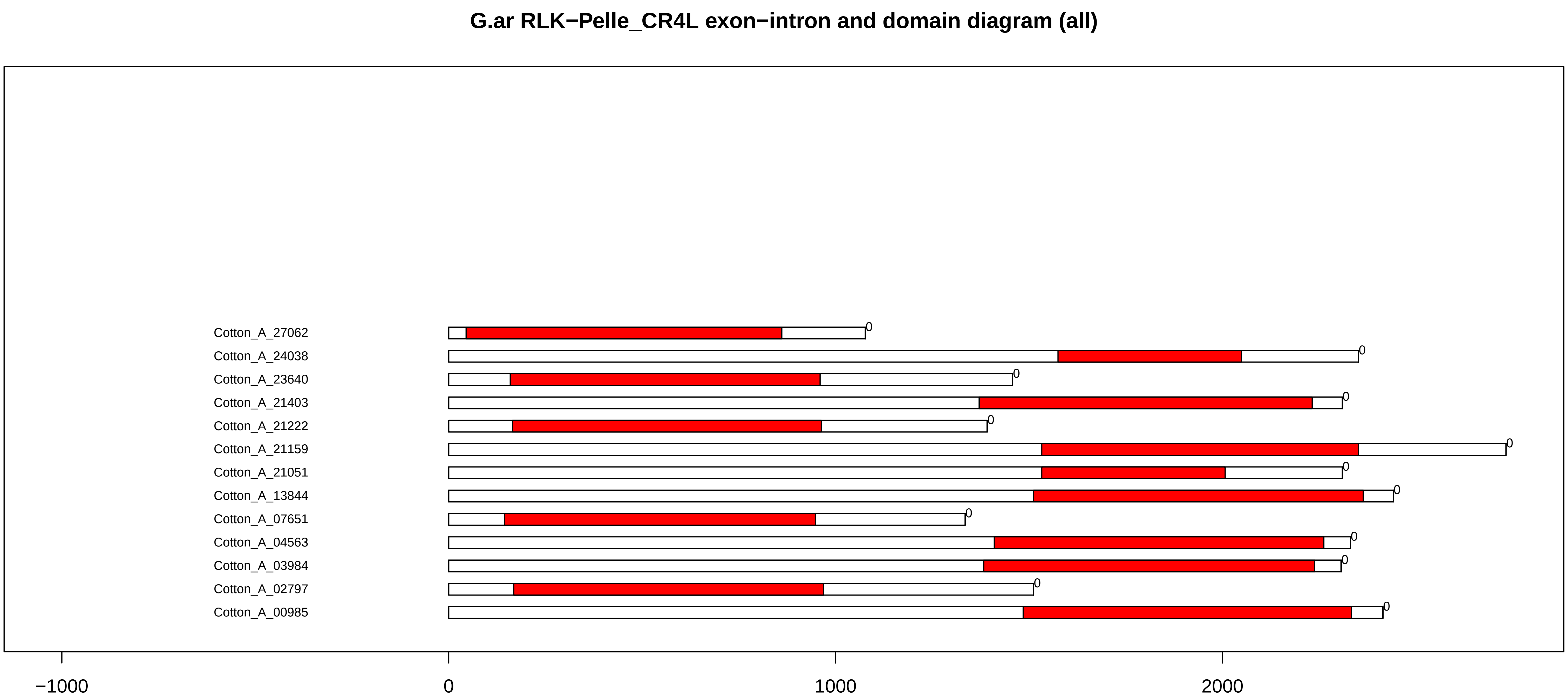

G.ar RLK−Pelle_CR4L exon−intron and domain diagram (all)
Cotton_A_27062
Cotton_A_24038
Cotton_A_23640
Cotton_A_21403
Cotton_A_21222
Cotton_A_21159
Cotton_A_21051
Cotton_A_13844
Cotton_A_07651
Cotton_A_04563
Cotton_A_03984
Cotton_A_02797
Cotton_A_00985
0
0
0
0
0
0
0
0
0
0
0
0
0
−1000
0
1000
2000

G.ar RLK−Pelle_CrRLK1L−1 exon−intron and domain diagram (part 1)

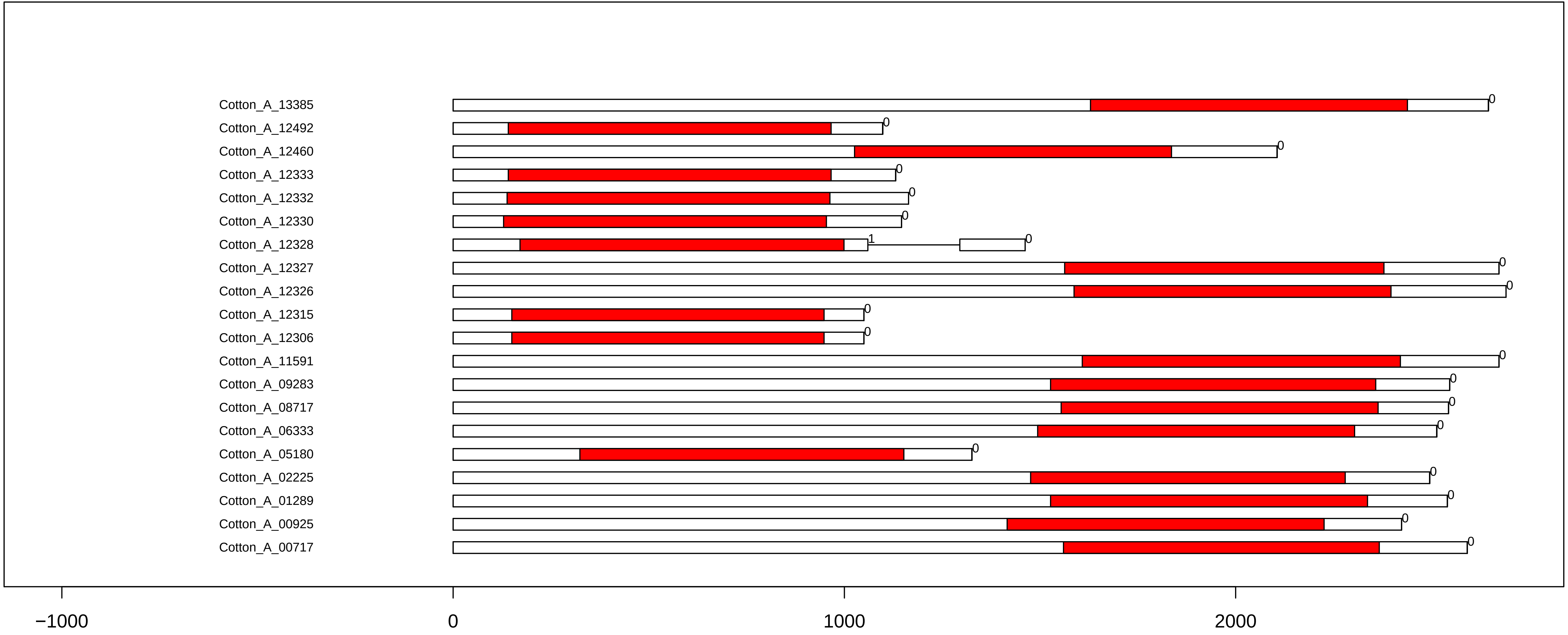

G.ar RLK−Pelle_CrRLK1L−1 exon−intron and domain diagram (part 2)

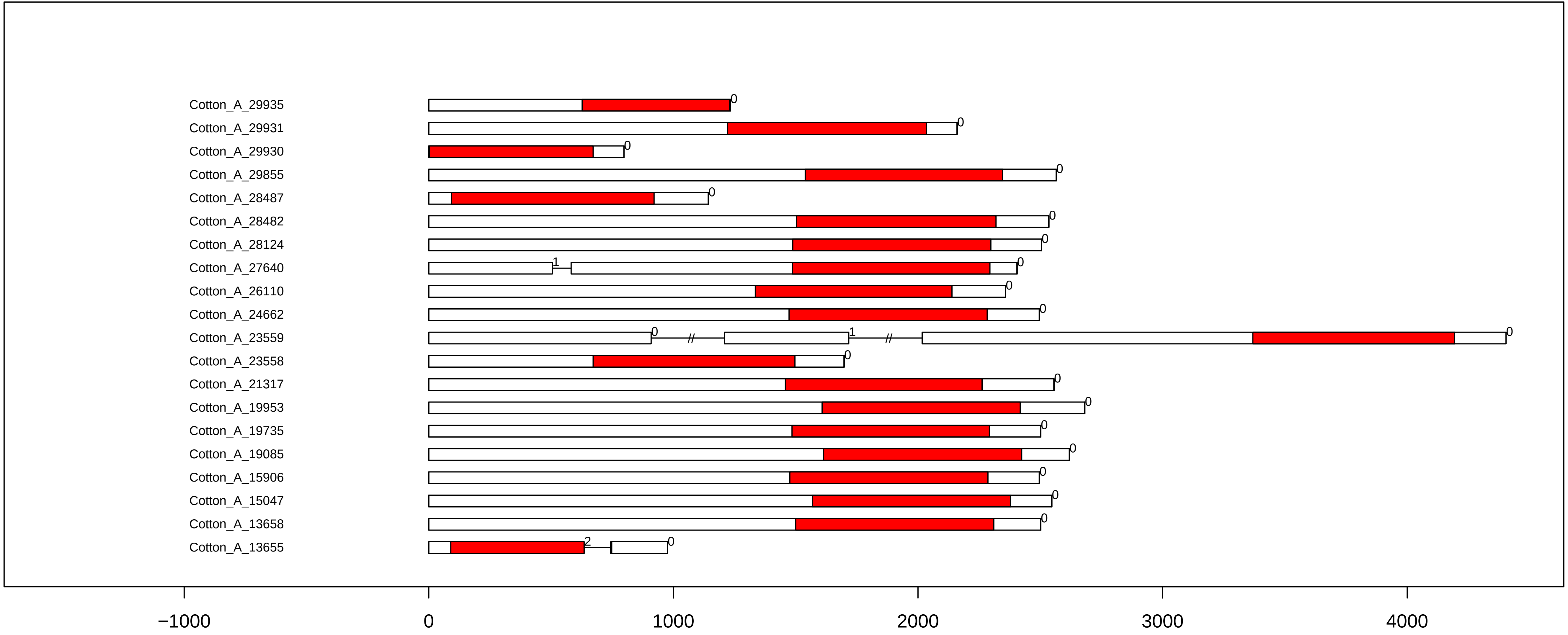

G.ar RLK−Pelle_CrRLK1L−1 exon−intron and domain diagram (part 3)

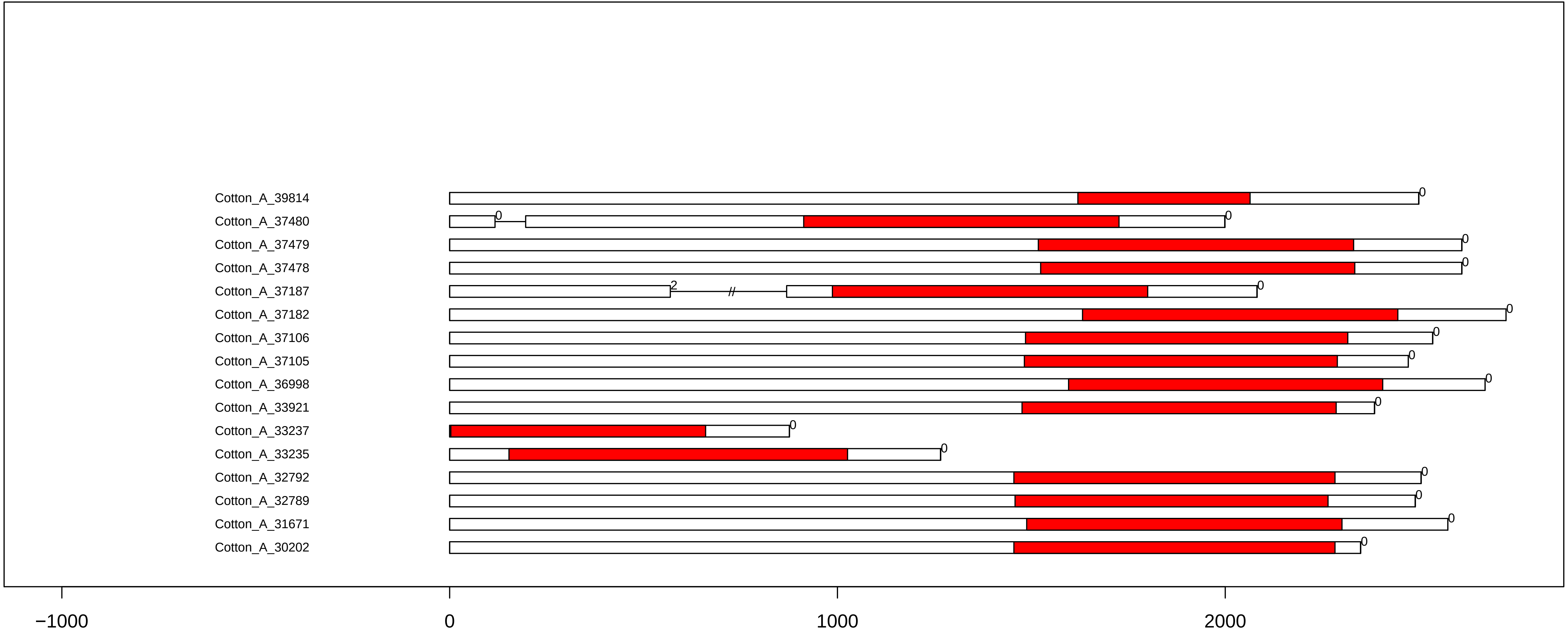

G.ar RLK−Pelle_DLSV exon−intron and domain diagram (part 1)

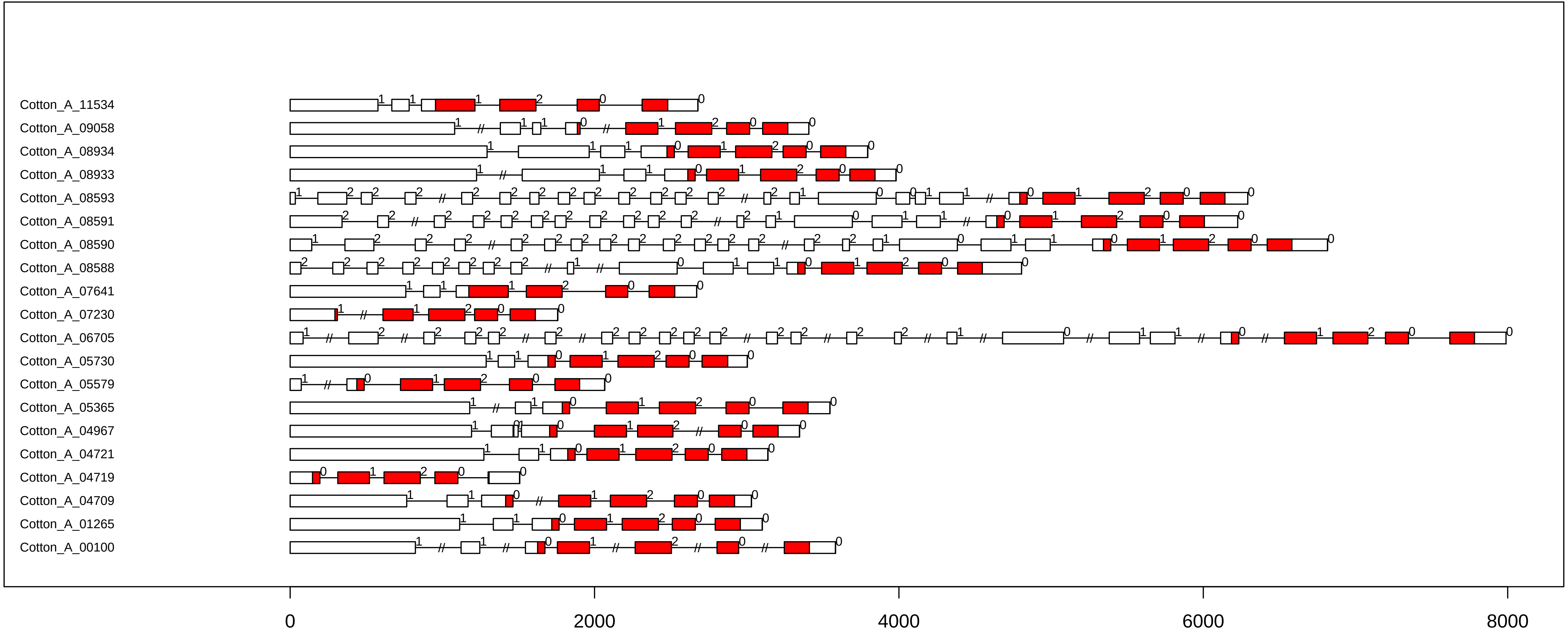

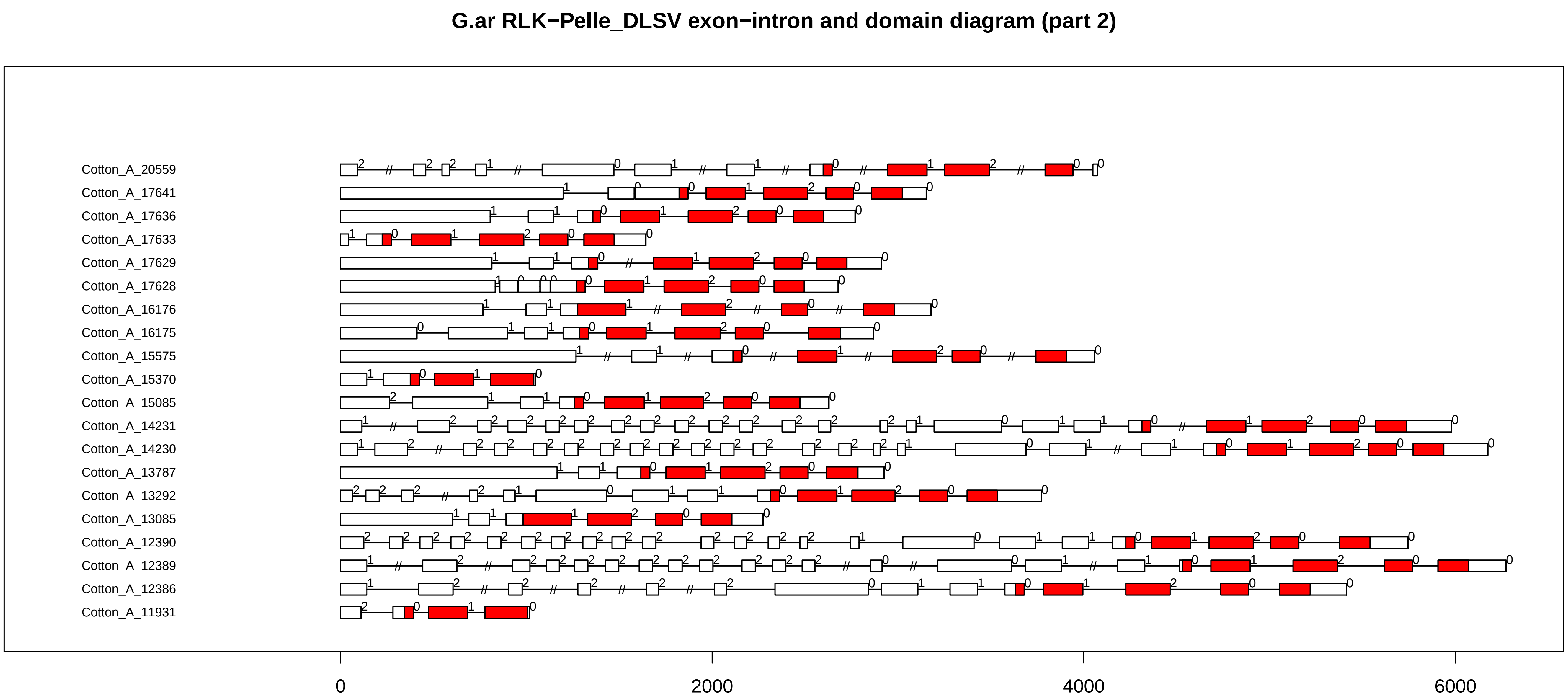
G.ar RLK−Pelle_DLSV exon−intron and domain diagram (part 2)
Cotton_A_20559
Cotton_A_17641
Cotton_A_17636
Cotton_A_17633
Cotton_A_17629
Cotton_A_17628
Cotton_A_16176
Cotton_A_16175
Cotton_A_15575
Cotton_A_15370
Cotton_A_15085
Cotton_A_14231
Cotton_A_14230
Cotton_A_13787
Cotton_A_13292
Cotton_A_13085
Cotton_A_12390
Cotton_A_12389
Cotton_A_12386
Cotton_A_11931
0
2000
4000
6000

G.ar RLK−Pelle_DLSV exon−intron and domain diagram (part 3)

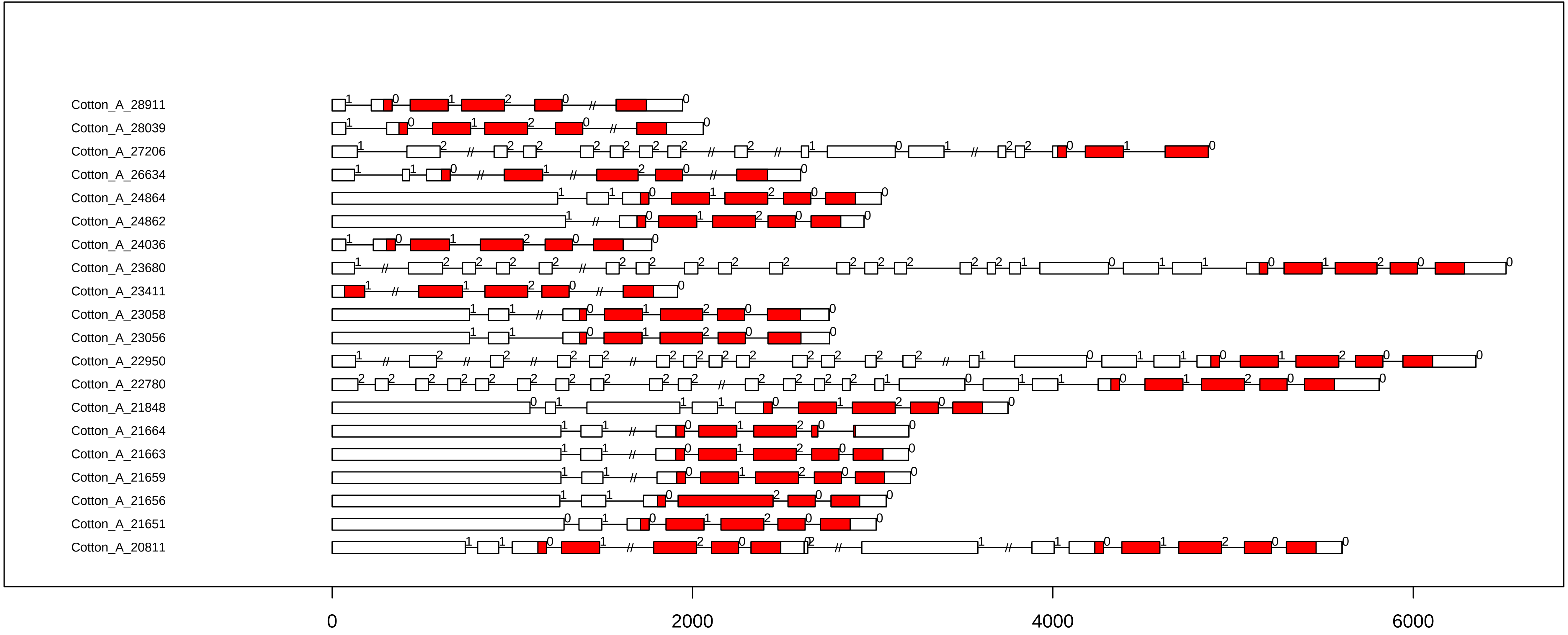

G.ar RLK−Pelle_DLSV exon−intron and domain diagram (part 4)

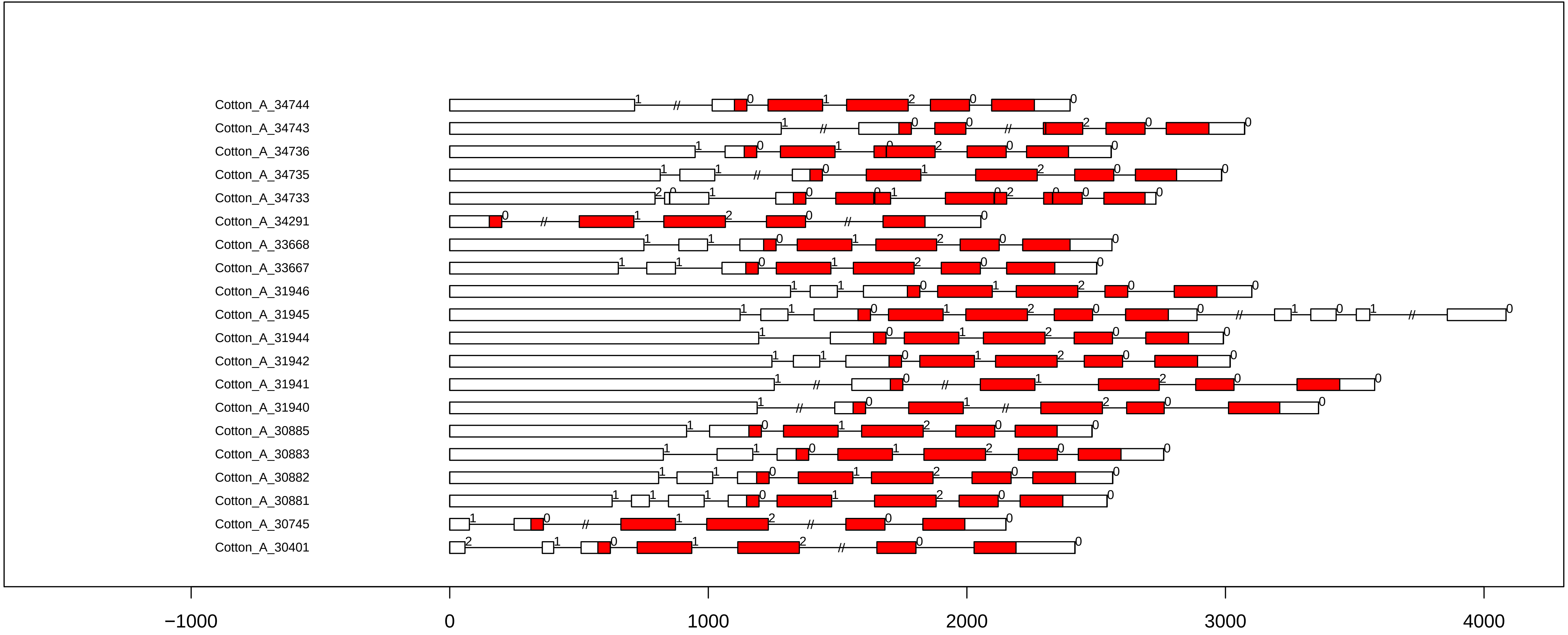

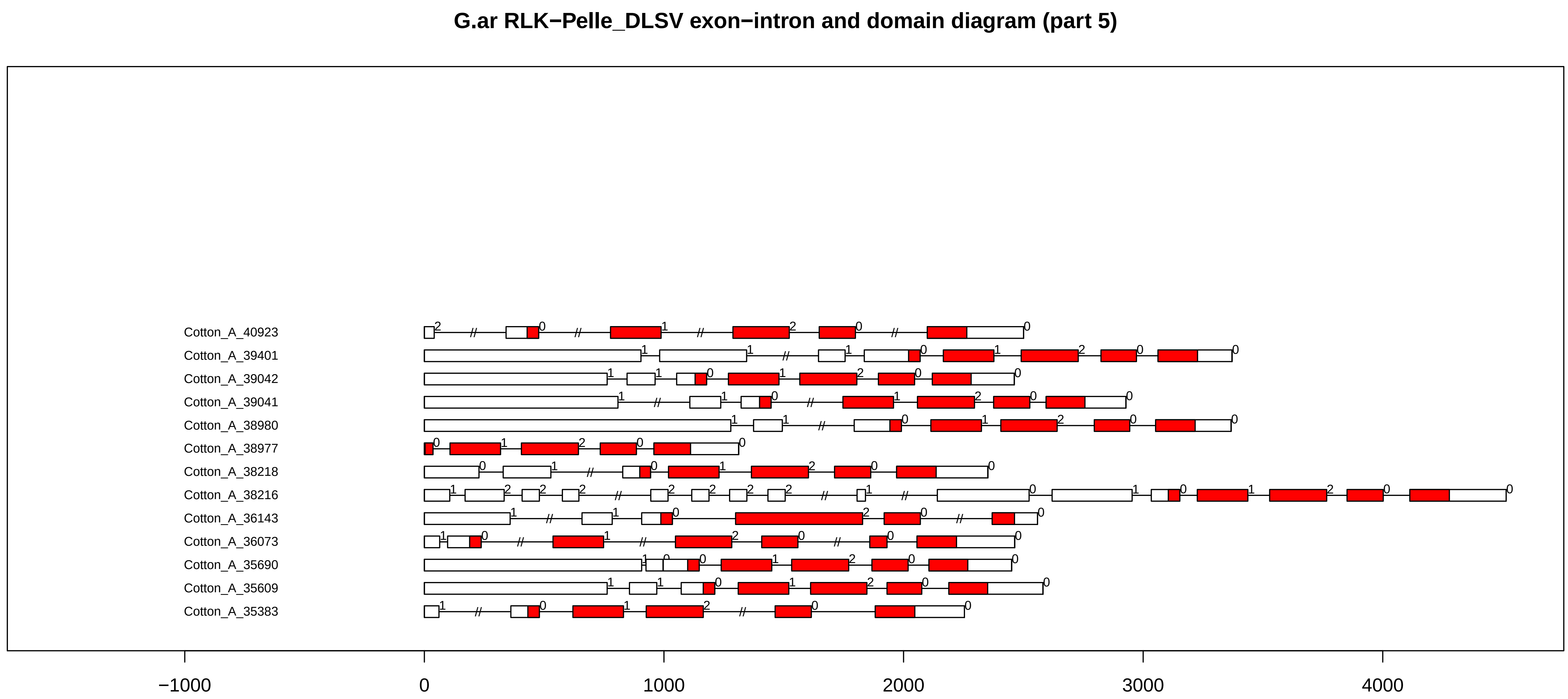
G.ar RLK−Pelle_DLSV exon−intron and domain diagram (part 5)
Cotton_A_40923
Cotton_A_39401
Cotton_A_39042
Cotton_A_39041
Cotton_A_38980
Cotton_A_38977
Cotton_A_38218
Cotton_A_38216
Cotton_A_36143
Cotton_A_36073
Cotton_A_35690
Cotton_A_35609
Cotton_A_35383
−1000
0
1000
2000
3000
4000

G.ar RLK−Pelle_Extensin exon−intron and domain diagram (all)

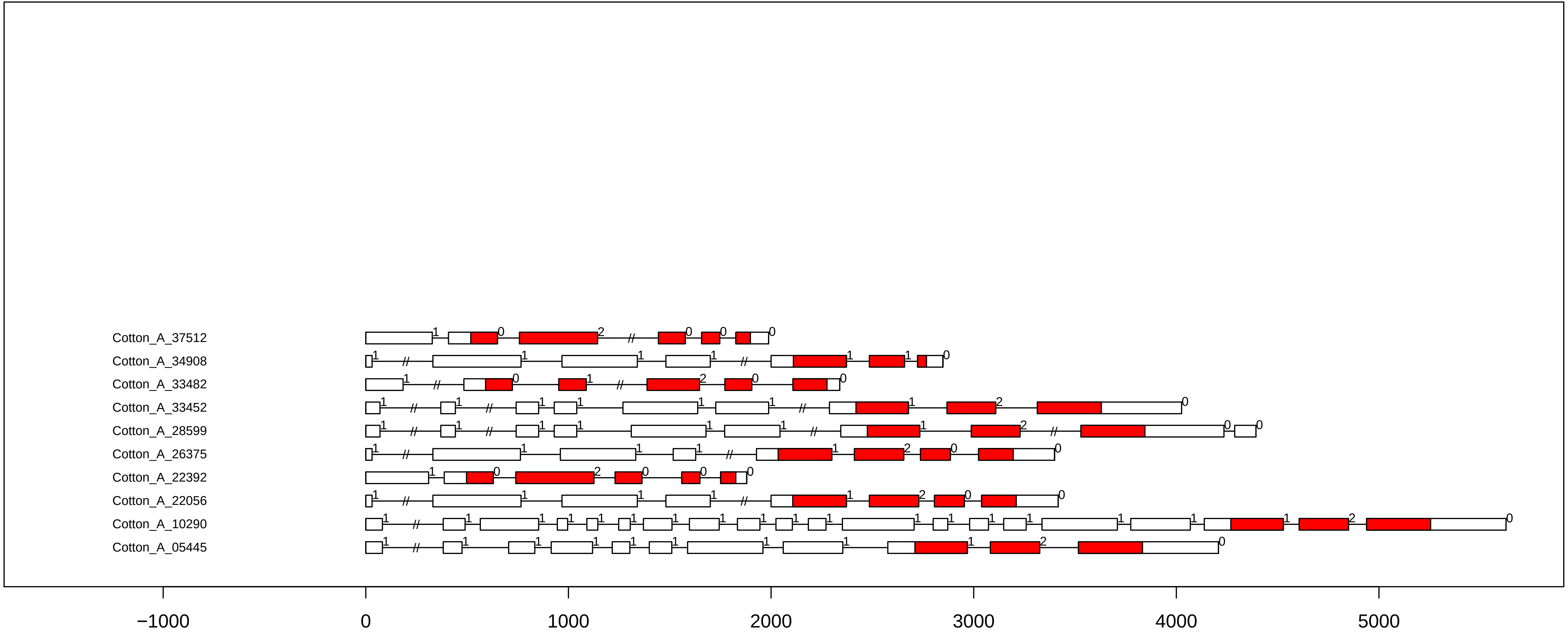

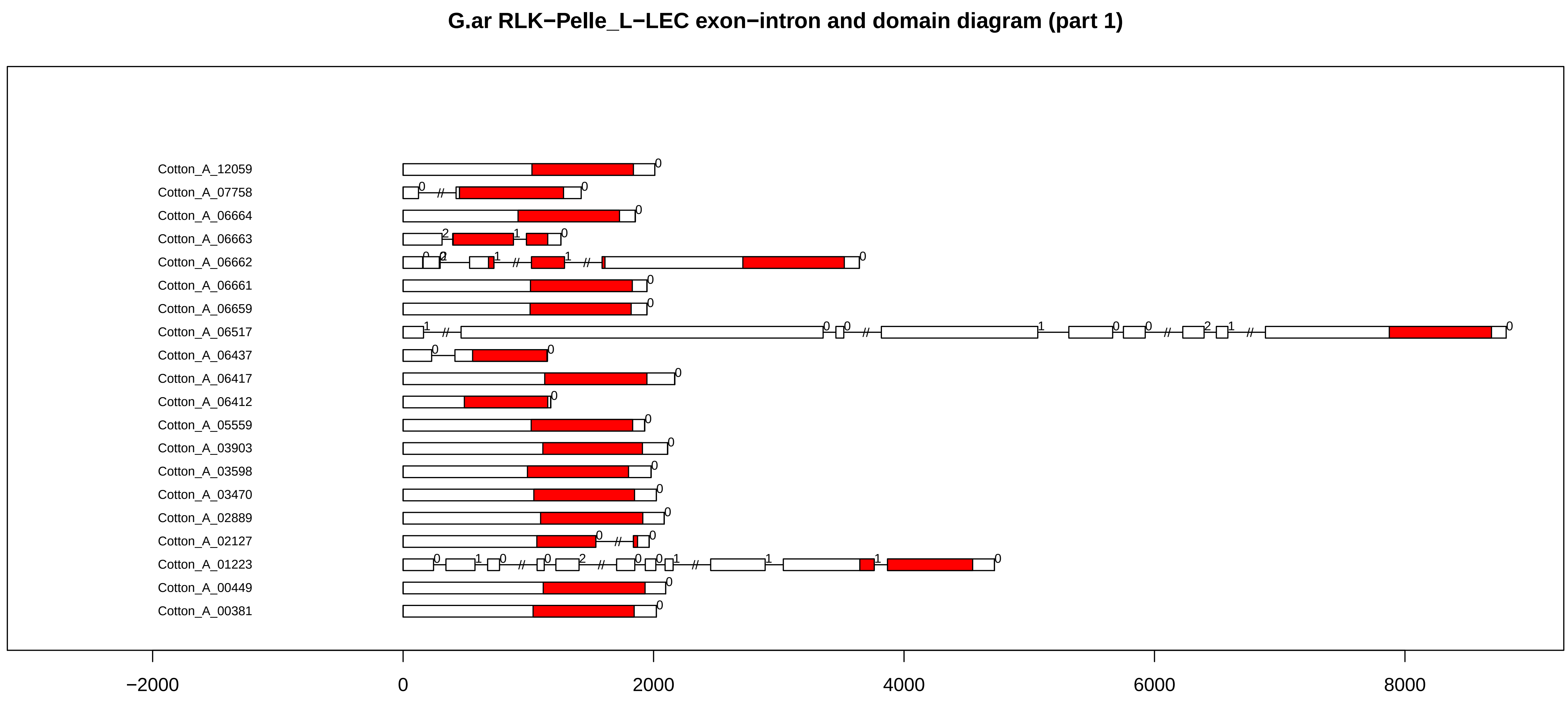

G.ar RLK−Pelle_L−LEC exon−intron and domain diagram (part 1)
Cotton_A_12059
Cotton_A_07758
Cotton_A_06664
Cotton_A_06663
Cotton_A_06662
Cotton_A_06661
Cotton_A_06659
Cotton_A_06517
Cotton_A_06437
Cotton_A_06417
Cotton_A_06412
Cotton_A_05559
Cotton_A_03903
Cotton_A_03598
Cotton_A_03470
Cotton_A_02889
Cotton_A_02127
Cotton_A_01223
Cotton_A_00449
Cotton_A_00381
−2000
0
2000
4000
6000
8000

G.ar RLK−Pelle_L−LEC exon−intron and domain diagram (part 2)

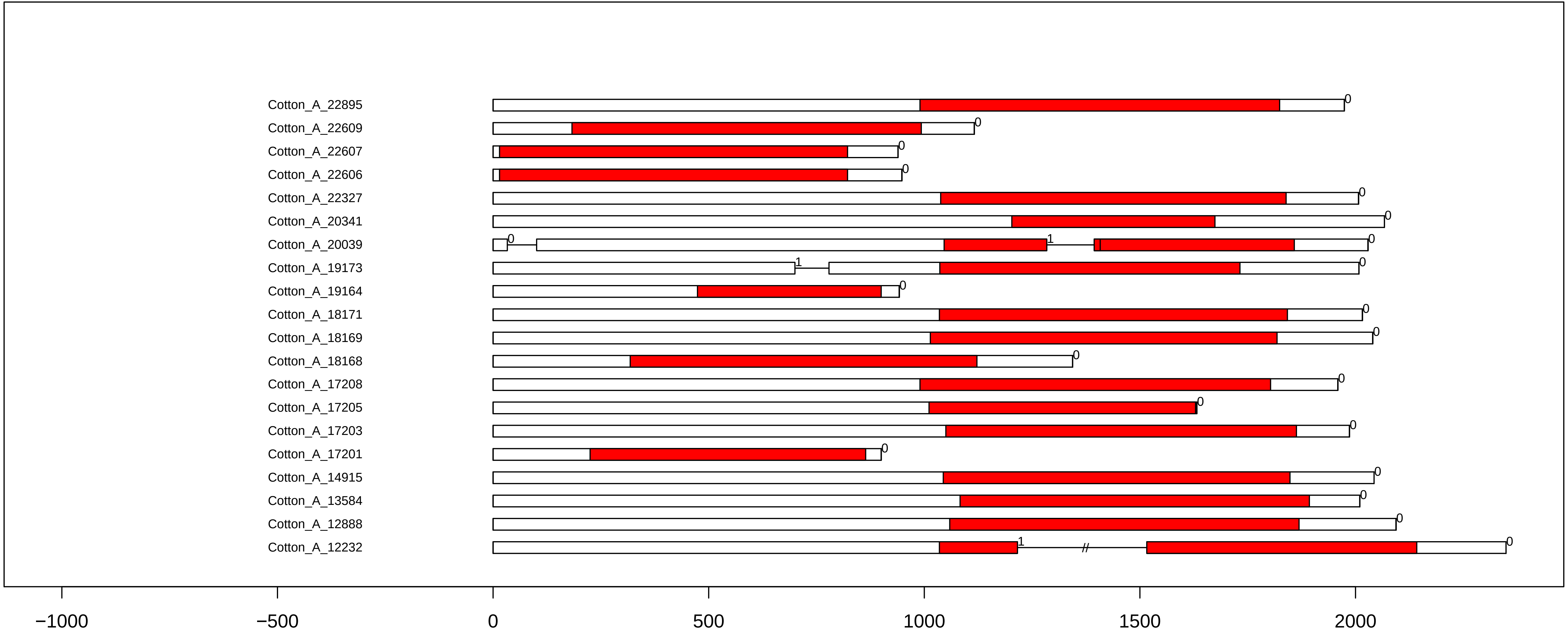

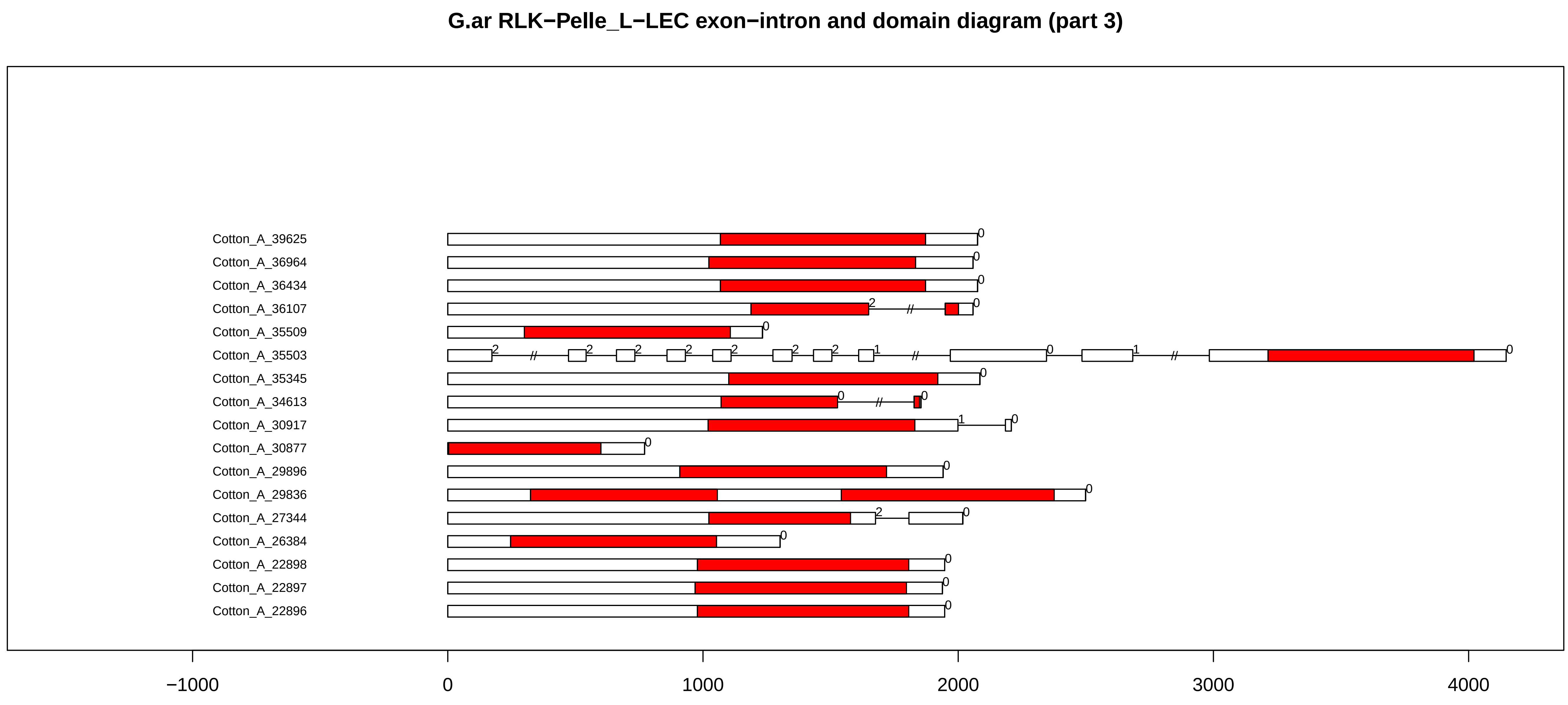

G.ar RLK−Pelle_LRK10L−2 exon−intron and domain diagram (part 1)

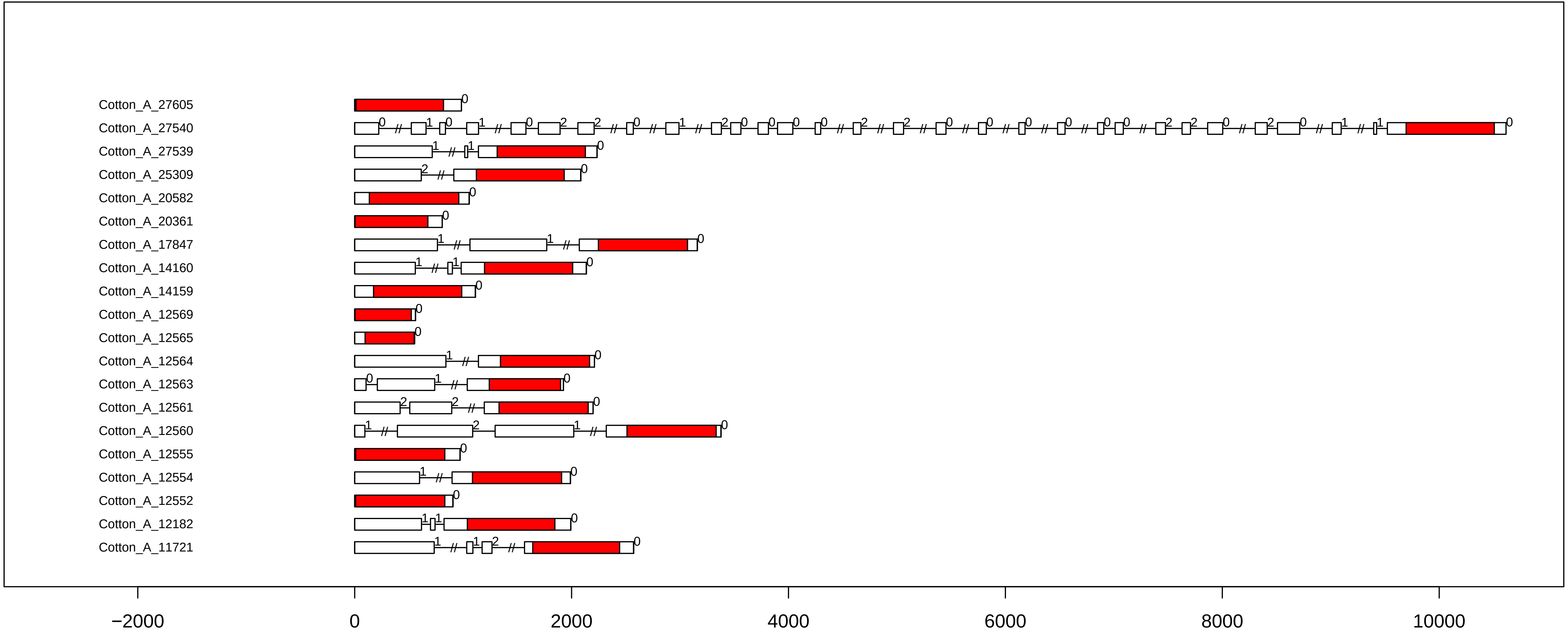

G.ar RLK−Pelle_LRK10L−2 exon−intron and domain diagram (part 2)

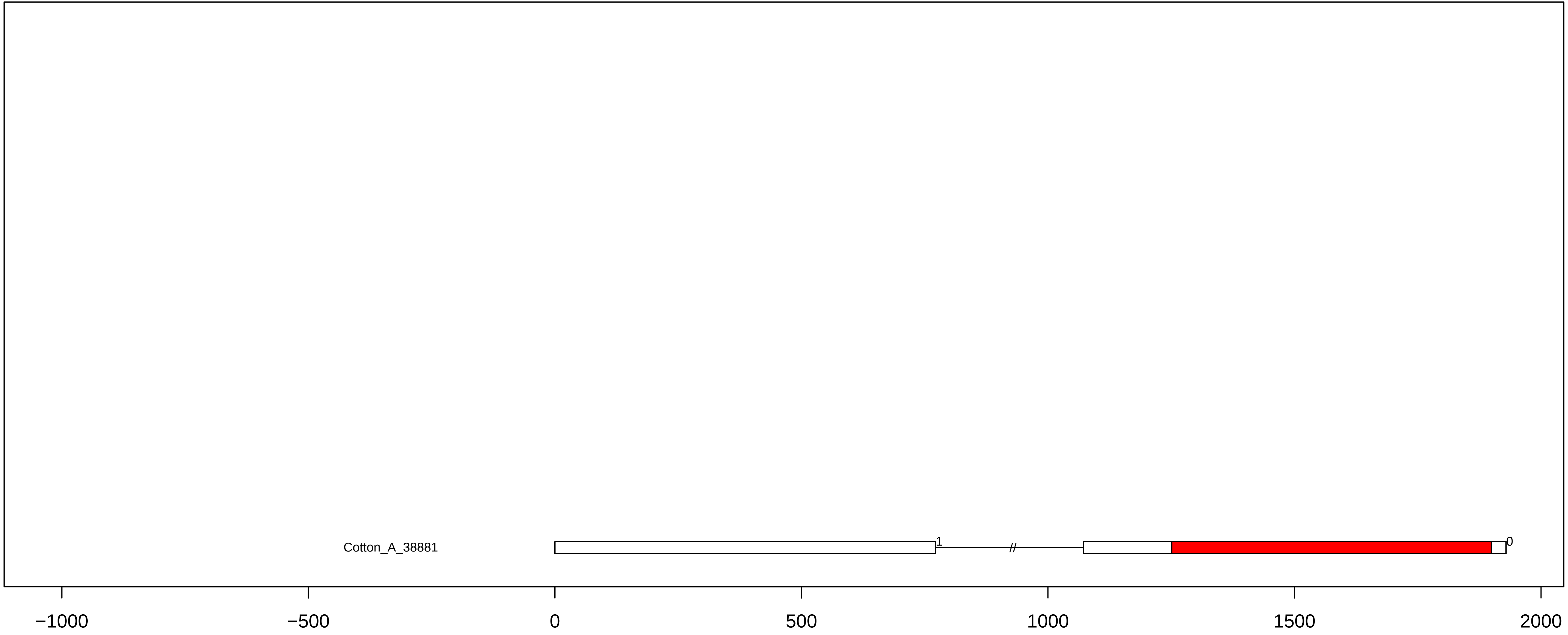

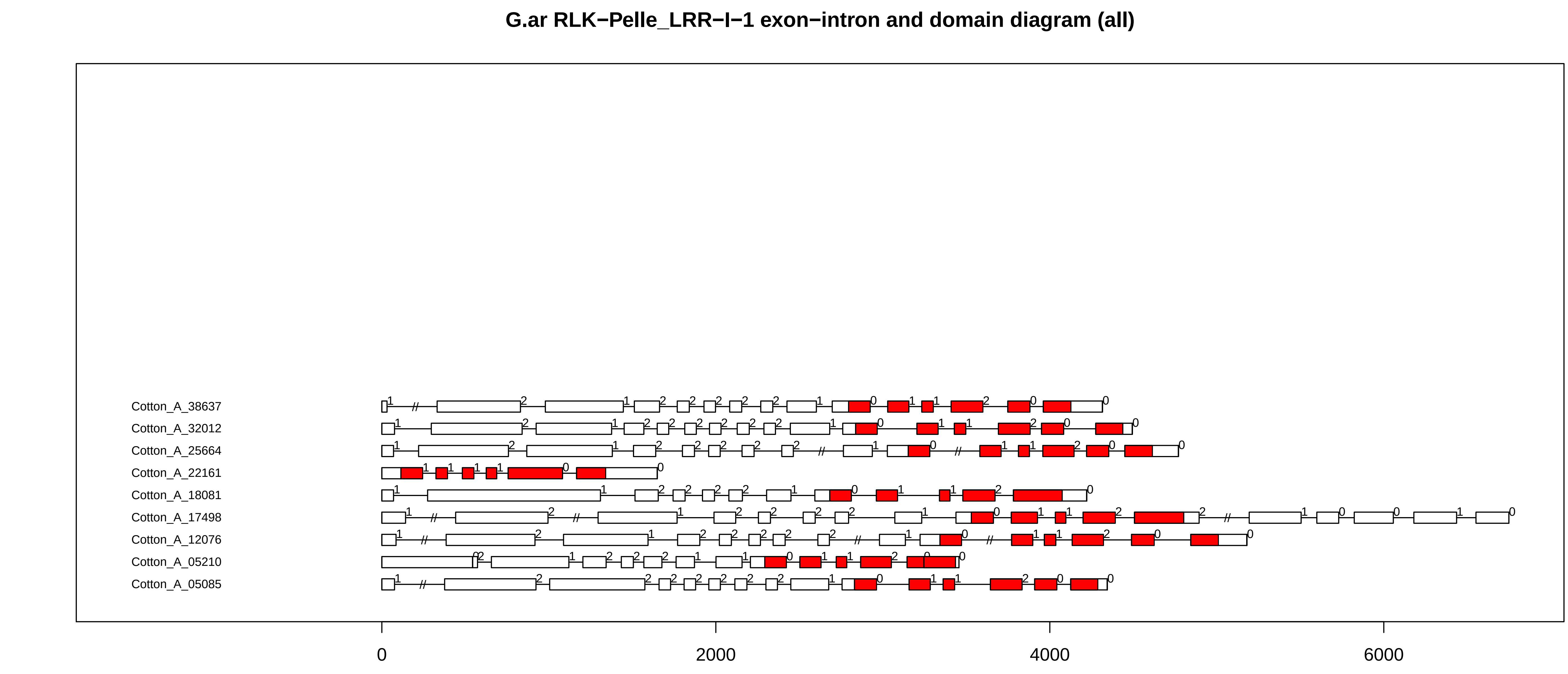
G.ar RLK−Pelle_LRR−I−1 exon−intron and domain diagram (all)
Cotton_A_38637
Cotton_A_32012
Cotton_A_25664
Cotton_A_22161
Cotton_A_18081
Cotton_A_17498
Cotton_A_12076
Cotton_A_05210
Cotton_A_05085
0
2000
4000
6000

G.ar RLK−Pelle_LRR−I−2 exon−intron and domain diagram (all)

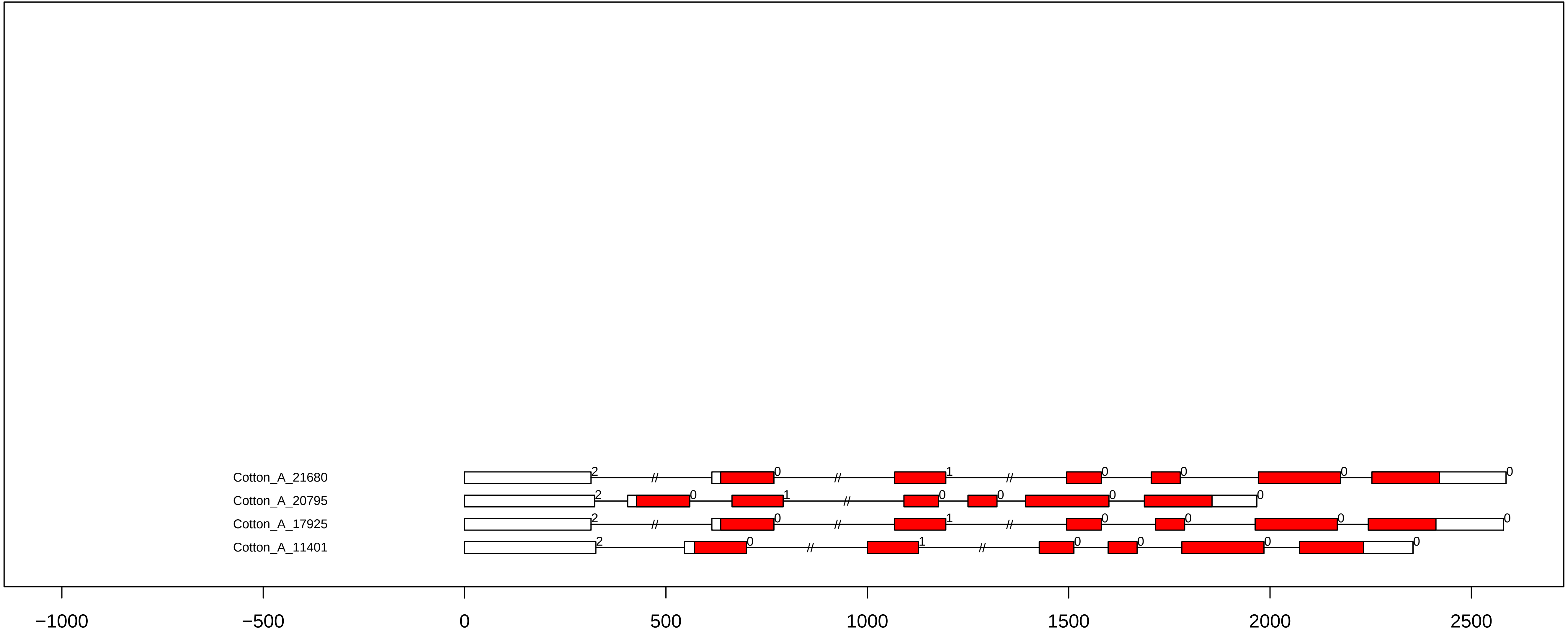

G.ar RLK−Pelle_LRR−III exon−intron and domain diagram (part 1)

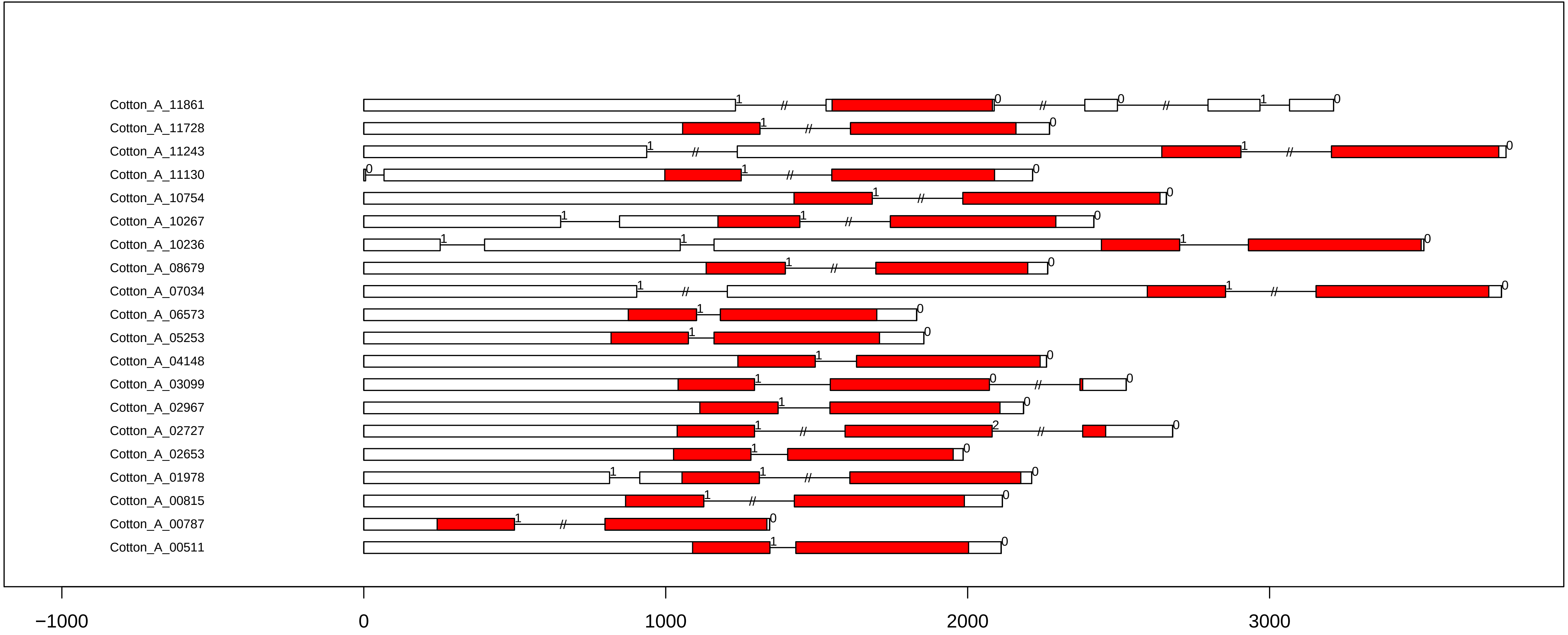

G.ar RLK−Pelle_LRR−III exon−intron and domain diagram (part 2)

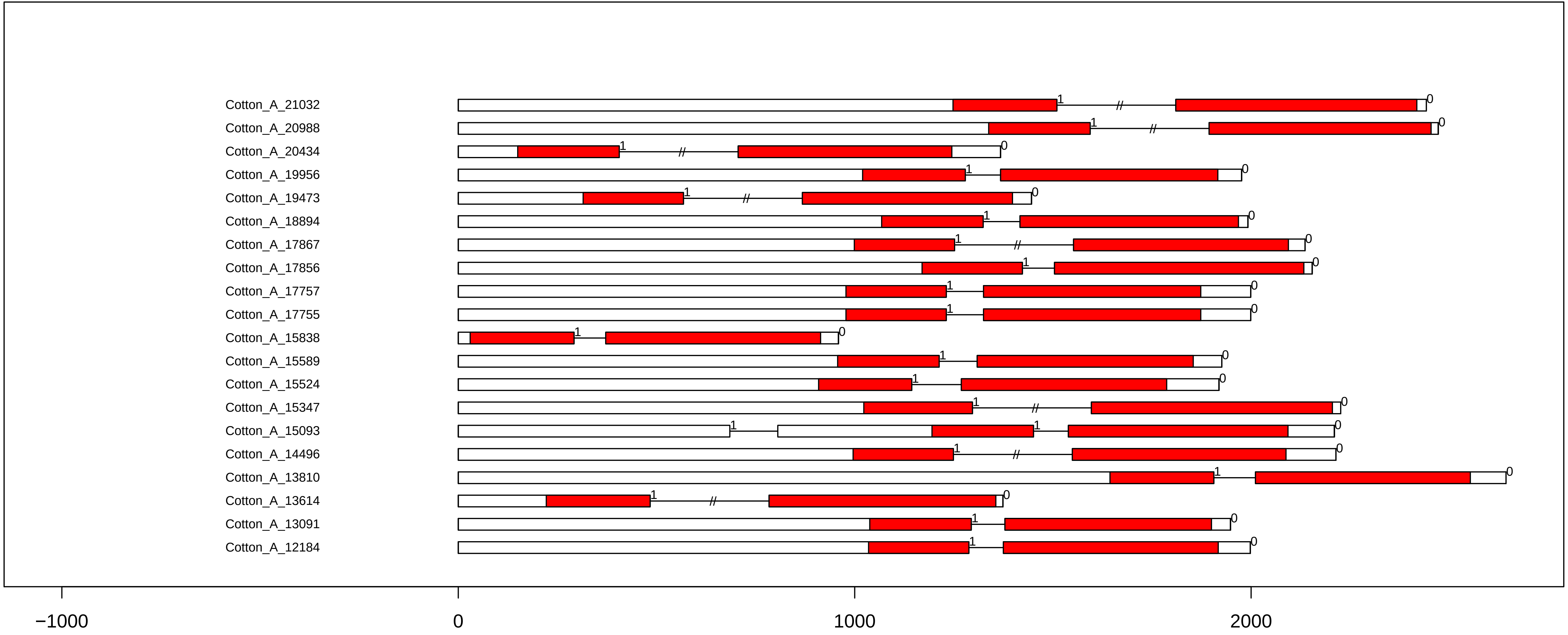

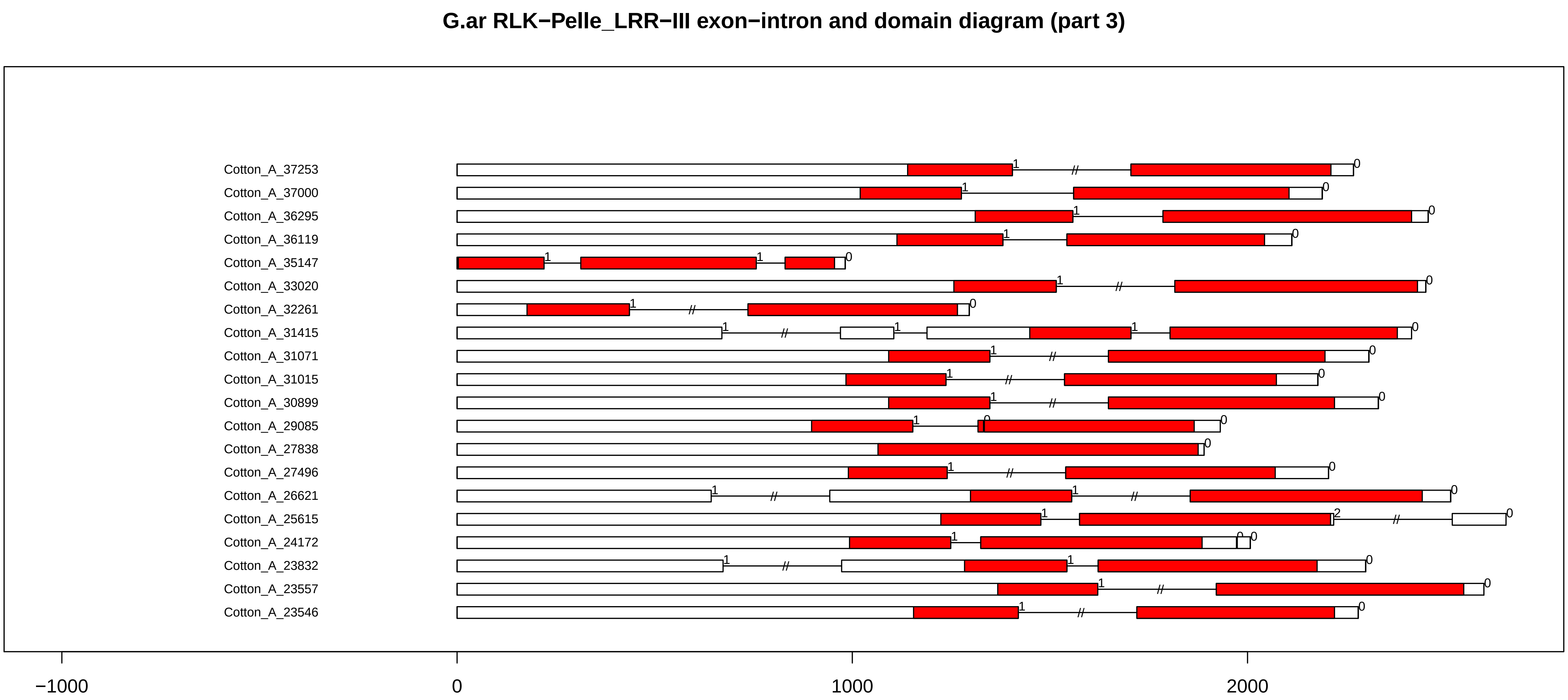

G.ar RLK−Pelle_LRR−III exon−intron and domain diagram (part 3)
Cotton_A_37253
Cotton_A_37000
Cotton_A_36295
Cotton_A_36119
Cotton_A_35147
Cotton_A_33020
Cotton_A_32261
Cotton_A_31415
Cotton_A_31071
Cotton_A_31015
Cotton_A_30899
Cotton_A_29085
Cotton_A_27838
Cotton_A_27496
Cotton_A_26621
Cotton_A_25615
Cotton_A_24172
Cotton_A_23832
Cotton_A_23557
Cotton_A_23546
−1000
0
1000
2000

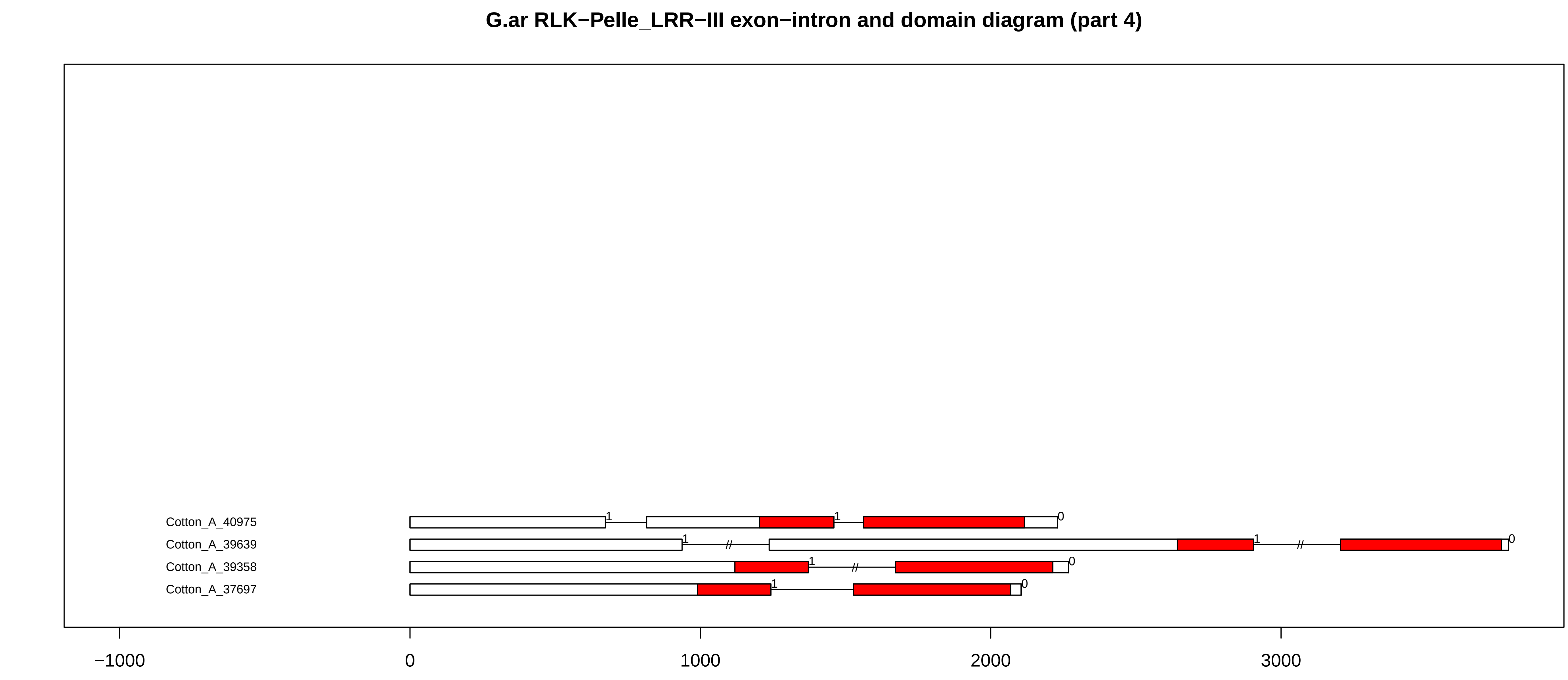

G.ar RLK−Pelle_LRR−III exon−intron and domain diagram (part 4)
Cotton_A_40975
Cotton_A_39639
Cotton_A_39358
Cotton_A_37697
1
1
0
1
1
0
1
0
1
0
−1000
0
1000
2000
3000

G.ar RLK−Pelle_LRR−II exon−intron and domain diagram (all)

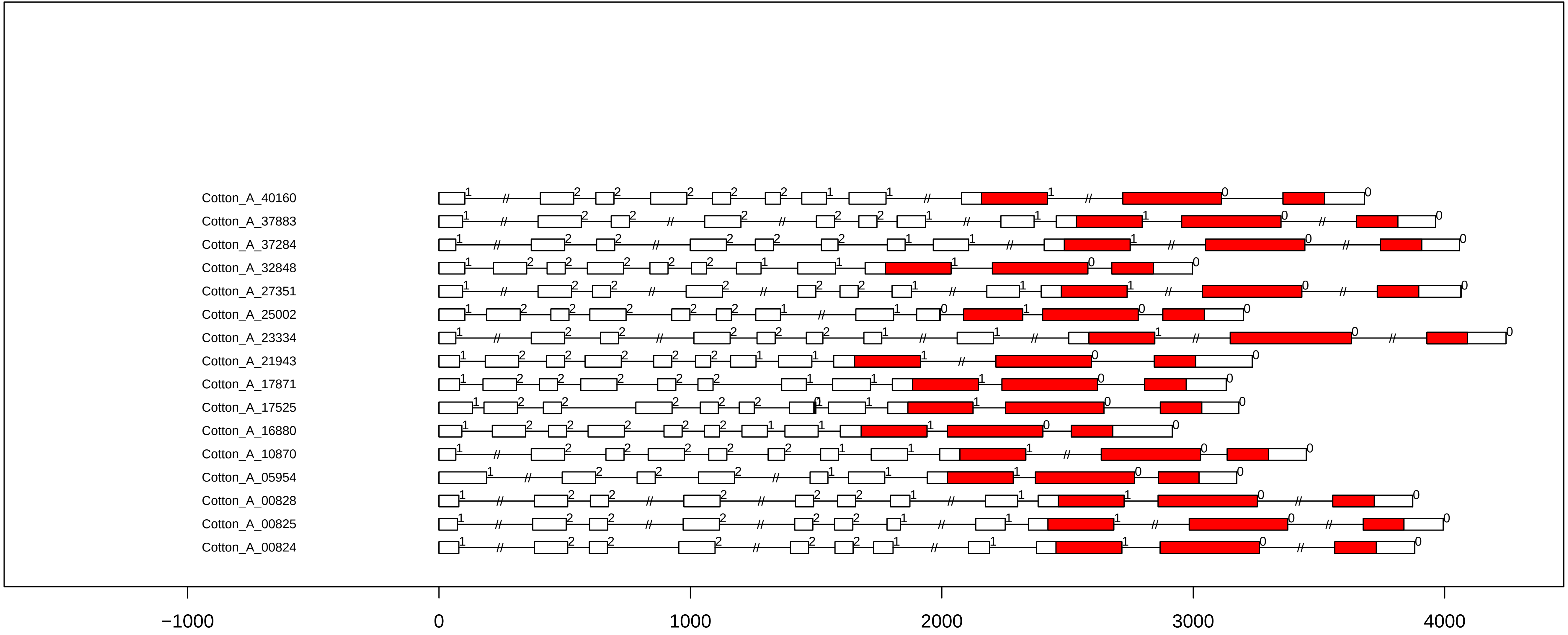

G.ar RLK−Pelle_LRR−IV exon−intron and domain diagram (all)

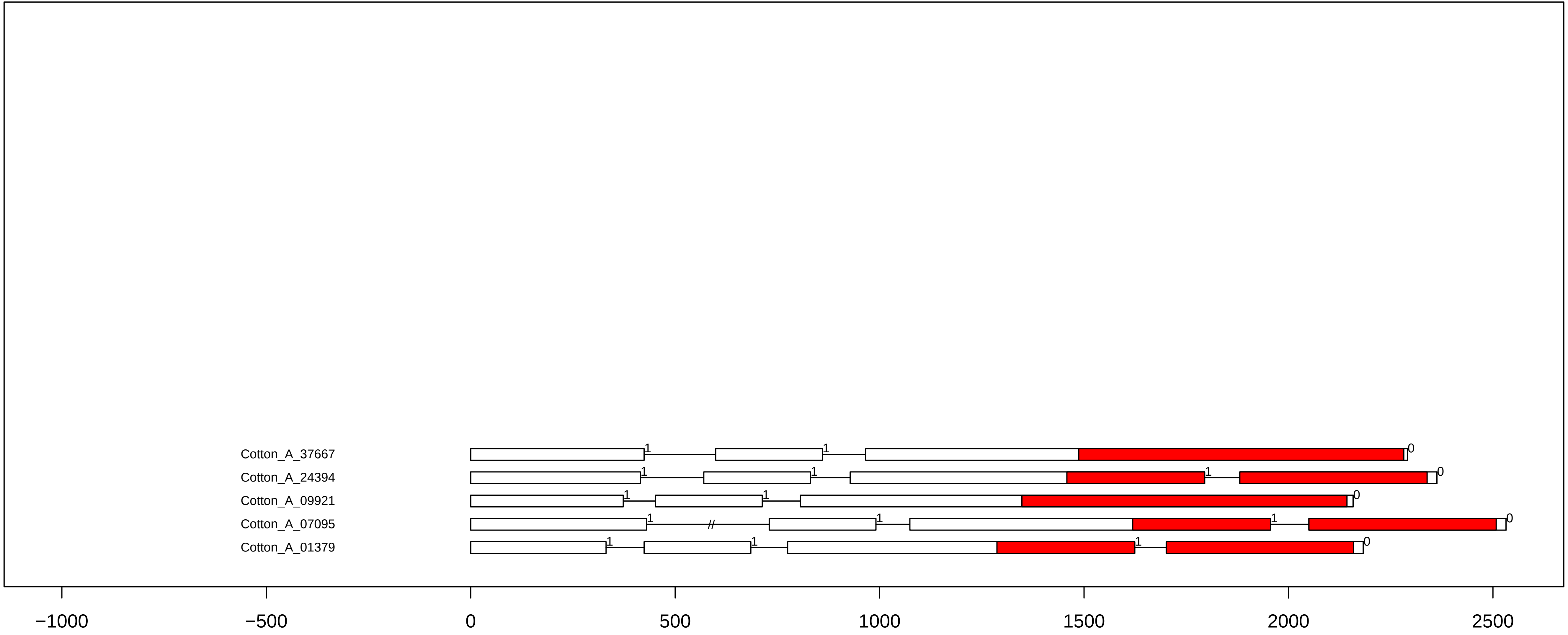

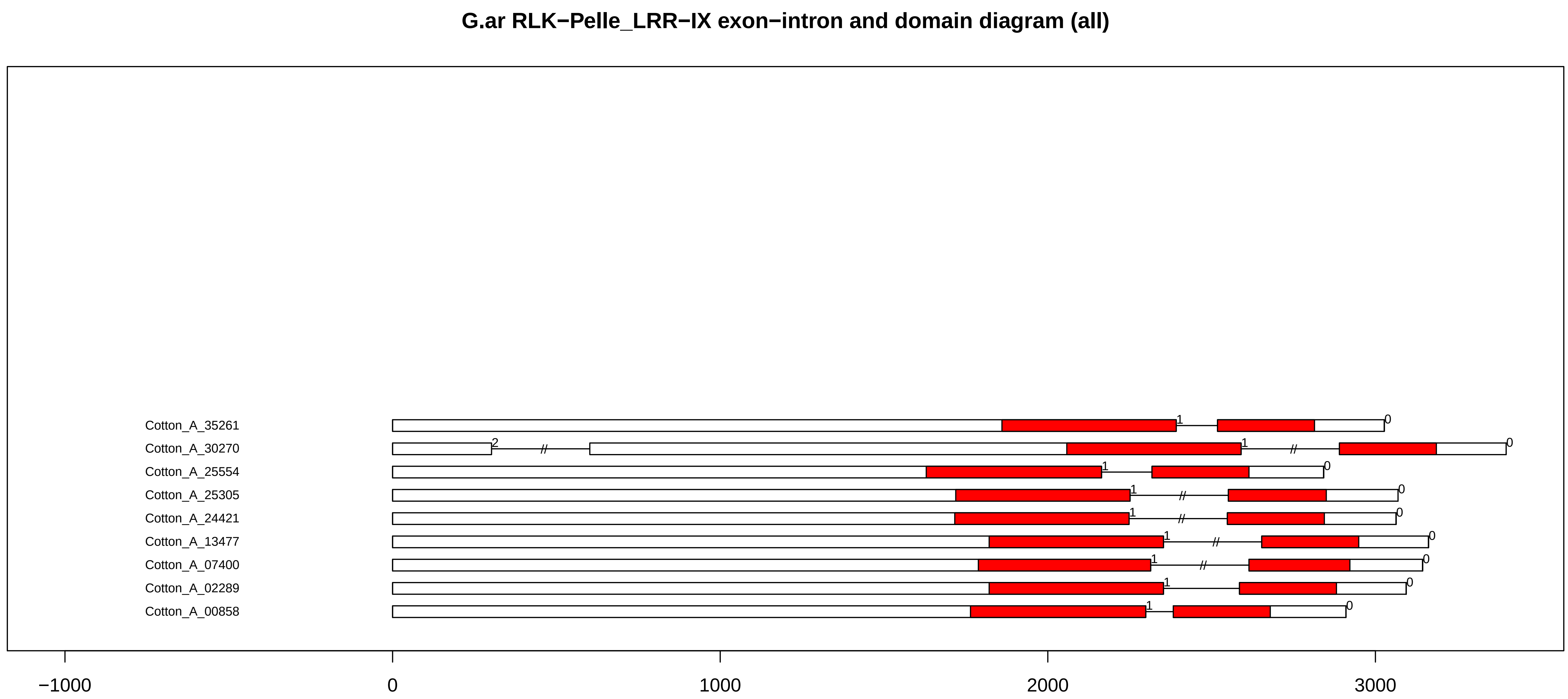
G.ar RLK−Pelle_LRR−IX exon−intron and domain diagram (all)
Cotton_A_35261
Cotton_A_30270
Cotton_A_25554
Cotton_A_25305
Cotton_A_24421
Cotton_A_13477
Cotton_A_07400
Cotton_A_02289
Cotton_A_00858
−1000
0
1000
2000
3000

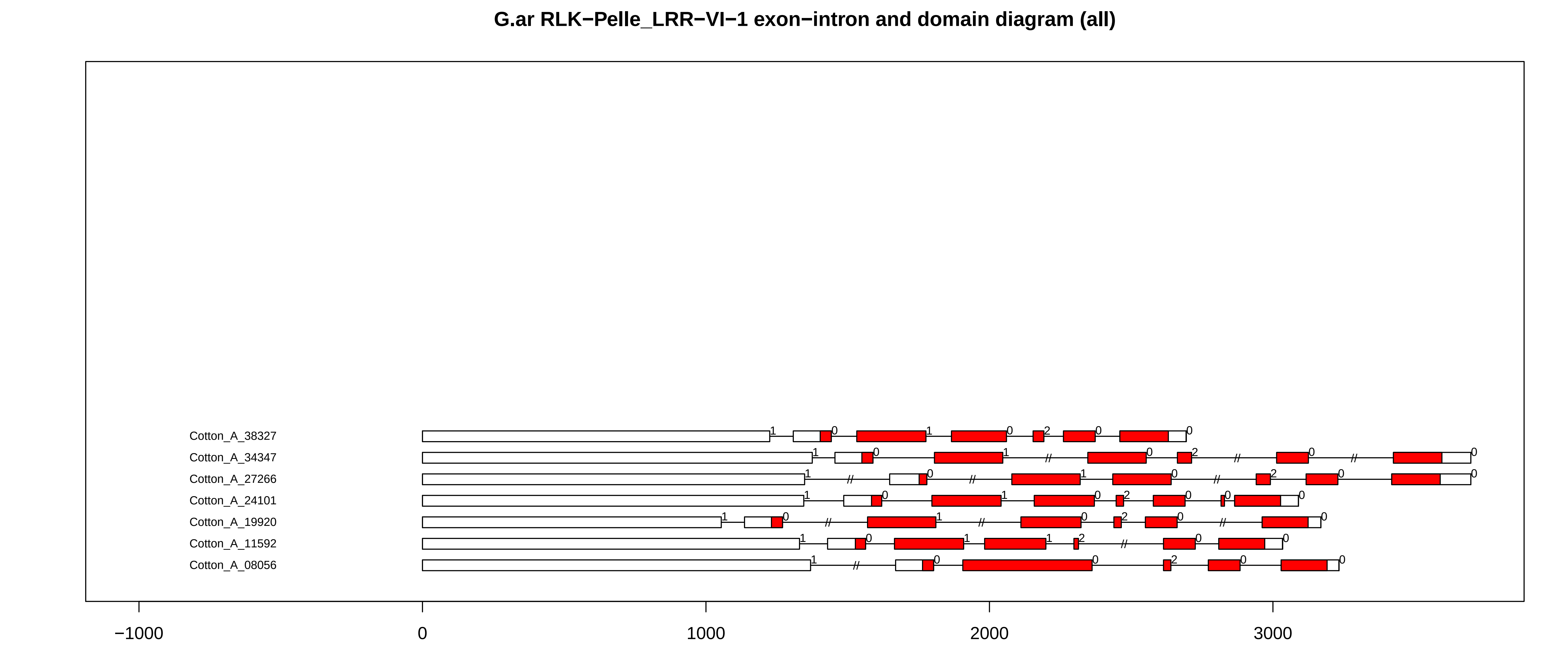
G.ar RLK−Pelle_LRR−VI−1 exon−intron and domain diagram (all)
Cotton_A_38327
Cotton_A_34347
Cotton_A_27266
Cotton_A_24101
Cotton_A_19920
Cotton_A_11592
Cotton_A_08056
−1000
0
1000
2000
3000

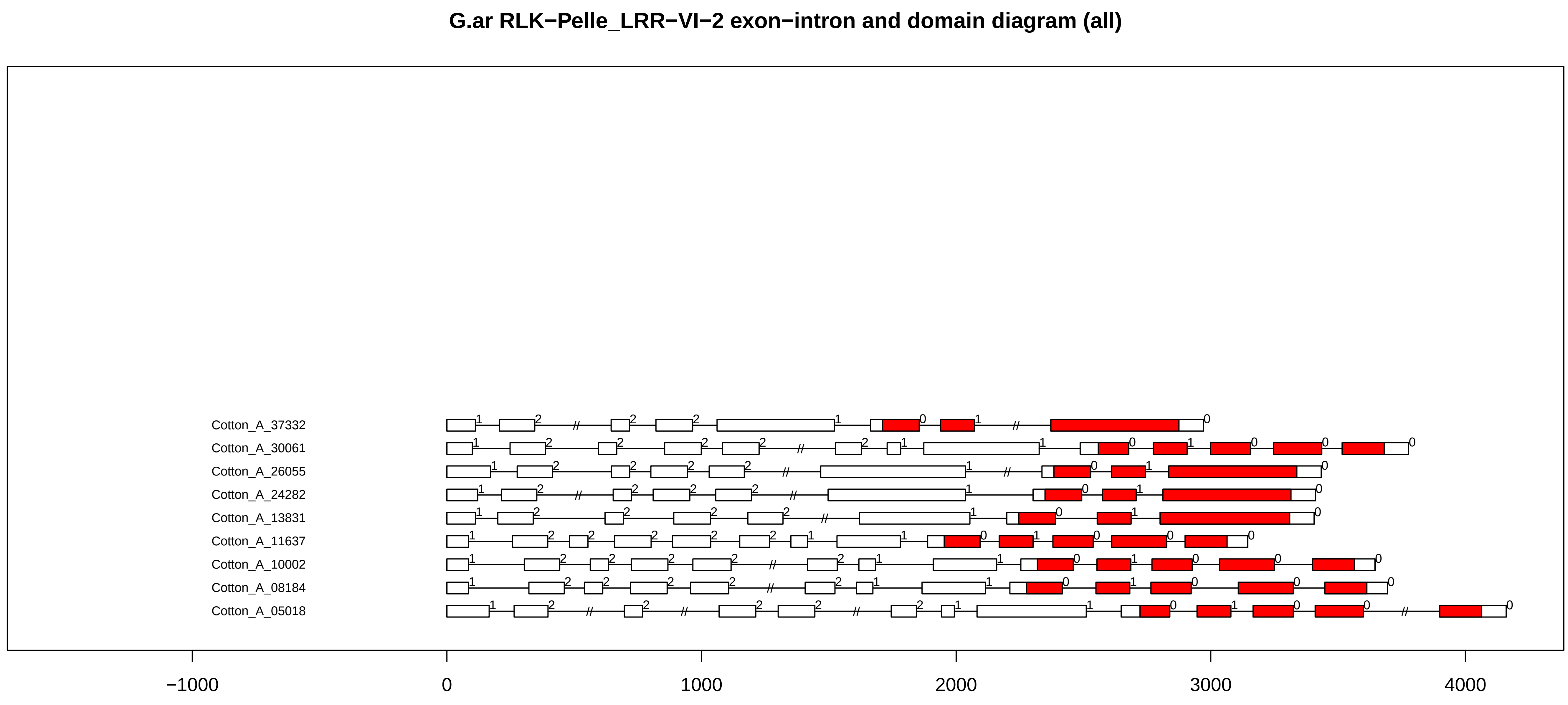
G.ar RLK−Pelle_LRR−VI−2 exon−intron and domain diagram (all)
Cotton_A_37332
Cotton_A_30061
Cotton_A_26055
Cotton_A_24282
Cotton_A_13831
Cotton_A_11637
Cotton_A_10002
Cotton_A_08184
Cotton_A_05018
−1000
0
1000
2000
3000
4000

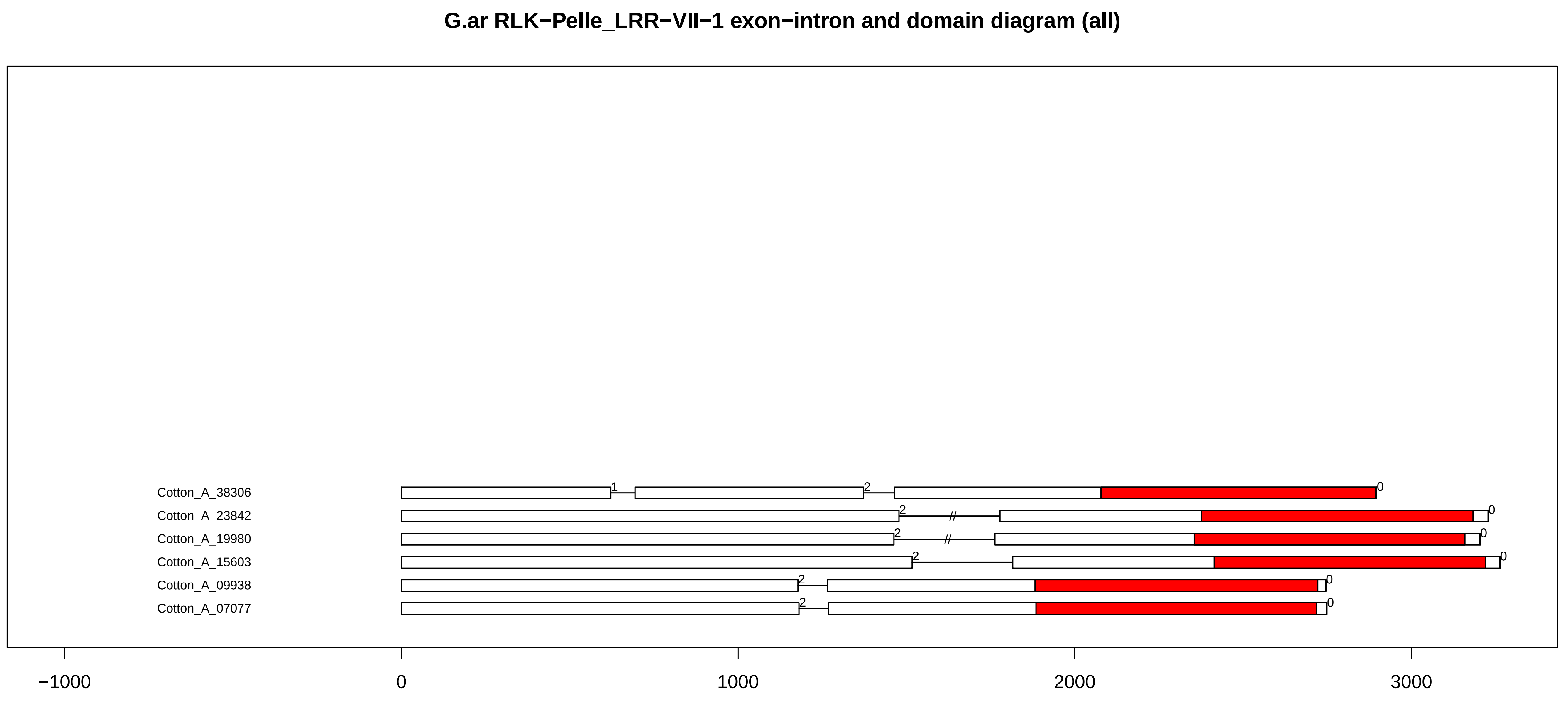
G.ar RLK−Pelle_LRR−VII−1 exon−intron and domain diagram (all)
Cotton_A_38306
Cotton_A_23842
Cotton_A_19980
Cotton_A_15603
Cotton_A_09938
Cotton_A_07077
−1000
0
1000
2000
3000

G.ar RLK−Pelle_LRR−VII−2 exon−intron and domain diagram (all)

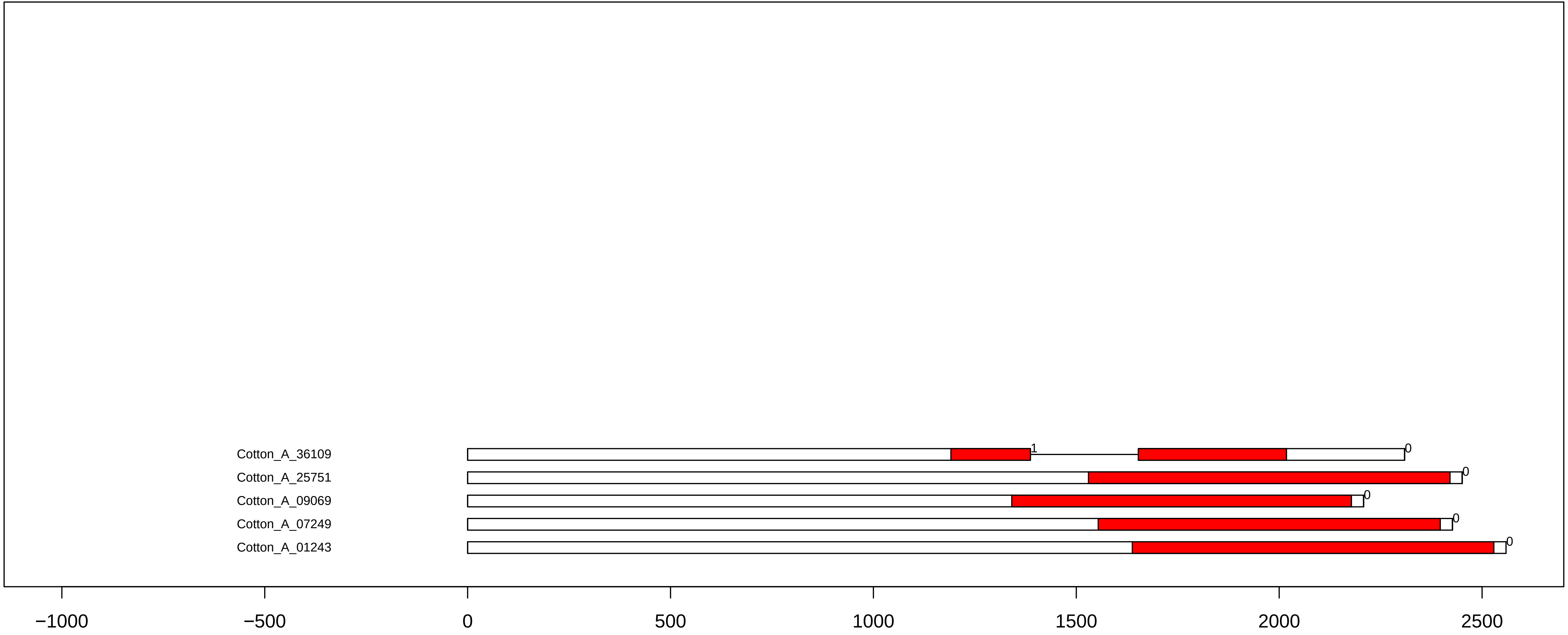

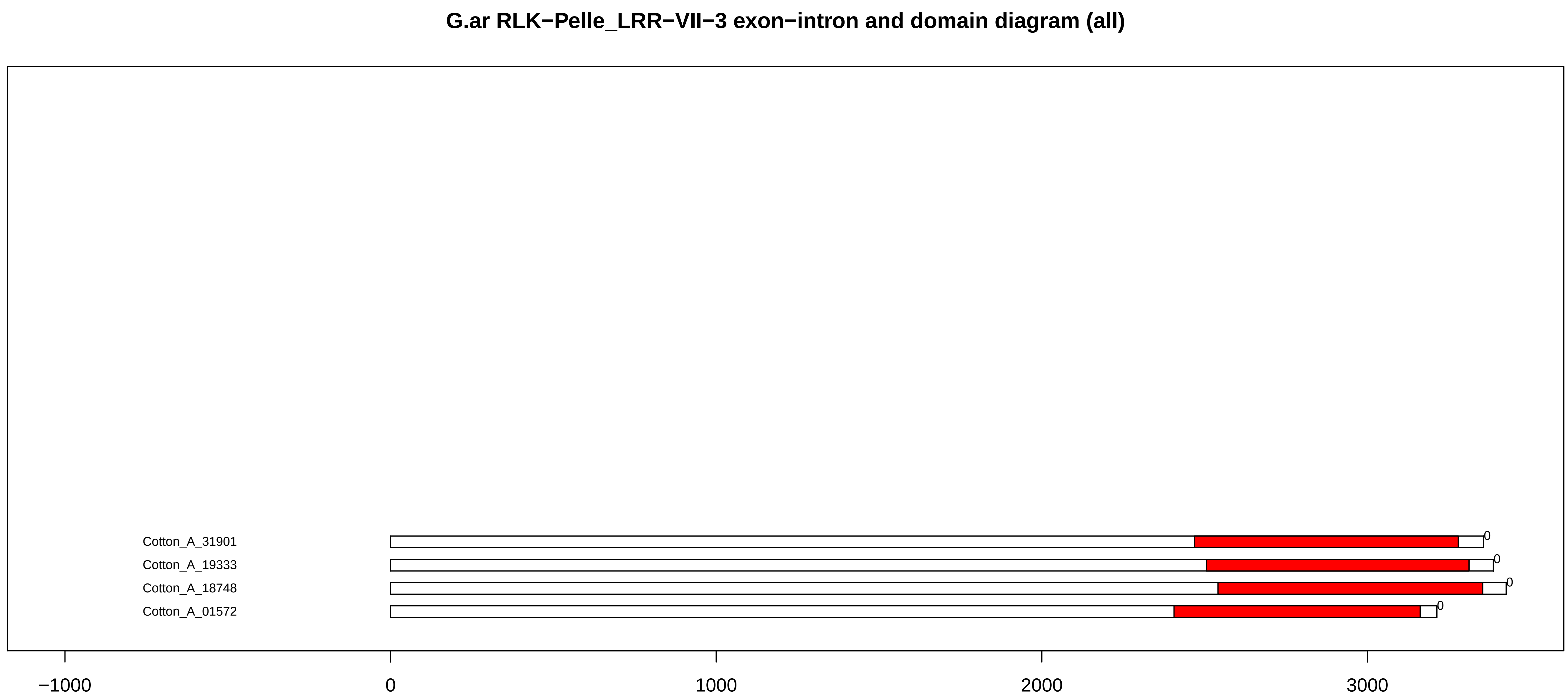
G.ar RLK−Pelle_LRR−VII−3 exon−intron and domain diagram (all)
Cotton_A_31901
Cotton_A_19333
Cotton_A_18748
Cotton_A_01572
0
0
0
0
−1000
0
1000
2000
3000

G.ar RLK−Pelle_LRR−VIII−1 exon−intron and domain diagram (all)

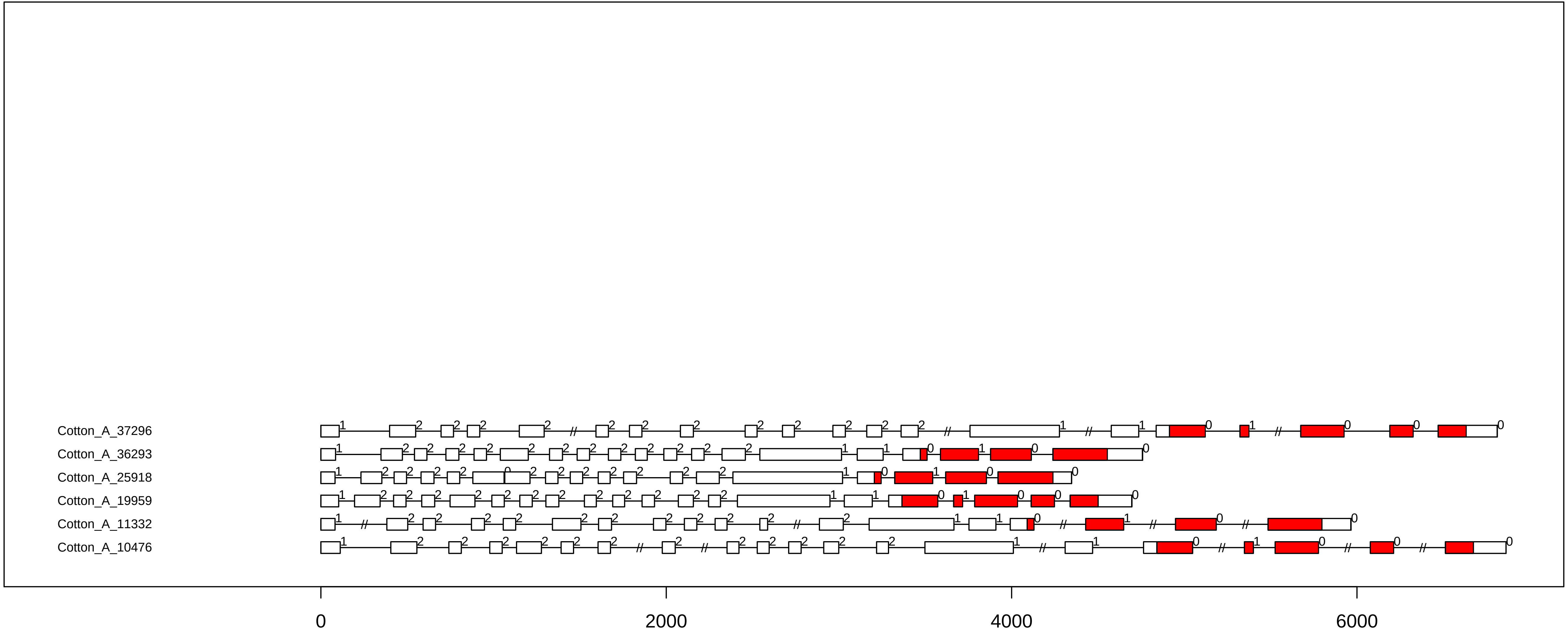

G.ar RLK−Pelle_LRR−V exon−intron and domain diagram (all)

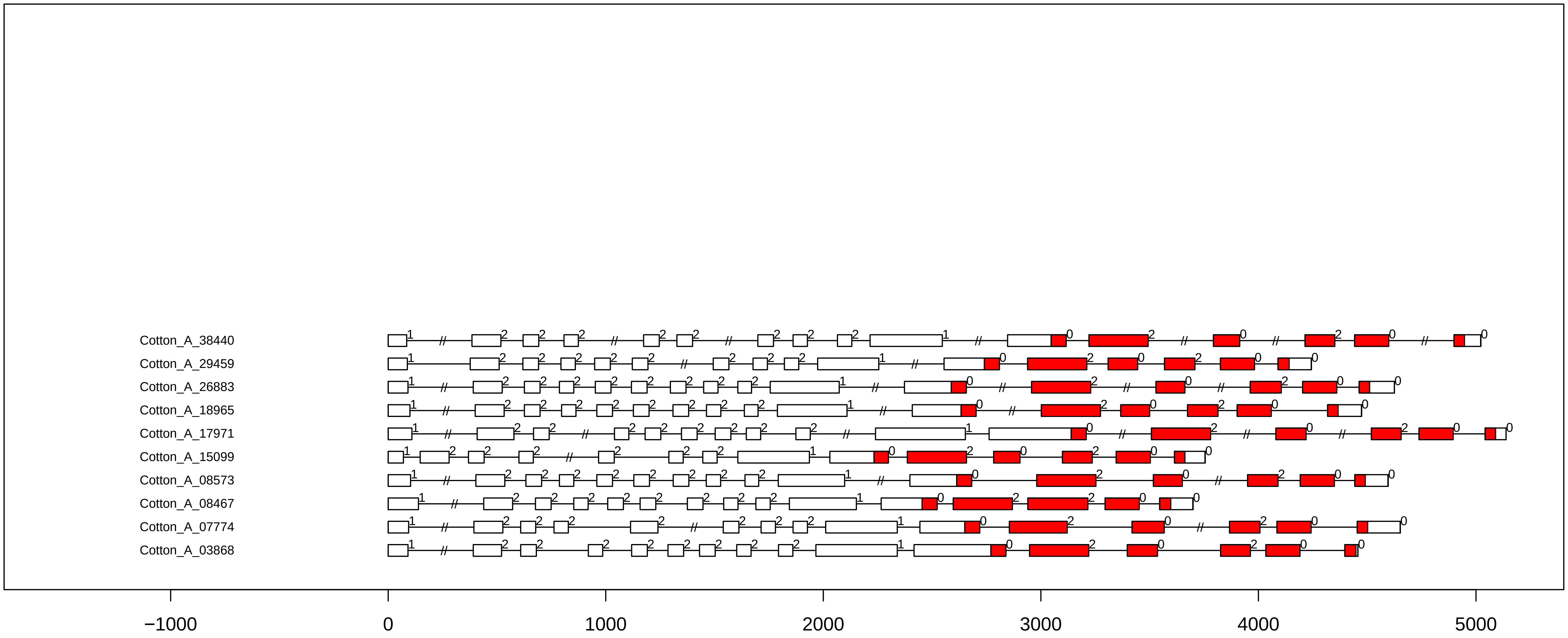

G.ar RLK−Pelle_LRR−Xa exon−intron and domain diagram (all)

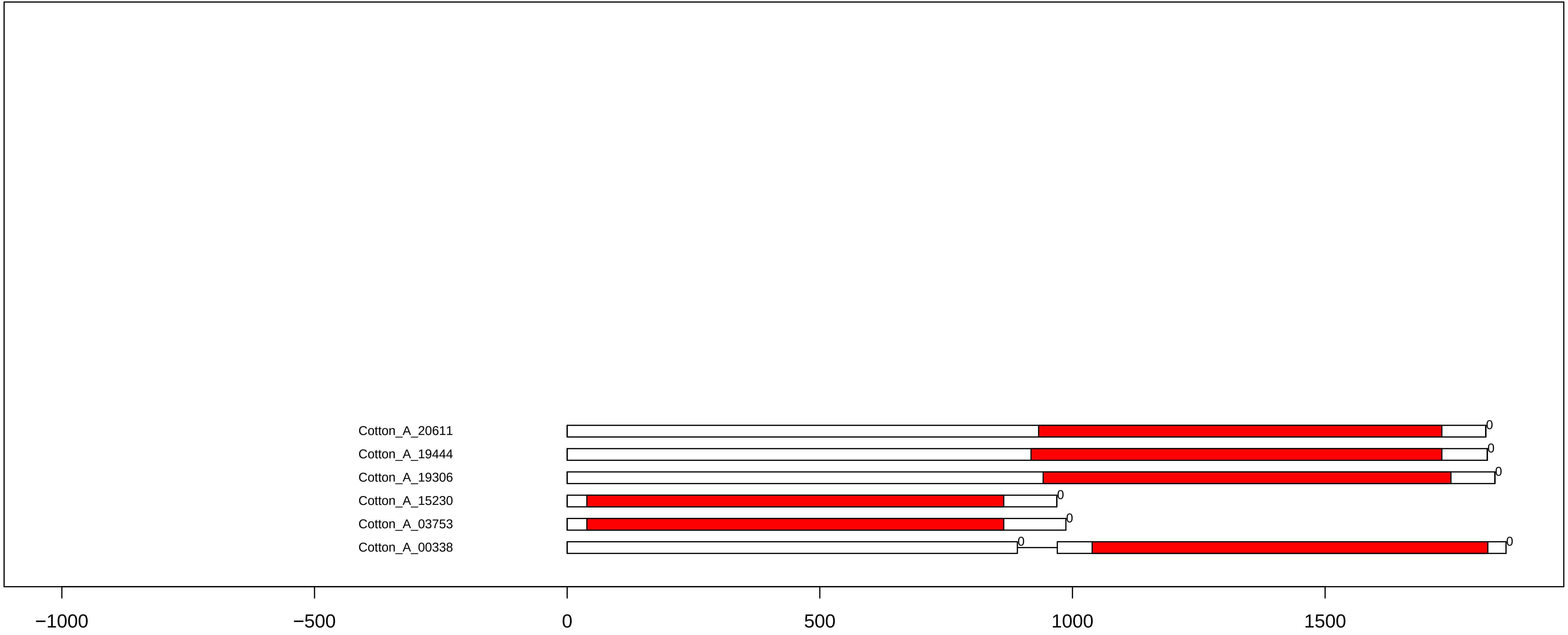

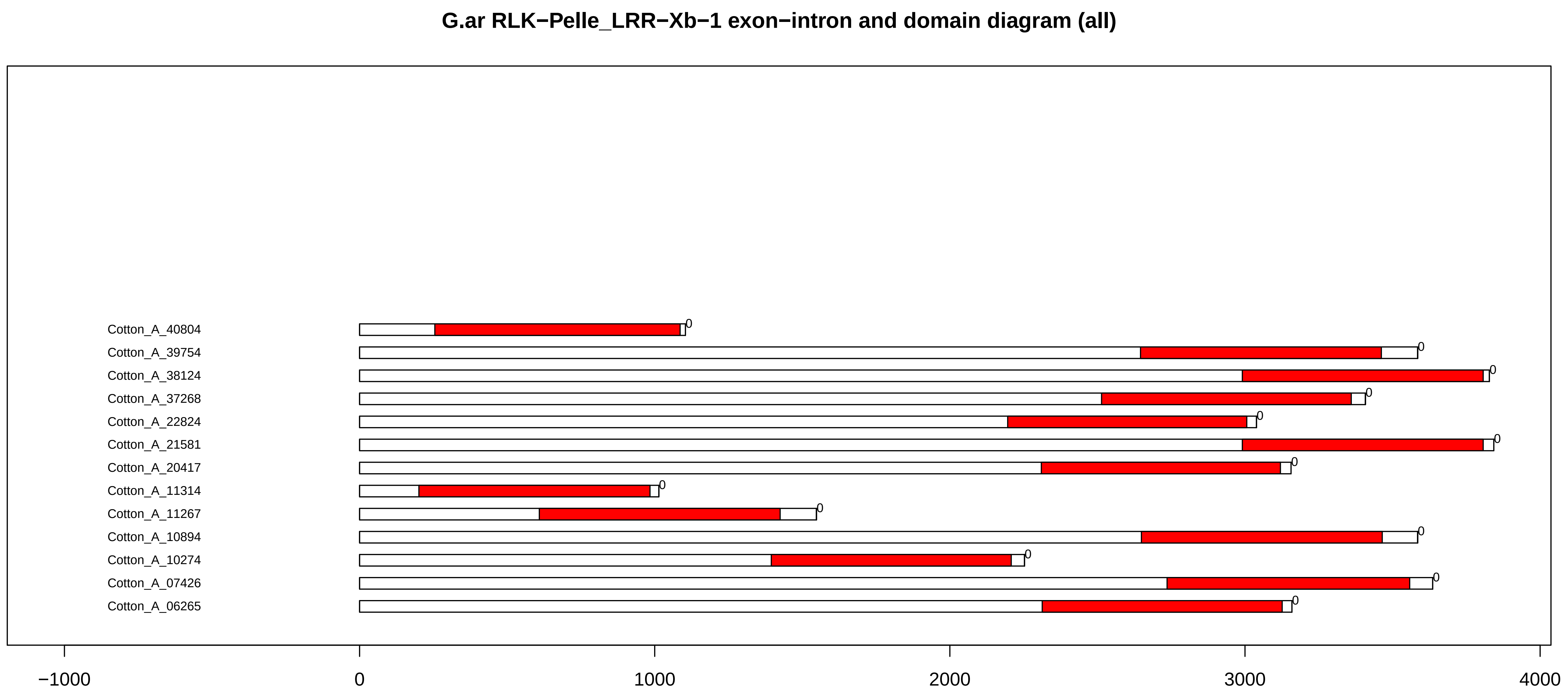
G.ar RLK−Pelle_LRR−Xb−1 exon−intron and domain diagram (all)
Cotton_A_40804
Cotton_A_39754
Cotton_A_38124
Cotton_A_37268
Cotton_A_22824
Cotton_A_21581
Cotton_A_20417
Cotton_A_11314
Cotton_A_11267
Cotton_A_10894
Cotton_A_10274
Cotton_A_07426
Cotton_A_06265
0
0
0
0
0
0
0
0
0
0
0
0
0
−1000
0
1000
2000
3000
4000

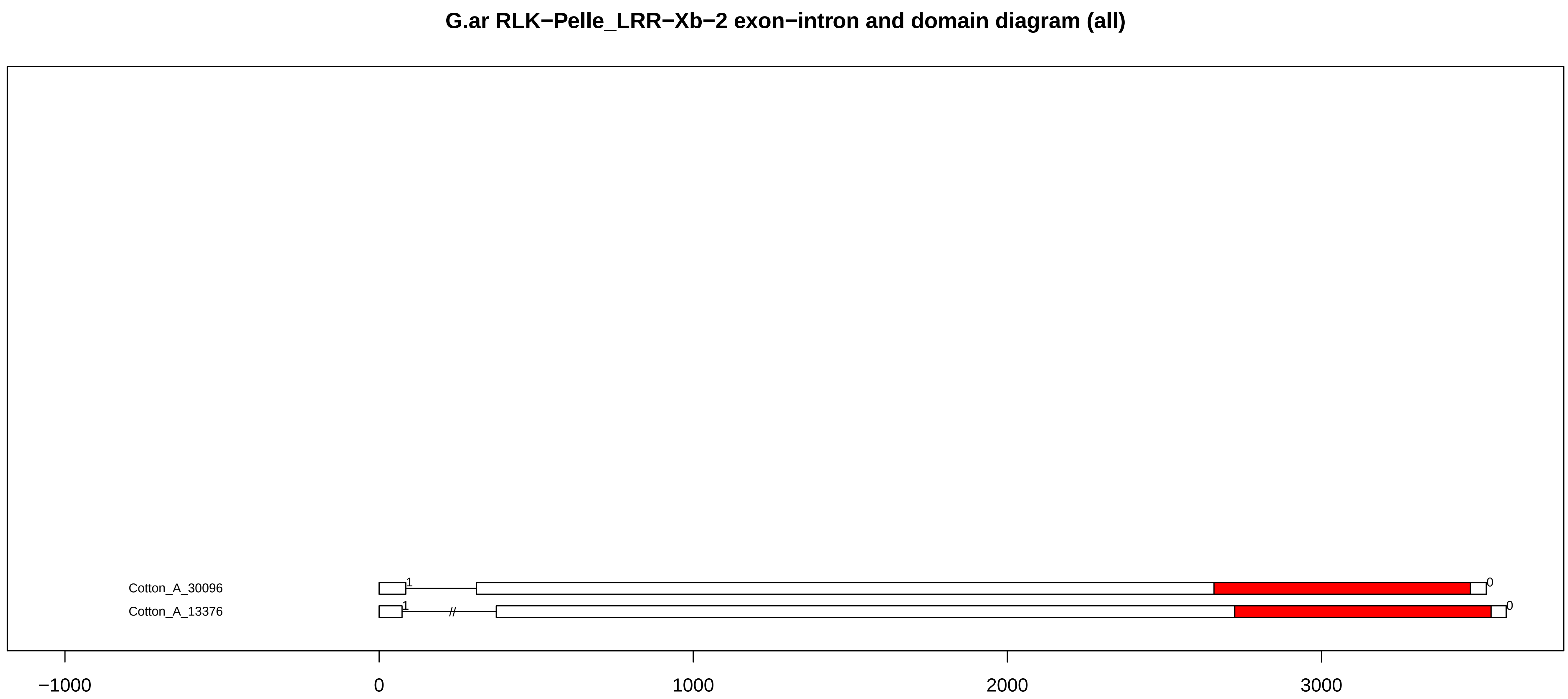
G.ar RLK−Pelle_LRR−Xb−2 exon−intron and domain diagram (all)
Cotton_A_30096
Cotton_A_13376
1
1
0
0
−1000
0
1000
2000
3000

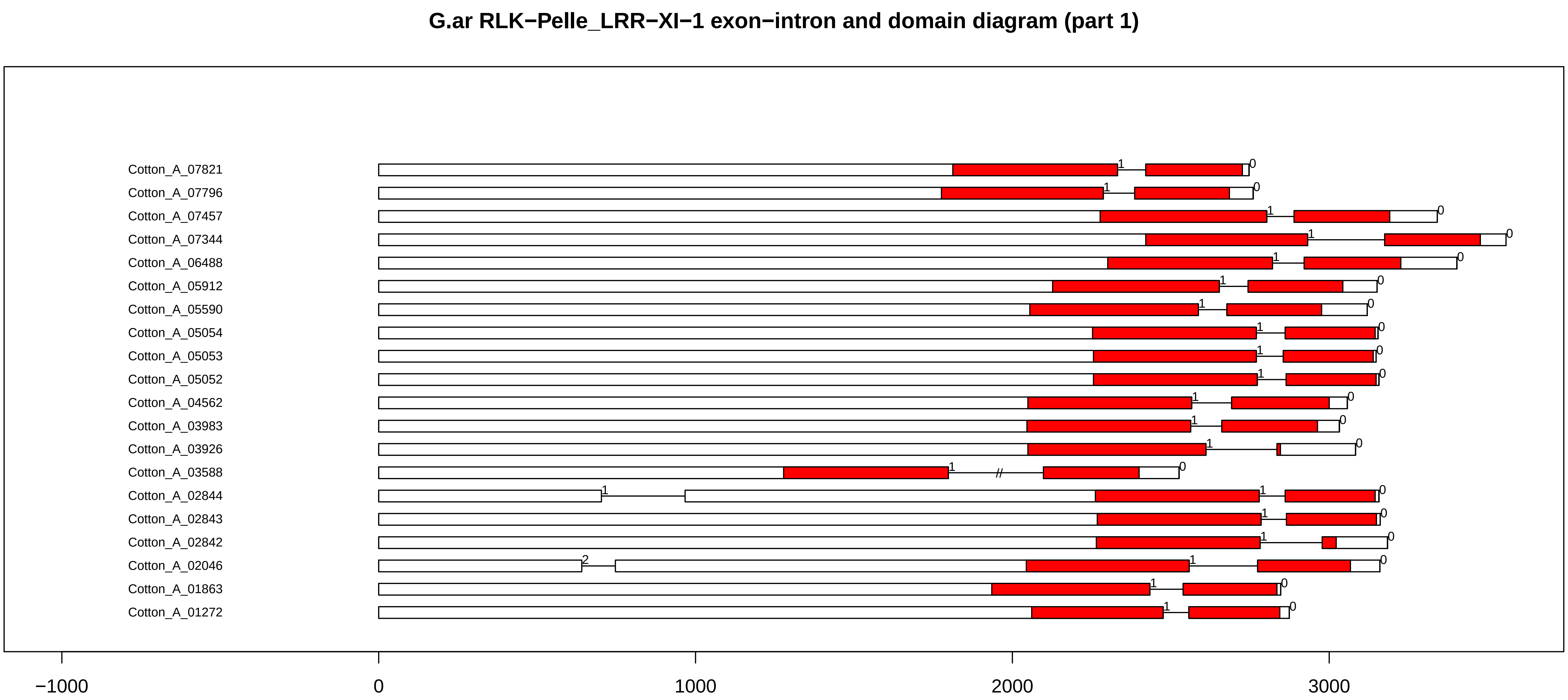
G.ar RLK−Pelle_LRR−XI−1 exon−intron and domain diagram (part 1)
Cotton_A_07821
Cotton_A_07796
Cotton_A_07457
Cotton_A_07344
Cotton_A_06488
Cotton_A_05912
Cotton_A_05590
Cotton_A_05054
Cotton_A_05053
Cotton_A_05052
Cotton_A_04562
Cotton_A_03983
Cotton_A_03926
Cotton_A_03588
Cotton_A_02844
Cotton_A_02843
Cotton_A_02842
Cotton_A_02046
Cotton_A_01863
Cotton_A_01272
−1000
0
1000
2000
3000

G.ar RLK−Pelle_LRR−XI−1 exon−intron and domain diagram (part 2)

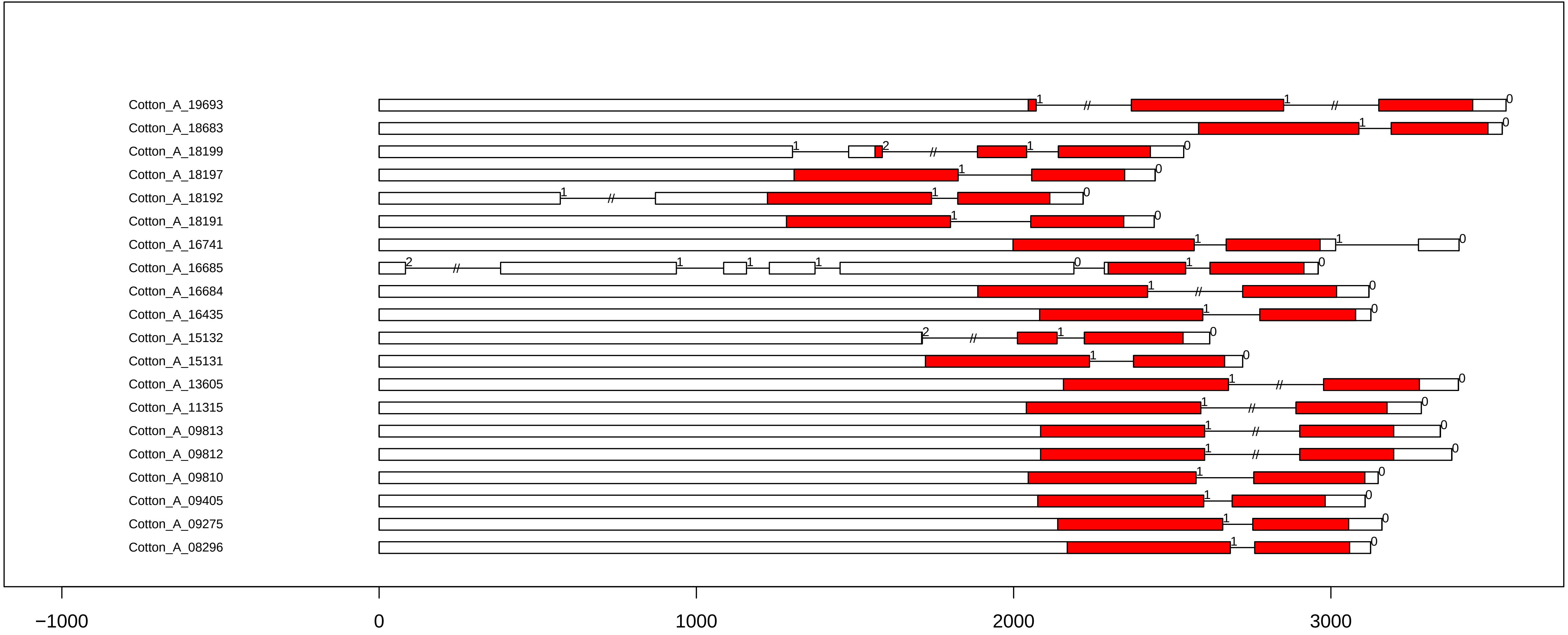

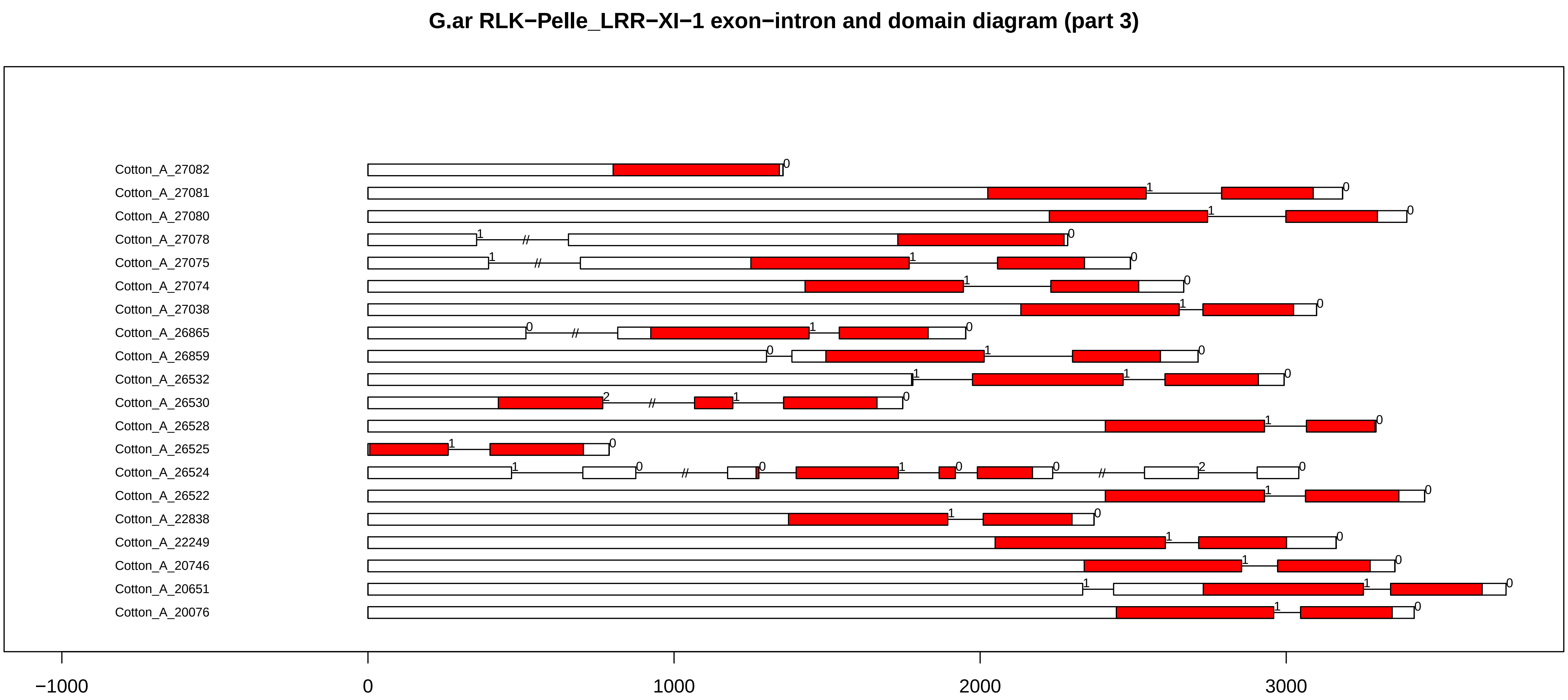
G.ar RLK−Pelle_LRR−XI−1 exon−intron and domain diagram (part 3)
Cotton_A_27082
Cotton_A_27081
Cotton_A_27080
Cotton_A_27078
Cotton_A_27075
Cotton_A_27074
Cotton_A_27038
Cotton_A_26865
Cotton_A_26859
Cotton_A_26532
Cotton_A_26530
Cotton_A_26528
Cotton_A_26525
Cotton_A_26524
Cotton_A_26522
Cotton_A_22838
Cotton_A_22249
Cotton_A_20746
Cotton_A_20651
Cotton_A_20076
−1000
0
1000
2000
3000

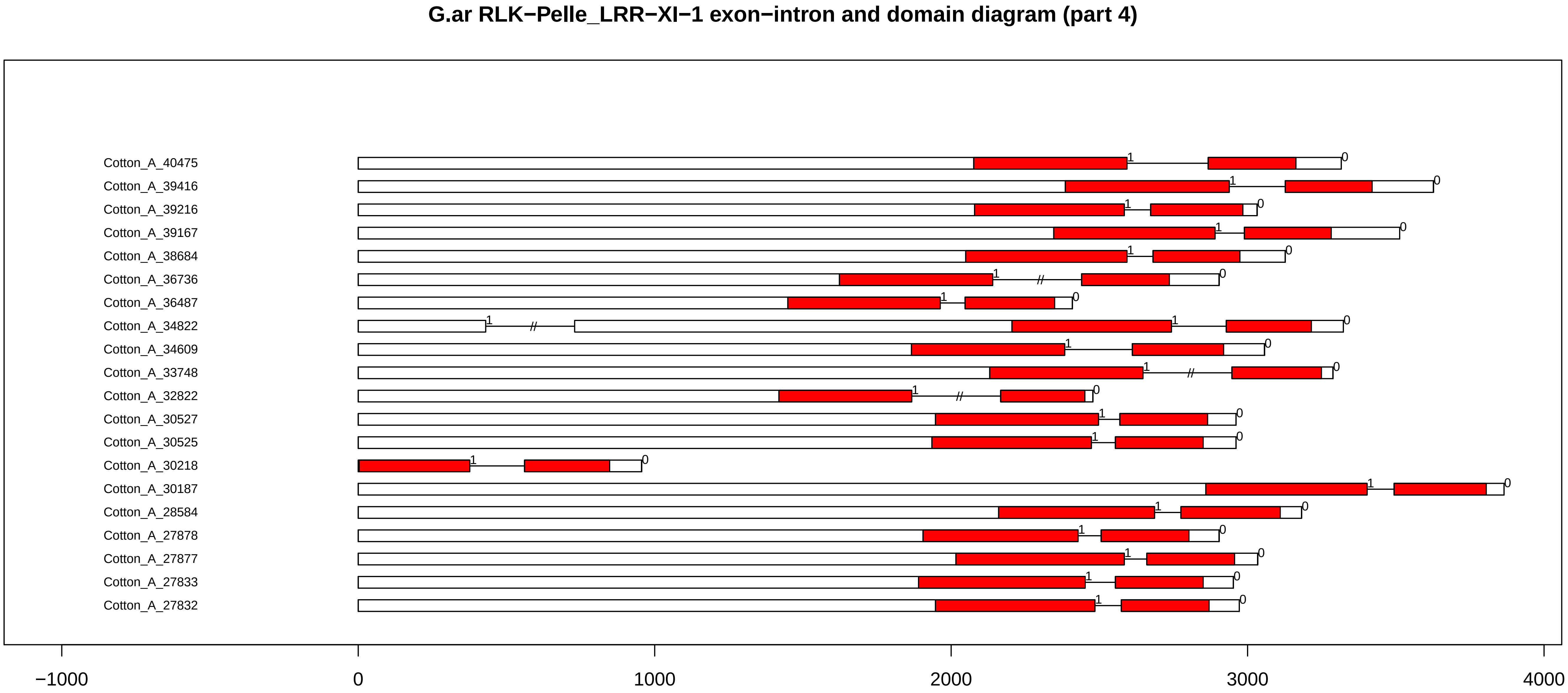
G.ar RLK−Pelle_LRR−XI−1 exon−intron and domain diagram (part 4)
Cotton_A_40475
Cotton_A_39416
Cotton_A_39216
Cotton_A_39167
Cotton_A_38684
Cotton_A_36736
Cotton_A_36487
Cotton_A_34822
Cotton_A_34609
Cotton_A_33748
Cotton_A_32822
Cotton_A_30527
Cotton_A_30525
Cotton_A_30218
Cotton_A_30187
Cotton_A_28584
Cotton_A_27878
Cotton_A_27877
Cotton_A_27833
Cotton_A_27832
−1000
0
1000
2000
3000
4000

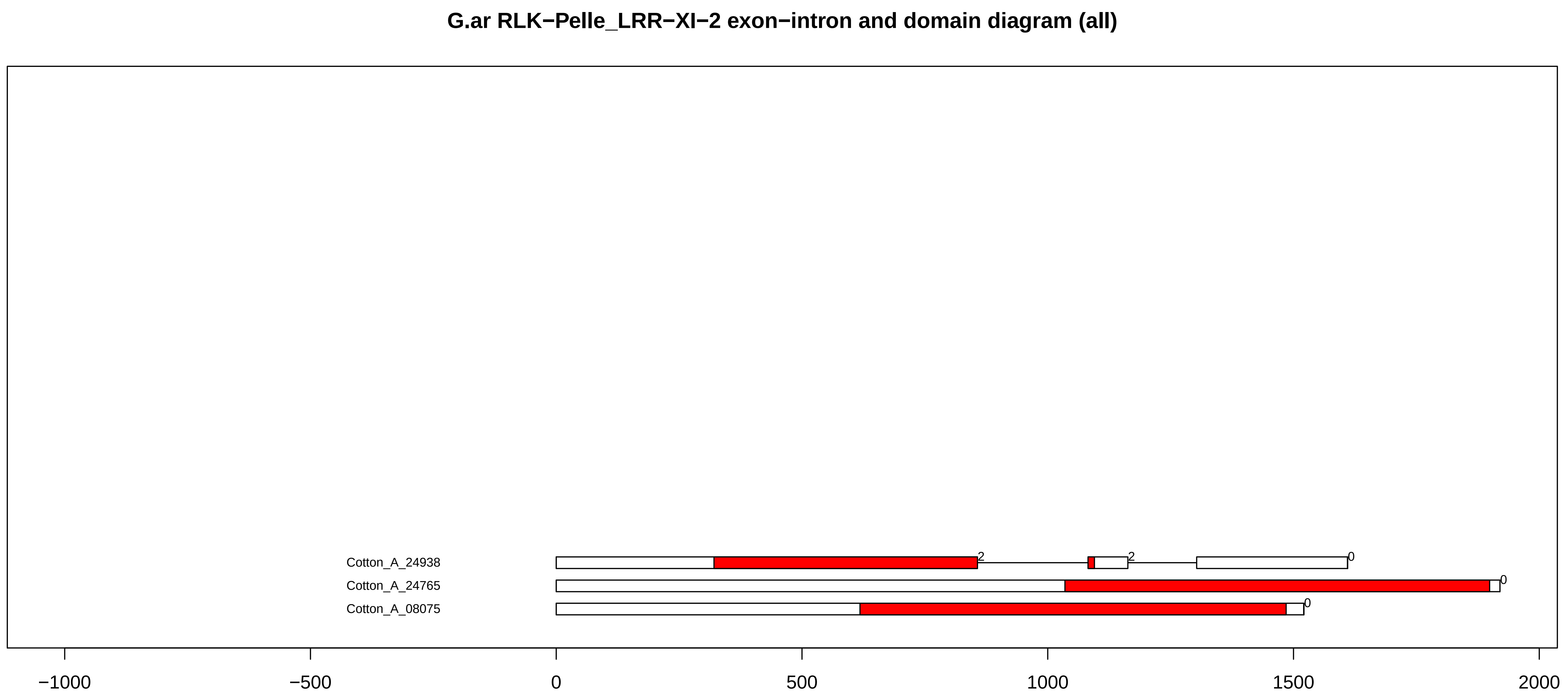
G.ar RLK−Pelle_LRR−XI−2 exon−intron and domain diagram (all)
Cotton_A_24938
Cotton_A_24765
Cotton_A_08075
2
2
0
0
0
−1000
−500
0
500
1000
1500
2000

G.ar RLK−Pelle_LRR−XII−1 exon−intron and domain diagram (part 1)

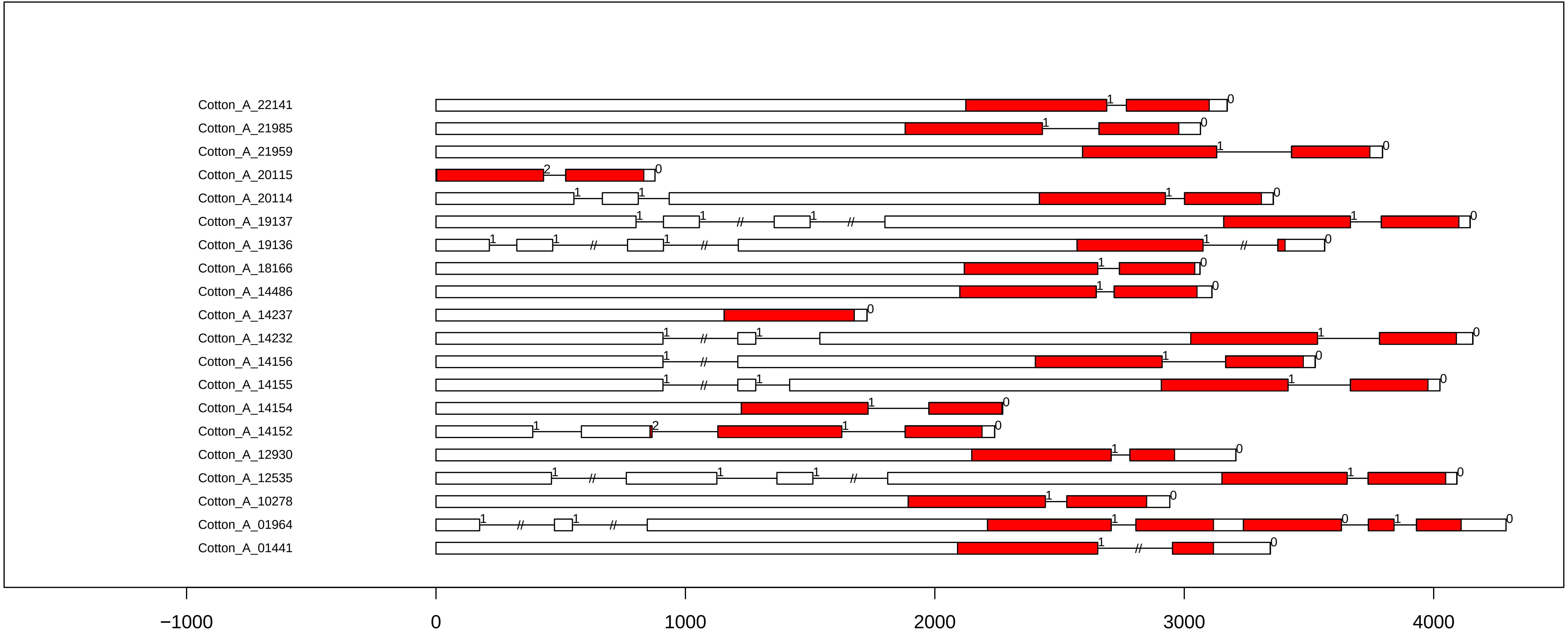

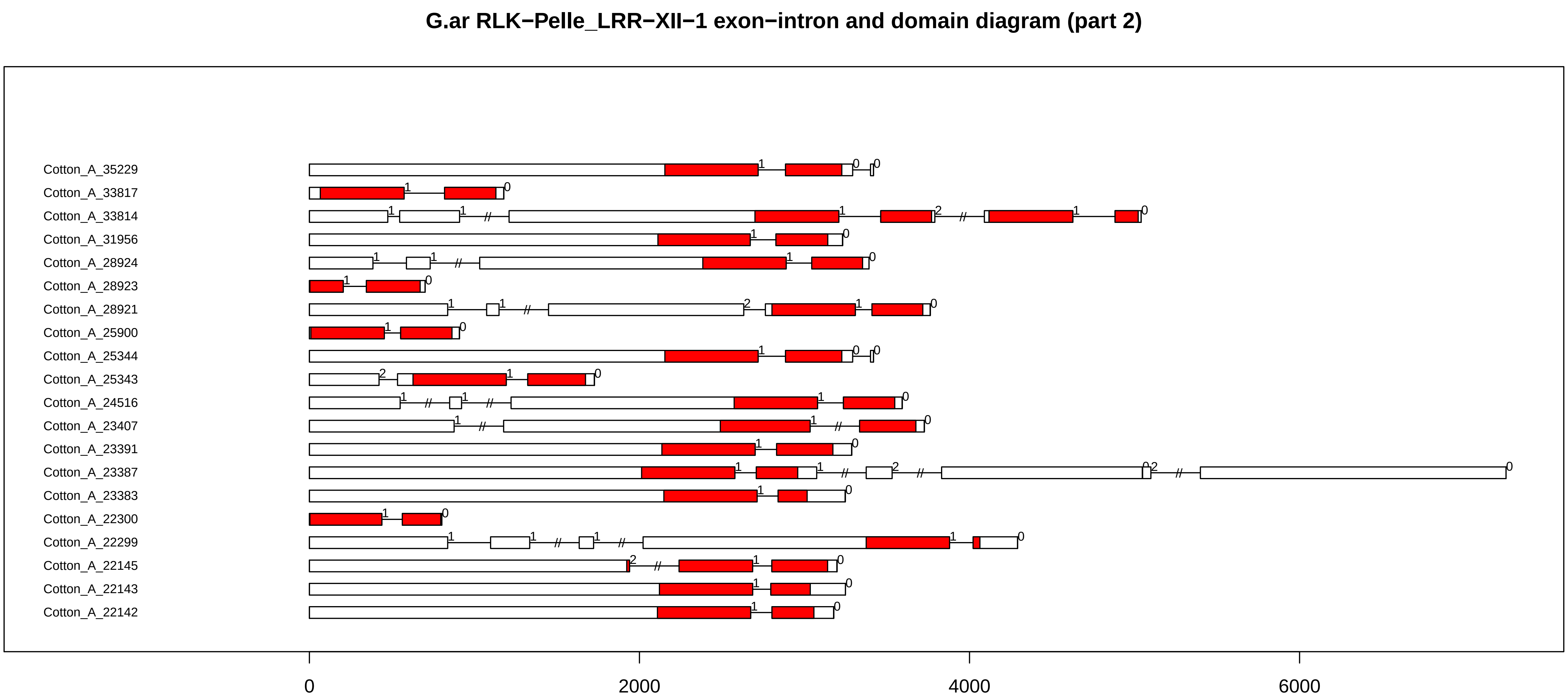
G.ar RLK−Pelle_LRR−XII−1 exon−intron and domain diagram (part 2)
Cotton_A_35229
Cotton_A_33817
Cotton_A_33814
Cotton_A_31956
Cotton_A_28924
Cotton_A_28923
Cotton_A_28921
Cotton_A_25900
Cotton_A_25344
Cotton_A_25343
Cotton_A_24516
Cotton_A_23407
Cotton_A_23391
Cotton_A_23387
Cotton_A_23383
Cotton_A_22300
Cotton_A_22299
Cotton_A_22145
Cotton_A_22143
Cotton_A_22142
0
2000
4000
6000

G.ar RLK−Pelle_LRR−XII−1 exon−intron and domain diagram (part 3)

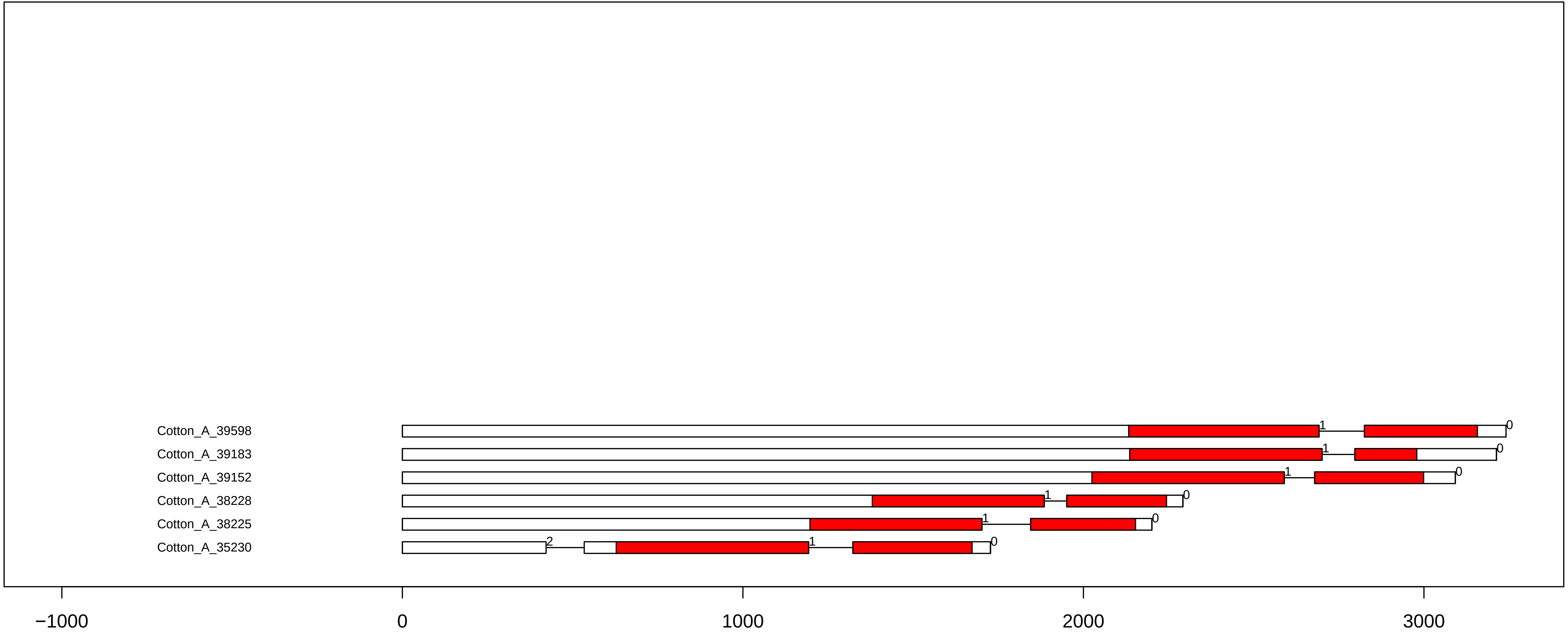

G.ar RLK−Pelle_LRR−XIIIa exon−intron and domain diagram (all)

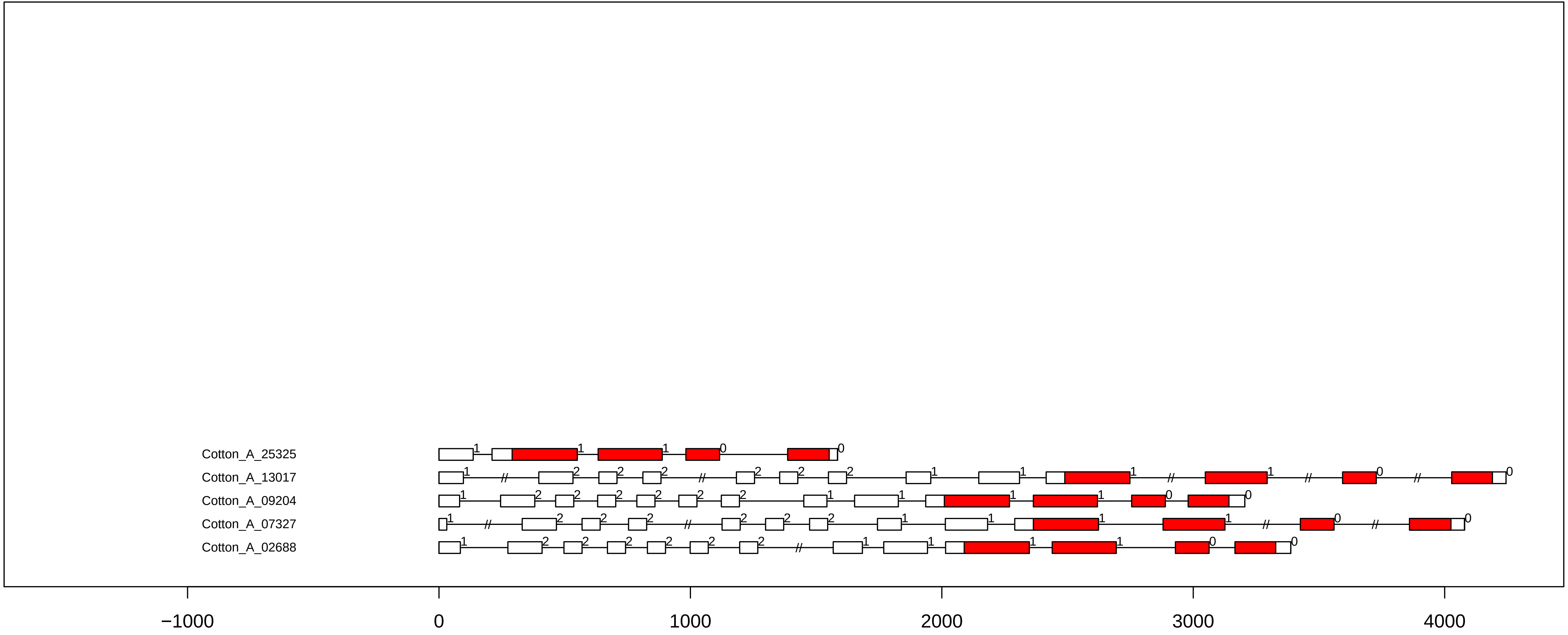

G.ar RLK−Pelle_LRR−XIIIb exon−intron and domain diagram (all)

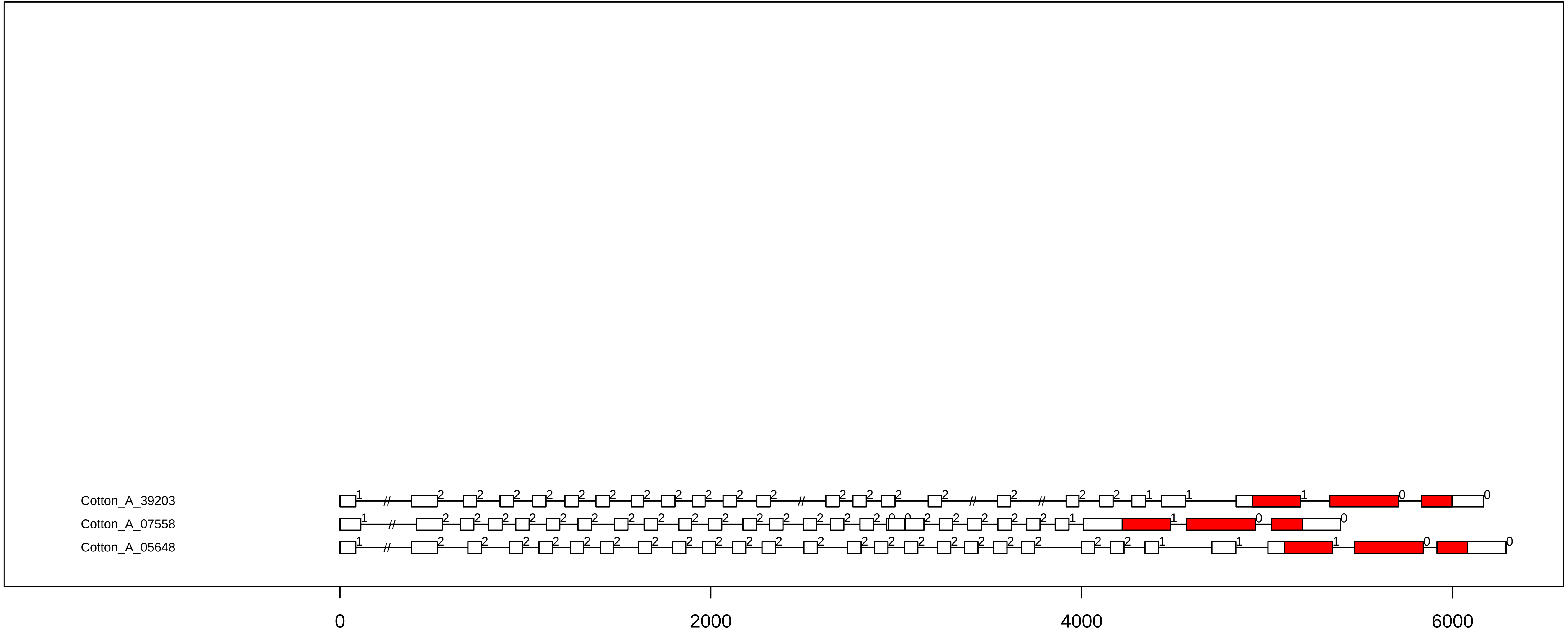

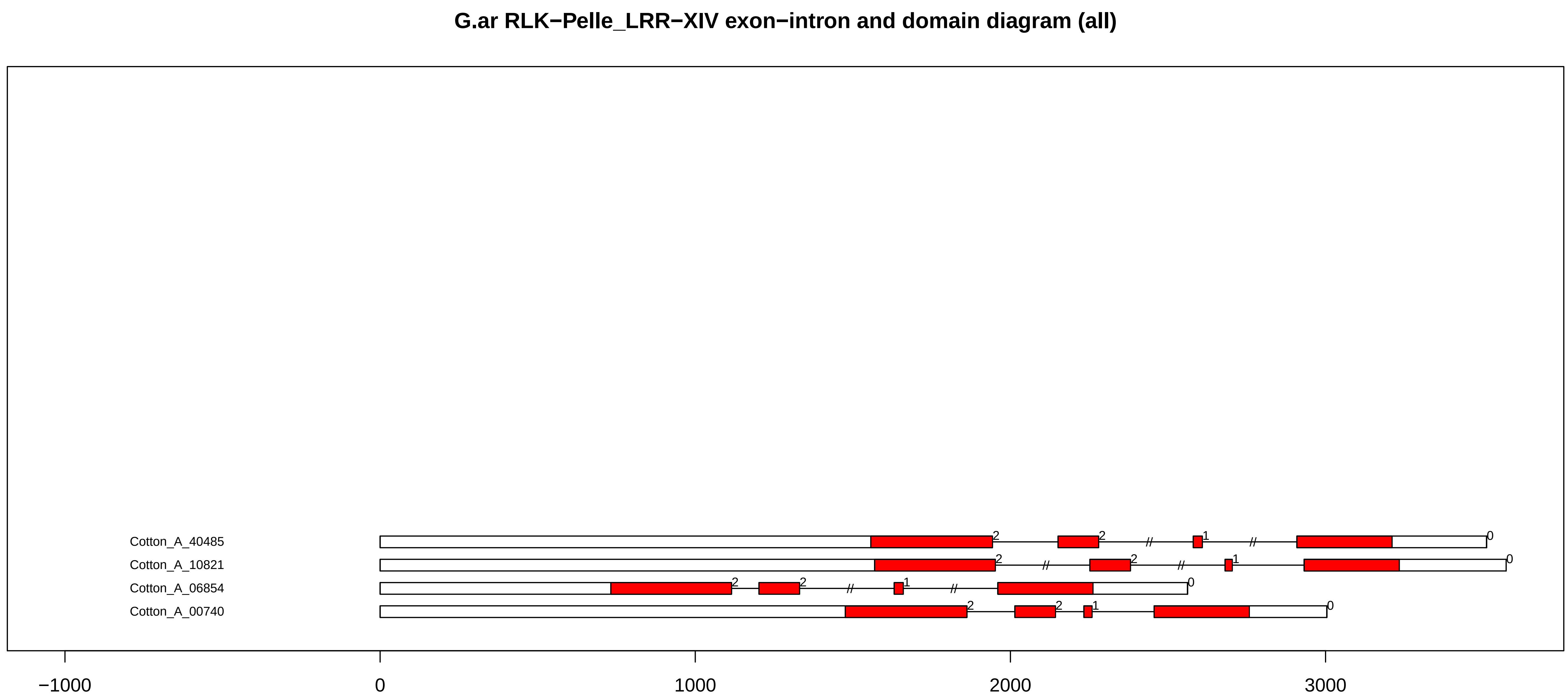
G.ar RLK−Pelle_LRR−XIV exon−intron and domain diagram (all)
Cotton_A_40485
Cotton_A_10821
Cotton_A_06854
Cotton_A_00740
−1000
0
1000
2000
3000

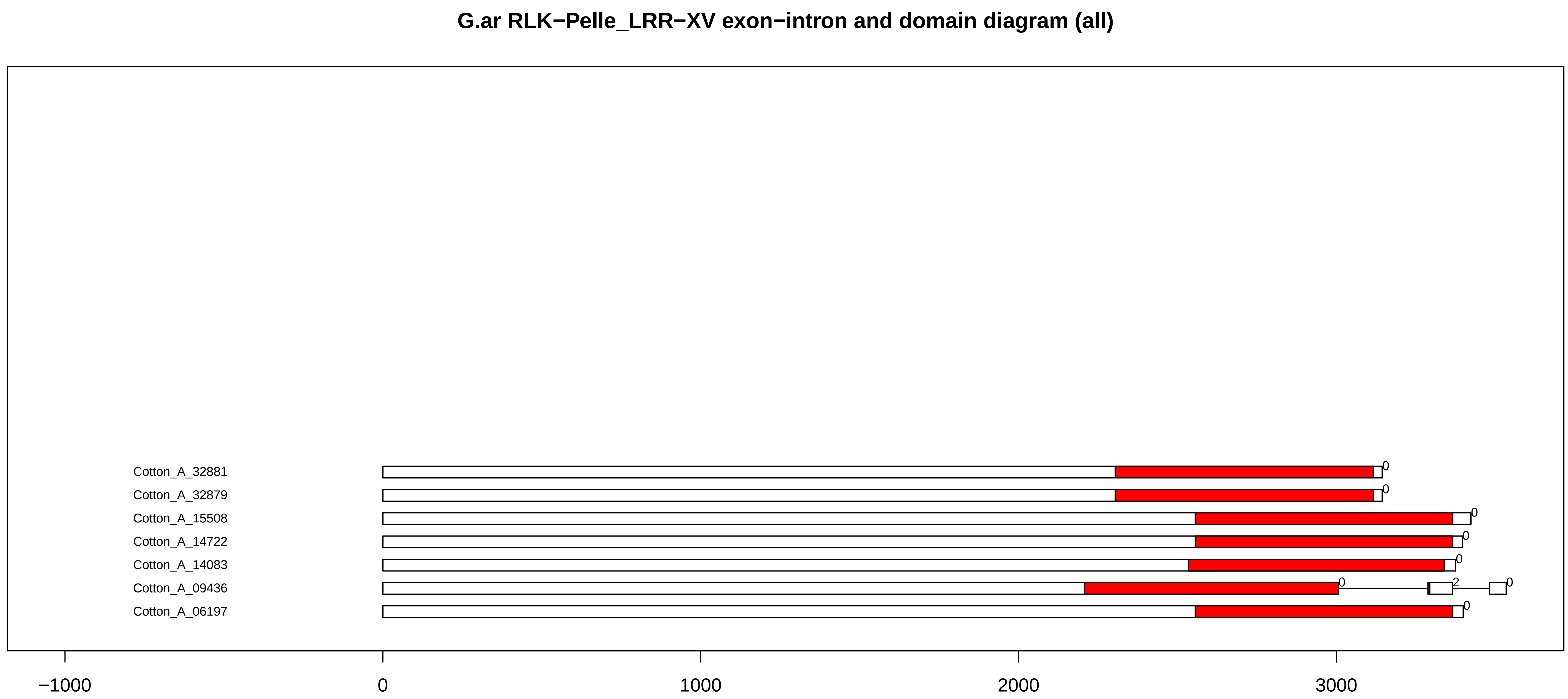

G.ar RLK−Pelle_LRR−XV exon−intron and domain diagram (all)
Cotton_A_32881
Cotton_A_32879
Cotton_A_15508
Cotton_A_14722
Cotton_A_14083
Cotton_A_09436
Cotton_A_06197
0
2
−1000
0
1000
2000
3000

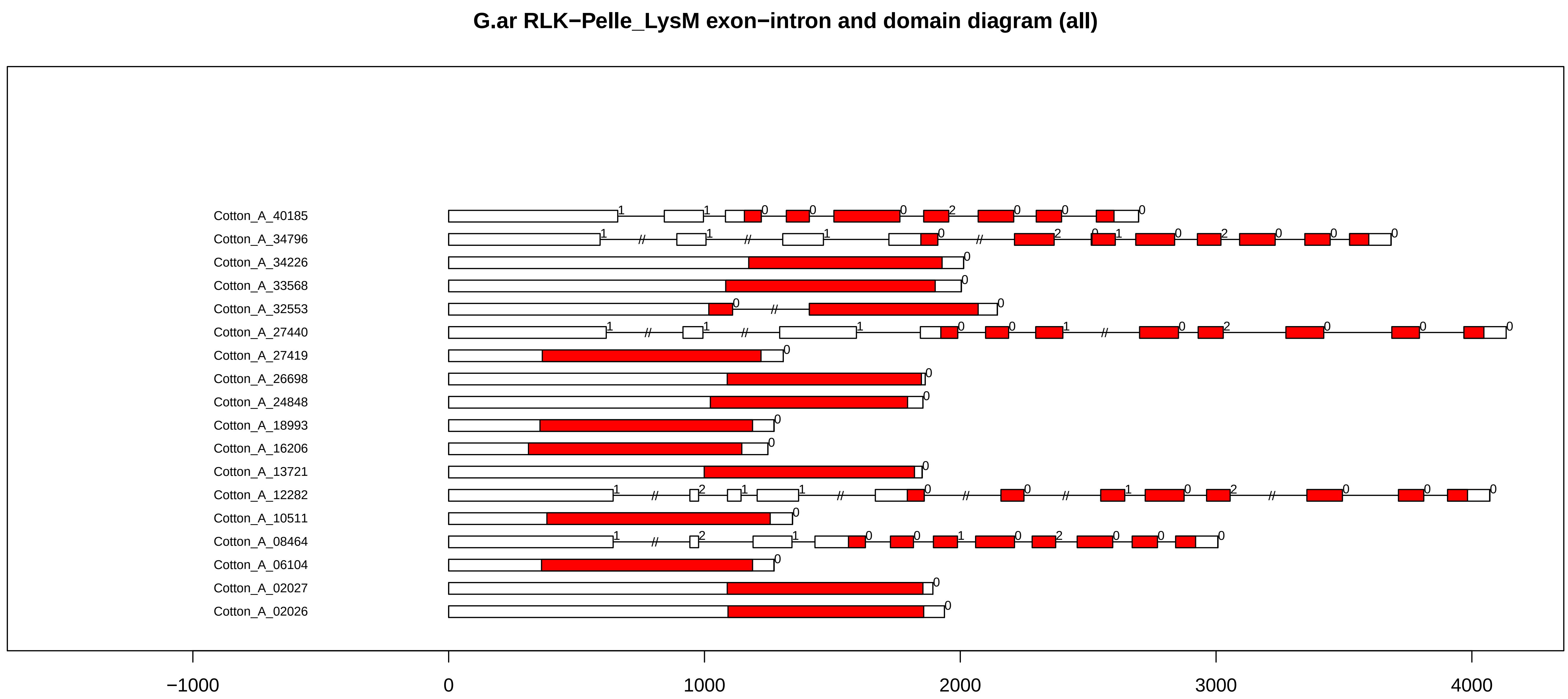
G.ar RLK−Pelle_LysM exon−intron and domain diagram (all)
Cotton_A_40185
Cotton_A_34796
Cotton_A_34226
Cotton_A_33568
Cotton_A_32553
Cotton_A_27440
Cotton_A_27419
Cotton_A_26698
Cotton_A_24848
Cotton_A_18993
Cotton_A_16206
Cotton_A_13721
Cotton_A_12282
Cotton_A_10511
Cotton_A_08464
Cotton_A_06104
Cotton_A_02027
Cotton_A_02026
−1000
0
1000
2000
3000
4000

G.ar RLK−Pelle_PERK−1 exon−intron and domain diagram (all)

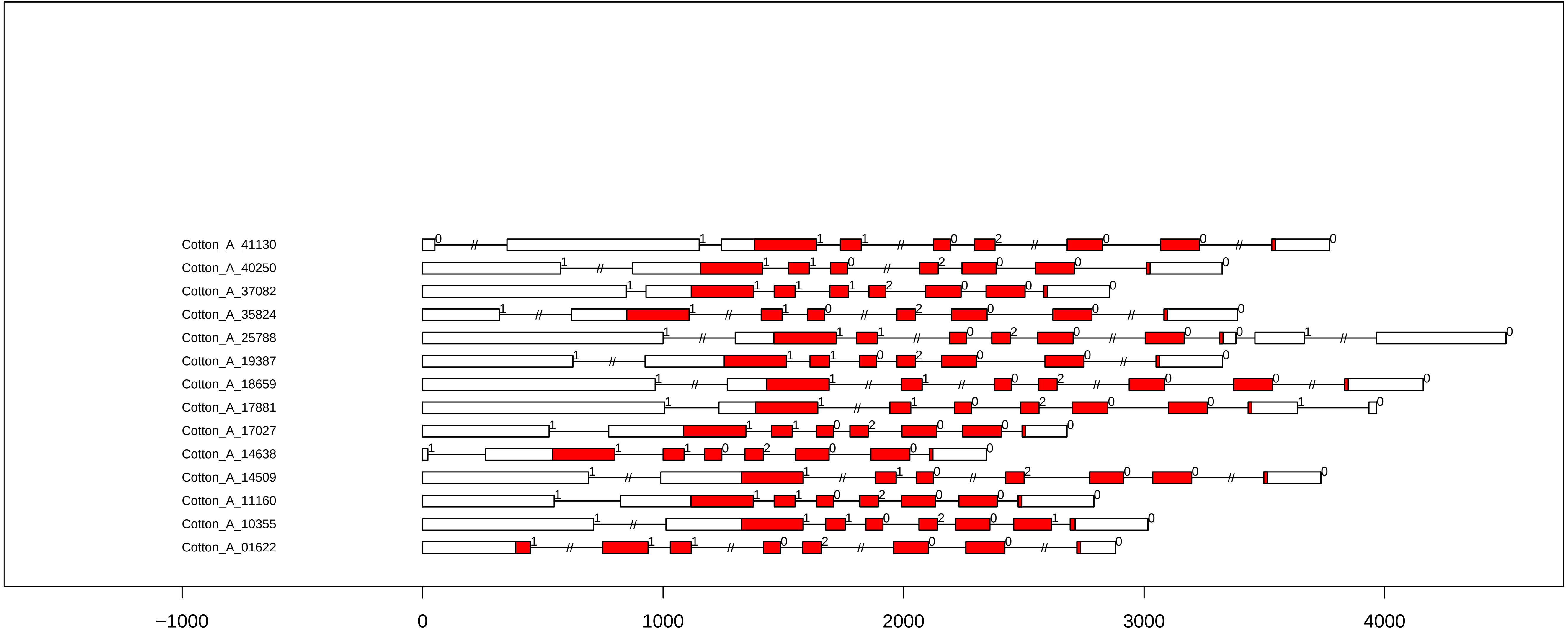

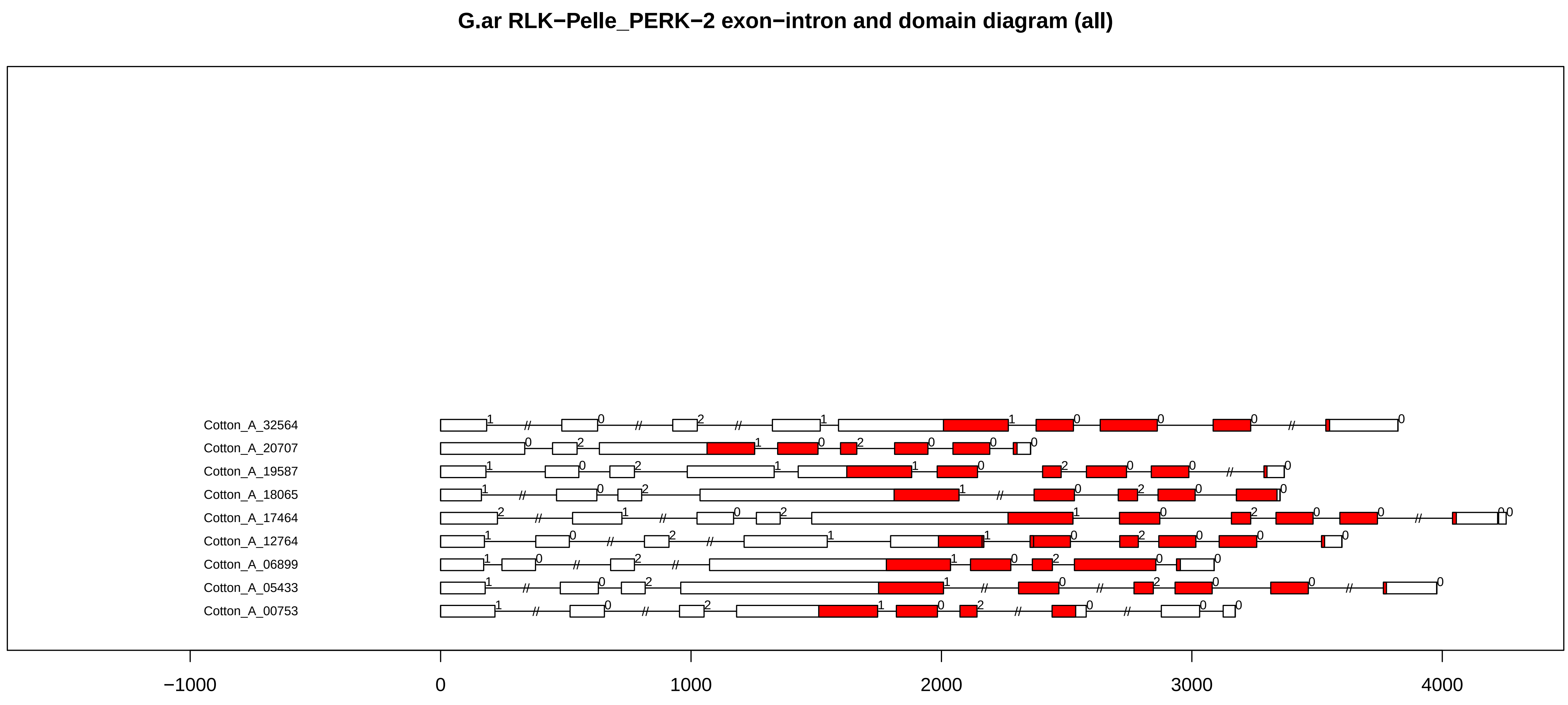
G.ar RLK−Pelle_PERK−2 exon−intron and domain diagram (all)
Cotton_A_32564
Cotton_A_20707
Cotton_A_19587
Cotton_A_18065
Cotton_A_17464
Cotton_A_12764
Cotton_A_06899
Cotton_A_05433
Cotton_A_00753
−1000
0
1000
2000
3000
4000

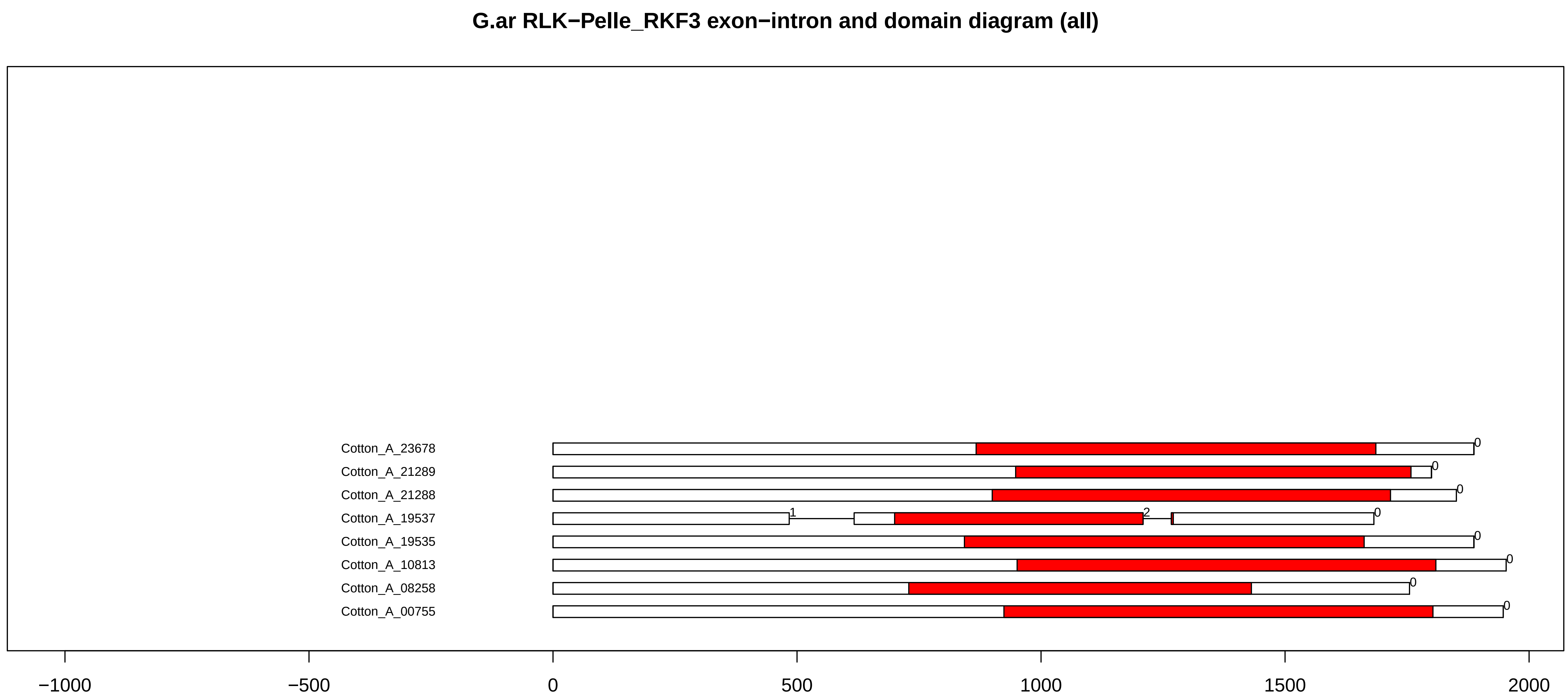

G.ar RLK−Pelle_RKF3 exon−intron and domain diagram (all)
Cotton_A_23678
Cotton_A_21289
Cotton_A_21288
Cotton_A_19537
Cotton_A_19535
Cotton_A_10813
Cotton_A_08258
Cotton_A_00755
0
0
0
1
2
0
0
0
0
0
−1000
−500
0
500
1000
1500
2000

G.ar RLK−Pelle_RLCK−II exon−intron and domain diagram (all)

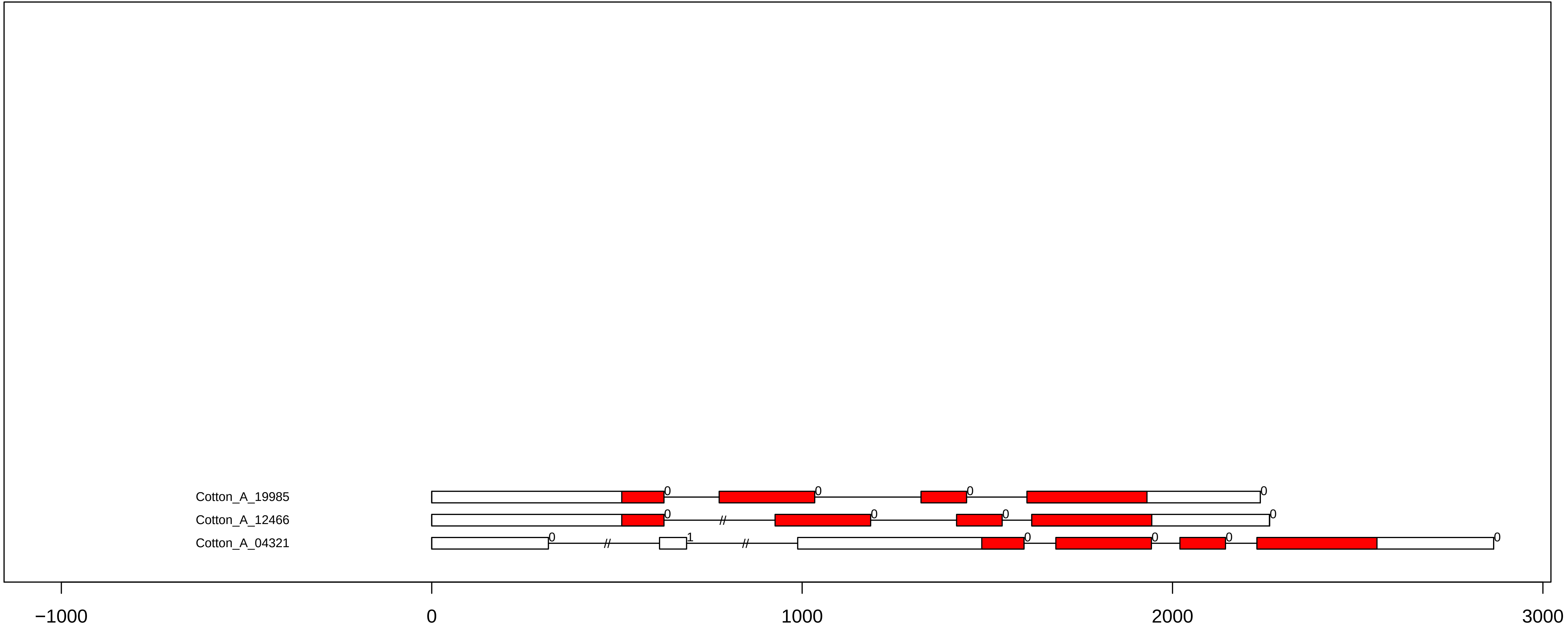

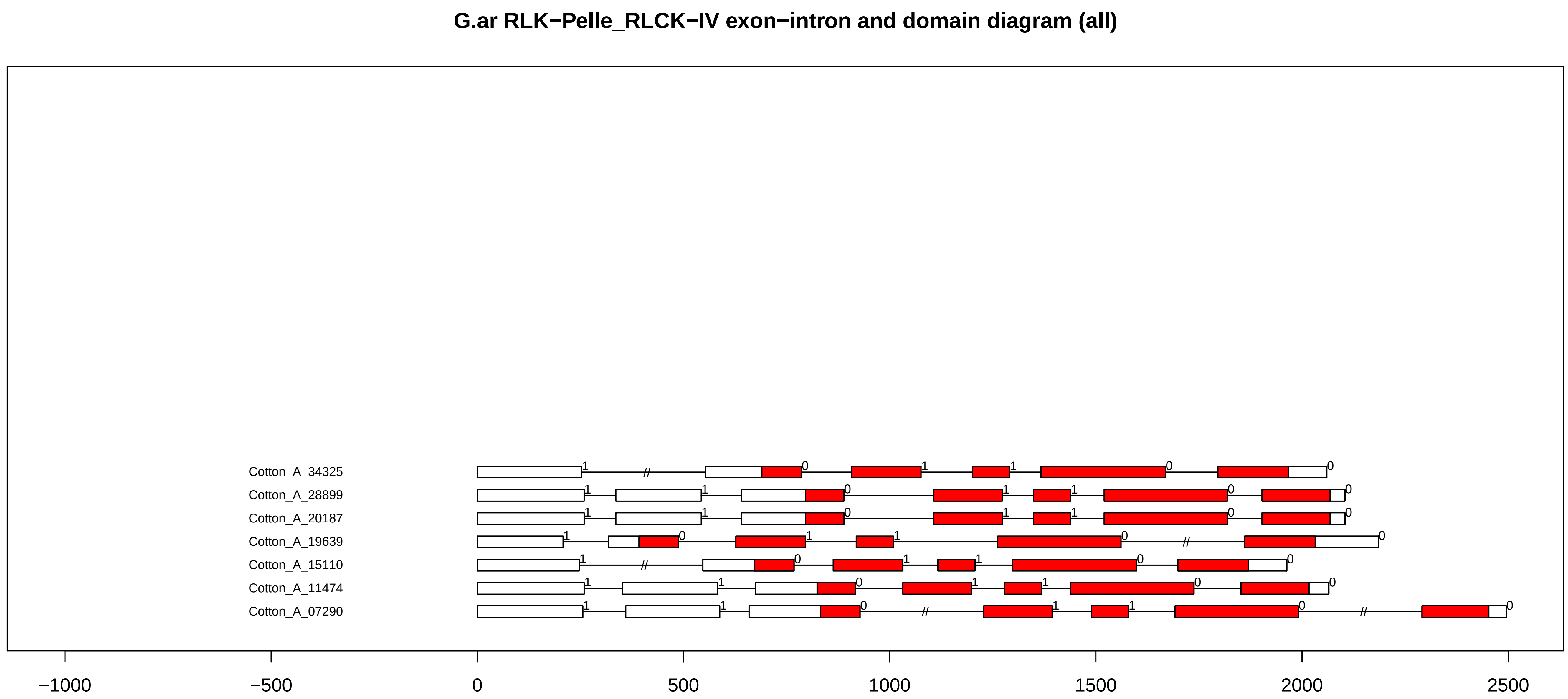
G.ar RLK−Pelle_RLCK−IV exon−intron and domain diagram (all)
Cotton_A_34325
Cotton_A_28899
Cotton_A_20187
Cotton_A_19639
Cotton_A_15110
Cotton_A_11474
Cotton_A_07290
−1000
−500
0
500
1000
1500
2000
2500

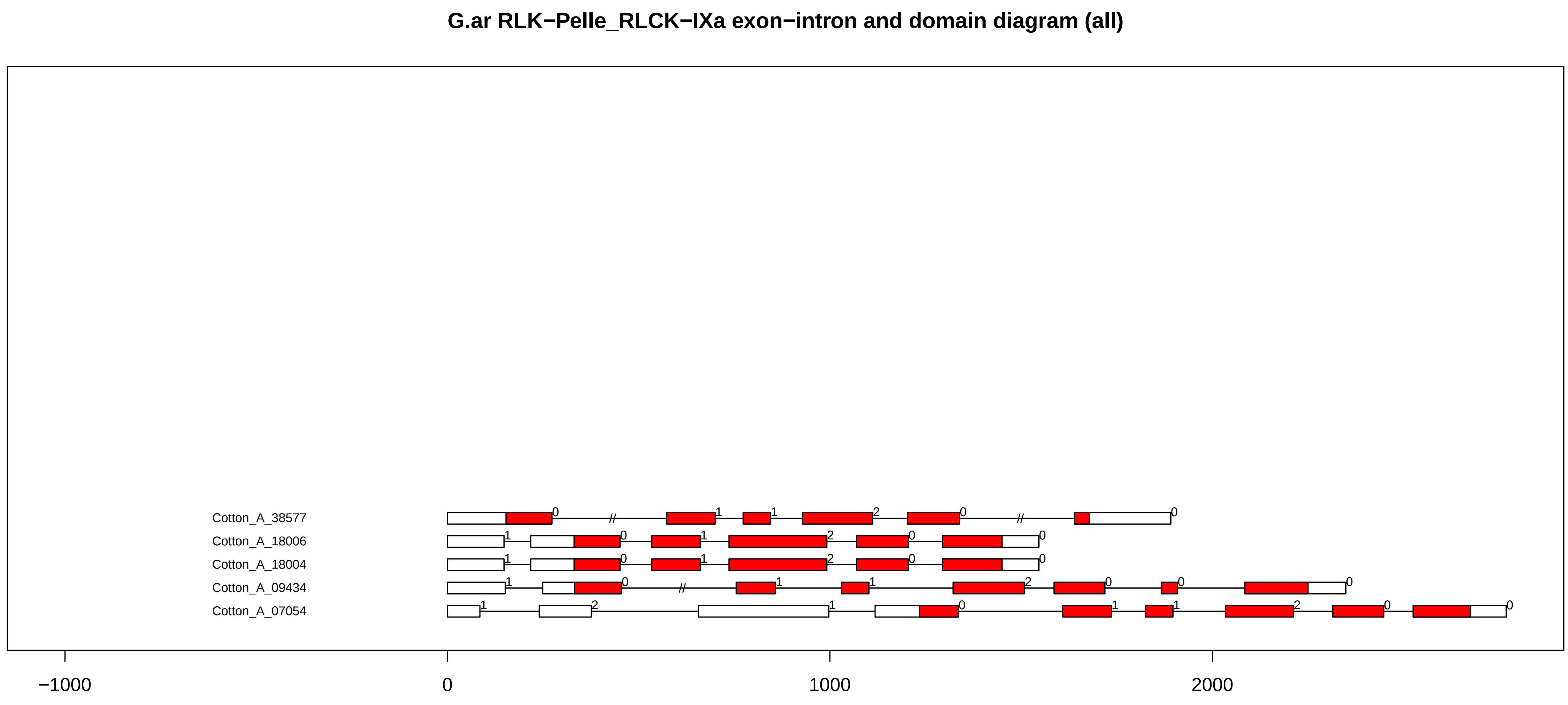
G.ar RLK−Pelle_RLCK−IXa exon−intron and domain diagram (all)
Cotton_A_38577
Cotton_A_18006
Cotton_A_18004
Cotton_A_09434
Cotton_A_07054
−1000
0
1000
2000

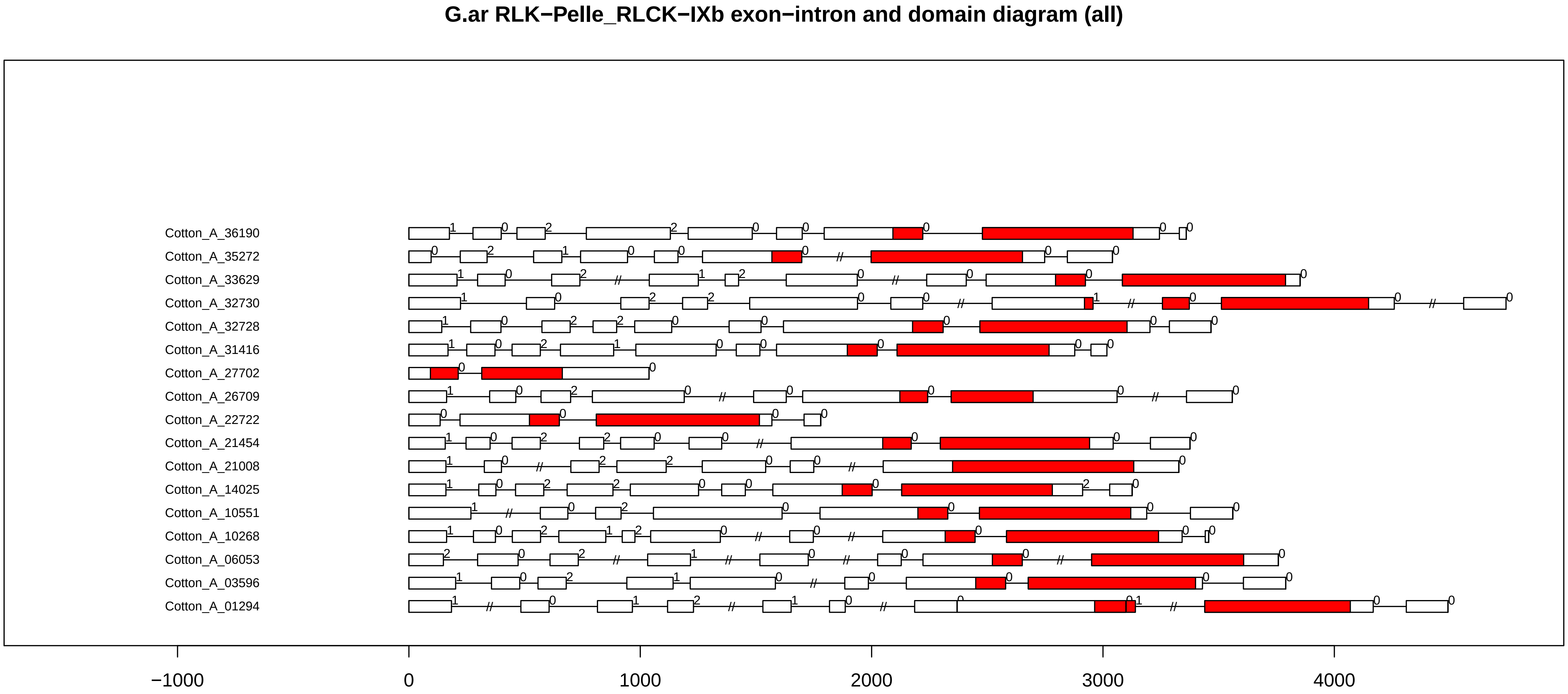
G.ar RLK−Pelle_RLCK−IXb exon−intron and domain diagram (all)
Cotton_A_36190
Cotton_A_35272
Cotton_A_33629
Cotton_A_32730
Cotton_A_32728
Cotton_A_31416
Cotton_A_27702
Cotton_A_26709
Cotton_A_22722
Cotton_A_21454
Cotton_A_21008
Cotton_A_14025
Cotton_A_10551
Cotton_A_10268
Cotton_A_06053
Cotton_A_03596
Cotton_A_01294
−1000
0
1000
2000
3000
4000

G.ar RLK−Pelle_RLCK−Os exon−intron and domain diagram (all)

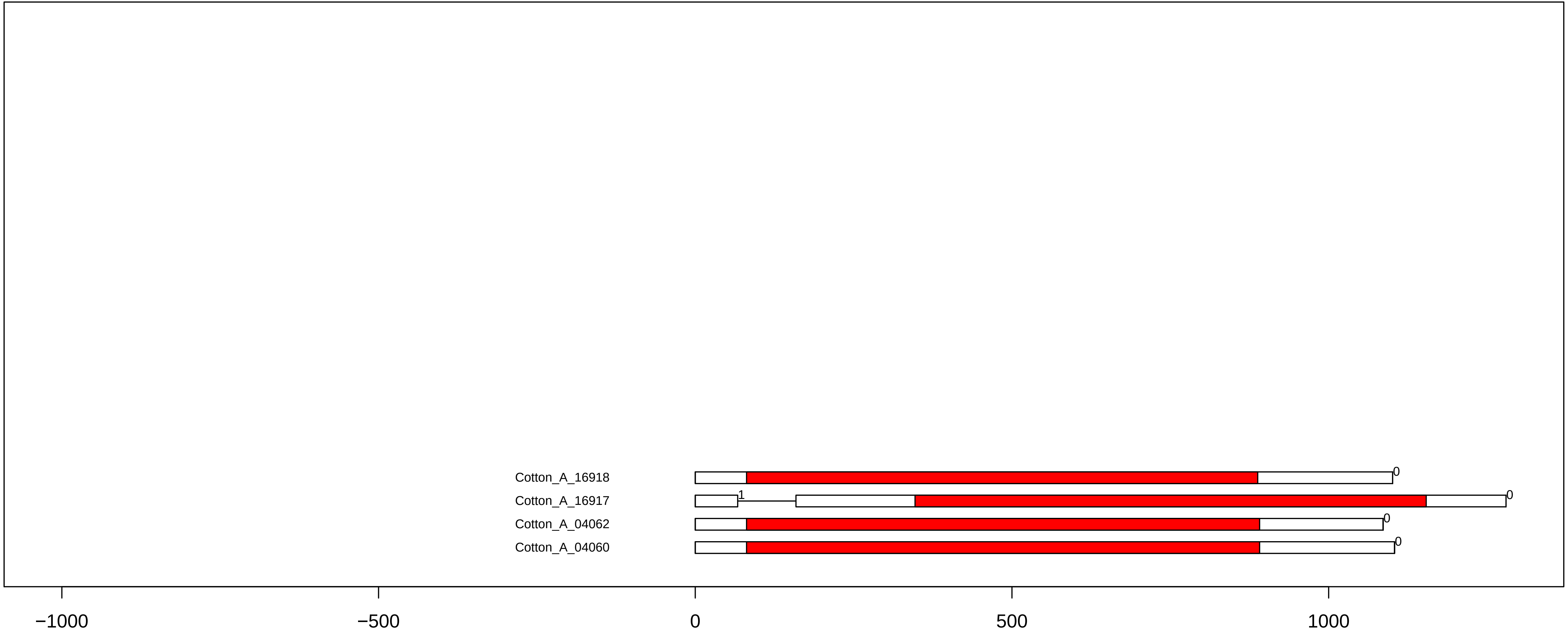

G.ar RLK−Pelle_RLCK−VIIa−1 exon−intron and domain diagram (all)

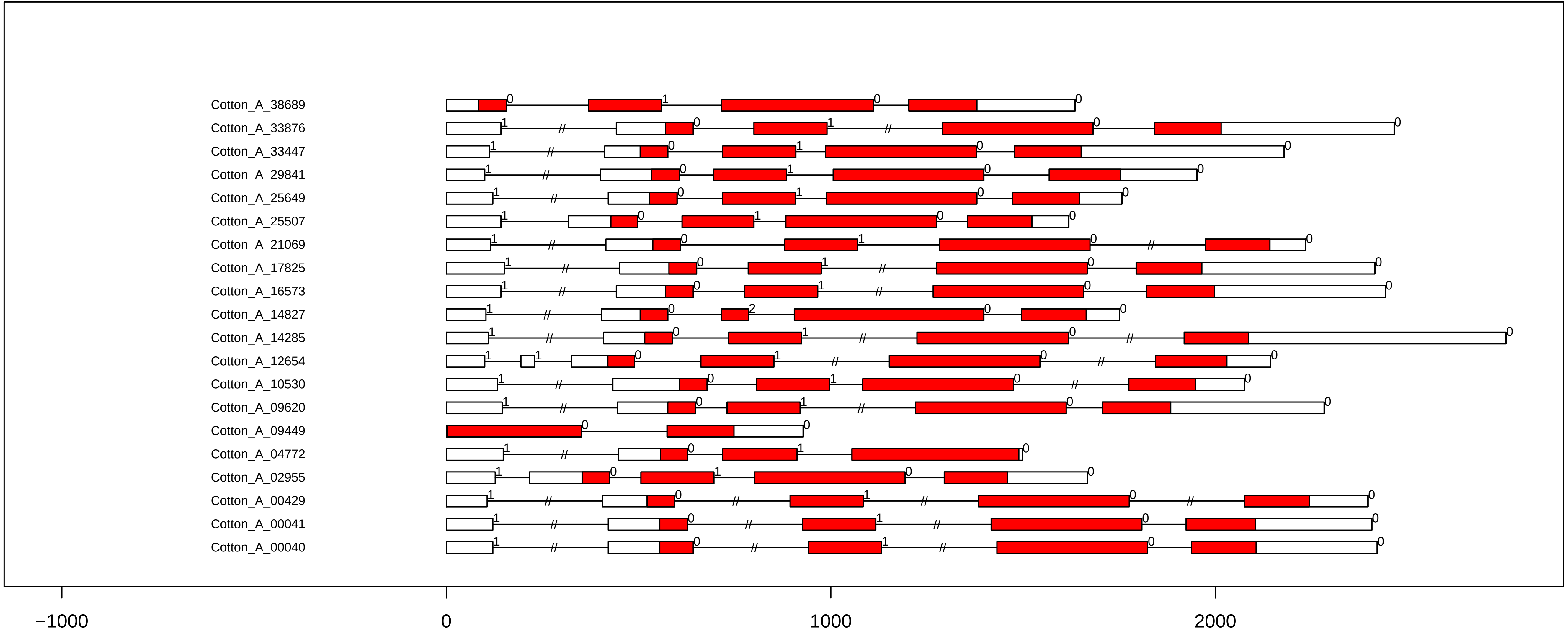

G.ar RLK−Pelle_RLCK−VIIa−2 exon−intron and domain diagram (part 1)

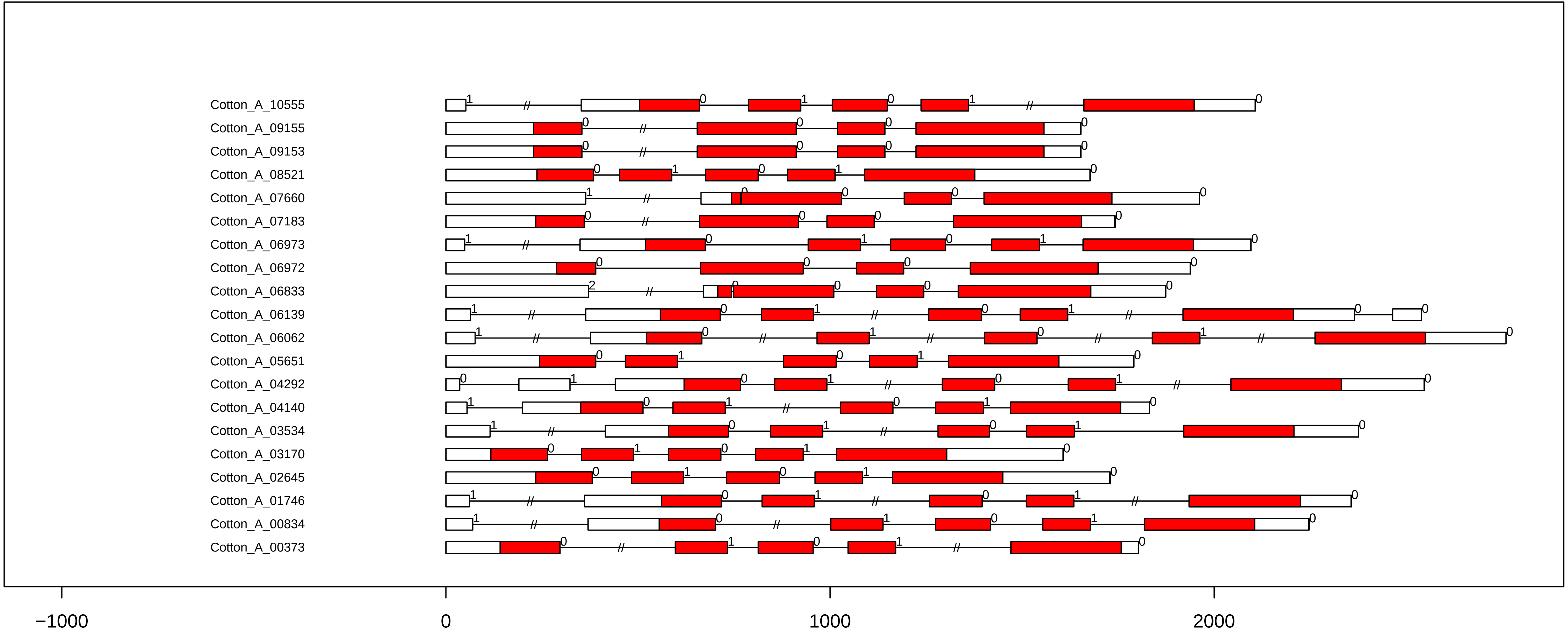

G.ar RLK−Pelle_RLCK−VIIa−2 exon−intron and domain diagram (part 2)

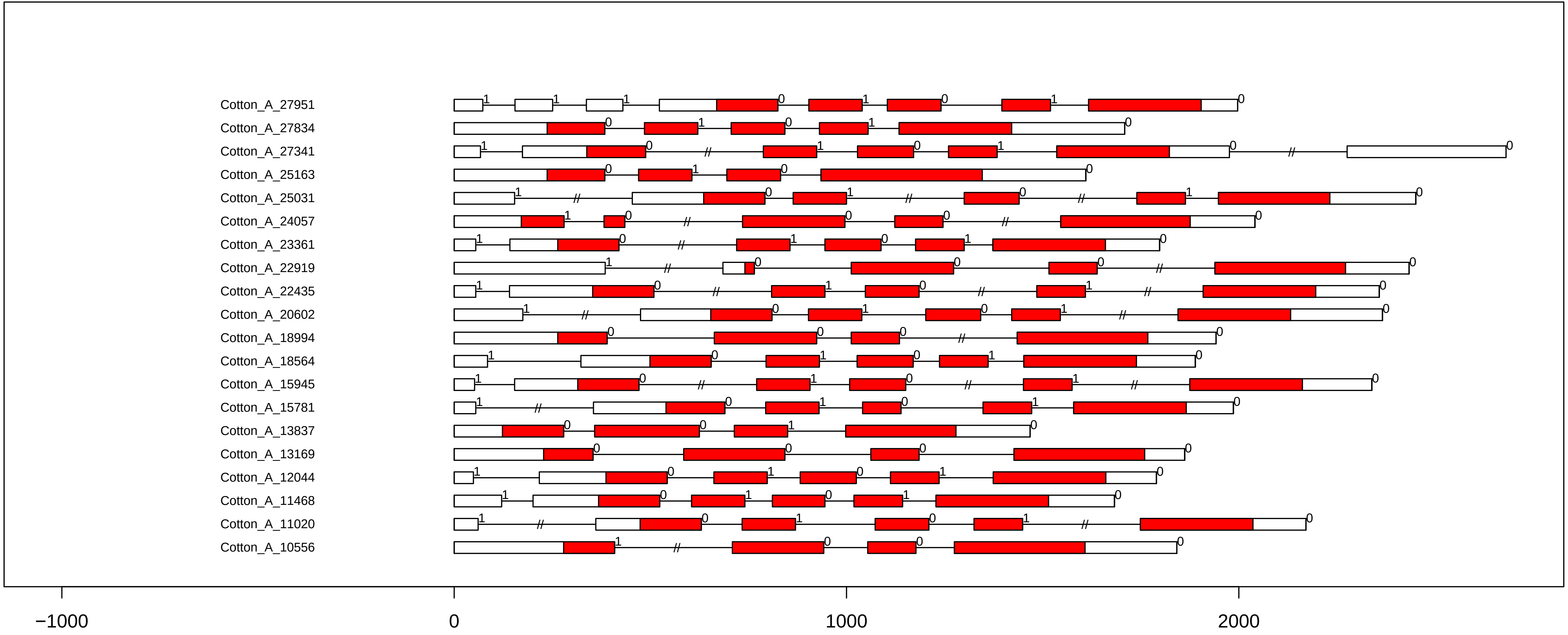

G.ar RLK−Pelle_RLCK−VIIa−2 exon−intron and domain diagram (part 3)

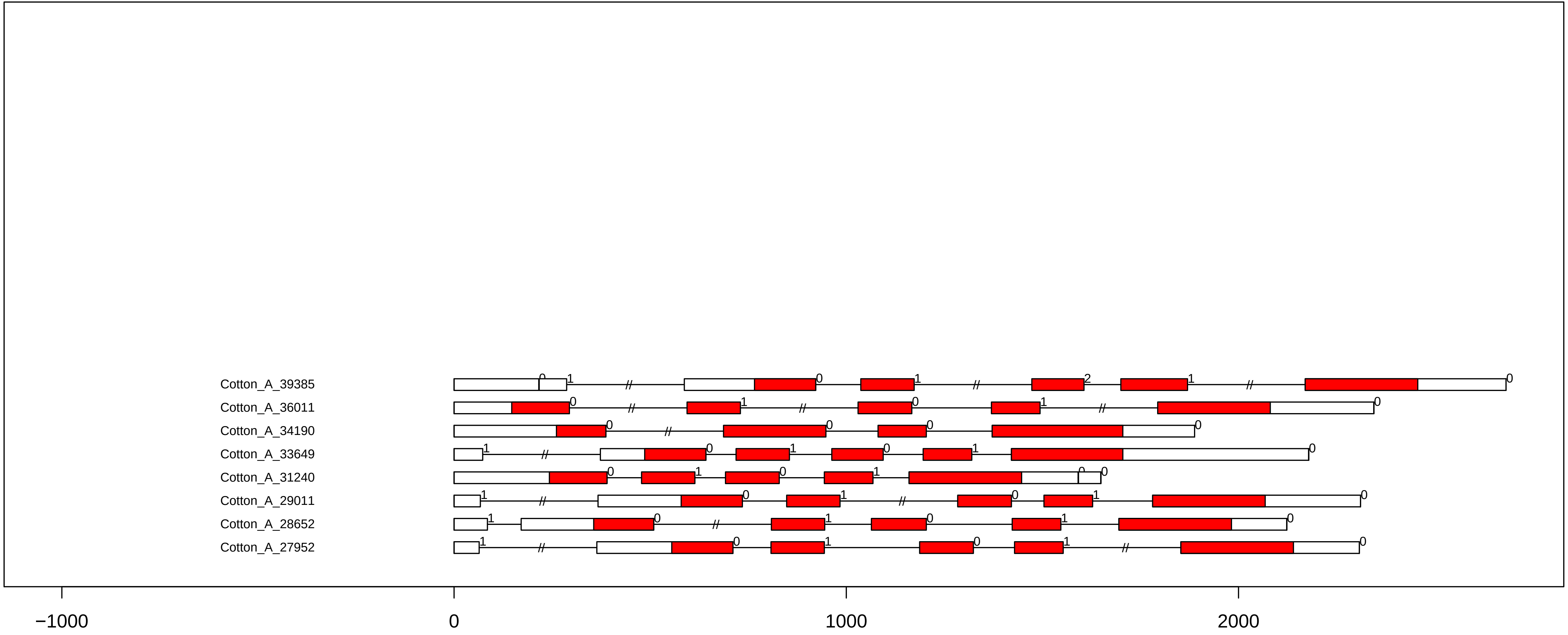

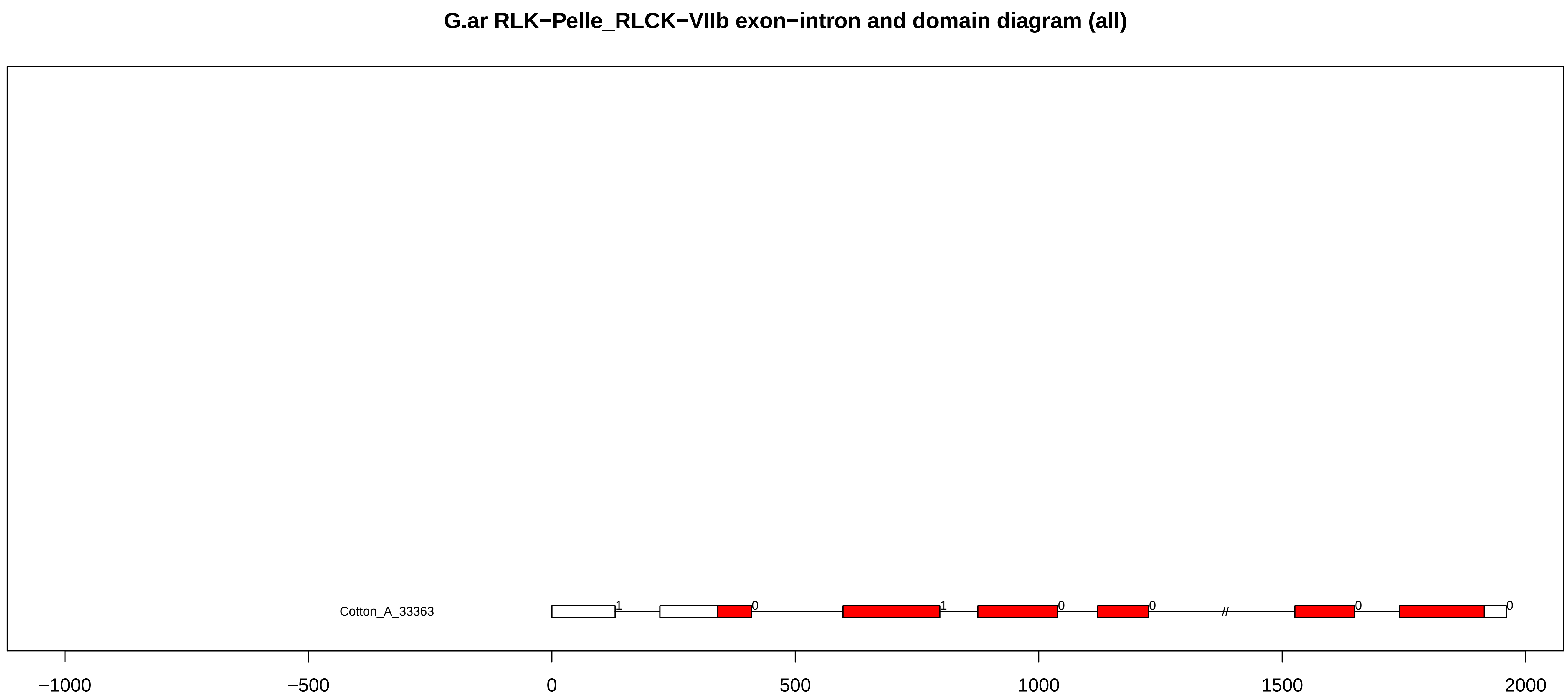

G.ar RLK−Pelle_RLCK−VIIb exon−intron and domain diagram (all)
Cotton_A_33363
1
0
1
0
0
0
0
−1000
−500
0
500
1000
1500
2000

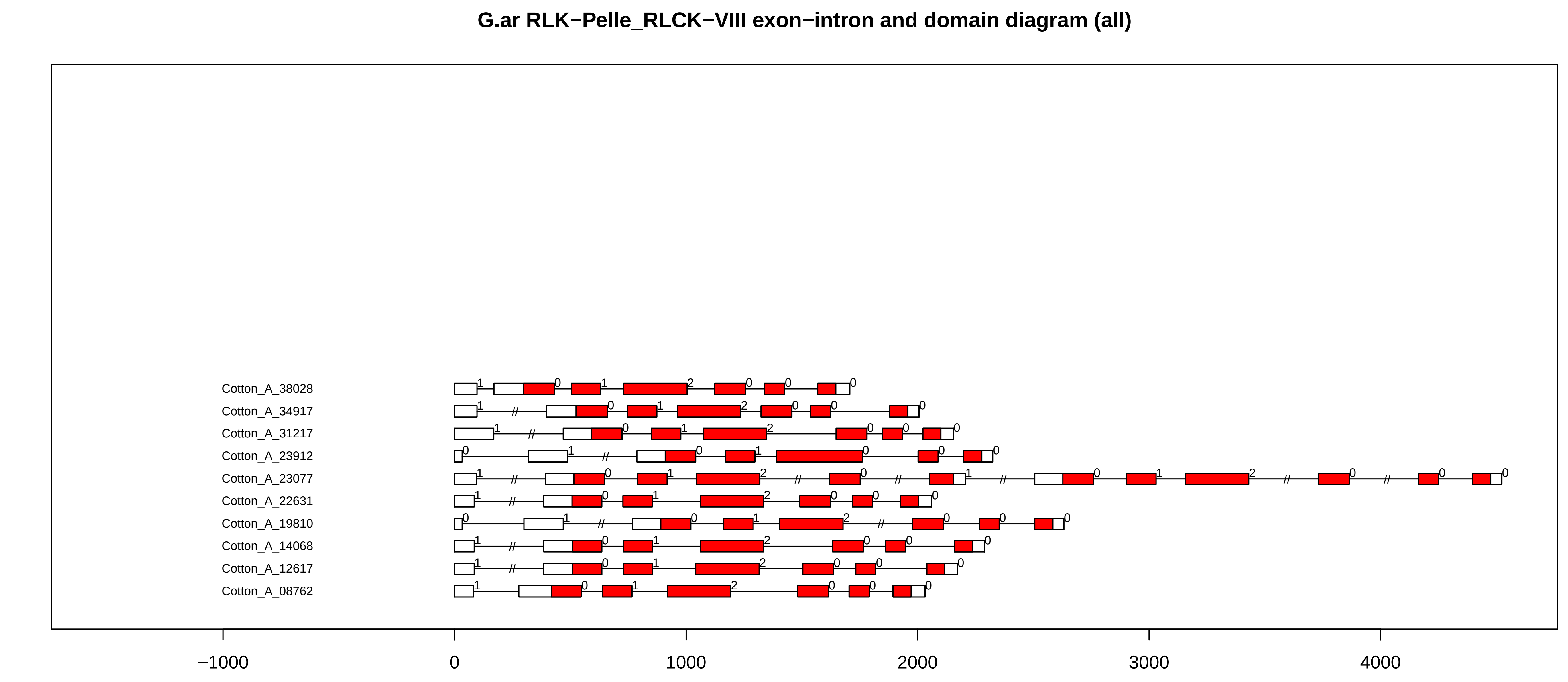
G.ar RLK−Pelle_RLCK−VIII exon−intron and domain diagram (all)
Cotton_A_38028
Cotton_A_34917
Cotton_A_31217
Cotton_A_23912
Cotton_A_23077
Cotton_A_22631
Cotton_A_19810
Cotton_A_14068
Cotton_A_12617
Cotton_A_08762
−1000
0
1000
2000
3000
4000

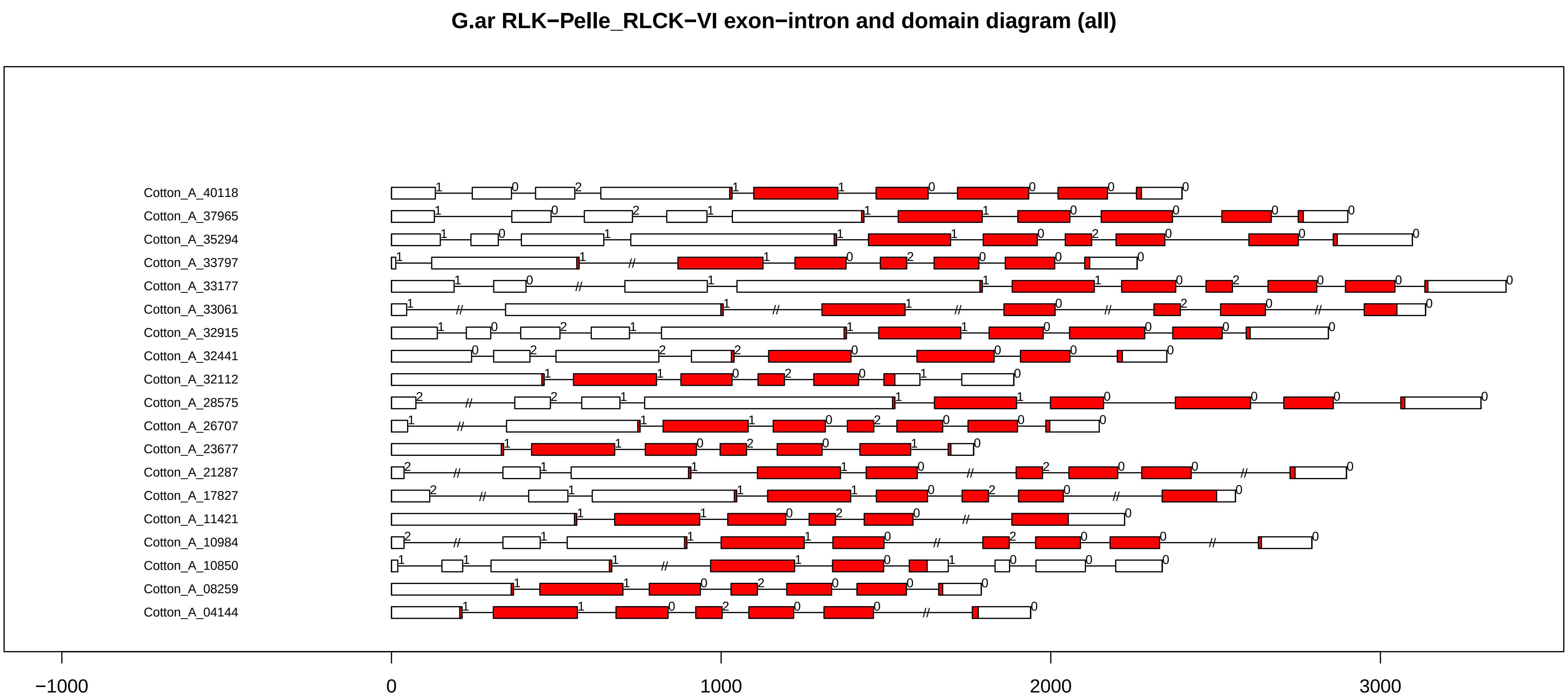
G.ar RLK−Pelle_RLCK−VI exon−intron and domain diagram (all)
Cotton_A_40118
Cotton_A_37965
Cotton_A_35294
Cotton_A_33797
Cotton_A_33177
Cotton_A_33061
Cotton_A_32915
Cotton_A_32441
Cotton_A_32112
Cotton_A_28575
Cotton_A_26707
Cotton_A_23677
Cotton_A_21287
Cotton_A_17827
Cotton_A_11421
Cotton_A_10984
Cotton_A_10850
Cotton_A_08259
Cotton_A_04144
−1000
0
1000
2000
3000

G.ar RLK−Pelle_RLCK−V exon−intron and domain diagram (all)

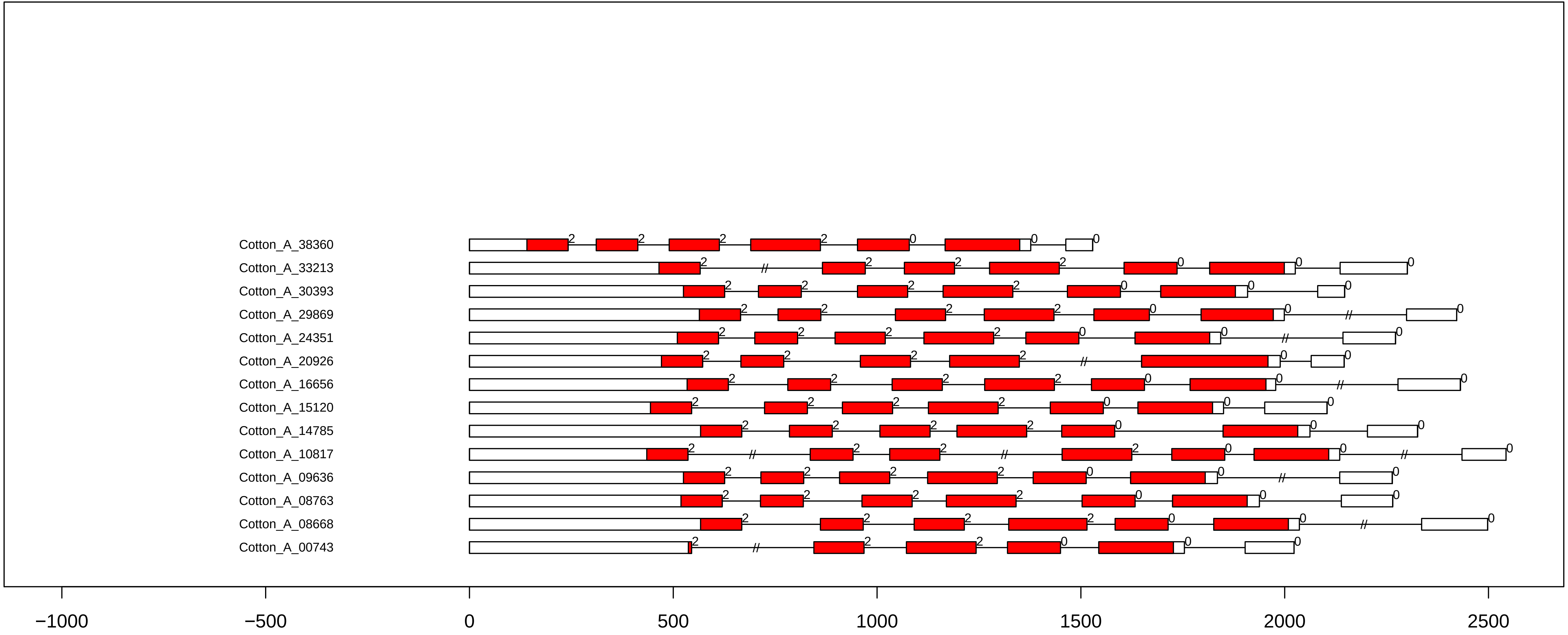

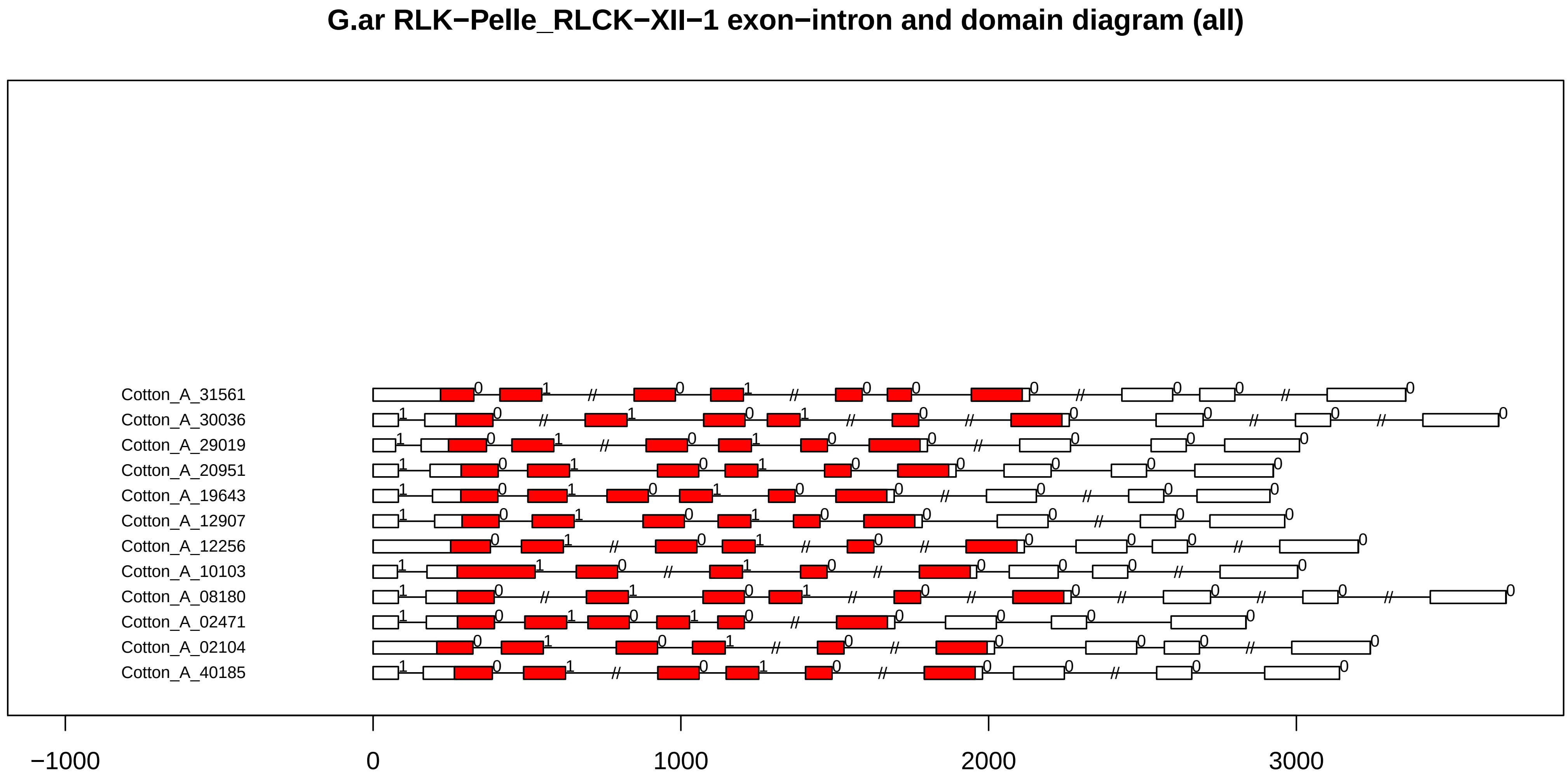
G.ar RLK−Pelle_RLCK−XII−1 exon−intron and domain diagram (all)
Cotton_A_31561
Cotton_A_30036
Cotton_A_29019
Cotton_A_20951
Cotton_A_19643
Cotton_A_12907
Cotton_A_12256
Cotton_A_10103
Cotton_A_08180
Cotton_A_02471
Cotton_A_02104
Cotton_A_40185
−1000
0
1000
2000
3000

G.ar RLK−Pelle_RLCK−XII−2 exon−intron and domain diagram (all)

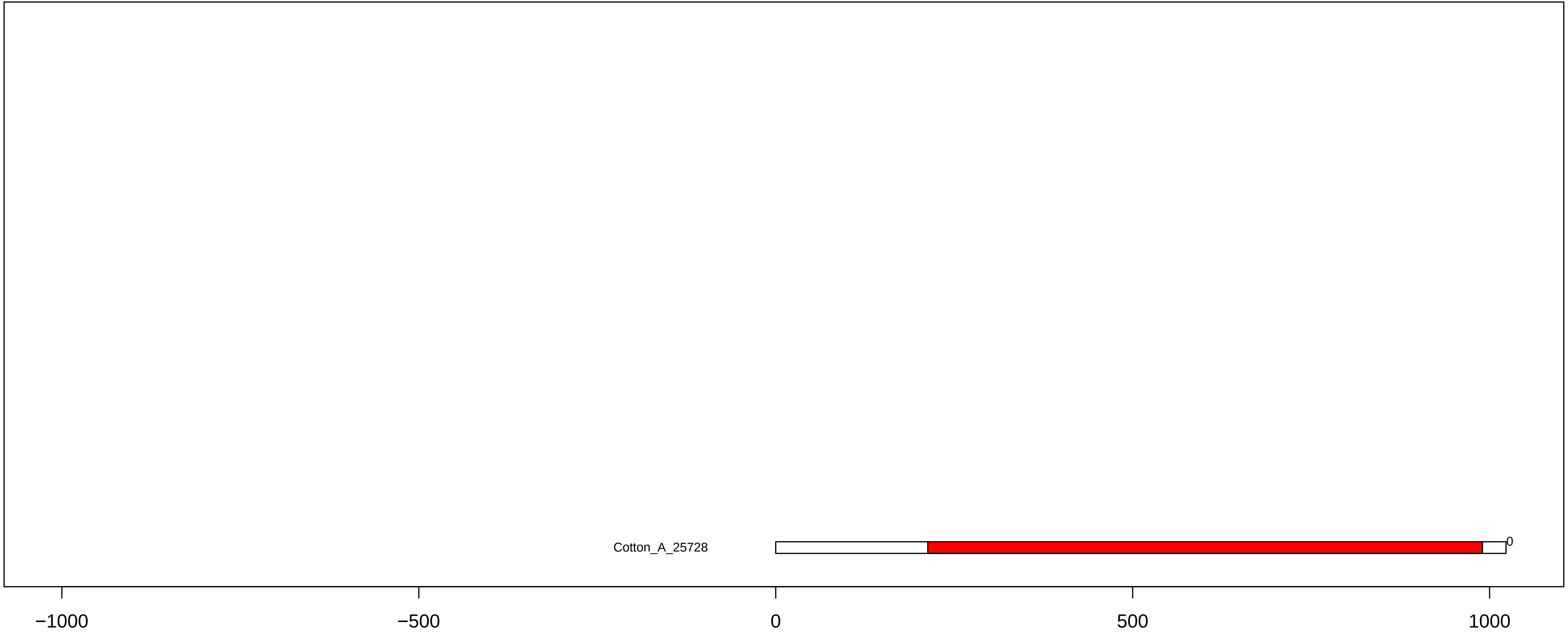

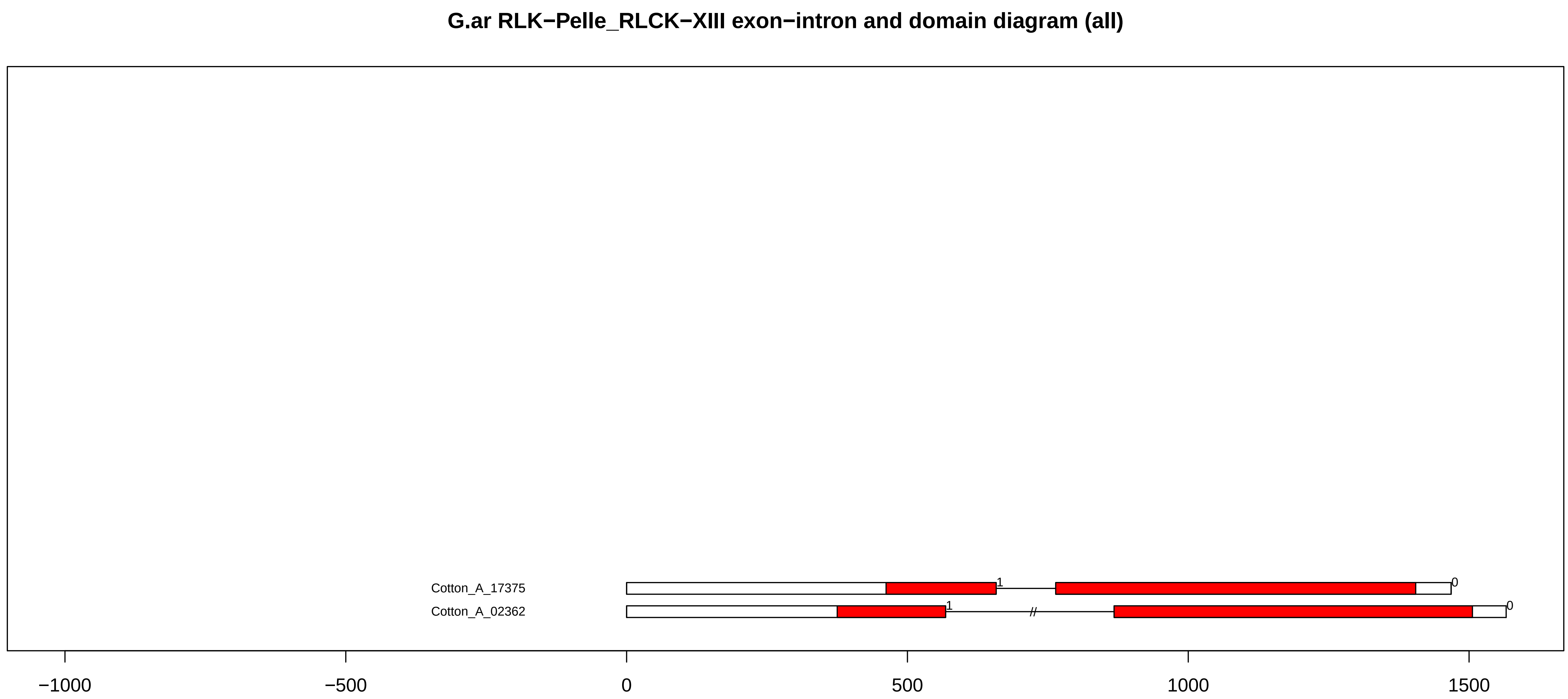
G.ar RLK−Pelle_RLCK−XIII exon−intron and domain diagram (all)
Cotton_A_17375
Cotton_A_02362
1
0
1
0
−1000
−500
0
500
1000
1500

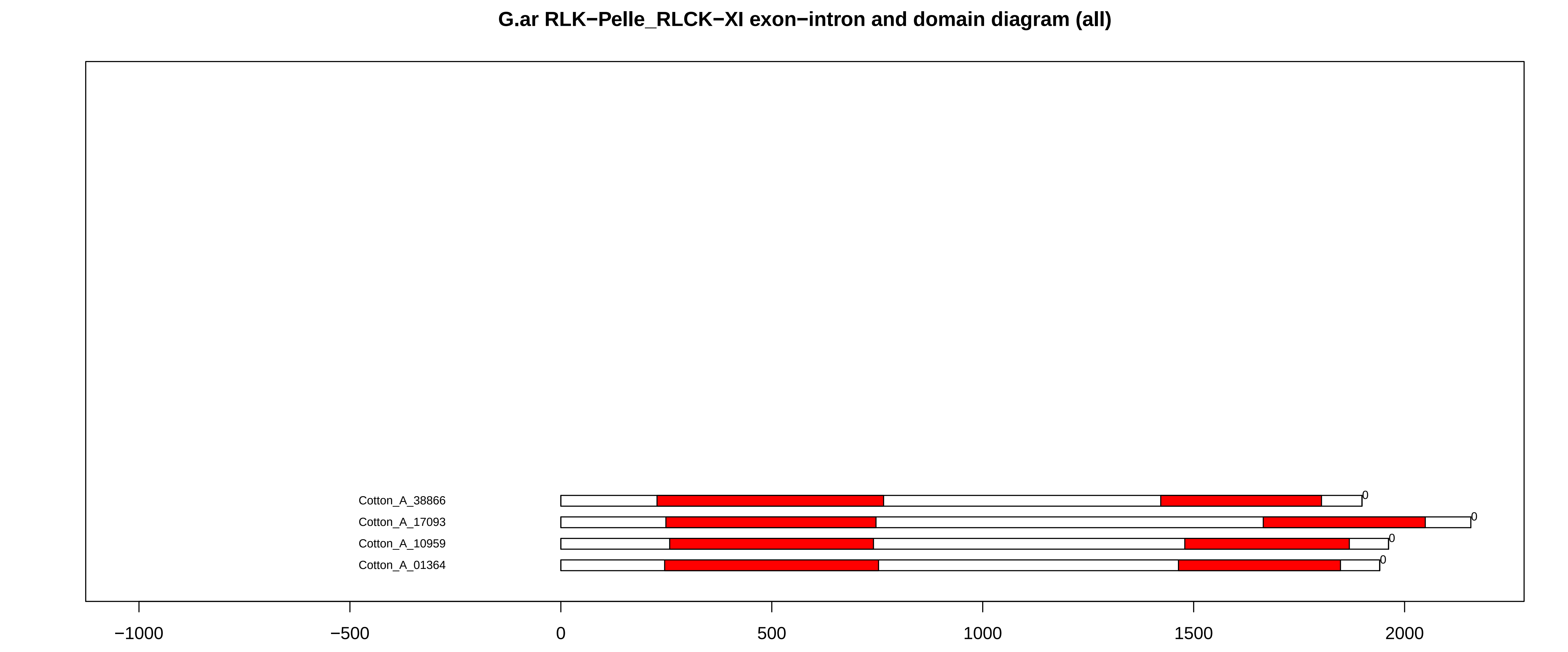
G.ar RLK−Pelle_RLCK−XI exon−intron and domain diagram (all)
Cotton_A_38866
Cotton_A_17093
Cotton_A_10959
Cotton_A_01364
0
0
0
0
−1000
−500
0
500
1000
1500
2000

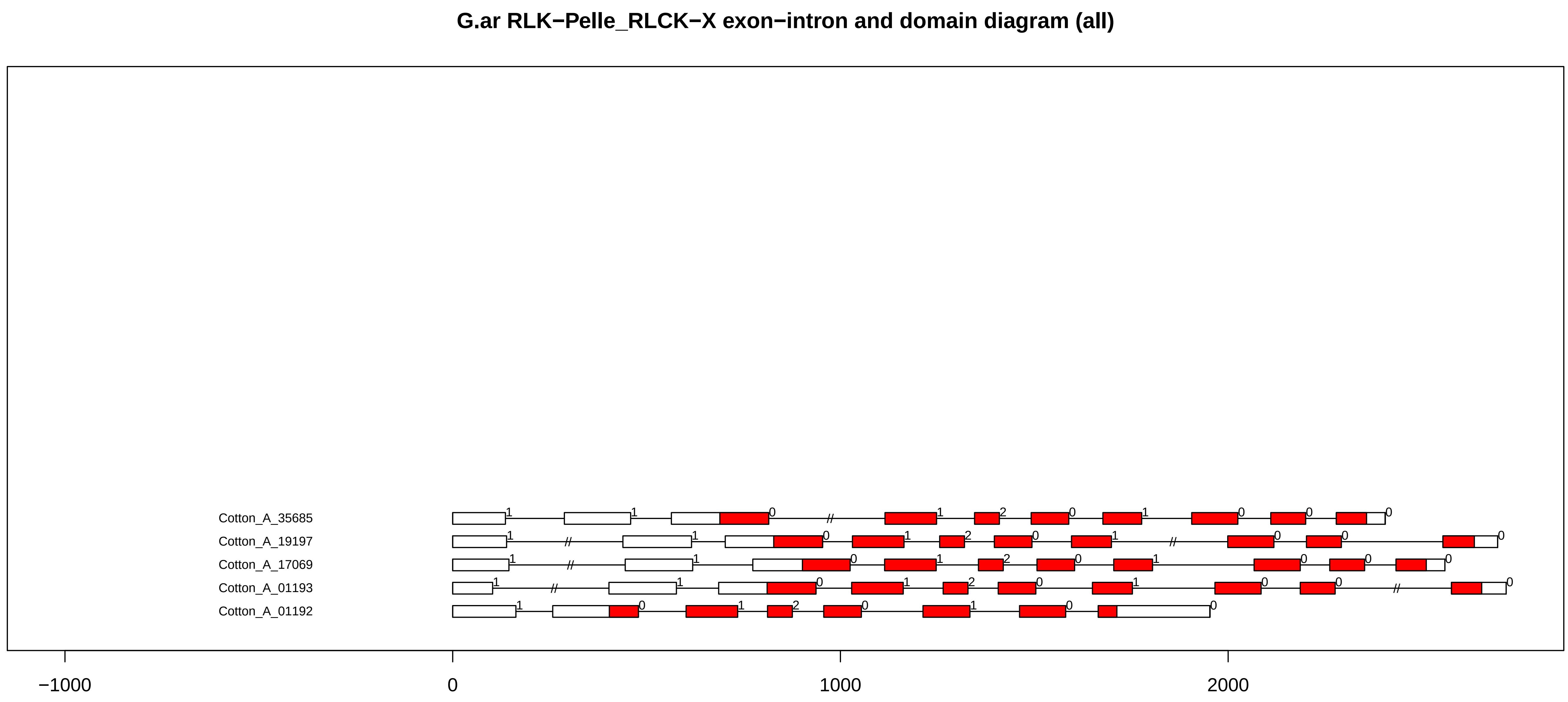
G.ar RLK−Pelle_RLCK−X exon−intron and domain diagram (all)
Cotton_A_35685
Cotton_A_19197
Cotton_A_17069
Cotton_A_01193
Cotton_A_01192
−1000
0
1000
2000

G.ar RLK−Pelle_RLCK−XVI exon−intron and domain diagram (all)

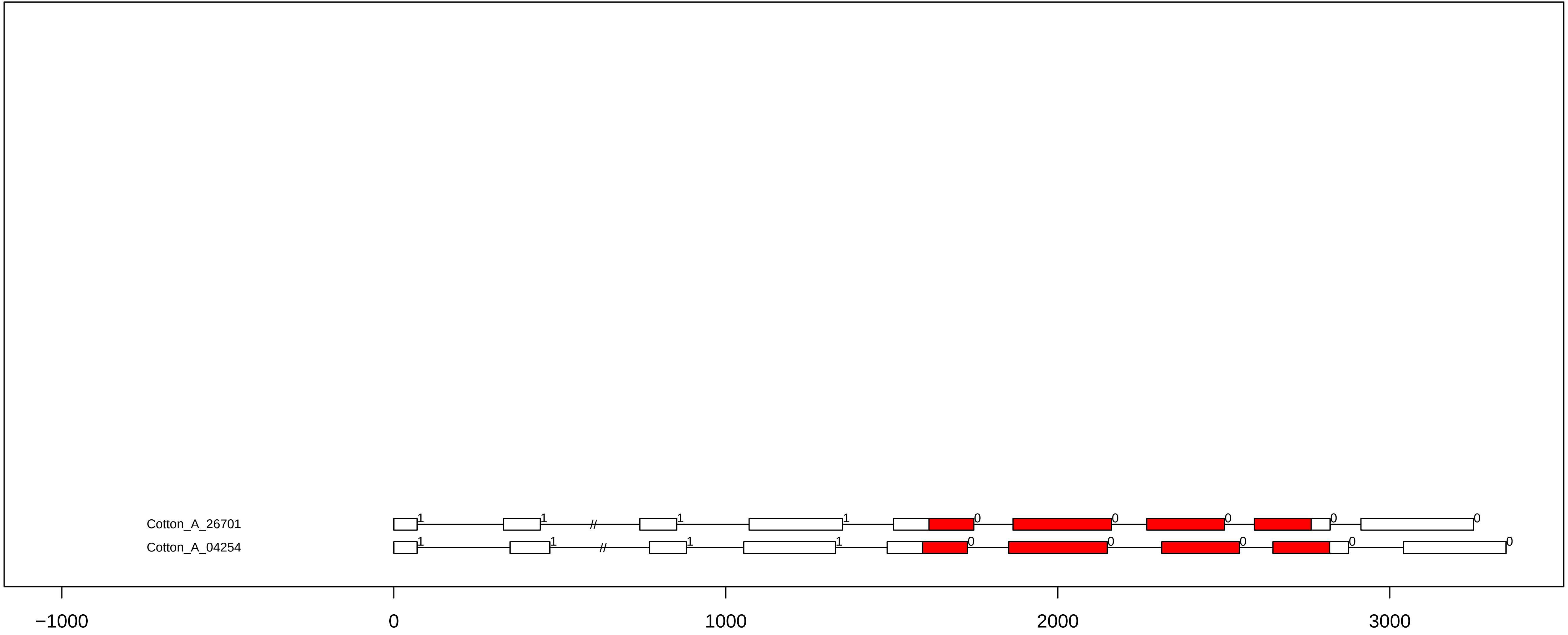

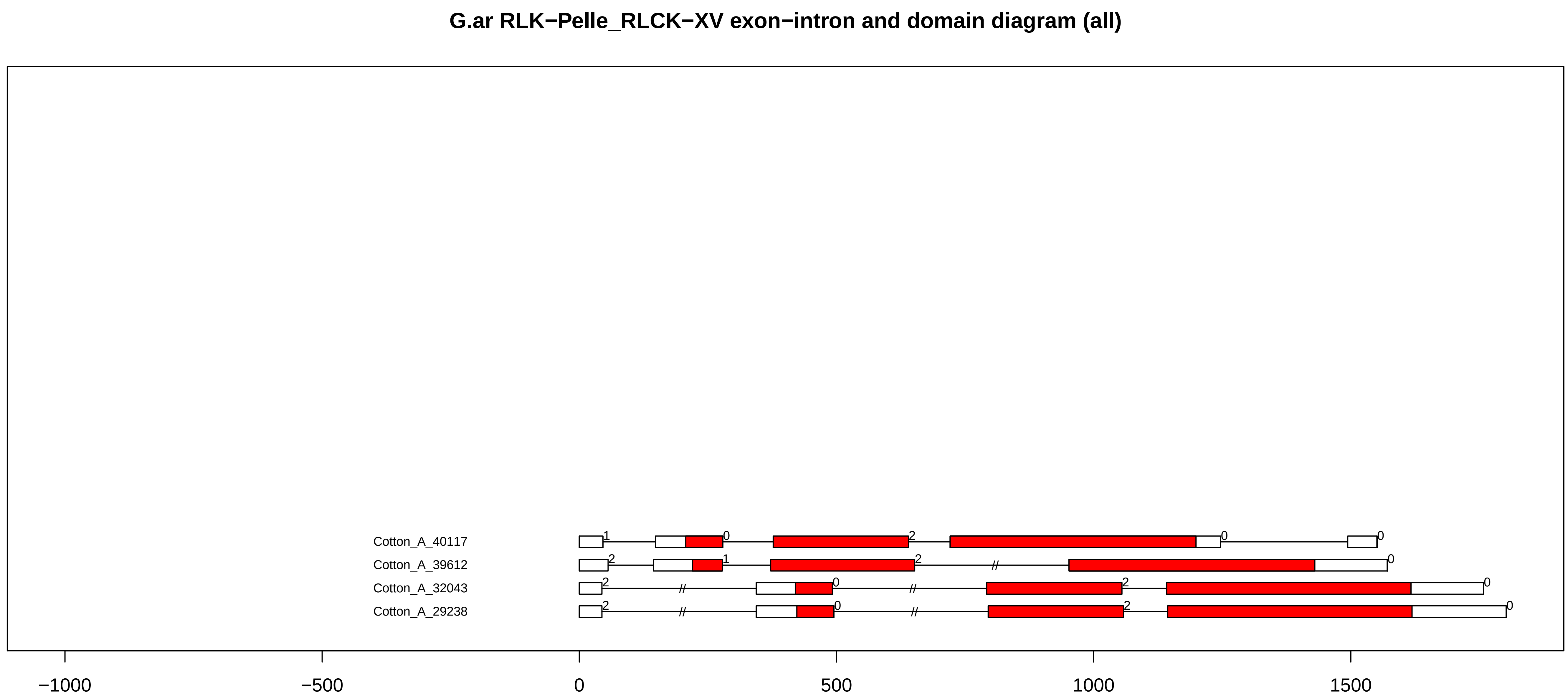

G.ar RLK−Pelle_RLCK−XV exon−intron and domain diagram (all)
Cotton_A_40117
Cotton_A_39612
Cotton_A_32043
Cotton_A_29238
−1000
−500
0
500
1000
1500

G.ar RLK−Pelle_SD−2b exon−intron and domain diagram (part 1)

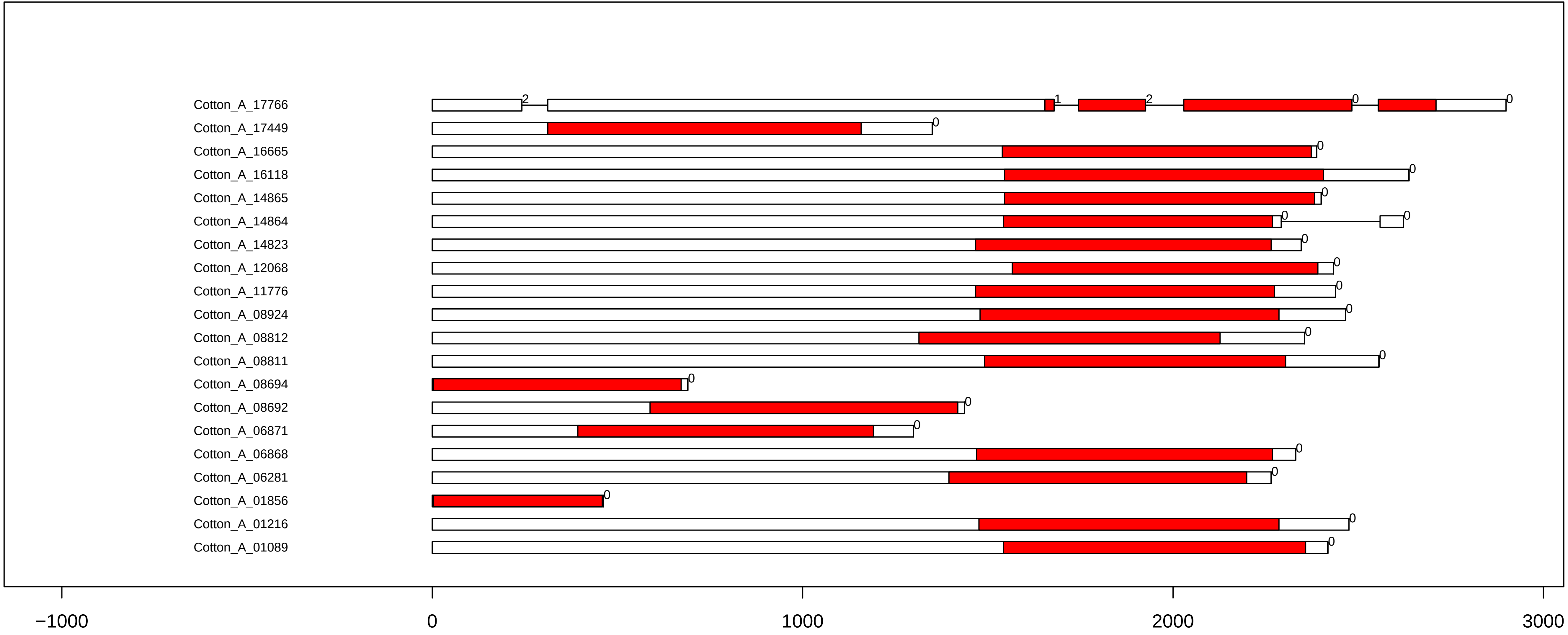

G.ar RLK−Pelle_SD−2b exon−intron and domain diagram (part 2)

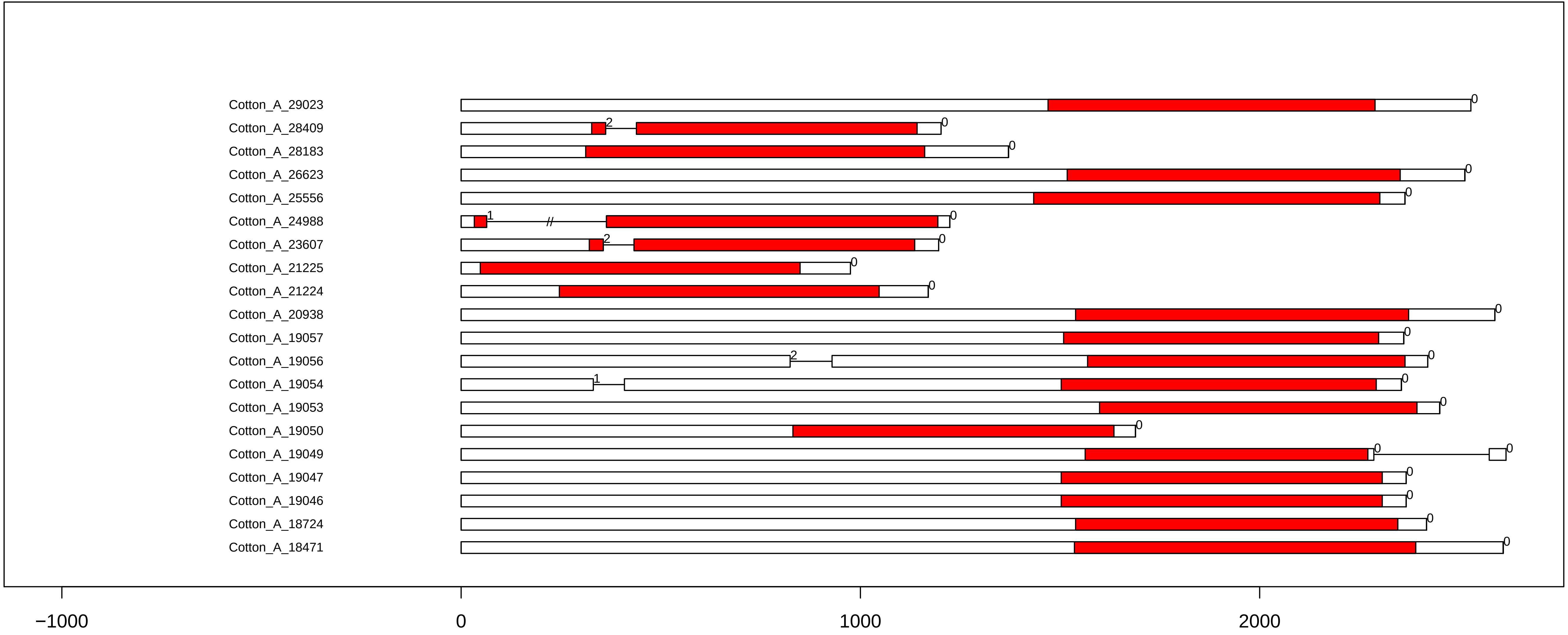

G.ar RLK−Pelle_SD−2b exon−intron and domain diagram (part 3)

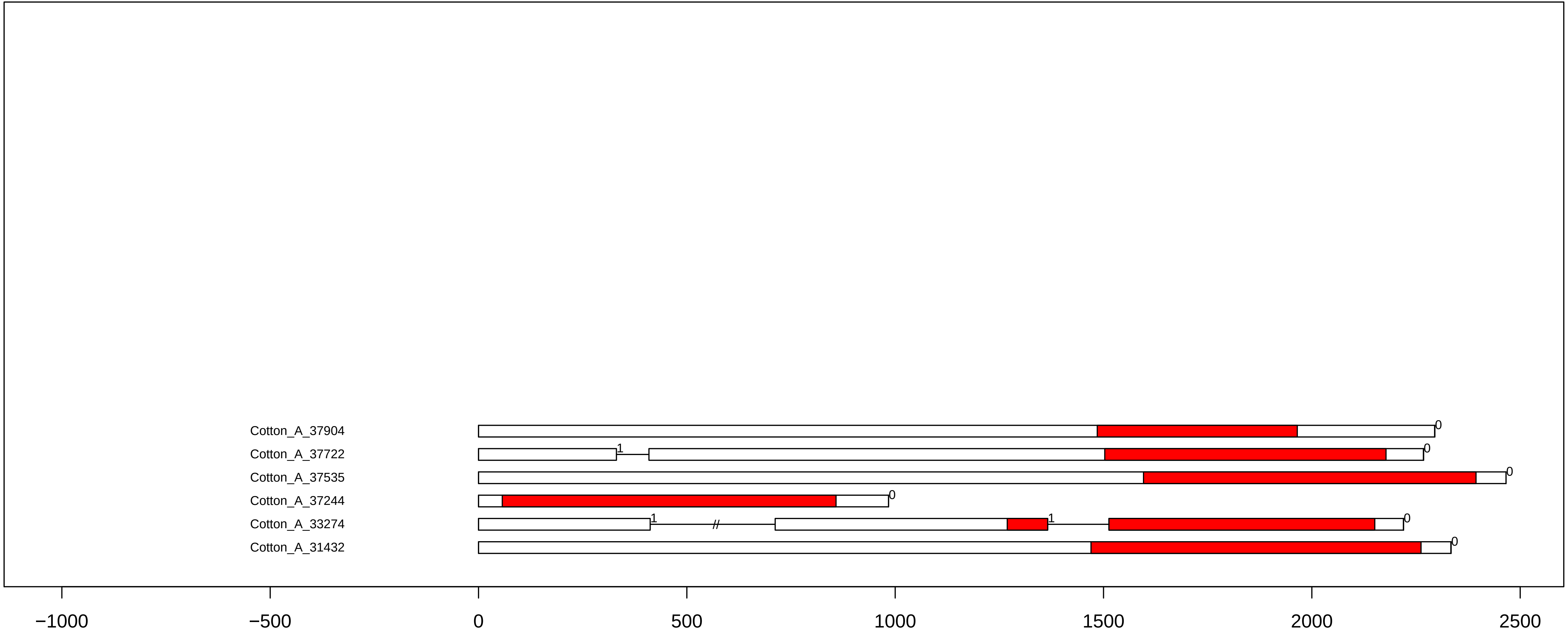

G.ar RLK−Pelle_Singleton exon−intron and domain diagram (all)

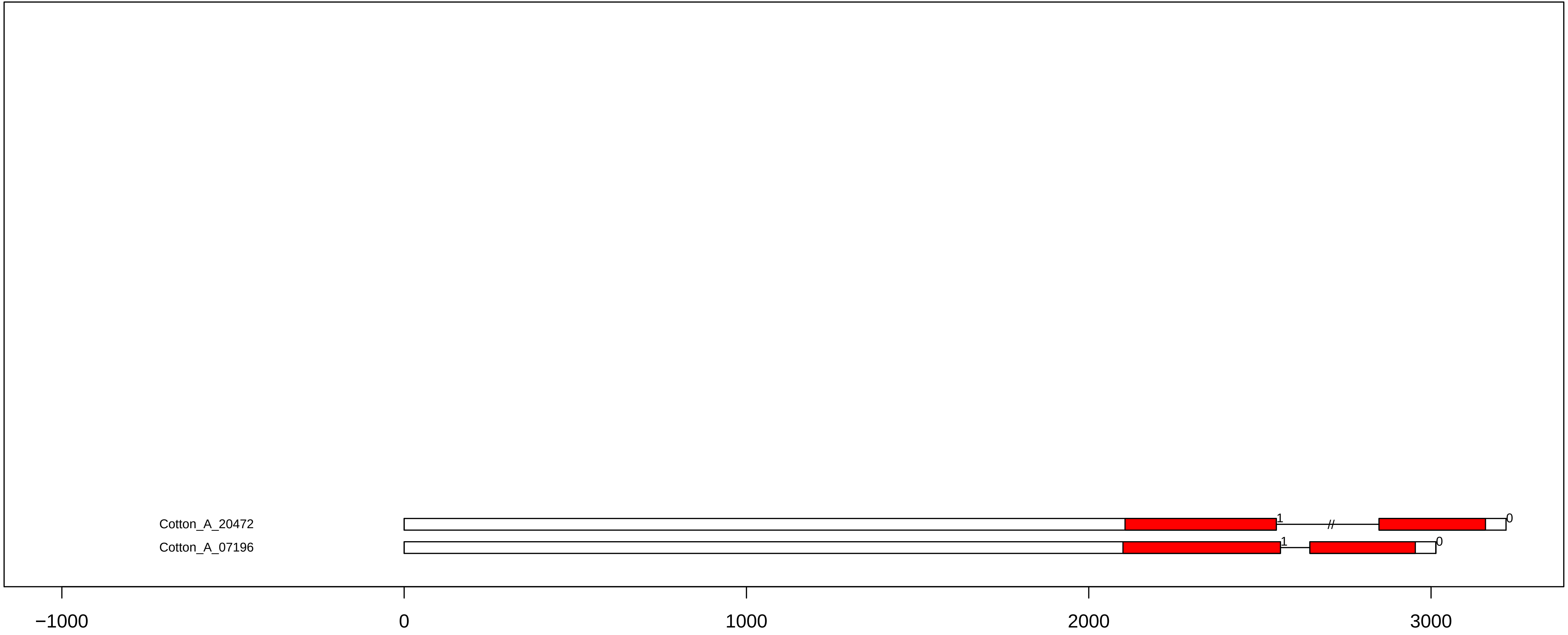

G.ar RLK−Pelle_URK−1 exon−intron and domain diagram (all)

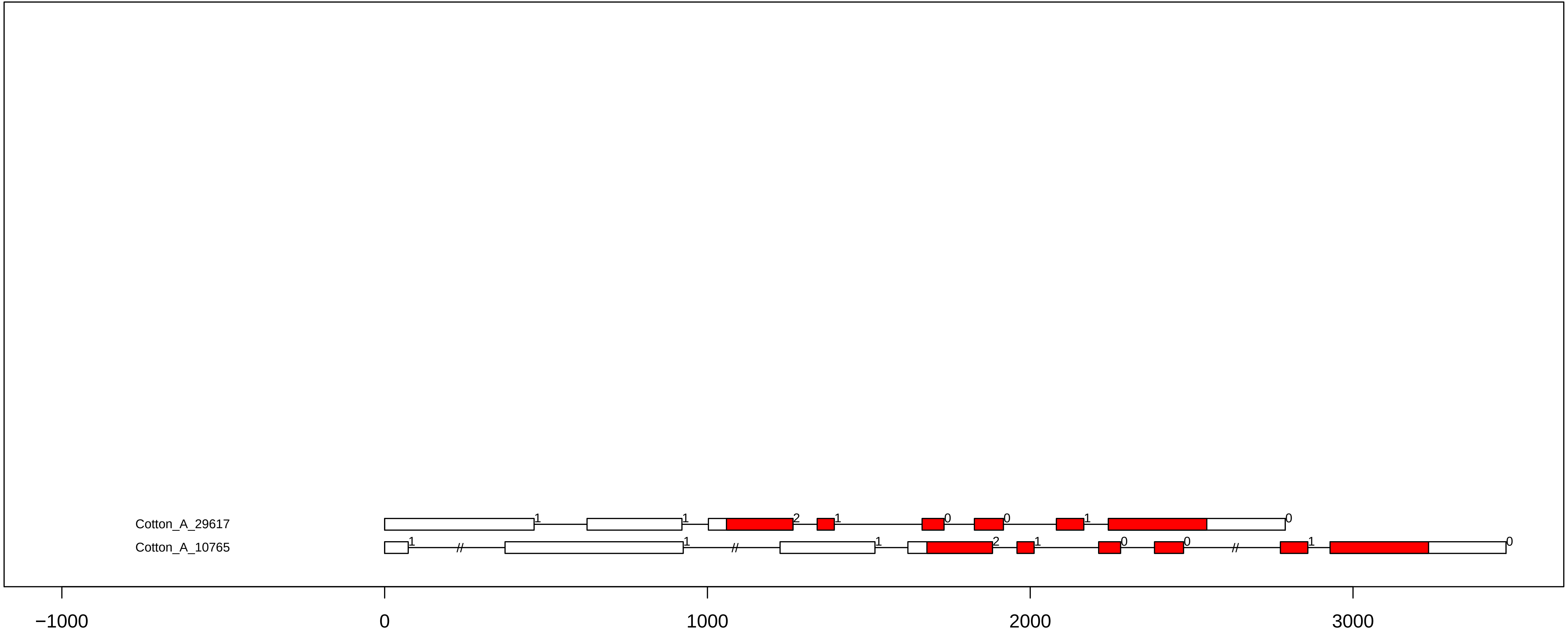

G.ar RLK−Pelle_URK−2 exon−intron and domain diagram (all)

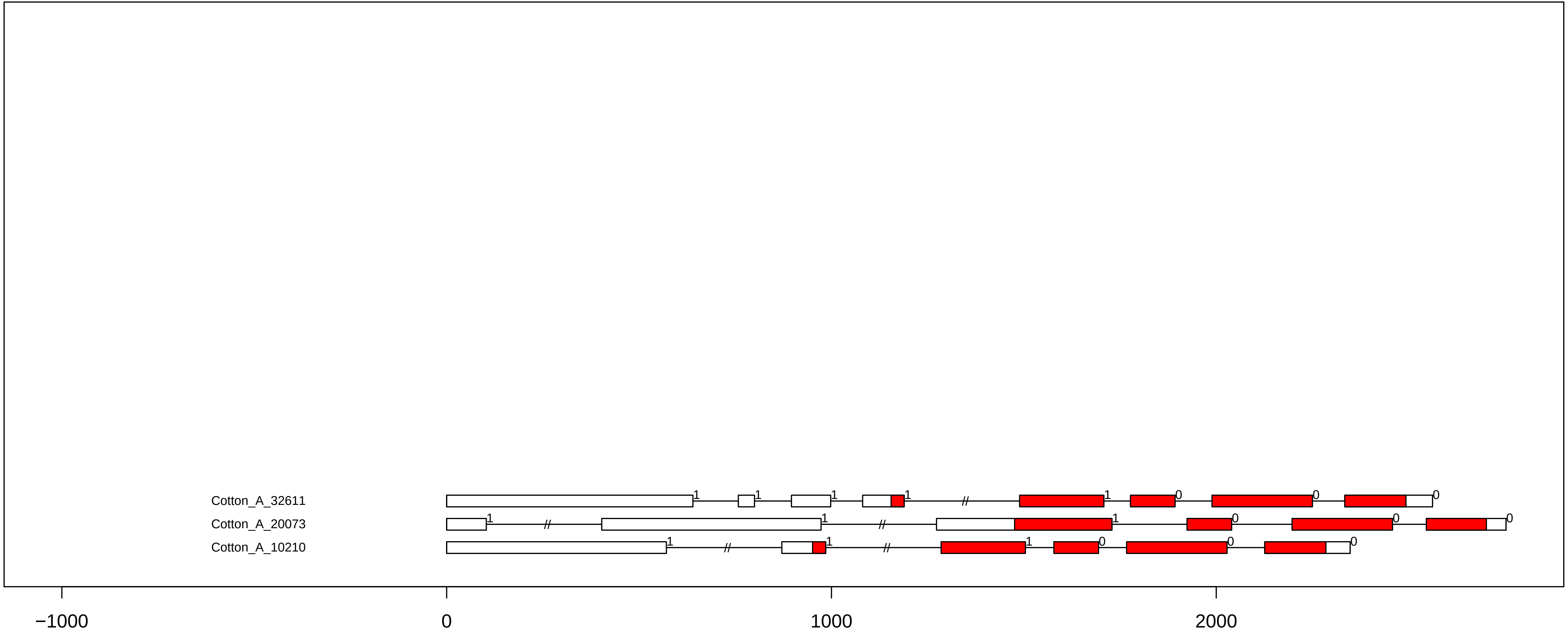

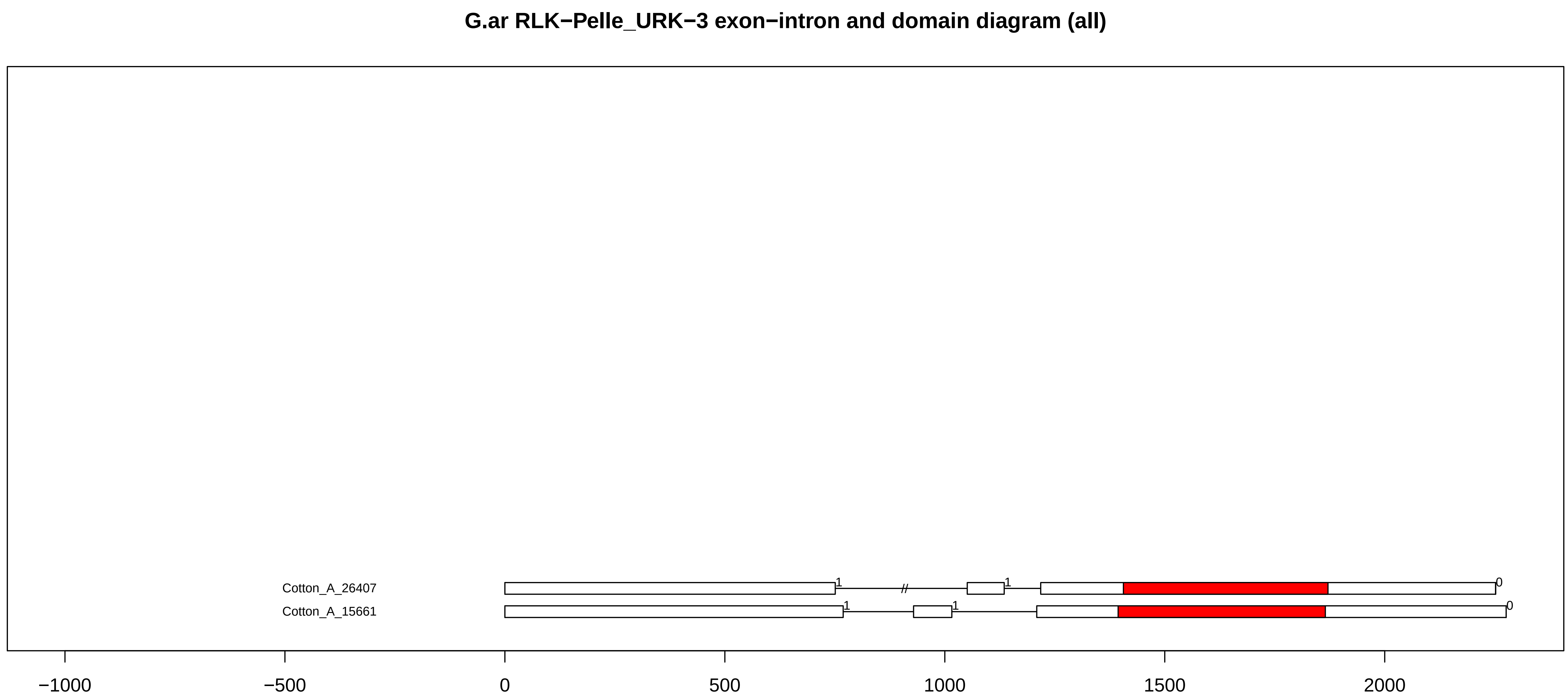
G.ar RLK−Pelle_URK−3 exon−intron and domain diagram (all)
Cotton_A_26407
Cotton_A_15661
1
1
0
1
1
0
−1000
−500
0
500
1000
1500
2000

G.ar RLK−Pelle_WAK_LRK10L−1 exon−intron and domain diagram (all)

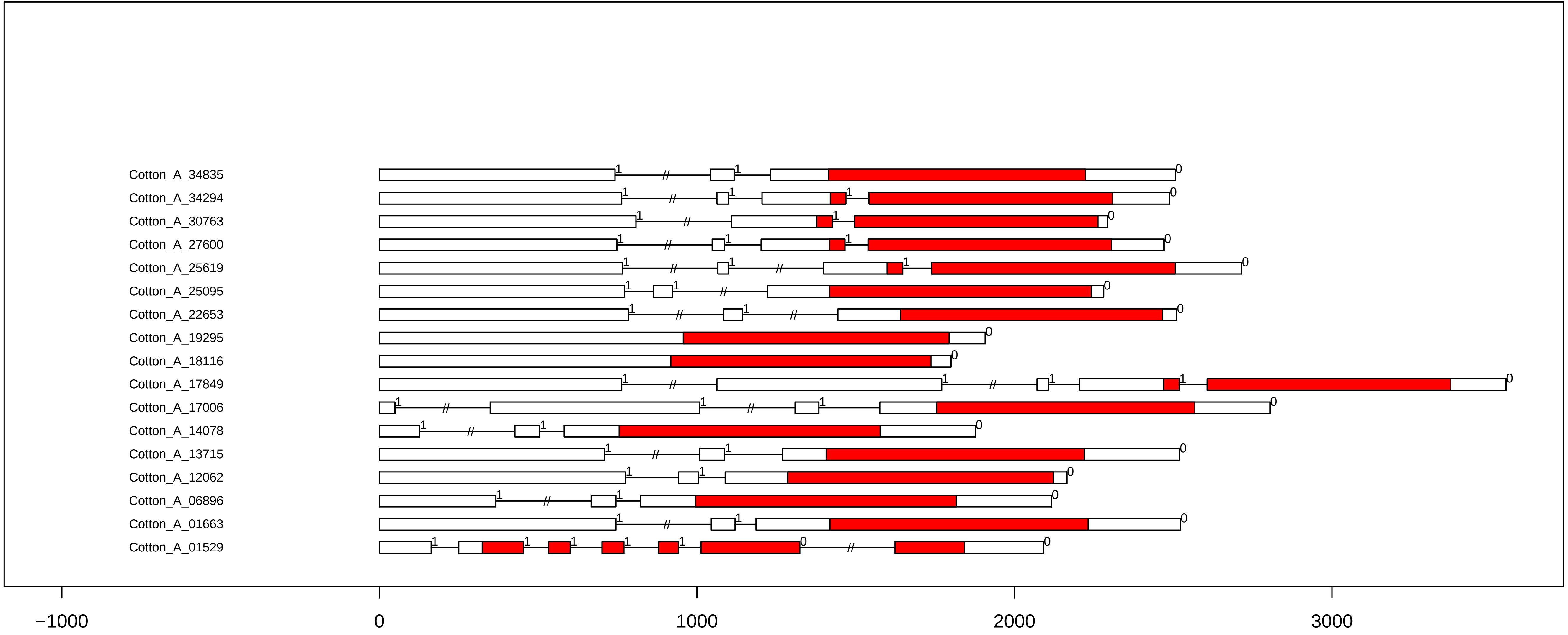

G.ar RLK−Pelle_WAK exon−intron and domain diagram (part 1)

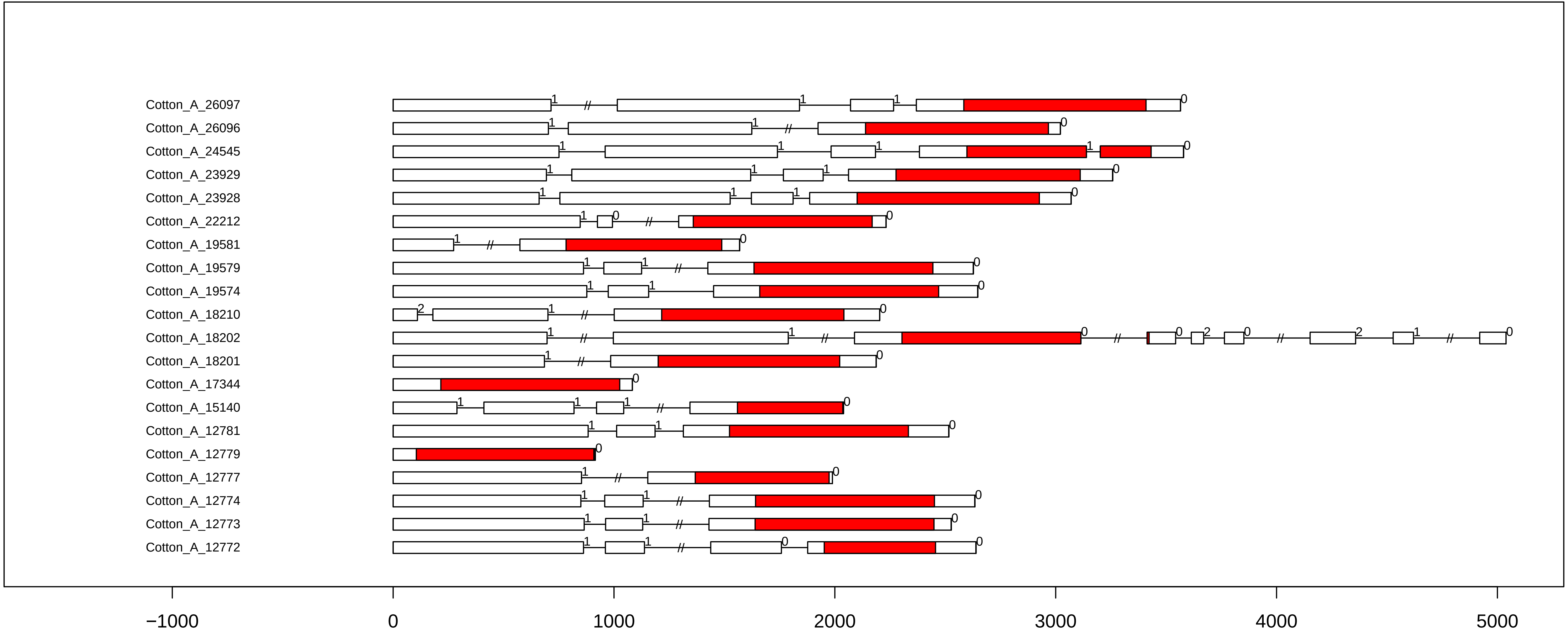

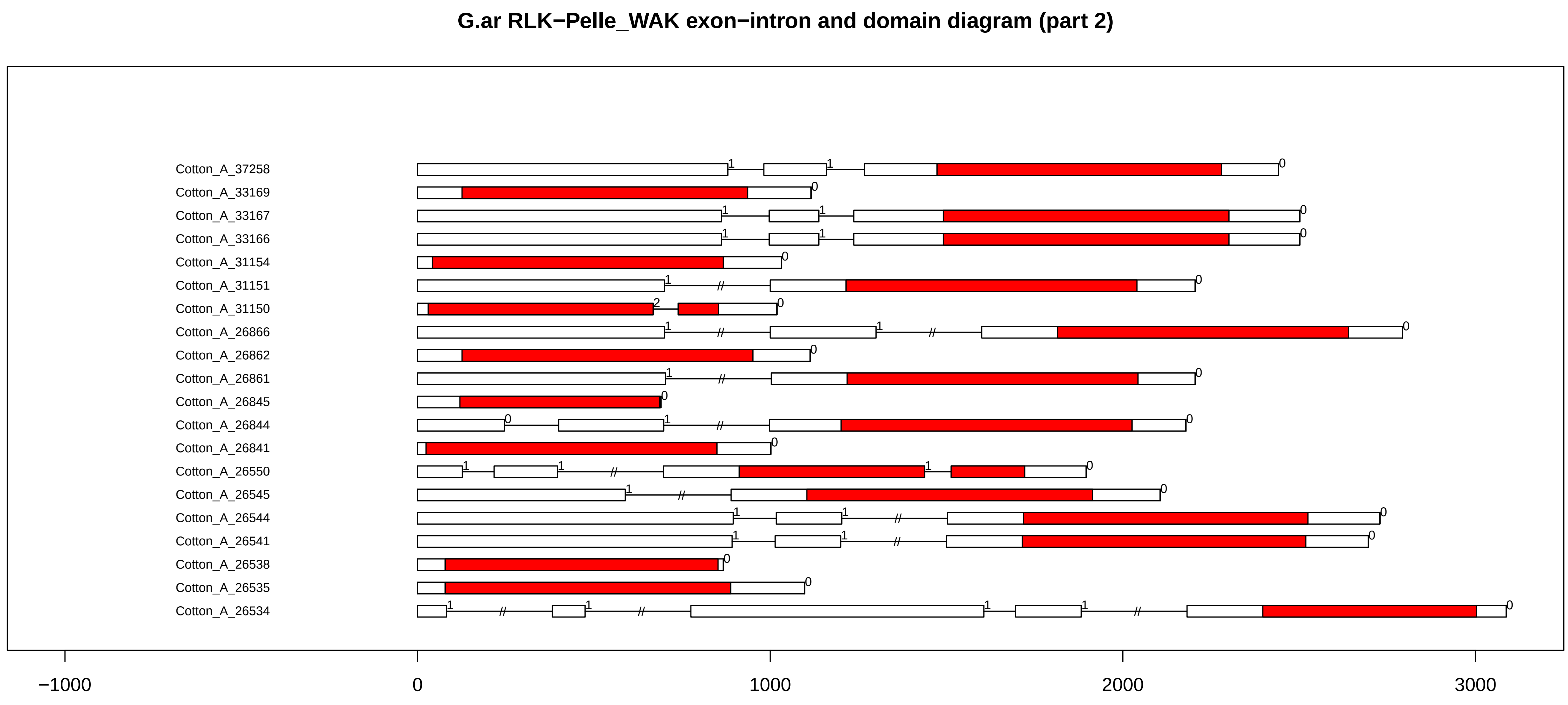

G.ar RLK−Pelle_WAK exon−intron and domain diagram (part 2)
Cotton_A_37258
Cotton_A_33169
Cotton_A_33167
Cotton_A_33166
Cotton_A_31154
Cotton_A_31151
Cotton_A_31150
Cotton_A_26866
Cotton_A_26862
Cotton_A_26861
Cotton_A_26845
Cotton_A_26844
Cotton_A_26841
Cotton_A_26550
Cotton_A_26545
Cotton_A_26544
Cotton_A_26541
Cotton_A_26538
Cotton_A_26535
Cotton_A_26534
−1000
0
1000
2000
3000

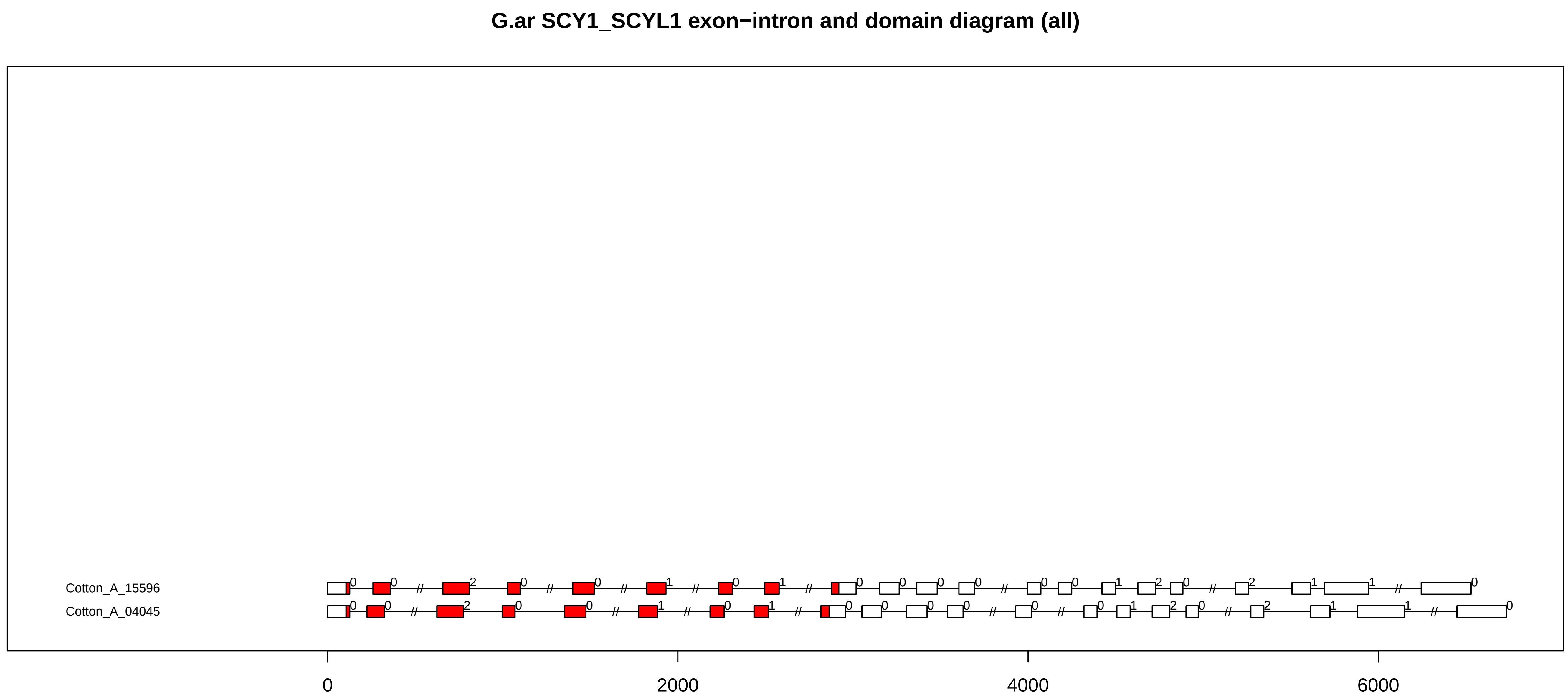

G.ar SCY1_SCYL1 exon−intron and domain diagram (all)
Cotton_A_15596
Cotton_A_04045
0
2000
4000
6000

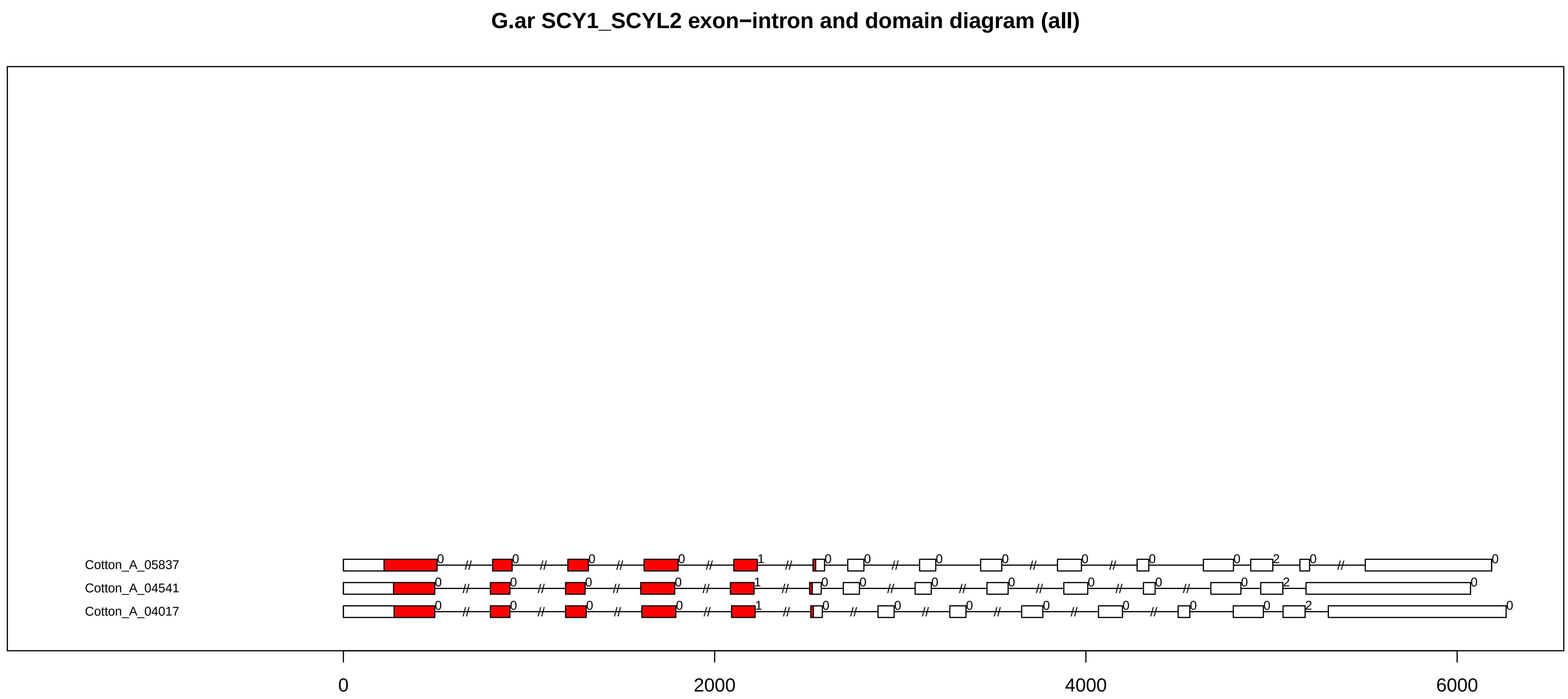
G.ar SCY1_SCYL2 exon−intron and domain diagram (all)
Cotton_A_05837
Cotton_A_04541
Cotton_A_04017
0
2000
4000
6000

G.ar STE_STE11 exon−intron and domain diagram (part 1)

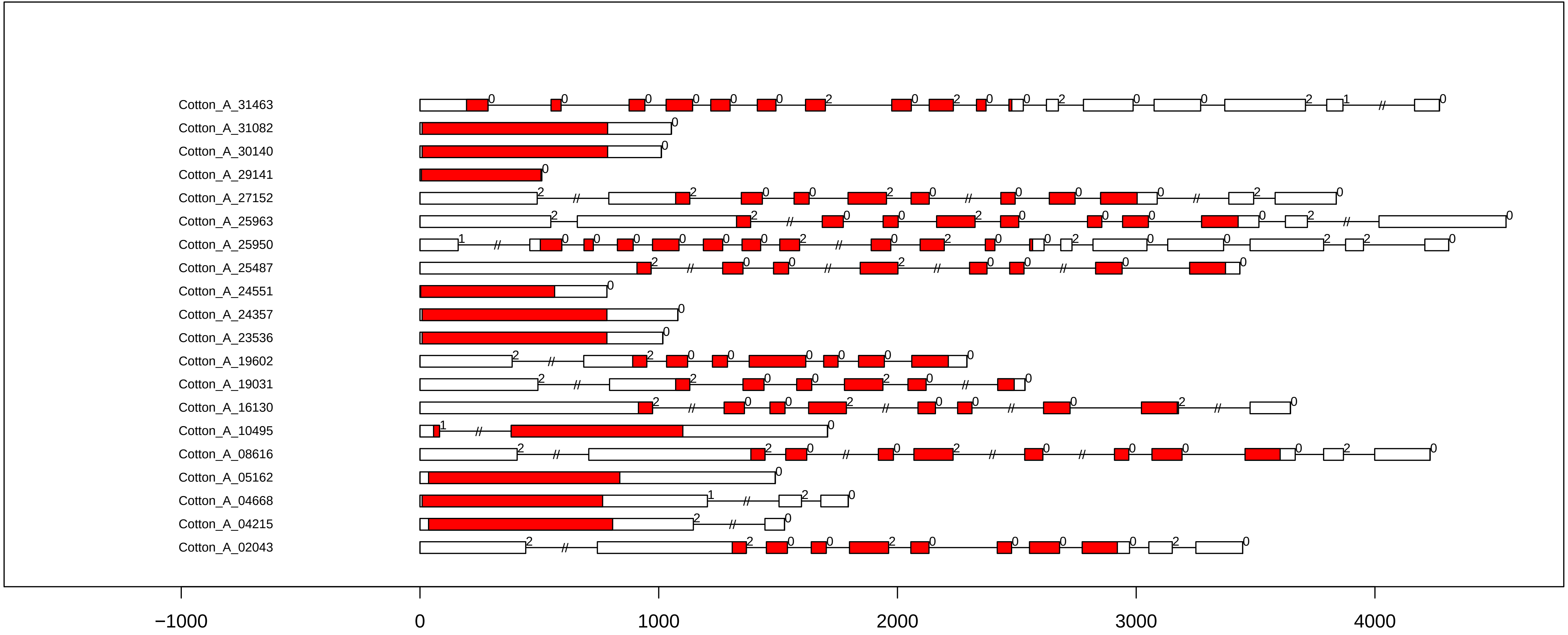

G.ar STE_STE11 exon−intron and domain diagram (part 2)

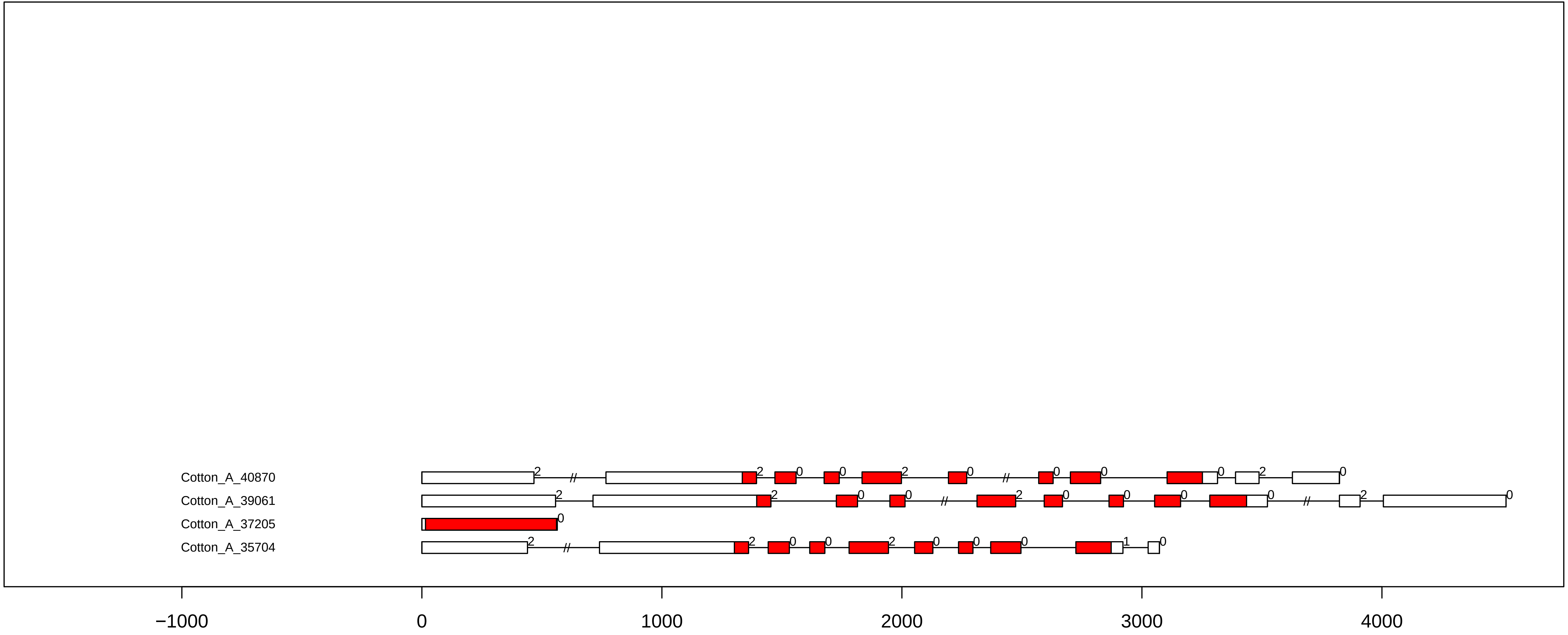

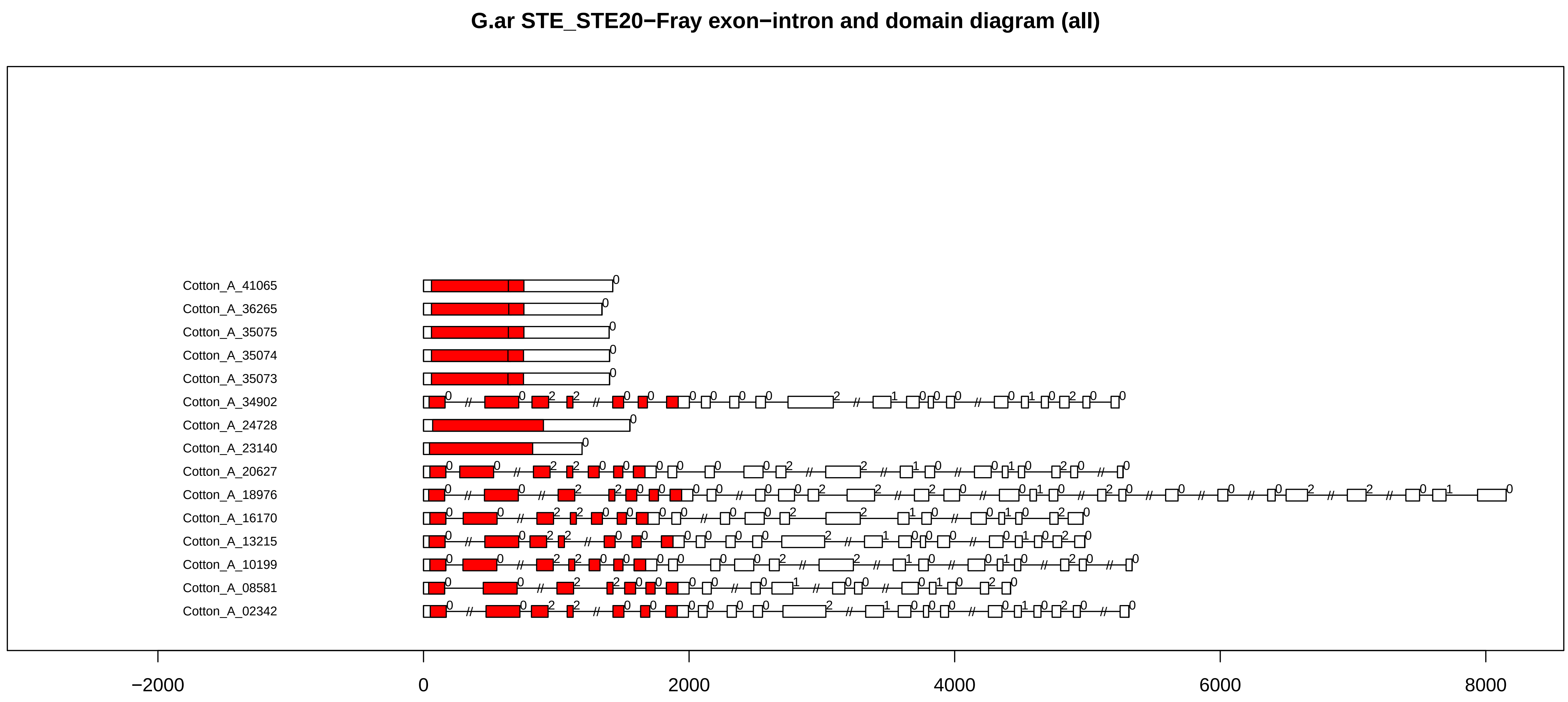
G.ar STE_STE20−Fray exon−intron and domain diagram (all)
Cotton_A_41065
Cotton_A_36265
Cotton_A_35075
Cotton_A_35074
Cotton_A_35073
Cotton_A_34902
Cotton_A_24728
Cotton_A_23140
Cotton_A_20627
Cotton_A_18976
Cotton_A_16170
Cotton_A_13215
Cotton_A_10199
Cotton_A_08581
Cotton_A_02342
−2000
0
2000
4000
6000
8000

G.ar STE_STE20−Pl exon−intron and domain diagram (all)

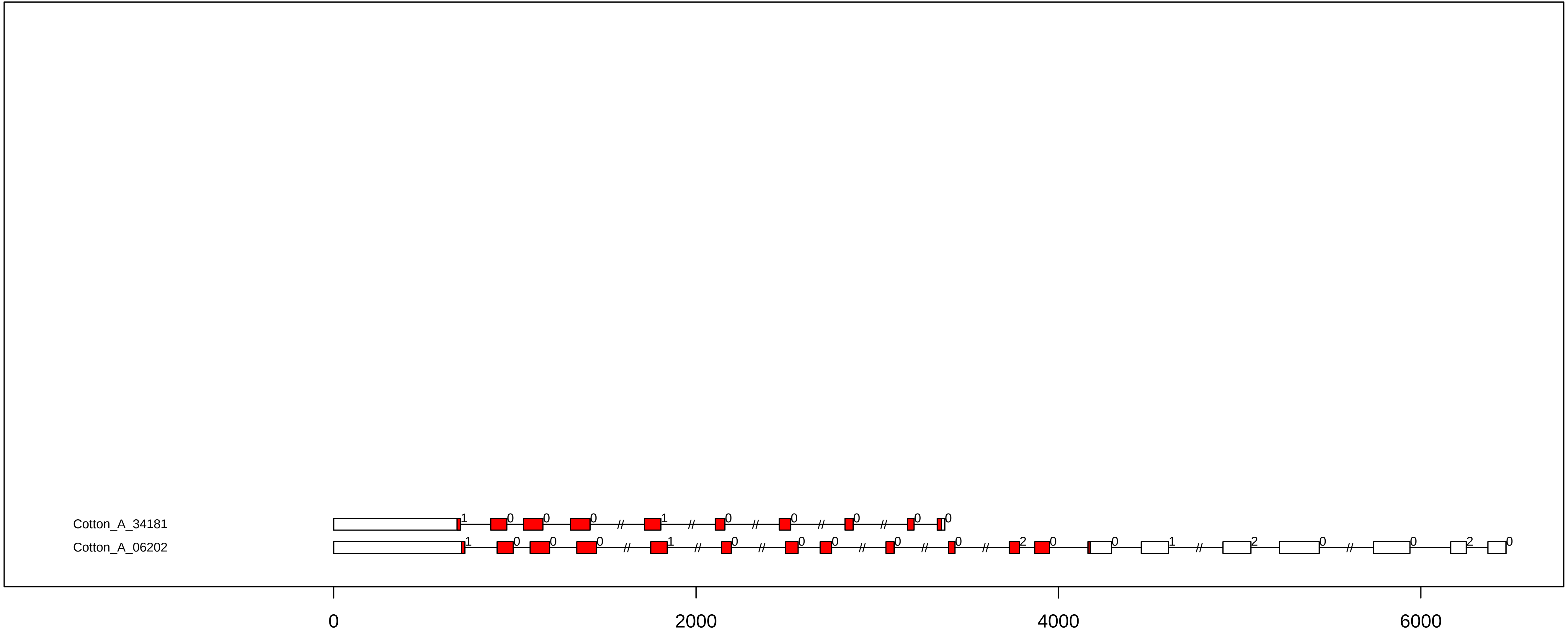

G.ar STE_STE20−YSK exon−intron and domain diagram (all)

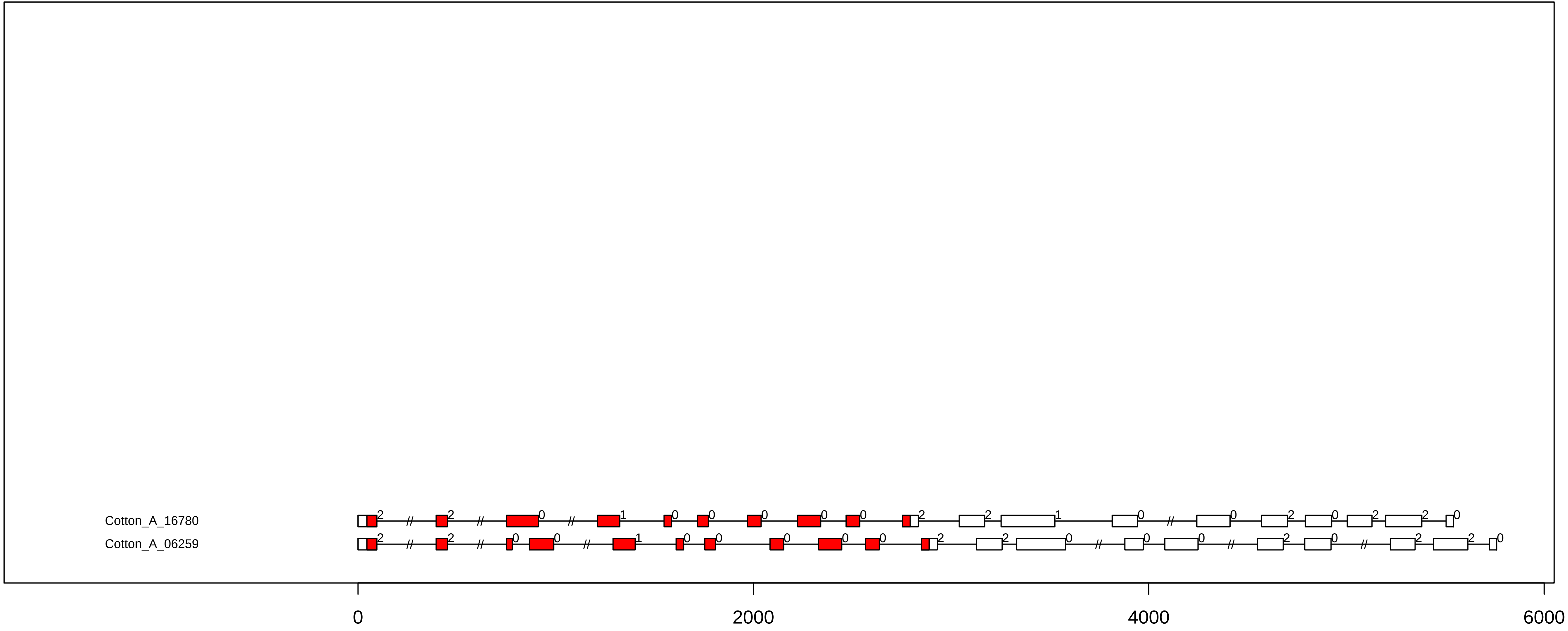

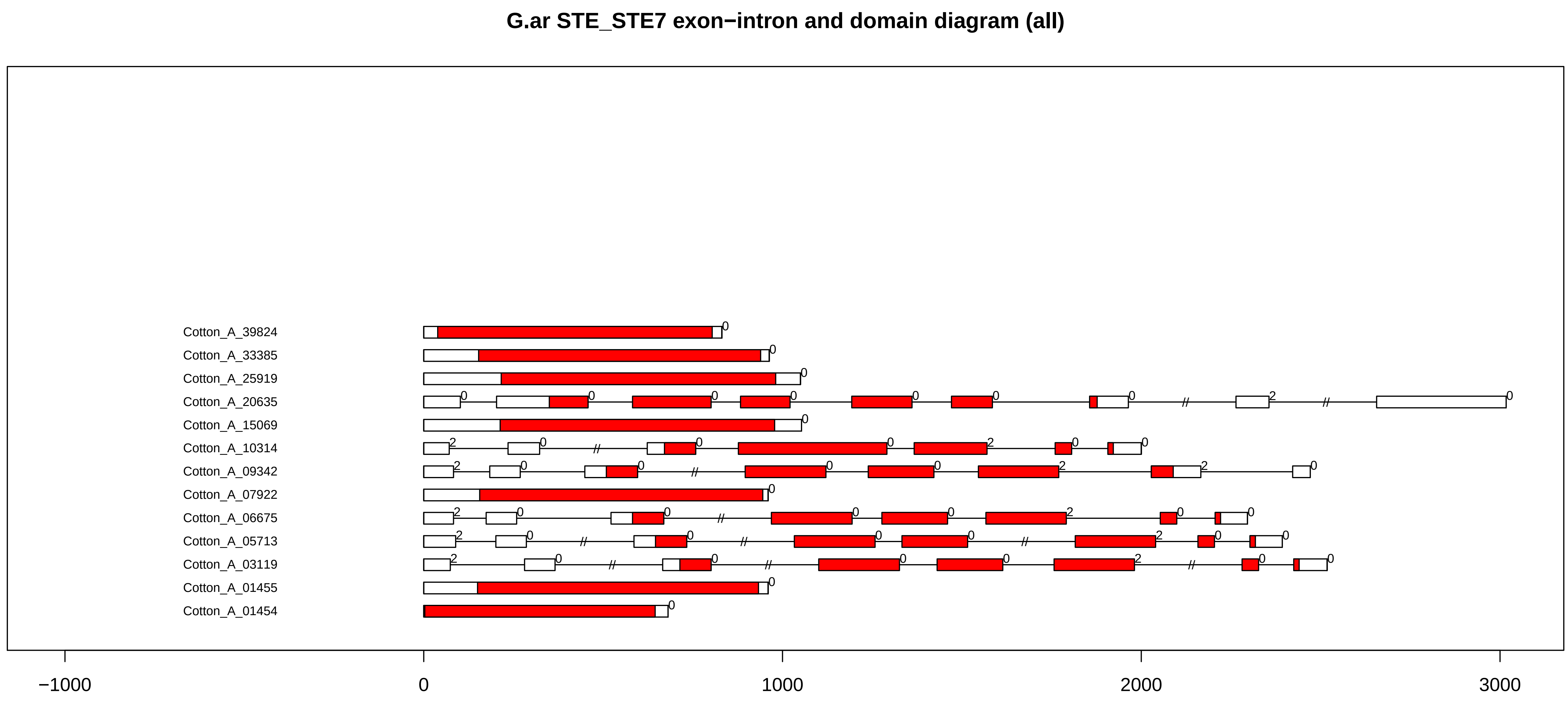
G.ar STE_STE7 exon−intron and domain diagram (all)
Cotton_A_39824
Cotton_A_33385
Cotton_A_25919
Cotton_A_20635
Cotton_A_15069
Cotton_A_10314
Cotton_A_09342
Cotton_A_07922
Cotton_A_06675
Cotton_A_05713
Cotton_A_03119
Cotton_A_01455
Cotton_A_01454
−1000
0
1000
2000
3000

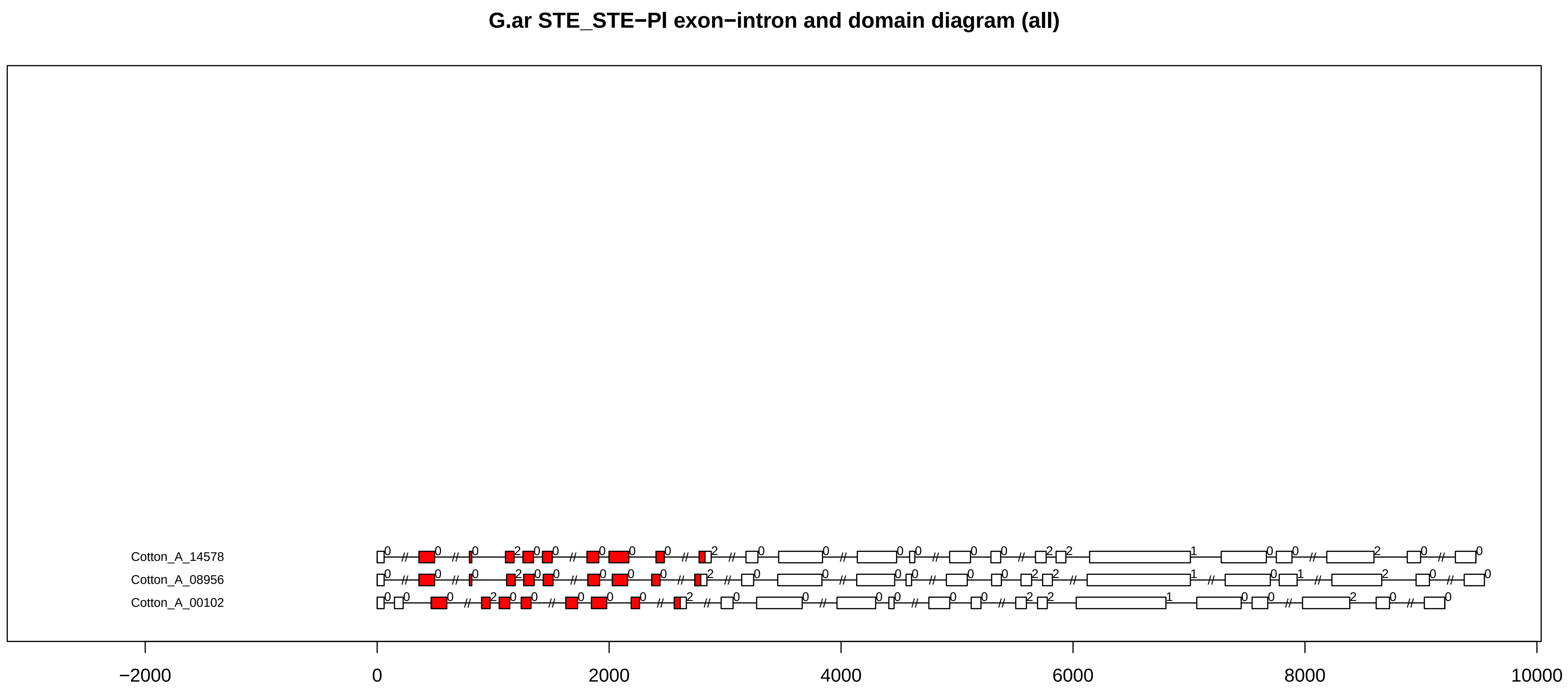
G.ar STE_STE−PI exon−intron and domain diagram (all)
Cotton_A_14578
Cotton_A_08956
Cotton_A_00102
−2000
0
2000
4000
6000
8000
10000

G.ar TKL_CTR1−DRK−1 exon−intron and domain diagram (all)

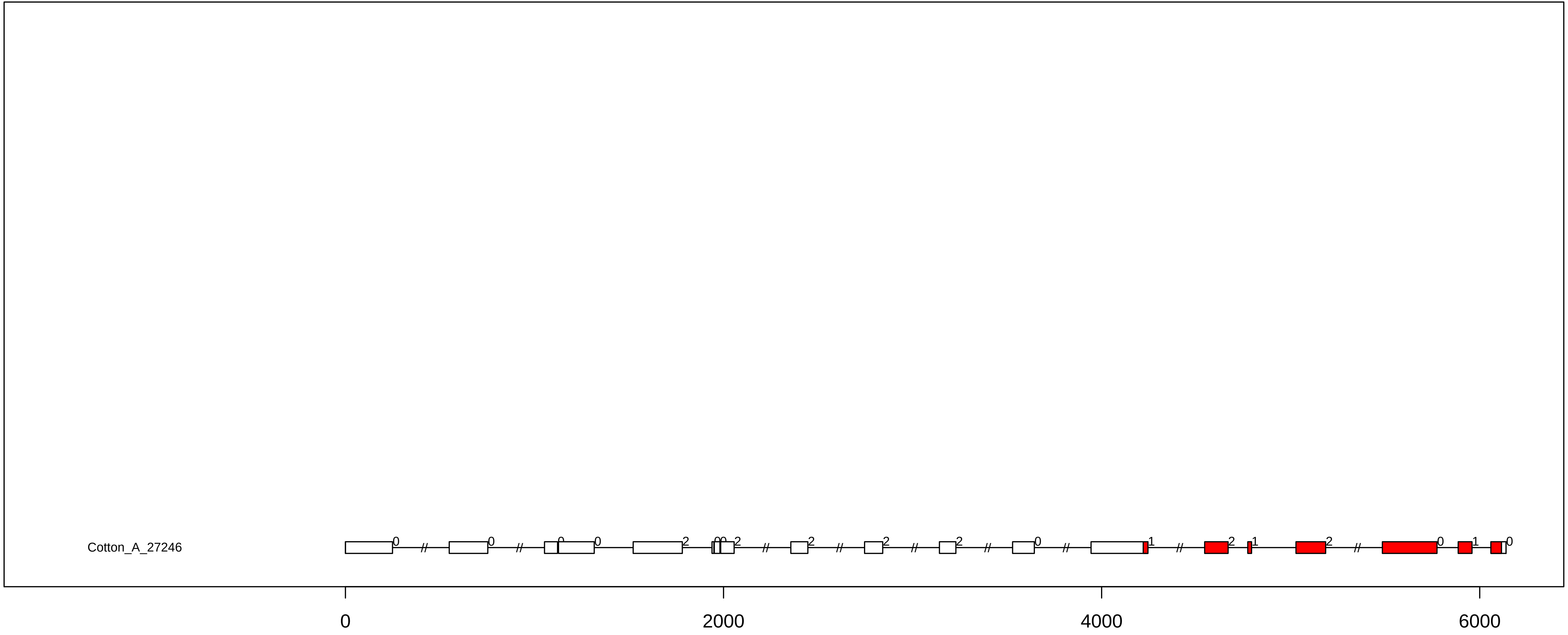

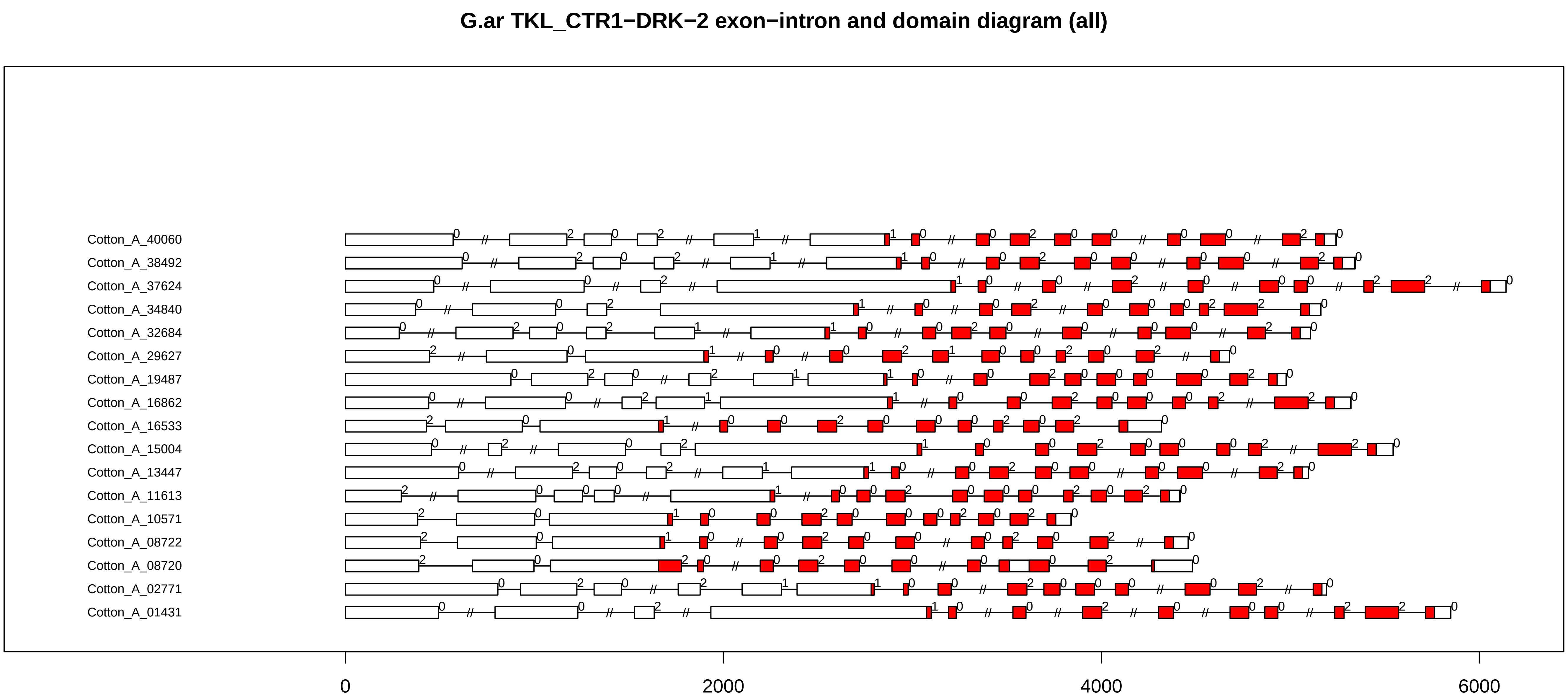
G.ar TKL_CTR1−DRK−2 exon−intron and domain diagram (all)
Cotton_A_40060
Cotton_A_38492
Cotton_A_37624
Cotton_A_34840
Cotton_A_32684
Cotton_A_29627
Cotton_A_19487
Cotton_A_16862
Cotton_A_16533
Cotton_A_15004
Cotton_A_13447
Cotton_A_11613
Cotton_A_10571
Cotton_A_08722
Cotton_A_08720
Cotton_A_02771
Cotton_A_01431
0
2000
4000
6000

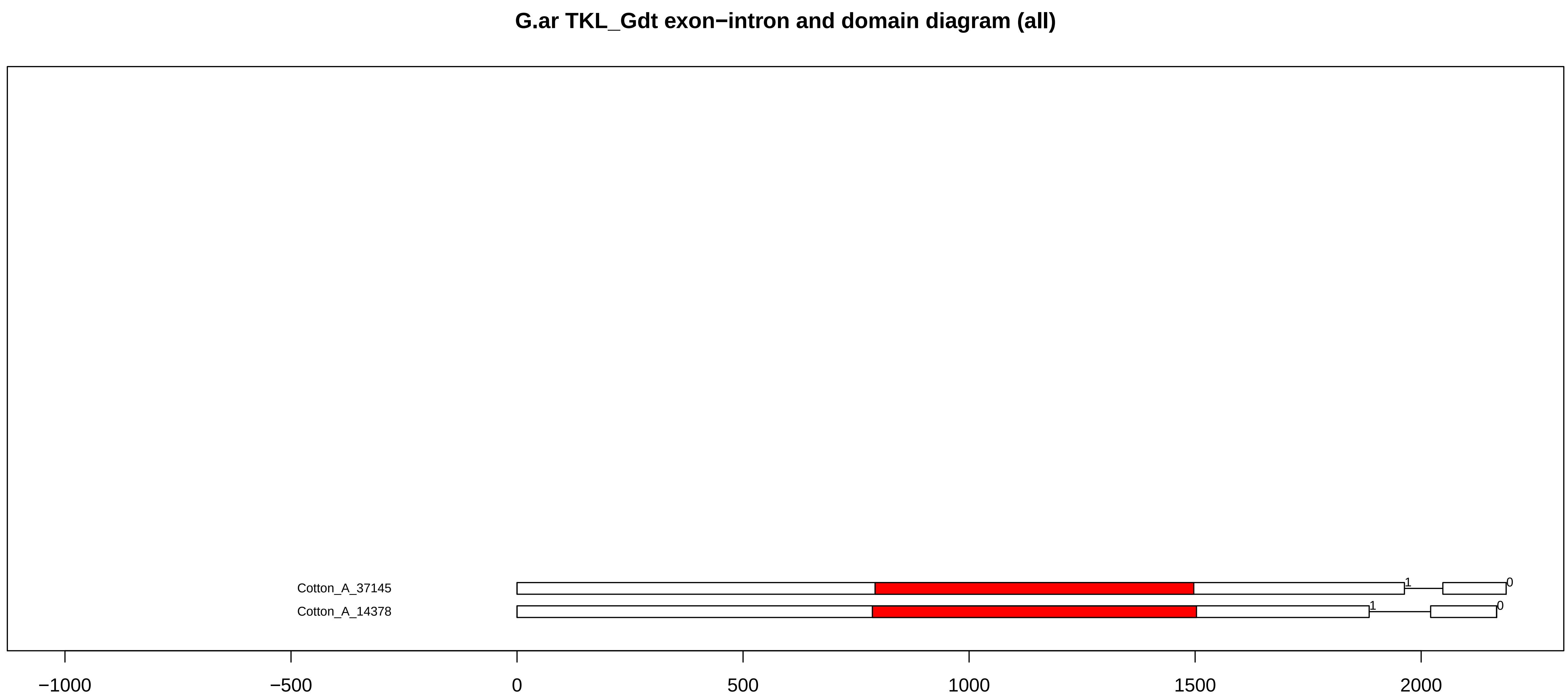
G.ar TKL_Gdt exon−intron and domain diagram (all)
Cotton_A_37145
Cotton_A_14378
1
0
1
0
−1000
−500
0
500
1000
1500
2000

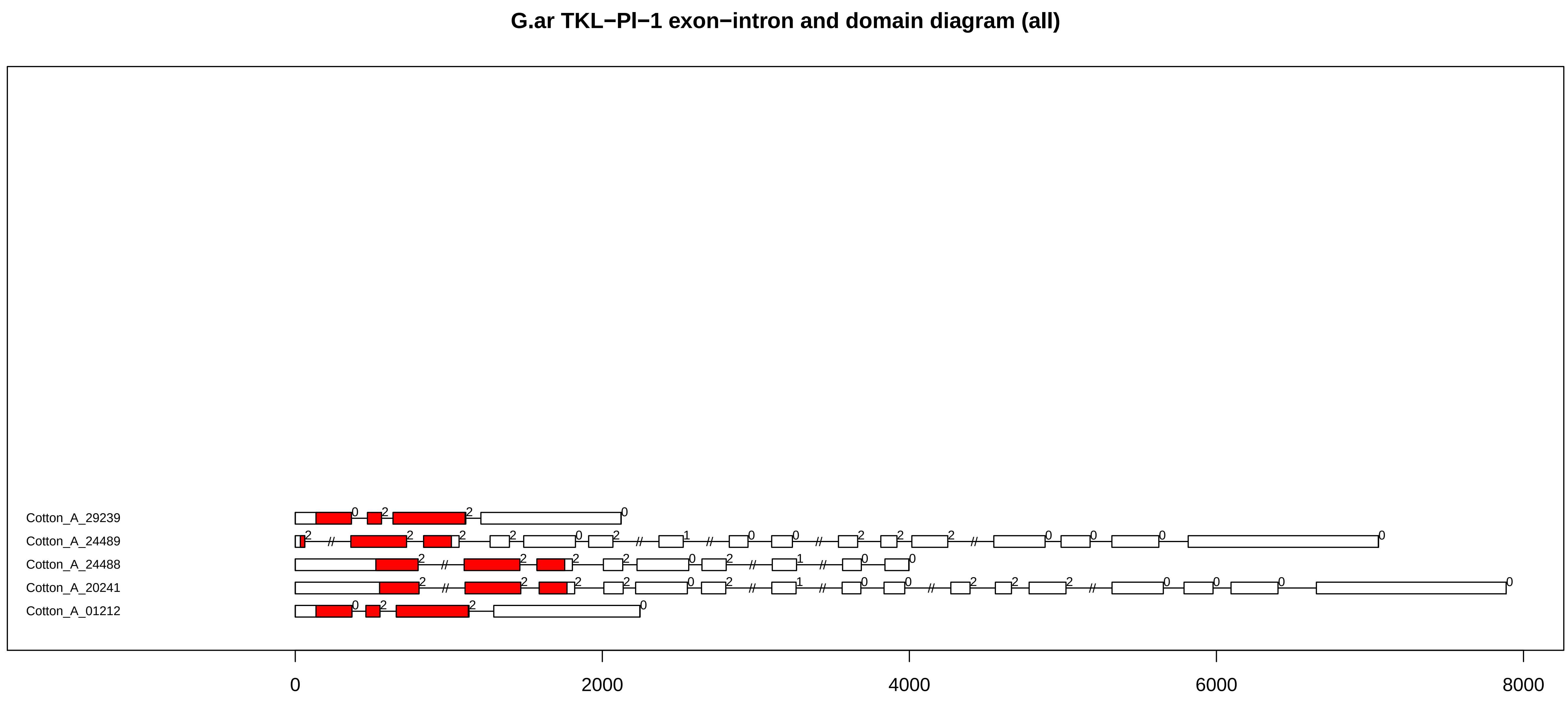
G.ar TKL−Pl−1 exon−intron and domain diagram (all)
Cotton_A_29239
Cotton_A_24489
Cotton_A_24488
Cotton_A_20241
Cotton_A_01212
0
2000
4000
6000
8000

G.ar TKL−Pl−2 exon−intron and domain diagram (all)

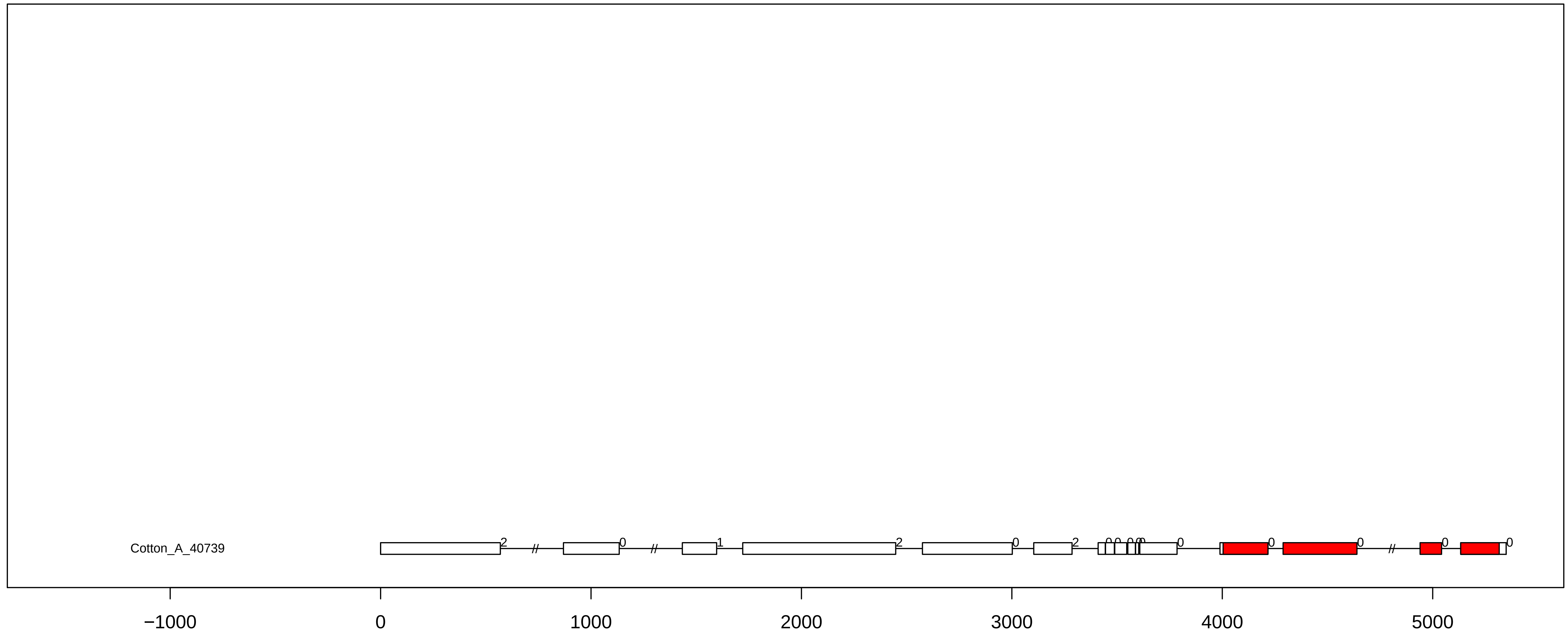

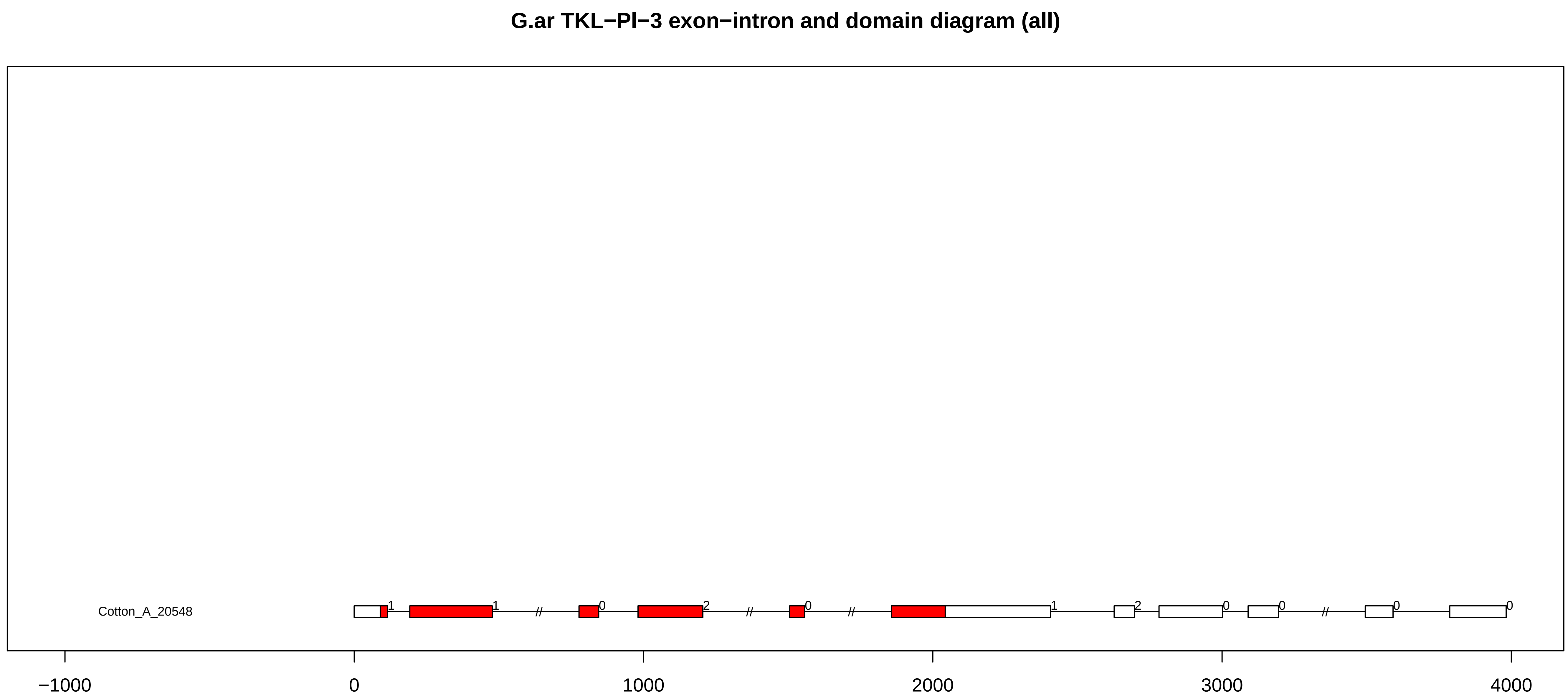
G.ar TKL−Pl−3 exon−intron and domain diagram (all)
Cotton_A_20548
1
1
0
2
0
1
2
0
0
0
0
−1000
0
1000
2000
3000
4000

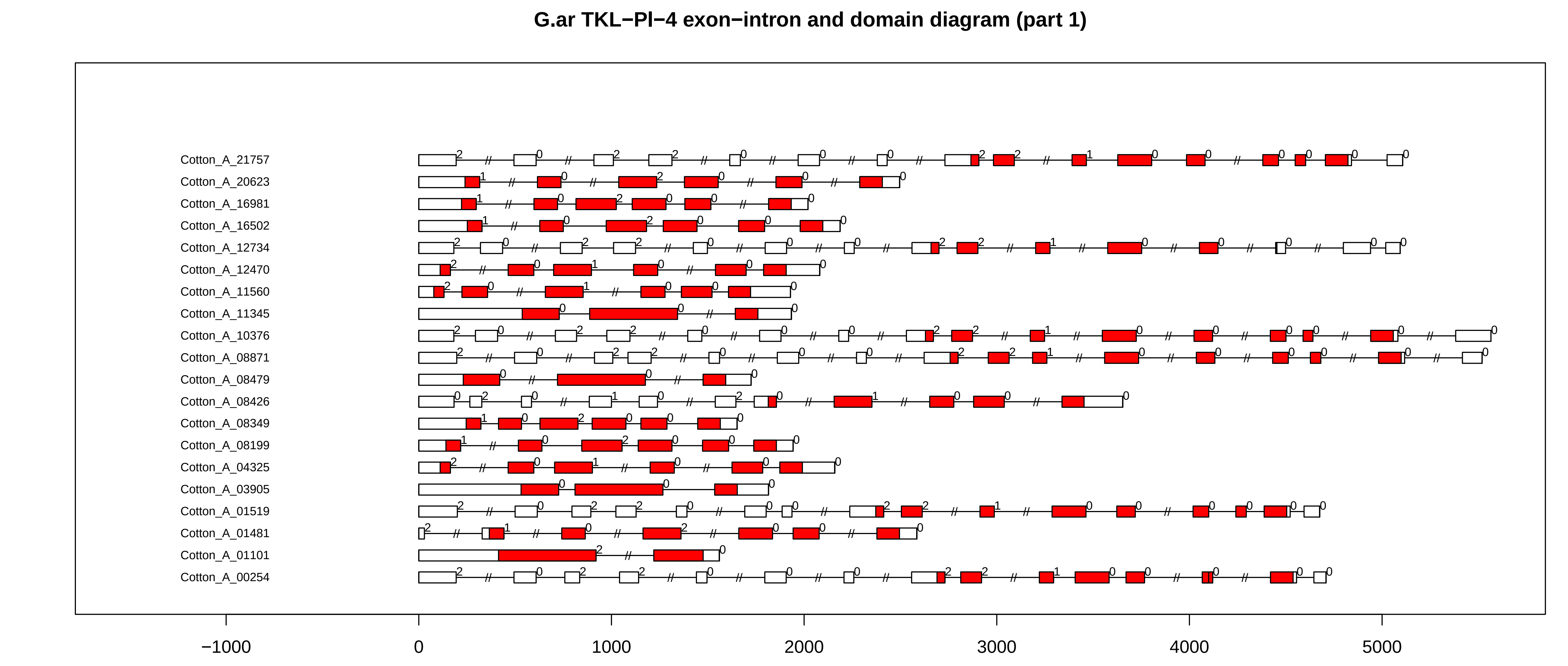
G.ar TKL−Pl−4 exon−intron and domain diagram (part 1)
Cotton_A_21757
Cotton_A_20623
Cotton_A_16981
Cotton_A_16502
Cotton_A_12734
Cotton_A_12470
Cotton_A_11560
Cotton_A_11345
Cotton_A_10376
Cotton_A_08871
Cotton_A_08479
Cotton_A_08426
Cotton_A_08349
Cotton_A_08199
Cotton_A_04325
Cotton_A_03905
Cotton_A_01519
Cotton_A_01481
Cotton_A_01101
Cotton_A_00254
−1000
0
1000
2000
3000
4000
5000

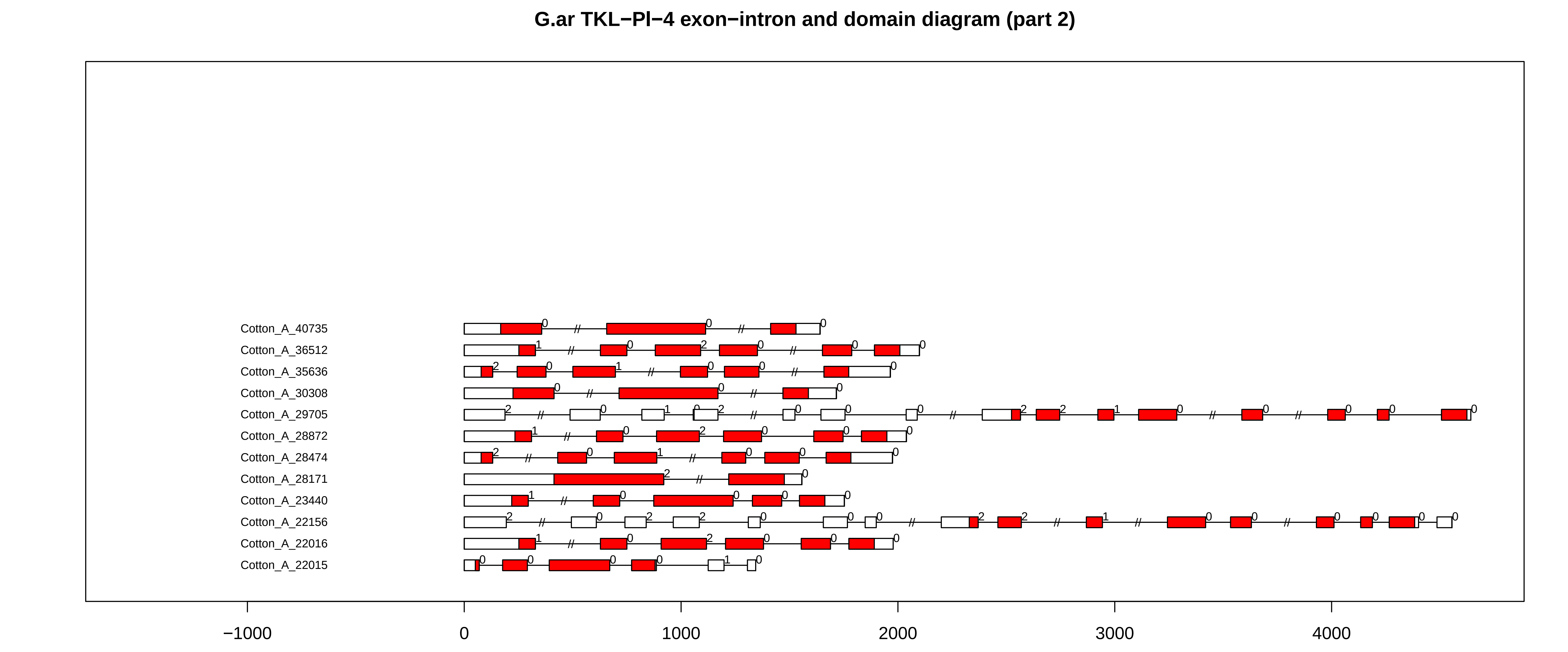

G.ar TKL−Pl−4 exon−intron and domain diagram (part 2)
Cotton_A_40735
Cotton_A_36512
Cotton_A_35636
Cotton_A_30308
Cotton_A_29705
Cotton_A_28872
Cotton_A_28474
Cotton_A_28171
Cotton_A_23440
Cotton_A_22156
Cotton_A_22016
Cotton_A_22015
−1000
0
1000
2000
3000
4000

G.ar TKL−Pl−5 exon−intron and domain diagram (all)

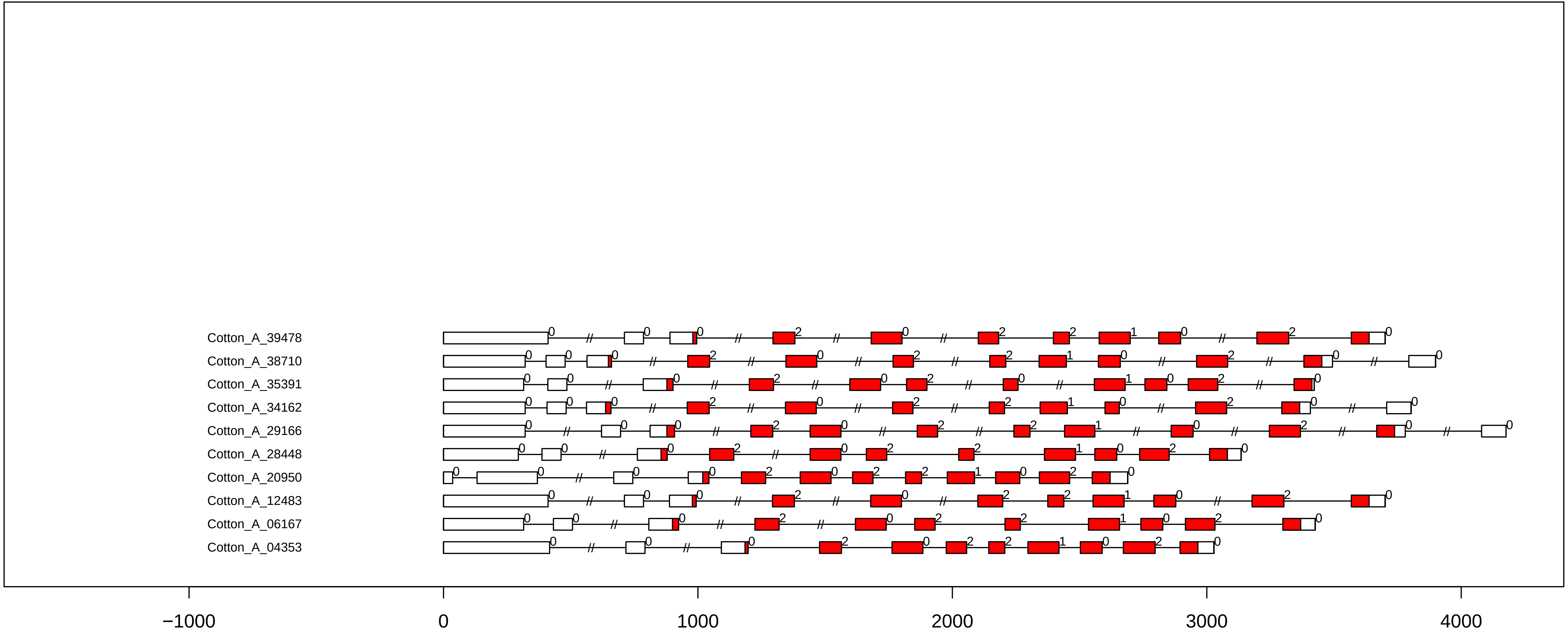

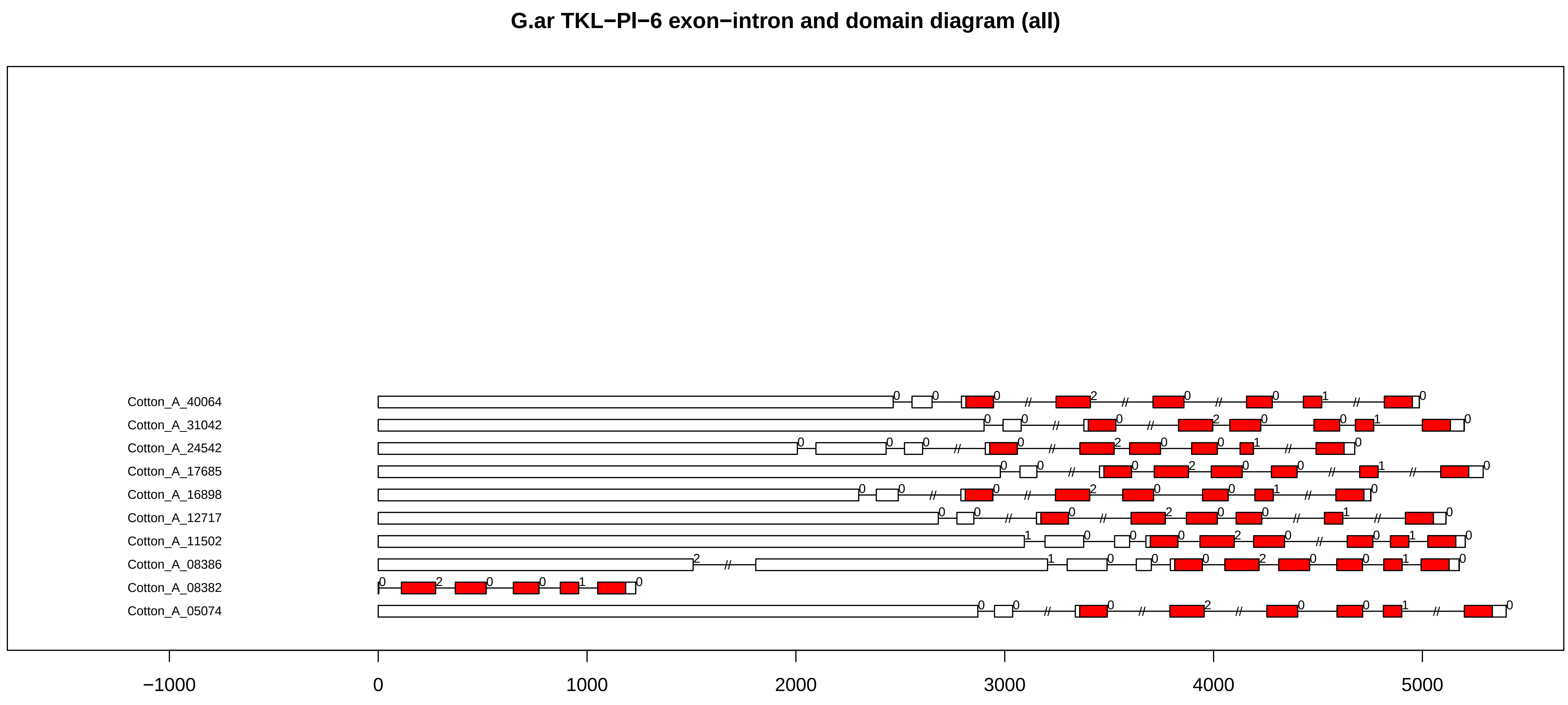

G.ar TKL−Pl−6 exon−intron and domain diagram (all)
Cotton_A_40064
Cotton_A_31042
Cotton_A_24542
Cotton_A_17685
Cotton_A_16898
Cotton_A_12717
Cotton_A_11502
Cotton_A_08386
Cotton_A_08382
Cotton_A_05074
−1000
0
1000
2000
3000
4000
5000

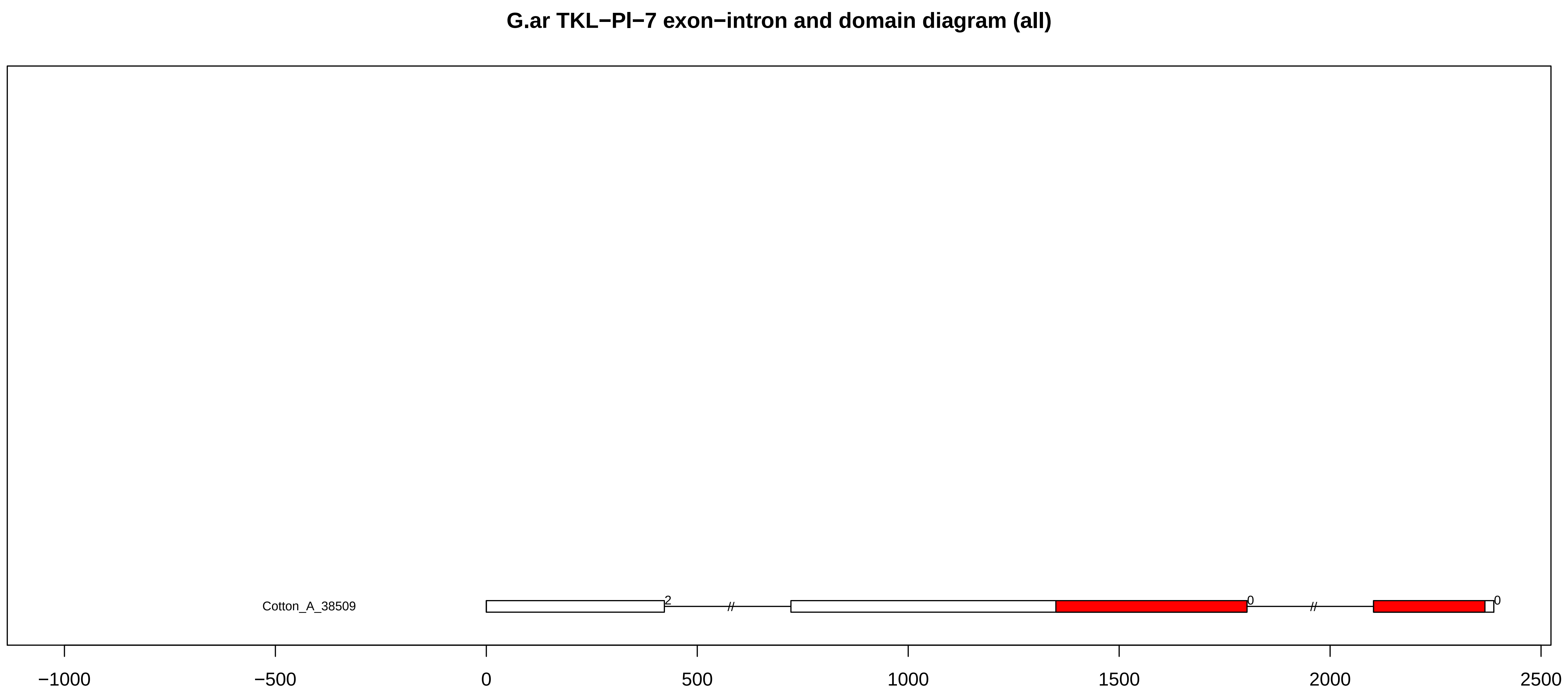
G.ar TKL−Pl−7 exon−intron and domain diagram (all)
Cotton_A_38509
2
0
0
−1000
−500
0
500
1000
1500
2000
2500

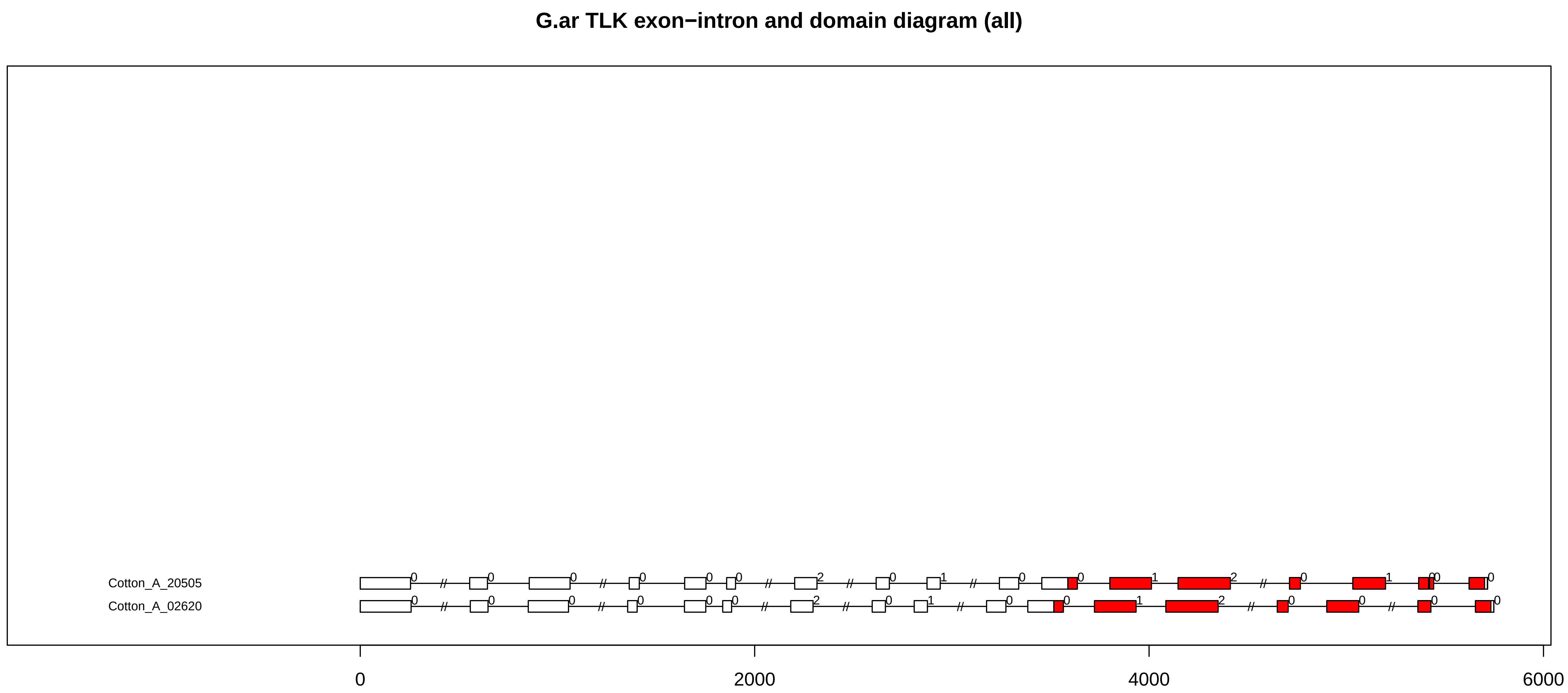

G.ar TLK exon−intron and domain diagram (all)
Cotton_A_20505
Cotton_A_02620
0
2000
4000
6000

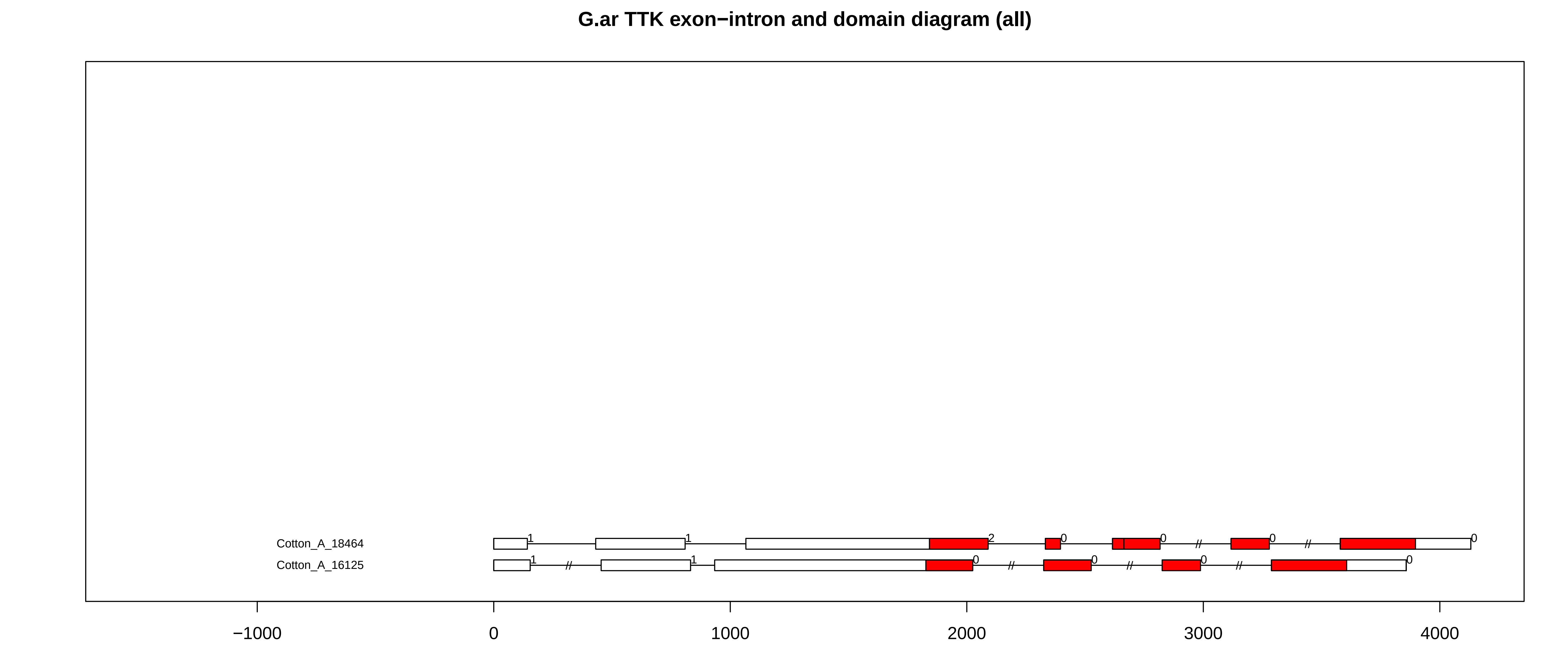
G.ar TTK exon−intron and domain diagram (all)
Cotton_A_18464
Cotton_A_16125
1
1
2
0
0
0
0
1
1
0
0
0
0
−1000
0
1000
2000
3000
4000

G.ar ULK_Fused exon−intron and domain diagram (all)

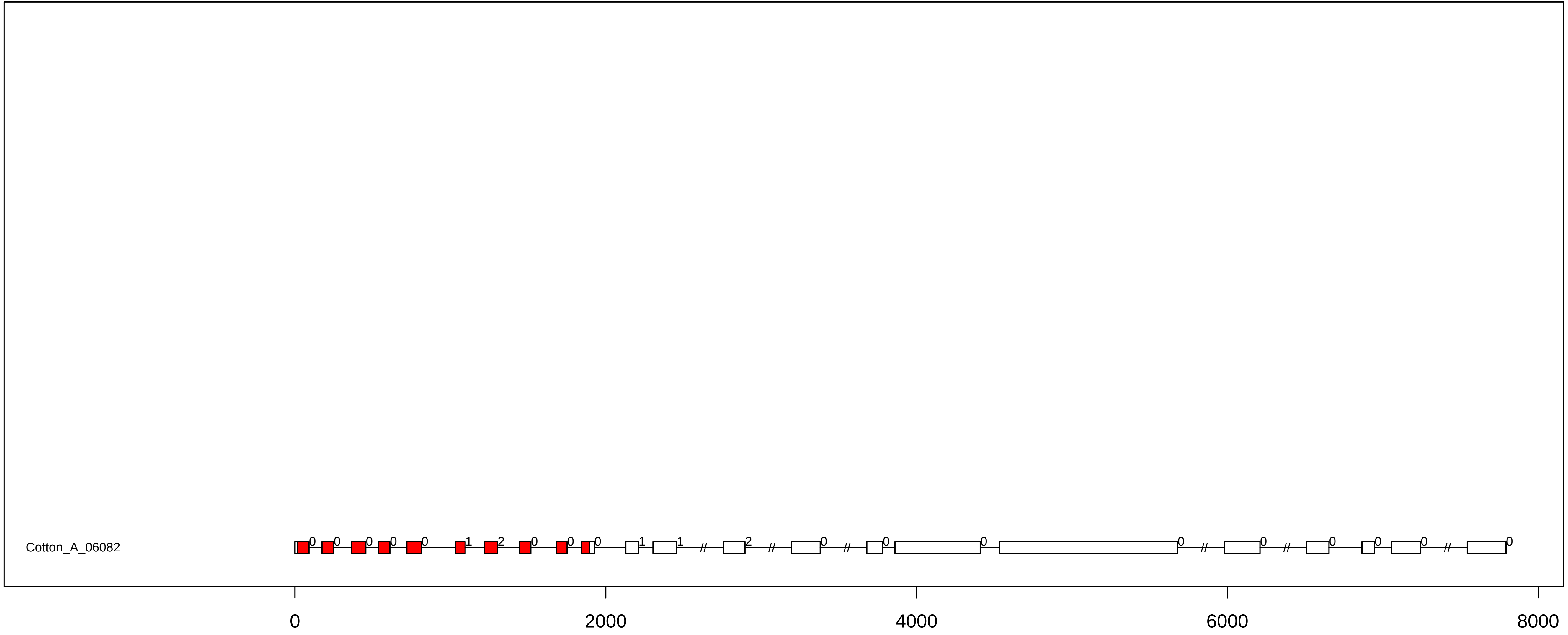

G.ar ULK_ULK4 exon−intron and domain diagram (all)

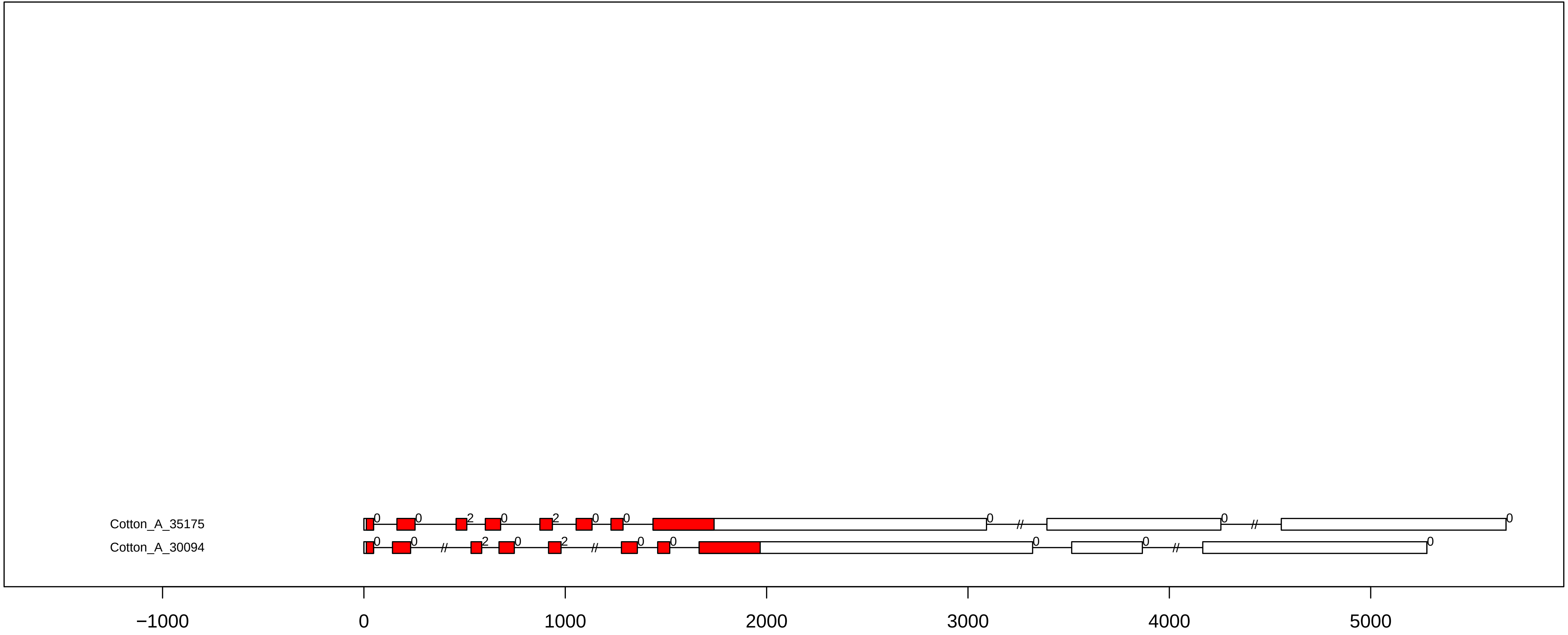

G.ar WEE exon−intron and domain diagram (all)

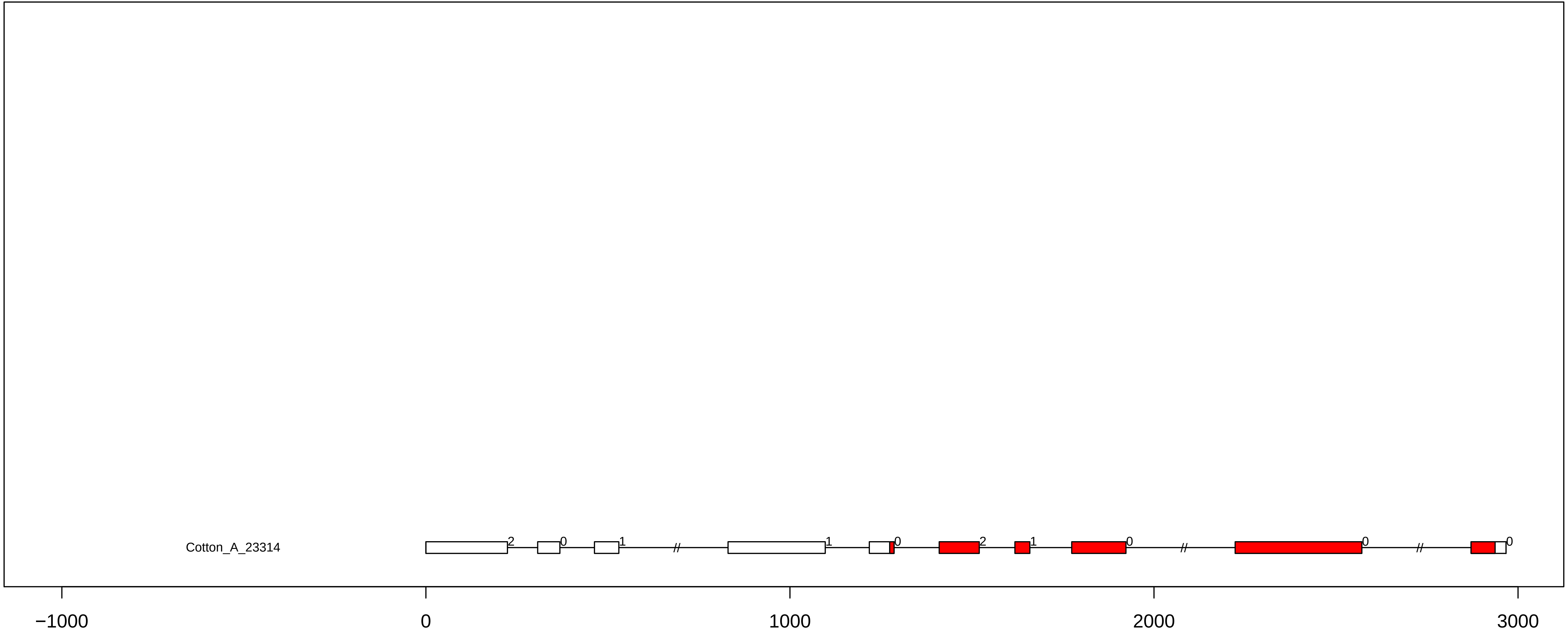

G.ar WNK_NRBP exon−intron and domain diagram (all)

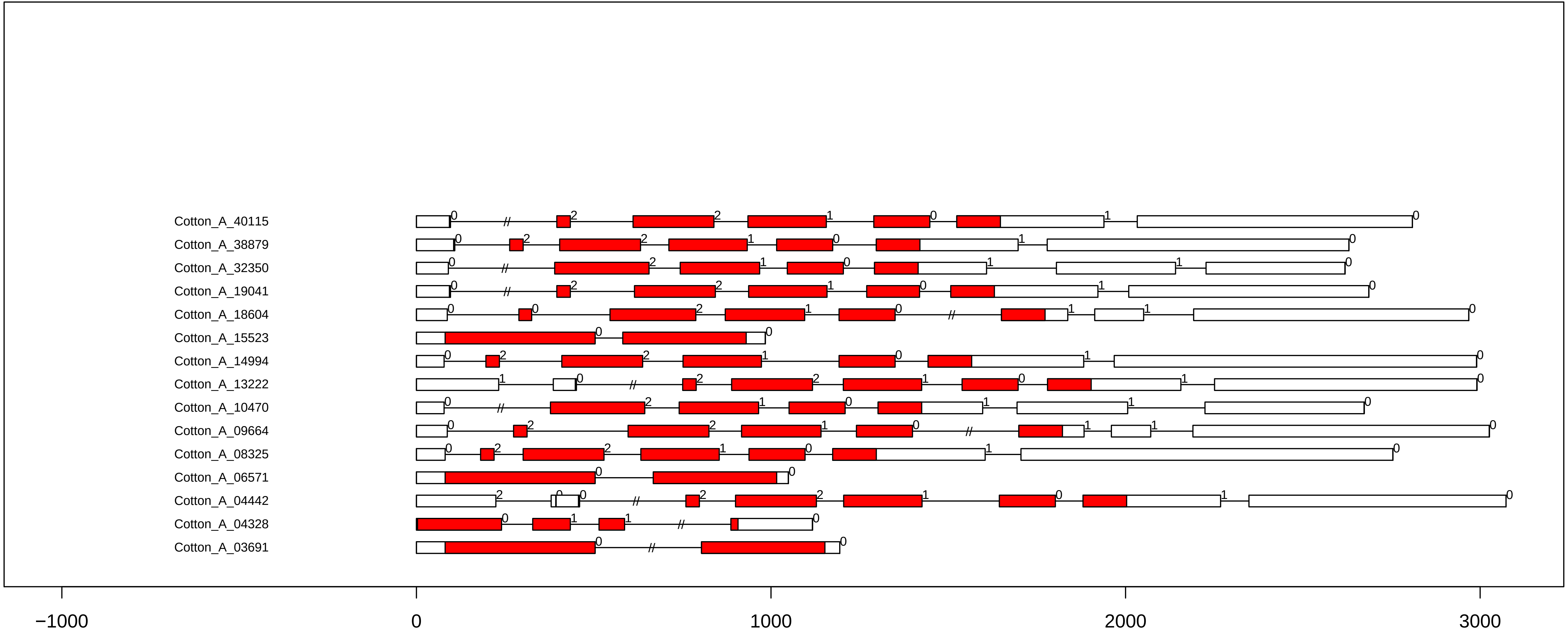

G.ra AGC_MAST exon−intron and domain diagram (all)

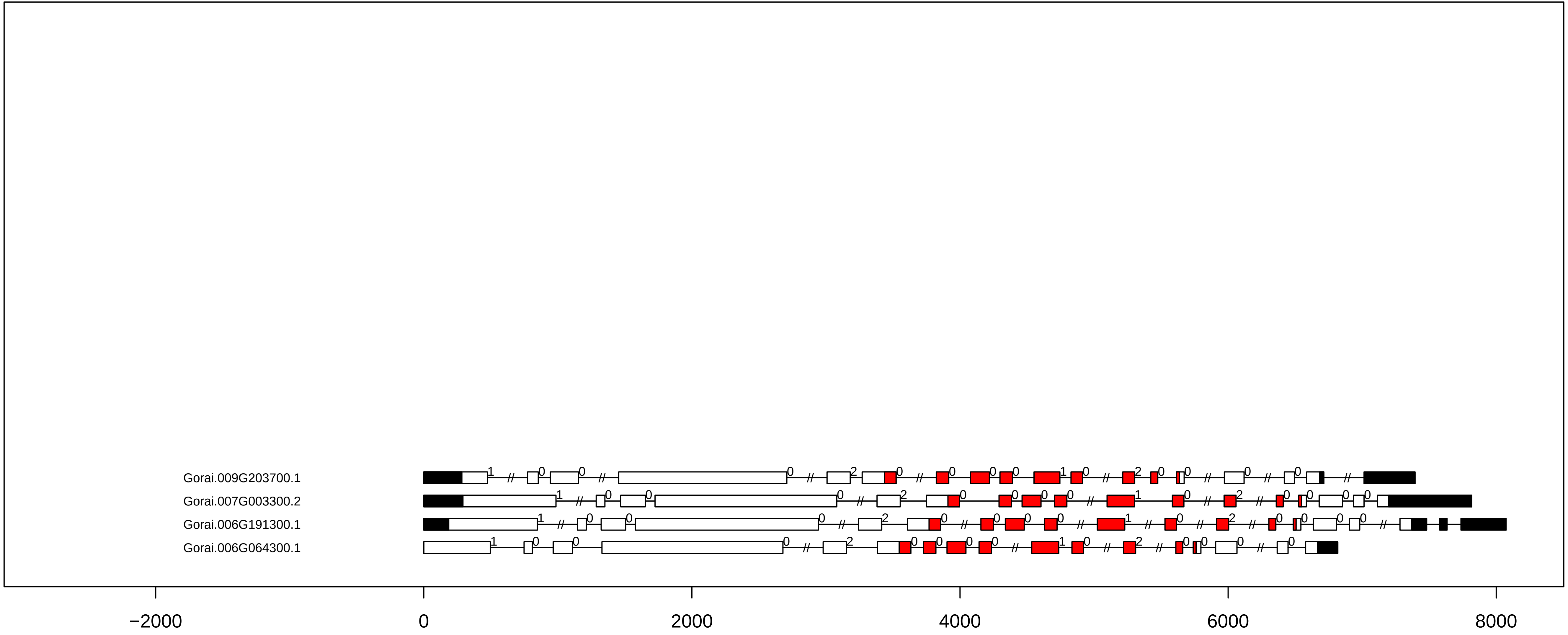

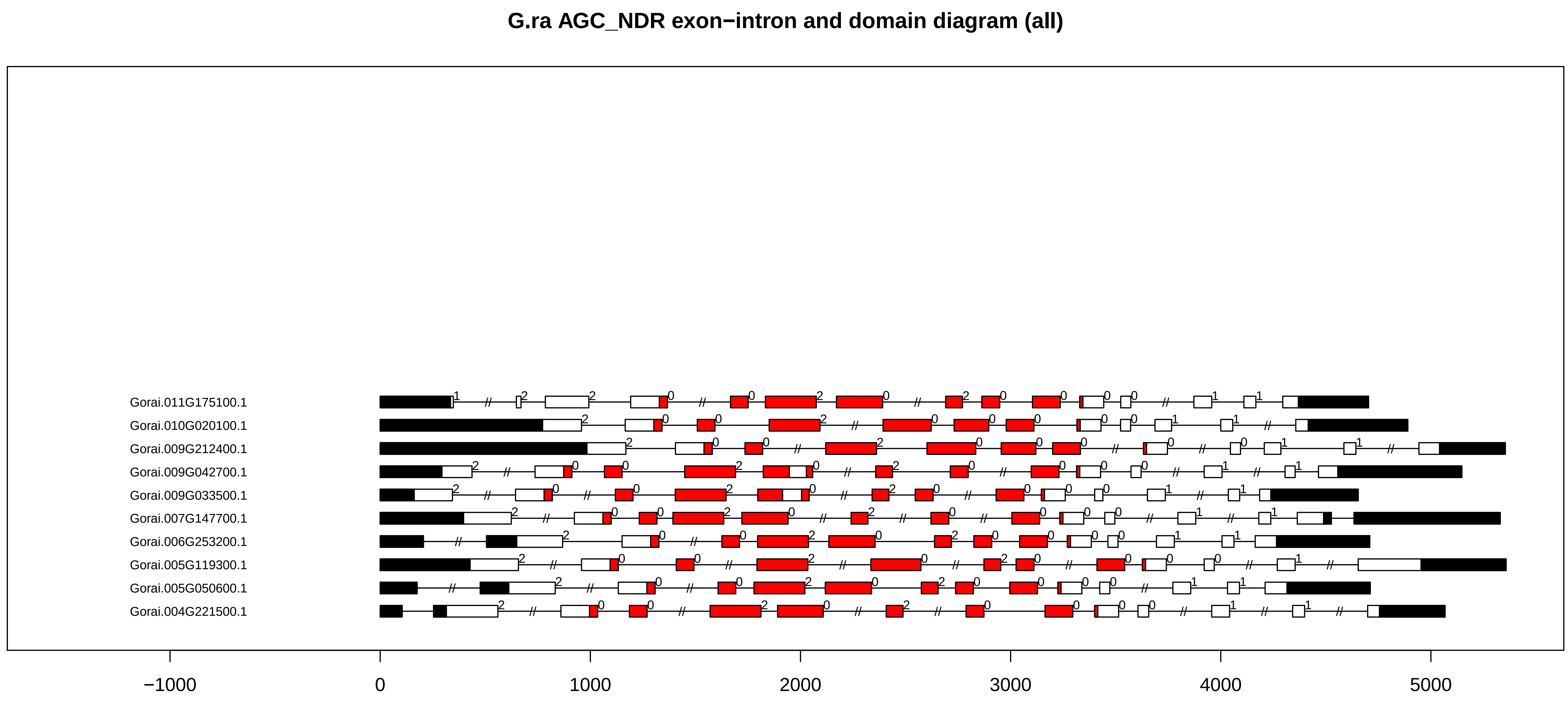
G.ra AGC_NDR exon−intron and domain diagram (all)
Gorai.011G175100.1
Gorai.010G020100.1
Gorai.009G212400.1
Gorai.009G042700.1
Gorai.009G033500.1
Gorai.007G147700.1
Gorai.006G253200.1
Gorai.005G119300.1
Gorai.005G050600.1
Gorai.004G221500.1
−1000
0
1000
2000
3000
4000
5000

G.ra AGC_PDK1 exon−intron and domain diagram (all)

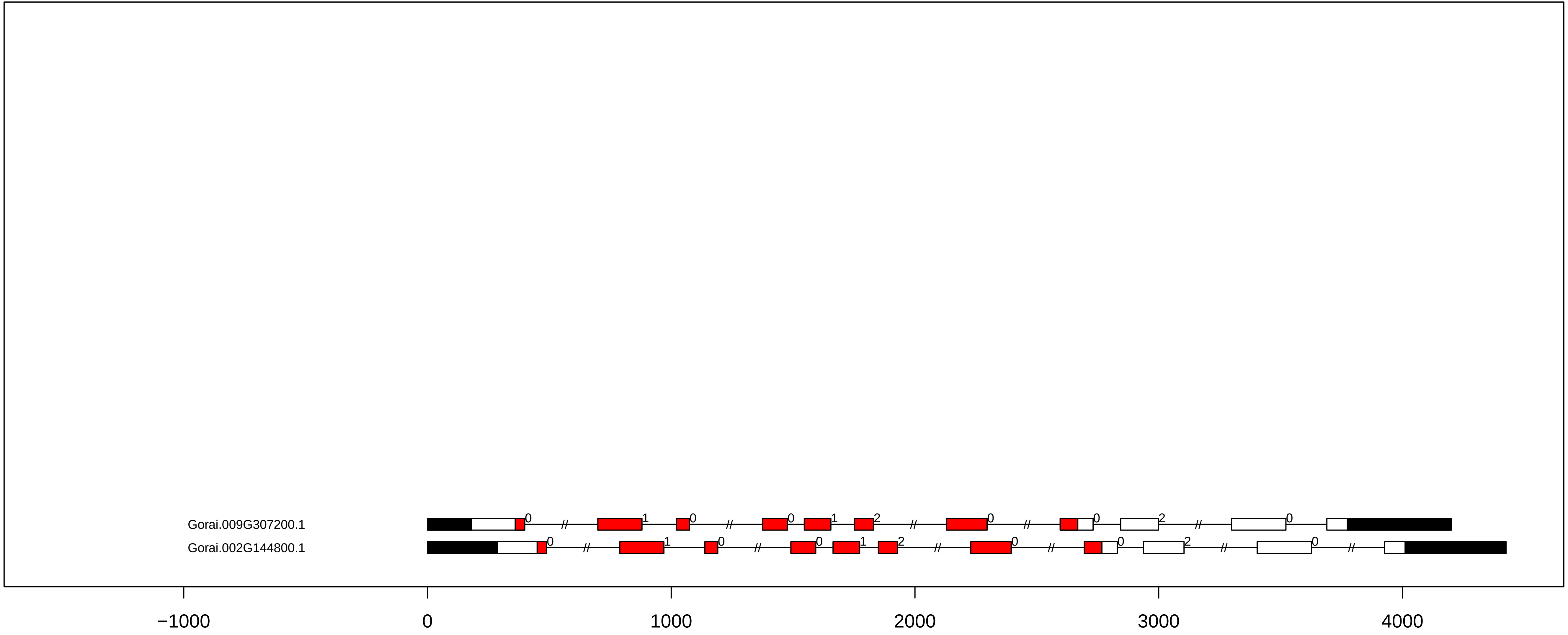

G.ra AGC_PKA−PKG exon−intron and domain diagram (all)

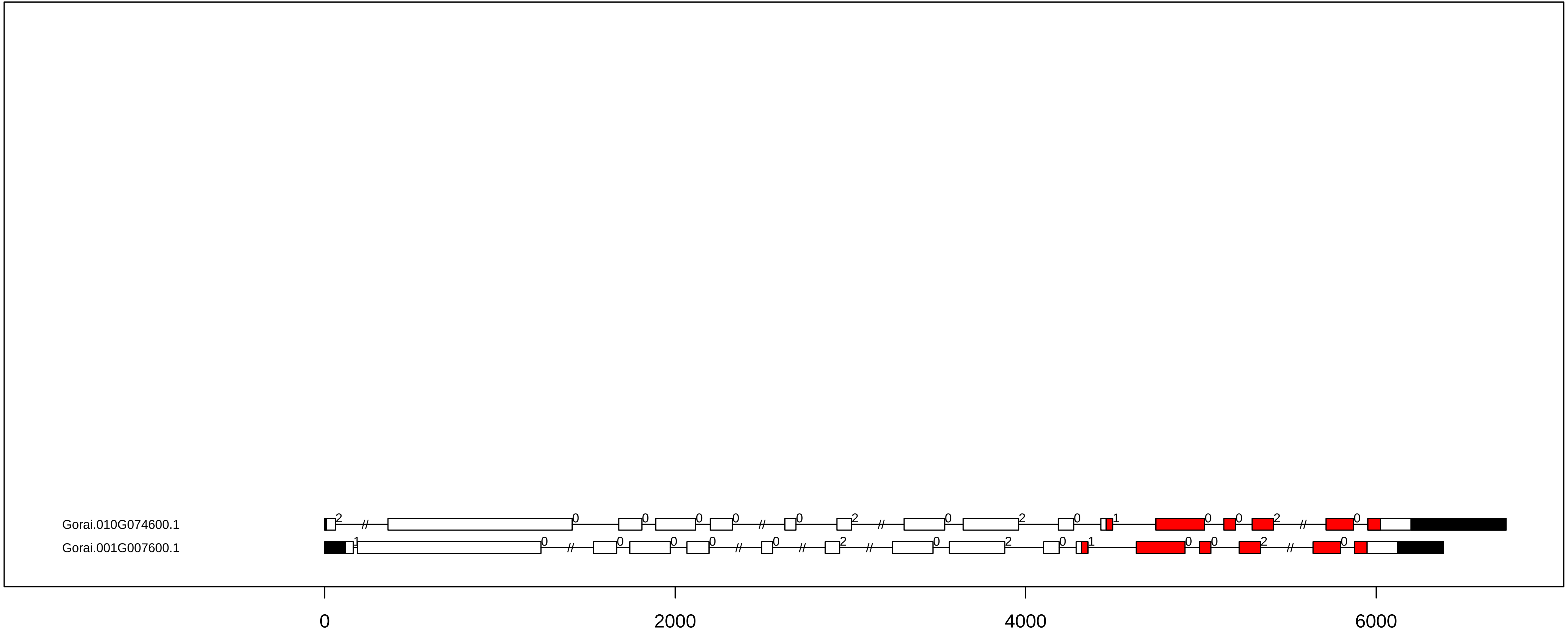

G.ra AGC_RSK−2 exon−intron and domain diagram (part 1)

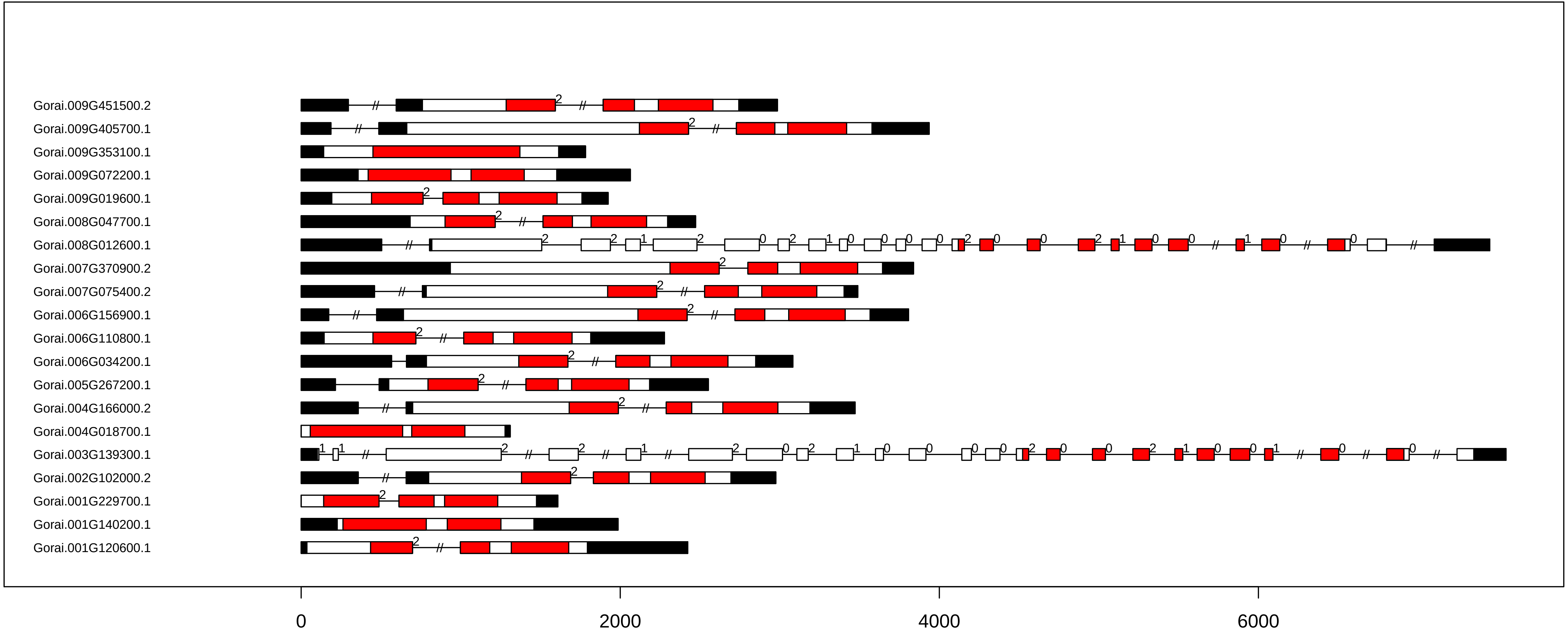

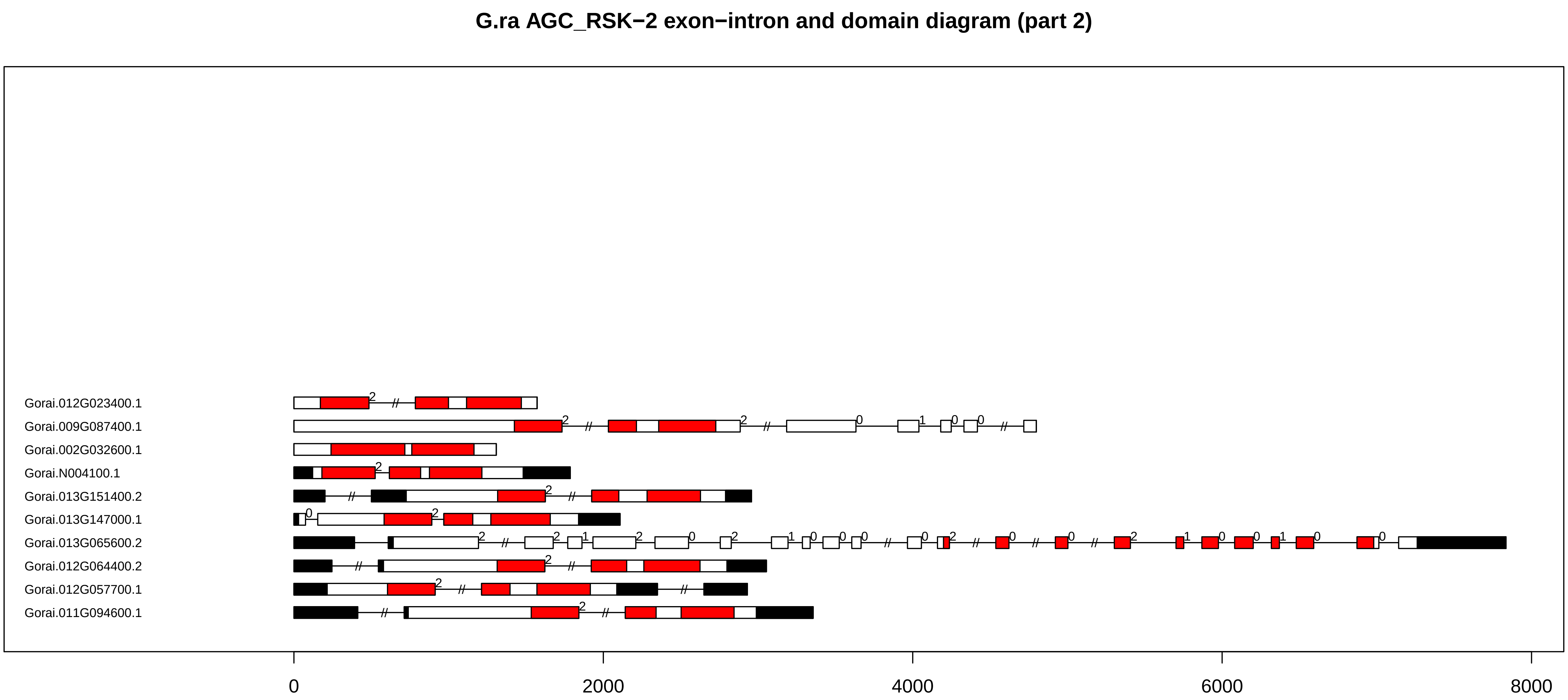
G.ra AGC_RSK−2 exon−intron and domain diagram (part 2)
Gorai.012G023400.1
Gorai.009G087400.1
Gorai.002G032600.1
Gorai.N004100.1
Gorai.013G151400.2
Gorai.013G147000.1
Gorai.013G065600.2
Gorai.012G064400.2
Gorai.012G057700.1
Gorai.011G094600.1
0
2000
4000
6000
8000

G.ra AGC−PI exon−intron and domain diagram (all)

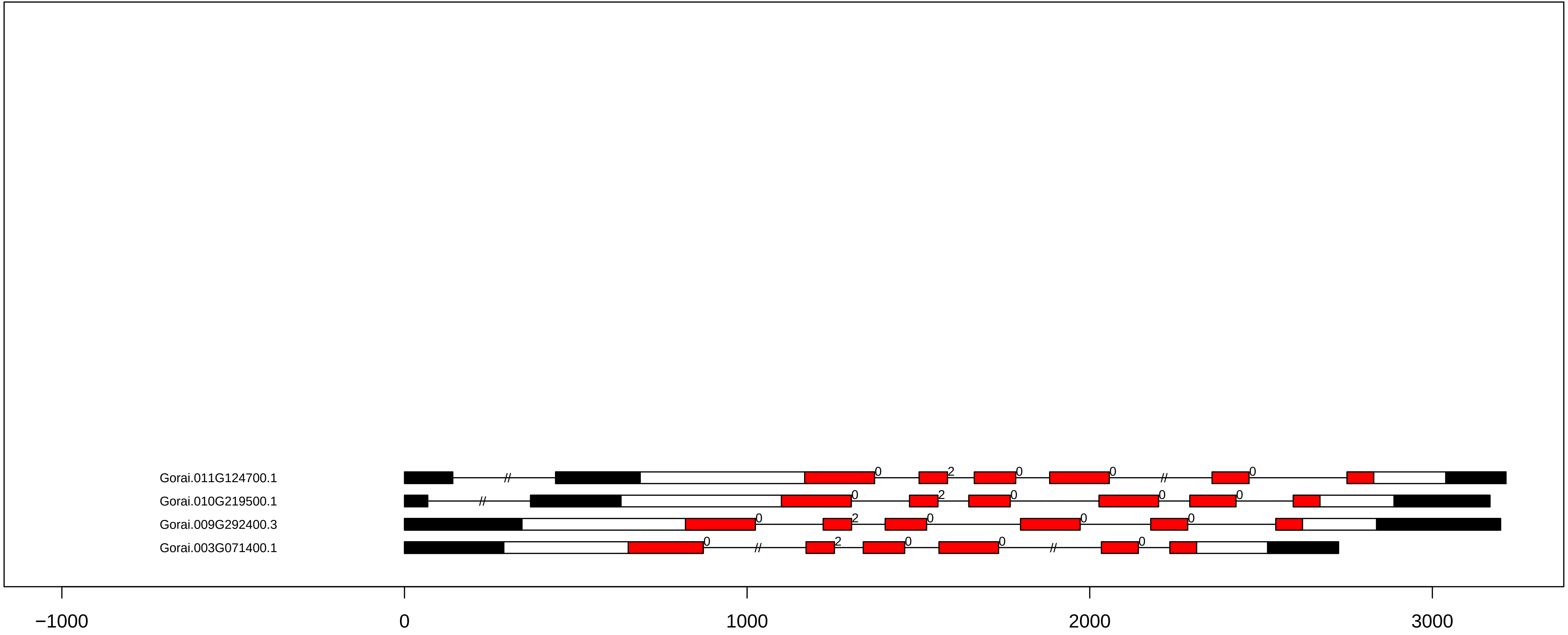

G.ra Aur exon−intron and domain diagram (all)

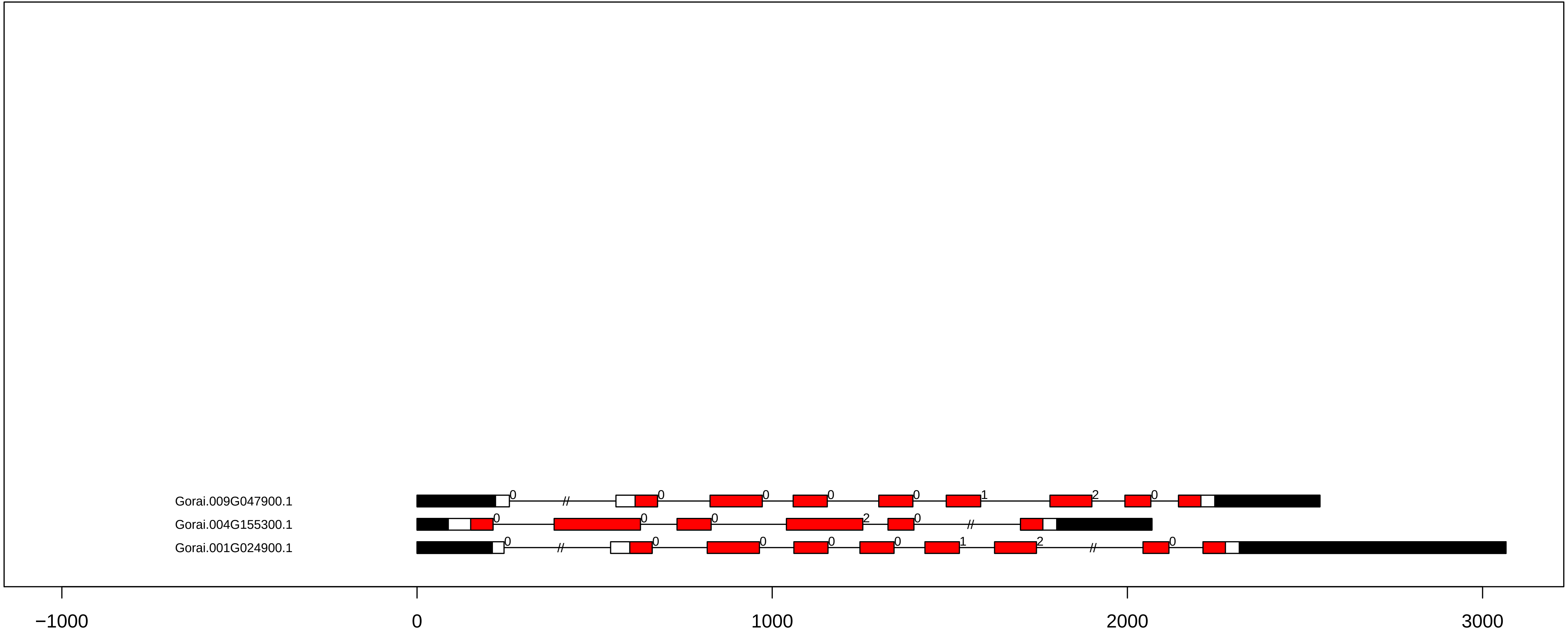

G.ra BUB exon−intron and domain diagram (all)

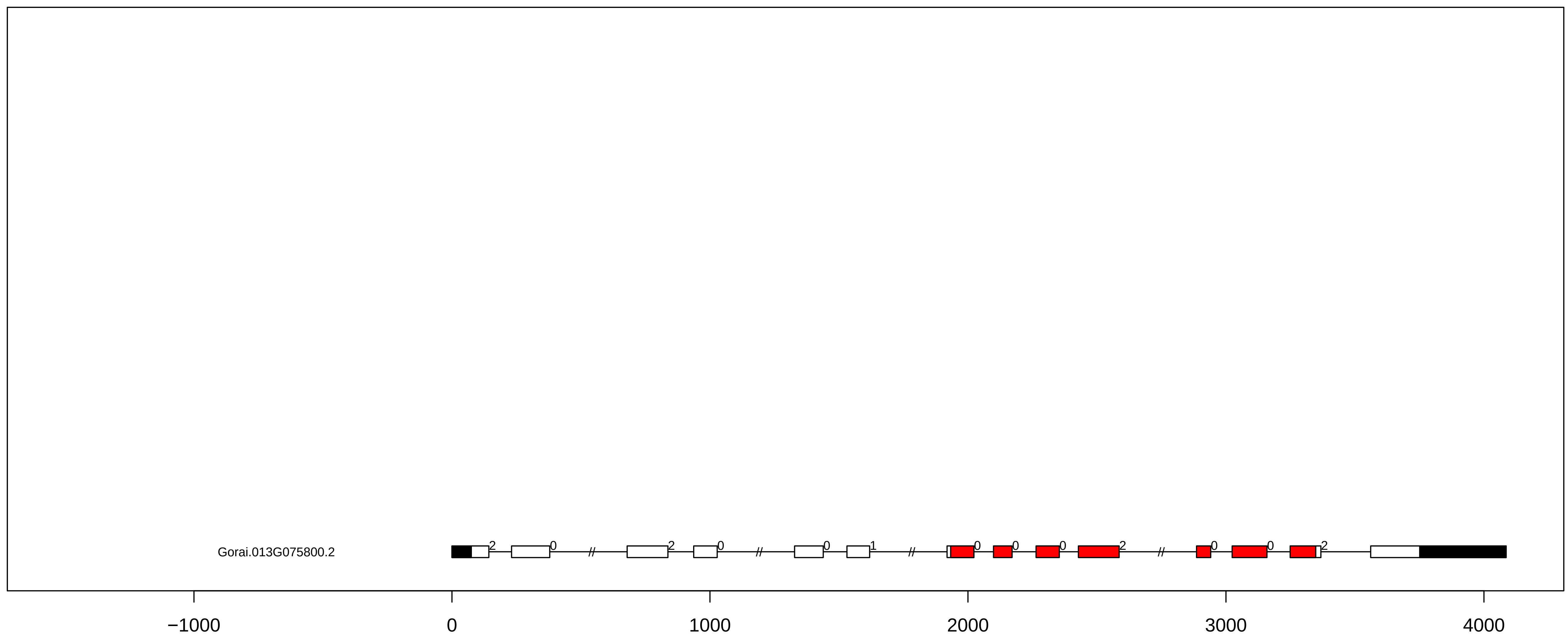

G.ra CAMK_AMPK exon−intron and domain diagram (all)

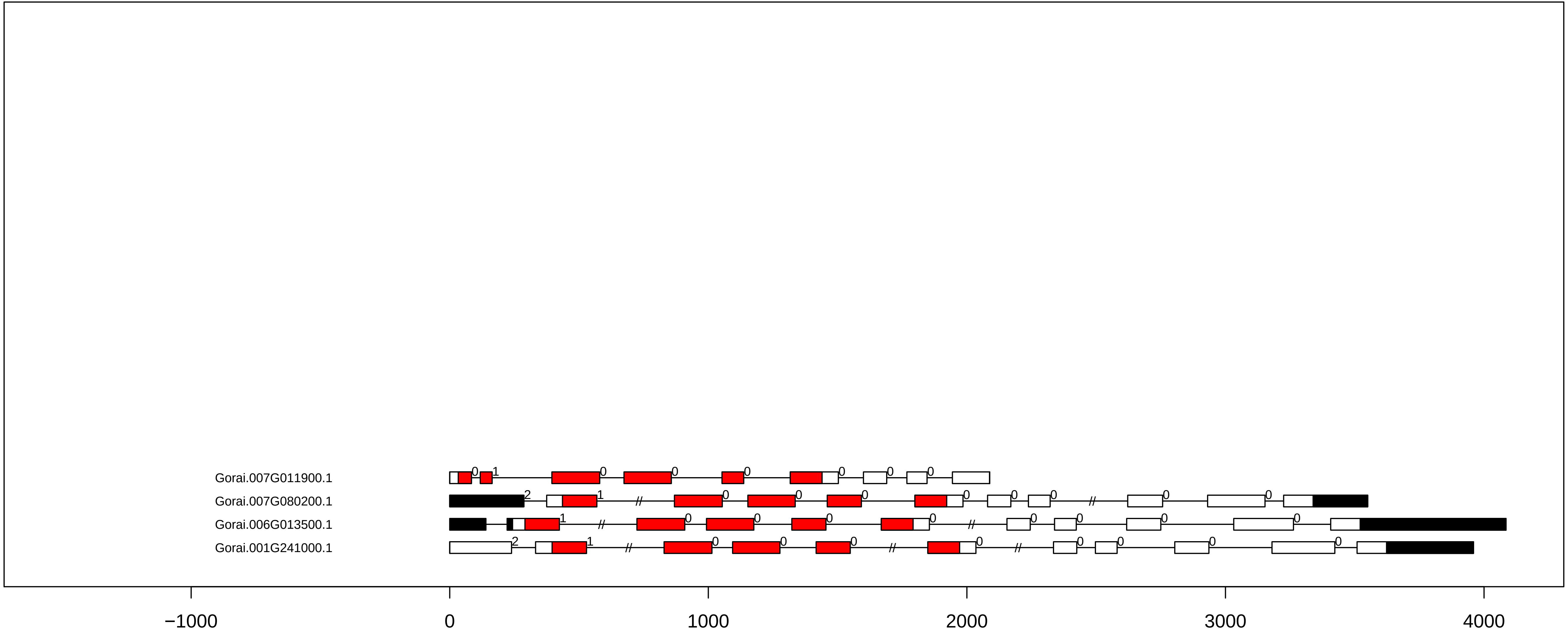

G.ra CAMK_CAMK1−DCAMKL exon−intron and domain diagram (all)

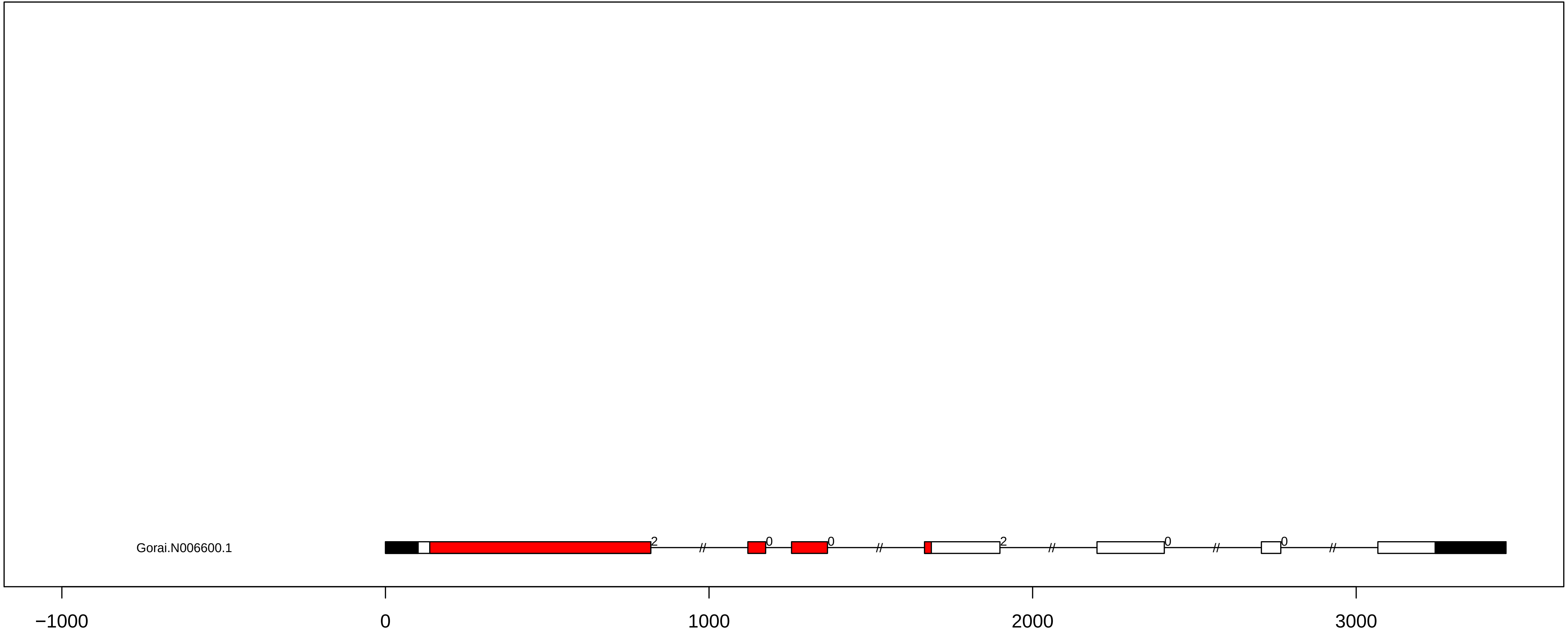

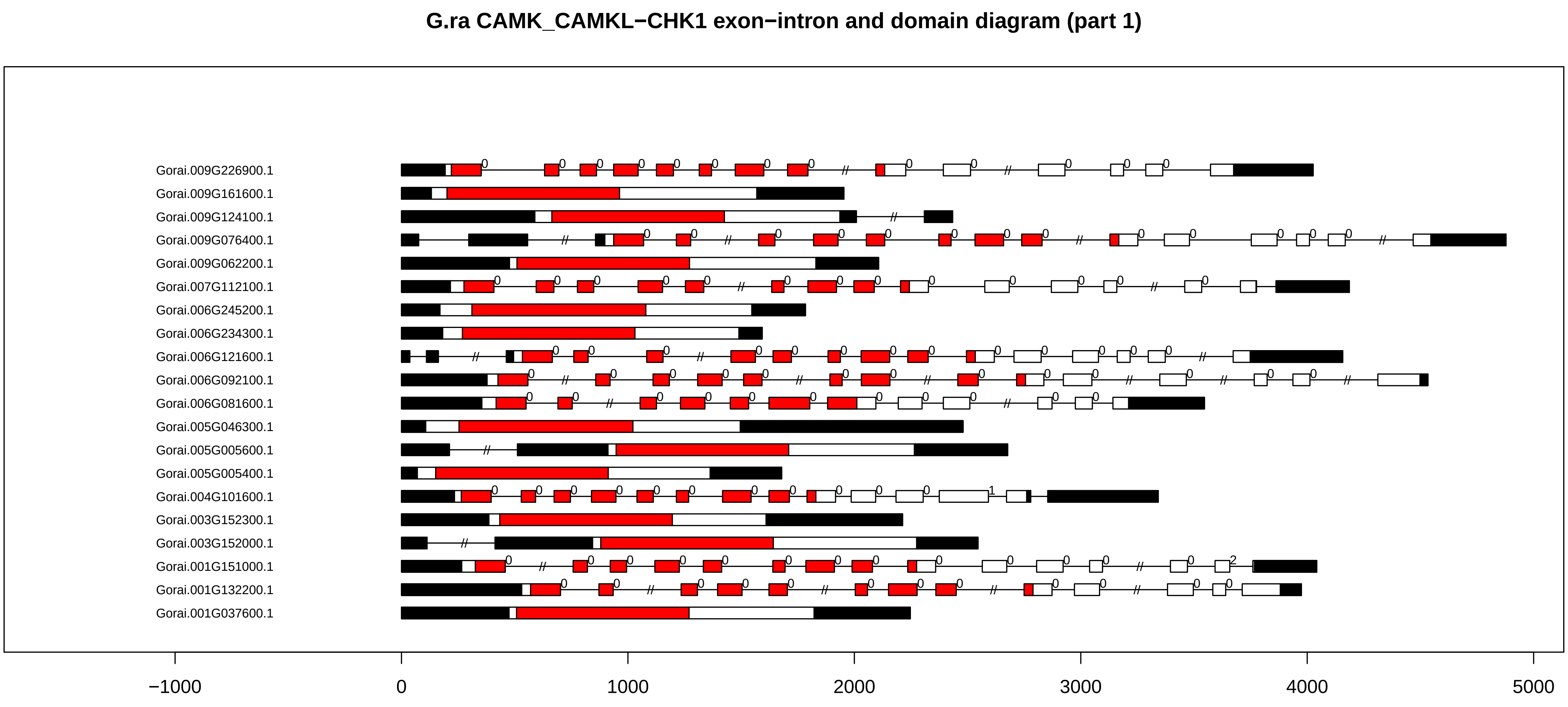
G.ra CAMK_CAMKL−CHK1 exon−intron and domain diagram (part 1)
Gorai.009G226900.1
Gorai.009G161600.1
Gorai.009G124100.1
Gorai.009G076400.1
Gorai.009G062200.1
Gorai.007G112100.1
Gorai.006G245200.1
Gorai.006G234300.1
Gorai.006G121600.1
Gorai.006G092100.1
Gorai.006G081600.1
Gorai.005G046300.1
Gorai.005G005600.1
Gorai.005G005400.1
Gorai.004G101600.1
Gorai.003G152300.1
Gorai.003G152000.1
Gorai.001G151000.1
Gorai.001G132200.1
Gorai.001G037600.1
−1000
0
1000
2000
3000
4000
5000

G.ra CAMK_CAMKL−CHK1 exon−intron and domain diagram (part 2)

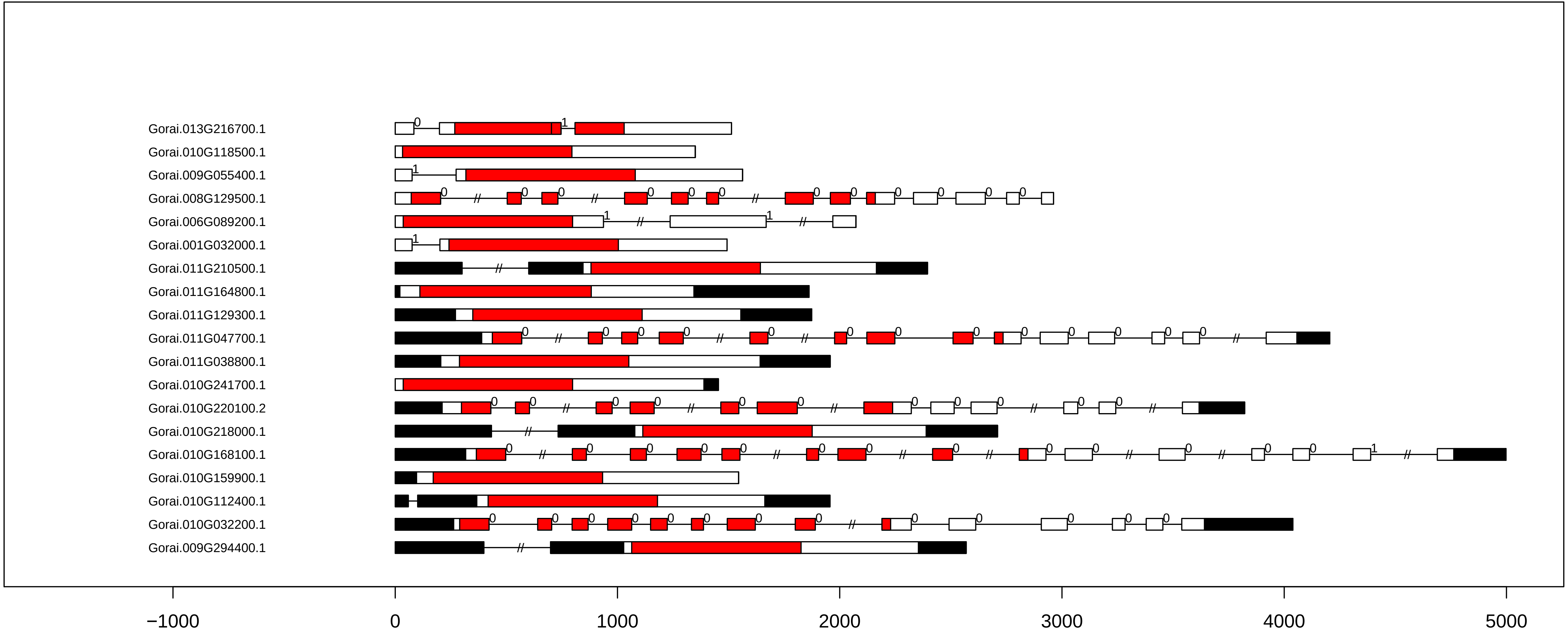

G.ra CAMK_CAMKL−LKB exon−intron and domain diagram (all)

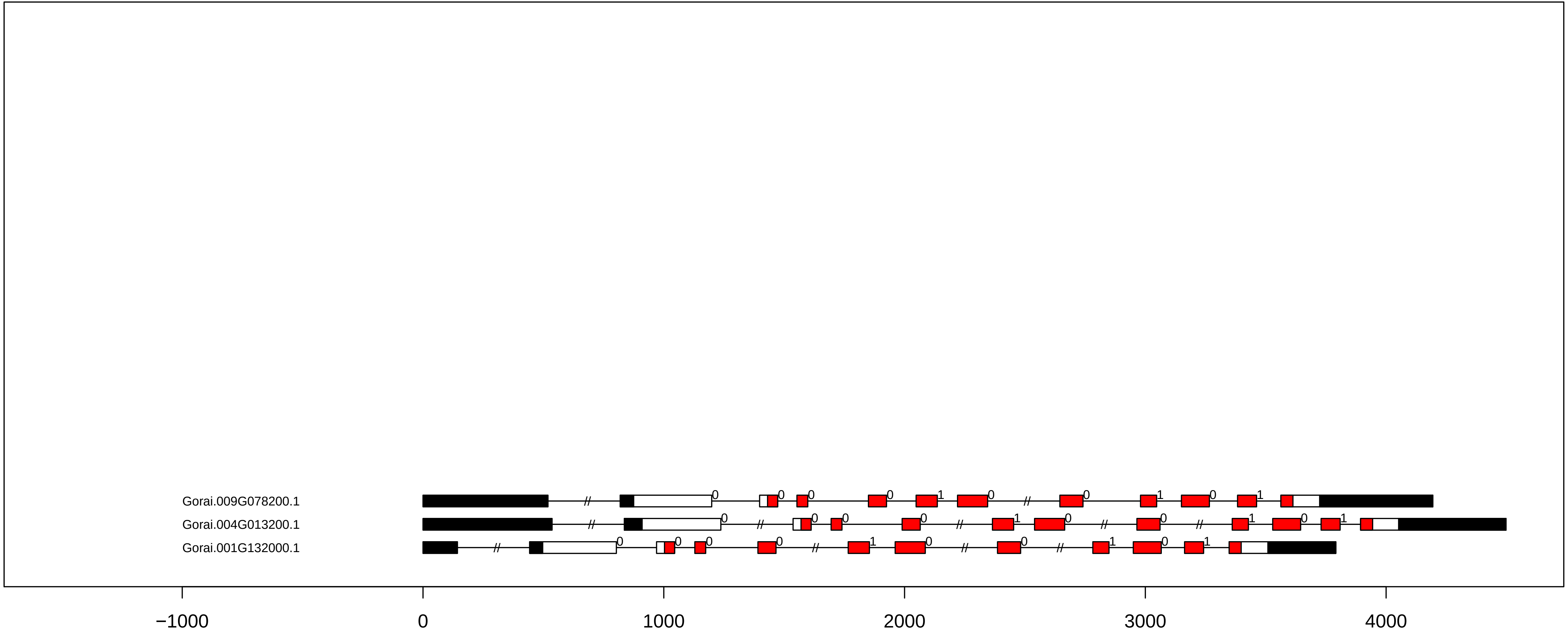

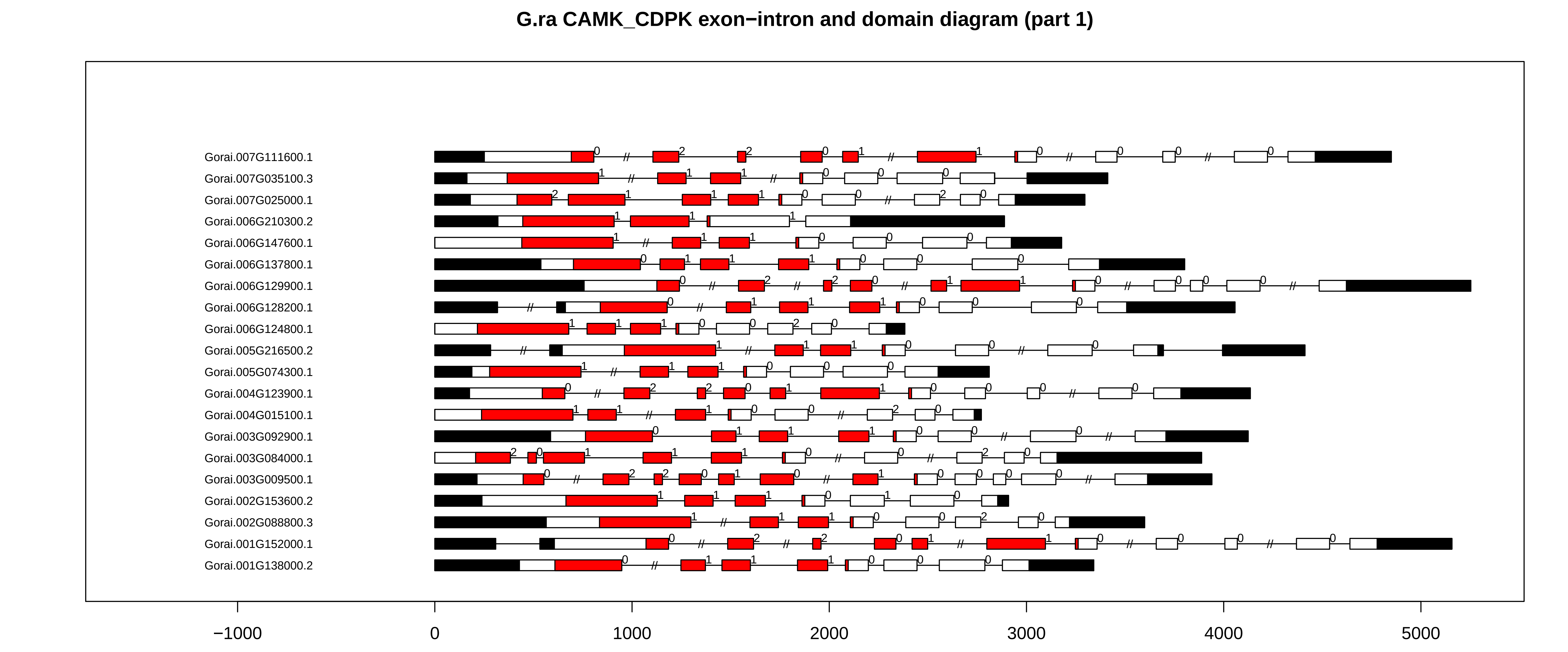
G.ra CAMK_CDPK exon−intron and domain diagram (part 1)
Gorai.007G111600.1
Gorai.007G035100.3
Gorai.007G025000.1
Gorai.006G210300.2
Gorai.006G147600.1
Gorai.006G137800.1
Gorai.006G129900.1
Gorai.006G128200.1
Gorai.006G124800.1
Gorai.005G216500.2
Gorai.005G074300.1
Gorai.004G123900.1
Gorai.004G015100.1
Gorai.003G092900.1
Gorai.003G084000.1
Gorai.003G009500.1
Gorai.002G153600.2
Gorai.002G088800.3
Gorai.001G152000.1
Gorai.001G138000.2
−1000
0
1000
2000
3000
4000
5000

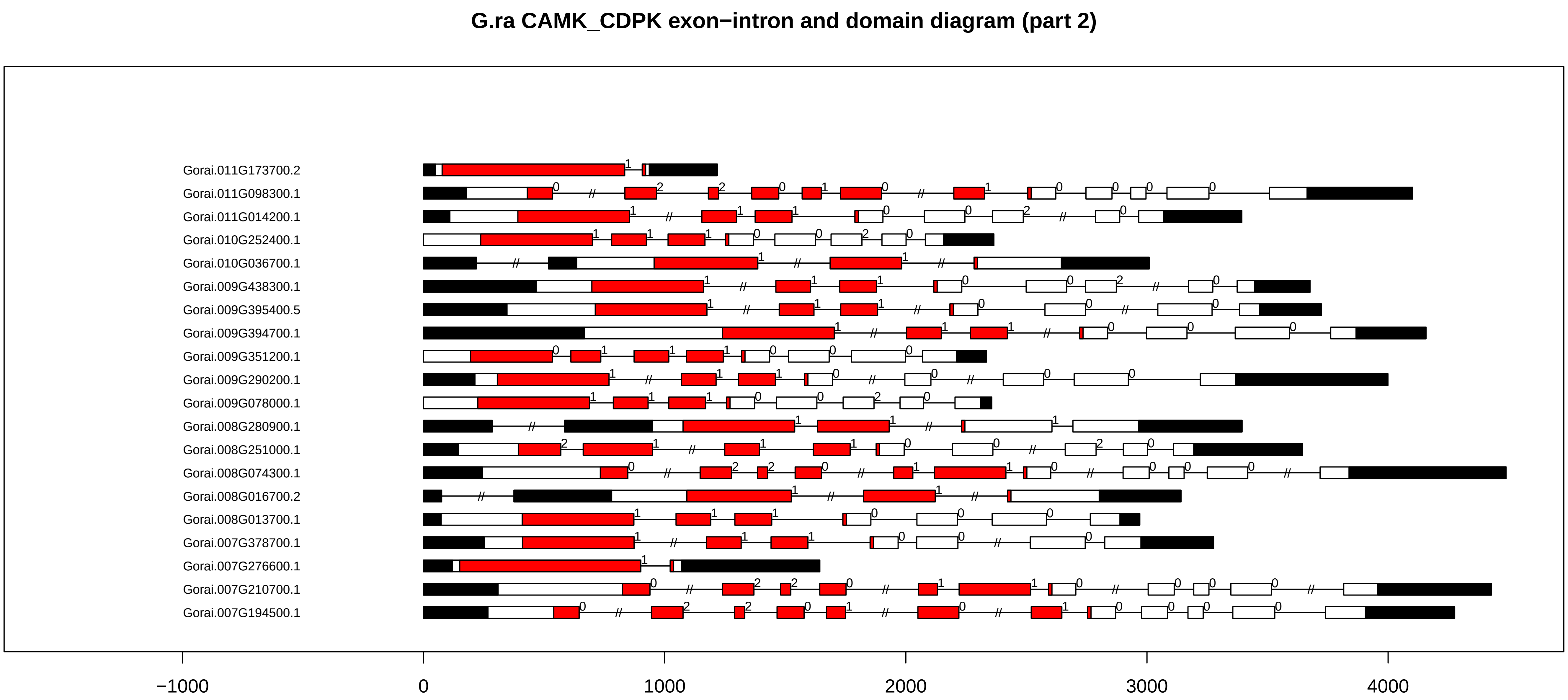
G.ra CAMK_CDPK exon−intron and domain diagram (part 2)
Gorai.011G173700.2
Gorai.011G098300.1
Gorai.011G014200.1
Gorai.010G252400.1
Gorai.010G036700.1
Gorai.009G438300.1
Gorai.009G395400.5
Gorai.009G394700.1
Gorai.009G351200.1
Gorai.009G290200.1
Gorai.009G078000.1
Gorai.008G280900.1
Gorai.008G251000.1
Gorai.008G074300.1
Gorai.008G016700.2
Gorai.008G013700.1
Gorai.007G378700.1
Gorai.007G276600.1
Gorai.007G210700.1
Gorai.007G194500.1
−1000
0
1000
2000
3000
4000

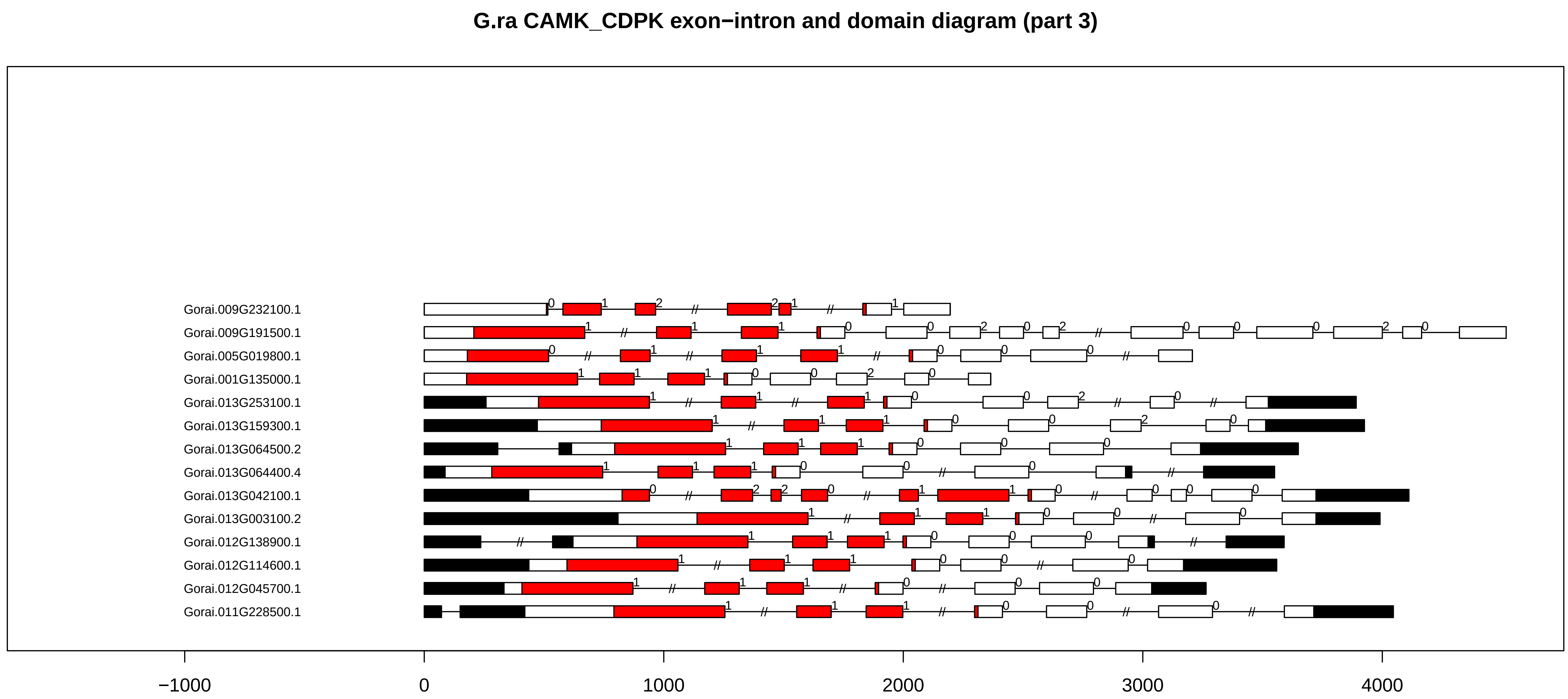

G.ra CAMK_CDPK exon−intron and domain diagram (part 3)
Gorai.009G232100.1
Gorai.009G191500.1
Gorai.005G019800.1
Gorai.001G135000.1
Gorai.013G253100.1
Gorai.013G159300.1
Gorai.013G064500.2
Gorai.013G064400.4
Gorai.013G042100.1
Gorai.013G003100.2
Gorai.012G138900.1
Gorai.012G114600.1
Gorai.012G045700.1
Gorai.011G228500.1
−1000
0
1000
2000
3000
4000

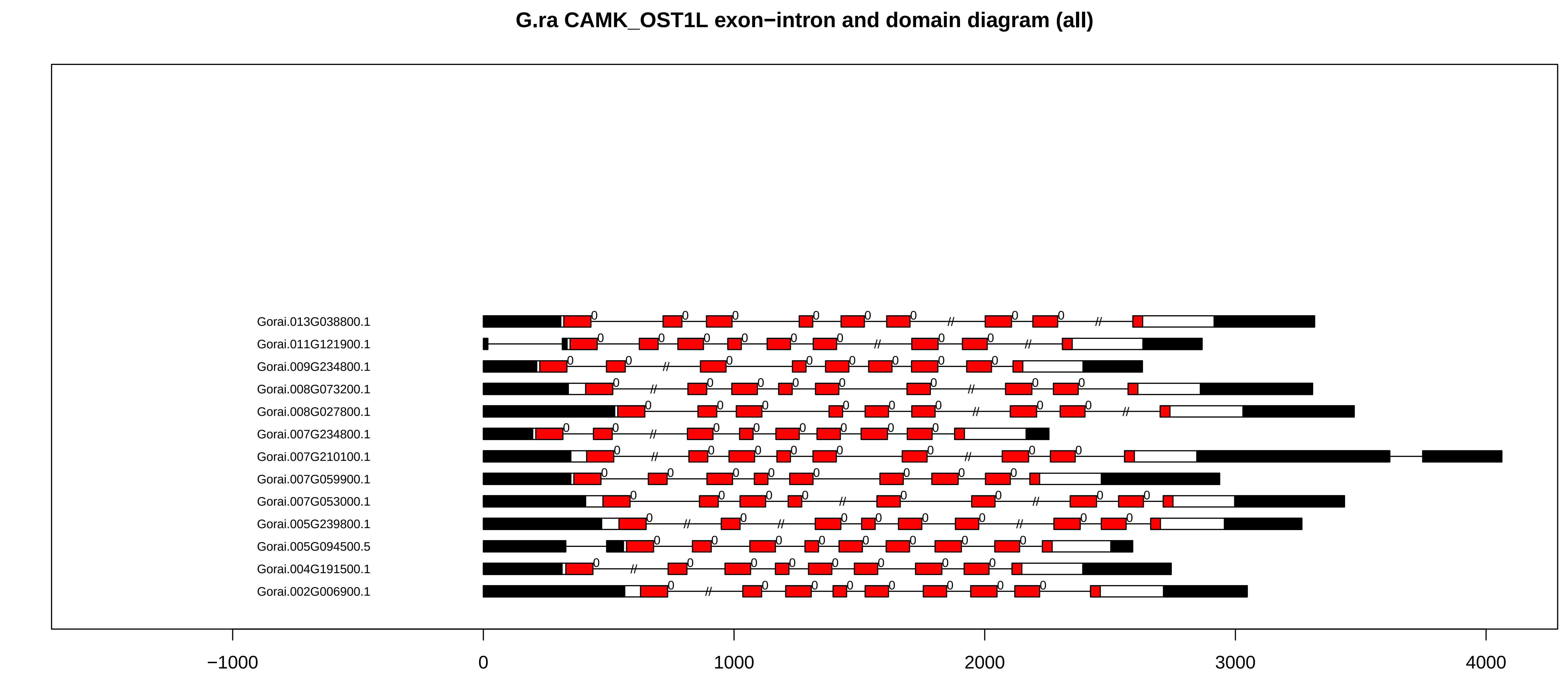
G.ra CAMK_OST1L exon−intron and domain diagram (all)
Gorai.013G038800.1
Gorai.011G121900.1
Gorai.009G234800.1
Gorai.008G073200.1
Gorai.008G027800.1
Gorai.007G234800.1
Gorai.007G210100.1
Gorai.007G059900.1
Gorai.007G053000.1
Gorai.005G239800.1
Gorai.005G094500.5
Gorai.004G191500.1
Gorai.002G006900.1
−1000
0
1000
2000
3000
4000

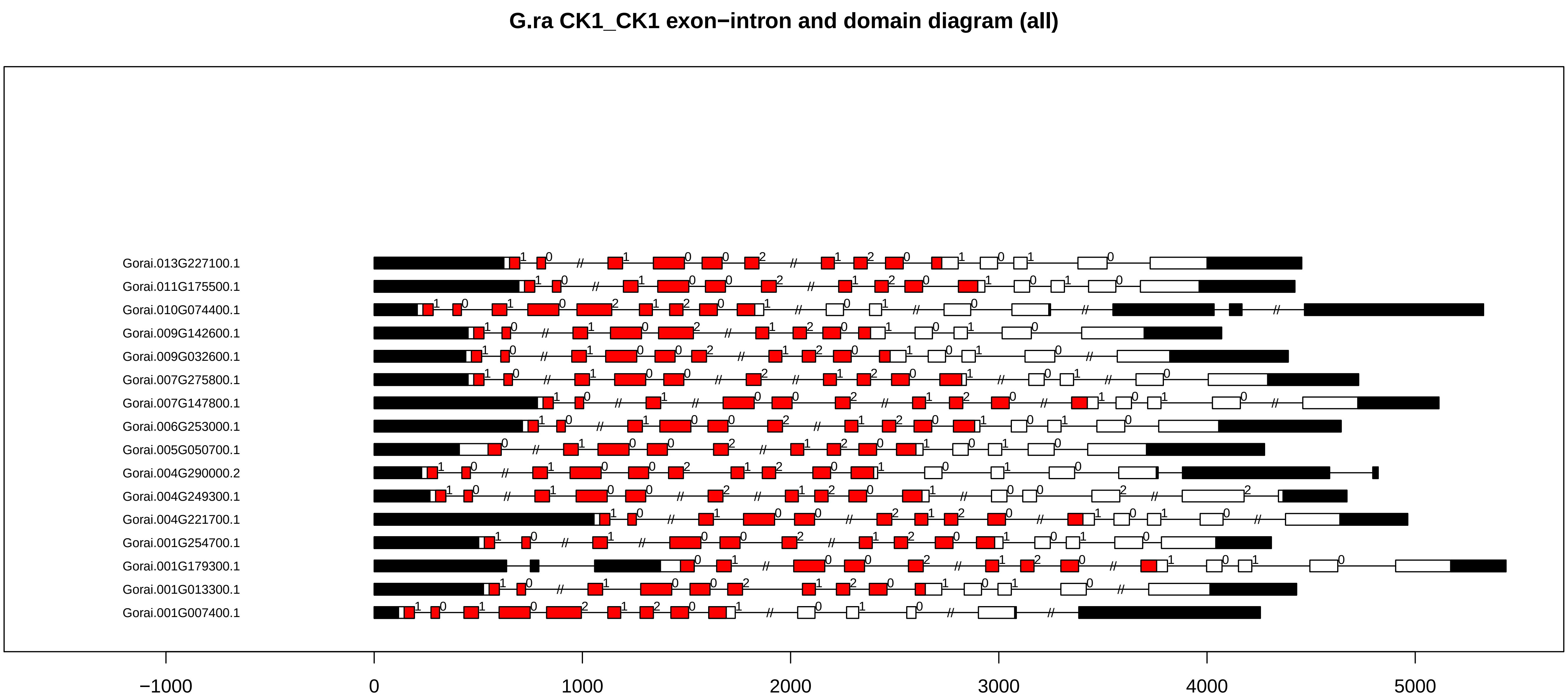
G.ra CK1_CK1 exon−intron and domain diagram (all)
Gorai.013G227100.1
Gorai.011G175500.1
Gorai.010G074400.1
Gorai.009G142600.1
Gorai.009G032600.1
Gorai.007G275800.1
Gorai.007G147800.1
Gorai.006G253000.1
Gorai.005G050700.1
Gorai.004G290000.2
Gorai.004G249300.1
Gorai.004G221700.1
Gorai.001G254700.1
Gorai.001G179300.1
Gorai.001G013300.1
Gorai.001G007400.1
−1000
0
1000
2000
3000
4000
5000

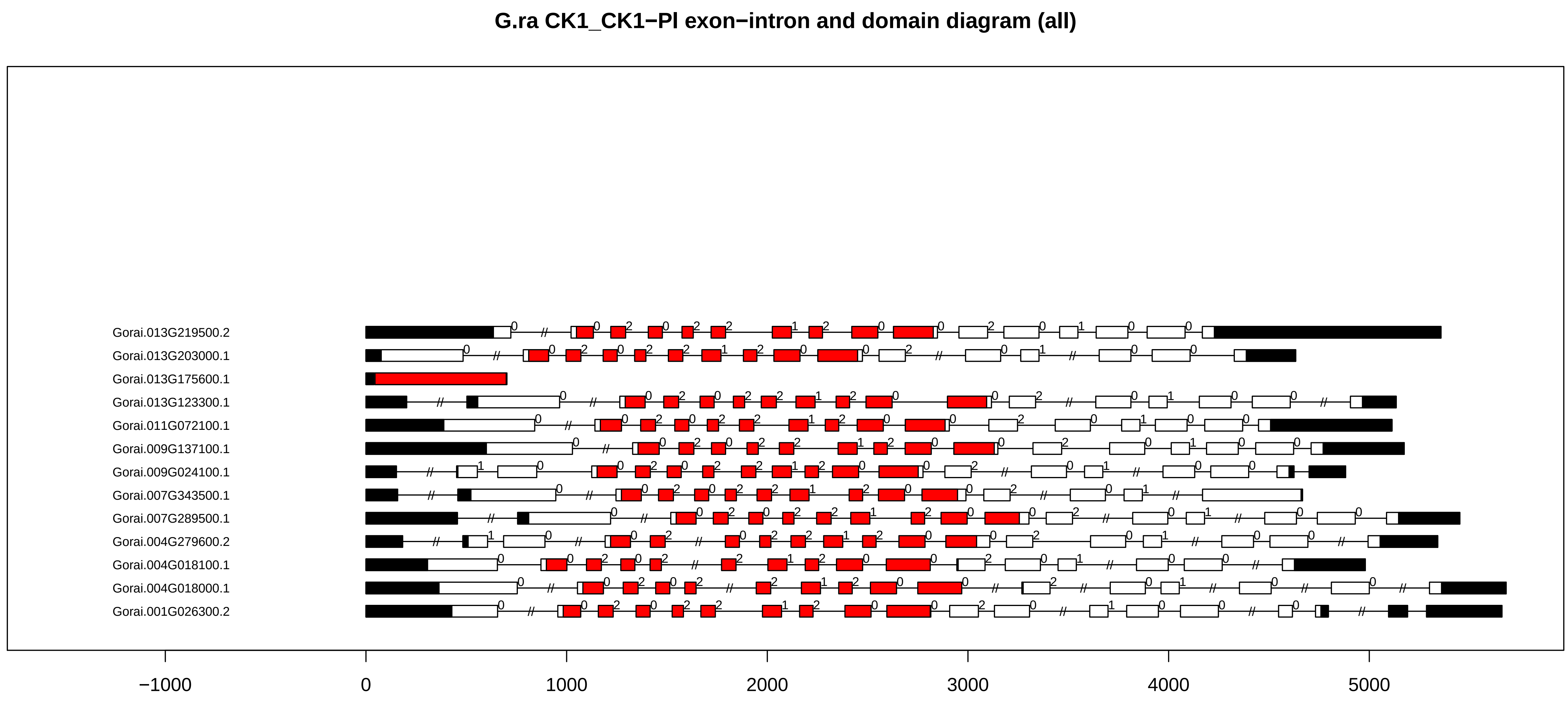
G.ra CK1_CK1−PI exon−intron and domain diagram (all)
Gorai.013G219500.2
Gorai.013G203000.1
Gorai.013G175600.1
Gorai.013G123300.1
Gorai.011G072100.1
Gorai.009G137100.1
Gorai.009G024100.1
Gorai.007G343500.1
Gorai.007G289500.1
Gorai.004G279600.2
Gorai.004G018100.1
Gorai.004G018000.1
Gorai.001G026300.2
−1000
0
1000
2000
3000
4000
5000

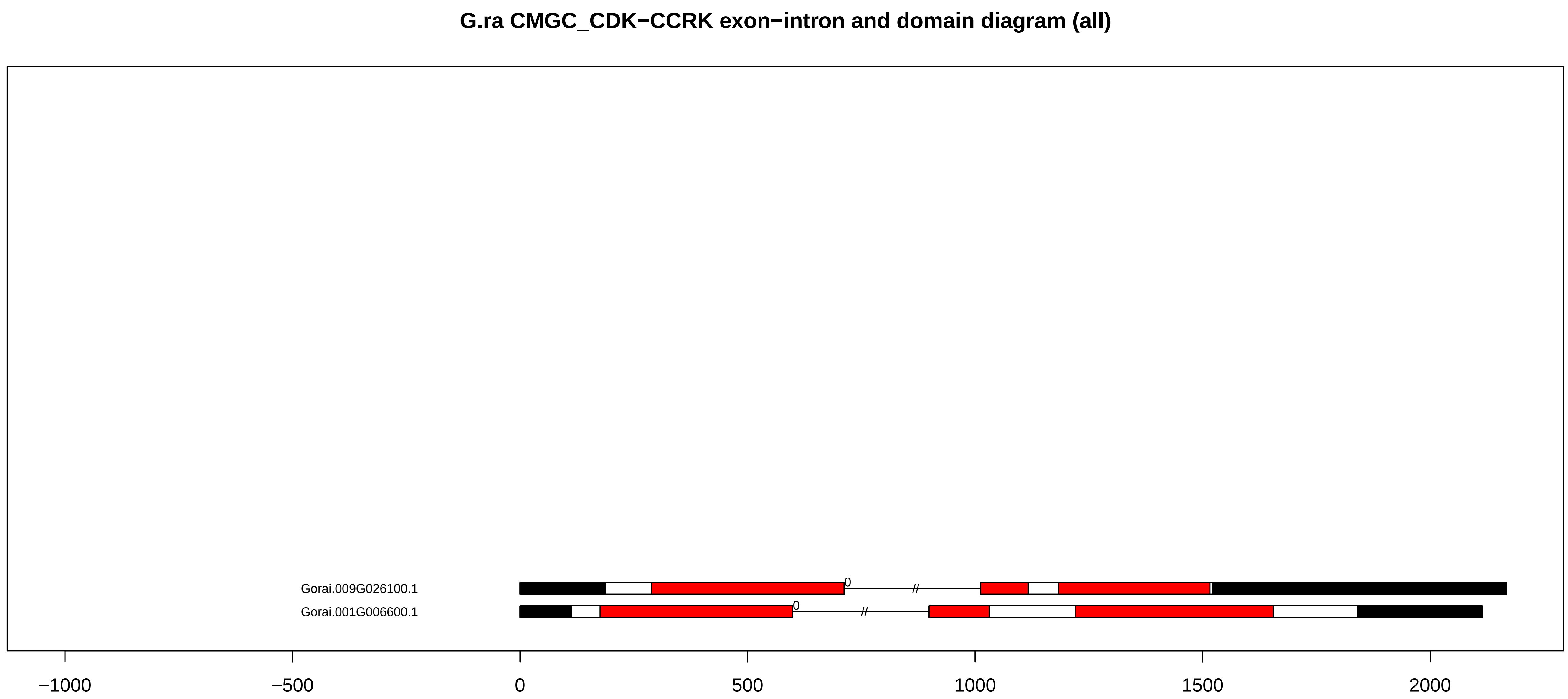
G.ra CMGC_CDK−CCRK exon−intron and domain diagram (all)
Gorai.009G026100.1
Gorai.001G006600.1
0
0
−1000
−500
0
500
1000
1500
2000

G.ra CMGC_CDK−CDK7 exon−intron and domain diagram (all)

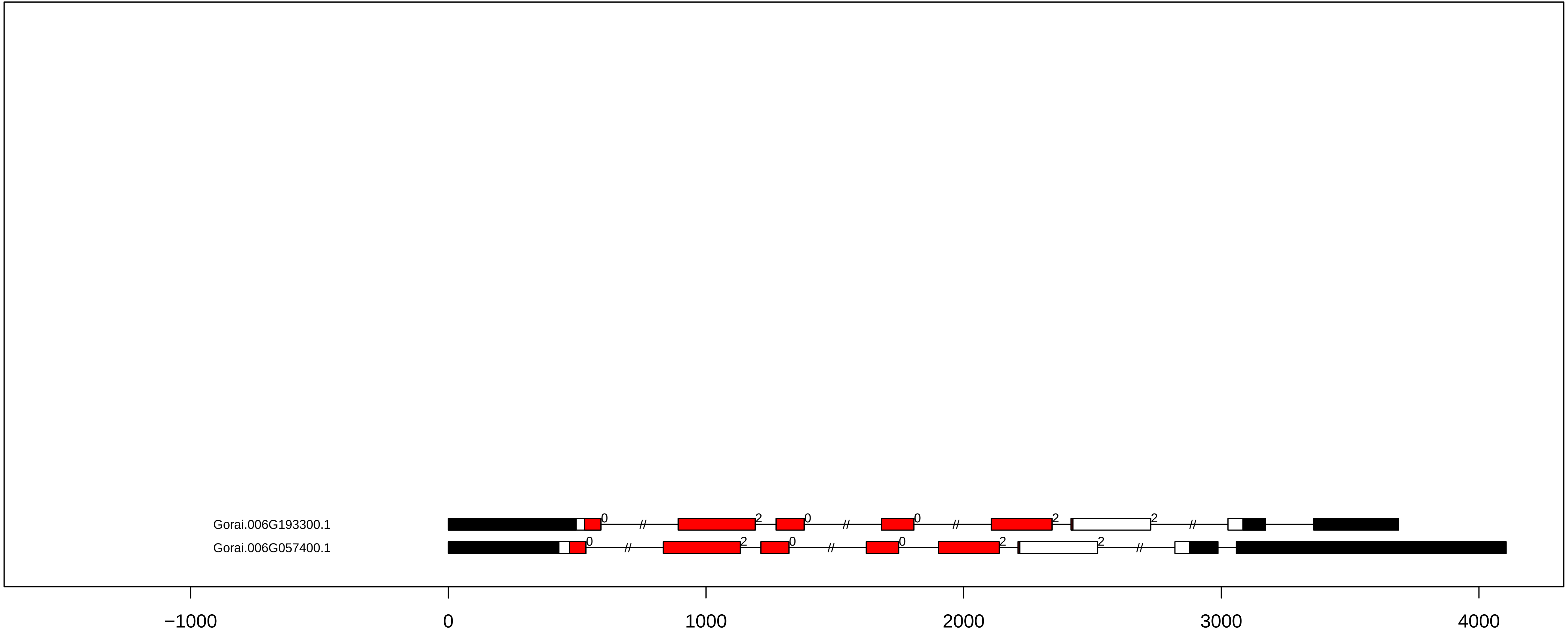

G.ra CMGC_CDK−CDK8 exon−intron and domain diagram (all)

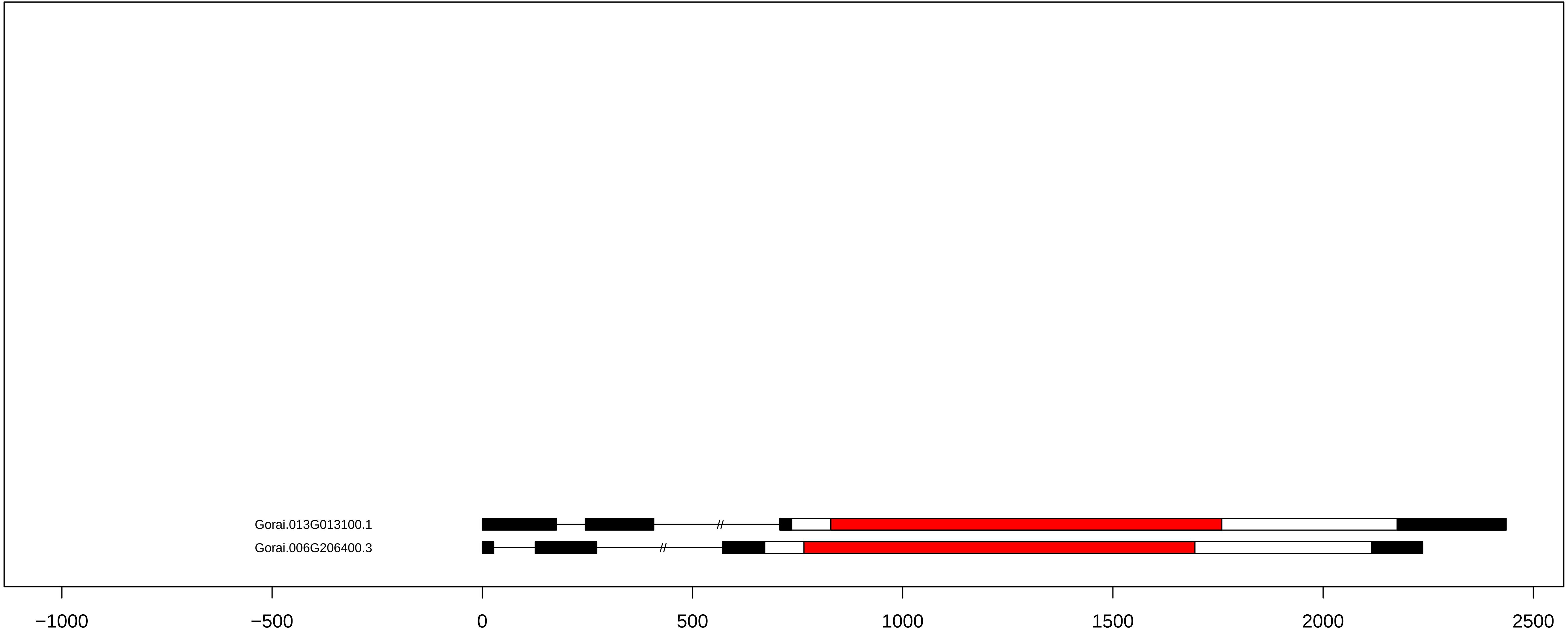

G.ra CMGC_CDK−CRK7−CDK9 exon−intron and domain diagram (part 1)

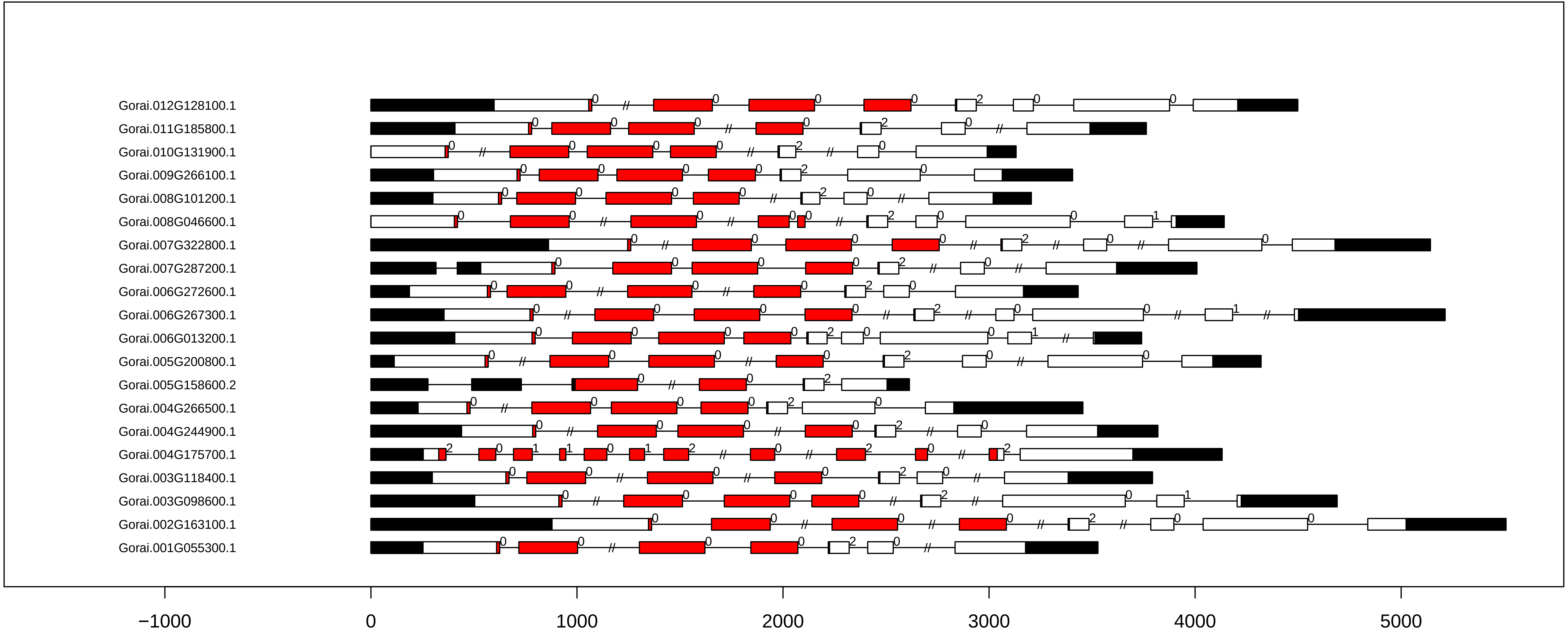

G.ra CMGC_CDK−CRK7−CDK9 exon−intron and domain diagram (part 2)

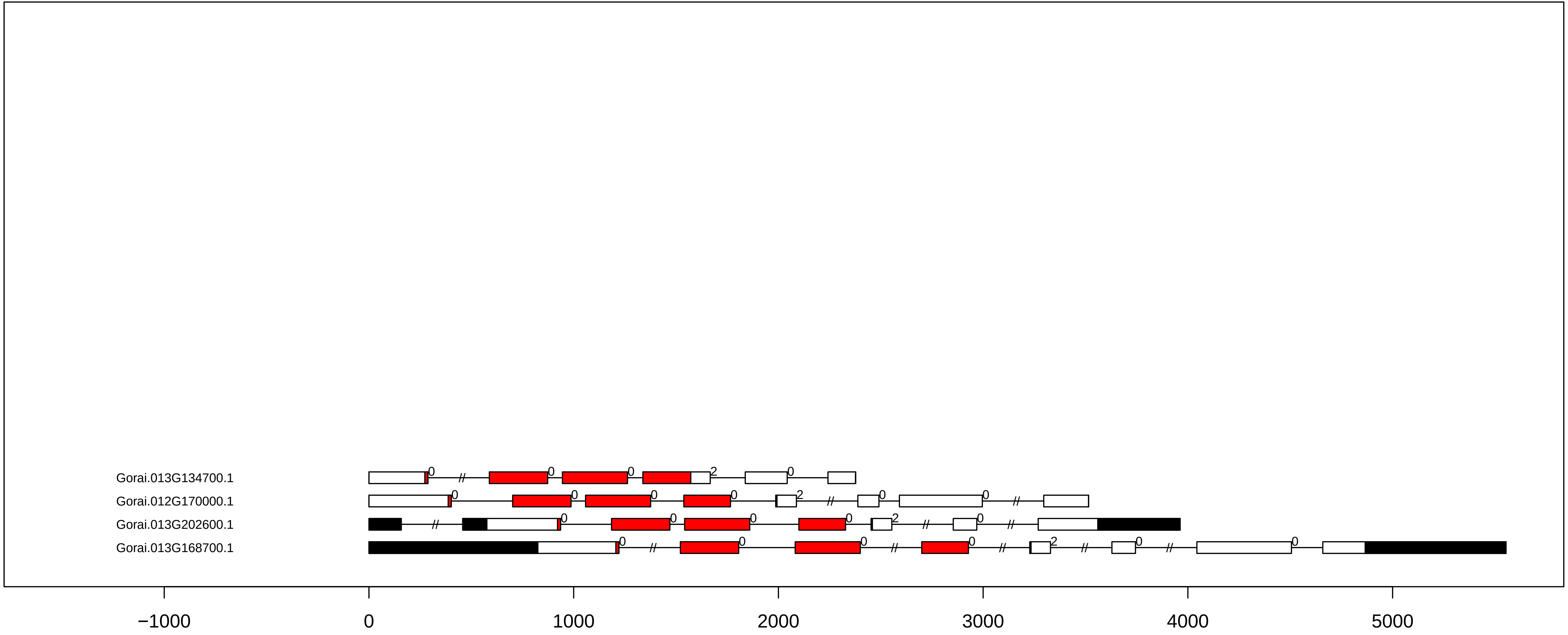

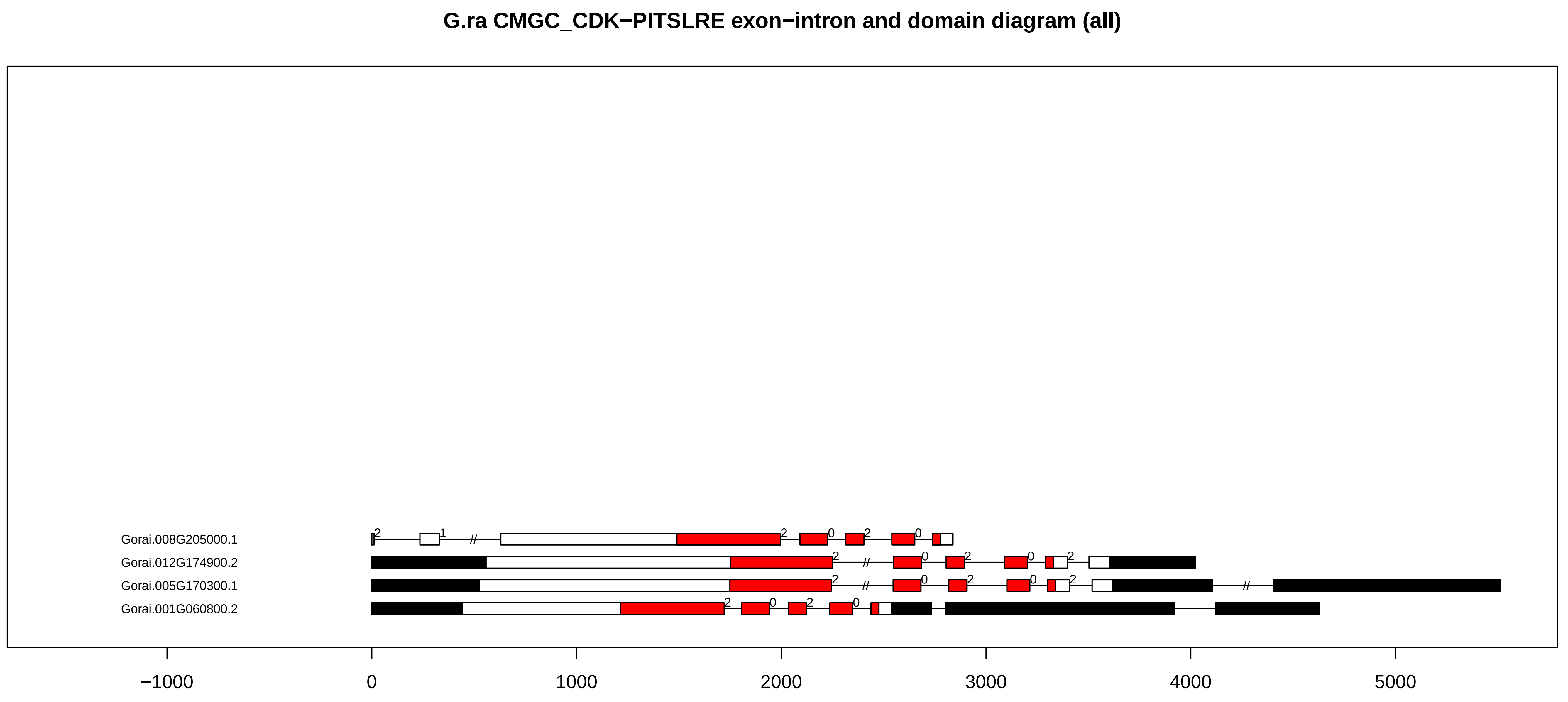
G.ra CMGC_CDK−PITSLRE exon−intron and domain diagram (all)
Gorai.008G205000.1
Gorai.012G174900.2
Gorai.005G170300.1
Gorai.001G060800.2
−1000
0
1000
2000
3000
4000
5000

G.ra CMGC_CDK−Pl exon−intron and domain diagram (all)

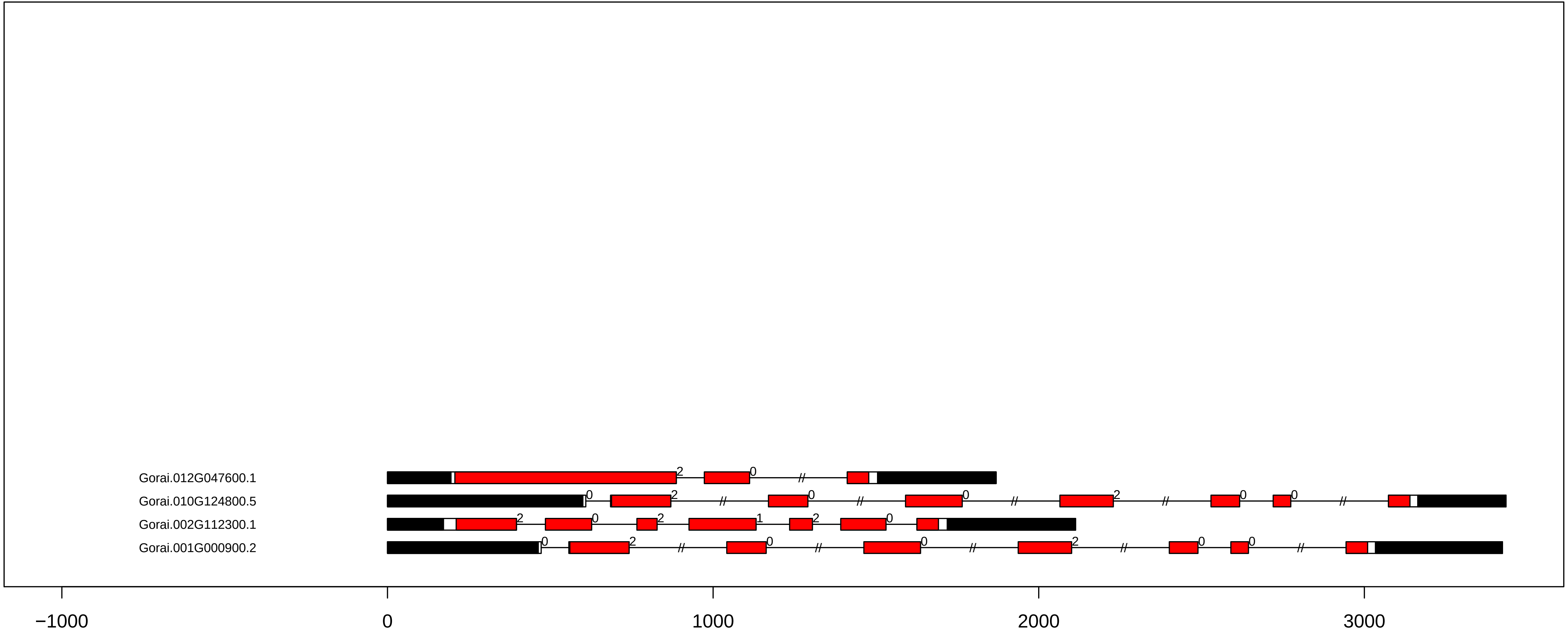

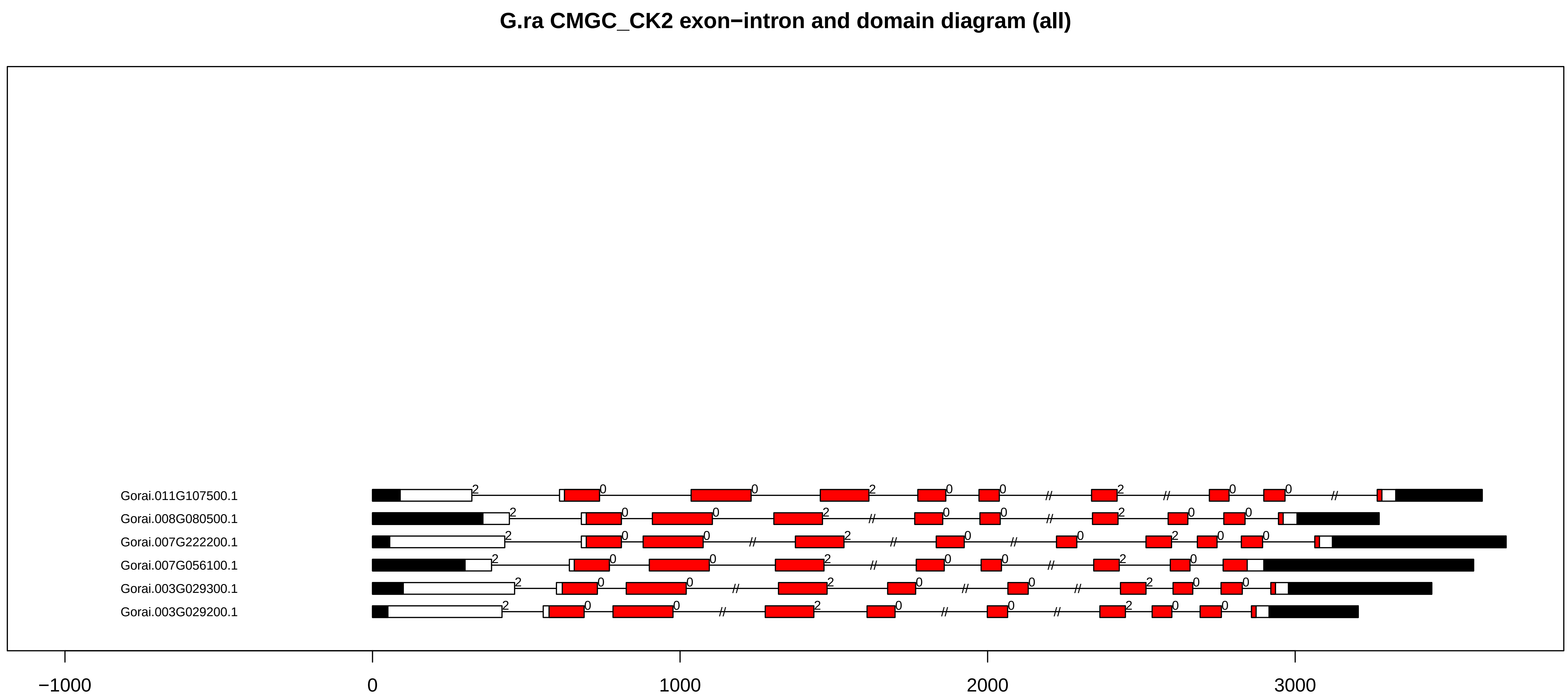

G.ra CMGC_CK2 exon−intron and domain diagram (all)
Gorai.011G107500.1
Gorai.008G080500.1
Gorai.007G222200.1
Gorai.007G056100.1
Gorai.003G029300.1
Gorai.003G029200.1
−1000
0
1000
2000
3000

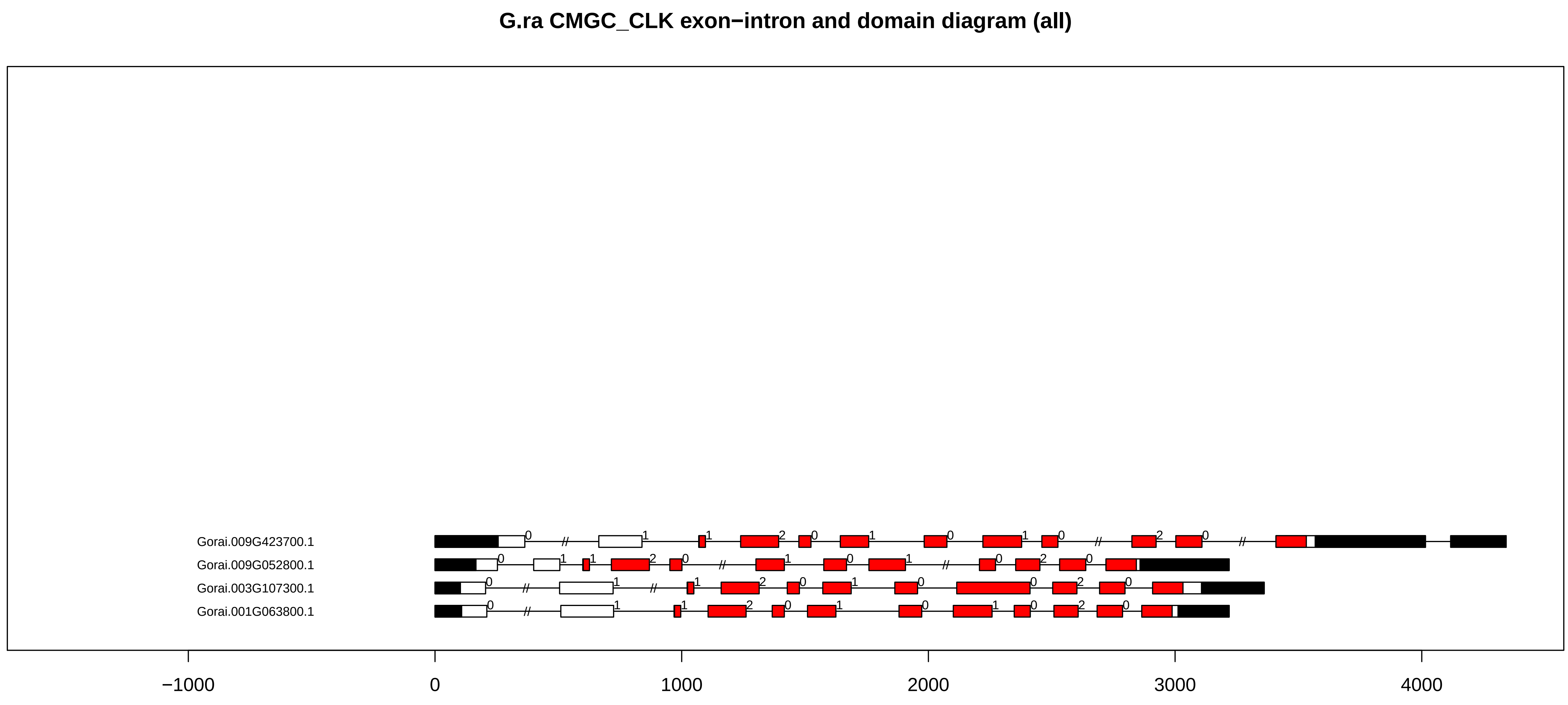
G.ra CMGC_CLK exon−intron and domain diagram (all)
Gorai.009G423700.1
Gorai.009G052800.1
Gorai.003G107300.1
Gorai.001G063800.1
−1000
0
1000
2000
3000
4000

G.ra CMGC_DYRK−PRP4 exon−intron and domain diagram (all)

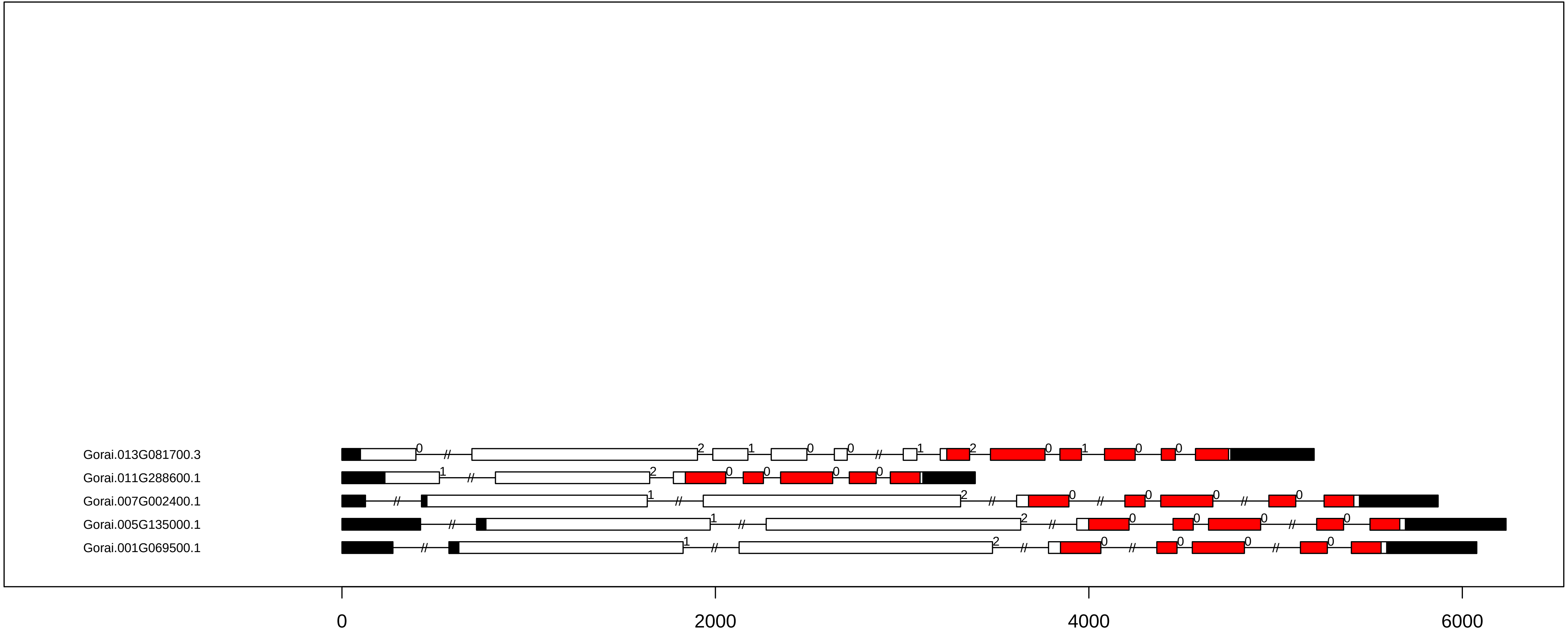

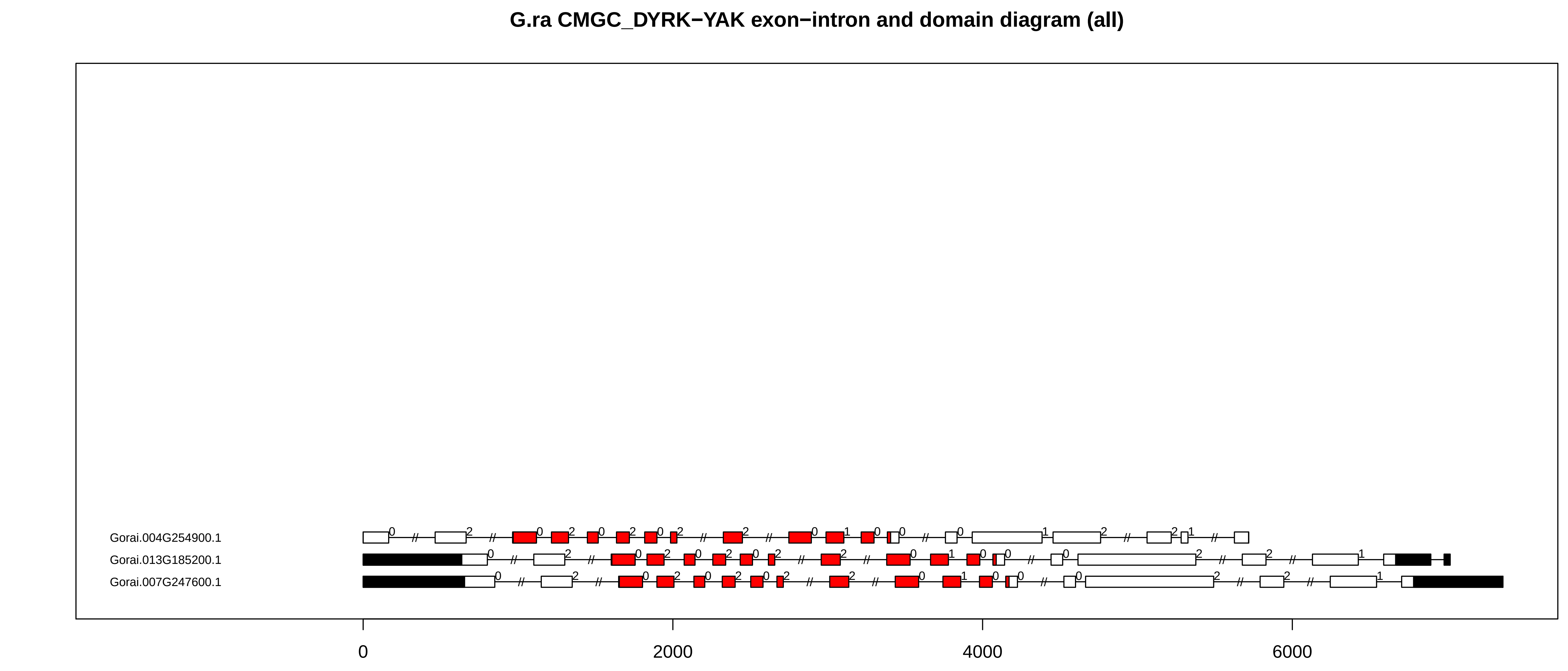
G.ra CMGC_DYRK−YAK exon−intron and domain diagram (all)
Gorai.004G254900.1
Gorai.013G185200.1
Gorai.007G247600.1
0
2000
4000
6000

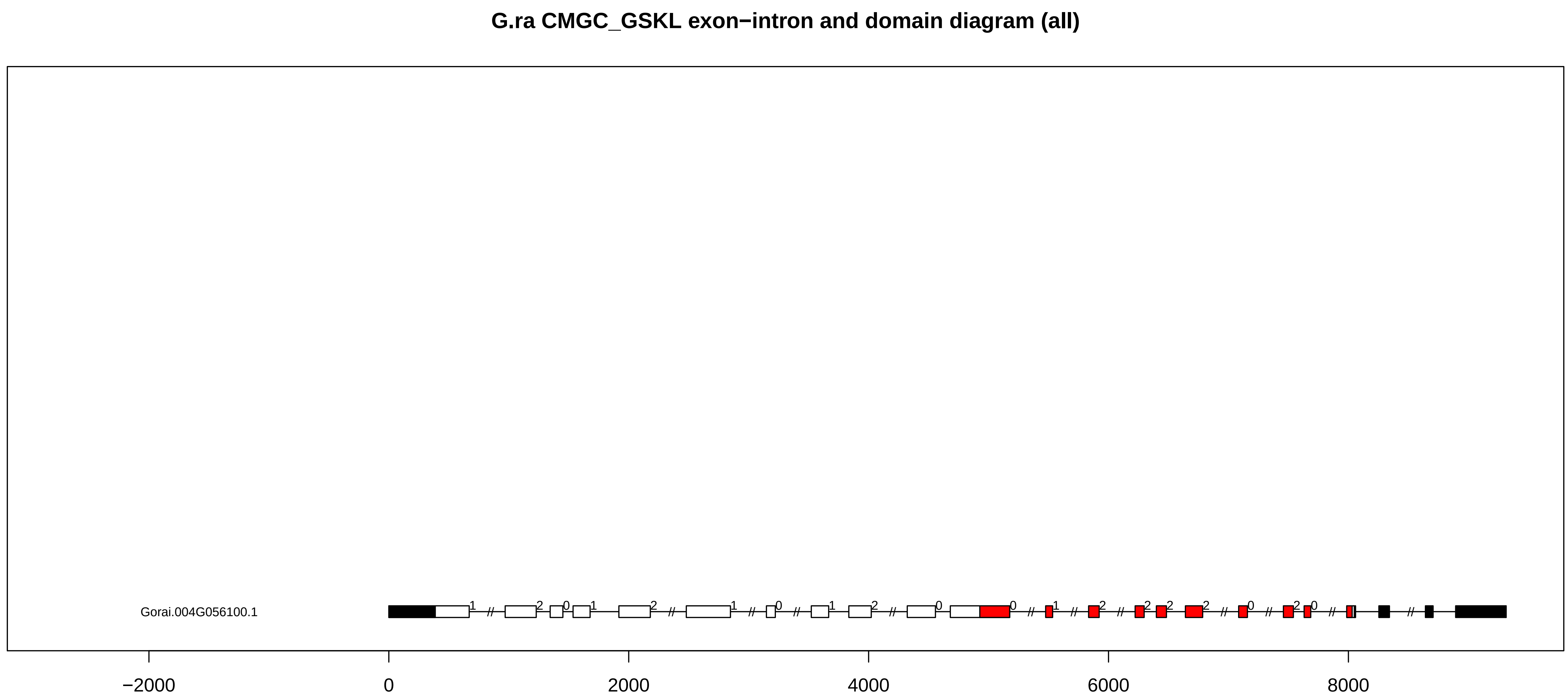

G.ra CMGC_GSKL exon−intron and domain diagram (all)
Gorai.004G056100.1
−2000
0
2000
4000
6000
8000

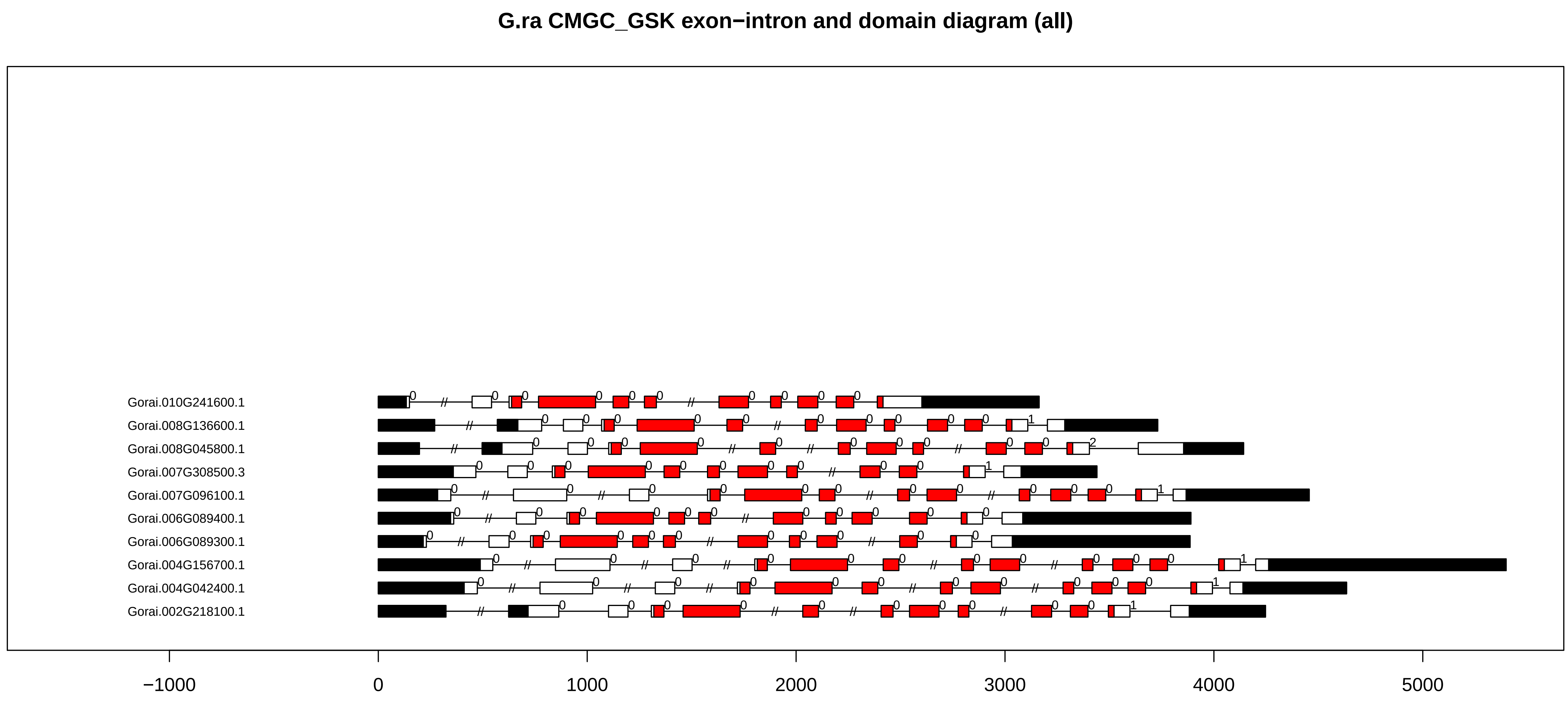
G.ra CMGC_GSK exon−intron and domain diagram (all)
Gorai.010G241600.1
Gorai.008G136600.1
Gorai.008G045800.1
Gorai.007G308500.3
Gorai.007G096100.1
Gorai.006G089400.1
Gorai.006G089300.1
Gorai.004G156700.1
Gorai.004G042400.1
Gorai.002G218100.1
−1000
0
1000
2000
3000
4000
5000

G.ra CMGC_MAPK exon−intron and domain diagram (part 1)

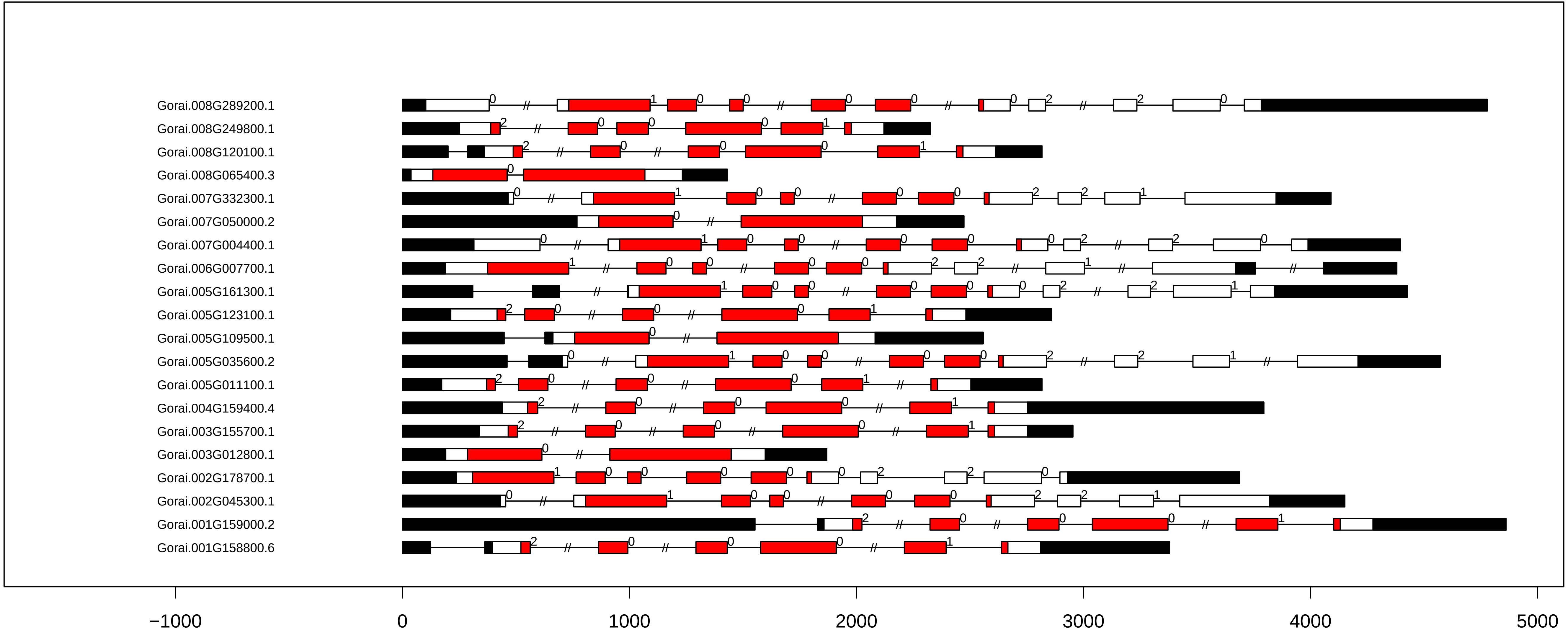

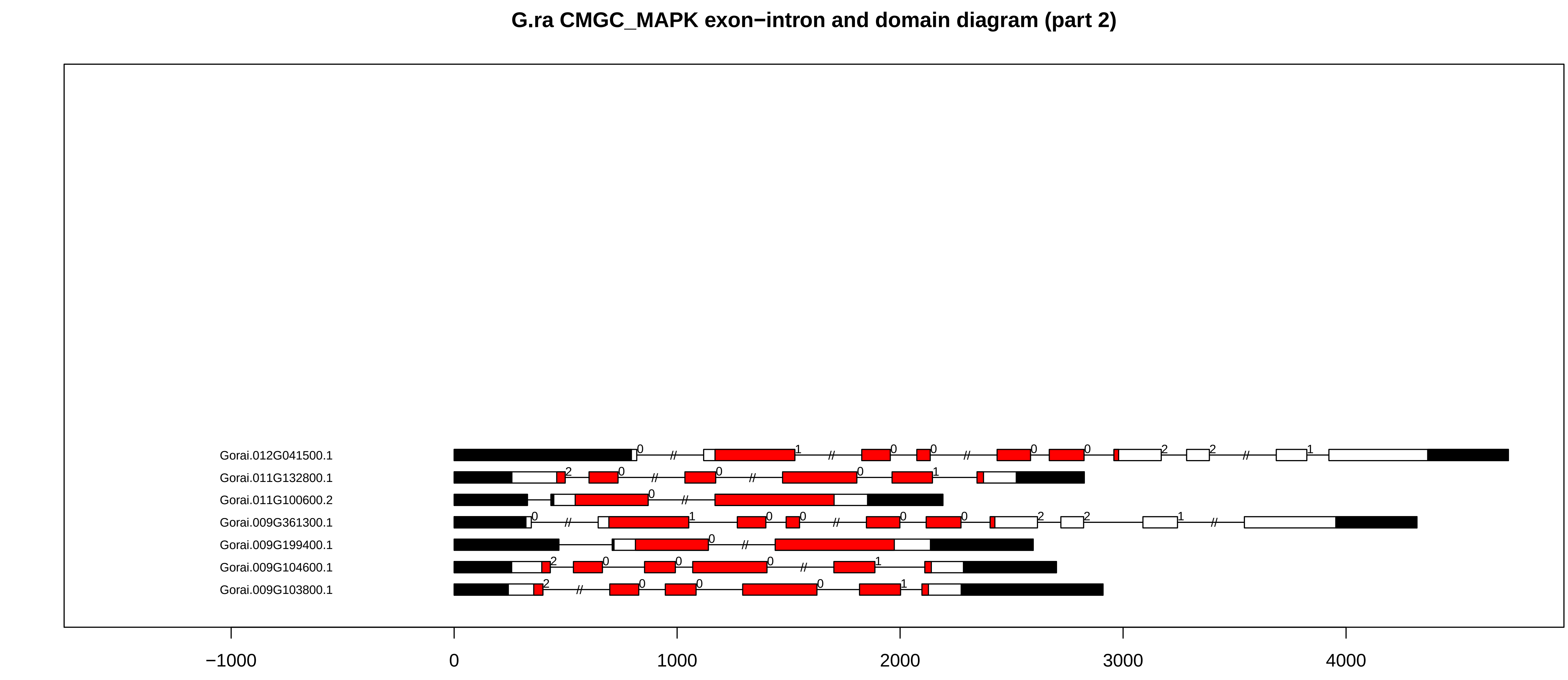

G.ra CMGC_MAPK exon−intron and domain diagram (part 2)
Gorai.012G041500.1
Gorai.011G132800.1
Gorai.011G100600.2
Gorai.009G361300.1
Gorai.009G199400.1
Gorai.009G104600.1
Gorai.009G103800.1
−1000
0
1000
2000
3000
4000

G.ra CMGC_Pl−Tthe exon−intron and domain diagram (all)

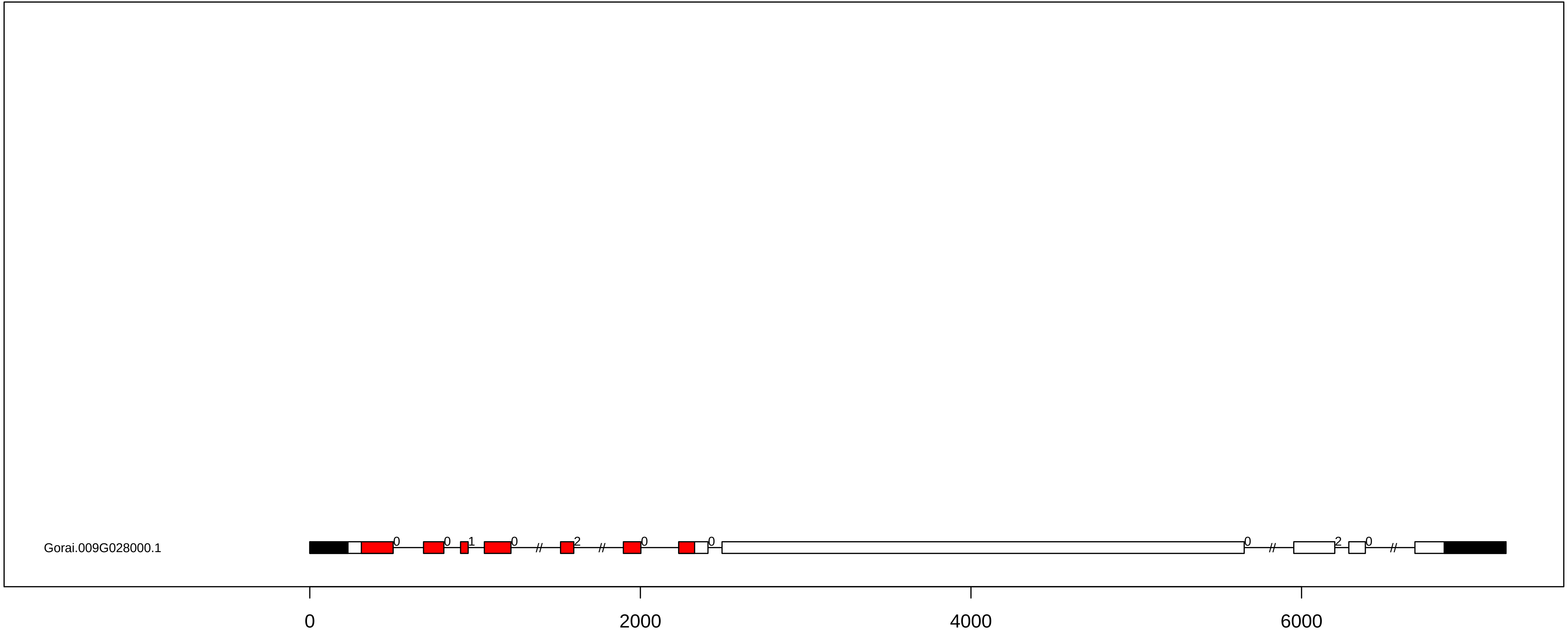

G.ra CMGC_RCK exon−intron and domain diagram (all)

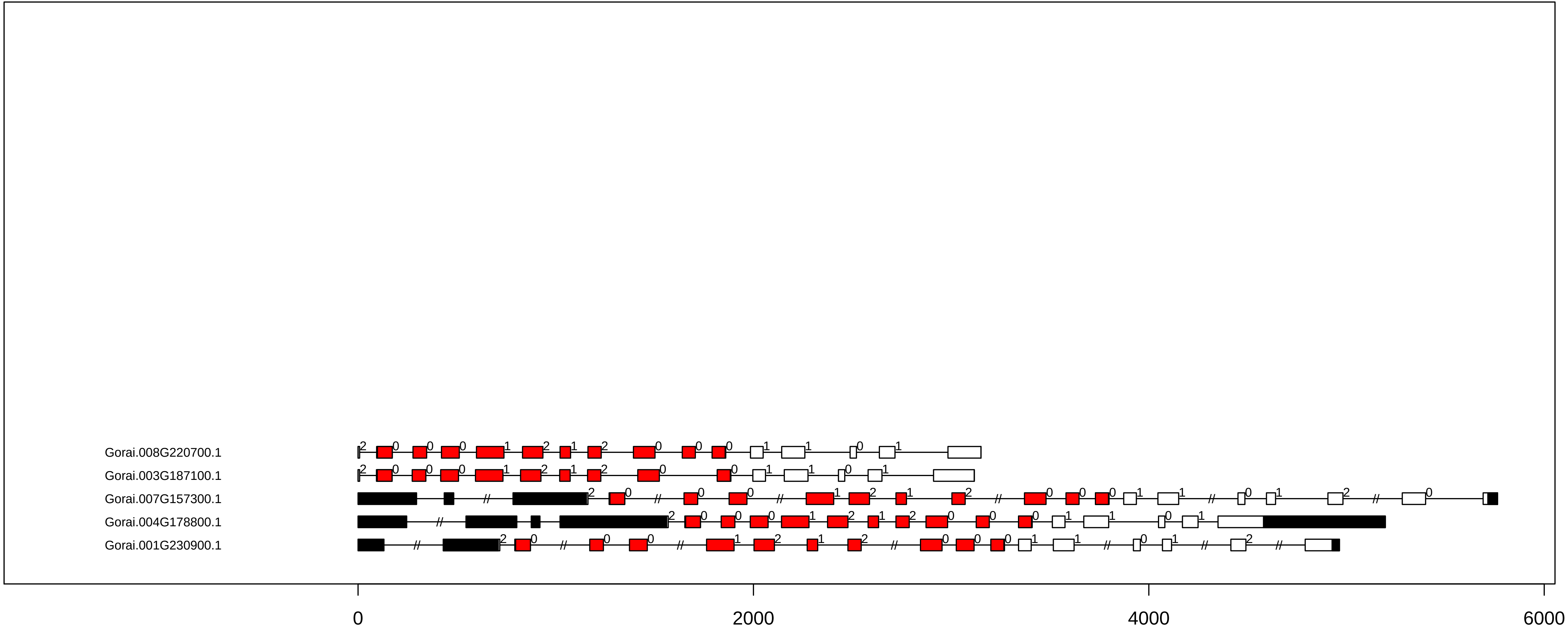

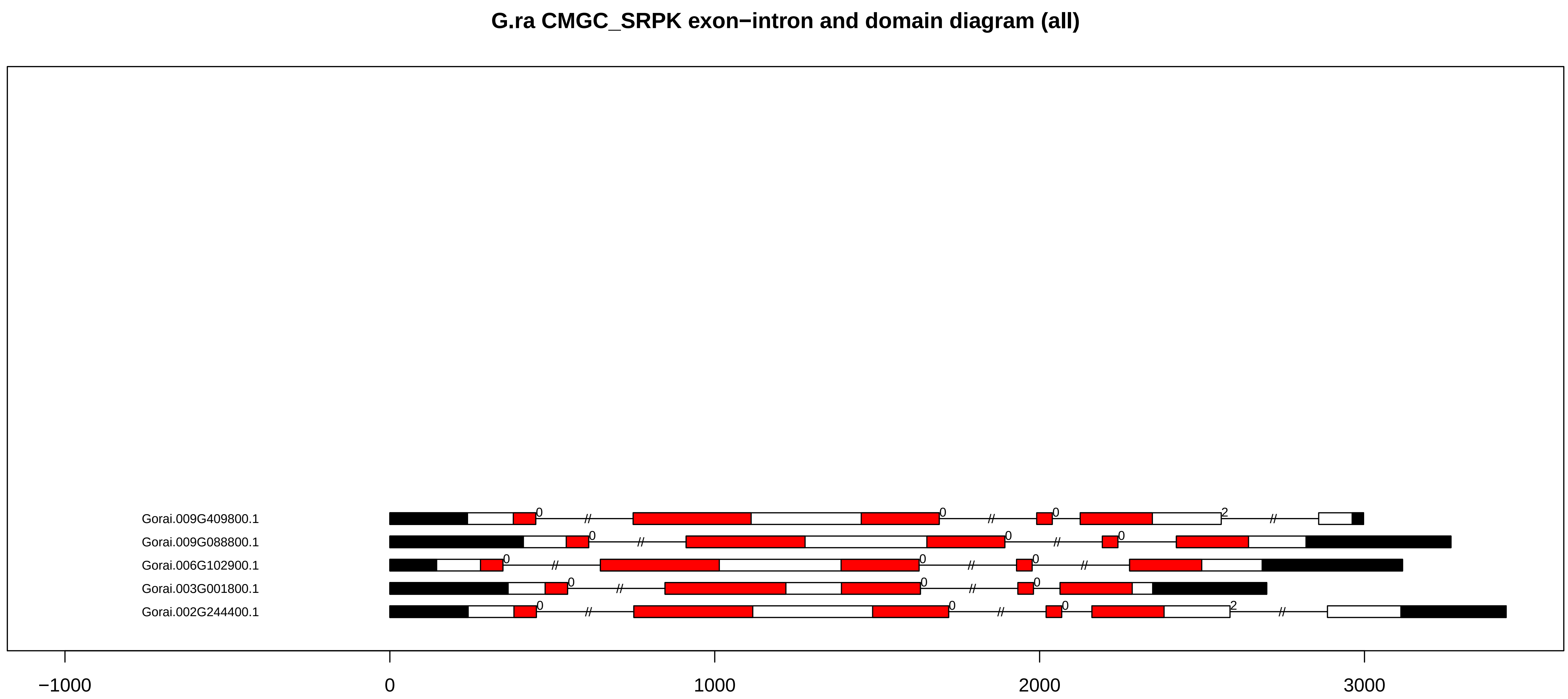
G.ra CMGC_SRPK exon−intron and domain diagram (all)
Gorai.009G409800.1
Gorai.009G088800.1
Gorai.006G102900.1
Gorai.003G001800.1
Gorai.002G244400.1
−1000
0
1000
2000
3000

G.ra Group−PI−2 exon−intron and domain diagram (all)

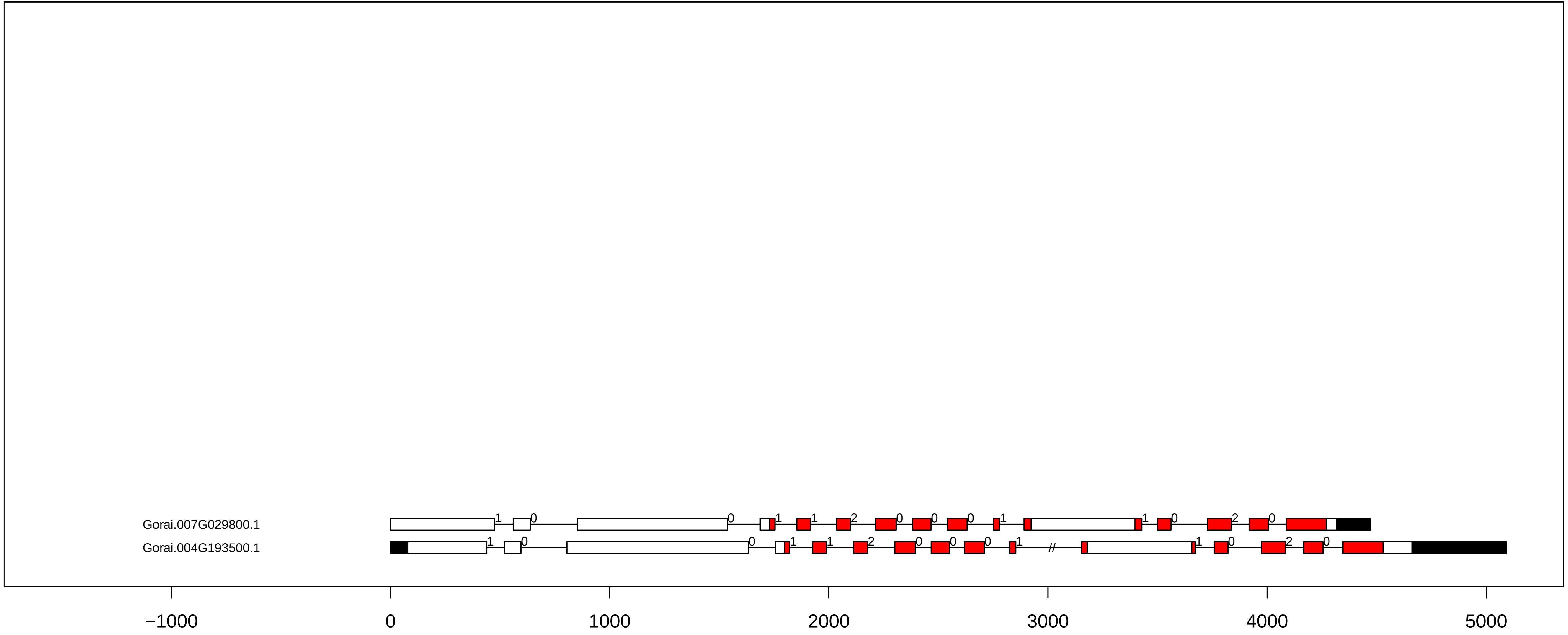

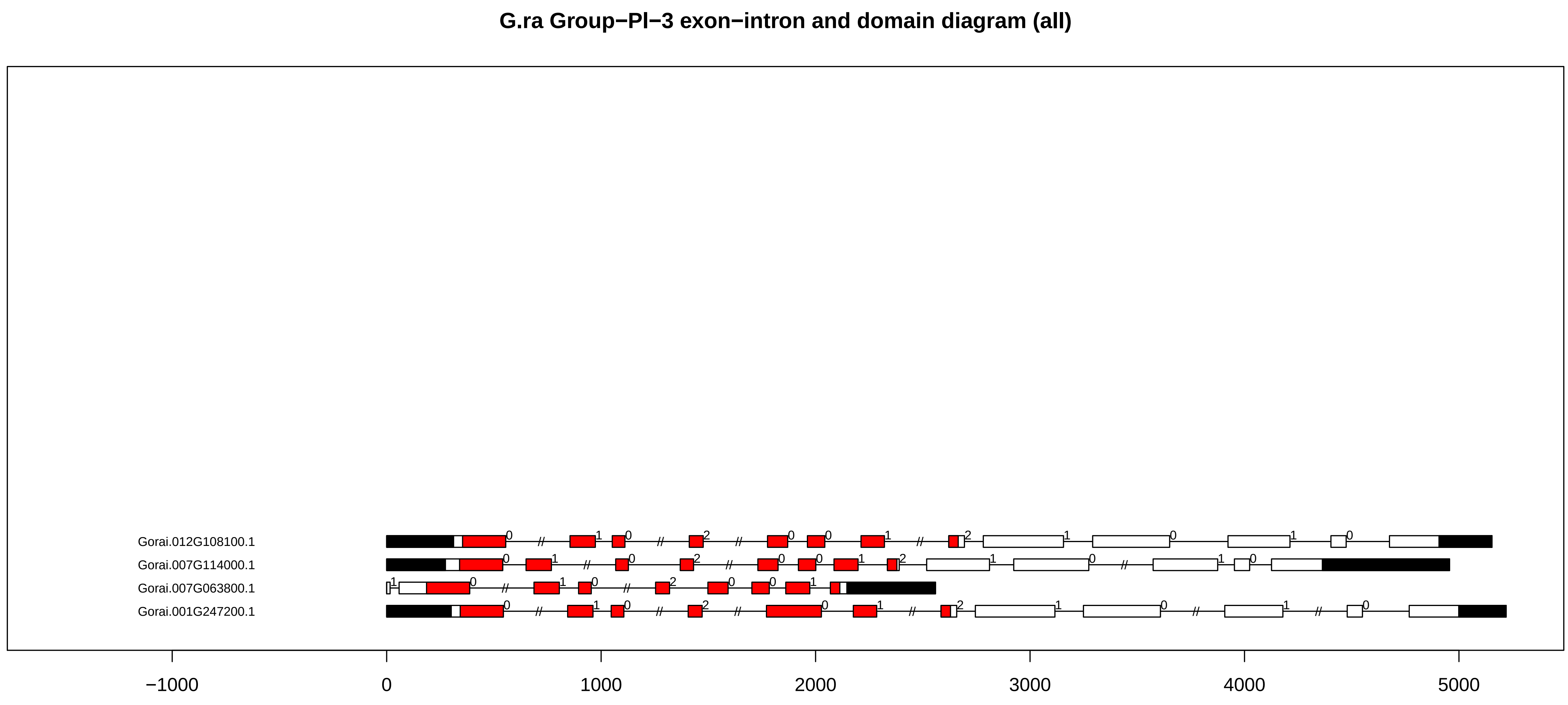
G.ra Group−PI−3 exon−intron and domain diagram (all)
Gorai.012G108100.1
Gorai.007G114000.1
Gorai.007G063800.1
Gorai.001G247200.1
−1000
0
1000
2000
3000
4000
5000

G.ra Group−PI−4 exon−intron and domain diagram (all)

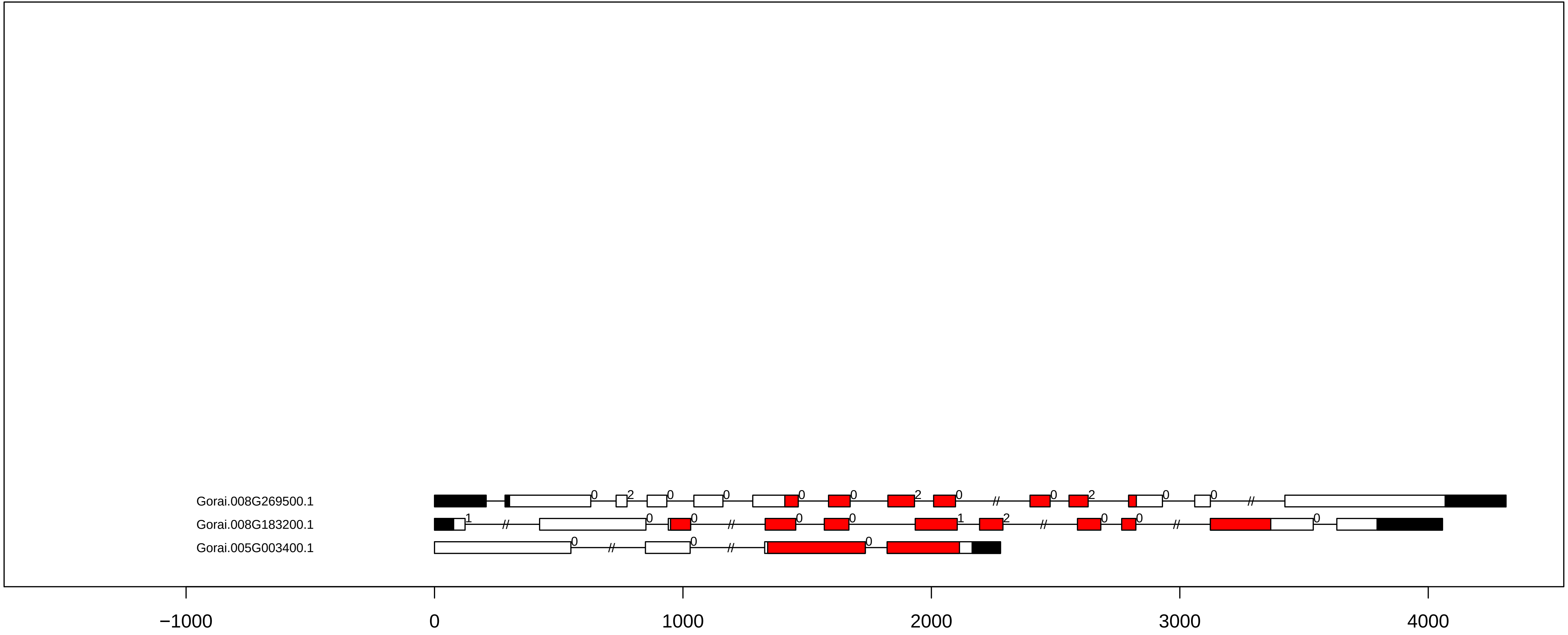

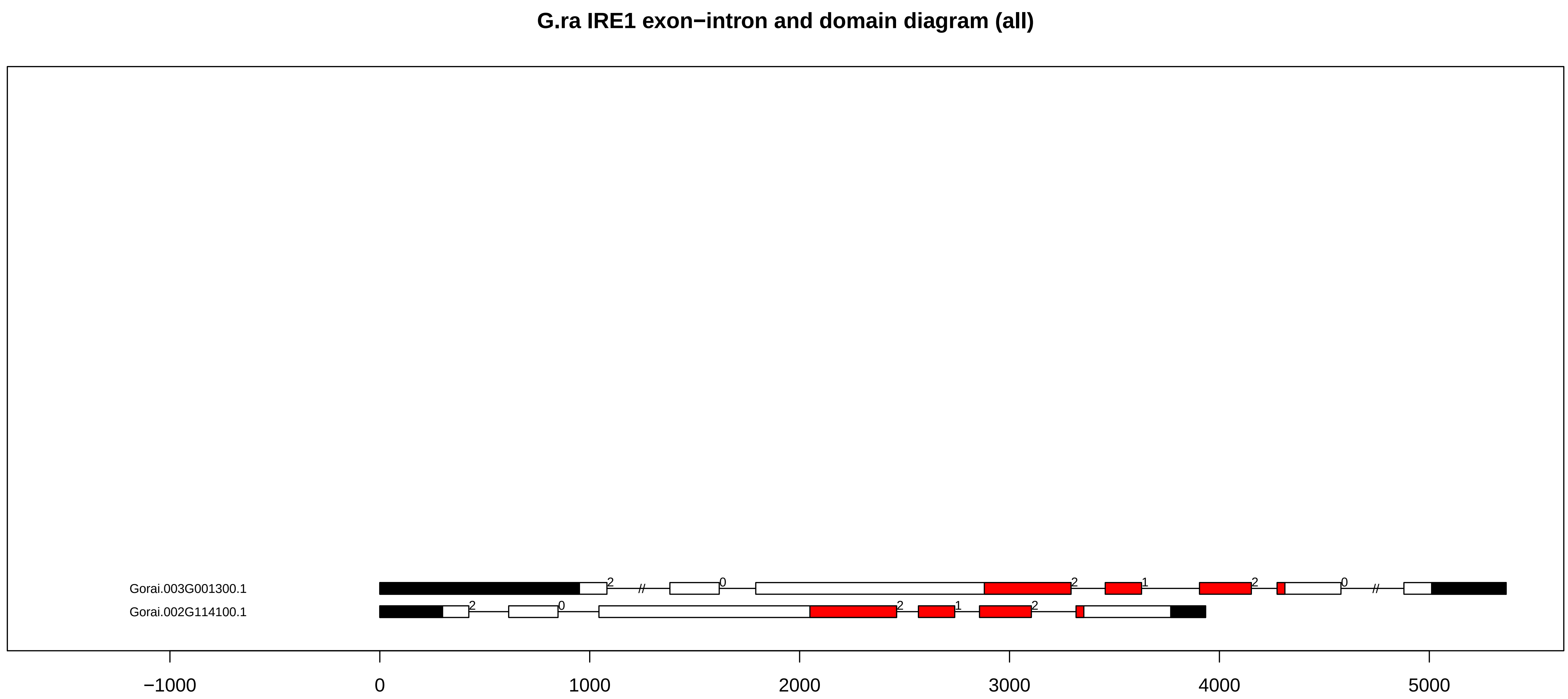
G.ra IRE1 exon−intron and domain diagram (all)
Gorai.003G001300.1
Gorai.002G114100.1
2
0
2
1
2
0
2
0
2
1
2
−1000
0
1000
2000
3000
4000
5000

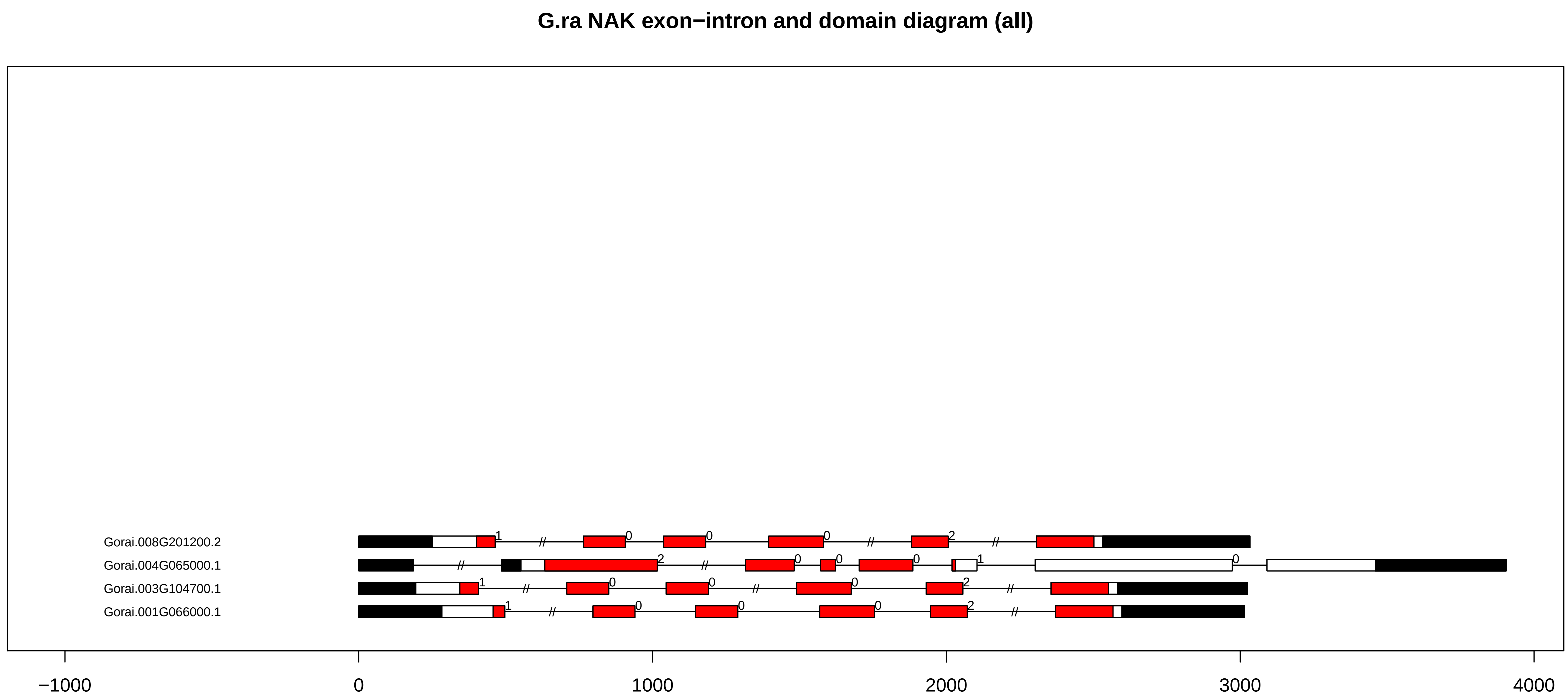

G.ra NAK exon−intron and domain diagram (all)
Gorai.008G201200.2
Gorai.004G065000.1
Gorai.003G104700.1
Gorai.001G066000.1
−1000
0
1000
2000
3000
4000

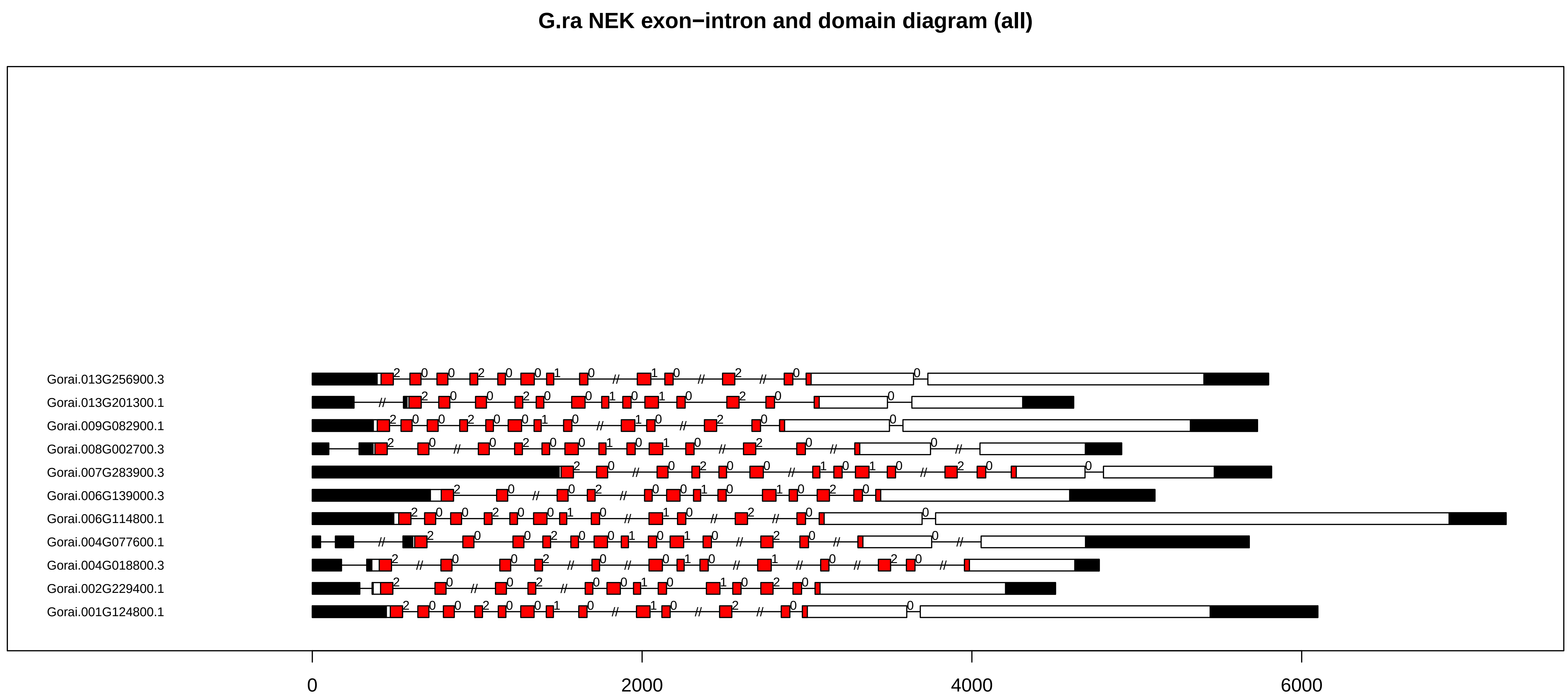
G.ra NEK exon−intron and domain diagram (all)
Gorai.013G256900.3
Gorai.013G201300.1
Gorai.009G082900.1
Gorai.008G002700.3
Gorai.007G283900.3
Gorai.006G139000.3
Gorai.006G114800.1
Gorai.004G077600.1
Gorai.004G018800.3
Gorai.002G229400.1
Gorai.001G124800.1
0
2000
4000
6000

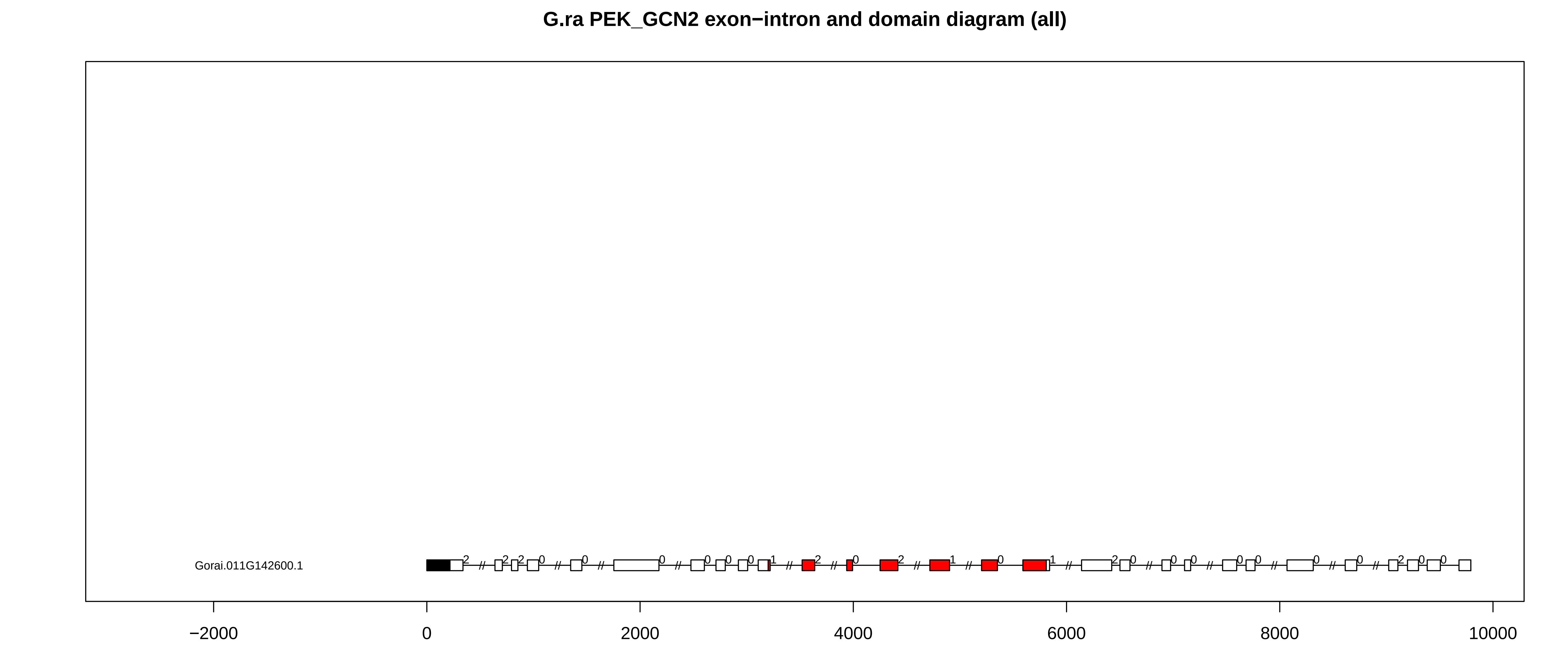
G.ra PEK_GCN2 exon−intron and domain diagram (all)
Gorai.011G142600.1
−2000
0
2000
4000
6000
8000
10000

G.ra PEK_PEK exon−intron and domain diagram (all)

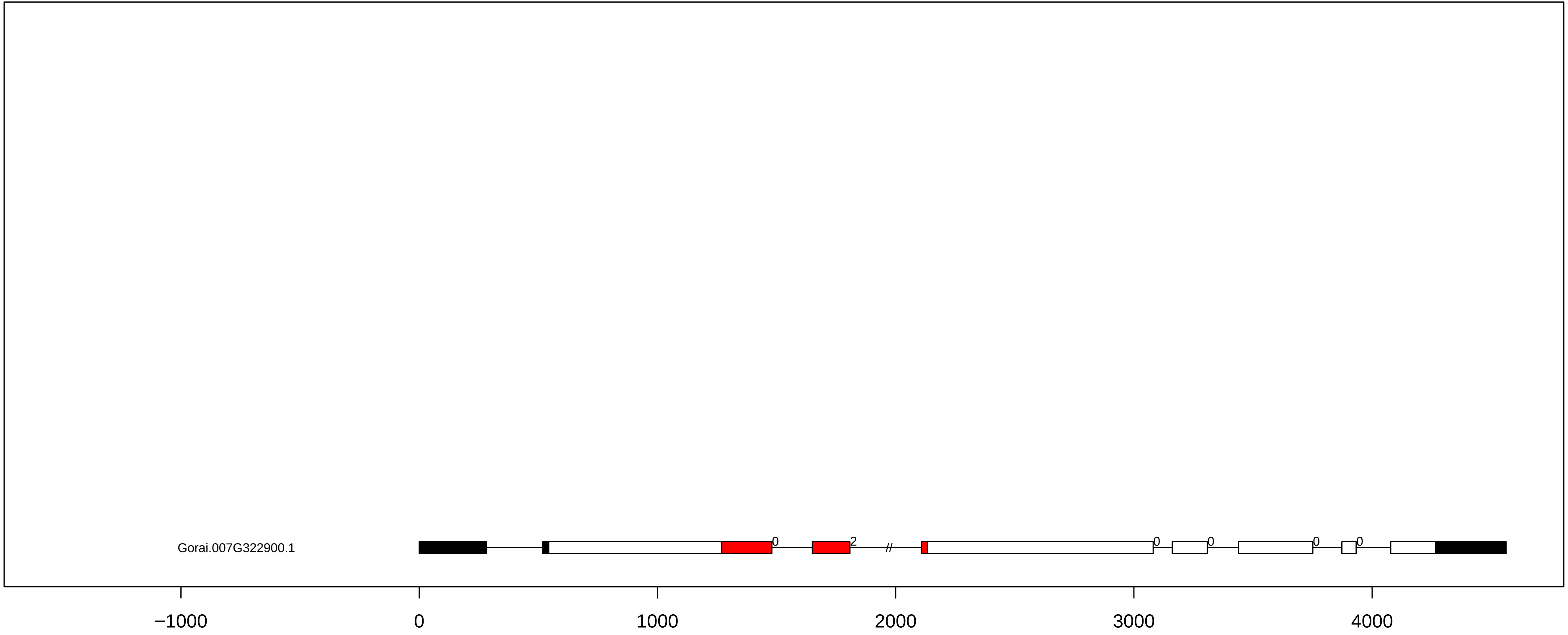

G.ra RLK−Pelle_C−LEC exon−intron and domain diagram (all)

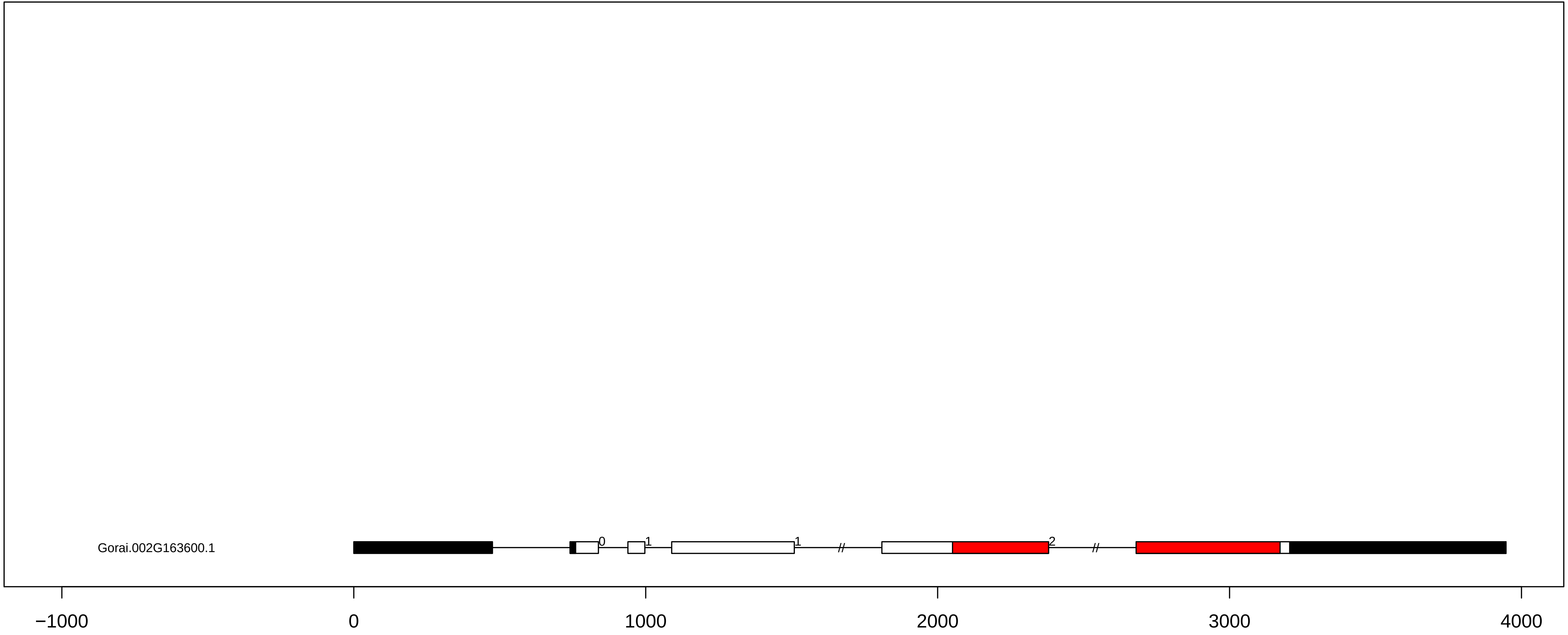

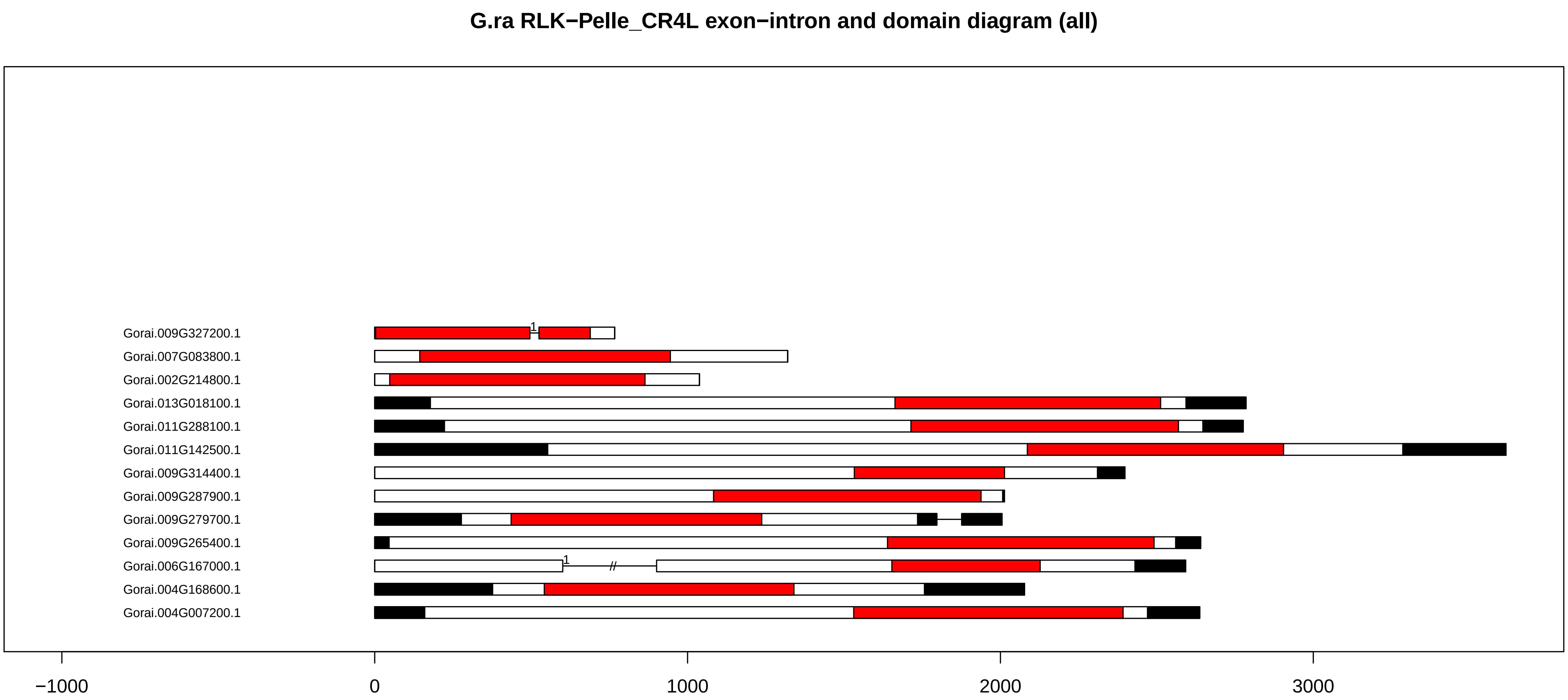
G.ra RLK−Pelle_CR4L exon−intron and domain diagram (all)
Gorai.009G327200.1
Gorai.007G083800.1
Gorai.002G214800.1
Gorai.013G018100.1
Gorai.011G288100.1
Gorai.011G142500.1
Gorai.009G314400.1
Gorai.009G287900.1
Gorai.009G279700.1
Gorai.009G265400.1
Gorai.006G167000.1
Gorai.004G168600.1
Gorai.004G007200.1
1
1
−1000
0
1000
2000
3000

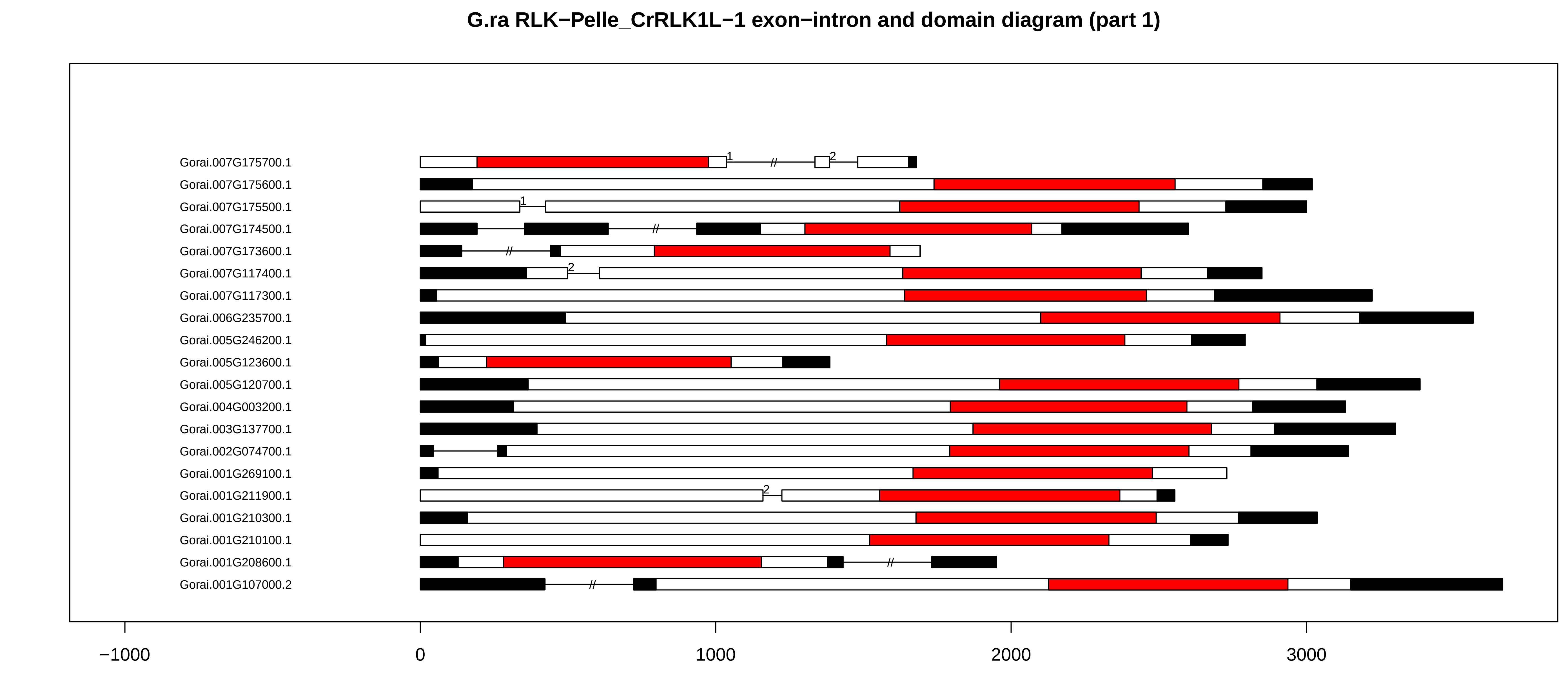
G.ra RLK−Pelle_CrRLK1L−1 exon−intron and domain diagram (part 1)
Gorai.007G175700.1
Gorai.007G175600.1
Gorai.007G175500.1
Gorai.007G174500.1
Gorai.007G173600.1
Gorai.007G117400.1
Gorai.007G117300.1
Gorai.006G235700.1
Gorai.005G246200.1
Gorai.005G123600.1
Gorai.005G120700.1
Gorai.004G003200.1
Gorai.003G137700.1
Gorai.002G074700.1
Gorai.001G269100.1
Gorai.001G211900.1
Gorai.001G210300.1
Gorai.001G210100.1
Gorai.001G208600.1
Gorai.001G107000.2
−1000
0
1000
2000
3000

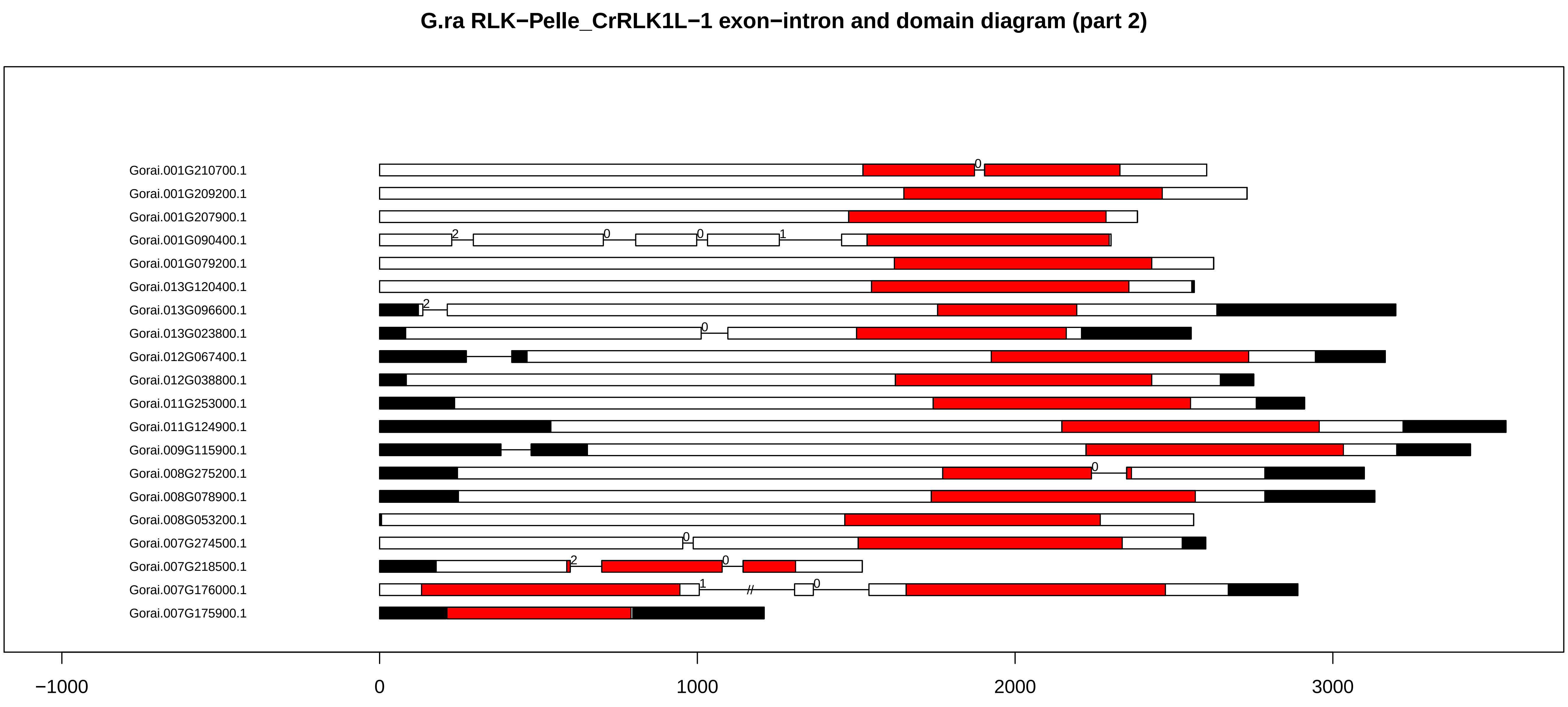
G.ra RLK−Pelle_CrRLK1L−1 exon−intron and domain diagram (part 2)
Gorai.001G210700.1
Gorai.001G209200.1
Gorai.001G207900.1
Gorai.001G090400.1
Gorai.001G079200.1
Gorai.013G120400.1
Gorai.013G096600.1
Gorai.013G023800.1
Gorai.012G067400.1
Gorai.012G038800.1
Gorai.011G253000.1
Gorai.011G124900.1
Gorai.009G115900.1
Gorai.008G275200.1
Gorai.008G078900.1
Gorai.008G053200.1
Gorai.007G274500.1
Gorai.007G218500.1
Gorai.007G176000.1
Gorai.007G175900.1
−1000
0
1000
2000
3000

G.ra RLK−Pelle_CrRLK1L−1 exon−intron and domain diagram (part 3)

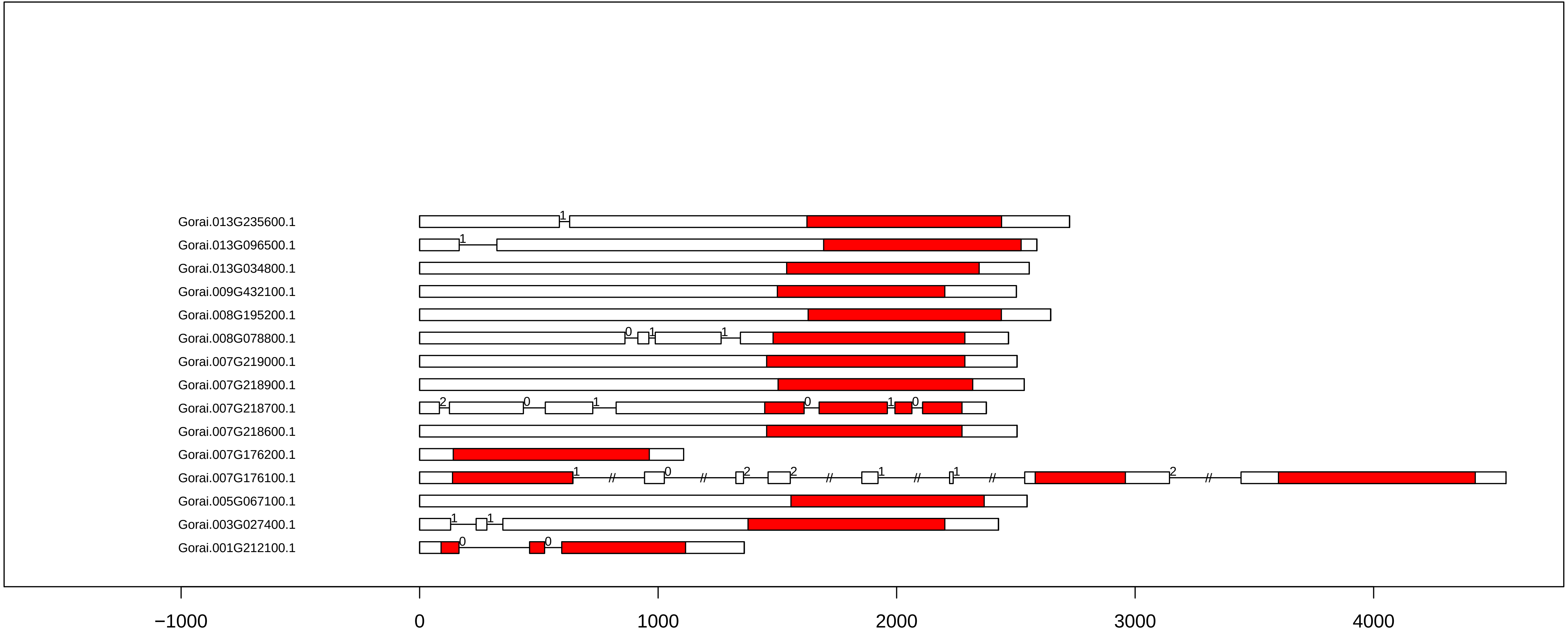

G.ra RLK−Pelle_DLSV exon−intron and domain diagram (part 1)

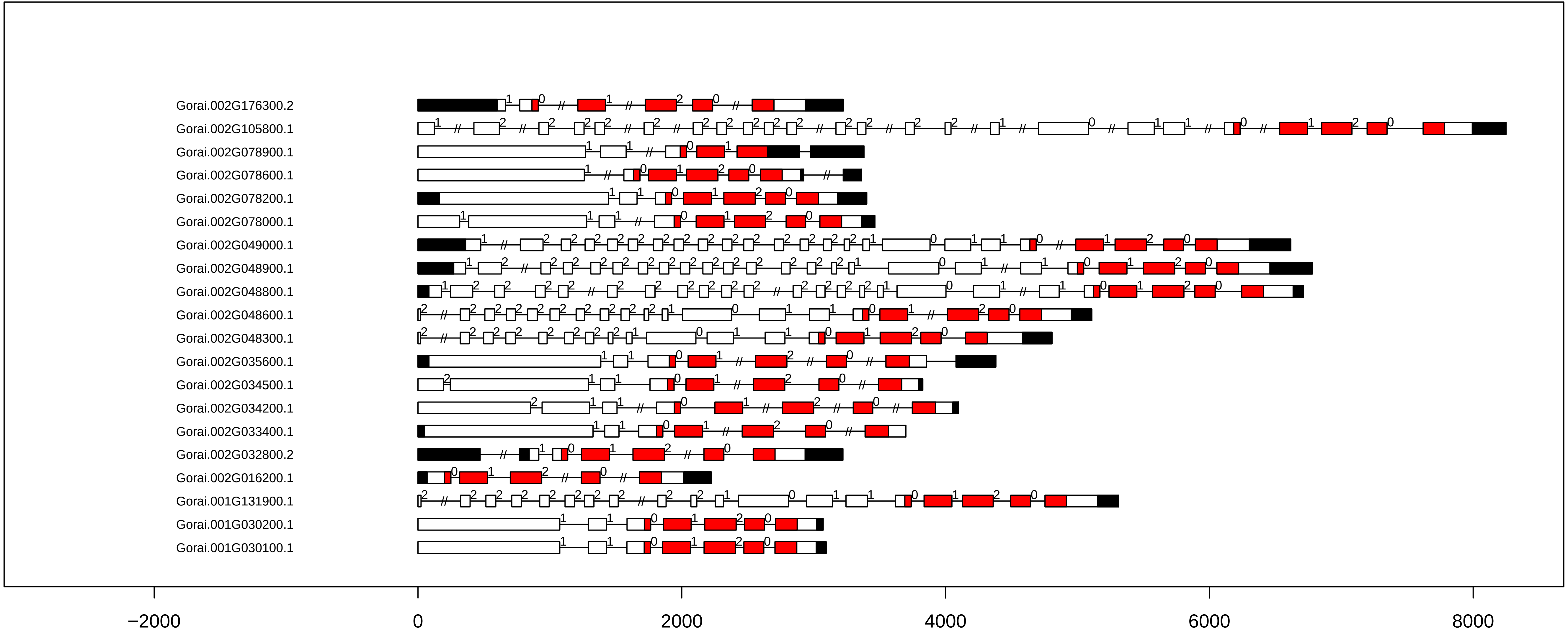

G.ra RLK−Pelle_DLSV exon−intron and domain diagram (part 2)

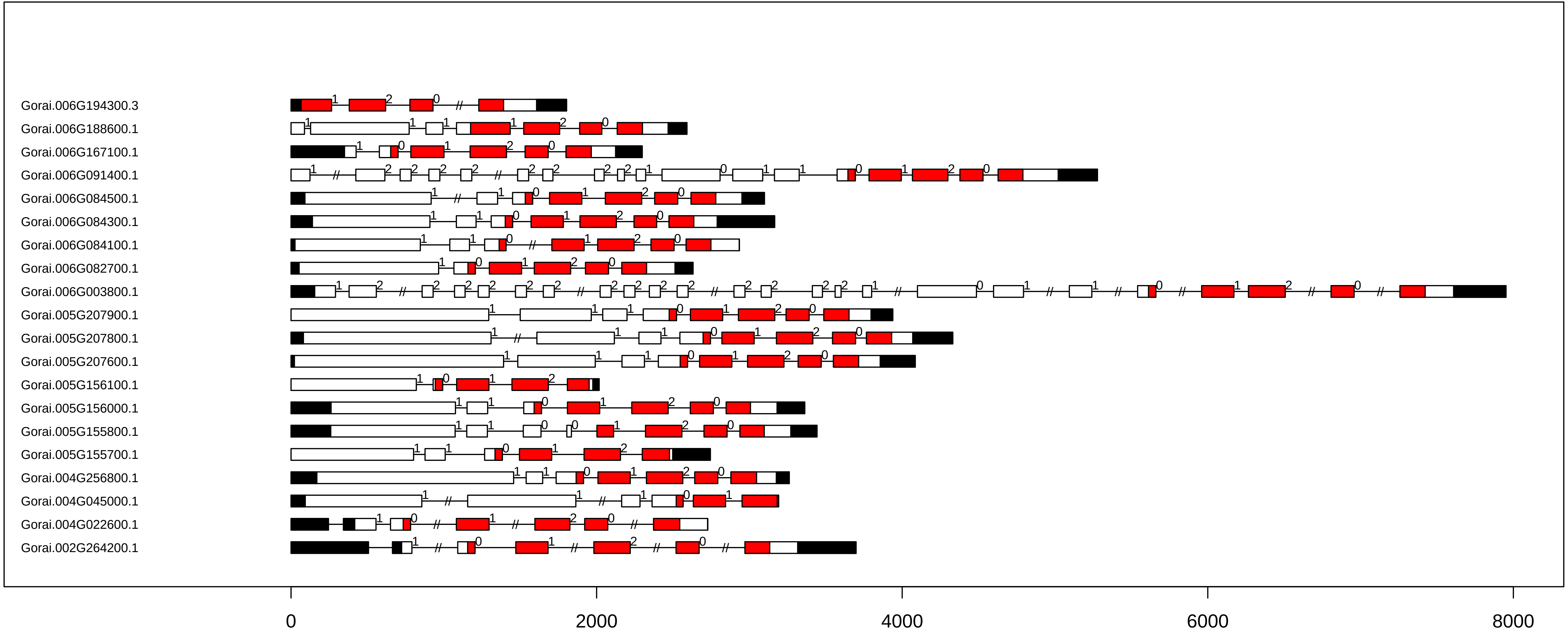

G.ra RLK−Pelle_DLSV exon−intron and domain diagram (part 3)

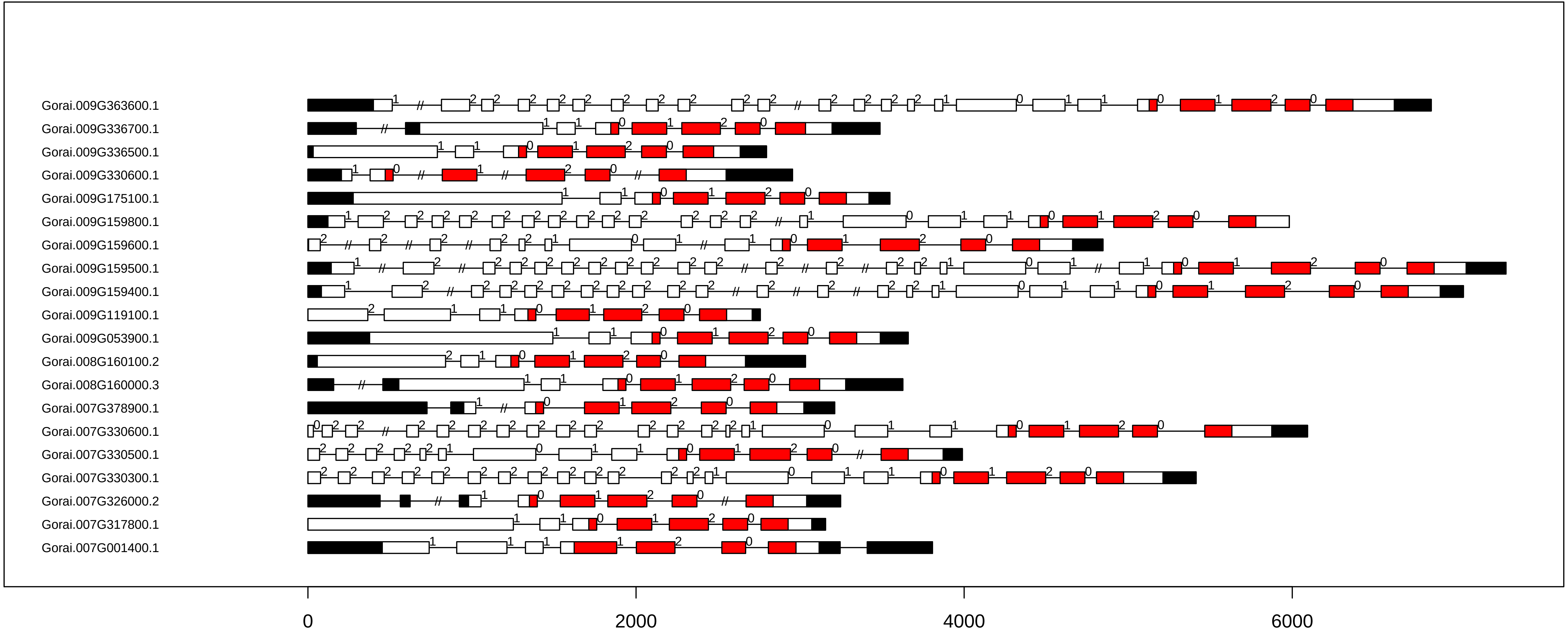

G.ra RLK−Pelle_DLSV exon−intron and domain diagram (part 4)

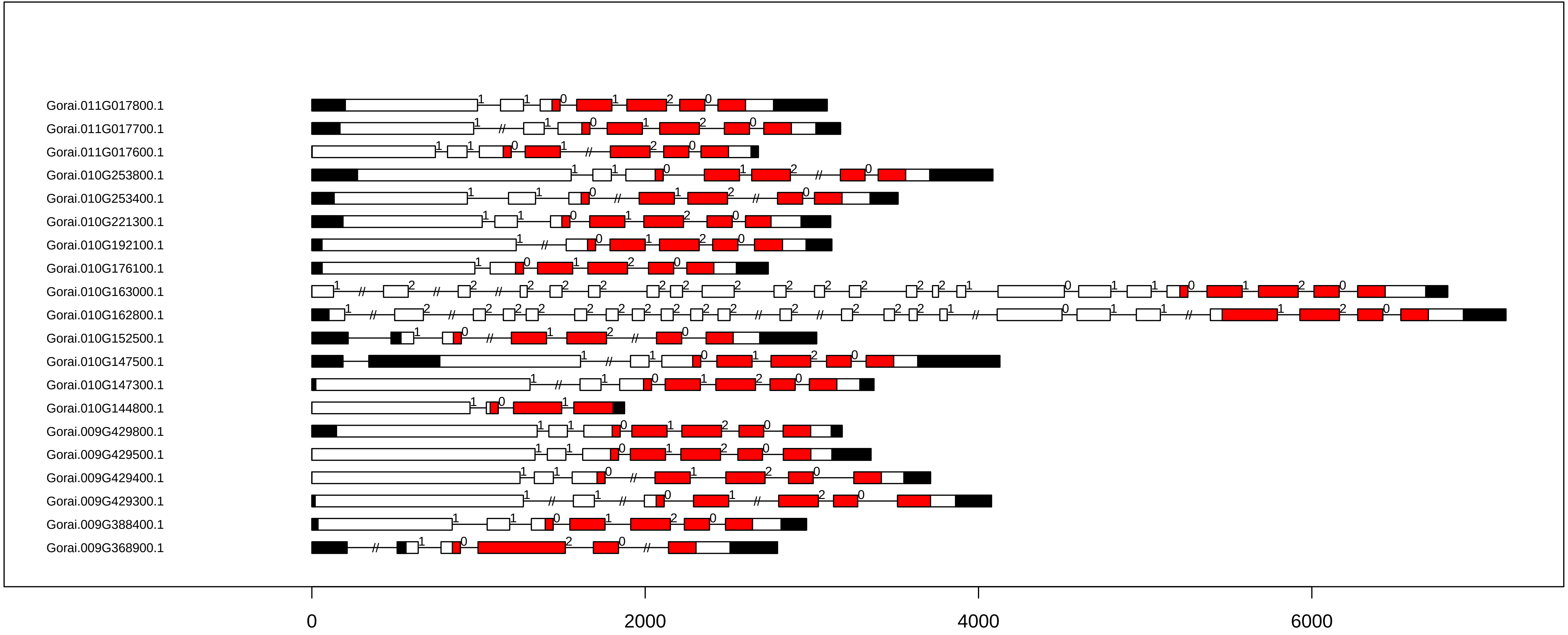

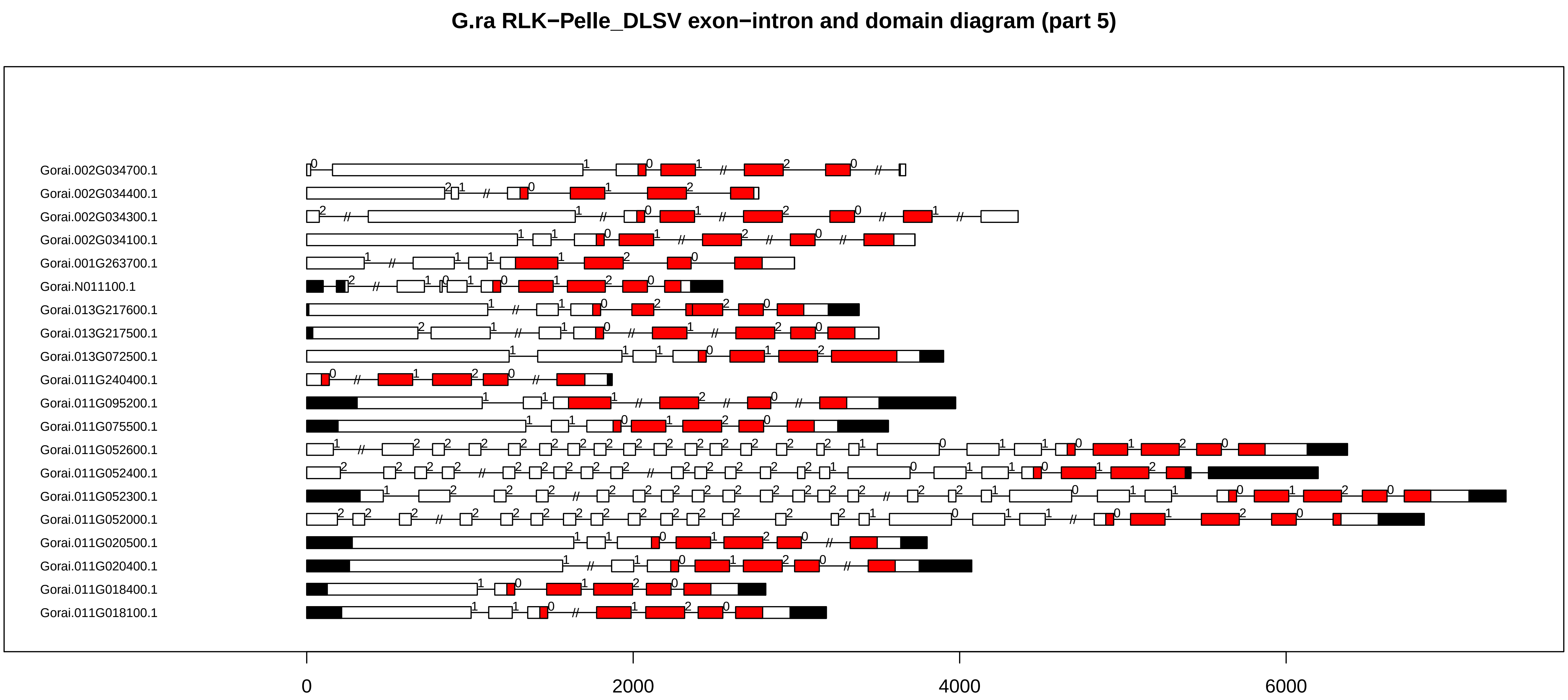
G.ra RLK−Pelle_DLSV exon−intron and domain diagram (part 5)
Gorai.002G034700.1
Gorai.002G034400.1
Gorai.002G034300.1
Gorai.002G034100.1
Gorai.001G263700.1
Gorai.N011100.1
Gorai.013G217600.1
Gorai.013G217500.1
Gorai.013G072500.1
Gorai.011G240400.1
Gorai.011G095200.1
Gorai.011G075500.1
Gorai.011G052600.1
Gorai.011G052400.1
Gorai.011G052300.1
Gorai.011G052000.1
Gorai.011G020500.1
Gorai.011G020400.1
Gorai.011G018400.1
Gorai.011G018100.1
0
2000
4000
6000

G.ra RLK−Pelle_DLSV exon−intron and domain diagram (part 6)

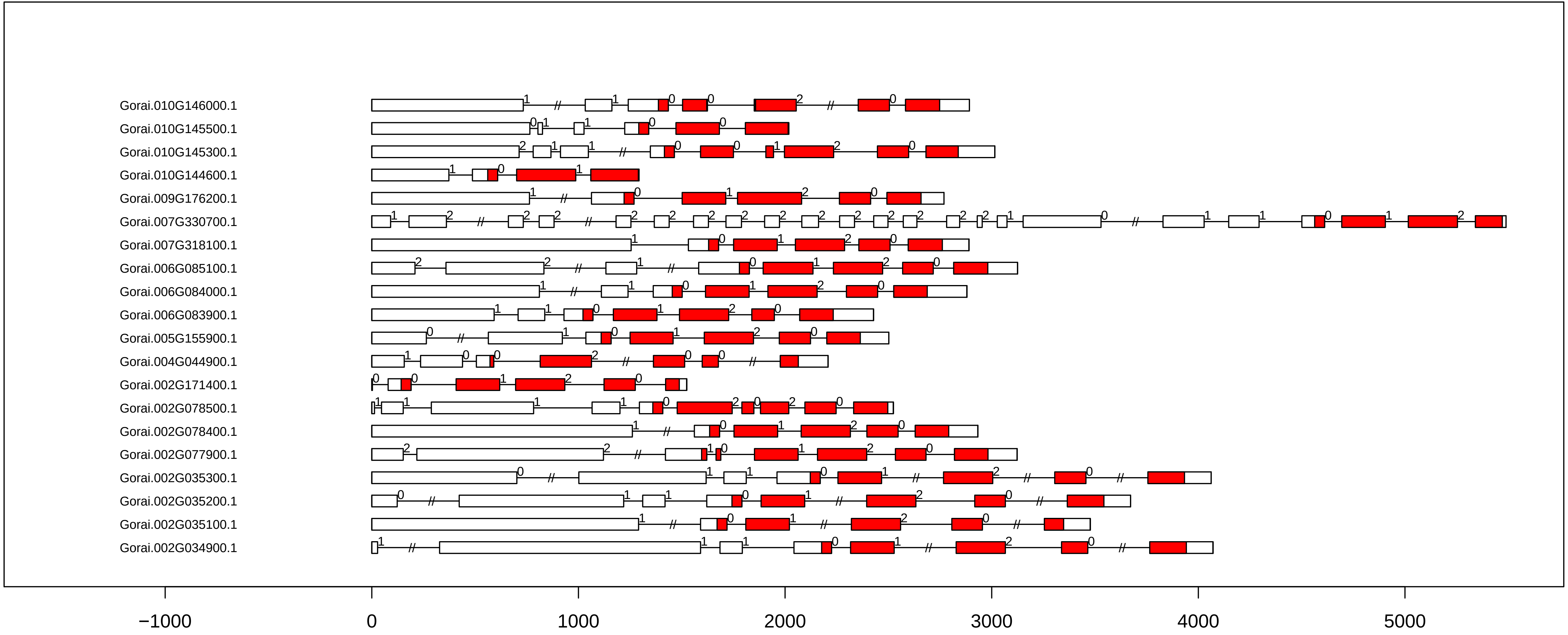

G.ra RLK−Pelle_DLSV exon−intron and domain diagram (part 7)

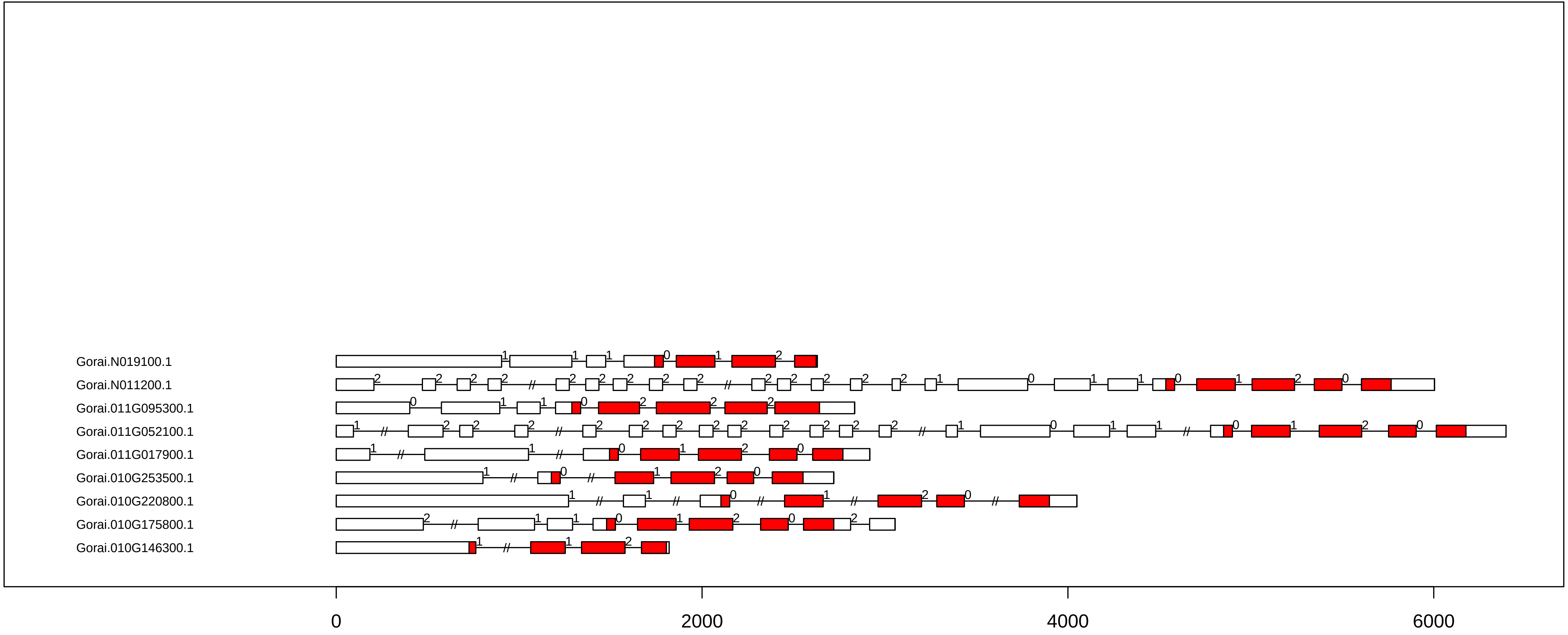

G.ra RLK−Pelle_Extensin exon−intron and domain diagram (all)

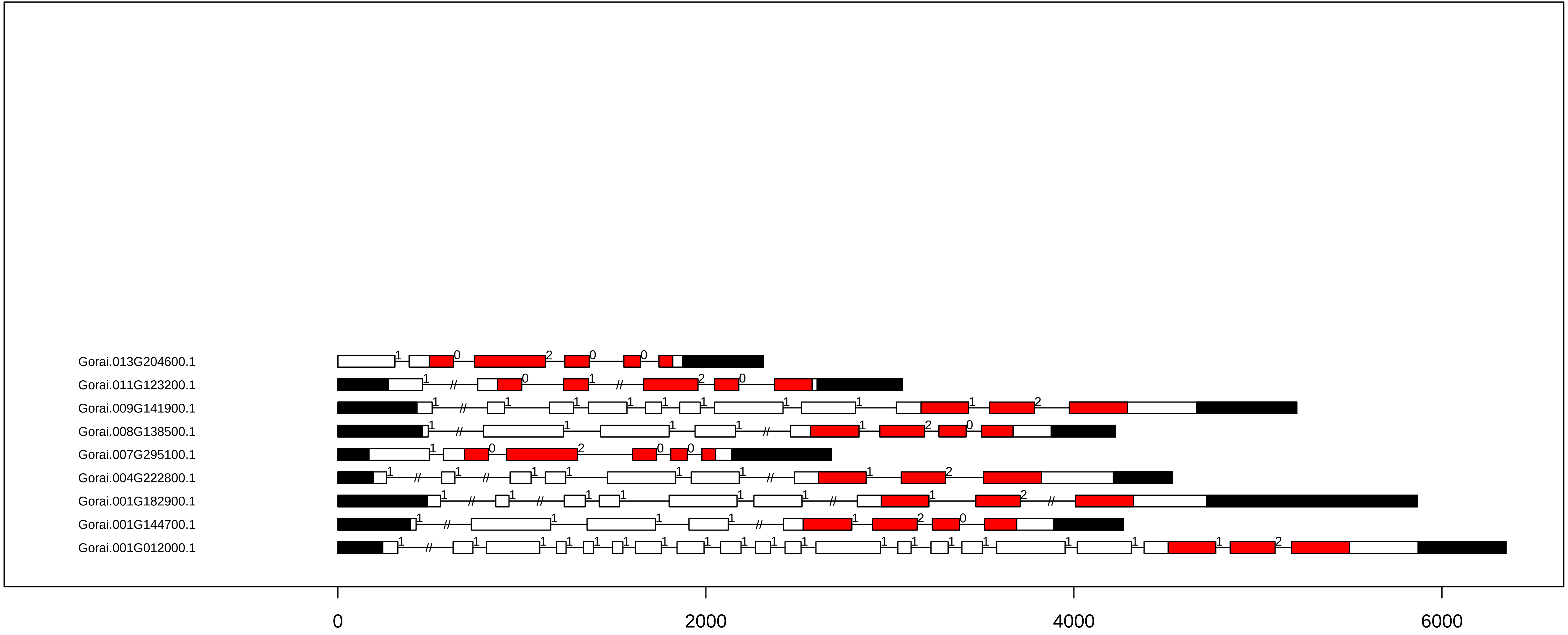

G.ra RLK−Pelle_L−LEC exon−intron and domain diagram (part 1)

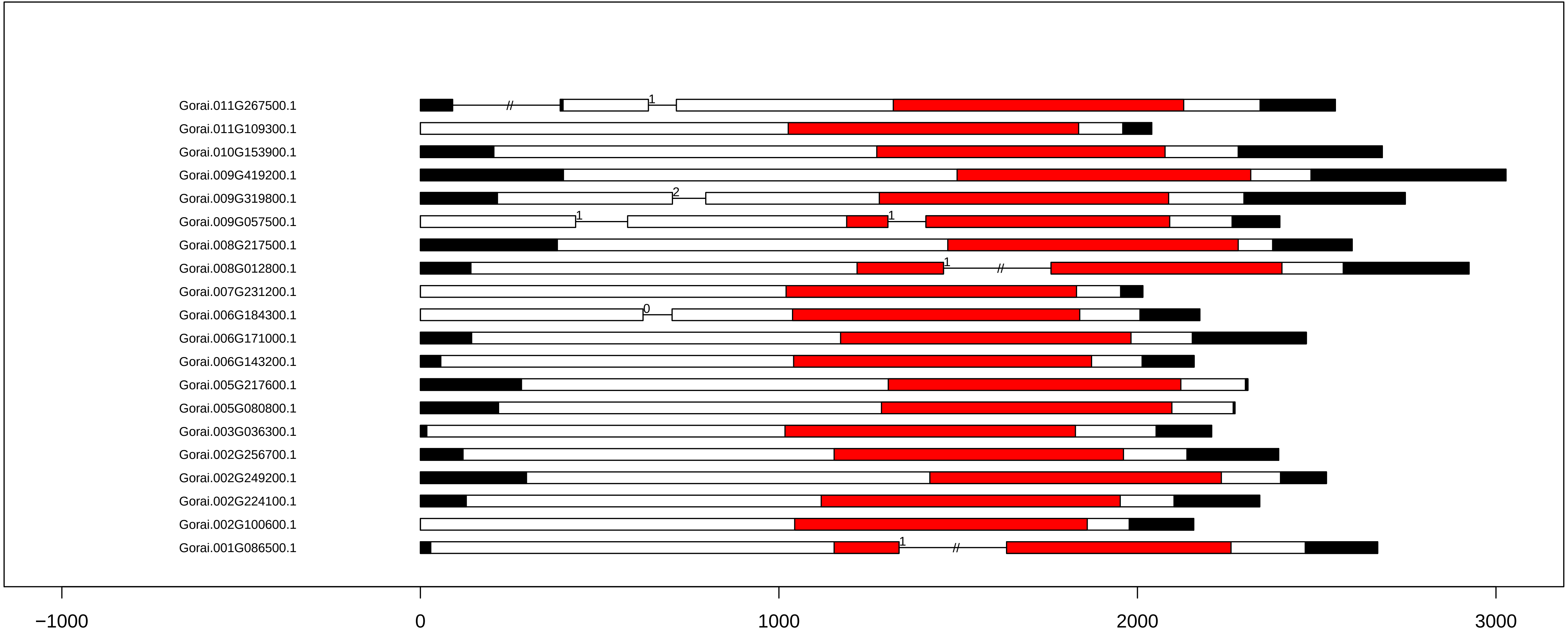

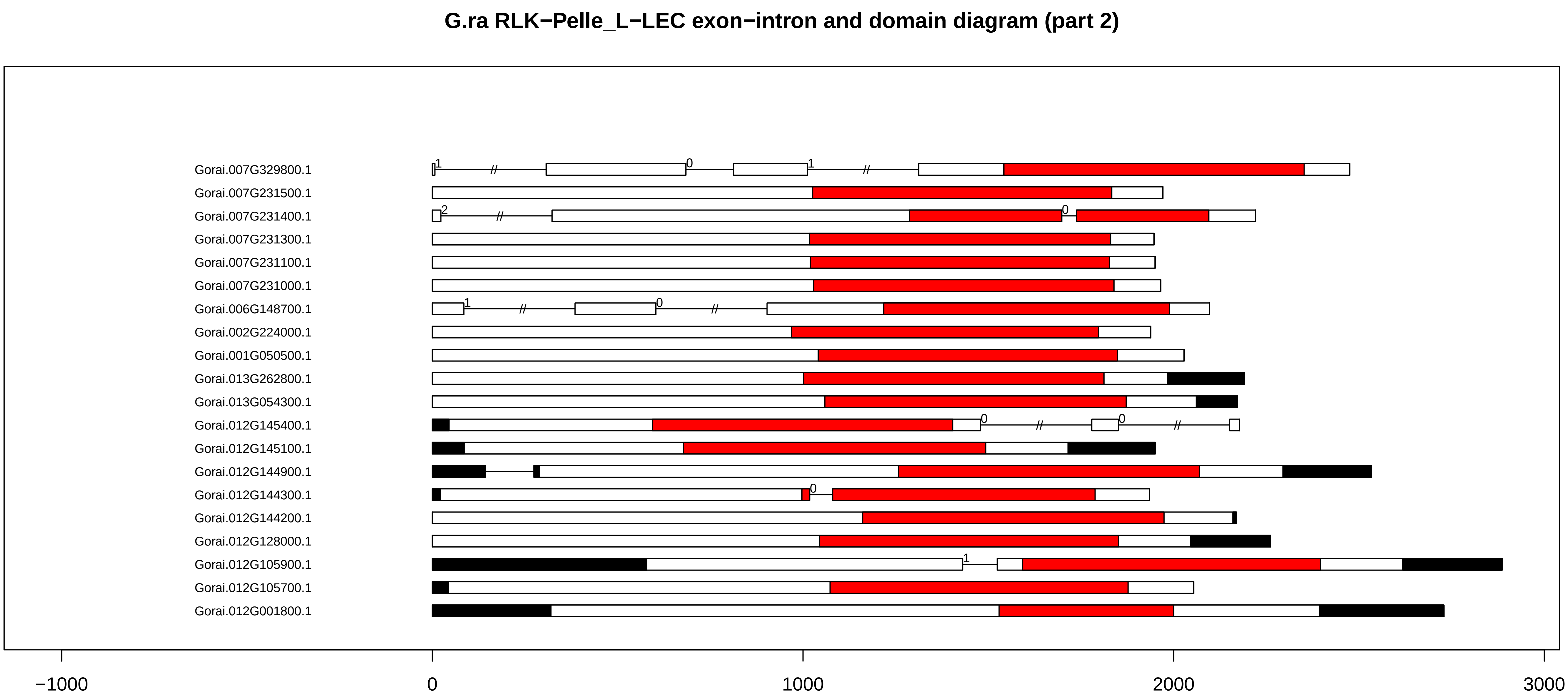

G.ra RLK−Pelle_L−LEC exon−intron and domain diagram (part 2)
Gorai.007G329800.1
Gorai.007G231500.1
Gorai.007G231400.1
Gorai.007G231300.1
Gorai.007G231100.1
Gorai.007G231000.1
Gorai.006G148700.1
Gorai.002G224000.1
Gorai.001G050500.1
Gorai.013G262800.1
Gorai.013G054300.1
Gorai.012G145400.1
Gorai.012G145100.1
Gorai.012G144900.1
Gorai.012G144300.1
Gorai.012G144200.1
Gorai.012G128000.1
Gorai.012G105900.1
Gorai.012G105700.1
Gorai.012G001800.1
−1000
0
1000
2000
3000

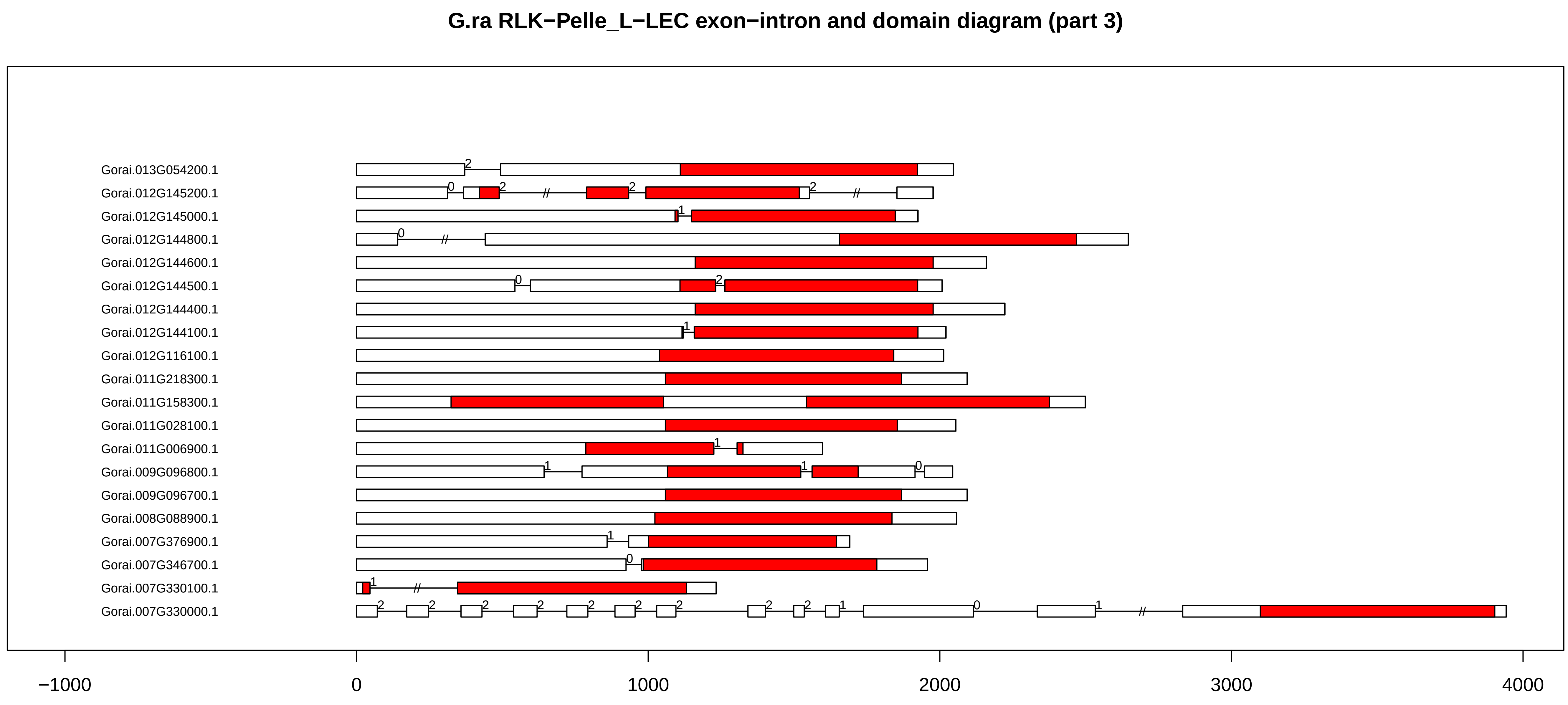
G.ra RLK−Pelle_L−LEC exon−intron and domain diagram (part 3)
Gorai.013G054200.1
Gorai.012G145200.1
Gorai.012G145000.1
Gorai.012G144800.1
Gorai.012G144600.1
Gorai.012G144500.1
Gorai.012G144400.1
Gorai.012G144100.1
Gorai.012G116100.1
Gorai.011G218300.1
Gorai.011G158300.1
Gorai.011G028100.1
Gorai.011G006900.1
Gorai.009G096800.1
Gorai.009G096700.1
Gorai.008G088900.1
Gorai.007G376900.1
Gorai.007G346700.1
Gorai.007G330100.1
Gorai.007G330000.1
−1000
0
1000
2000
3000
4000

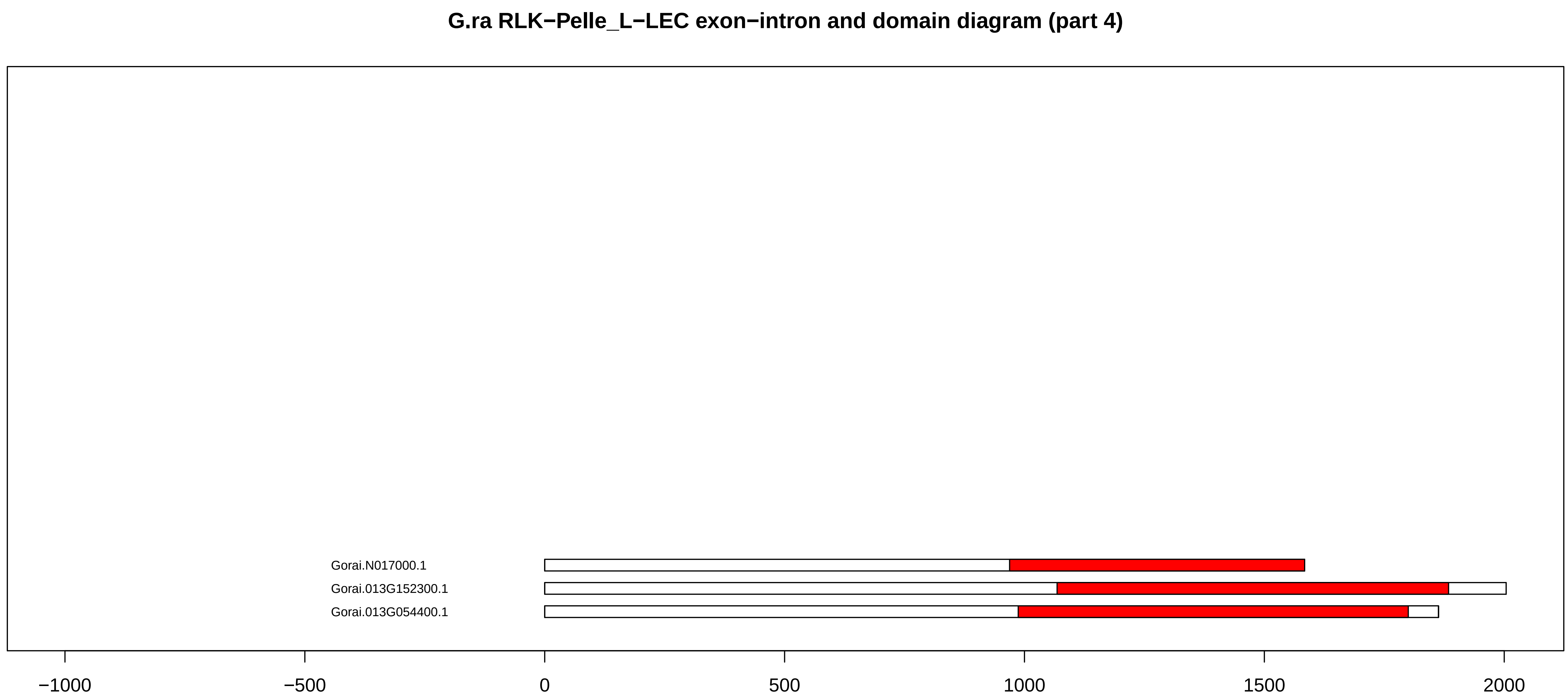
G.ra RLK−Pelle_L−LEC exon−intron and domain diagram (part 4)
Gorai.N017000.1
Gorai.013G152300.1
Gorai.013G054400.1
−1000
−500
0
500
1000
1500
2000

G.ra RLK−Pelle_LRK10L−2 exon−intron and domain diagram (part 1)

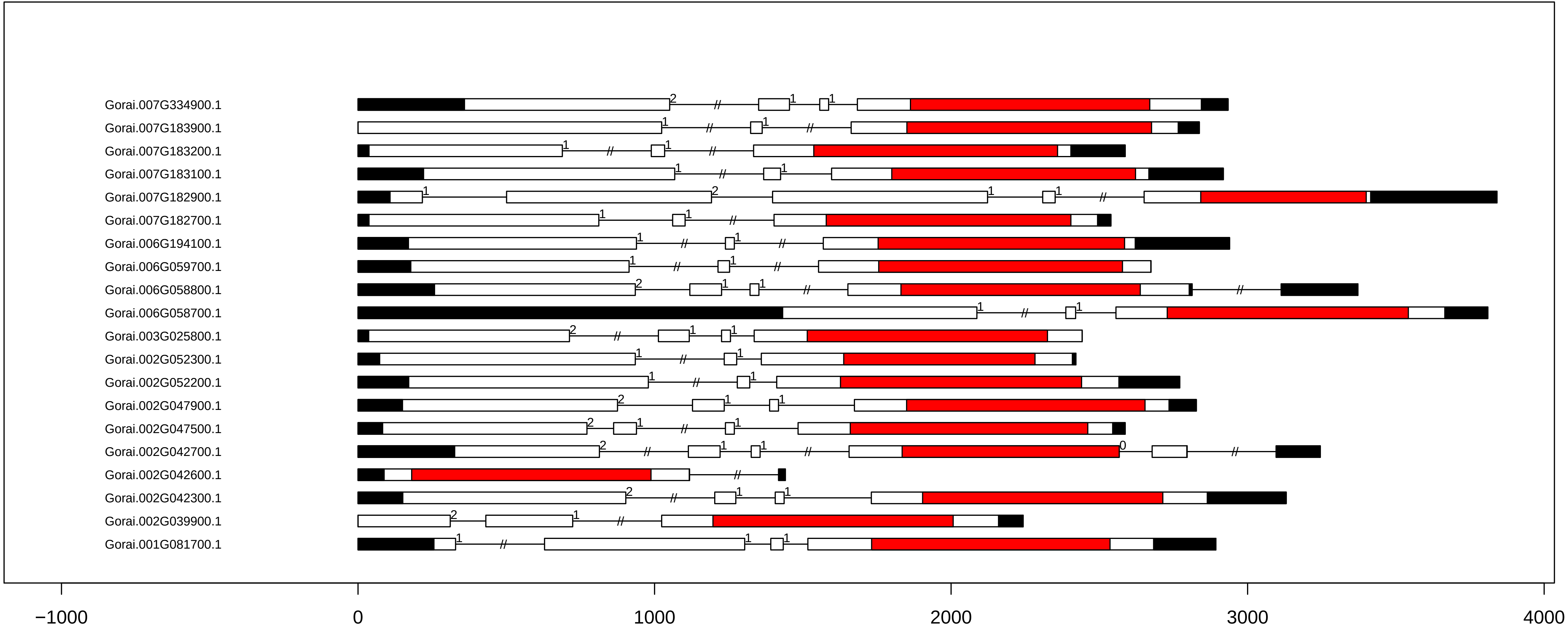

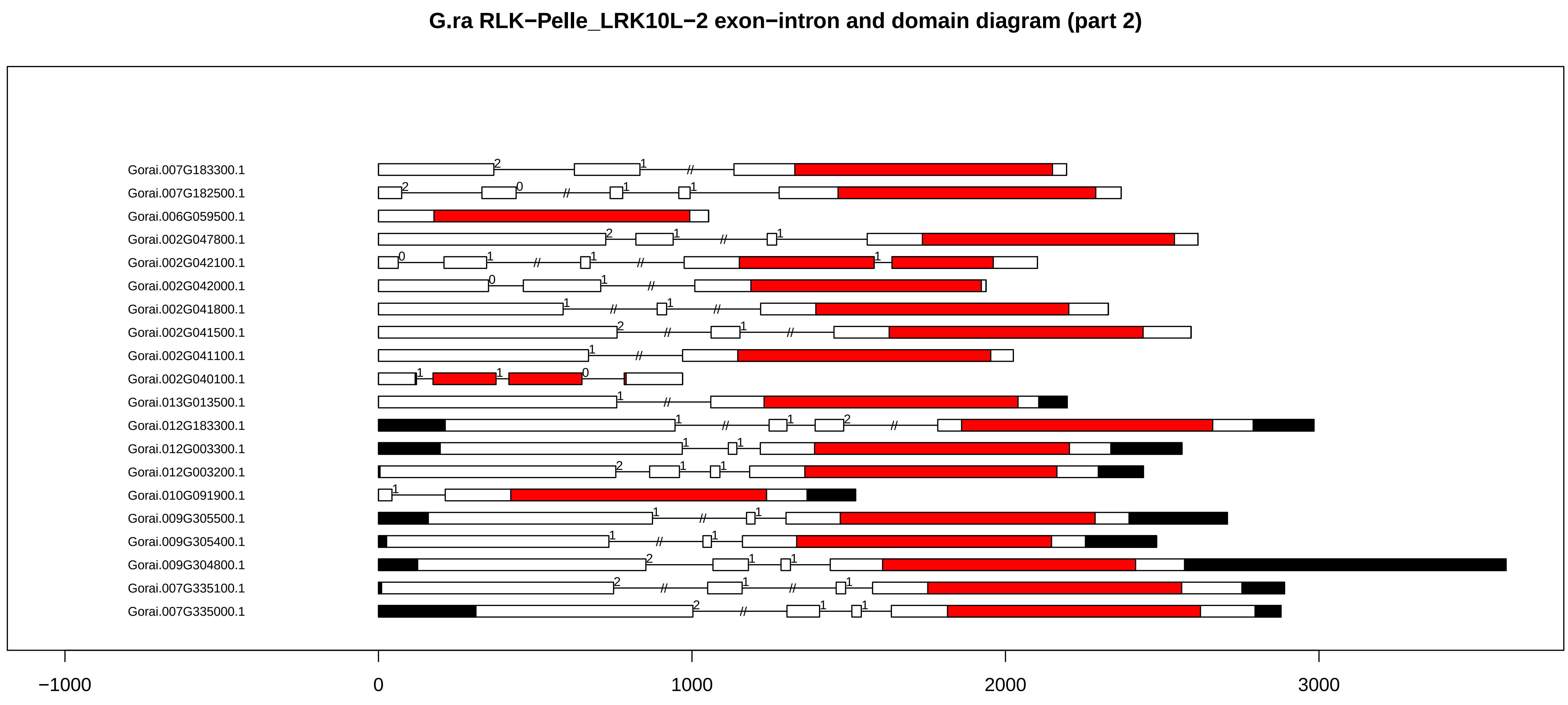

G.ra RLK−Pelle_LRK10L−2 exon−intron and domain diagram (part 2)
Gorai.007G183300.1
Gorai.007G182500.1
Gorai.006G059500.1
Gorai.002G047800.1
Gorai.002G042100.1
Gorai.002G042000.1
Gorai.002G041800.1
Gorai.002G041500.1
Gorai.002G041100.1
Gorai.002G040100.1
Gorai.013G013500.1
Gorai.012G183300.1
Gorai.012G003300.1
Gorai.012G003200.1
Gorai.010G091900.1
Gorai.009G305500.1
Gorai.009G305400.1
Gorai.009G304800.1
Gorai.007G335100.1
Gorai.007G335000.1
−1000
0
1000
2000
3000

G.ra RLK−Pelle_LRK10L−2 exon−intron and domain diagram (part 3)

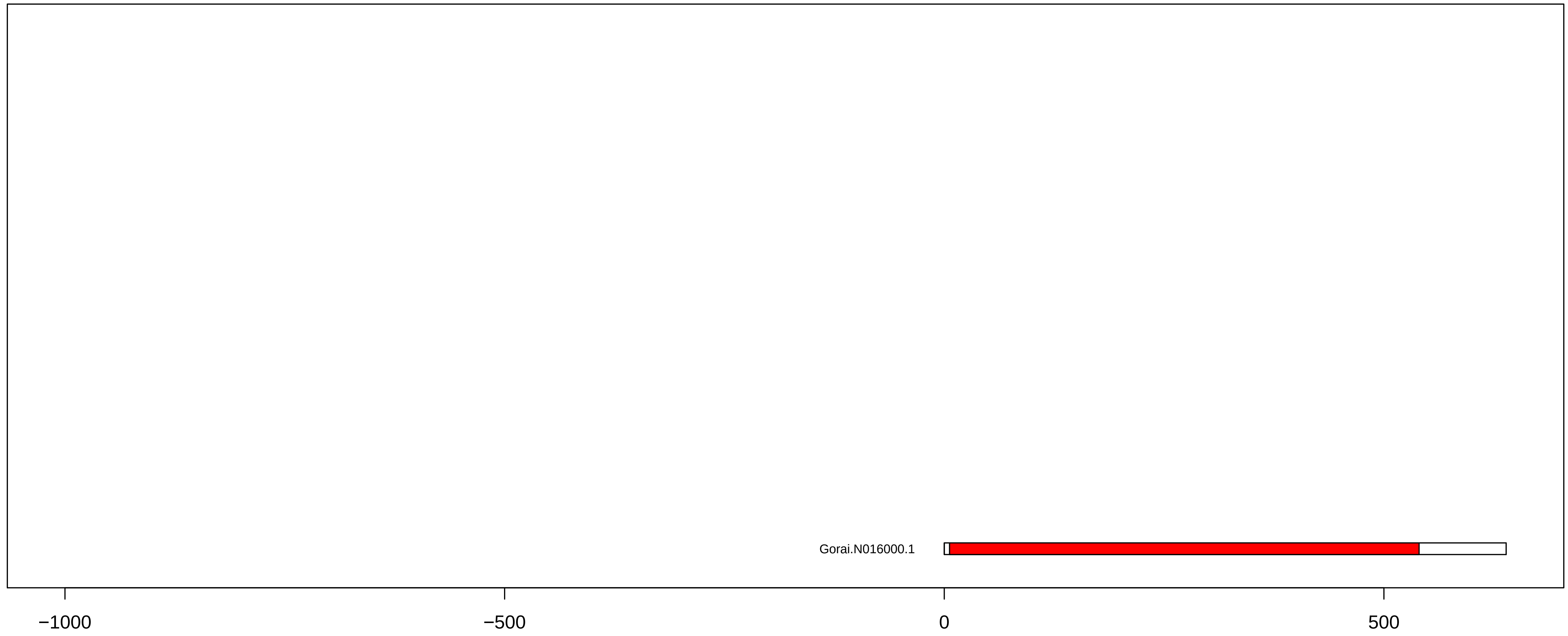

G.ra RLK−Pelle_LRR−I−1 exon−intron and domain diagram (all)

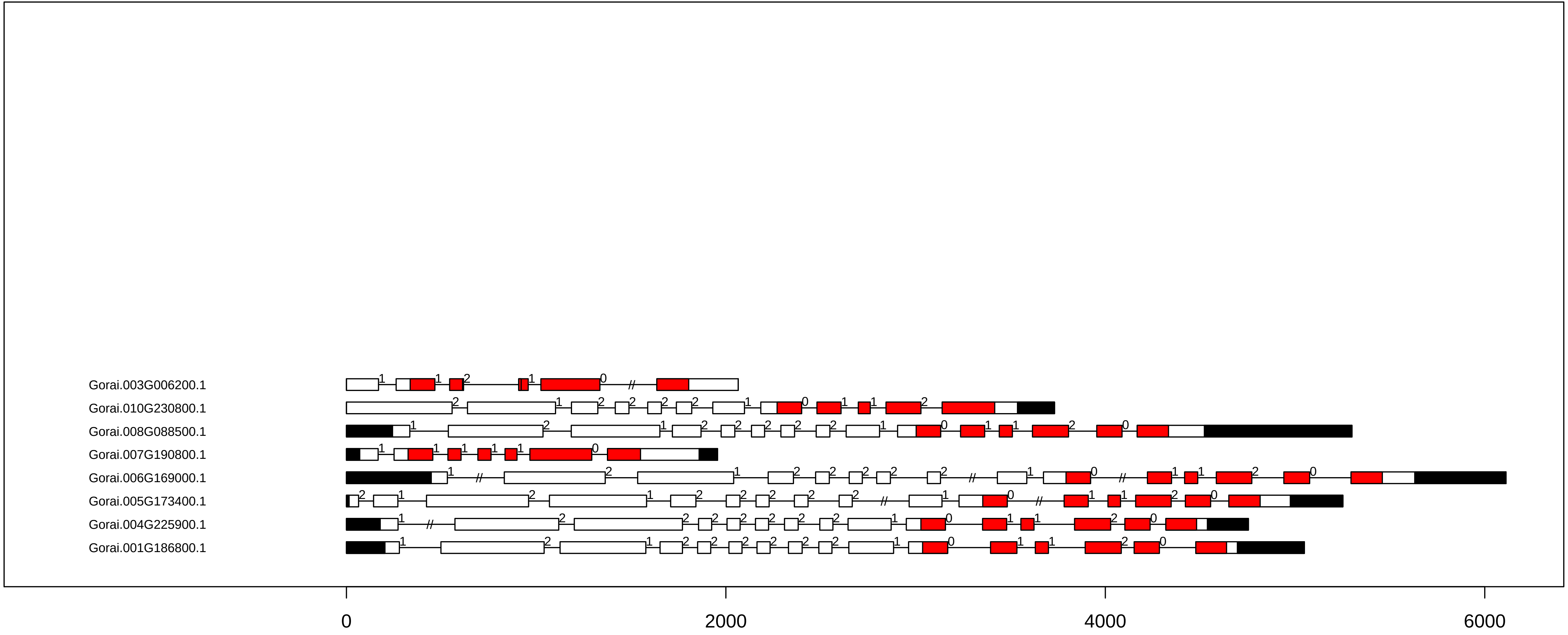

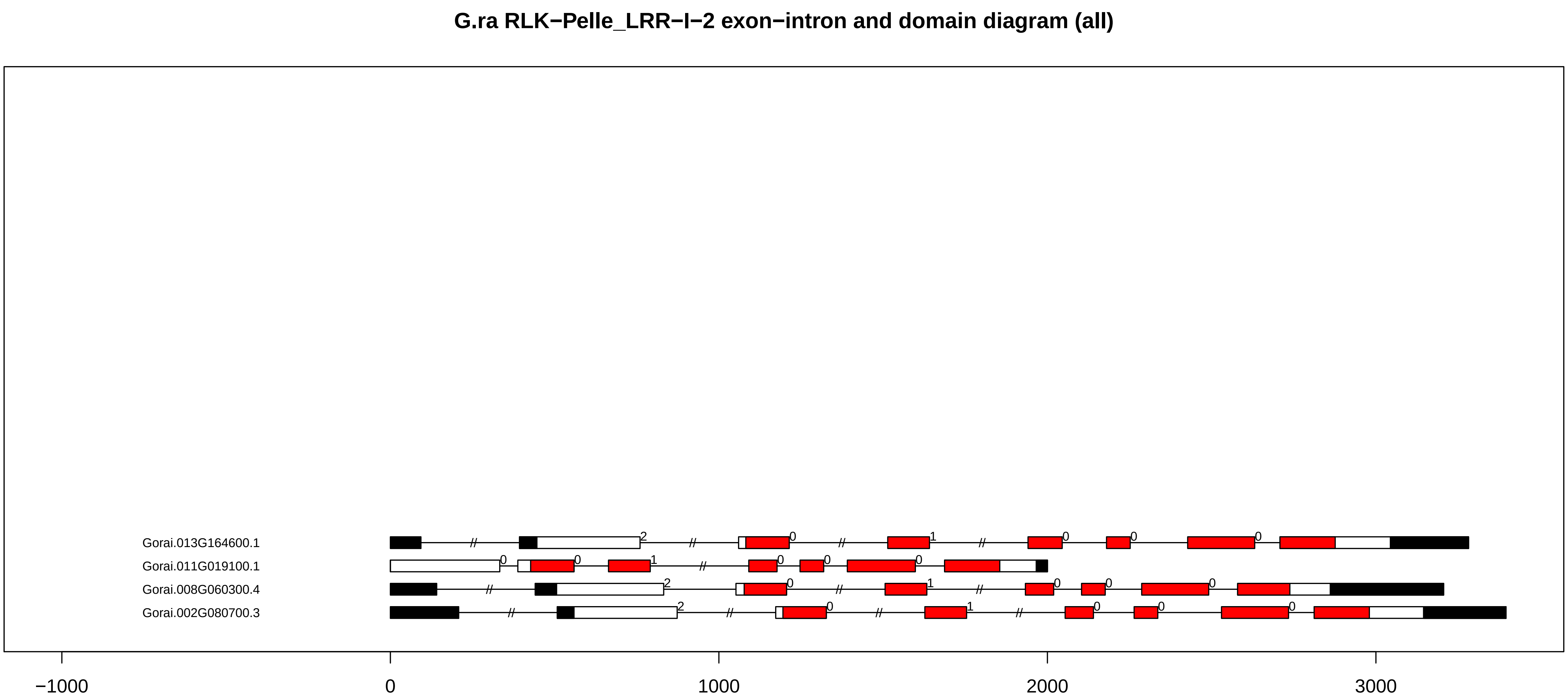
G.ra RLK−Pelle_LRR−I−2 exon−intron and domain diagram (all)
Gorai.013G164600.1
Gorai.011G019100.1
Gorai.008G060300.4
Gorai.002G080700.3
−1000
0
1000
2000
3000

G.ra RLK−Pelle_LRR−III exon−intron and domain diagram (part 1)

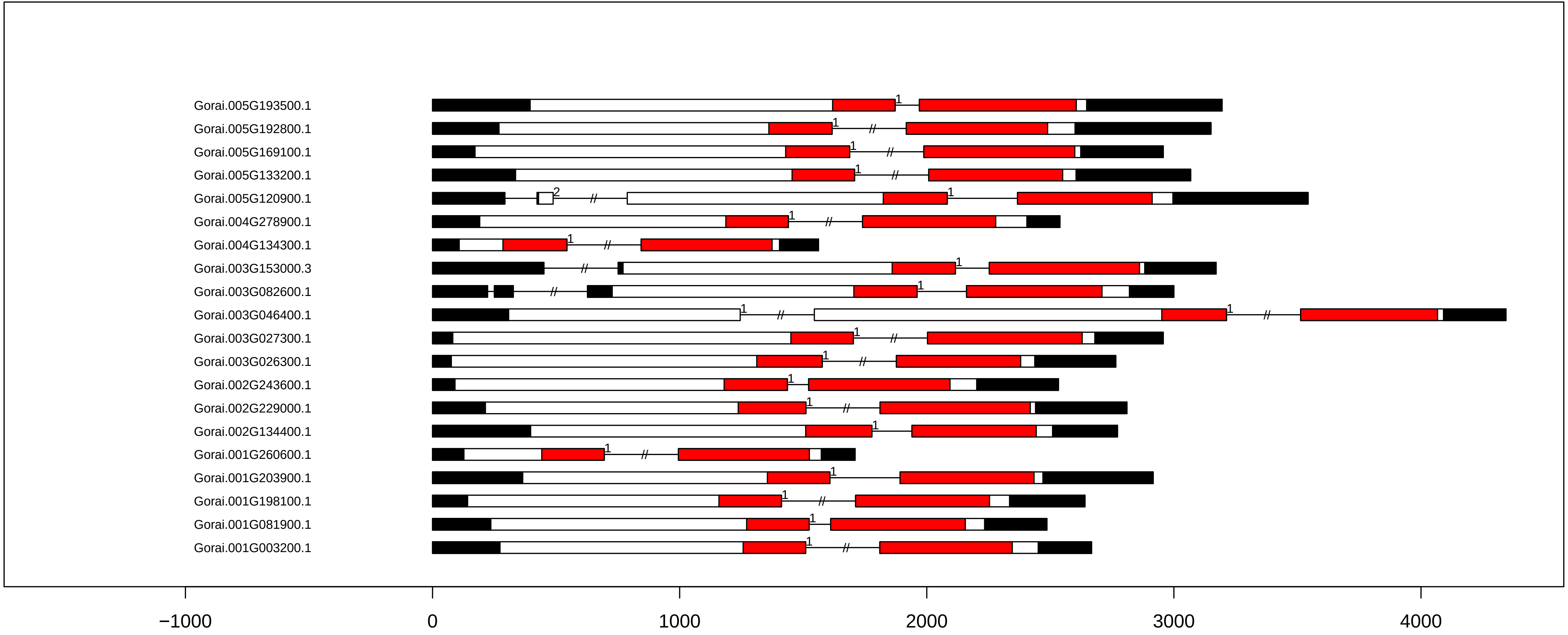

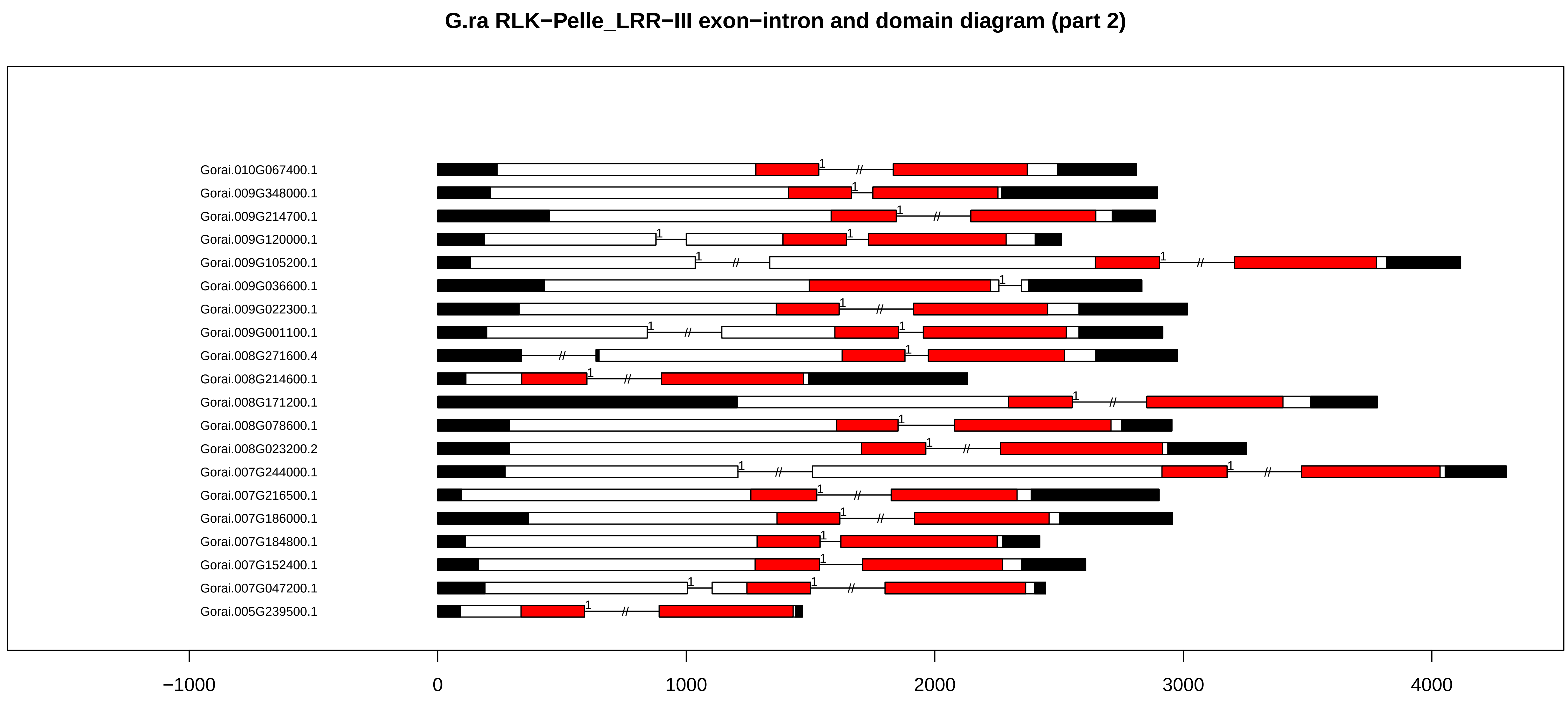
G.ra RLK−Pelle_LRR−III exon−intron and domain diagram (part 2)
Gorai.010G067400.1
Gorai.009G348000.1
Gorai.009G214700.1
Gorai.009G120000.1
Gorai.009G105200.1
Gorai.009G036600.1
Gorai.009G022300.1
Gorai.009G001100.1
Gorai.008G271600.4
Gorai.008G214600.1
Gorai.008G171200.1
Gorai.008G078600.1
Gorai.008G023200.2
Gorai.007G244000.1
Gorai.007G216500.1
Gorai.007G186000.1
Gorai.007G184800.1
Gorai.007G152400.1
Gorai.007G047200.1
Gorai.005G239500.1
−1000
0
1000
2000
3000
4000

G.ra RLK−Pelle_LRR−III exon−intron and domain diagram (part 3)

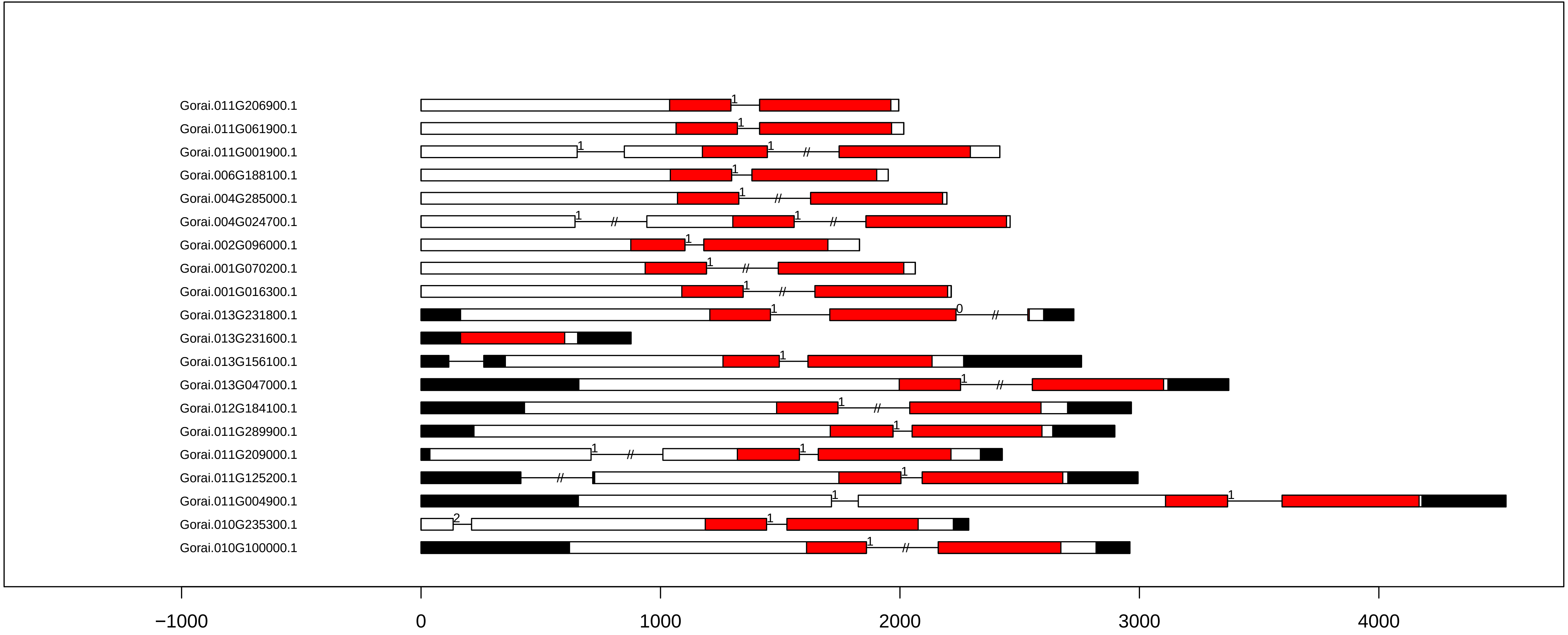

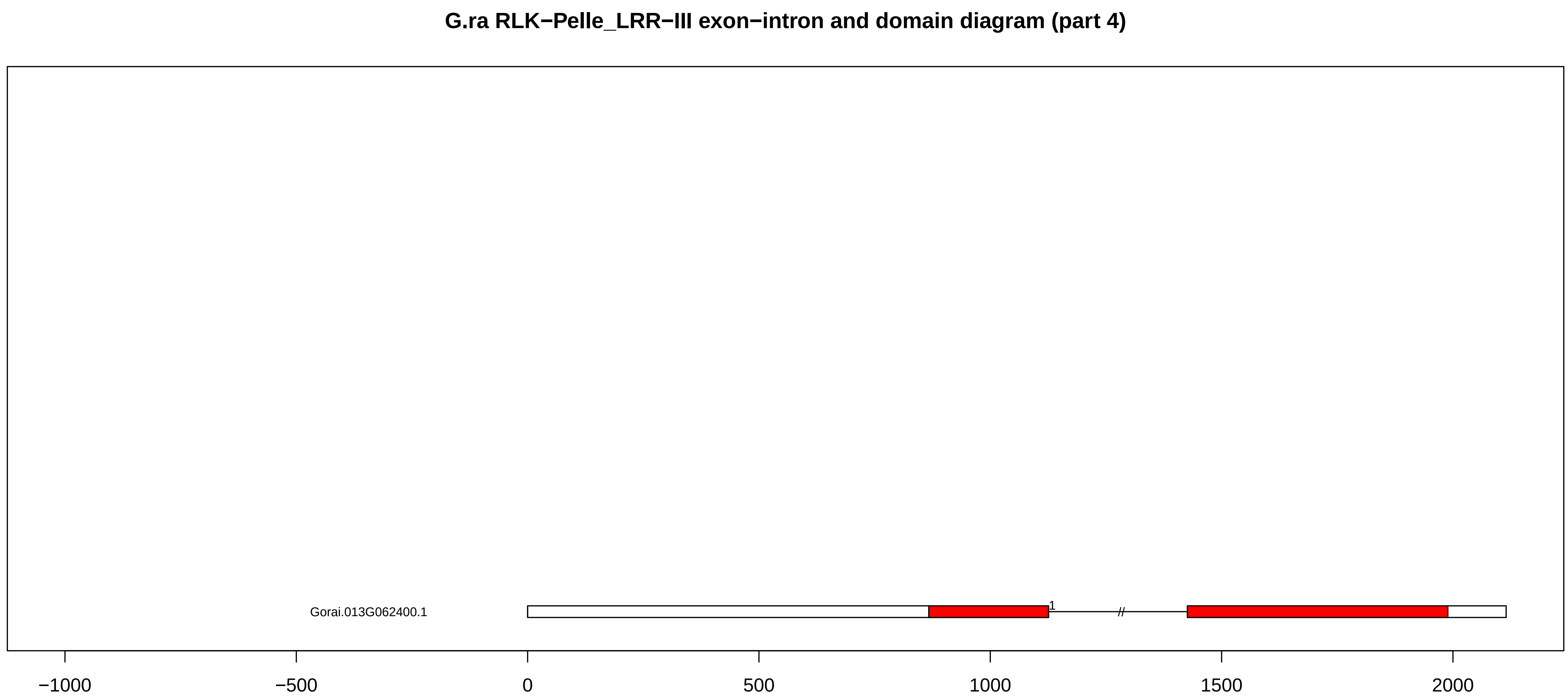
G.ra RLK−Pelle_LRR−III exon−intron and domain diagram (part 4)
Gorai.013G062400.1
1
−1000
−500
0
500
1000
1500
2000

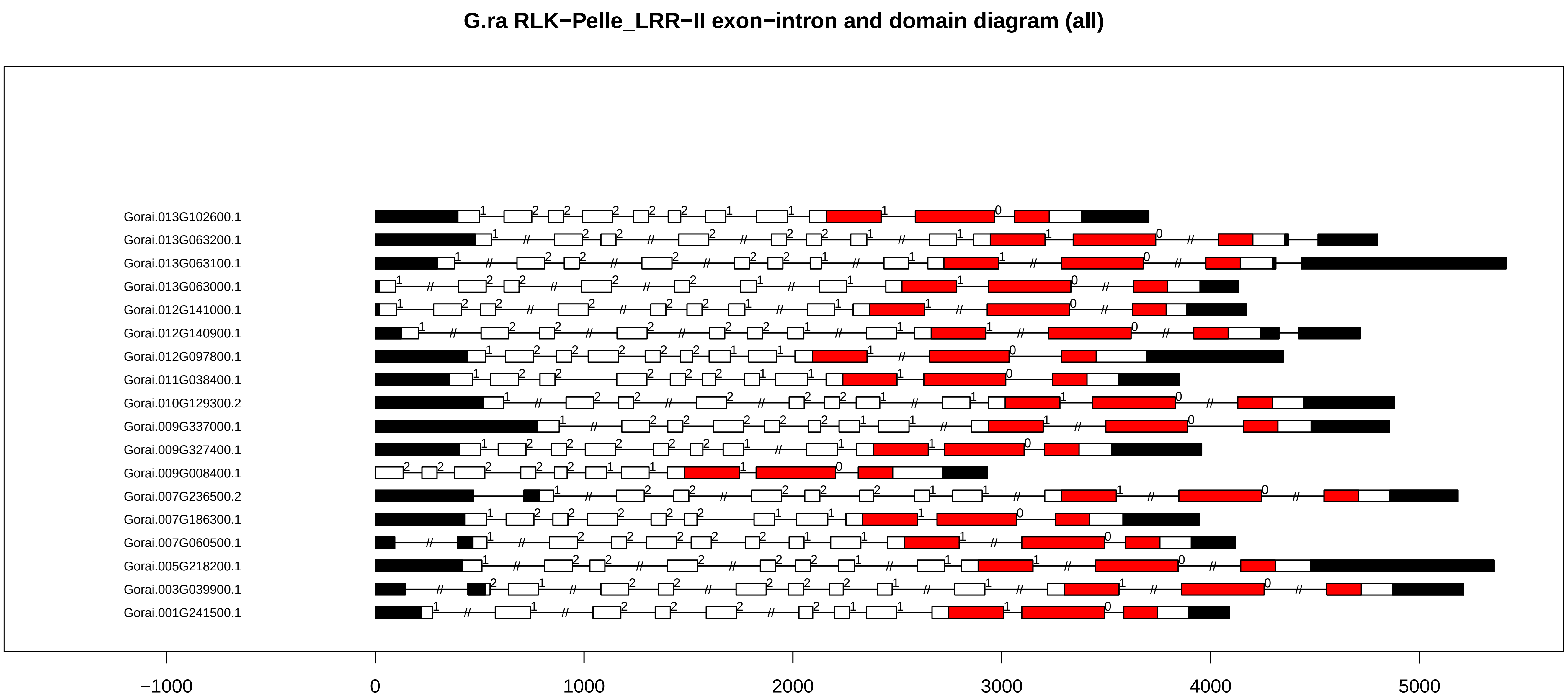
G.ra RLK−Pelle_LRR−II exon−intron and domain diagram (all)
Gorai.013G102600.1
Gorai.013G063200.1
Gorai.013G063100.1
Gorai.013G063000.1
Gorai.012G141000.1
Gorai.012G140900.1
Gorai.012G097800.1
Gorai.011G038400.1
Gorai.010G129300.2
Gorai.009G337000.1
Gorai.009G327400.1
Gorai.009G008400.1
Gorai.007G236500.2
Gorai.007G186300.1
Gorai.007G060500.1
Gorai.005G218200.1
Gorai.003G039900.1
Gorai.001G241500.1
−1000
0
1000
2000
3000
4000
5000

G.ra RLK−Pelle_LRR−IV exon−intron and domain diagram (all)

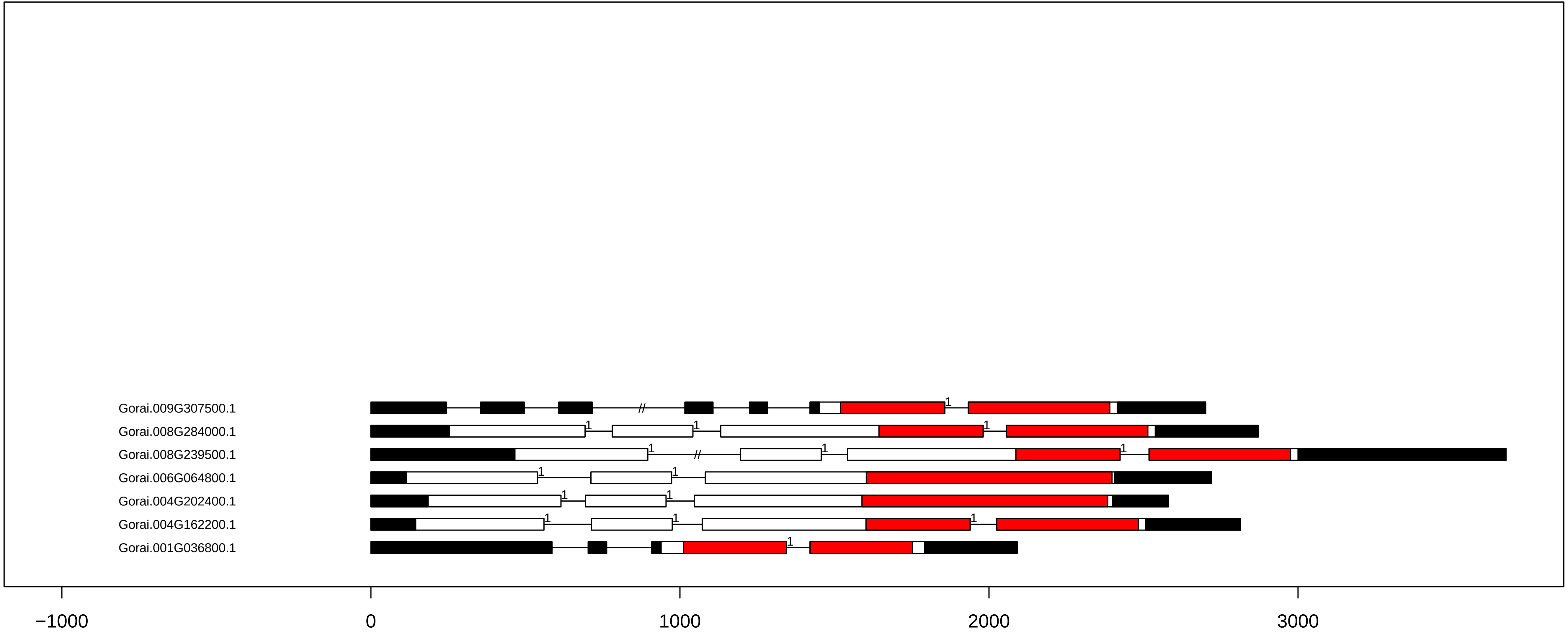

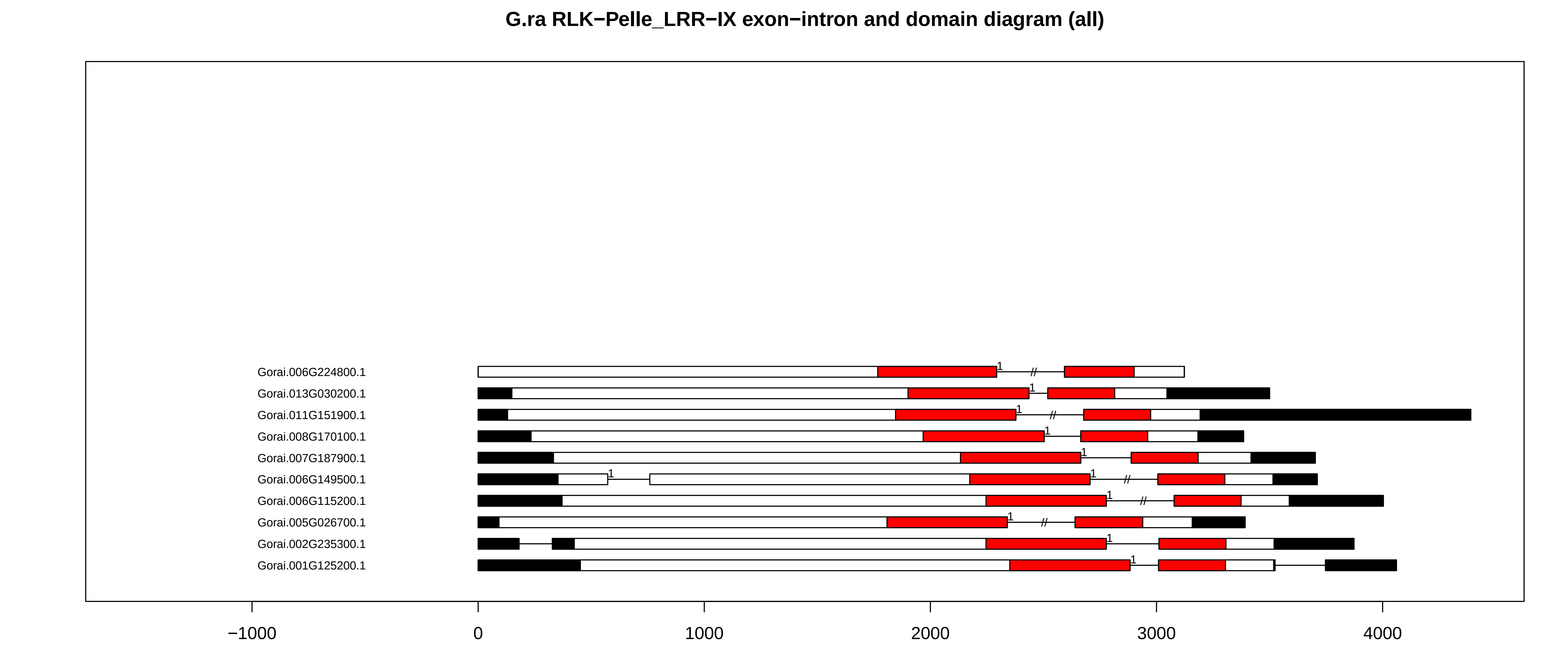
G.ra RLK−Pelle_LRR−IX exon−intron and domain diagram (all)
Gorai.006G224800.1
Gorai.013G030200.1
Gorai.011G151900.1
Gorai.008G170100.1
Gorai.007G187900.1
Gorai.006G149500.1
Gorai.006G115200.1
Gorai.005G026700.1
Gorai.002G235300.1
Gorai.001G125200.1
−1000
0
1000
2000
3000
4000

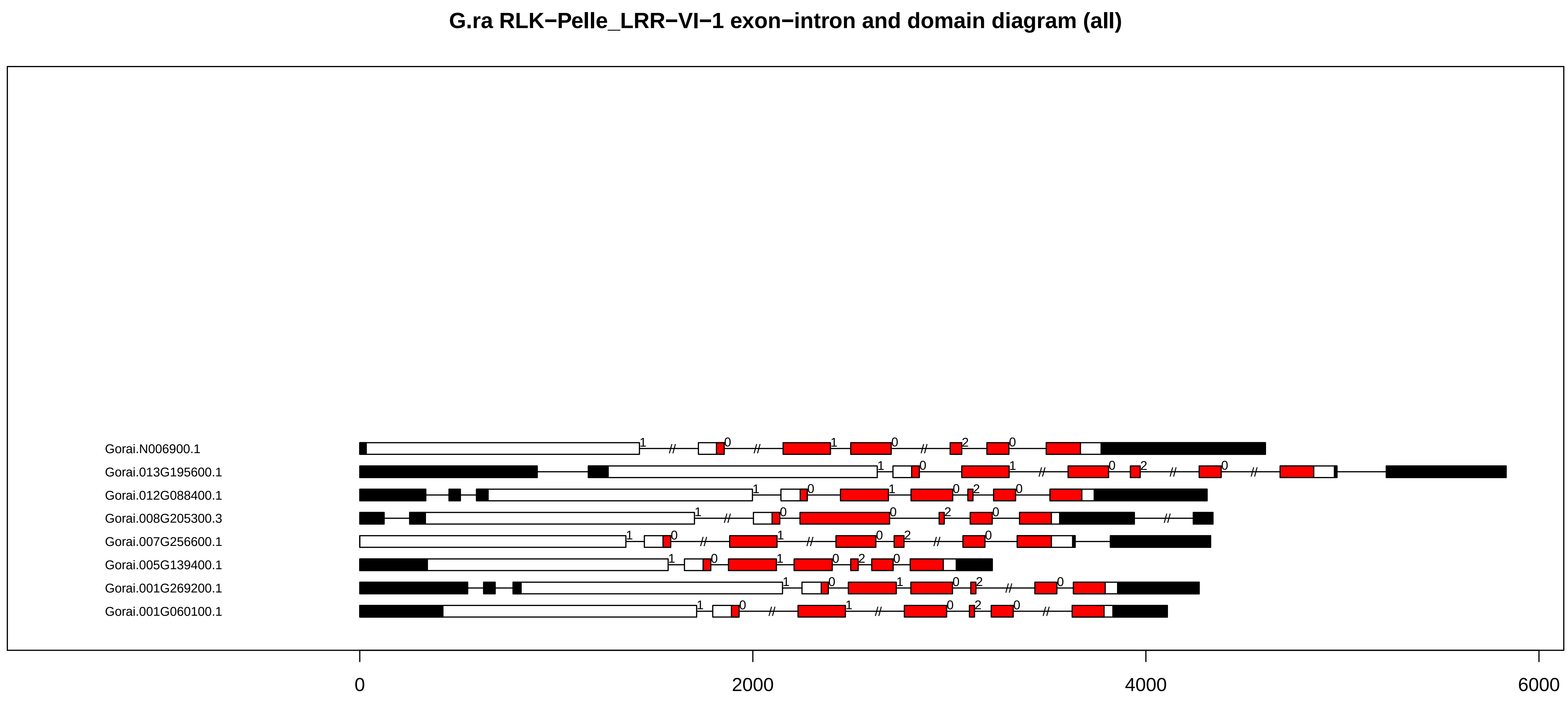
G.ra RLK−Pelle_LRR−VI−1 exon−intron and domain diagram (all)
Gorai.N006900.1
Gorai.013G195600.1
Gorai.012G088400.1
Gorai.008G205300.3
Gorai.007G256600.1
Gorai.005G139400.1
Gorai.001G269200.1
Gorai.001G060100.1
0
2000
4000
6000

G.ra RLK−Pelle_LRR−VI−2 exon−intron and domain diagram (all)

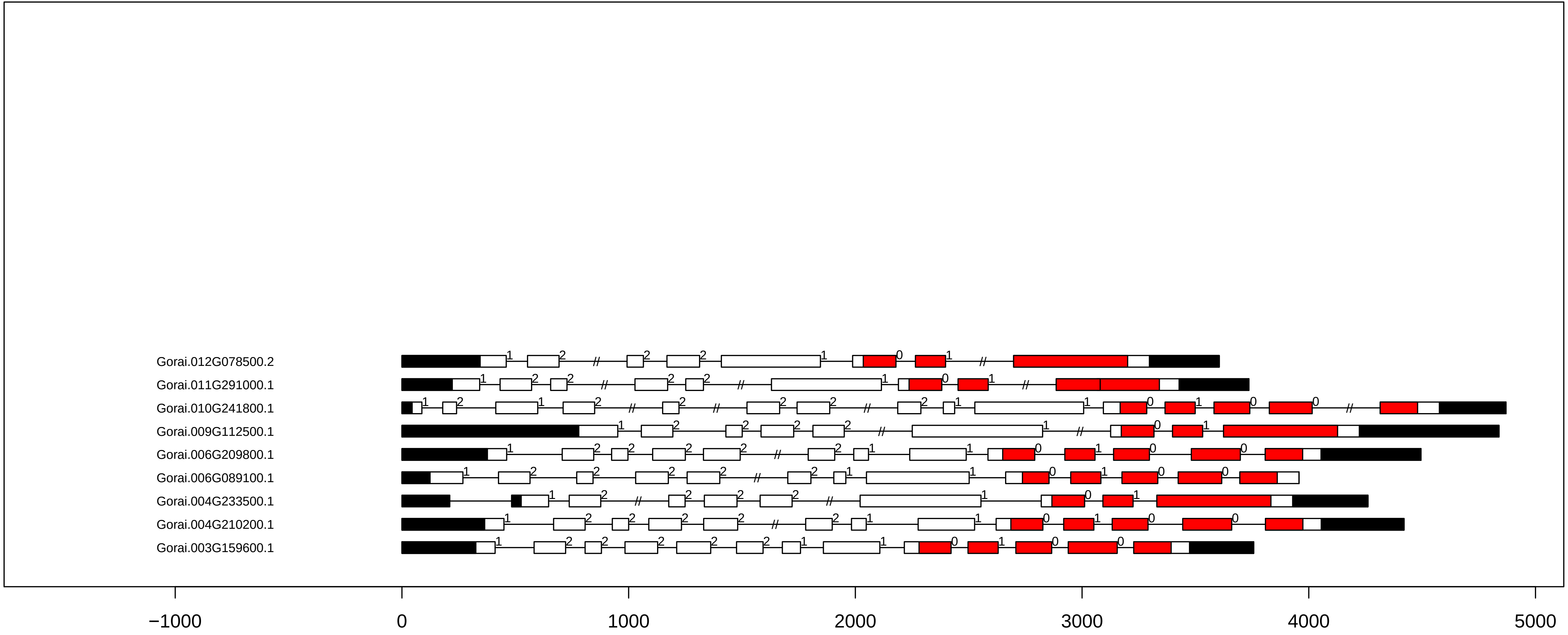

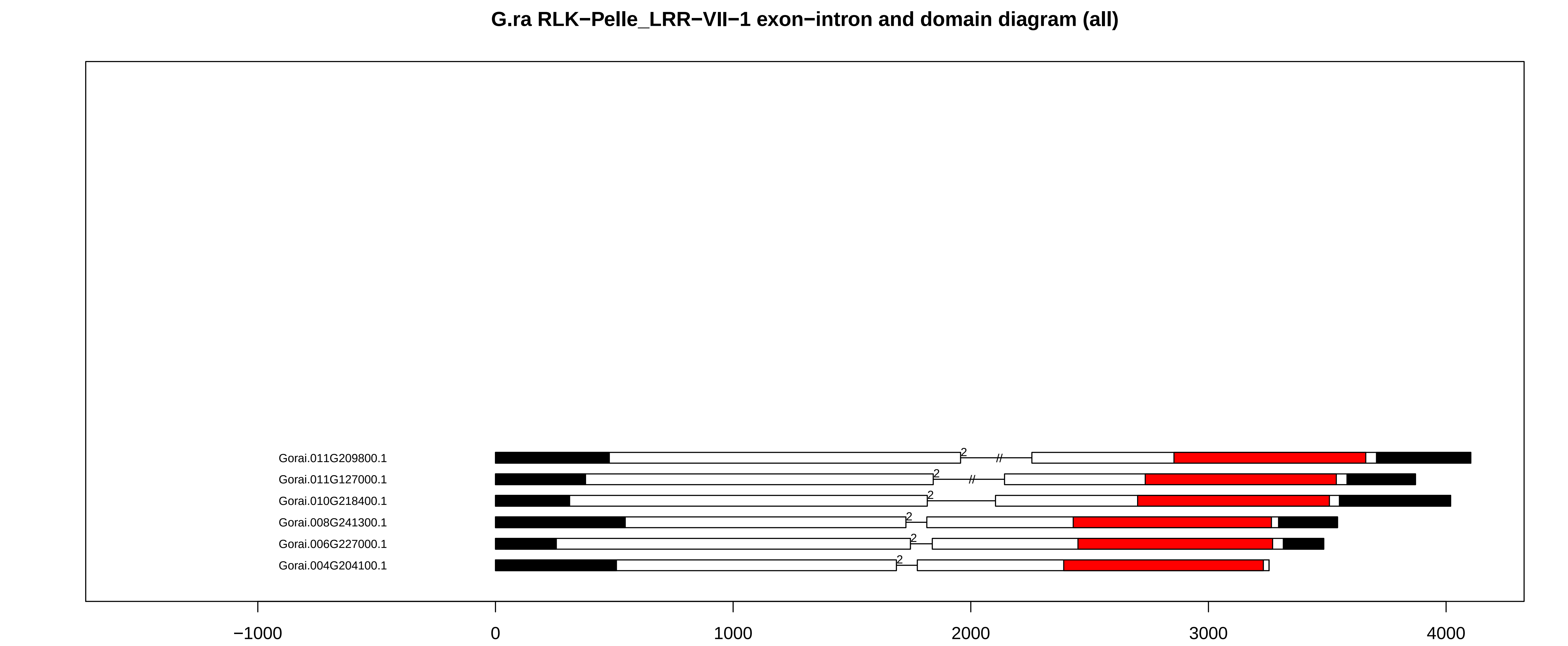
G.ra RLK−Pelle_LRR−VII−1 exon−intron and domain diagram (all)
Gorai.011G209800.1
Gorai.011G127000.1
Gorai.010G218400.1
Gorai.008G241300.1
Gorai.006G227000.1
Gorai.004G204100.1
2
2
2
2
2
2
−1000
0
1000
2000
3000
4000

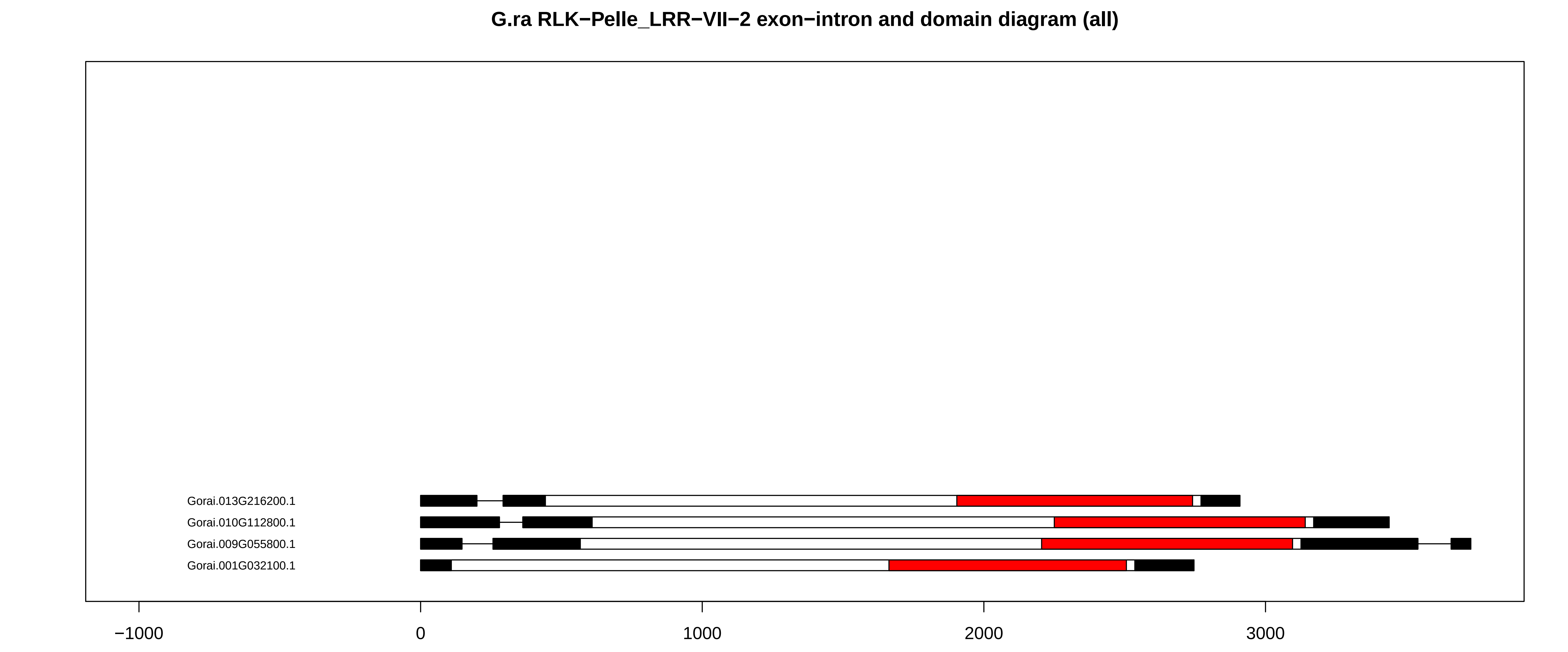
G.ra RLK−Pelle_LRR−VII−2 exon−intron and domain diagram (all)
Gorai.013G216200.1
Gorai.010G112800.1
Gorai.009G055800.1
Gorai.001G032100.1
−1000
0
1000
2000
3000

G.ra RLK−Pelle_LRR−VII−3 exon−intron and domain diagram (all)

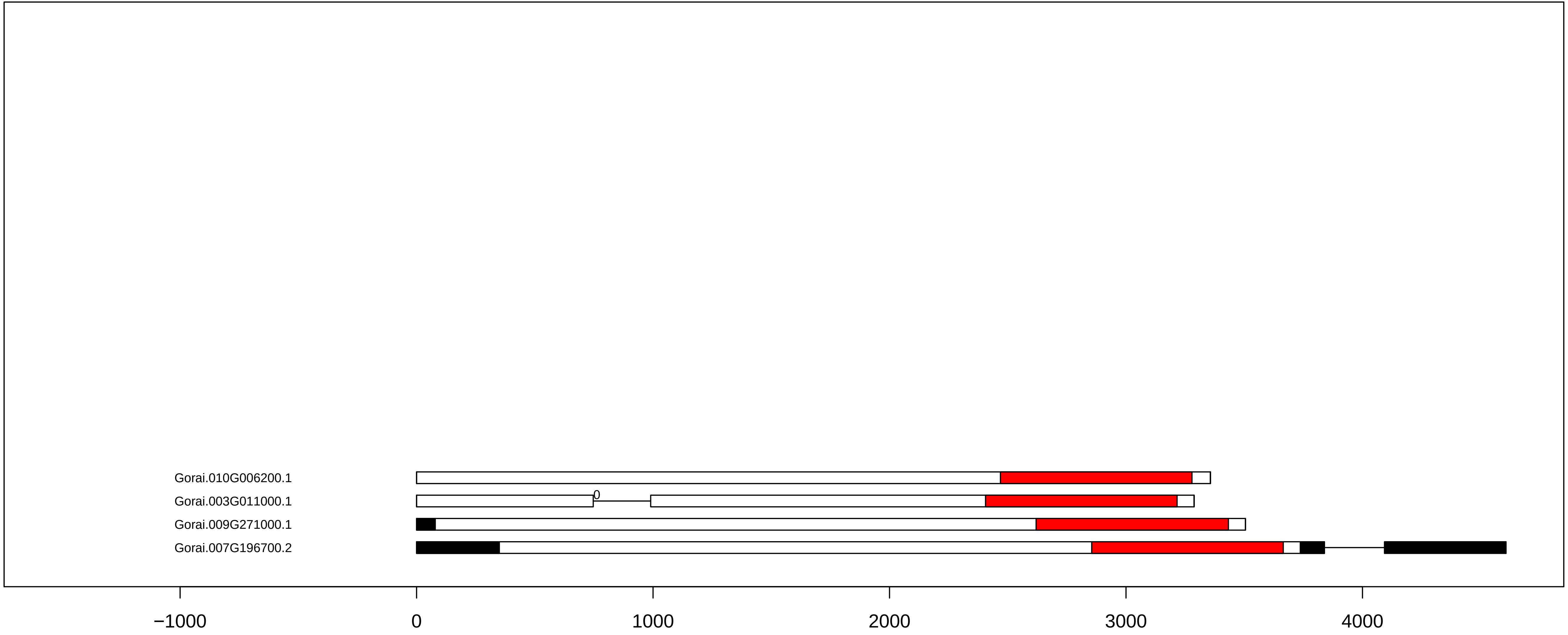

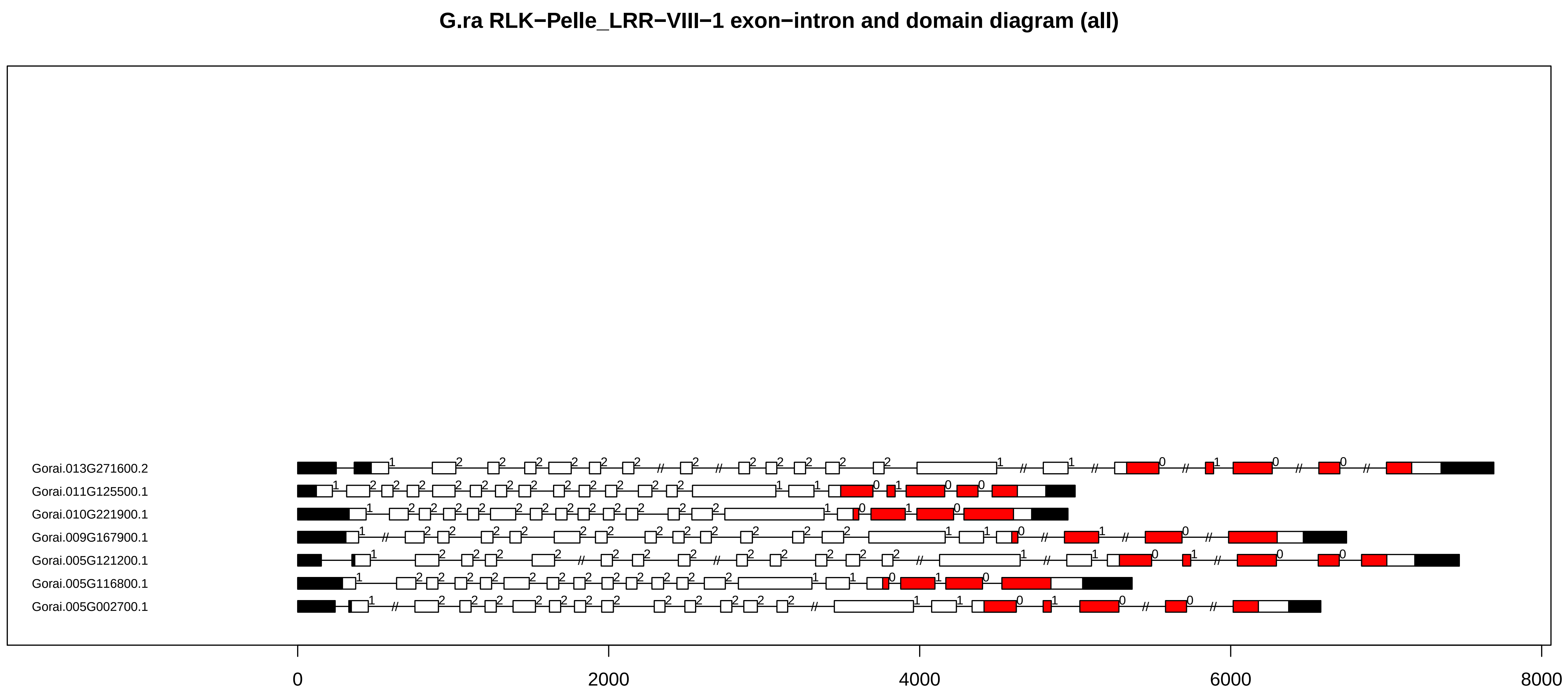
G.ra RLK−Pelle_LRR−VIII−1 exon−intron and domain diagram (all)
Gorai.013G271600.2
Gorai.011G125500.1
Gorai.010G221900.1
Gorai.009G167900.1
Gorai.005G121200.1
Gorai.005G116800.1
Gorai.005G002700.1
0
2000
4000
6000
8000

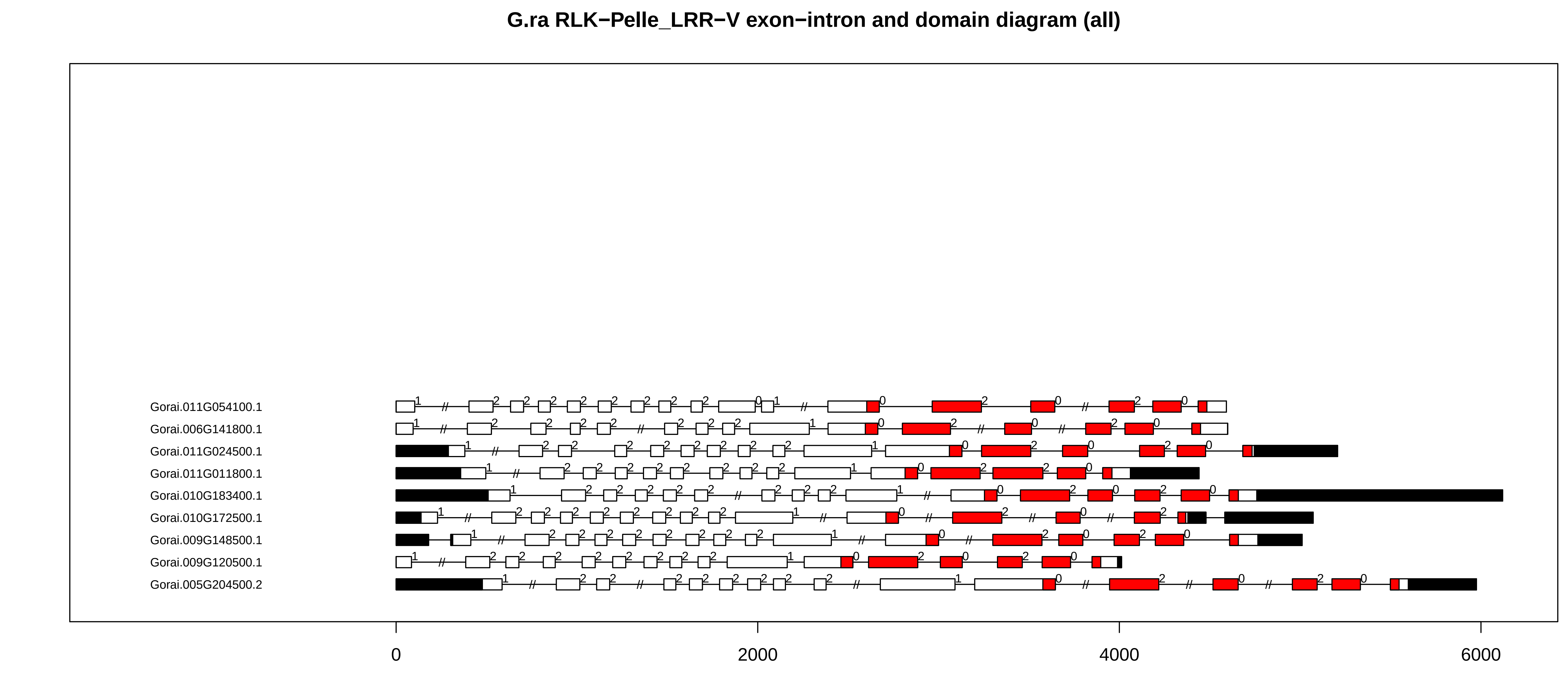
G.ra RLK−Pelle_LRR−V exon−intron and domain diagram (all)
Gorai.011G054100.1
Gorai.006G141800.1
Gorai.011G024500.1
Gorai.011G011800.1
Gorai.010G183400.1
Gorai.010G172500.1
Gorai.009G148500.1
Gorai.009G120500.1
Gorai.005G204500.2
0
2000
4000
6000

G.ra RLK−Pelle_LRR−Xa exon−intron and domain diagram (all)

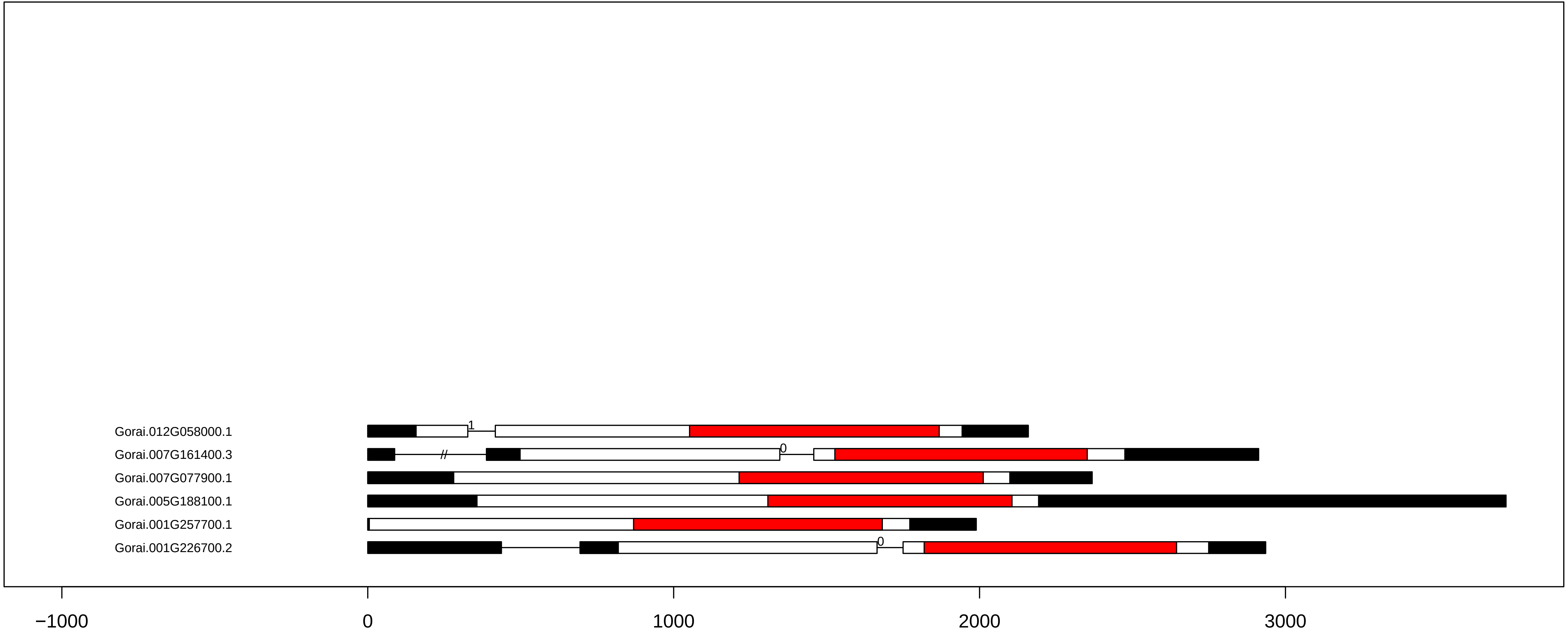

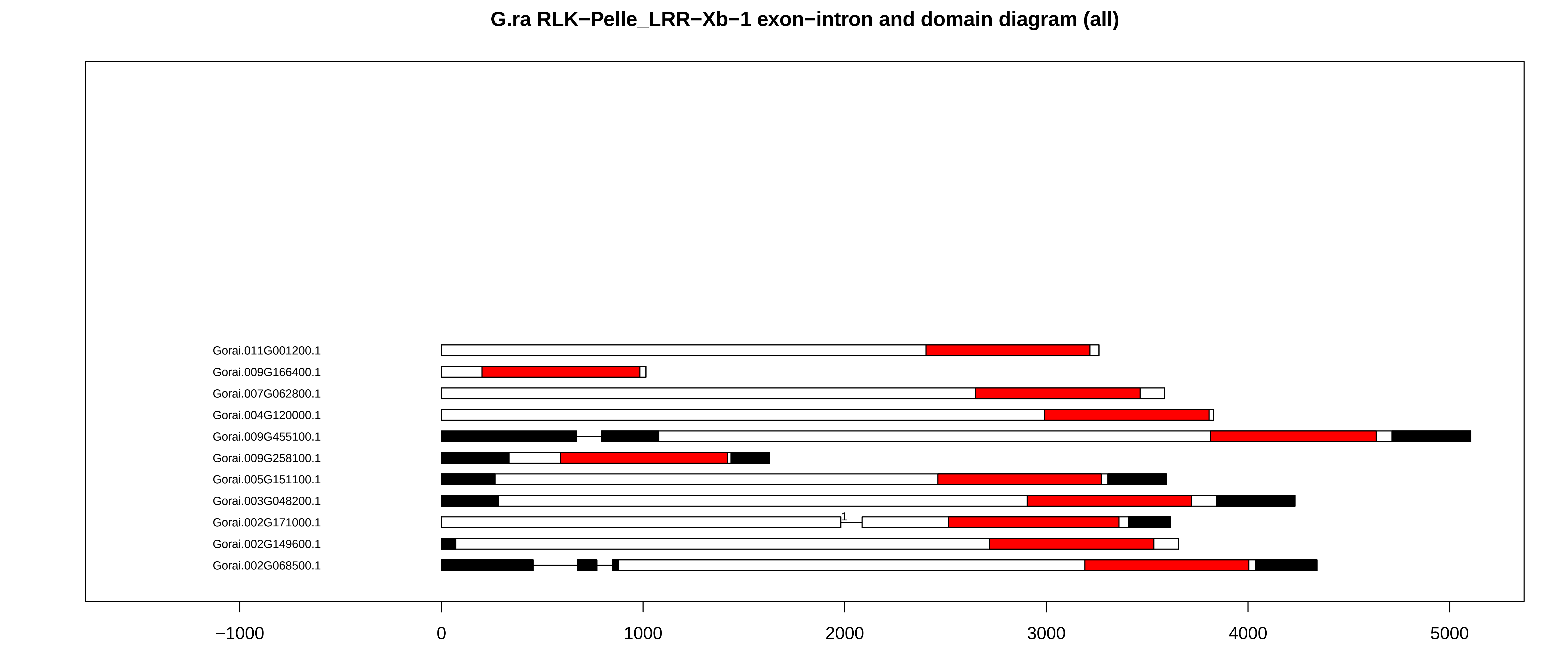
G.ra RLK−Pelle_LRR−Xb−1 exon−intron and domain diagram (all)
Gorai.011G001200.1
Gorai.009G166400.1
Gorai.007G062800.1
Gorai.004G120000.1
Gorai.009G455100.1
Gorai.009G258100.1
Gorai.005G151100.1
Gorai.003G048200.1
Gorai.002G171000.1
Gorai.002G149600.1
Gorai.002G068500.1
1
−1000
0
1000
2000
3000
4000
5000

G.ra RLK−Pelle_LRR−Xb−2 exon−intron and domain diagram (all)

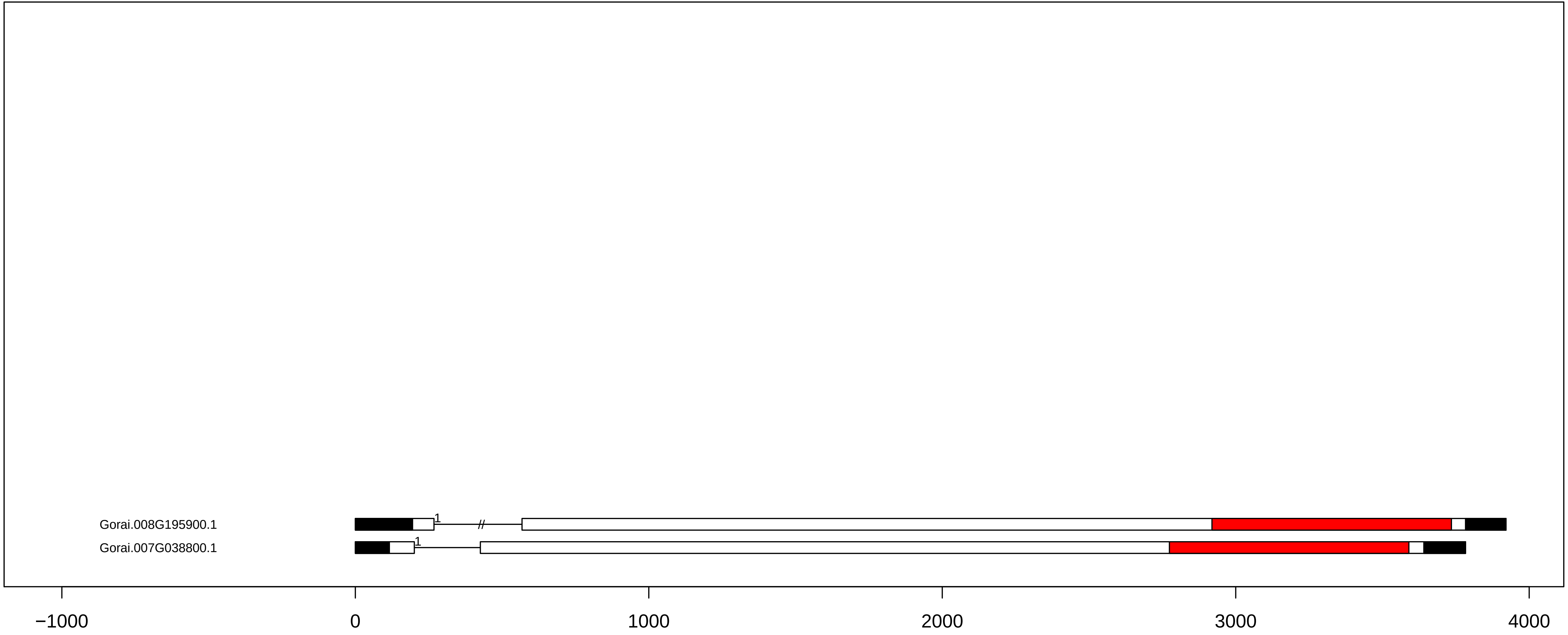

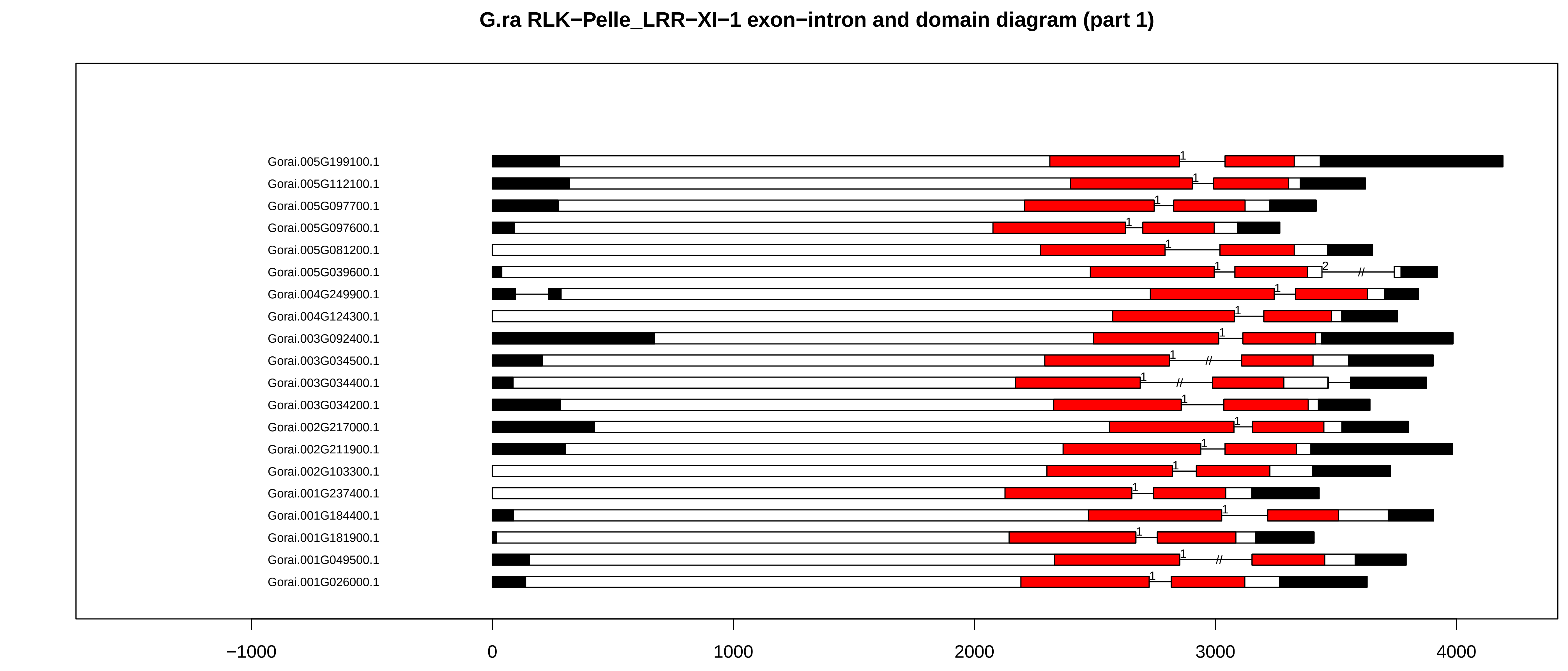
G.ra RLK−Pelle_LRR−XI−1 exon−intron and domain diagram (part 1)
Gorai.005G199100.1
Gorai.005G112100.1
Gorai.005G097700.1
Gorai.005G097600.1
Gorai.005G081200.1
Gorai.005G039600.1
Gorai.004G249900.1
Gorai.004G124300.1
Gorai.003G092400.1
Gorai.003G034500.1
Gorai.003G034400.1
Gorai.003G034200.1
Gorai.002G217000.1
Gorai.002G211900.1
Gorai.002G103300.1
Gorai.001G237400.1
Gorai.001G184400.1
Gorai.001G181900.1
Gorai.001G049500.1
Gorai.001G026000.1
−1000
0
1000
2000
3000
4000

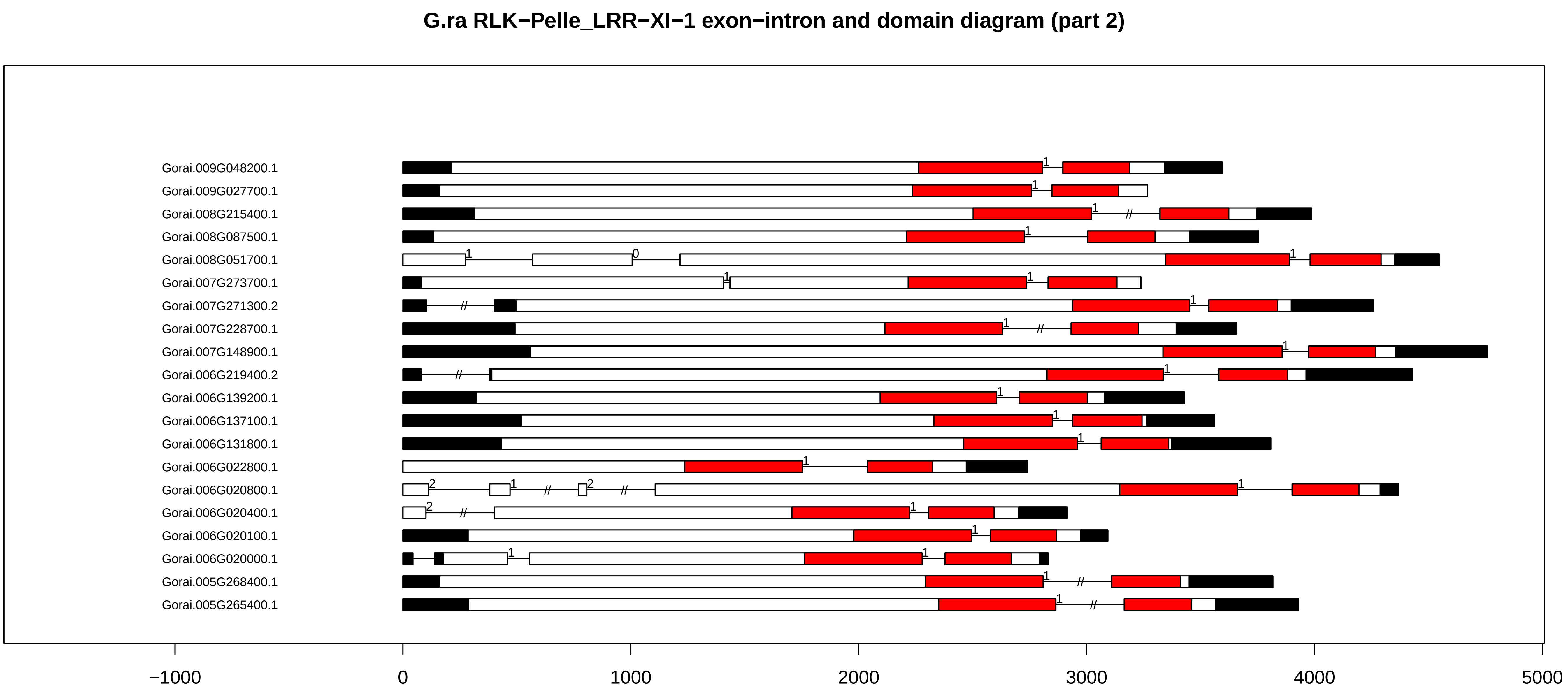
G.ra RLK−Pelle_LRR−XI−1 exon−intron and domain diagram (part 2)
Gorai.009G048200.1
Gorai.009G027700.1
Gorai.008G215400.1
Gorai.008G087500.1
Gorai.008G051700.1
Gorai.007G273700.1
Gorai.007G271300.2
Gorai.007G228700.1
Gorai.007G148900.1
Gorai.006G219400.2
Gorai.006G139200.1
Gorai.006G137100.1
Gorai.006G131800.1
Gorai.006G022800.1
Gorai.006G020800.1
Gorai.006G020400.1
Gorai.006G020100.1
Gorai.006G020000.1
Gorai.005G268400.1
Gorai.005G265400.1
−1000
0
1000
2000
3000
4000
5000

G.ra RLK−Pelle_LRR−XI−1 exon−intron and domain diagram (part 3)

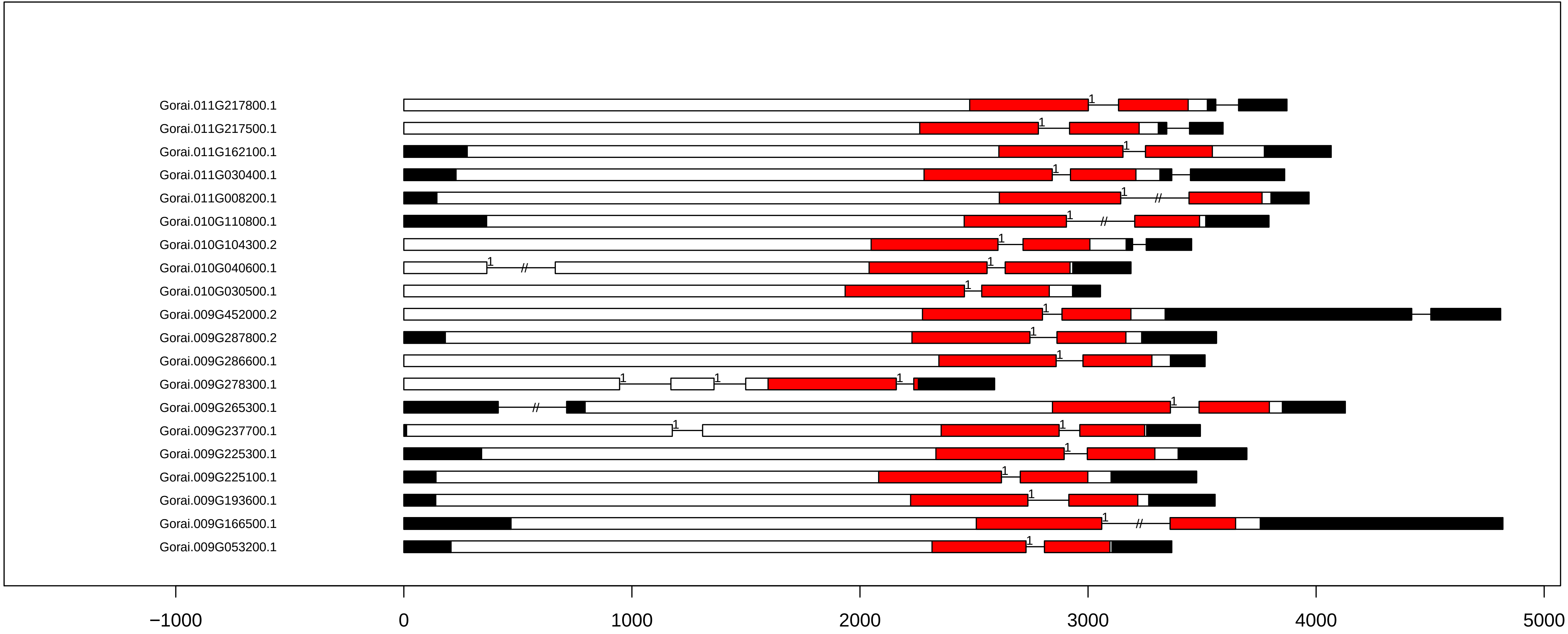

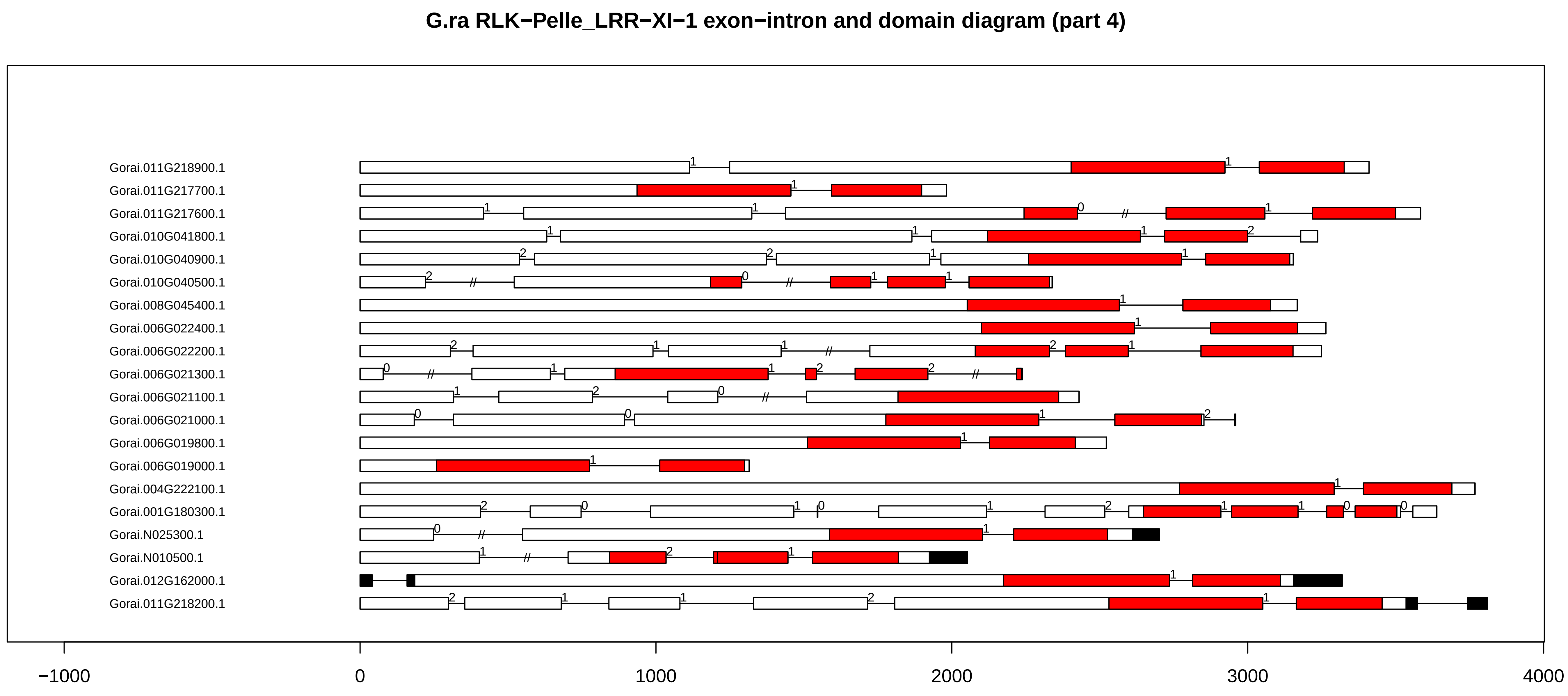

G.ra RLK−Pelle_LRR−XI−1 exon−intron and domain diagram (part 4)
Gorai.011G218900.1
Gorai.011G217700.1
Gorai.011G217600.1
Gorai.010G041800.1
Gorai.010G040900.1
Gorai.010G040500.1
Gorai.008G045400.1
Gorai.006G022400.1
Gorai.006G022200.1
Gorai.006G021300.1
Gorai.006G021100.1
Gorai.006G021000.1
Gorai.006G019800.1
Gorai.006G019000.1
Gorai.004G222100.1
Gorai.001G180300.1
Gorai.N025300.1
Gorai.N010500.1
Gorai.012G162000.1
Gorai.011G218200.1
−1000
0
1000
2000
3000
4000

G.ra RLK−Pelle_LRR−XI−1 exon−intron and domain diagram (part 5)

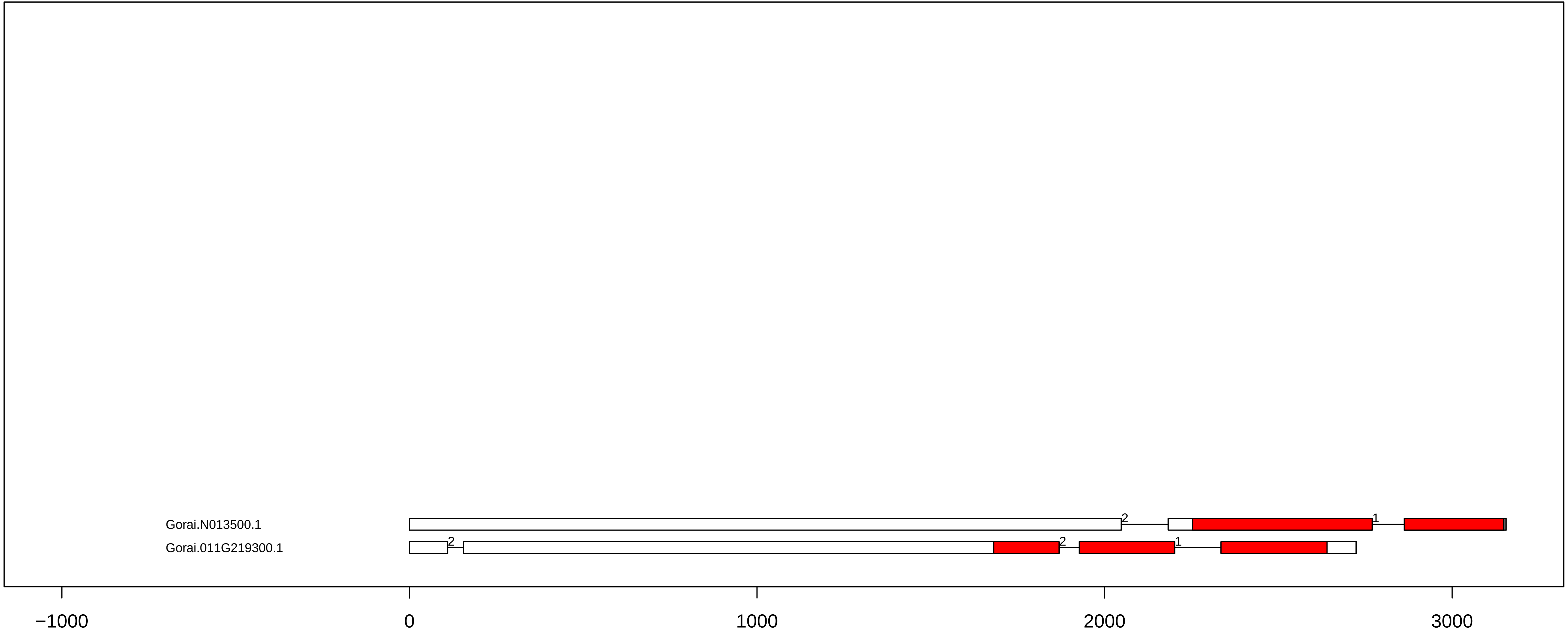

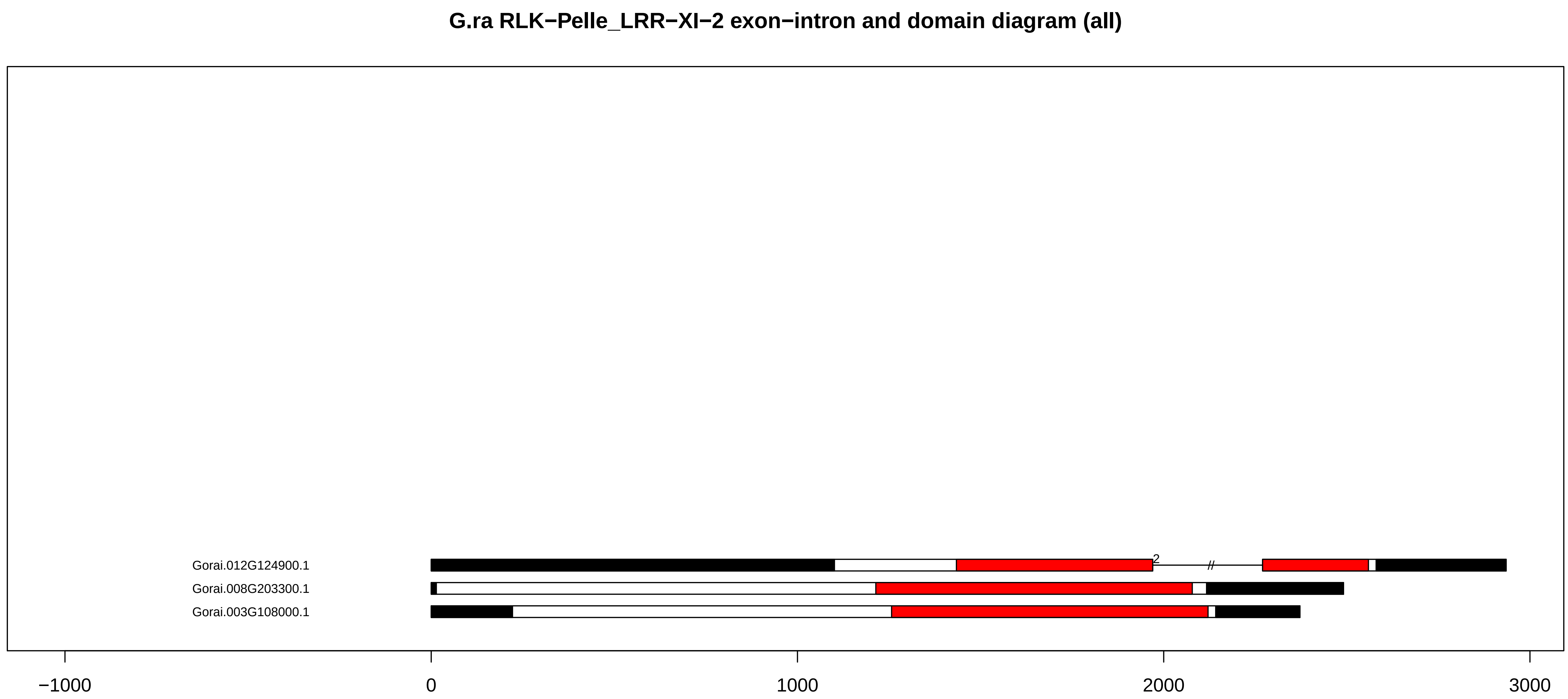
G.ra RLK−Pelle_LRR−XI−2 exon−intron and domain diagram (all)
Gorai.012G124900.1
Gorai.008G203300.1
Gorai.003G108000.1
2
//
−1000
0
1000
2000
3000

G.ra RLK−Pelle_LRR−XII−1 exon−intron and domain diagram (part 1)

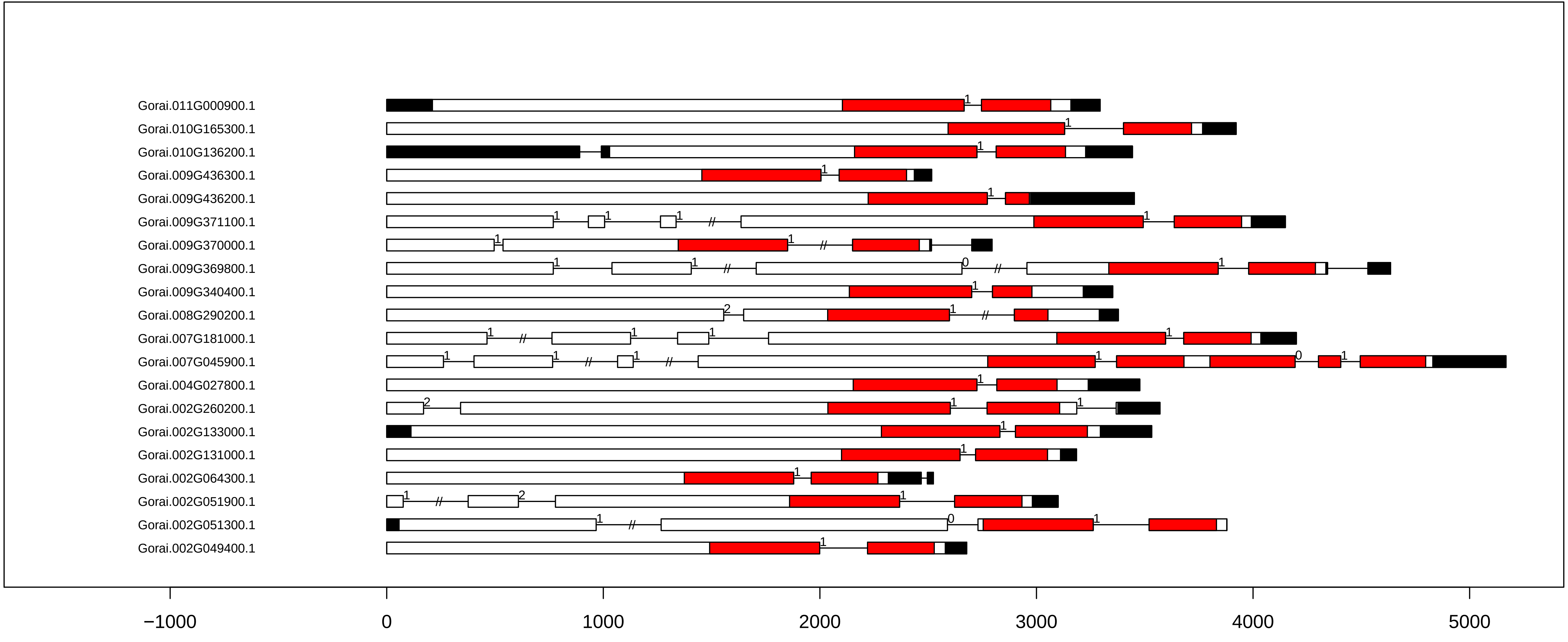

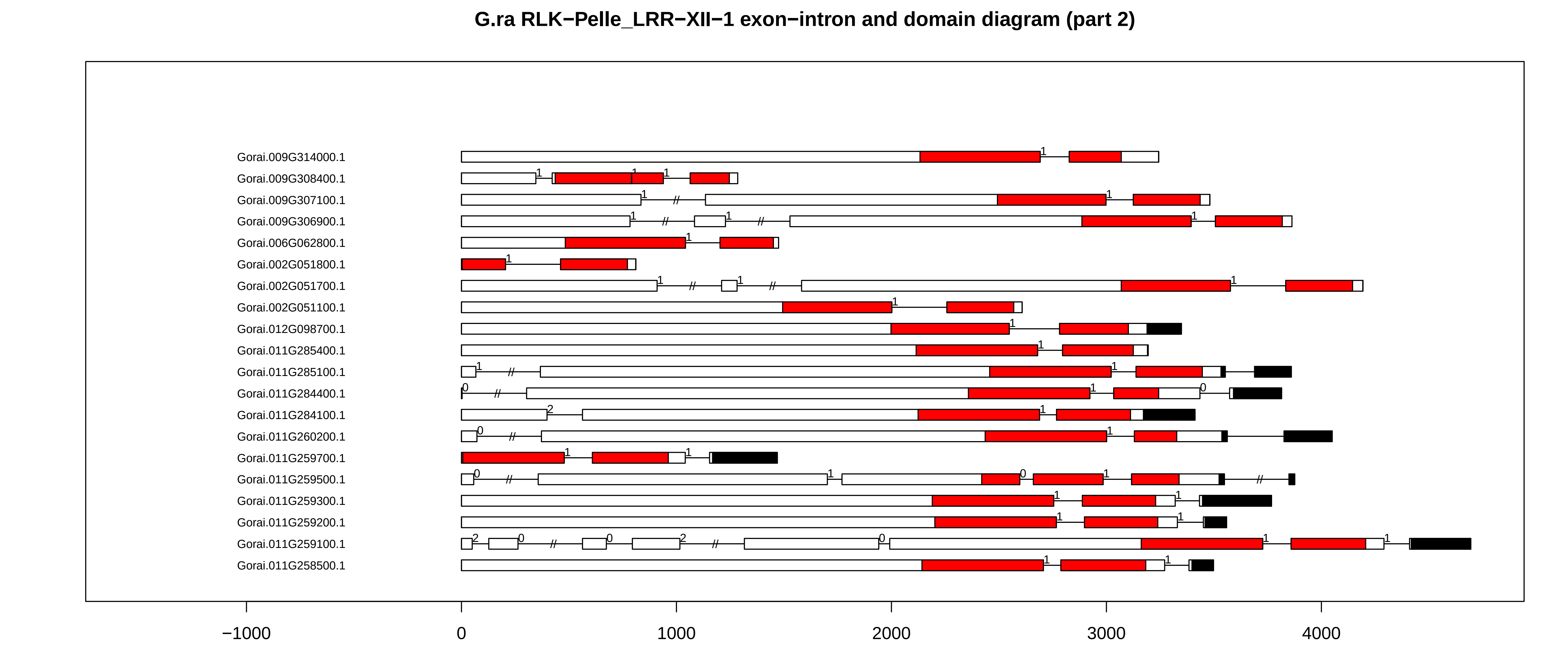
G.ra RLK−Pelle_LRR−XII−1 exon−intron and domain diagram (part 2)
Gorai.009G314000.1
Gorai.009G308400.1
Gorai.009G307100.1
Gorai.009G306900.1
Gorai.006G062800.1
Gorai.002G051800.1
Gorai.002G051700.1
Gorai.002G051100.1
Gorai.012G098700.1
Gorai.011G285400.1
Gorai.011G285100.1
Gorai.011G284400.1
Gorai.011G284100.1
Gorai.011G260200.1
Gorai.011G259700.1
Gorai.011G259500.1
Gorai.011G259300.1
Gorai.011G259200.1
Gorai.011G259100.1
Gorai.011G258500.1
−1000
0
1000
2000
3000
4000

G.ra RLK−Pelle_LRR−XII−1 exon−intron and domain diagram (part 3)

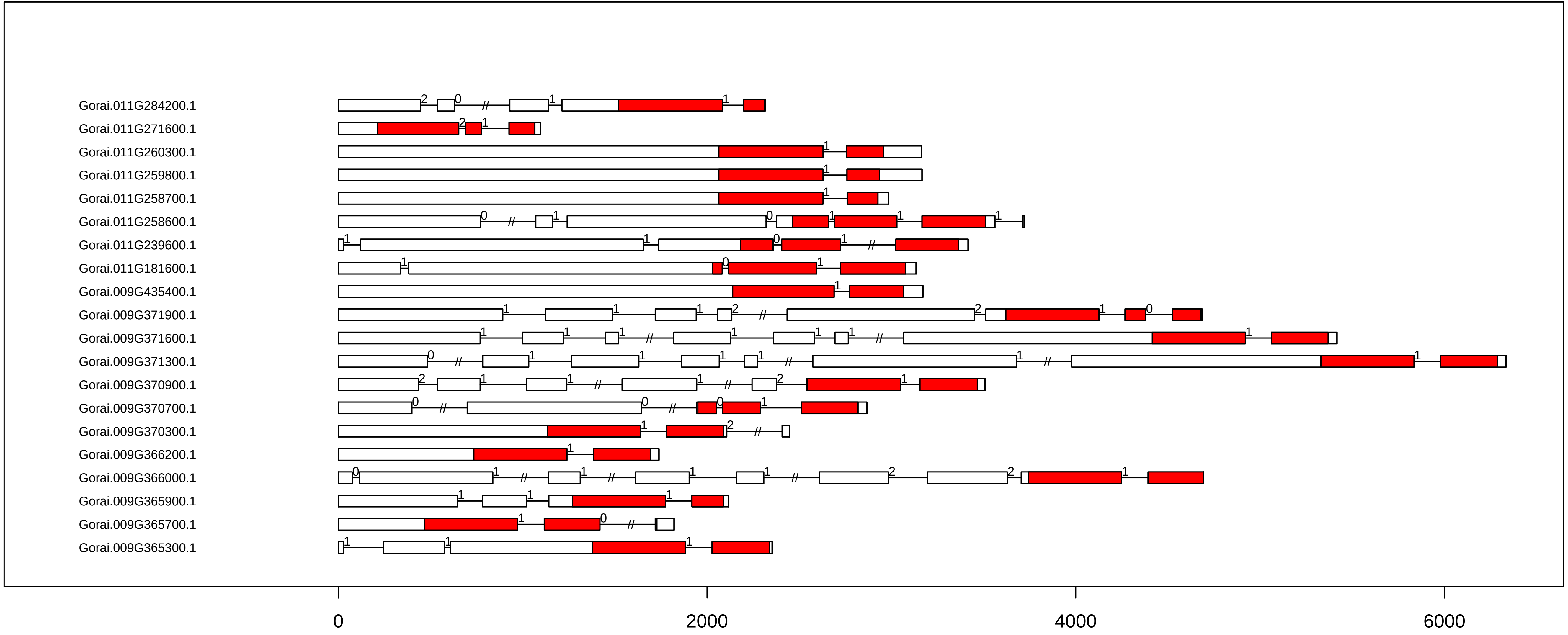

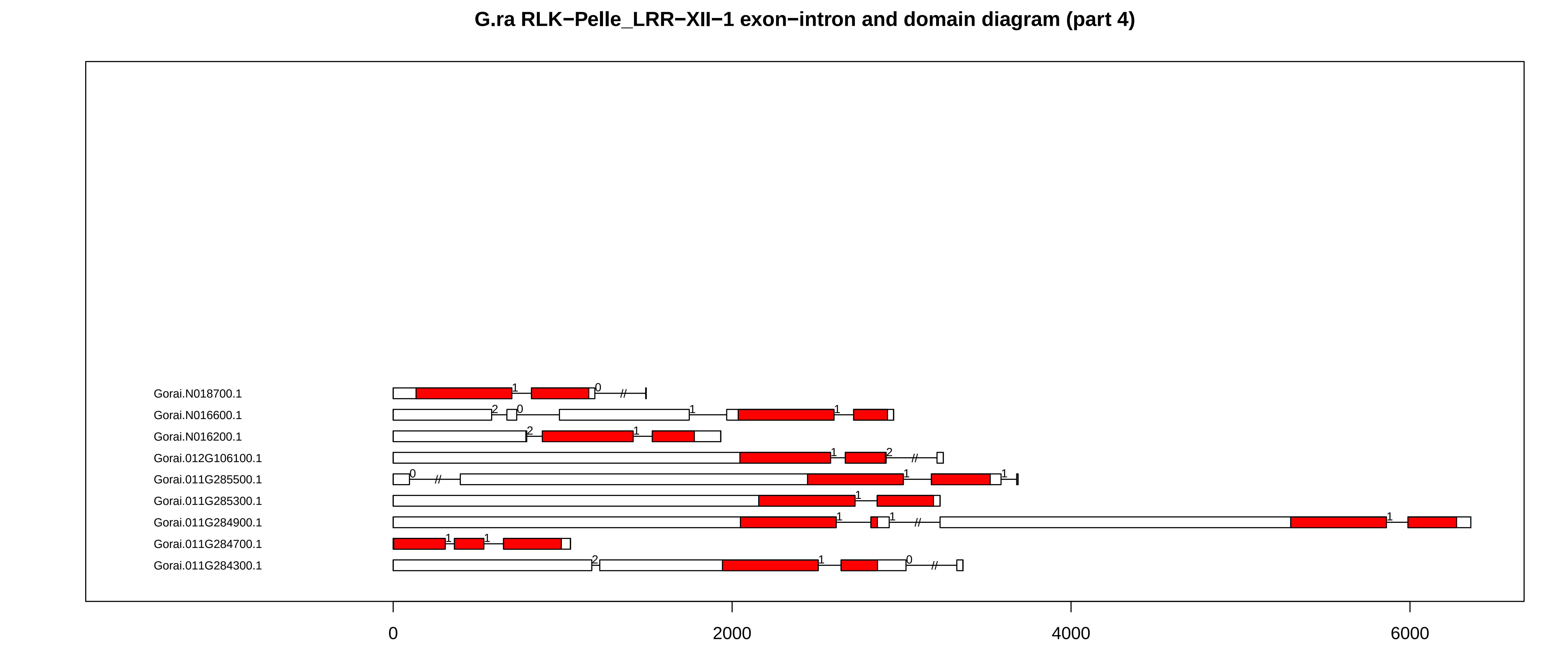
G.ra RLK−Pelle_LRR−XII−1 exon−intron and domain diagram (part 4)
Gorai.N018700.1
Gorai.N016600.1
Gorai.N016200.1
Gorai.012G106100.1
Gorai.011G285500.1
Gorai.011G285300.1
Gorai.011G284900.1
Gorai.011G284700.1
Gorai.011G284300.1
0
2000
4000
6000

G.ra RLK−Pelle_LRR−XIIIa exon−intron and domain diagram (all)

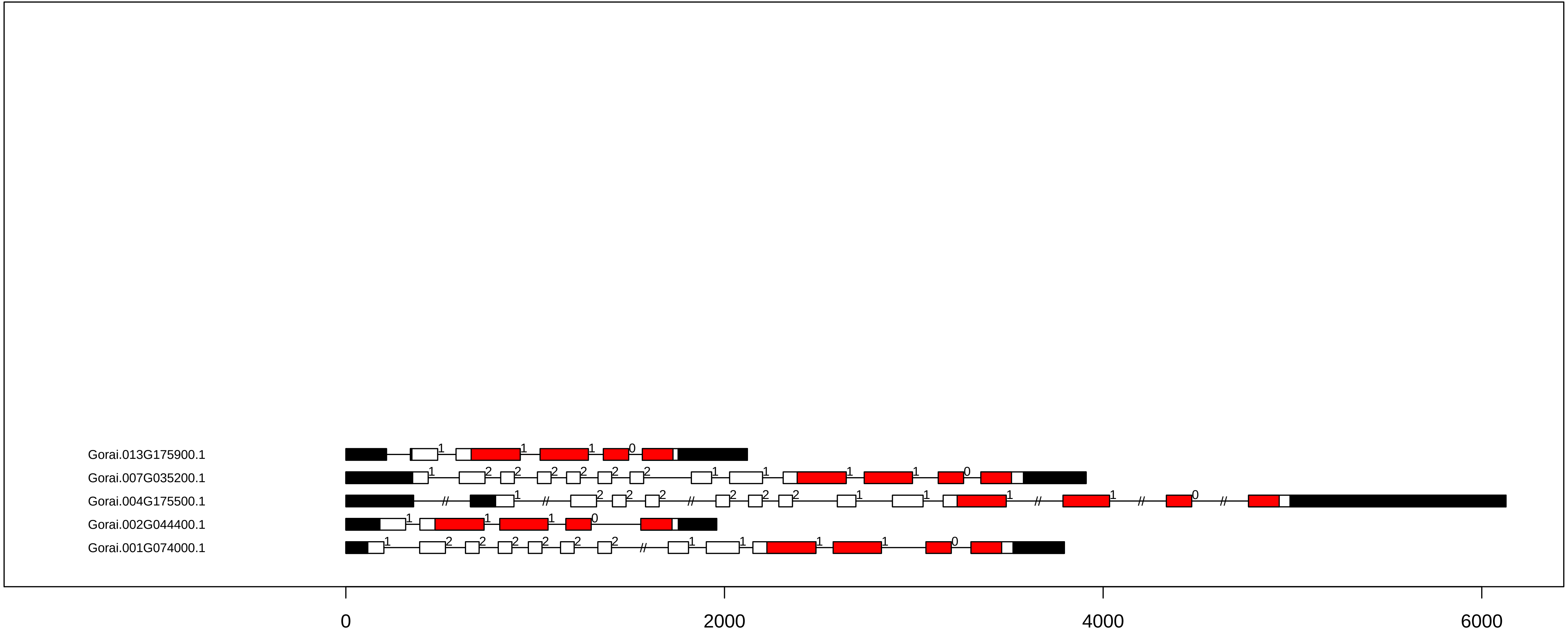

G.ra RLK−Pelle_LRR−XIIIb exon−intron and domain diagram (all)

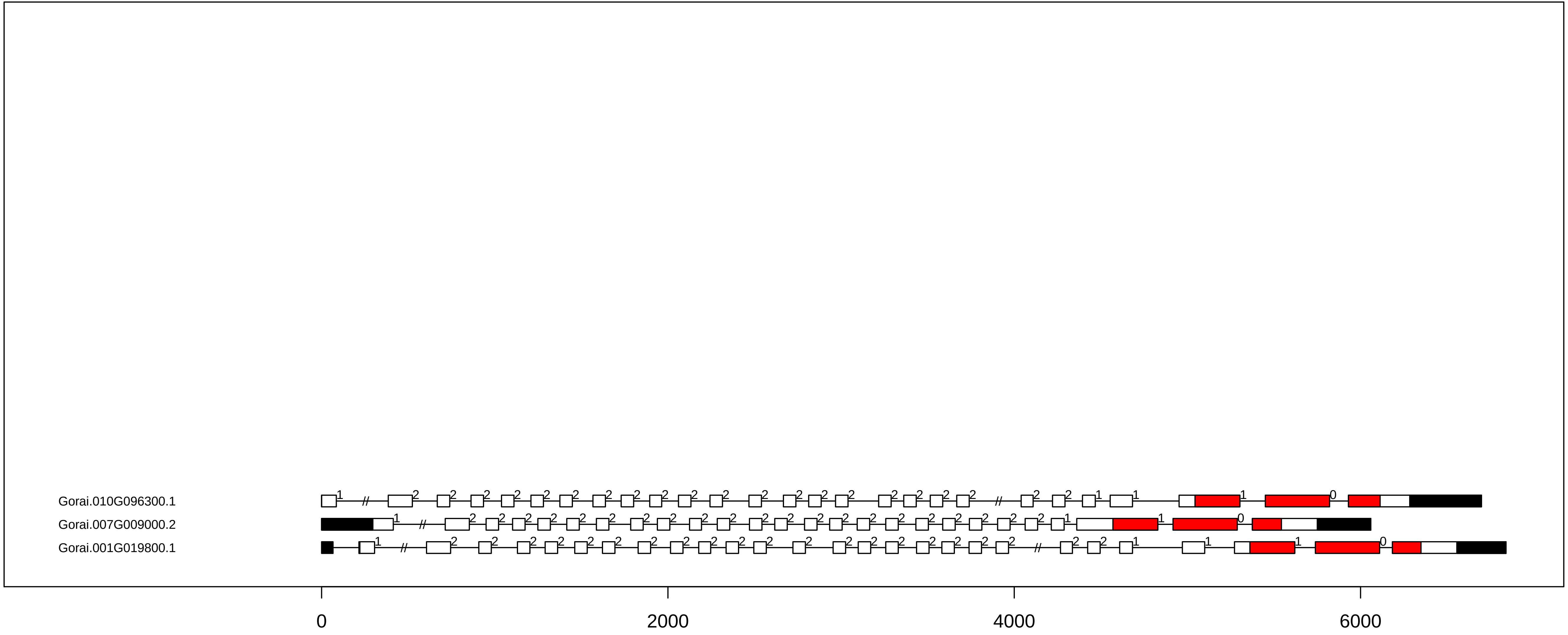

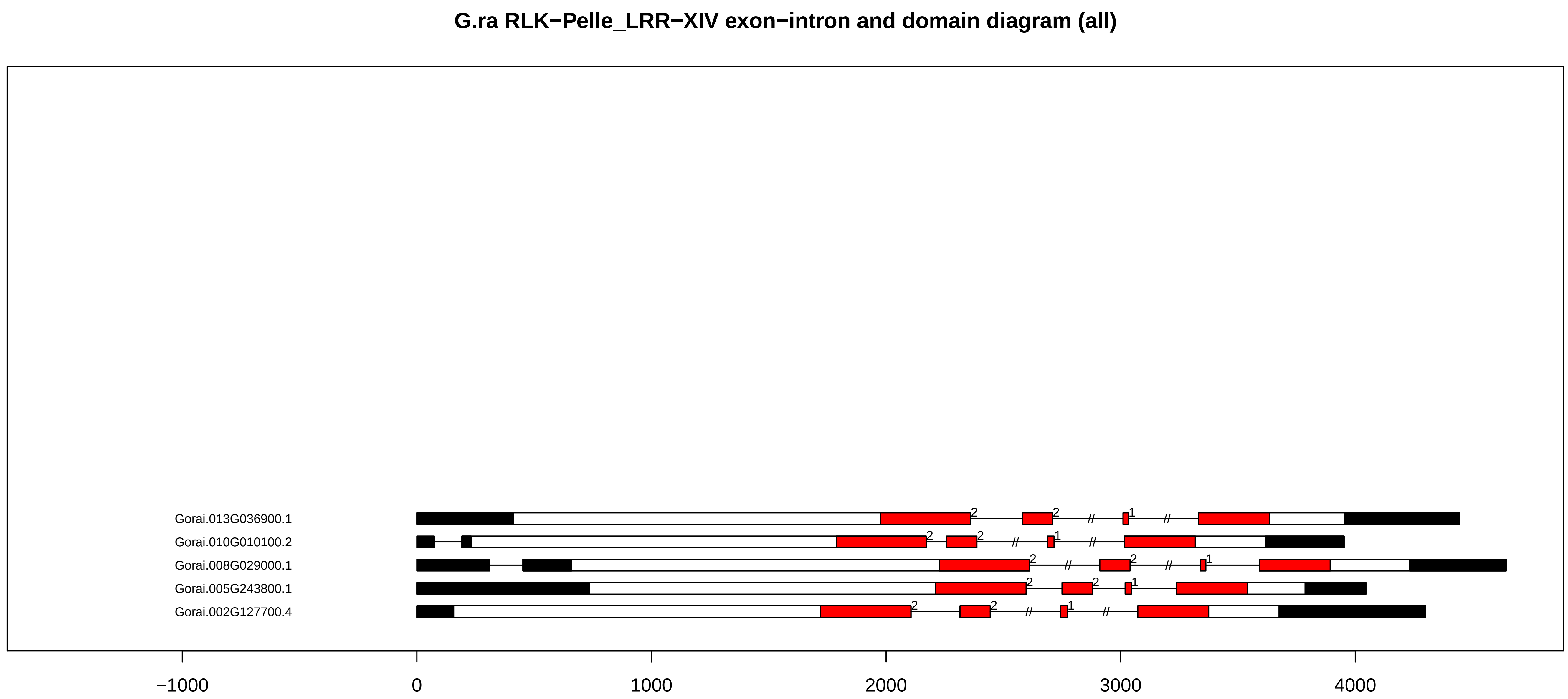
G.ra RLK−Pelle_LRR−XIV exon−intron and domain diagram (all)
Gorai.013G036900.1
Gorai.010G010100.2
Gorai.008G029000.1
Gorai.005G243800.1
Gorai.002G127700.4
−1000
0
1000
2000
3000
4000

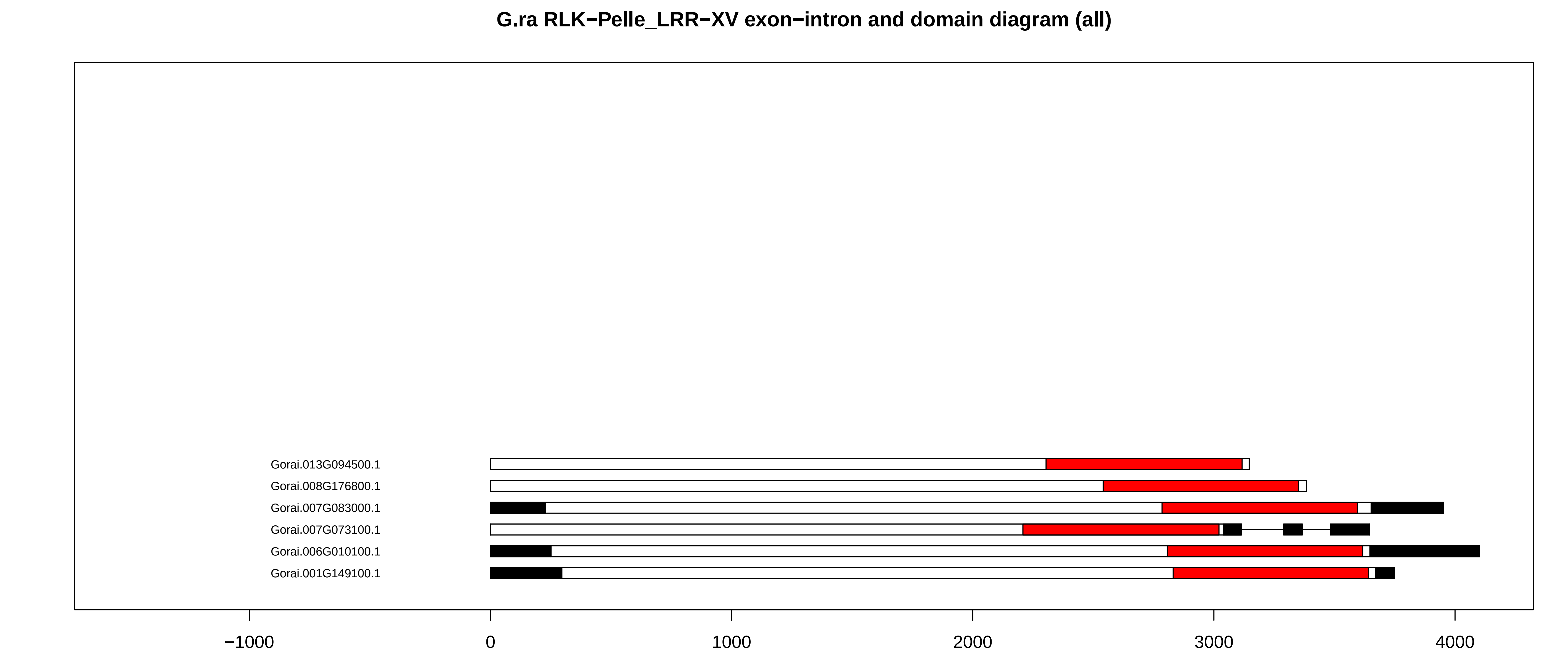
G.ra RLK−Pelle_LRR−XV exon−intron and domain diagram (all)
Gorai.013G094500.1
Gorai.008G176800.1
Gorai.007G083000.1
Gorai.007G073100.1
Gorai.006G010100.1
Gorai.001G149100.1
−1000
0
1000
2000
3000
4000

G.ra RLK−Pelle_LysM exon−intron and domain diagram (all)

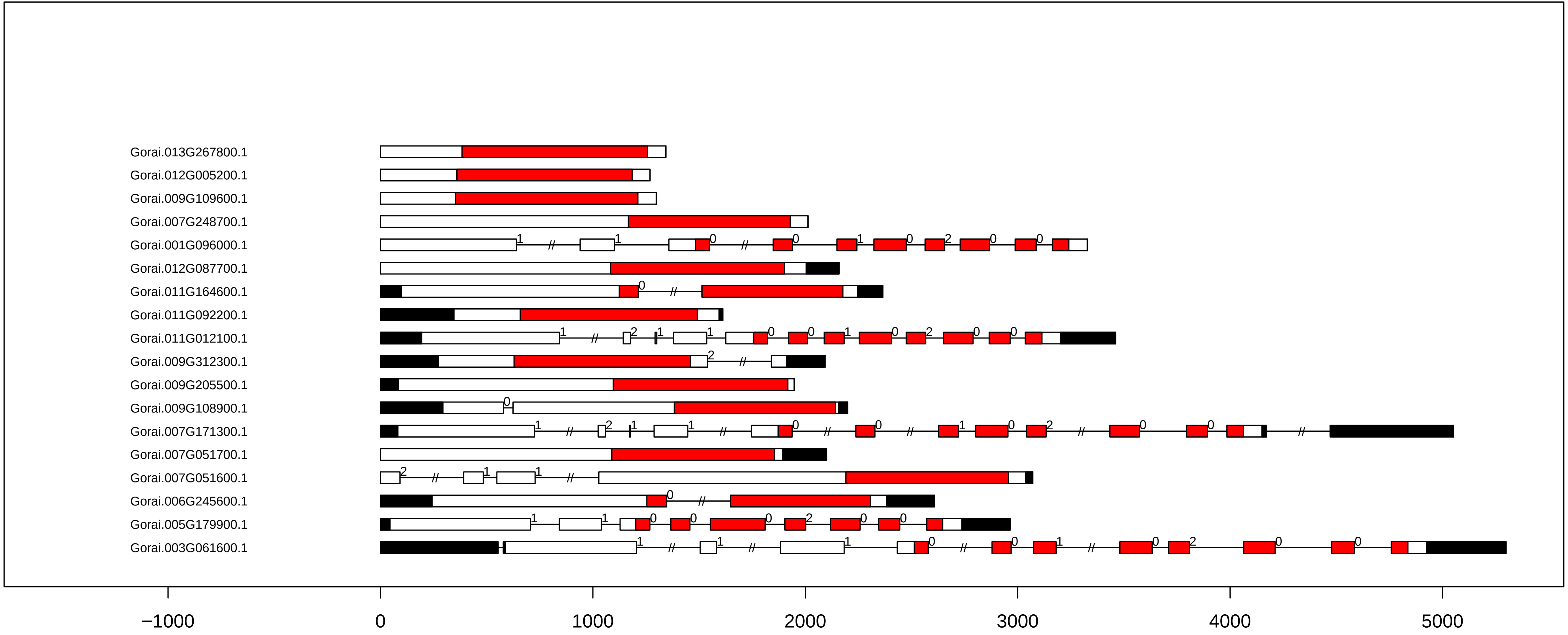

G.ra RLK−Pelle_PERK−1 exon−intron and domain diagram (all)

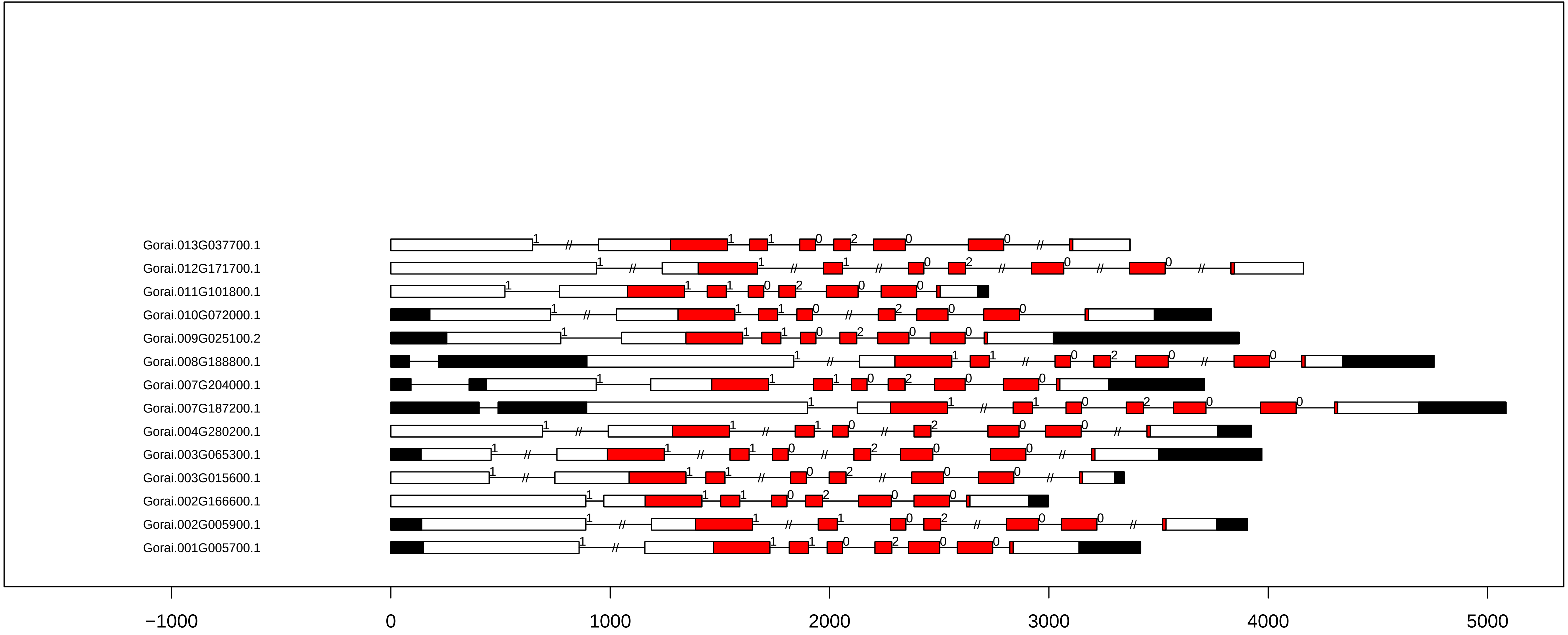

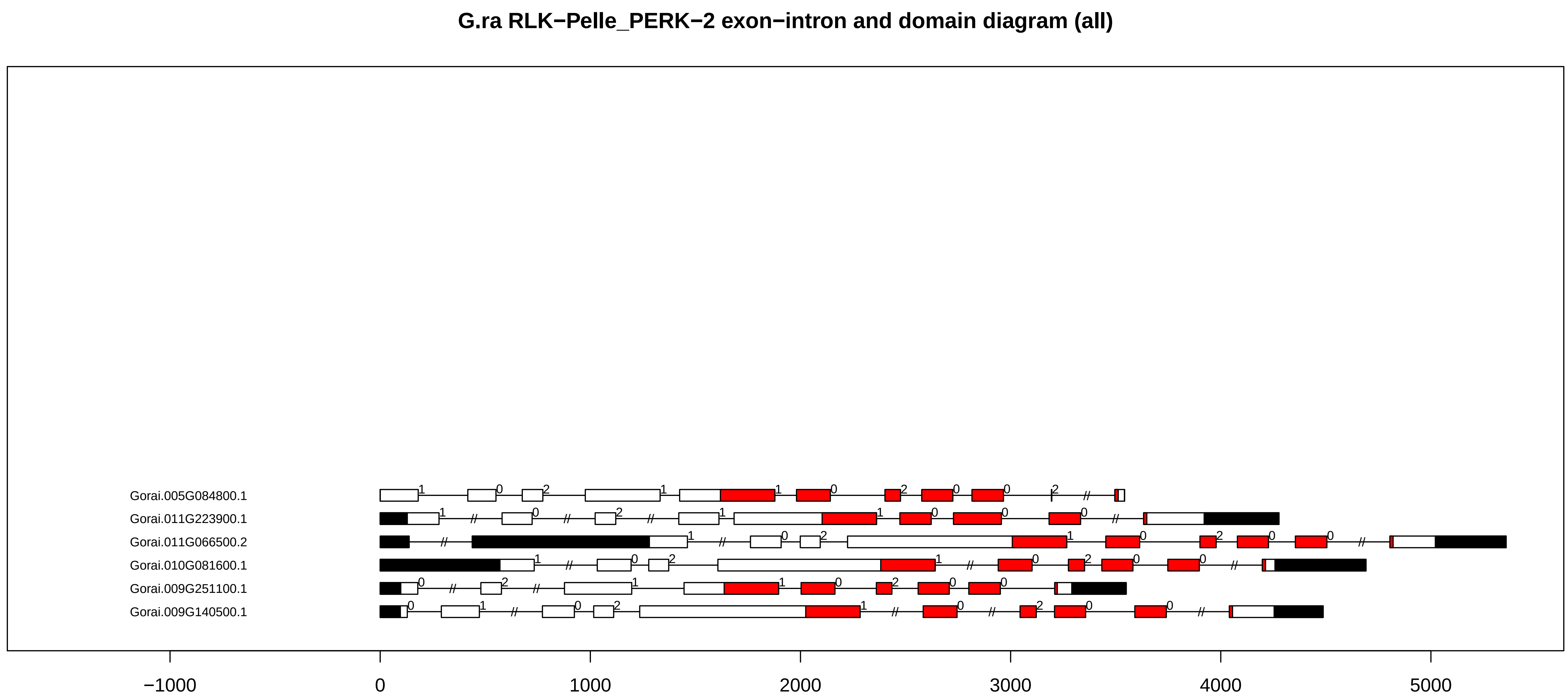
G.ra RLK−Pelle_PERK−2 exon−intron and domain diagram (all)
Gorai.005G084800.1
Gorai.011G223900.1
Gorai.011G066500.2
Gorai.010G081600.1
Gorai.009G251100.1
Gorai.009G140500.1
−1000
0
1000
2000
3000
4000
5000

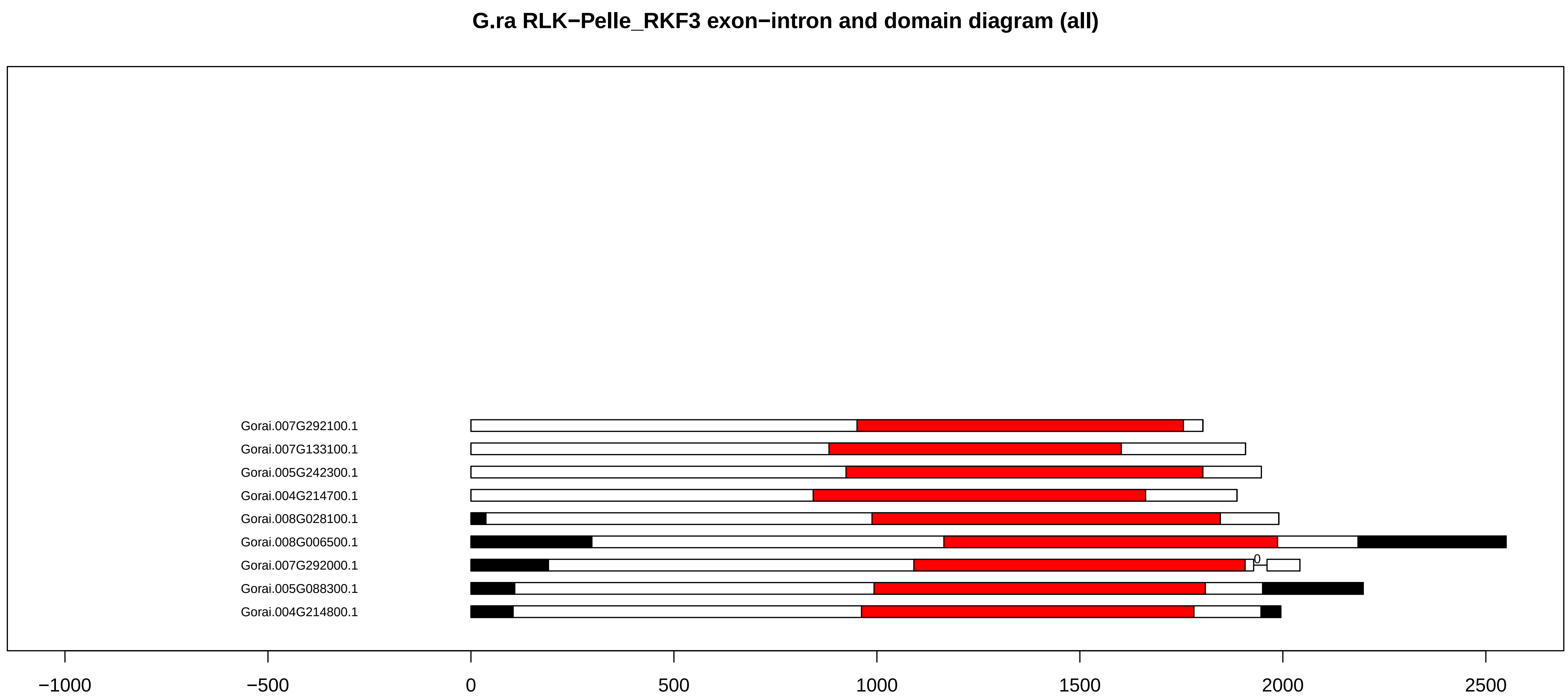
G.ra RLK−Pelle_RKF3 exon−intron and domain diagram (all)
Gorai.007G292100.1
Gorai.007G133100.1
Gorai.005G242300.1
Gorai.004G214700.1
Gorai.008G028100.1
Gorai.008G006500.1
Gorai.007G292000.1
Gorai.005G088300.1
Gorai.004G214800.1
0
−1000
−500
0
500
1000
1500
2000
2500

G.ra RLK−Pelle_RLCK−II exon−intron and domain diagram (all)

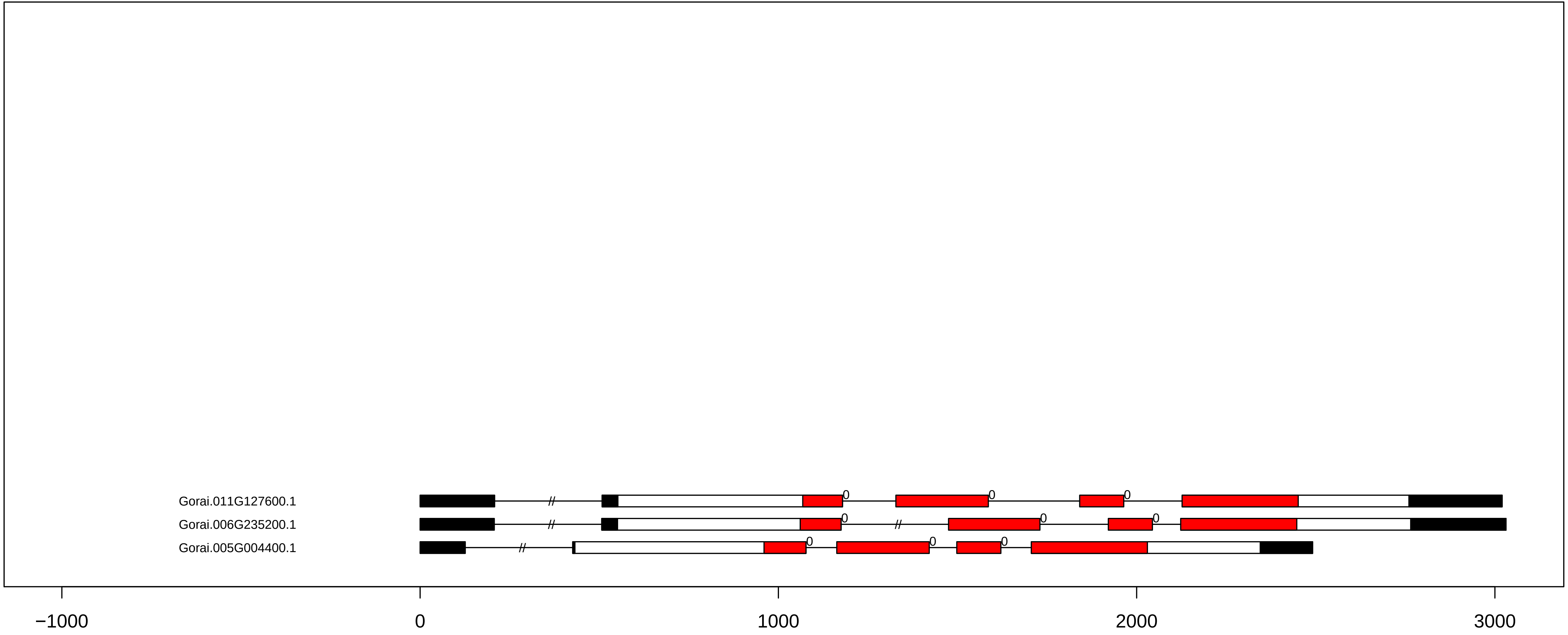

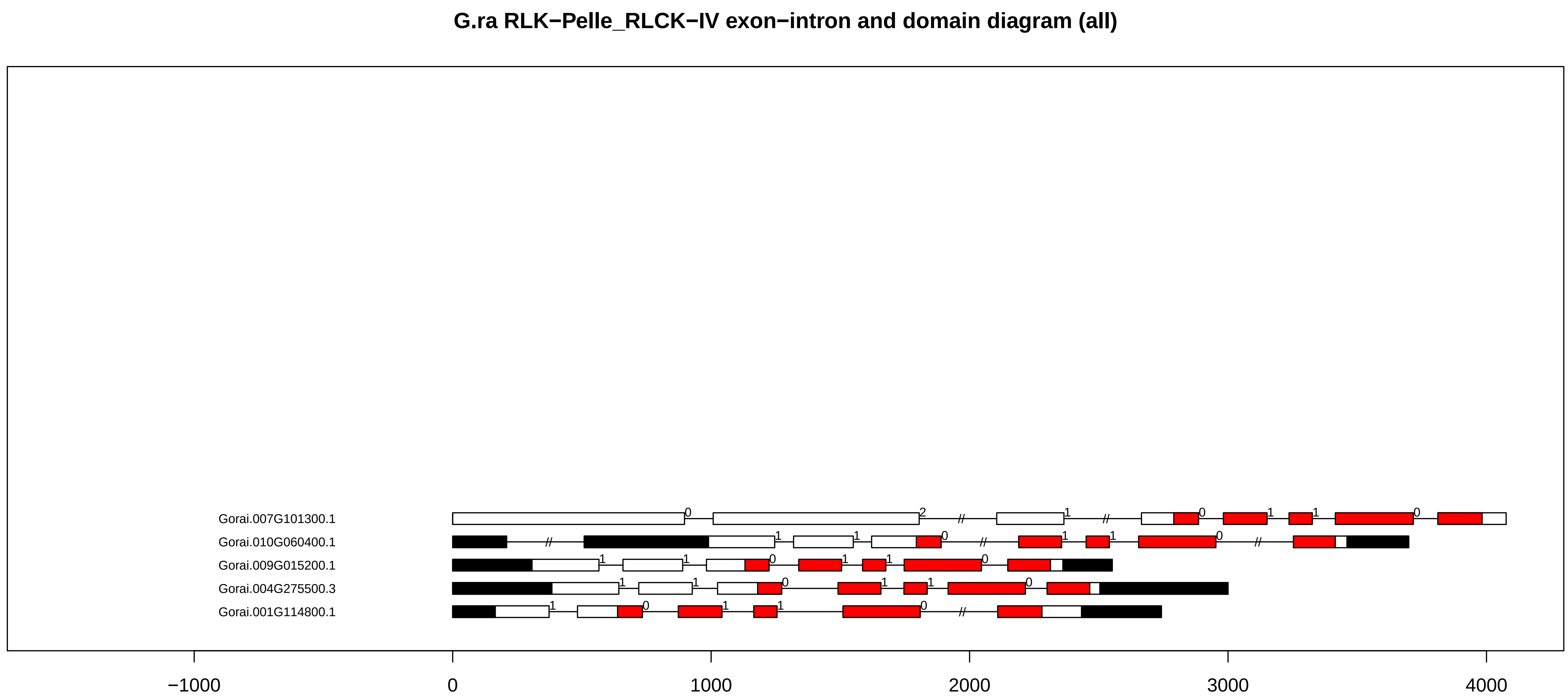

G.ra RLK−Pelle_RLCK−IV exon−intron and domain diagram (all)
Gorai.007G101300.1
Gorai.010G060400.1
Gorai.009G015200.1
Gorai.004G275500.3
Gorai.001G114800.1
−1000
0
1000
2000
3000
4000

G.ra RLK−Pelle_RLCK−IXa exon−intron and domain diagram (all)

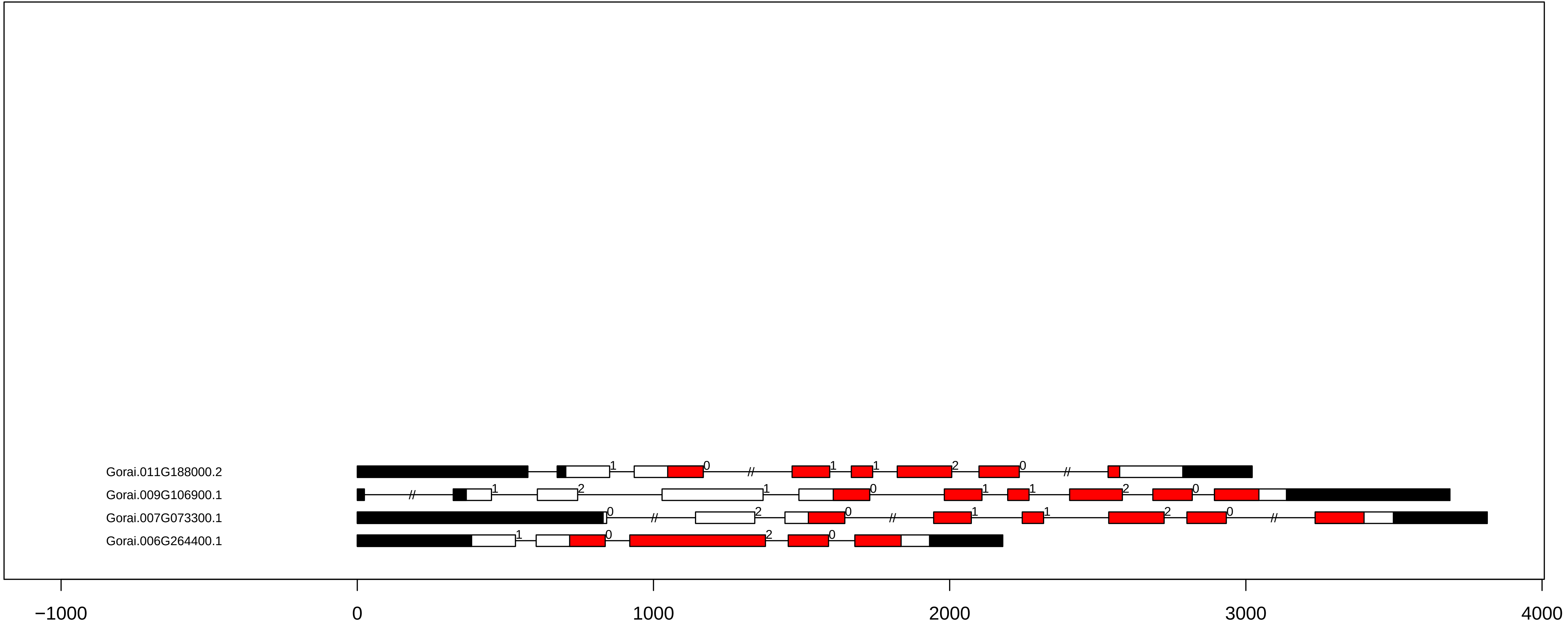

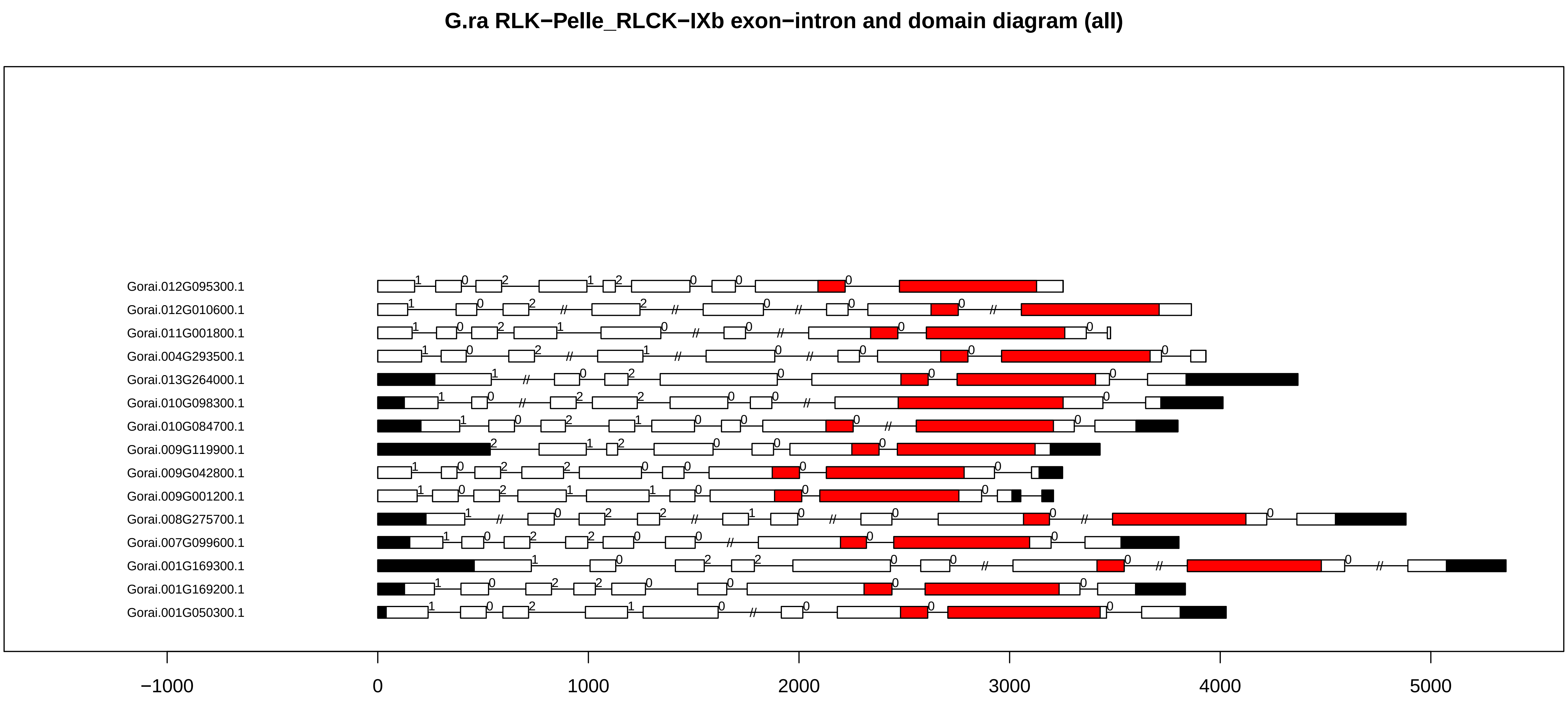
G.ra RLK−Pelle_RLCK−IXb exon−intron and domain diagram (all)
Gorai.012G095300.1
Gorai.012G010600.1
Gorai.011G001800.1
Gorai.004G293500.1
Gorai.013G264000.1
Gorai.010G098300.1
Gorai.010G084700.1
Gorai.009G119900.1
Gorai.009G042800.1
Gorai.009G001200.1
Gorai.008G275700.1
Gorai.007G099600.1
Gorai.001G169300.1
Gorai.001G169200.1
Gorai.001G050300.1
−1000
0
1000
2000
3000
4000
5000

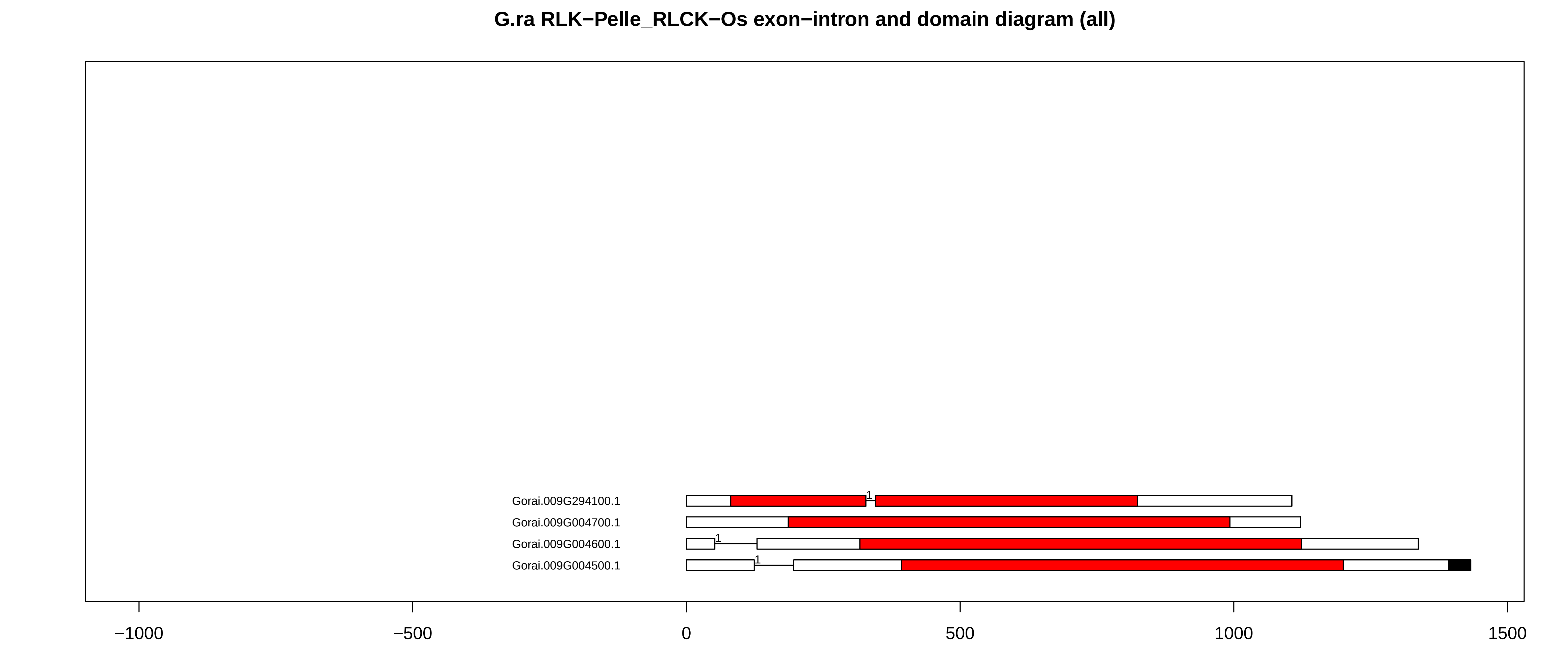
G.ra RLK−Pelle_RLCK−Os exon−intron and domain diagram (all)
Gorai.009G294100.1
Gorai.009G004700.1
Gorai.009G004600.1
Gorai.009G004500.1
1
1
1
−1000
−500
0
500
1000
1500

G.ra RLK−Pelle_RLCK−VIIa−1 exon−intron and domain diagram (part 1)

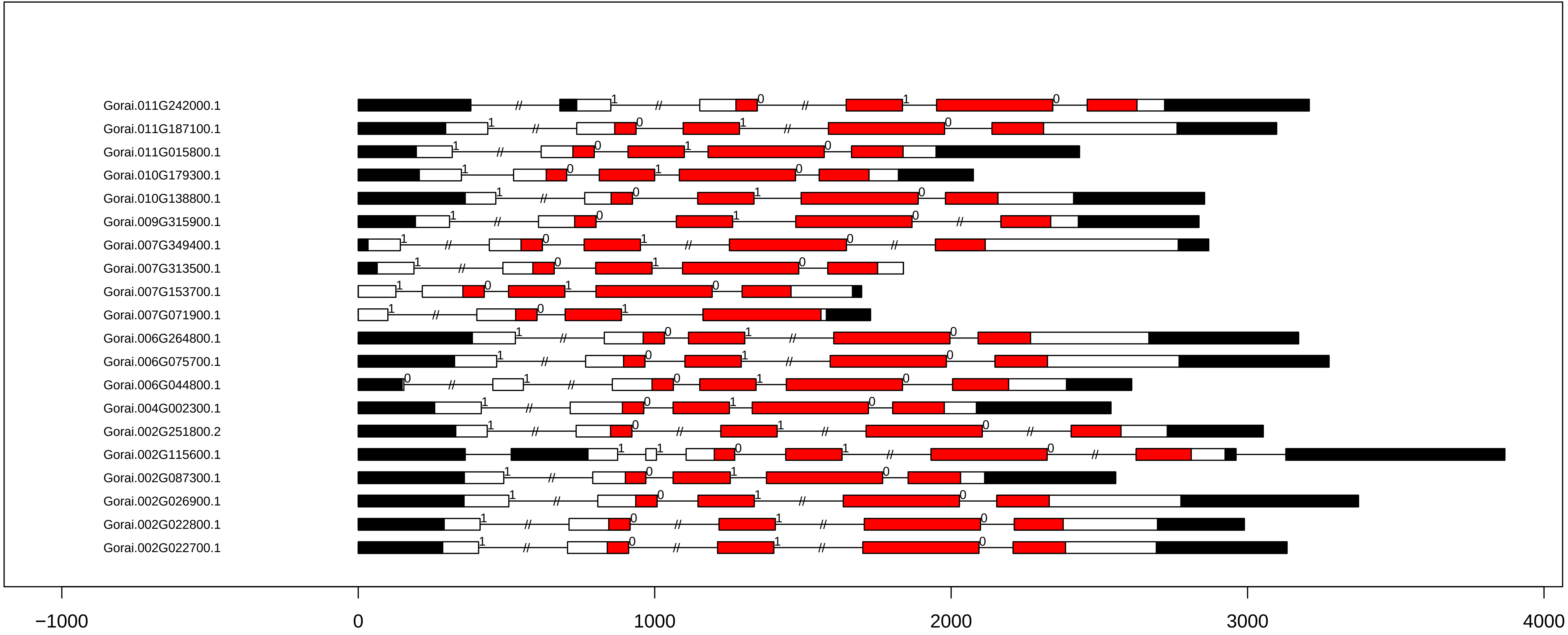

G.ra RLK−Pelle_RLCK−VIIa−1 exon−intron and domain diagram (part 2)

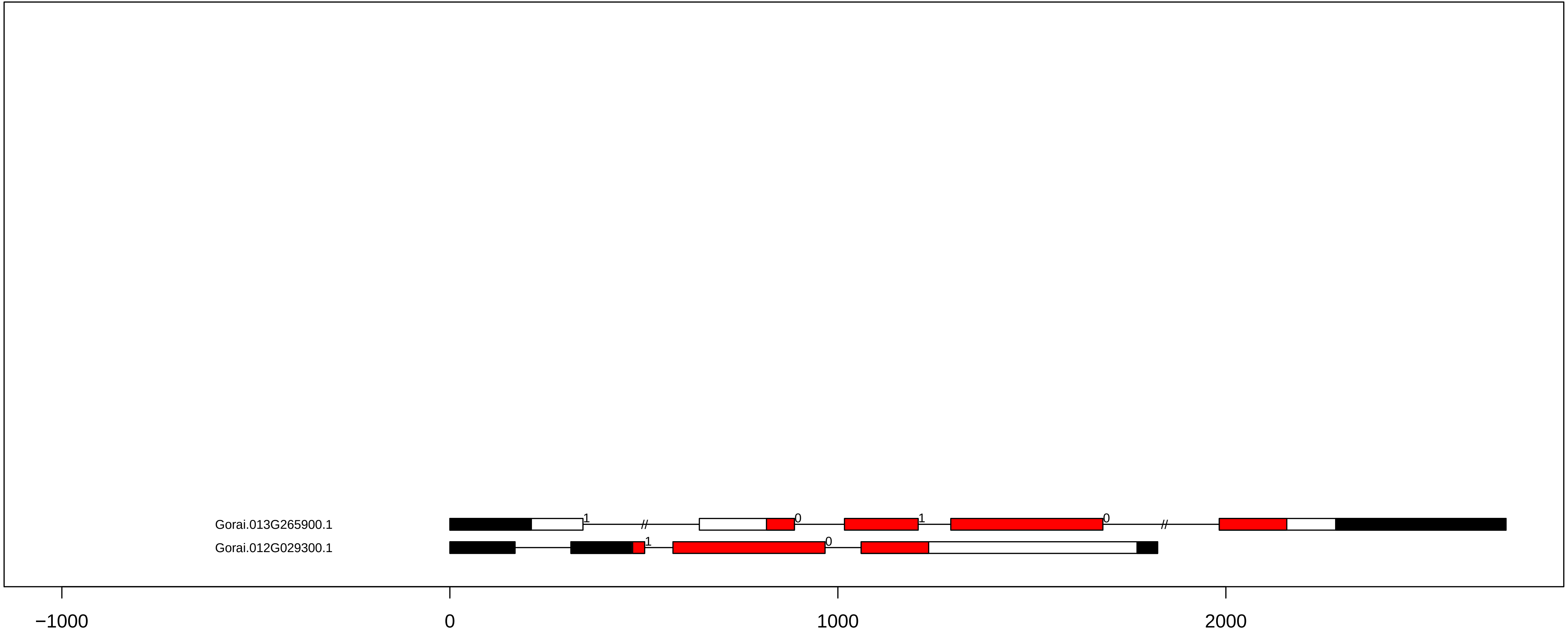

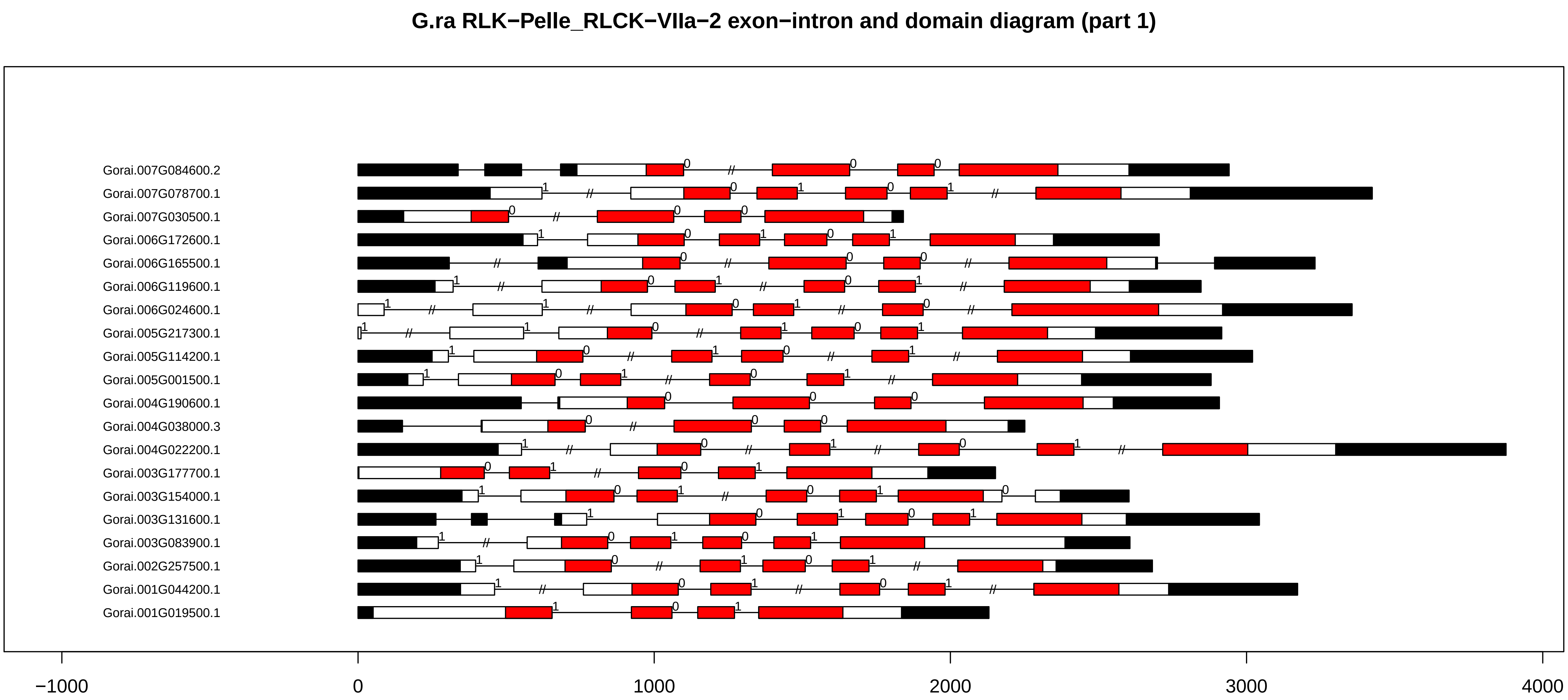

G.ra RLK−Pelle_RLCK−VIIa−2 exon−intron and domain diagram (part 1)
Gorai.007G084600.2
Gorai.007G078700.1
Gorai.007G030500.1
Gorai.006G172600.1
Gorai.006G165500.1
Gorai.006G119600.1
Gorai.006G024600.1
Gorai.005G217300.1
Gorai.005G114200.1
Gorai.005G001500.1
Gorai.004G190600.1
Gorai.004G038000.3
Gorai.004G022200.1
Gorai.003G177700.1
Gorai.003G154000.1
Gorai.003G131600.1
Gorai.003G083900.1
Gorai.002G257500.1
Gorai.001G044200.1
Gorai.001G019500.1
−1000
0
1000
2000
3000
4000

G.ra RLK−Pelle_RLCK−VIIa−2 exon−intron and domain diagram (part 2)

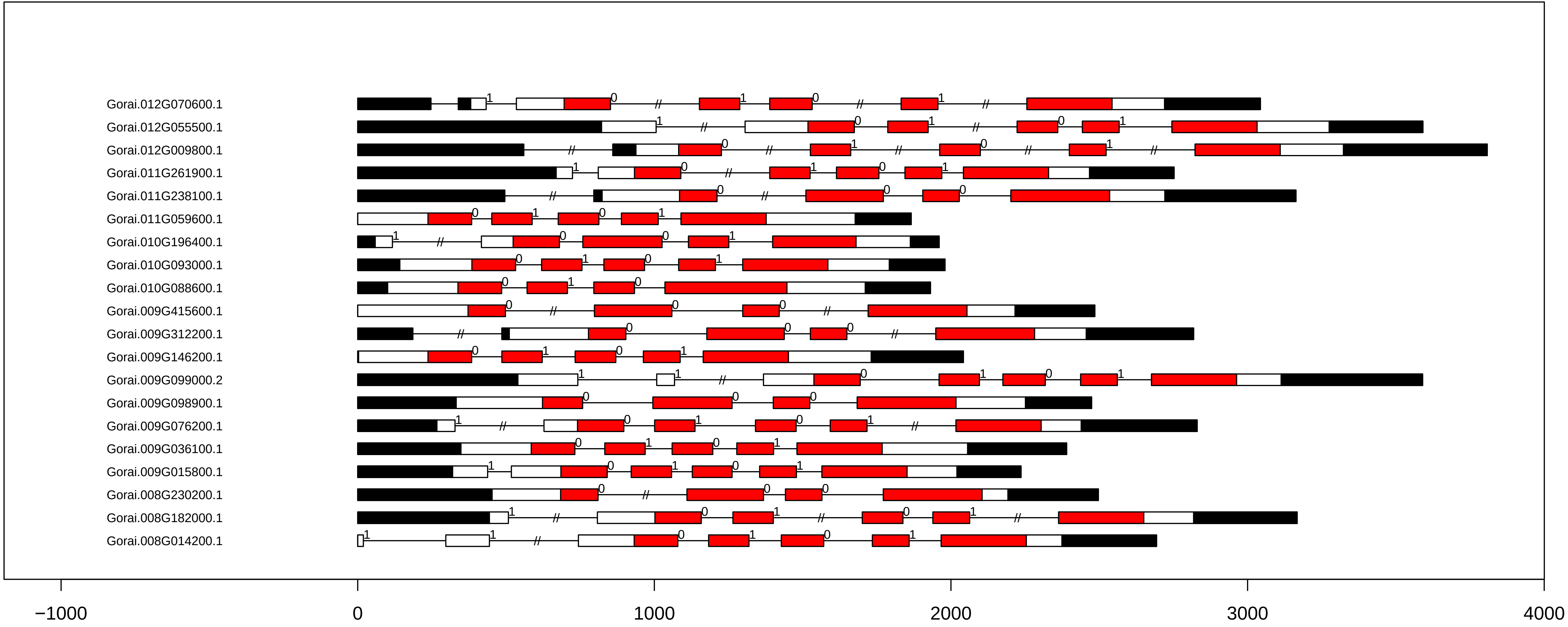

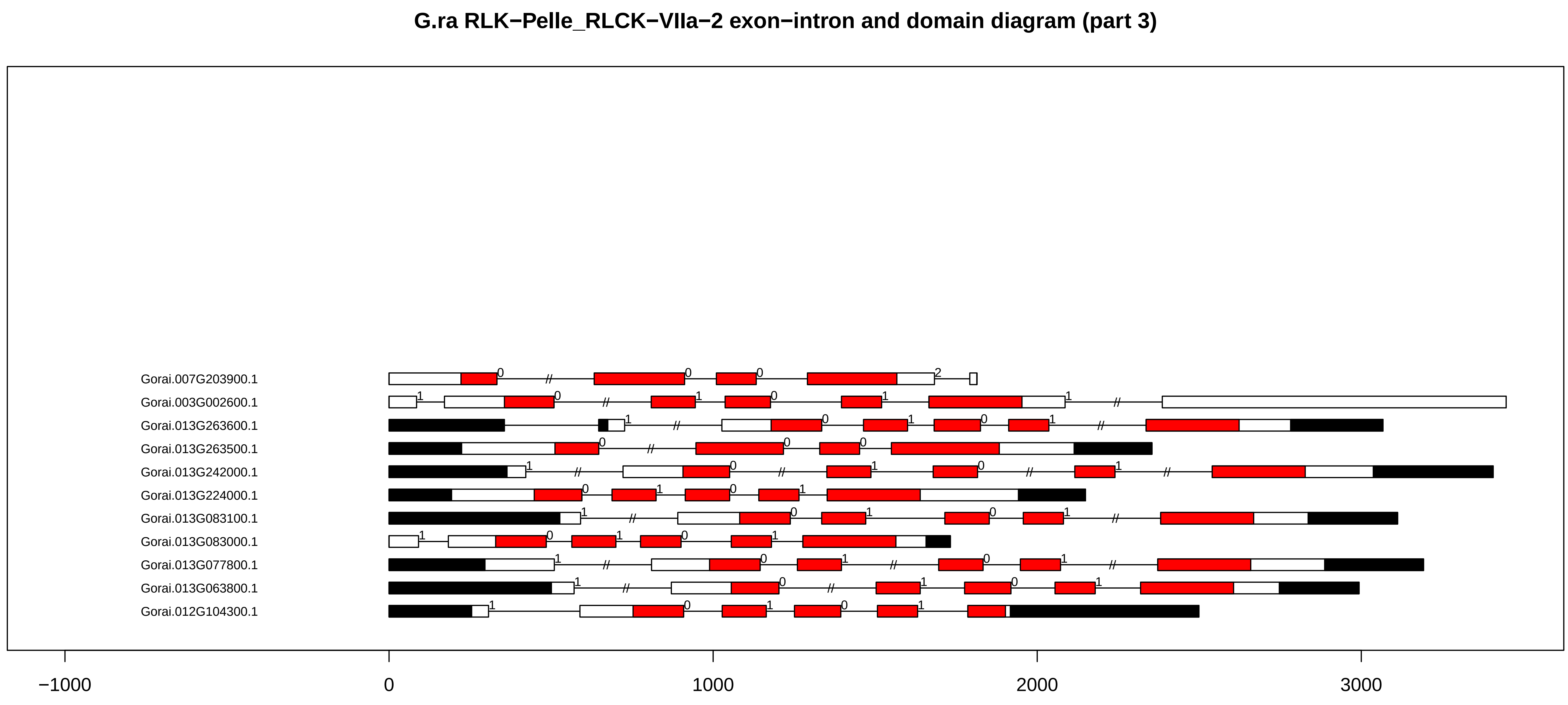
G.ra RLK−Pelle_RLCK−VIIa−2 exon−intron and domain diagram (part 3)
Gorai.007G203900.1
Gorai.003G002600.1
Gorai.013G263600.1
Gorai.013G263500.1
Gorai.013G242000.1
Gorai.013G224000.1
Gorai.013G083100.1
Gorai.013G083000.1
Gorai.013G077800.1
Gorai.013G063800.1
Gorai.012G104300.1
−1000
0
1000
2000
3000

G.ra RLK−Pelle_RLCK−VIIb exon−intron and domain diagram (all)

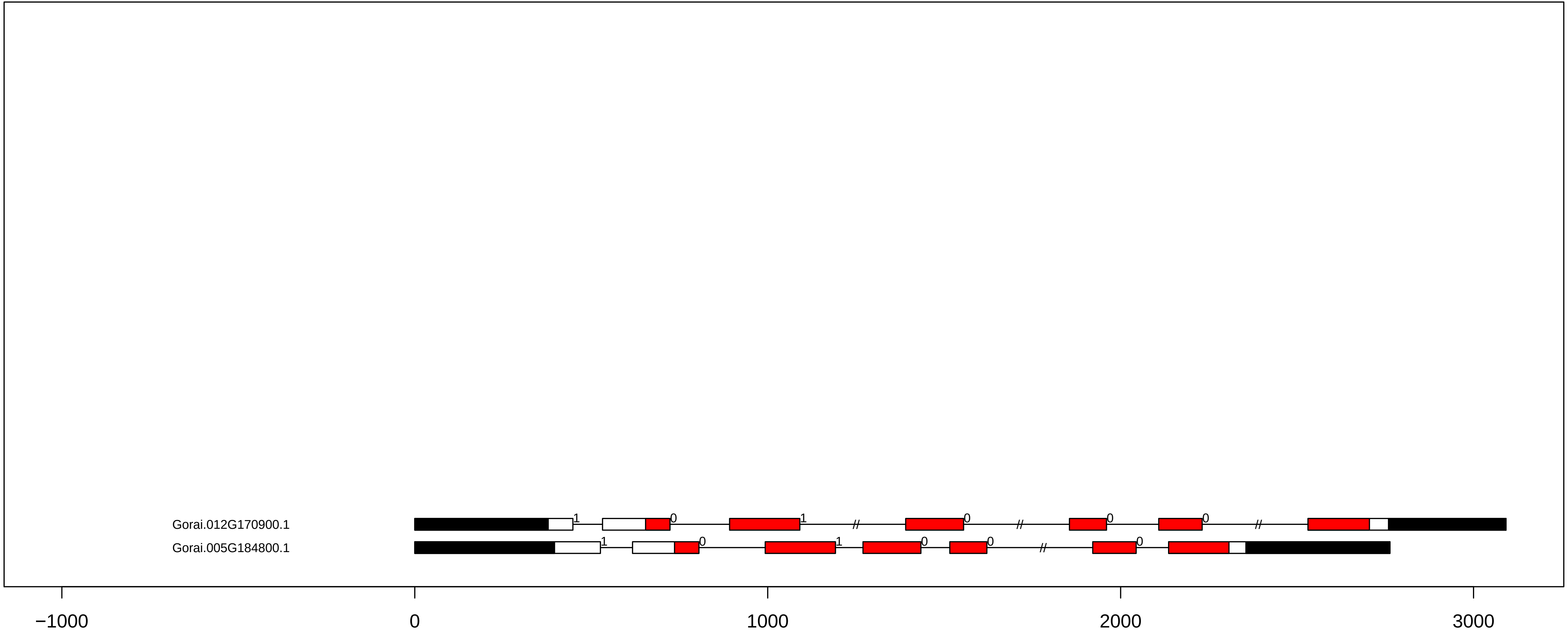

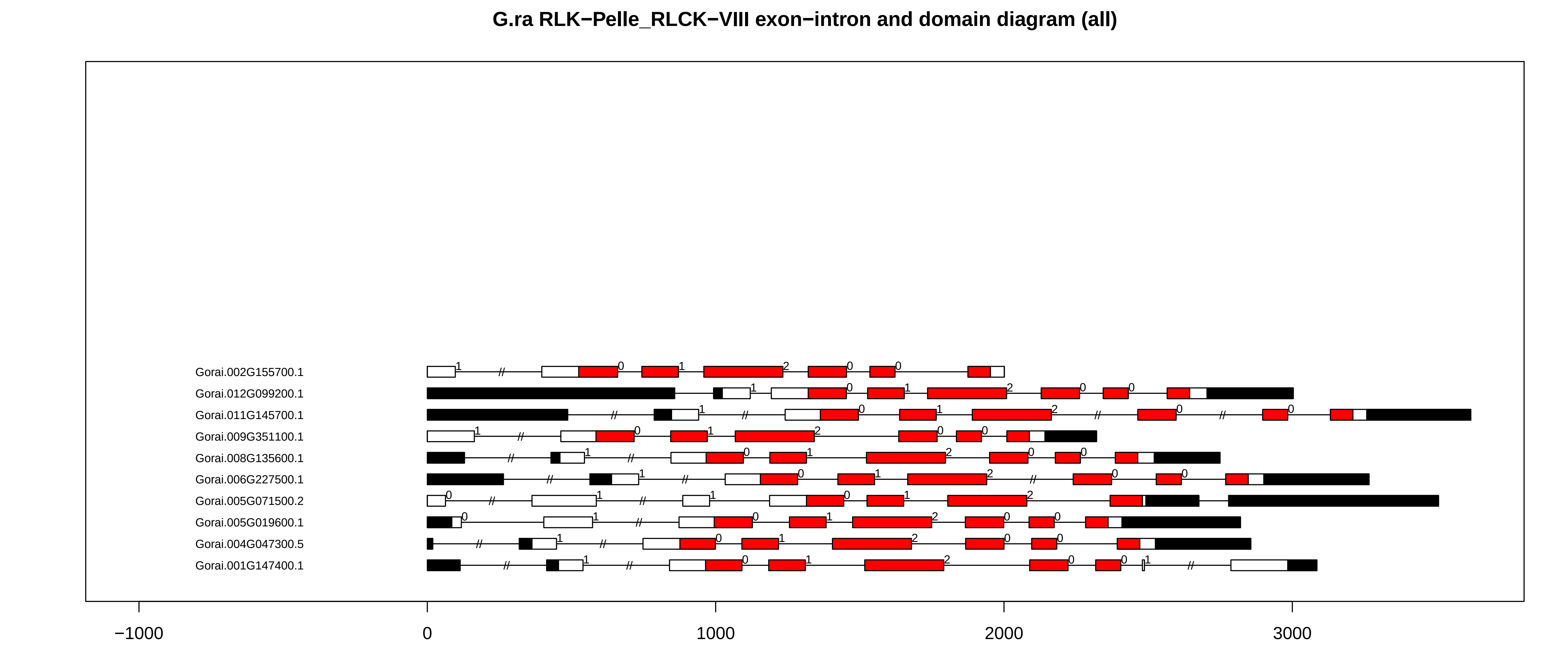

G.ra RLK−Pelle_RLCK−VIII exon−intron and domain diagram (all)
Gorai.002G155700.1
Gorai.012G099200.1
Gorai.011G145700.1
Gorai.009G351100.1
Gorai.008G135600.1
Gorai.006G227500.1
Gorai.005G071500.2
Gorai.005G019600.1
Gorai.004G047300.5
Gorai.001G147400.1
−1000
0
1000
2000
3000

G.ra RLK−Pelle_RLCK−VI exon−intron and domain diagram (part 1)

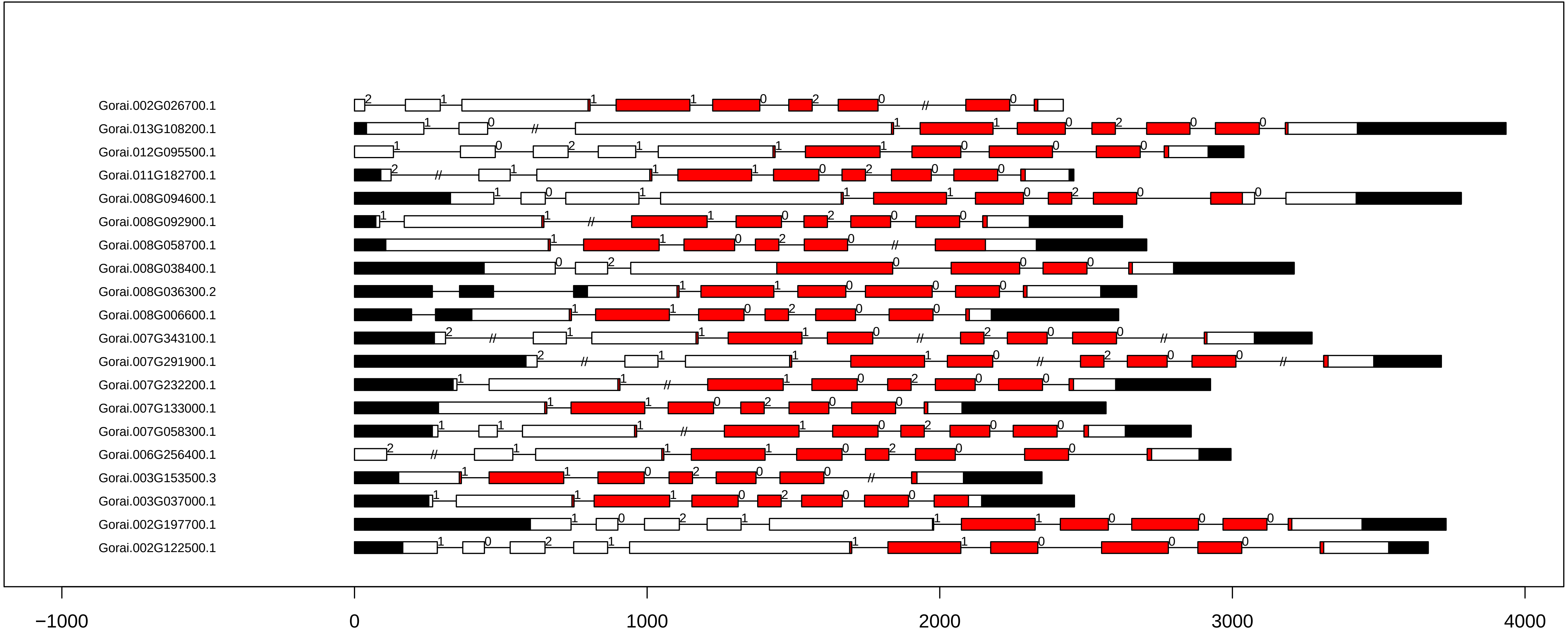

G.ra RLK−Pelle_RLCK−VI exon−intron and domain diagram (part 2)

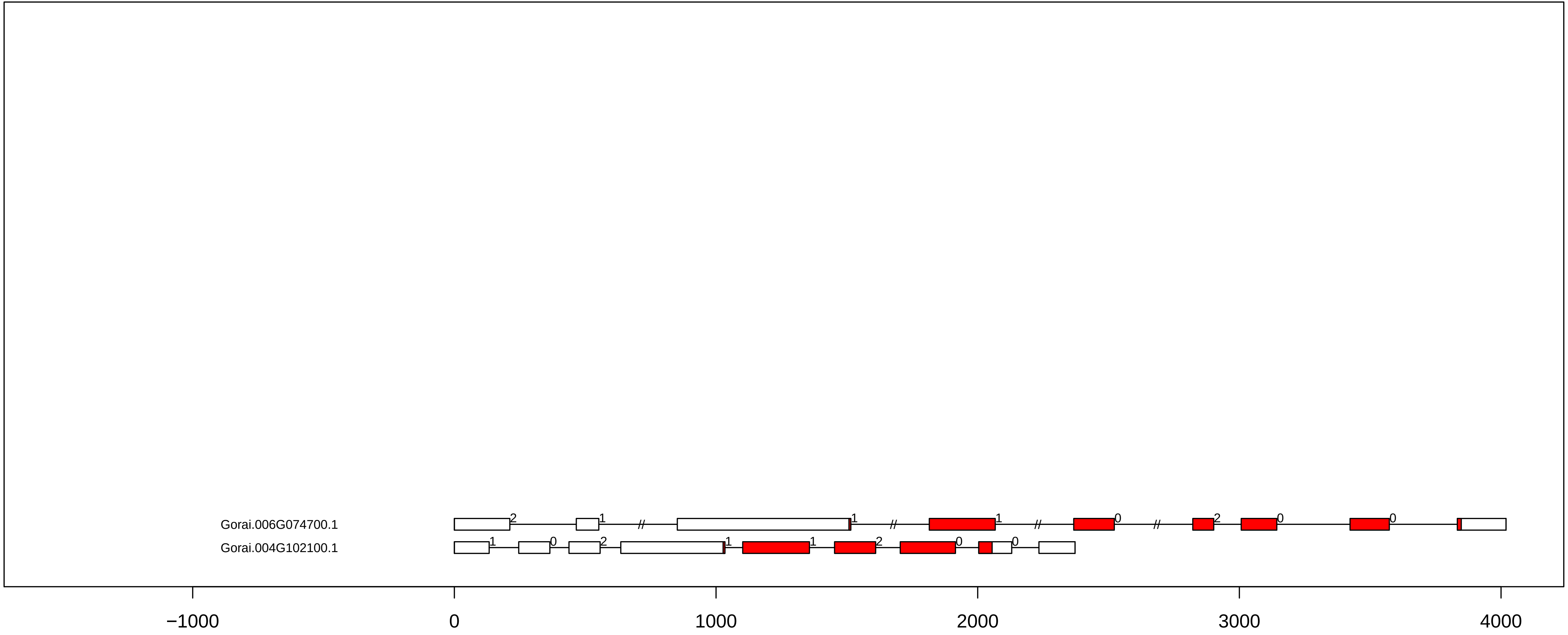

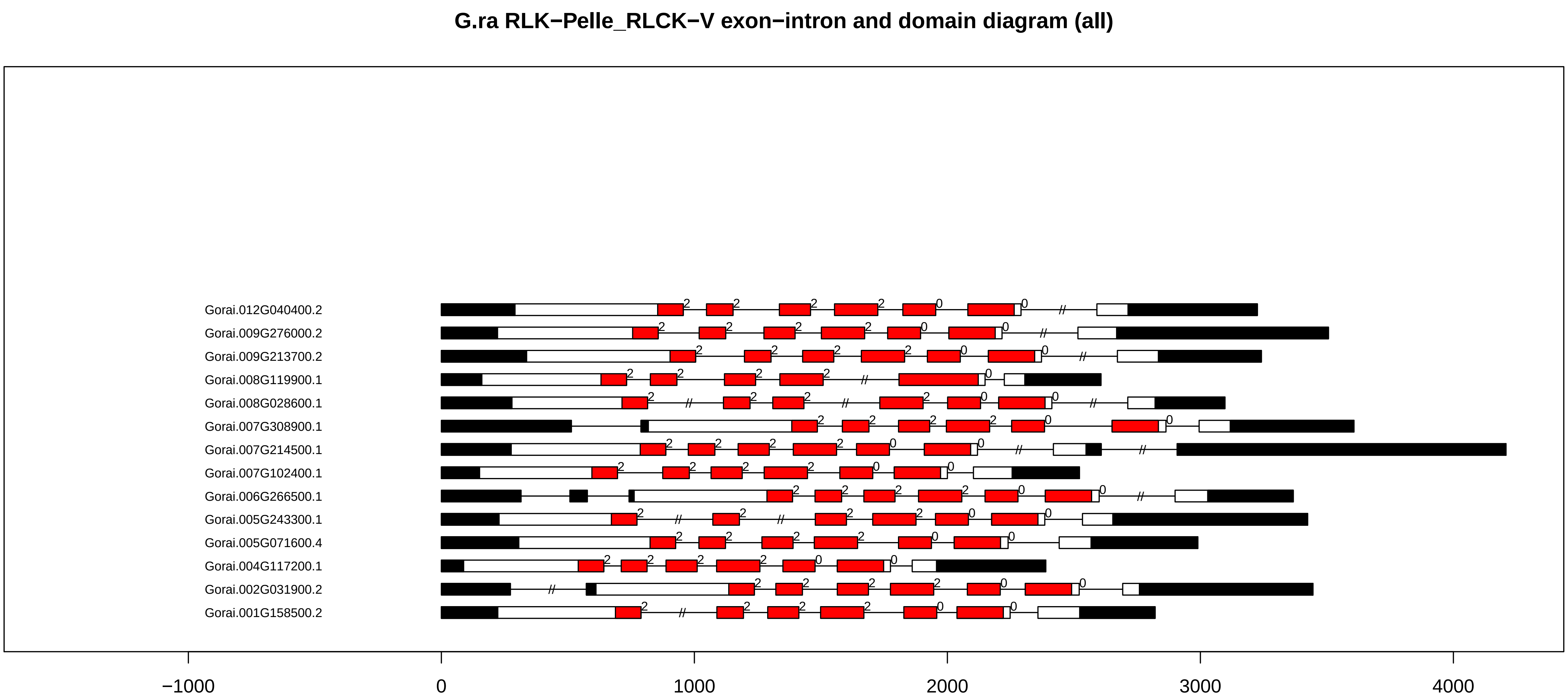

G.ra RLK−Pelle_RLCK−V exon−intron and domain diagram (all)
Gorai.012G040400.2
Gorai.009G276000.2
Gorai.009G213700.2
Gorai.008G119900.1
Gorai.008G028600.1
Gorai.007G308900.1
Gorai.007G214500.1
Gorai.007G102400.1
Gorai.006G266500.1
Gorai.005G243300.1
Gorai.005G071600.4
Gorai.004G117200.1
Gorai.002G031900.2
Gorai.001G158500.2
−1000
0
1000
2000
3000
4000

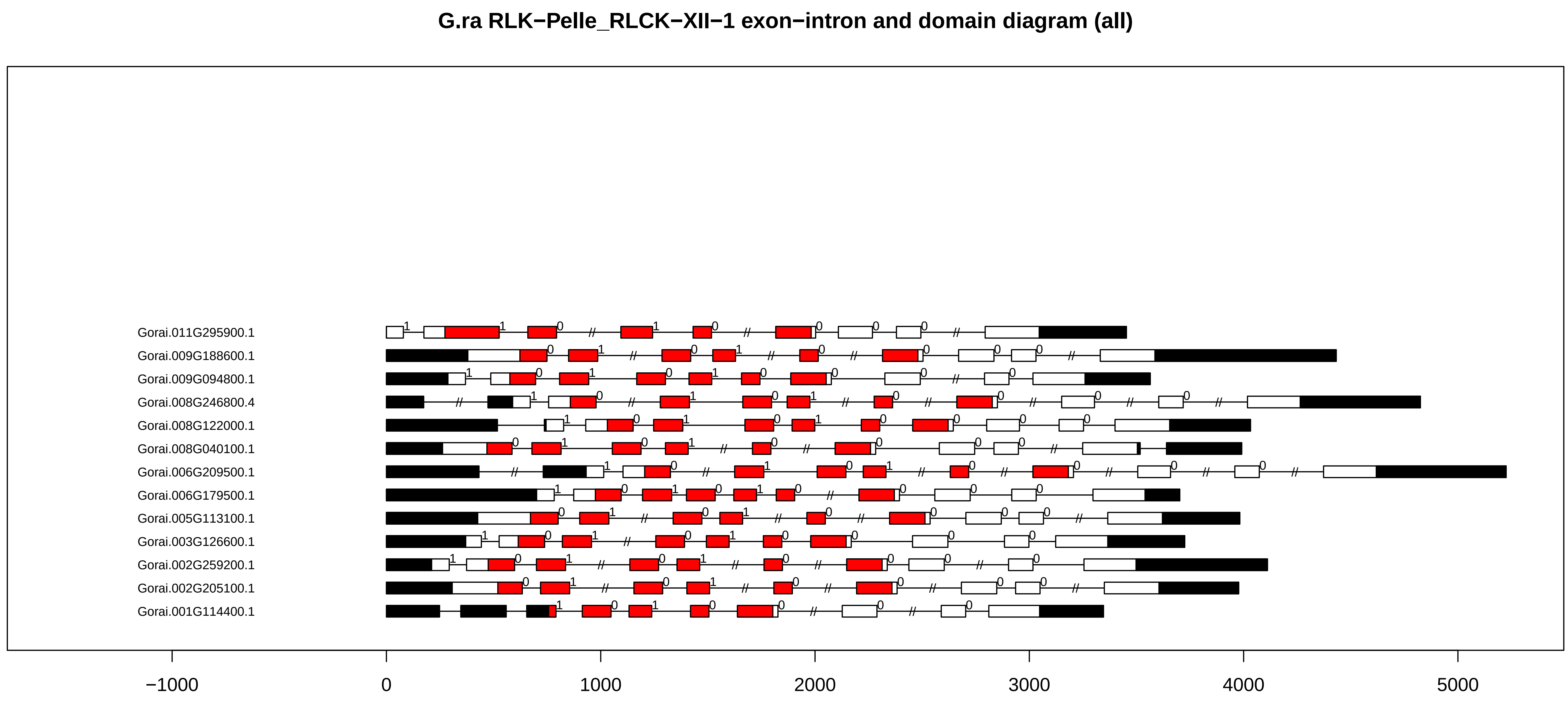

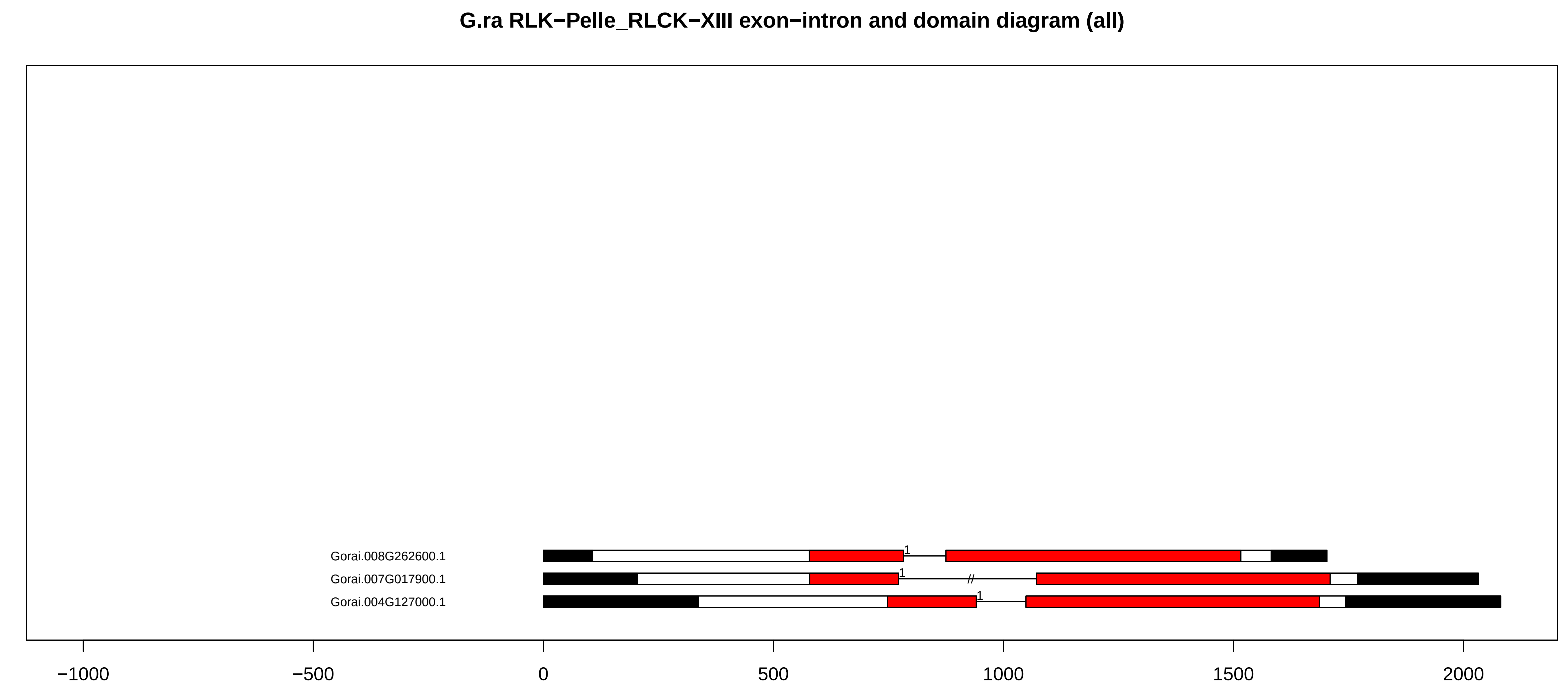
G.ra RLK−Pelle_RLCK−XIII exon−intron and domain diagram (all)
Gorai.008G262600.1
Gorai.007G017900.1
Gorai.004G127000.1
1
1
1
−1000
−500
0
500
1000
1500
2000

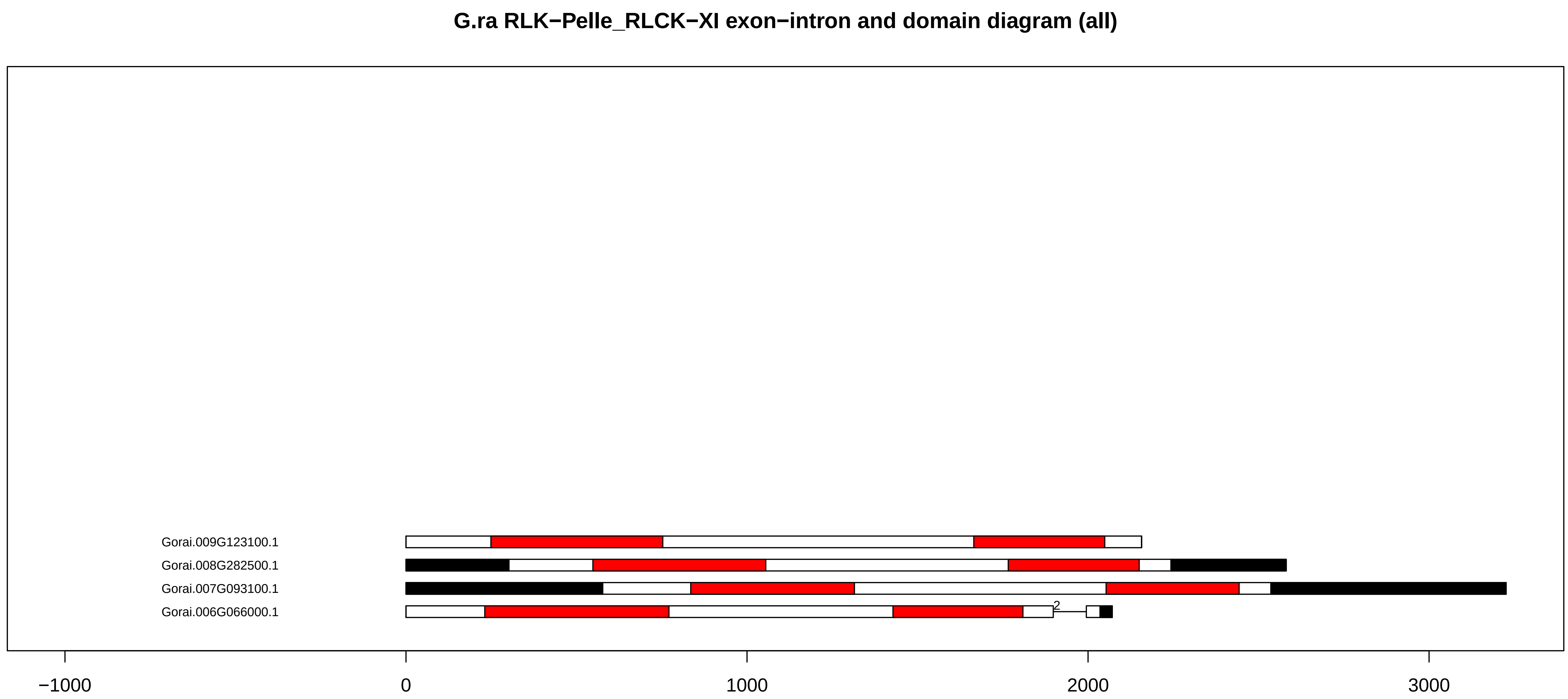

G.ra RLK−Pelle_RLCK−XI exon−intron and domain diagram (all)
Gorai.009G123100.1
Gorai.008G282500.1
Gorai.007G093100.1
Gorai.006G066000.1
2
−1000
0
1000
2000
3000

G.ra RLK−Pelle_RLCK−X exon−intron and domain diagram (all)

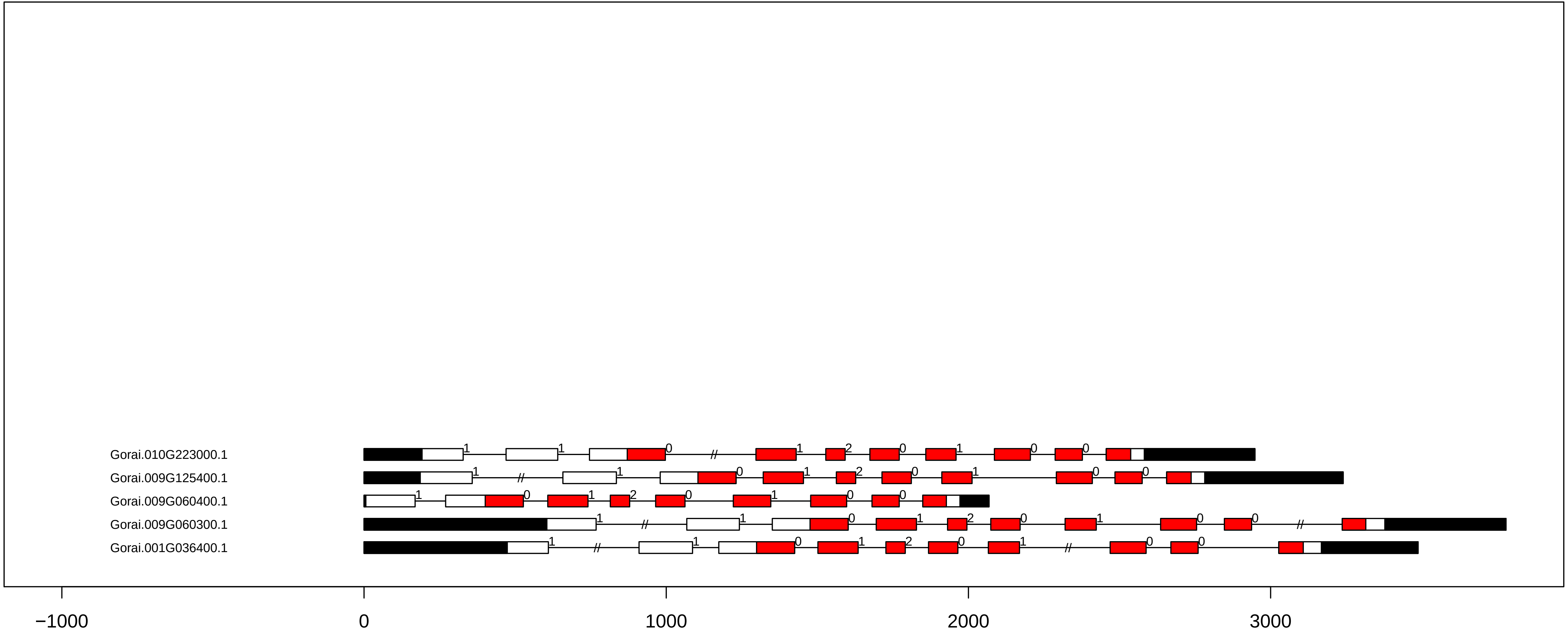

G.ra RLK−Pelle_RLCK−XVI exon−intron and domain diagram (all)

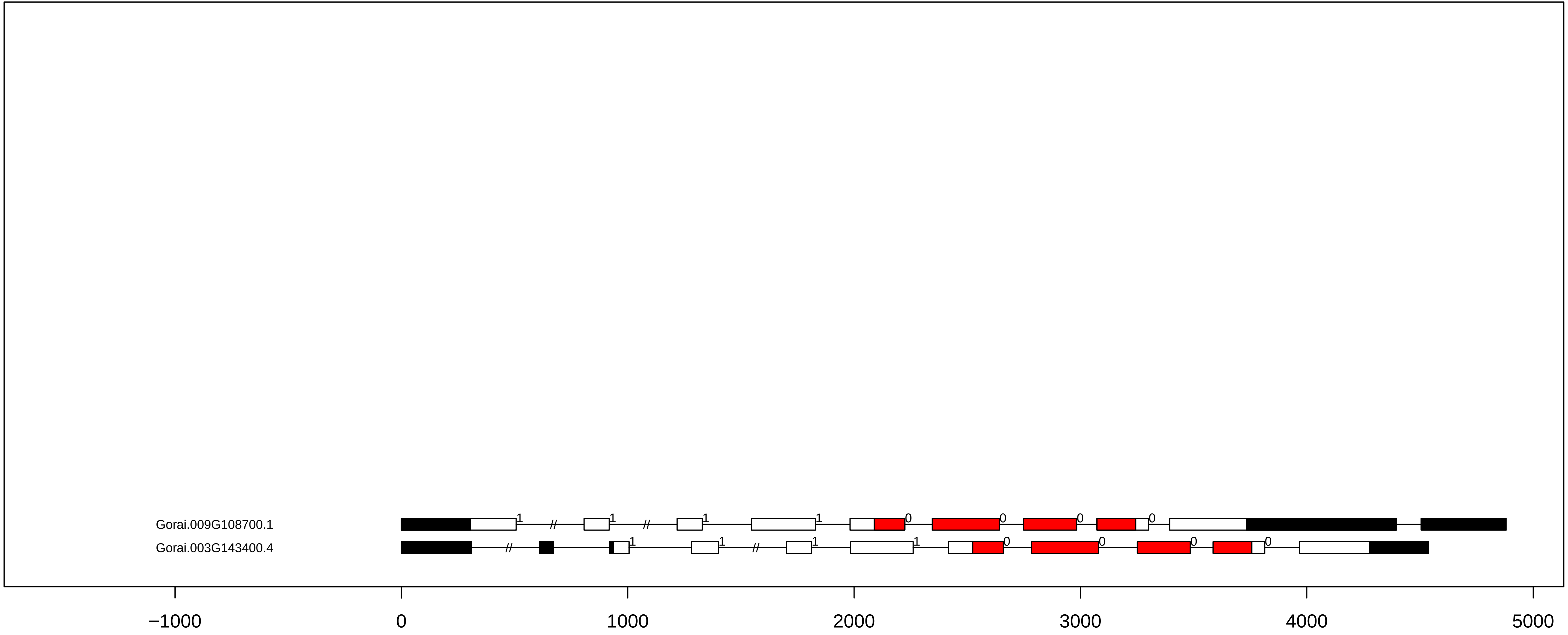

G.ra RLK−Pelle_RLCK−XV exon−intron and domain diagram (all)

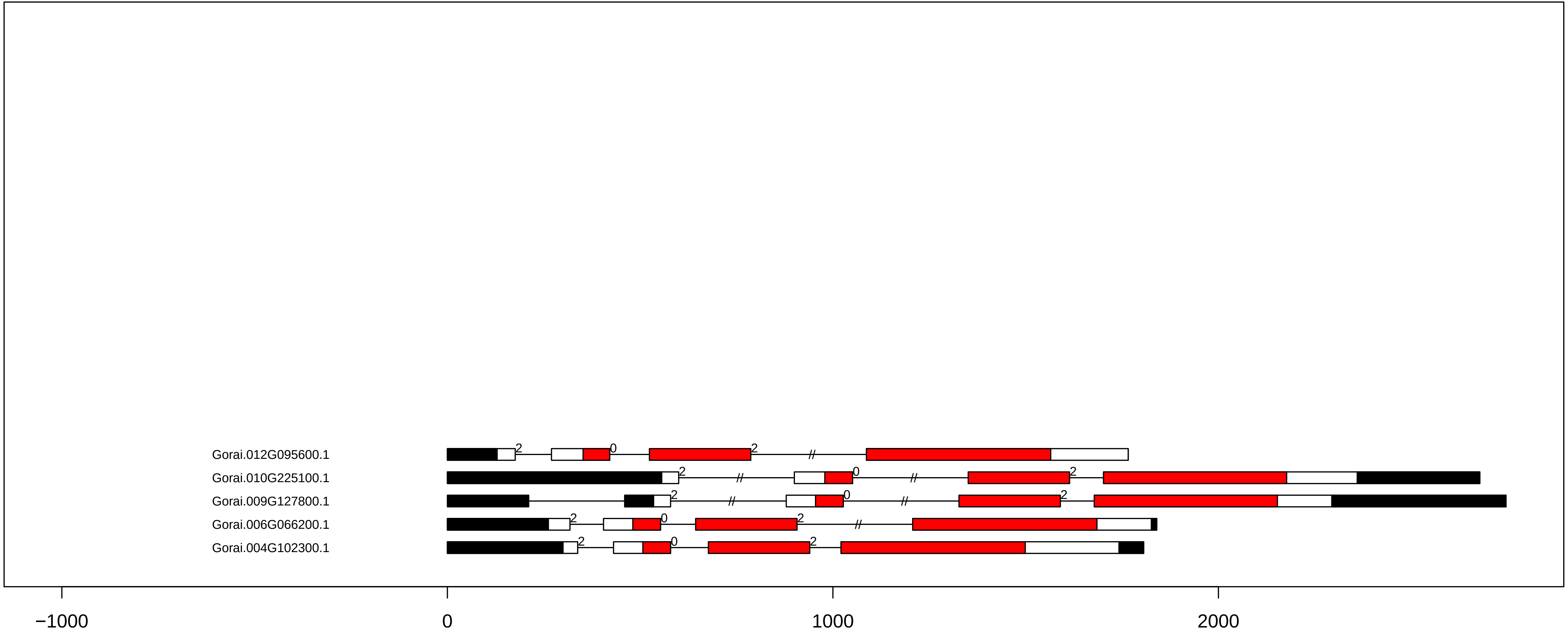

G.ra RLK−Pelle_SD−2b exon−intron and domain diagram (part 1)

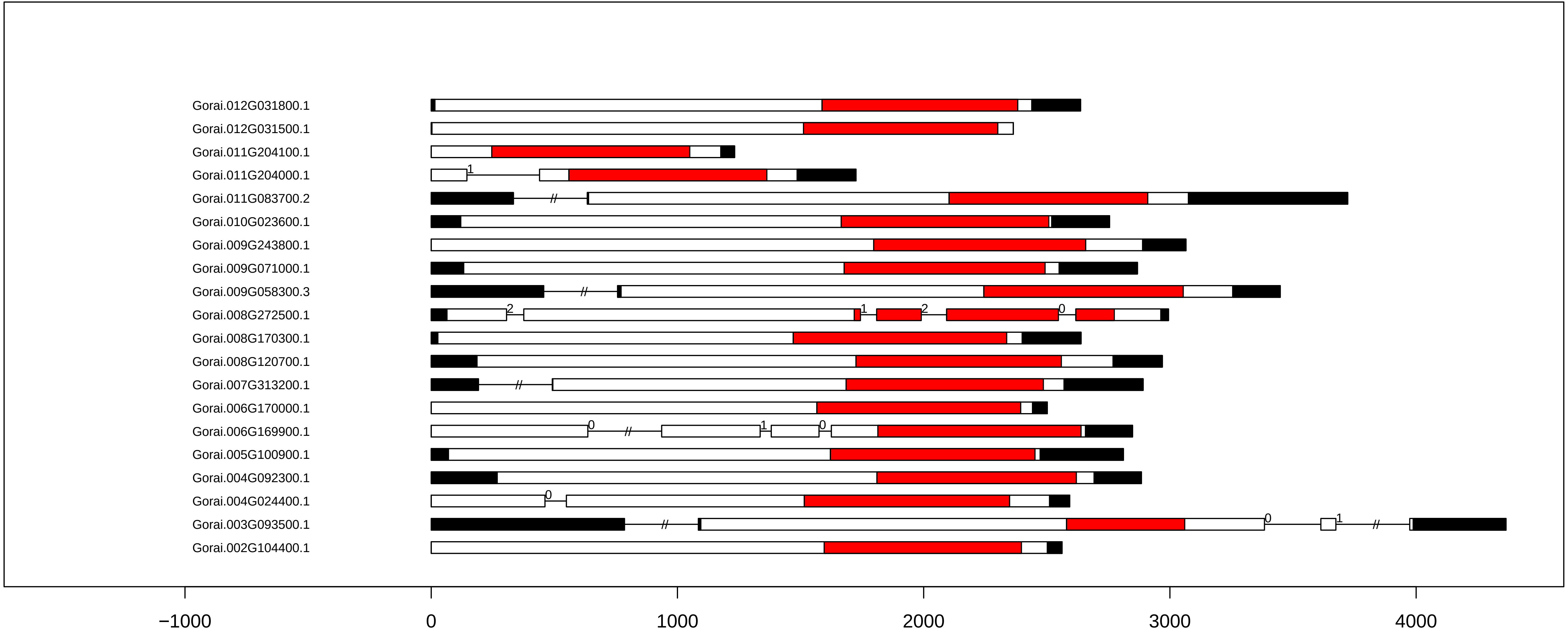

G.ra RLK−Pelle_SD−2b exon−intron and domain diagram (part 2)

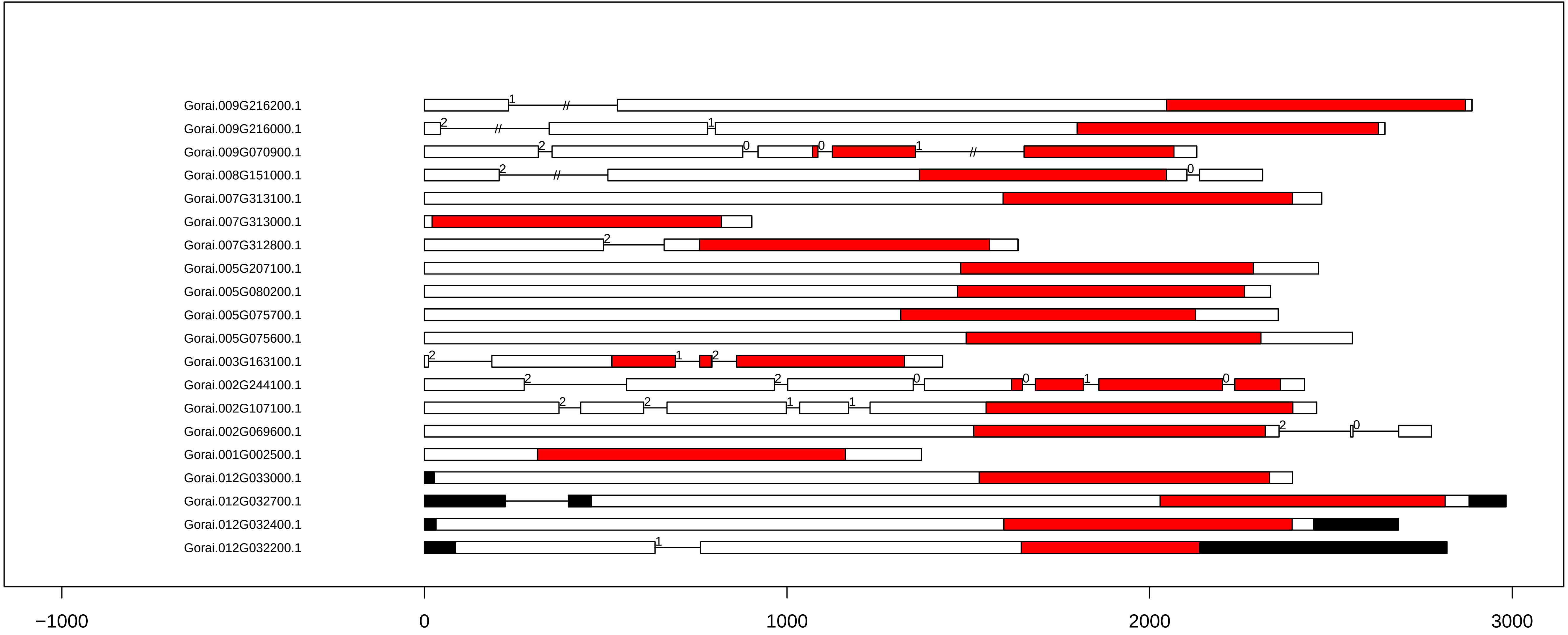

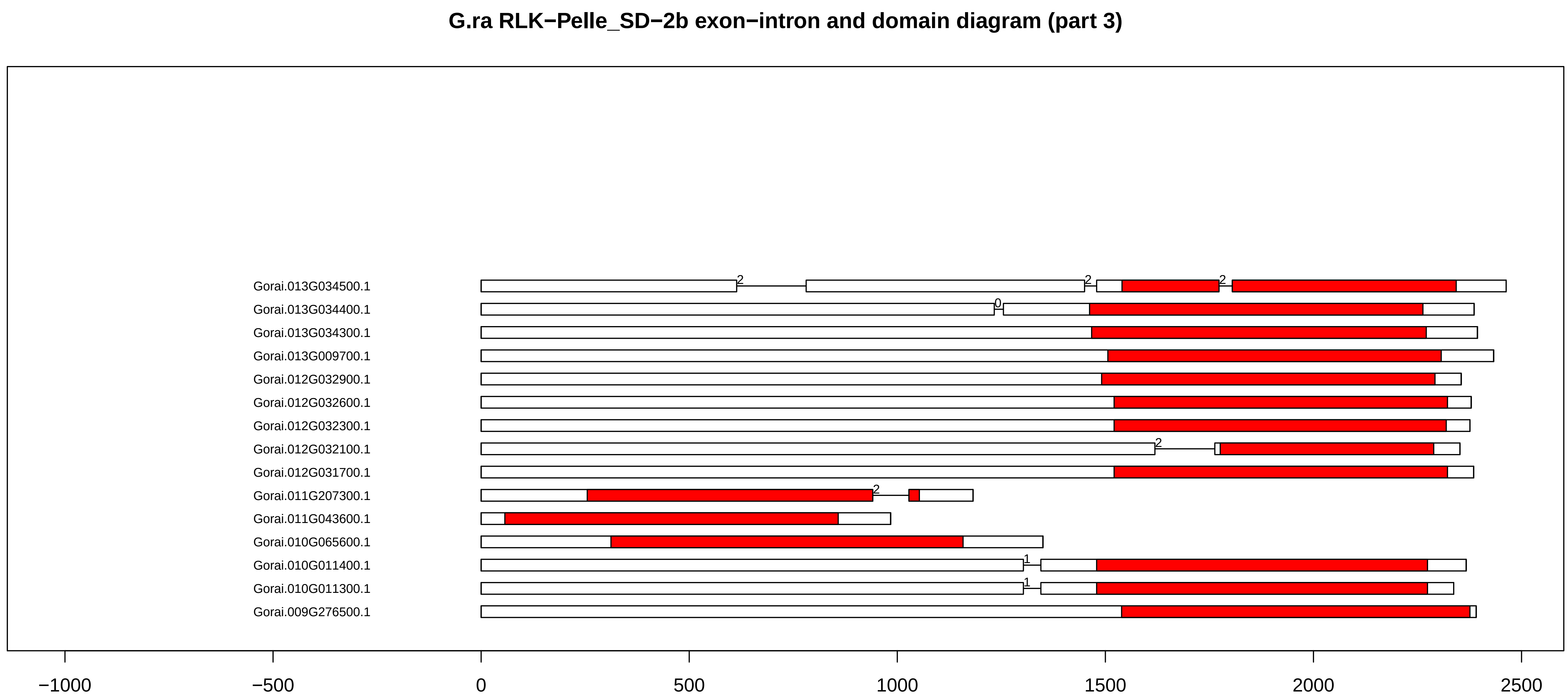
G.ra RLK−Pelle_SD−2b exon−intron and domain diagram (part 3)
Gorai.013G034500.1
Gorai.013G034400.1
Gorai.013G034300.1
Gorai.013G009700.1
Gorai.012G032900.1
Gorai.012G032600.1
Gorai.012G032300.1
Gorai.012G032100.1
Gorai.012G031700.1
Gorai.011G207300.1
Gorai.011G043600.1
Gorai.010G065600.1
Gorai.010G011400.1
Gorai.010G011300.1
Gorai.009G276500.1
−1000
−500
0
500
1000
1500
2000
2500

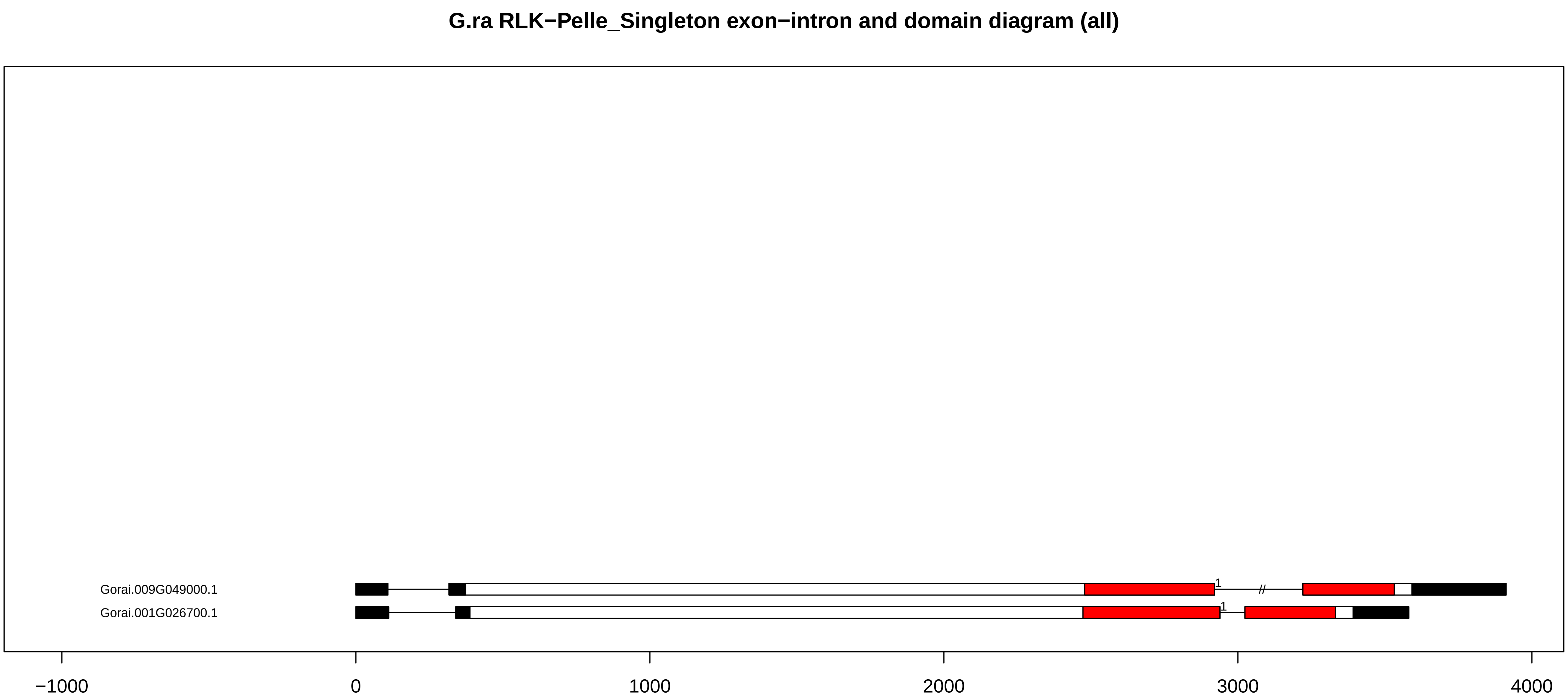
G.ra RLK−Pelle_Singleton exon−intron and domain diagram (all)
Gorai.009G049000.1
Gorai.001G026700.1
1
1
−1000
0
1000
2000
3000
4000

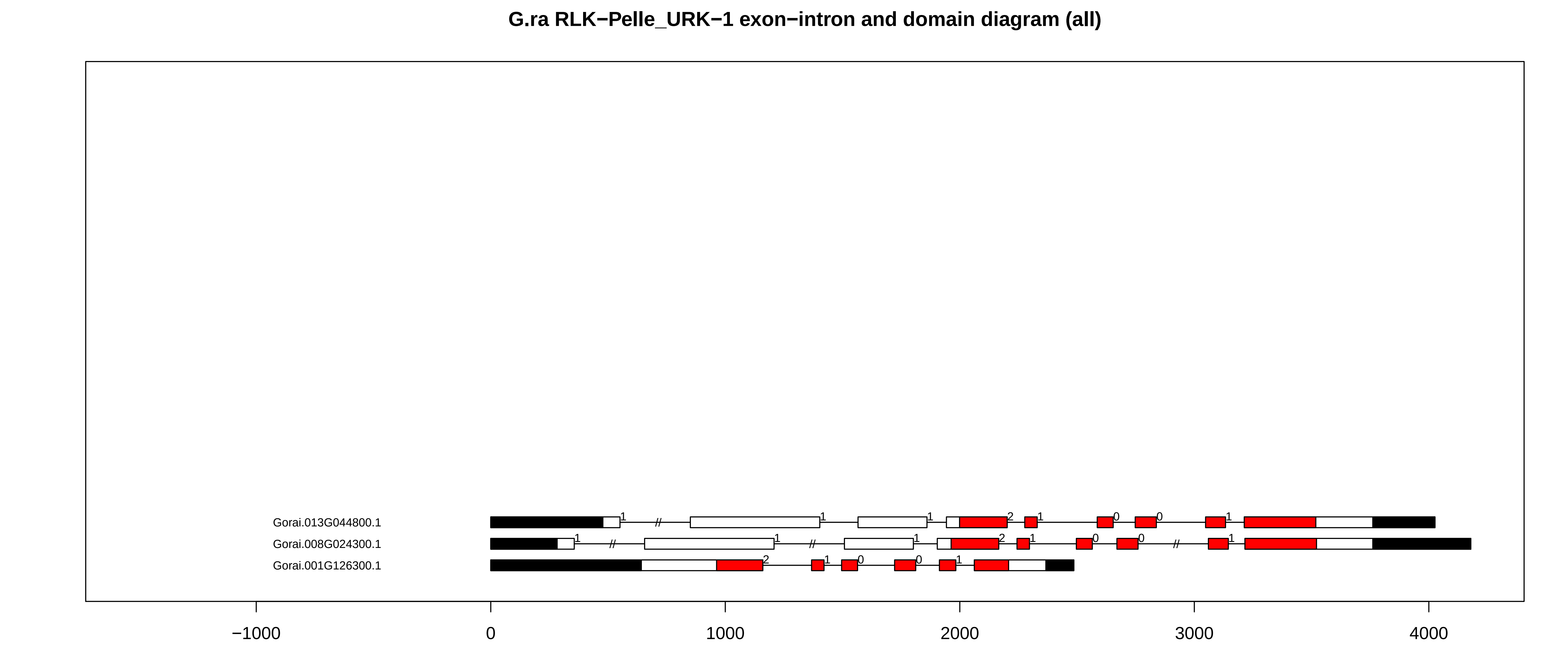
G.ra RLK−Pelle_URK−1 exon−intron and domain diagram (all)
Gorai.013G044800.1
Gorai.008G024300.1
Gorai.001G126300.1
−1000
0
1000
2000
3000
4000

G.ra RLK−Pelle_URK−2 exon−intron and domain diagram (all)

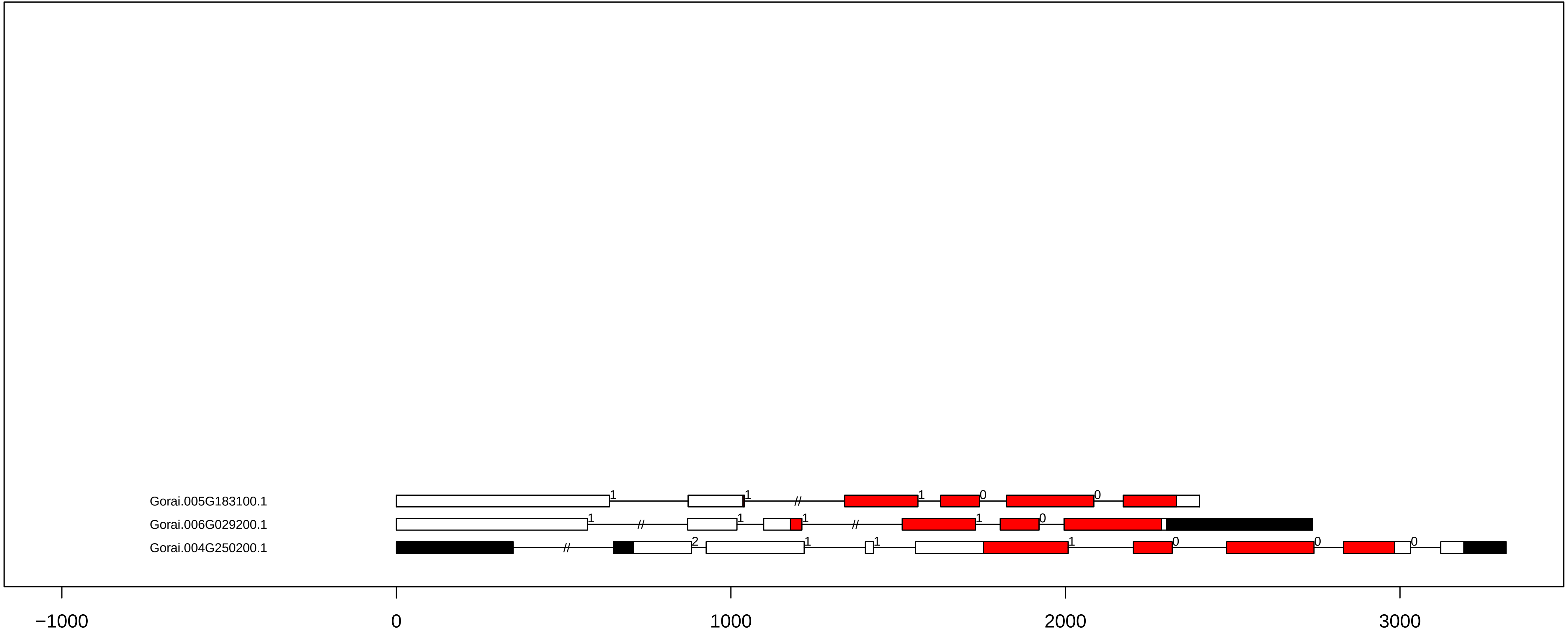

G.ra RLK−Pelle_URK−3 exon−intron and domain diagram (all)

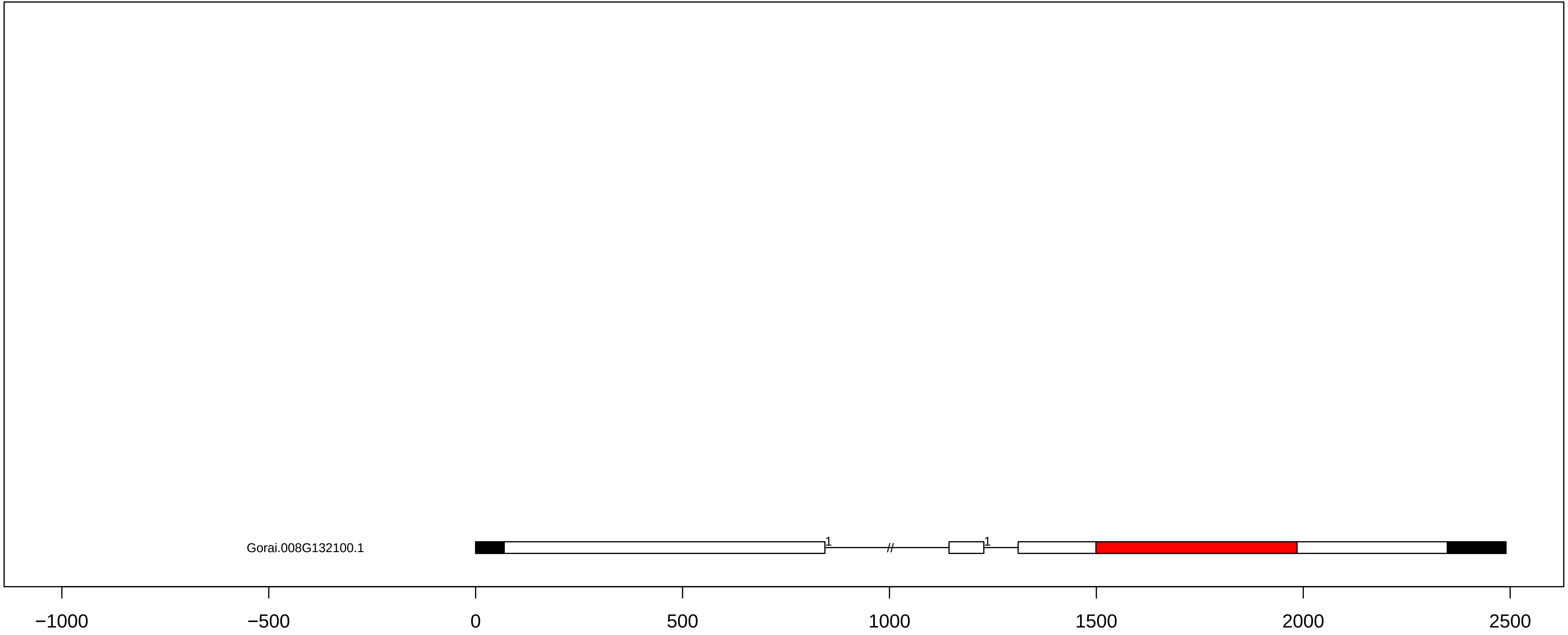

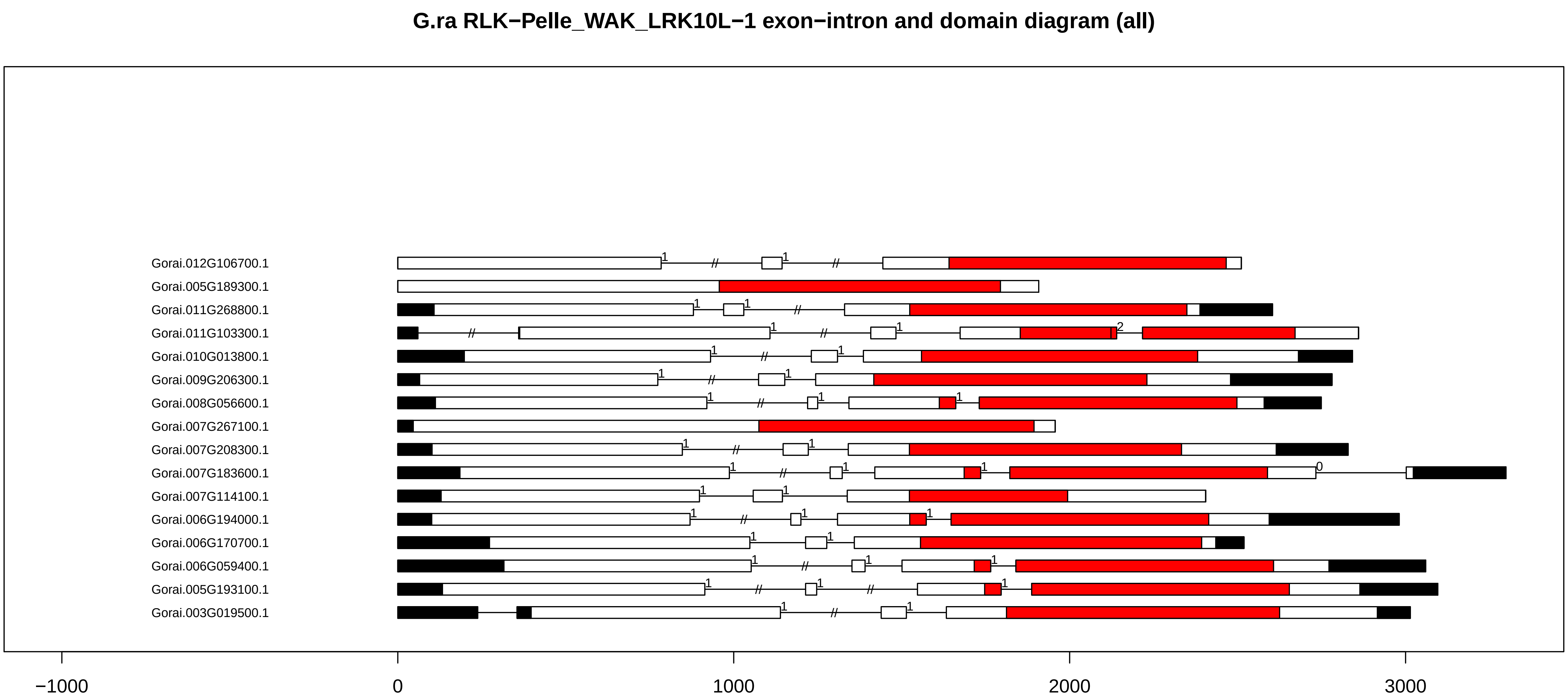

G.ra RLK−Pelle_WAK_LRK10L−1 exon−intron and domain diagram (all)
Gorai.012G106700.1
Gorai.005G189300.1
Gorai.011G268800.1
Gorai.011G103300.1
Gorai.010G013800.1
Gorai.009G206300.1
Gorai.008G056600.1
Gorai.007G267100.1
Gorai.007G208300.1
Gorai.007G183600.1
Gorai.007G114100.1
Gorai.006G194000.1
Gorai.006G170700.1
Gorai.006G059400.1
Gorai.005G193100.1
Gorai.003G019500.1
−1000
0
1000
2000
3000

G.ra RLK−Pelle_WAK exon−intron and domain diagram (part 1)

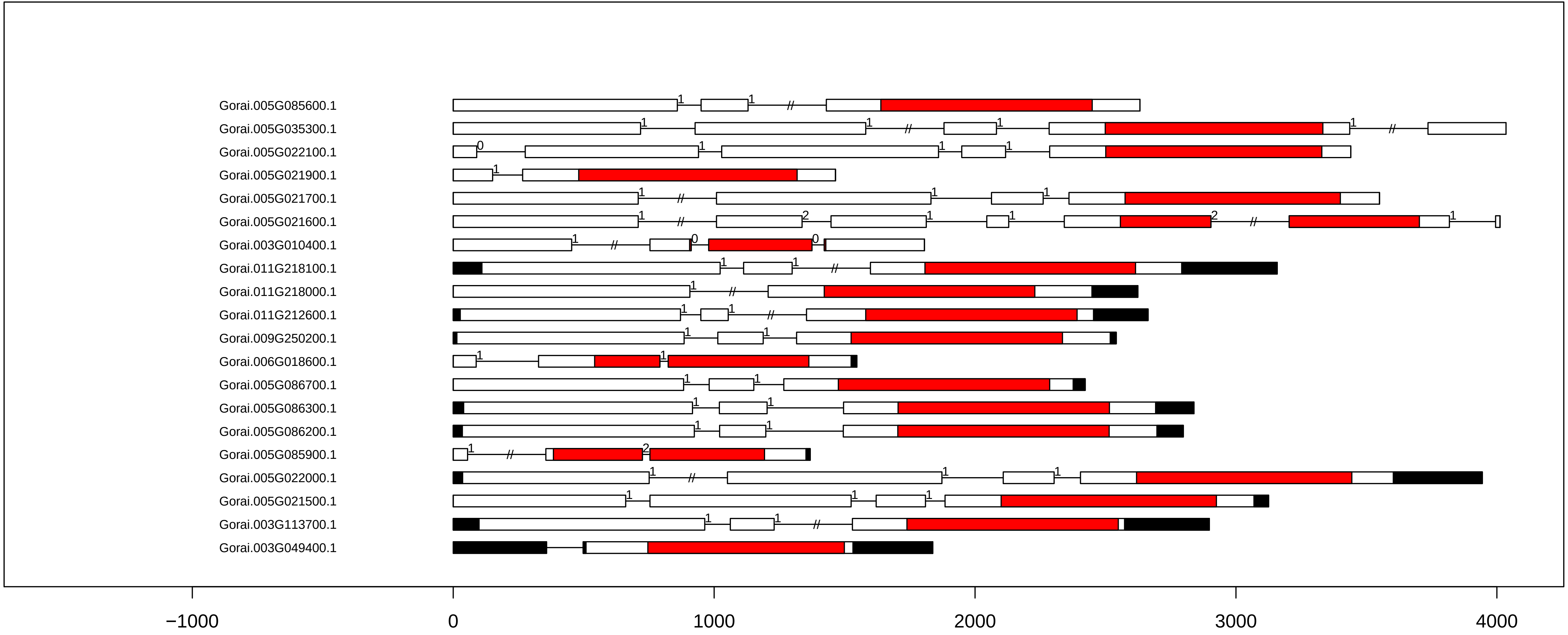

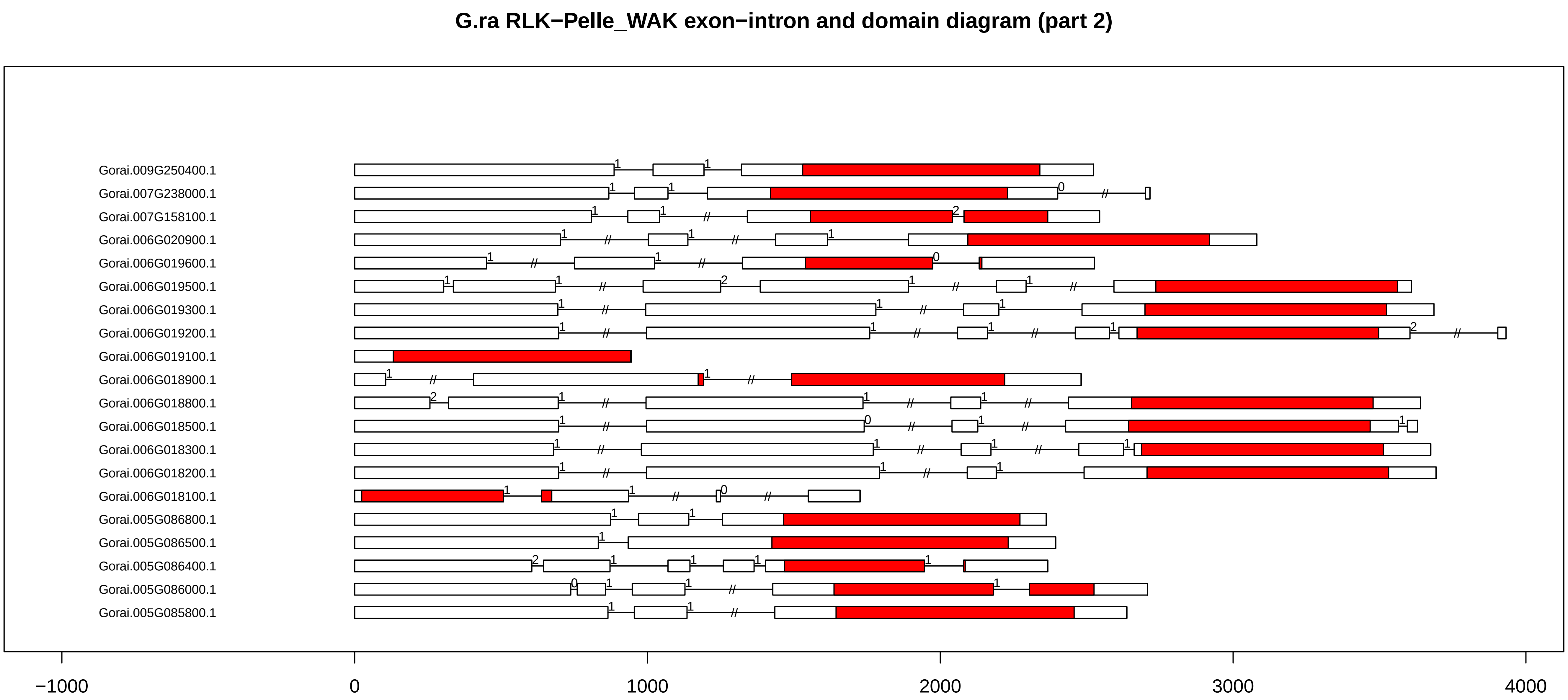
G.ra RLK−Pelle_WAK exon−intron and domain diagram (part 2)
Gorai.009G250400.1
Gorai.007G238000.1
Gorai.007G158100.1
Gorai.006G020900.1
Gorai.006G019600.1
Gorai.006G019500.1
Gorai.006G019300.1
Gorai.006G019200.1
Gorai.006G019100.1
Gorai.006G018900.1
Gorai.006G018800.1
Gorai.006G018500.1
Gorai.006G018300.1
Gorai.006G018200.1
Gorai.006G018100.1
Gorai.005G086800.1
Gorai.005G086500.1
Gorai.005G086400.1
Gorai.005G086000.1
Gorai.005G085800.1
−1000
0
1000
2000
3000
4000

G.ra RLK−Pelle_WAK exon−intron and domain diagram (part 3)

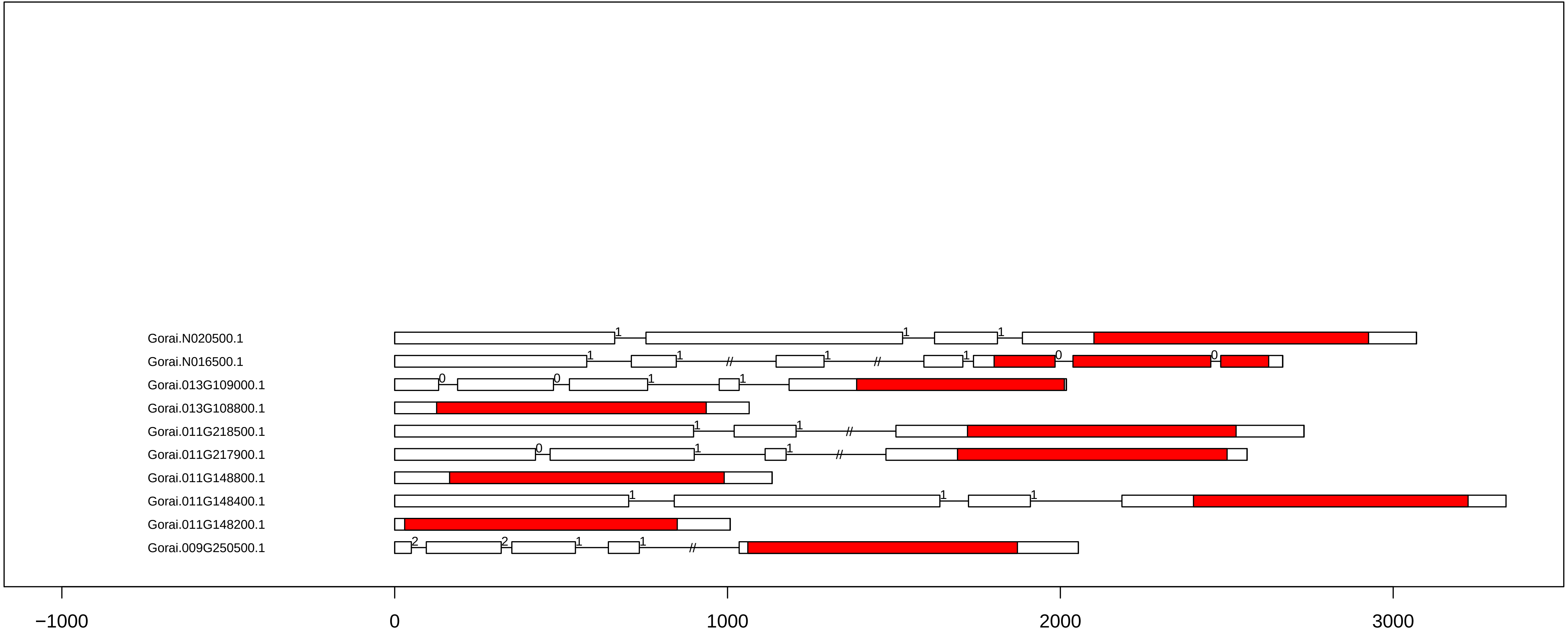

G.ra SCY1_SCYL1 exon−intron and domain diagram (all)

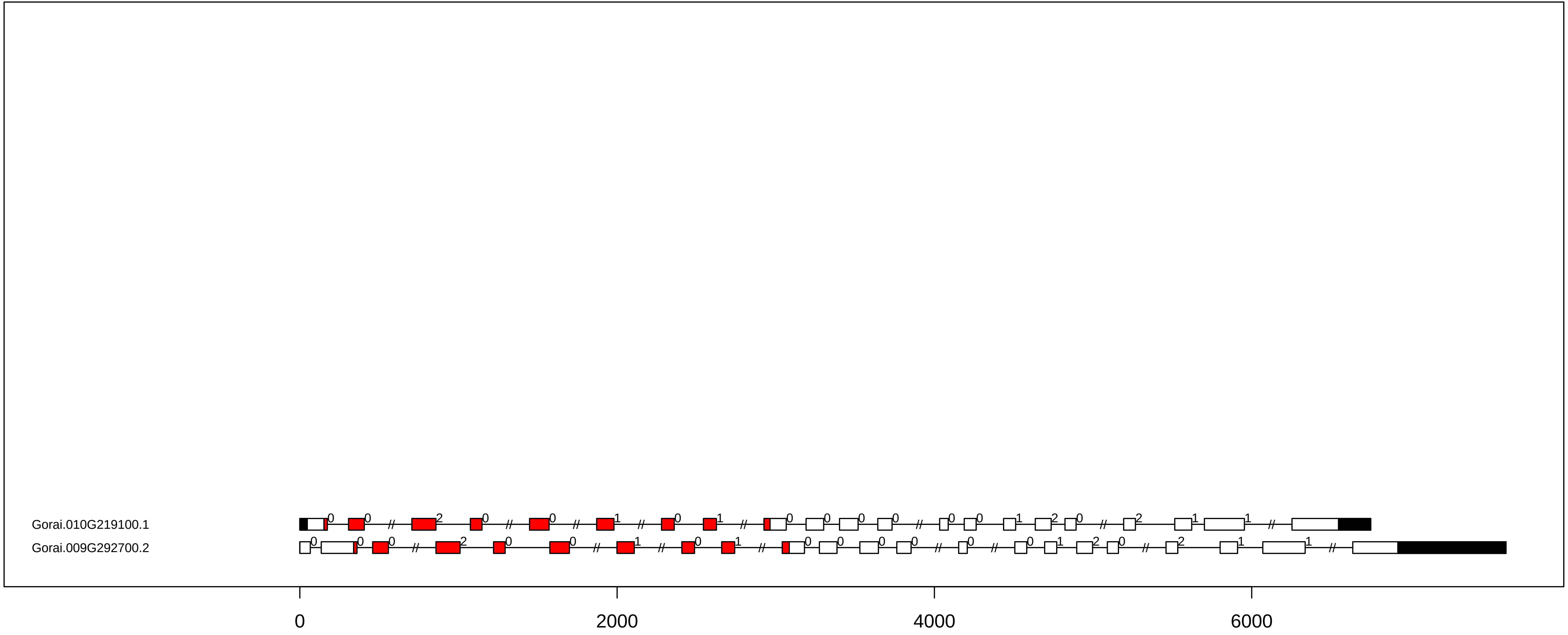

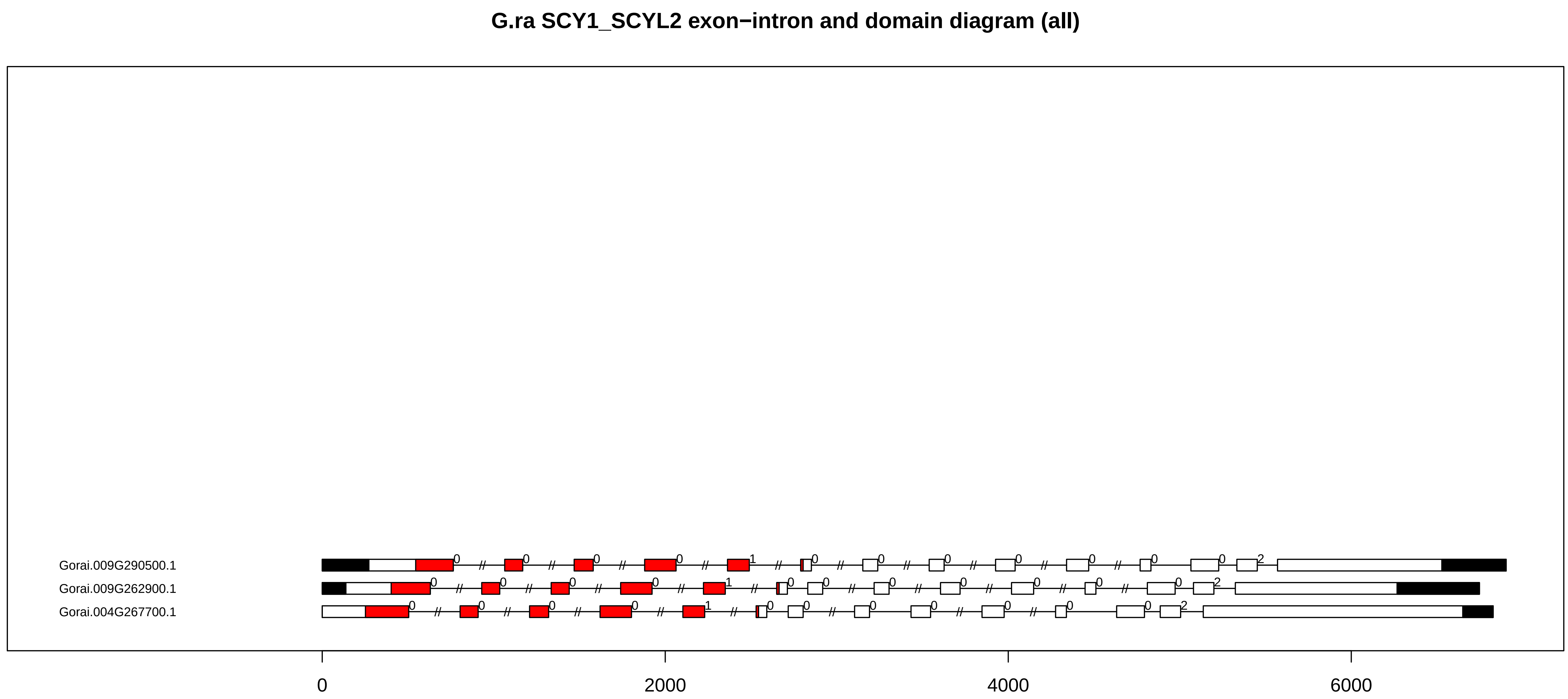

G.ra SCY1_SCYL2 exon−intron and domain diagram (all)
Gorai.009G290500.1
Gorai.009G262900.1
Gorai.004G267700.1
0
2000
4000
6000

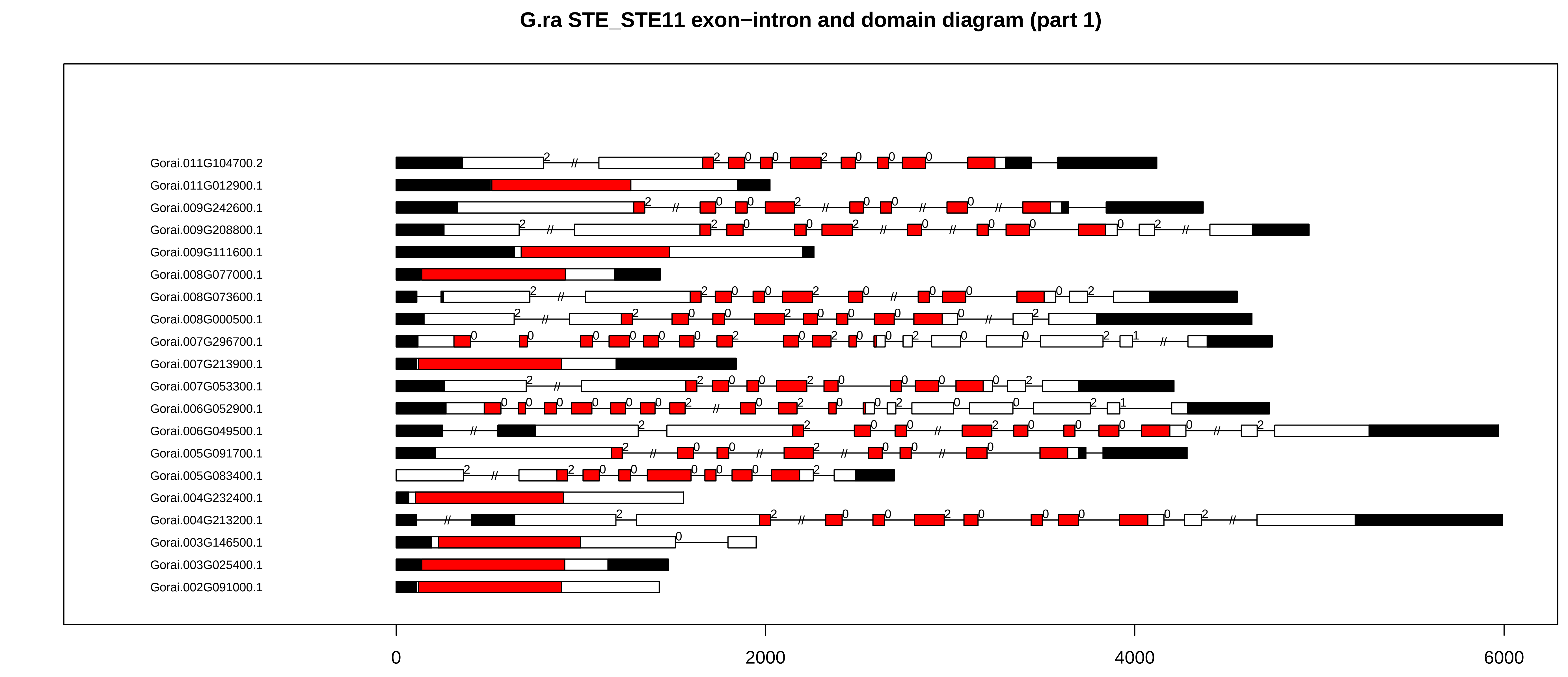
G.ra STE_STE11 exon−intron and domain diagram (part 1)
Gorai.011G104700.2
Gorai.011G012900.1
Gorai.009G242600.1
Gorai.009G208800.1
Gorai.009G111600.1
Gorai.008G077000.1
Gorai.008G073600.1
Gorai.008G000500.1
Gorai.007G296700.1
Gorai.007G213900.1
Gorai.007G053300.1
Gorai.006G052900.1
Gorai.006G049500.1
Gorai.005G091700.1
Gorai.005G083400.1
Gorai.004G232400.1
Gorai.004G213200.1
Gorai.003G146500.1
Gorai.003G025400.1
Gorai.002G091000.1
0
2000
4000
6000

G.ra STE_STE11 exon−intron and domain diagram (part 2)

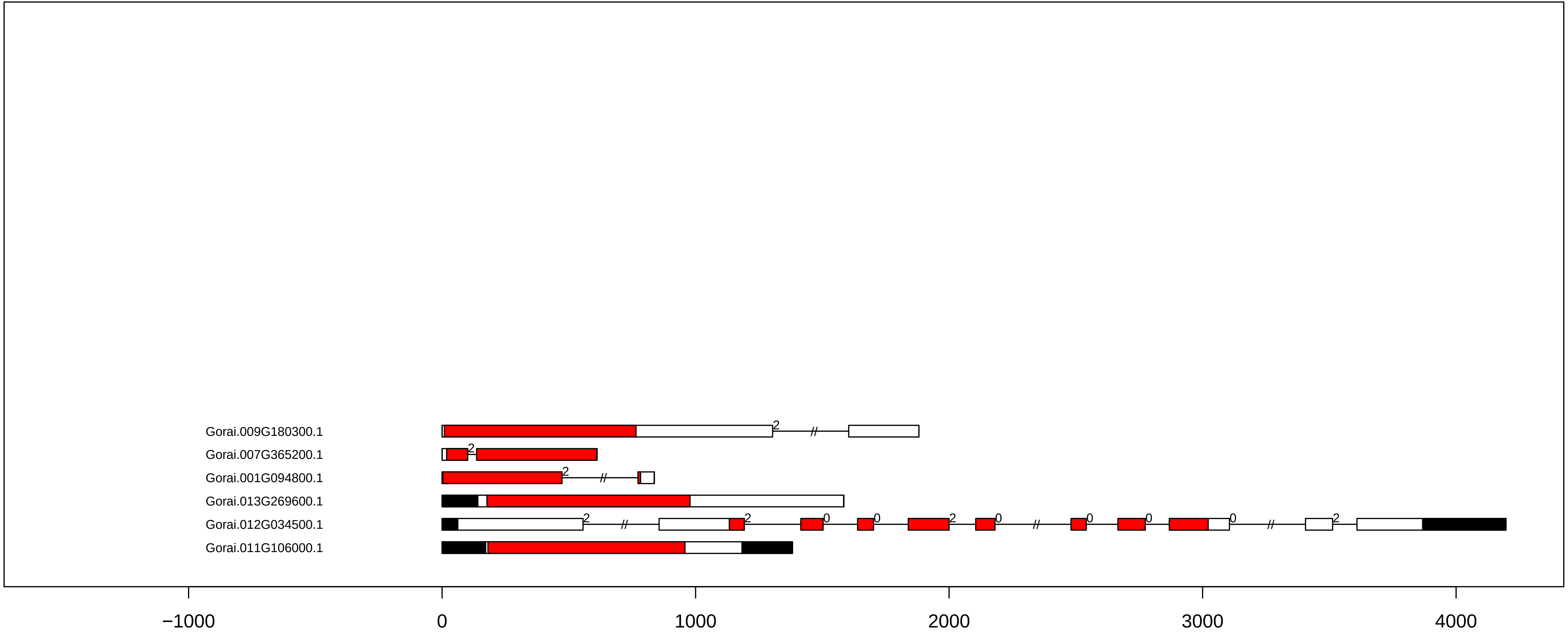

G.ra STE_STE20−Fray exon−intron and domain diagram (all)

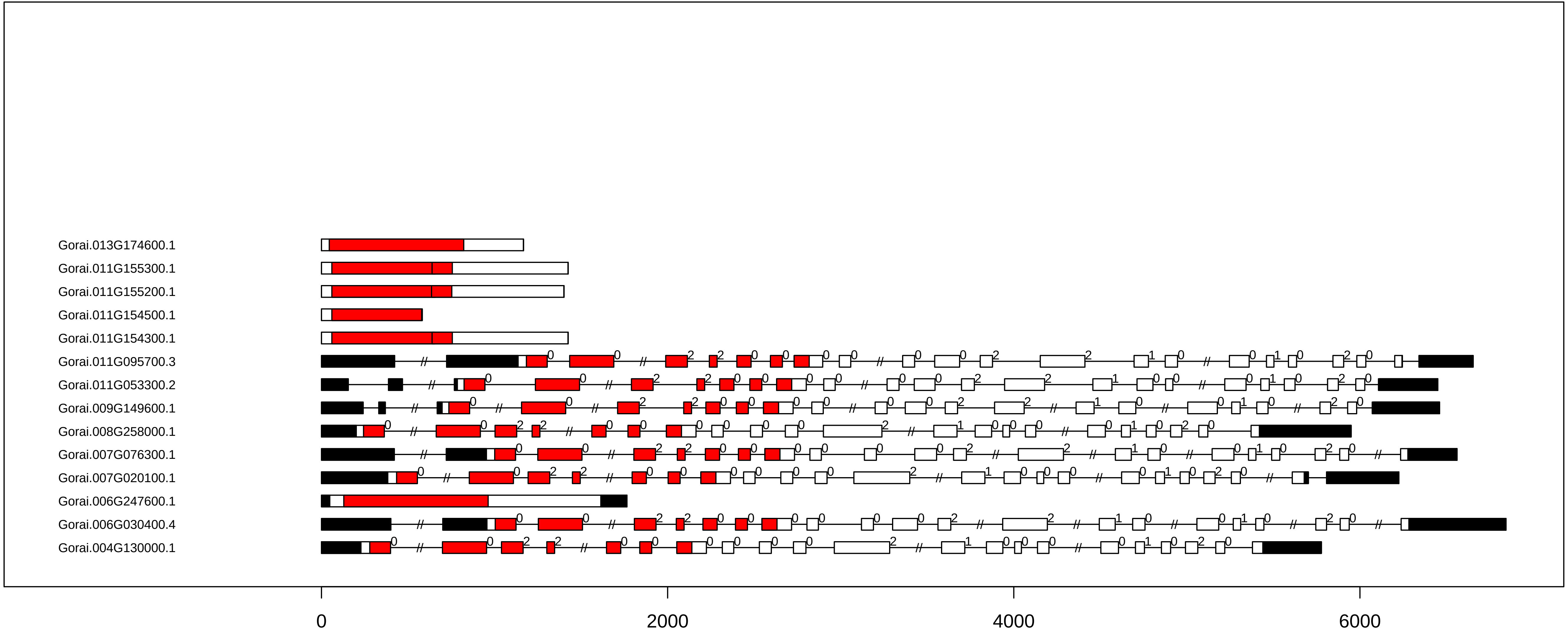

G.ra STE_STE20−PI exon−intron and domain diagram (all)

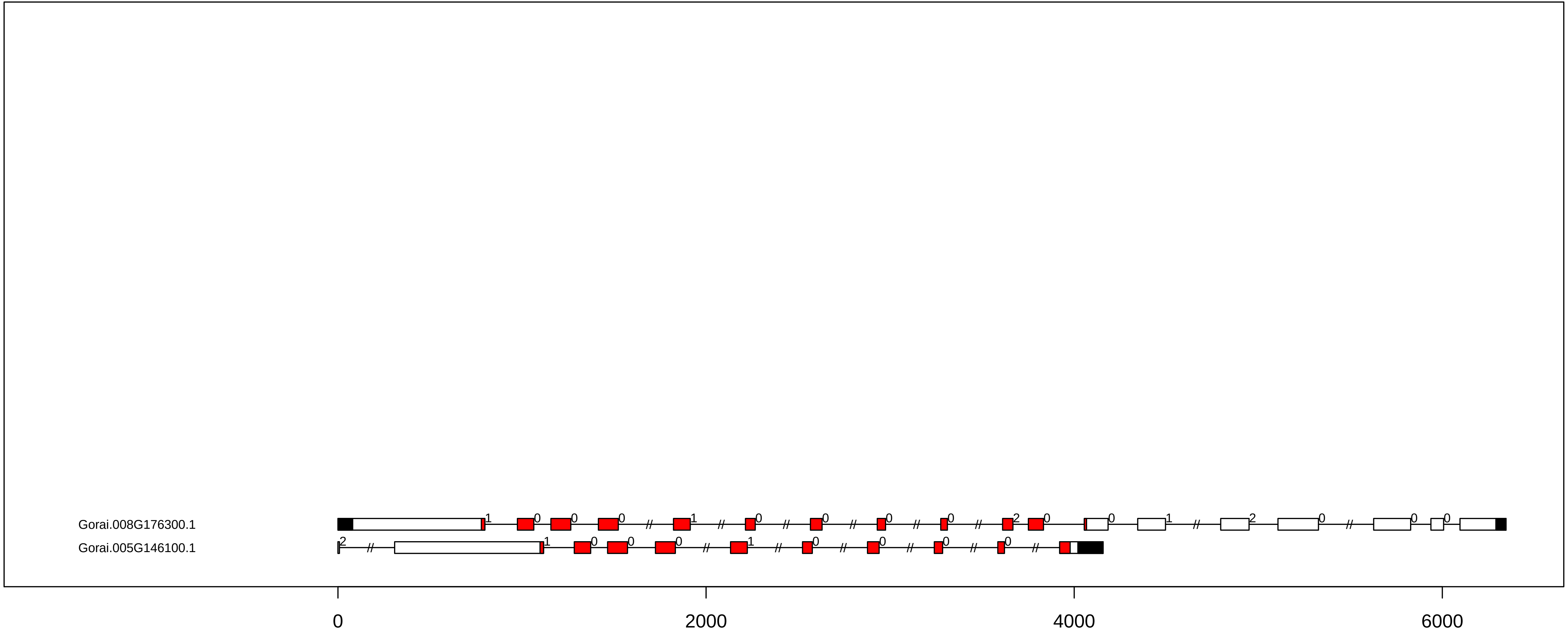

G.ra STE_STE20−YSK exon−intron and domain diagram (all)

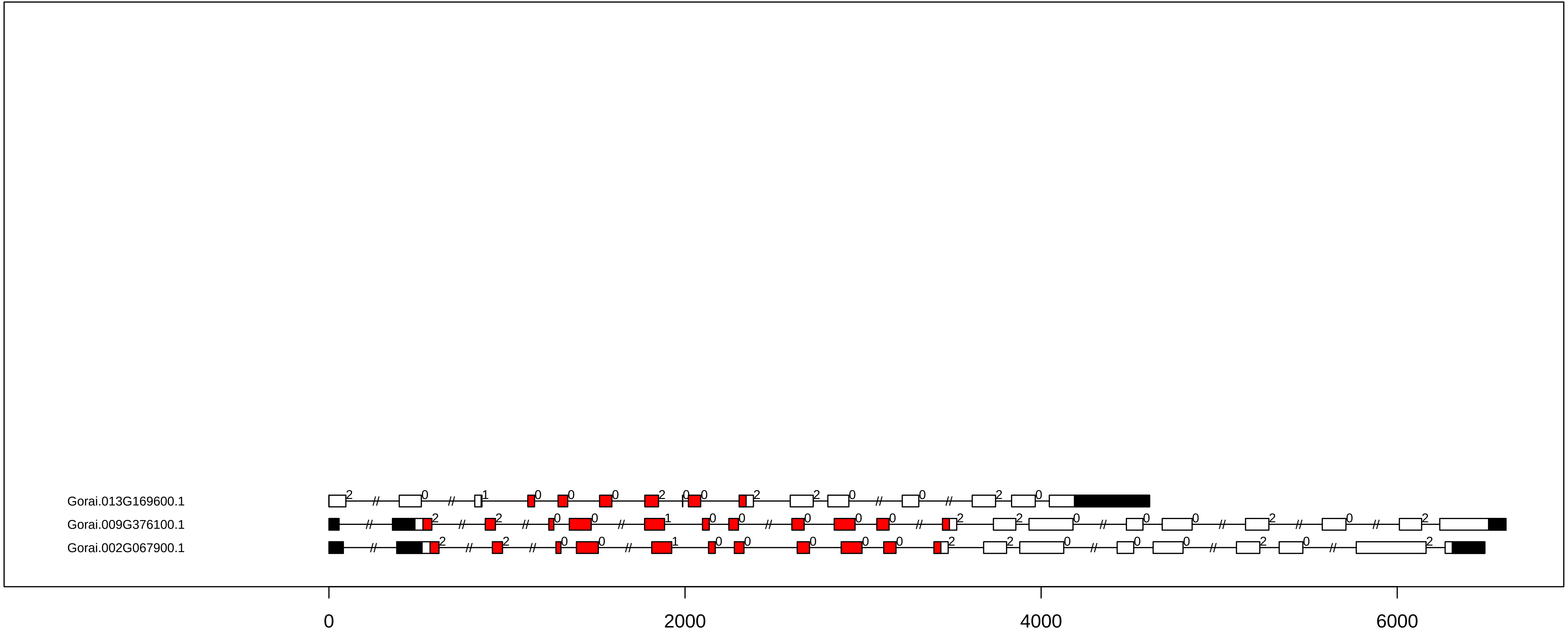

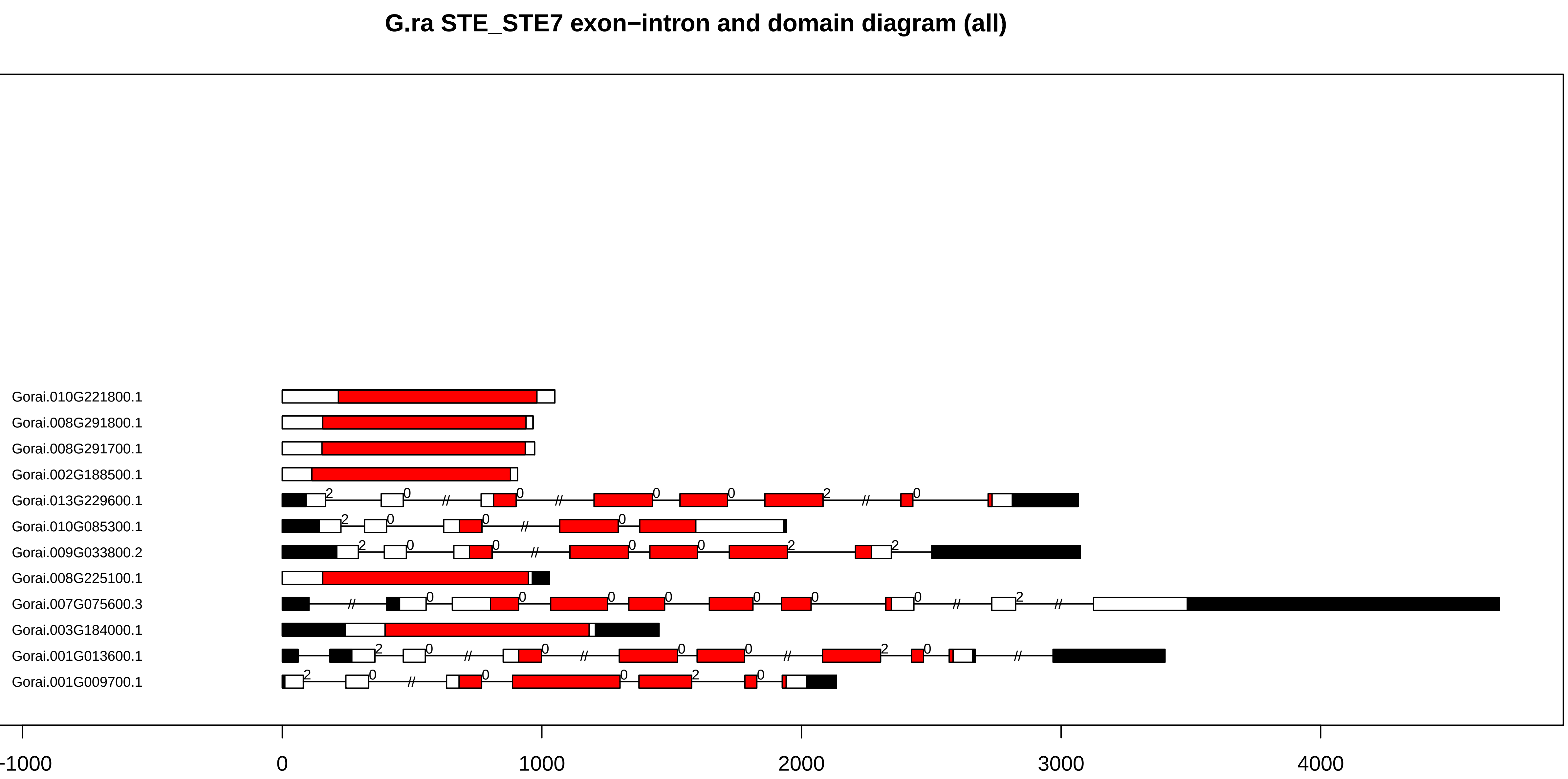
G.ra STE_STE7 exon−intron and domain diagram (all)
Gorai.010G221800.1
Gorai.008G291800.1
Gorai.008G291700.1
Gorai.002G188500.1
Gorai.013G229600.1
Gorai.010G085300.1
Gorai.009G033800.2
Gorai.008G225100.1
Gorai.007G075600.3
Gorai.003G184000.1
Gorai.001G013600.1
Gorai.001G009700.1
−1000
0
1000
2000
3000
4000

G.ra STE_STE−PI exon−intron and domain diagram (all)

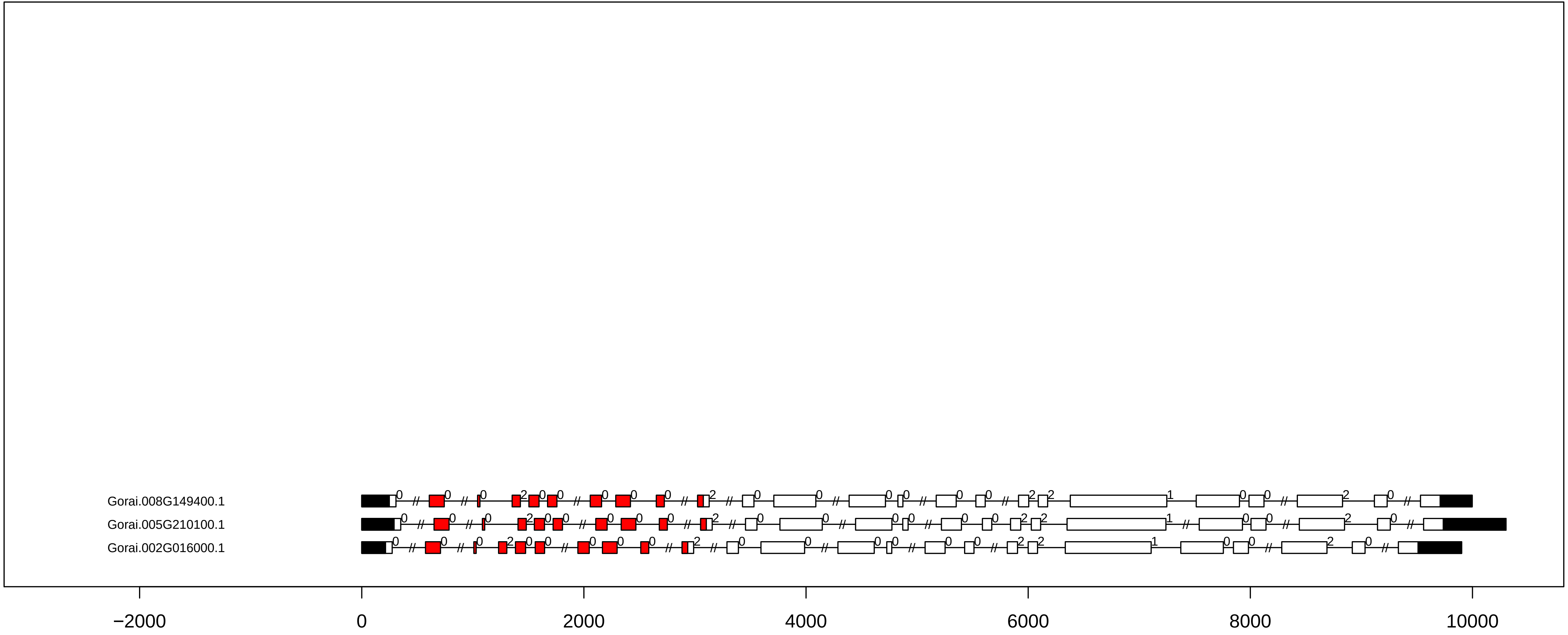

G.ra TKL_CTR1−DRK−1 exon−intron and domain diagram (all)

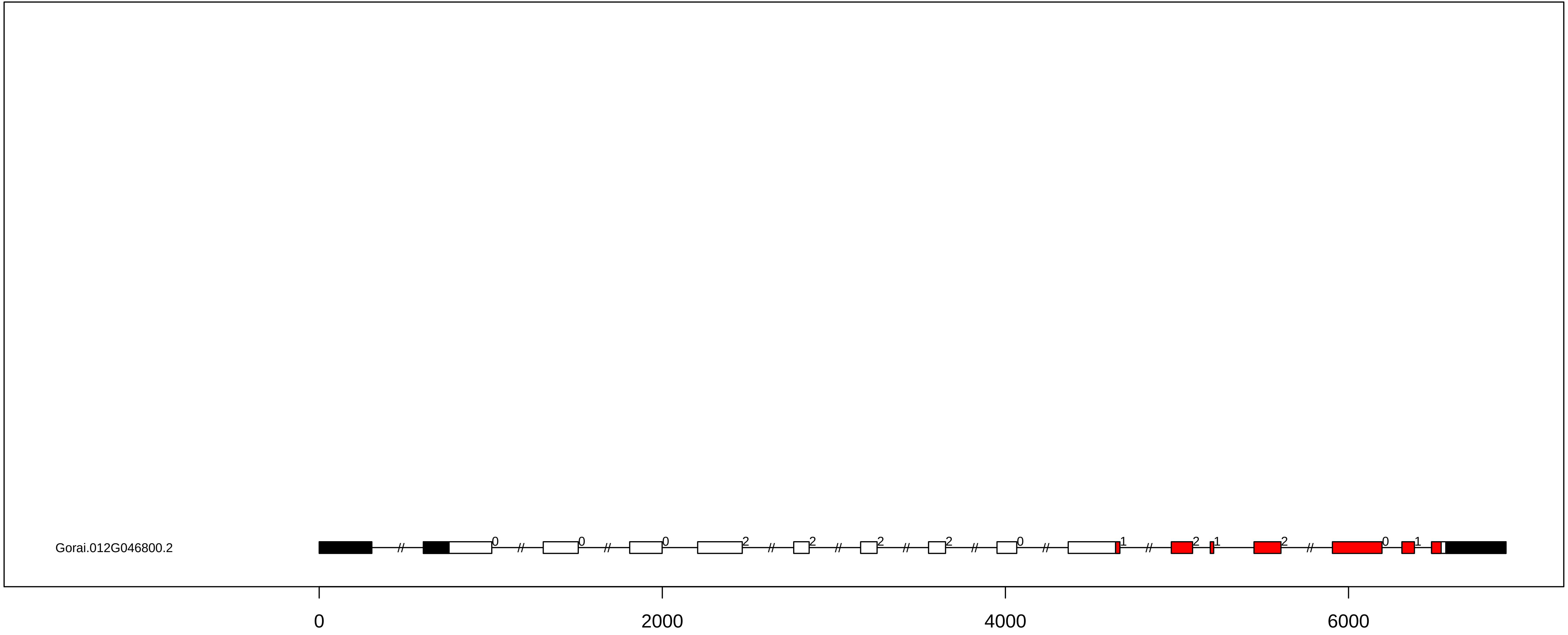

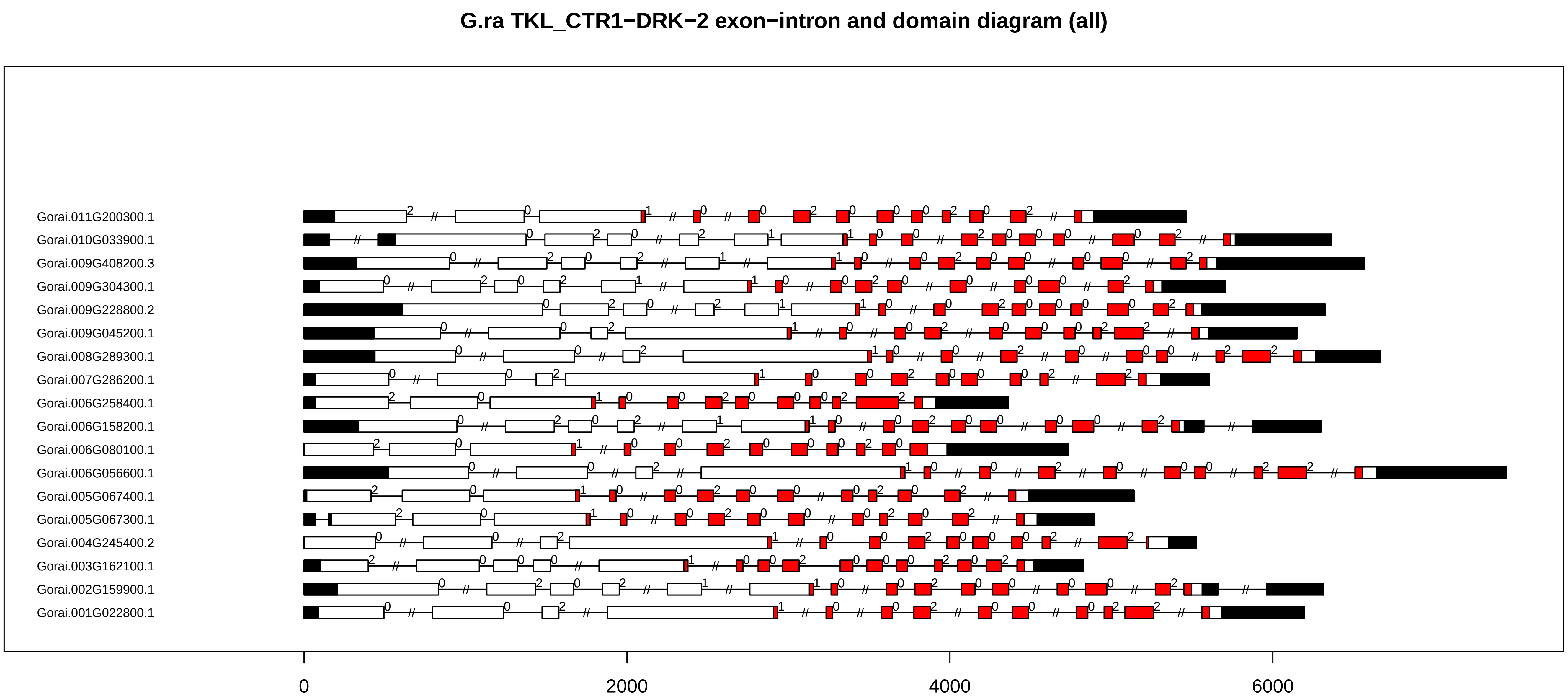
G.ra TKL_CTR1−DRK−2 exon−intron and domain diagram (all)
Gorai.011G200300.1
Gorai.010G033900.1
Gorai.009G408200.3
Gorai.009G304300.1
Gorai.009G228800.2
Gorai.009G045200.1
Gorai.008G289300.1
Gorai.007G286200.1
Gorai.006G258400.1
Gorai.006G158200.1
Gorai.006G080100.1
Gorai.006G056600.1
Gorai.005G067400.1
Gorai.005G067300.1
Gorai.004G245400.2
Gorai.003G162100.1
Gorai.002G159900.1
Gorai.001G022800.1
0
2000
4000
6000

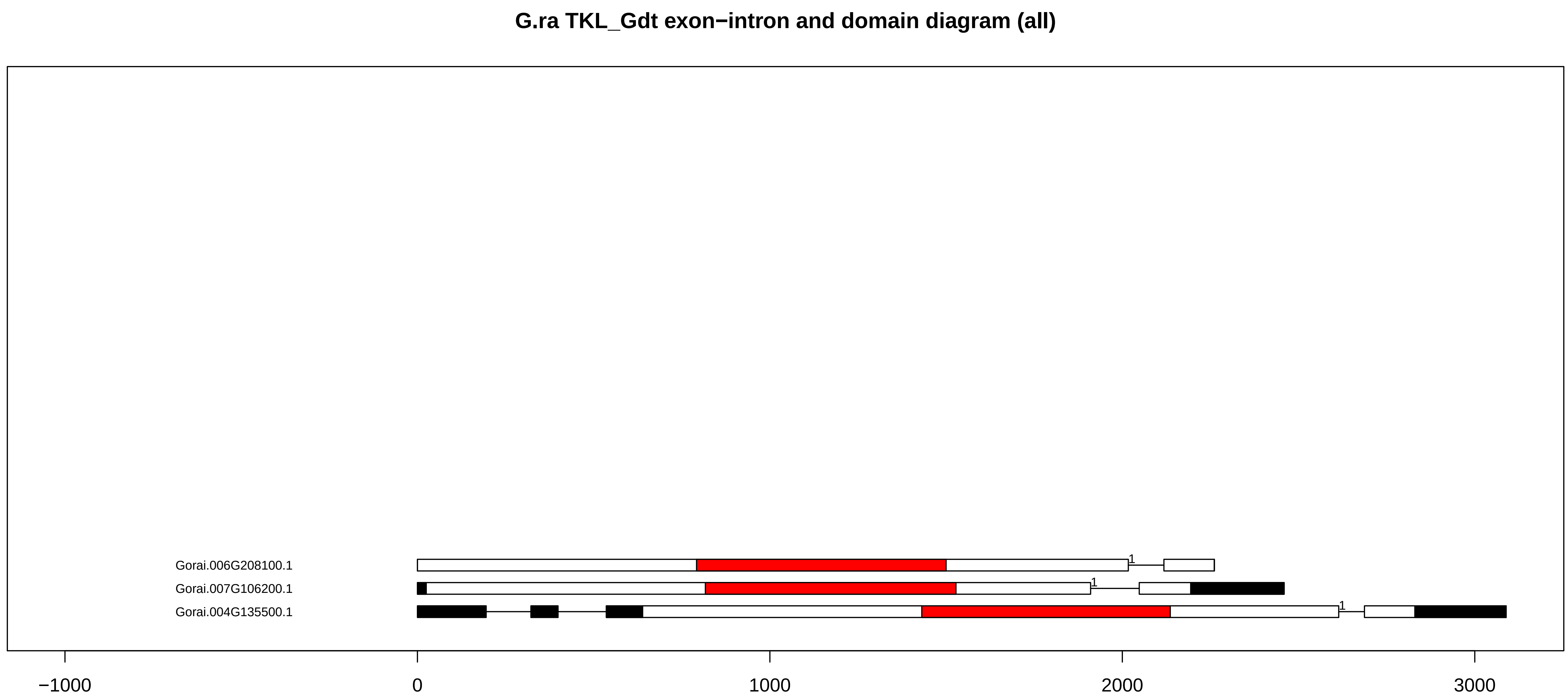
G.ra TKL_Gdt exon−intron and domain diagram (all)
Gorai.006G208100.1
Gorai.007G106200.1
Gorai.004G135500.1
1
1
1
−1000
0
1000
2000
3000

G.ra TKL−Pl−1 exon−intron and domain diagram (all)

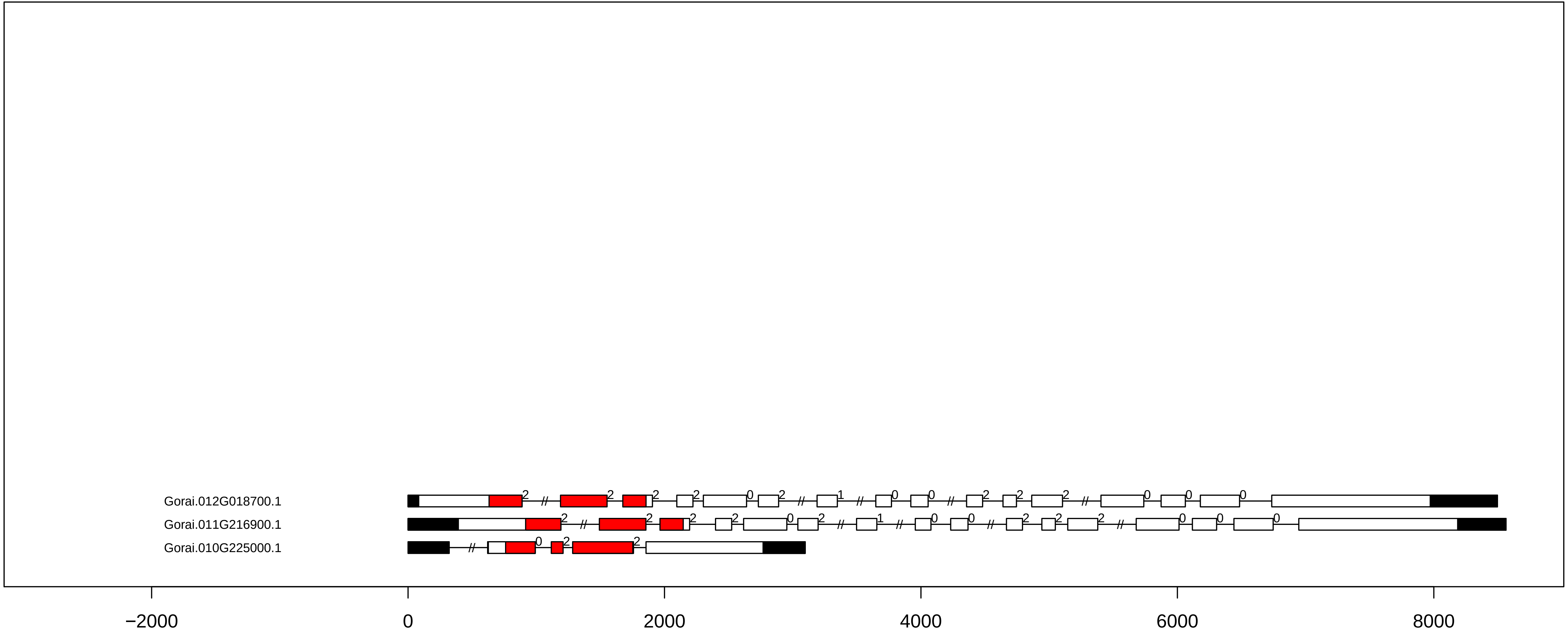

G.ra TKL−Pl−2 exon−intron and domain diagram (all)

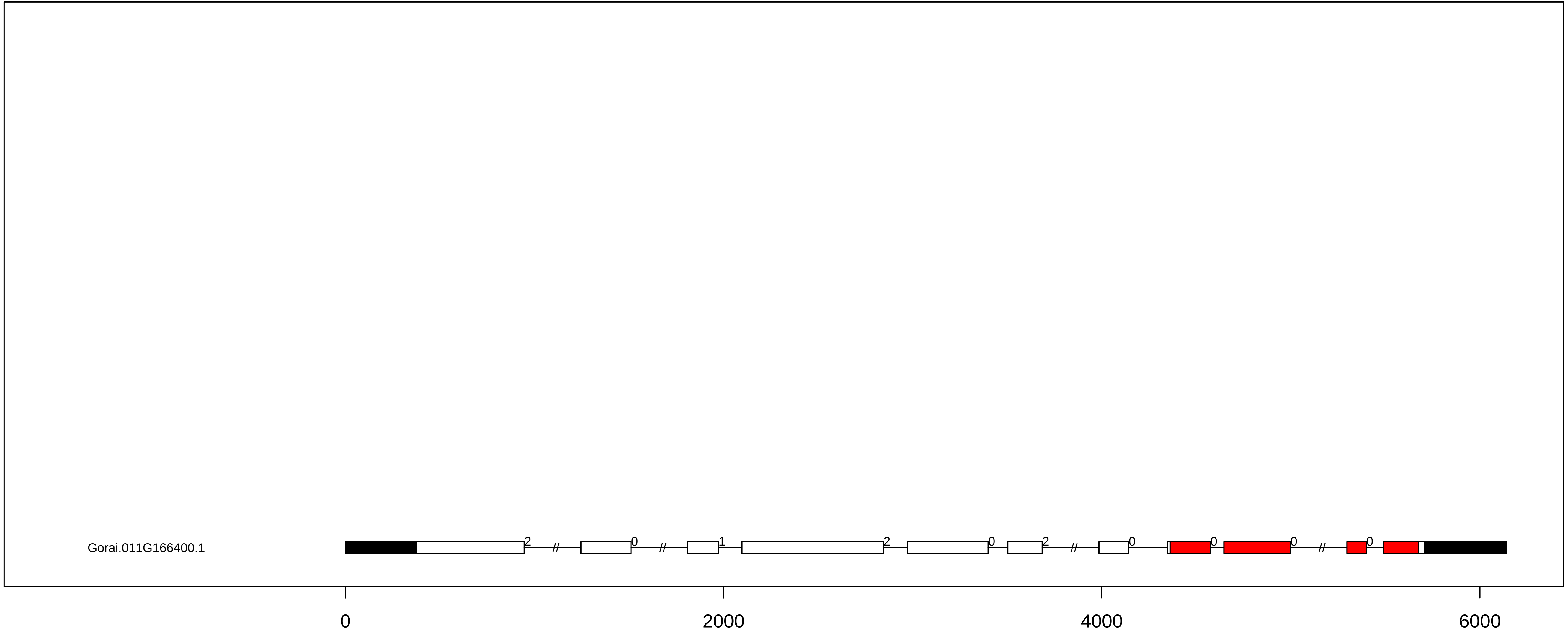

G.ra TKL−Pl−3 exon−intron and domain diagram (all)

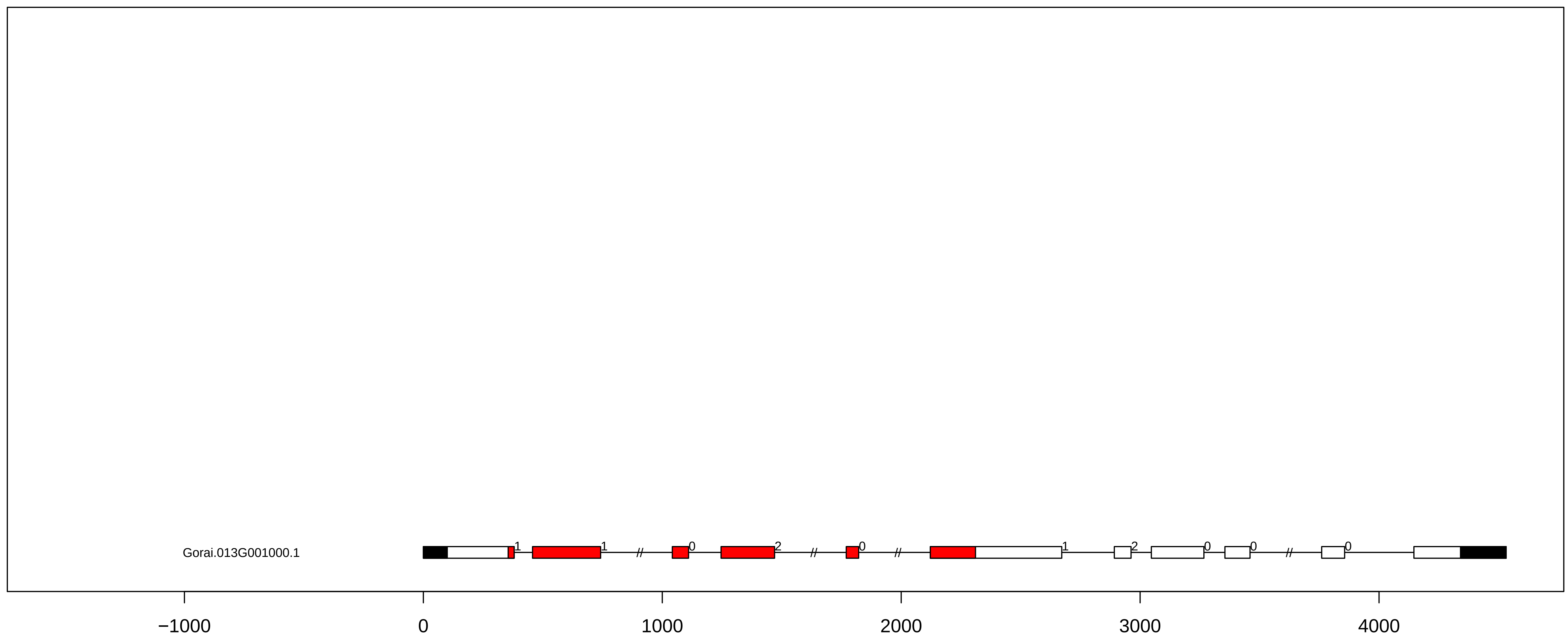

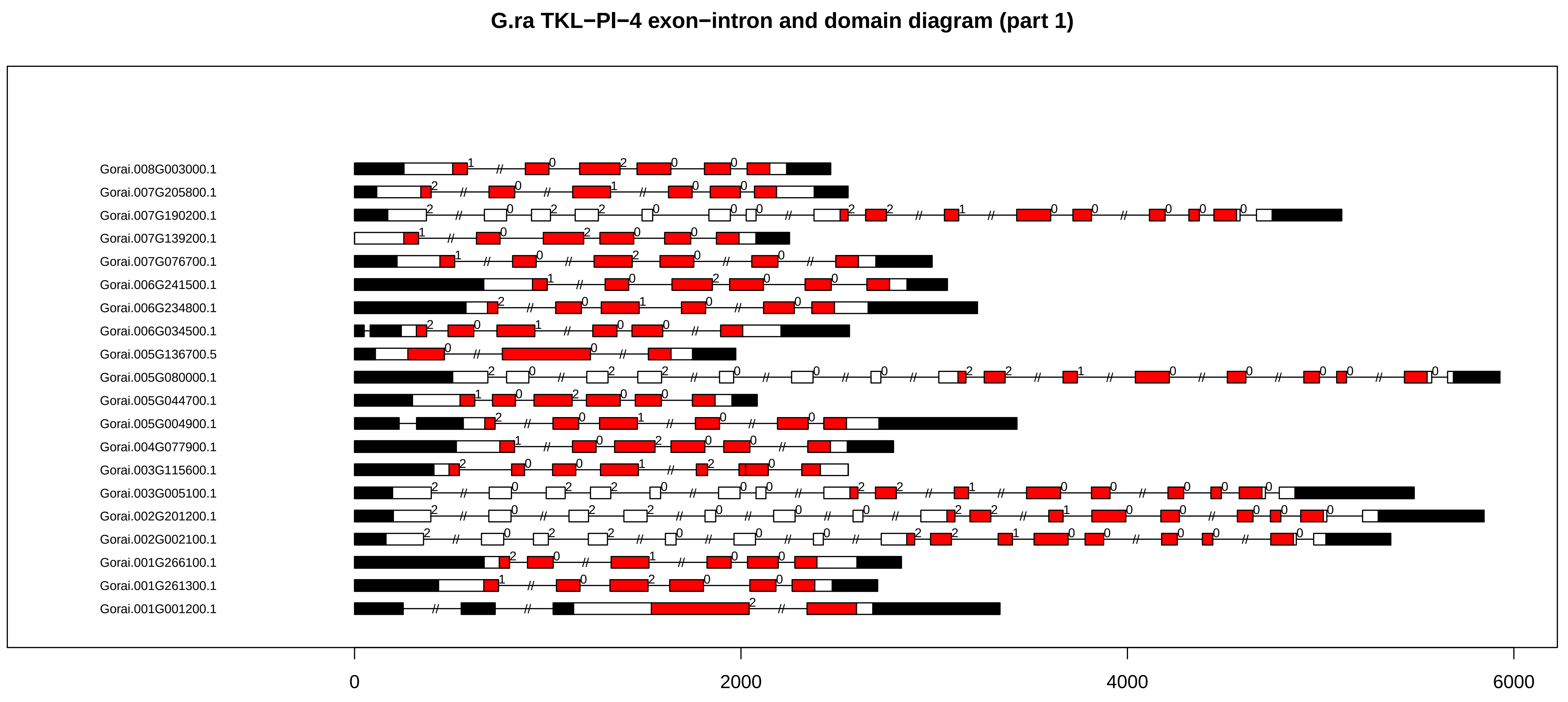
G.ra TKL−Pl−4 exon−intron and domain diagram (part 1)
Gorai.008G003000.1
Gorai.007G205800.1
Gorai.007G190200.1
Gorai.007G139200.1
Gorai.007G076700.1
Gorai.006G241500.1
Gorai.006G234800.1
Gorai.006G034500.1
Gorai.005G136700.5
Gorai.005G080000.1
Gorai.005G044700.1
Gorai.005G004900.1
Gorai.004G077900.1
Gorai.003G115600.1
Gorai.003G005100.1
Gorai.002G201200.1
Gorai.002G002100.1
Gorai.001G266100.1
Gorai.001G261300.1
Gorai.001G001200.1
0
2000
4000
6000

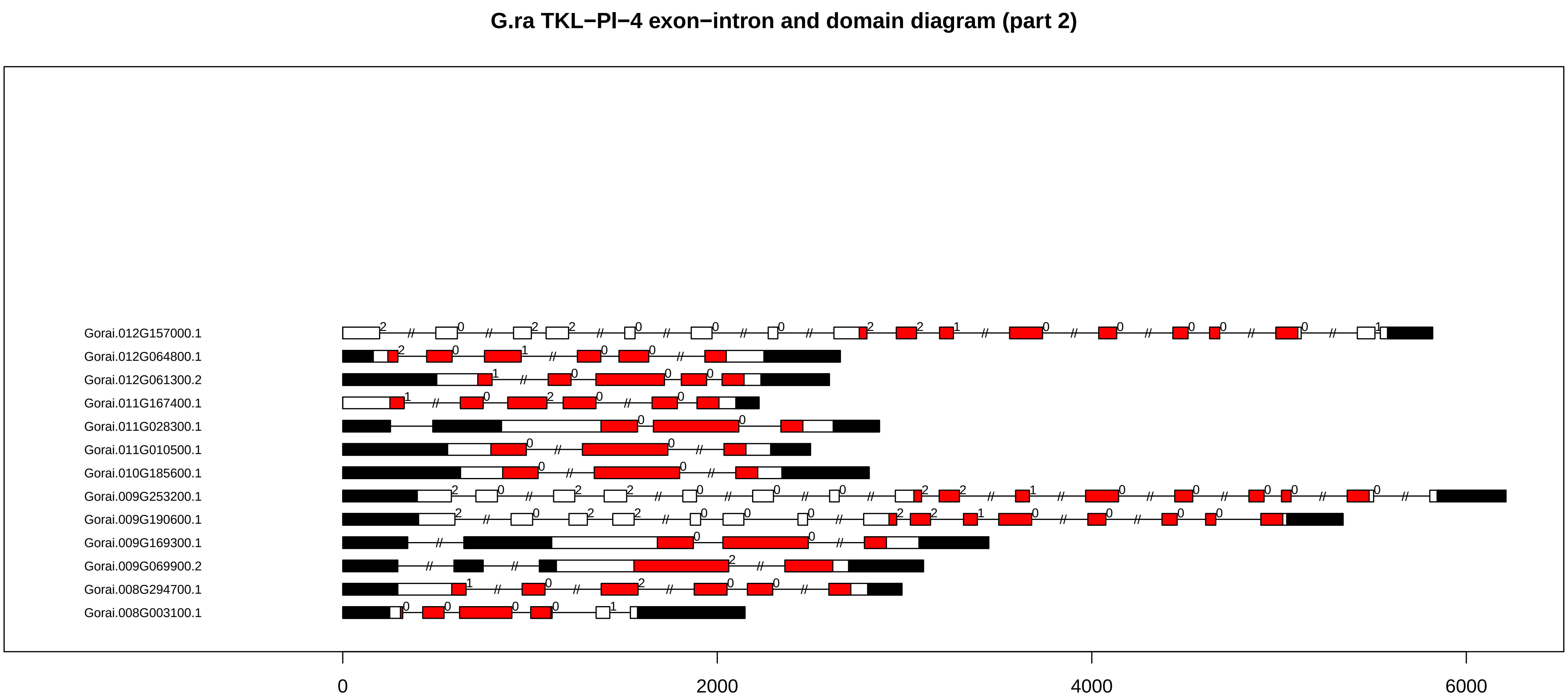
G.ra TKL−Pl−4 exon−intron and domain diagram (part 2)
Gorai.012G157000.1
Gorai.012G064800.1
Gorai.012G061300.2
Gorai.011G167400.1
Gorai.011G028300.1
Gorai.011G010500.1
Gorai.010G185600.1
Gorai.009G253200.1
Gorai.009G190600.1
Gorai.009G169300.1
Gorai.009G069900.2
Gorai.008G294700.1
Gorai.008G003100.1
0
2000
4000
6000

G.ra TKL−Pl−5 exon−intron and domain diagram (all)

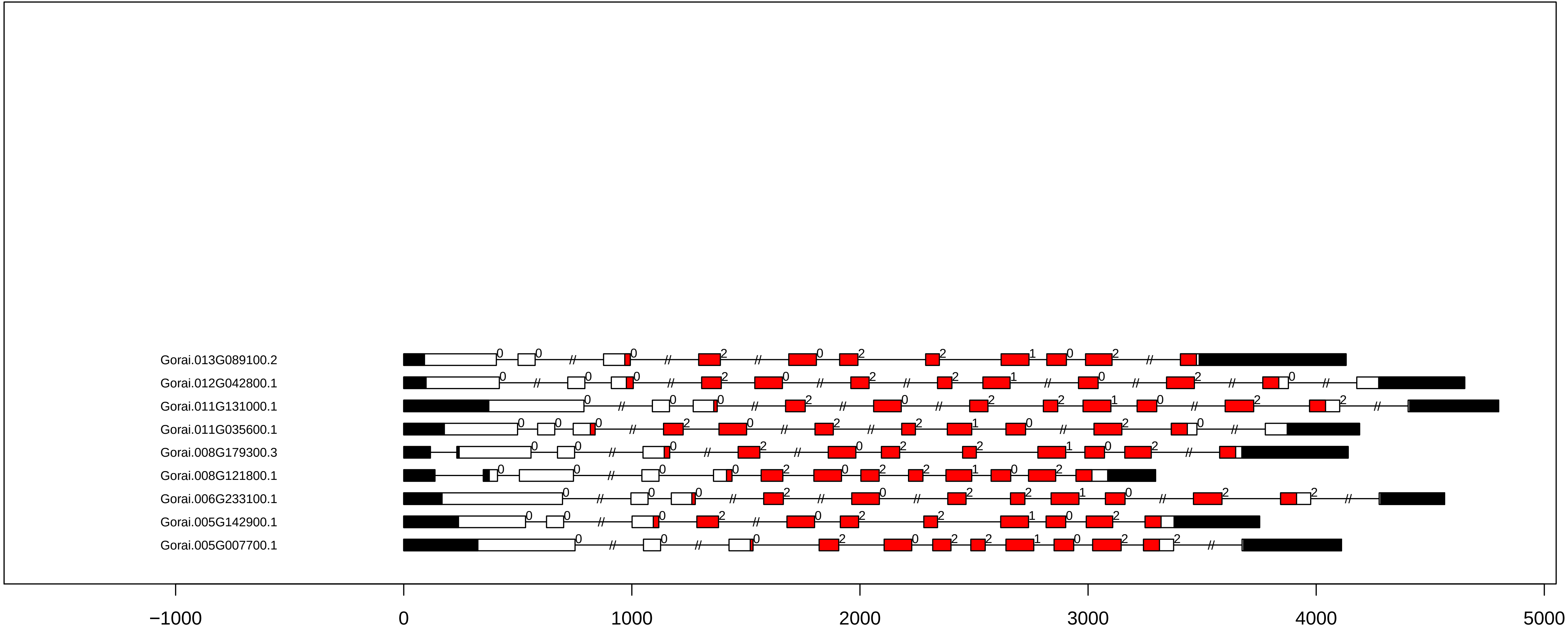

G.ra TKL−Pl−6 exon−intron and domain diagram (all)

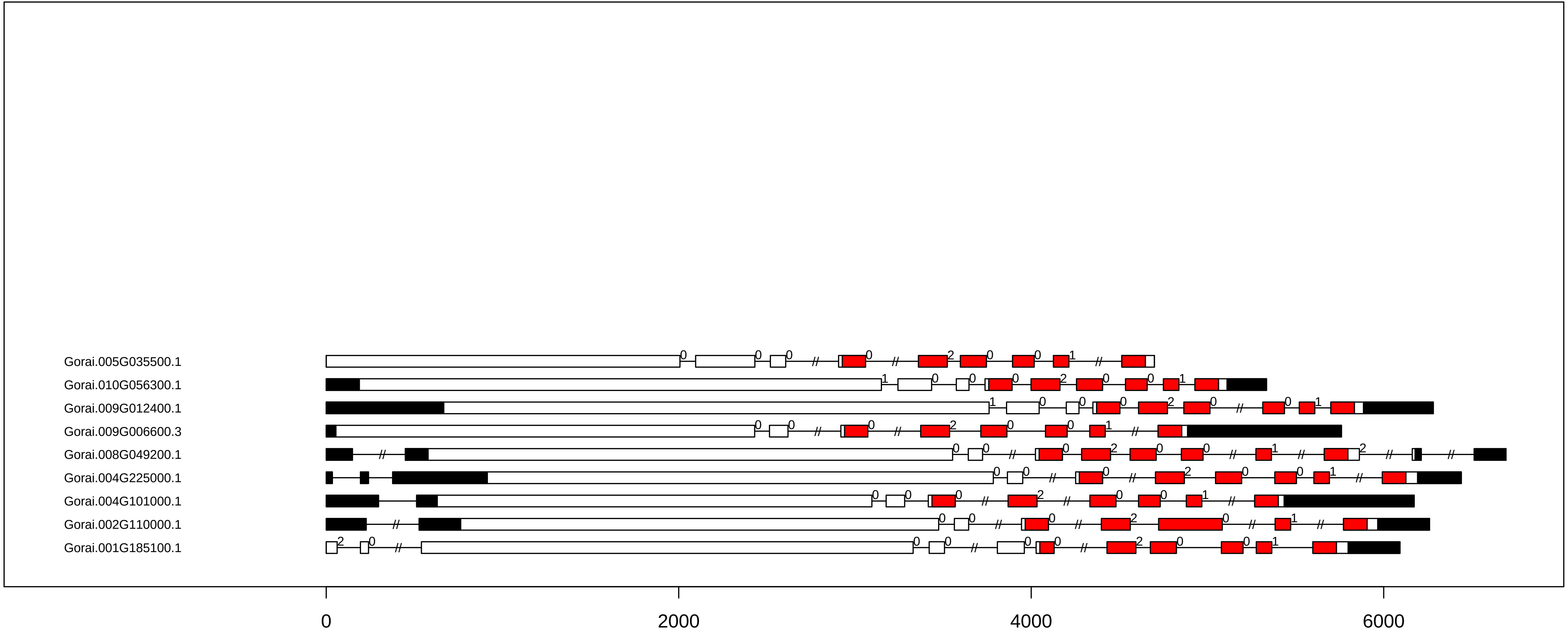

G.ra TKL−Pl−7 exon−intron and domain diagram (all)

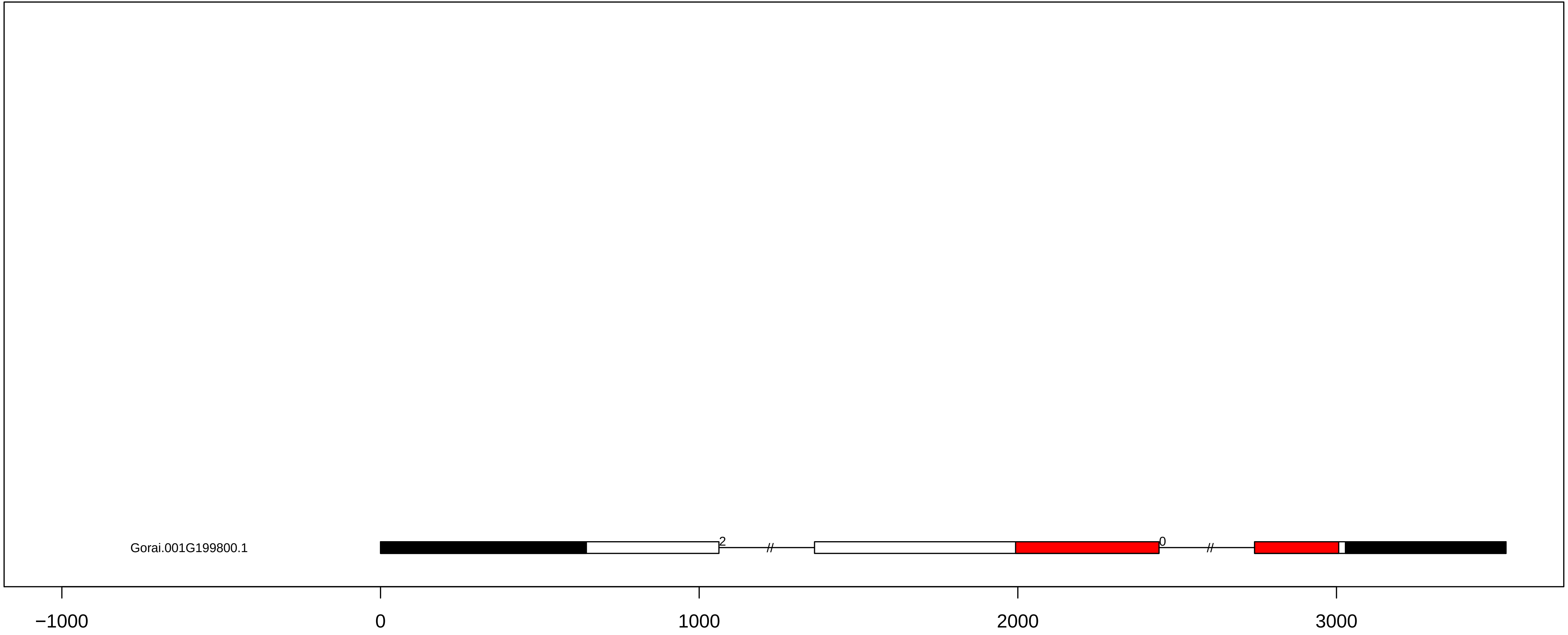

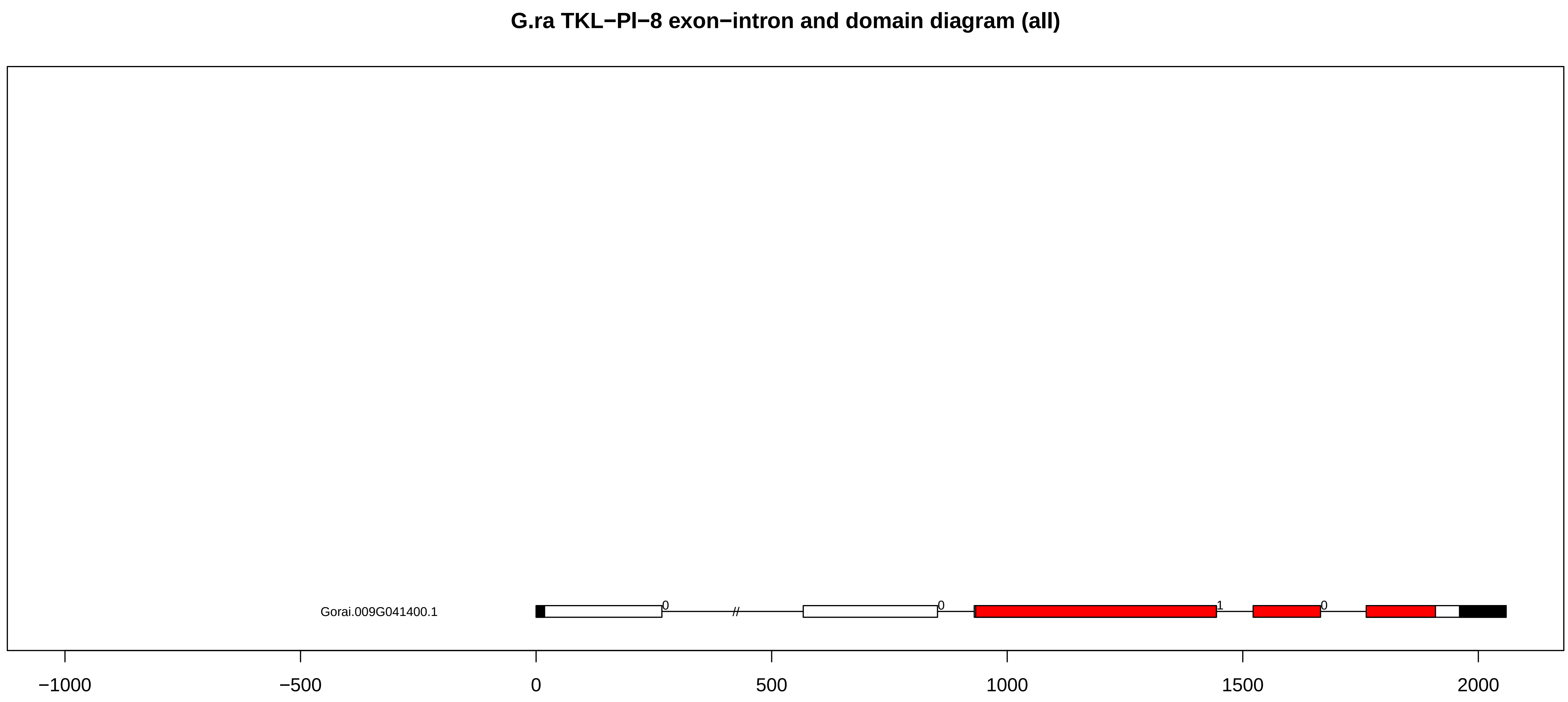
G.ra TKL−Pl−8 exon−intron and domain diagram (all)
Gorai.009G041400.1
0
0
1
0
−1000
−500
0
500
1000
1500
2000

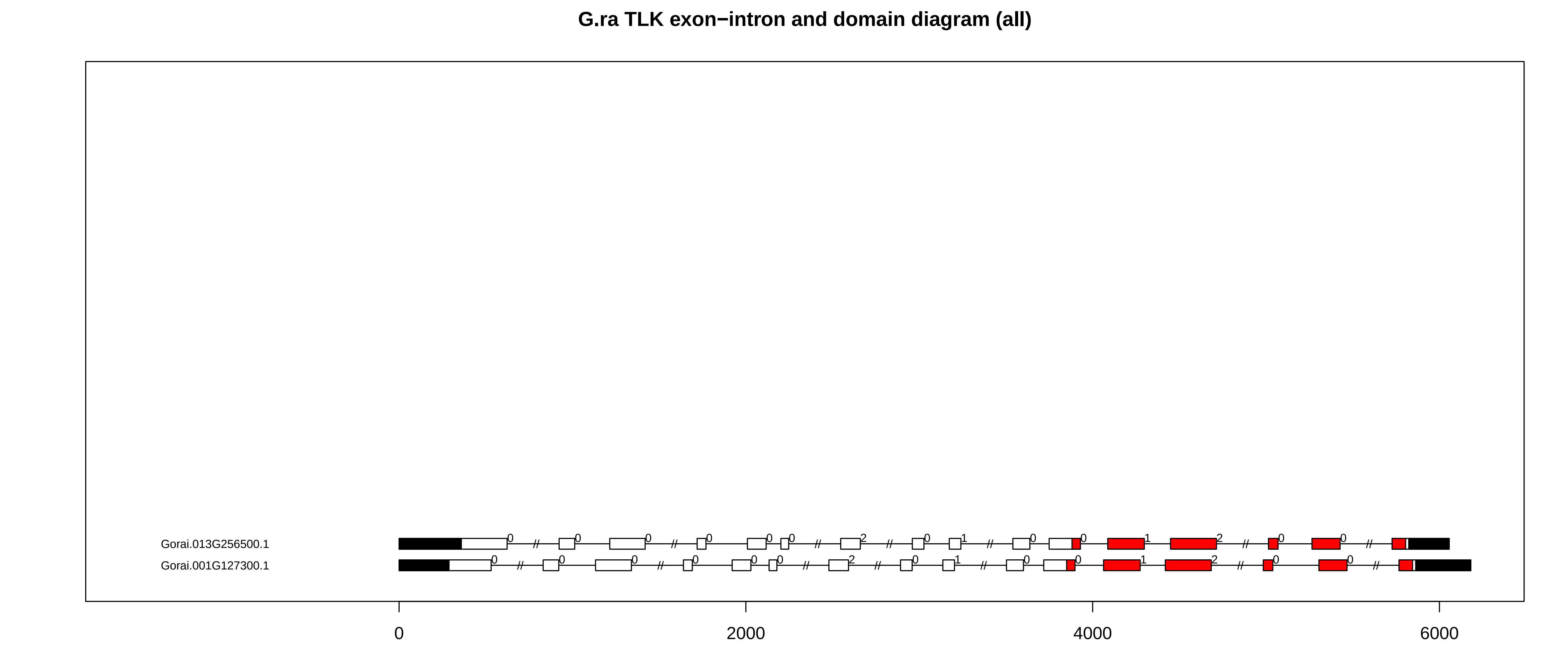
G.ra TLK exon−intron and domain diagram (all)
Gorai.013G256500.1
Gorai.001G127300.1
0
2000
4000
6000

G.ra TTK exon−intron and domain diagram (all)

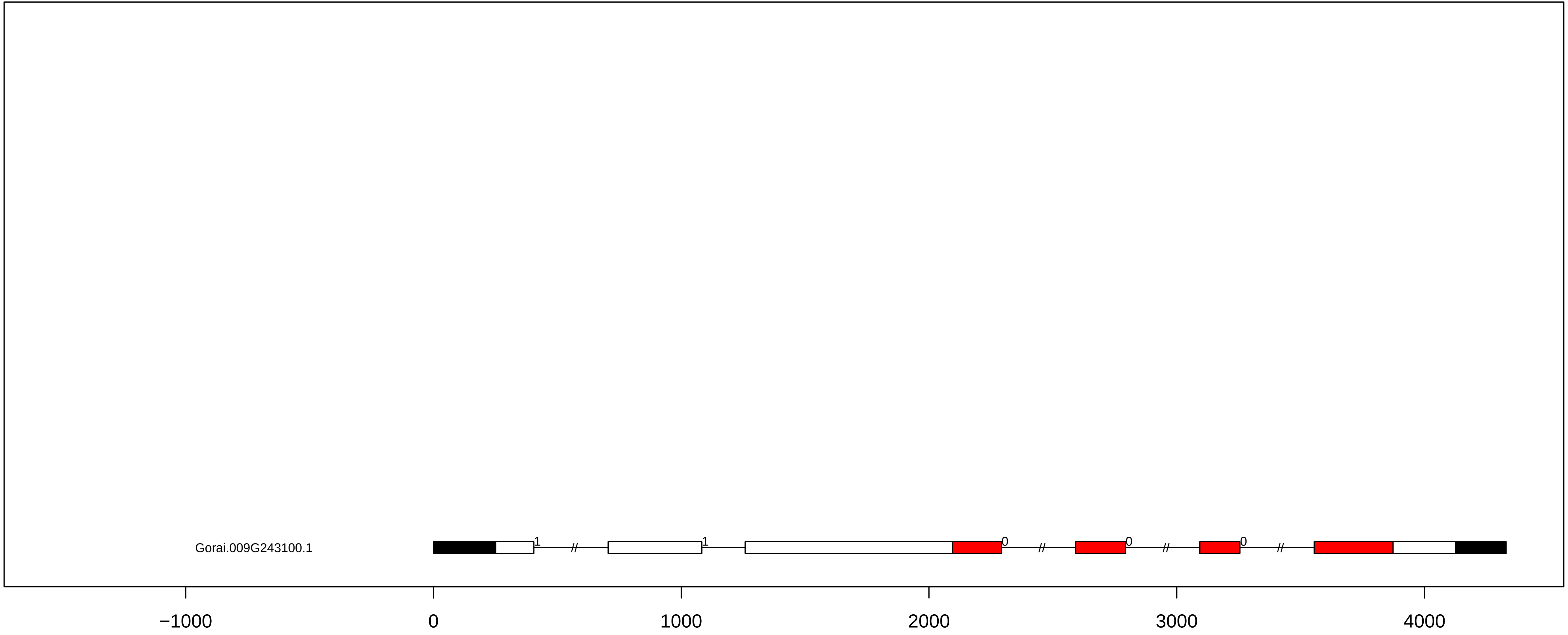

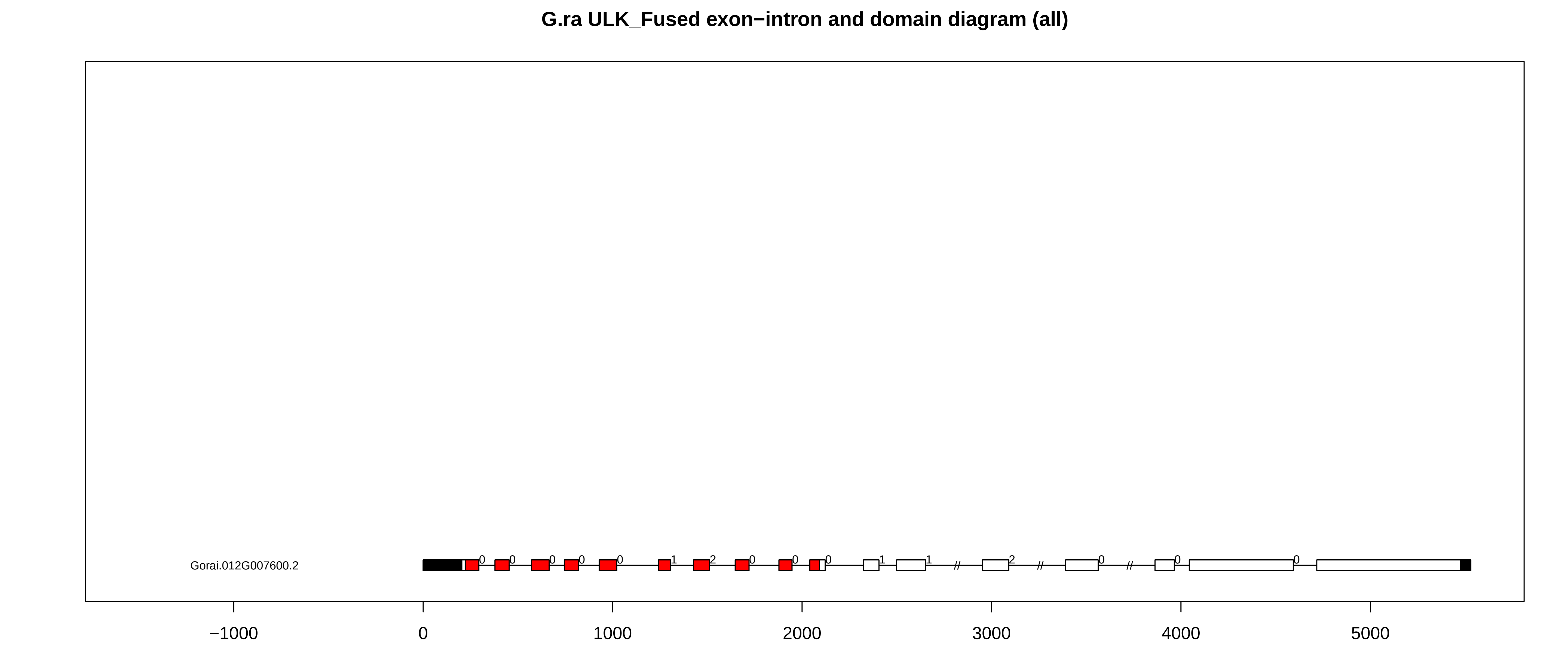
G.ra ULK_Fused exon−intron and domain diagram (all)
Gorai.012G007600.2
0
0
0
0
0
1
2
0
0
0
1
1
2
0
0
0
−1000
0
1000
2000
3000
4000
5000

G.ra ULK_ULK4 exon−intron and domain diagram (all)

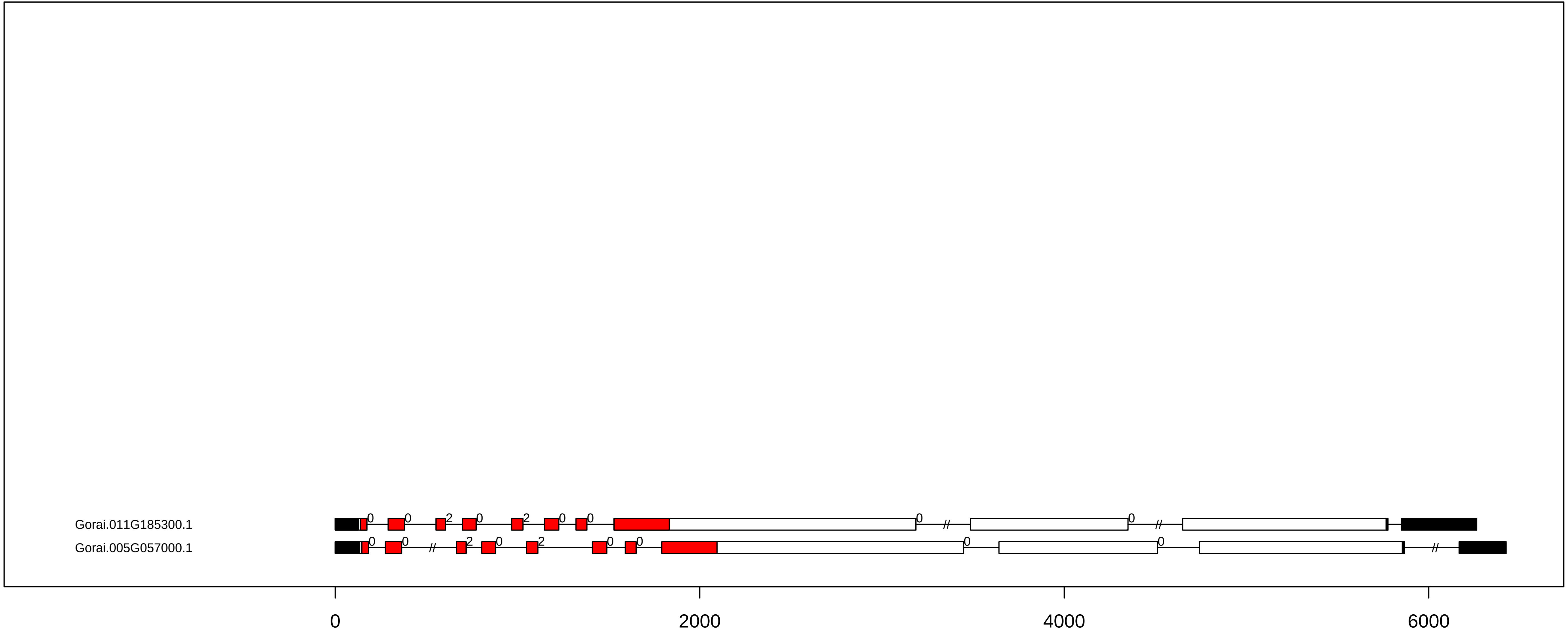

G.ra WEE exon−intron and domain diagram (all)

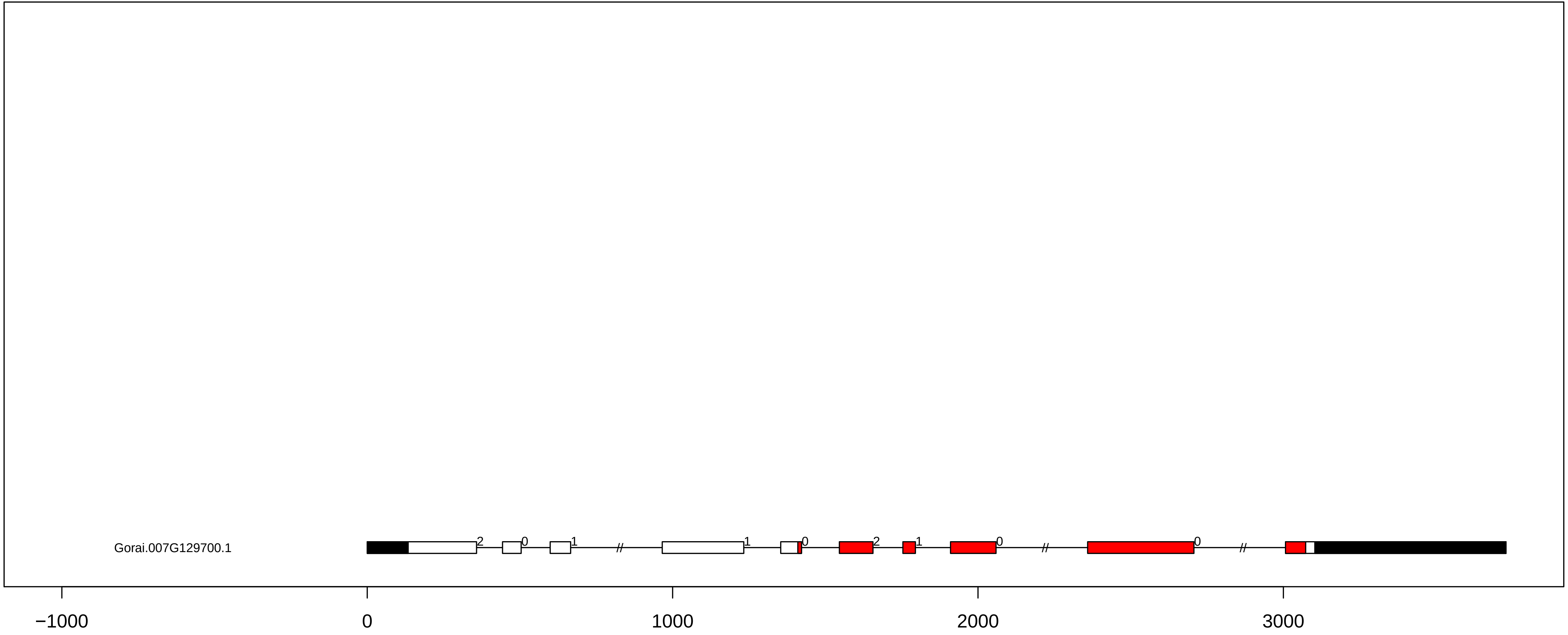

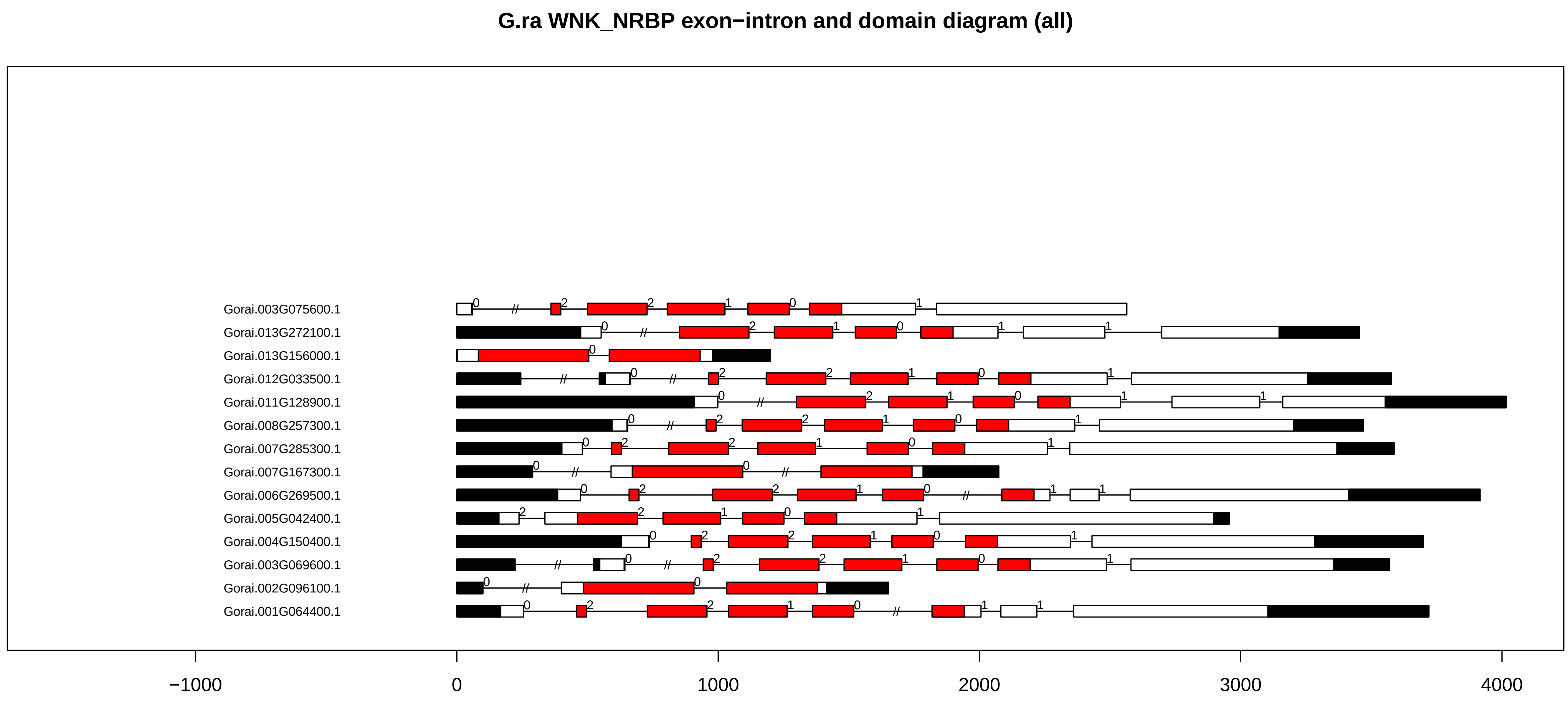

G.ra WNK_NRBP exon−intron and domain diagram (all)
Gorai.003G075600.1
Gorai.013G272100.1
Gorai.013G156000.1
Gorai.012G033500.1
Gorai.011G128900.1
Gorai.008G257300.1
Gorai.007G285300.1
Gorai.007G167300.1
Gorai.006G269500.1
Gorai.005G042400.1
Gorai.004G150400.1
Gorai.003G069600.1
Gorai.002G096100.1
Gorai.001G064400.1
−1000
0
1000
2000
3000
4000

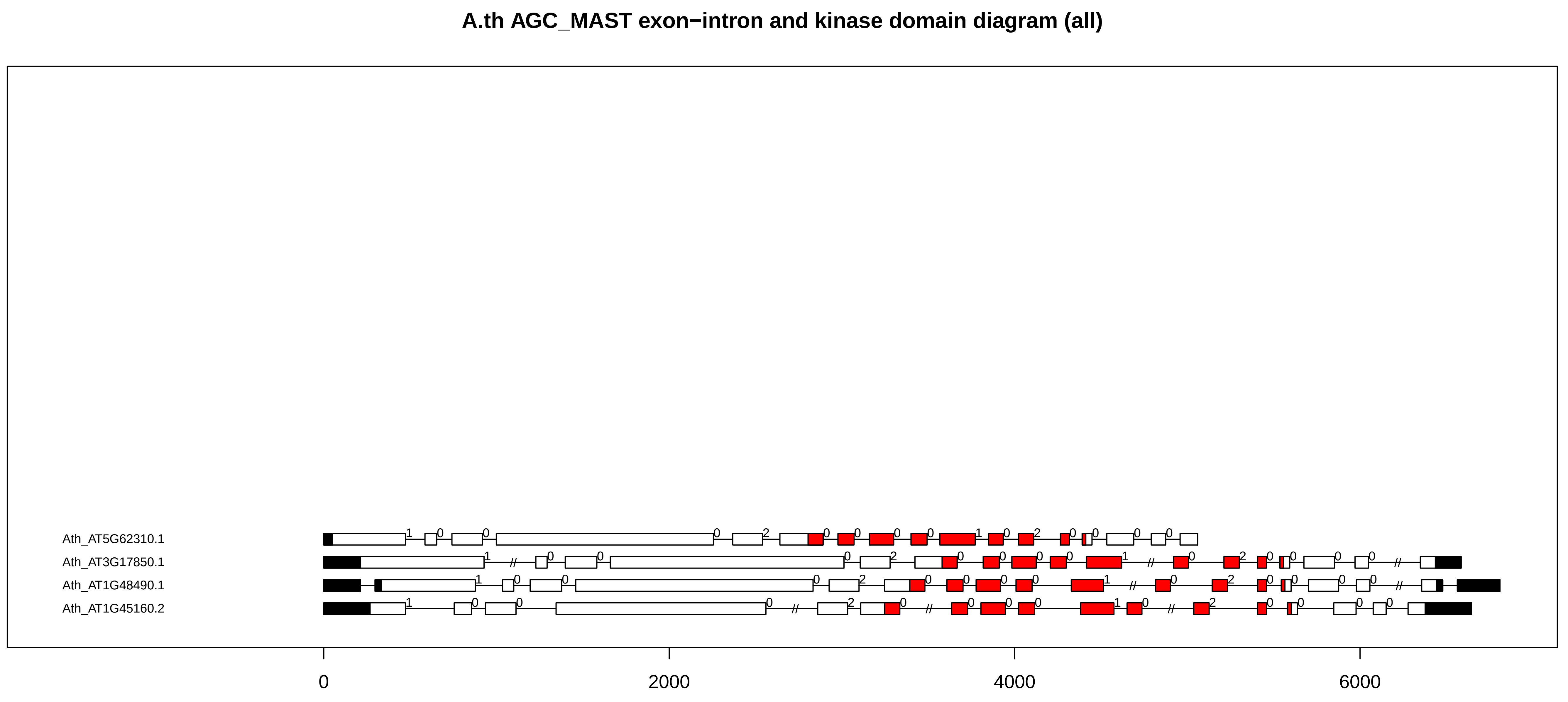
A.th AGC_MAST exon−intron and kinase domain diagram (all)
Ath_AT5G62310.1
Ath_AT3G17850.1
Ath_AT1G48490.1
Ath_AT1G45160.2
0
2000
4000
6000

A.th AGC_NDR exon−intron and kinase domain diagram (all)

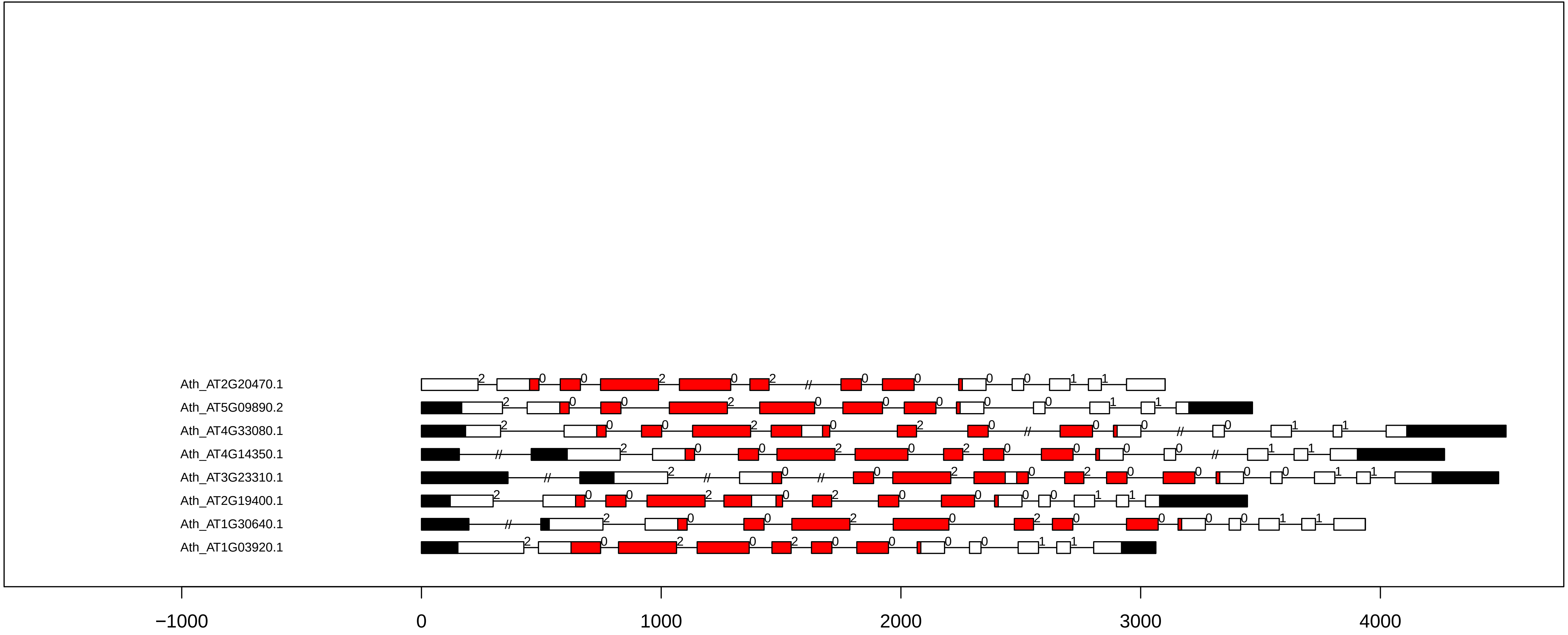

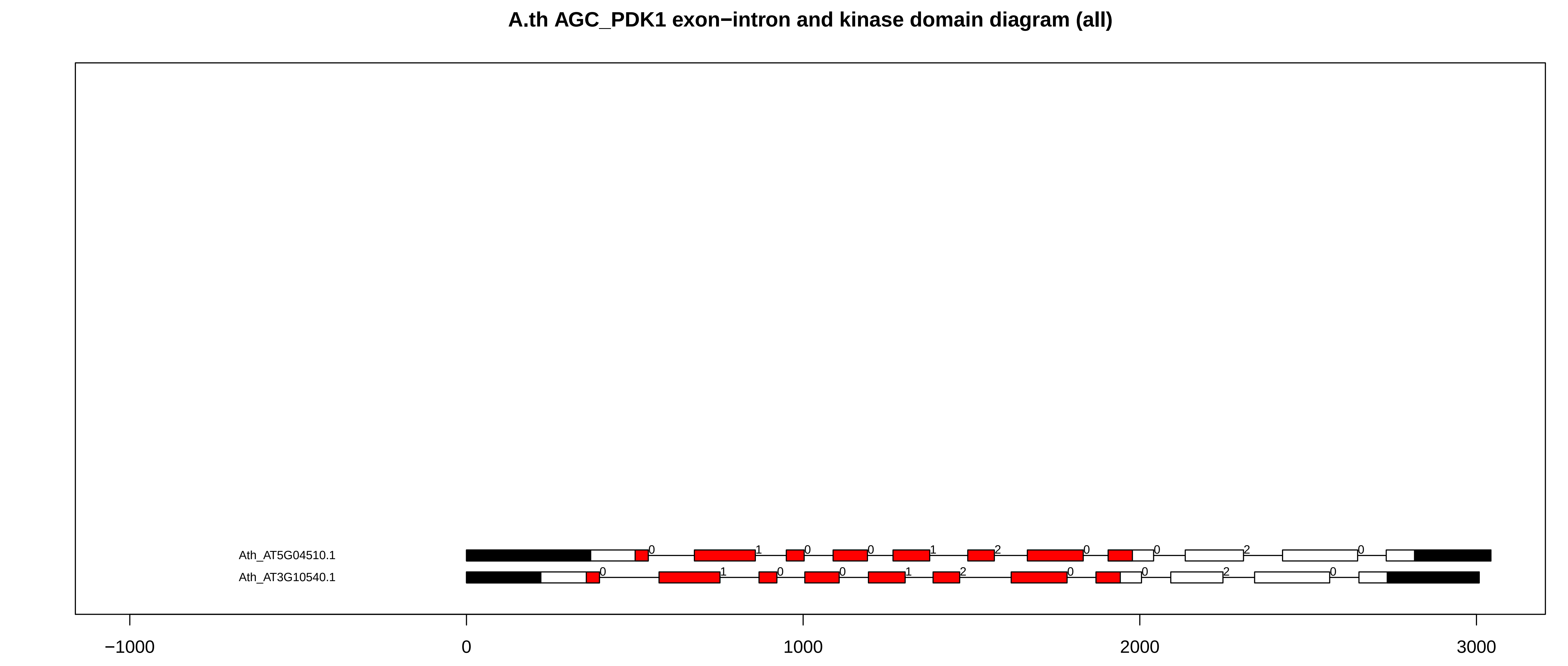
A.th AGC_PDK1 exon−intron and kinase domain diagram (all)
Ath_AT5G04510.1
Ath_AT3G10540.1
0
1
0
0
1
2
0
0
2
0
−1000
0
1000
2000
3000

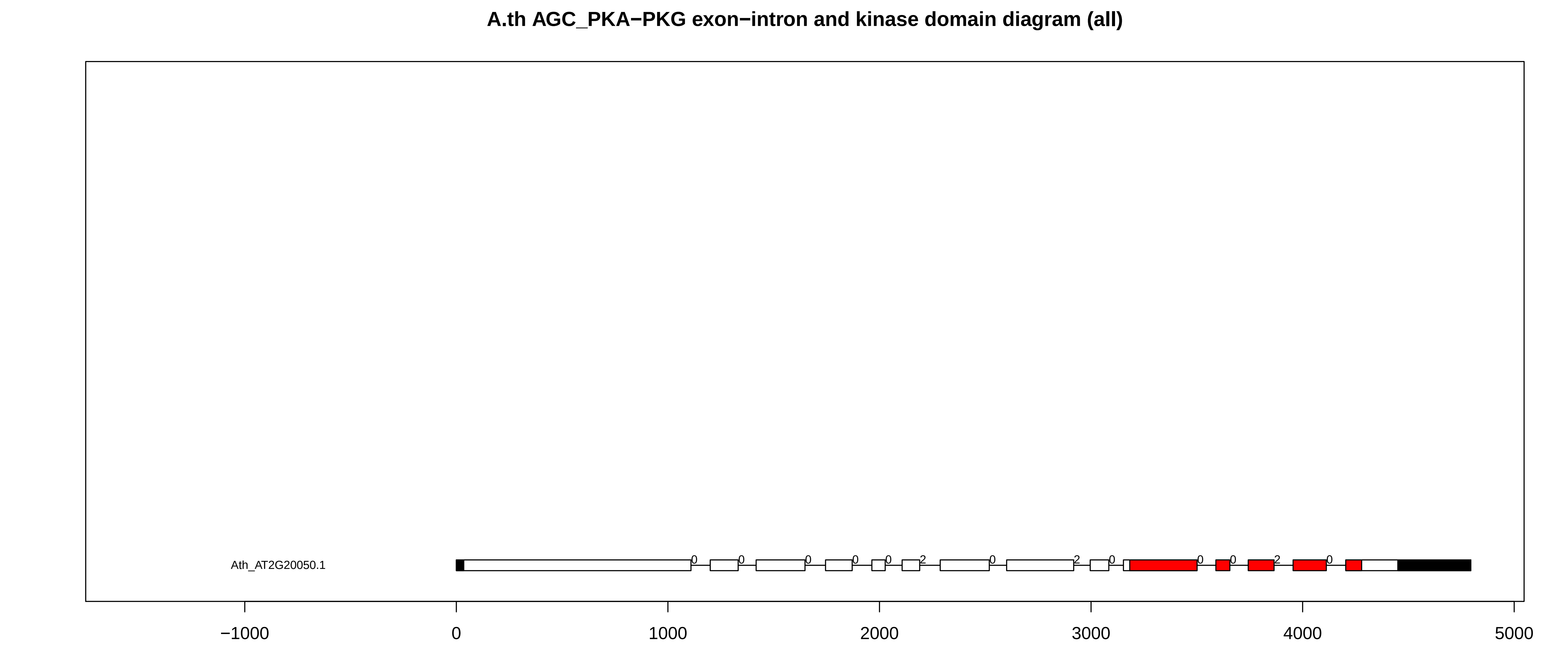
A.th AGC_PKA−PKG exon−intron and kinase domain diagram (all)
Ath_AT2G20050.1
0
0
0
0
0
2
0
2
0
0
0
2
0
−1000
0
1000
2000
3000
4000
5000

A.th AGC_RSK−2 exon−intron and kinase domain diagram (part 1)

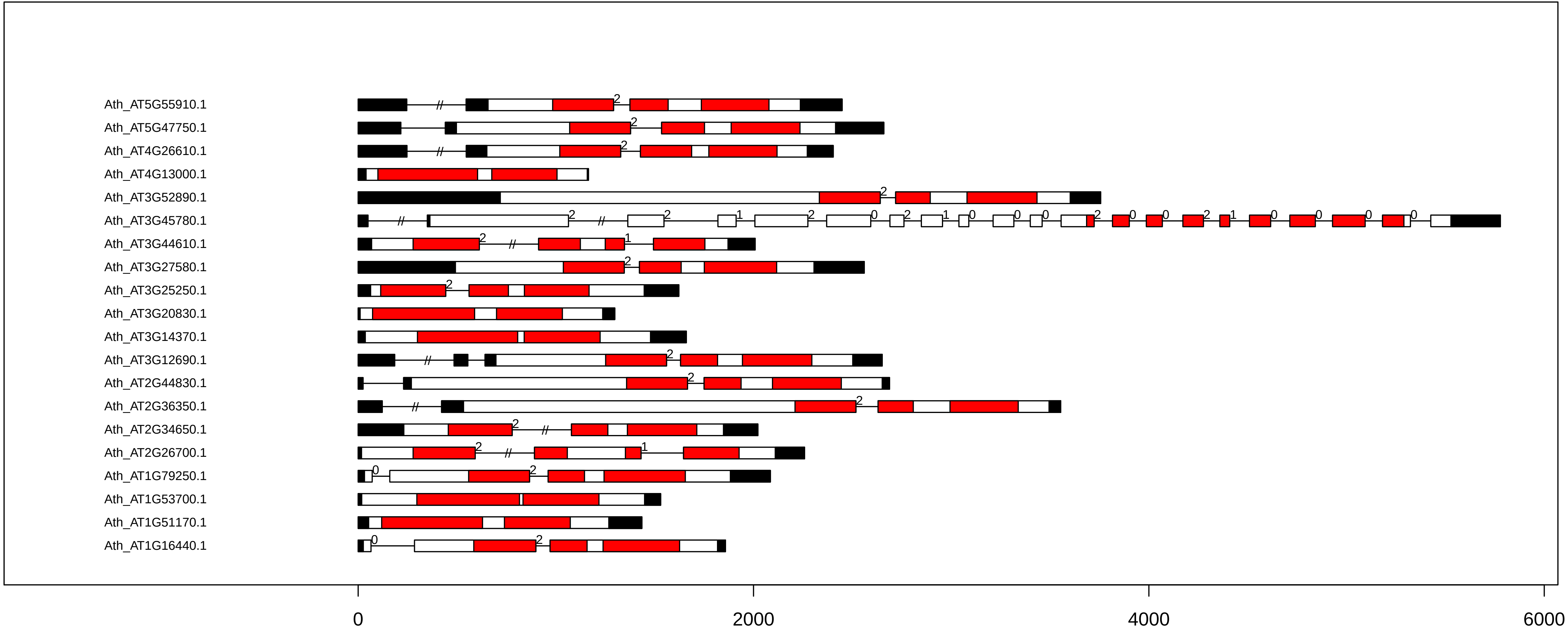

A.th AGC_RSK−2 exon−intron and kinase domain diagram (part 2)

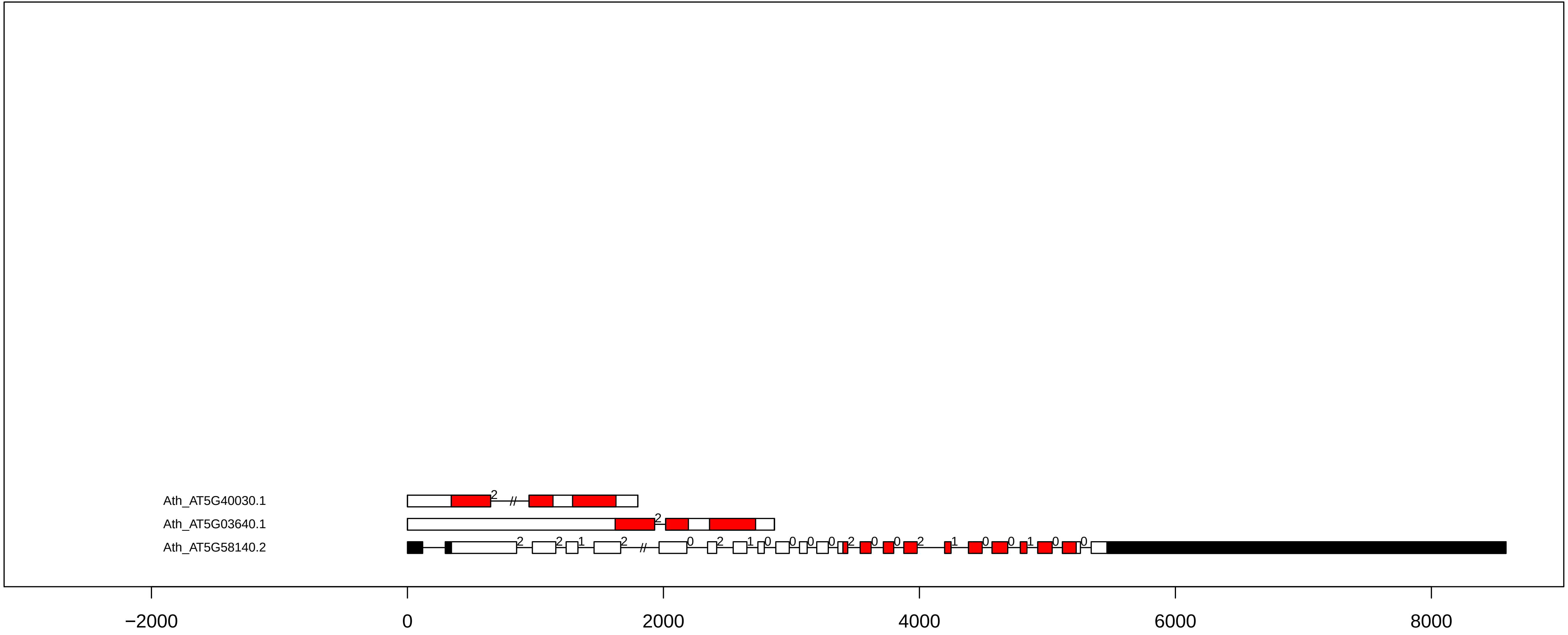

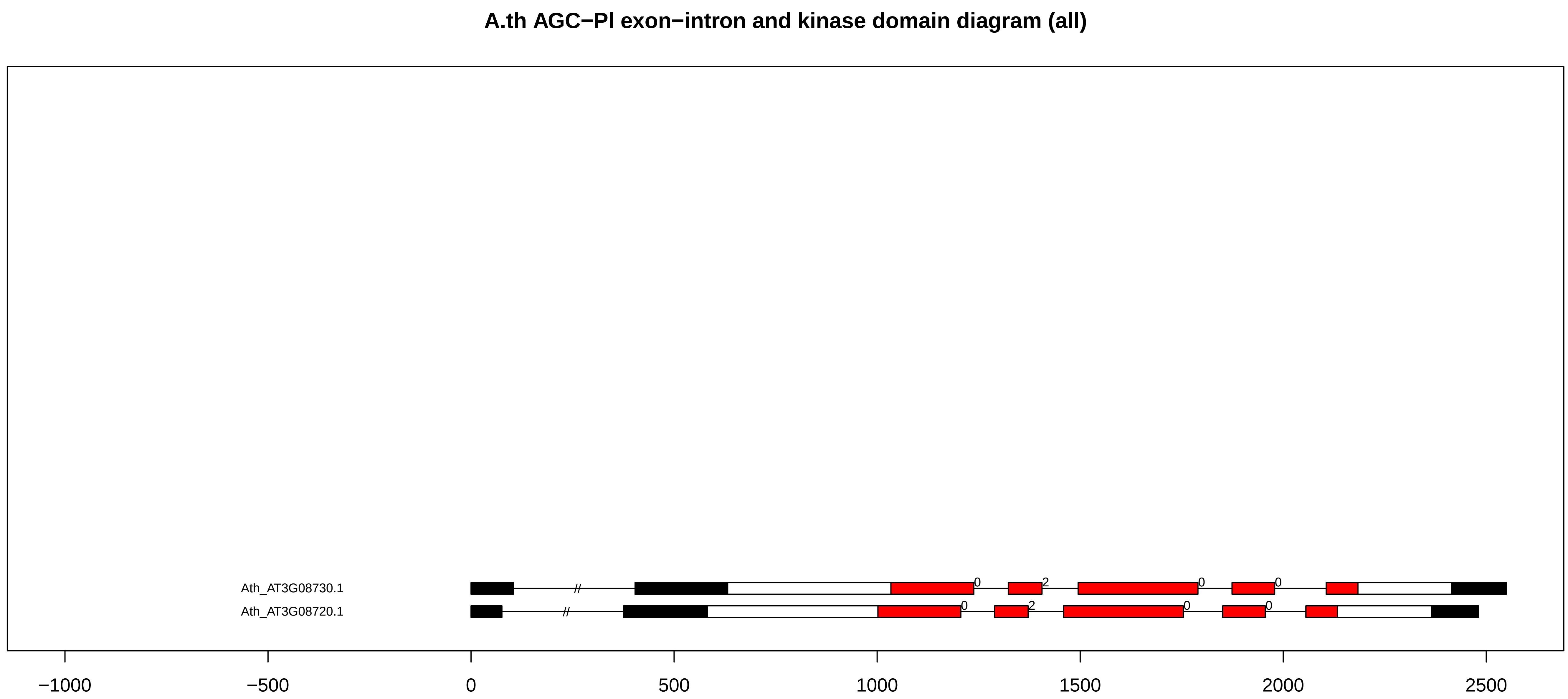
A.th AGC−PI exon−intron and kinase domain diagram (all)
Ath_AT3G08730.1
Ath_AT3G08720.1
0
2
0
0
0
2
0
0
−1000
−500
0
500
1000
1500
2000
2500

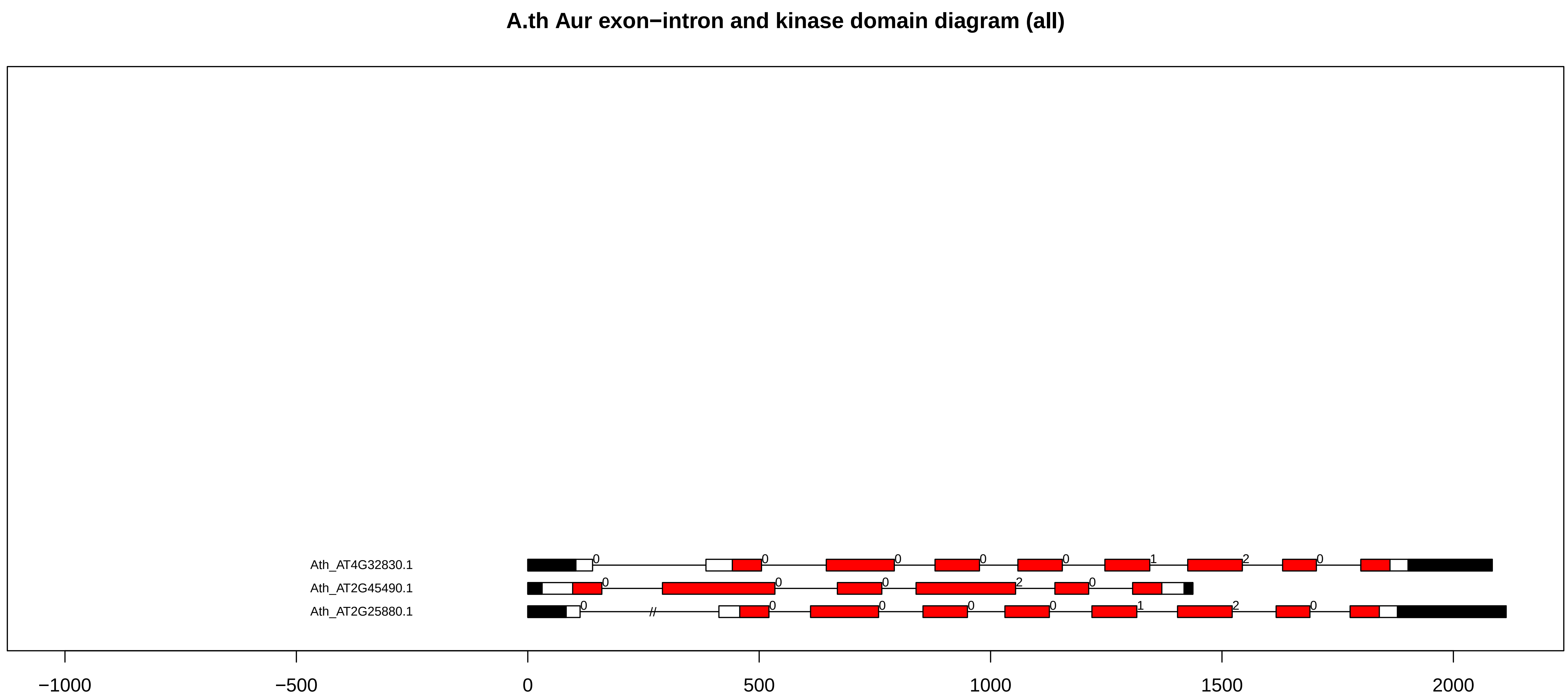
A.th Aur exon−intron and kinase domain diagram (all)
Ath_AT4G32830.1
Ath_AT2G45490.1
Ath_AT2G25880.1
0
0
0
0
0
1
2
0
0
0
0
2
0
0
0
0
0
0
1
2
0
−1000
−500
0
500
1000
1500
2000

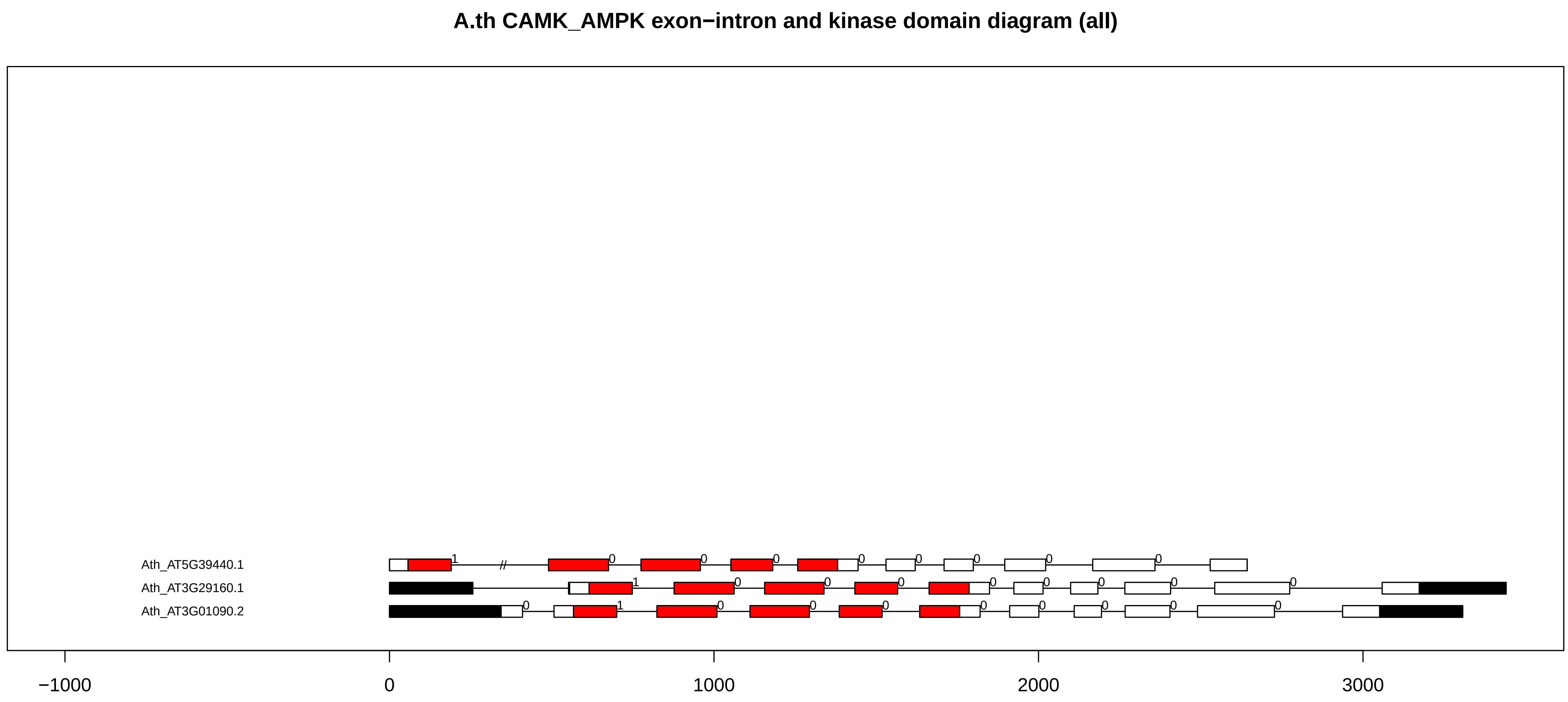
A.th CAMK_AMPK exon−intron and kinase domain diagram (all)
Ath_AT5G39440.1
Ath_AT3G29160.1
Ath_AT3G01090.2
−1000
0
1000
2000
3000

A.th CAMK_CAMKL−CHK1 exon−intron and kinase domain diagram (part 1)

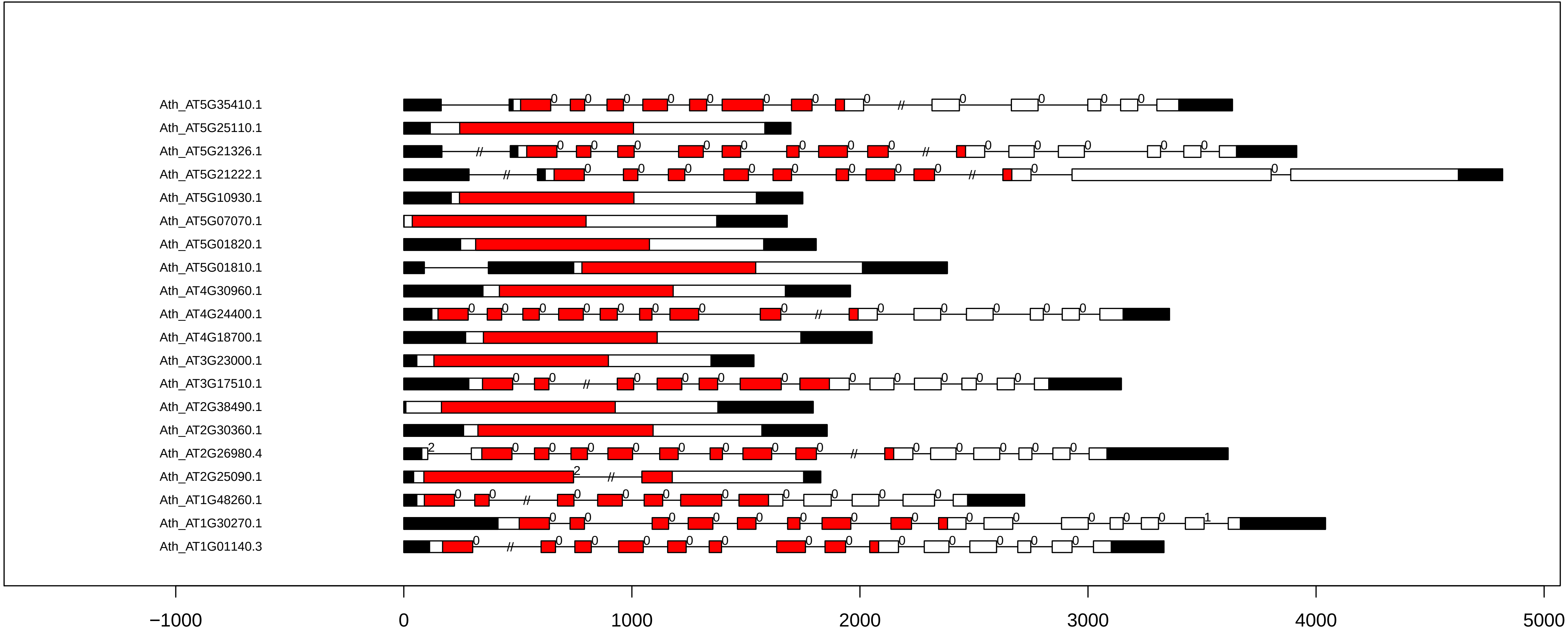

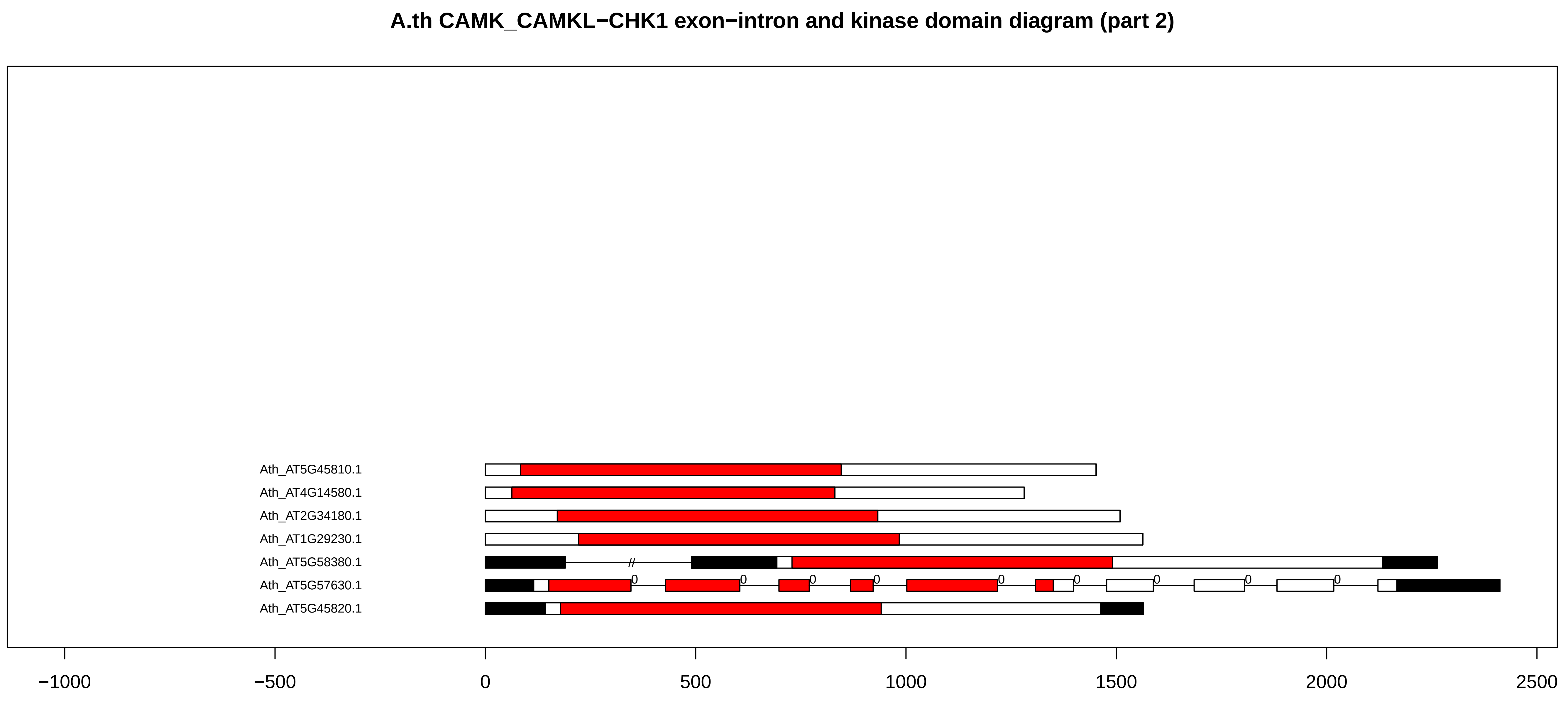
A.th CAMK_CAMKL−CHK1 exon−intron and kinase domain diagram (part 2)
Ath_AT5G45810.1
Ath_AT4G14580.1
Ath_AT2G34180.1
Ath_AT1G29230.1
Ath_AT5G58380.1
Ath_AT5G57630.1
Ath_AT5G45820.1
−1000
−500
0
500
1000
1500
2000
2500

A.th CAMK_CAMKL−LKB exon−intron and kinase domain diagram (all)

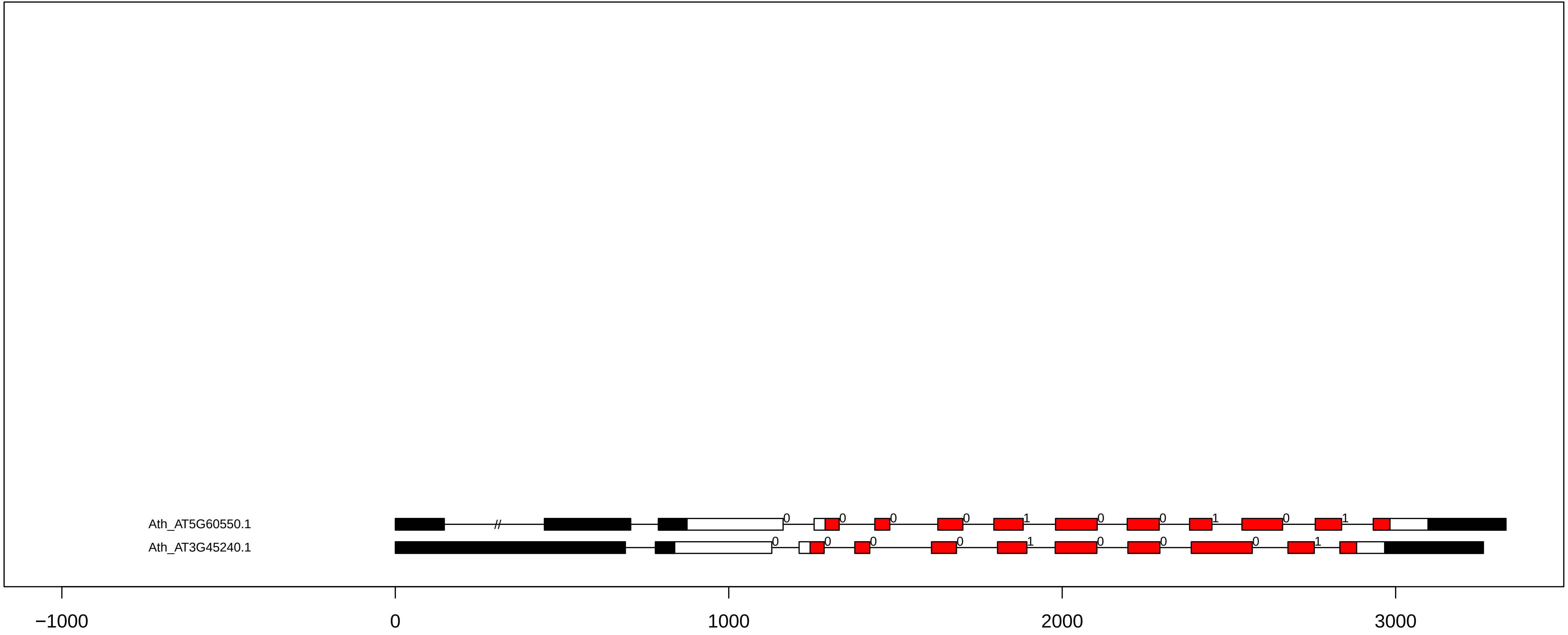

A.th CAMK_CDPK exon−intron and kinase domain diagram (part 1)

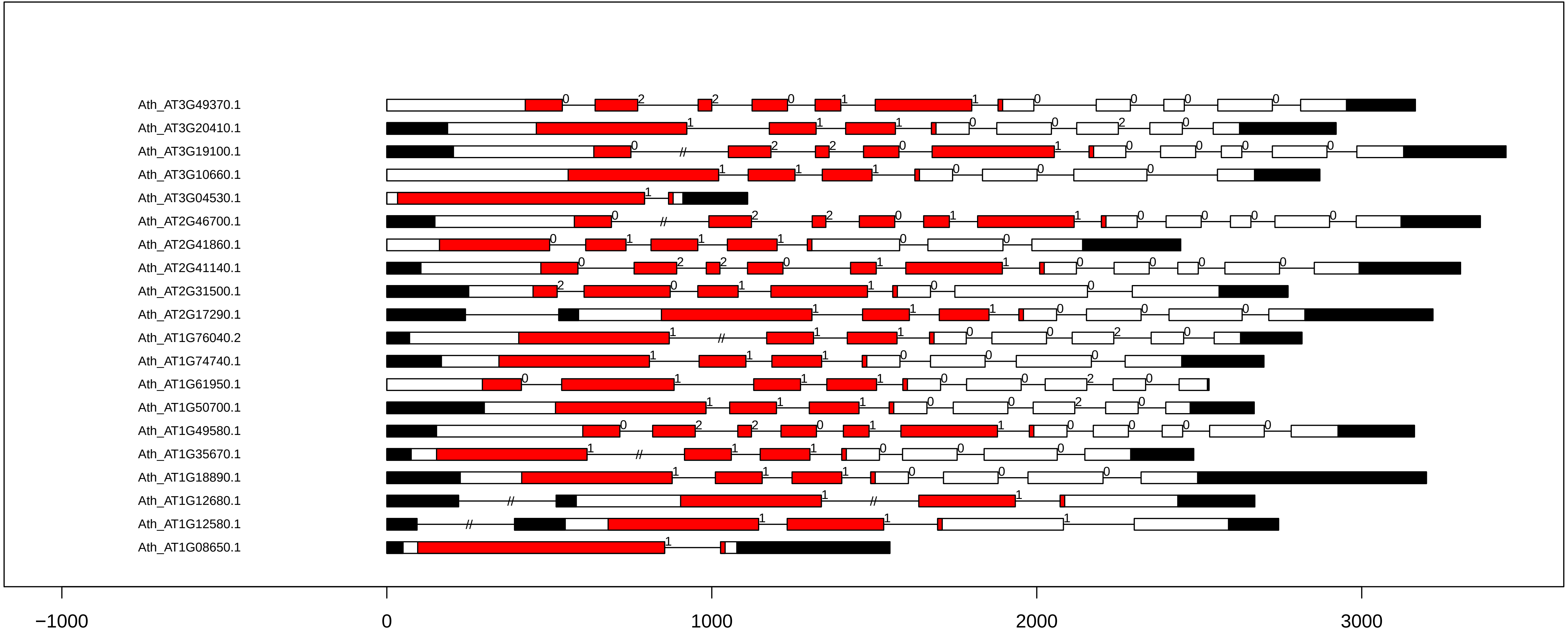

A.th CAMK_CDPK exon−intron and kinase domain diagram (part 2)

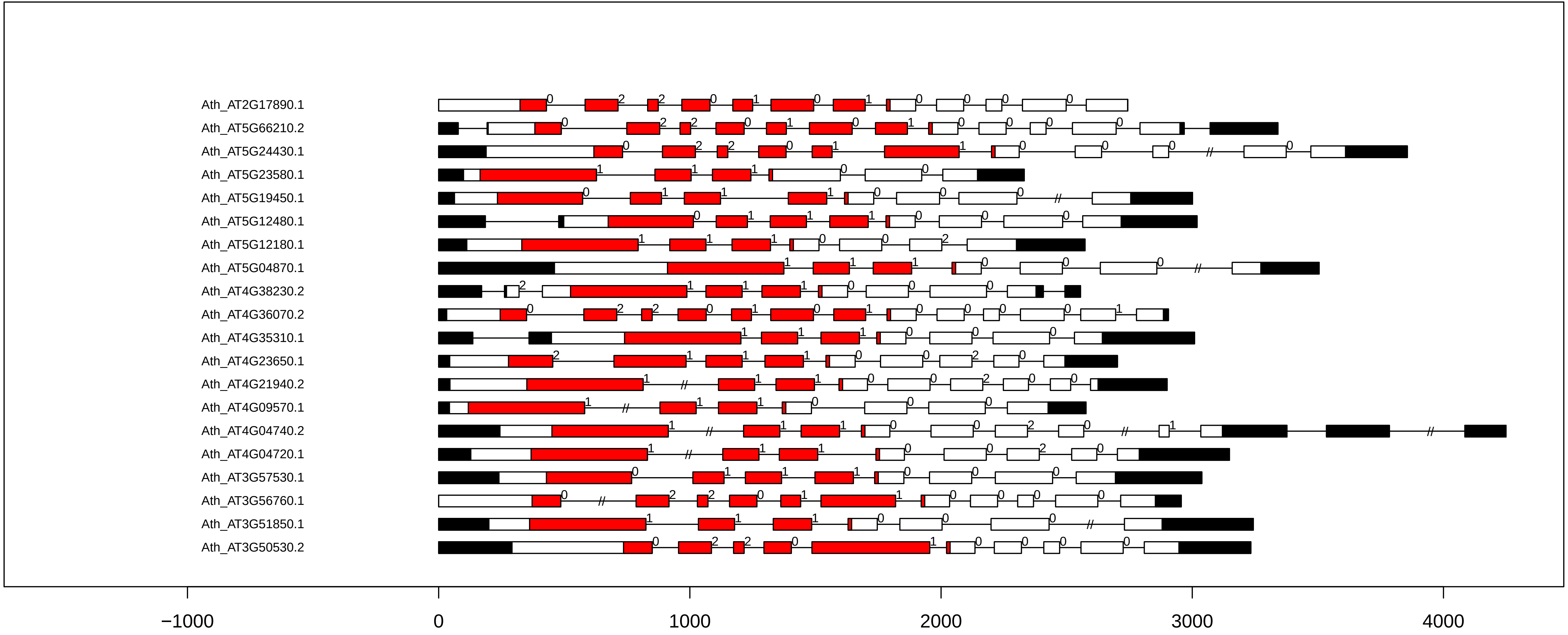

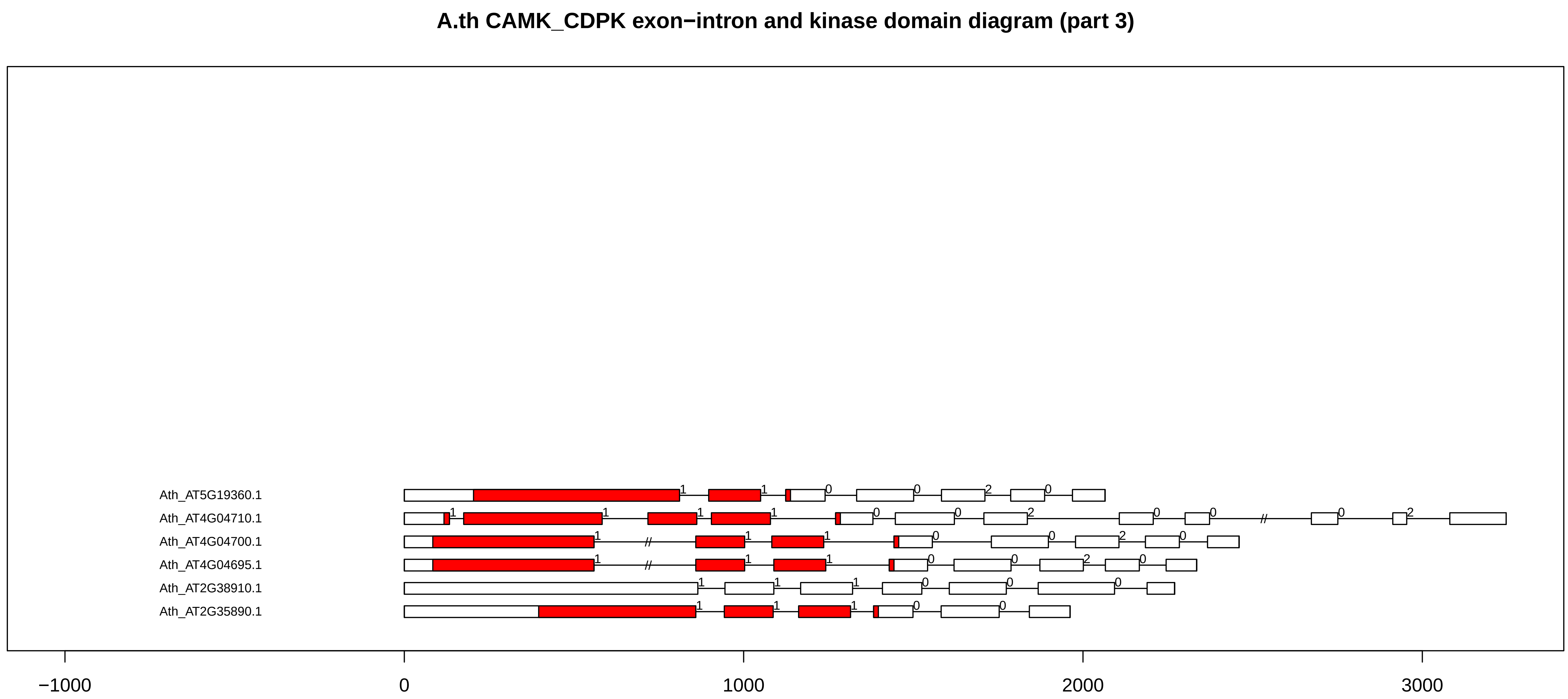
A.th CAMK_CDPK exon−intron and kinase domain diagram (part 3)
Ath_AT5G19360.1
Ath_AT4G04710.1
Ath_AT4G04700.1
Ath_AT4G04695.1
Ath_AT2G38910.1
Ath_AT2G35890.1
−1000
0
1000
2000
3000

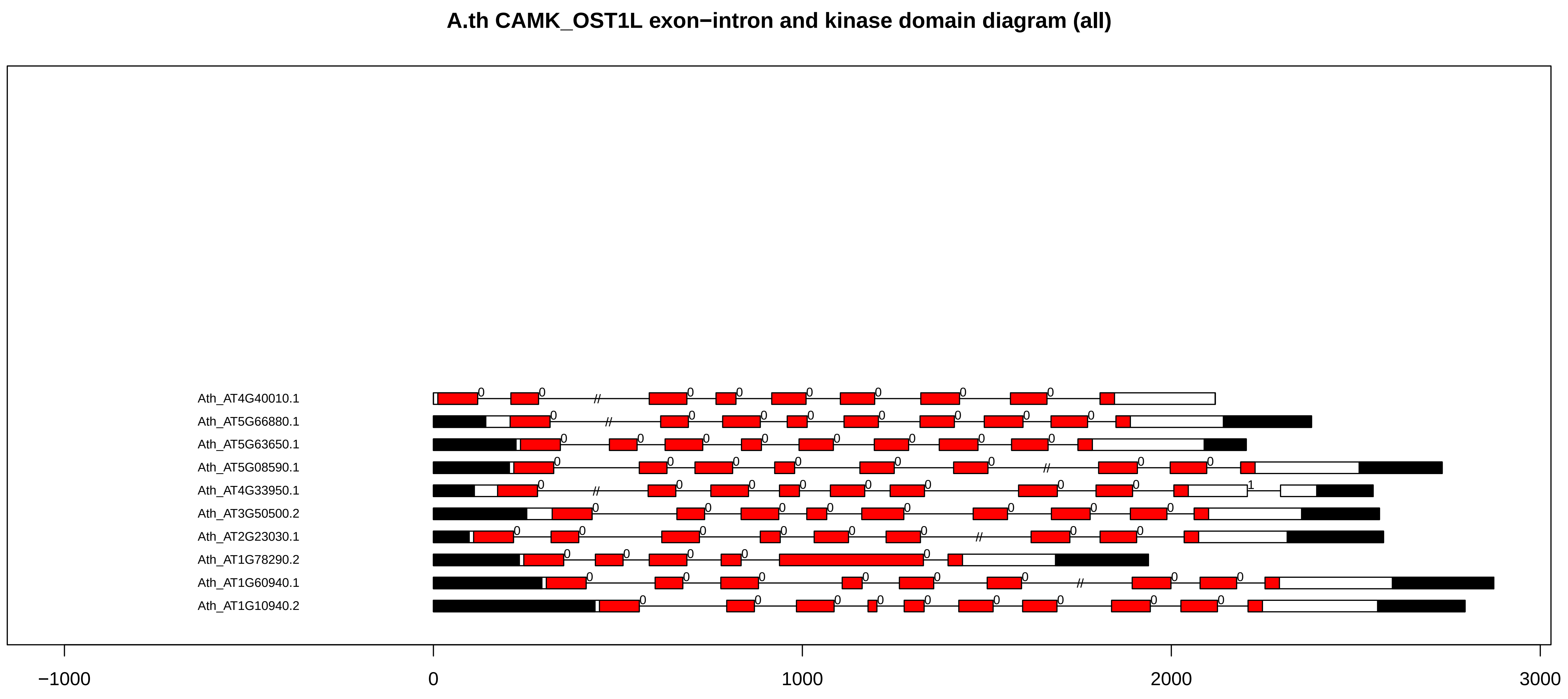

A.th CAMK_OST1L exon−intron and kinase domain diagram (all)
Ath_AT4G40010.1
Ath_AT5G66880.1
Ath_AT5G63650.1
Ath_AT5G08590.1
Ath_AT4G33950.1
Ath_AT3G50500.2
Ath_AT2G23030.1
Ath_AT1G78290.2
Ath_AT1G60940.1
Ath_AT1G10940.2
−1000
0
1000
2000
3000

A.th CK1_CK1 exon−intron and kinase domain diagram (all)

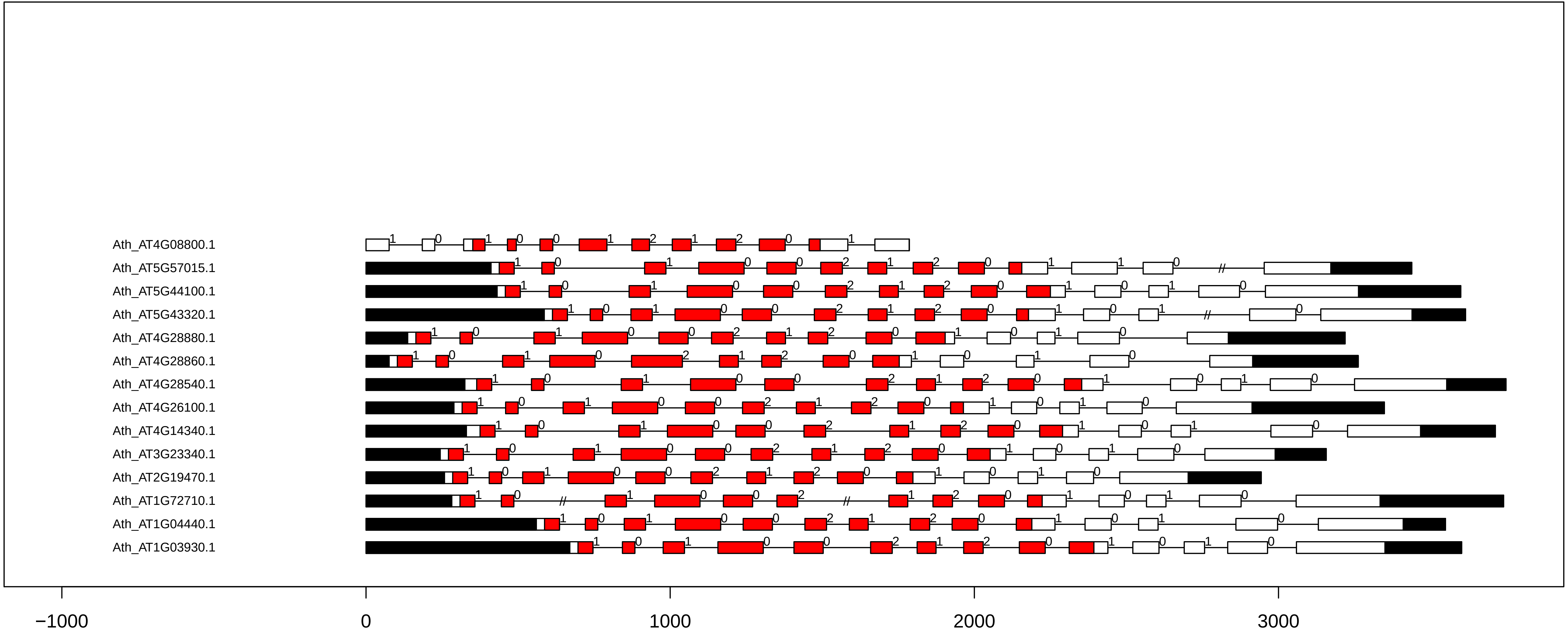

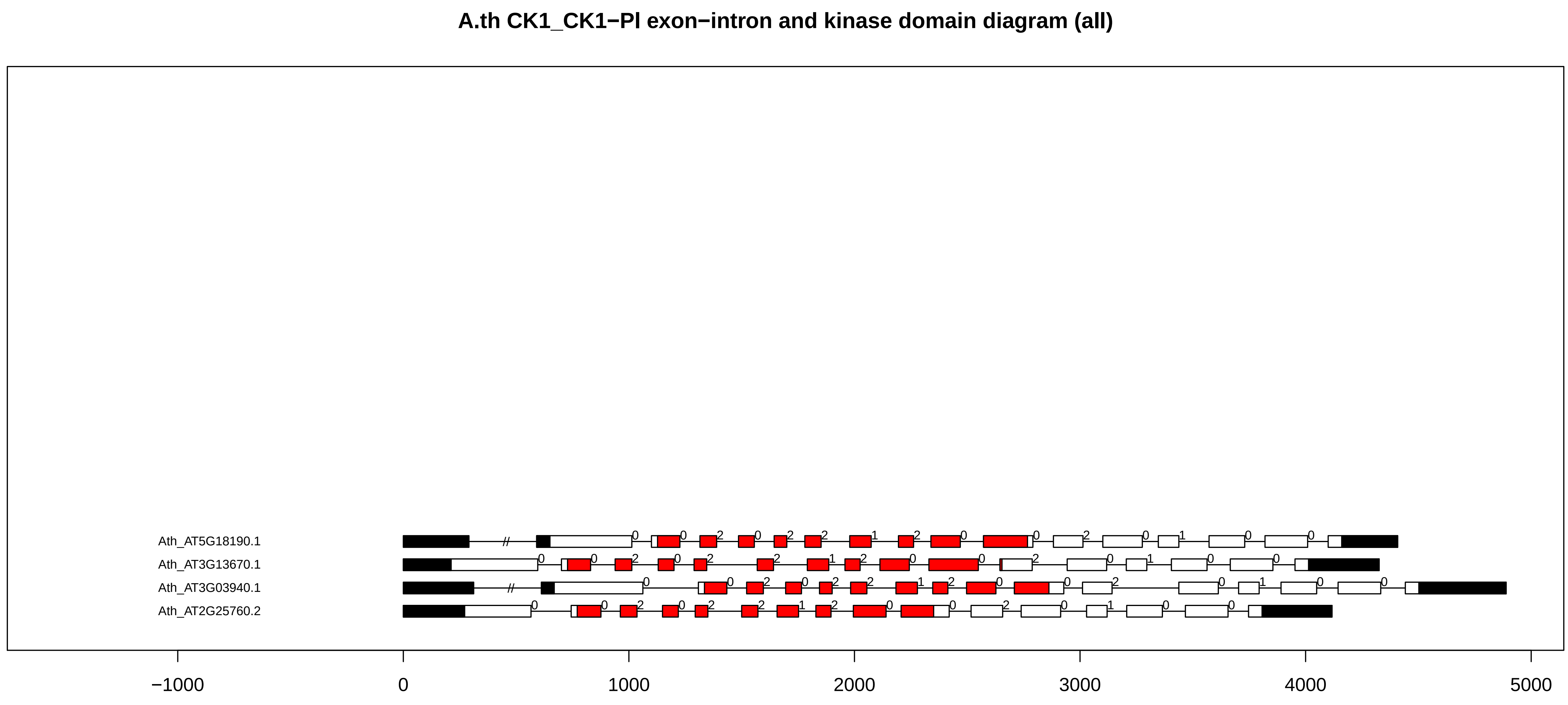
A.th CK1_CK1−PI exon−intron and kinase domain diagram (all)
Ath_AT5G18190.1
Ath_AT3G13670.1
Ath_AT3G03940.1
Ath_AT2G25760.2
−1000
0
1000
2000
3000
4000
5000

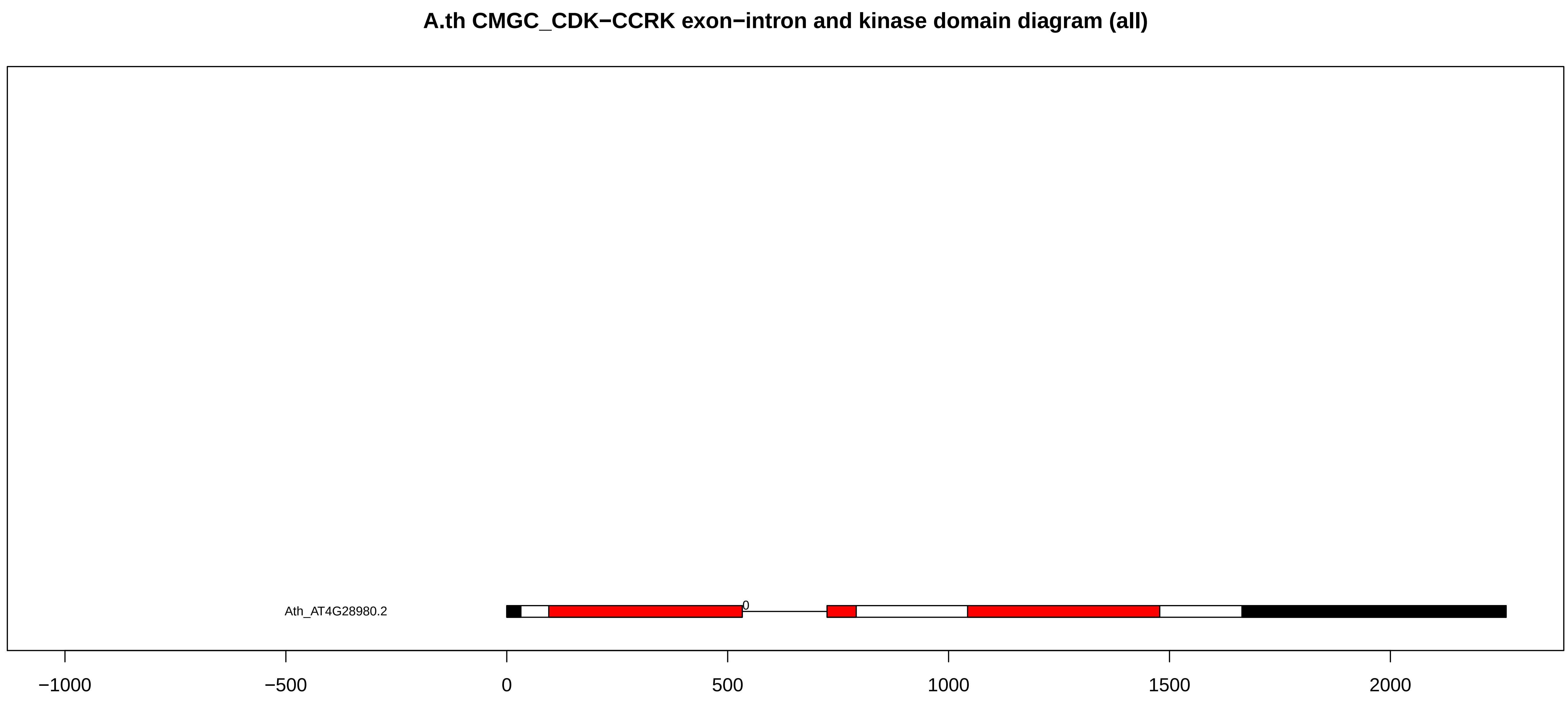

A.th CMGC_CDK−CCRK exon−intron and kinase domain diagram (all)
Ath_AT4G28980.2
0
−1000
−500
0
500
1000
1500
2000

A.th CMGC_CDK−CDK7 exon−intron and kinase domain diagram (all)

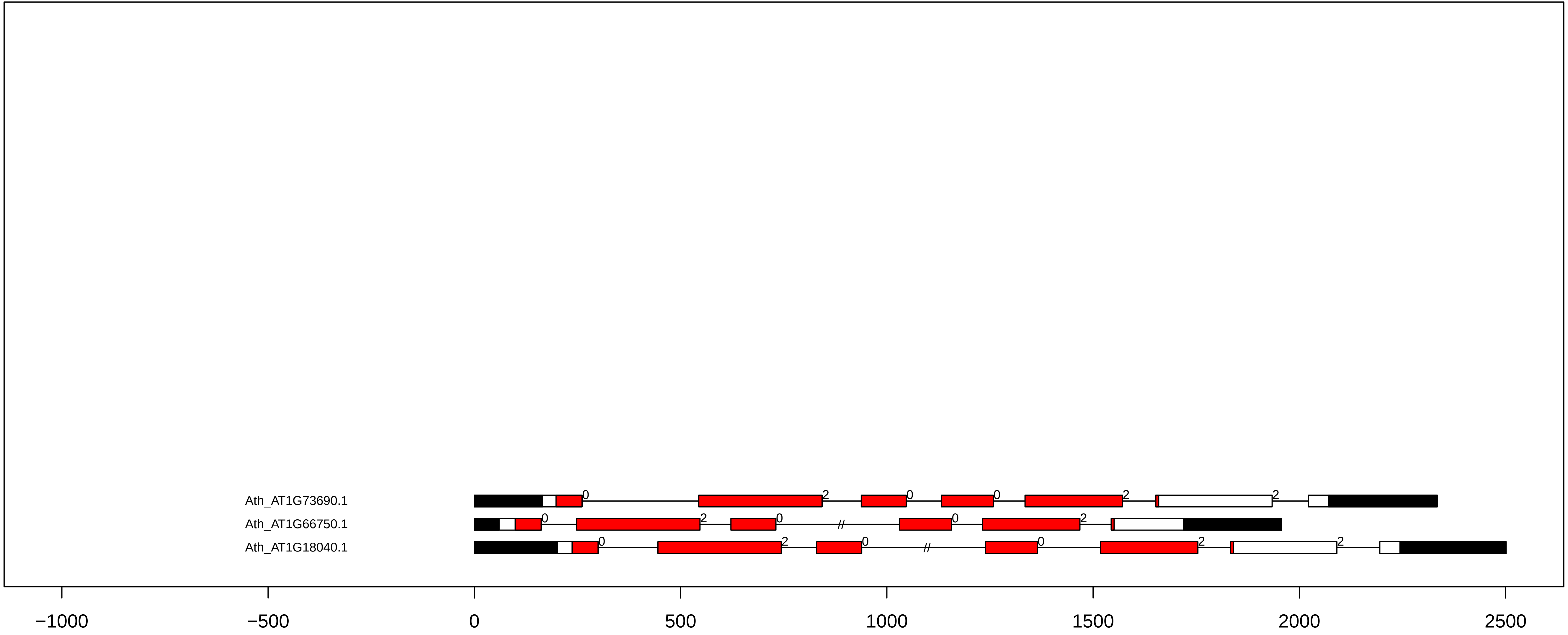

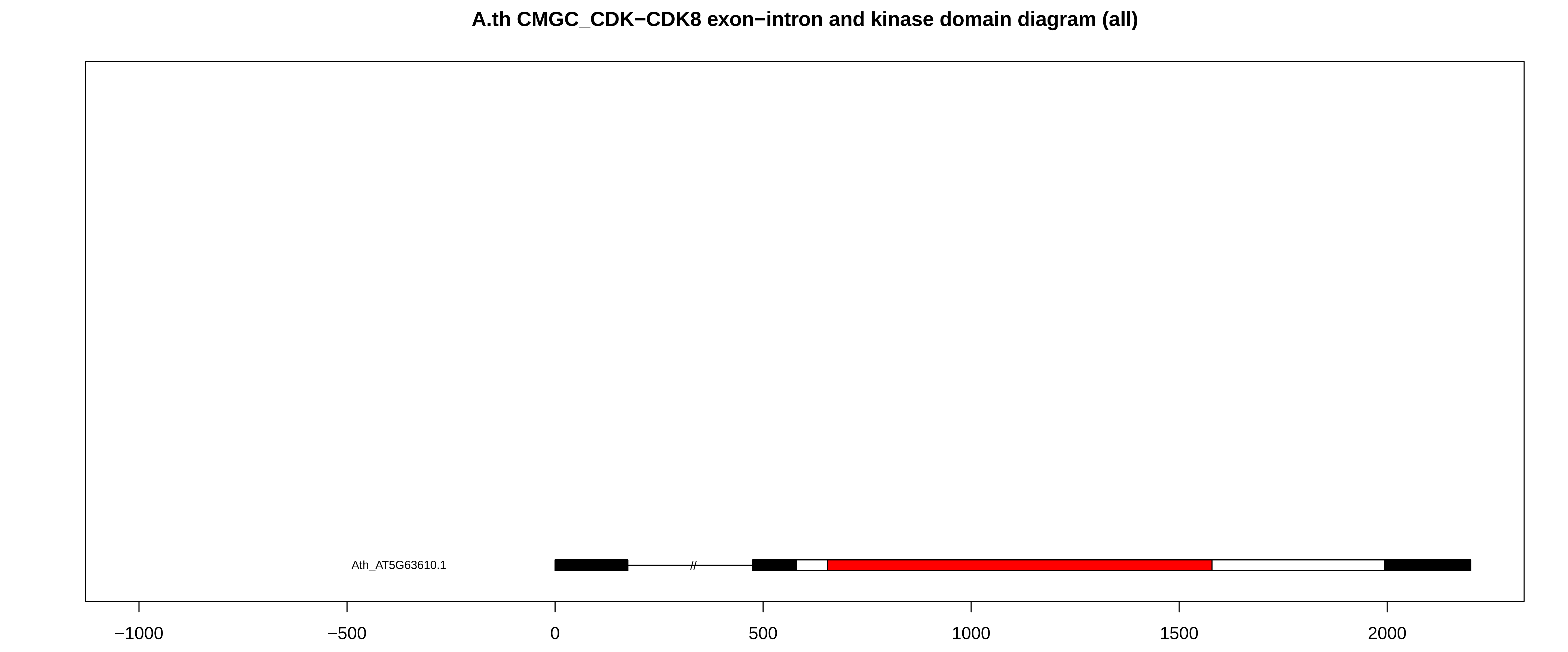
A.th CMGC_CDK−CDK8 exon−intron and kinase domain diagram (all)
Ath_AT5G63610.1
−1000
−500
0
500
1000
1500
2000

A.th CMGC_CDK−CRK7−CDK9 exon−intron and kinase domain diagram (all)

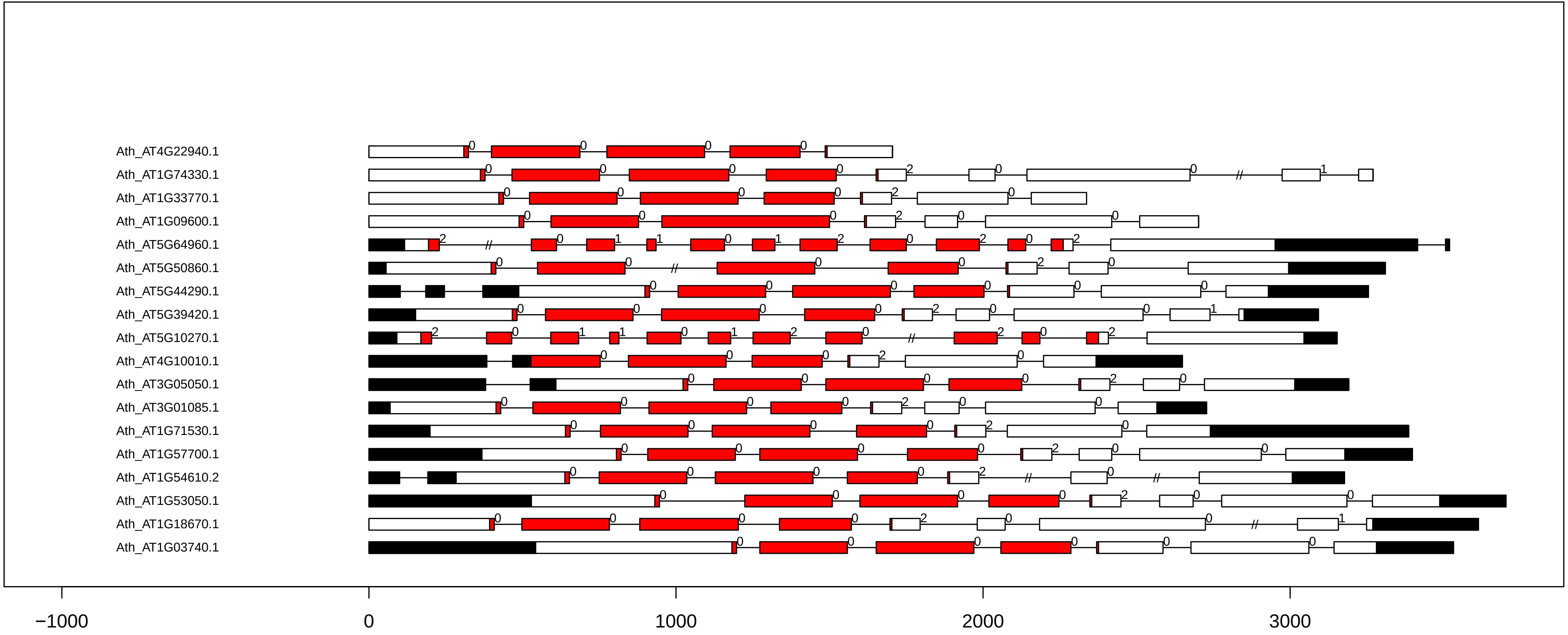

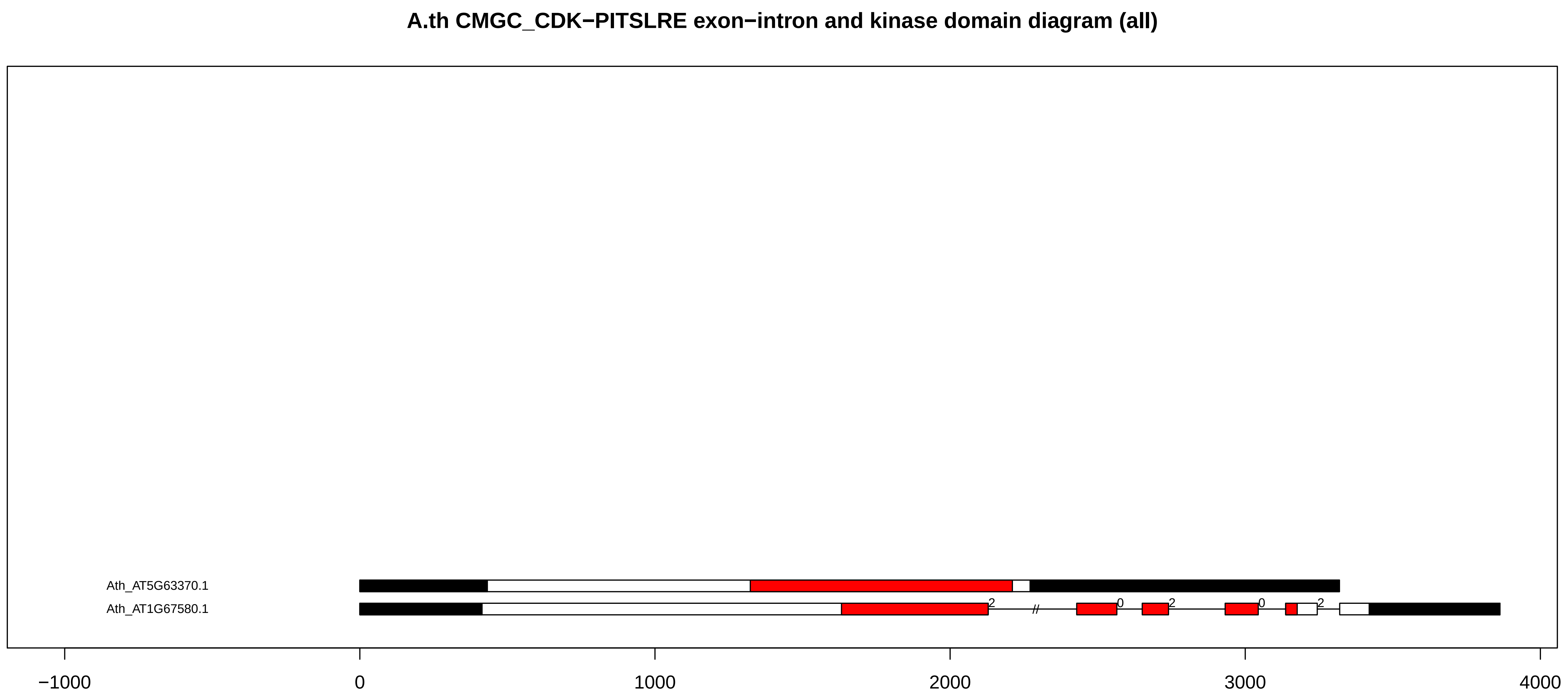
A.th CMGC_CDK−PITSLRE exon−intron and kinase domain diagram (all)
Ath_AT5G63370.1
Ath_AT1G67580.1
2
0
2
0
2
//
−1000
0
1000
2000
3000
4000

A.th CMGC_CDK−Pl exon−intron and kinase domain diagram (all)

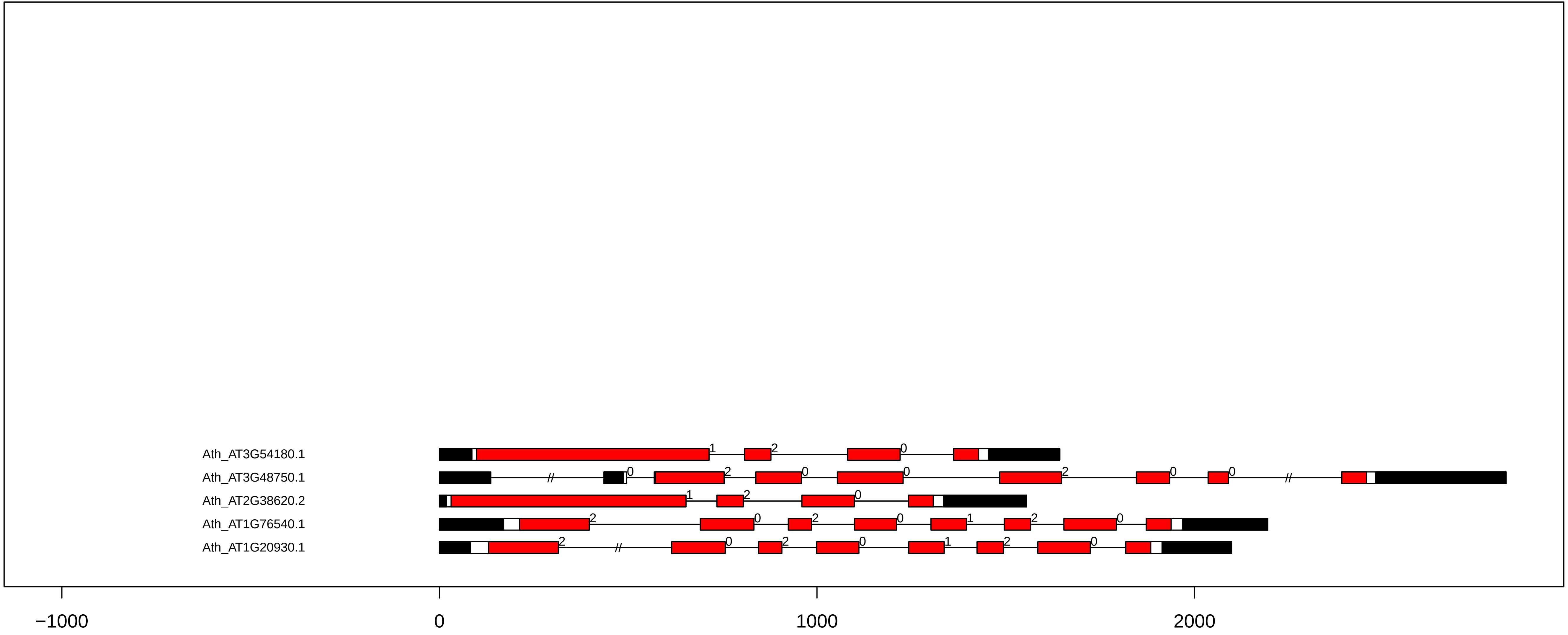

A.th CMGC_CK2 exon−intron and kinase domain diagram (all)

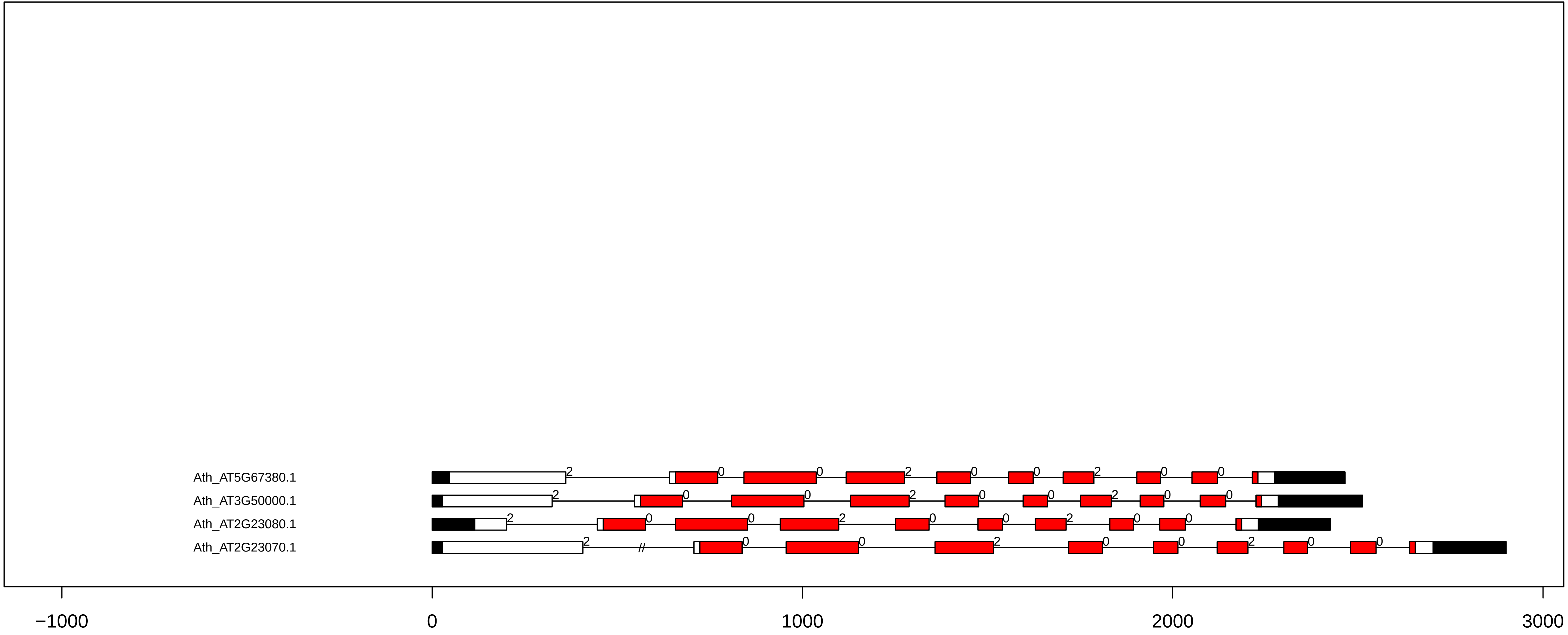

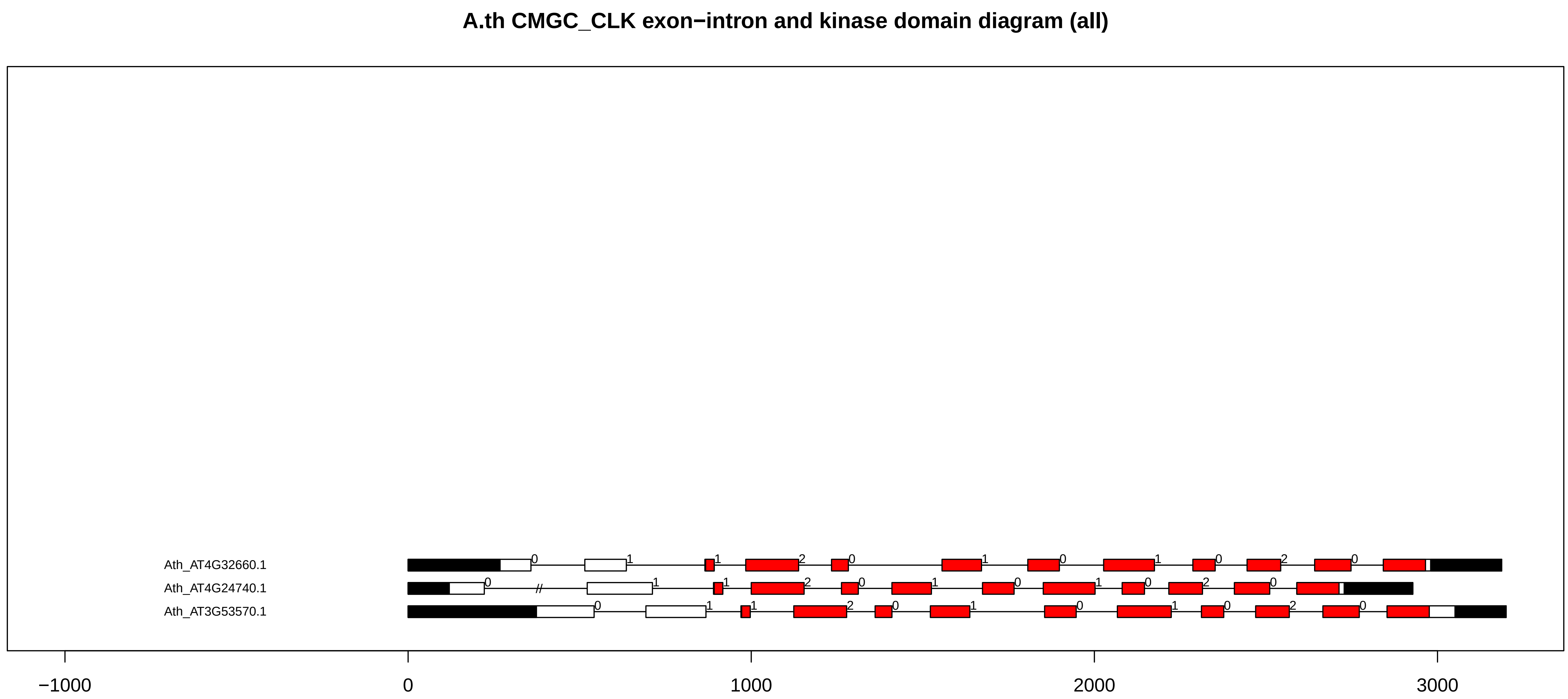

A.th CMGC_CLK exon−intron and kinase domain diagram (all)
Ath_AT4G32660.1
Ath_AT4G24740.1
Ath_AT3G53570.1
−1000
0
1000
2000
3000

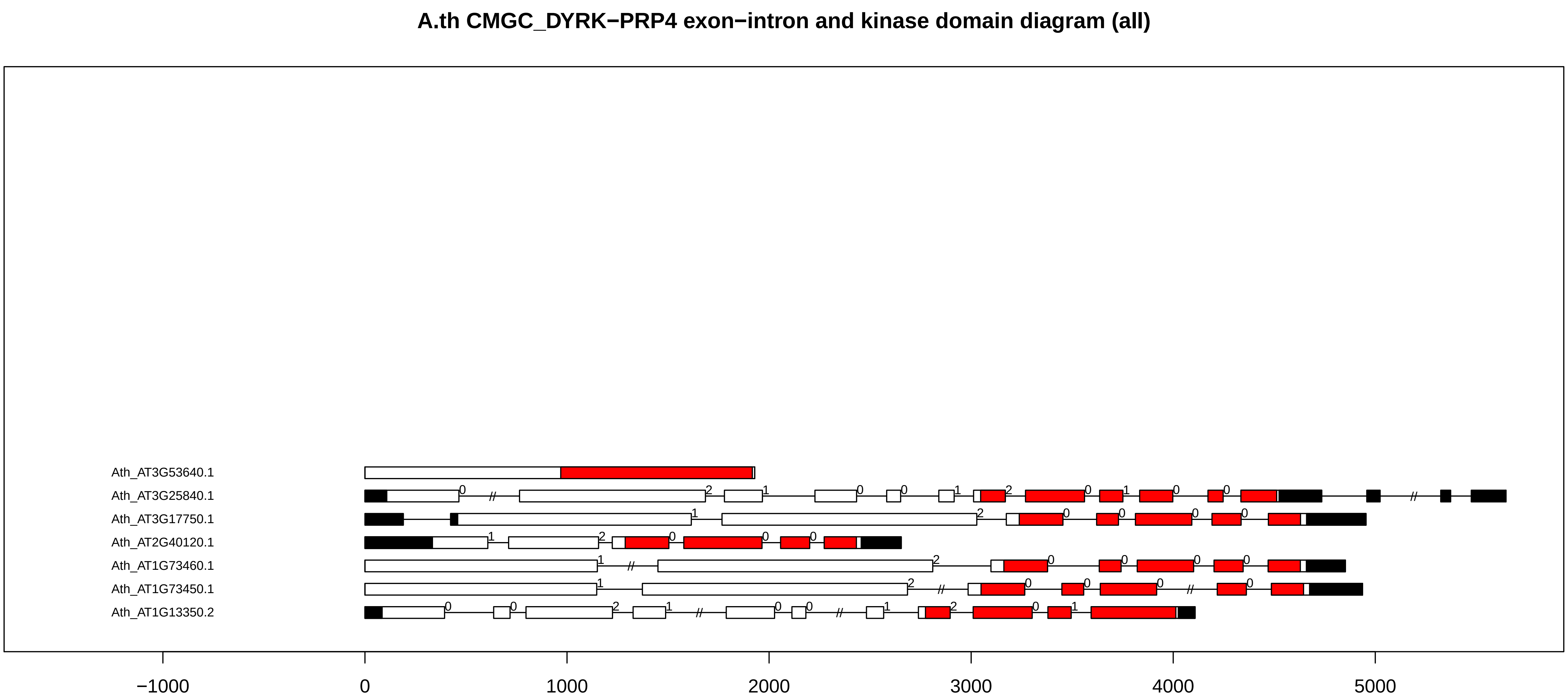

A.th CMGC_DYRK−PRP4 exon−intron and kinase domain diagram (all)
Ath_AT3G53640.1
Ath_AT3G25840.1
Ath_AT3G17750.1
Ath_AT2G40120.1
Ath_AT1G73460.1
Ath_AT1G73450.1
Ath_AT1G13350.2
−1000
0
1000
2000
3000
4000
5000

A.th CMGC_DYRK−YAK exon−intron and kinase domain diagram (all)

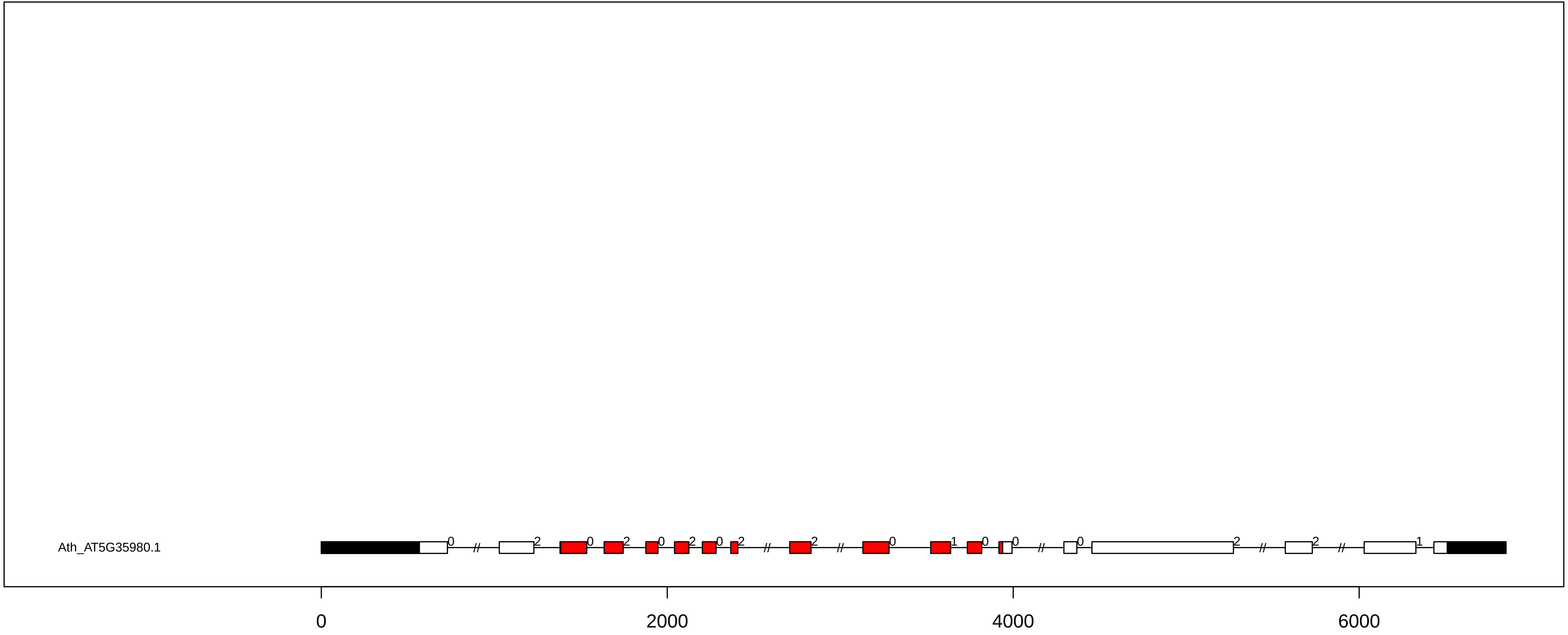

A.th CMGC_GSKL exon−intron and kinase domain diagram (all)

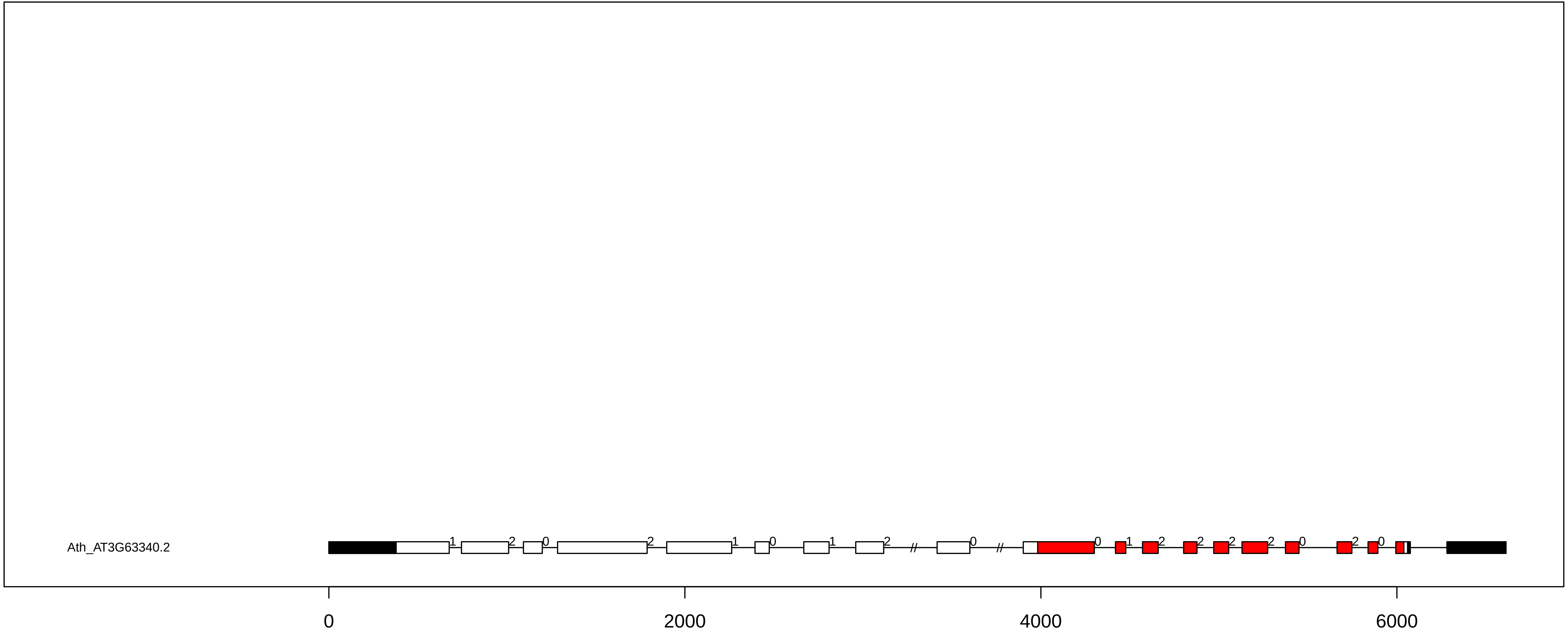

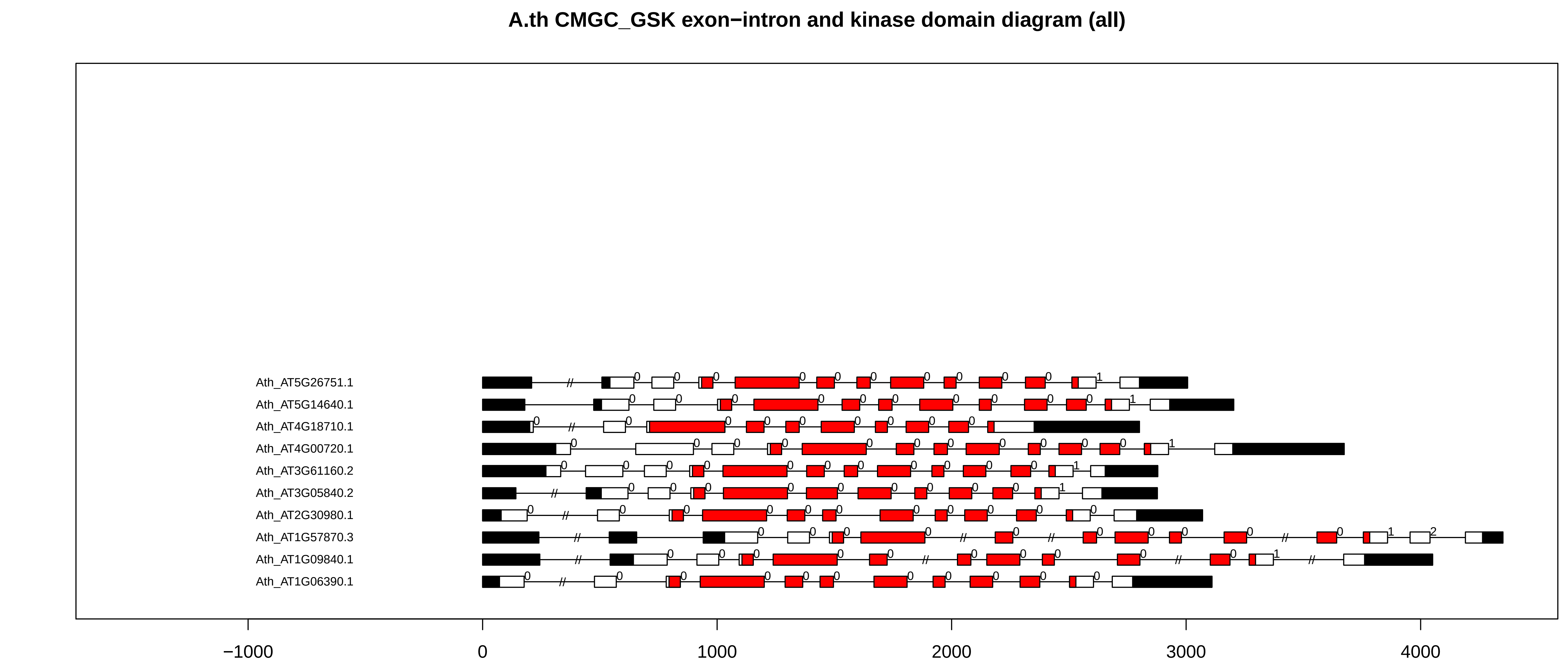
A.th CMGC_GSK exon−intron and kinase domain diagram (all)
Ath_AT5G26751.1
Ath_AT5G14640.1
Ath_AT4G18710.1
Ath_AT4G00720.1
Ath_AT3G61160.2
Ath_AT3G05840.2
Ath_AT2G30980.1
Ath_AT1G57870.3
Ath_AT1G09840.1
Ath_AT1G06390.1
−1000
0
1000
2000
3000
4000

A.th CMGC_MAPK exon−intron and kinase domain diagram (all)

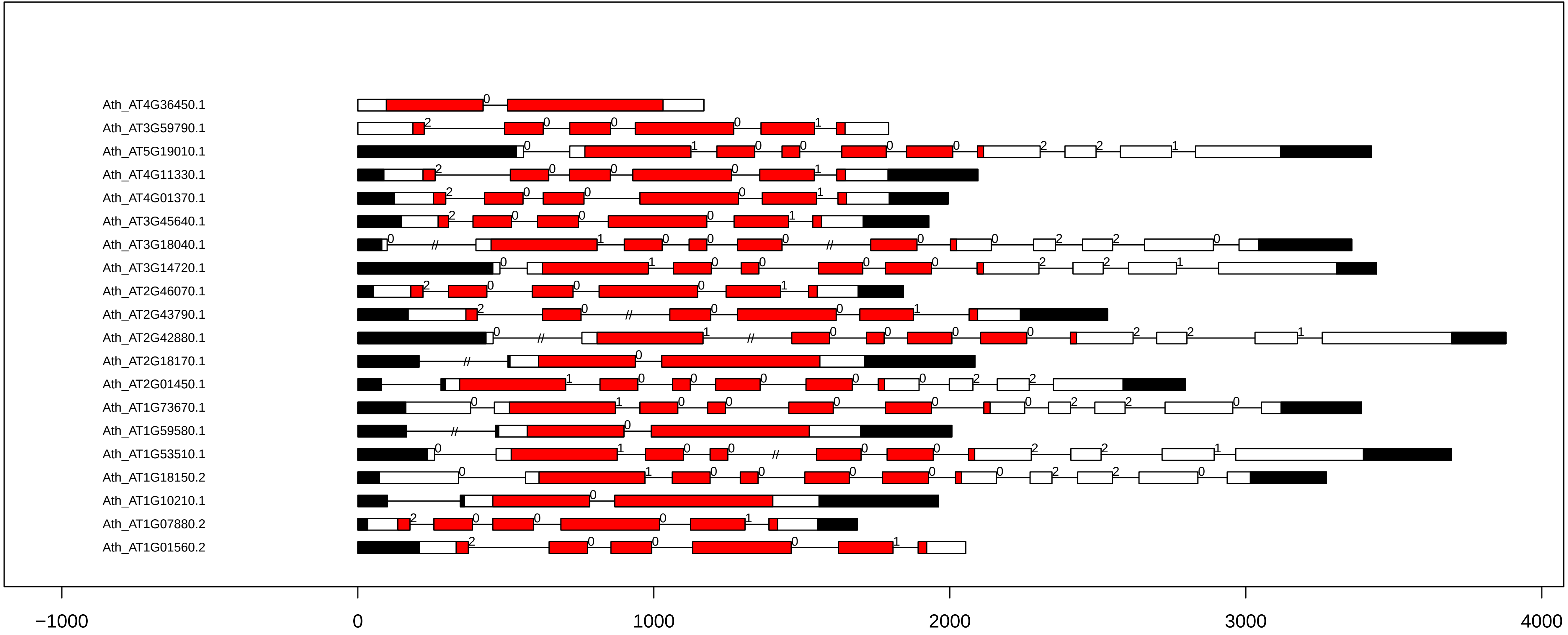

A.th CMGC_Pl−Tthe exon−intron and kinase domain diagram (all)

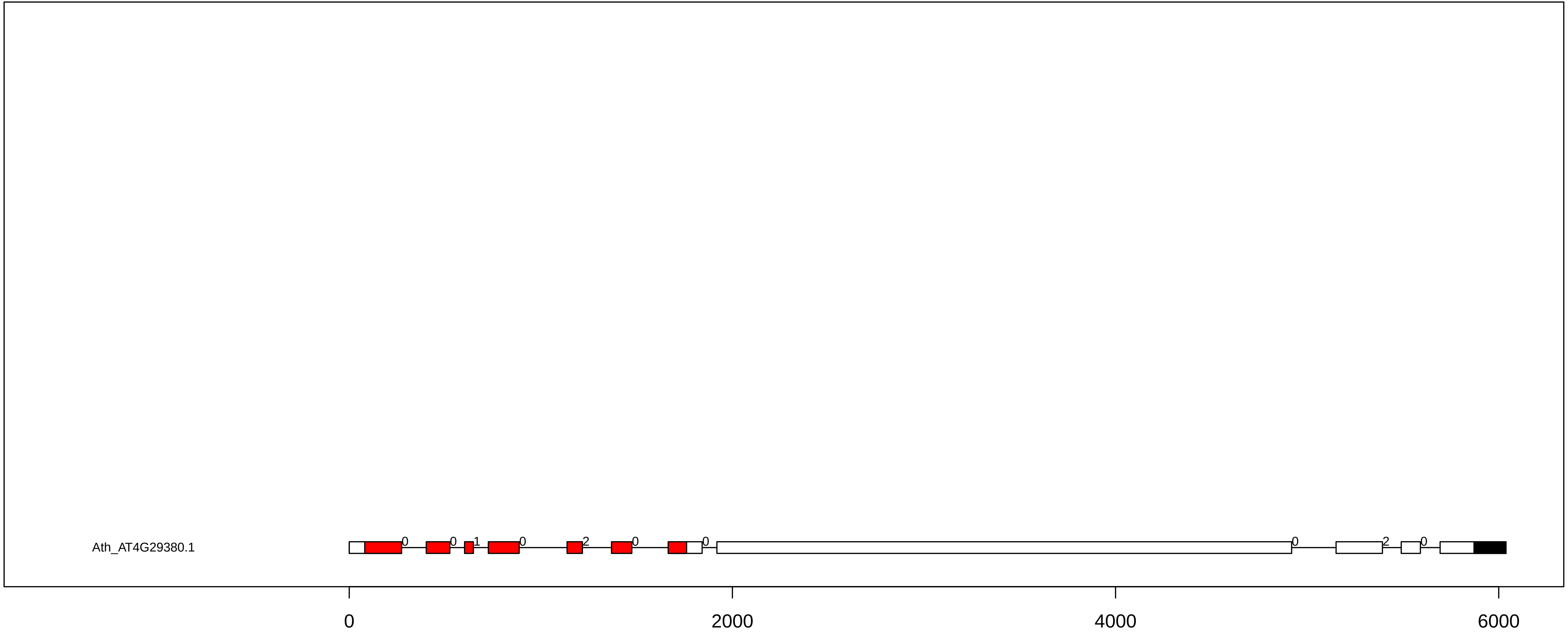

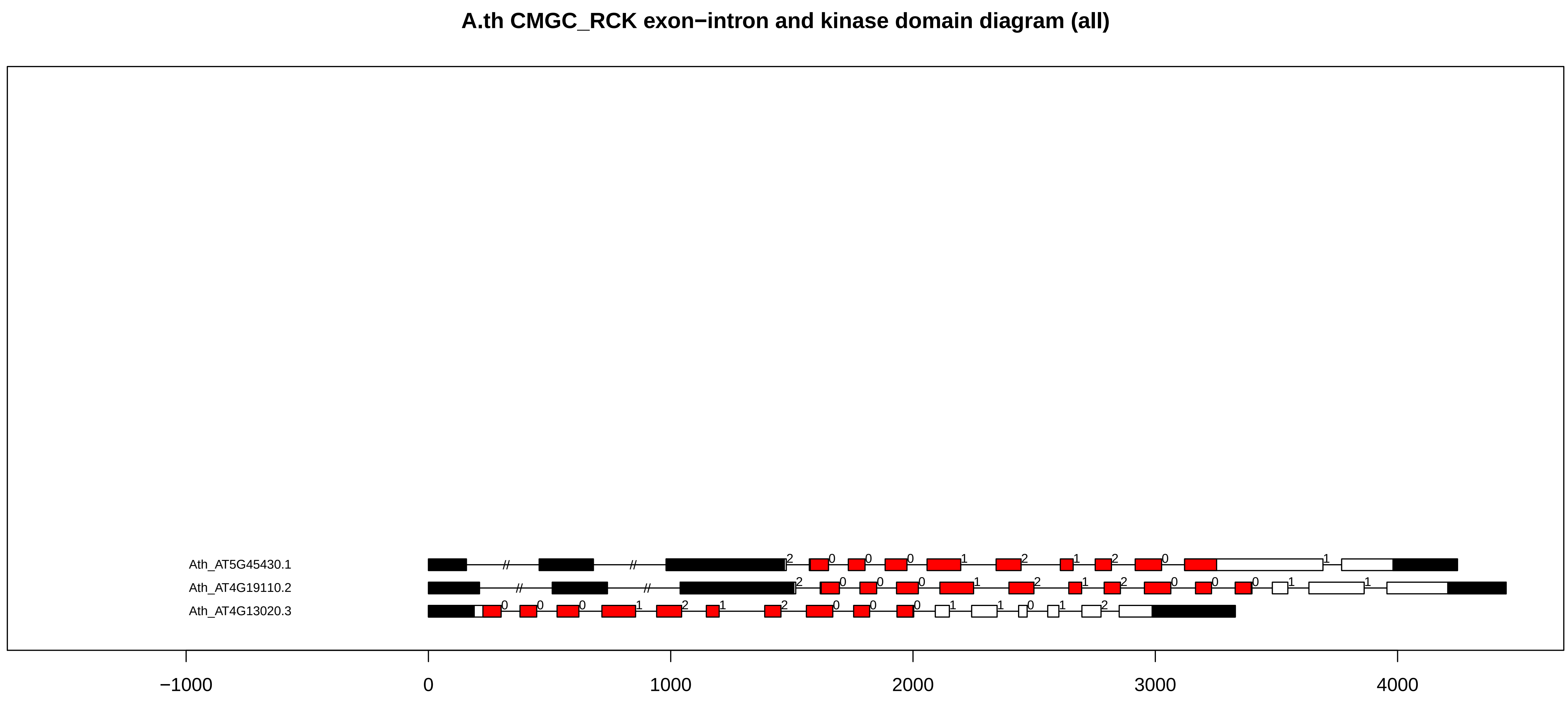
A.th CMGC_RCK exon−intron and kinase domain diagram (all)
Ath_AT5G45430.1
Ath_AT4G19110.2
Ath_AT4G13020.3
−1000
0
1000
2000
3000
4000

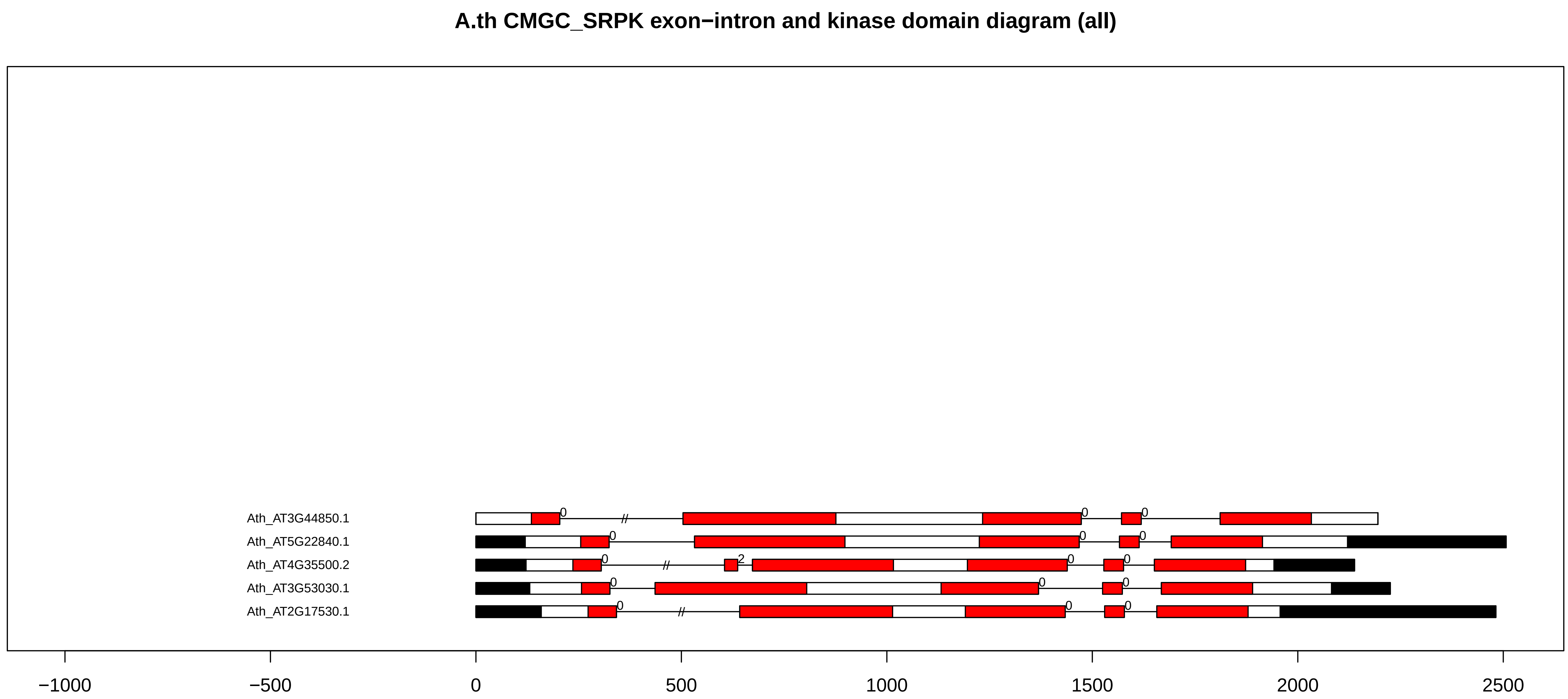
A.th CMGC_SRPK exon−intron and kinase domain diagram (all)
Ath_AT3G44850.1
Ath_AT5G22840.1
Ath_AT4G35500.2
Ath_AT3G53030.1
Ath_AT2G17530.1
−1000
−500
0
500
1000
1500
2000
2500

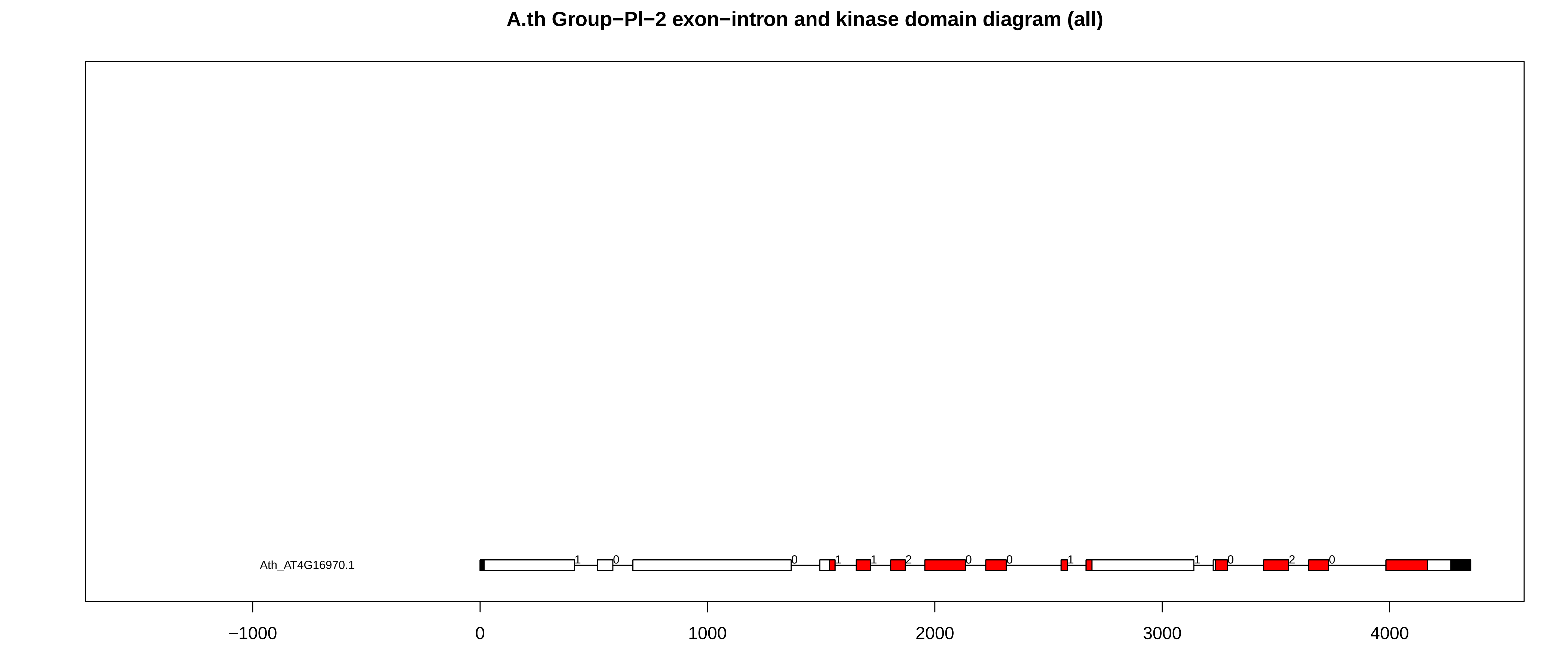
A.th Group−PI−2 exon−intron and kinase domain diagram (all)
Ath_AT4G16970.1
1
0
0
1
1
2
0
0
1
1
0
2
0
−1000
0
1000
2000
3000
4000

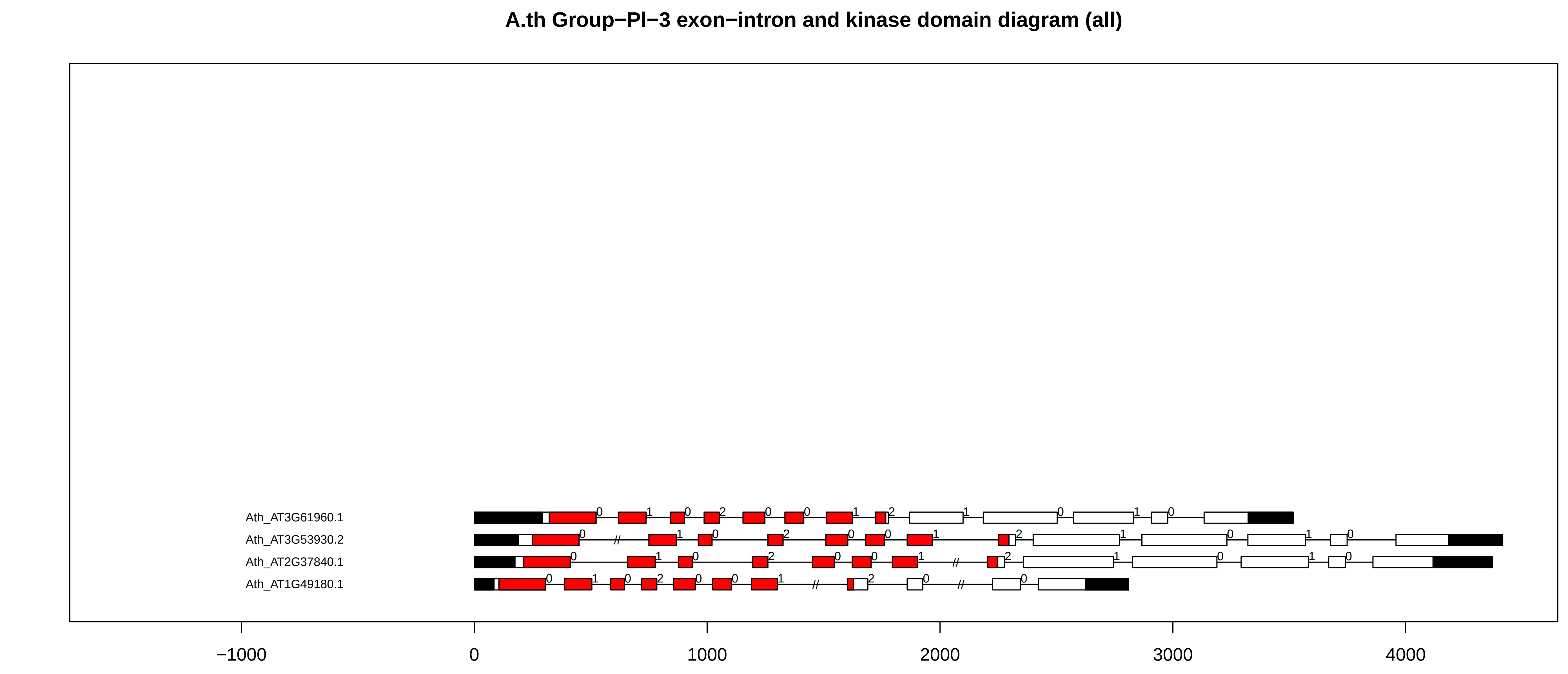
A.th Group−PI−3 exon−intron and kinase domain diagram (all)
Ath_AT3G61960.1
Ath_AT3G53930.2
Ath_AT2G37840.1
Ath_AT1G49180.1
−1000
0
1000
2000
3000
4000

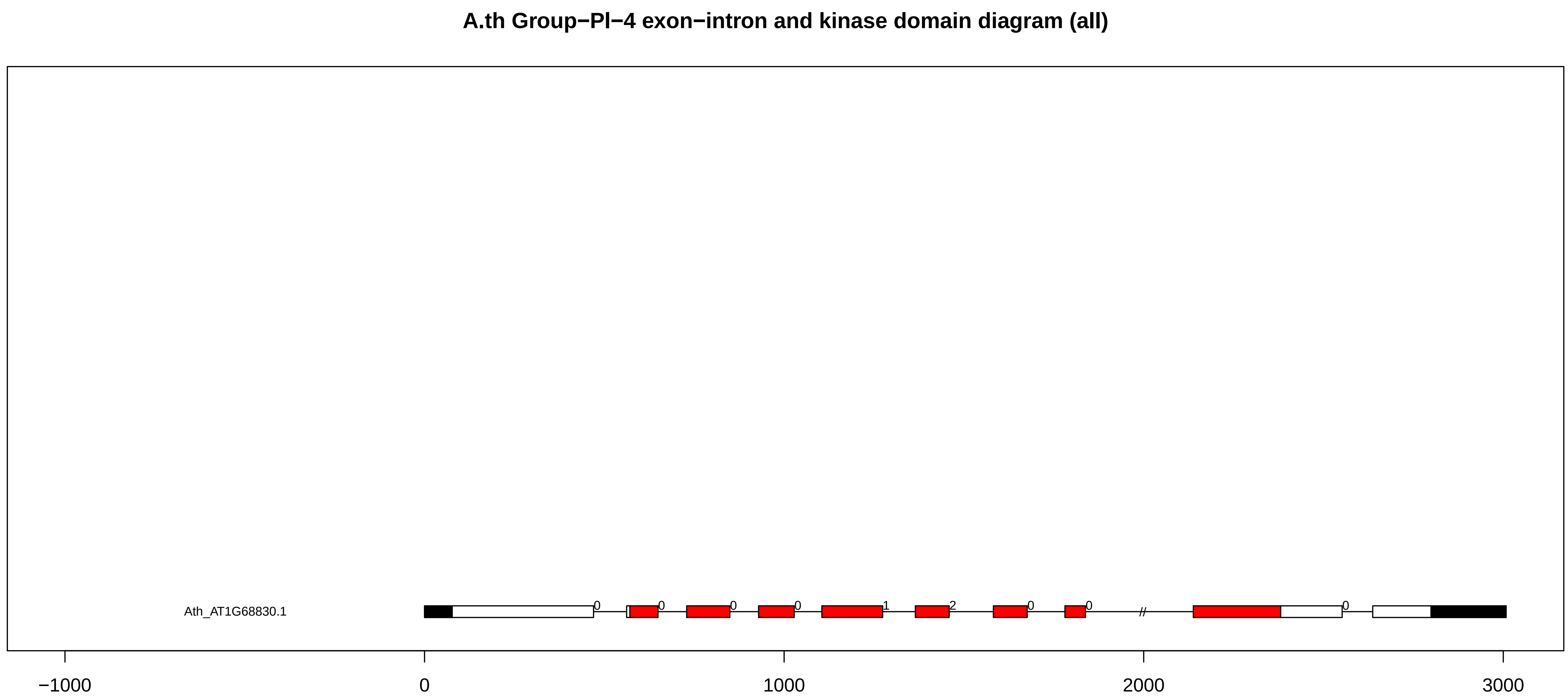
A.th Group−PI−4 exon−intron and kinase domain diagram (all)
Ath_AT1G68830.1
0
0
0
0
1
2
0
0
0
−1000
0
1000
2000
3000

A.th IRE1 exon−intron and kinase domain diagram (all)

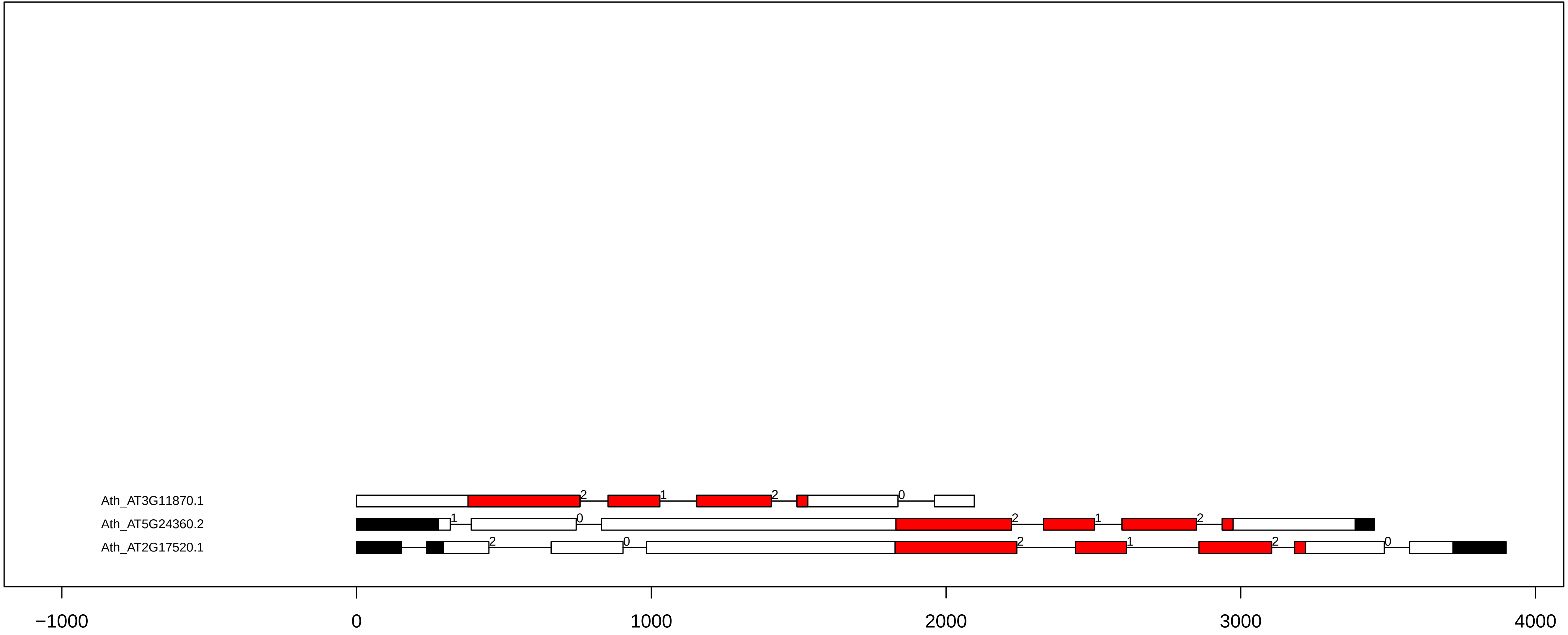

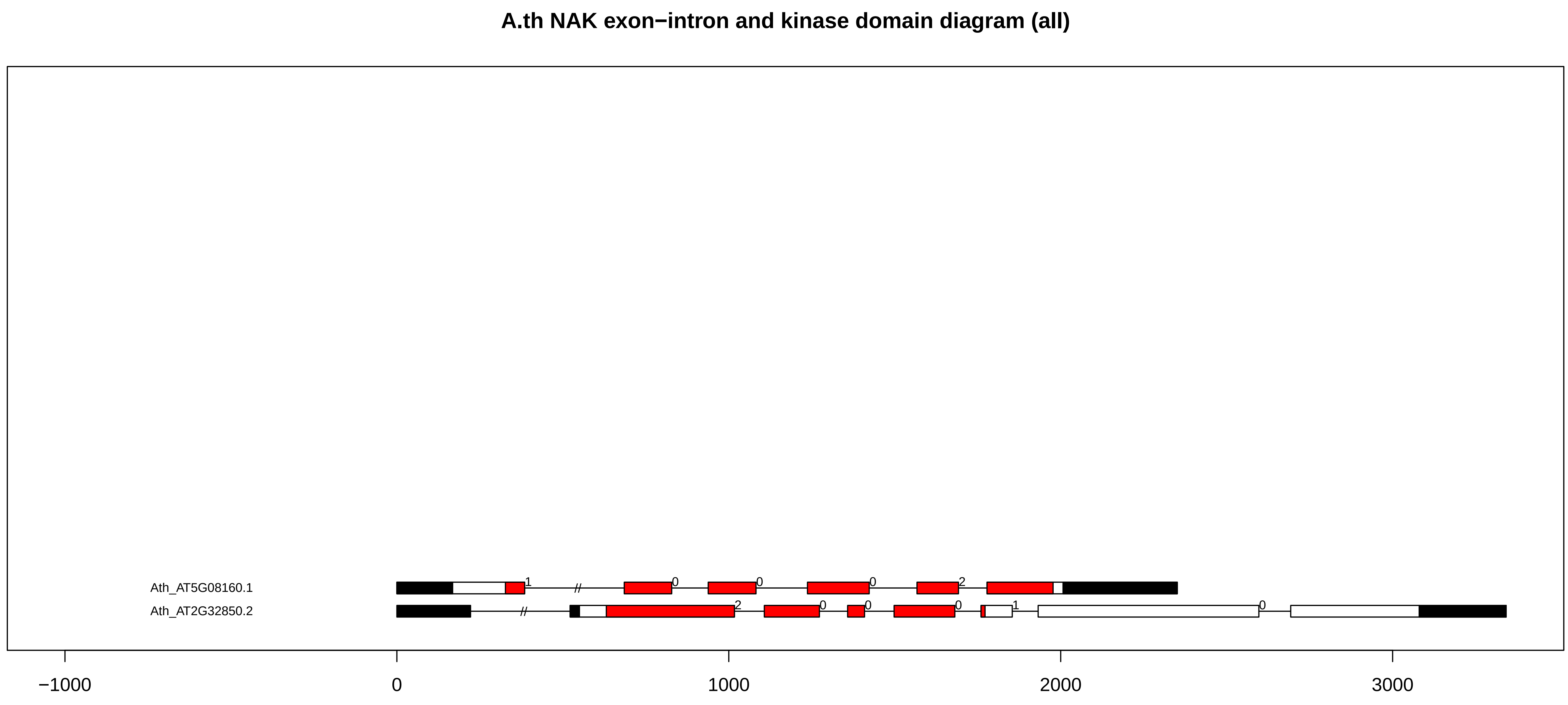
A.th NAK exon−intron and kinase domain diagram (all)
Ath_AT5G08160.1
Ath_AT2G32850.2
1
0
0
0
2
2
0
0
0
1
0
−1000
0
1000
2000
3000

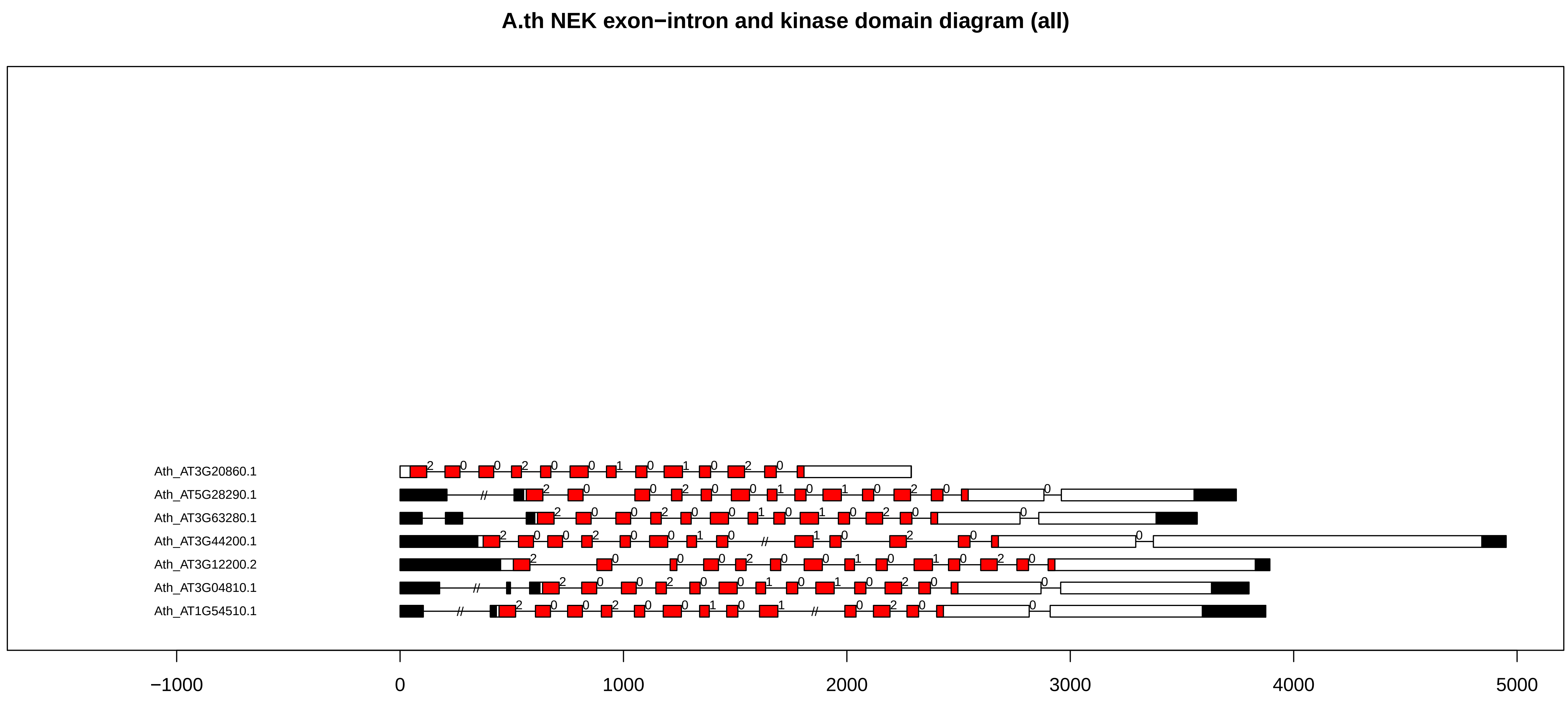
A.th NEK exon−intron and kinase domain diagram (all)
Ath_AT3G20860.1
Ath_AT5G28290.1
Ath_AT3G63280.1
Ath_AT3G44200.1
Ath_AT3G12200.2
Ath_AT3G04810.1
Ath_AT1G54510.1
−1000
0
1000
2000
3000
4000
5000

A.th PEK_GCN2 exon−intron and kinase domain diagram (all)

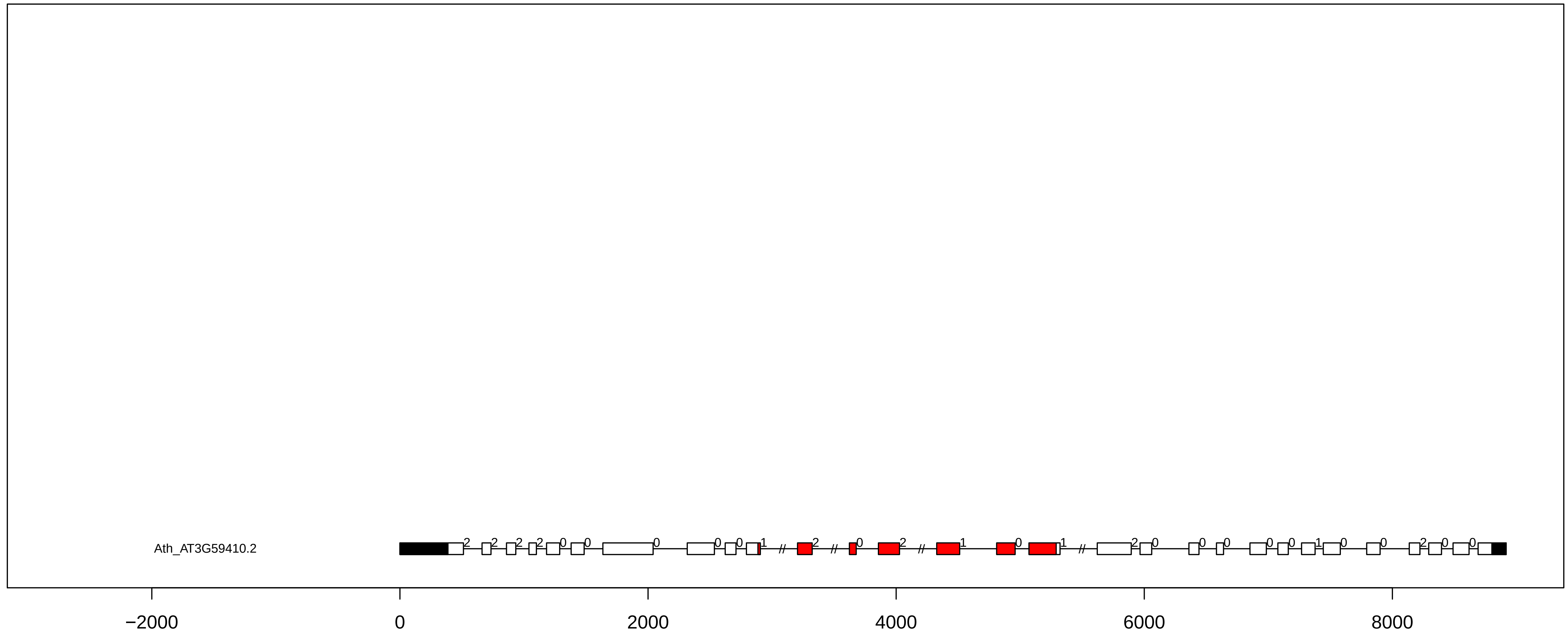

A.th RLK−Pelle_C−LEC exon−intron and kinase domain diagram (all)

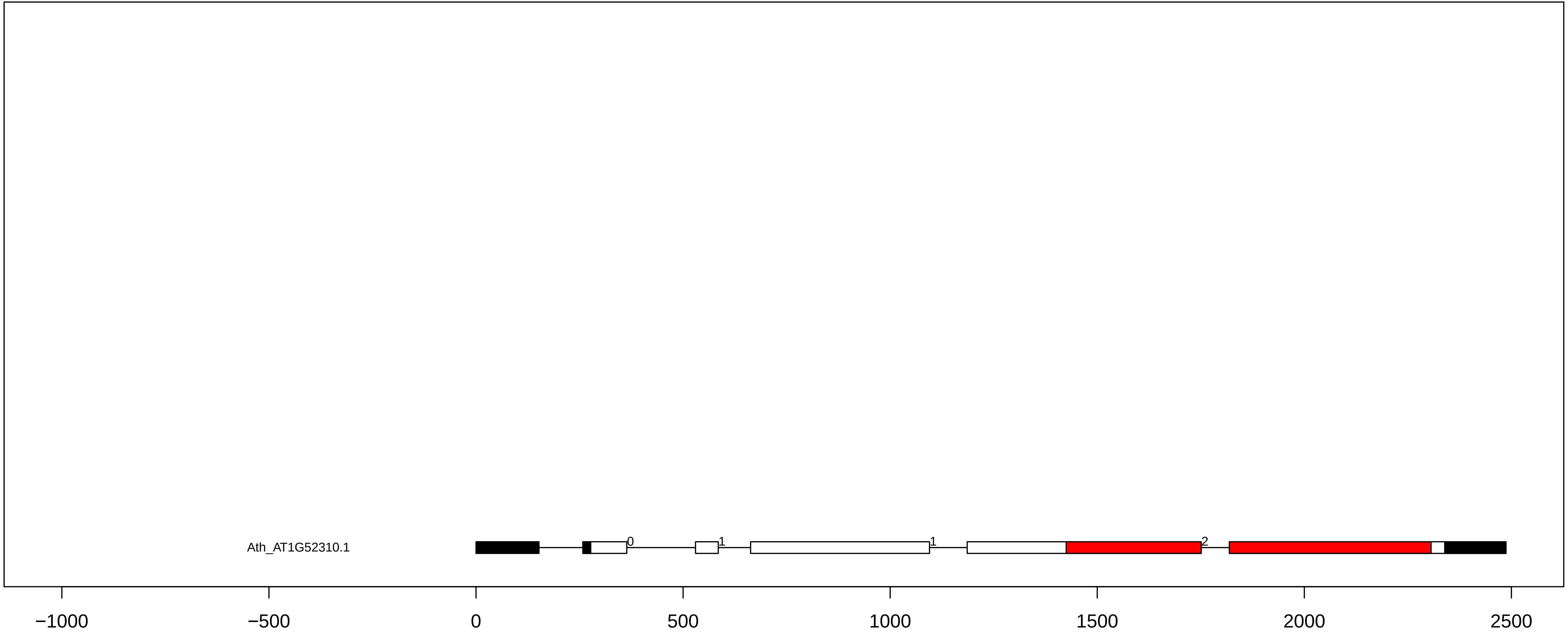

A.th RLK−Pelle_CR4L exon−intron and kinase domain diagram (all)

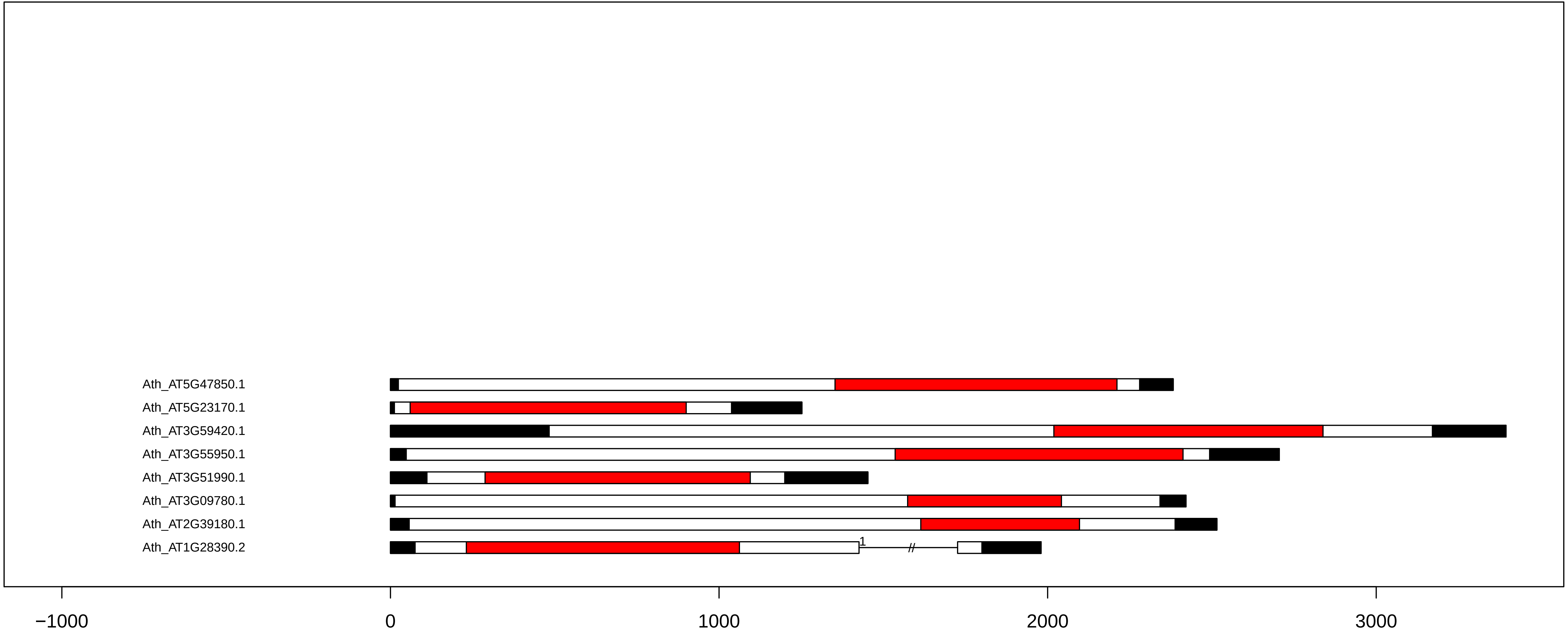

A.th RLK−Pelle_CrRLK1L−1 exon−intron and kinase domain diagram (all)

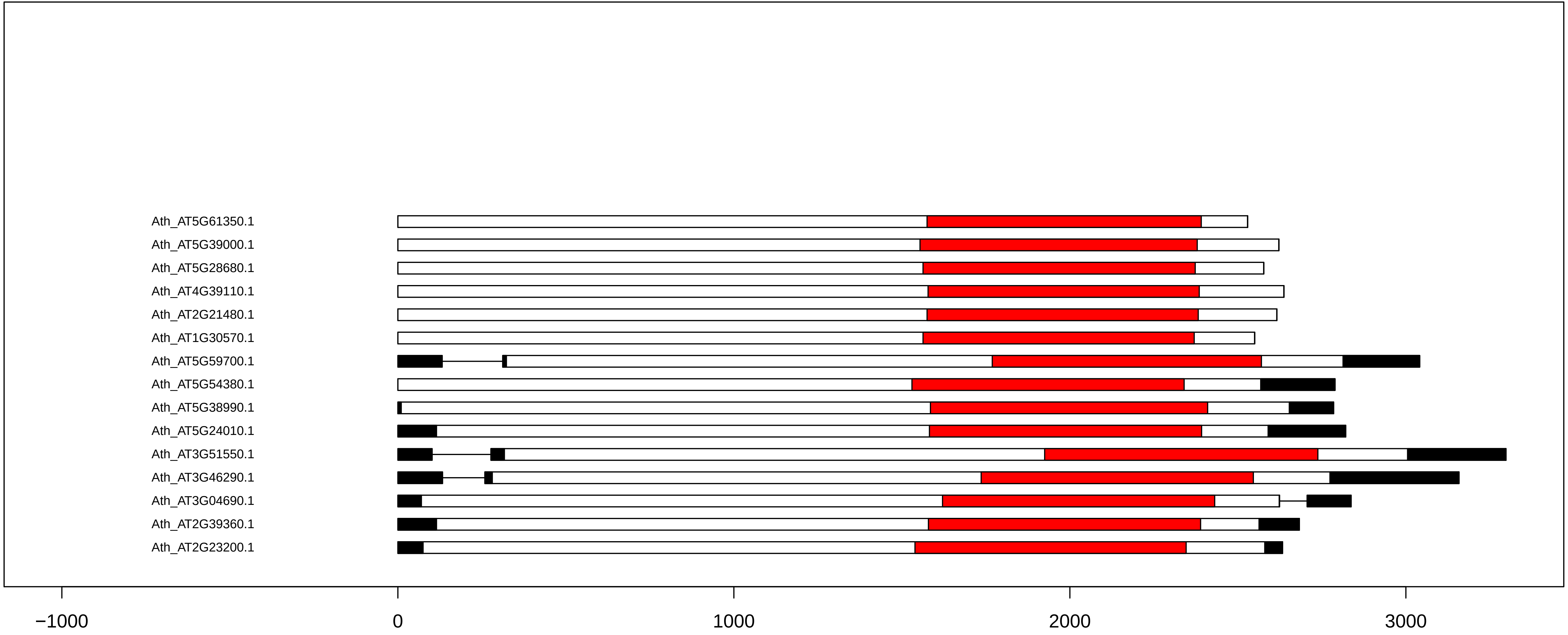

A.th RLK−Pelle_DLSV exon−intron and kinase domain diagram (part 1)

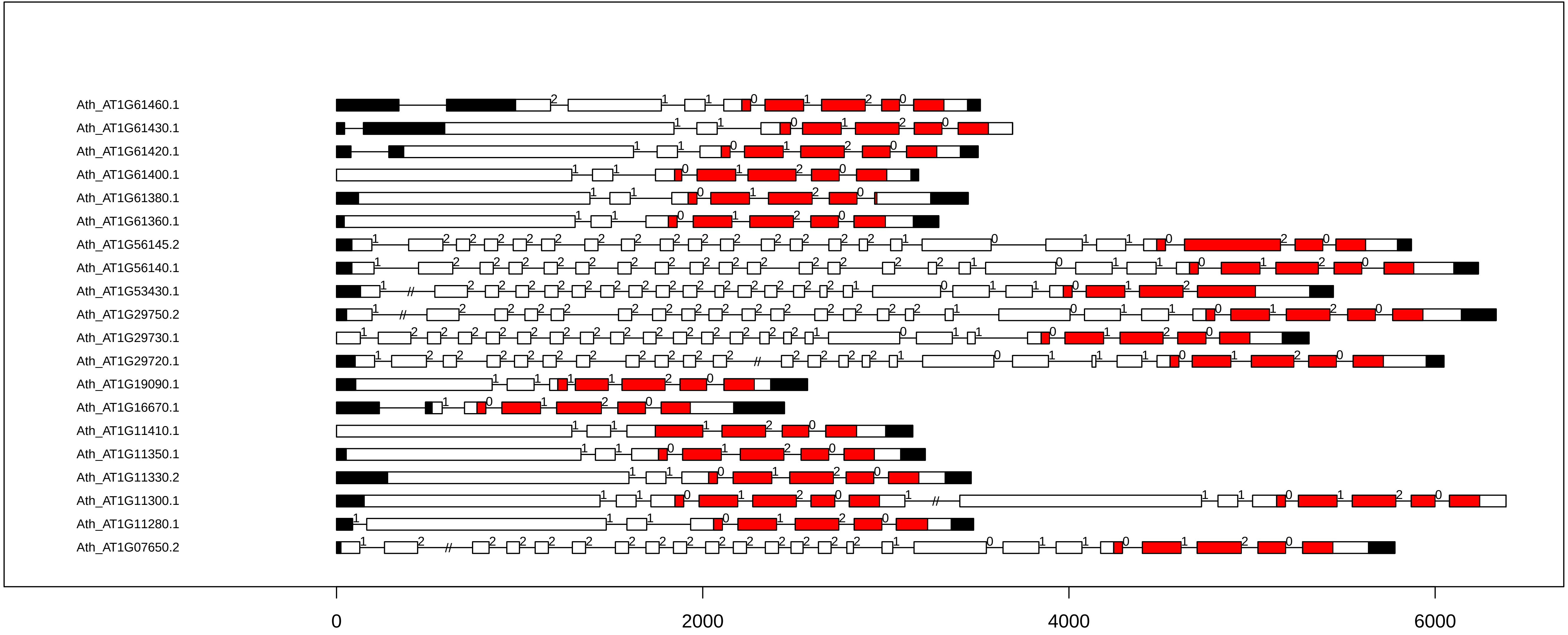

A.th RLK−Pelle_DLSV exon−intron and kinase domain diagram (part 2)

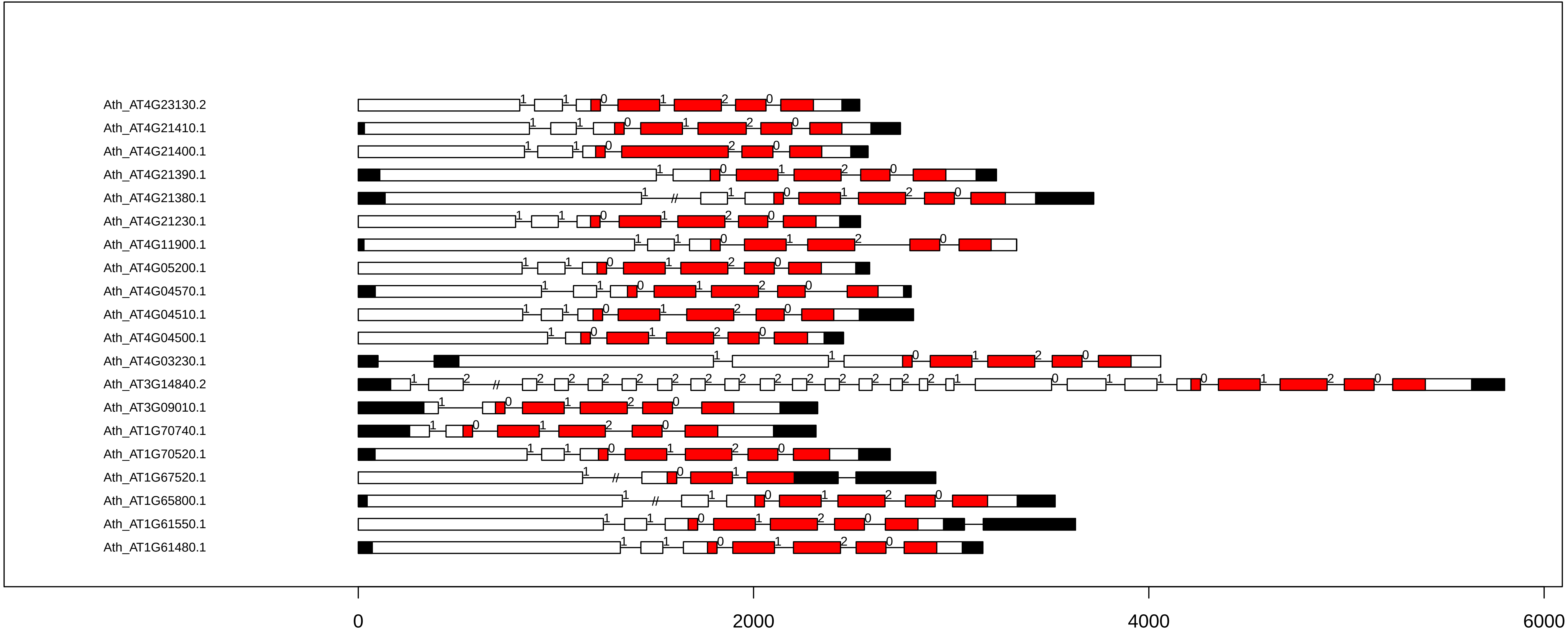

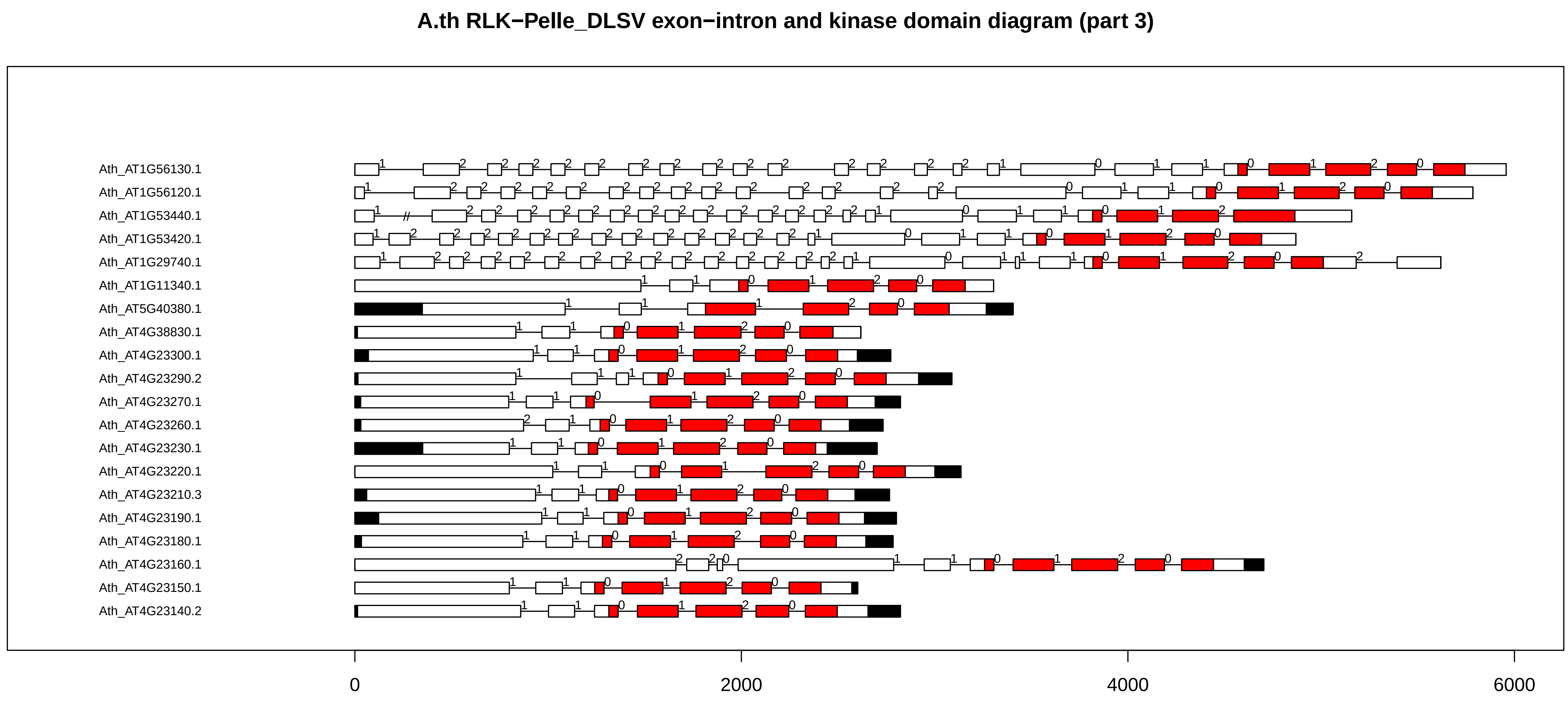
A.th RLK−Pelle_DLSV exon−intron and kinase domain diagram (part 3)
Ath_AT1G56130.1
Ath_AT1G56120.1
Ath_AT1G53440.1
Ath_AT1G53420.1
Ath_AT1G29740.1
Ath_AT1G11340.1
Ath_AT5G40380.1
Ath_AT4G38830.1
Ath_AT4G23300.1
Ath_AT4G23290.2
Ath_AT4G23270.1
Ath_AT4G23260.1
Ath_AT4G23230.1
Ath_AT4G23220.1
Ath_AT4G23210.3
Ath_AT4G23190.1
Ath_AT4G23180.1
Ath_AT4G23160.1
Ath_AT4G23150.1
Ath_AT4G23140.2
0
2000
4000
6000

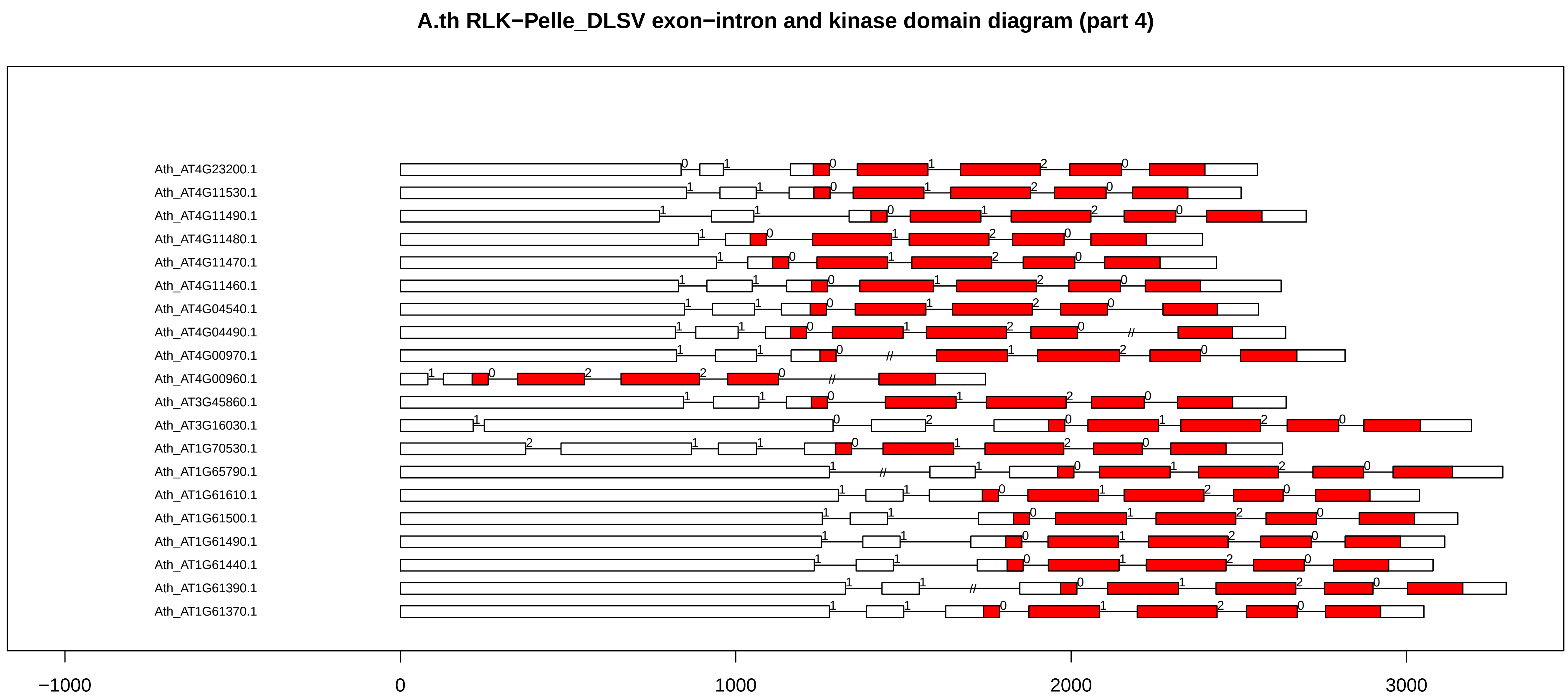

A.th RLK−Pelle_DLSV exon−intron and kinase domain diagram (part 4)
Ath_AT4G23200.1
Ath_AT4G11530.1
Ath_AT4G11490.1
Ath_AT4G11480.1
Ath_AT4G11470.1
Ath_AT4G11460.1
Ath_AT4G04540.1
Ath_AT4G04490.1
Ath_AT4G00970.1
Ath_AT4G00960.1
Ath_AT3G45860.1
Ath_AT3G16030.1
Ath_AT1G70530.1
Ath_AT1G65790.1
Ath_AT1G61610.1
Ath_AT1G61500.1
Ath_AT1G61490.1
Ath_AT1G61440.1
Ath_AT1G61390.1
Ath_AT1G61370.1
−1000
0
1000
2000
3000

A.th RLK−Pelle_DLSV exon−intron and kinase domain diagram (part 5)

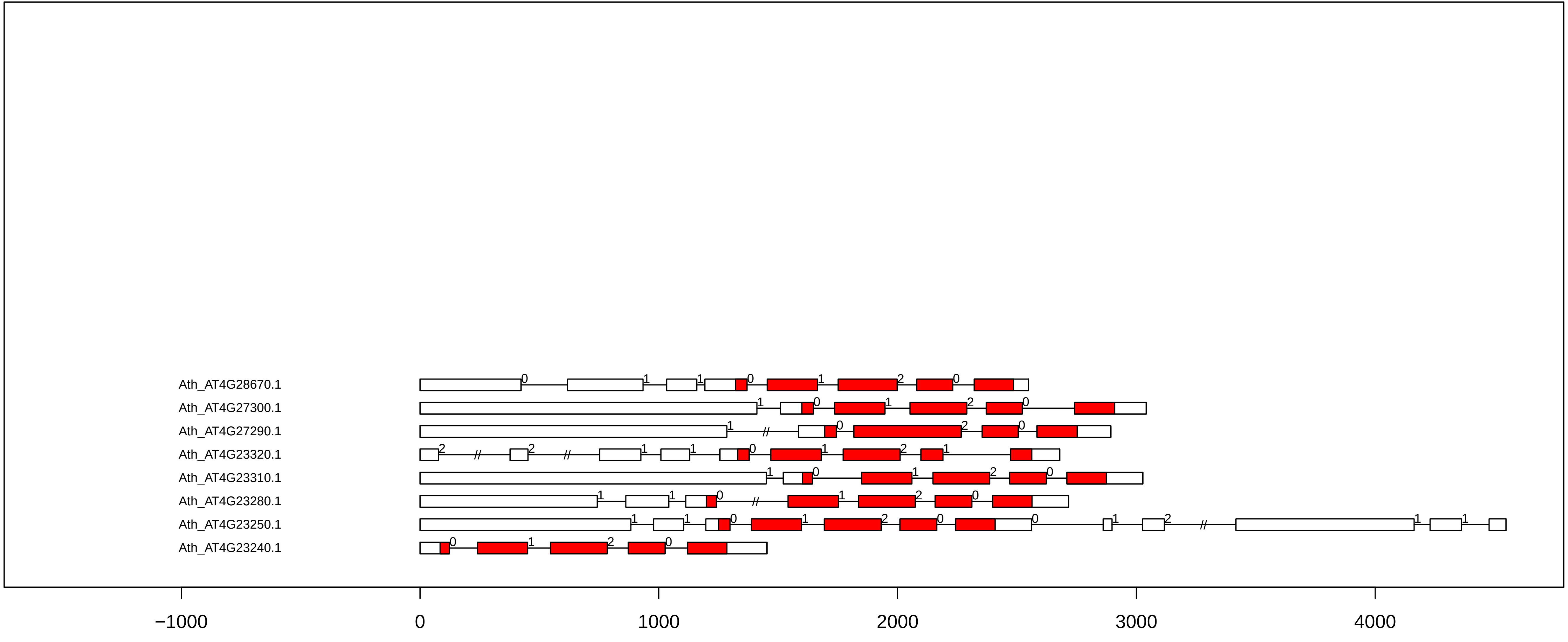

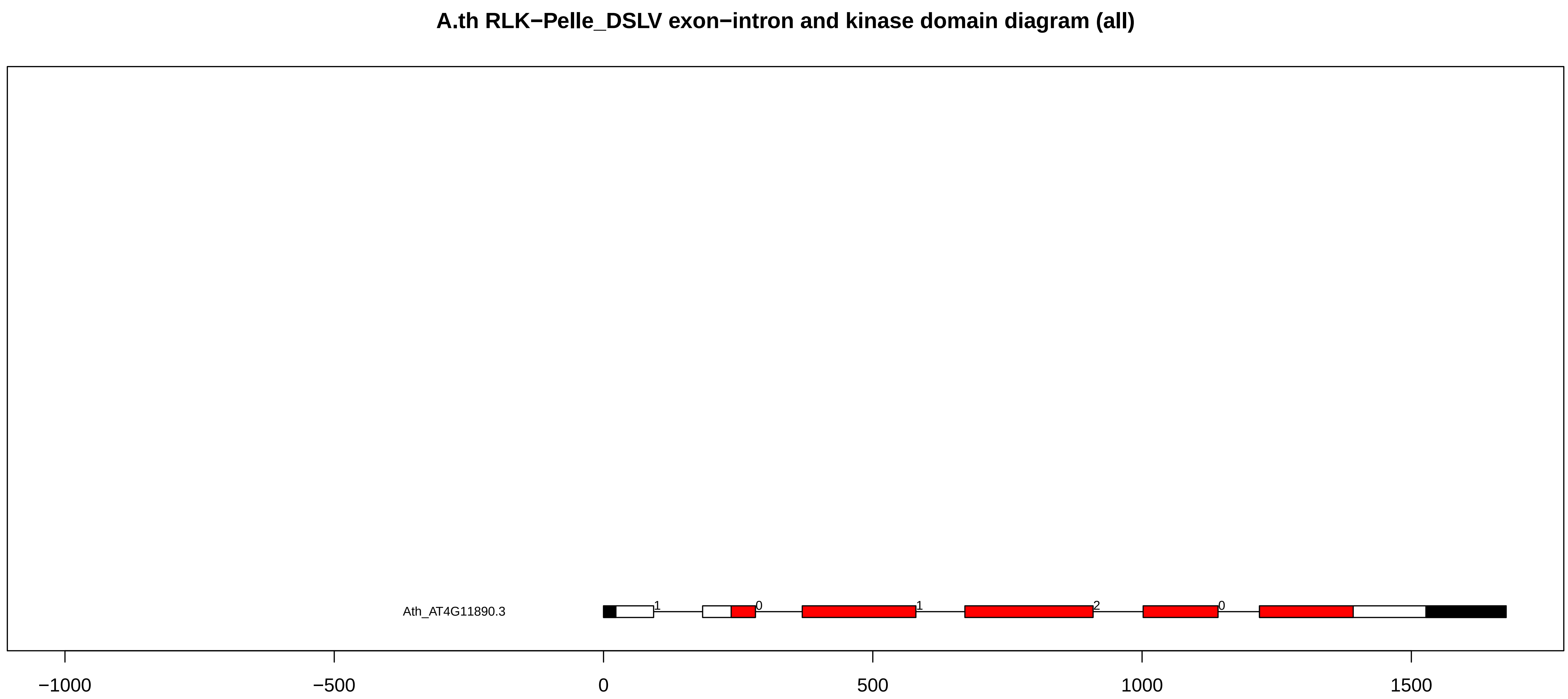
A.th RLK−Pelle_DSLV exon−intron and kinase domain diagram (all)
Ath_AT4G11890.3
1
0
1
2
0
−1000
−500
0
500
1000
1500

A.th RLK−Pelle_Extensin exon−intron and kinase domain diagram (all)

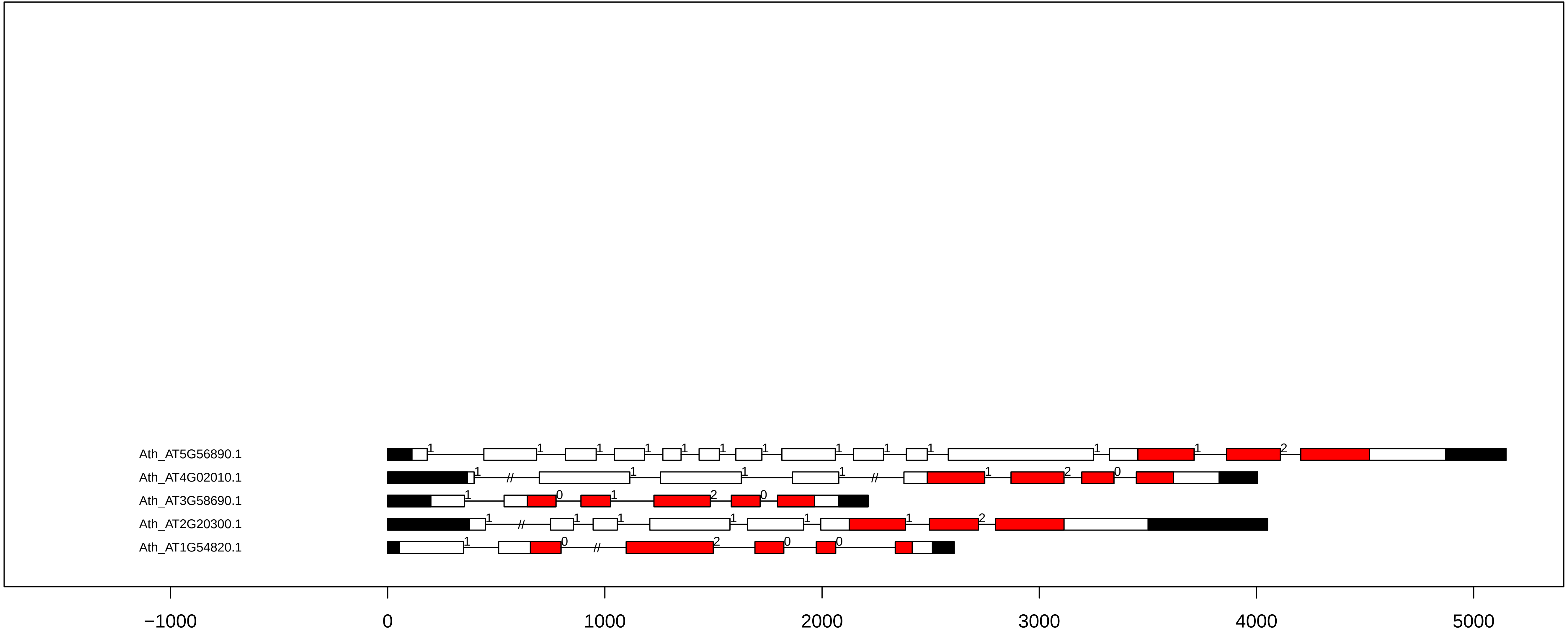

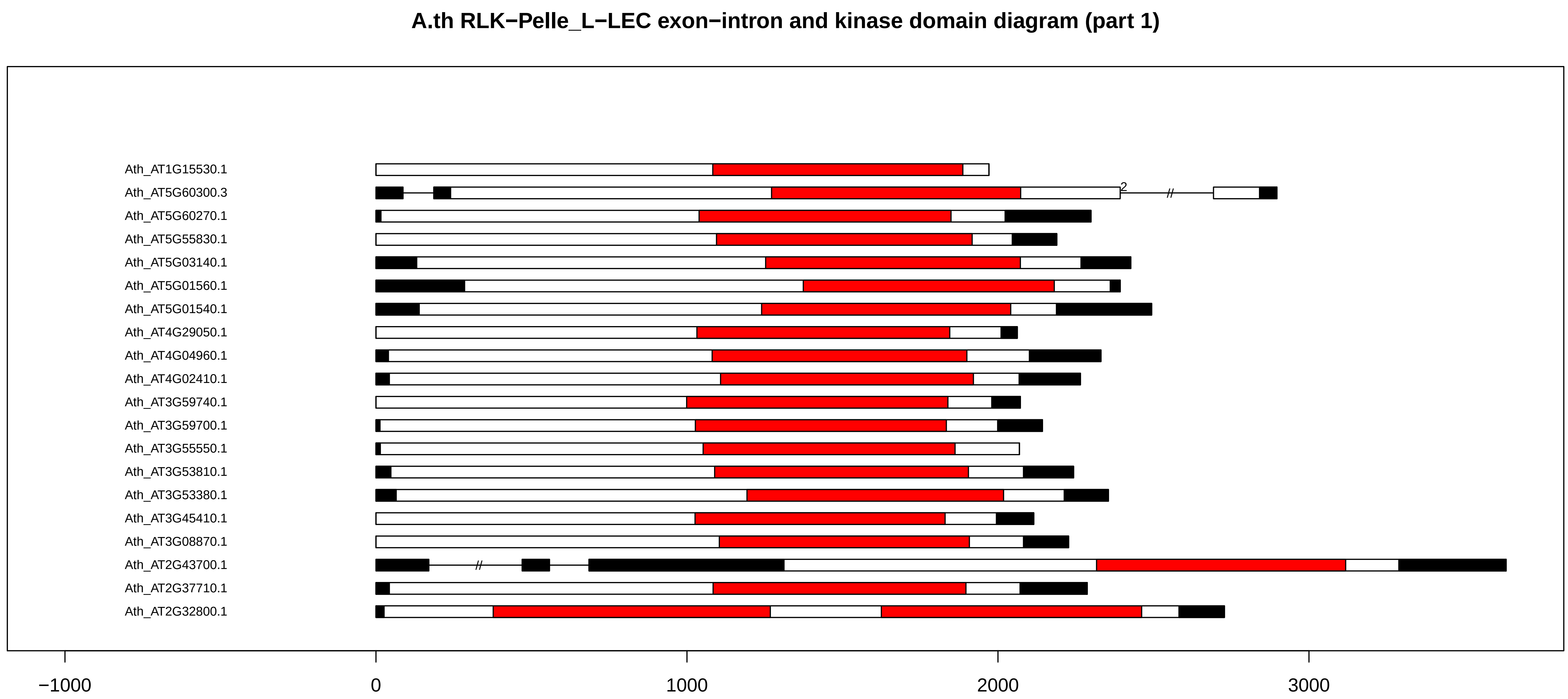

A.th RLK−Pelle_L−LEC exon−intron and kinase domain diagram (part 1)
Ath_AT1G15530.1
Ath_AT5G60300.3
Ath_AT5G60270.1
Ath_AT5G55830.1
Ath_AT5G03140.1
Ath_AT5G01560.1
Ath_AT5G01540.1
Ath_AT4G29050.1
Ath_AT4G04960.1
Ath_AT4G02410.1
Ath_AT3G59740.1
Ath_AT3G59700.1
Ath_AT3G55550.1
Ath_AT3G53810.1
Ath_AT3G53380.1
Ath_AT3G45410.1
Ath_AT3G08870.1
Ath_AT2G43700.1
Ath_AT2G37710.1
Ath_AT2G32800.1
2
−1000
0
1000
2000
3000

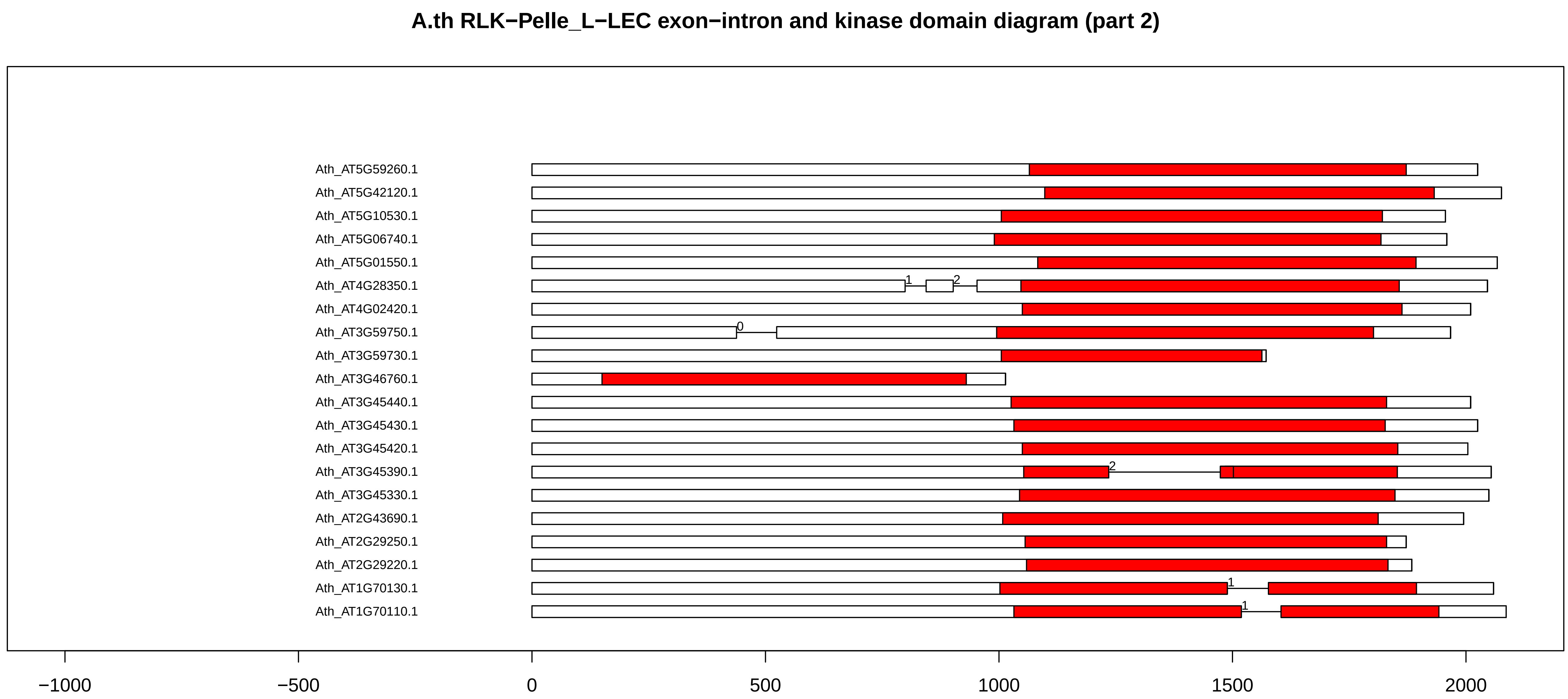

A.th RLK−Pelle_L−LEC exon−intron and kinase domain diagram (part 2)
Ath_AT5G59260.1
Ath_AT5G42120.1
Ath_AT5G10530.1
Ath_AT5G06740.1
Ath_AT5G01550.1
Ath_AT4G28350.1
Ath_AT4G02420.1
Ath_AT3G59750.1
Ath_AT3G59730.1
Ath_AT3G46760.1
Ath_AT3G45440.1
Ath_AT3G45430.1
Ath_AT3G45420.1
Ath_AT3G45390.1
Ath_AT3G45330.1
Ath_AT2G43690.1
Ath_AT2G29250.1
Ath_AT2G29220.1
Ath_AT1G70130.1
Ath_AT1G70110.1
1
2
0
2
1
1
−1000
−500
0
500
1000
1500
2000

A.th RLK−Pelle_L−LEC exon−intron and kinase domain diagram (part 3)

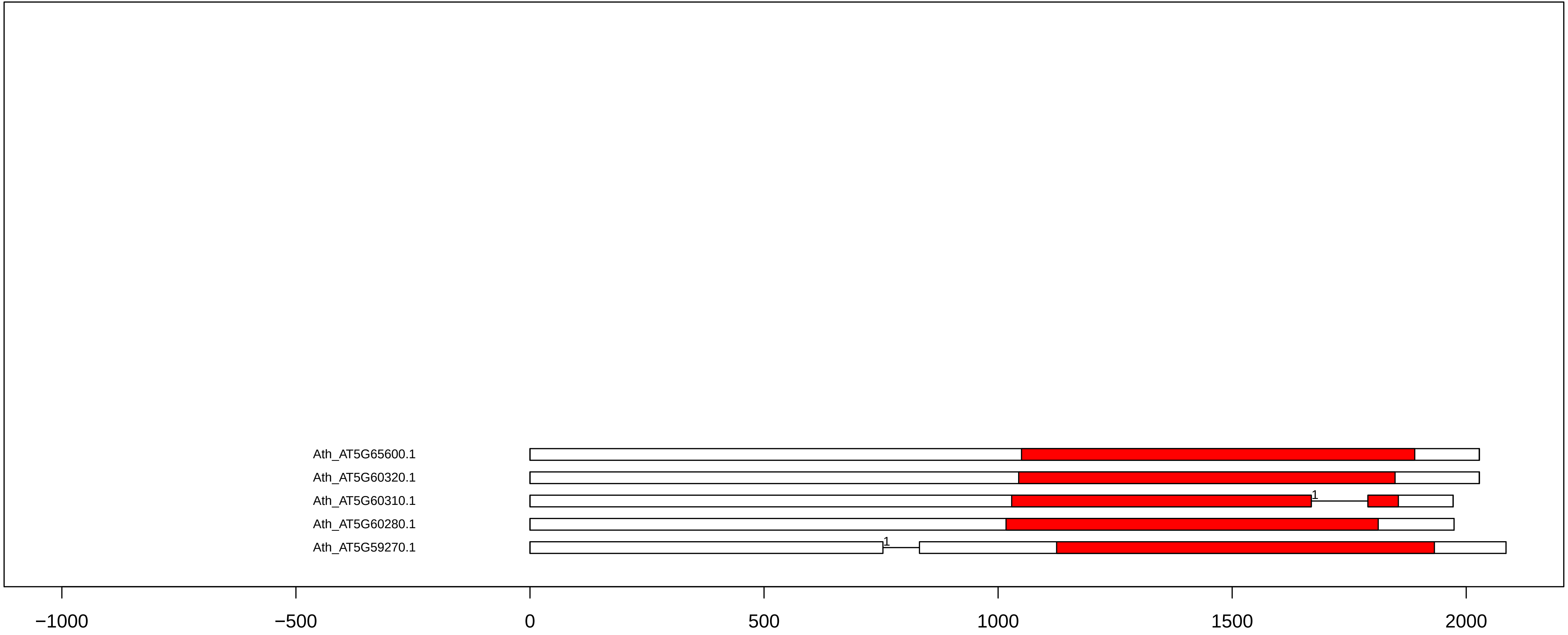

A.th RLK−Pelle_LRK10L−2 exon−intron and kinase domain diagram (all)

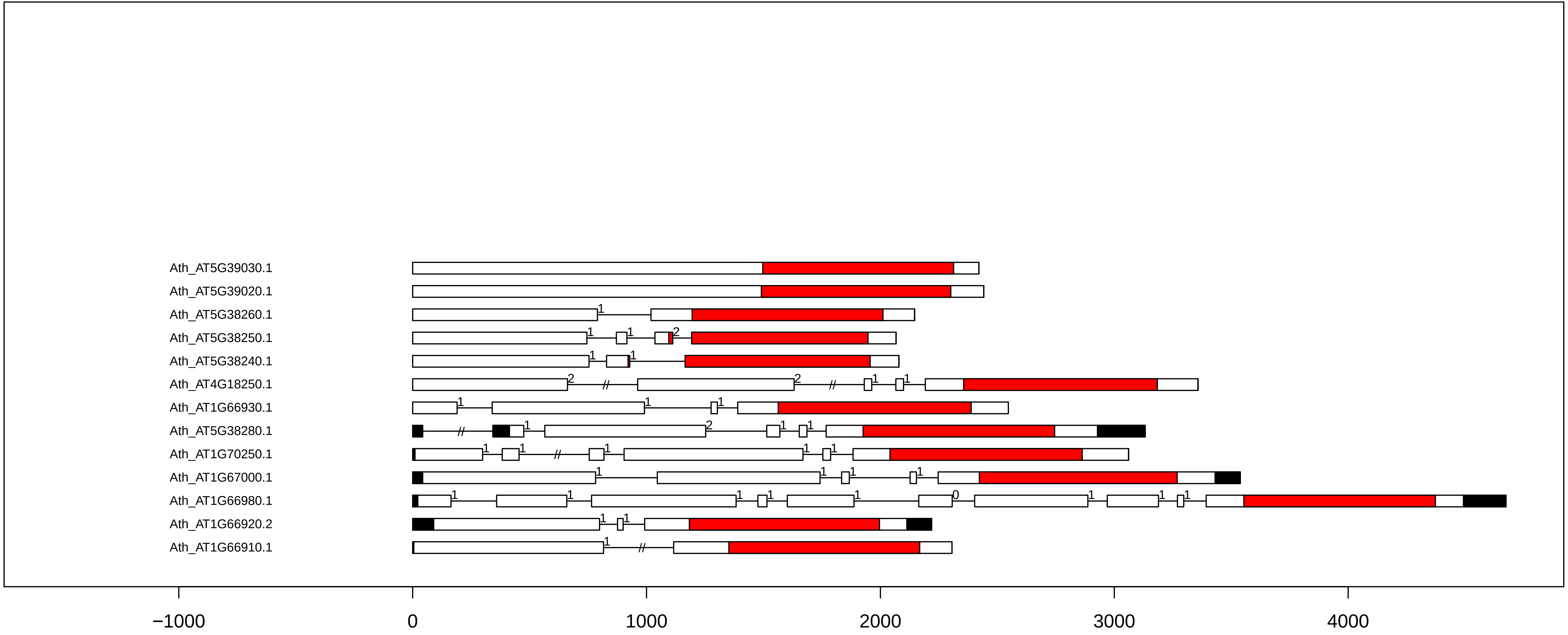

A.th RLK−Pelle_LRR−I−1 exon−intron and kinase domain diagram (part 1)

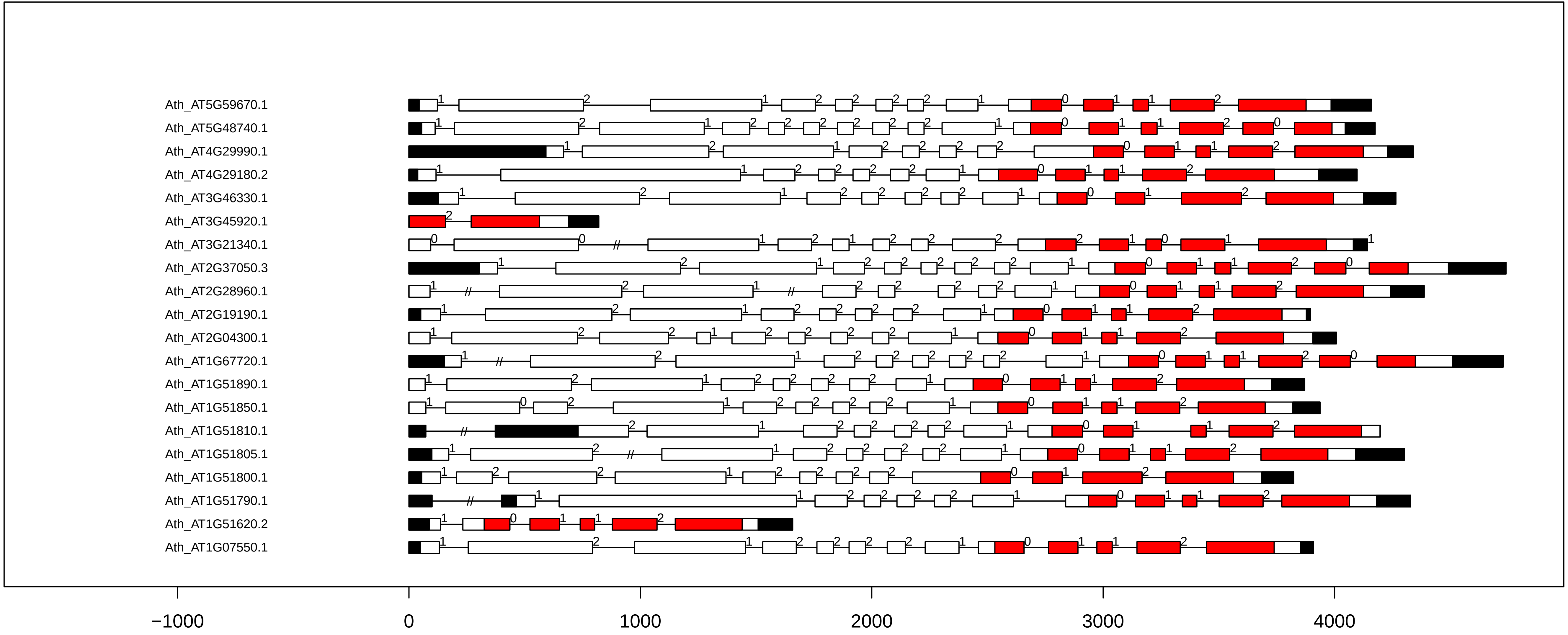

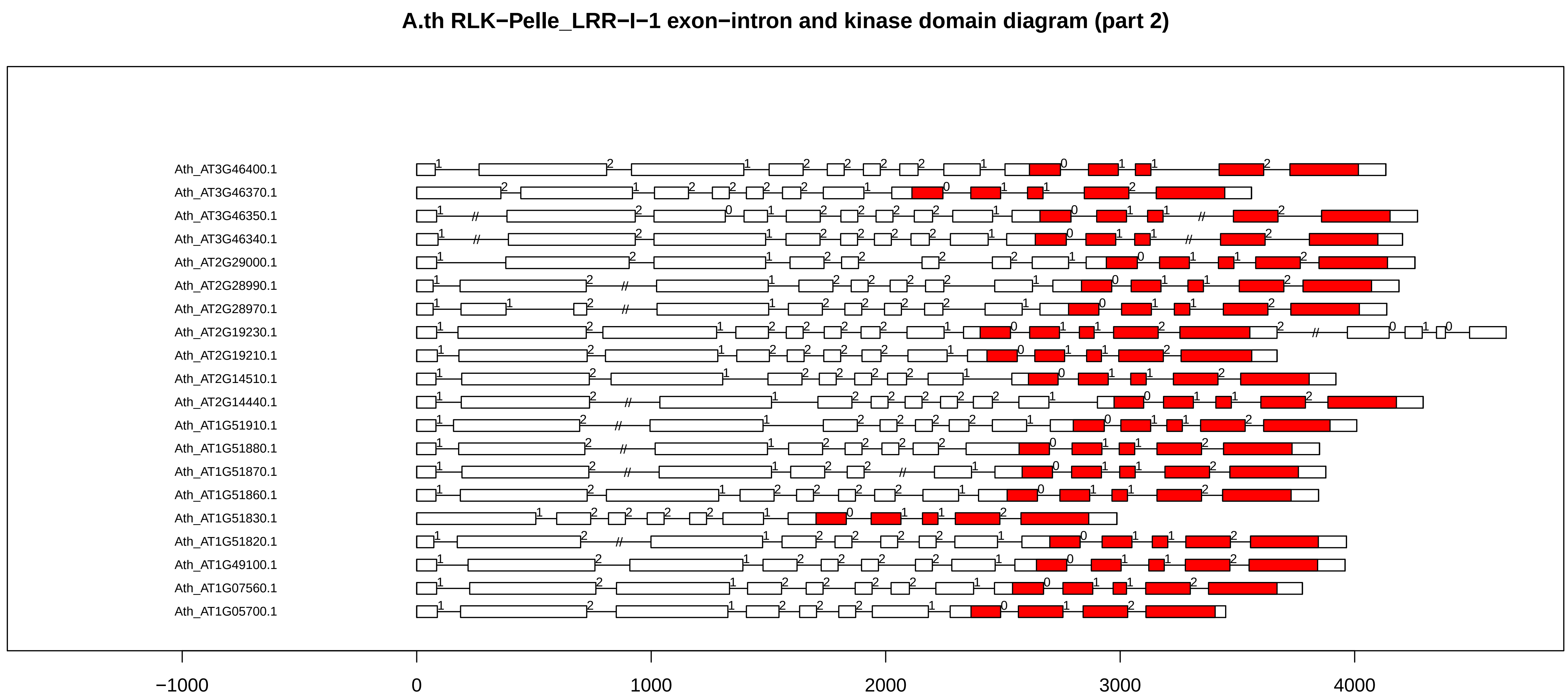
A.th RLK−Pelle_LRR−I−1 exon−intron and kinase domain diagram (part 2)
Ath_AT3G46400.1
Ath_AT3G46370.1
Ath_AT3G46350.1
Ath_AT3G46340.1
Ath_AT2G29000.1
Ath_AT2G28990.1
Ath_AT2G28970.1
Ath_AT2G19230.1
Ath_AT2G19210.1
Ath_AT2G14510.1
Ath_AT2G14440.1
Ath_AT1G51910.1
Ath_AT1G51880.1
Ath_AT1G51870.1
Ath_AT1G51860.1
Ath_AT1G51830.1
Ath_AT1G51820.1
Ath_AT1G49100.1
Ath_AT1G07560.1
Ath_AT1G05700.1
−1000
0
1000
2000
3000
4000

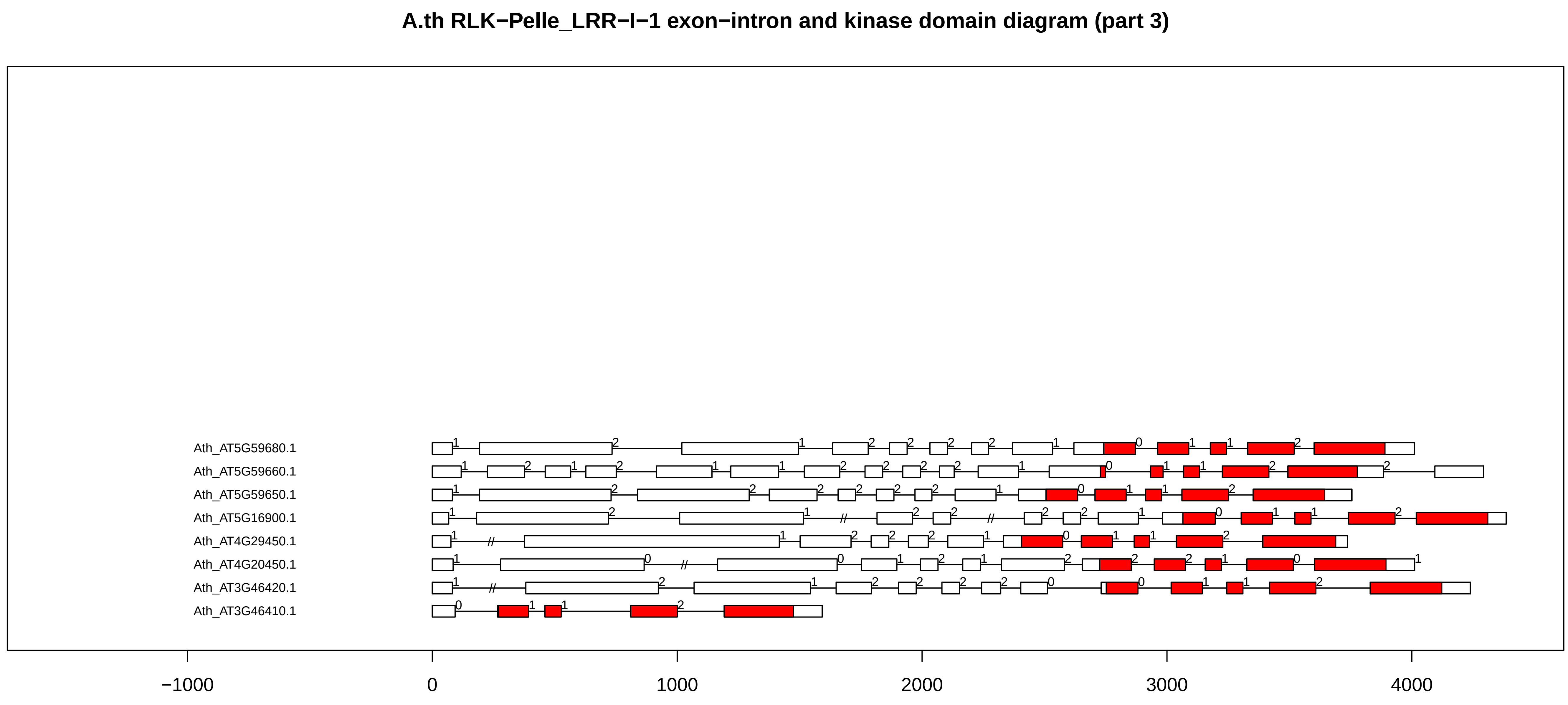
A.th RLK−Pelle_LRR−I−1 exon−intron and kinase domain diagram (part 3)
Ath_AT5G59680.1
Ath_AT5G59660.1
Ath_AT5G59650.1
Ath_AT5G16900.1
Ath_AT4G29450.1
Ath_AT4G20450.1
Ath_AT3G46420.1
Ath_AT3G46410.1
−1000
0
1000
2000
3000
4000

A.th RLK−Pelle_LRR−I−2 exon−intron and kinase domain diagram (all)

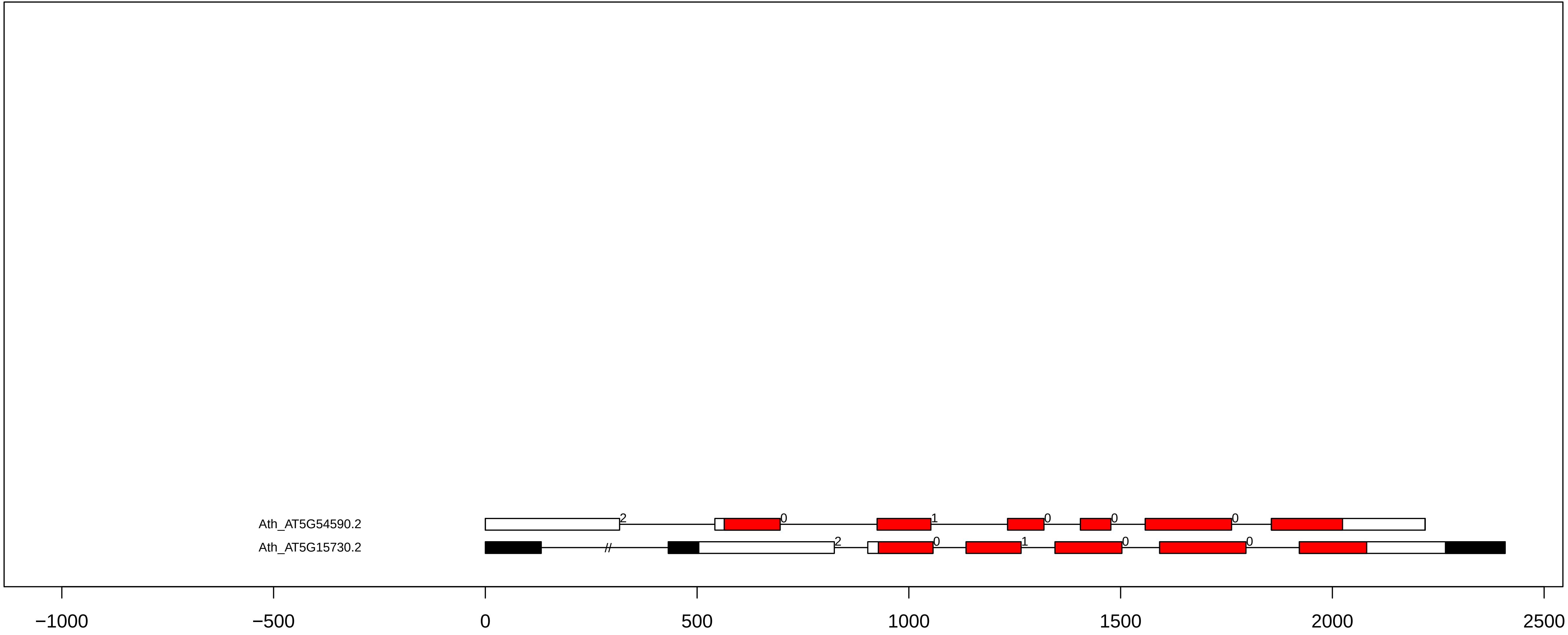

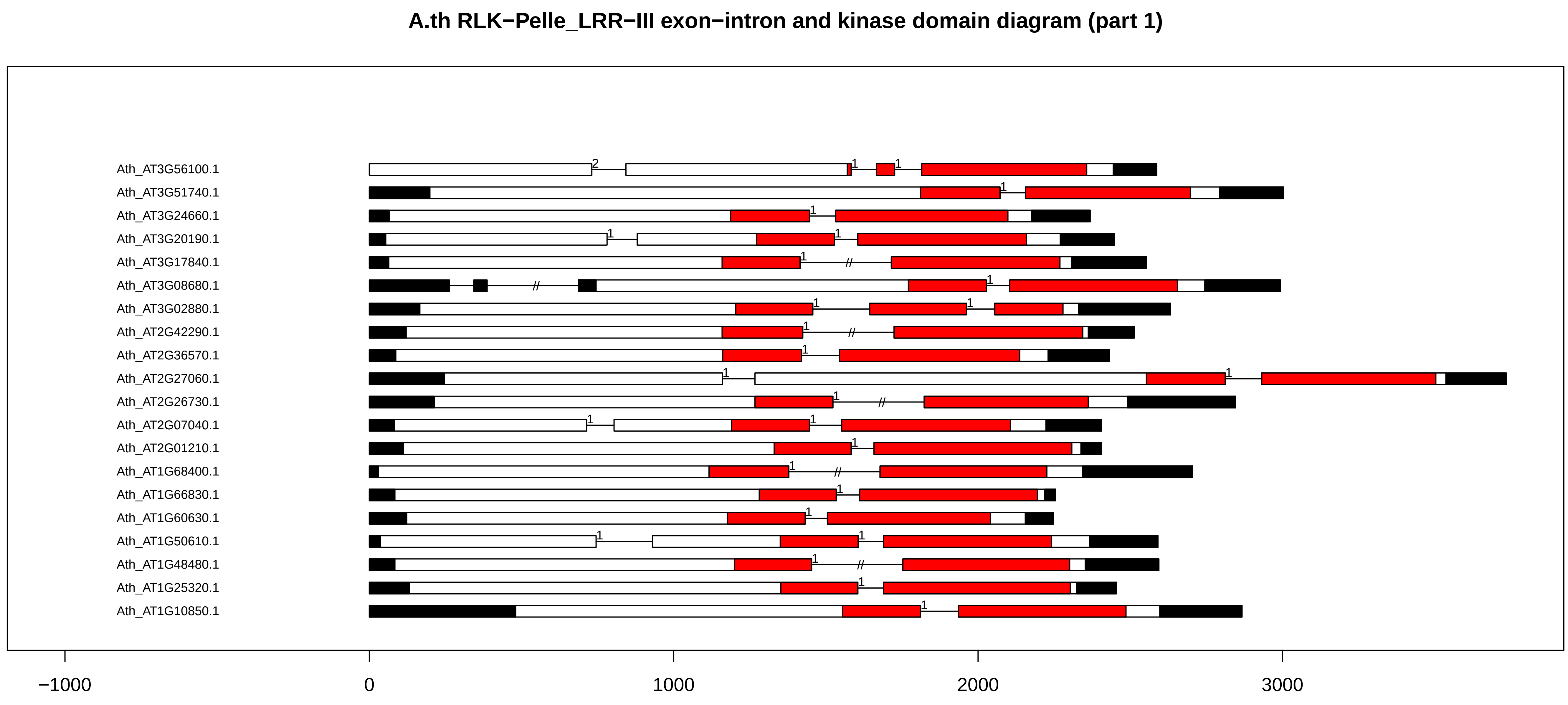

A.th RLK−Pelle_LRR−III exon−intron and kinase domain diagram (part 1)
Ath_AT3G56100.1
Ath_AT3G51740.1
Ath_AT3G24660.1
Ath_AT3G20190.1
Ath_AT3G17840.1
Ath_AT3G08680.1
Ath_AT3G02880.1
Ath_AT2G42290.1
Ath_AT2G36570.1
Ath_AT2G27060.1
Ath_AT2G26730.1
Ath_AT2G07040.1
Ath_AT2G01210.1
Ath_AT1G68400.1
Ath_AT1G66830.1
Ath_AT1G60630.1
Ath_AT1G50610.1
Ath_AT1G48480.1
Ath_AT1G25320.1
Ath_AT1G10850.1
−1000
0
1000
2000
3000

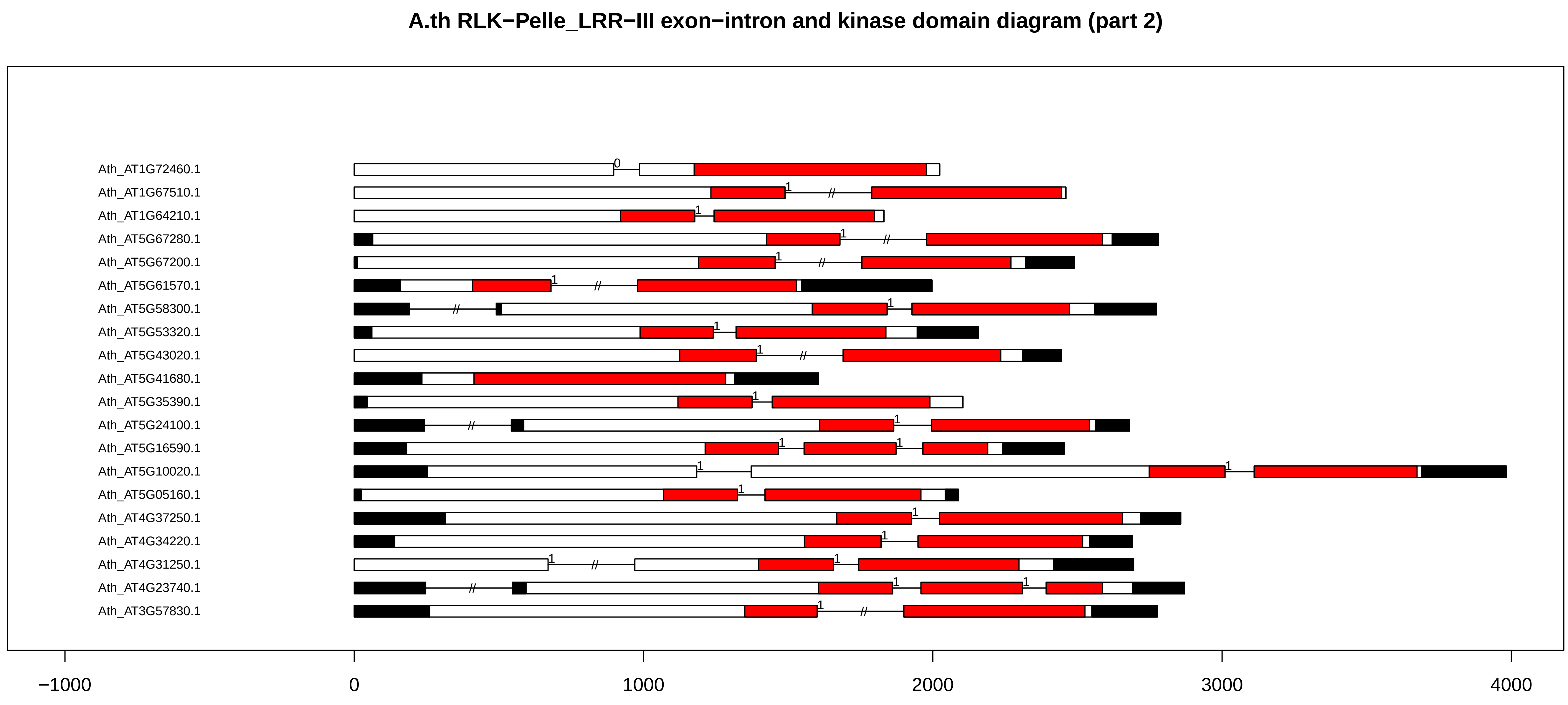
A.th RLK−Pelle_LRR−III exon−intron and kinase domain diagram (part 2)
Ath_AT1G72460.1
Ath_AT1G67510.1
Ath_AT1G64210.1
Ath_AT5G67280.1
Ath_AT5G67200.1
Ath_AT5G61570.1
Ath_AT5G58300.1
Ath_AT5G53320.1
Ath_AT5G43020.1
Ath_AT5G41680.1
Ath_AT5G35390.1
Ath_AT5G24100.1
Ath_AT5G16590.1
Ath_AT5G10020.1
Ath_AT5G05160.1
Ath_AT4G37250.1
Ath_AT4G34220.1
Ath_AT4G31250.1
Ath_AT4G23740.1
Ath_AT3G57830.1
−1000
0
1000
2000
3000
4000

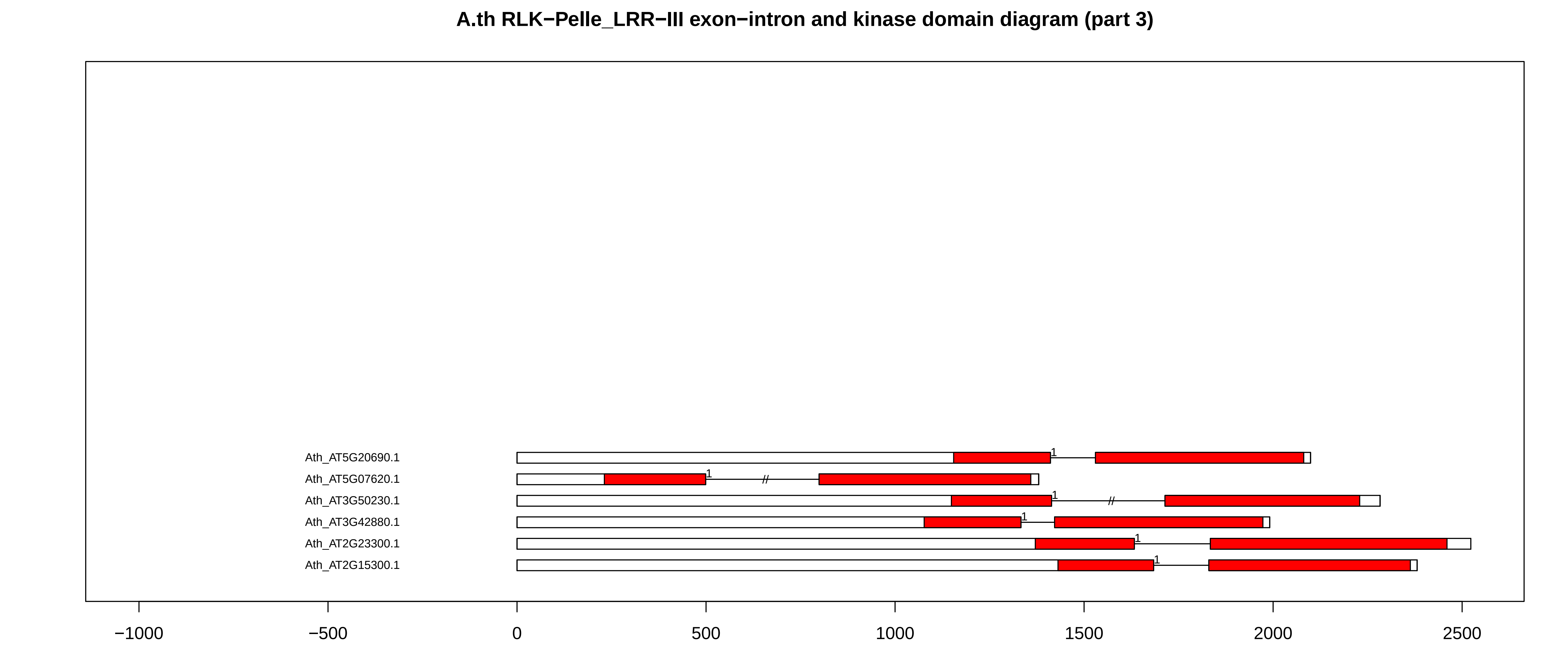
A.th RLK−Pelle_LRR−III exon−intron and kinase domain diagram (part 3)
Ath_AT5G20690.1
Ath_AT5G07620.1
Ath_AT3G50230.1
Ath_AT3G42880.1
Ath_AT2G23300.1
Ath_AT2G15300.1
1
1
1
1
1
1
−1000
−500
0
500
1000
1500
2000
2500

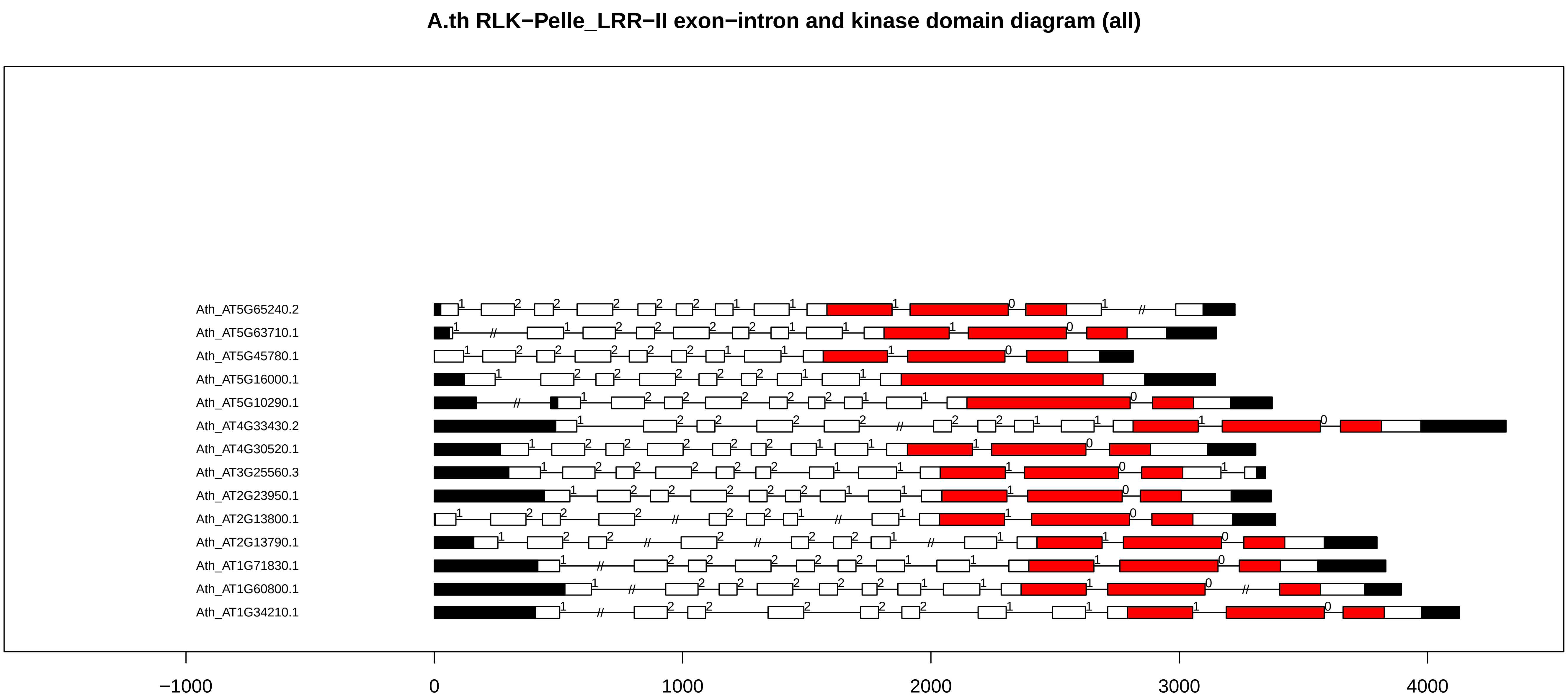
A.th RLK−Pelle_LRR−II exon−intron and kinase domain diagram (all)
Ath_AT5G65240.2
Ath_AT5G63710.1
Ath_AT5G45780.1
Ath_AT5G16000.1
Ath_AT5G10290.1
Ath_AT4G33430.2
Ath_AT4G30520.1
Ath_AT3G25560.3
Ath_AT2G23950.1
Ath_AT2G13800.1
Ath_AT2G13790.1
Ath_AT1G71830.1
Ath_AT1G60800.1
Ath_AT1G34210.1
−1000
0
1000
2000
3000
4000

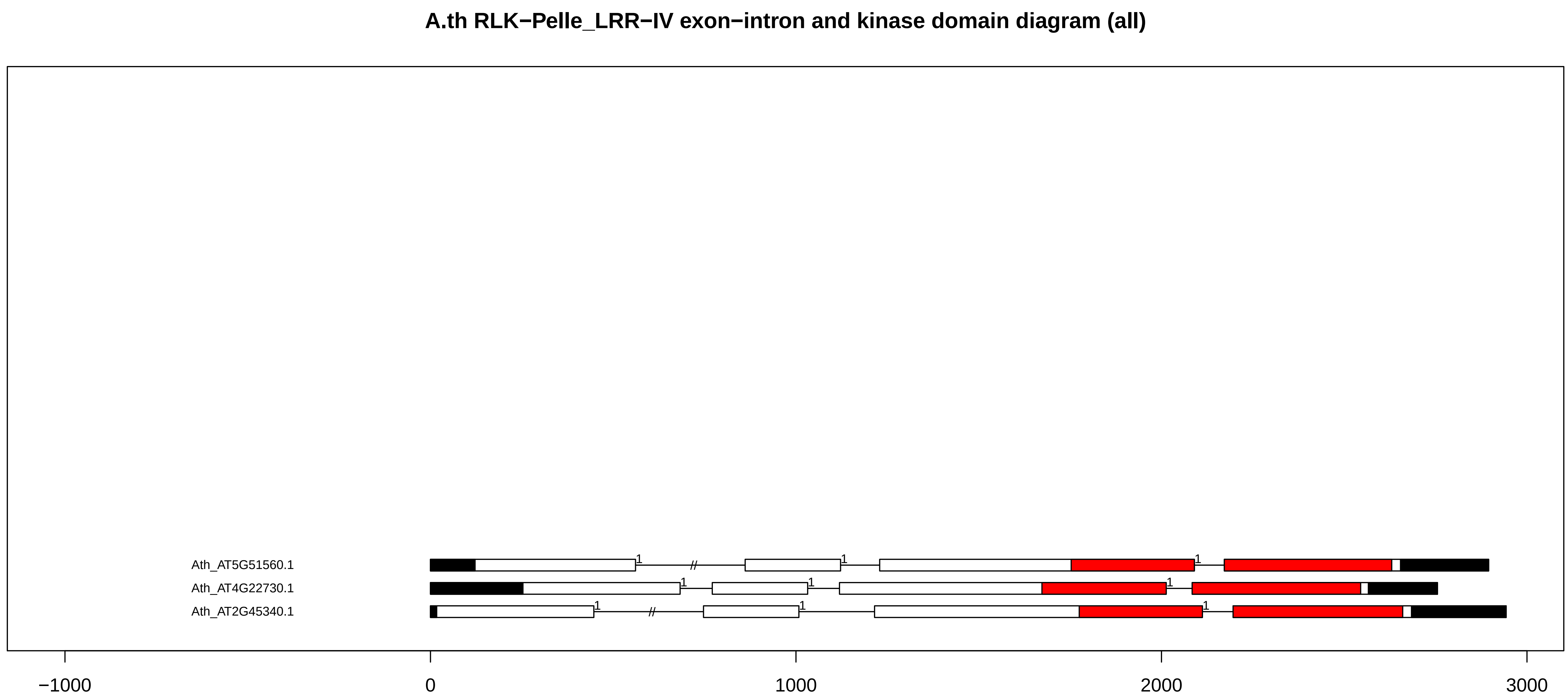

A.th RLK−Pelle_LRR−IV exon−intron and kinase domain diagram (all)
Ath_AT5G51560.1
Ath_AT4G22730.1
Ath_AT2G45340.1
−1000
0
1000
2000
3000

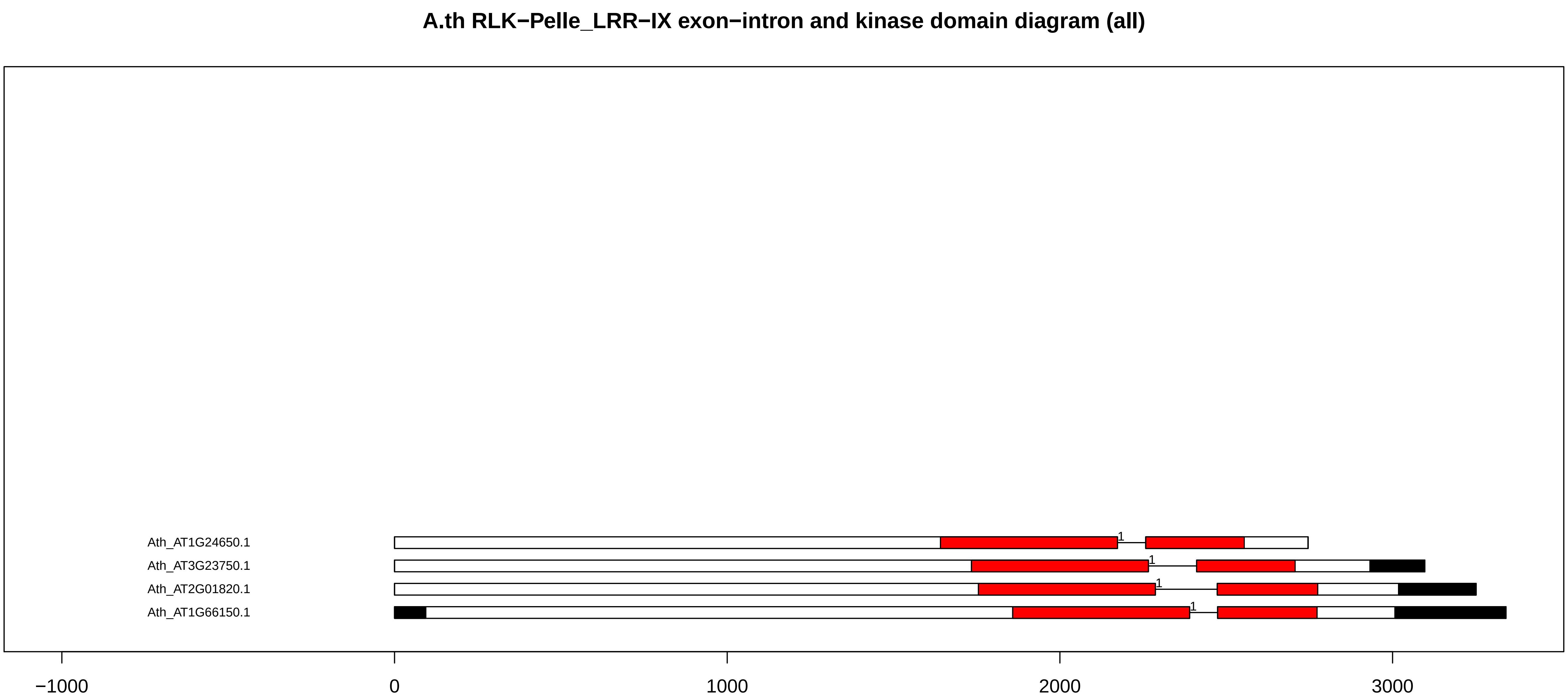
A.th RLK−Pelle_LRR−IX exon−intron and kinase domain diagram (all)
Ath_AT1G24650.1
Ath_AT3G23750.1
Ath_AT2G01820.1
Ath_AT1G66150.1
1
1
1
1
−1000
0
1000
2000
3000

A.th RLK−Pelle_LRR−VI−1 exon−intron and kinase domain diagram (all)

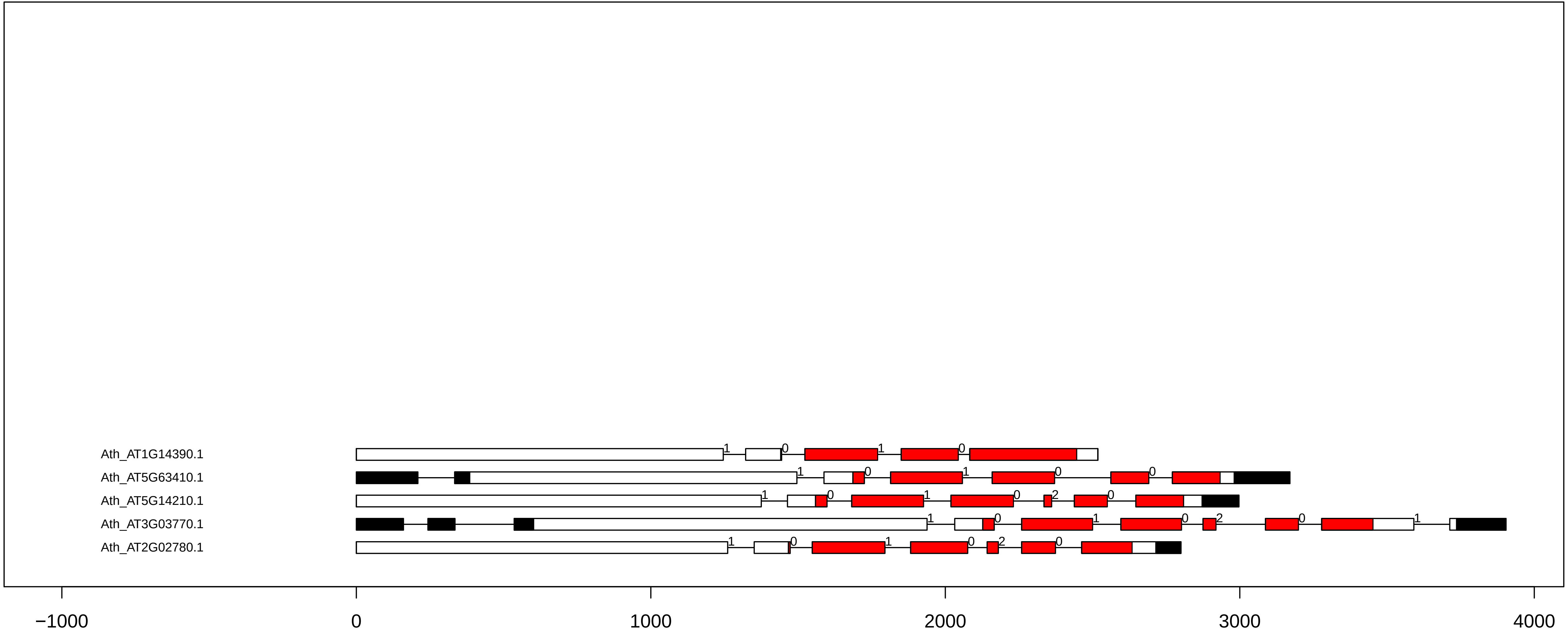

A.th RLK−Pelle_LRR−VI−2 exon−intron and kinase domain diagram (all)

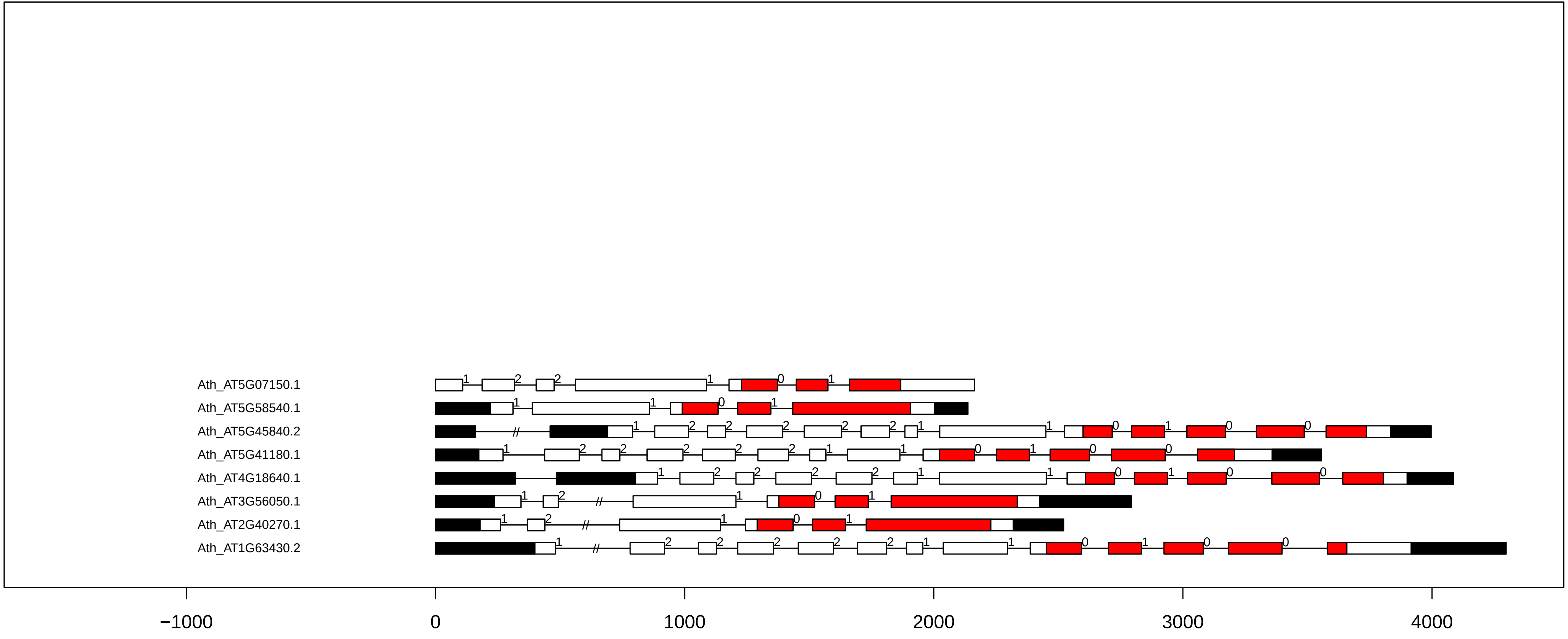

A.th RLK−Pelle_LRR−VII−1 exon−intron and kinase domain diagram (all)

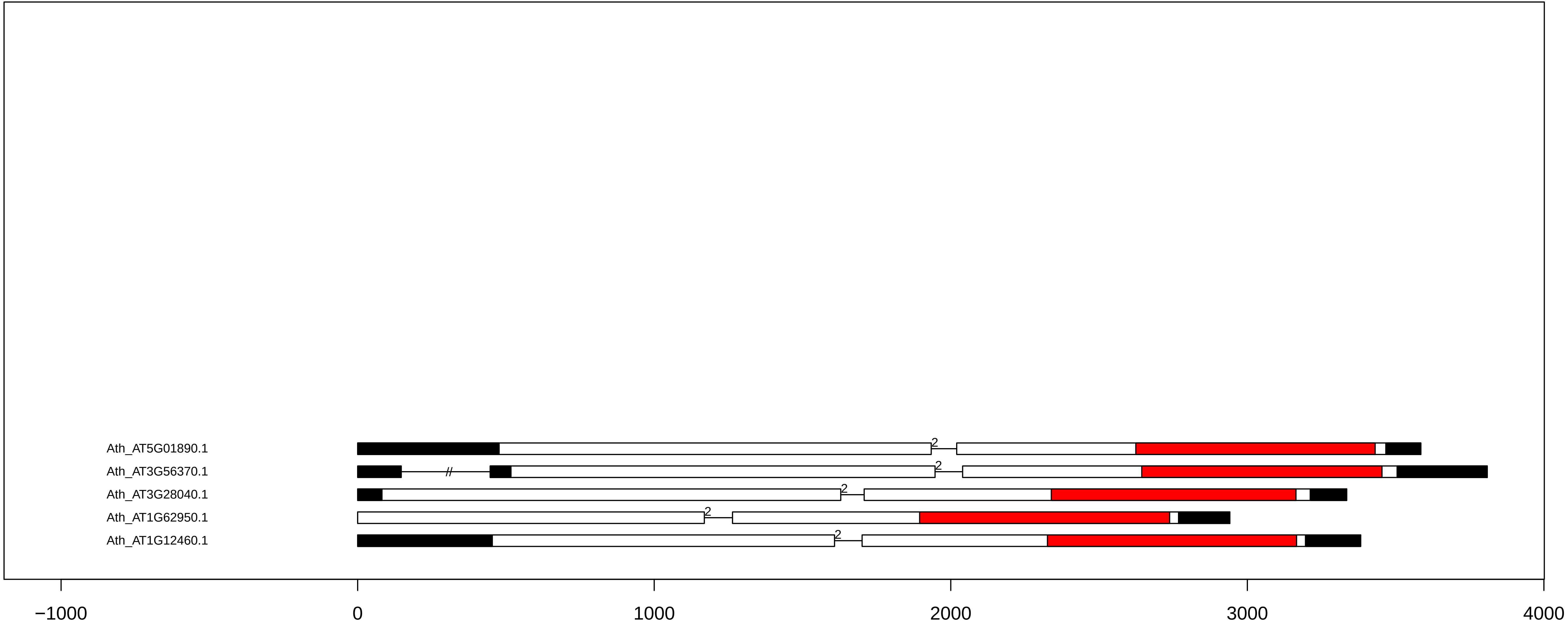

A.th RLK−Pelle_LRR−VII−2 exon−intron and kinase domain diagram (all)

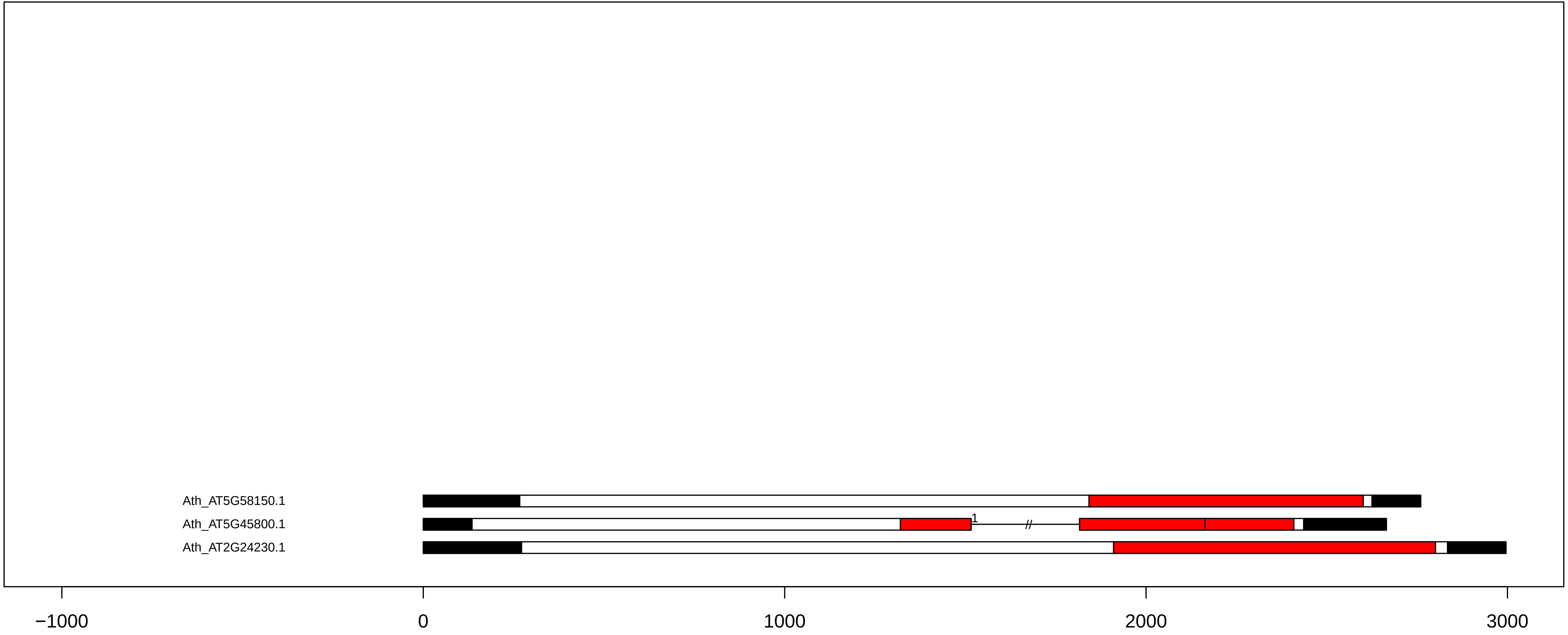

A.th RLK−Pelle_LRR−VII−3 exon−intron and kinase domain diagram (all)

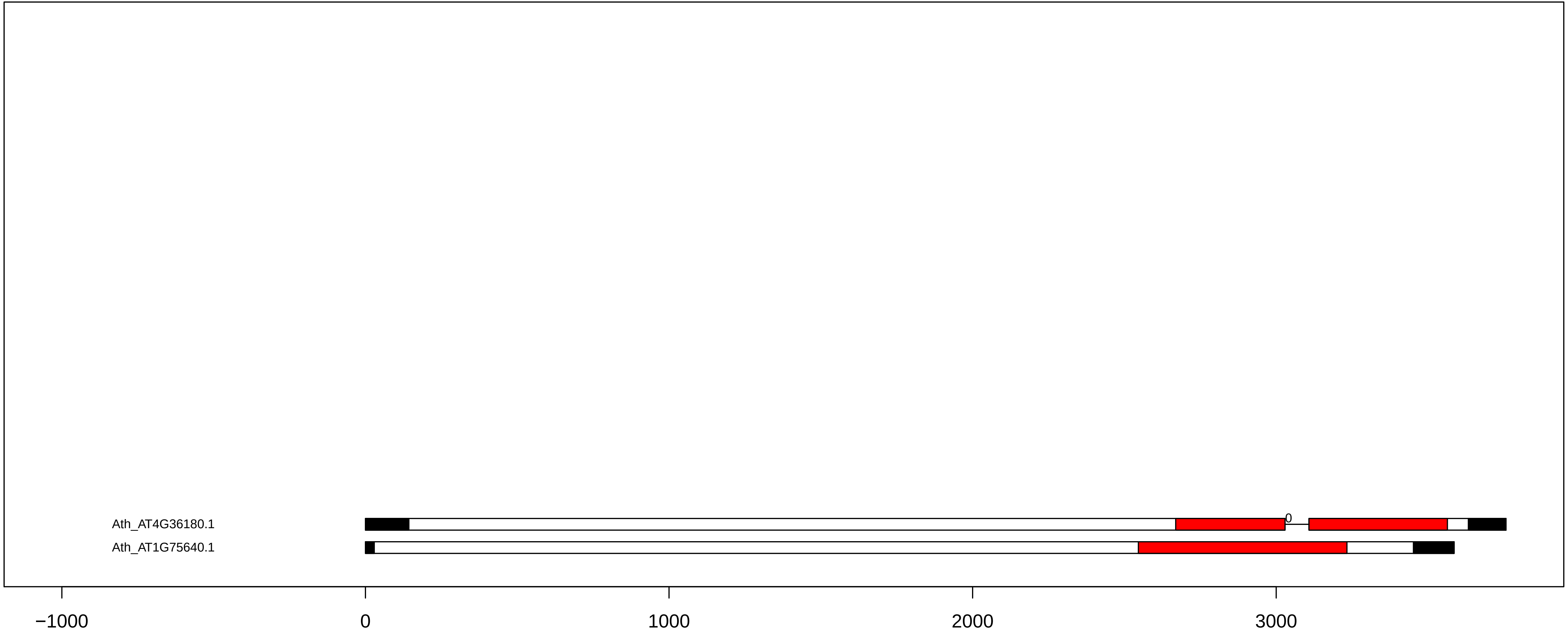

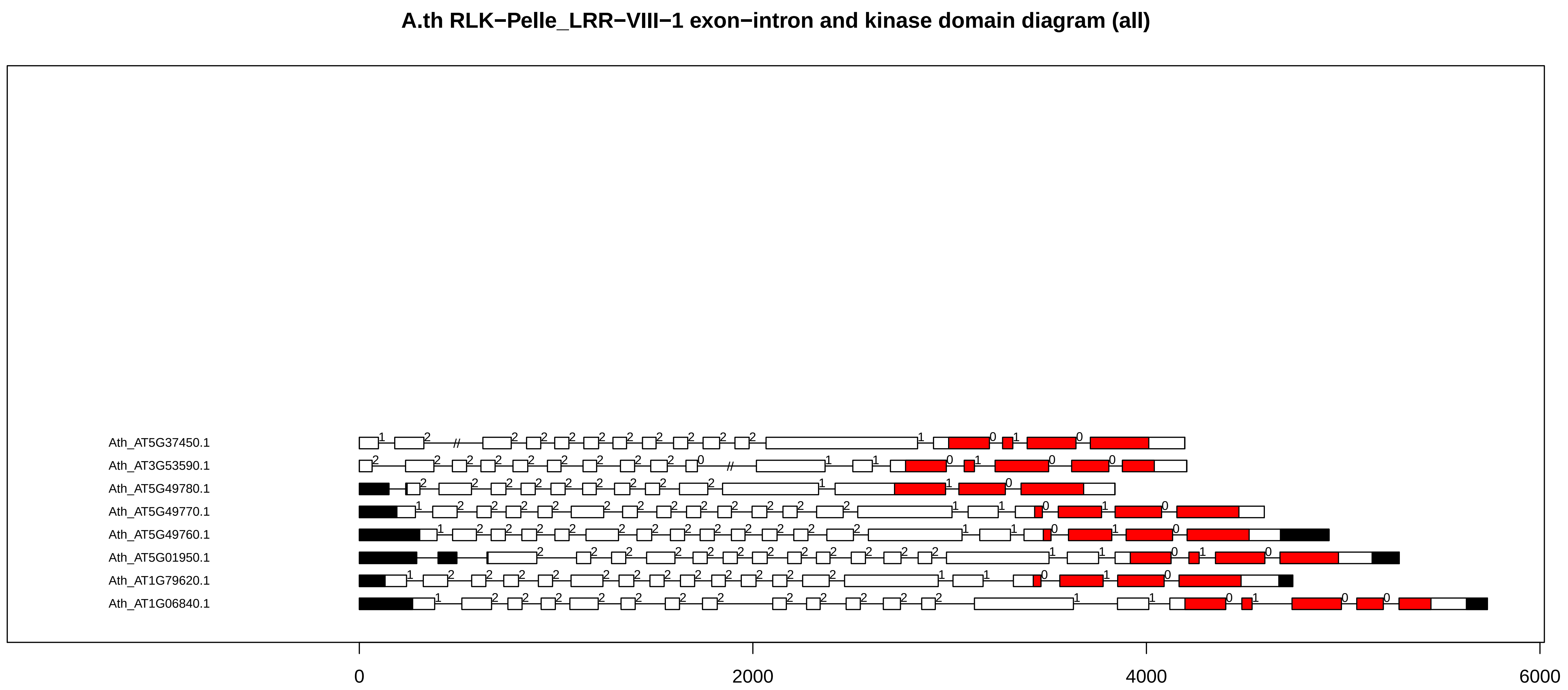
A.th RLK−Pelle_LRR−VIII−1 exon−intron and kinase domain diagram (all)
Ath_AT5G37450.1
Ath_AT3G53590.1
Ath_AT5G49780.1
Ath_AT5G49770.1
Ath_AT5G49760.1
Ath_AT5G01950.1
Ath_AT1G79620.1
Ath_AT1G06840.1
0
2000
4000
6000

A.th RLK−Pelle_LRR−V exon−intron and kinase domain diagram (all)

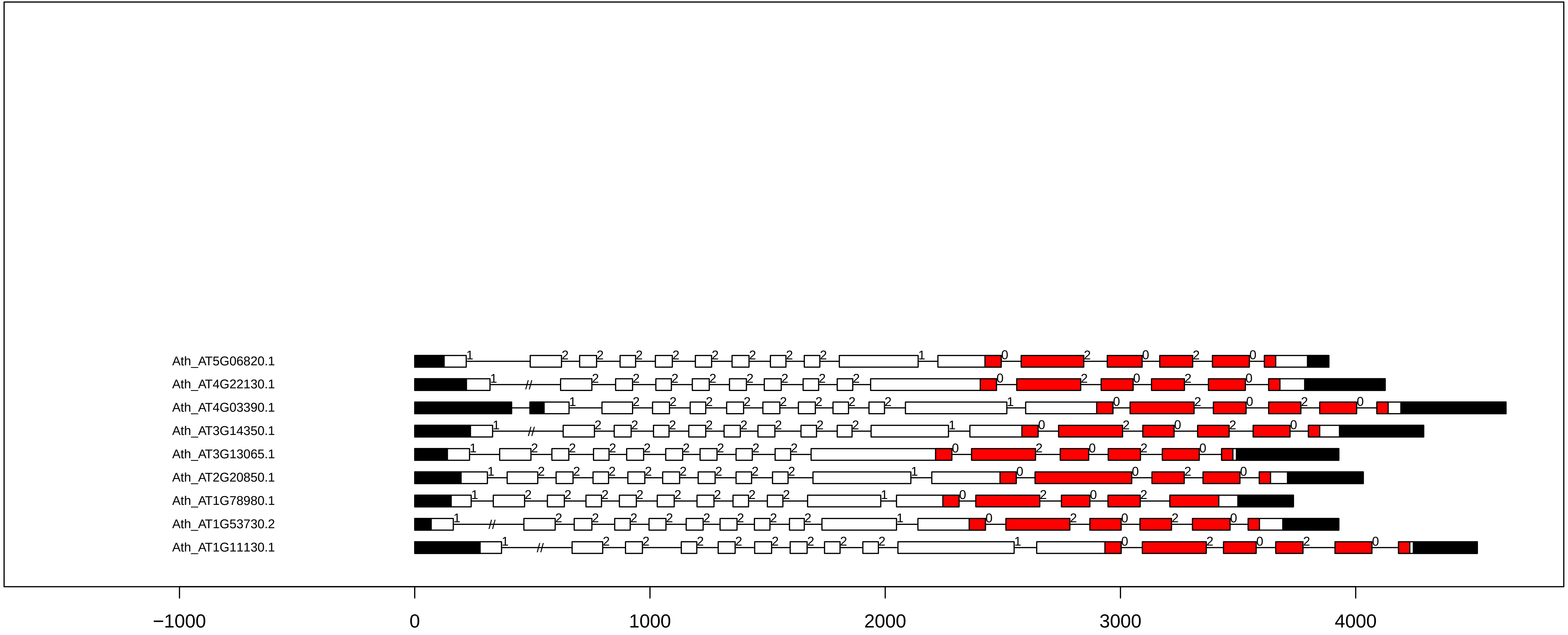

A.th RLK−Pelle_LRR−Xa exon−intron and kinase domain diagram (all)

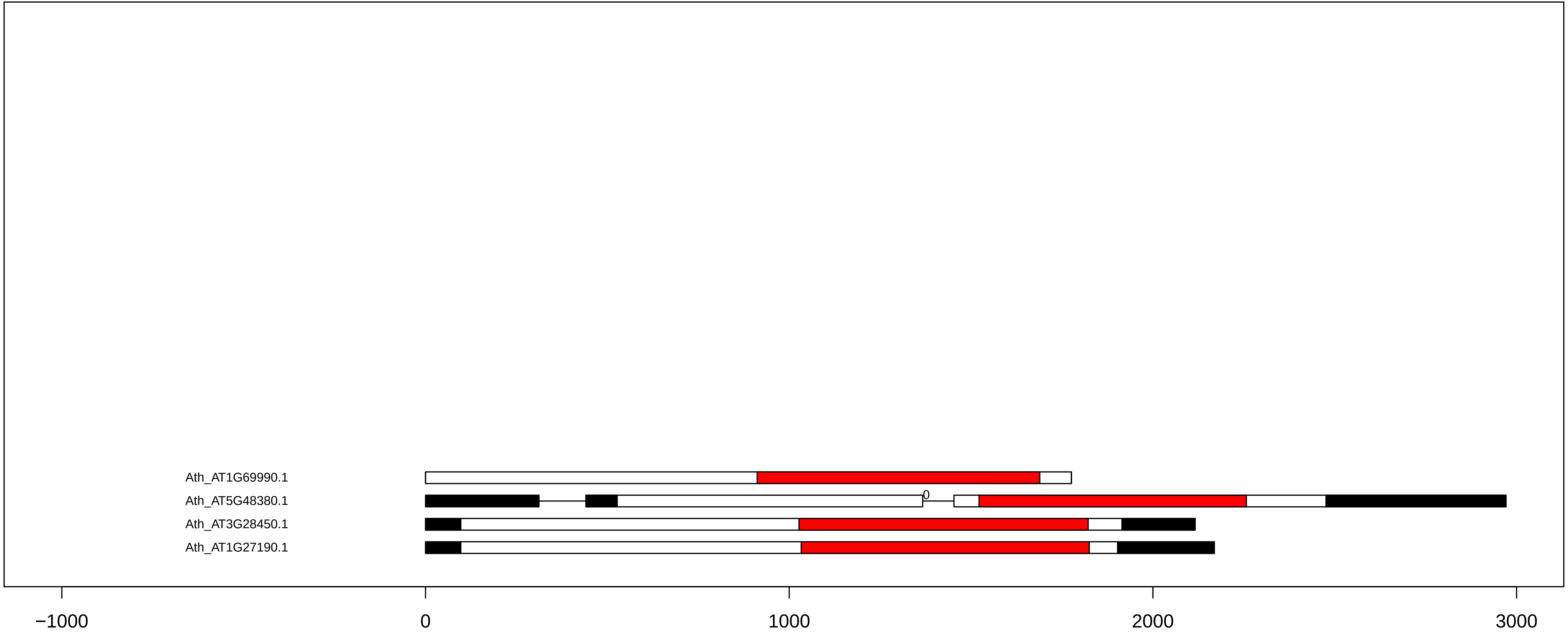

A.th RLK−Pelle_LRR−Xb−1 exon−intron and kinase domain diagram (all)

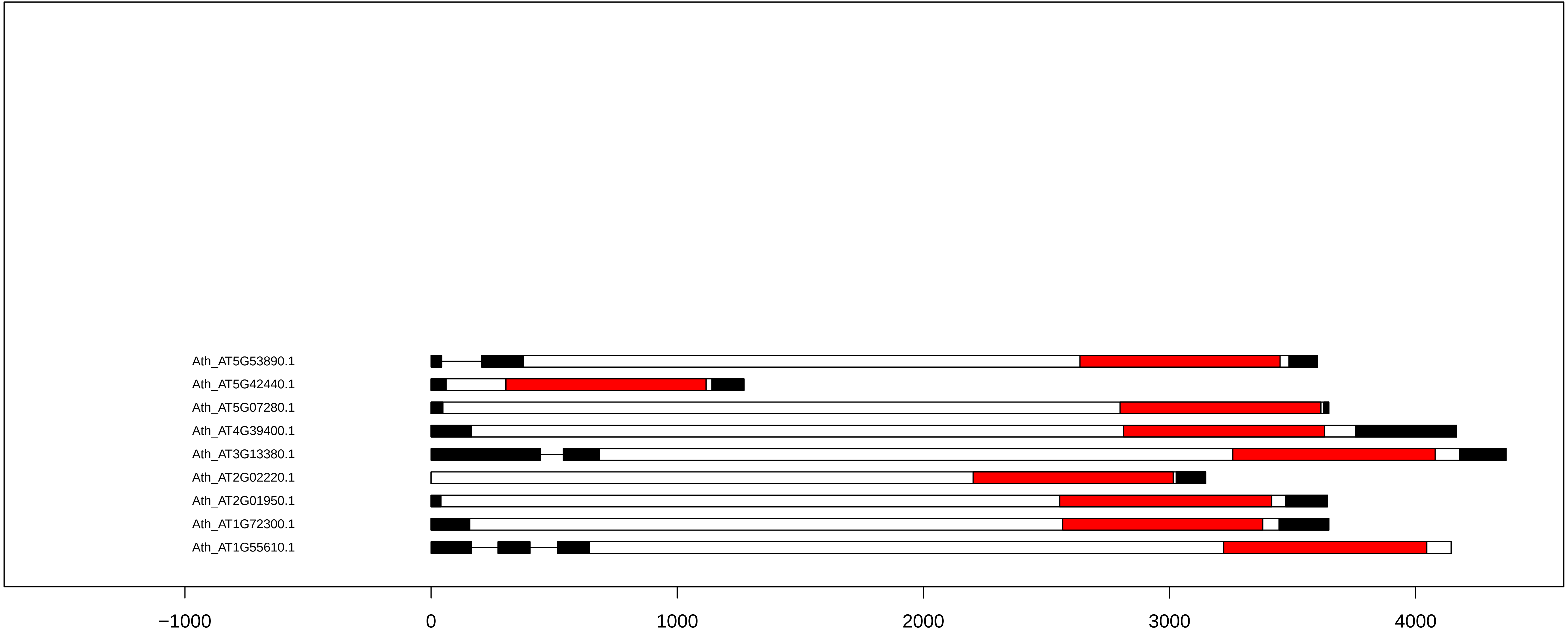

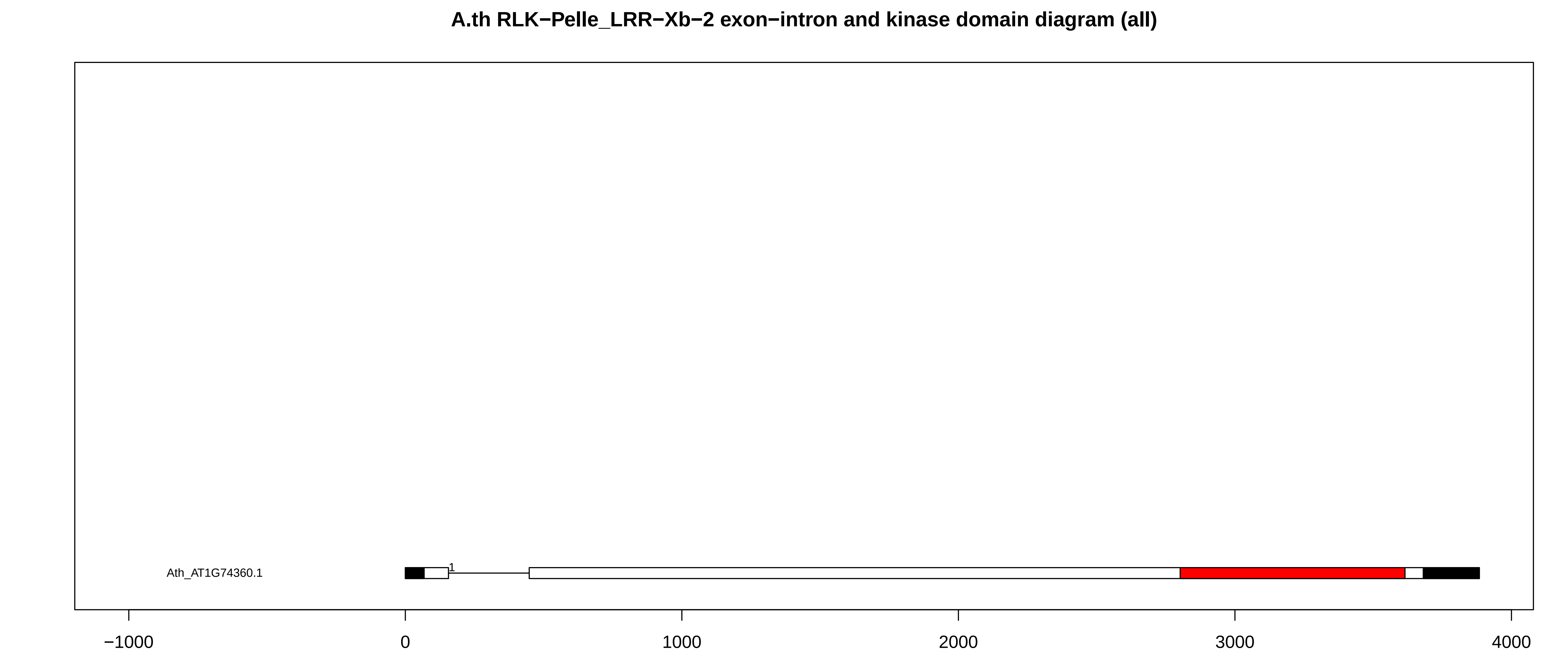

A.th RLK−Pelle_LRR−Xb−2 exon−intron and kinase domain diagram (all)
Ath_AT1G74360.1
1
−1000
0
1000
2000
3000
4000

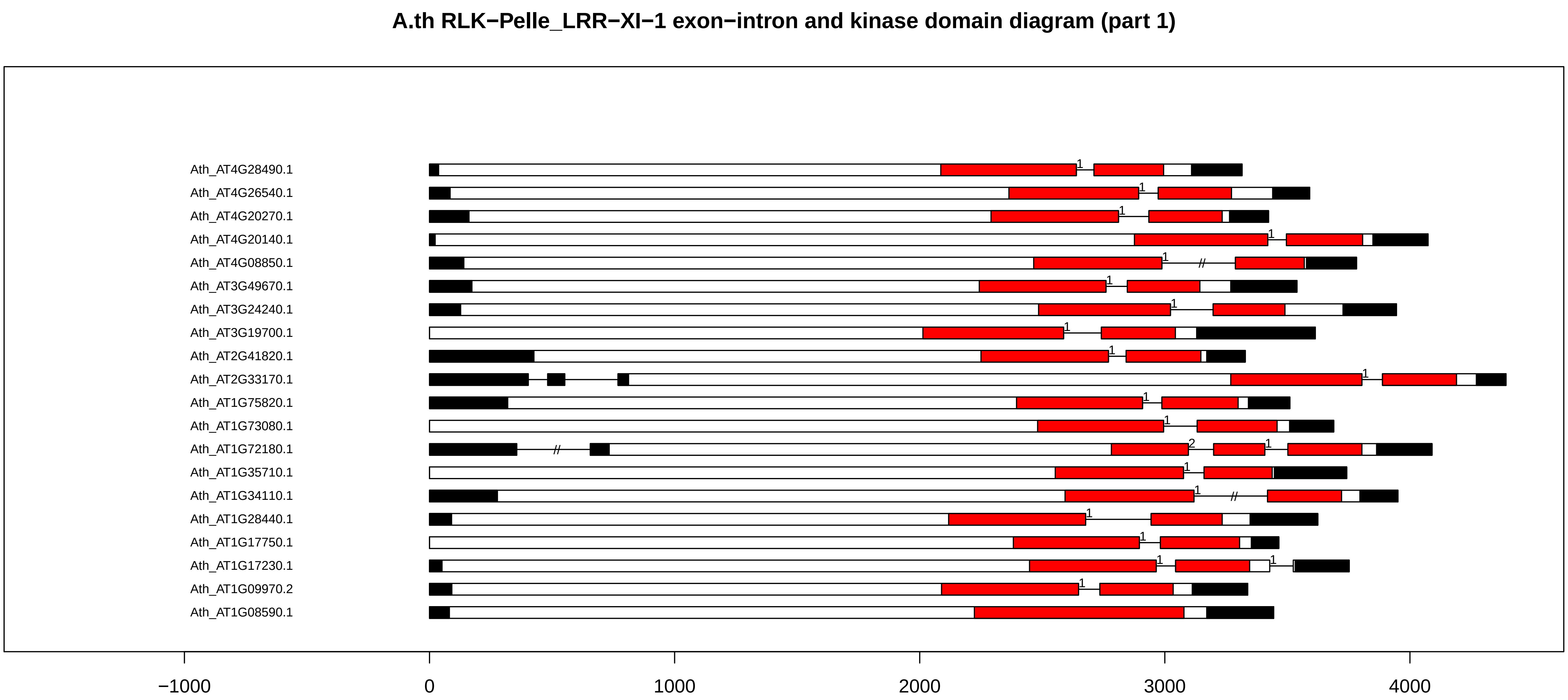

A.th RLK−Pelle_LRR−XI−1 exon−intron and kinase domain diagram (part 1)
Ath_AT4G28490.1
Ath_AT4G26540.1
Ath_AT4G20270.1
Ath_AT4G20140.1
Ath_AT4G08850.1
Ath_AT3G49670.1
Ath_AT3G24240.1
Ath_AT3G19700.1
Ath_AT2G41820.1
Ath_AT2G33170.1
Ath_AT1G75820.1
Ath_AT1G73080.1
Ath_AT1G72180.1
Ath_AT1G35710.1
Ath_AT1G34110.1
Ath_AT1G28440.1
Ath_AT1G17750.1
Ath_AT1G17230.1
Ath_AT1G09970.2
Ath_AT1G08590.1
−1000
0
1000
2000
3000
4000

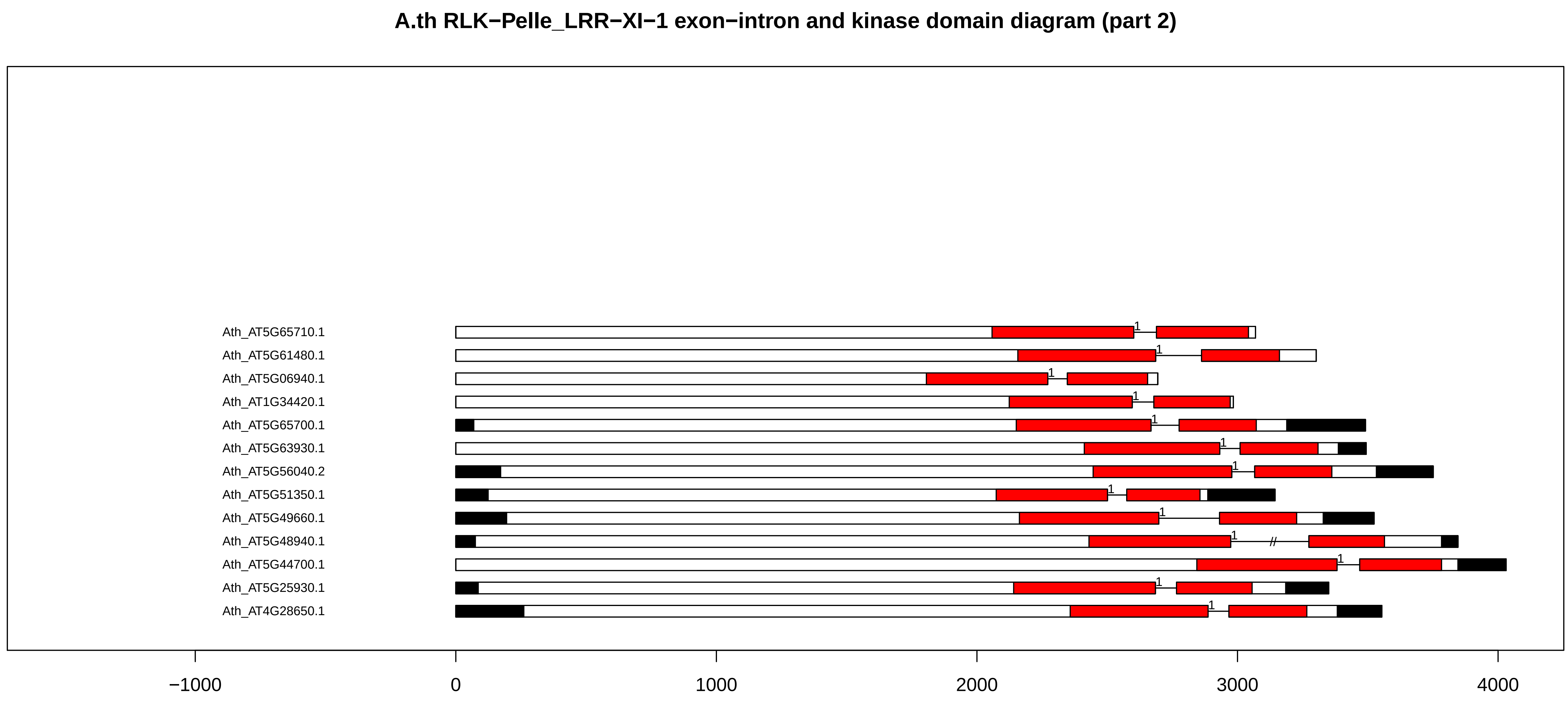
A.th RLK−Pelle_LRR−XI−1 exon−intron and kinase domain diagram (part 2)
Ath_AT5G65710.1
Ath_AT5G61480.1
Ath_AT5G06940.1
Ath_AT1G34420.1
Ath_AT5G65700.1
Ath_AT5G63930.1
Ath_AT5G56040.2
Ath_AT5G51350.1
Ath_AT5G49660.1
Ath_AT5G48940.1
Ath_AT5G44700.1
Ath_AT5G25930.1
Ath_AT4G28650.1
−1000
0
1000
2000
3000
4000

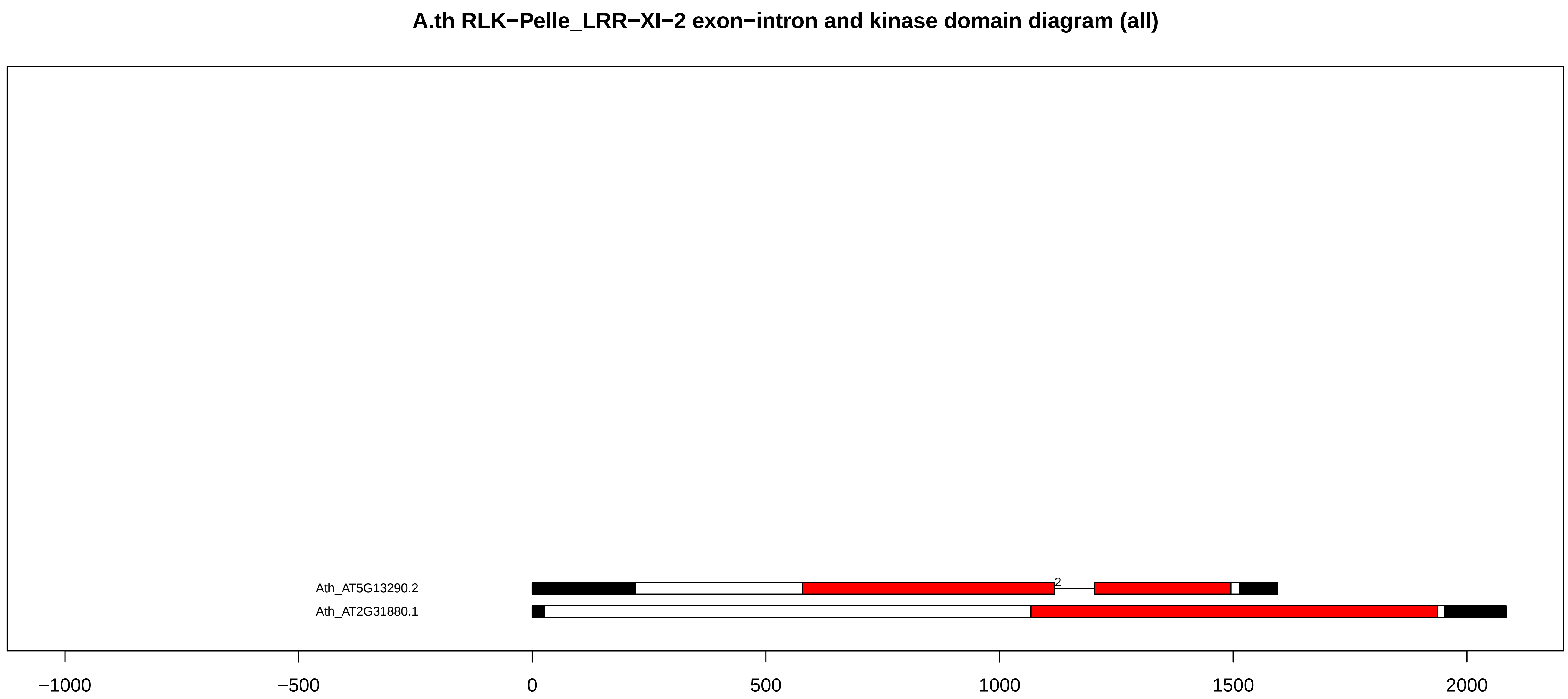
A.th RLK−Pelle_LRR−XI−2 exon−intron and kinase domain diagram (all)
Ath_AT5G13290.2
Ath_AT2G31880.1
2
−1000
−500
0
500
1000
1500
2000

A.th RLK−Pelle_LRR−XII−1 exon−intron and kinase domain diagram (all)

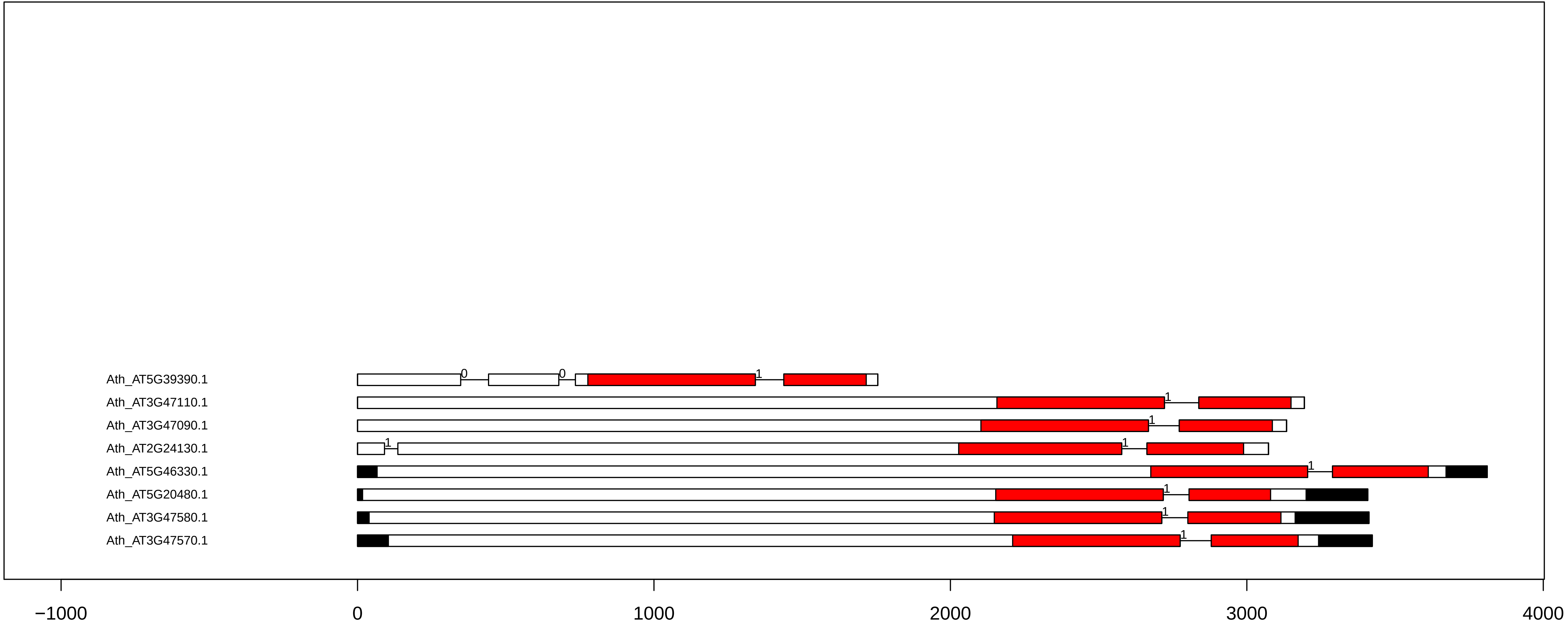

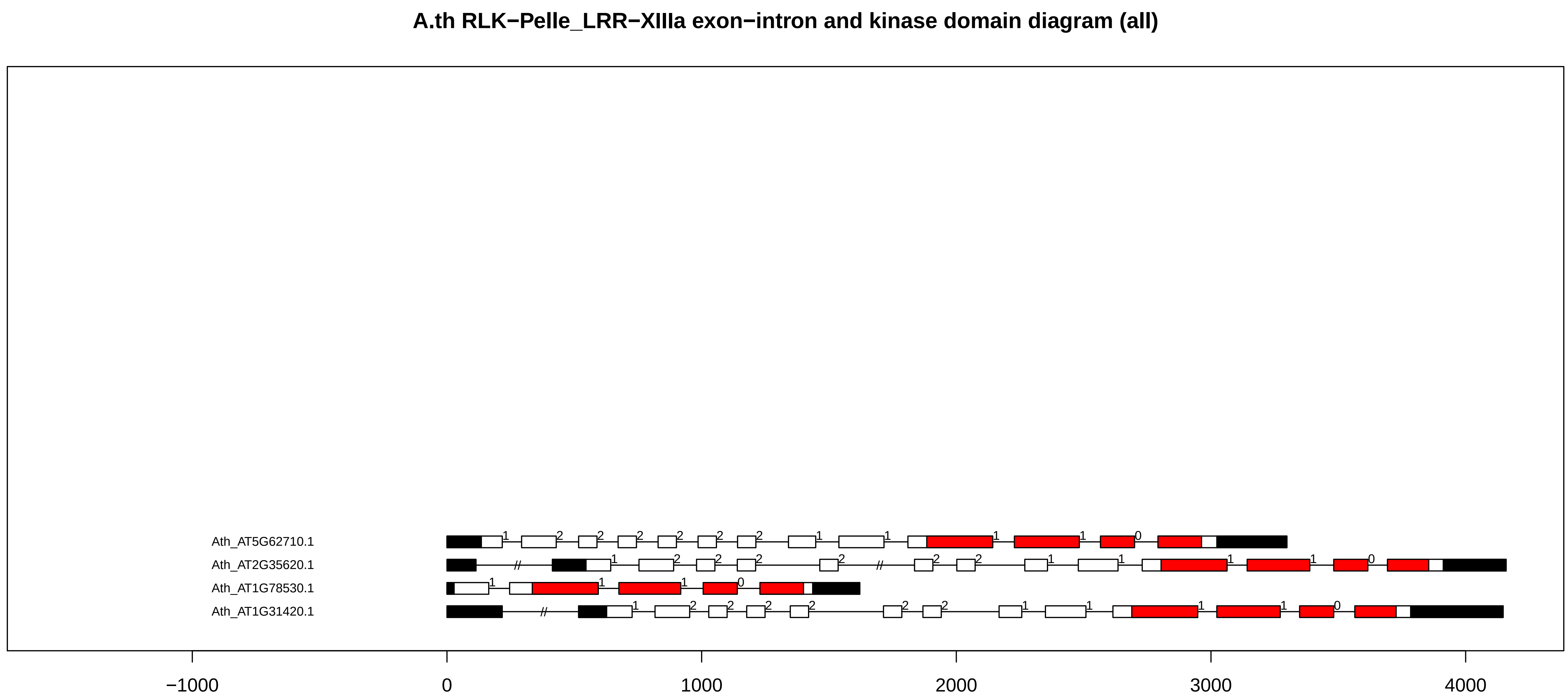
A.th RLK−Pelle_LRR−XIIIa exon−intron and kinase domain diagram (all)
Ath_AT5G62710.1
Ath_AT2G35620.1
Ath_AT1G78530.1
Ath_AT1G31420.1
−1000
0
1000
2000
3000
4000

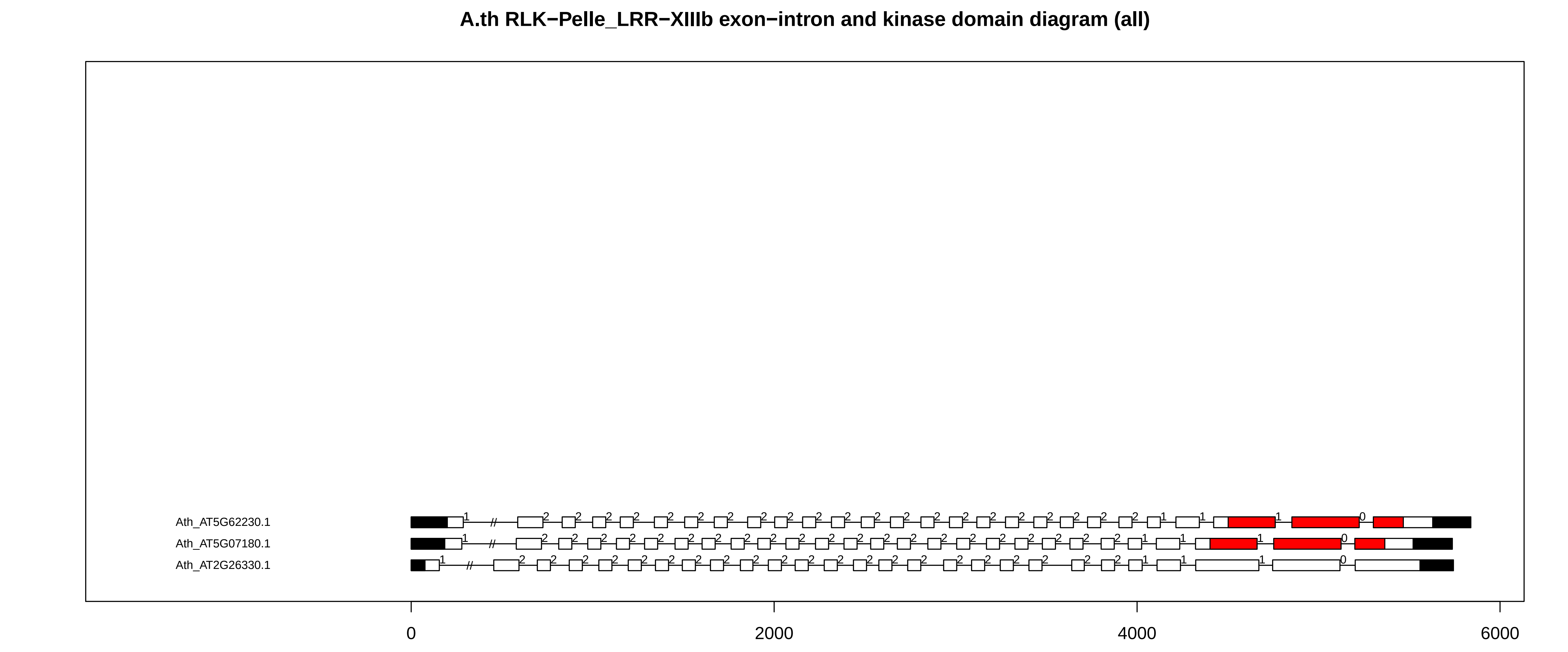
A.th RLK−Pelle_LRR−XIIIb exon−intron and kinase domain diagram (all)
Ath_AT5G62230.1
Ath_AT5G07180.1
Ath_AT2G26330.1
0
2000
4000
6000

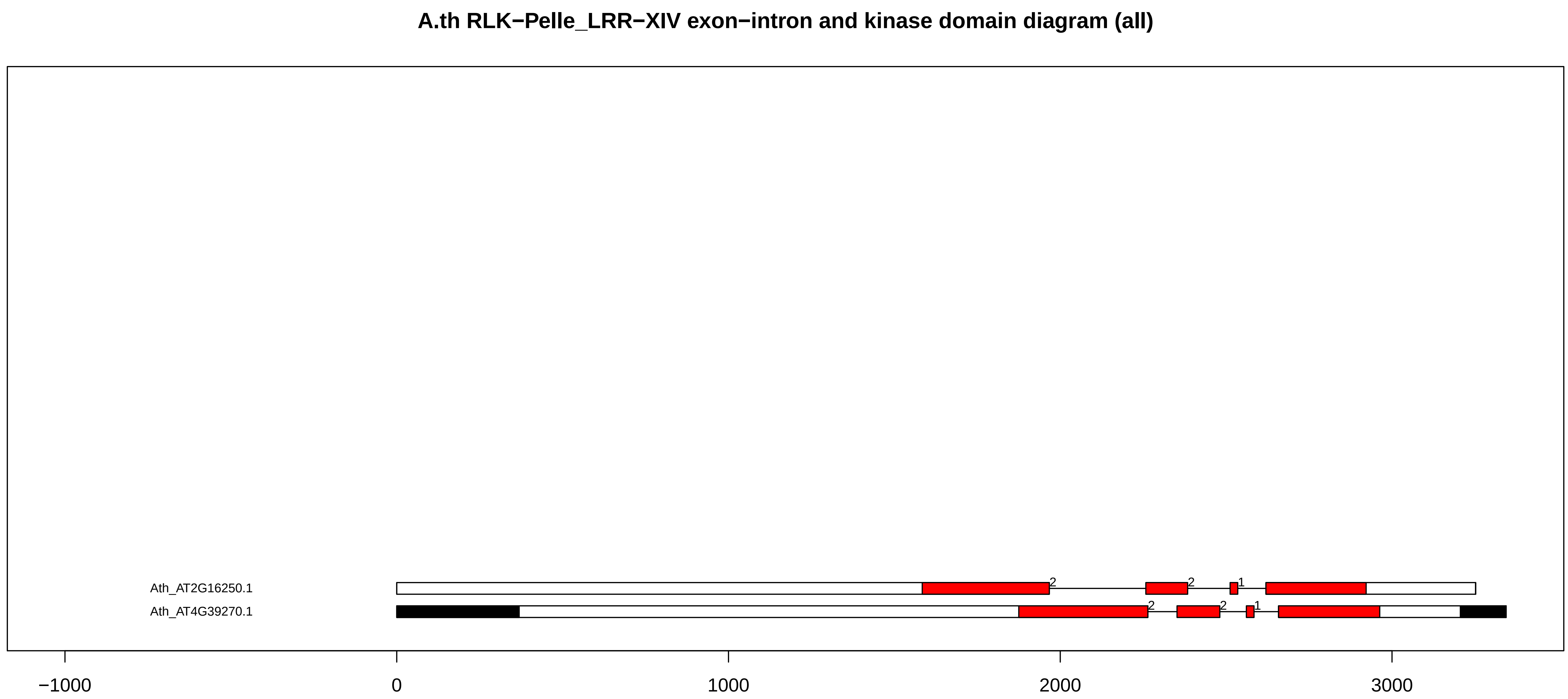
A.th RLK−Pelle_LRR−XIV exon−intron and kinase domain diagram (all)
Ath_AT2G16250.1
Ath_AT4G39270.1
2
2
1
2
2
1
−1000
0
1000
2000
3000

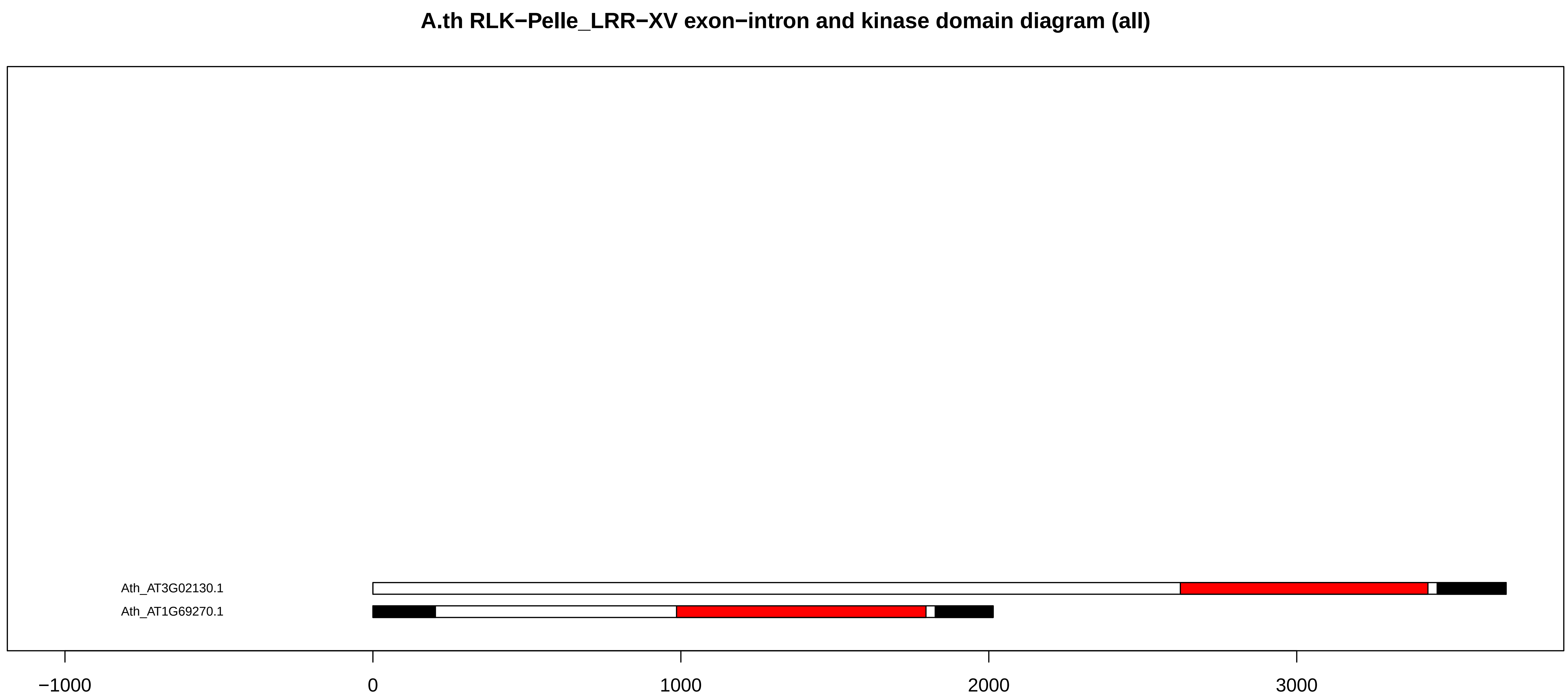
A.th RLK−Pelle_LRR−XV exon−intron and kinase domain diagram (all)
Ath_AT3G02130.1
Ath_AT1G69270.1
−1000
0
1000
2000
3000

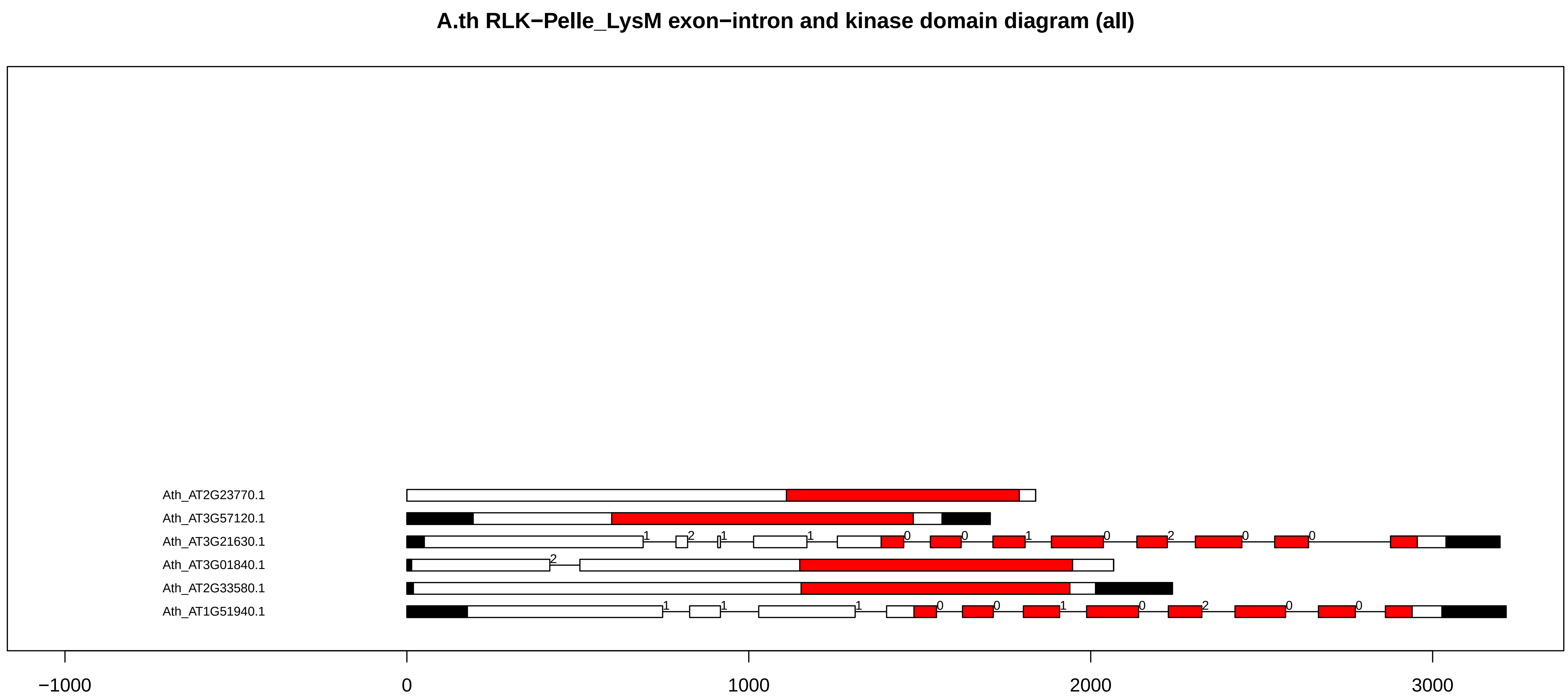

A.th RLK−Pelle_LysM exon−intron and kinase domain diagram (all)
Ath_AT2G23770.1
Ath_AT3G57120.1
Ath_AT3G21630.1
Ath_AT3G01840.1
Ath_AT2G33580.1
Ath_AT1G51940.1
−1000
0
1000
2000
3000

A.th RLK−Pelle_PERK−1 exon−intron and kinase domain diagram (all)

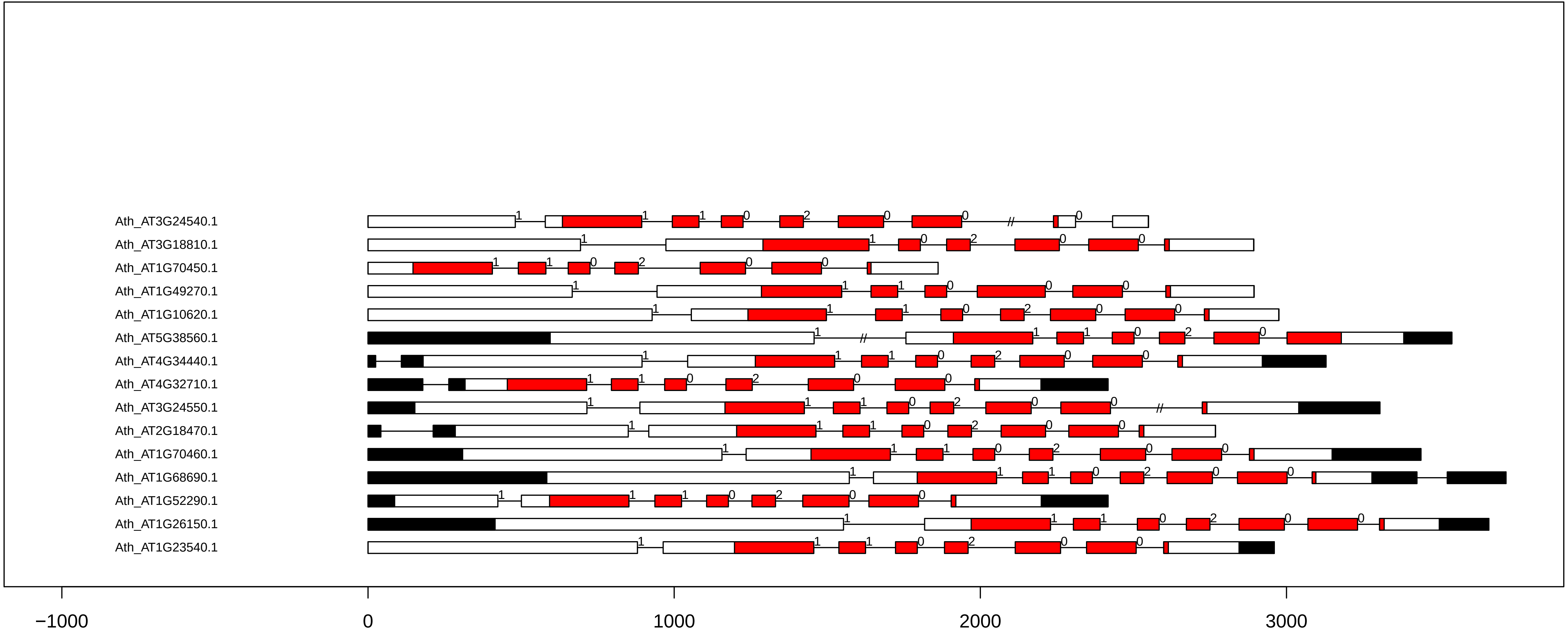

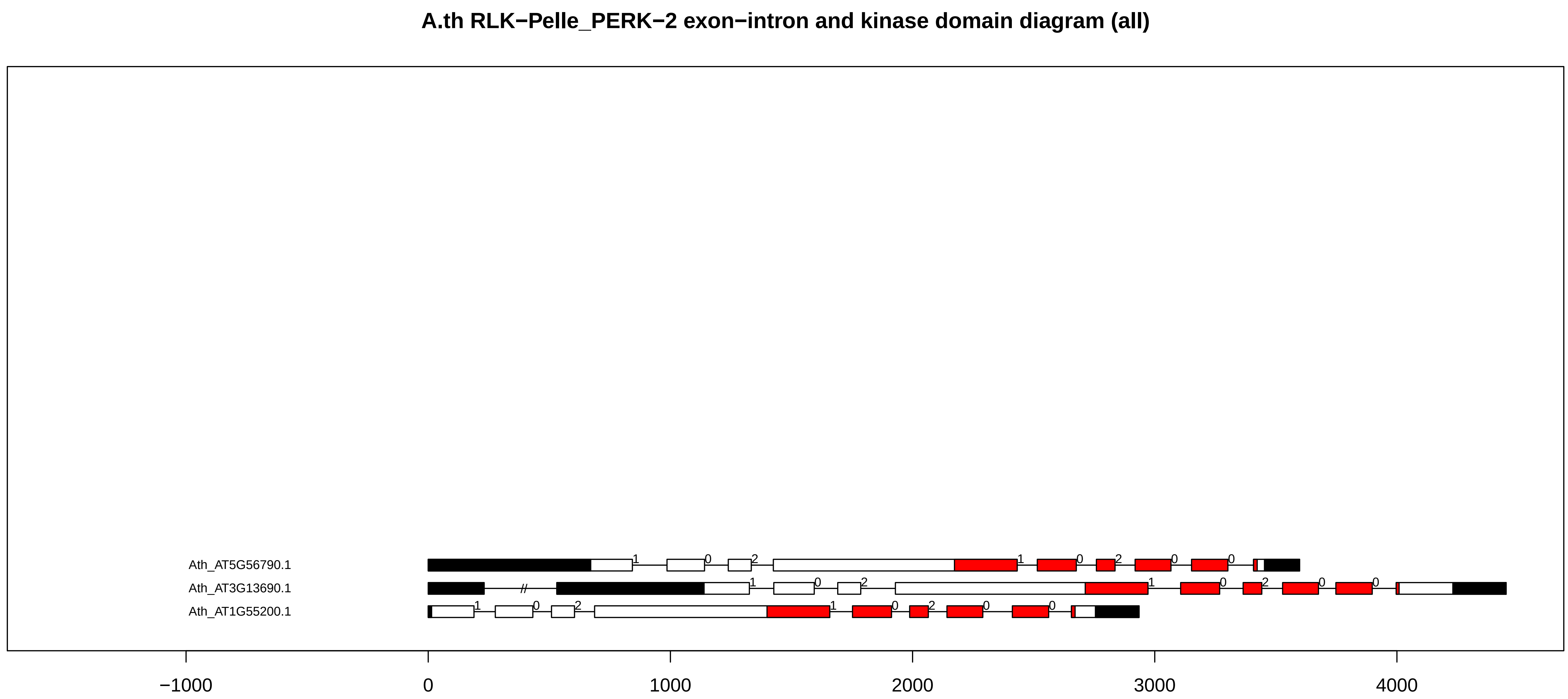

A.th RLK−Pelle_PERK−2 exon−intron and kinase domain diagram (all)
Ath_AT5G56790.1
Ath_AT3G13690.1
Ath_AT1G55200.1
−1000
0
1000
2000
3000
4000

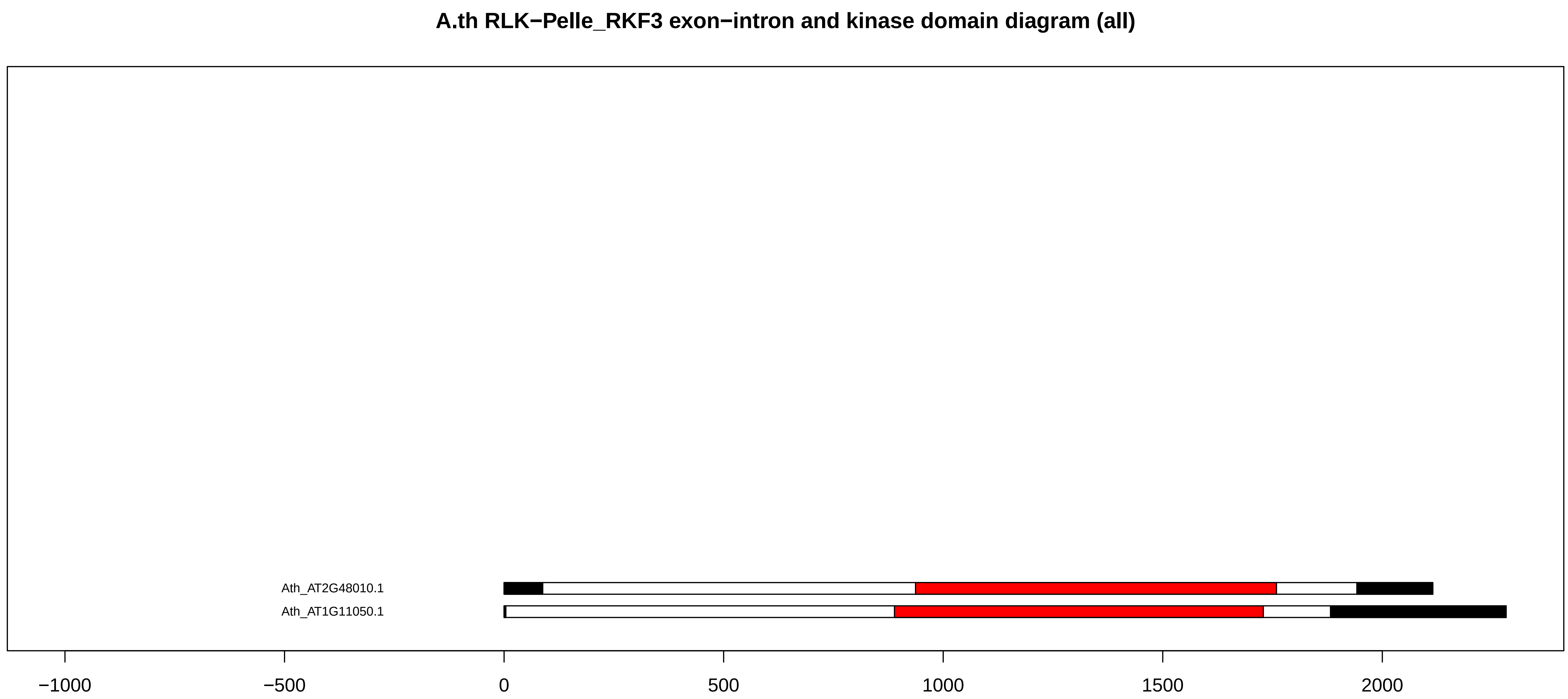
A.th RLK−Pelle_RKF3 exon−intron and kinase domain diagram (all)
Ath_AT2G48010.1
Ath_AT1G11050.1
−1000
−500
0
500
1000
1500
2000

A.th RLK−Pelle_RLCK−II exon−intron and kinase domain diagram (all)

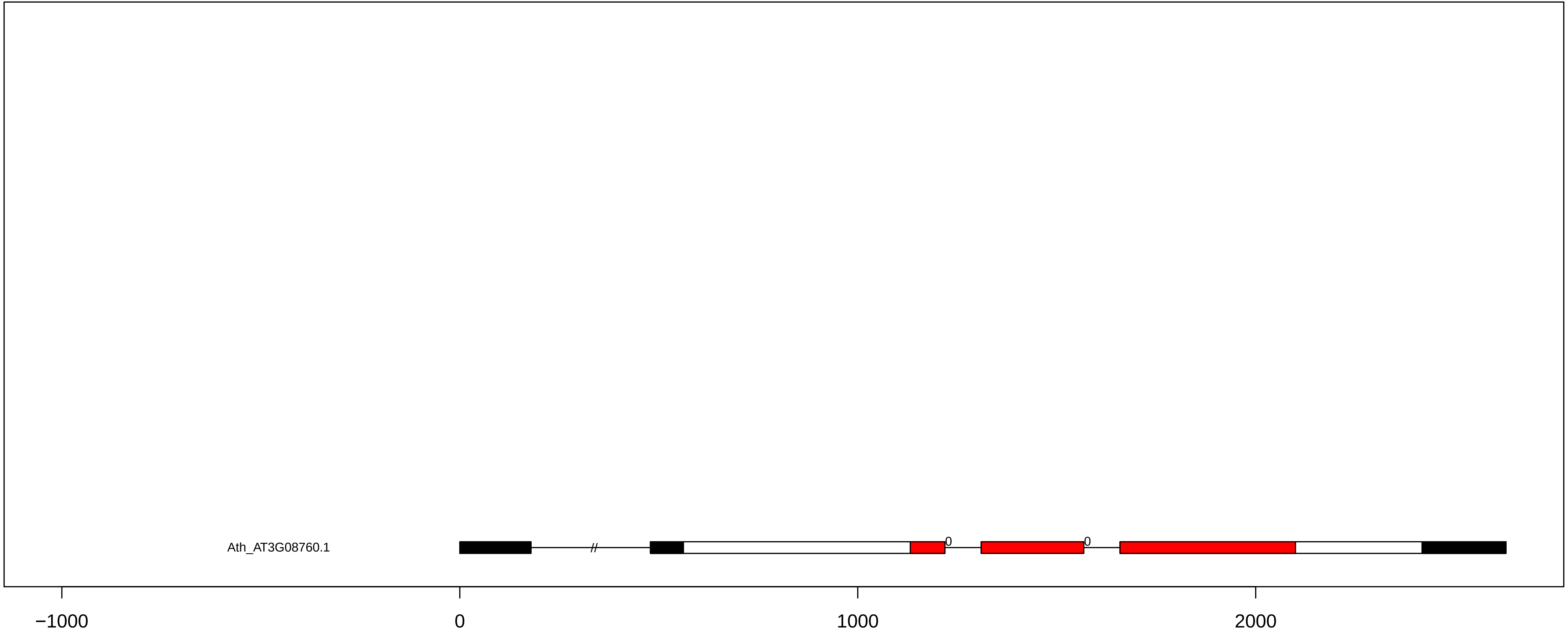

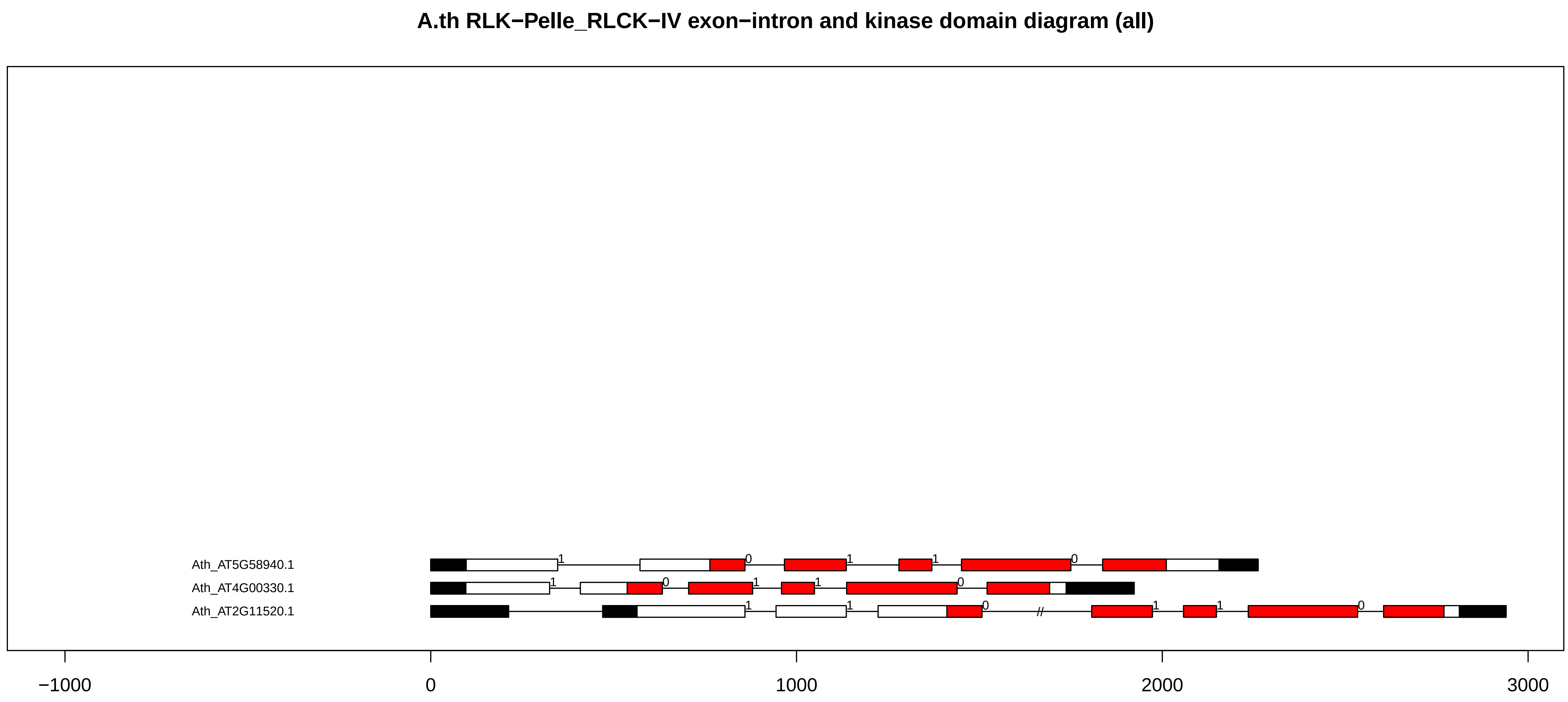
A.th RLK−Pelle_RLCK−IV exon−intron and kinase domain diagram (all)
Ath_AT5G58940.1
Ath_AT4G00330.1
Ath_AT2G11520.1
−1000
0
1000
2000
3000

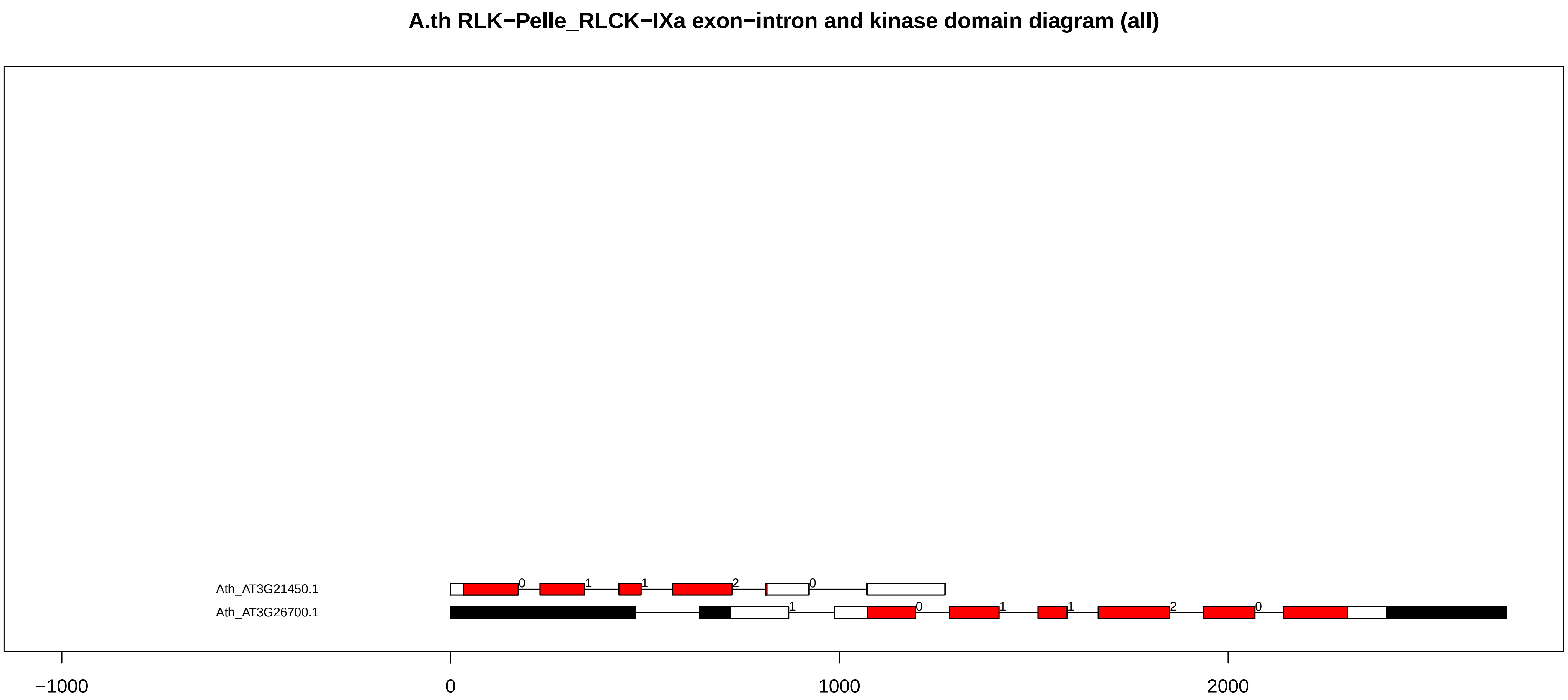
A.th RLK−Pelle_RLCK−IXa exon−intron and kinase domain diagram (all)
Ath_AT3G21450.1
Ath_AT3G26700.1
0
1
1
2
0
1
0
1
1
2
0
−1000
0
1000
2000

A.th RLK−Pelle_RLCK−IXb exon−intron and kinase domain diagram (all)

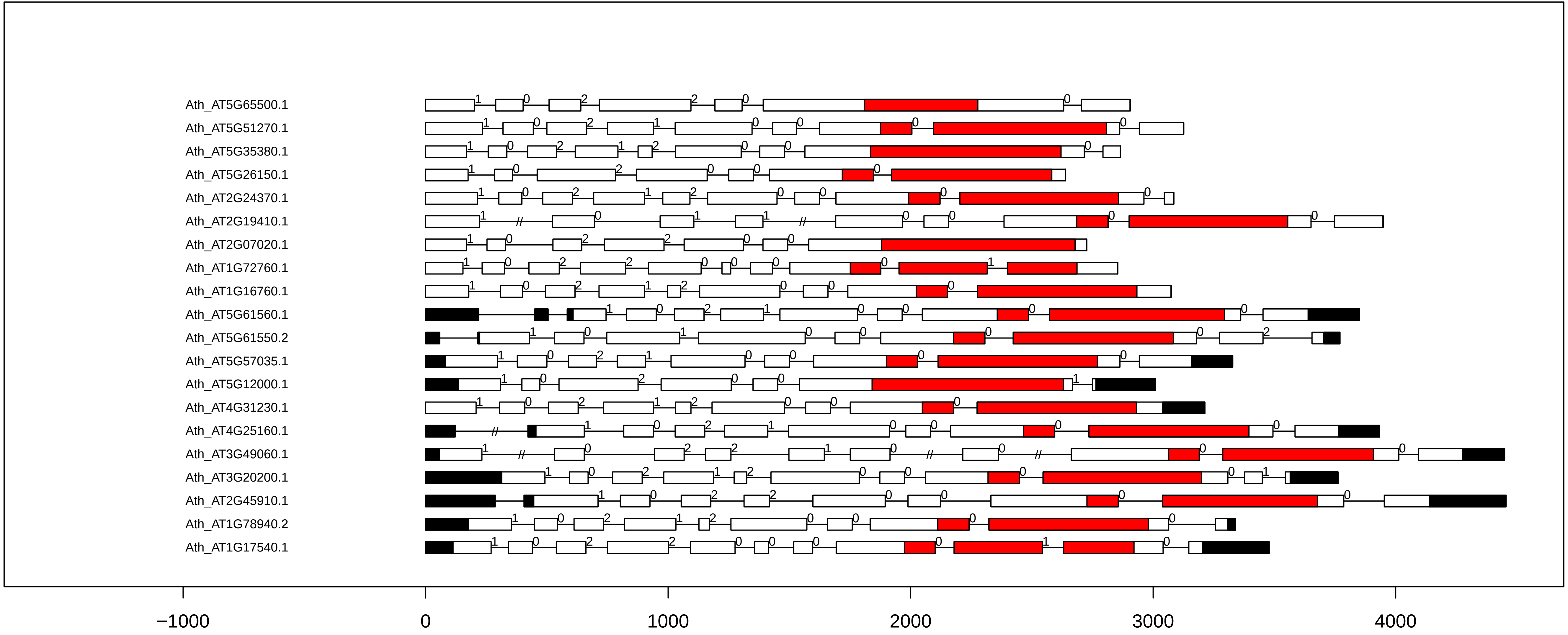

A.th RLK−Pelle_RLCK−VIIa−1 exon−intron and kinase domain diagram (all)

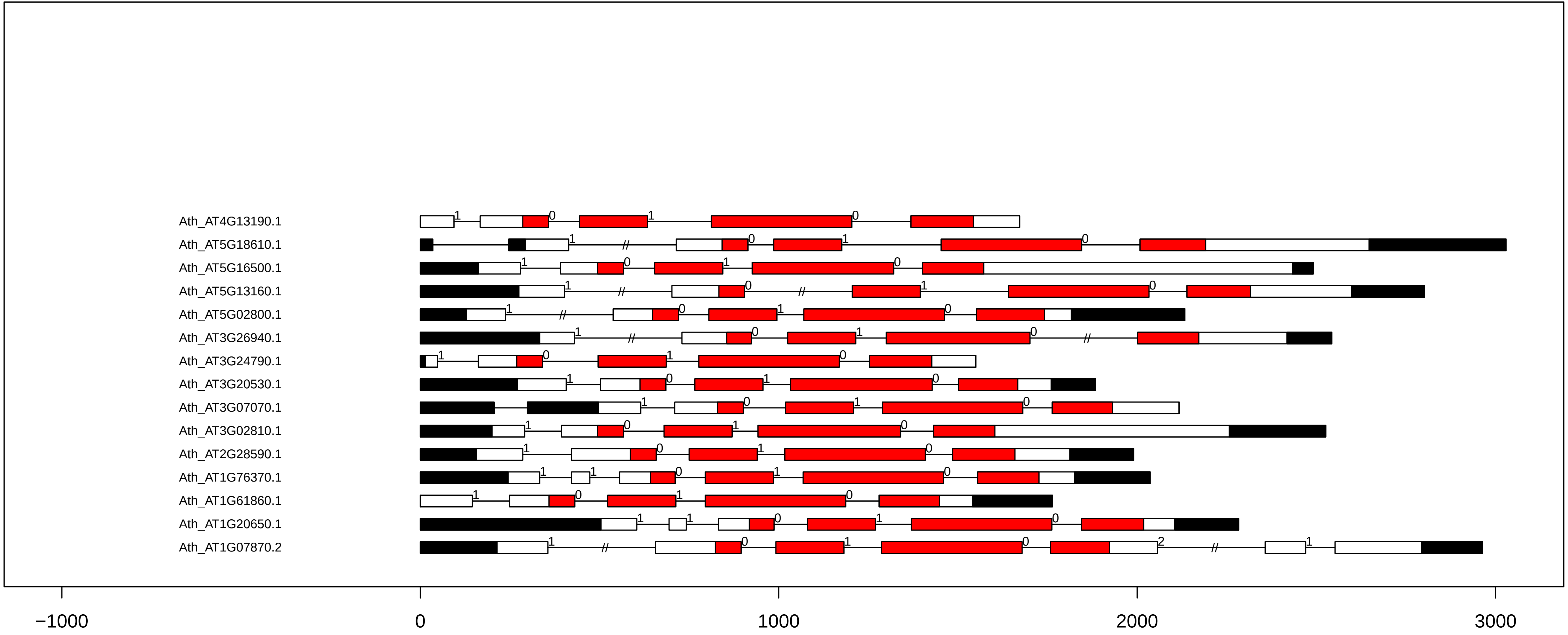

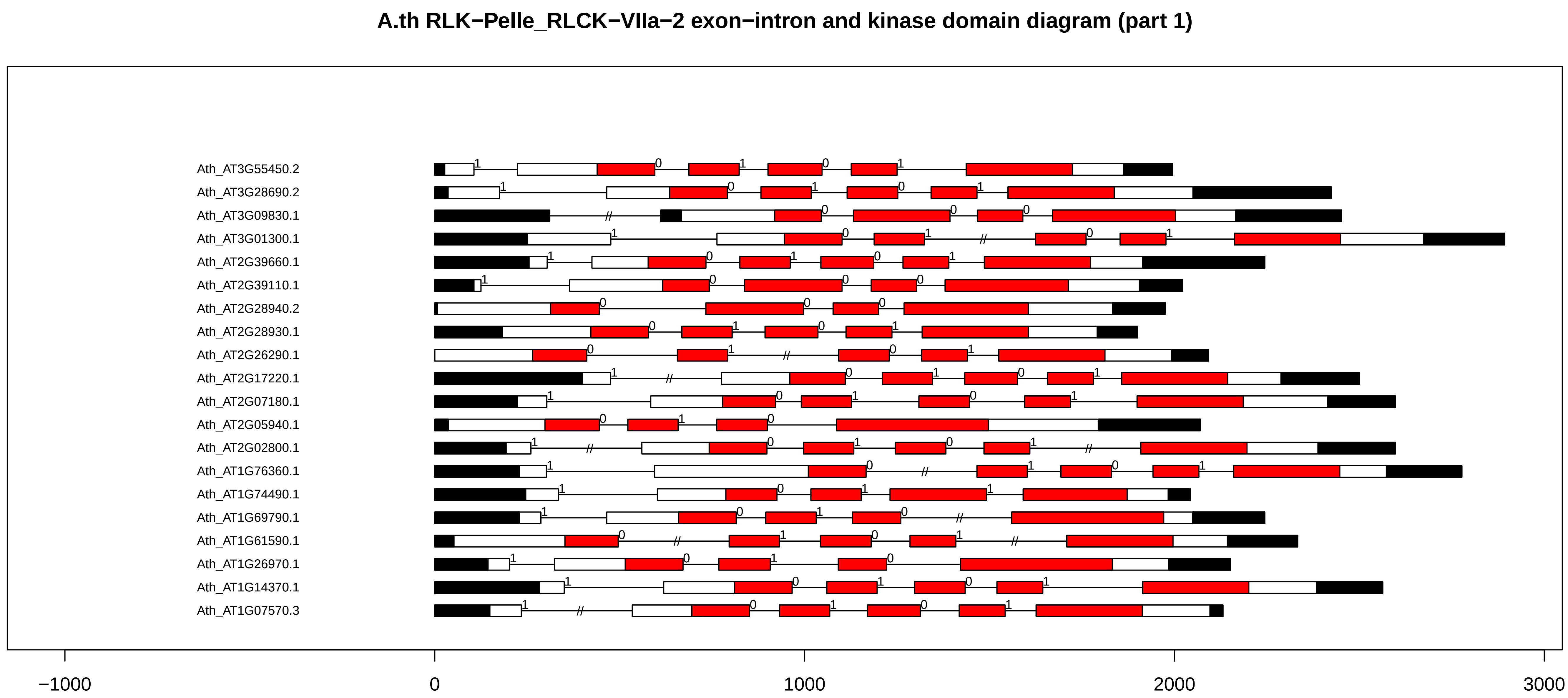
A.th RLK−Pelle_RLCK−VIIa−2 exon−intron and kinase domain diagram (part 1)
Ath_AT3G55450.2
Ath_AT3G28690.2
Ath_AT3G09830.1
Ath_AT3G01300.1
Ath_AT2G39660.1
Ath_AT2G39110.1
Ath_AT2G28940.2
Ath_AT2G28930.1
Ath_AT2G26290.1
Ath_AT2G17220.1
Ath_AT2G07180.1
Ath_AT2G05940.1
Ath_AT2G02800.1
Ath_AT1G76360.1
Ath_AT1G74490.1
Ath_AT1G69790.1
Ath_AT1G61590.1
Ath_AT1G26970.1
Ath_AT1G14370.1
Ath_AT1G07570.3
−1000
0
1000
2000
3000

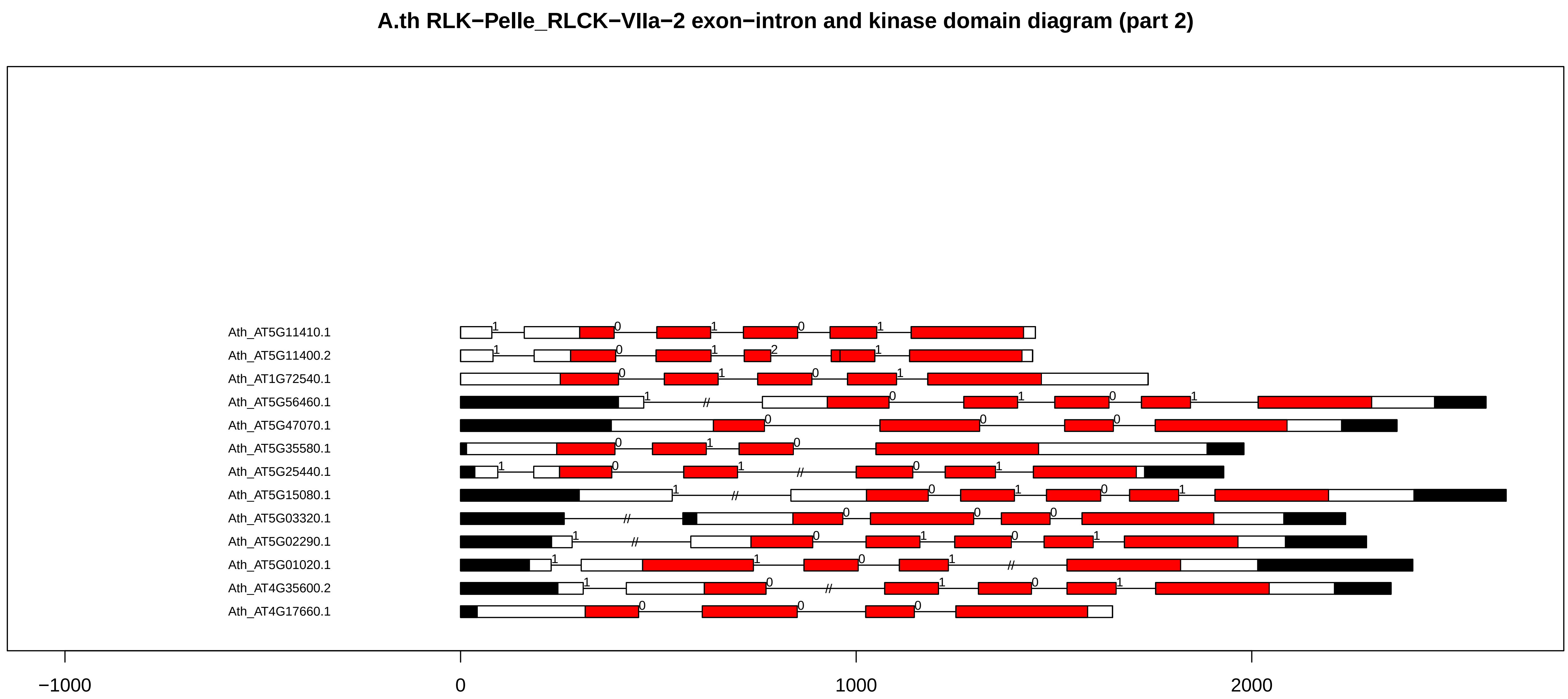
A.th RLK−Pelle_RLCK−VIIa−2 exon−intron and kinase domain diagram (part 2)
Ath_AT5G11410.1
Ath_AT5G11400.2
Ath_AT1G72540.1
Ath_AT5G56460.1
Ath_AT5G47070.1
Ath_AT5G35580.1
Ath_AT5G25440.1
Ath_AT5G15080.1
Ath_AT5G03320.1
Ath_AT5G02290.1
Ath_AT5G01020.1
Ath_AT4G35600.2
Ath_AT4G17660.1
−1000
0
1000
2000

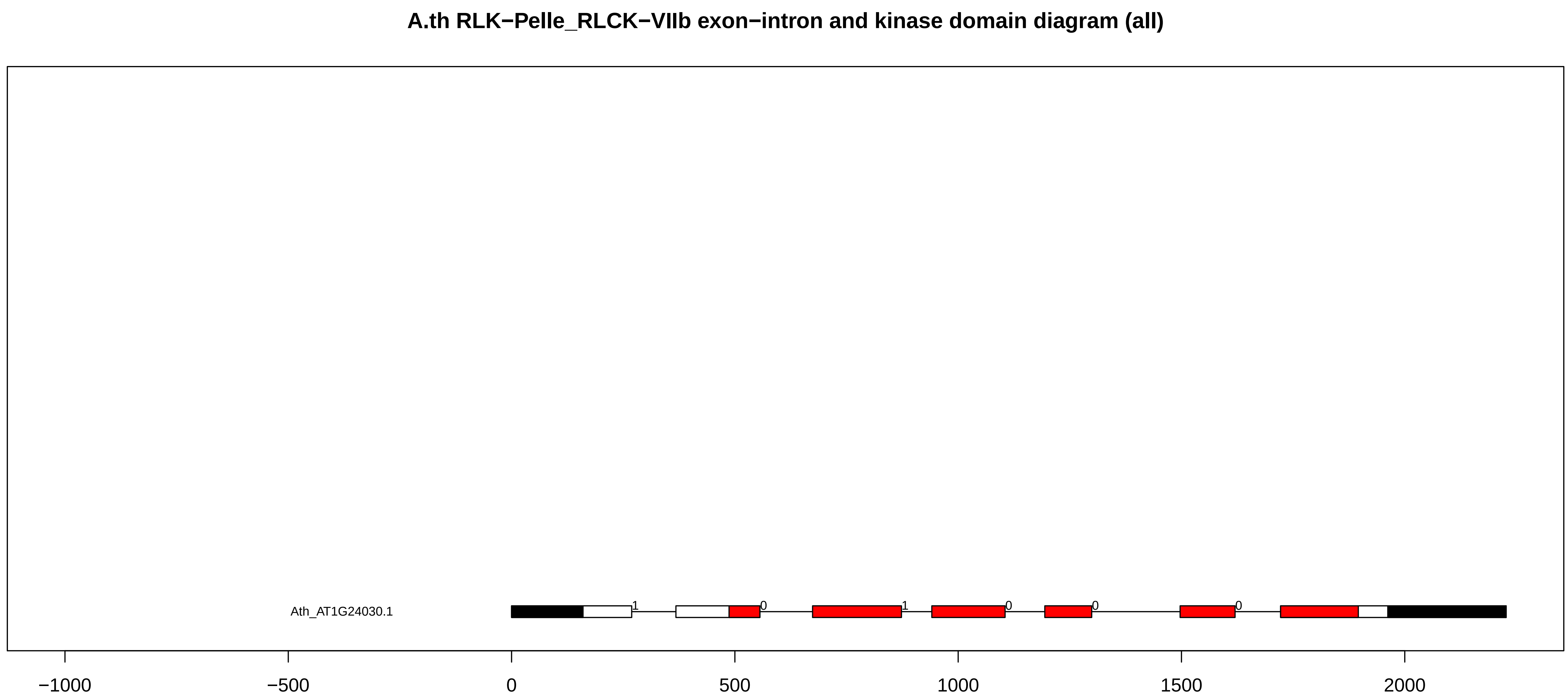
A.th RLK−Pelle_RLCK−VIIb exon−intron and kinase domain diagram (all)
Ath_AT1G24030.1
1
0
1
0
0
0
−1000
−500
0
500
1000
1500
2000

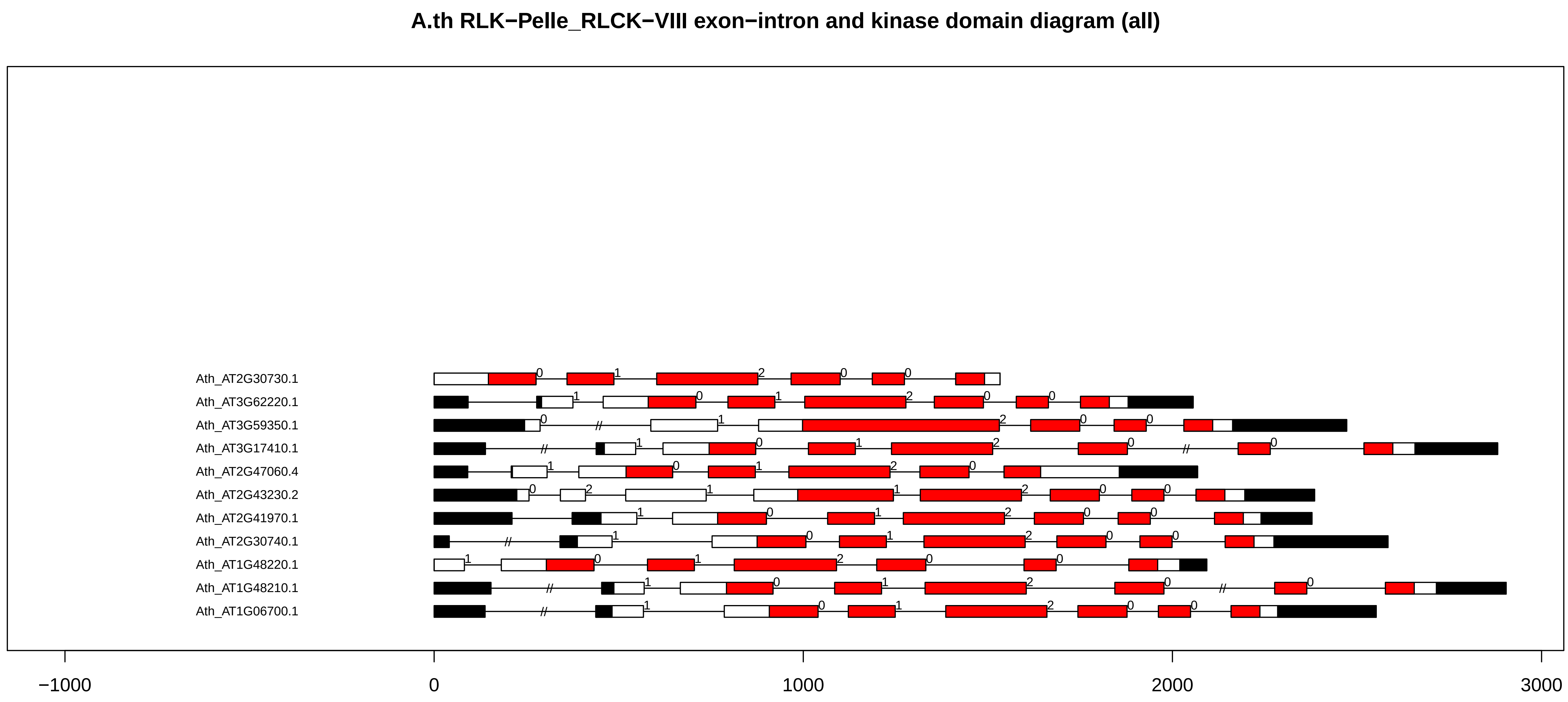

A.th RLK−Pelle_RLCK−VIII exon−intron and kinase domain diagram (all)
Ath_AT2G30730.1
Ath_AT3G62220.1
Ath_AT3G59350.1
Ath_AT3G17410.1
Ath_AT2G47060.4
Ath_AT2G43230.2
Ath_AT2G41970.1
Ath_AT2G30740.1
Ath_AT1G48220.1
Ath_AT1G48210.1
Ath_AT1G06700.1
−1000
0
1000
2000
3000

A.th RLK−Pelle_RLCK−VI exon−intron and kinase domain diagram (all)

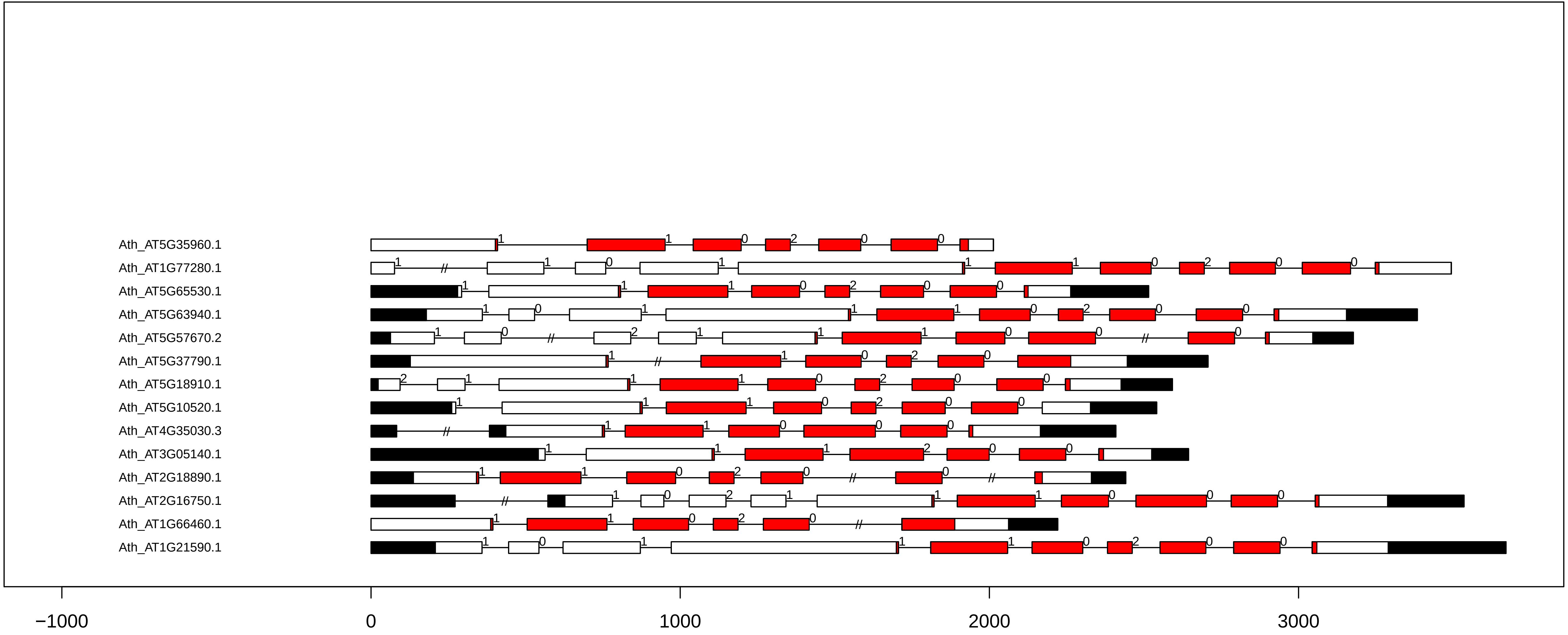

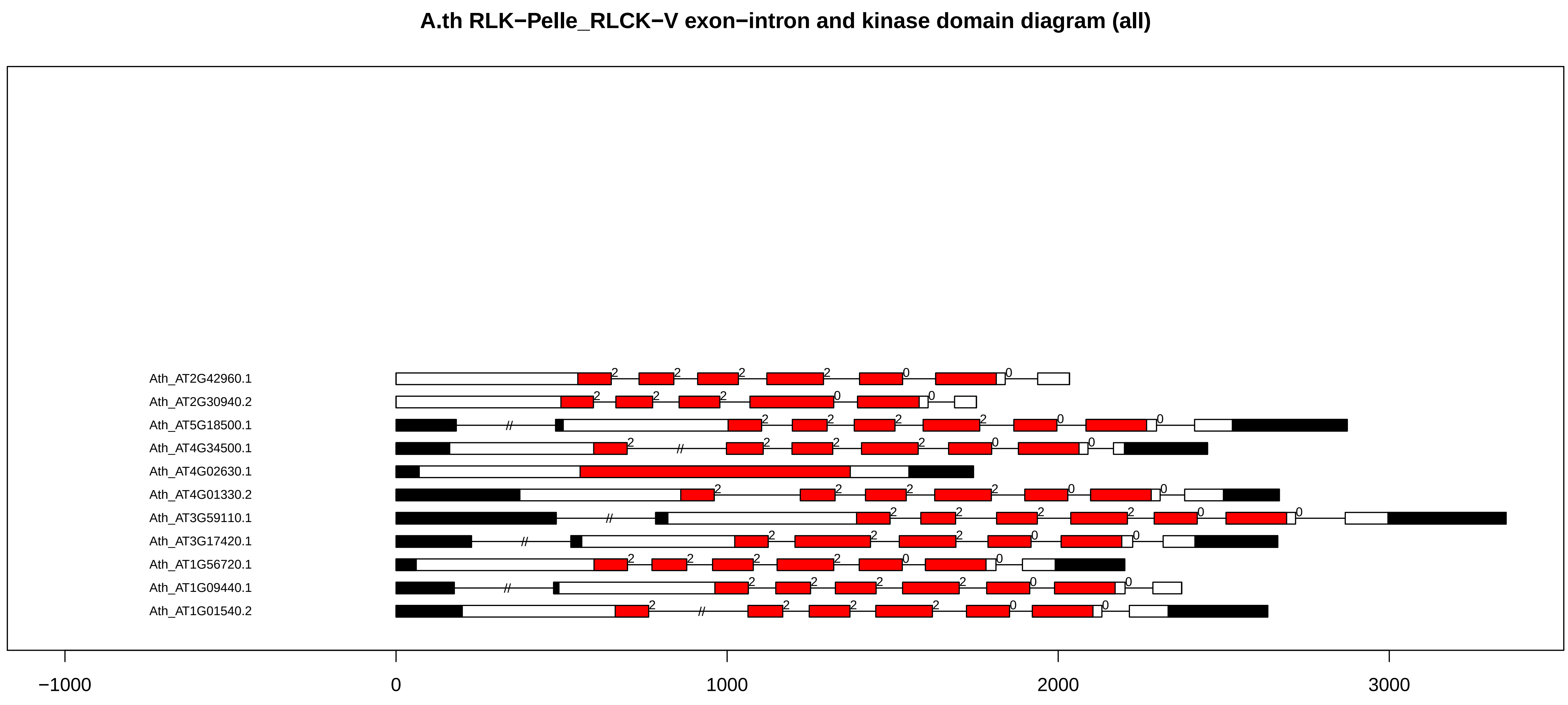
A.th RLK−Pelle_RLCK−V exon−intron and kinase domain diagram (all)
Ath_AT2G42960.1
Ath_AT2G30940.2
Ath_AT5G18500.1
Ath_AT4G34500.1
Ath_AT4G02630.1
Ath_AT4G01330.2
Ath_AT3G59110.1
Ath_AT3G17420.1
Ath_AT1G56720.1
Ath_AT1G09440.1
Ath_AT1G01540.2
−1000
0
1000
2000
3000

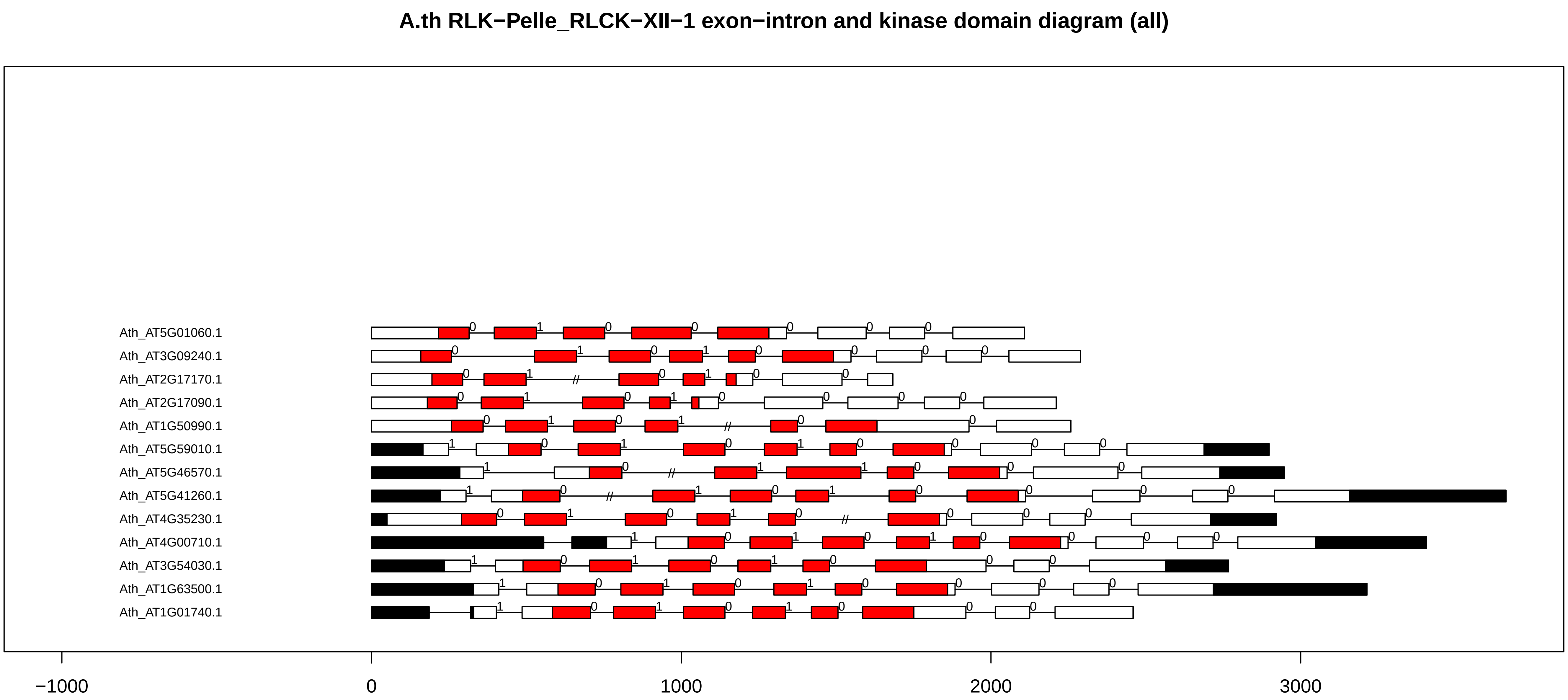
A.th RLK−Pelle_RLCK−XII−1 exon−intron and kinase domain diagram (all)
Ath_AT5G01060.1
Ath_AT3G09240.1
Ath_AT2G17170.1
Ath_AT2G17090.1
Ath_AT1G50990.1
Ath_AT5G59010.1
Ath_AT5G46570.1
Ath_AT5G41260.1
Ath_AT4G35230.1
Ath_AT4G00710.1
Ath_AT3G54030.1
Ath_AT1G63500.1
Ath_AT1G01740.1
−1000
0
1000
2000
3000

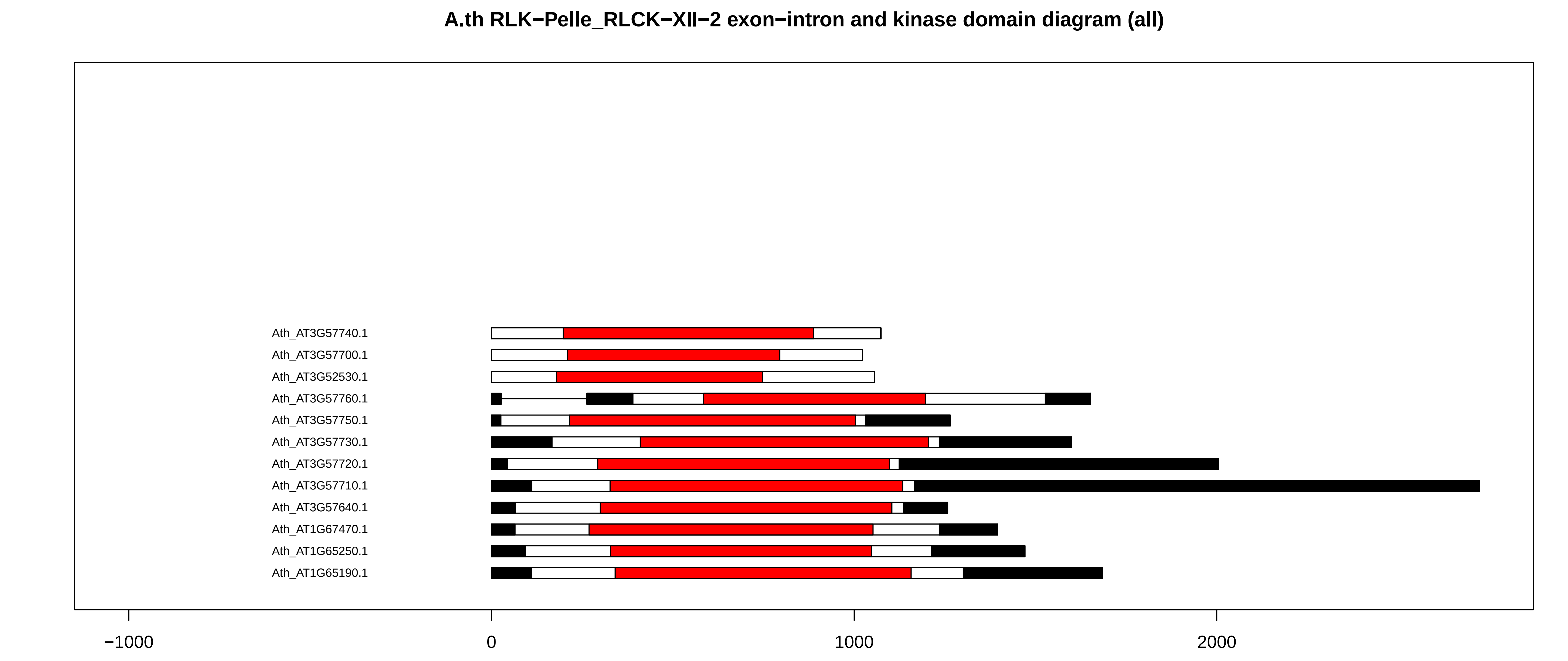
A.th RLK−Pelle_RLCK−XII−2 exon−intron and kinase domain diagram (all)
Ath_AT3G57740.1
Ath_AT3G57700.1
Ath_AT3G52530.1
Ath_AT3G57760.1
Ath_AT3G57750.1
Ath_AT3G57730.1
Ath_AT3G57720.1
Ath_AT3G57710.1
Ath_AT3G57640.1
Ath_AT1G67470.1
Ath_AT1G65250.1
Ath_AT1G65190.1
−1000
0
1000
2000

A.th RLK−Pelle_RLCK−XIII exon−intron and kinase domain diagram (all)

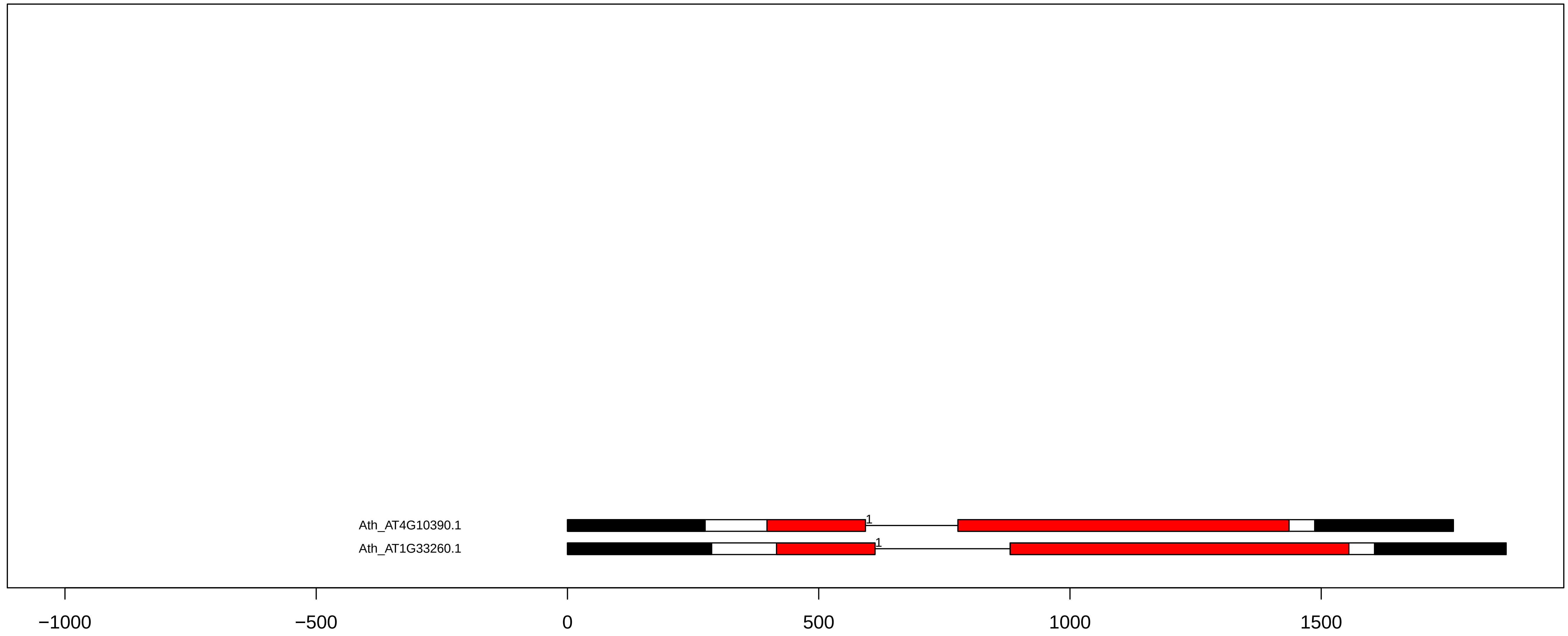

A.th RLK−Pelle_RLCK−XI exon−intron and kinase domain diagram (all)

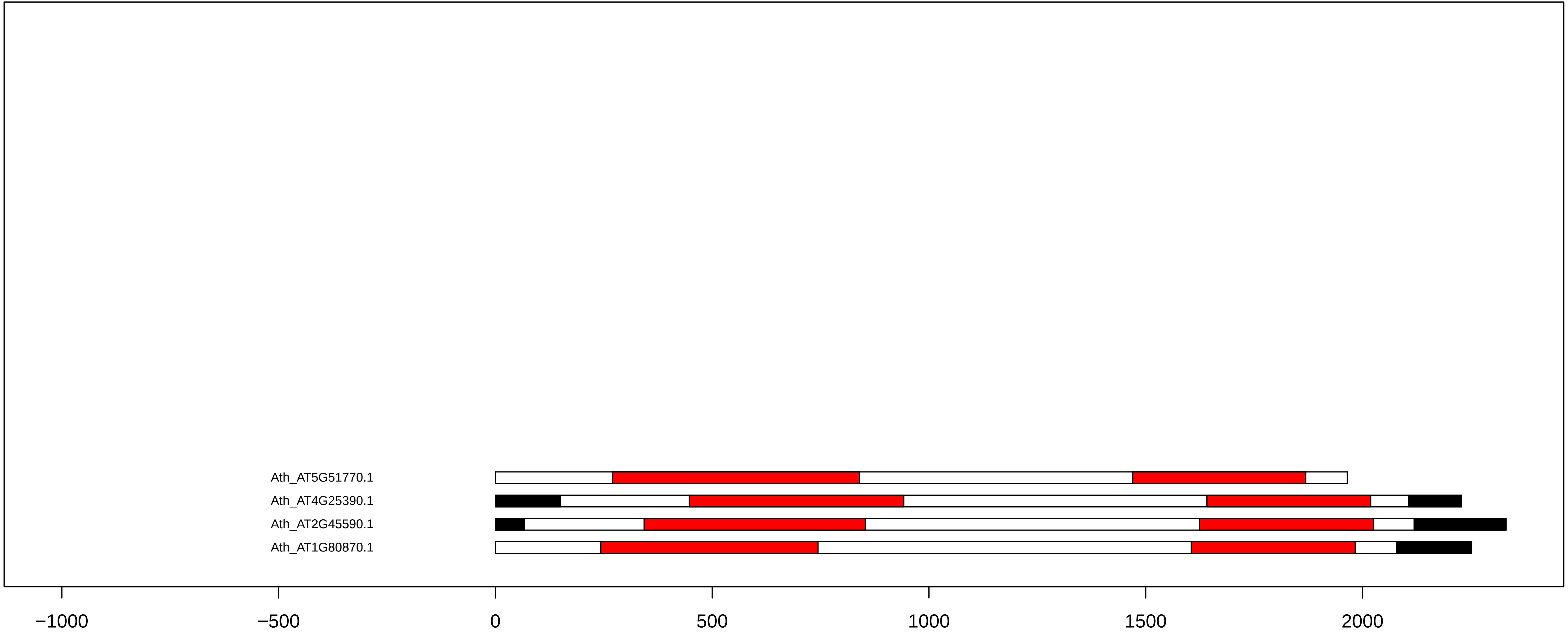

A.th RLK−Pelle_RLCK−X exon−intron and kinase domain diagram (all)

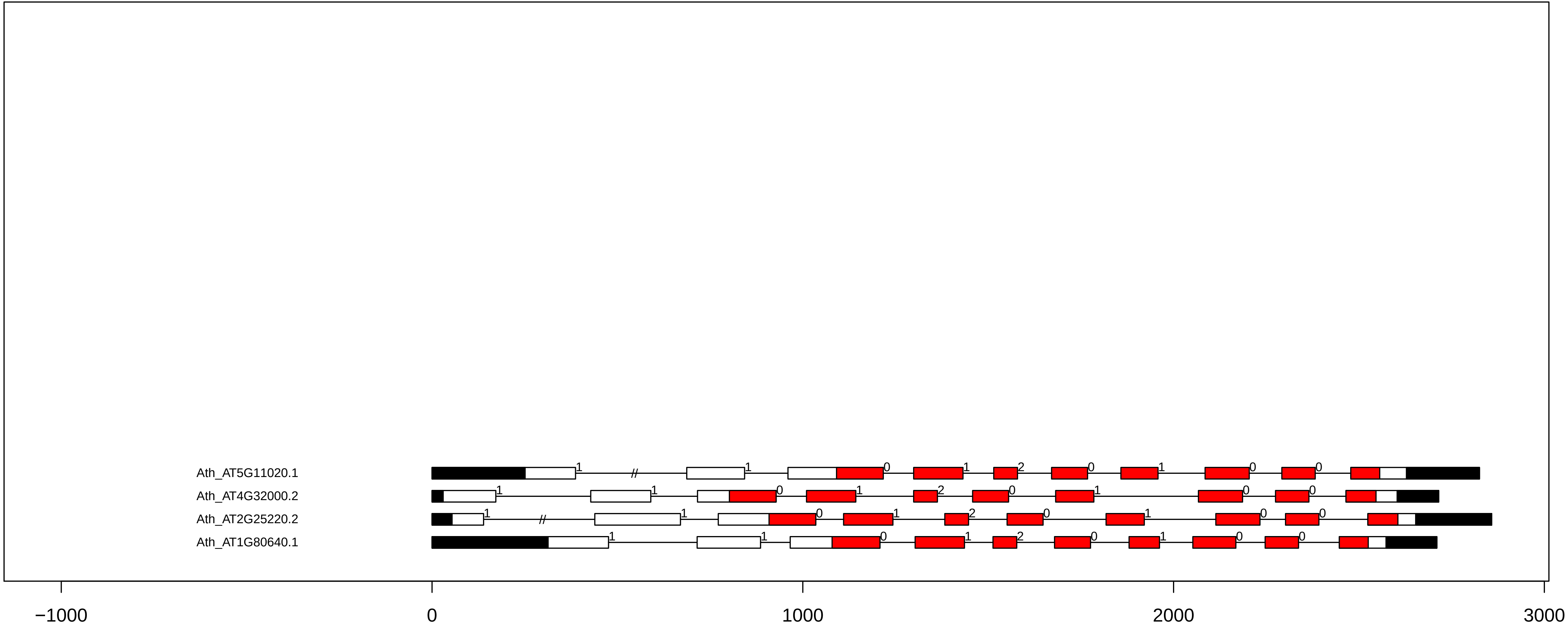

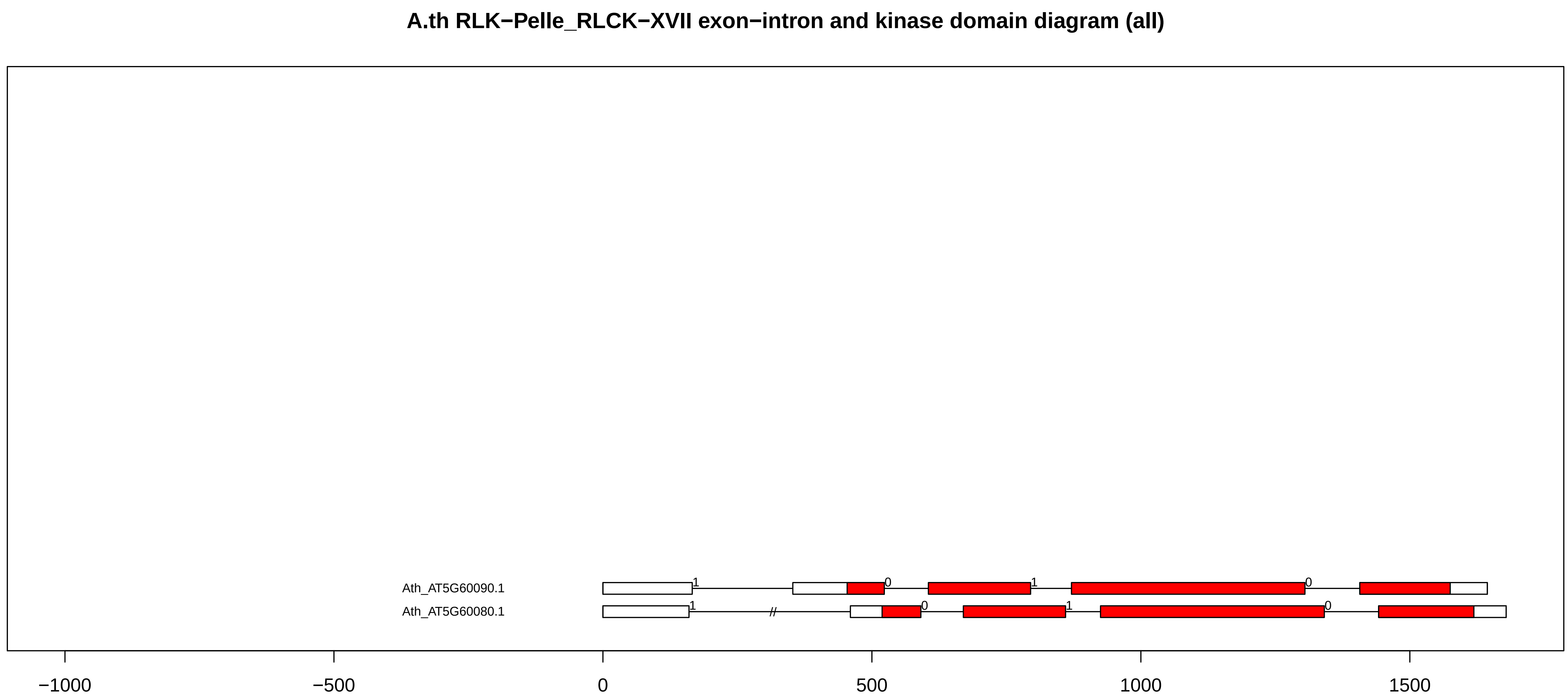
A.th RLK−Pelle_RLCK−XVII exon−intron and kinase domain diagram (all)
Ath_AT5G60090.1
Ath_AT5G60080.1
1
0
1
0
1
//
0
1
0
−1000
−500
0
500
1000
1500

A.th RLK−Pelle_RLCK−XVI exon−intron and kinase domain diagram (all)

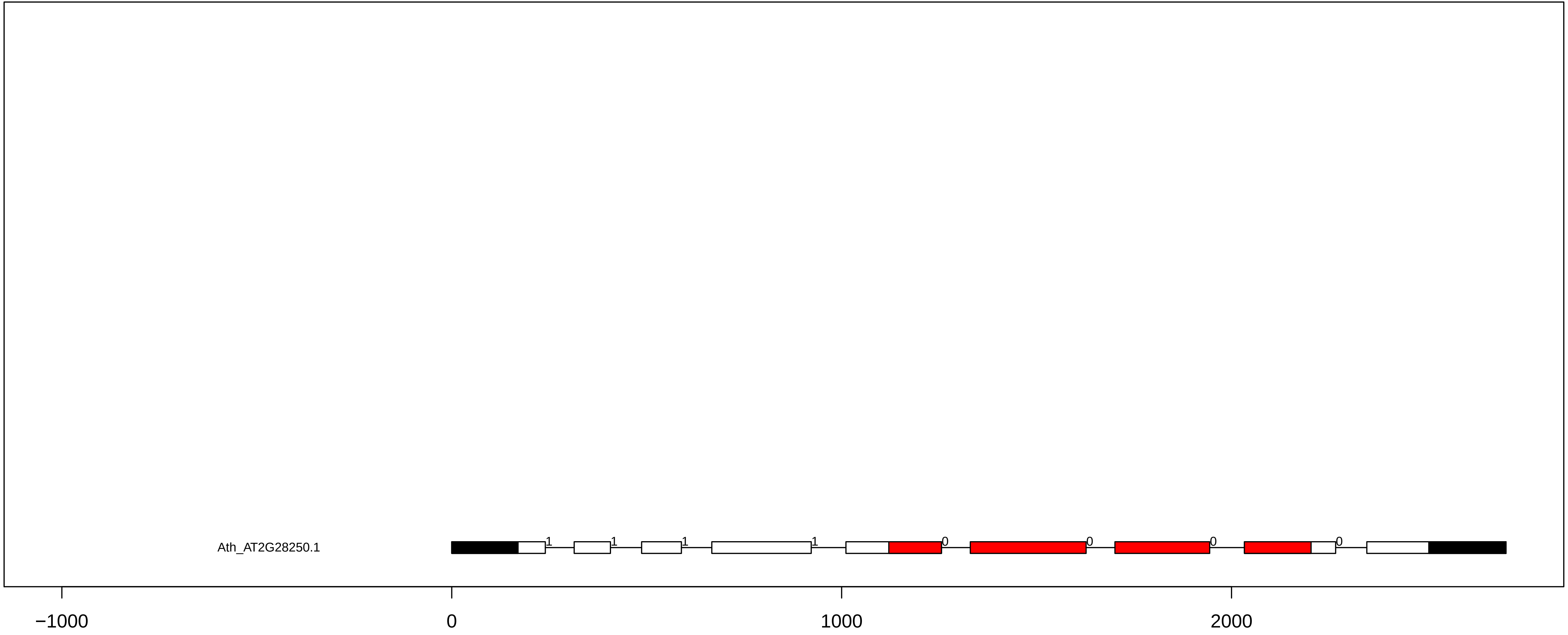

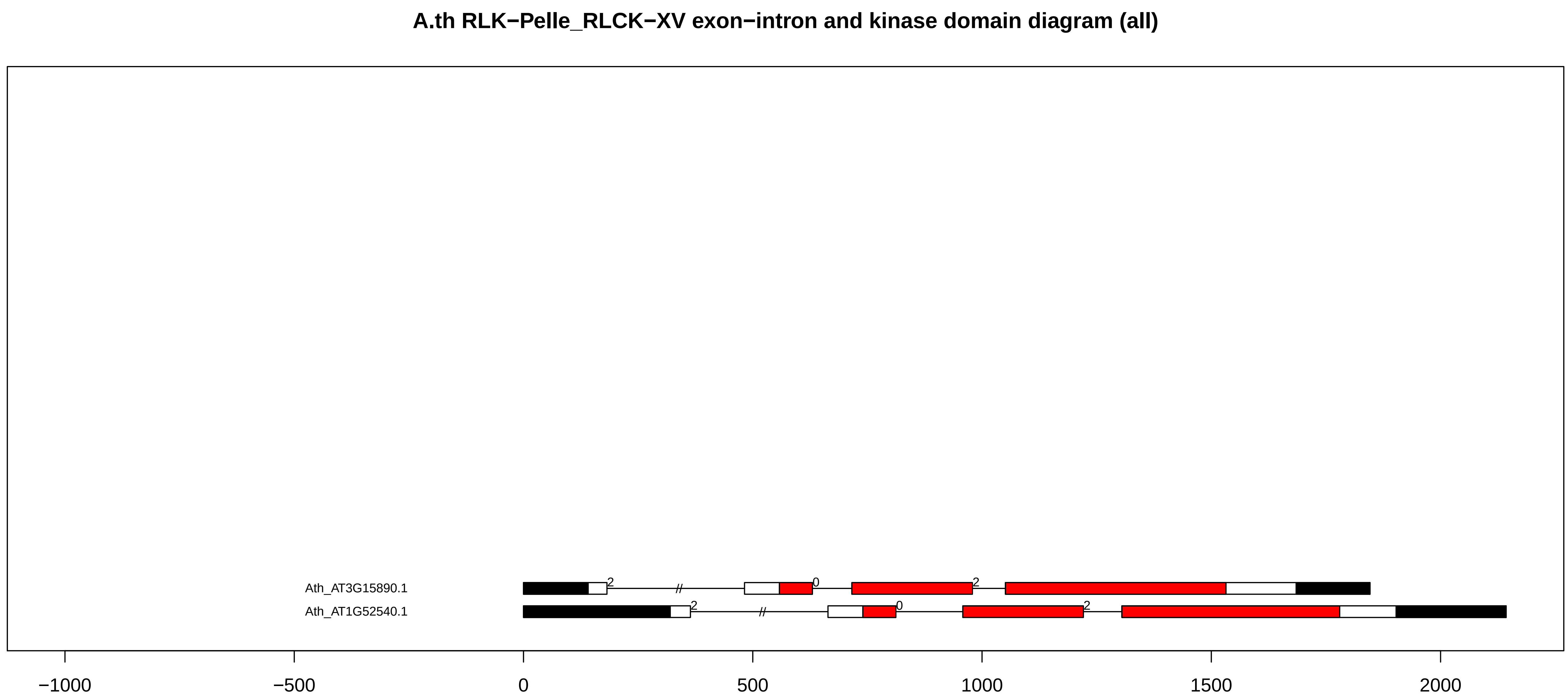

A.th RLK−Pelle_RLCK−XV exon−intron and kinase domain diagram (all)
Ath_AT3G15890.1
Ath_AT1G52540.1
2
0
2
2
0
2
−1000
−500
0
500
1000
1500
2000

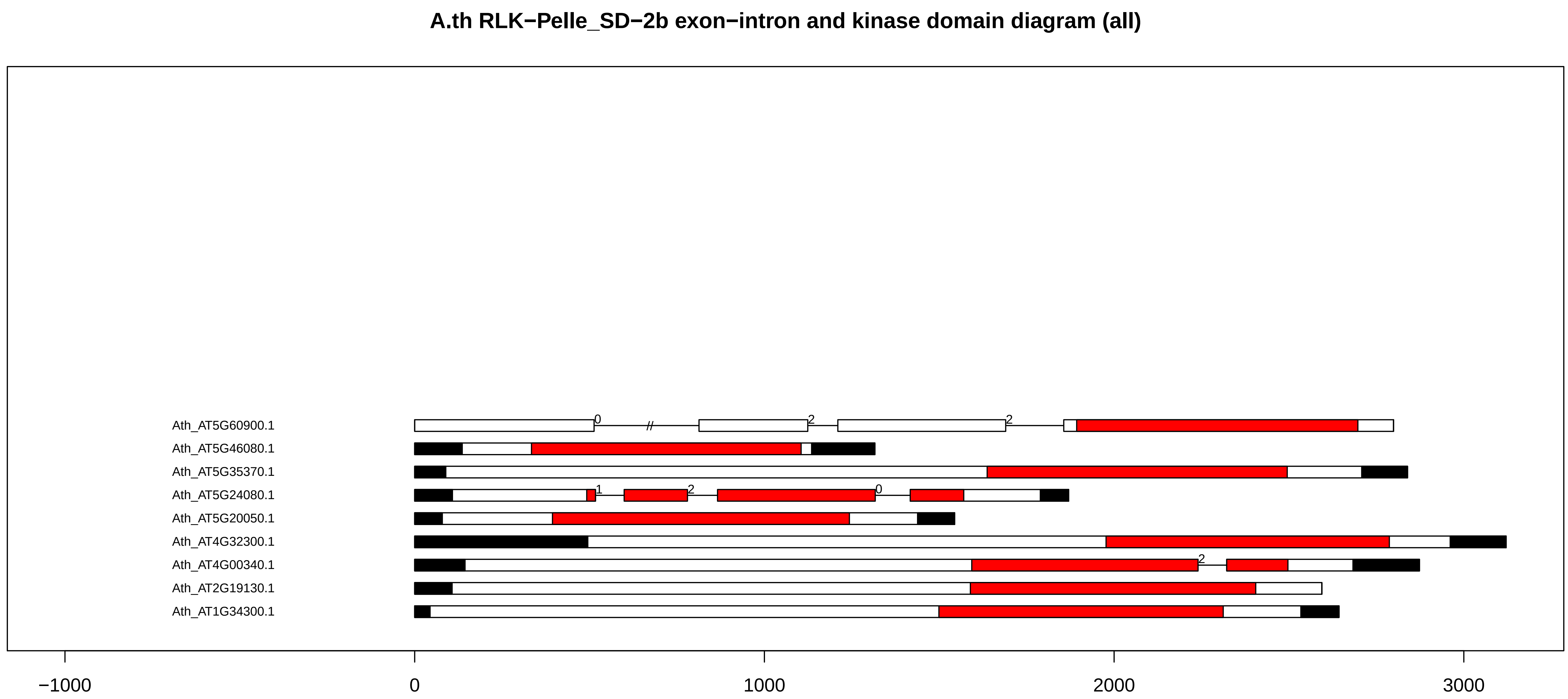

A.th RLK−Pelle_SD−2b exon−intron and kinase domain diagram (all)
Ath_AT5G60900.1
Ath_AT5G46080.1
Ath_AT5G35370.1
Ath_AT5G24080.1
Ath_AT5G20050.1
Ath_AT4G32300.1
Ath_AT4G00340.1
Ath_AT2G19130.1
Ath_AT1G34300.1
0
2
2
1
2
0
2
−1000
0
1000
2000
3000

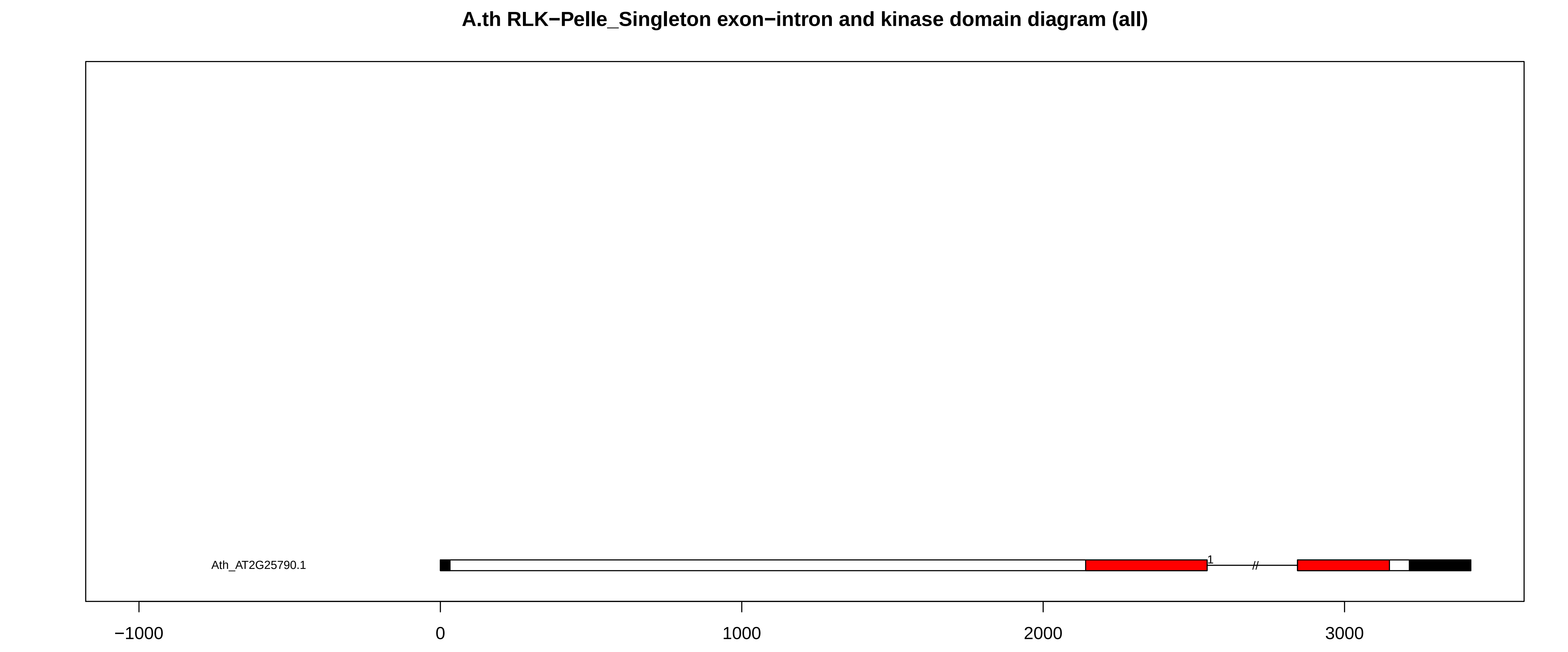
A.th RLK−Pelle_Singleton exon−intron and kinase domain diagram (all)
Ath_AT2G25790.1
1
−1000
0
1000
2000
3000

A.th RLK−Pelle_URK−1 exon−intron and kinase domain diagram (all)

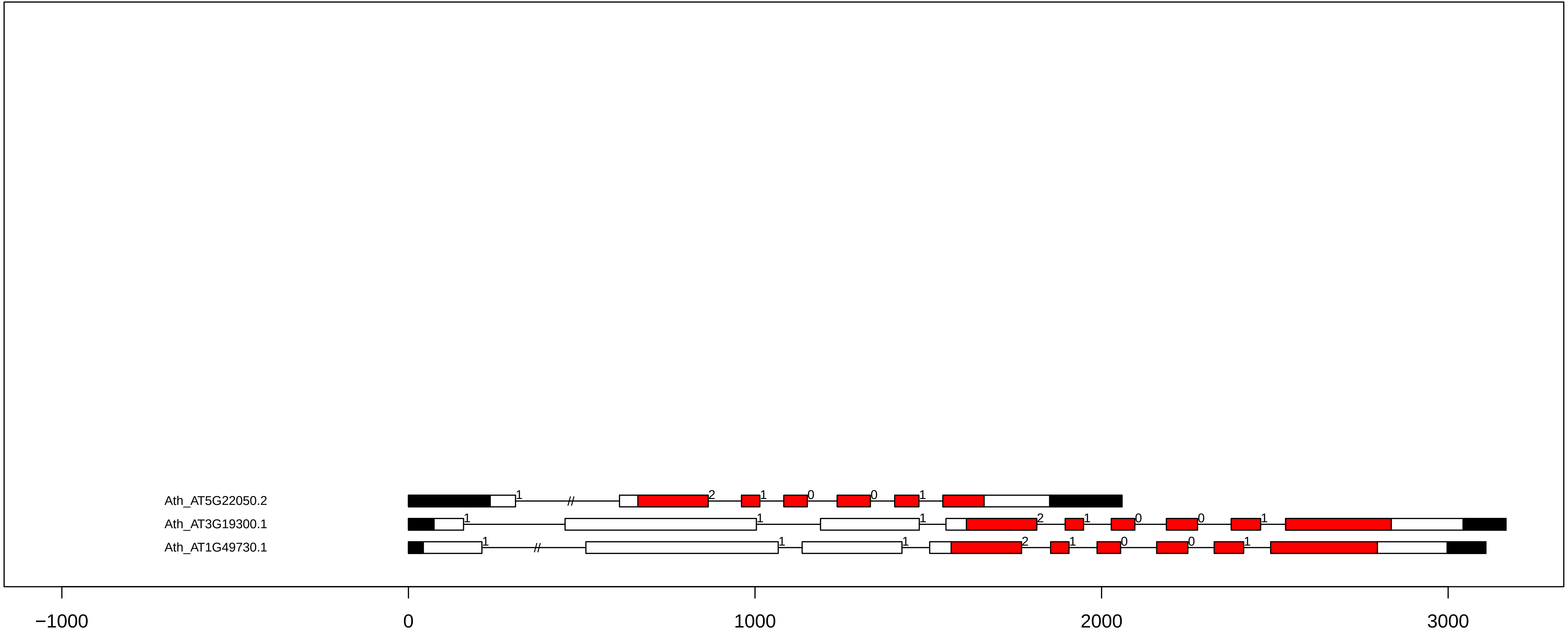

A.th RLK−Pelle_URK−3 exon−intron and kinase domain diagram (all)

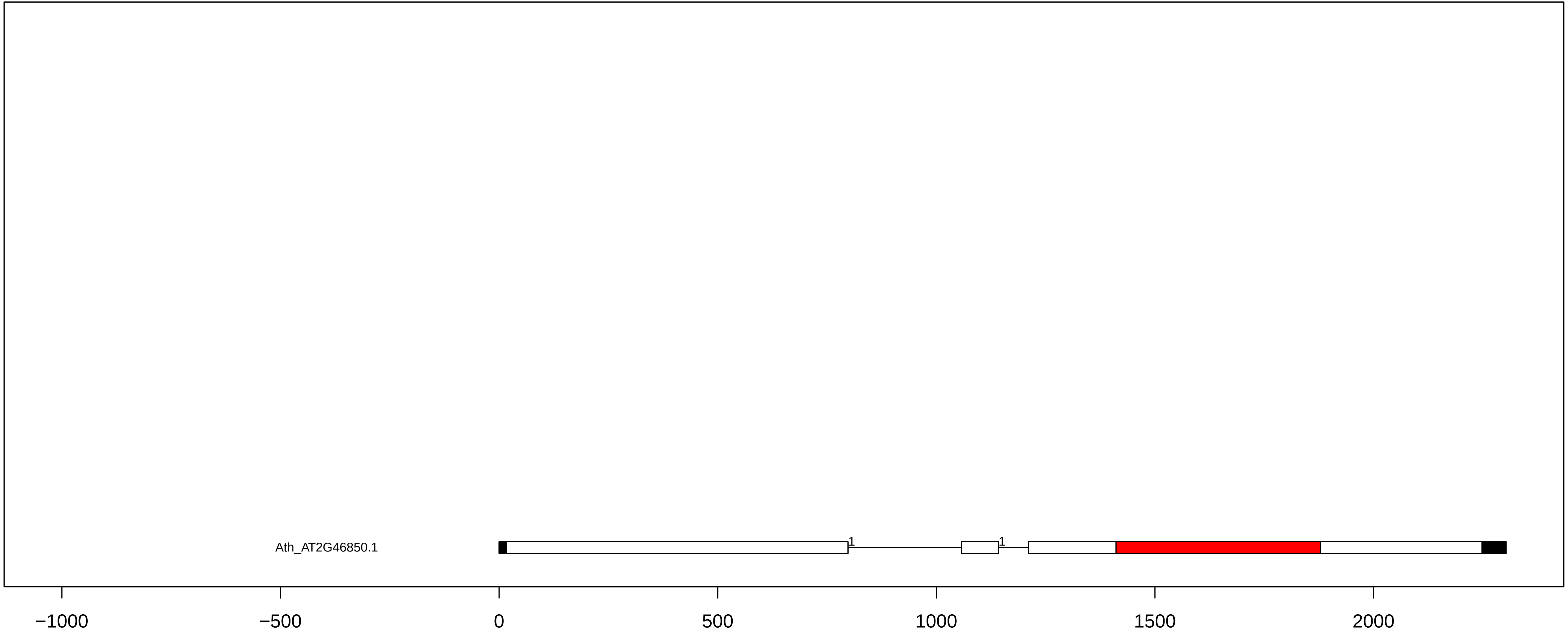

A.th RLK−Pelle_WAK_LRK10L−1 exon−intron and kinase domain diagram (all)

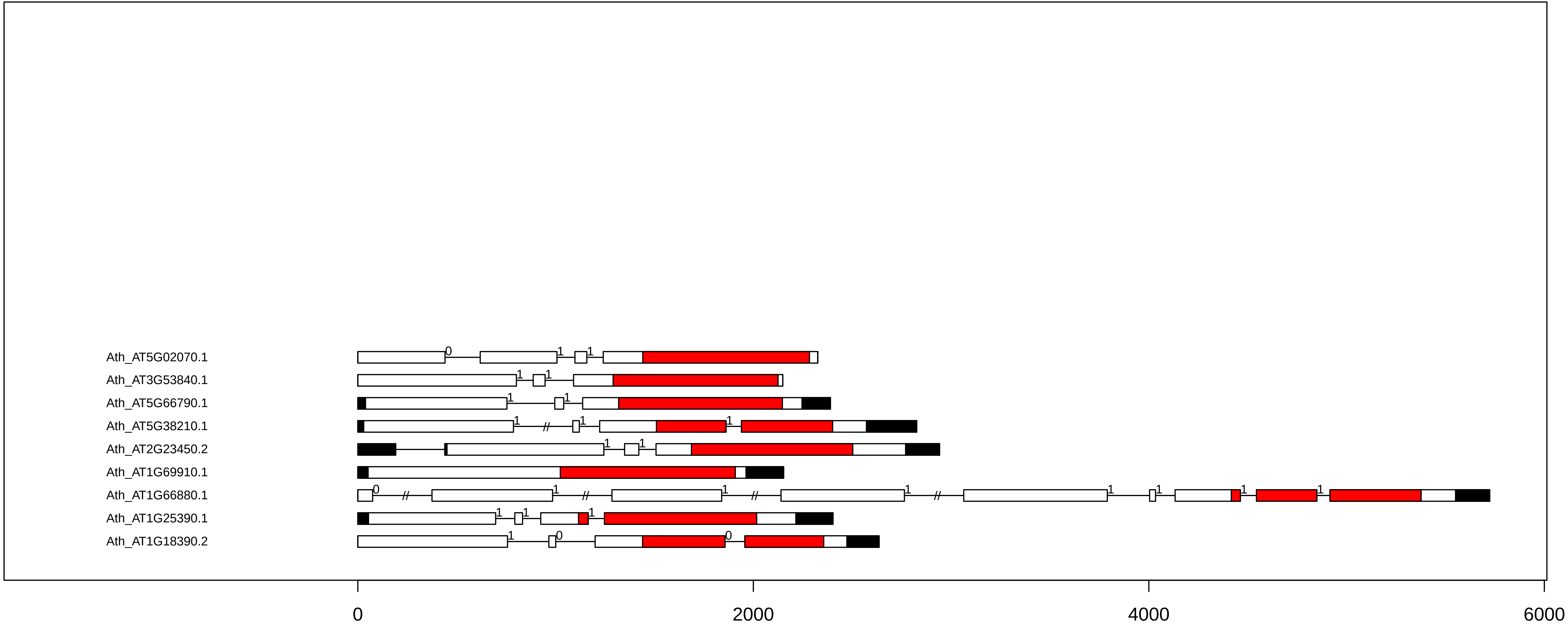

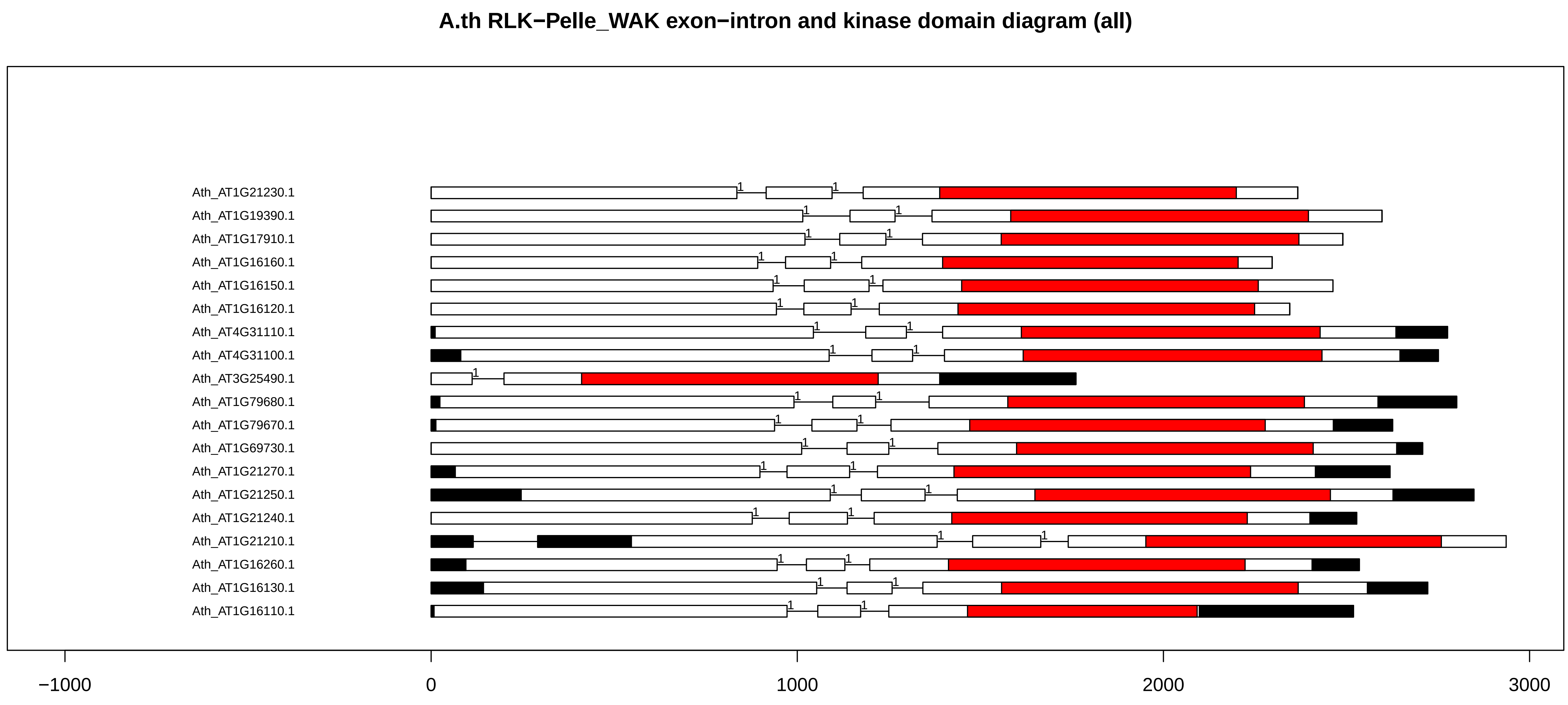

A.th RLK−Pelle_WAK exon−intron and kinase domain diagram (all)
Ath_AT1G21230.1
Ath_AT1G19390.1
Ath_AT1G17910.1
Ath_AT1G16160.1
Ath_AT1G16150.1
Ath_AT1G16120.1
Ath_AT4G31110.1
Ath_AT4G31100.1
Ath_AT3G25490.1
Ath_AT1G79680.1
Ath_AT1G79670.1
Ath_AT1G69730.1
Ath_AT1G21270.1
Ath_AT1G21250.1
Ath_AT1G21240.1
Ath_AT1G21210.1
Ath_AT1G16260.1
Ath_AT1G16130.1
Ath_AT1G16110.1
−1000
0
1000
2000
3000

A.th SCY1_SCYL1 exon−intron and kinase domain diagram (all)

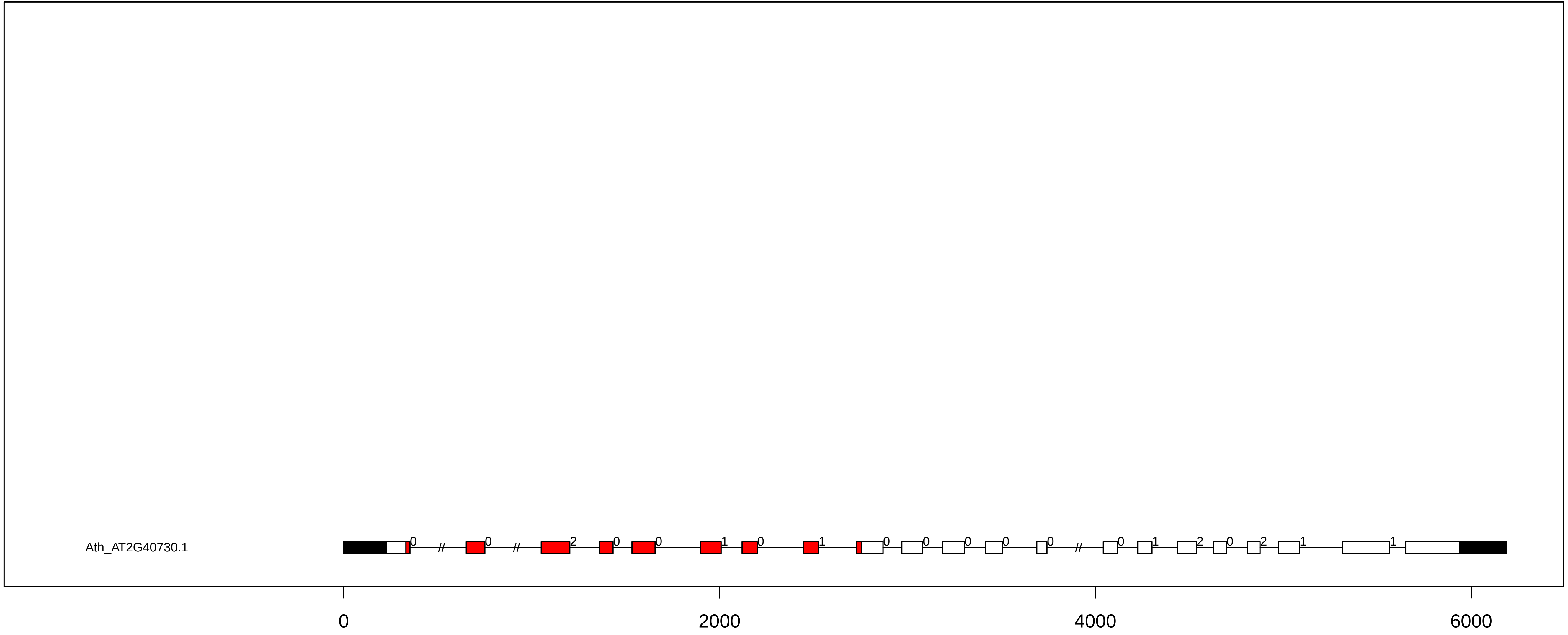

A.th SCY1_SCYL2 exon−intron and kinase domain diagram (all)

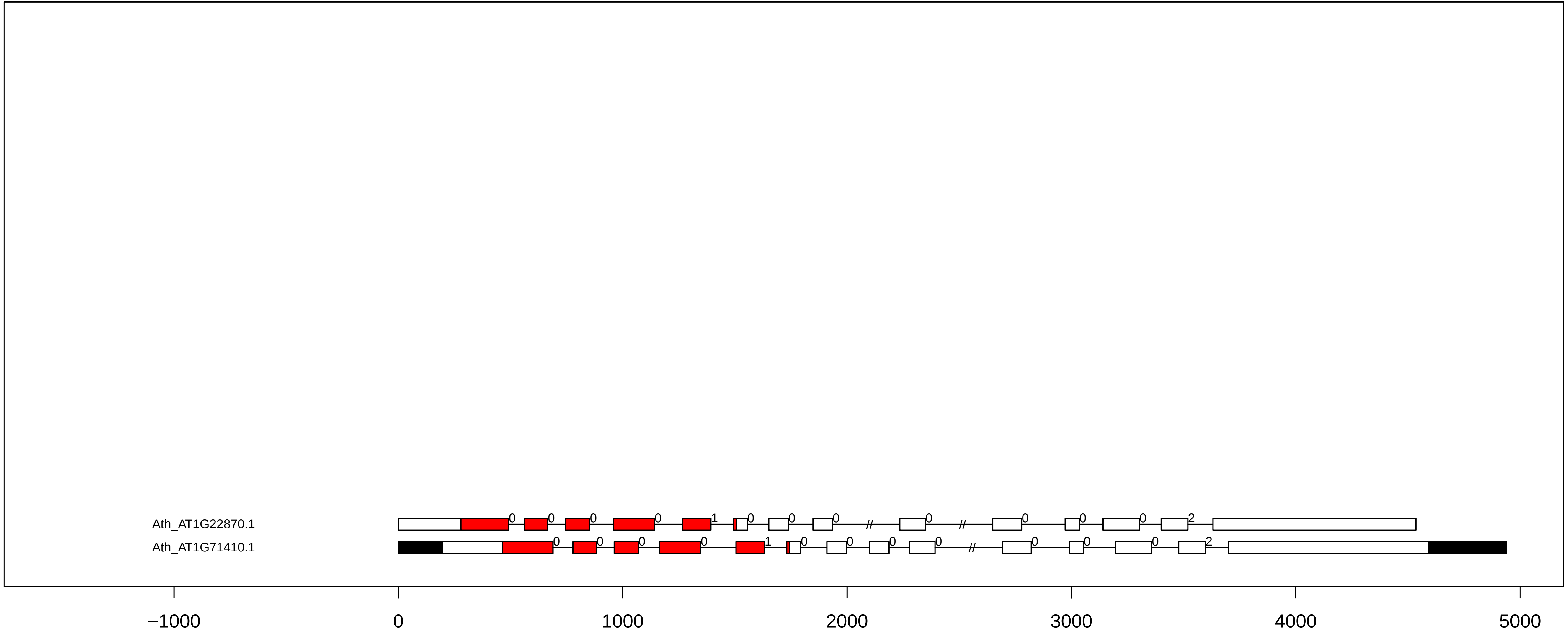

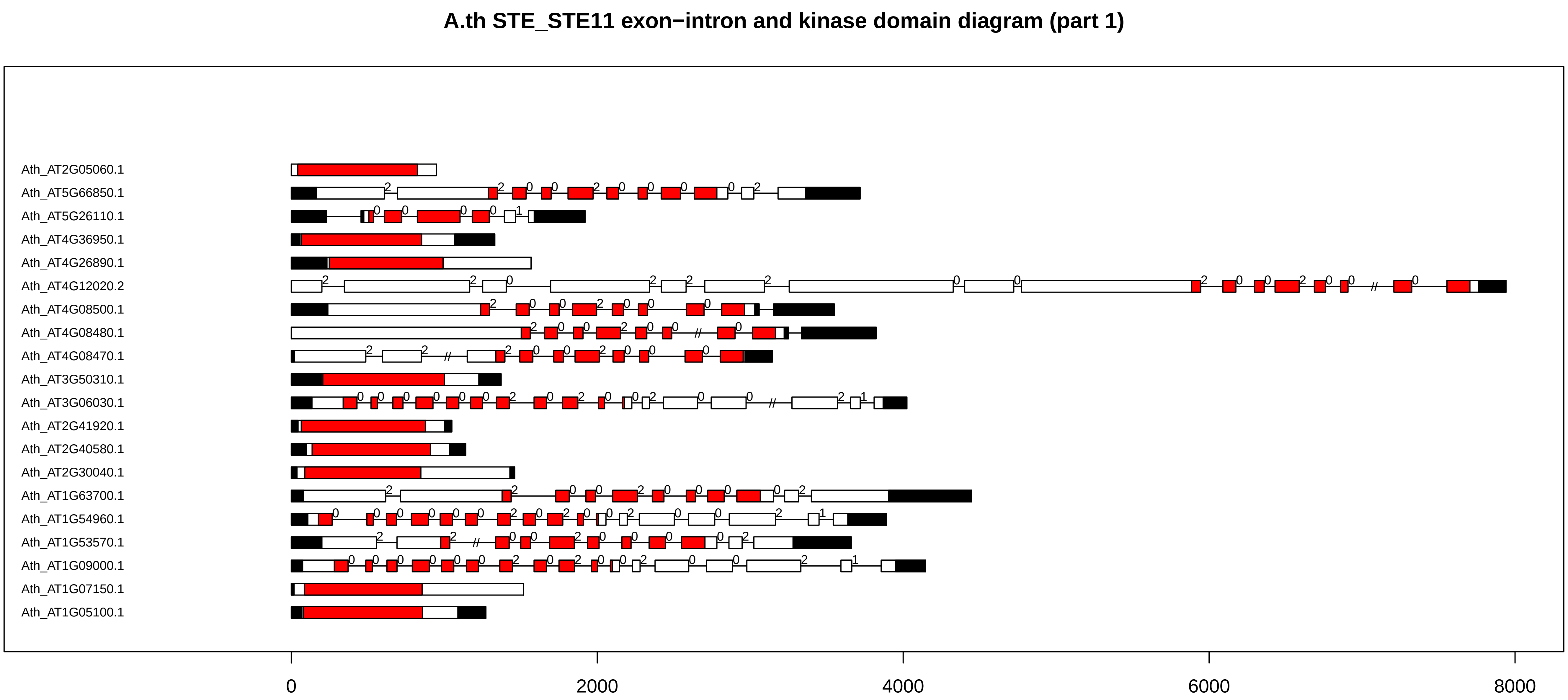
A.th STE_STE11 exon−intron and kinase domain diagram (part 1)
Ath_AT2G05060.1
Ath_AT5G66850.1
Ath_AT5G26110.1
Ath_AT4G36950.1
Ath_AT4G26890.1
Ath_AT4G12020.2
Ath_AT4G08500.1
Ath_AT4G08480.1
Ath_AT4G08470.1
Ath_AT3G50310.1
Ath_AT3G06030.1
Ath_AT2G41920.1
Ath_AT2G40580.1
Ath_AT2G30040.1
Ath_AT1G63700.1
Ath_AT1G54960.1
Ath_AT1G53570.1
Ath_AT1G09000.1
Ath_AT1G07150.1
Ath_AT1G05100.1
0
2000
4000
6000
8000

A.th STE_STE11 exon−intron and kinase domain diagram (part 2)

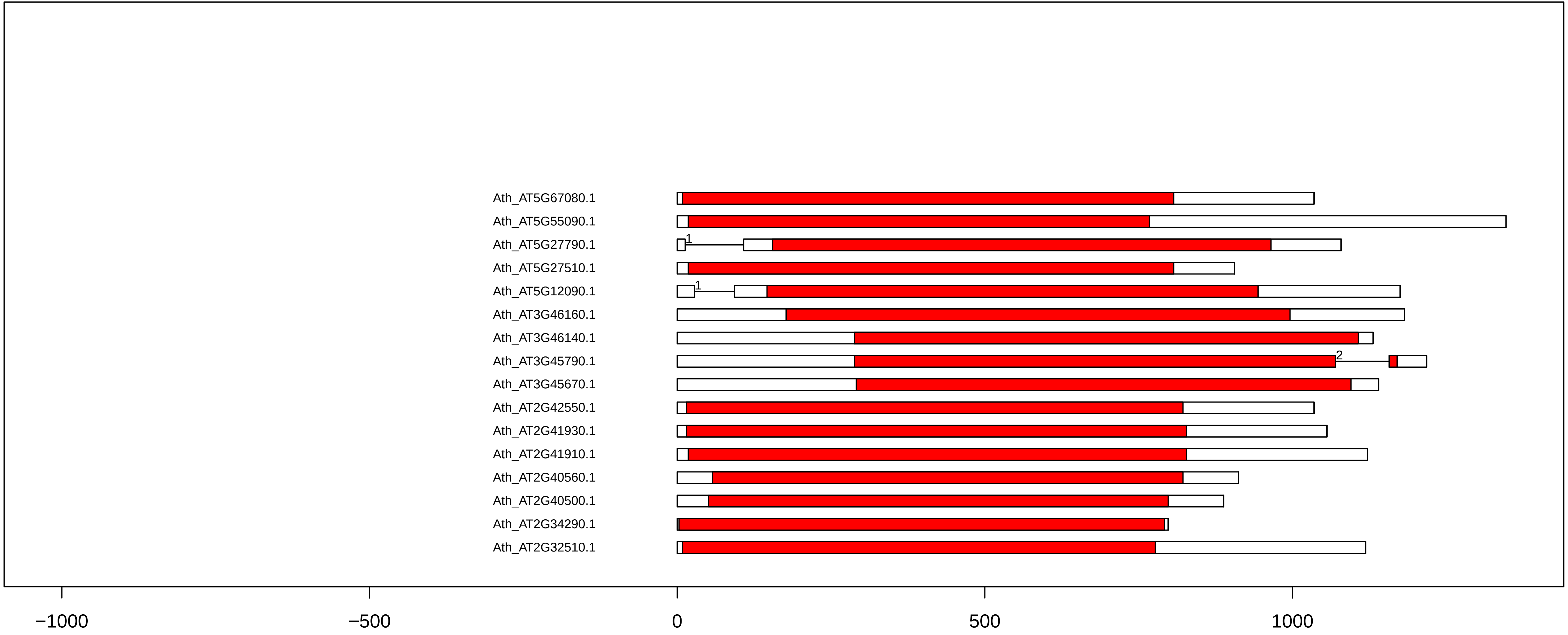

A.th STE_STE20−Fray exon−intron and kinase domain diagram (all)

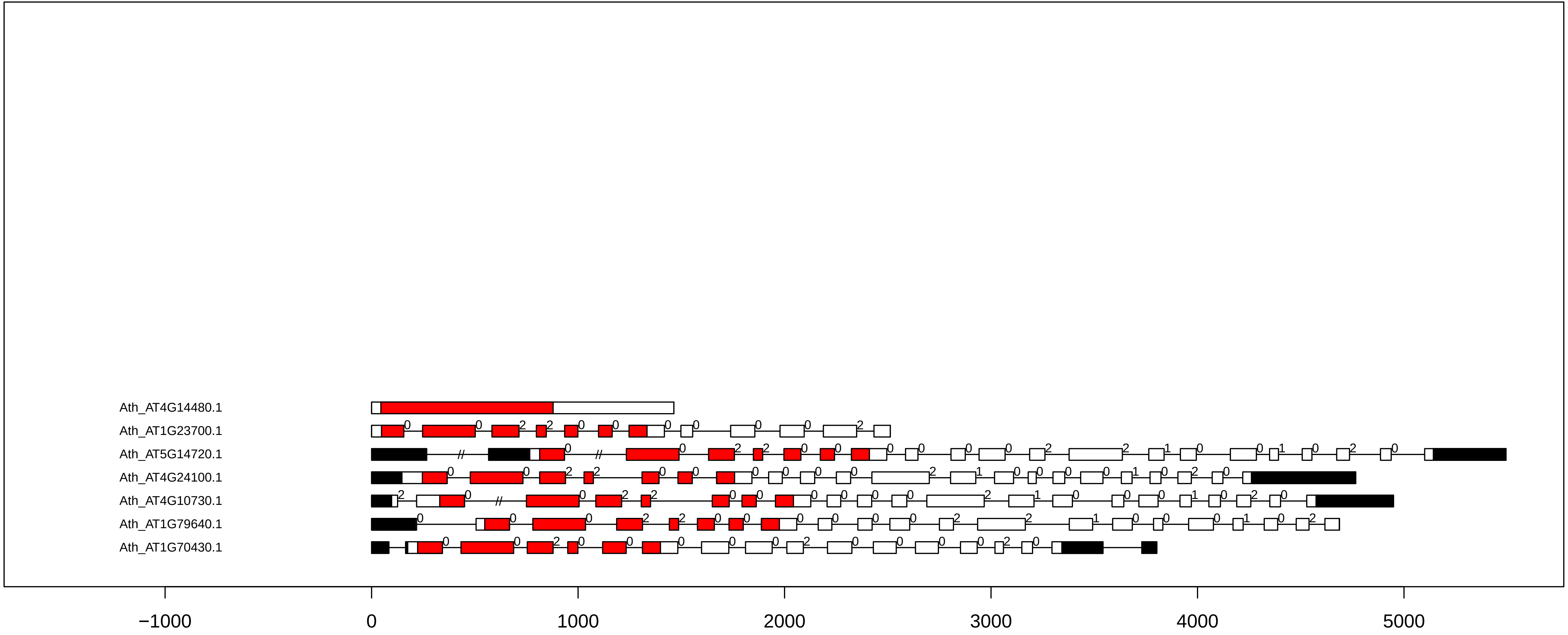

A.th STE_STE20−PI exon−intron and kinase domain diagram (all)

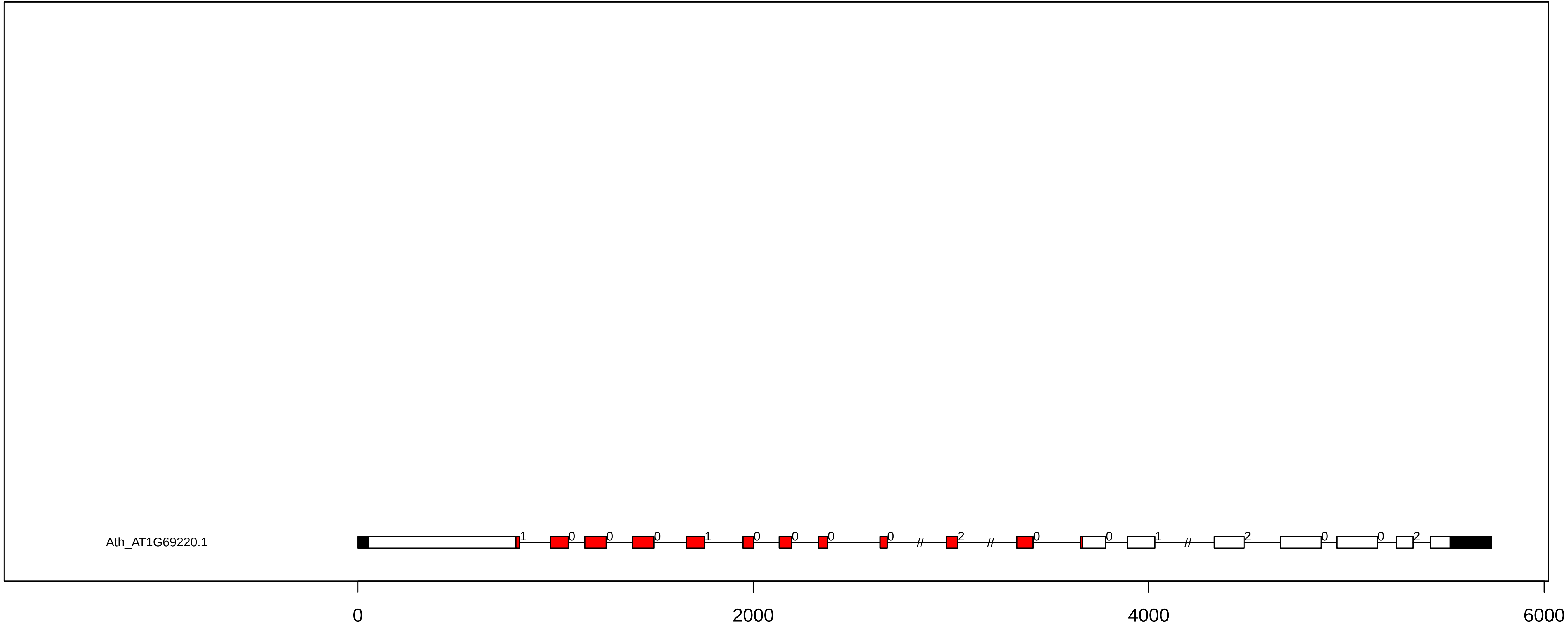

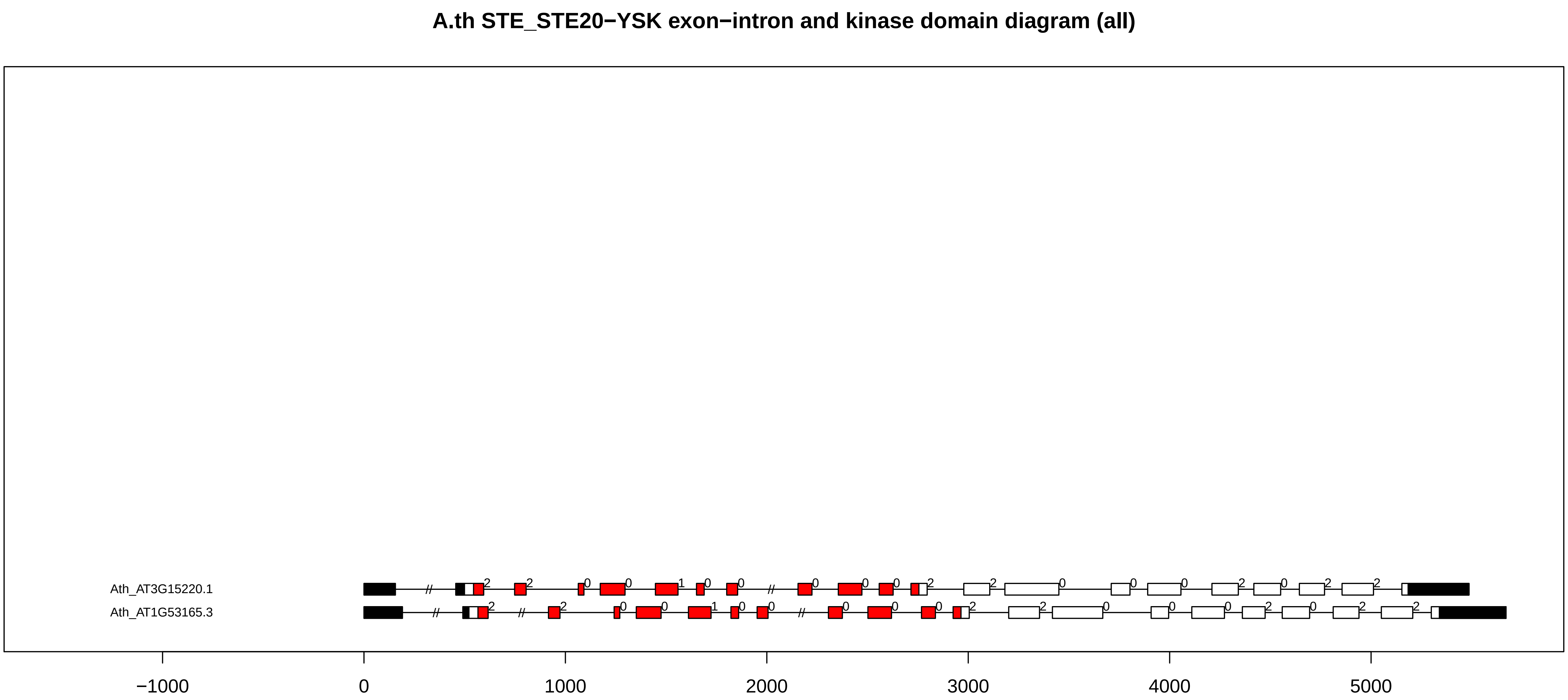

A.th STE_STE20−YSK exon−intron and kinase domain diagram (all)
Ath_AT3G15220.1
Ath_AT1G53165.3
−1000
0
1000
2000
3000
4000
5000

A.th STE_STE7 exon−intron and kinase domain diagram (all)

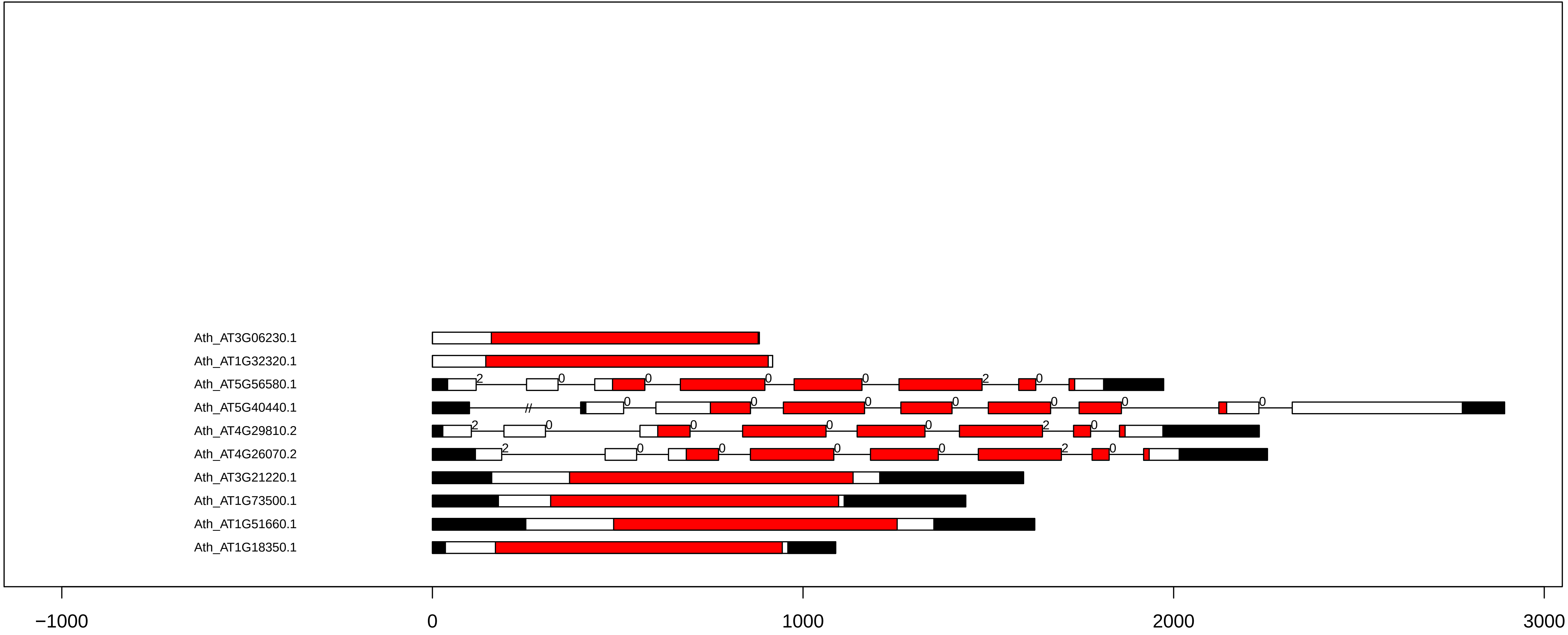

A.th STE_STE−Pl exon−intron and kinase domain diagram (all)

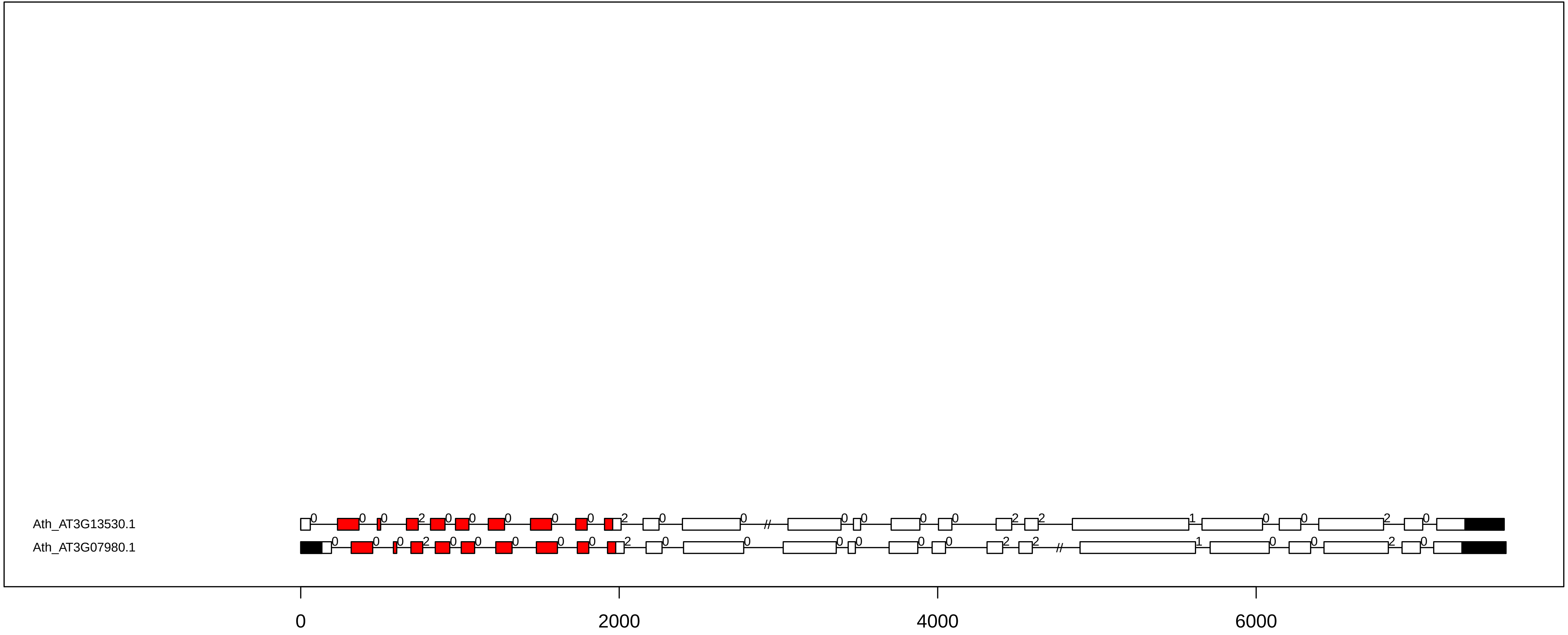

A.th TKL_CTR1−DRK−1 exon−intron and kinase domain diagram (all)

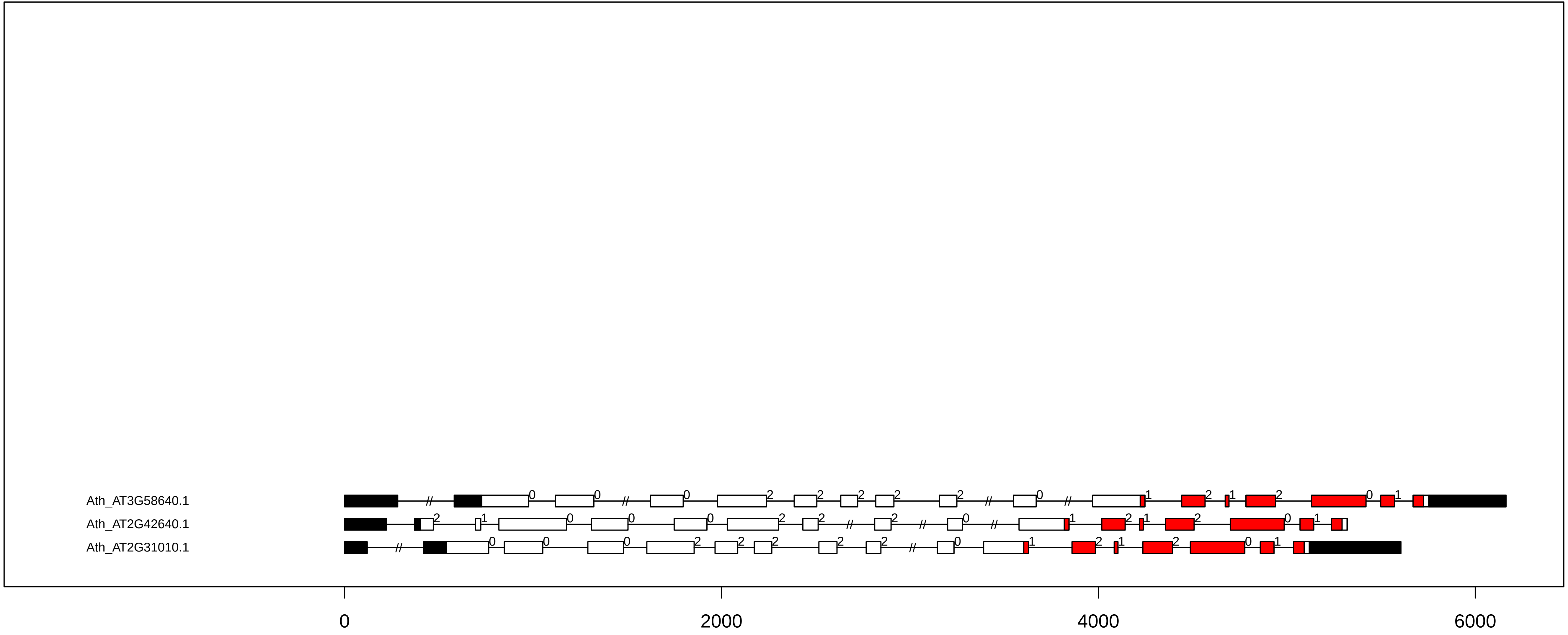

A.th TKL_CTR1−DRK−2 exon−intron and kinase domain diagram (all)

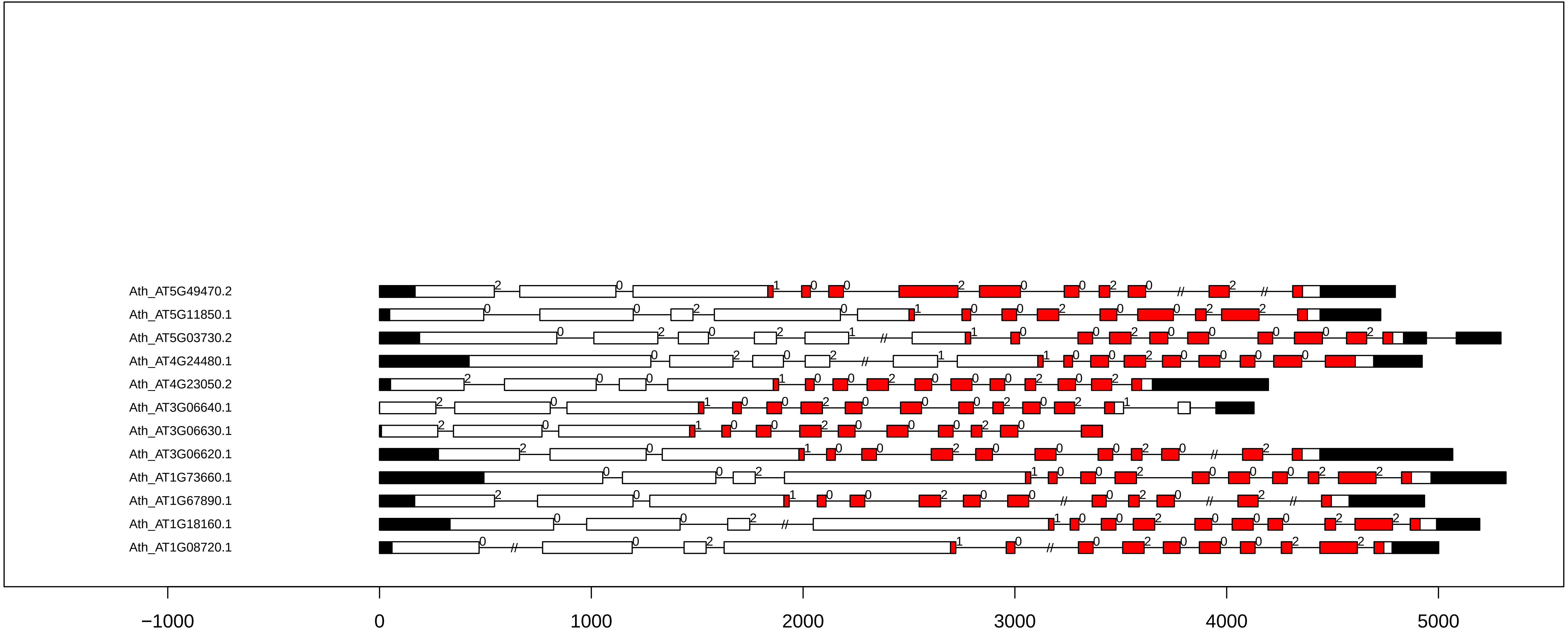

A.th TKL_Gdt exon−intron and kinase domain diagram (all)

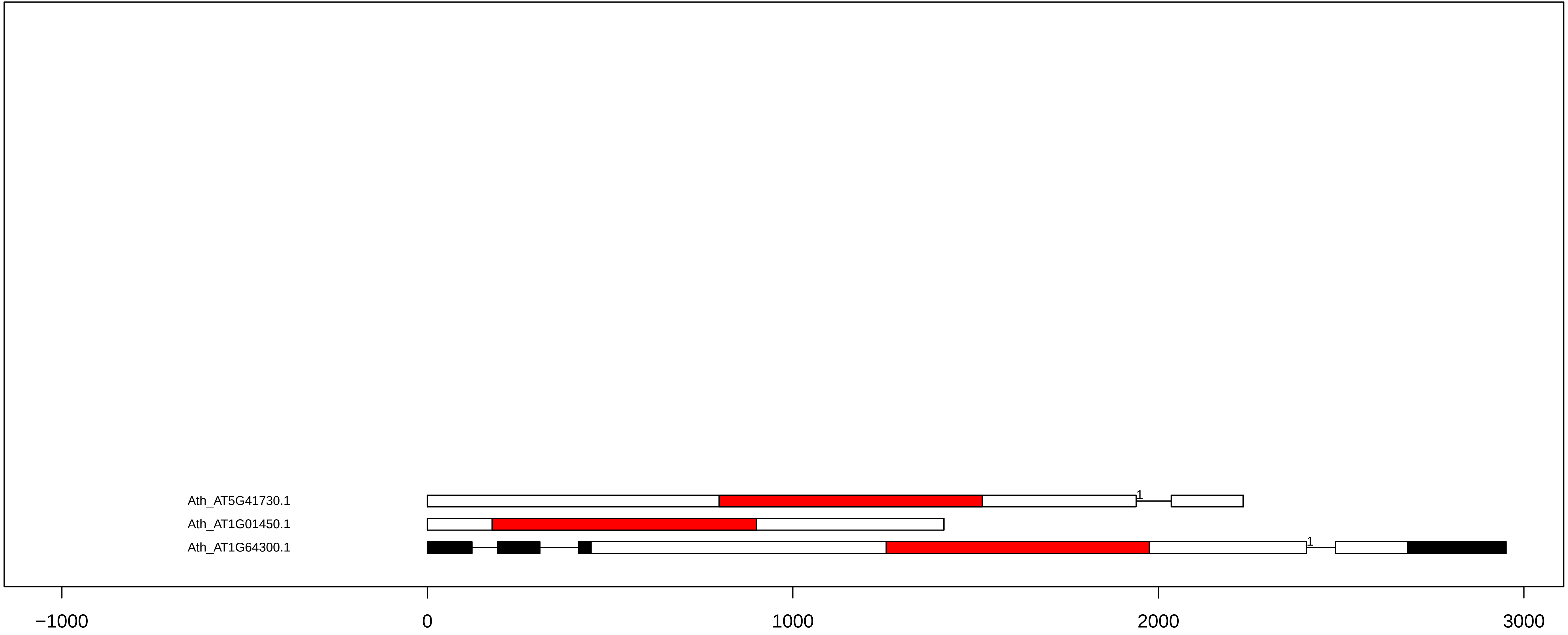

A.th TKL−Pl−1 exon−intron and kinase domain diagram (all)

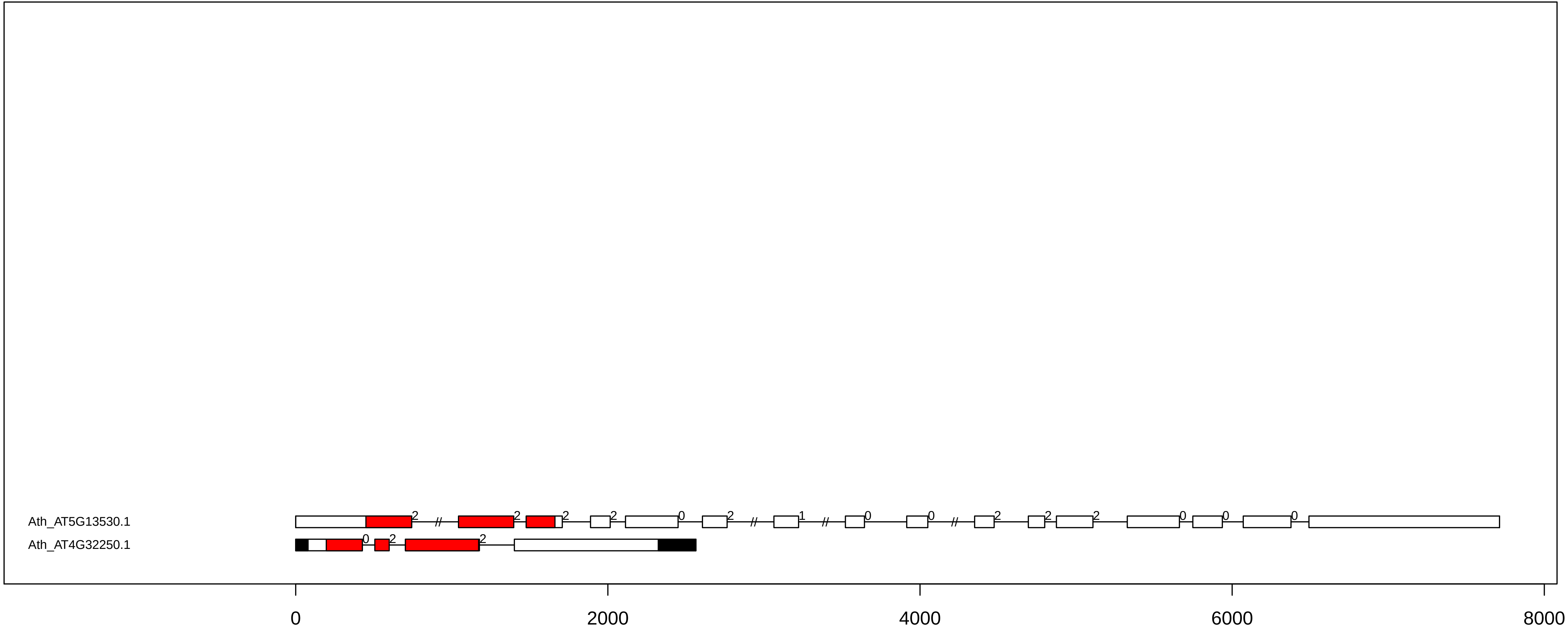

A.th TKL−Pl−2 exon−intron and kinase domain diagram (all)

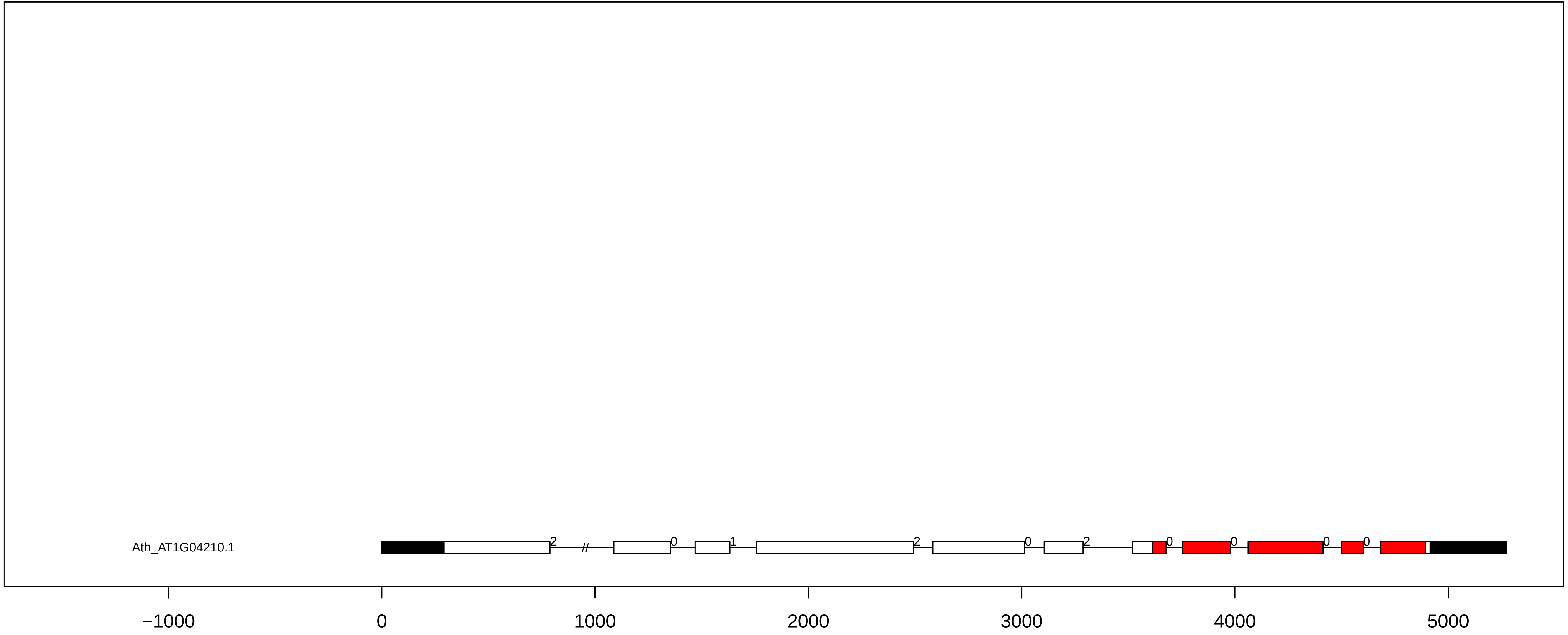

A.th TKL−Pl−3 exon−intron and kinase domain diagram (all)

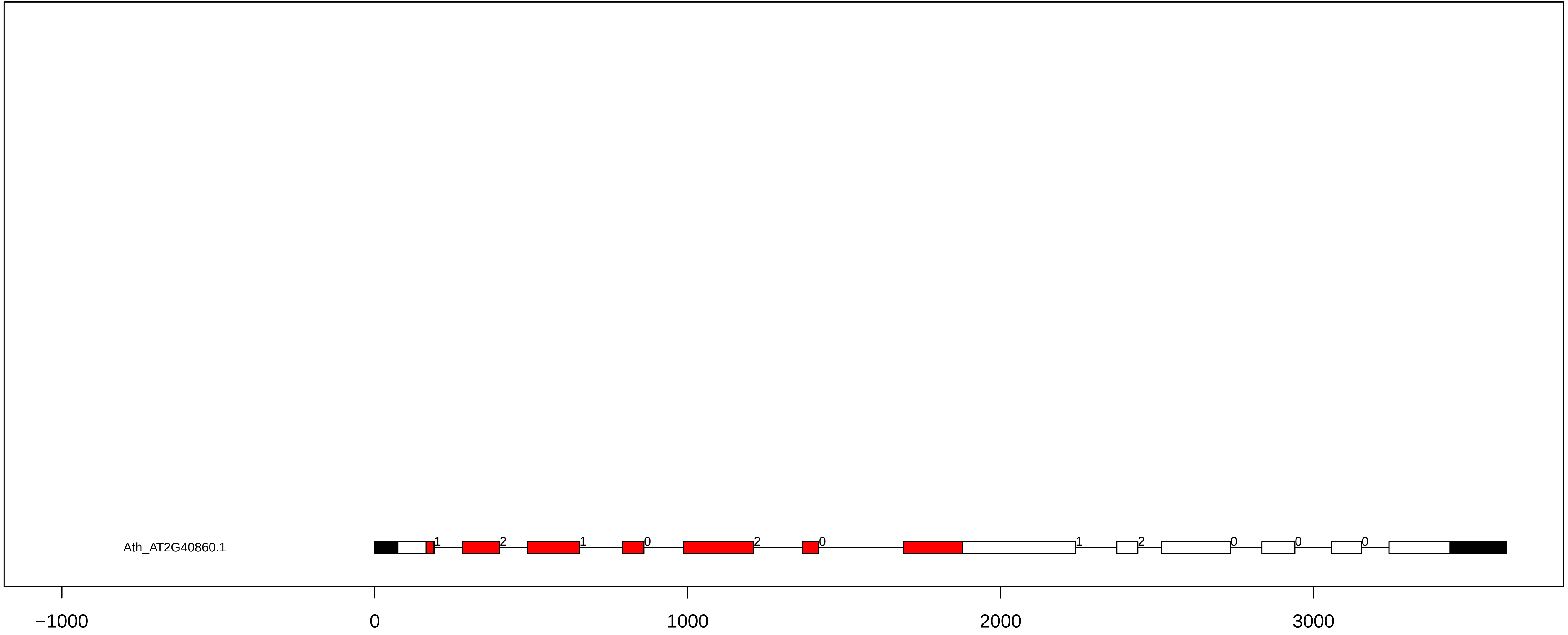

A.th TKL−PI−4 exon−intron and kinase domain diagram (all)

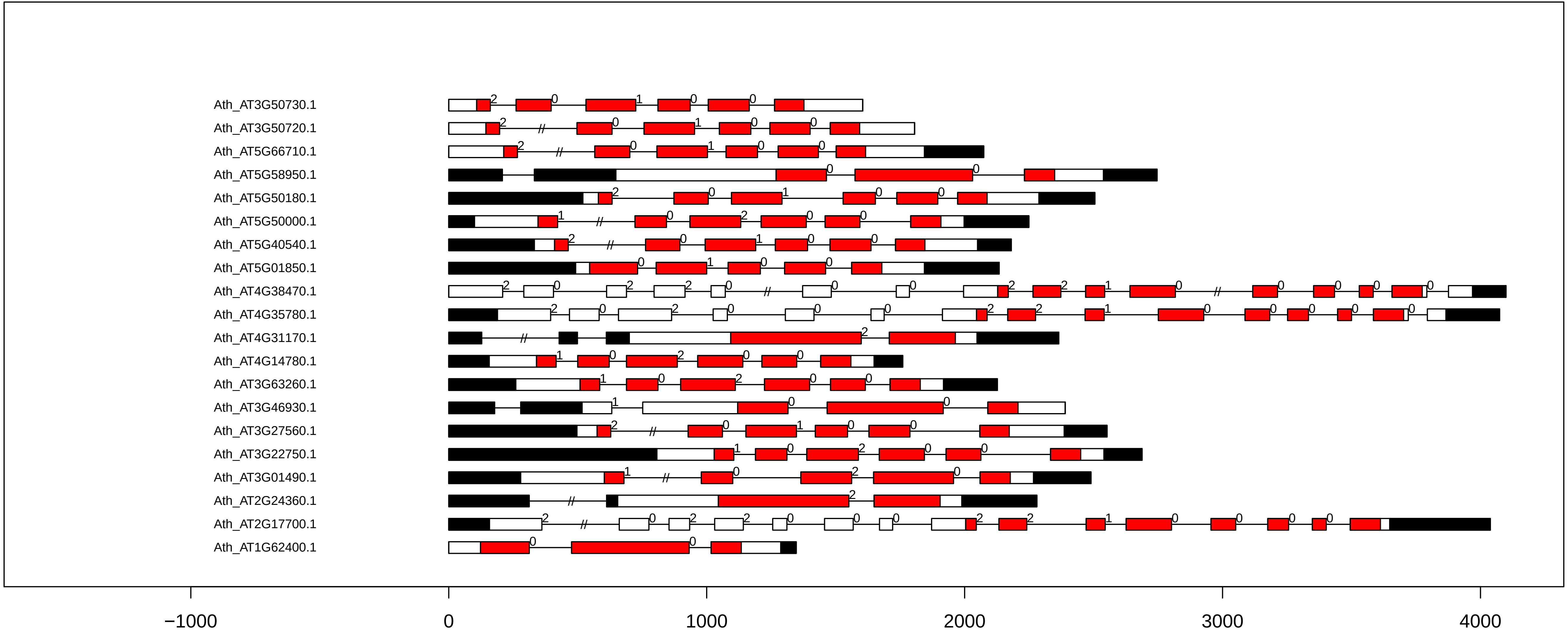

A.th TKL−Pl−5 exon−intron and kinase domain diagram (all)

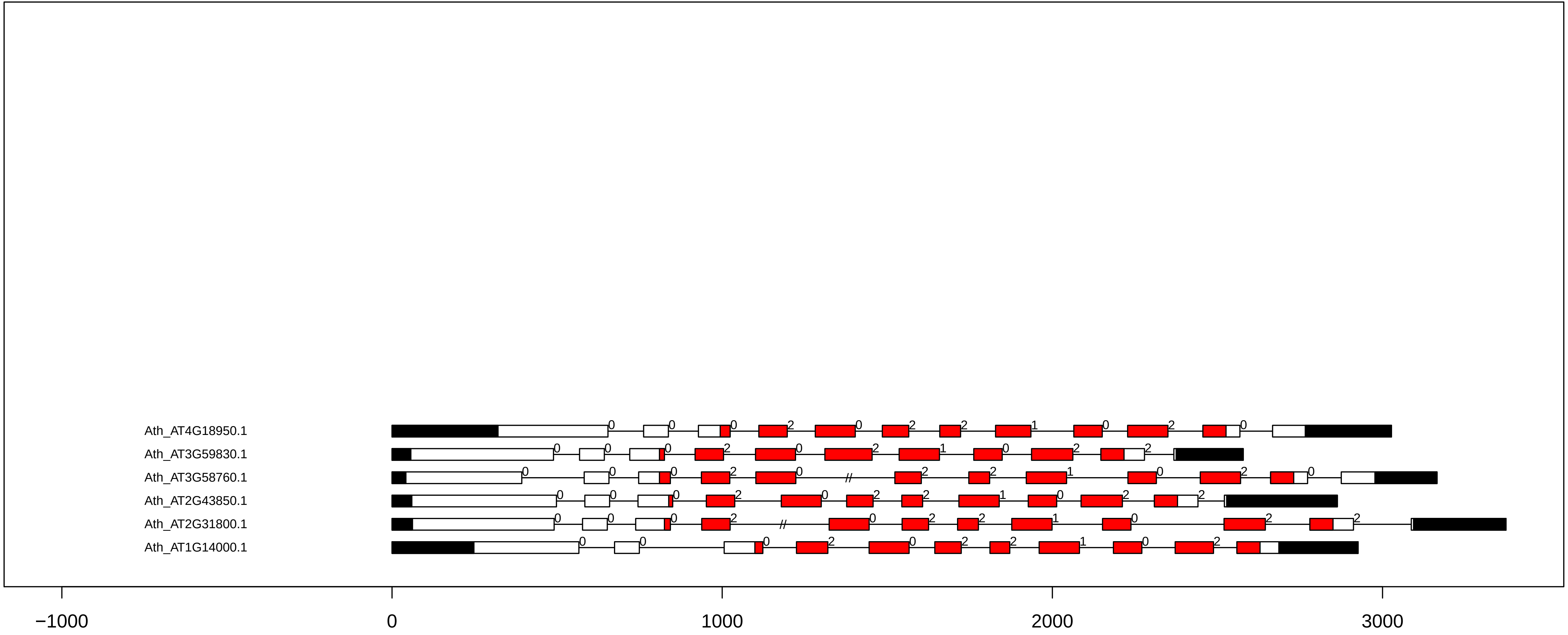

A.th TKL−Pl−6 exon−intron and kinase domain diagram (all)

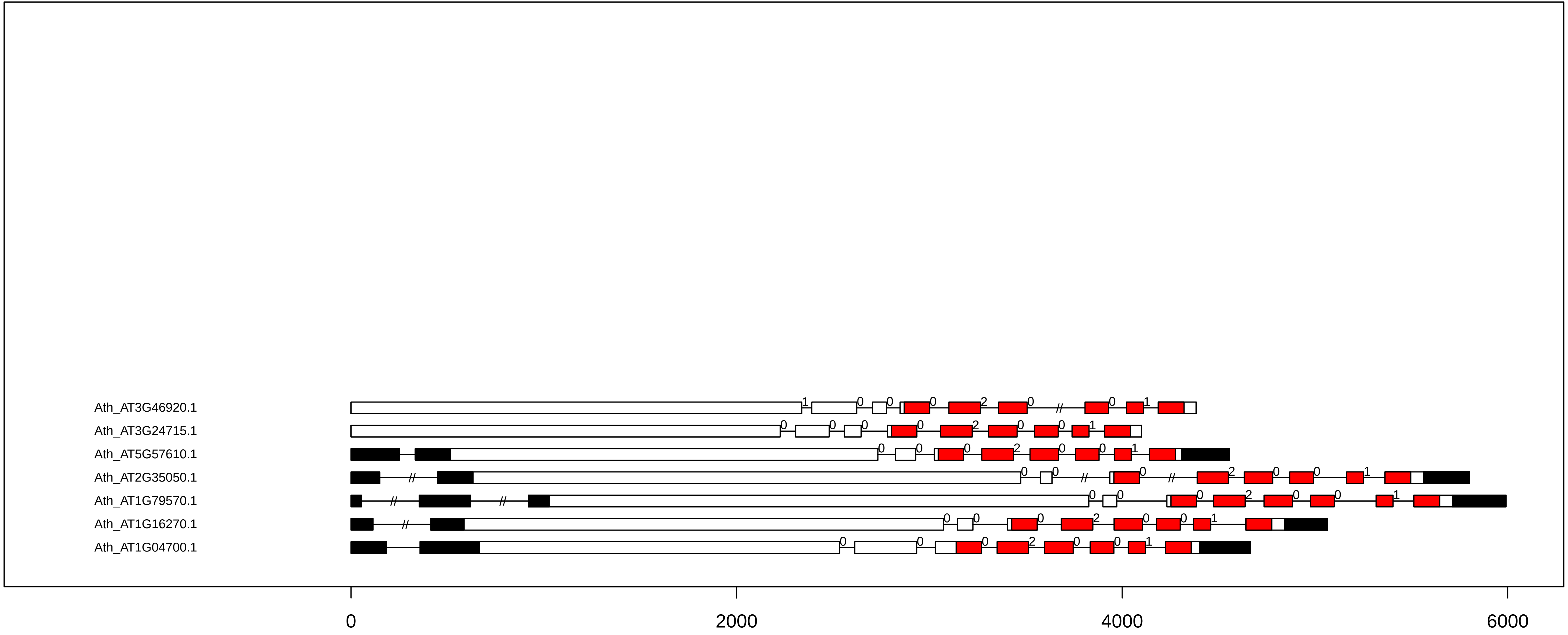

A.th TKL−Pl−7 exon−intron and kinase domain diagram (all)

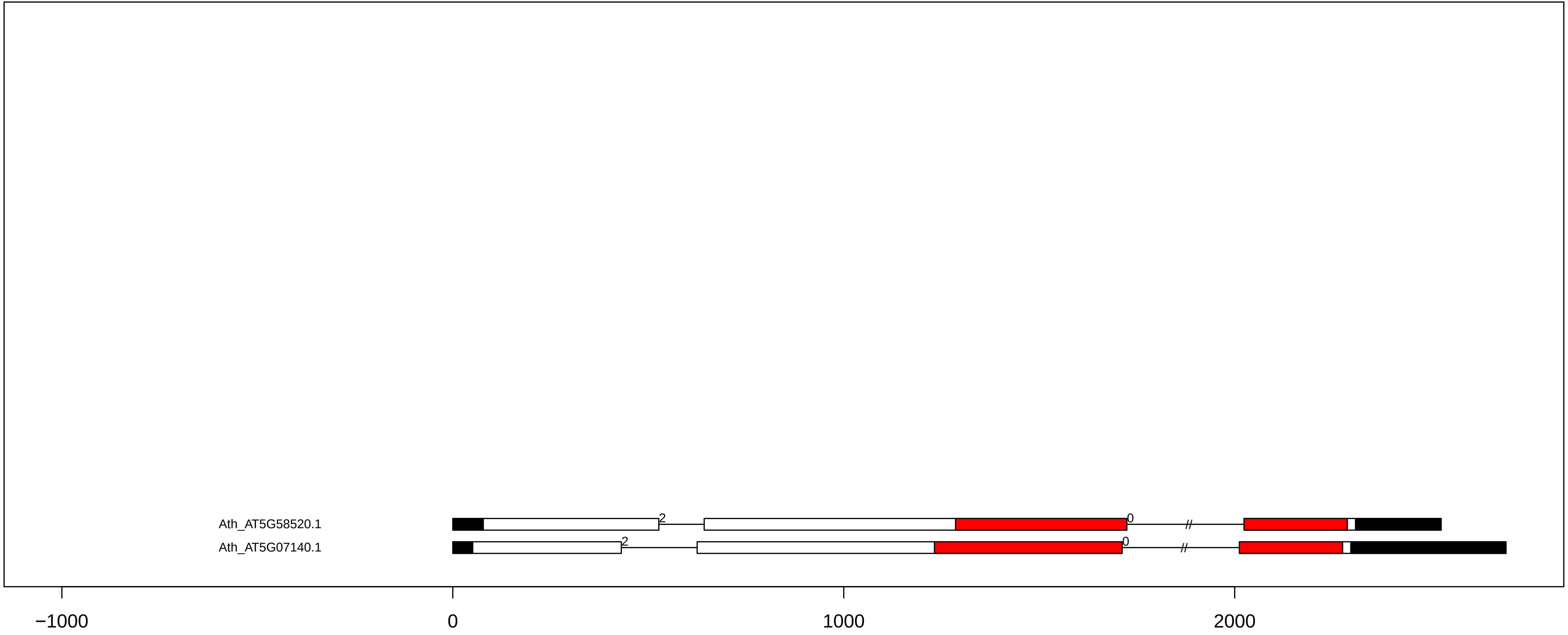

A.th TLK exon−intron and kinase domain diagram (all)

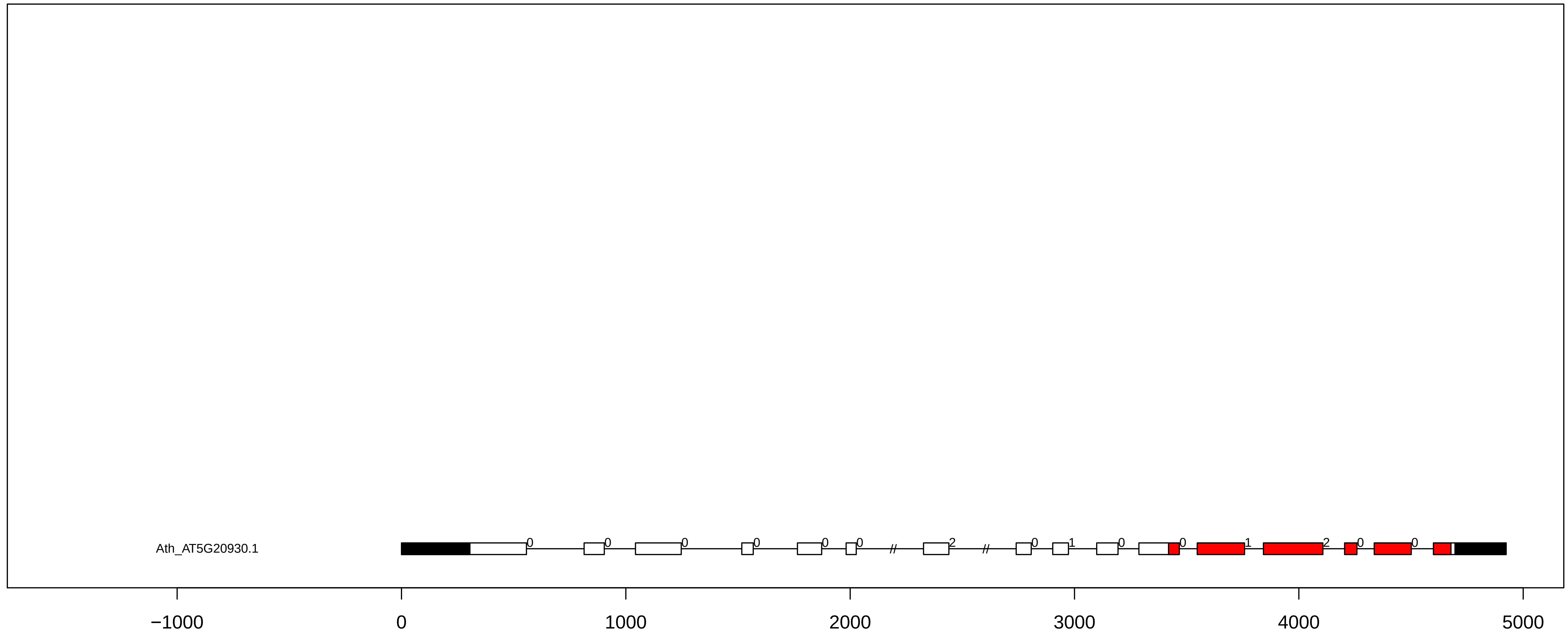

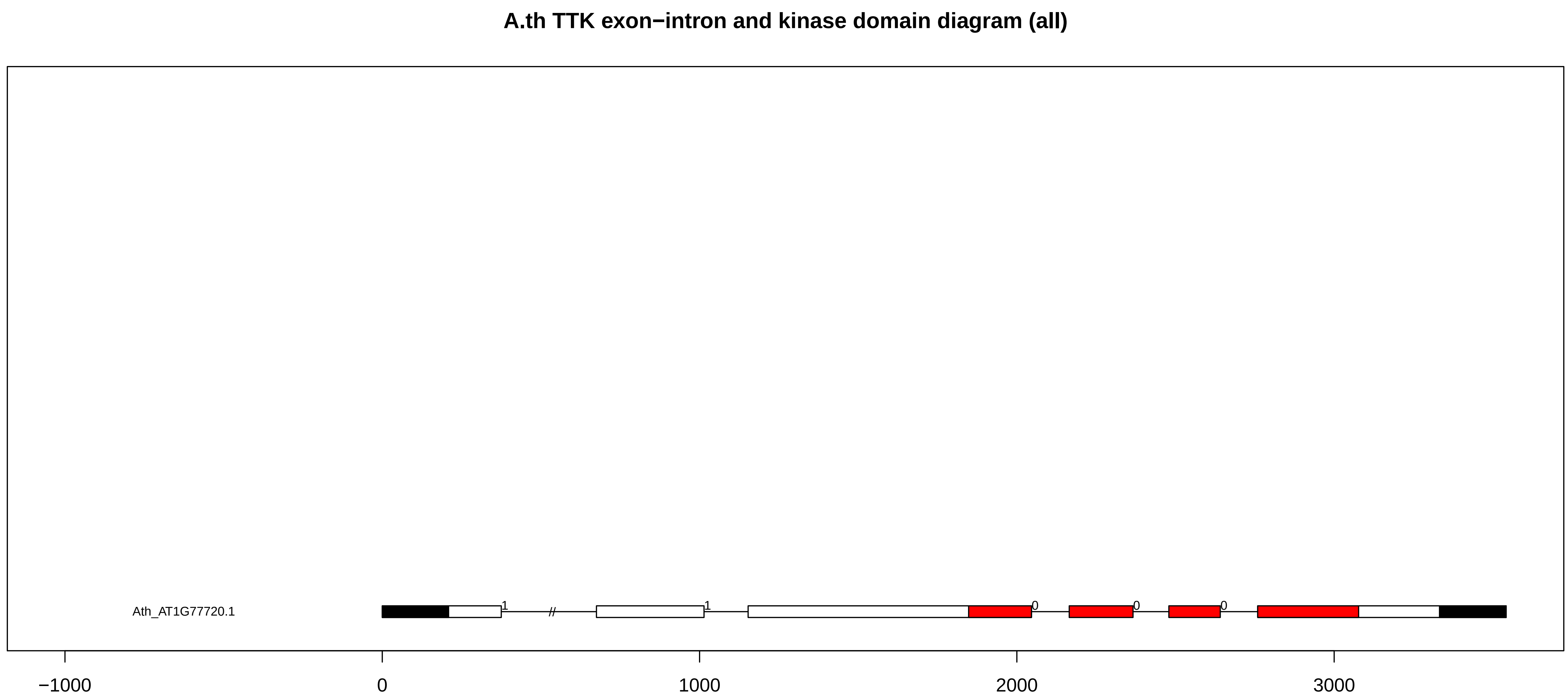
A.th TTK exon−intron and kinase domain diagram (all)
Ath_AT1G77720.1
1
1
0
0
0
−1000
0
1000
2000
3000

A.th ULK_Fused exon−intron and kinase domain diagram (all)

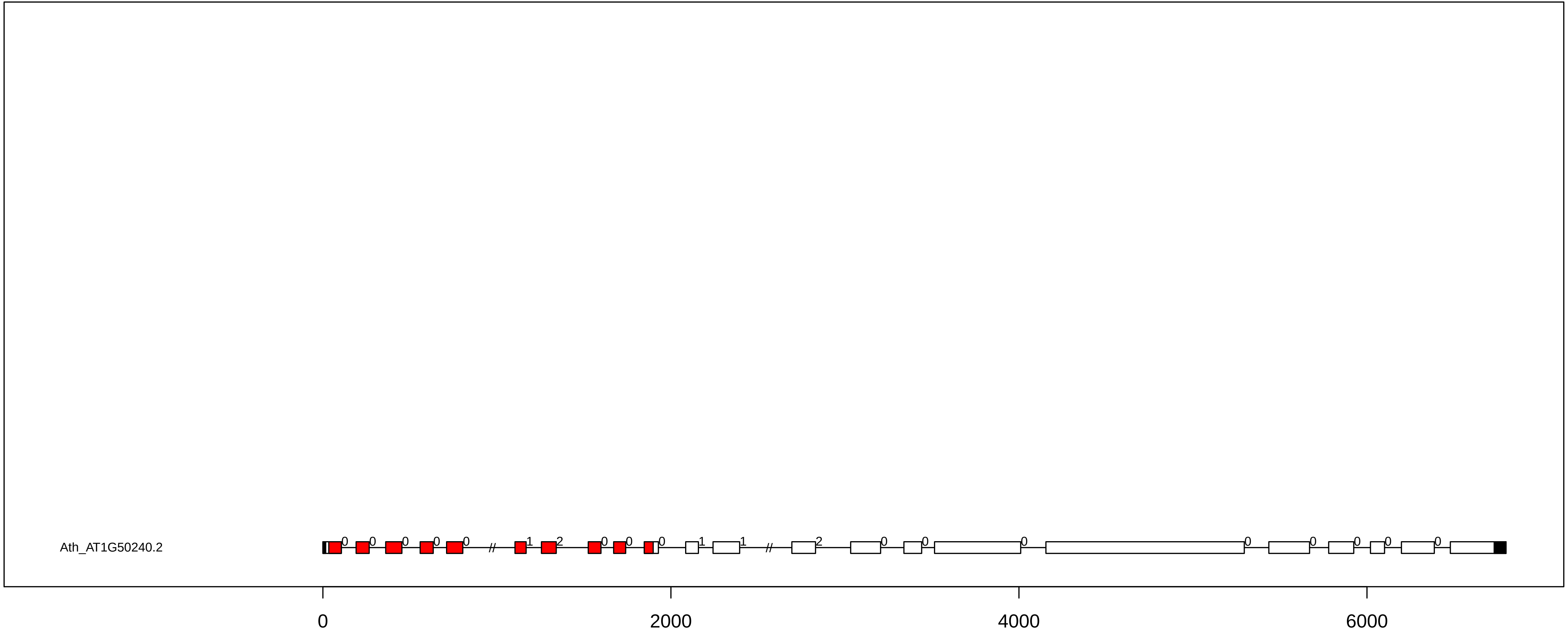

A.th ULK_ULK4 exon−intron and kinase domain diagram (all)

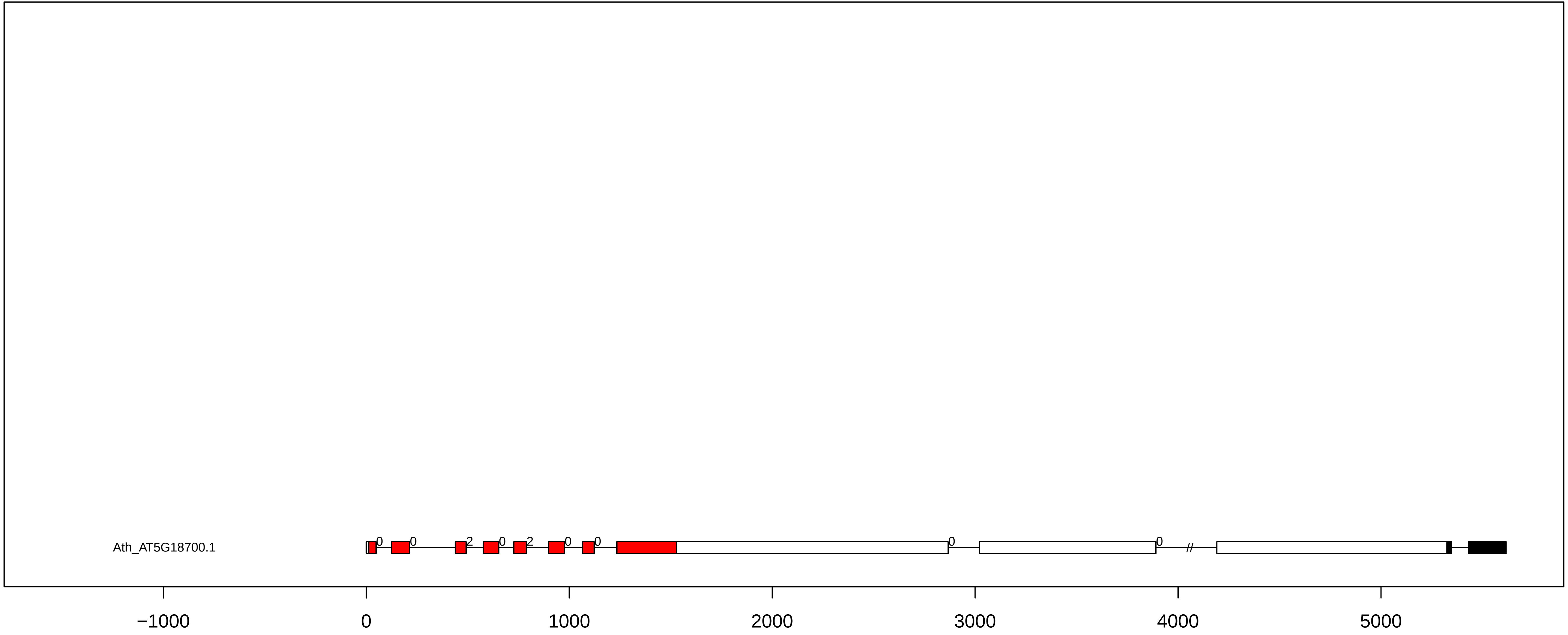

A.th WEE exon−intron and kinase domain diagram (all)

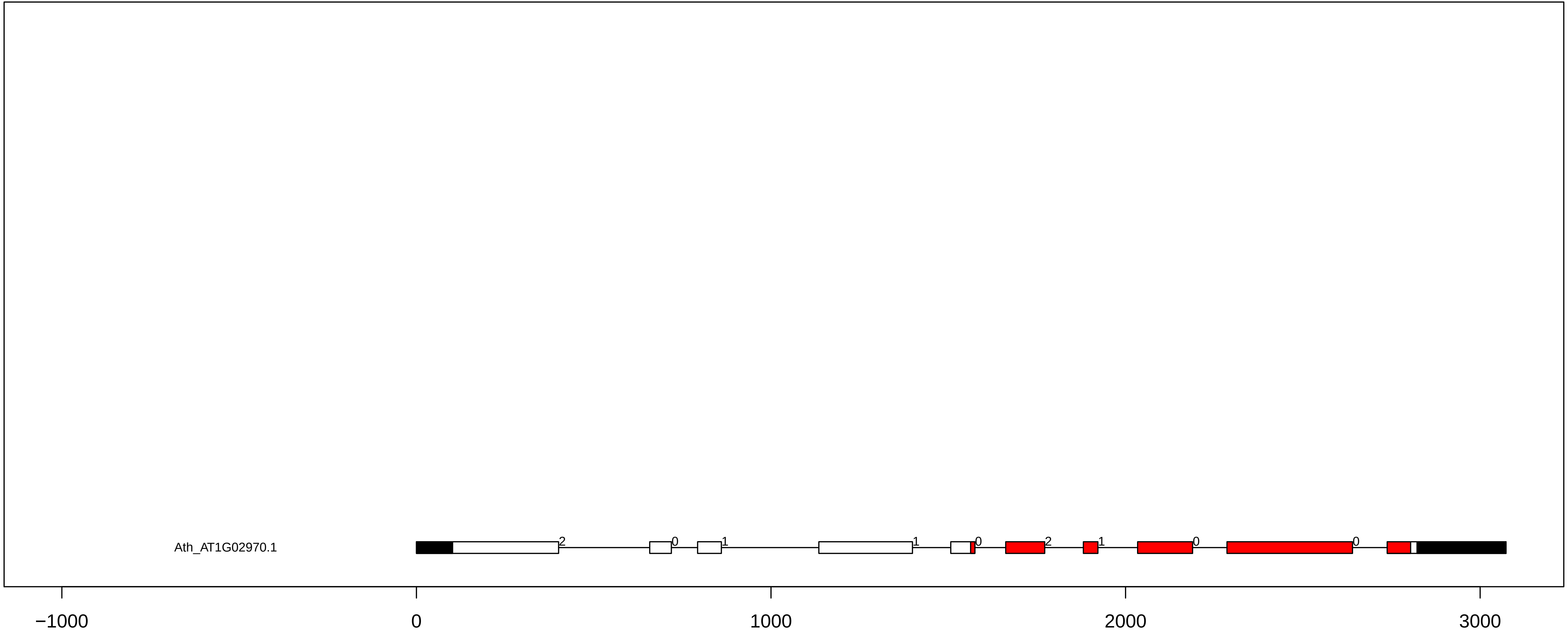

A.th WNK_NRBP exon−intron and kinase domain diagram (all)

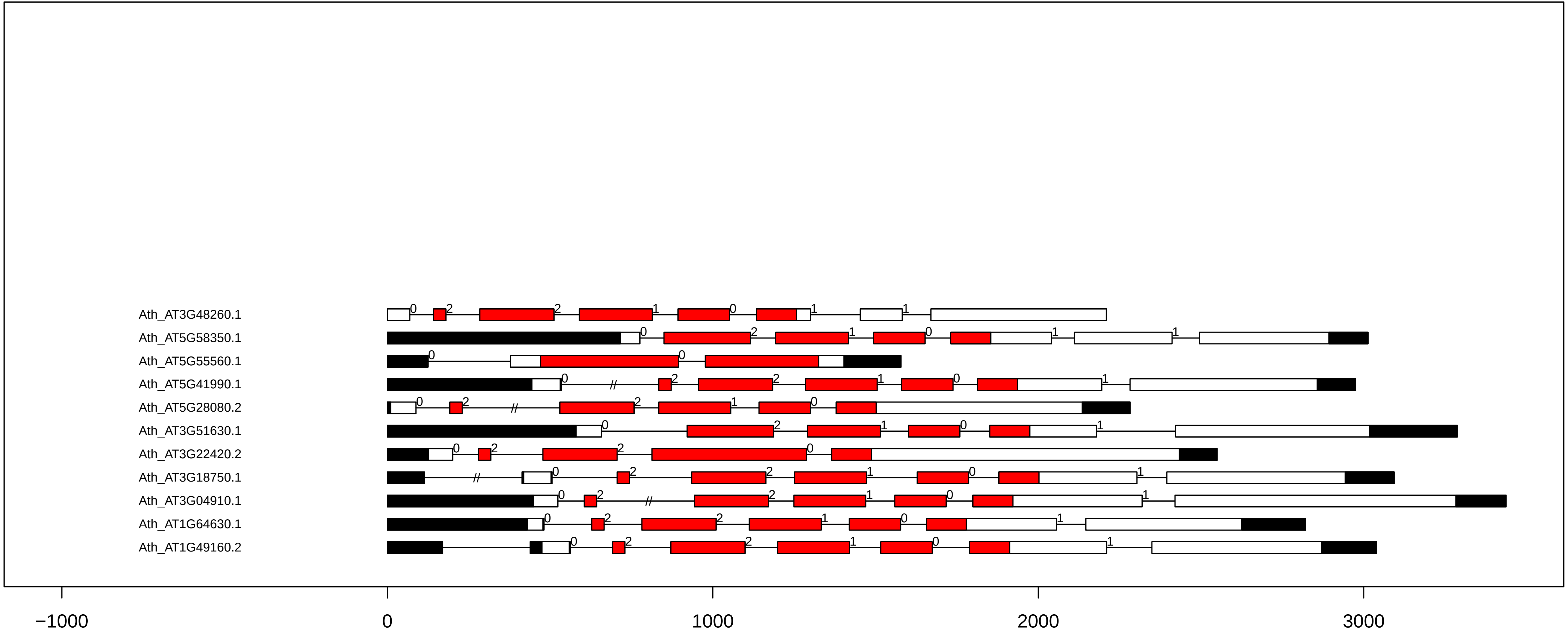

V.vi AGC_MAST exon−intron and domain diagram (all)

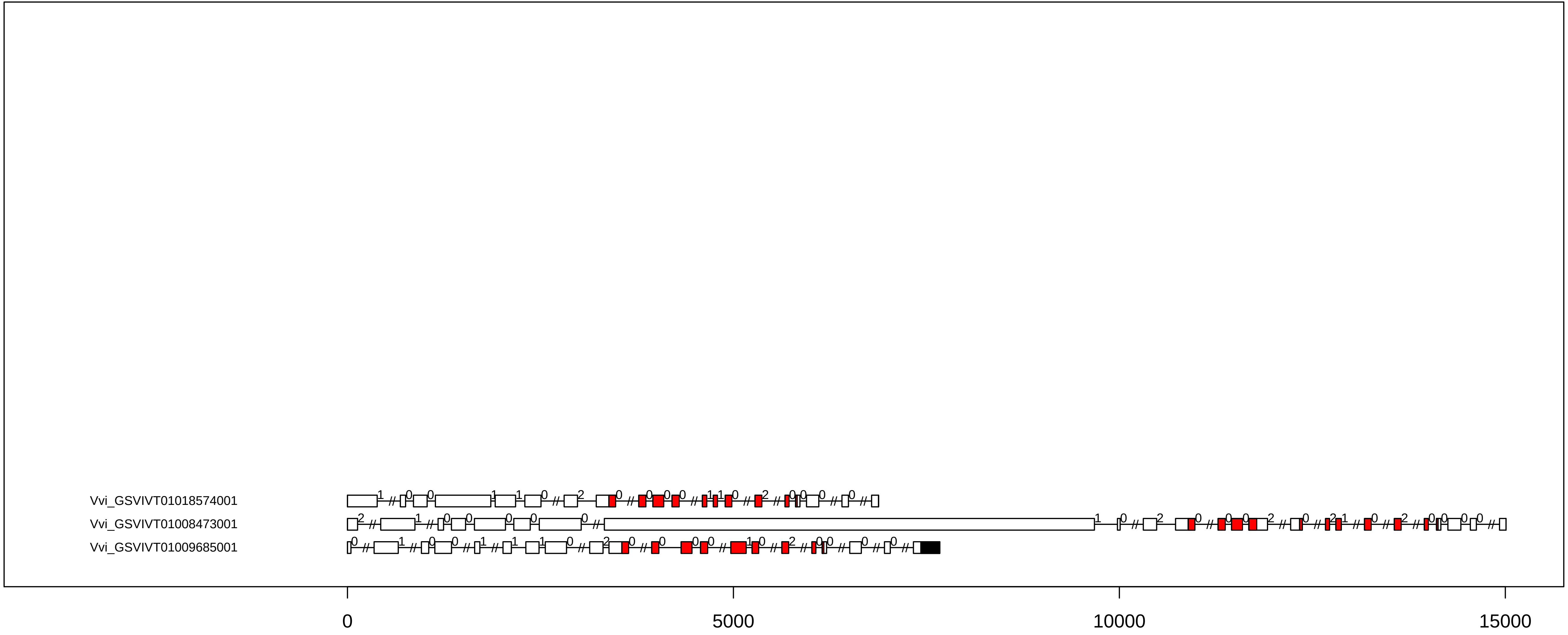

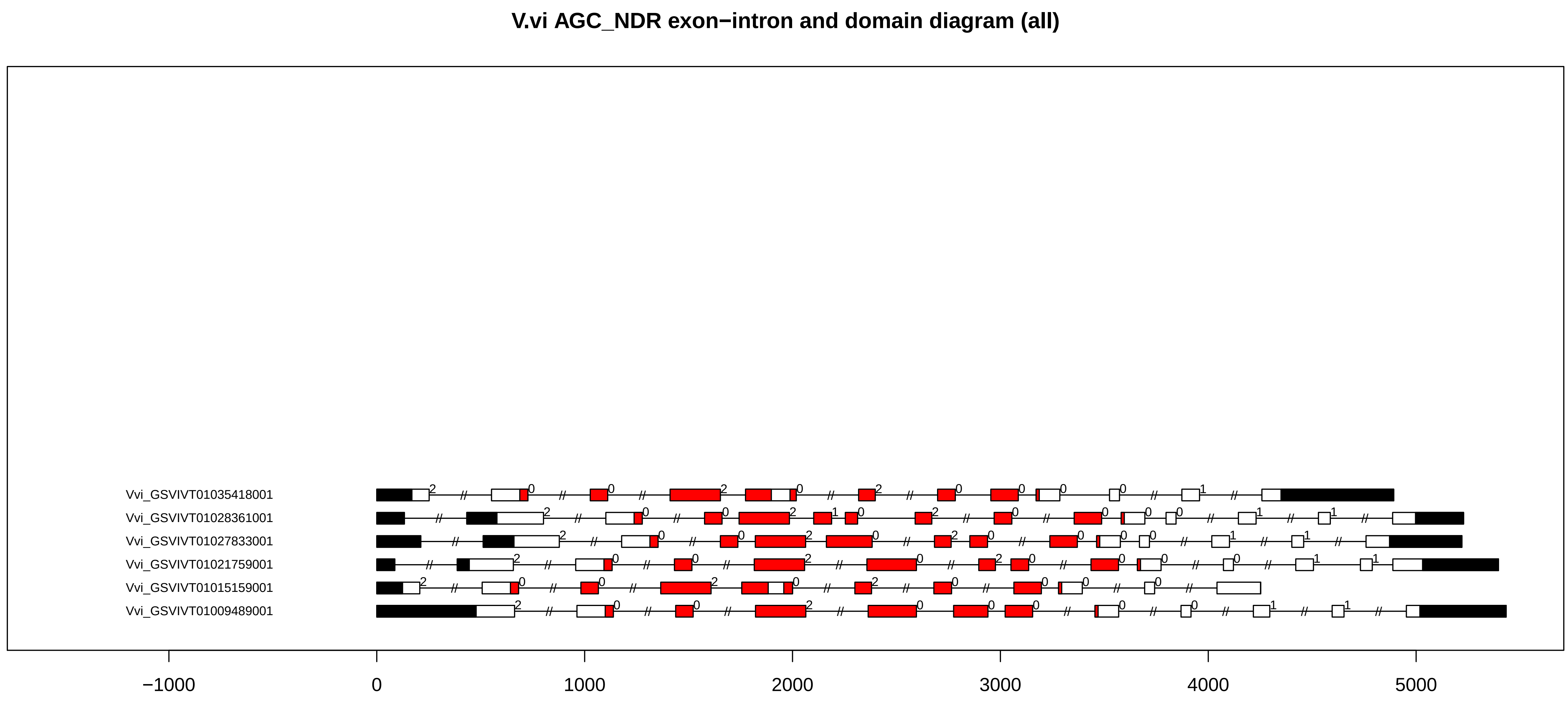
V.vi AGC_NDR exon−intron and domain diagram (all)
Vvi_GSVIVT01035418001
Vvi_GSVIVT01028361001
Vvi_GSVIVT01027833001
Vvi_GSVIVT01021759001
Vvi_GSVIVT01015159001
Vvi_GSVIVT01009489001
−1000
0
1000
2000
3000
4000
5000

V.vi AGC_PDK1 exon−intron and domain diagram (all)

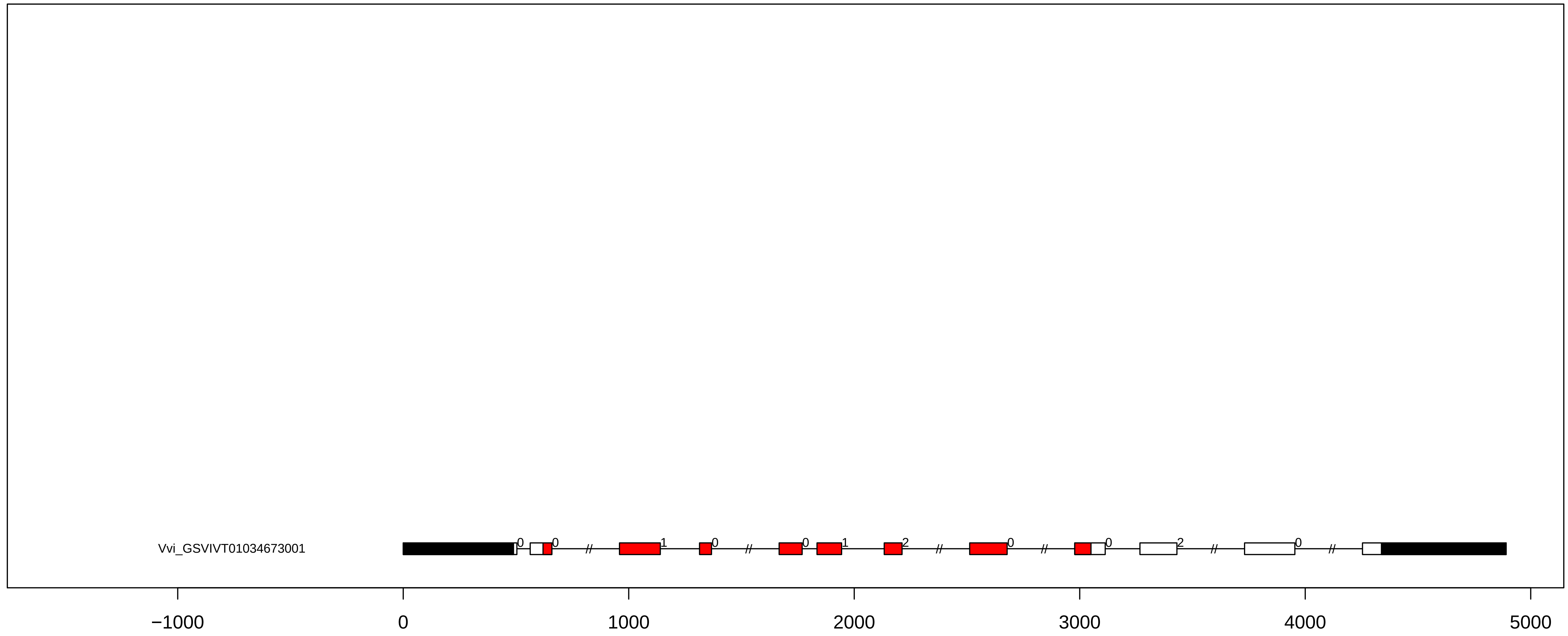

V.vi AGC_PKA−PKG exon−intron and domain diagram (all)

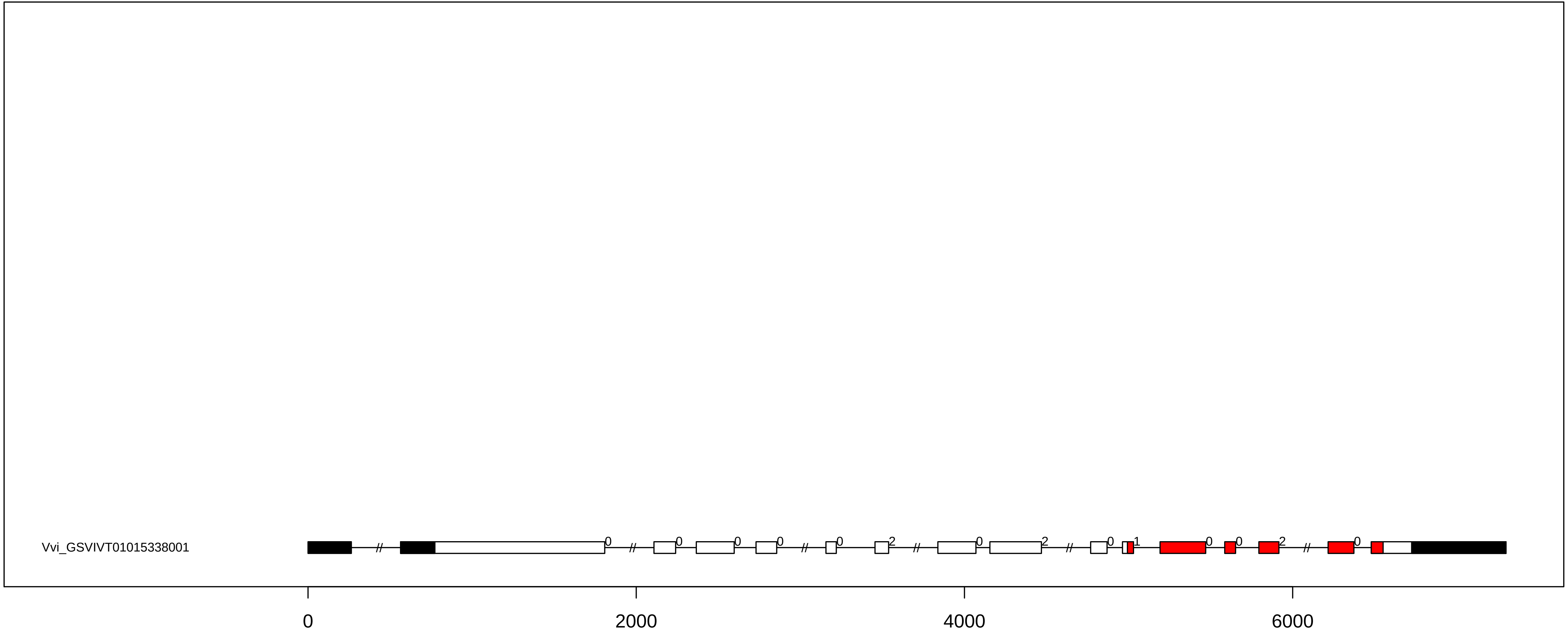

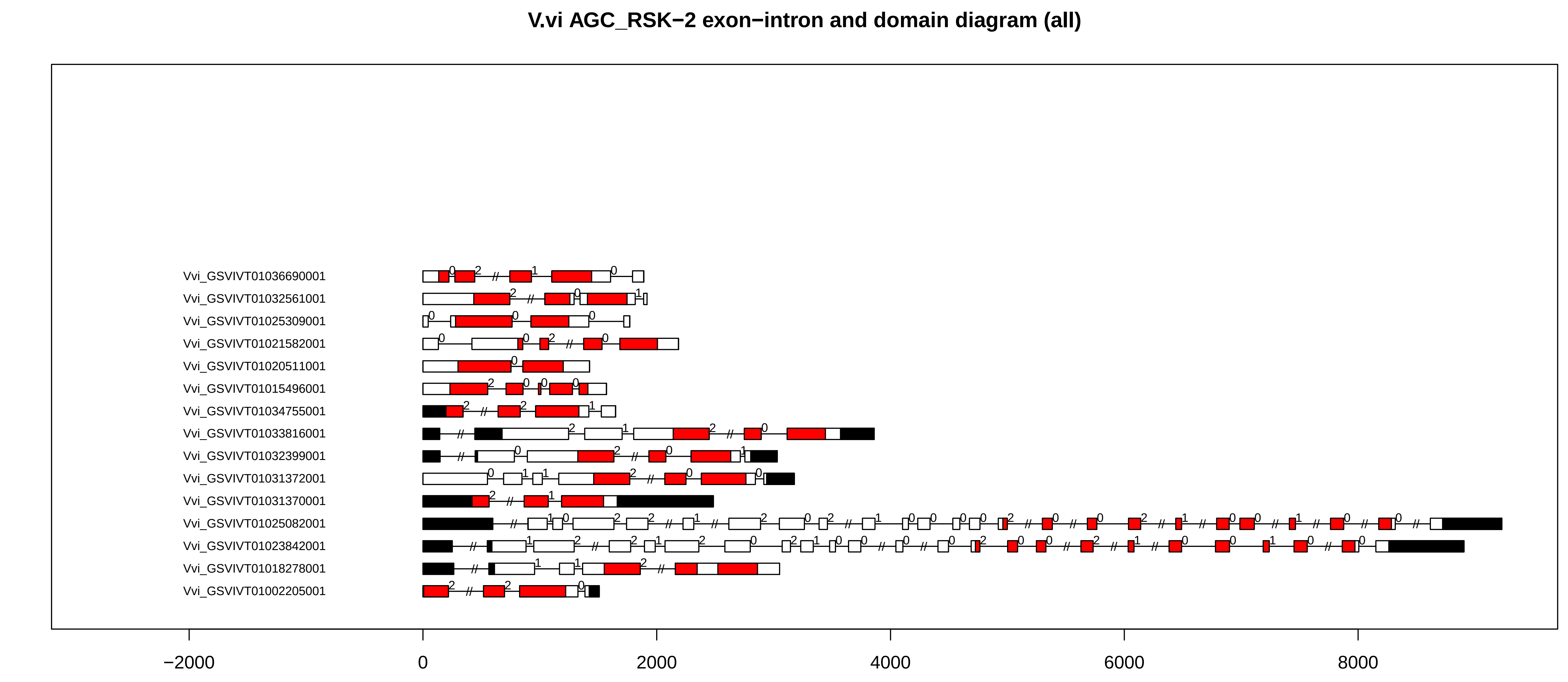
V.vi AGC_RSK−2 exon−intron and domain diagram (all)
Vvi_GSVIVT01036690001
Vvi_GSVIVT01032561001
Vvi_GSVIVT01025309001
Vvi_GSVIVT01021582001
Vvi_GSVIVT01020511001
Vvi_GSVIVT01015496001
Vvi_GSVIVT01034755001
Vvi_GSVIVT01033816001
Vvi_GSVIVT01032399001
Vvi_GSVIVT01031372001
Vvi_GSVIVT01031370001
Vvi_GSVIVT01025082001
Vvi_GSVIVT01023842001
Vvi_GSVIVT01018278001
Vvi_GSVIVT01002205001
−2000
0
2000
4000
6000
8000

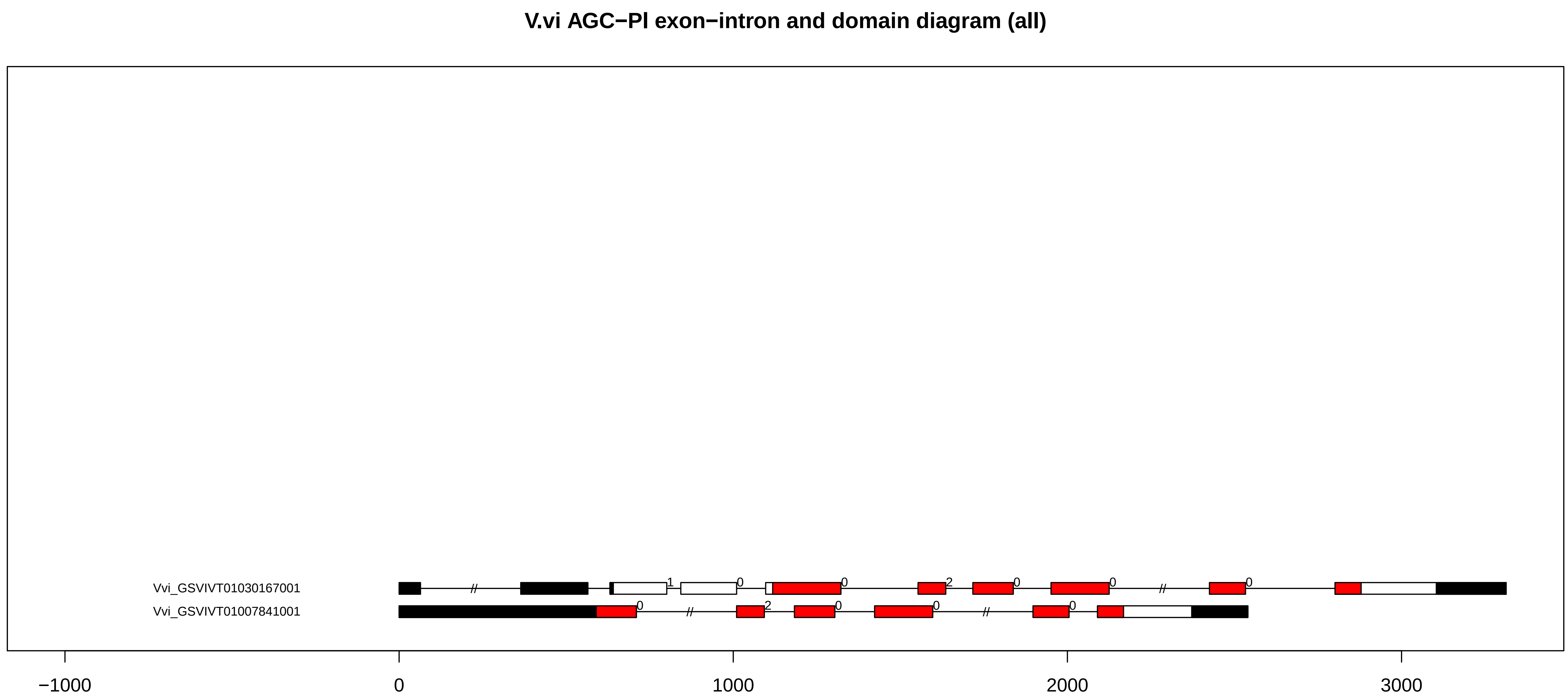
V.vi AGC−PI exon−intron and domain diagram (all)
Vvi_GSVIVT01030167001
Vvi_GSVIVT01007841001
−1000
0
1000
2000
3000

V.vi Aur exon−intron and domain diagram (all)

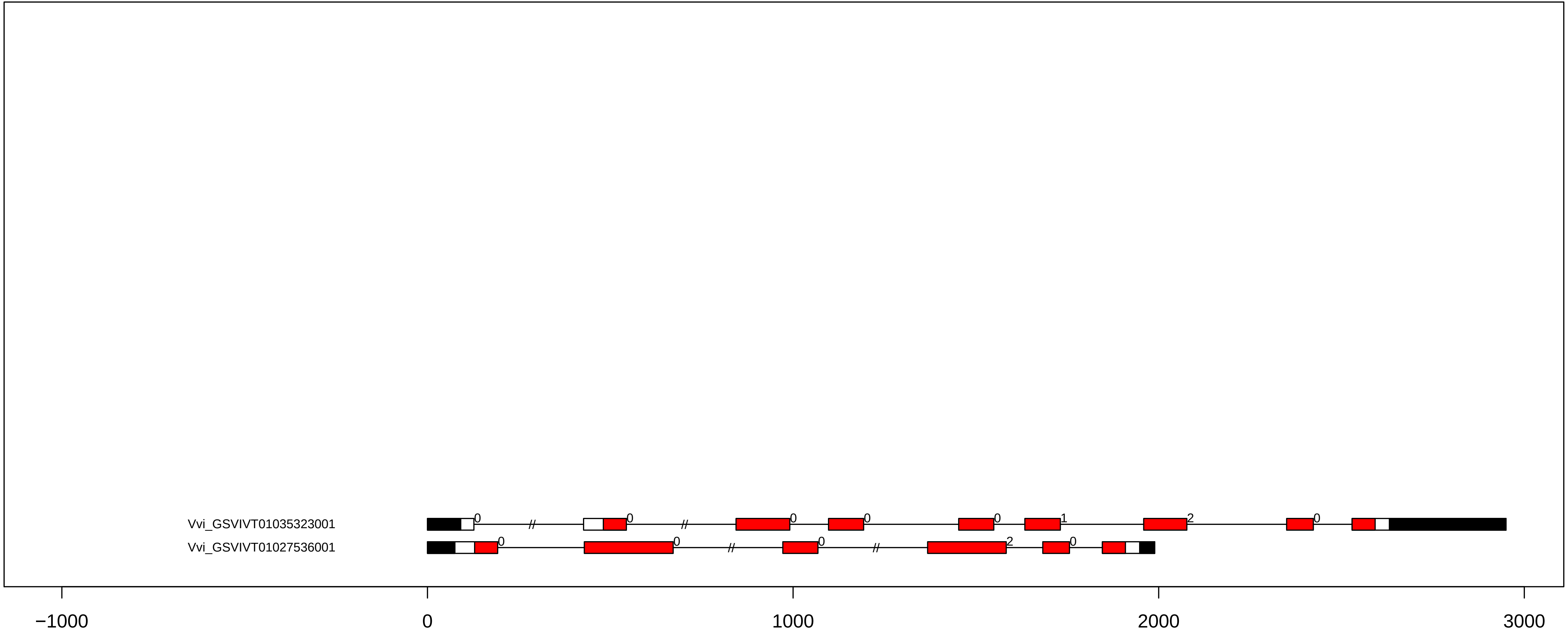

V.vi CAMK_AMPK exon−intron and domain diagram (all)

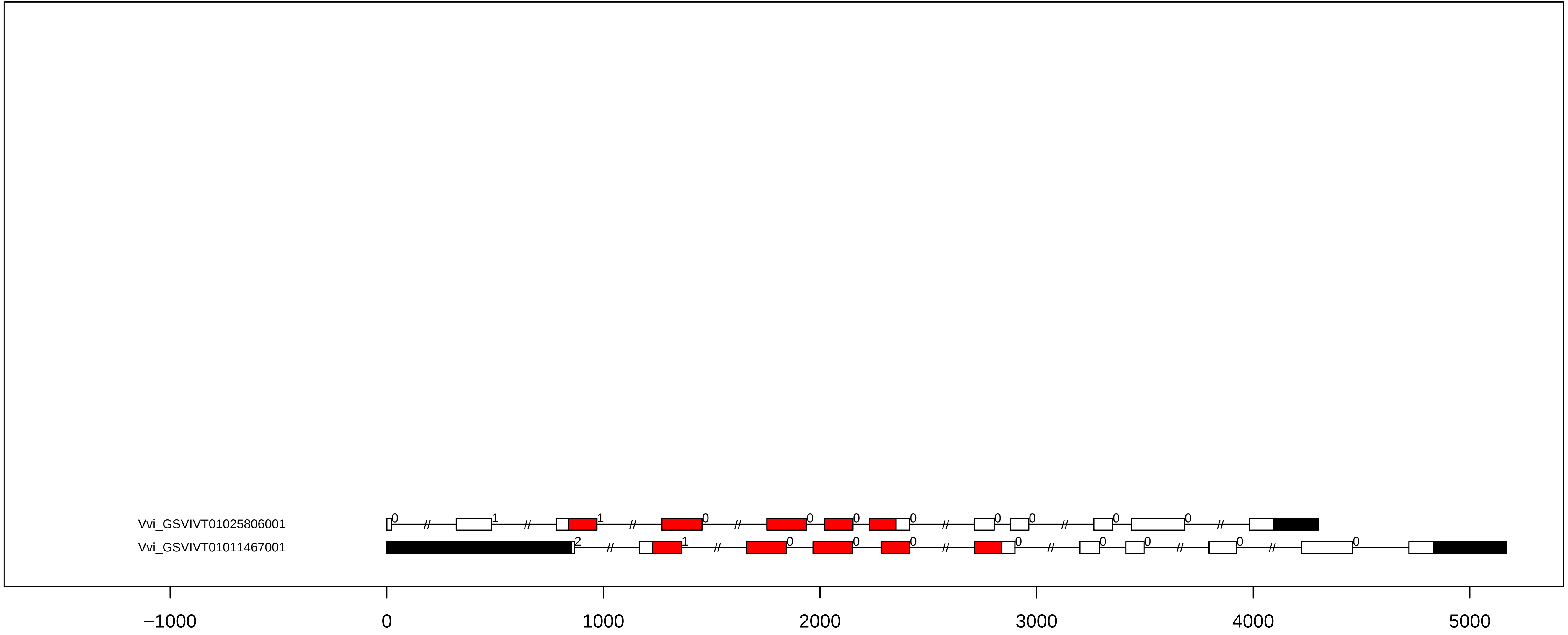

V.vi CAMK_CAMK1−DCAMKL exon−intron and domain diagram (all)

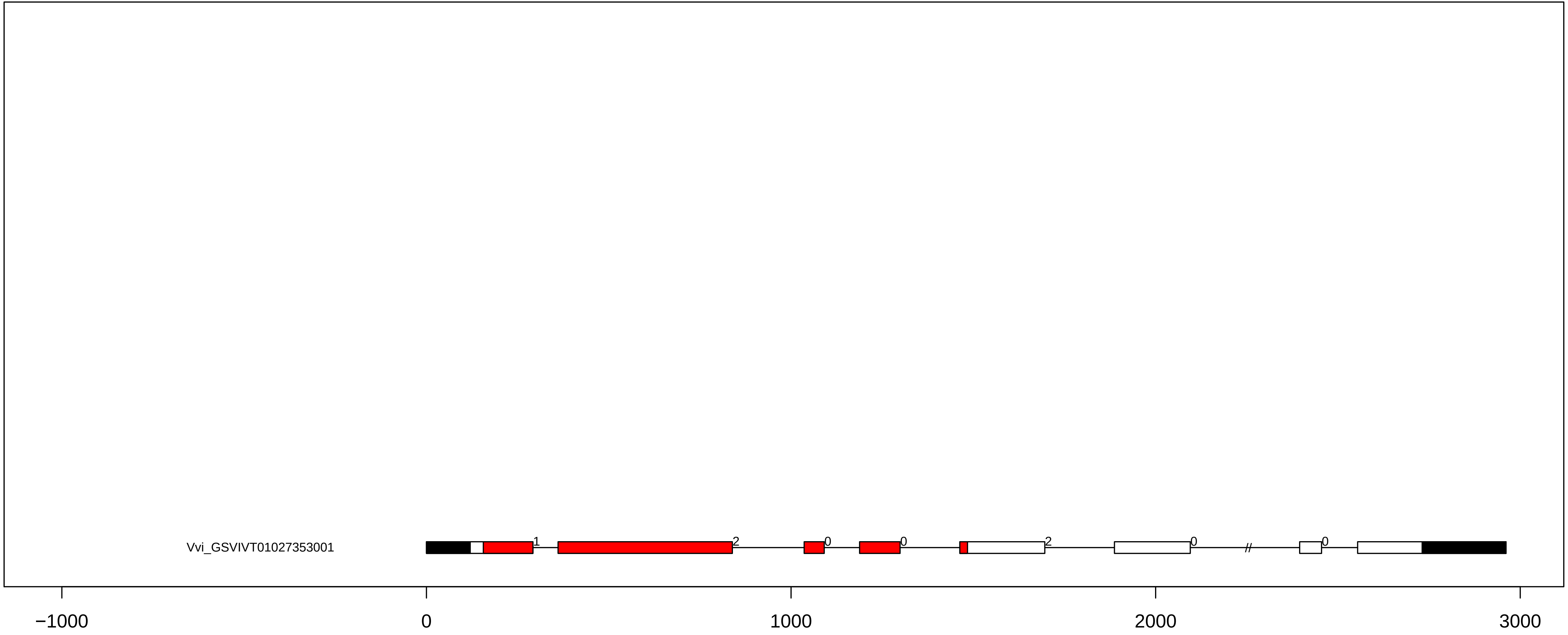

V.vi CAMK_CAMKL−CHK1 exon−intron and domain diagram (all)

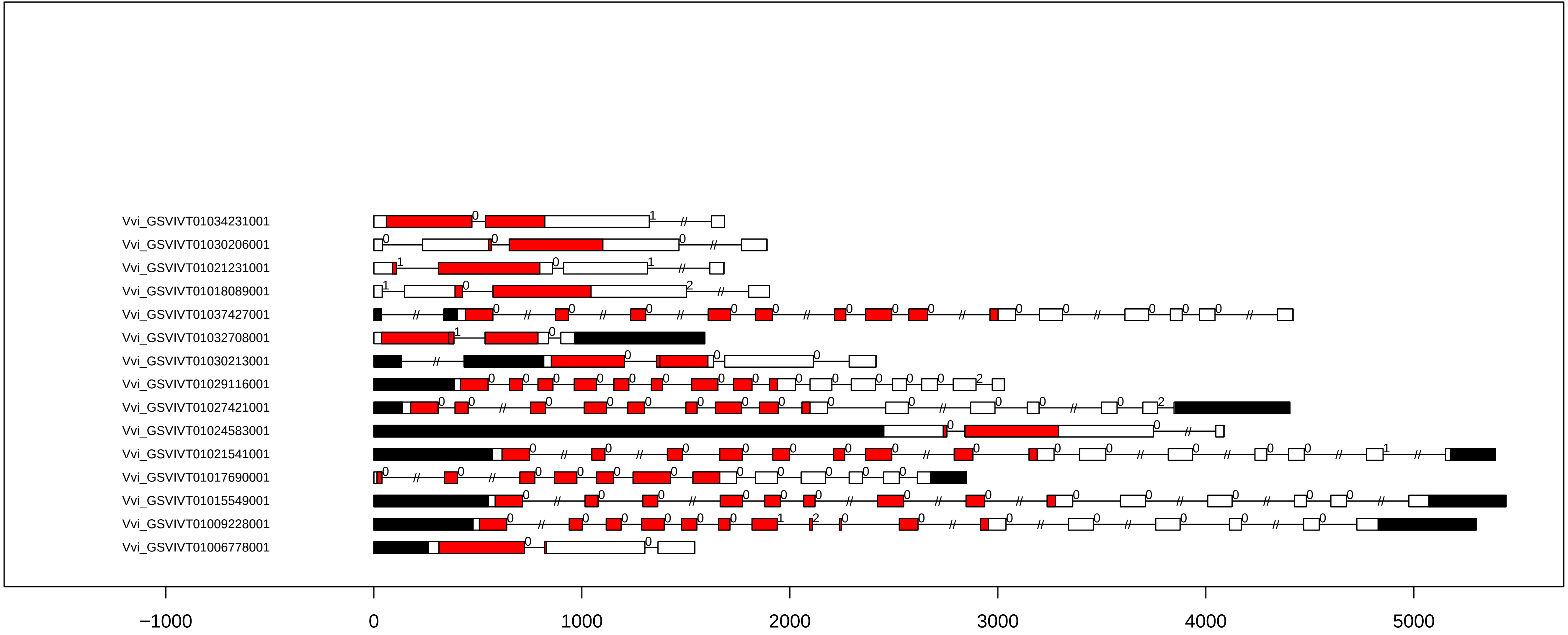

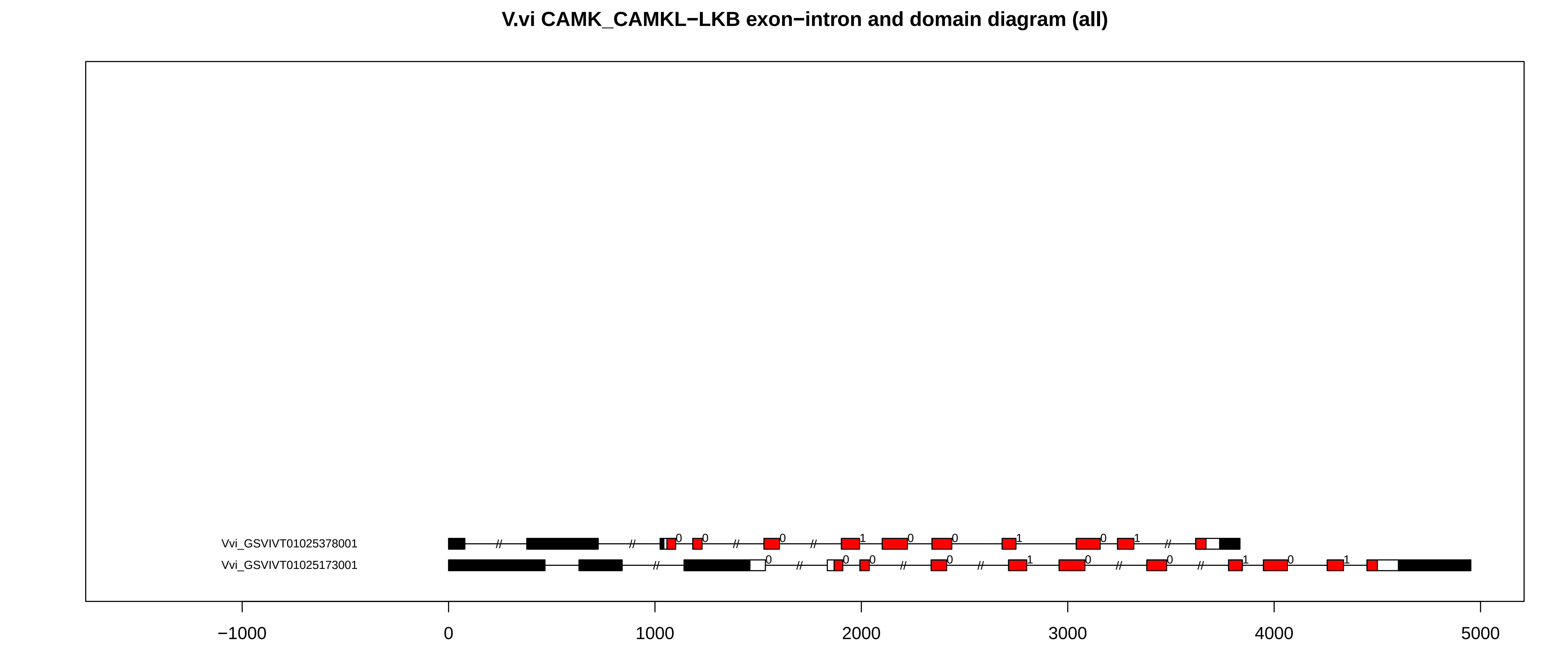
V.vi CAMK_CAMKL−LKB exon−intron and domain diagram (all)
Vvi_GSVIVT01025378001
Vvi_GSVIVT01025173001
−1000
0
1000
2000
3000
4000
5000

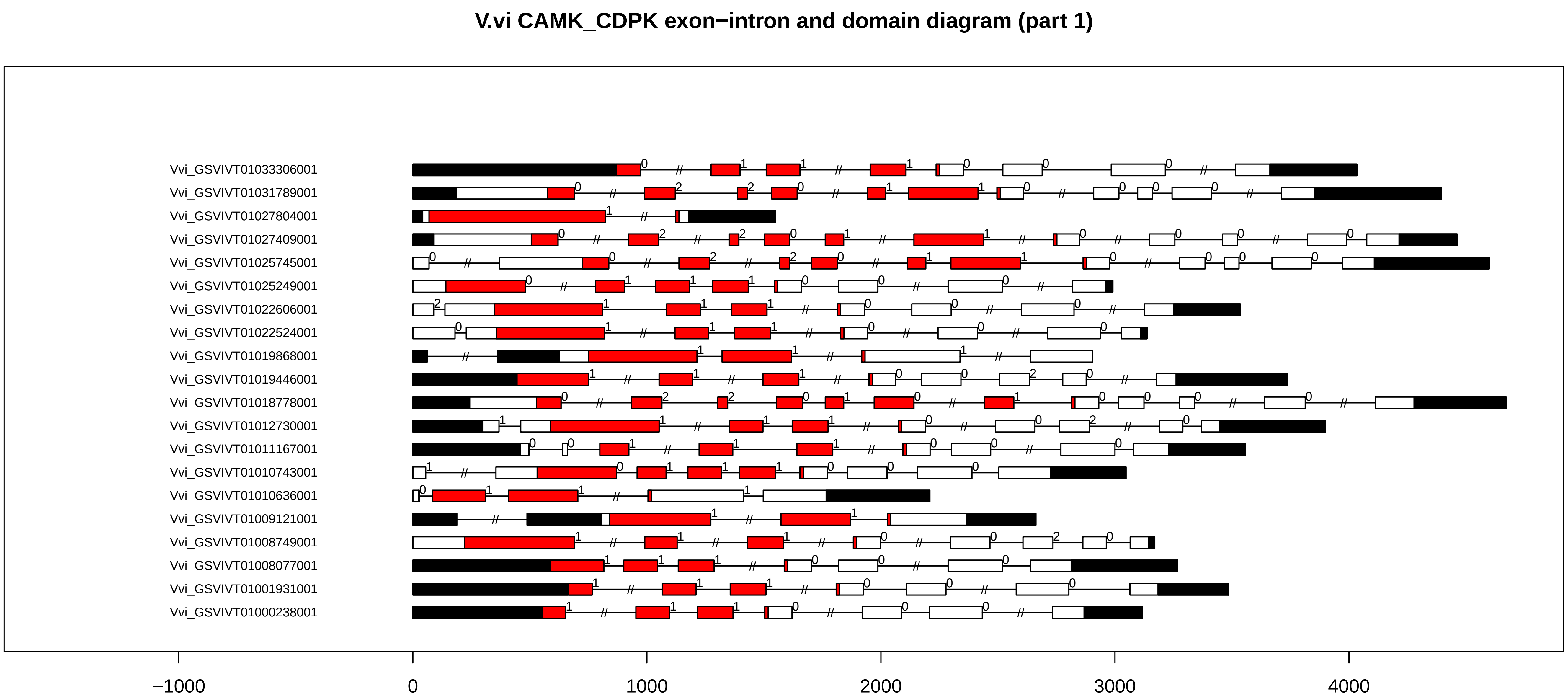
V.vi CAMK_CDPK exon−intron and domain diagram (part 1)
Vvi_GSVIVT01033306001
Vvi_GSVIVT01031789001
Vvi_GSVIVT01027804001
Vvi_GSVIVT01027409001
Vvi_GSVIVT01025745001
Vvi_GSVIVT01025249001
Vvi_GSVIVT01022606001
Vvi_GSVIVT01022524001
Vvi_GSVIVT01019868001
Vvi_GSVIVT01019446001
Vvi_GSVIVT01018778001
Vvi_GSVIVT01012730001
Vvi_GSVIVT01011167001
Vvi_GSVIVT01010743001
Vvi_GSVIVT01010636001
Vvi_GSVIVT01009121001
Vvi_GSVIVT01008749001
Vvi_GSVIVT01008077001
Vvi_GSVIVT01001931001
Vvi_GSVIVT01000238001
−1000
0
1000
2000
3000
4000

V.vi CAMK_CDPK exon−intron and domain diagram (part 2)

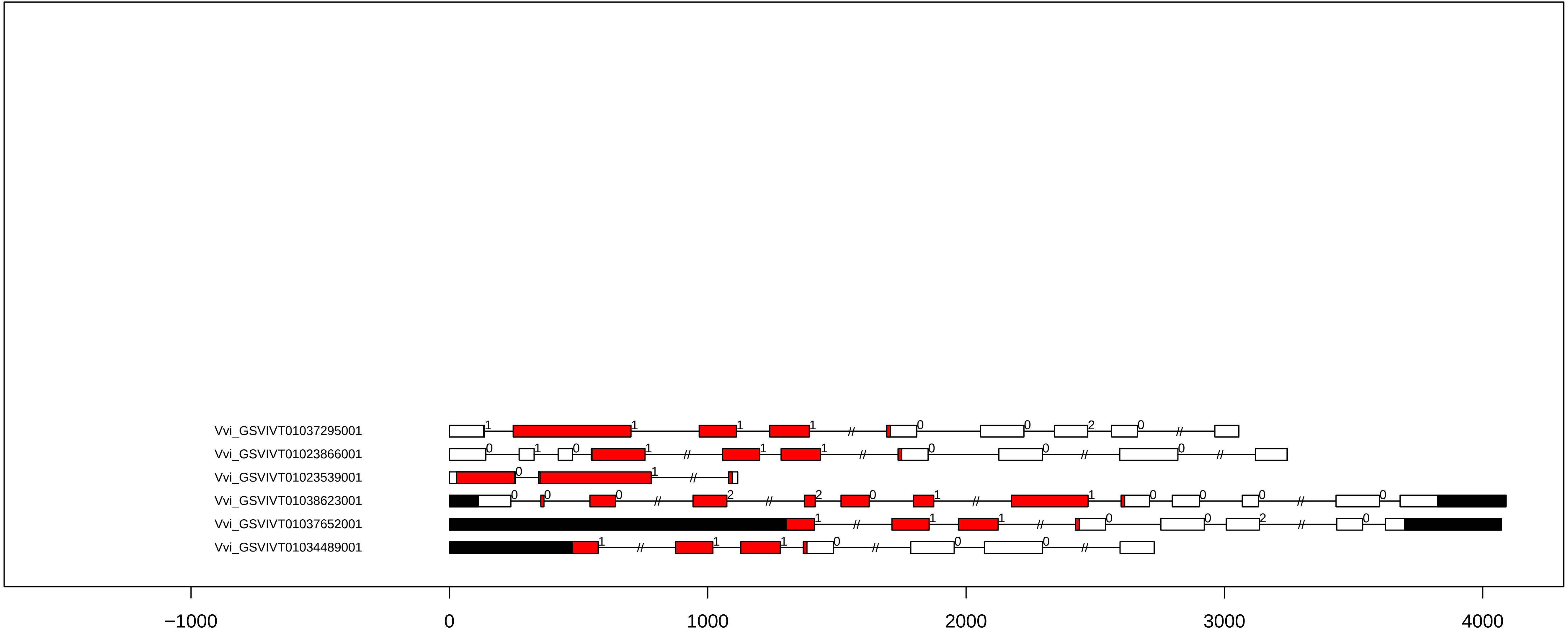

V.vi CAMK_OST1L exon−intron and domain diagram (all)

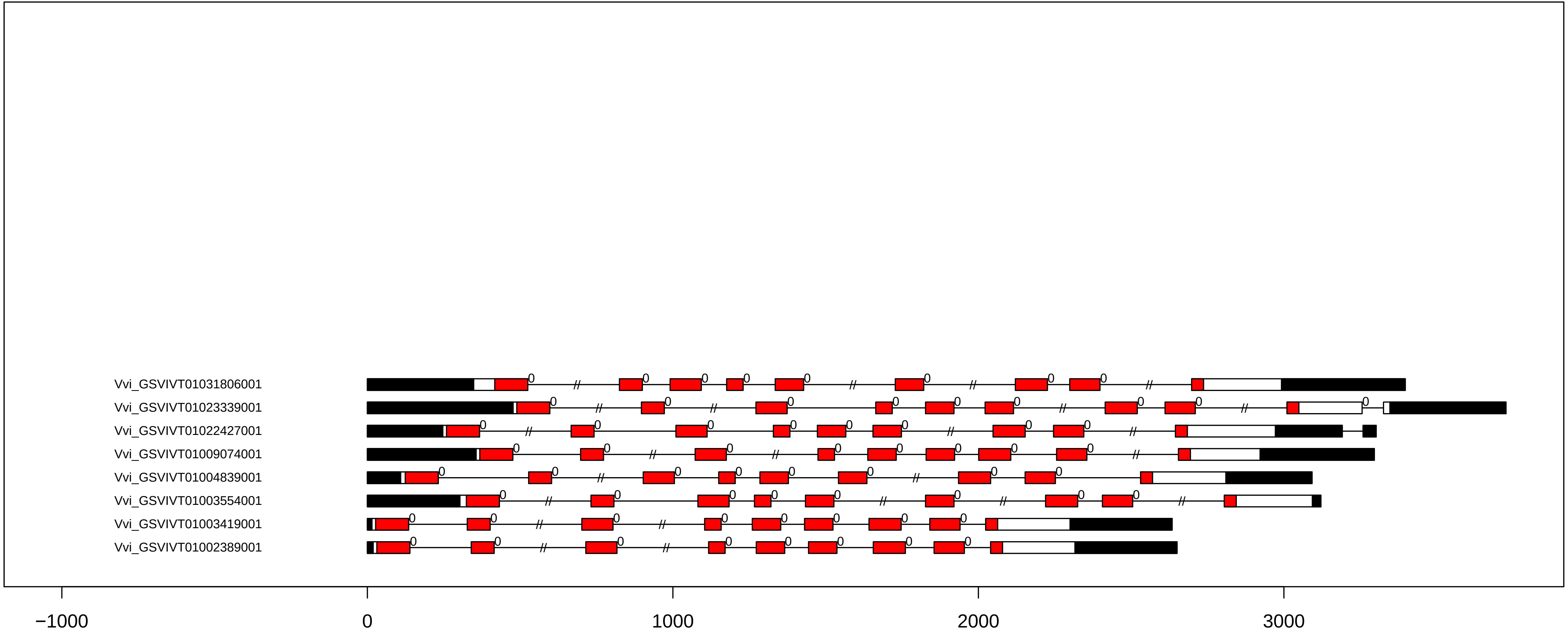

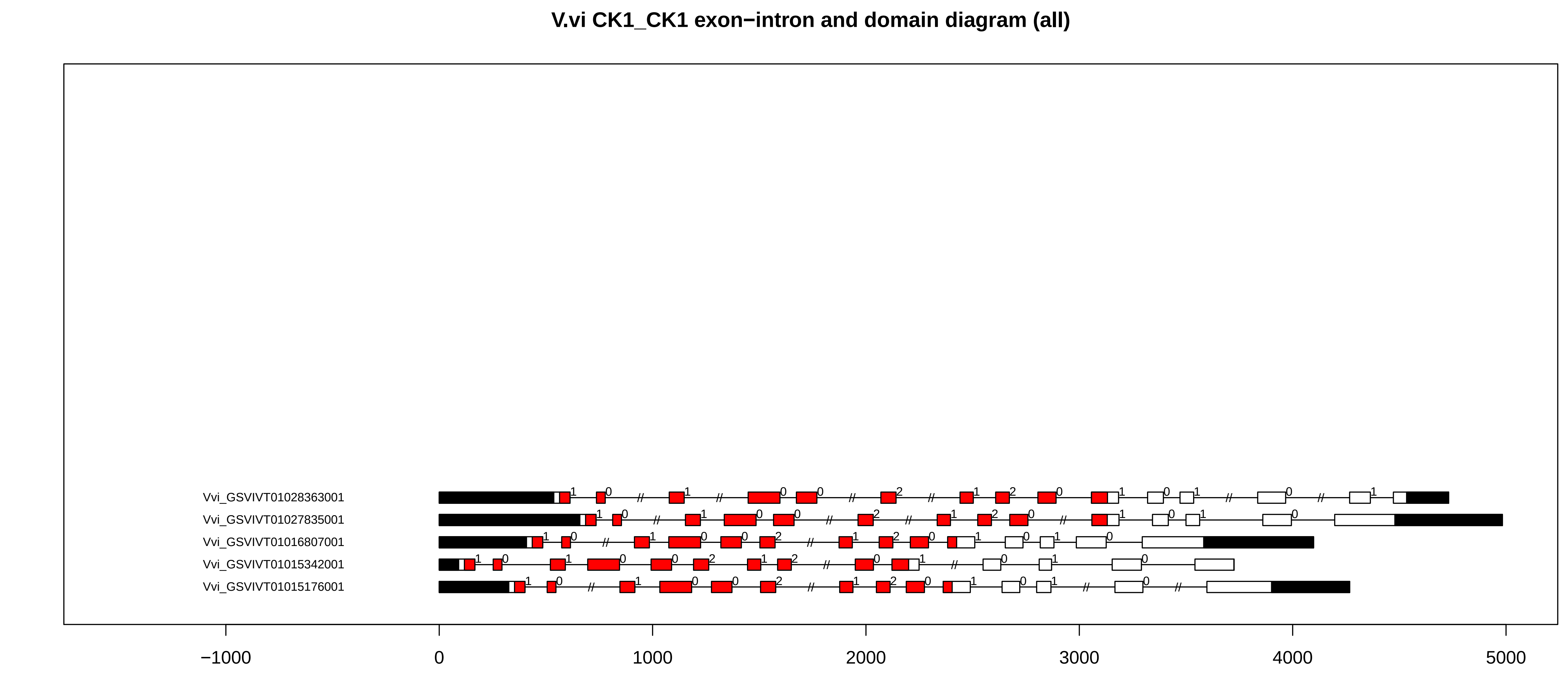
V.vi CK1_CK1 exon−intron and domain diagram (all)
Vvi_GSVIVT01028363001
Vvi_GSVIVT01027835001
Vvi_GSVIVT01016807001
Vvi_GSVIVT01015342001
Vvi_GSVIVT01015176001
−1000
0
1000
2000
3000
4000
5000

V.vi CK1_CK1−Pl exon−intron and domain diagram (all)

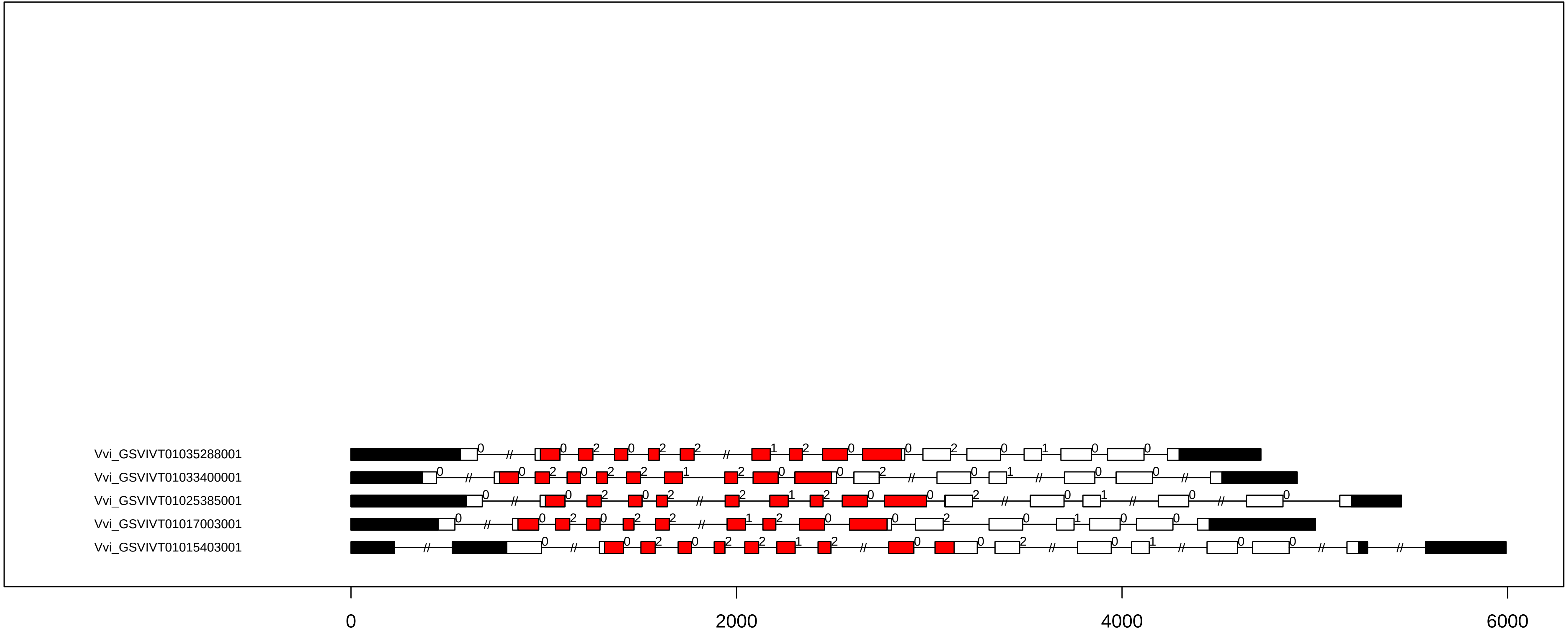

V.vi CMGC_CDK−CDK7 exon−intron and domain diagram (all)

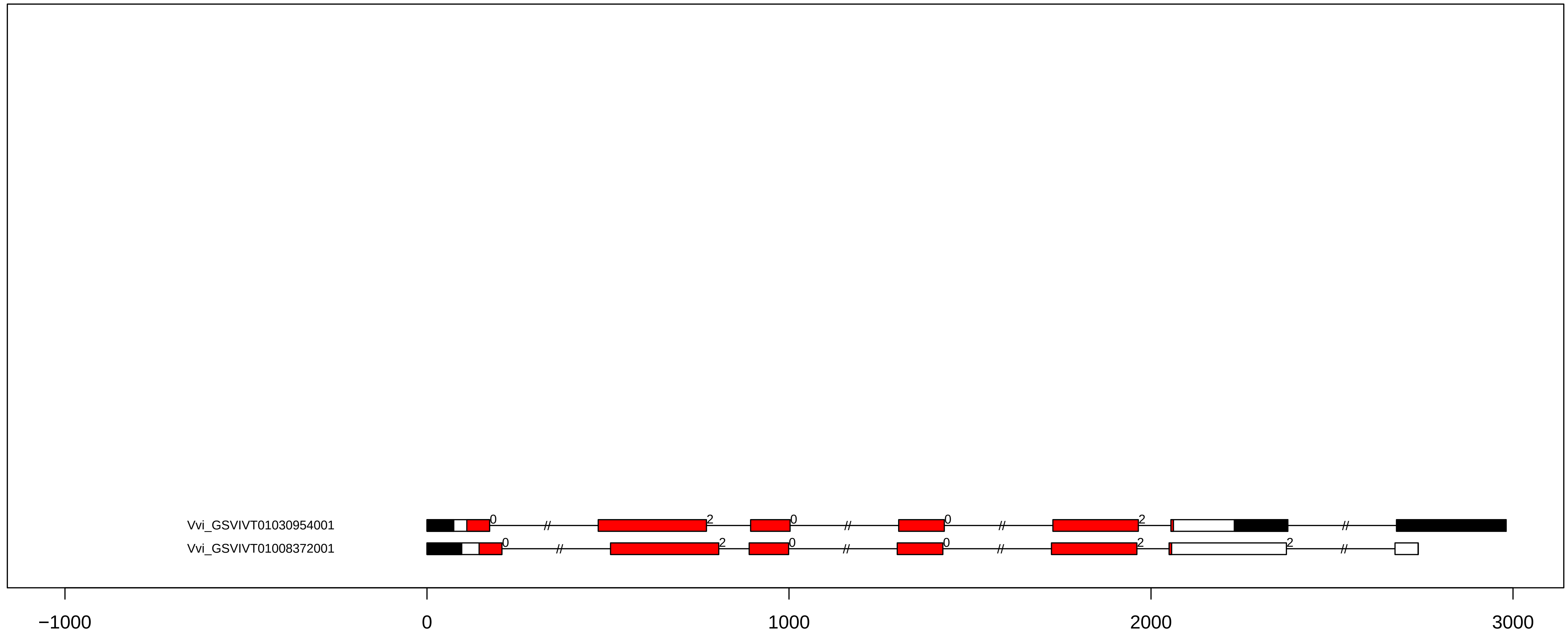

V.vi CMGC_CDK−CDK8 exon−intron and domain diagram (all)

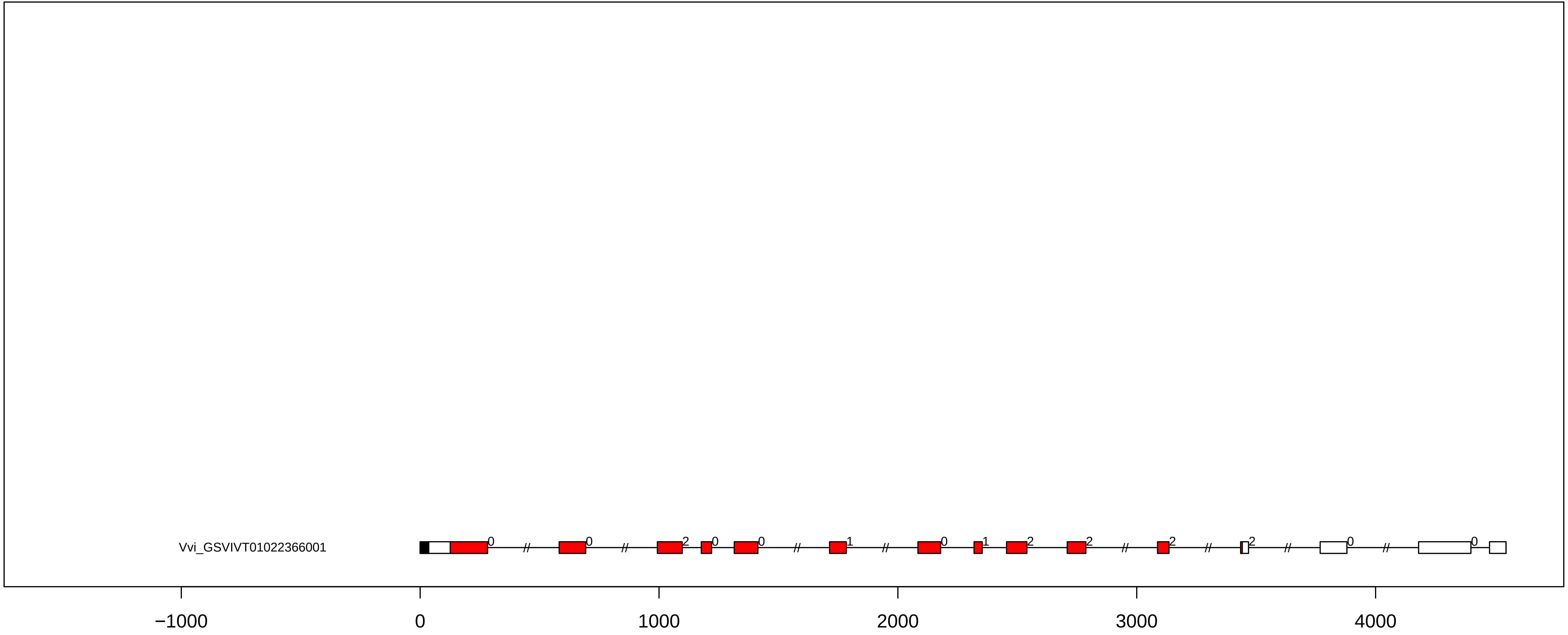

V.vi CMGC_CDK−CRK7−CDK9 exon−intron and domain diagram (all)

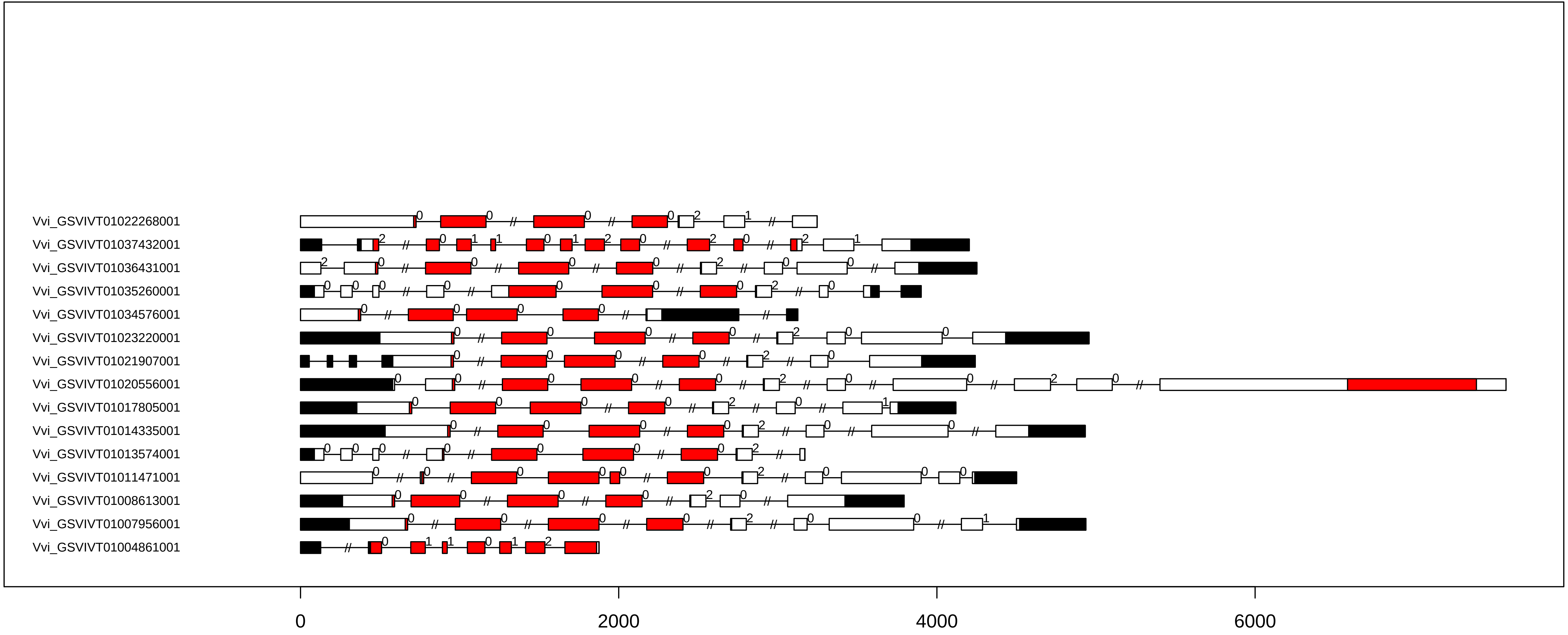

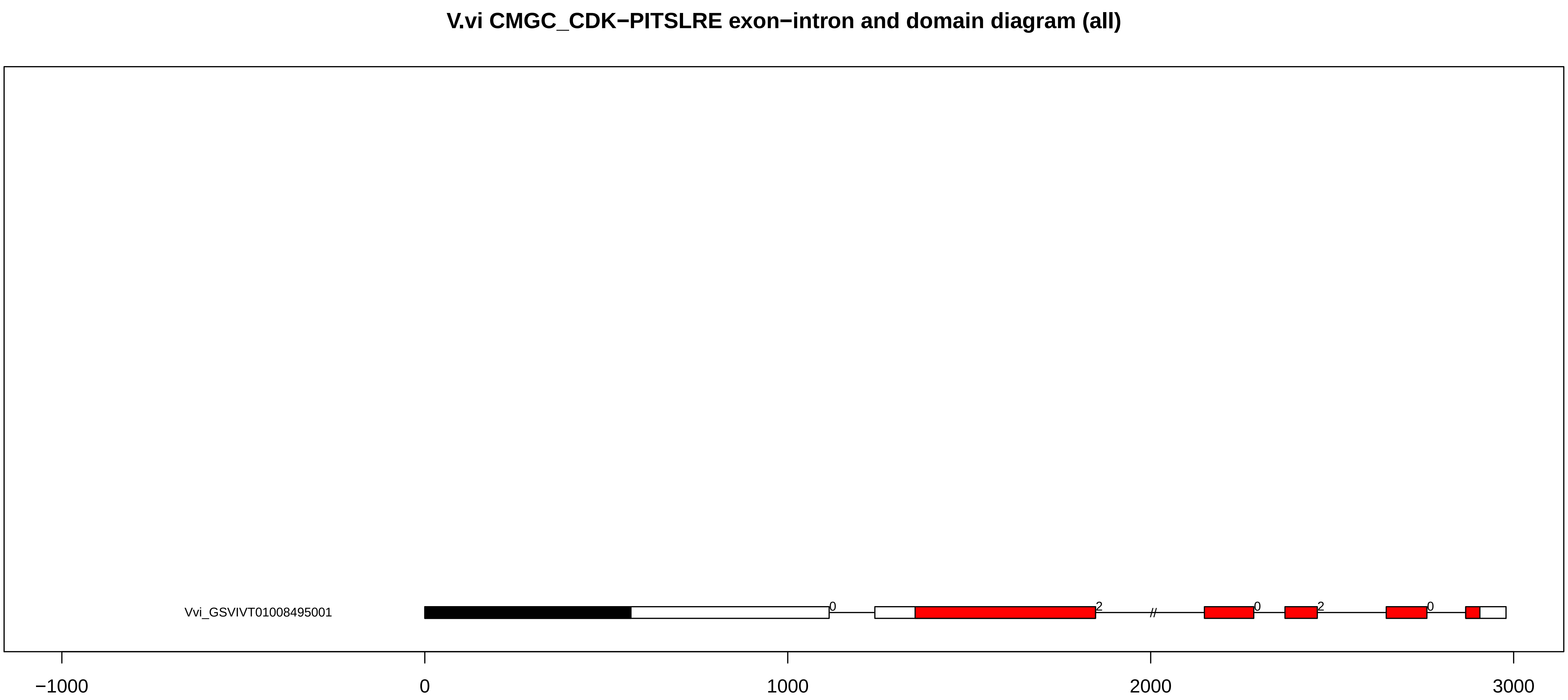
V.vi CMGC_CDK−PITSLRE exon−intron and domain diagram (all)
Vvi_GSVIVT01008495001
0
2
//
0
2
0
−1000
0
1000
2000
3000

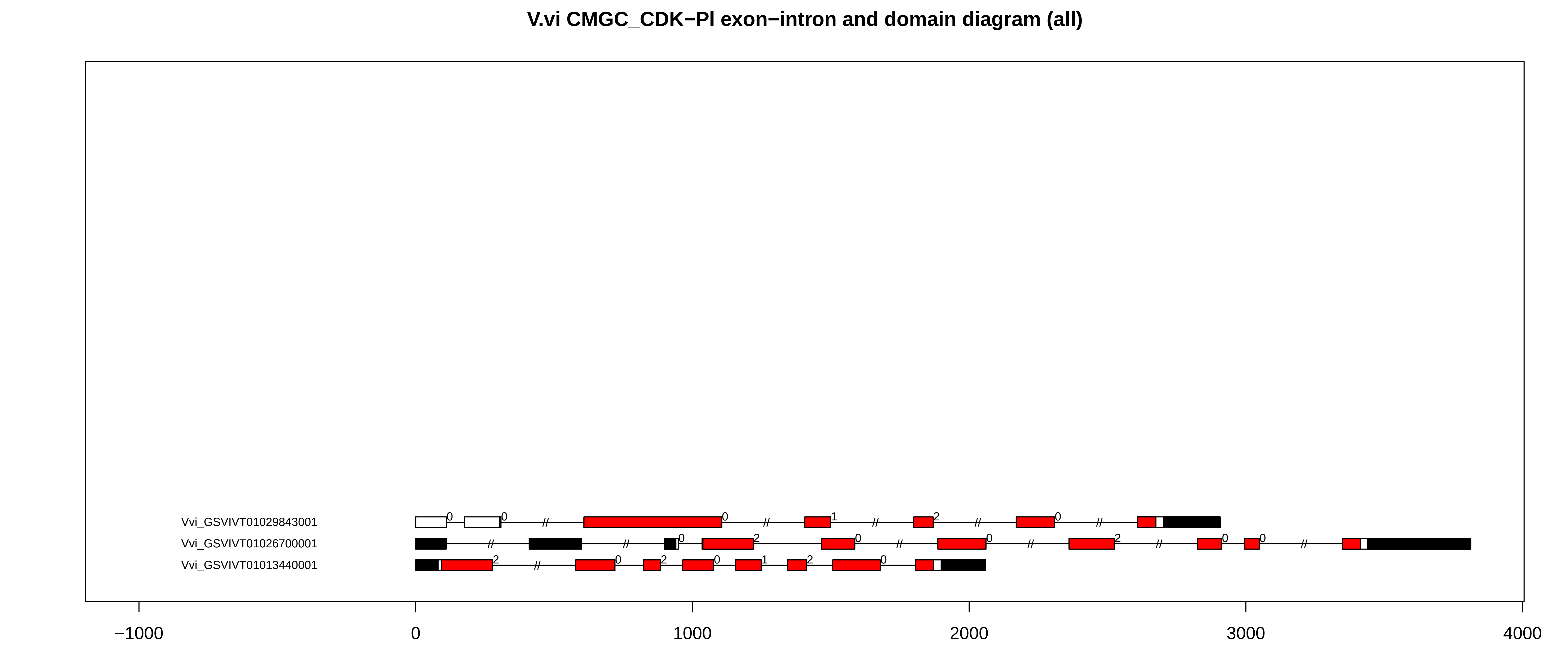
V.vi CMGC_CDK−PI exon−intron and domain diagram (all)
Vvi_GSVIVT01029843001
Vvi_GSVIVT01026700001
Vvi_GSVIVT01013440001
−1000
0
1000
2000
3000
4000

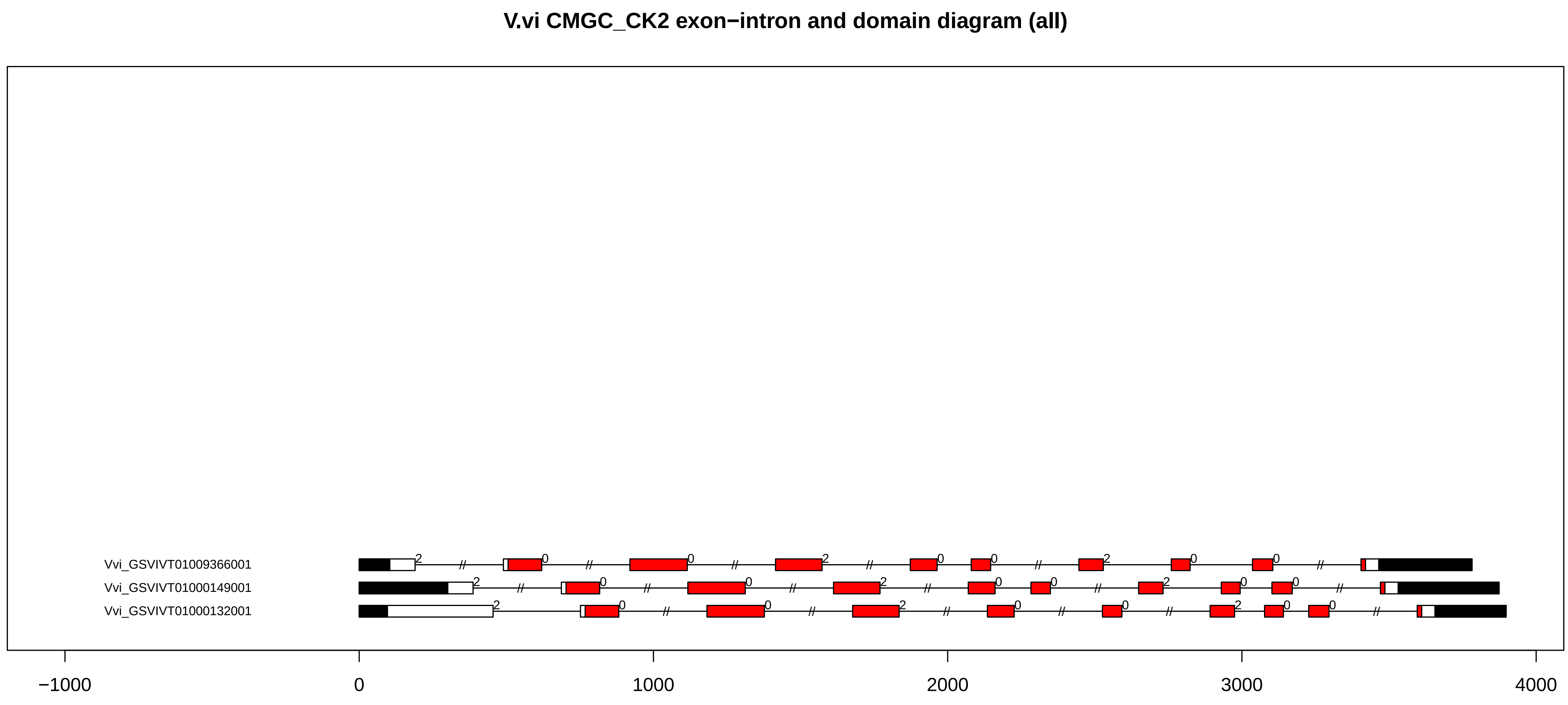
V.vi CMGC_CK2 exon−intron and domain diagram (all)
Vvi_GSVIVT01009366001
Vvi_GSVIVT01000149001
Vvi_GSVIVT01000132001
−1000
0
1000
2000
3000
4000

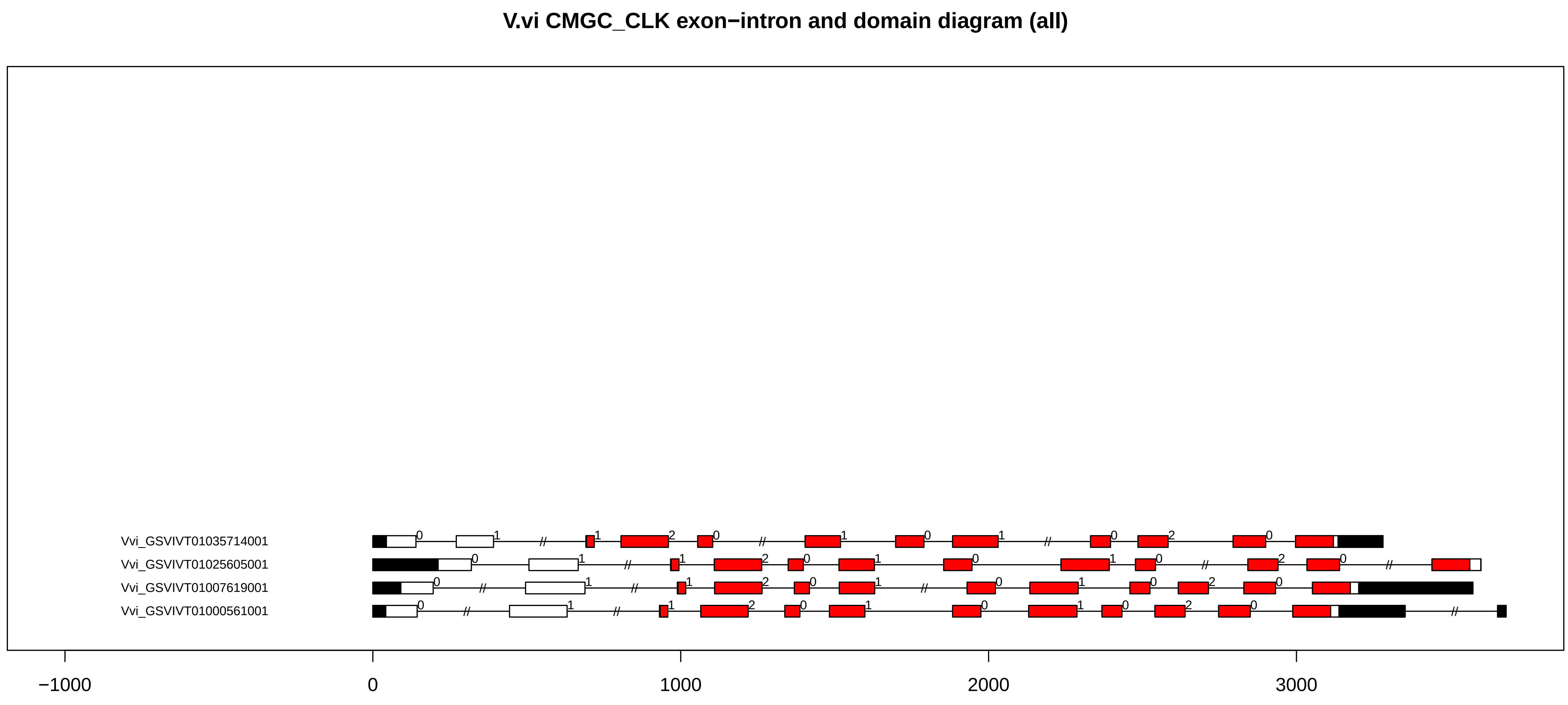
V.vi CMGC_CLK exon−intron and domain diagram (all)
Vvi_GSVIVT01035714001
Vvi_GSVIVT01025605001
Vvi_GSVIVT01007619001
Vvi_GSVIVT01000561001
−1000
0
1000
2000
3000

V.vi CMGC_DYRK−PRP4 exon−intron and domain diagram (all)

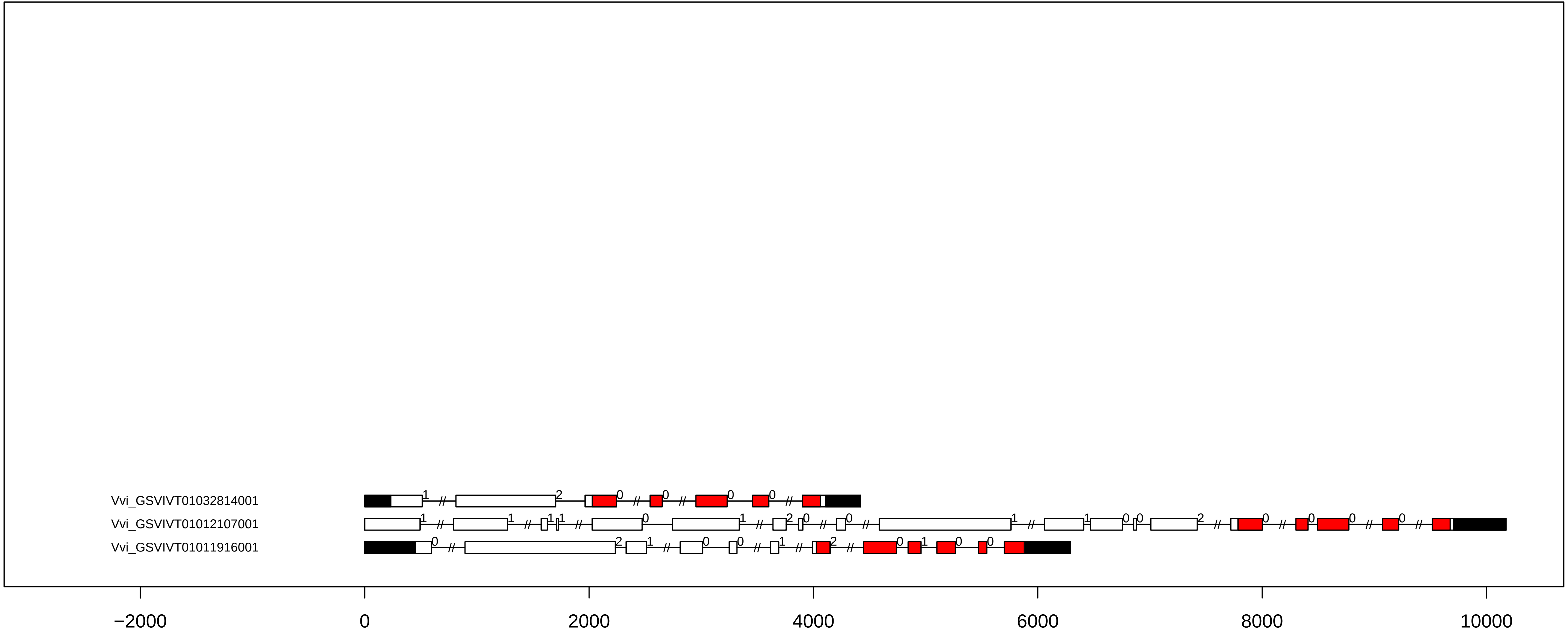

V.vi CMGC_DYRK−YAK exon−intron and domain diagram (all)

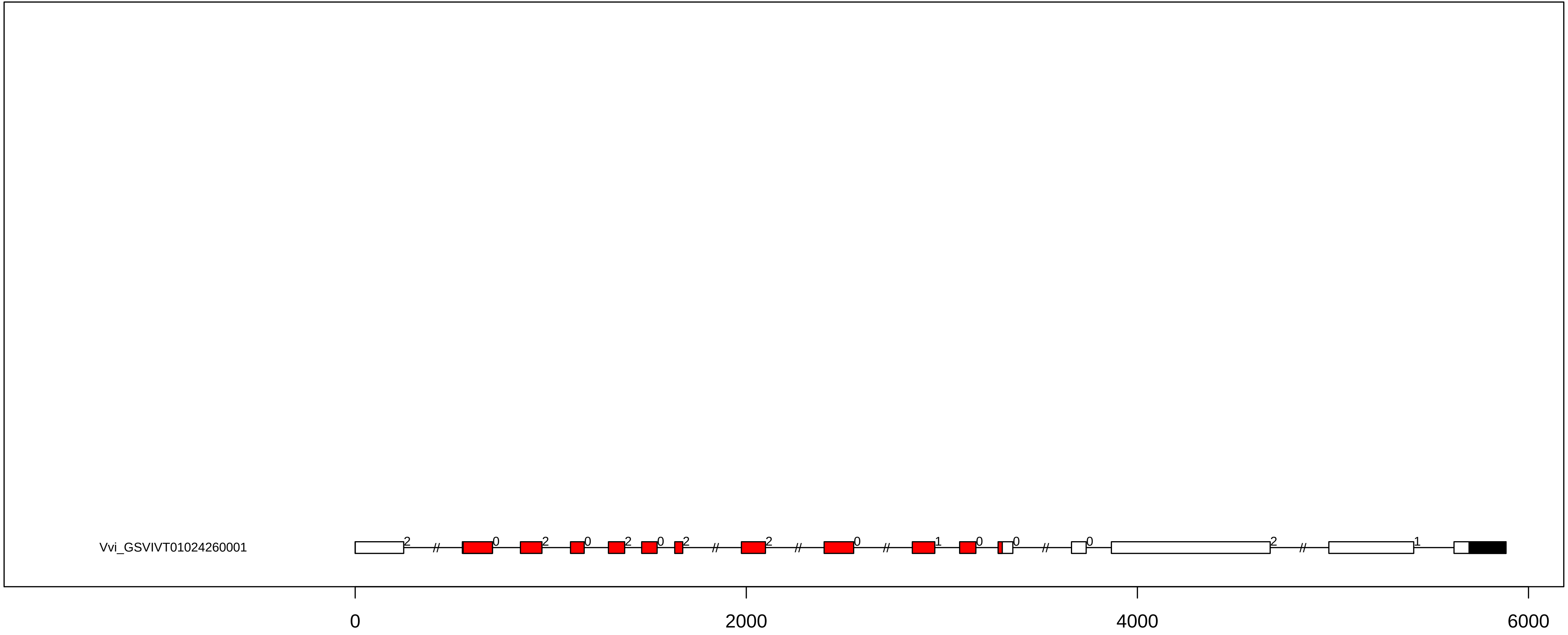

V.vi CMGC_GSK exon−intron and domain diagram (all)

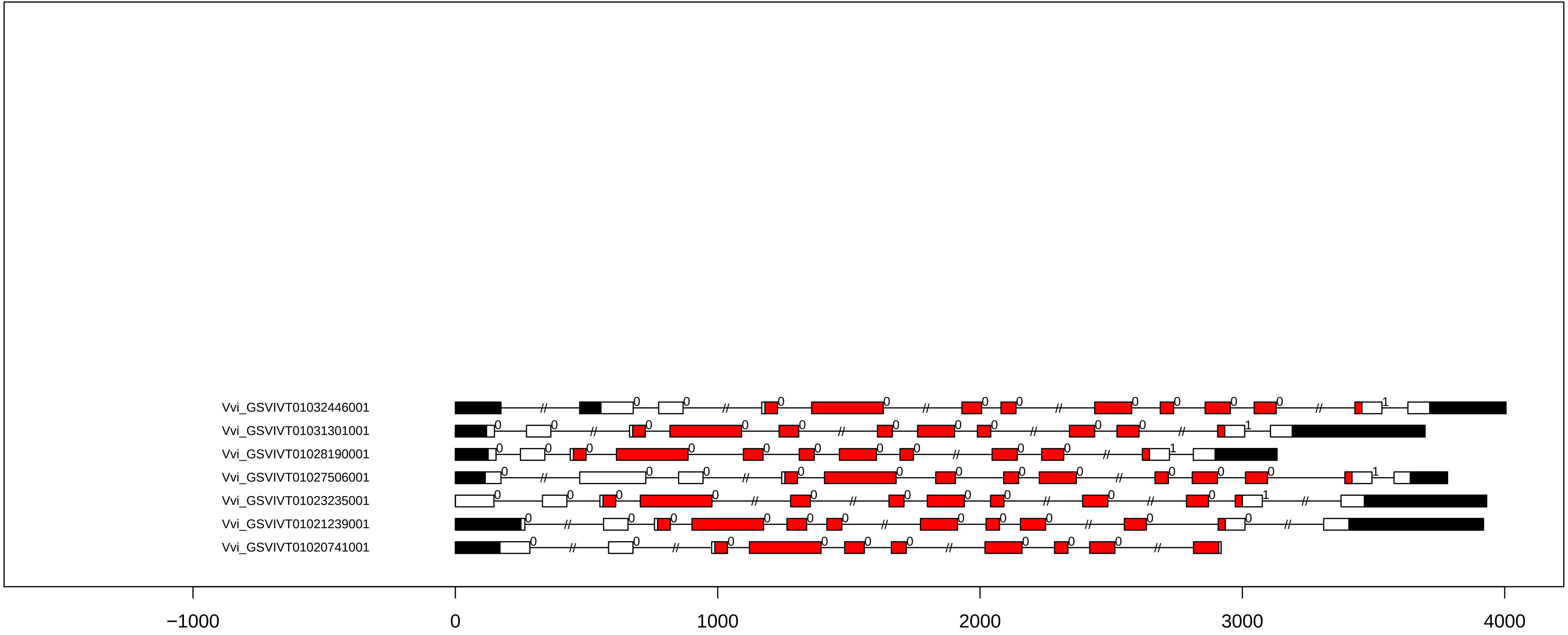

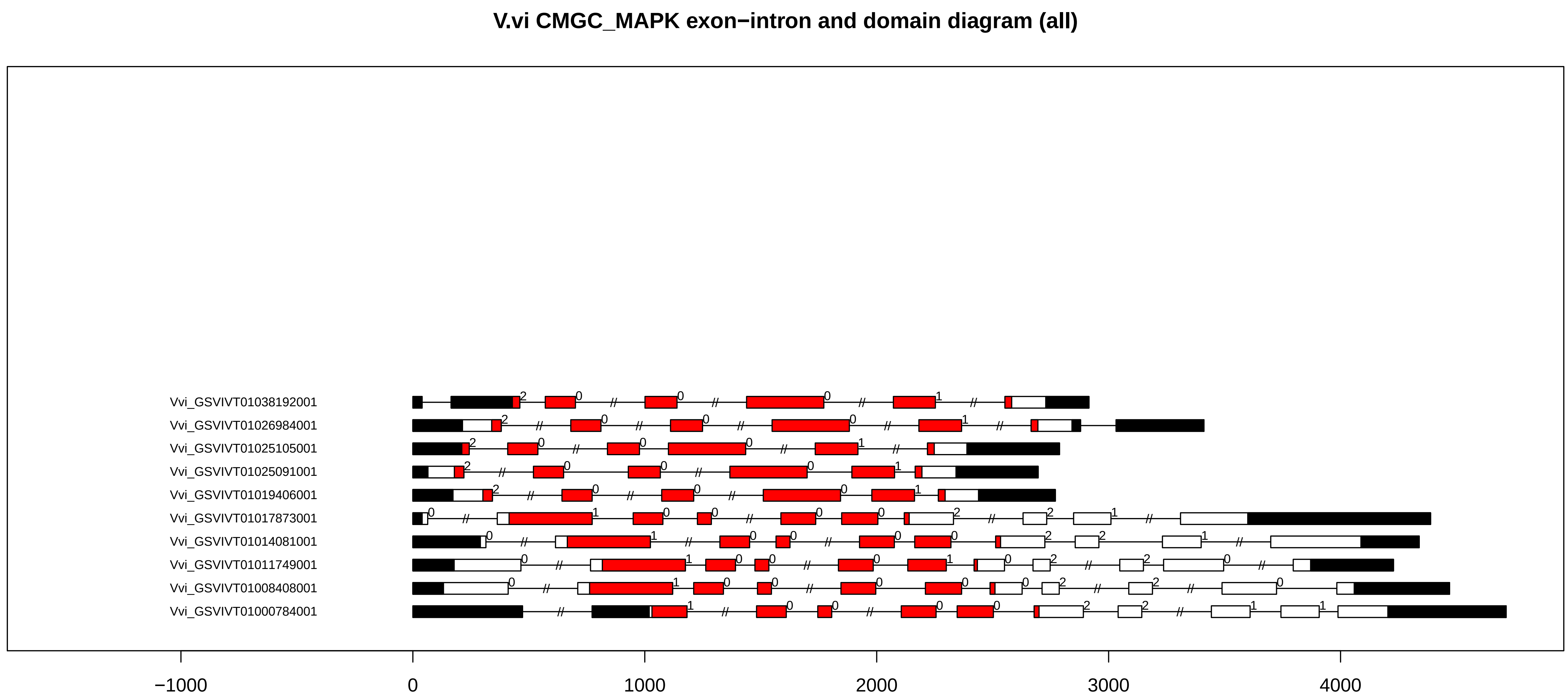

V.vi CMGC_MAPK exon−intron and domain diagram (all)
Vvi_GSVIVT01038192001
Vvi_GSVIVT01026984001
Vvi_GSVIVT01025105001
Vvi_GSVIVT01025091001
Vvi_GSVIVT01019406001
Vvi_GSVIVT01017873001
Vvi_GSVIVT01014081001
Vvi_GSVIVT01011749001
Vvi_GSVIVT01008408001
Vvi_GSVIVT01000784001
−1000
0
1000
2000
3000
4000

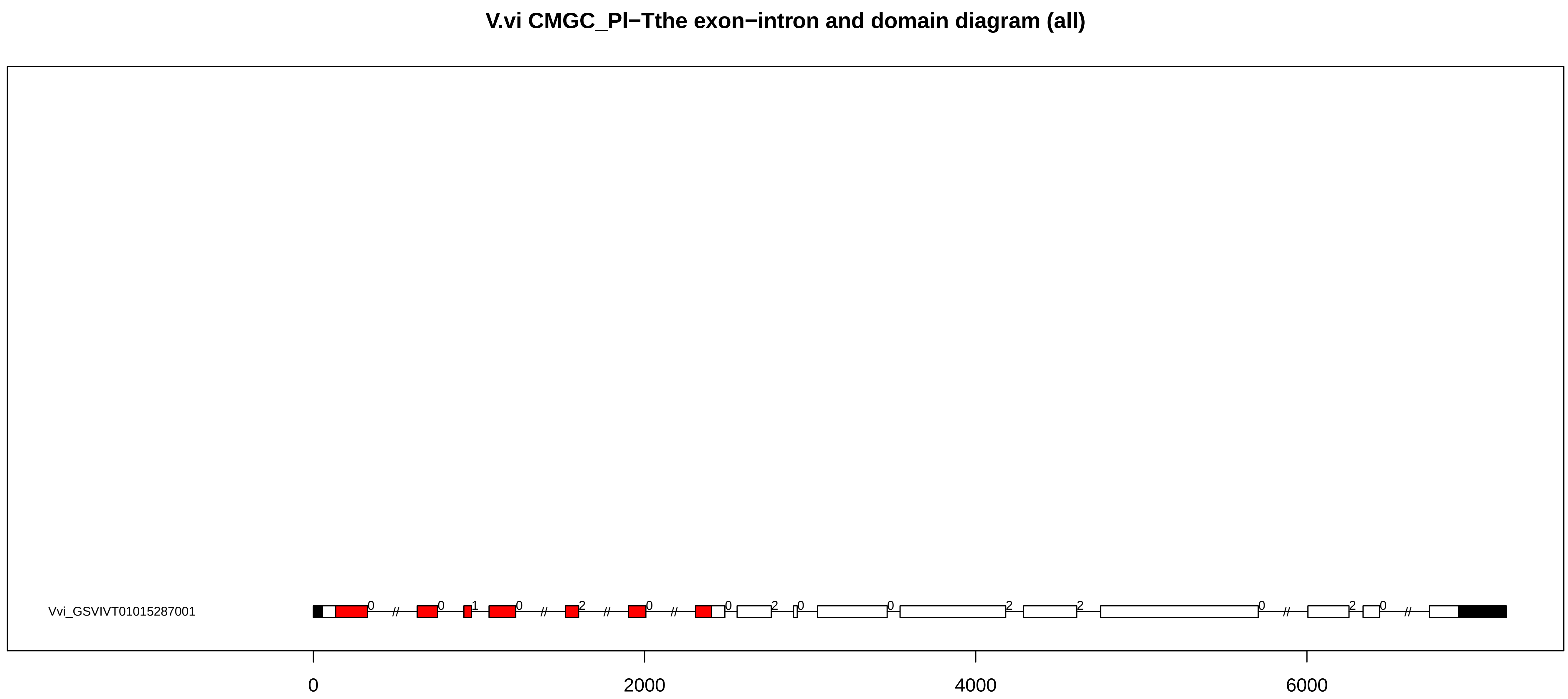
V.vi CMGC_Pl−Tthe exon−intron and domain diagram (all)
Vvi_GSVIVT01015287001
0
1
2
0
2
0
0
2
0
2
0
2
0
0
2000
4000
6000

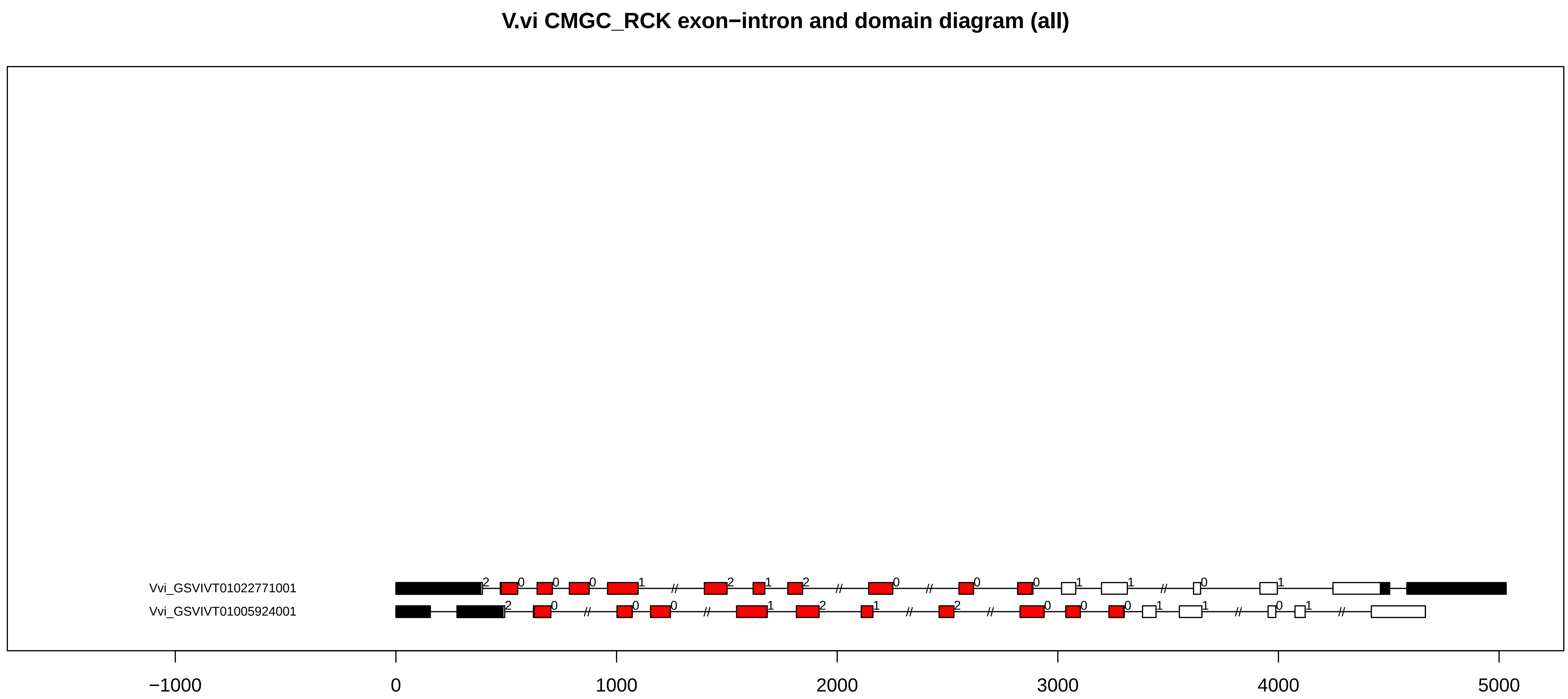
V.vi CMGC_RCK exon−intron and domain diagram (all)
Vvi_GSVIVT01022771001
Vvi_GSVIVT01005924001
−1000
0
1000
2000
3000
4000
5000

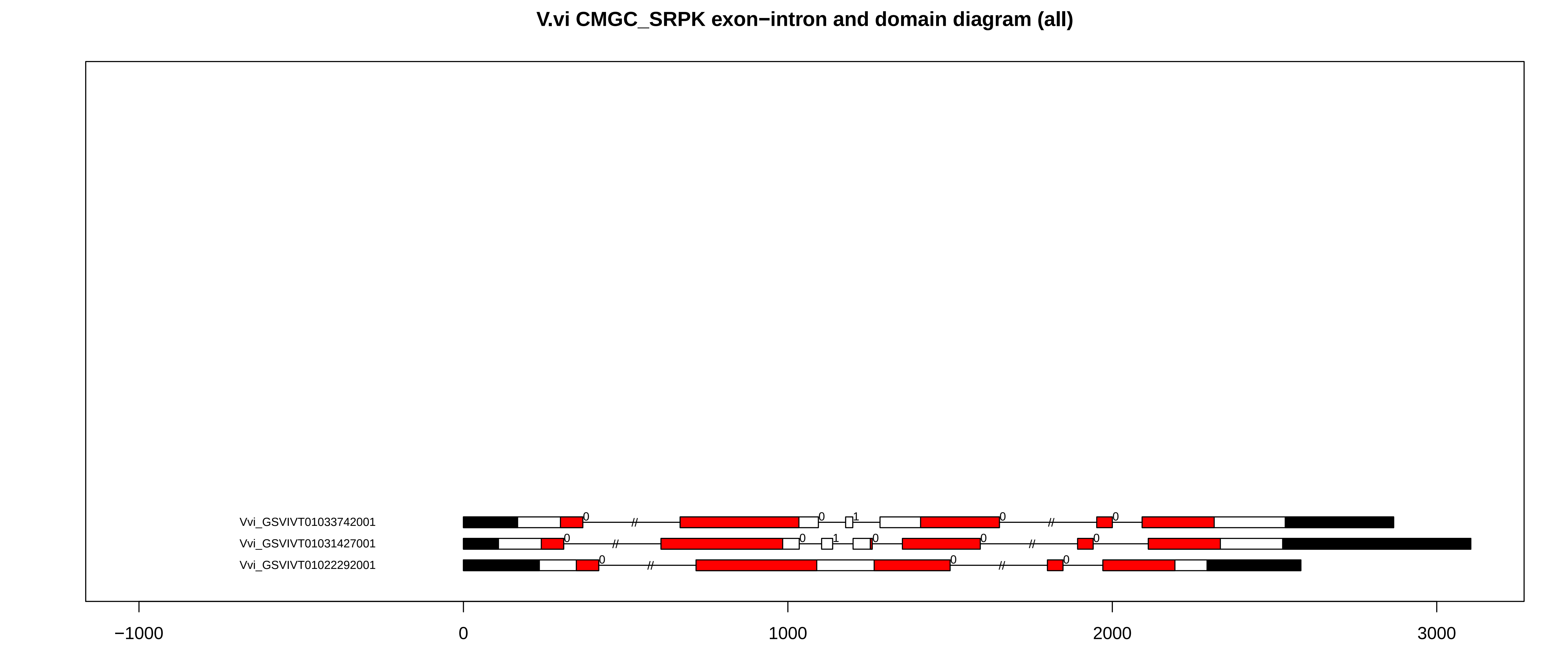
V.vi CMGC_SRPK exon−intron and domain diagram (all)
Vvi_GSVIVT01033742001
Vvi_GSVIVT01031427001
Vvi_GSVIVT01022292001
−1000
0
1000
2000
3000

V.vi Group−PI−2 exon−intron and domain diagram (all)

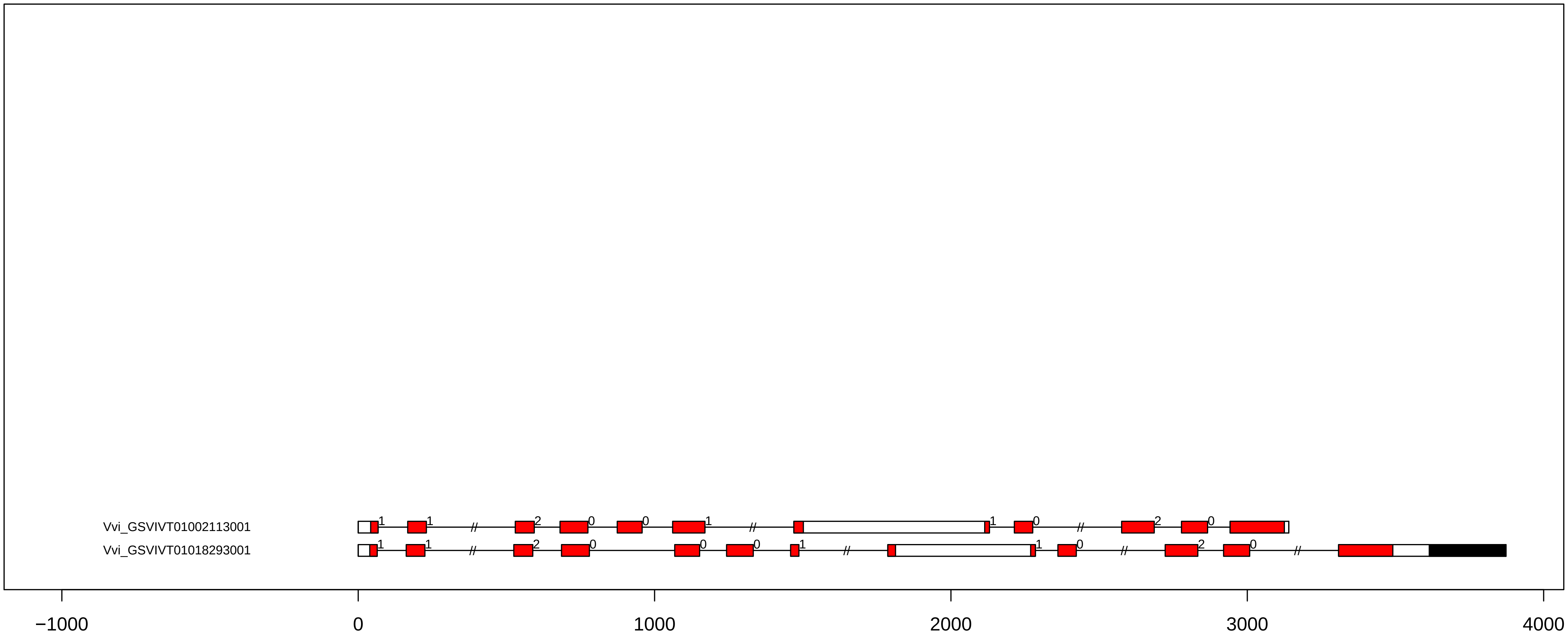

V.vi Group−PI−3 exon−intron and domain diagram (all)

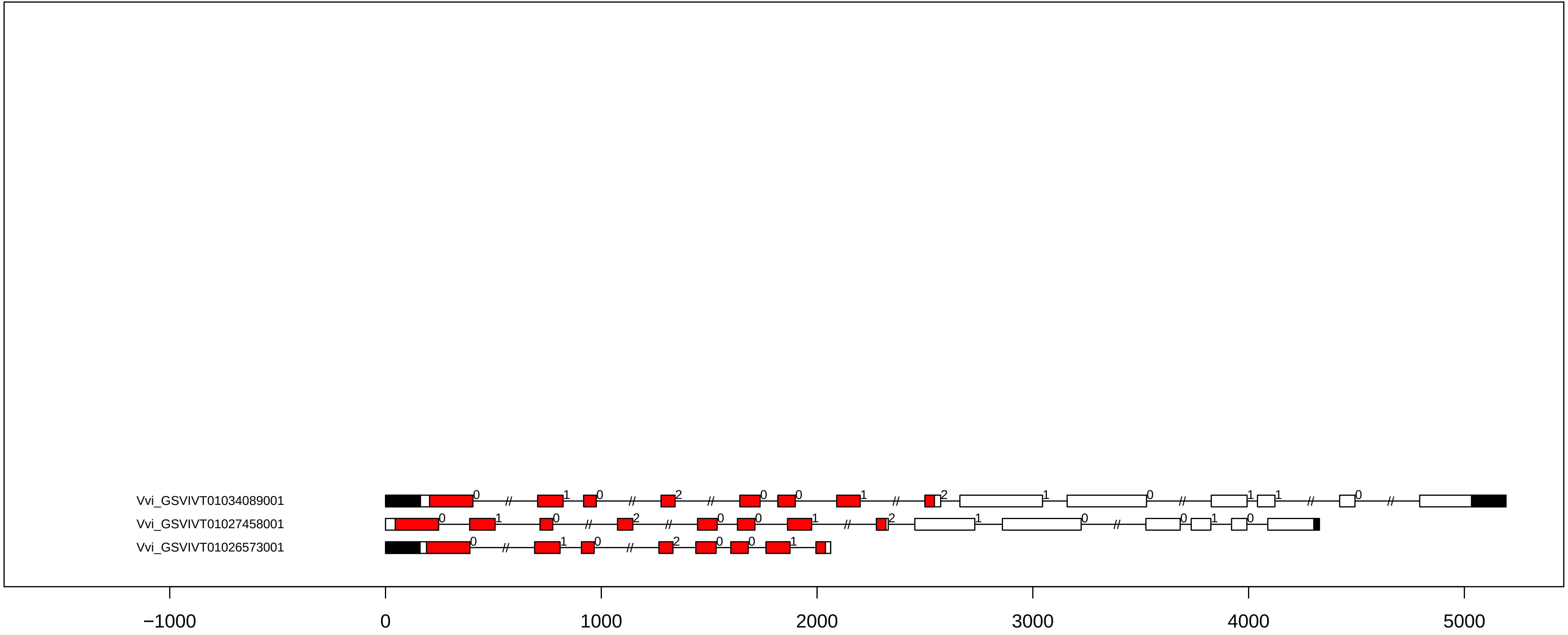

V.vi Group−PI−4 exon−intron and domain diagram (all)

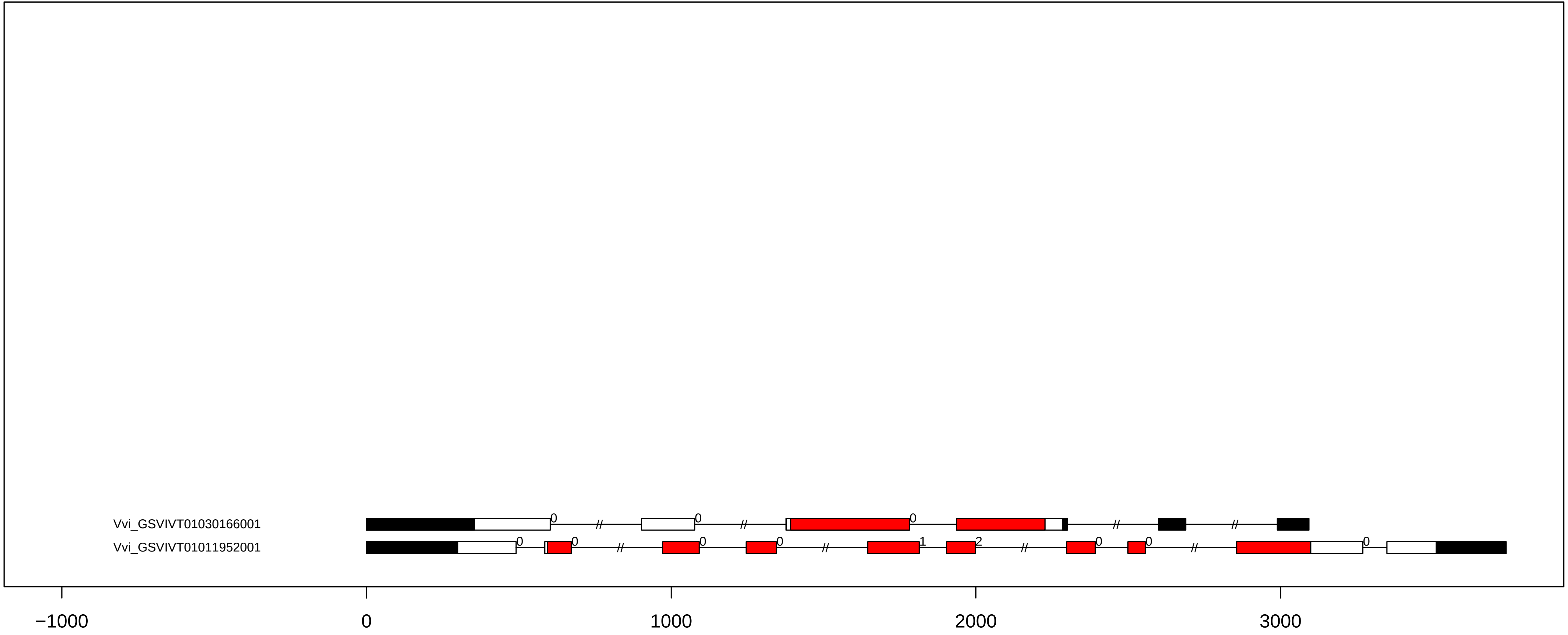

V.vi IRE1 exon−intron and domain diagram (all)

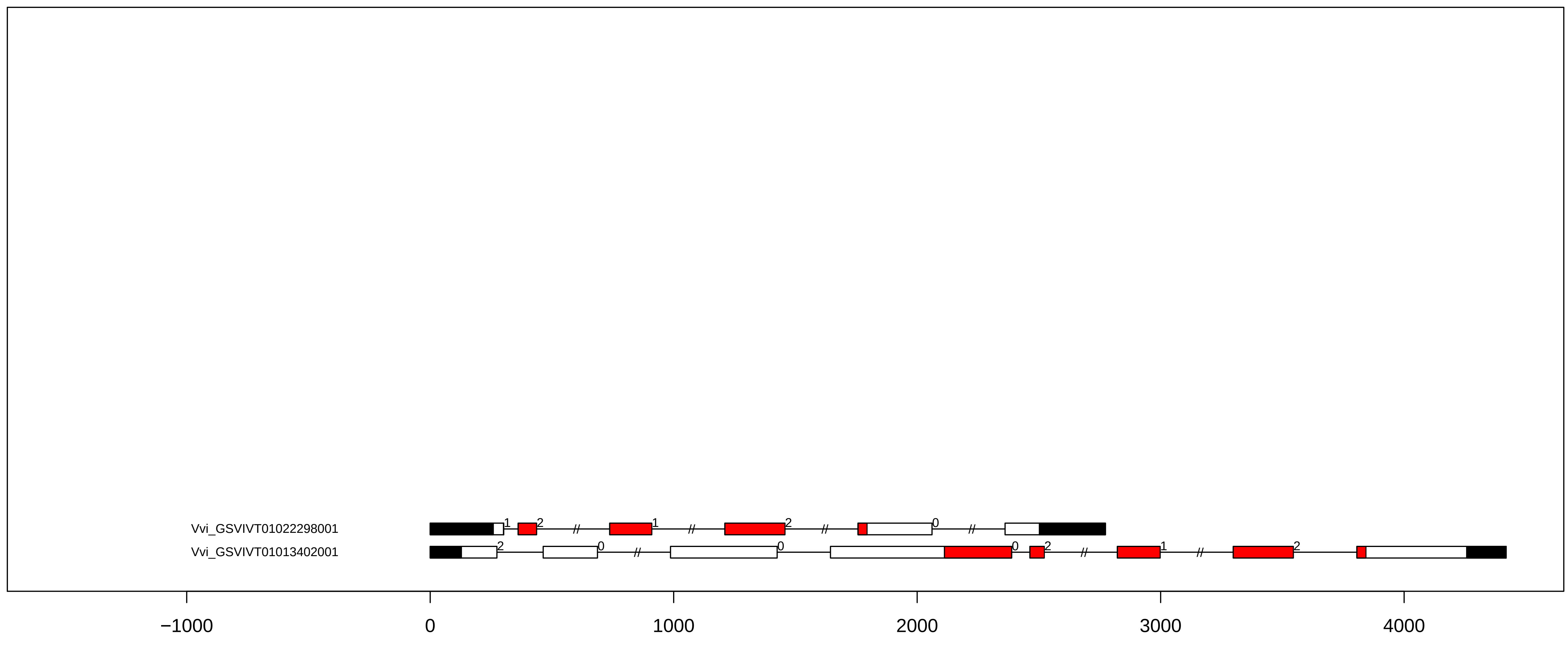

V.vi NAK exon−intron and domain diagram (all)

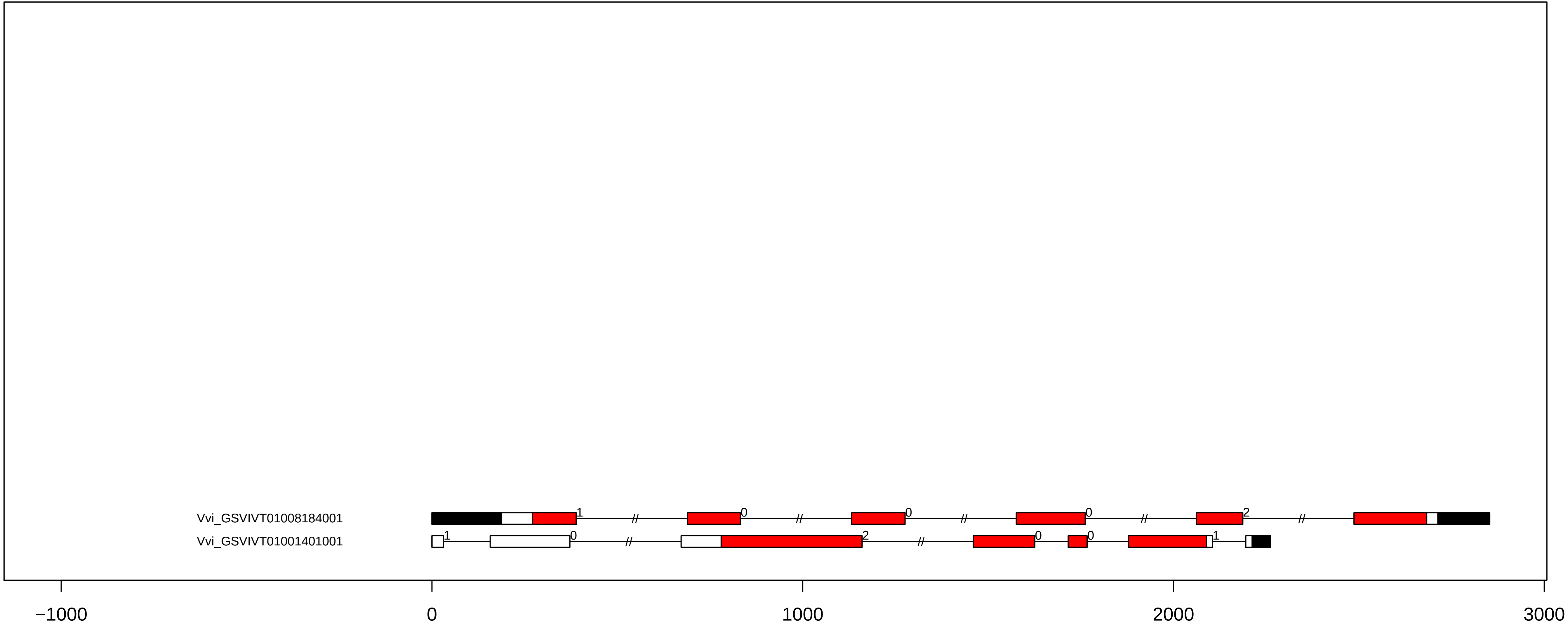

V.vi NEK exon−intron and domain diagram (all)

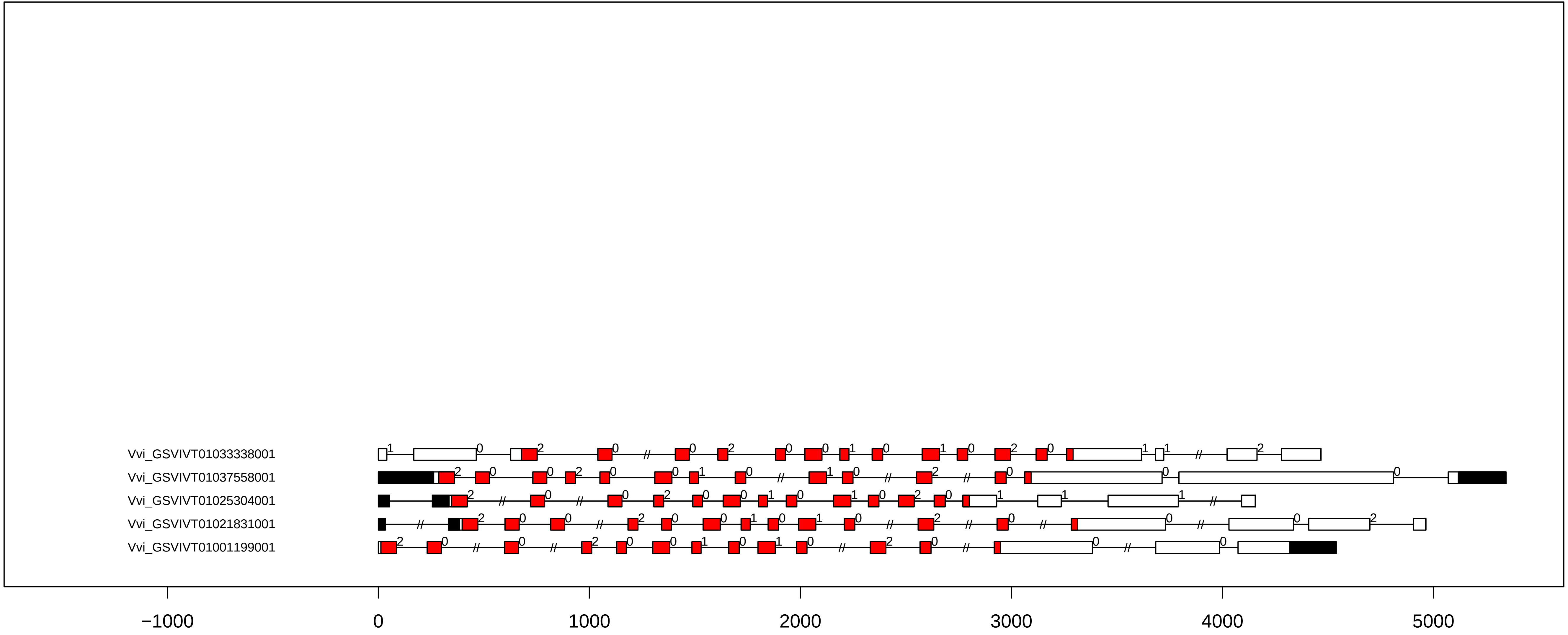

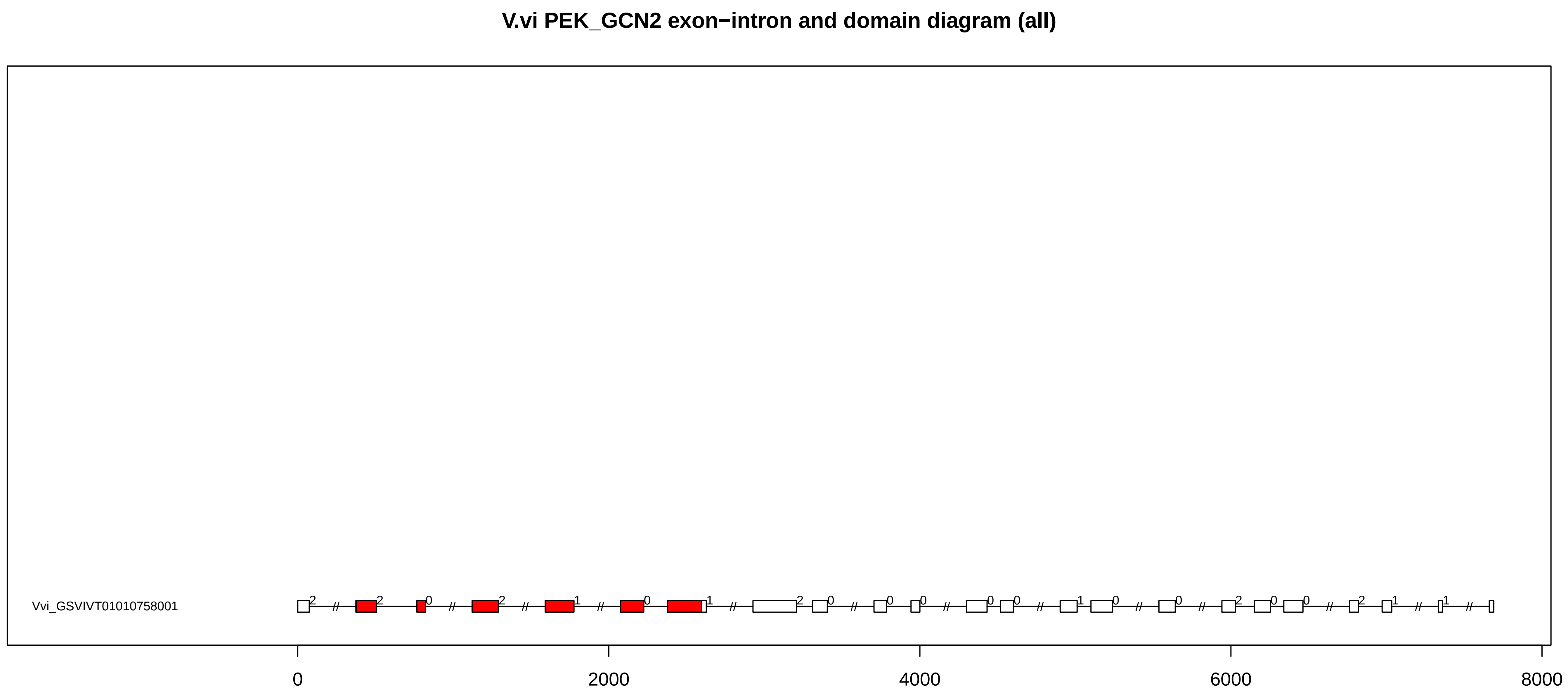
V.vi PEK_GCN2 exon−intron and domain diagram (all)
Vvi_GSVIVT01010758001
2
2
0
2
1
0
1
2
0
0
0
0
0
1
0
0
2
0
0
2
1
1
0
2000
4000
6000
8000

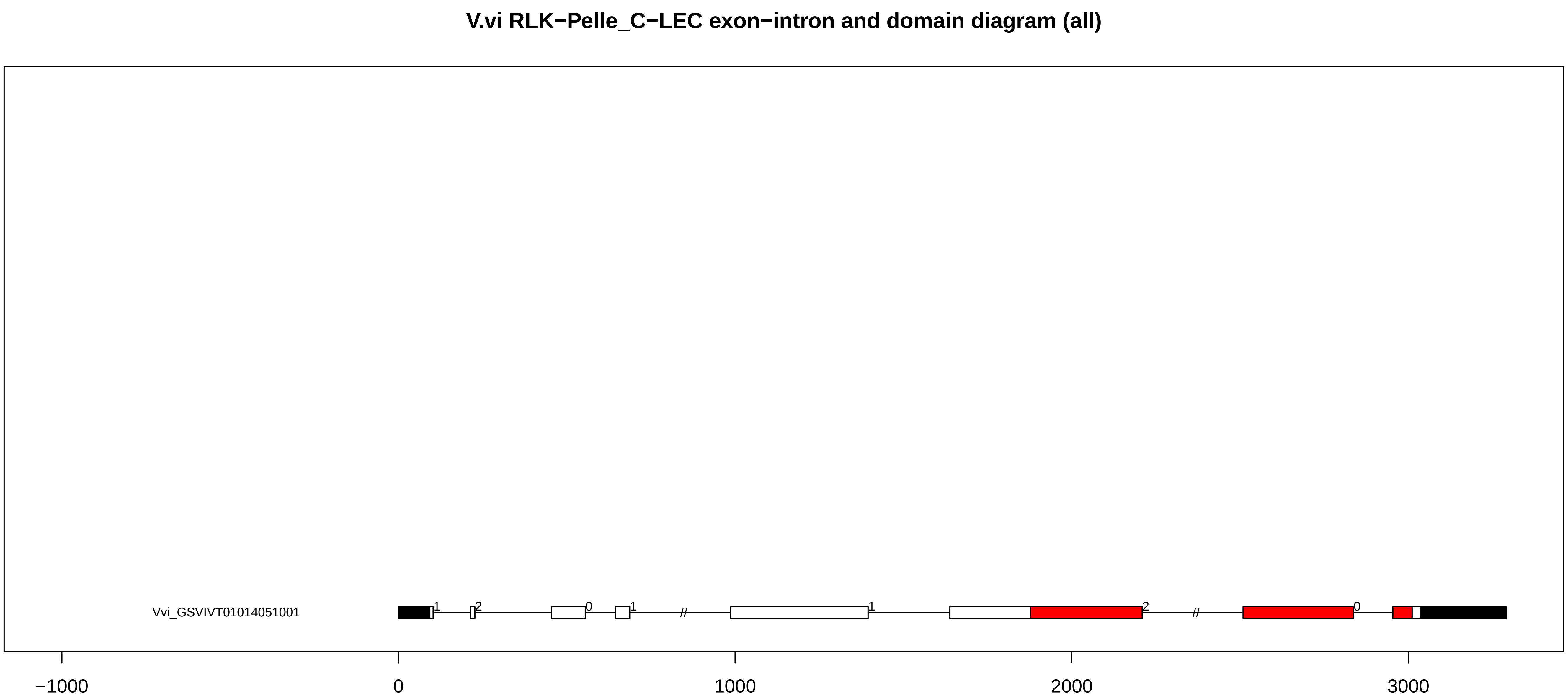
V.vi RLK−Pelle_C−LEC exon−intron and domain diagram (all)
Vvi_GSVIVT01014051001
1
2
0
1
1
2
0
−1000
0
1000
2000
3000

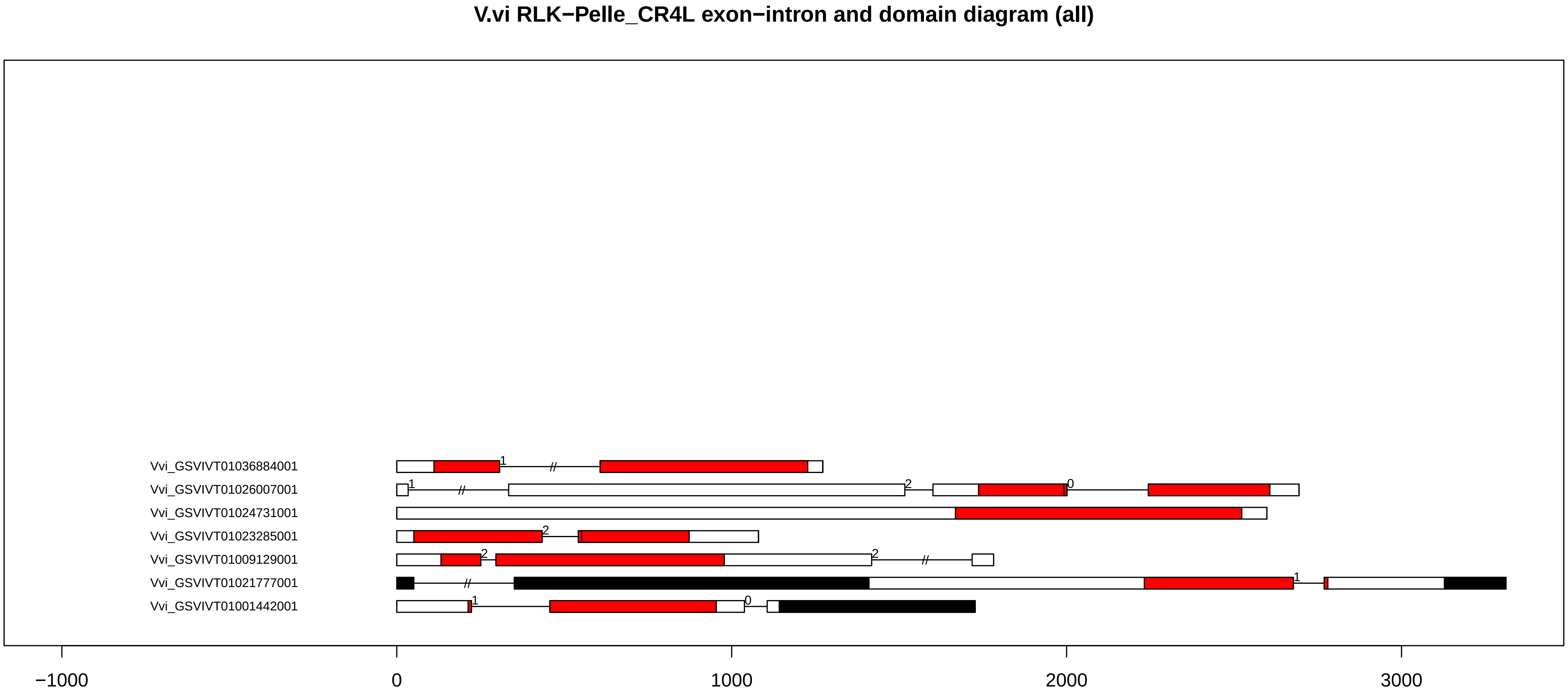

V.vi RLK−Pelle_CR4L exon−intron and domain diagram (all)
Vvi_GSVIVT01036884001
Vvi_GSVIVT01026007001
Vvi_GSVIVT01024731001
Vvi_GSVIVT01023285001
Vvi_GSVIVT01009129001
Vvi_GSVIVT01021777001
Vvi_GSVIVT01001442001
−1000
0
1000
2000
3000

V.vi RLK−Pelle_CrRLK1L−1 exon−intron and domain diagram (all)

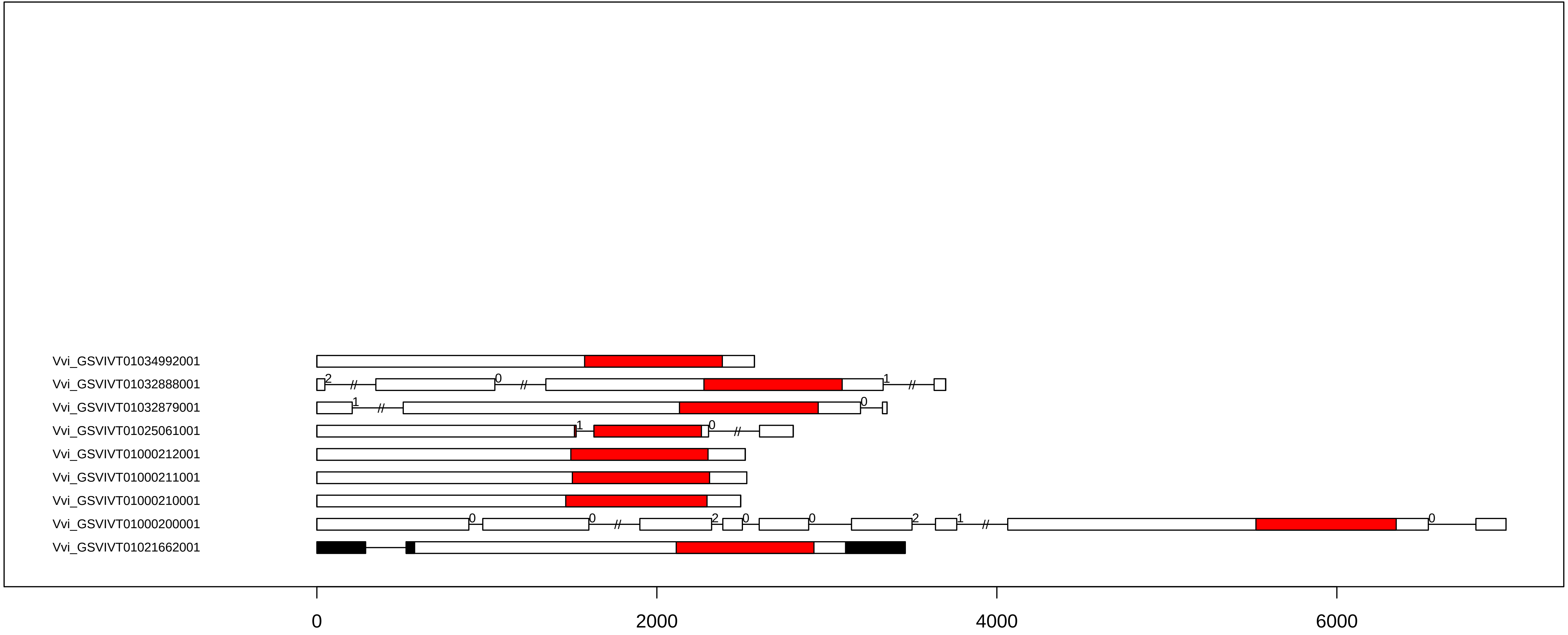

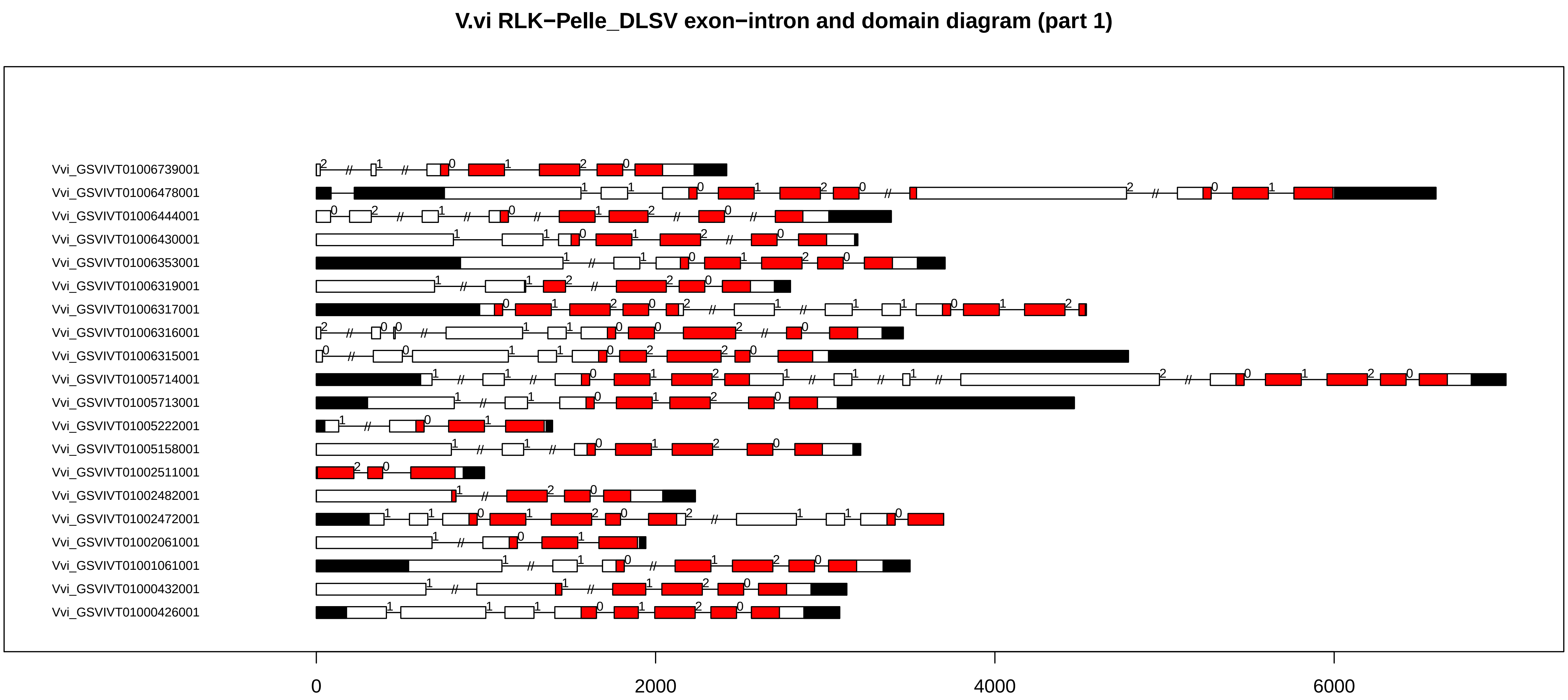

V.vi RLK−Pelle_DLSV exon−intron and domain diagram (part 1)
Vvi_GSVIVT01006739001
Vvi_GSVIVT01006478001
Vvi_GSVIVT01006444001
Vvi_GSVIVT01006430001
Vvi_GSVIVT01006353001
Vvi_GSVIVT01006319001
Vvi_GSVIVT01006317001
Vvi_GSVIVT01006316001
Vvi_GSVIVT01006315001
Vvi_GSVIVT01005714001
Vvi_GSVIVT01005713001
Vvi_GSVIVT01005222001
Vvi_GSVIVT01005158001
Vvi_GSVIVT01002511001
Vvi_GSVIVT01002482001
Vvi_GSVIVT01002472001
Vvi_GSVIVT01002061001
Vvi_GSVIVT01001061001
Vvi_GSVIVT01000432001
Vvi_GSVIVT01000426001
0
2000
4000
6000

V.vi RLK−Pelle_DLSV exon−intron and domain diagram (part 2)

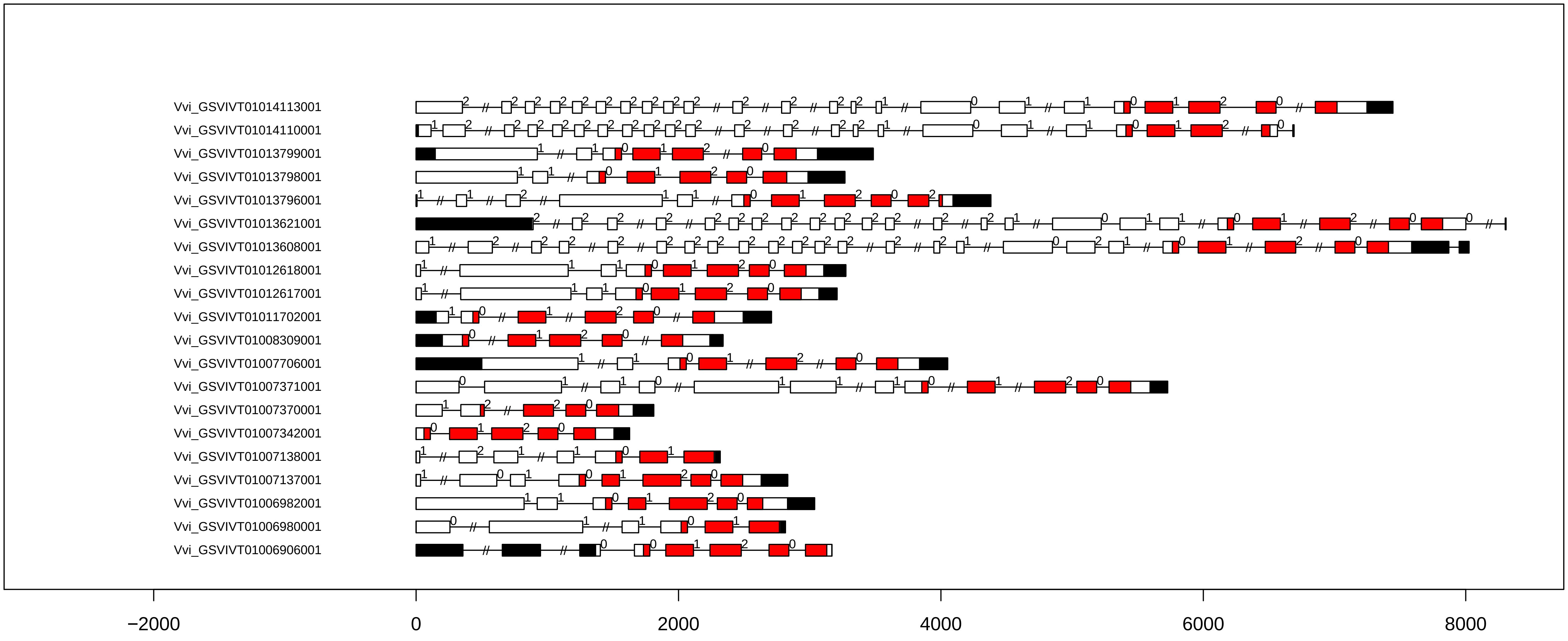

V.vi RLK−Pelle_DLSV exon−intron and domain diagram (part 3)

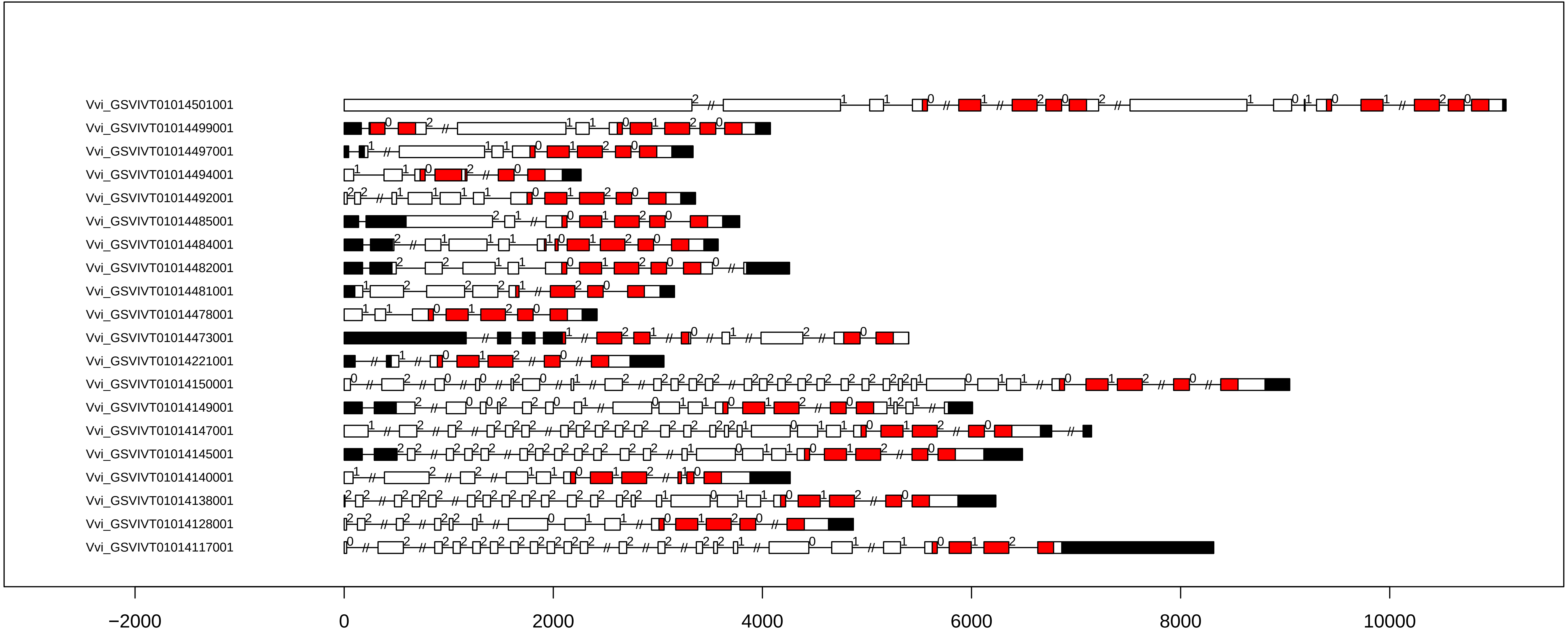

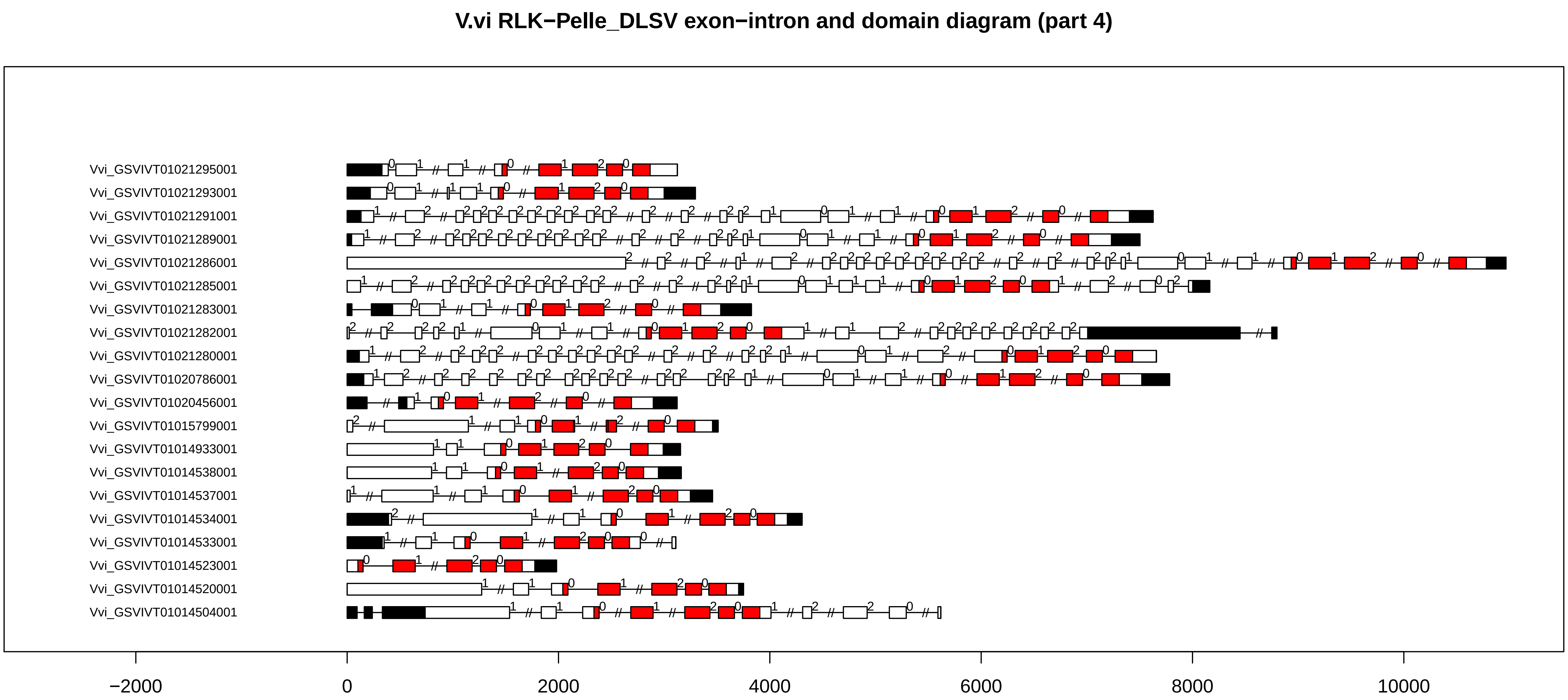
V.vi RLK−Pelle_DLSV exon−intron and domain diagram (part 4)
Vvi_GSVIVT01021295001
Vvi_GSVIVT01021293001
Vvi_GSVIVT01021291001
Vvi_GSVIVT01021289001
Vvi_GSVIVT01021286001
Vvi_GSVIVT01021285001
Vvi_GSVIVT01021283001
Vvi_GSVIVT01021282001
Vvi_GSVIVT01021280001
Vvi_GSVIVT01020786001
Vvi_GSVIVT01020456001
Vvi_GSVIVT01015799001
Vvi_GSVIVT01014933001
Vvi_GSVIVT01014538001
Vvi_GSVIVT01014537001
Vvi_GSVIVT01014534001
Vvi_GSVIVT01014533001
Vvi_GSVIVT01014523001
Vvi_GSVIVT01014520001
Vvi_GSVIVT01014504001
−2000
0
2000
4000
6000
8000
10000

V.vi RLK−Pelle_DLSV exon−intron and domain diagram (part 5)

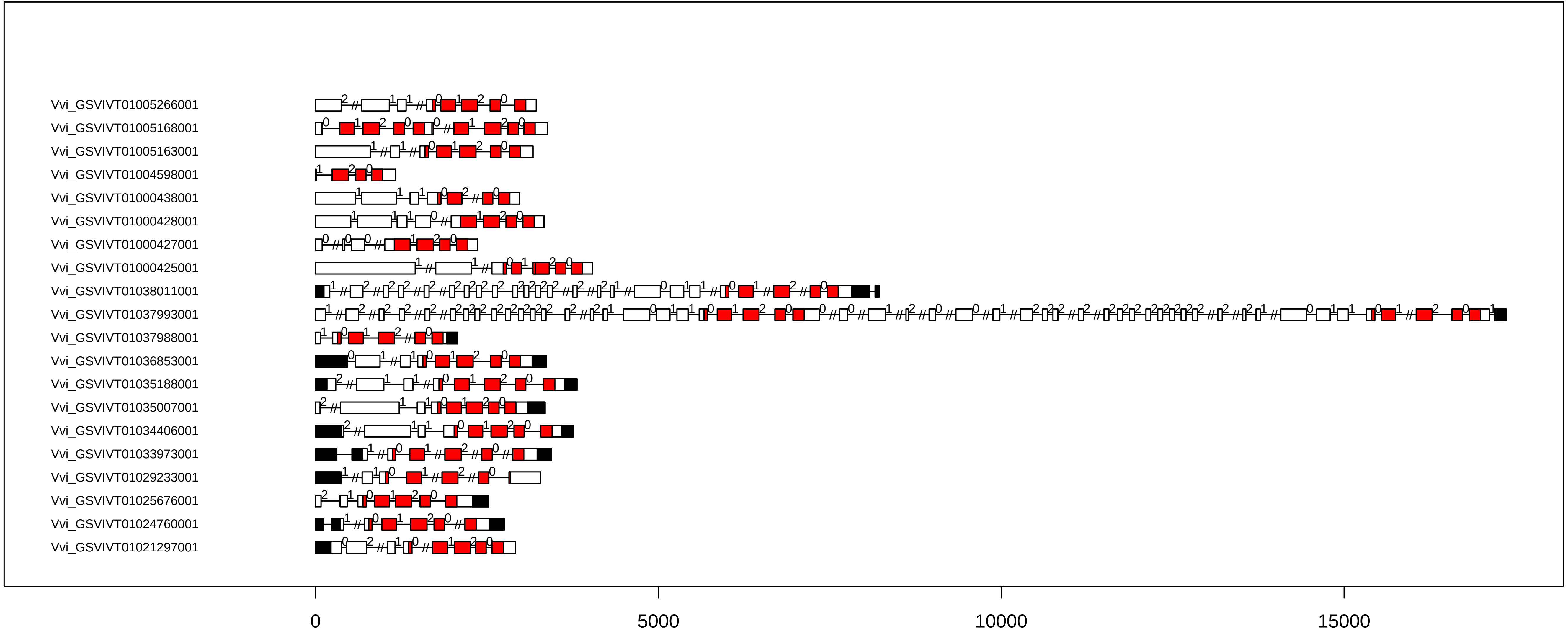

V.vi RLK−Pelle_DLSV exon−intron and domain diagram (part 6)

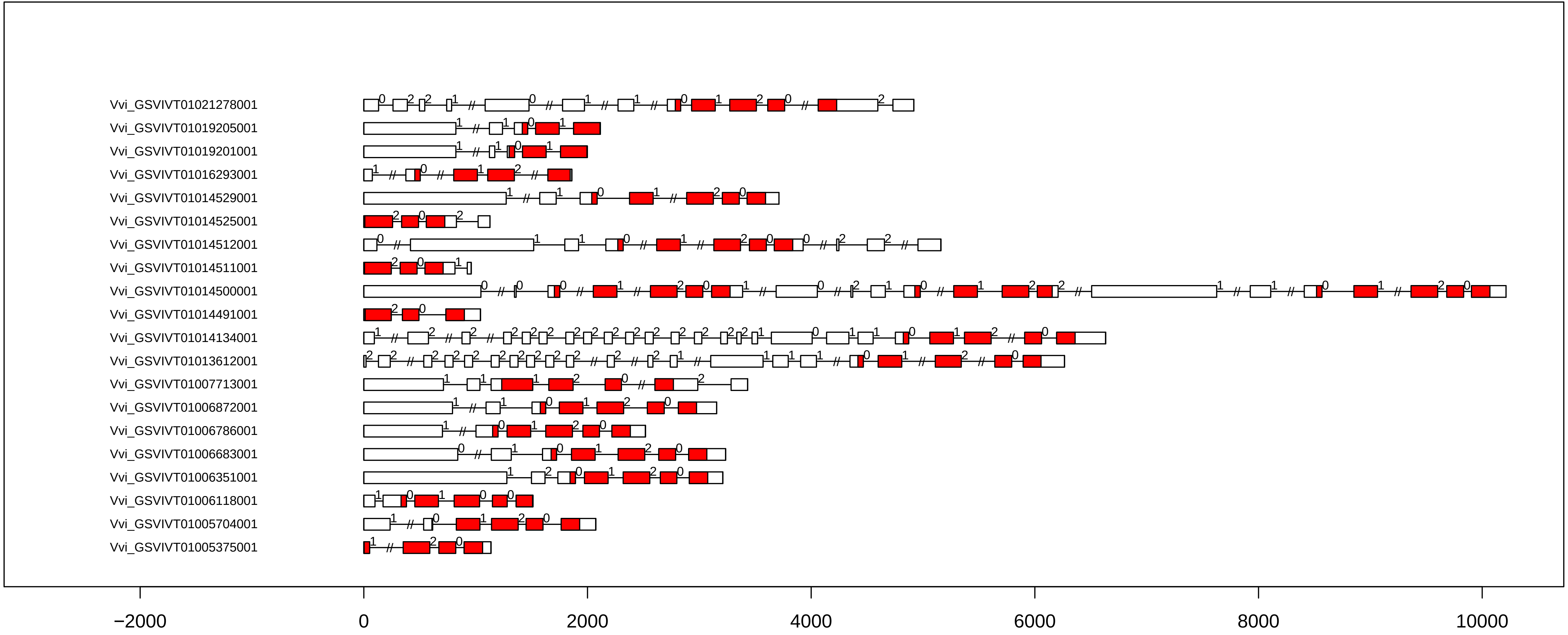

V.vi RLK−Pelle_DLSV exon−intron and domain diagram (part 7)

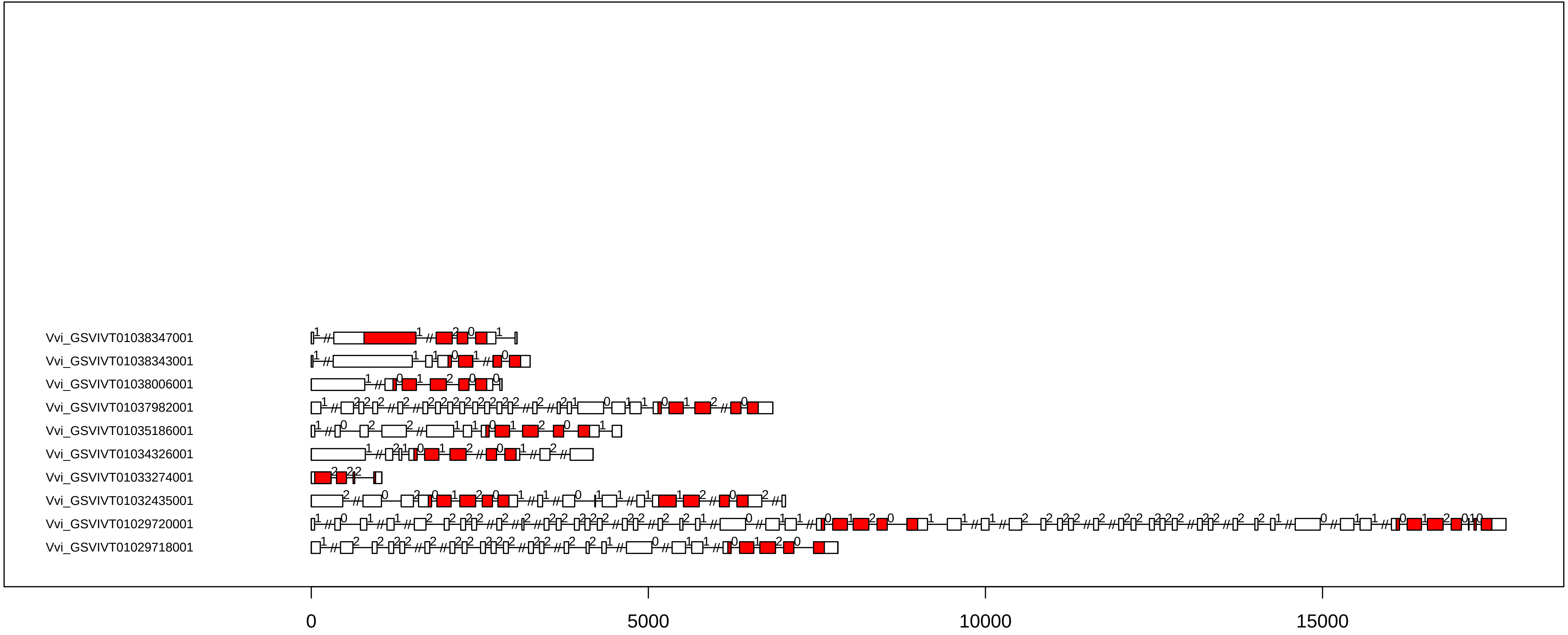

V.vi RLK−Pelle_Extensin exon−intron and domain diagram (all)

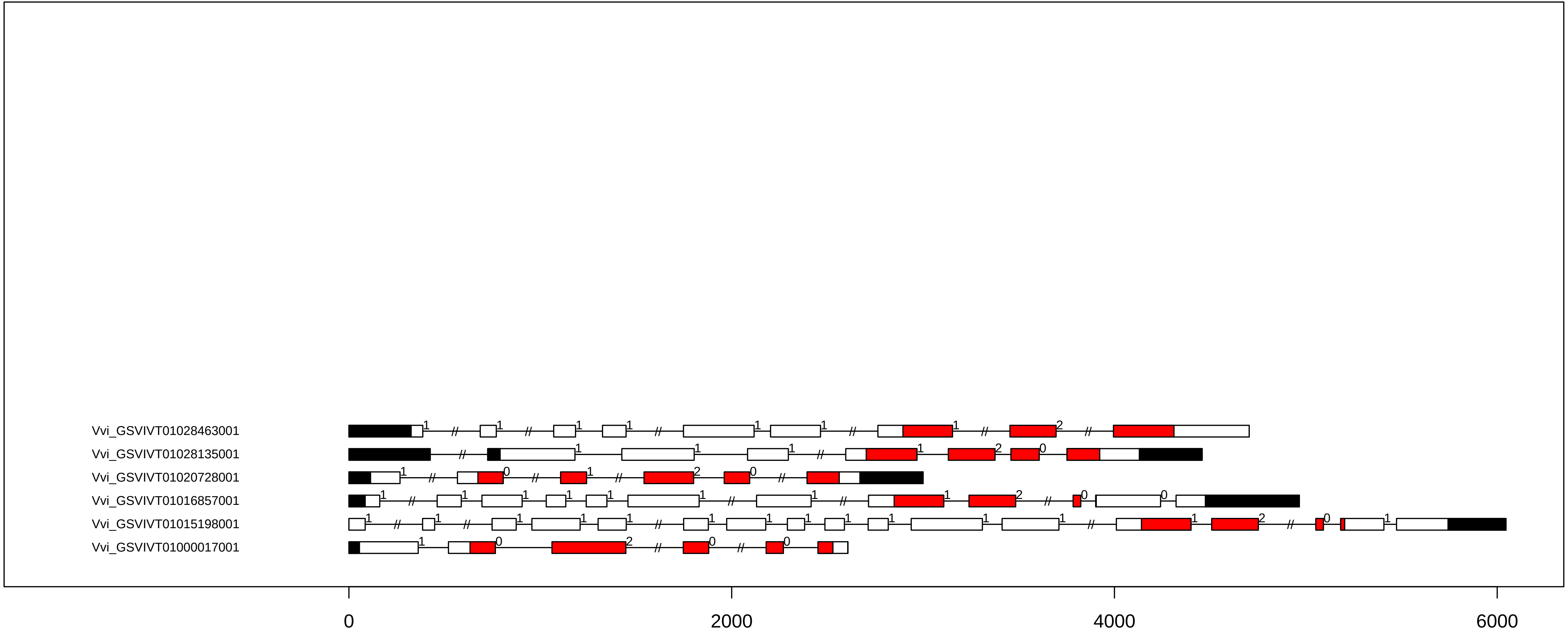

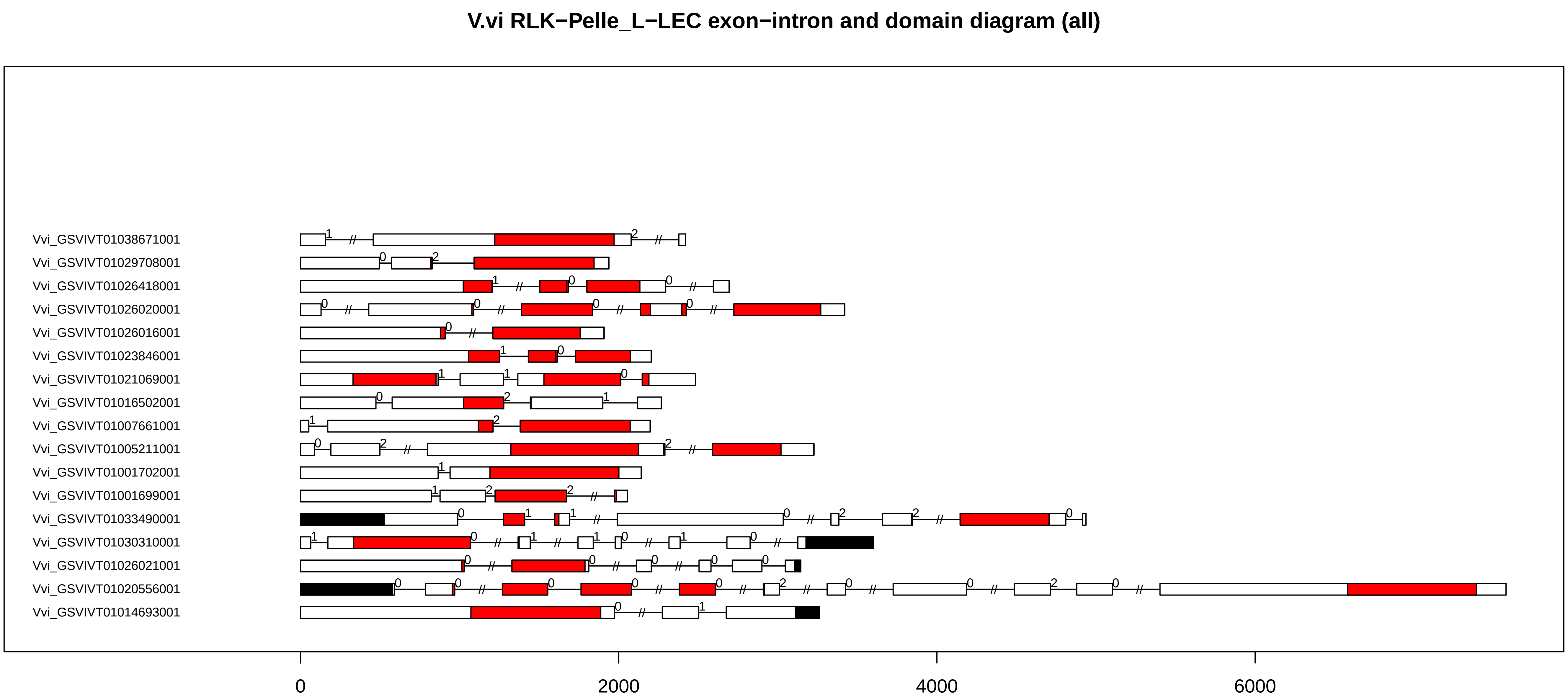
V.vi RLK−Pelle_L−LEC exon−intron and domain diagram (all)
Vvi_GSVIVT01038671001
Vvi_GSVIVT01029708001
Vvi_GSVIVT01026418001
Vvi_GSVIVT01026020001
Vvi_GSVIVT01026016001
Vvi_GSVIVT01023846001
Vvi_GSVIVT01021069001
Vvi_GSVIVT01016502001
Vvi_GSVIVT01007661001
Vvi_GSVIVT01005211001
Vvi_GSVIVT01001702001
Vvi_GSVIVT01001699001
Vvi_GSVIVT01033490001
Vvi_GSVIVT01030310001
Vvi_GSVIVT01026021001
Vvi_GSVIVT01020556001
Vvi_GSVIVT01014693001
0
2000
4000
6000

V.vi RLK−Pelle_LRK10L−2 exon−intron and domain diagram (all)

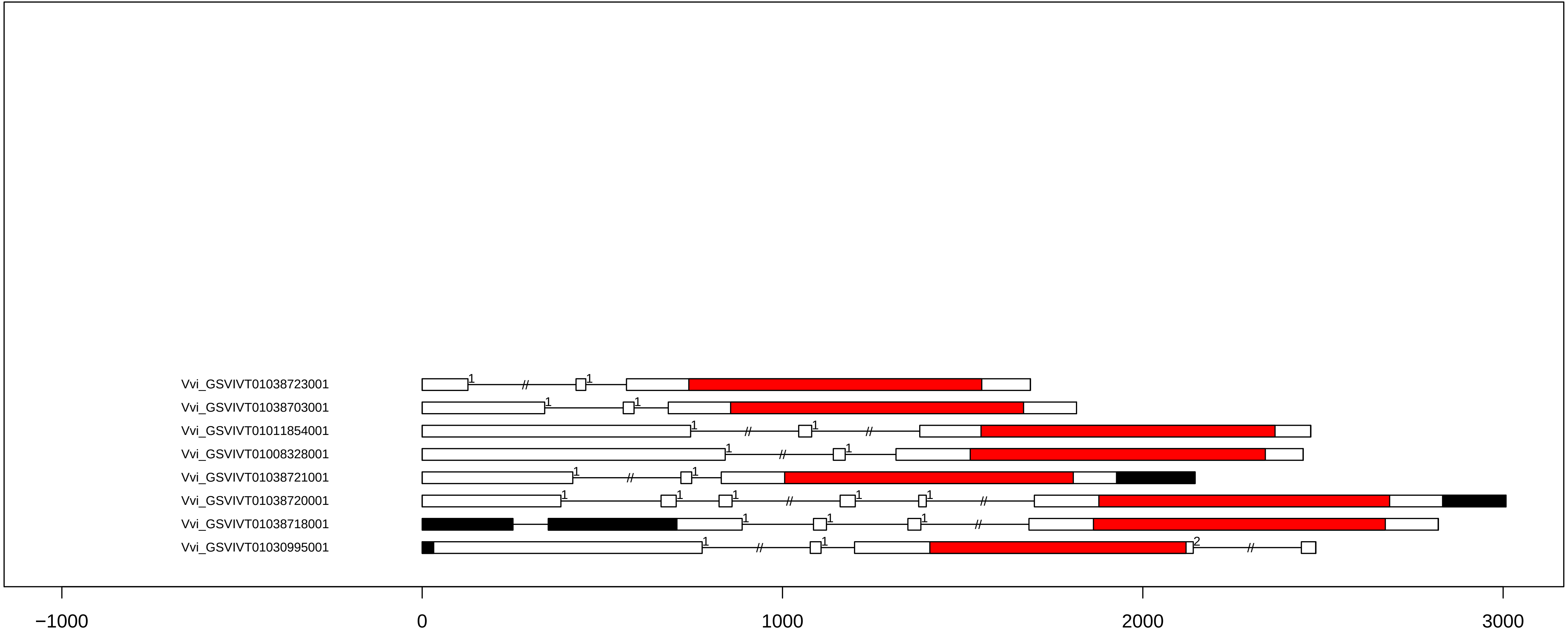

V.vi RLK−Pelle_LRR−I−1 exon−intron and domain diagram (all)

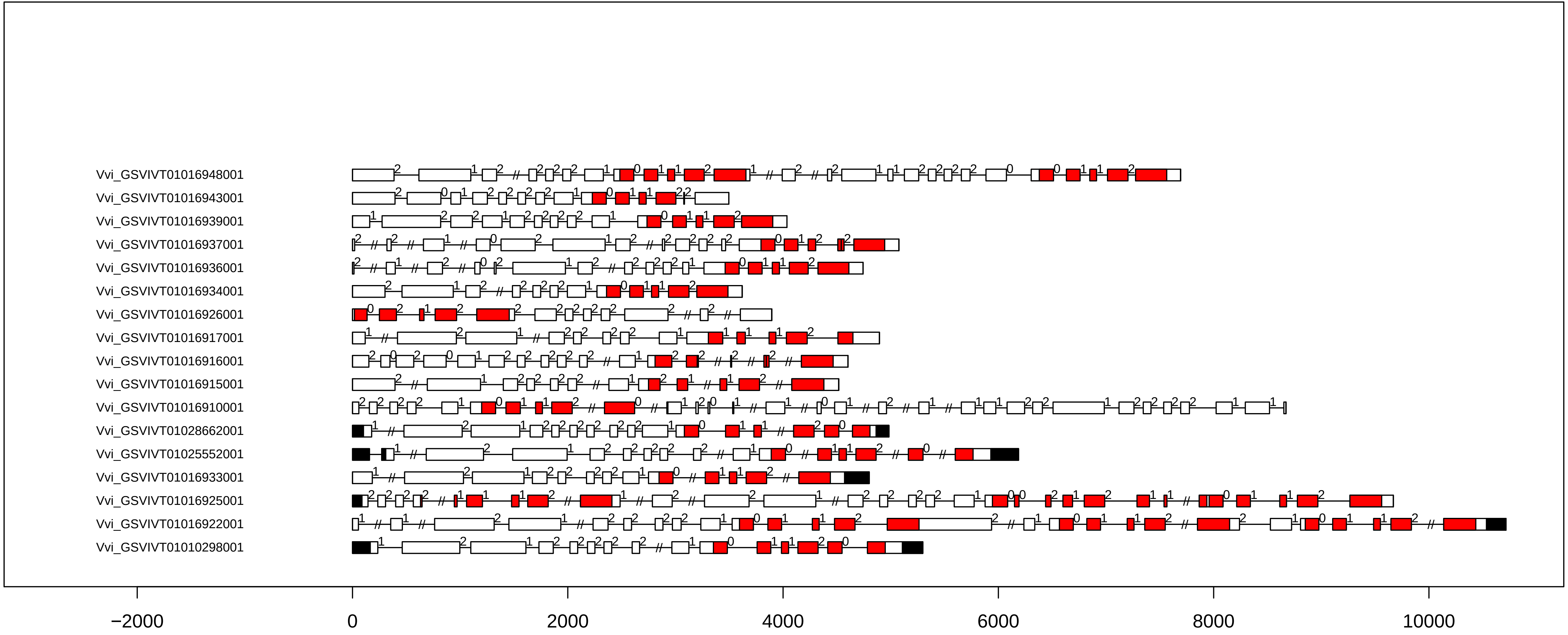

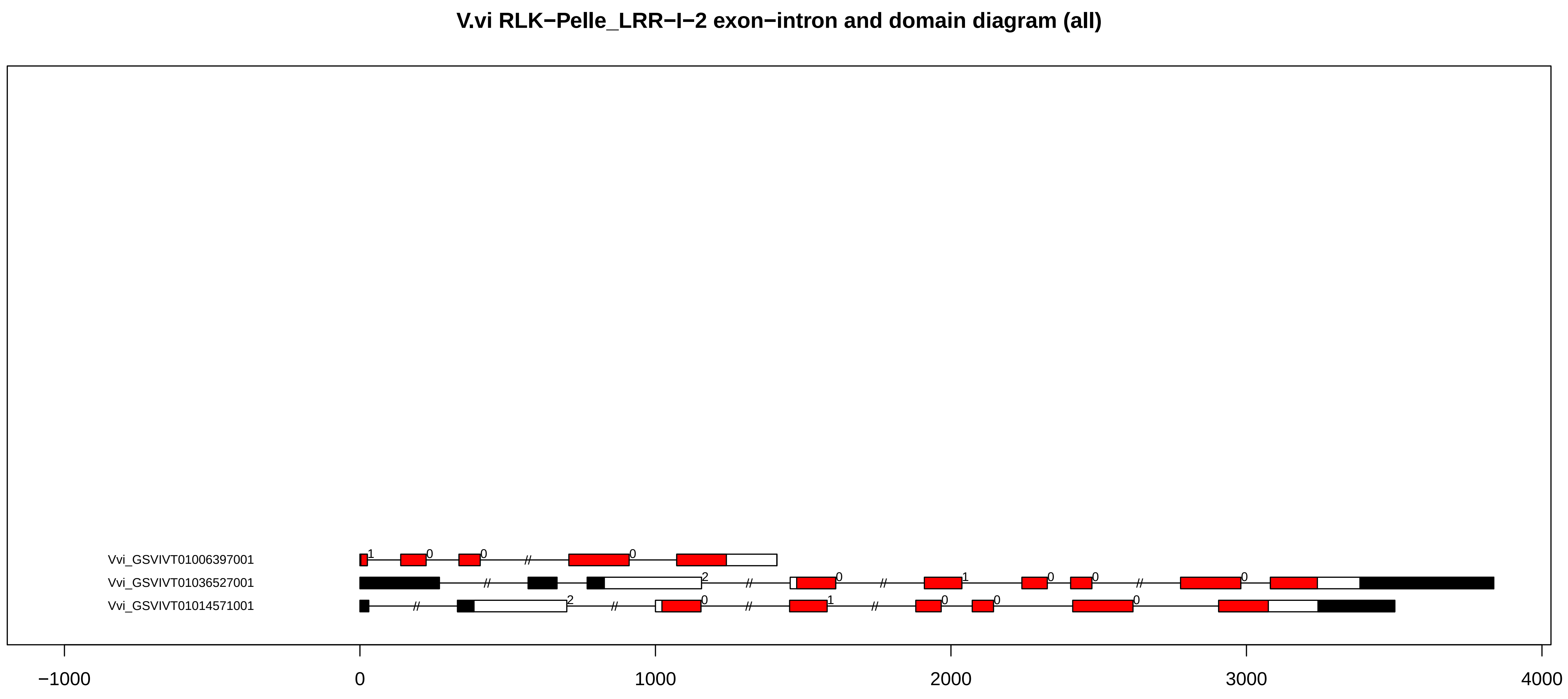
V.vi RLK−Pelle_LRR−I−2 exon−intron and domain diagram (all)
Vvi_GSVIVT01006397001
Vvi_GSVIVT01036527001
Vvi_GSVIVT01014571001
−1000
0
1000
2000
3000
4000

V.vi RLK−Pelle_LRR−III exon−intron and domain diagram (part 1)

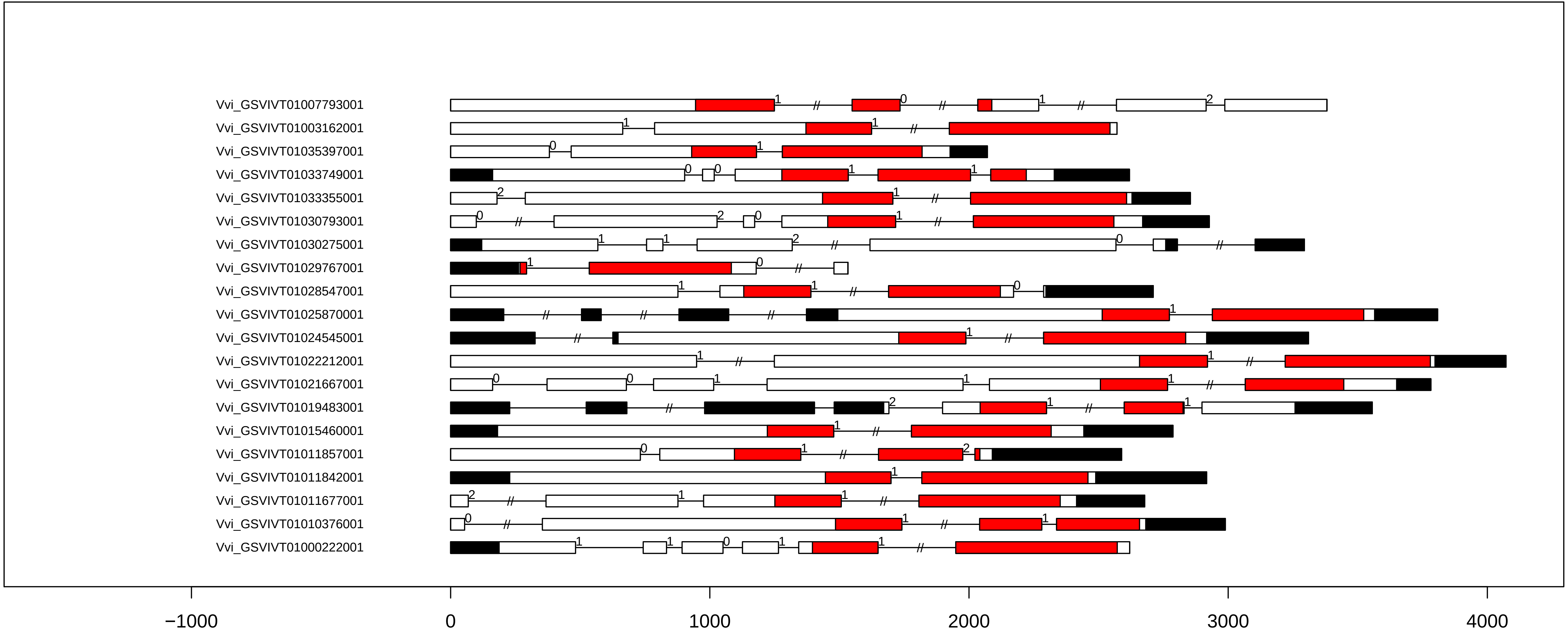

V.vi RLK−Pelle_LRR−III exon−intron and domain diagram (part 2)

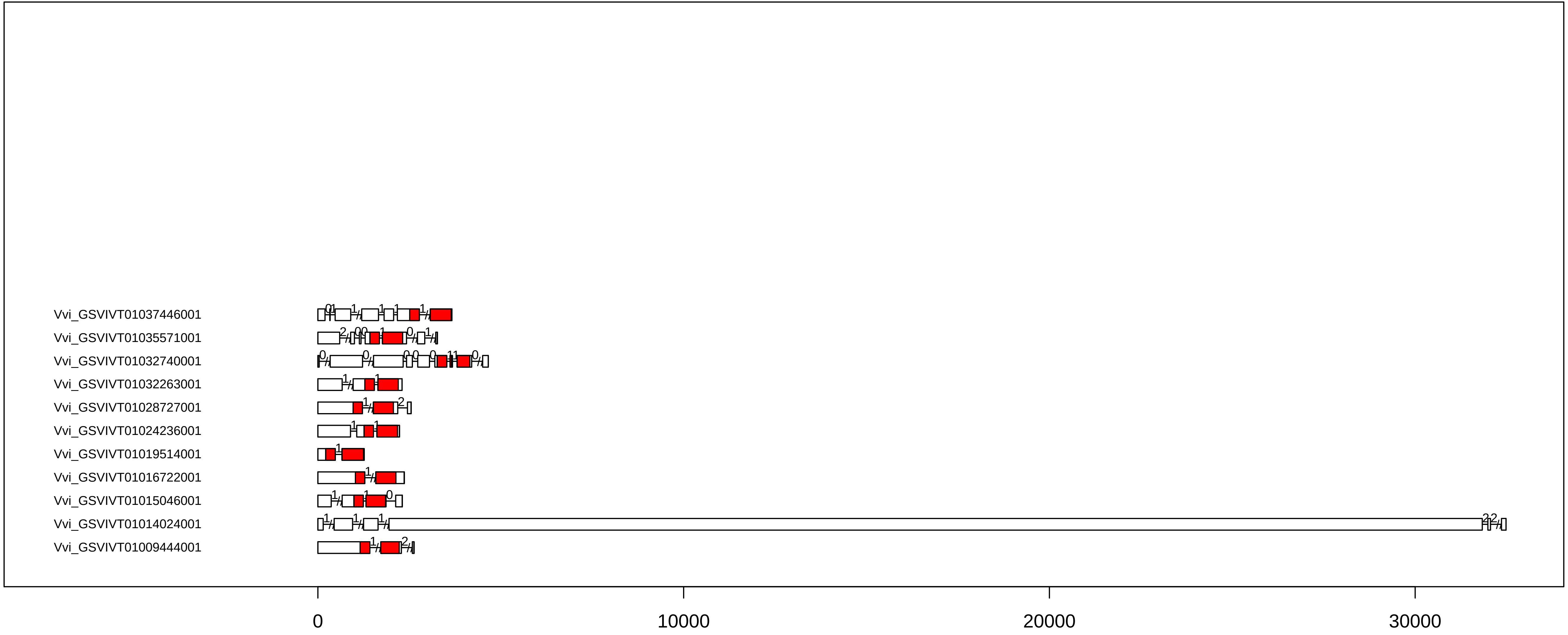

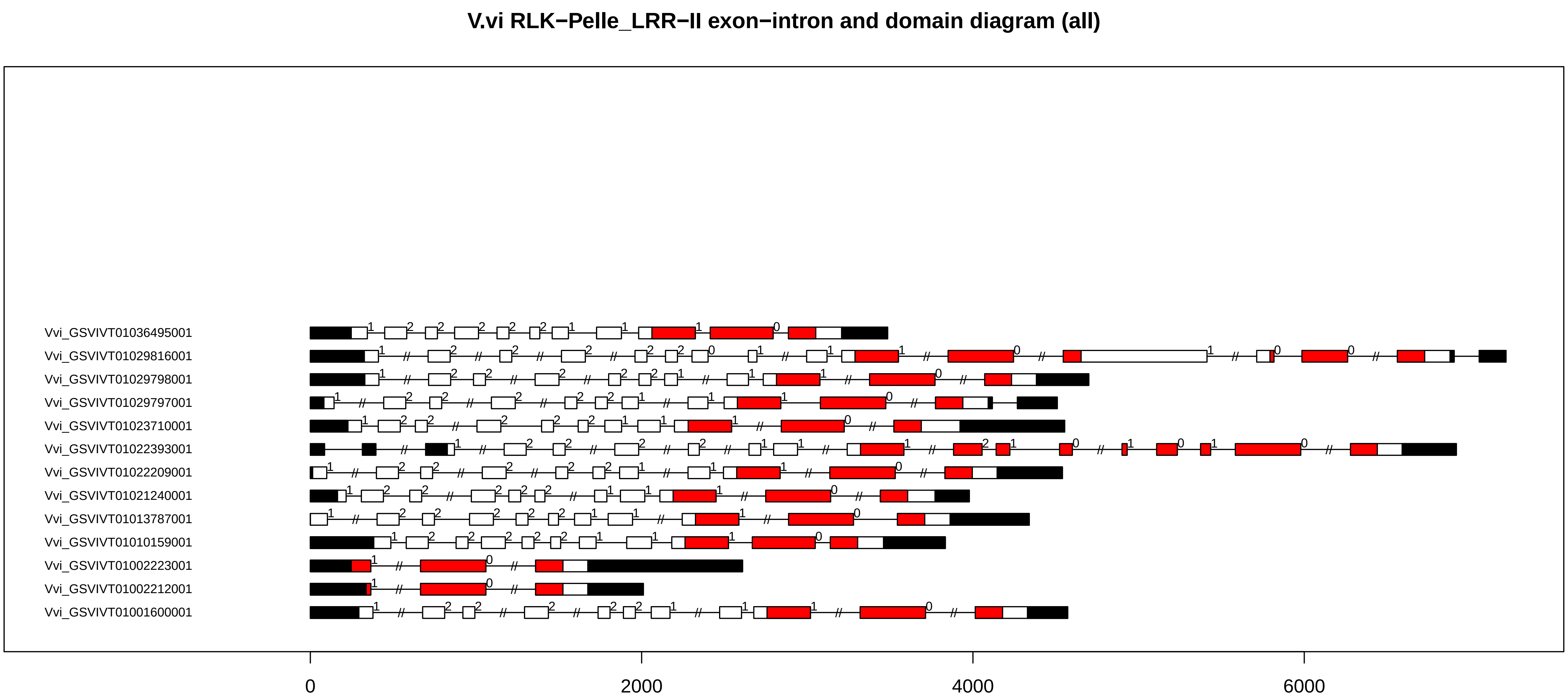

V.vi RLK−Pelle_LRR−II exon−intron and domain diagram (all)
Vvi_GSVIVT01036495001
Vvi_GSVIVT01029816001
Vvi_GSVIVT01029798001
Vvi_GSVIVT01029797001
Vvi_GSVIVT01023710001
Vvi_GSVIVT01022393001
Vvi_GSVIVT01022209001
Vvi_GSVIVT01021240001
Vvi_GSVIVT01013787001
Vvi_GSVIVT01010159001
Vvi_GSVIVT01002223001
Vvi_GSVIVT01002212001
Vvi_GSVIVT01001600001
0
2000
4000
6000

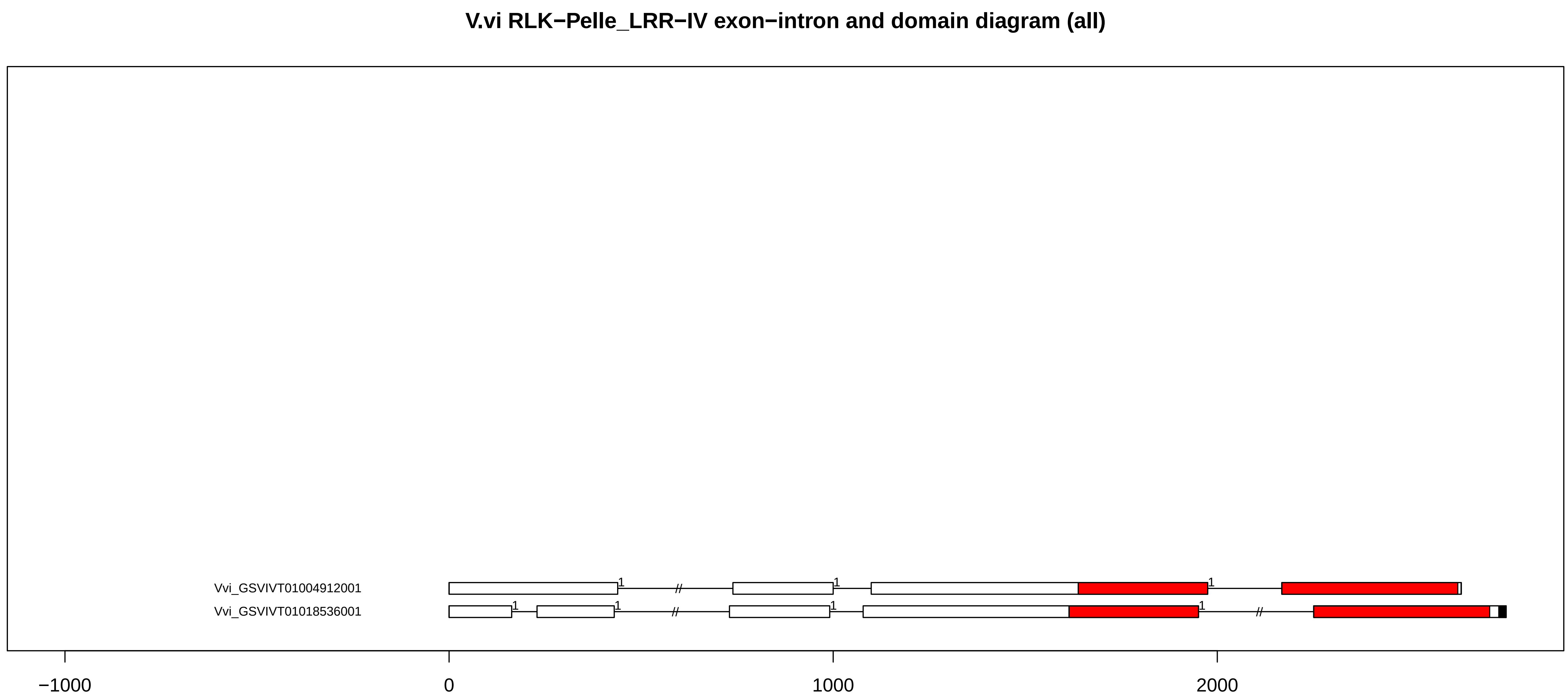
V.vi RLK−Pelle_LRR−IV exon−intron and domain diagram (all)
Vvi_GSVIVT01004912001
Vvi_GSVIVT01018536001
1
1
1
1
1
1
1
−1000
0
1000
2000

V.vi RLK−Pelle_LRR−IX exon−intron and domain diagram (all)

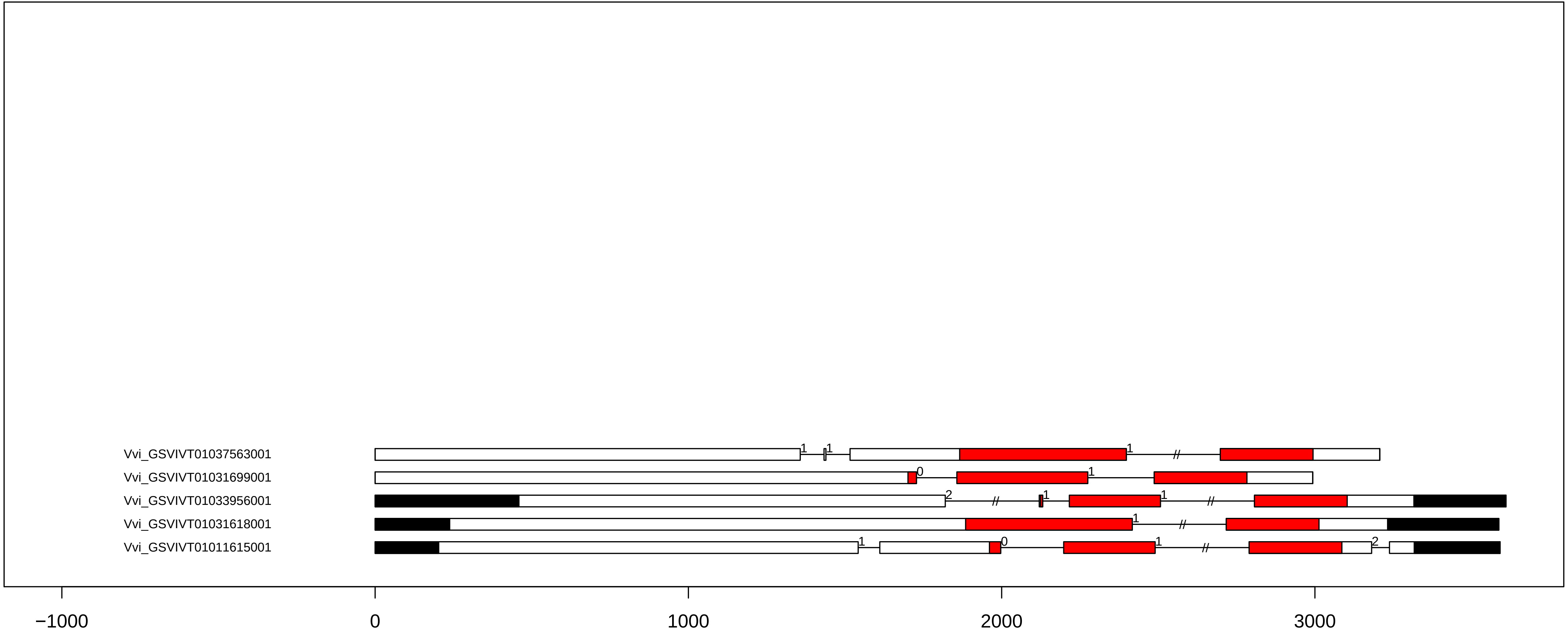

V.vi RLK−Pelle_LRR−VI−1 exon−intron and domain diagram (all)

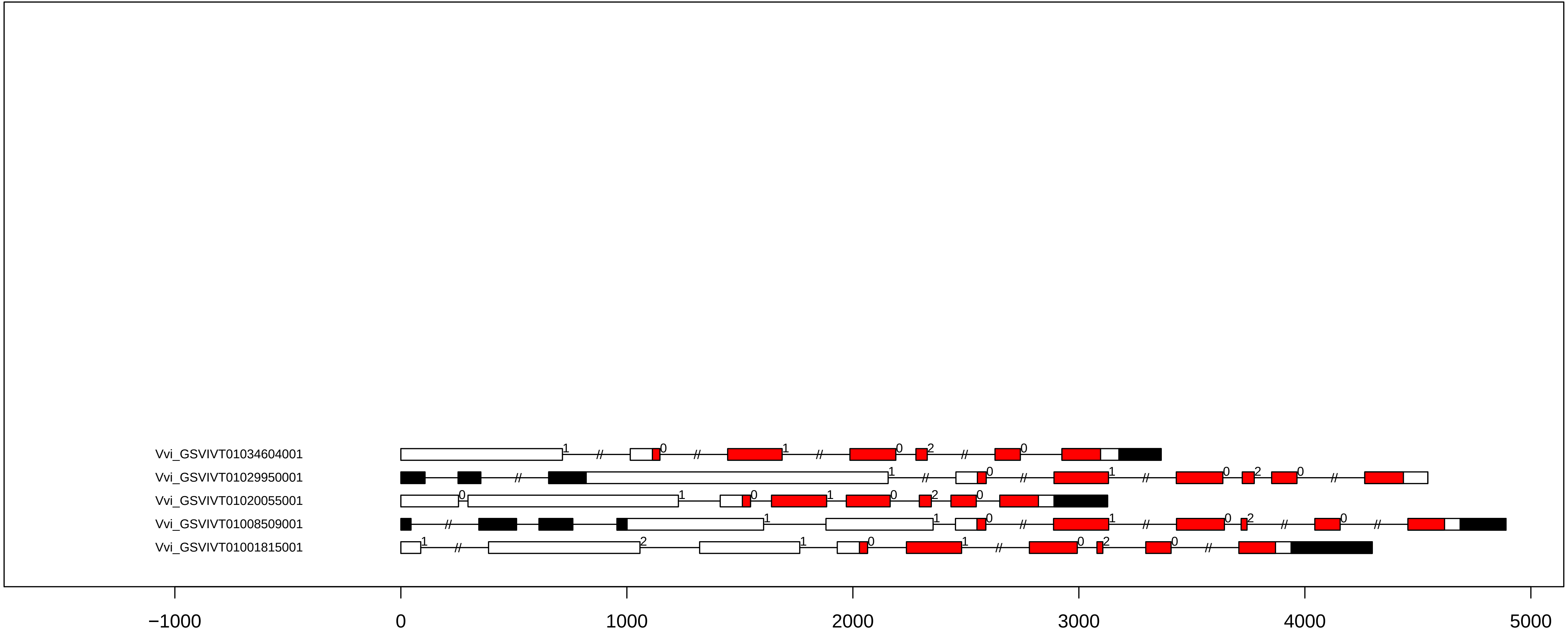

V.vi RLK−Pelle_LRR−VI−2 exon−intron and domain diagram (all)

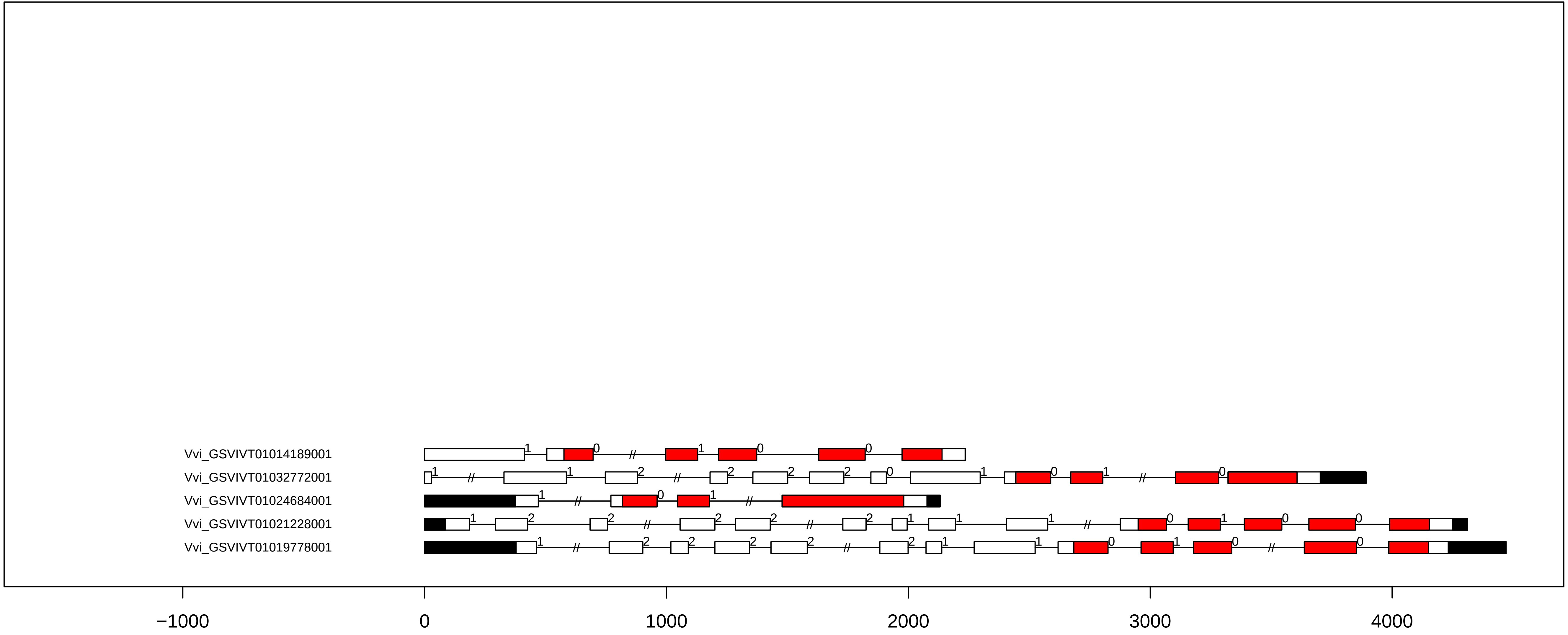

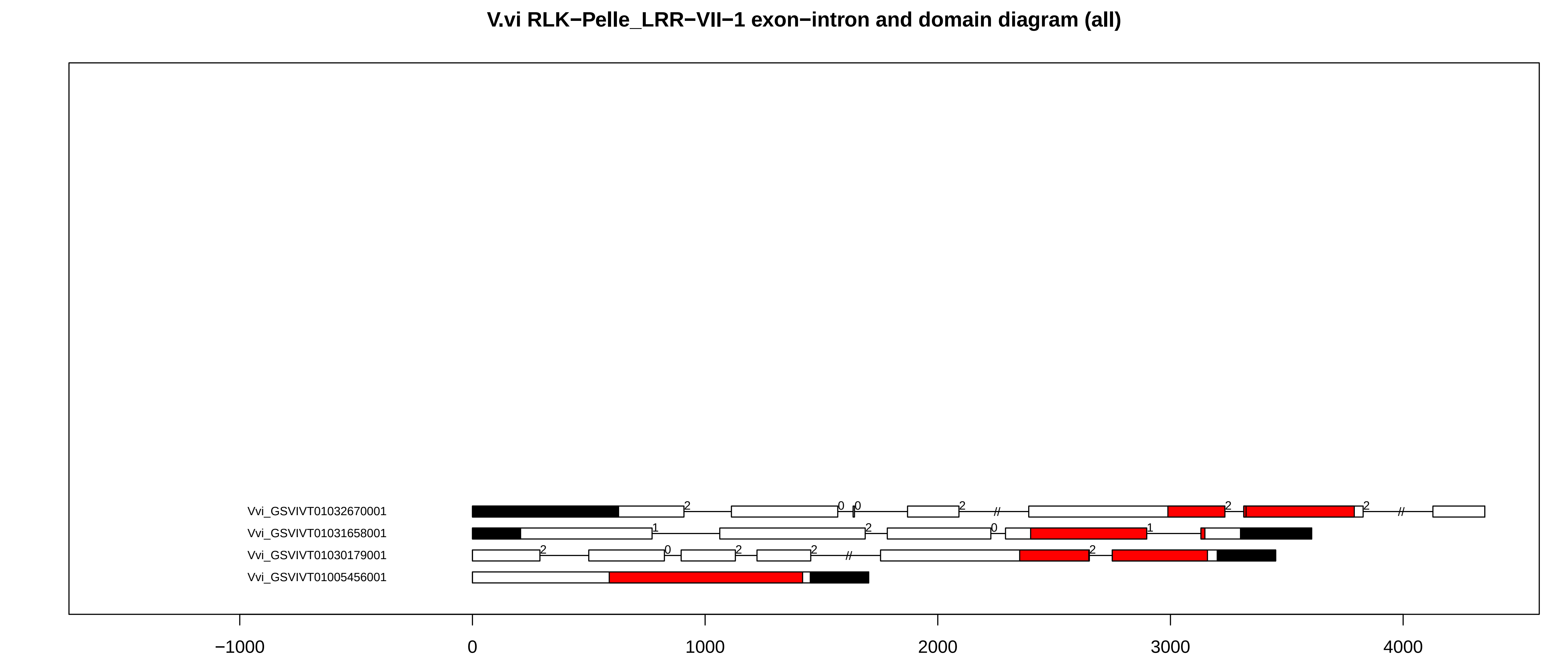

V.vi RLK−Pelle_LRR−VII−1 exon−intron and domain diagram (all)
Vvi_GSVIVT01032670001
Vvi_GSVIVT01031658001
Vvi_GSVIVT01030179001
Vvi_GSVIVT01005456001
−1000
0
1000
2000
3000
4000

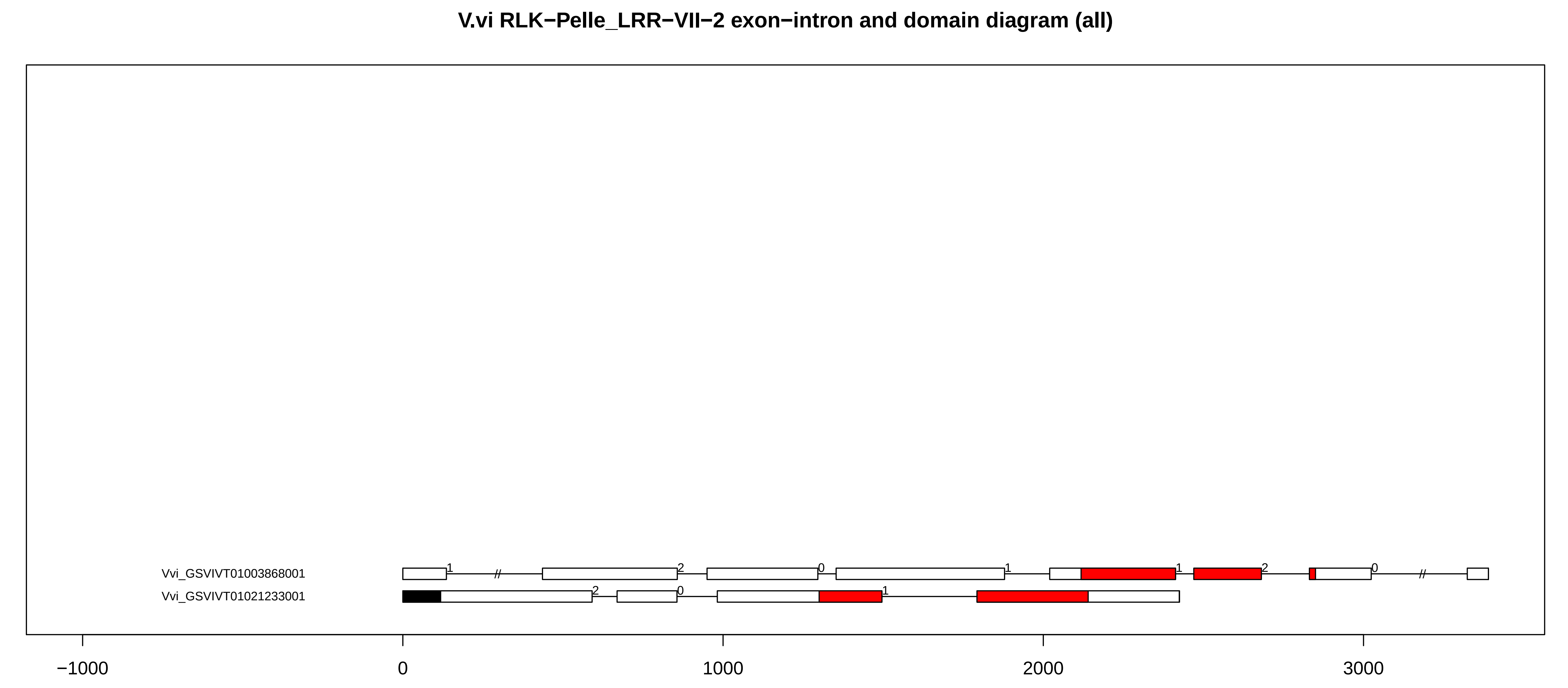

V.vi RLK−Pelle_LRR−VII−2 exon−intron and domain diagram (all)
Vvi_GSVIVT01003868001
Vvi_GSVIVT01021233001
1
2
0
1
1
2
0
2
0
1
−1000
0
1000
2000
3000

V.vi RLK−Pelle_LRR−VII−3 exon−intron and domain diagram (all)

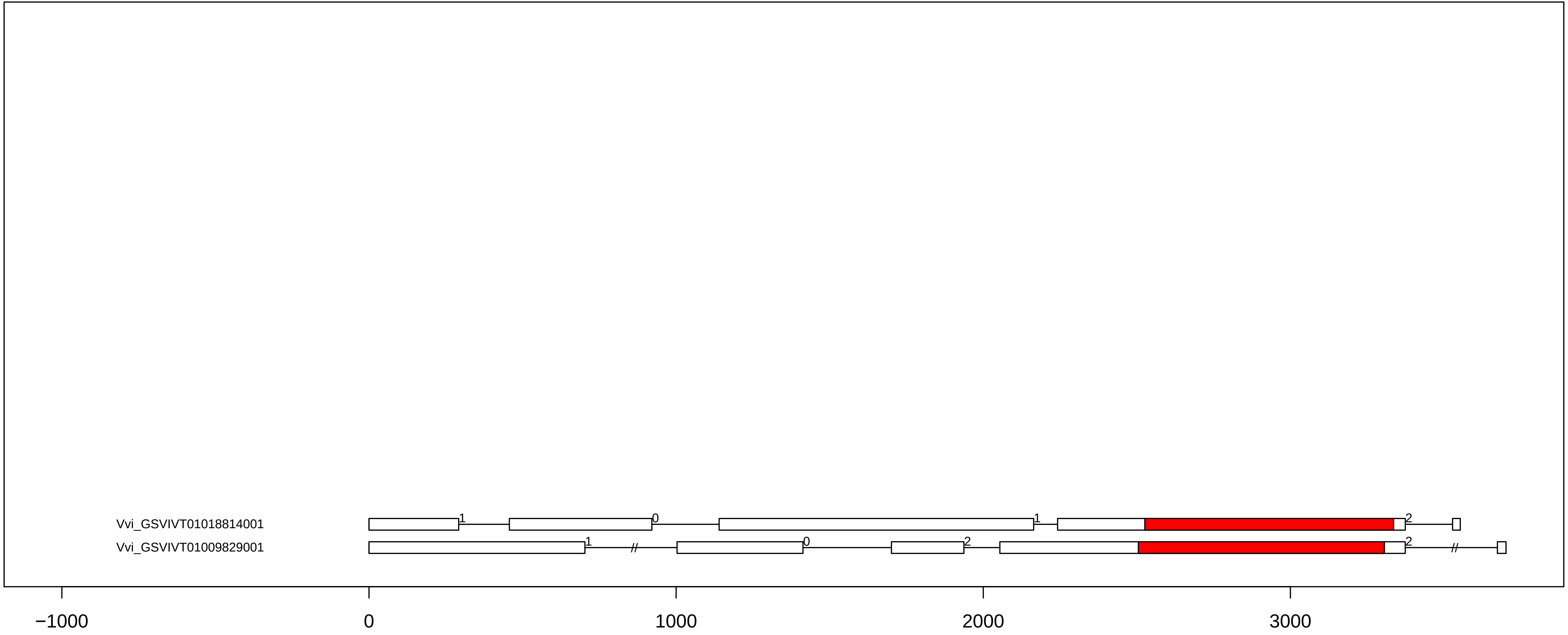

V.vi RLK−Pelle_LRR−VIII−1 exon−intron and domain diagram (all)

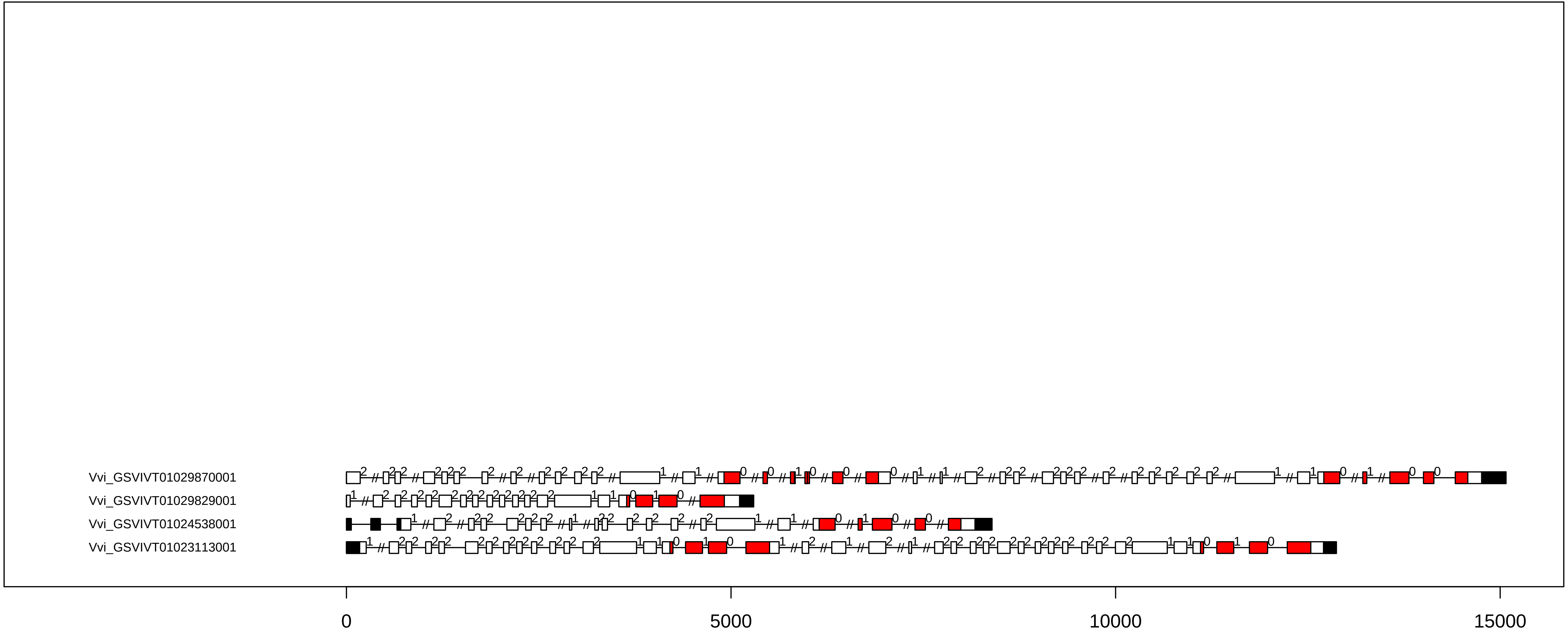

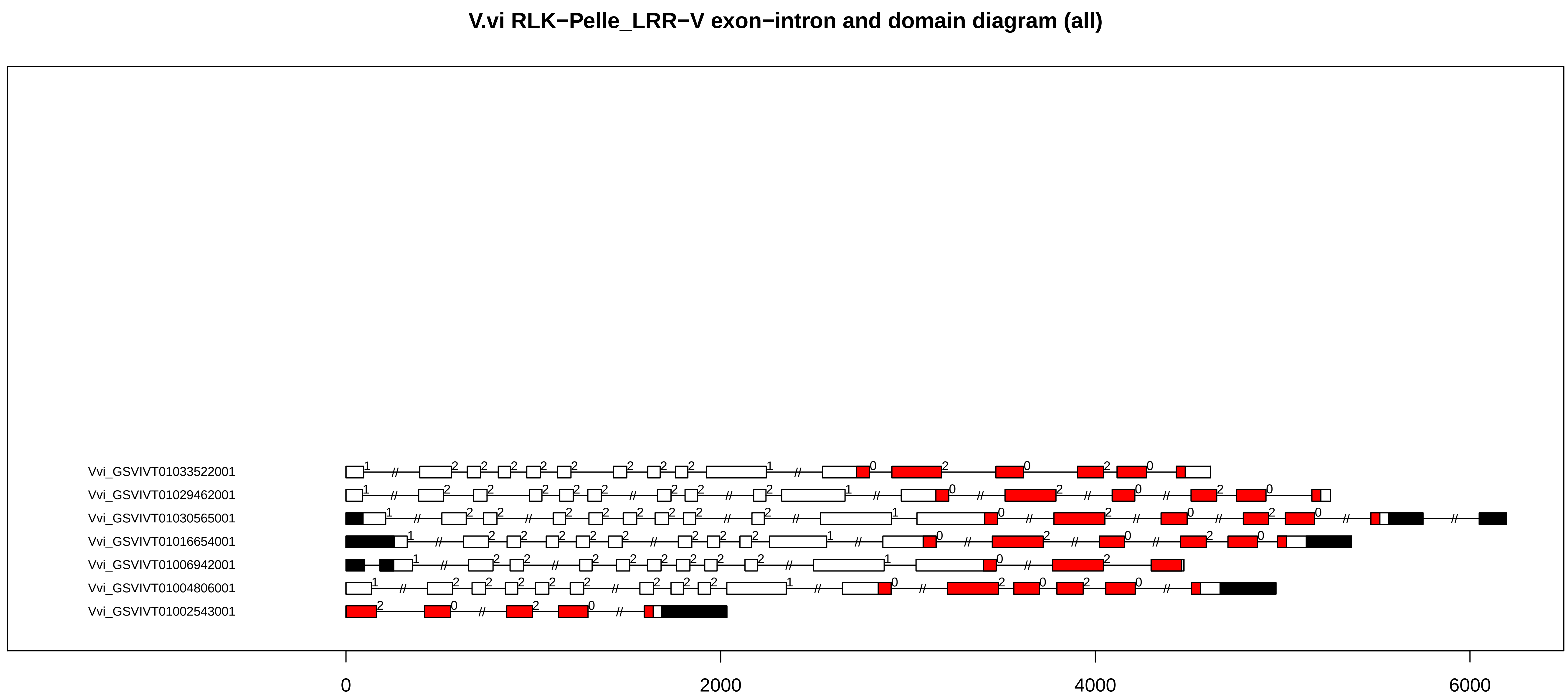
V.vi RLK−Pelle_LRR−V exon−intron and domain diagram (all)
Vvi_GSVIVT01033522001
Vvi_GSVIVT01029462001
Vvi_GSVIVT01030565001
Vvi_GSVIVT01016654001
Vvi_GSVIVT01006942001
Vvi_GSVIVT01004806001
Vvi_GSVIVT01002543001
0
2000
4000
6000

V.vi RLK−Pelle_LRR−Xa exon−intron and domain diagram (all)

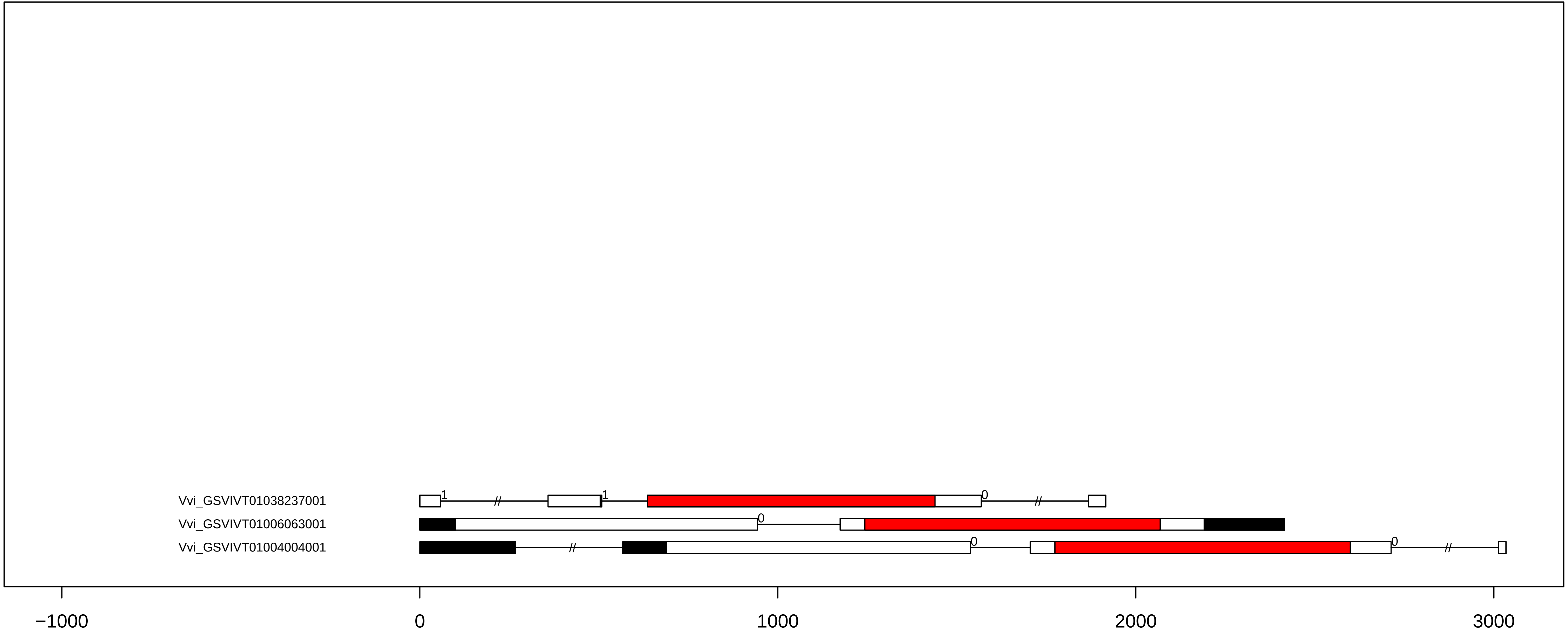

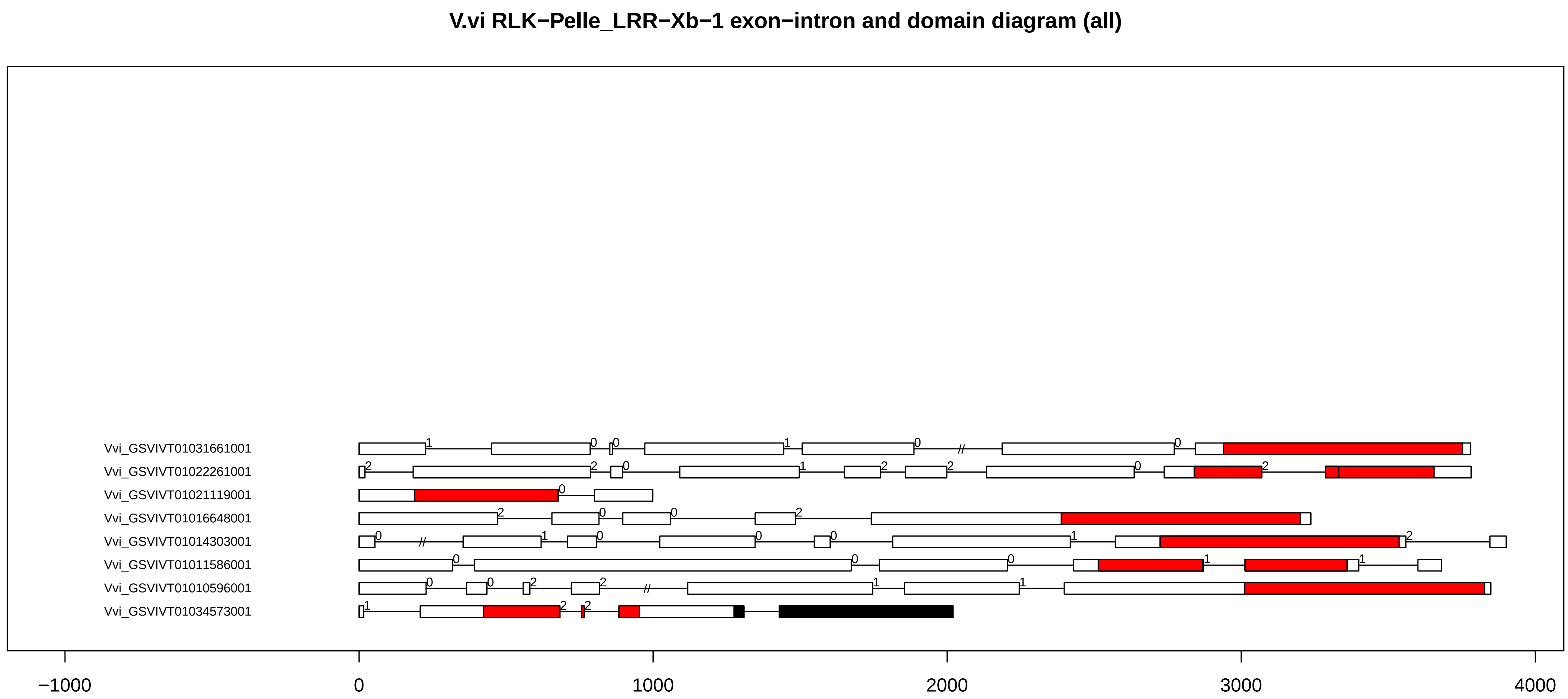

V.vi RLK−Pelle_LRR−Xb−1 exon−intron and domain diagram (all)
Vvi_GSVIVT01031661001
Vvi_GSVIVT01022261001
Vvi_GSVIVT01021119001
Vvi_GSVIVT01016648001
Vvi_GSVIVT01014303001
Vvi_GSVIVT01011586001
Vvi_GSVIVT01010596001
Vvi_GSVIVT01034573001
−1000
0
1000
2000
3000
4000

V.vi RLK−Pelle_LRR−XI−1 exon−intron and domain diagram (part 1)

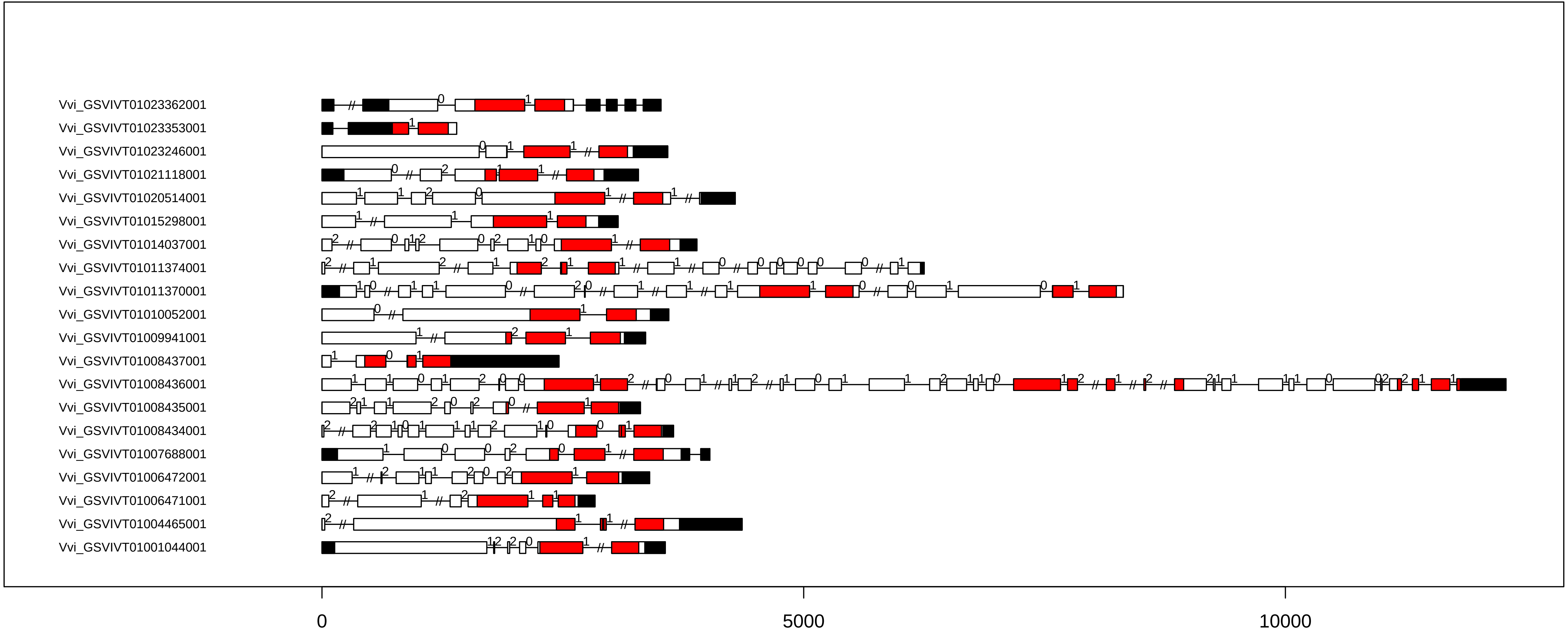

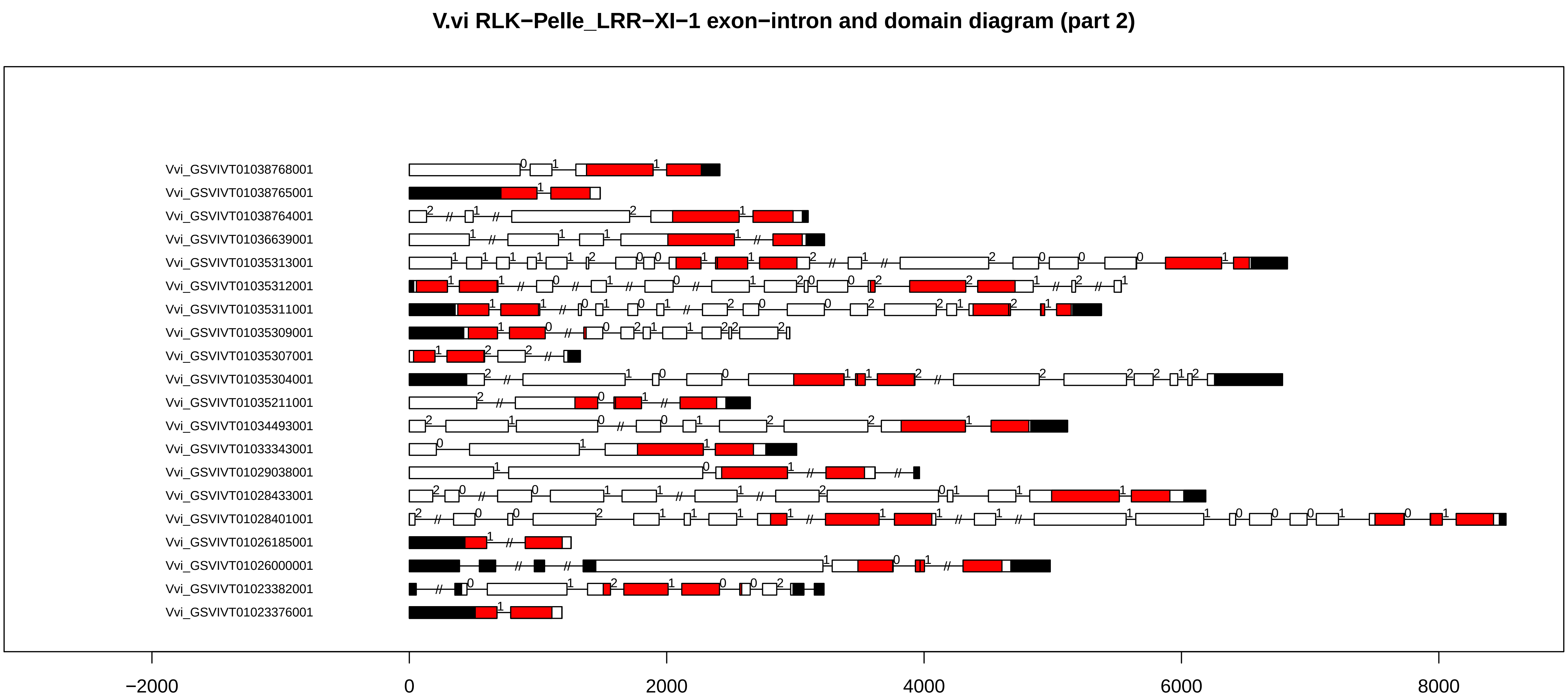
V.vi RLK−Pelle_LRR−XI−1 exon−intron and domain diagram (part 2)
Vvi_GSVIVT01038768001
Vvi_GSVIVT01038765001
Vvi_GSVIVT01038764001
Vvi_GSVIVT01036639001
Vvi_GSVIVT01035313001
Vvi_GSVIVT01035312001
Vvi_GSVIVT01035311001
Vvi_GSVIVT01035309001
Vvi_GSVIVT01035307001
Vvi_GSVIVT01035304001
Vvi_GSVIVT01035211001
Vvi_GSVIVT01034493001
Vvi_GSVIVT01033343001
Vvi_GSVIVT01029038001
Vvi_GSVIVT01028433001
Vvi_GSVIVT01028401001
Vvi_GSVIVT01026185001
Vvi_GSVIVT01026000001
Vvi_GSVIVT01023382001
Vvi_GSVIVT01023376001
−2000
0
2000
4000
6000
8000

V.vi RLK−Pelle_LRR−XI−1 exon−intron and domain diagram (part 3)

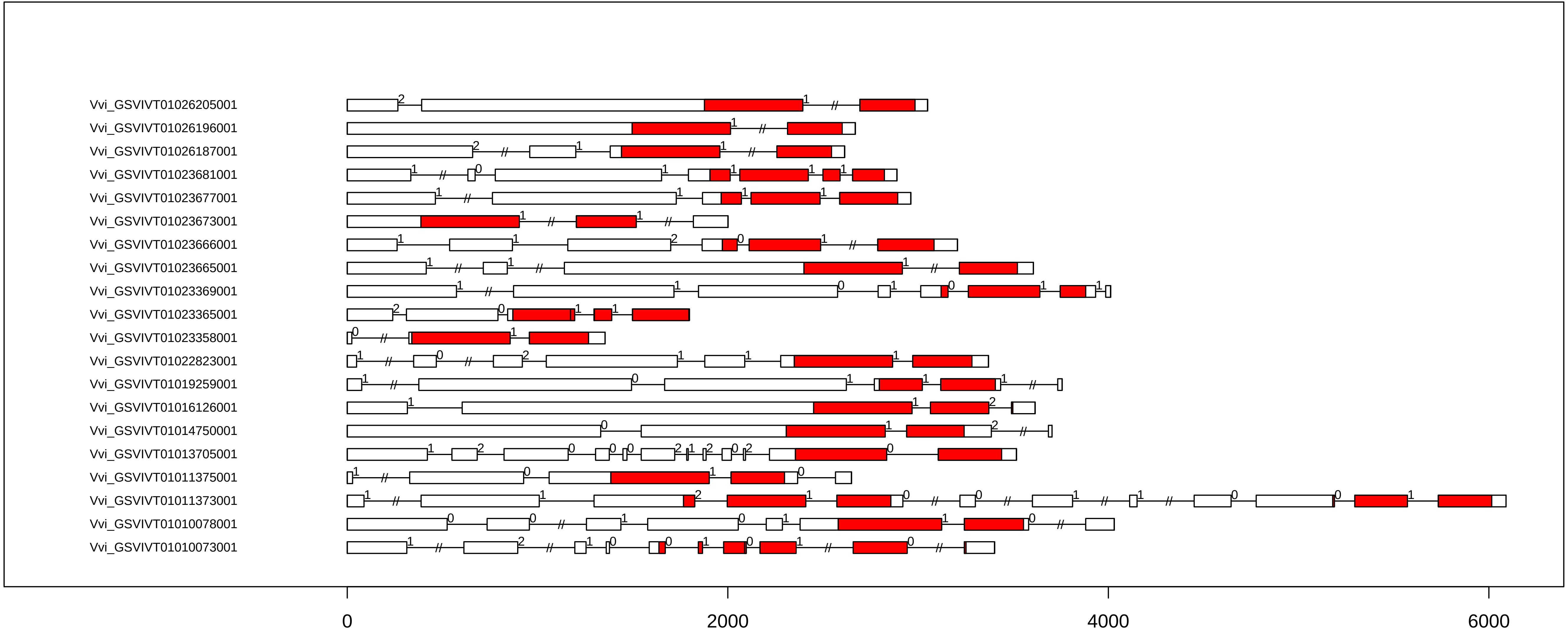

V.vi RLK−Pelle_LRR−XI−1 exon−intron and domain diagram (part 4)

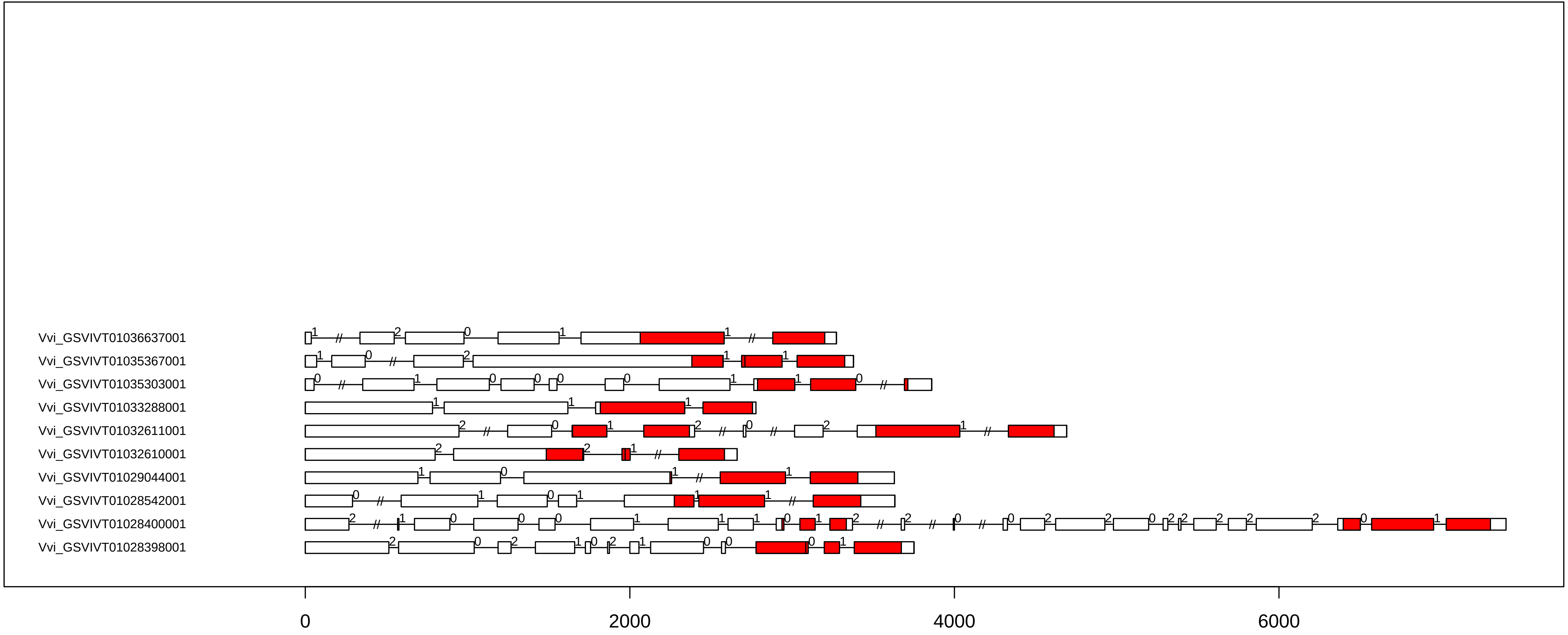

V.vi RLK−Pelle_LRR−XI−2 exon−intron and domain diagram (all)

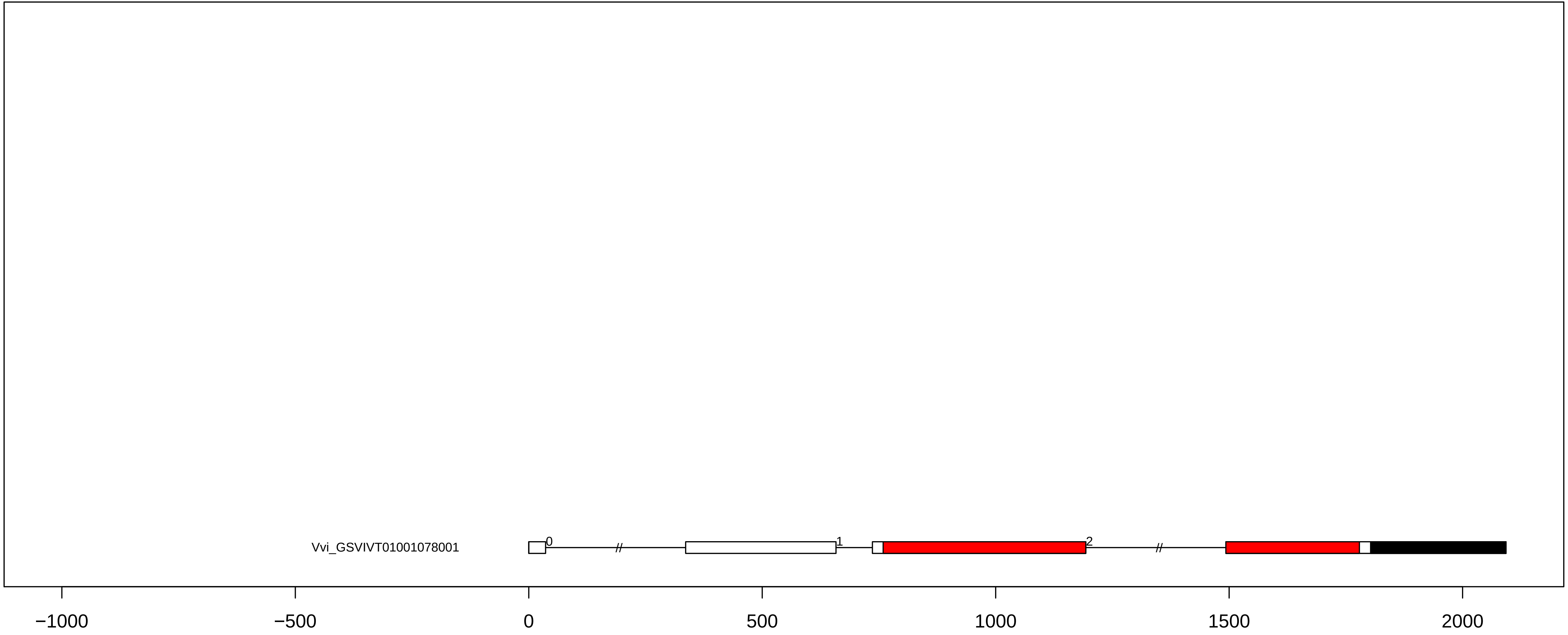

V.vi RLK−Pelle_LRR−XII−1 exon−intron and domain diagram (part 1)

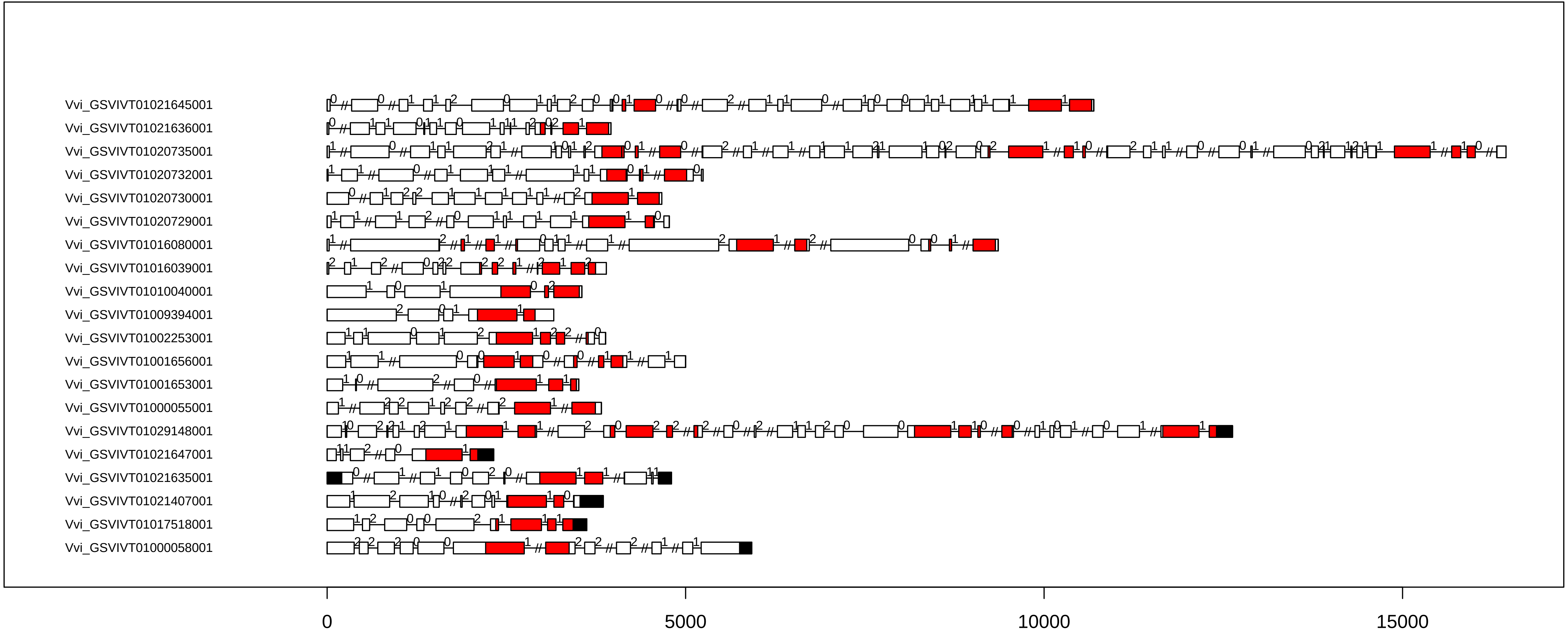

V.vi RLK−Pelle_LRR−XII−1 exon−intron and domain diagram (part 2)

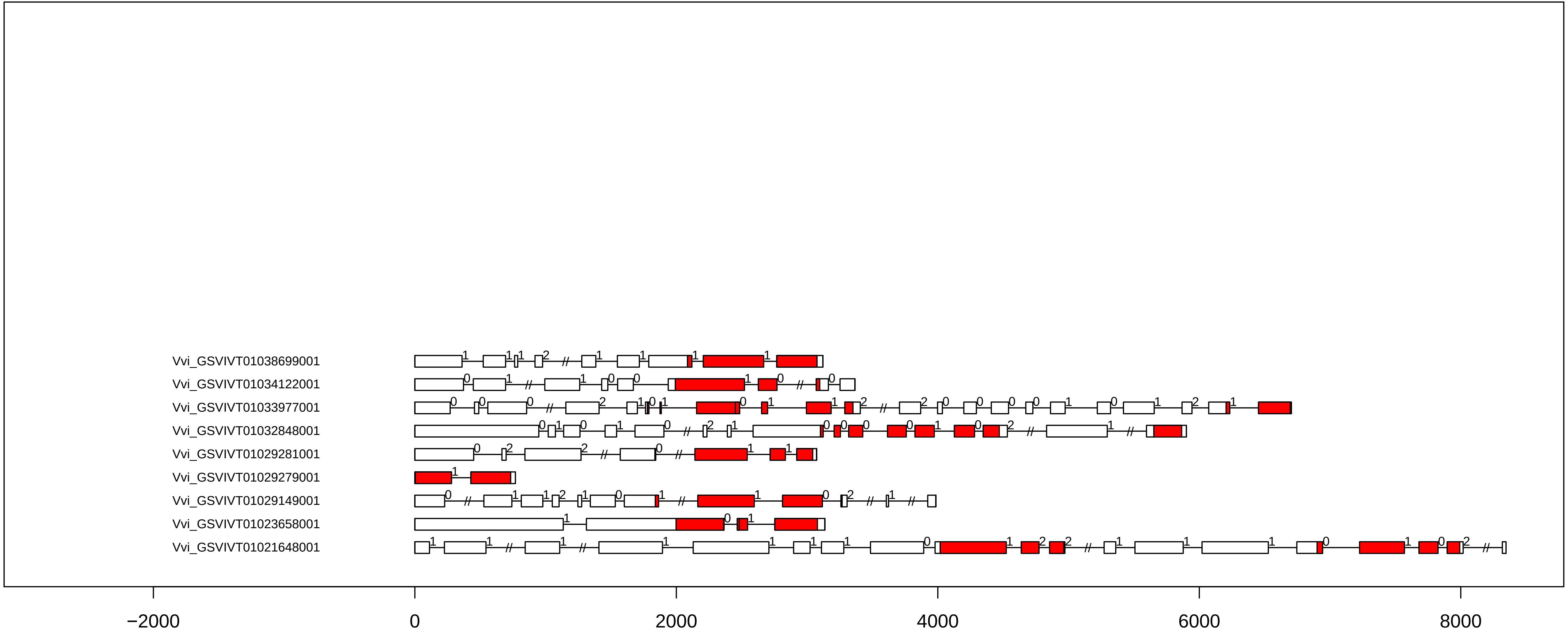

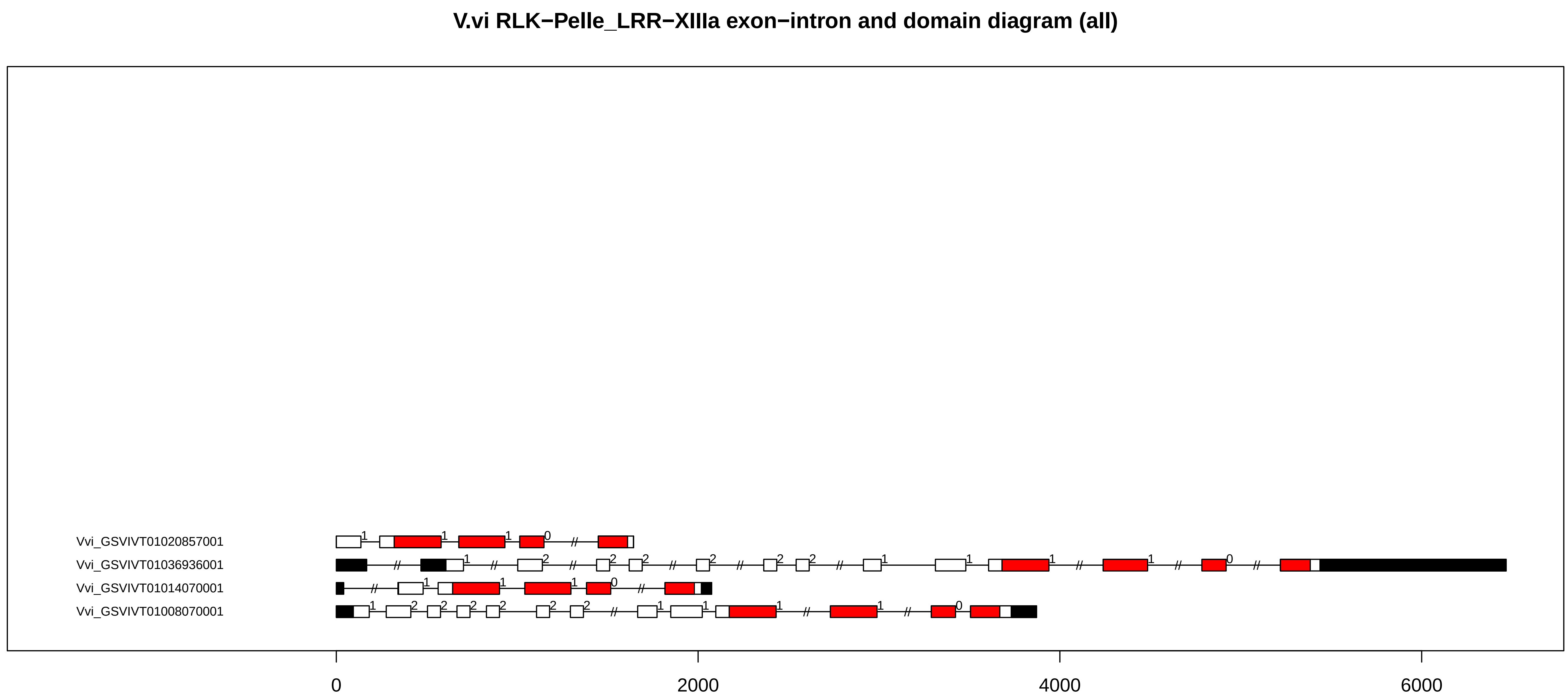

V.vi RLK−Pelle_LRR−XIIIa exon−intron and domain diagram (all)
Vvi_GSVIVT01020857001
Vvi_GSVIVT01036936001
Vvi_GSVIVT01014070001
Vvi_GSVIVT01008070001
0
2000
4000
6000

V.vi RLK−Pelle_LRR−XIIIb exon−intron and domain diagram (all)

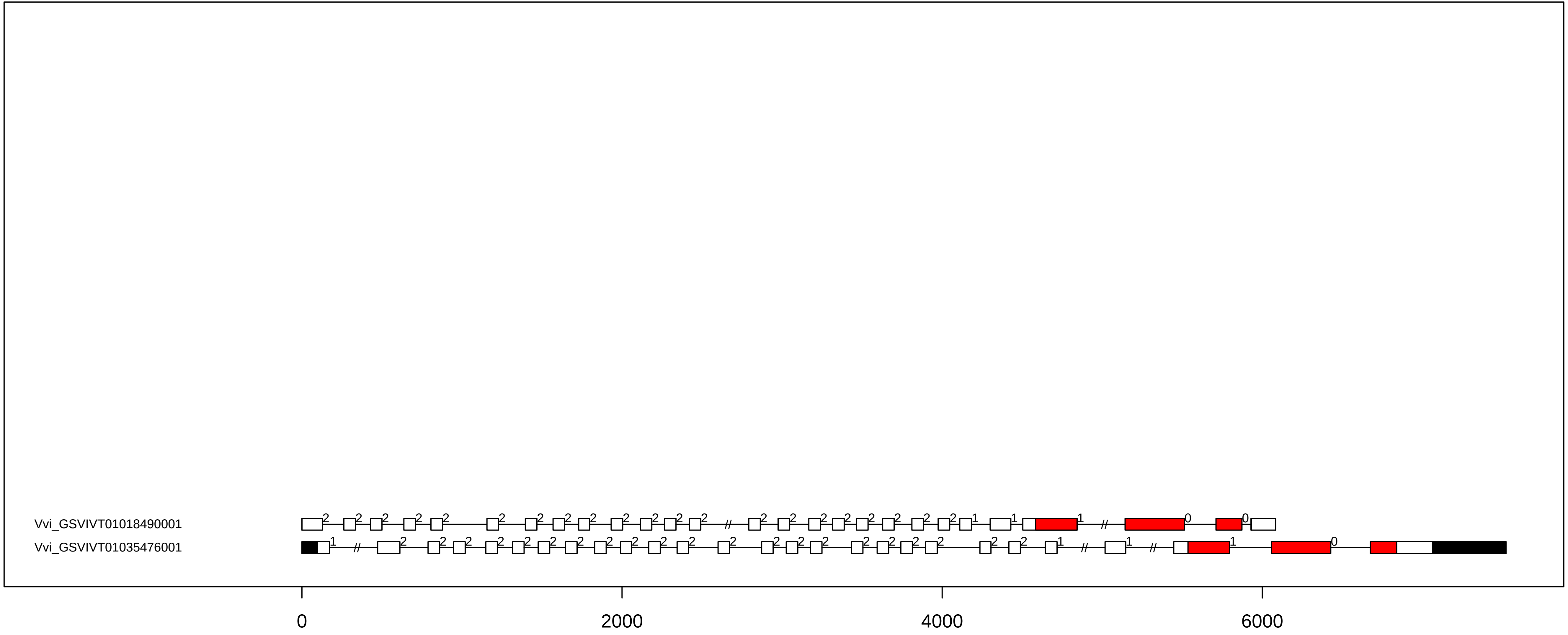

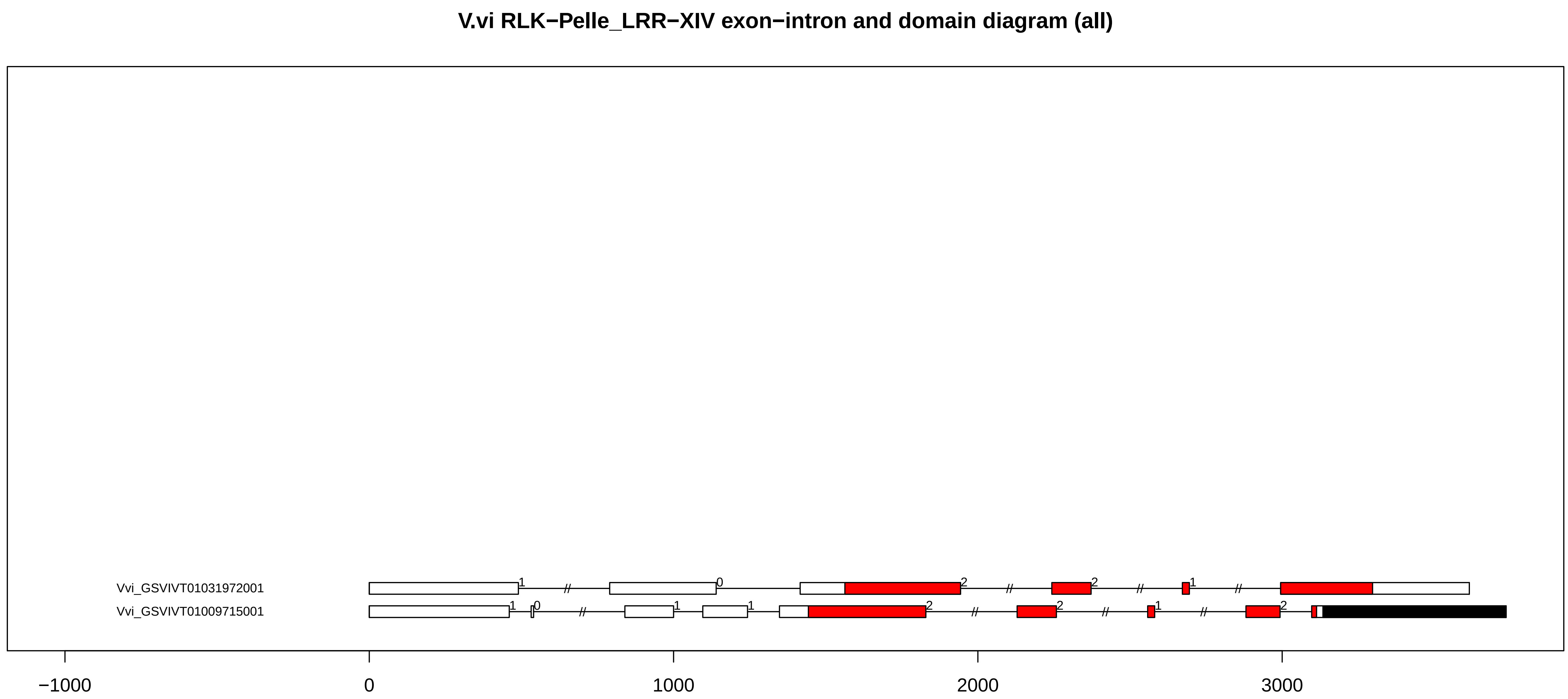

V.vi RLK−Pelle_LRR−XIV exon−intron and domain diagram (all)
Vvi_GSVIVT01031972001
Vvi_GSVIVT01009715001
−1000
0
1000
2000
3000

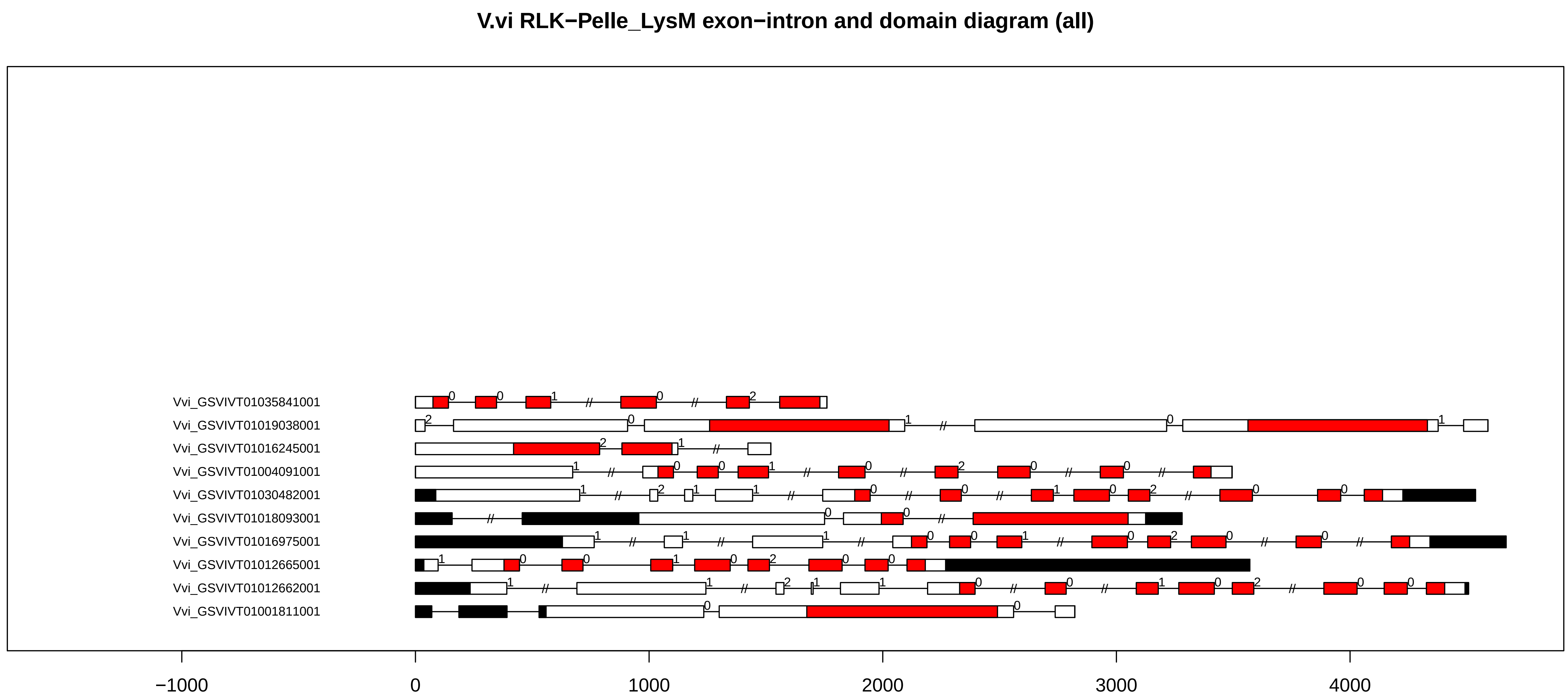

V.vi RLK−Pelle_LysM exon−intron and domain diagram (all)
Vvi_GSVIVT01035841001
Vvi_GSVIVT01019038001
Vvi_GSVIVT01016245001
Vvi_GSVIVT01004091001
Vvi_GSVIVT01030482001
Vvi_GSVIVT01018093001
Vvi_GSVIVT01016975001
Vvi_GSVIVT01012665001
Vvi_GSVIVT01012662001
Vvi_GSVIVT01001811001
−1000
0
1000
2000
3000
4000

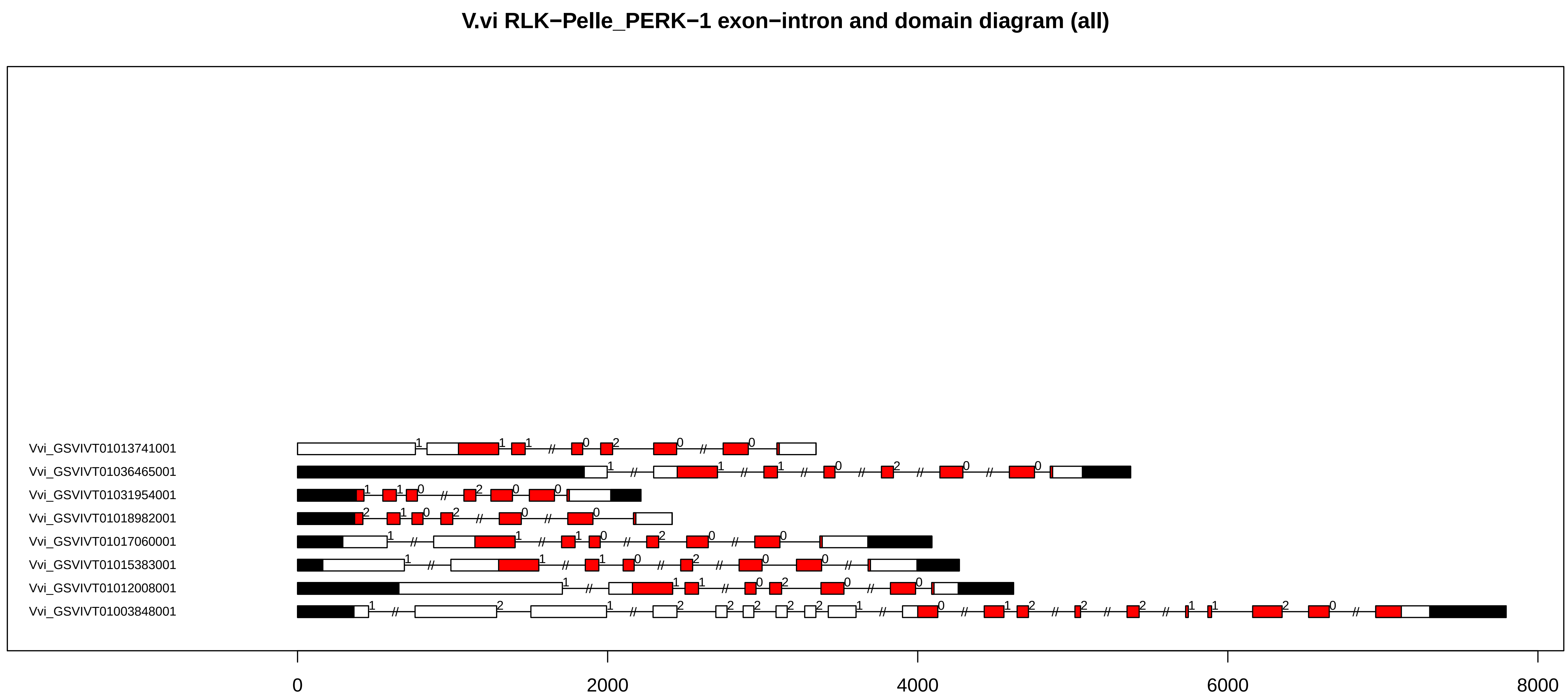
V.vi RLK−Pelle_PERK−1 exon−intron and domain diagram (all)
Vvi_GSVIVT01013741001
Vvi_GSVIVT01036465001
Vvi_GSVIVT01031954001
Vvi_GSVIVT01018982001
Vvi_GSVIVT01017060001
Vvi_GSVIVT01015383001
Vvi_GSVIVT01012008001
Vvi_GSVIVT01003848001
0
2000
4000
6000
8000

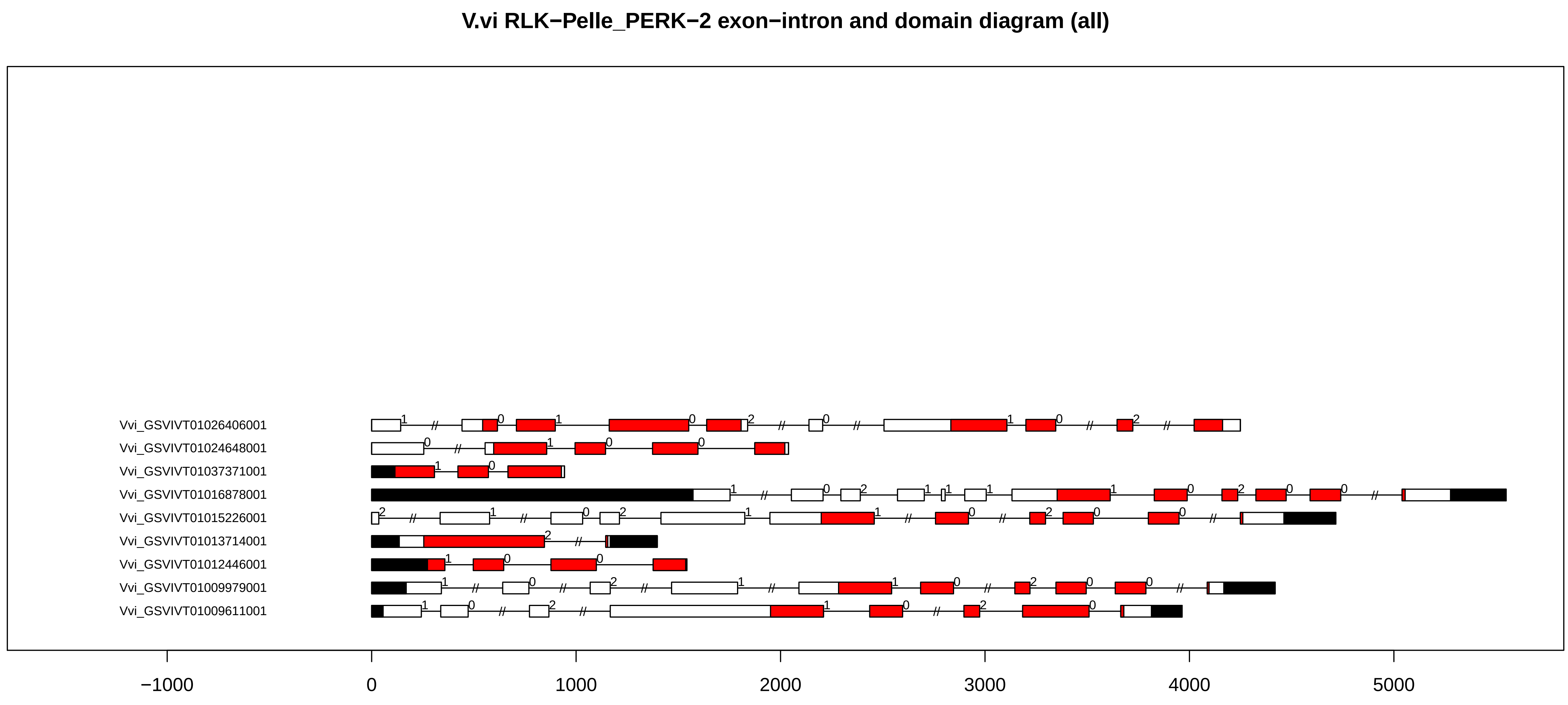
V.vi RLK−Pelle_PERK−2 exon−intron and domain diagram (all)
Vvi_GSVIVT01026406001
Vvi_GSVIVT01024648001
Vvi_GSVIVT01037371001
Vvi_GSVIVT01016878001
Vvi_GSVIVT01015226001
Vvi_GSVIVT01013714001
Vvi_GSVIVT01012446001
Vvi_GSVIVT01009979001
Vvi_GSVIVT01009611001
−1000
0
1000
2000
3000
4000
5000

V.vi RLK−Pelle_RKF3 exon−intron and domain diagram (all)

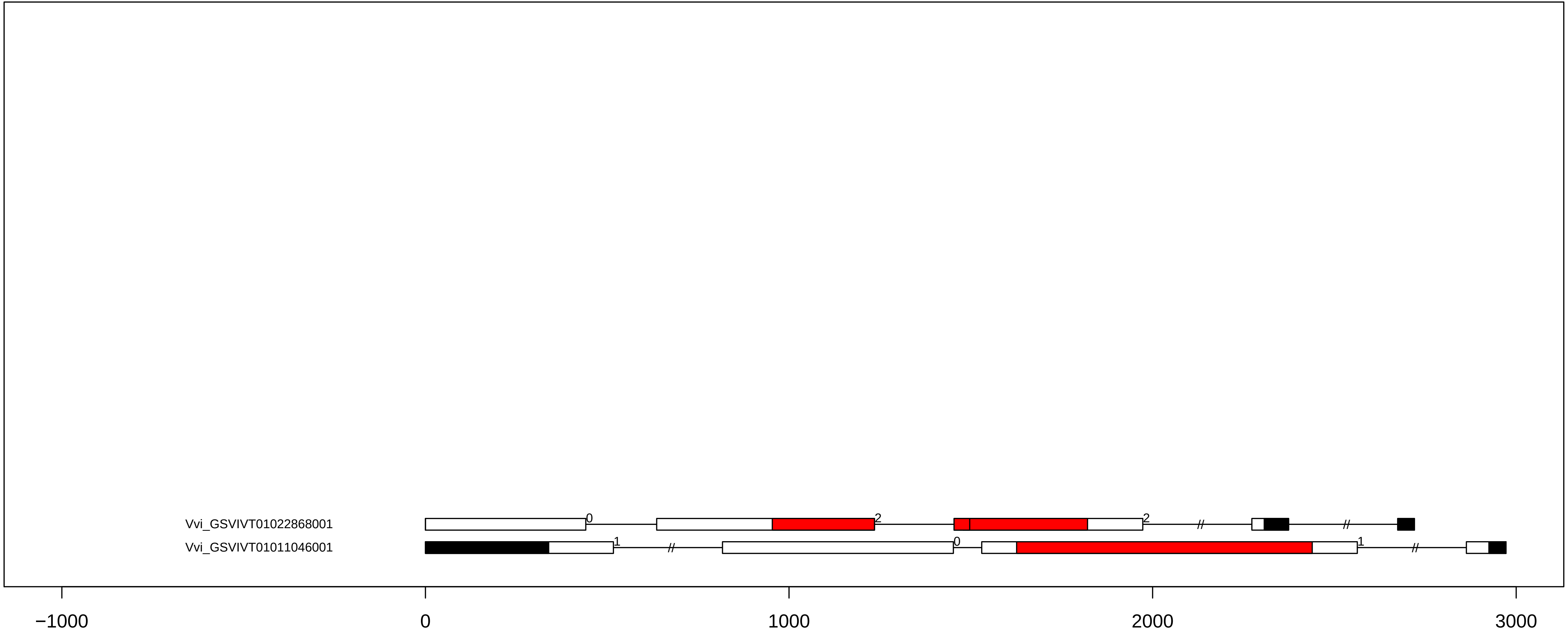

V.vi RLK−Pelle_RLCK−II exon−intron and domain diagram (all)

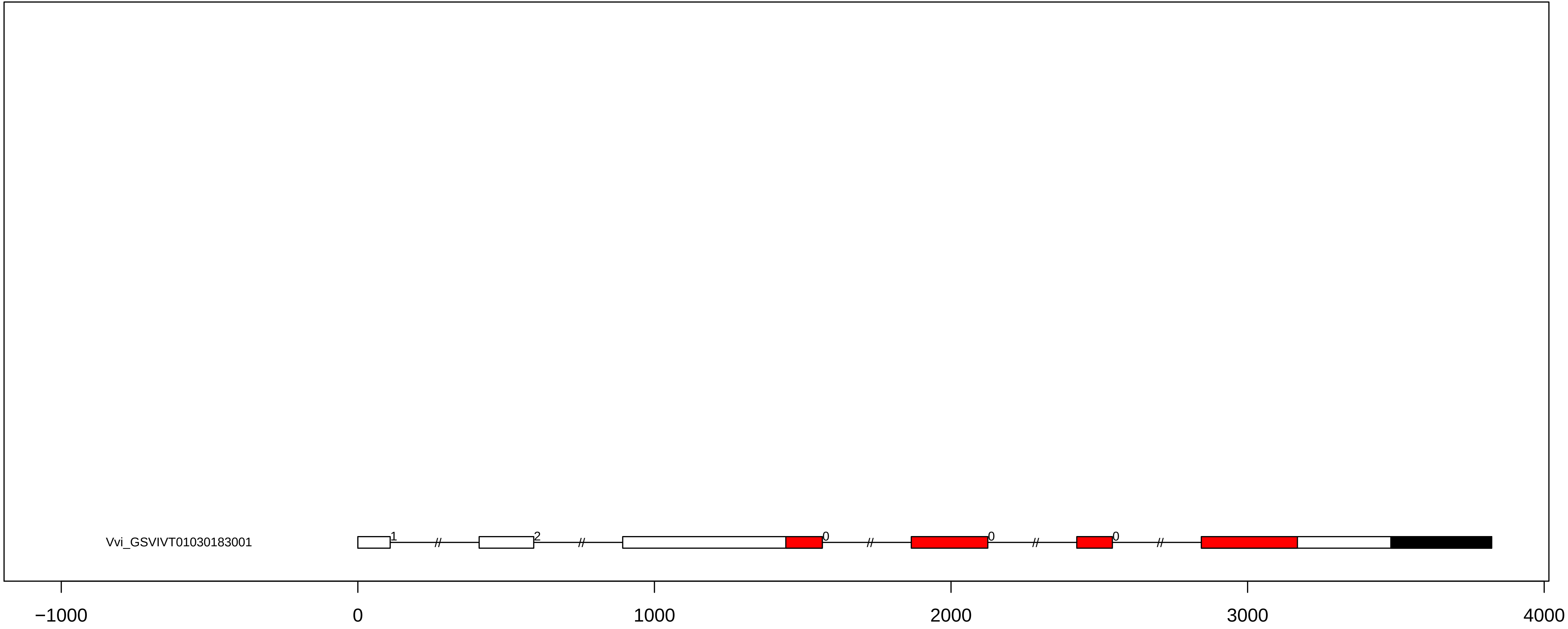

V.vi RLK−Pelle_RLCK−IV exon−intron and domain diagram (all)

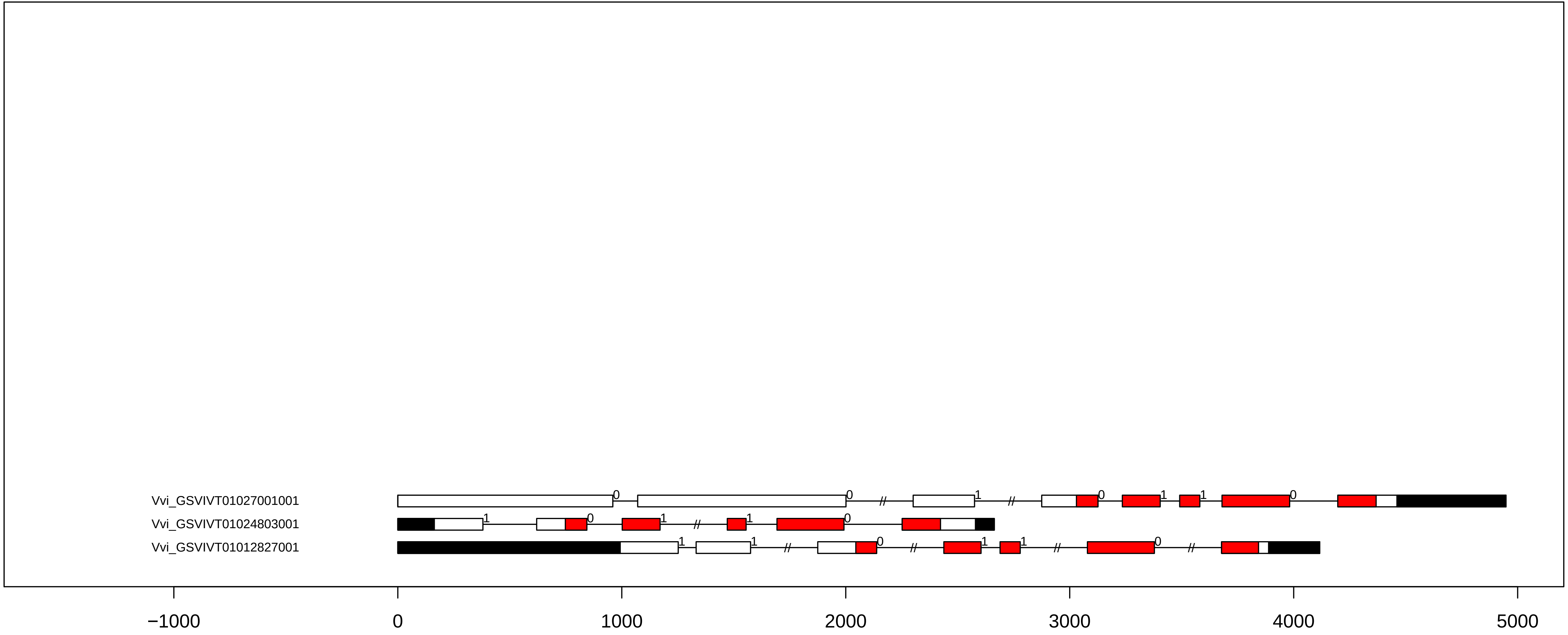

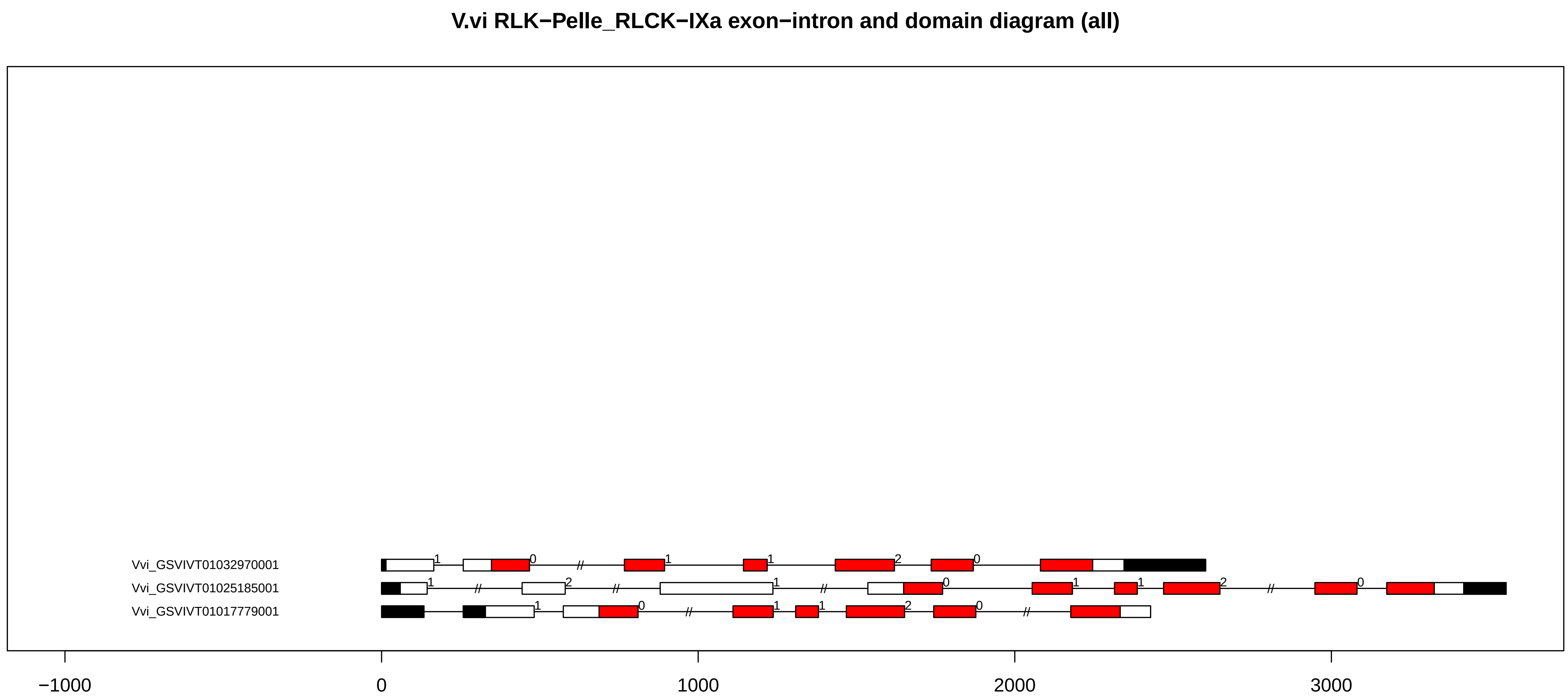
V.vi RLK−Pelle_RLCK−IXa exon−intron and domain diagram (all)
Vvi_GSVIVT01032970001
Vvi_GSVIVT01025185001
Vvi_GSVIVT01017779001
−1000
0
1000
2000
3000

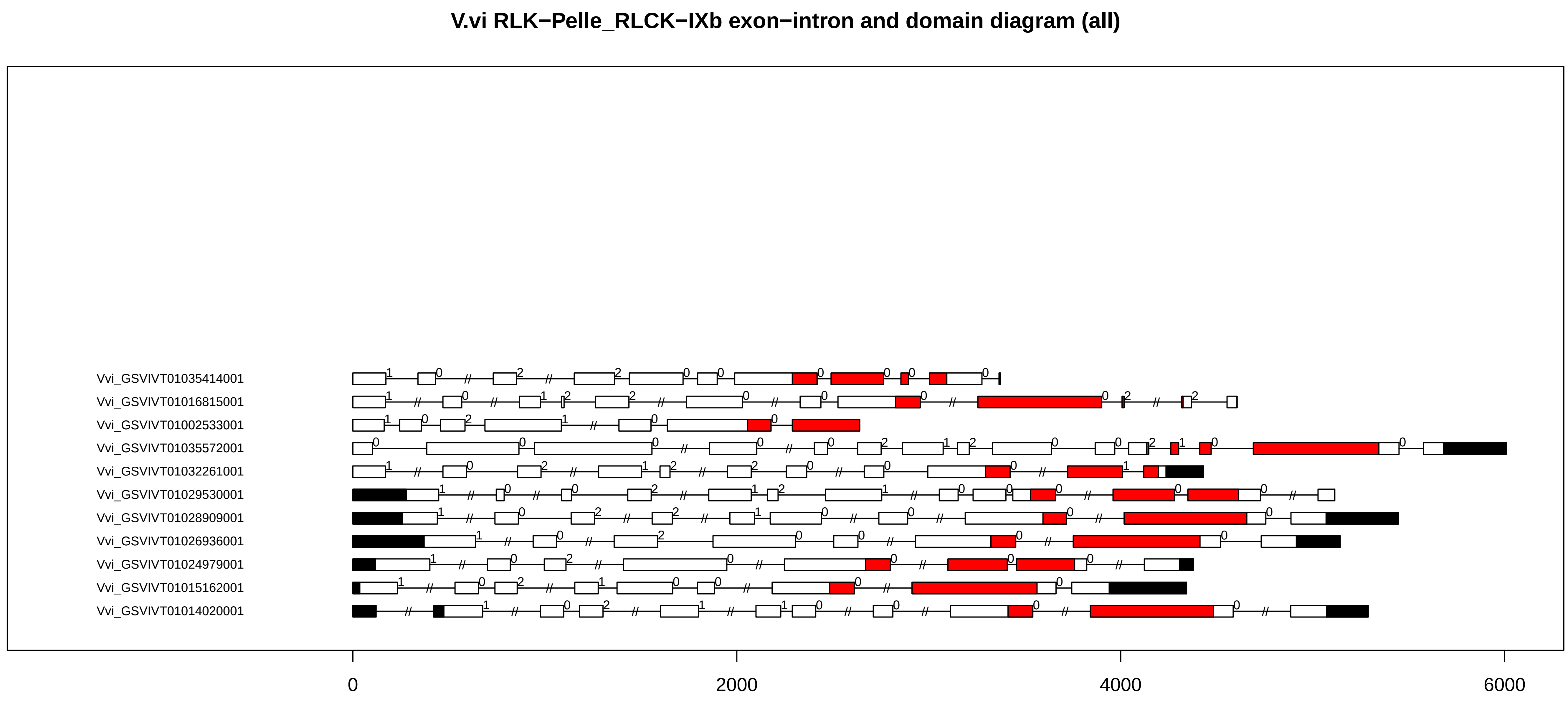

V.vi RLK−Pelle_RLCK−IXb exon−intron and domain diagram (all)
Vvi_GSVIVT01035414001
Vvi_GSVIVT01016815001
Vvi_GSVIVT01002533001
Vvi_GSVIVT01035572001
Vvi_GSVIVT01032261001
Vvi_GSVIVT01029530001
Vvi_GSVIVT01028909001
Vvi_GSVIVT01026936001
Vvi_GSVIVT01024979001
Vvi_GSVIVT01015162001
Vvi_GSVIVT01014020001
0
2000
4000
6000

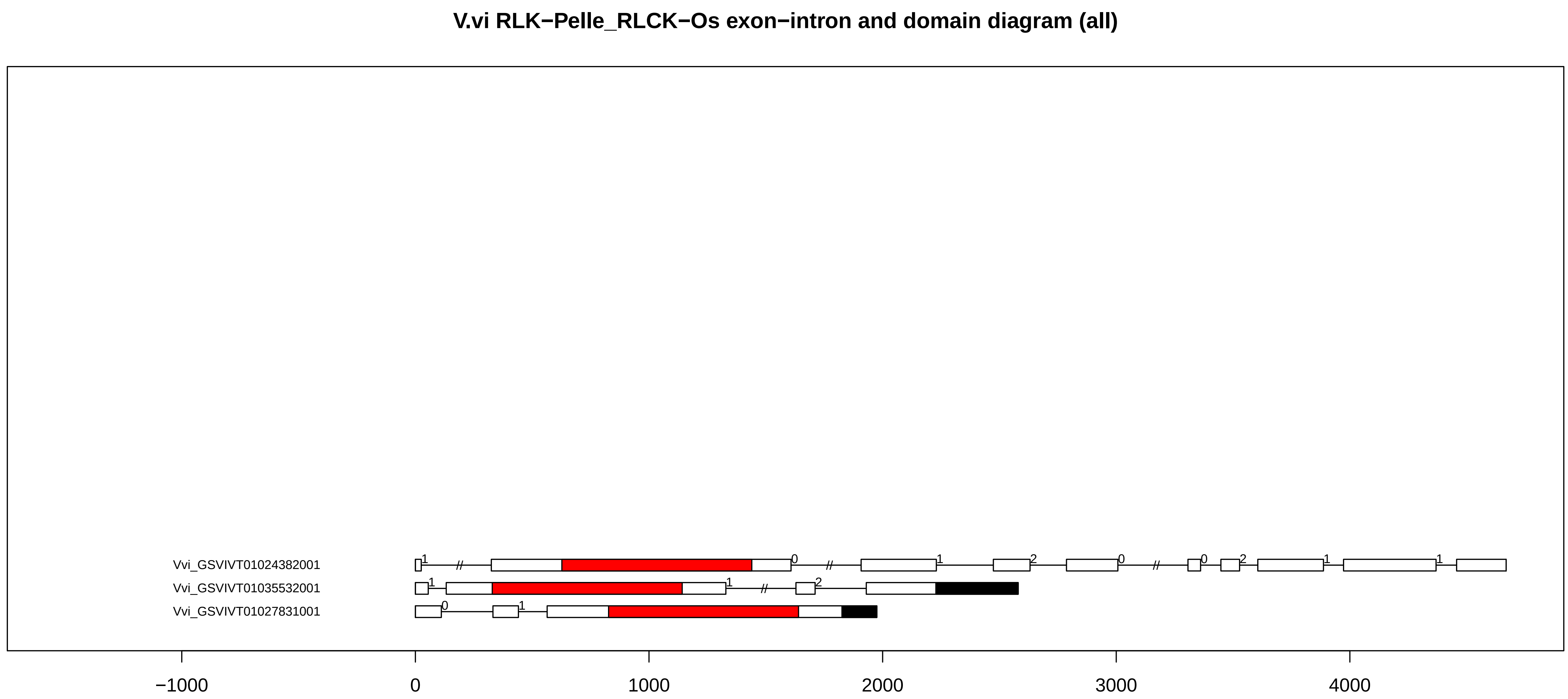
V.vi RLK−Pelle_RLCK−Os exon−intron and domain diagram (all)
Vvi_GSVIVT01024382001
Vvi_GSVIVT01035532001
Vvi_GSVIVT01027831001
−1000
0
1000
2000
3000
4000

V.vi RLK−Pelle_RLCK−VIIa−1 exon−intron and domain diagram (all)

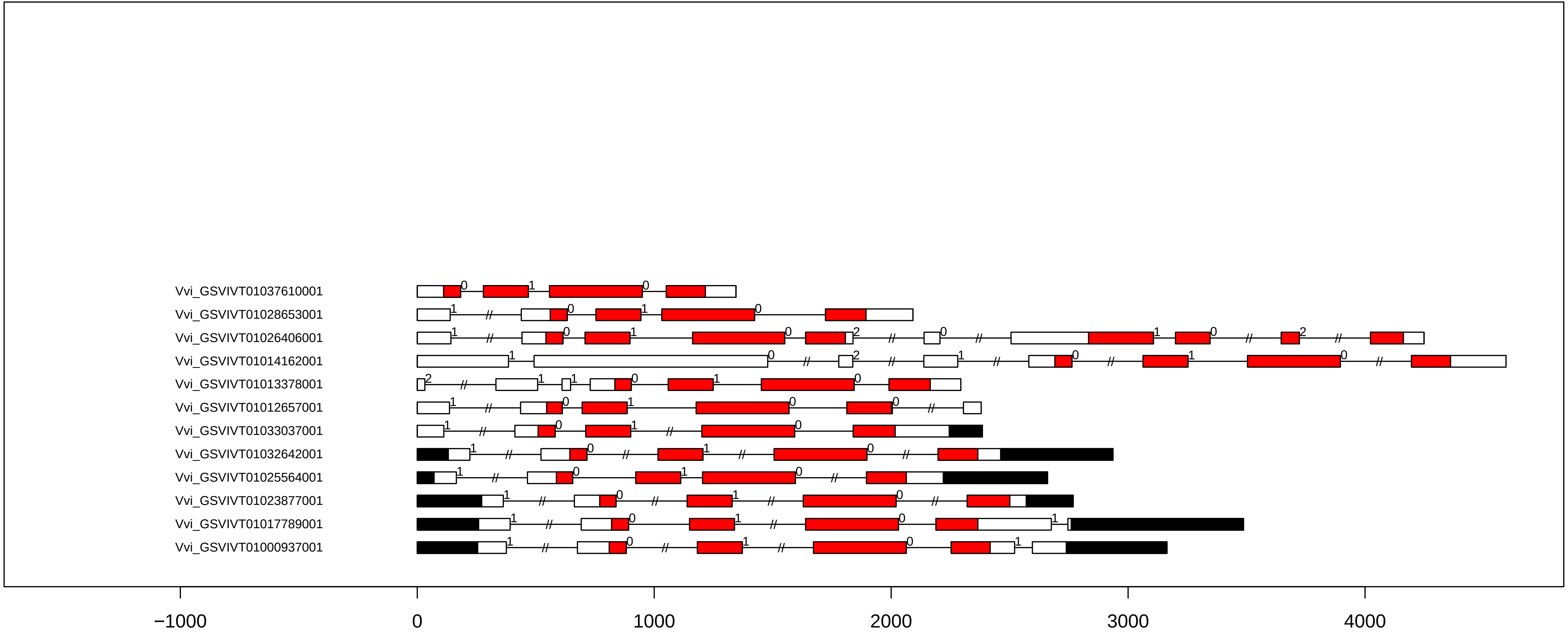

V.vi RLK−Pelle_RLCK−VIIa−2 exon−intron and domain diagram (part 1)

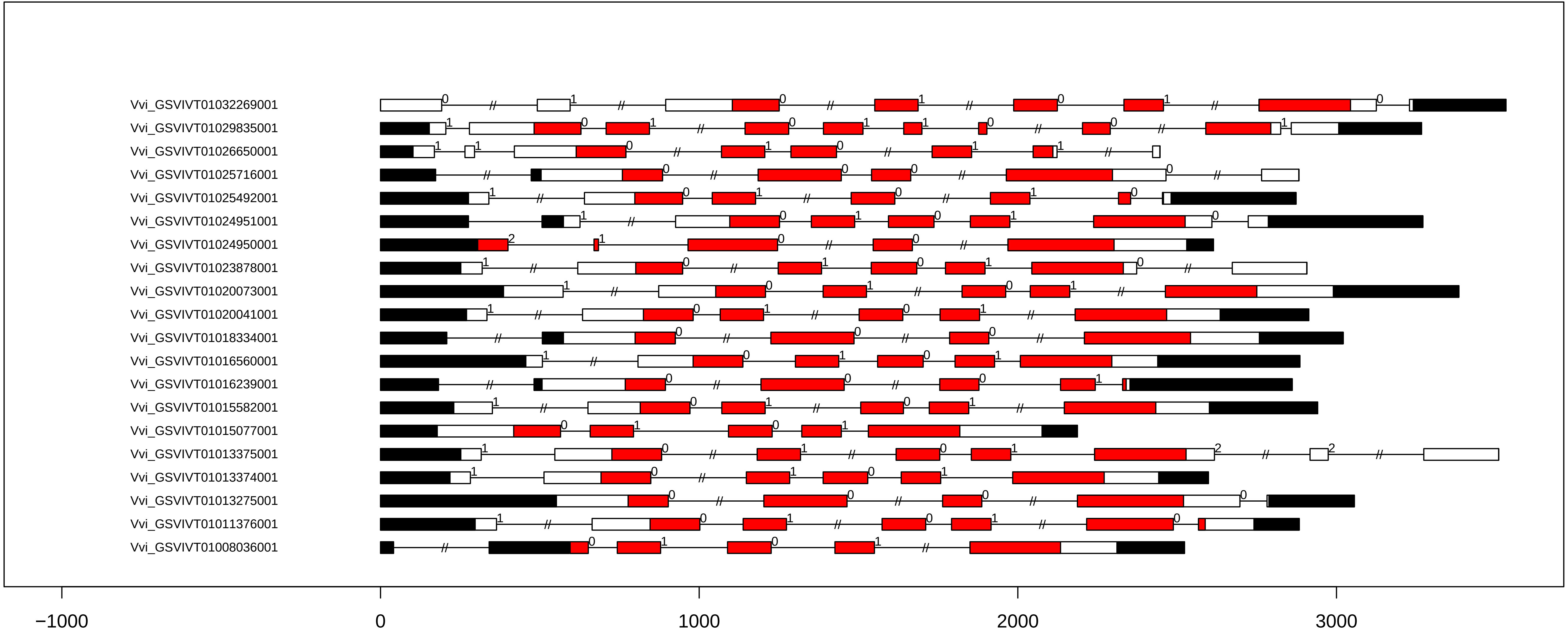

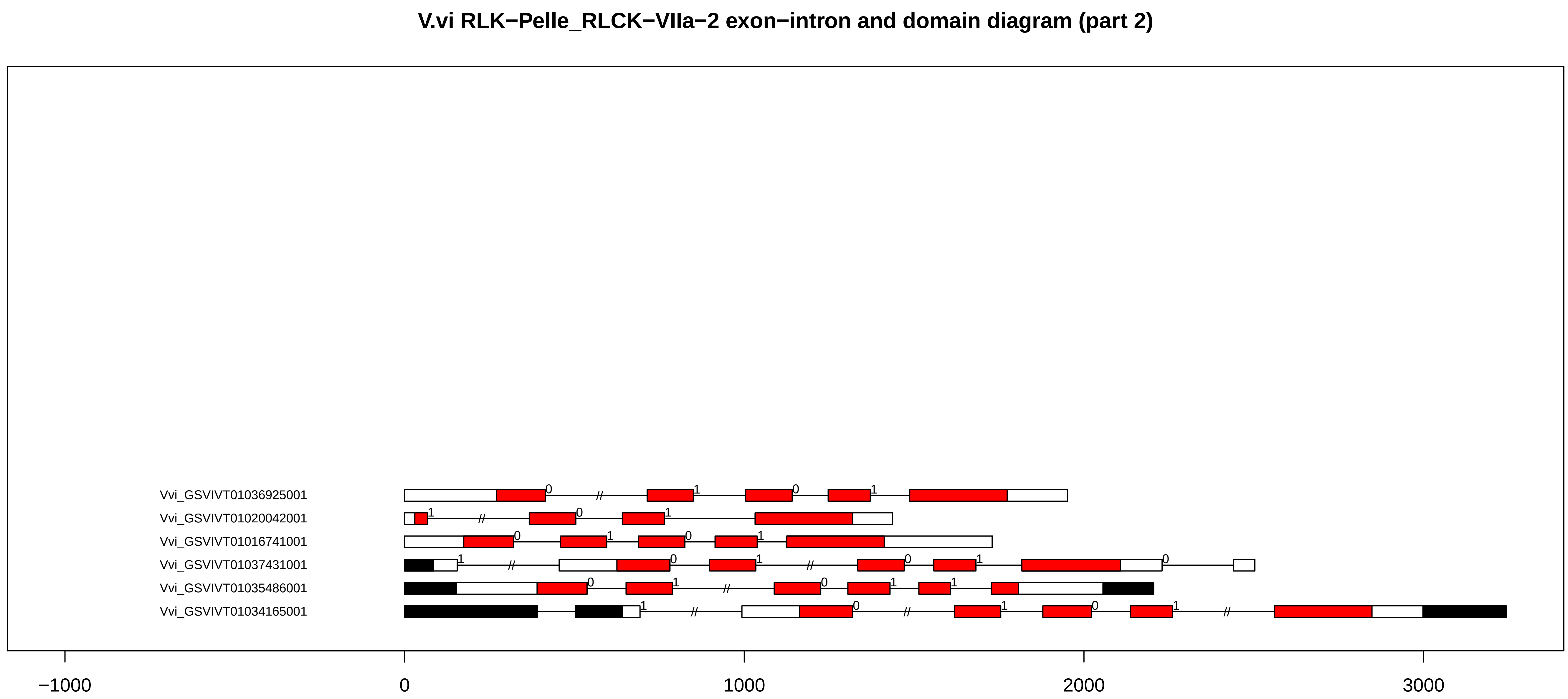
V.vi RLK−Pelle_RLCK−VIIa−2 exon−intron and domain diagram (part 2)
Vvi_GSVIVT01036925001
Vvi_GSVIVT01020042001
Vvi_GSVIVT01016741001
Vvi_GSVIVT01037431001
Vvi_GSVIVT01035486001
Vvi_GSVIVT01034165001
−1000
0
1000
2000
3000

V.vi RLK−Pelle_RLCK−VIIb exon−intron and domain diagram (all)

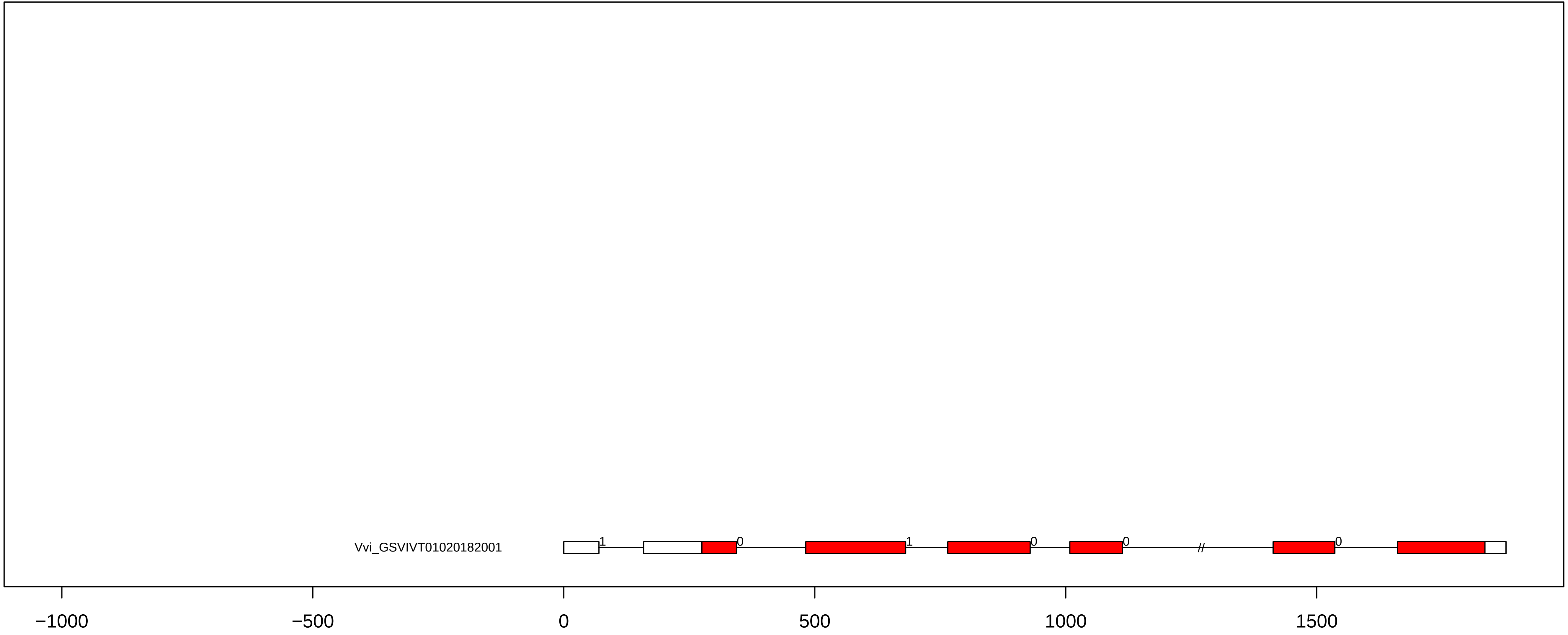

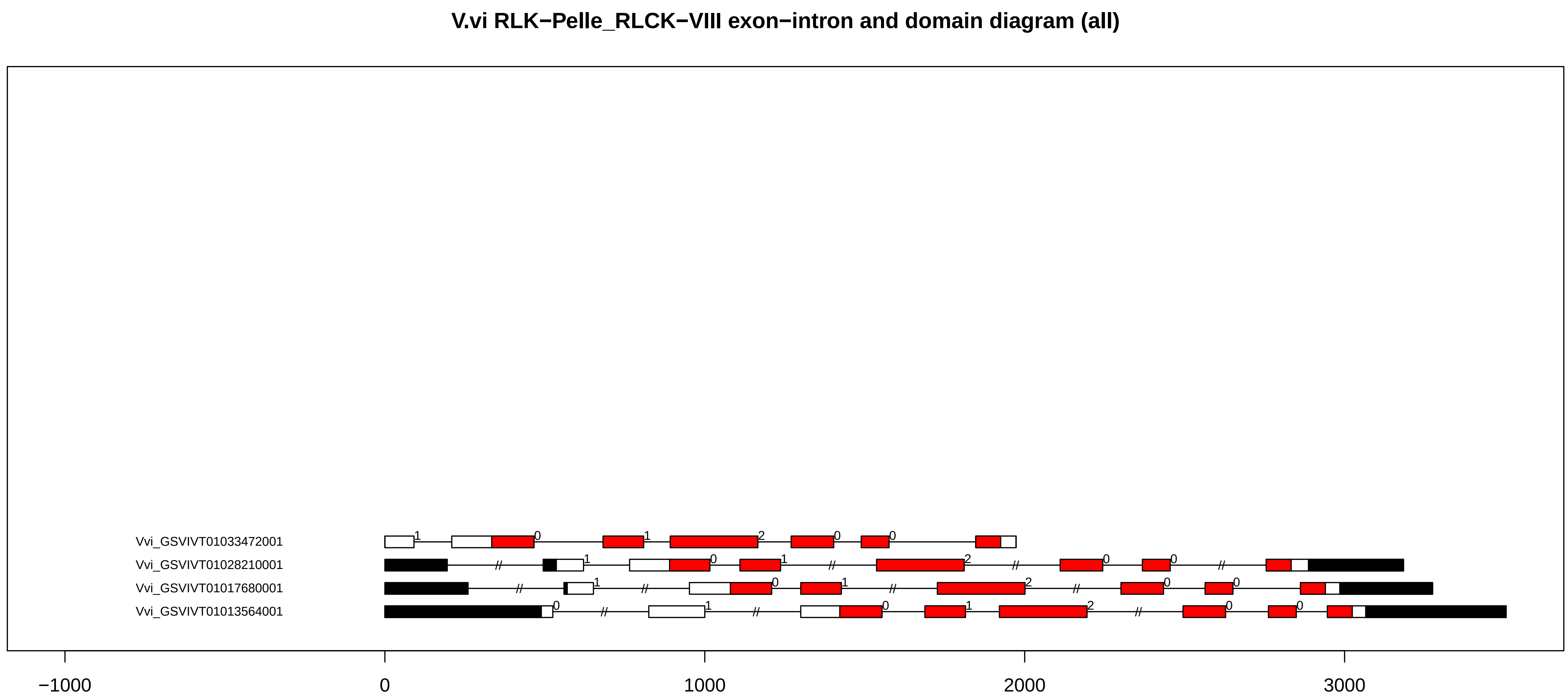
V.vi RLK−Pelle_RLCK−VIII exon−intron and domain diagram (all)
Vvi_GSVIVT01033472001
Vvi_GSVIVT01028210001
Vvi_GSVIVT01017680001
Vvi_GSVIVT01013564001
−1000
0
1000
2000
3000

V.vi RLK−Pelle_RLCK−VI exon−intron and domain diagram (all)

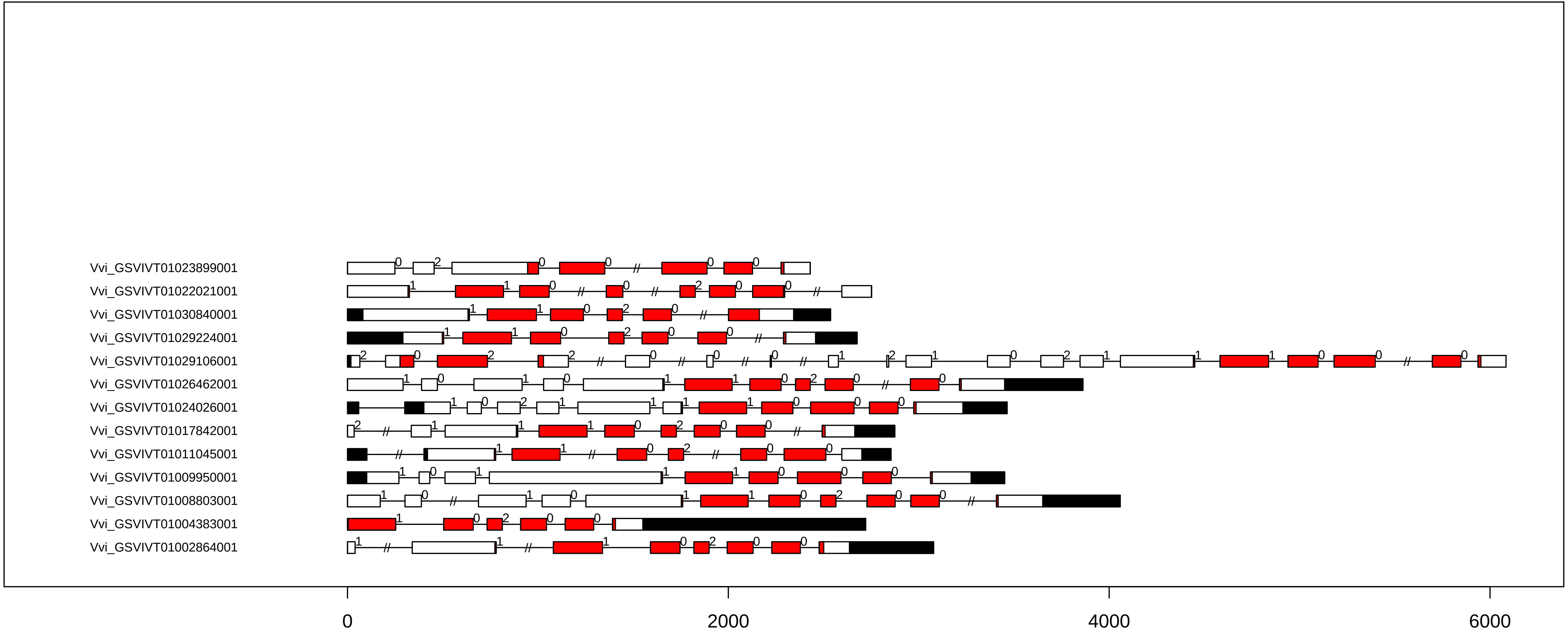

V.vi RLK−Pelle_RLCK−V exon−intron and domain diagram (all)

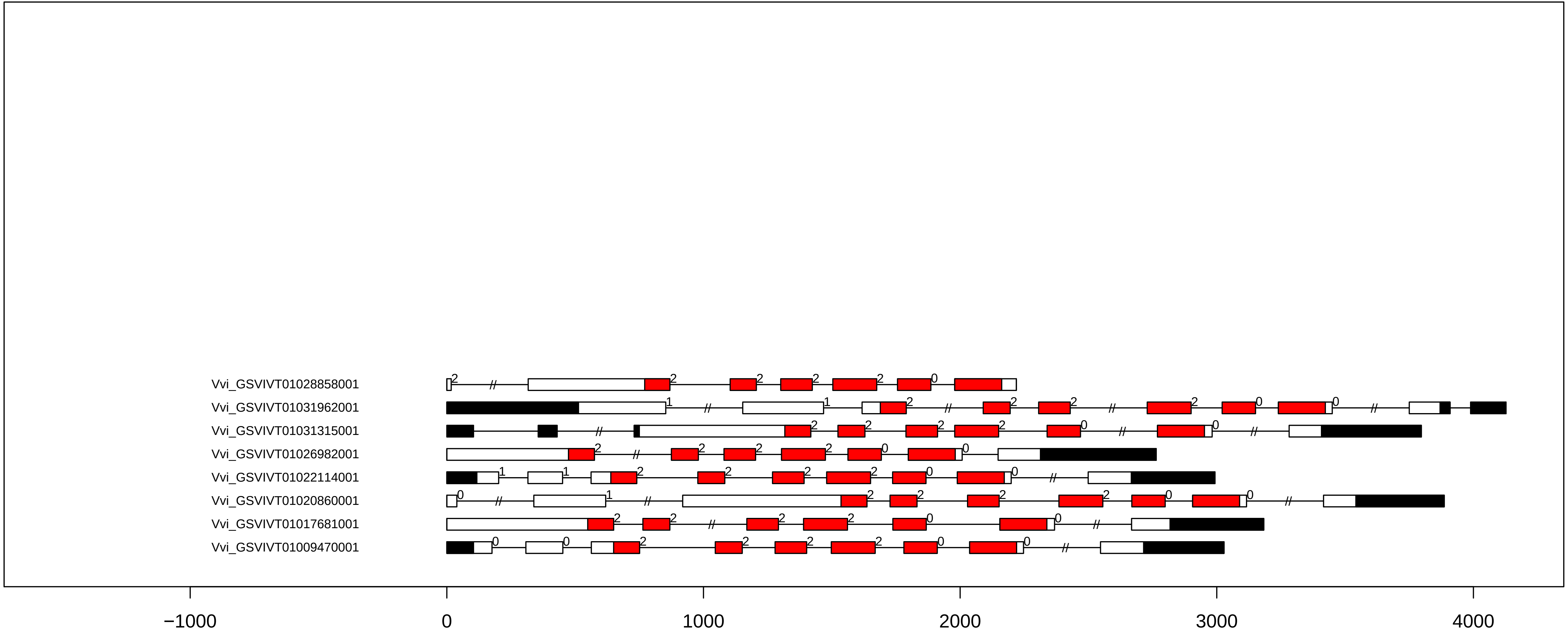

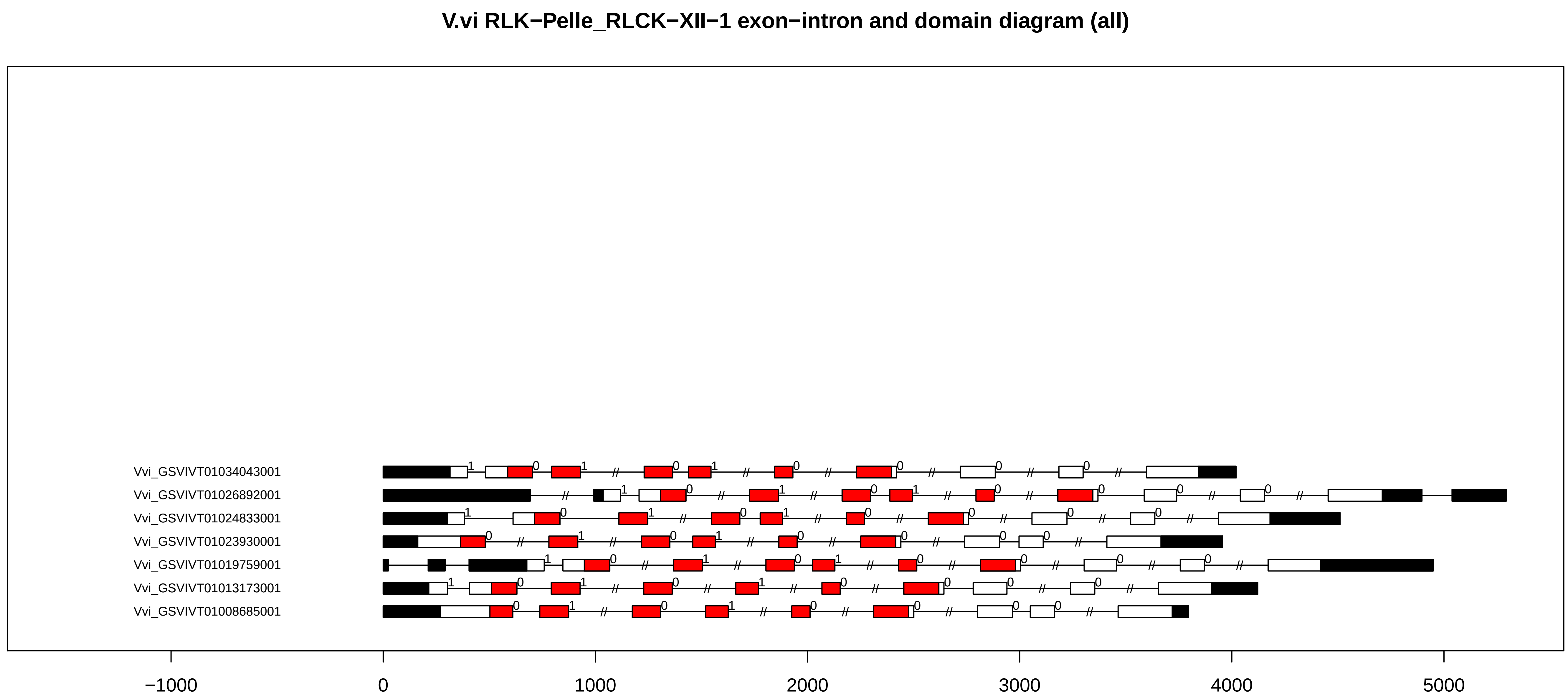
V.vi RLK−Pelle_RLCK−XII−1 exon−intron and domain diagram (all)
Vvi_GSVIVT01034043001
Vvi_GSVIVT01026892001
Vvi_GSVIVT01024833001
Vvi_GSVIVT01023930001
Vvi_GSVIVT01019759001
Vvi_GSVIVT01013173001
Vvi_GSVIVT01008685001
−1000
0
1000
2000
3000
4000
5000

V.vi RLK−Pelle_RLCK−XII−2 exon−intron and domain diagram (all)

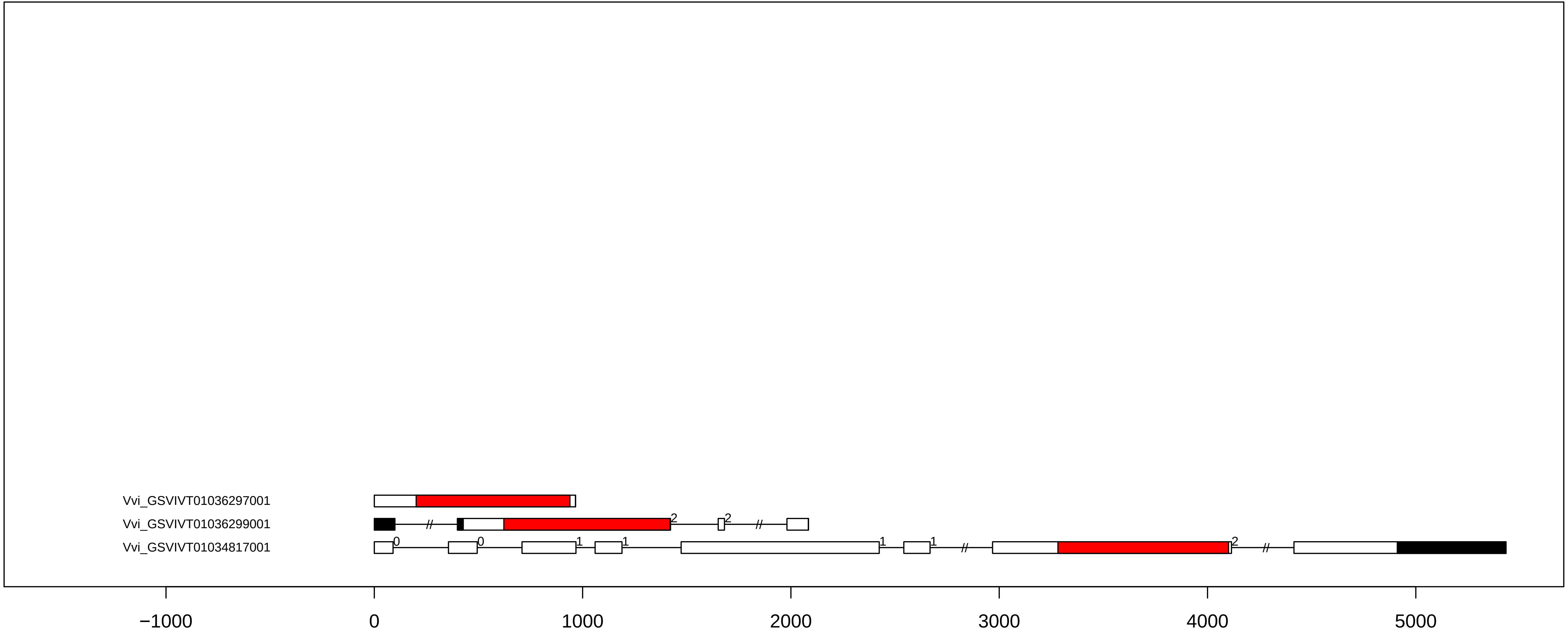

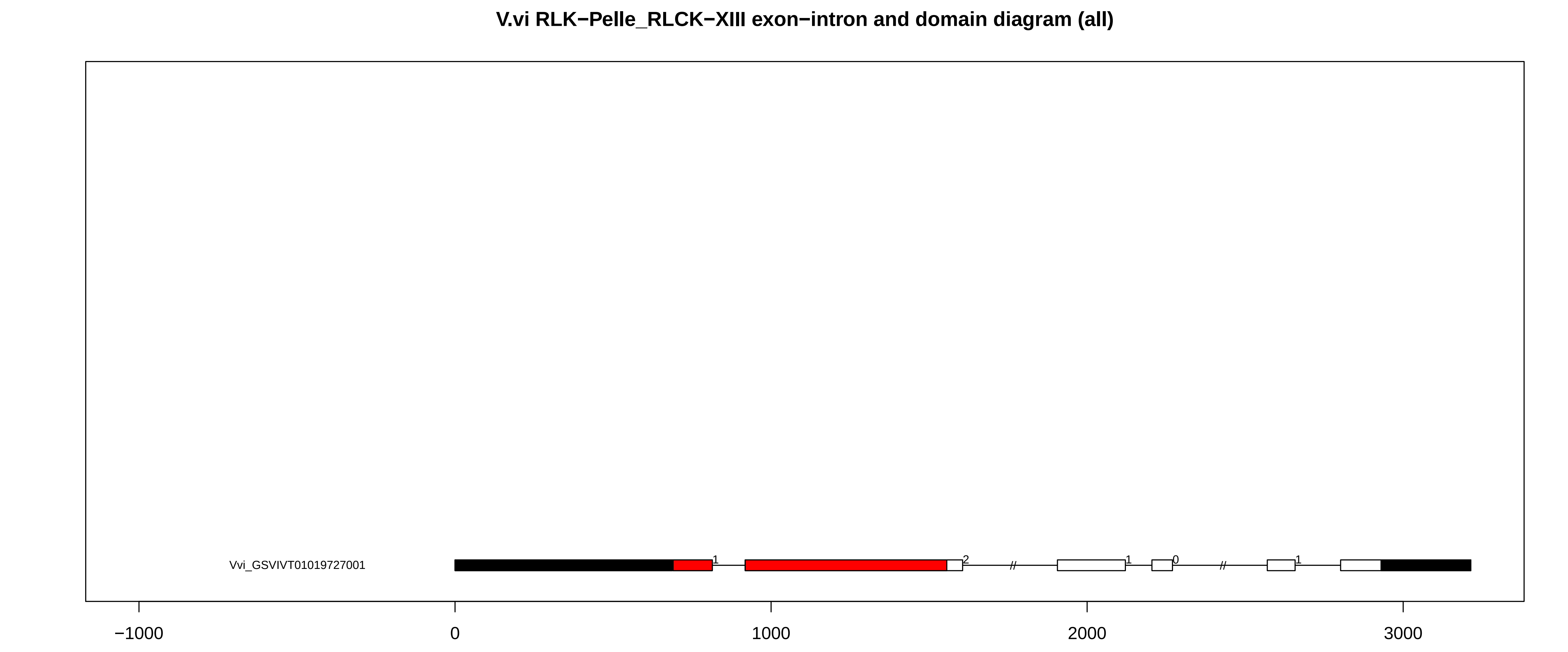
V.vi RLK−Pelle_RLCK−XIII exon−intron and domain diagram (all)
Vvi_GSVIVT01019727001
1
2
1
0
1
−1000
0
1000
2000
3000

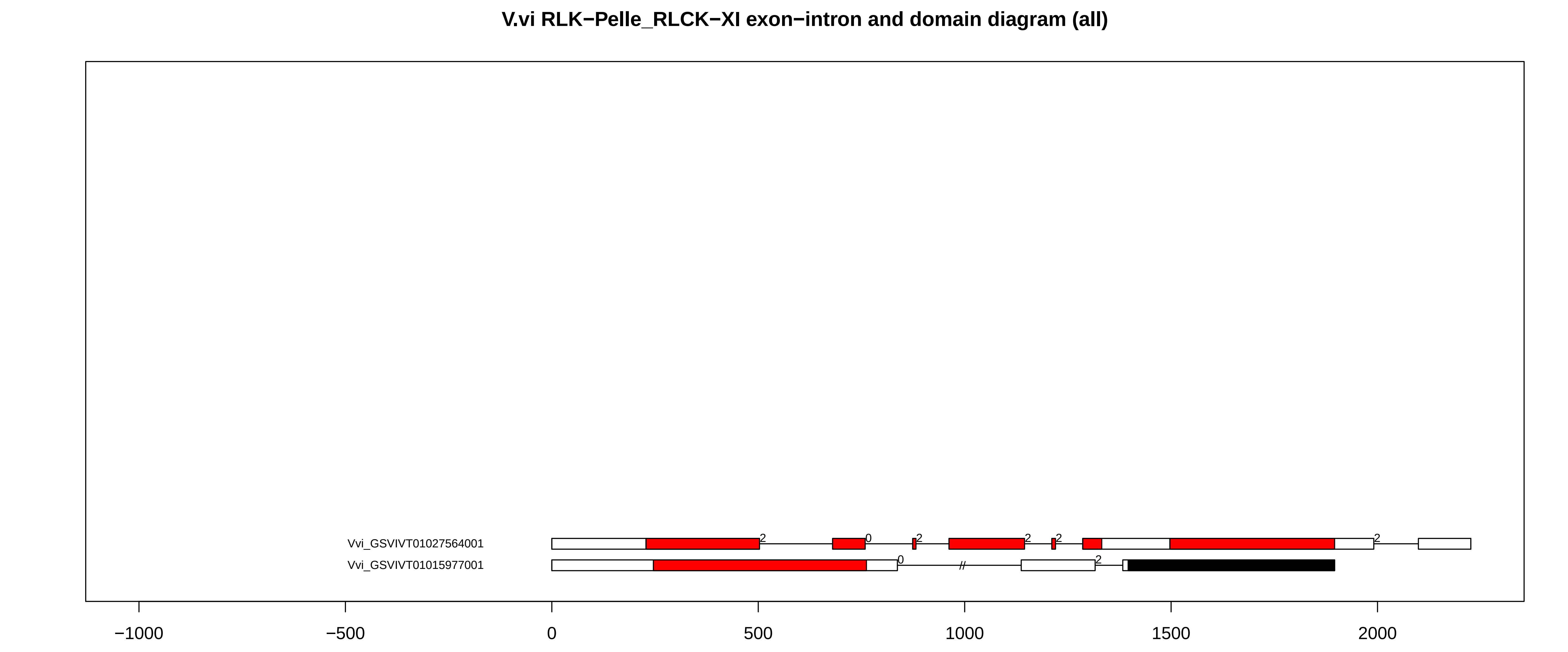
V.vi RLK−Pelle_RLCK−XI exon−intron and domain diagram (all)
Vvi_GSVIVT01027564001
Vvi_GSVIVT01015977001
2
0
2
2
2
2
0
2
−1000
−500
0
500
1000
1500
2000

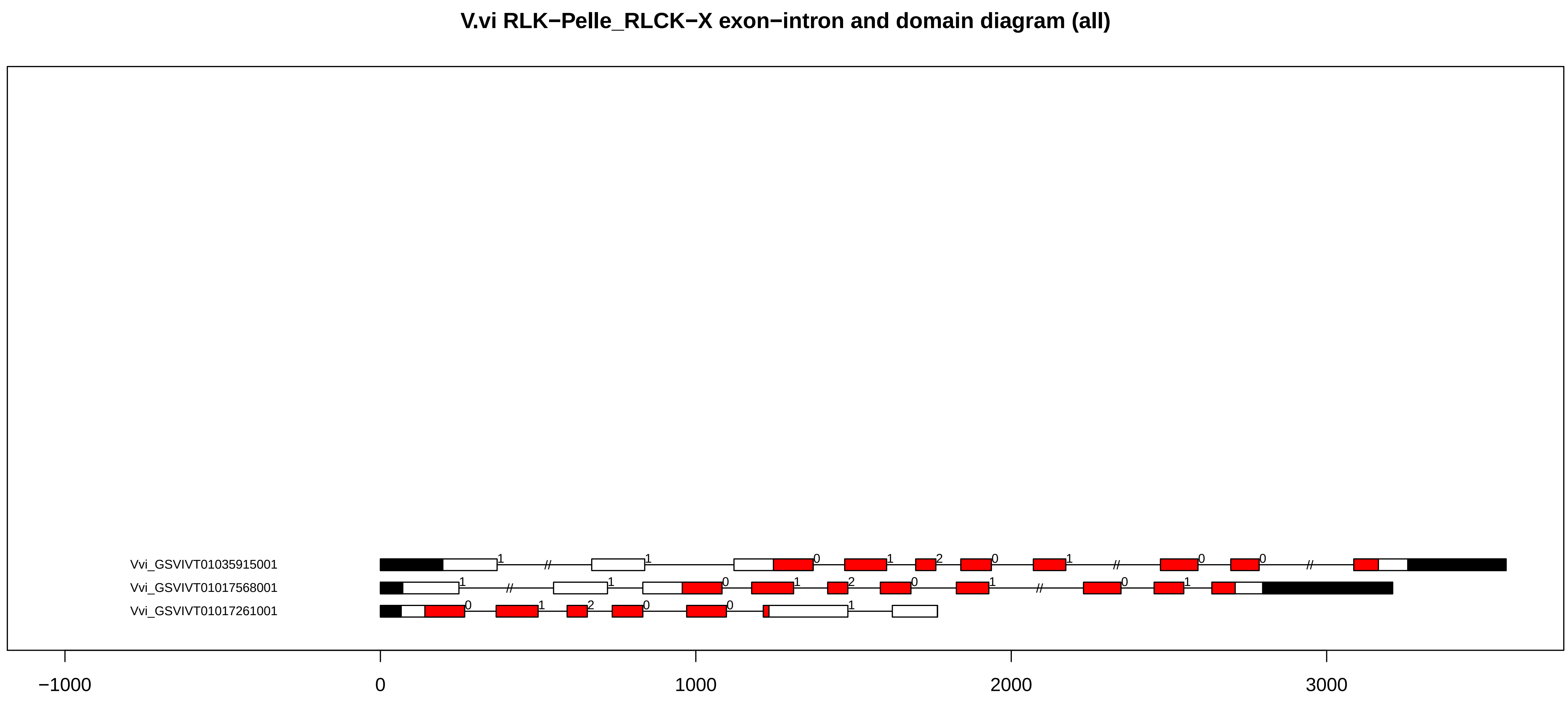
V.vi RLK−Pelle_RLCK−X exon−intron and domain diagram (all)
Vvi_GSVIVT01035915001
Vvi_GSVIVT01017568001
Vvi_GSVIVT01017261001
−1000
0
1000
2000
3000

V.vi RLK−Pelle_RLCK−XVI exon−intron and domain diagram (all)

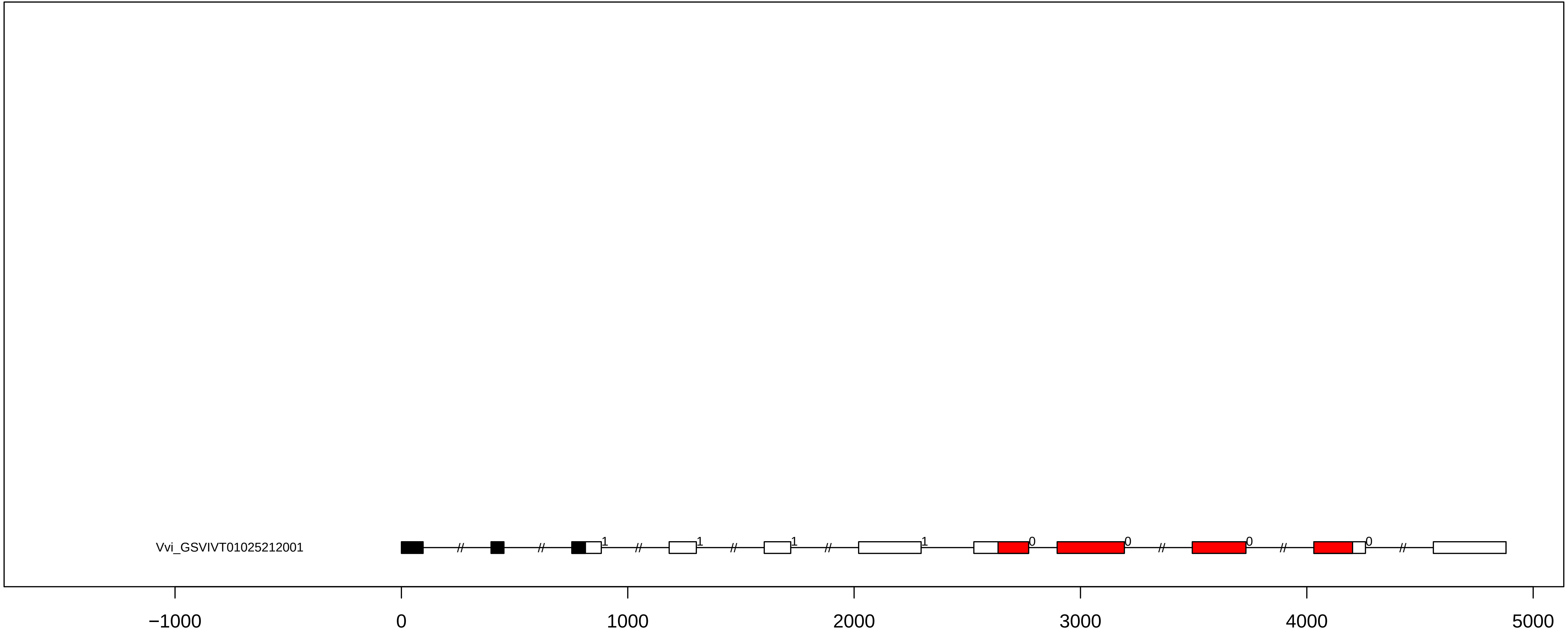

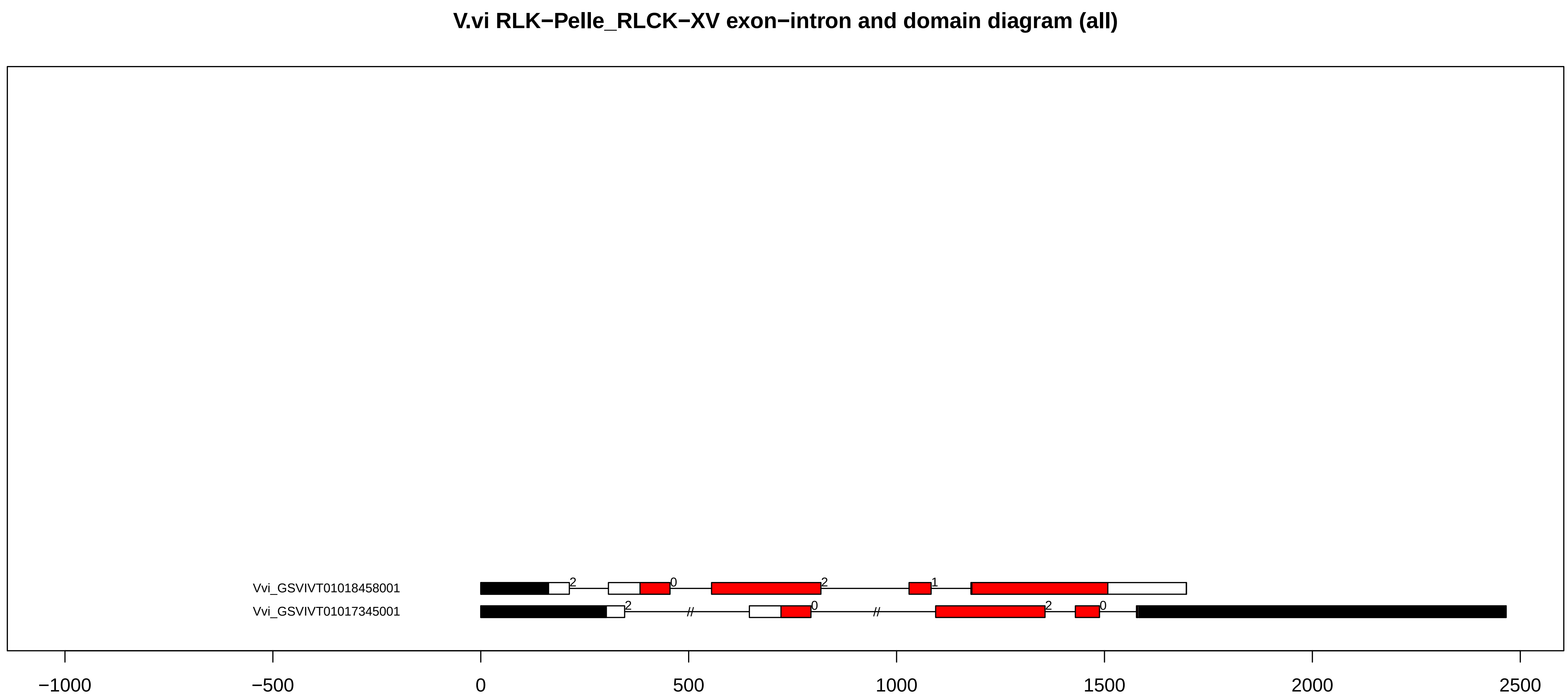
V.vi RLK−Pelle_RLCK−XV exon−intron and domain diagram (all)
Vvi_GSVIVT01018458001
Vvi_GSVIVT01017345001
2
0
2
1
2
0
2
0
−1000
−500
0
500
1000
1500
2000
2500

V.vi RLK−Pelle_SD−2b exon−intron and domain diagram (part 1)

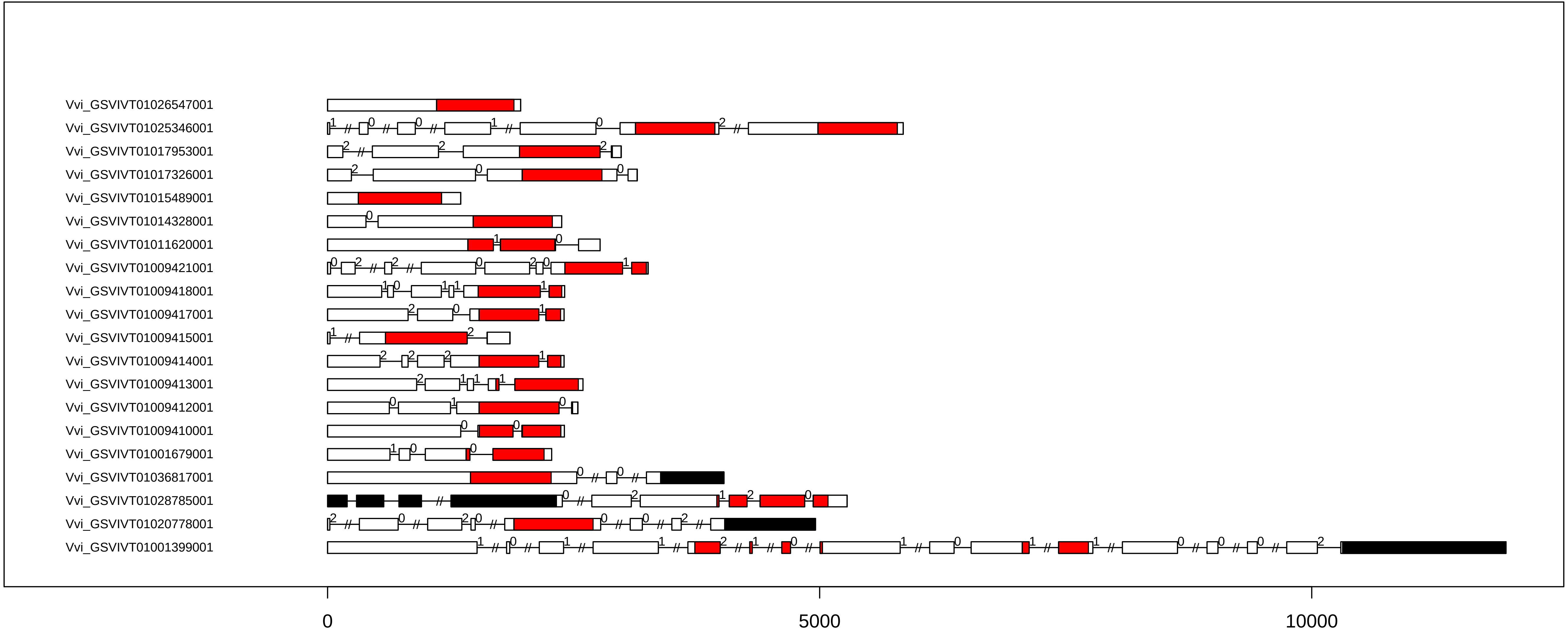

V.vi RLK−Pelle_SD−2b exon−intron and domain diagram (part 2)

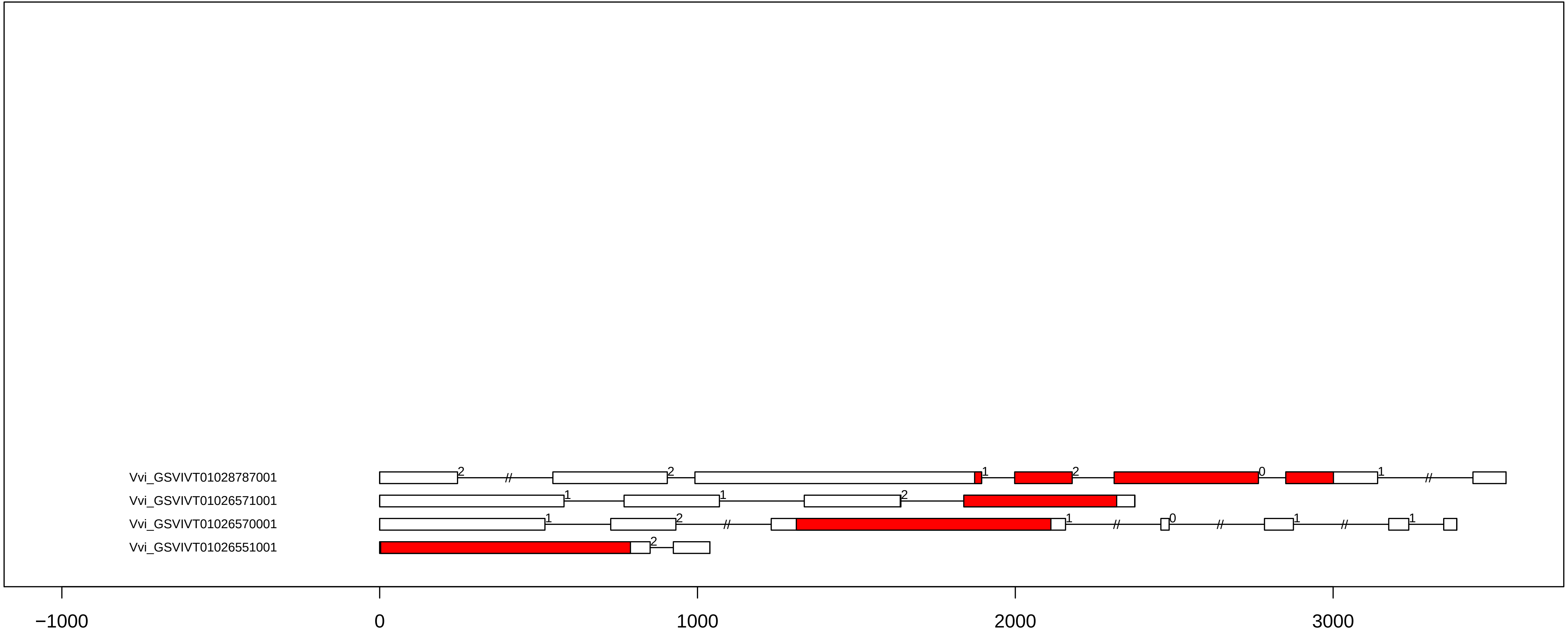

V.vi RLK−Pelle_Singleton exon−intron and domain diagram (all)

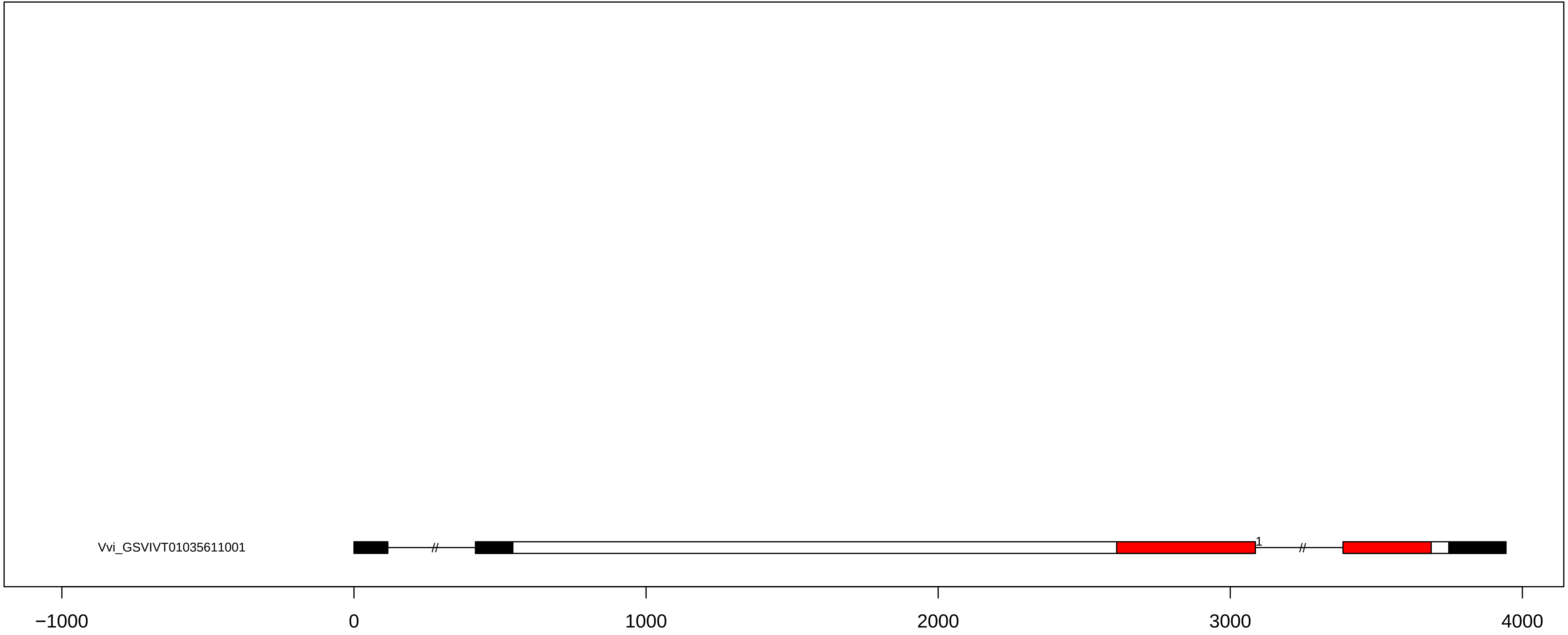

V.vi RLK−Pelle_URK−1 exon−intron and domain diagram (all)

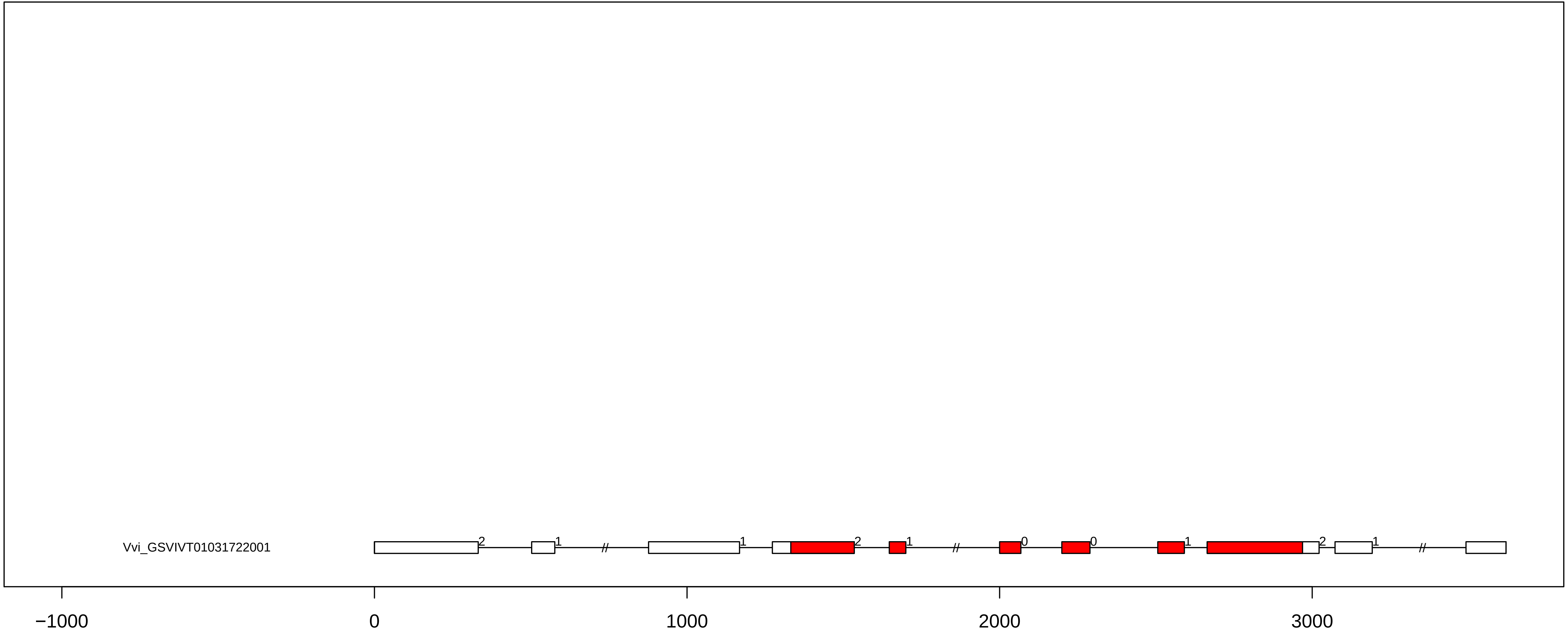

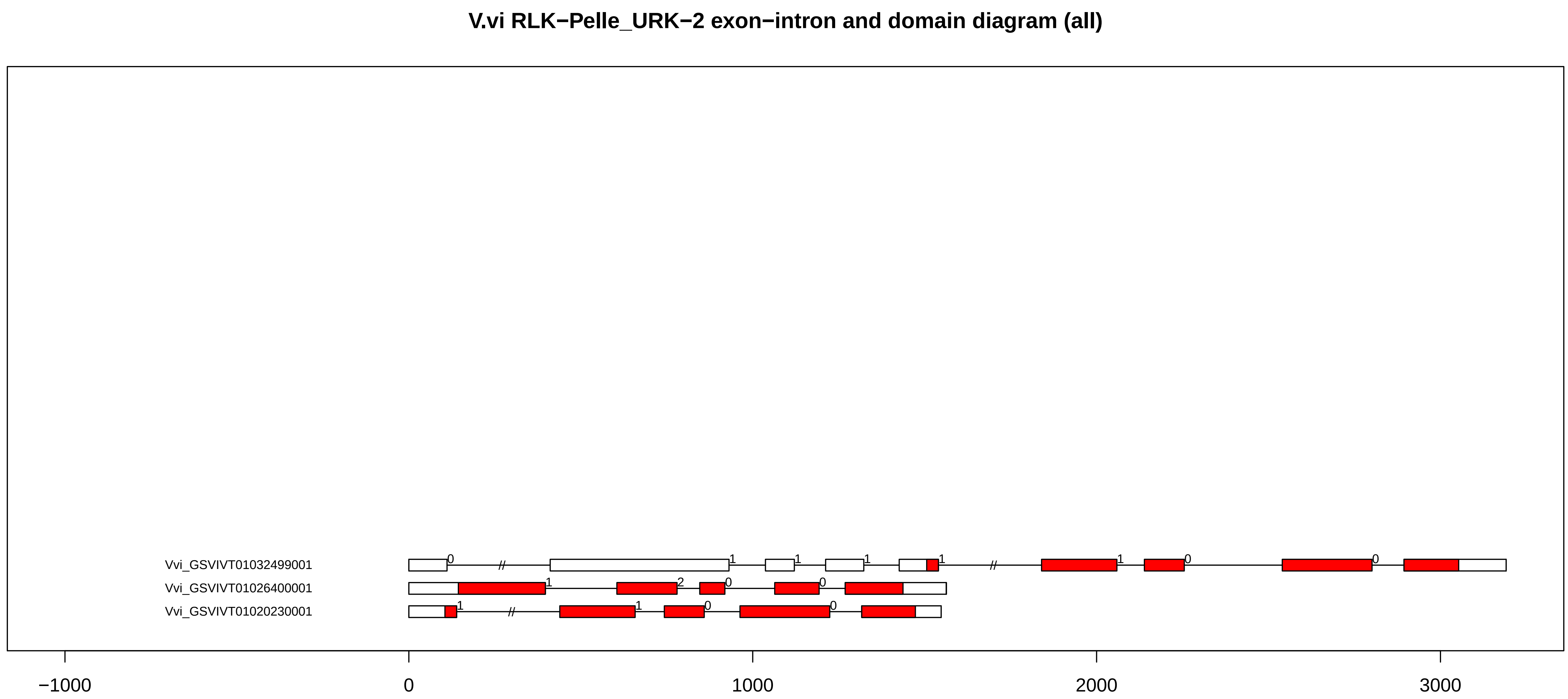

V.vi RLK−Pelle_URK−2 exon−intron and domain diagram (all)
Vvi_GSVIVT01032499001
Vvi_GSVIVT01026400001
Vvi_GSVIVT01020230001
0
1
1
1
1
1
0
0
1
2
0
0
1
1
0
0
−1000
0
1000
2000
3000

V.vi RLK−Pelle_URK−3 exon−intron and domain diagram (all)

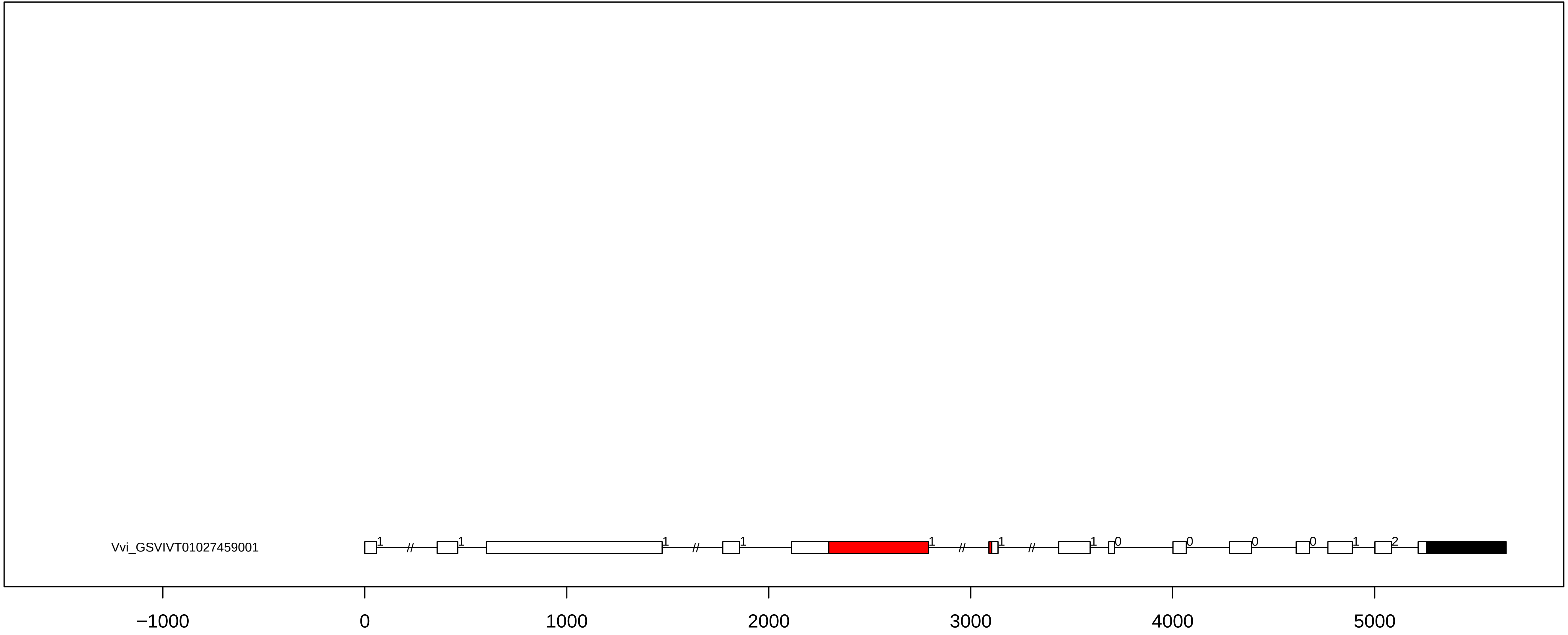

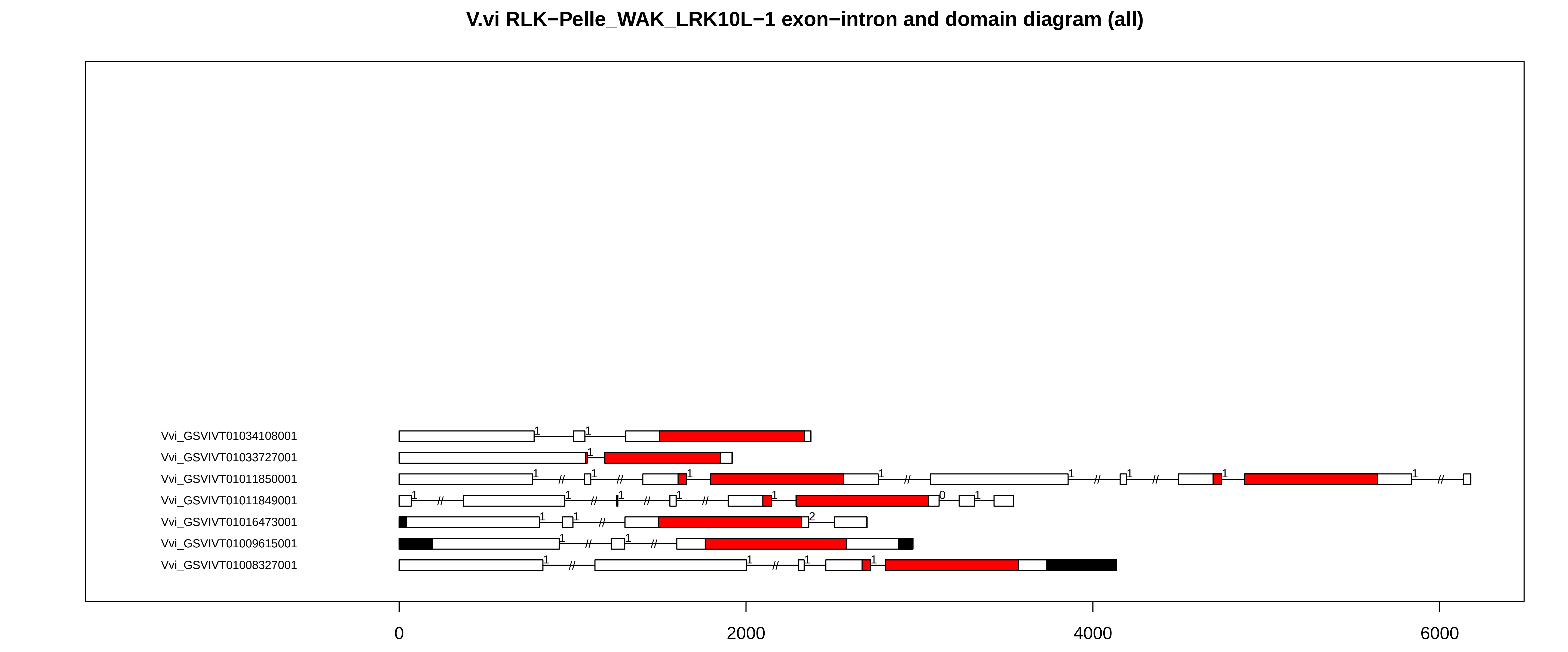

V.vi RLK−Pelle_WAK_LRK10L−1 exon−intron and domain diagram (all)
Vvi_GSVIVT01034108001
Vvi_GSVIVT01033727001
Vvi_GSVIVT01011850001
Vvi_GSVIVT01011849001
Vvi_GSVIVT01016473001
Vvi_GSVIVT01009615001
Vvi_GSVIVT01008327001
0
2000
4000
6000

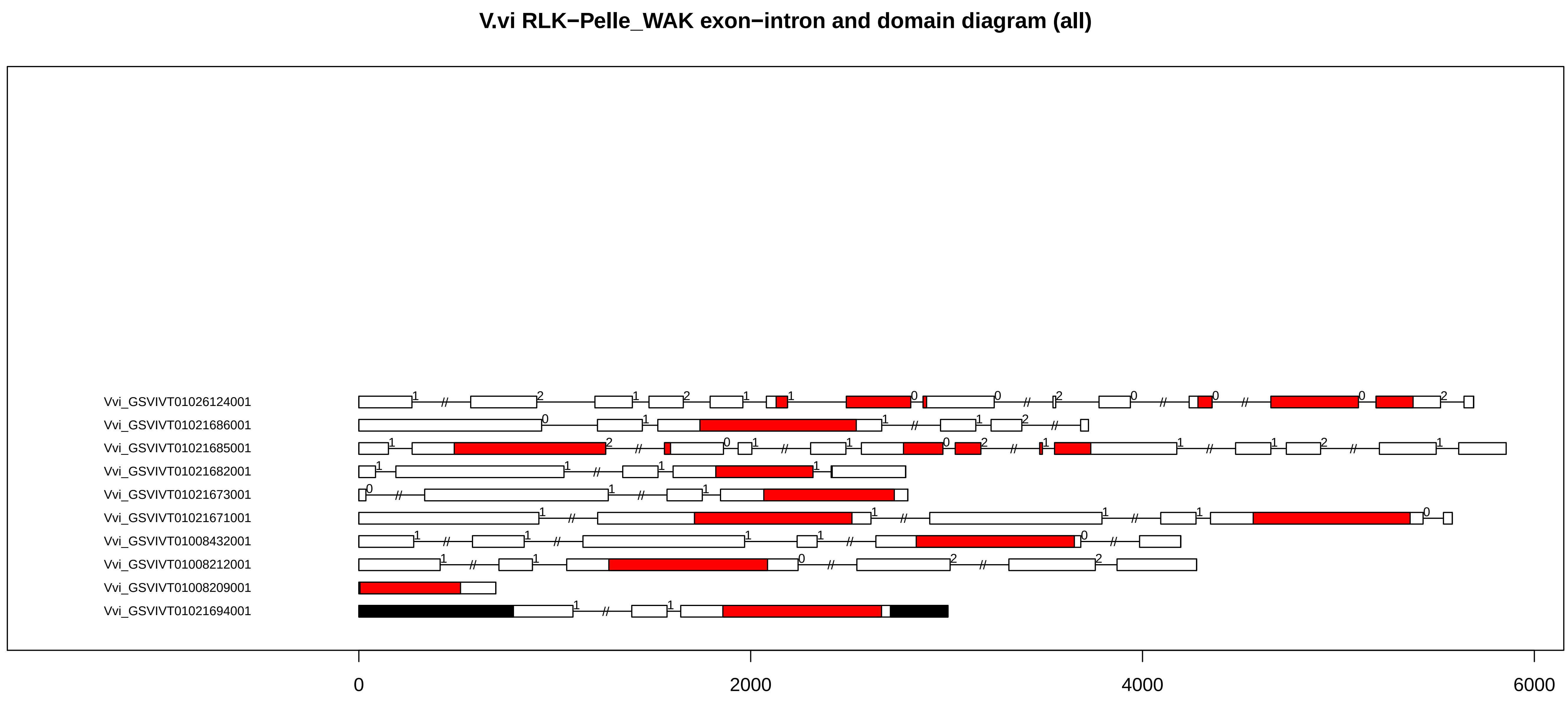
V.vi RLK−Pelle_WAK exon−intron and domain diagram (all)
Vvi_GSVIVT01026124001
Vvi_GSVIVT01021686001
Vvi_GSVIVT01021685001
Vvi_GSVIVT01021682001
Vvi_GSVIVT01021673001
Vvi_GSVIVT01021671001
Vvi_GSVIVT01008432001
Vvi_GSVIVT01008212001
Vvi_GSVIVT01008209001
Vvi_GSVIVT01021694001
0
2000
4000
6000

V.vi SCY1_SCYL1 exon−intron and domain diagram (all)

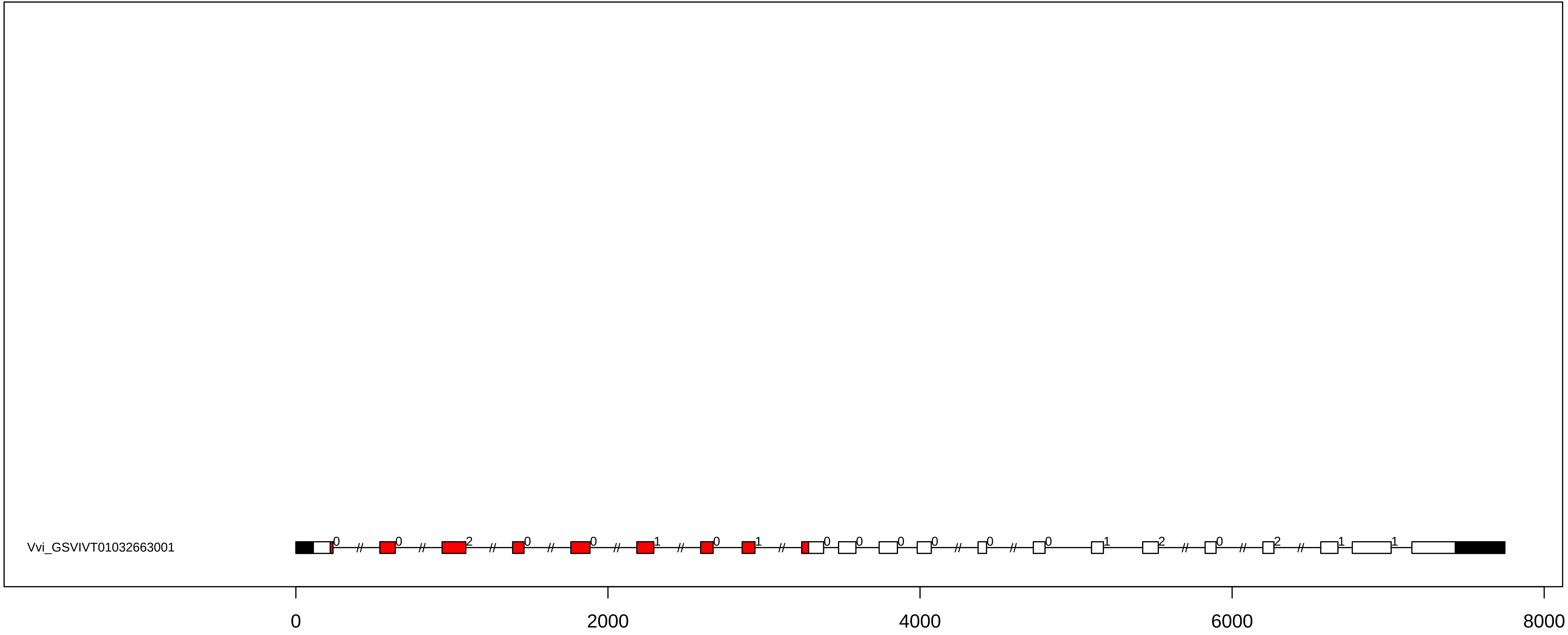

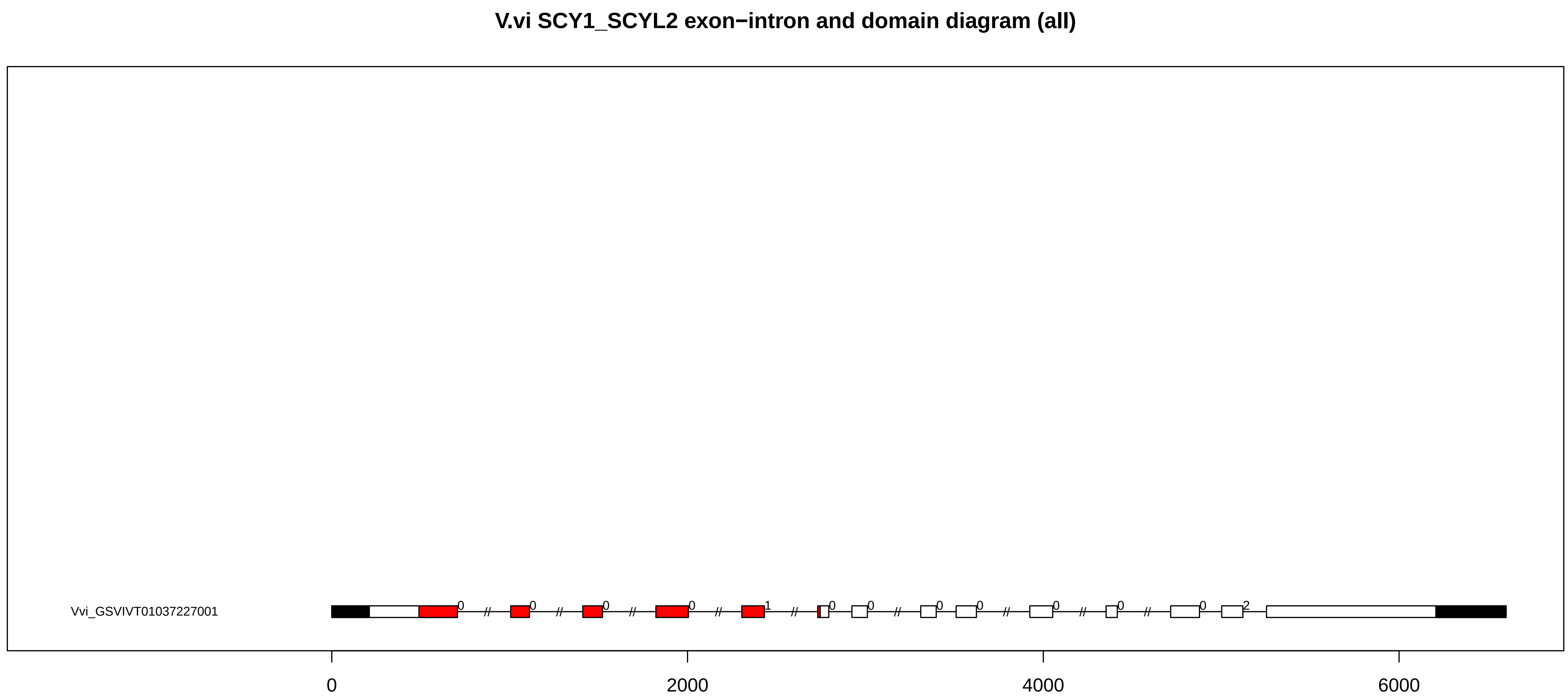
V.vi SCY1_SCYL2 exon−intron and domain diagram (all)
Vvi_GSVIVT01037227001
0
0
0
0
1
0
0
0
0
0
0
0
2
0
2000
4000
6000

V.vi STE_STE11 exon−intron and domain diagram (part 1)

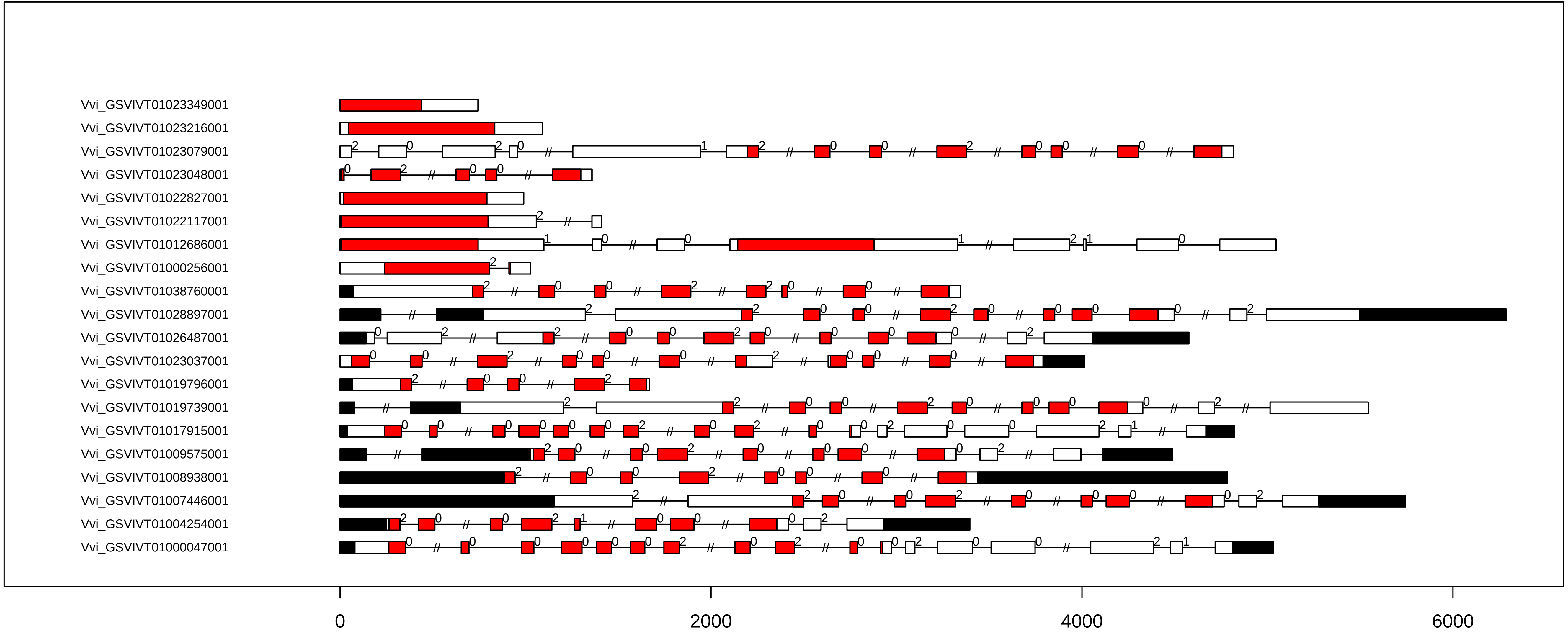

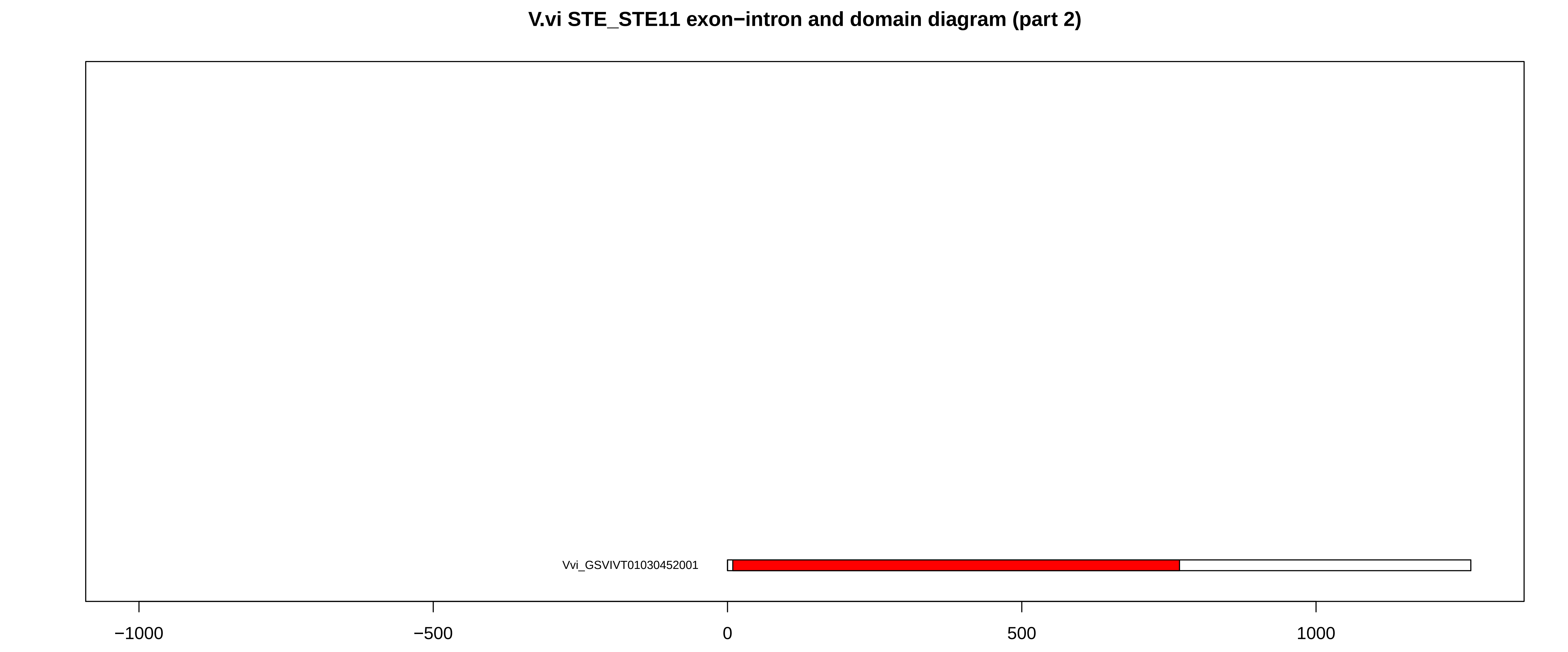
V.vi STE_STE11 exon−intron and domain diagram (part 2)
Vvi_GSVIVT01030452001
−1000
−500
0
500
1000

V.vi STE_STE20−Fray exon−intron and domain diagram (all)

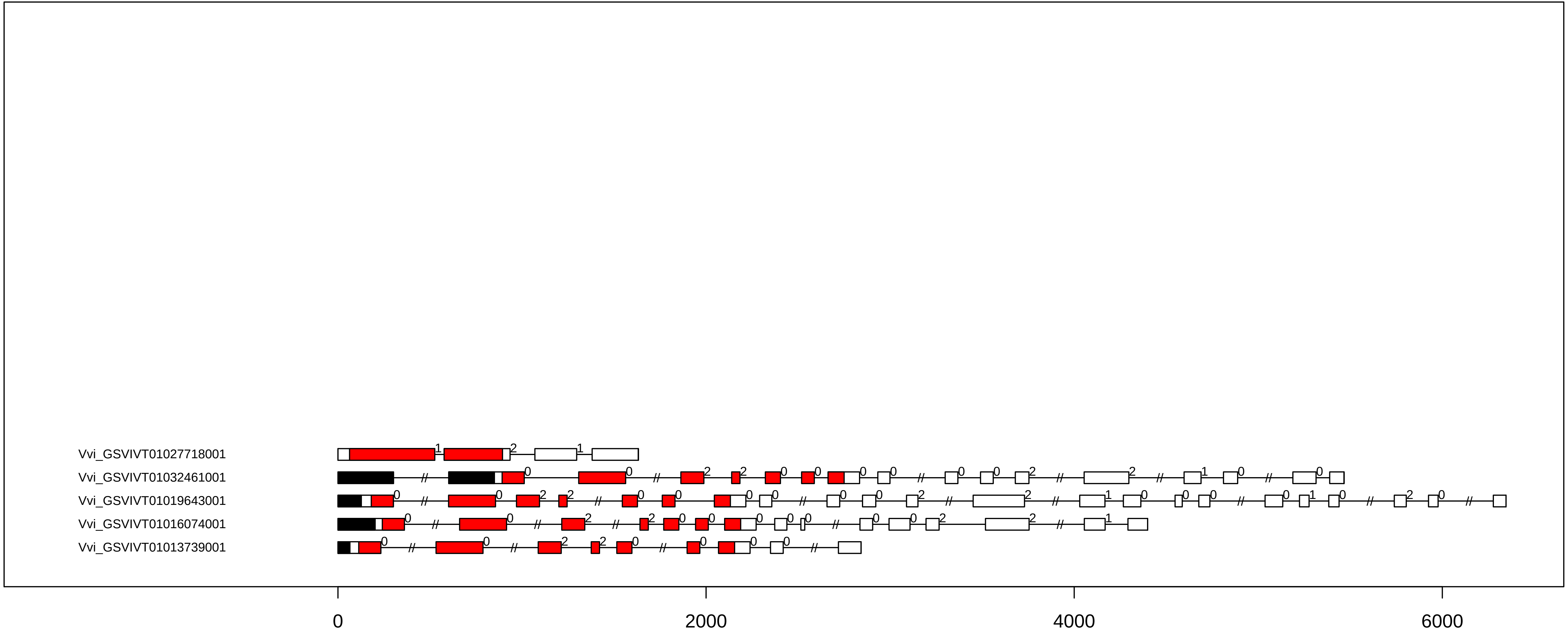

V.vi STE_STE20−PI exon−intron and domain diagram (all)

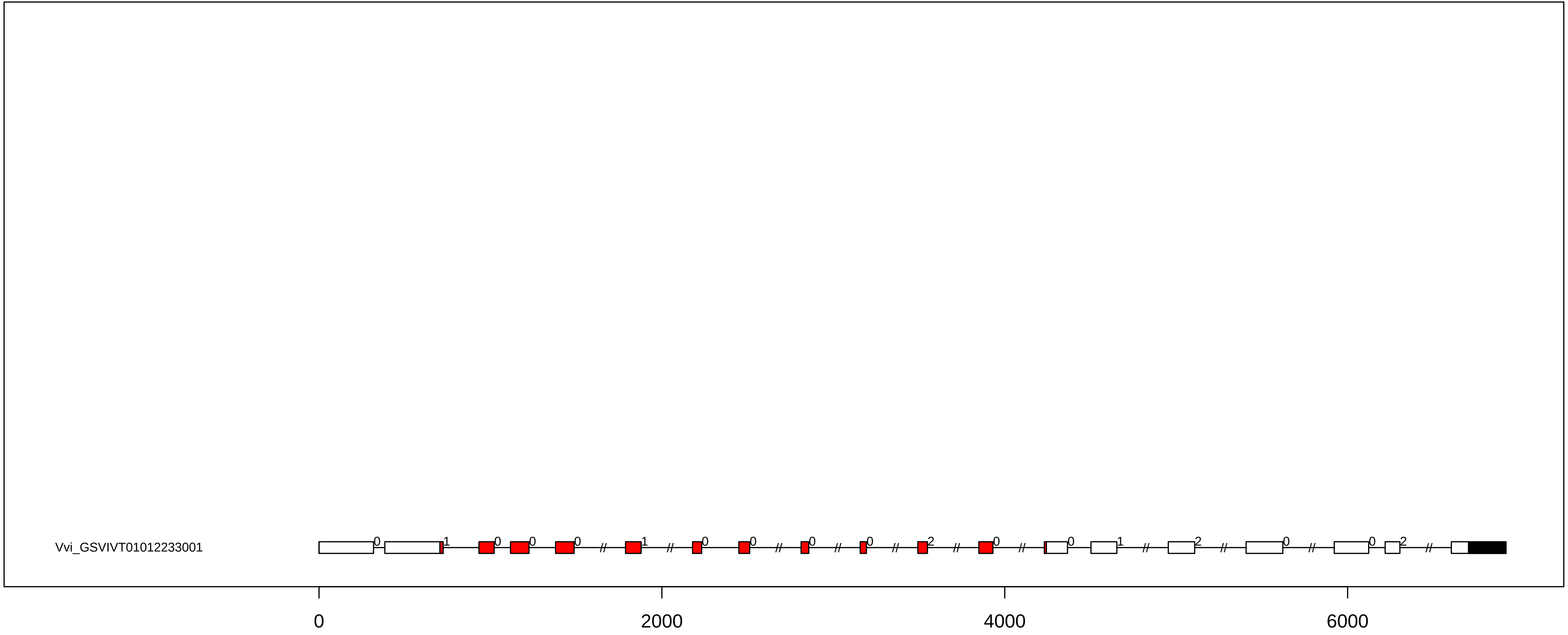

V.vi STE_STE20−YSK exon−intron and domain diagram (all)

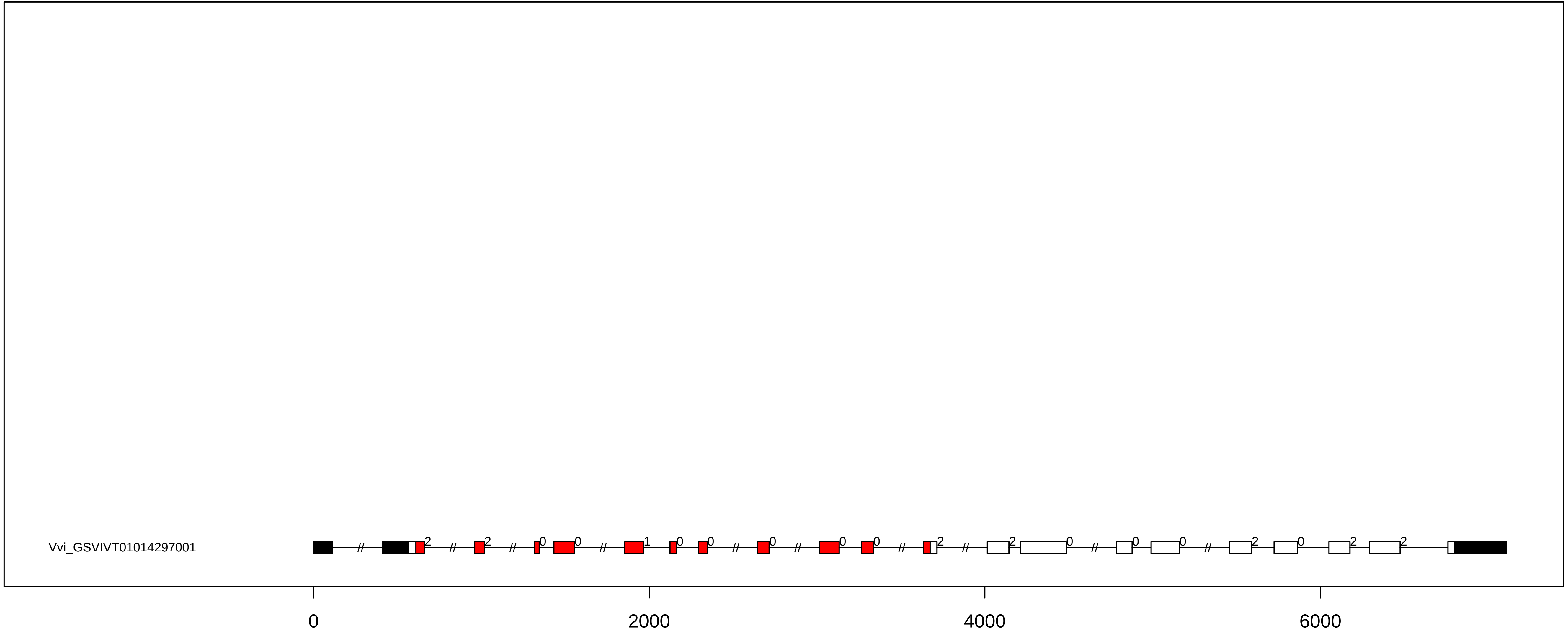

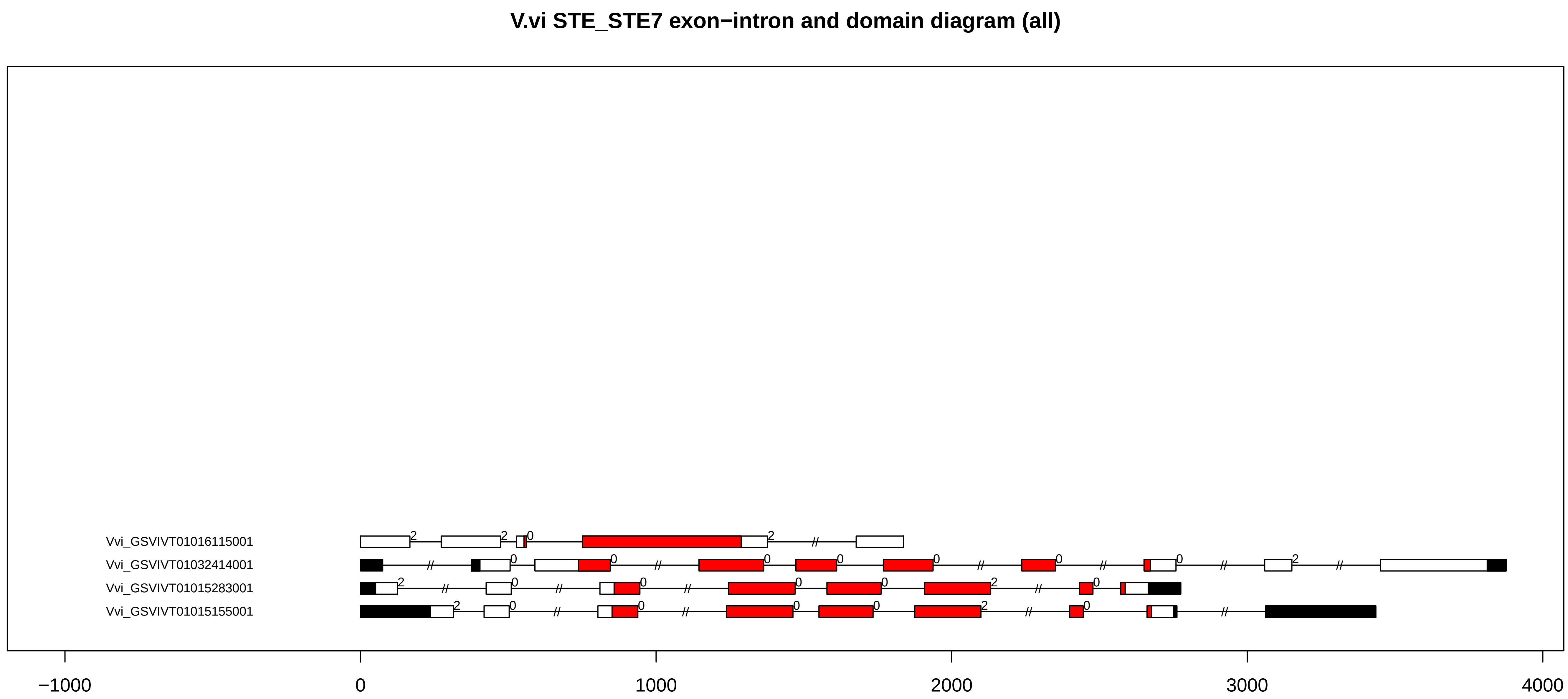

V.vi STE_STE7 exon−intron and domain diagram (all)
Vvi_GSVIVT01016115001
Vvi_GSVIVT01032414001
Vvi_GSVIVT01015283001
Vvi_GSVIVT01015155001
−1000
0
1000
2000
3000
4000

V.vi STE_STE−PI exon−intron and domain diagram (all)

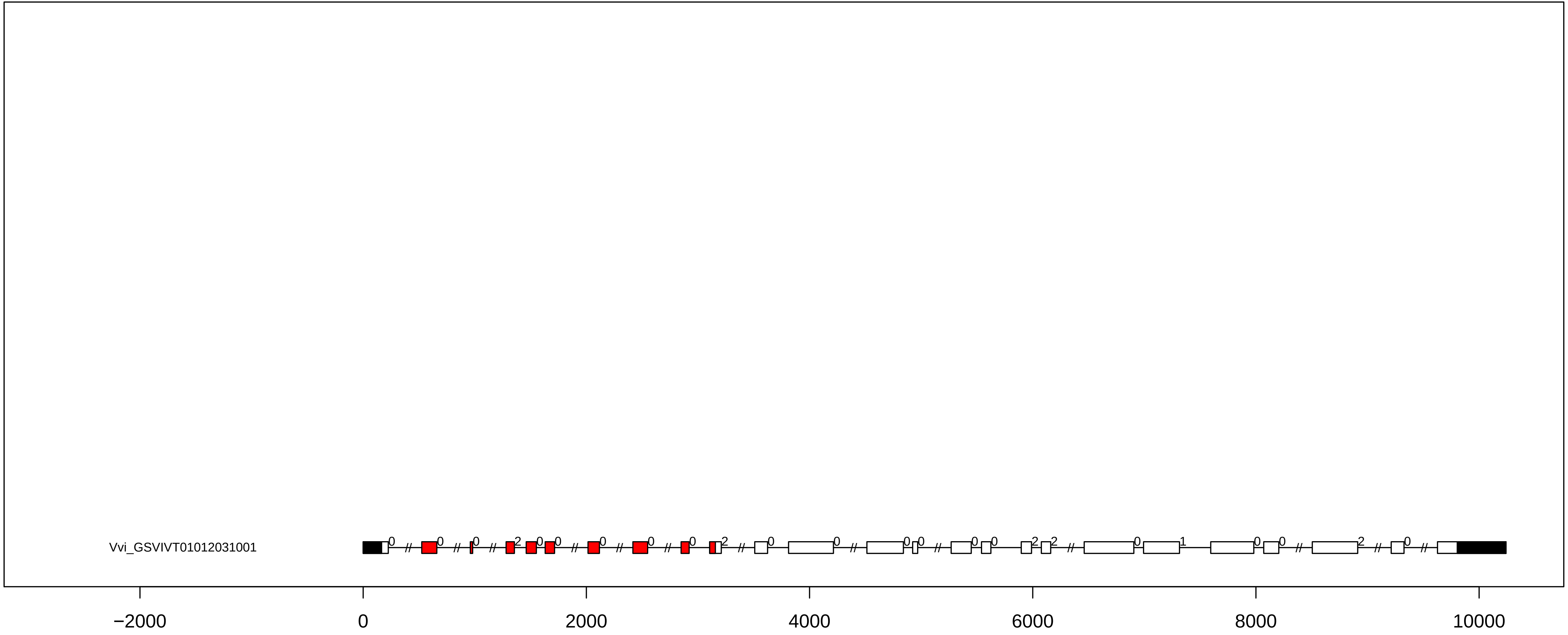

V.vi TKL_CTR1−DRK−1 exon−intron and domain diagram (all)

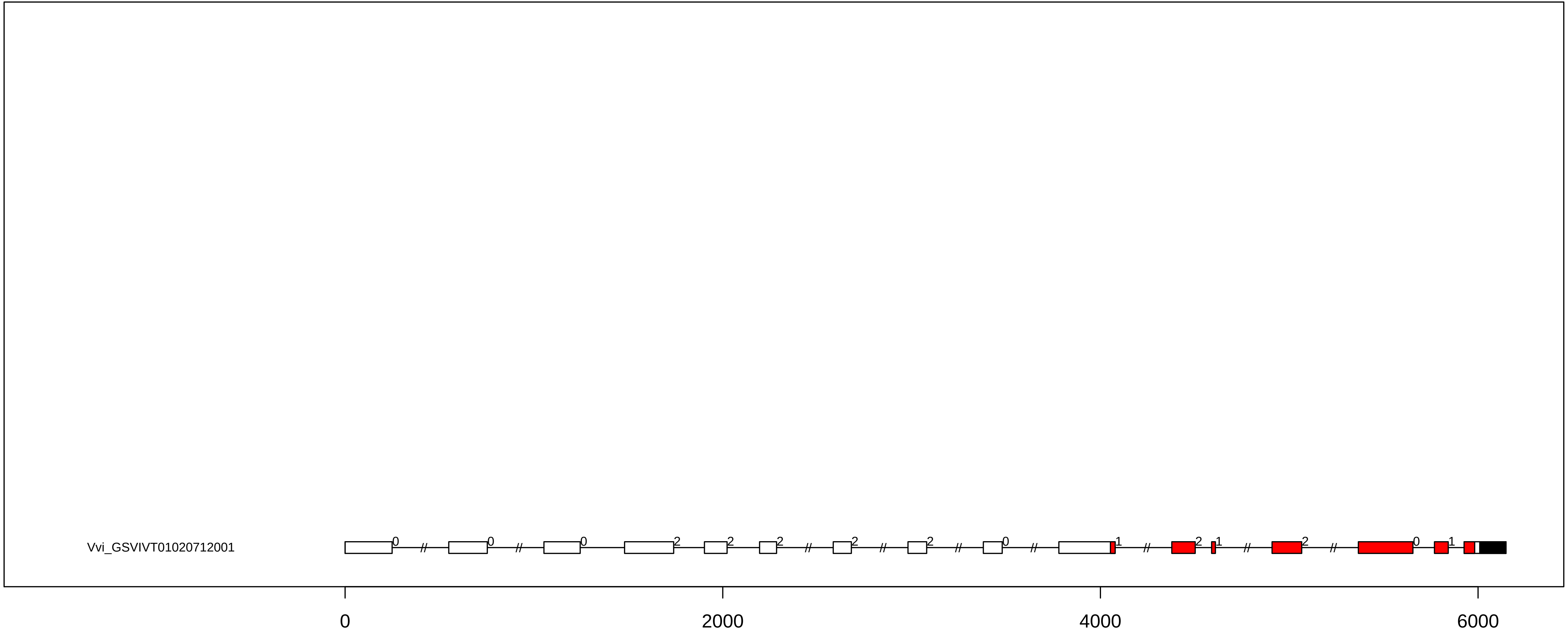

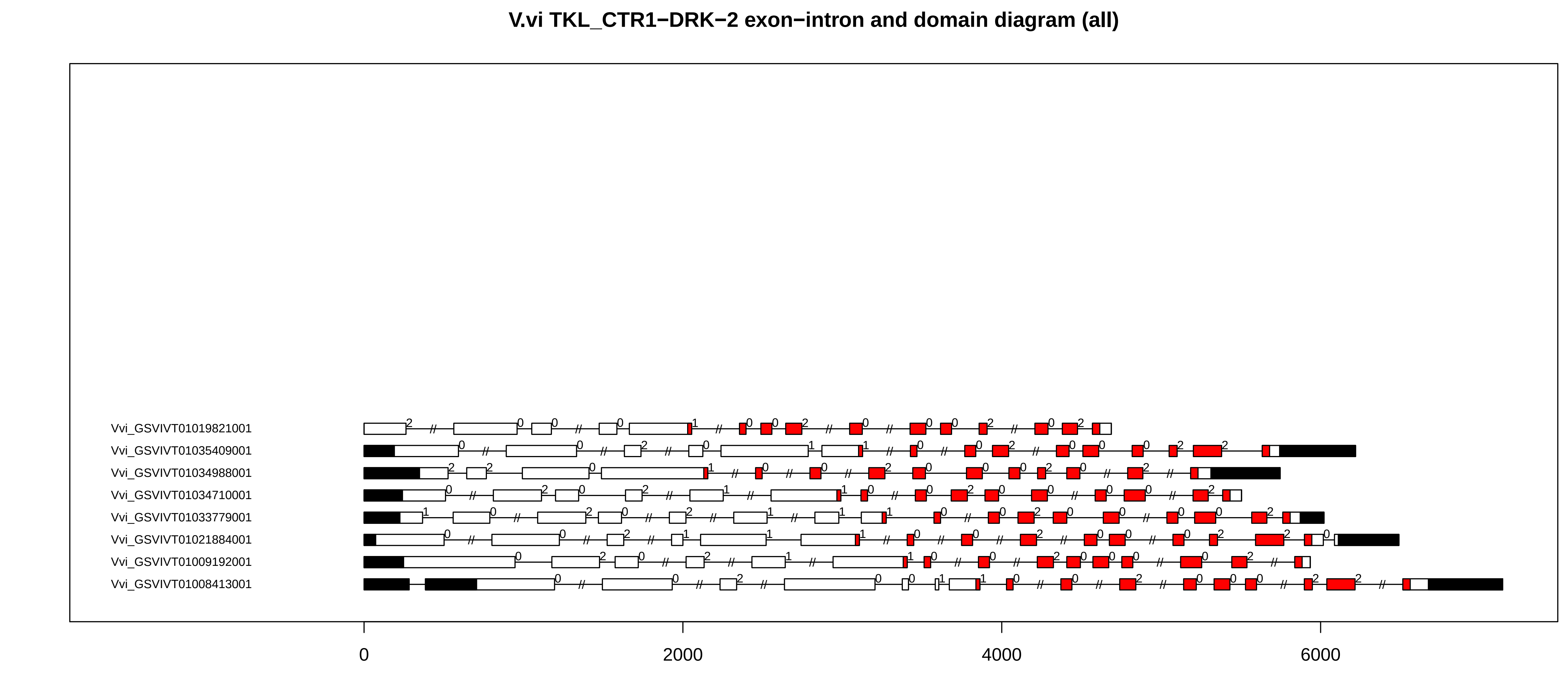
V.vi TKL_CTR1−DRK−2 exon−intron and domain diagram (all)
Vvi_GSVIVT01019821001
Vvi_GSVIVT01035409001
Vvi_GSVIVT01034988001
Vvi_GSVIVT01034710001
Vvi_GSVIVT01033779001
Vvi_GSVIVT01021884001
Vvi_GSVIVT01009192001
Vvi_GSVIVT01008413001
0
2000
4000
6000

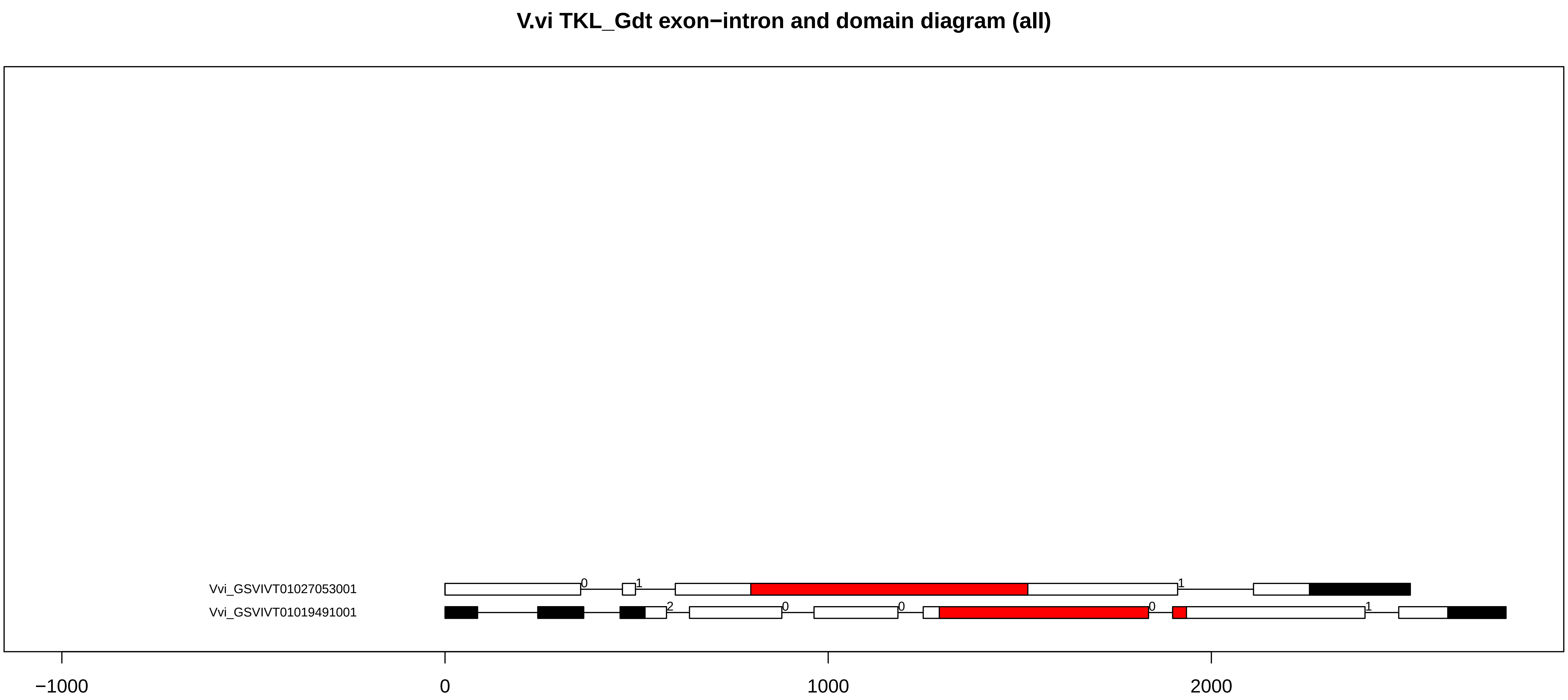

V.vi TKL_Gdt exon−intron and domain diagram (all)
Vvi_GSVIVT01027053001
Vvi_GSVIVT01019491001
0
1
1
2
0
0
0
1
−1000
0
1000
2000

V.vi TKL−Pl−1 exon−intron and domain diagram (all)

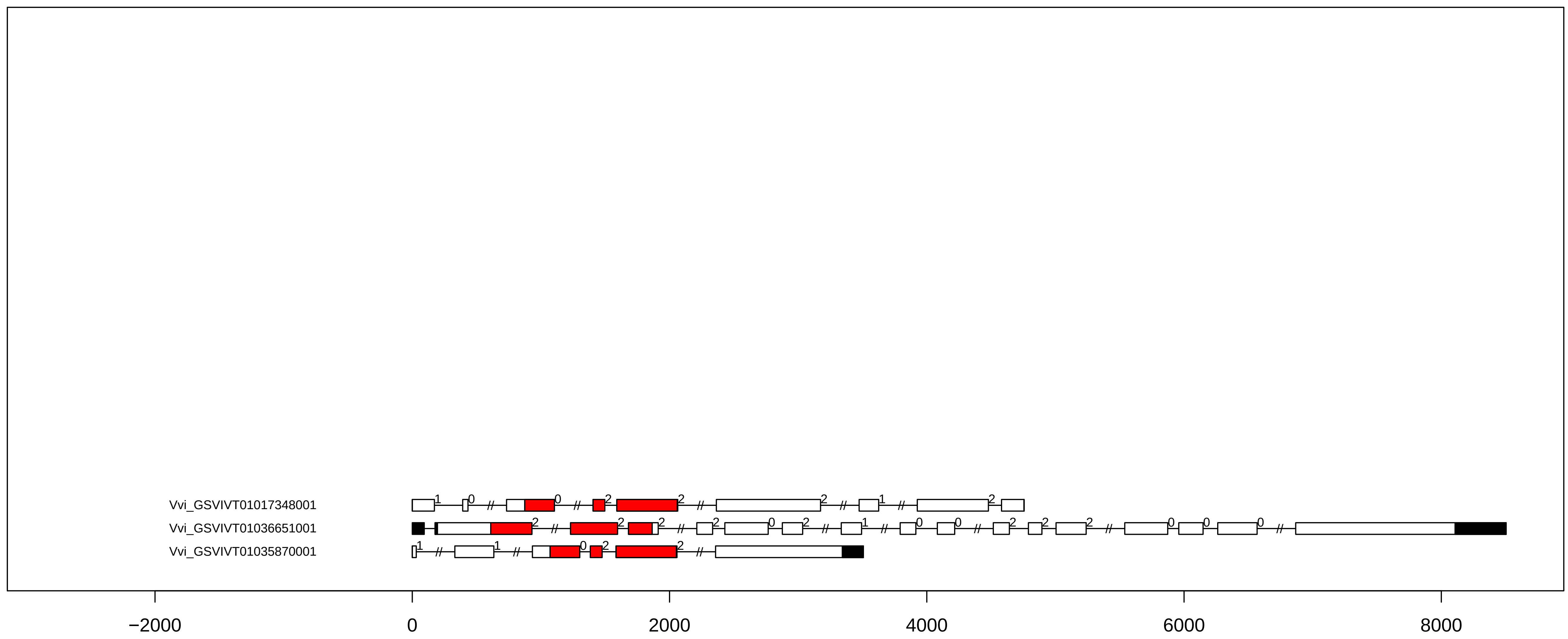

V.vi TKL−Pl−2 exon−intron and domain diagram (all)

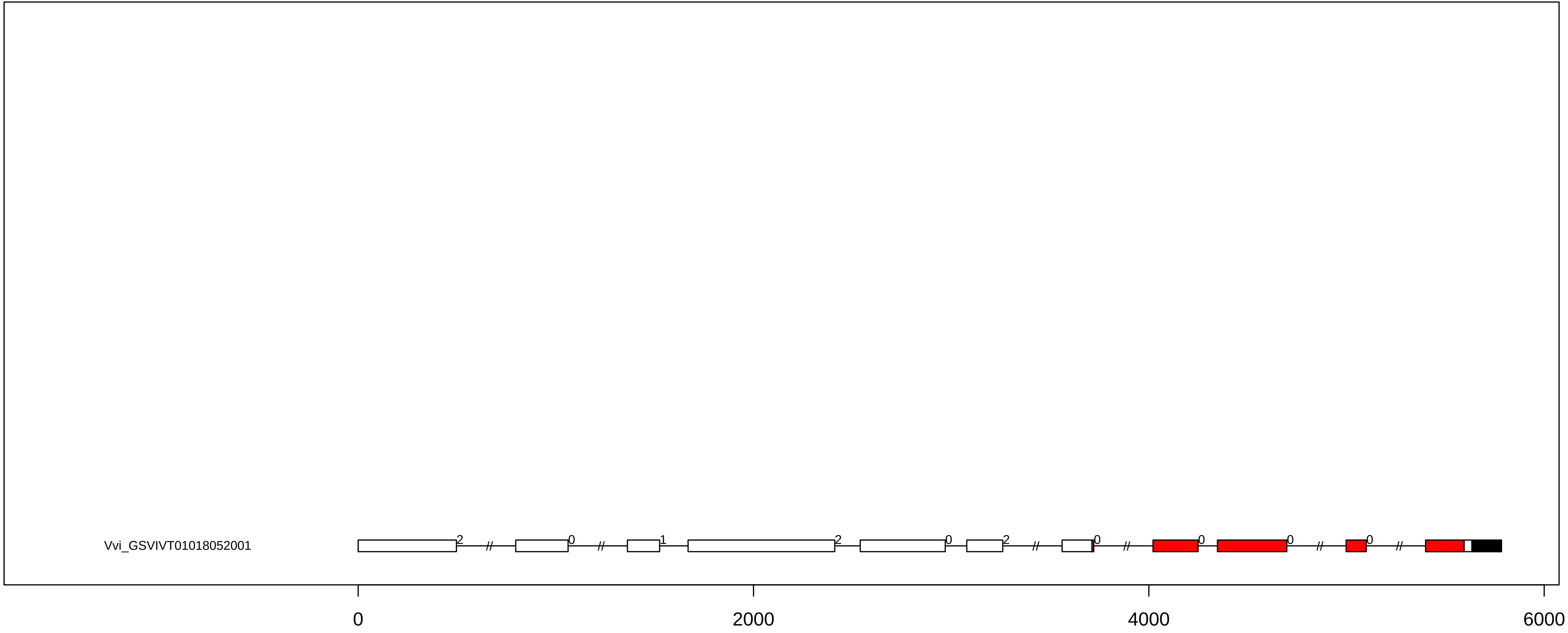

V.vi TKL−Pl−3 exon−intron and domain diagram (all)

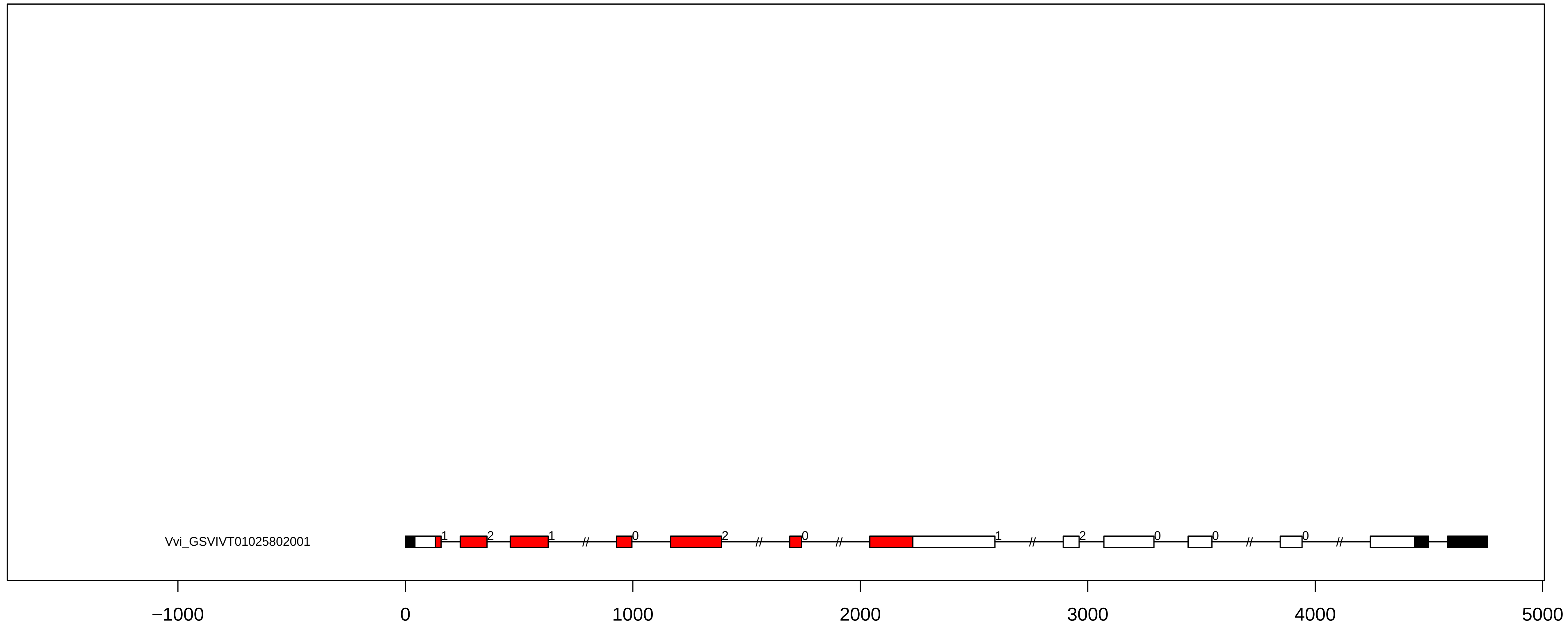

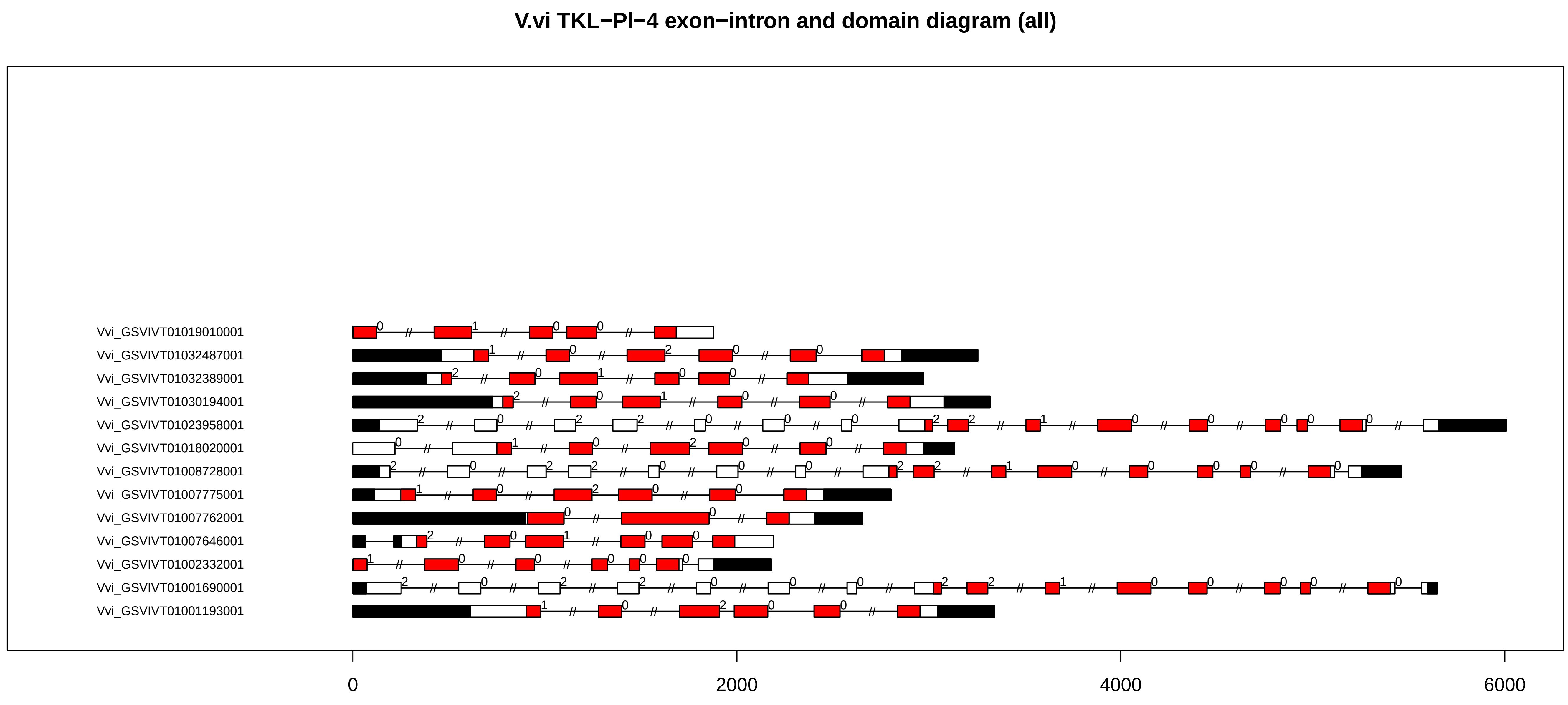
V.vi TKL−Pl−4 exon−intron and domain diagram (all)
Vvi_GSVIVT01019010001
Vvi_GSVIVT01032487001
Vvi_GSVIVT01032389001
Vvi_GSVIVT01030194001
Vvi_GSVIVT01023958001
Vvi_GSVIVT01018020001
Vvi_GSVIVT01008728001
Vvi_GSVIVT01007775001
Vvi_GSVIVT01007762001
Vvi_GSVIVT01007646001
Vvi_GSVIVT01002332001
Vvi_GSVIVT01001690001
Vvi_GSVIVT01001193001
0
2000
4000
6000

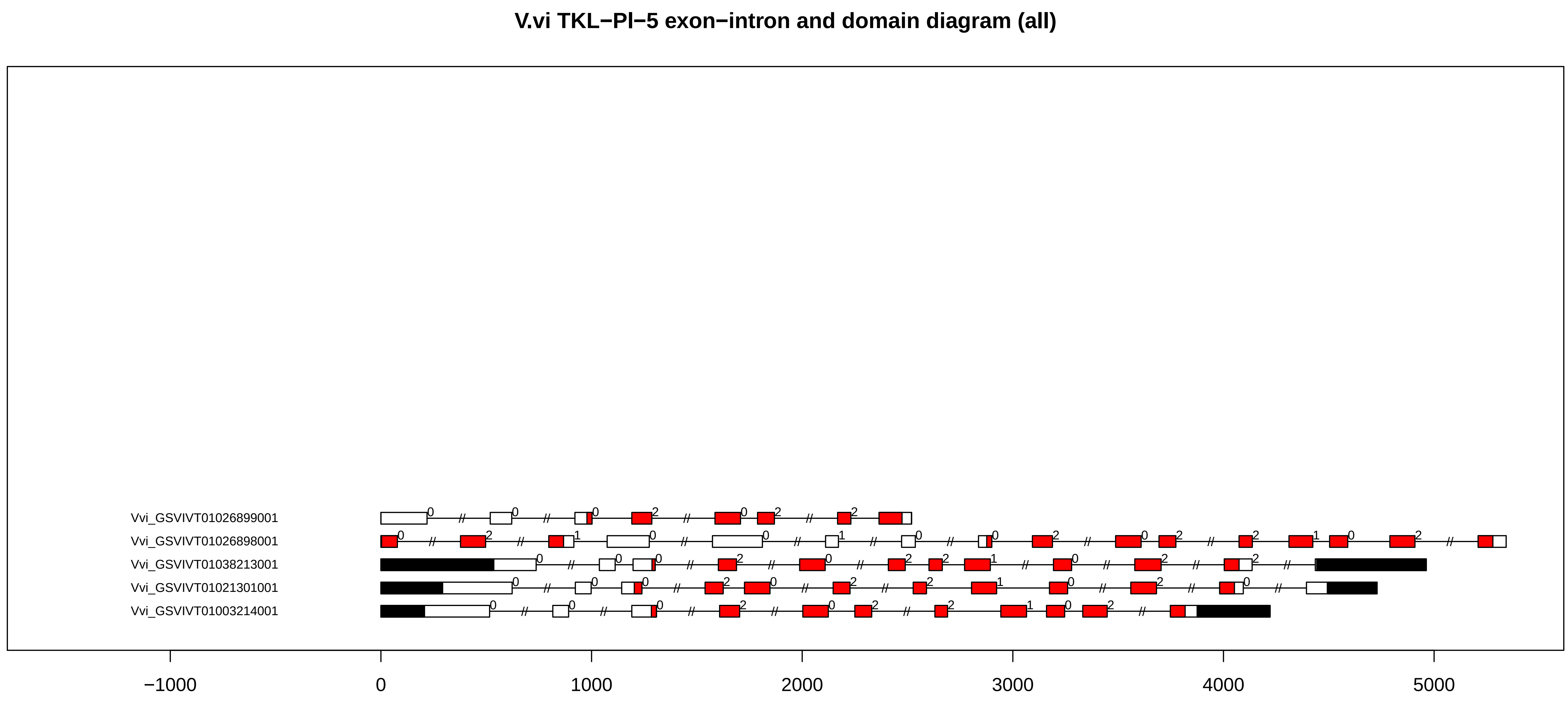

V.vi TKL−Pl−5 exon−intron and domain diagram (all)
Vvi_GSVIVT01026899001
Vvi_GSVIVT01026898001
Vvi_GSVIVT01038213001
Vvi_GSVIVT01021301001
Vvi_GSVIVT01003214001
−1000
0
1000
2000
3000
4000
5000

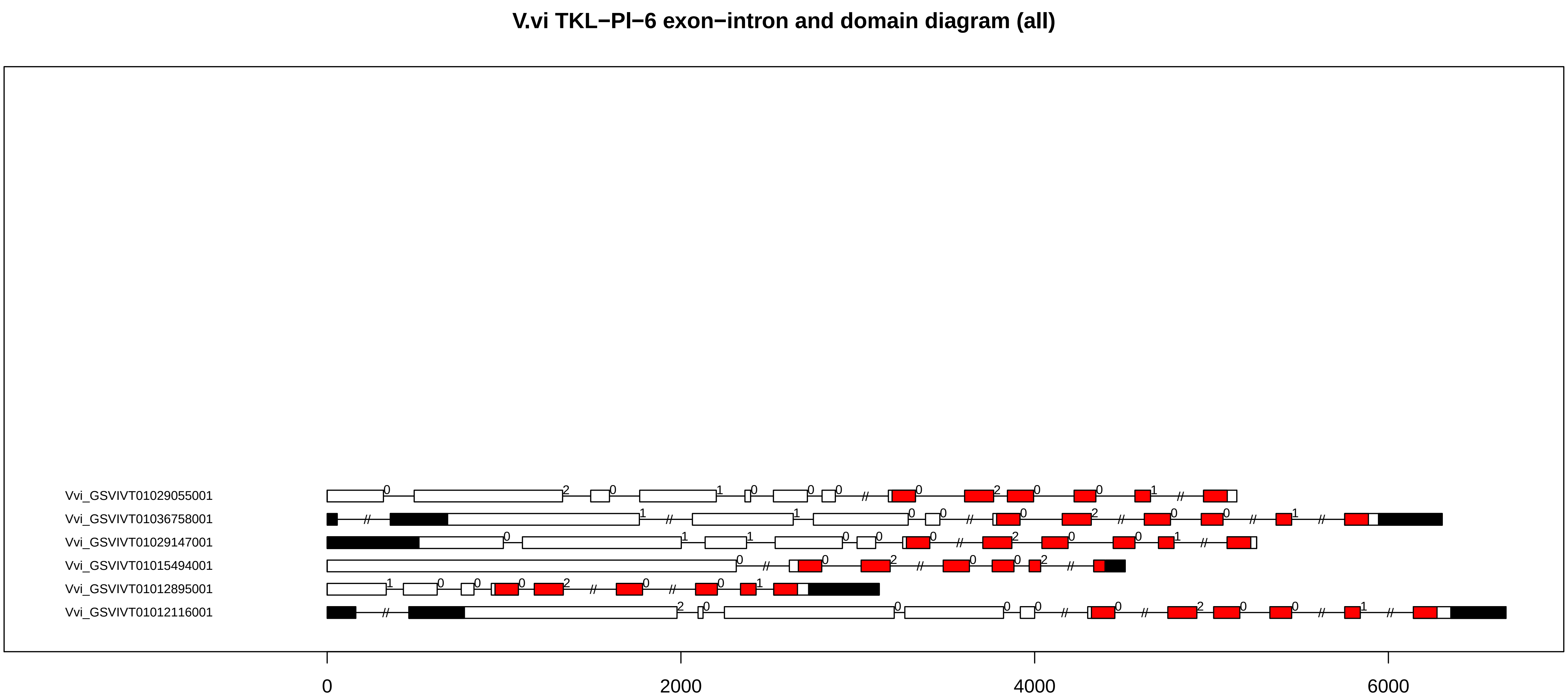

V.vi TKL−Pl−6 exon−intron and domain diagram (all)
Vvi_GSVIVT01029055001
Vvi_GSVIVT01036758001
Vvi_GSVIVT01029147001
Vvi_GSVIVT01015494001
Vvi_GSVIVT01012895001
Vvi_GSVIVT01012116001
0
2000
4000
6000

V.vi TKL−Pl−7 exon−intron and domain diagram (all)

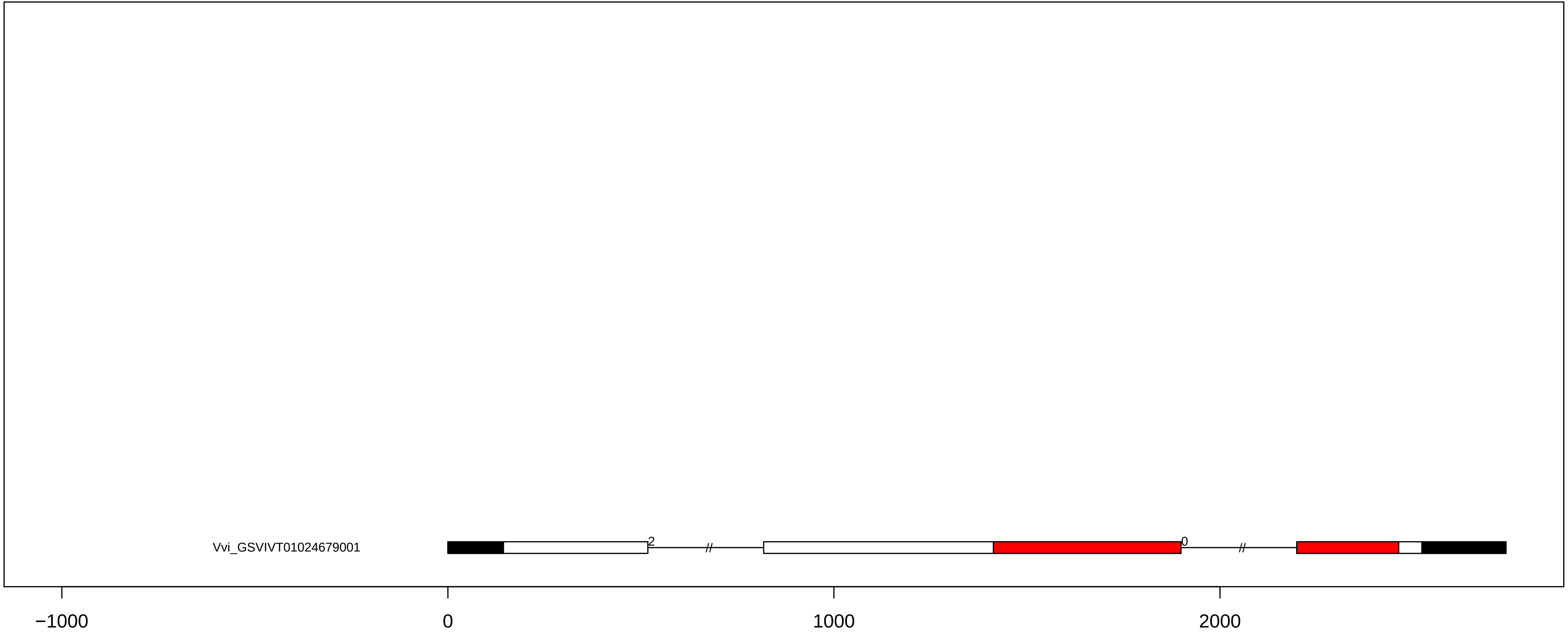

V.vi TKL−Pl−8 exon−intron and domain diagram (all)

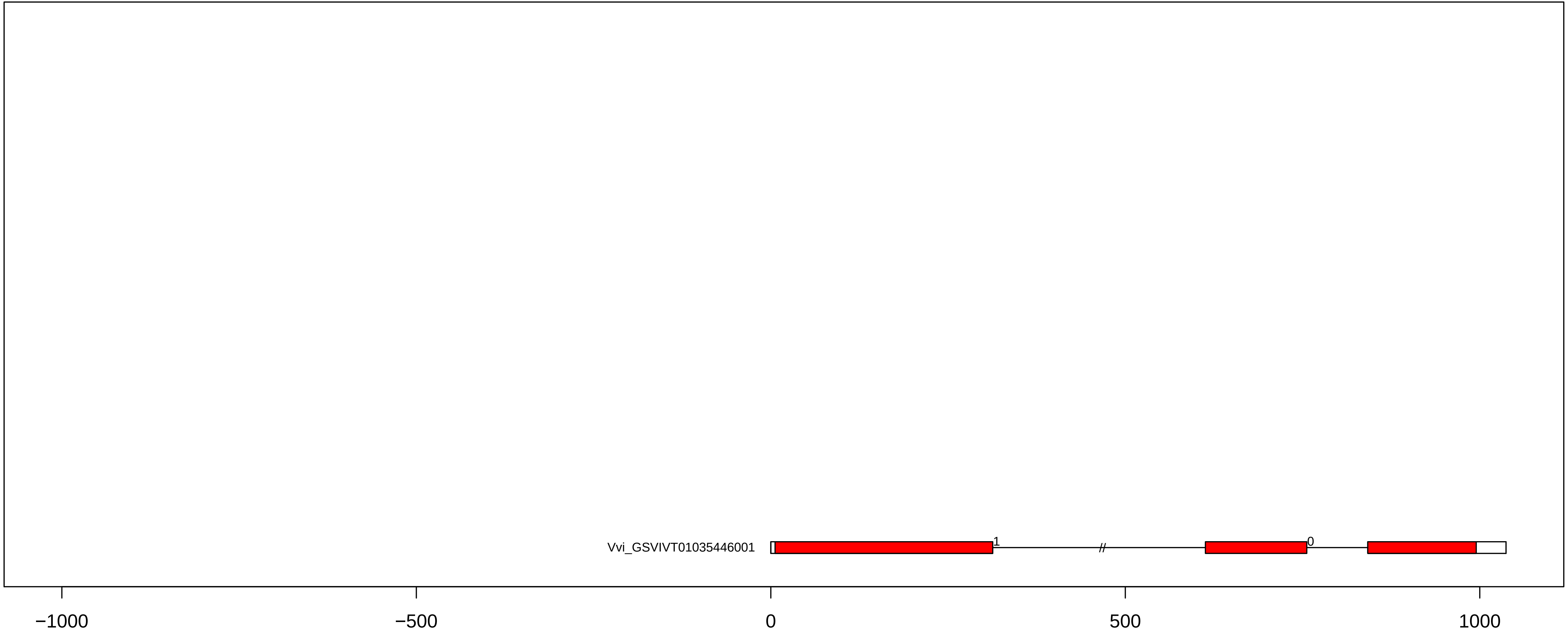

V.vi TLK exon−intron and domain diagram (all)

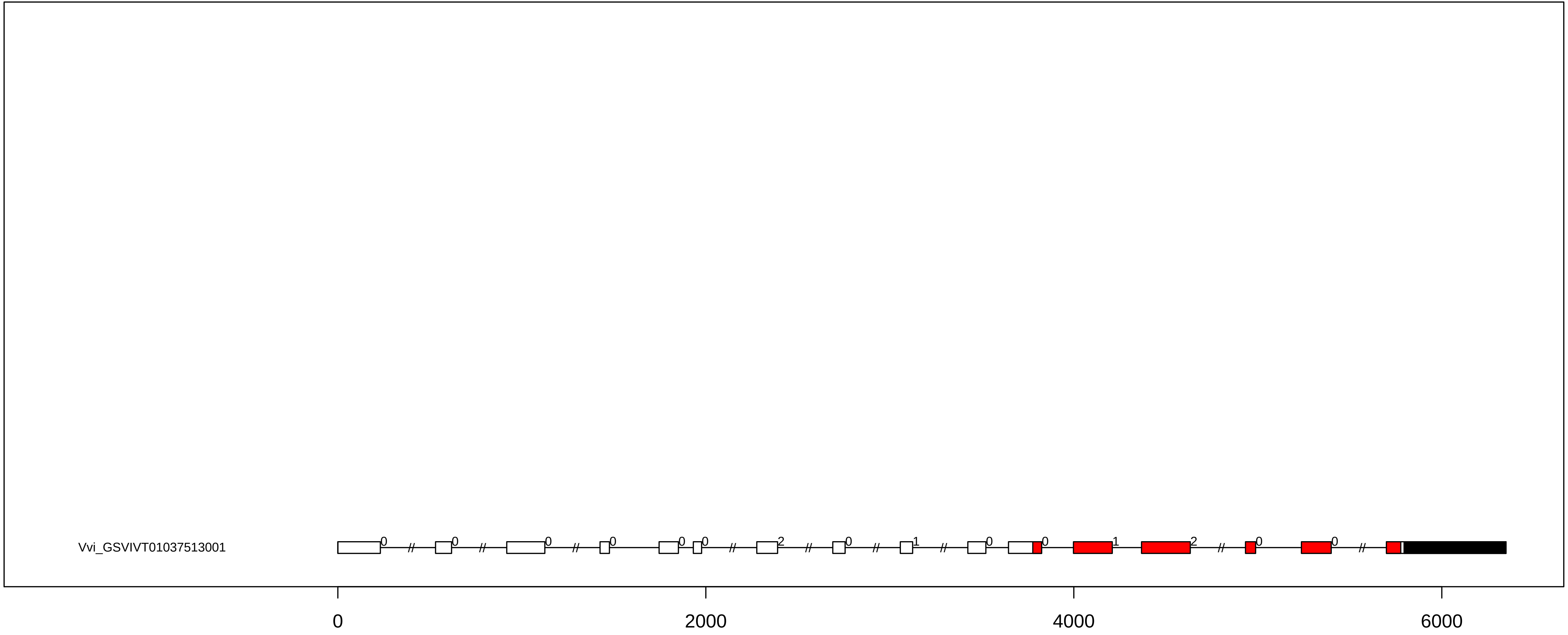

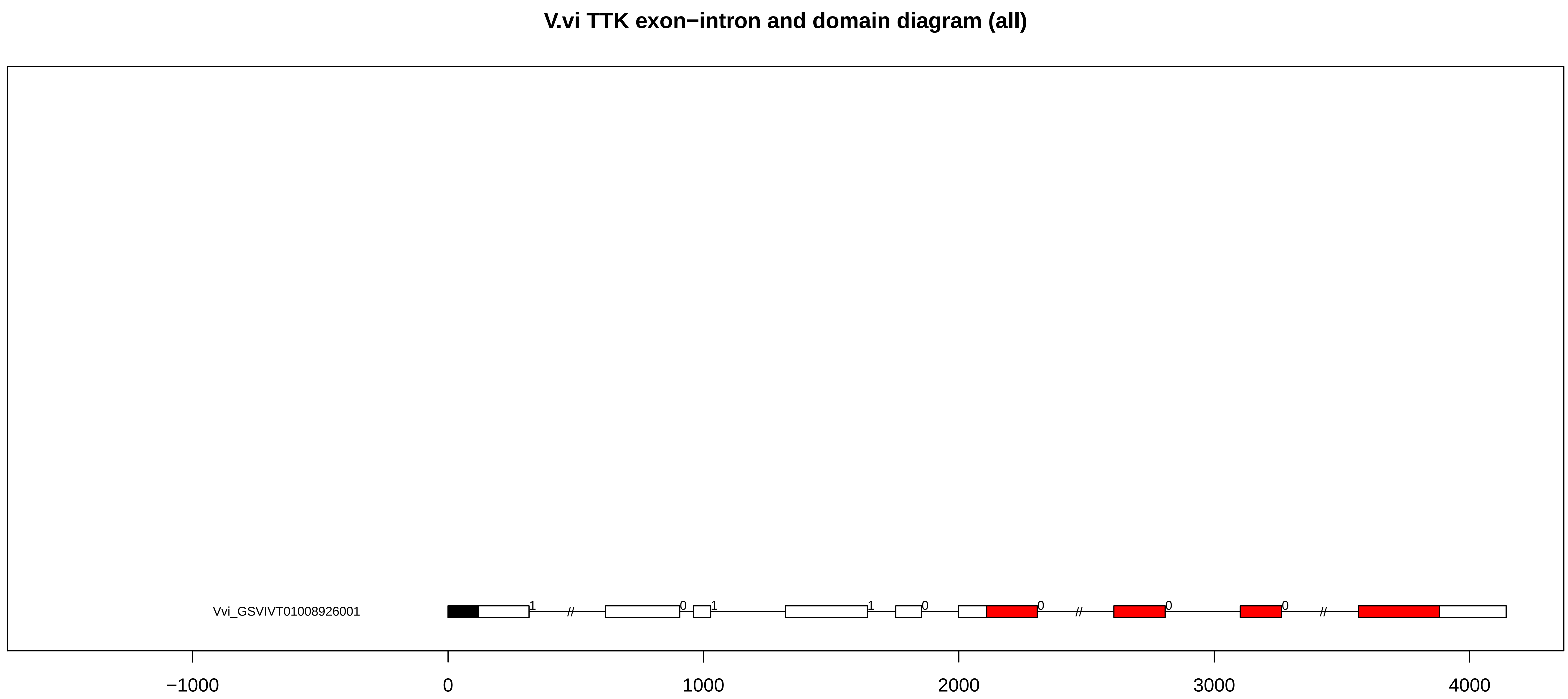
V.vi TTK exon−intron and domain diagram (all)
Vvi_GSVIVT01008926001
1
0
1
1
0
0
0
0
−1000
0
1000
2000
3000
4000

V.vi ULK_Fused exon−intron and domain diagram (all)

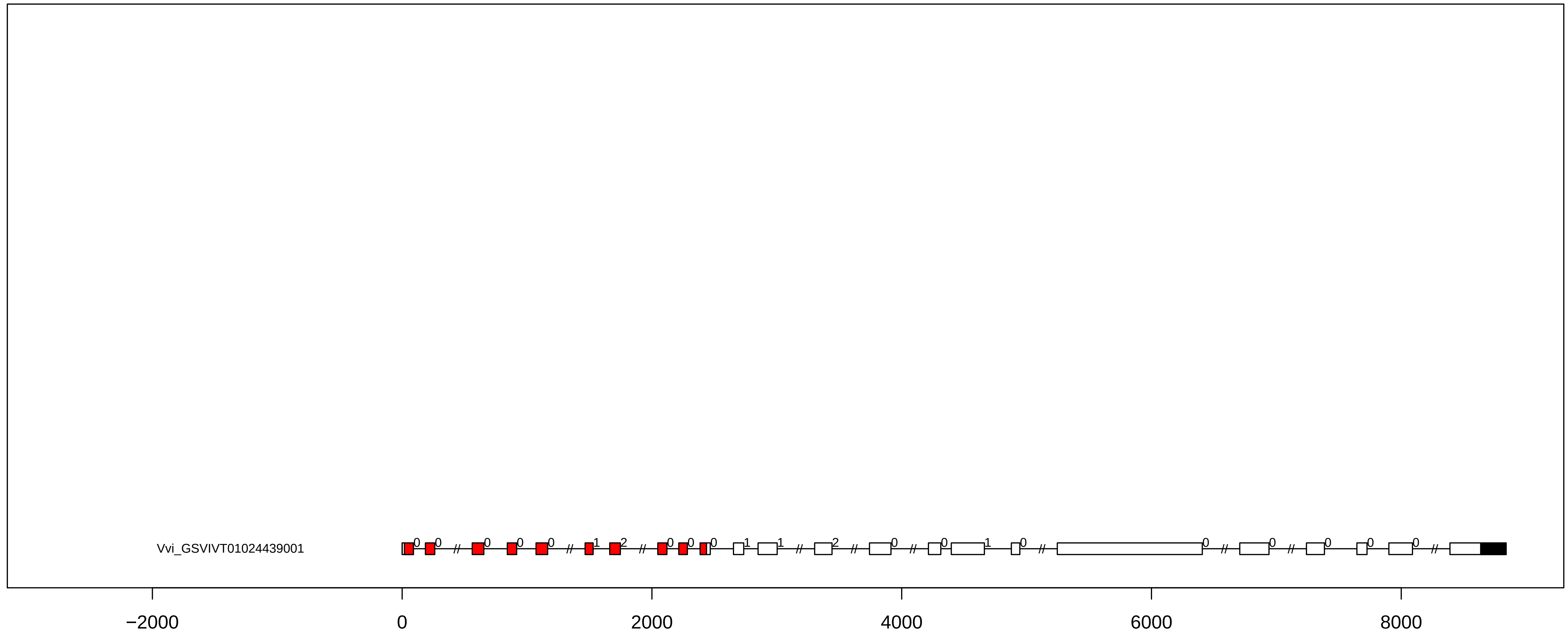

V.vi ULK_ULK4 exon−intron and domain diagram (all)

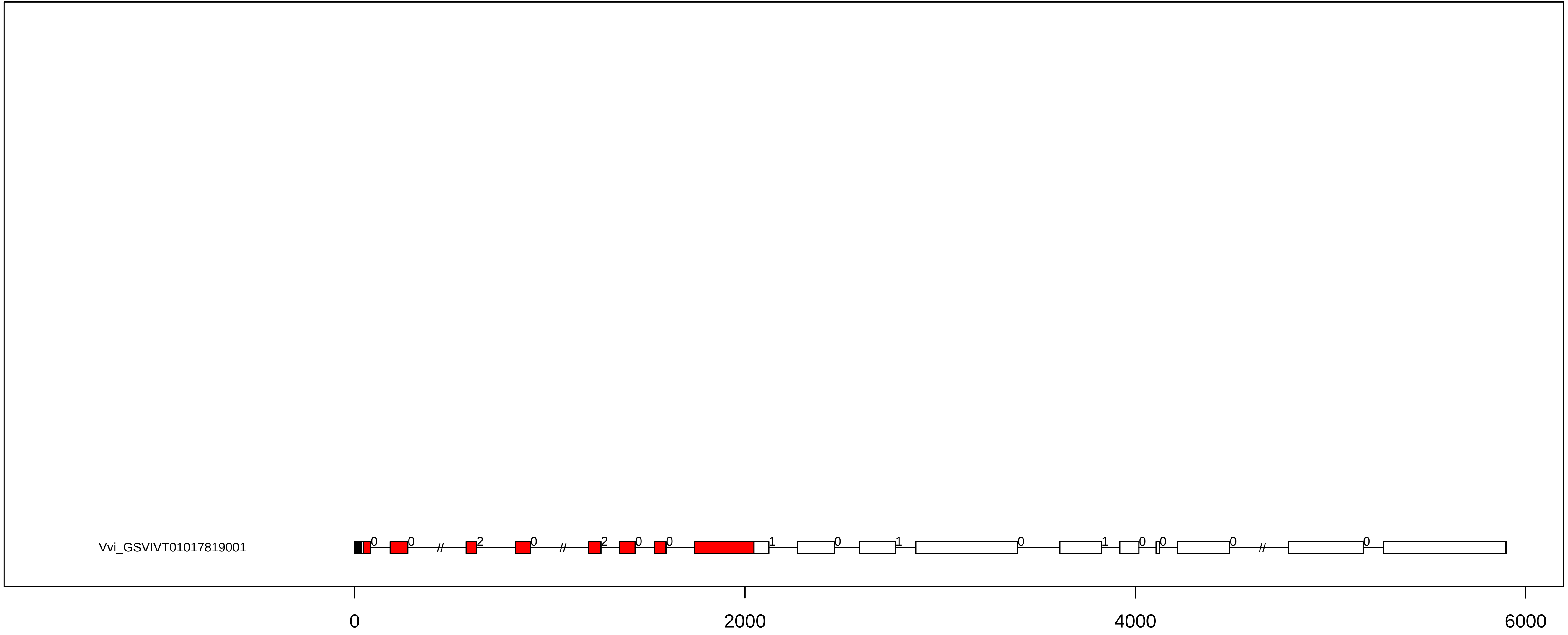

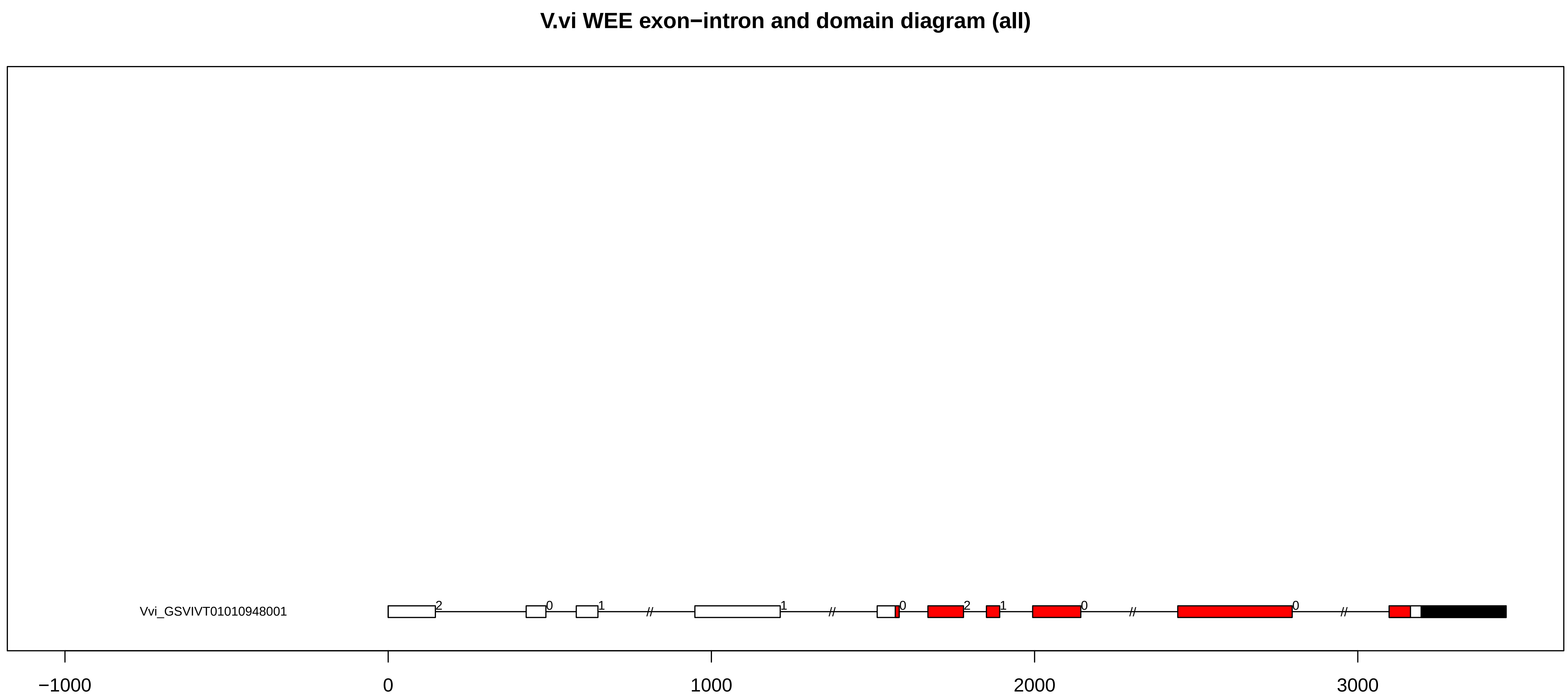

V.vi WEE exon−intron and domain diagram (all)
Vvi_GSVIVT01010948001
2
0
1
1
0
2
1
0
0
−1000
0
1000
2000
3000

V.vi WNK_NRBP exon−intron and domain diagram (all)

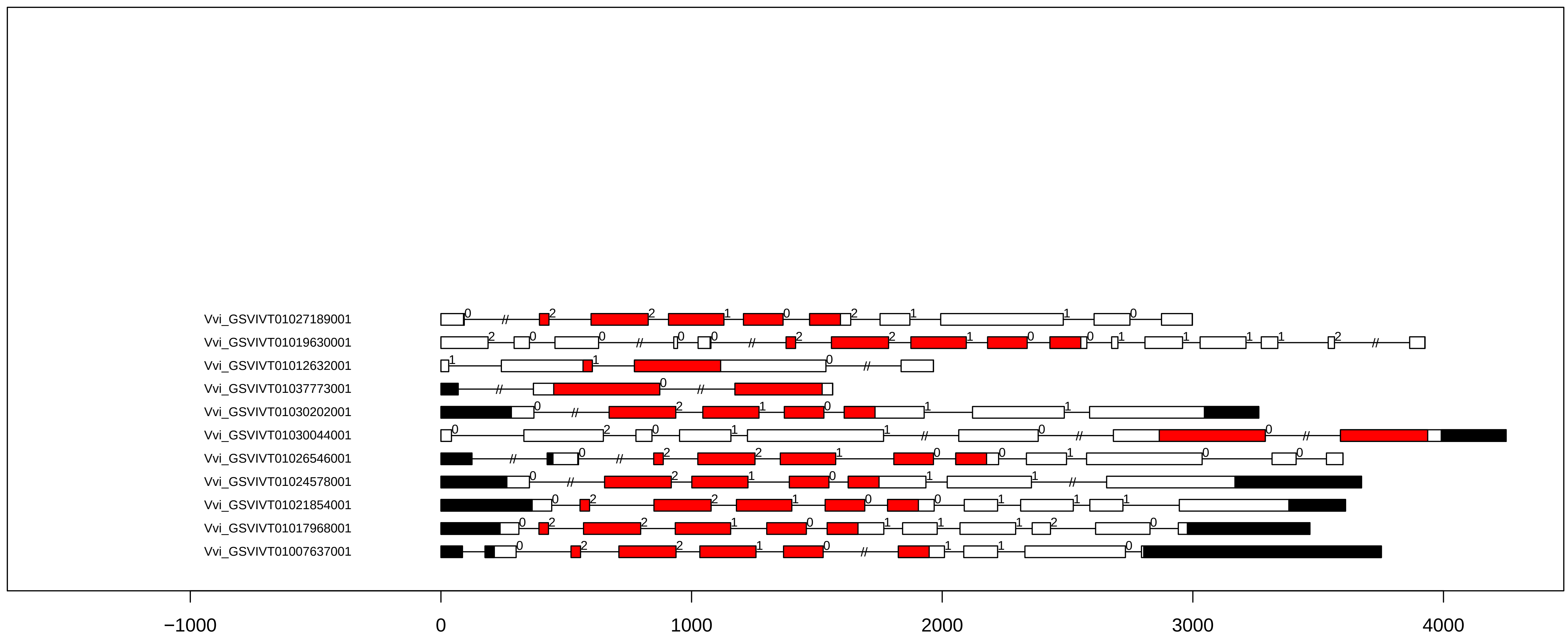

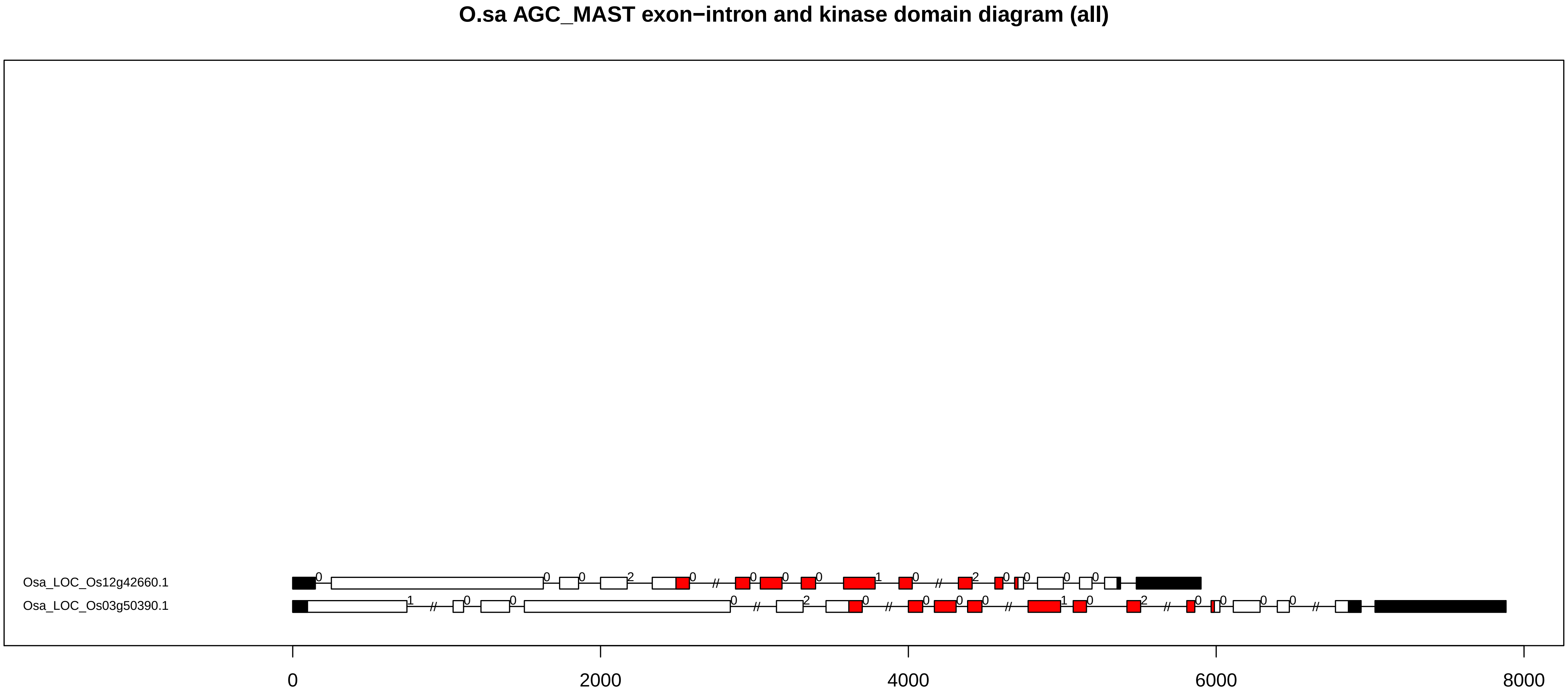

O.sa AGC_MAST exon−intron and kinase domain diagram (all)
Osa_LOC_Os12g42660.1
Osa_LOC_Os03g50390.1
0
2000
4000
6000
8000

O.sa AGC_NDR exon−intron and kinase domain diagram (all)

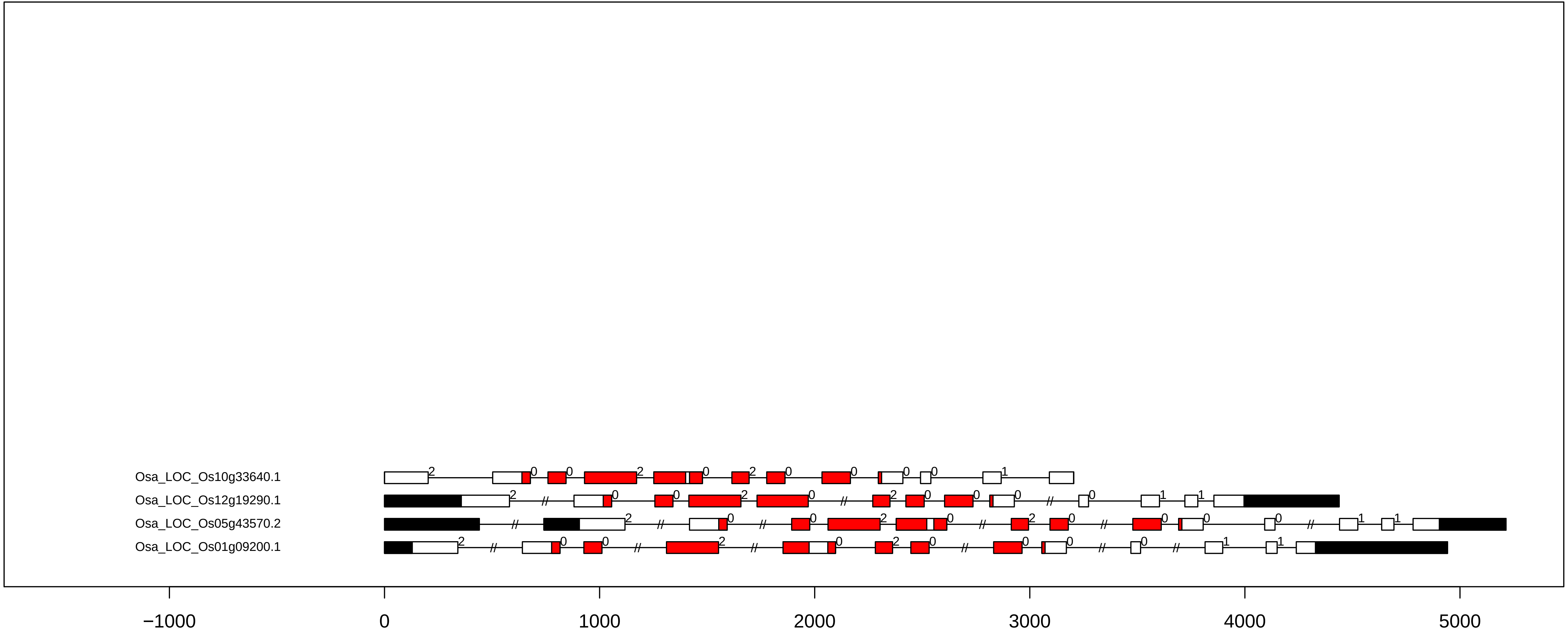

O.sa AGC_PDK1 exon−intron and kinase domain diagram (all)

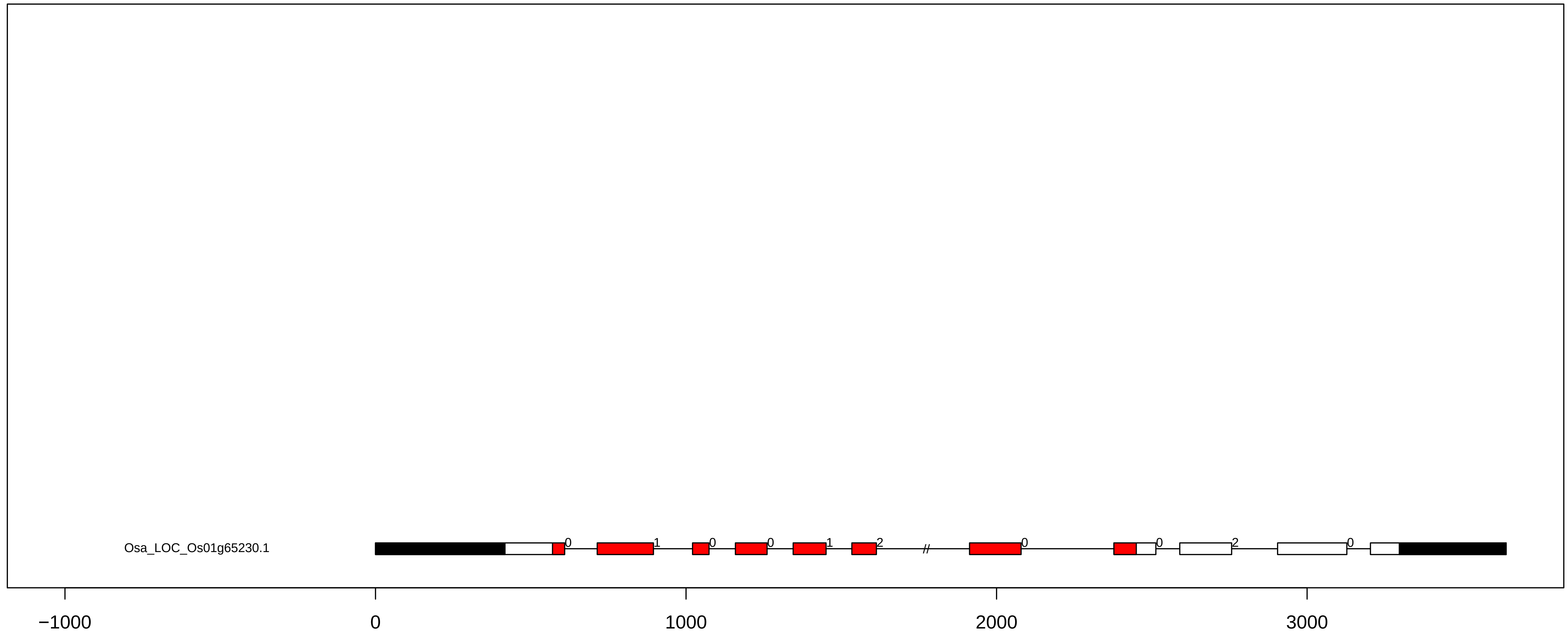

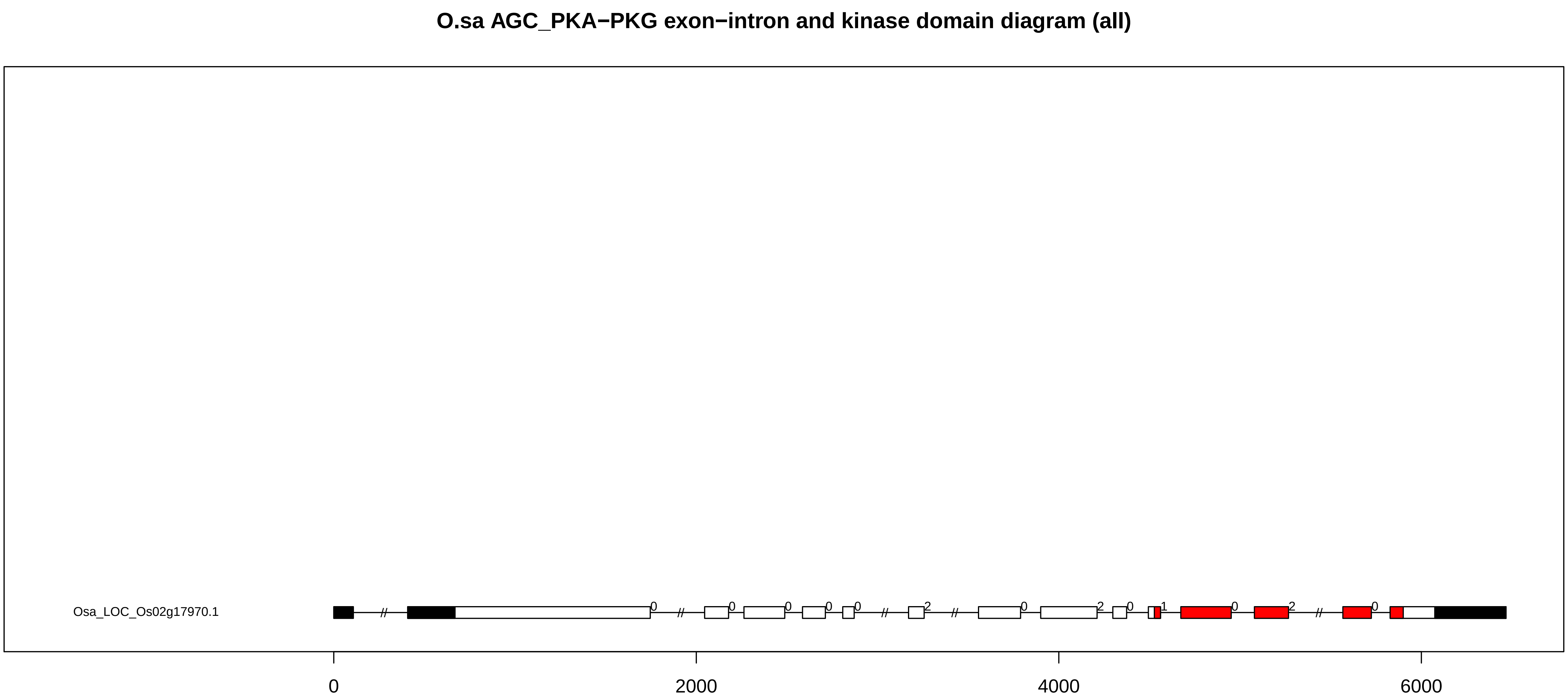

O.sa AGC_PKA−PKG exon−intron and kinase domain diagram (all)
Osa_LOC_Os02g17970.1
0
0
0
0
0
2
0
2
0
1
0
2
0
0
2000
4000
6000

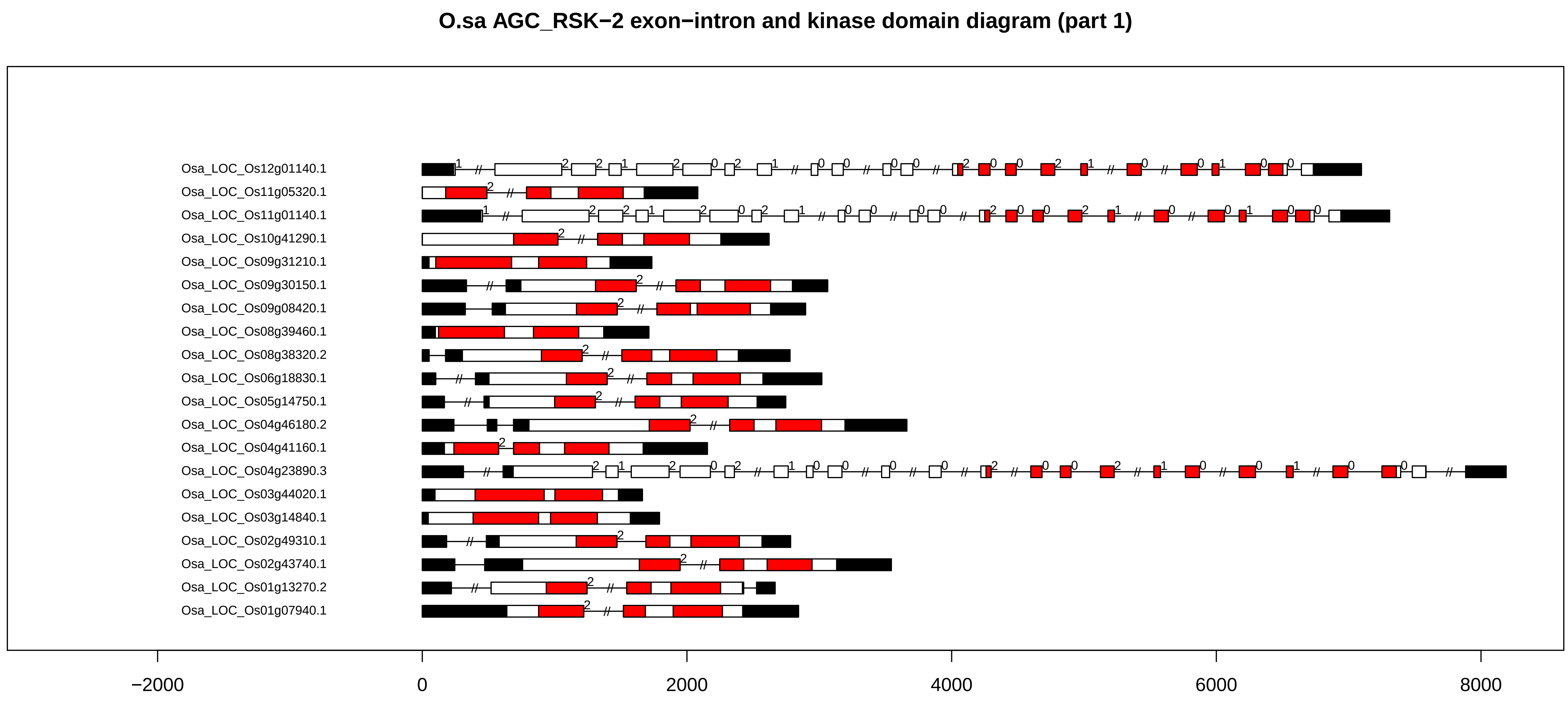
O.sa AGC_RSK−2 exon−intron and kinase domain diagram (part 1)
Osa_LOC_Os12g01140.1
Osa_LOC_Os11g05320.1
Osa_LOC_Os11g01140.1
Osa_LOC_Os10g41290.1
Osa_LOC_Os09g31210.1
Osa_LOC_Os09g30150.1
Osa_LOC_Os09g08420.1
Osa_LOC_Os08g39460.1
Osa_LOC_Os08g38320.2
Osa_LOC_Os06g18830.1
Osa_LOC_Os05g14750.1
Osa_LOC_Os04g46180.2
Osa_LOC_Os04g41160.1
Osa_LOC_Os04g23890.3
Osa_LOC_Os03g44020.1
Osa_LOC_Os03g14840.1
Osa_LOC_Os02g49310.1
Osa_LOC_Os02g43740.1
Osa_LOC_Os01g13270.2
Osa_LOC_Os01g07940.1
−2000
0
2000
4000
6000
8000

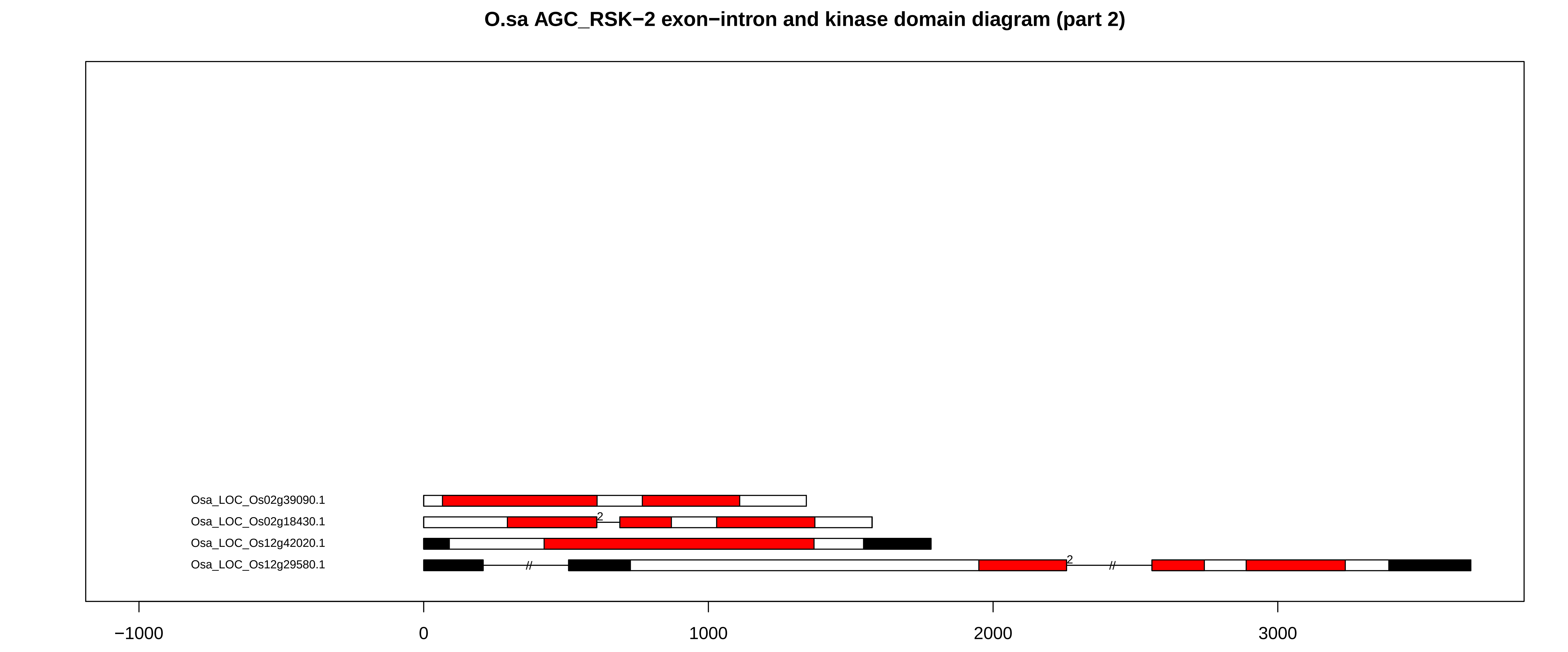

O.sa AGC_RSK−2 exon−intron and kinase domain diagram (part 2)
Osa_LOC_Os02g39090.1
Osa_LOC_Os02g18430.1
Osa_LOC_Os12g42020.1
Osa_LOC_Os12g29580.1
2
2
−1000
0
1000
2000
3000

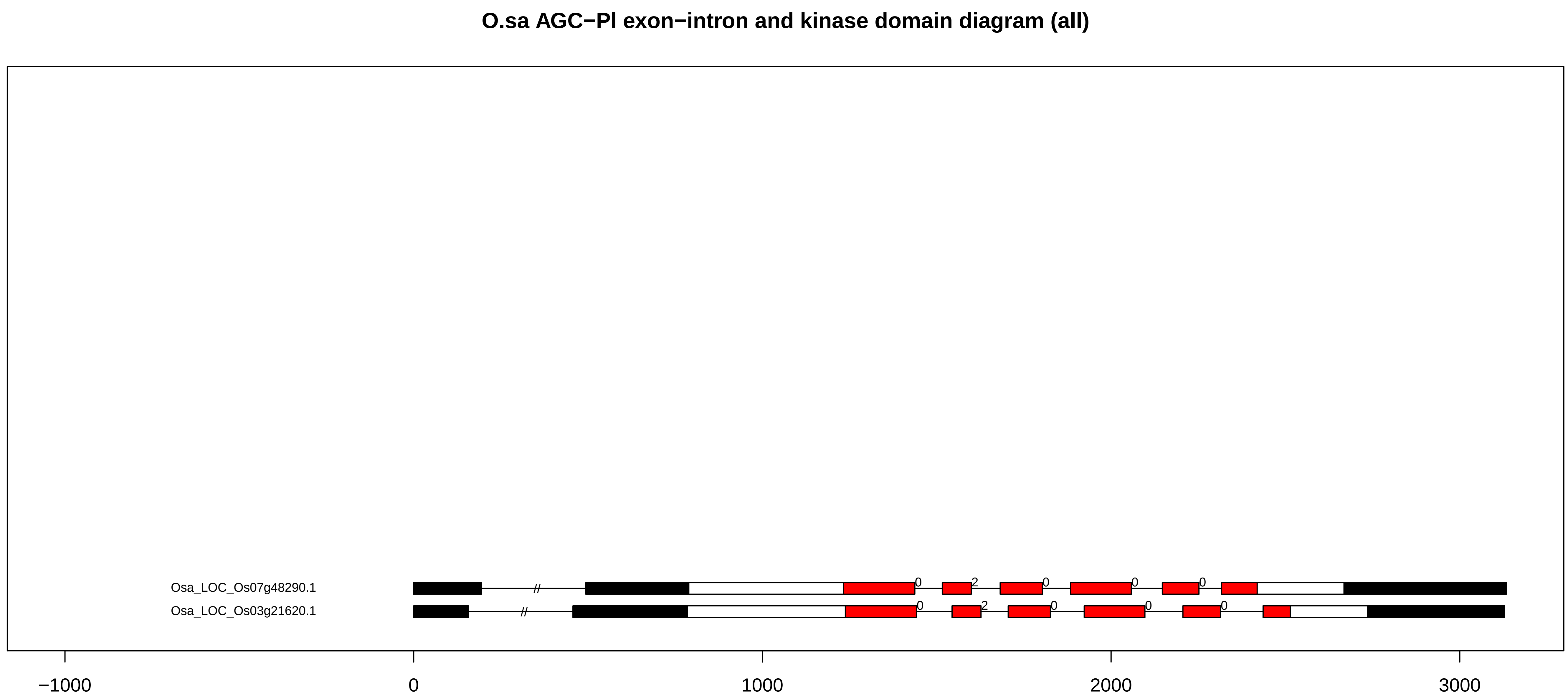

O.sa AGC−PI exon−intron and kinase domain diagram (all)
Osa_LOC_Os07g48290.1
Osa_LOC_Os03g21620.1
0
2
0
0
0
0
2
0
0
0
−1000
0
1000
2000
3000

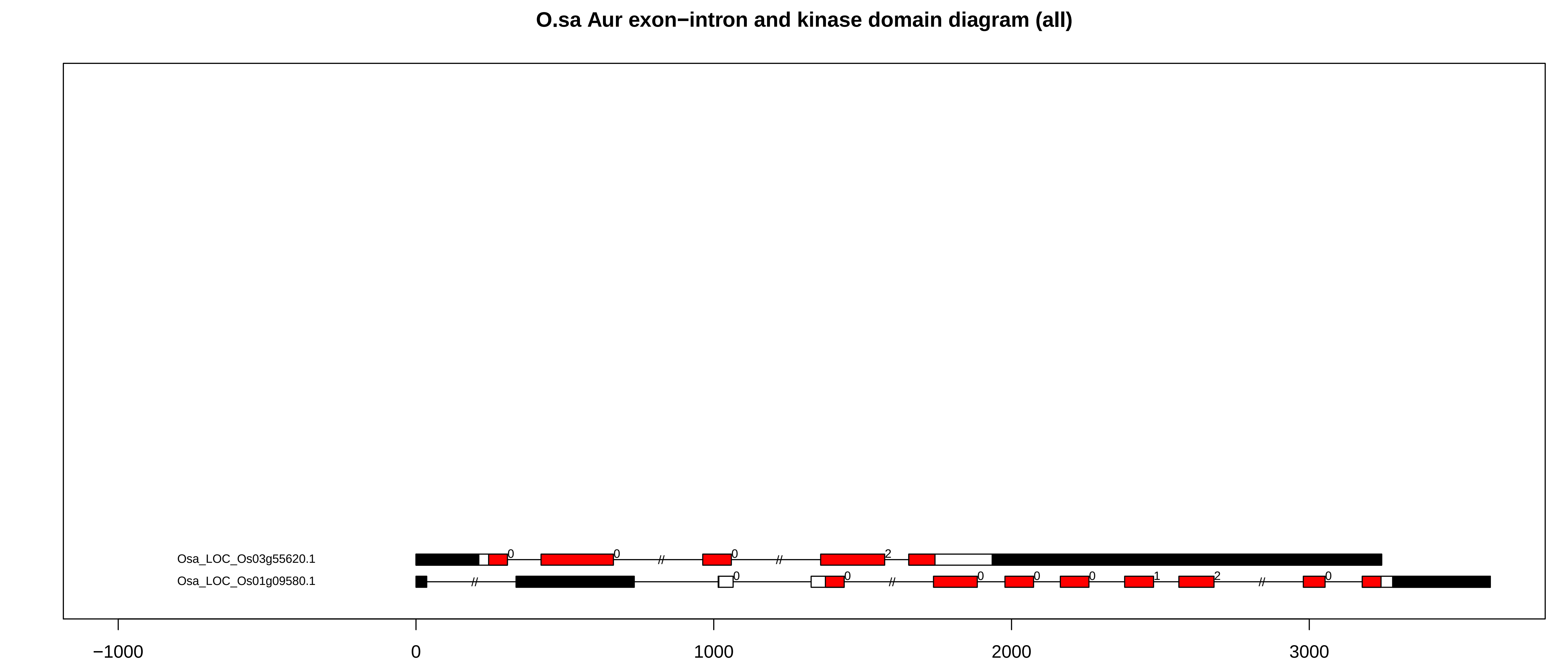

O.sa Aur exon−intron and kinase domain diagram (all)
Osa_LOC_Os03g55620.1
Osa_LOC_Os01g09580.1
0
0
0
2
0
0
0
0
0
1
2
0
−1000
0
1000
2000
3000

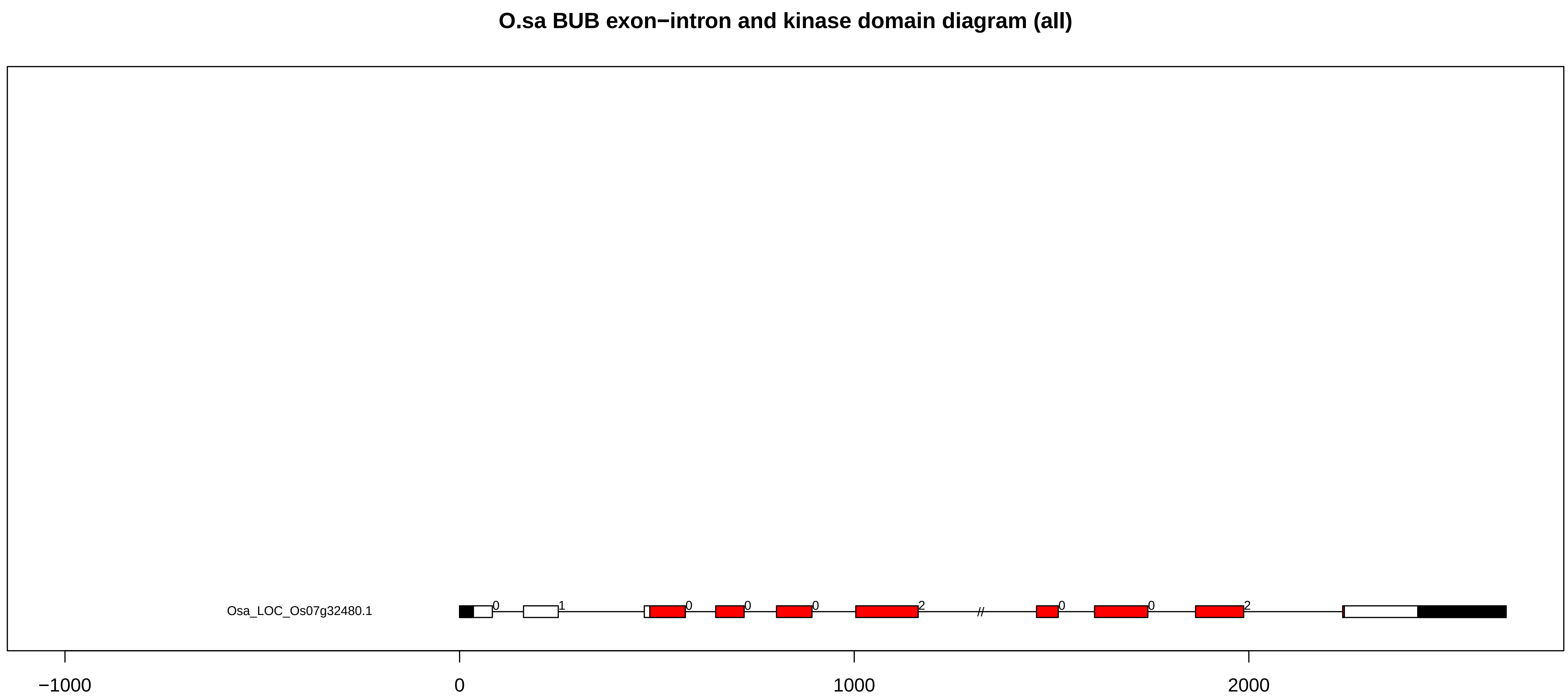
O.sa BUB exon−intron and kinase domain diagram (all)
Osa_LOC_Os07g32480.1
0
1
0
0
0
2
//
0
0
2
−1000
0
1000
2000

O.sa CAMK_AMPK exon−intron and kinase domain diagram (all)

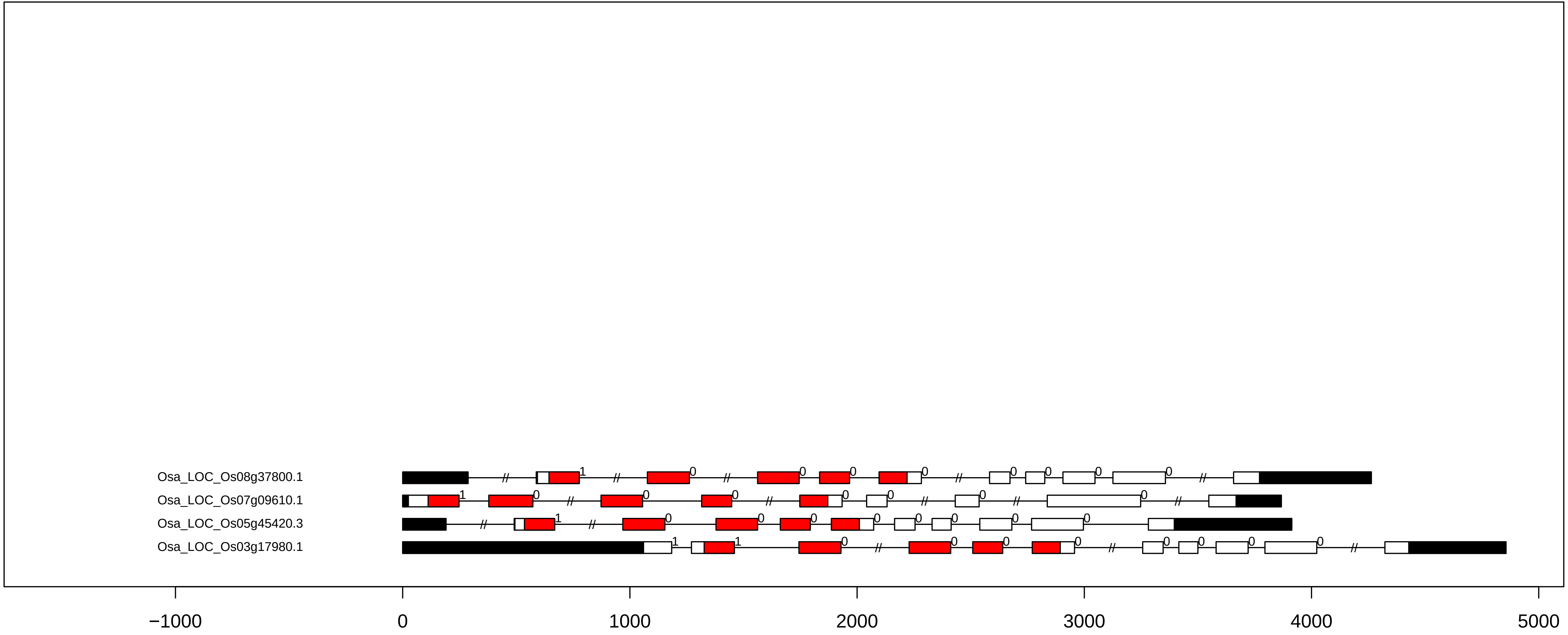

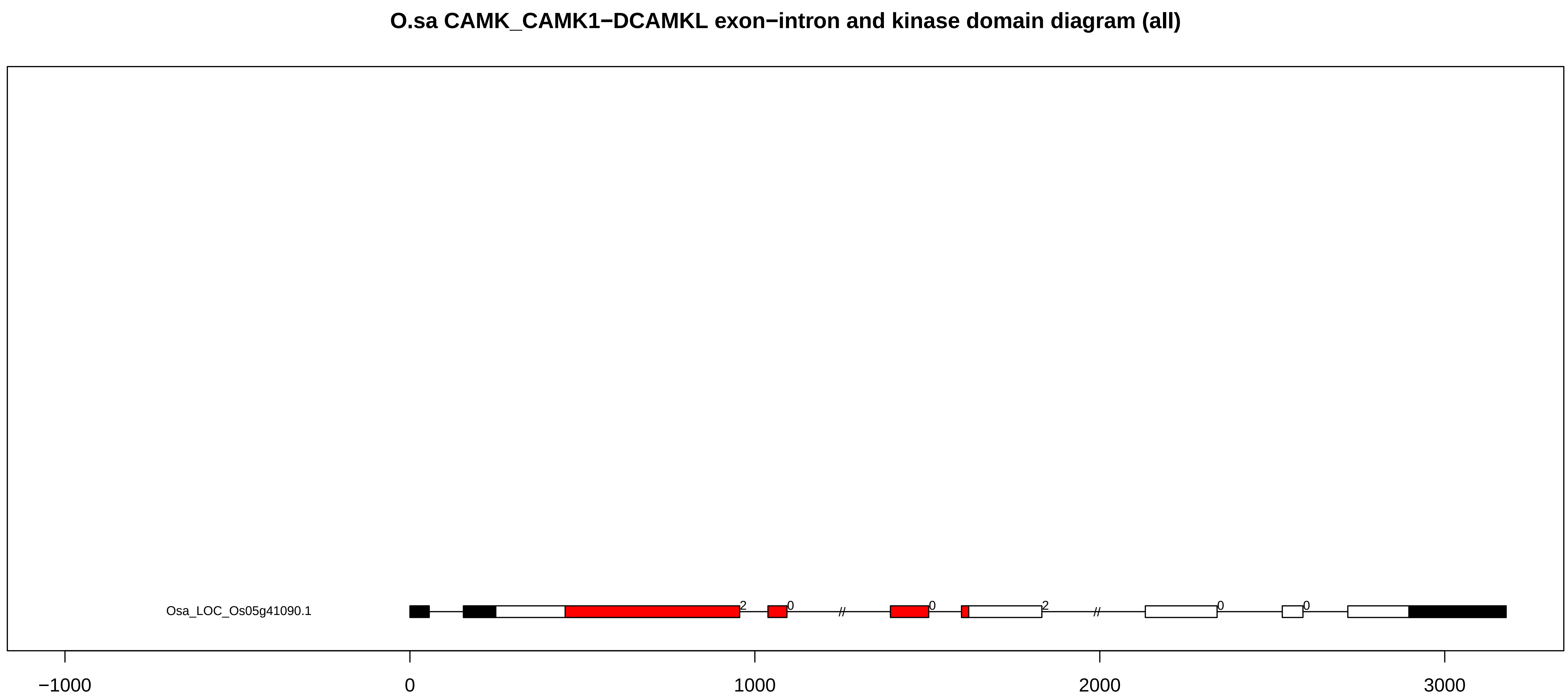
O.sa CAMK_CAMK1−DCAMKL exon−intron and kinase domain diagram (all)
Osa_LOC_Os05g41090.1
2
0
0
2
0
0
−1000
0
1000
2000
3000

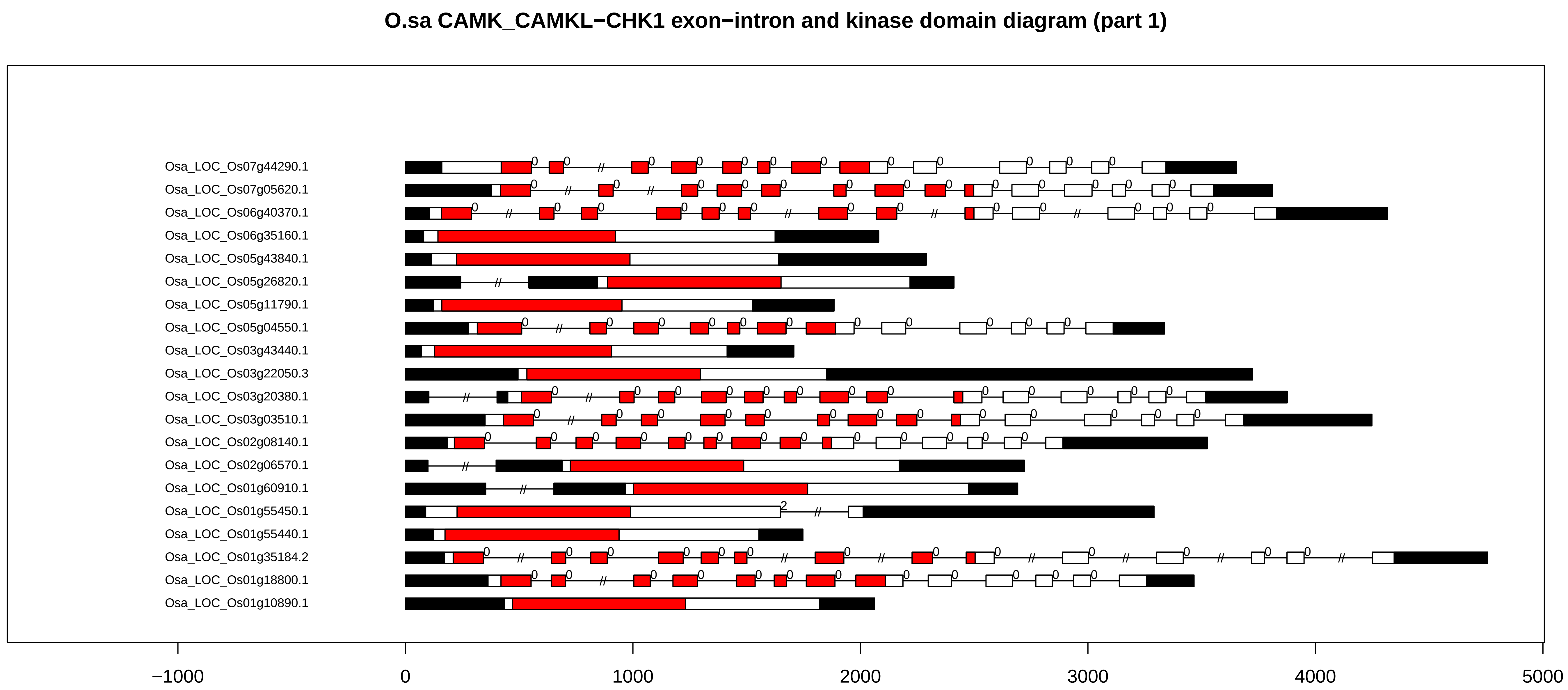

O.sa CAMK_CAMKL−CHK1 exon−intron and kinase domain diagram (part 1)
Osa_LOC_Os07g44290.1
Osa_LOC_Os07g05620.1
Osa_LOC_Os06g40370.1
Osa_LOC_Os06g35160.1
Osa_LOC_Os05g43840.1
Osa_LOC_Os05g26820.1
Osa_LOC_Os05g11790.1
Osa_LOC_Os05g04550.1
Osa_LOC_Os03g43440.1
Osa_LOC_Os03g22050.3
Osa_LOC_Os03g20380.1
Osa_LOC_Os03g03510.1
Osa_LOC_Os02g08140.1
Osa_LOC_Os02g06570.1
Osa_LOC_Os01g60910.1
Osa_LOC_Os01g55450.1
Osa_LOC_Os01g55440.1
Osa_LOC_Os01g35184.2
Osa_LOC_Os01g18800.1
Osa_LOC_Os01g10890.1
−1000
0
1000
2000
3000
4000
5000

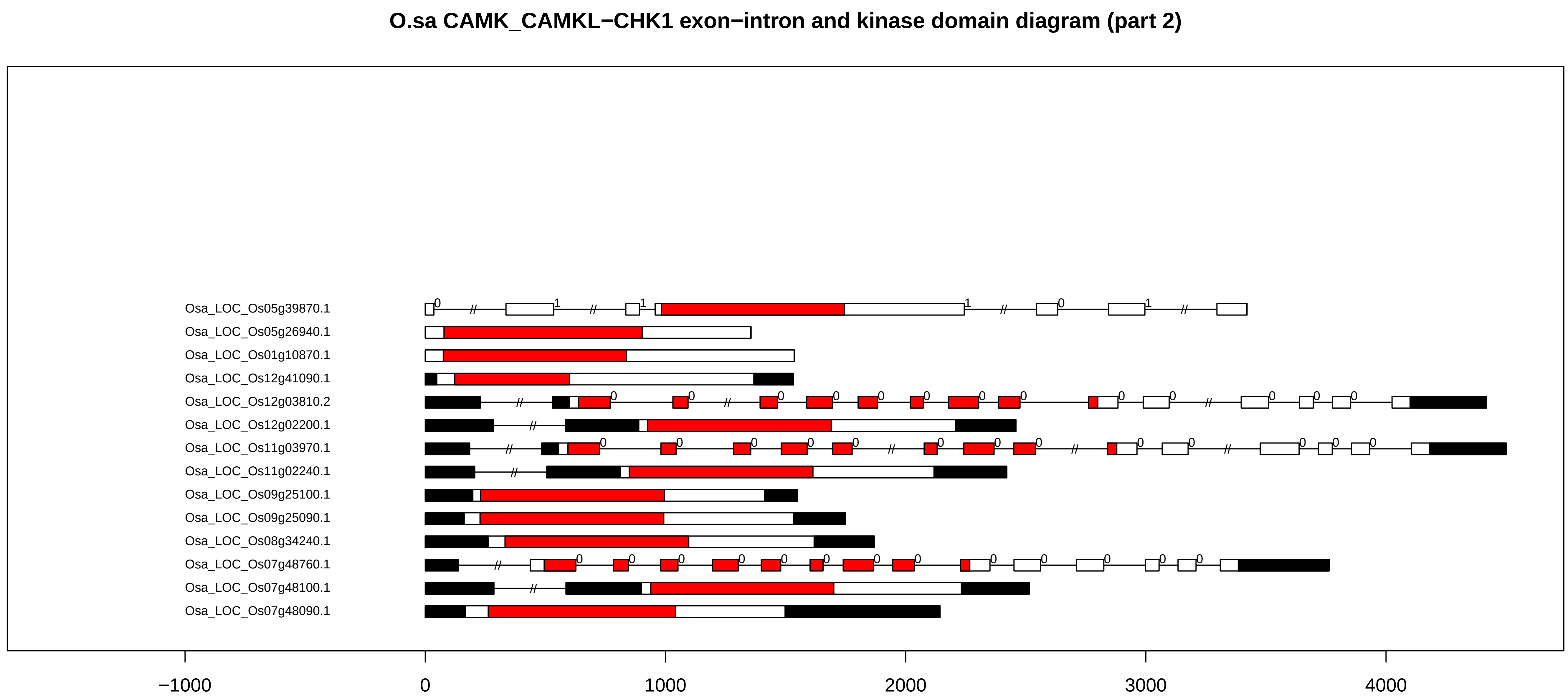
O.sa CAMK_CAMKL−CHK1 exon−intron and kinase domain diagram (part 2)
Osa_LOC_Os05g39870.1
Osa_LOC_Os05g26940.1
Osa_LOC_Os01g10870.1
Osa_LOC_Os12g41090.1
Osa_LOC_Os12g03810.2
Osa_LOC_Os12g02200.1
Osa_LOC_Os11g03970.1
Osa_LOC_Os11g02240.1
Osa_LOC_Os09g25100.1
Osa_LOC_Os09g25090.1
Osa_LOC_Os08g34240.1
Osa_LOC_Os07g48760.1
Osa_LOC_Os07g48100.1
Osa_LOC_Os07g48090.1
−1000
0
1000
2000
3000
4000

O.sa CAMK_CAMKL−LKB exon−intron and kinase domain diagram (all)

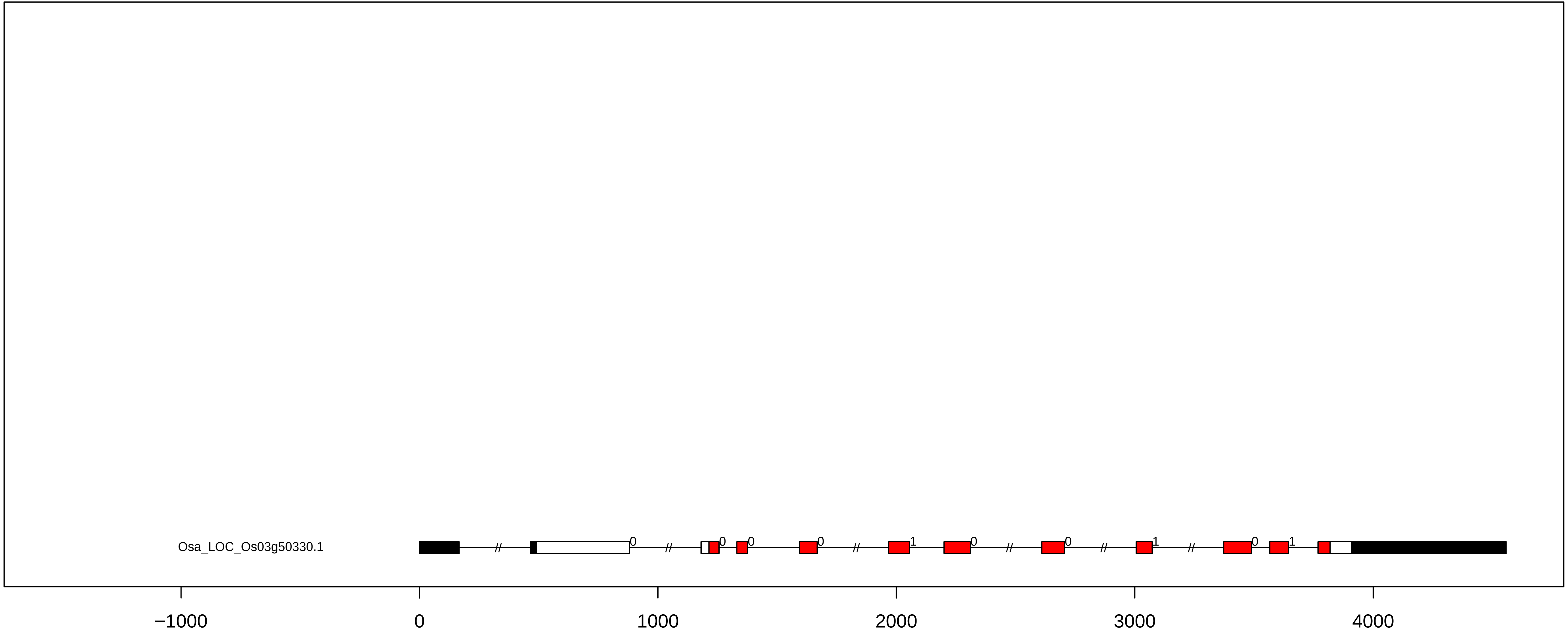

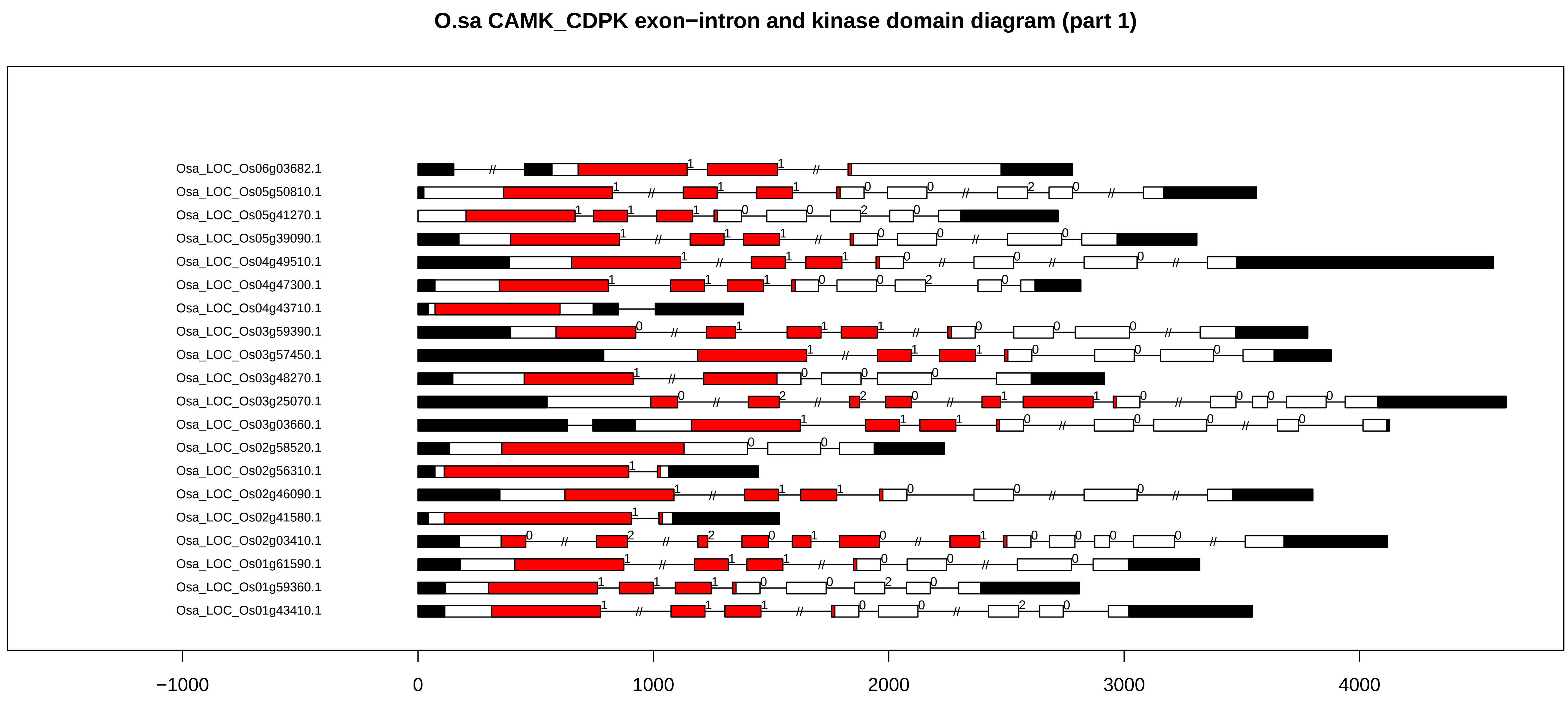
O.sa CAMK_CDPK exon−intron and kinase domain diagram (part 1)
Osa_LOC_Os06g03682.1
Osa_LOC_Os05g50810.1
Osa_LOC_Os05g41270.1
Osa_LOC_Os05g39090.1
Osa_LOC_Os04g49510.1
Osa_LOC_Os04g47300.1
Osa_LOC_Os04g43710.1
Osa_LOC_Os03g59390.1
Osa_LOC_Os03g57450.1
Osa_LOC_Os03g48270.1
Osa_LOC_Os03g25070.1
Osa_LOC_Os03g03660.1
Osa_LOC_Os02g58520.1
Osa_LOC_Os02g56310.1
Osa_LOC_Os02g46090.1
Osa_LOC_Os02g41580.1
Osa_LOC_Os02g03410.1
Osa_LOC_Os01g61590.1
Osa_LOC_Os01g59360.1
Osa_LOC_Os01g43410.1
−1000
0
1000
2000
3000
4000

O.sa CAMK_CDPK exon−intron and kinase domain diagram (part 2)

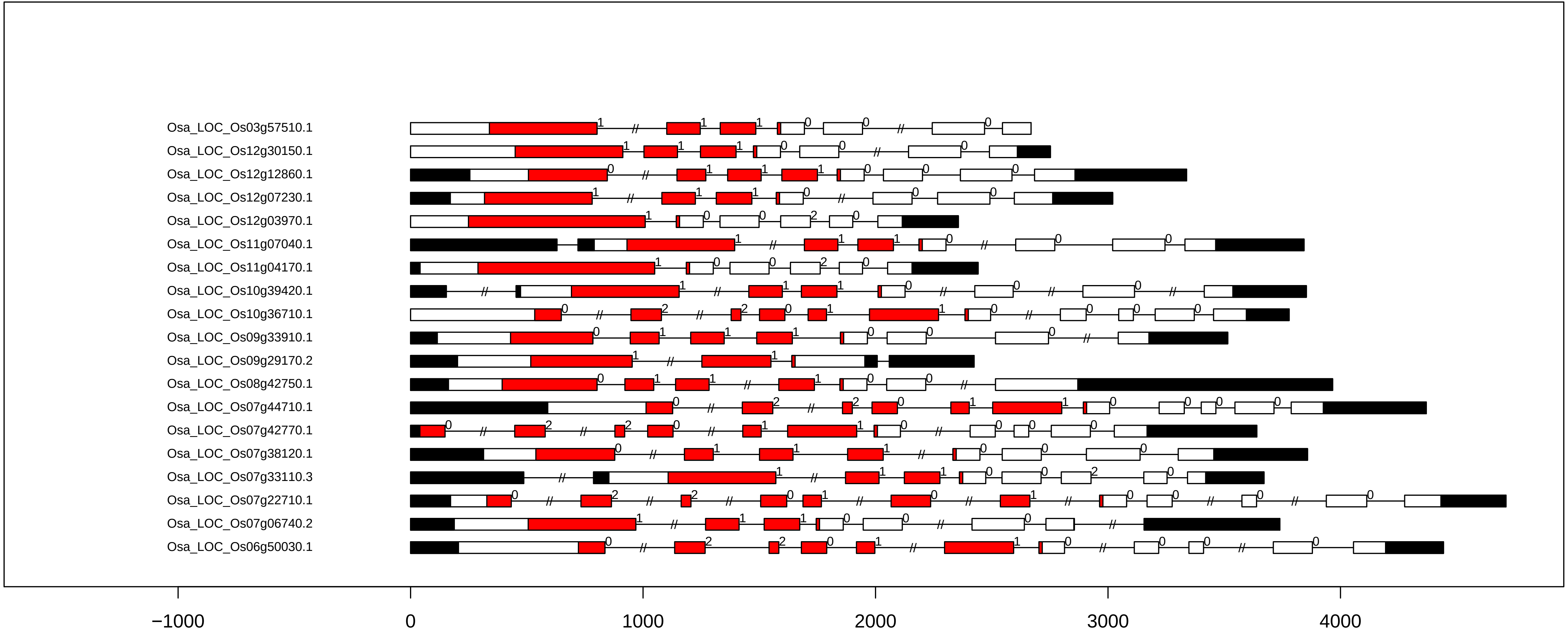

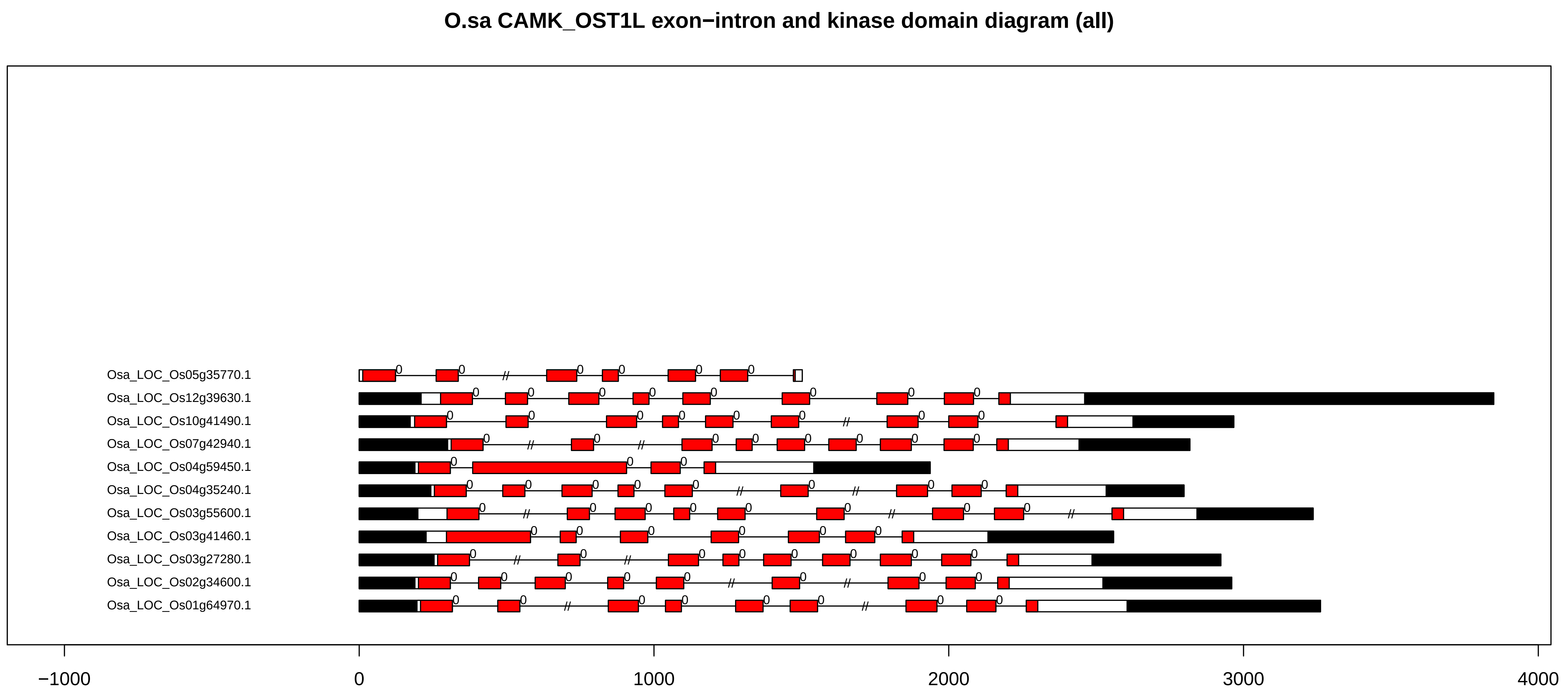

O.sa CAMK_OST1L exon−intron and kinase domain diagram (all)
Osa_LOC_Os05g35770.1
Osa_LOC_Os12g39630.1
Osa_LOC_Os10g41490.1
Osa_LOC_Os07g42940.1
Osa_LOC_Os04g59450.1
Osa_LOC_Os04g35240.1
Osa_LOC_Os03g55600.1
Osa_LOC_Os03g41460.1
Osa_LOC_Os03g27280.1
Osa_LOC_Os02g34600.1
Osa_LOC_Os01g64970.1
−1000
0
1000
2000
3000
4000

O.sa CK1_CK1 exon−intron and kinase domain diagram (all)

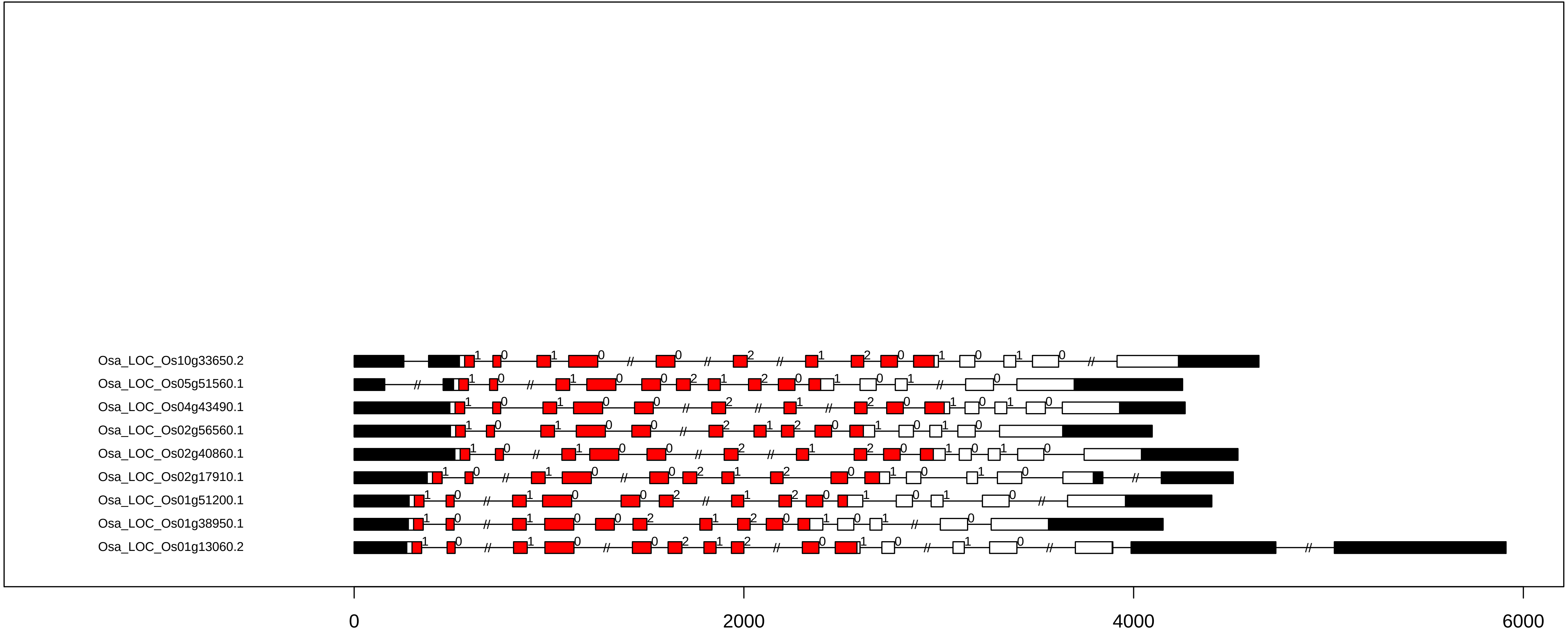

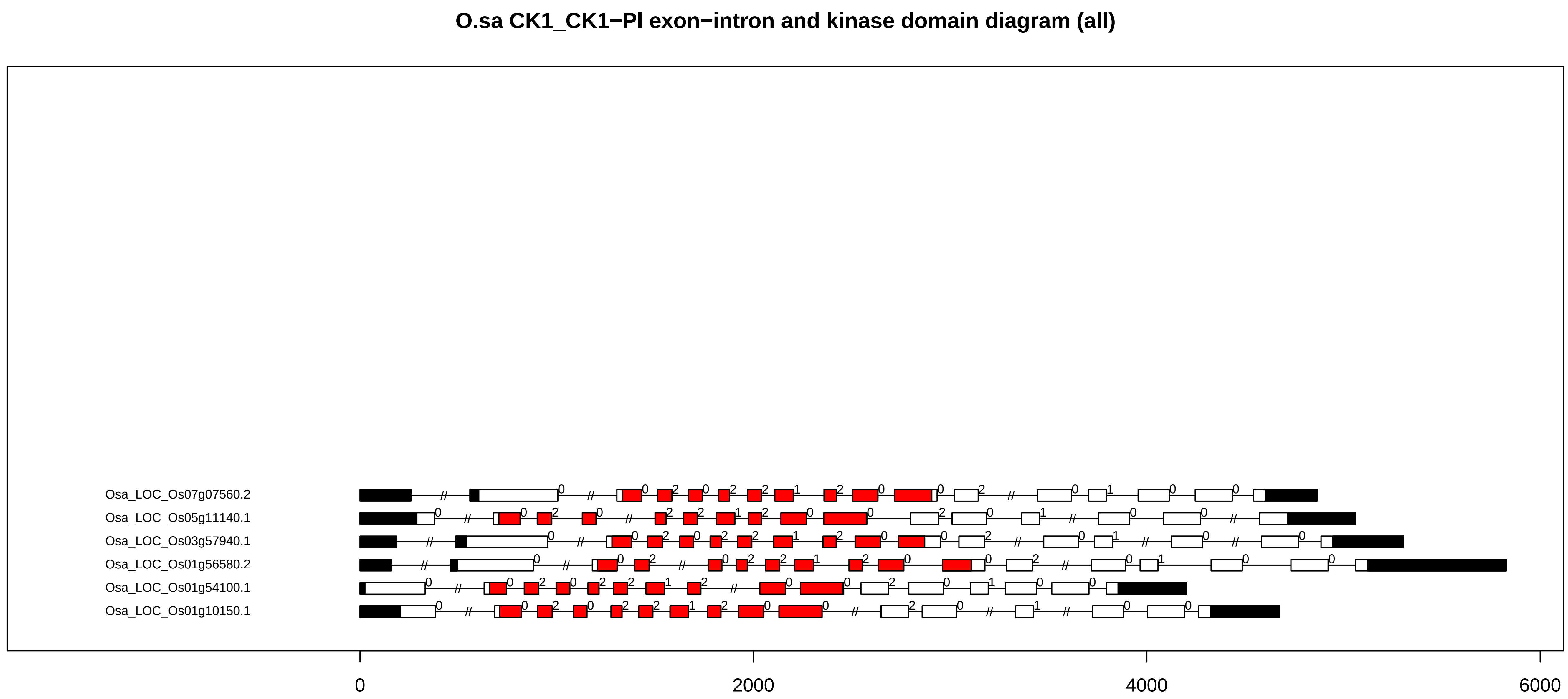
O.sa CK1_CK1−Pl exon−intron and kinase domain diagram (all)
Osa_LOC_Os07g07560.2
Osa_LOC_Os05g11140.1
Osa_LOC_Os03g57940.1
Osa_LOC_Os01g56580.2
Osa_LOC_Os01g54100.1
Osa_LOC_Os01g10150.1
0
2000
4000
6000

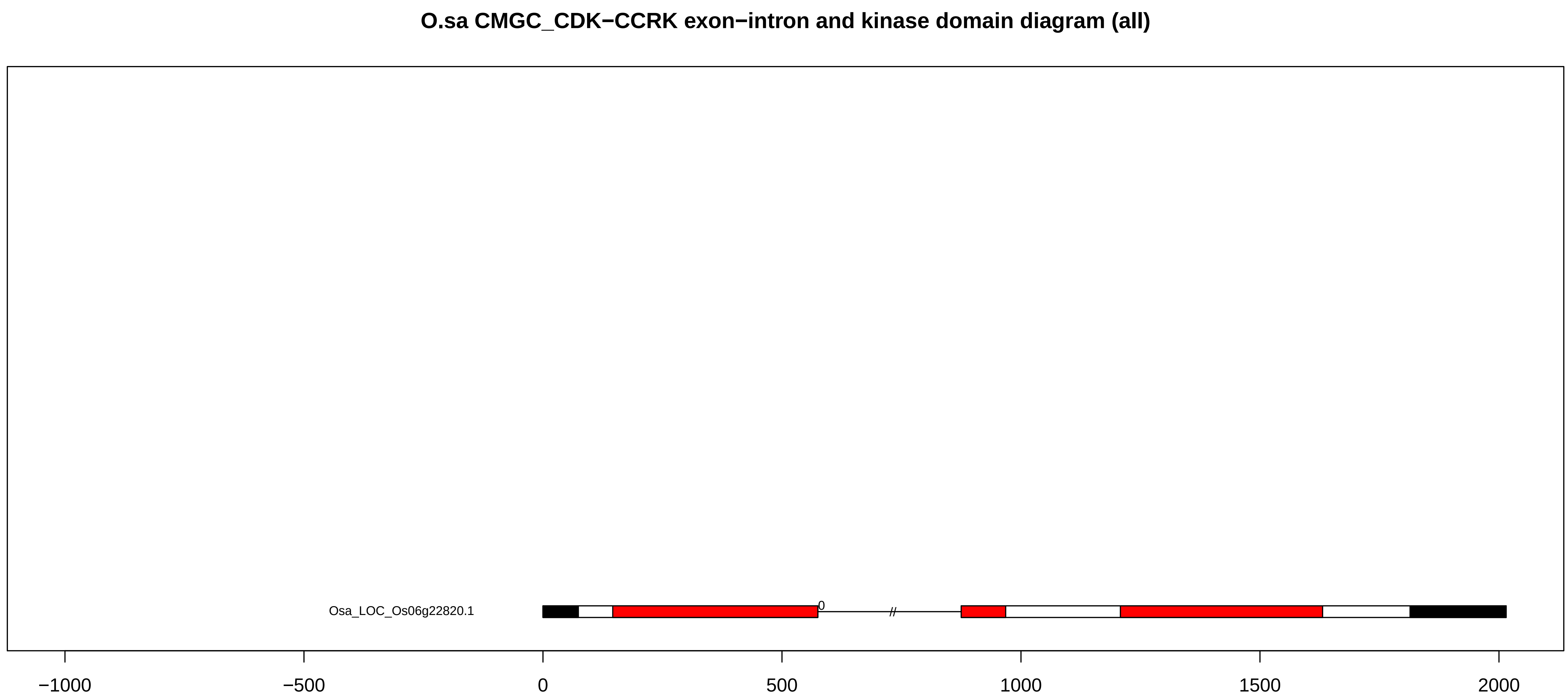
O.sa CMGC_CDK−CCRK exon−intron and kinase domain diagram (all)
Osa_LOC_Os06g22820.1
0
−1000
−500
0
500
1000
1500
2000

O.sa CMGC_CDK−CDK7 exon−intron and kinase domain diagram (all)

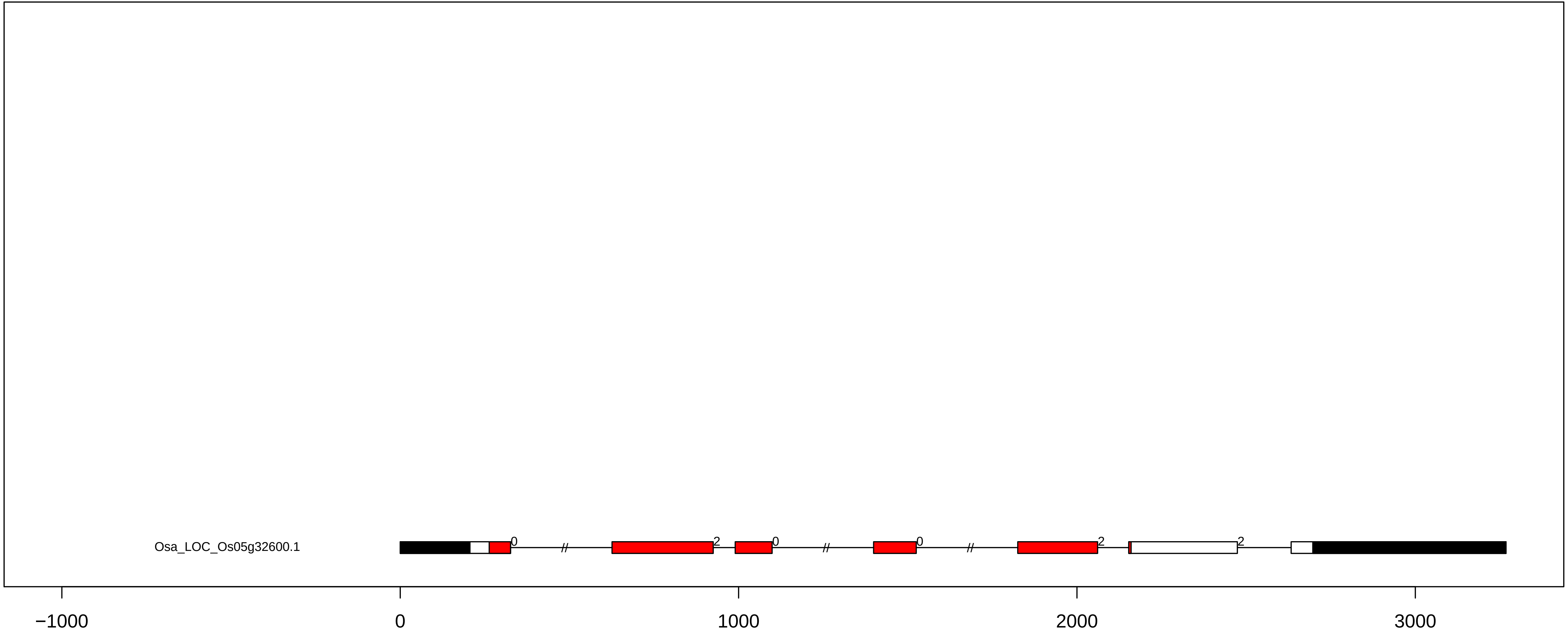

O.sa CMGC_CDK−CDK8 exon−intron and kinase domain diagram (all)

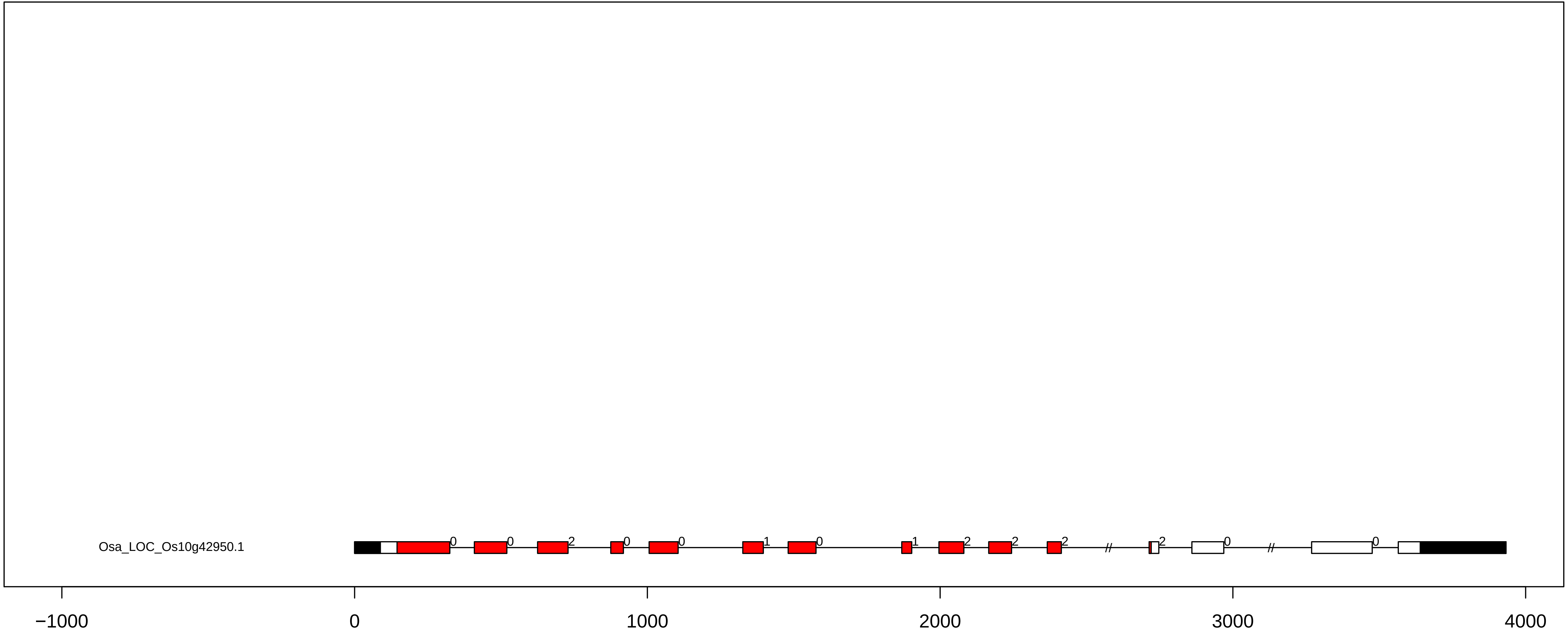

O.sa CMGC_CDK−CRK7−CDK9 exon−intron and kinase domain diagram (all)

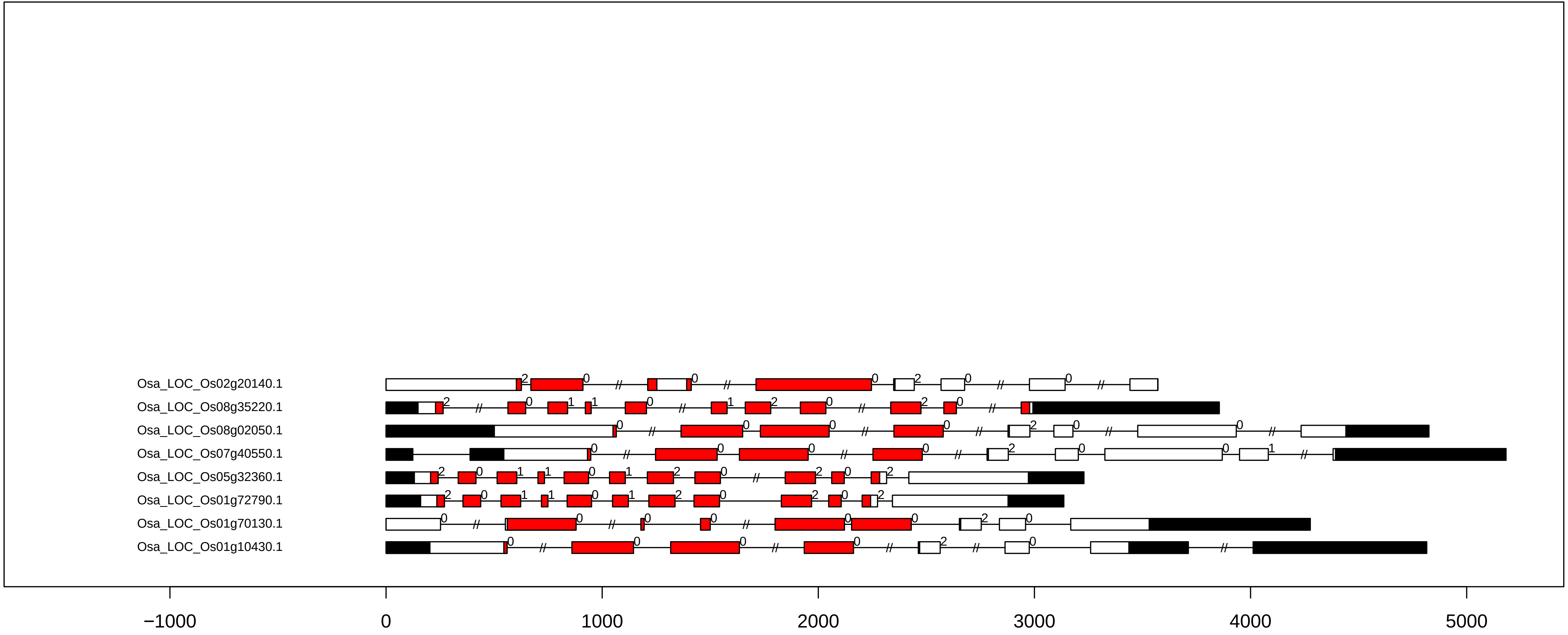

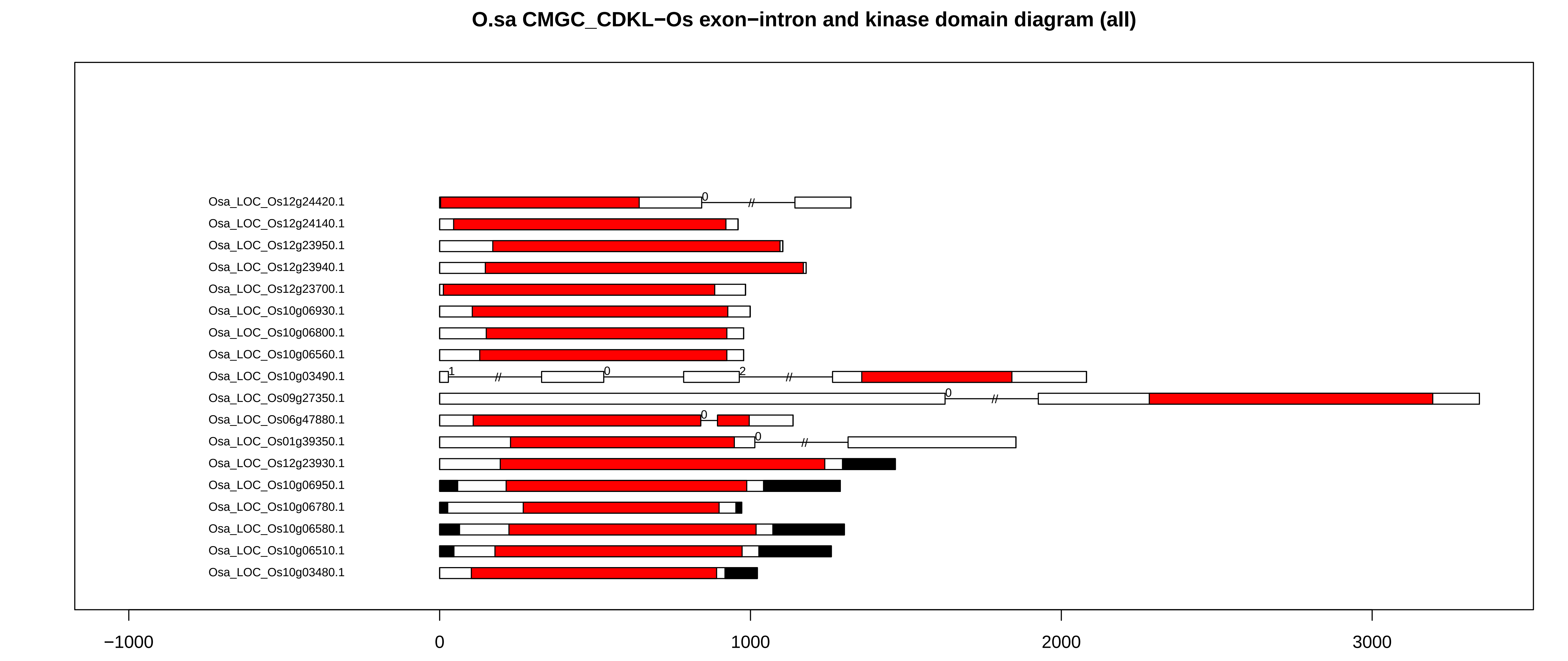

O.sa CMGC_CDKL−Os exon−intron and kinase domain diagram (all)
Osa_LOC_Os12g24420.1
Osa_LOC_Os12g24140.1
Osa_LOC_Os12g23950.1
Osa_LOC_Os12g23940.1
Osa_LOC_Os12g23700.1
Osa_LOC_Os10g06930.1
Osa_LOC_Os10g06800.1
Osa_LOC_Os10g06560.1
Osa_LOC_Os10g03490.1
Osa_LOC_Os09g27350.1
Osa_LOC_Os06g47880.1
Osa_LOC_Os01g39350.1
Osa_LOC_Os12g23930.1
Osa_LOC_Os10g06950.1
Osa_LOC_Os10g06780.1
Osa_LOC_Os10g06580.1
Osa_LOC_Os10g06510.1
Osa_LOC_Os10g03480.1
−1000
0
1000
2000
3000

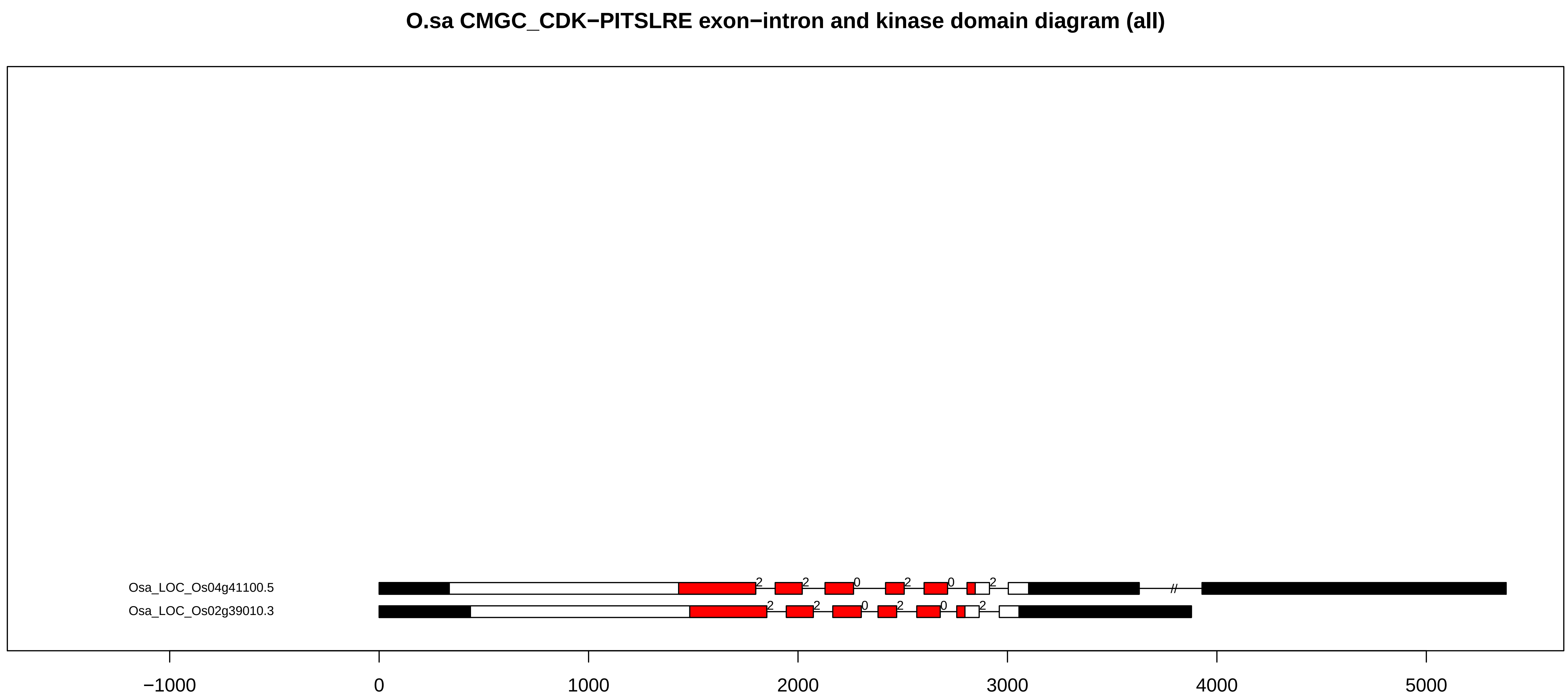
O.sa CMGC_CDK−PITSLRE exon−intron and kinase domain diagram (all)
Osa_LOC_Os04g41100.5
Osa_LOC_Os02g39010.3
2
2
0
2
0
2
−1000
0
1000
2000
3000
4000
5000

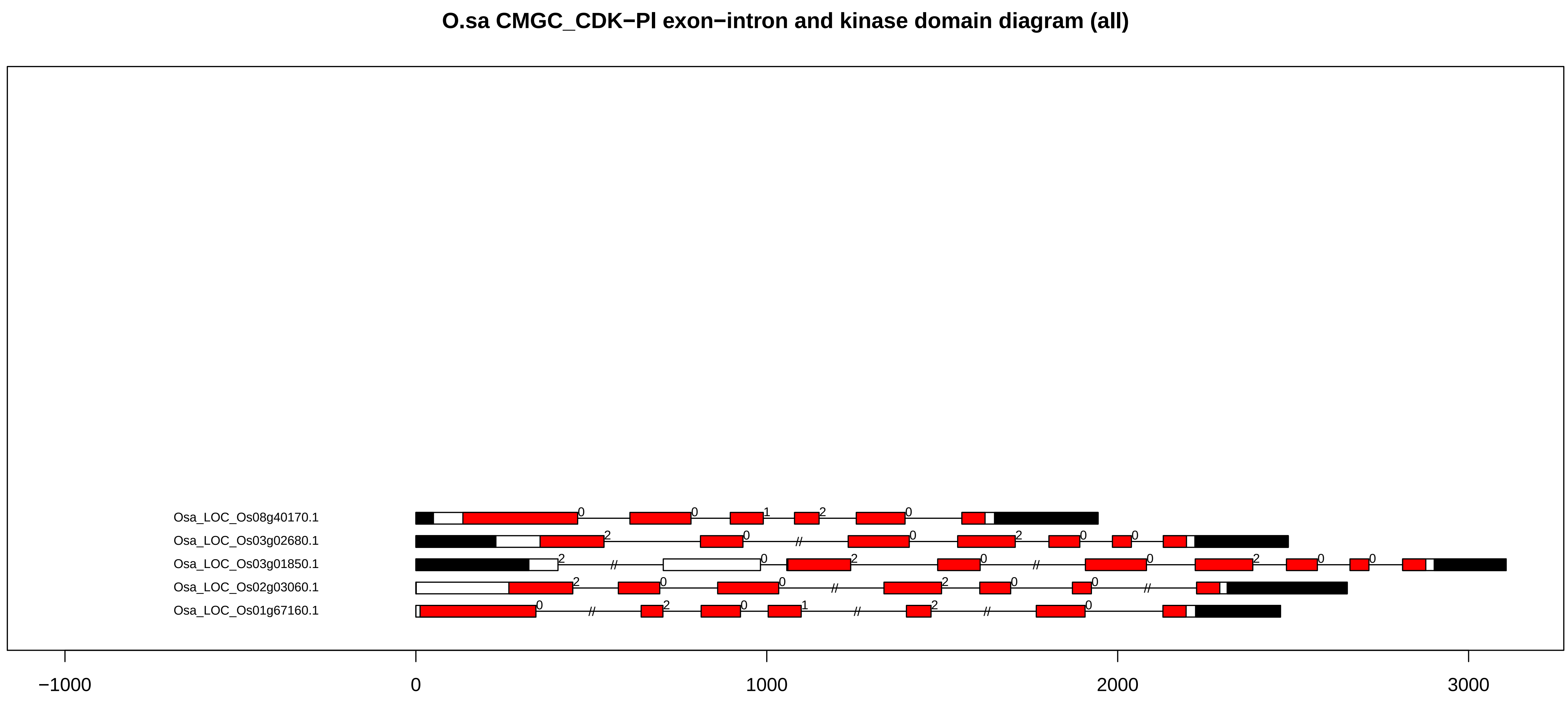

O.sa CMGC_CDK−Pl exon−intron and kinase domain diagram (all)
Osa_LOC_Os08g40170.1
Osa_LOC_Os03g02680.1
Osa_LOC_Os03g01850.1
Osa_LOC_Os02g03060.1
Osa_LOC_Os01g67160.1
−1000
0
1000
2000
3000

O.sa CMGC_CK2 exon−intron and kinase domain diagram (all)

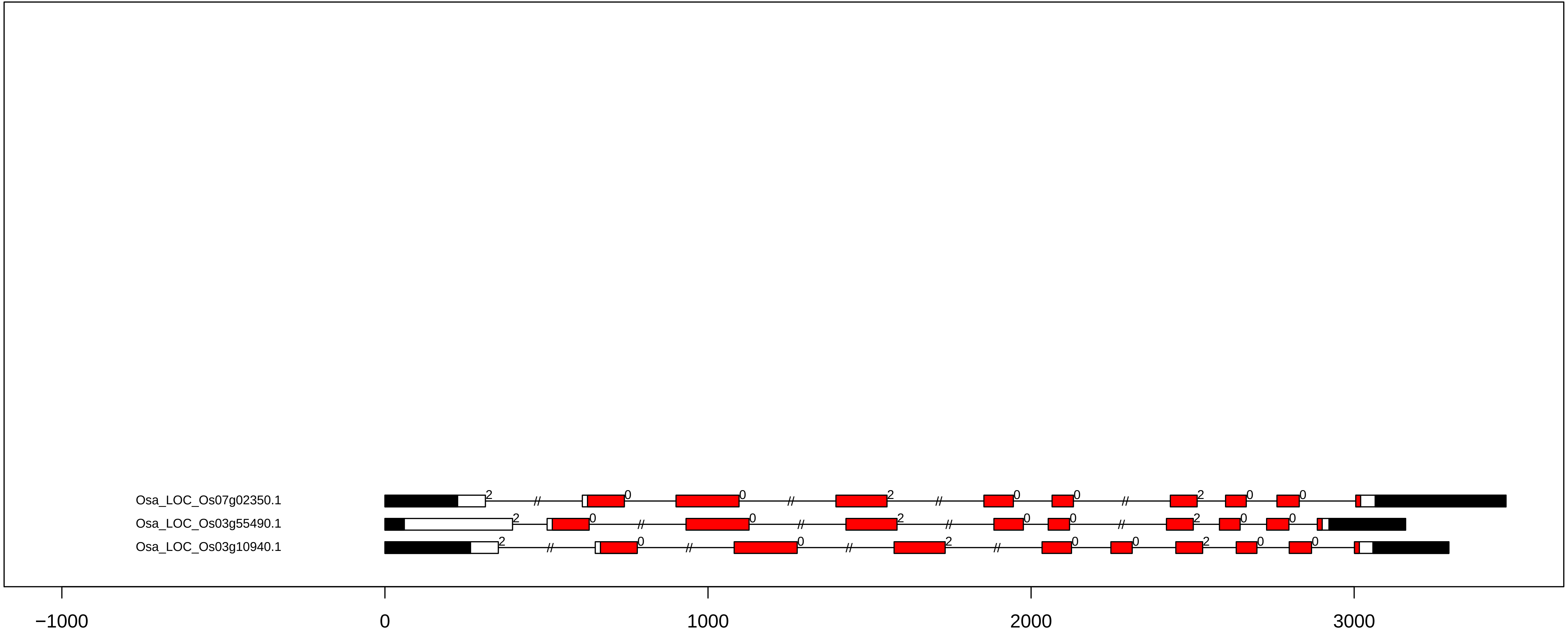

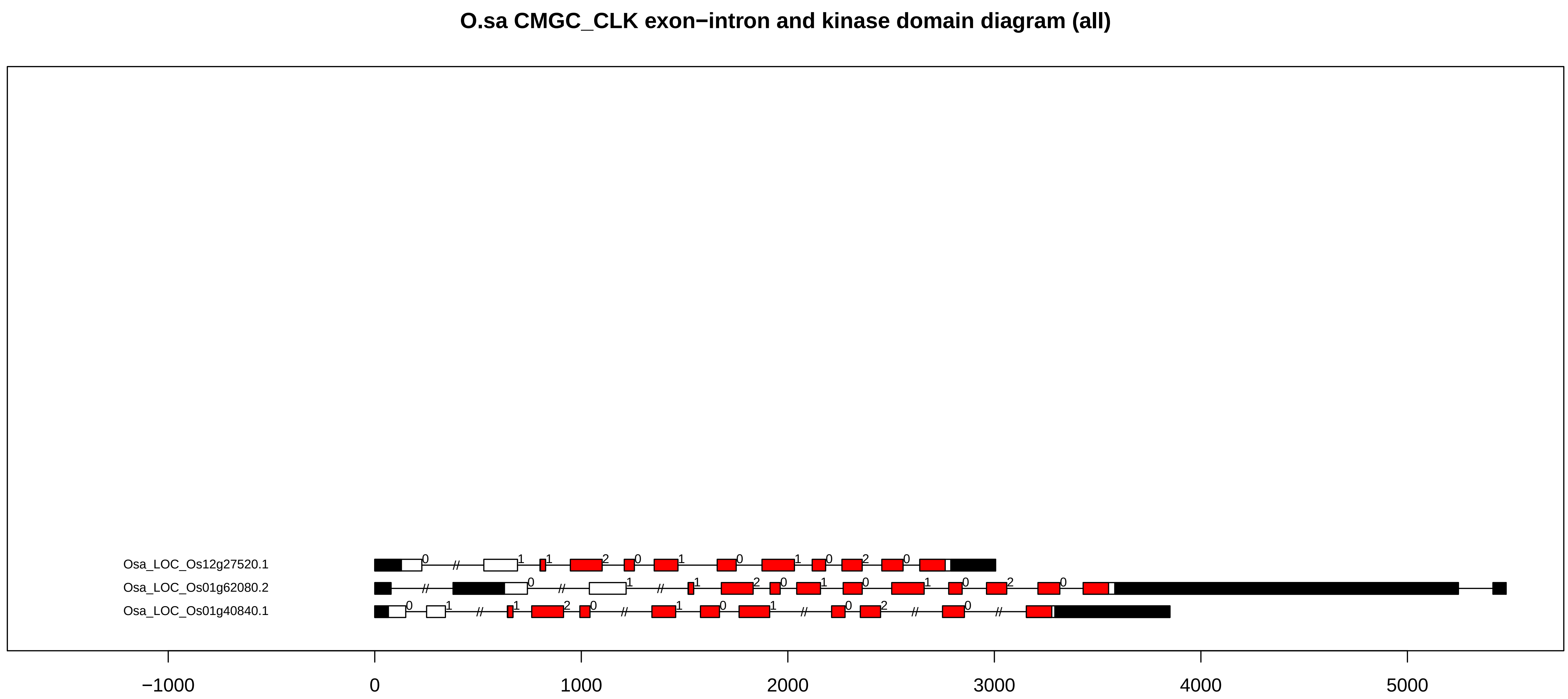
O.sa CMGC_CLK exon−intron and kinase domain diagram (all)
Osa_LOC_Os12g27520.1
Osa_LOC_Os01g62080.2
Osa_LOC_Os01g40840.1
−1000
0
1000
2000
3000
4000
5000

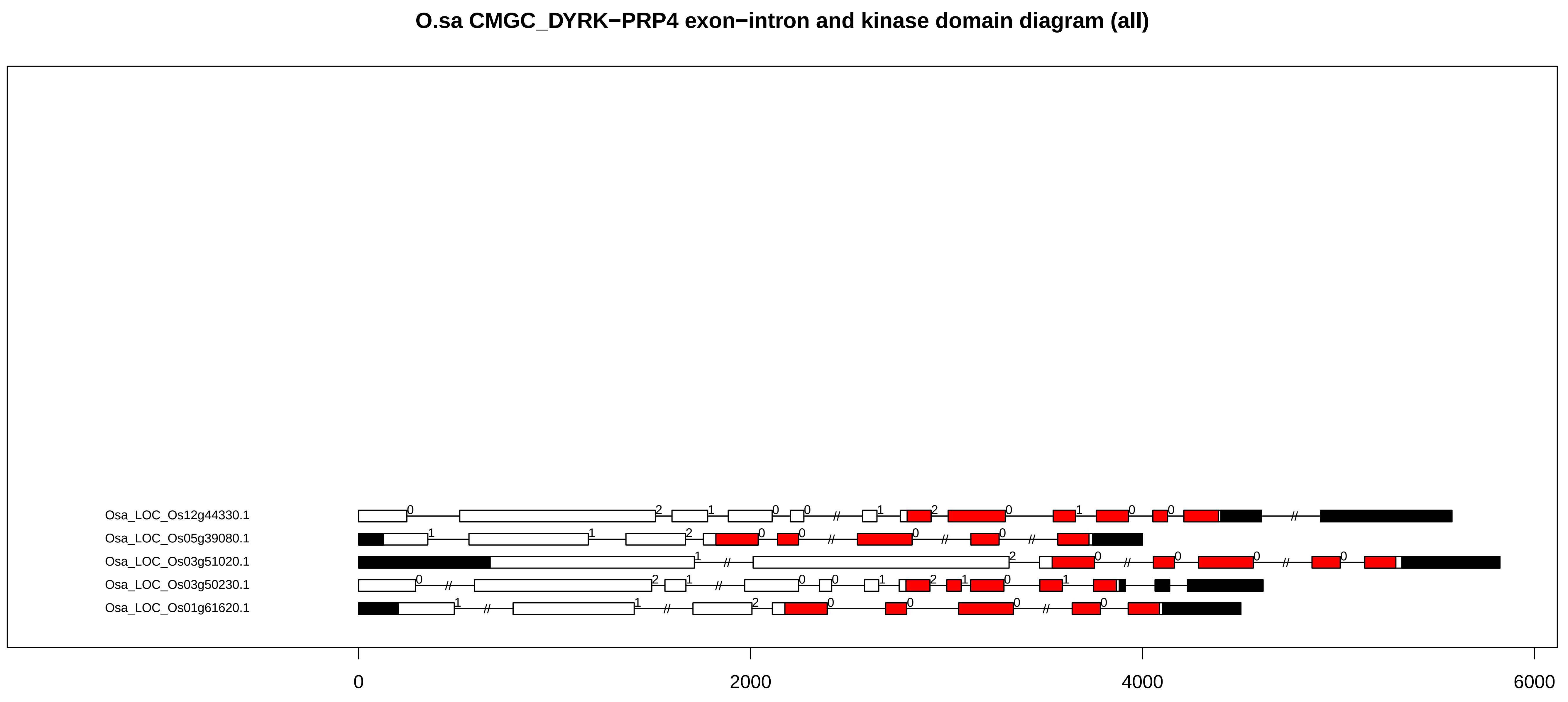
O.sa CMGC_DYRK−PRP4 exon−intron and kinase domain diagram (all)
Osa_LOC_Os12g44330.1
Osa_LOC_Os05g39080.1
Osa_LOC_Os03g51020.1
Osa_LOC_Os03g50230.1
Osa_LOC_Os01g61620.1
0
2000
4000
6000

O.sa CMGC_DYRK−YAK exon−intron and kinase domain diagram (all)

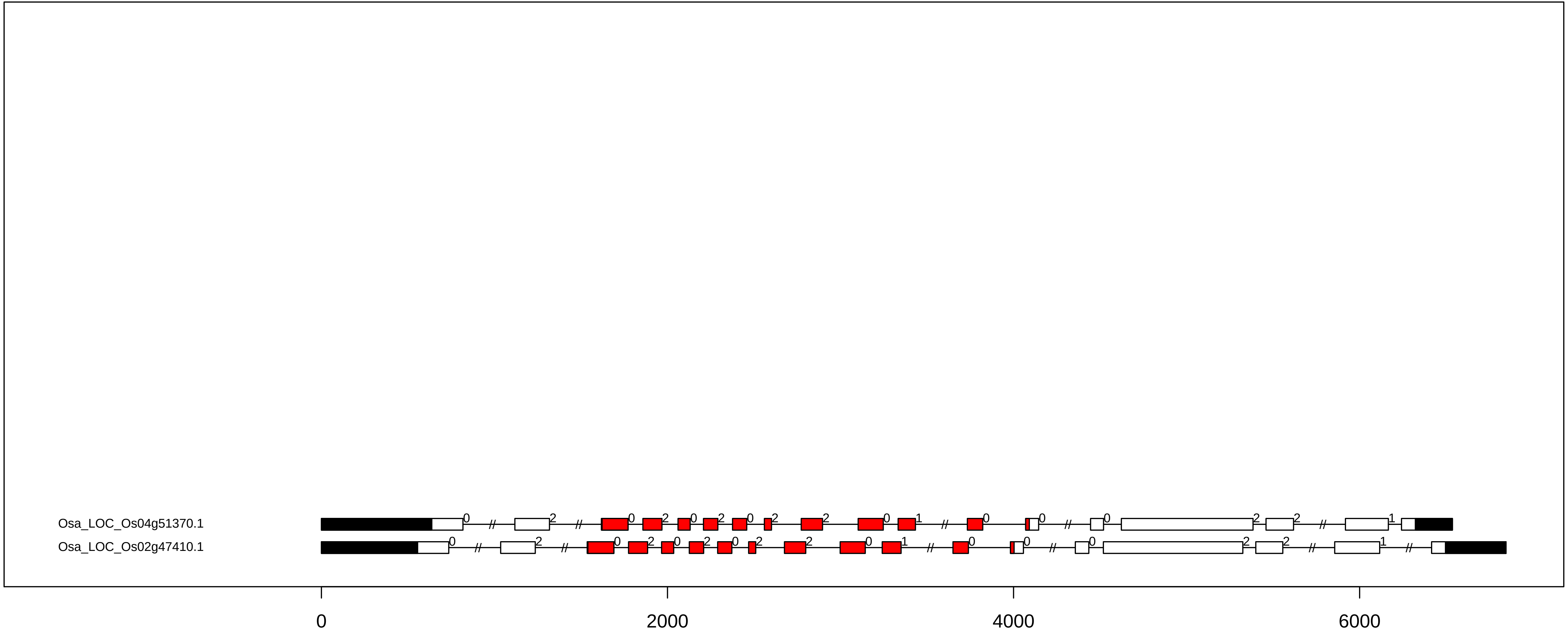

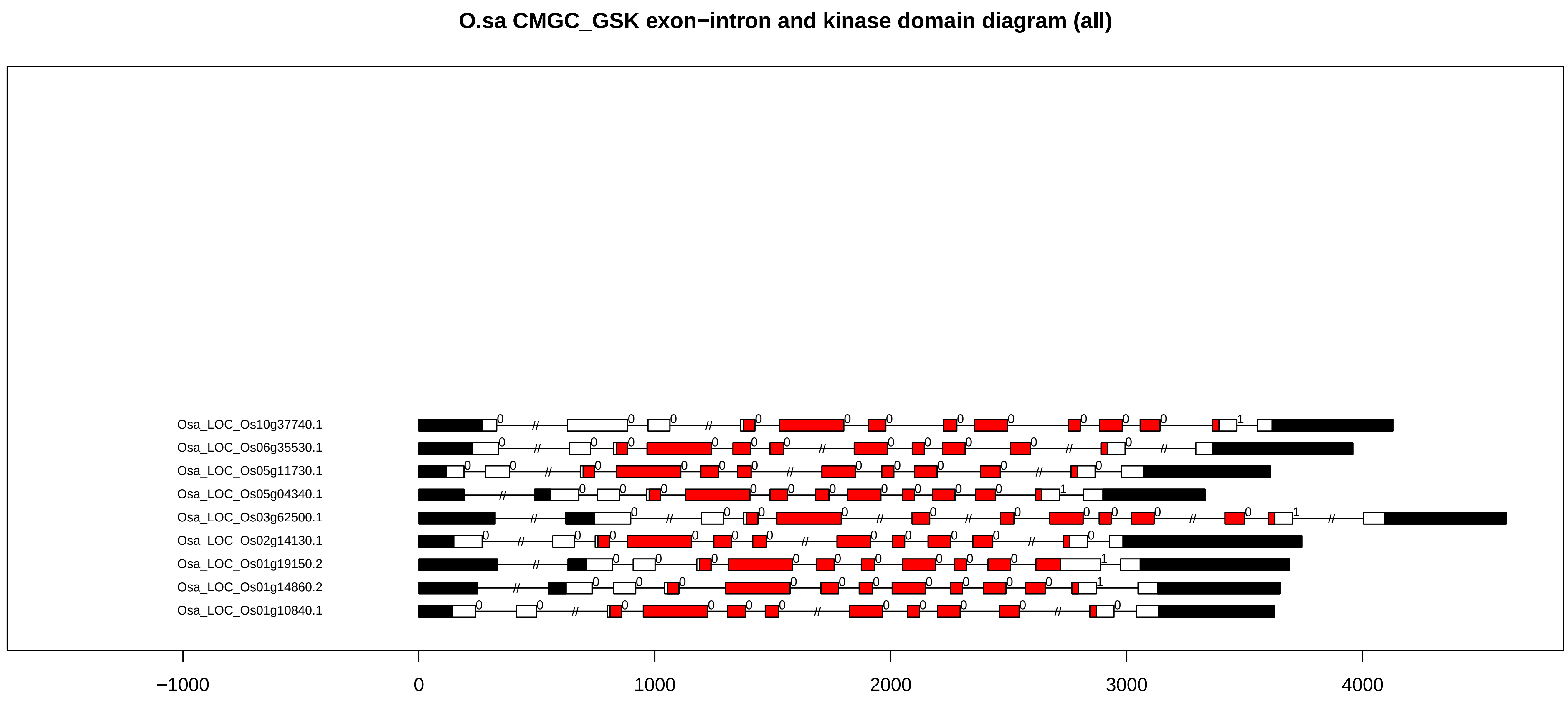

O.sa CMGC_GSK exon−intron and kinase domain diagram (all)
Osa_LOC_Os10g37740.1
Osa_LOC_Os06g35530.1
Osa_LOC_Os05g11730.1
Osa_LOC_Os05g04340.1
Osa_LOC_Os03g62500.1
Osa_LOC_Os02g14130.1
Osa_LOC_Os01g19150.2
Osa_LOC_Os01g14860.2
Osa_LOC_Os01g10840.1
−1000
0
1000
2000
3000
4000

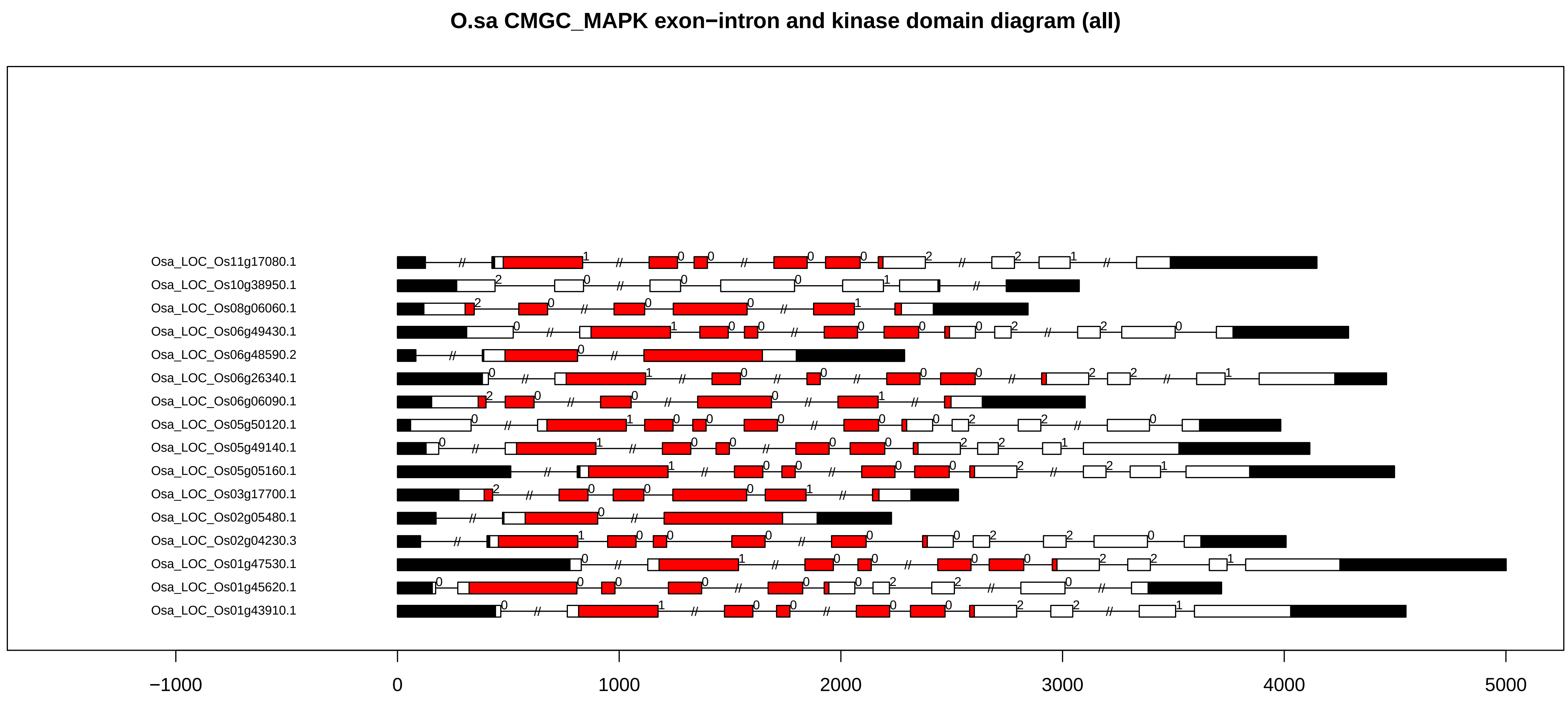
O.sa CMGC_MAPK exon−intron and kinase domain diagram (all)
Osa_LOC_Os11g17080.1
Osa_LOC_Os10g38950.1
Osa_LOC_Os08g06060.1
Osa_LOC_Os06g49430.1
Osa_LOC_Os06g48590.2
Osa_LOC_Os06g26340.1
Osa_LOC_Os06g06090.1
Osa_LOC_Os05g50120.1
Osa_LOC_Os05g49140.1
Osa_LOC_Os05g05160.1
Osa_LOC_Os03g17700.1
Osa_LOC_Os02g05480.1
Osa_LOC_Os02g04230.3
Osa_LOC_Os01g47530.1
Osa_LOC_Os01g45620.1
Osa_LOC_Os01g43910.1
−1000
0
1000
2000
3000
4000
5000

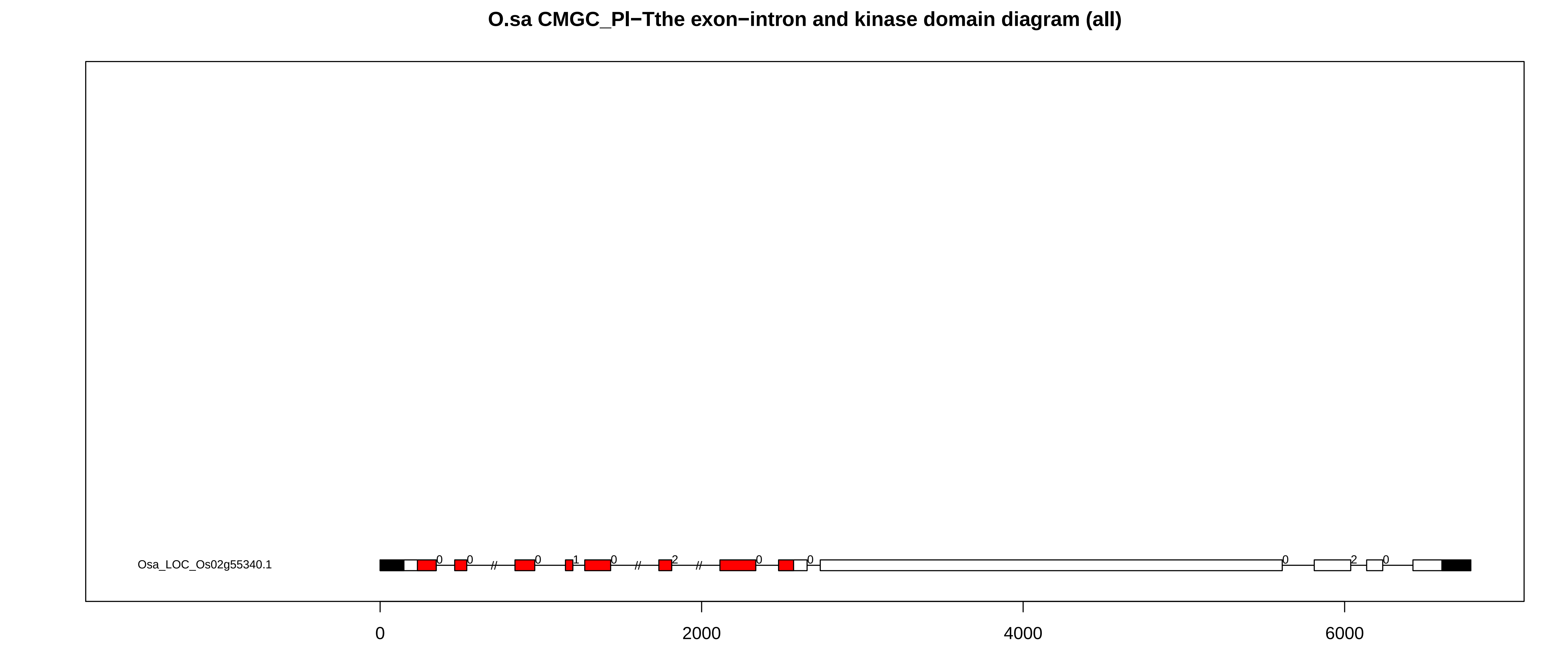
O.sa CMGC_Pl−Tthe exon−intron and kinase domain diagram (all)
Osa_LOC_Os02g55340.1
0
0
0
1
0
2
0
0
0
2
0
0
2000
4000
6000

O.sa CMGC_RCK exon−intron and kinase domain diagram (all)

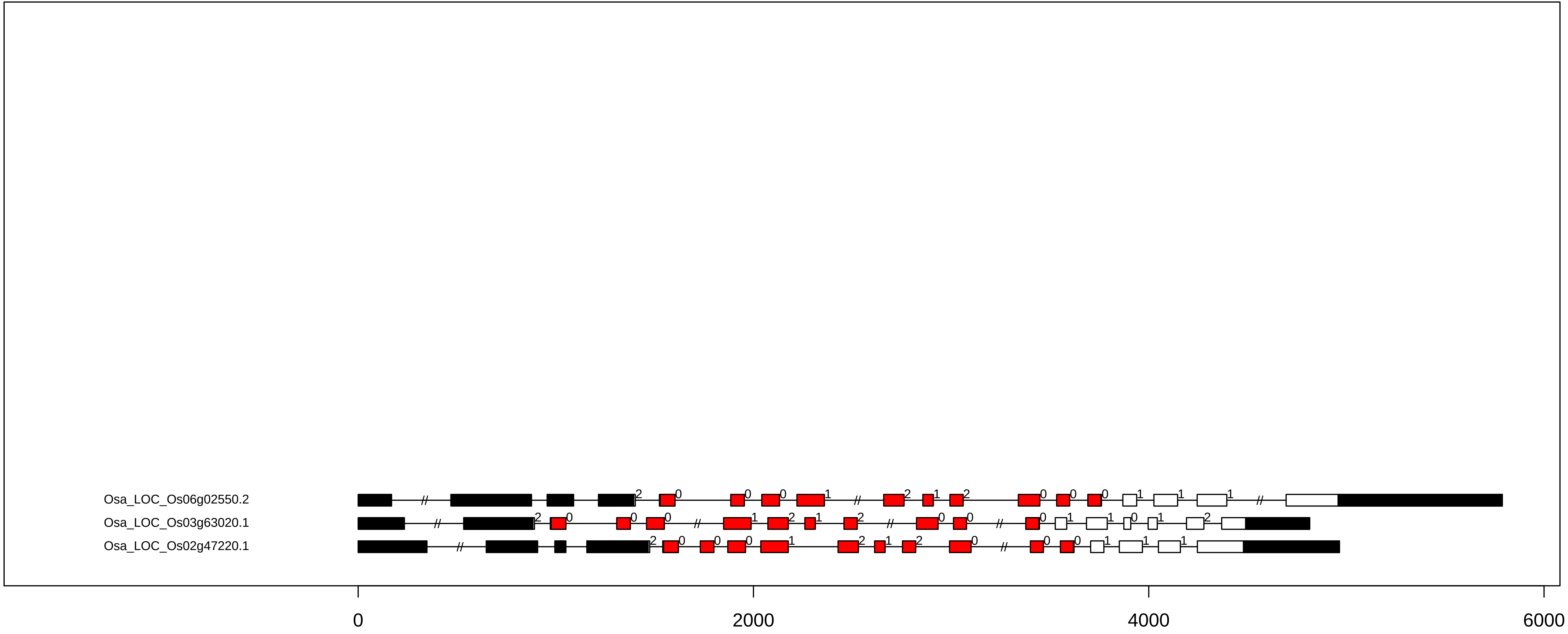

O.sa CMGC_SRPK exon−intron and kinase domain diagram (all)

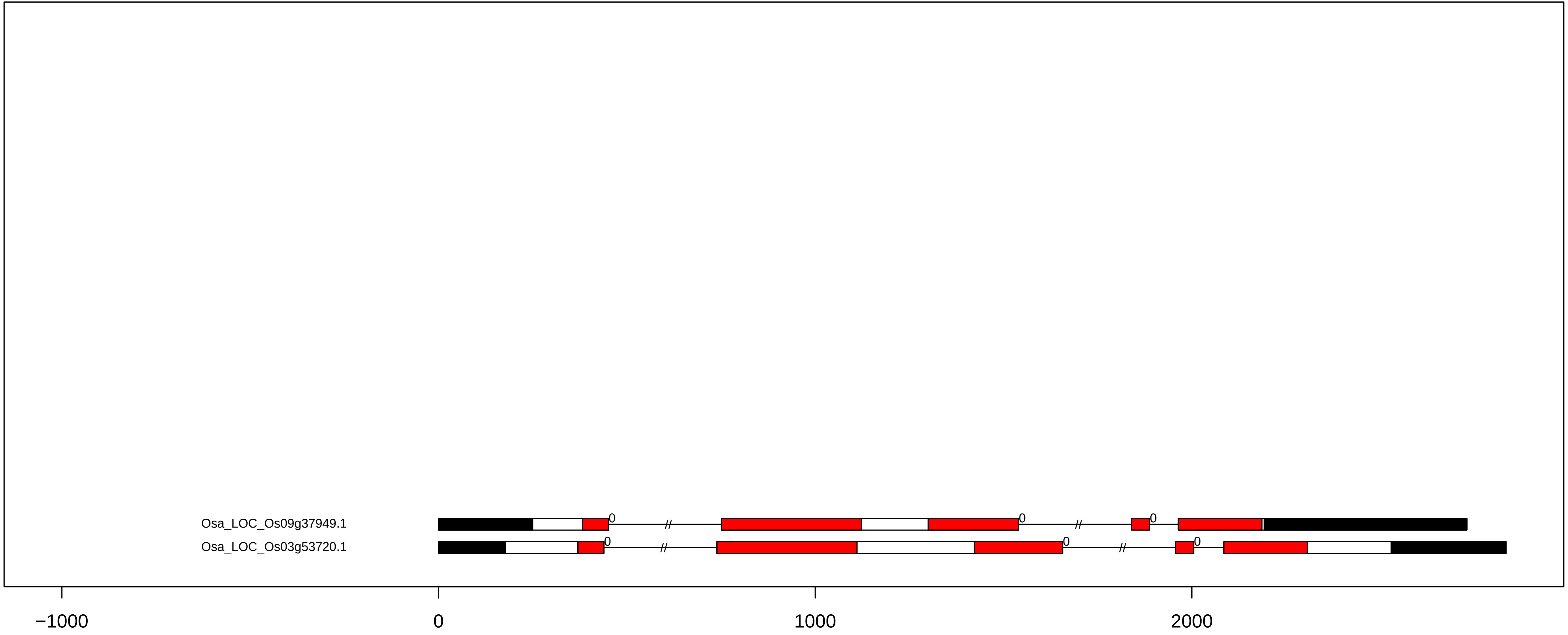

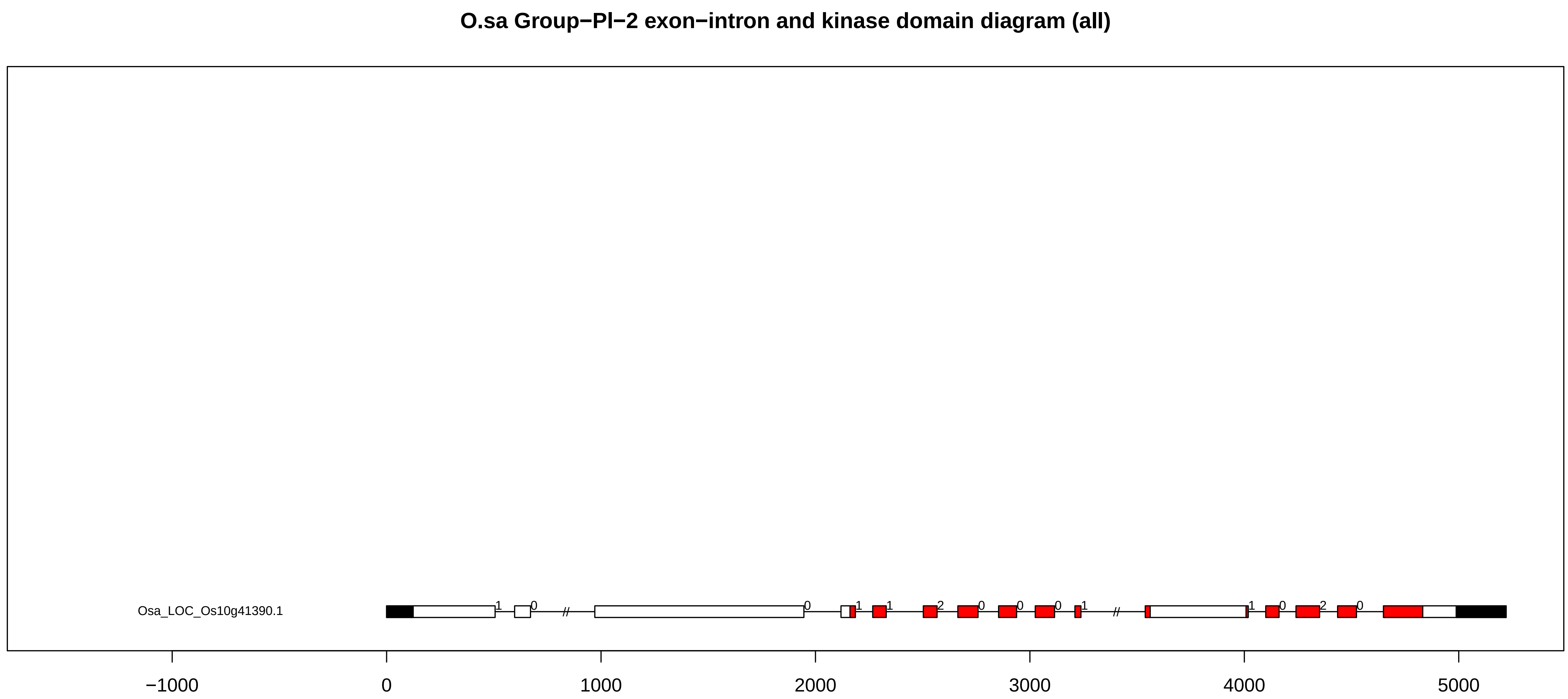
O.sa Group−PI−2 exon−intron and kinase domain diagram (all)
Osa_LOC_Os10g41390.1
1
0
0
1
1
2
0
0
0
1
1
0
2
0
−1000
0
1000
2000
3000
4000
5000

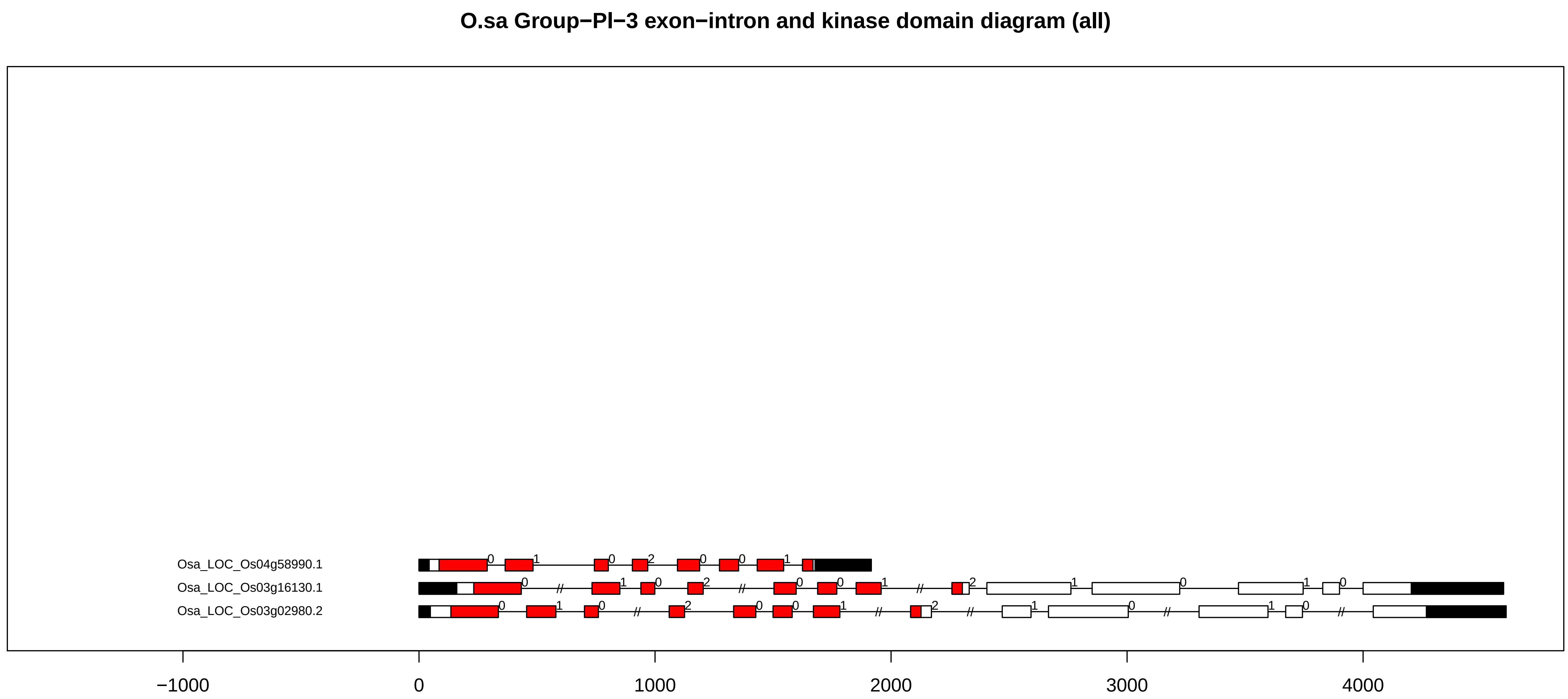

O.sa Group−PI−3 exon−intron and kinase domain diagram (all)
Osa_LOC_Os04g58990.1
Osa_LOC_Os03g16130.1
Osa_LOC_Os03g02980.2
−1000
0
1000
2000
3000
4000

O.sa Group−PI−4 exon−intron and kinase domain diagram (all)

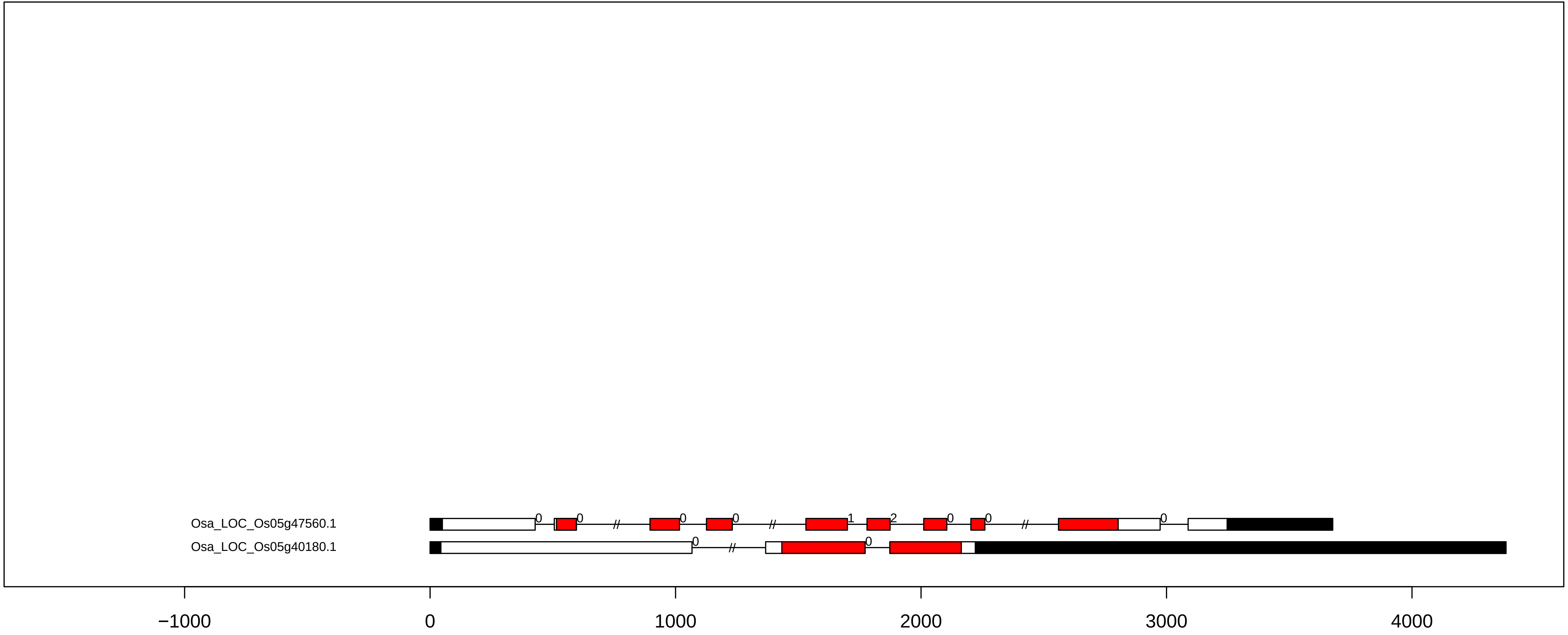

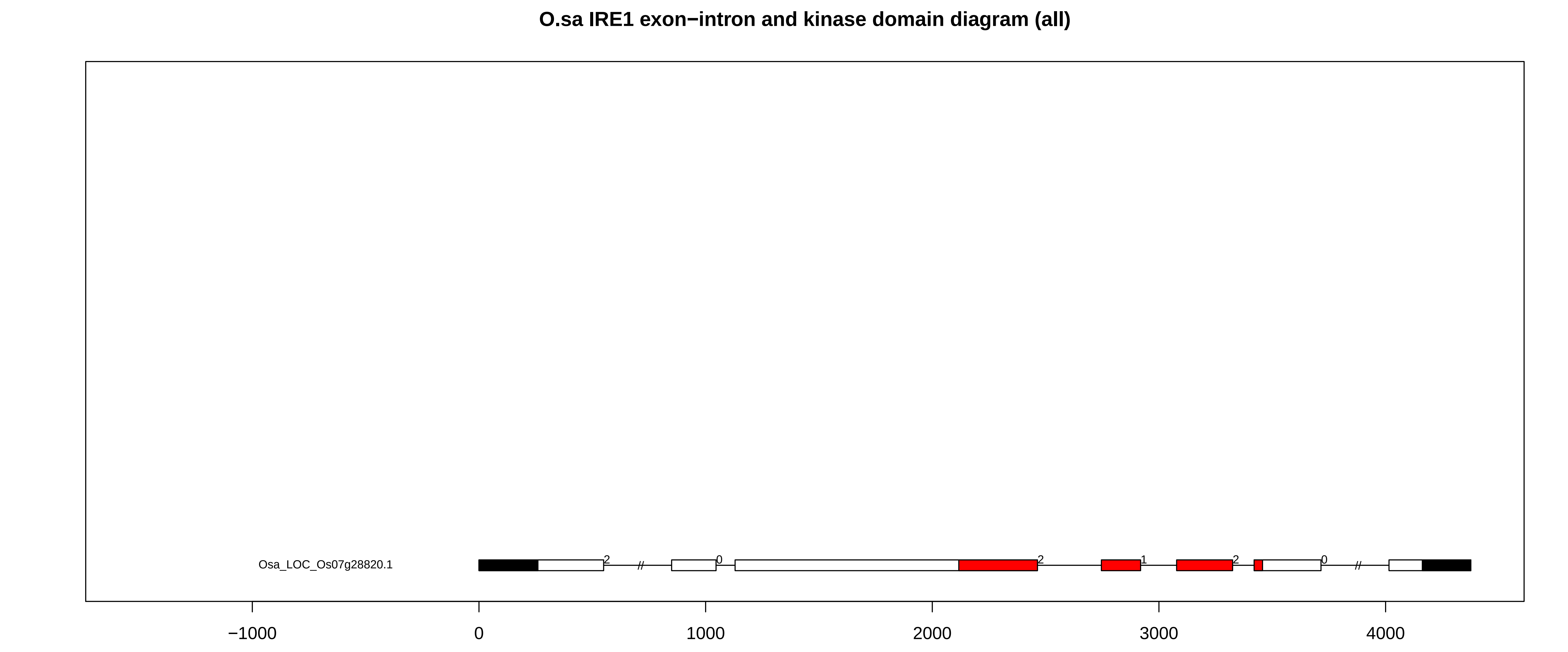

O.sa IRE1 exon−intron and kinase domain diagram (all)
Osa_LOC_Os07g28820.1
2
0
2
1
2
0
−1000
0
1000
2000
3000
4000

O.sa NAK exon−intron and kinase domain diagram (all)

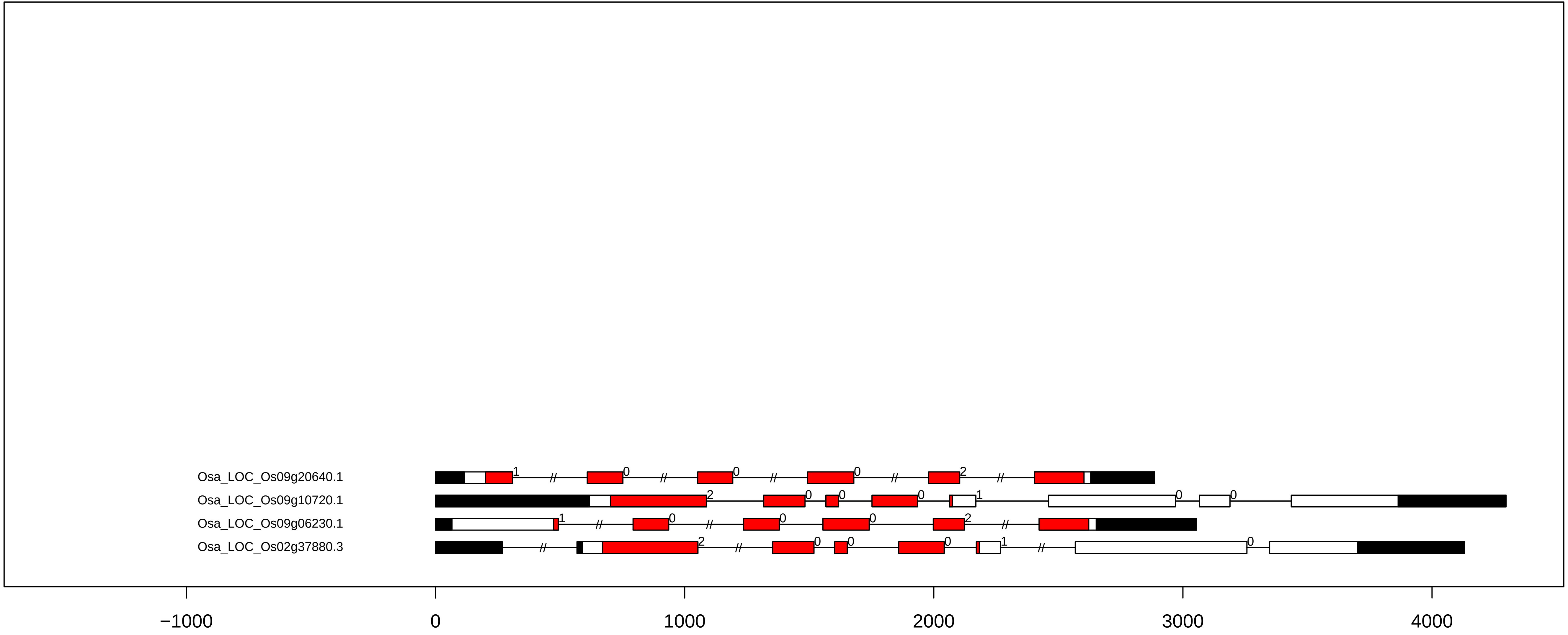

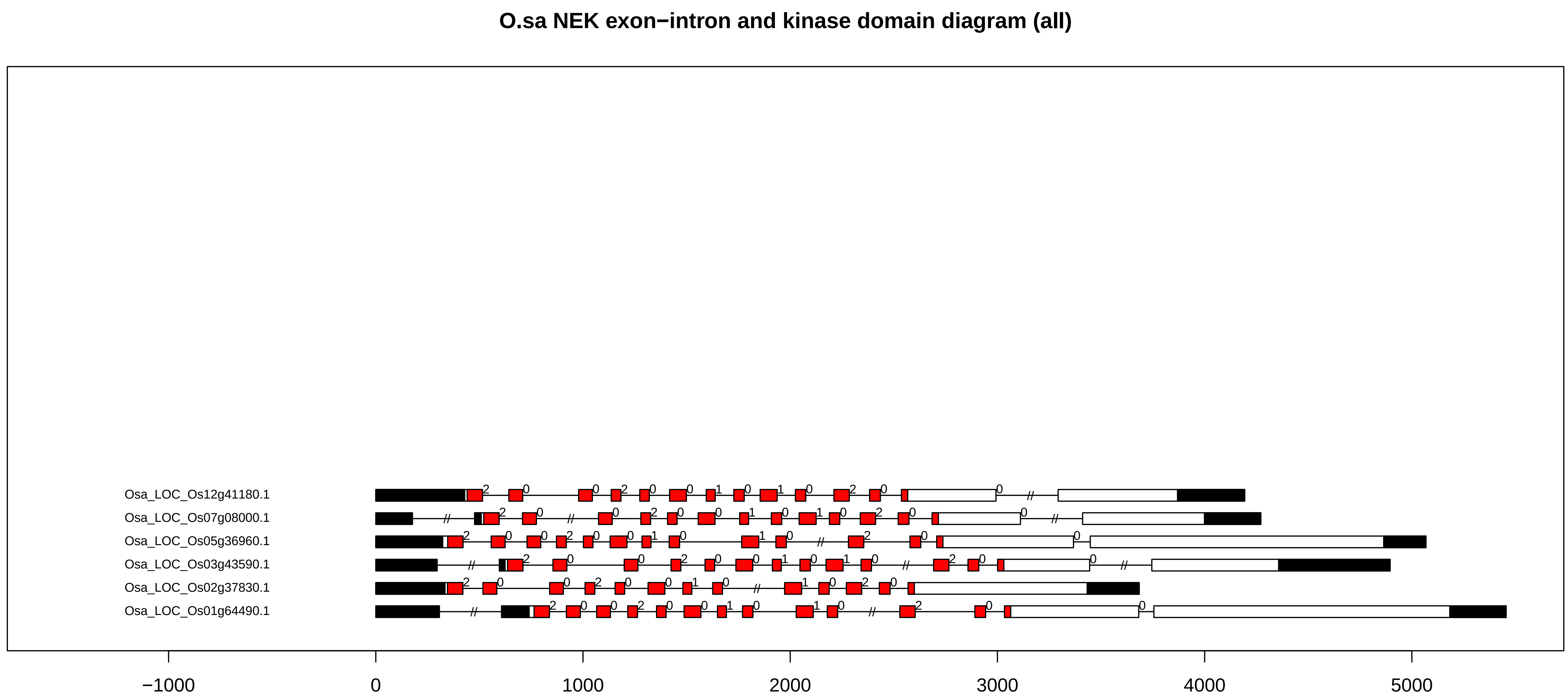
O.sa NEK exon−intron and kinase domain diagram (all)
Osa_LOC_Os12g41180.1
Osa_LOC_Os07g08000.1
Osa_LOC_Os05g36960.1
Osa_LOC_Os03g43590.1
Osa_LOC_Os02g37830.1
Osa_LOC_Os01g64490.1
−1000
0
1000
2000
3000
4000
5000

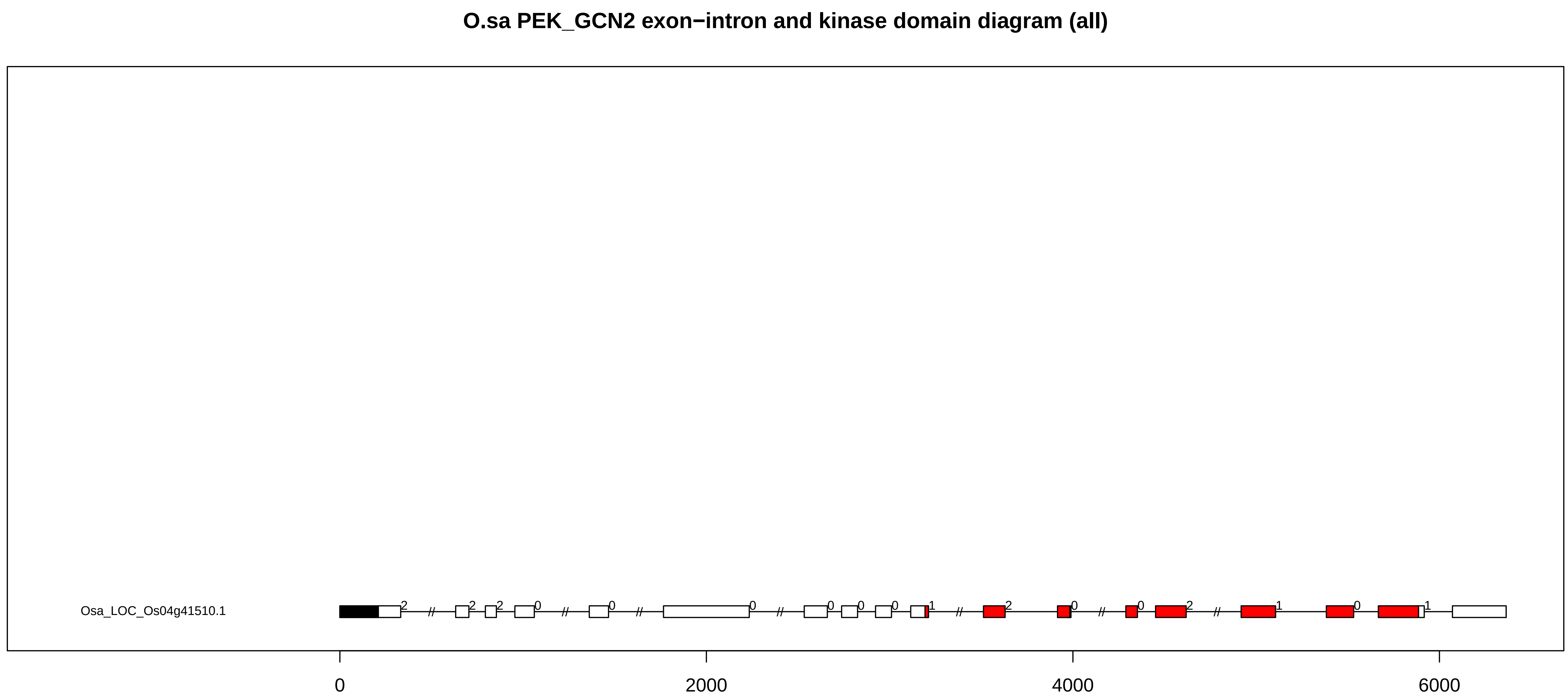
O.sa PEK_GCN2 exon−intron and kinase domain diagram (all)
Osa_LOC_Os04g41510.1
2
2
2
0
0
0
0
0
0
1
2
0
0
2
1
0
1
0
2000
4000
6000

O.sa RLK−Pelle_C−LEC exon−intron and kinase domain diagram (all)

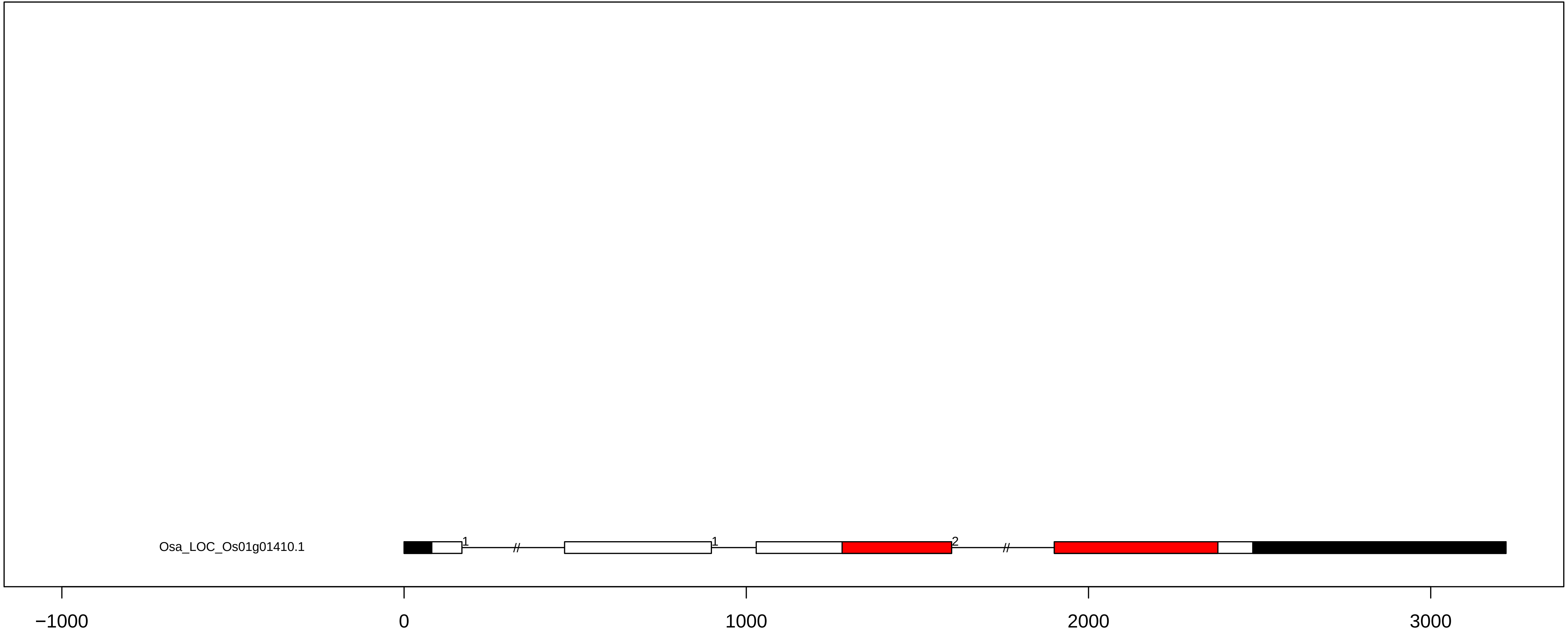

O.sa RLK−Pelle_CR4L exon−intron and kinase domain diagram (all)

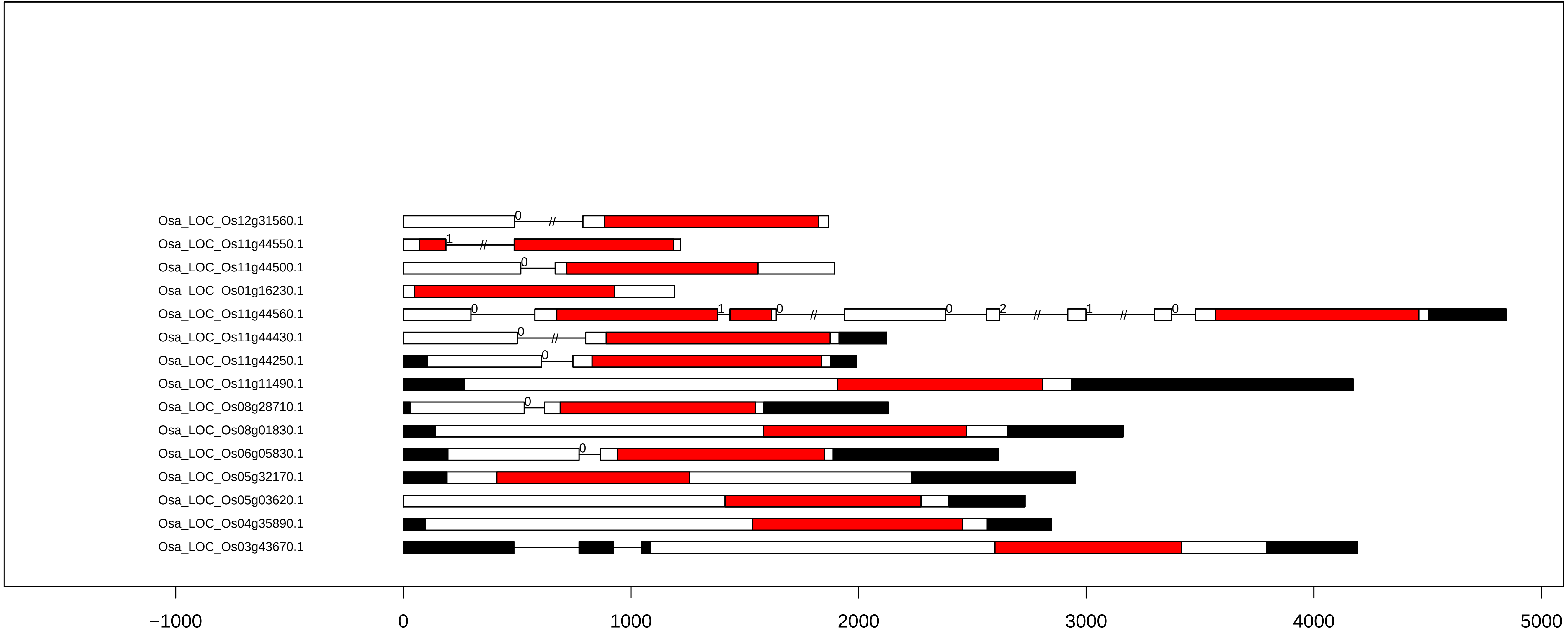

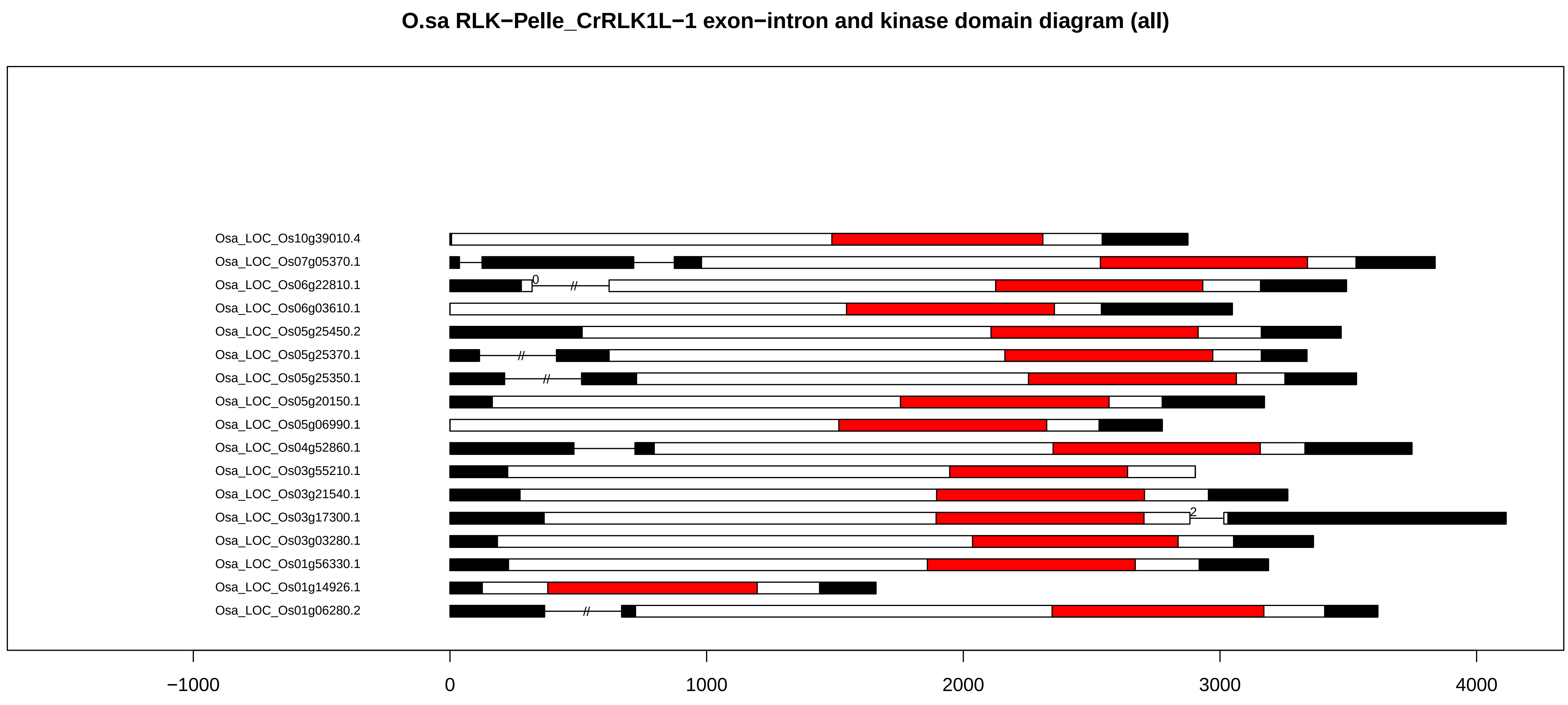
O.sa RLK−Pelle_CrRLK1L−1 exon−intron and kinase domain diagram (all)
Osa_LOC_Os10g39010.4
Osa_LOC_Os07g05370.1
Osa_LOC_Os06g22810.1
Osa_LOC_Os06g03610.1
Osa_LOC_Os05g25450.2
Osa_LOC_Os05g25370.1
Osa_LOC_Os05g25350.1
Osa_LOC_Os05g20150.1
Osa_LOC_Os05g06990.1
Osa_LOC_Os04g52860.1
Osa_LOC_Os03g55210.1
Osa_LOC_Os03g21540.1
Osa_LOC_Os03g17300.1
Osa_LOC_Os03g03280.1
Osa_LOC_Os01g56330.1
Osa_LOC_Os01g14926.1
Osa_LOC_Os01g06280.2
0
2
−1000
0
1000
2000
3000
4000

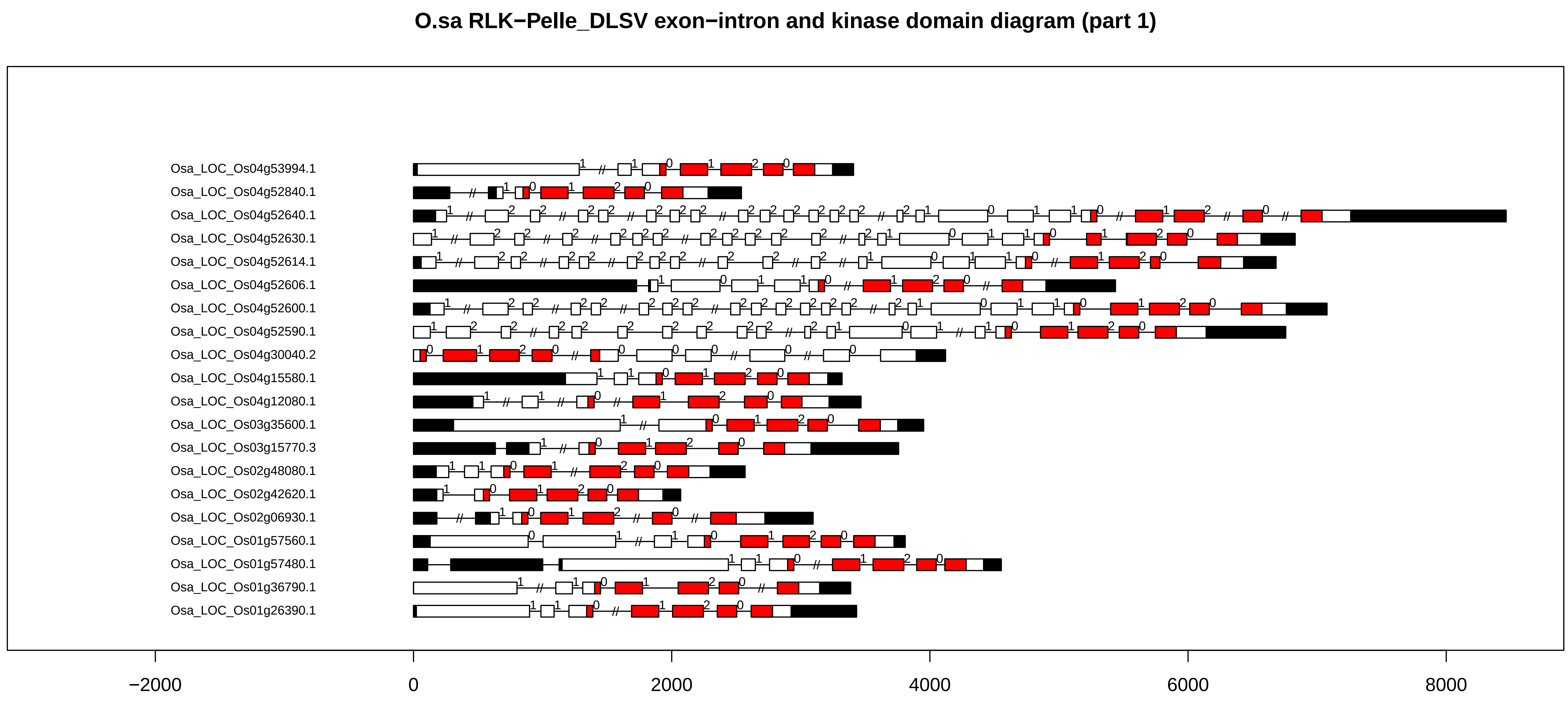
O.sa RLK−Pelle_DLSV exon−intron and kinase domain diagram (part 1)
Osa_LOC_Os04g53994.1
Osa_LOC_Os04g52840.1
Osa_LOC_Os04g52640.1
Osa_LOC_Os04g52630.1
Osa_LOC_Os04g52614.1
Osa_LOC_Os04g52606.1
Osa_LOC_Os04g52600.1
Osa_LOC_Os04g52590.1
Osa_LOC_Os04g30040.2
Osa_LOC_Os04g15580.1
Osa_LOC_Os04g12080.1
Osa_LOC_Os03g35600.1
Osa_LOC_Os03g15770.3
Osa_LOC_Os02g48080.1
Osa_LOC_Os02g42620.1
Osa_LOC_Os02g06930.1
Osa_LOC_Os01g57560.1
Osa_LOC_Os01g57480.1
Osa_LOC_Os01g36790.1
Osa_LOC_Os01g26390.1
−2000
0
2000
4000
6000
8000

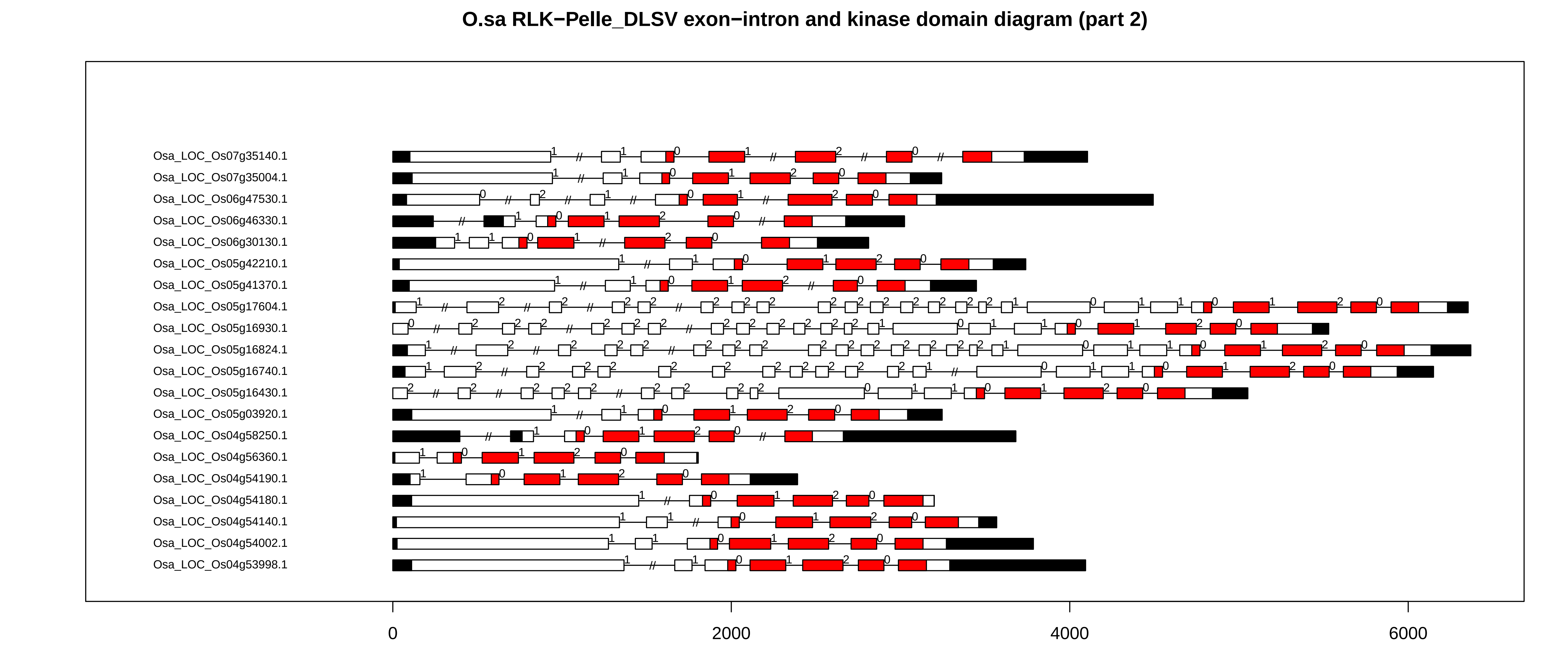
O.sa RLK−Pelle_DLSV exon−intron and kinase domain diagram (part 2)
Osa_LOC_Os07g35140.1
Osa_LOC_Os07g35004.1
Osa_LOC_Os06g47530.1
Osa_LOC_Os06g46330.1
Osa_LOC_Os06g30130.1
Osa_LOC_Os05g42210.1
Osa_LOC_Os05g41370.1
Osa_LOC_Os05g17604.1
Osa_LOC_Os05g16930.1
Osa_LOC_Os05g16824.1
Osa_LOC_Os05g16740.1
Osa_LOC_Os05g16430.1
Osa_LOC_Os05g03920.1
Osa_LOC_Os04g58250.1
Osa_LOC_Os04g56360.1
Osa_LOC_Os04g54190.1
Osa_LOC_Os04g54180.1
Osa_LOC_Os04g54140.1
Osa_LOC_Os04g54002.1
Osa_LOC_Os04g53998.1
0
2000
4000
6000

O.sa RLK−Pelle_DLSV exon−intron and kinase domain diagram (part 3)

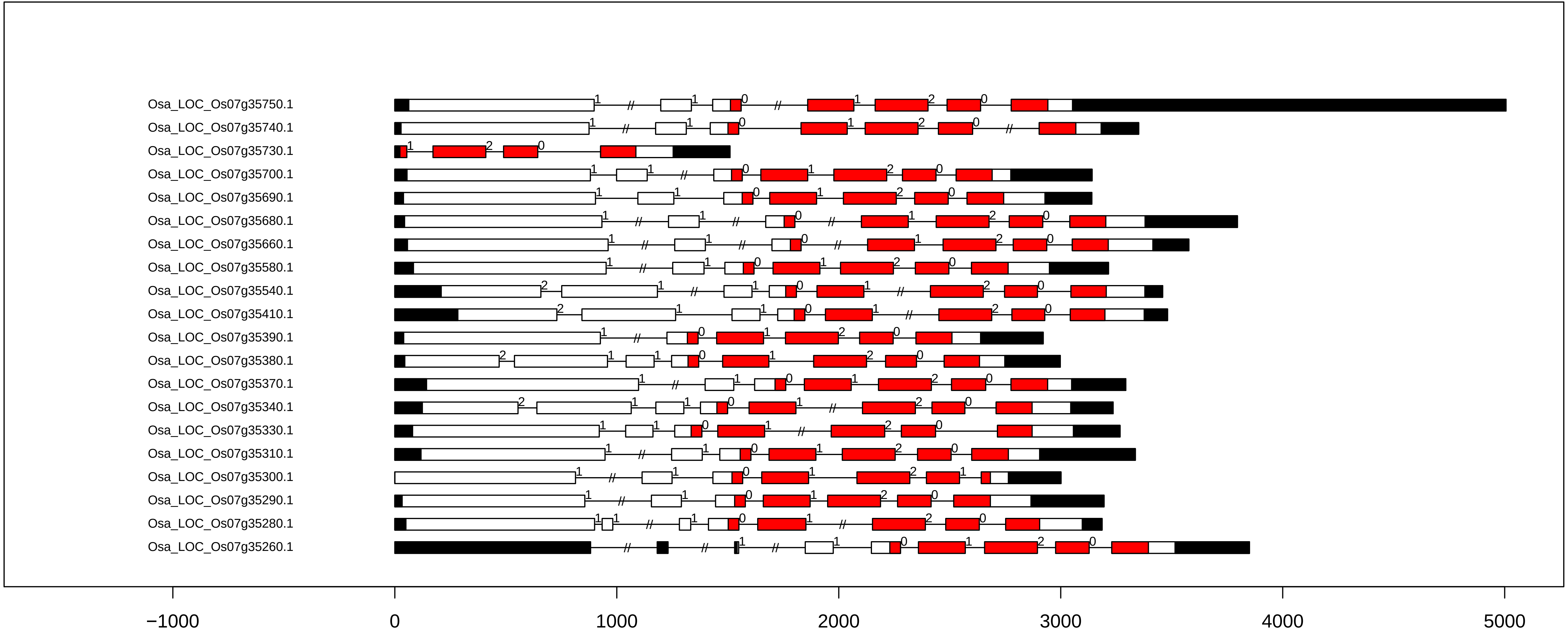

O.sa RLK−Pelle_DLSV exon−intron and kinase domain diagram (part 4)

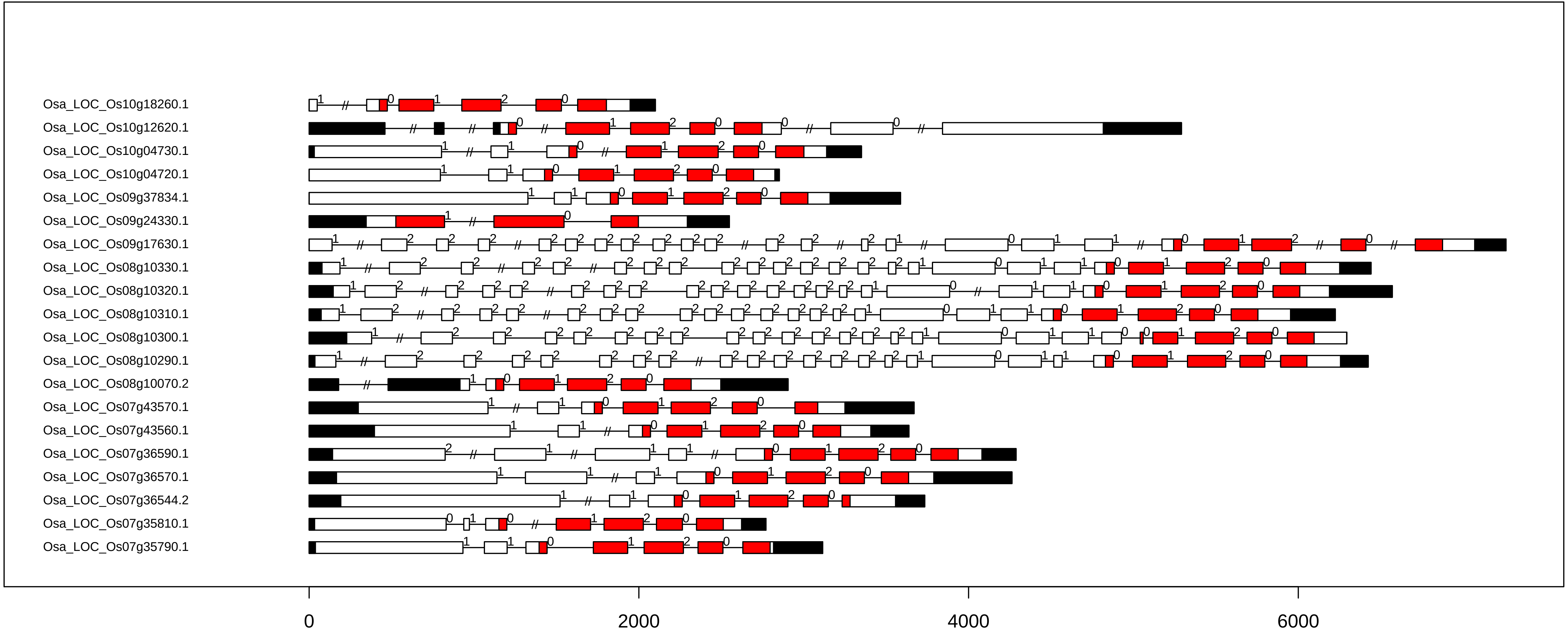

O.sa RLK−Pelle_DLSV exon−intron and kinase domain diagram (part 5)

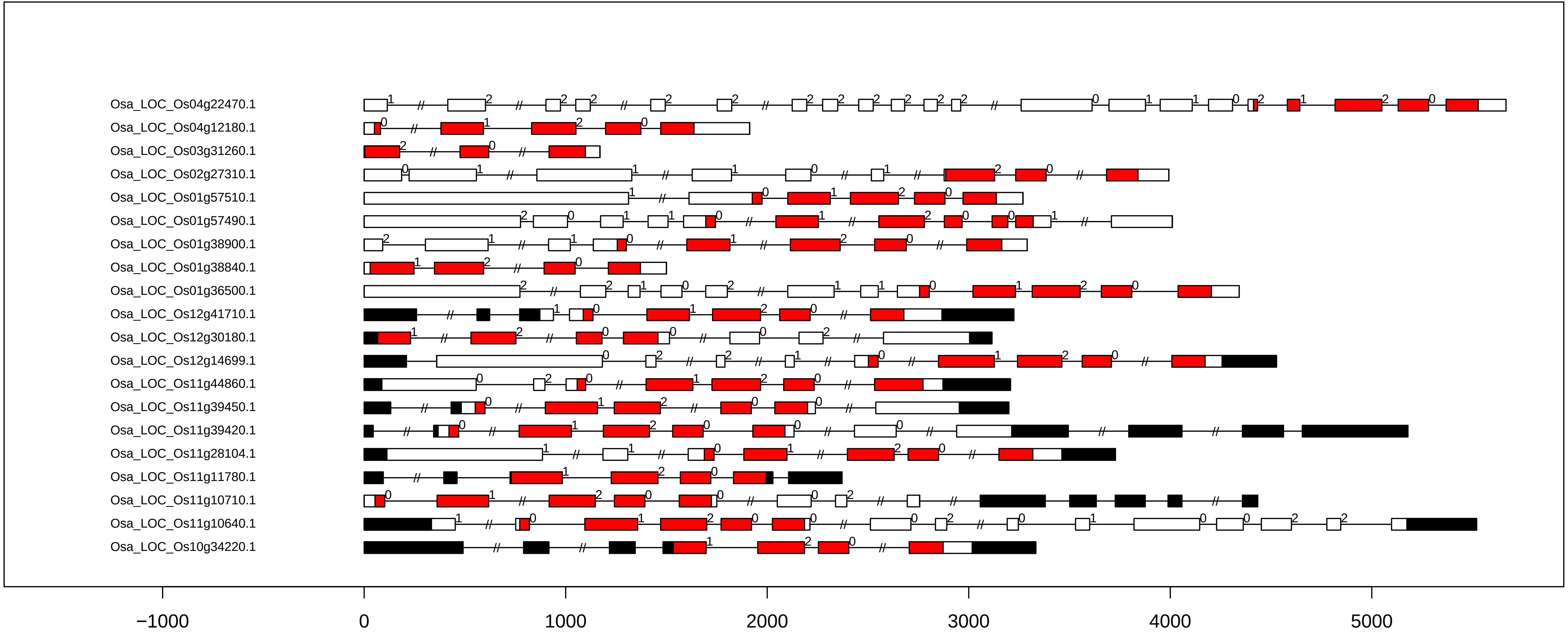

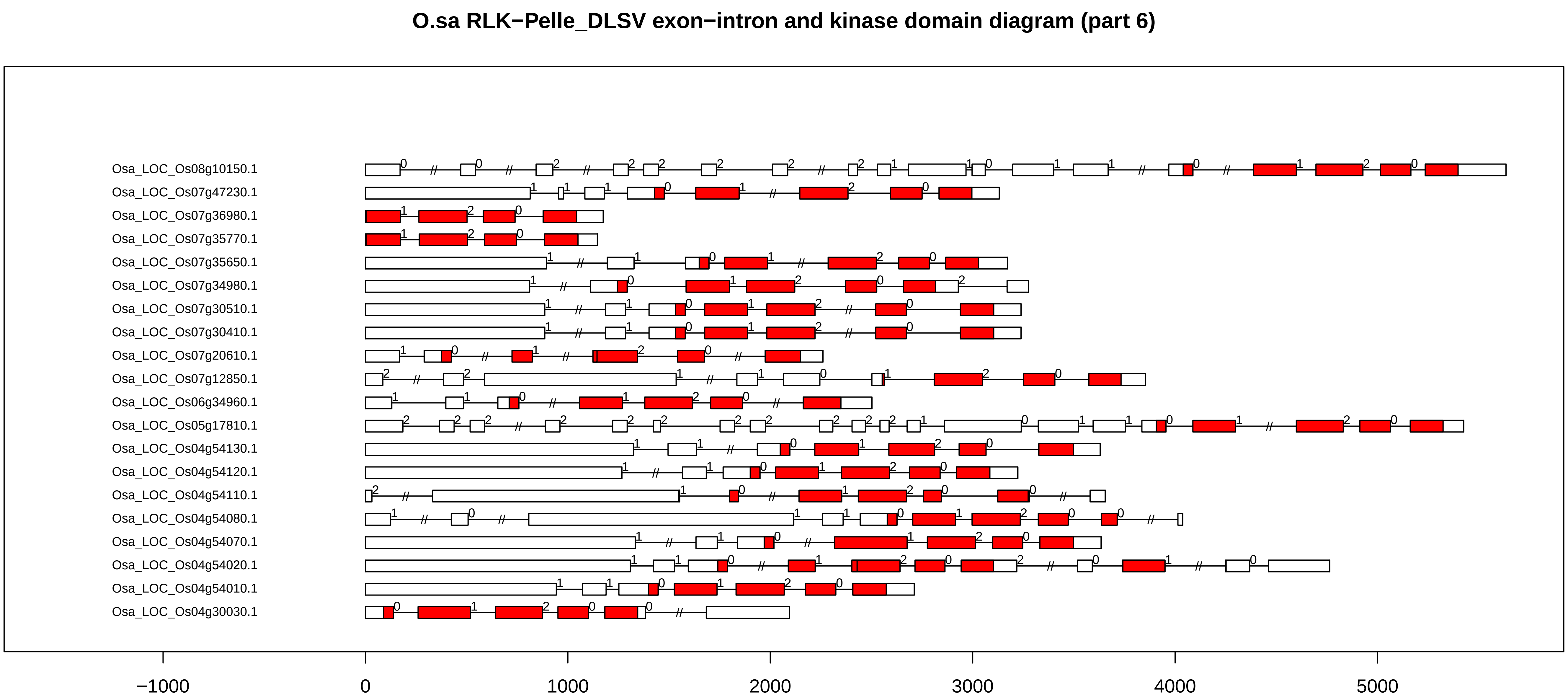
O.sa RLK−Pelle_DLSV exon−intron and kinase domain diagram (part 6)
Osa_LOC_Os08g10150.1
Osa_LOC_Os07g47230.1
Osa_LOC_Os07g36980.1
Osa_LOC_Os07g35770.1
Osa_LOC_Os07g35650.1
Osa_LOC_Os07g34980.1
Osa_LOC_Os07g30510.1
Osa_LOC_Os07g30410.1
Osa_LOC_Os07g20610.1
Osa_LOC_Os07g12850.1
Osa_LOC_Os06g34960.1
Osa_LOC_Os05g17810.1
Osa_LOC_Os04g54130.1
Osa_LOC_Os04g54120.1
Osa_LOC_Os04g54110.1
Osa_LOC_Os04g54080.1
Osa_LOC_Os04g54070.1
Osa_LOC_Os04g54020.1
Osa_LOC_Os04g54010.1
Osa_LOC_Os04g30030.1
−1000
0
1000
2000
3000
4000
5000

O.sa RLK−Pelle_DLSV exon−intron and kinase domain diagram (part 7)

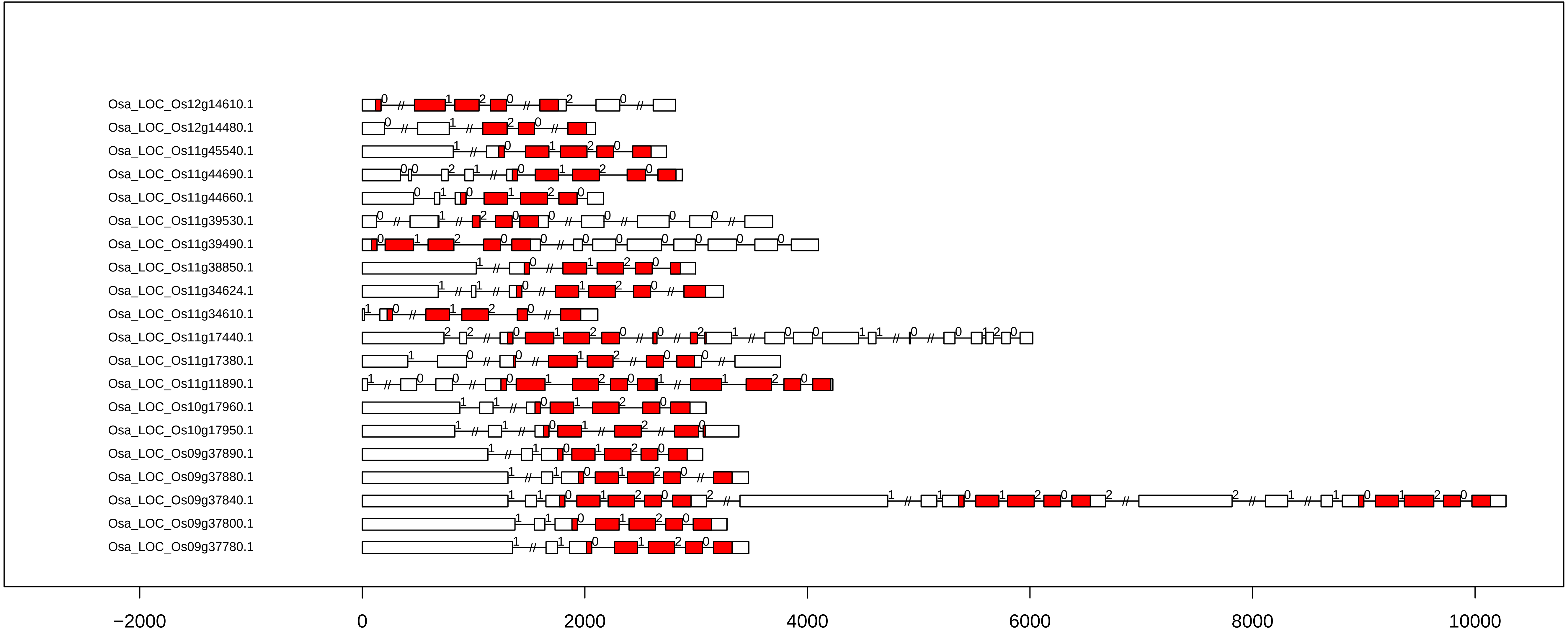

O.sa RLK−Pelle_Extensin exon−intron and kinase domain diagram (all)

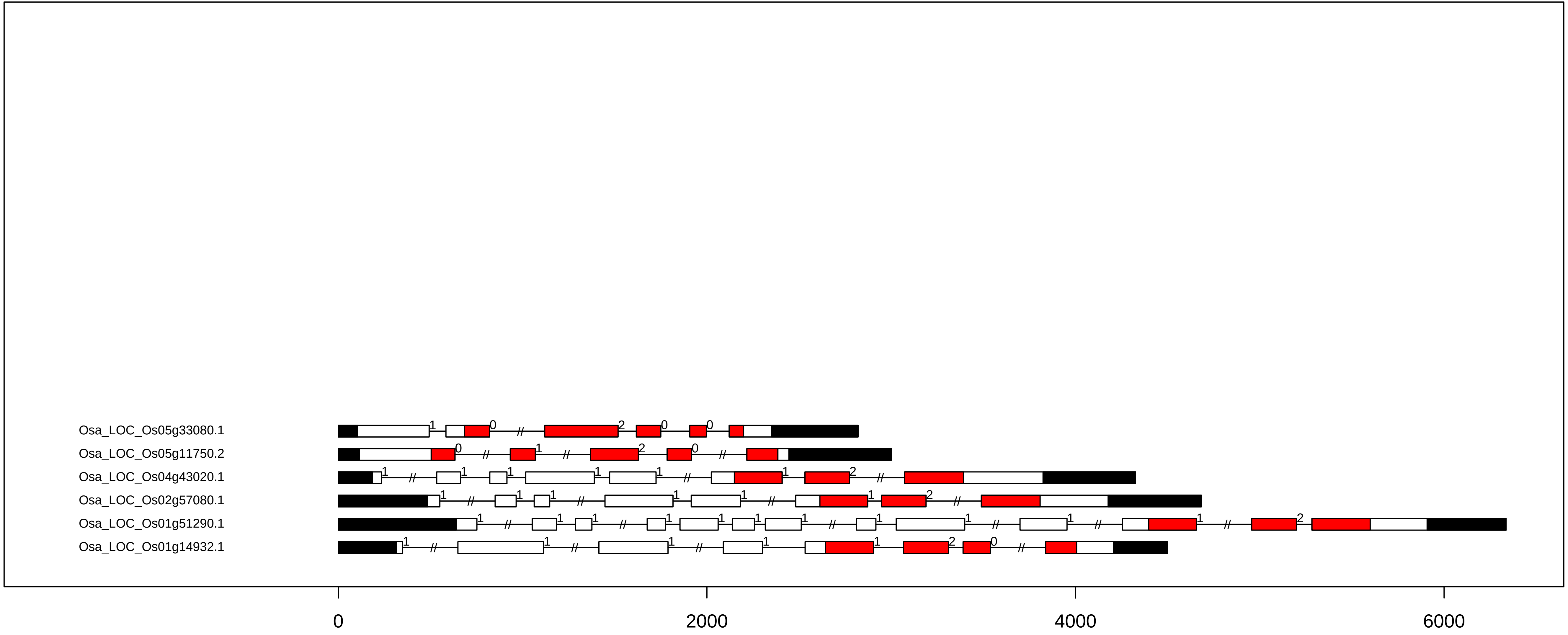

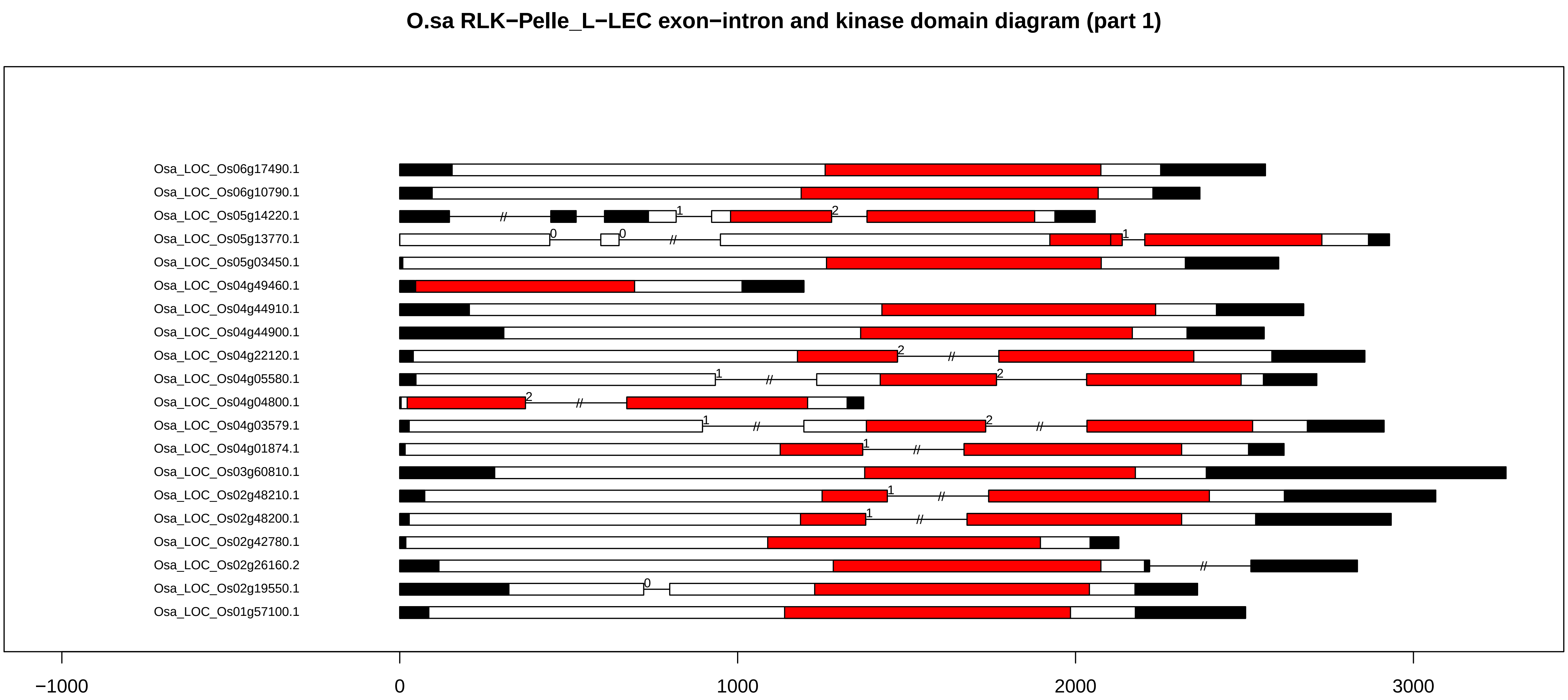

O.sa RLK−Pelle_L−LEC exon−intron and kinase domain diagram (part 1)
Osa_LOC_Os06g17490.1
Osa_LOC_Os06g10790.1
Osa_LOC_Os05g14220.1
Osa_LOC_Os05g13770.1
Osa_LOC_Os05g03450.1
Osa_LOC_Os04g49460.1
Osa_LOC_Os04g44910.1
Osa_LOC_Os04g44900.1
Osa_LOC_Os04g22120.1
Osa_LOC_Os04g05580.1
Osa_LOC_Os04g04800.1
Osa_LOC_Os04g03579.1
Osa_LOC_Os04g01874.1
Osa_LOC_Os03g60810.1
Osa_LOC_Os02g48210.1
Osa_LOC_Os02g48200.1
Osa_LOC_Os02g42780.1
Osa_LOC_Os02g26160.2
Osa_LOC_Os02g19550.1
Osa_LOC_Os01g57100.1
−1000
0
1000
2000
3000

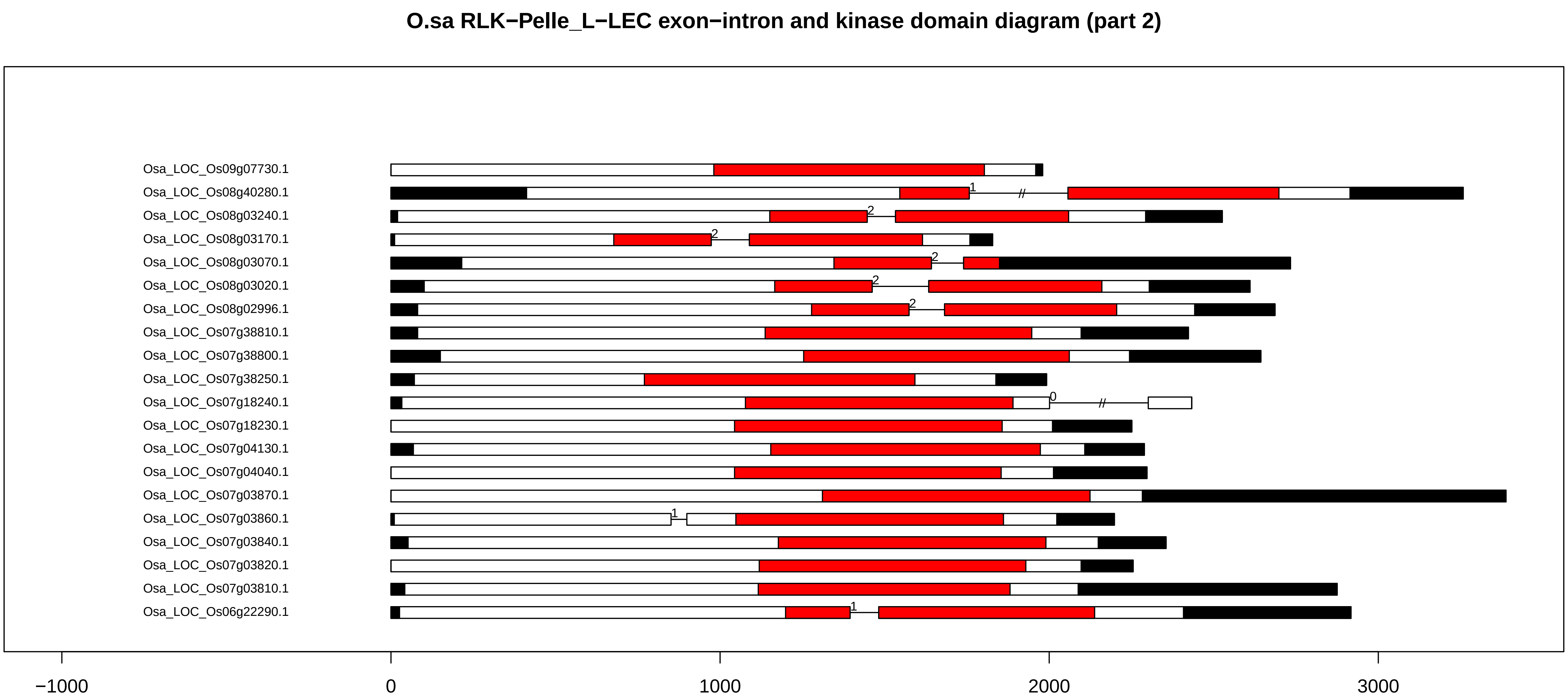
O.sa RLK−Pelle_L−LEC exon−intron and kinase domain diagram (part 2)
Osa_LOC_Os09g07730.1
Osa_LOC_Os08g40280.1
Osa_LOC_Os08g03240.1
Osa_LOC_Os08g03170.1
Osa_LOC_Os08g03070.1
Osa_LOC_Os08g03020.1
Osa_LOC_Os08g02996.1
Osa_LOC_Os07g38810.1
Osa_LOC_Os07g38800.1
Osa_LOC_Os07g38250.1
Osa_LOC_Os07g18240.1
Osa_LOC_Os07g18230.1
Osa_LOC_Os07g04130.1
Osa_LOC_Os07g04040.1
Osa_LOC_Os07g03870.1
Osa_LOC_Os07g03860.1
Osa_LOC_Os07g03840.1
Osa_LOC_Os07g03820.1
Osa_LOC_Os07g03810.1
Osa_LOC_Os06g22290.1
−1000
0
1000
2000
3000

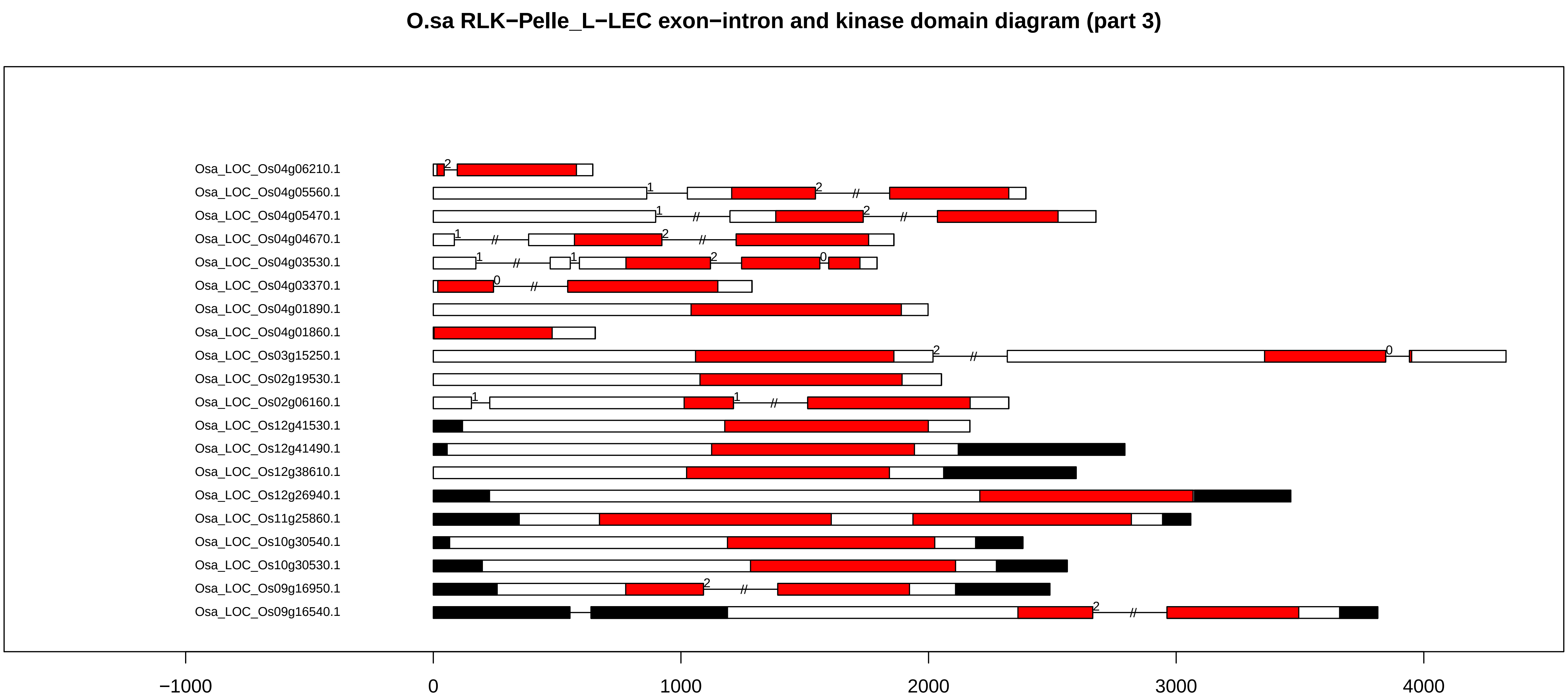
O.sa RLK−Pelle_L−LEC exon−intron and kinase domain diagram (part 3)
Osa_LOC_Os04g06210.1
Osa_LOC_Os04g05560.1
Osa_LOC_Os04g05470.1
Osa_LOC_Os04g04670.1
Osa_LOC_Os04g03530.1
Osa_LOC_Os04g03370.1
Osa_LOC_Os04g01890.1
Osa_LOC_Os04g01860.1
Osa_LOC_Os03g15250.1
Osa_LOC_Os02g19530.1
Osa_LOC_Os02g06160.1
Osa_LOC_Os12g41530.1
Osa_LOC_Os12g41490.1
Osa_LOC_Os12g38610.1
Osa_LOC_Os12g26940.1
Osa_LOC_Os11g25860.1
Osa_LOC_Os10g30540.1
Osa_LOC_Os10g30530.1
Osa_LOC_Os09g16950.1
Osa_LOC_Os09g16540.1
−1000
0
1000
2000
3000
4000

O.sa RLK−Pelle_L−LEC exon−intron and kinase domain diagram (part 4)

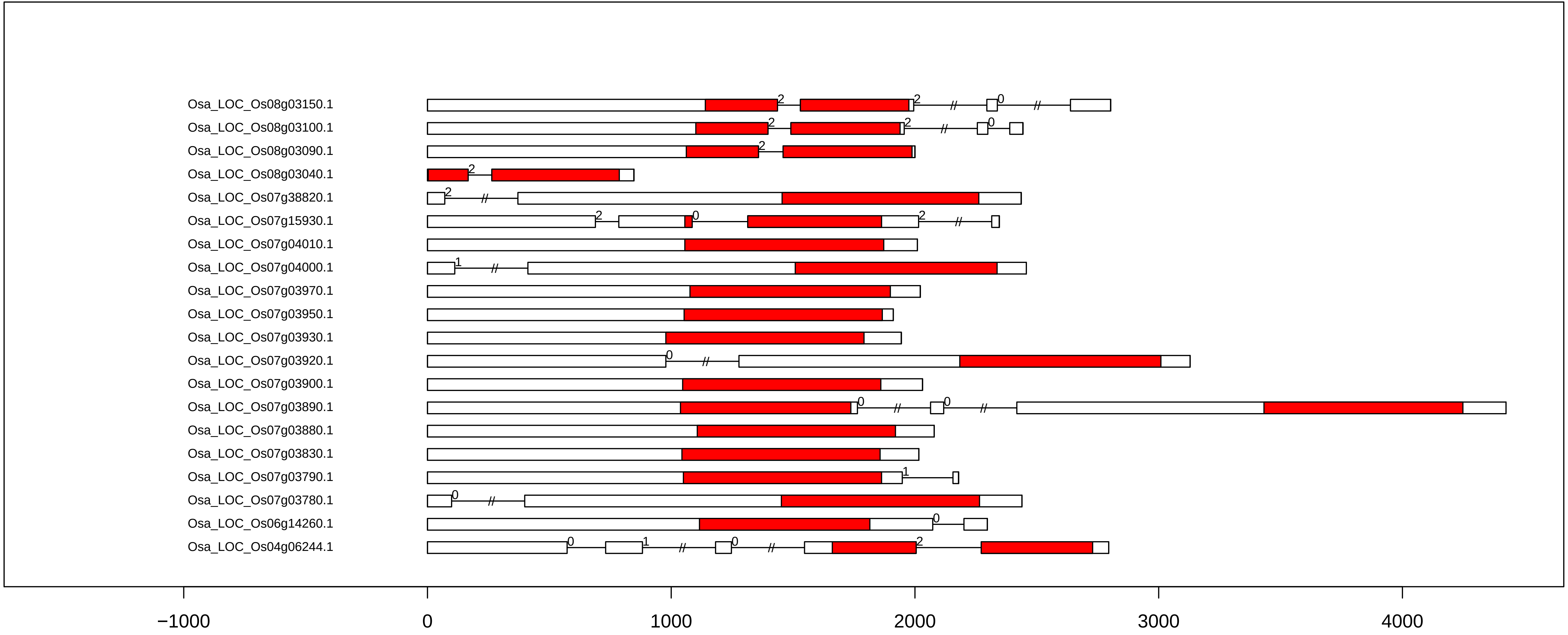

O.sa RLK−Pelle_L−LEC exon−intron and kinase domain diagram (part 5)

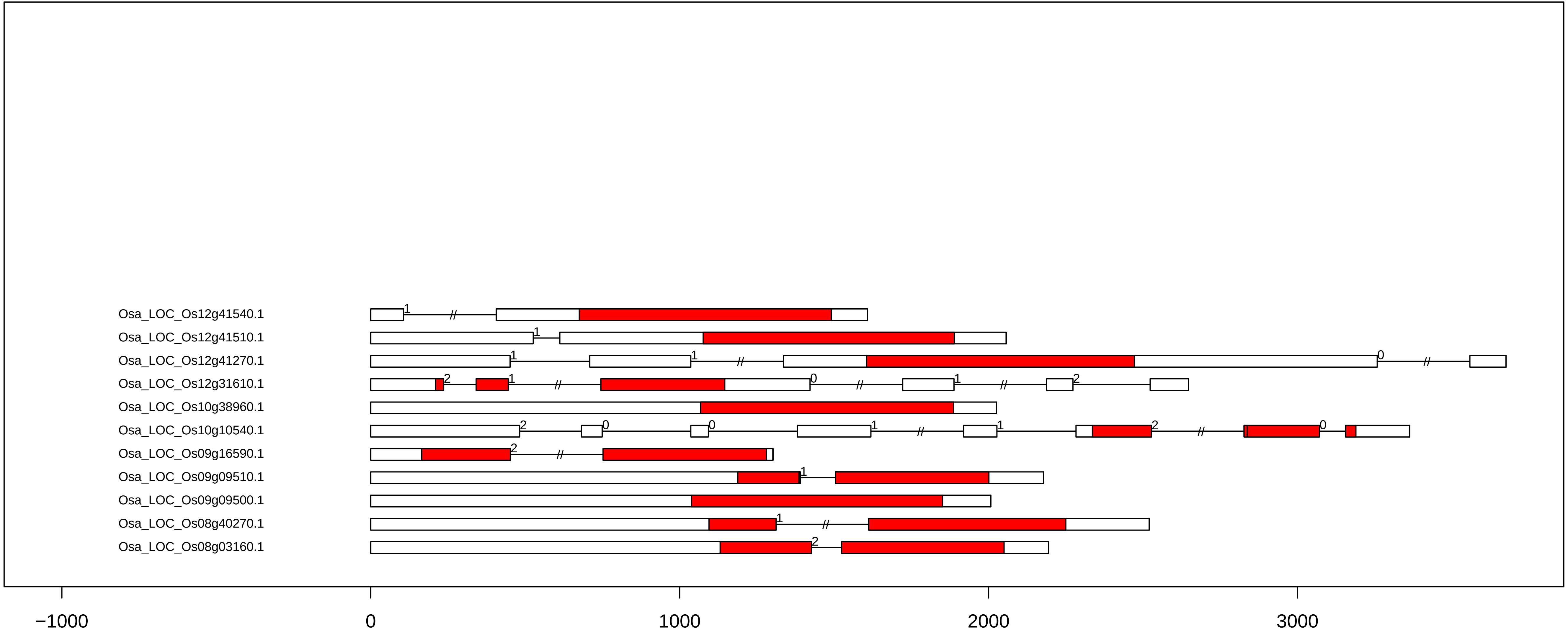

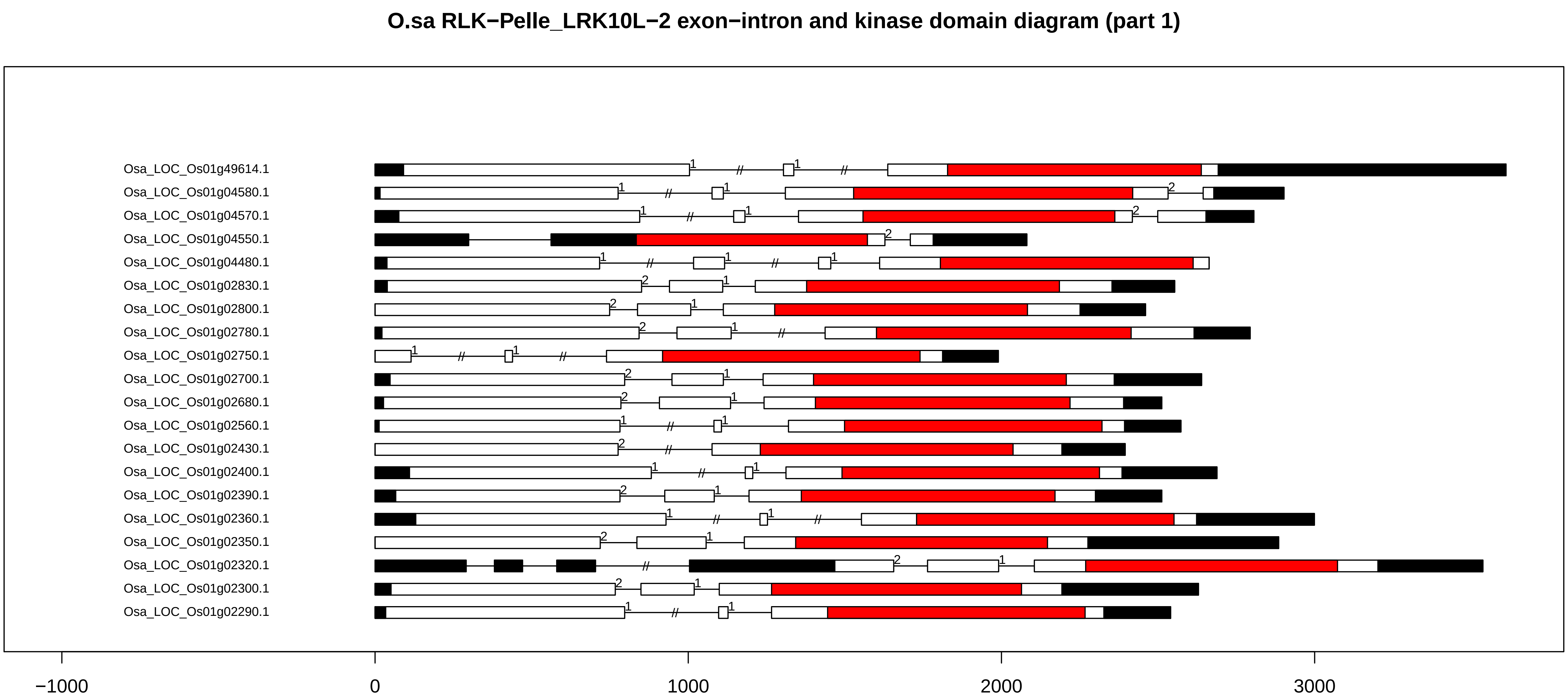
O.sa RLK−Pelle_LRK10L−2 exon−intron and kinase domain diagram (part 1)
Osa_LOC_Os01g49614.1
Osa_LOC_Os01g04580.1
Osa_LOC_Os01g04570.1
Osa_LOC_Os01g04550.1
Osa_LOC_Os01g04480.1
Osa_LOC_Os01g02830.1
Osa_LOC_Os01g02800.1
Osa_LOC_Os01g02780.1
Osa_LOC_Os01g02750.1
Osa_LOC_Os01g02700.1
Osa_LOC_Os01g02680.1
Osa_LOC_Os01g02560.1
Osa_LOC_Os01g02430.1
Osa_LOC_Os01g02400.1
Osa_LOC_Os01g02390.1
Osa_LOC_Os01g02360.1
Osa_LOC_Os01g02350.1
Osa_LOC_Os01g02320.1
Osa_LOC_Os01g02300.1
Osa_LOC_Os01g02290.1
−1000
0
1000
2000
3000

O.sa RLK−Pelle_LRK10L−2 exon−intron and kinase domain diagram (part 2)

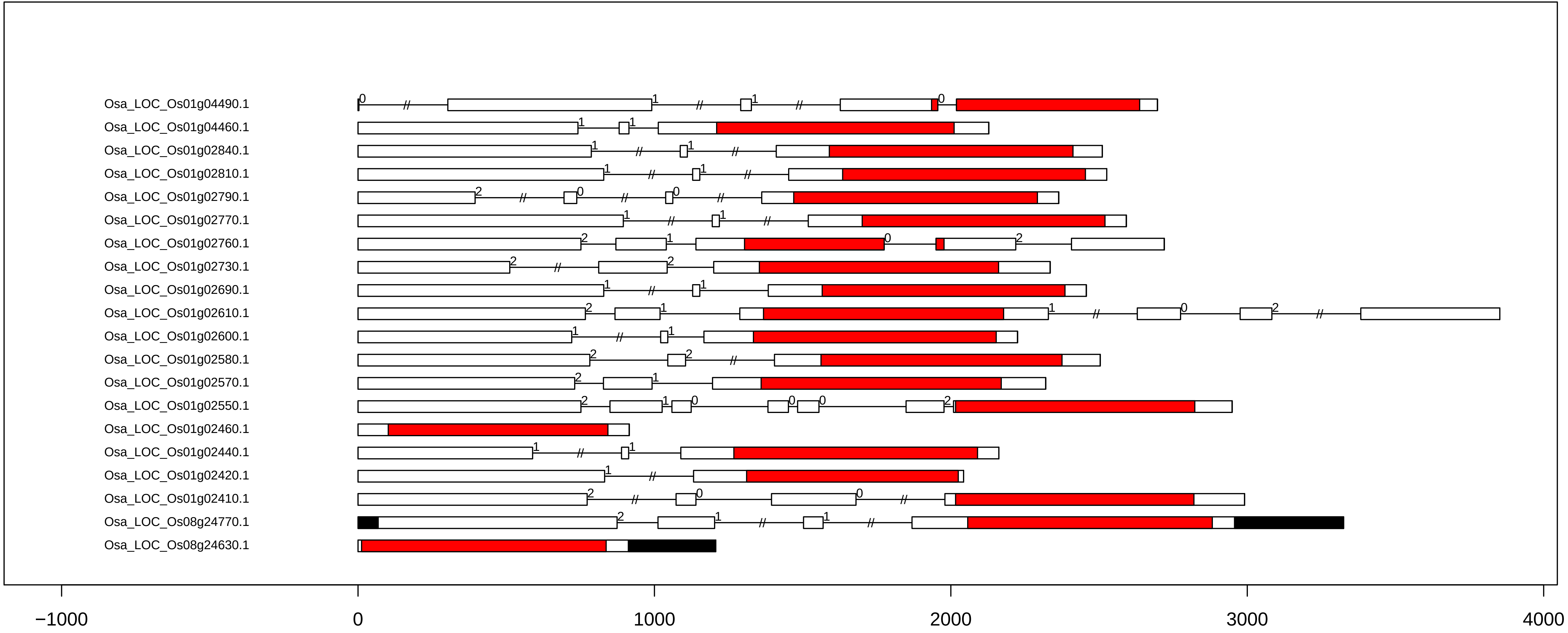

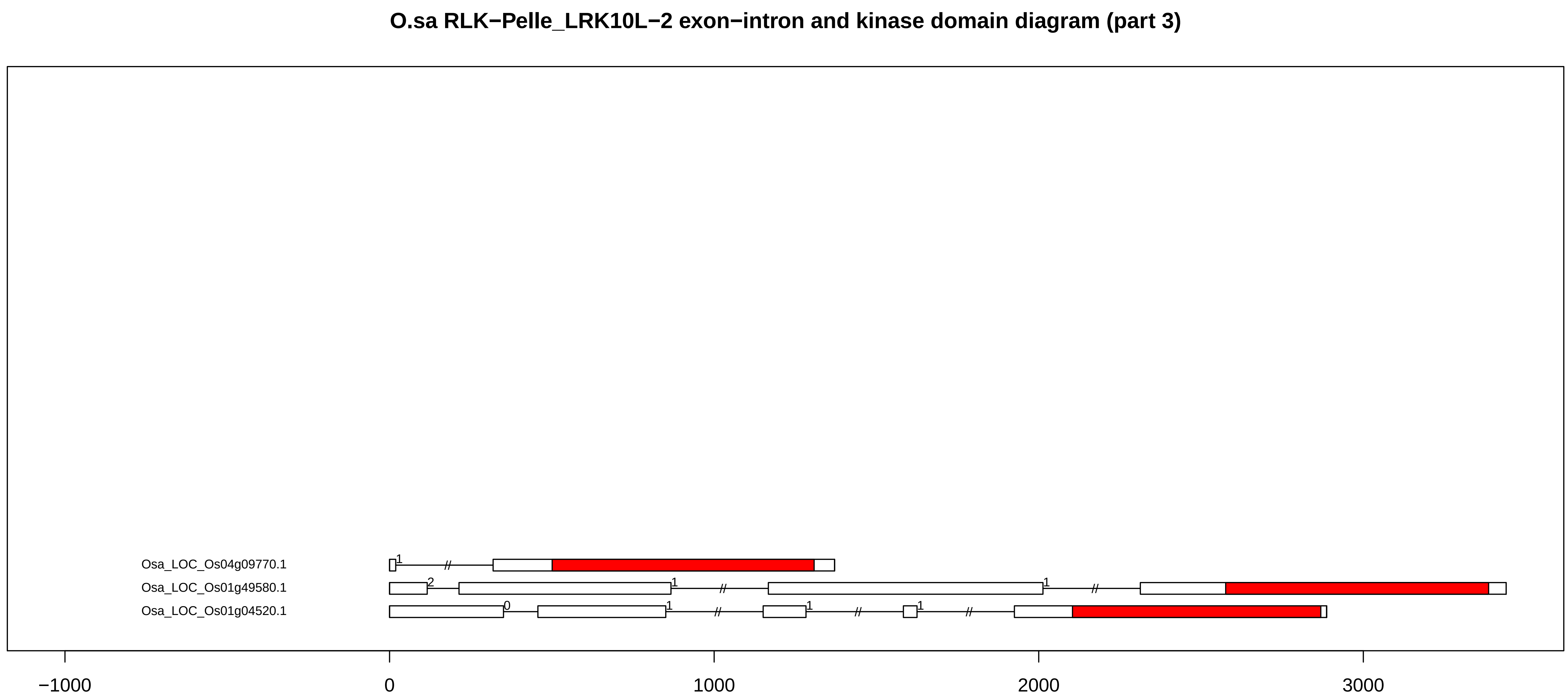

O.sa RLK−Pelle_LRK10L−2 exon−intron and kinase domain diagram (part 3)
Osa_LOC_Os04g09770.1
Osa_LOC_Os01g49580.1
Osa_LOC_Os01g04520.1
1
2
1
1
0
1
1
1
−1000
0
1000
2000
3000

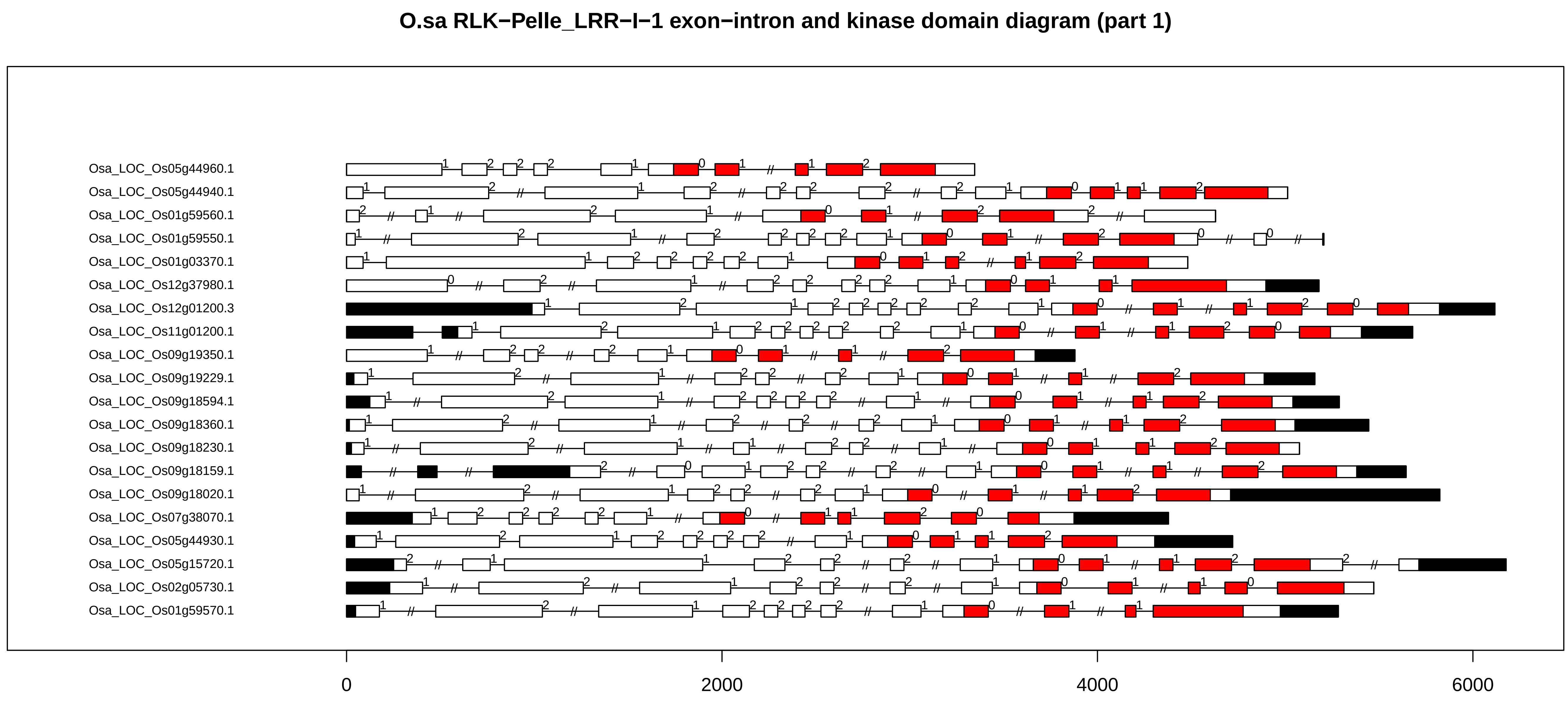

O.sa RLK−Pelle_LRR−I−1 exon−intron and kinase domain diagram (part 1)
Osa_LOC_Os05g44960.1
Osa_LOC_Os05g44940.1
Osa_LOC_Os01g59560.1
Osa_LOC_Os01g59550.1
Osa_LOC_Os01g03370.1
Osa_LOC_Os12g37980.1
Osa_LOC_Os12g01200.3
Osa_LOC_Os11g01200.1
Osa_LOC_Os09g19350.1
Osa_LOC_Os09g19229.1
Osa_LOC_Os09g18594.1
Osa_LOC_Os09g18360.1
Osa_LOC_Os09g18230.1
Osa_LOC_Os09g18159.1
Osa_LOC_Os09g18020.1
Osa_LOC_Os07g38070.1
Osa_LOC_Os05g44930.1
Osa_LOC_Os05g15720.1
Osa_LOC_Os02g05730.1
Osa_LOC_Os01g59570.1
0
2000
4000
6000

O.sa RLK−Pelle_LRR−I−1 exon−intron and kinase domain diagram (part 2)

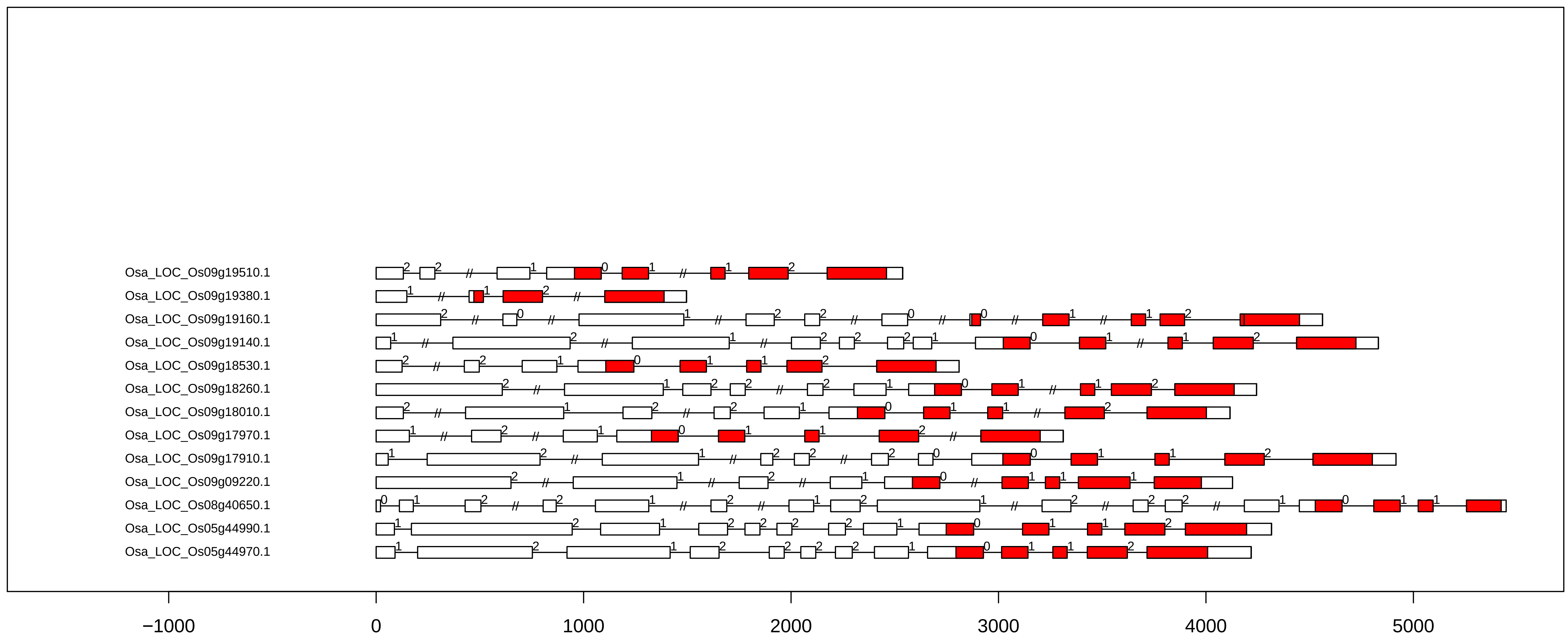

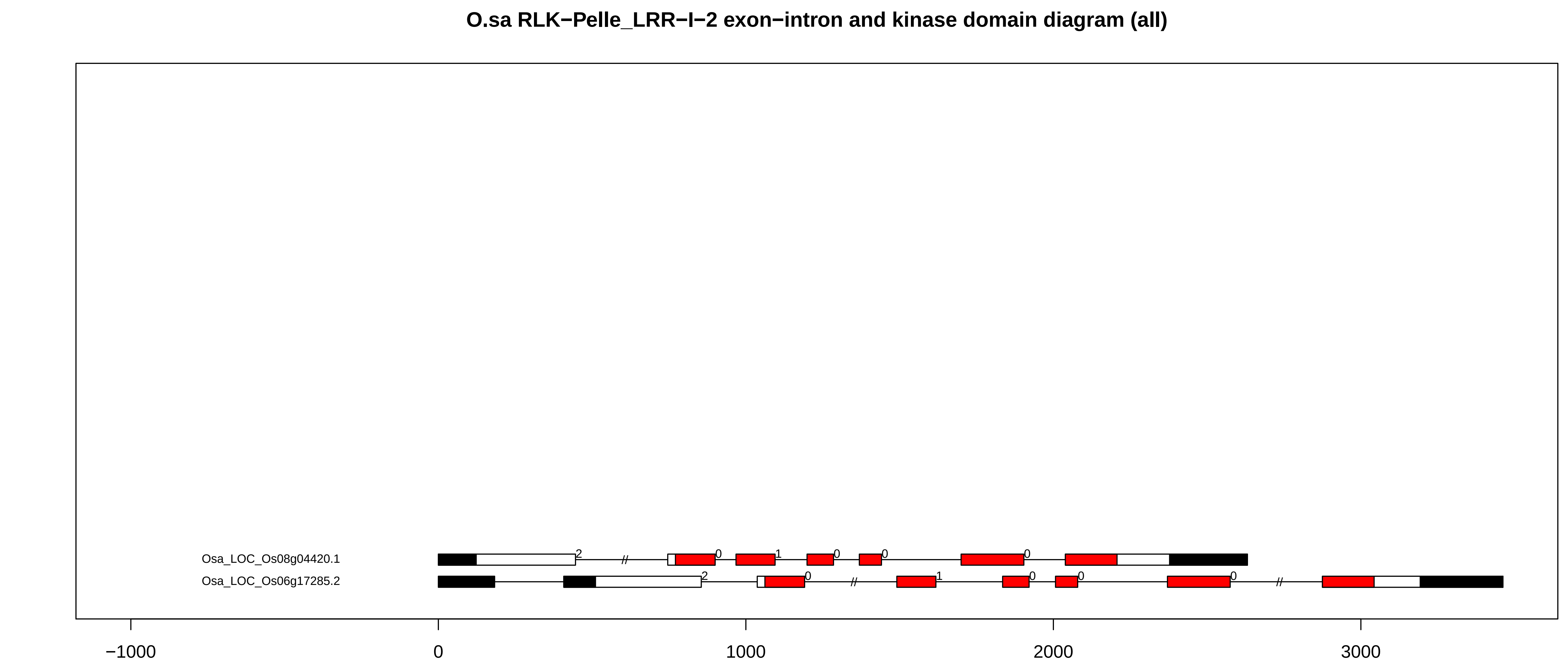

O.sa RLK−Pelle_LRR−I−2 exon−intron and kinase domain diagram (all)
Osa_LOC_Os08g04420.1
Osa_LOC_Os06g17285.2
2
0
1
0
0
0
2
0
1
0
0
0
−1000
0
1000
2000
3000

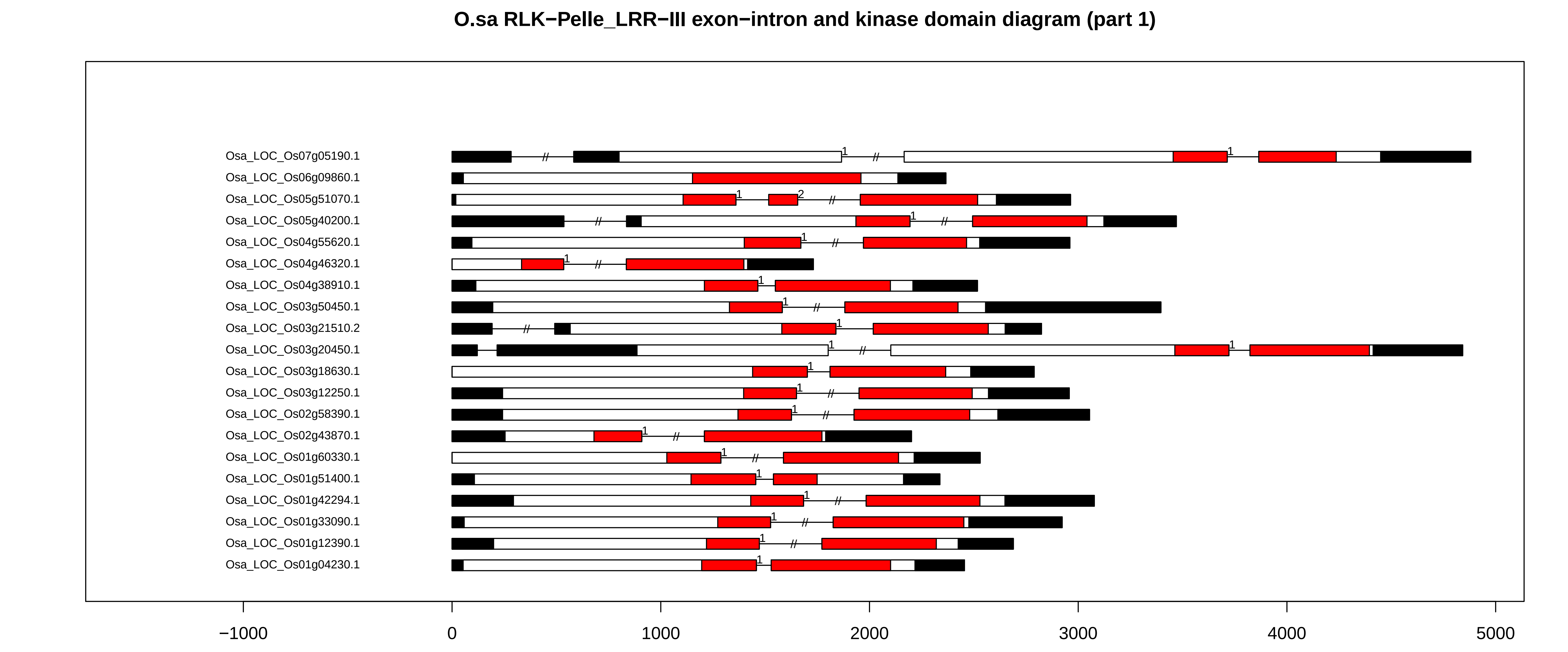
O.sa RLK−Pelle_LRR−III exon−intron and kinase domain diagram (part 1)
Osa_LOC_Os07g05190.1
Osa_LOC_Os06g09860.1
Osa_LOC_Os05g51070.1
Osa_LOC_Os05g40200.1
Osa_LOC_Os04g55620.1
Osa_LOC_Os04g46320.1
Osa_LOC_Os04g38910.1
Osa_LOC_Os03g50450.1
Osa_LOC_Os03g21510.2
Osa_LOC_Os03g20450.1
Osa_LOC_Os03g18630.1
Osa_LOC_Os03g12250.1
Osa_LOC_Os02g58390.1
Osa_LOC_Os02g43870.1
Osa_LOC_Os01g60330.1
Osa_LOC_Os01g51400.1
Osa_LOC_Os01g42294.1
Osa_LOC_Os01g33090.1
Osa_LOC_Os01g12390.1
Osa_LOC_Os01g04230.1
−1000
0
1000
2000
3000
4000
5000

O.sa RLK−Pelle_LRR−III exon−intron and kinase domain diagram (part 2)

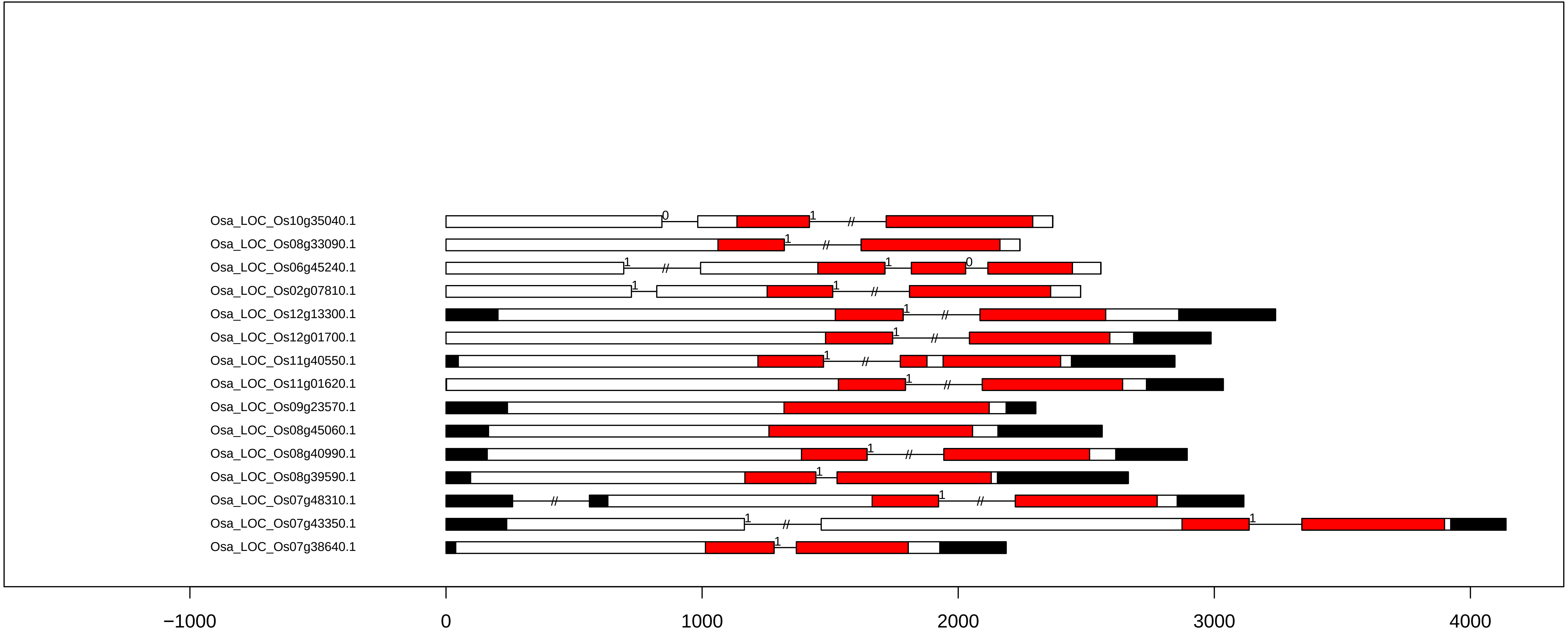

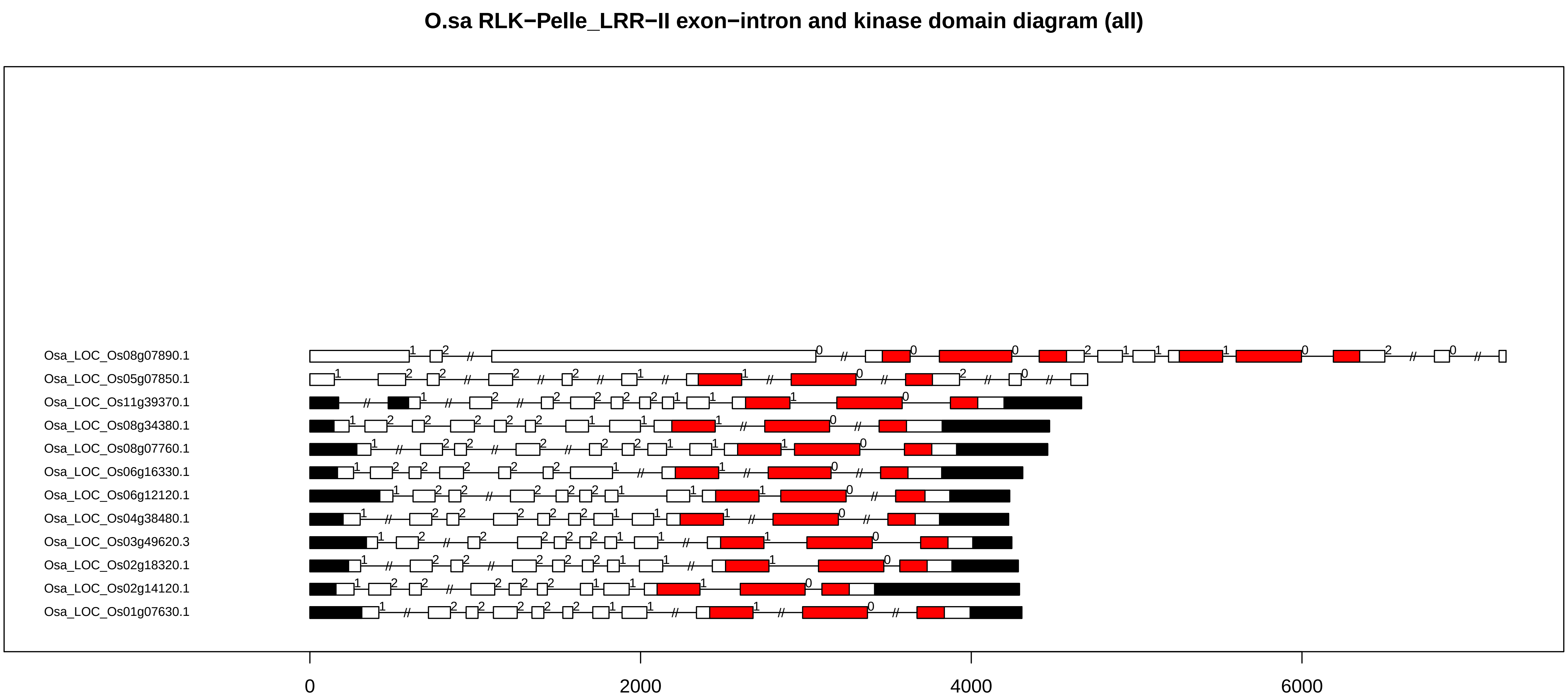

O.sa RLK−Pelle_LRR−II exon−intron and kinase domain diagram (all)
Osa_LOC_Os08g07890.1
Osa_LOC_Os05g07850.1
Osa_LOC_Os11g39370.1
Osa_LOC_Os08g34380.1
Osa_LOC_Os08g07760.1
Osa_LOC_Os06g16330.1
Osa_LOC_Os06g12120.1
Osa_LOC_Os04g38480.1
Osa_LOC_Os03g49620.3
Osa_LOC_Os02g18320.1
Osa_LOC_Os02g14120.1
Osa_LOC_Os01g07630.1
0
2000
4000
6000

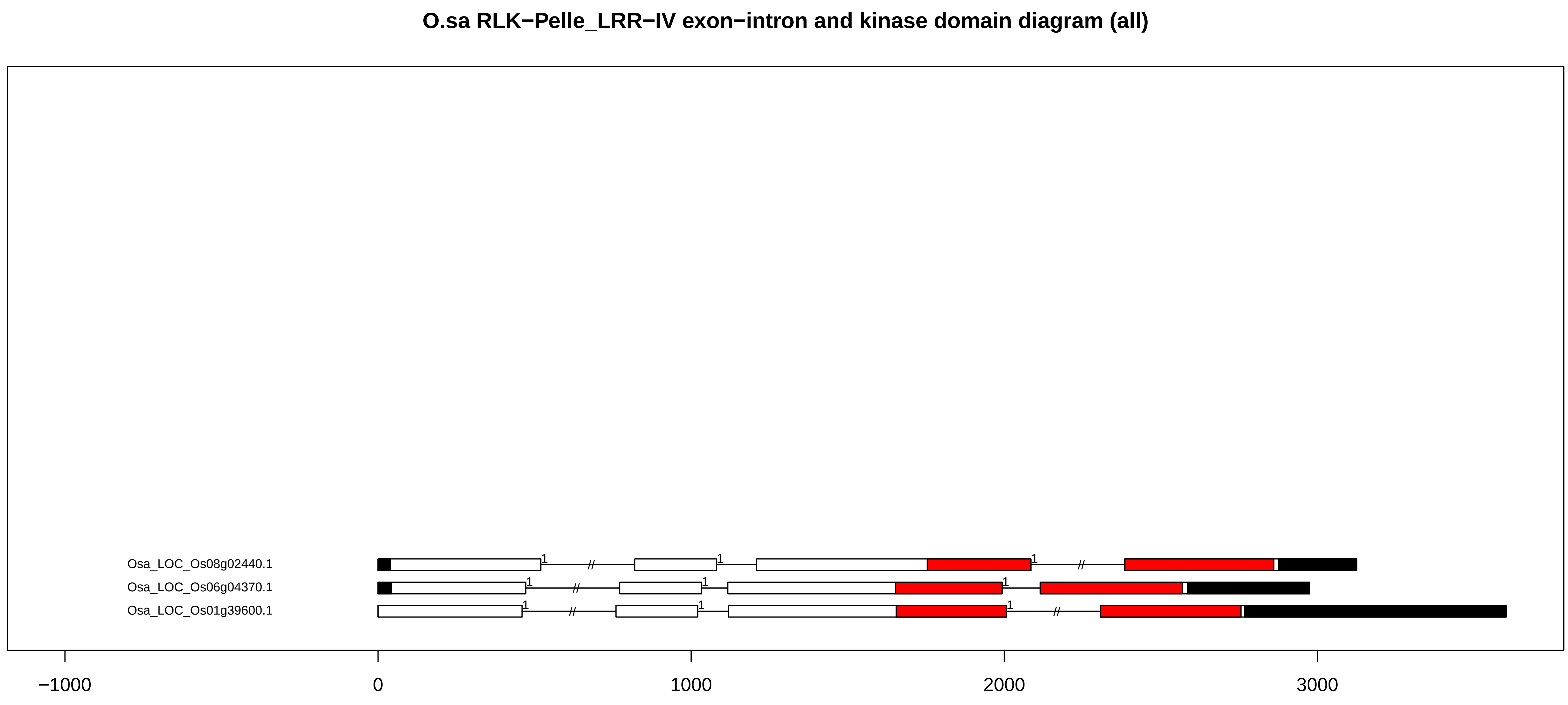
O.sa RLK−Pelle_LRR−IV exon−intron and kinase domain diagram (all)
Osa_LOC_Os08g02440.1
Osa_LOC_Os06g04370.1
Osa_LOC_Os01g39600.1
−1000
0
1000
2000
3000

O.sa RLK−Pelle_LRR−IX exon−intron and kinase domain diagram (all)

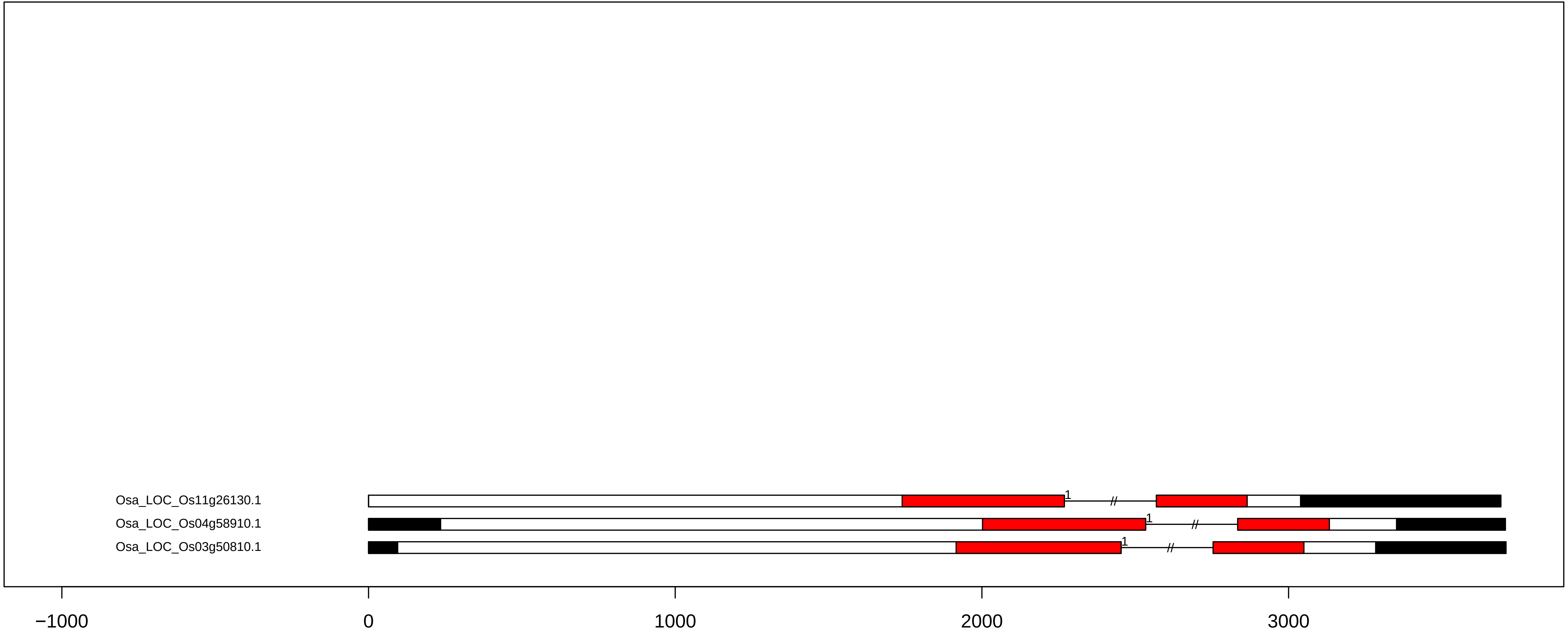

O.sa RLK−Pelle_LRR−VI−1 exon−intron and kinase domain diagram (all)

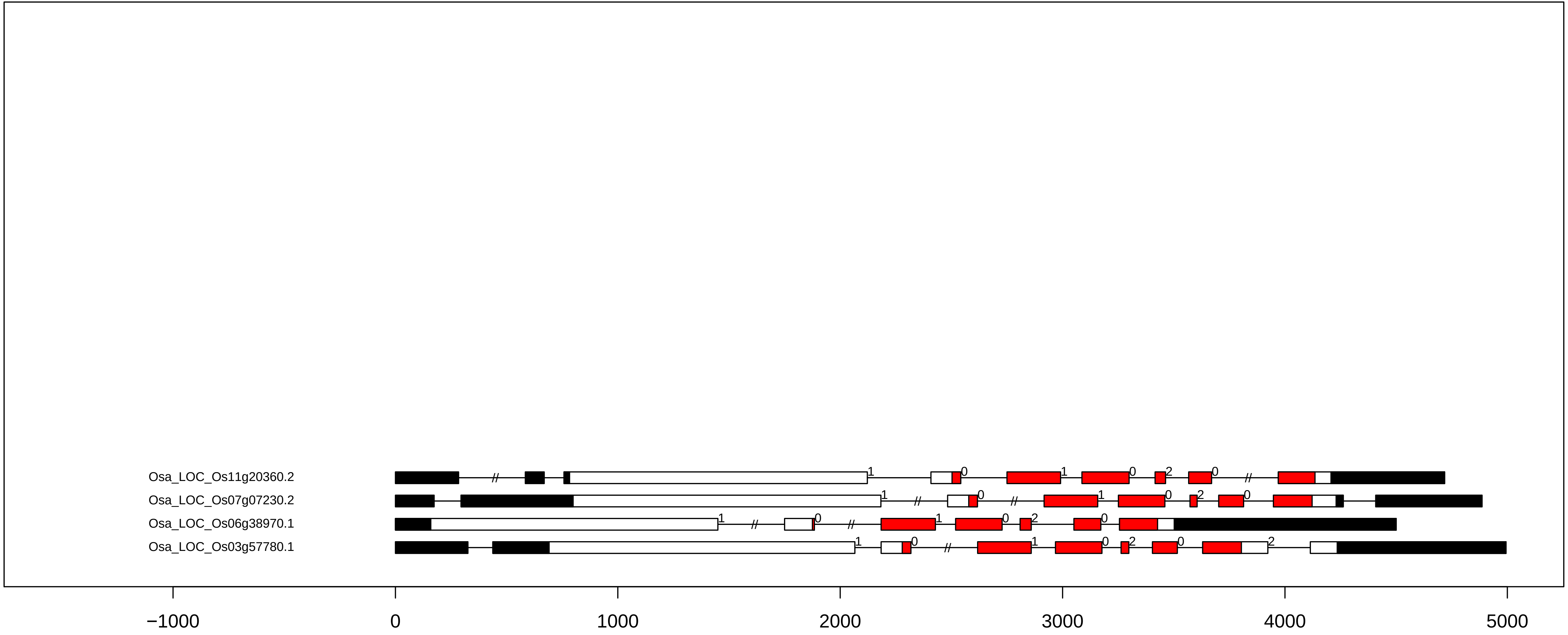

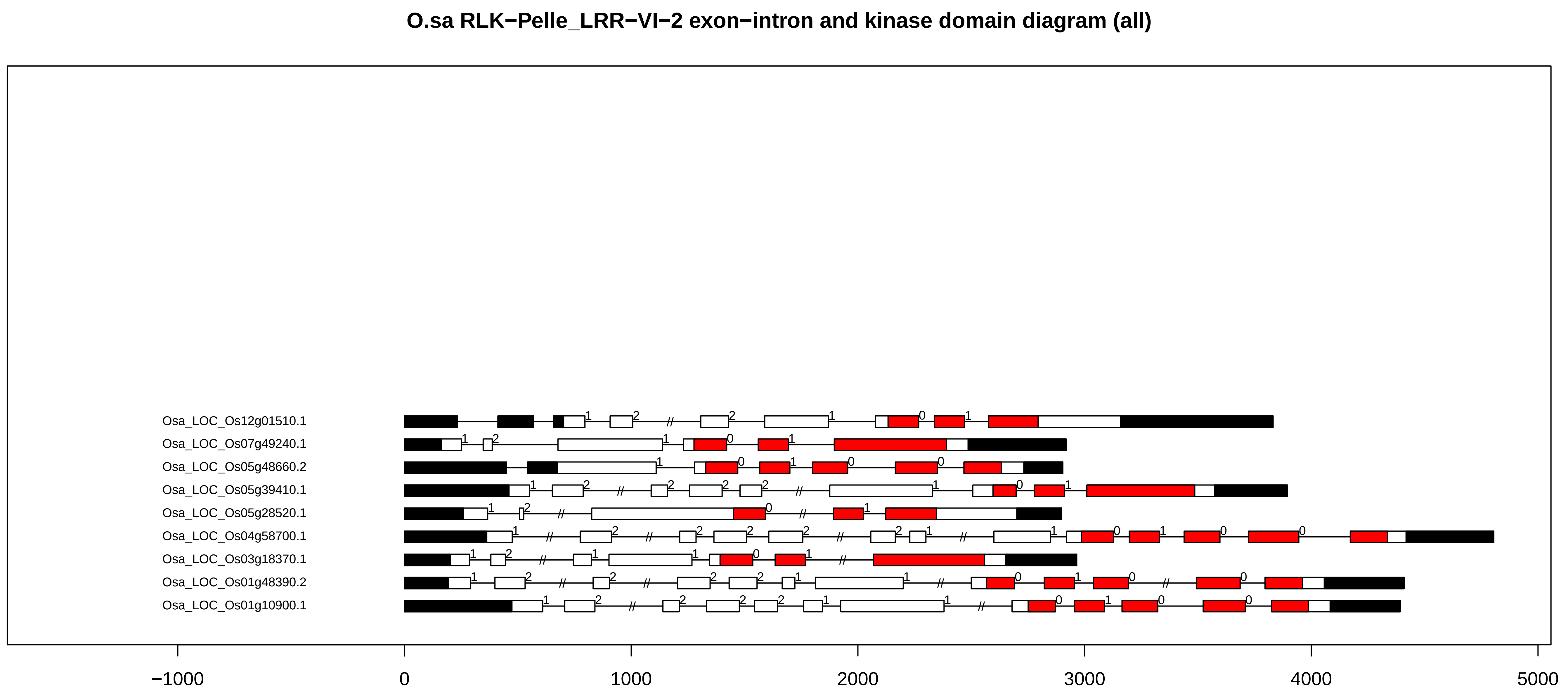
O.sa RLK−Pelle_LRR−VI−2 exon−intron and kinase domain diagram (all)
Osa_LOC_Os12g01510.1
Osa_LOC_Os07g49240.1
Osa_LOC_Os05g48660.2
Osa_LOC_Os05g39410.1
Osa_LOC_Os05g28520.1
Osa_LOC_Os04g58700.1
Osa_LOC_Os03g18370.1
Osa_LOC_Os01g48390.2
Osa_LOC_Os01g10900.1
−1000
0
1000
2000
3000
4000
5000

O.sa RLK−Pelle_LRR−VII−1 exon−intron and kinase domain diagram (all)

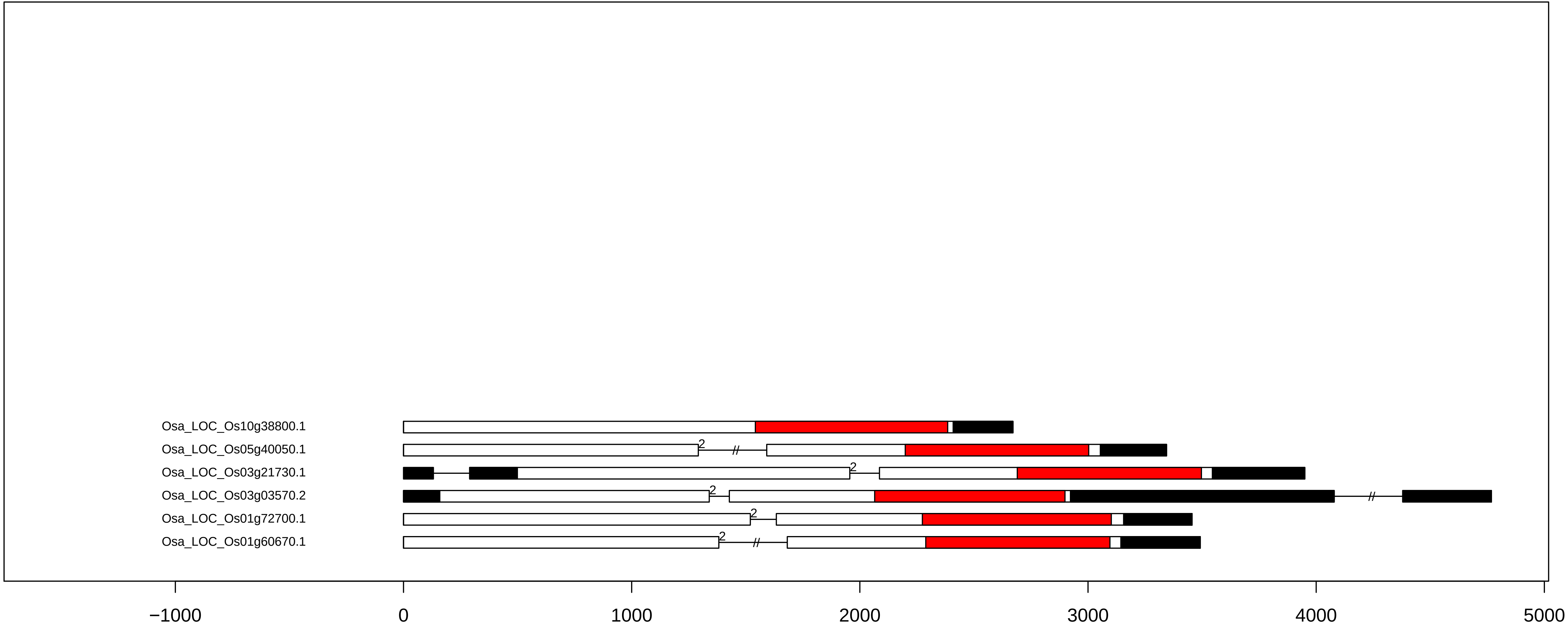

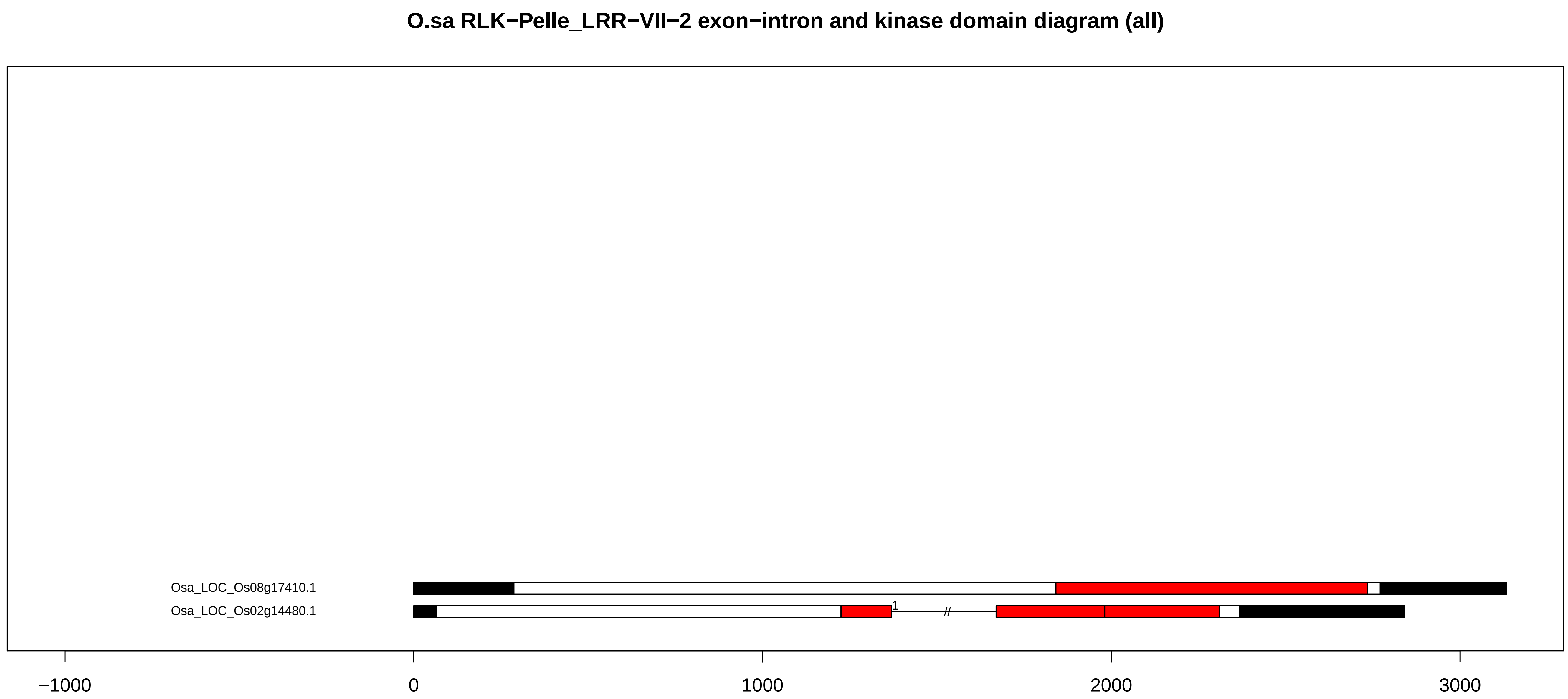
O.sa RLK−Pelle_LRR−VII−2 exon−intron and kinase domain diagram (all)
Osa_LOC_Os08g17410.1
Osa_LOC_Os02g14480.1
1
−1000
0
1000
2000
3000

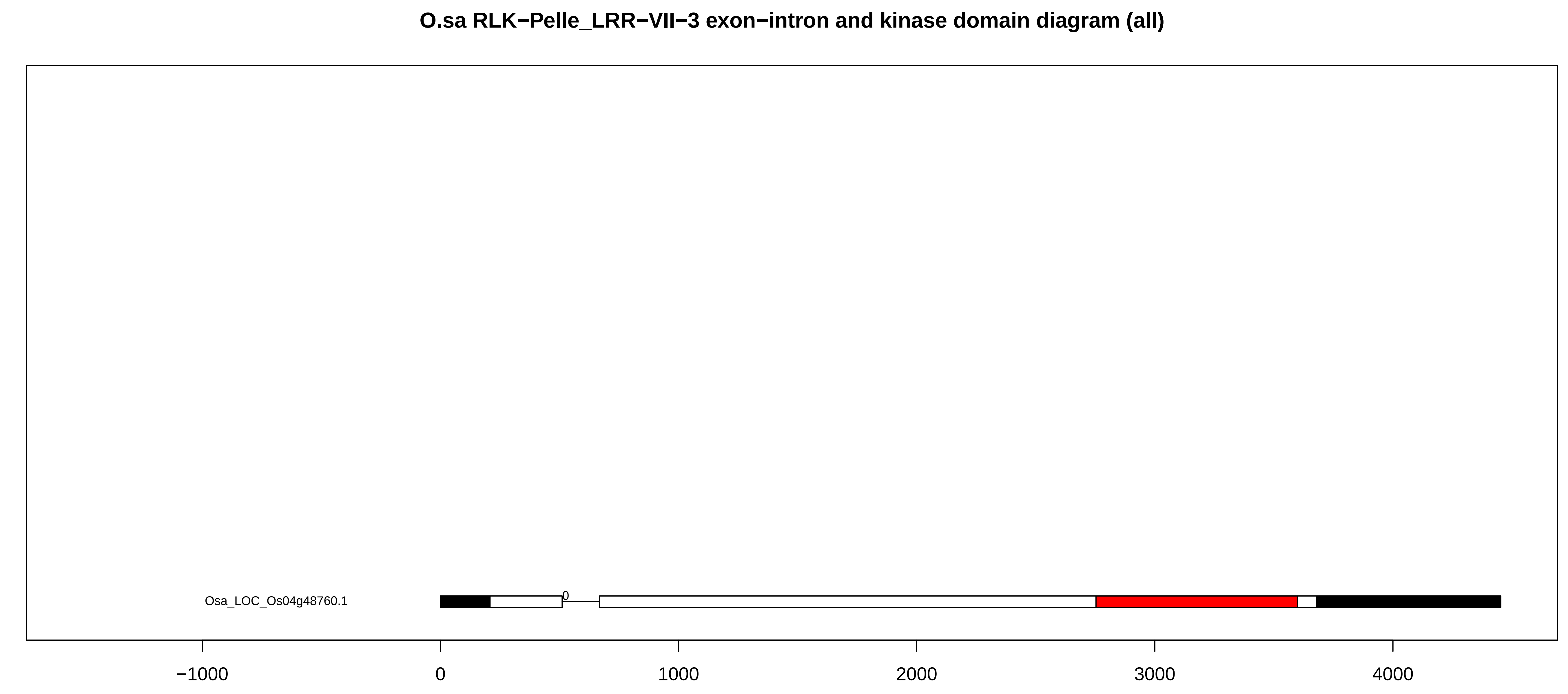

O.sa RLK−Pelle_LRR−VII−3 exon−intron and kinase domain diagram (all)
Osa_LOC_Os04g48760.1
0
−1000
0
1000
2000
3000
4000

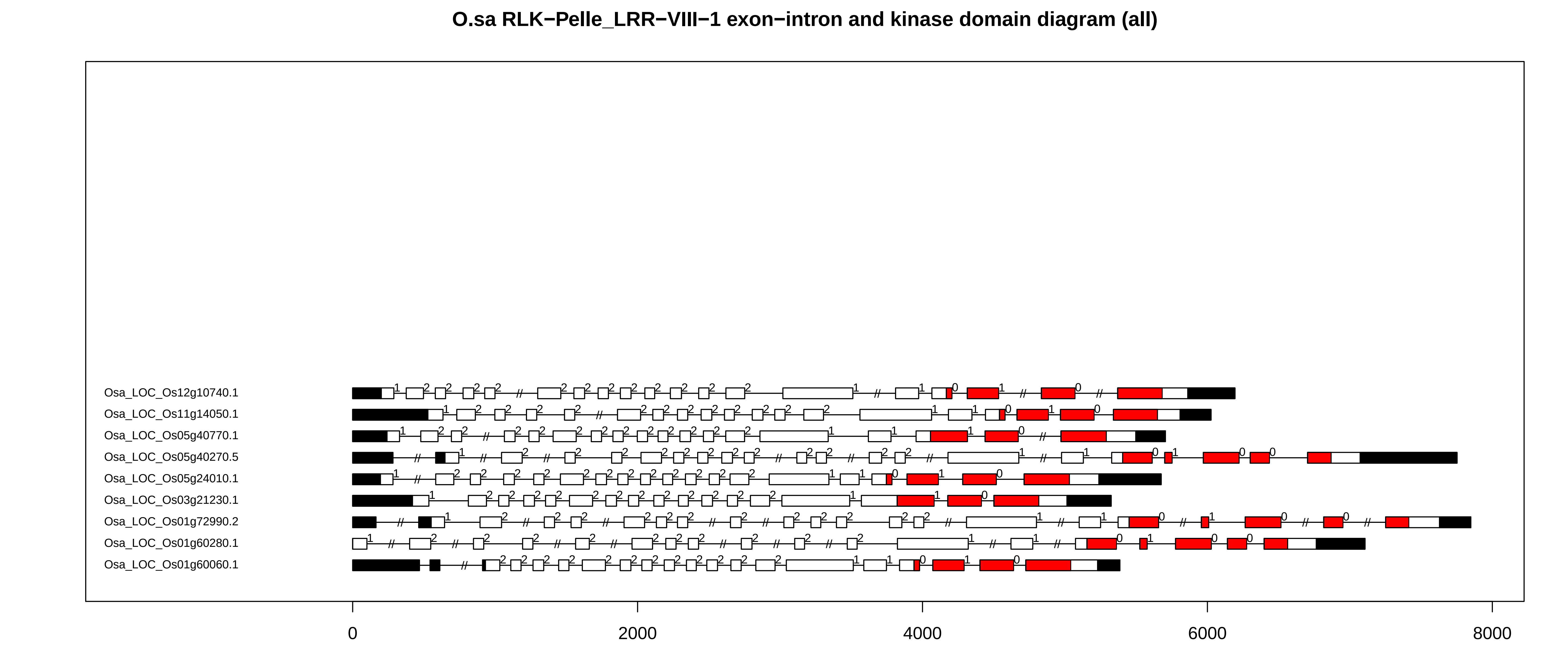

O.sa RLK−Pelle_LRR−VIII−1 exon−intron and kinase domain diagram (all)
Osa_LOC_Os12g10740.1
Osa_LOC_Os11g14050.1
Osa_LOC_Os05g40770.1
Osa_LOC_Os05g40270.5
Osa_LOC_Os05g24010.1
Osa_LOC_Os03g21230.1
Osa_LOC_Os01g72990.2
Osa_LOC_Os01g60280.1
Osa_LOC_Os01g60060.1
0
2000
4000
6000
8000

O.sa RLK−Pelle_LRR−V exon−intron and kinase domain diagram (all)

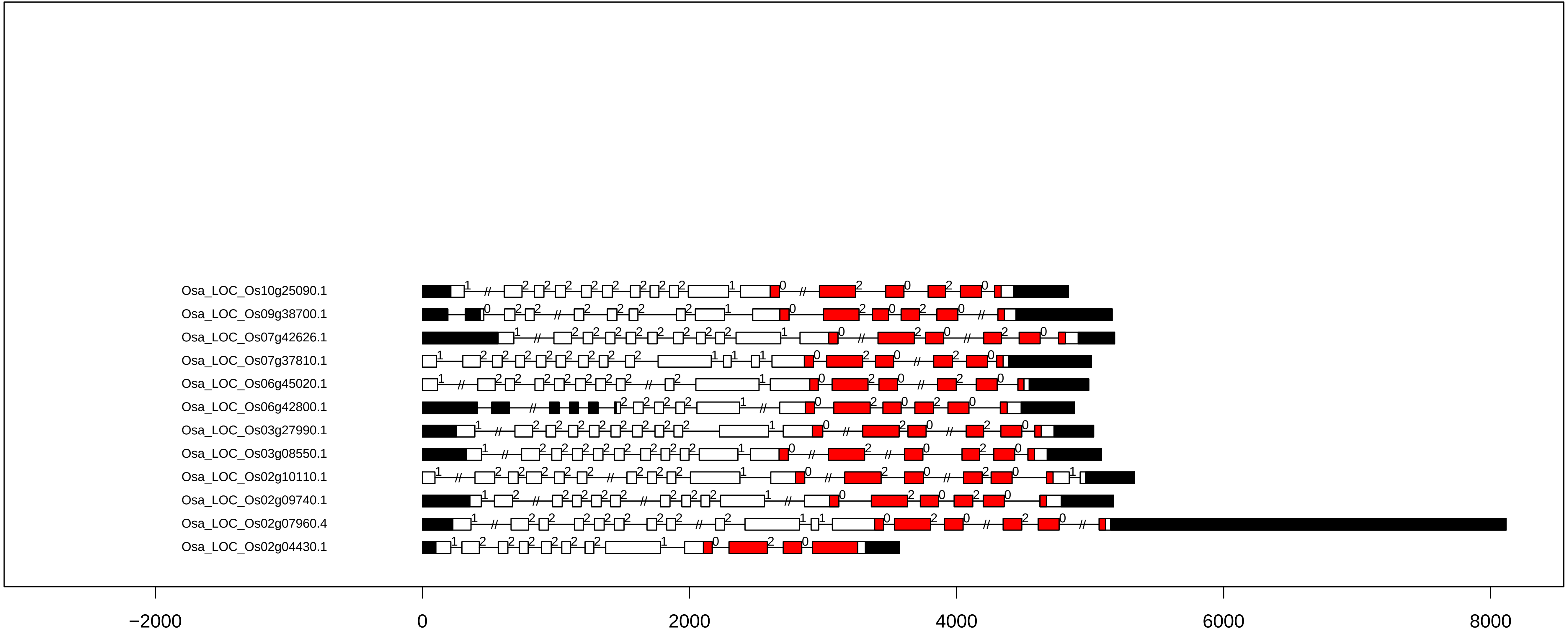

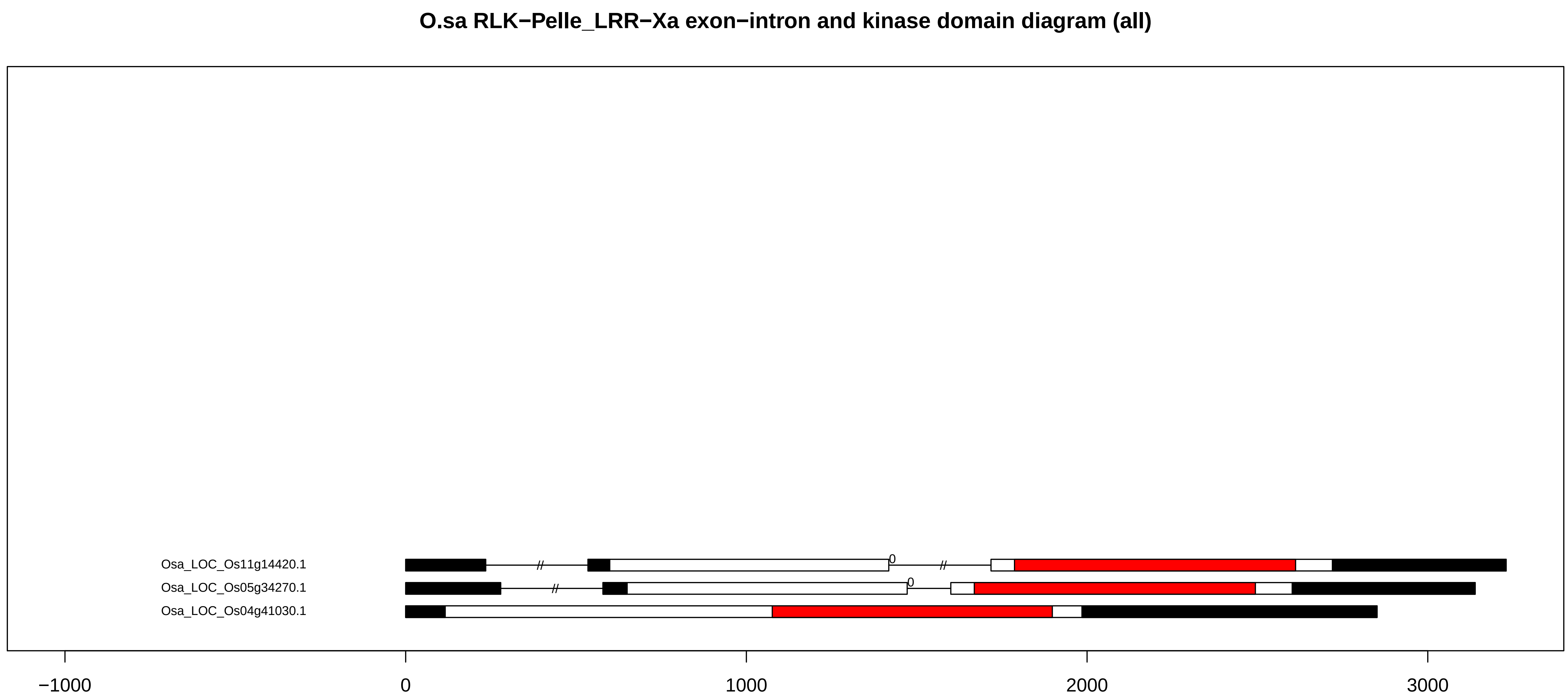
O.sa RLK−Pelle_LRR−Xa exon−intron and kinase domain diagram (all)
Osa_LOC_Os11g14420.1
Osa_LOC_Os05g34270.1
Osa_LOC_Os04g41030.1
0
0
−1000
0
1000
2000
3000

O.sa RLK−Pelle_LRR−Xb−1 exon−intron and kinase domain diagram (part 1)

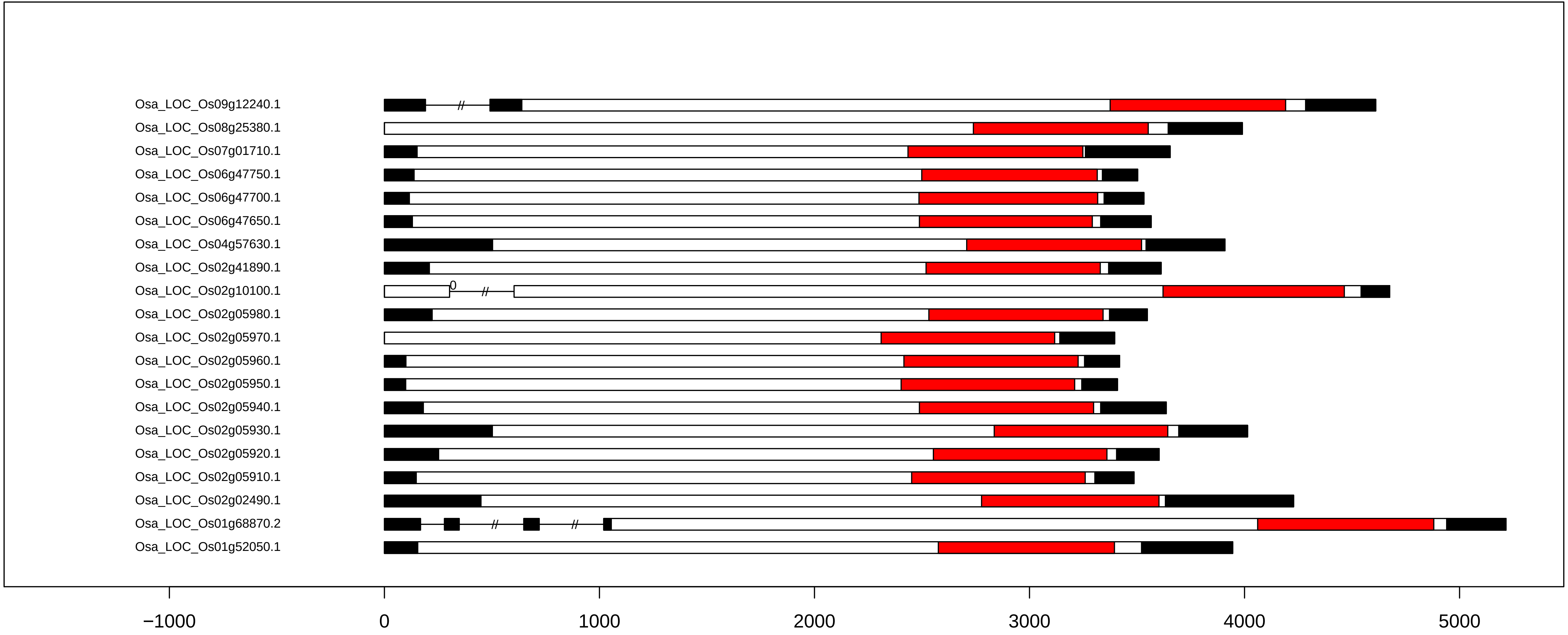

O.sa RLK−Pelle_LRR−Xb−1 exon−intron and kinase domain diagram (part 2)

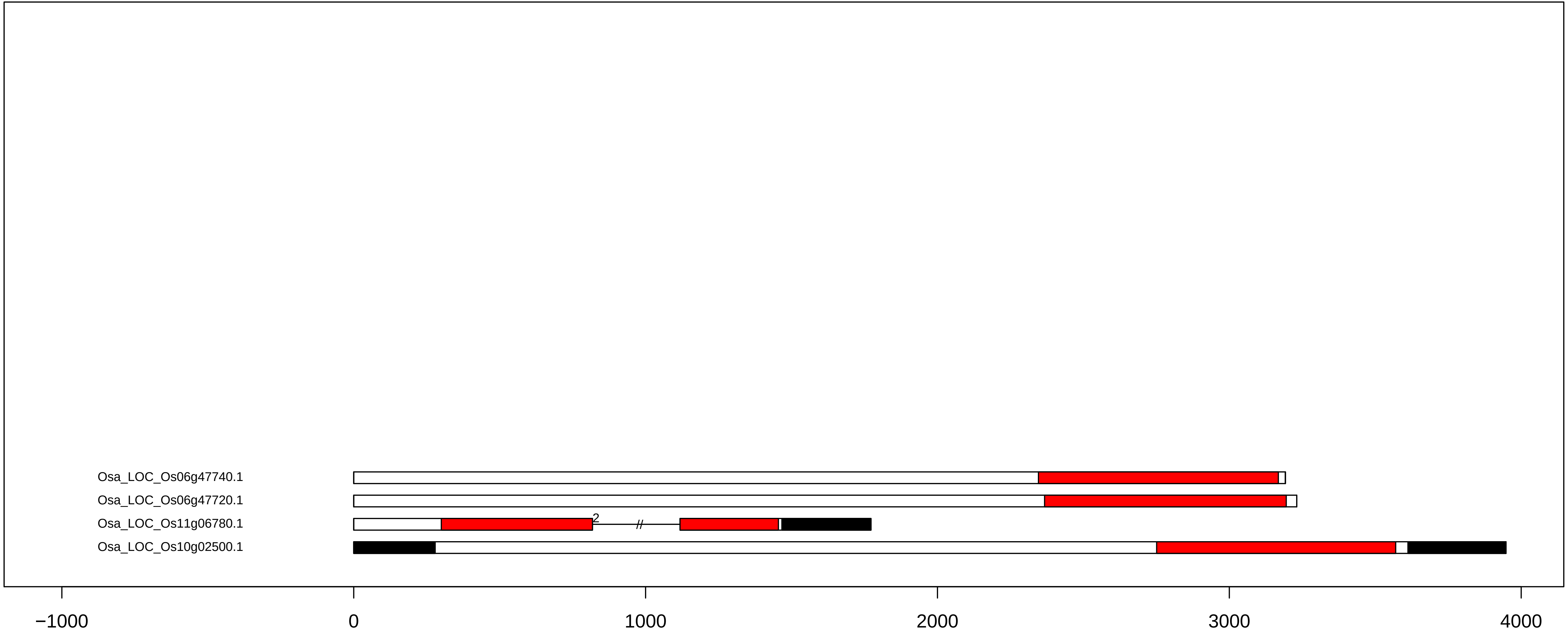

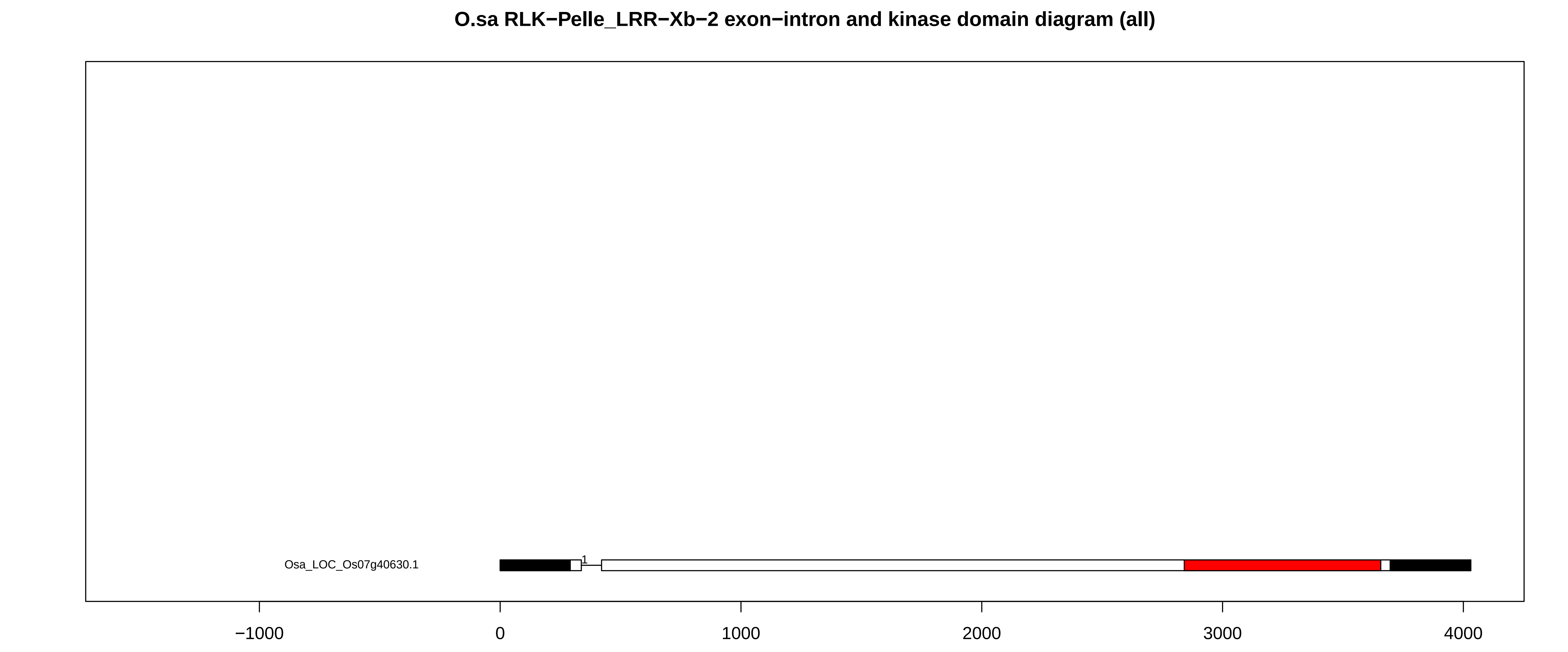
O.sa RLK−Pelle_LRR−Xb−2 exon−intron and kinase domain diagram (all)
Osa_LOC_Os07g40630.1
1
−1000
0
1000
2000
3000
4000

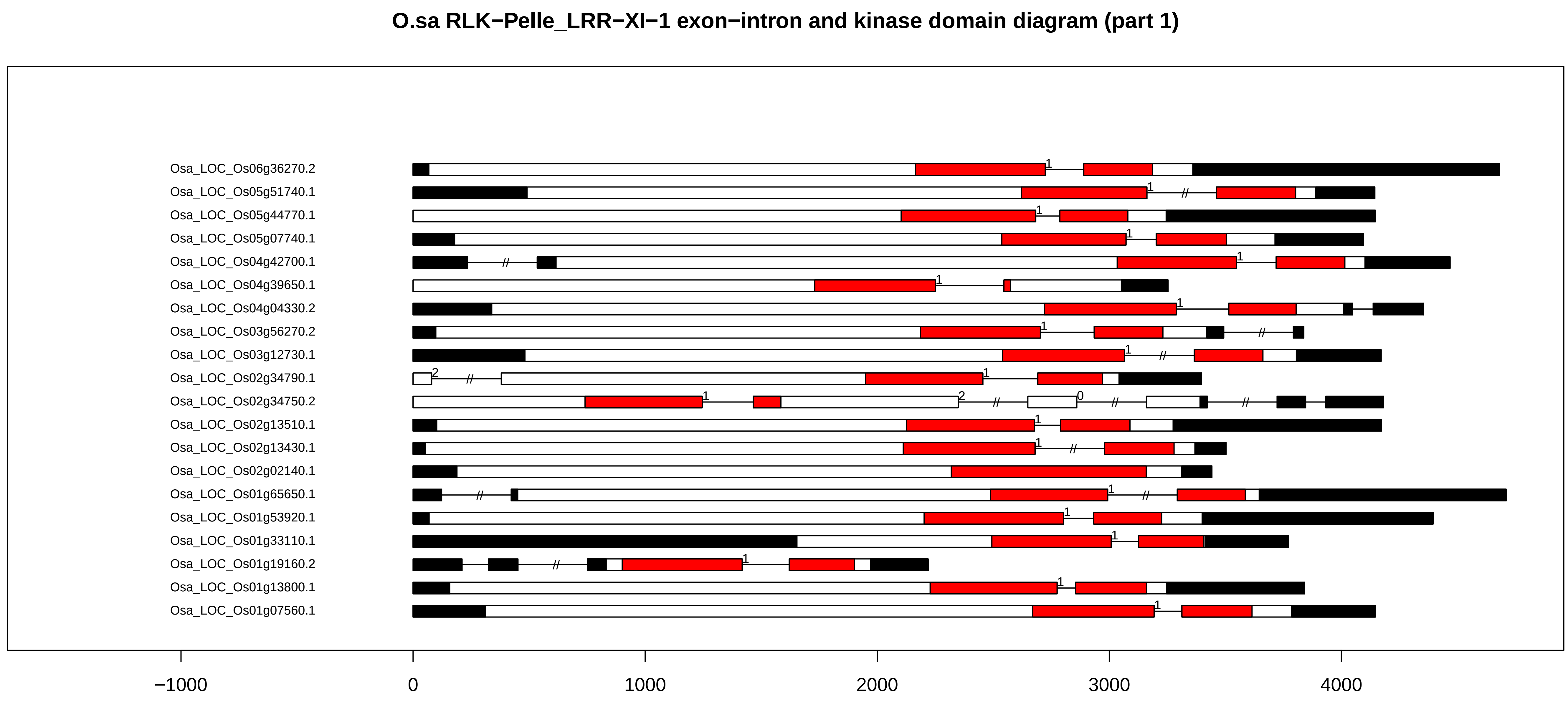

O.sa RLK−Pelle_LRR−XI−1 exon−intron and kinase domain diagram (part 1)
Osa_LOC_Os06g36270.2
Osa_LOC_Os05g51740.1
Osa_LOC_Os05g44770.1
Osa_LOC_Os05g07740.1
Osa_LOC_Os04g42700.1
Osa_LOC_Os04g39650.1
Osa_LOC_Os04g04330.2
Osa_LOC_Os03g56270.2
Osa_LOC_Os03g12730.1
Osa_LOC_Os02g34790.1
Osa_LOC_Os02g34750.2
Osa_LOC_Os02g13510.1
Osa_LOC_Os02g13430.1
Osa_LOC_Os02g02140.1
Osa_LOC_Os01g65650.1
Osa_LOC_Os01g53920.1
Osa_LOC_Os01g33110.1
Osa_LOC_Os01g19160.2
Osa_LOC_Os01g13800.1
Osa_LOC_Os01g07560.1
−1000
0
1000
2000
3000
4000

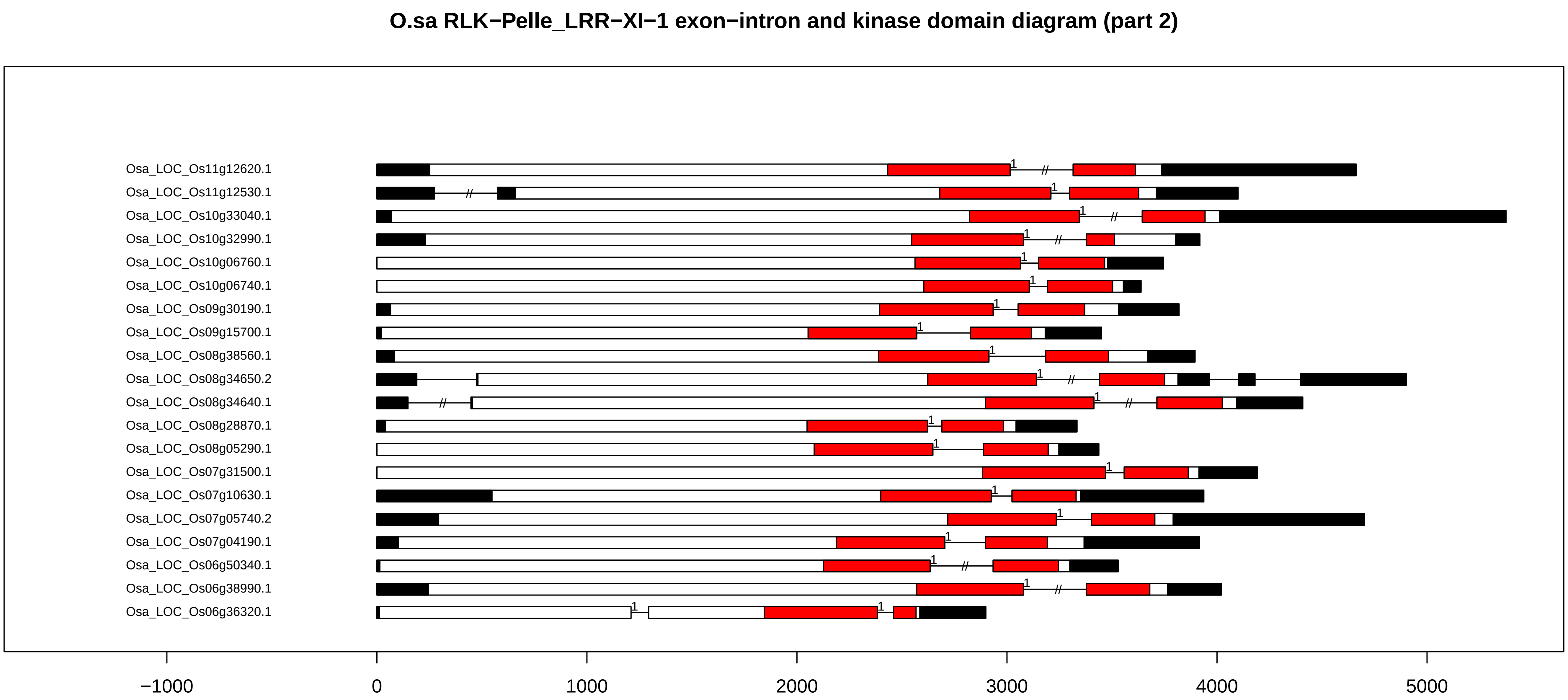
O.sa RLK−Pelle_LRR−XI−1 exon−intron and kinase domain diagram (part 2)
Osa_LOC_Os11g12620.1
Osa_LOC_Os11g12530.1
Osa_LOC_Os10g33040.1
Osa_LOC_Os10g32990.1
Osa_LOC_Os10g06760.1
Osa_LOC_Os10g06740.1
Osa_LOC_Os09g30190.1
Osa_LOC_Os09g15700.1
Osa_LOC_Os08g38560.1
Osa_LOC_Os08g34650.2
Osa_LOC_Os08g34640.1
Osa_LOC_Os08g28870.1
Osa_LOC_Os08g05290.1
Osa_LOC_Os07g31500.1
Osa_LOC_Os07g10630.1
Osa_LOC_Os07g05740.2
Osa_LOC_Os07g04190.1
Osa_LOC_Os06g50340.1
Osa_LOC_Os06g38990.1
Osa_LOC_Os06g36320.1
−1000
0
1000
2000
3000
4000
5000

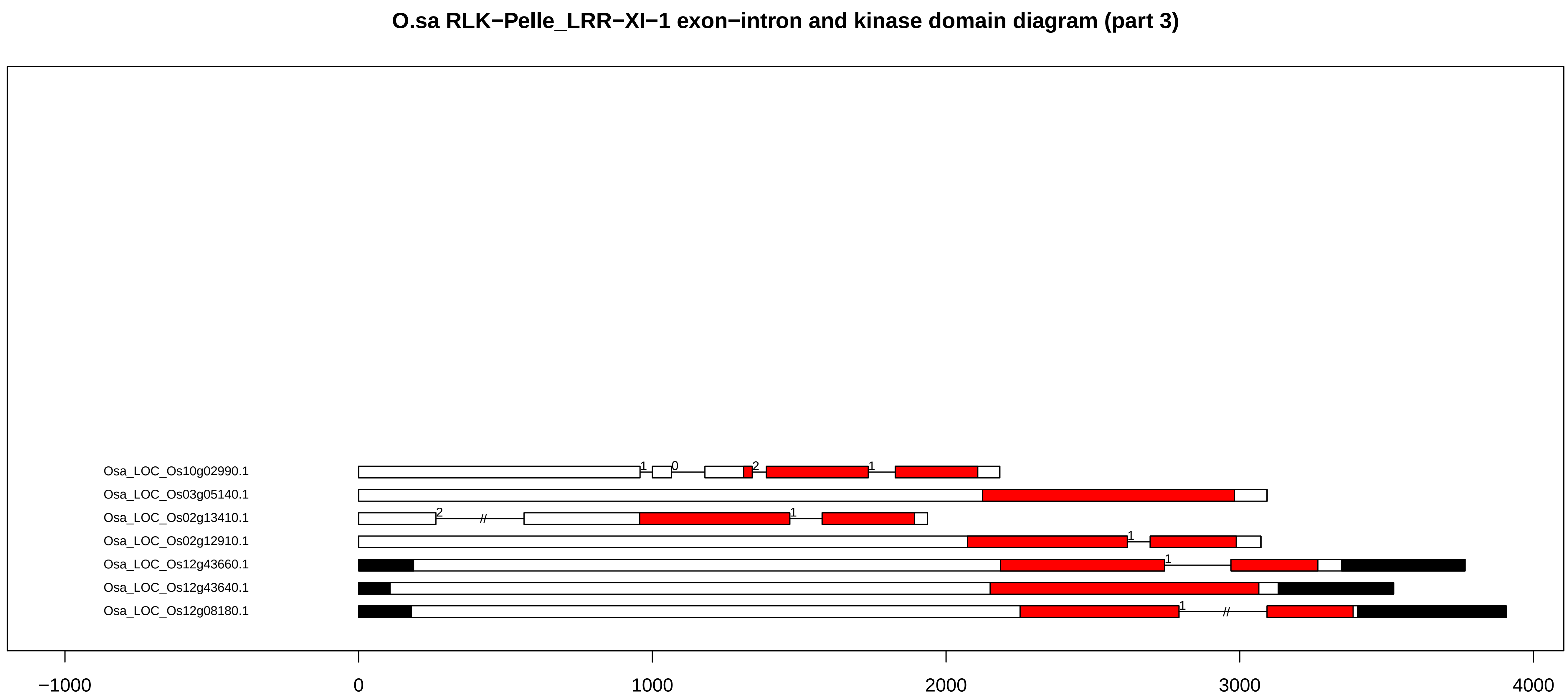

O.sa RLK−Pelle_LRR−XI−1 exon−intron and kinase domain diagram (part 3)
Osa_LOC_Os10g02990.1
Osa_LOC_Os03g05140.1
Osa_LOC_Os02g13410.1
Osa_LOC_Os02g12910.1
Osa_LOC_Os12g43660.1
Osa_LOC_Os12g43640.1
Osa_LOC_Os12g08180.1
1
0
2
1
2
1
1
1
1
−1000
0
1000
2000
3000
4000

O.sa RLK−Pelle_LRR−XI−2 exon−intron and kinase domain diagram (all)

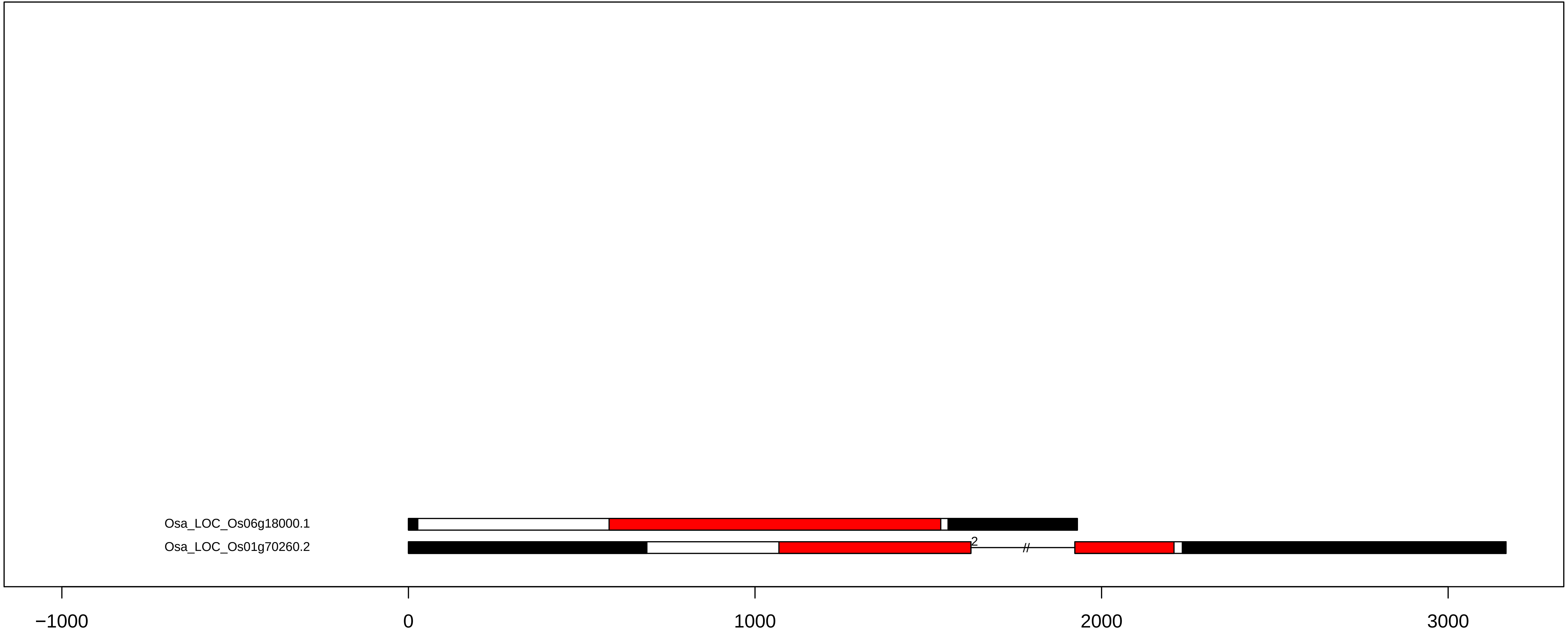

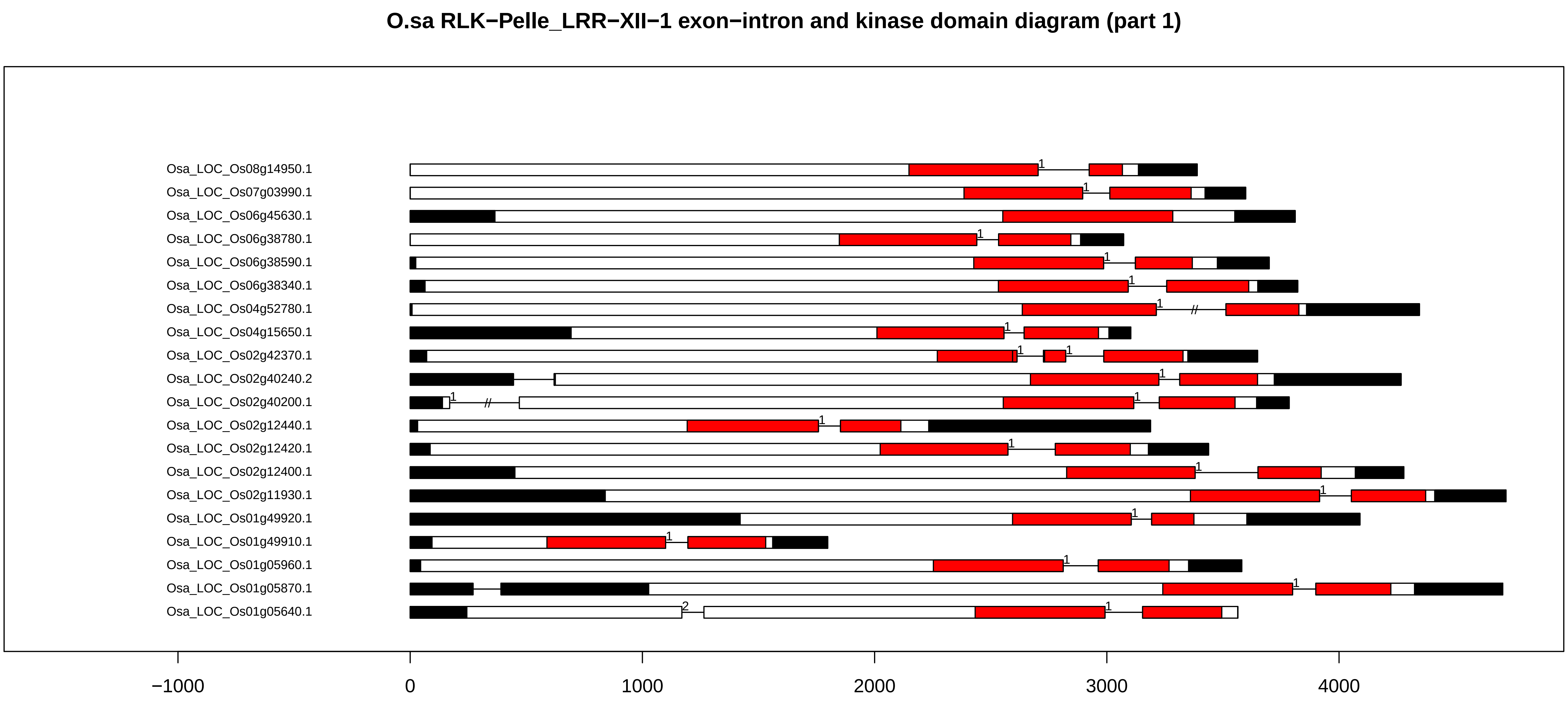
O.sa RLK−Pelle_LRR−XII−1 exon−intron and kinase domain diagram (part 1)
Osa_LOC_Os08g14950.1
Osa_LOC_Os07g03990.1
Osa_LOC_Os06g45630.1
Osa_LOC_Os06g38780.1
Osa_LOC_Os06g38590.1
Osa_LOC_Os06g38340.1
Osa_LOC_Os04g52780.1
Osa_LOC_Os04g15650.1
Osa_LOC_Os02g42370.1
Osa_LOC_Os02g40240.2
Osa_LOC_Os02g40200.1
Osa_LOC_Os02g12440.1
Osa_LOC_Os02g12420.1
Osa_LOC_Os02g12400.1
Osa_LOC_Os02g11930.1
Osa_LOC_Os01g49920.1
Osa_LOC_Os01g49910.1
Osa_LOC_Os01g05960.1
Osa_LOC_Os01g05870.1
Osa_LOC_Os01g05640.1
−1000
0
1000
2000
3000
4000

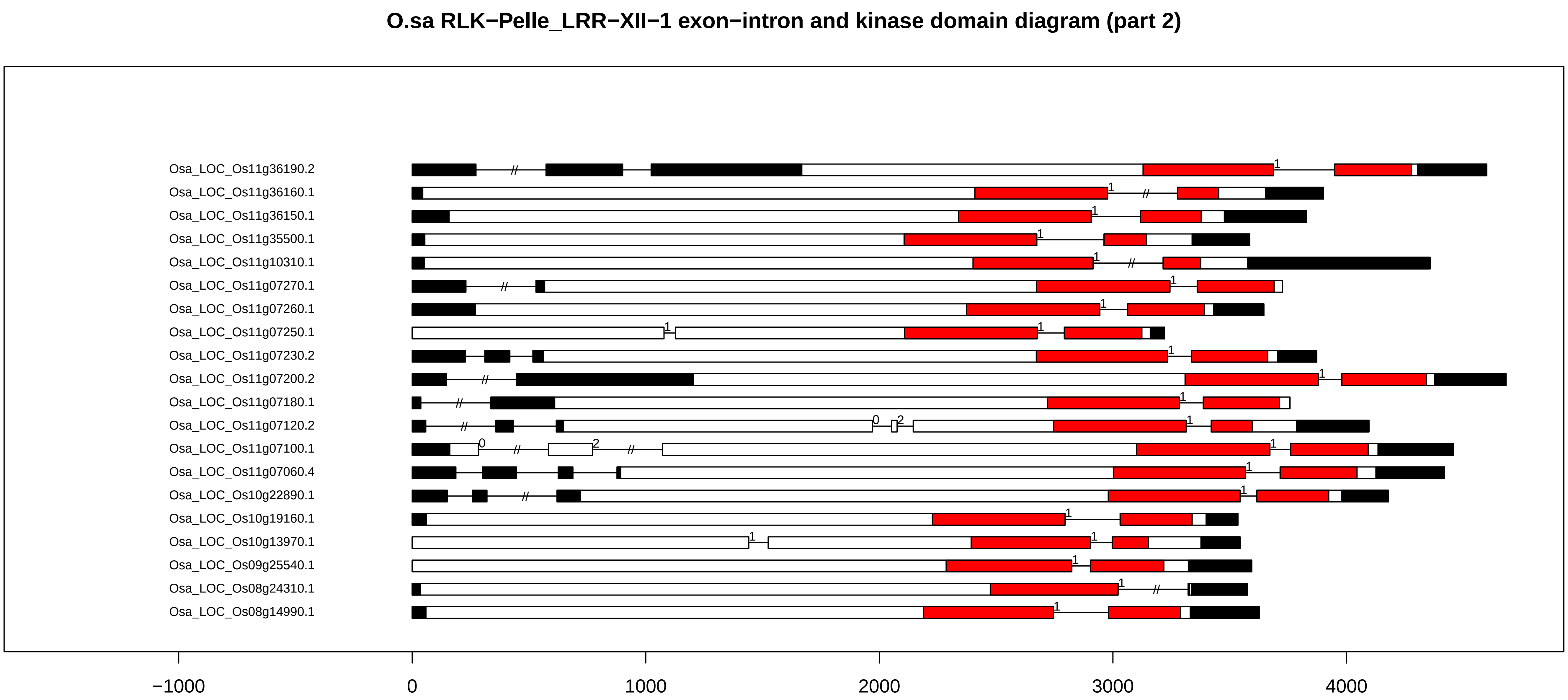
O.sa RLK−Pelle_LRR−XII−1 exon−intron and kinase domain diagram (part 2)
Osa_LOC_Os11g36190.2
Osa_LOC_Os11g36160.1
Osa_LOC_Os11g36150.1
Osa_LOC_Os11g35500.1
Osa_LOC_Os11g10310.1
Osa_LOC_Os11g07270.1
Osa_LOC_Os11g07260.1
Osa_LOC_Os11g07250.1
Osa_LOC_Os11g07230.2
Osa_LOC_Os11g07200.2
Osa_LOC_Os11g07180.1
Osa_LOC_Os11g07120.2
Osa_LOC_Os11g07100.1
Osa_LOC_Os11g07060.4
Osa_LOC_Os10g22890.1
Osa_LOC_Os10g19160.1
Osa_LOC_Os10g13970.1
Osa_LOC_Os09g25540.1
Osa_LOC_Os08g24310.1
Osa_LOC_Os08g14990.1
0
1
2
−1000
0
1000
2000
3000
4000

O.sa RLK−Pelle_LRR−XII−1 exon−intron and kinase domain diagram (part 3)

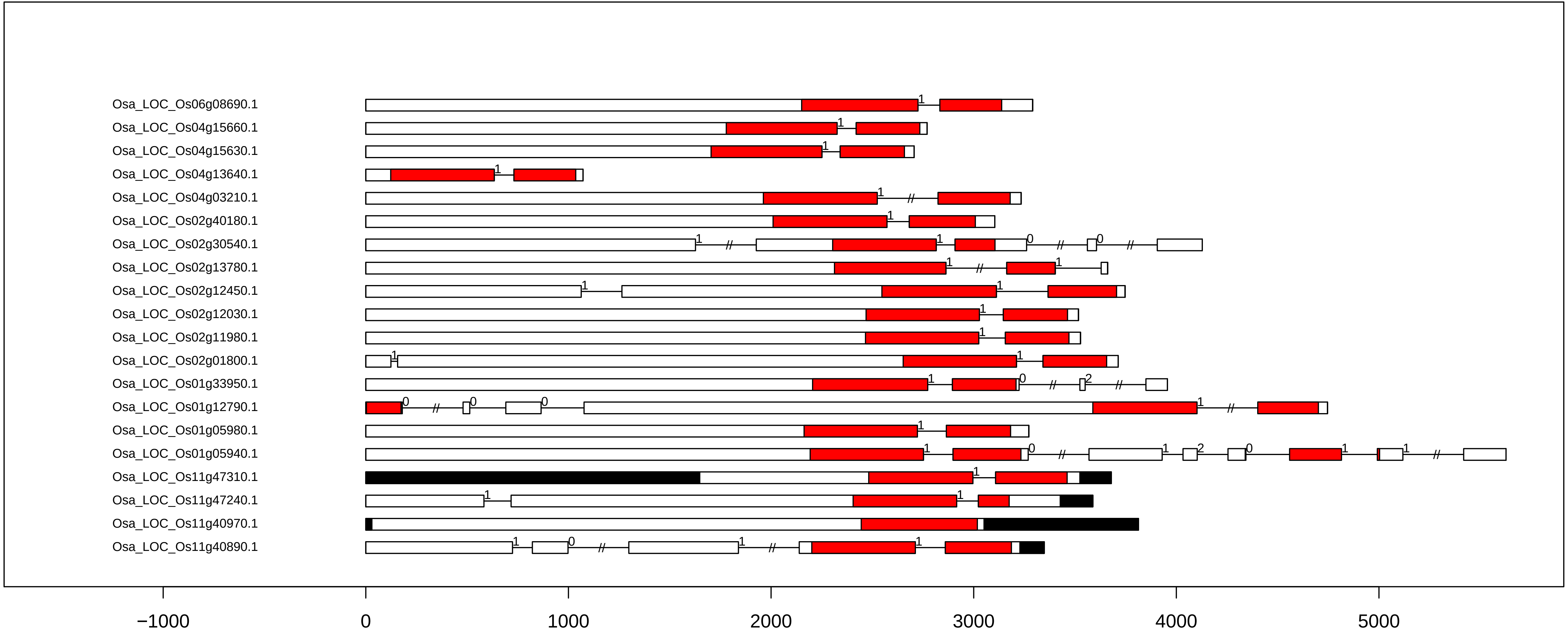

O.sa RLK−Pelle_LRR−XII−1 exon−intron and kinase domain diagram (part 4)

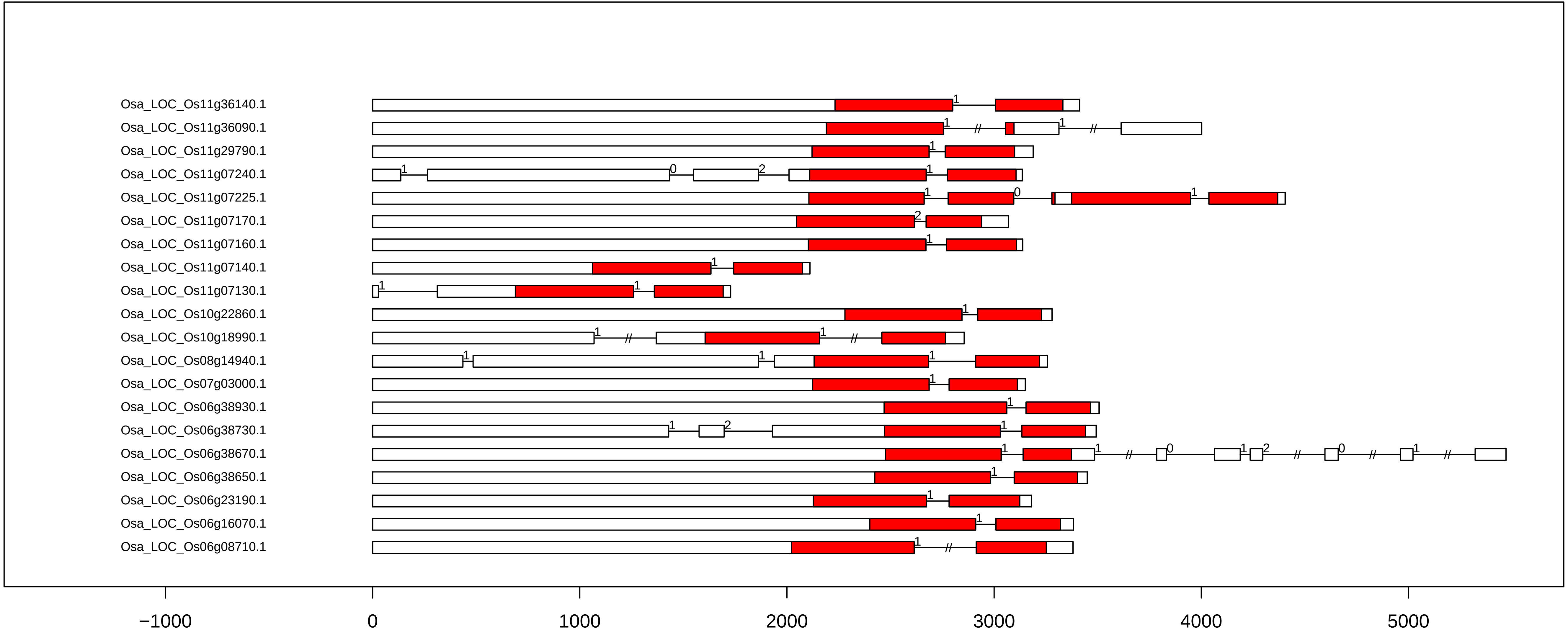

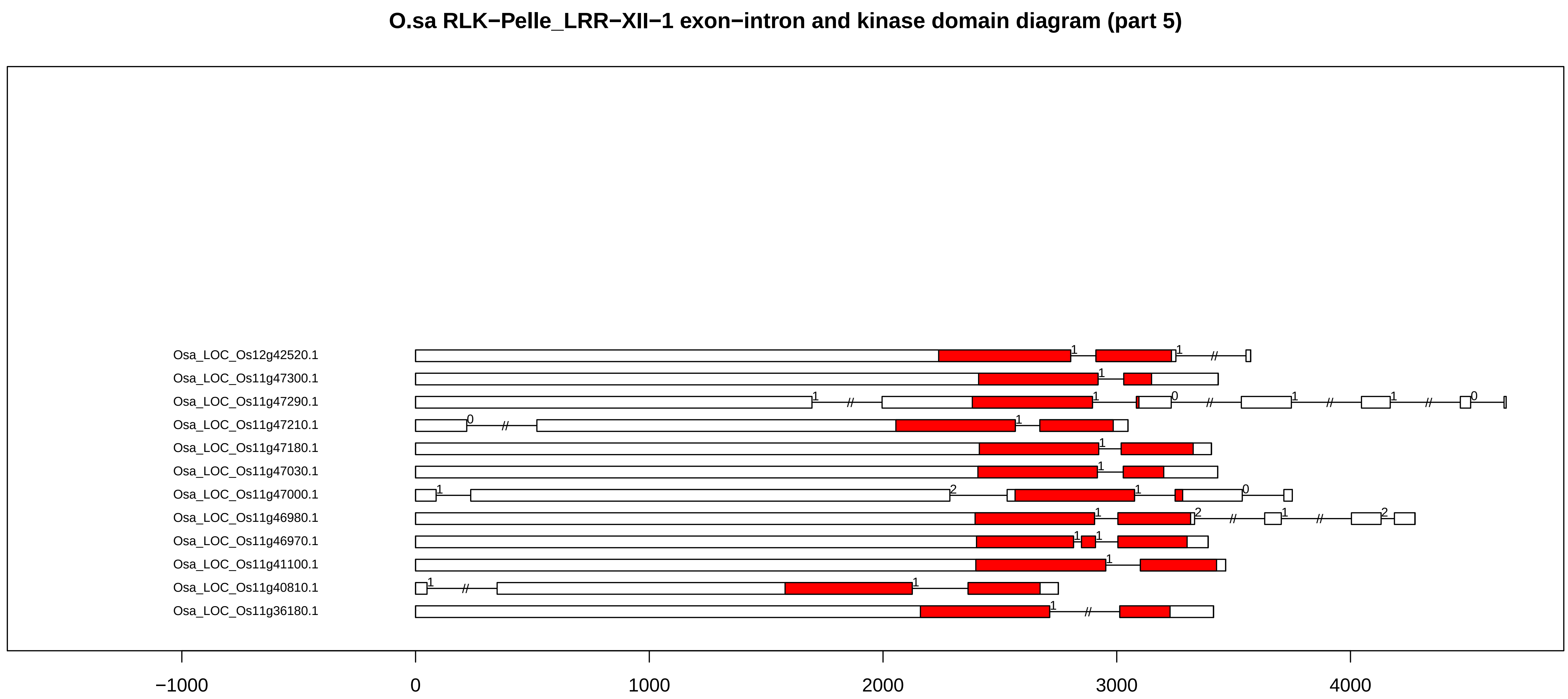

O.sa RLK−Pelle_LRR−XII−1 exon−intron and kinase domain diagram (part 5)
Osa_LOC_Os12g42520.1
Osa_LOC_Os11g47300.1
Osa_LOC_Os11g47290.1
Osa_LOC_Os11g47210.1
Osa_LOC_Os11g47180.1
Osa_LOC_Os11g47030.1
Osa_LOC_Os11g47000.1
Osa_LOC_Os11g46980.1
Osa_LOC_Os11g46970.1
Osa_LOC_Os11g41100.1
Osa_LOC_Os11g40810.1
Osa_LOC_Os11g36180.1
−1000
0
1000
2000
3000
4000

O.sa RLK−Pelle_LRR−XIIIa exon−intron and kinase domain diagram (all)

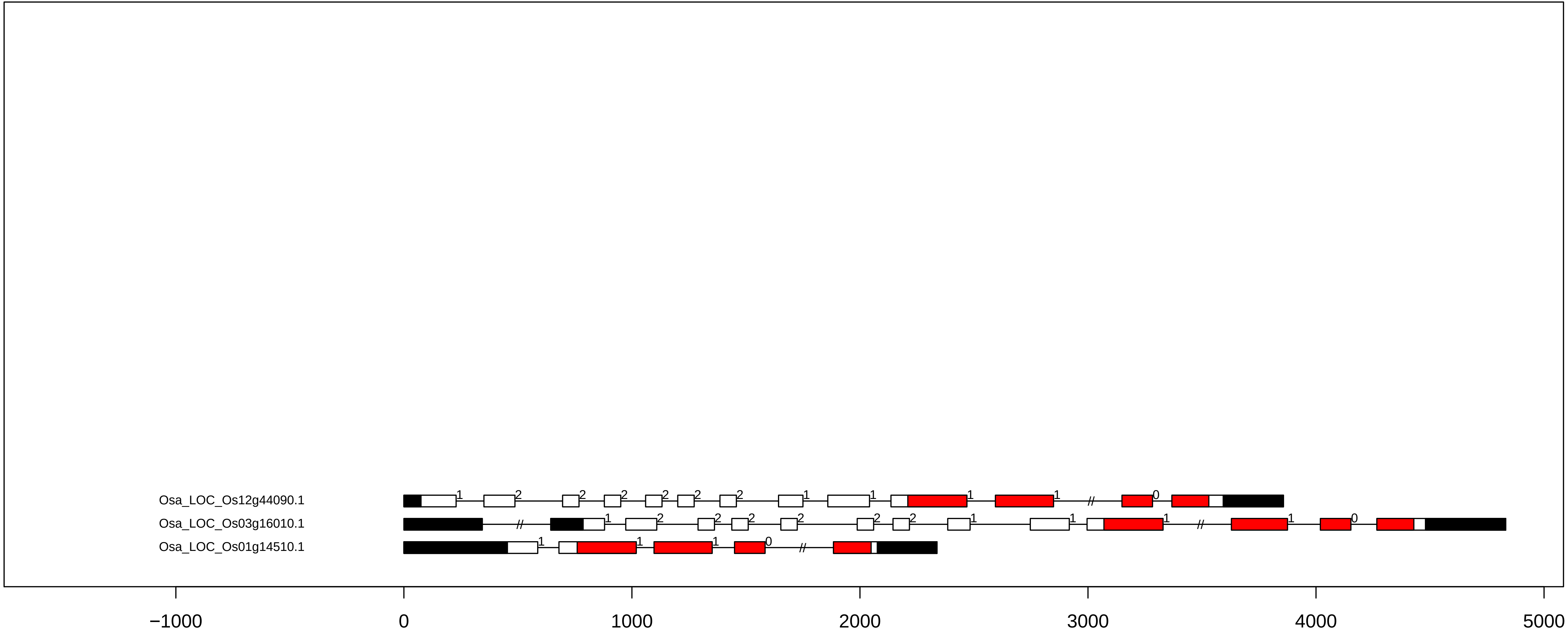

O.sa RLK−Pelle_LRR−XIIIb exon−intron and kinase domain diagram (all)

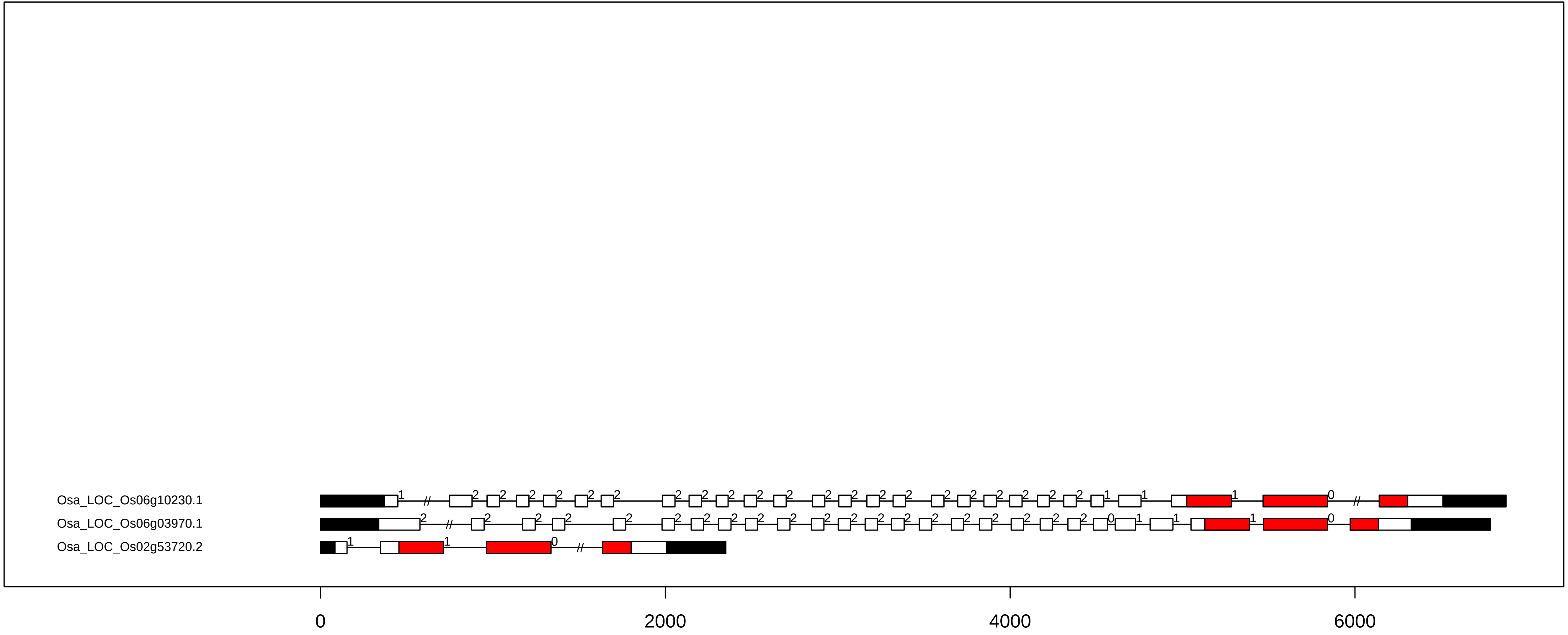

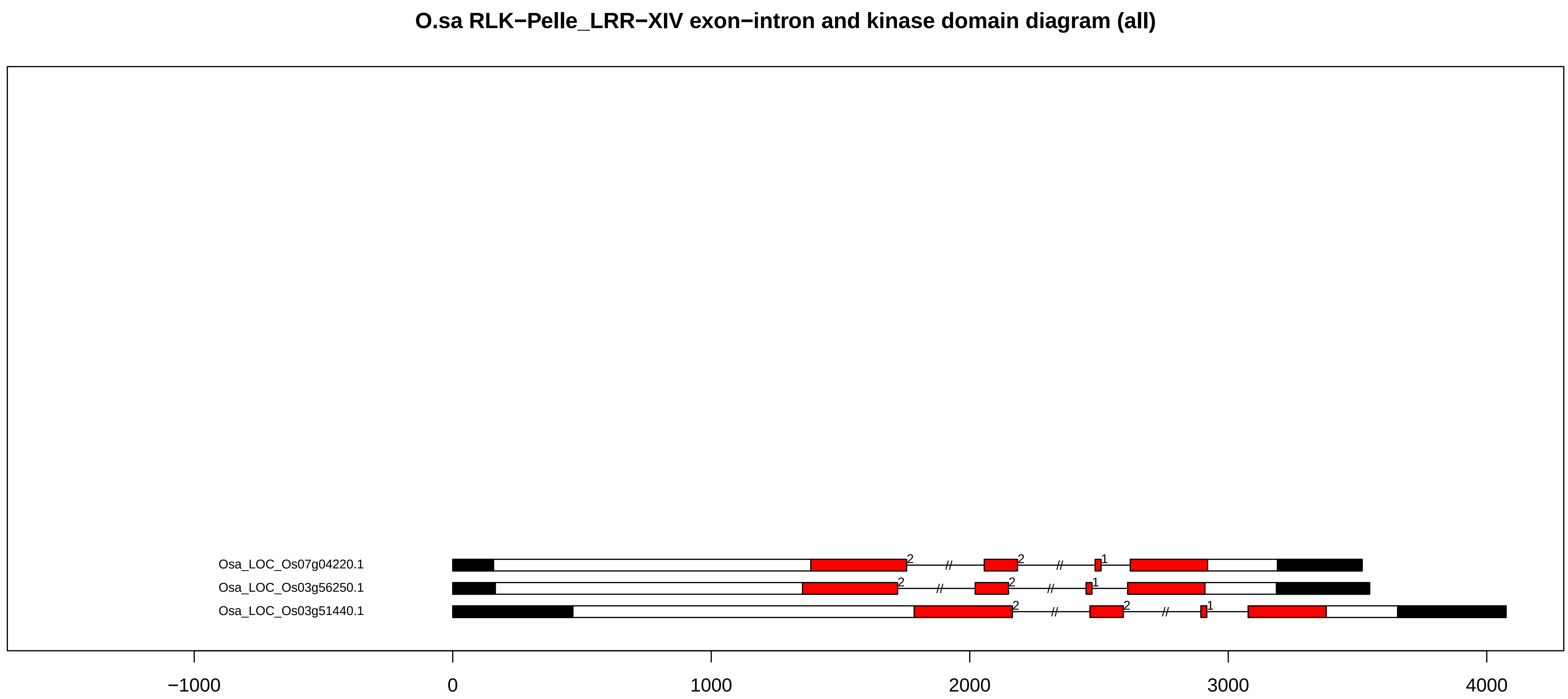
O.sa RLK−Pelle_LRR−XIV exon−intron and kinase domain diagram (all)
Osa_LOC_Os07g04220.1
Osa_LOC_Os03g56250.1
Osa_LOC_Os03g51440.1
2
2
1
−1000
0
1000
2000
3000
4000

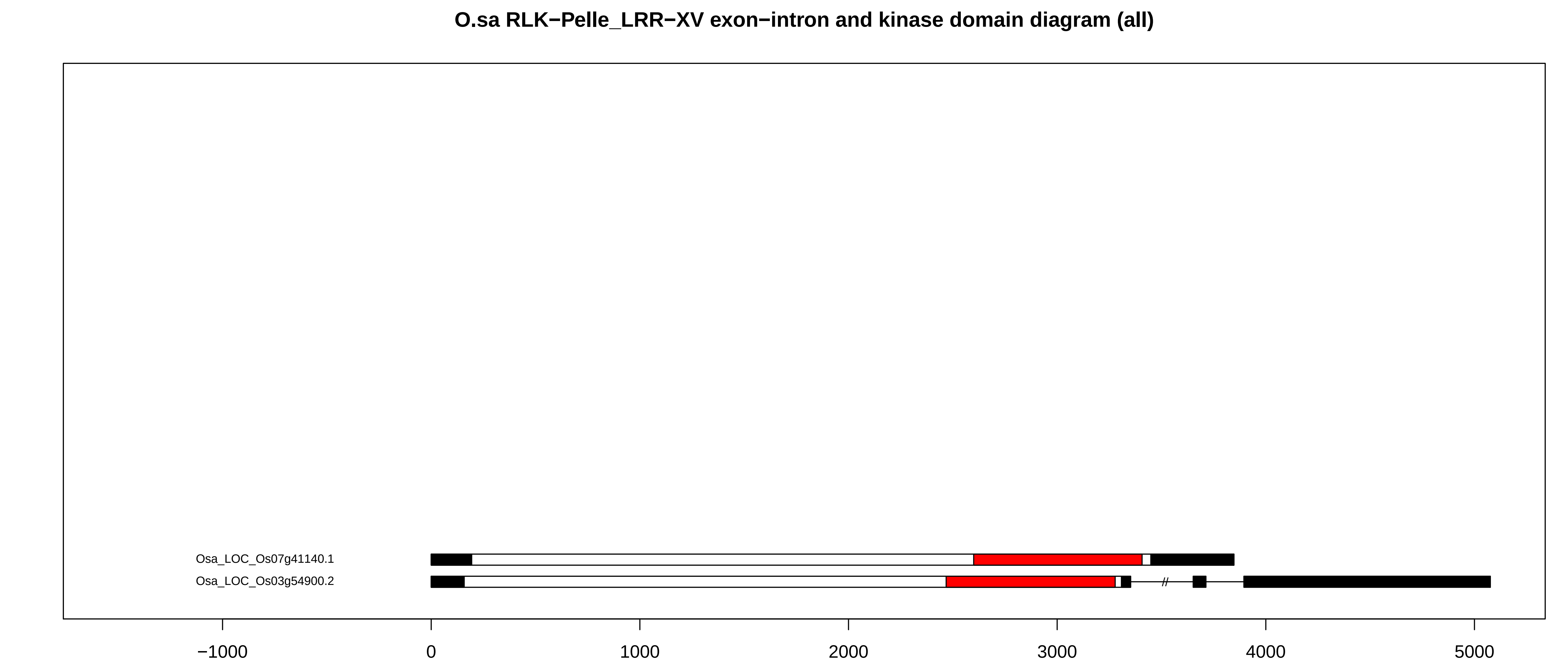
O.sa RLK−Pelle_LRR−XV exon−intron and kinase domain diagram (all)
Osa_LOC_Os07g41140.1
Osa_LOC_Os03g54900.2
−1000
0
1000
2000
3000
4000
5000

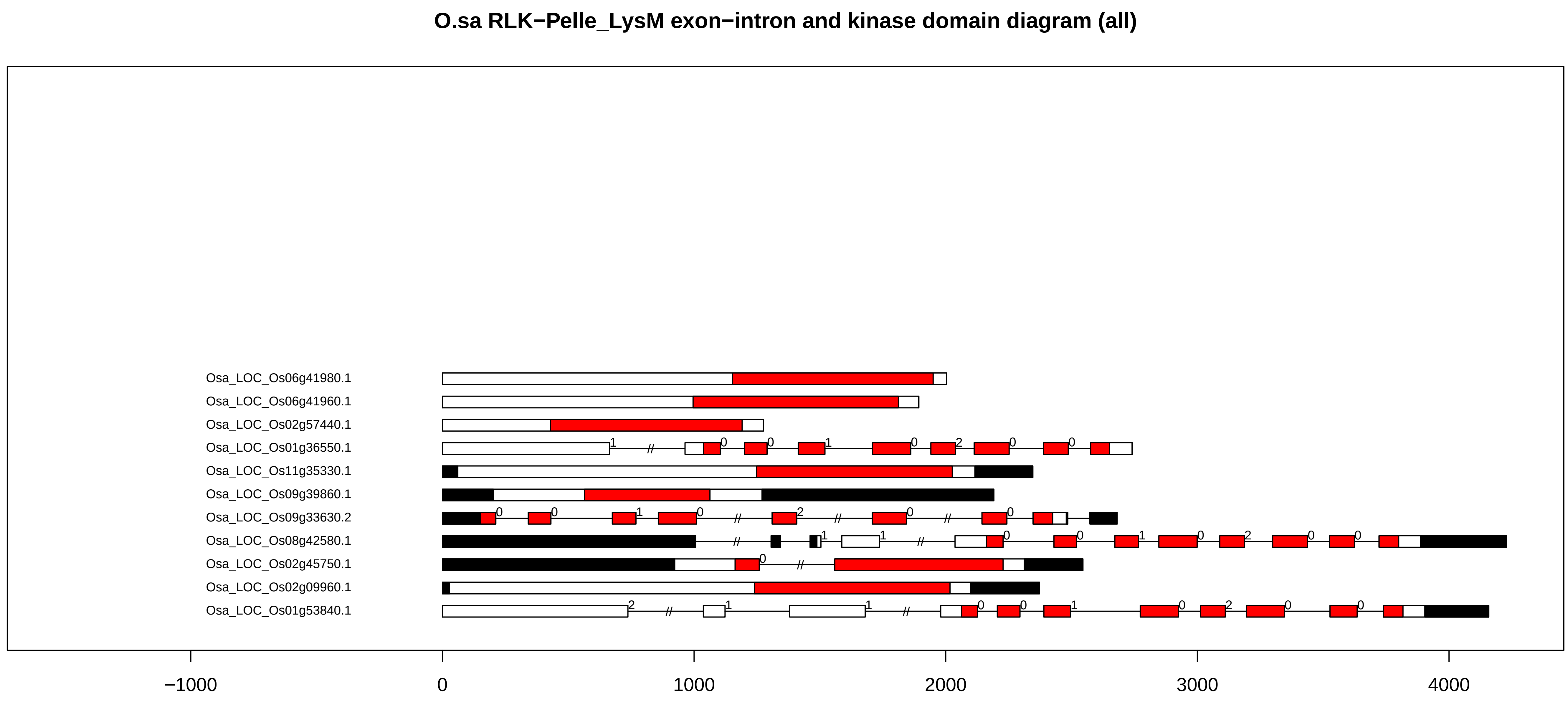
O.sa RLK−Pelle_LysM exon−intron and kinase domain diagram (all)
Osa_LOC_Os06g41980.1
Osa_LOC_Os06g41960.1
Osa_LOC_Os02g57440.1
Osa_LOC_Os01g36550.1
Osa_LOC_Os11g35330.1
Osa_LOC_Os09g39860.1
Osa_LOC_Os09g33630.2
Osa_LOC_Os08g42580.1
Osa_LOC_Os02g45750.1
Osa_LOC_Os02g09960.1
Osa_LOC_Os01g53840.1
−1000
0
1000
2000
3000
4000

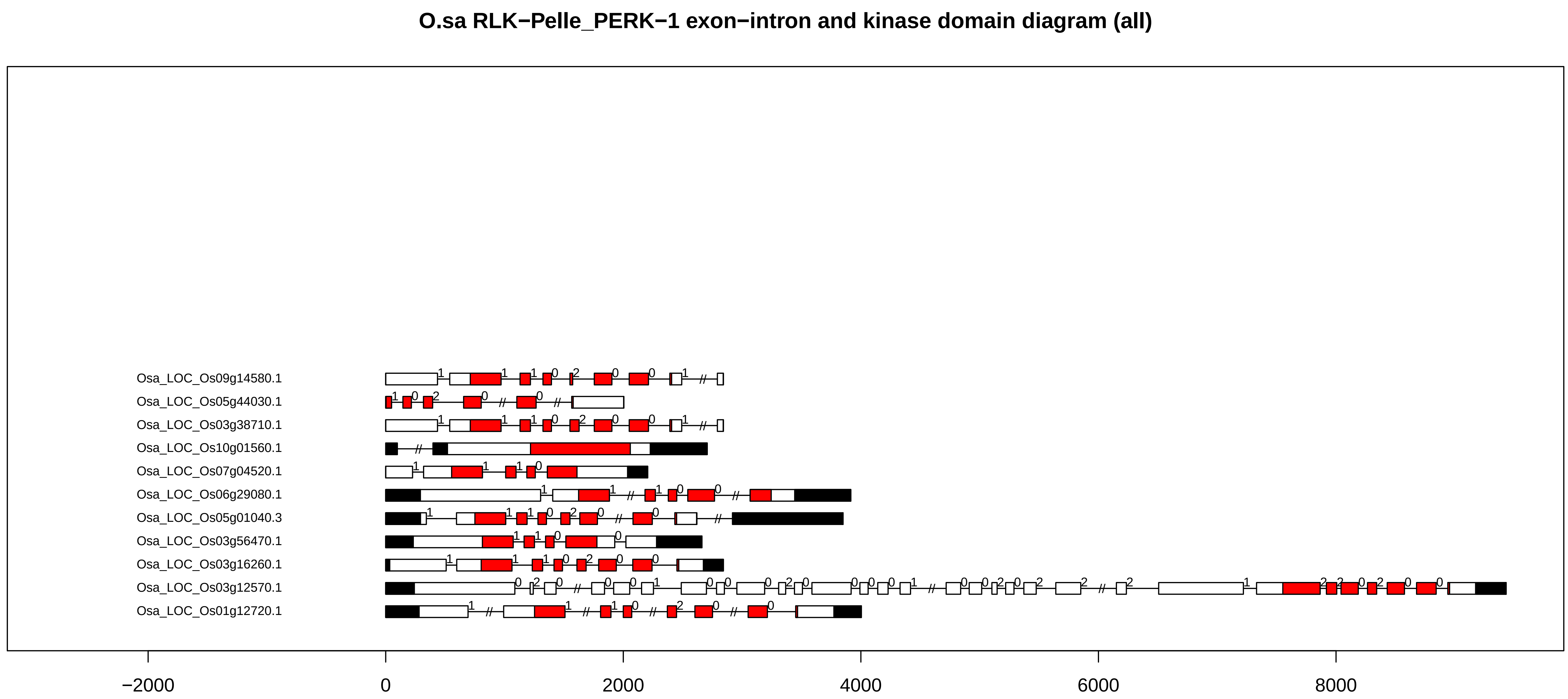
O.sa RLK−Pelle_PERK−1 exon−intron and kinase domain diagram (all)
Osa_LOC_Os09g14580.1
Osa_LOC_Os05g44030.1
Osa_LOC_Os03g38710.1
Osa_LOC_Os10g01560.1
Osa_LOC_Os07g04520.1
Osa_LOC_Os06g29080.1
Osa_LOC_Os05g01040.3
Osa_LOC_Os03g56470.1
Osa_LOC_Os03g16260.1
Osa_LOC_Os03g12570.1
Osa_LOC_Os01g12720.1
−2000
0
2000
4000
6000
8000

O.sa RLK−Pelle_PERK−2 exon−intron and kinase domain diagram (all)

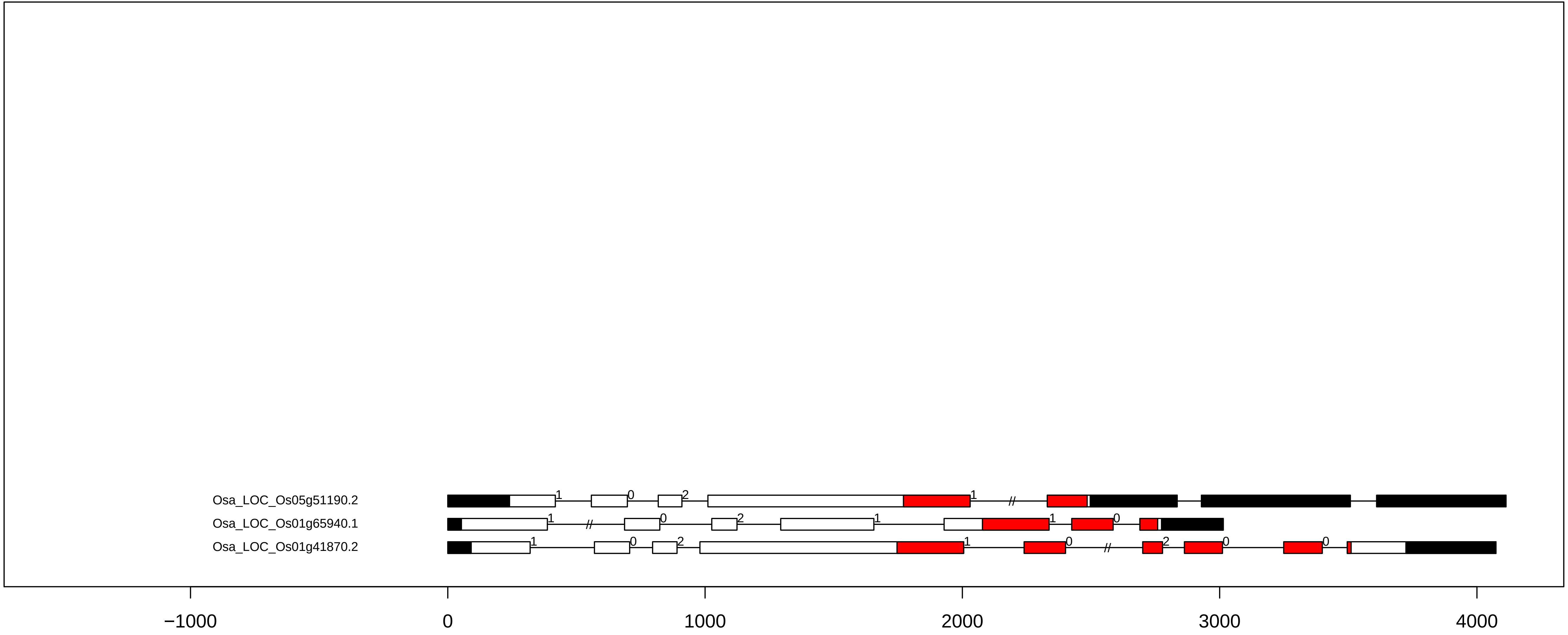

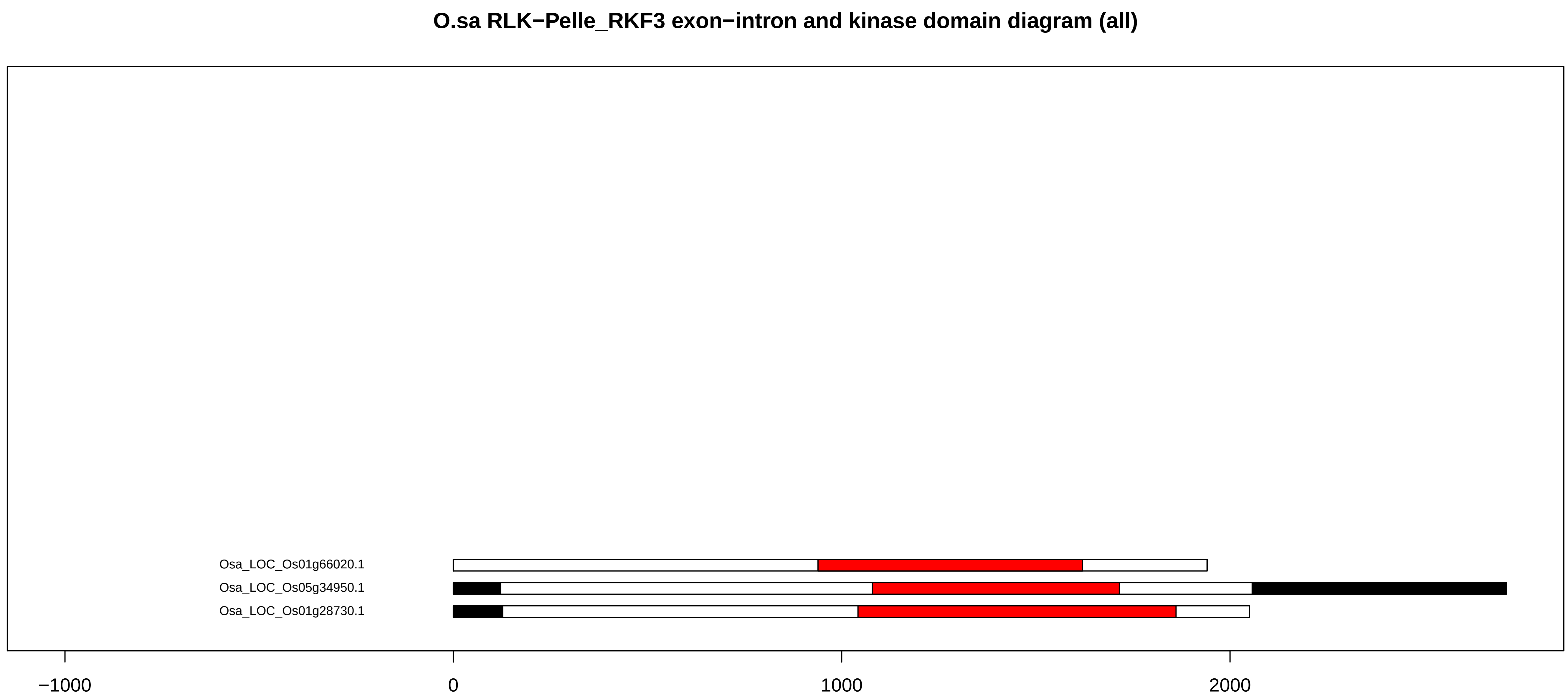

O.sa RLK−Pelle_RKF3 exon−intron and kinase domain diagram (all)
Osa_LOC_Os01g66020.1
Osa_LOC_Os05g34950.1
Osa_LOC_Os01g28730.1
−1000
0
1000
2000

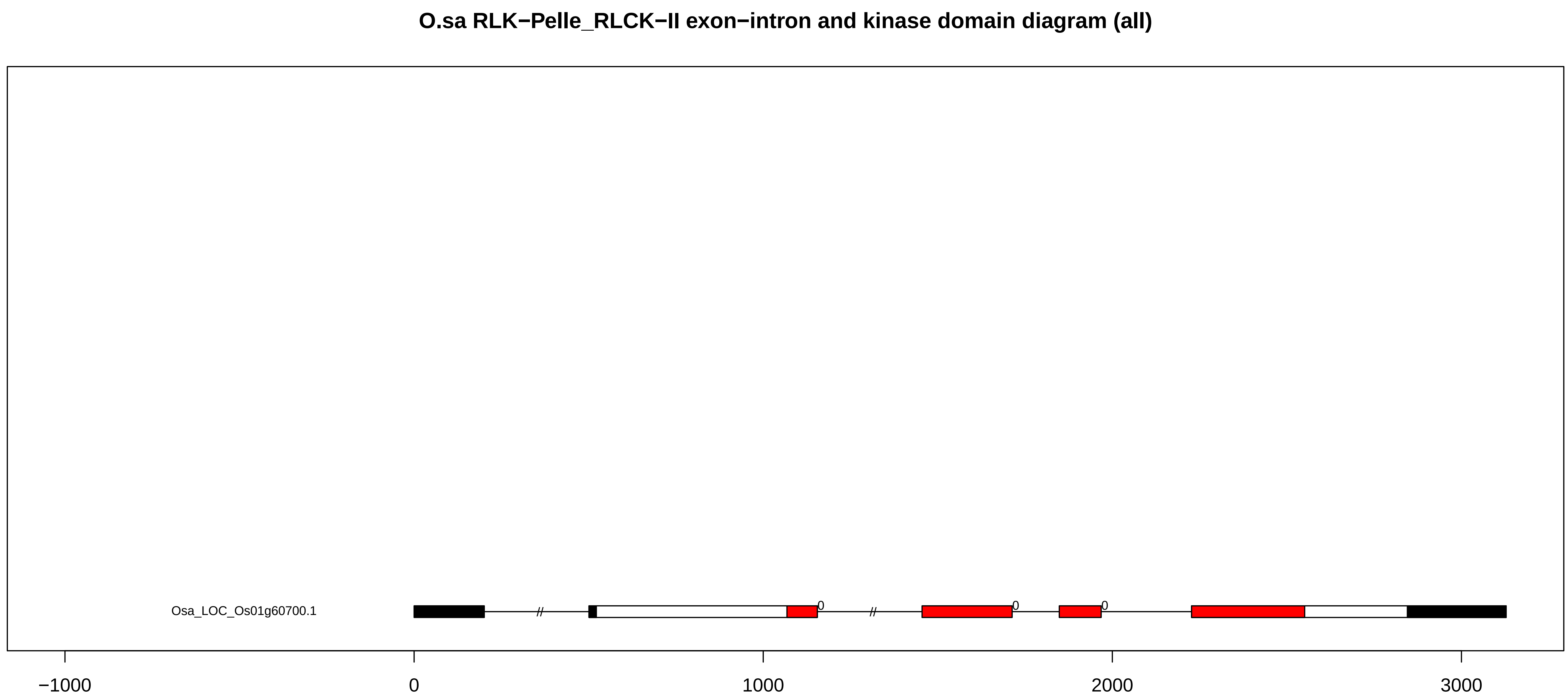

O.sa RLK−Pelle_RLCK−II exon−intron and kinase domain diagram (all)
Osa_LOC_Os01g60700.1
0
0
0
−1000
0
1000
2000
3000

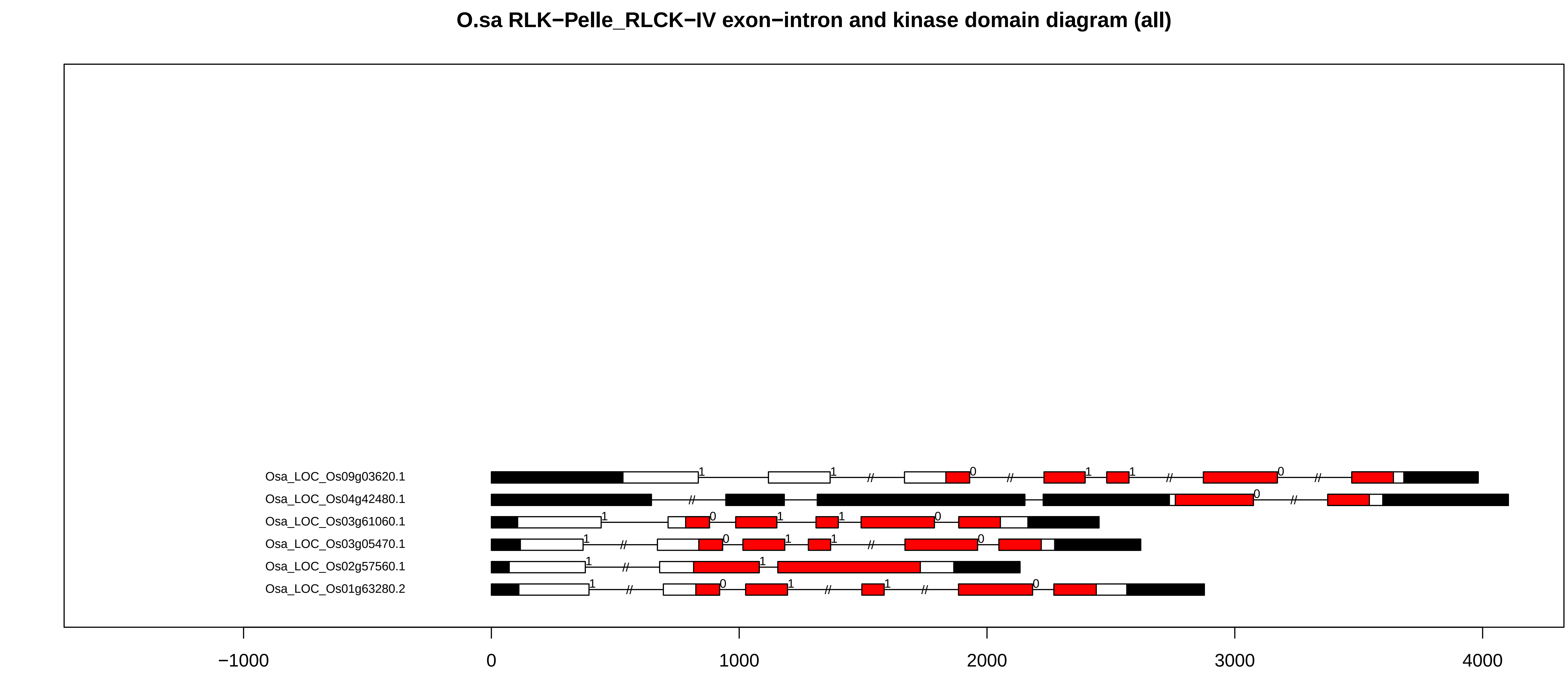

O.sa RLK−Pelle_RLCK−IV exon−intron and kinase domain diagram (all)
Osa_LOC_Os09g03620.1
Osa_LOC_Os04g42480.1
Osa_LOC_Os03g61060.1
Osa_LOC_Os03g05470.1
Osa_LOC_Os02g57560.1
Osa_LOC_Os01g63280.2
−1000
0
1000
2000
3000
4000

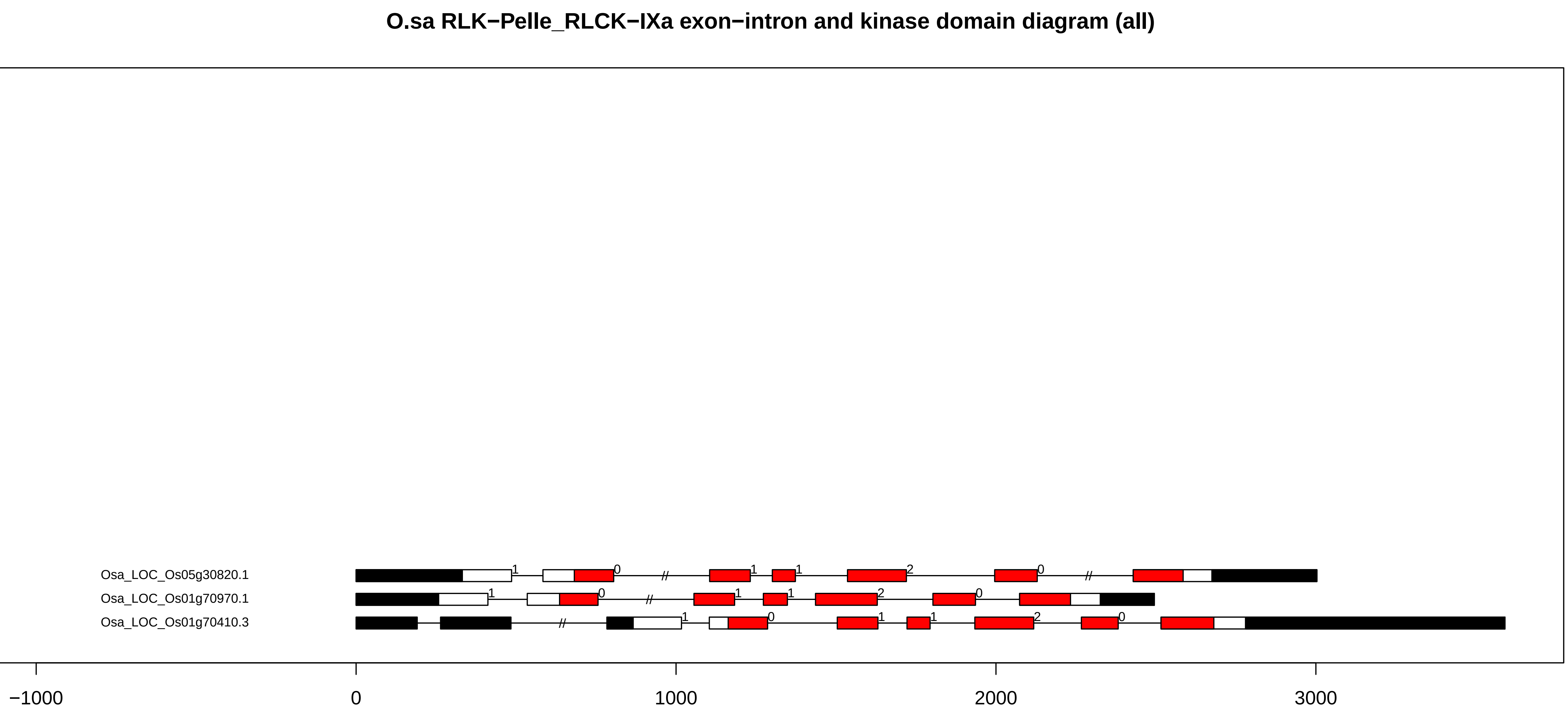

O.sa RLK−Pelle_RLCK−IXa exon−intron and kinase domain diagram (all)
Osa_LOC_Os05g30820.1
Osa_LOC_Os01g70970.1
Osa_LOC_Os01g70410.3
1
0
1
1
2
0
−1000
0
1000
2000
3000

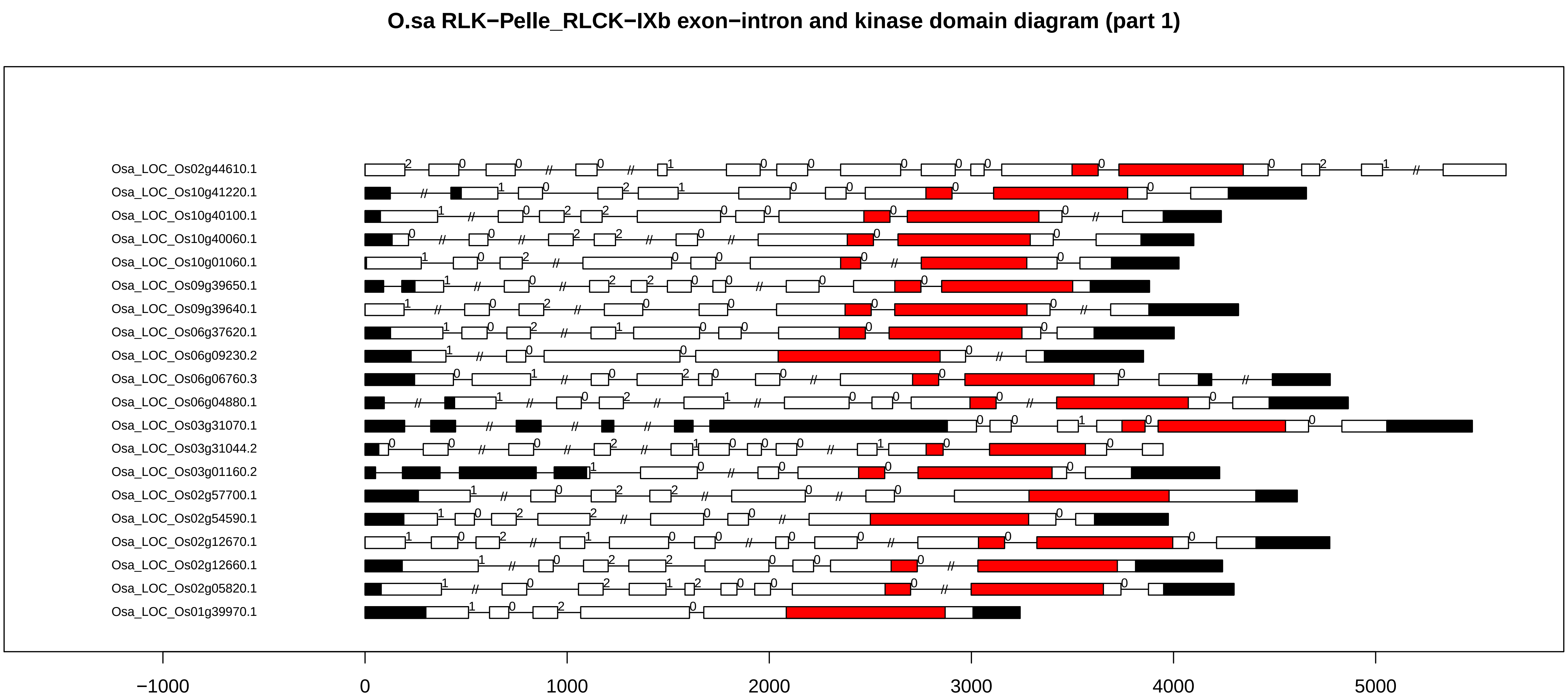

O.sa RLK−Pelle_RLCK−IXb exon−intron and kinase domain diagram (part 1)
Osa_LOC_Os02g44610.1
Osa_LOC_Os10g41220.1
Osa_LOC_Os10g40100.1
Osa_LOC_Os10g40060.1
Osa_LOC_Os10g01060.1
Osa_LOC_Os09g39650.1
Osa_LOC_Os09g39640.1
Osa_LOC_Os06g37620.1
Osa_LOC_Os06g09230.2
Osa_LOC_Os06g06760.3
Osa_LOC_Os06g04880.1
Osa_LOC_Os03g31070.1
Osa_LOC_Os03g31044.2
Osa_LOC_Os03g01160.2
Osa_LOC_Os02g57700.1
Osa_LOC_Os02g54590.1
Osa_LOC_Os02g12670.1
Osa_LOC_Os02g12660.1
Osa_LOC_Os02g05820.1
Osa_LOC_Os01g39970.1
−1000
0
1000
2000
3000
4000
5000

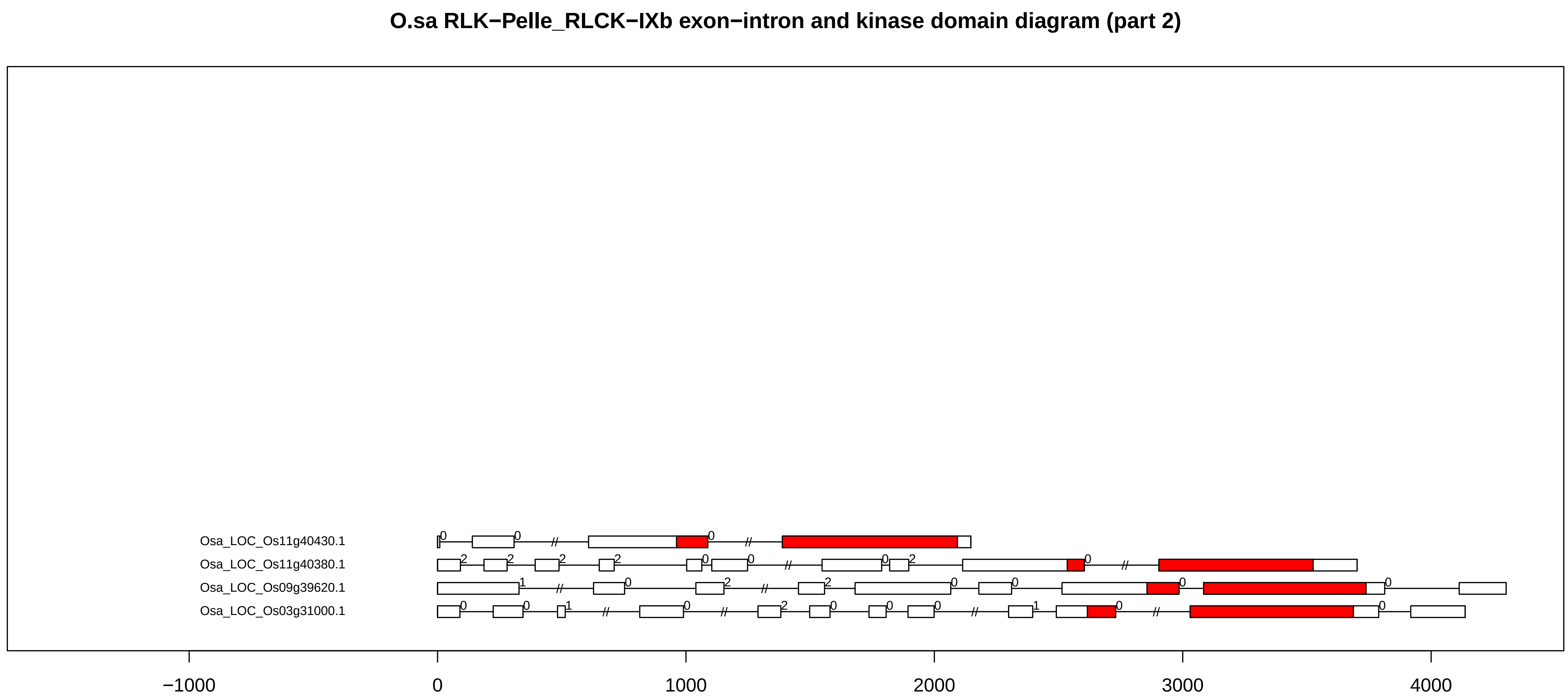

O.sa RLK−Pelle_RLCK−IXb exon−intron and kinase domain diagram (part 2)
Osa_LOC_Os11g40430.1
Osa_LOC_Os11g40380.1
Osa_LOC_Os09g39620.1
Osa_LOC_Os03g31000.1
−1000
0
1000
2000
3000
4000

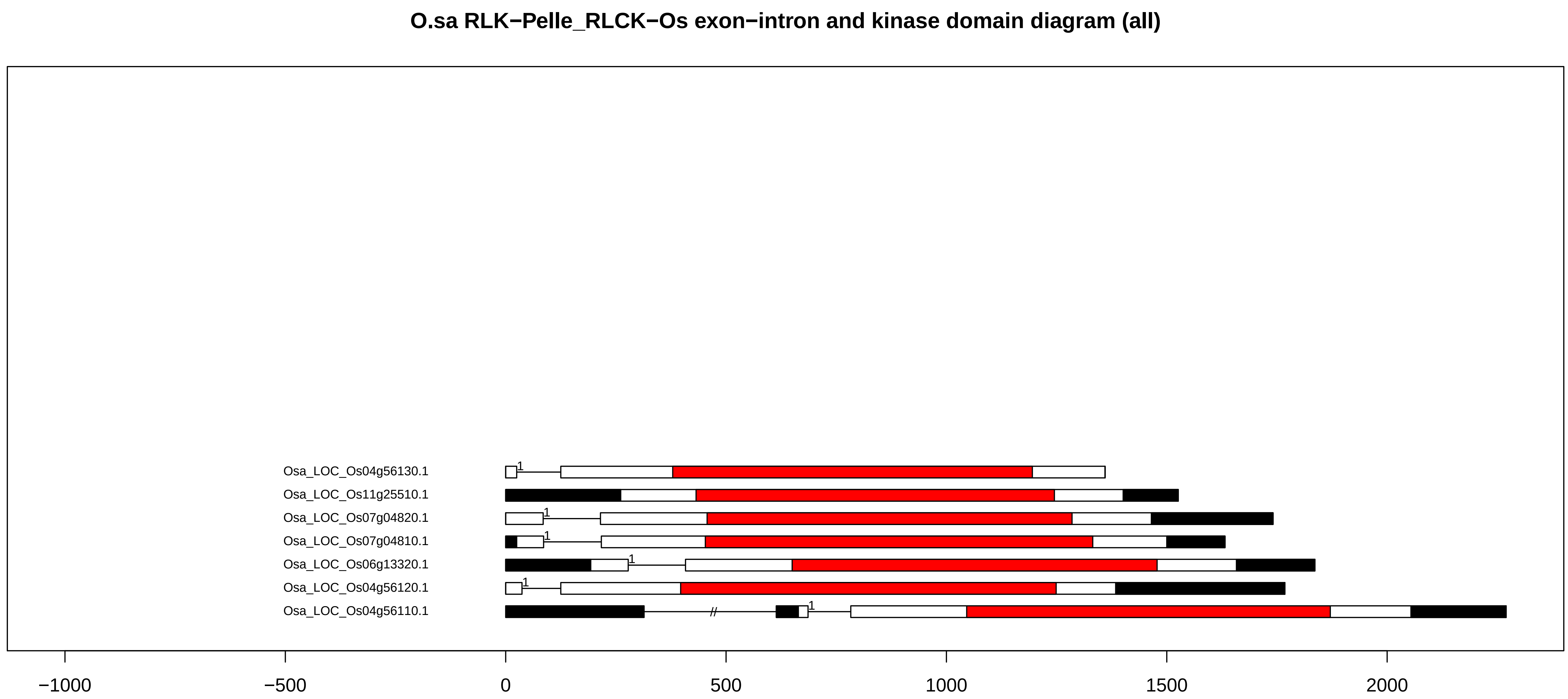
O.sa RLK−Pelle_RLCK−Os exon−intron and kinase domain diagram (all)
Osa_LOC_Os04g56130.1
Osa_LOC_Os11g25510.1
Osa_LOC_Os07g04820.1
Osa_LOC_Os07g04810.1
Osa_LOC_Os06g13320.1
Osa_LOC_Os04g56120.1
Osa_LOC_Os04g56110.1
1
−1000
−500
0
500
1000
1500
2000

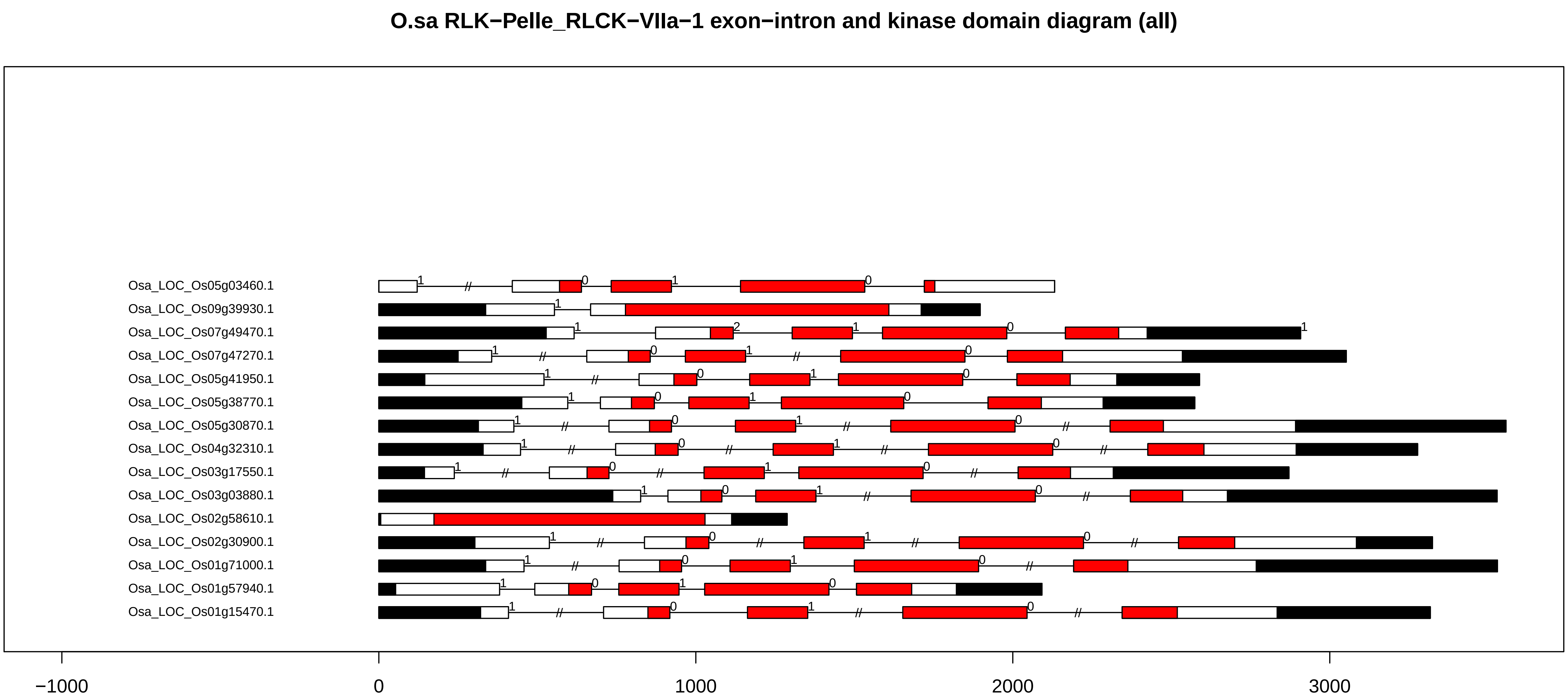
O.sa RLK−Pelle_RLCK−VIIa−1 exon−intron and kinase domain diagram (all)
Osa_LOC_Os05g03460.1
Osa_LOC_Os09g39930.1
Osa_LOC_Os07g49470.1
Osa_LOC_Os07g47270.1
Osa_LOC_Os05g41950.1
Osa_LOC_Os05g38770.1
Osa_LOC_Os05g30870.1
Osa_LOC_Os04g32310.1
Osa_LOC_Os03g17550.1
Osa_LOC_Os03g03880.1
Osa_LOC_Os02g58610.1
Osa_LOC_Os02g30900.1
Osa_LOC_Os01g71000.1
Osa_LOC_Os01g57940.1
Osa_LOC_Os01g15470.1
−1000
0
1000
2000
3000

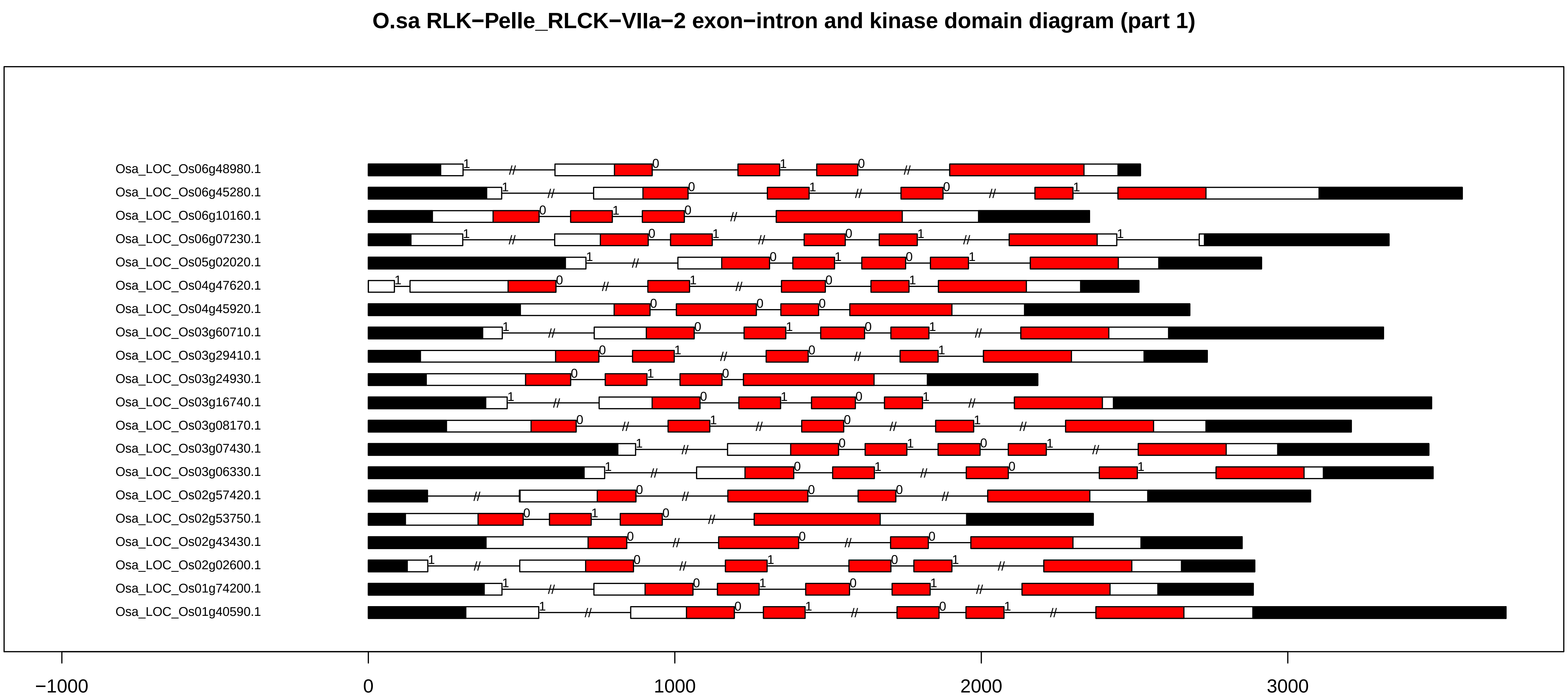
O.sa RLK−Pelle_RLCK−VIIa−2 exon−intron and kinase domain diagram (part 1)
Osa_LOC_Os06g48980.1
Osa_LOC_Os06g45280.1
Osa_LOC_Os06g10160.1
Osa_LOC_Os06g07230.1
Osa_LOC_Os05g02020.1
Osa_LOC_Os04g47620.1
Osa_LOC_Os04g45920.1
Osa_LOC_Os03g60710.1
Osa_LOC_Os03g29410.1
Osa_LOC_Os03g24930.1
Osa_LOC_Os03g16740.1
Osa_LOC_Os03g08170.1
Osa_LOC_Os03g07430.1
Osa_LOC_Os03g06330.1
Osa_LOC_Os02g57420.1
Osa_LOC_Os02g53750.1
Osa_LOC_Os02g43430.1
Osa_LOC_Os02g02600.1
Osa_LOC_Os01g74200.1
Osa_LOC_Os01g40590.1
−1000
0
1000
2000
3000

O.sa RLK−Pelle_RLCK−VIIa−2 exon−intron and kinase domain diagram (part 2)

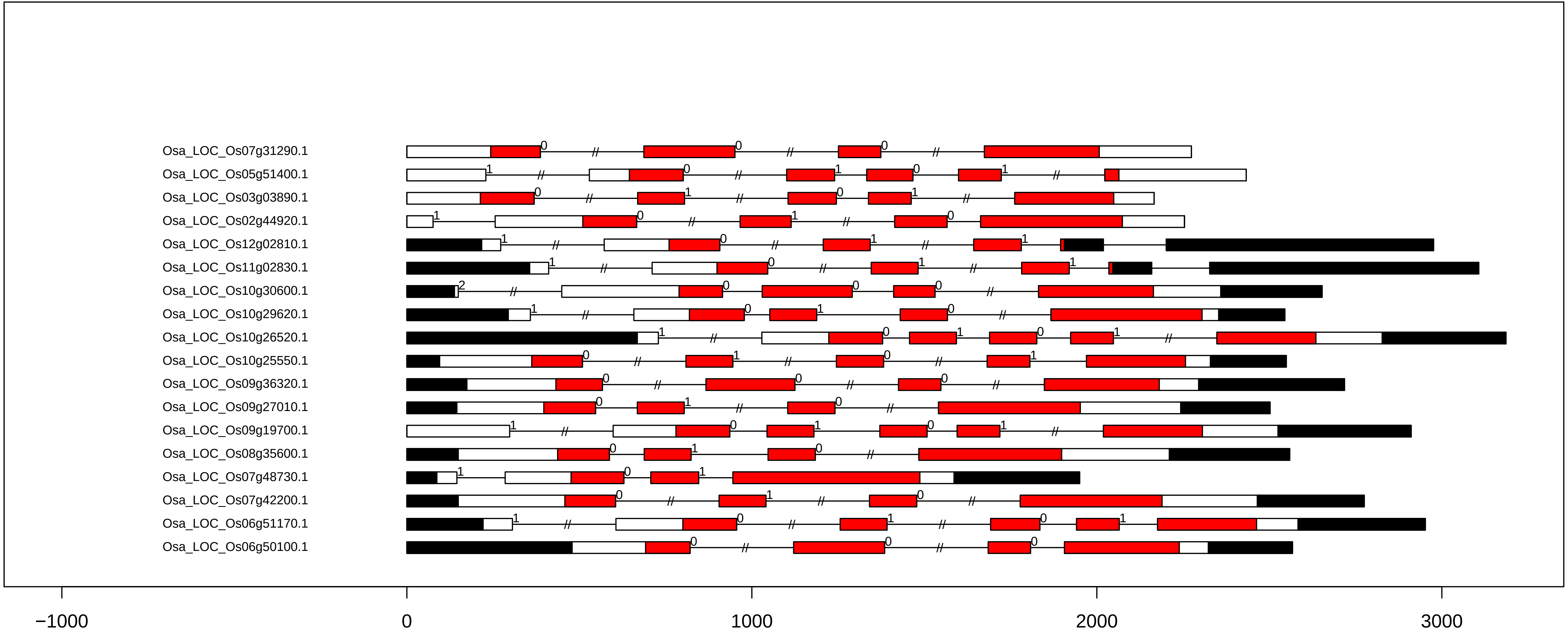

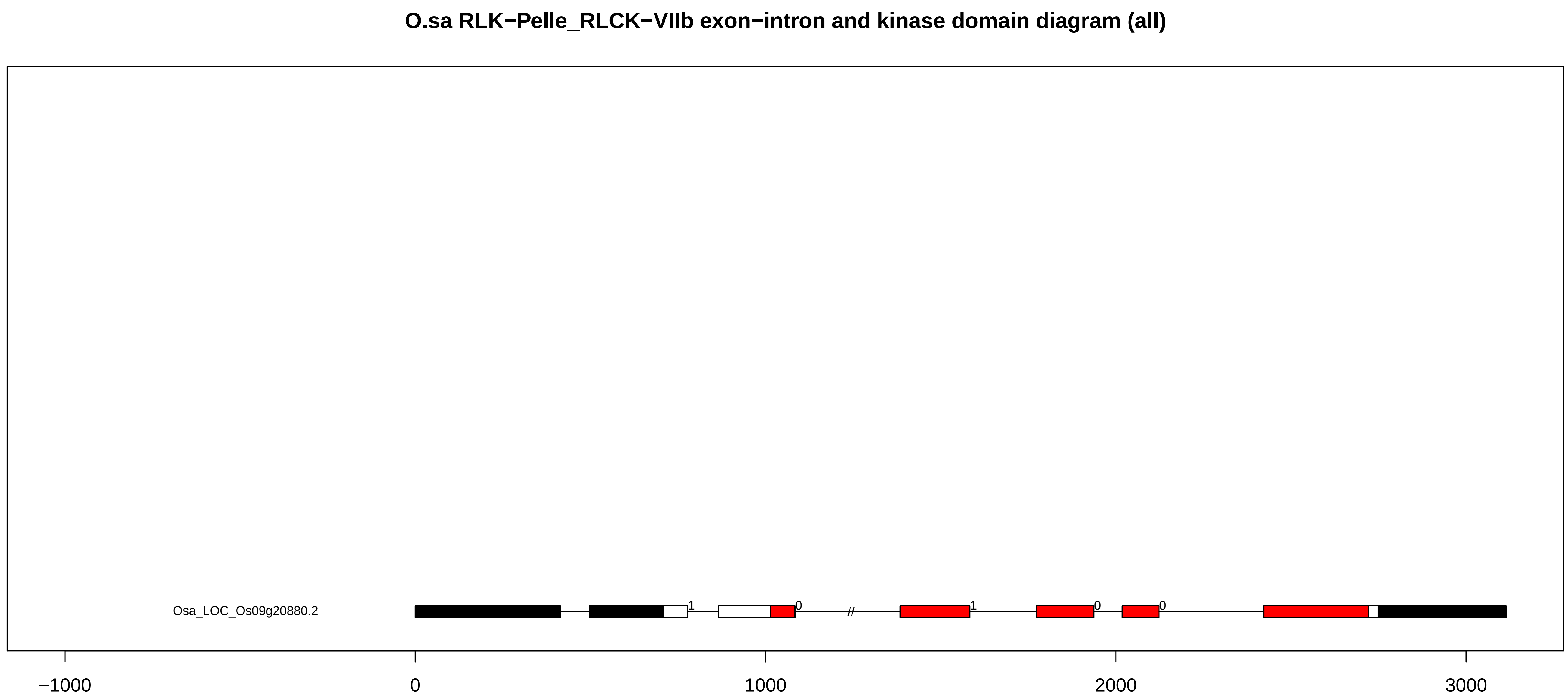

O.sa RLK−Pelle_RLCK−VIIb exon−intron and kinase domain diagram (all)
Osa_LOC_Os09g20880.2
1
0
//
1
0
0
−1000
0
1000
2000
3000

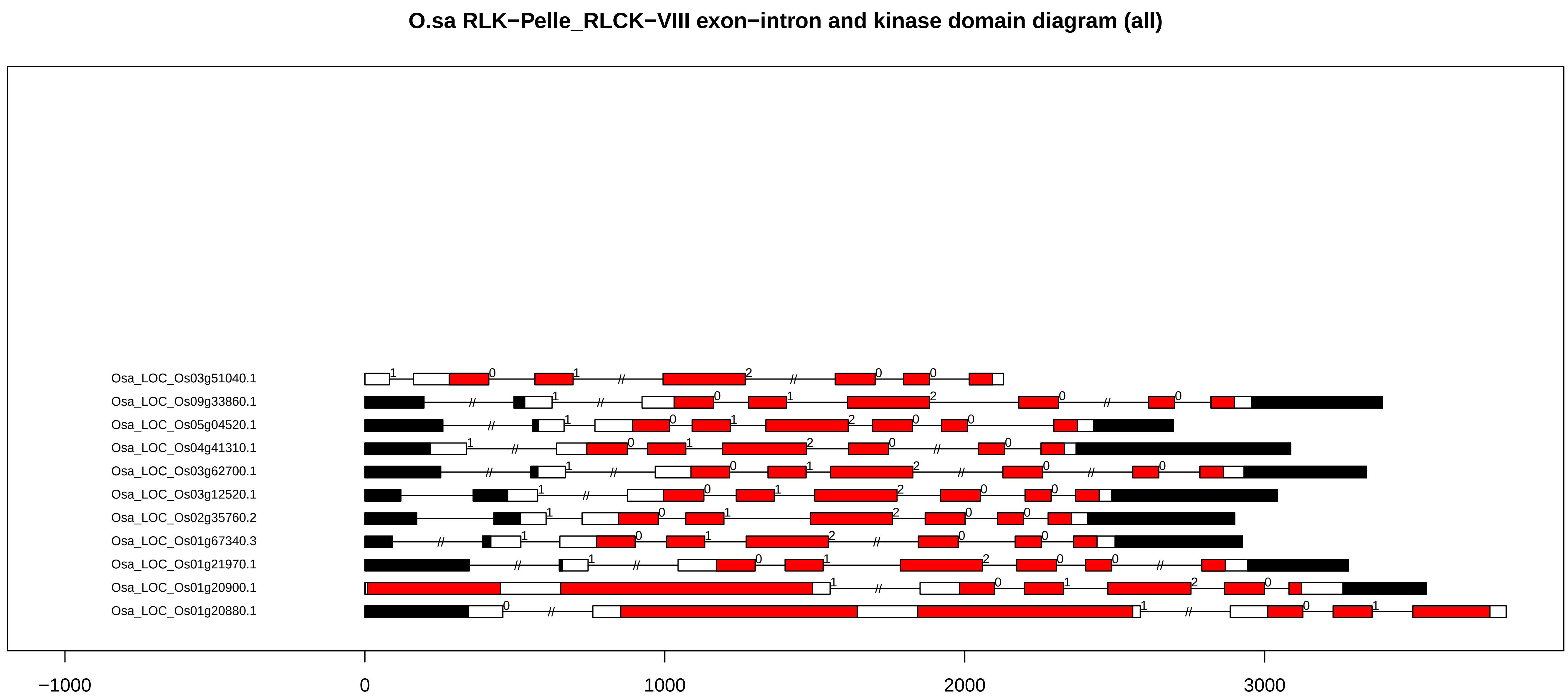

O.sa RLK−Pelle_RLCK−VIII exon−intron and kinase domain diagram (all)
Osa_LOC_Os03g51040.1
Osa_LOC_Os09g33860.1
Osa_LOC_Os05g04520.1
Osa_LOC_Os04g41310.1
Osa_LOC_Os03g62700.1
Osa_LOC_Os03g12520.1
Osa_LOC_Os02g35760.2
Osa_LOC_Os01g67340.3
Osa_LOC_Os01g21970.1
Osa_LOC_Os01g20900.1
Osa_LOC_Os01g20880.1
−1000
0
1000
2000
3000

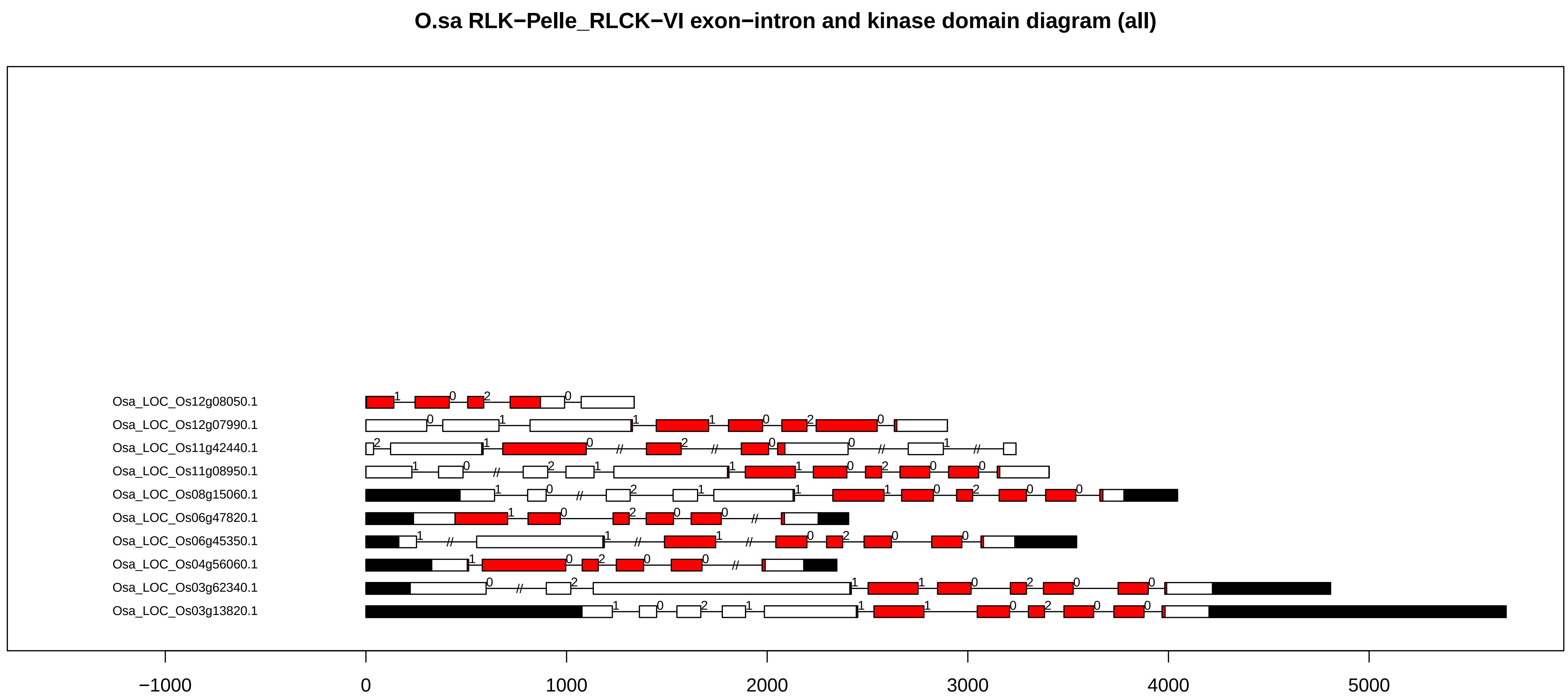

O.sa RLK−Pelle_RLCK−VI exon−intron and kinase domain diagram (all)
Osa_LOC_Os12g08050.1
Osa_LOC_Os12g07990.1
Osa_LOC_Os11g42440.1
Osa_LOC_Os11g08950.1
Osa_LOC_Os08g15060.1
Osa_LOC_Os06g47820.1
Osa_LOC_Os06g45350.1
Osa_LOC_Os04g56060.1
Osa_LOC_Os03g62340.1
Osa_LOC_Os03g13820.1
−1000
0
1000
2000
3000
4000
5000

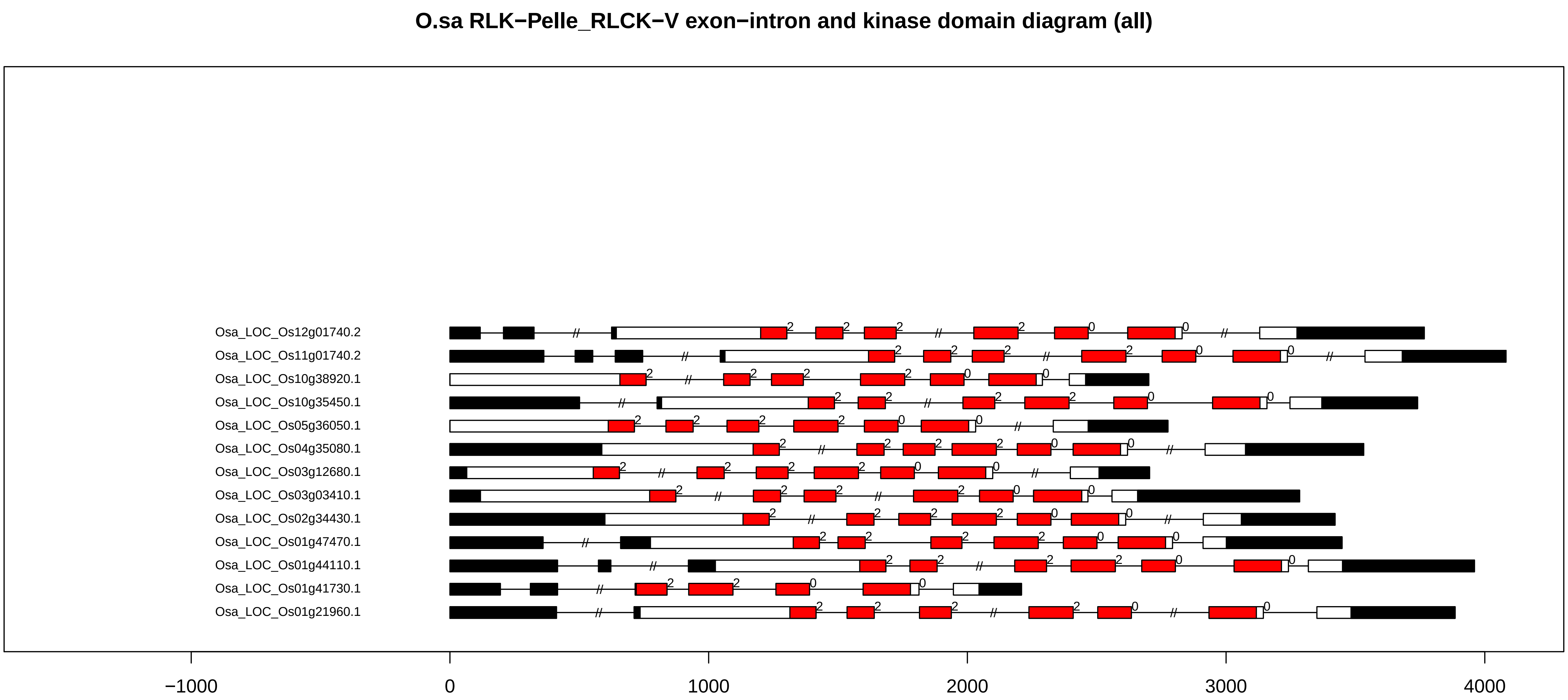

O.sa RLK−Pelle_RLCK−V exon−intron and kinase domain diagram (all)
Osa_LOC_Os12g01740.2
Osa_LOC_Os11g01740.2
Osa_LOC_Os10g38920.1
Osa_LOC_Os10g35450.1
Osa_LOC_Os05g36050.1
Osa_LOC_Os04g35080.1
Osa_LOC_Os03g12680.1
Osa_LOC_Os03g03410.1
Osa_LOC_Os02g34430.1
Osa_LOC_Os01g47470.1
Osa_LOC_Os01g44110.1
Osa_LOC_Os01g41730.1
Osa_LOC_Os01g21960.1
−1000
0
1000
2000
3000
4000

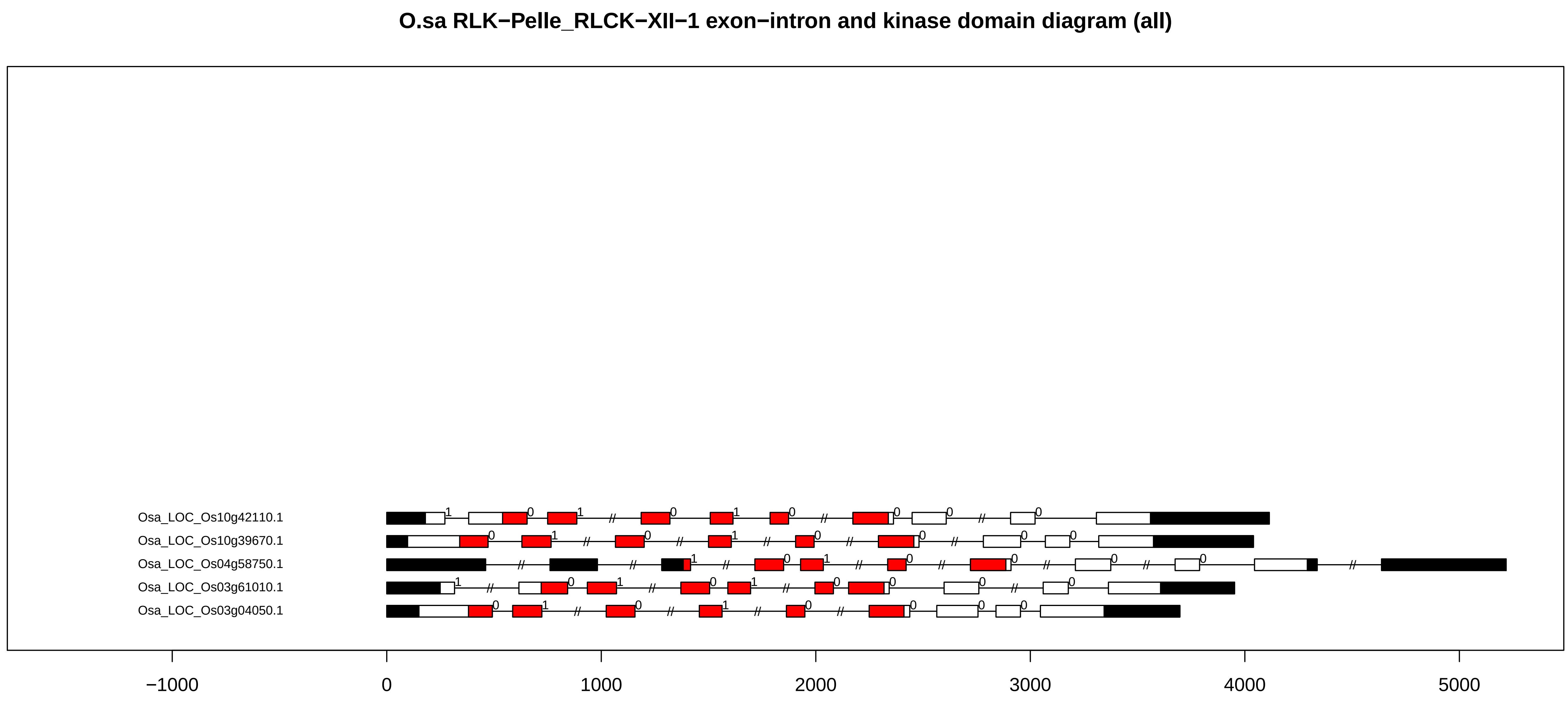
O.sa RLK−Pelle_RLCK−XII−1 exon−intron and kinase domain diagram (all)
Osa_LOC_Os10g42110.1
Osa_LOC_Os10g39670.1
Osa_LOC_Os04g58750.1
Osa_LOC_Os03g61010.1
Osa_LOC_Os03g04050.1
−1000
0
1000
2000
3000
4000
5000

O.sa RLK−Pelle_RLCK−XIII exon−intron and kinase domain diagram (all)

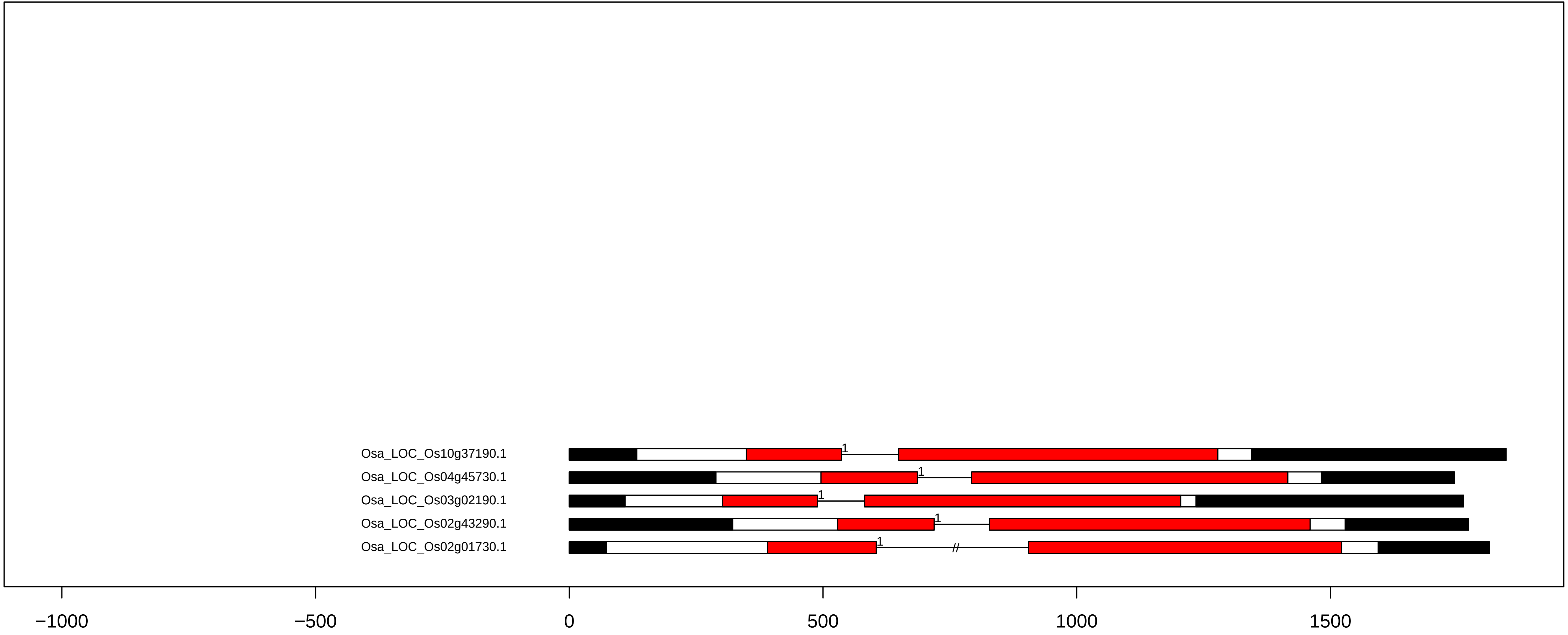

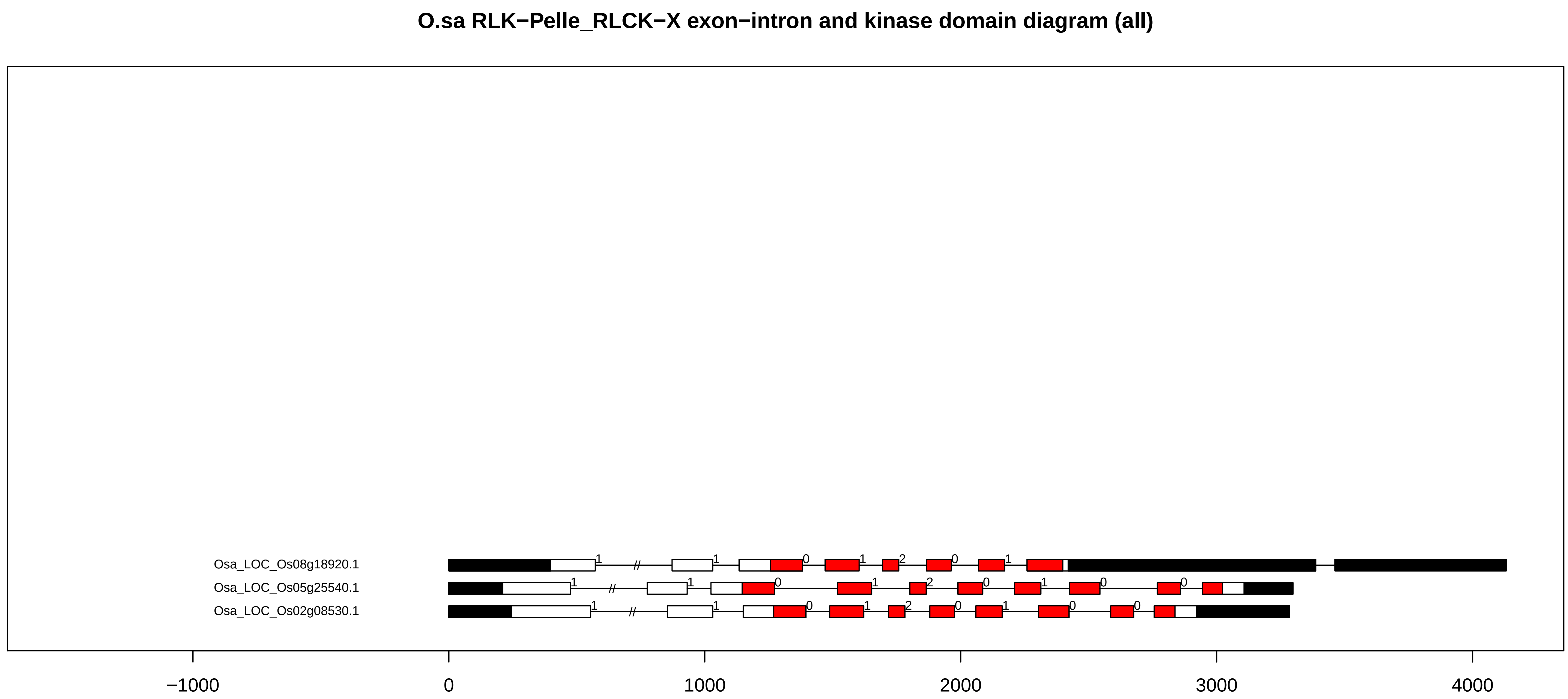
O.sa RLK−Pelle_RLCK−X exon−intron and kinase domain diagram (all)
Osa_LOC_Os08g18920.1
Osa_LOC_Os05g25540.1
Osa_LOC_Os02g08530.1
−1000
0
1000
2000
3000
4000

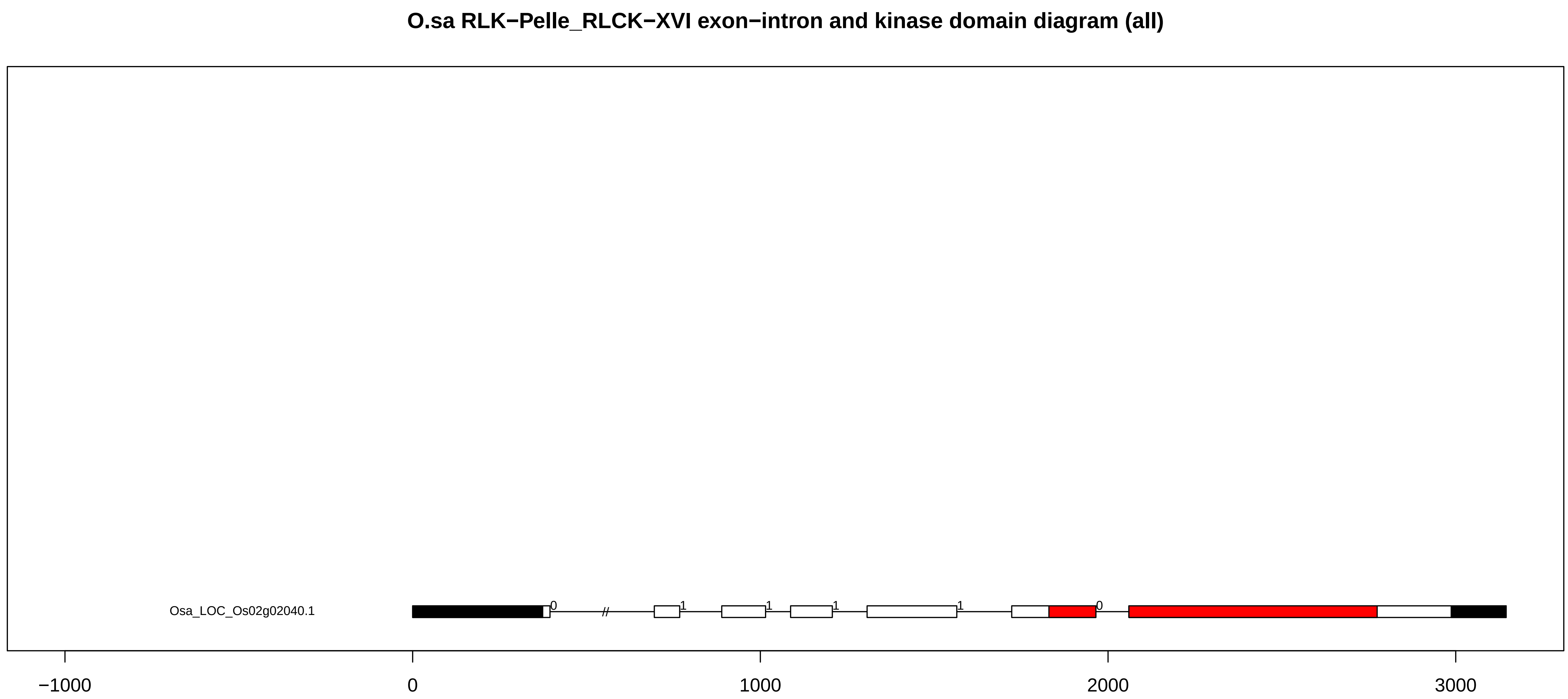

O.sa RLK−Pelle_RLCK−XVI exon−intron and kinase domain diagram (all)
Osa_LOC_Os02g02040.1
0
1
1
1
1
0
−1000
0
1000
2000
3000

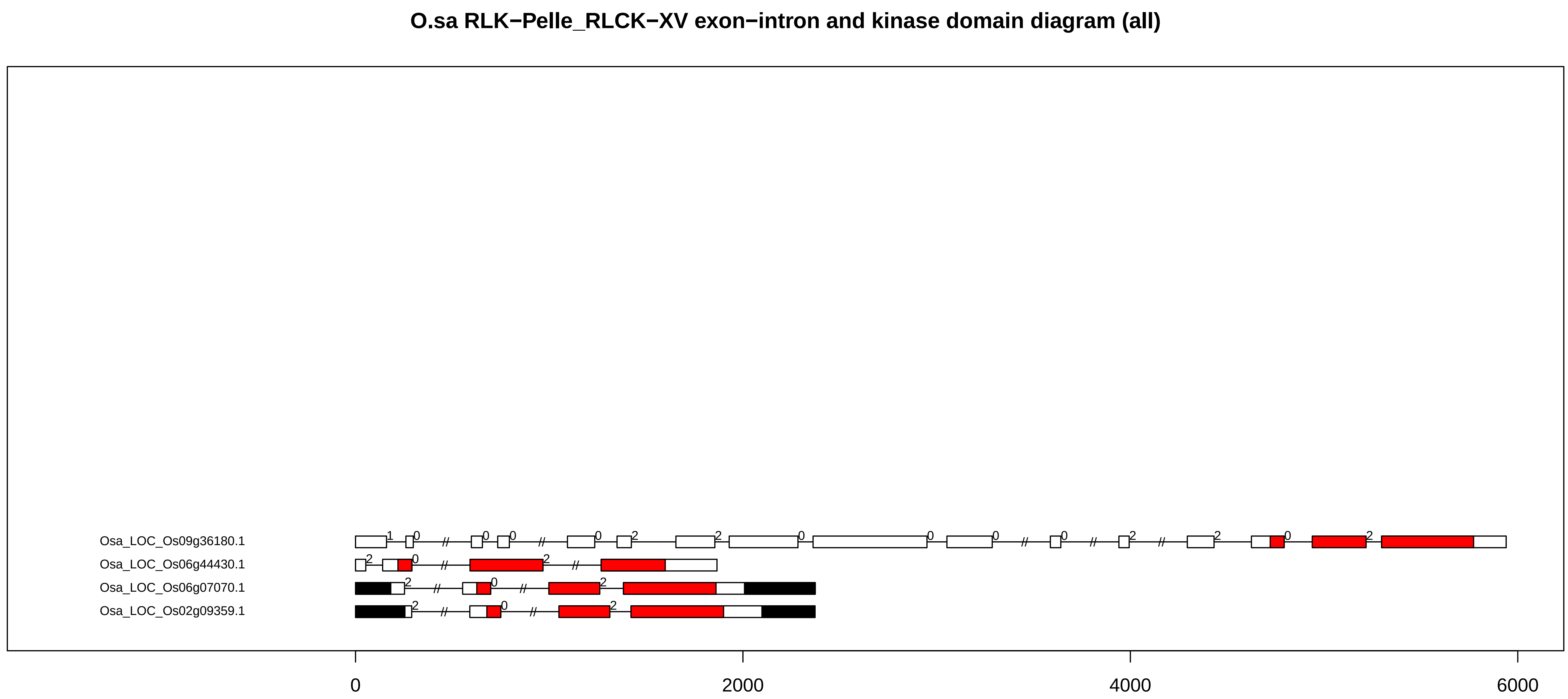

O.sa RLK−Pelle_RLCK−XV exon−intron and kinase domain diagram (all)
Osa_LOC_Os09g36180.1
Osa_LOC_Os06g44430.1
Osa_LOC_Os06g07070.1
Osa_LOC_Os02g09359.1
0
2000
4000
6000

O.sa RLK−Pelle_SD−2b exon−intron and kinase domain diagram (part 1)

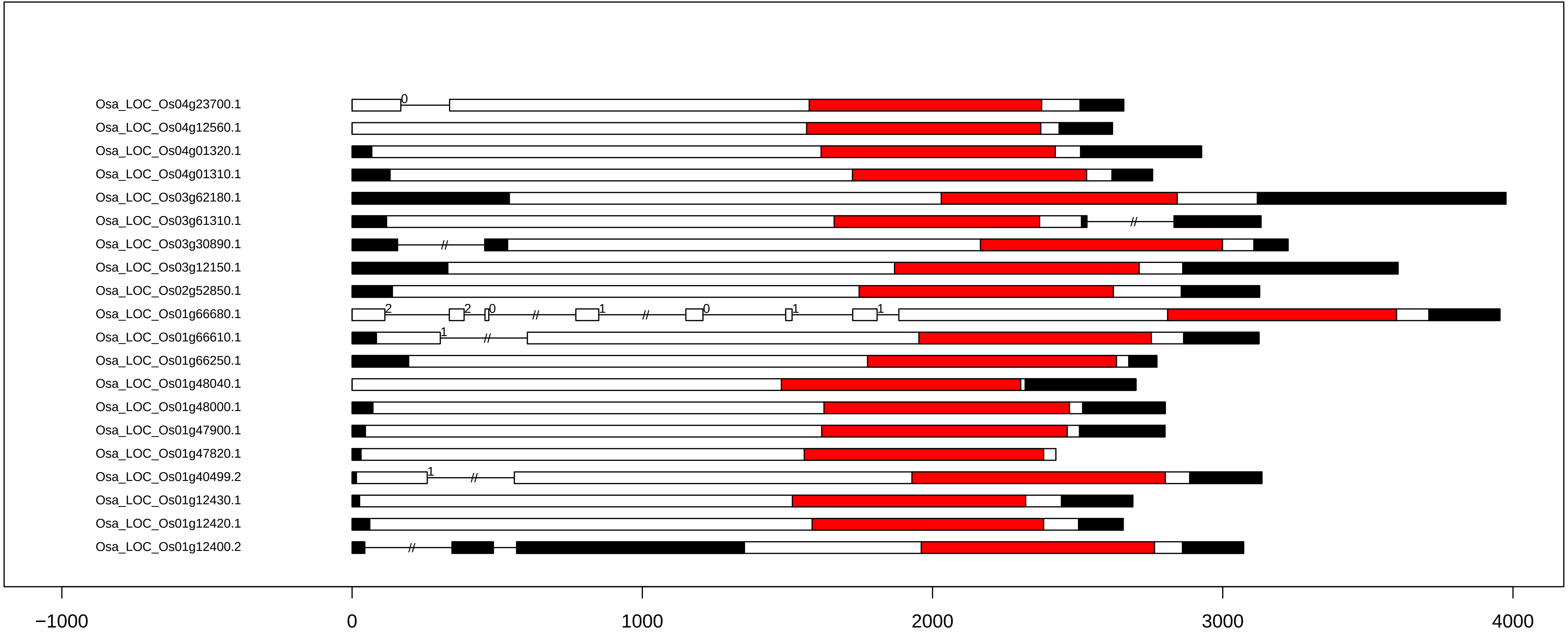

O.sa RLK−Pelle_SD−2b exon−intron and kinase domain diagram (part 2)

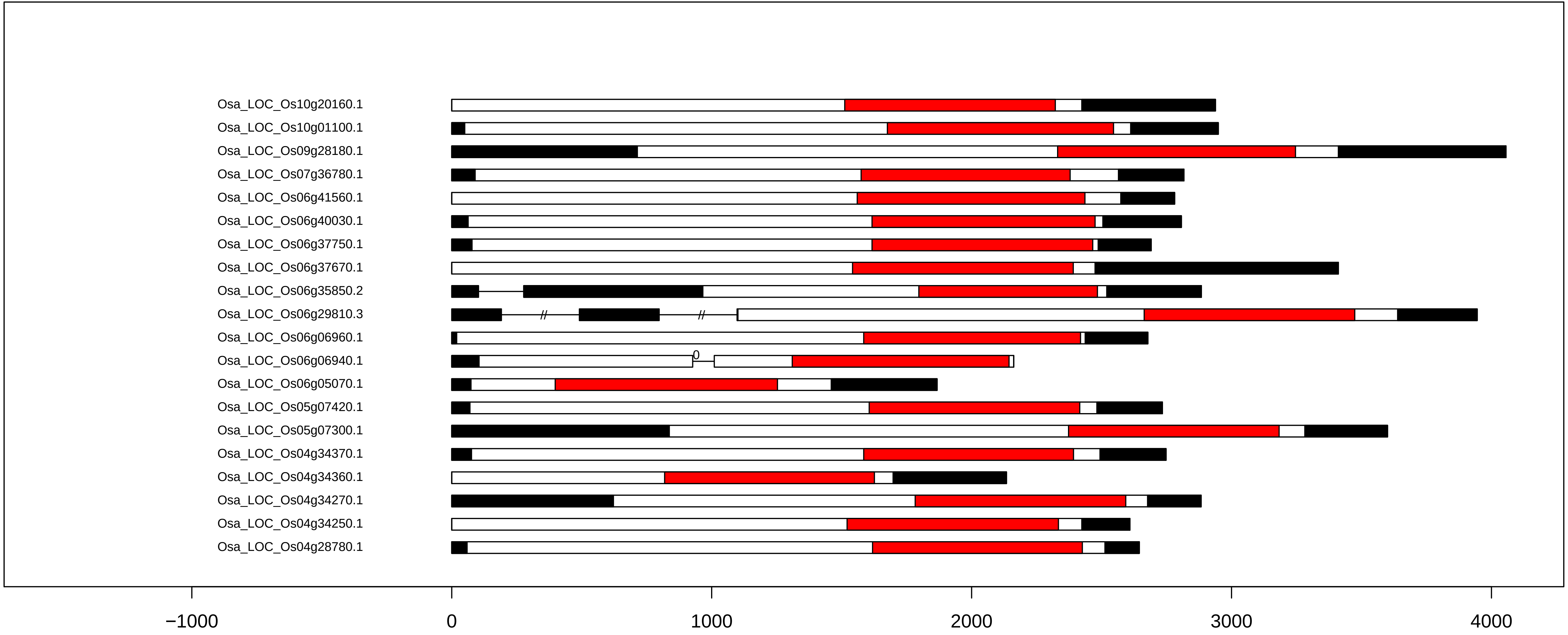

O.sa RLK−Pelle_SD−2b exon−intron and kinase domain diagram (part 3)

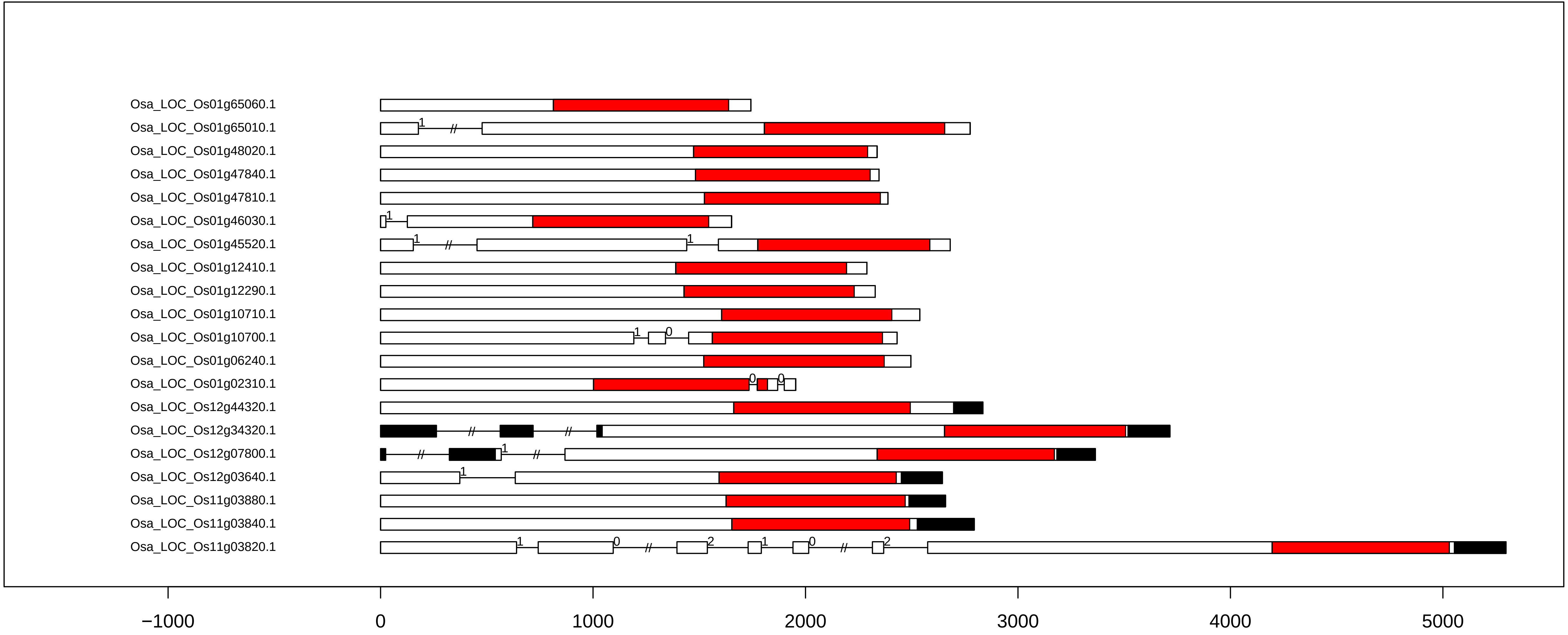

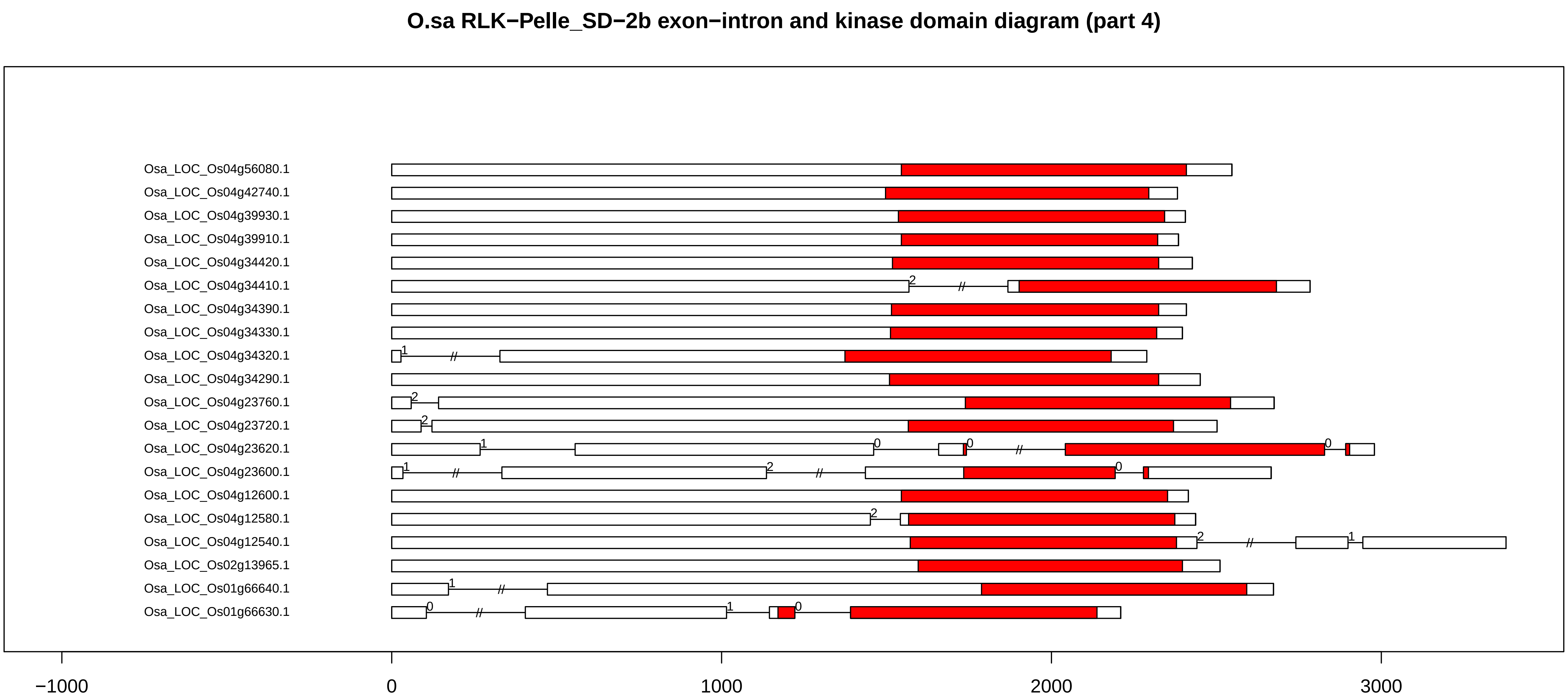

O.sa RLK−Pelle_SD−2b exon−intron and kinase domain diagram (part 4)
Osa_LOC_Os04g56080.1
Osa_LOC_Os04g42740.1
Osa_LOC_Os04g39930.1
Osa_LOC_Os04g39910.1
Osa_LOC_Os04g34420.1
Osa_LOC_Os04g34410.1
Osa_LOC_Os04g34390.1
Osa_LOC_Os04g34330.1
Osa_LOC_Os04g34320.1
Osa_LOC_Os04g34290.1
Osa_LOC_Os04g23760.1
Osa_LOC_Os04g23720.1
Osa_LOC_Os04g23620.1
Osa_LOC_Os04g23600.1
Osa_LOC_Os04g12600.1
Osa_LOC_Os04g12580.1
Osa_LOC_Os04g12540.1
Osa_LOC_Os02g13965.1
Osa_LOC_Os01g66640.1
Osa_LOC_Os01g66630.1
−1000
0
1000
2000
3000

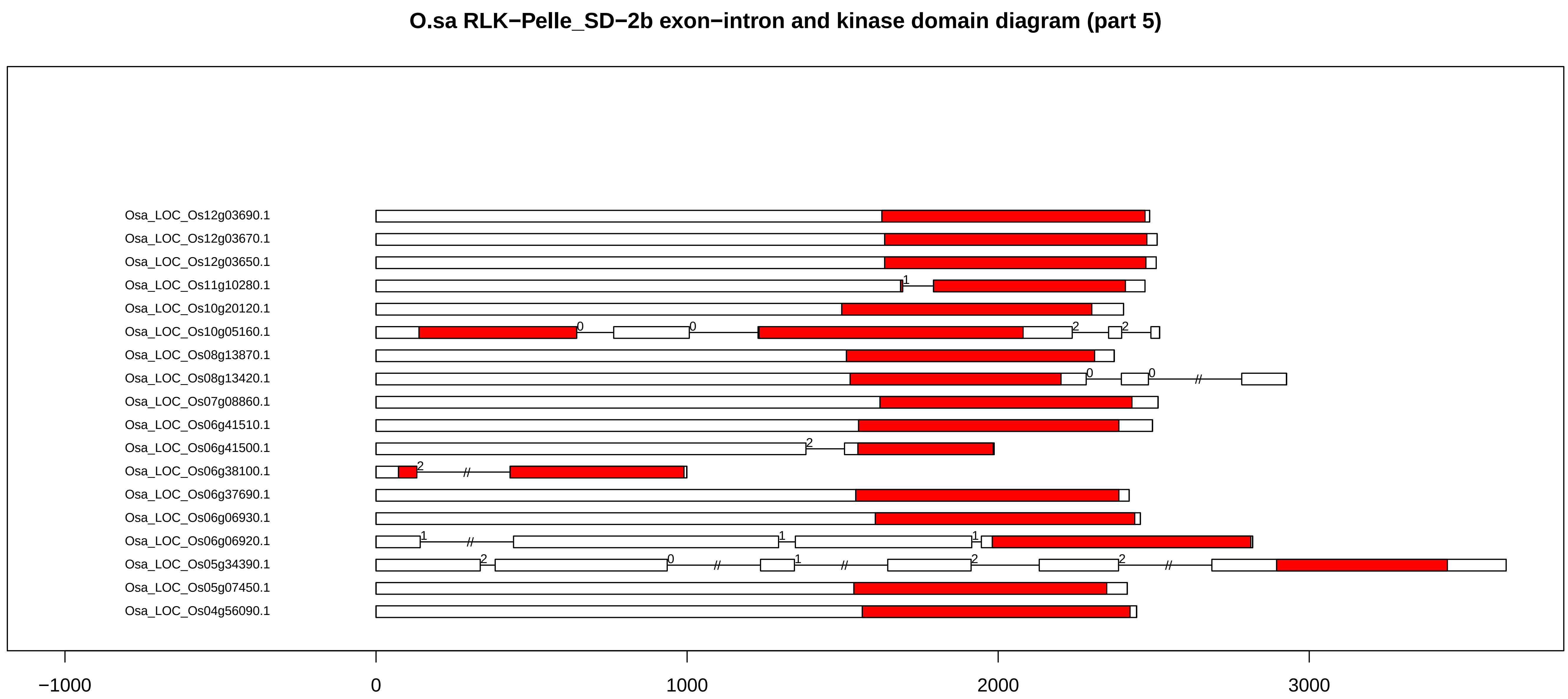
O.sa RLK−Pelle_SD−2b exon−intron and kinase domain diagram (part 5)
Osa_LOC_Os12g03690.1
Osa_LOC_Os12g03670.1
Osa_LOC_Os12g03650.1
Osa_LOC_Os11g10280.1
Osa_LOC_Os10g20120.1
Osa_LOC_Os10g05160.1
Osa_LOC_Os08g13870.1
Osa_LOC_Os08g13420.1
Osa_LOC_Os07g08860.1
Osa_LOC_Os06g41510.1
Osa_LOC_Os06g41500.1
Osa_LOC_Os06g38100.1
Osa_LOC_Os06g37690.1
Osa_LOC_Os06g06930.1
Osa_LOC_Os06g06920.1
Osa_LOC_Os05g34390.1
Osa_LOC_Os05g07450.1
Osa_LOC_Os04g56090.1
−1000
0
1000
2000
3000

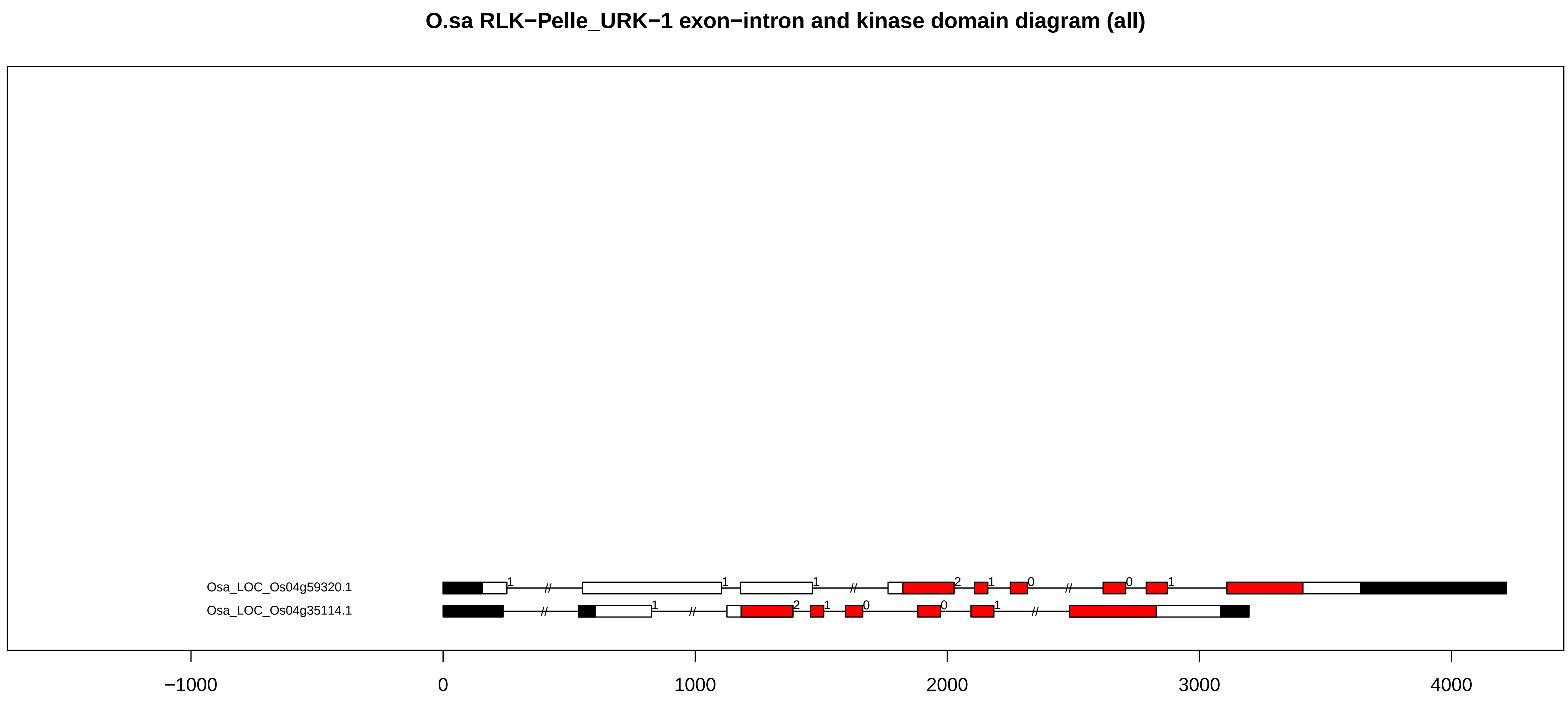
O.sa RLK−Pelle_URK−1 exon−intron and kinase domain diagram (all)
Osa_LOC_Os04g59320.1
Osa_LOC_Os04g35114.1
−1000
0
1000
2000
3000
4000

O.sa RLK−Pelle_URK−2 exon−intron and kinase domain diagram (all)

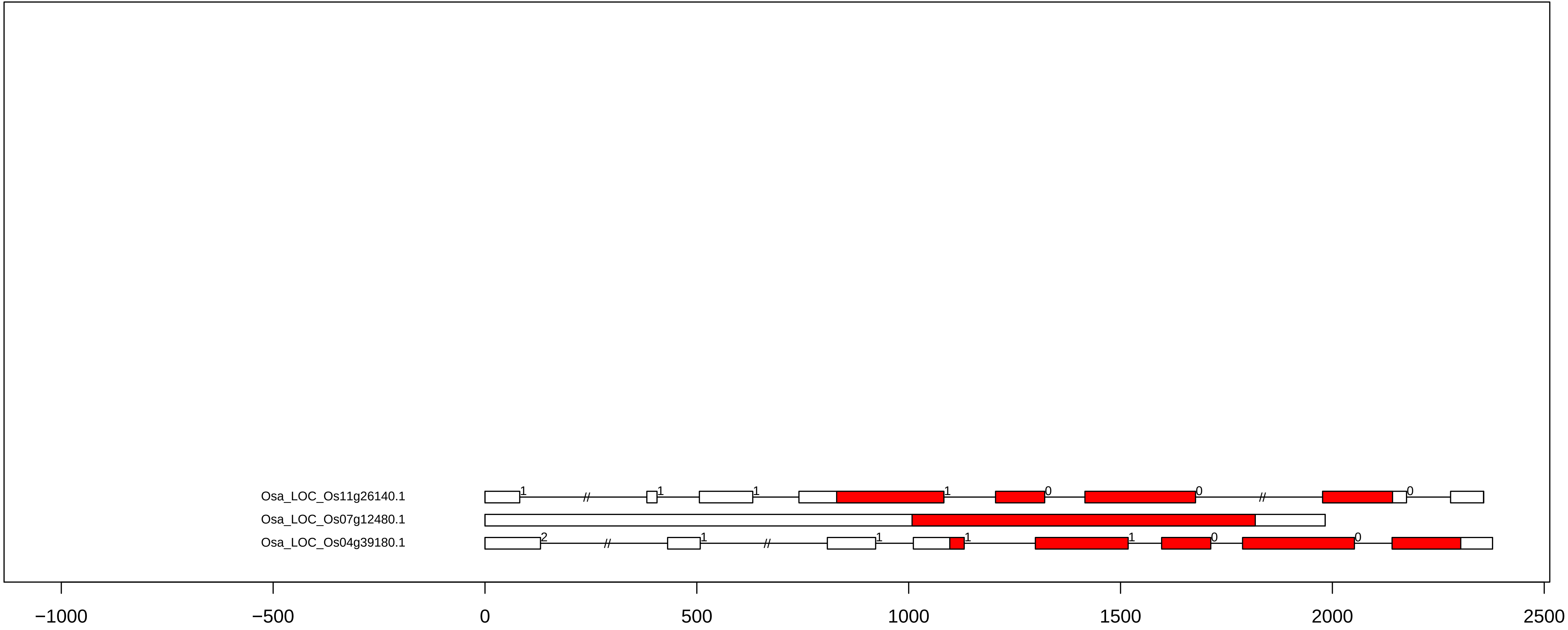

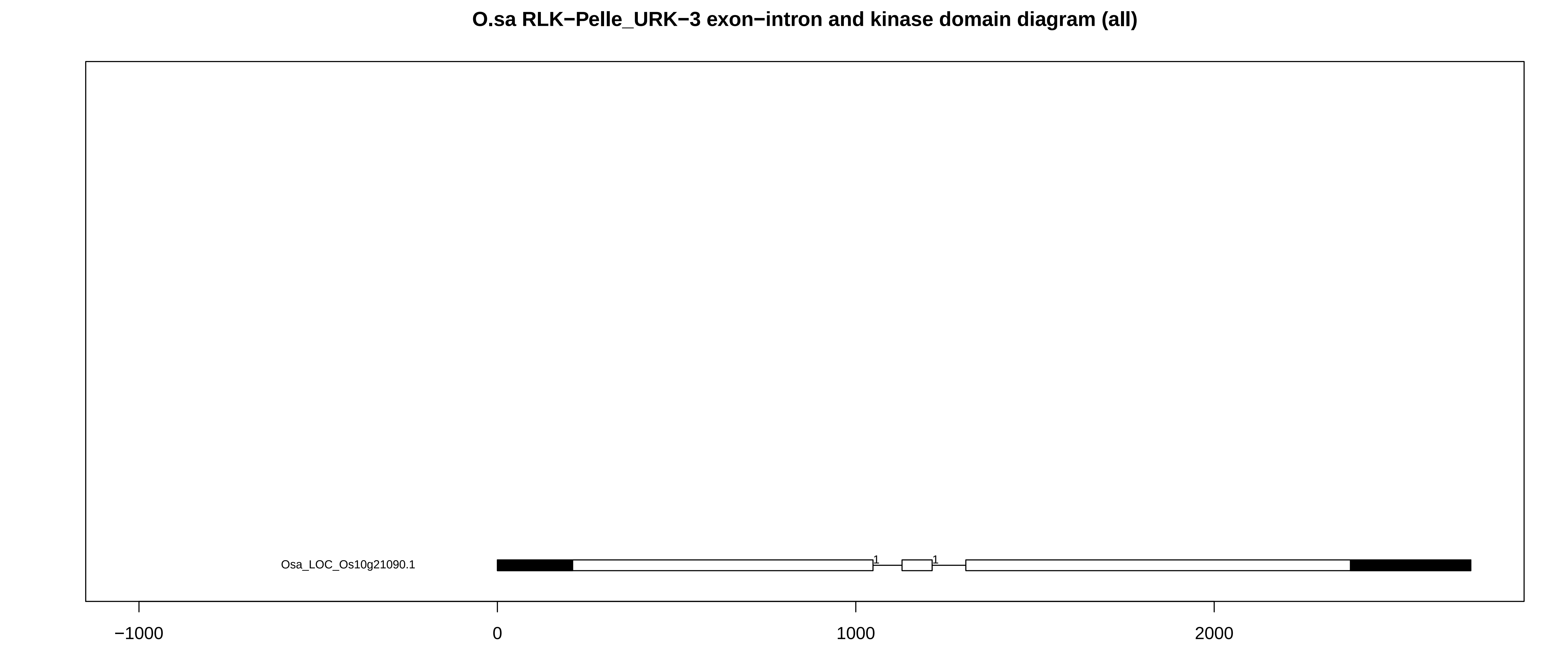
O.sa RLK−Pelle_URK−3 exon−intron and kinase domain diagram (all)
Osa_LOC_Os10g21090.1
1
1
−1000
0
1000
2000

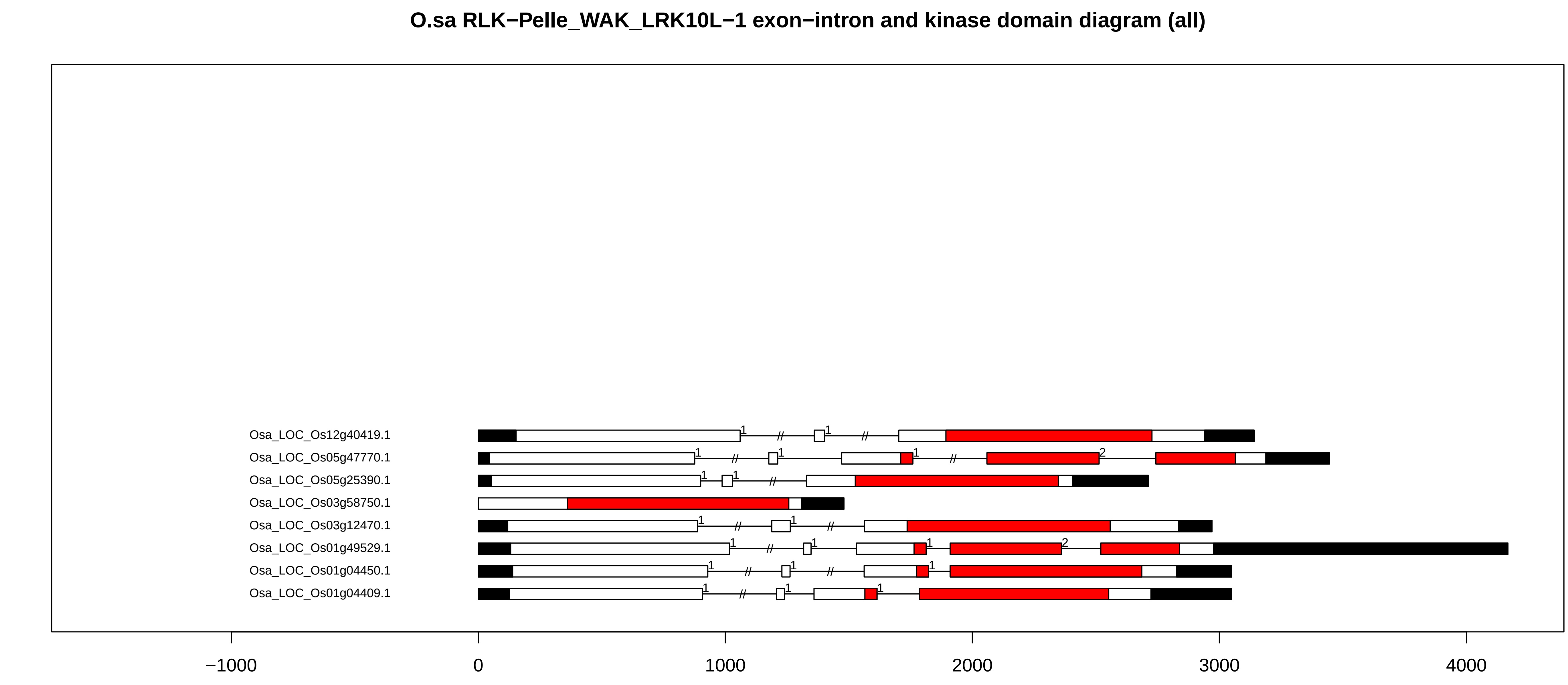
O.sa RLK−Pelle_WAK_LRK10L−1 exon−intron and kinase domain diagram (all)
Osa_LOC_Os12g40419.1
Osa_LOC_Os05g47770.1
Osa_LOC_Os05g25390.1
Osa_LOC_Os03g58750.1
Osa_LOC_Os03g12470.1
Osa_LOC_Os01g49529.1
Osa_LOC_Os01g04450.1
Osa_LOC_Os01g04409.1
−1000
0
1000
2000
3000
4000

O.sa RLK−Pelle_WAK exon−intron and kinase domain diagram (part 1)

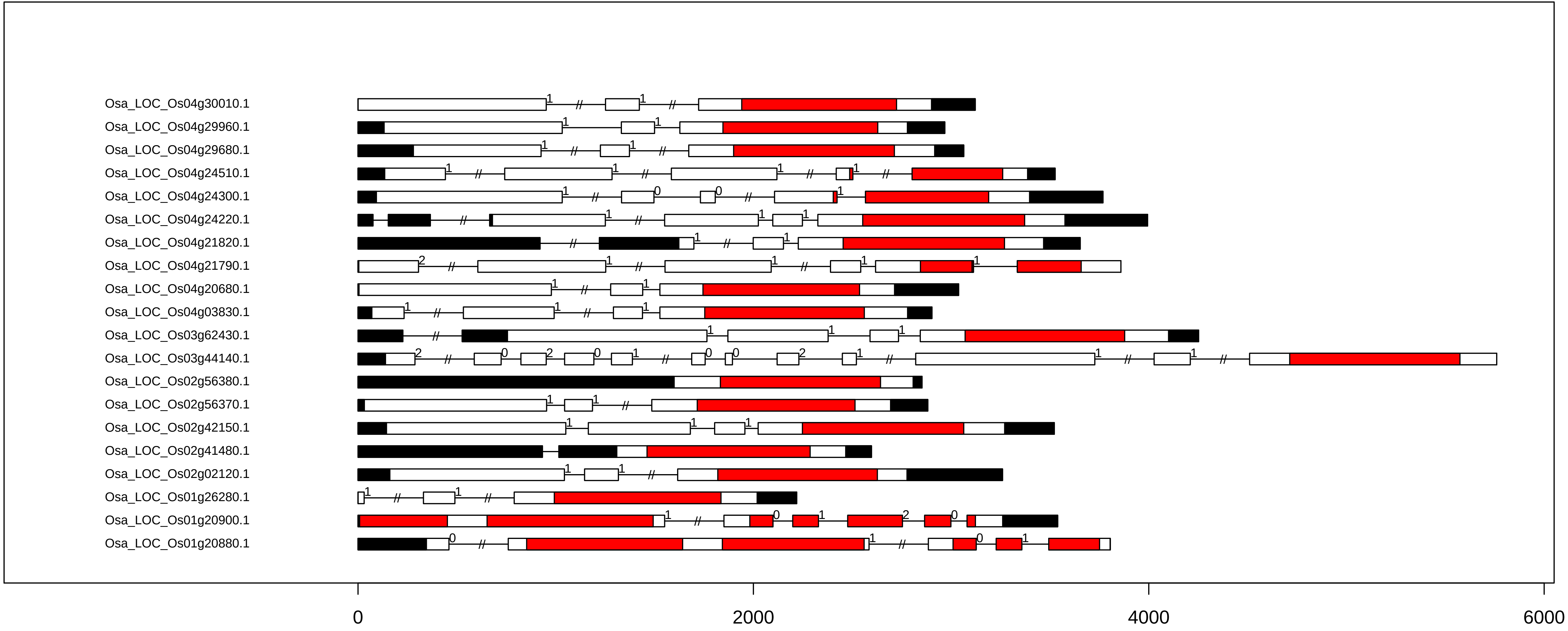

O.sa RLK−Pelle_WAK exon−intron and kinase domain diagram (part 2)

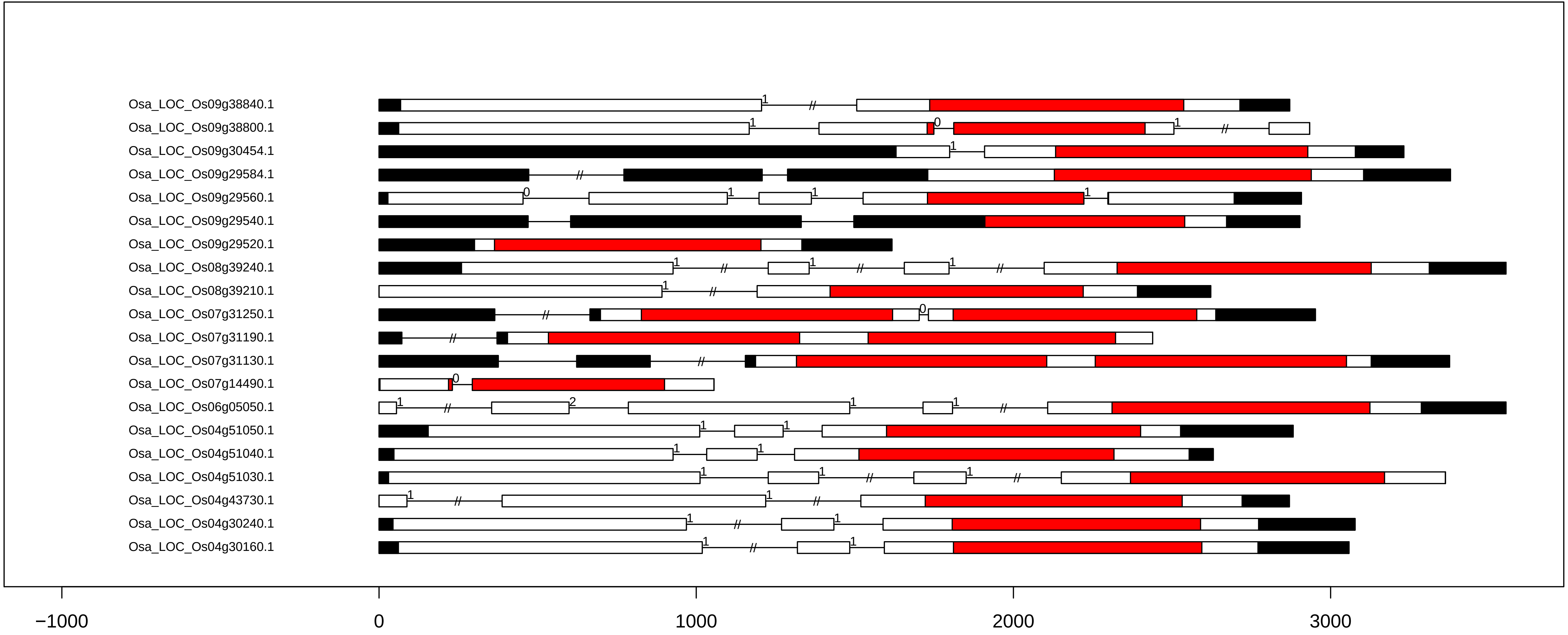

O.sa RLK−Pelle_WAK exon−intron and kinase domain diagram (part 3)

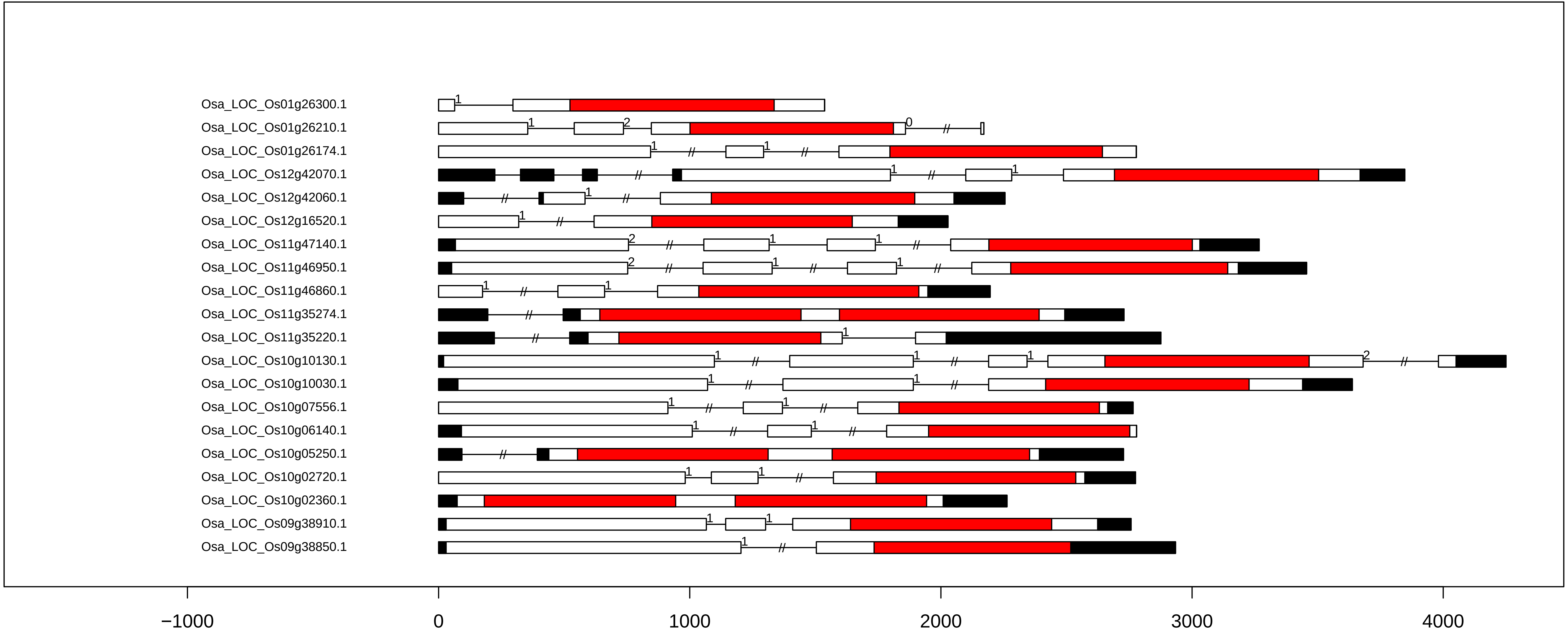

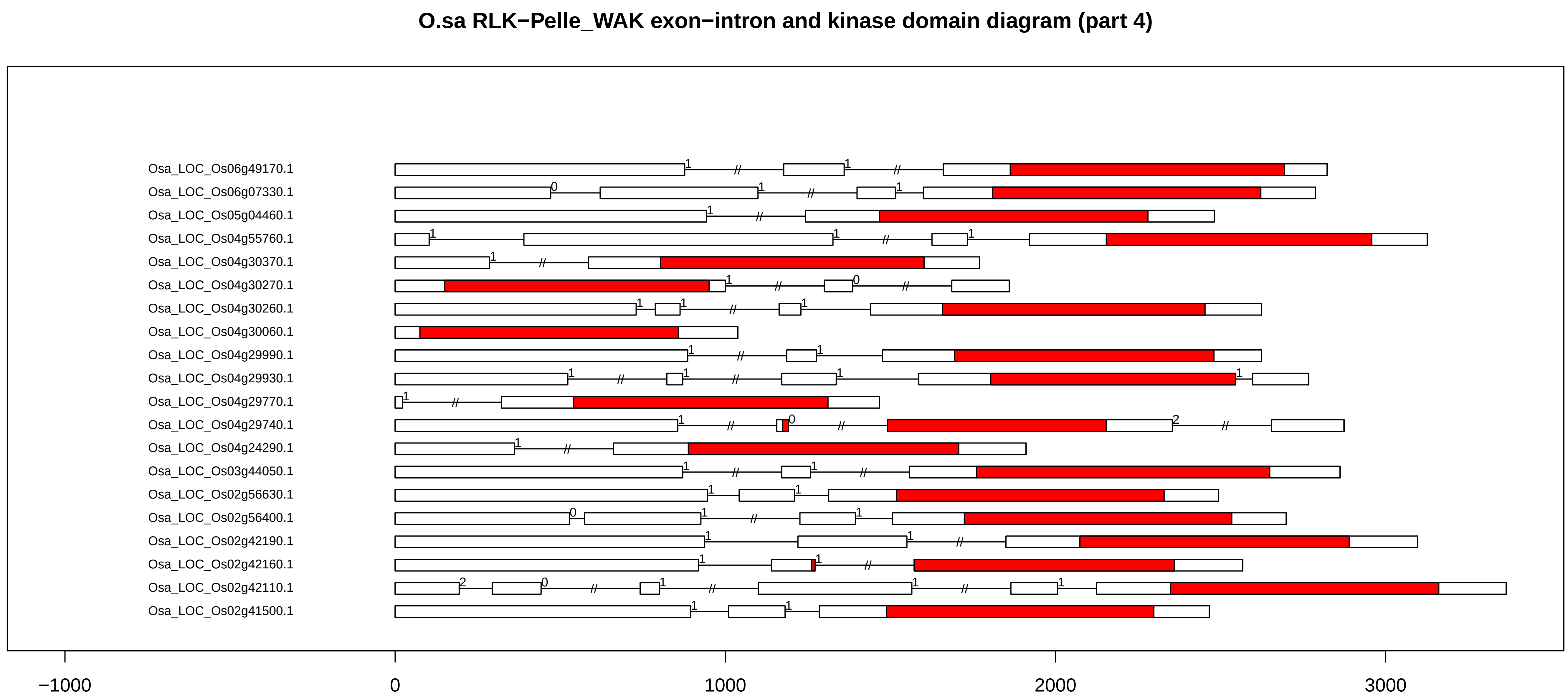
O.sa RLK−Pelle_WAK exon−intron and kinase domain diagram (part 4)
Osa_LOC_Os06g49170.1
Osa_LOC_Os06g07330.1
Osa_LOC_Os05g04460.1
Osa_LOC_Os04g55760.1
Osa_LOC_Os04g30370.1
Osa_LOC_Os04g30270.1
Osa_LOC_Os04g30260.1
Osa_LOC_Os04g30060.1
Osa_LOC_Os04g29990.1
Osa_LOC_Os04g29930.1
Osa_LOC_Os04g29770.1
Osa_LOC_Os04g29740.1
Osa_LOC_Os04g24290.1
Osa_LOC_Os03g44050.1
Osa_LOC_Os02g56630.1
Osa_LOC_Os02g56400.1
Osa_LOC_Os02g42190.1
Osa_LOC_Os02g42160.1
Osa_LOC_Os02g42110.1
Osa_LOC_Os02g41500.1
−1000
0
1000
2000
3000

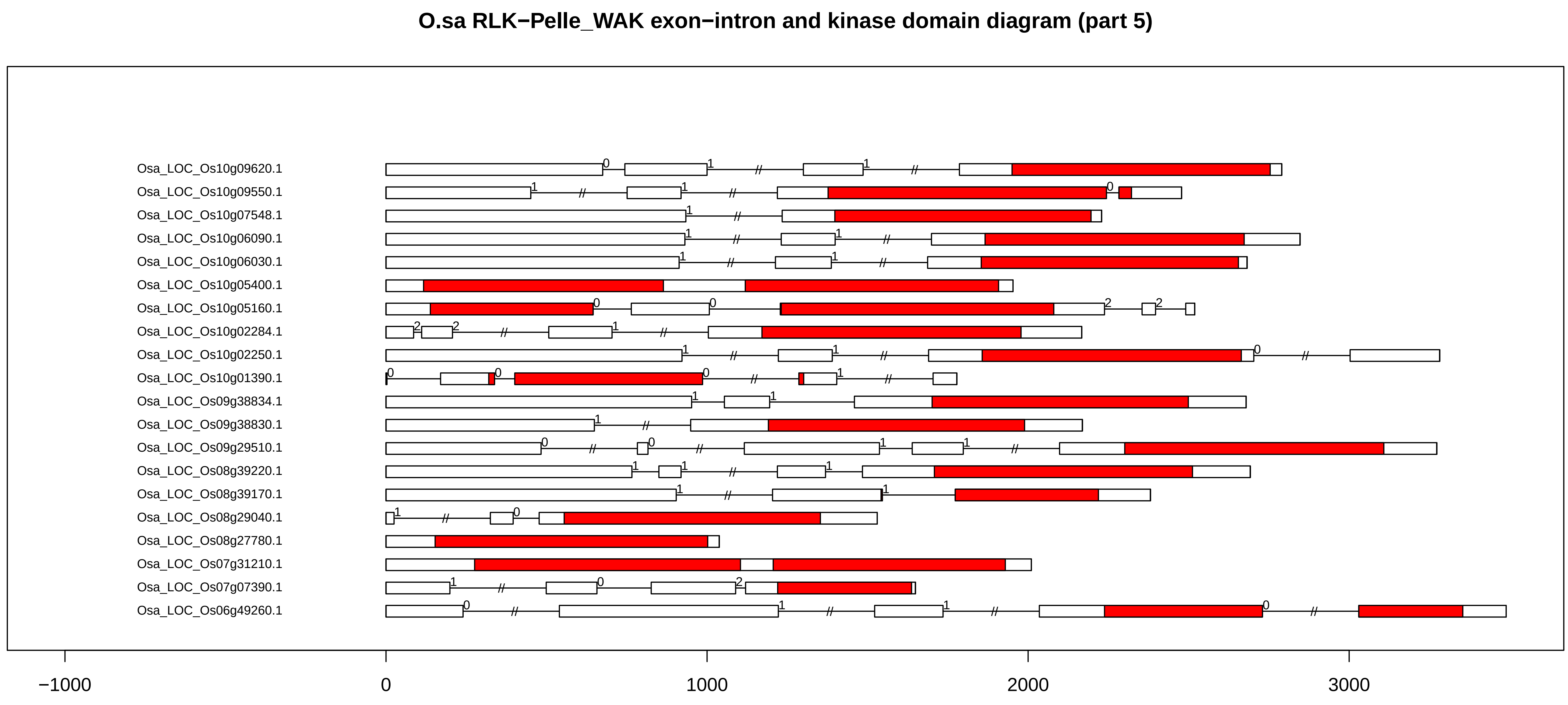

O.sa RLK−Pelle_WAK exon−intron and kinase domain diagram (part 5)
Osa_LOC_Os10g09620.1
Osa_LOC_Os10g09550.1
Osa_LOC_Os10g07548.1
Osa_LOC_Os10g06090.1
Osa_LOC_Os10g06030.1
Osa_LOC_Os10g05400.1
Osa_LOC_Os10g05160.1
Osa_LOC_Os10g02284.1
Osa_LOC_Os10g02250.1
Osa_LOC_Os10g01390.1
Osa_LOC_Os09g38834.1
Osa_LOC_Os09g38830.1
Osa_LOC_Os09g29510.1
Osa_LOC_Os08g39220.1
Osa_LOC_Os08g39170.1
Osa_LOC_Os08g29040.1
Osa_LOC_Os08g27780.1
Osa_LOC_Os07g31210.1
Osa_LOC_Os07g07390.1
Osa_LOC_Os06g49260.1
−1000
0
1000
2000
3000

O.sa RLK−Pelle_WAK exon−intron and kinase domain diagram (part 6)

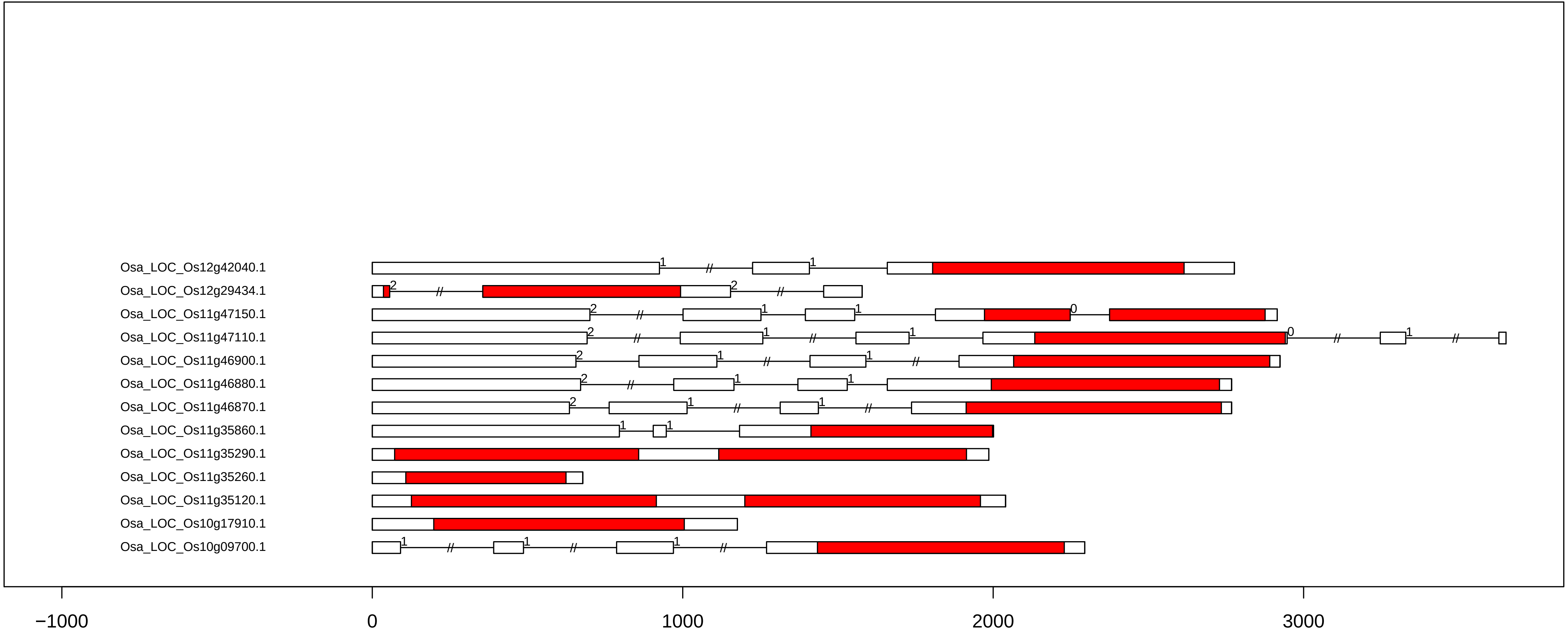

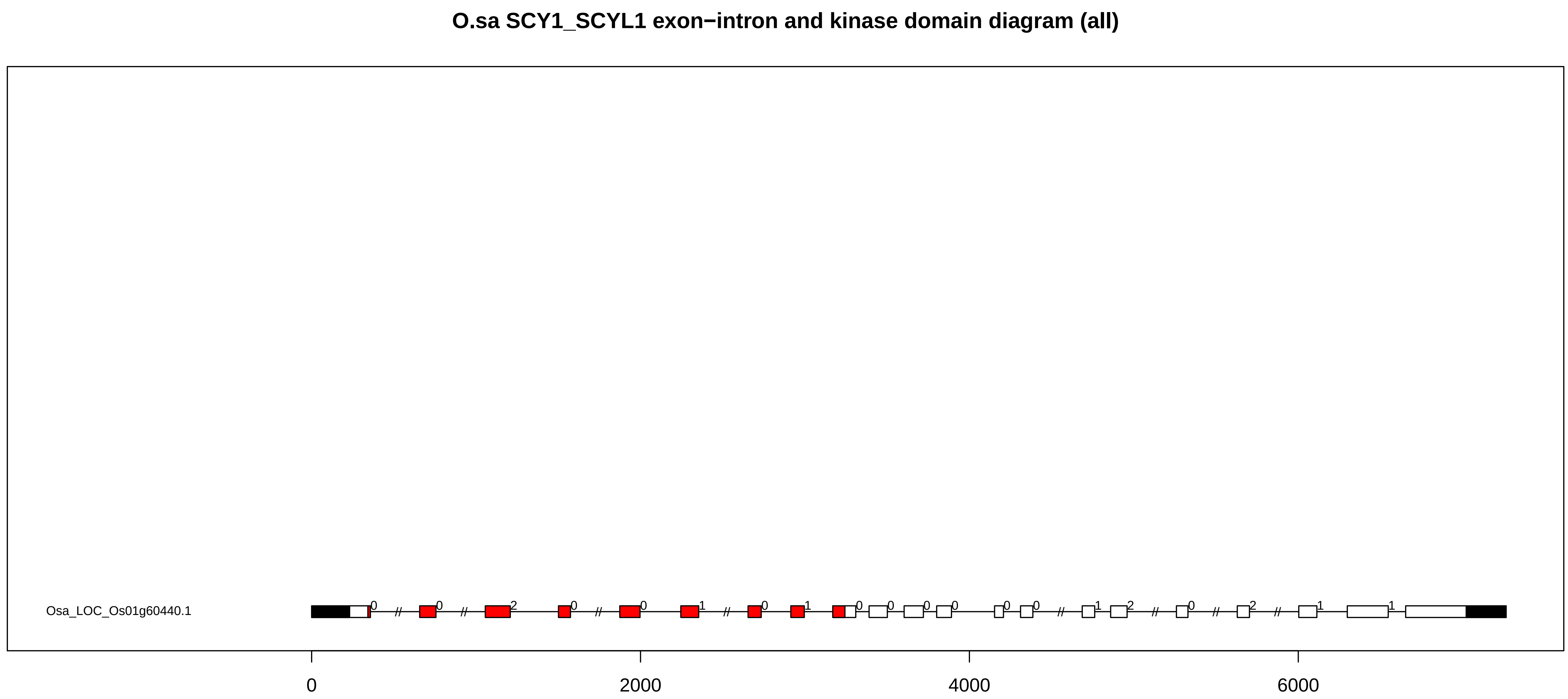
O.sa SCY1_SCYL1 exon−intron and kinase domain diagram (all)
Osa_LOC_Os01g60440.1
0
0
2
0
0
1
0
1
0
0
0
0
0
0
1
2
0
2
1
1
0
2000
4000
6000

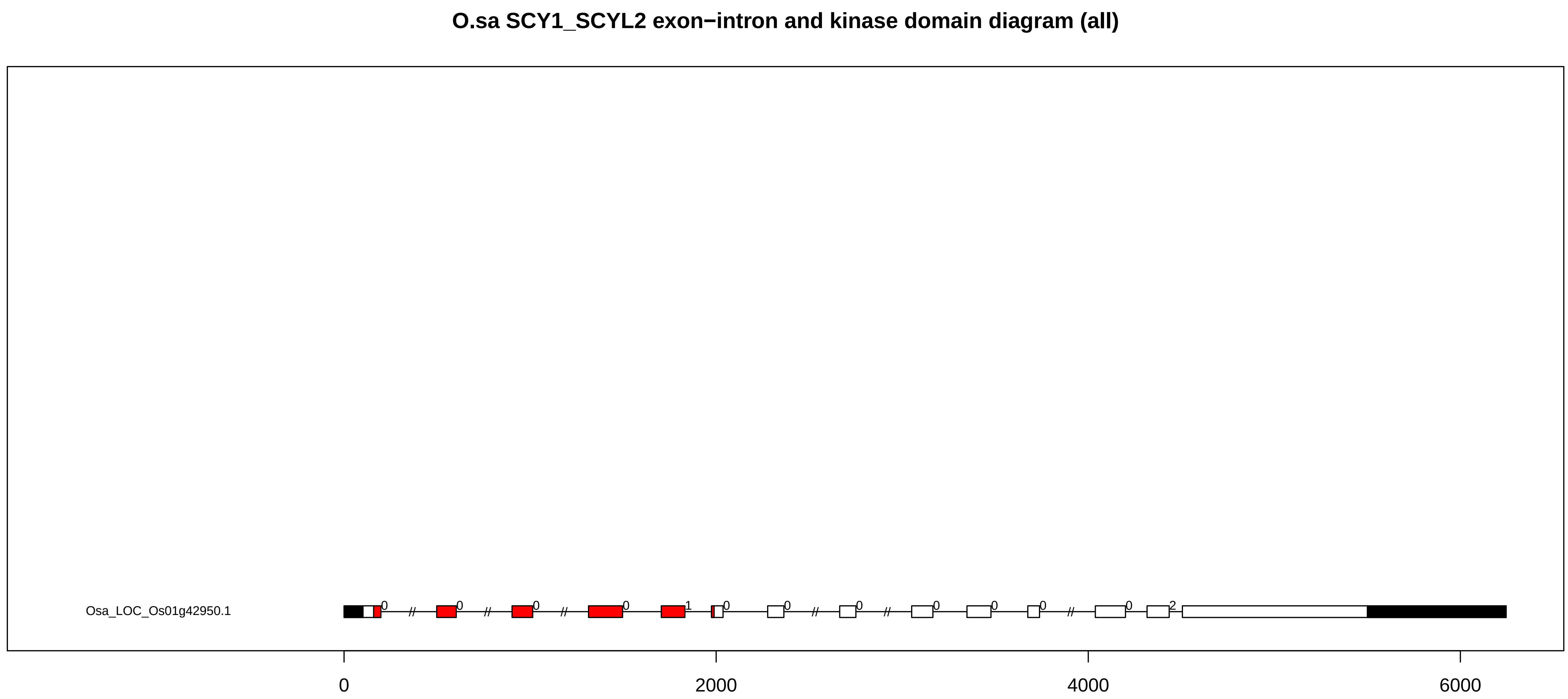
O.sa SCY1_SCYL2 exon−intron and kinase domain diagram (all)
Osa_LOC_Os01g42950.1
0
0
0
0
1
0
0
0
0
0
0
0
2
0
2000
4000
6000

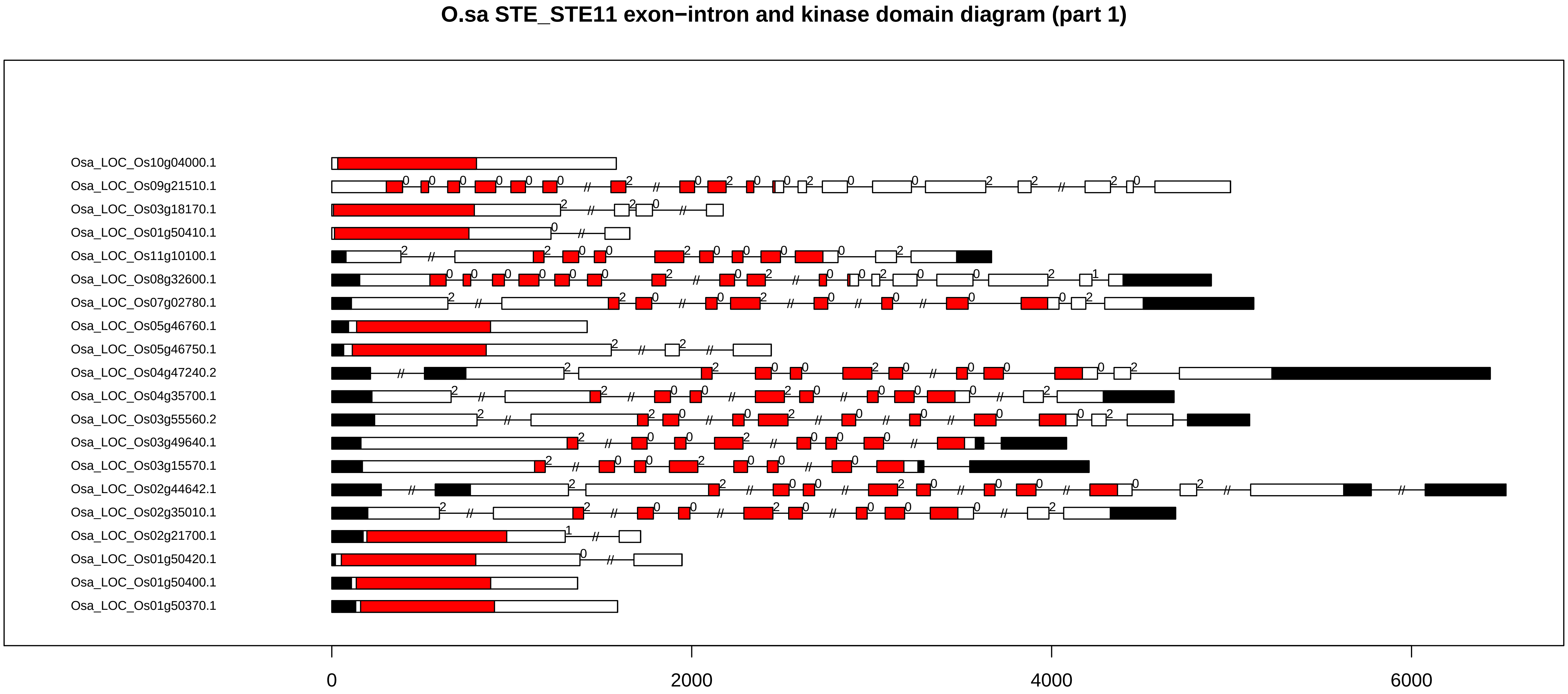
O.sa STE_STE11 exon−intron and kinase domain diagram (part 1)
Osa_LOC_Os10g04000.1
Osa_LOC_Os09g21510.1
Osa_LOC_Os03g18170.1
Osa_LOC_Os01g50410.1
Osa_LOC_Os11g10100.1
Osa_LOC_Os08g32600.1
Osa_LOC_Os07g02780.1
Osa_LOC_Os05g46760.1
Osa_LOC_Os05g46750.1
Osa_LOC_Os04g47240.2
Osa_LOC_Os04g35700.1
Osa_LOC_Os03g55560.2
Osa_LOC_Os03g49640.1
Osa_LOC_Os03g15570.1
Osa_LOC_Os02g44642.1
Osa_LOC_Os02g35010.1
Osa_LOC_Os02g21700.1
Osa_LOC_Os01g50420.1
Osa_LOC_Os01g50400.1
Osa_LOC_Os01g50370.1
0
2000
4000
6000

O.sa STE_STE11 exon−intron and kinase domain diagram (part 2)

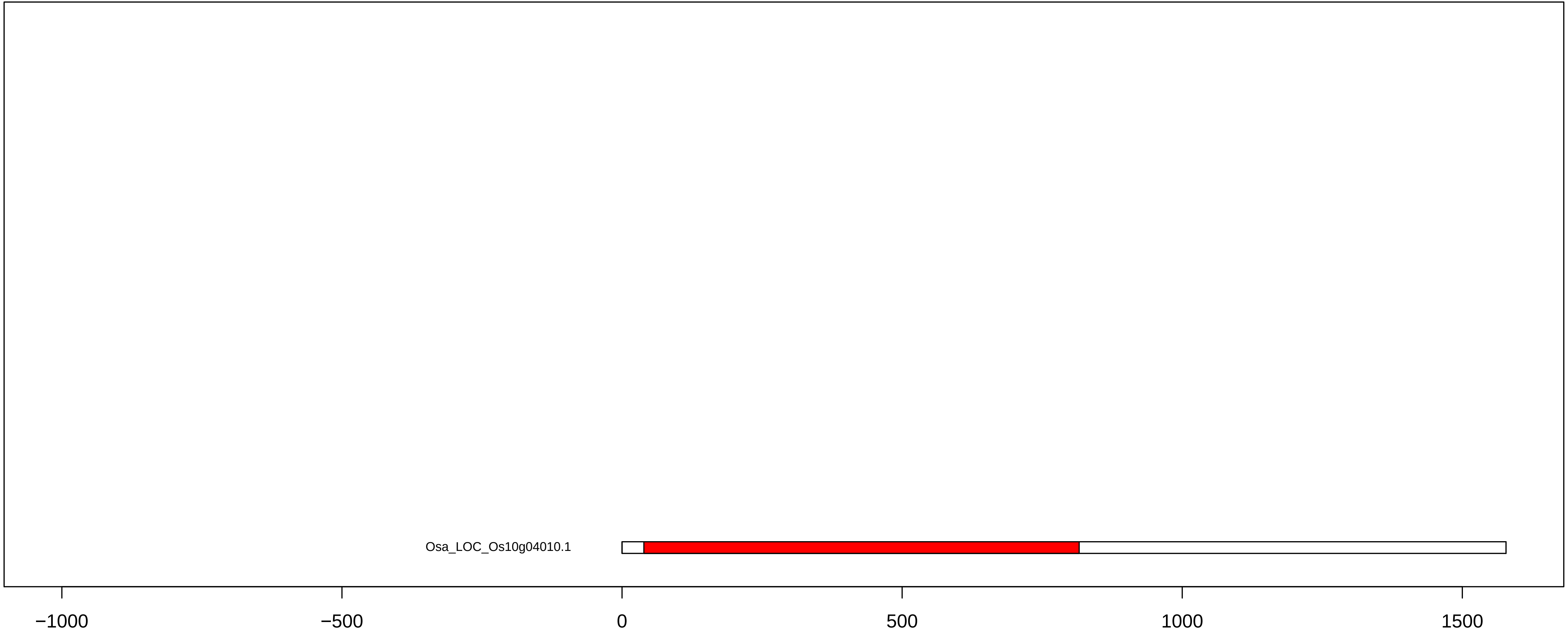

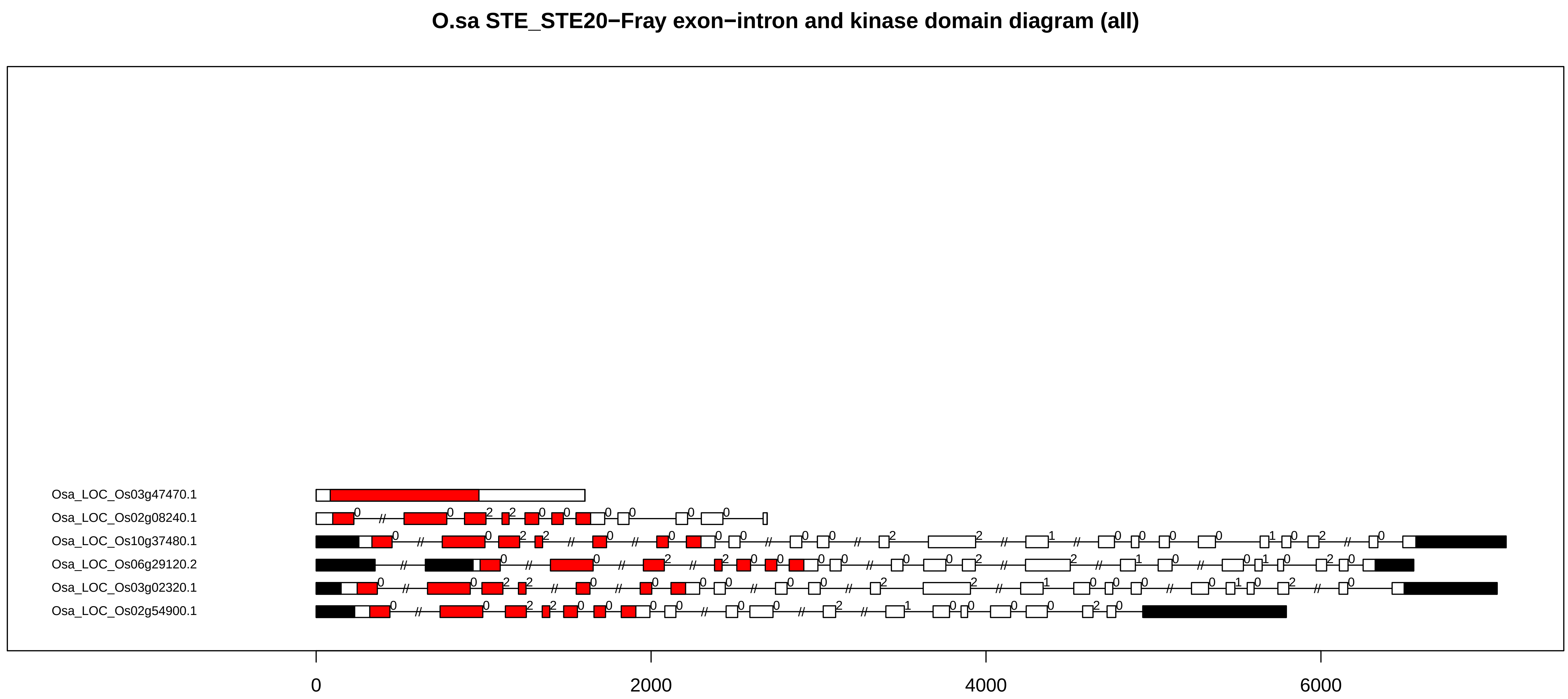
O.sa STE_STE20−Fray exon−intron and kinase domain diagram (all)
Osa_LOC_Os03g47470.1
Osa_LOC_Os02g08240.1
Osa_LOC_Os10g37480.1
Osa_LOC_Os06g29120.2
Osa_LOC_Os03g02320.1
Osa_LOC_Os02g54900.1
0
2000
4000
6000

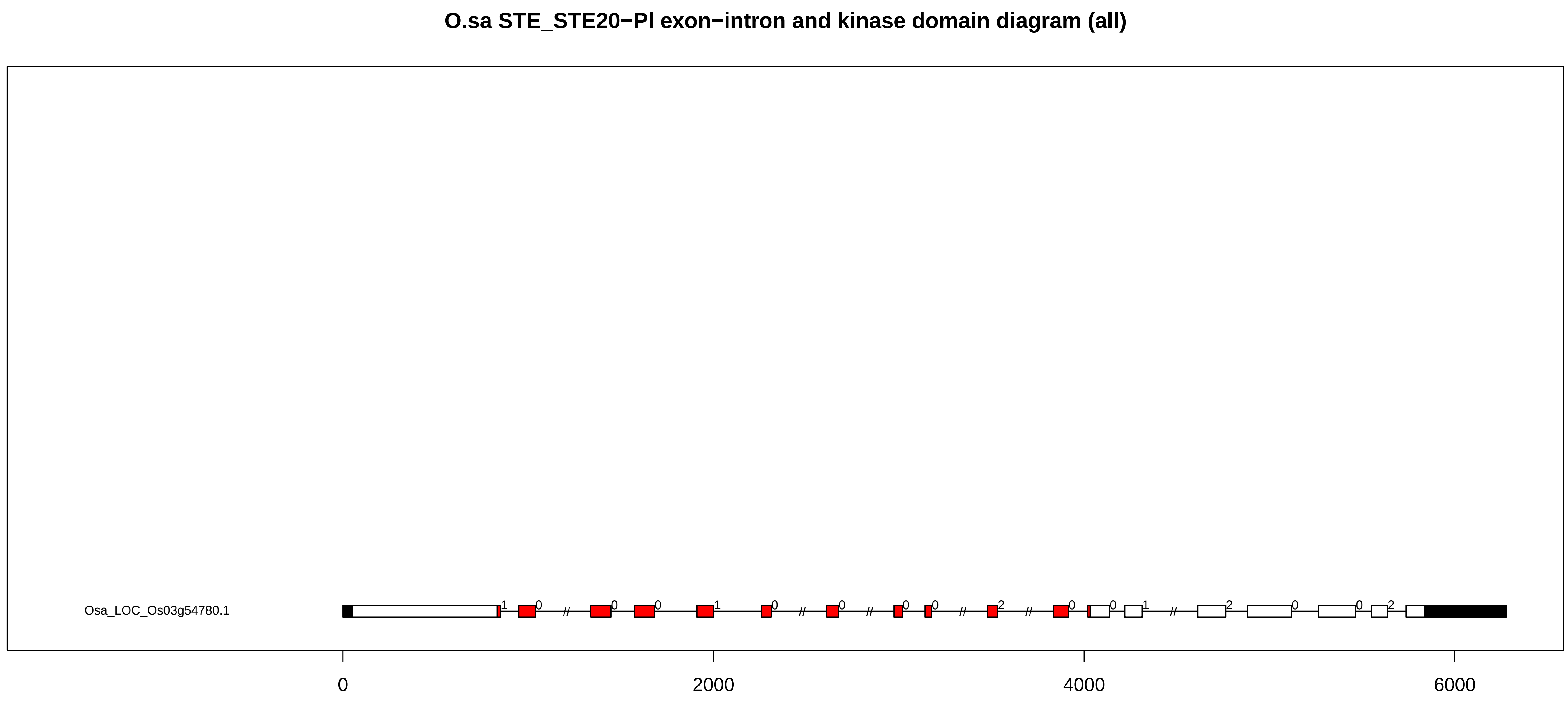
O.sa STE_STE20−PI exon−intron and kinase domain diagram (all)
Osa_LOC_Os03g54780.1
1
0
0
0
1
0
0
0
0
2
0
0
1
2
0
0
2
0
2000
4000
6000

O.sa STE_STE20−YSK exon−intron and kinase domain diagram (all)

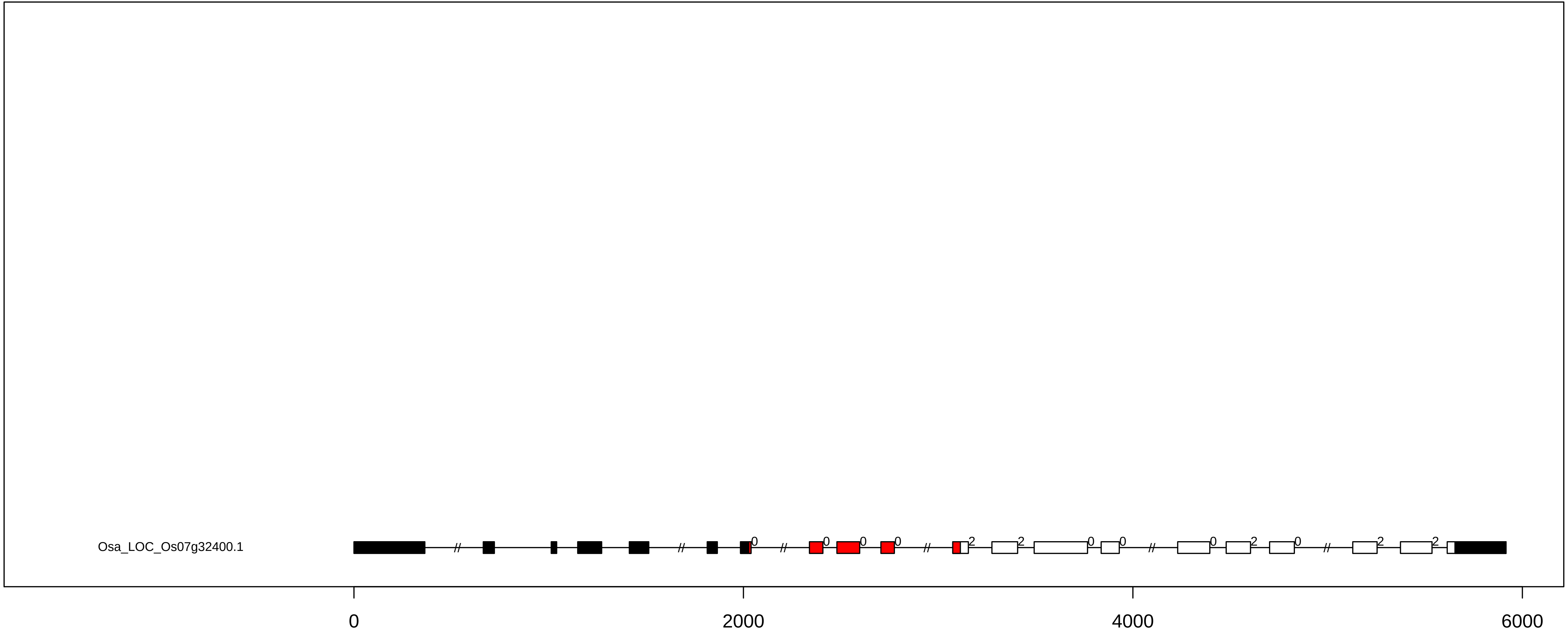

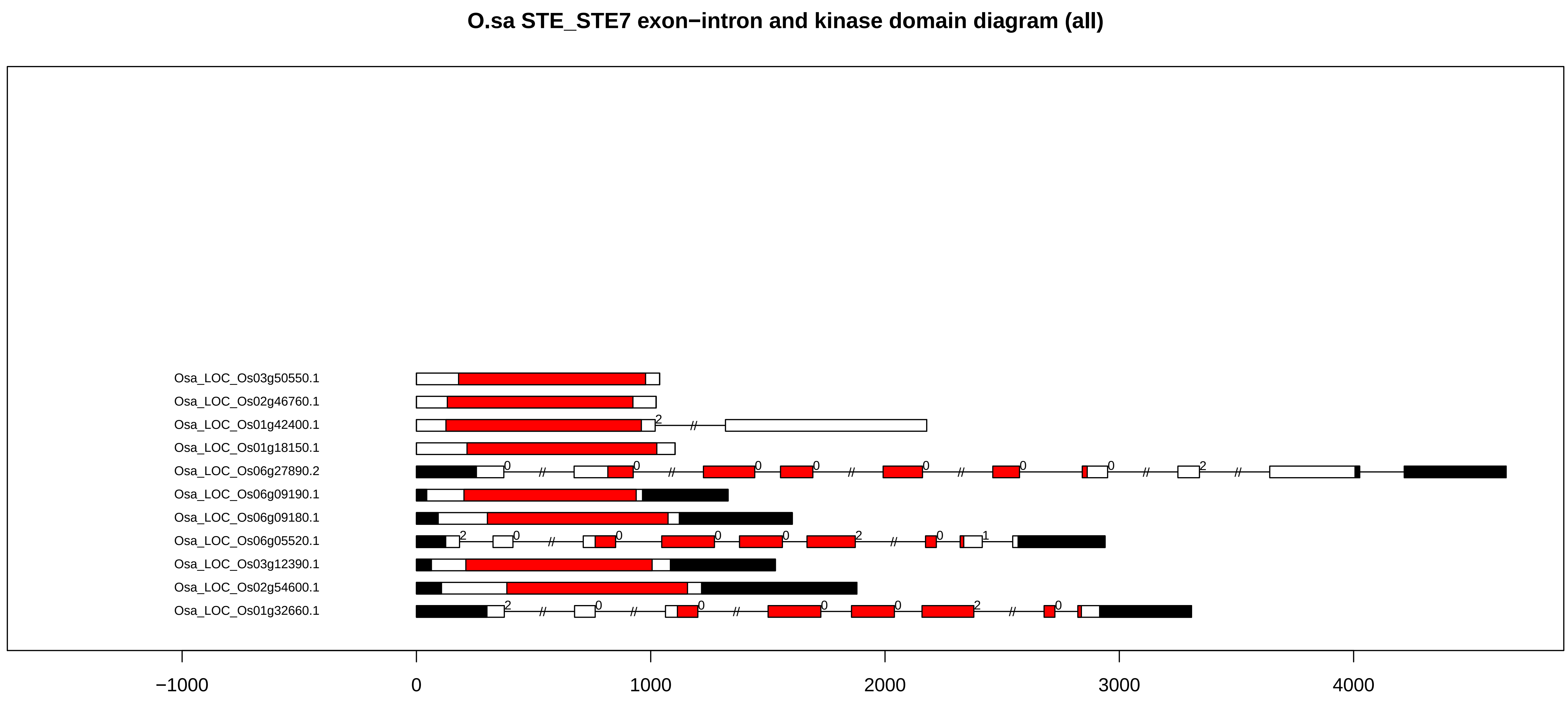

O.sa STE_STE7 exon−intron and kinase domain diagram (all)
Osa_LOC_Os03g50550.1
Osa_LOC_Os02g46760.1
Osa_LOC_Os01g42400.1
Osa_LOC_Os01g18150.1
Osa_LOC_Os06g27890.2
Osa_LOC_Os06g09190.1
Osa_LOC_Os06g09180.1
Osa_LOC_Os06g05520.1
Osa_LOC_Os03g12390.1
Osa_LOC_Os02g54600.1
Osa_LOC_Os01g32660.1
−1000
0
1000
2000
3000
4000

O.sa STE_STE−PI exon−intron and kinase domain diagram (all)

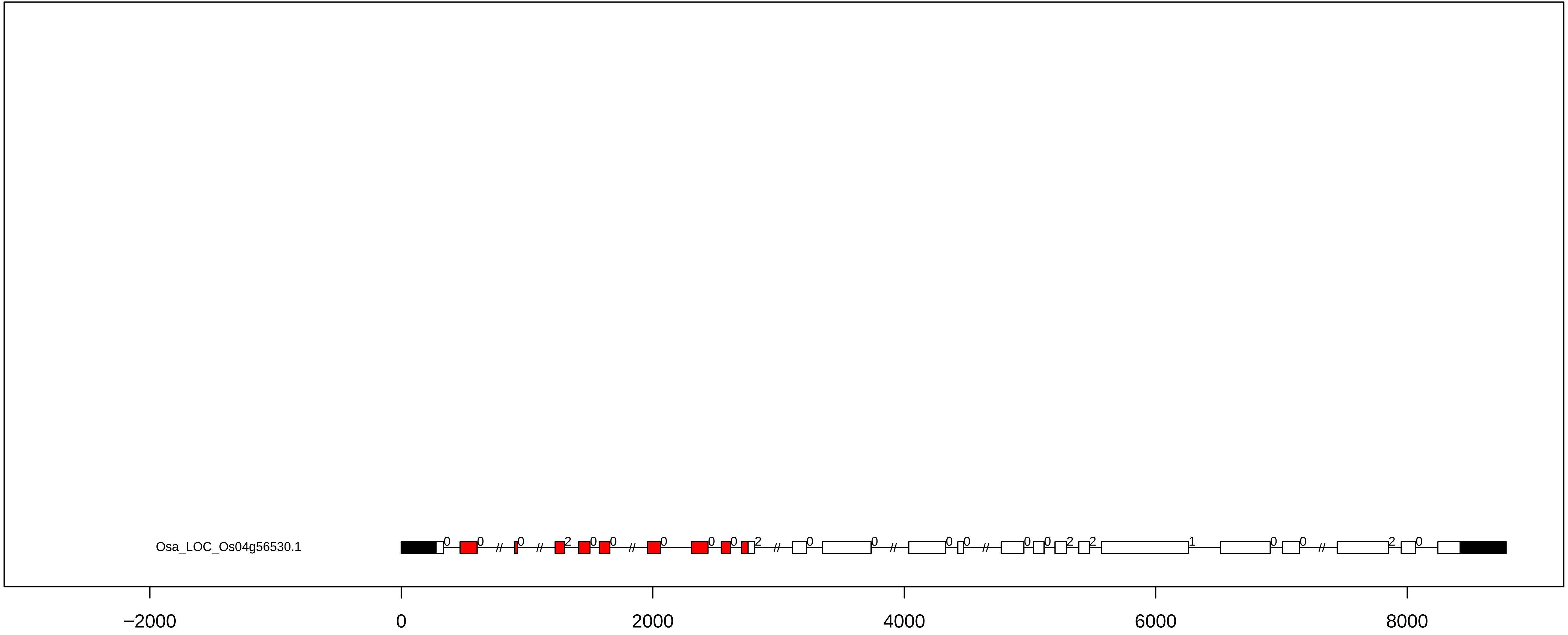

O.sa TKL_CTR1−DRK−1 exon−intron and kinase domain diagram (all)

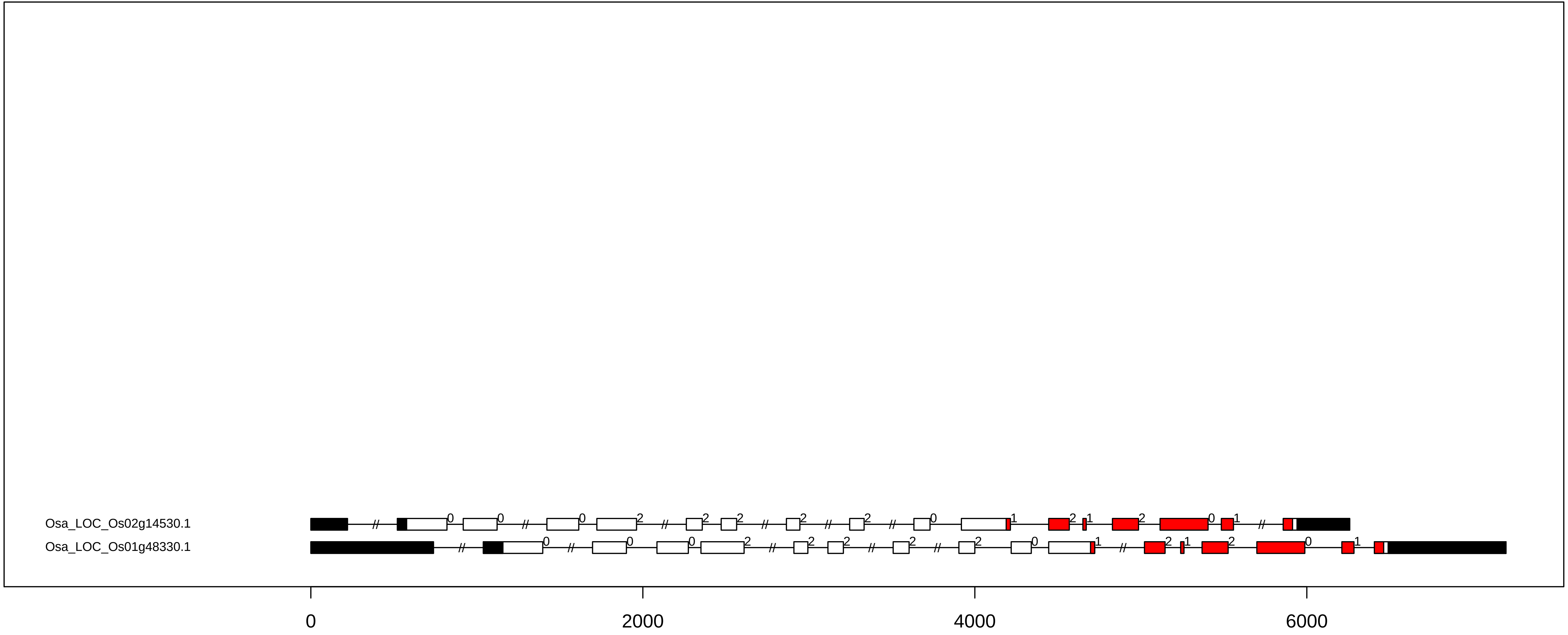

O.sa TKL_CTR1−DRK−2 exon−intron and kinase domain diagram (all)

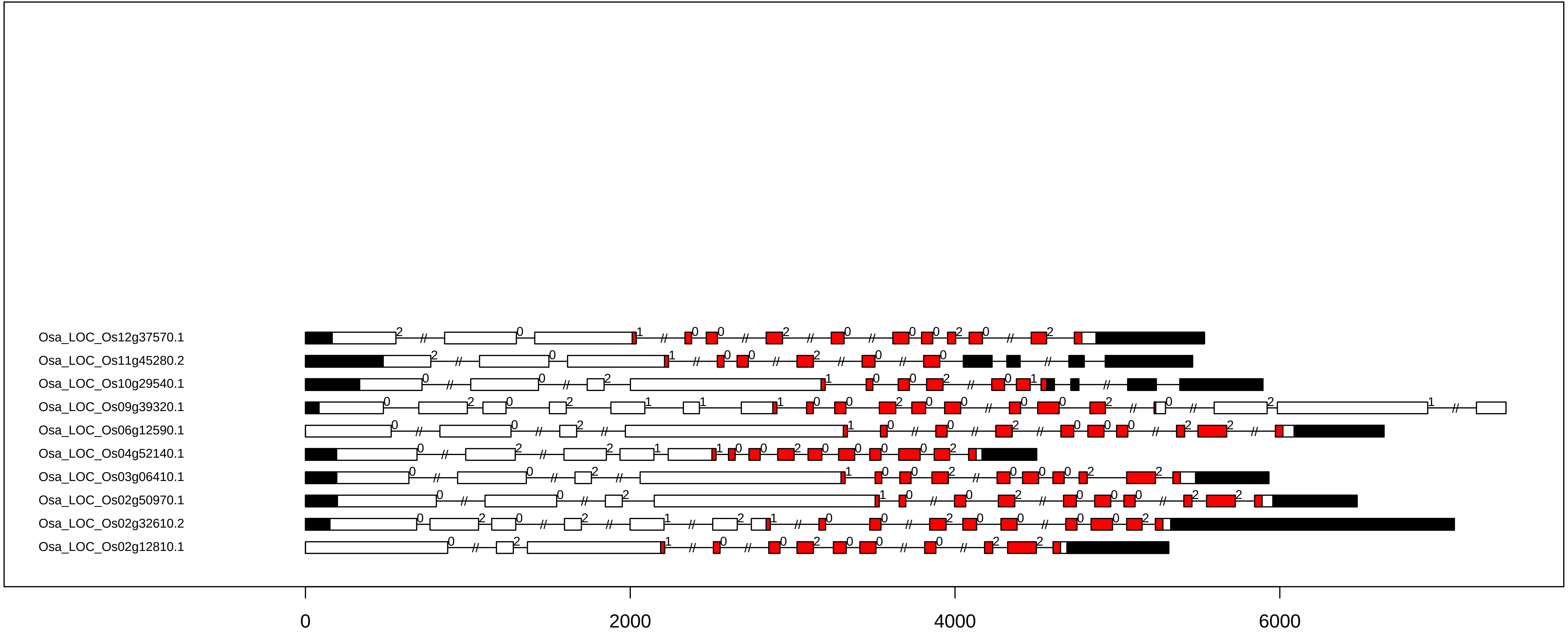

O.sa TKL_Gdt exon−intron and kinase domain diagram (all)

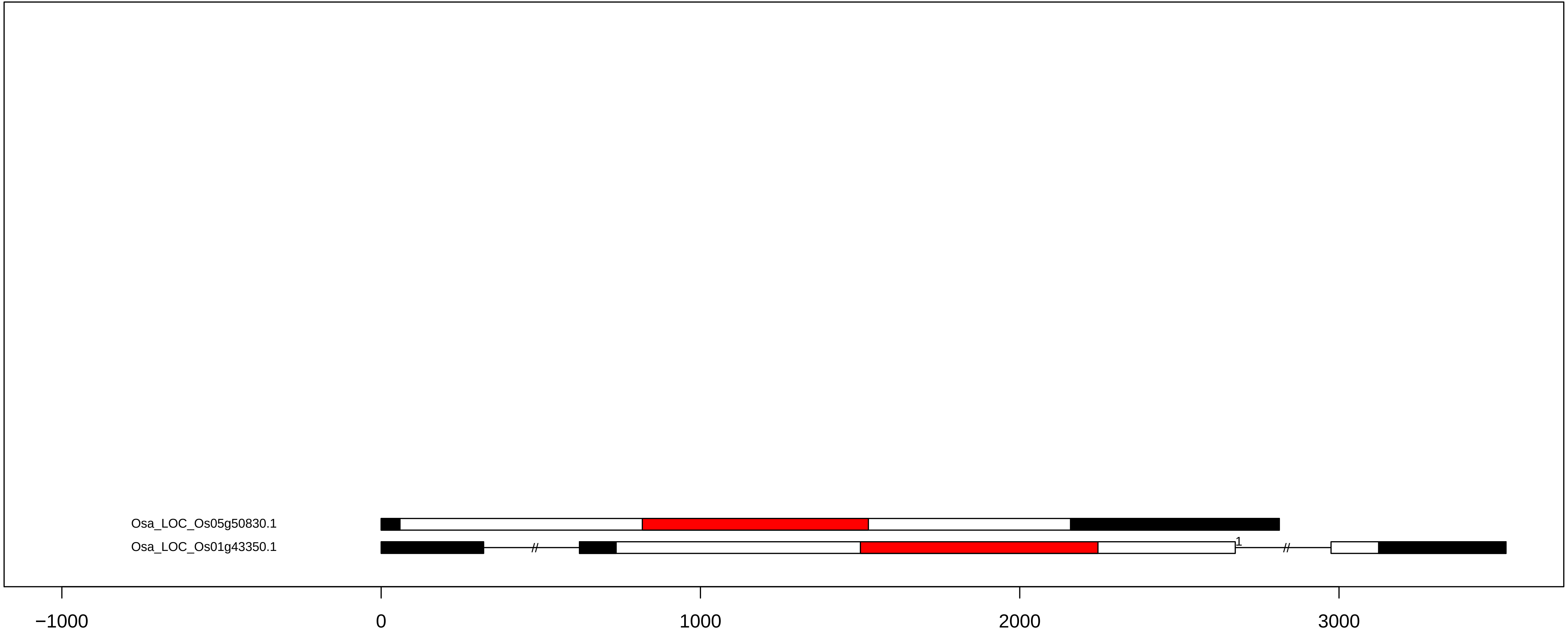

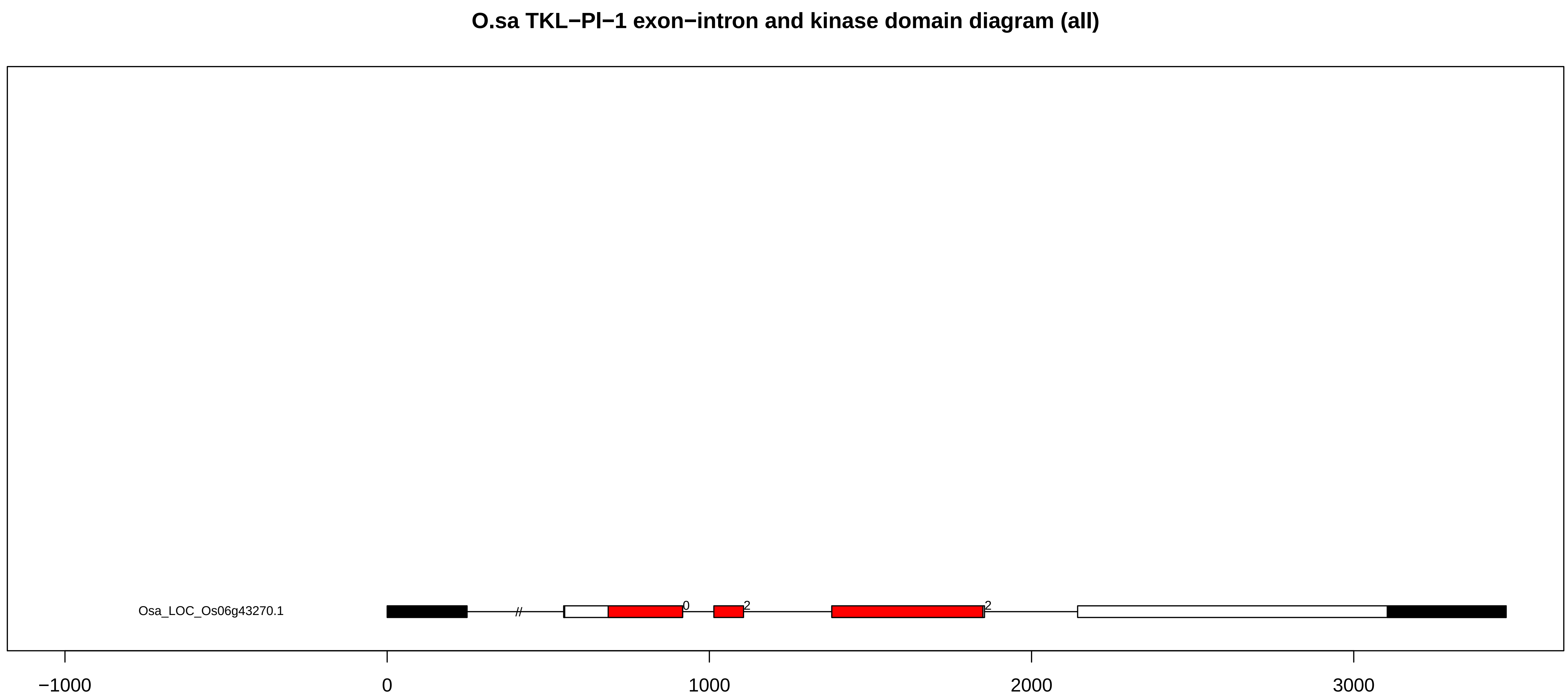

O.sa TKL−Pl−1 exon−intron and kinase domain diagram (all)
Osa_LOC_Os06g43270.1
0
2
2
−1000
0
1000
2000
3000

O.sa TKL−Pl−2 exon−intron and kinase domain diagram (all)

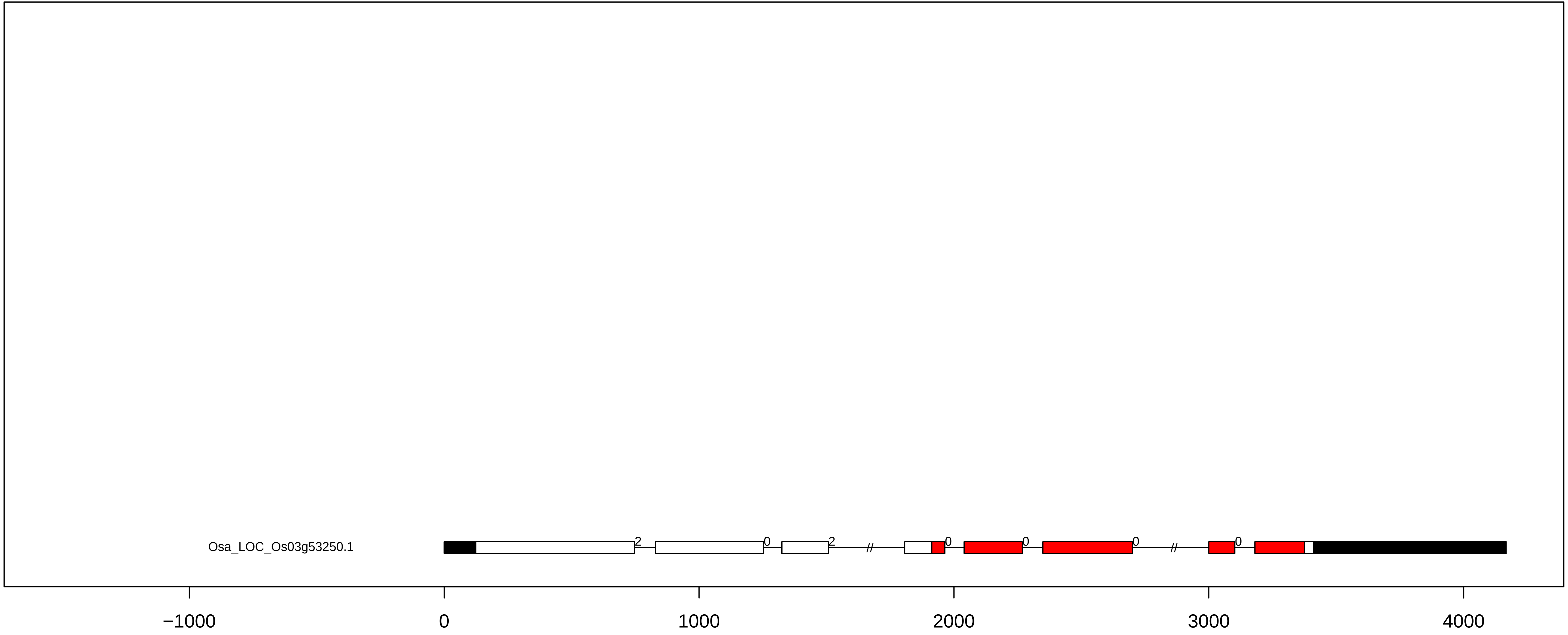

O.sa TKL−Pl−4 exon−intron and kinase domain diagram (part 1)

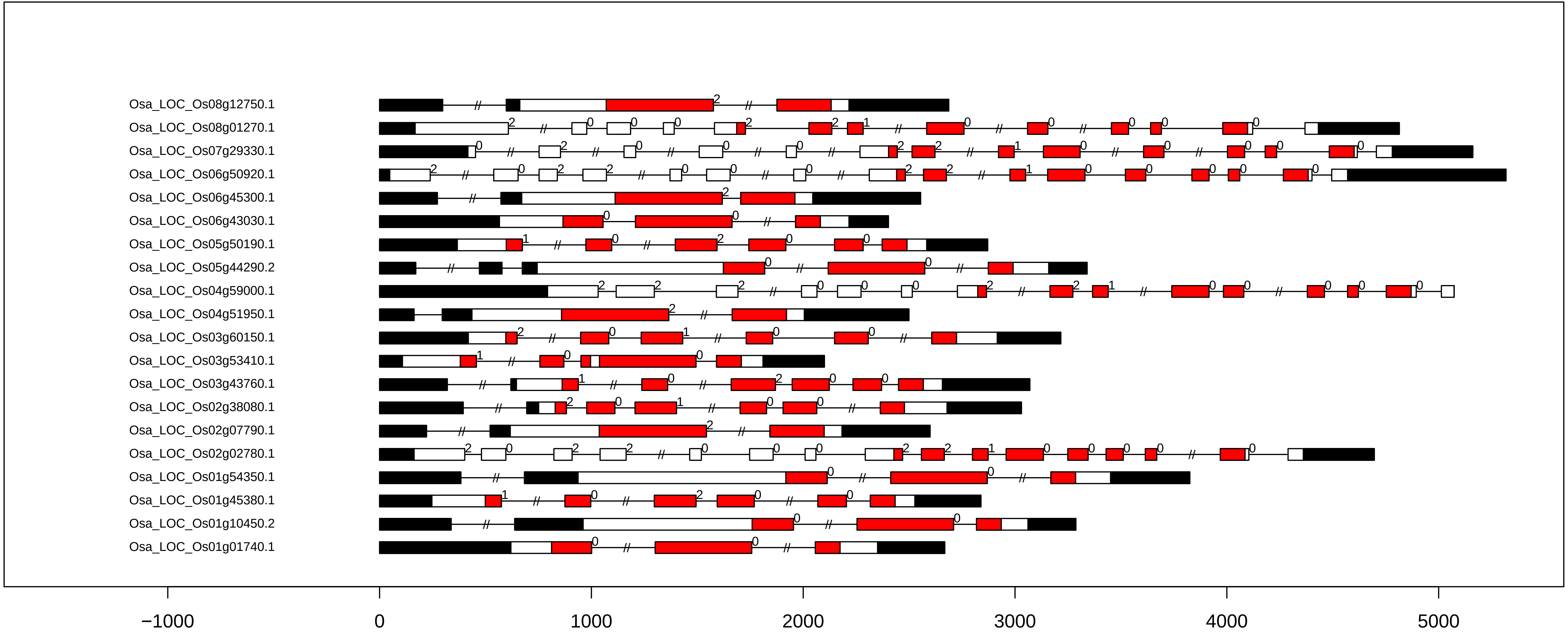

O.sa TKL−Pl−4 exon−intron and kinase domain diagram (part 2)

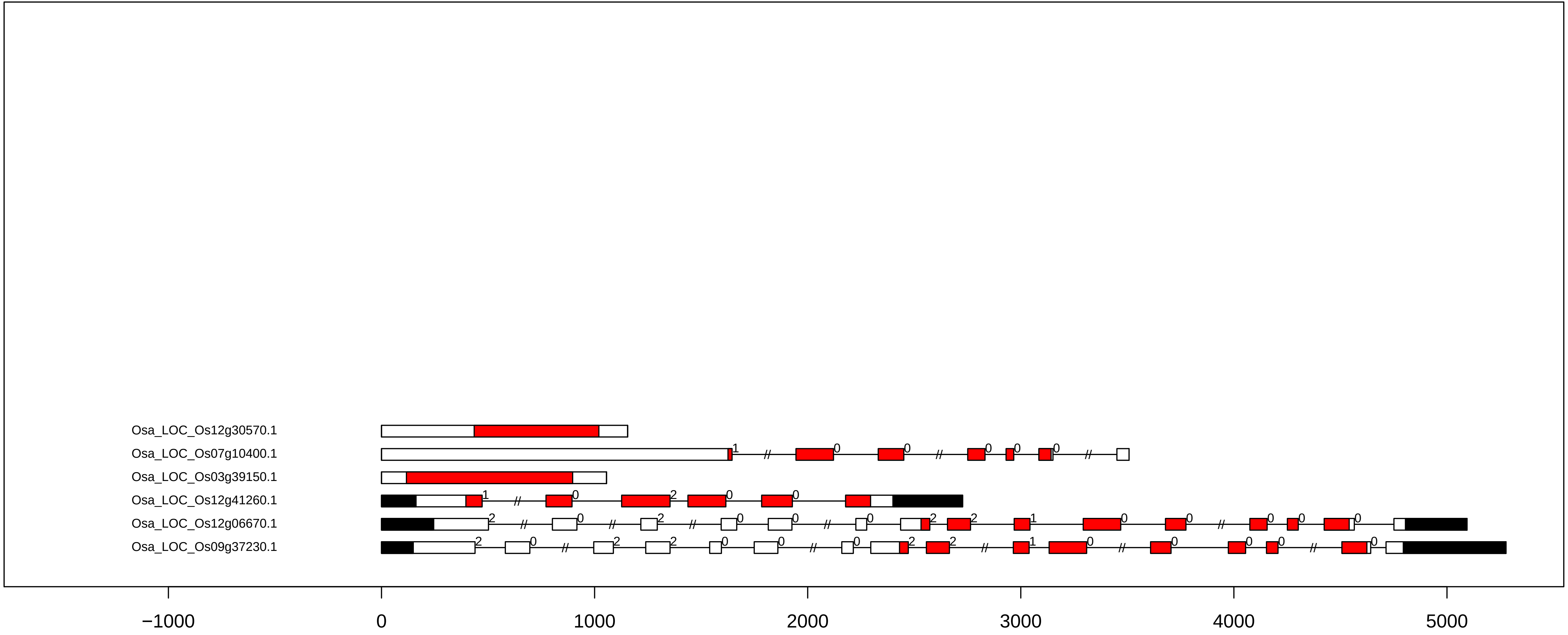

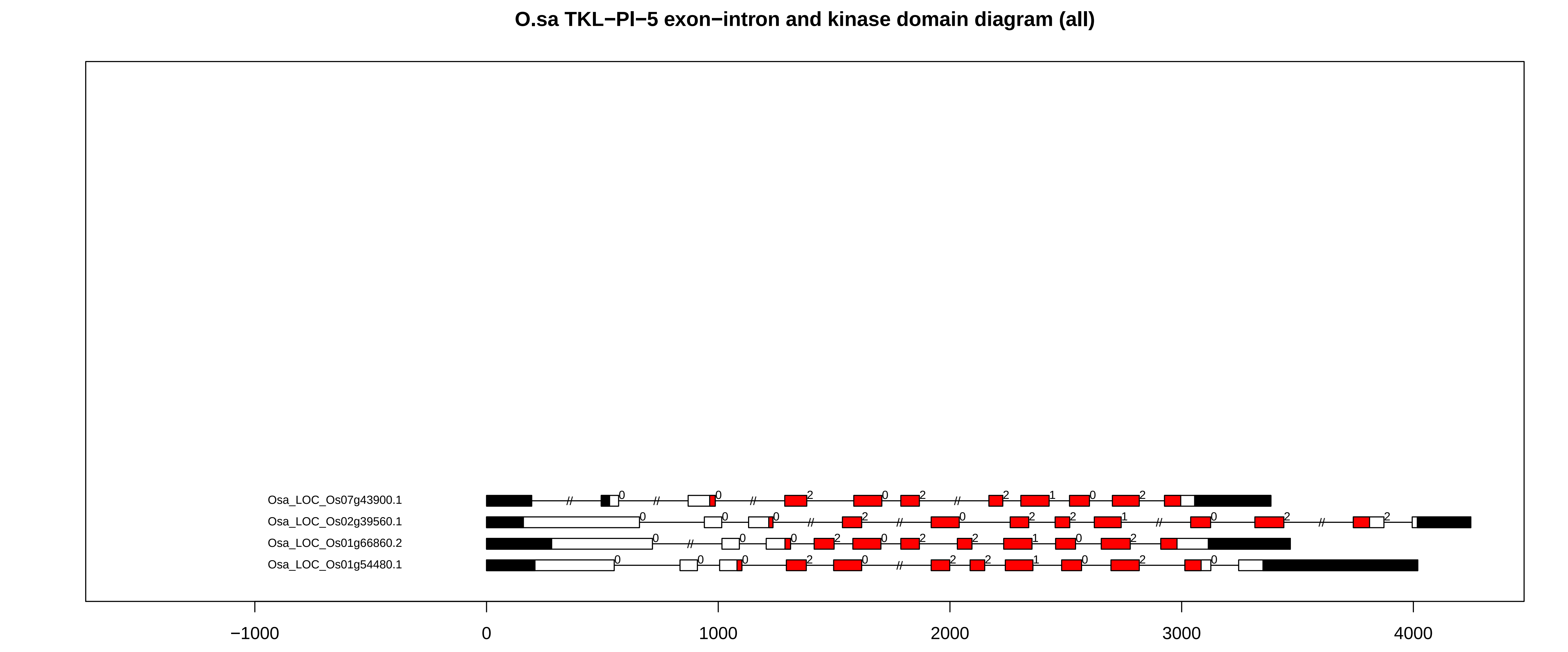
O.sa TKL−Pl−5 exon−intron and kinase domain diagram (all)
Osa_LOC_Os07g43900.1
Osa_LOC_Os02g39560.1
Osa_LOC_Os01g66860.2
Osa_LOC_Os01g54480.1
−1000
0
1000
2000
3000
4000

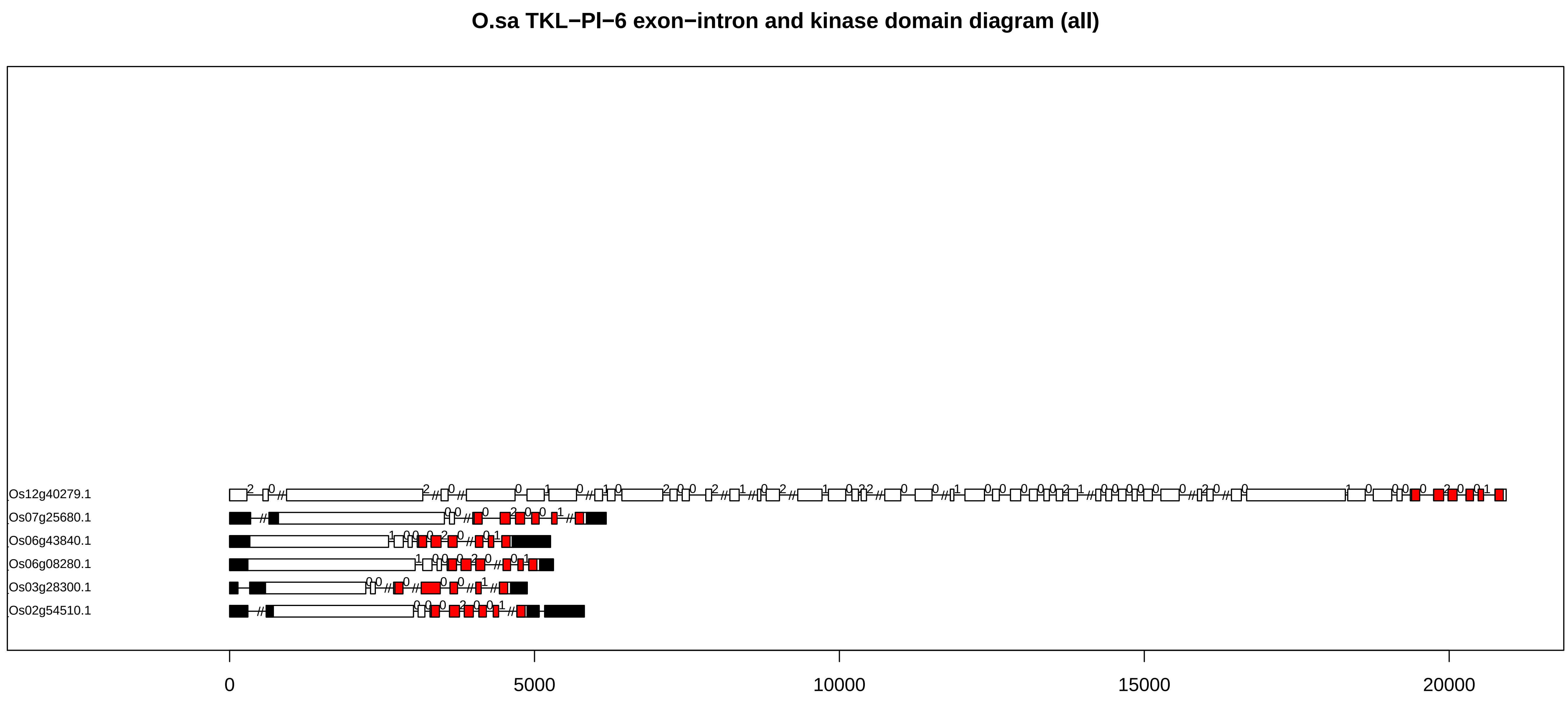
O.sa TKL−Pl−6 exon−intron and kinase domain diagram (all)
Os12g40279.1
Os07g25680.1
Os06g43840.1
Os06g08280.1
Os03g28300.1
Os02g54510.1
0
5000
10000
15000
20000

O.sa TKL−Pl−7 exon−intron and kinase domain diagram (all)

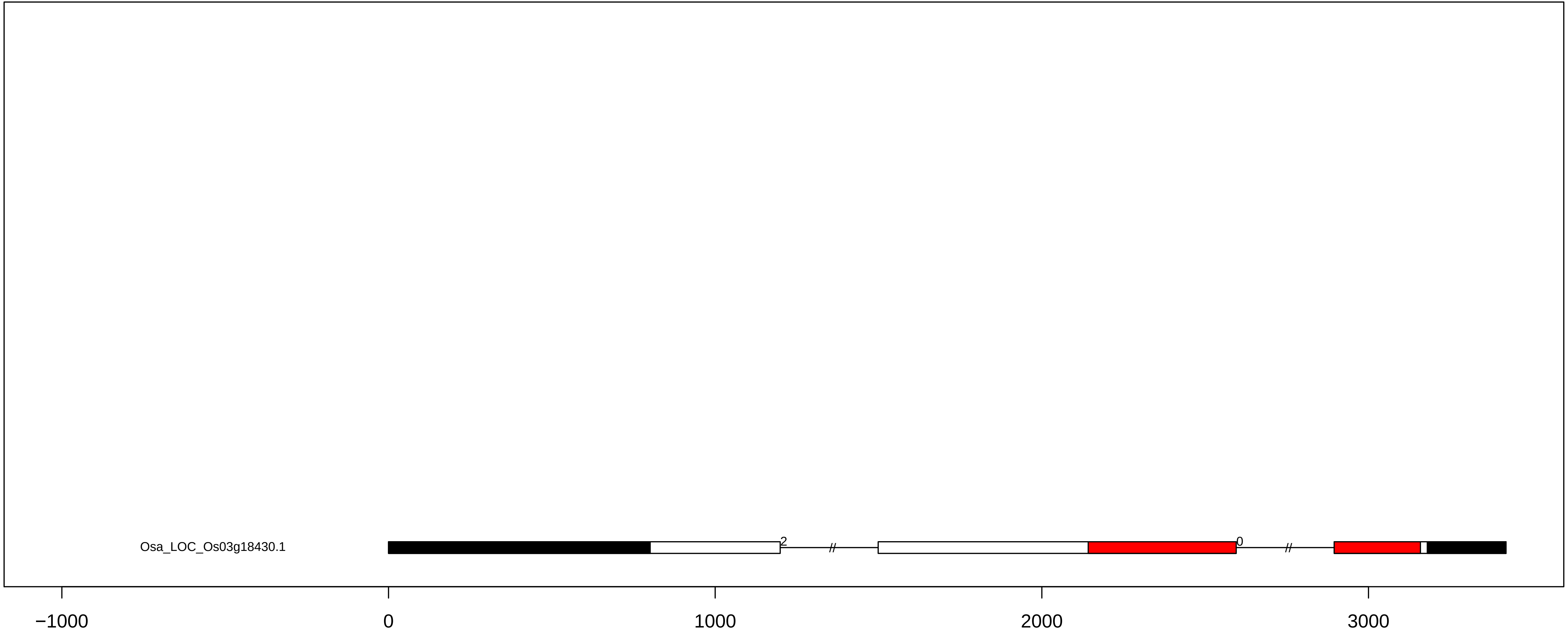

O.sa TKL−Pl−8 exon−intron and kinase domain diagram (all)

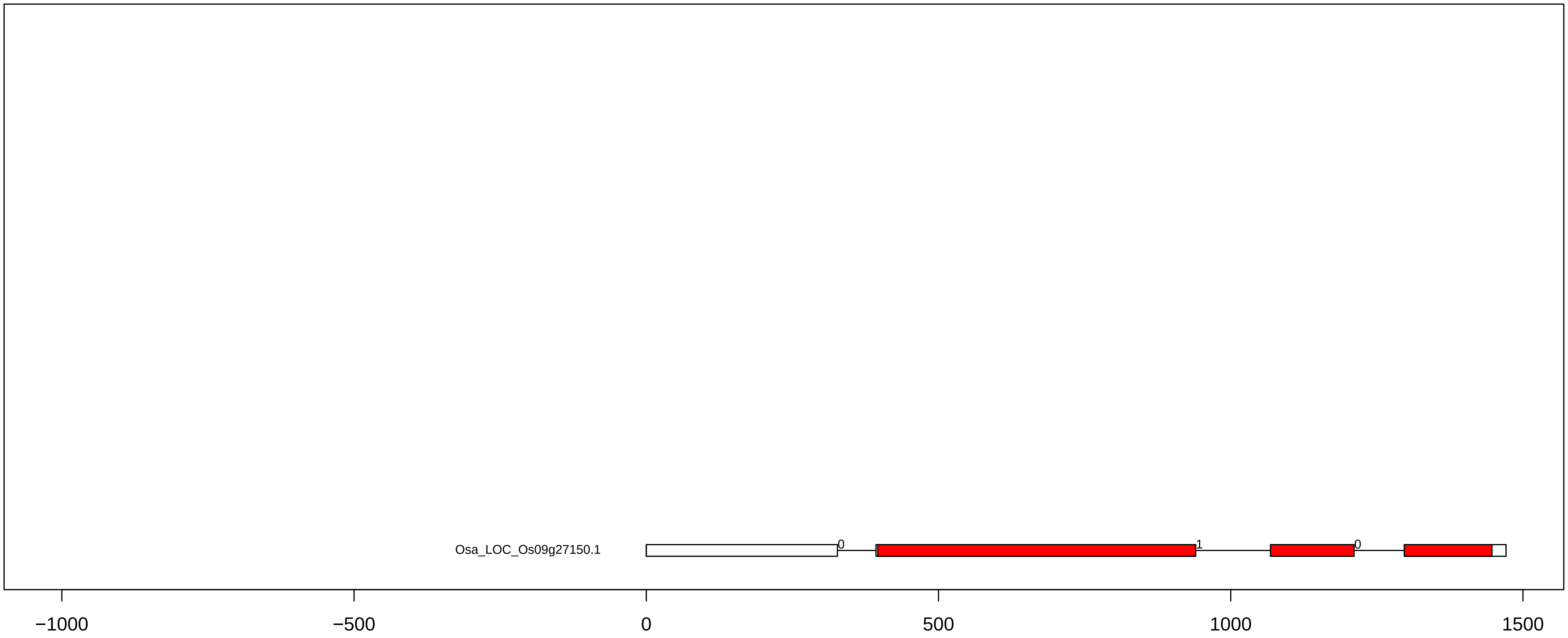

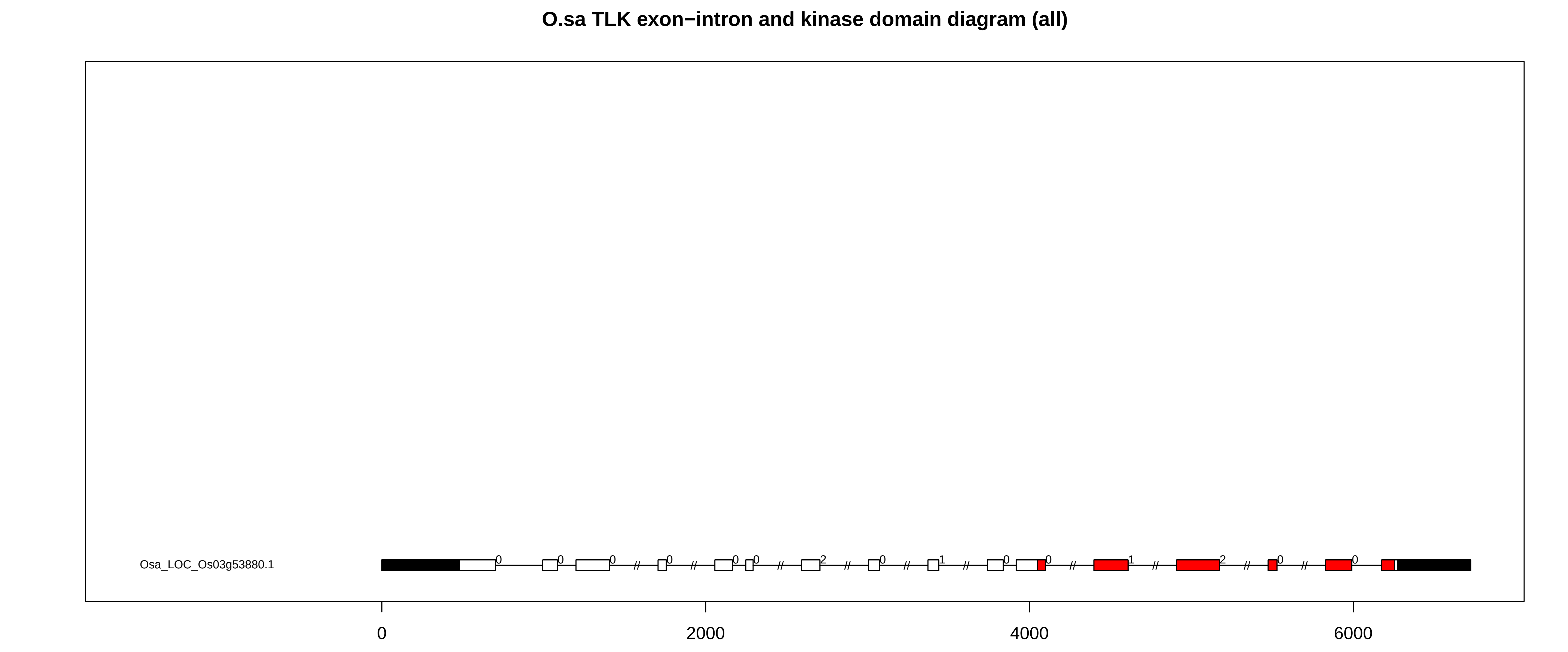

O.sa TLK exon−intron and kinase domain diagram (all)
Osa_LOC_Os03g53880.1
0
0
0
0
0
0
2
0
1
0
0
1
2
0
0
0
2000
4000
6000

O.sa TTK exon−intron and kinase domain diagram (all)

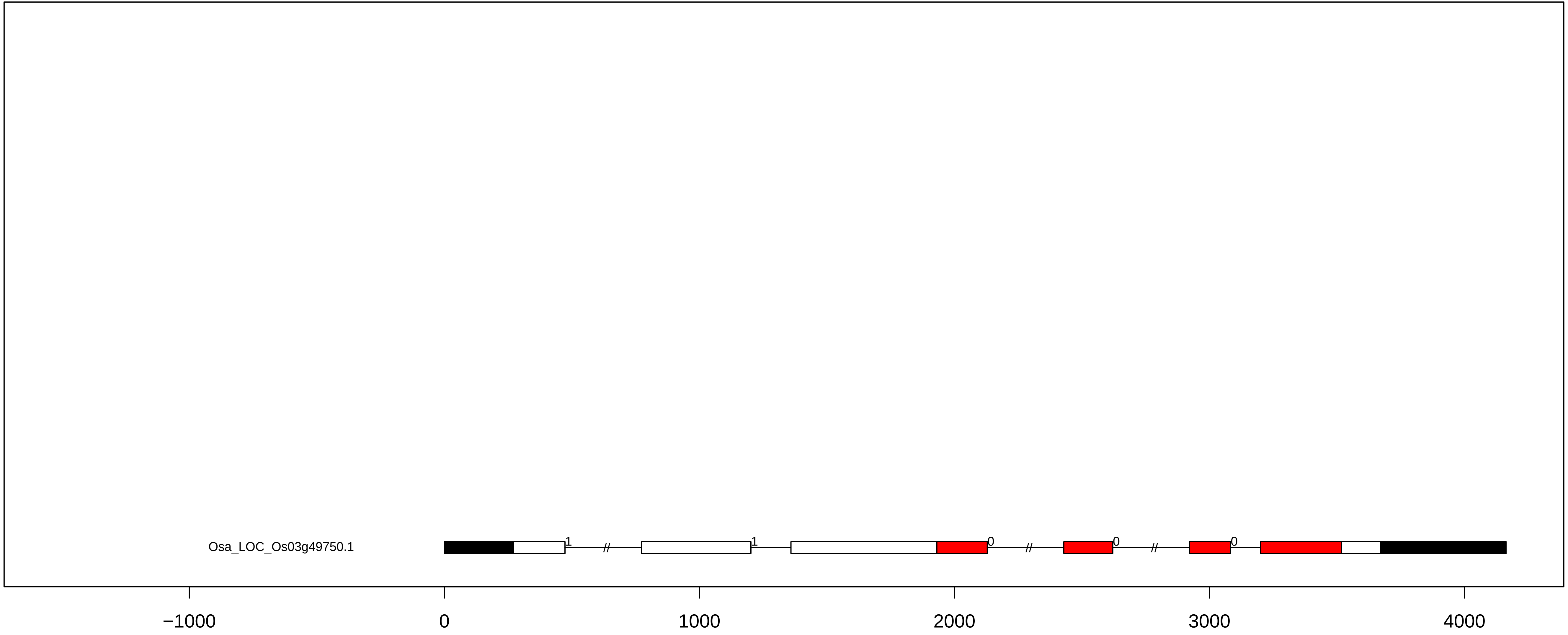

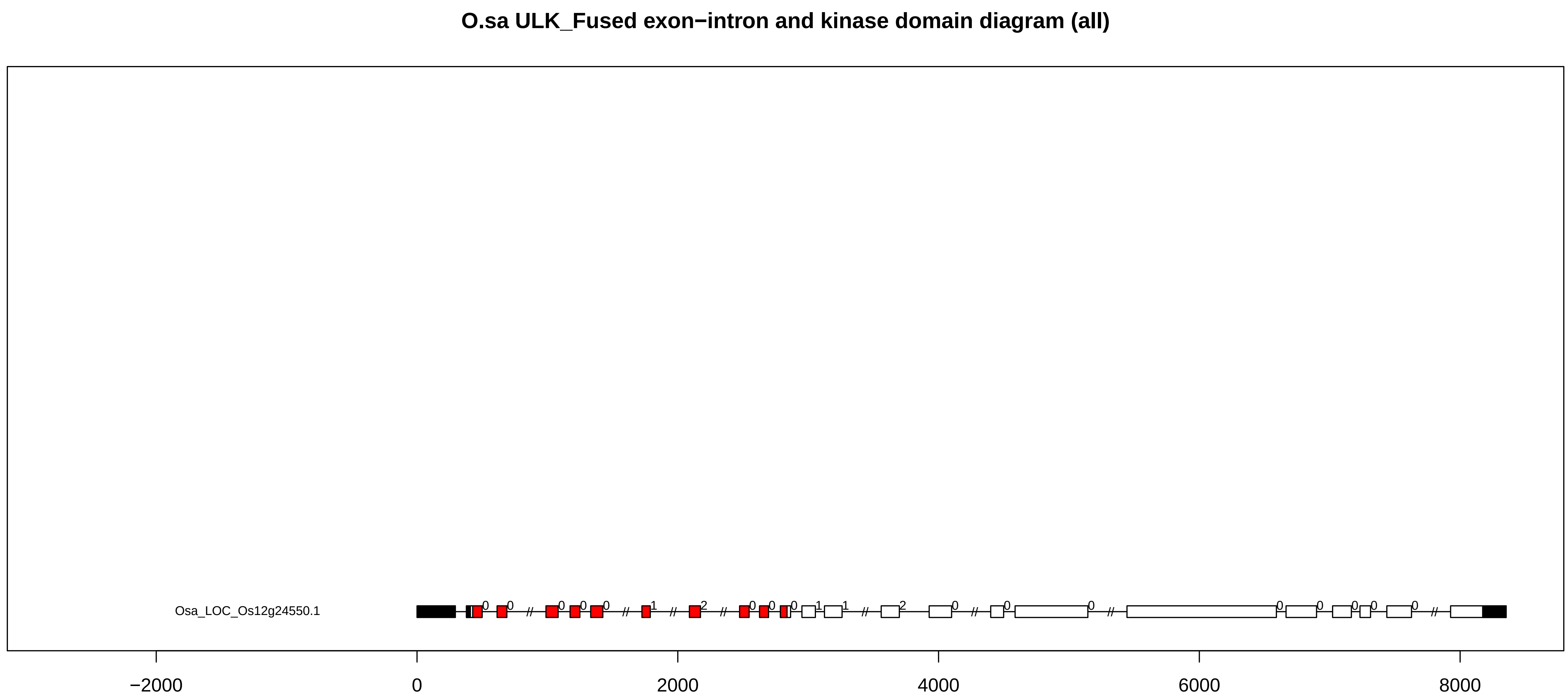

O.sa ULK_ULK4 exon−intron and kinase domain diagram (all)

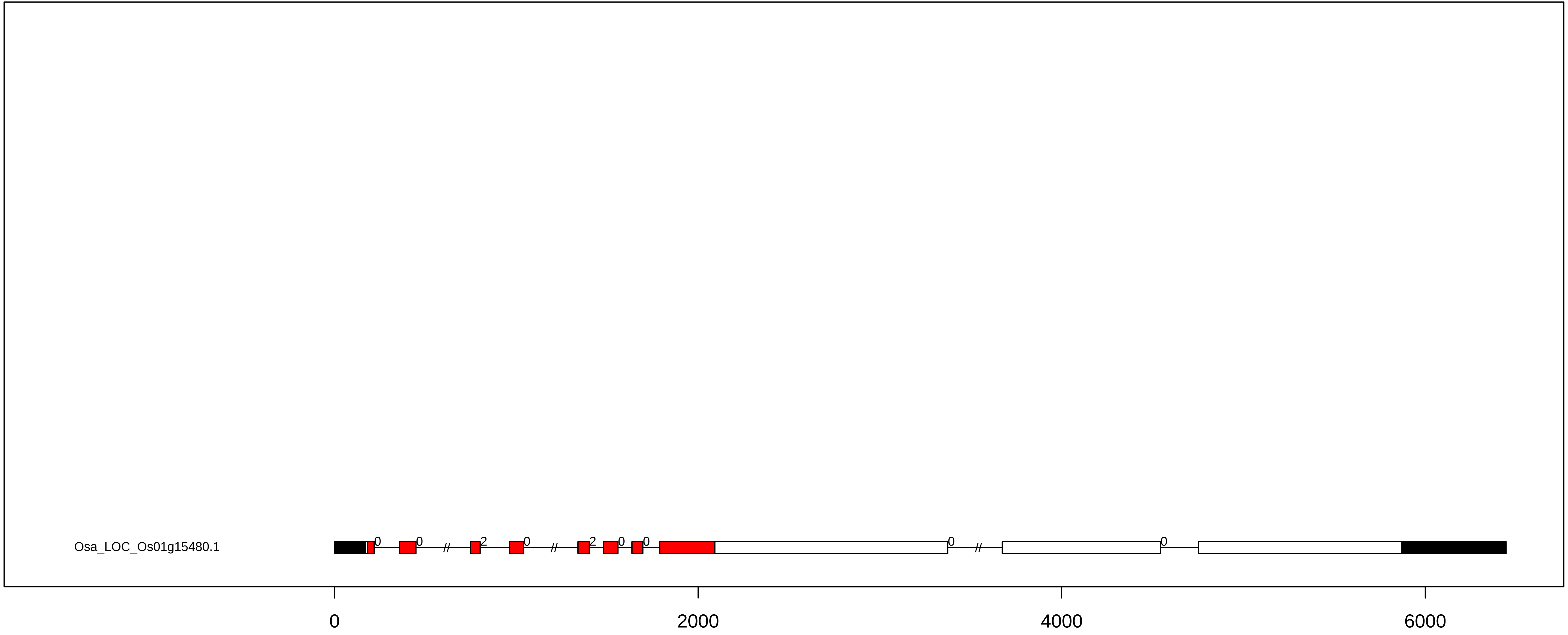

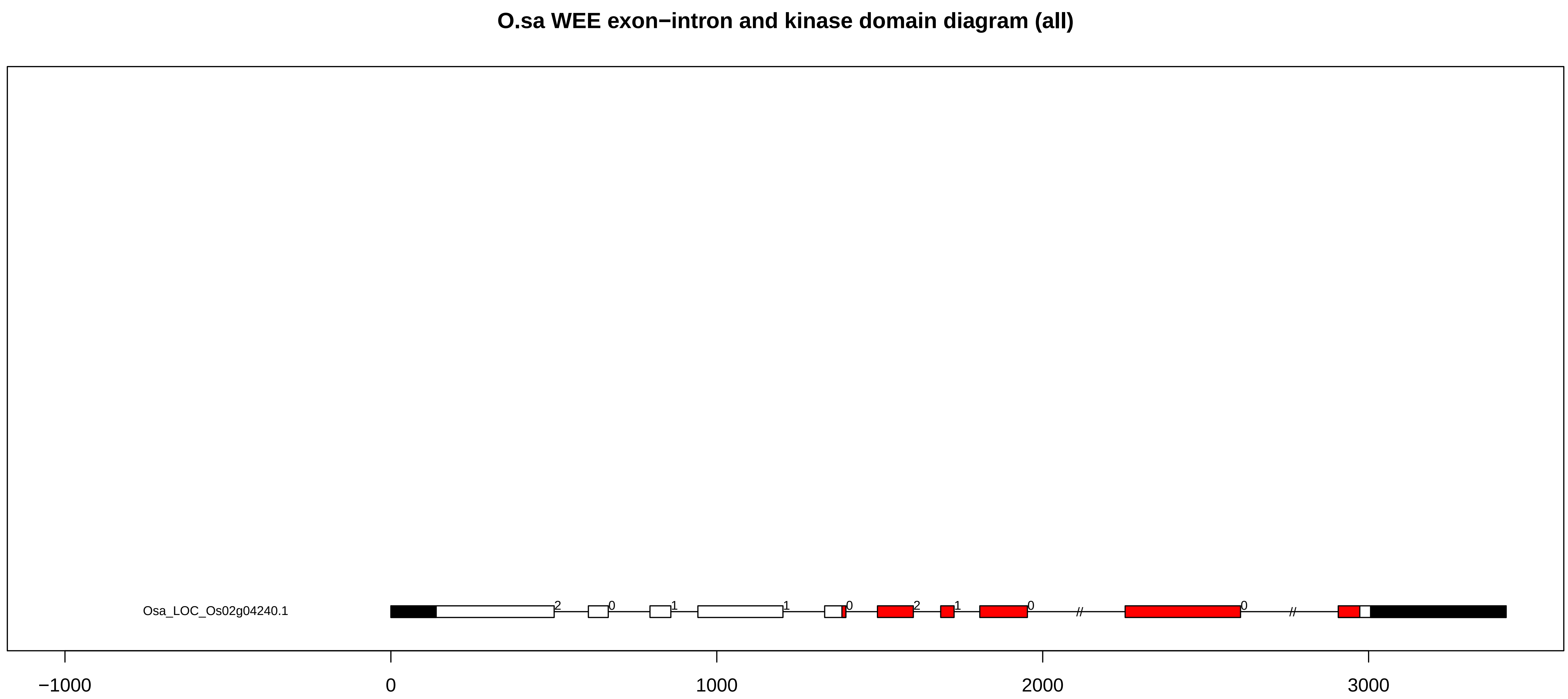
O.sa WEE exon−intron and kinase domain diagram (all)
Osa_LOC_Os02g04240.1
2
0
1
1
0
2
1
0
0
−1000
0
1000
2000
3000

O.sa WNK_NRBP exon−intron and kinase domain diagram (all)

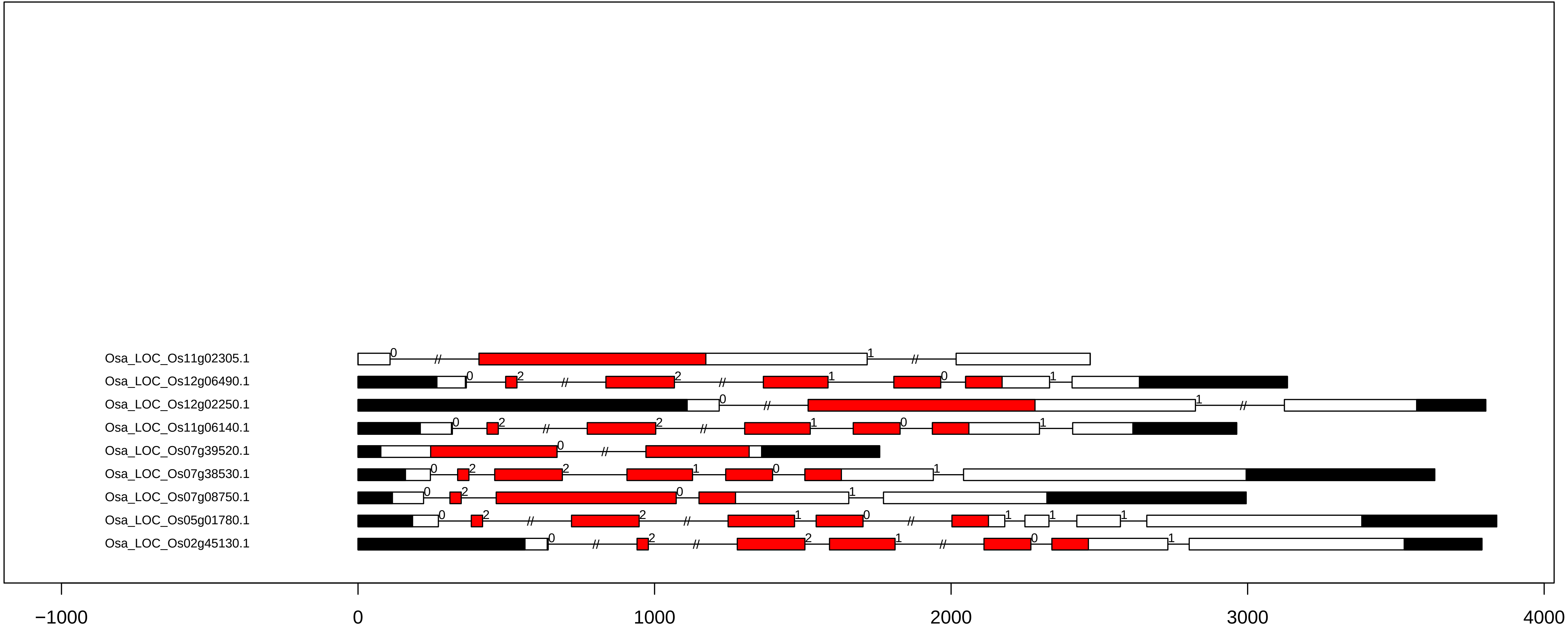

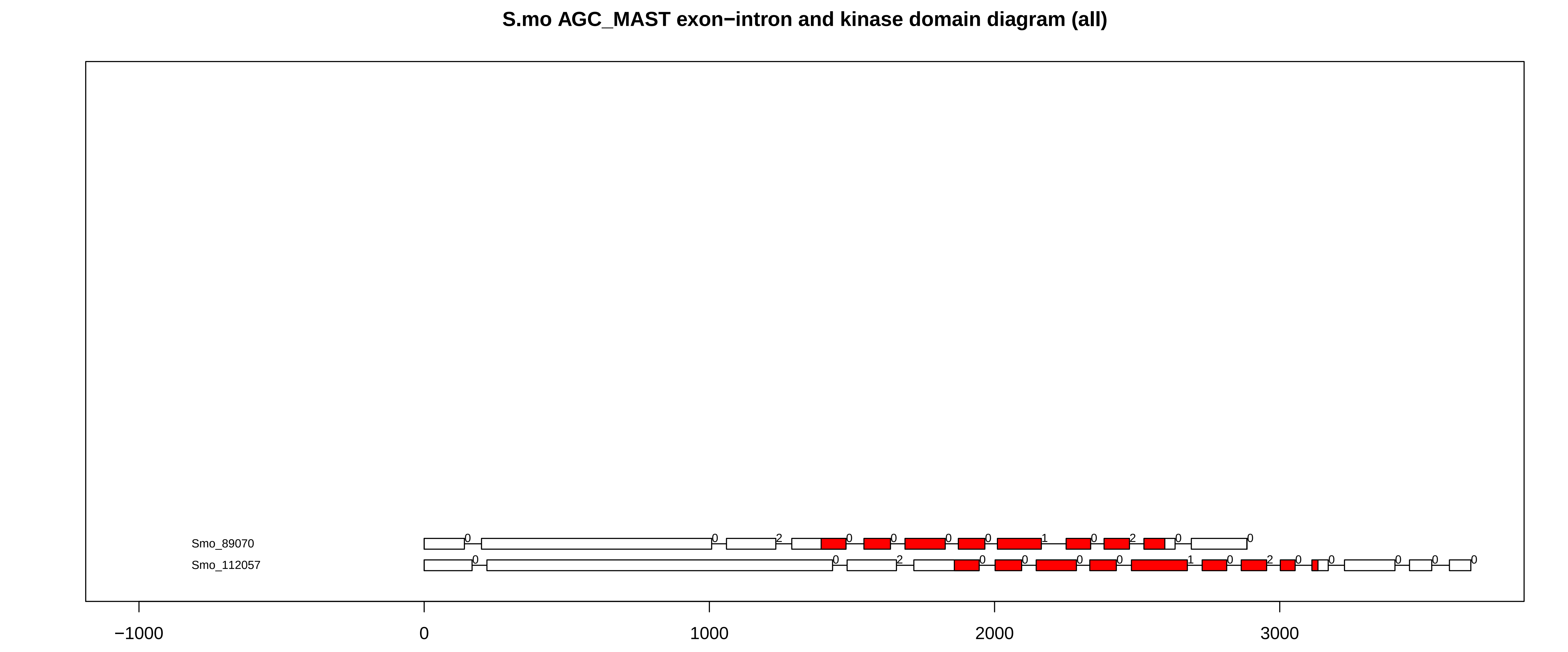
S.mo AGC_MAST exon−intron and kinase domain diagram (all)
Smo_89070
Smo_112057
−1000
0
1000
2000
3000

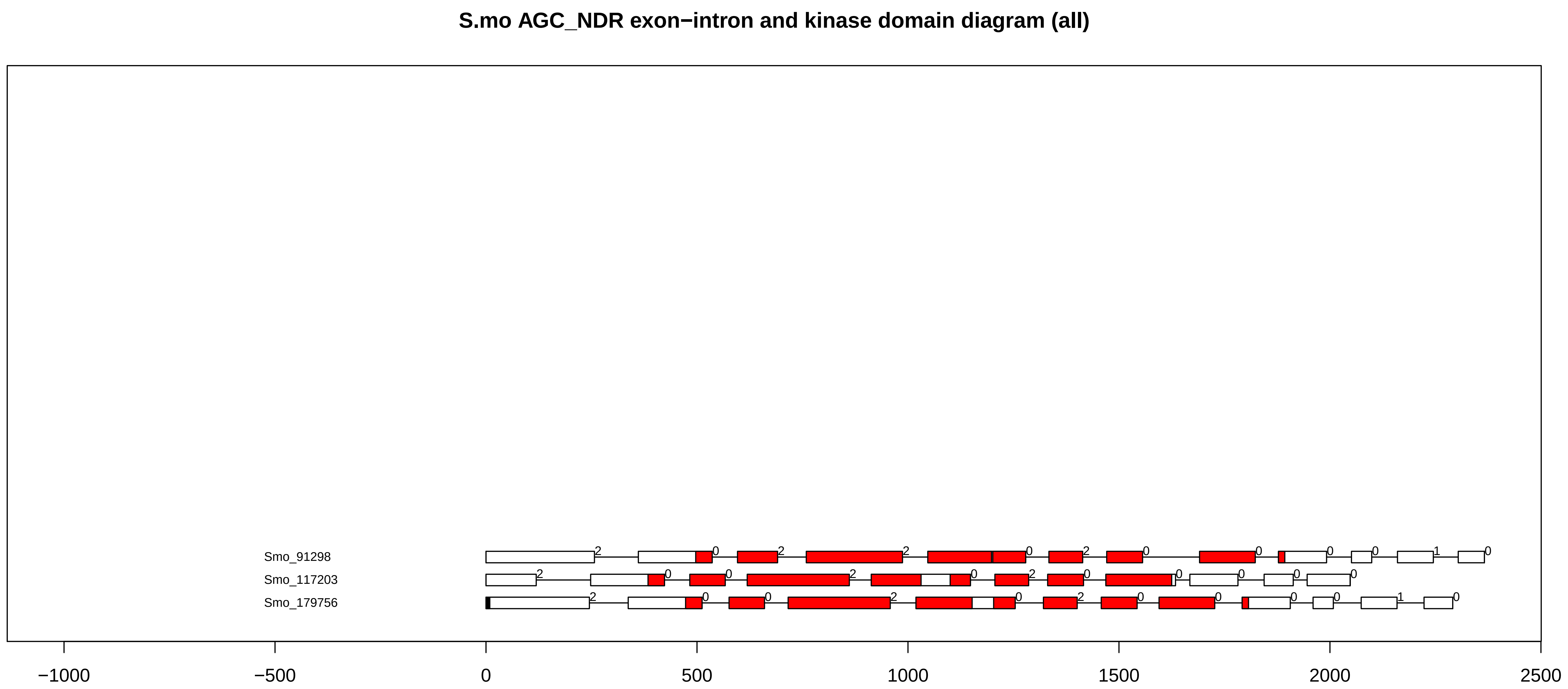
S.mo AGC_NDR exon−intron and kinase domain diagram (all)
Smo_91298
Smo_117203
Smo_179756
−1000
−500
0
500
1000
1500
2000
2500

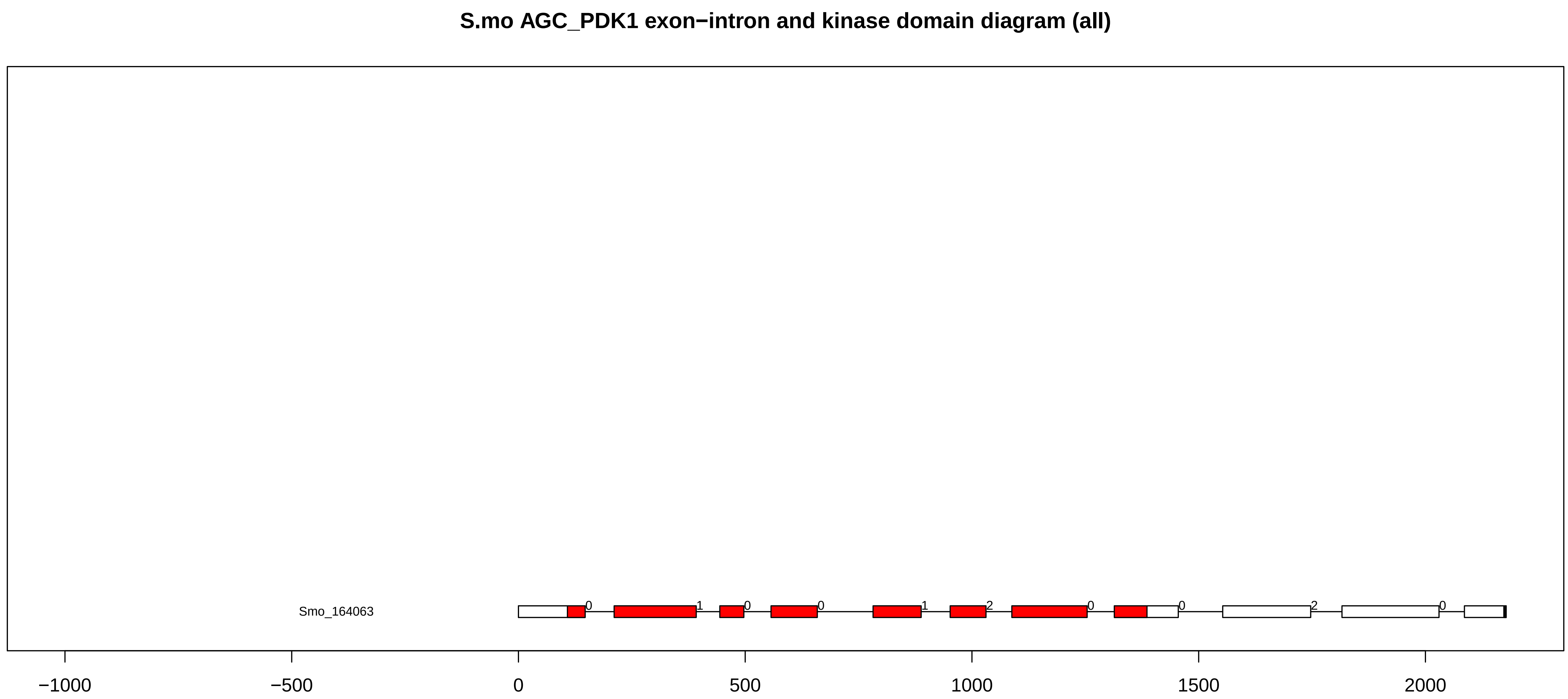
S.mo AGC_PDK1 exon−intron and kinase domain diagram (all)
Smo_164063
0
1
0
0
1
2
0
0
2
0
−1000
−500
0
500
1000
1500
2000

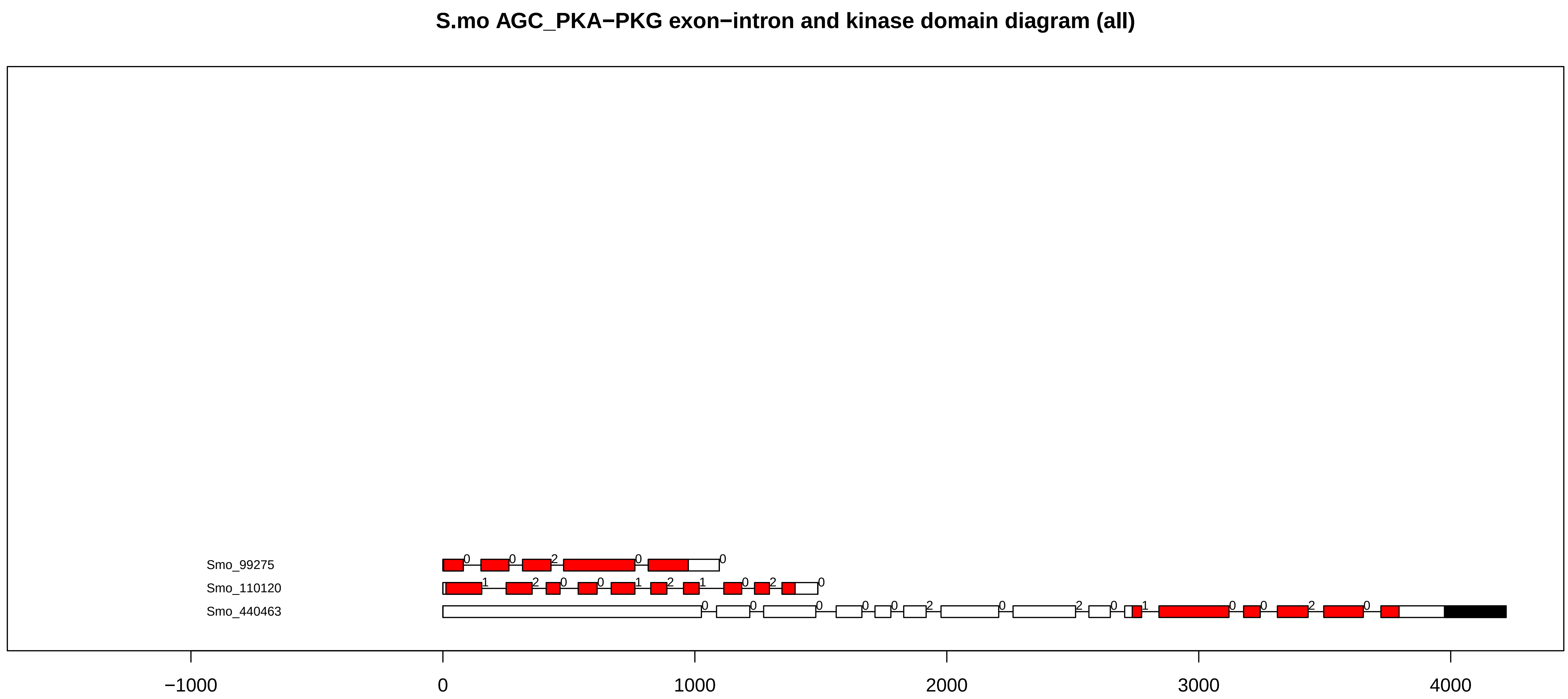
S.mo AGC_PKA−PKG exon−intron and kinase domain diagram (all)
Smo_99275
Smo_110120
Smo_440463
−1000
0
1000
2000
3000
4000

S.mo AGC_RSK−2 exon−intron and kinase domain diagram (all)

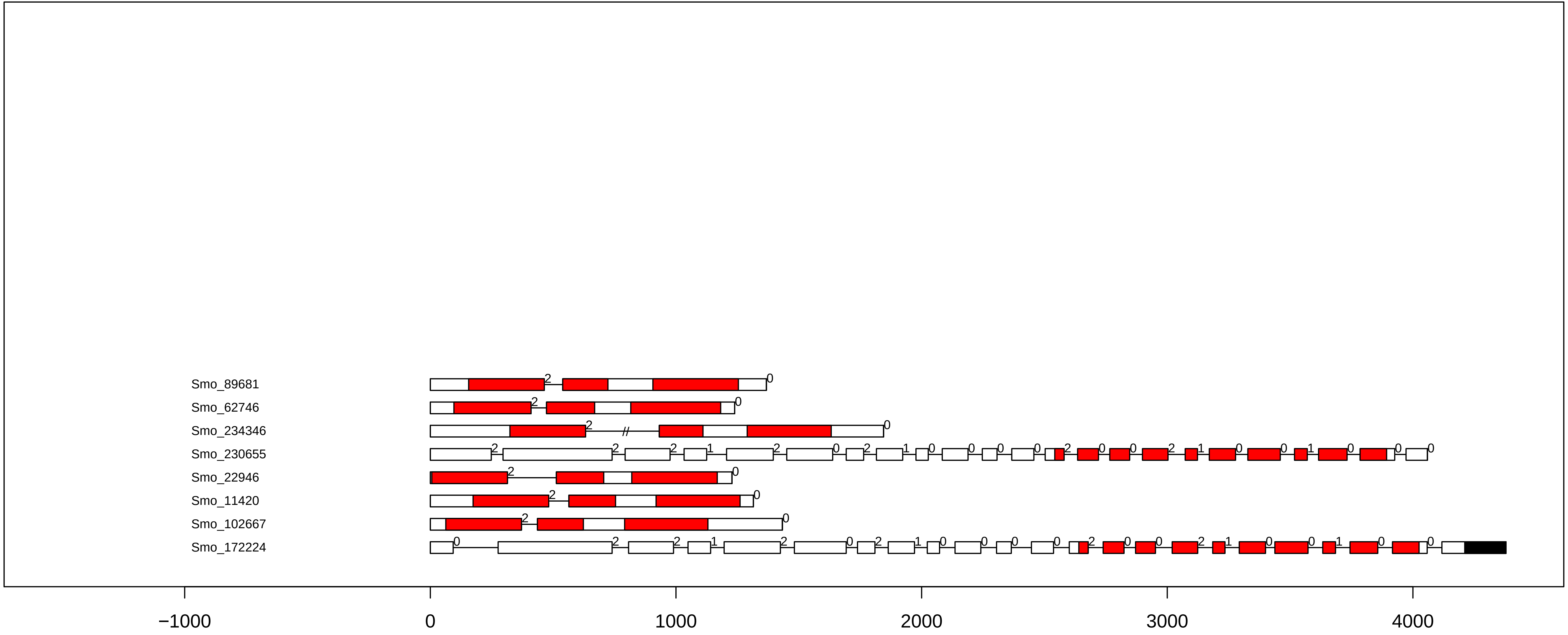

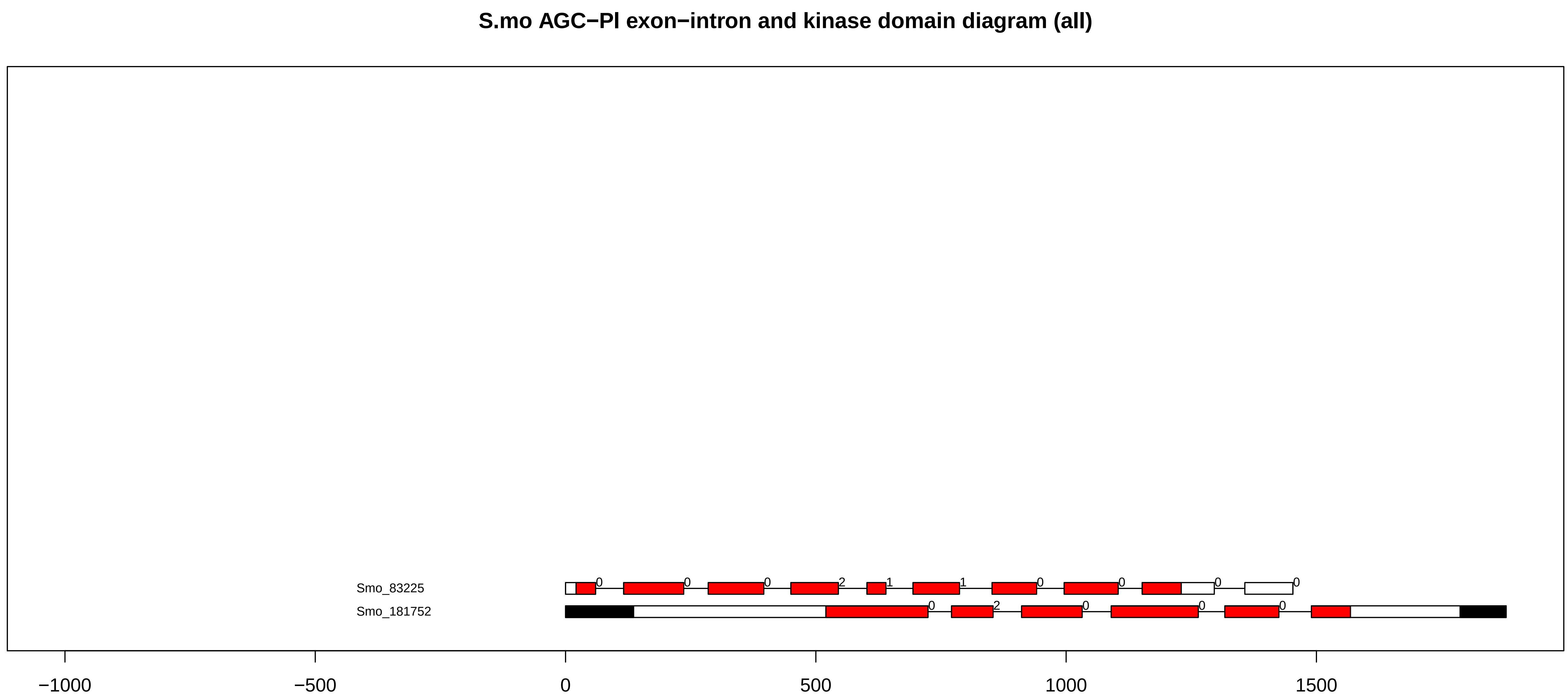
S.mo AGC−PI exon−intron and kinase domain diagram (all)
Smo_83225
Smo_181752
0
0
0
2
1
1
0
0
0
0
0
2
0
0
0
−1000
−500
0
500
1000
1500

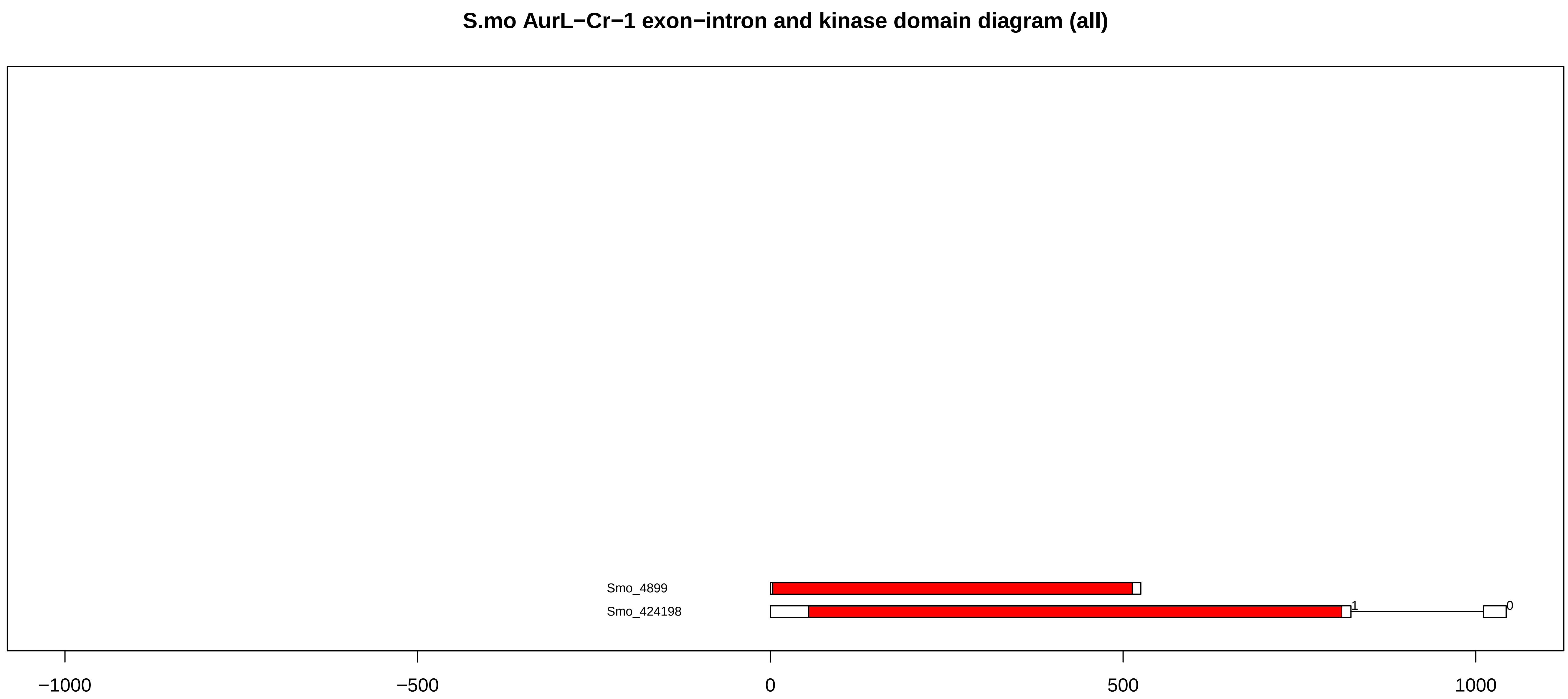
S.mo AurL−Cr−1 exon−intron and kinase domain diagram (all)
Smo_4899
Smo_424198
1
0
−1000
−500
0
500
1000

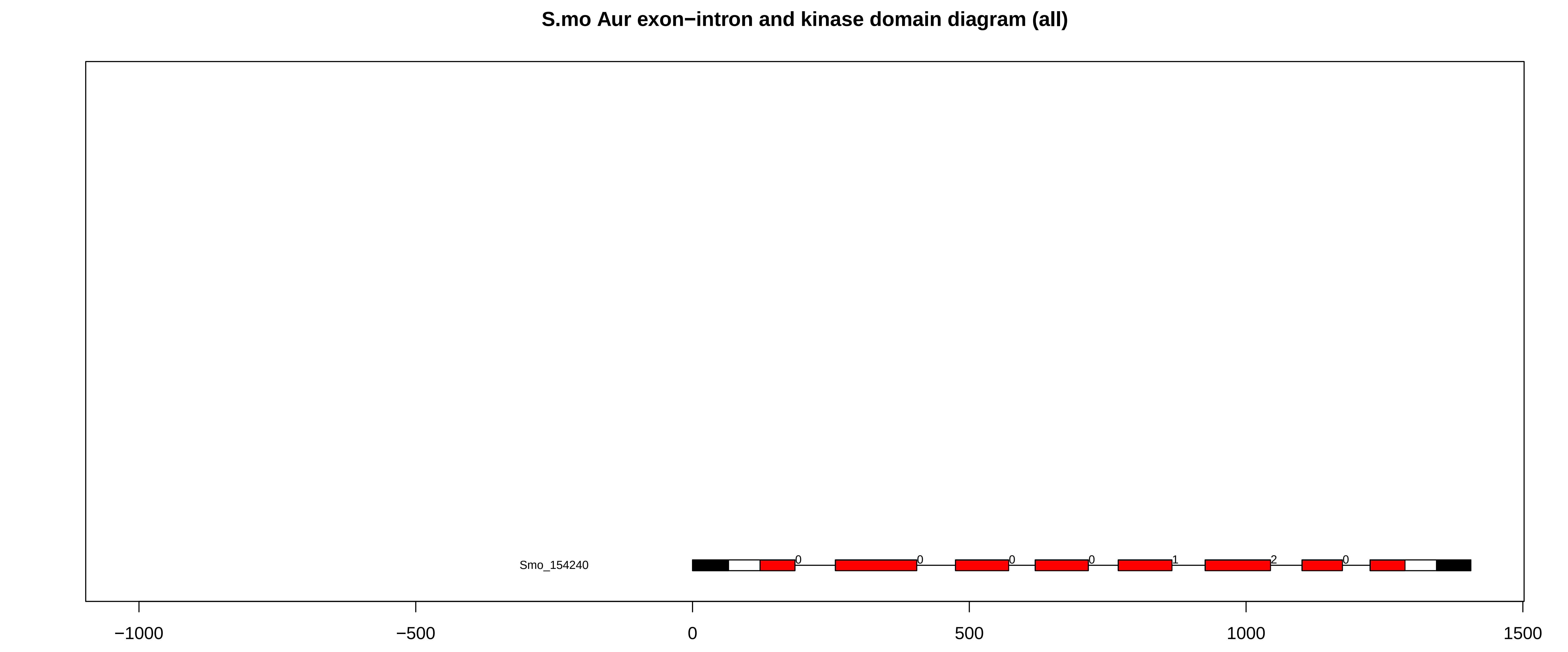
S.mo Aur exon−intron and kinase domain diagram (all)
Smo_154240
0
0
0
0
1
2
0
−1000
−500
0
500
1000
1500

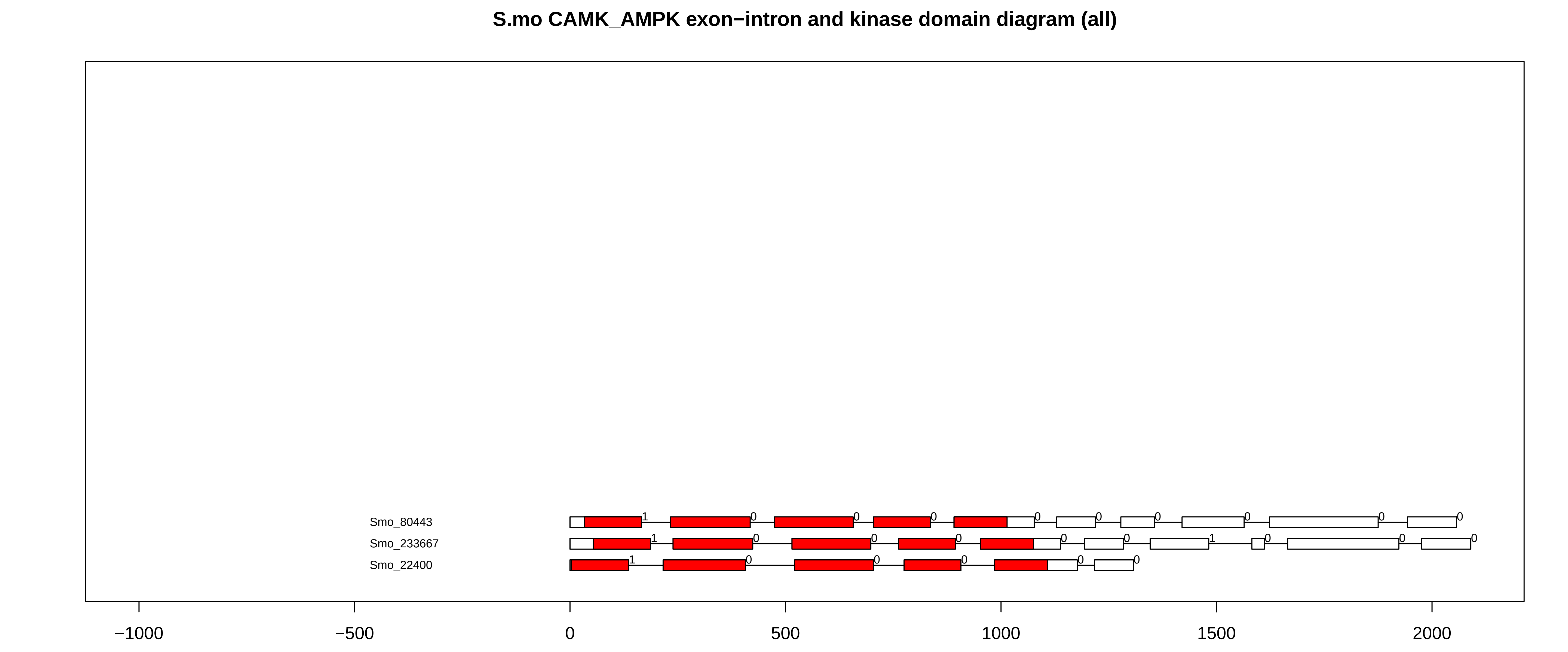
S.mo CAMK_AMPK exon−intron and kinase domain diagram (all)
Smo_80443
Smo_233667
Smo_22400
−1000
−500
0
500
1000
1500
2000

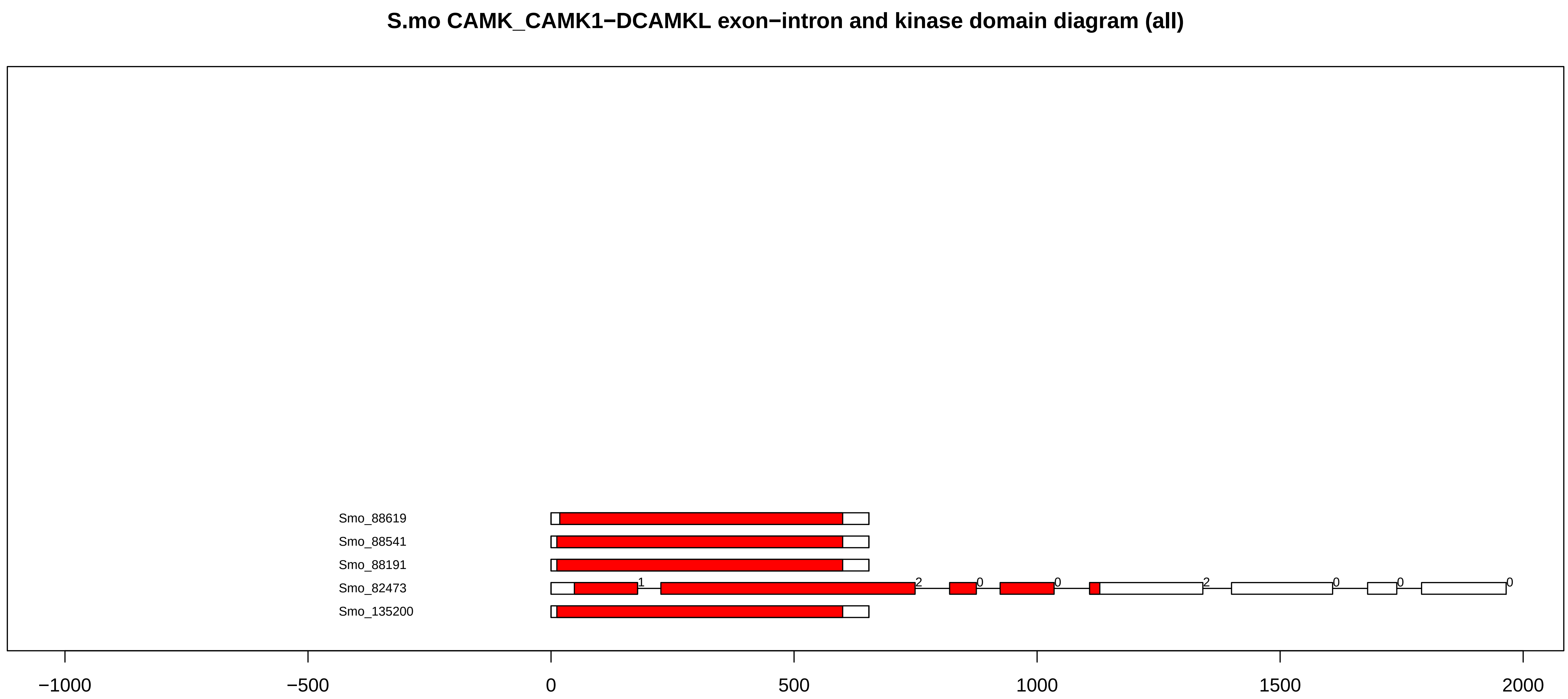
S.mo CAMK_CAMK1−DCAMKL exon−intron and kinase domain diagram (all)
Smo_88619
Smo_88541
Smo_88191
Smo_82473
Smo_135200
1
2
0
0
2
0
0
0
−1000
−500
0
500
1000
1500
2000

S.mo CAMK_CAMK1−Tthe exon−intron and kinase domain diagram (all)

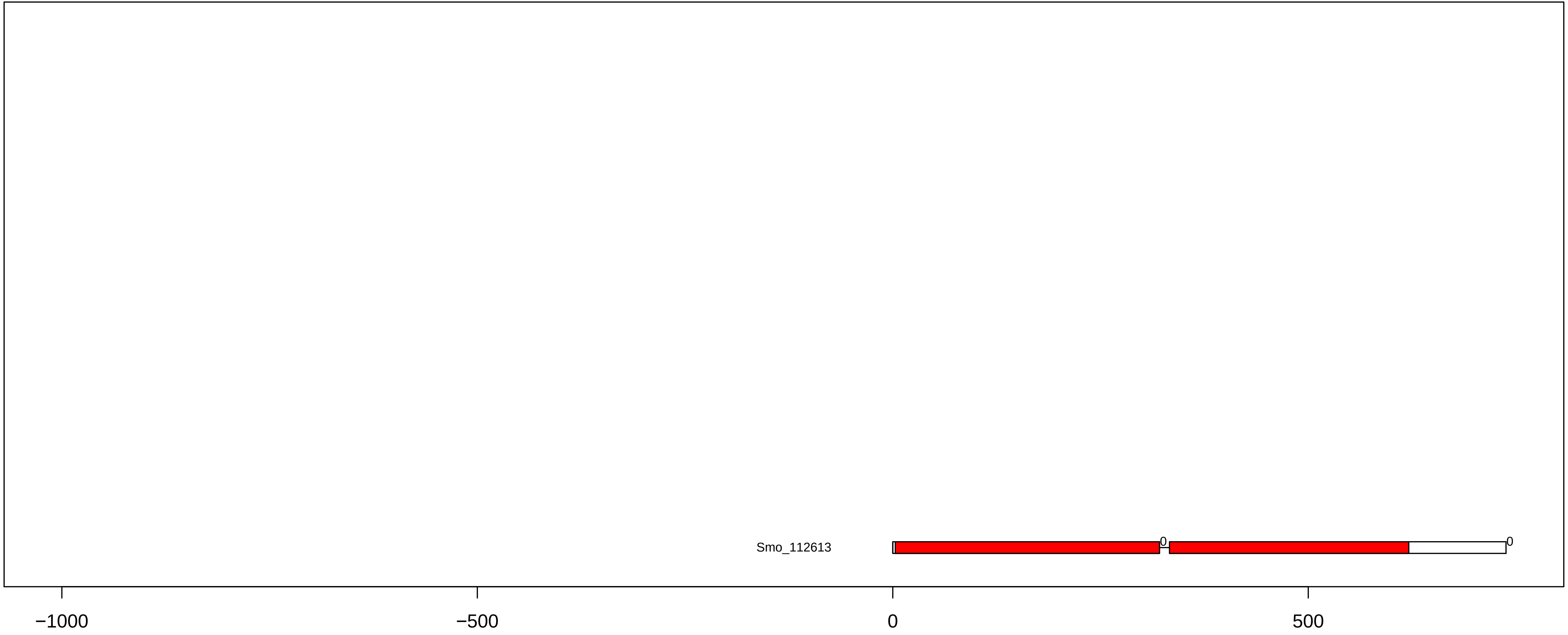

S.mo CAMK_CAMKL−CHK1 exon−intron and kinase domain diagram (all)

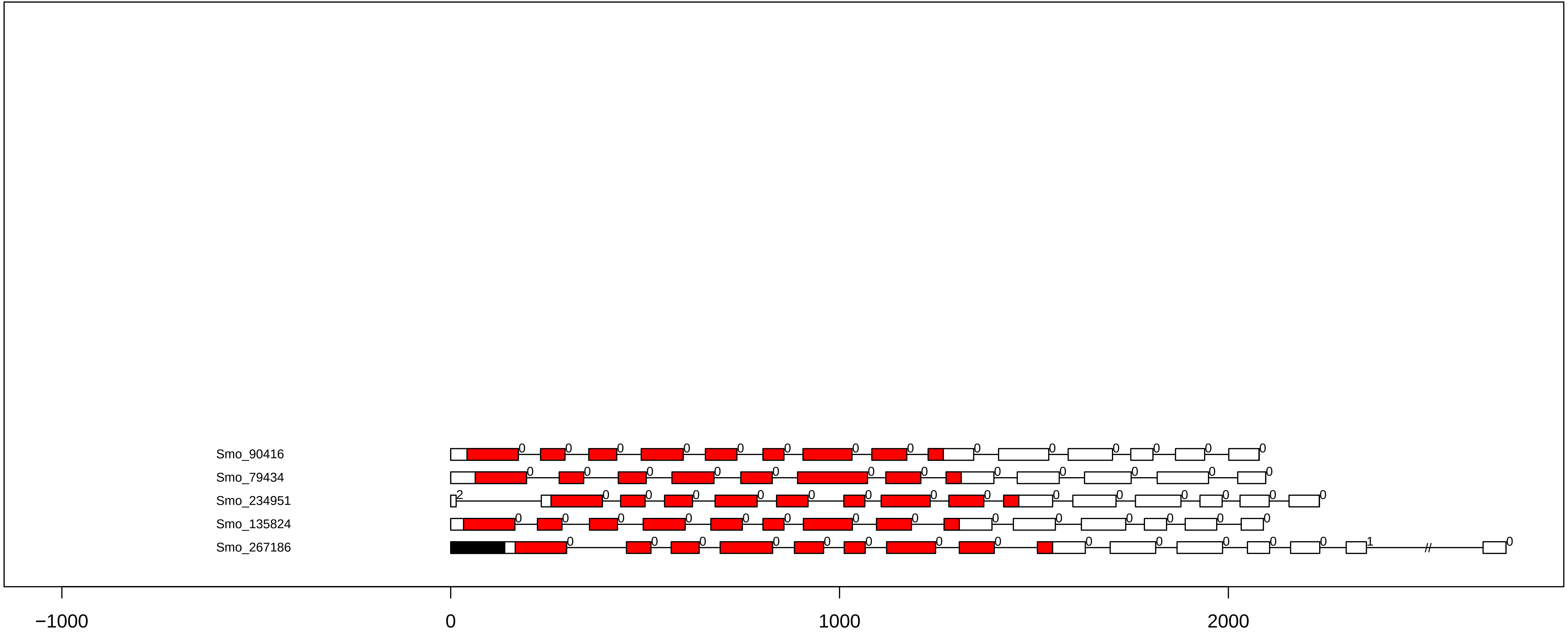

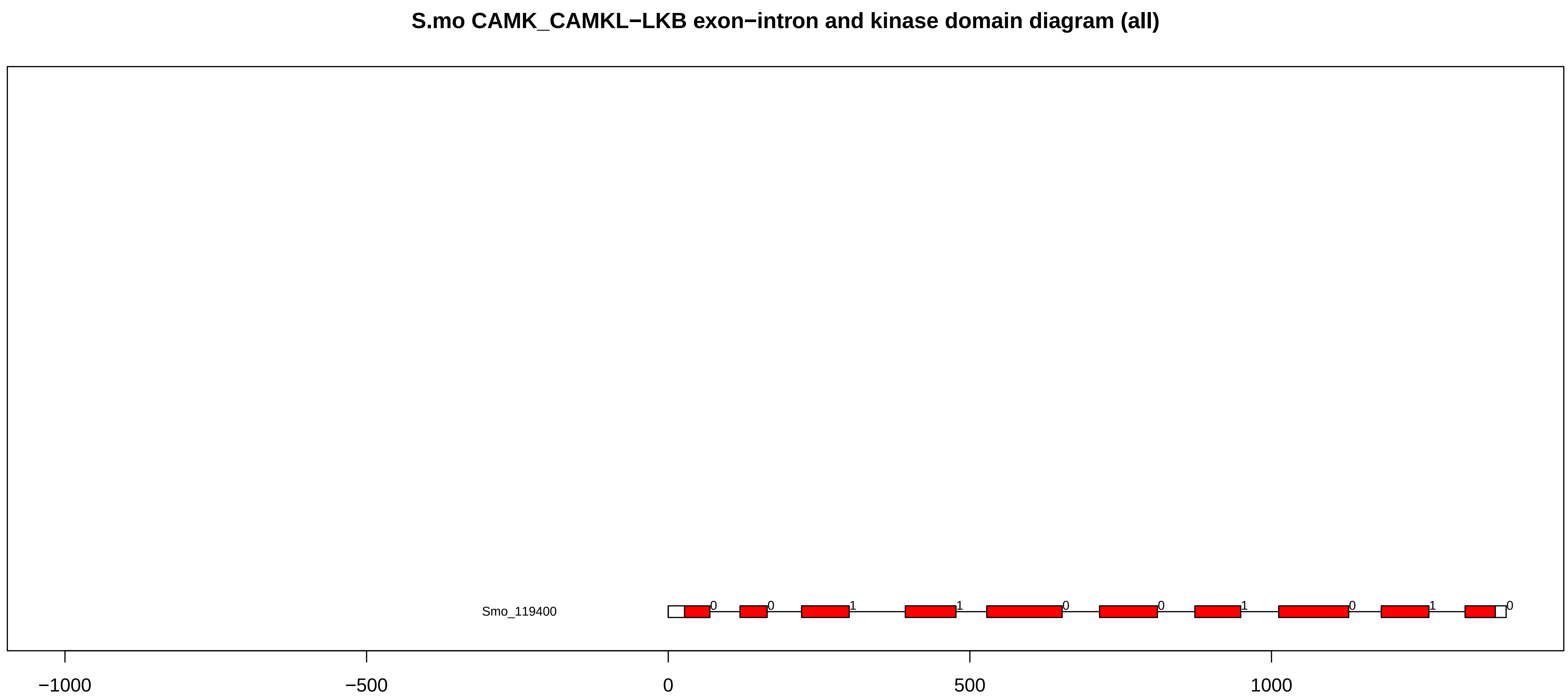

S.mo CAMK_CAMKL−LKB exon−intron and kinase domain diagram (all)
Smo_119400
0
0
1
1
0
0
1
0
1
0
−1000
−500
0
500
1000

S.mo CAMK_CDPK exon−intron and kinase domain diagram (part 1)

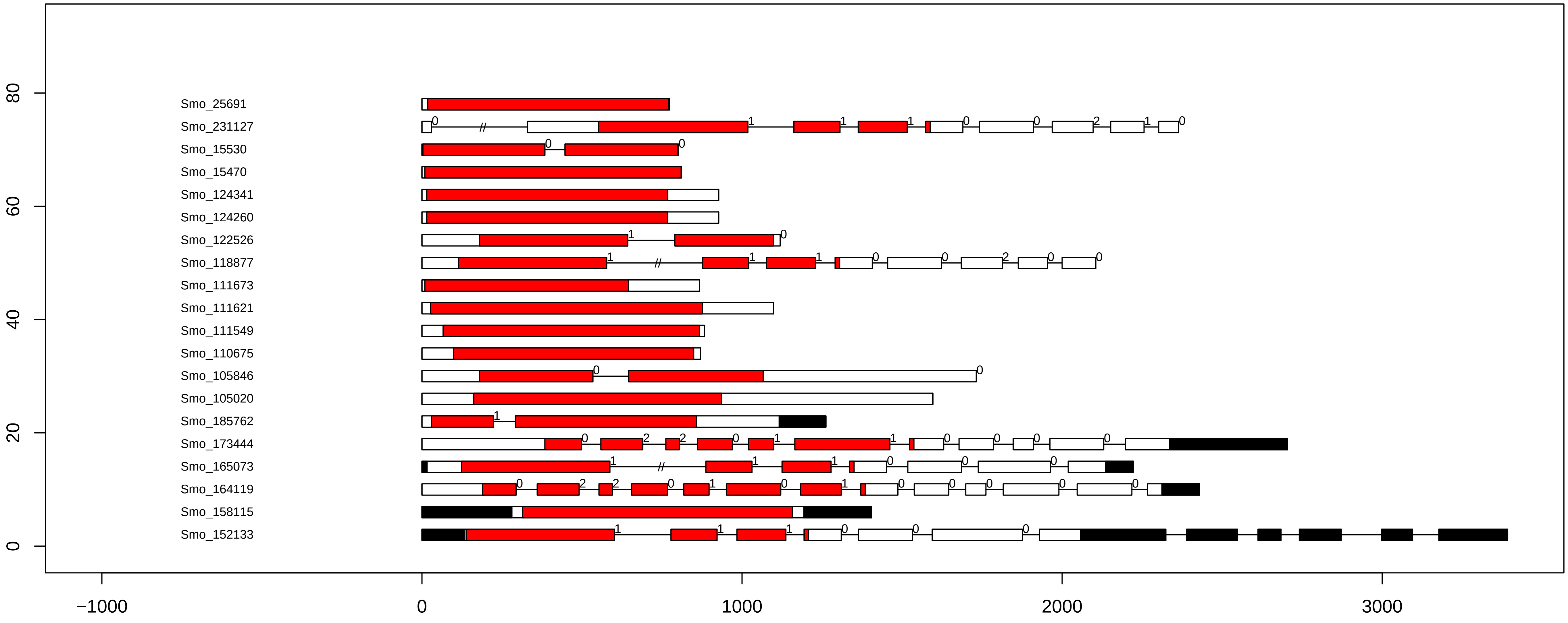

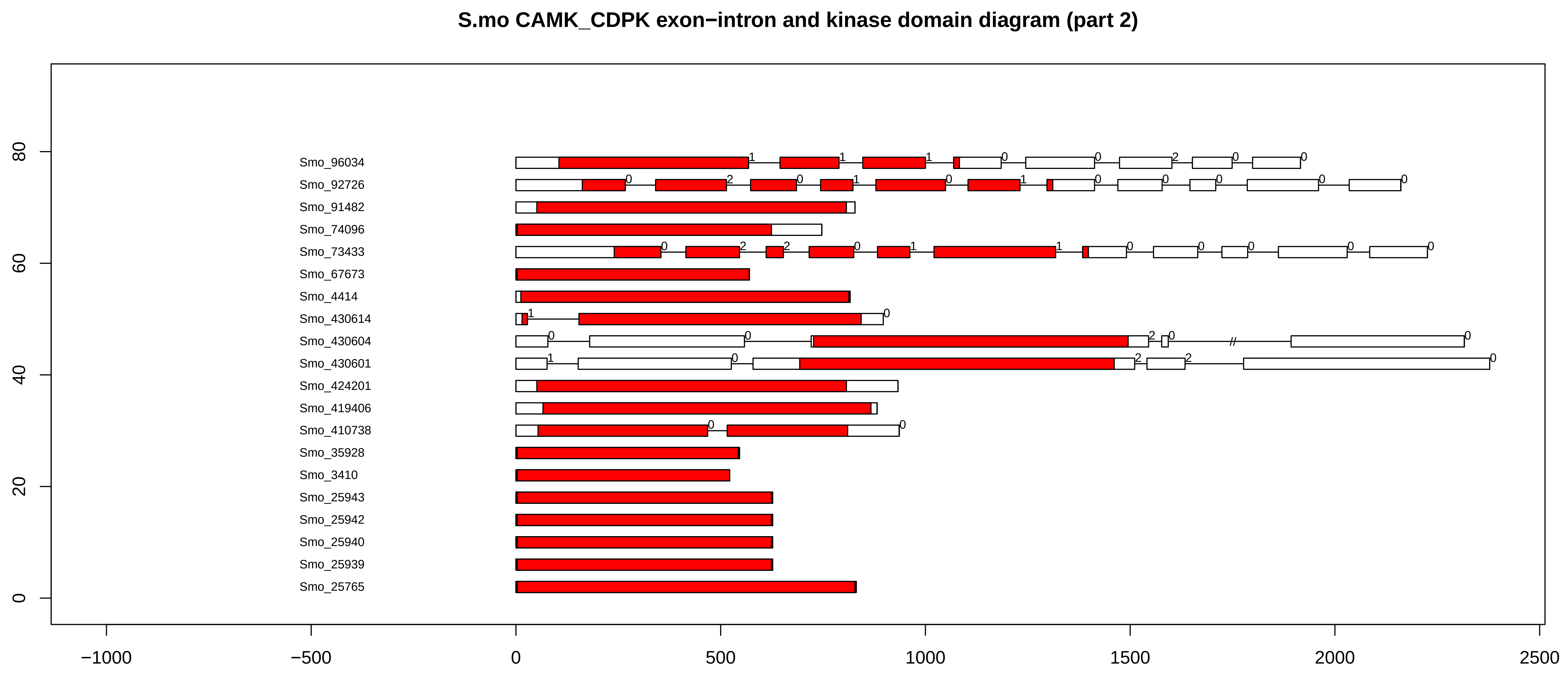

S.mo CAMK_CDPK exon−intron and kinase domain diagram (part 2)
Smo_96034
Smo_92726
Smo_91482
Smo_74096
Smo_73433
Smo_67673
Smo_4414
Smo_430614
Smo_430604
Smo_430601
Smo_424201
Smo_419406
Smo_410738
Smo_35928
Smo_3410
Smo_25943
Smo_25942
Smo_25940
Smo_25939
Smo_25765
0
20
40
60
80
−1000
−500
0
500
1000
1500
2000
2500

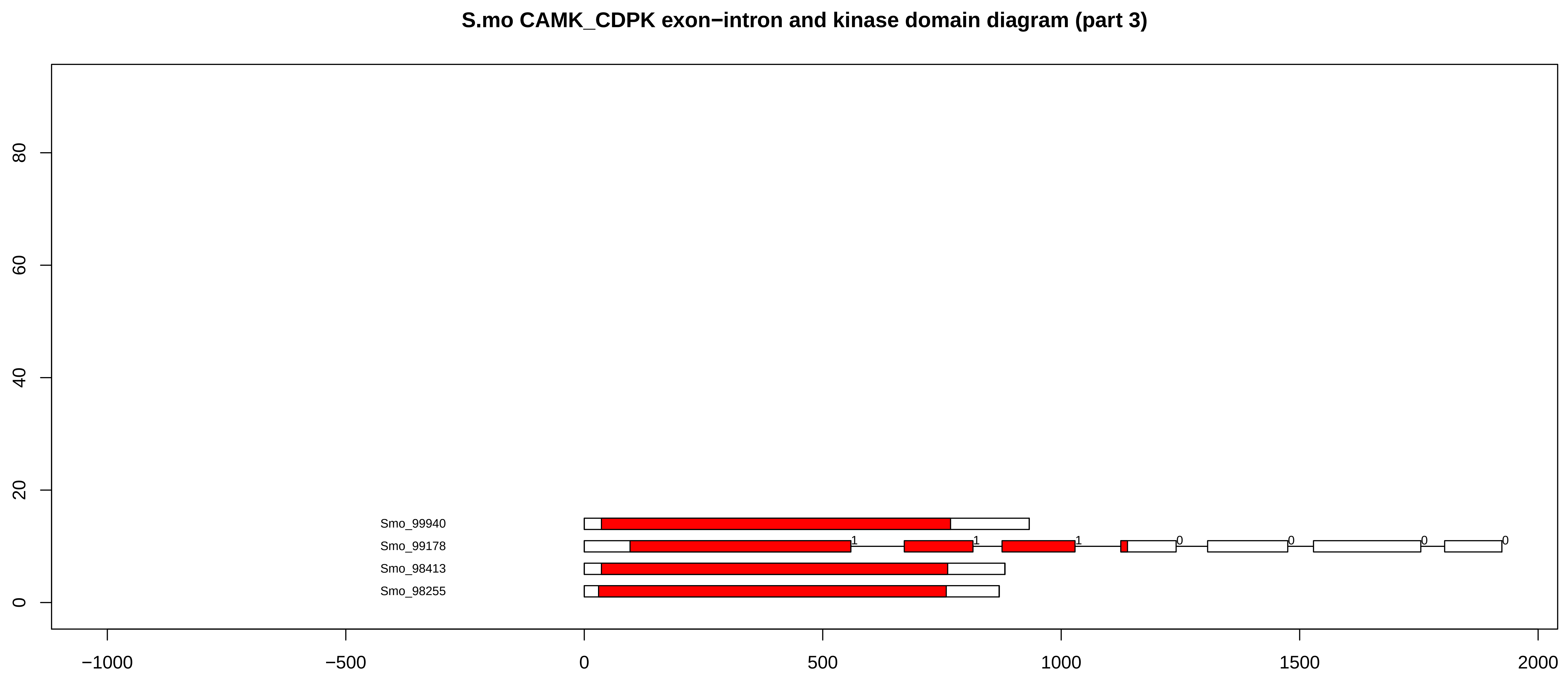

S.mo CAMK_CDPK exon−intron and kinase domain diagram (part 3)
Smo_99940
Smo_99178
Smo_98413
Smo_98255
1
1
1
0
0
0
0
0
20
40
60
80
−1000
−500
0
500
1000
1500
2000

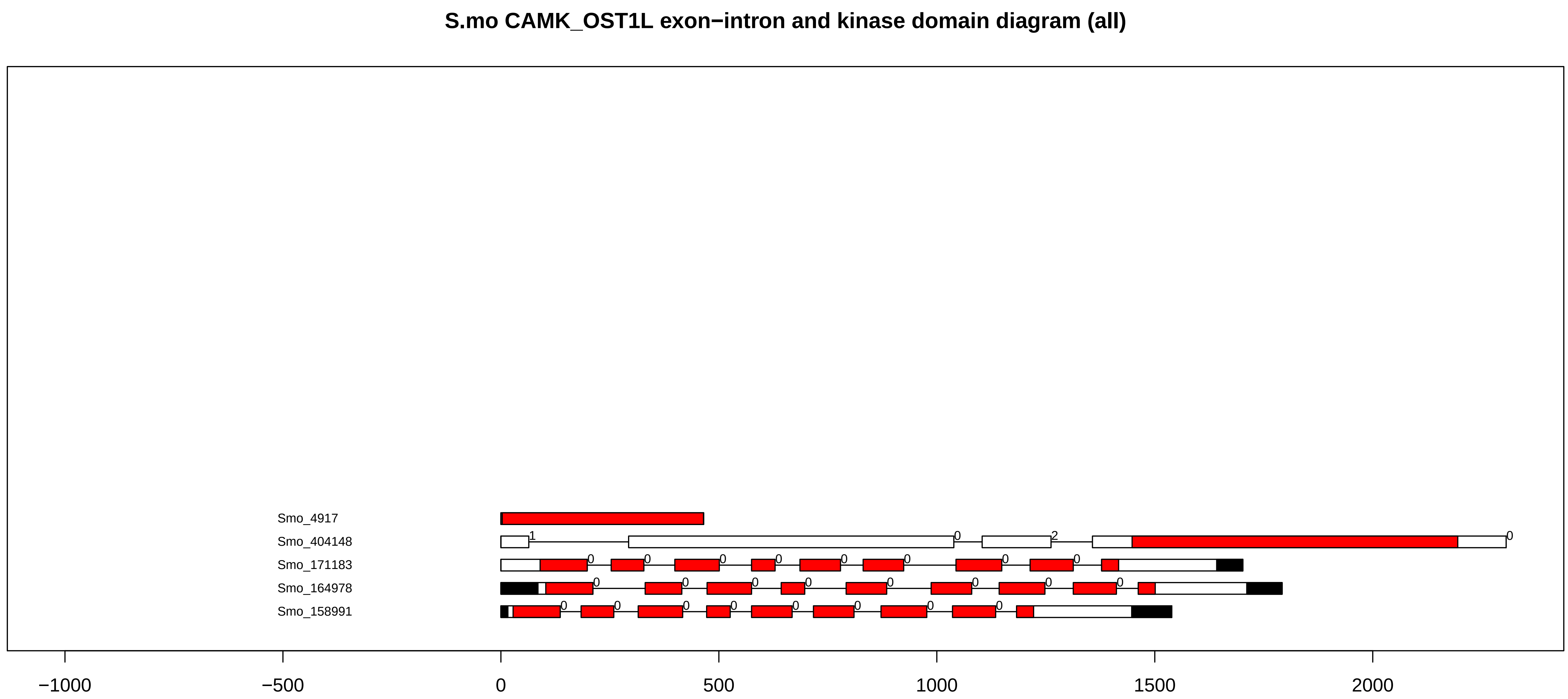

S.mo CAMK_OST1L exon−intron and kinase domain diagram (all)
Smo_4917
Smo_404148
Smo_171183
Smo_164978
Smo_158991
−1000
−500
0
500
1000
1500
2000

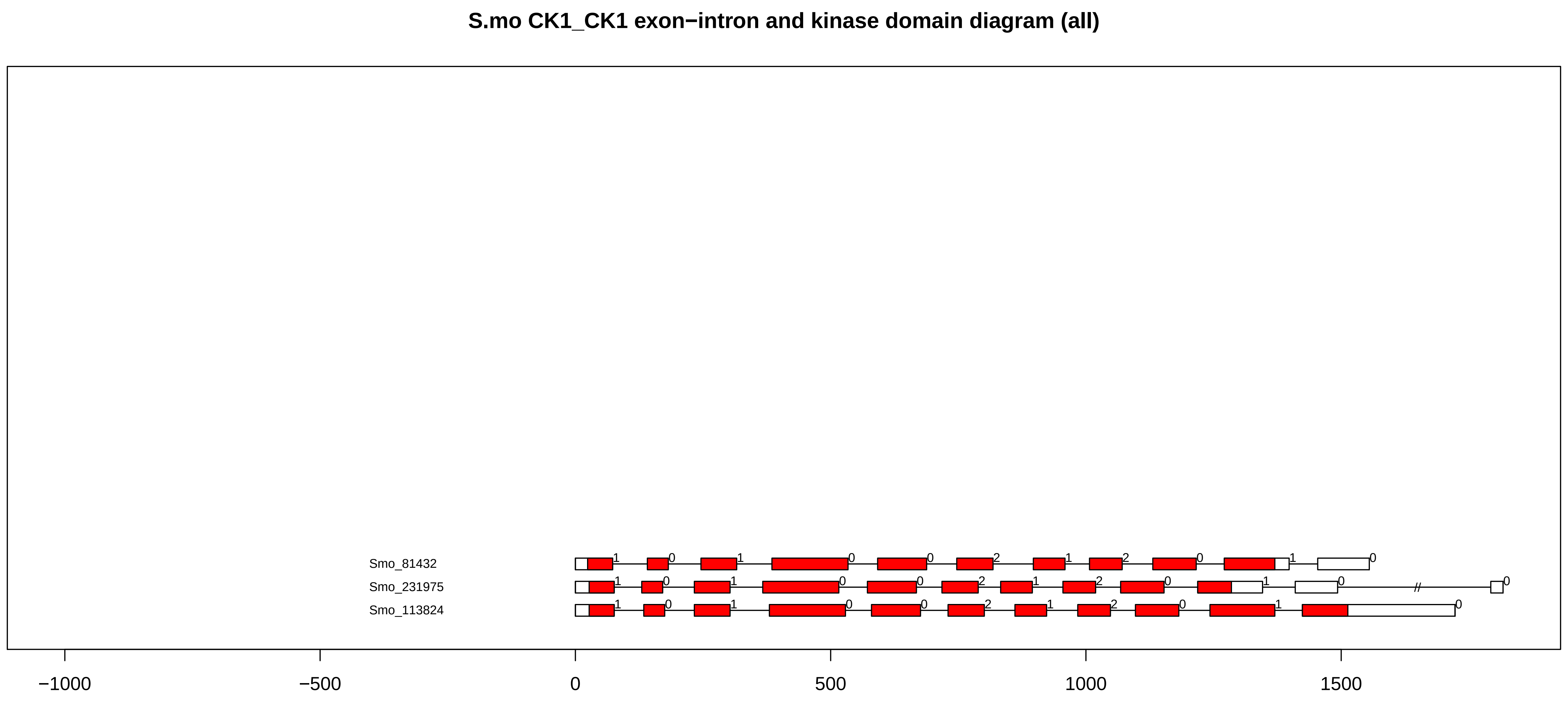
S.mo CK1_CK1 exon−intron and kinase domain diagram (all)
Smo_81432
Smo_231975
Smo_113824
−1000
−500
0
500
1000
1500

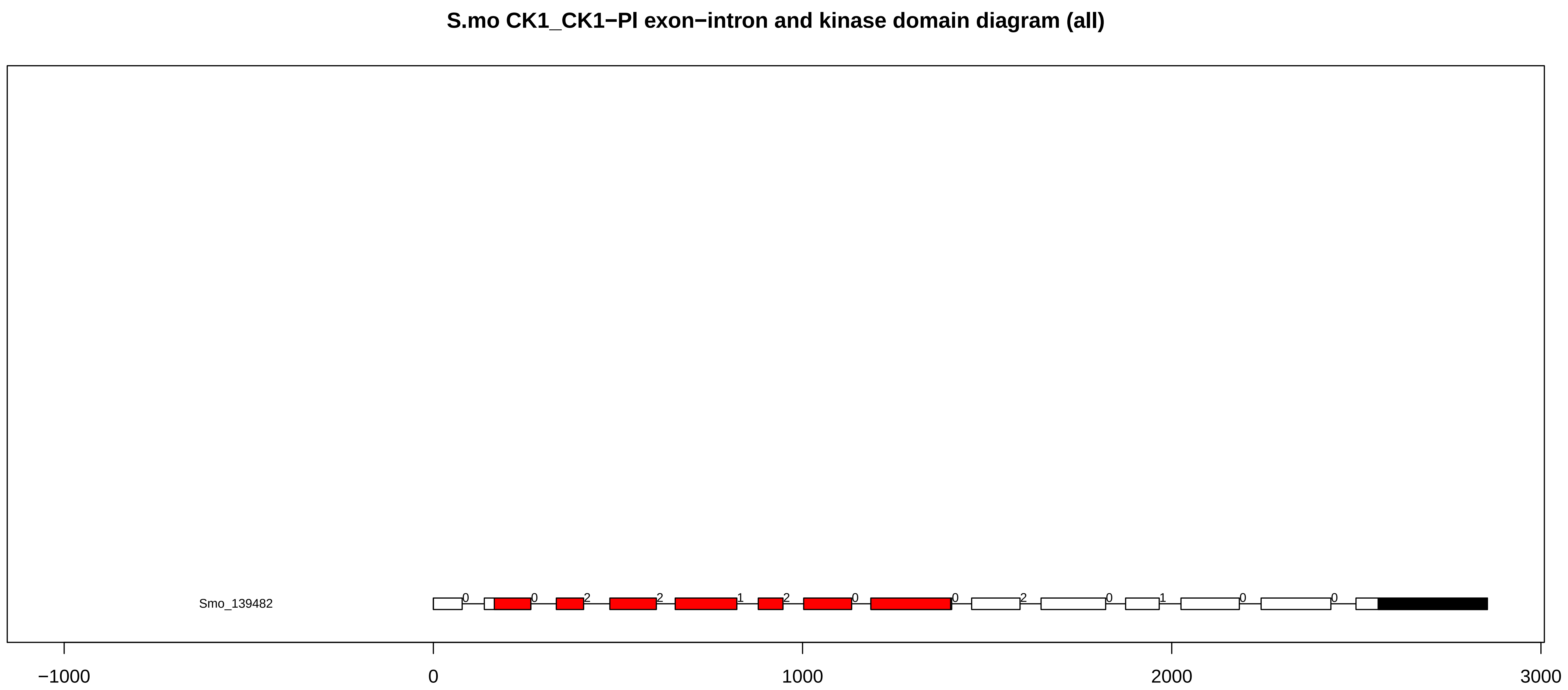
S.mo CK1_CK1−Pl exon−intron and kinase domain diagram (all)
Smo_139482
0
0
2
2
1
2
0
0
2
0
1
0
0
−1000
0
1000
2000
3000

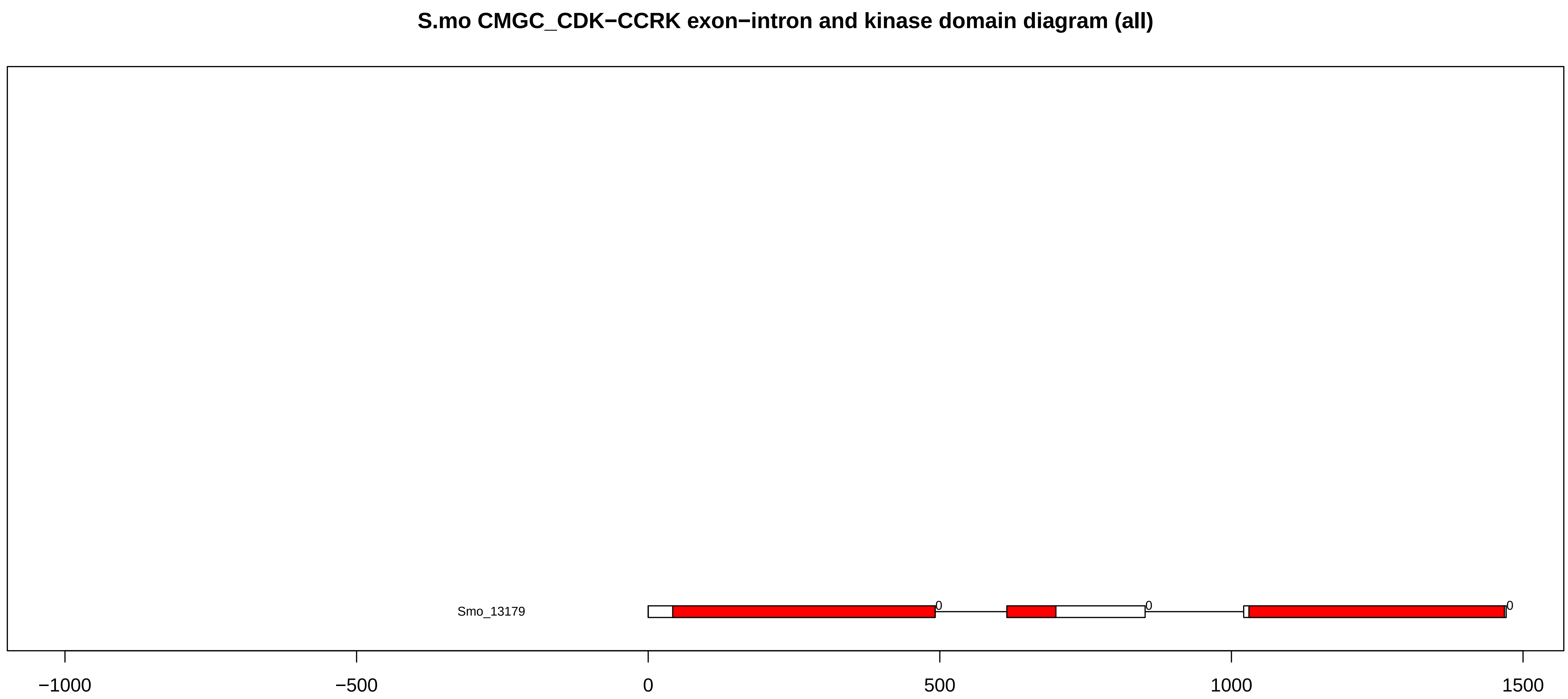

S.mo CMGC_CDK−CCRK exon−intron and kinase domain diagram (all)
Smo_13179
0
0
0
−1000
−500
0
500
1000
1500

S.mo CMGC_CDK−CDK7 exon−intron and kinase domain diagram (all)

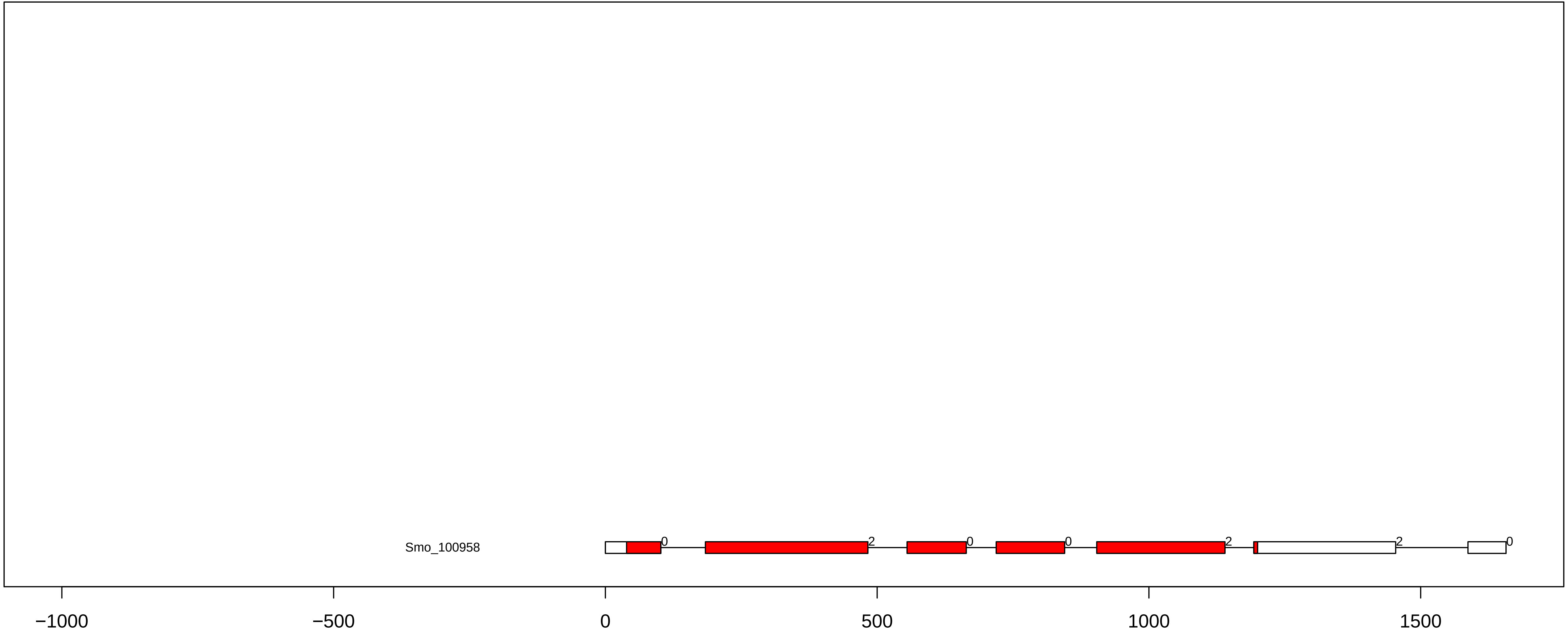

S.mo CMGC_CDK−CDK8 exon−intron and kinase domain diagram (all)

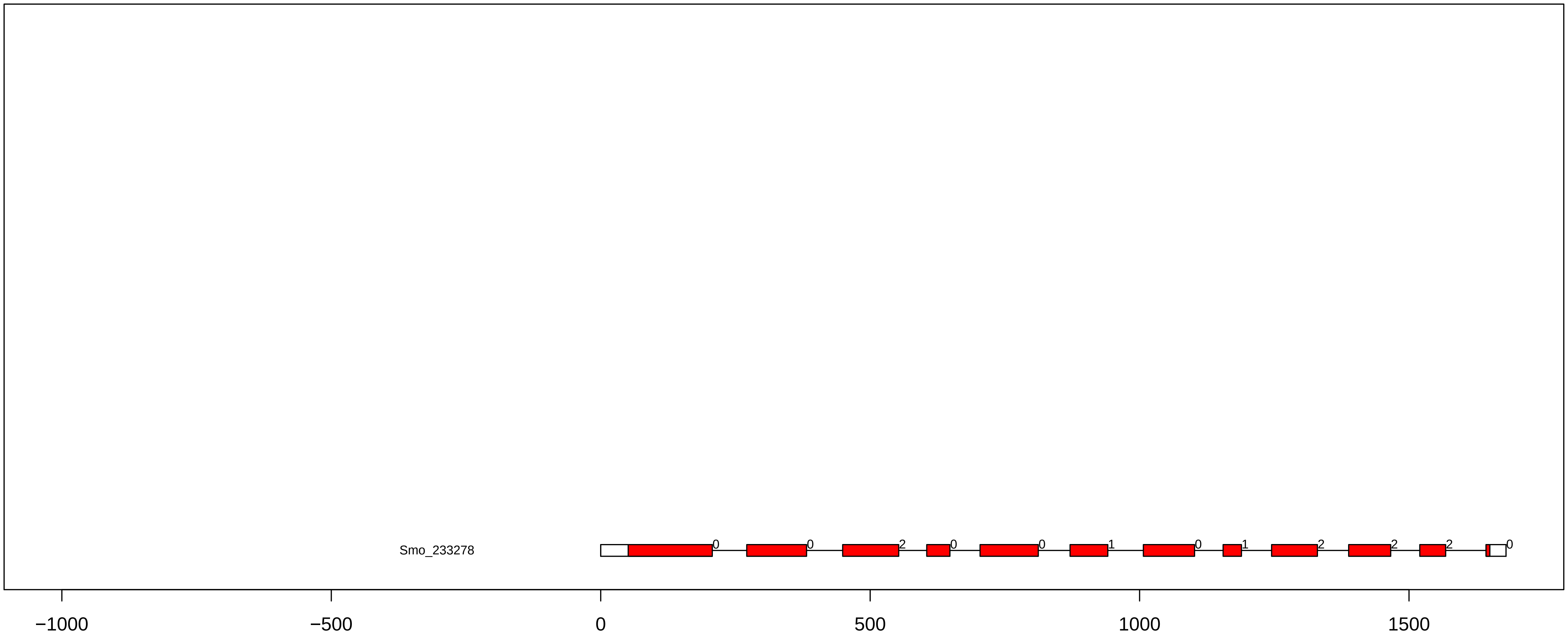

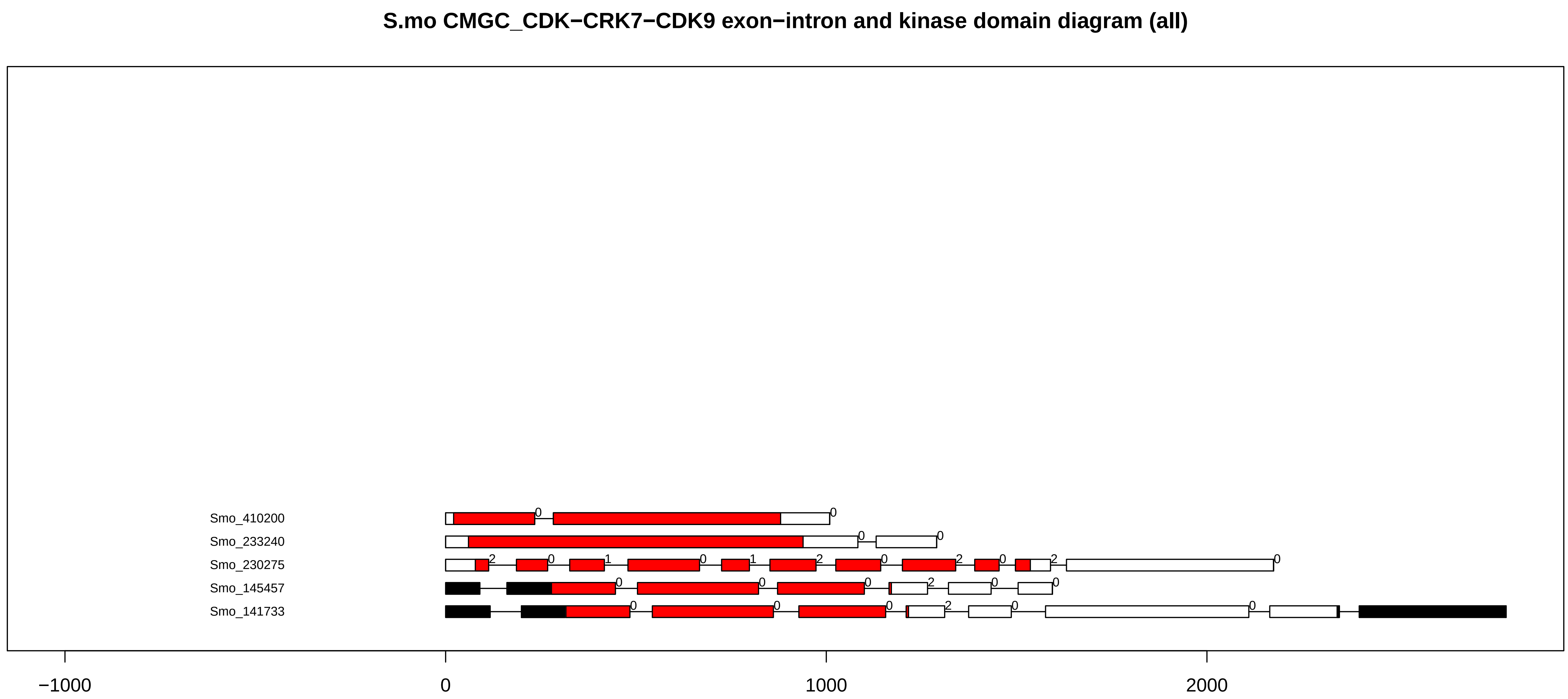

S.mo CMGC_CDK−CRK7−CDK9 exon−intron and kinase domain diagram (all)
Smo_410200
Smo_233240
Smo_230275
Smo_145457
Smo_141733
−1000
0
1000
2000

S.mo CMGC_CDKL−Cr exon−intron and kinase domain diagram (all)

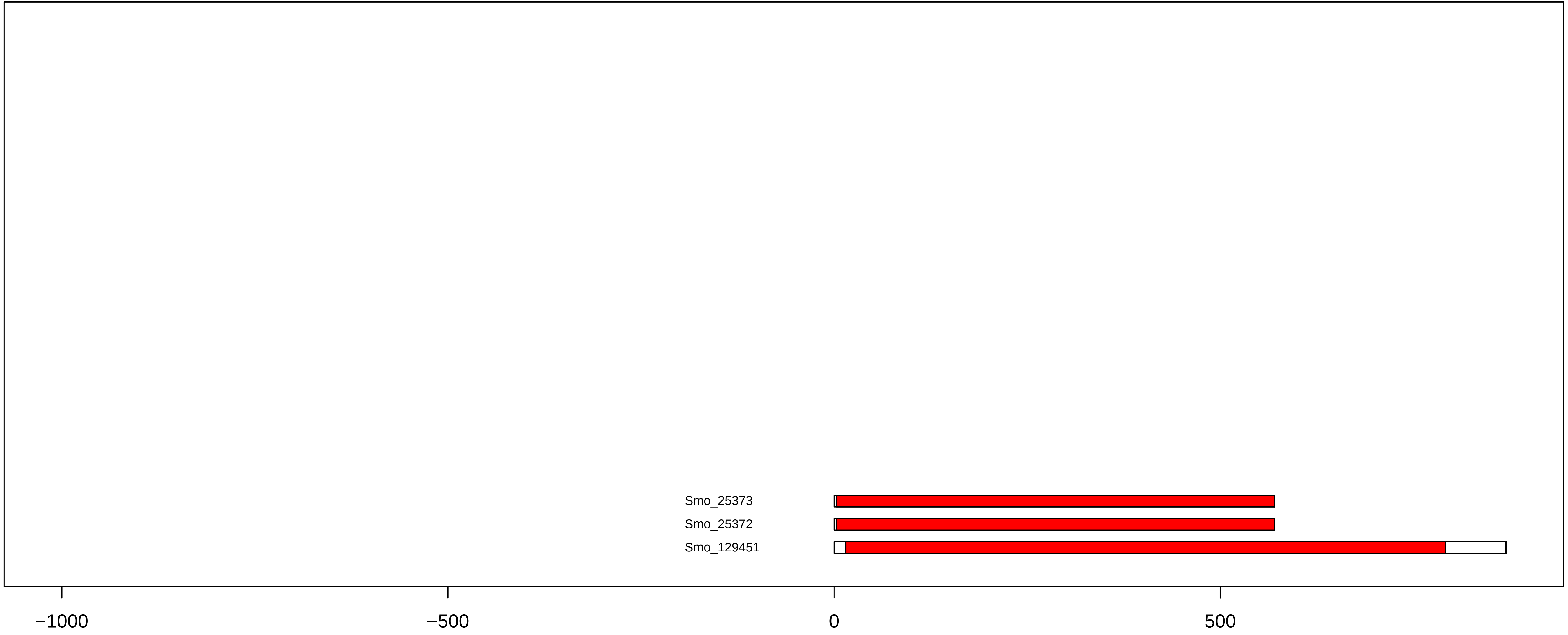

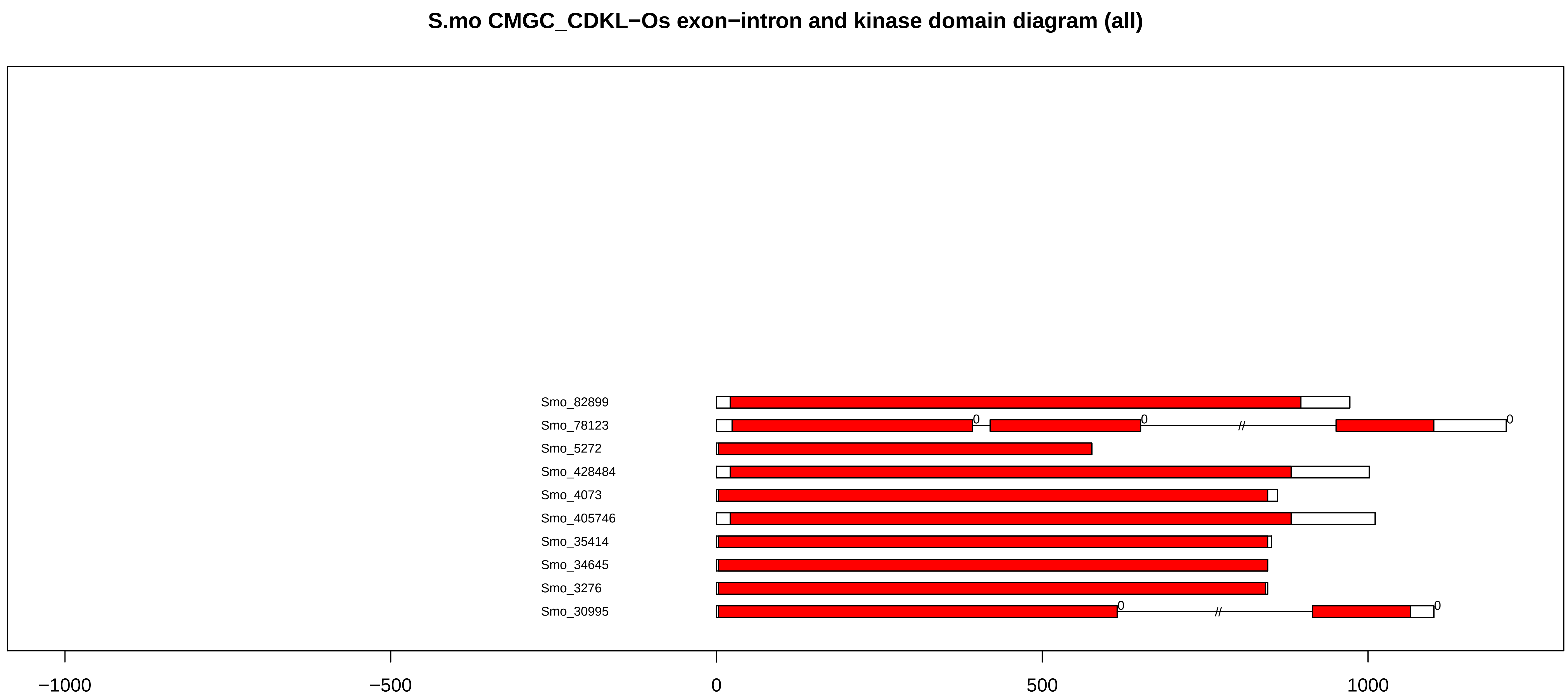

S.mo CMGC_CDKL−Os exon−intron and kinase domain diagram (all)
Smo_82899
Smo_78123
Smo_5272
Smo_428484
Smo_4073
Smo_405746
Smo_35414
Smo_34645
Smo_3276
Smo_30995
0
0
0
0
0
−1000
−500
0
500
1000

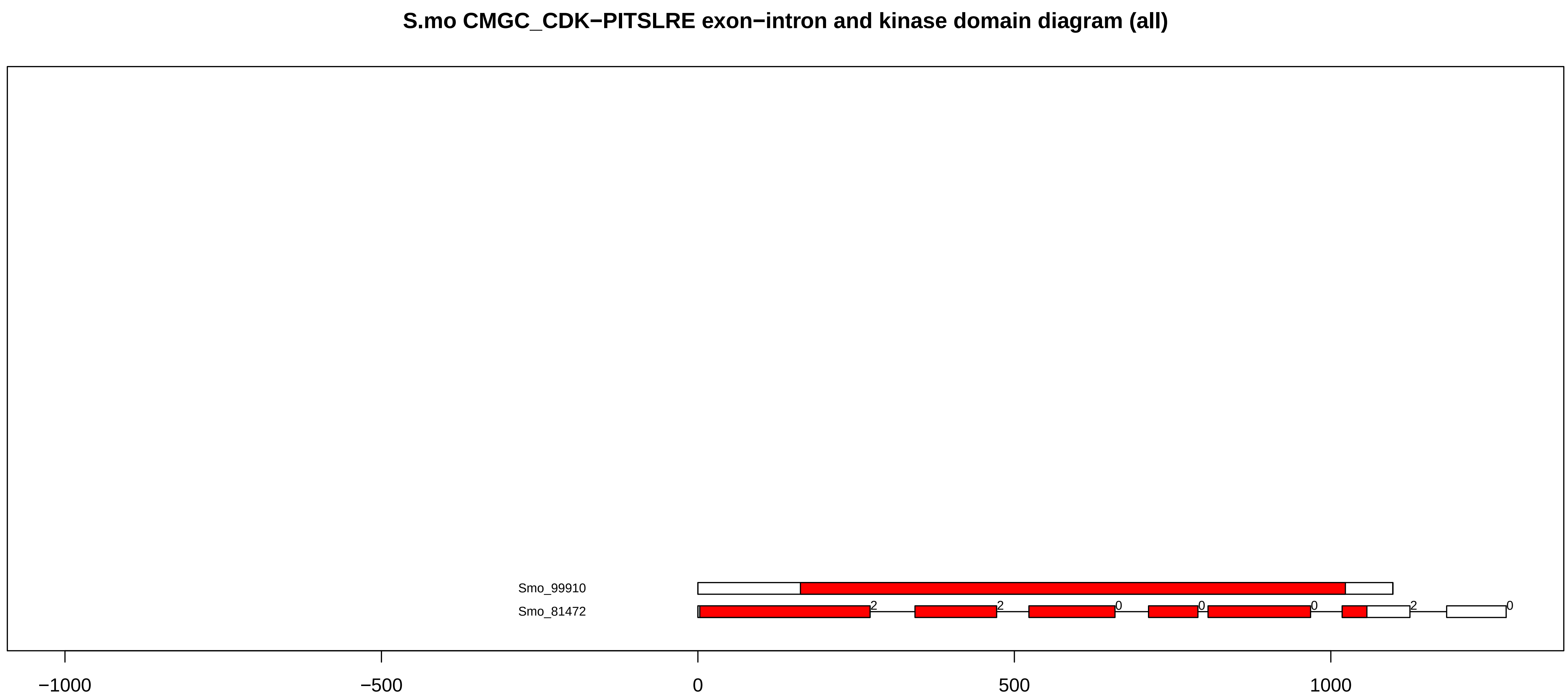

S.mo CMGC_CDK−PITSLRE exon−intron and kinase domain diagram (all)
Smo_99910
Smo_81472
2
2
0
0
0
2
0
−1000
−500
0
500
1000

S.mo CMGC_CDK−Pl exon−intron and kinase domain diagram (all)

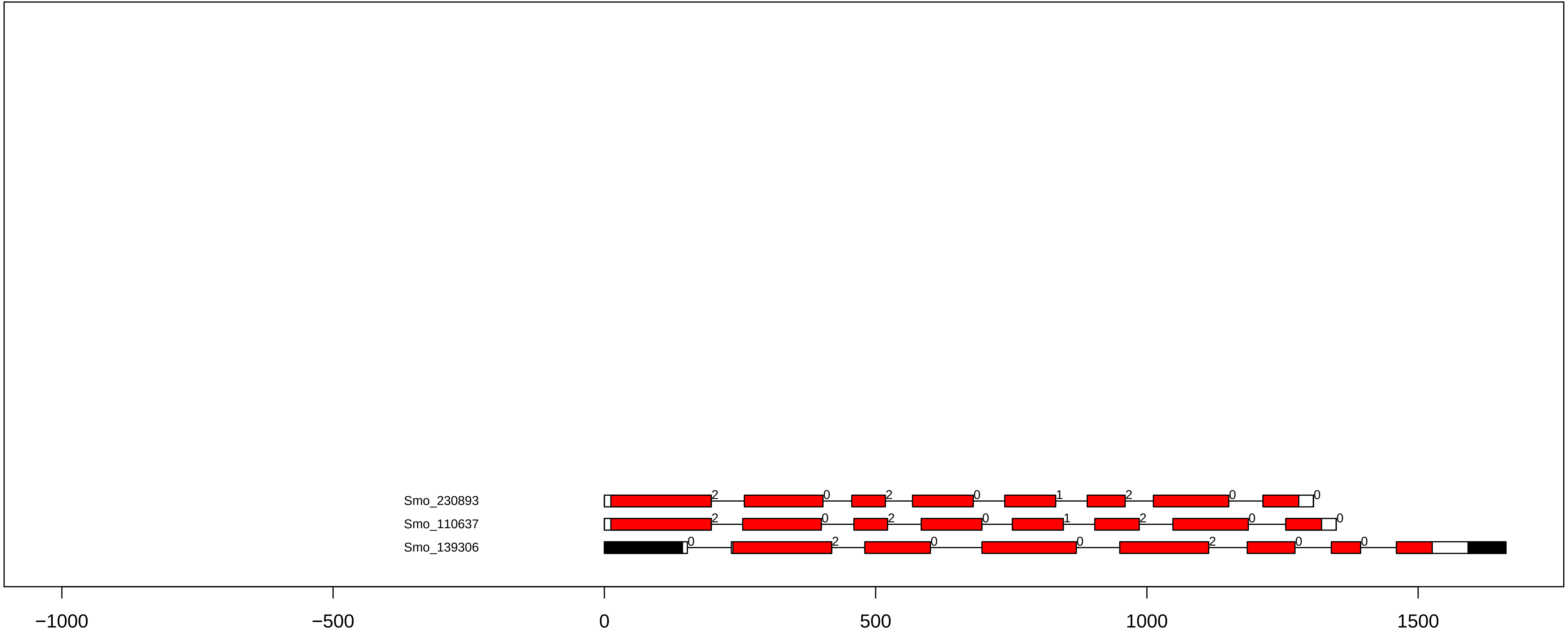

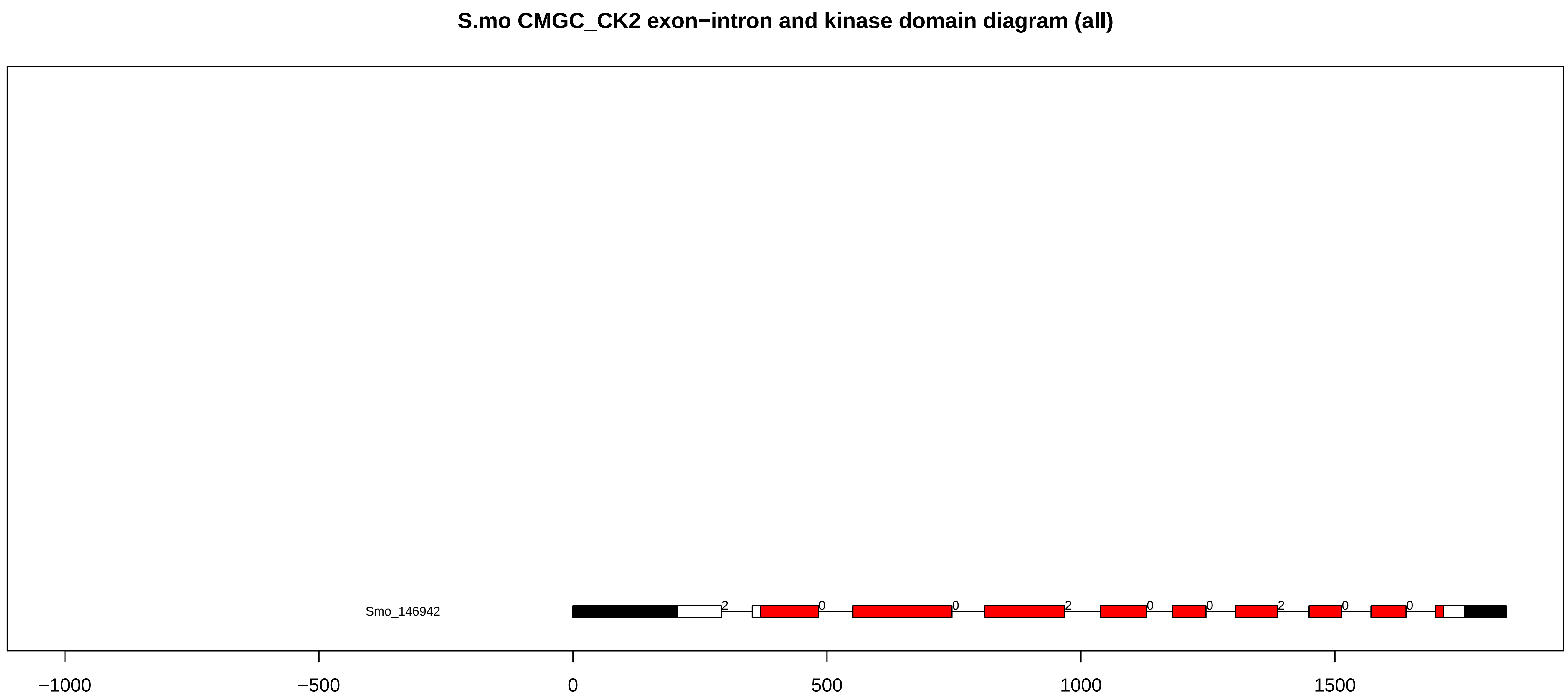
S.mo CMGC_CK2 exon−intron and kinase domain diagram (all)
Smo_146942
2
0
0
2
0
0
2
0
0
−1000
−500
0
500
1000
1500

S.mo CMGC_CLK exon−intron and kinase domain diagram (all)

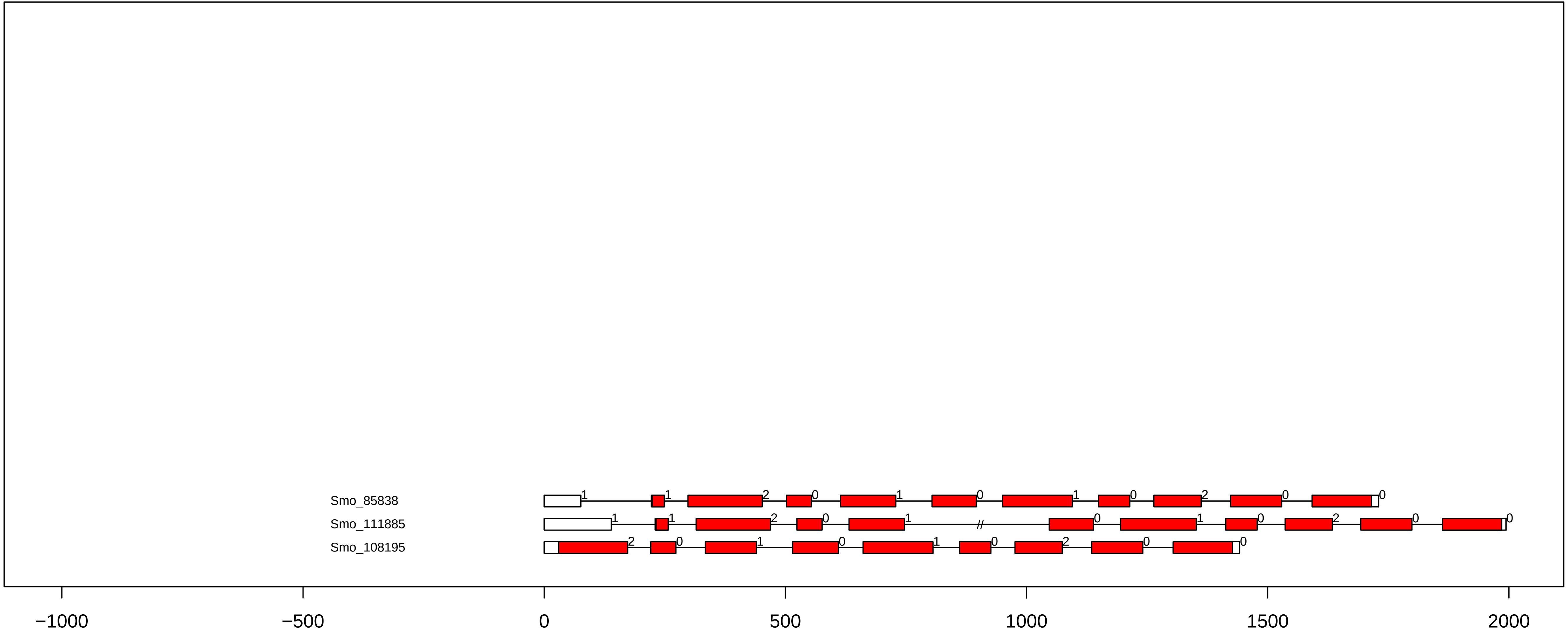

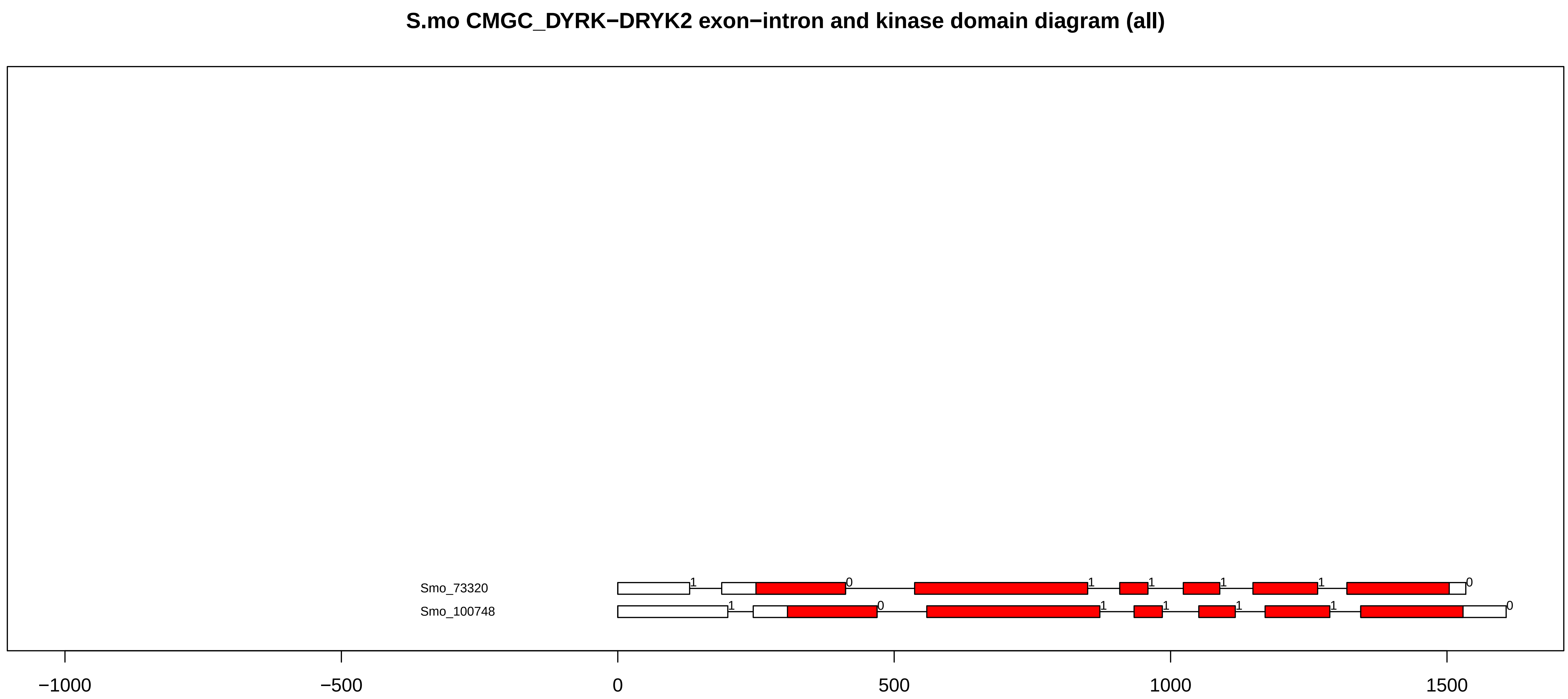
S.mo CMGC_DYRK−DRYK2 exon−intron and kinase domain diagram (all)
Smo_73320
Smo_100748
1
0
−1000
−500
0
500
1000
1500

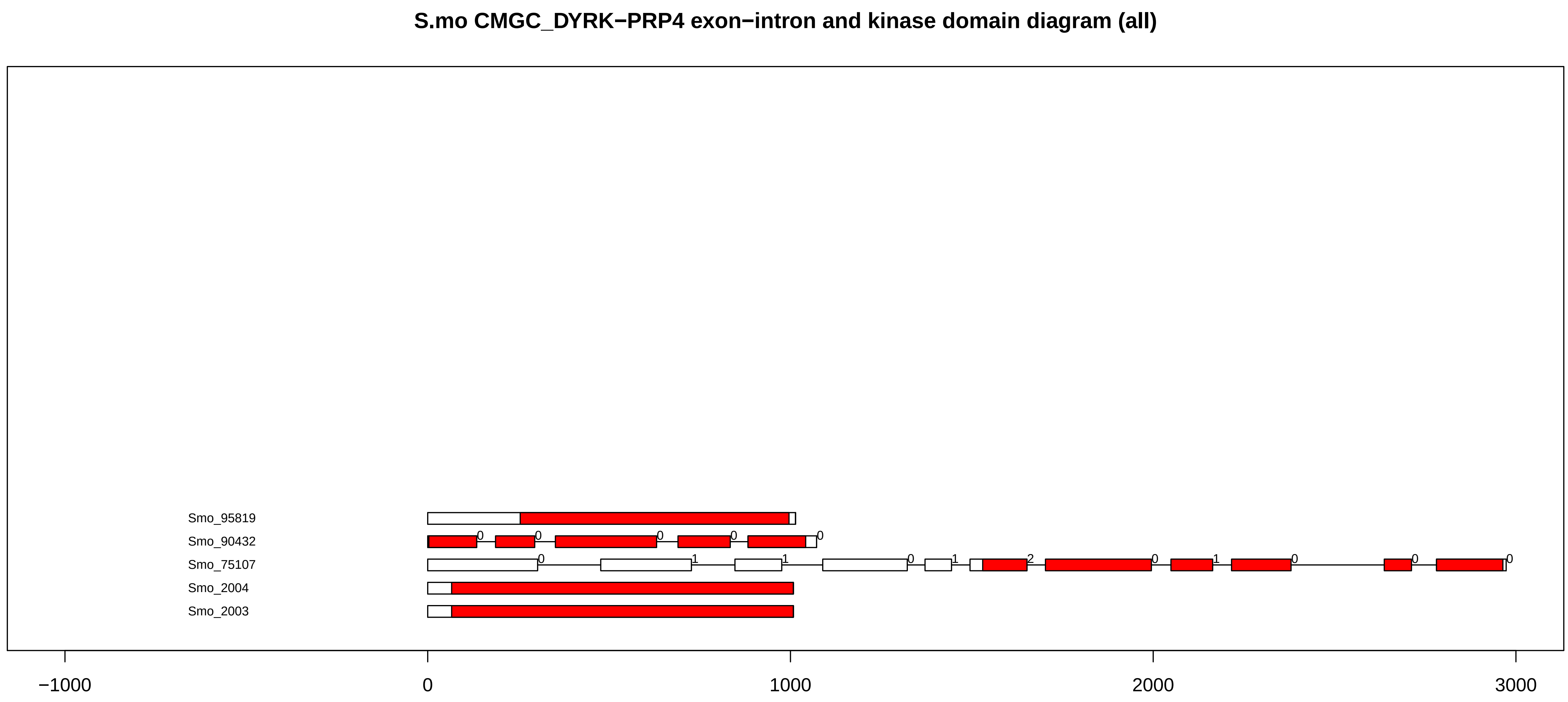
S.mo CMGC_DYRK−PRP4 exon−intron and kinase domain diagram (all)
Smo_95819
Smo_90432
Smo_75107
Smo_2004
Smo_2003
−1000
0
1000
2000
3000

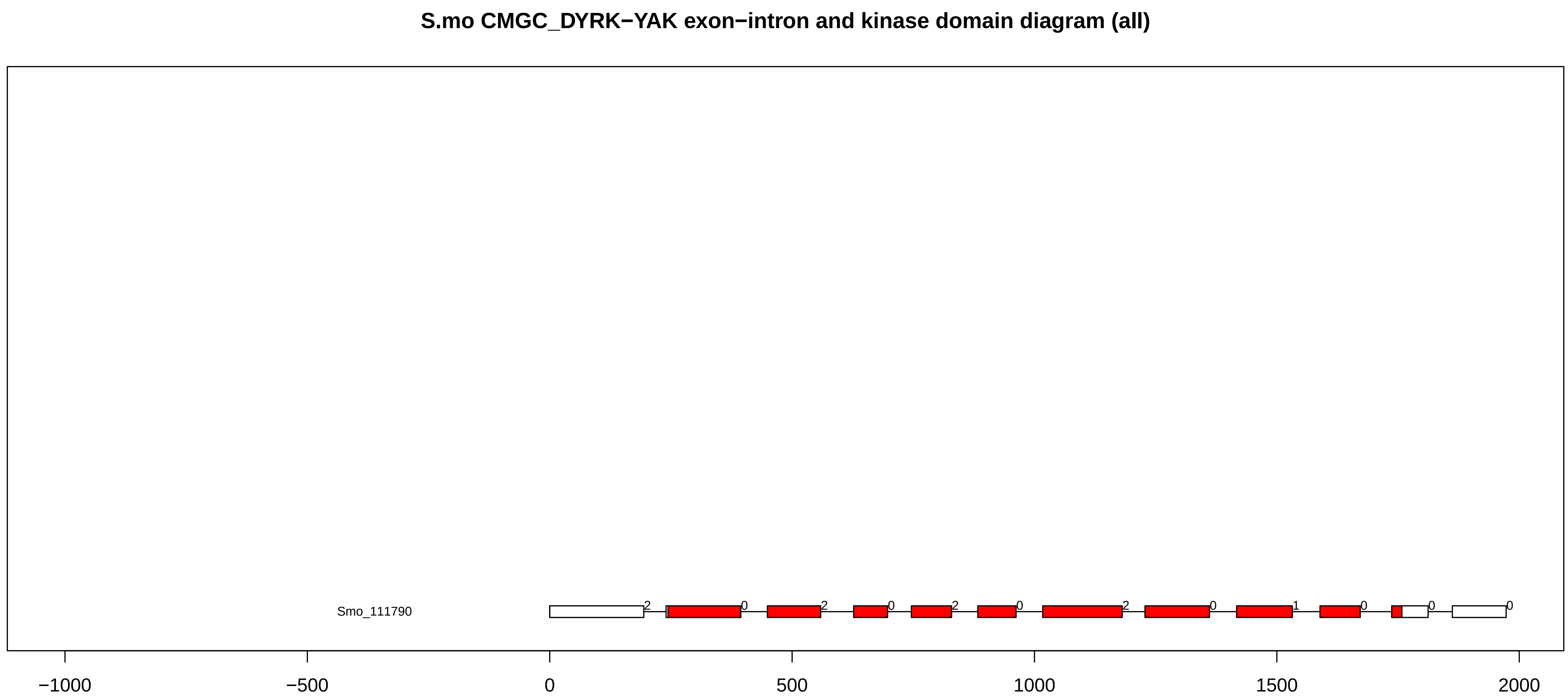
S.mo CMGC_DYRK−YAK exon−intron and kinase domain diagram (all)
Smo_111790
2
0
2
0
2
0
2
0
1
0
0
0
−1000
−500
0
500
1000
1500
2000

S.mo CMGC_GSKL exon−intron and kinase domain diagram (all)

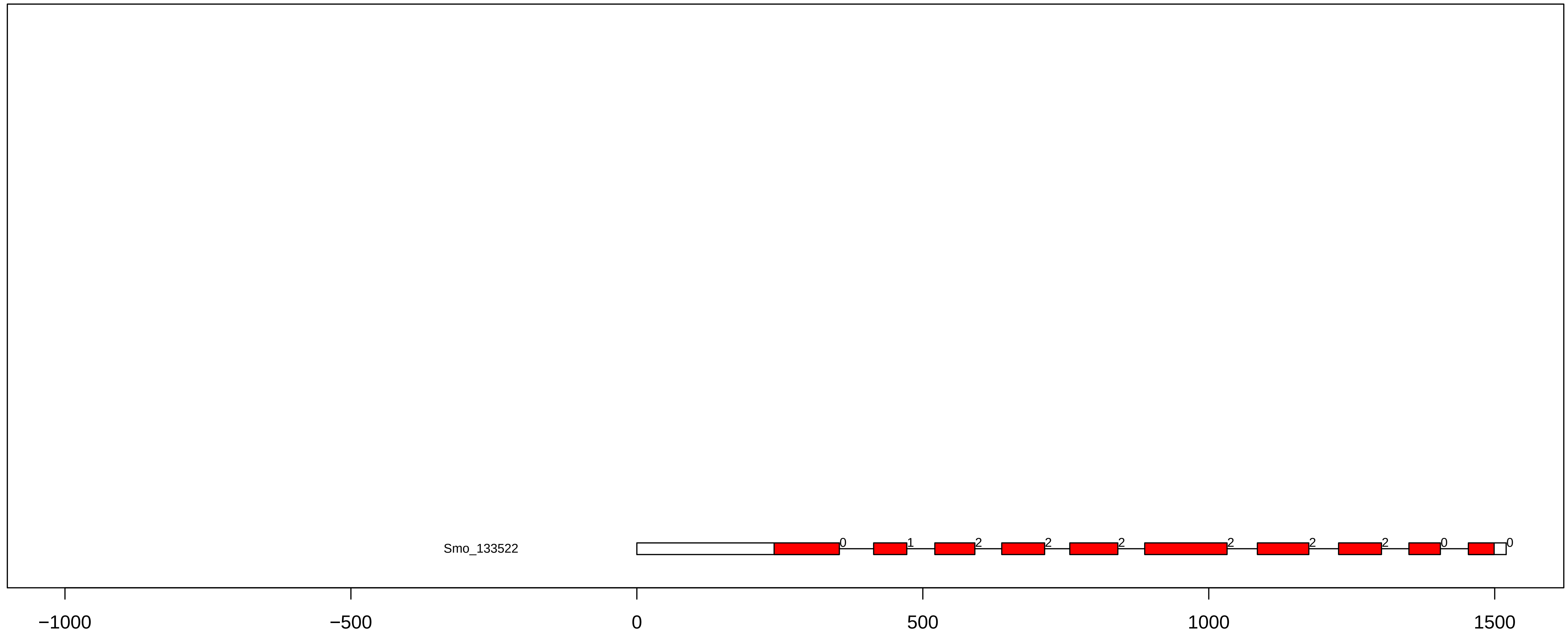

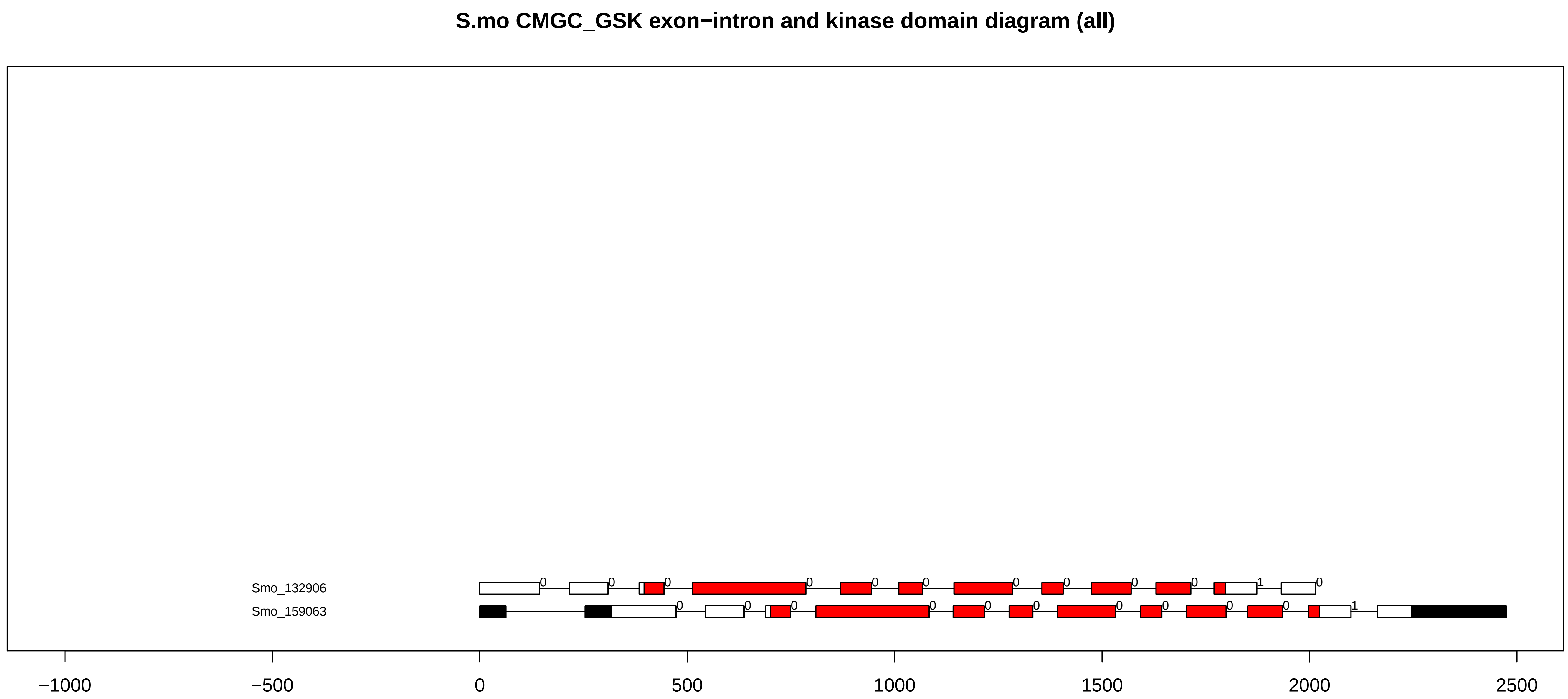

S.mo CMGC_GSK exon−intron and kinase domain diagram (all)
Smo_132906
Smo_159063
−1000
−500
0
500
1000
1500
2000
2500

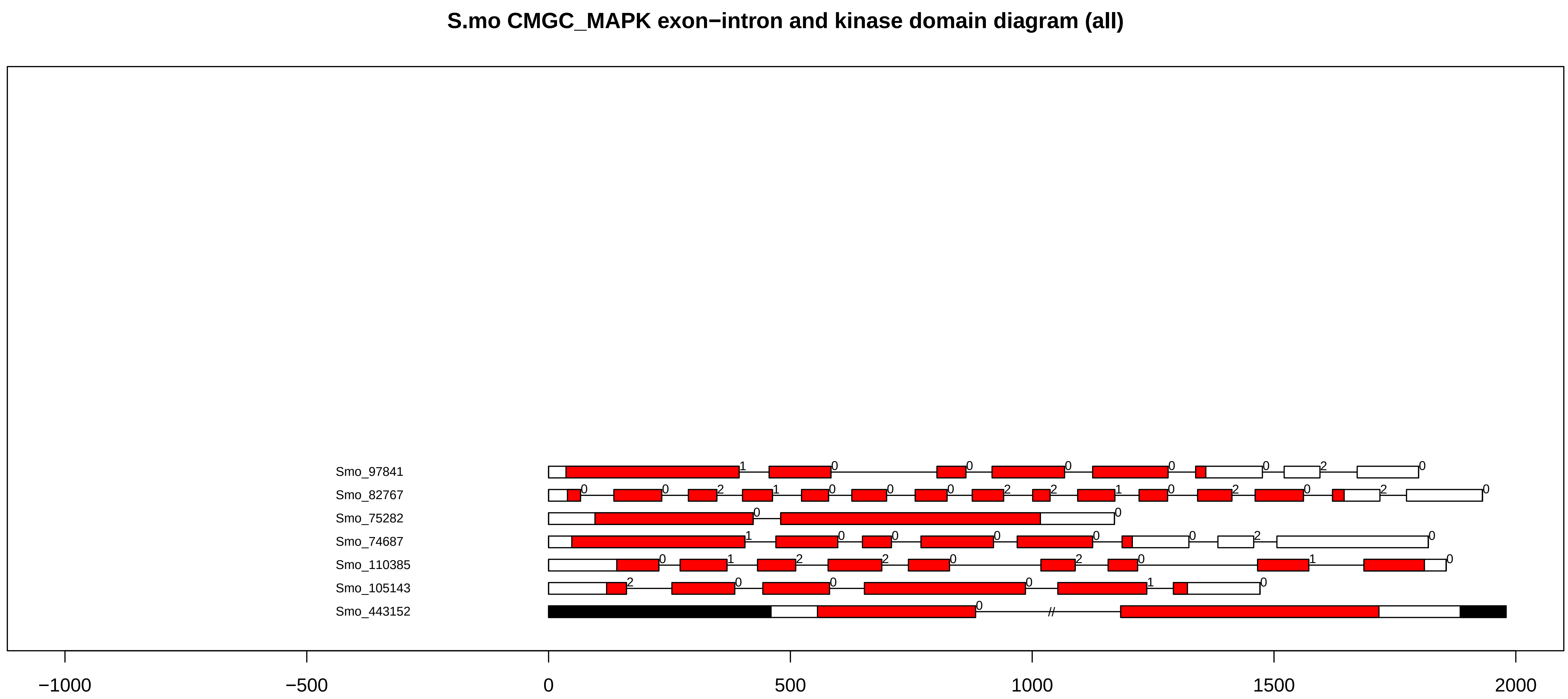
S.mo CMGC_MAPK exon−intron and kinase domain diagram (all)
Smo_97841
Smo_82767
Smo_75282
Smo_74687
Smo_110385
Smo_105143
Smo_443152
−1000
−500
0
500
1000
1500
2000

S.mo CMGC_RCK exon−intron and kinase domain diagram (all)

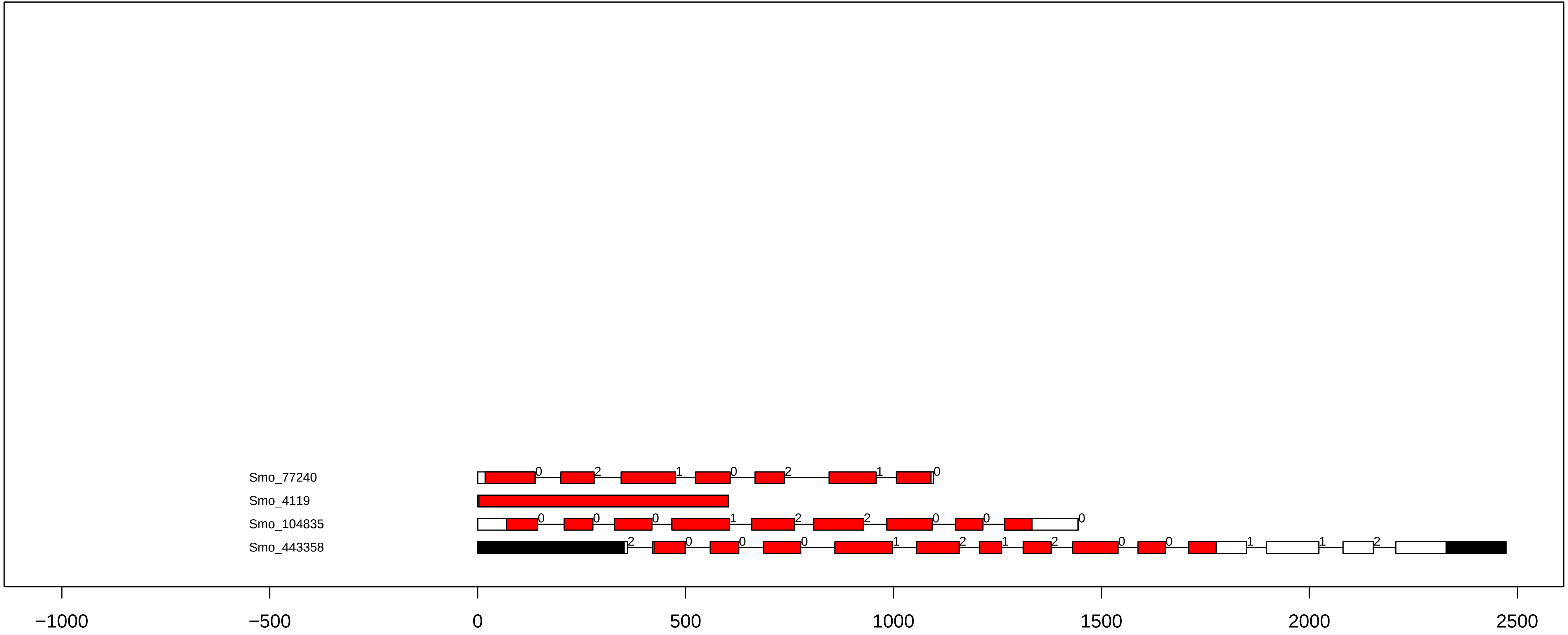

S.mo CMGC_SRPK exon−intron and kinase domain diagram (all)

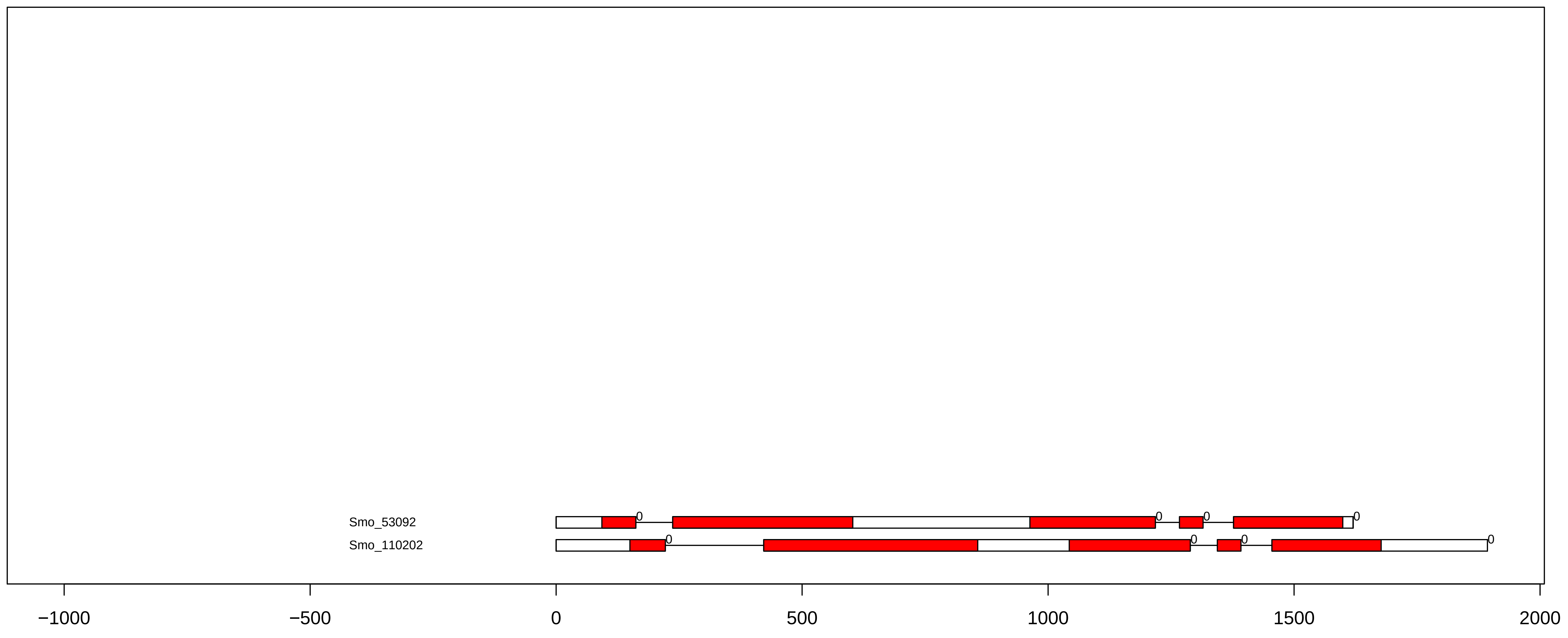

S.mo Group−Cr−1 exon−intron and kinase domain diagram (all)

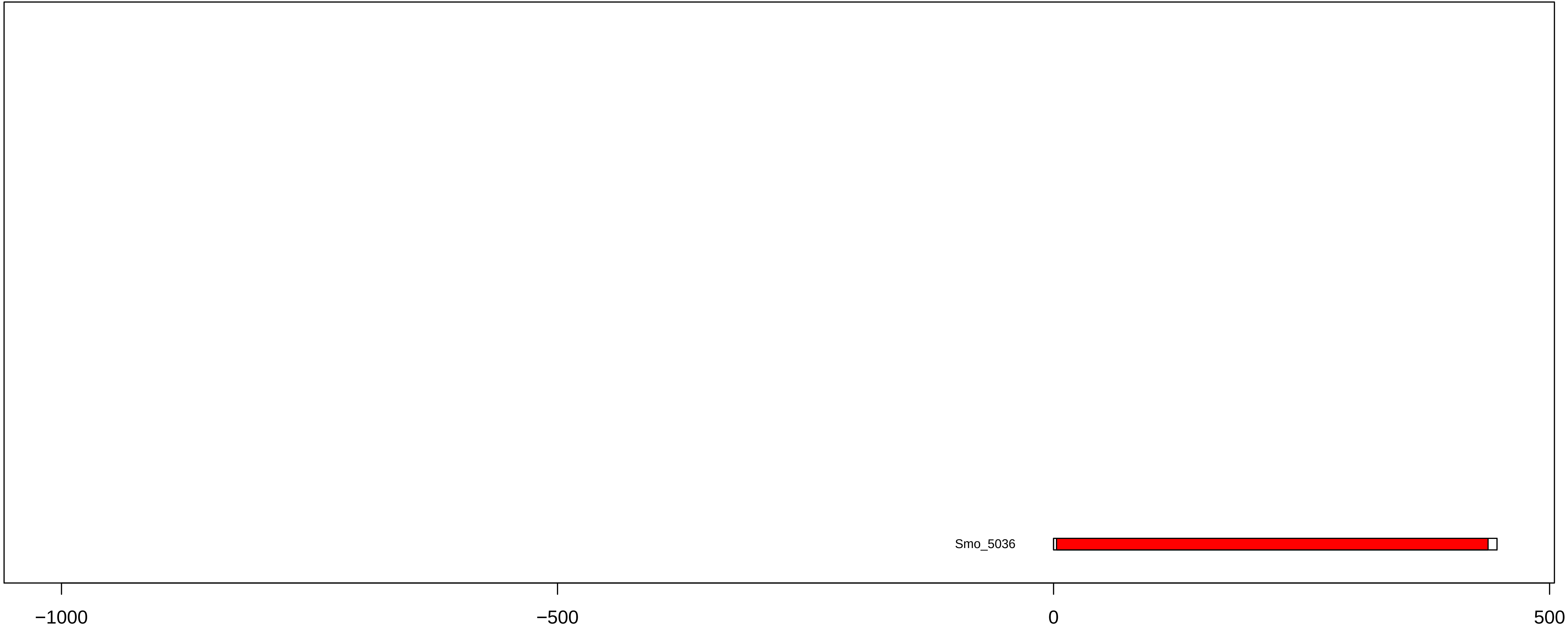

S.mo Group−PI−1 exon−intron and kinase domain diagram (all)

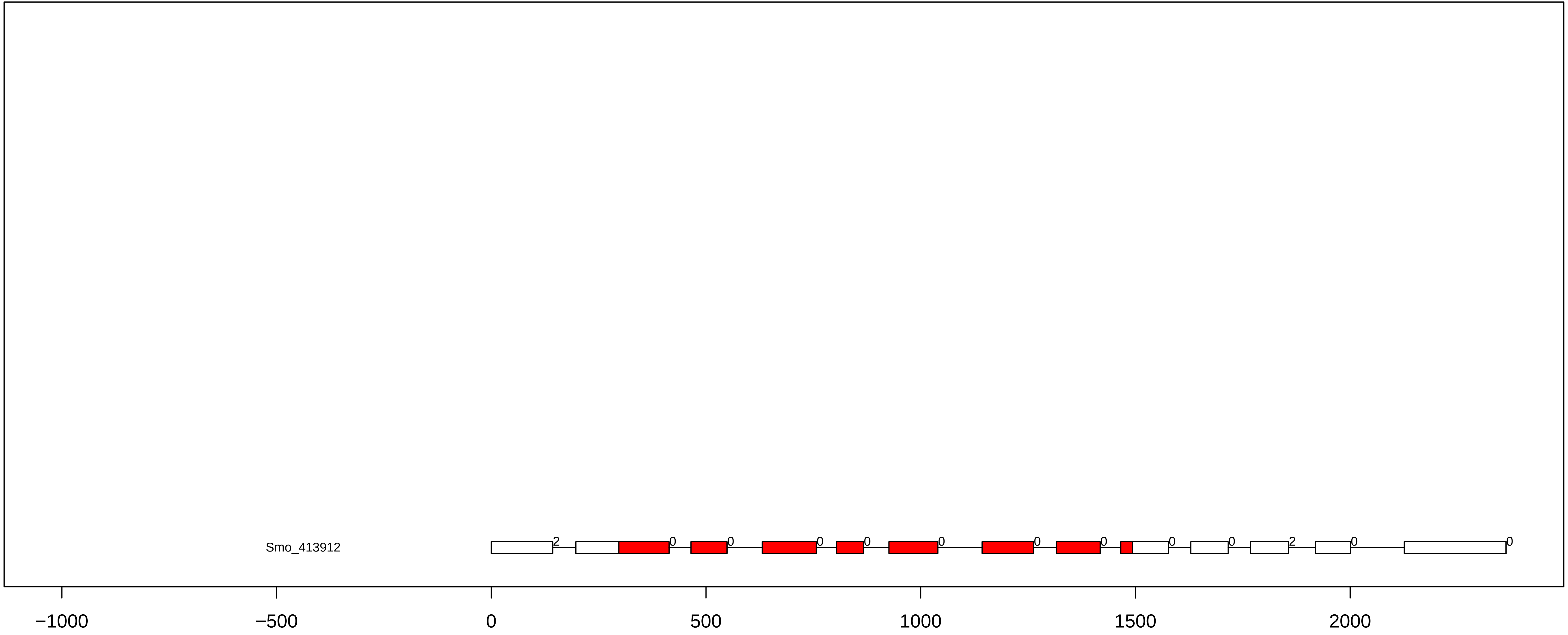

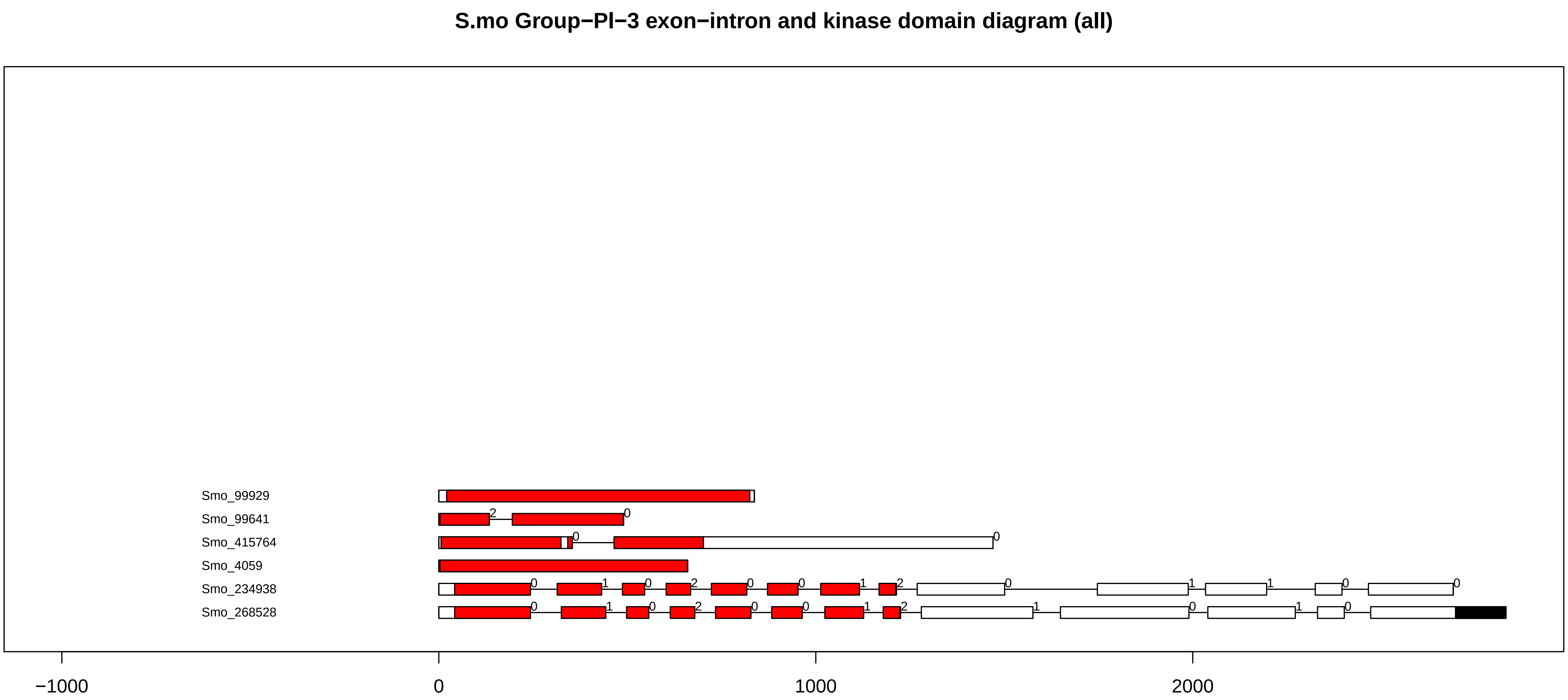

S.mo Group−PI−3 exon−intron and kinase domain diagram (all)
Smo_99929
Smo_99641
Smo_415764
Smo_4059
Smo_234938
Smo_268528
−1000
0
1000
2000

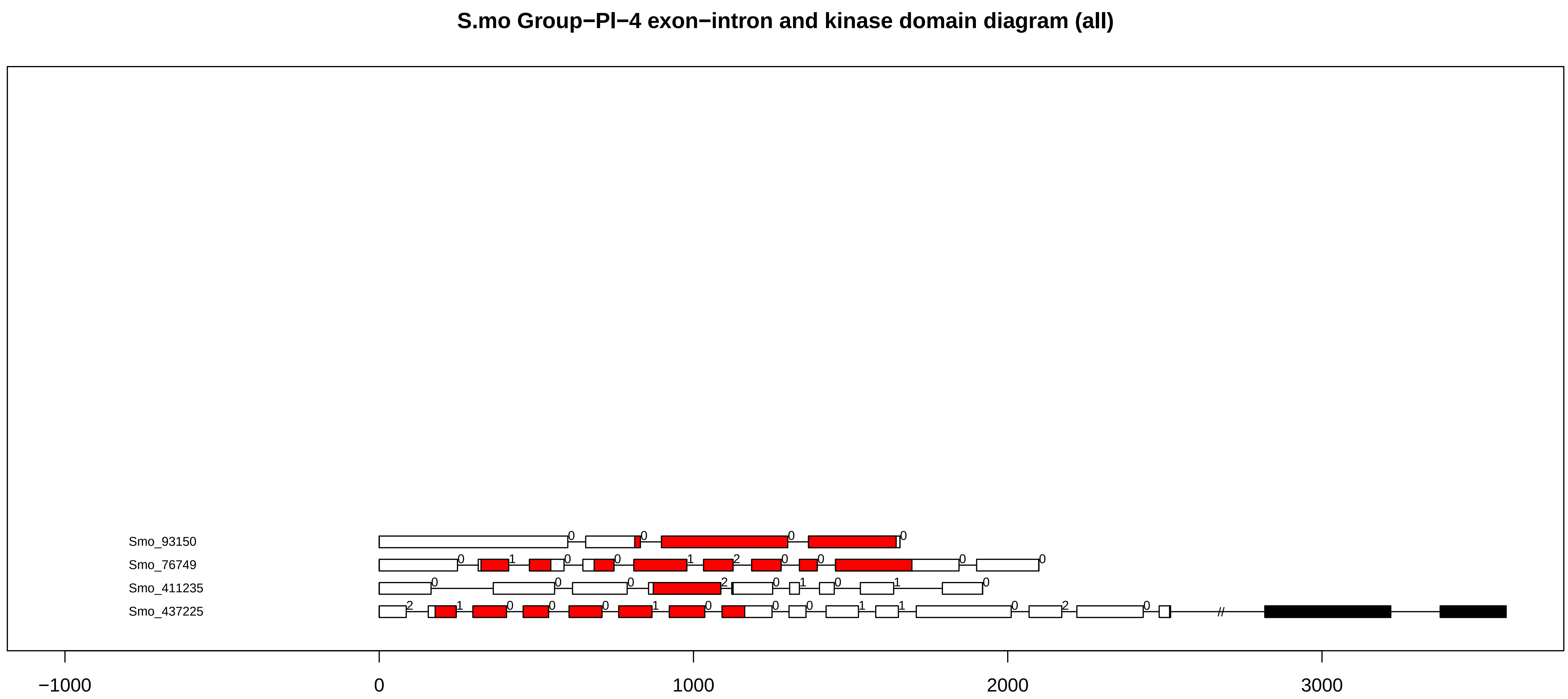

S.mo Group−PI−4 exon−intron and kinase domain diagram (all)
Smo_93150
Smo_76749
Smo_411235
Smo_437225
−1000
0
1000
2000
3000

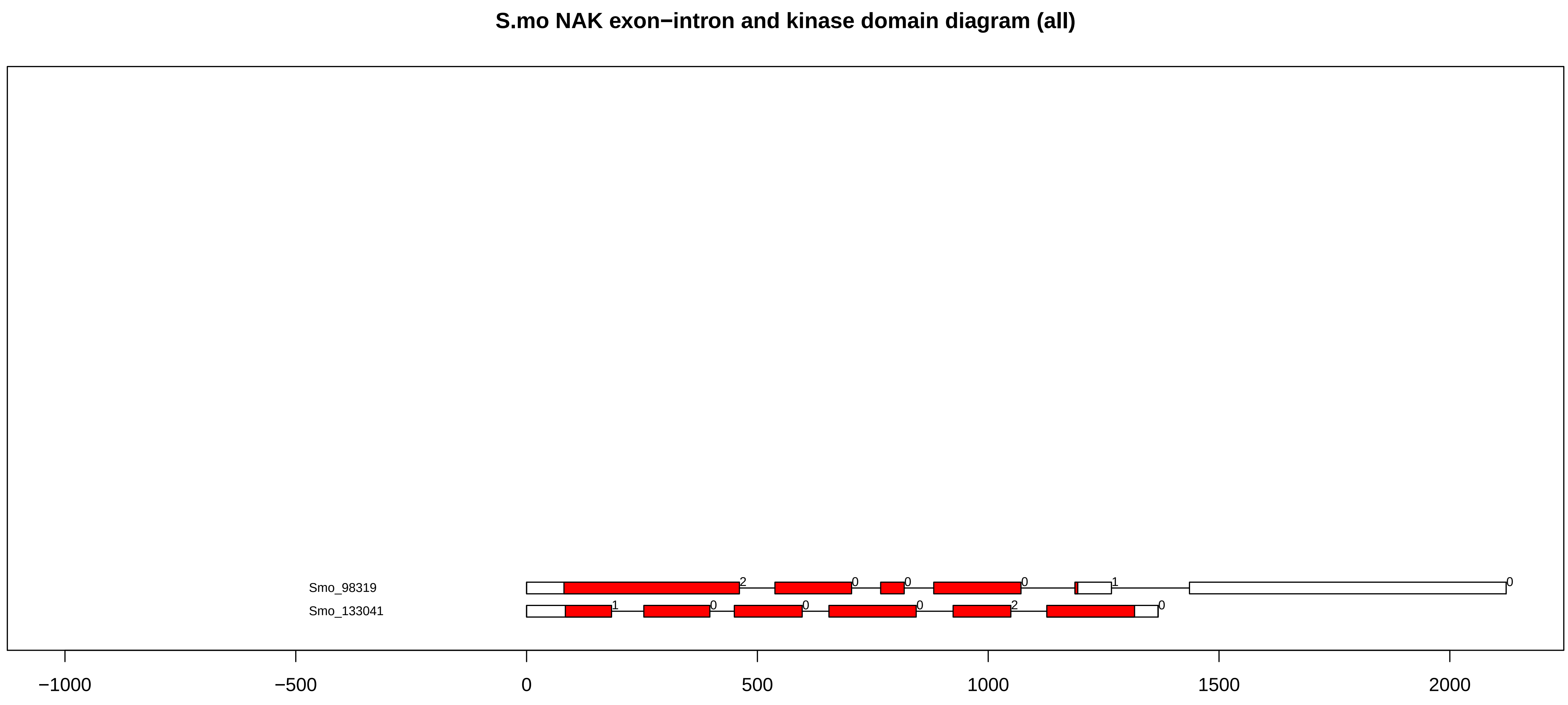
S.mo NAK exon−intron and kinase domain diagram (all)
Smo_98319
Smo_133041
2
0
0
0
1
0
1
0
0
0
2
0
−1000
−500
0
500
1000
1500
2000

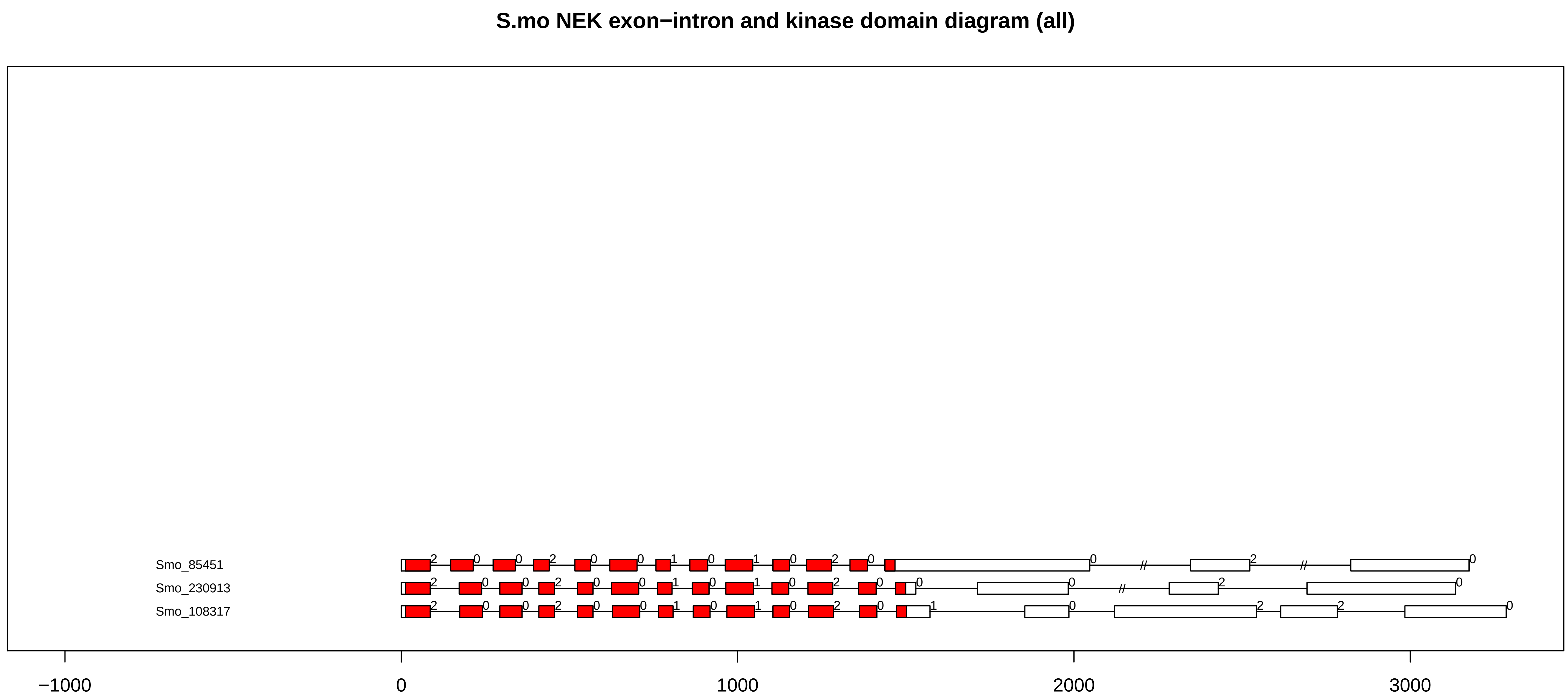
S.mo NEK exon−intron and kinase domain diagram (all)
Smo_85451
Smo_230913
Smo_108317
−1000
0
1000
2000
3000

S.mo PEK_GCN2 exon−intron and kinase domain diagram (all)

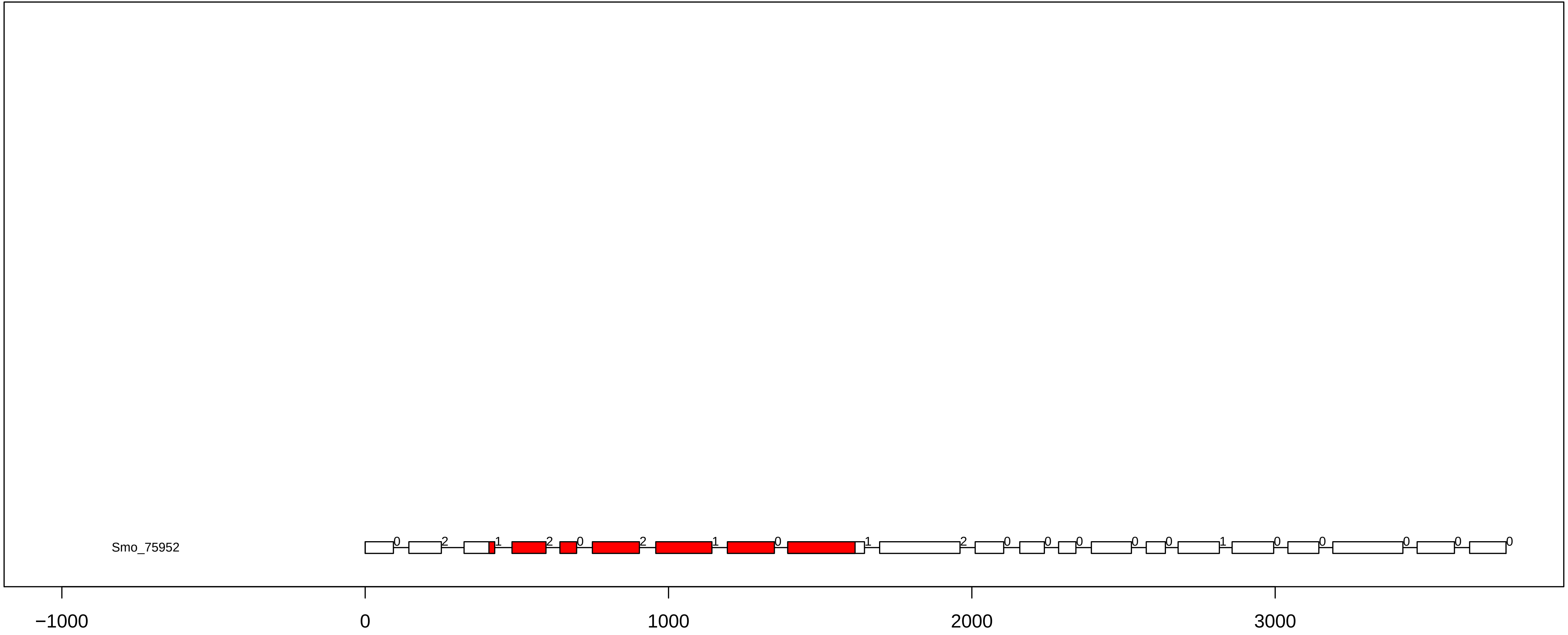

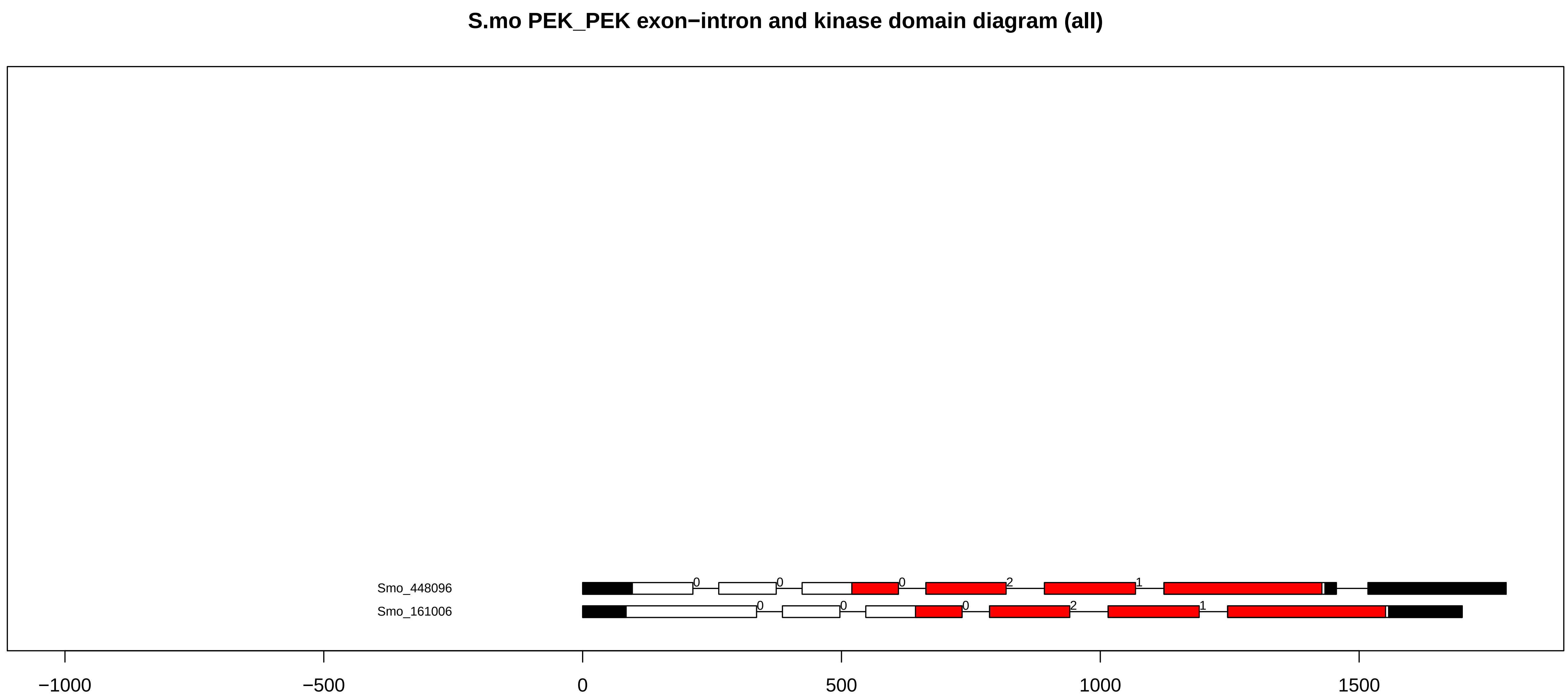
S.mo PEK_PEK exon−intron and kinase domain diagram (all)
Smo_448096
Smo_161006
0
0
0
2
1
0
0
0
2
1
−1000
−500
0
500
1000
1500

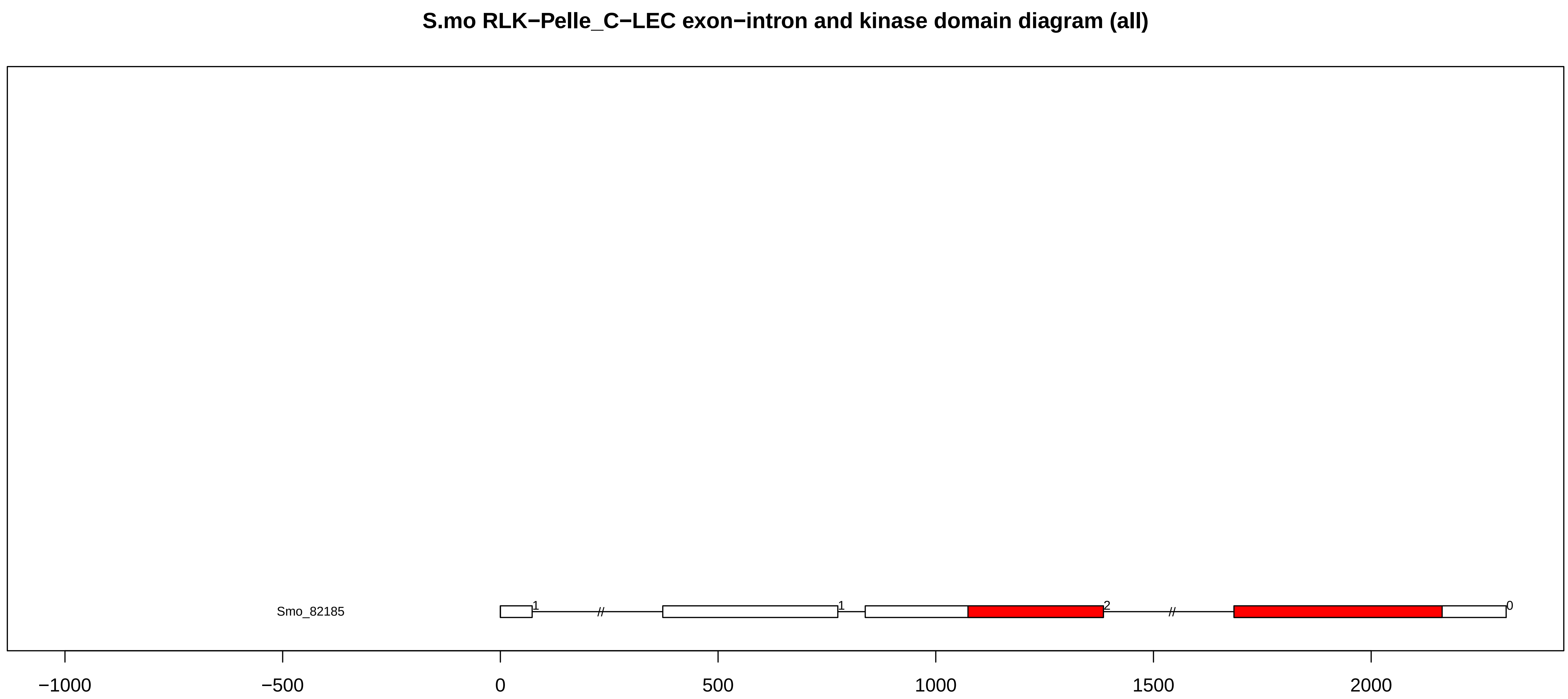
S.mo RLK−Pelle_C−LEC exon−intron and kinase domain diagram (all)
Smo_82185
1
1
2
0
−1000
−500
0
500
1000
1500
2000

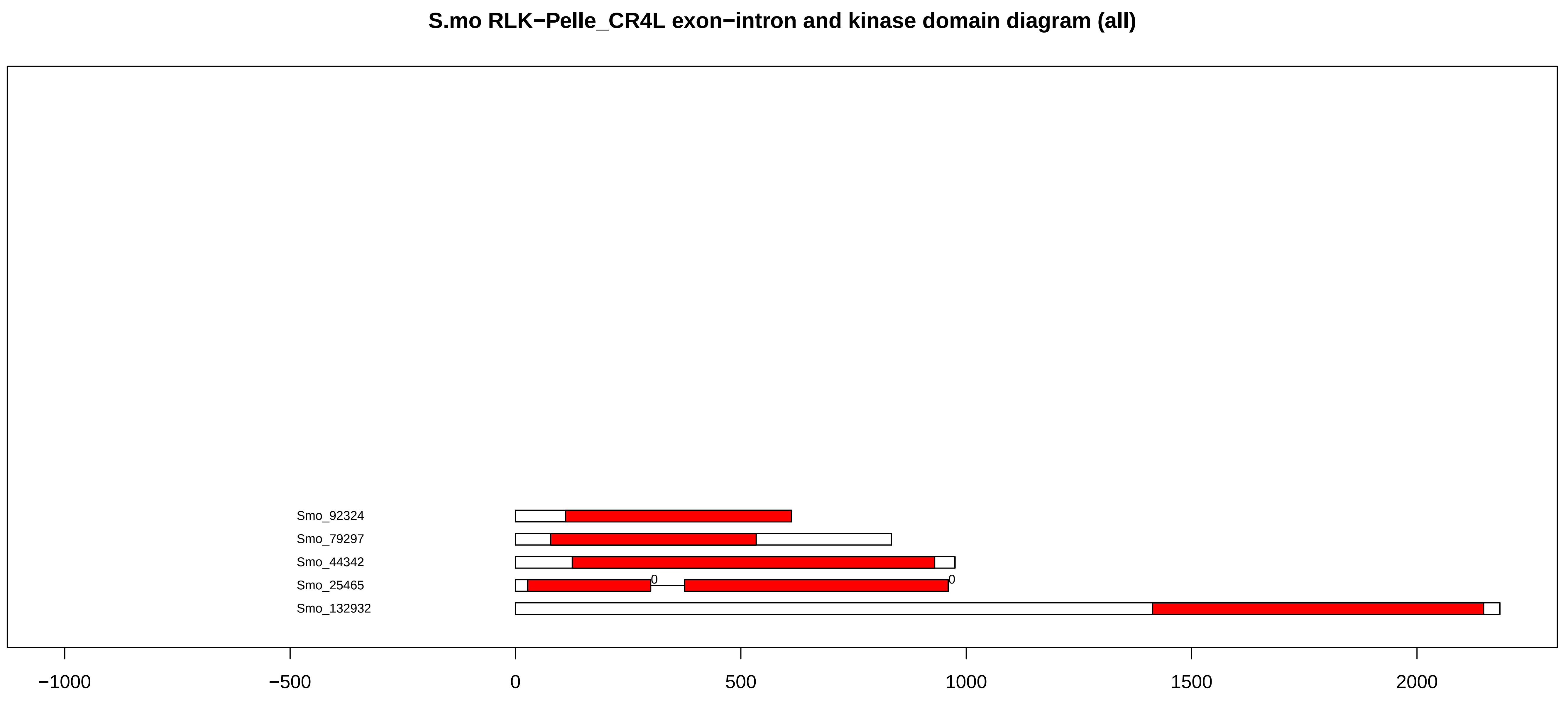
S.mo RLK−Pelle_CR4L exon−intron and kinase domain diagram (all)
Smo_92324
Smo_79297
Smo_44342
Smo_25465
Smo_132932
0
0
−1000
−500
0
500
1000
1500
2000

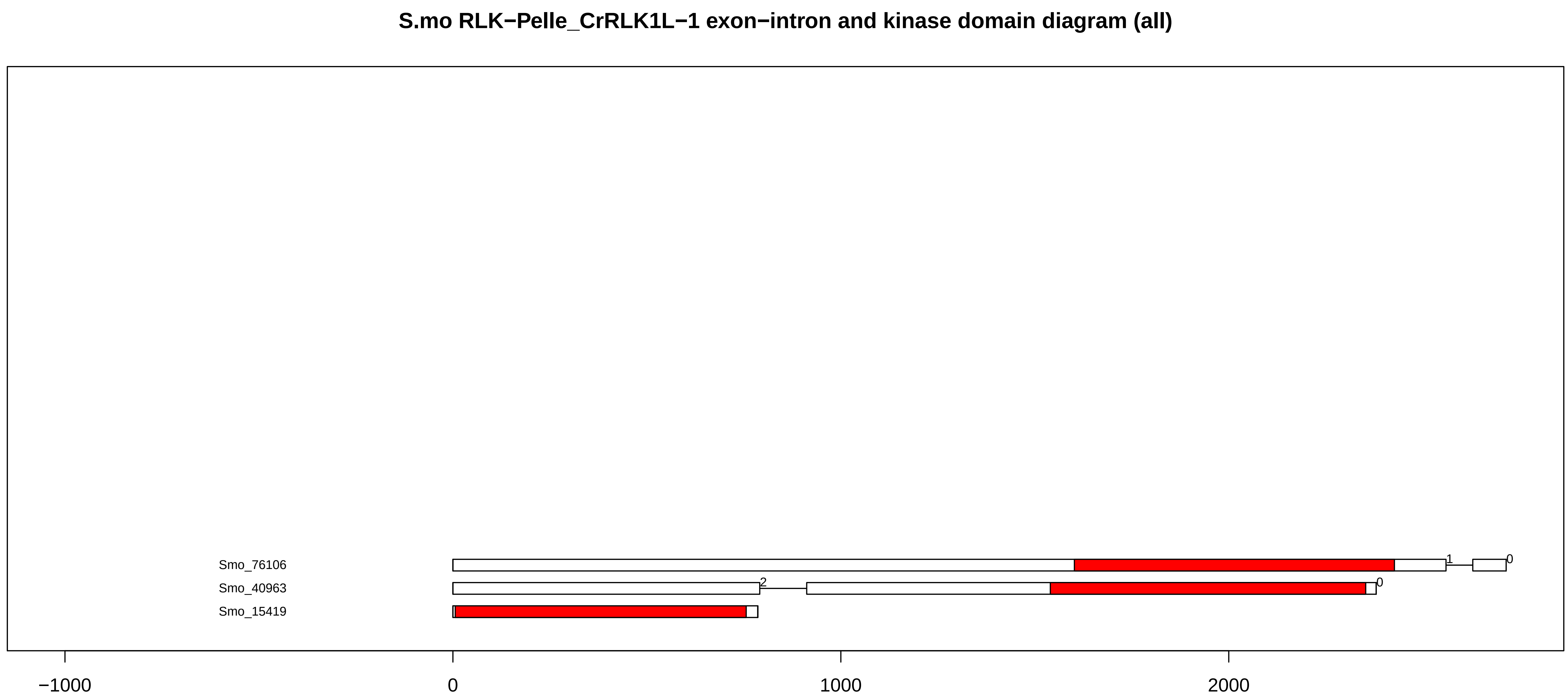
S.mo RLK−Pelle_CrRLK1L−1 exon−intron and kinase domain diagram (all)
Smo_76106
Smo_40963
Smo_15419
1
0
2
0
−1000
0
1000
2000

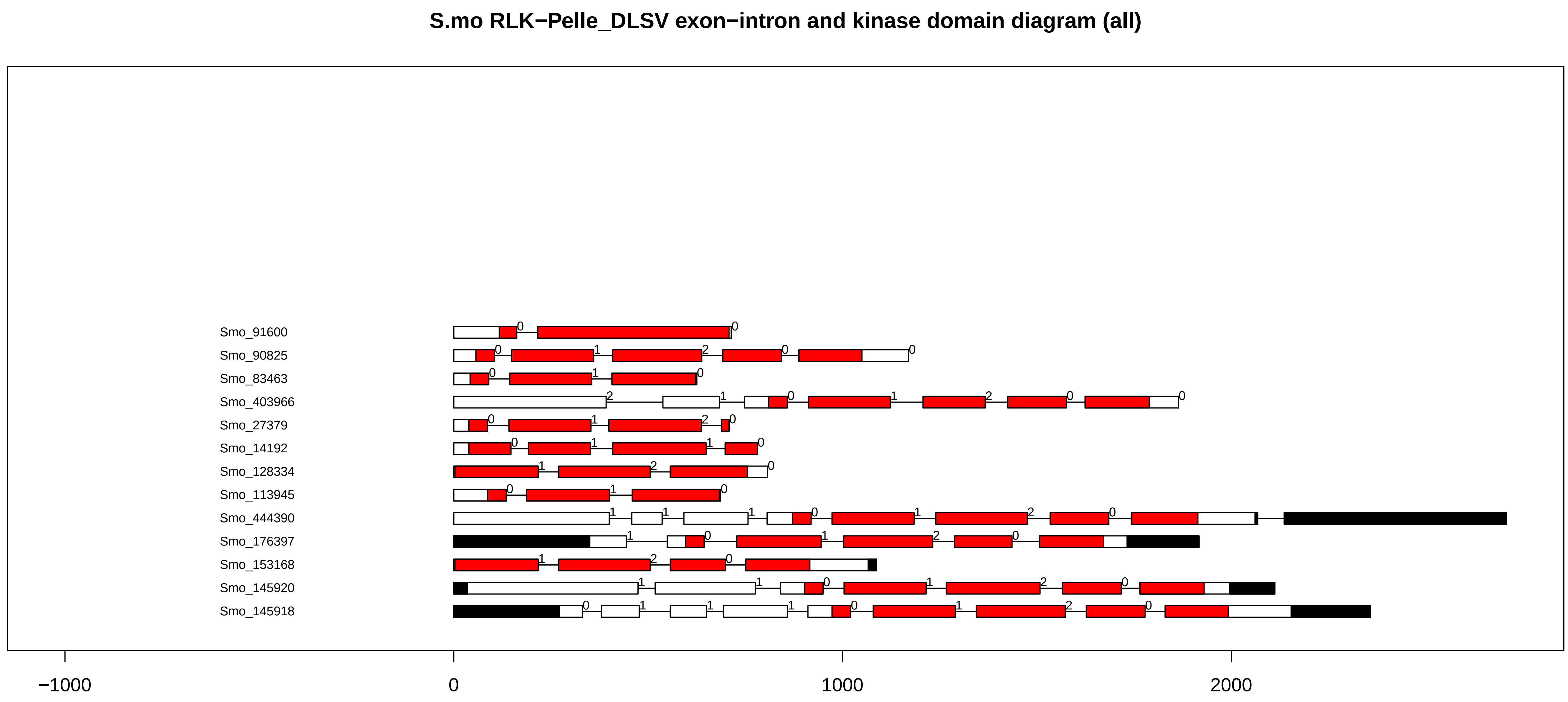
S.mo RLK−Pelle_DLSV exon−intron and kinase domain diagram (all)
Smo_91600
Smo_90825
Smo_83463
Smo_403966
Smo_27379
Smo_14192
Smo_128334
Smo_113945
Smo_444390
Smo_176397
Smo_153168
Smo_145920
Smo_145918
−1000
0
1000
2000

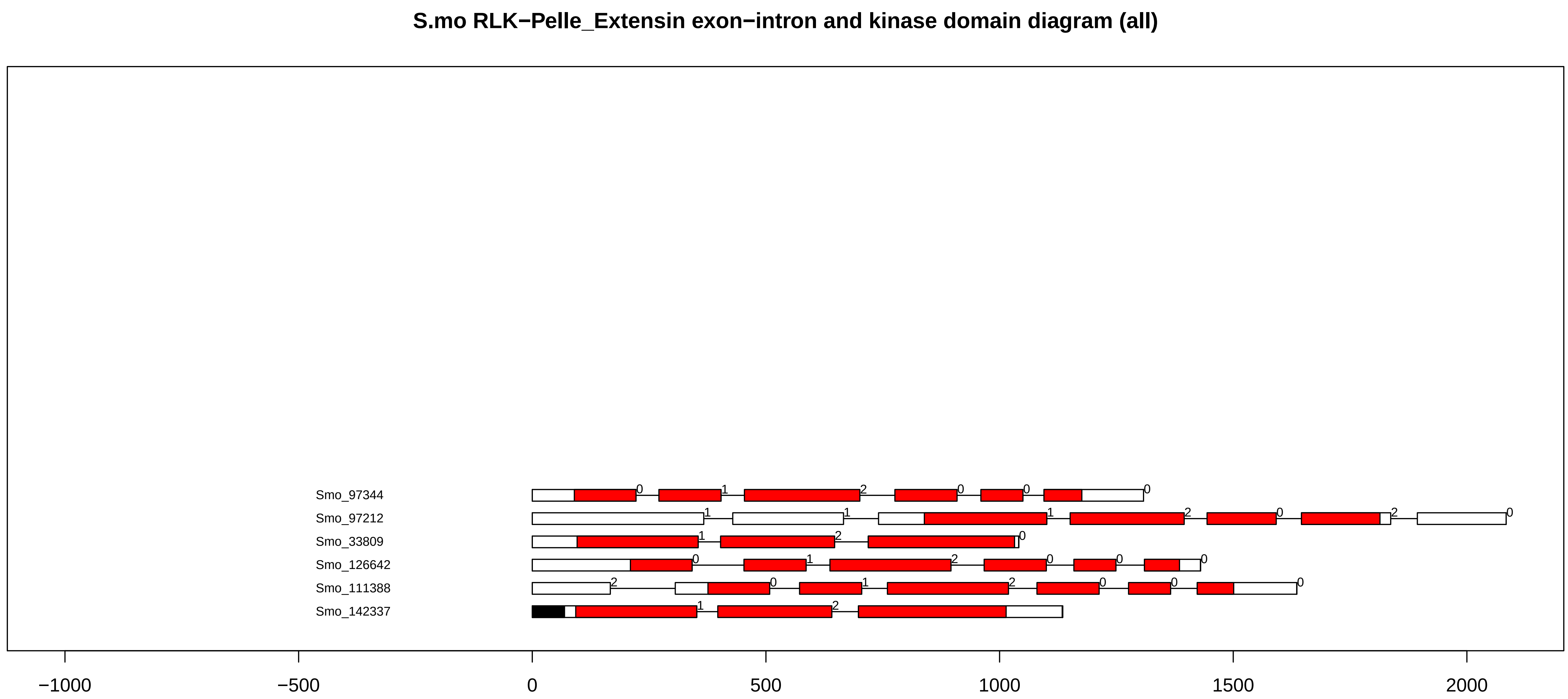

S.mo RLK−Pelle_Extensin exon−intron and kinase domain diagram (all)
Smo_97344
Smo_97212
Smo_33809
Smo_126642
Smo_111388
Smo_142337
−1000
−500
0
500
1000
1500
2000

S.mo RLK−Pelle_L−LEC exon−intron and kinase domain diagram (all)

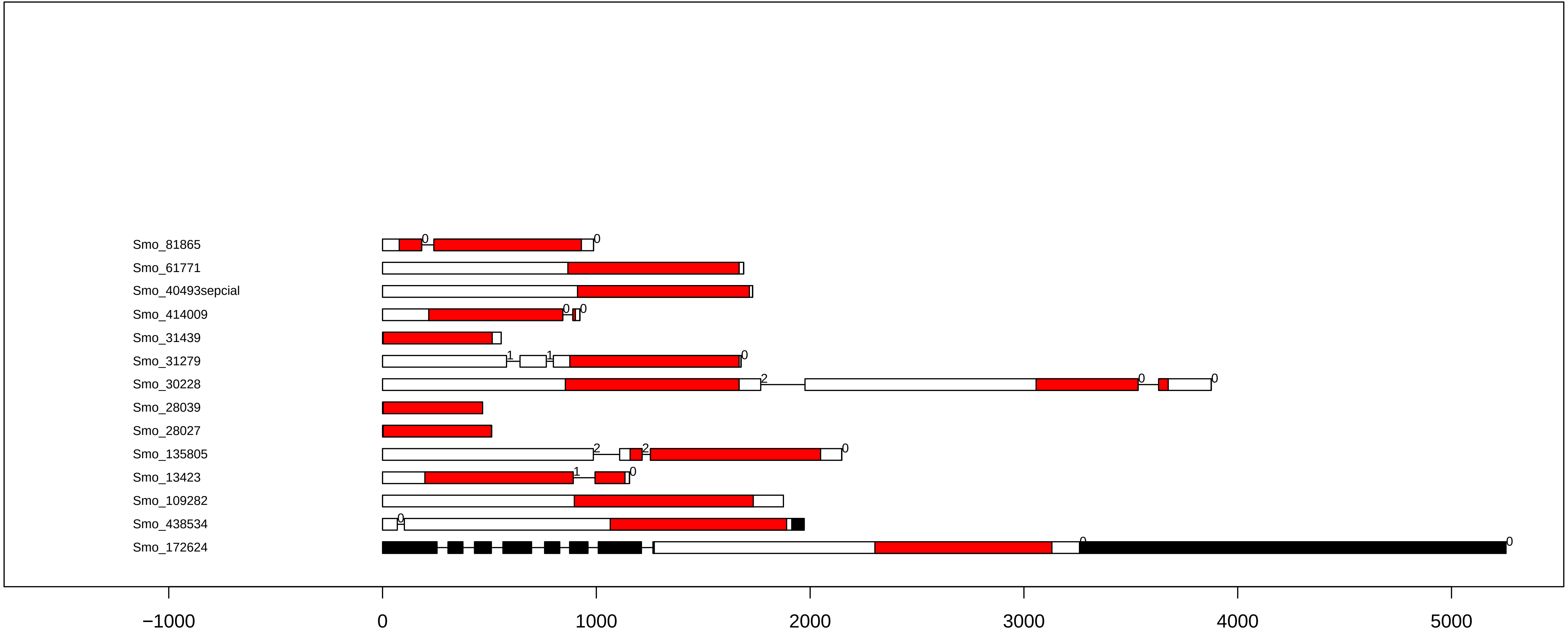

S.mo RLK−Pelle_LRR−I−1 exon−intron and kinase domain diagram (all)

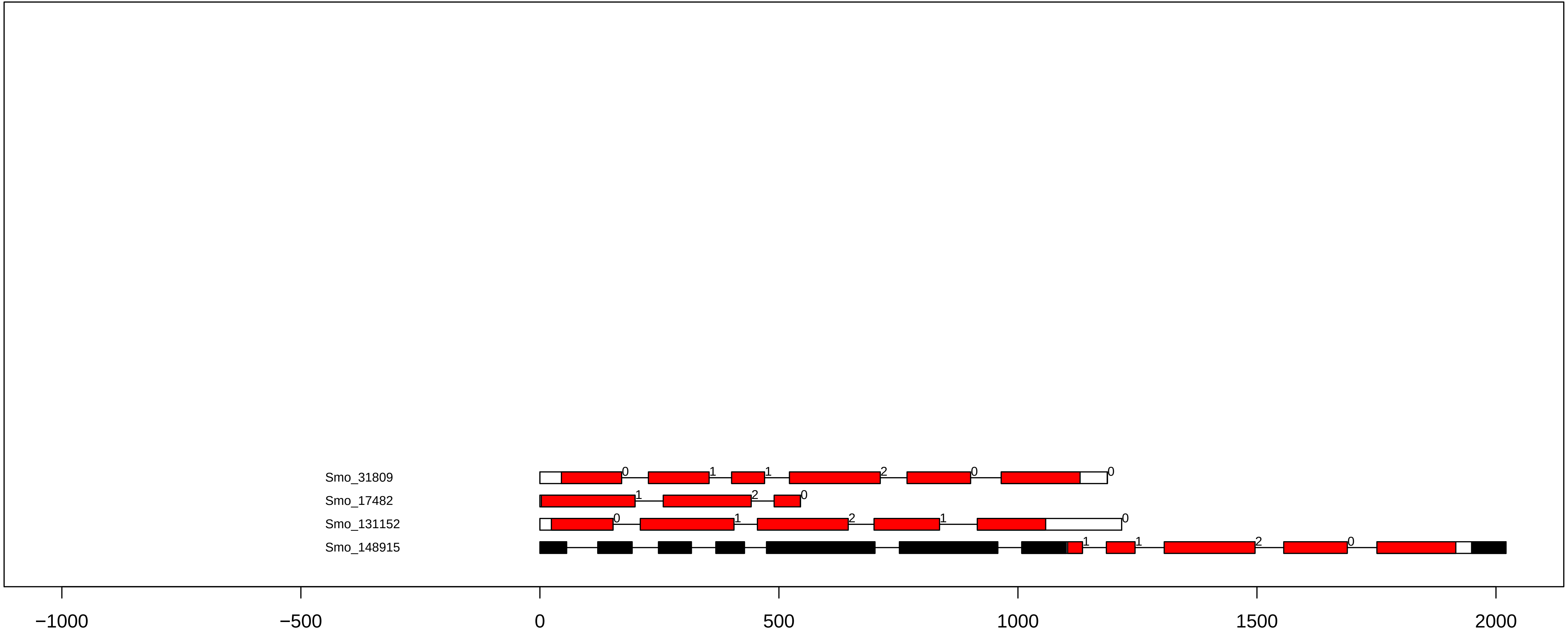

S.mo RLK−Pelle_LRR−I−2 exon−intron and kinase domain diagram (all)

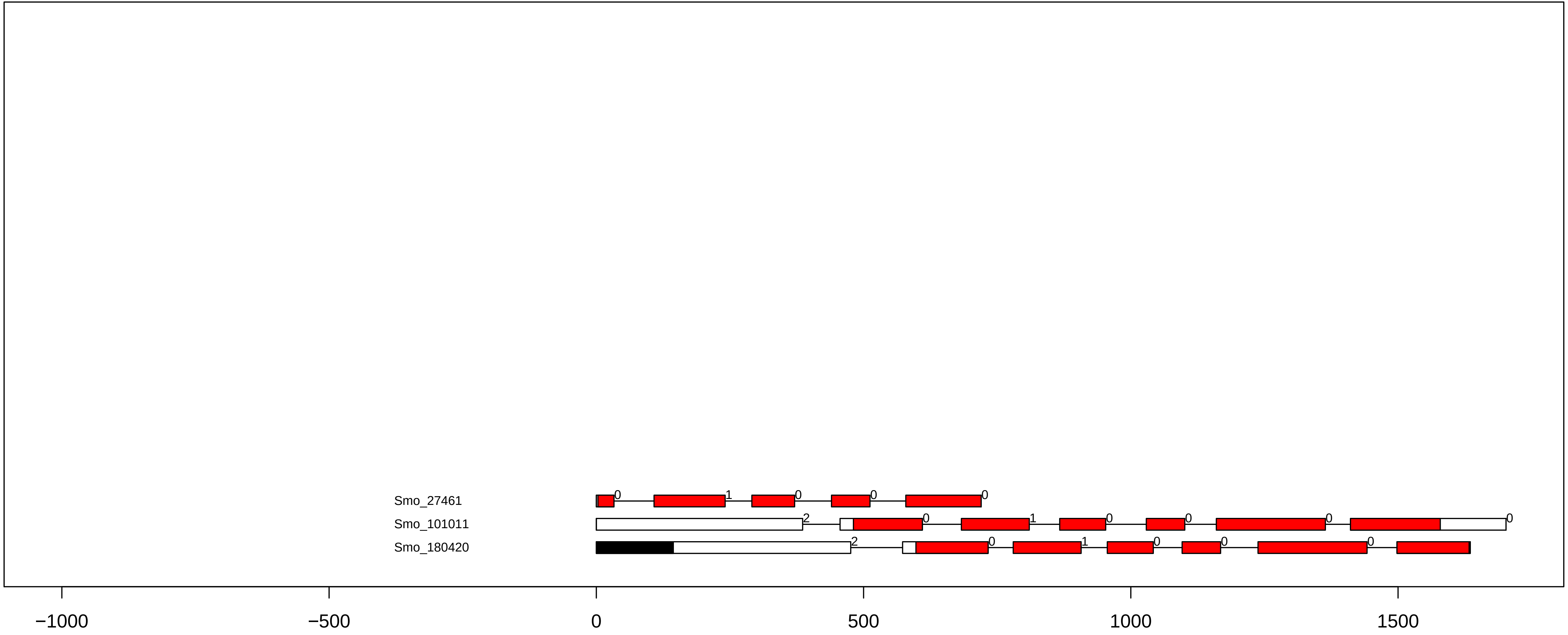

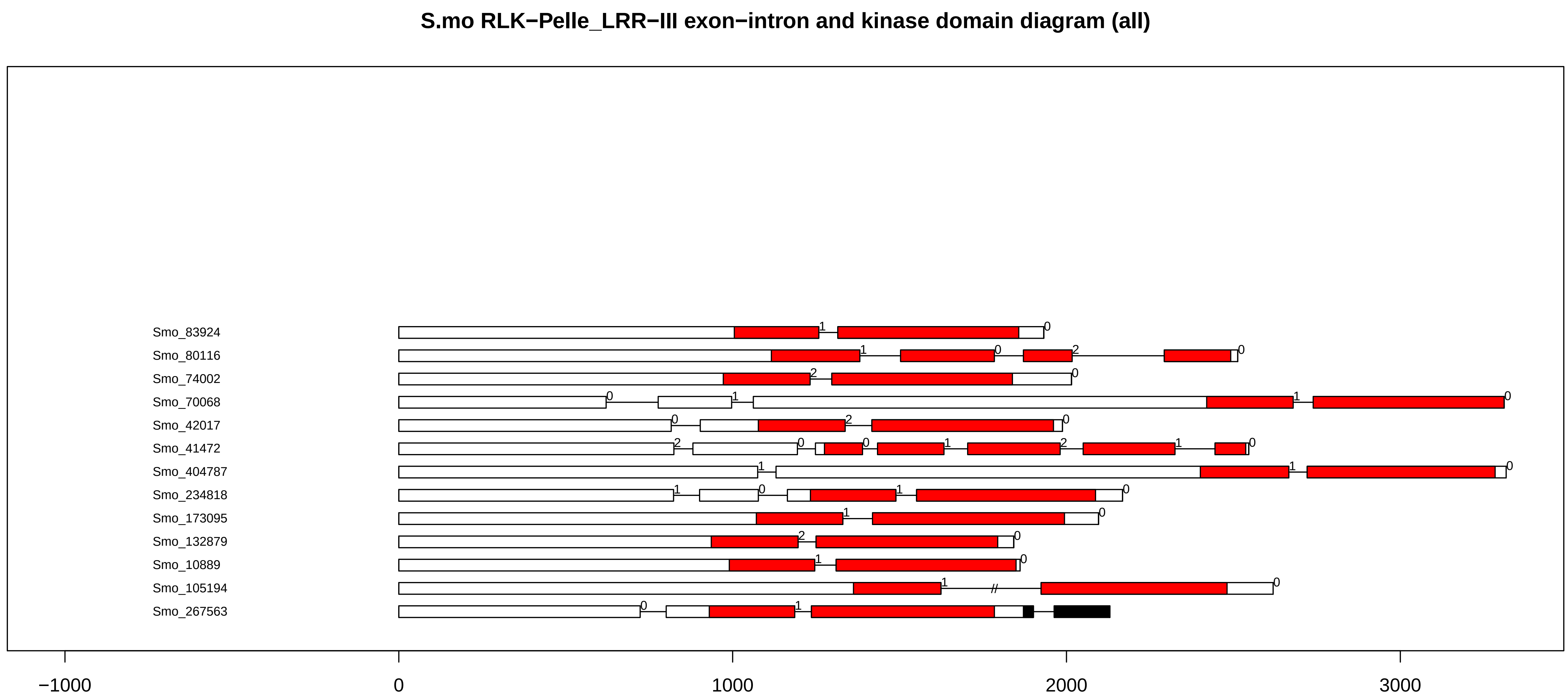
S.mo RLK−Pelle_LRR−III exon−intron and kinase domain diagram (all)
Smo_83924
Smo_80116
Smo_74002
Smo_70068
Smo_42017
Smo_41472
Smo_404787
Smo_234818
Smo_173095
Smo_132879
Smo_10889
Smo_105194
Smo_267563
−1000
0
1000
2000
3000

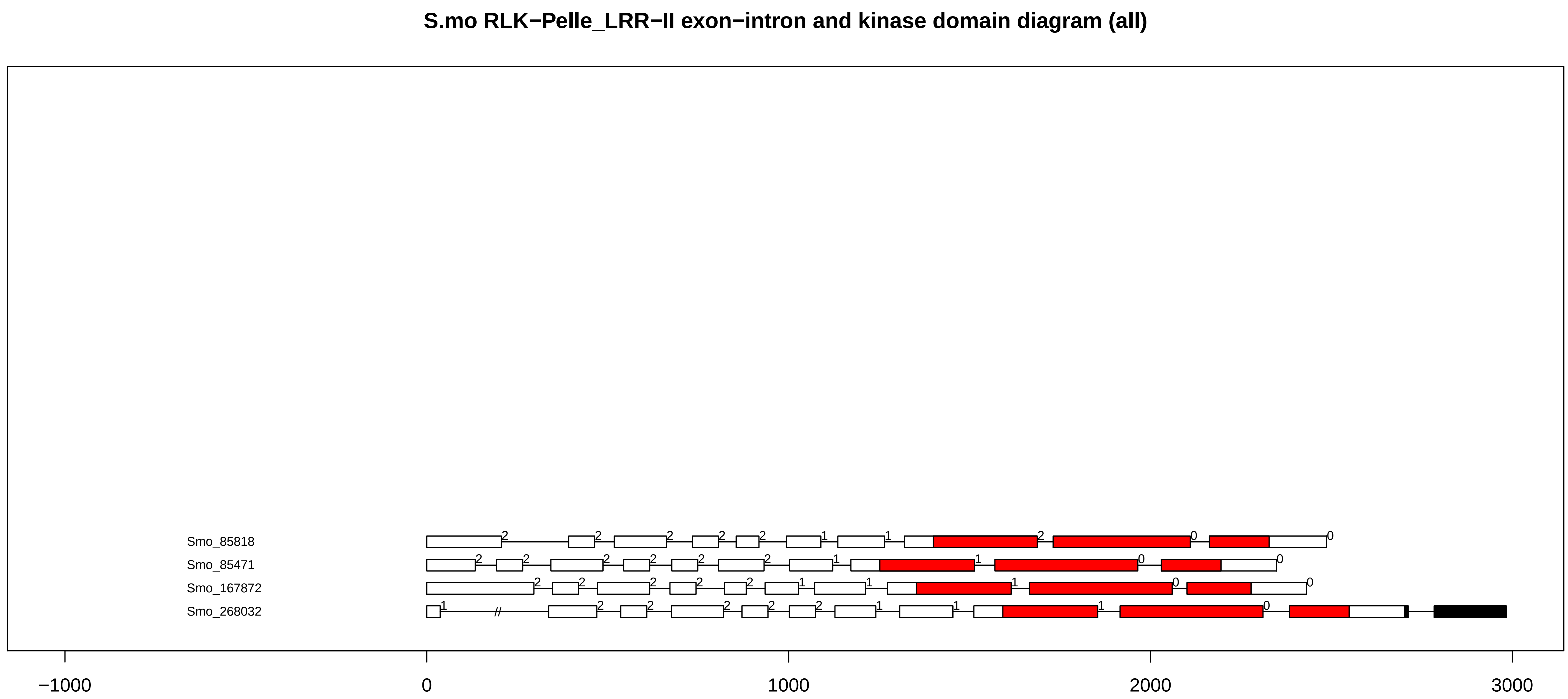
S.mo RLK−Pelle_LRR−II exon−intron and kinase domain diagram (all)
Smo_85818
Smo_85471
Smo_167872
Smo_268032
−1000
0
1000
2000
3000

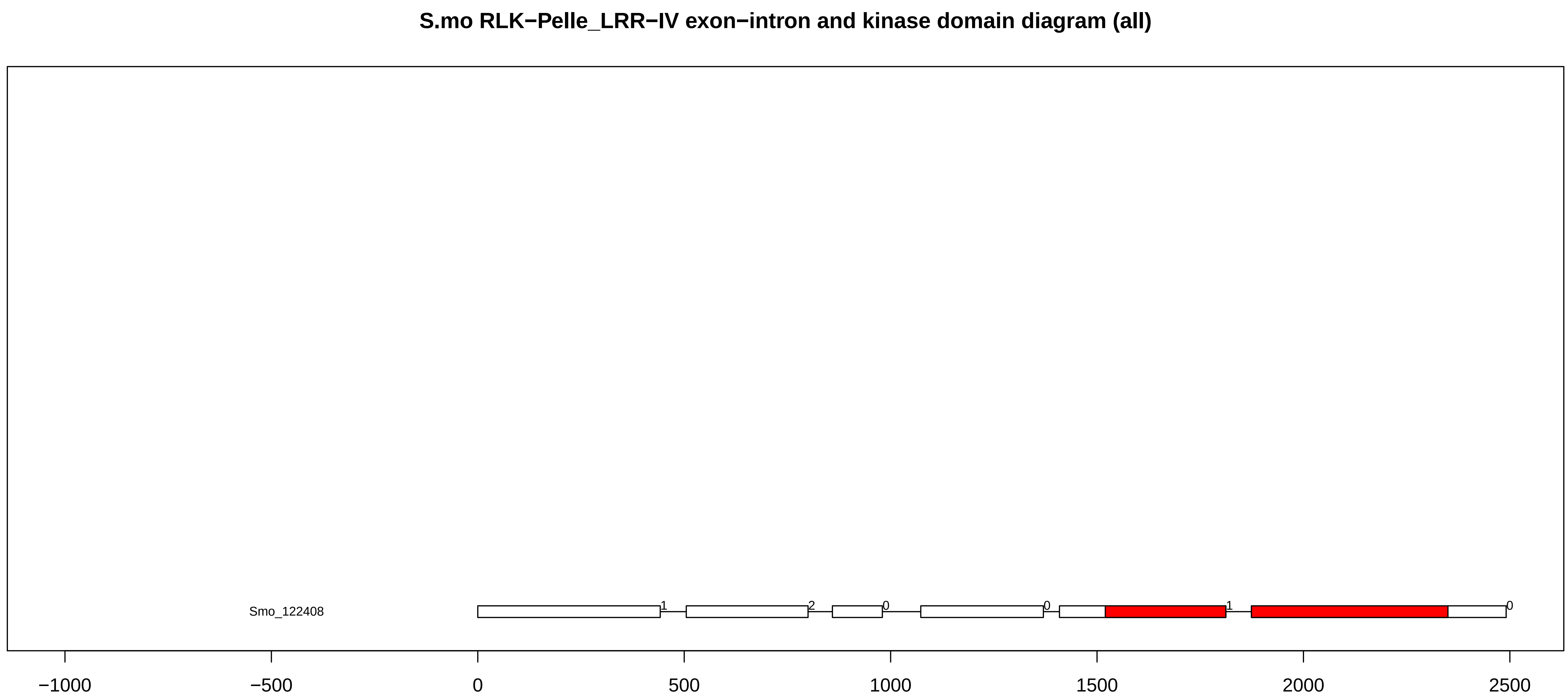

S.mo RLK−Pelle_LRR−IV exon−intron and kinase domain diagram (all)
Smo_122408
1
2
0
0
1
0
−1000
−500
0
500
1000
1500
2000
2500

S.mo RLK−Pelle_LRR−IX exon−intron and kinase domain diagram (all)

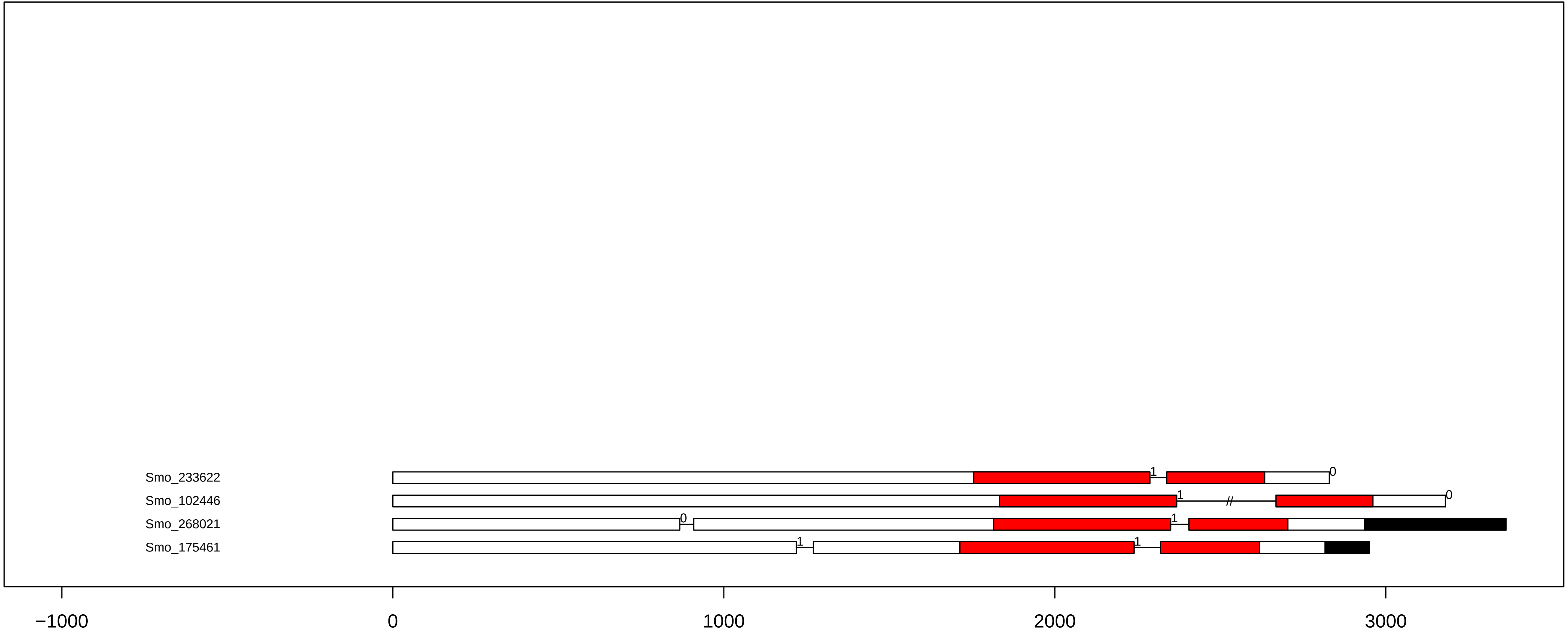

S.mo RLK−Pelle_LRR−VI−1 exon−intron and kinase domain diagram (all)

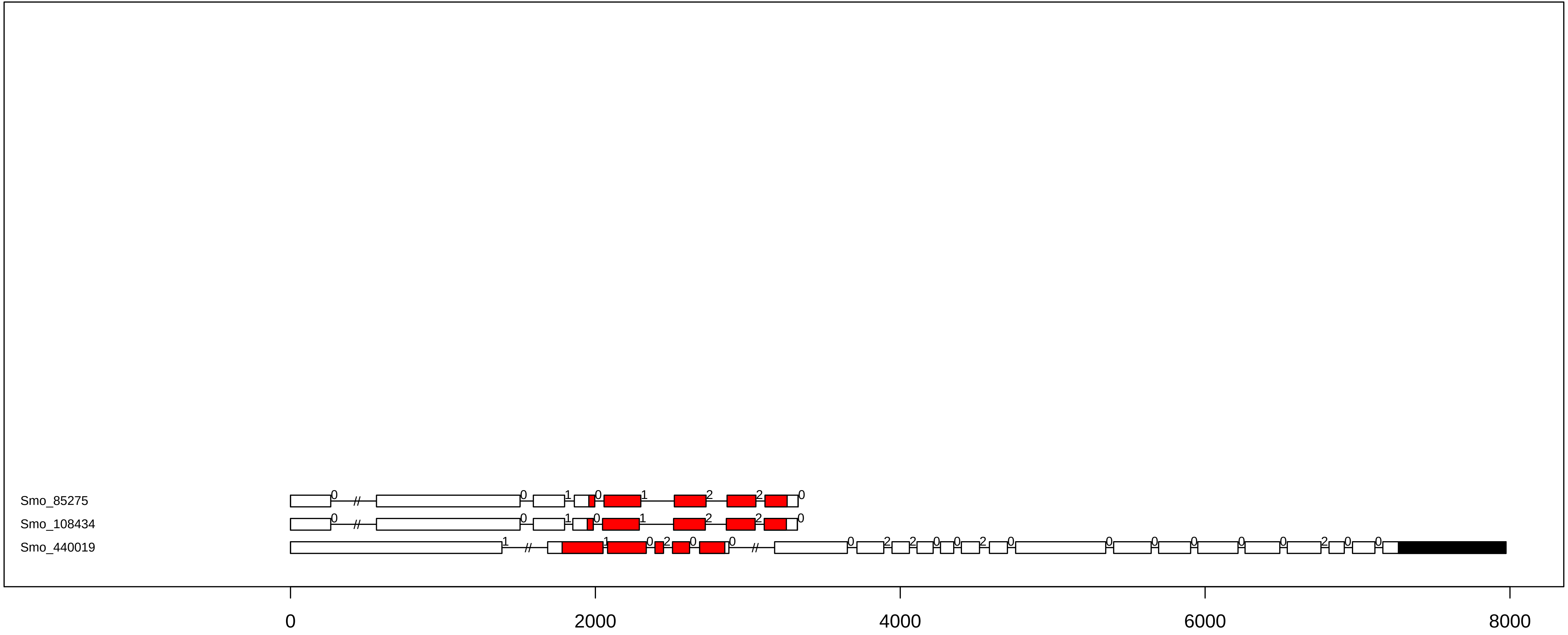

S.mo RLK−Pelle_LRR−VI−2 exon−intron and kinase domain diagram (all)

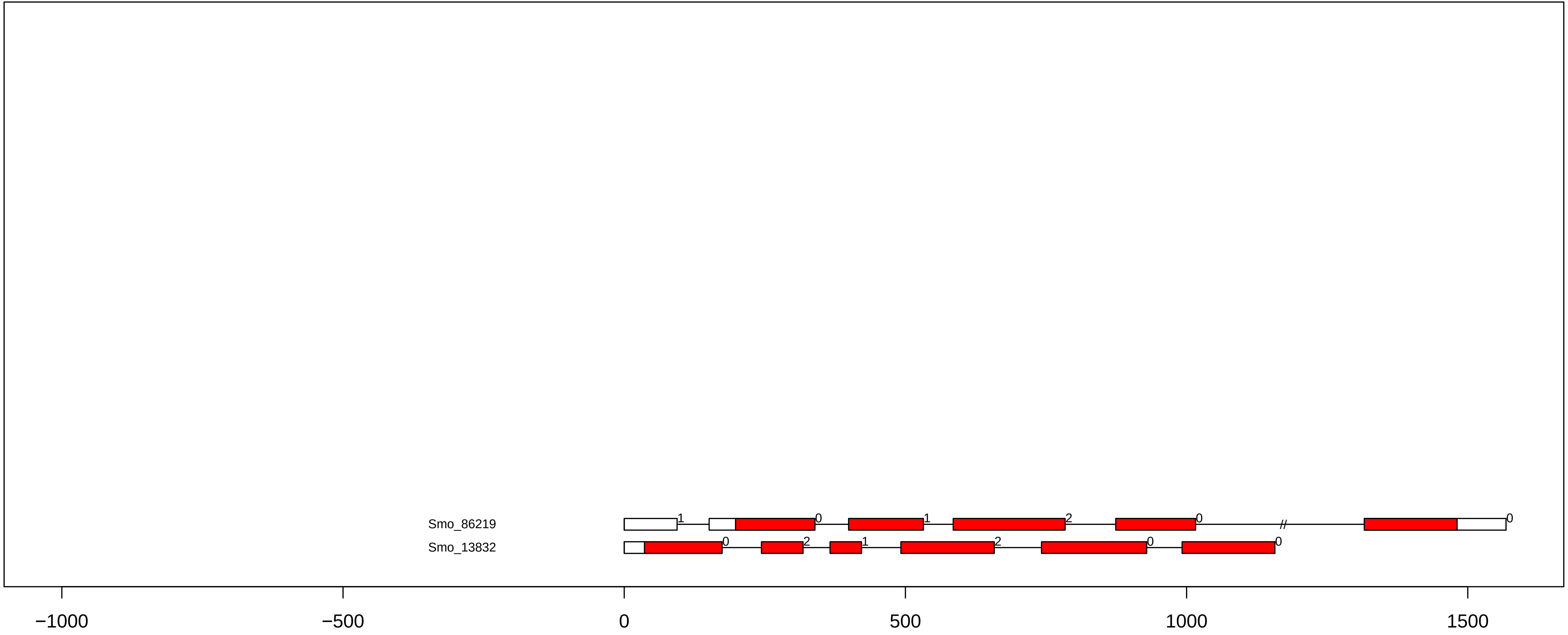

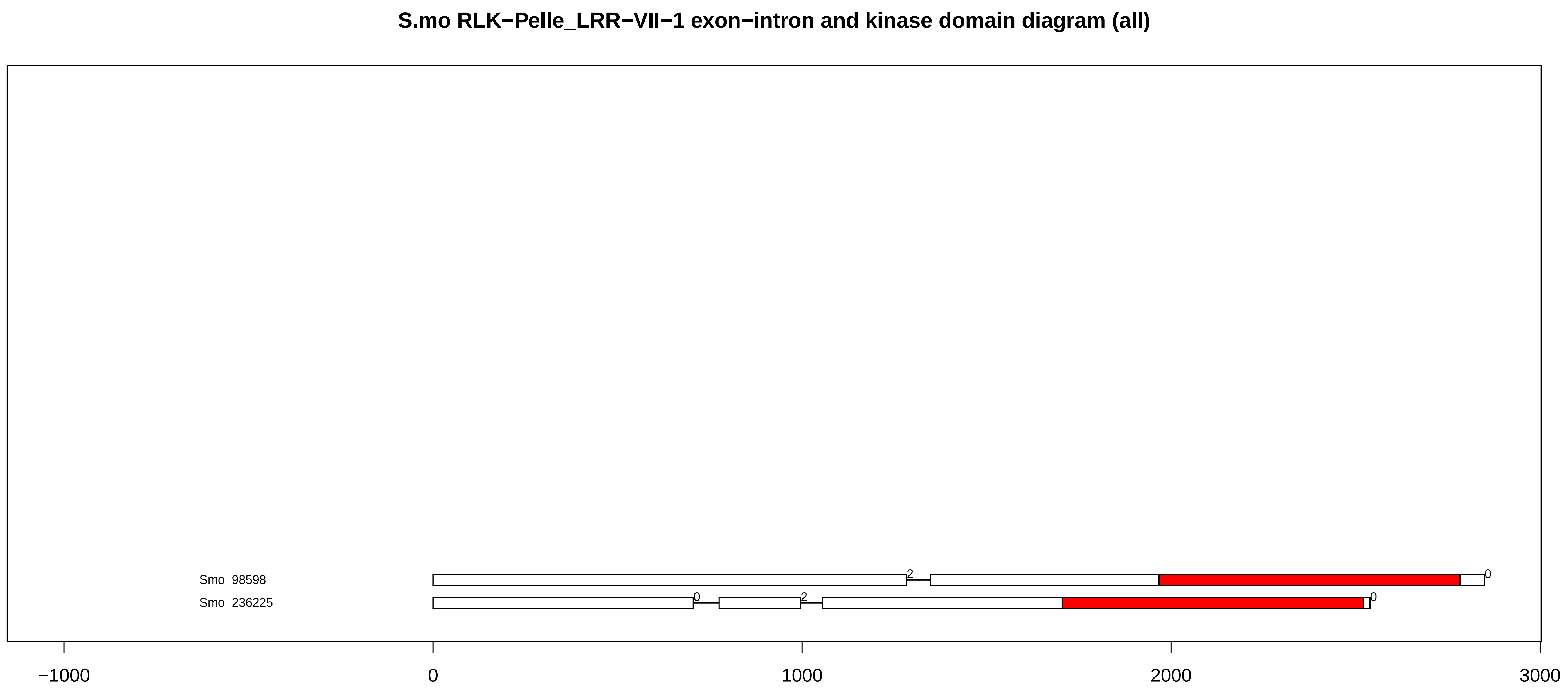

S.mo RLK−Pelle_LRR−VII−1 exon−intron and kinase domain diagram (all)
Smo_98598
Smo_236225
2
0
0
2
0
−1000
0
1000
2000
3000

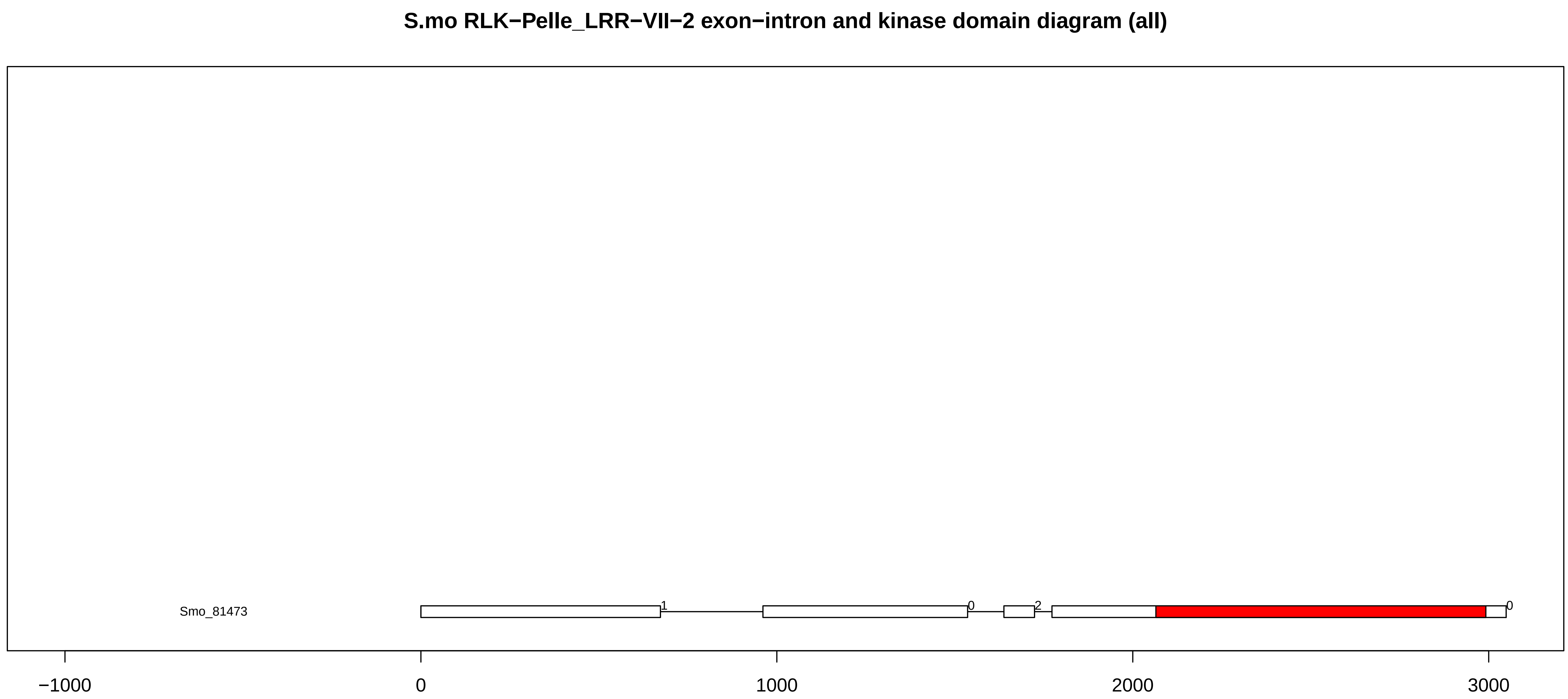

S.mo RLK−Pelle_LRR−VII−2 exon−intron and kinase domain diagram (all)
Smo_81473
1
0
2
0
−1000
0
1000
2000
3000

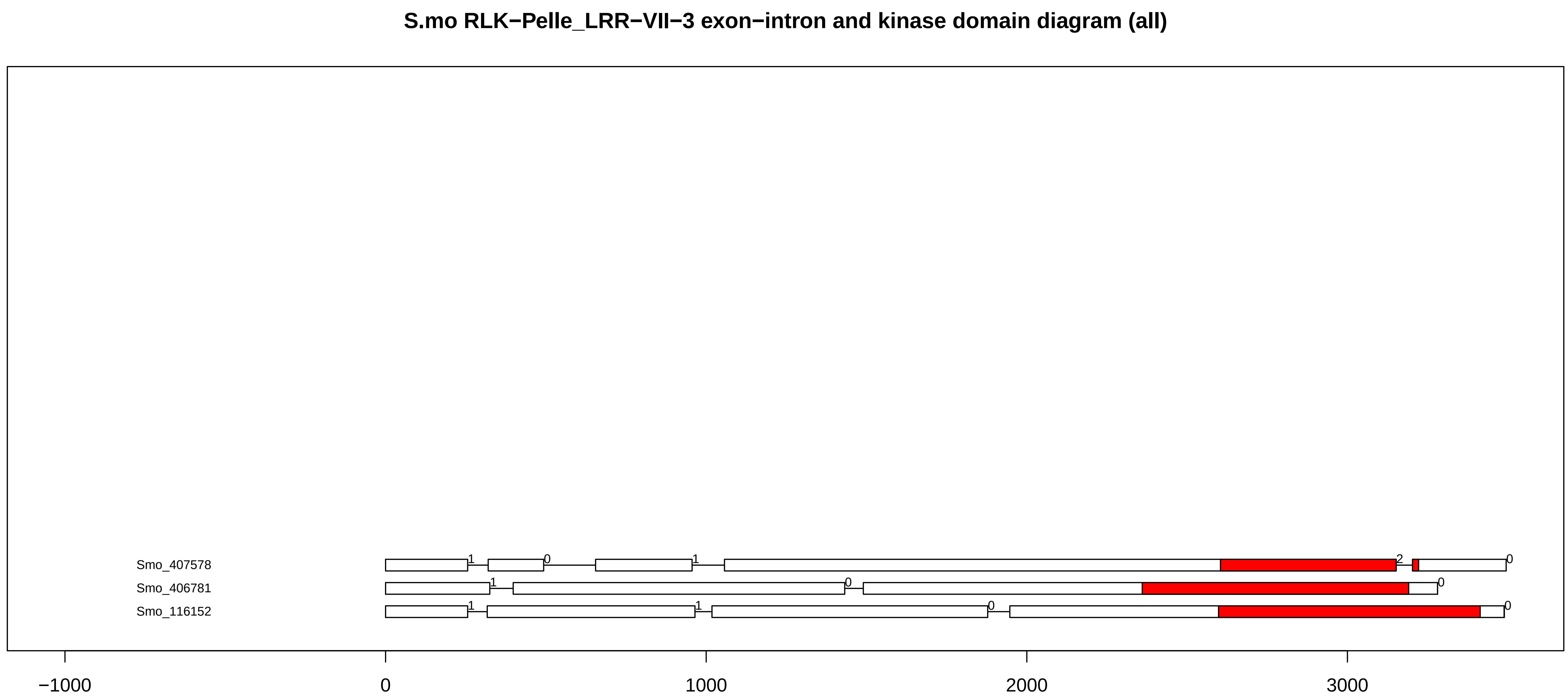

S.mo RLK−Pelle_LRR−VII−3 exon−intron and kinase domain diagram (all)
Smo_407578
Smo_406781
Smo_116152
−1000
0
1000
2000
3000

S.mo RLK−Pelle_LRR−VIII−1 exon−intron and kinase domain diagram (all)

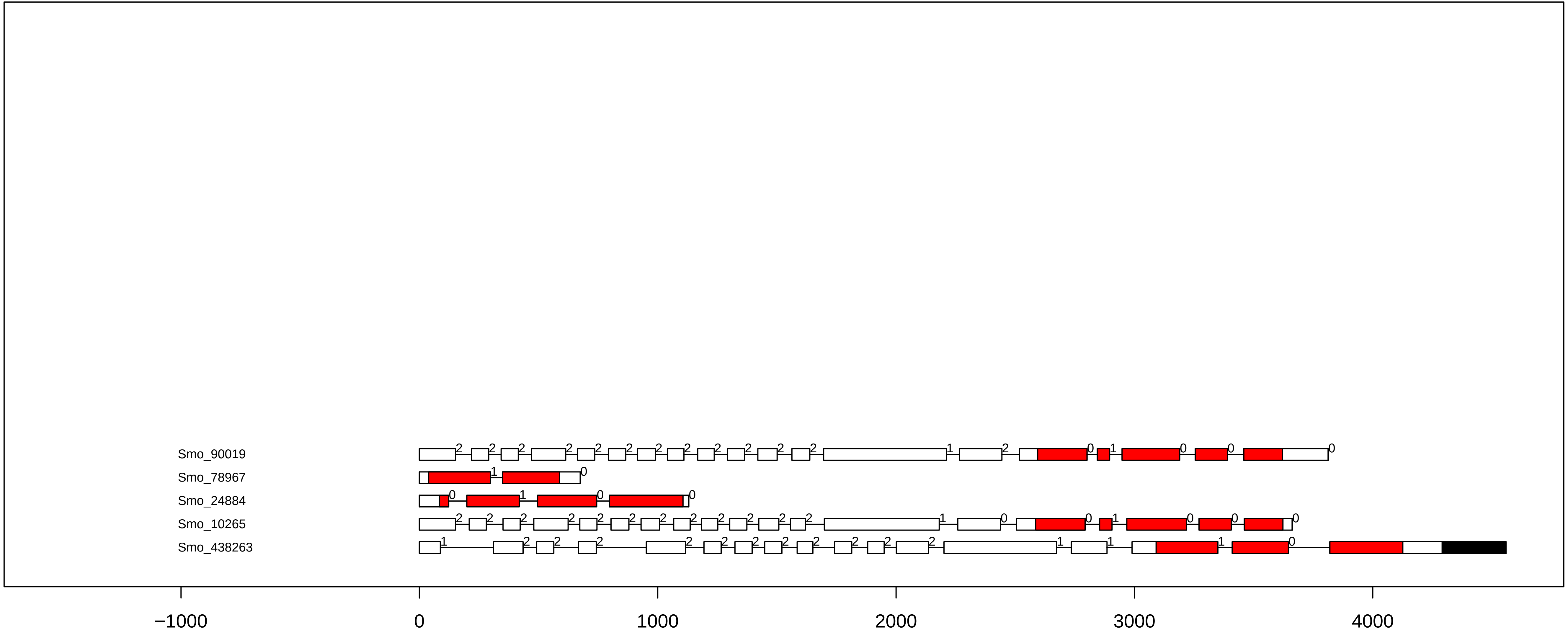

S.mo RLK−Pelle_LRR−V exon−intron and kinase domain diagram (all)

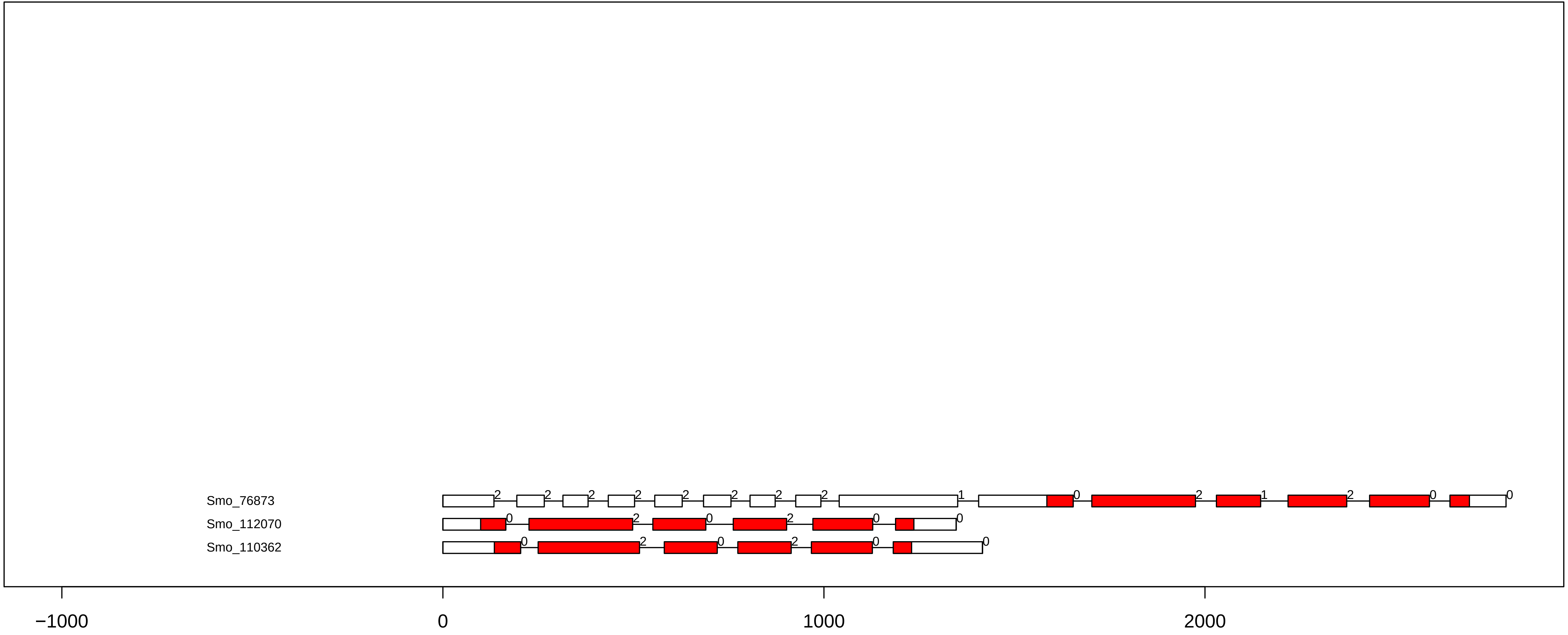

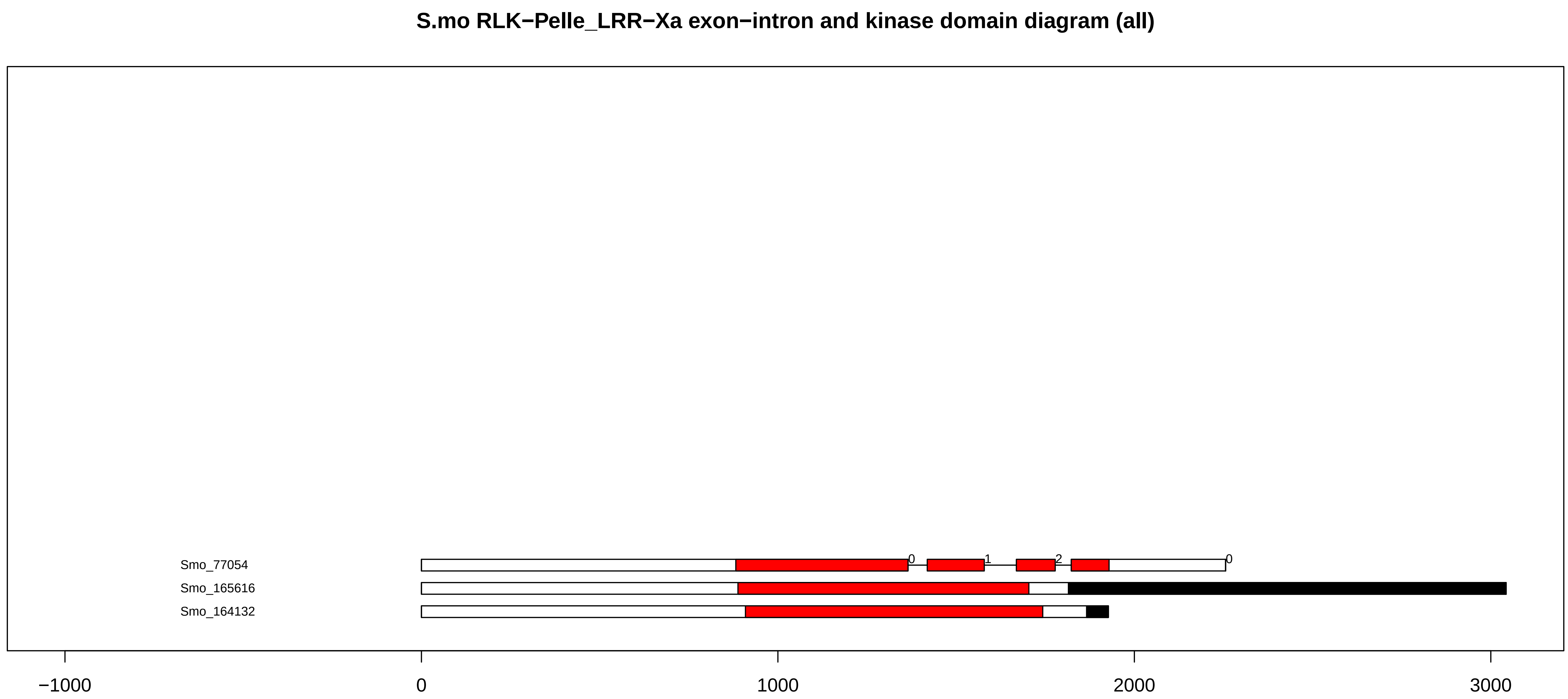

S.mo RLK−Pelle_LRR−Xa exon−intron and kinase domain diagram (all)
Smo_77054
Smo_165616
Smo_164132
0
1
2
0
−1000
0
1000
2000
3000

S.mo RLK−Pelle_LRR−Xb−1 exon−intron and kinase domain diagram (all)

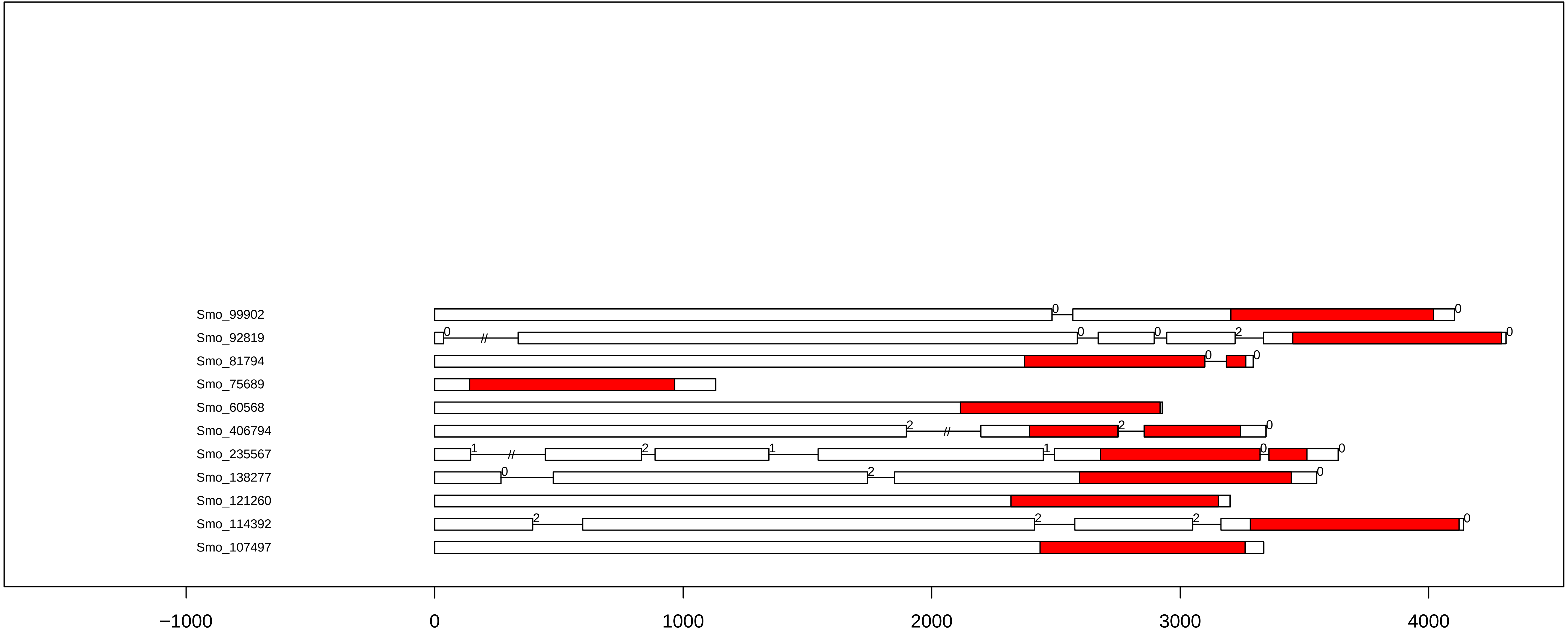

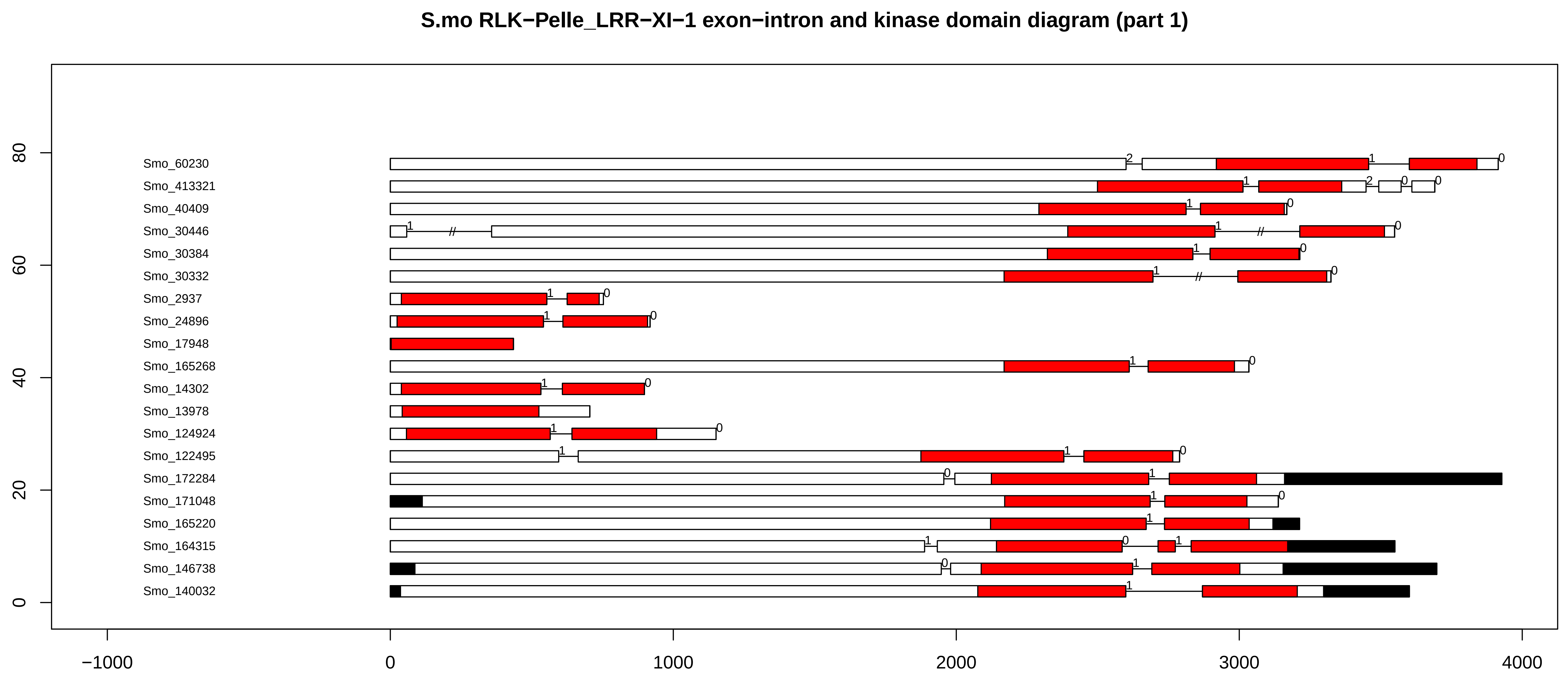
S.mo RLK−Pelle_LRR−XI−1 exon−intron and kinase domain diagram (part 1)
Smo_60230
Smo_413321
Smo_40409
Smo_30446
Smo_30384
Smo_30332
Smo_2937
Smo_24896
Smo_17948
Smo_165268
Smo_14302
Smo_13978
Smo_124924
Smo_122495
Smo_172284
Smo_171048
Smo_165220
Smo_164315
Smo_146738
Smo_140032
0
20
40
60
80
−1000
0
1000
2000
3000
4000

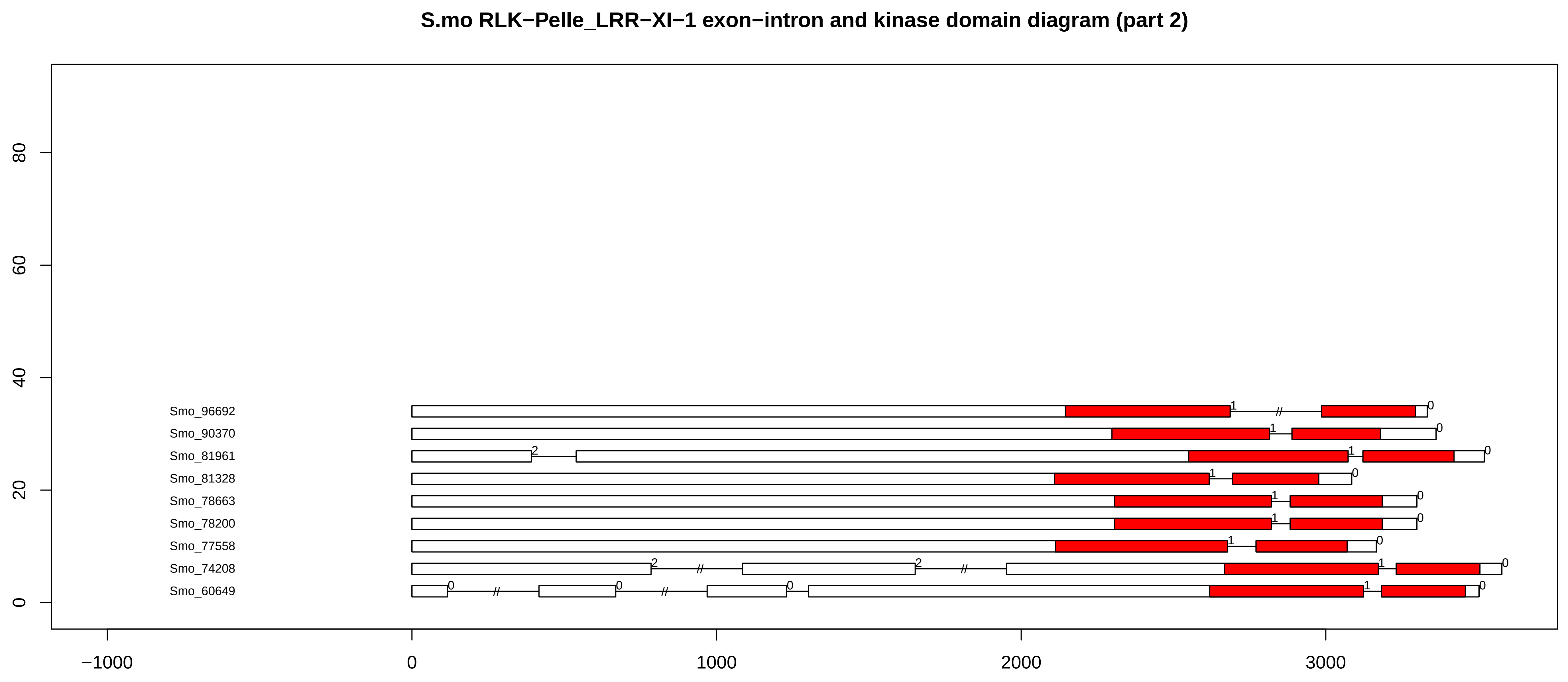

S.mo RLK−Pelle_LRR−XI−1 exon−intron and kinase domain diagram (part 2)
Smo_96692
Smo_90370
Smo_81961
Smo_81328
Smo_78663
Smo_78200
Smo_77558
Smo_74208
Smo_60649
0
20
40
60
80
−1000
0
1000
2000
3000

S.mo RLK−Pelle_LRR−XI−2 exon−intron and kinase domain diagram (all)

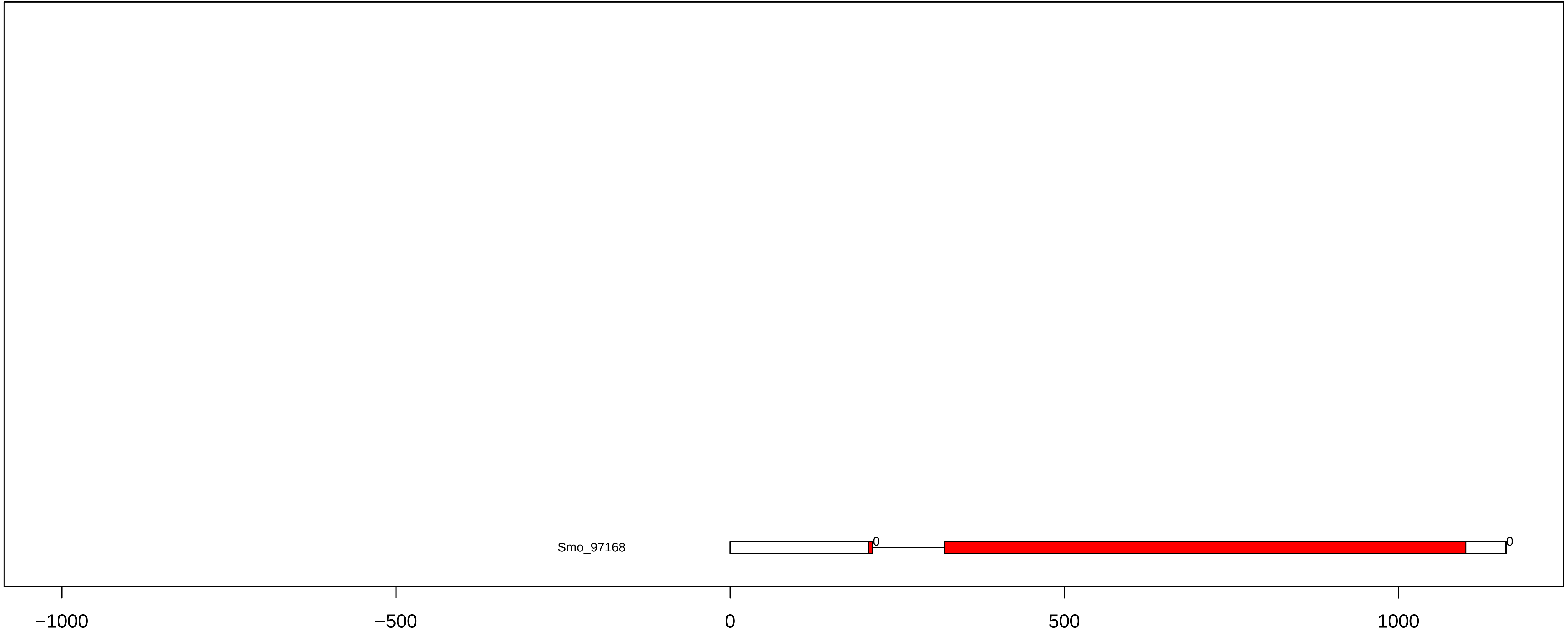

S.mo RLK−Pelle_LRR−XII−1 exon−intron and kinase domain diagram (all)

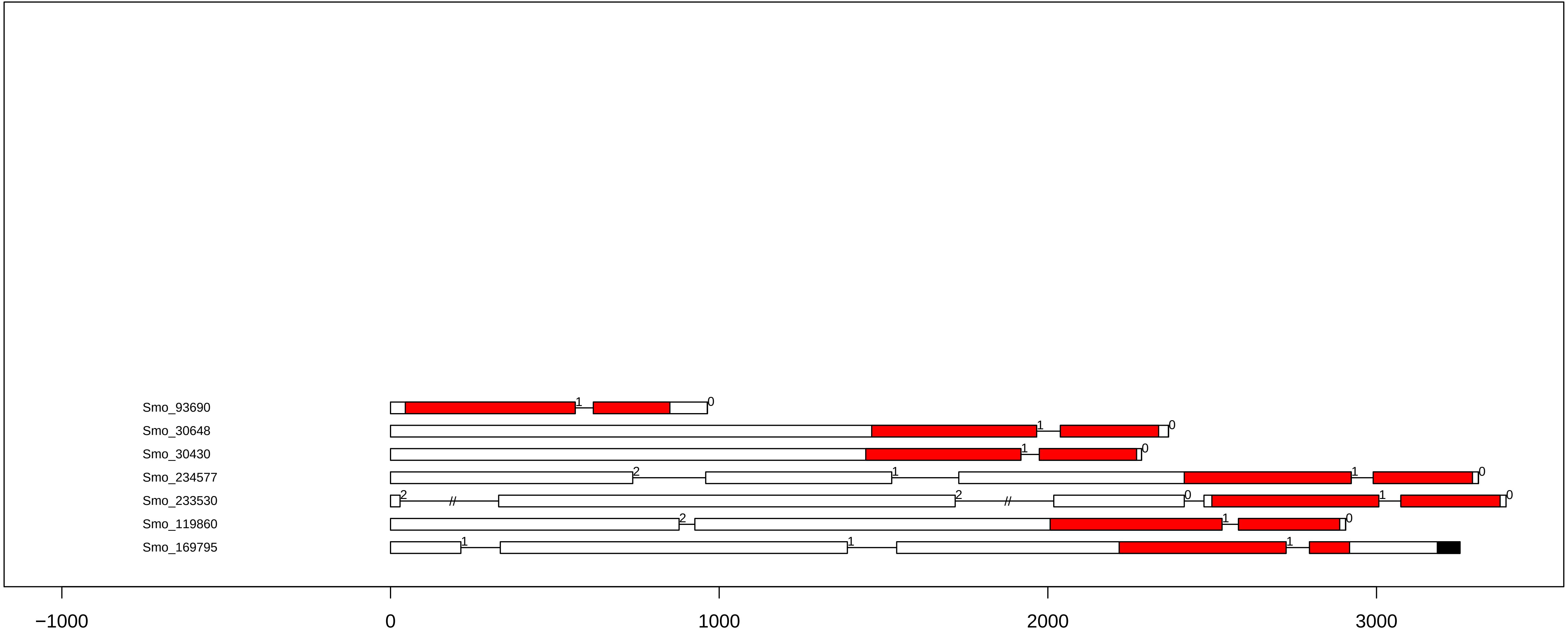

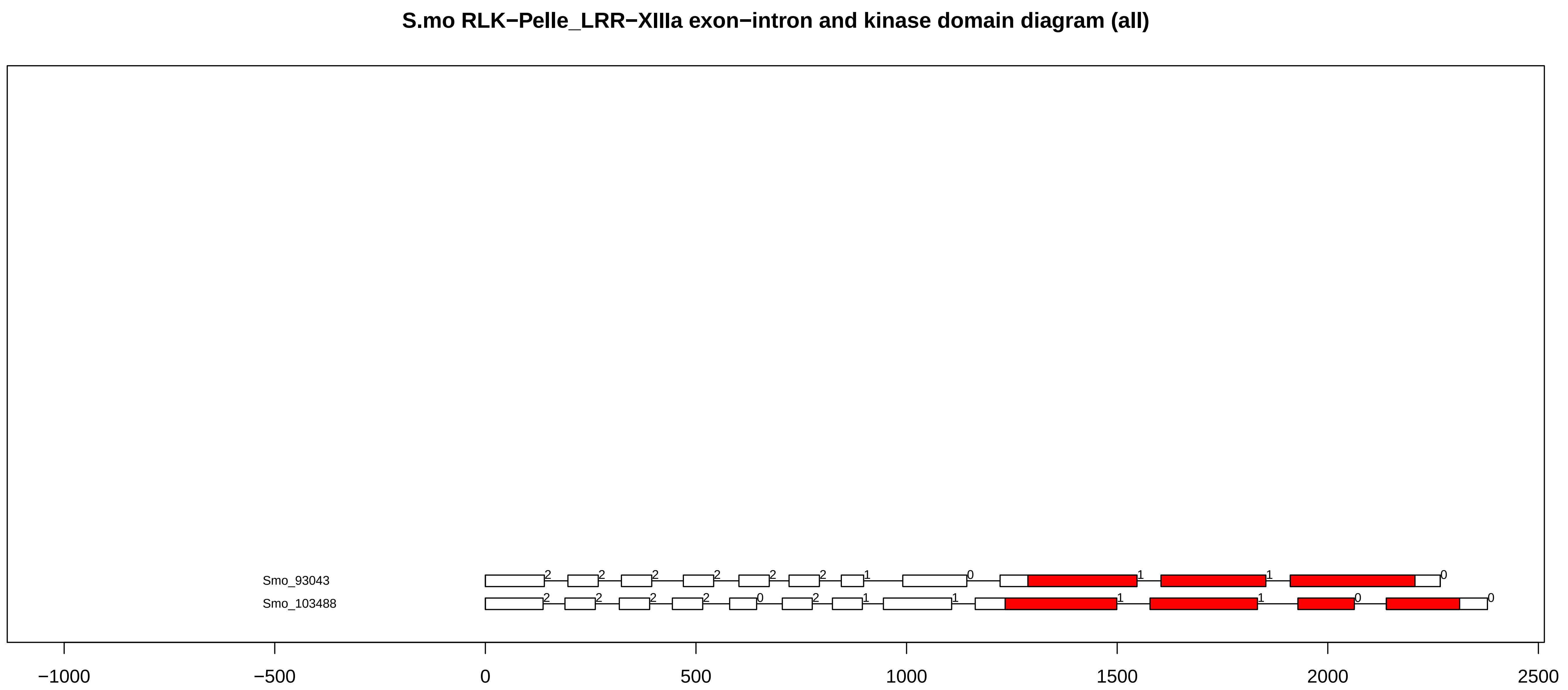
S.mo RLK−Pelle_LRR−XIIIa exon−intron and kinase domain diagram (all)
Smo_93043
Smo_103488
−1000
−500
0
500
1000
1500
2000
2500

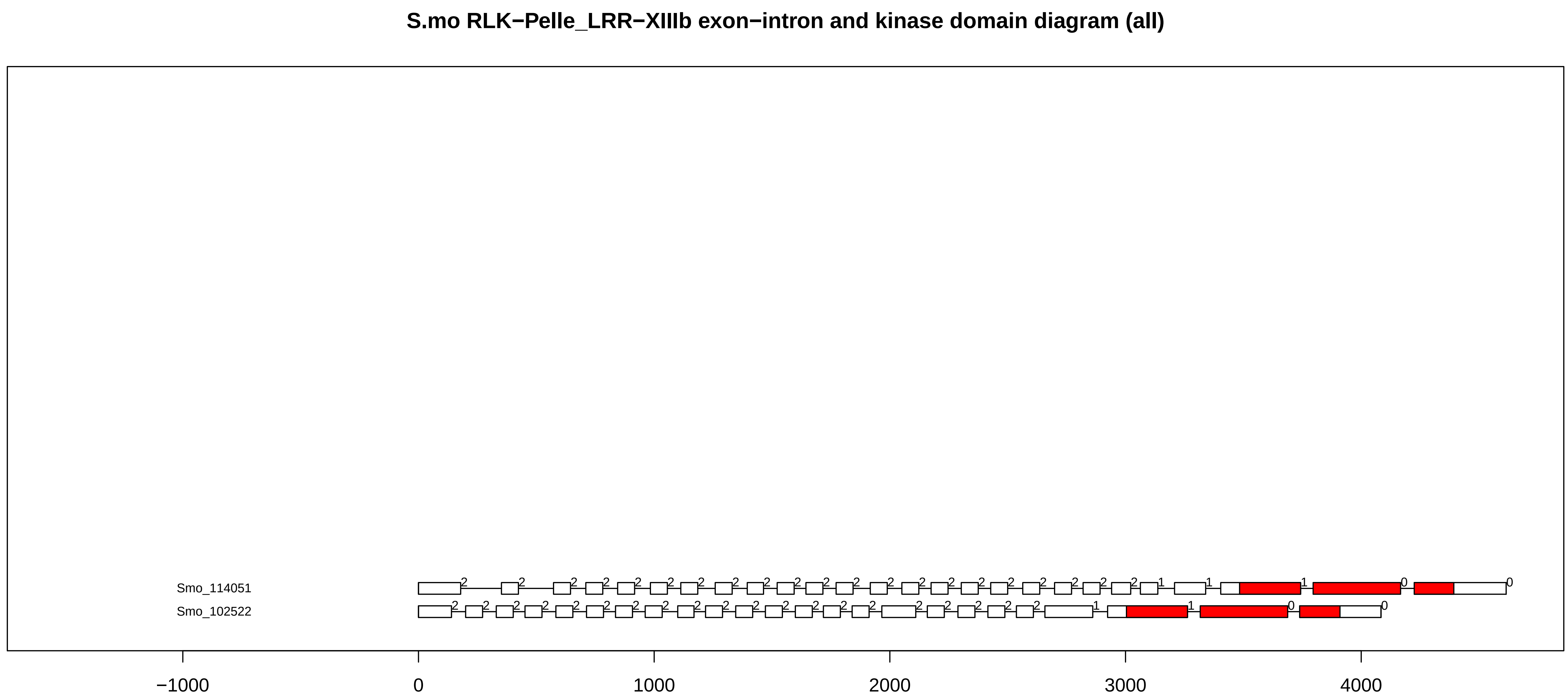

S.mo RLK−Pelle_LRR−XIIIb exon−intron and kinase domain diagram (all)
Smo_114051
Smo_102522
−1000
0
1000
2000
3000
4000

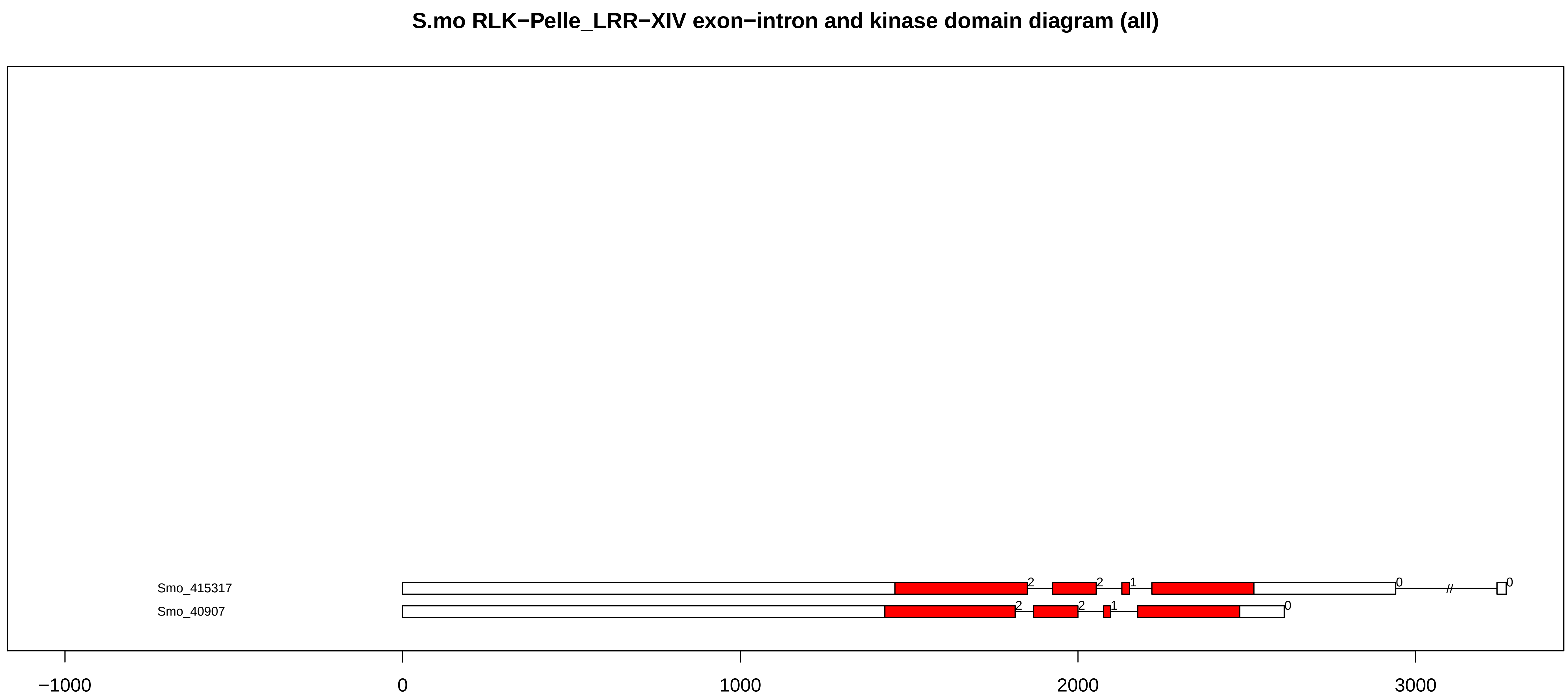
S.mo RLK−Pelle_LRR−XIV exon−intron and kinase domain diagram (all)
Smo_415317
Smo_40907
2
2
1
0
0
2
2
1
0
−1000
0
1000
2000
3000

S.mo RLK−Pelle_LRR−XV exon−intron and kinase domain diagram (all)

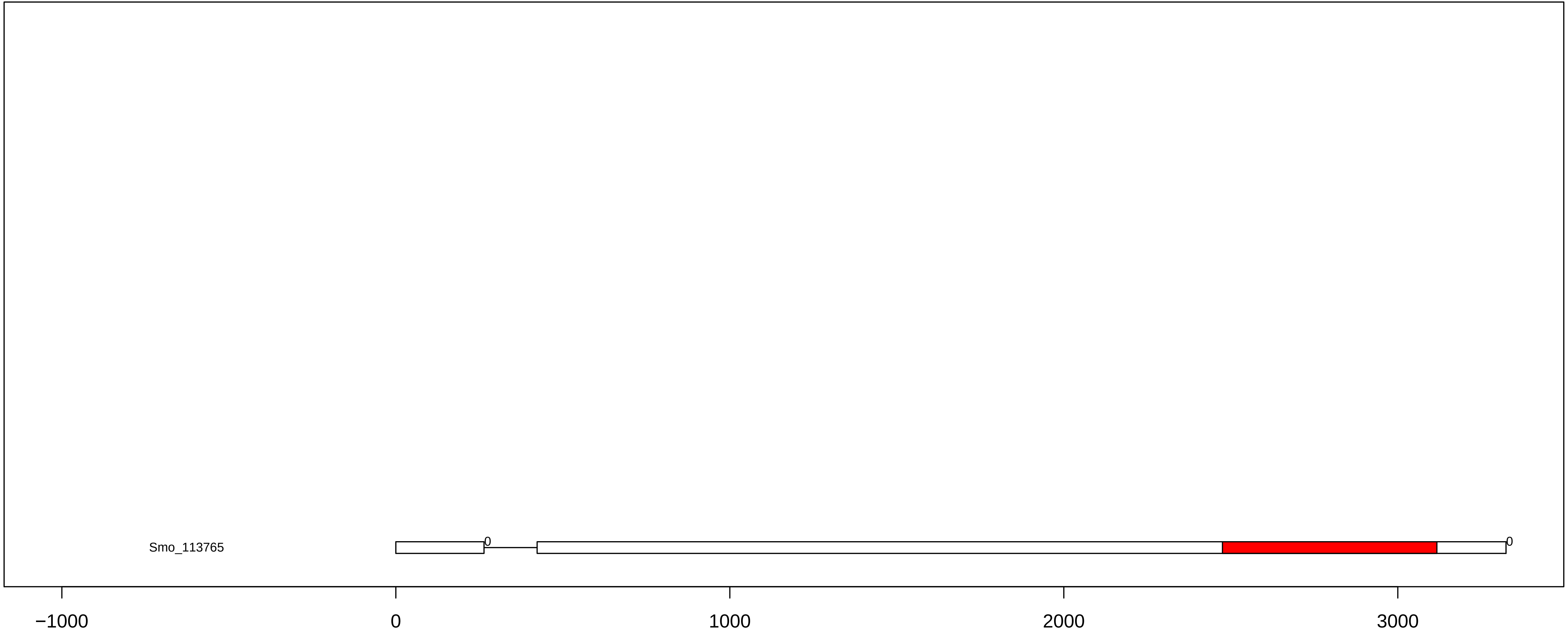

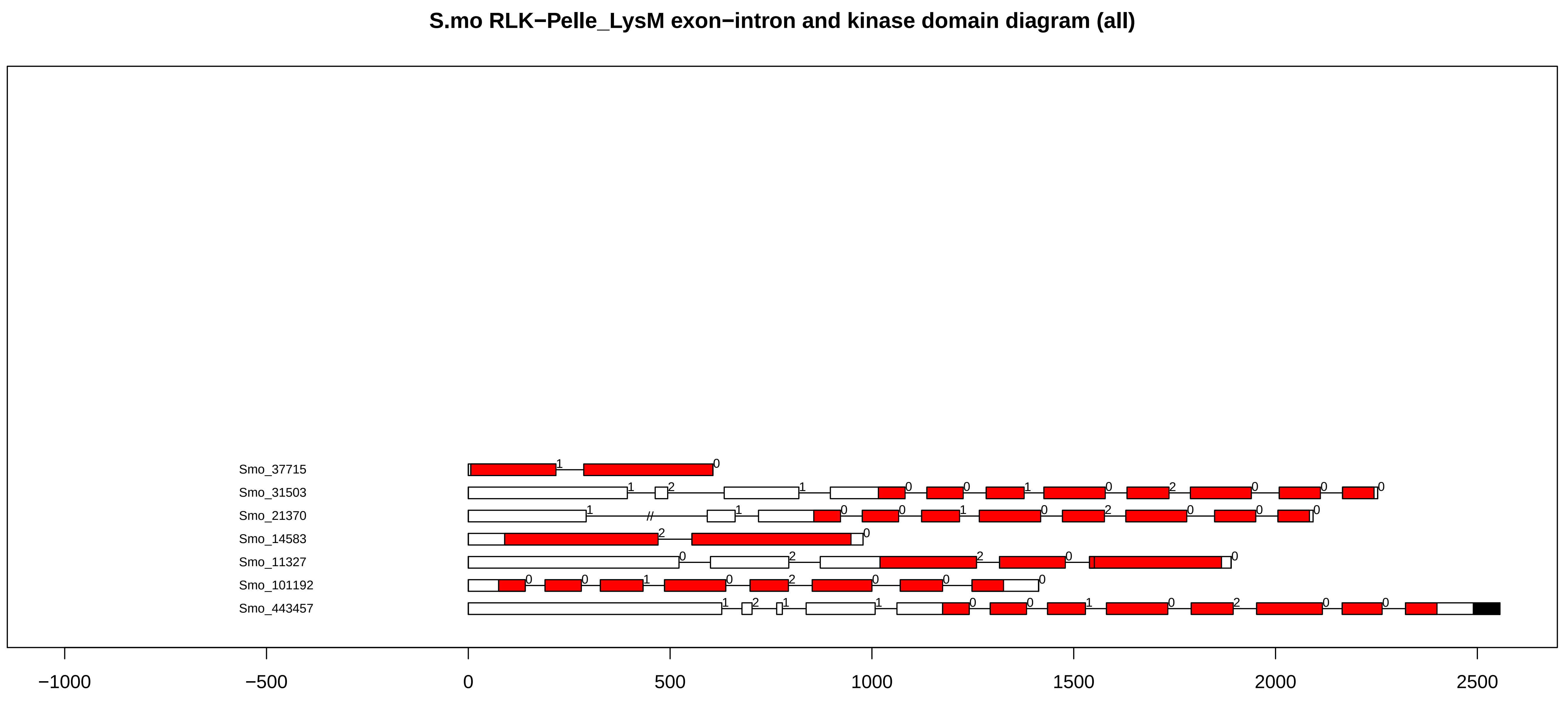
S.mo RLK−Pelle_LysM exon−intron and kinase domain diagram (all)
Smo_37715
Smo_31503
Smo_21370
Smo_14583
Smo_11327
Smo_101192
Smo_443457
−1000
−500
0
500
1000
1500
2000
2500

S.mo RLK−Pelle_PERK−1 exon−intron and kinase domain diagram (all)

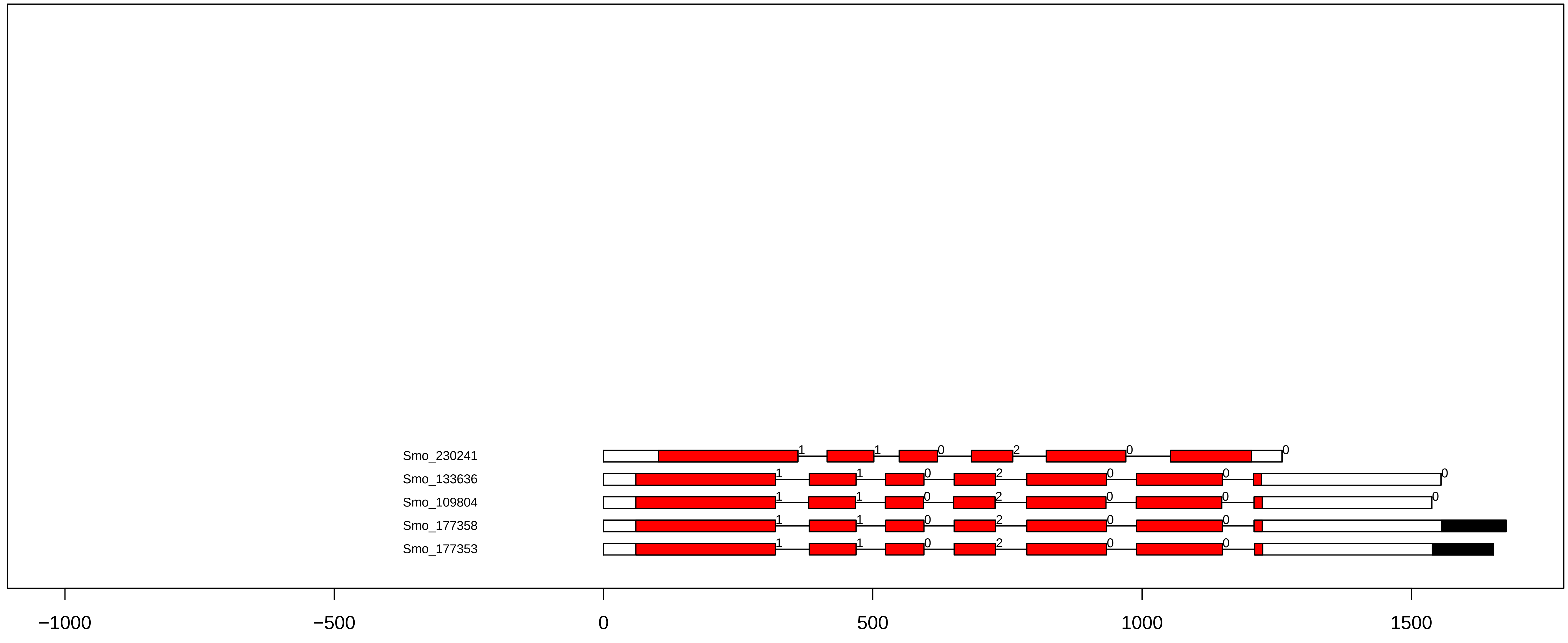

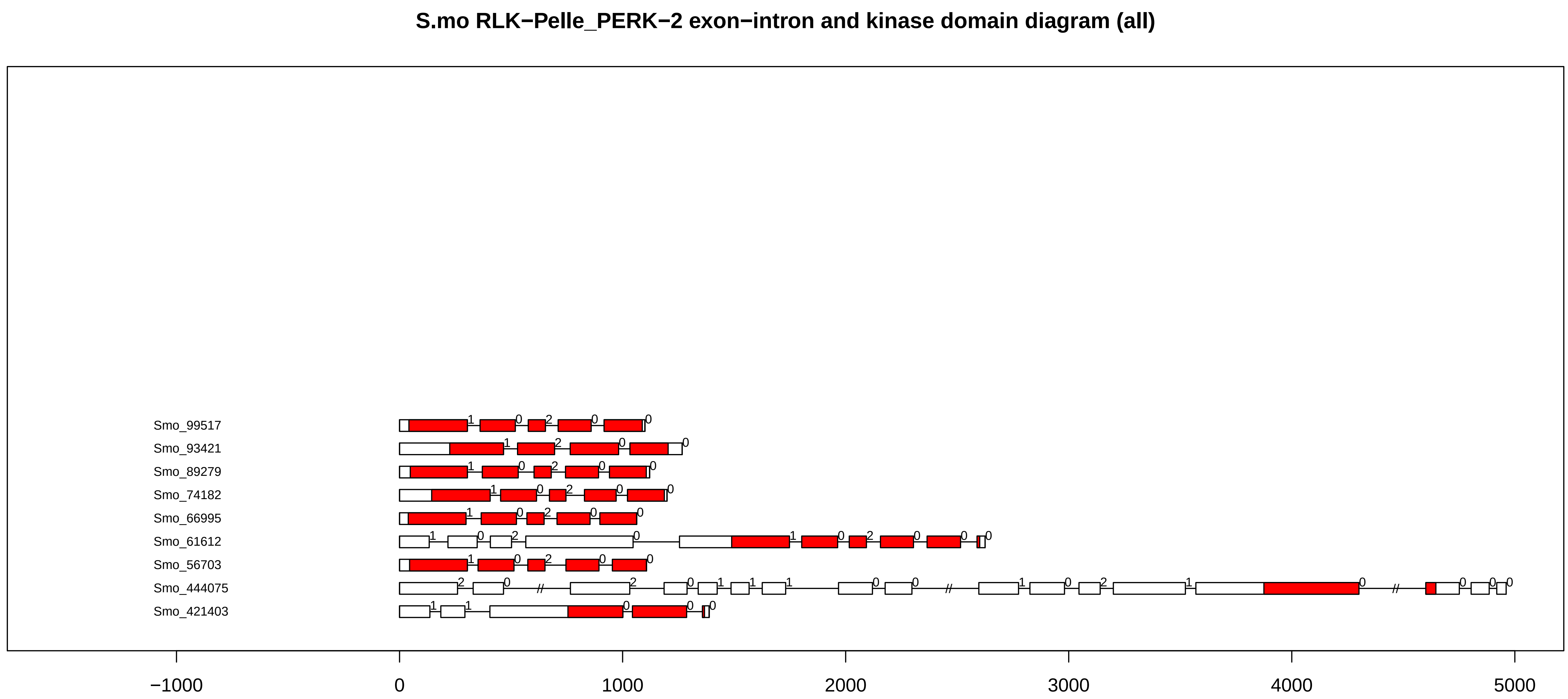

S.mo RLK−Pelle_PERK−2 exon−intron and kinase domain diagram (all)
Smo_99517
Smo_93421
Smo_89279
Smo_74182
Smo_66995
Smo_61612
Smo_56703
Smo_444075
Smo_421403
−1000
0
1000
2000
3000
4000
5000

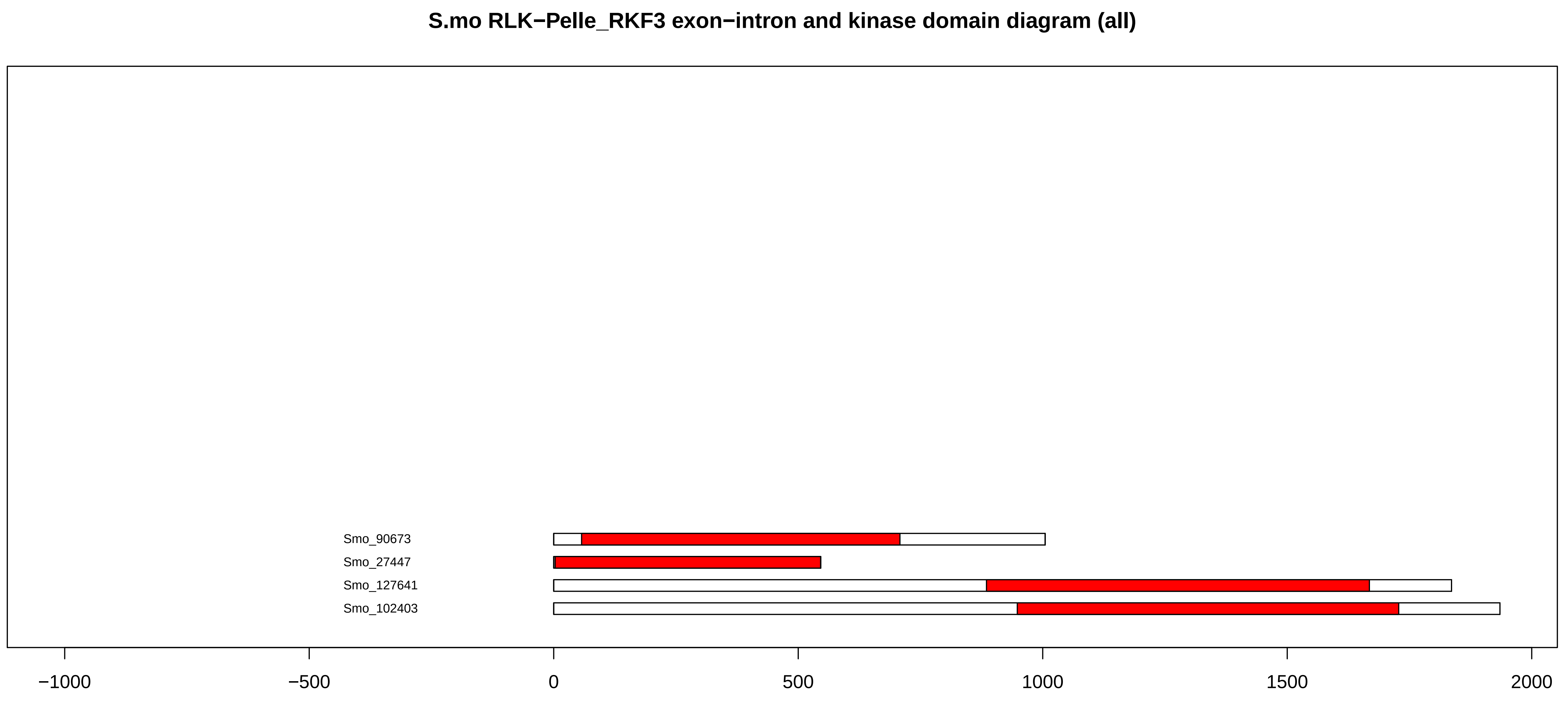

S.mo RLK−Pelle_RKF3 exon−intron and kinase domain diagram (all)
Smo_90673
Smo_27447
Smo_127641
Smo_102403
−1000
−500
0
500
1000
1500
2000

S.mo RLK−Pelle_RLCK−II exon−intron and kinase domain diagram (all)

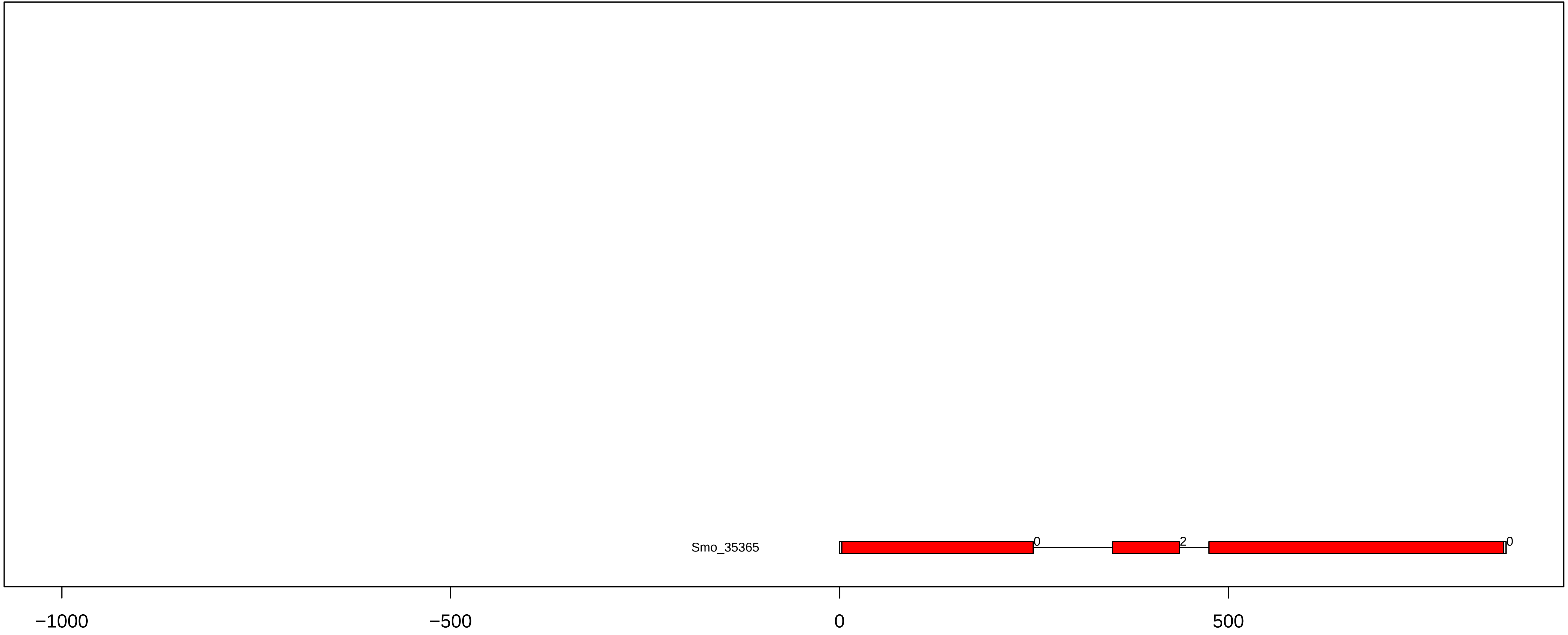

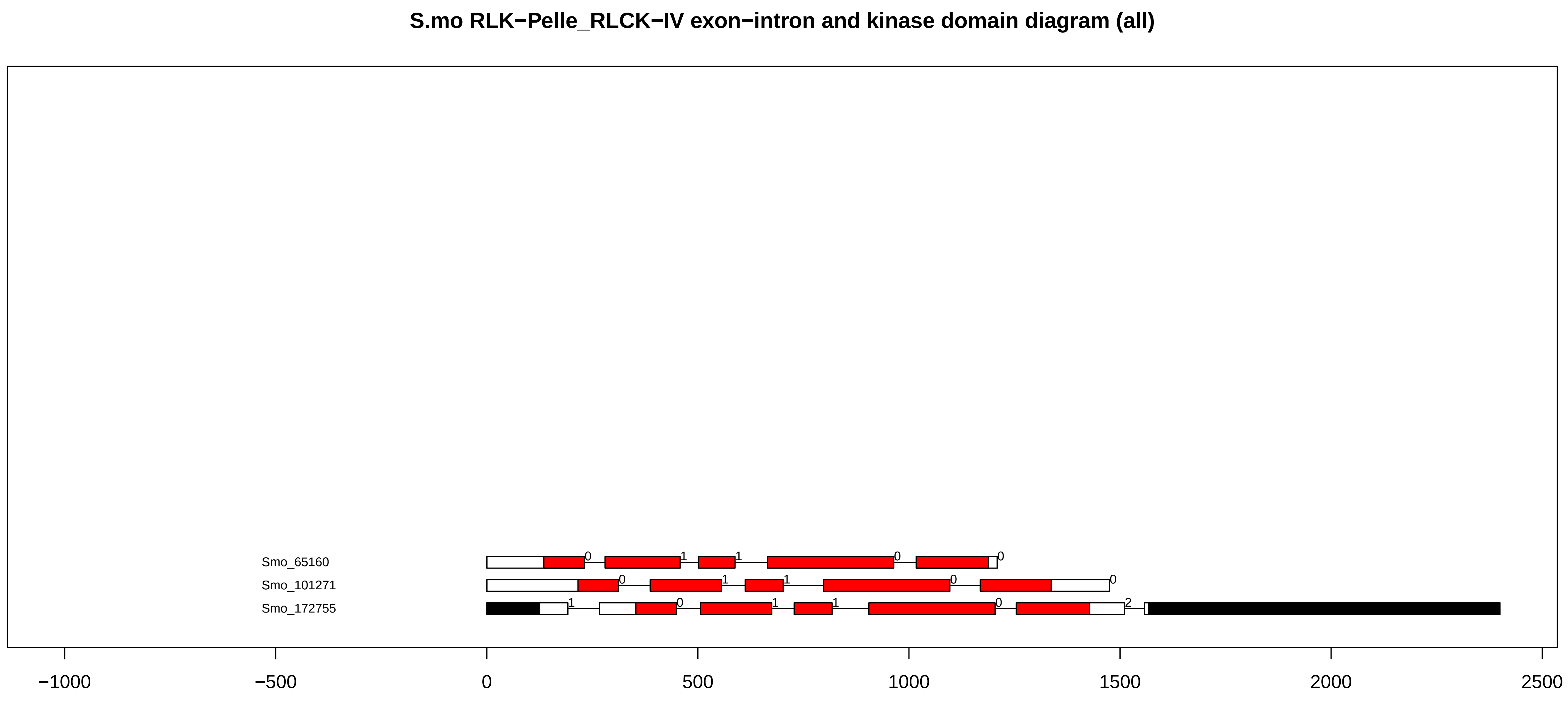
S.mo RLK−Pelle_RLCK−IV exon−intron and kinase domain diagram (all)
Smo_65160
Smo_101271
Smo_172755
−1000
−500
0
500
1000
1500
2000
2500

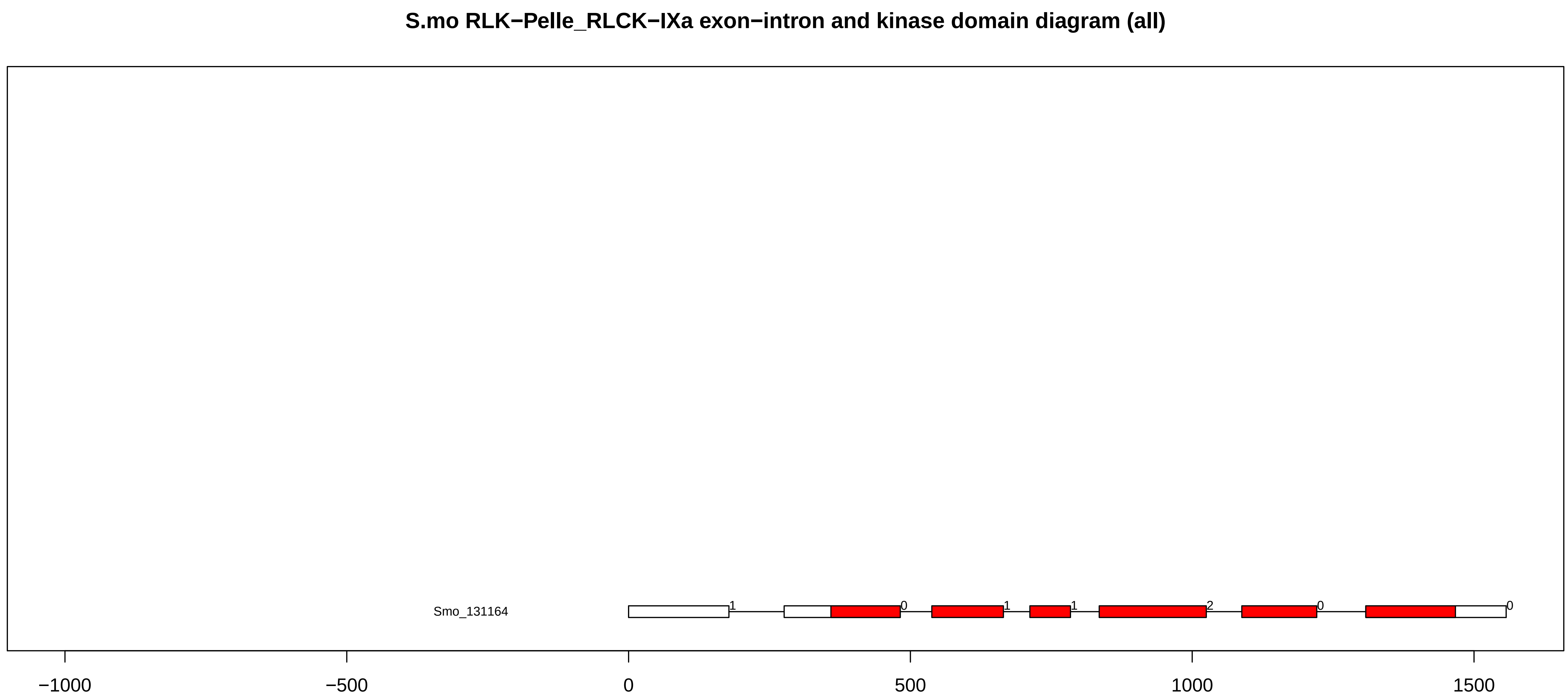

S.mo RLK−Pelle_RLCK−IXa exon−intron and kinase domain diagram (all)
Smo_131164
1
0
1
1
2
0
0
−1000
−500
0
500
1000
1500

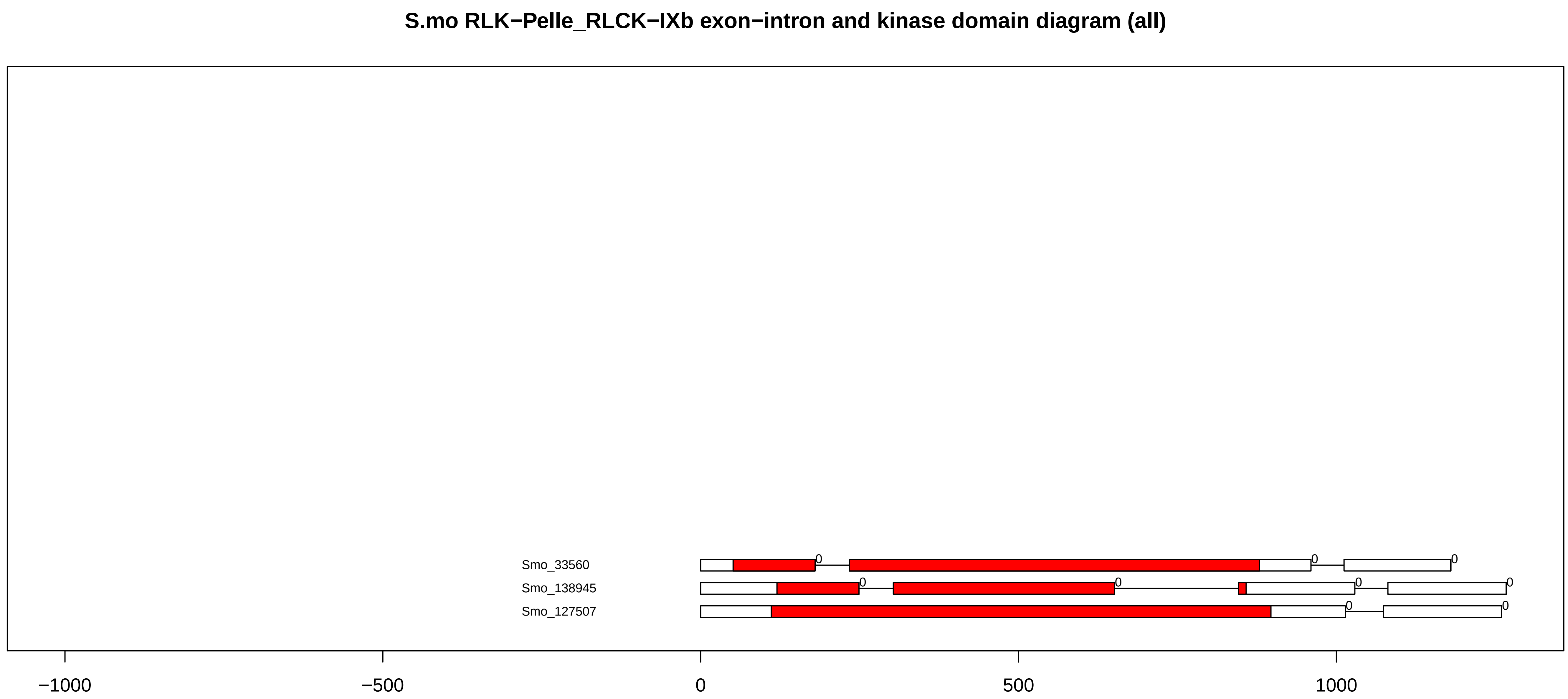
S.mo RLK−Pelle_RLCK−IXb exon−intron and kinase domain diagram (all)
Smo_33560
Smo_138945
Smo_127507
0
0
0
0
0
0
0
0
0
−1000
−500
0
500
1000

S.mo RLK−Pelle_RLCK−VIIa−1 exon−intron and kinase domain diagram (all)

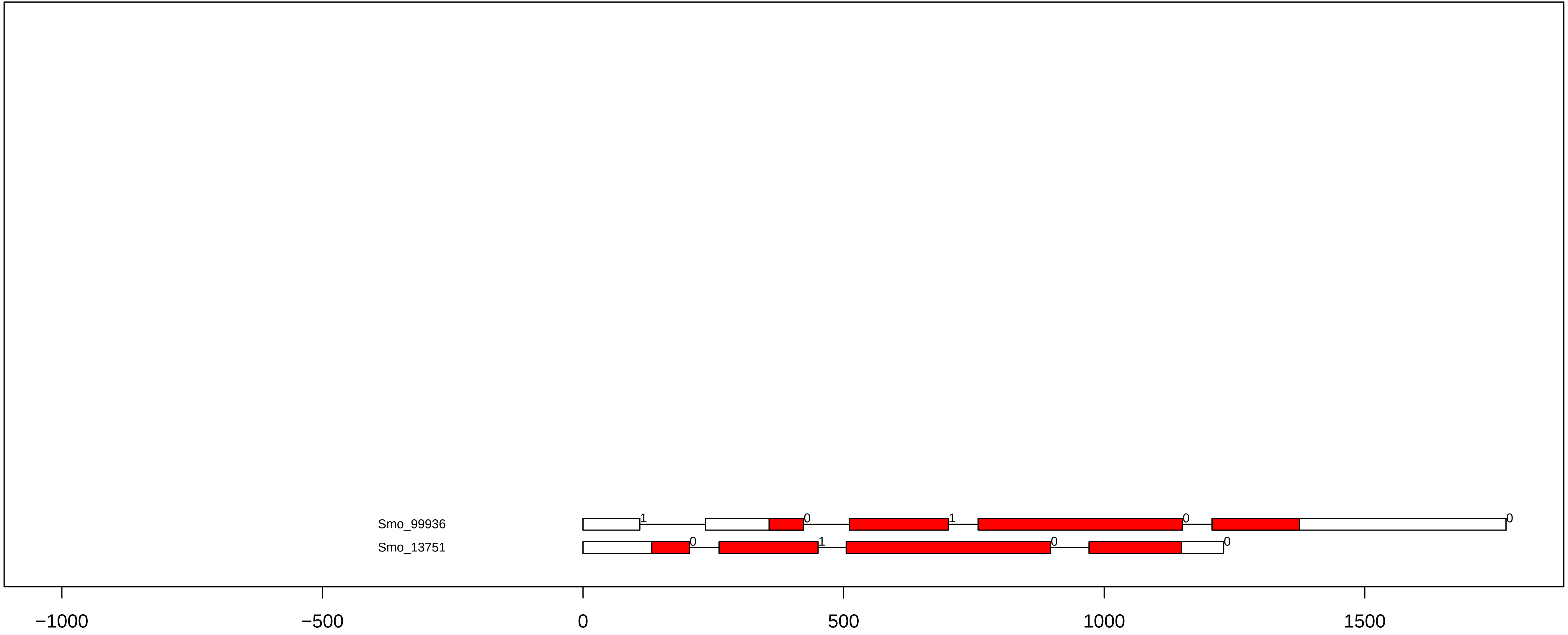

S.mo RLK−Pelle_RLCK−VIIa−2 exon−intron and kinase domain diagram (all)

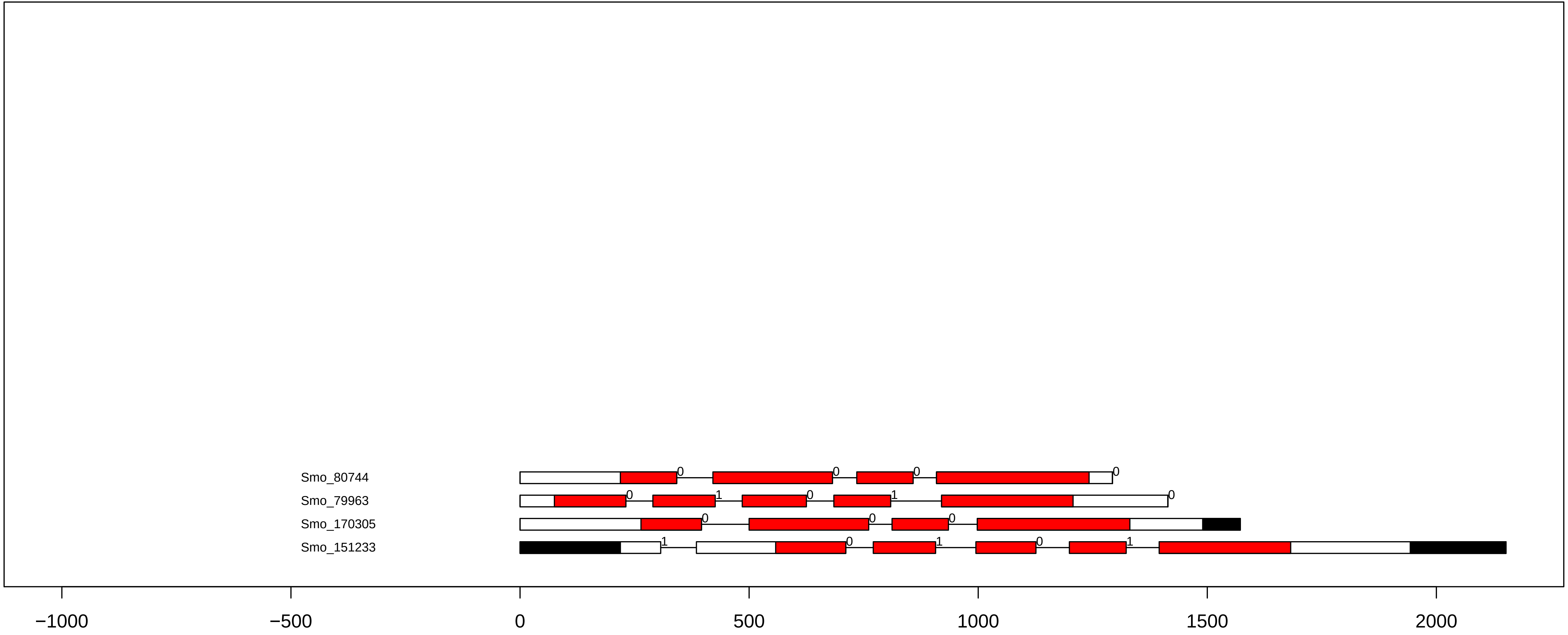

S.mo RLK−Pelle_RLCK−VIIb exon−intron and kinase domain diagram (all)

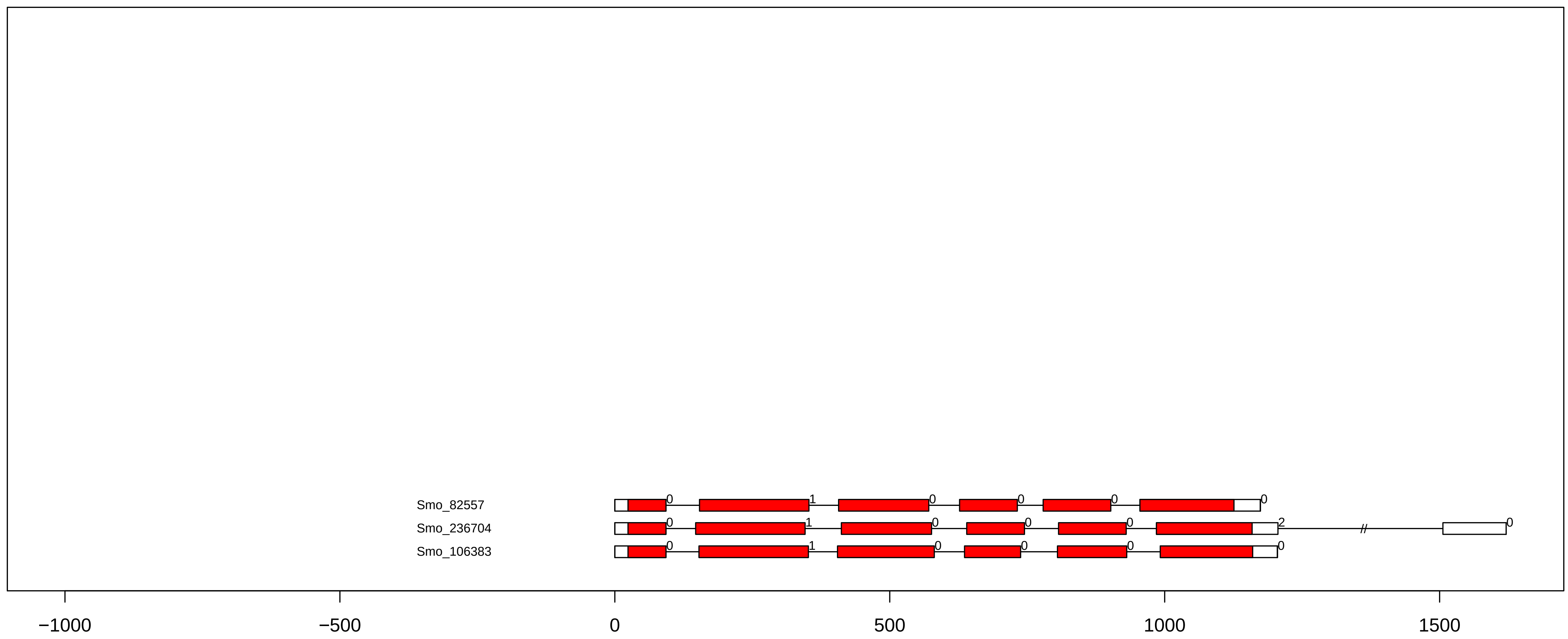

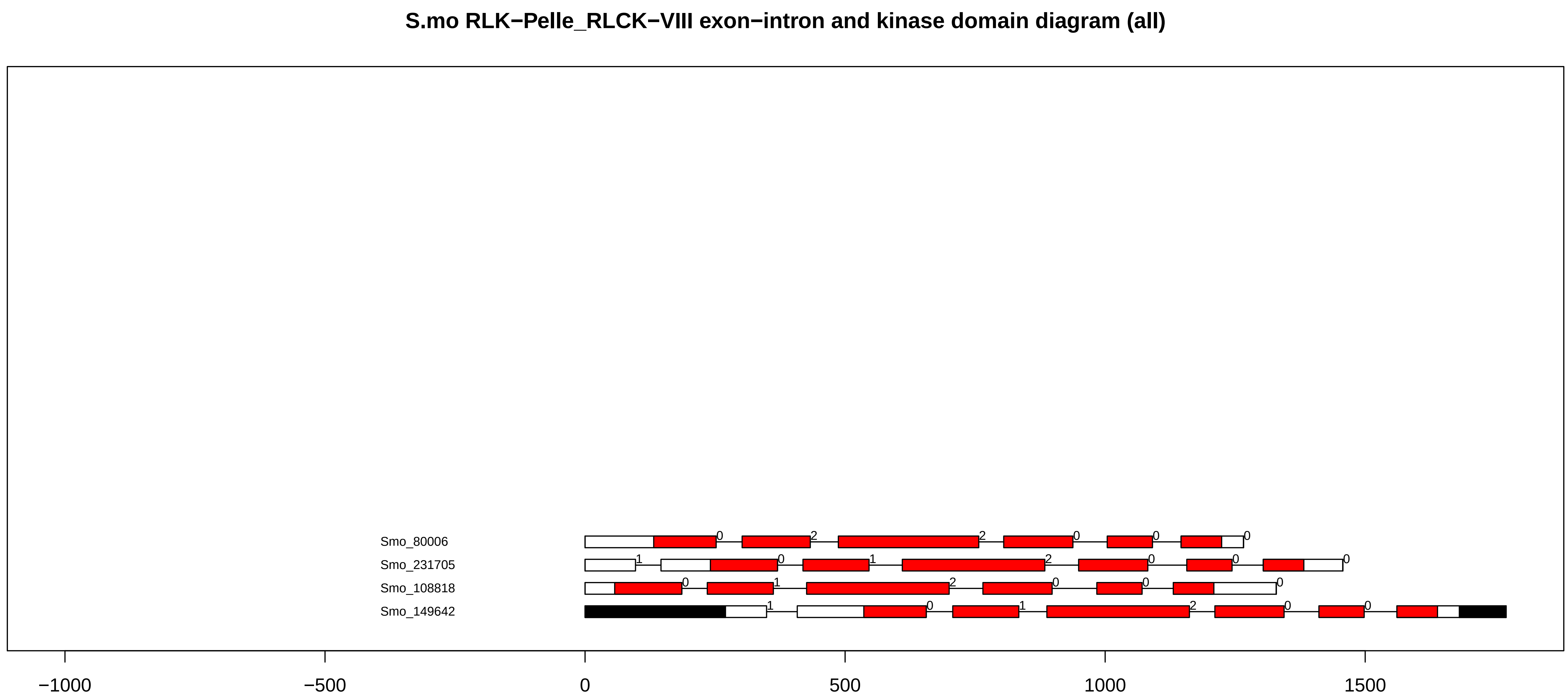
S.mo RLK−Pelle_RLCK−VIII exon−intron and kinase domain diagram (all)
Smo_80006
Smo_231705
Smo_108818
Smo_149642
0
2
2
0
0
0
1
0
1
2
0
0
0
0
1
2
0
0
0
1
0
1
2
0
0
−1000
−500
0
500
1000
1500

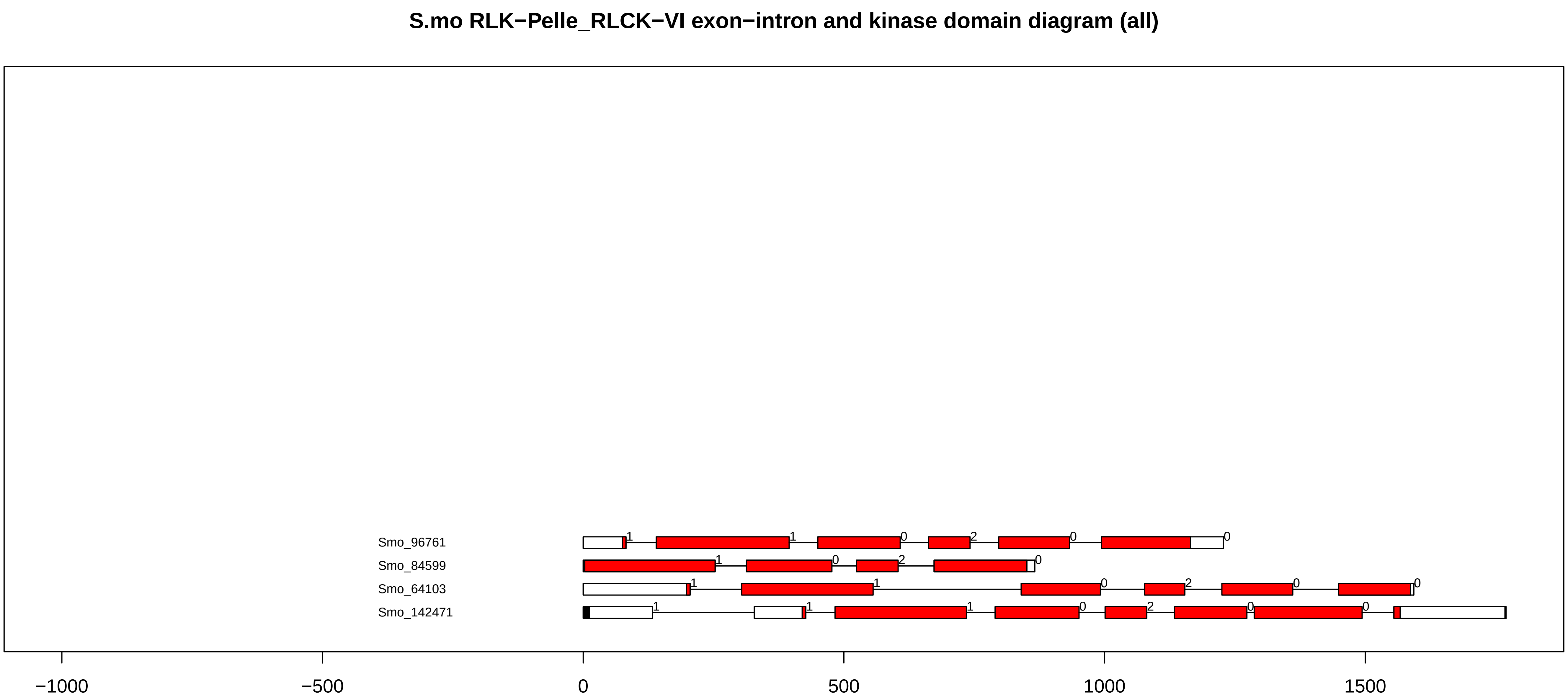
S.mo RLK−Pelle_RLCK−VI exon−intron and kinase domain diagram (all)
Smo_96761
Smo_84599
Smo_64103
Smo_142471
1
1
0
2
0
0
1
0
2
0
1
1
0
2
0
0
1
1
1
0
2
0
0
−1000
−500
0
500
1000
1500

S.mo RLK−Pelle_RLCK−V exon−intron and kinase domain diagram (all)

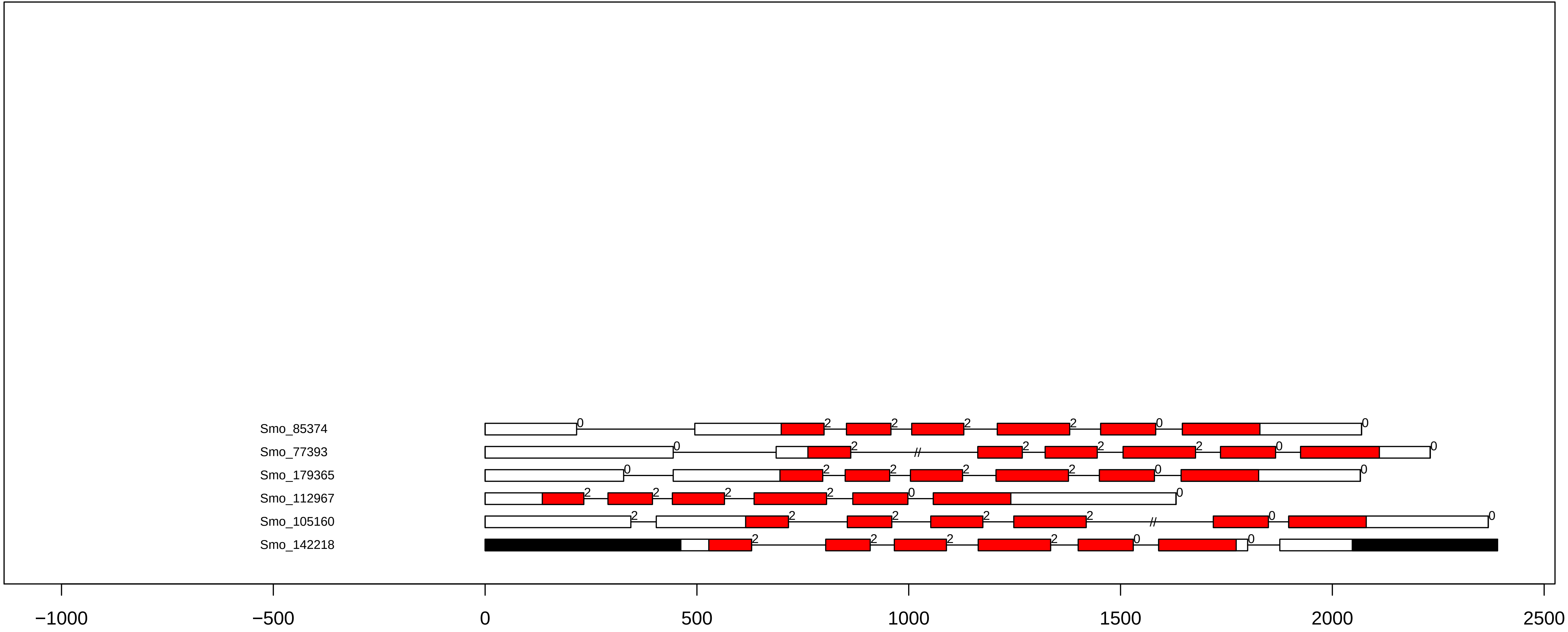

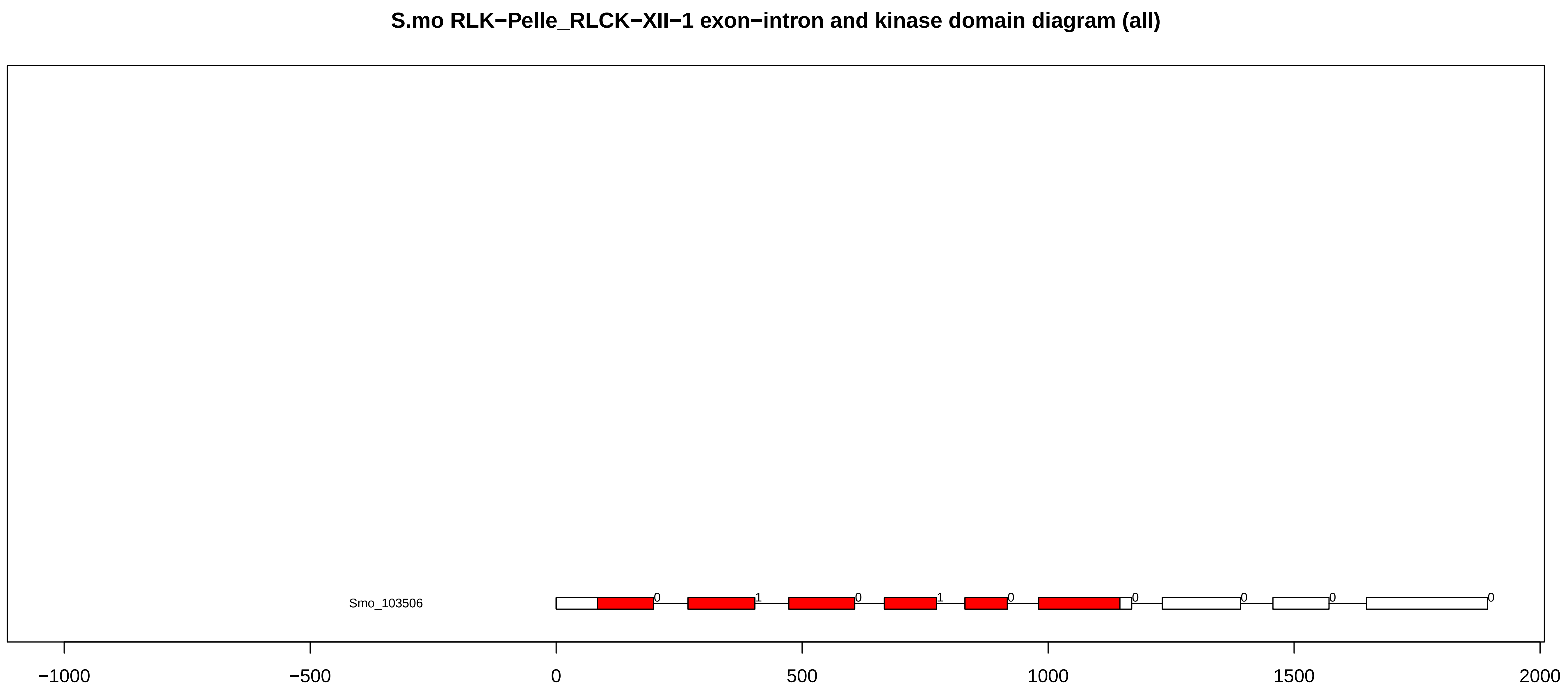

S.mo RLK−Pelle_RLCK−XII−1 exon−intron and kinase domain diagram (all)
Smo_103506
0
1
0
1
0
0
0
0
0
−1000
−500
0
500
1000
1500
2000

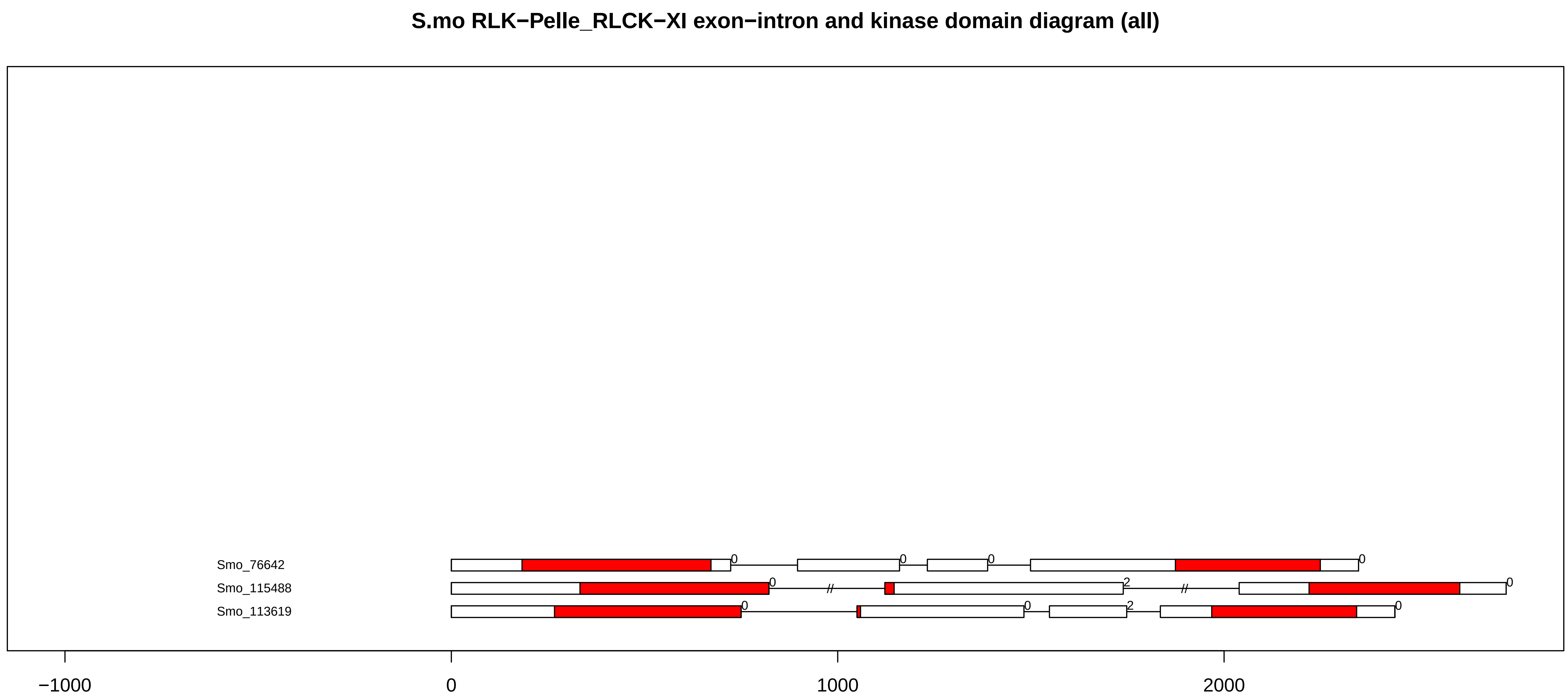
S.mo RLK−Pelle_RLCK−XI exon−intron and kinase domain diagram (all)
Smo_76642
Smo_115488
Smo_113619
0
0
0
0
0
2
0
0
0
2
0
−1000
0
1000
2000

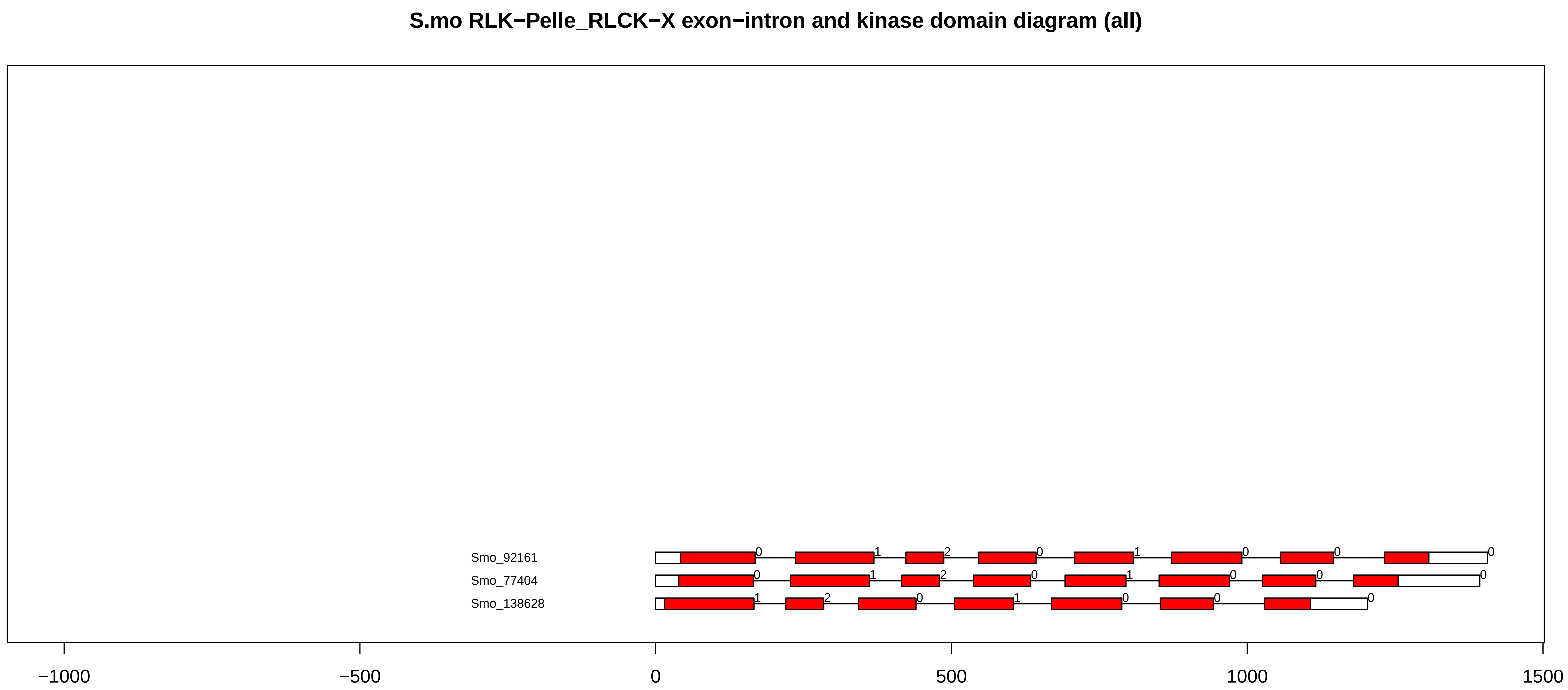

S.mo RLK−Pelle_RLCK−X exon−intron and kinase domain diagram (all)
Smo_92161
Smo_77404
Smo_138628
−1000
−500
0
500
1000
1500

S.mo RLK−Pelle_RLCK−XV exon−intron and kinase domain diagram (all)

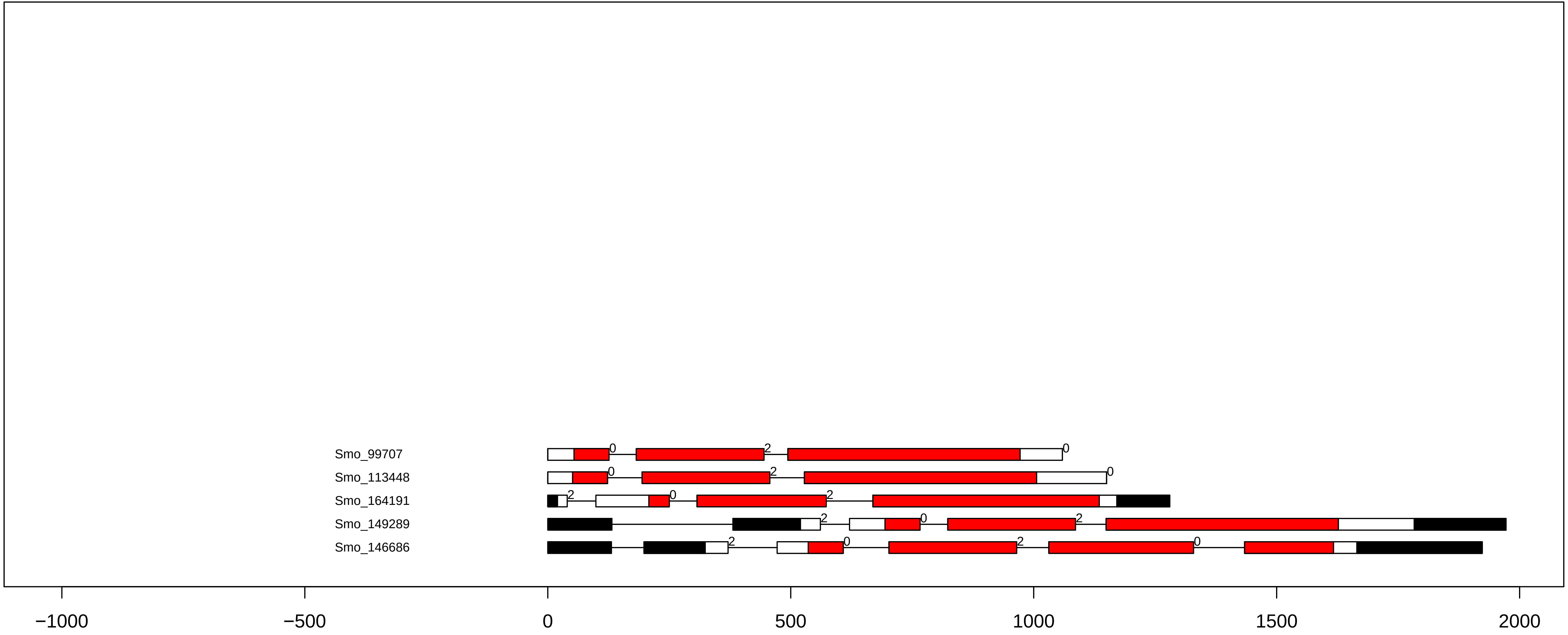

S.mo RLK−Pelle_SD−2b exon−intron and kinase domain diagram (part 1)

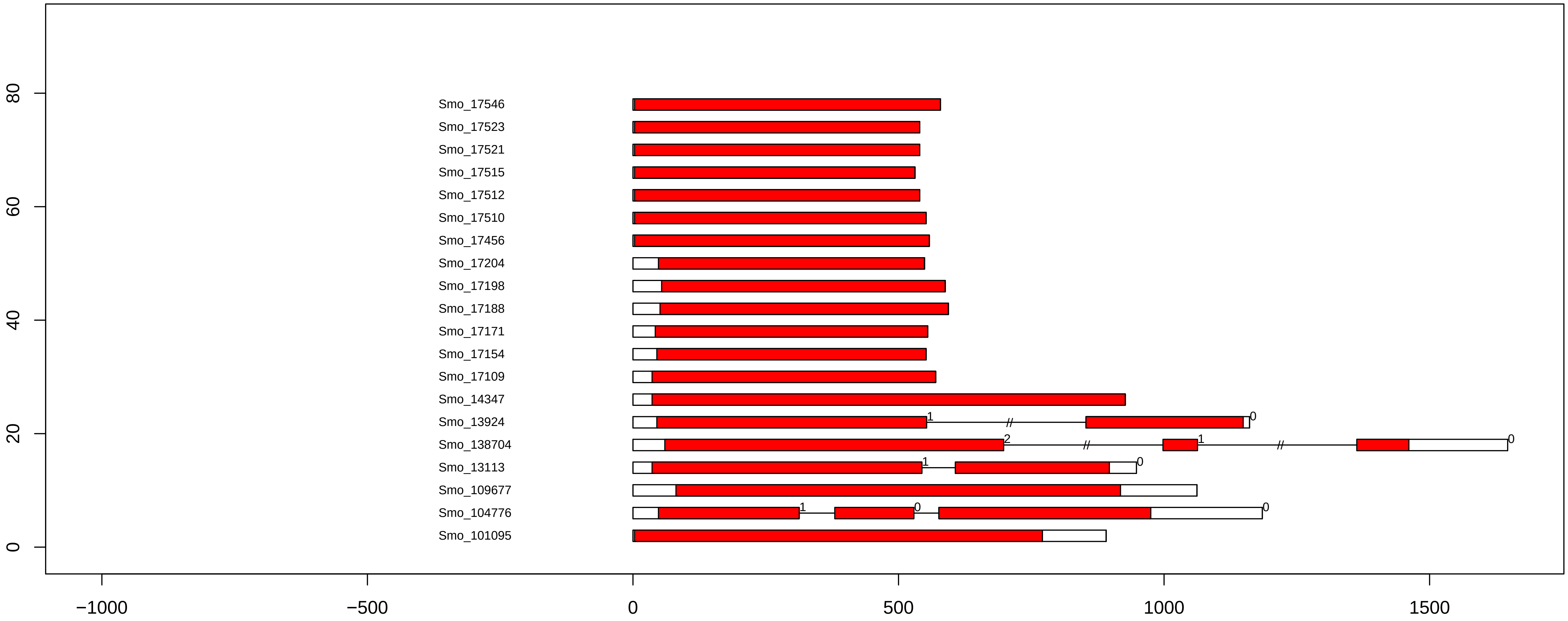

S.mo RLK−Pelle_SD−2b exon−intron and kinase domain diagram (part 2)

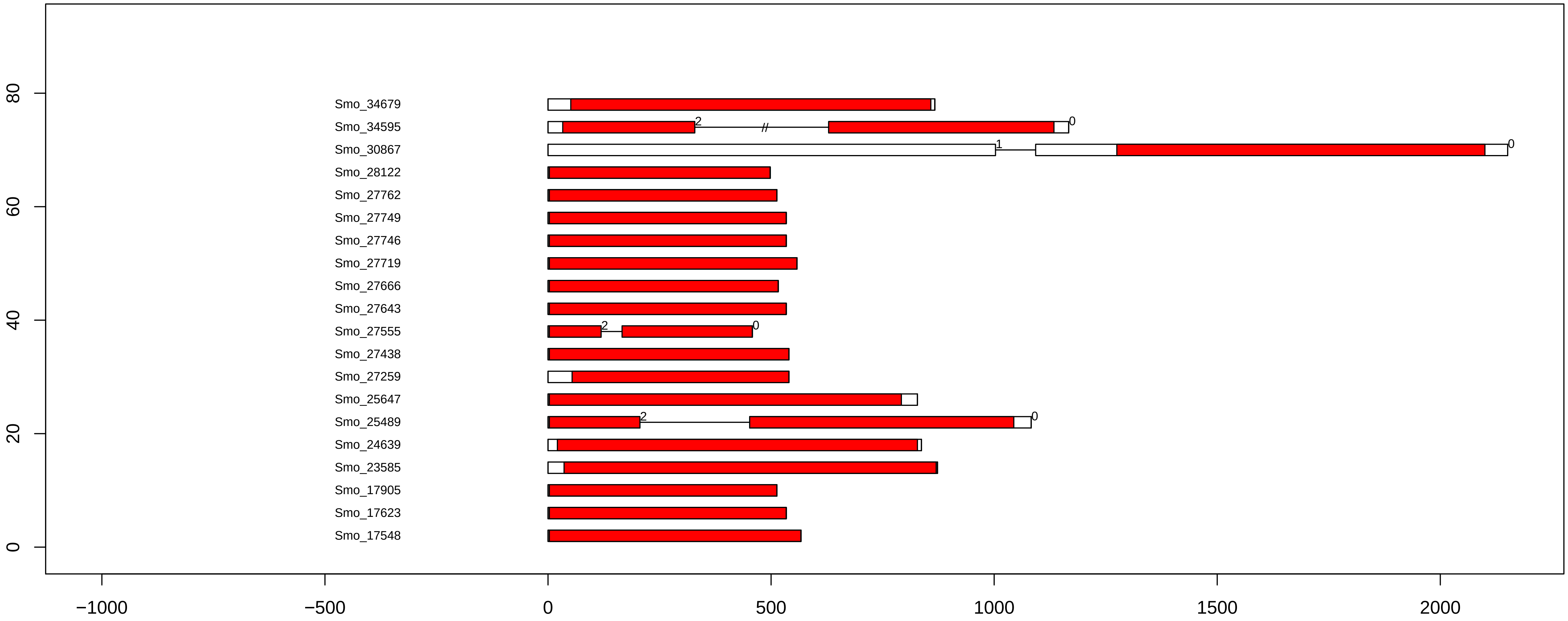

S.mo RLK−Pelle_SD−2b exon−intron and kinase domain diagram (part 3)

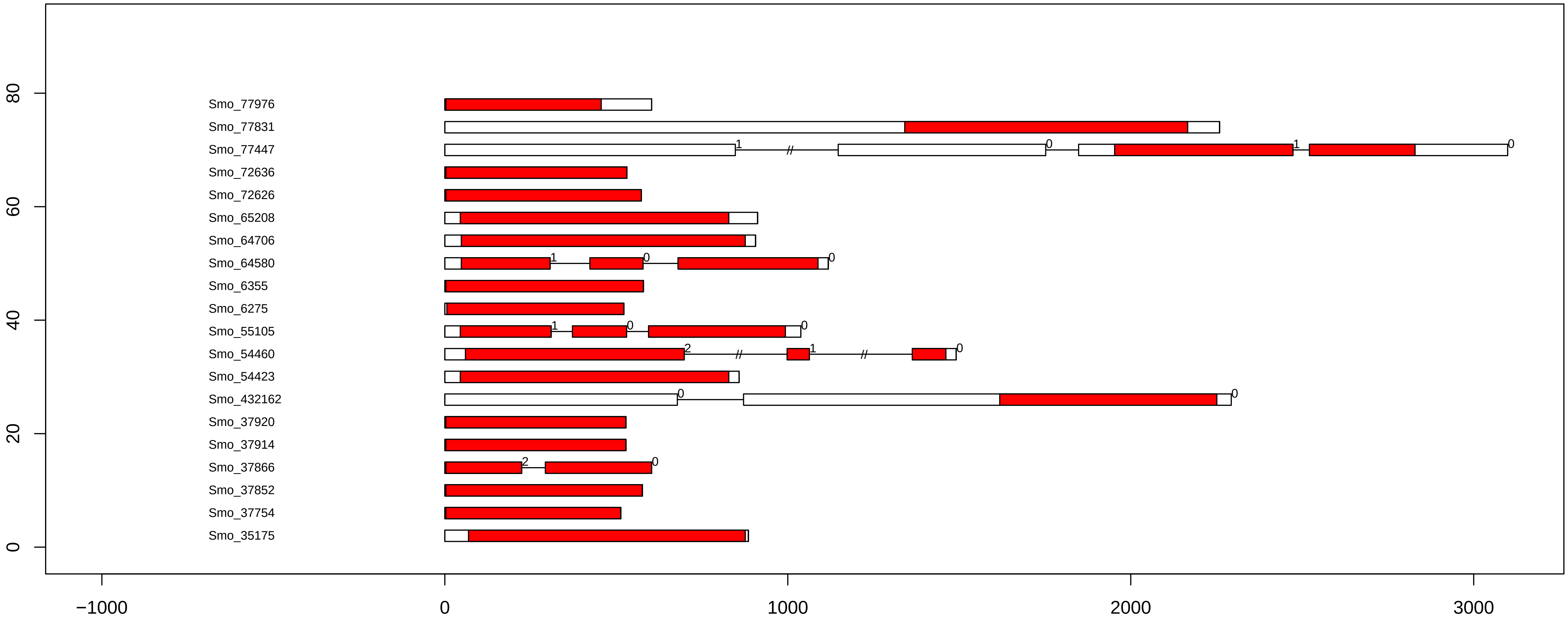

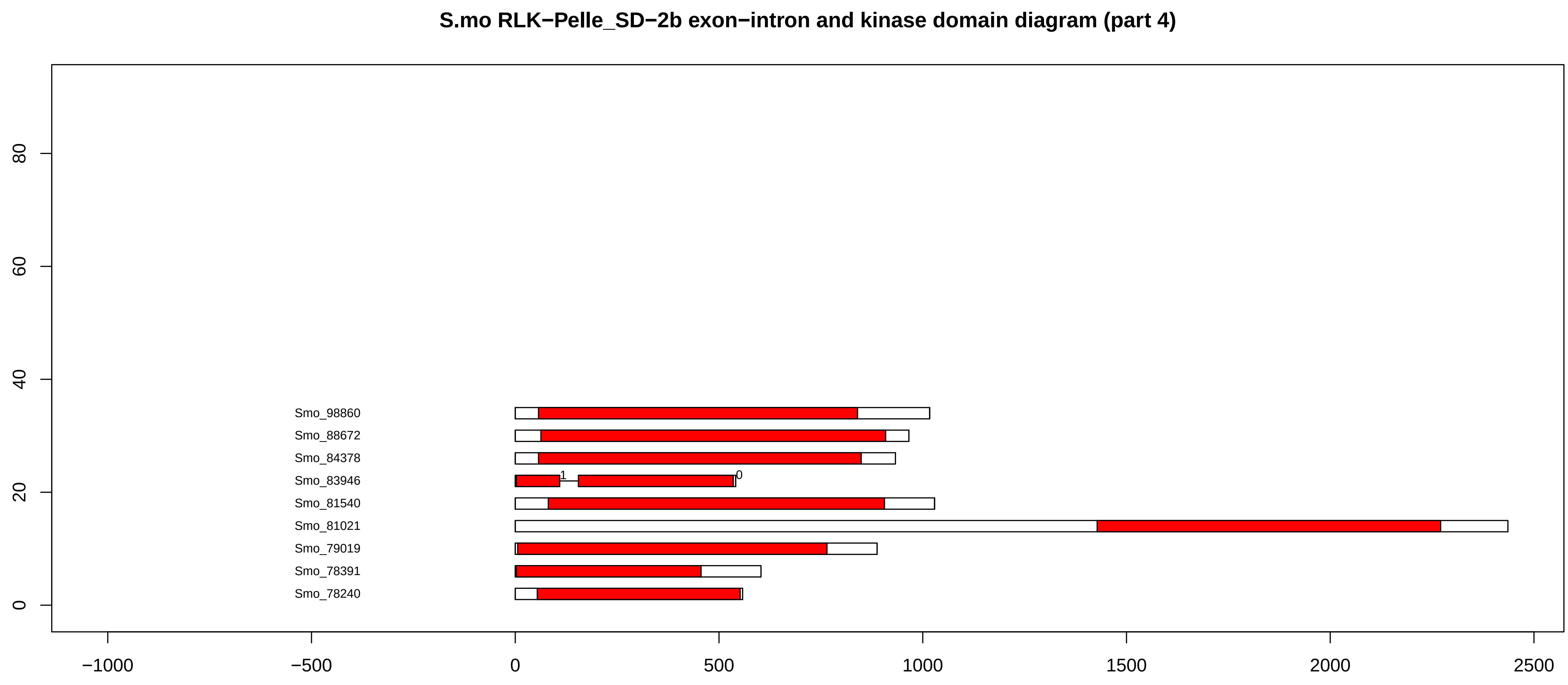

S.mo RLK−Pelle_SD−2b exon−intron and kinase domain diagram (part 4)
Smo_98860
Smo_88672
Smo_84378
Smo_83946
Smo_81540
Smo_81021
Smo_79019
Smo_78391
Smo_78240
1
0
0
20
40
60
80
−1000
−500
0
500
1000
1500
2000
2500

S.mo RLK−Pelle_URK−1 exon−intron and kinase domain diagram (all)

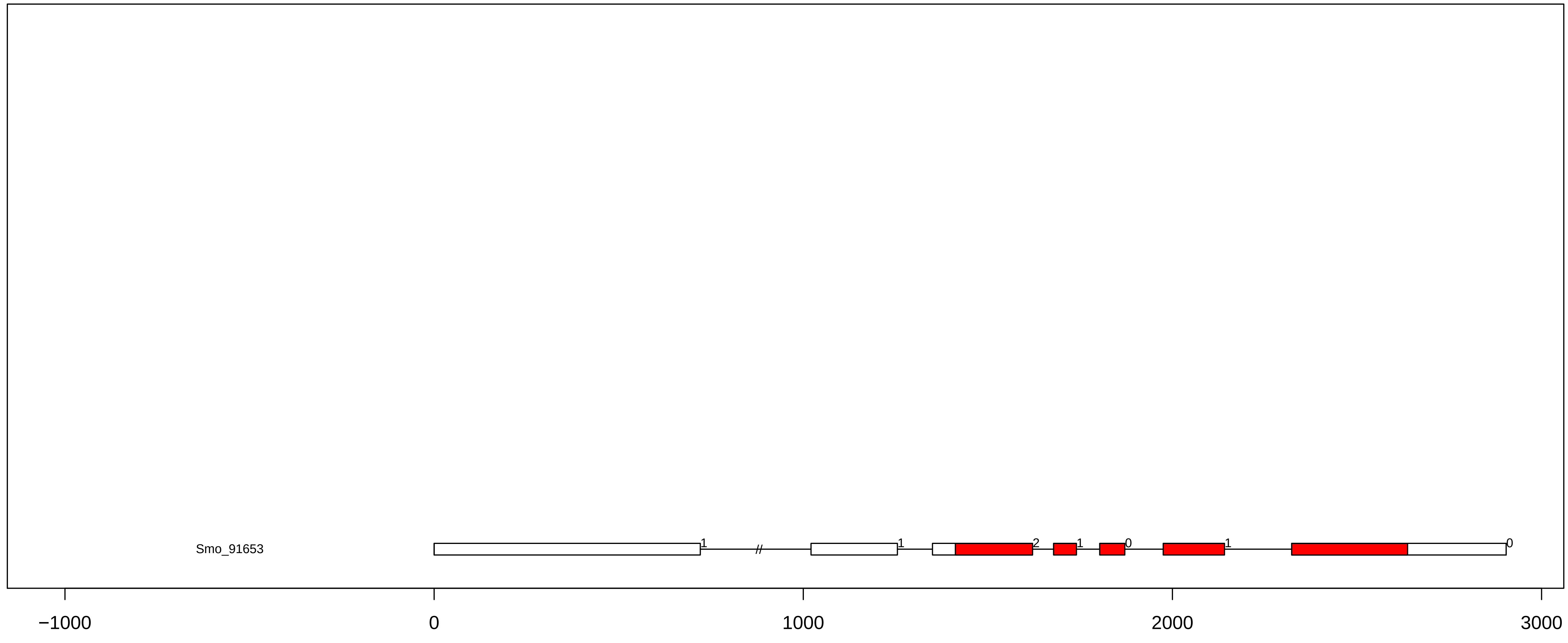

S.mo RLK−Pelle_URK−2 exon−intron and kinase domain diagram (all)

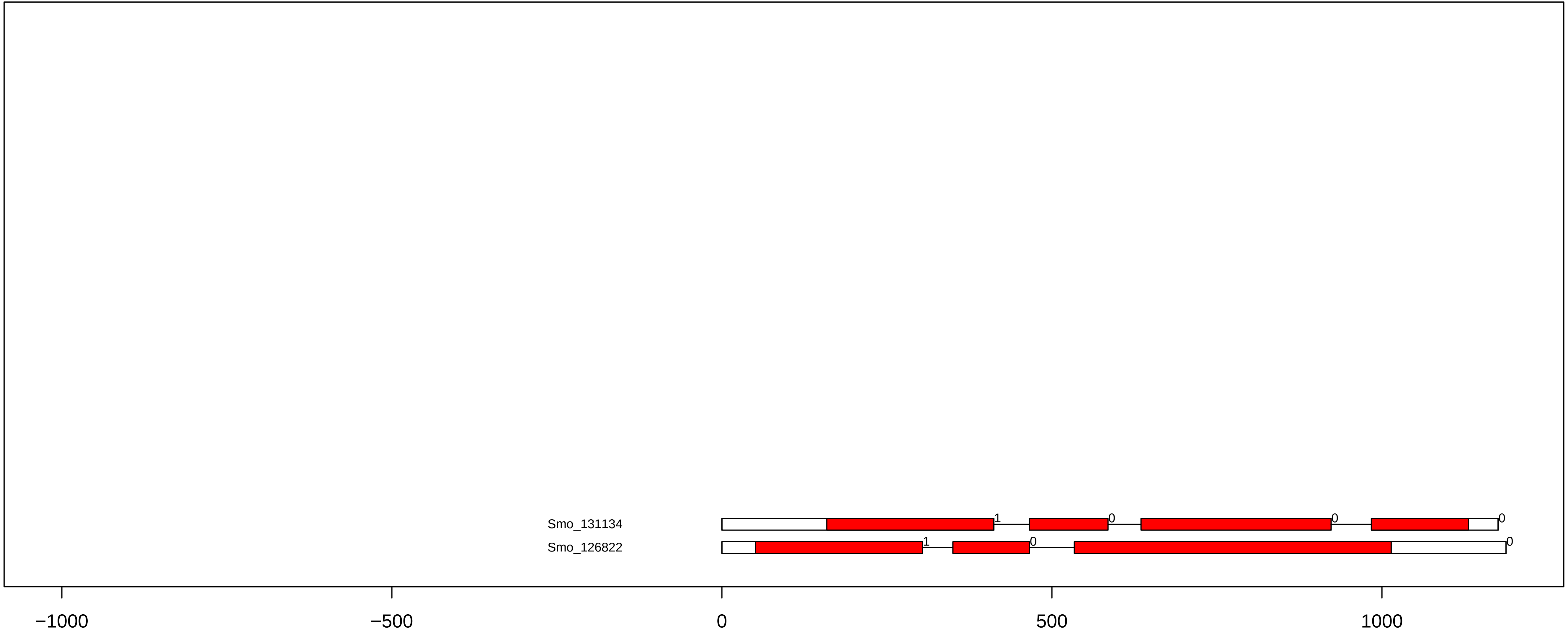

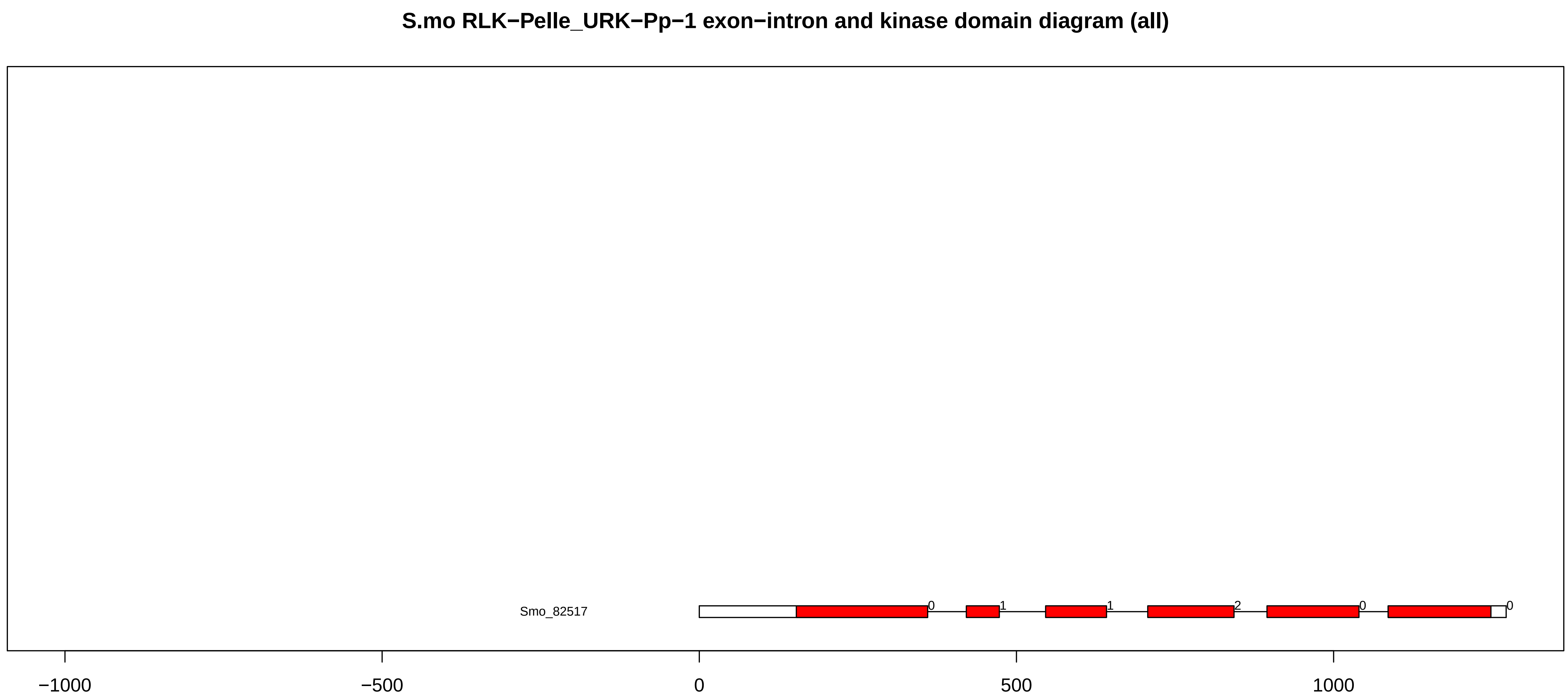
S.mo RLK−Pelle_URK−Pp−1 exon−intron and kinase domain diagram (all)
Smo_82517
0
1
1
2
0
0
−1000
−500
0
500
1000

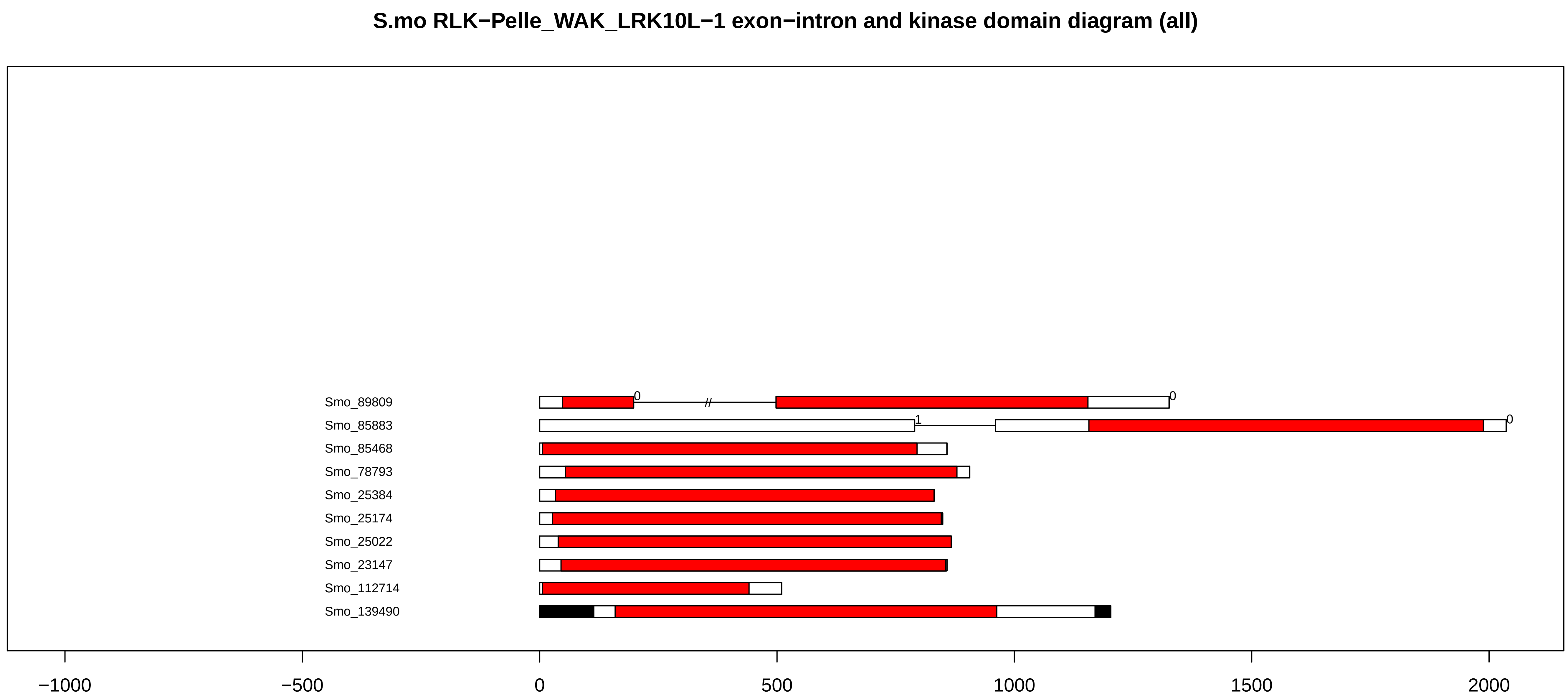

S.mo RLK−Pelle_WAK_LRK10L−1 exon−intron and kinase domain diagram (all)
Smo_89809
Smo_85883
Smo_85468
Smo_78793
Smo_25384
Smo_25174
Smo_25022
Smo_23147
Smo_112714
Smo_139490
0
0
1
0
−1000
−500
0
500
1000
1500
2000

S.mo RLK−Pelle_WAK exon−intron and kinase domain diagram (all)

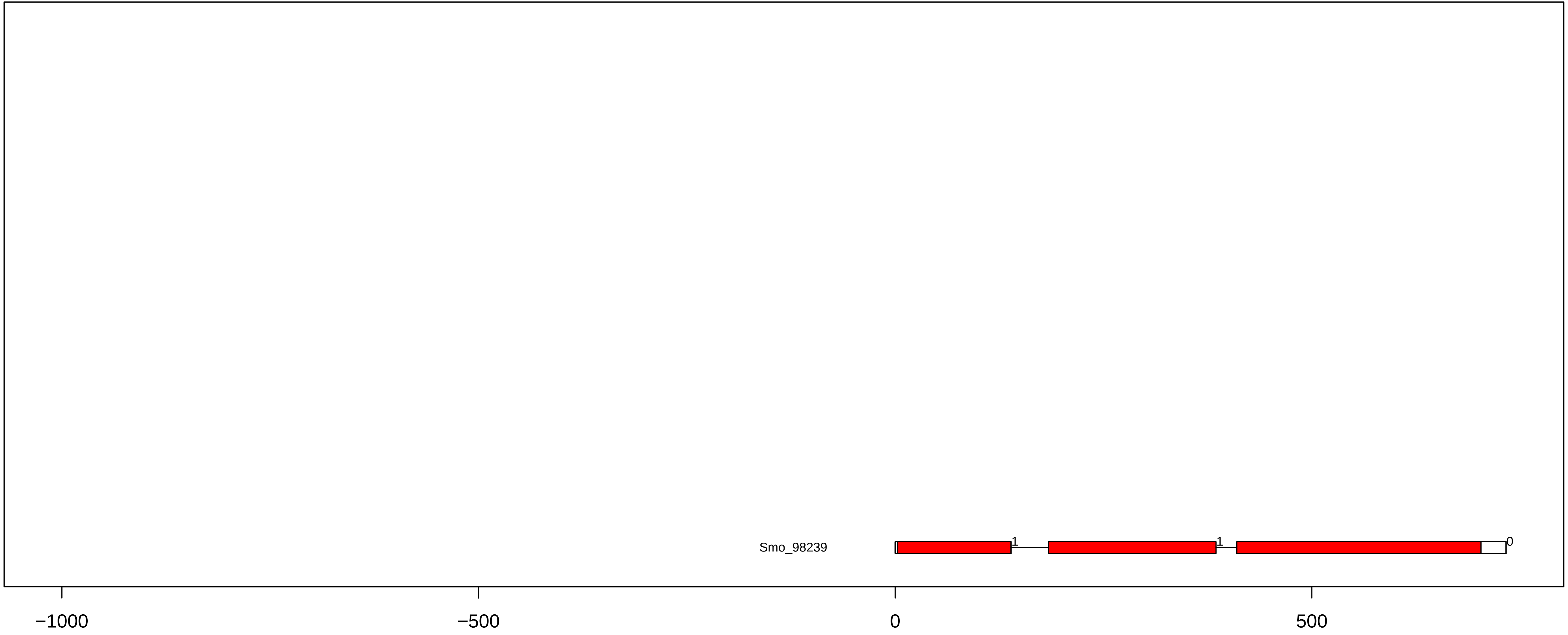

S.mo SCY1_SCYL1 exon−intron and kinase domain diagram (all)

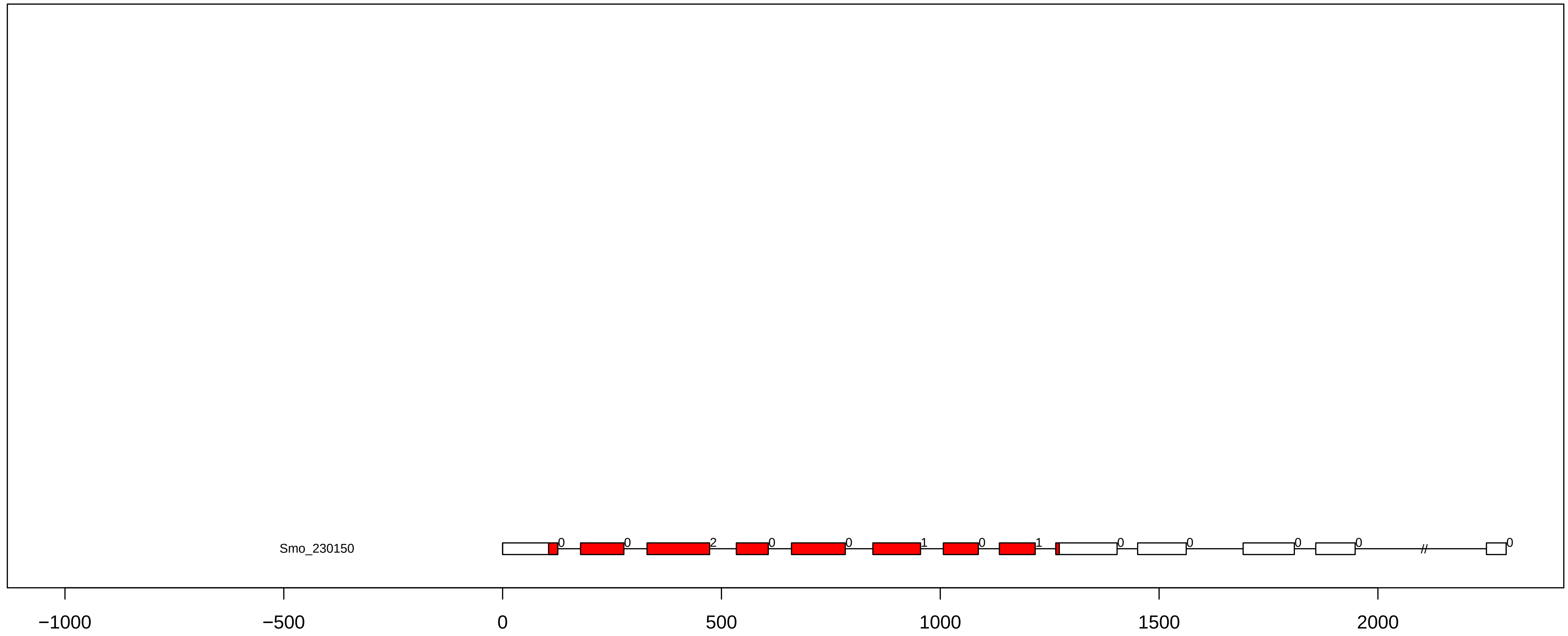

S.mo SCY1_SCYL2 exon−intron and kinase domain diagram (all)

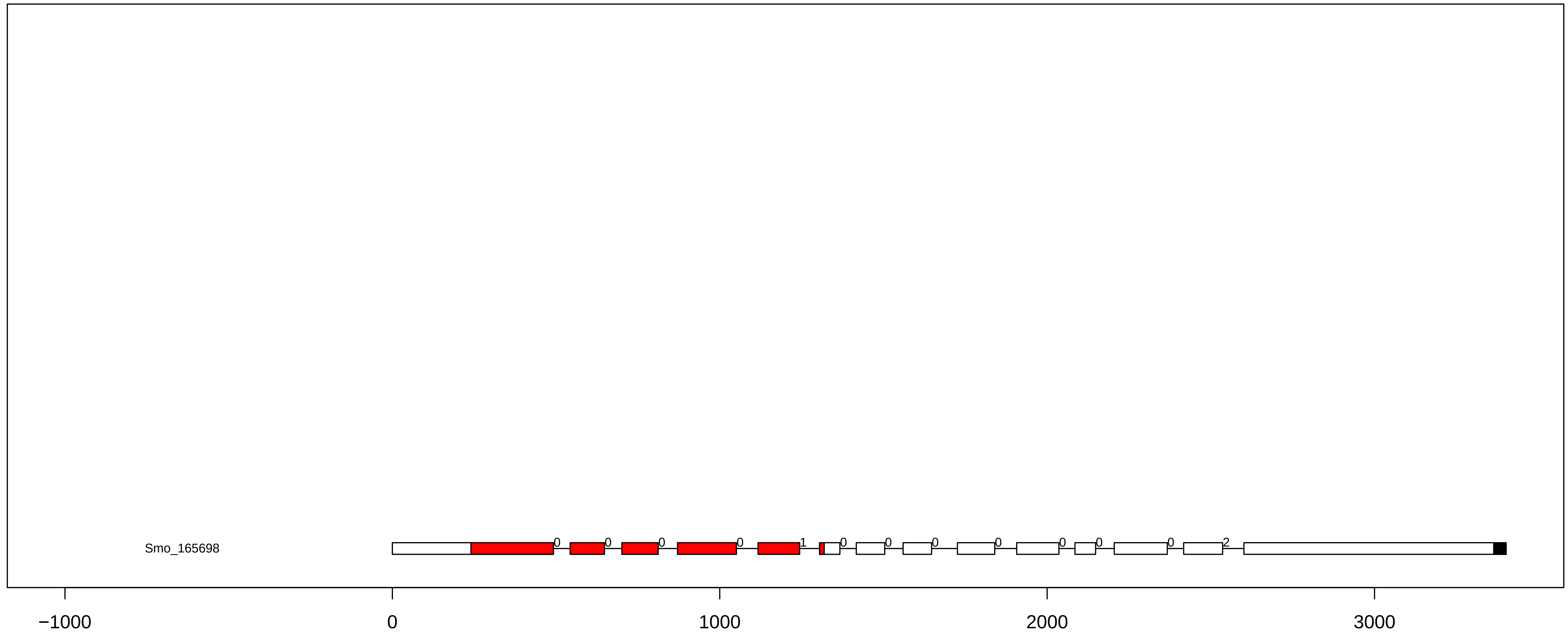

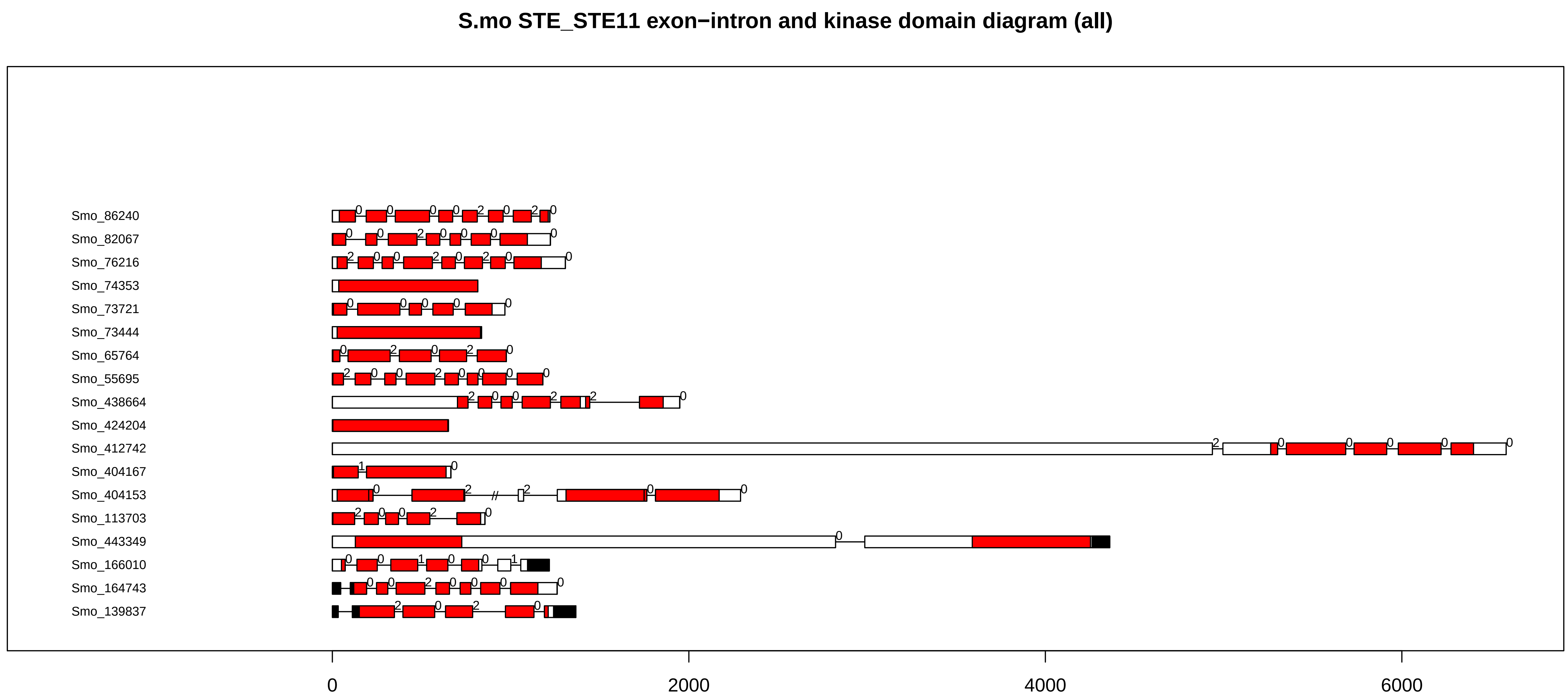

S.mo STE_STE11 exon−intron and kinase domain diagram (all)
Smo_86240
Smo_82067
Smo_76216
Smo_74353
Smo_73721
Smo_73444
Smo_65764
Smo_55695
Smo_438664
Smo_424204
Smo_412742
Smo_404167
Smo_404153
Smo_113703
Smo_443349
Smo_166010
Smo_164743
Smo_139837
0
2000
4000
6000

S.mo STE_STE20−Fray exon−intron and kinase domain diagram (all)

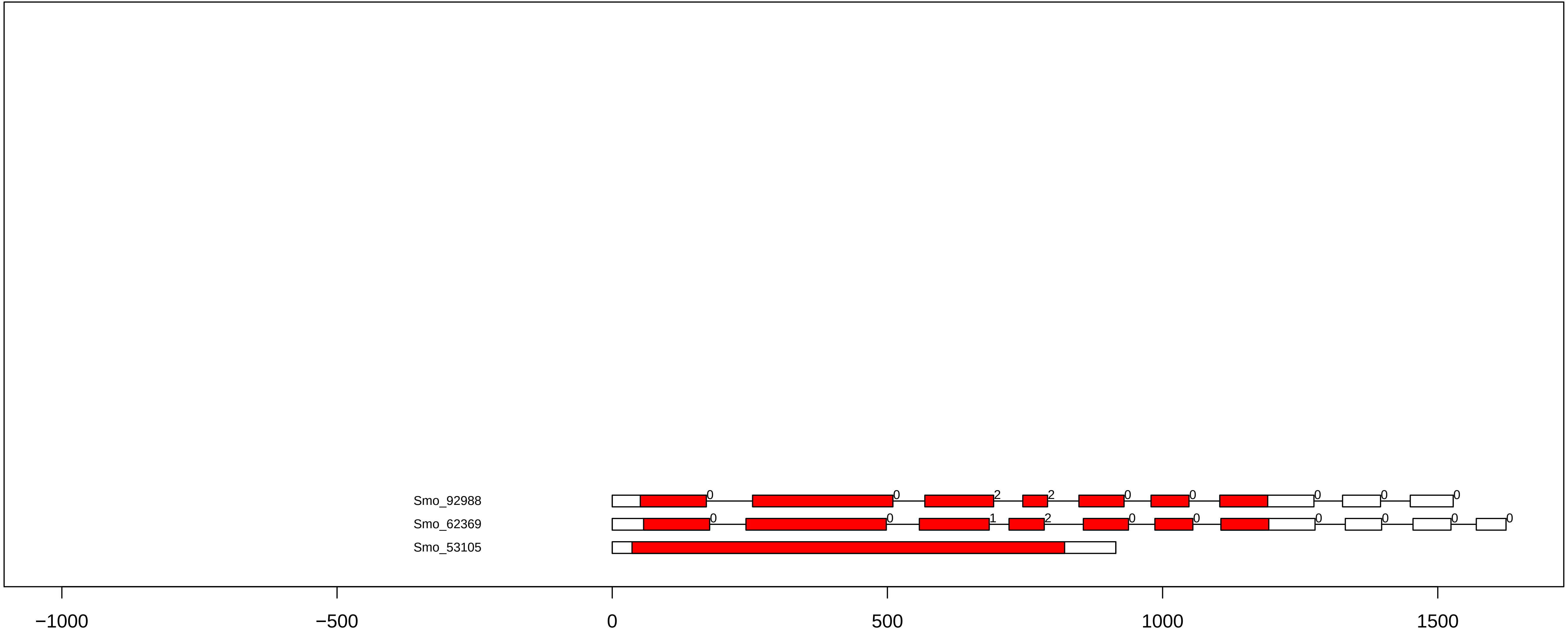

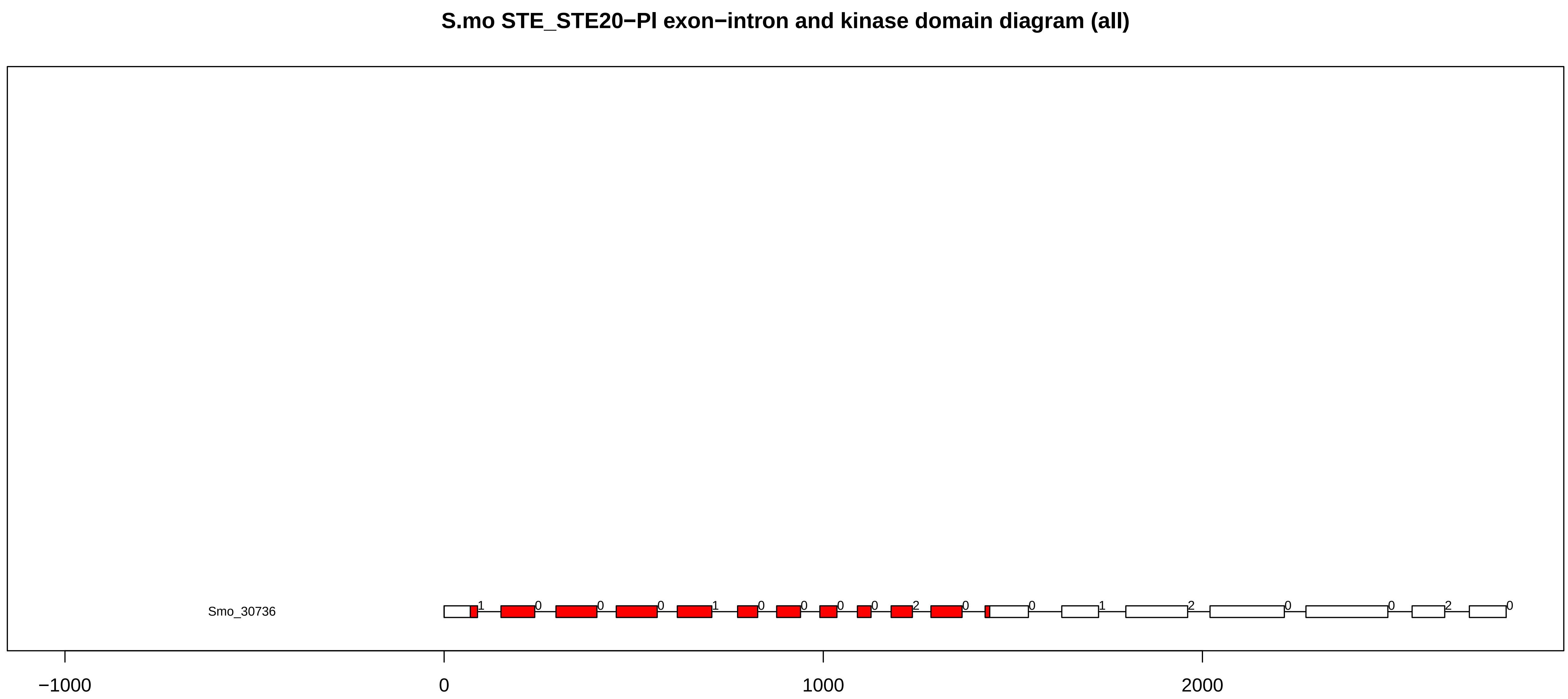
S.mo STE_STE20−Pl exon−intron and kinase domain diagram (all)
Smo_30736
1
0
0
0
1
0
0
0
0
2
0
0
1
2
0
0
2
0
−1000
0
1000
2000

S.mo STE_STE20−YSK exon−intron and kinase domain diagram (all)

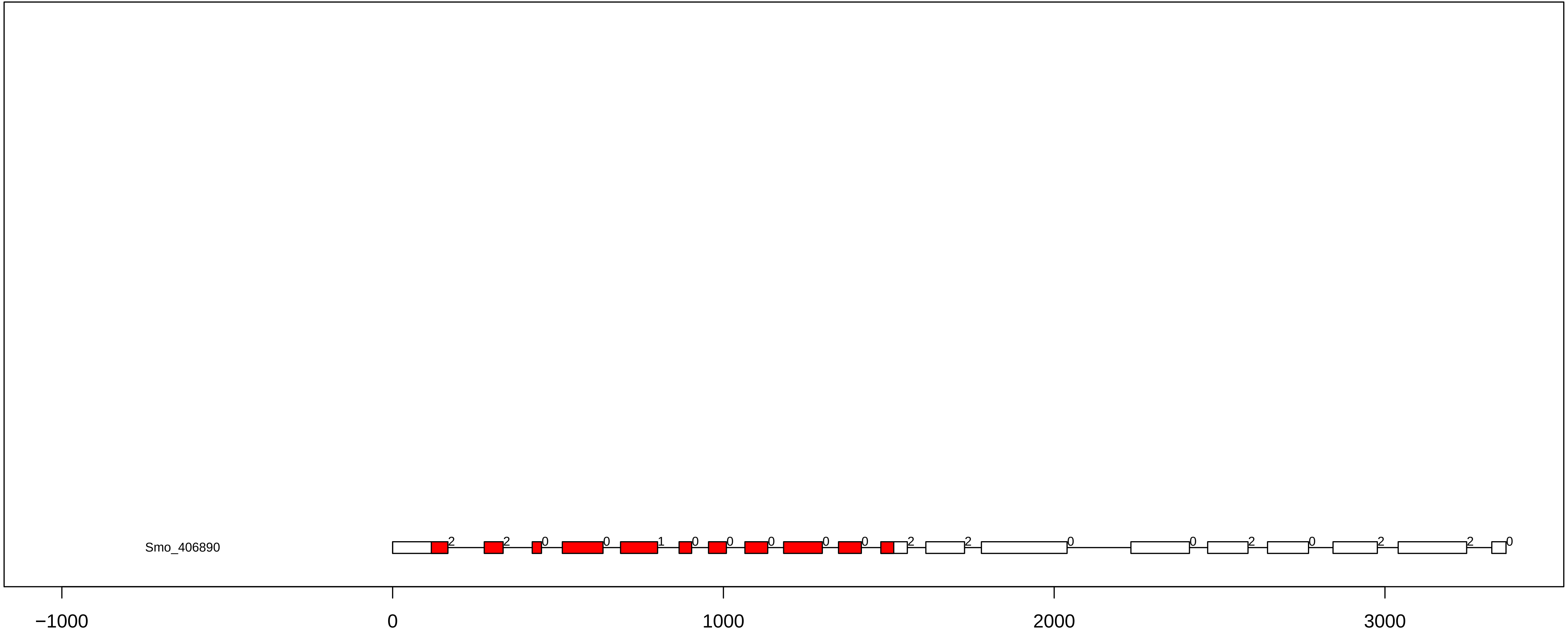

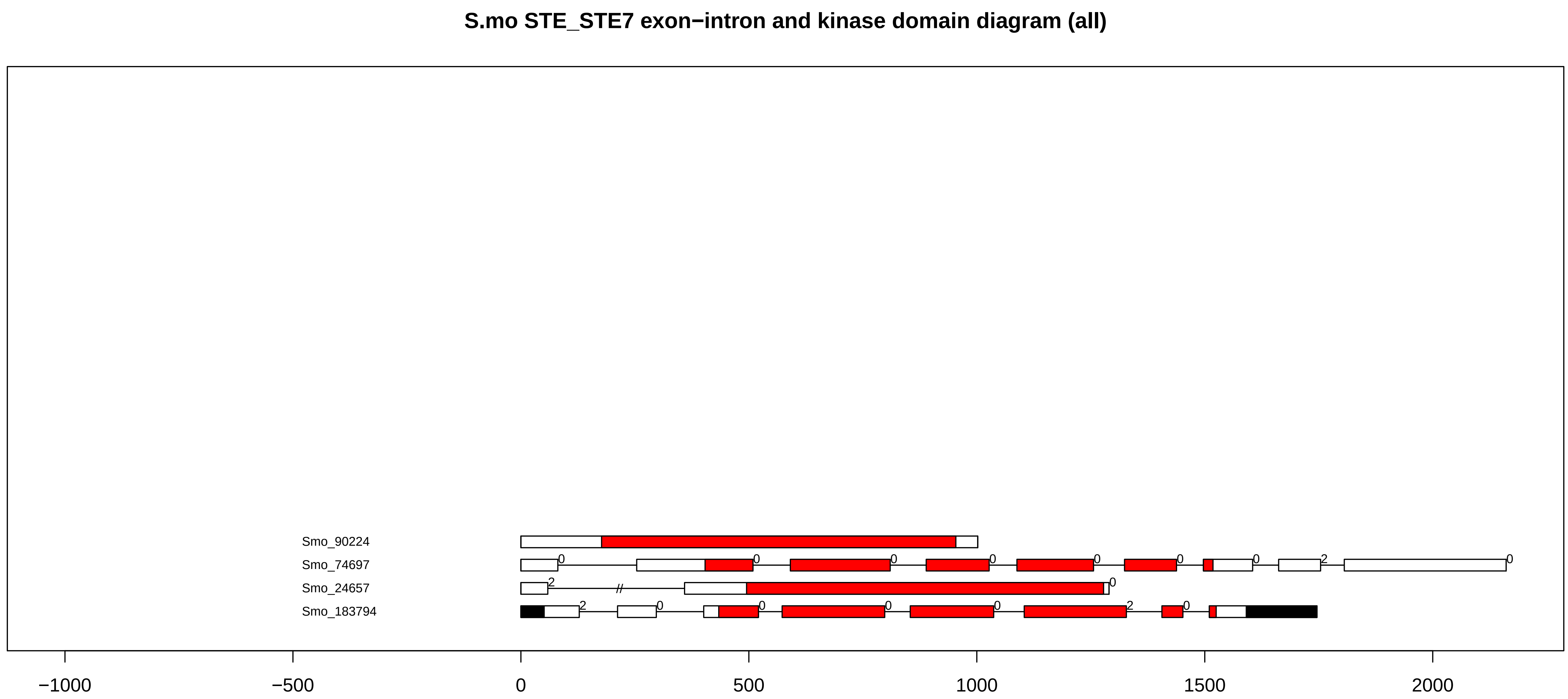
S.mo STE_STE7 exon−intron and kinase domain diagram (all)
Smo_90224
Smo_74697
Smo_24657
Smo_183794
−1000
−500
0
500
1000
1500
2000

S.mo STE_STE−Pl exon−intron and kinase domain diagram (all)

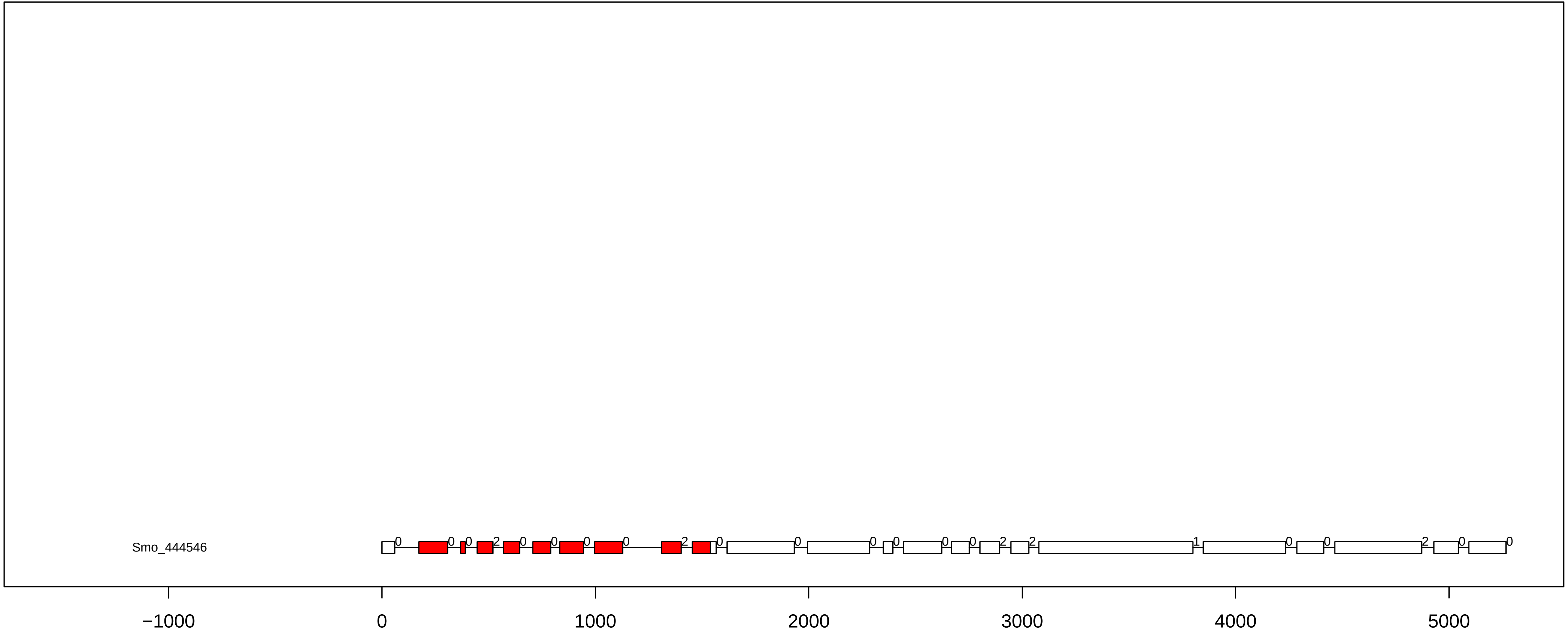

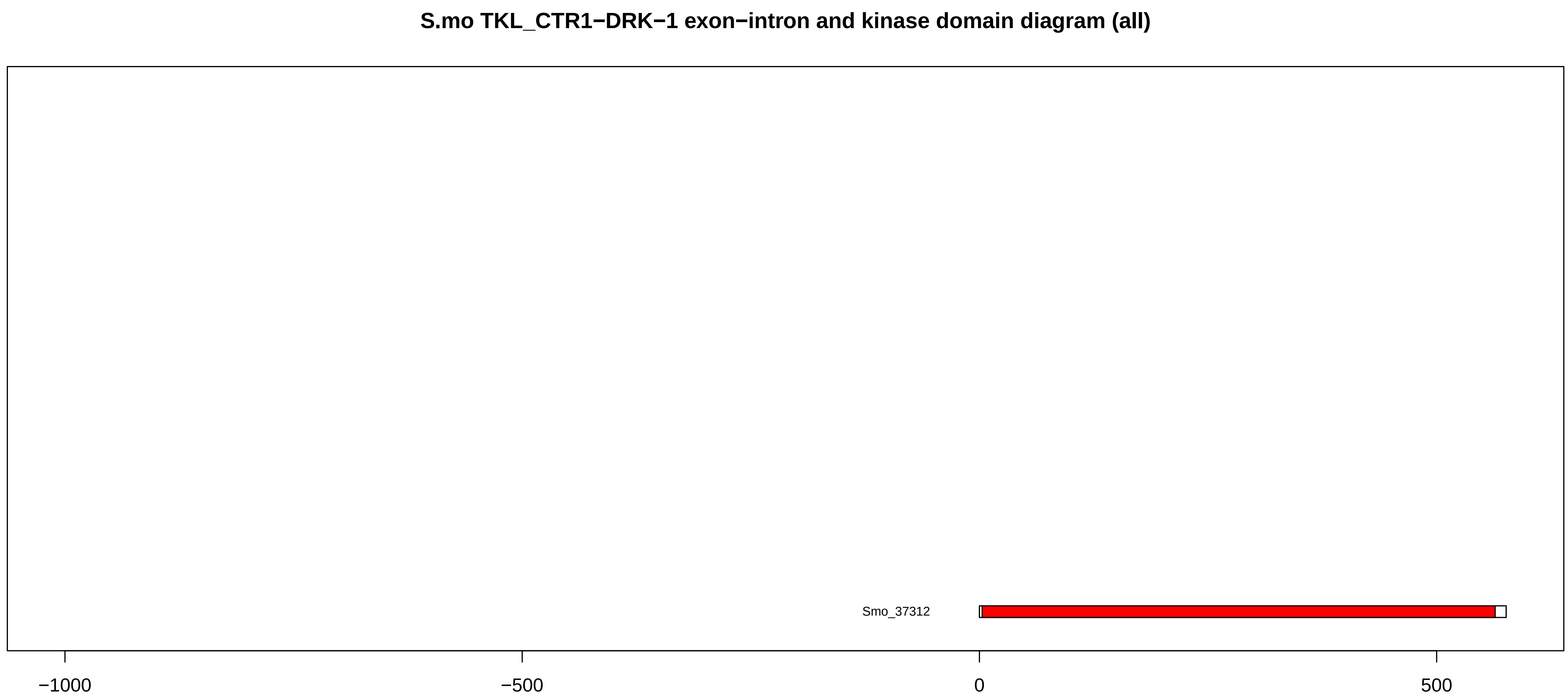
S.mo TKL_CTR1−DRK−1 exon−intron and kinase domain diagram (all)
Smo_37312
−1000
−500
0
500

S.mo TKL_CTR1−DRK−2 exon−intron and kinase domain diagram (all)

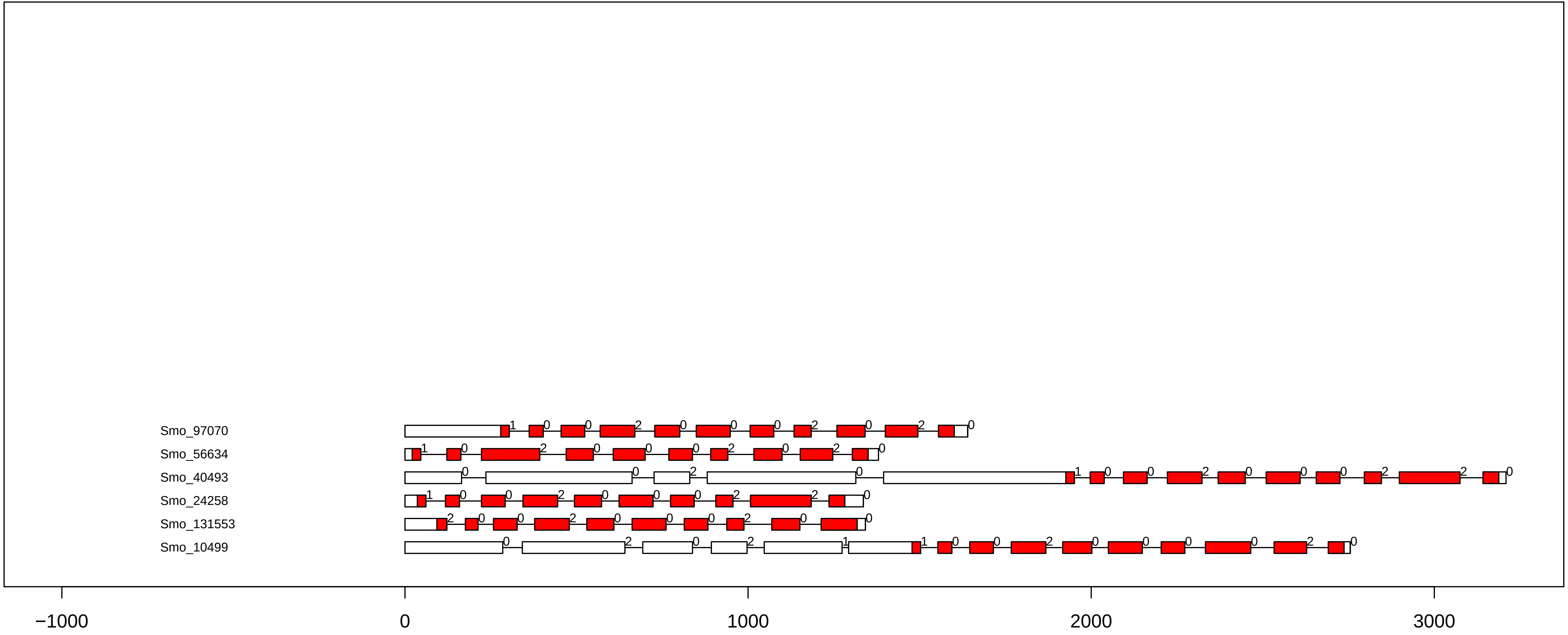

S.mo TKL_Gdt exon−intron and kinase domain diagram (all)

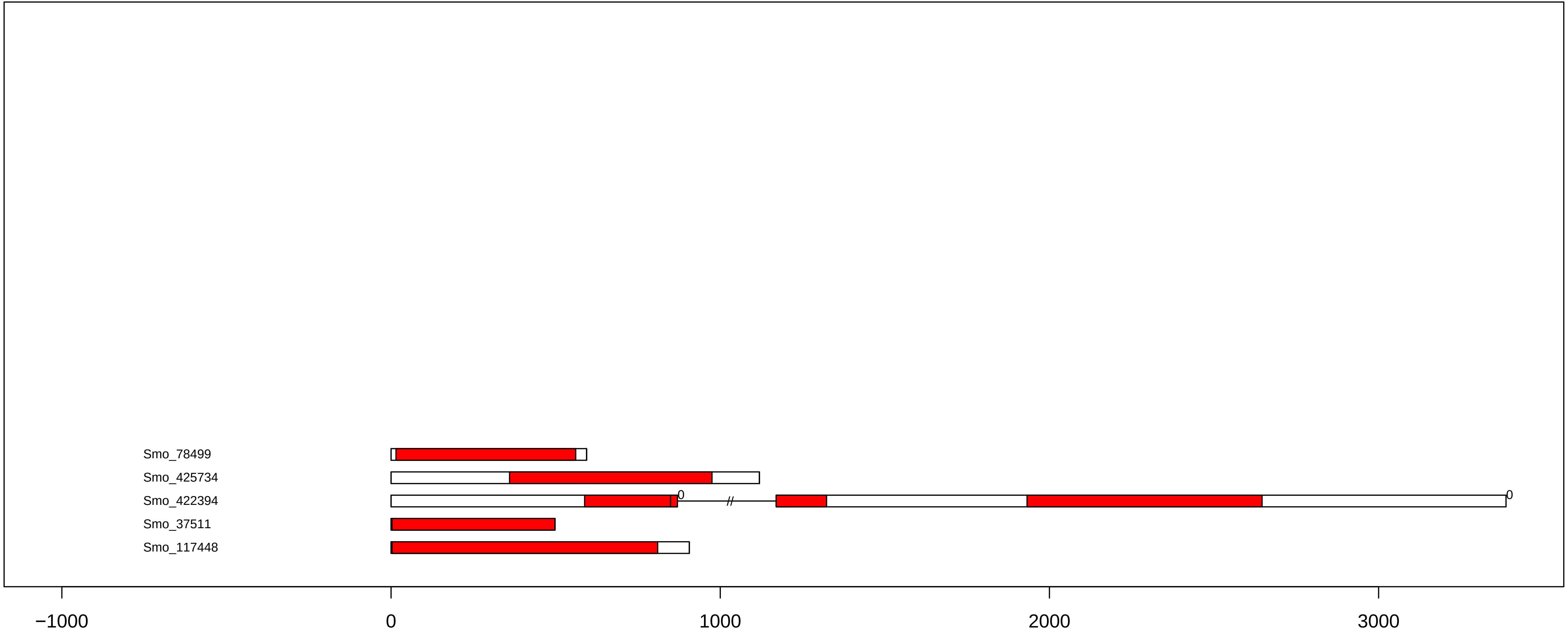

S.mo TKL−Cr−3 exon−intron and kinase domain diagram (all)

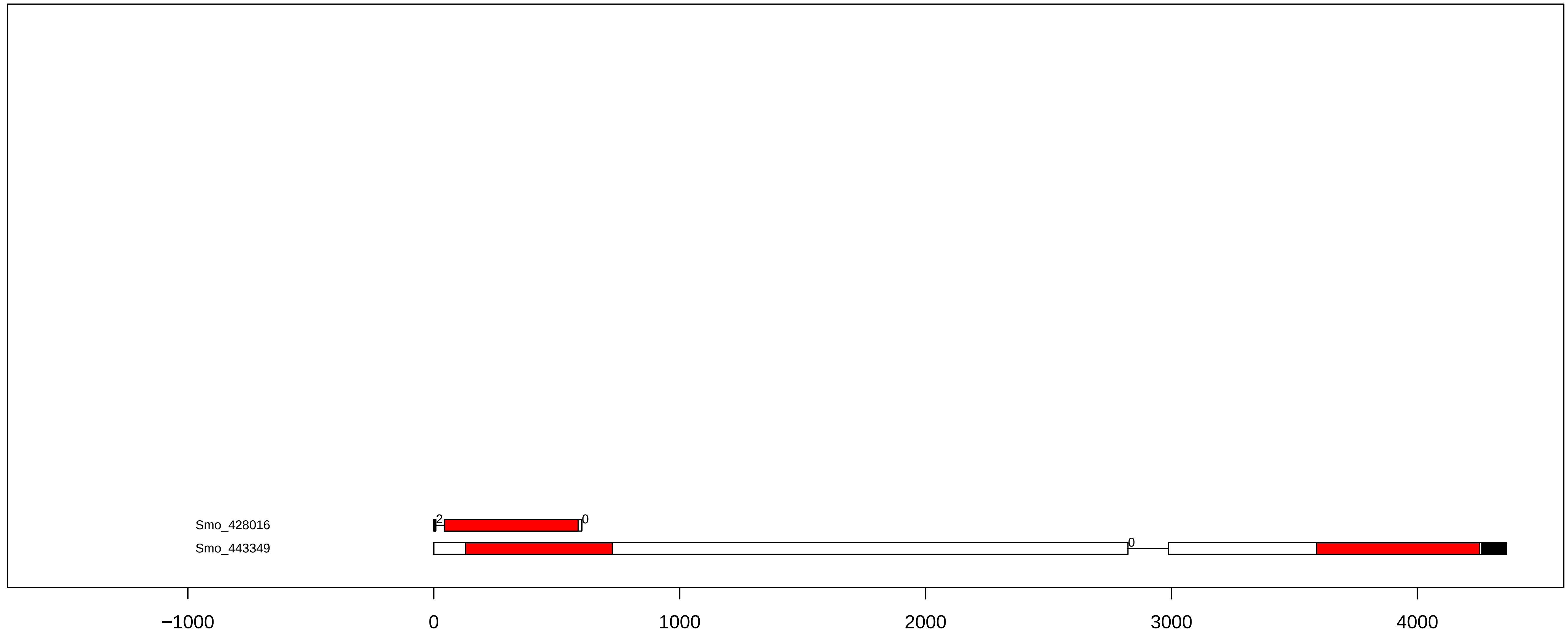

S.mo TKL−Pl−1 exon−intron and kinase domain diagram (all)

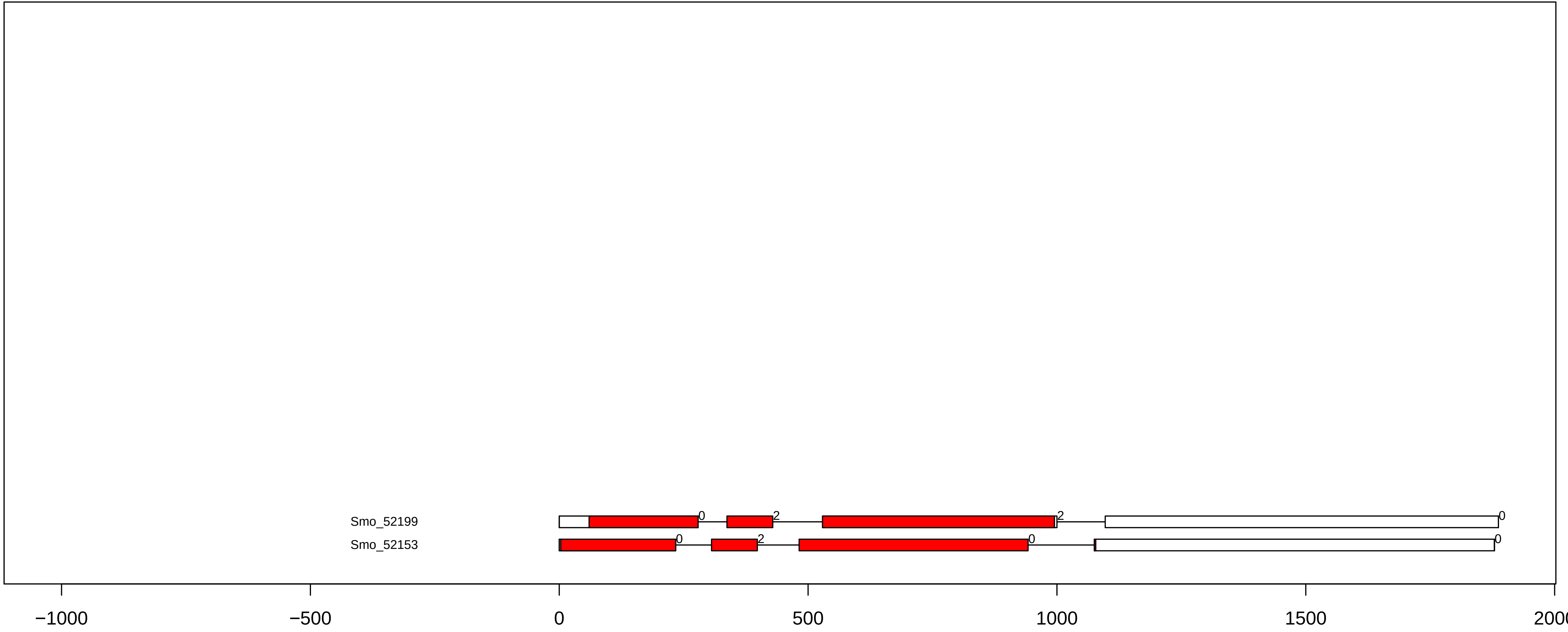

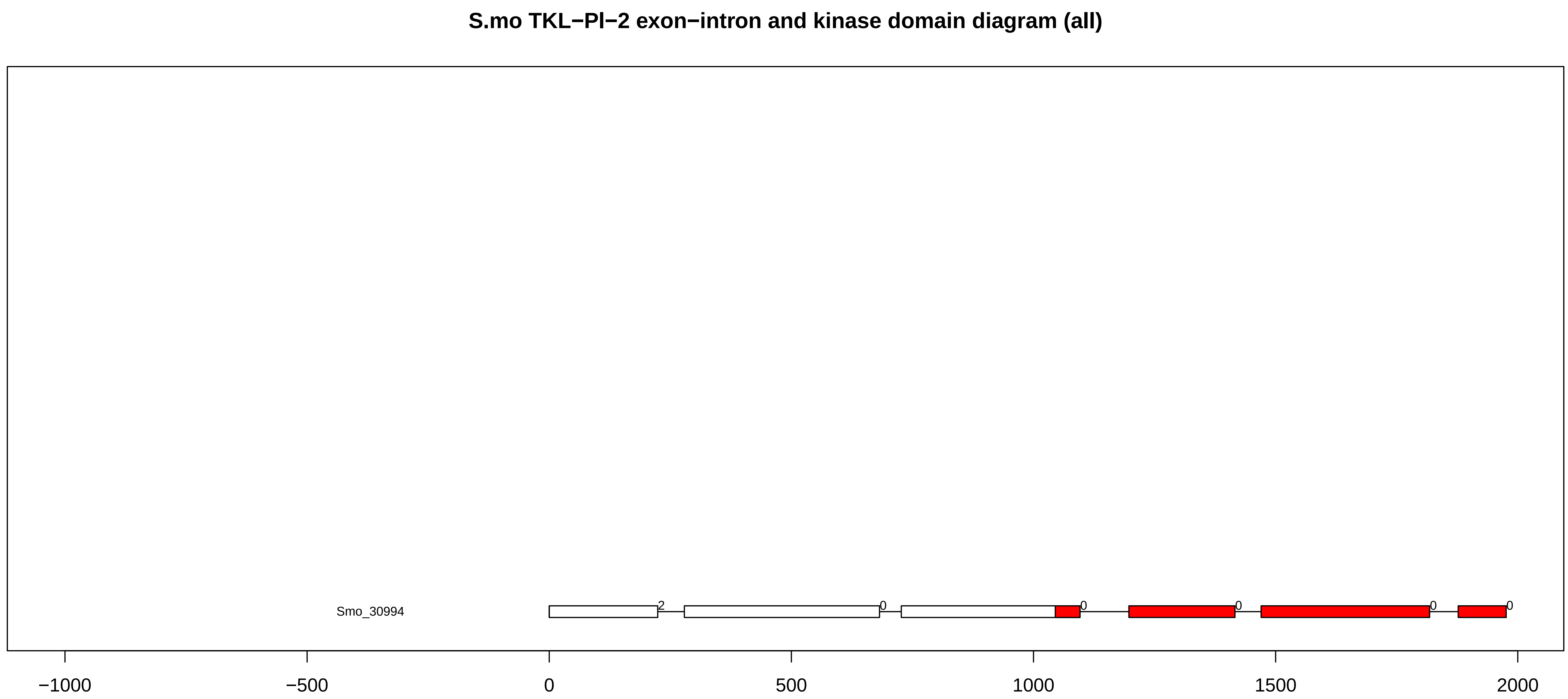
S.mo TKL−Pl−2 exon−intron and kinase domain diagram (all)
Smo_30994
2
0
0
0
0
0
−1000
−500
0
500
1000
1500
2000

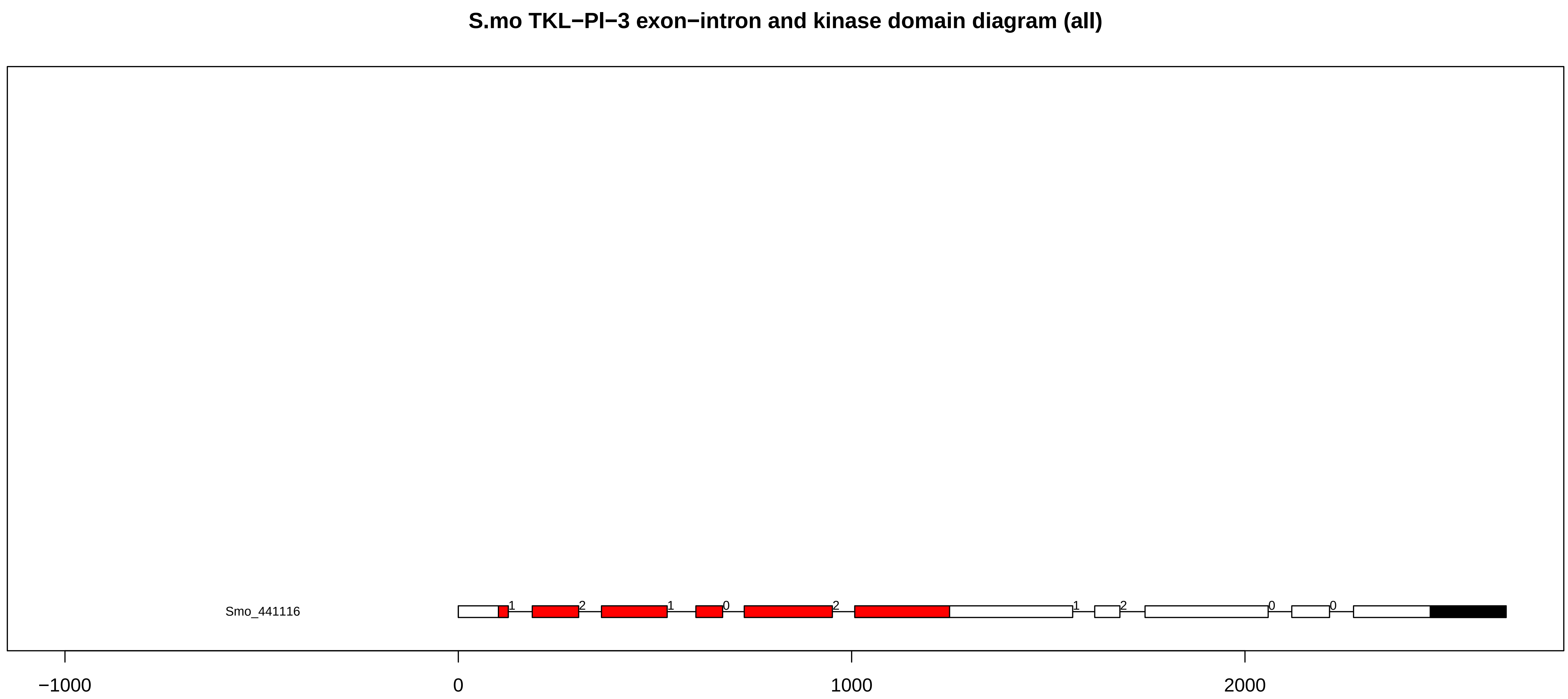
S.mo TKL−Pl−3 exon−intron and kinase domain diagram (all)
Smo_441116
1
2
1
0
2
1
2
0
0
−1000
0
1000
2000

S.mo TKL−Pl−4 exon−intron and kinase domain diagram (all)

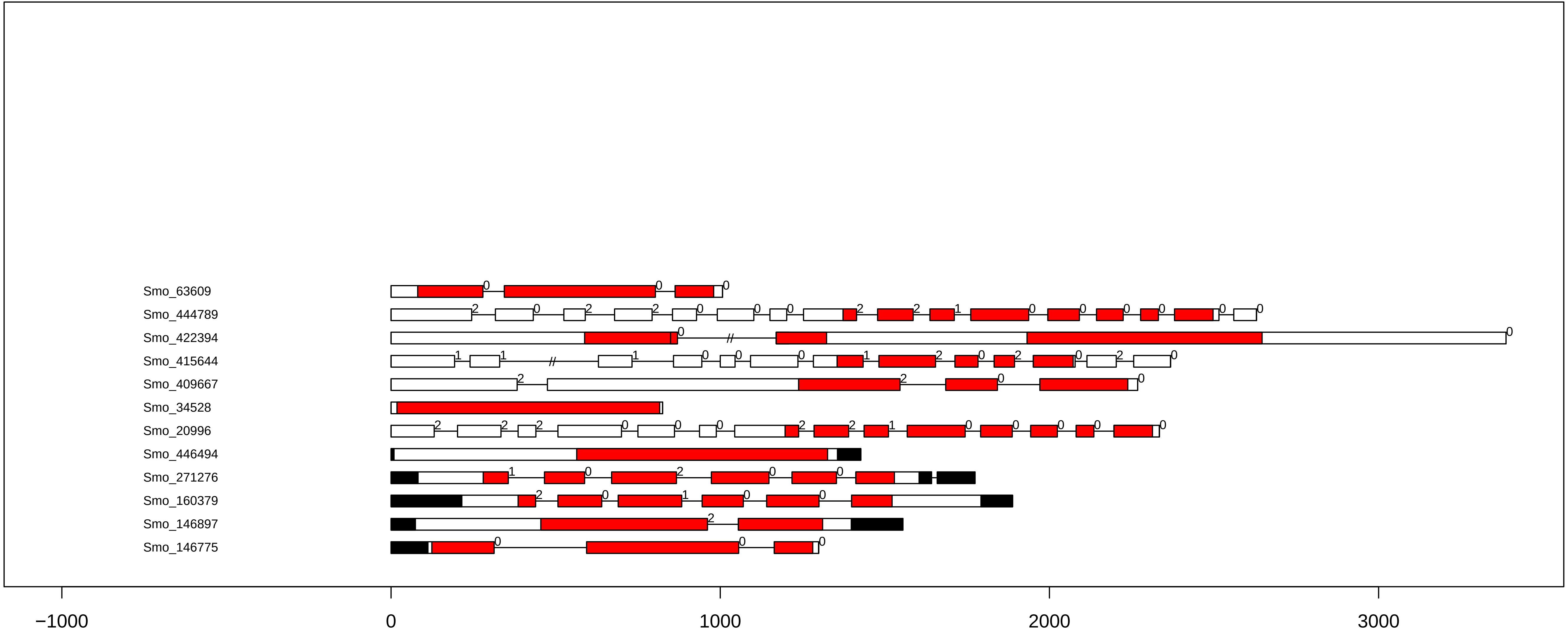

S.mo TKL−Pl−5 exon−intron and kinase domain diagram (all)

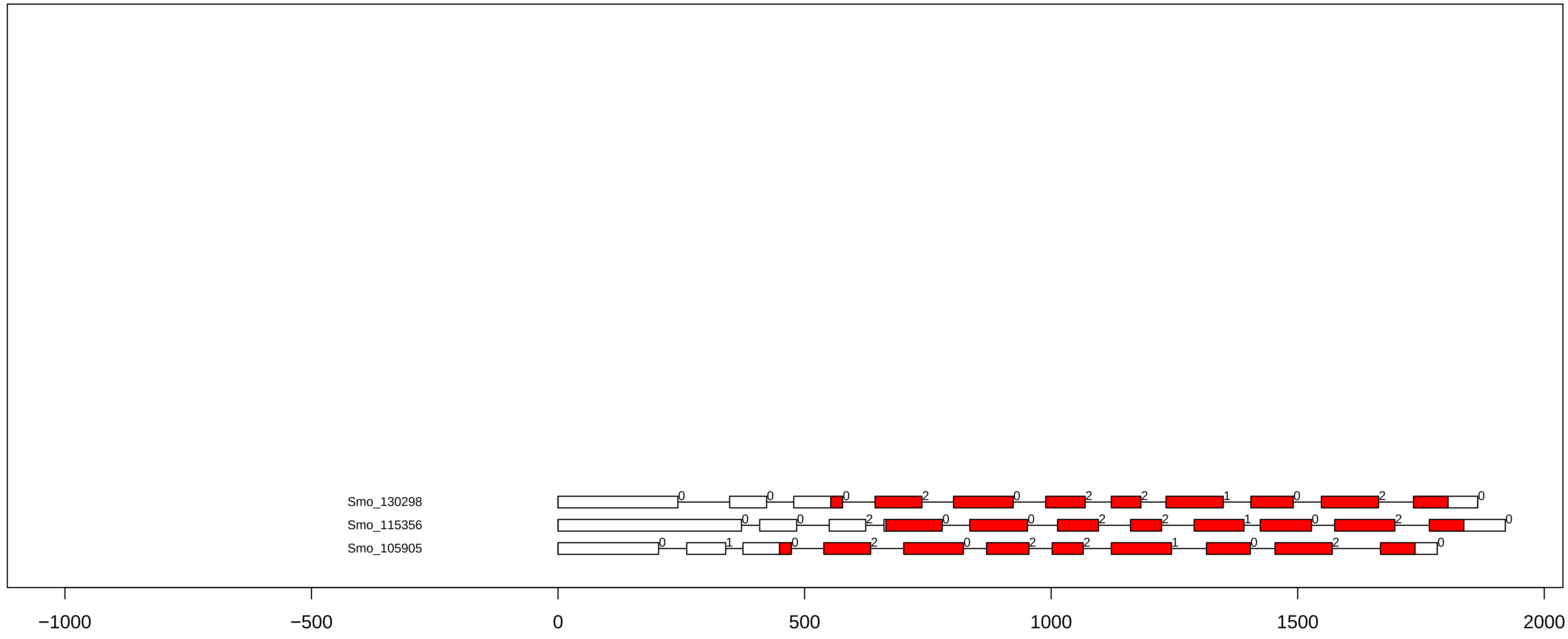

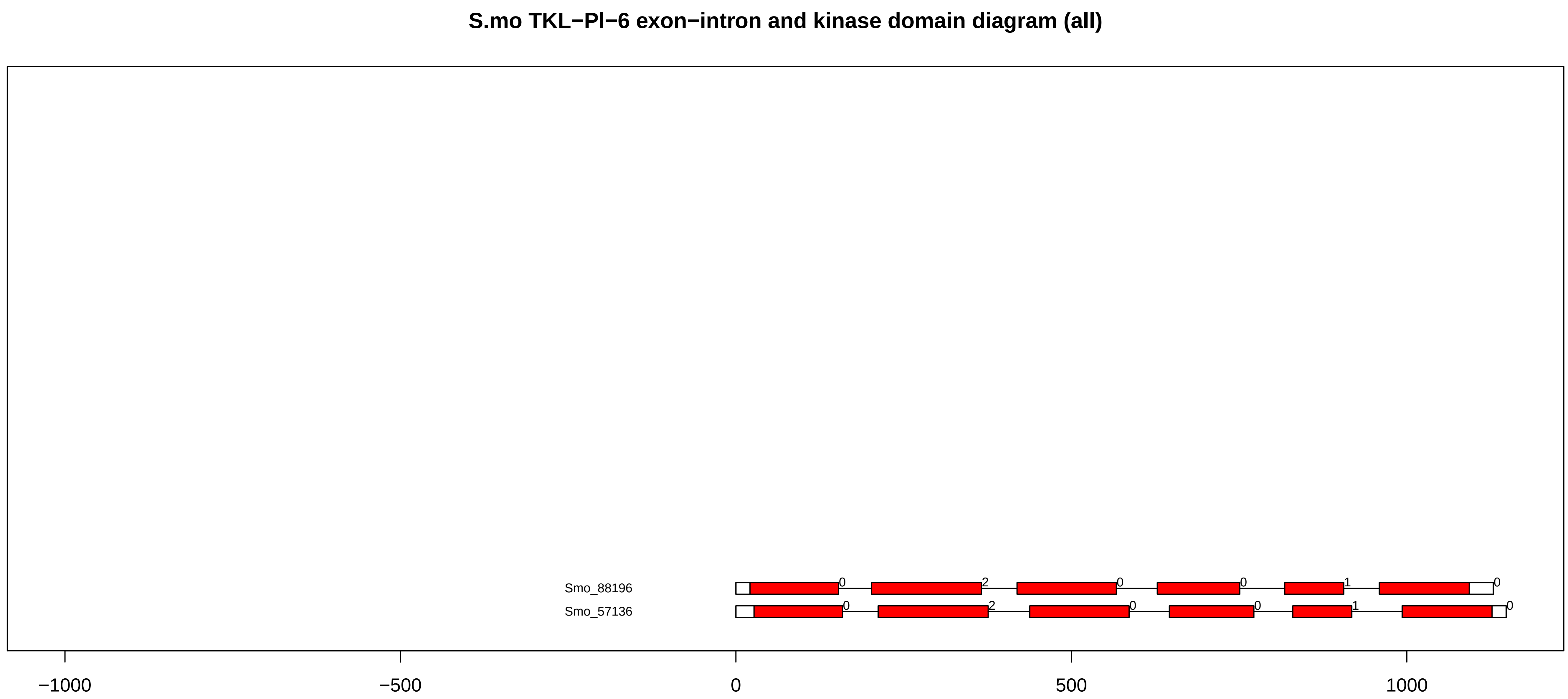
S.mo TKL−Pl−6 exon−intron and kinase domain diagram (all)
Smo_88196
Smo_57136
0
2
0
0
1
0
0
2
0
0
1
0
−1000
−500
0
500
1000

S.mo TKL−Pl−8 exon−intron and kinase domain diagram (all)

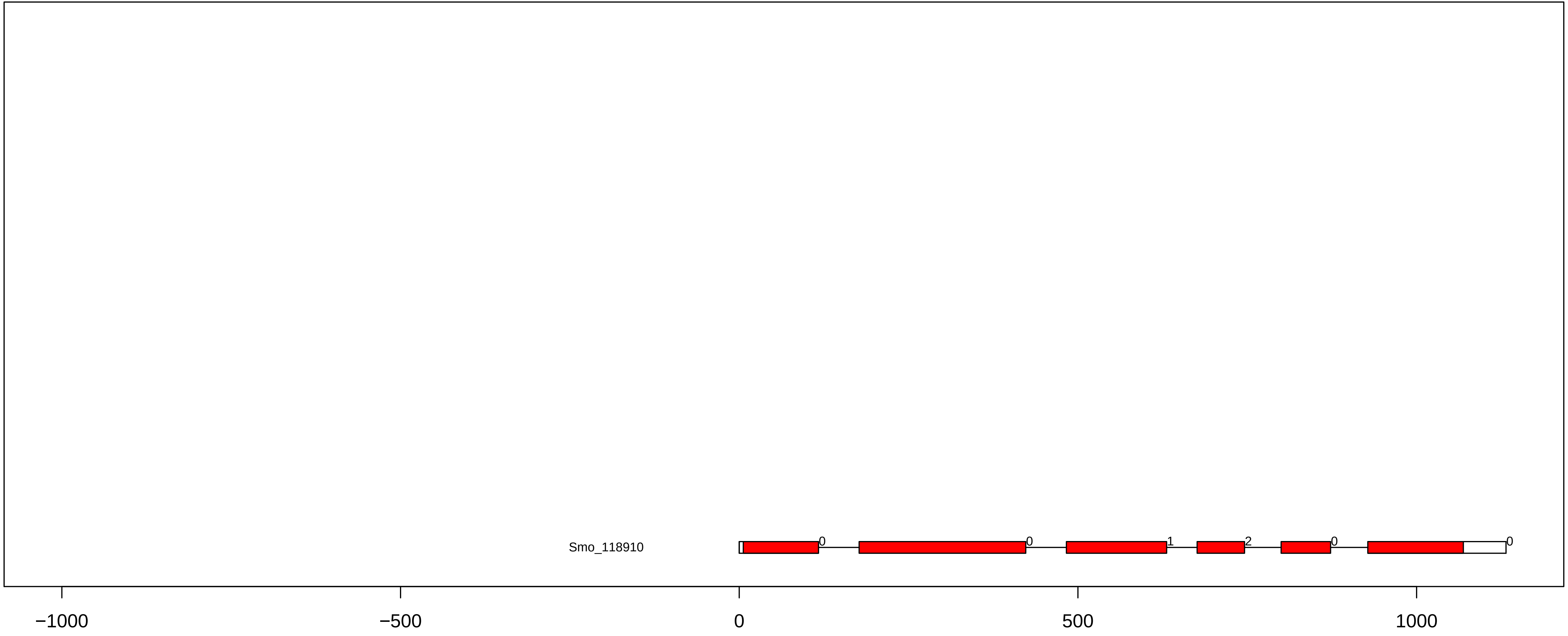

S.mo TLK exon−intron and kinase domain diagram (all)

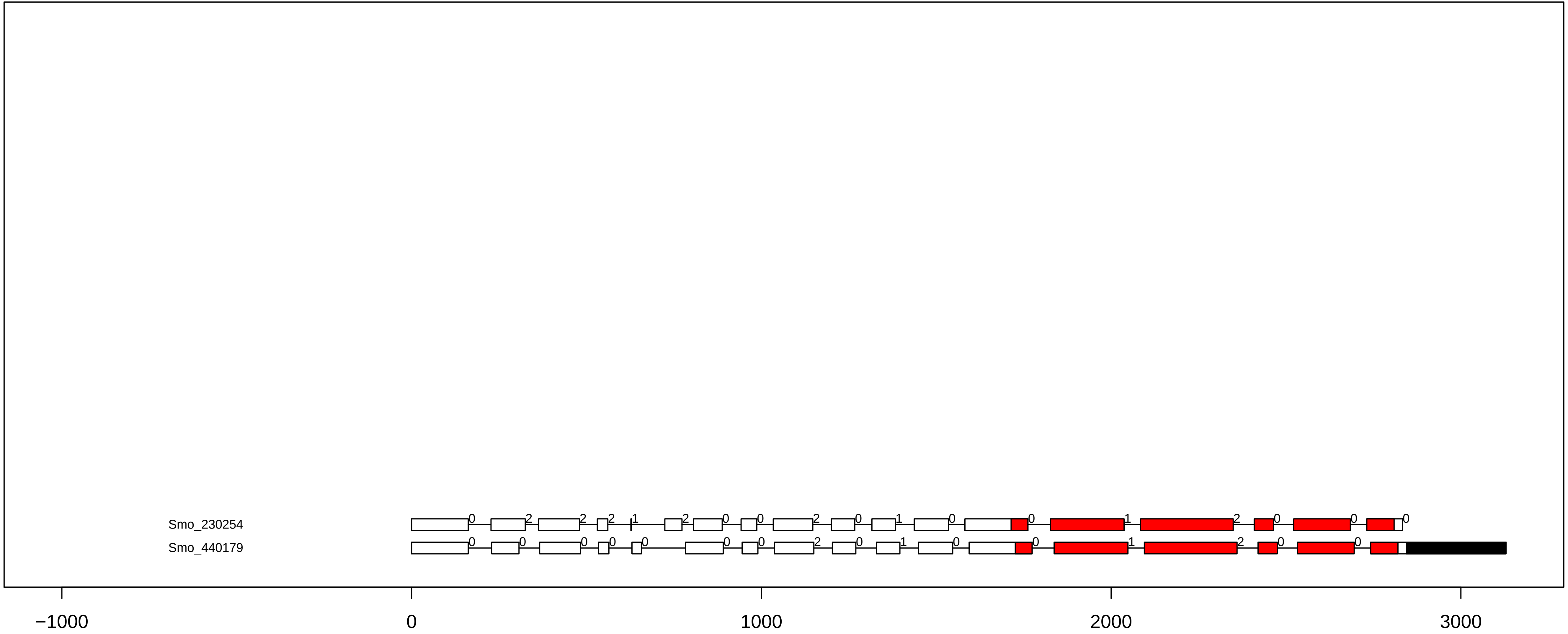

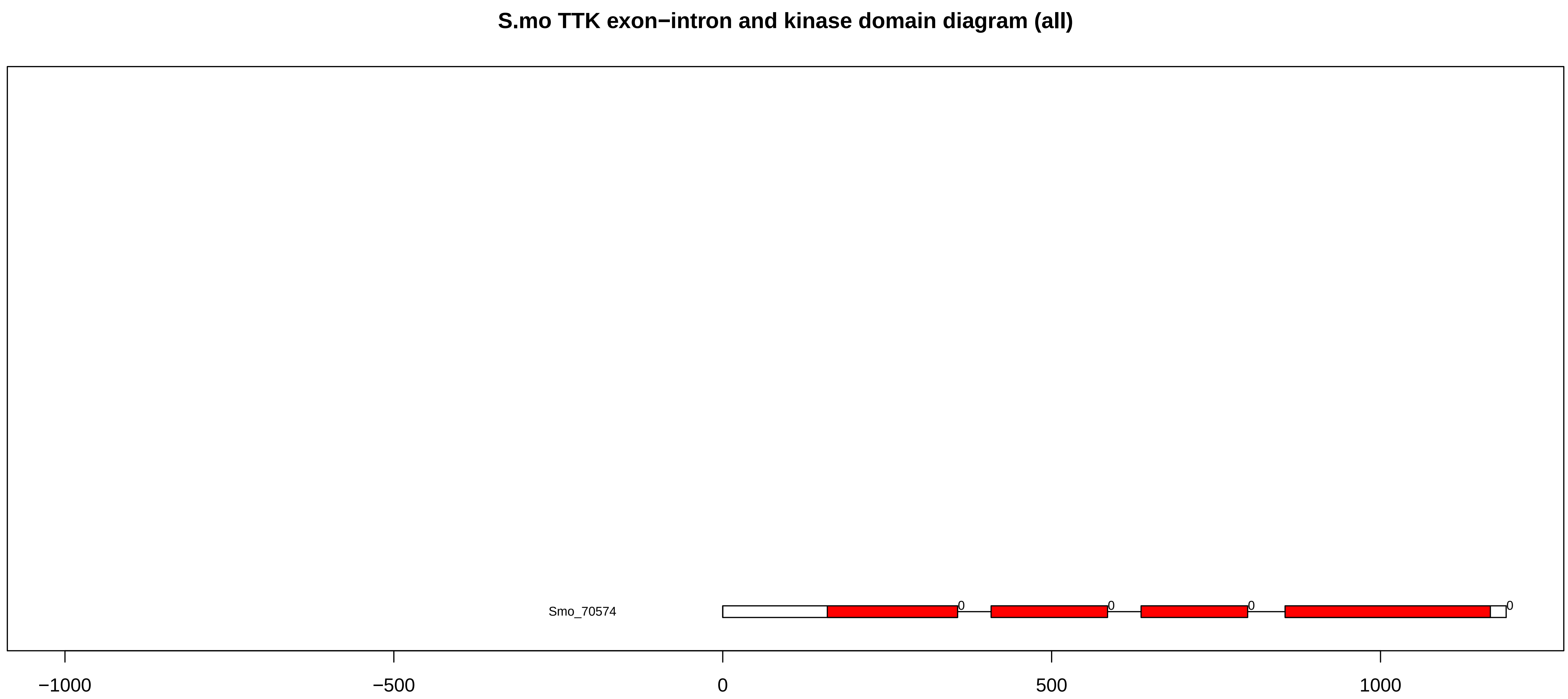
S.mo TTK exon−intron and kinase domain diagram (all)
Smo_70574
0
0
0
0
−1000
−500
0
500
1000

S.mo ULK_Fused exon−intron and kinase domain diagram (all)

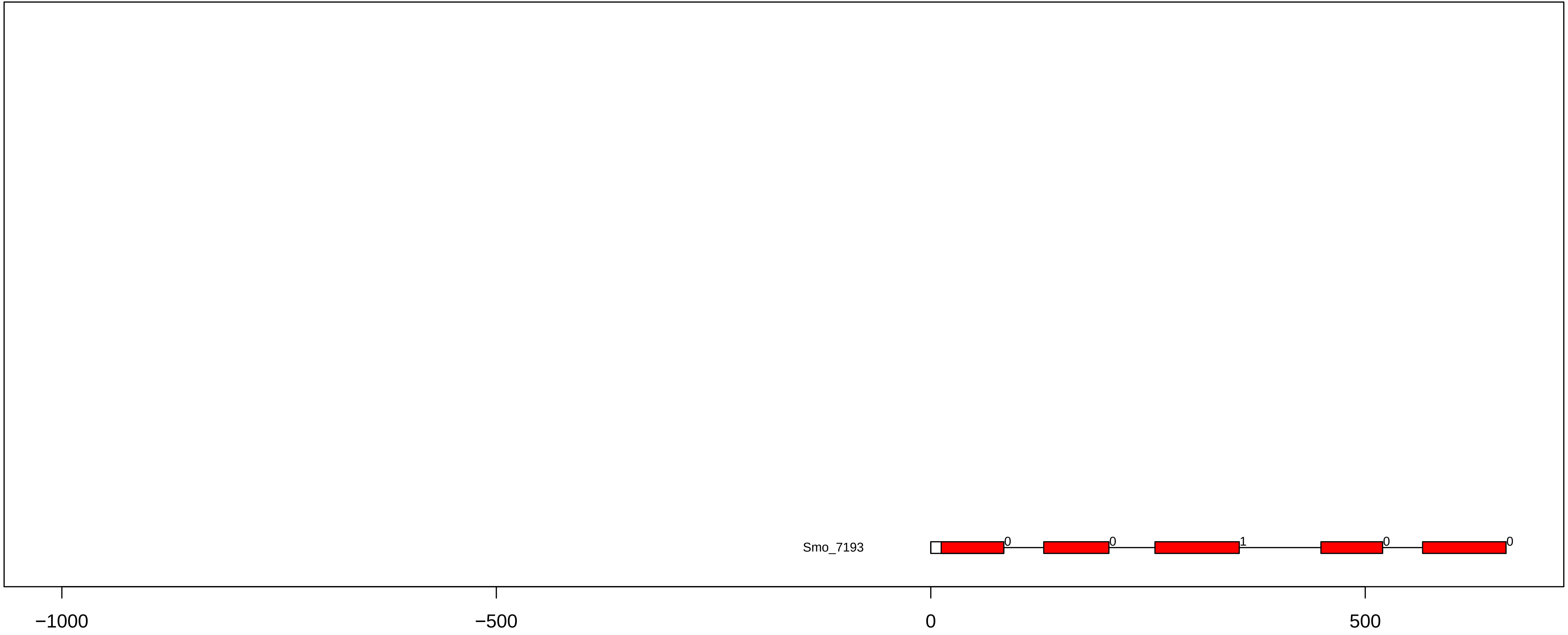

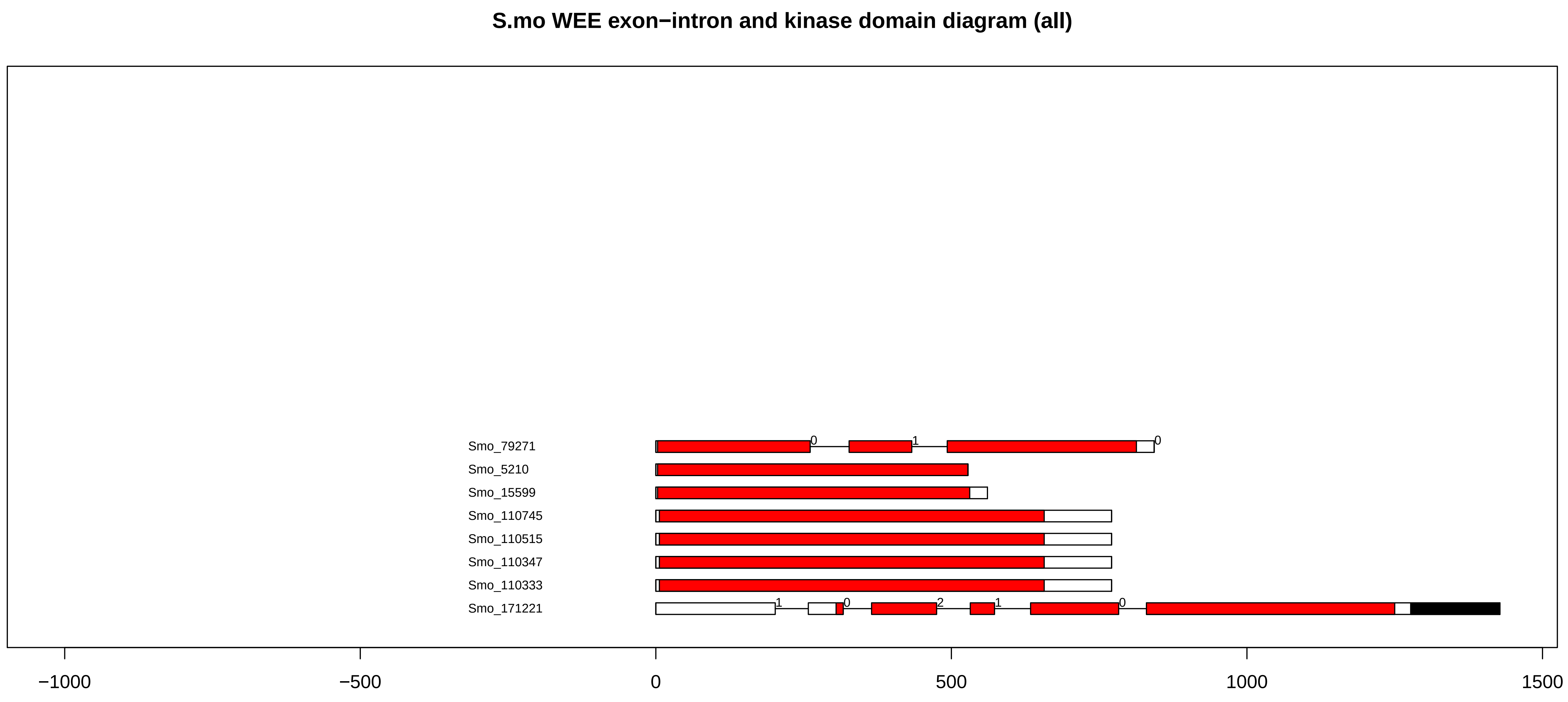
S.mo WEE exon−intron and kinase domain diagram (all)
Smo_79271
Smo_5210
Smo_15599
Smo_110745
Smo_110515
Smo_110347
Smo_110333
Smo_171221
0
1
0
1
0
2
1
0
−1000
−500
0
500
1000
1500

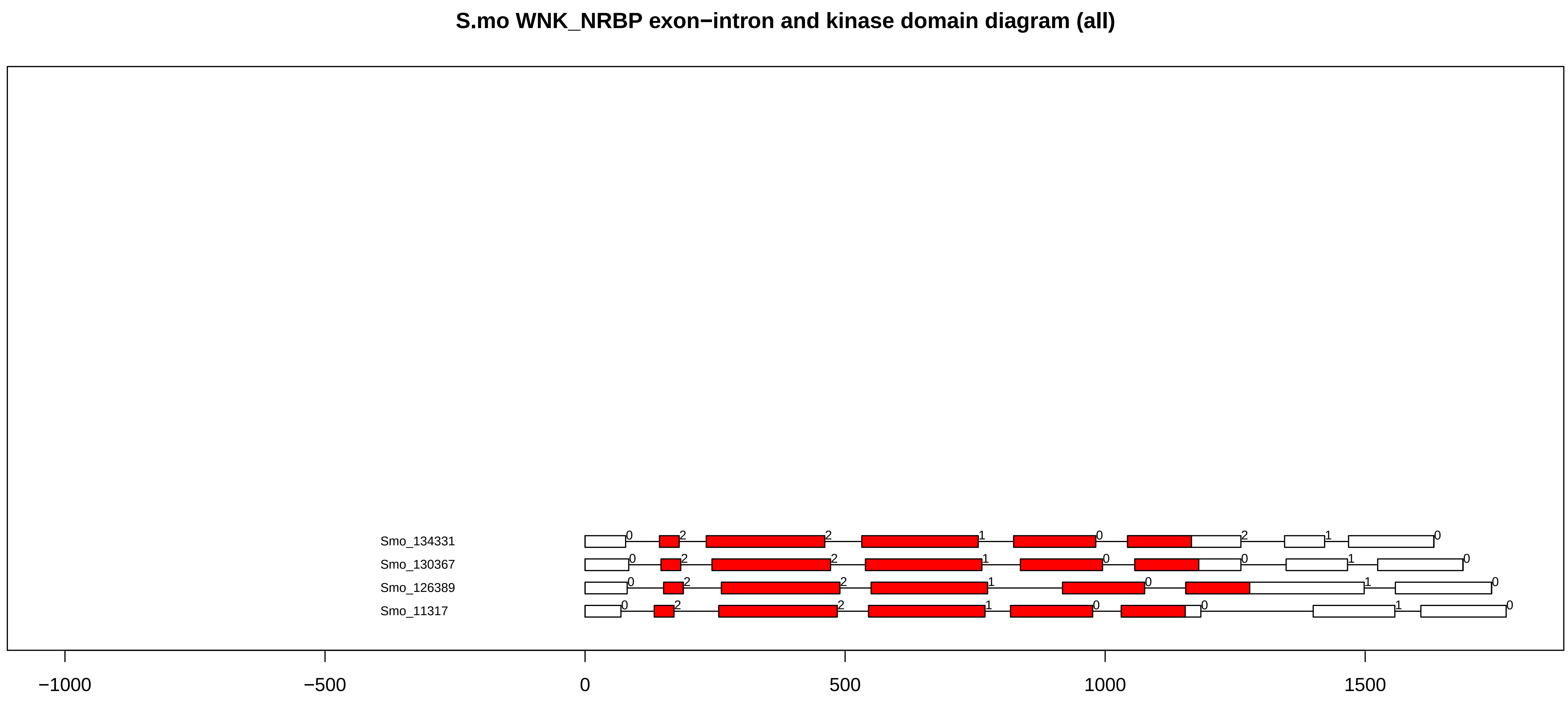

S.mo WNK_NRBP exon−intron and kinase domain diagram (all)
Smo_134331
Smo_130367
Smo_126389
Smo_11317
−1000
−500
0
500
1000
1500

P.pa AGC_MAST exon−intron and kinase domain diagram (all)

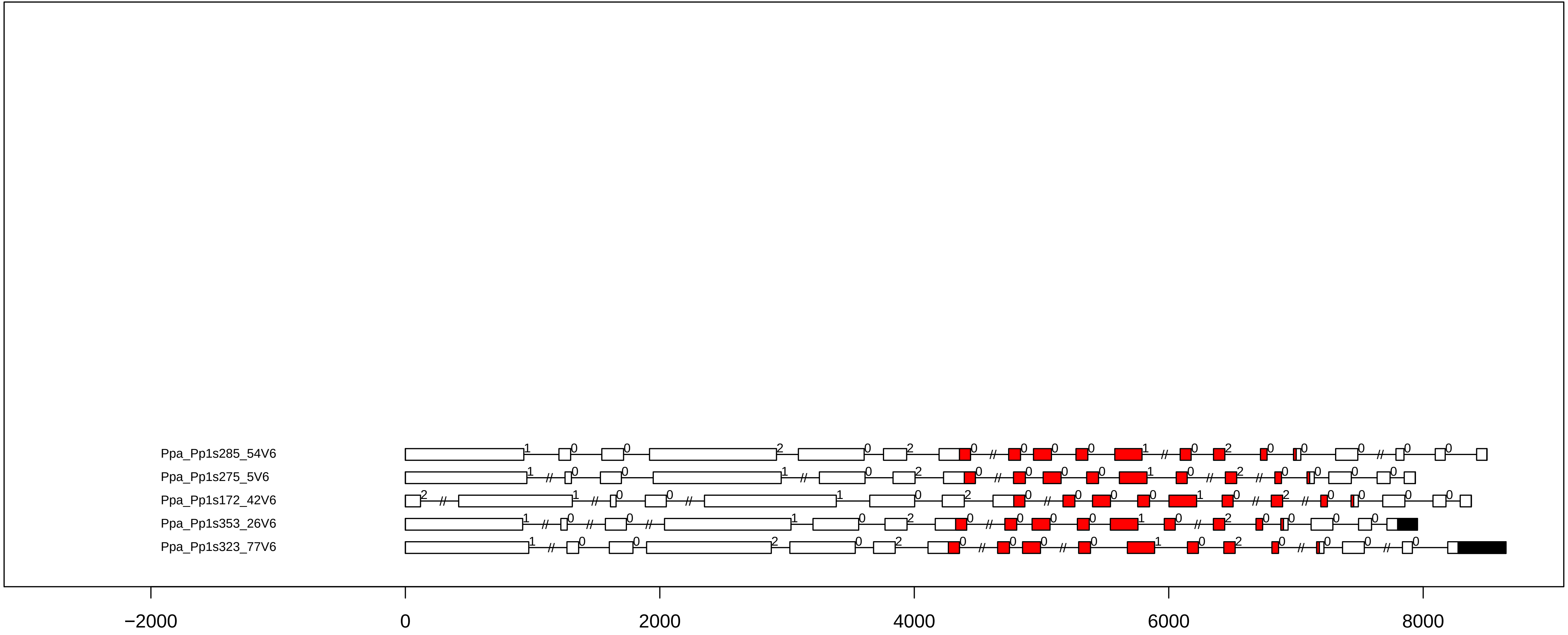

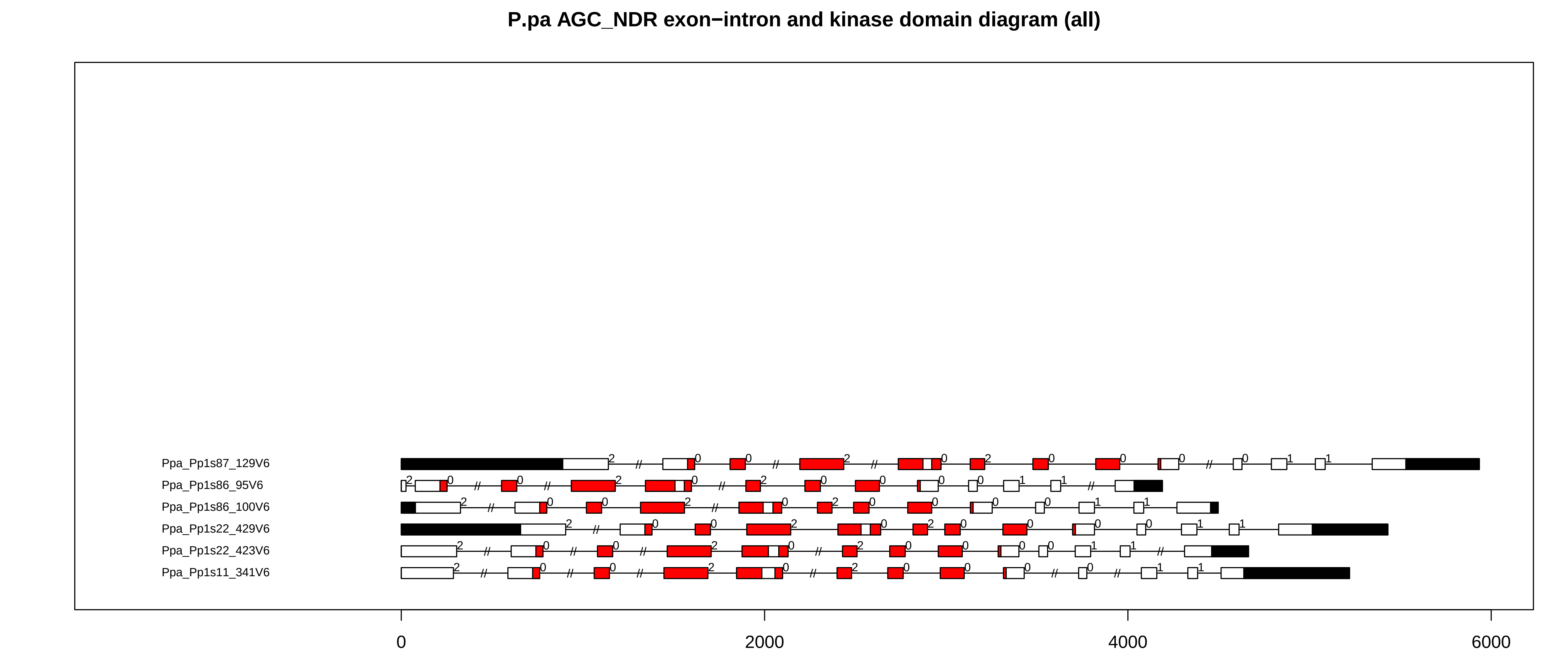
P.pa AGC_NDR exon−intron and kinase domain diagram (all)
Ppa_Pp1s87_129V6
Ppa_Pp1s86_95V6
Ppa_Pp1s86_100V6
Ppa_Pp1s22_429V6
Ppa_Pp1s22_423V6
Ppa_Pp1s11_341V6
0
2000
4000
6000

P.pa AGC_PDK1 exon−intron and kinase domain diagram (all)

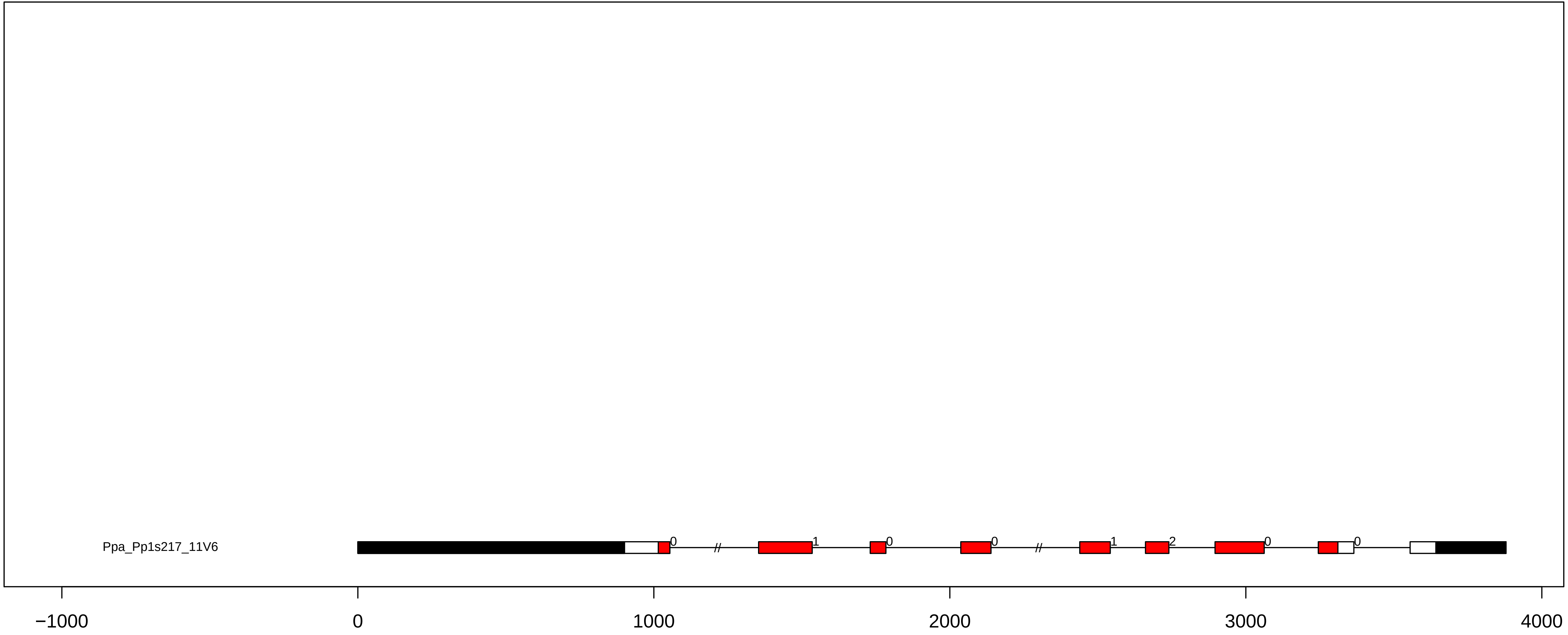

P.pa AGC_PKA−PKG exon−intron and kinase domain diagram (all)

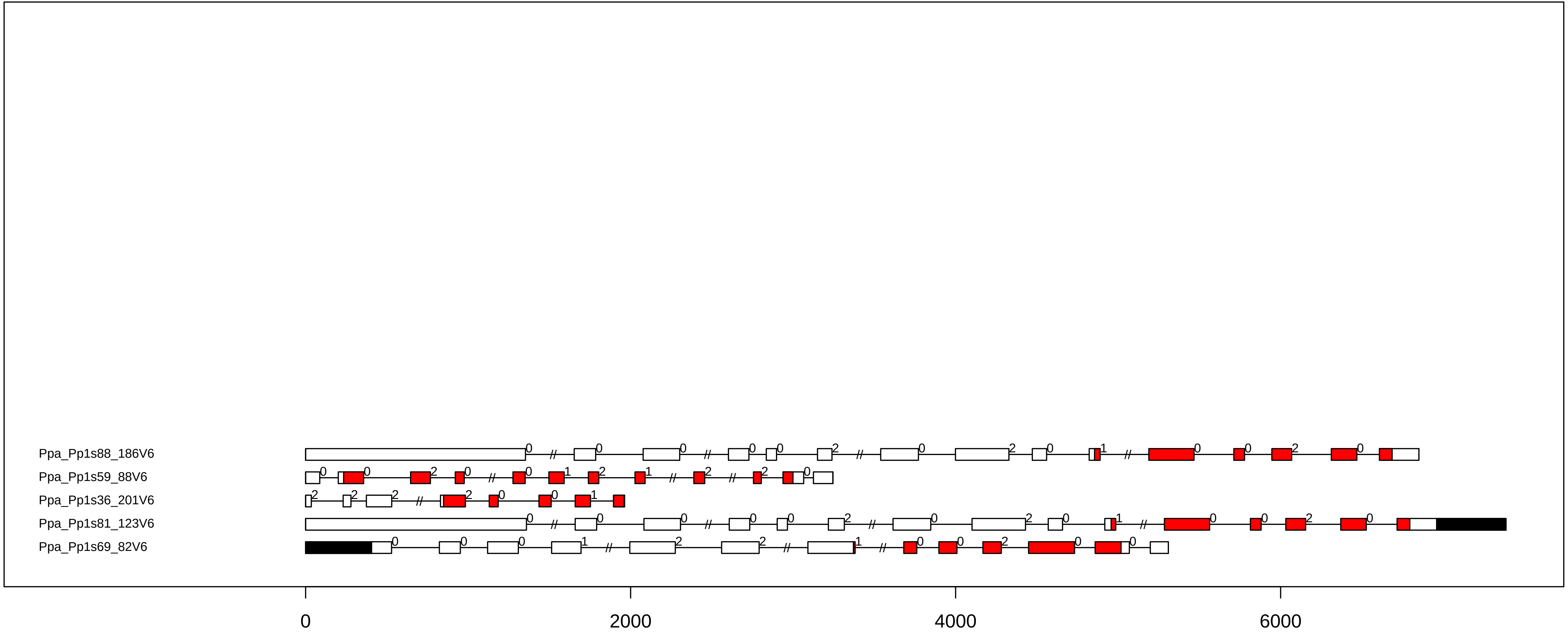

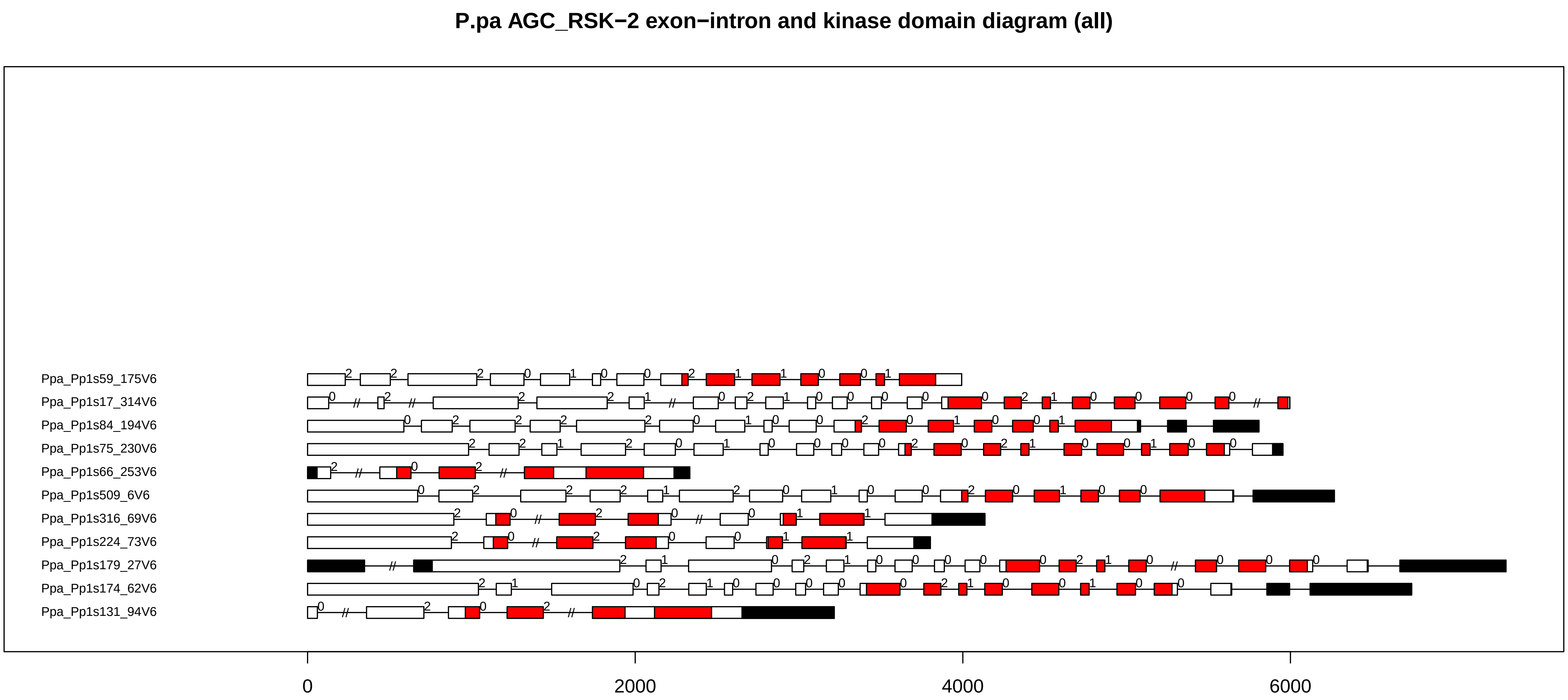
P.pa AGC_RSK−2 exon−intron and kinase domain diagram (all)
Ppa_Pp1s59_175V6
Ppa_Pp1s17_314V6
Ppa_Pp1s84_194V6
Ppa_Pp1s75_230V6
Ppa_Pp1s66_253V6
Ppa_Pp1s509_6V6
Ppa_Pp1s316_69V6
Ppa_Pp1s224_73V6
Ppa_Pp1s179_27V6
Ppa_Pp1s174_62V6
Ppa_Pp1s131_94V6
0
2000
4000
6000

P.pa AGC−PI exon−intron and kinase domain diagram (all)

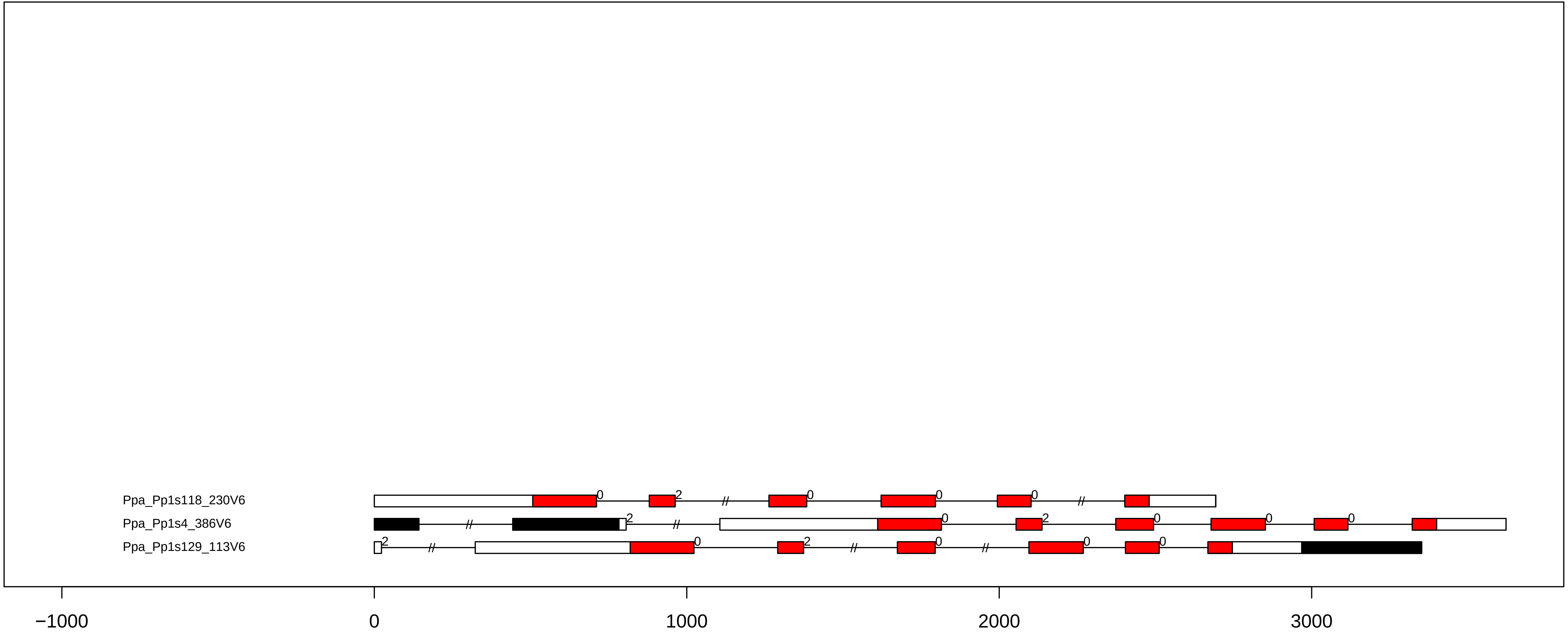

P.pa Aur exon−intron and kinase domain diagram (all)

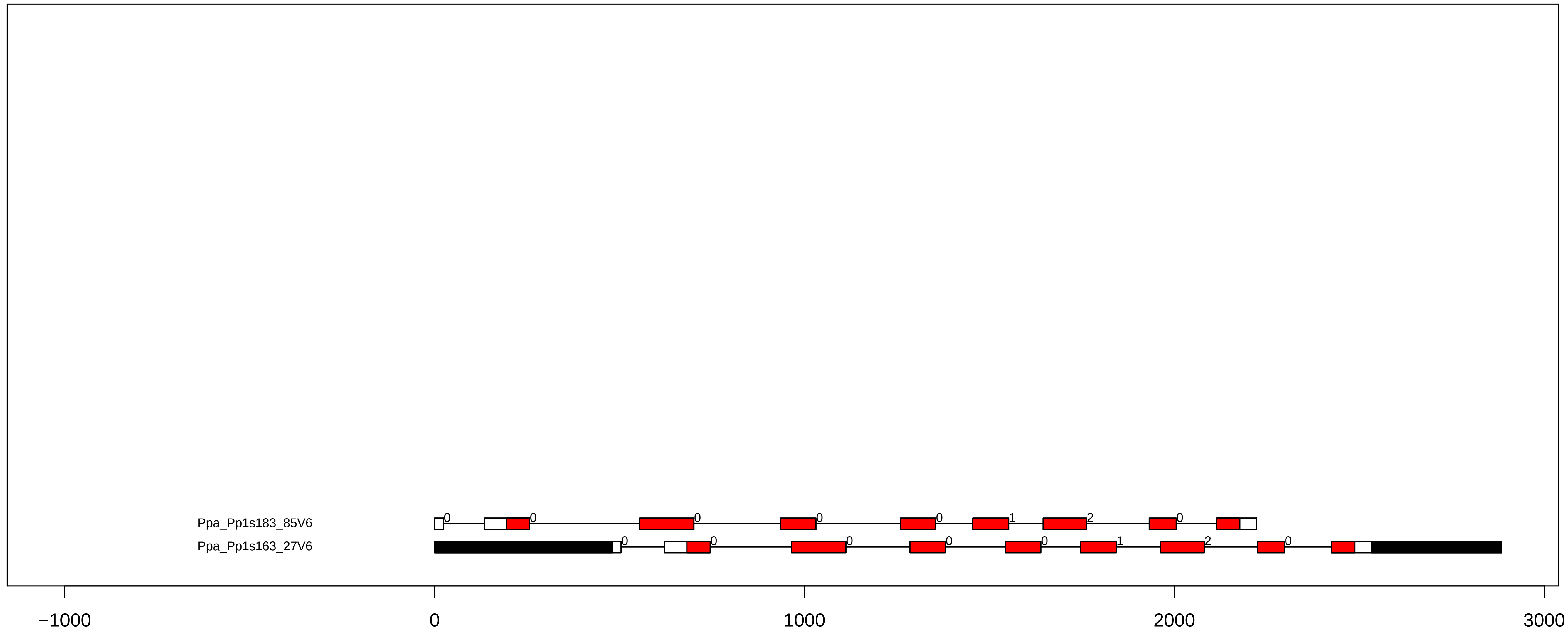

P.pa CAMK_AMPK exon−intron and kinase domain diagram (all)

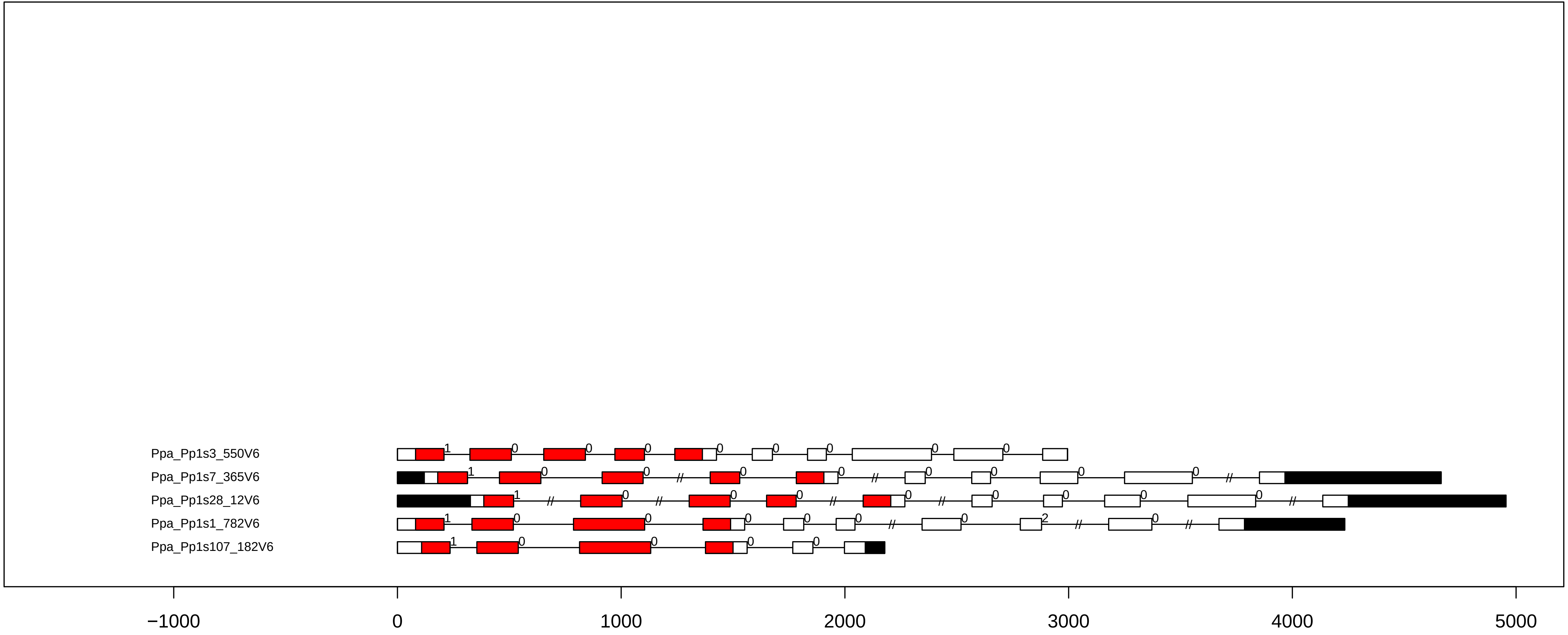

P.pa CAMK_CAMK1−DCAMKL exon−intron and kinase domain diagram (all)

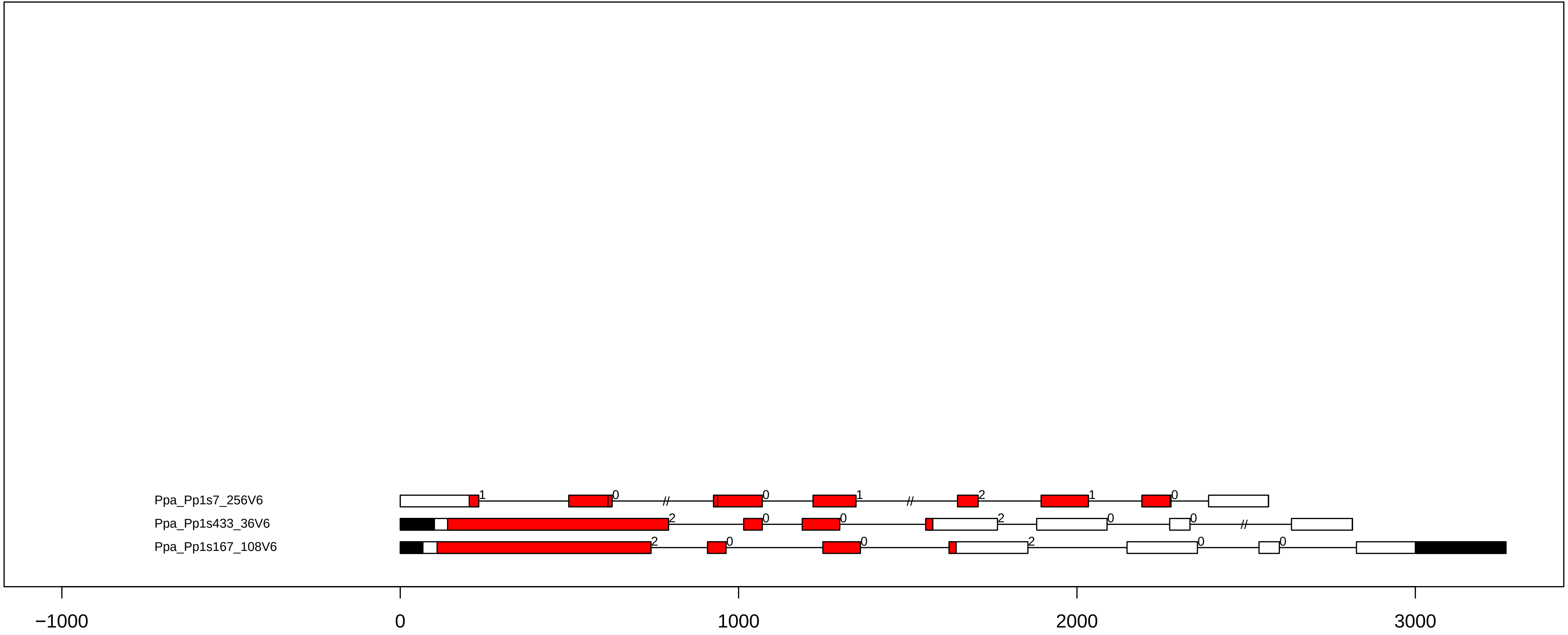

P.pa CAMK_CAMKL−CHK1 exon−intron and kinase domain diagram (all)

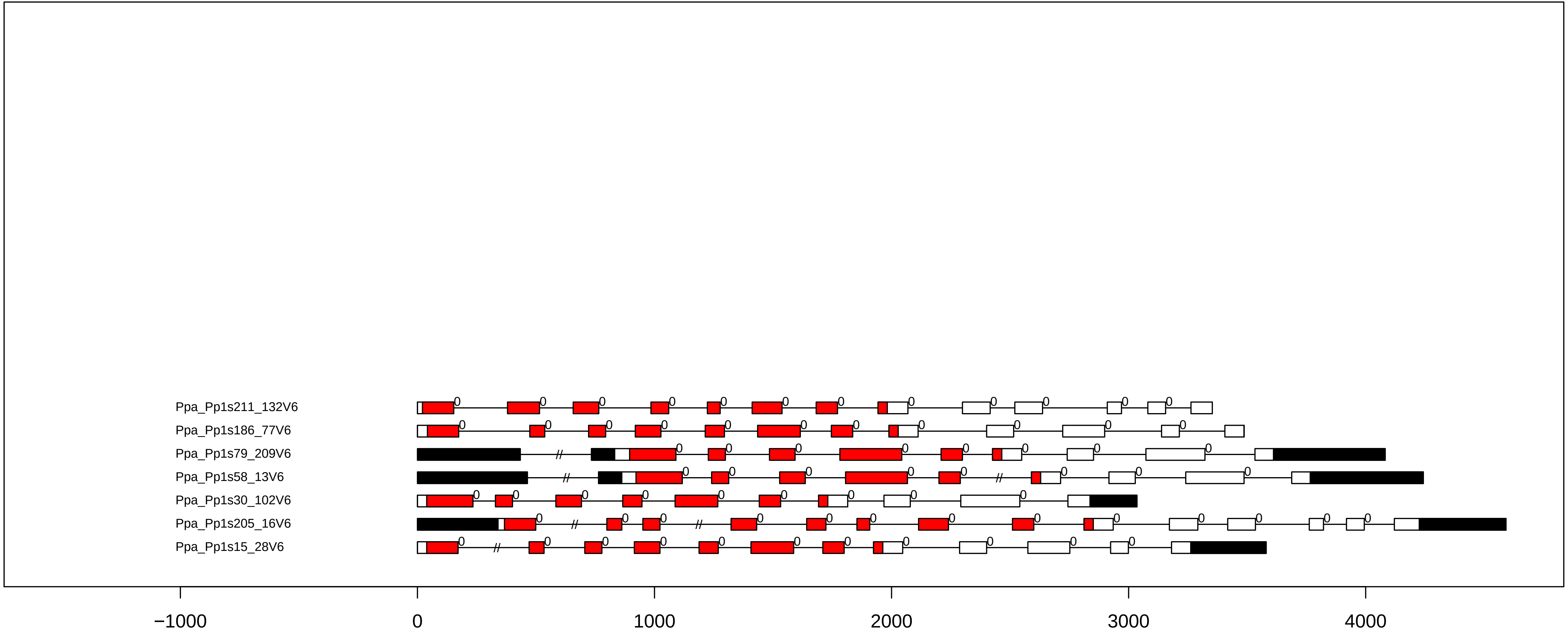

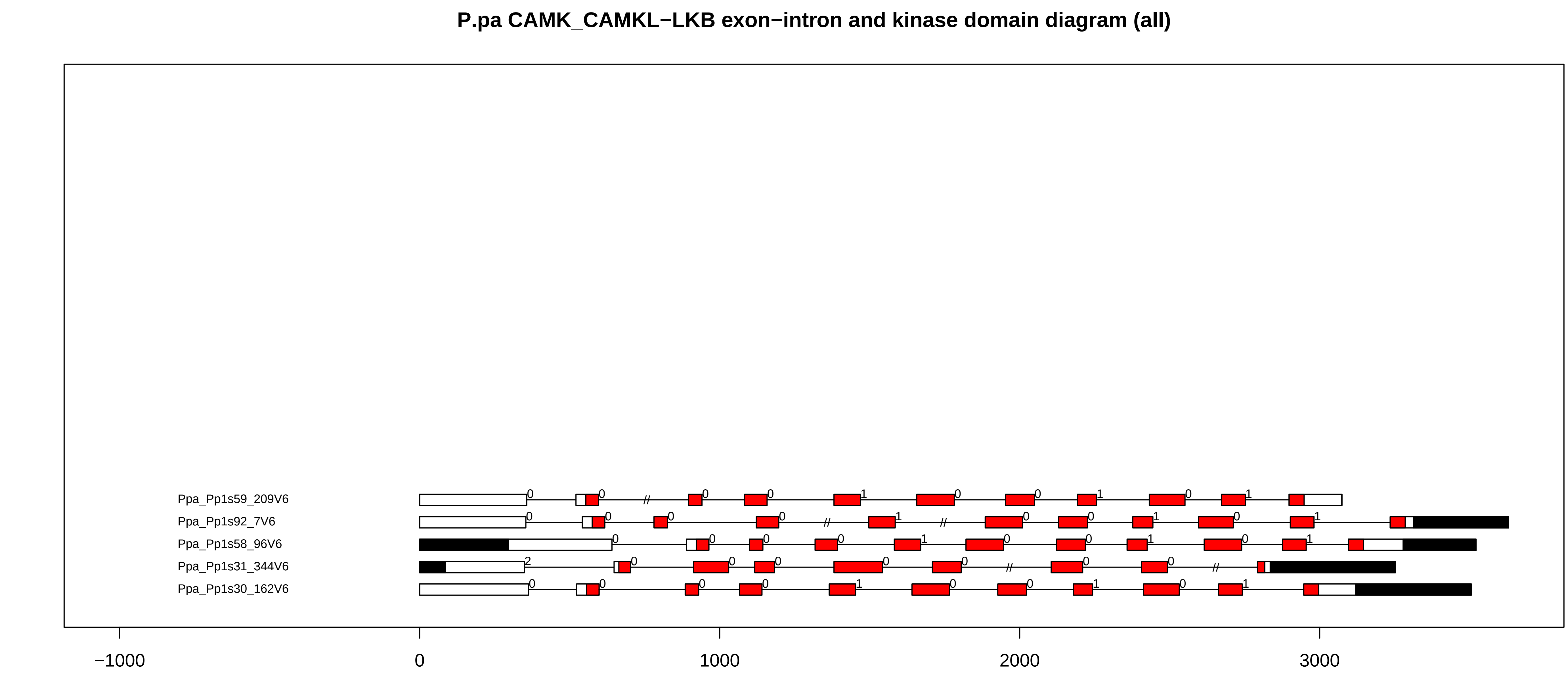
P.pa CAMK_CAMKL−LKB exon−intron and kinase domain diagram (all)
Ppa_Pp1s59_209V6
Ppa_Pp1s92_7V6
Ppa_Pp1s58_96V6
Ppa_Pp1s31_344V6
Ppa_Pp1s30_162V6
−1000
0
1000
2000
3000

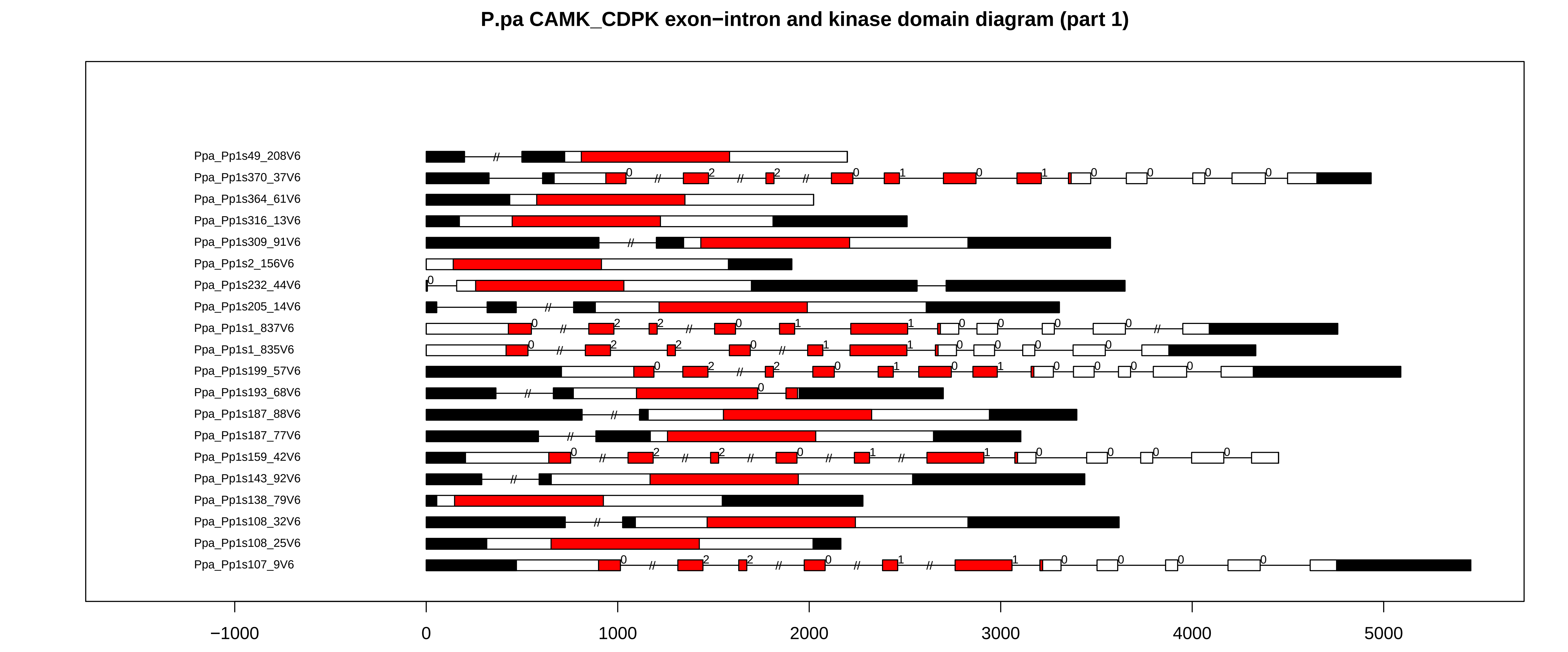
P.pa CAMK_CDPK exon−intron and kinase domain diagram (part 1)
Ppa_Pp1s49_208V6
Ppa_Pp1s370_37V6
Ppa_Pp1s364_61V6
Ppa_Pp1s316_13V6
Ppa_Pp1s309_91V6
Ppa_Pp1s2_156V6
Ppa_Pp1s232_44V6
Ppa_Pp1s205_14V6
Ppa_Pp1s1_837V6
Ppa_Pp1s1_835V6
Ppa_Pp1s199_57V6
Ppa_Pp1s193_68V6
Ppa_Pp1s187_88V6
Ppa_Pp1s187_77V6
Ppa_Pp1s159_42V6
Ppa_Pp1s143_92V6
Ppa_Pp1s138_79V6
Ppa_Pp1s108_32V6
Ppa_Pp1s108_25V6
Ppa_Pp1s107_9V6
−1000
0
1000
2000
3000
4000
5000

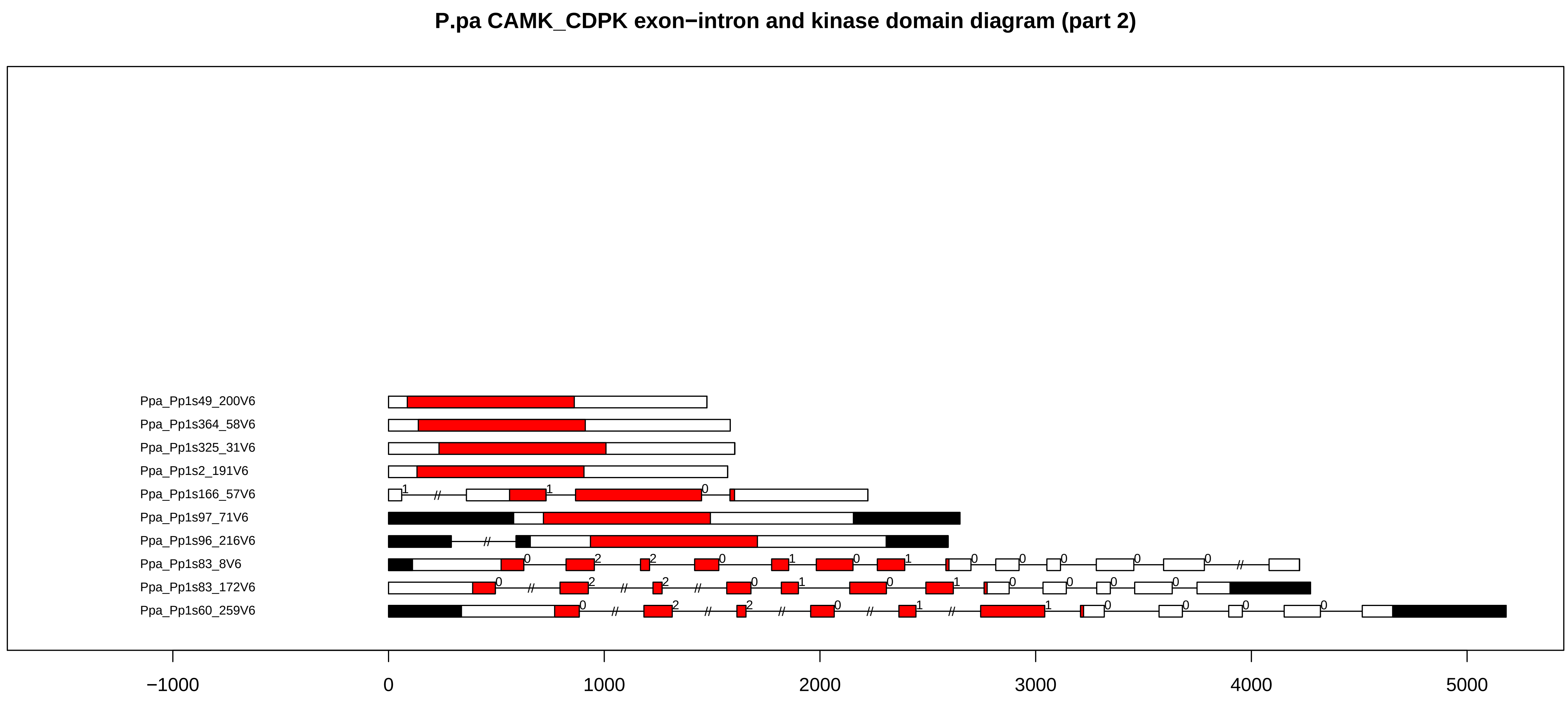

P.pa CAMK_CDPK exon−intron and kinase domain diagram (part 2)
Ppa_Pp1s49_200V6
Ppa_Pp1s364_58V6
Ppa_Pp1s325_31V6
Ppa_Pp1s2_191V6
Ppa_Pp1s166_57V6
Ppa_Pp1s97_71V6
Ppa_Pp1s96_216V6
Ppa_Pp1s83_8V6
Ppa_Pp1s83_172V6
Ppa_Pp1s60_259V6
−1000
0
1000
2000
3000
4000
5000

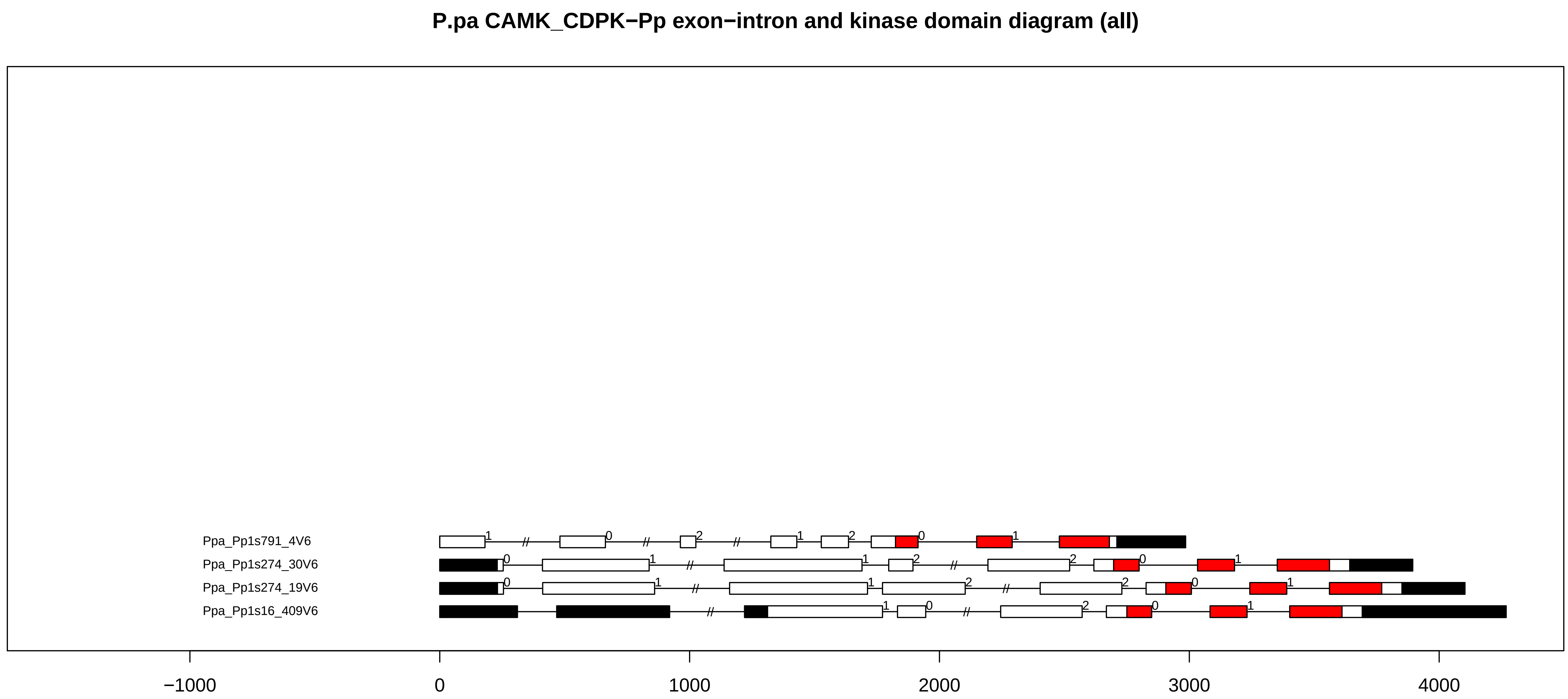
P.pa CAMK_CDPK−Pp exon−intron and kinase domain diagram (all)
Ppa_Pp1s791_4V6
Ppa_Pp1s274_30V6
Ppa_Pp1s274_19V6
Ppa_Pp1s16_409V6
−1000
0
1000
2000
3000
4000

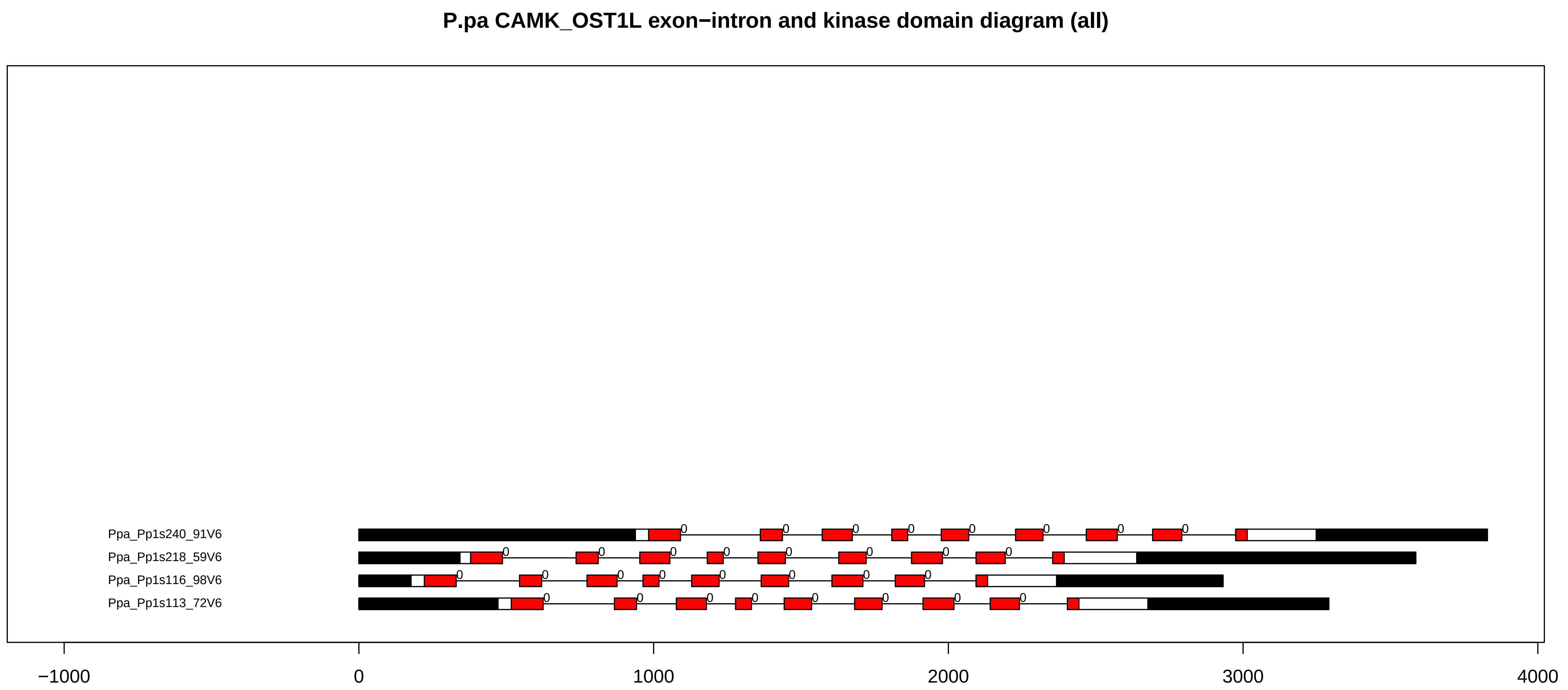
P.pa CAMK_OST1L exon−intron and kinase domain diagram (all)
Ppa_Pp1s240_91V6
Ppa_Pp1s218_59V6
Ppa_Pp1s116_98V6
Ppa_Pp1s113_72V6
−1000
0
1000
2000
3000
4000

P.pa CK1_CK1 exon−intron and kinase domain diagram (all)

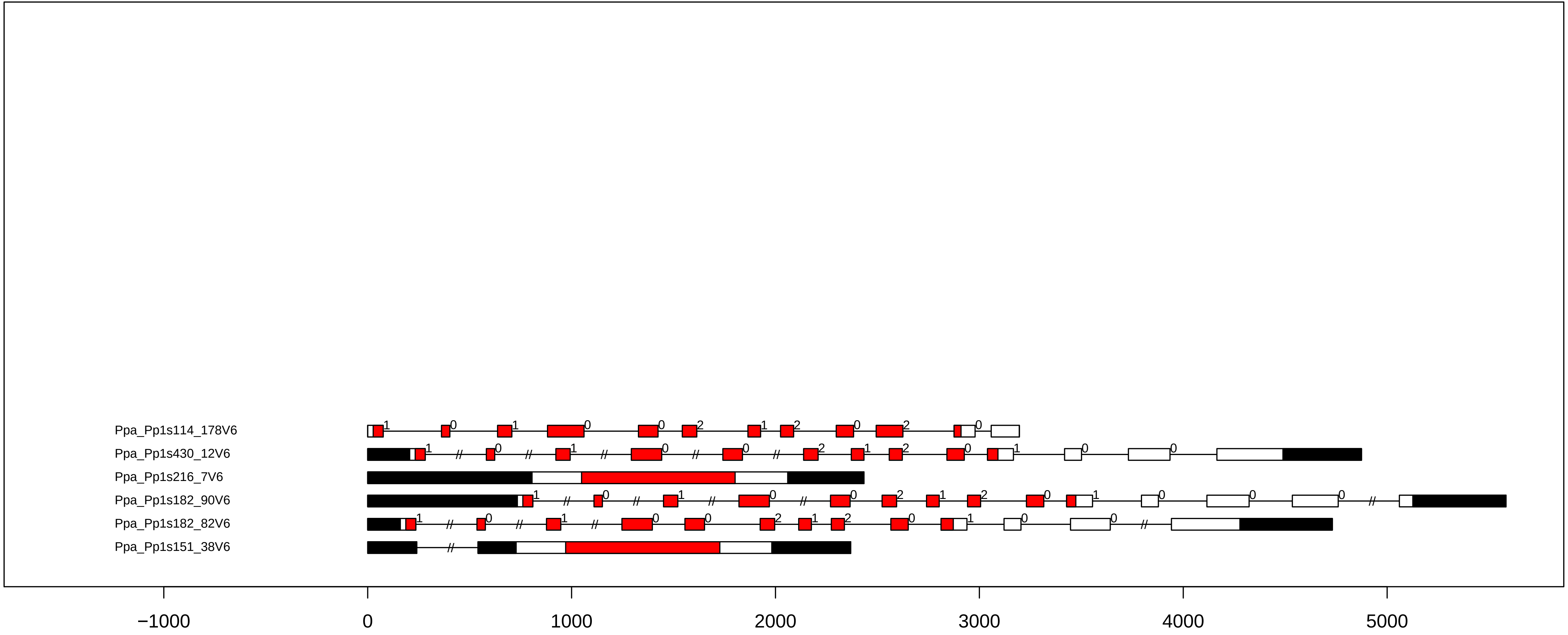

P.pa CK1_CK1−PI exon−intron and kinase domain diagram (all)

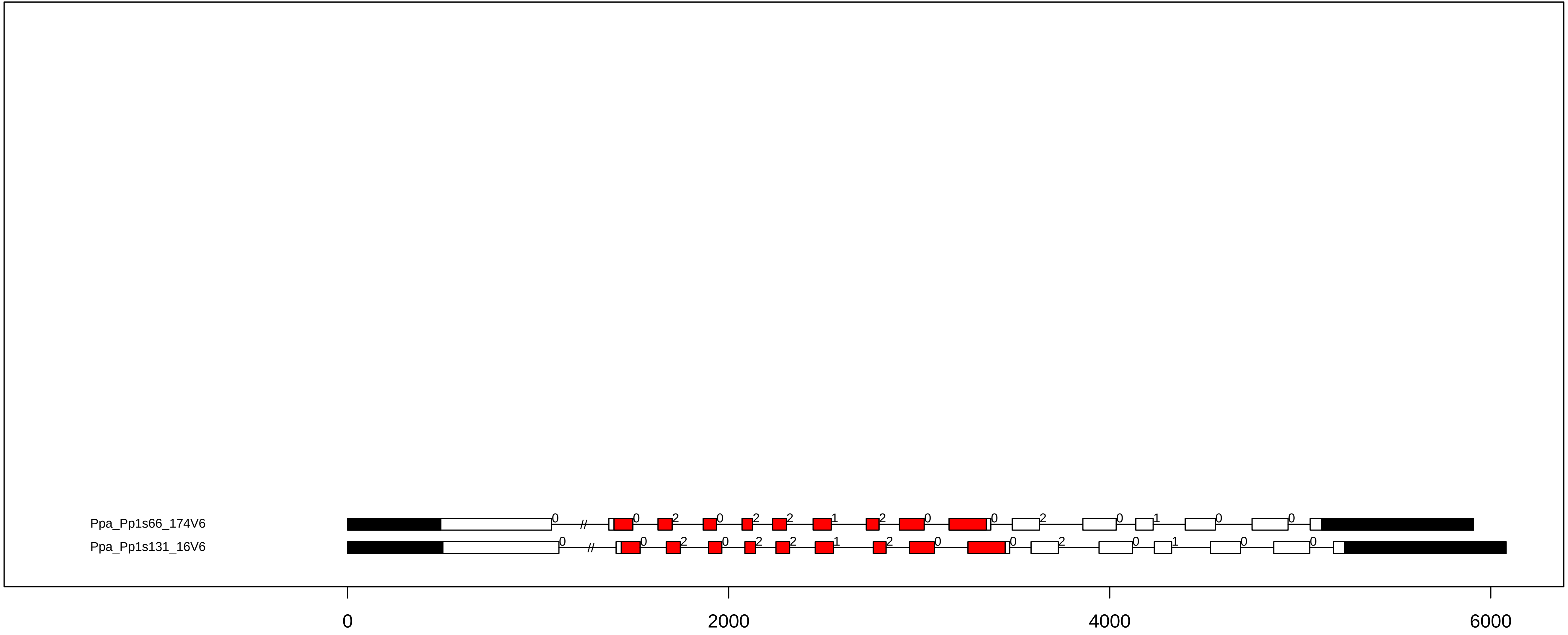

P.pa CMGC_CDK−CCRK exon−intron and kinase domain diagram (all)

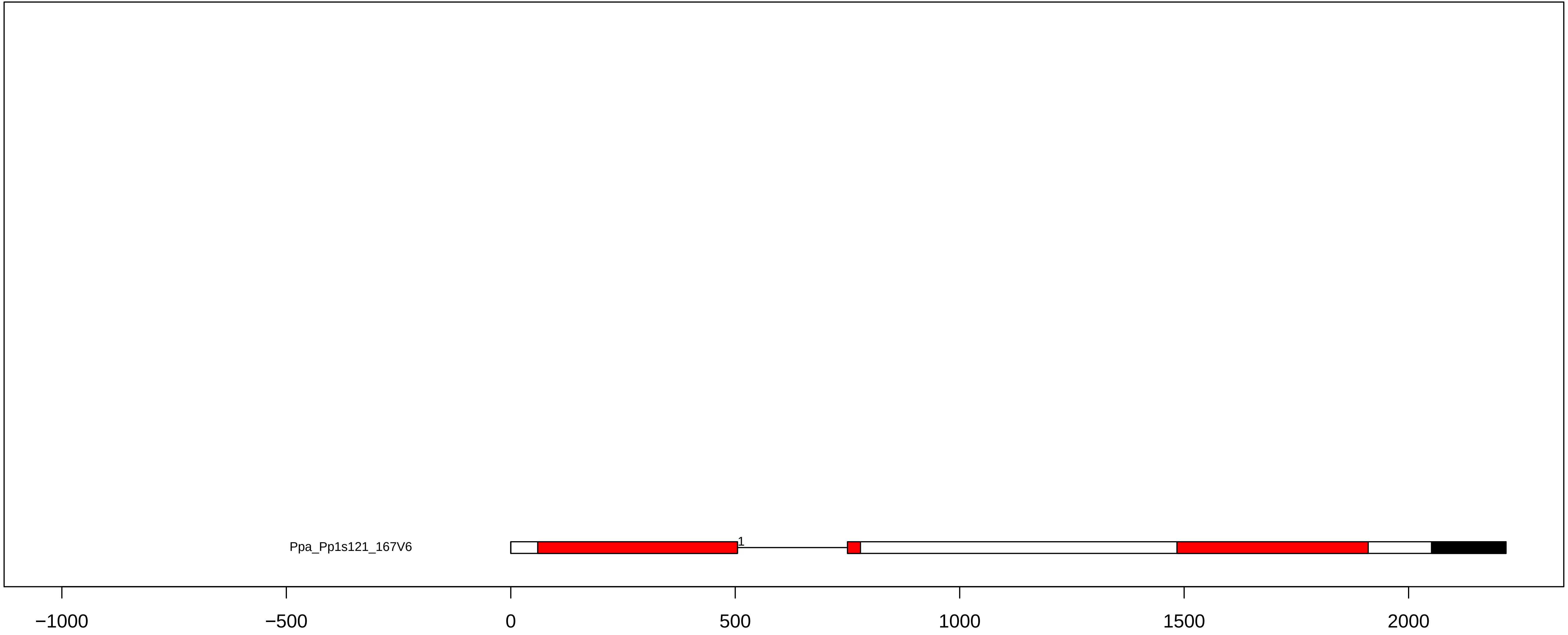

P.pa CMGC_CDK−CDK7 exon−intron and kinase domain diagram (all)

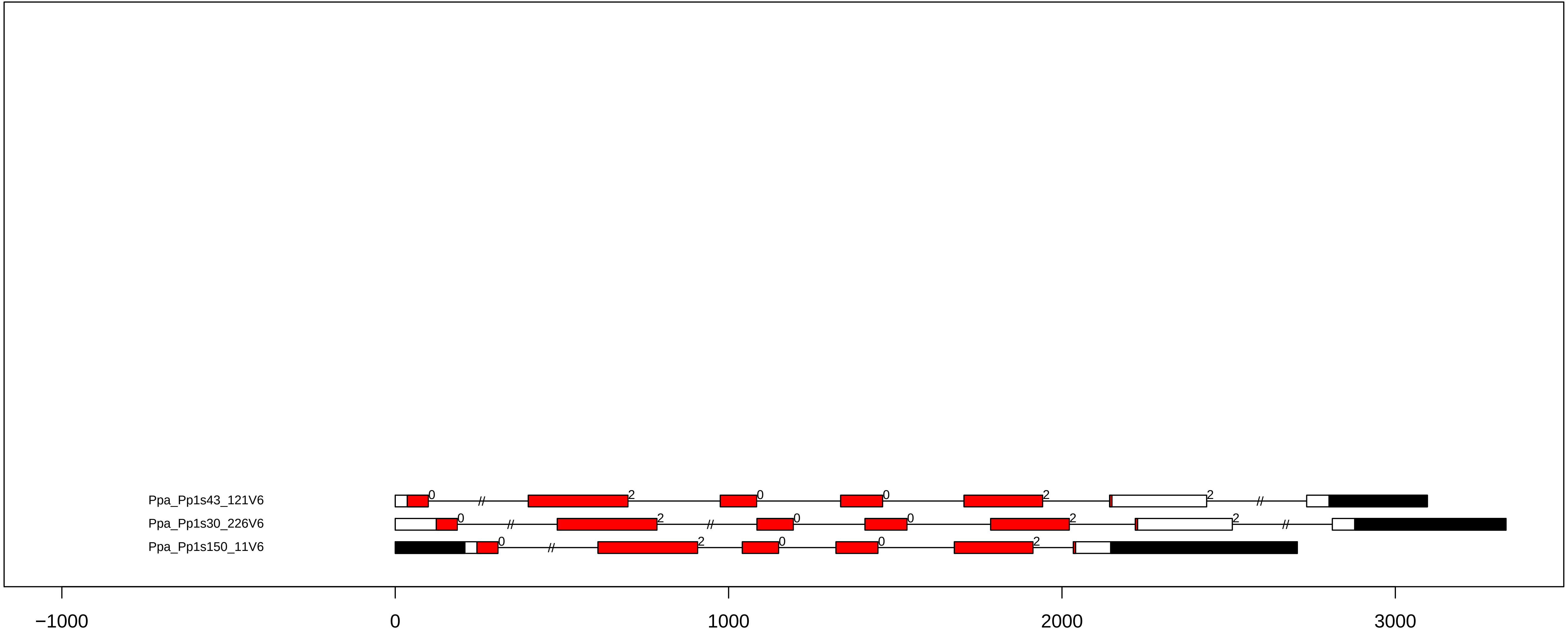

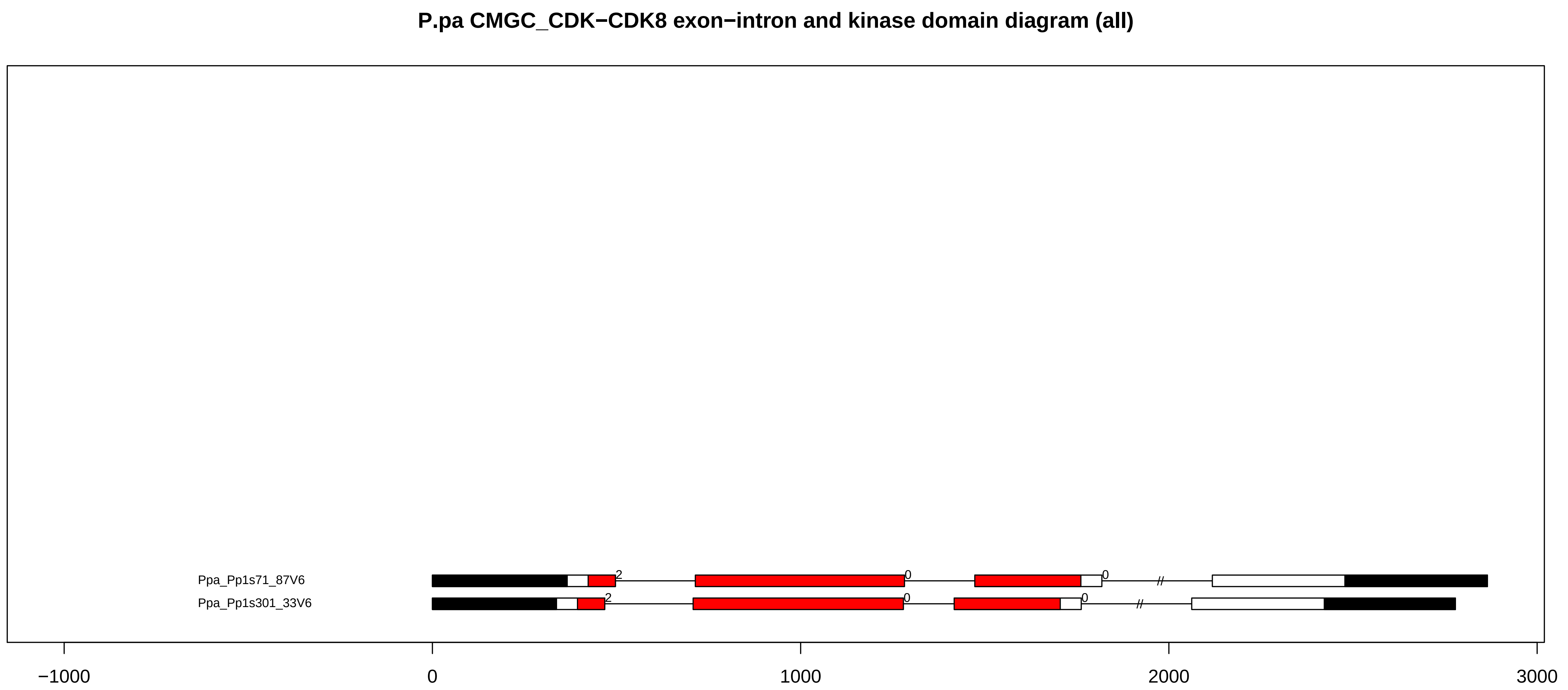
P.pa CMGC_CDK−CDK8 exon−intron and kinase domain diagram (all)
Ppa_Pp1s71_87V6
Ppa_Pp1s301_33V6
2
0
0
2
0
0
−1000
0
1000
2000
3000

P.pa CMGC_CDK−CRK7−CDK9 exon−intron and kinase domain diagram (all)

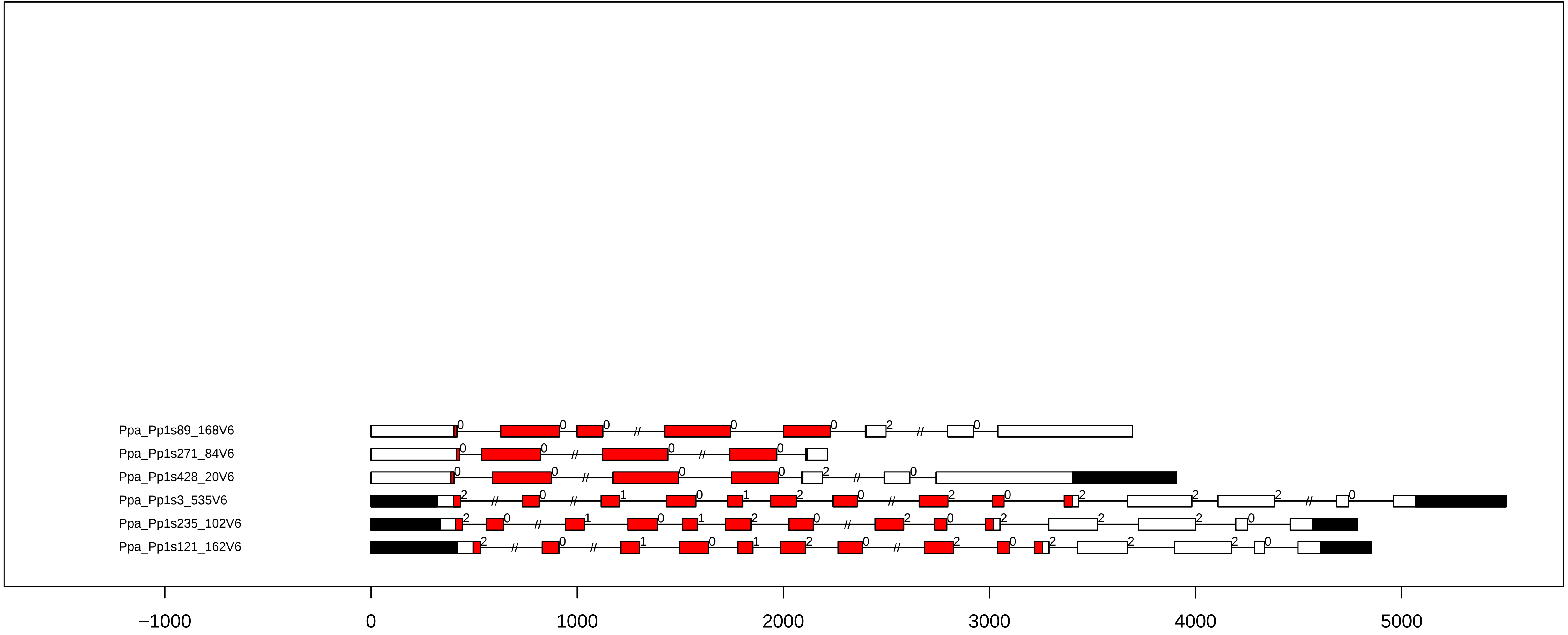

P.pa CMGC_CDK−PITSLRE exon−intron and kinase domain diagram (all)

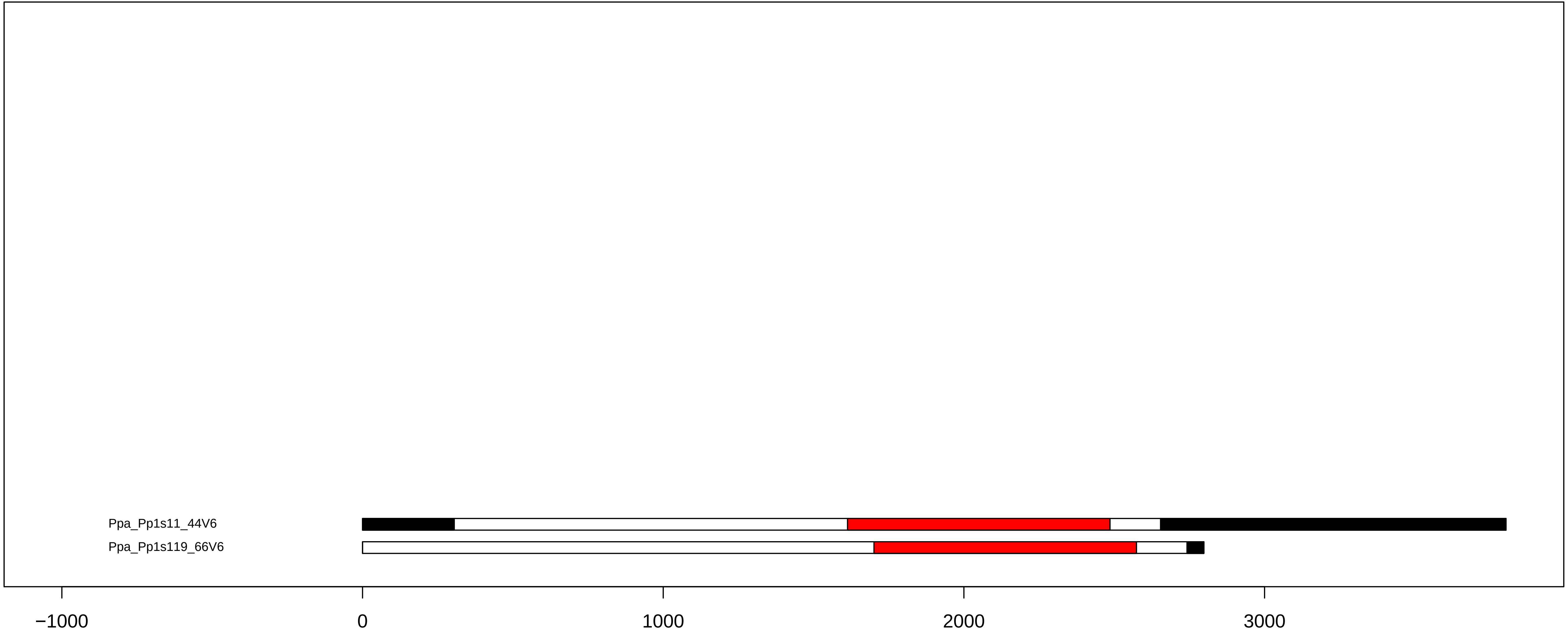

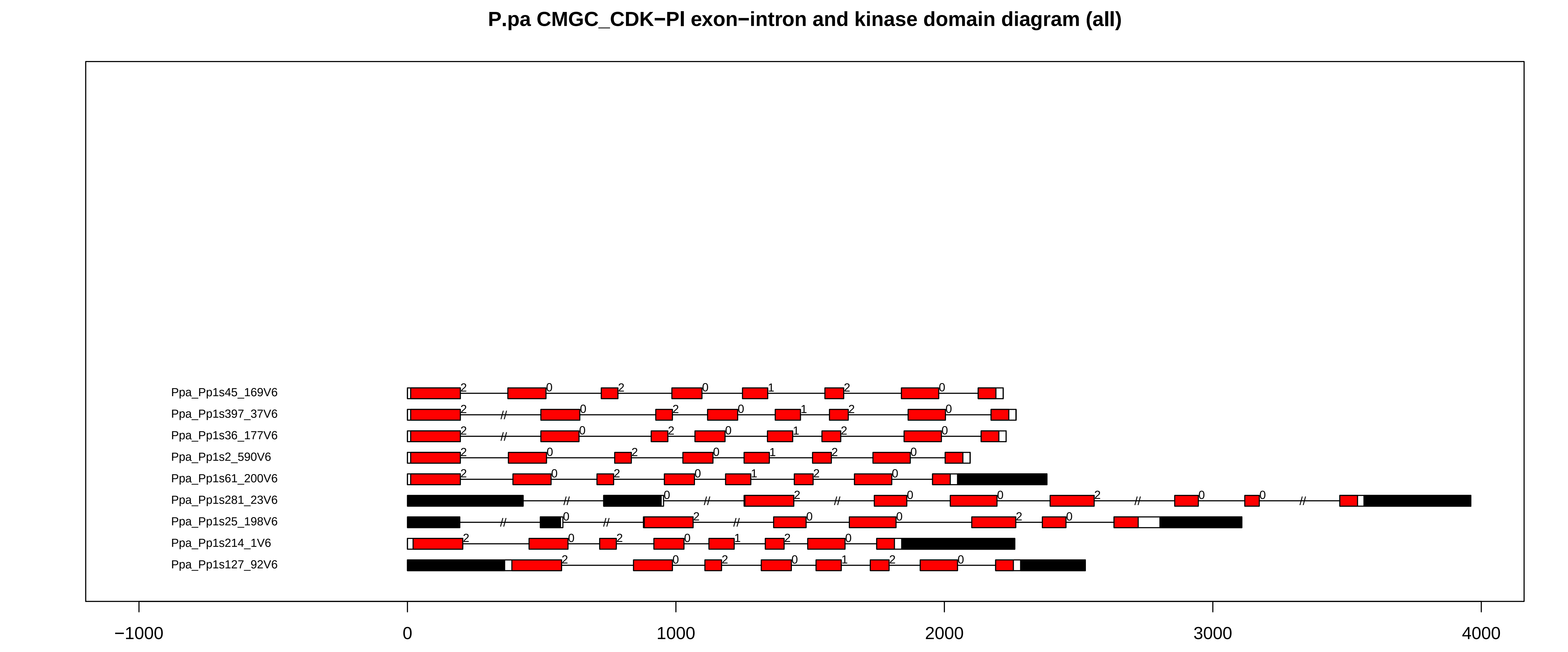
P.pa CMGC_CDK−Pl exon−intron and kinase domain diagram (all)
Ppa_Pp1s45_169V6
Ppa_Pp1s397_37V6
Ppa_Pp1s36_177V6
Ppa_Pp1s2_590V6
Ppa_Pp1s61_200V6
Ppa_Pp1s281_23V6
Ppa_Pp1s25_198V6
Ppa_Pp1s214_1V6
Ppa_Pp1s127_92V6
−1000
0
1000
2000
3000
4000

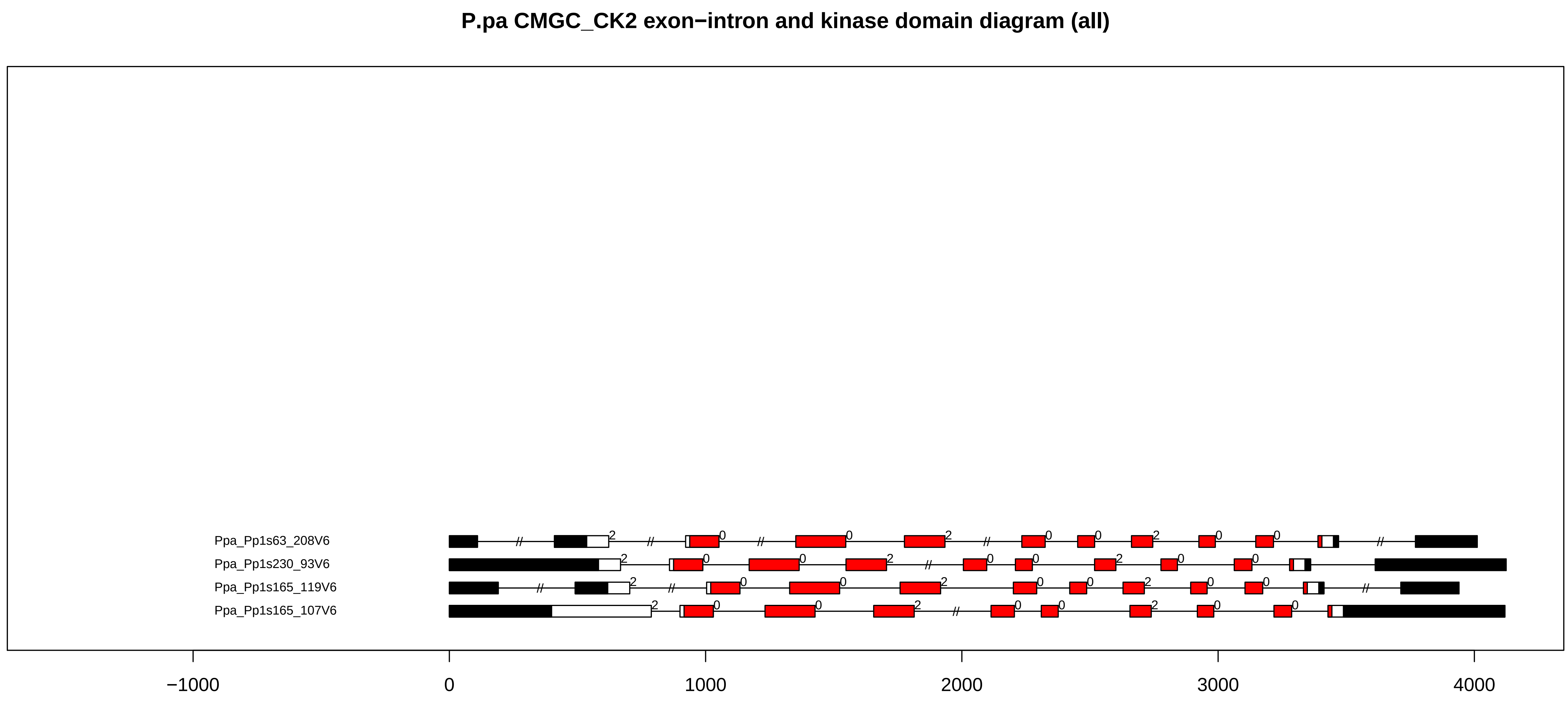
P.pa CMGC_CK2 exon−intron and kinase domain diagram (all)
Ppa_Pp1s63_208V6
Ppa_Pp1s230_93V6
Ppa_Pp1s165_119V6
Ppa_Pp1s165_107V6
−1000
0
1000
2000
3000
4000

P.pa CMGC_CLK exon−intron and kinase domain diagram (all)

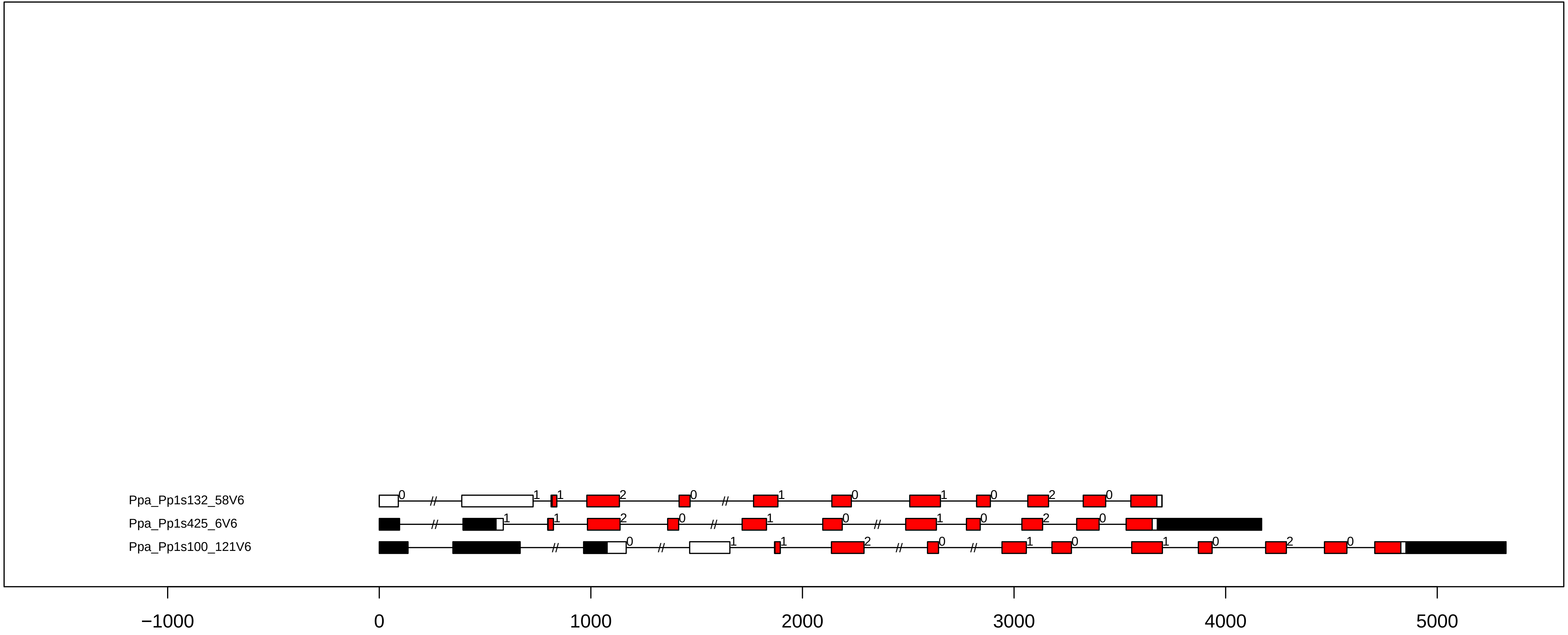

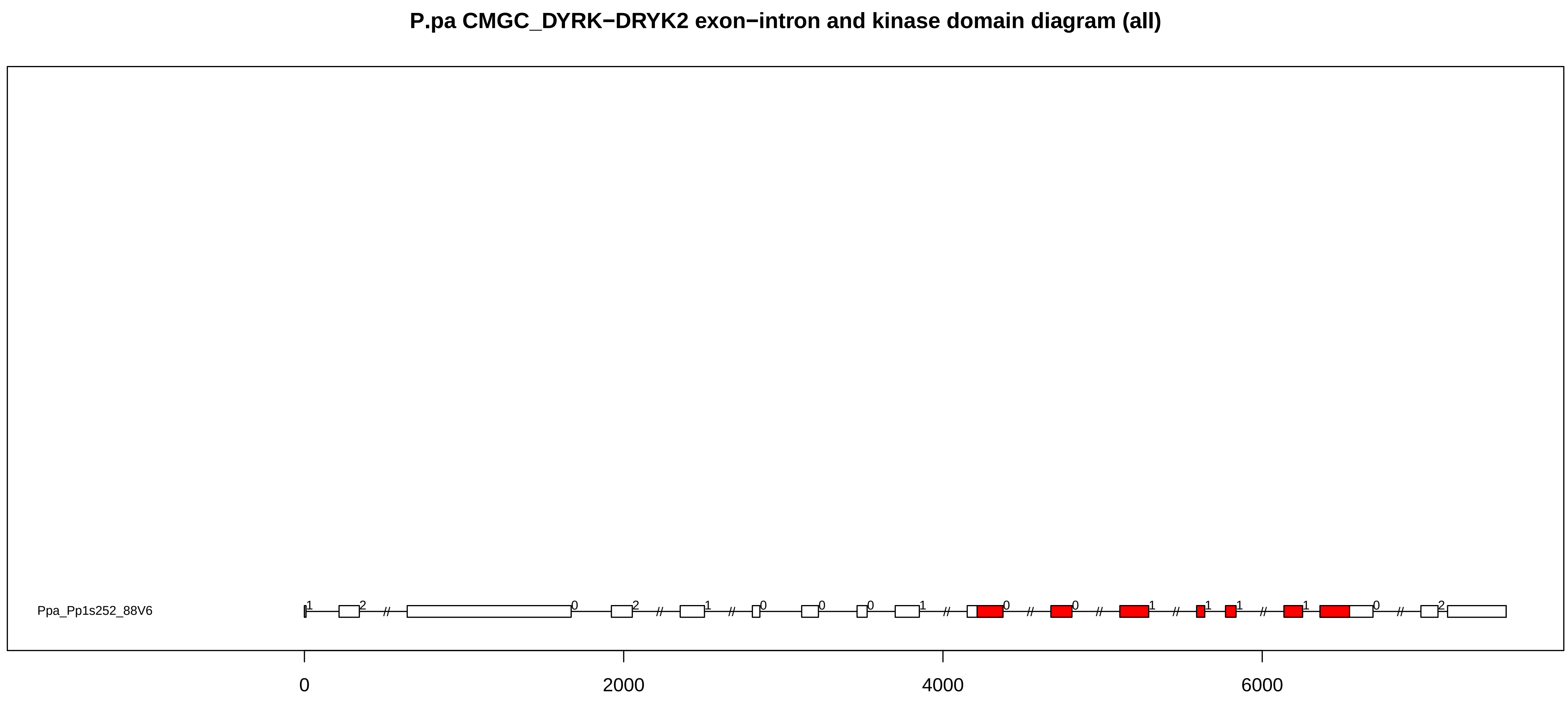
P.pa CMGC_DYRK−DRYK2 exon−intron and kinase domain diagram (all)
Ppa_Pp1s252_88V6
1
2
0
2
1
0
0
0
1
0
0
1
1
1
1
0
2
0
2000
4000
6000

P.pa CMGC_DYRK−PRP4 exon−intron and kinase domain diagram (all)

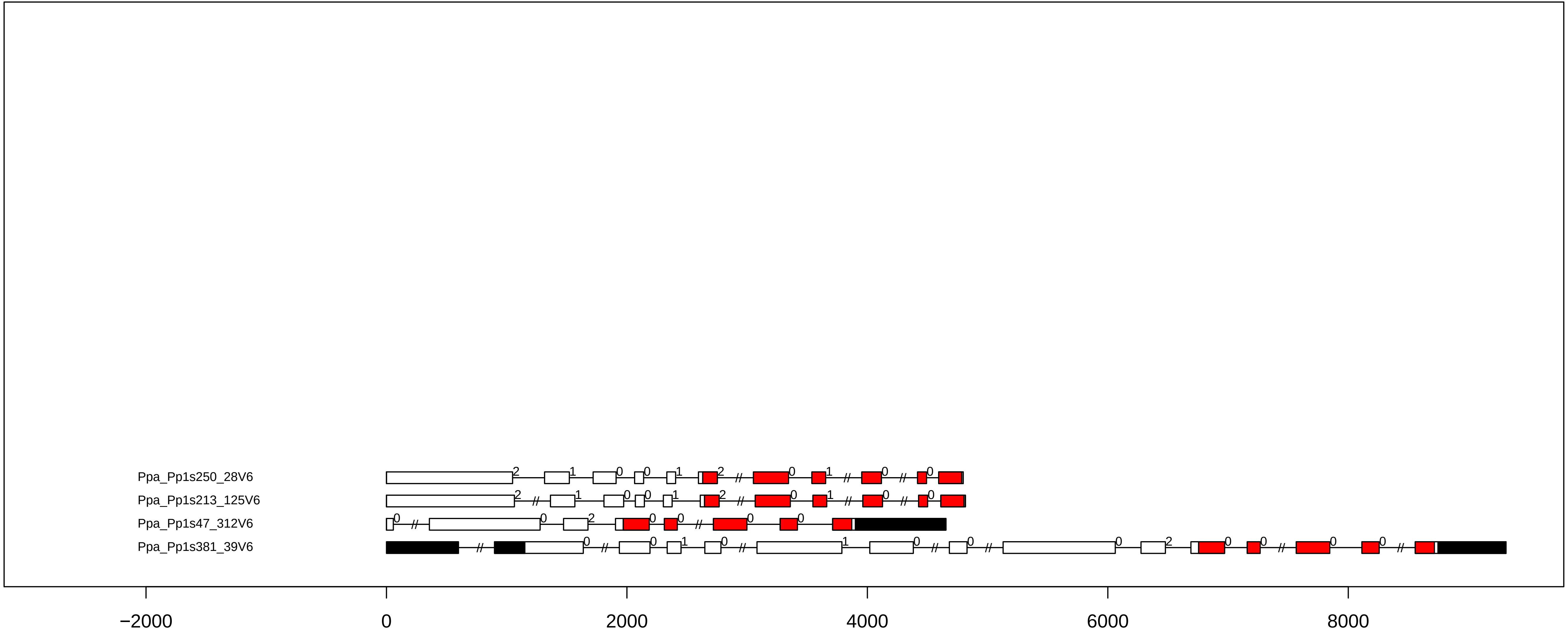

P.pa CMGC_DYRK−YAK exon−intron and kinase domain diagram (all)

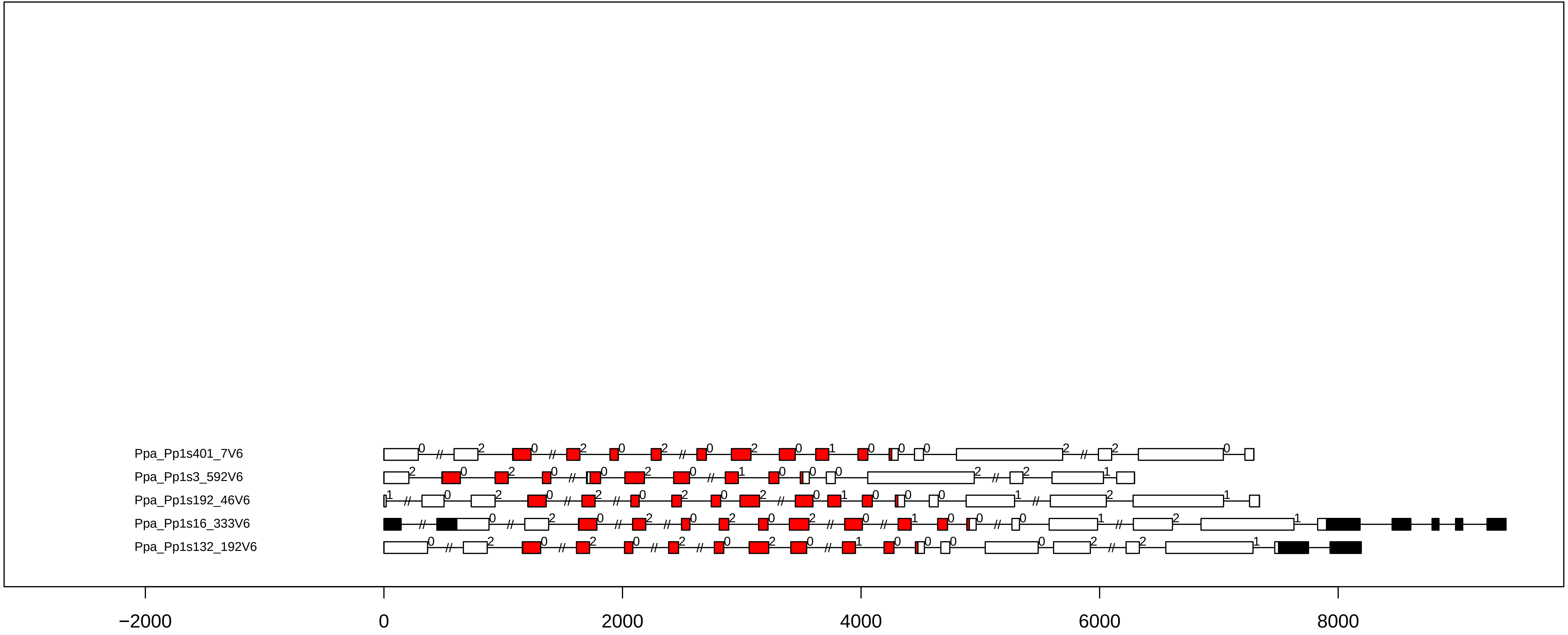

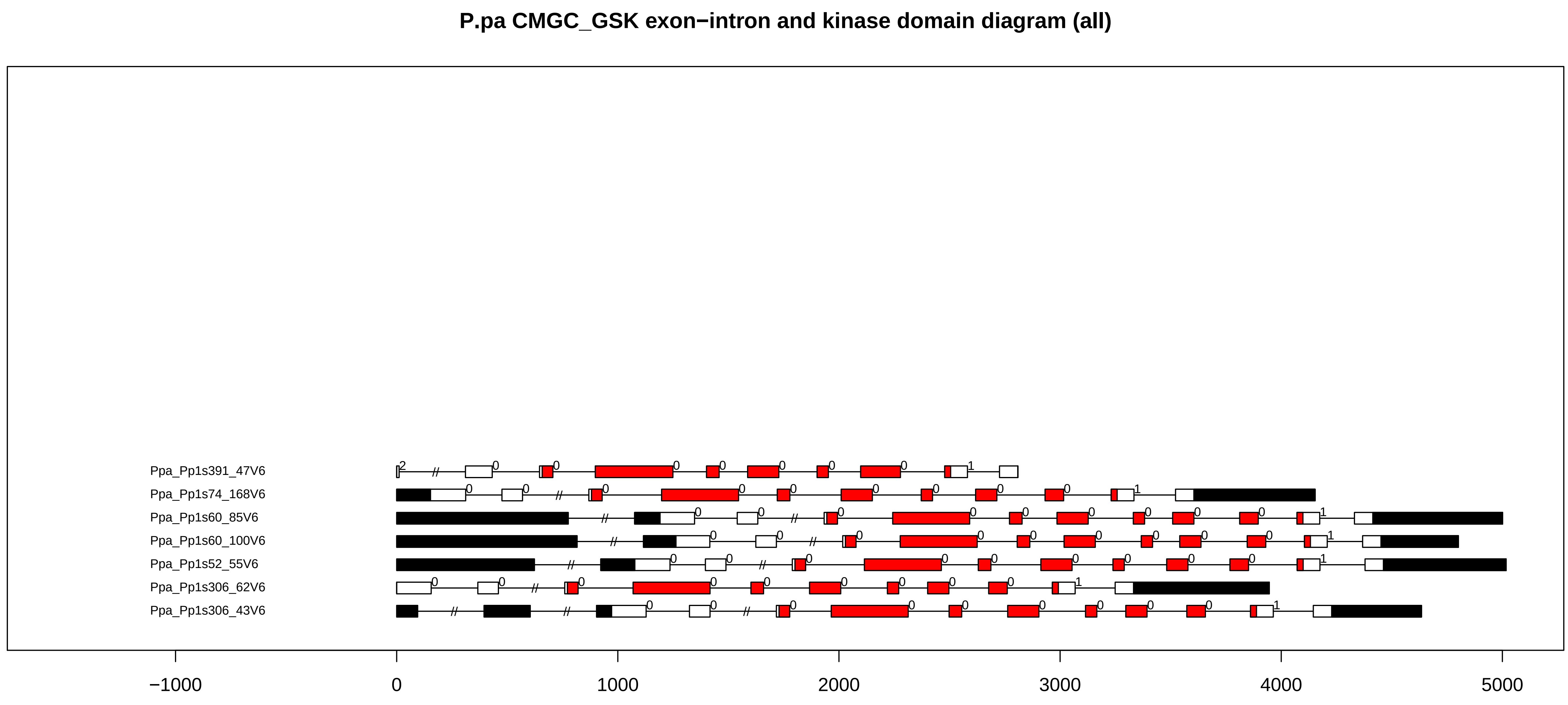

P.pa CMGC_GSK exon−intron and kinase domain diagram (all)
Ppa_Pp1s391_47V6
Ppa_Pp1s74_168V6
Ppa_Pp1s60_85V6
Ppa_Pp1s60_100V6
Ppa_Pp1s52_55V6
Ppa_Pp1s306_62V6
Ppa_Pp1s306_43V6
−1000
0
1000
2000
3000
4000
5000

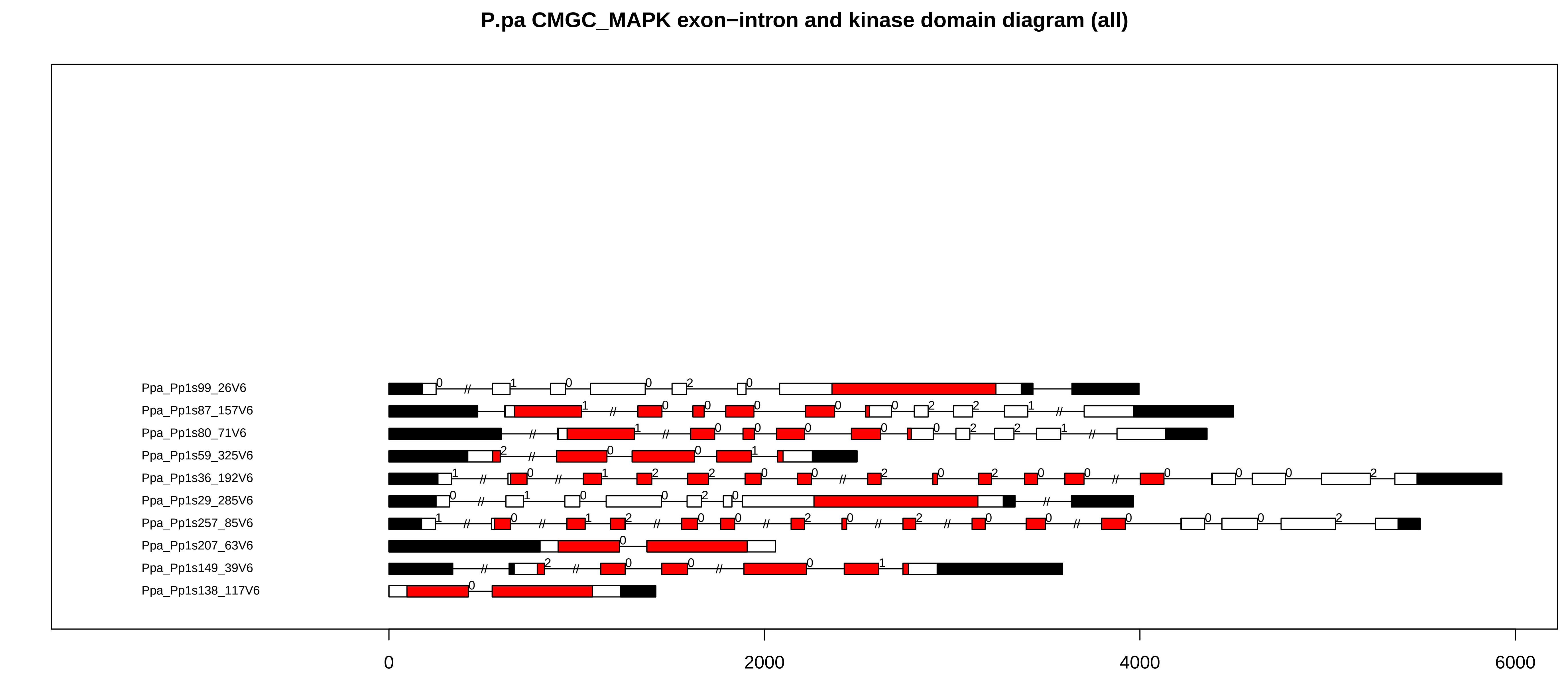
P.pa CMGC_MAPK exon−intron and kinase domain diagram (all)
Ppa_Pp1s99_26V6
Ppa_Pp1s87_157V6
Ppa_Pp1s80_71V6
Ppa_Pp1s59_325V6
Ppa_Pp1s36_192V6
Ppa_Pp1s29_285V6
Ppa_Pp1s257_85V6
Ppa_Pp1s207_63V6
Ppa_Pp1s149_39V6
Ppa_Pp1s138_117V6
0
2000
4000
6000

P.pa CMGC_Pl−Tthe exon−intron and kinase domain diagram (all)

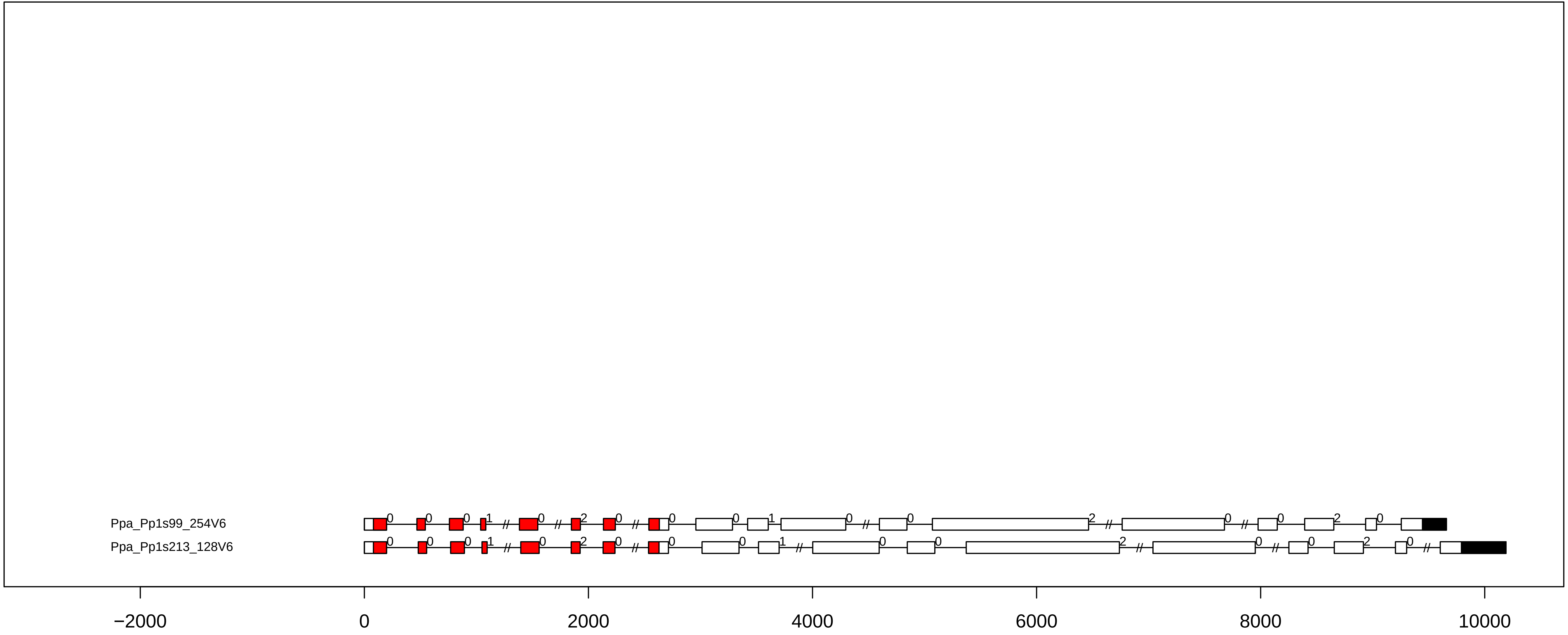

P.pa CMGC_RCK exon−intron and kinase domain diagram (all)

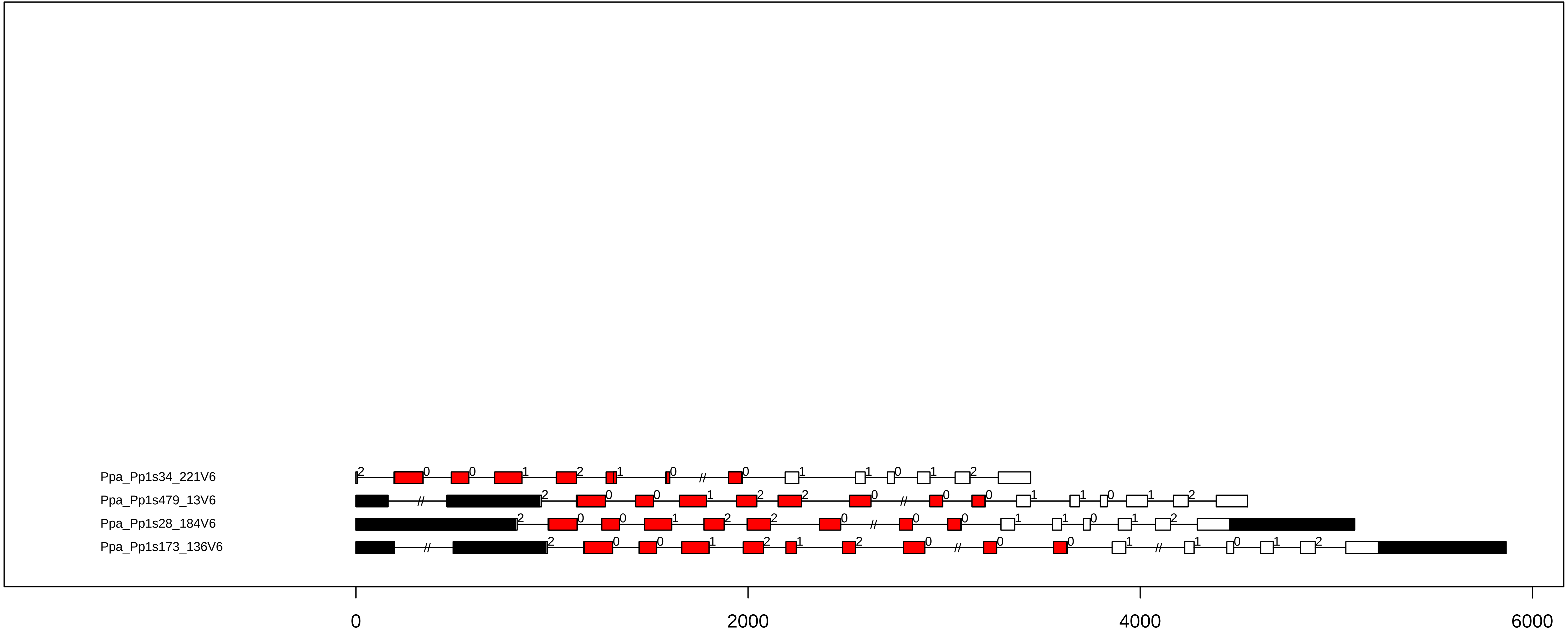

P.pa CMGC_SRPK exon−intron and kinase domain diagram (all)

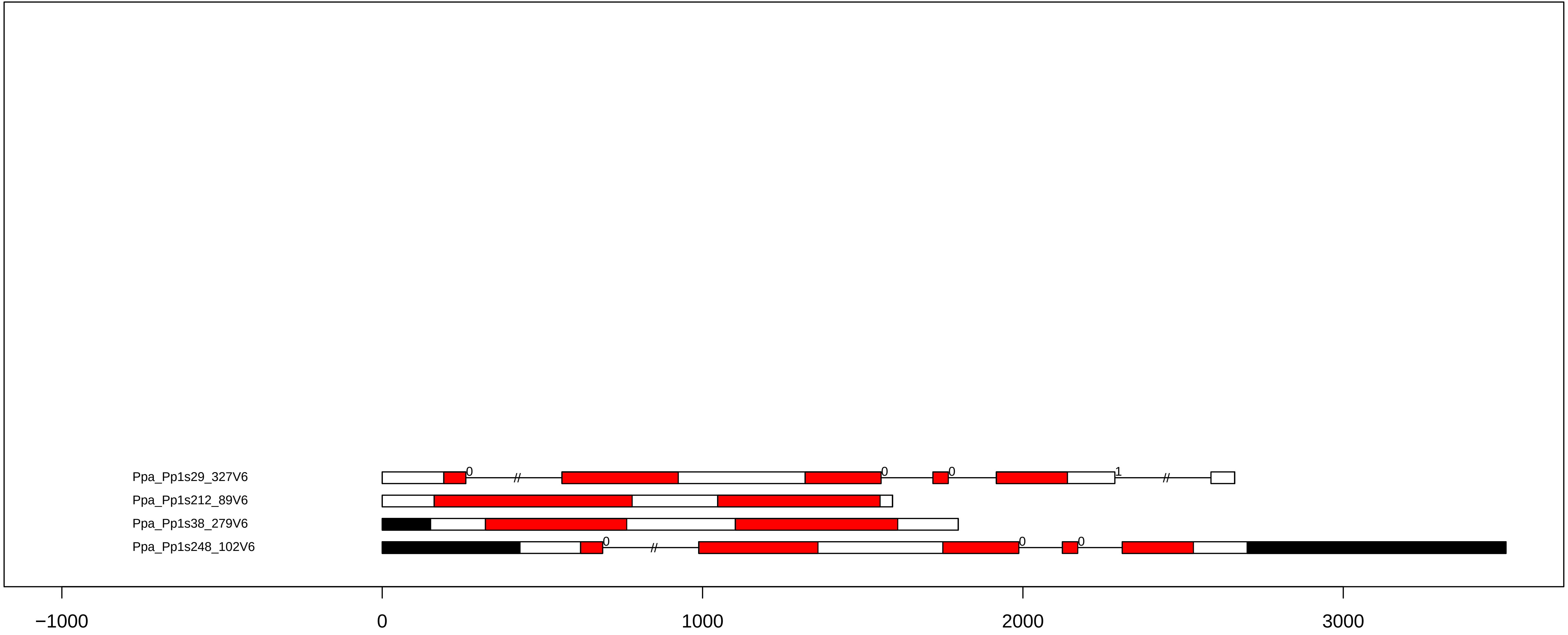

P.pa Group−PI−1 exon−intron and kinase domain diagram (all)

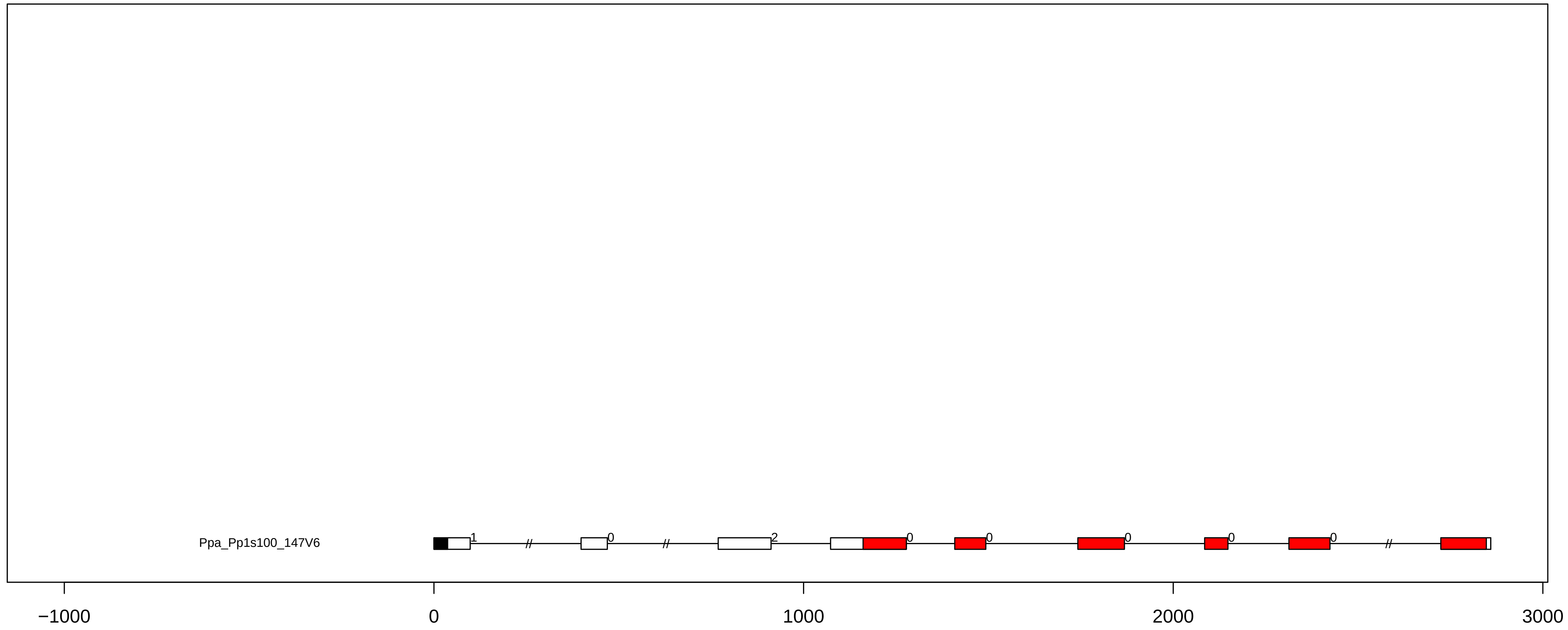

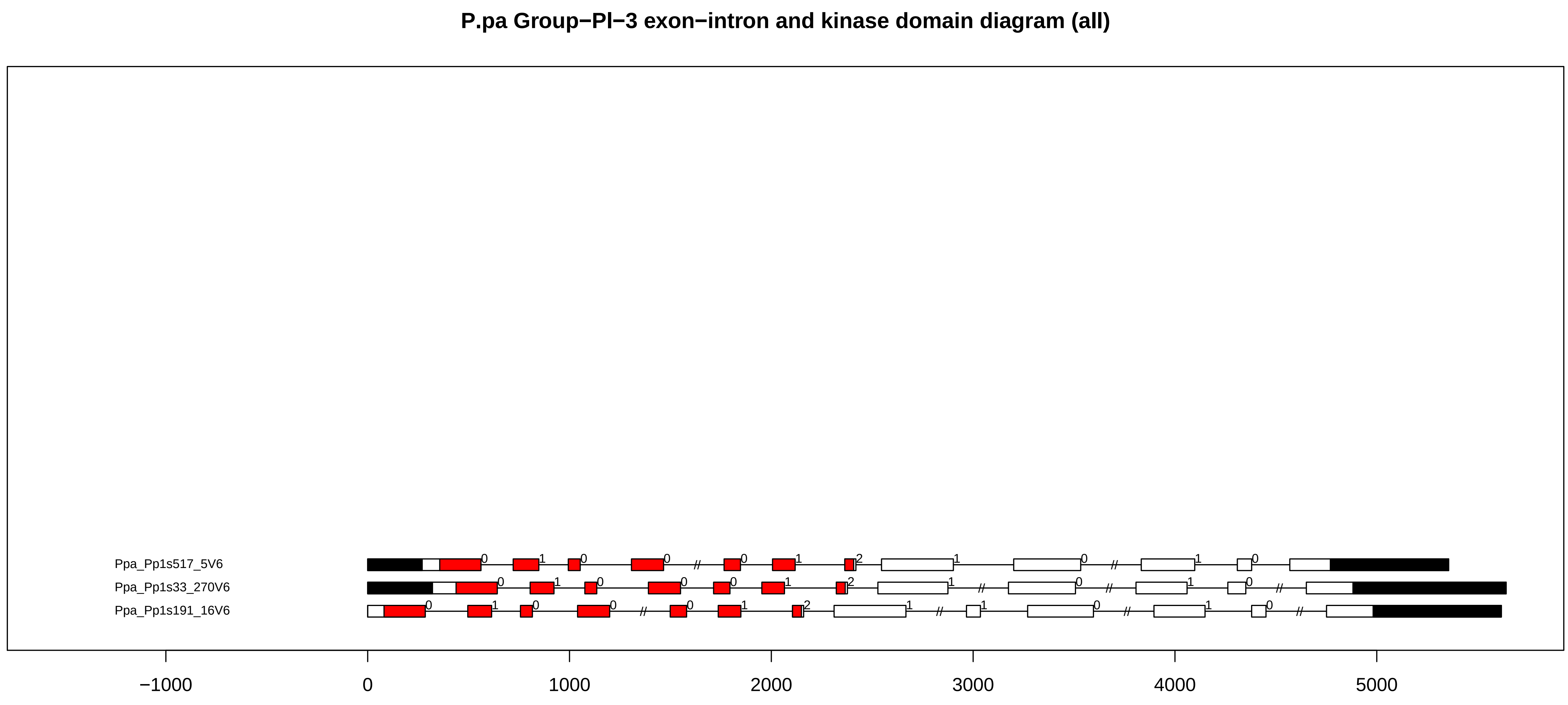
P.pa Group−PI−3 exon−intron and kinase domain diagram (all)
Ppa_Pp1s517_5V6
Ppa_Pp1s33_270V6
Ppa_Pp1s191_16V6
−1000
0
1000
2000
3000
4000
5000

P.pa Group−PI−4 exon−intron and kinase domain diagram (all)

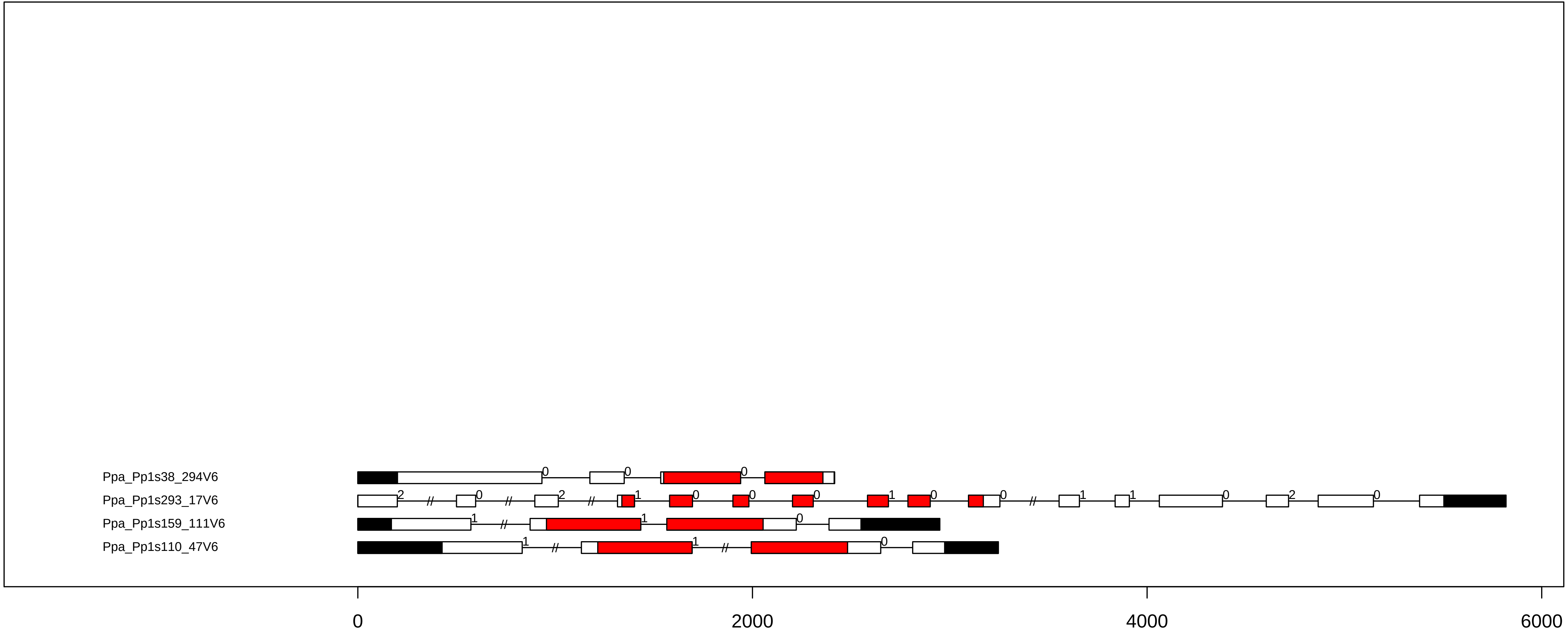

P.pa IRE1 exon−intron and kinase domain diagram (all)

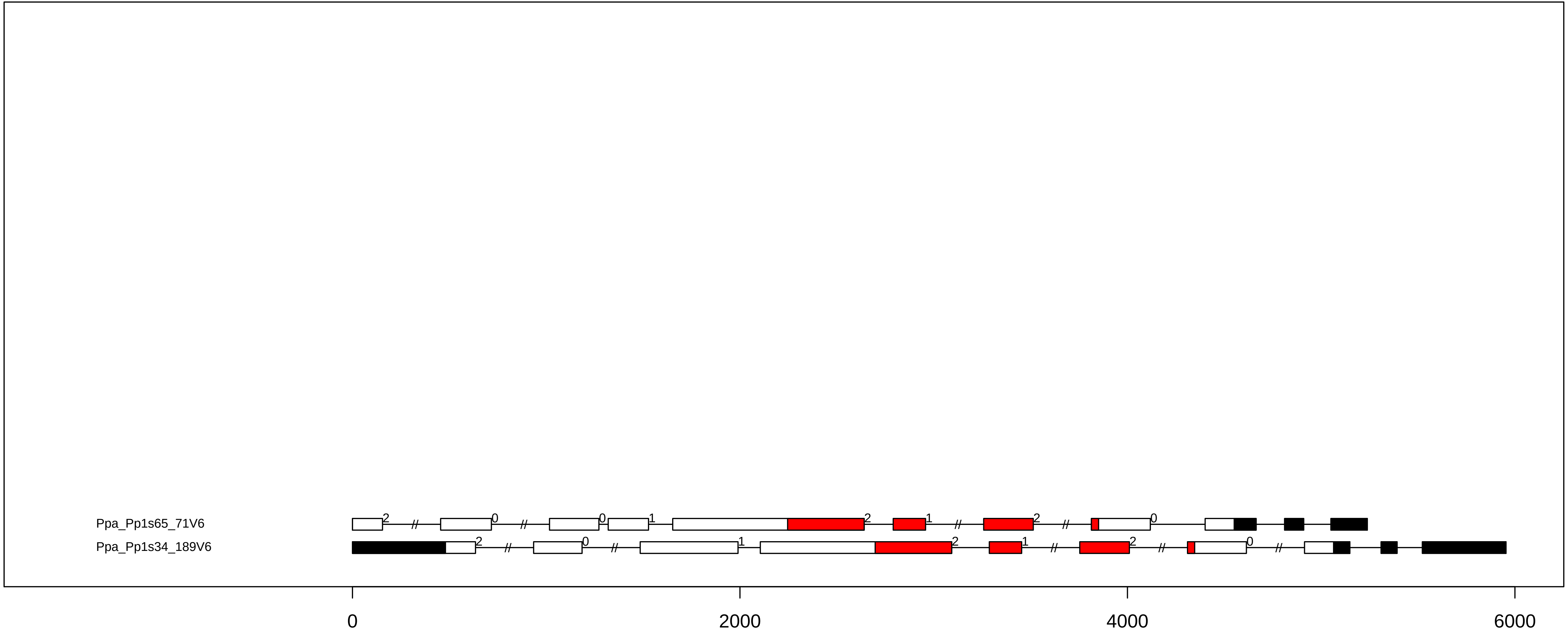

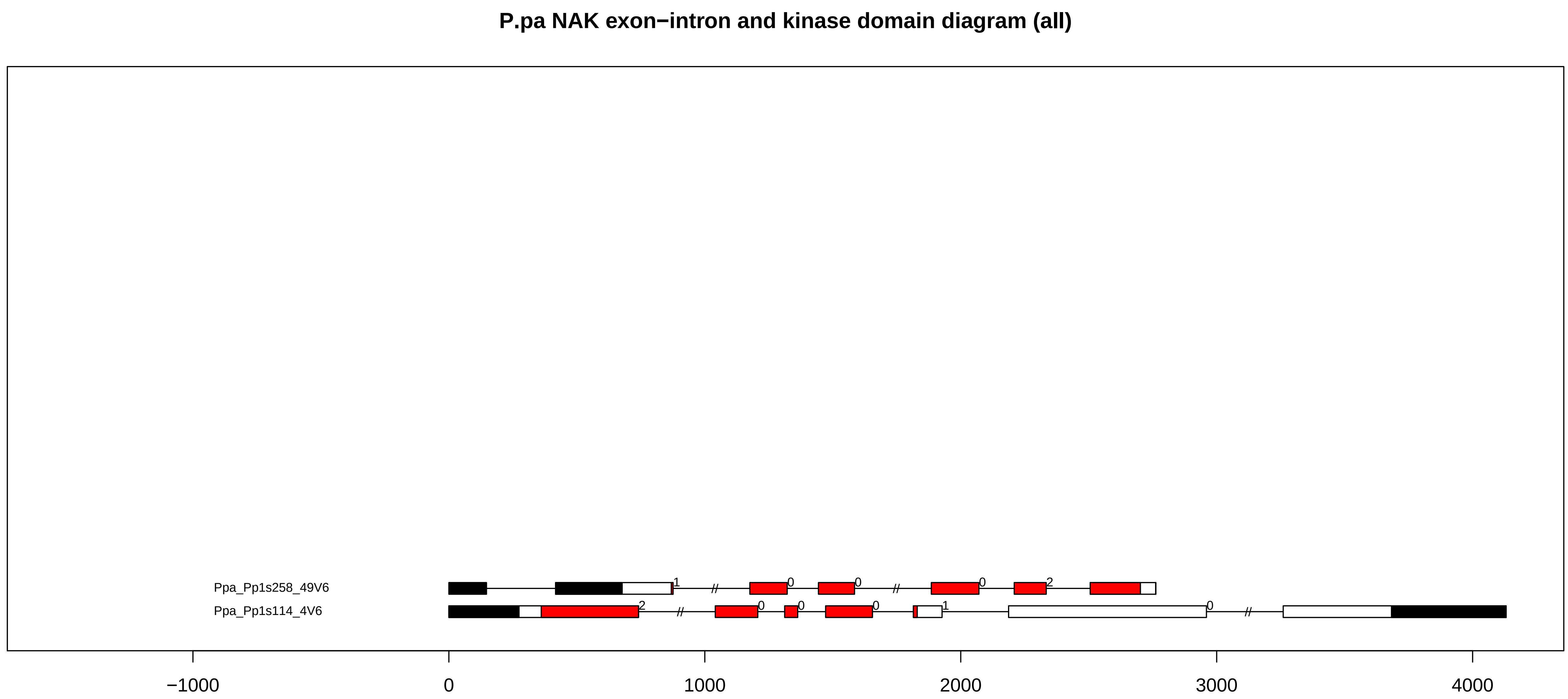

P.pa NAK exon−intron and kinase domain diagram (all)
Ppa_Pp1s258_49V6
Ppa_Pp1s114_4V6
1
0
0
0
2
2
0
0
0
1
0
−1000
0
1000
2000
3000
4000

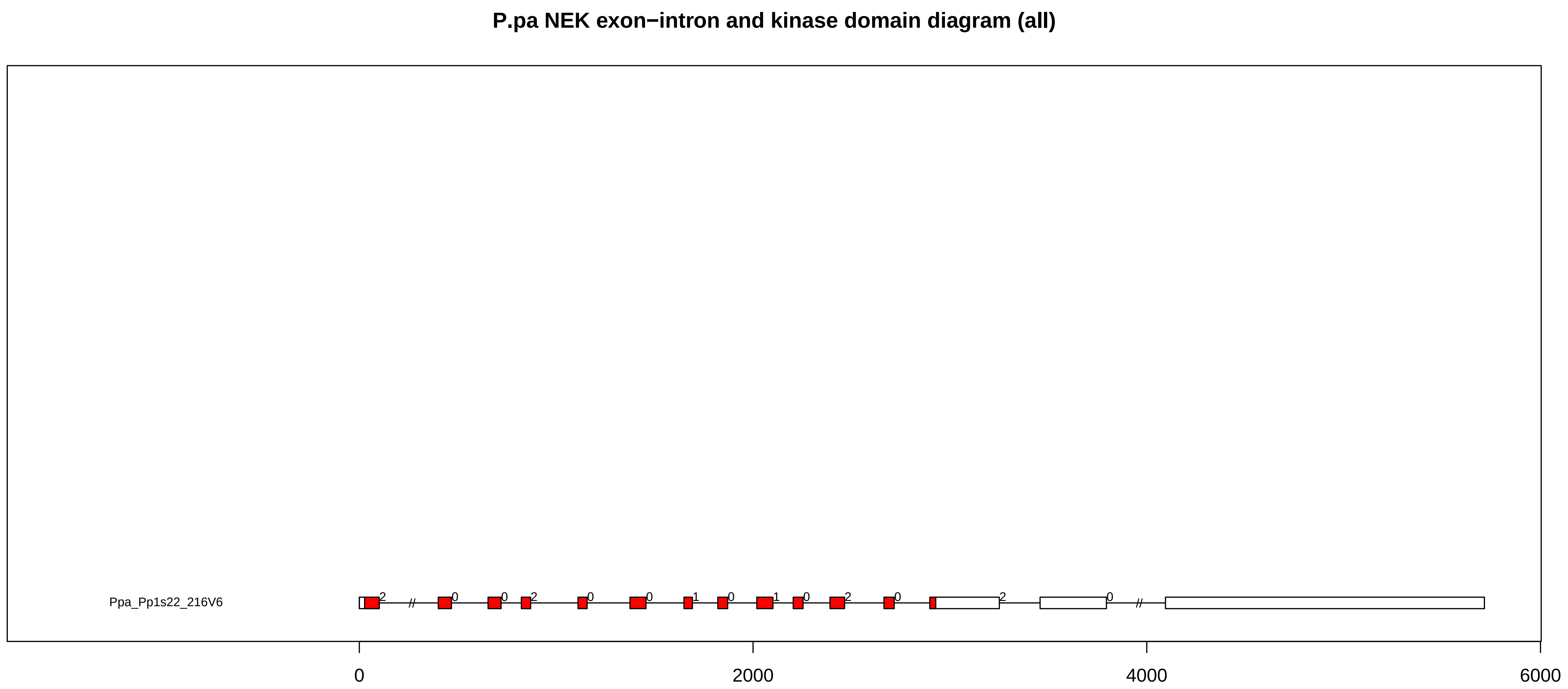
P.pa NEK exon−intron and kinase domain diagram (all)
Ppa_Pp1s22_216V6
2
0
0
2
0
0
1
0
1
0
2
0
2
0
0
2000
4000
6000

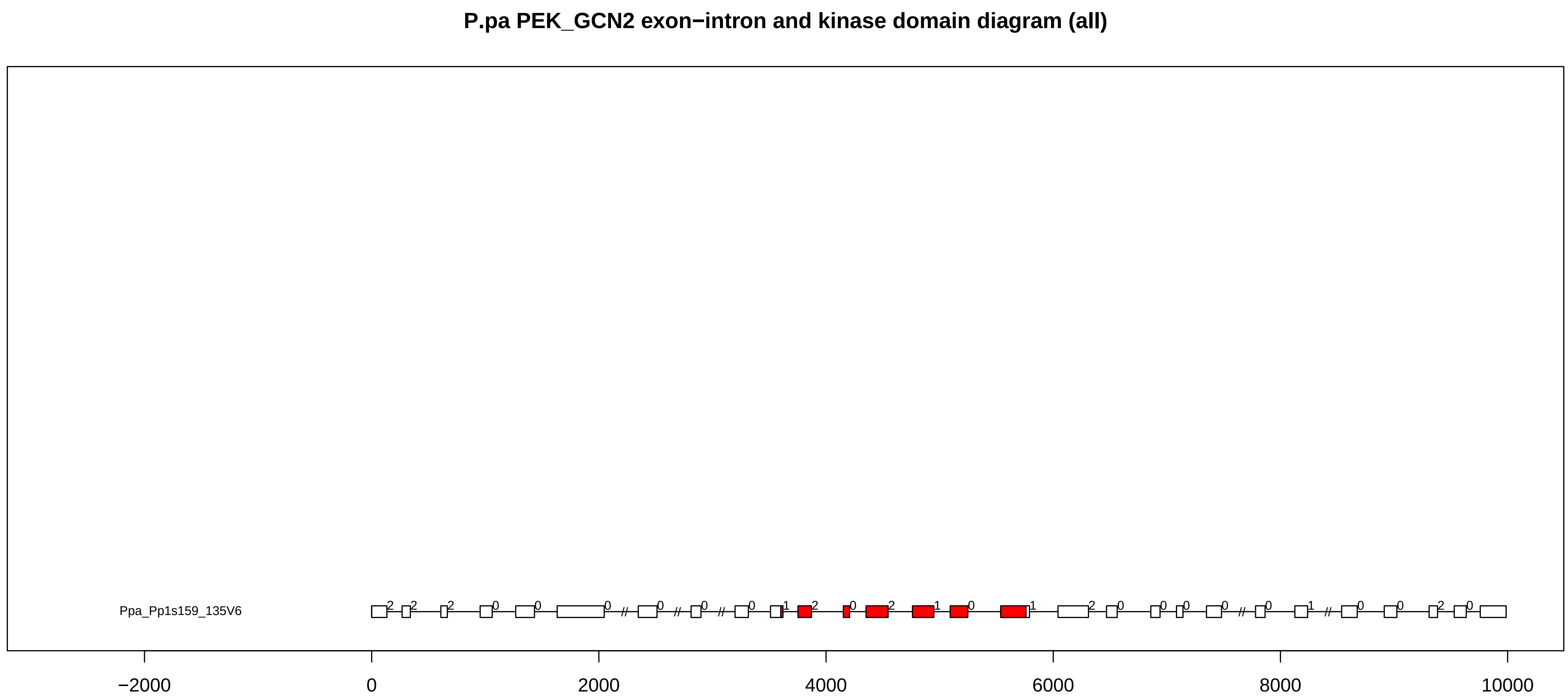
P.pa PEK_GCN2 exon−intron and kinase domain diagram (all)
Ppa_Pp1s159_135V6
−2000
0
2000
4000
6000
8000
10000

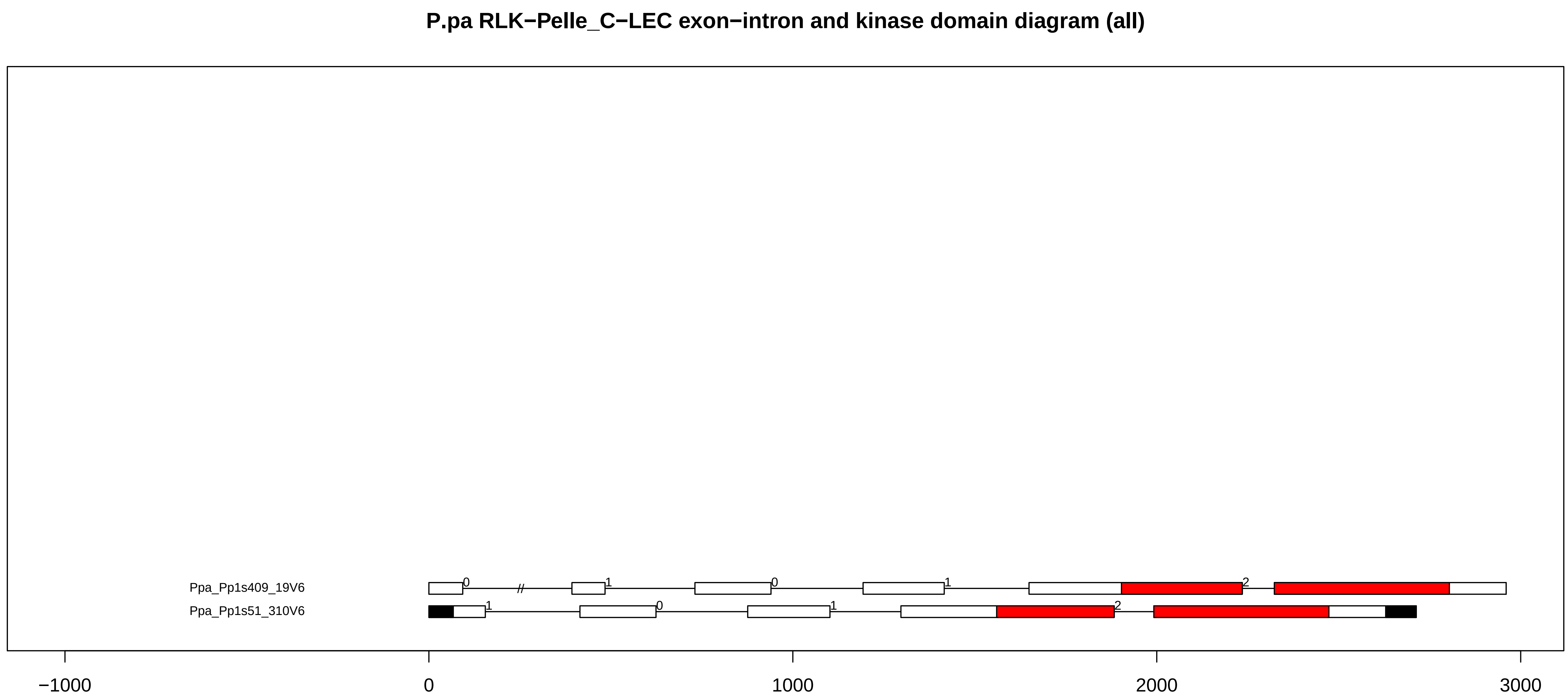

P.pa RLK−Pelle_C−LEC exon−intron and kinase domain diagram (all)
Ppa_Pp1s409_19V6
Ppa_Pp1s51_310V6
0
1
0
1
2
1
0
1
2
−1000
0
1000
2000
3000

P.pa RLK−Pelle_CR4L exon−intron and kinase domain diagram (all)

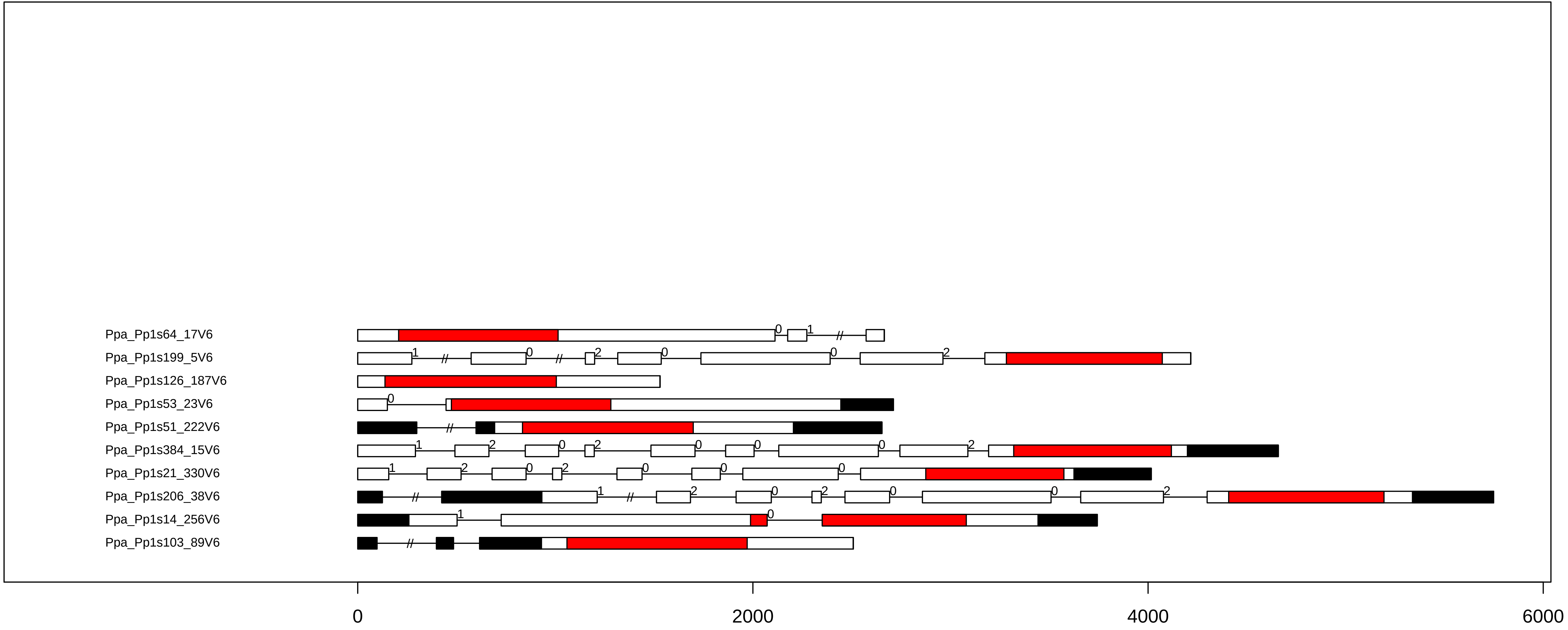

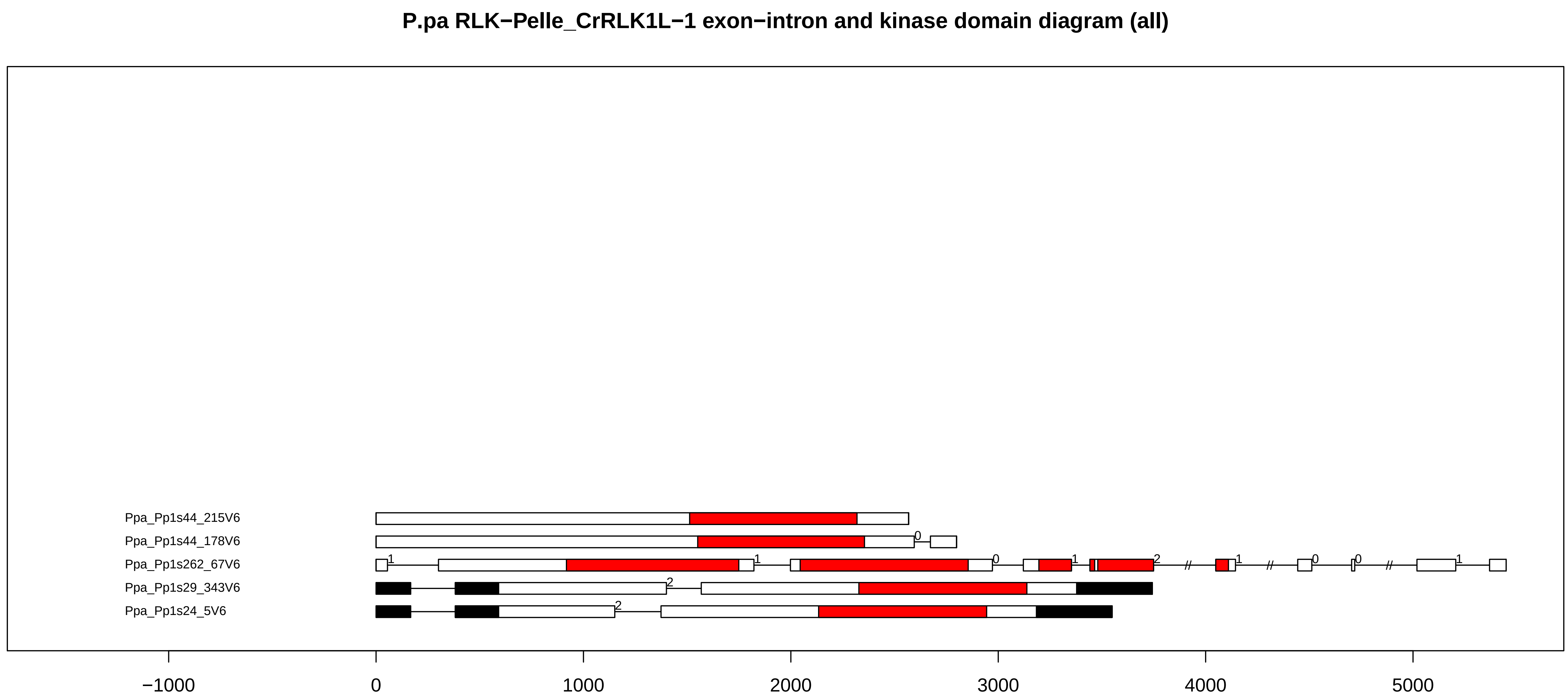
P.pa RLK−Pelle_CrRLK1L−1 exon−intron and kinase domain diagram (all)
Ppa_Pp1s44_215V6
Ppa_Pp1s44_178V6
Ppa_Pp1s262_67V6
Ppa_Pp1s29_343V6
Ppa_Pp1s24_5V6
−1000
0
1000
2000
3000
4000
5000

P.pa RLK−Pelle_DLSV exon−intron and kinase domain diagram (part 1)

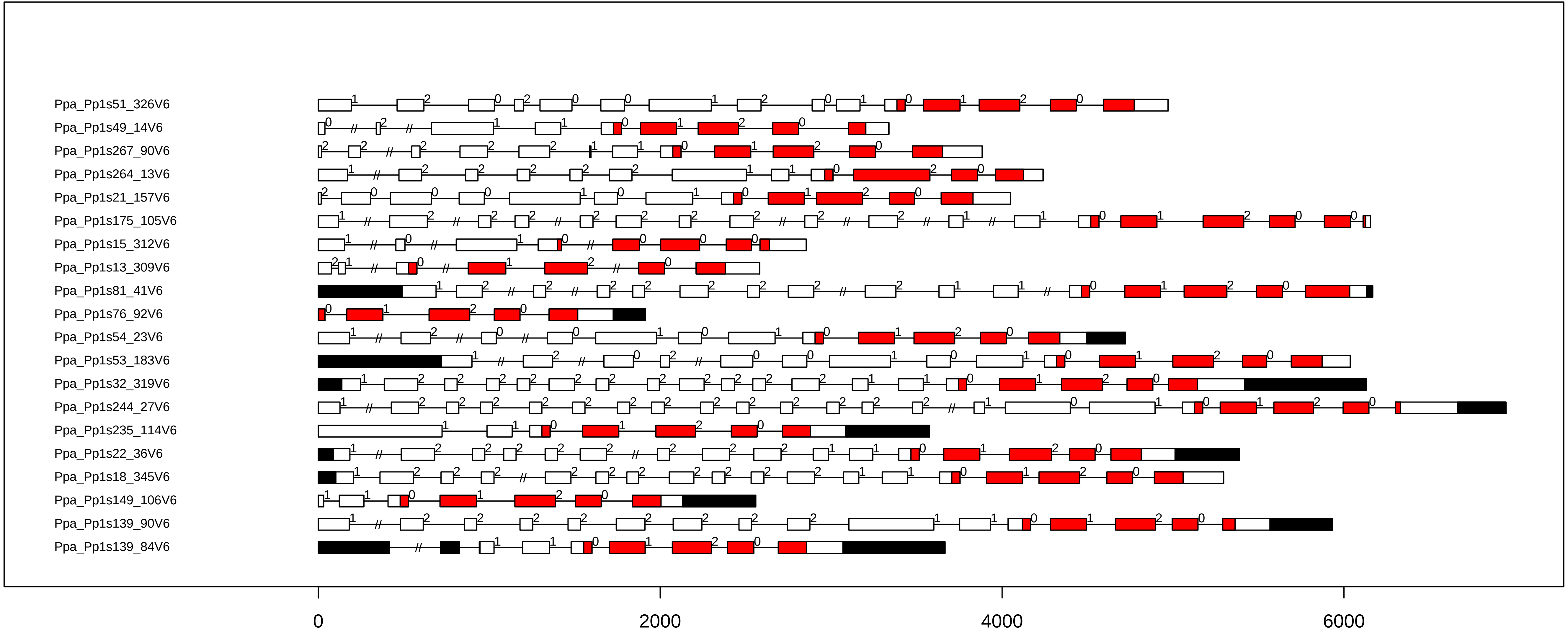

P.pa RLK−Pelle_DLSV exon−intron and kinase domain diagram (part 2)

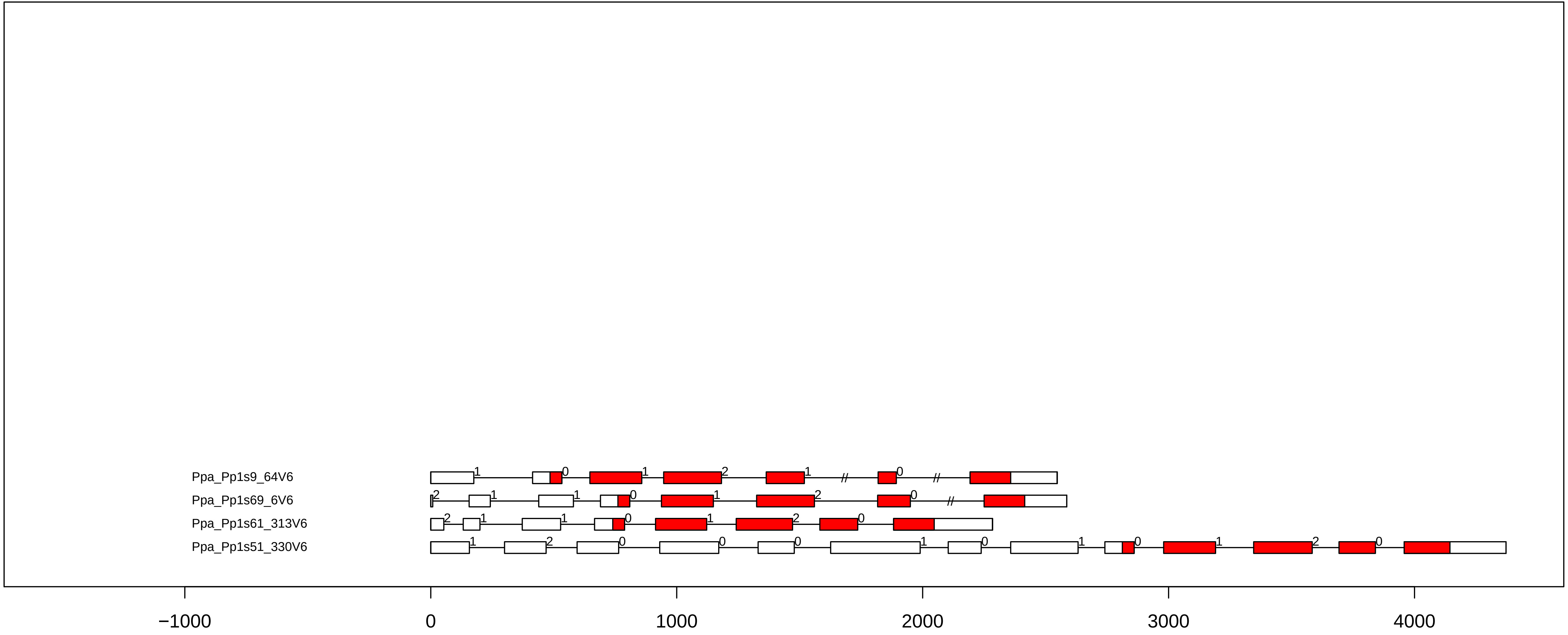

P.pa RLK−Pelle_DSLV exon−intron and kinase domain diagram (all)

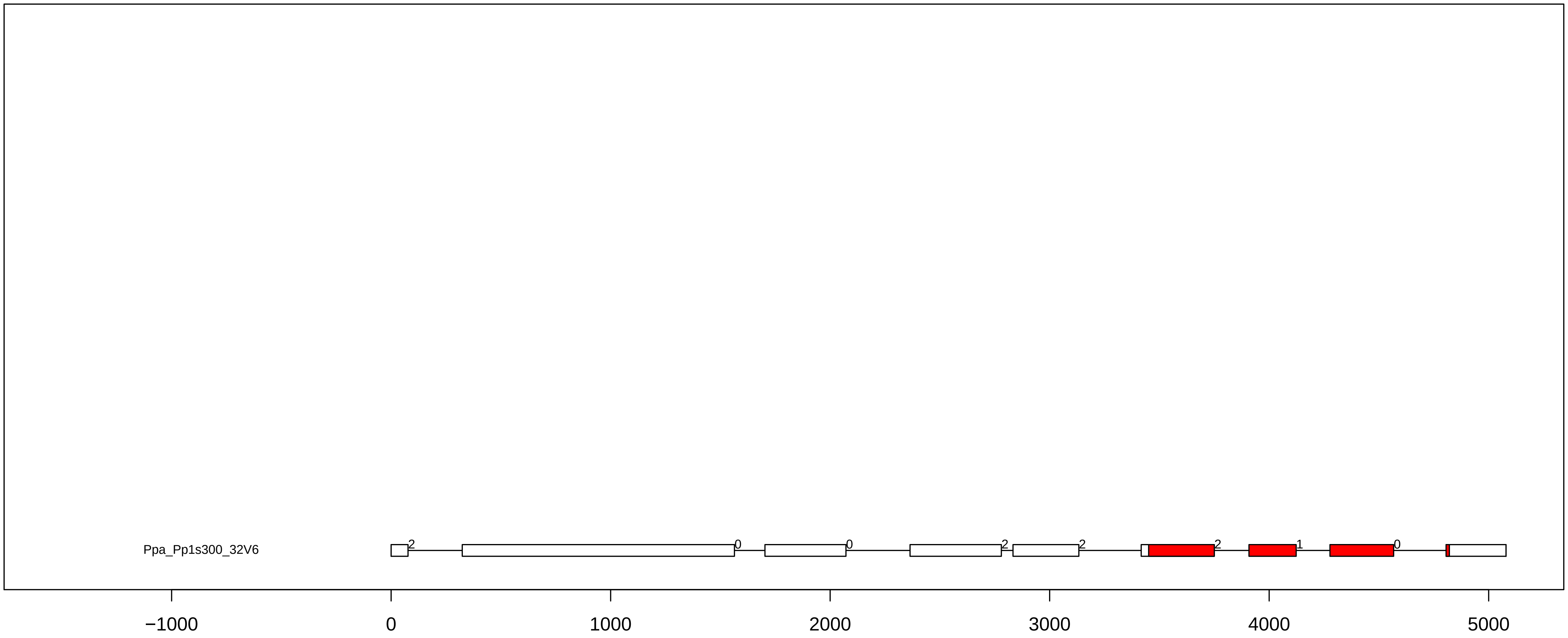

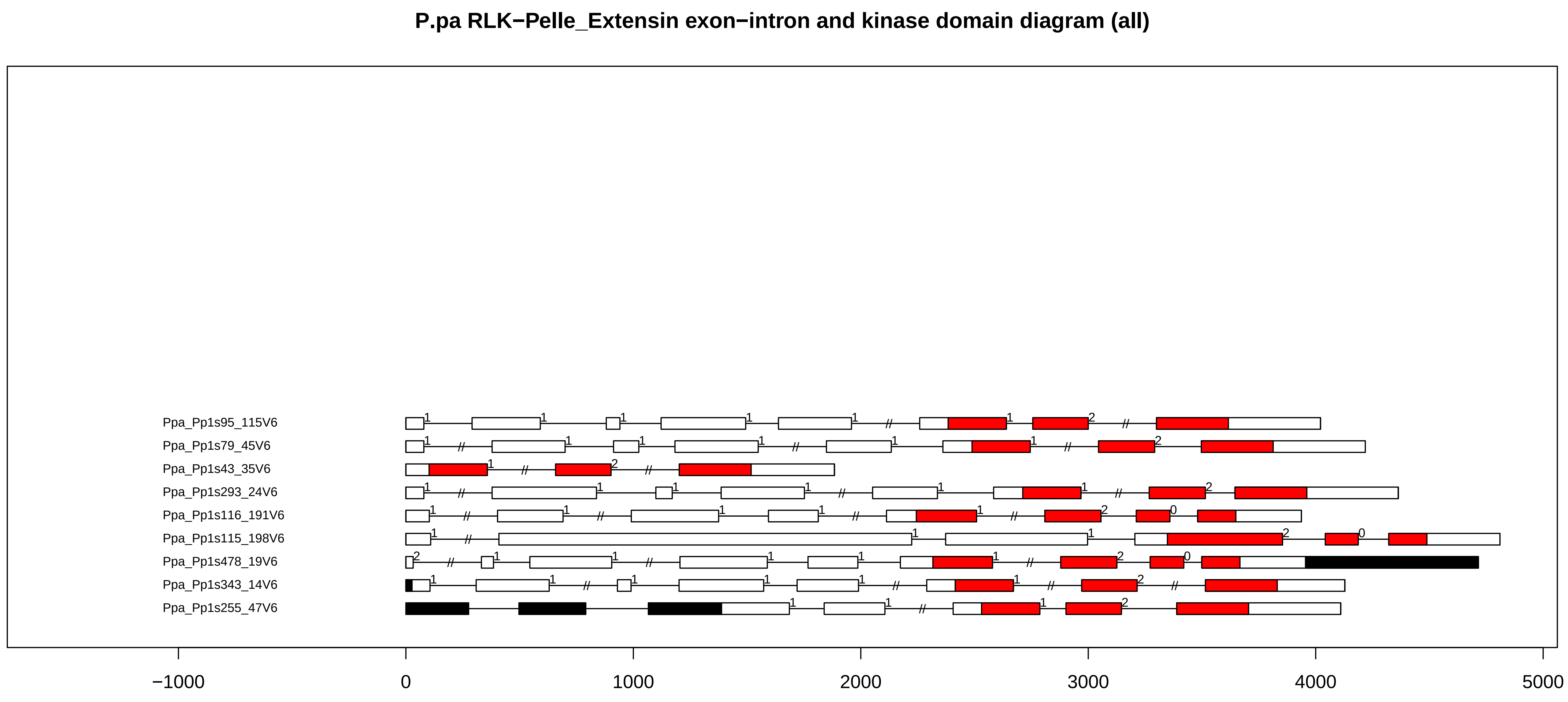
P.pa RLK−Pelle_Extensin exon−intron and kinase domain diagram (all)
Ppa_Pp1s95_115V6
Ppa_Pp1s79_45V6
Ppa_Pp1s43_35V6
Ppa_Pp1s293_24V6
Ppa_Pp1s116_191V6
Ppa_Pp1s115_198V6
Ppa_Pp1s478_19V6
Ppa_Pp1s343_14V6
Ppa_Pp1s255_47V6
−1000
0
1000
2000
3000
4000
5000

P.pa RLK−Pelle_L−LEC exon−intron and kinase domain diagram (all)

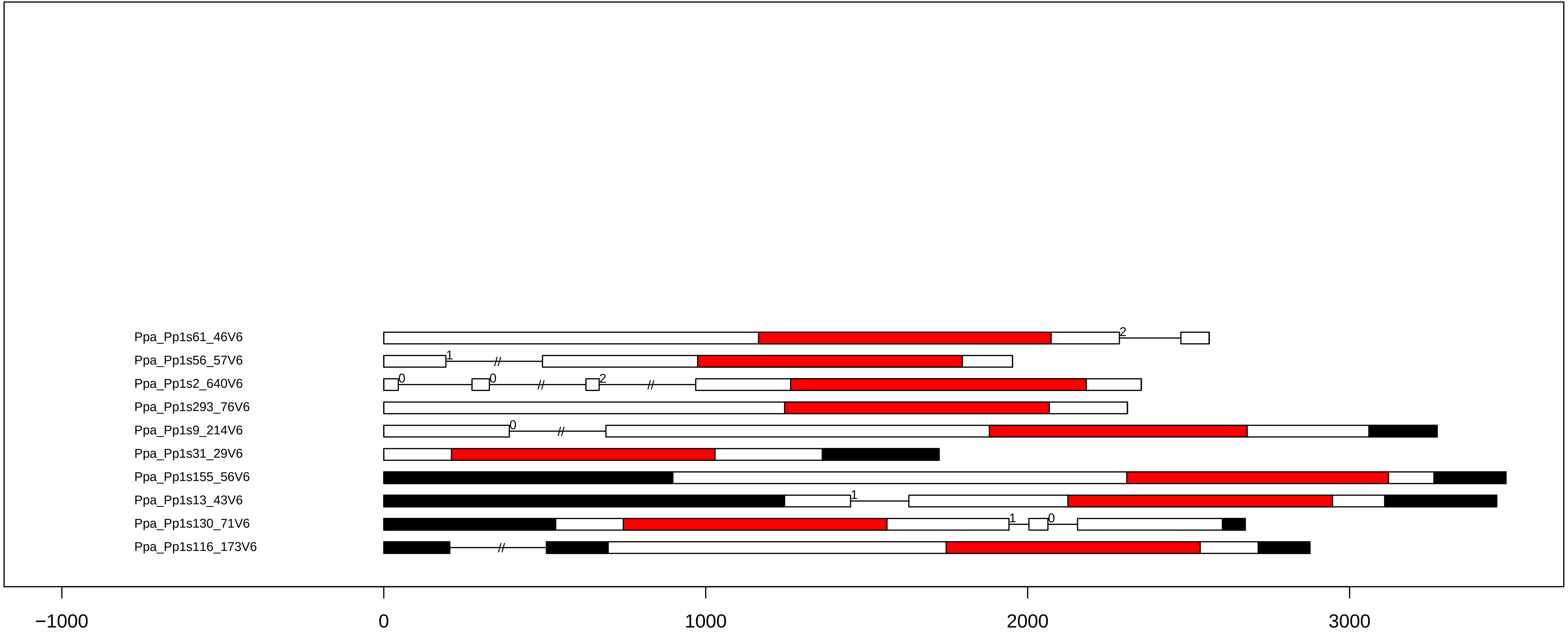

P.pa RLK−Pelle_LRR−I−1 exon−intron and kinase domain diagram (all)

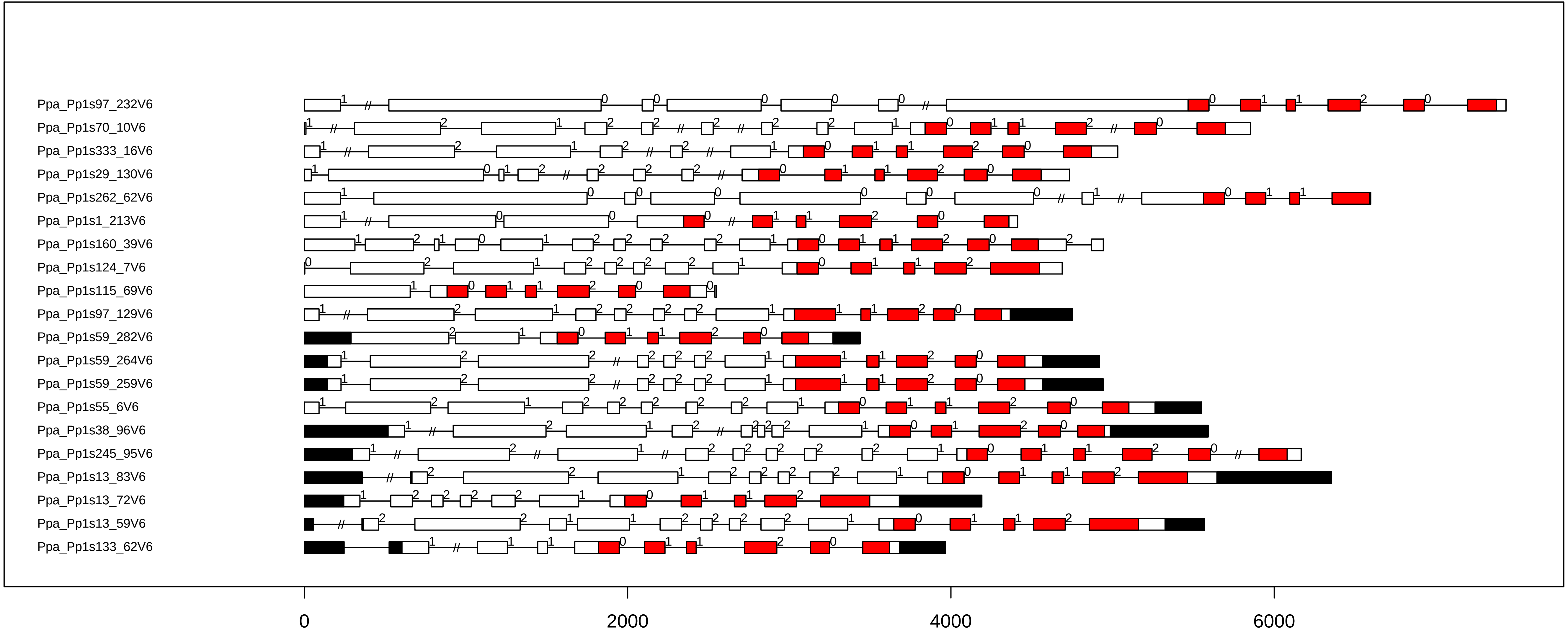

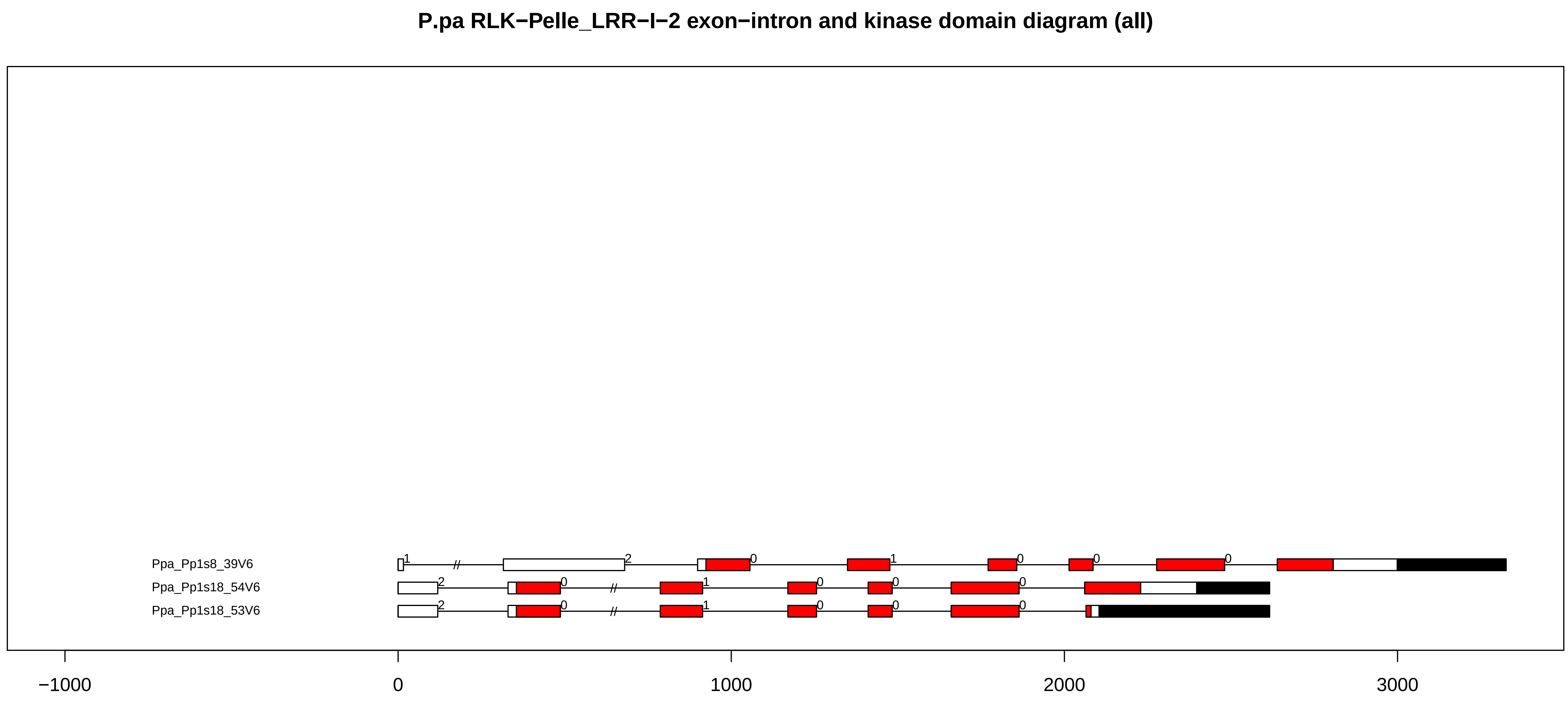

P.pa RLK−Pelle_LRR−I−2 exon−intron and kinase domain diagram (all)
Ppa_Pp1s8_39V6
Ppa_Pp1s18_54V6
Ppa_Pp1s18_53V6
1
2
0
1
0
0
0
2
0
1
0
0
0
2
0
1
0
0
0
−1000
0
1000
2000
3000

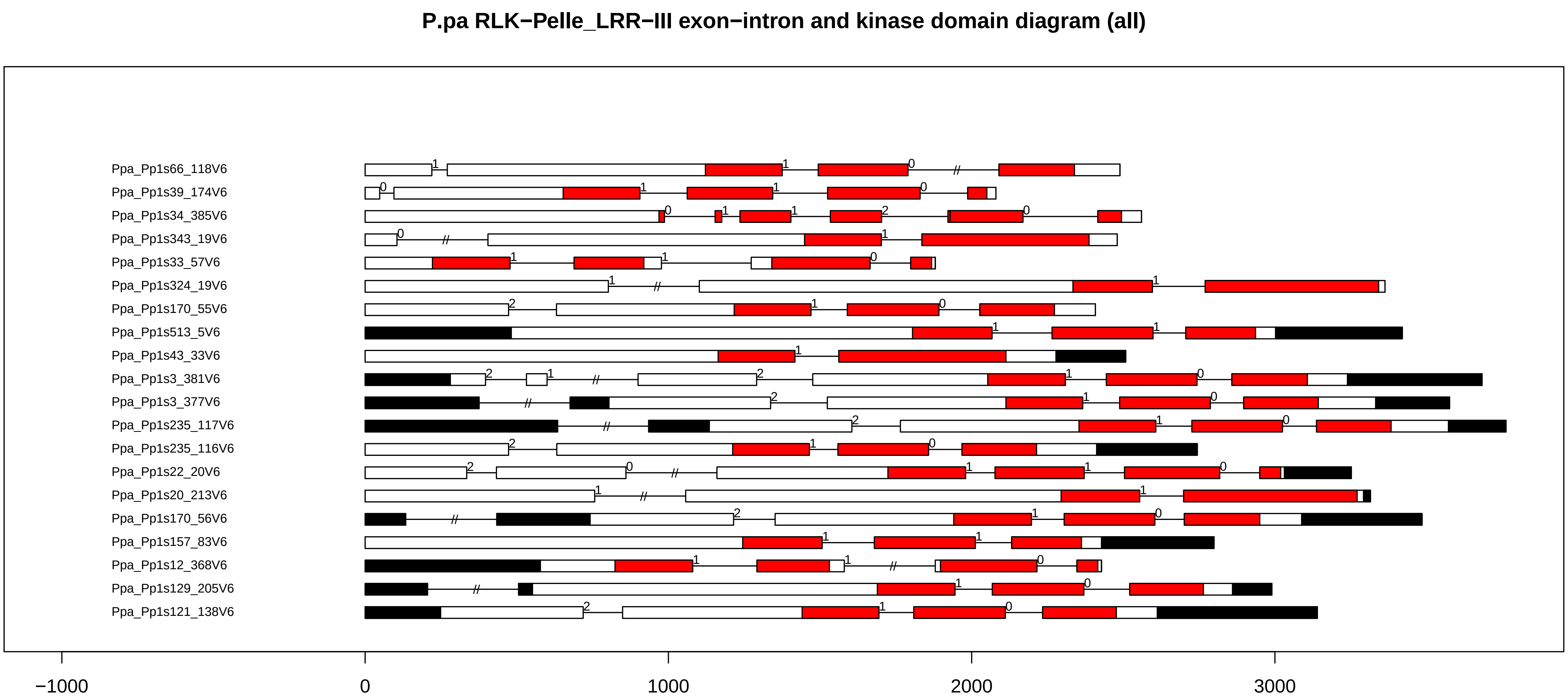
P.pa RLK−Pelle_LRR−III exon−intron and kinase domain diagram (all)
Ppa_Pp1s66_118V6
Ppa_Pp1s39_174V6
Ppa_Pp1s34_385V6
Ppa_Pp1s343_19V6
Ppa_Pp1s33_57V6
Ppa_Pp1s324_19V6
Ppa_Pp1s170_55V6
Ppa_Pp1s513_5V6
Ppa_Pp1s43_33V6
Ppa_Pp1s3_381V6
Ppa_Pp1s3_377V6
Ppa_Pp1s235_117V6
Ppa_Pp1s235_116V6
Ppa_Pp1s22_20V6
Ppa_Pp1s20_213V6
Ppa_Pp1s170_56V6
Ppa_Pp1s157_83V6
Ppa_Pp1s12_368V6
Ppa_Pp1s129_205V6
Ppa_Pp1s121_138V6
−1000
0
1000
2000
3000

P.pa RLK−Pelle_LRR−II exon−intron and kinase domain diagram (all)

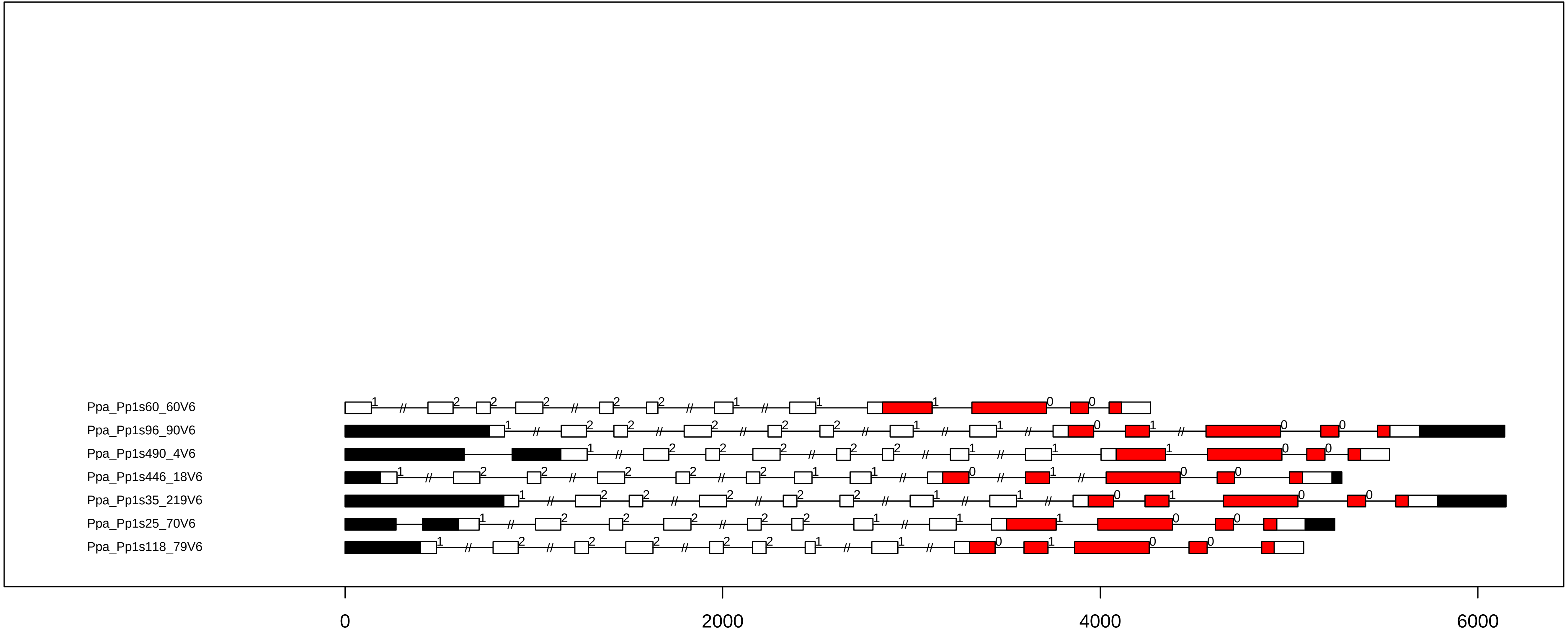

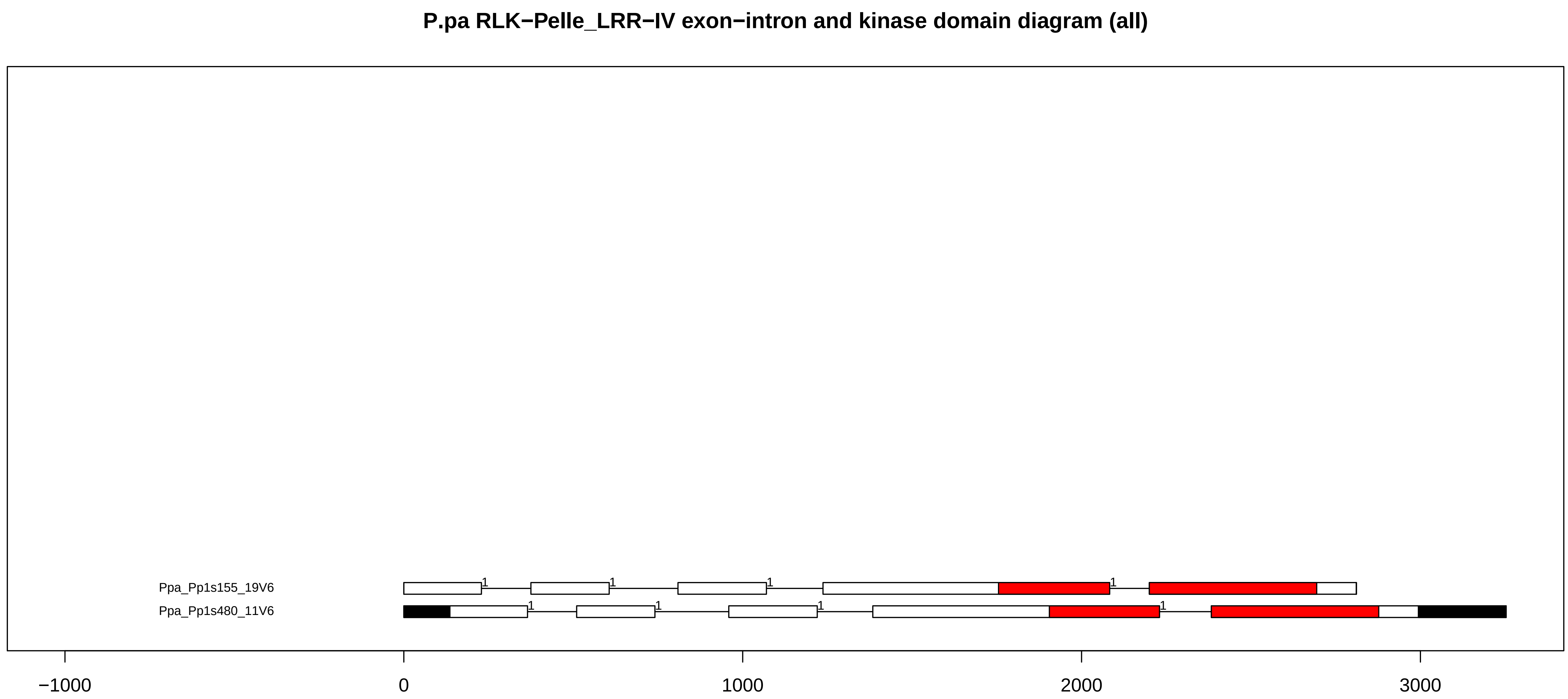
P.pa RLK−Pelle_LRR−IV exon−intron and kinase domain diagram (all)
Ppa_Pp1s155_19V6
Ppa_Pp1s480_11V6
1
1
1
1
1
1
1
1
−1000
0
1000
2000
3000

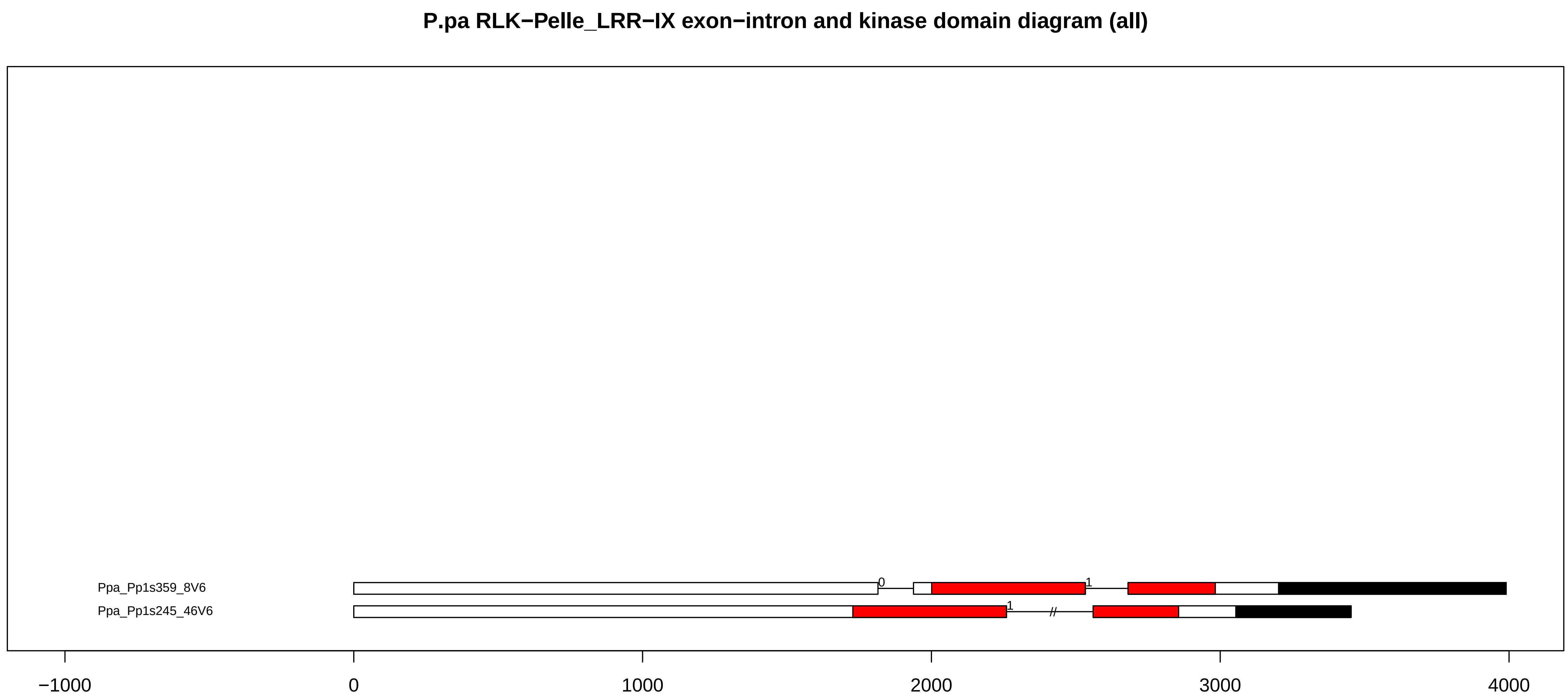
P.pa RLK−Pelle_LRR−IX exon−intron and kinase domain diagram (all)
Ppa_Pp1s359_8V6
Ppa_Pp1s245_46V6
0
1
1
//
−1000
0
1000
2000
3000
4000

P.pa RLK−Pelle_LRR−VI−1 exon−intron and kinase domain diagram (all)

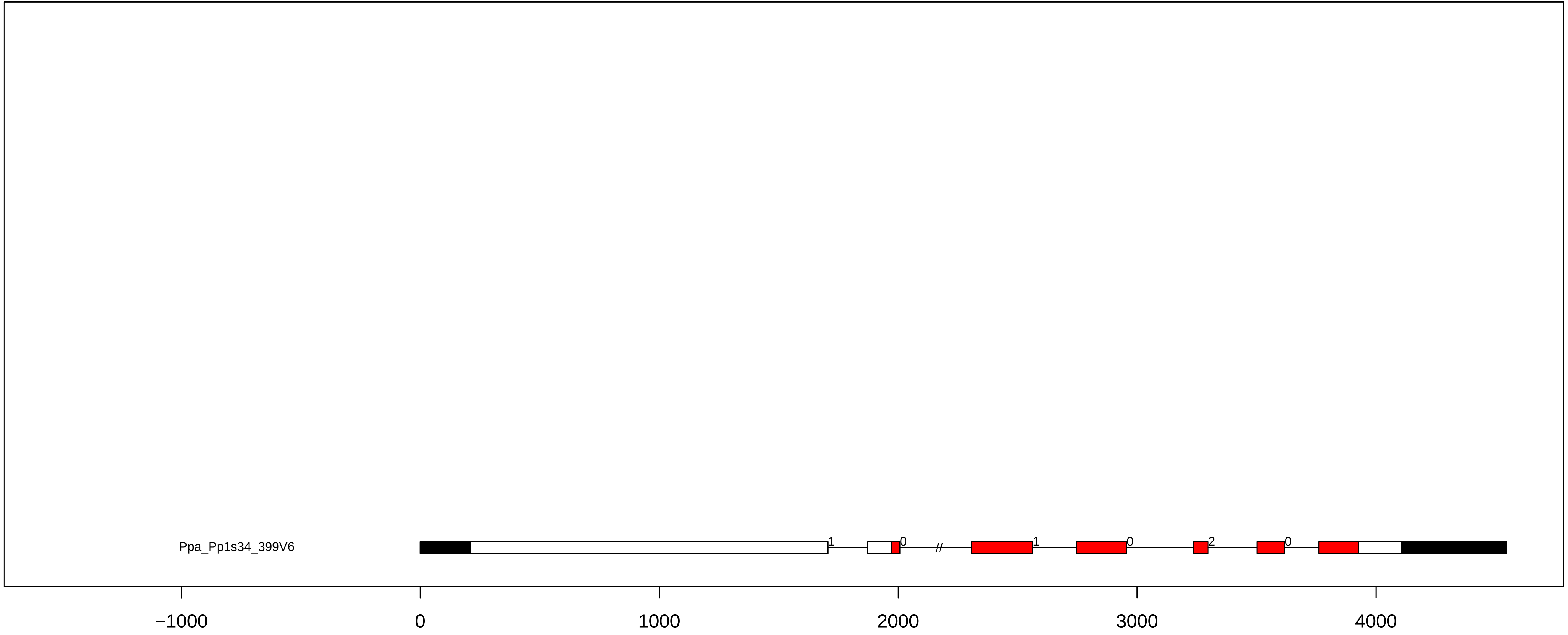

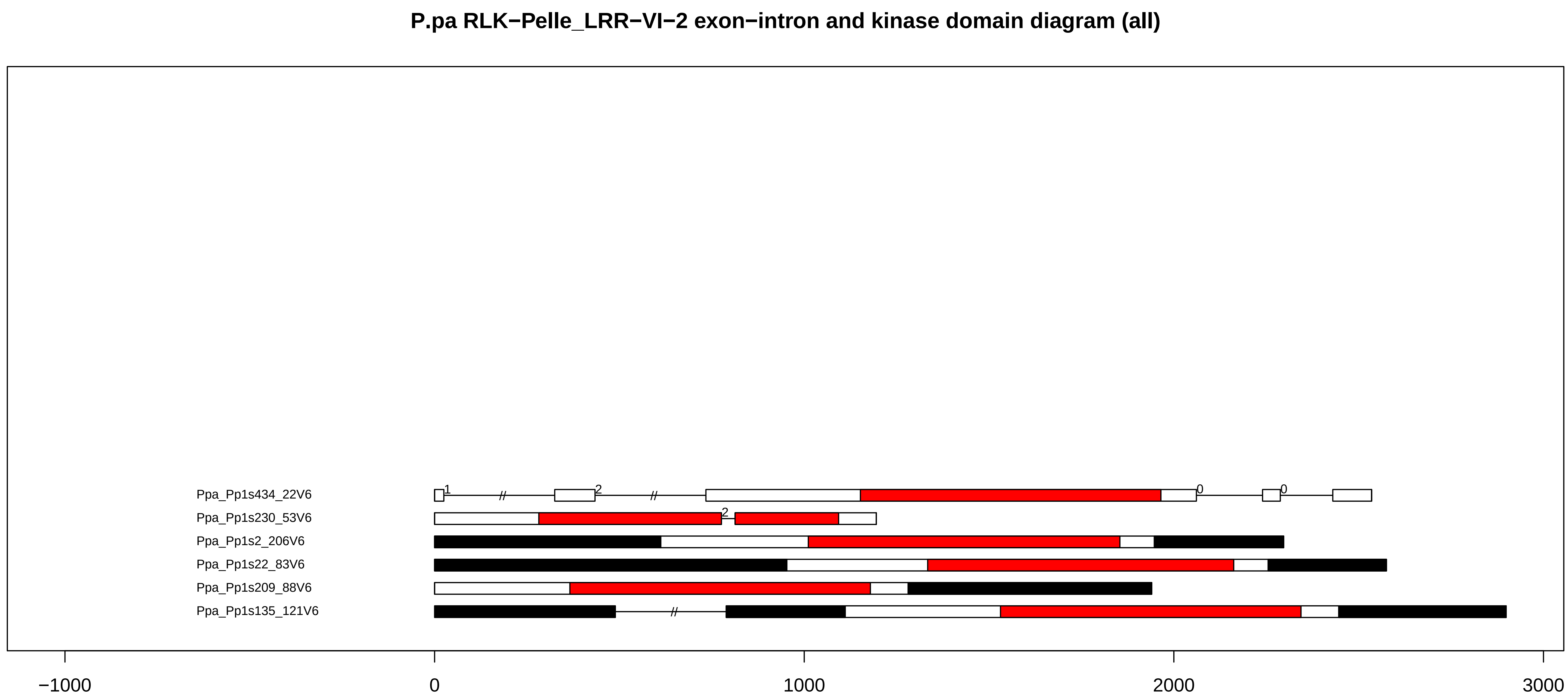
P.pa RLK−Pelle_LRR−VI−2 exon−intron and kinase domain diagram (all)
Ppa_Pp1s434_22V6
Ppa_Pp1s230_53V6
Ppa_Pp1s2_206V6
Ppa_Pp1s22_83V6
Ppa_Pp1s209_88V6
Ppa_Pp1s135_121V6
1
2
0
0
2
−1000
0
1000
2000
3000

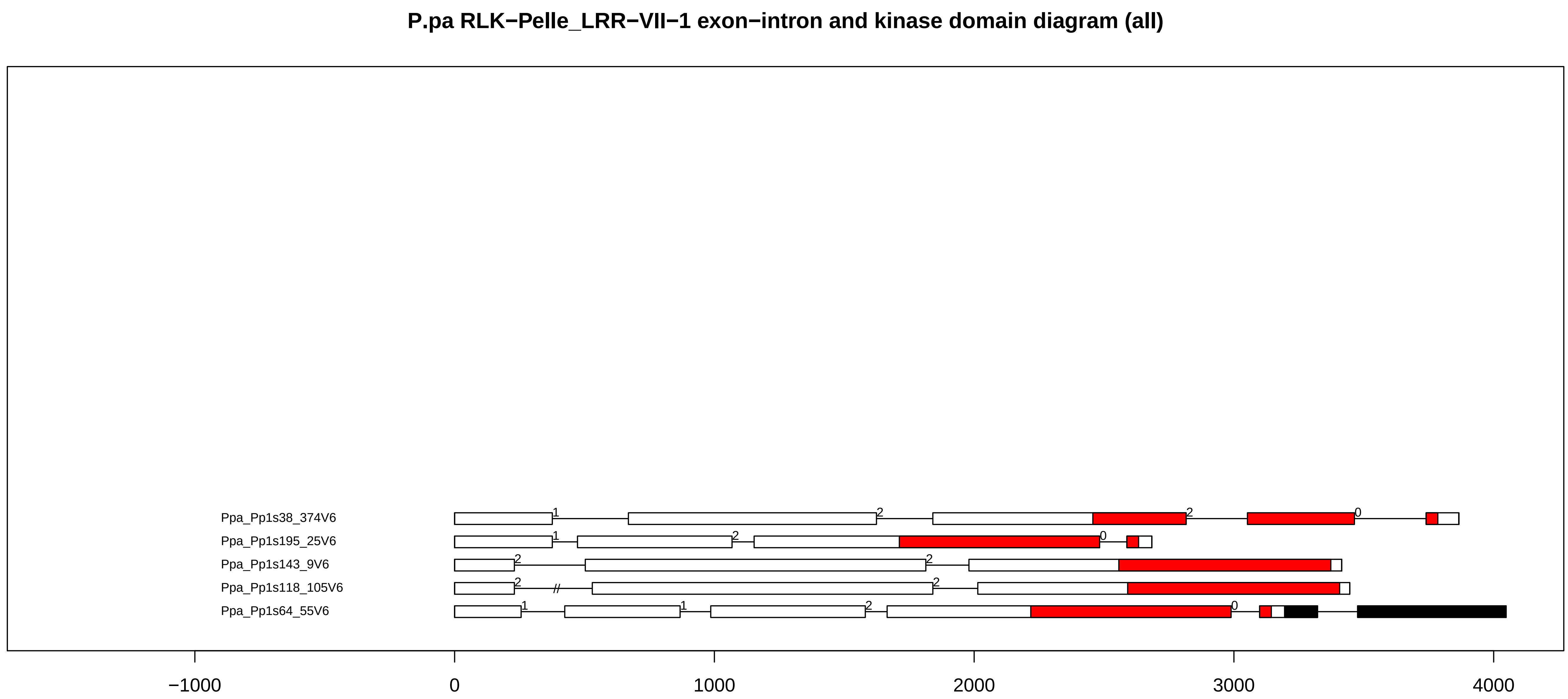

P.pa RLK−Pelle_LRR−VII−1 exon−intron and kinase domain diagram (all)
Ppa_Pp1s38_374V6
Ppa_Pp1s195_25V6
Ppa_Pp1s143_9V6
Ppa_Pp1s118_105V6
Ppa_Pp1s64_55V6
−1000
0
1000
2000
3000
4000

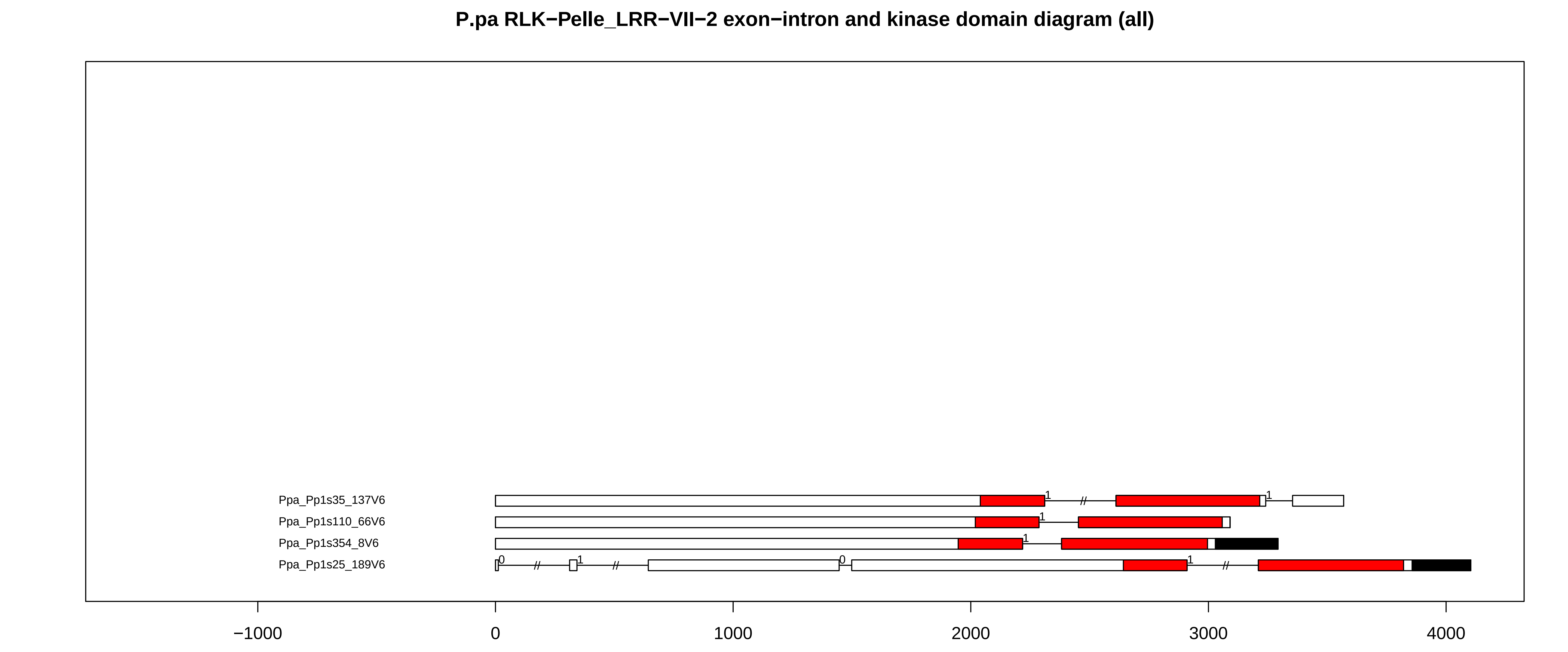
P.pa RLK−Pelle_LRR−VII−2 exon−intron and kinase domain diagram (all)
Ppa_Pp1s35_137V6
Ppa_Pp1s110_66V6
Ppa_Pp1s354_8V6
Ppa_Pp1s25_189V6
1
1
1
1
0
1
0
1
−1000
0
1000
2000
3000
4000

P.pa RLK−Pelle_LRR−VII−3 exon−intron and kinase domain diagram (all)

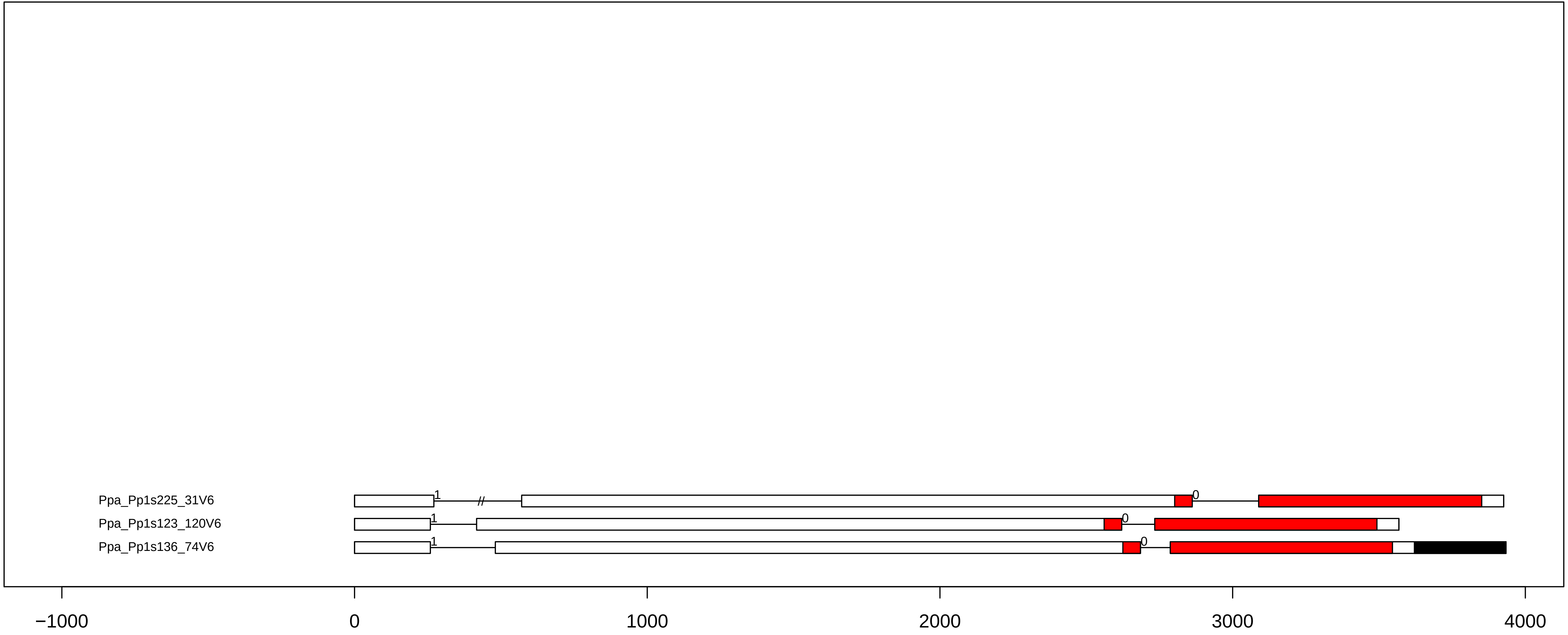

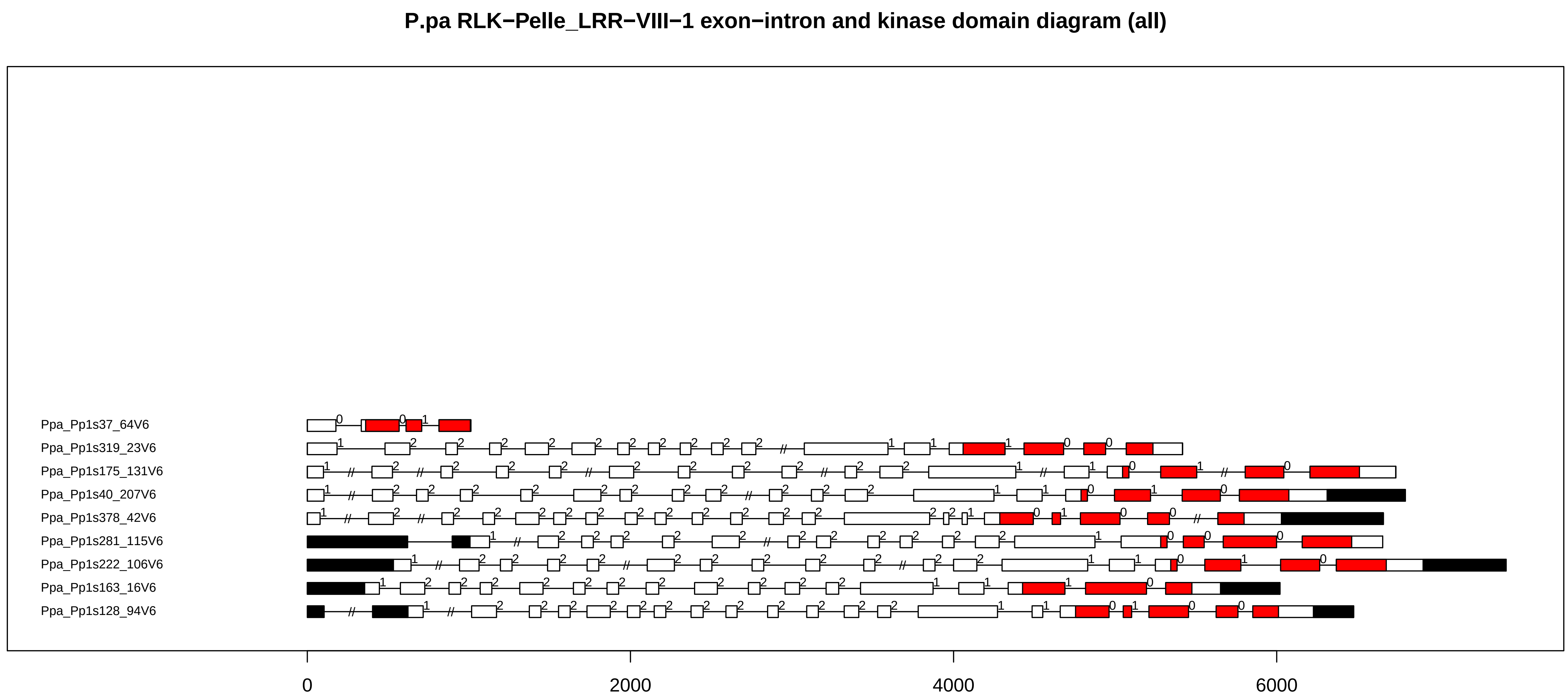
P.pa RLK−Pelle_LRR−VIII−1 exon−intron and kinase domain diagram (all)
Ppa_Pp1s37_64V6
Ppa_Pp1s319_23V6
Ppa_Pp1s175_131V6
Ppa_Pp1s40_207V6
Ppa_Pp1s378_42V6
Ppa_Pp1s281_115V6
Ppa_Pp1s222_106V6
Ppa_Pp1s163_16V6
Ppa_Pp1s128_94V6
0
2000
4000
6000

P.pa RLK−Pelle_LRR−V exon−intron and kinase domain diagram (all)

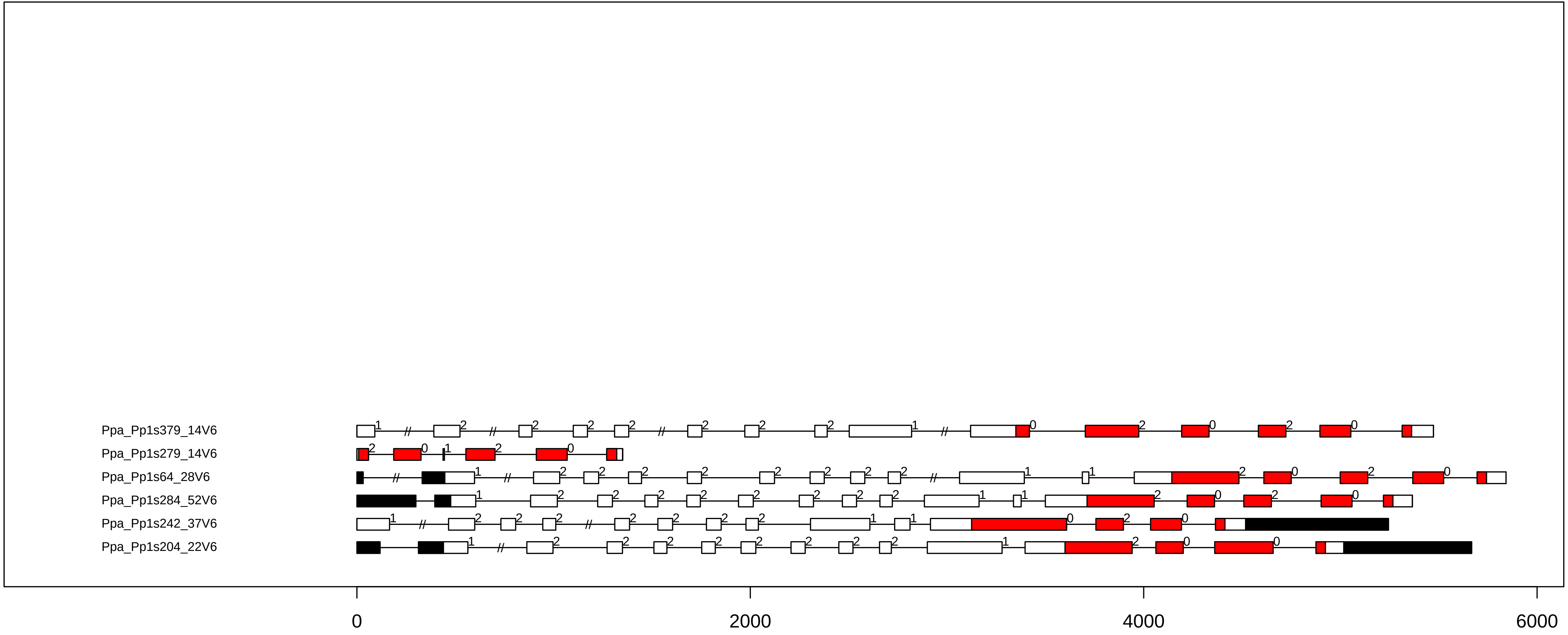

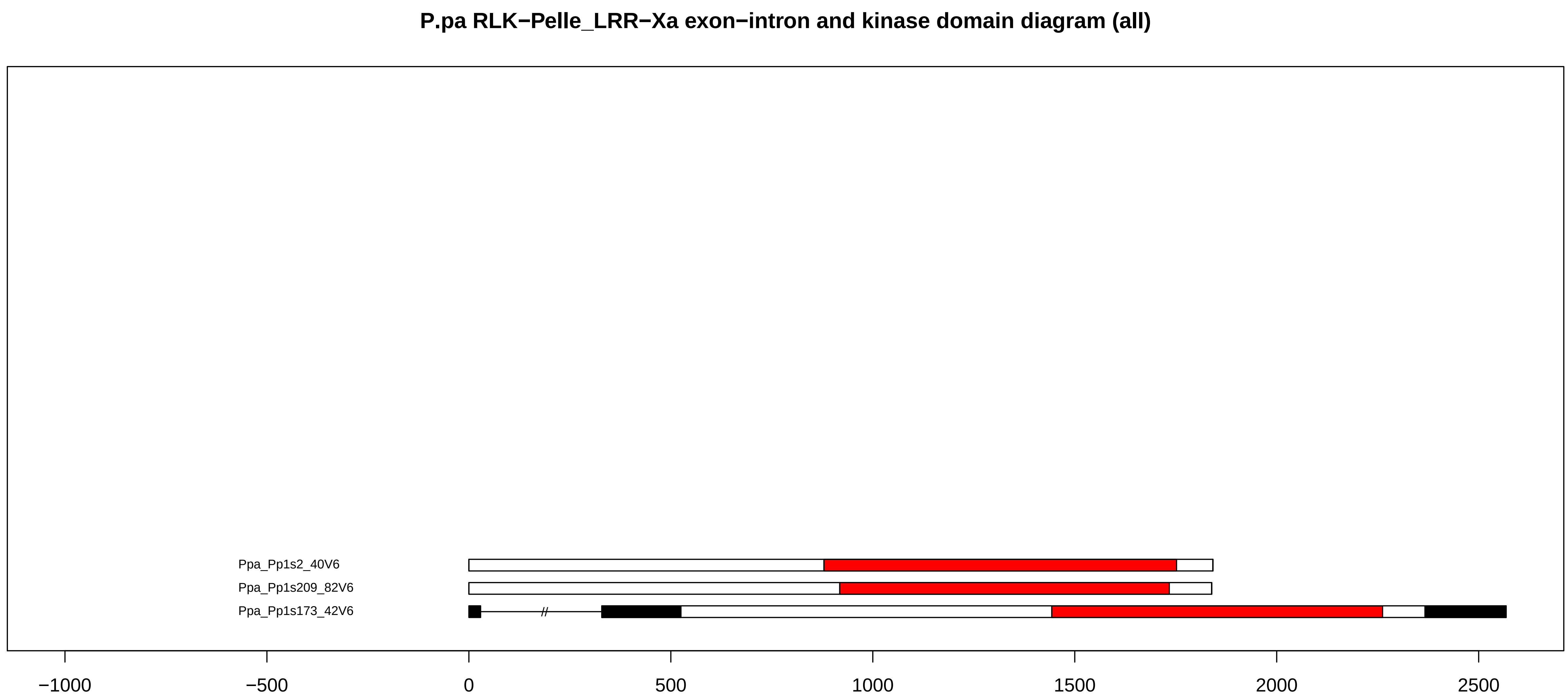
P.pa RLK−Pelle_LRR−Xa exon−intron and kinase domain diagram (all)
Ppa_Pp1s2_40V6
Ppa_Pp1s209_82V6
Ppa_Pp1s173_42V6
−1000
−500
0
500
1000
1500
2000
2500

P.pa RLK−Pelle_LRR−Xb−1 exon−intron and kinase domain diagram (all)

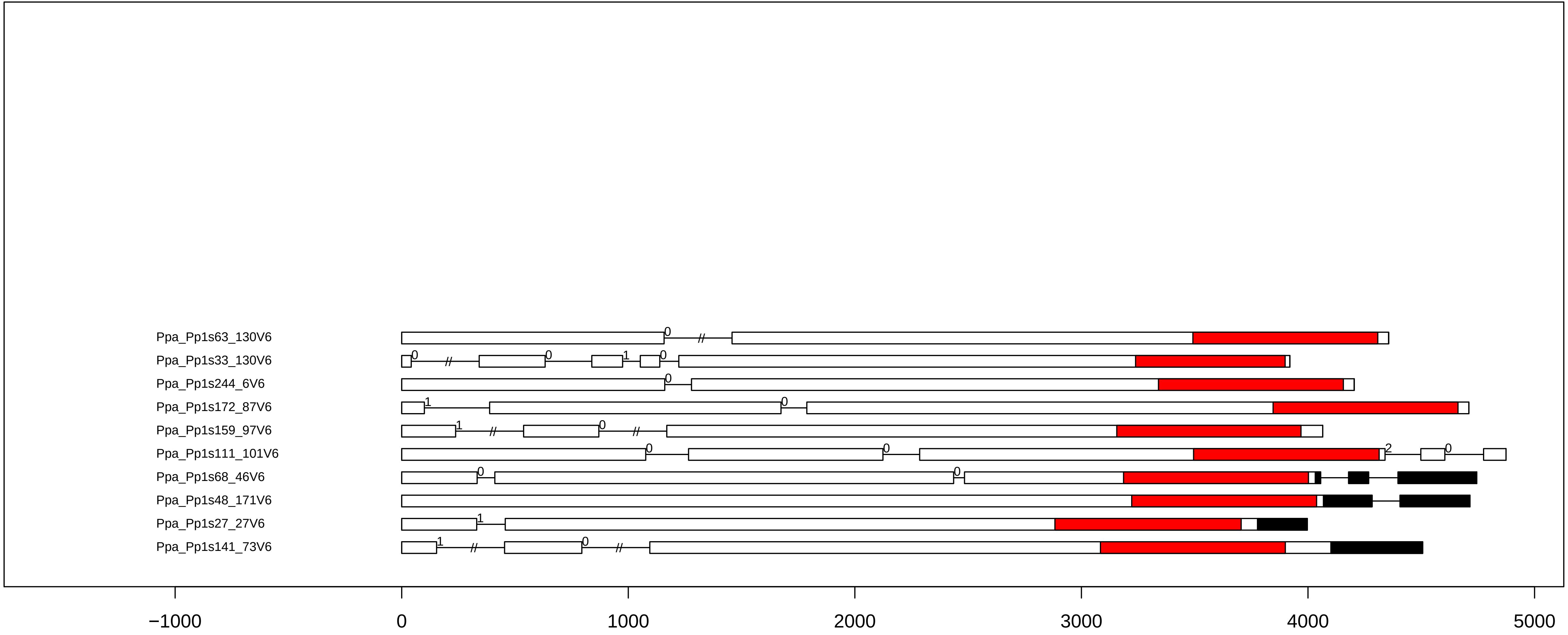

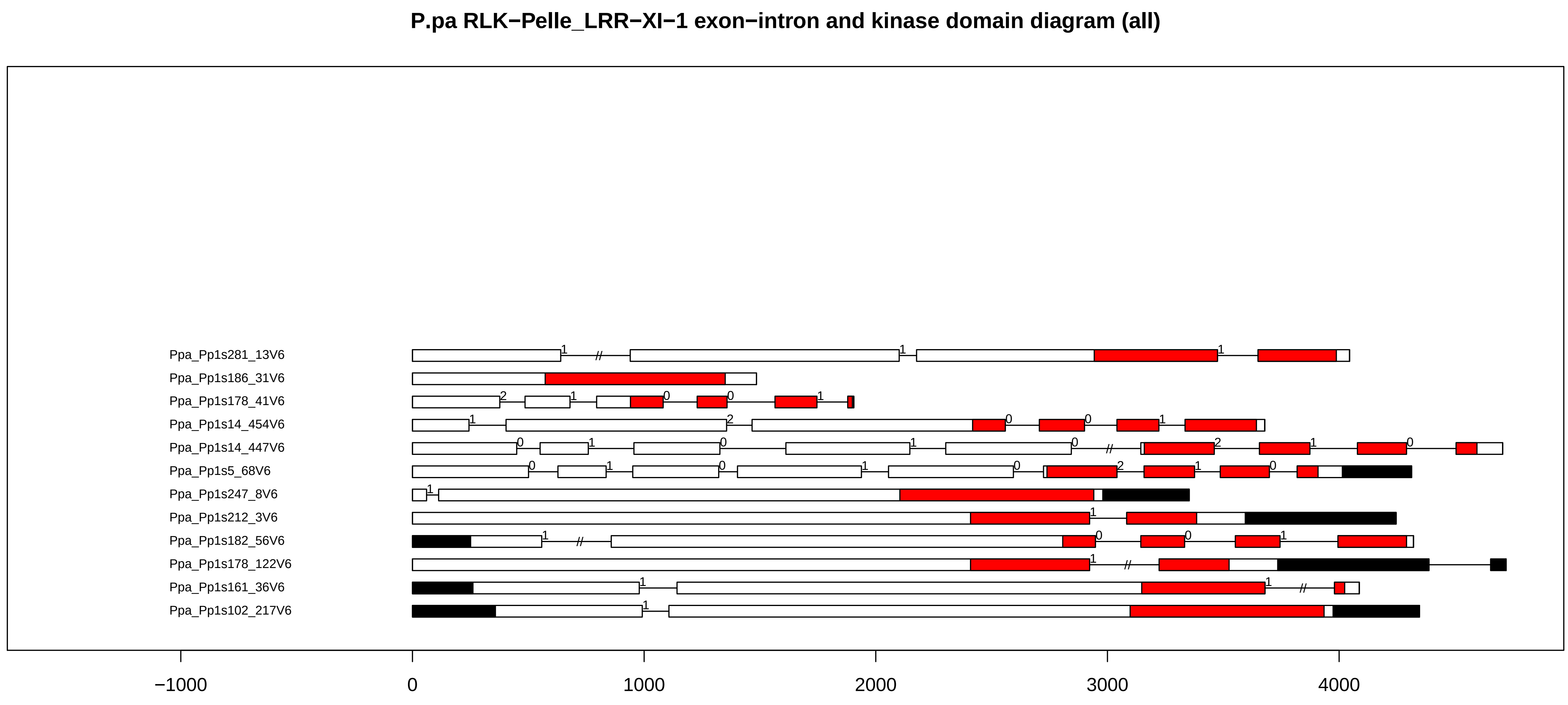
P.pa RLK−Pelle_LRR−XI−1 exon−intron and kinase domain diagram (all)
Ppa_Pp1s281_13V6
Ppa_Pp1s186_31V6
Ppa_Pp1s178_41V6
Ppa_Pp1s14_454V6
Ppa_Pp1s14_447V6
Ppa_Pp1s5_68V6
Ppa_Pp1s247_8V6
Ppa_Pp1s212_3V6
Ppa_Pp1s182_56V6
Ppa_Pp1s178_122V6
Ppa_Pp1s161_36V6
Ppa_Pp1s102_217V6
−1000
0
1000
2000
3000
4000

P.pa RLK−Pelle_LRR−XI−2 exon−intron and kinase domain diagram (all)

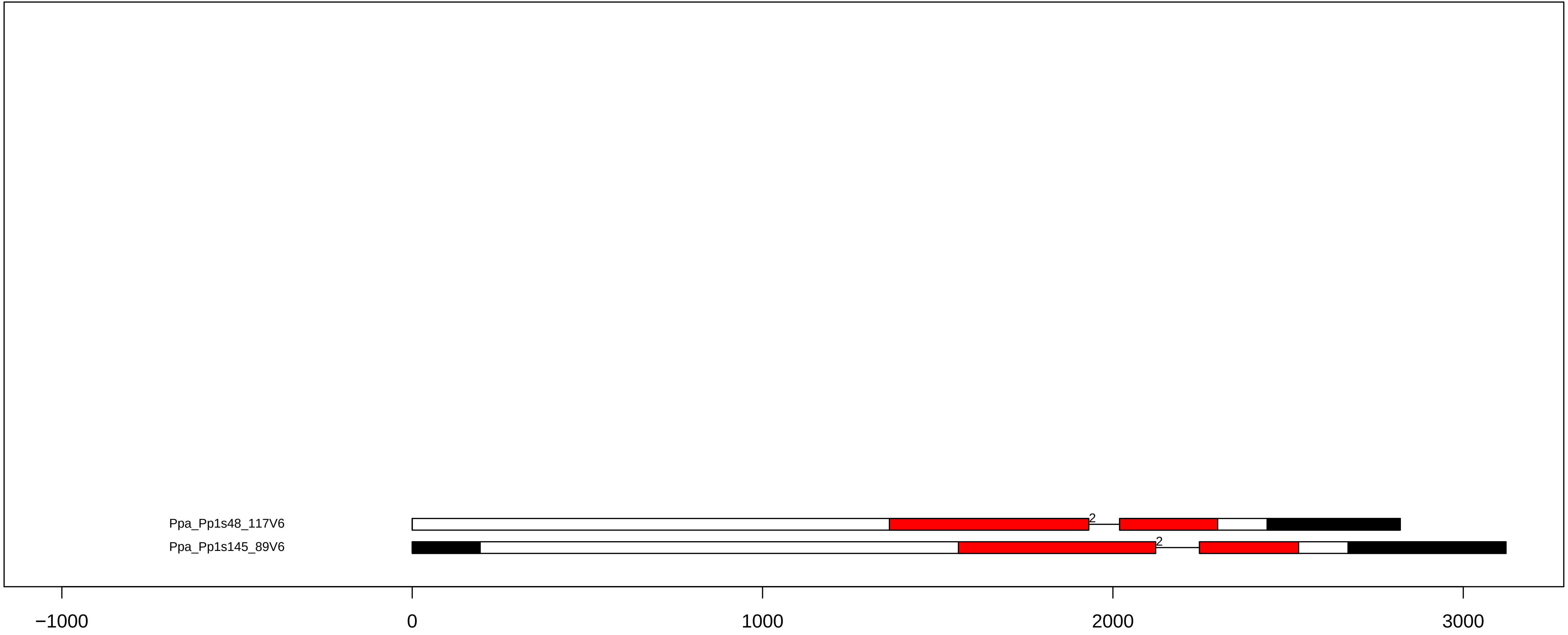

P.pa RLK−Pelle_LRR−XII−1 exon−intron and kinase domain diagram (all)

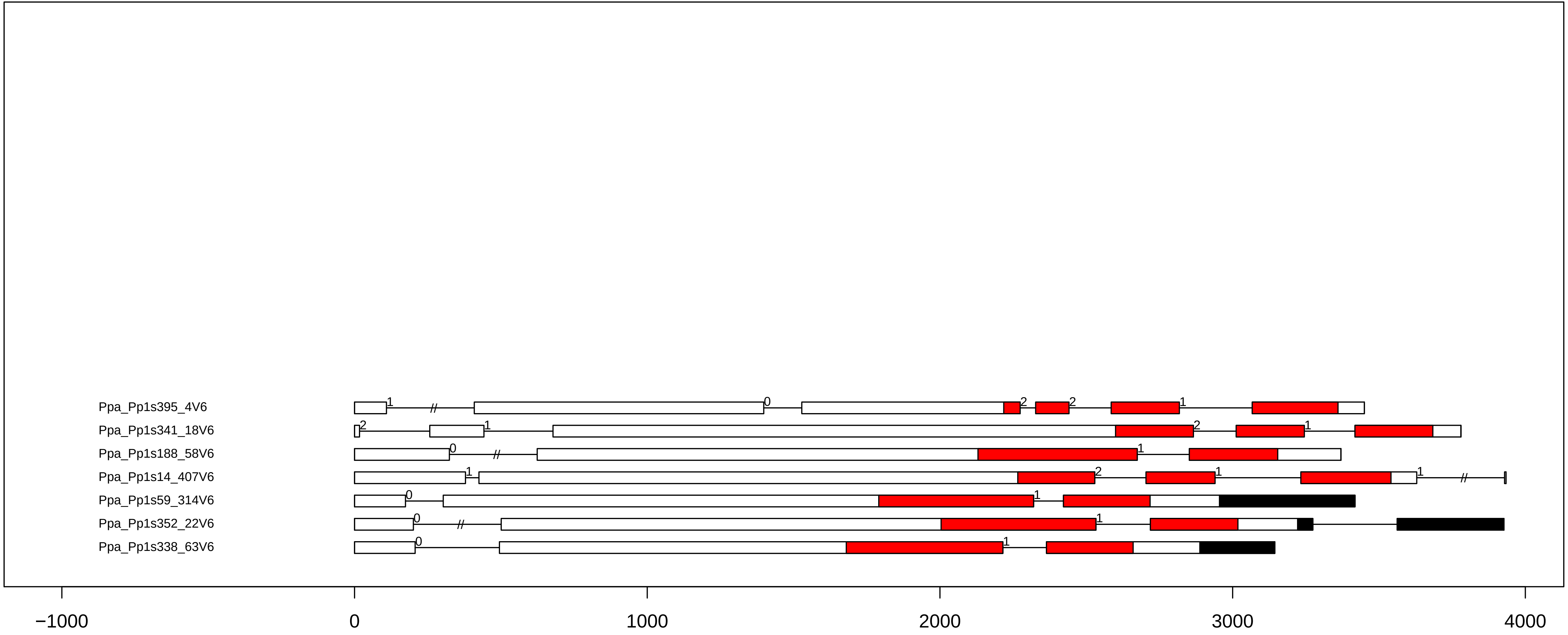

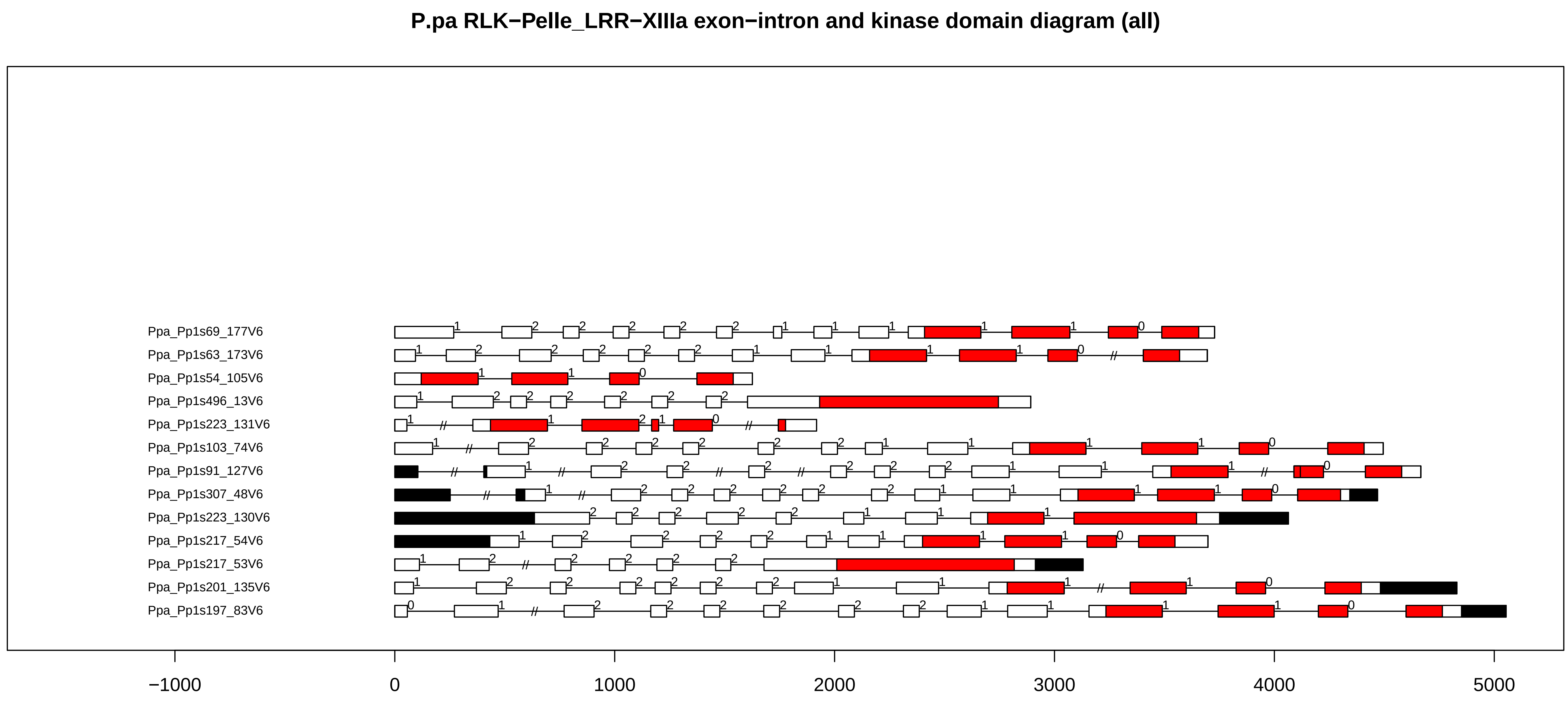
P.pa RLK−Pelle_LRR−XIIIa exon−intron and kinase domain diagram (all)
Ppa_Pp1s69_177V6
Ppa_Pp1s63_173V6
Ppa_Pp1s54_105V6
Ppa_Pp1s496_13V6
Ppa_Pp1s223_131V6
Ppa_Pp1s103_74V6
Ppa_Pp1s91_127V6
Ppa_Pp1s307_48V6
Ppa_Pp1s223_130V6
Ppa_Pp1s217_54V6
Ppa_Pp1s217_53V6
Ppa_Pp1s201_135V6
Ppa_Pp1s197_83V6
−1000
0
1000
2000
3000
4000
5000

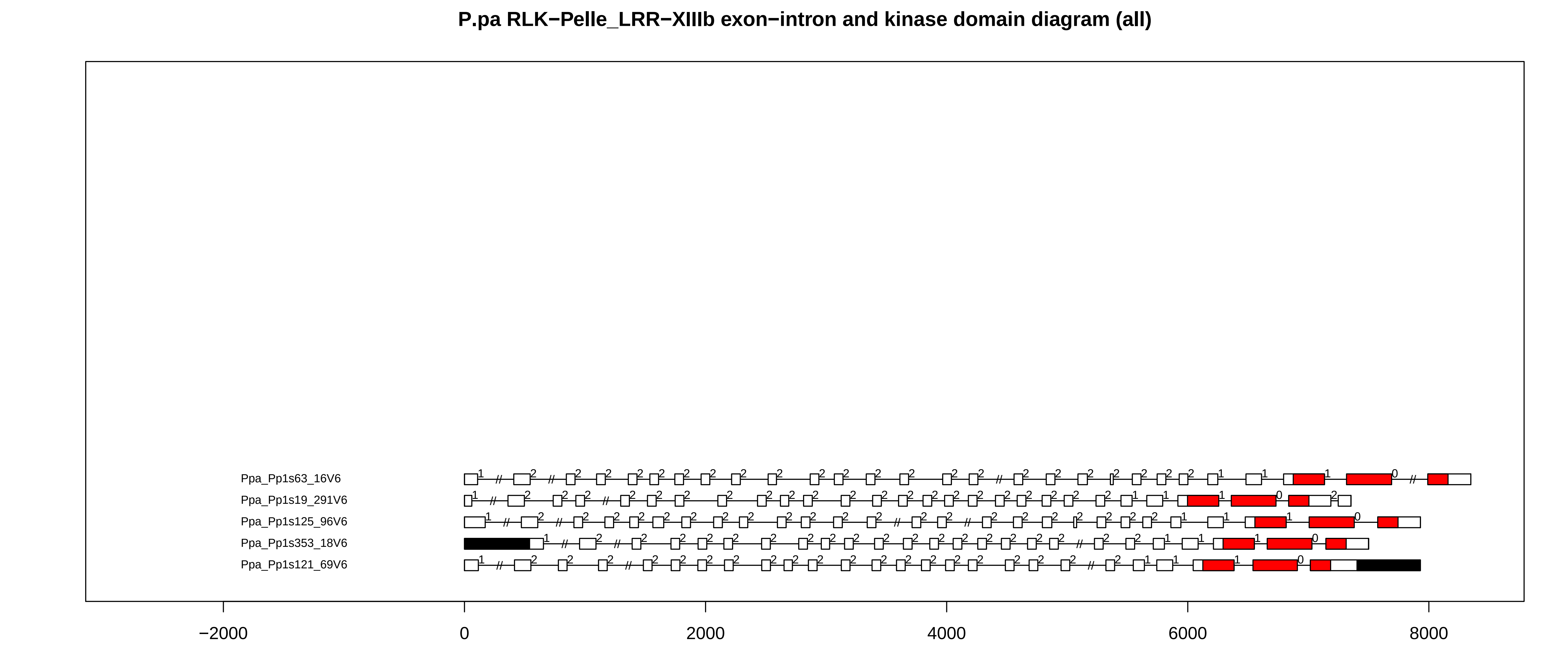

P.pa RLK−Pelle_LRR−XIIIb exon−intron and kinase domain diagram (all)
Ppa_Pp1s63_16V6
Ppa_Pp1s19_291V6
Ppa_Pp1s125_96V6
Ppa_Pp1s353_18V6
Ppa_Pp1s121_69V6
−2000
0
2000
4000
6000
8000

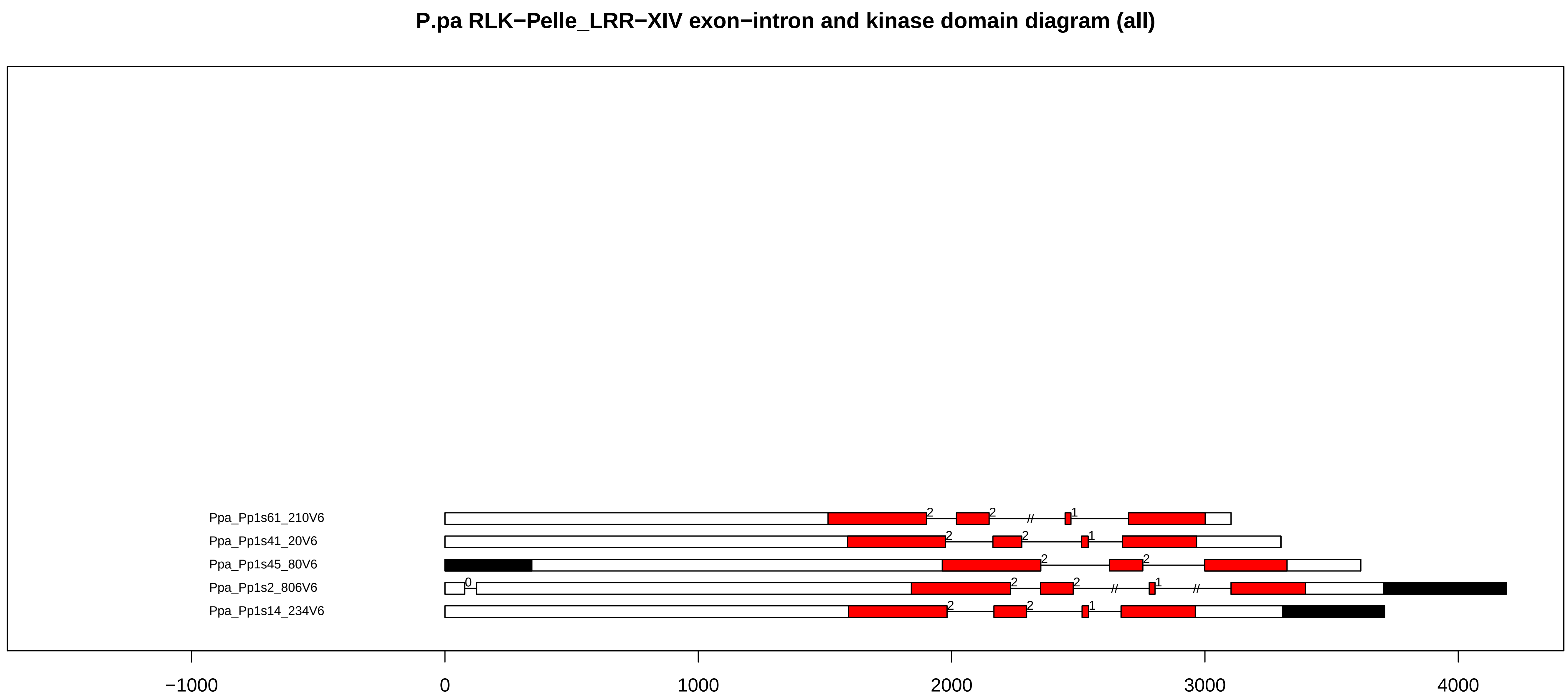

P.pa RLK−Pelle_LRR−XIV exon−intron and kinase domain diagram (all)
Ppa_Pp1s61_210V6
Ppa_Pp1s41_20V6
Ppa_Pp1s45_80V6
Ppa_Pp1s2_806V6
Ppa_Pp1s14_234V6
−1000
0
1000
2000
3000
4000

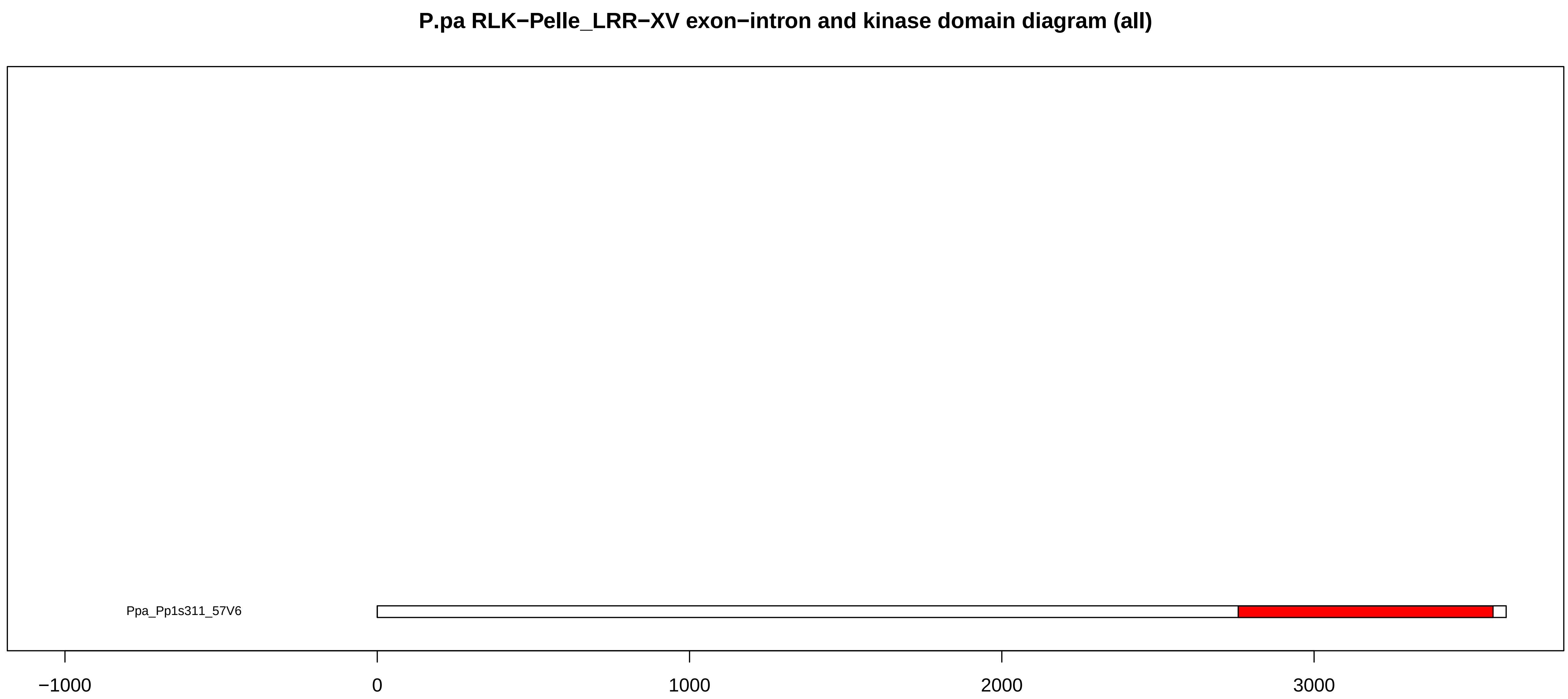
P.pa RLK−Pelle_LRR−XV exon−intron and kinase domain diagram (all)
Ppa_Pp1s311_57V6
−1000
0
1000
2000
3000

P.pa RLK−Pelle_LysM exon−intron and kinase domain diagram (all)

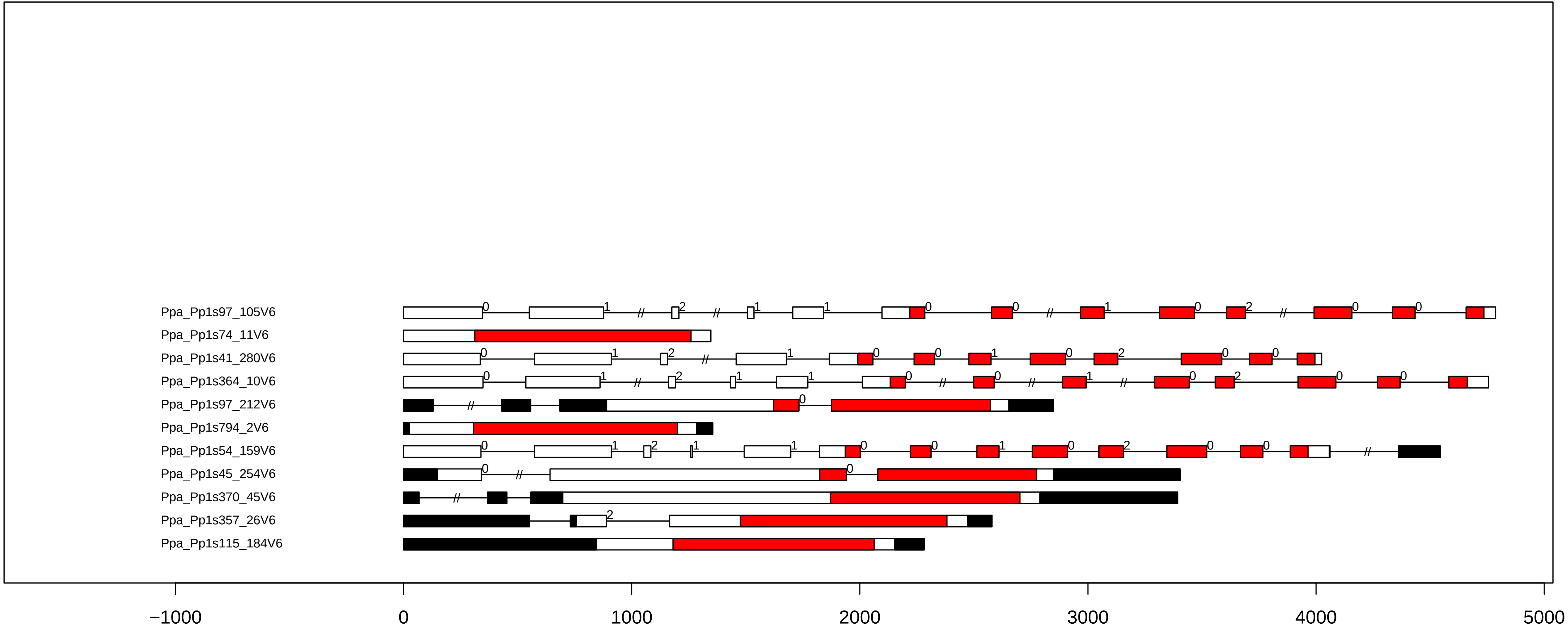

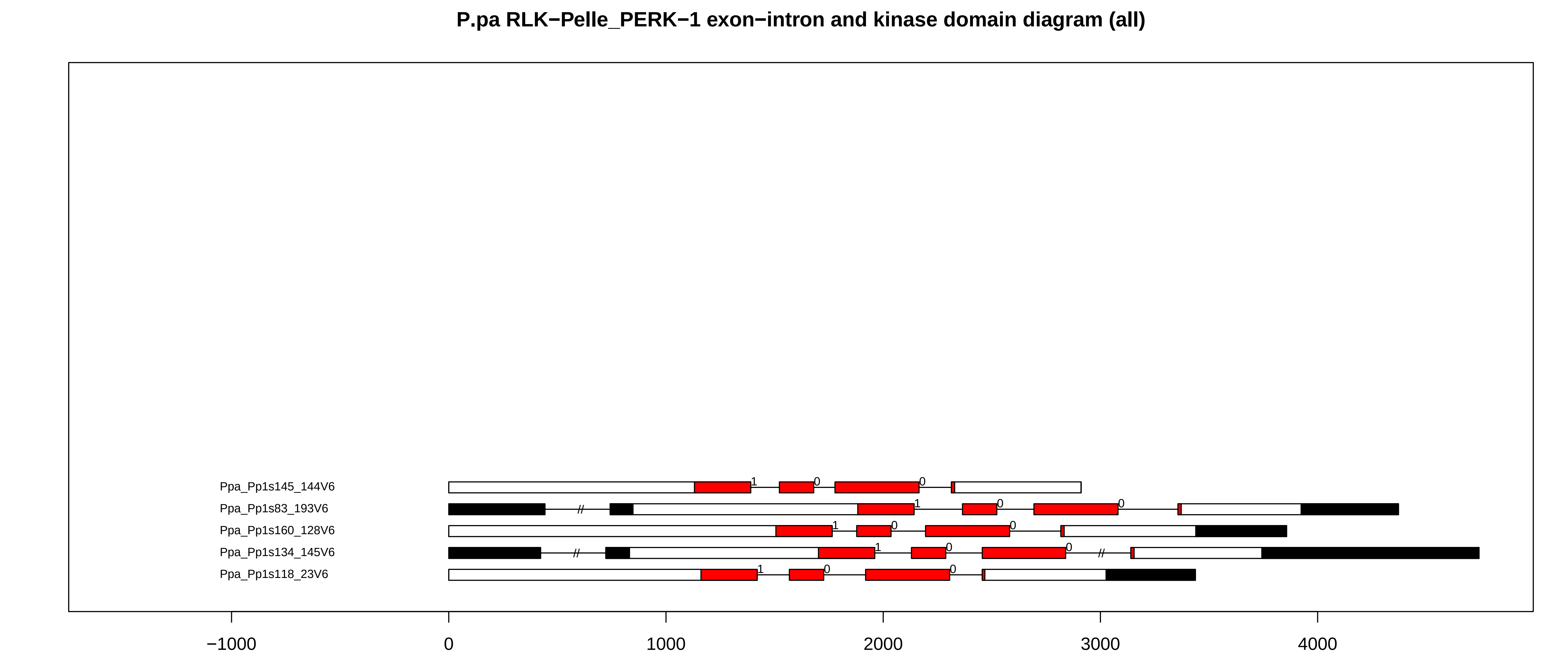
P.pa RLK−Pelle_PERK−1 exon−intron and kinase domain diagram (all)
Ppa_Pp1s145_144V6
Ppa_Pp1s83_193V6
Ppa_Pp1s160_128V6
Ppa_Pp1s134_145V6
Ppa_Pp1s118_23V6
−1000
0
1000
2000
3000
4000

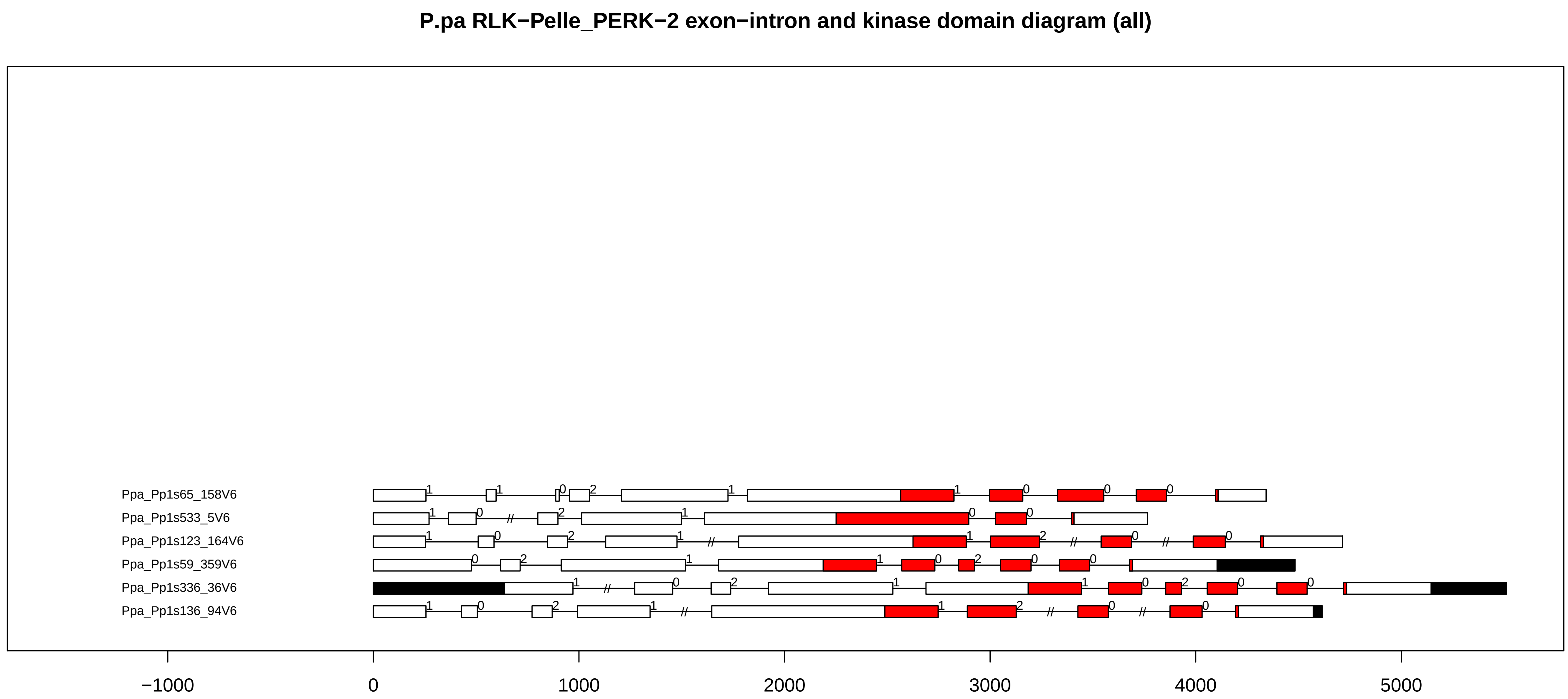

P.pa RLK−Pelle_PERK−2 exon−intron and kinase domain diagram (all)
Ppa_Pp1s65_158V6
Ppa_Pp1s533_5V6
Ppa_Pp1s123_164V6
Ppa_Pp1s59_359V6
Ppa_Pp1s336_36V6
Ppa_Pp1s136_94V6
−1000
0
1000
2000
3000
4000
5000

P.pa RLK−Pelle_Pp exon−intron and kinase domain diagram (all)

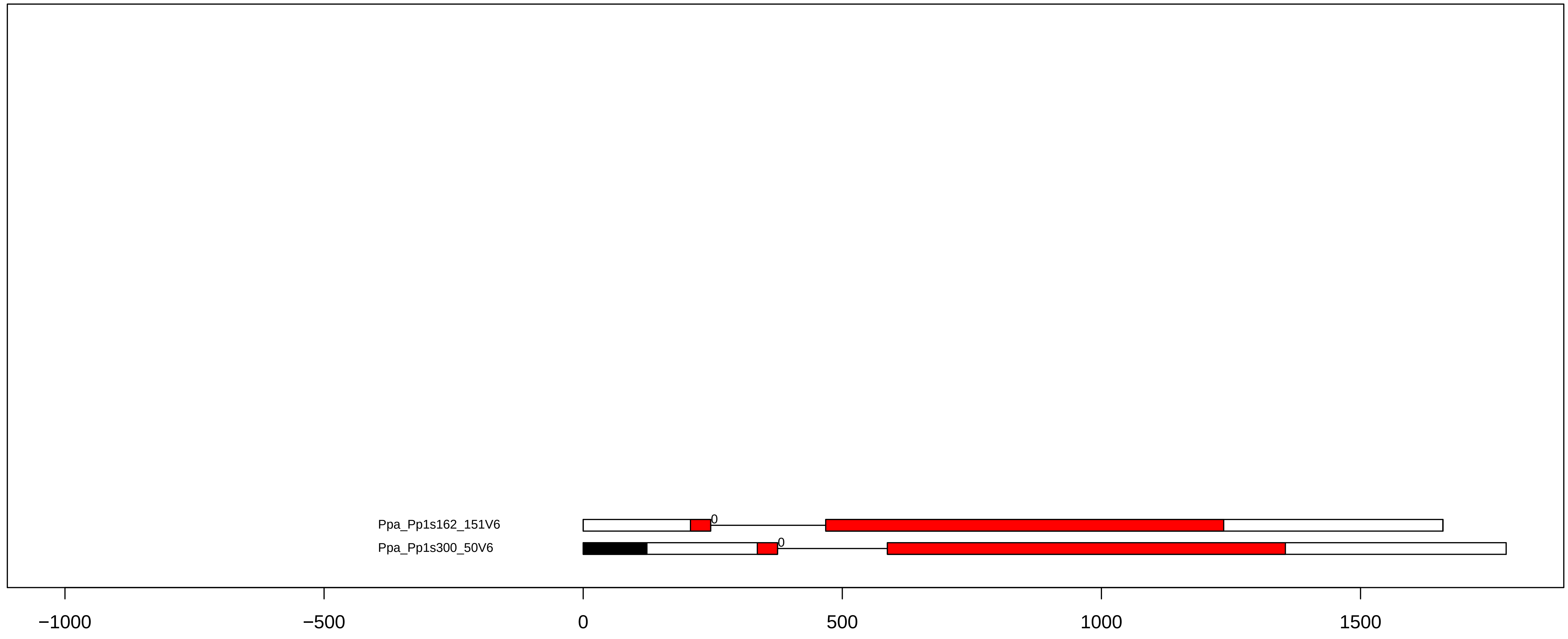

P.pa RLK−Pelle_RKF3 exon−intron and kinase domain diagram (all)

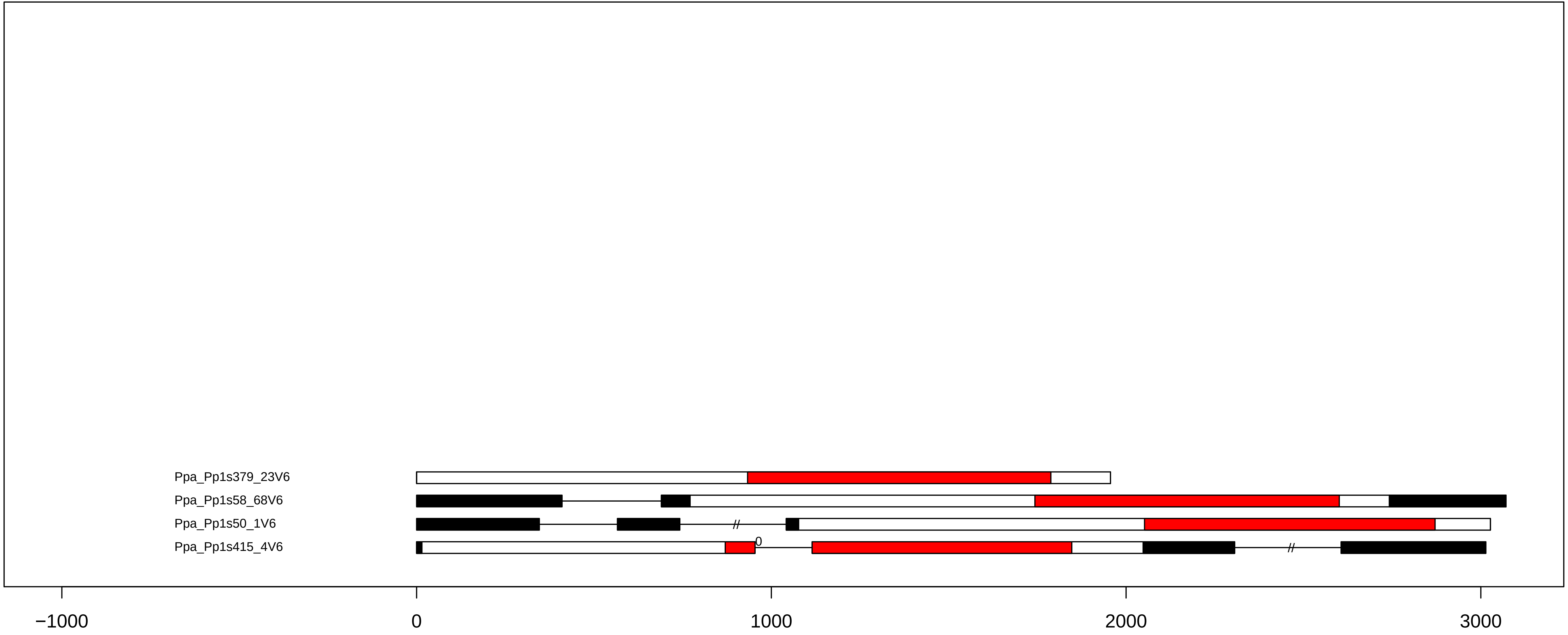

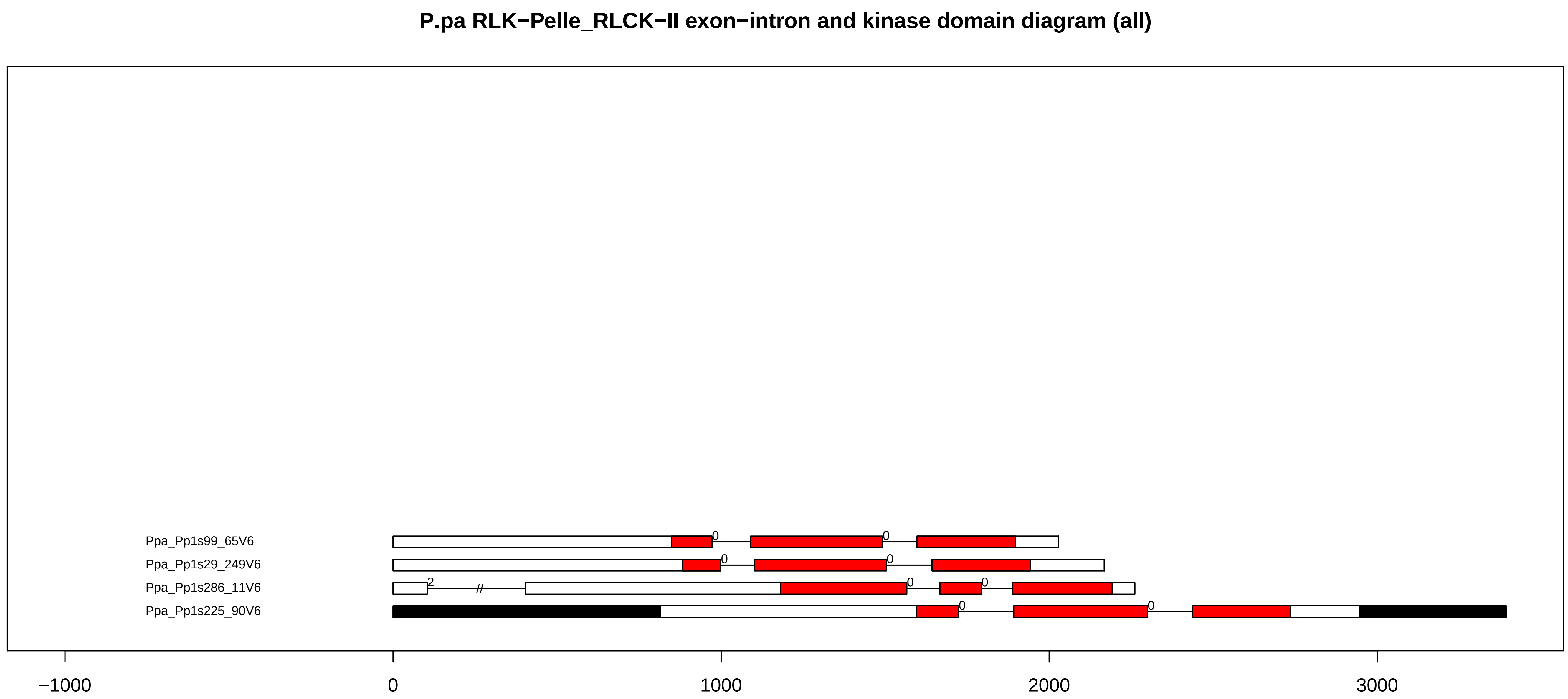
P.pa RLK−Pelle_RLCK−II exon−intron and kinase domain diagram (all)
Ppa_Pp1s99_65V6
Ppa_Pp1s29_249V6
Ppa_Pp1s286_11V6
Ppa_Pp1s225_90V6
0
0
0
0
2
0
0
0
0
−1000
0
1000
2000
3000

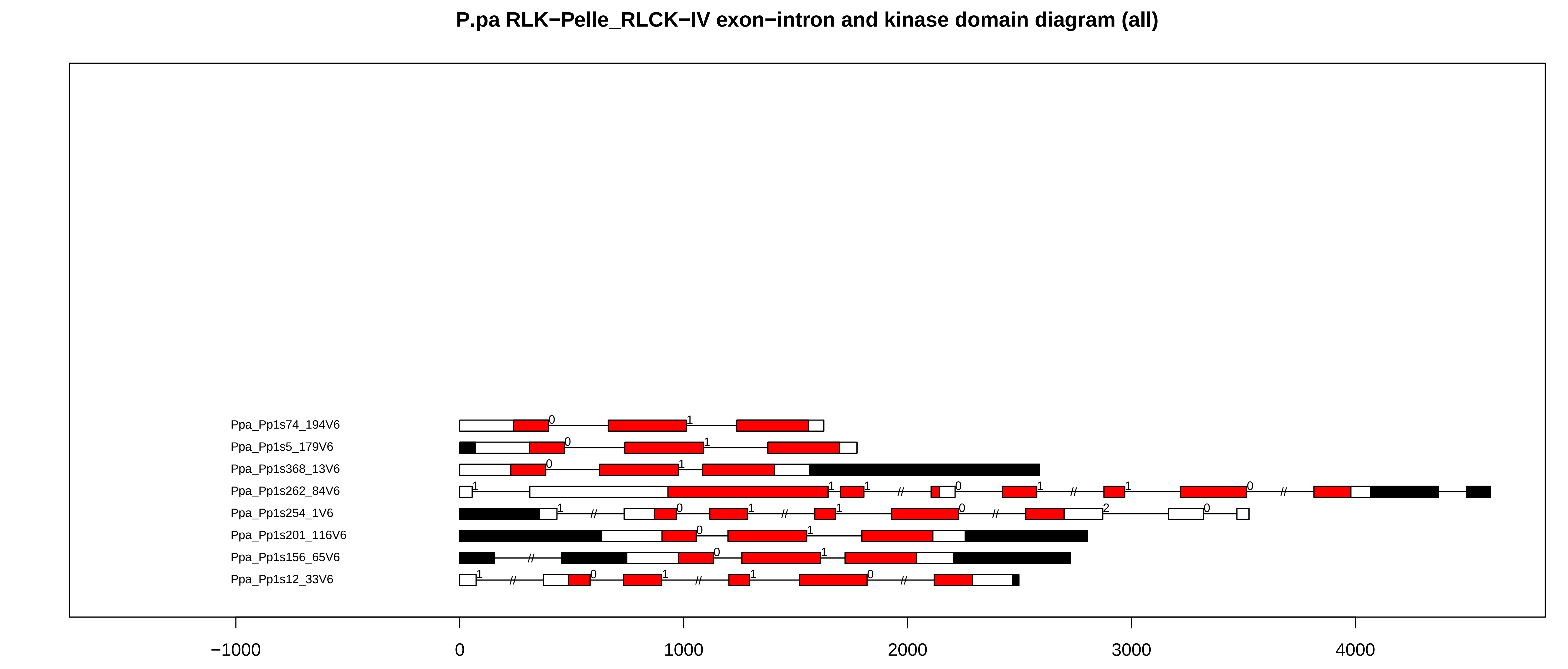

P.pa RLK−Pelle_RLCK−IV exon−intron and kinase domain diagram (all)
Ppa_Pp1s74_194V6
Ppa_Pp1s5_179V6
Ppa_Pp1s368_13V6
Ppa_Pp1s262_84V6
Ppa_Pp1s254_1V6
Ppa_Pp1s201_116V6
Ppa_Pp1s156_65V6
Ppa_Pp1s12_33V6
−1000
0
1000
2000
3000
4000

P.pa RLK−Pelle_RLCK−Pp exon−intron and kinase domain diagram (all)

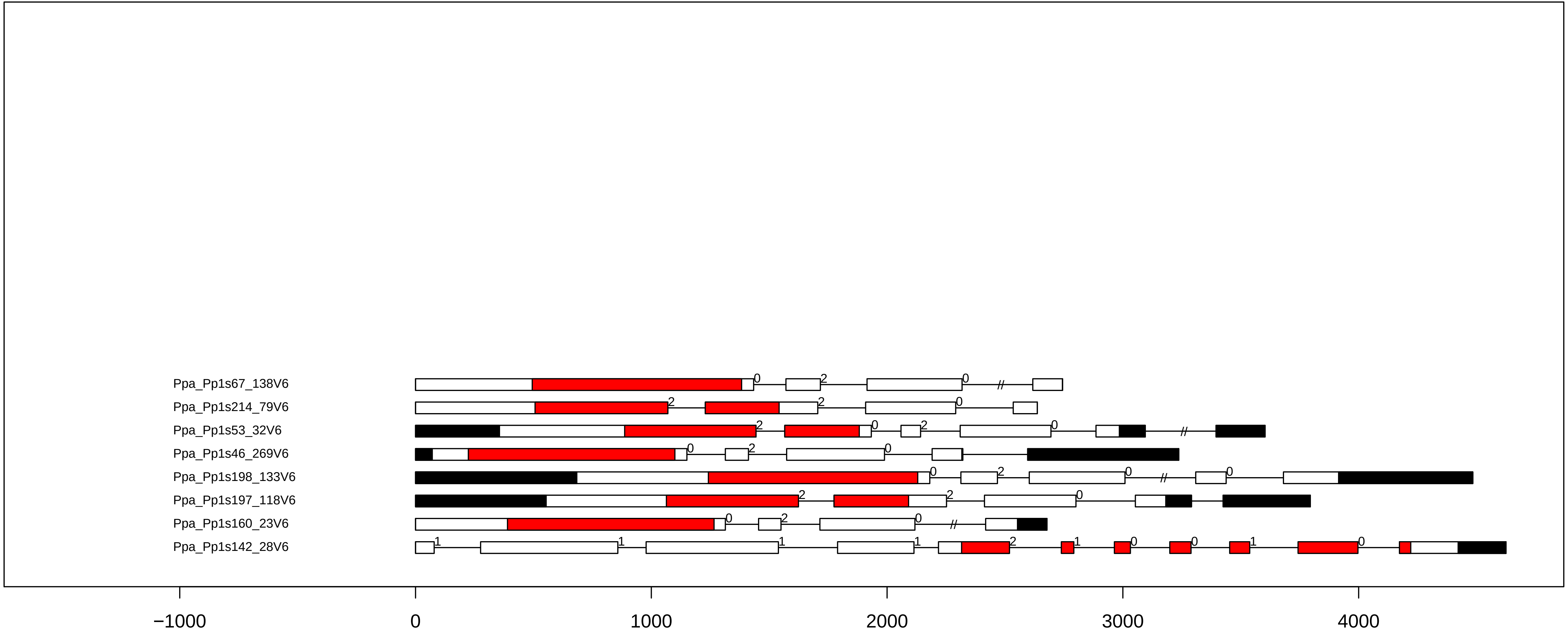

P.pa RLK−Pelle_RLCK−VIIa−1 exon−intron and kinase domain diagram (all)

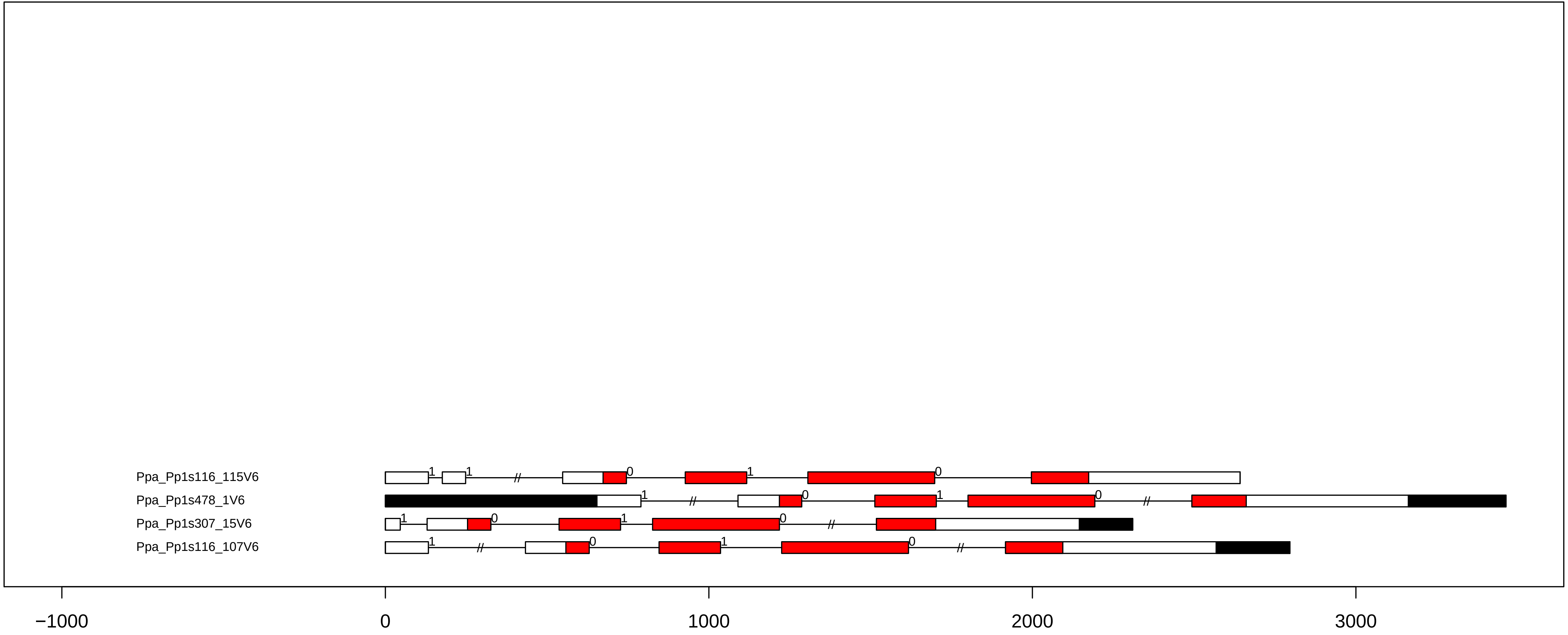

P.pa RLK−Pelle_RLCK−VIIa−2 exon−intron and kinase domain diagram (all)

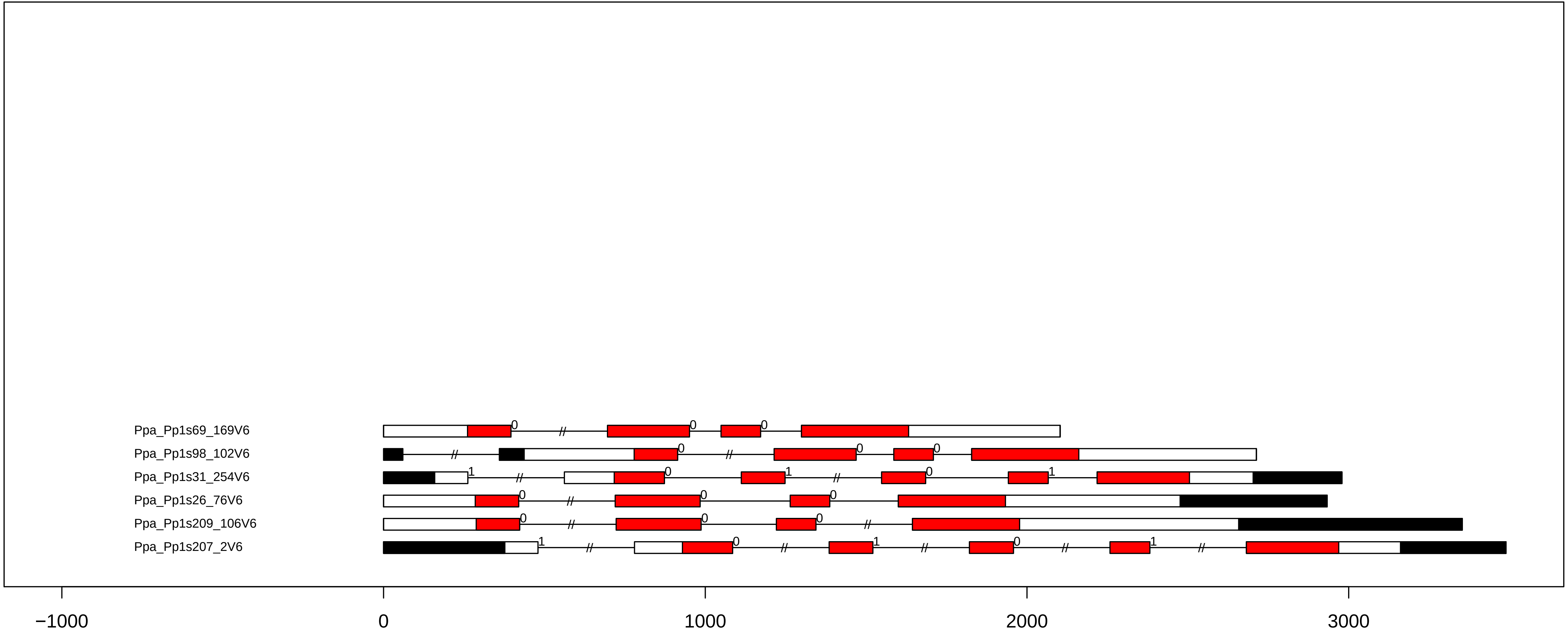

P.pa RLK−Pelle_RLCK−VIII exon−intron and kinase domain diagram (all)

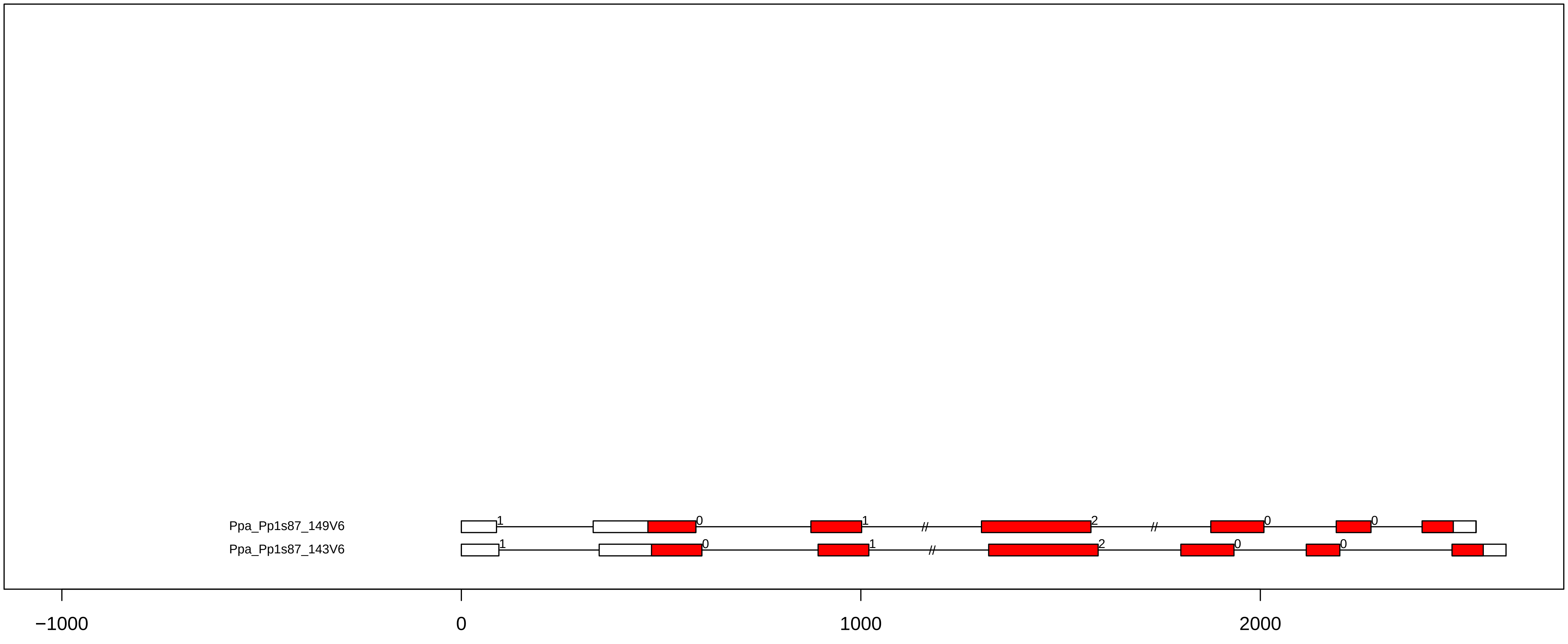

P.pa RLK−Pelle_RLCK−VI exon−intron and kinase domain diagram (all)

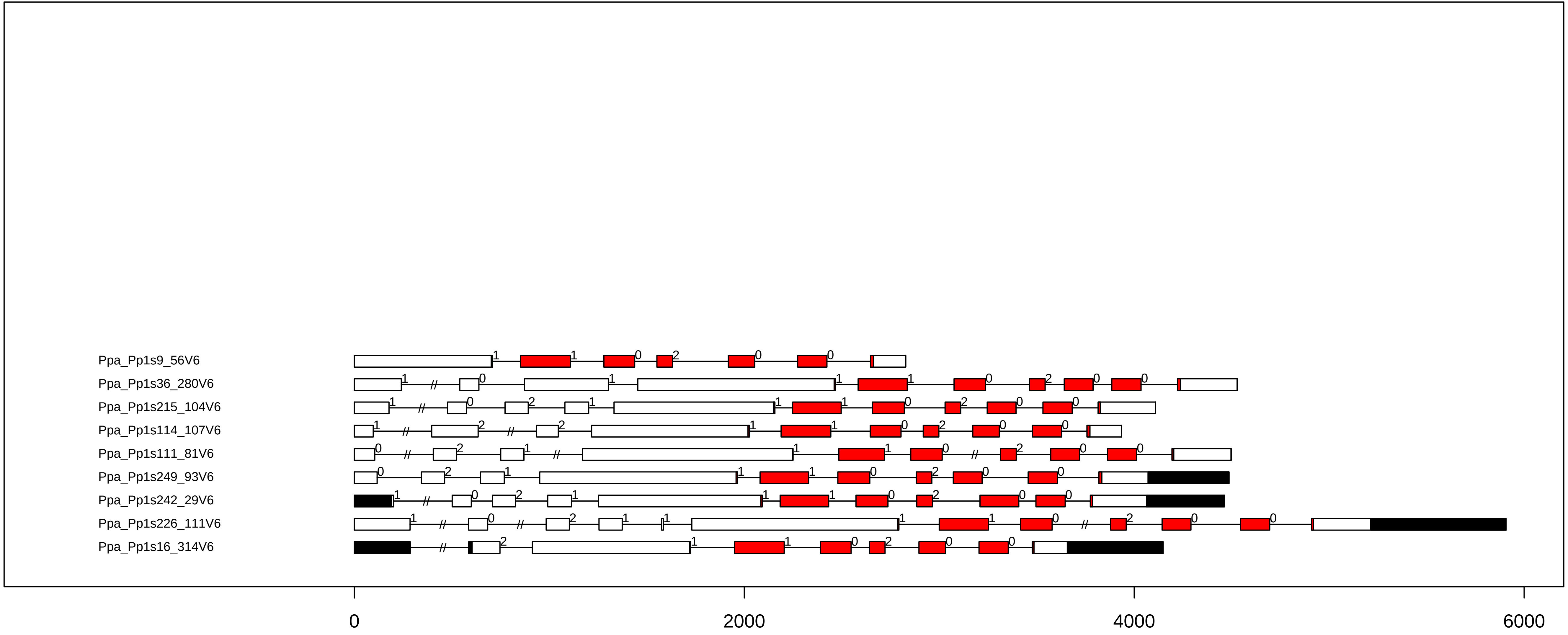

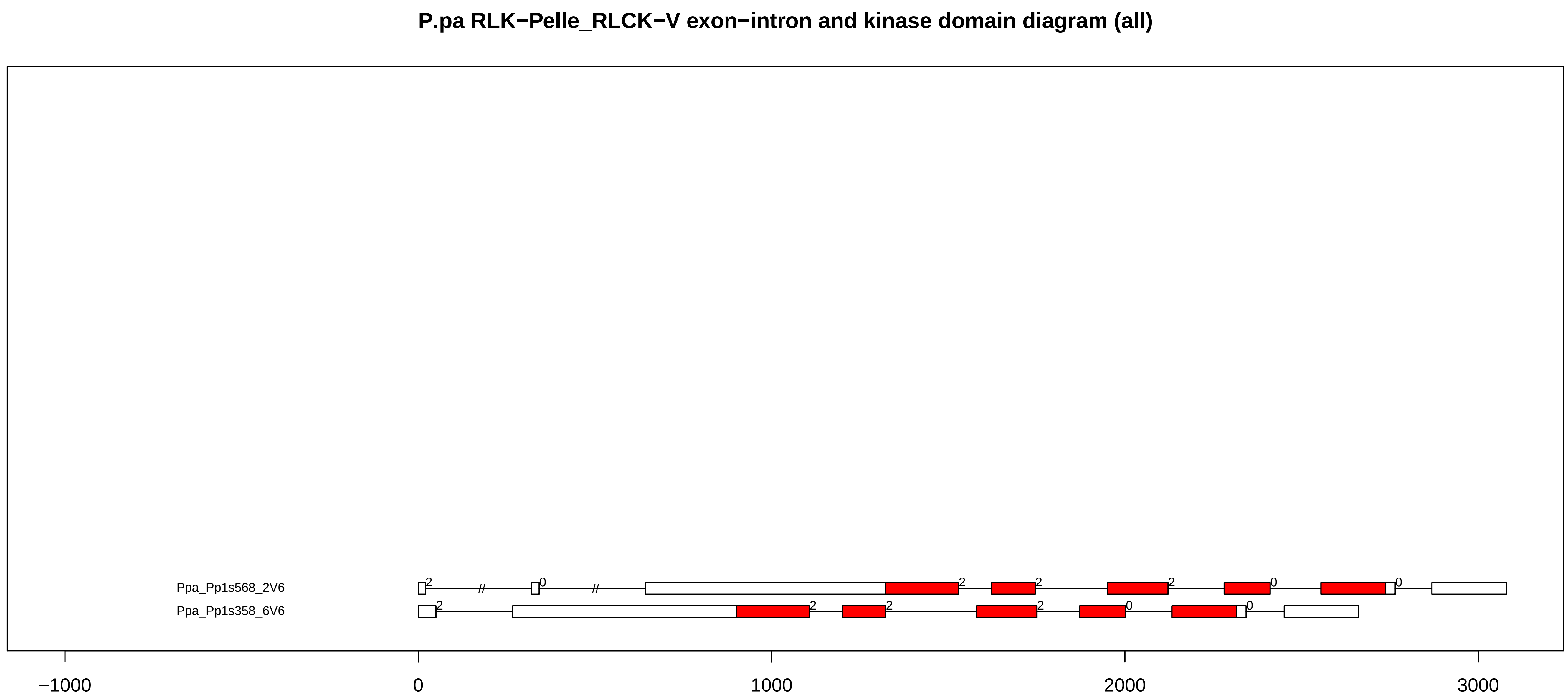
P.pa RLK−Pelle_RLCK−V exon−intron and kinase domain diagram (all)
Ppa_Pp1s568_2V6
Ppa_Pp1s358_6V6
−1000
0
1000
2000
3000

P.pa RLK−Pelle_RLCK−XII−1 exon−intron and kinase domain diagram (all)

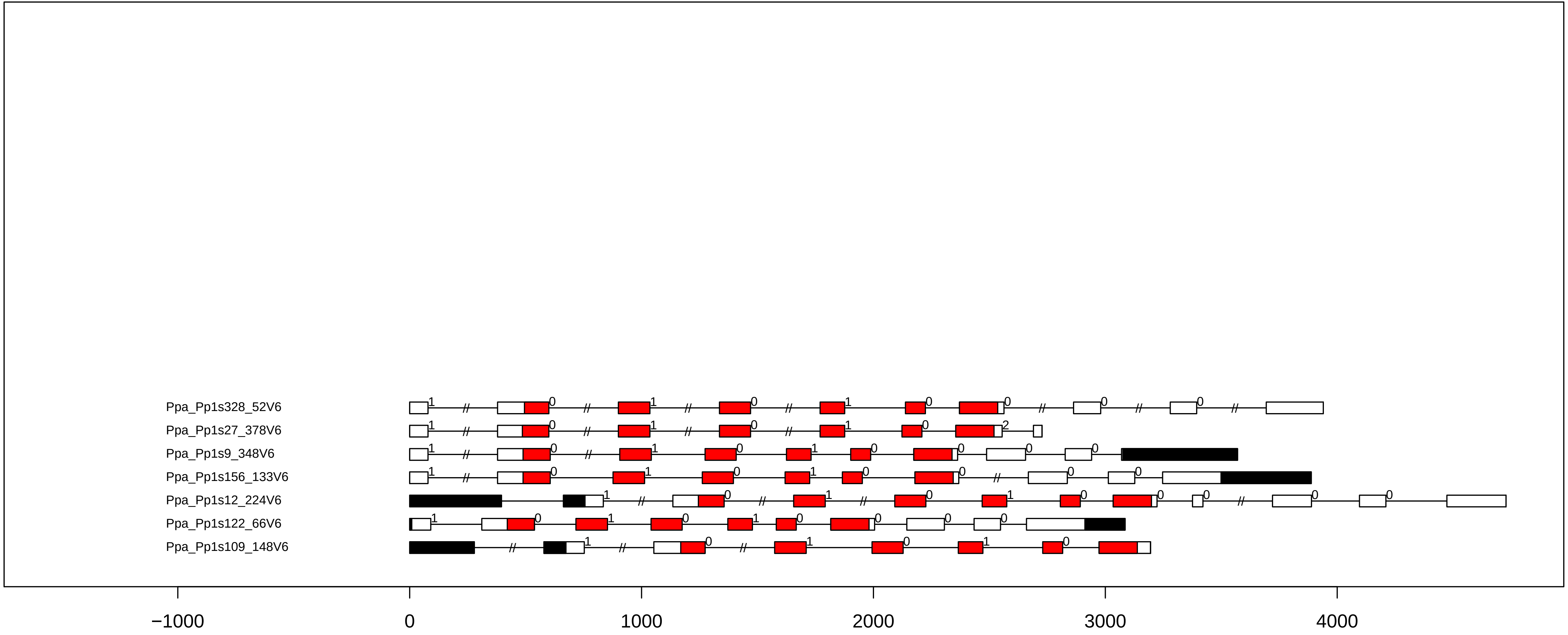

P.pa RLK−Pelle_RLCK−XI exon−intron and kinase domain diagram (all)

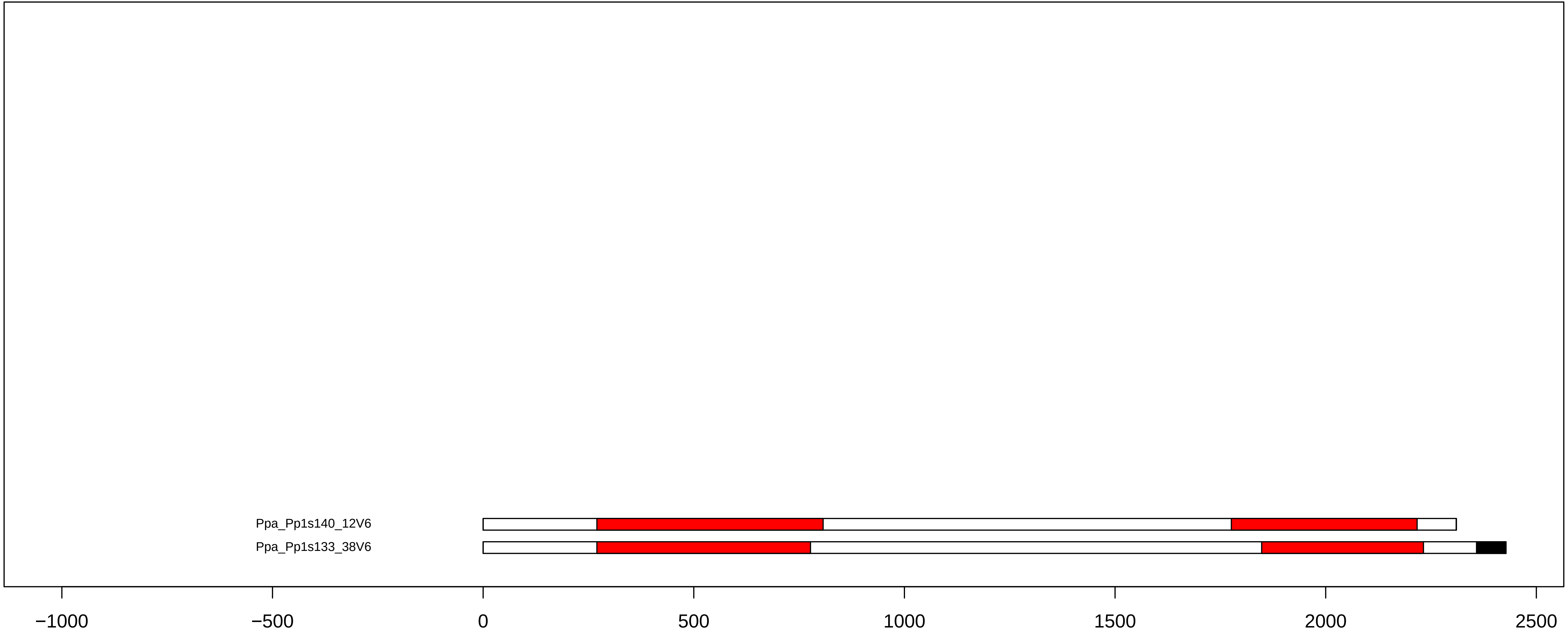

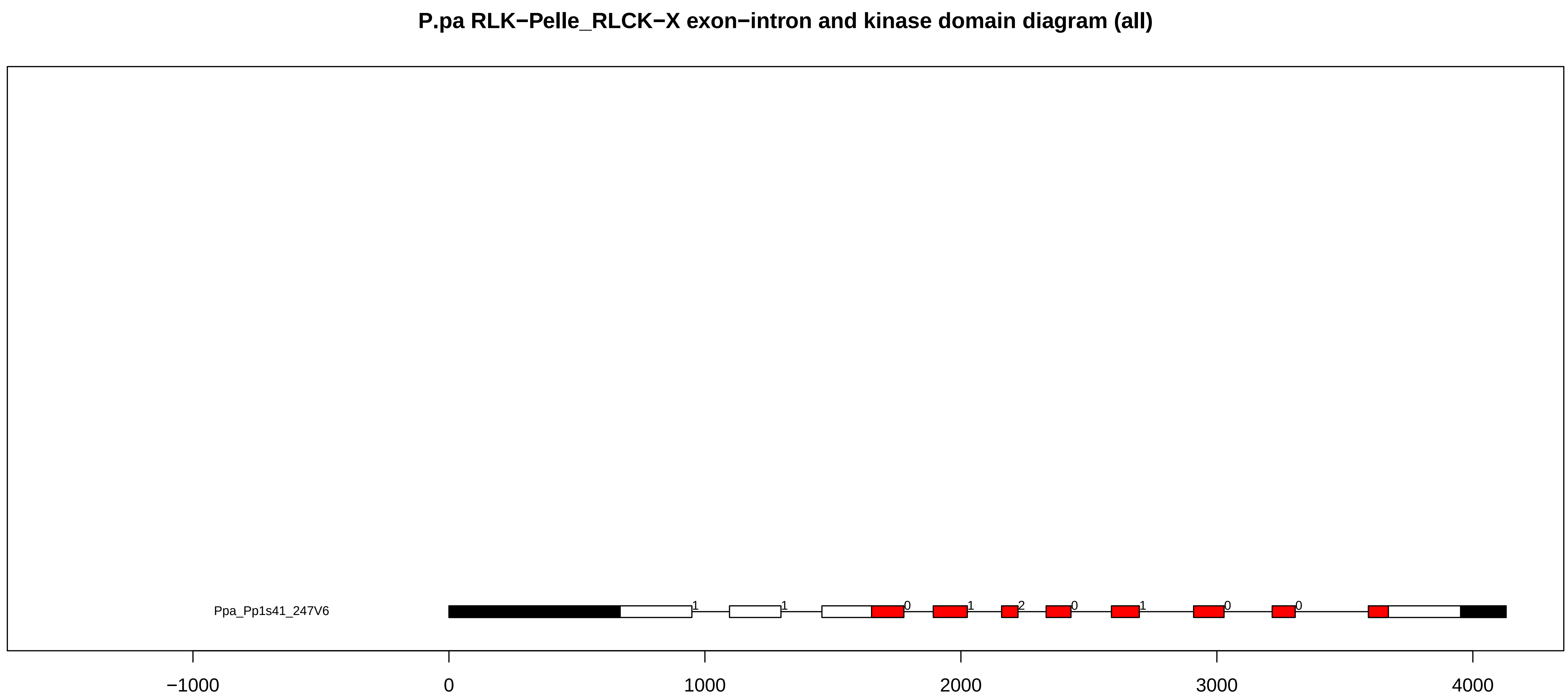
P.pa RLK−Pelle_RLCK−X exon−intron and kinase domain diagram (all)
Ppa_Pp1s41_247V6
1
1
0
1
2
0
1
0
0
−1000
0
1000
2000
3000
4000

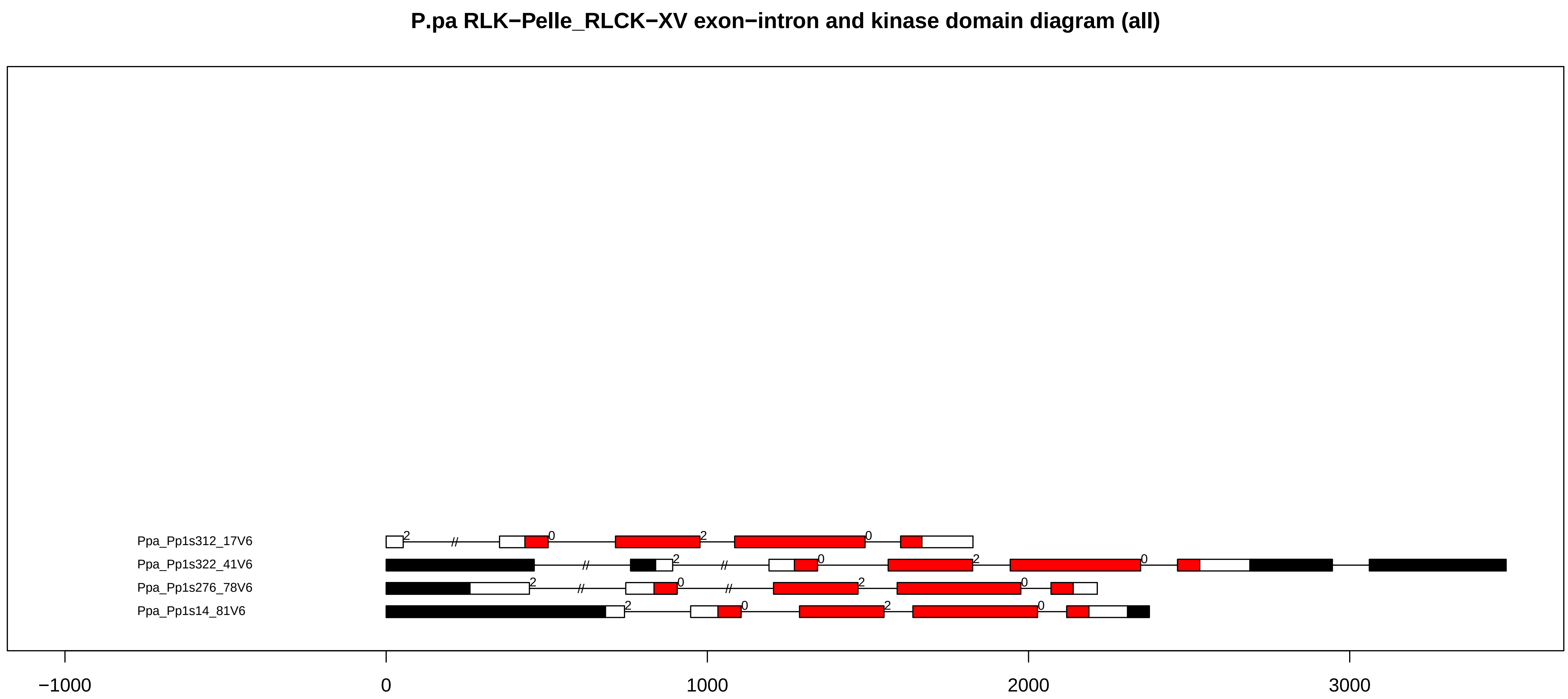
P.pa RLK−Pelle_RLCK−XV exon−intron and kinase domain diagram (all)
Ppa_Pp1s312_17V6
Ppa_Pp1s322_41V6
Ppa_Pp1s276_78V6
Ppa_Pp1s14_81V6
−1000
0
1000
2000
3000

P.pa RLK−Pelle_SD−2b exon−intron and kinase domain diagram (all)

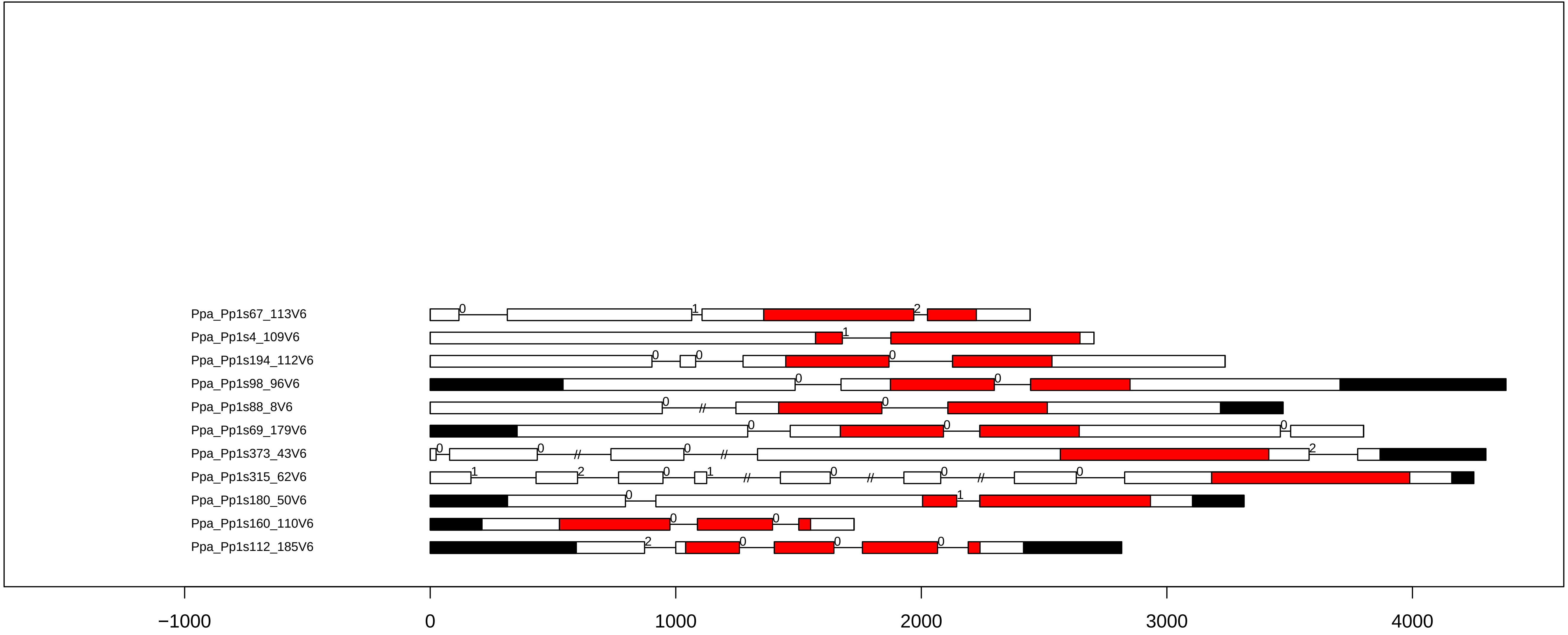

P.pa RLK−Pelle_Singleton exon−intron and kinase domain diagram (all)

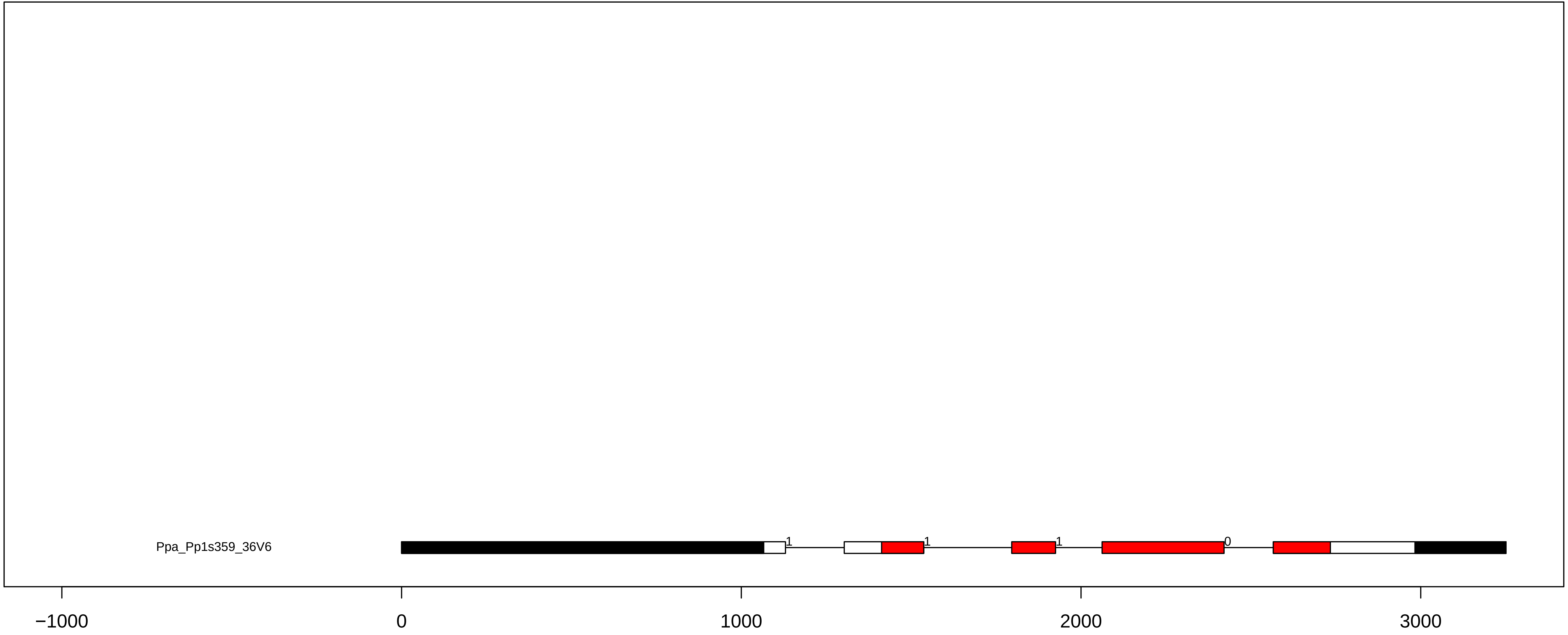

P.pa RLK−Pelle_URK−Pp−1 exon−intron and kinase domain diagram (all)

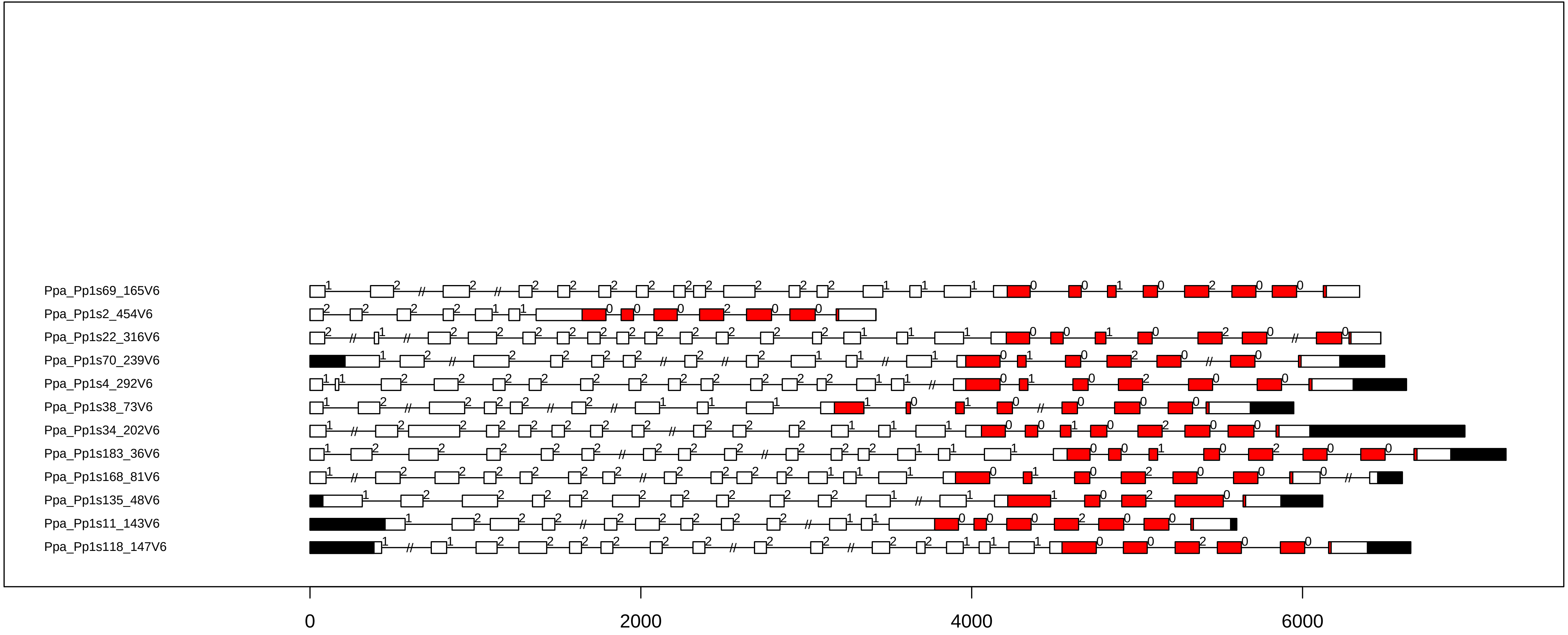

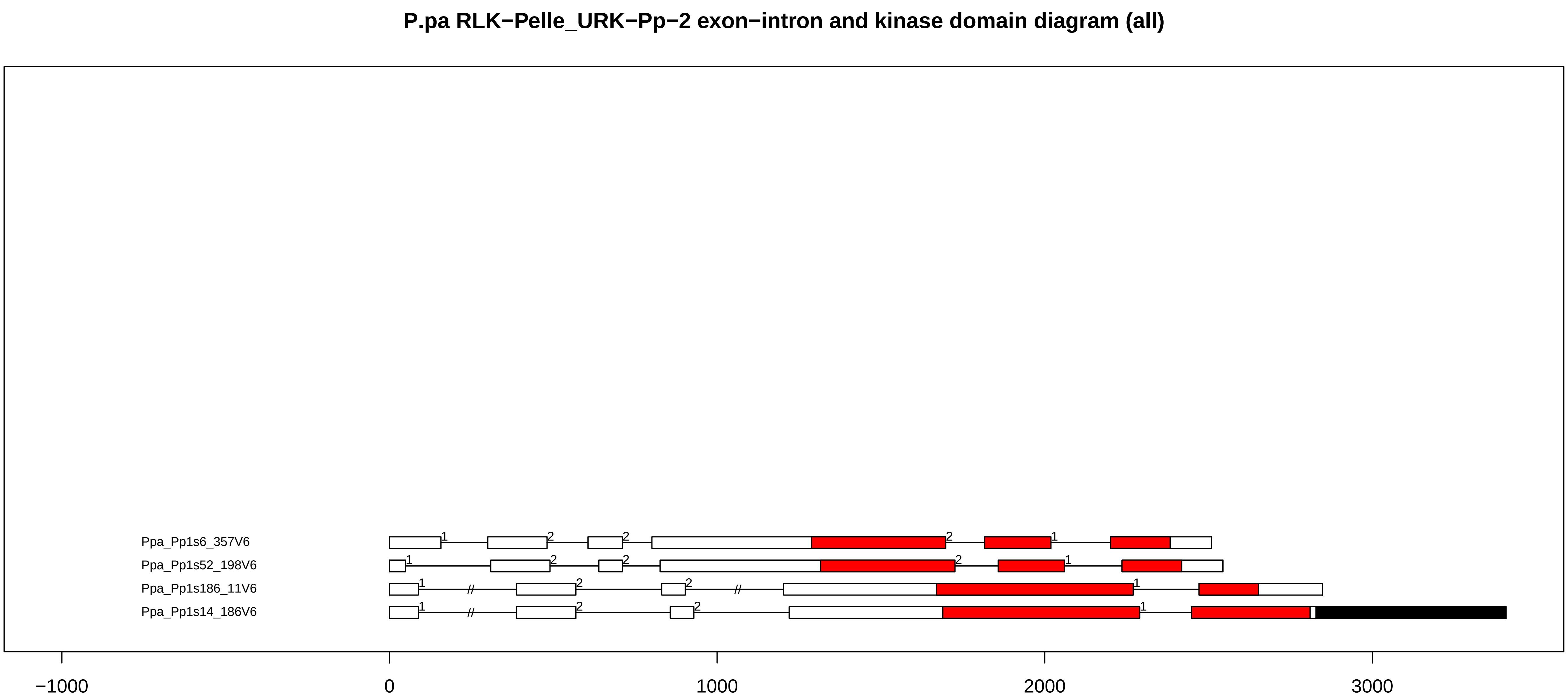

P.pa RLK−Pelle_URK−Pp−2 exon−intron and kinase domain diagram (all)
Ppa_Pp1s6_357V6
Ppa_Pp1s52_198V6
Ppa_Pp1s186_11V6
Ppa_Pp1s14_186V6
−1000
0
1000
2000
3000

P.pa RLK−Pelle_WAK_LRK10L−1 exon−intron and kinase domain diagram (all)

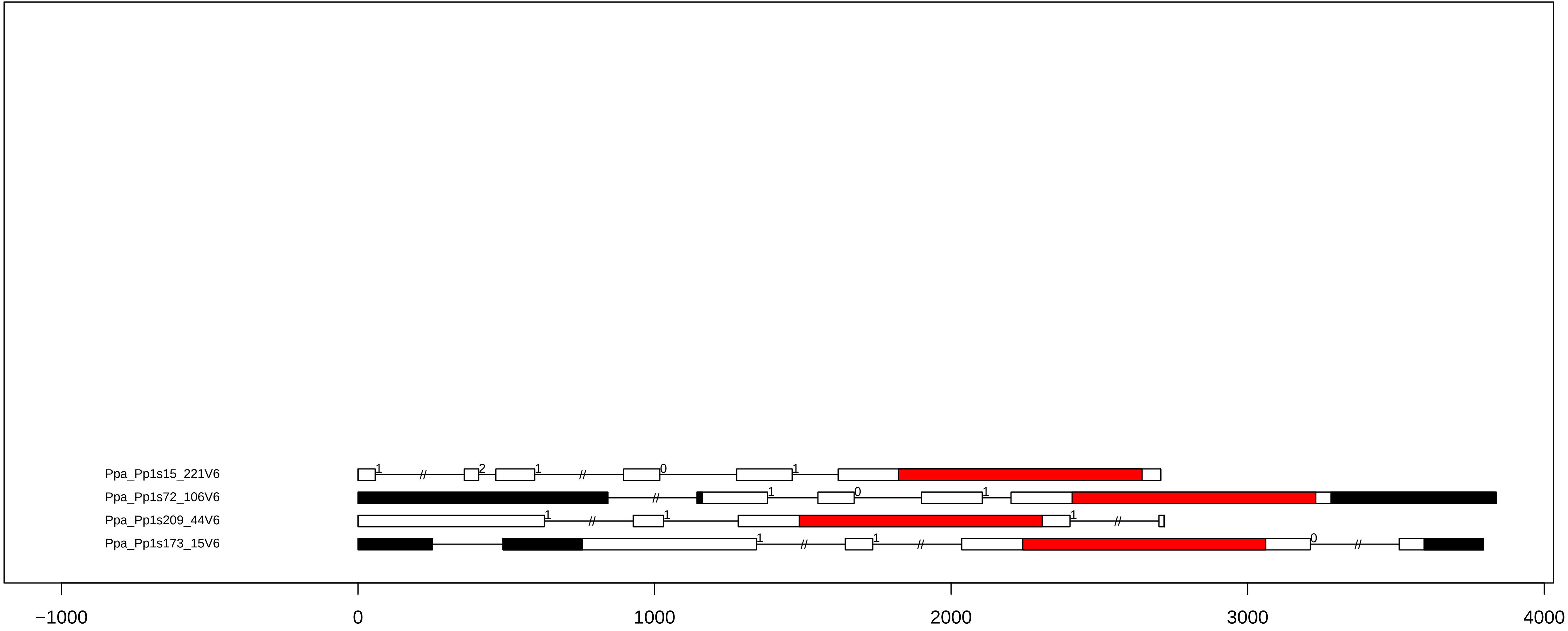

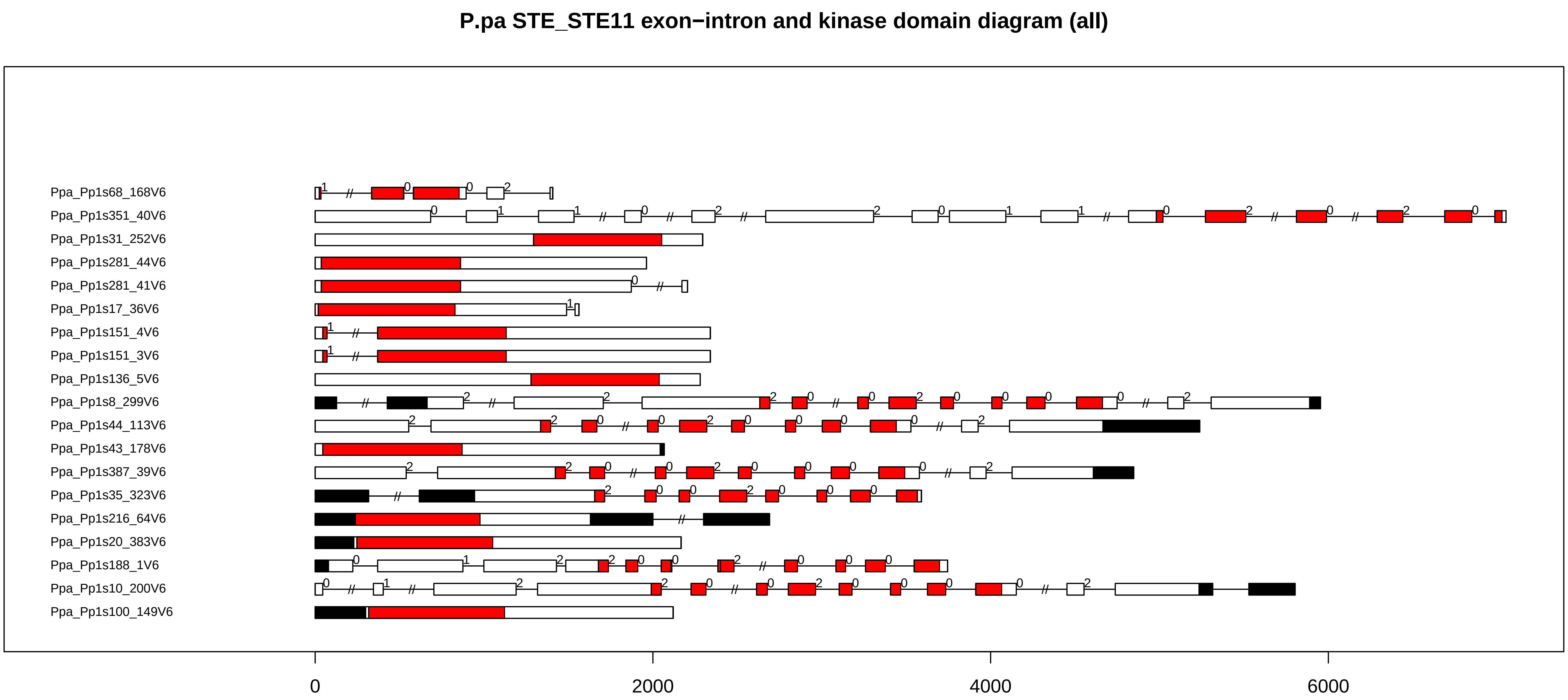

P.pa STE_STE11 exon−intron and kinase domain diagram (all)
Ppa_Pp1s68_168V6
Ppa_Pp1s351_40V6
Ppa_Pp1s31_252V6
Ppa_Pp1s281_44V6
Ppa_Pp1s281_41V6
Ppa_Pp1s17_36V6
Ppa_Pp1s151_4V6
Ppa_Pp1s151_3V6
Ppa_Pp1s136_5V6
Ppa_Pp1s8_299V6
Ppa_Pp1s44_113V6
Ppa_Pp1s43_178V6
Ppa_Pp1s387_39V6
Ppa_Pp1s35_323V6
Ppa_Pp1s216_64V6
Ppa_Pp1s20_383V6
Ppa_Pp1s188_1V6
Ppa_Pp1s10_200V6
Ppa_Pp1s100_149V6
0
2000
4000
6000

P.pa STE_STE20−Fray exon−intron and kinase domain diagram (all)

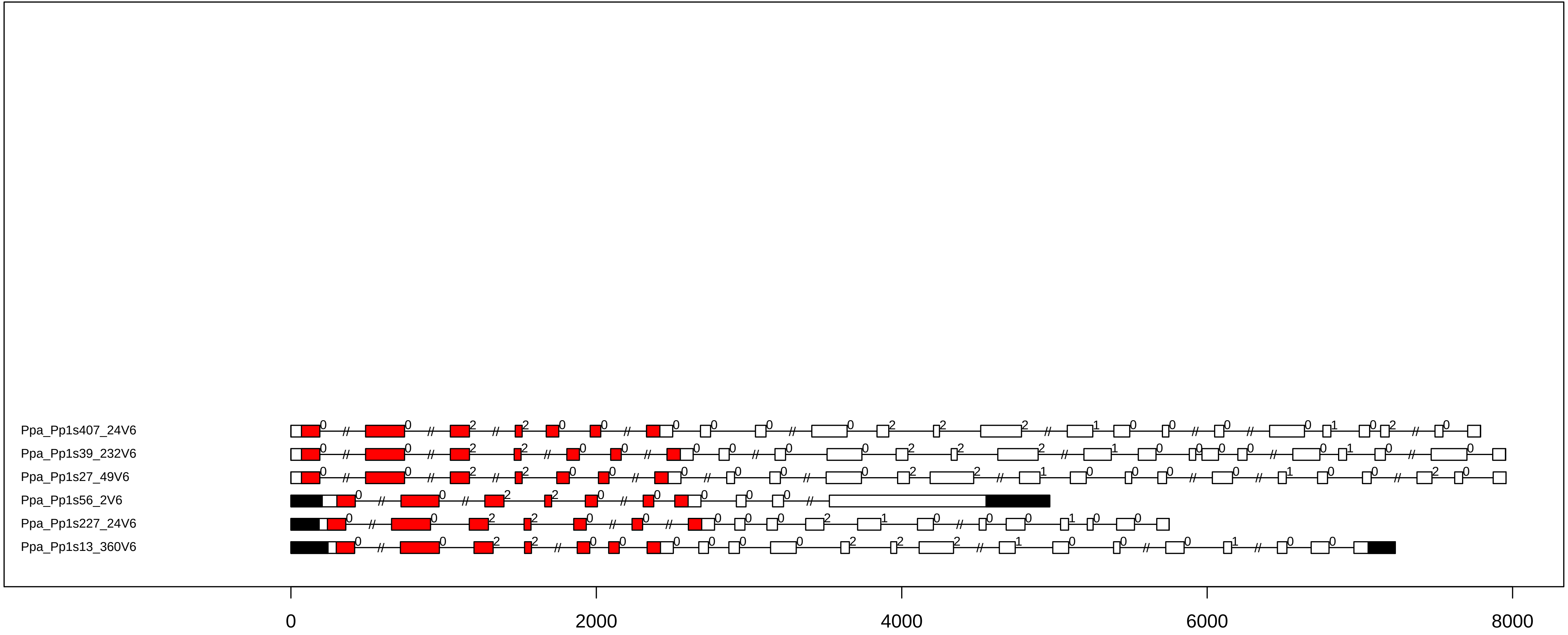

P.pa STE_STE20−PI exon−intron and kinase domain diagram (all)

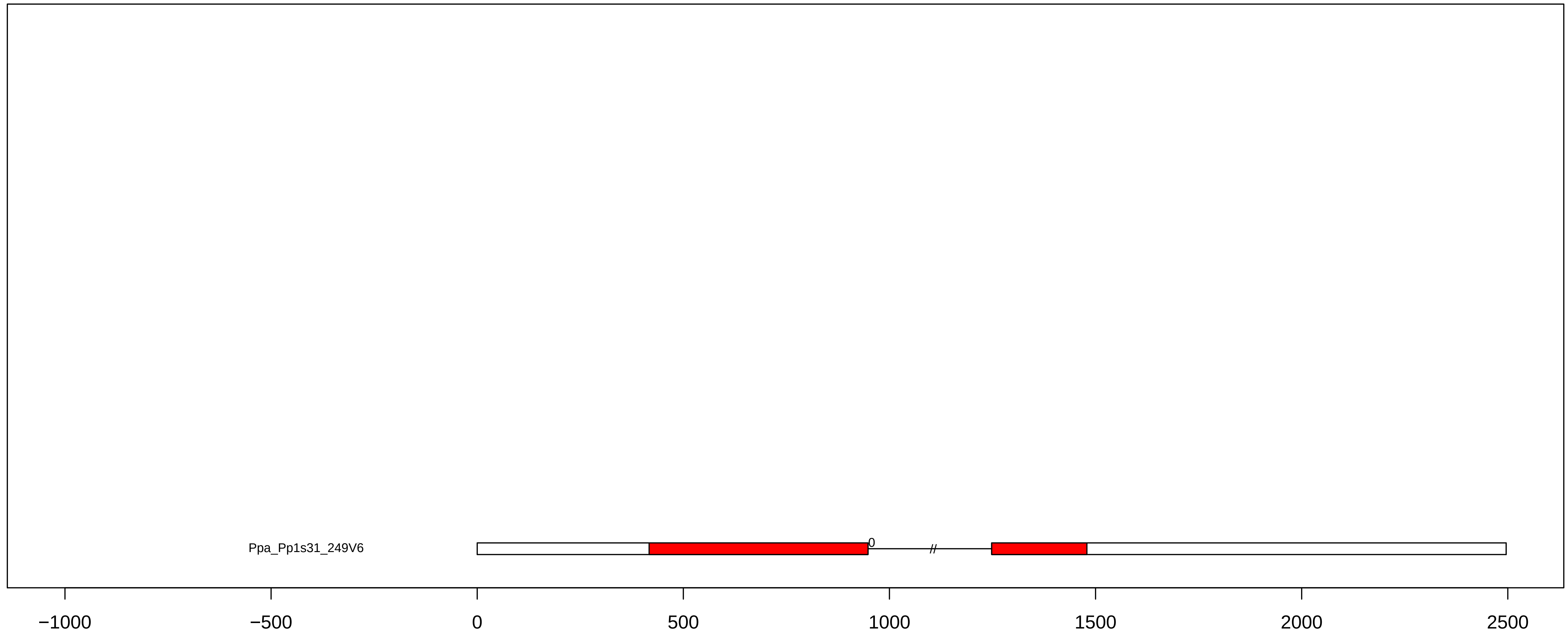

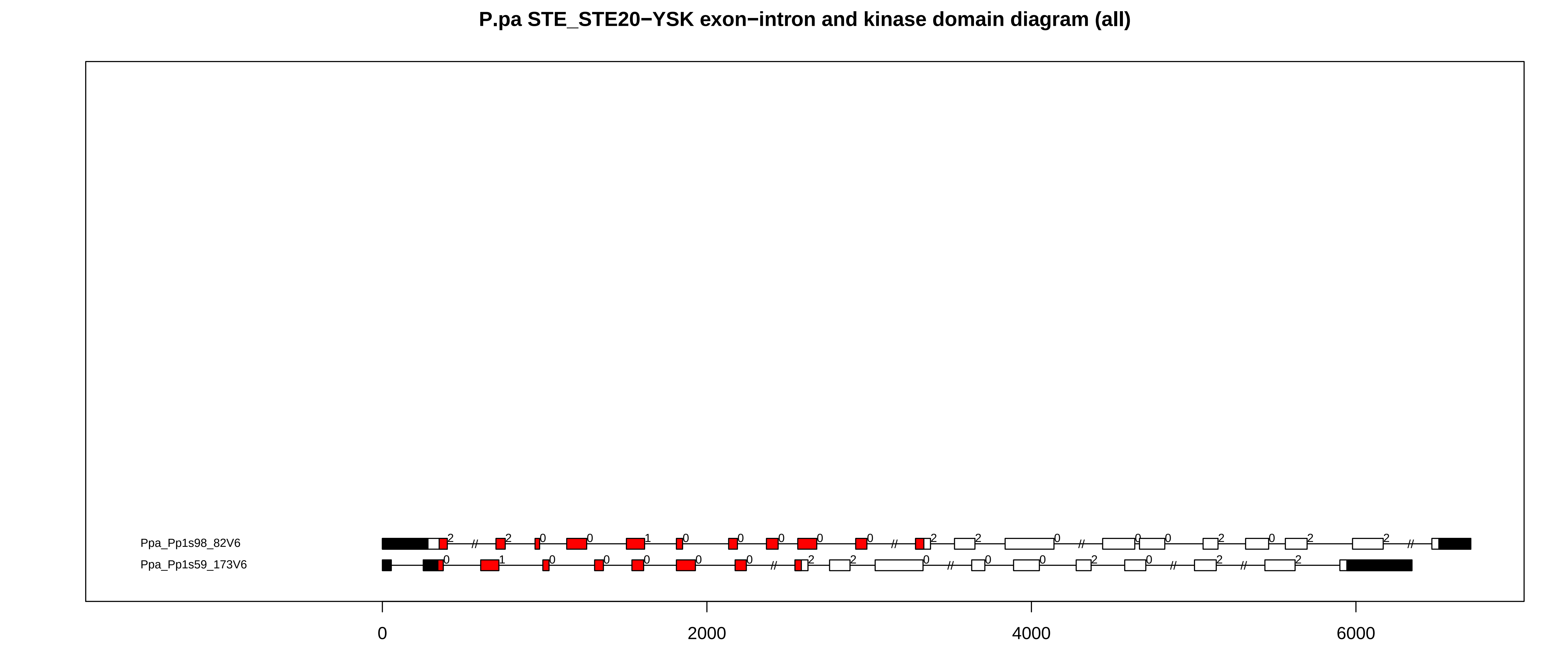

P.pa STE_STE20−YSK exon−intron and kinase domain diagram (all)
Ppa_Pp1s98_82V6
Ppa_Pp1s59_173V6
0
2000
4000
6000

P.pa STE_STE7 exon−intron and kinase domain diagram (all)

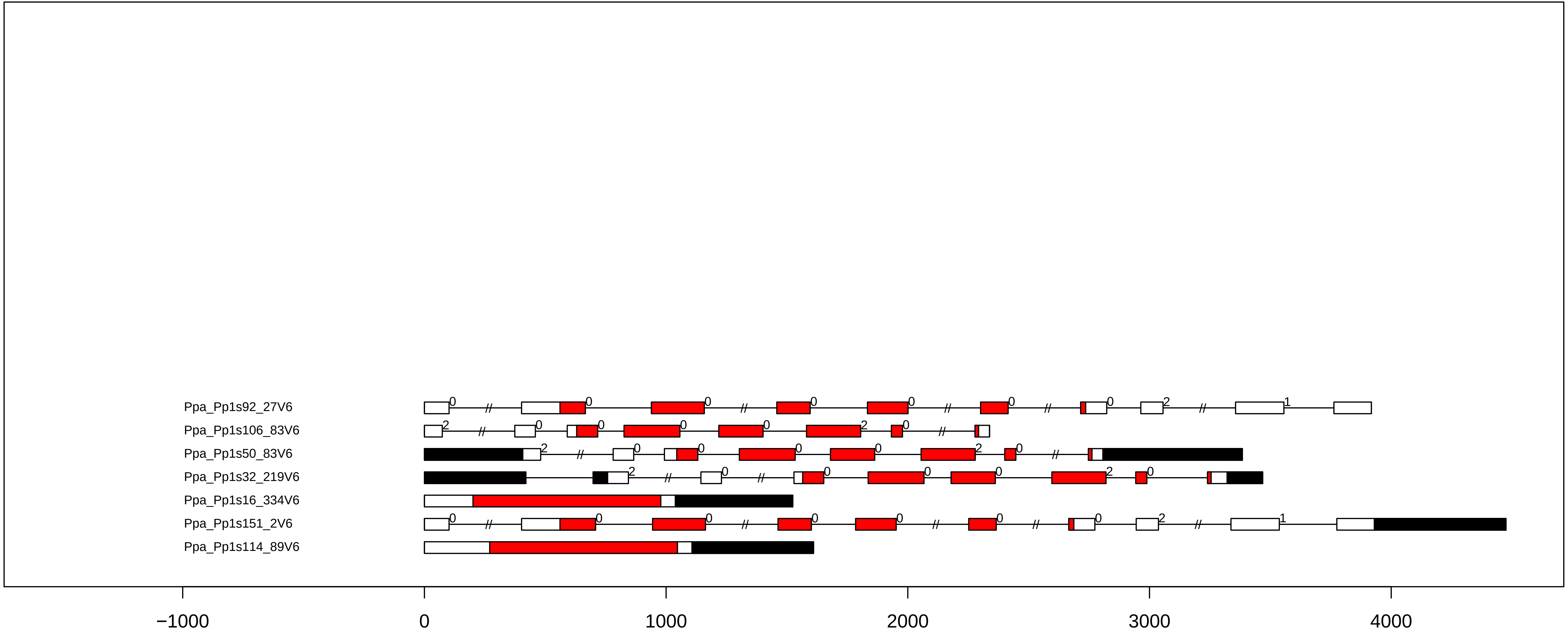

P.pa STE_STE−PI exon−intron and kinase domain diagram (all)

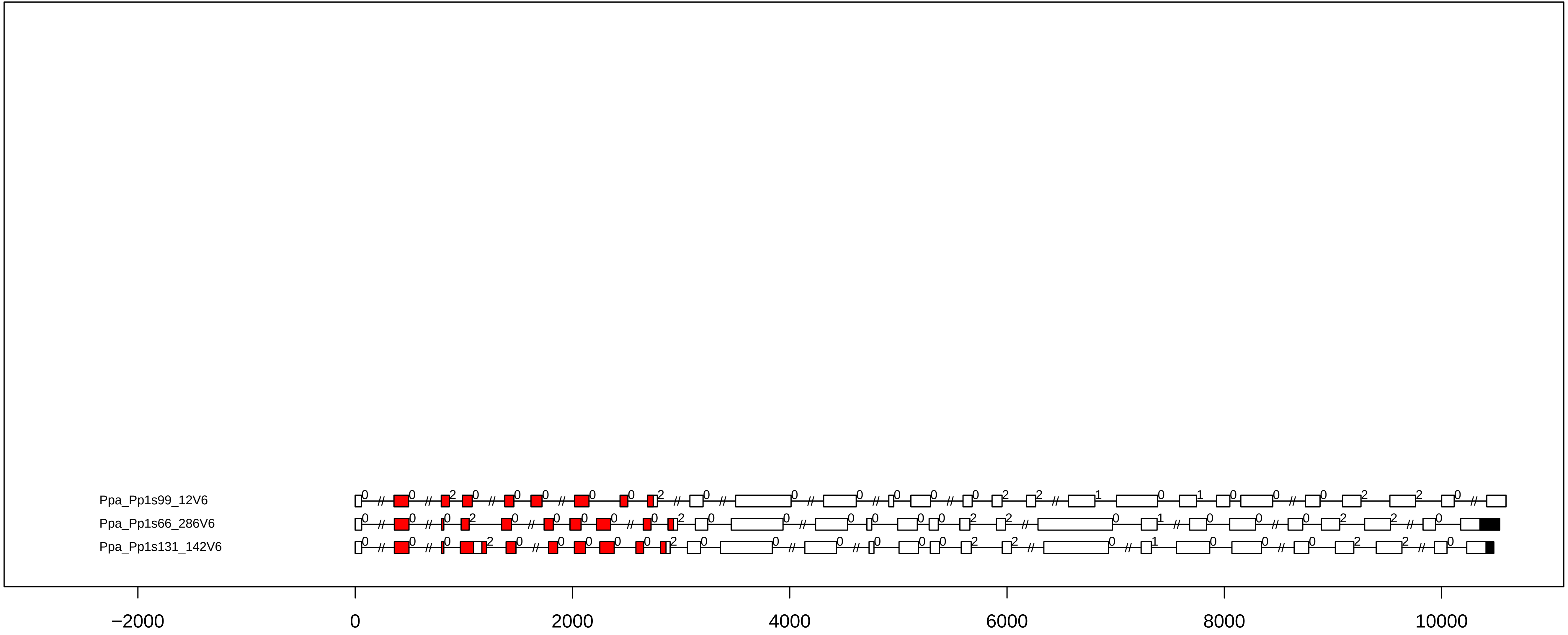

P.pa TKL_CTR1−DRK−1 exon−intron and kinase domain diagram (all)

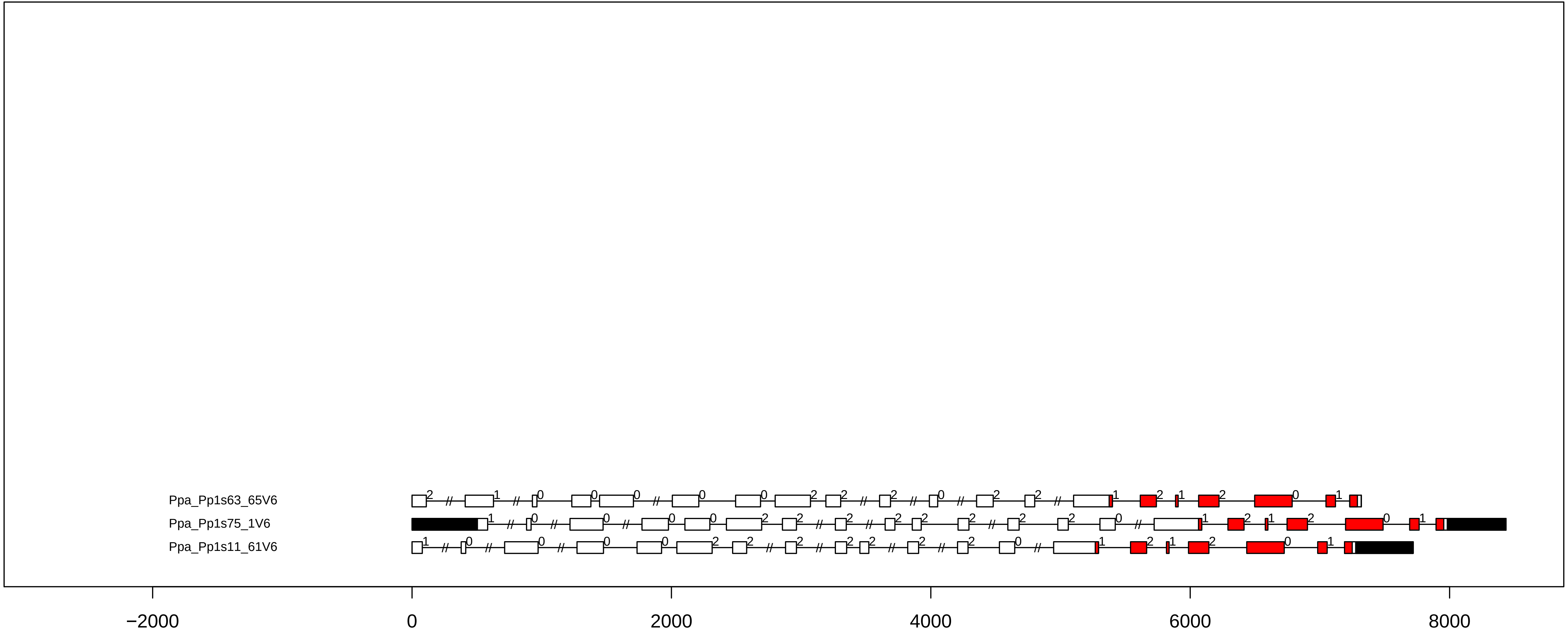

P.pa TKL_CTR1−DRK−2 exon−intron and kinase domain diagram (all)

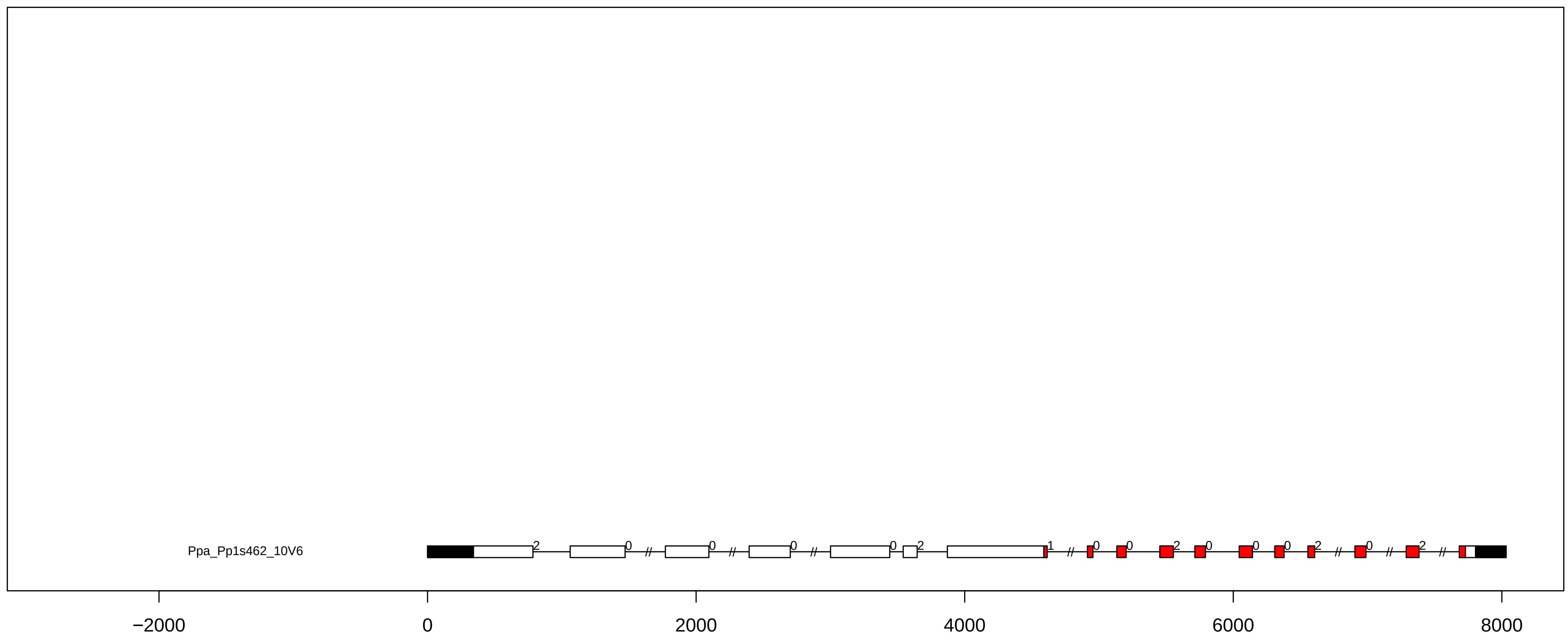

P.pa TKL_Gdt exon−intron and kinase domain diagram (part 1)

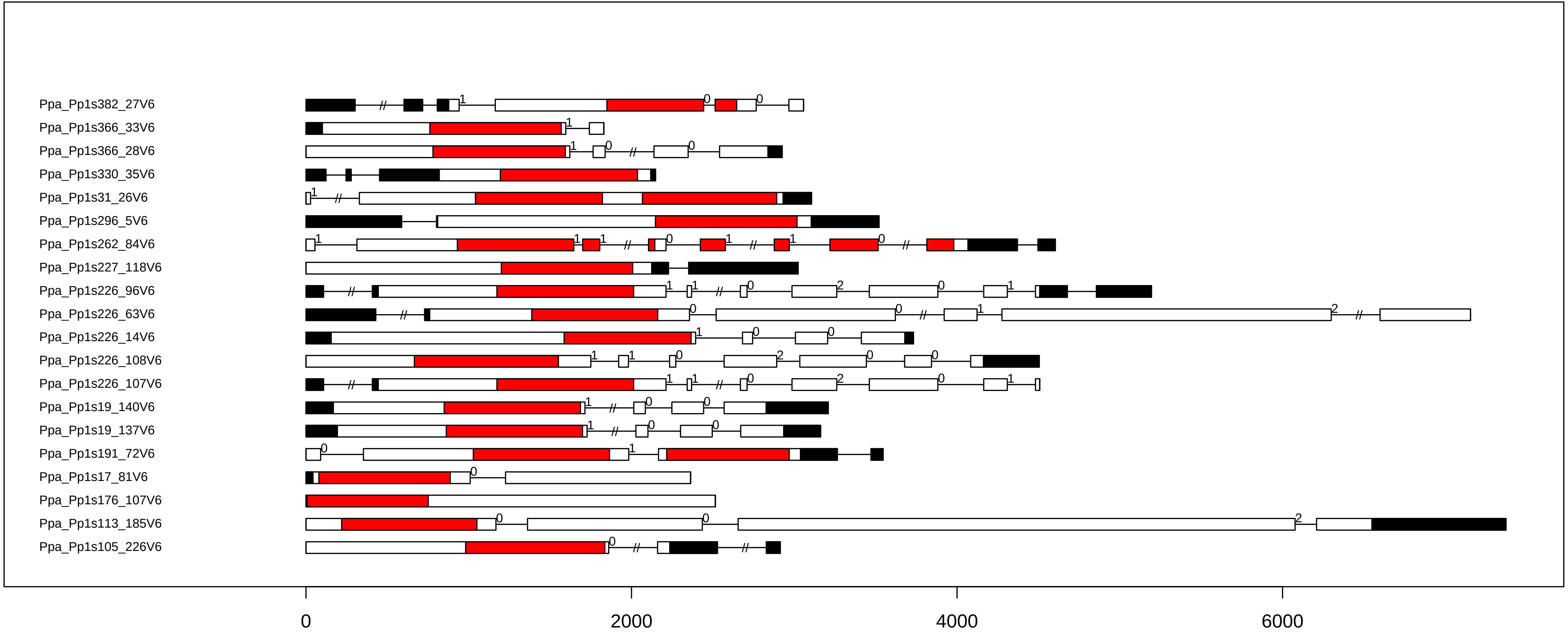

P.pa TKL_Gdt exon−intron and kinase domain diagram (part 2)

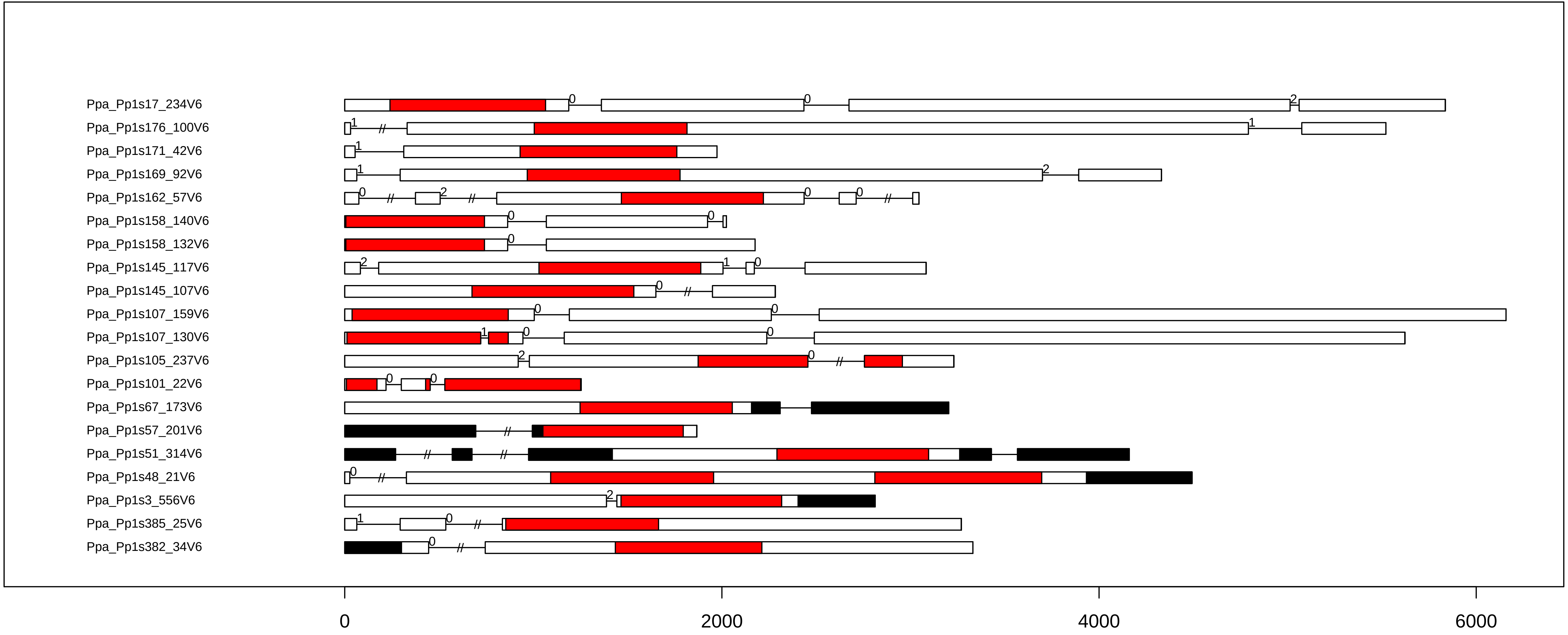

P.pa TKL_Gdt exon−intron and kinase domain diagram (part 3)

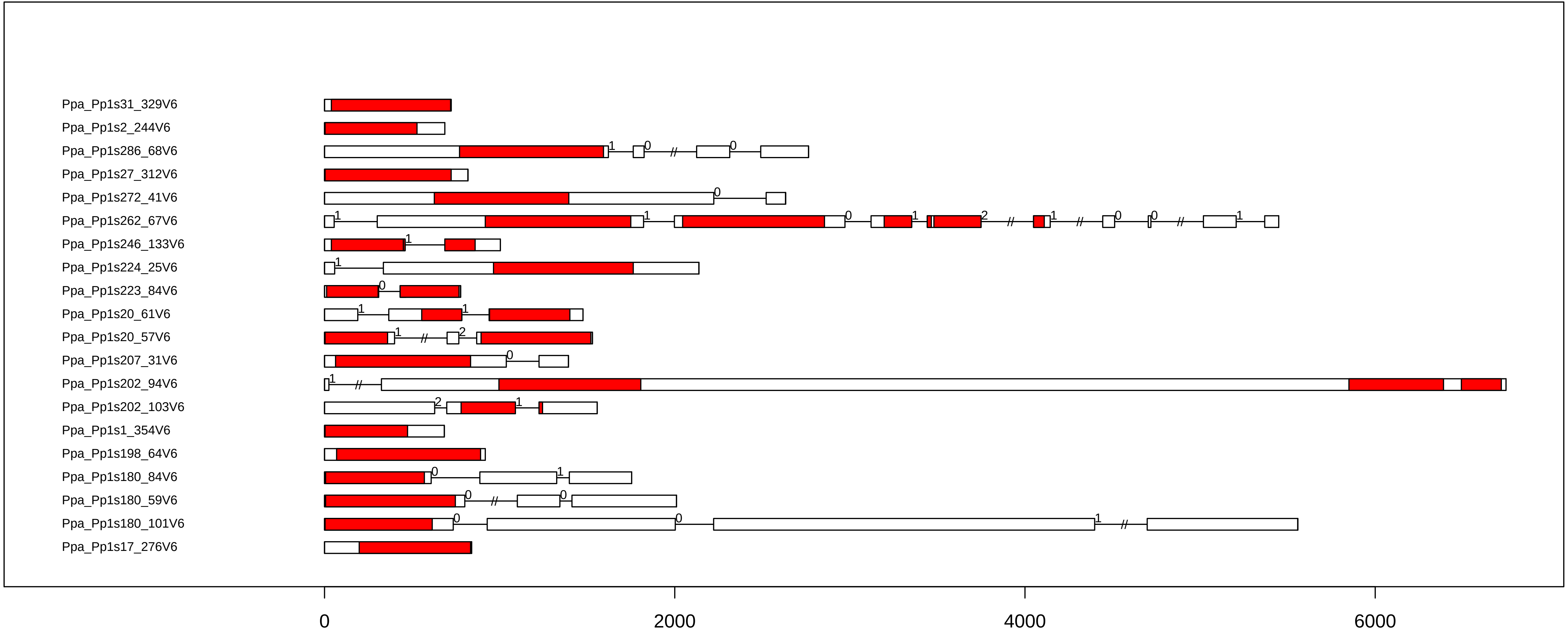

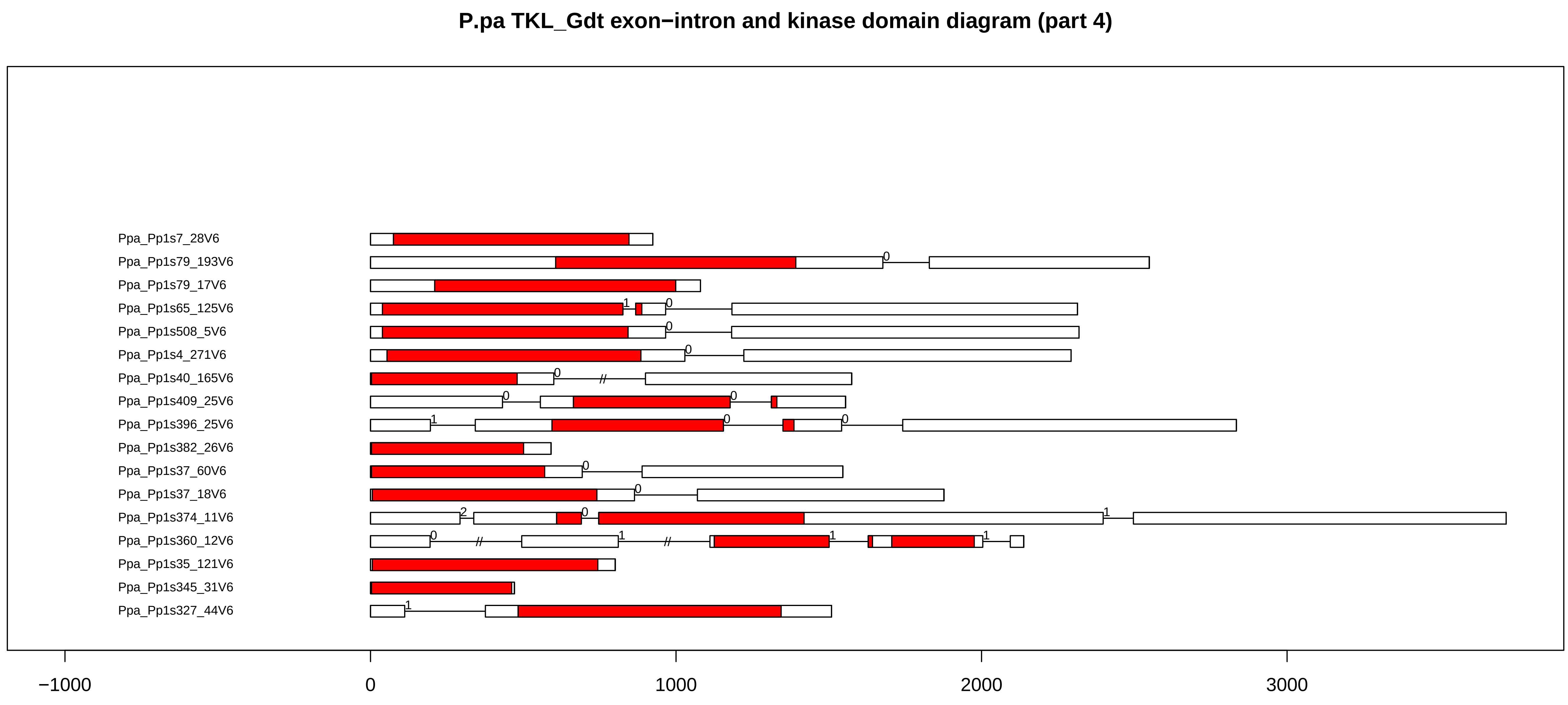

P.pa TKL_Gdt exon−intron and kinase domain diagram (part 4)
Ppa_Pp1s7_28V6
Ppa_Pp1s79_193V6
Ppa_Pp1s79_17V6
Ppa_Pp1s65_125V6
Ppa_Pp1s508_5V6
Ppa_Pp1s4_271V6
Ppa_Pp1s40_165V6
Ppa_Pp1s409_25V6
Ppa_Pp1s396_25V6
Ppa_Pp1s382_26V6
Ppa_Pp1s37_60V6
Ppa_Pp1s37_18V6
Ppa_Pp1s374_11V6
Ppa_Pp1s360_12V6
Ppa_Pp1s35_121V6
Ppa_Pp1s345_31V6
Ppa_Pp1s327_44V6
−1000
0
1000
2000
3000

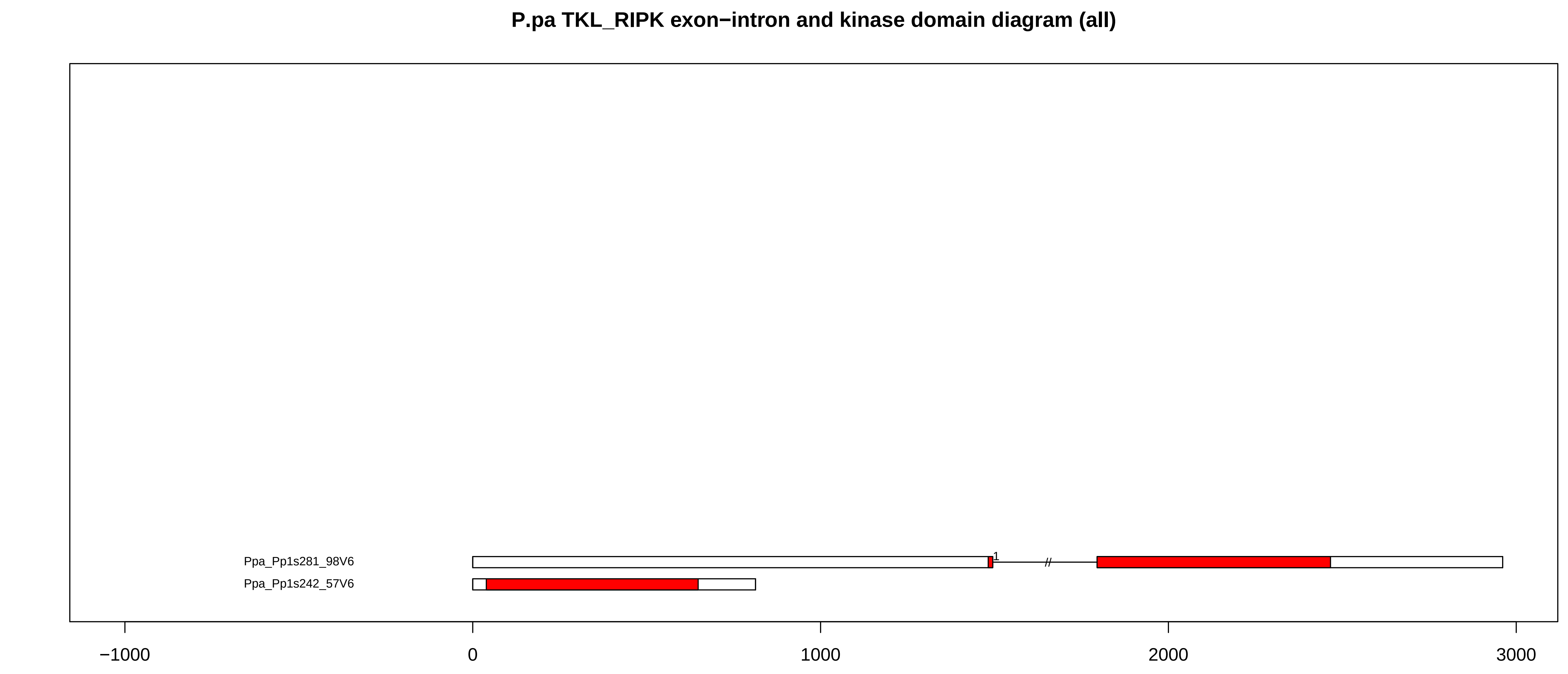

P.pa TKL_RIPK exon−intron and kinase domain diagram (all)
Ppa_Pp1s281_98V6
1
Ppa_Pp1s242_57V6
−1000
0
1000
2000
3000

P.pa TKL−Pl−1 exon−intron and kinase domain diagram (all)

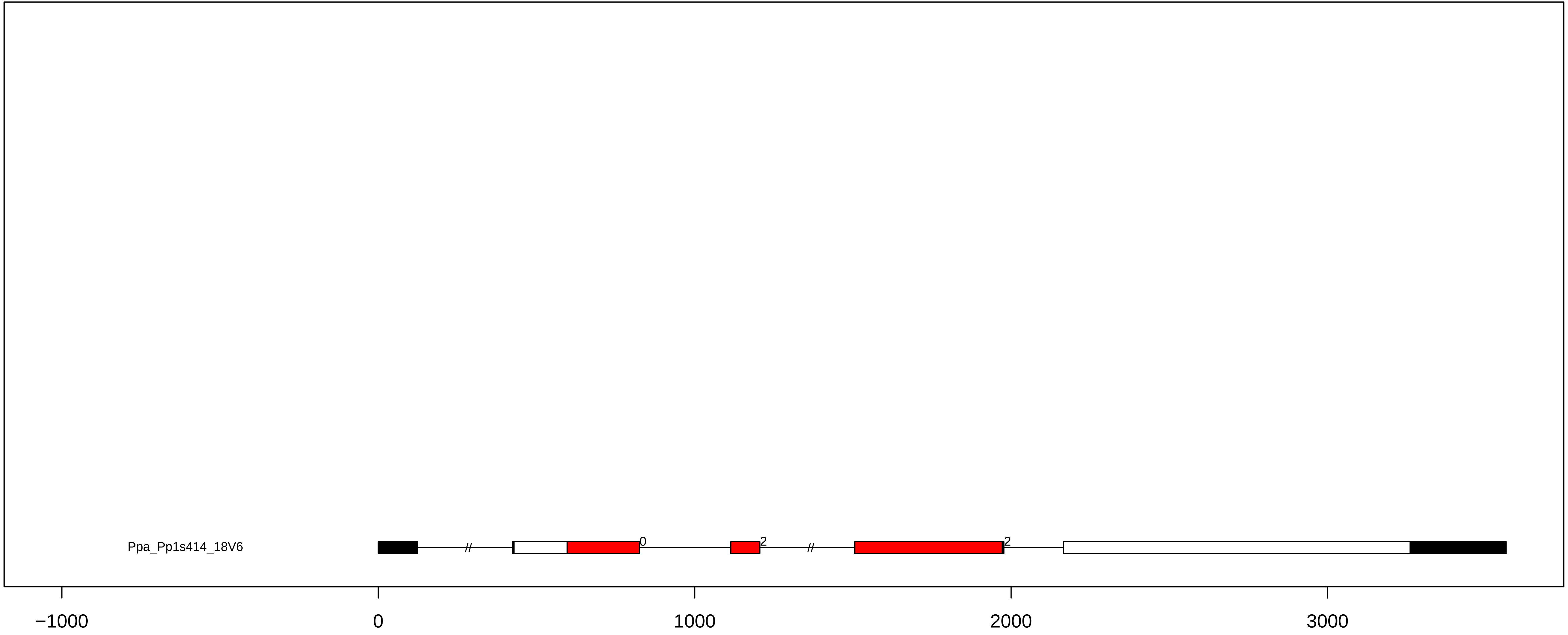

P.pa TKL−Pl−2 exon−intron and kinase domain diagram (all)

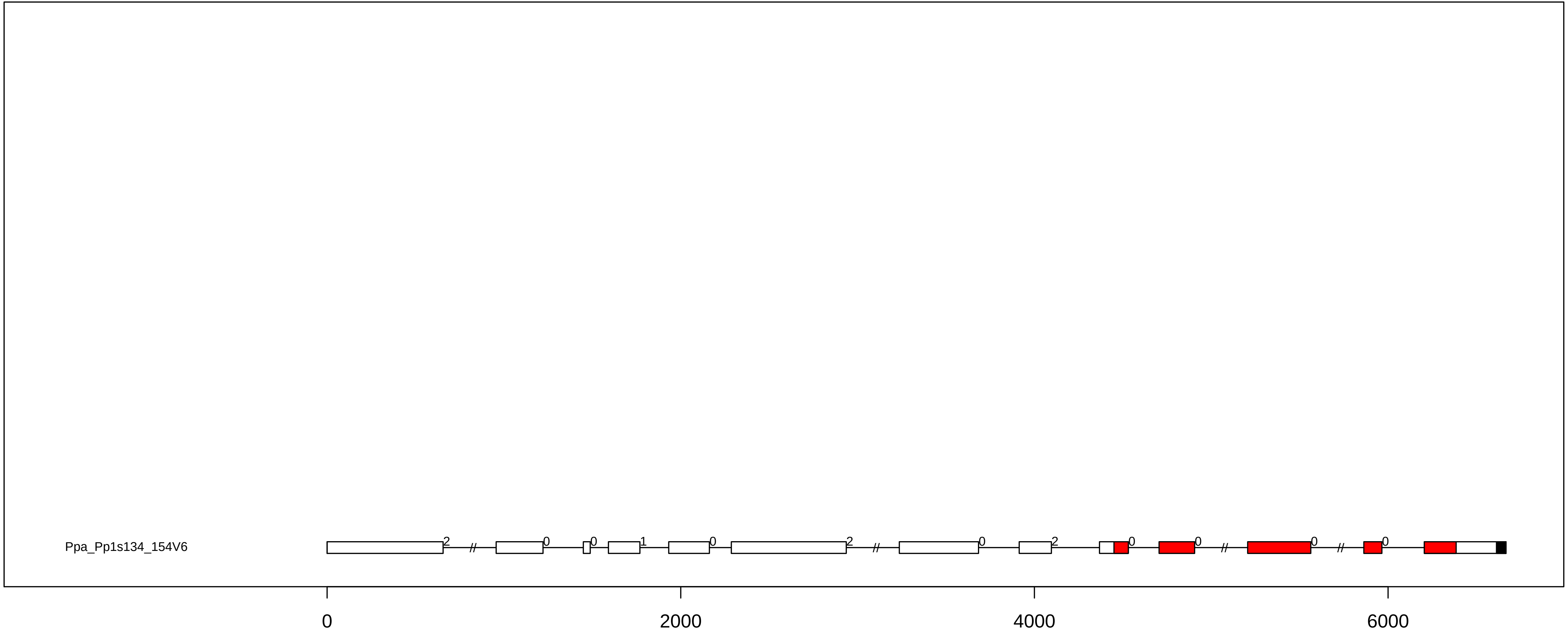

P.pa TKL−Pl−3 exon−intron and kinase domain diagram (all)

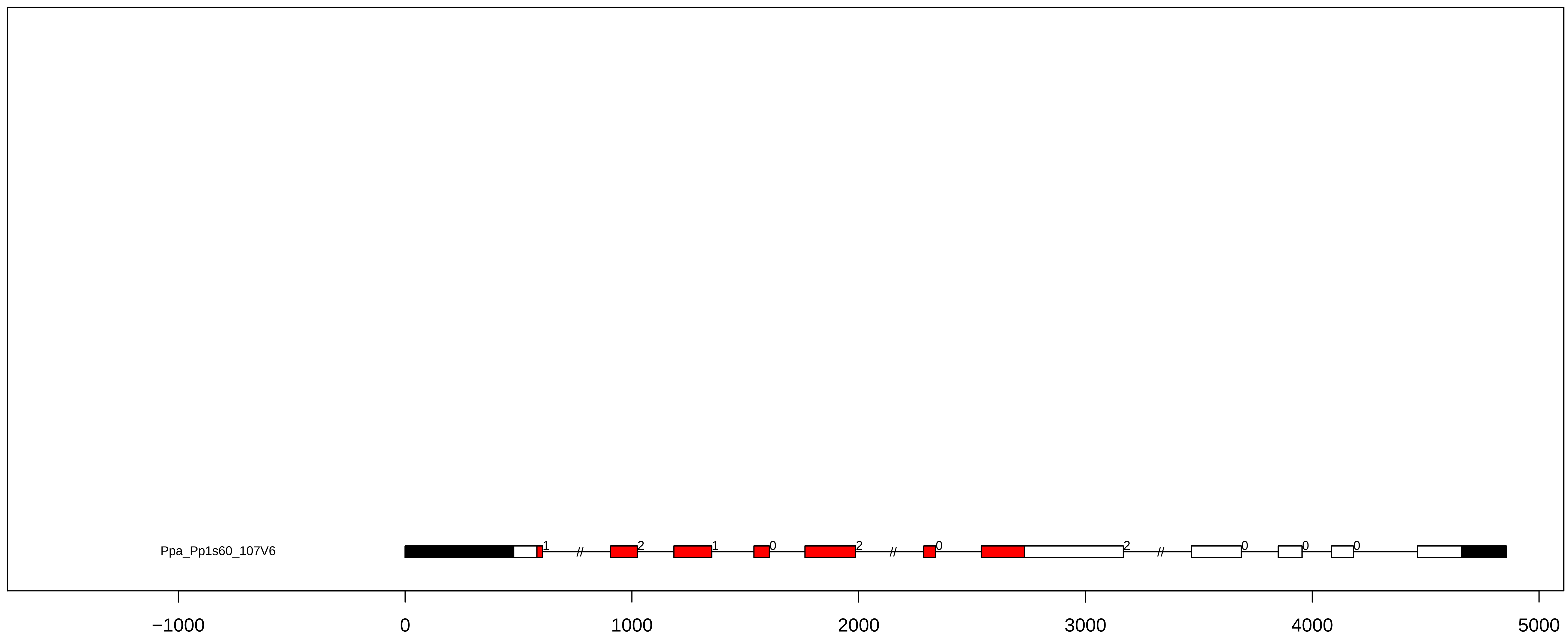

P.pa TKL−PI−4 exon−intron and kinase domain diagram (part 1)

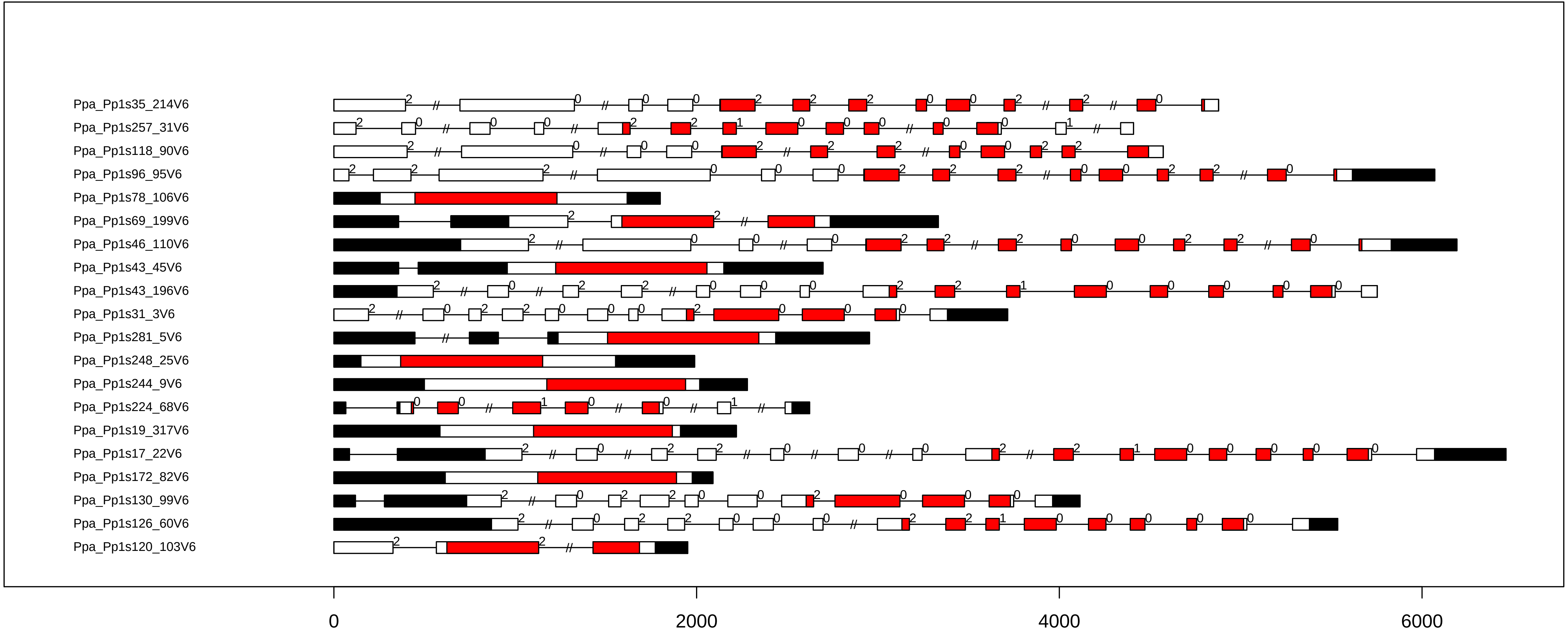

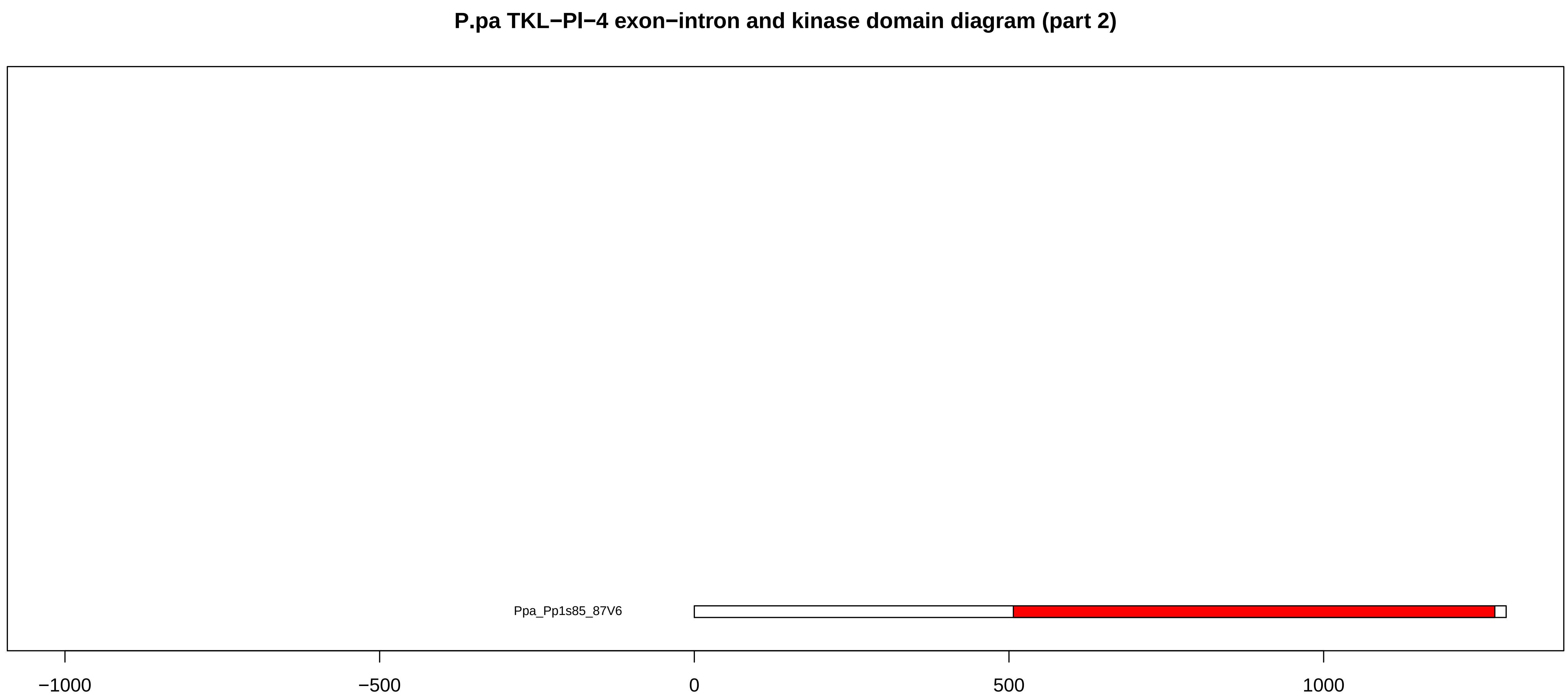
P.pa TKL−Pl−4 exon−intron and kinase domain diagram (part 2)
Ppa_Pp1s85_87V6
−1000
−500
0
500
1000

P.pa TKL−Pl−5 exon−intron and kinase domain diagram (all)

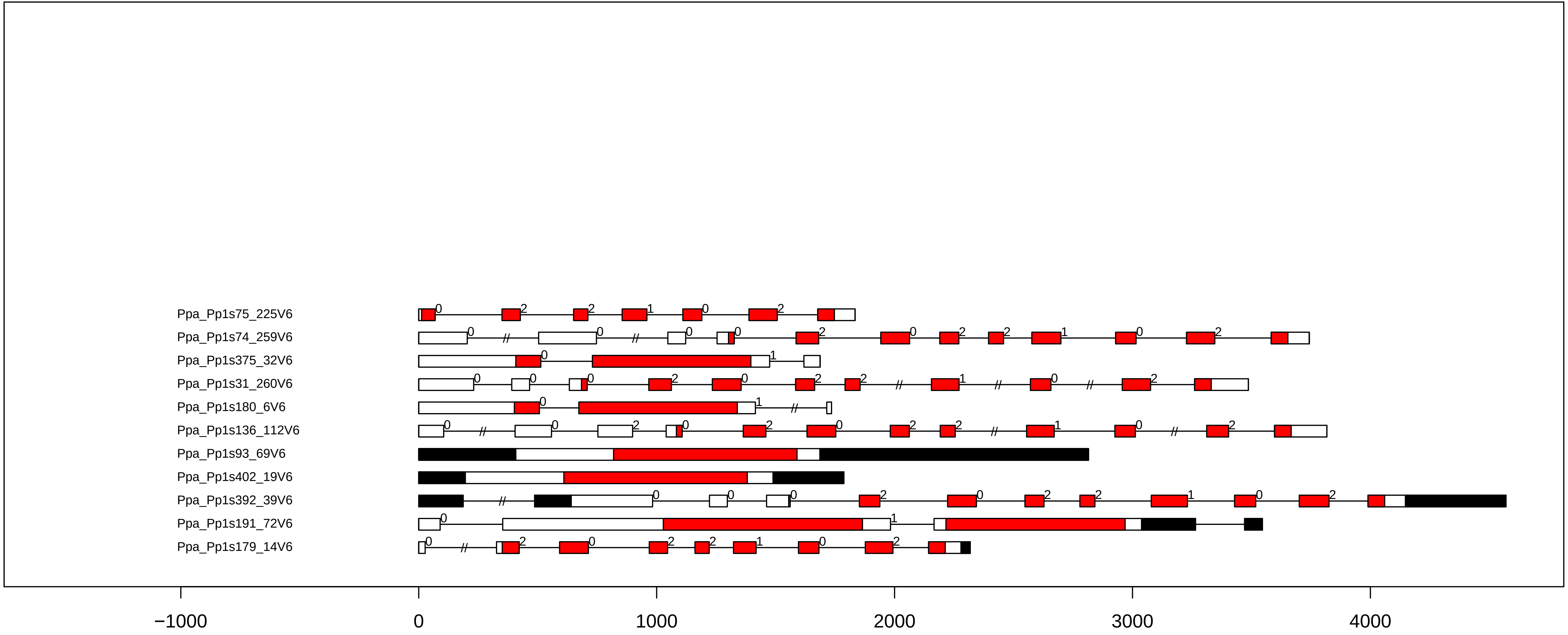

P.pa TKL−Pl−6 exon−intron and kinase domain diagram (all)

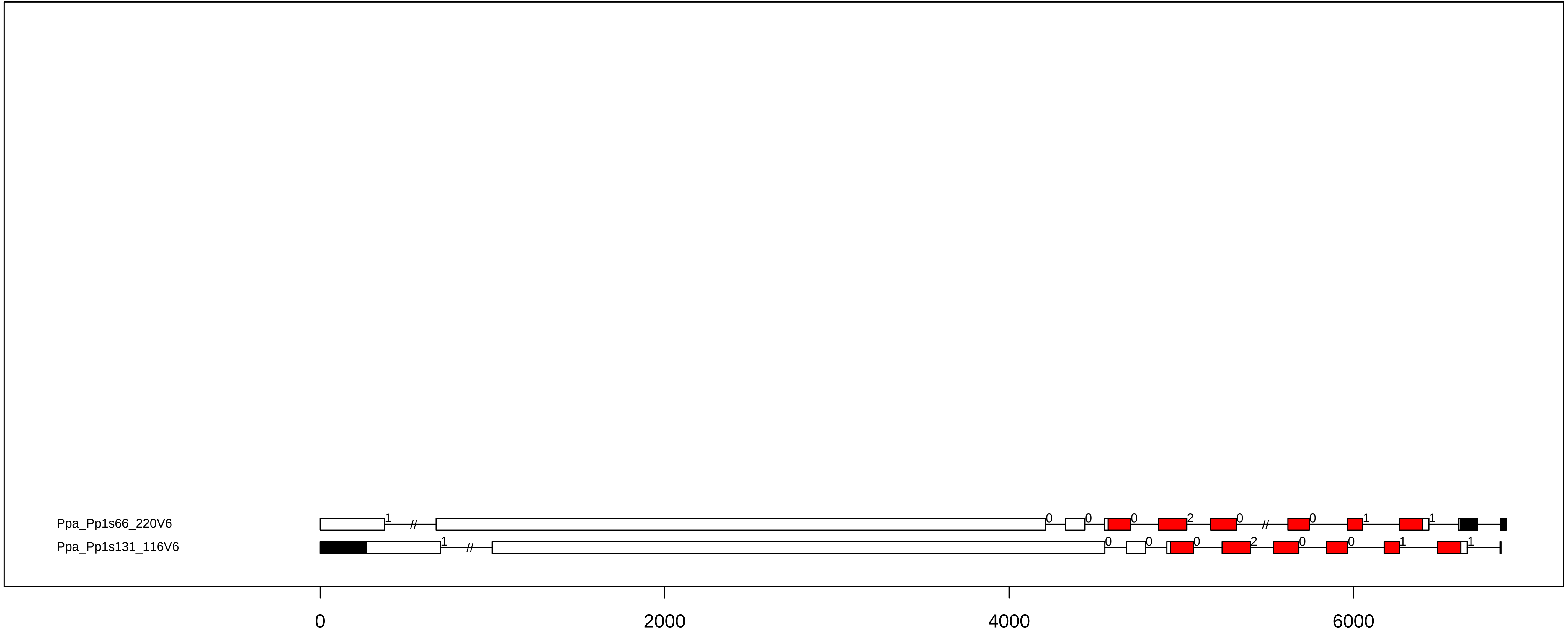

P.pa TLK exon−intron and kinase domain diagram (all)

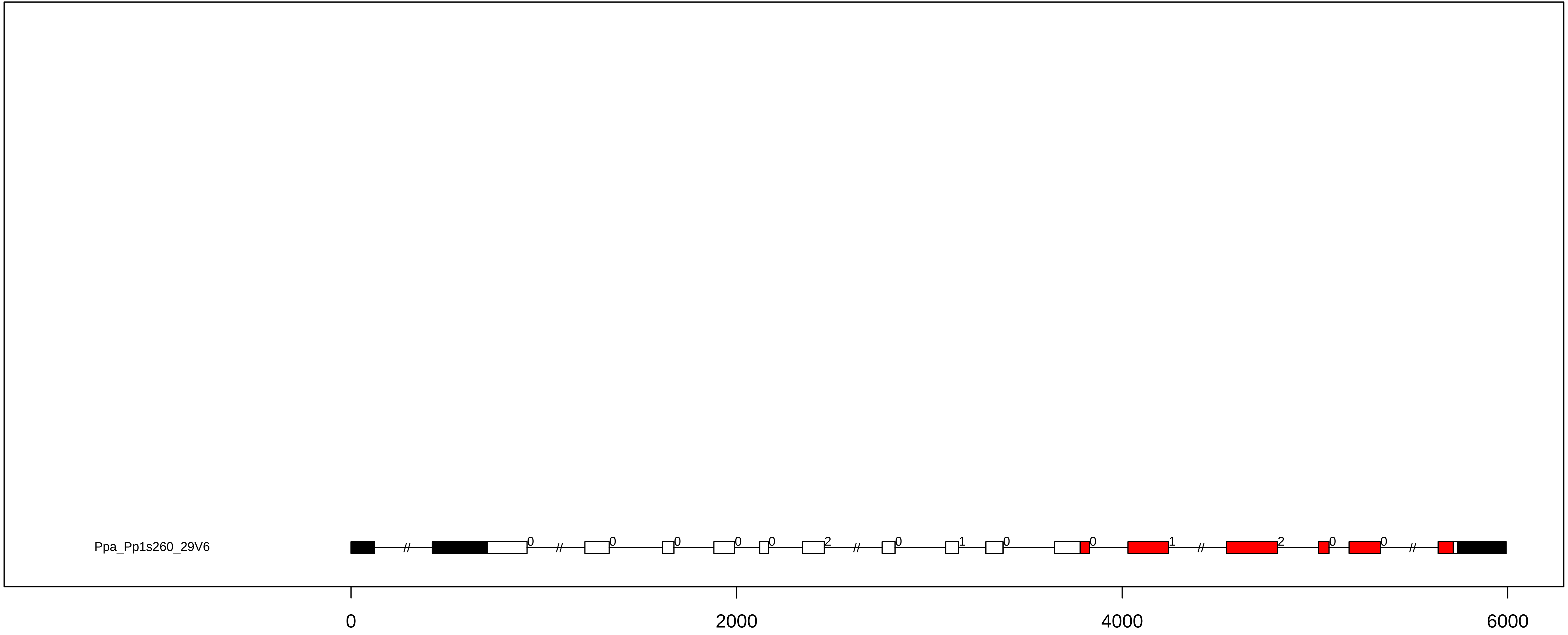

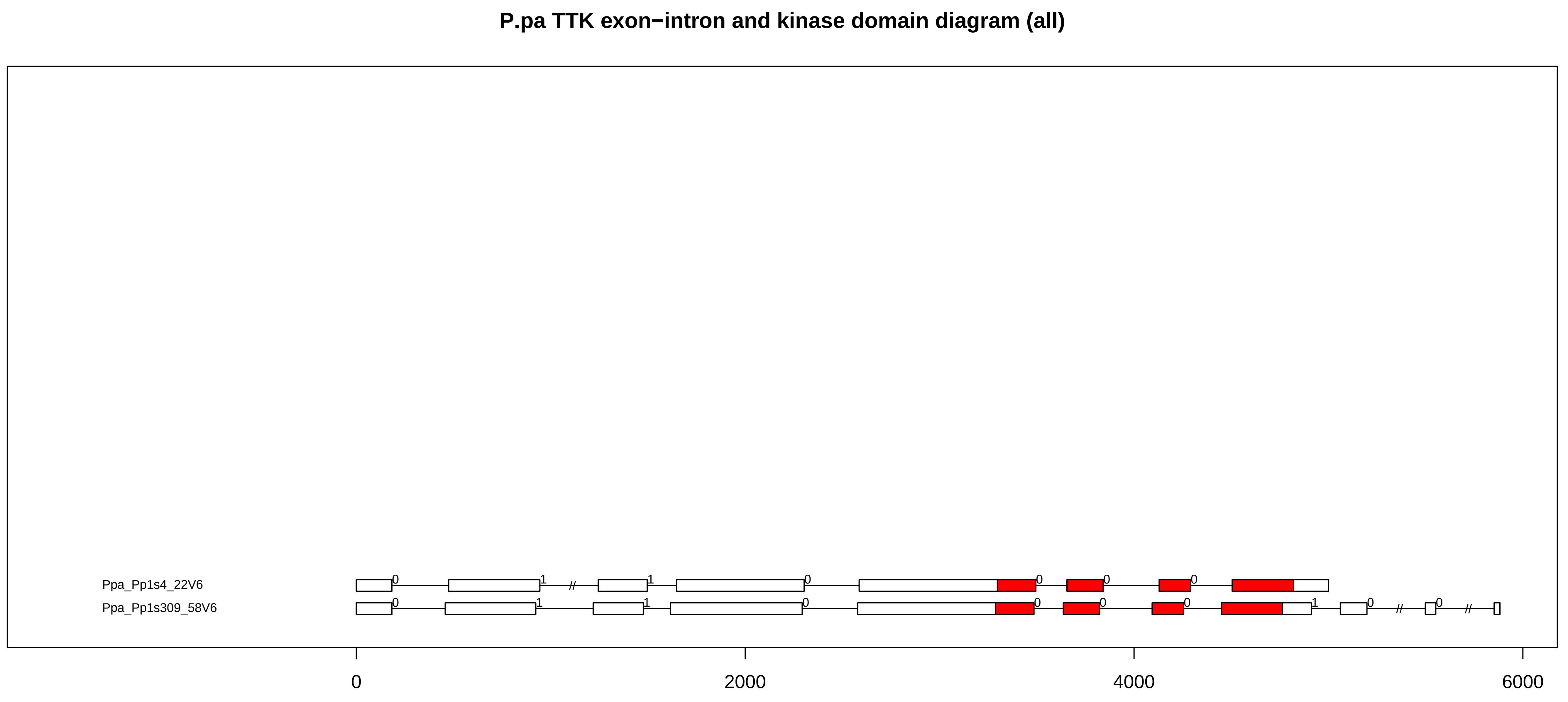

P.pa TTK exon−intron and kinase domain diagram (all)
Ppa_Pp1s4_22V6
Ppa_Pp1s309_58V6
0
2000
4000
6000

P.pa ULK_Fused exon−intron and kinase domain diagram (all)

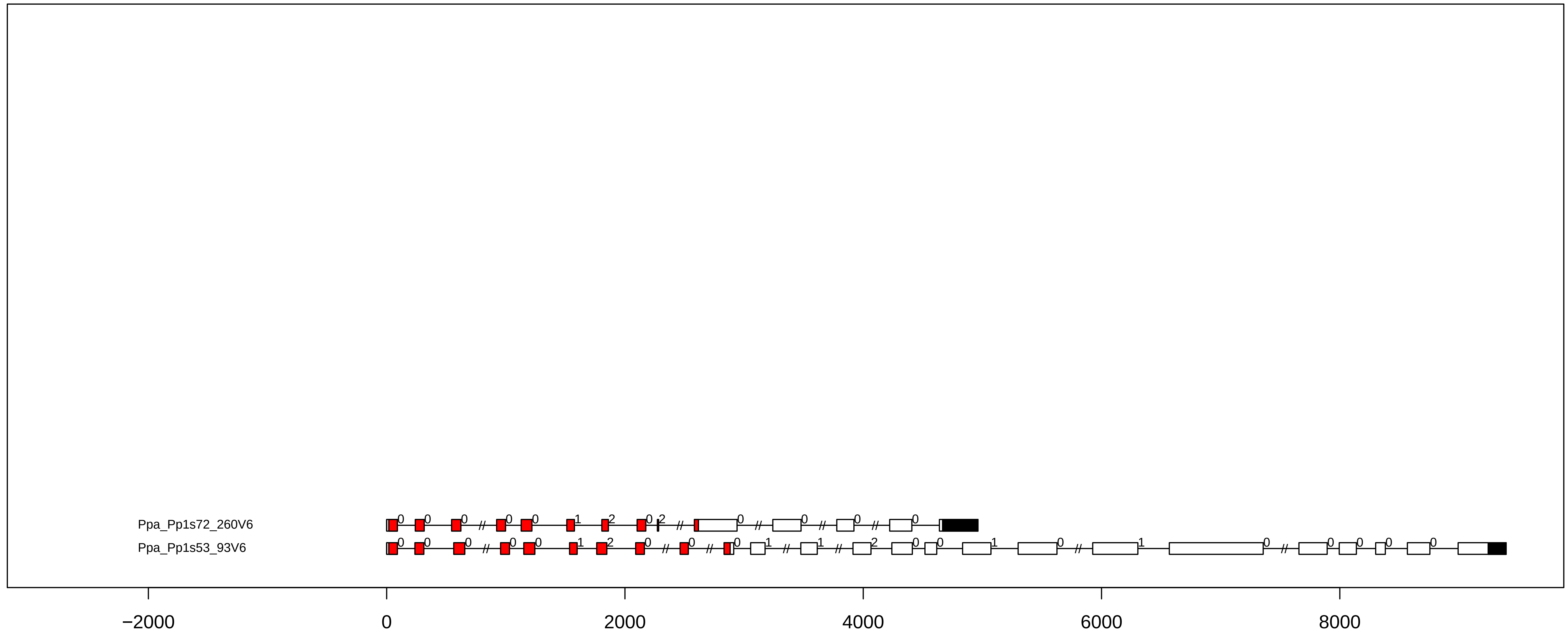

P.pa ULK_ULK4 exon−intron and kinase domain diagram (all)

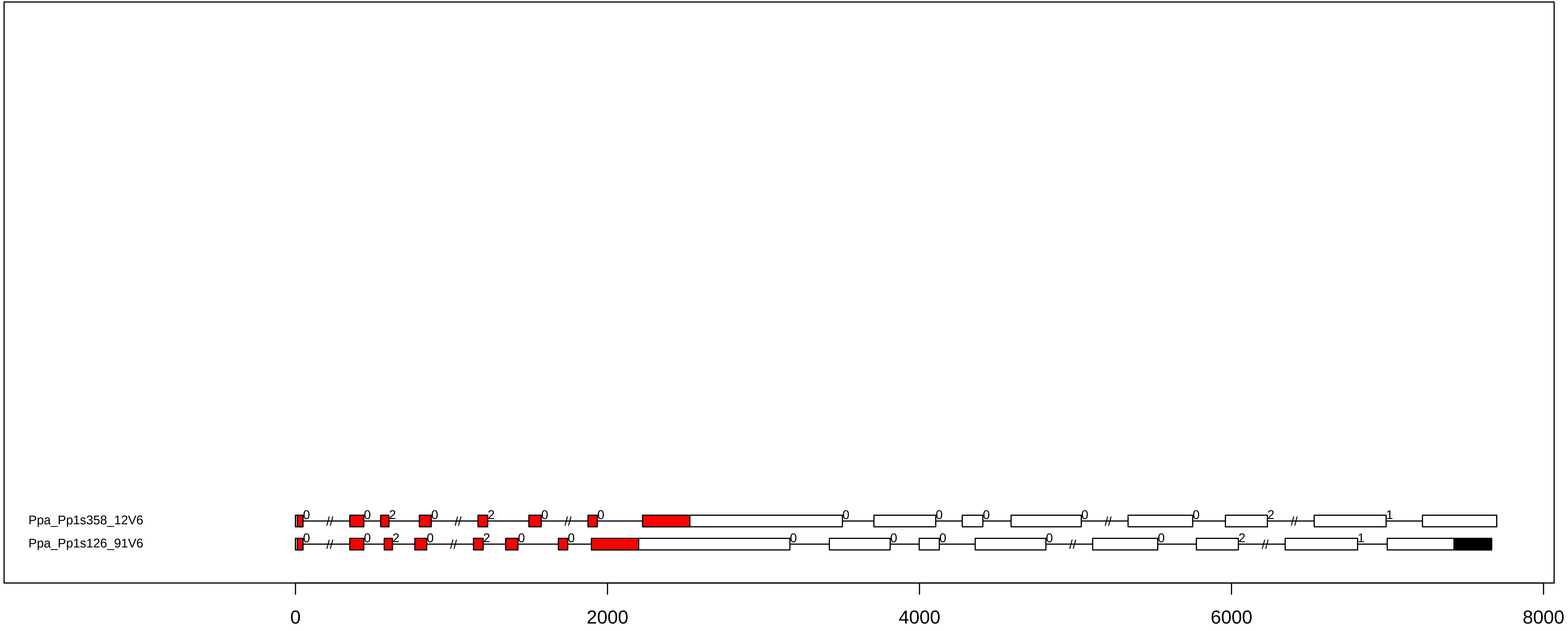

P.pa WEE exon−intron and kinase domain diagram (all)

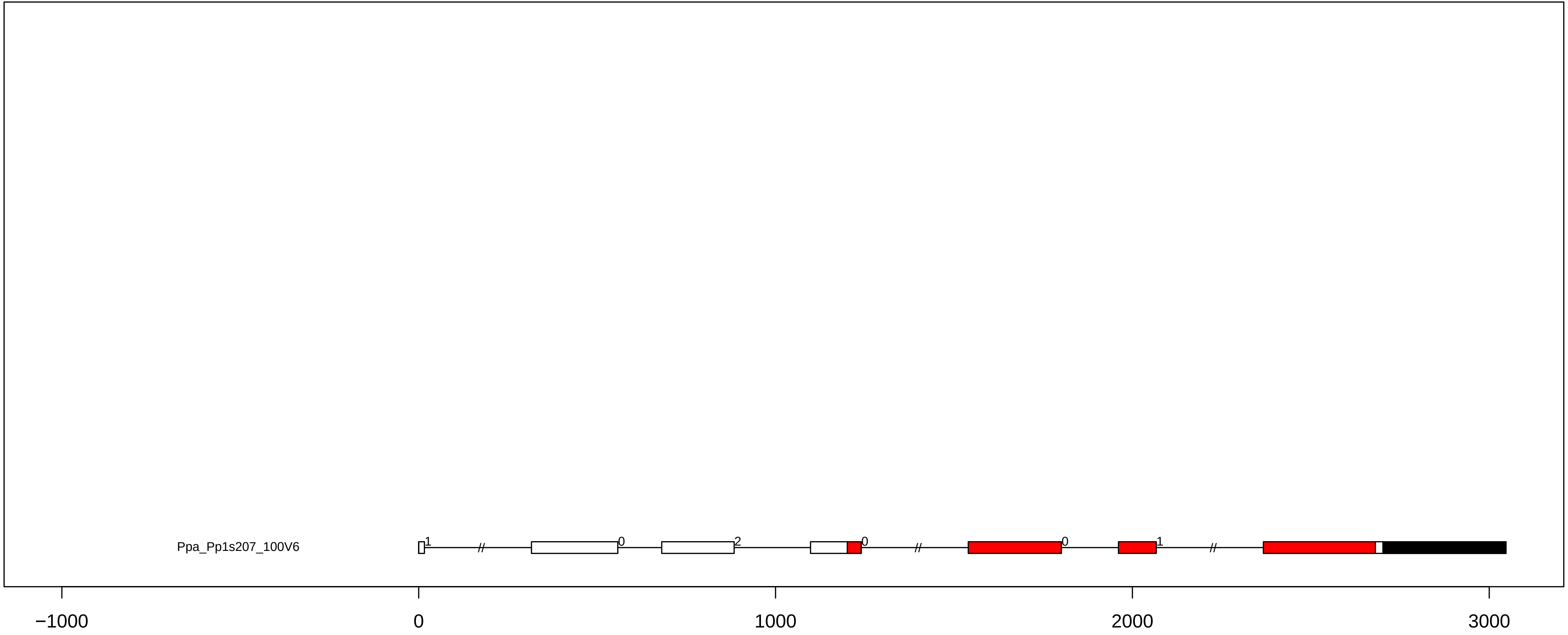

P.pa WNK_NRBP exon−intron and kinase domain diagram (all)

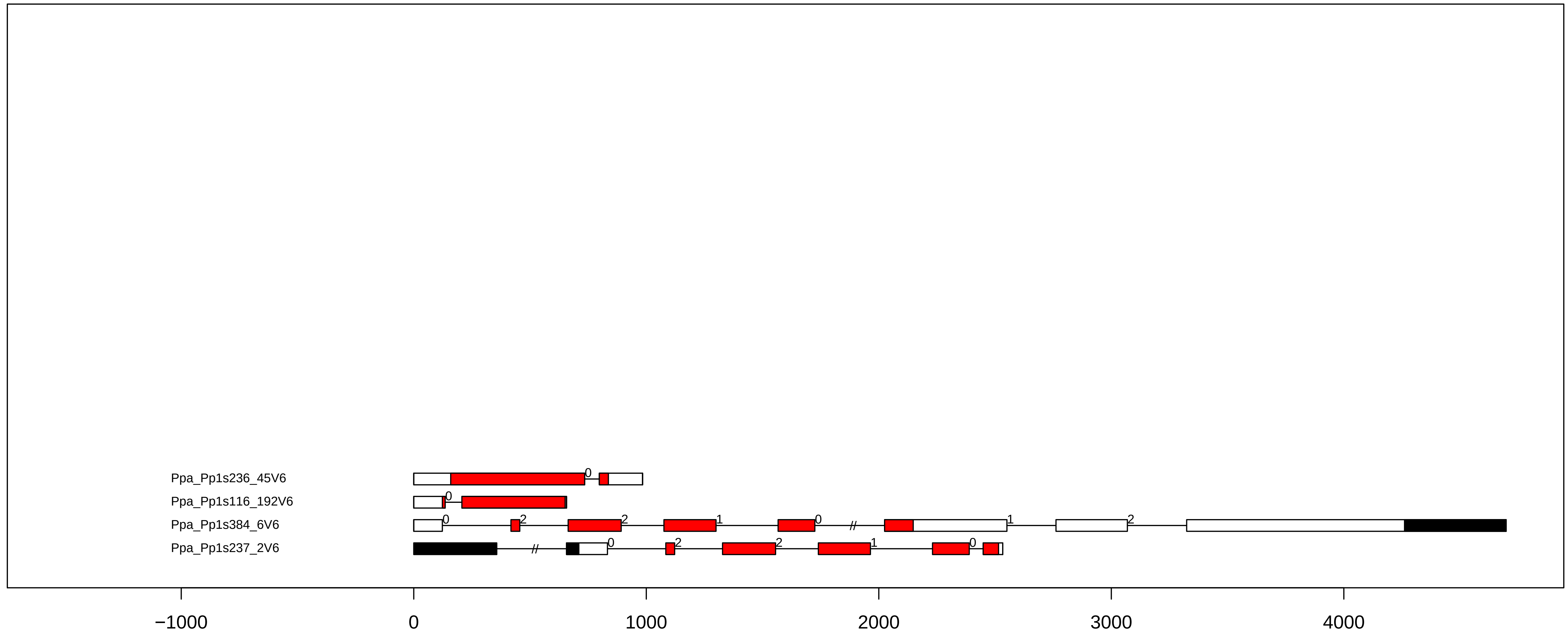

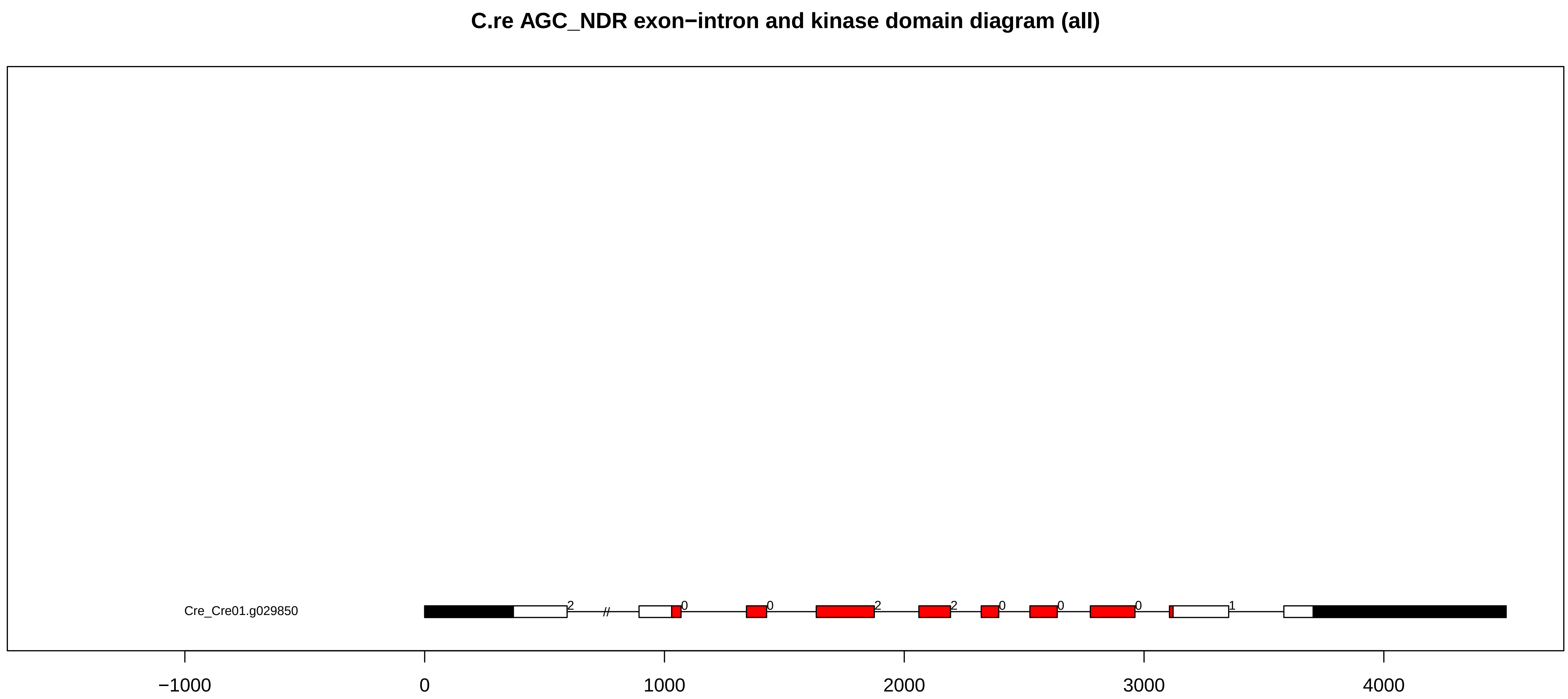
C.re AGC_NDR exon−intron and kinase domain diagram (all)
Cre_Cre01.g029850
2
0
0
2
2
0
0
0
1
−1000
0
1000
2000
3000
4000

C.re AGC_PKA−PKG exon−intron and kinase domain diagram (all)

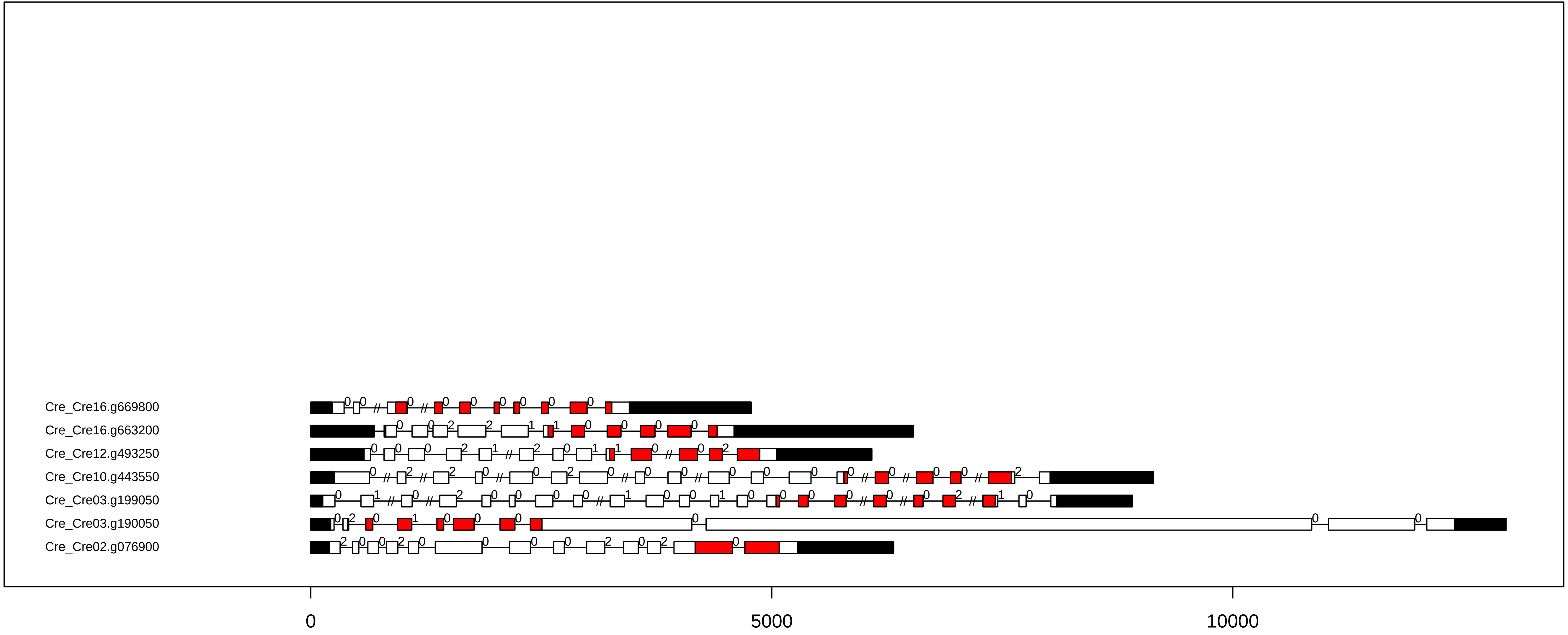

C.re AGC_RSK−1 exon−intron and kinase domain diagram (all)

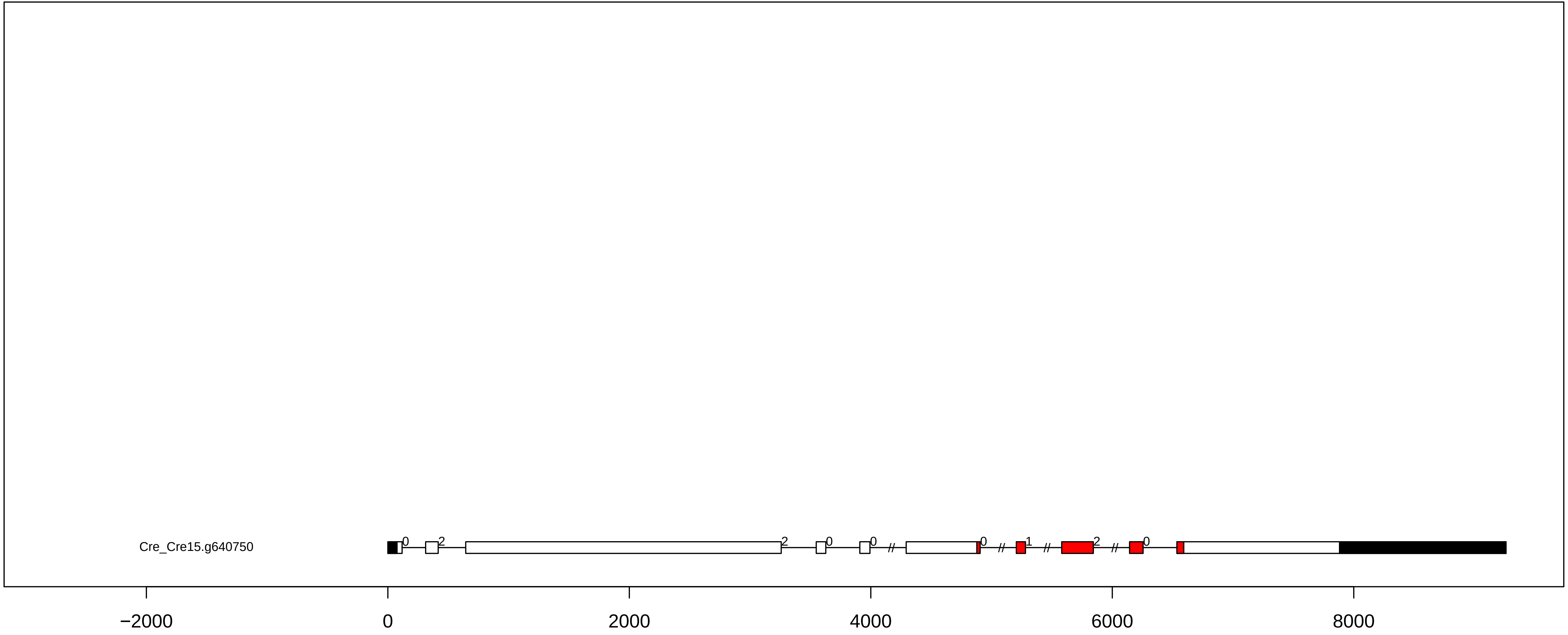

C.re AGC_RSK−2 exon−intron and kinase domain diagram (all)

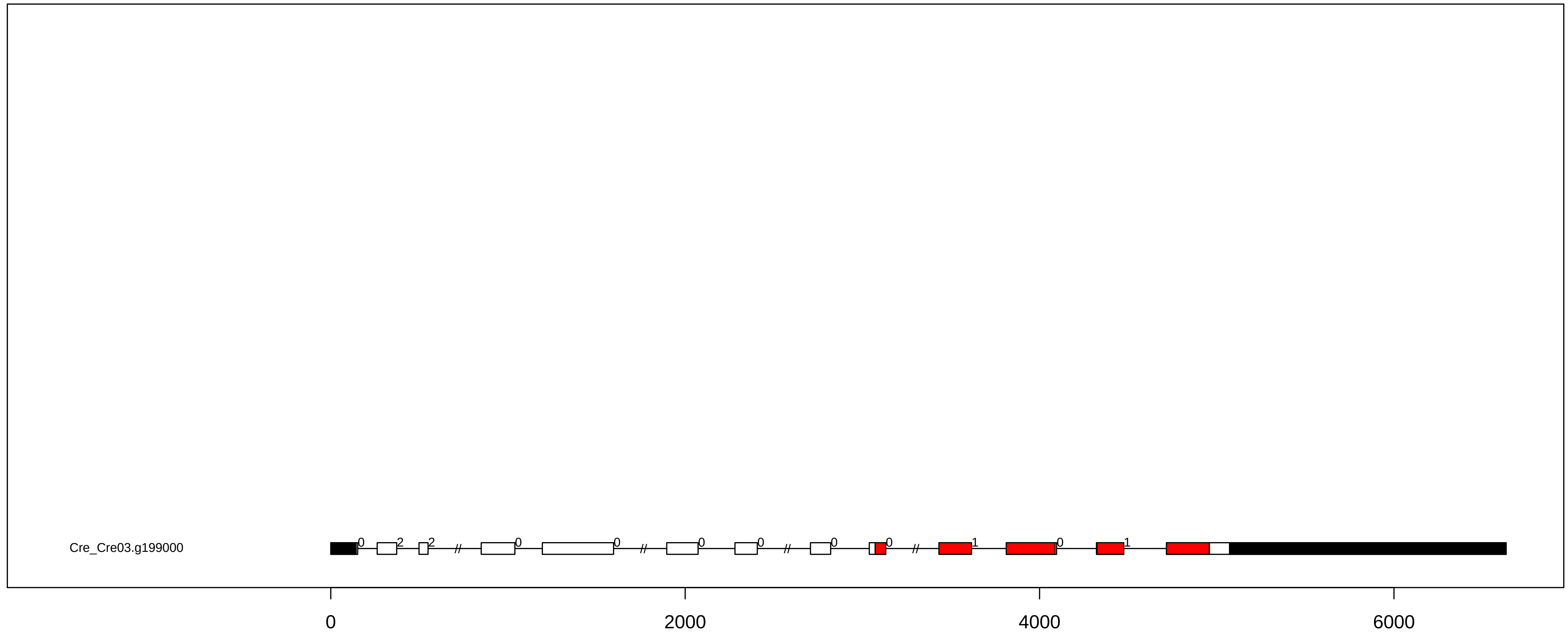

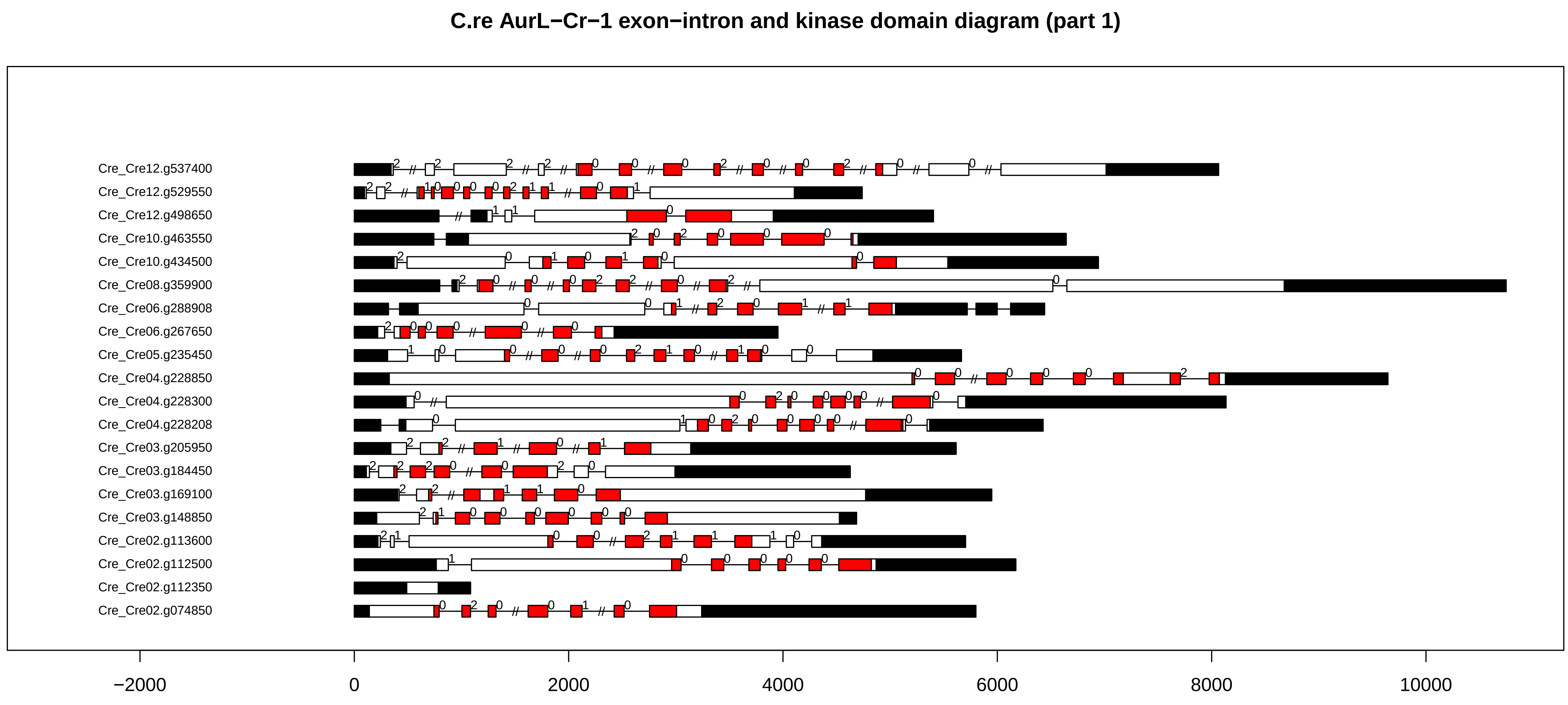
C.re AurL−Cr−1 exon−intron and kinase domain diagram (part 1)
Cre_Cre12.g537400
Cre_Cre12.g529550
Cre_Cre12.g498650
Cre_Cre10.g463550
Cre_Cre10.g434500
Cre_Cre08.g359900
Cre_Cre06.g288908
Cre_Cre06.g267650
Cre_Cre05.g235450
Cre_Cre04.g228850
Cre_Cre04.g228300
Cre_Cre04.g228208
Cre_Cre03.g205950
Cre_Cre03.g184450
Cre_Cre03.g169100
Cre_Cre03.g148850
Cre_Cre02.g113600
Cre_Cre02.g112500
Cre_Cre02.g112350
Cre_Cre02.g074850
−2000
0
2000
4000
6000
8000
10000

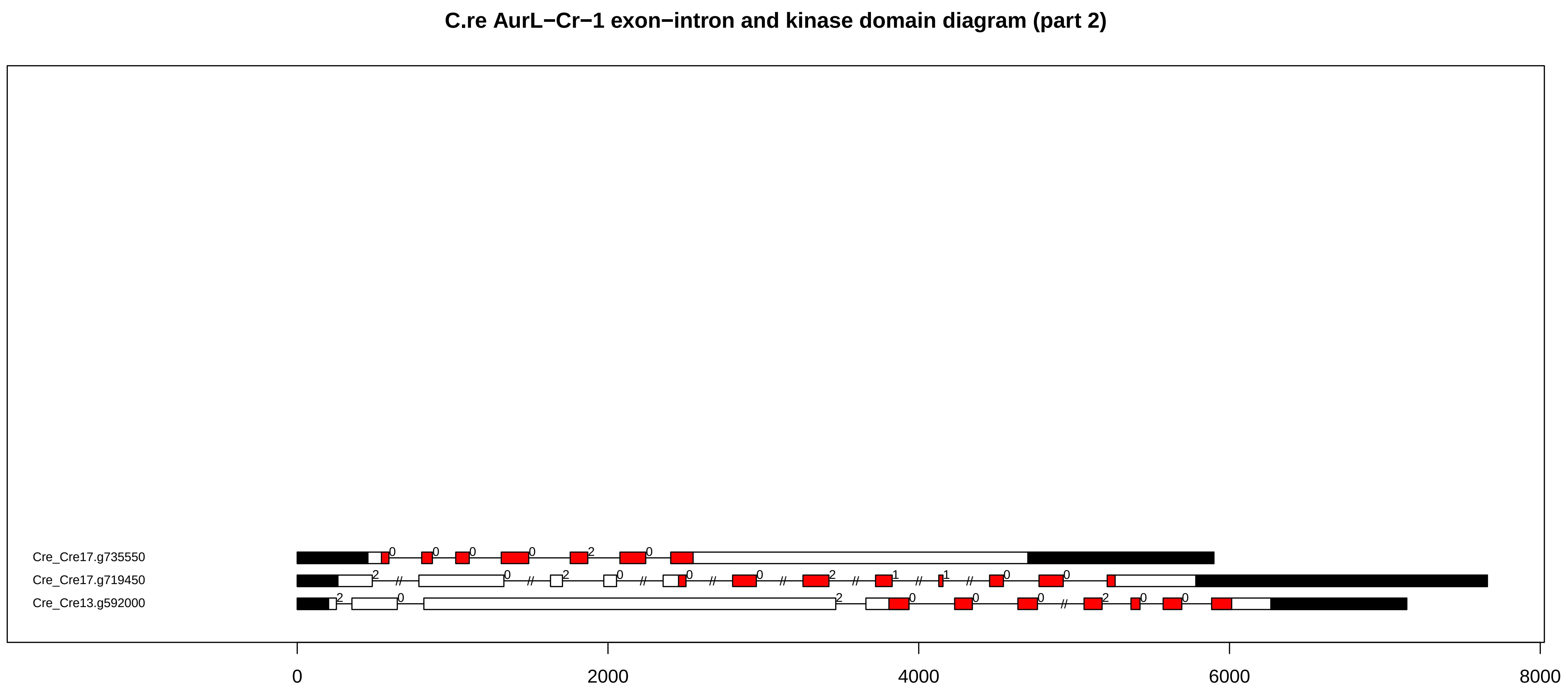

C.re AurL−Cr−1 exon−intron and kinase domain diagram (part 2)
Cre_Cre17.g735550
Cre_Cre17.g719450
Cre_Cre13.g592000
0
2000
4000
6000
8000

C.re AurL−Cr−2 exon−intron and kinase domain diagram (all)

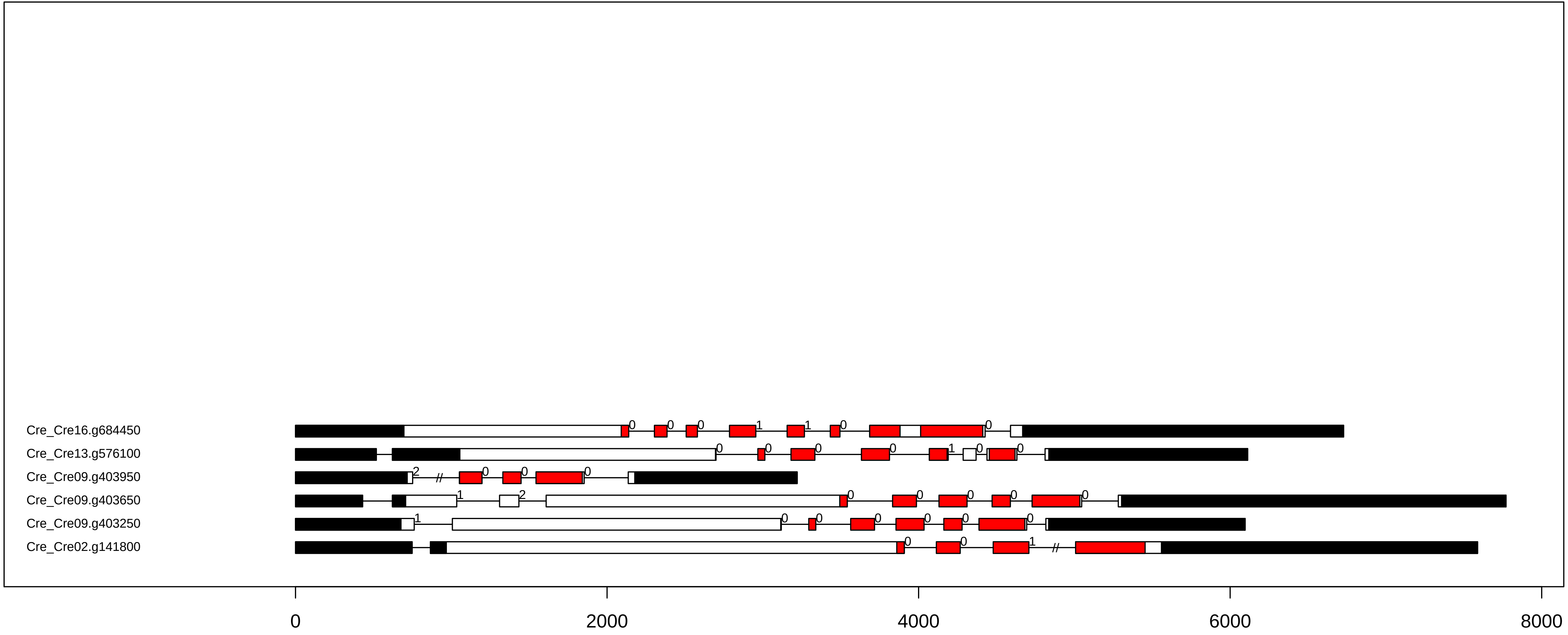

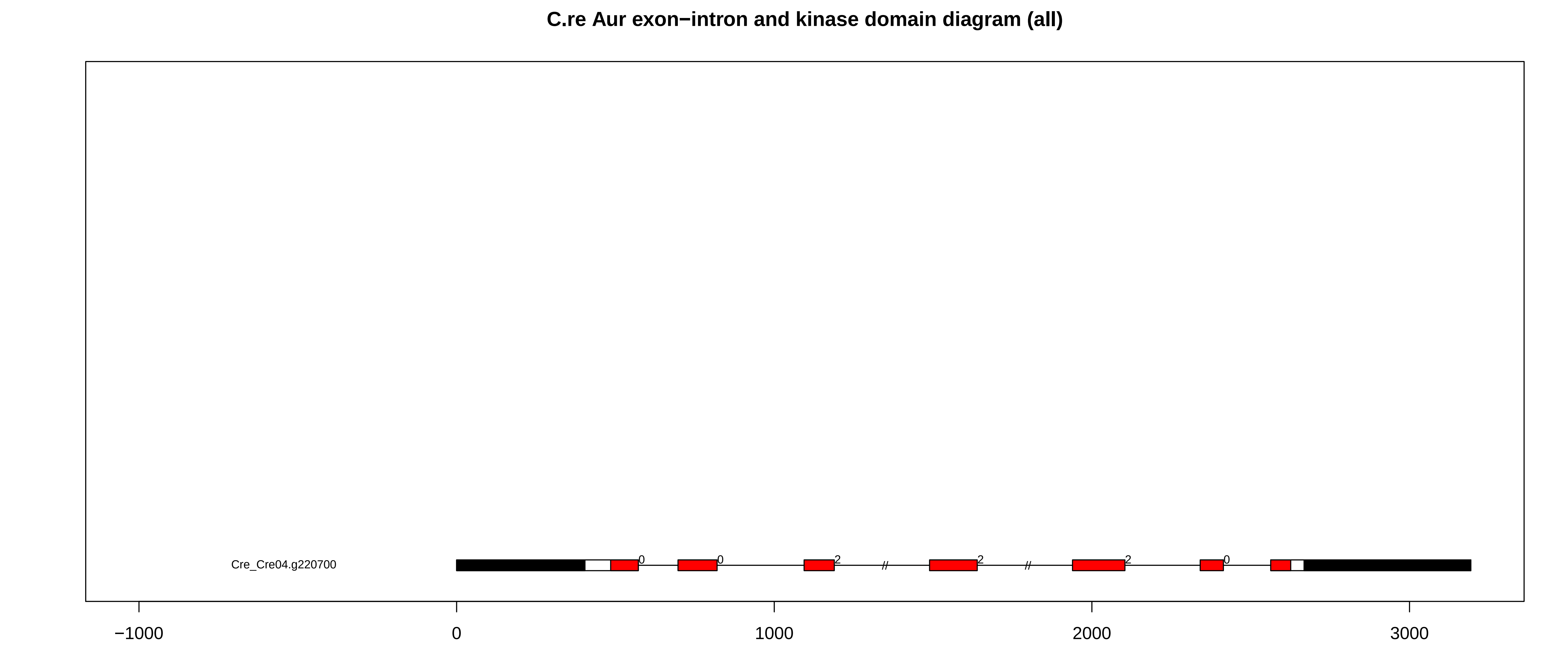
C.re Aur exon−intron and kinase domain diagram (all)
Cre_Cre04.g220700
0
0
2
2
2
0
−1000
0
1000
2000
3000

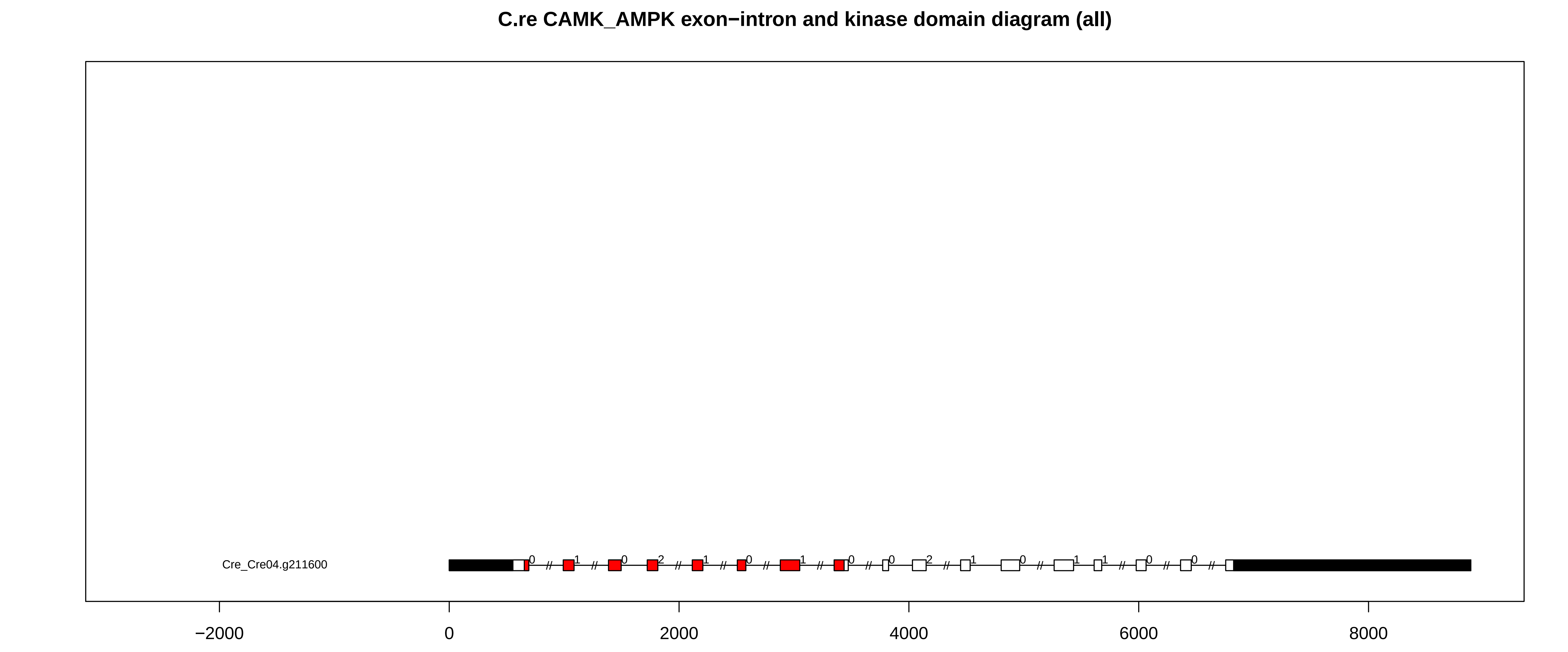
C.re CAMK_AMPK exon−intron and kinase domain diagram (all)
Cre_Cre04.g211600
0
1
0
2
1
0
1
0
0
2
1
0
1
1
0
0
−2000
0
2000
4000
6000
8000

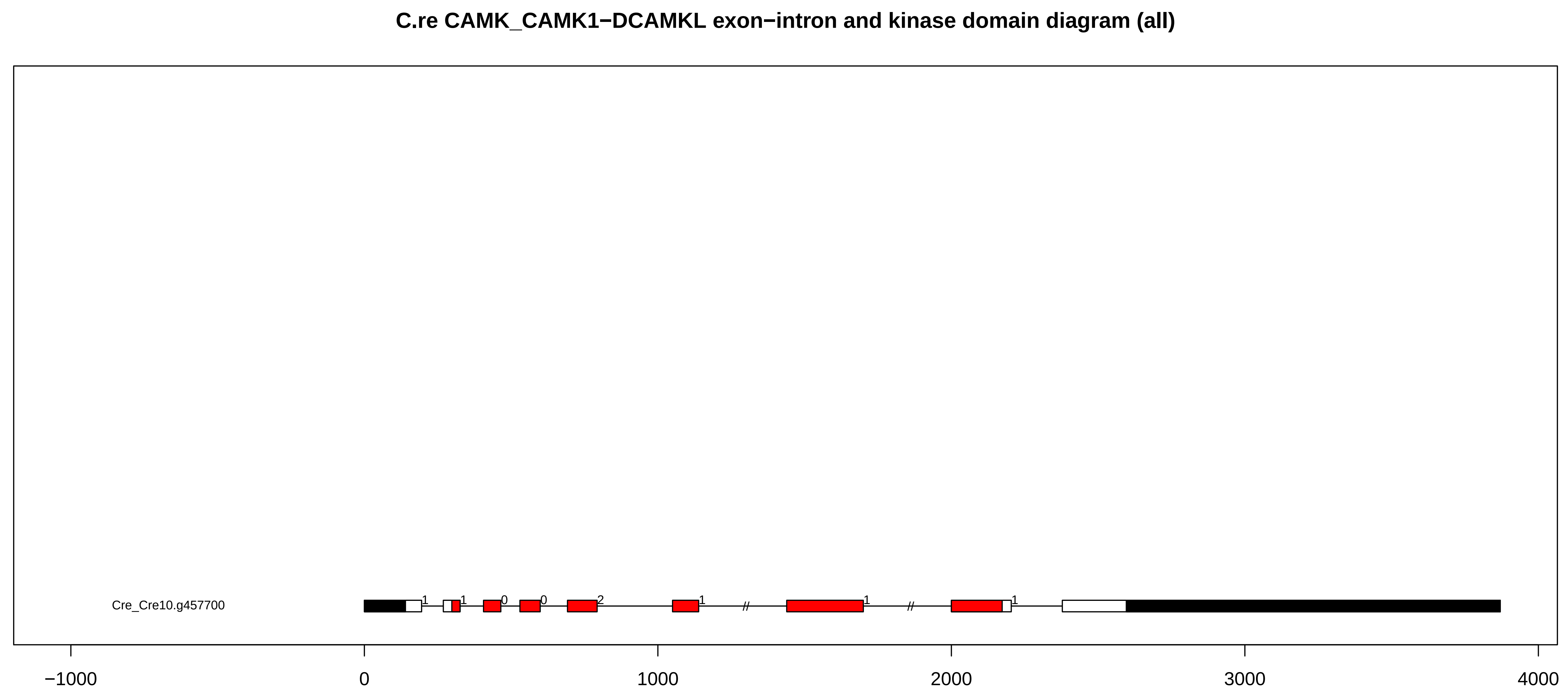

C.re CAMK_CAMK1−DCAMKL exon−intron and kinase domain diagram (all)
Cre_Cre10.g457700
1
1
0
0
2
1
1
1
−1000
0
1000
2000
3000
4000

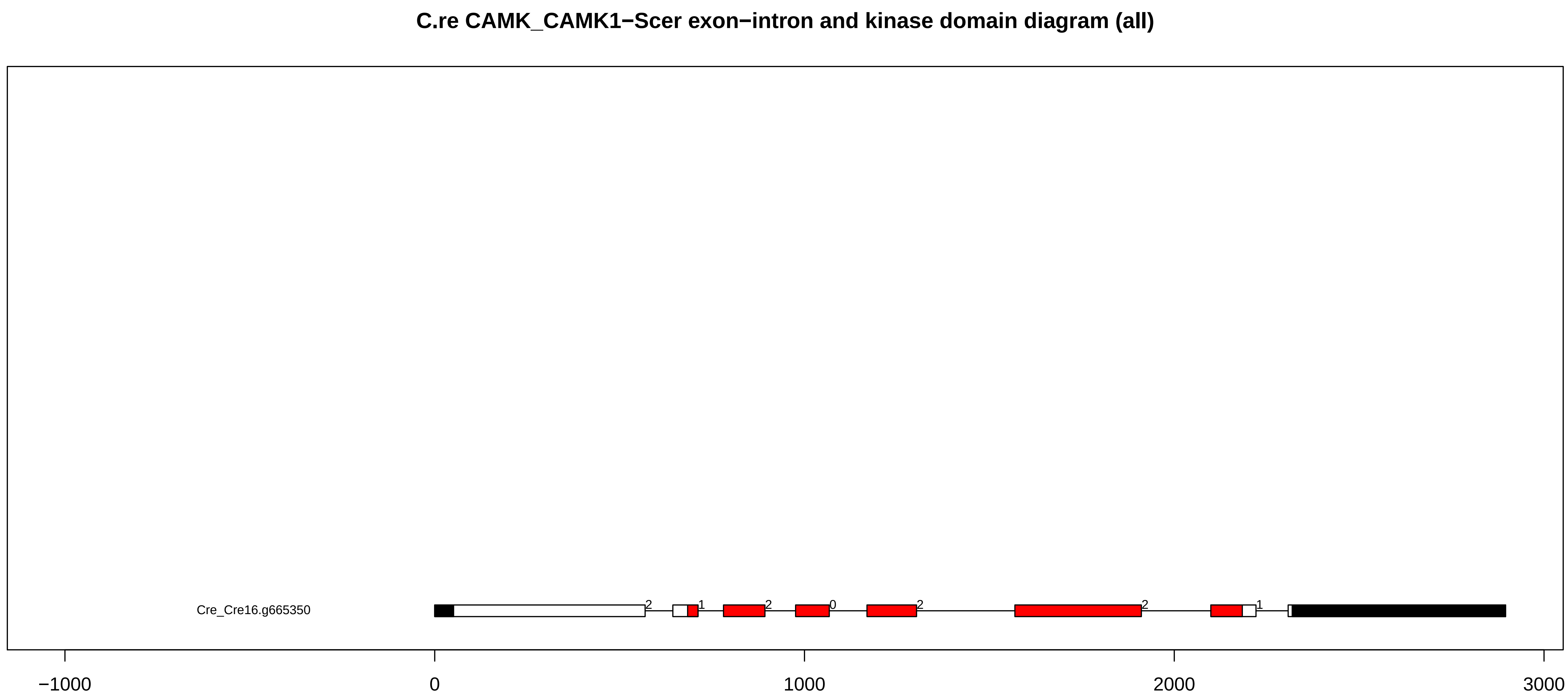
C.re CAMK_CAMK1−Scer exon−intron and kinase domain diagram (all)
Cre_Cre16.g665350
2
1
2
0
2
2
1
−1000
0
1000
2000
3000

C.re CAMK_CAMK1−Tthe exon−intron and kinase domain diagram (all)

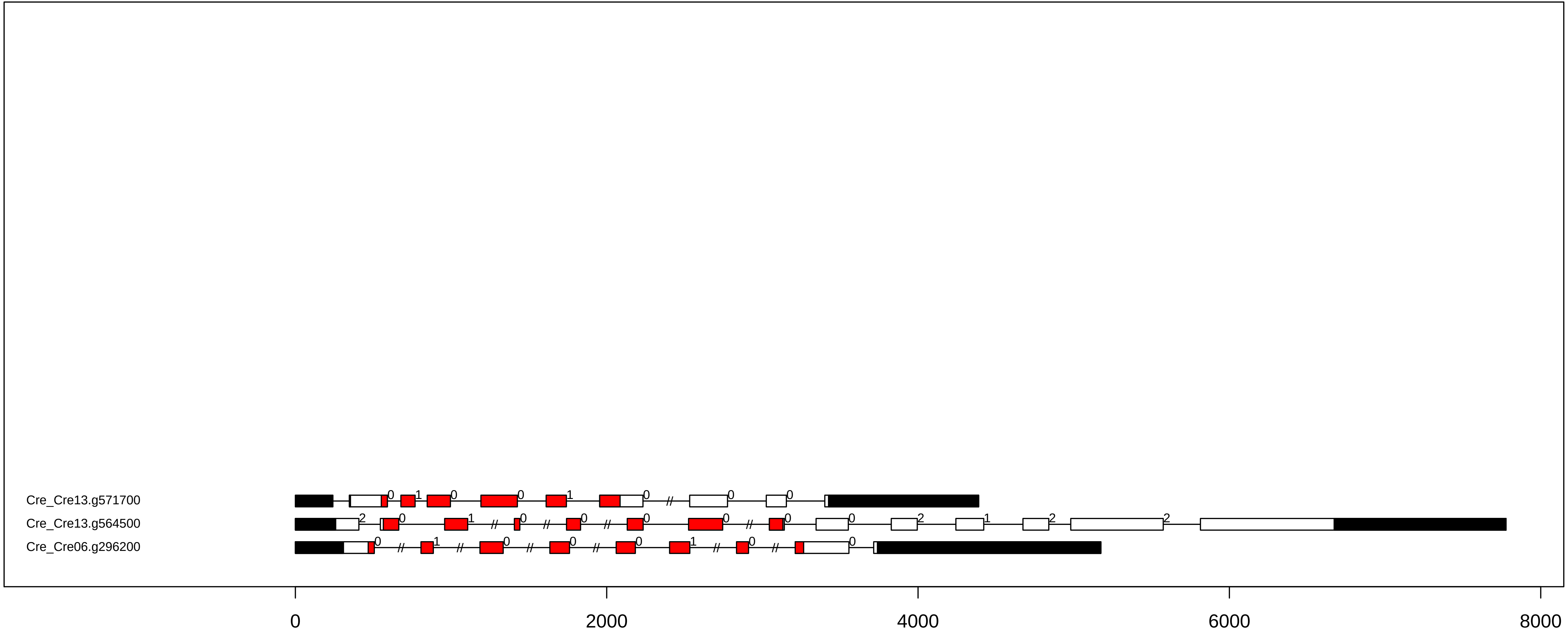

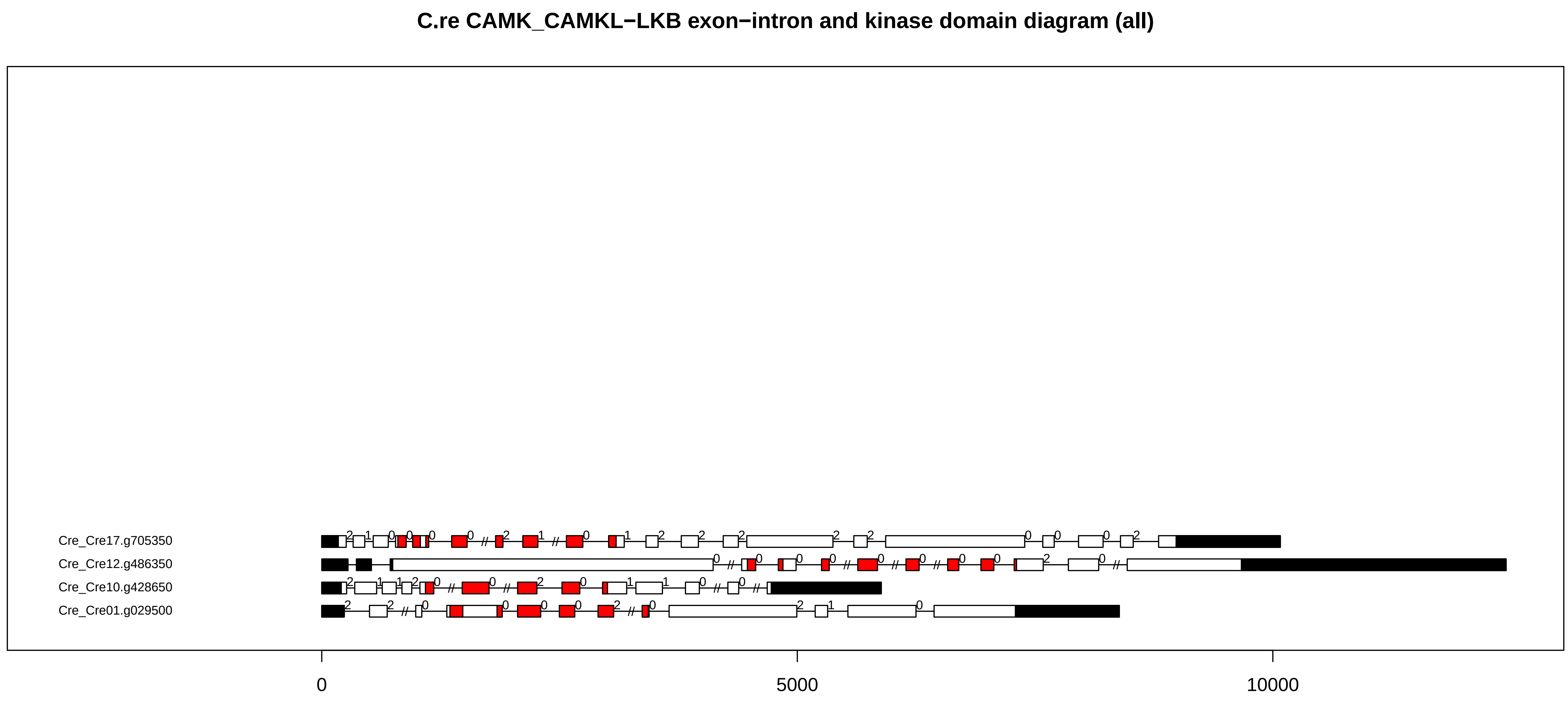

C.re CAMK_CAMKL−LKB exon−intron and kinase domain diagram (all)
Cre_Cre17.g705350
Cre_Cre12.g486350
Cre_Cre10.g428650
Cre_Cre01.g029500
0
5000
10000

C.re CAMK_CAMKL−MARK−Scer exon−intron and kinase domain diagram (all)

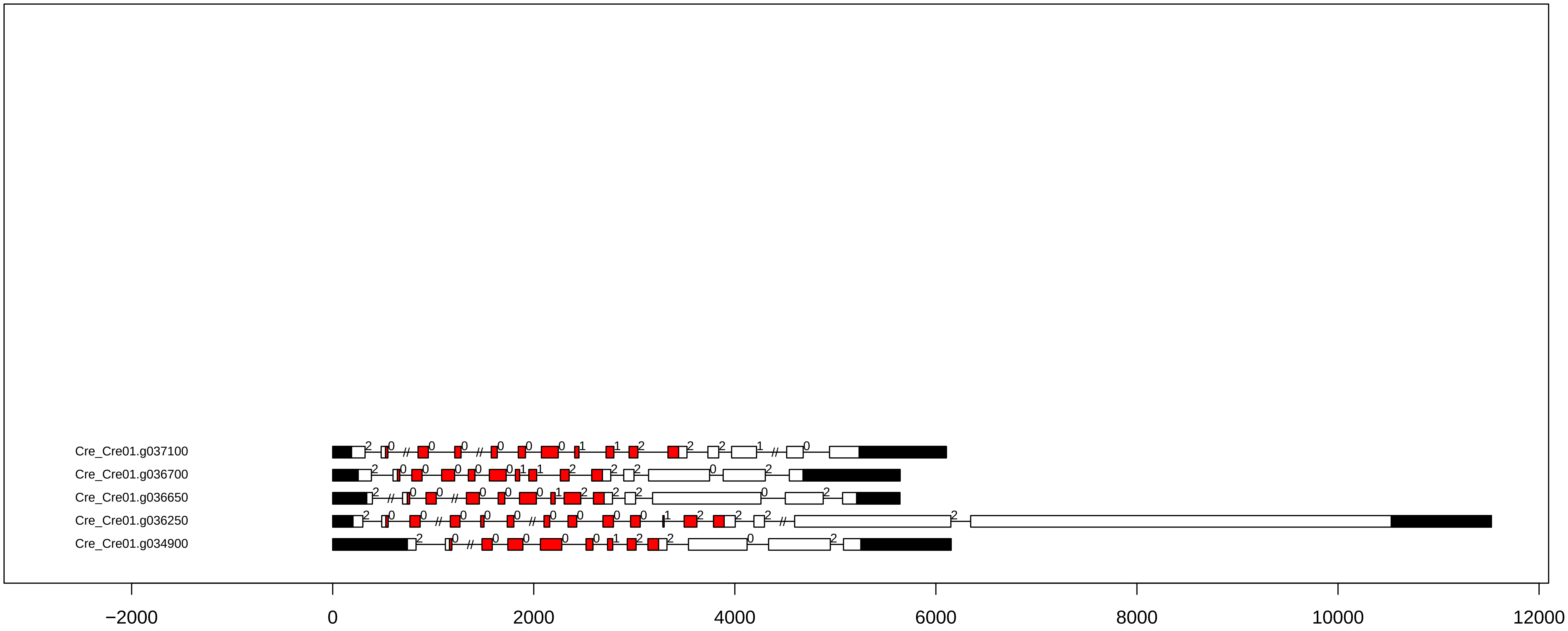

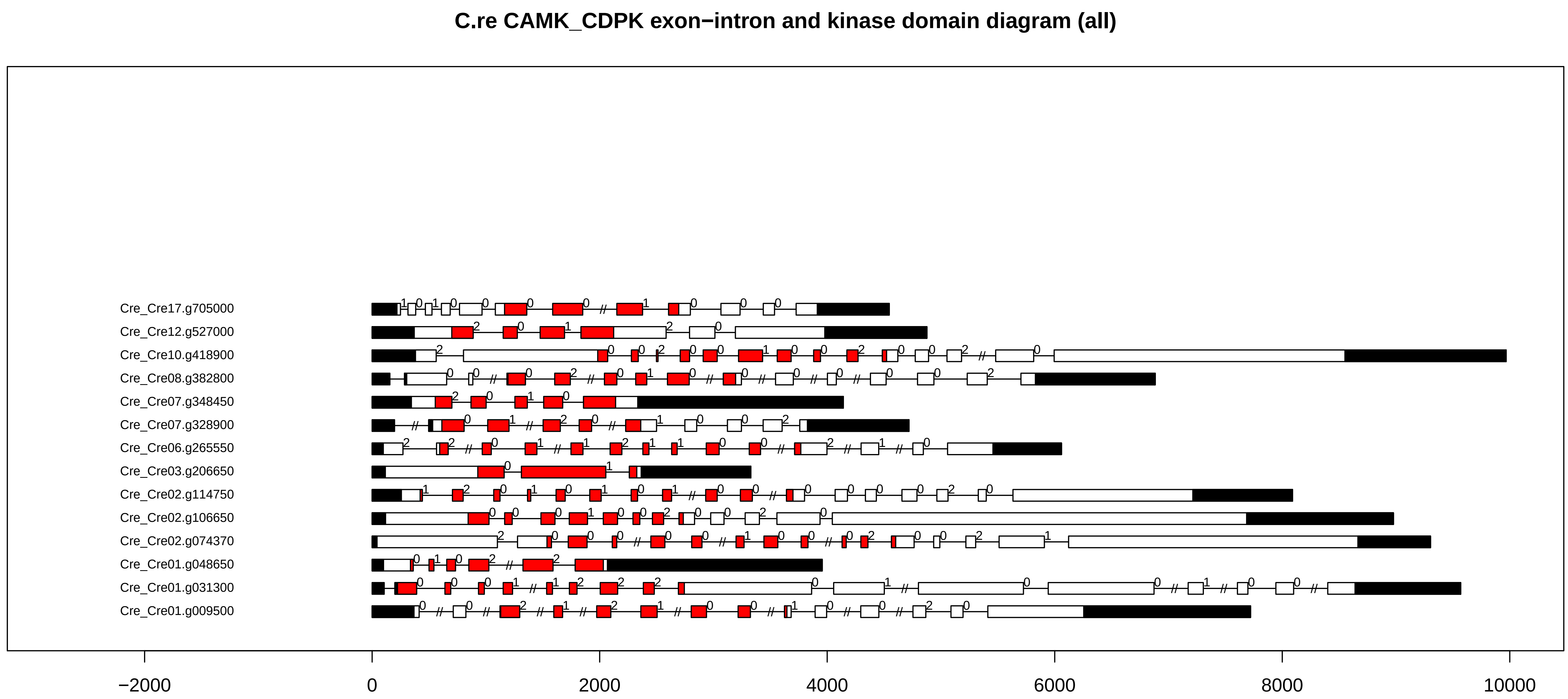

C.re CAMK_CDPK exon−intron and kinase domain diagram (all)
Cre_Cre17.g705000
Cre_Cre12.g527000
Cre_Cre10.g418900
Cre_Cre08.g382800
Cre_Cre07.g348450
Cre_Cre07.g328900
Cre_Cre06.g265550
Cre_Cre03.g206650
Cre_Cre02.g114750
Cre_Cre02.g106650
Cre_Cre02.g074370
Cre_Cre01.g048650
Cre_Cre01.g031300
Cre_Cre01.g009500
−2000
0
2000
4000
6000
8000
10000

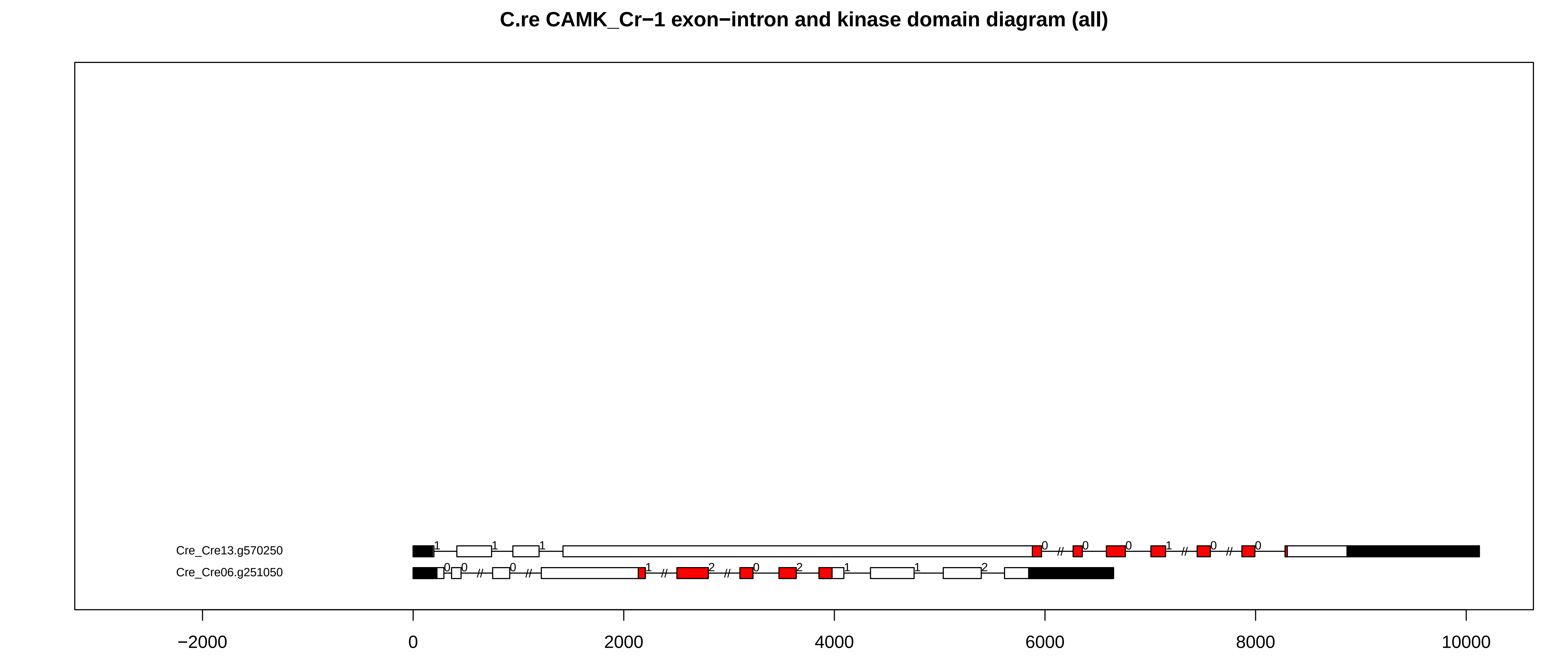
C.re CAMK_Cr−1 exon−intron and kinase domain diagram (all)
Cre_Cre13.g570250
Cre_Cre06.g251050
−2000
0
2000
4000
6000
8000
10000

C.re CAMK_Cr−single exon−intron and kinase domain diagram (all)

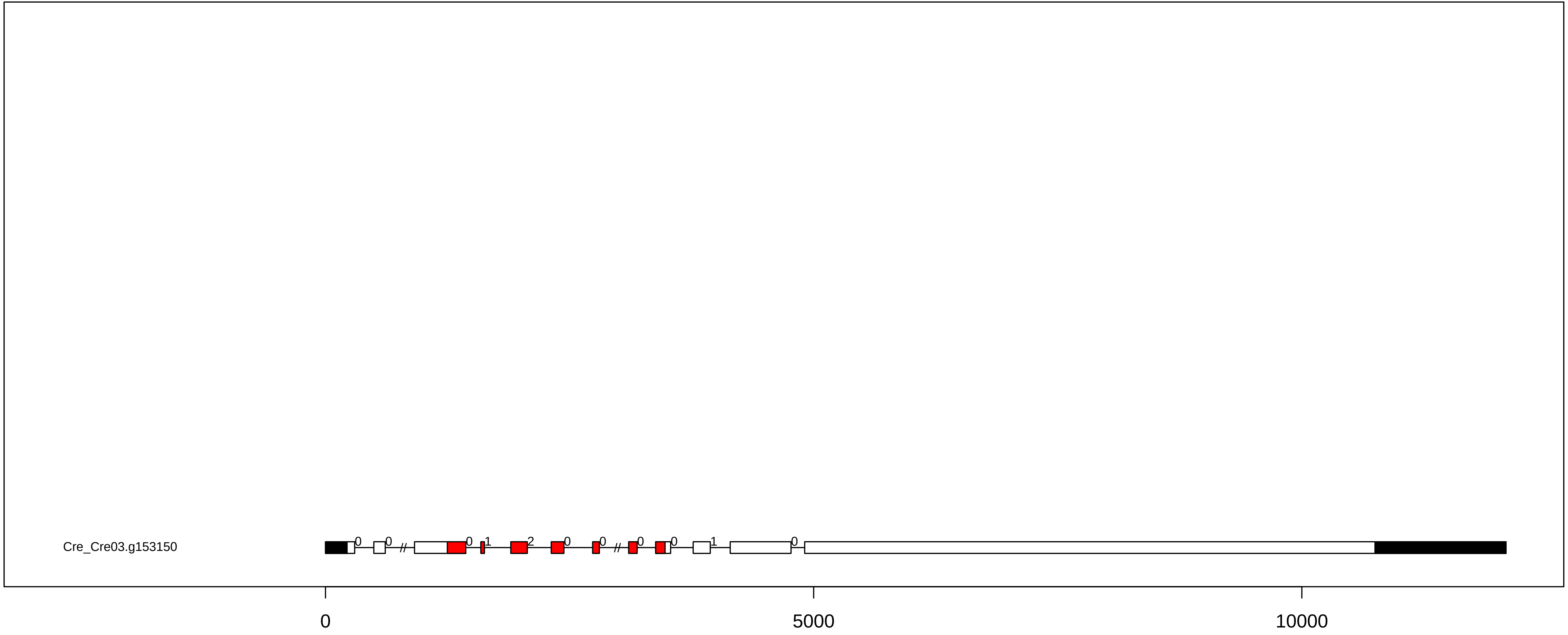

C.re CAMK_OST1L exon−intron and kinase domain diagram (all)

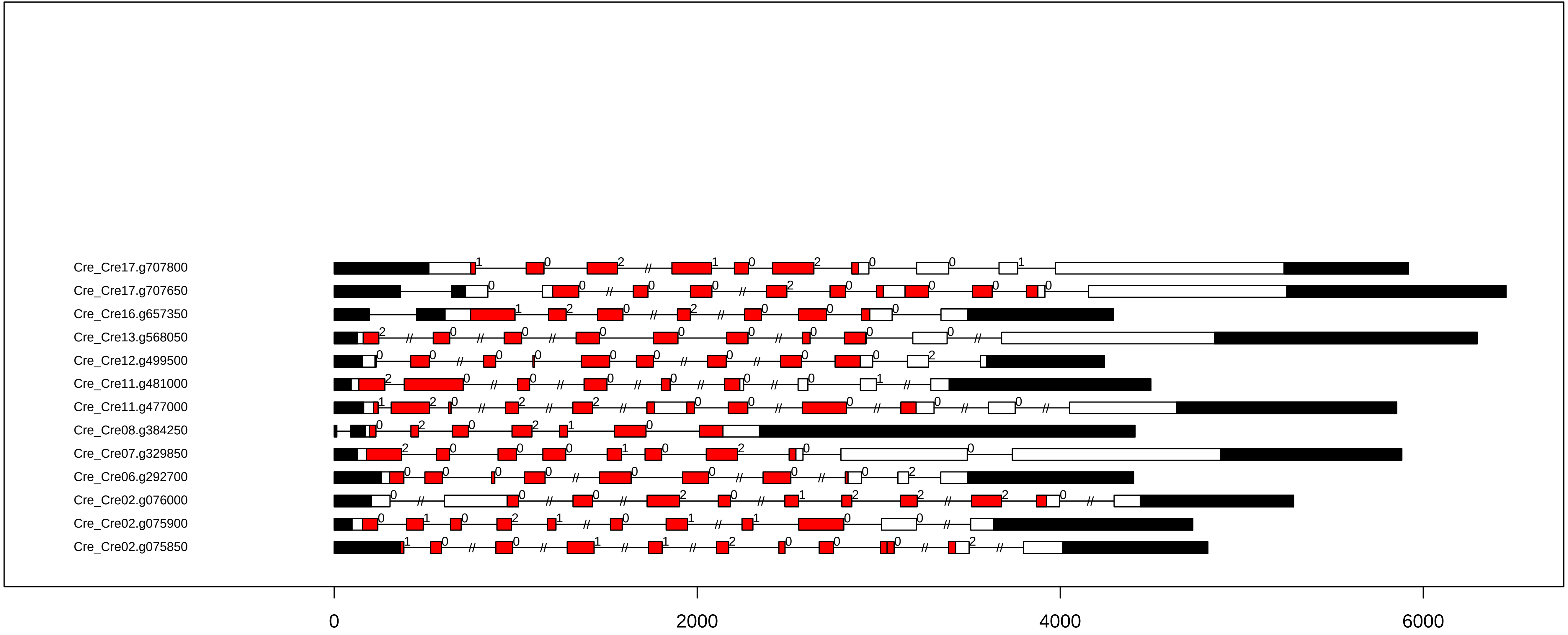

C.re CK1_CK1 exon−intron and kinase domain diagram (all)

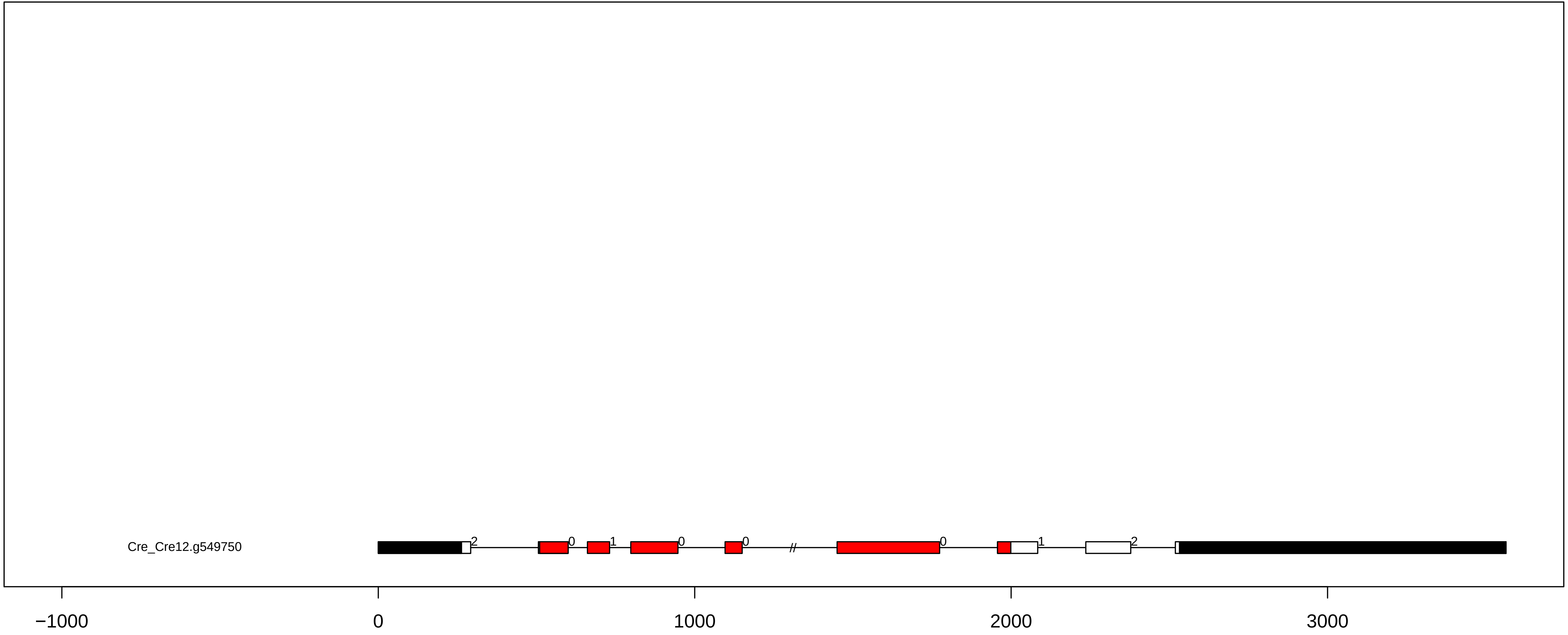

C.re CK1_CK1−PI exon−intron and kinase domain diagram (all)

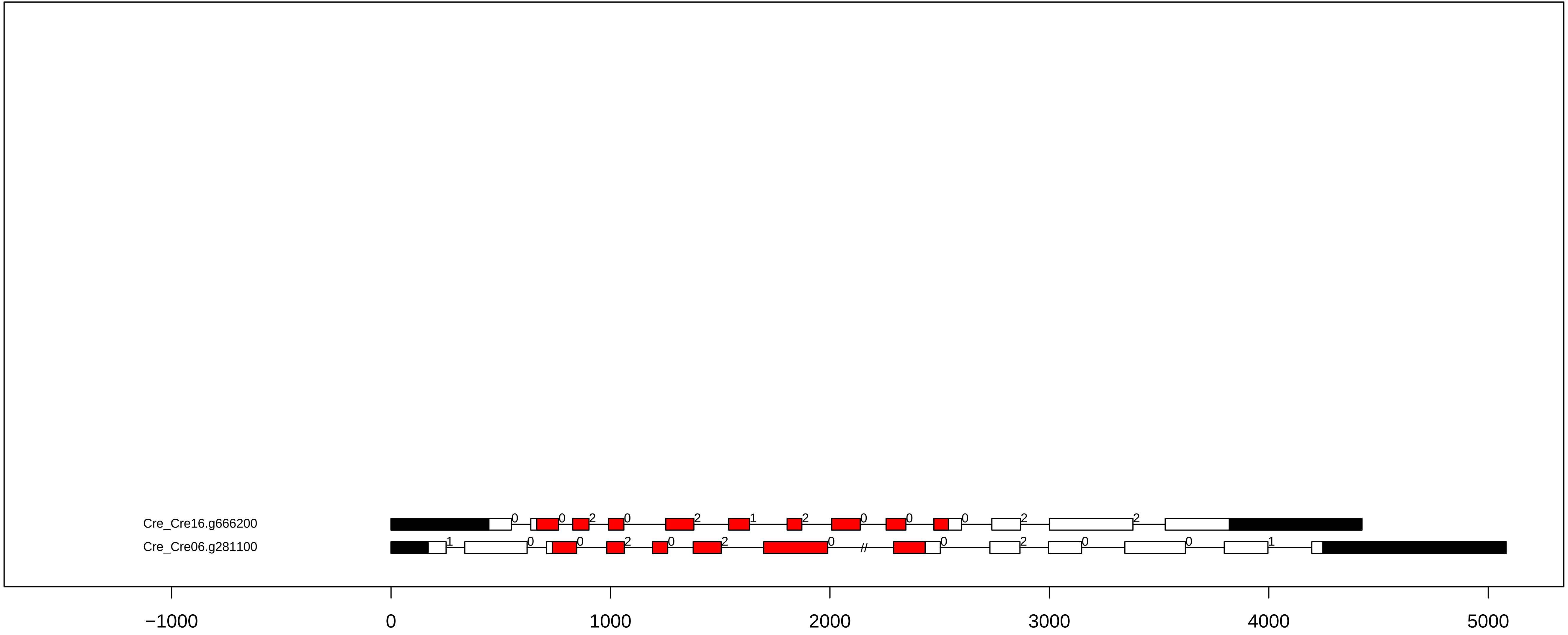

C.re CMGC_CDK−CCRK exon−intron and kinase domain diagram (all)

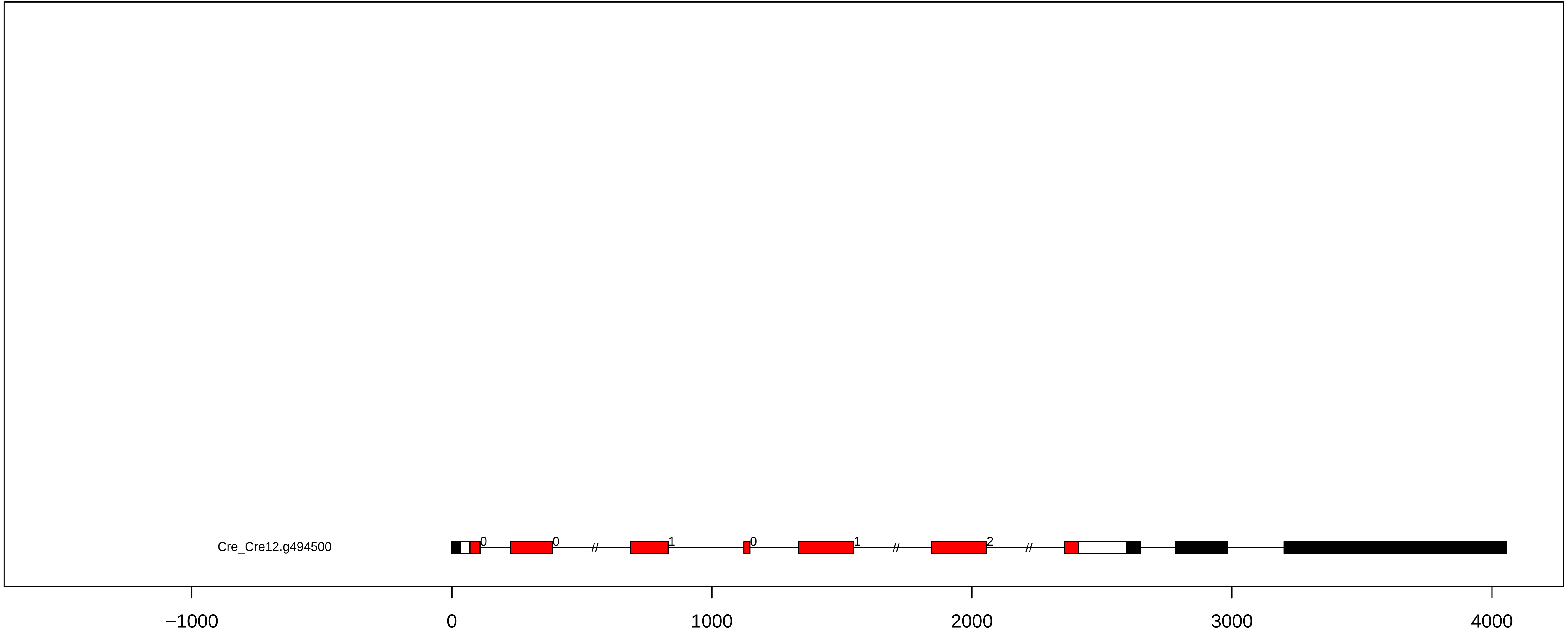

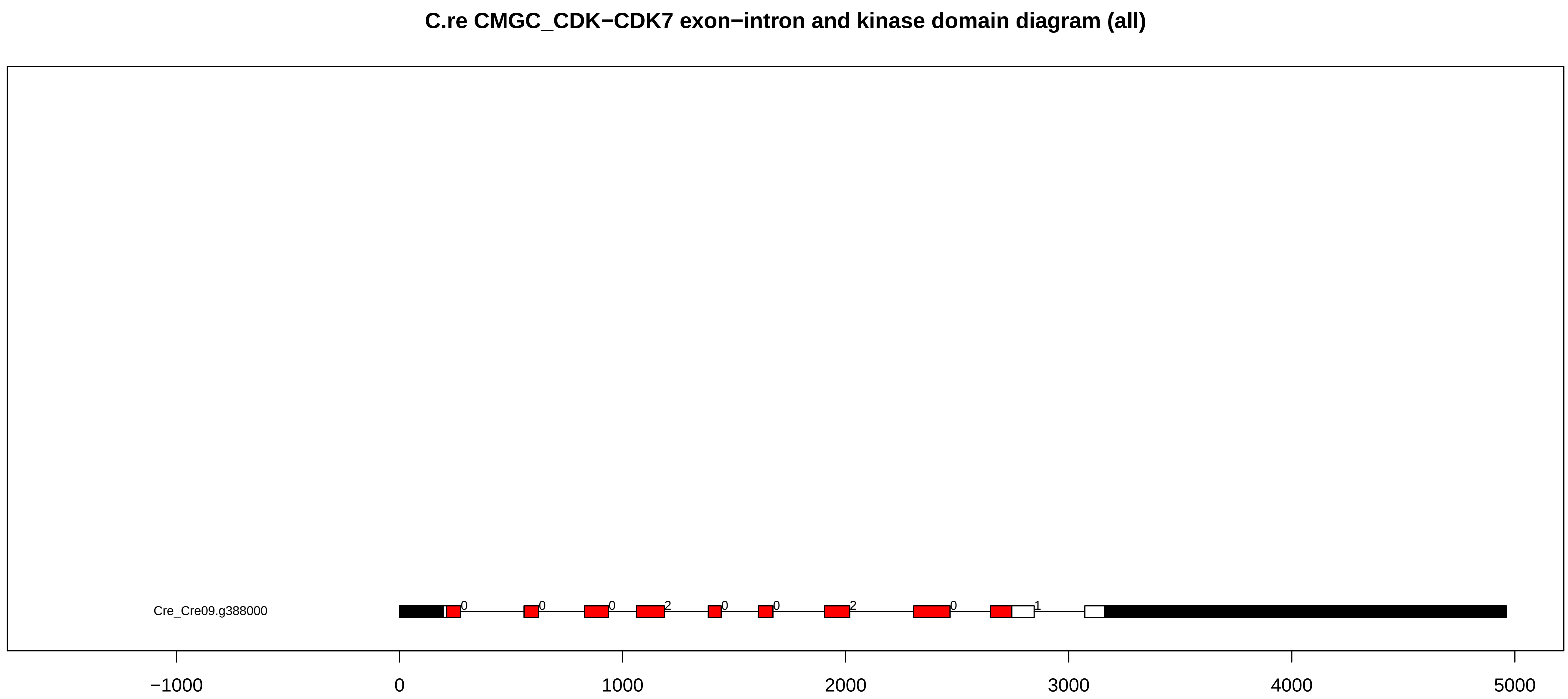

C.re CMGC_CDK−CDK7 exon−intron and kinase domain diagram (all)
Cre_Cre09.g388000
0
0
0
2
0
0
2
0
1
−1000
0
1000
2000
3000
4000
5000

C.re CMGC_CDK−CRK7−CDK9 exon−intron and kinase domain diagram (all)

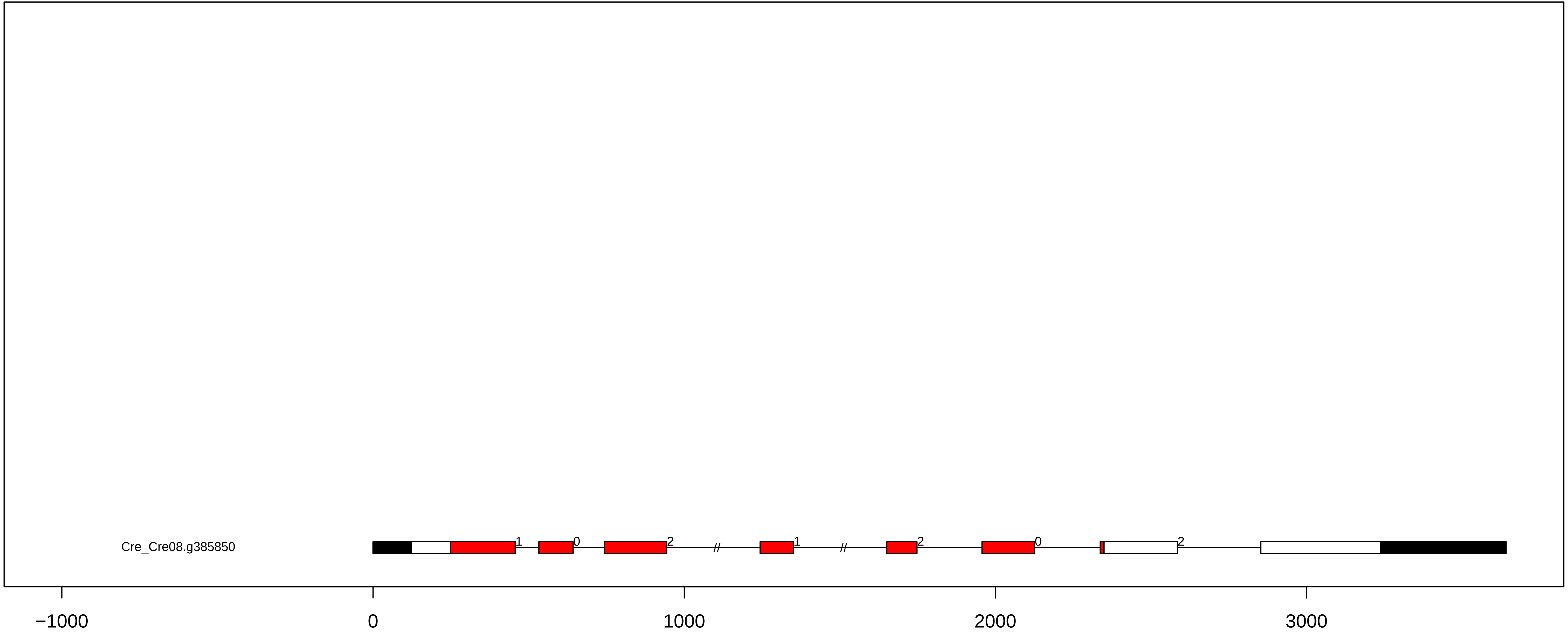

C.re CMGC_CDK−Cr exon−intron and kinase domain diagram (all)

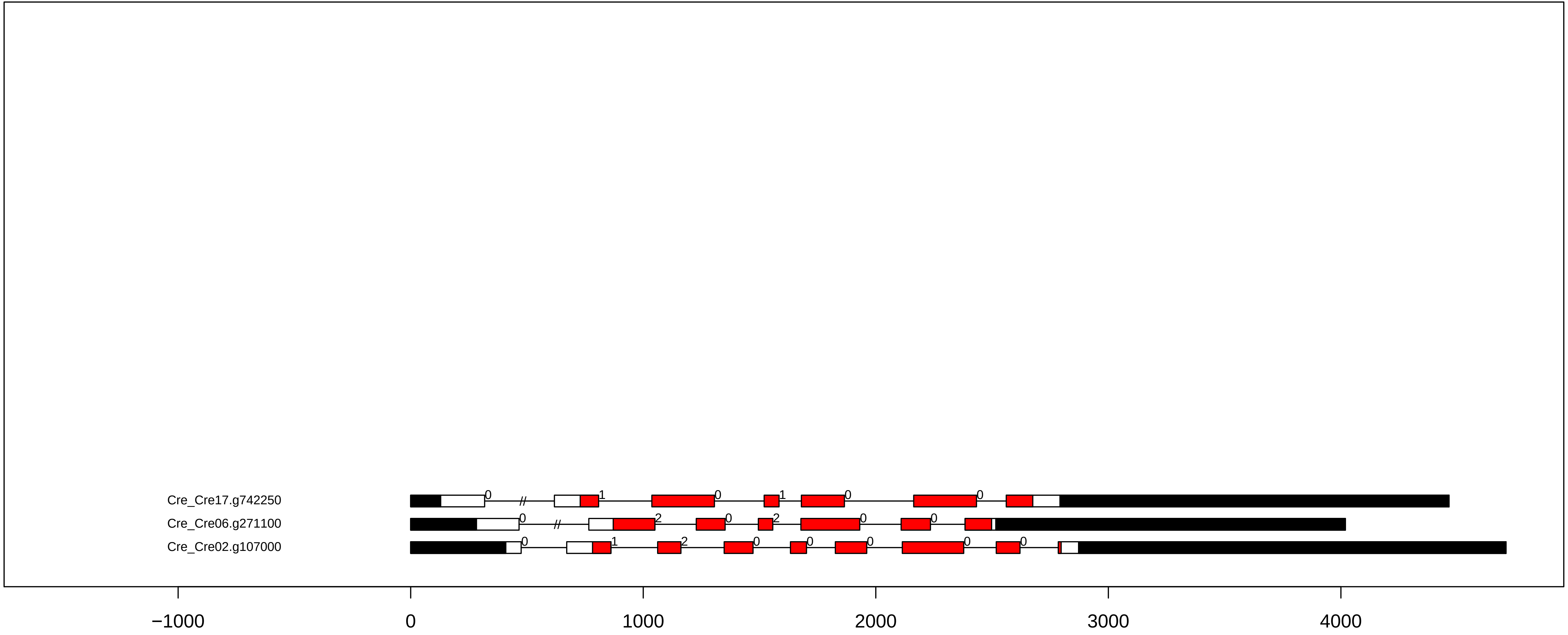

C.re CMGC_CDKL−Cr exon−intron and kinase domain diagram (part 1)

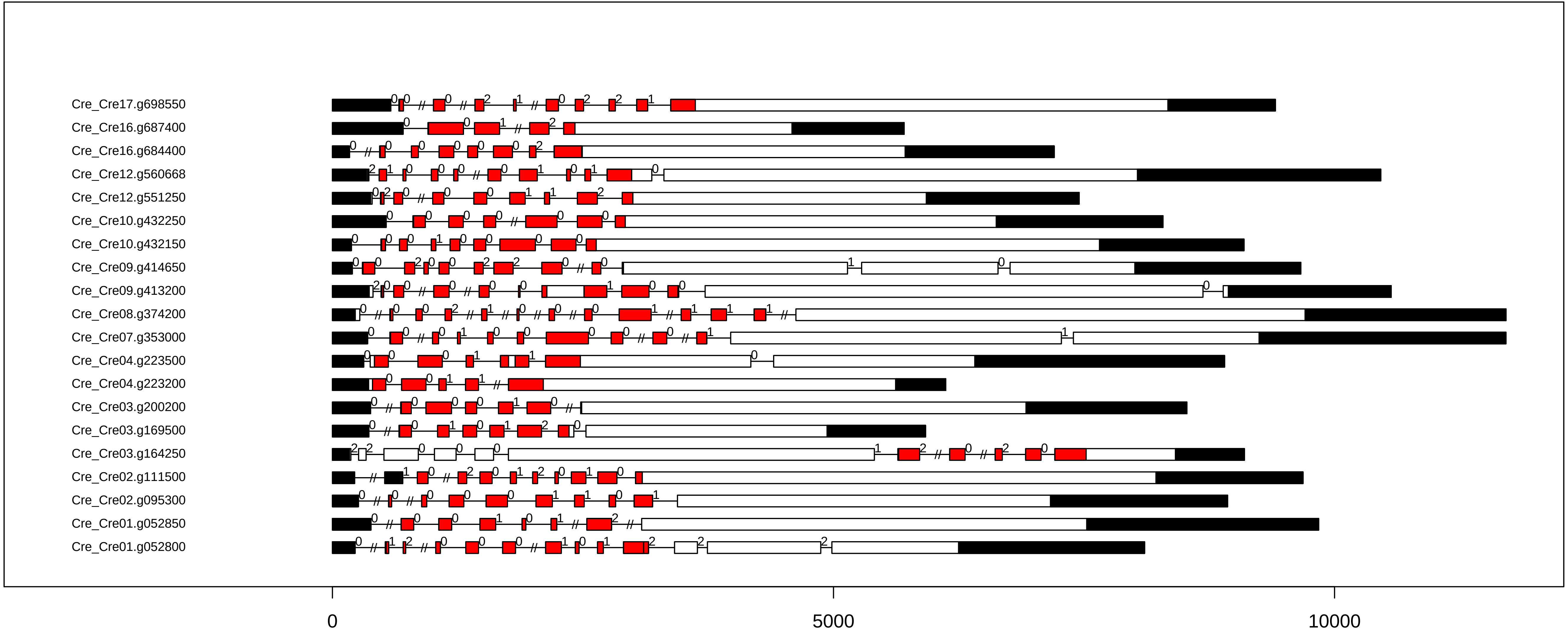

C.re CMGC_CDKL−Cr exon−intron and kinase domain diagram (part 2)

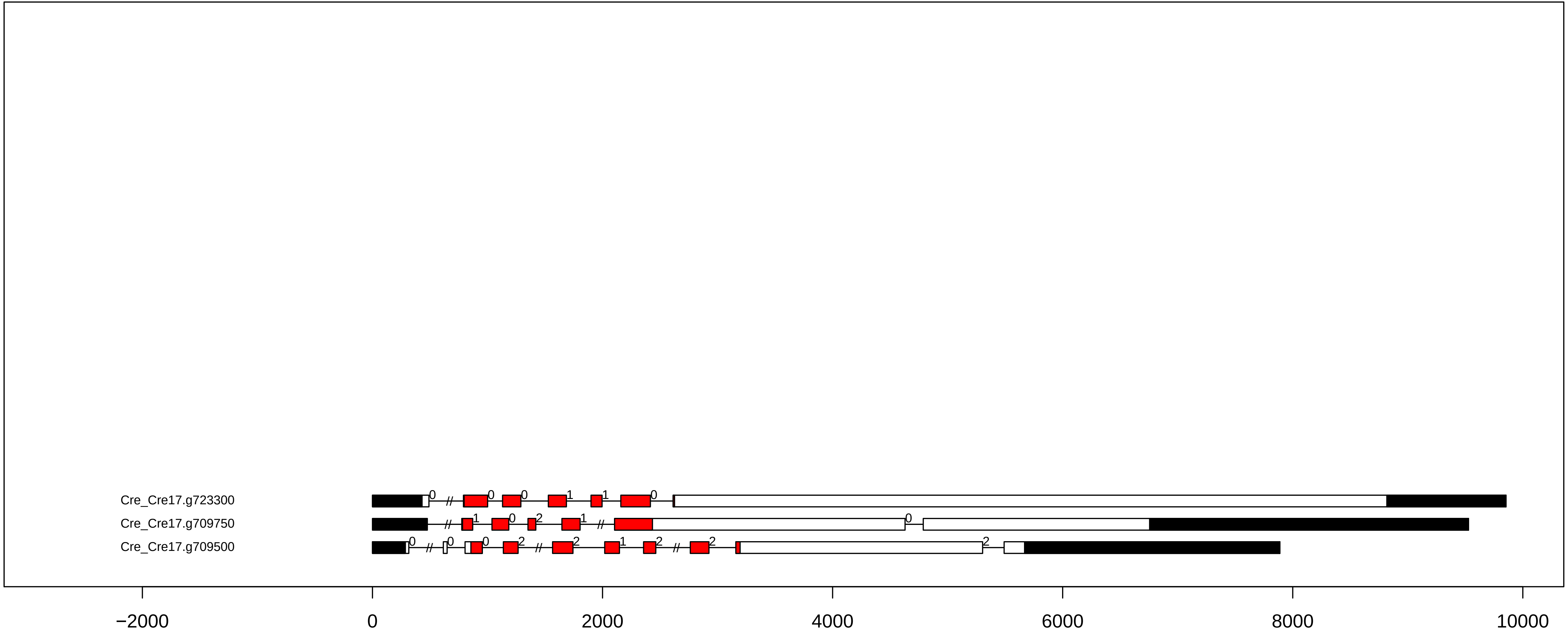

C.re CMGC_CDK−PITSLRE exon−intron and kinase domain diagram (all)

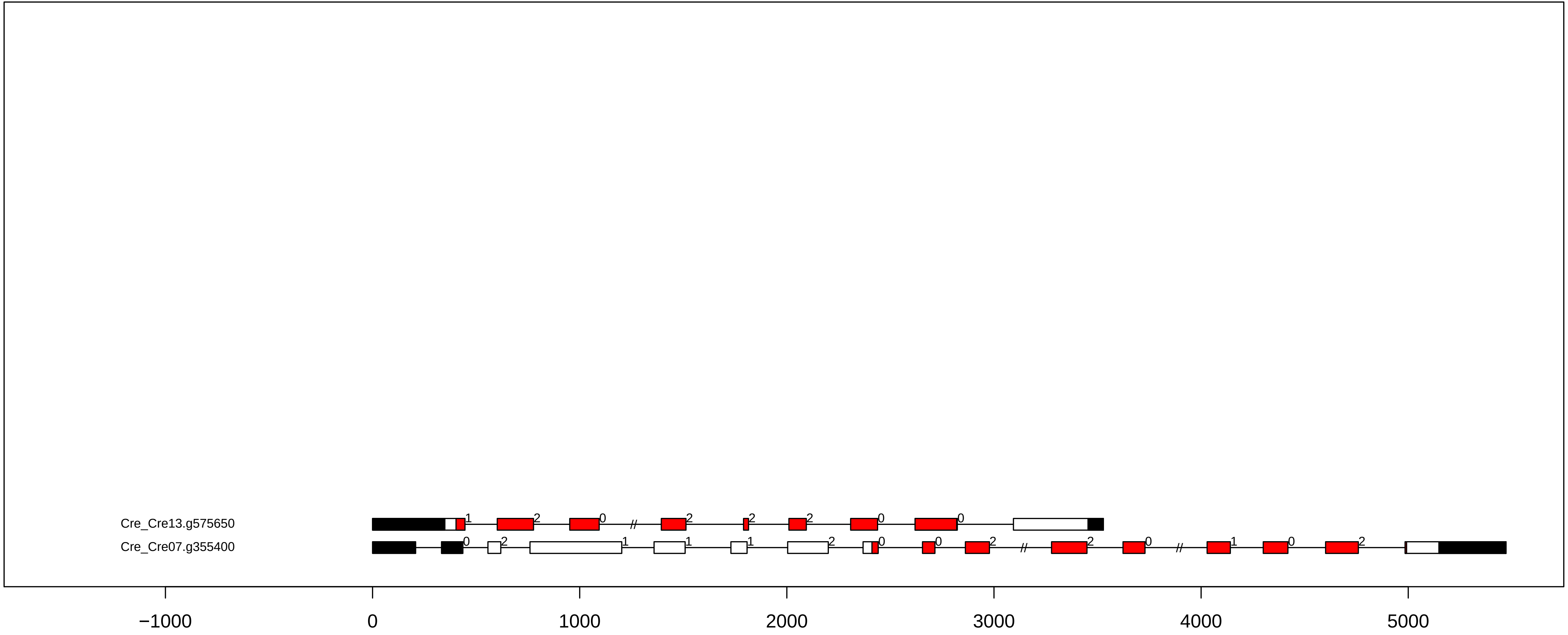

C.re CMGC_CDK−Pl exon−intron and kinase domain diagram (all)

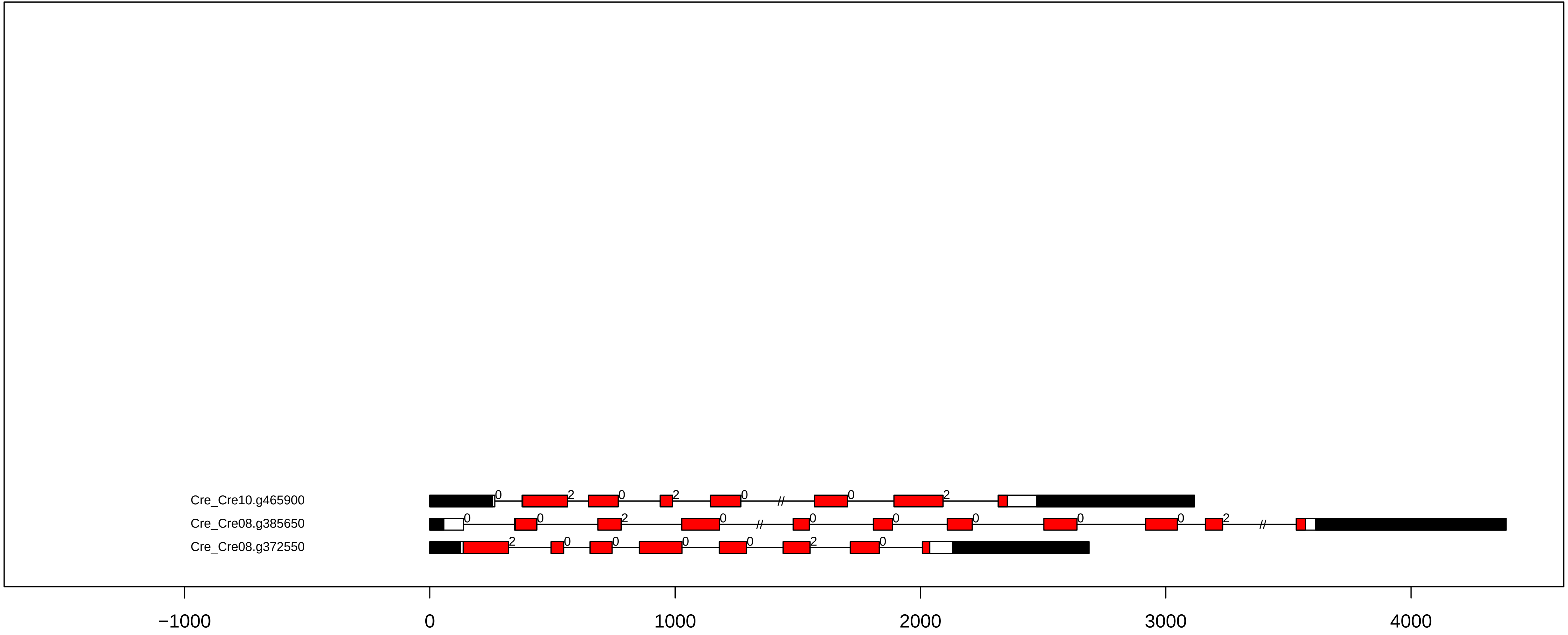

C.re CMGC_CK2 exon−intron and kinase domain diagram (all)

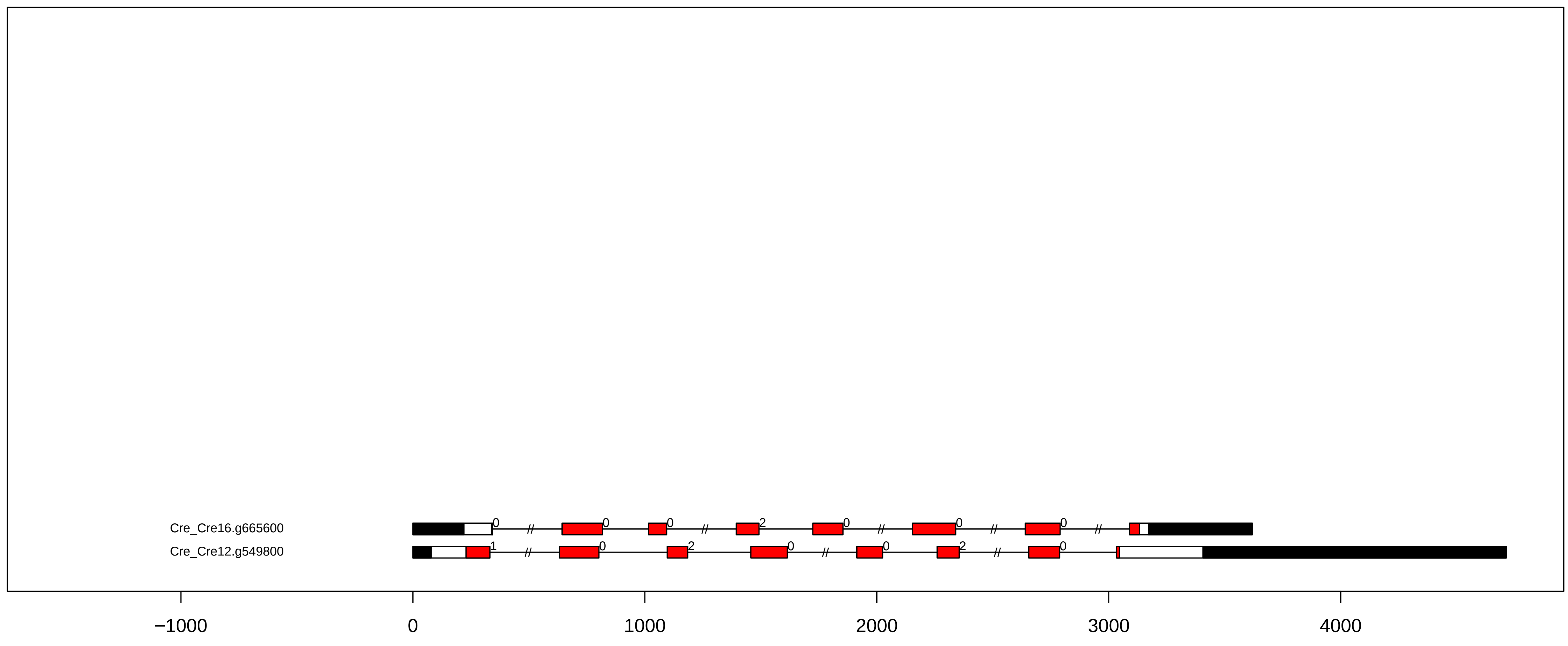

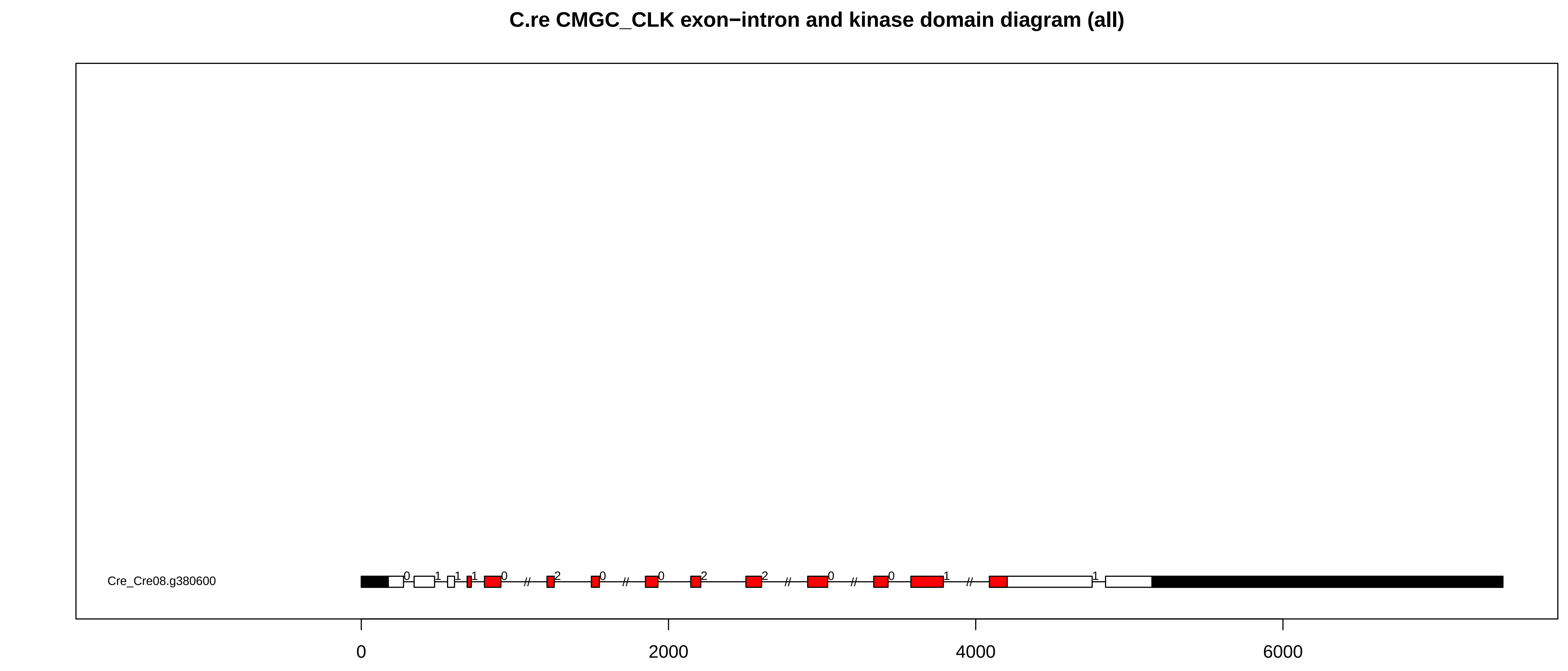
C.re CMGC_CLK exon−intron and kinase domain diagram (all)
Cre_Cre08.g380600
0
2000
4000
6000

C.re CMGC_DYRK−DRYK2 exon−intron and kinase domain diagram (all)

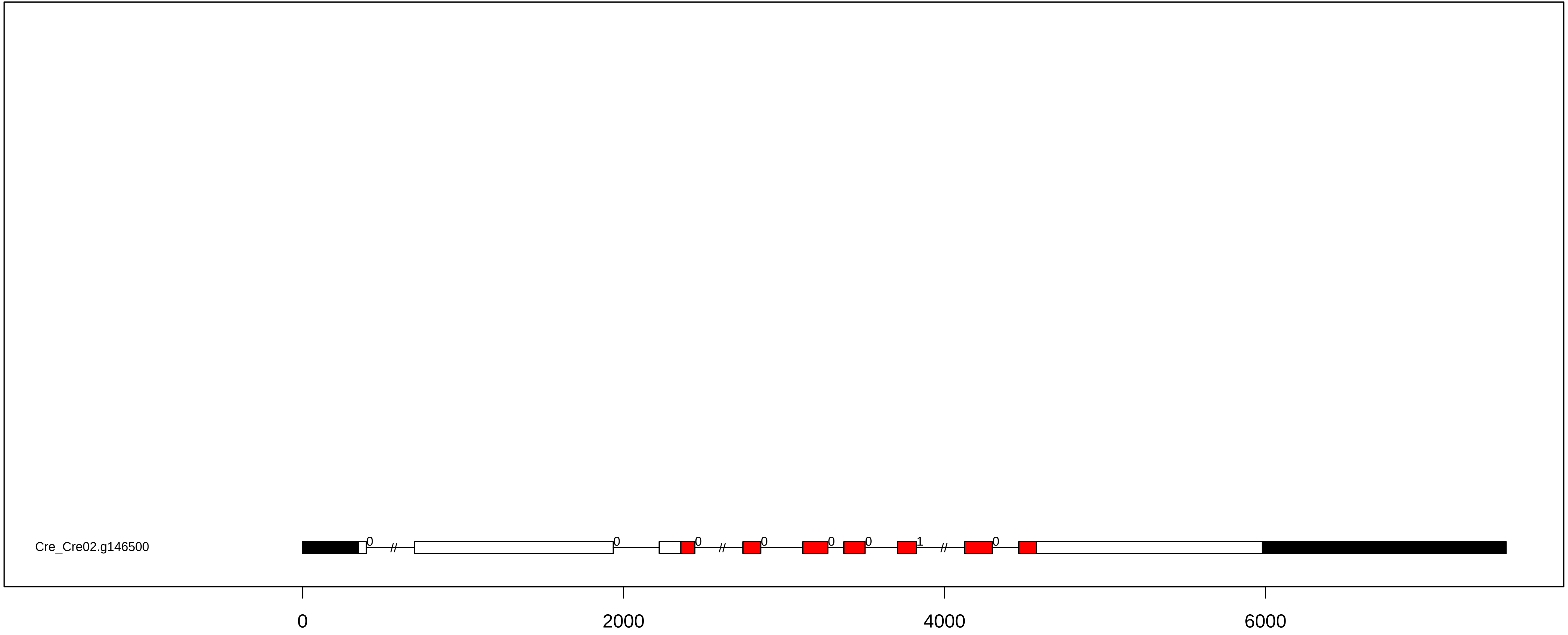

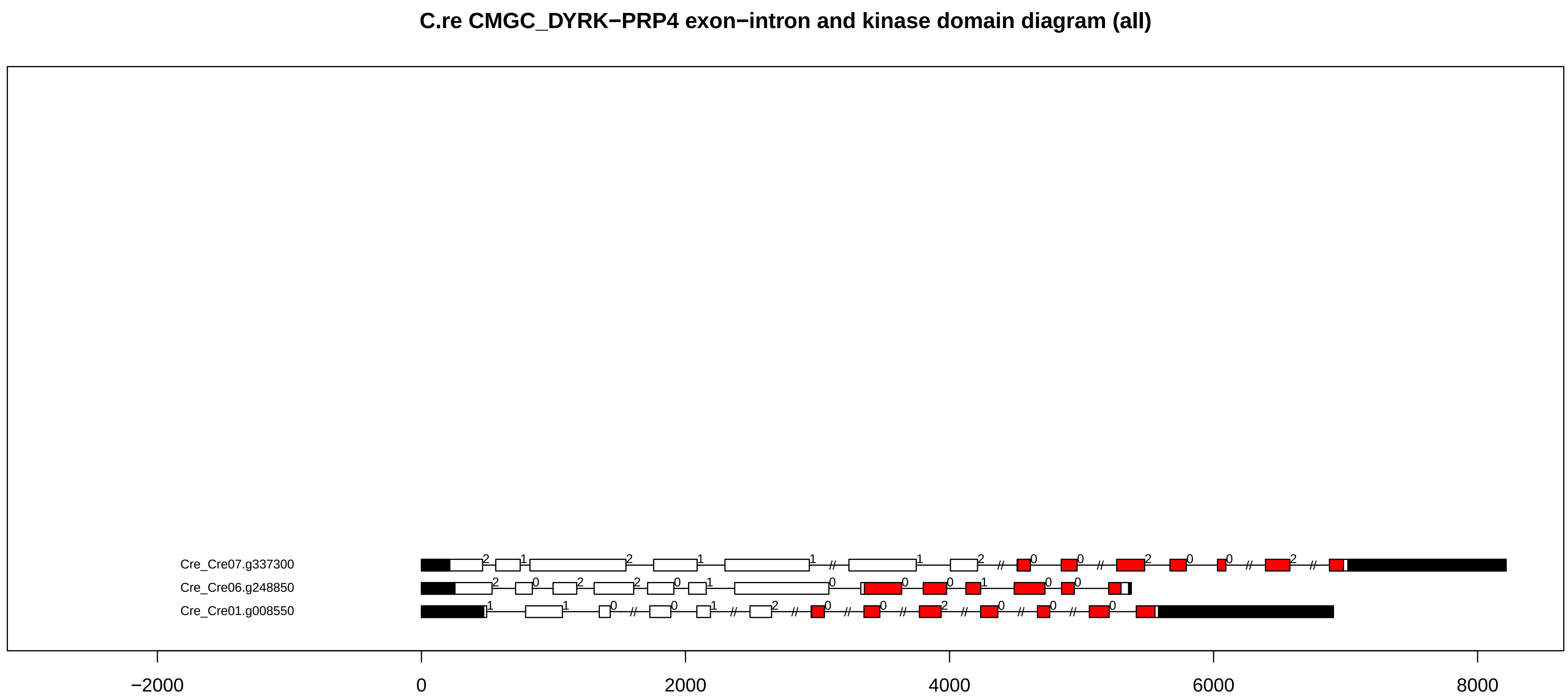

C.re CMGC_DYRK−PRP4 exon−intron and kinase domain diagram (all)
Cre_Cre07.g337300
Cre_Cre06.g248850
Cre_Cre01.g008550
−2000
0
2000
4000
6000
8000

C.re CMGC_DYRK−YAK exon−intron and kinase domain diagram (all)

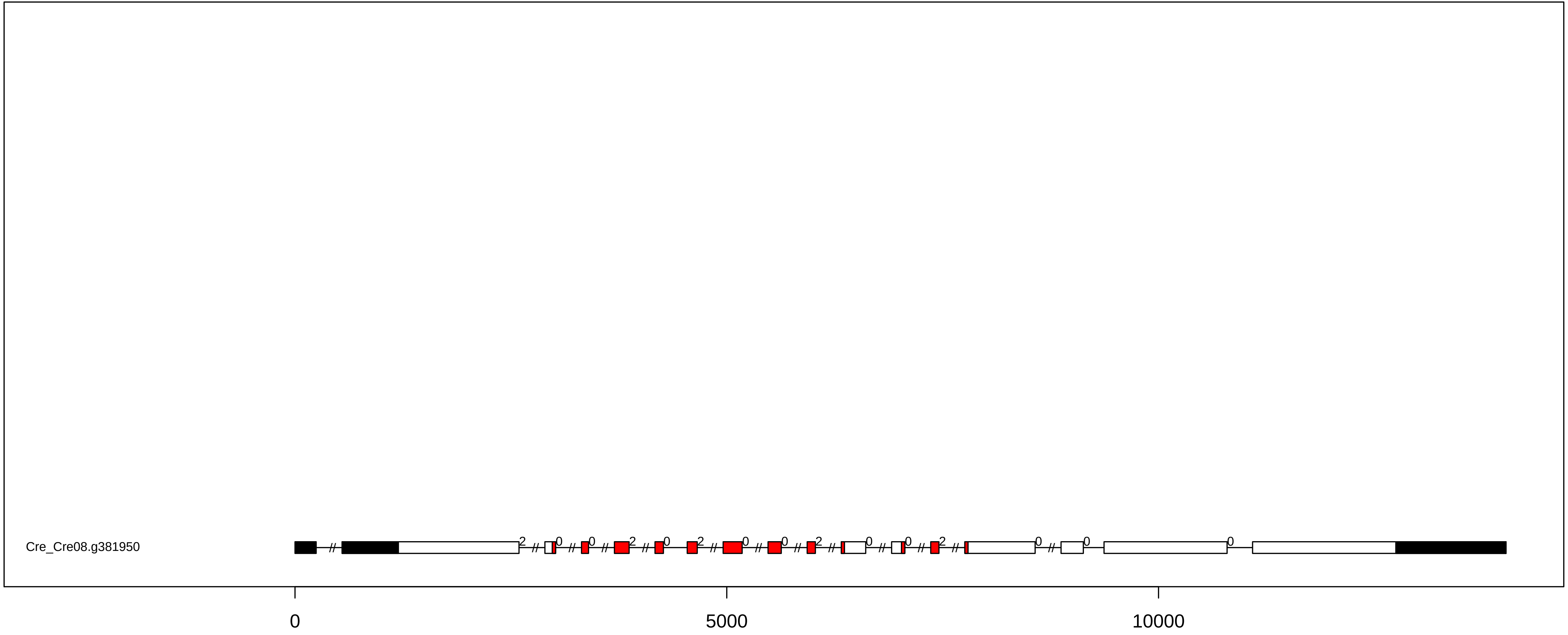

C.re CMGC_GSK exon−intron and kinase domain diagram (all)

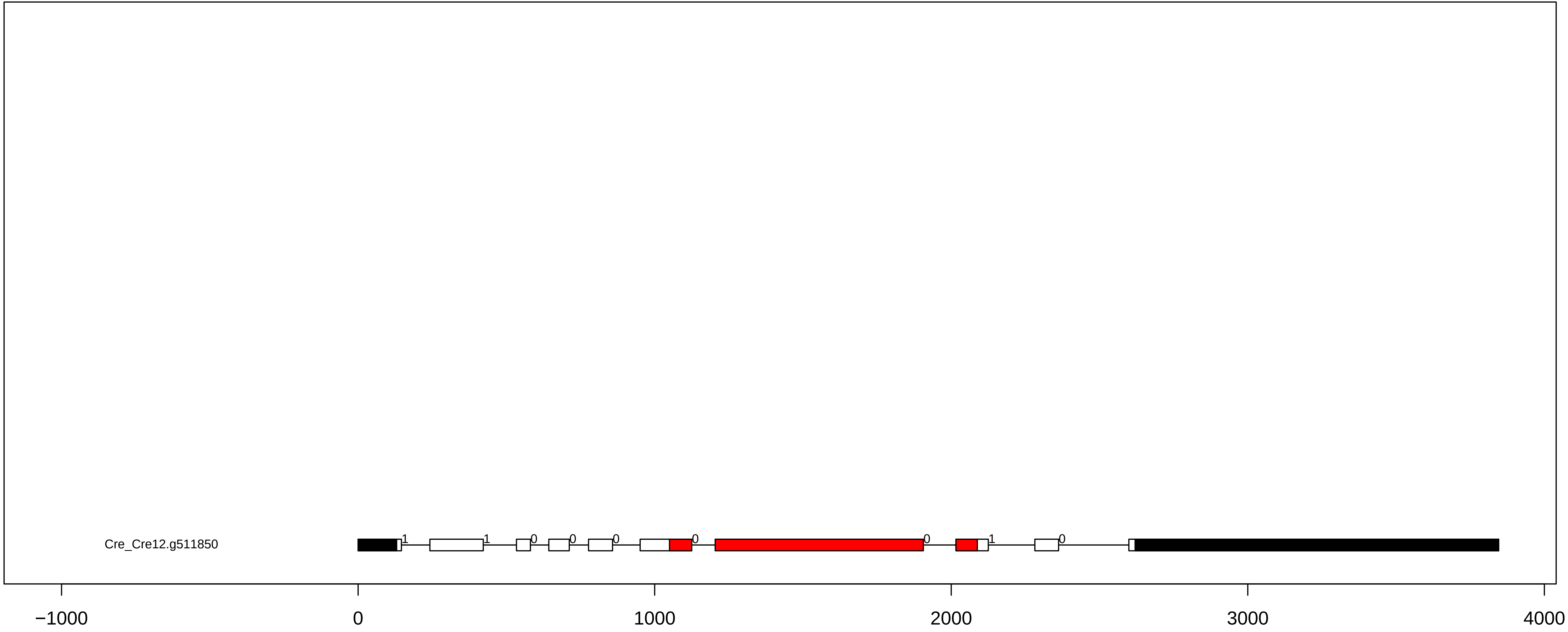

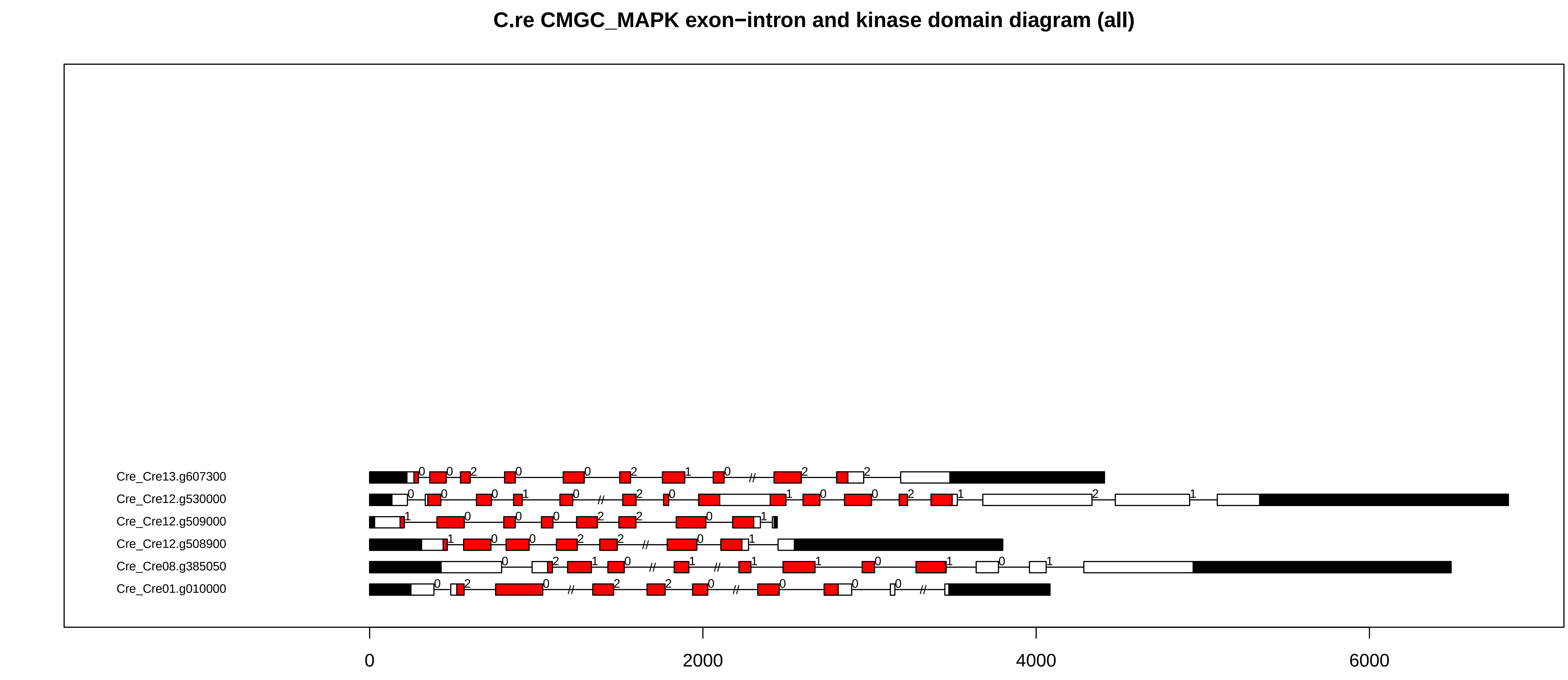
C.re CMGC_MAPK exon−intron and kinase domain diagram (all)
Cre_Cre13.g607300
Cre_Cre12.g530000
Cre_Cre12.g509000
Cre_Cre12.g508900
Cre_Cre08.g385050
Cre_Cre01.g010000
0
2000
4000
6000

C.re CMGC_RCK exon−intron and kinase domain diagram (all)

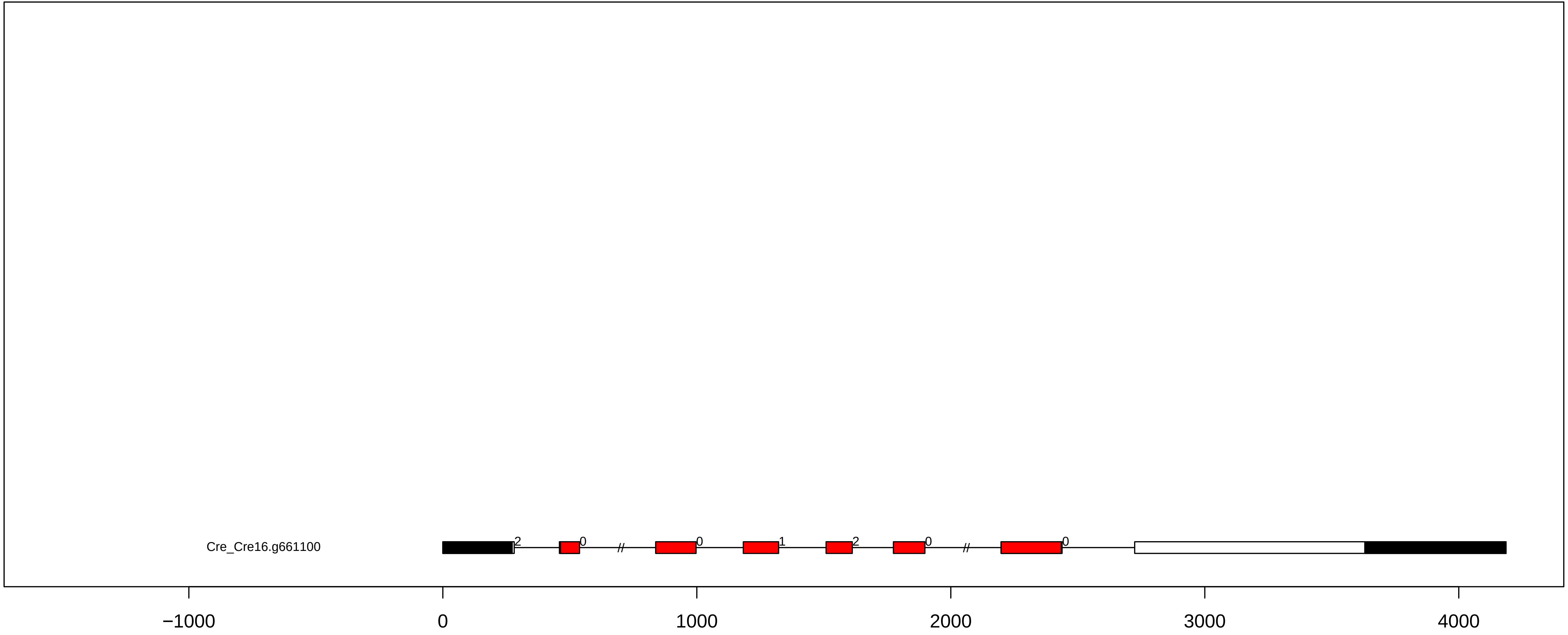

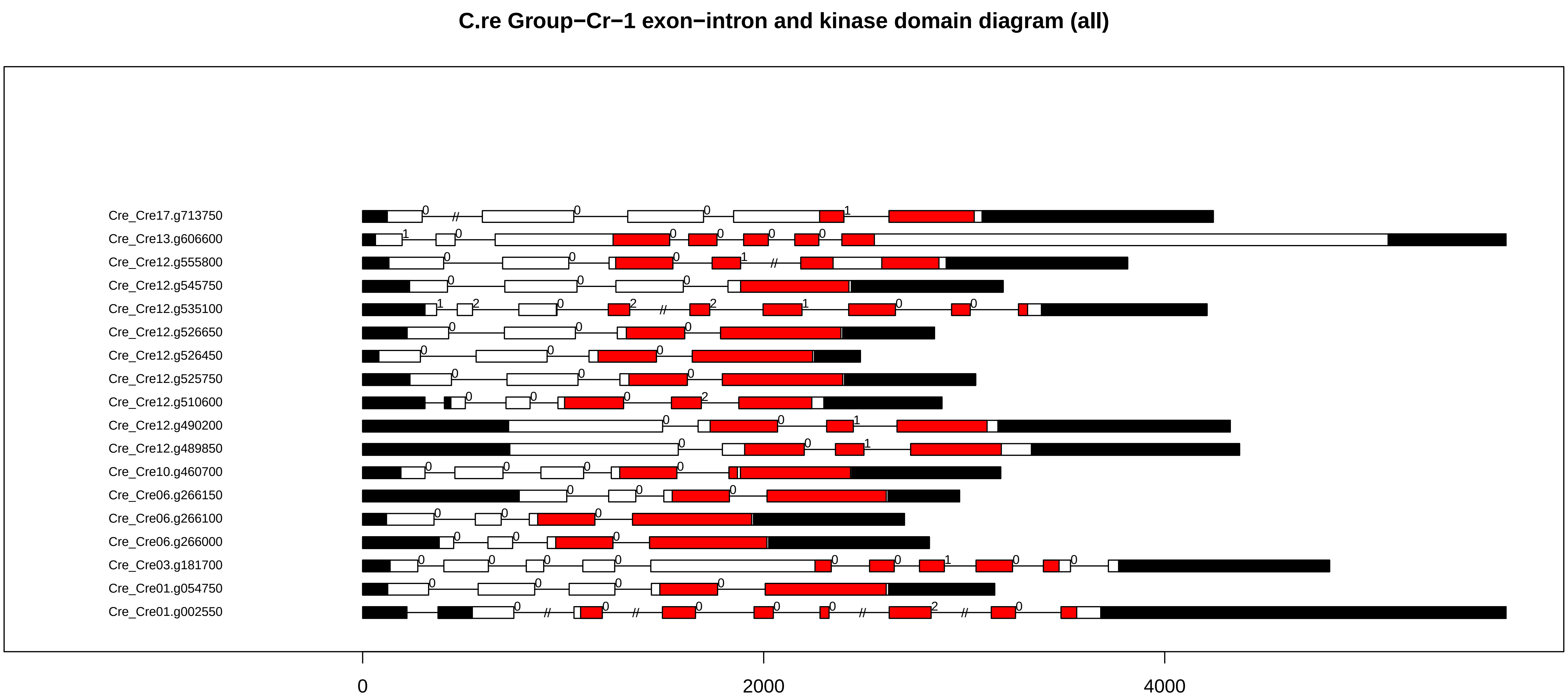
C.re Group−Cr−1 exon−intron and kinase domain diagram (all)
Cre_Cre17.g713750
Cre_Cre13.g606600
Cre_Cre12.g555800
Cre_Cre12.g545750
Cre_Cre12.g535100
Cre_Cre12.g526650
Cre_Cre12.g526450
Cre_Cre12.g525750
Cre_Cre12.g510600
Cre_Cre12.g490200
Cre_Cre12.g489850
Cre_Cre10.g460700
Cre_Cre06.g266150
Cre_Cre06.g266100
Cre_Cre06.g266000
Cre_Cre03.g181700
Cre_Cre01.g054750
Cre_Cre01.g002550
0
2000
4000

C.re Group−Cr−2 exon−intron and kinase domain diagram (all)

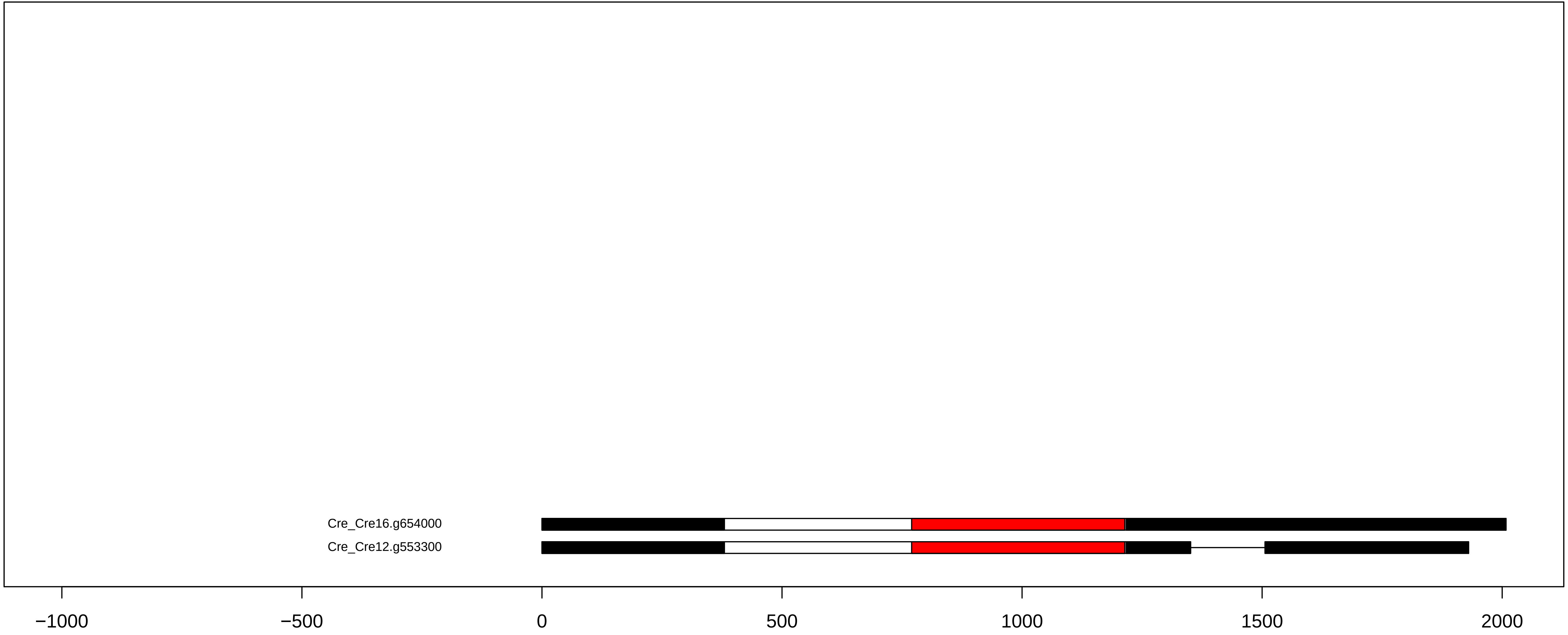

C.re Group−PI−1 exon−intron and kinase domain diagram (all)

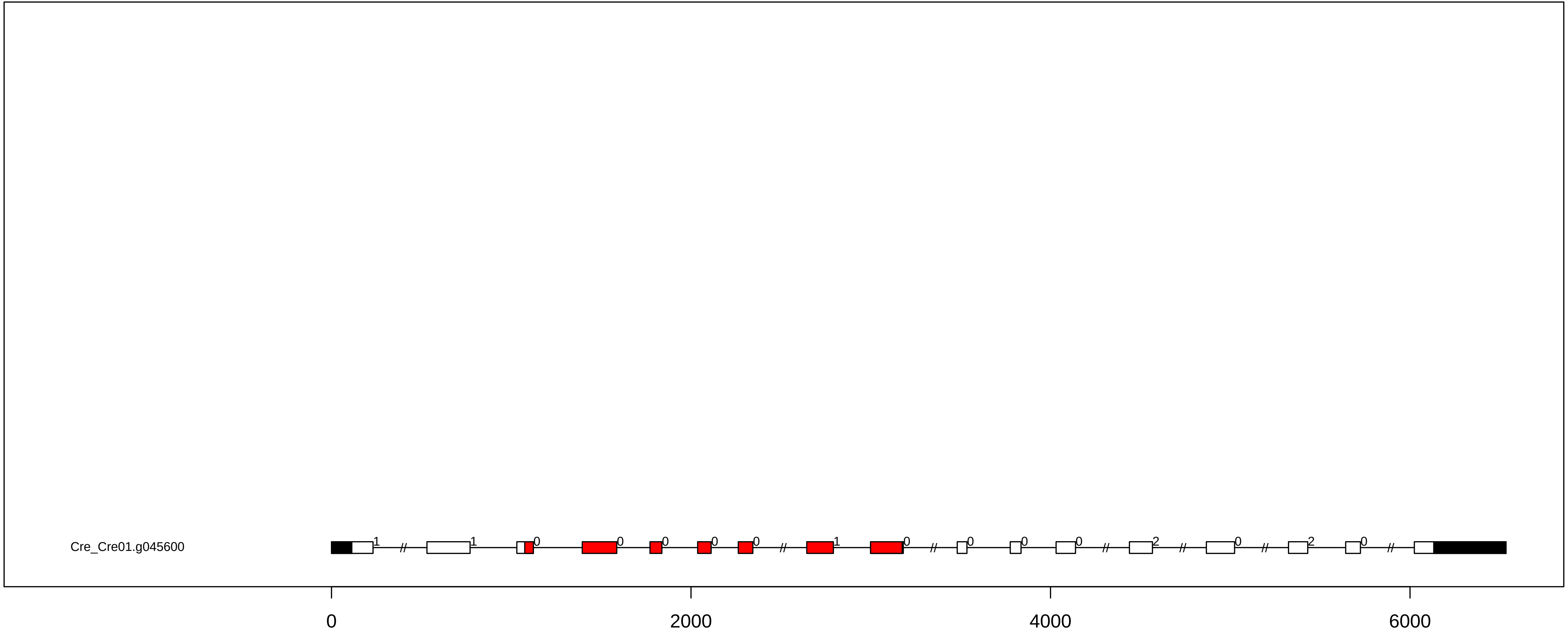

C.re Group−PI−4 exon−intron and kinase domain diagram (all)

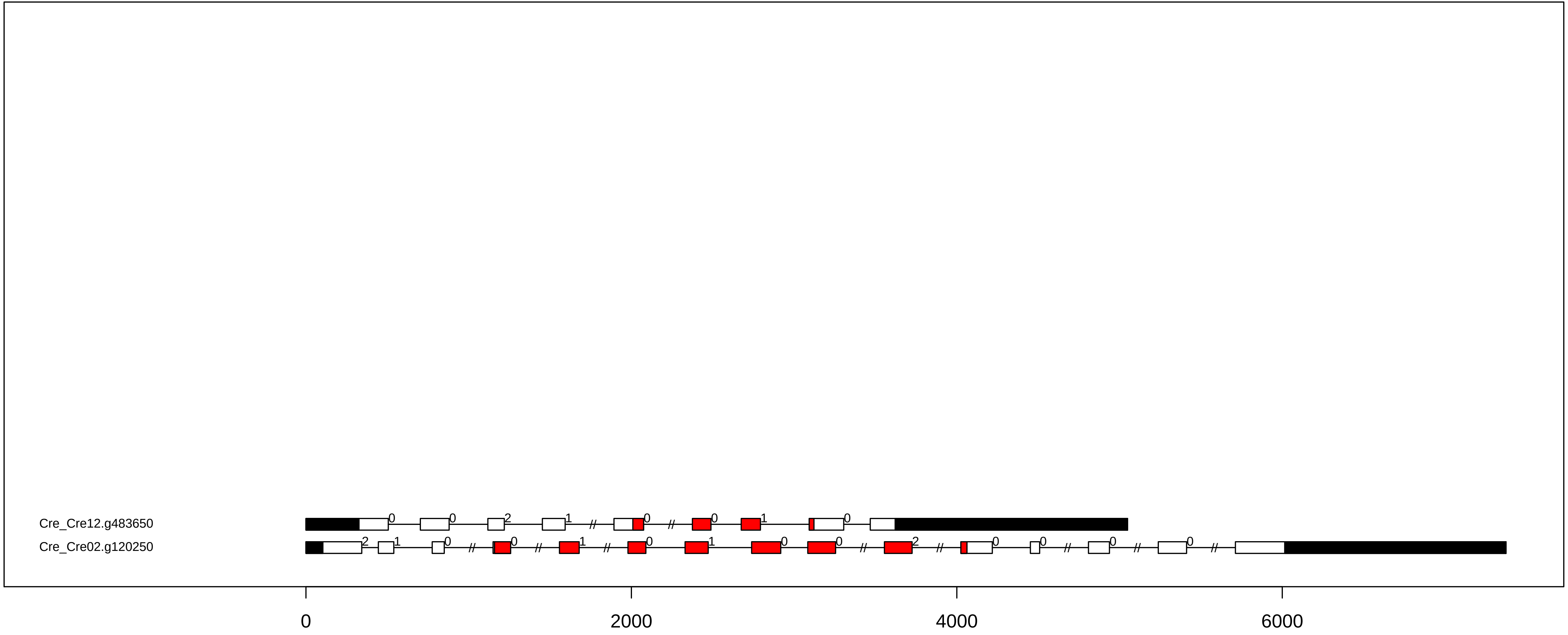

C.re NAK exon−intron and kinase domain diagram (all)

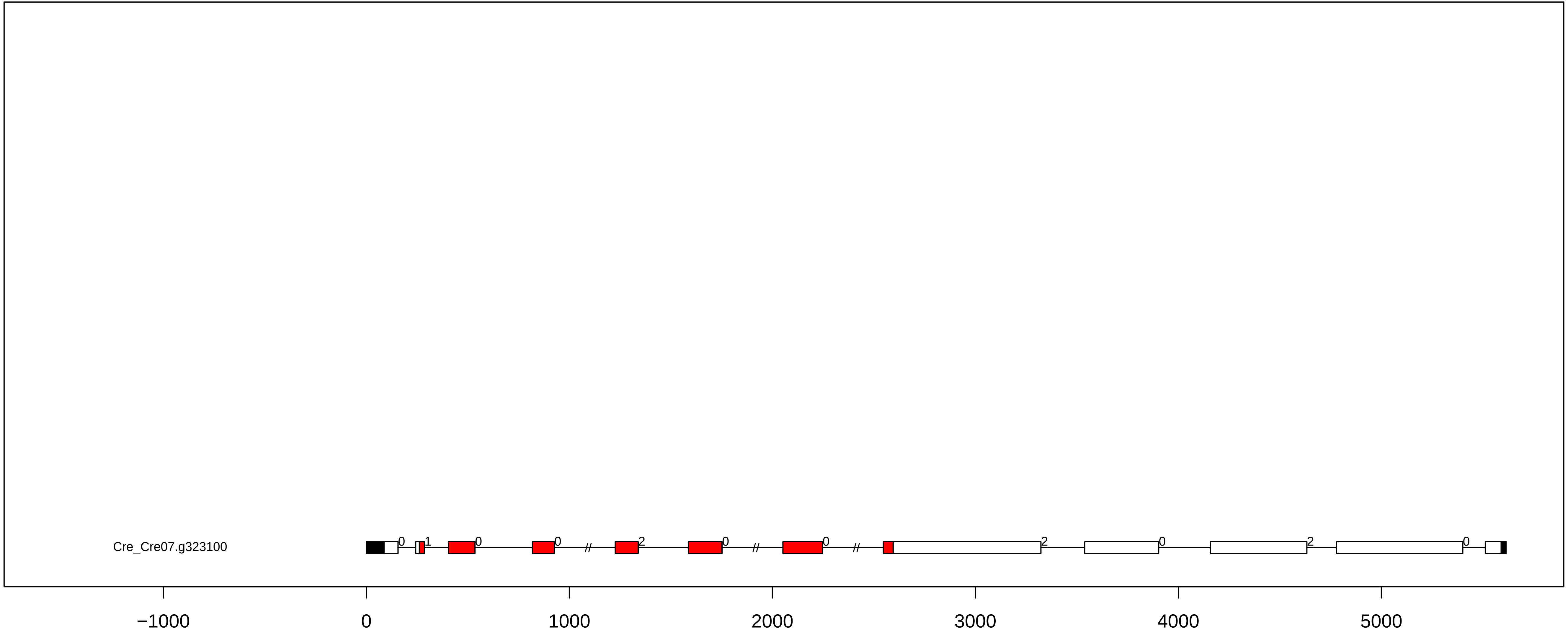

C.re NEK exon−intron and kinase domain diagram (all)

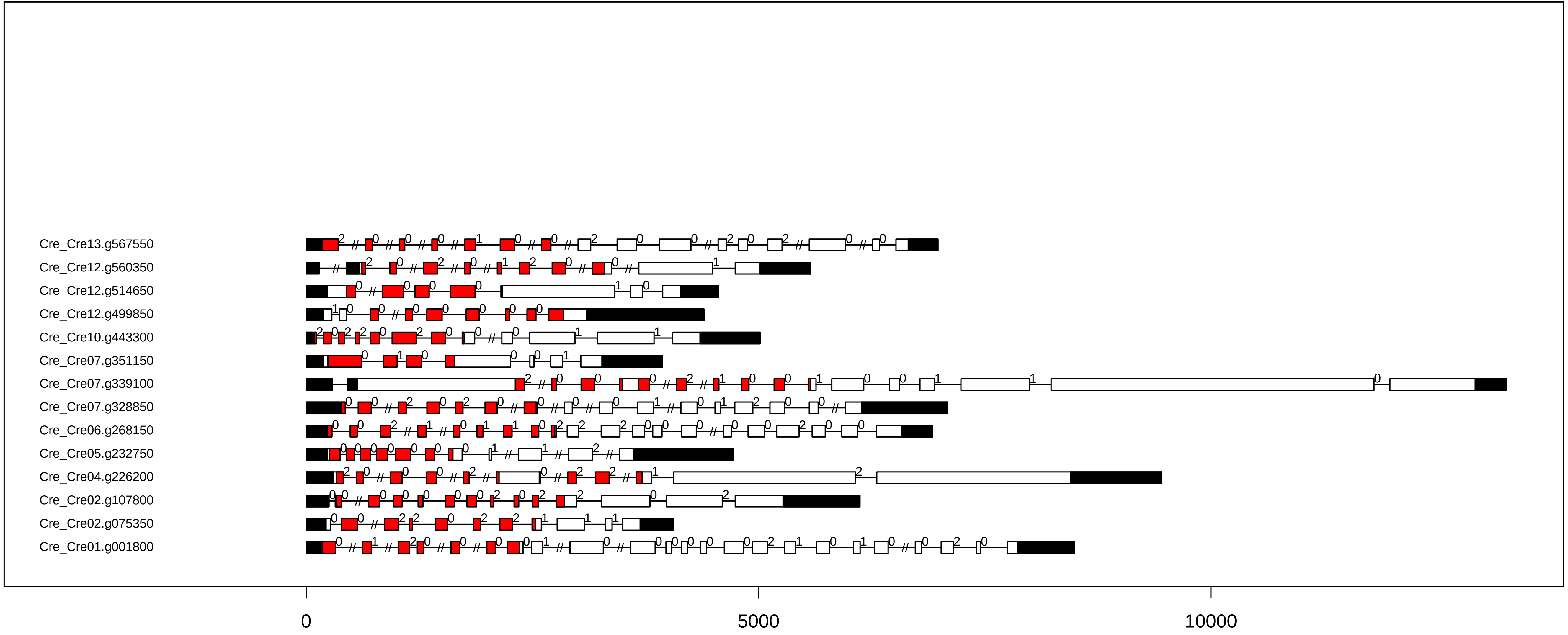

C.re PEK_GCN2 exon−intron and kinase domain diagram (all)

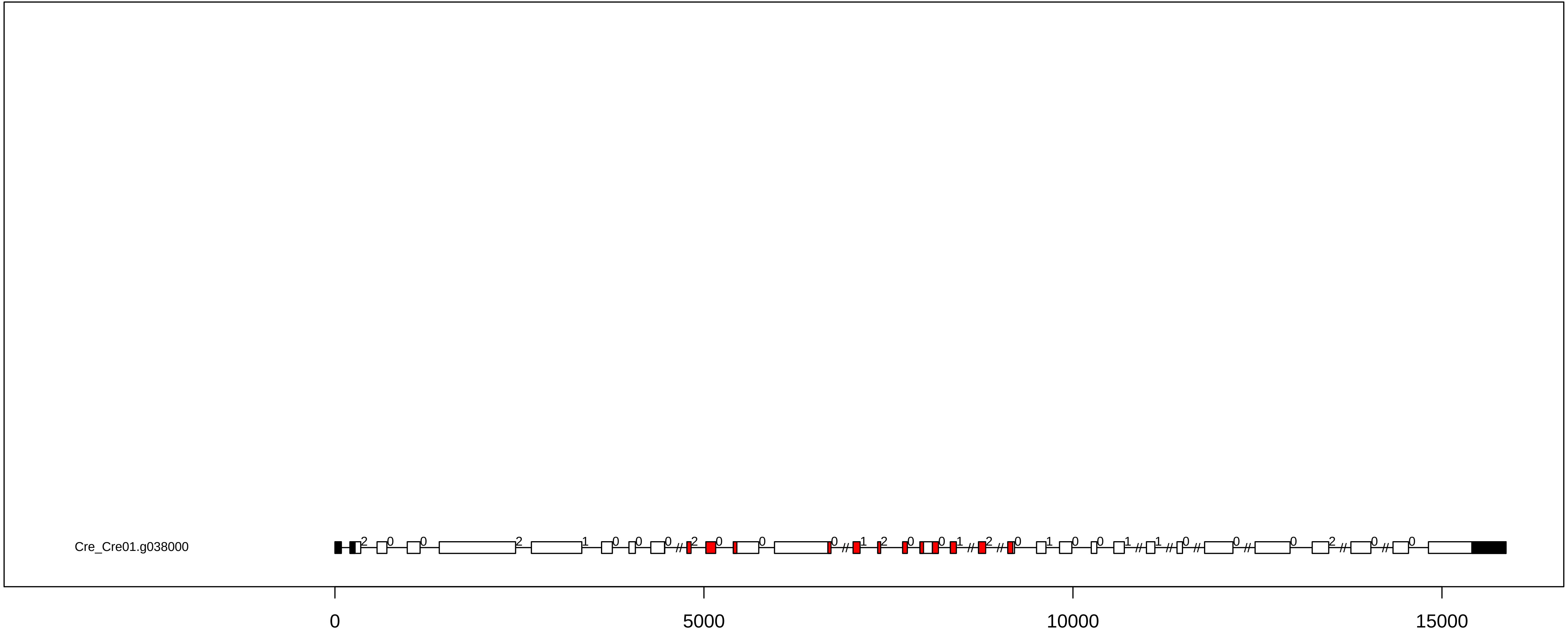

C.re RLK−Pelle_RLCK−IXb exon−intron and kinase domain diagram (all)

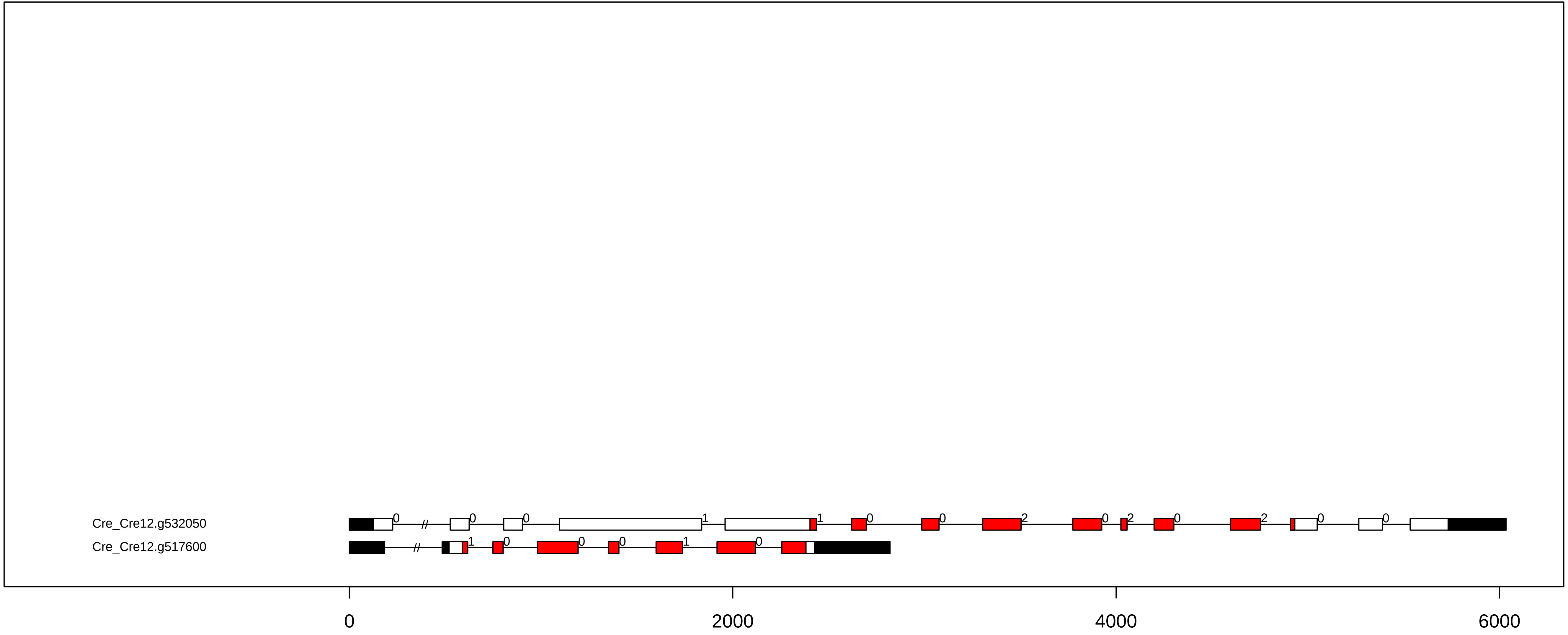

C.re SCY1_SCYL1 exon−intron and kinase domain diagram (all)

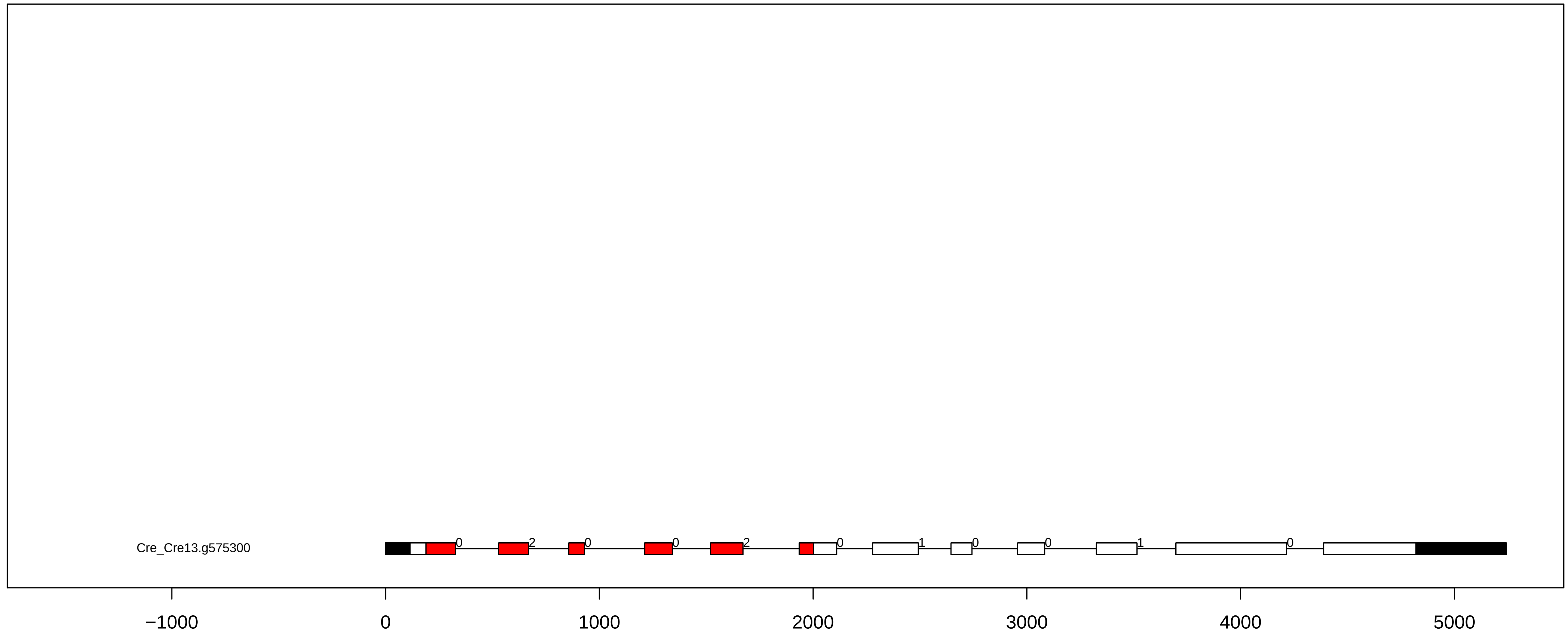

C.re STE_STE11 exon−intron and kinase domain diagram (all)

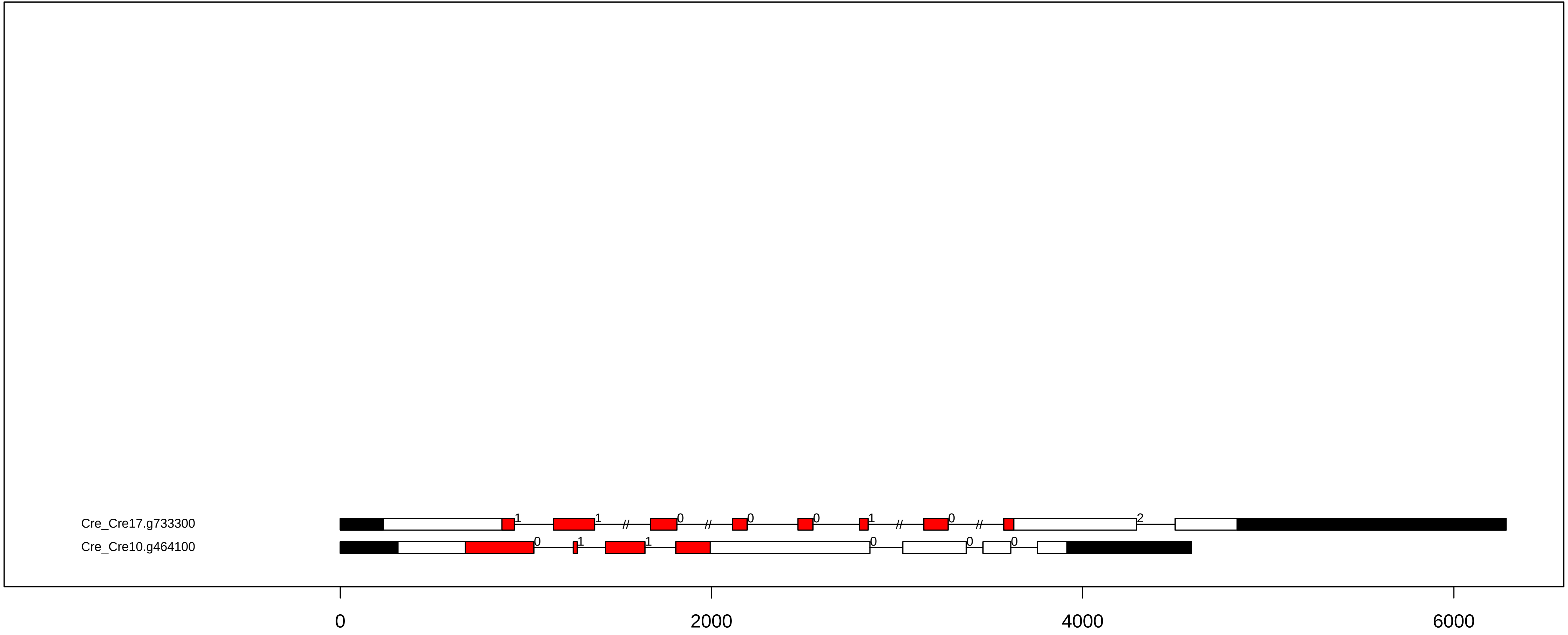

C.re STE_STE20−Fray exon−intron and kinase domain diagram (all)

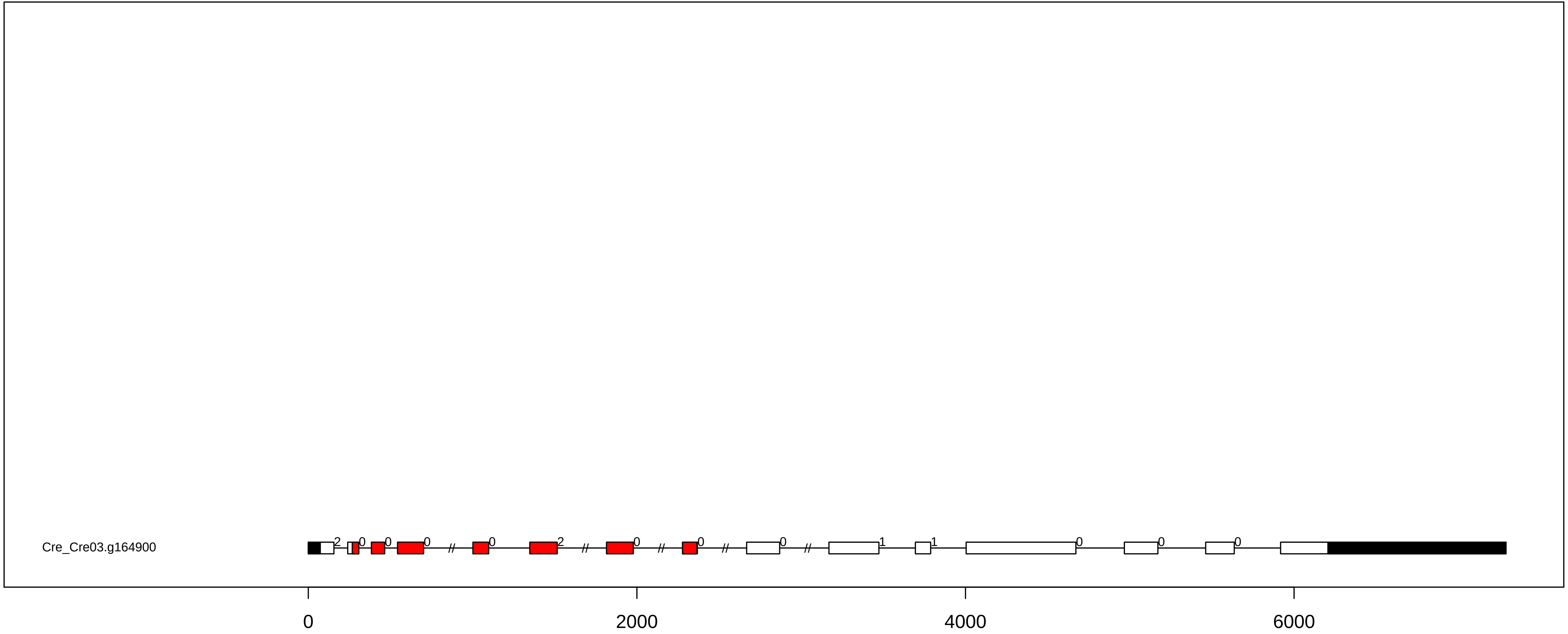

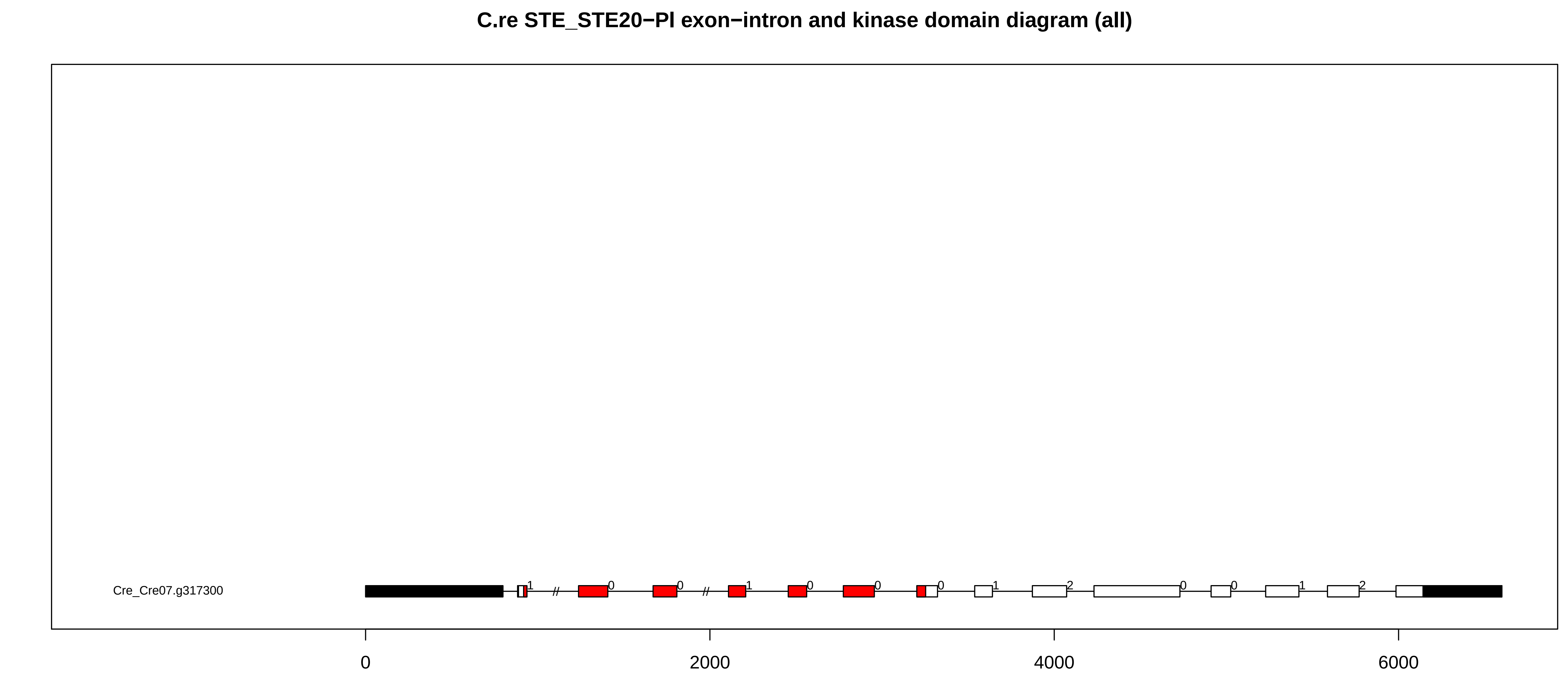
C.re STE_STE20−PI exon−intron and kinase domain diagram (all)
Cre_Cre07.g317300
1
0
0
1
0
0
0
1
2
0
0
1
2
0
2000
4000
6000

C.re STE_STE20−YSK exon−intron and kinase domain diagram (all)

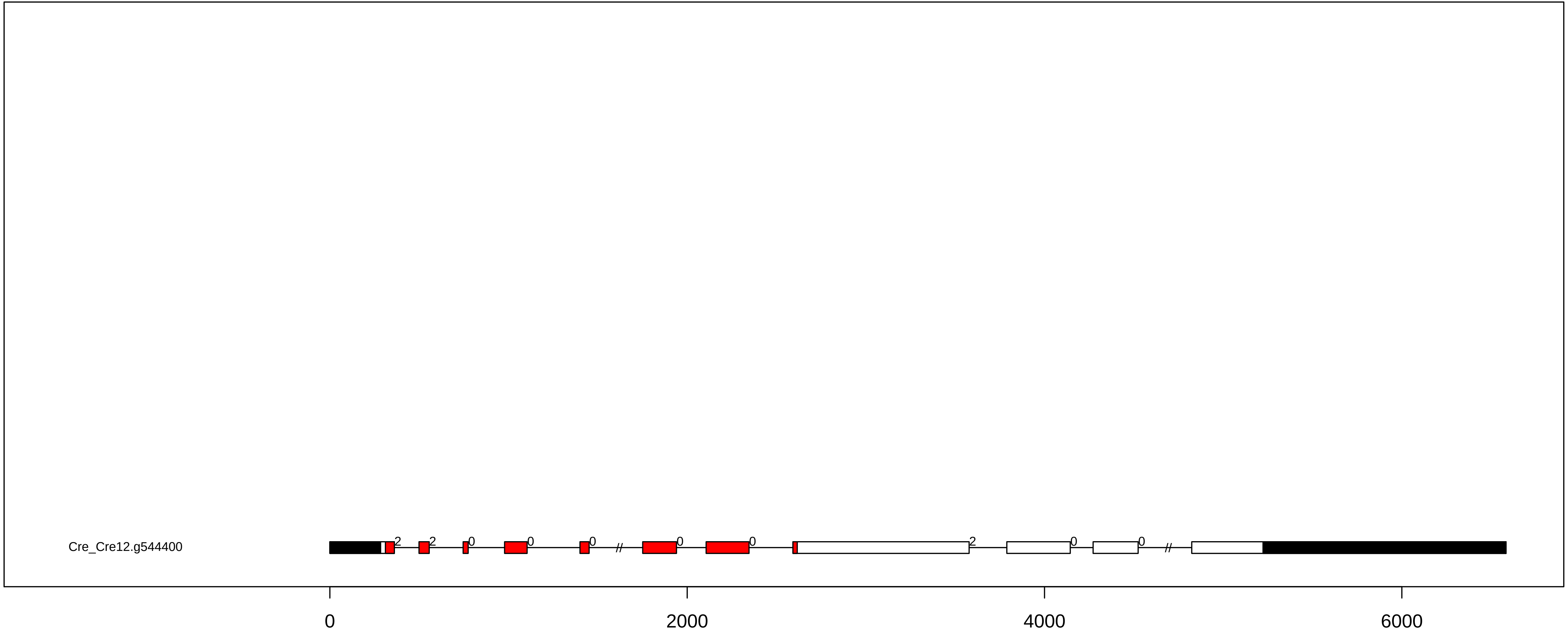

C.re STE_STE7 exon−intron and kinase domain diagram (all)

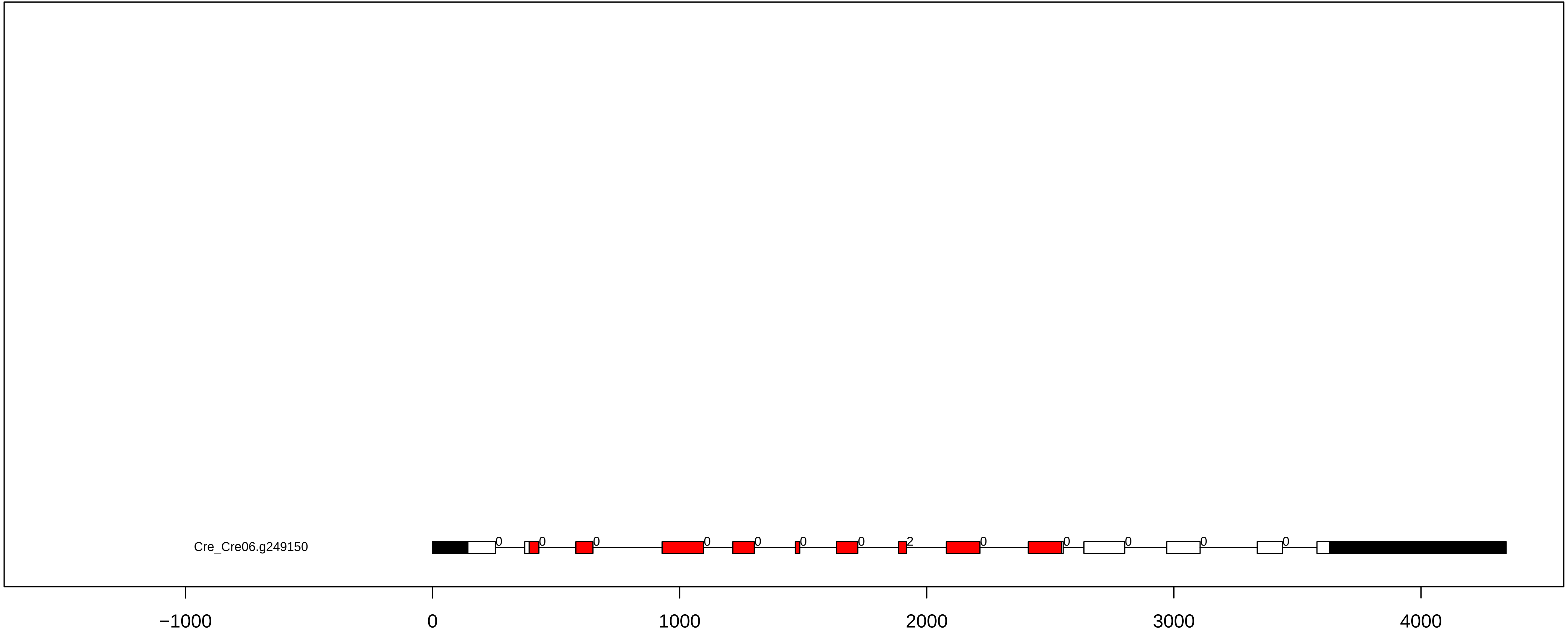

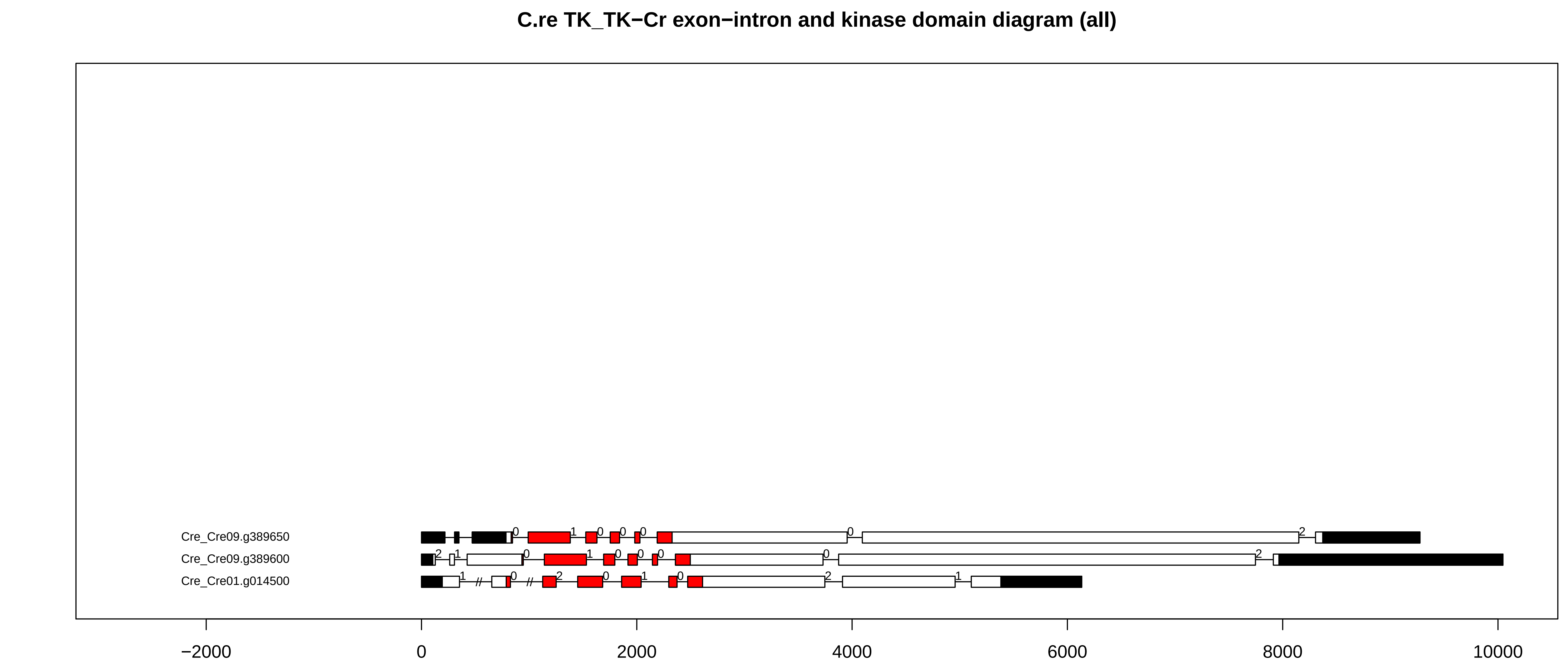
C.re TK_TK−Cr exon−intron and kinase domain diagram (all)
Cre_Cre09.g389650
Cre_Cre09.g389600
Cre_Cre01.g014500
−2000
0
2000
4000
6000
8000
10000

C.re TKL_CTR1−DRK−2 exon−intron and kinase domain diagram (all)

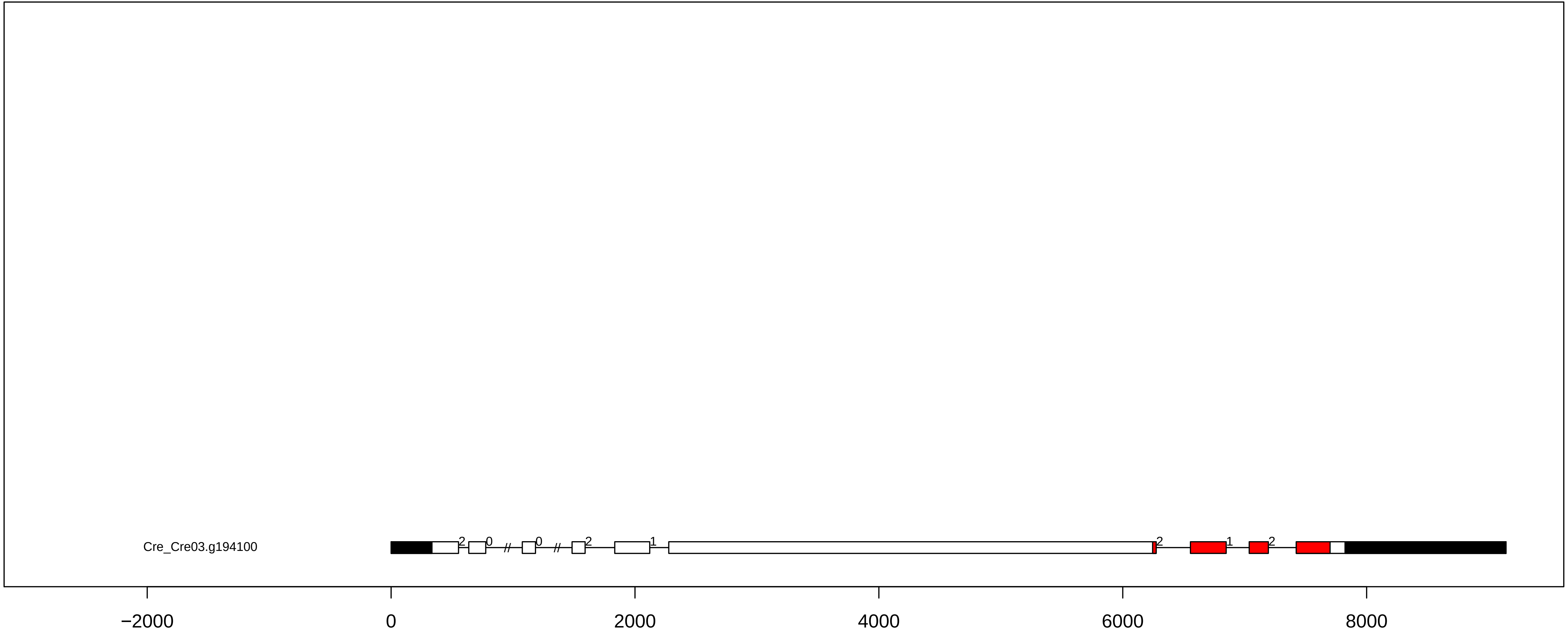

C.re TKL_CTR1−DRK−Cr exon−intron and kinase domain diagram (all)

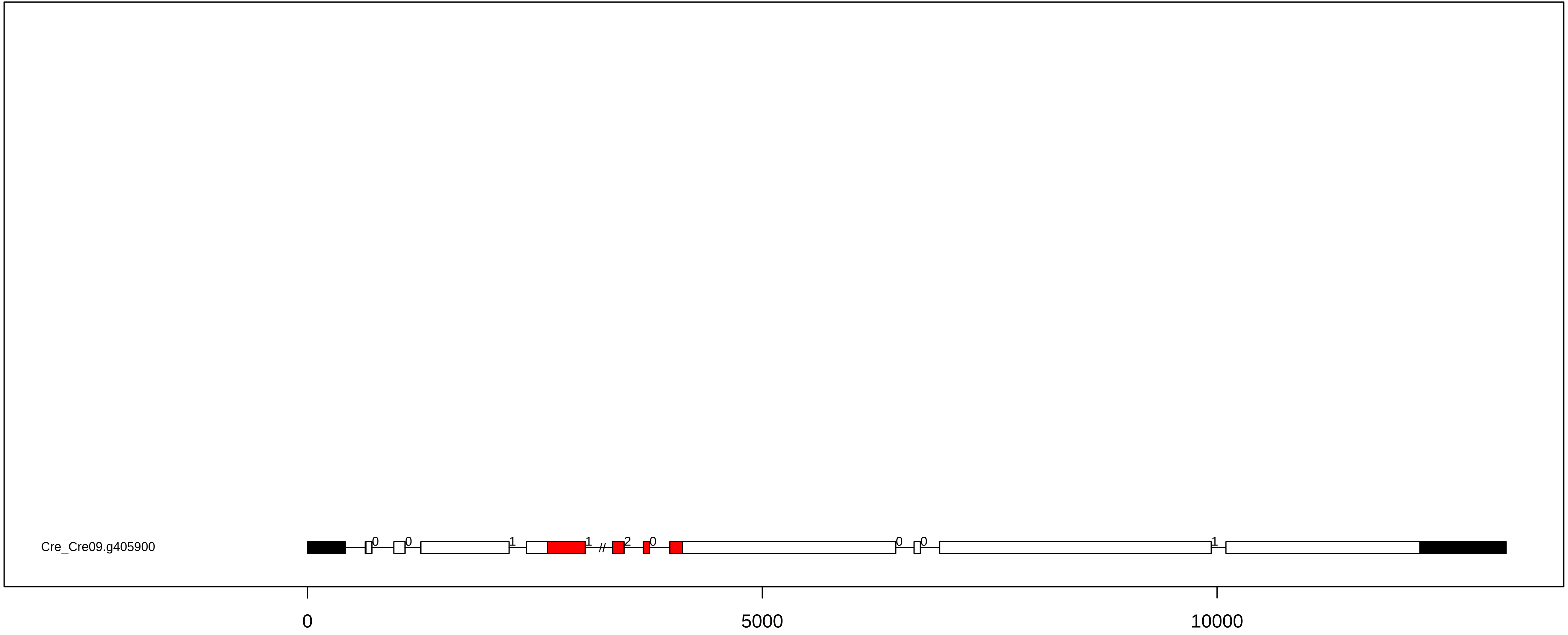

C.re TKL−Cr−1 exon−intron and kinase domain diagram (all)

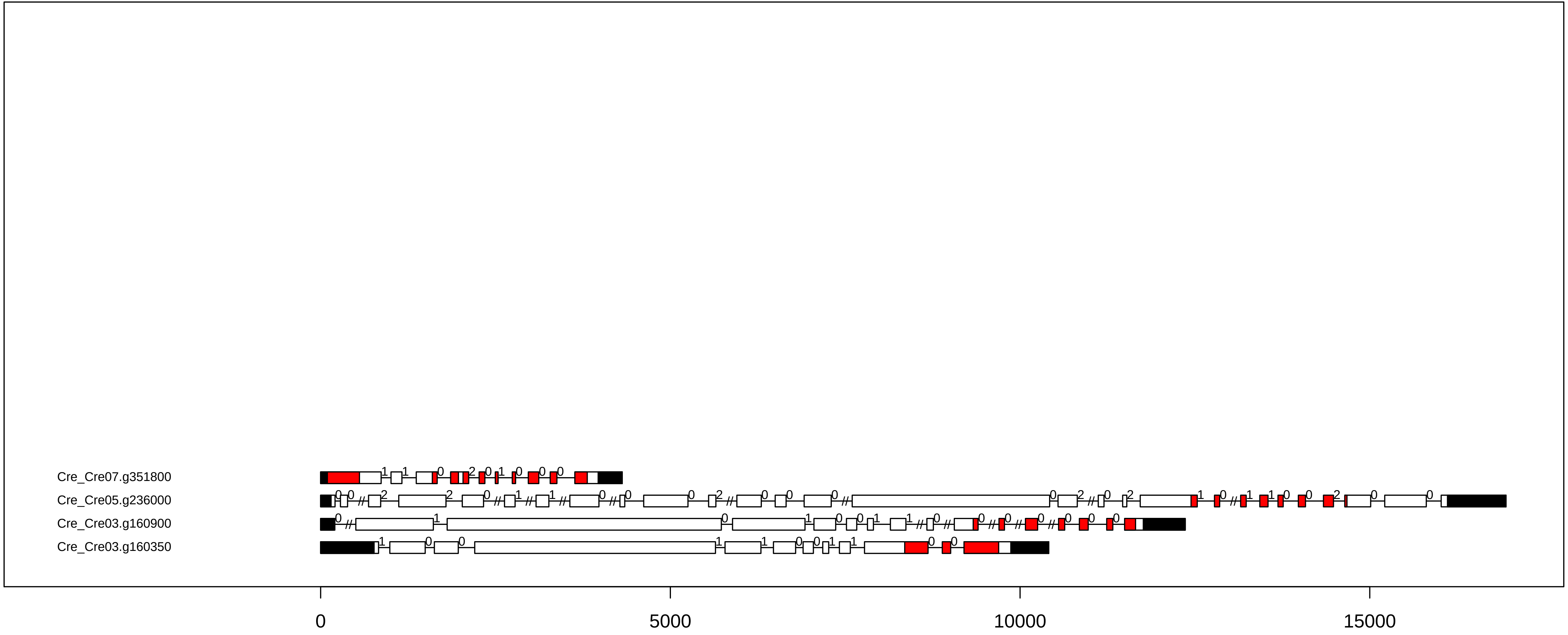

C.re TKL−Cr−2 exon−intron and kinase domain diagram (all)

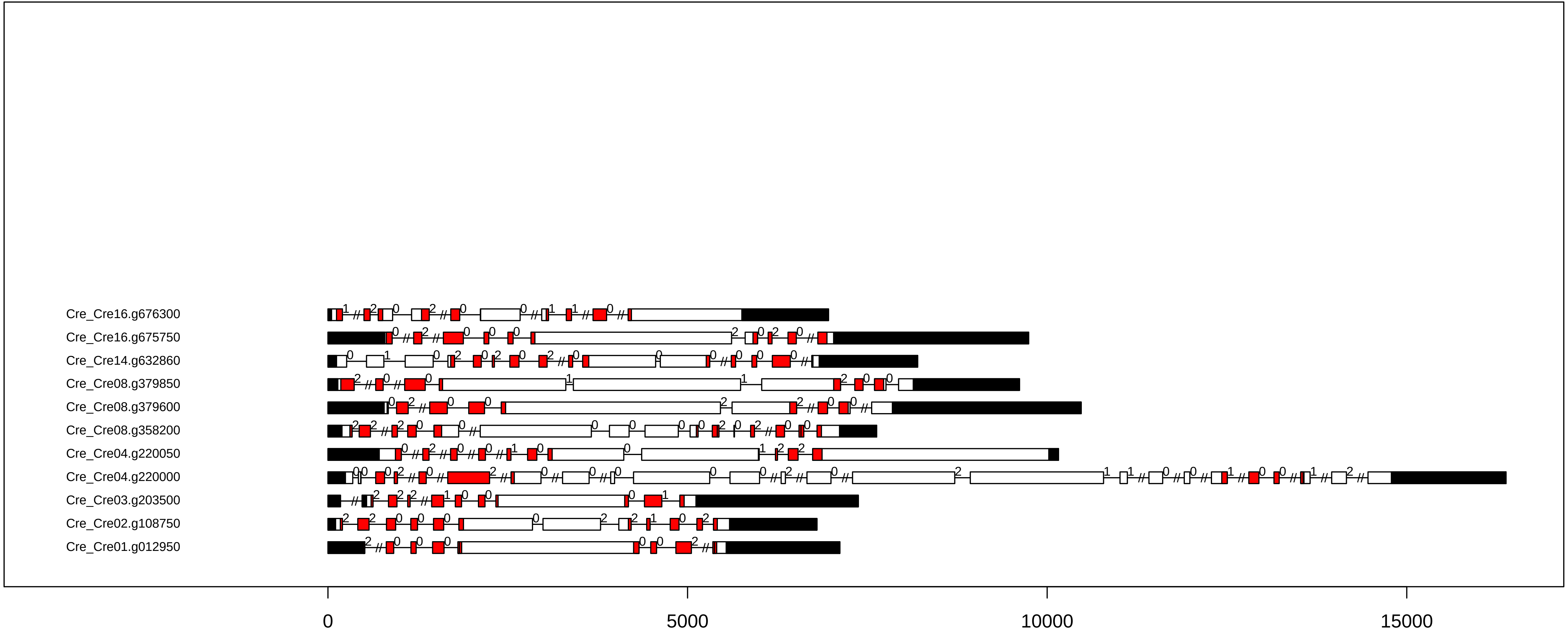

C.re TKL−Cr−3 exon−intron and kinase domain diagram (part 1)

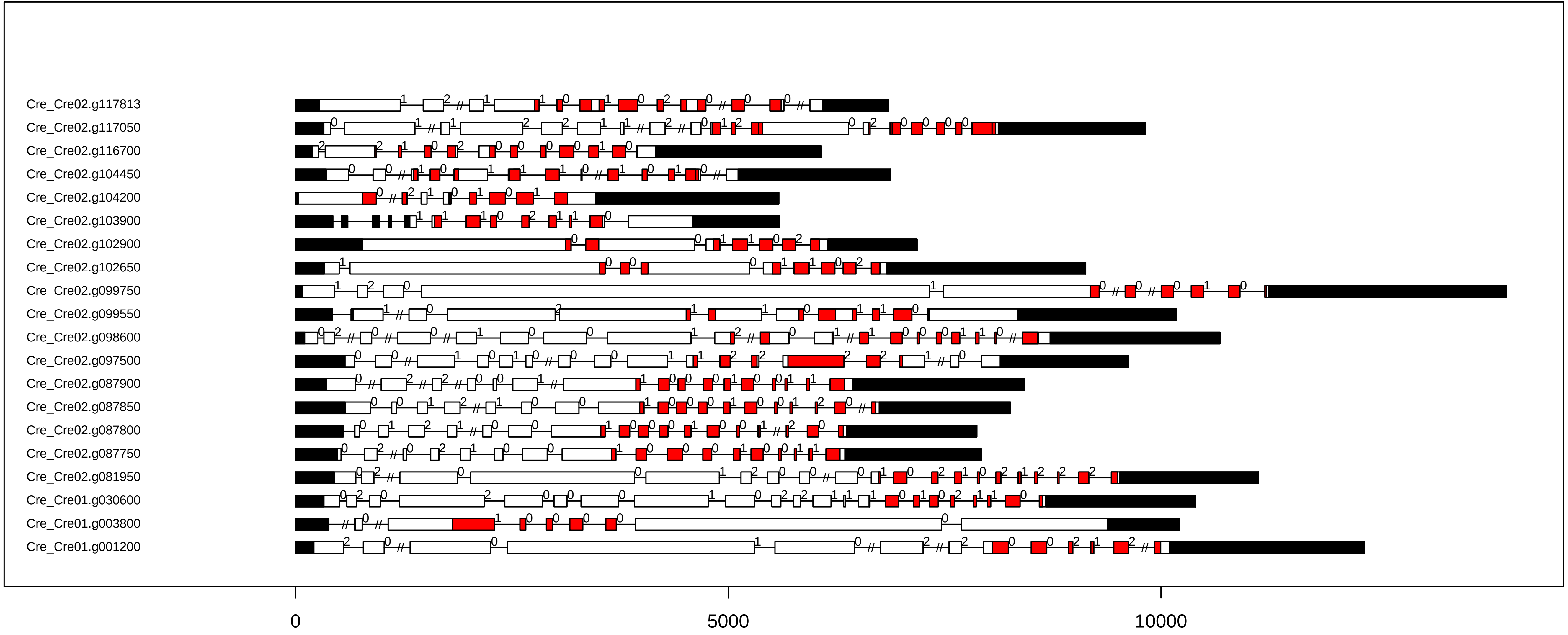

C.re TKL−Cr−3 exon−intron and kinase domain diagram (part 2)

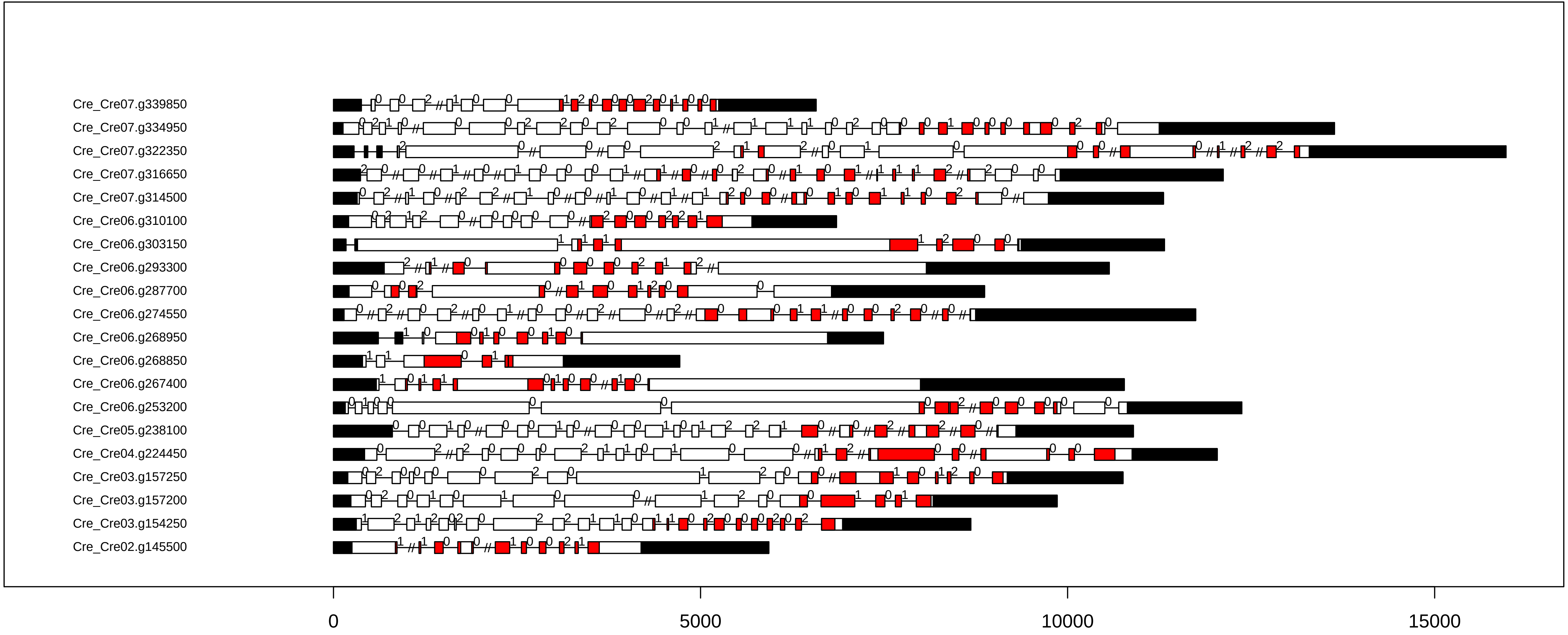

C.re TKL−Cr−3 exon−intron and kinase domain diagram (part 3)

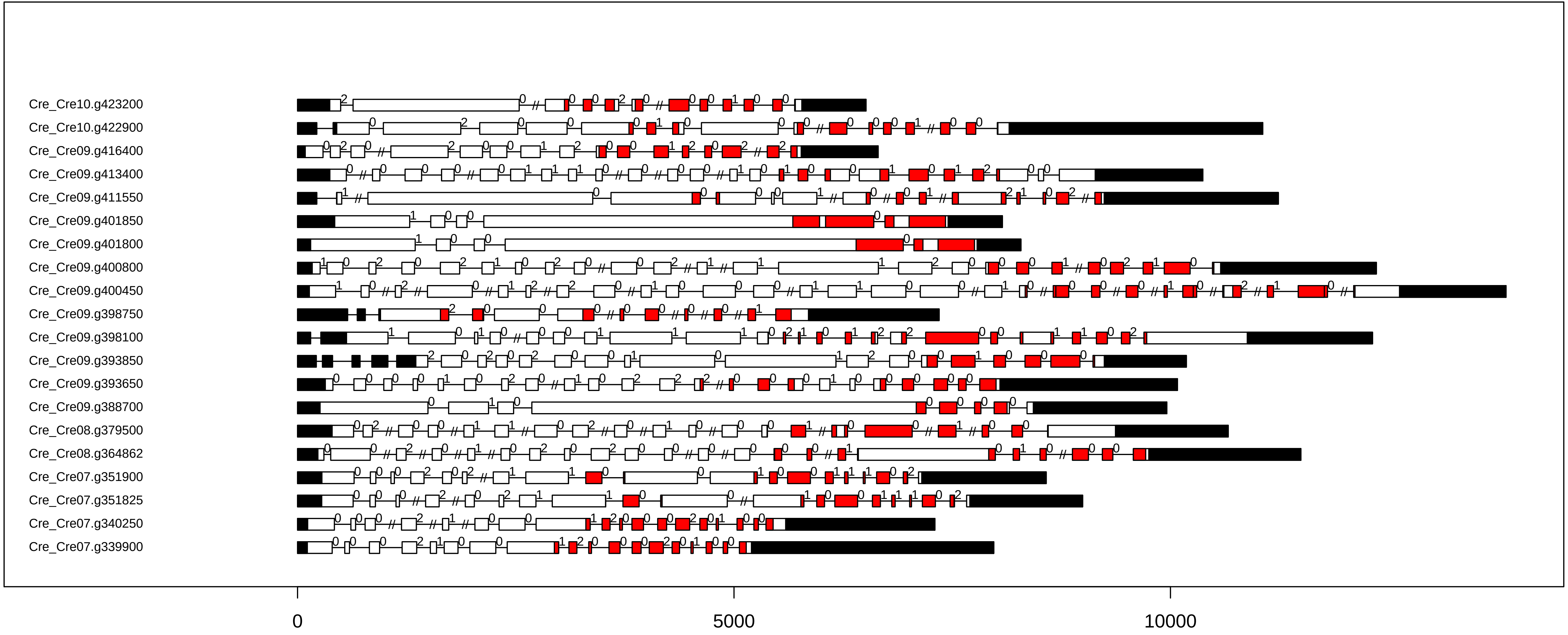

C.re TKL−Cr−3 exon−intron and kinase domain diagram (part 4)

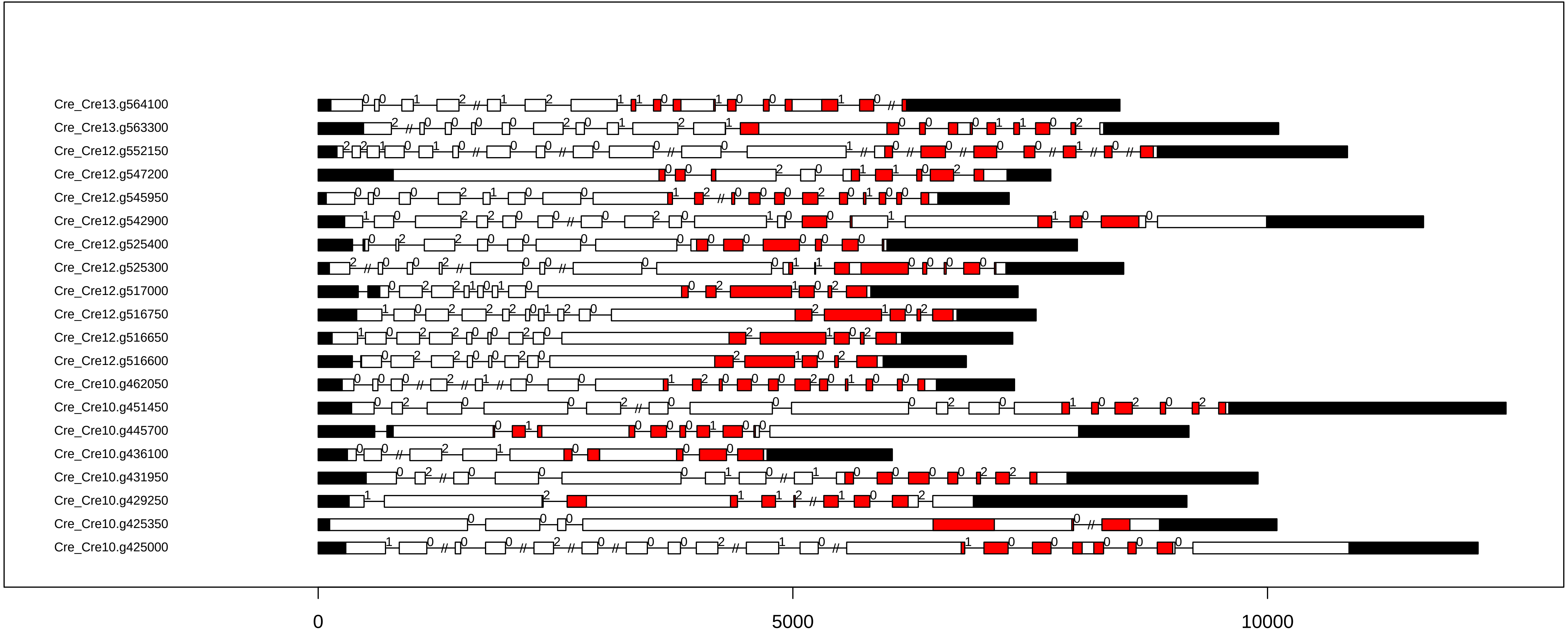

C.re TKL−Cr−3 exon−intron and kinase domain diagram (part 5)

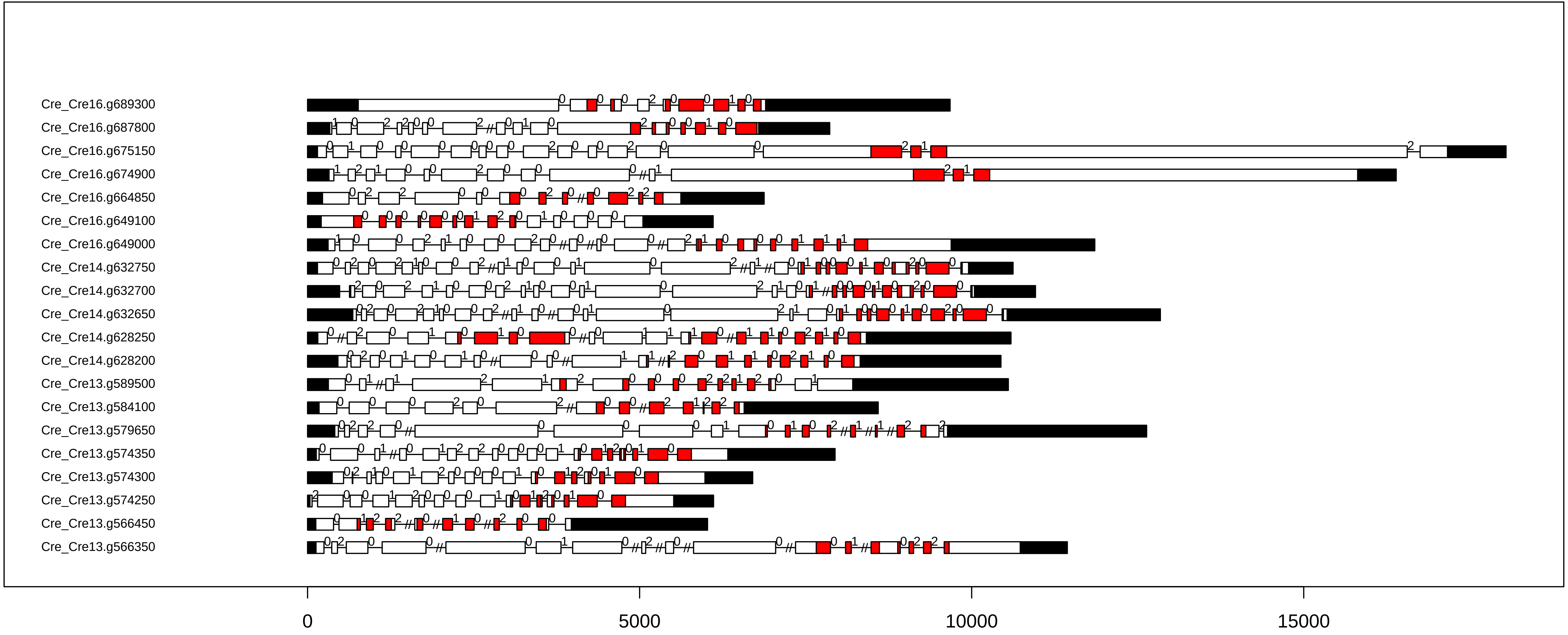

C.re TKL−Cr−3 exon−intron and kinase domain diagram (part 6)

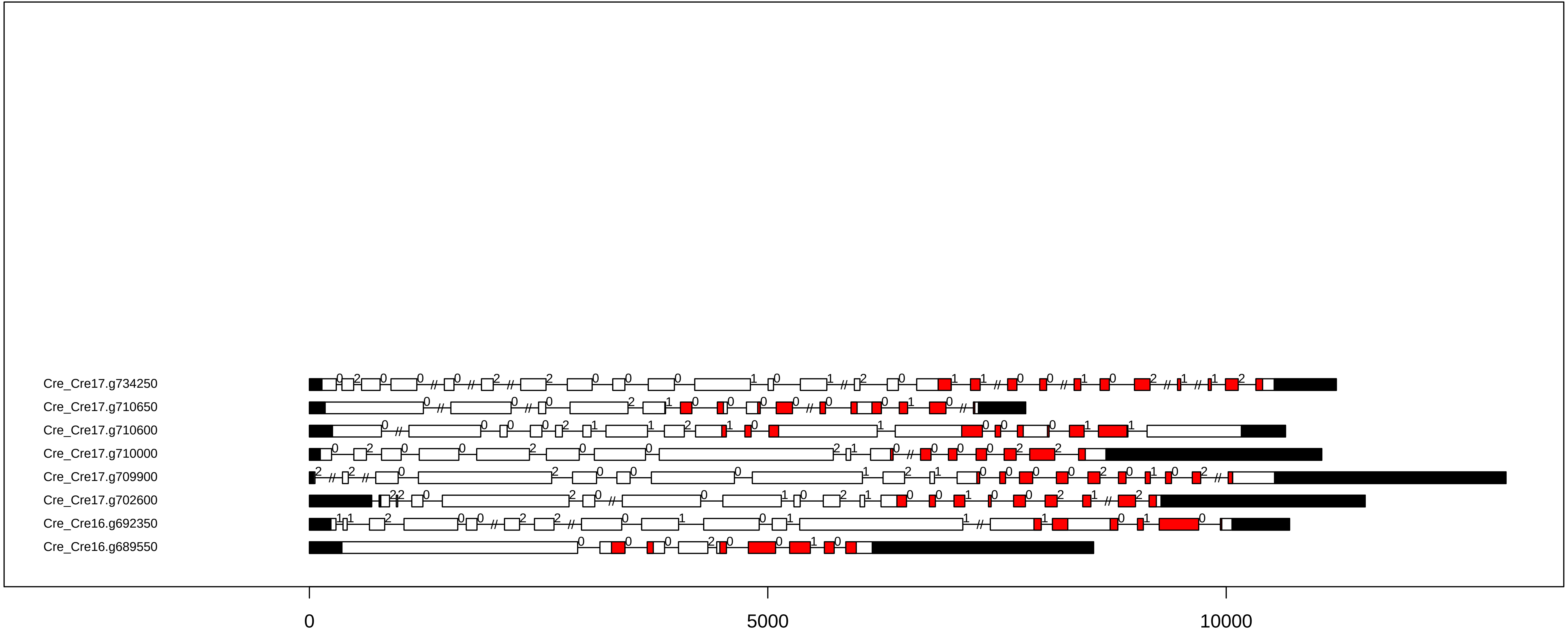

C.re TKL−Cr−4 exon−intron and kinase domain diagram (part 1)

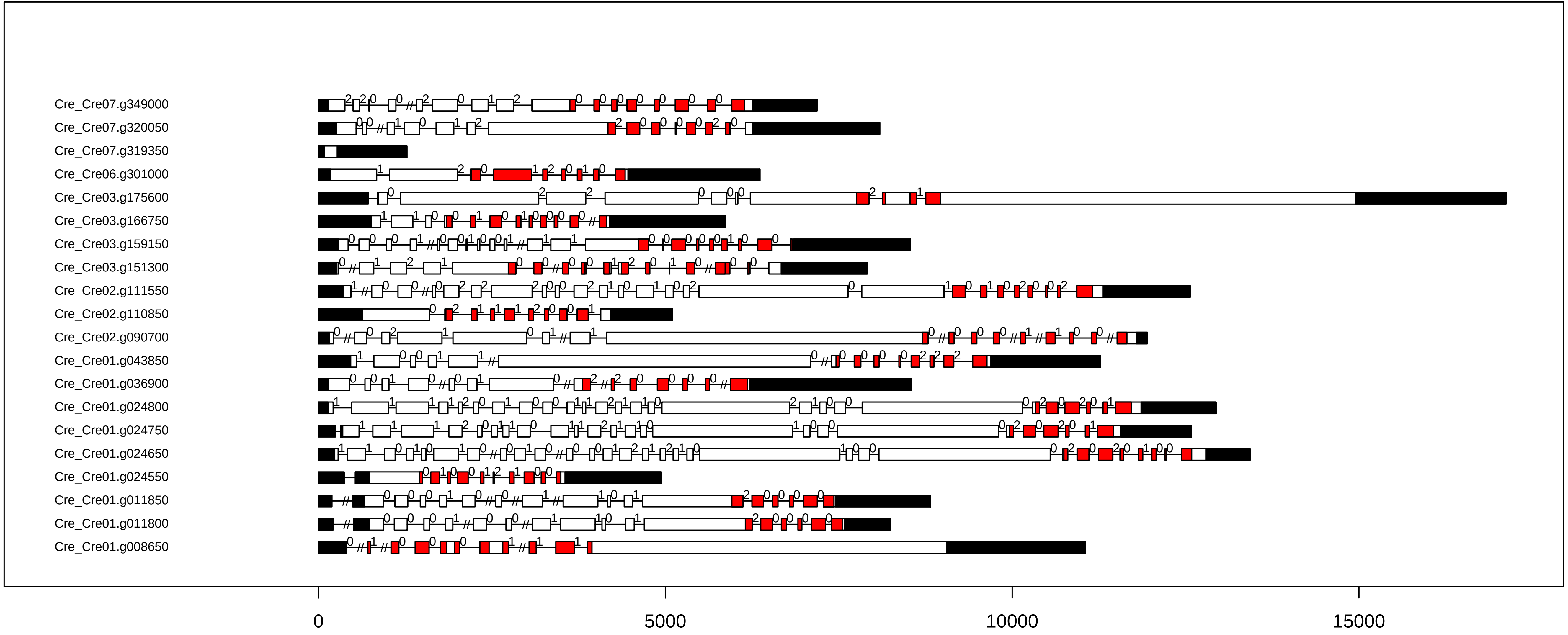

C.re TKL−Cr−4 exon−intron and kinase domain diagram (part 2)

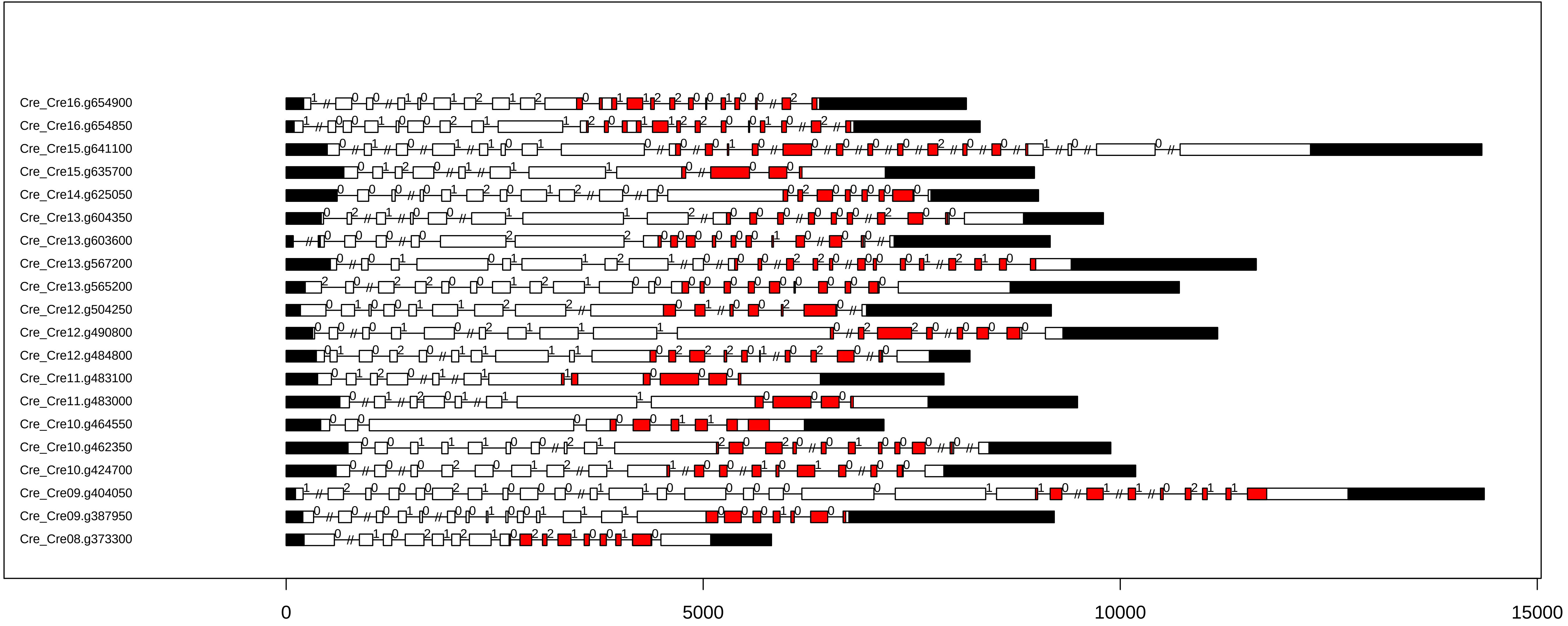

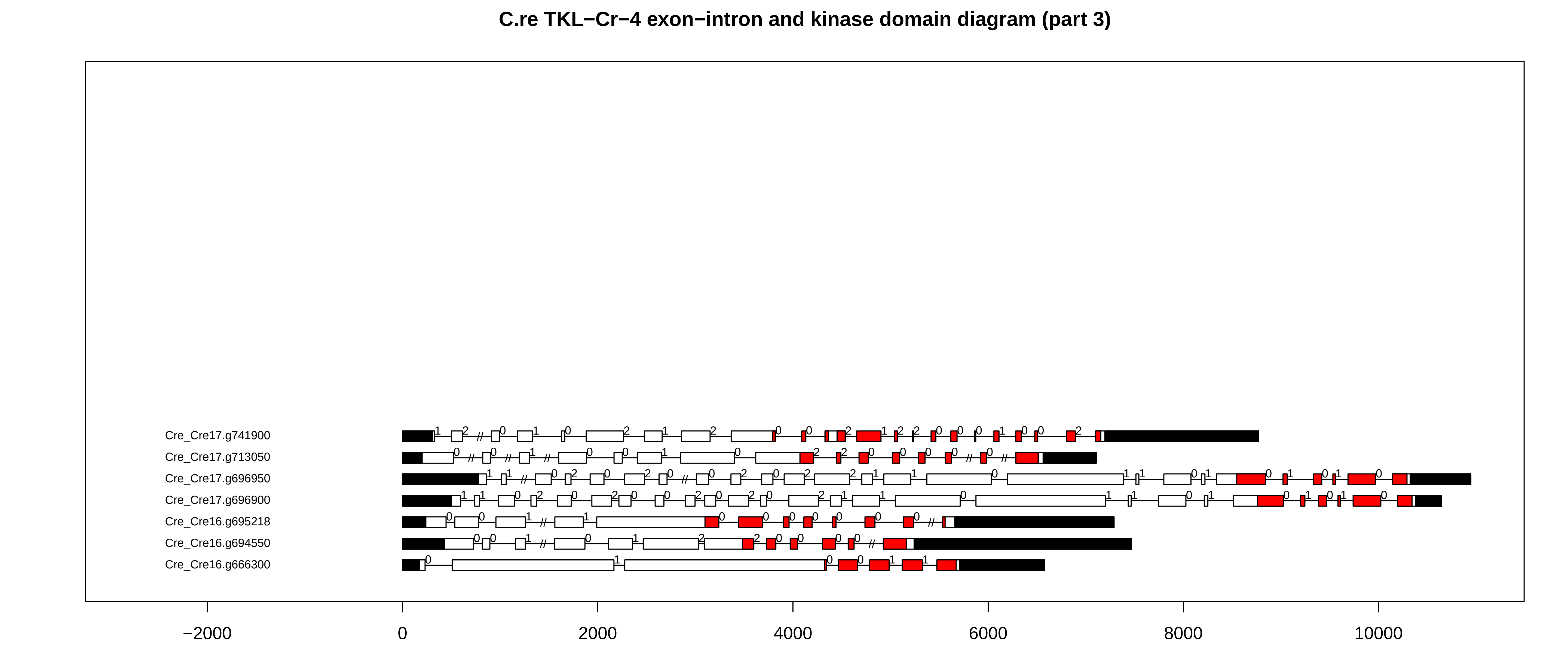

C.re TKL−Cr−4 exon−intron and kinase domain diagram (part 3)
Cre_Cre17.g741900
Cre_Cre17.g713050
Cre_Cre17.g696950
Cre_Cre17.g696900
Cre_Cre16.g695218
Cre_Cre16.g694550
Cre_Cre16.g666300
−2000
0
2000
4000
6000
8000
10000

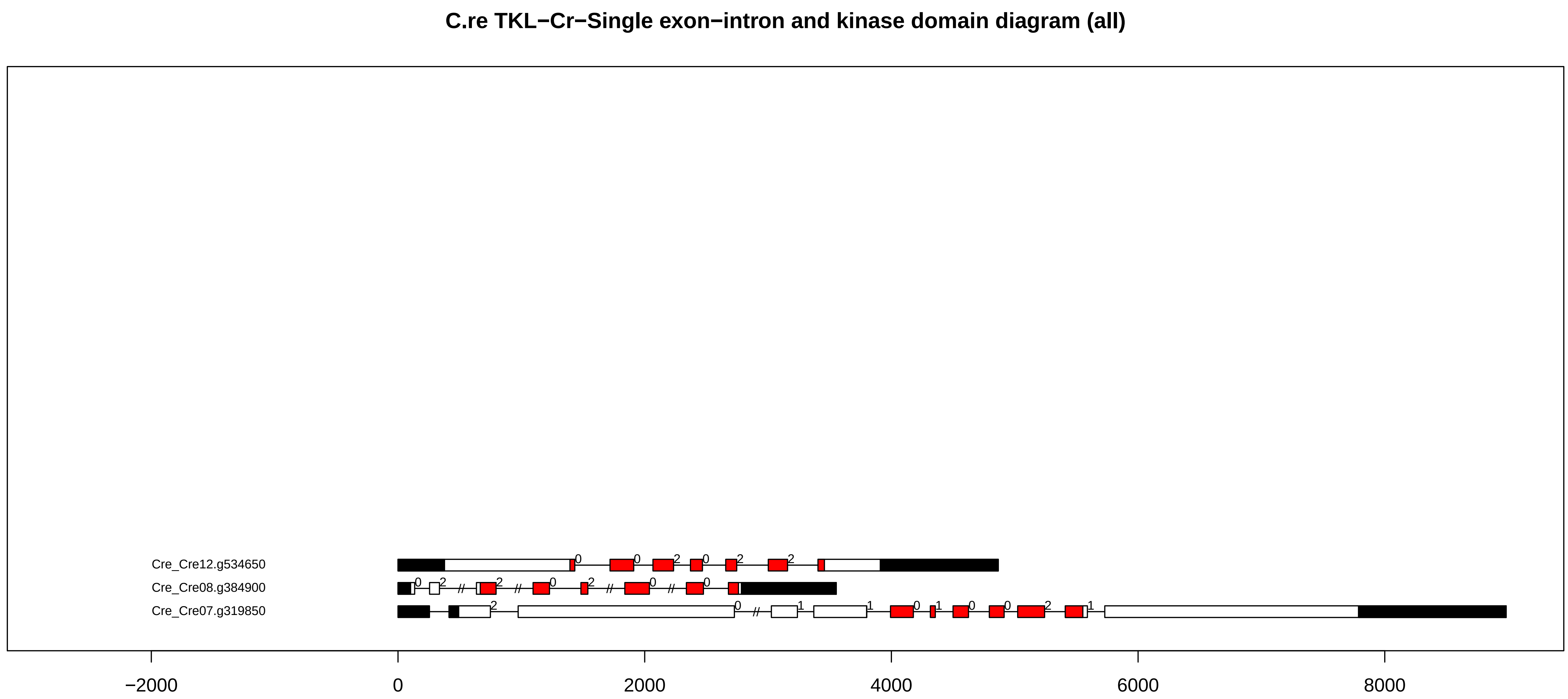
C.re TKL−Cr−Single exon−intron and kinase domain diagram (all)
Cre_Cre12.g534650
Cre_Cre08.g384900
Cre_Cre07.g319850
−2000
0
2000
4000
6000
8000

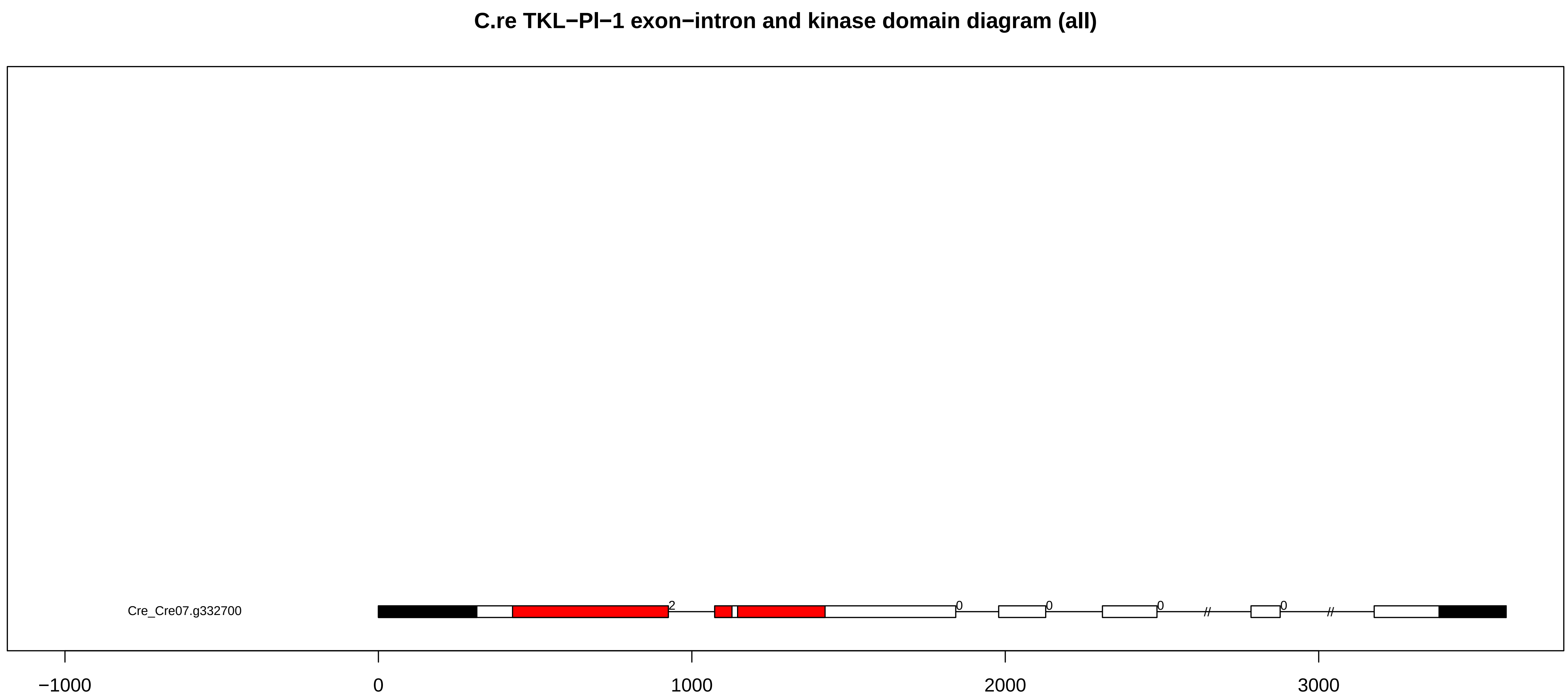
C.re TKL−Pl−1 exon−intron and kinase domain diagram (all)
Cre_Cre07.g332700
2
0
0
0
0
−1000
0
1000
2000
3000

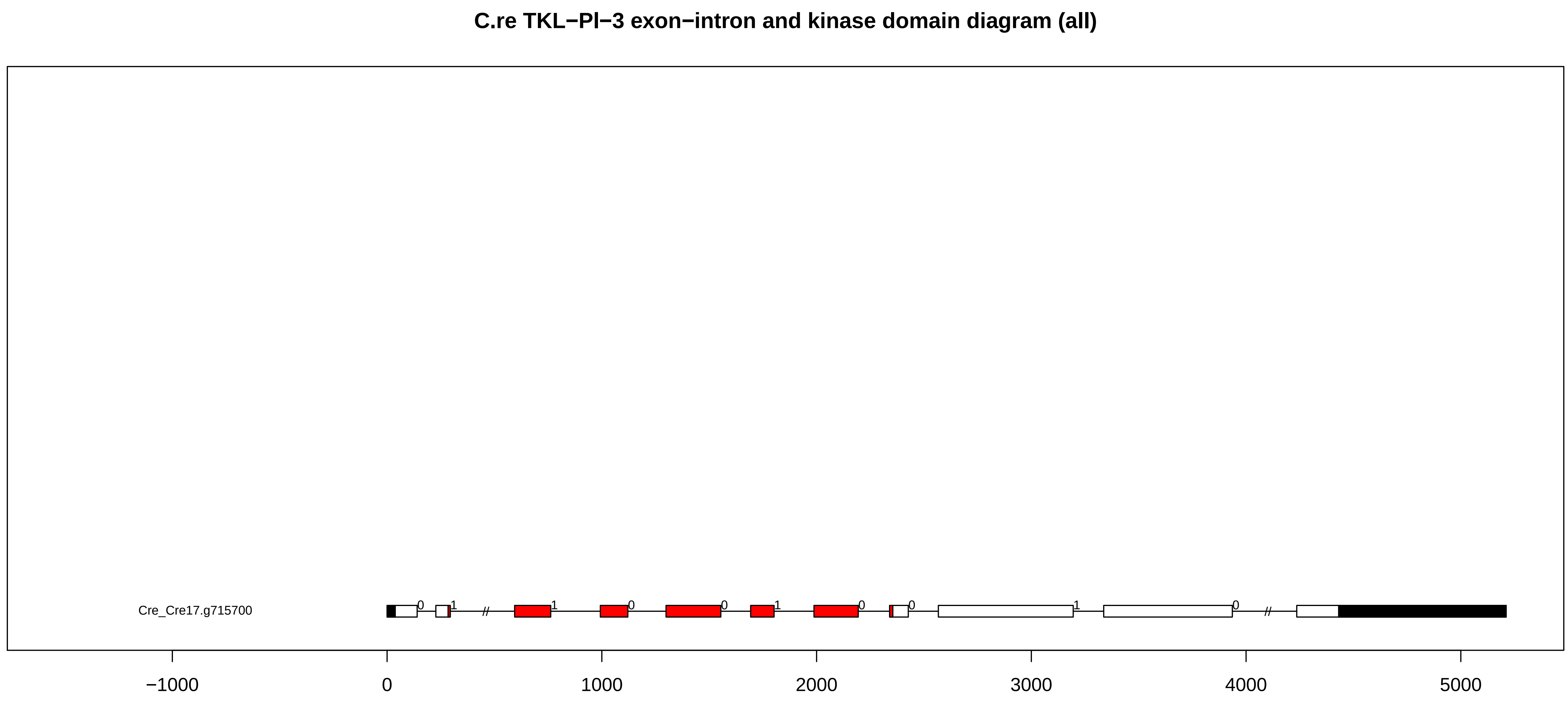
C.re TKL−Pl−3 exon−intron and kinase domain diagram (all)
Cre_Cre17.g715700
0
1
1
0
0
1
0
0
1
0
−1000
0
1000
2000
3000
4000
5000

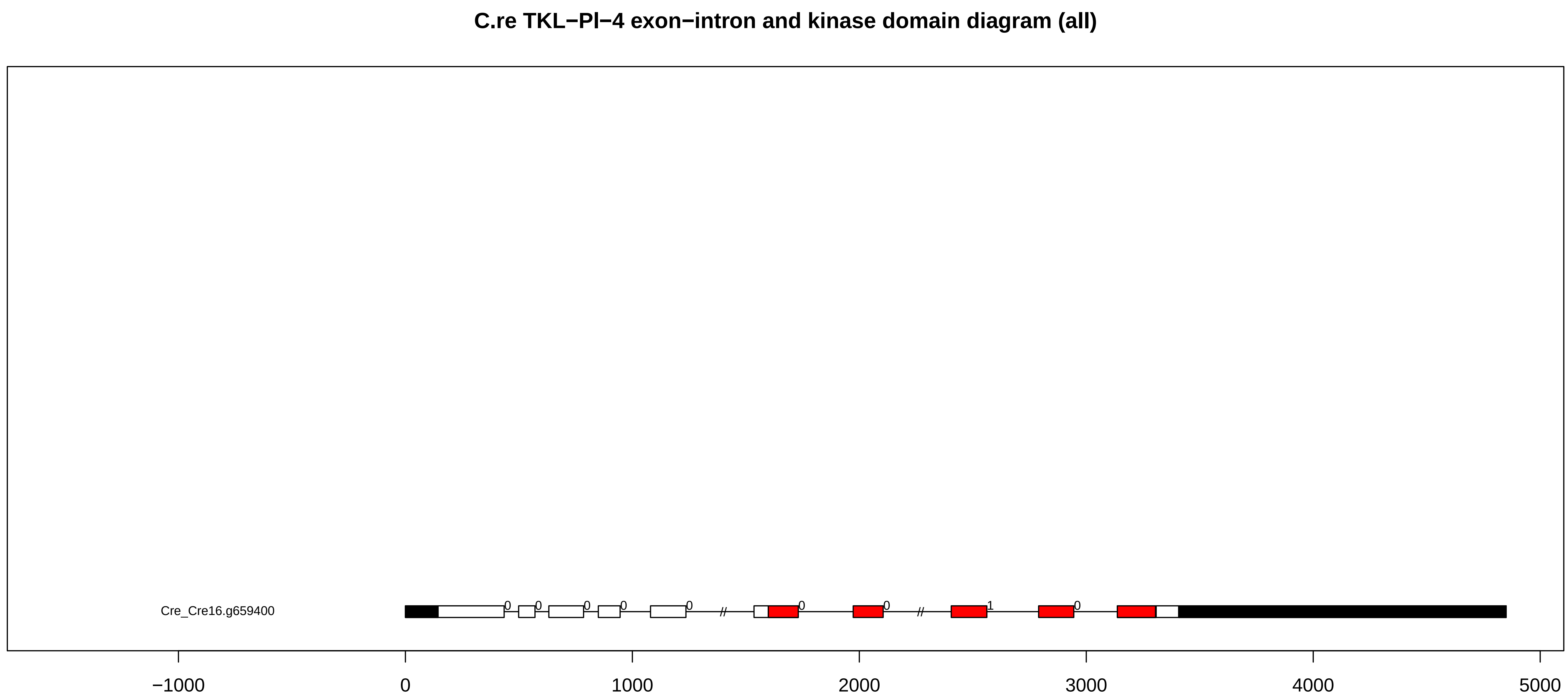

C.re TKL−Pl−4 exon−intron and kinase domain diagram (all)
Cre_Cre16.g659400
0
0
0
0
0
0
0
1
0
−1000
0
1000
2000
3000
4000
5000

C.re TKL−PI−5 exon−intron and kinase domain diagram (all)

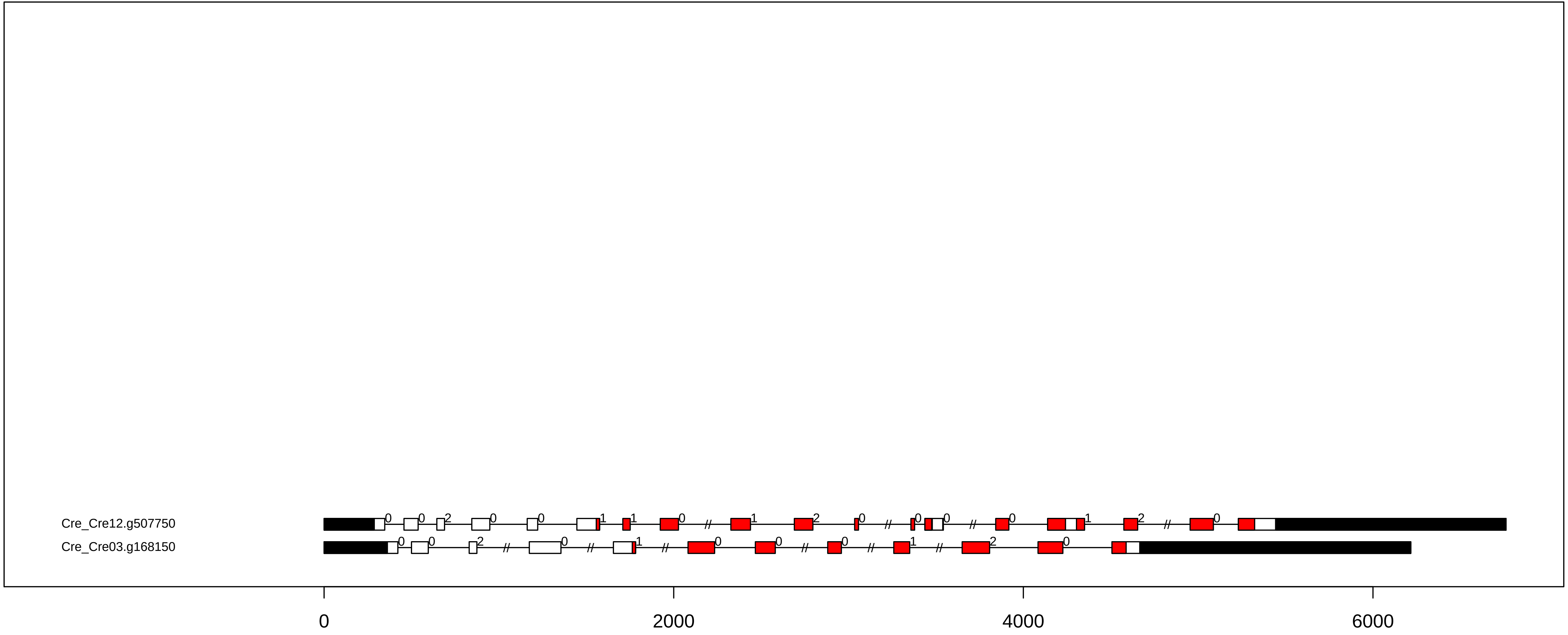

C.re TKL−Pl−6 exon−intron and kinase domain diagram (all)

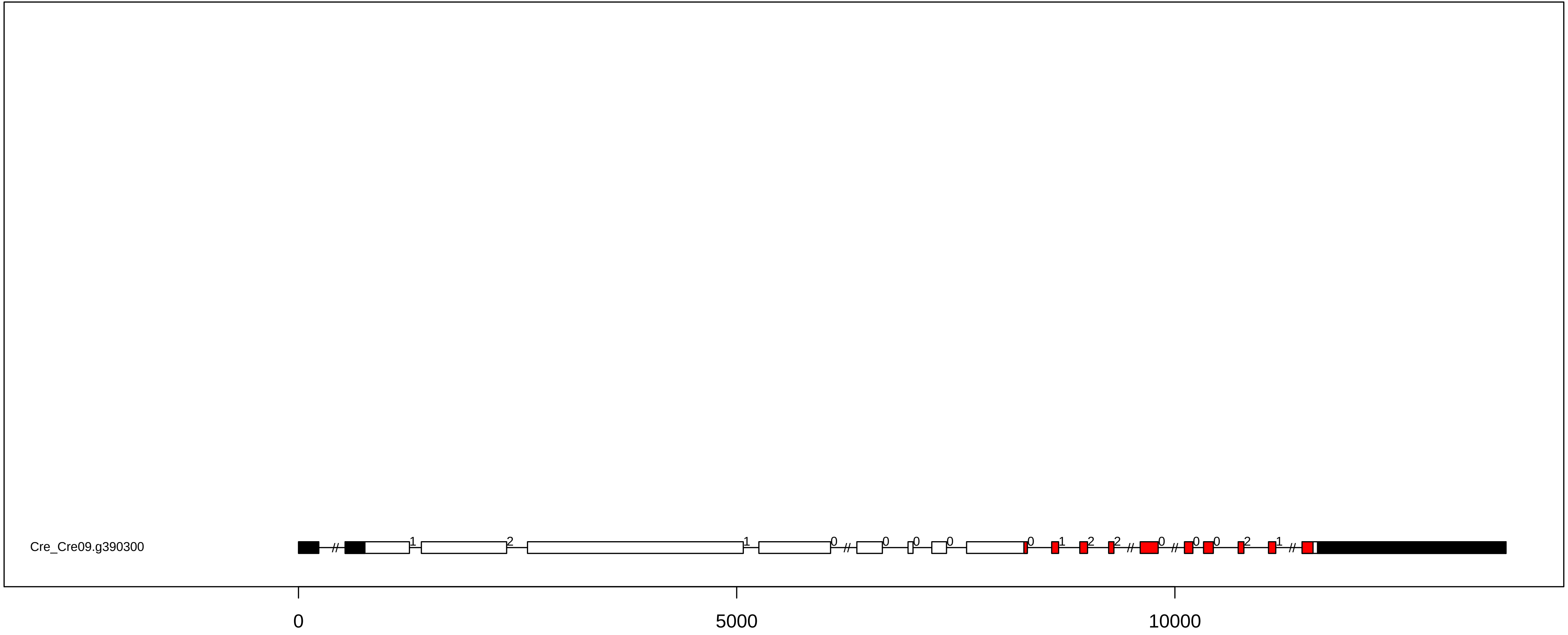

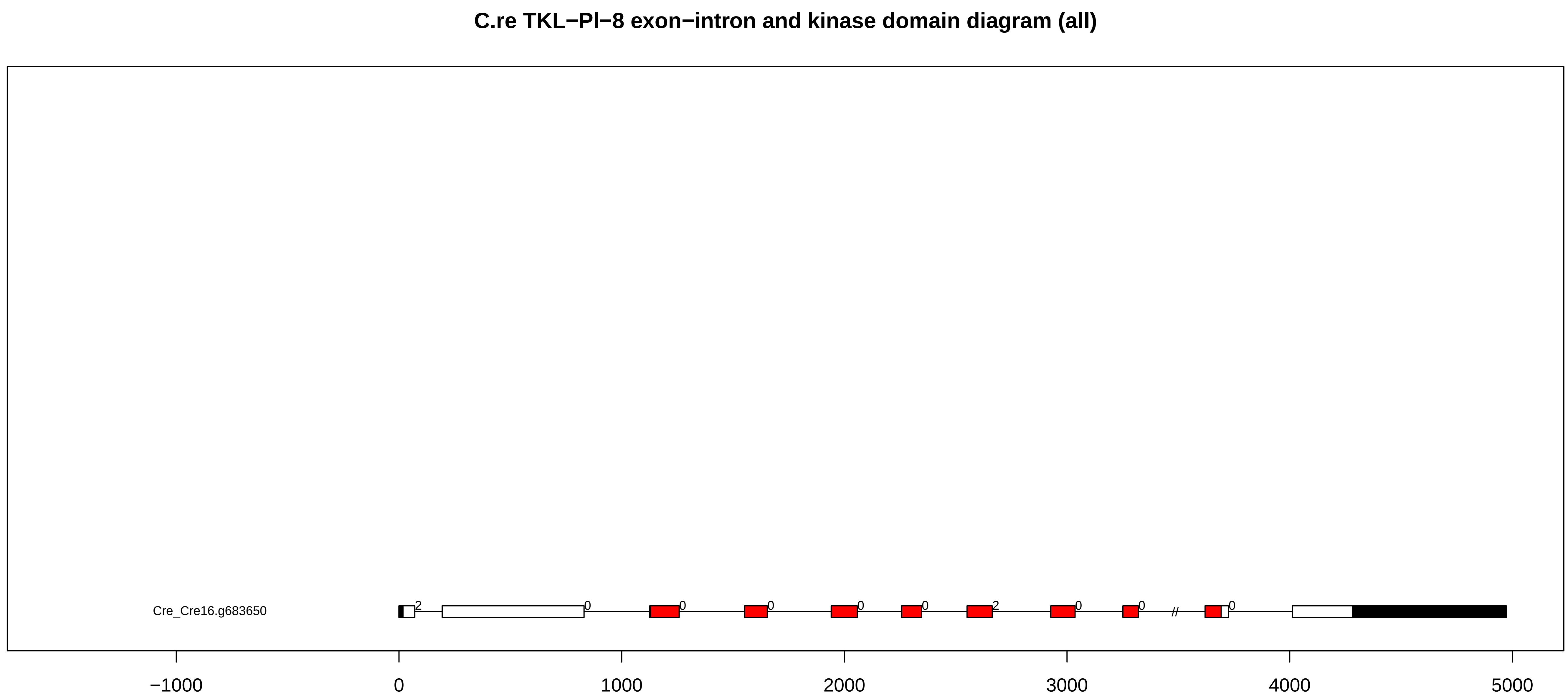
C.re TKL−Pl−8 exon−intron and kinase domain diagram (all)
Cre_Cre16.g683650
2
0
0
0
0
0
2
0
0
0
−1000
0
1000
2000
3000
4000
5000

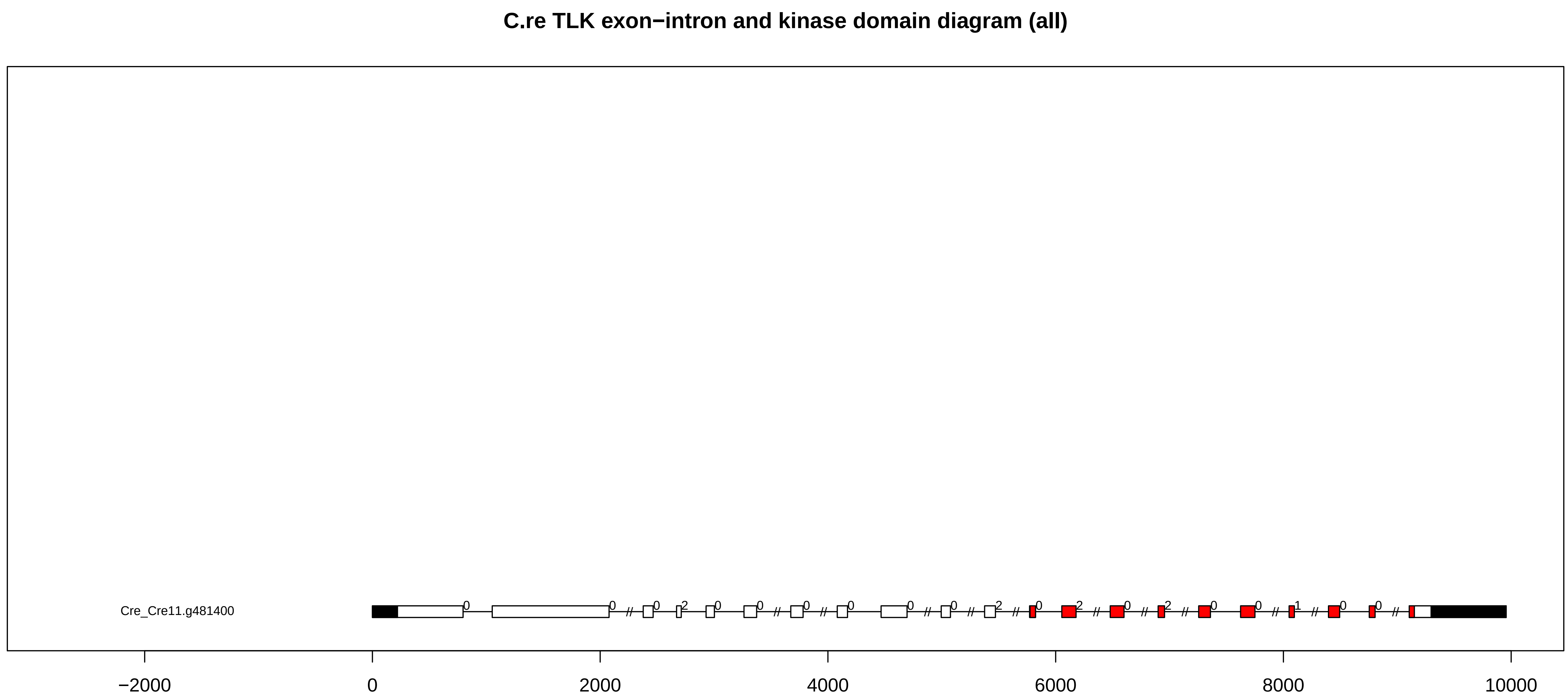
C.re TLK exon−intron and kinase domain diagram (all)
Cre_Cre11.g481400
0
0
0
2
0
0
0
0
0
0
2
0
2
0
2
0
0
1
0
0
−2000
0
2000
4000
6000
8000
10000

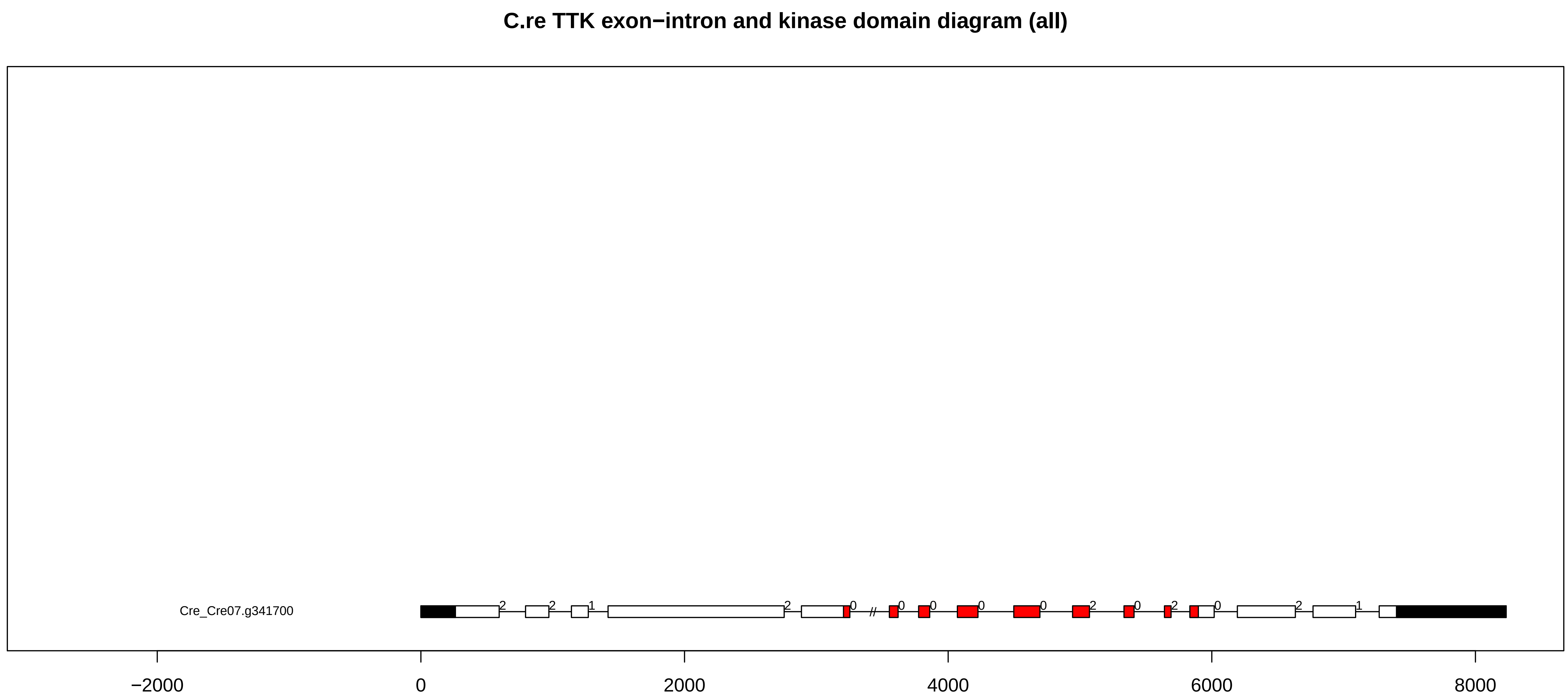

C.re TTK exon−intron and kinase domain diagram (all)
Cre_Cre07.g341700
2
2
1
2
0
0
0
0
0
2
0
2
0
2
1
−2000
0
2000
4000
6000
8000

C.re ULK_Fused exon−intron and kinase domain diagram (all)

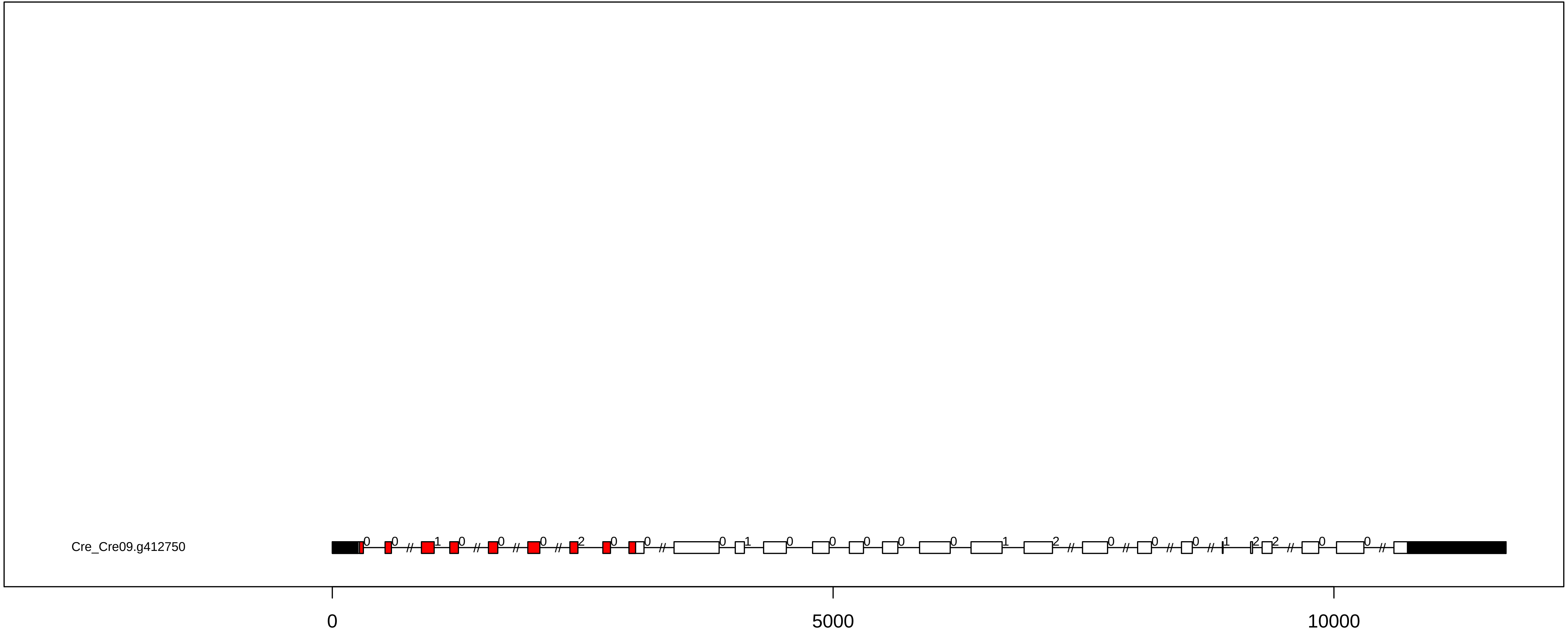

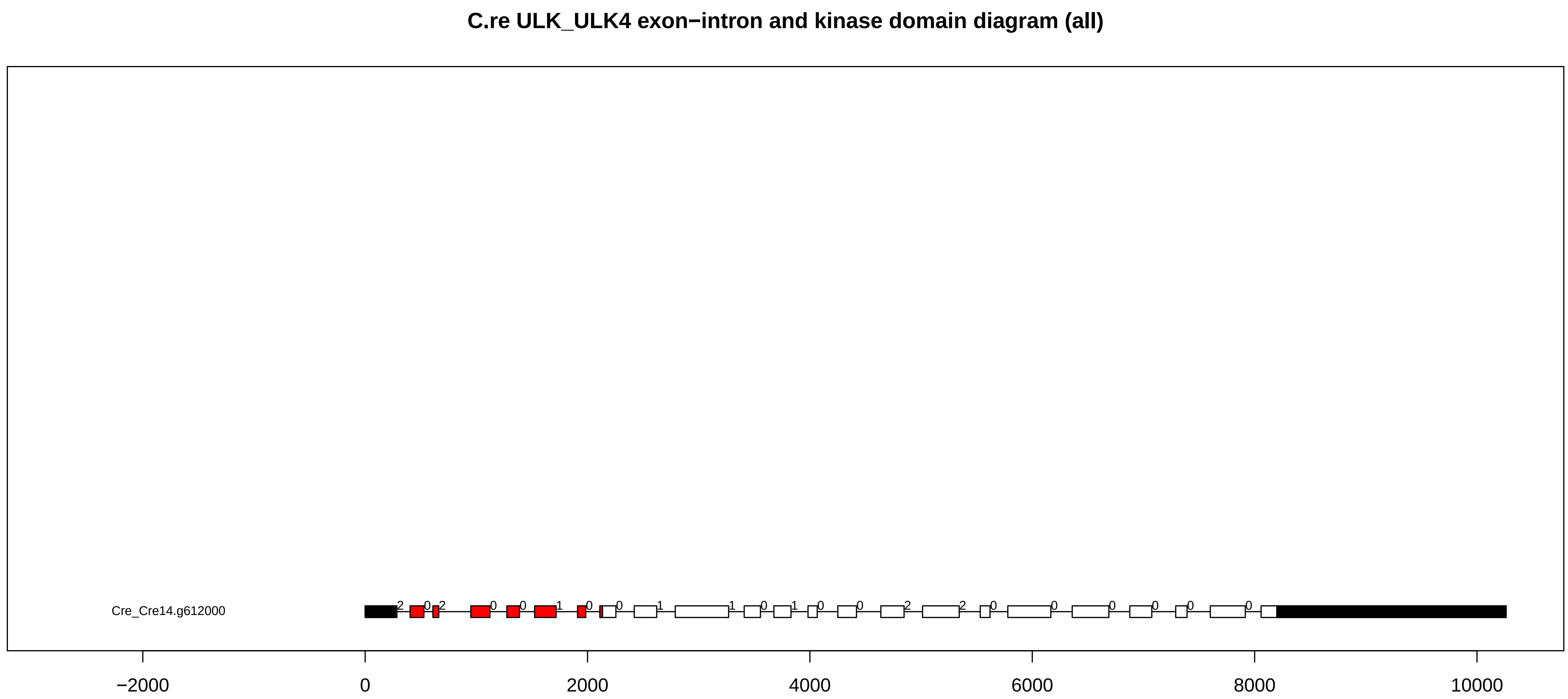
C.re ULK_ULK4 exon−intron and kinase domain diagram (all)
Cre_Cre14.g612000
2
0
2
0
0
1
0
0
1
1
0
1
0
0
2
2
0
0
0
0
0
0
−2000
0
2000
4000
6000
8000
10000

C.re WEE exon−intron and kinase domain diagram (all)

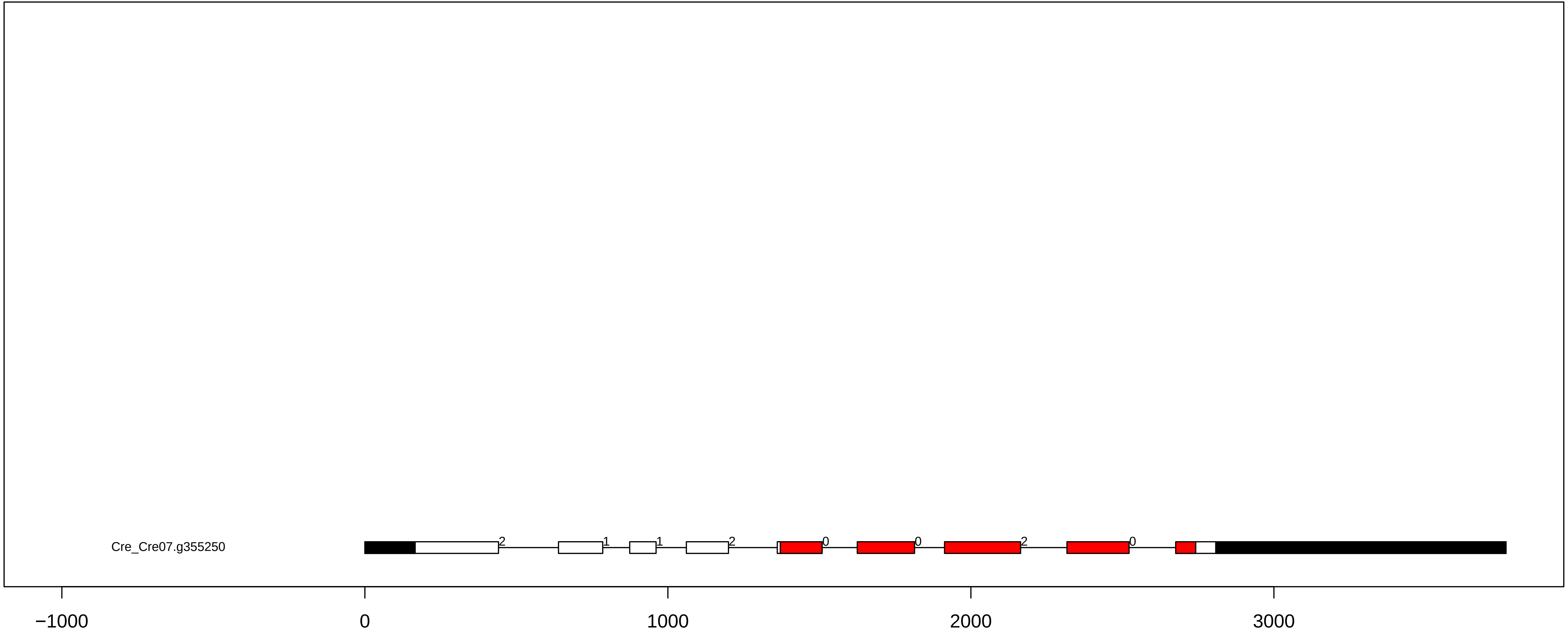

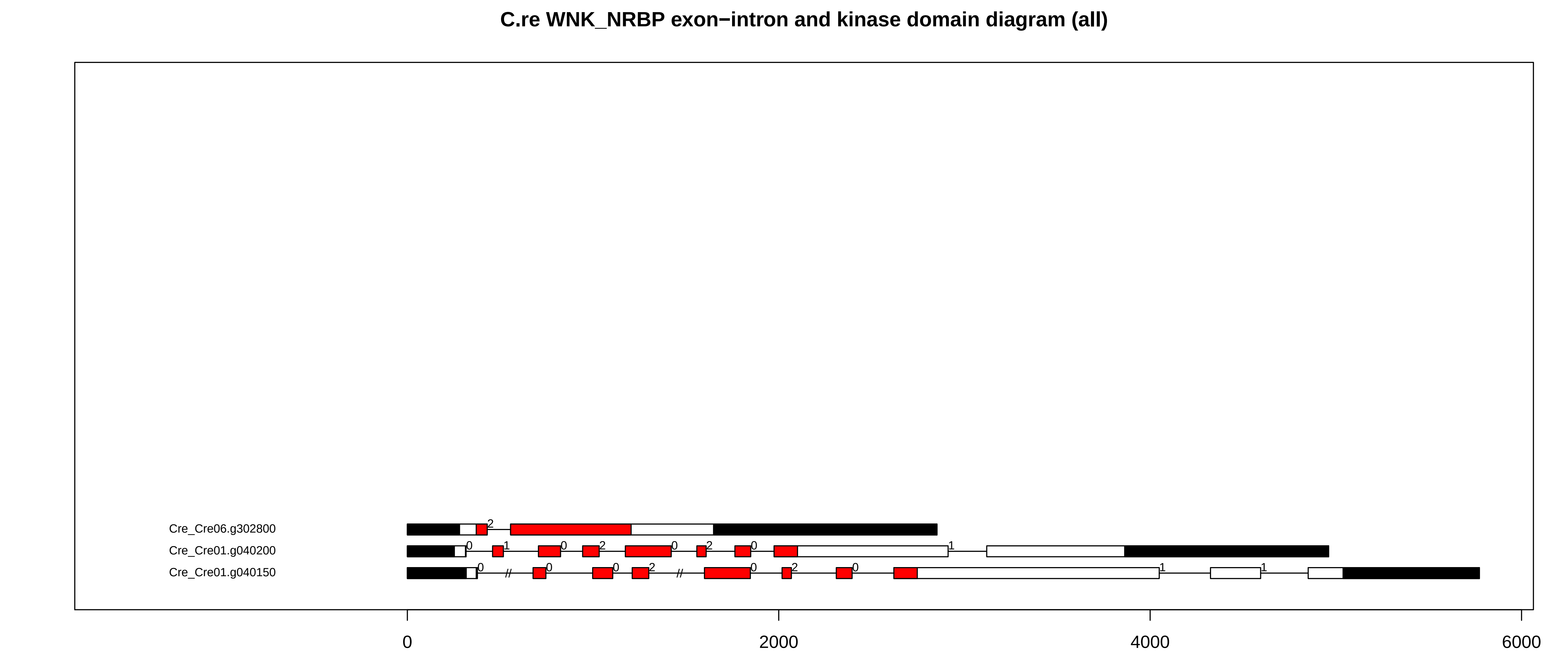

C.re WNK_NRBP exon−intron and kinase domain diagram (all)
Cre_Cre06.g302800
Cre_Cre01.g040200
Cre_Cre01.g040150
0
2000
4000
6000
