# Supplementary material for: Genome-wide classification, evolutionary analysis and gene expression patterns of the kinome in Gossypium
Source: PLoS One. 2018 May 16;13(5):e0197392. doi: 10.1371/journal.pone.0197392 (PMC5955557; doi:10.1371/journal.pone.0197392)
Supplement: S9 Fig — The expression patterns of 564 PK genes under abiotic stress treatments are presented. The heat maps were generated using MeV (Multiple Experiment Viewer) software, version 4.9. Red and green correspond to up-regulation and down-regulation, respectively. Normalized gene expression values are provided in Supplemental S11 Table. (PDF) [file pone.0197392.s009.pdf]

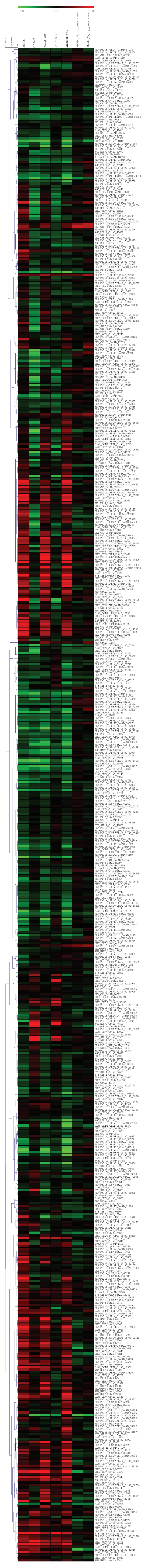

-2.0
0.0
2.0
-0.12092197
0.43953902
1.0
ABA/CK
Cold/CK
Drought/CK
Salinity/CK
Alkalinity/CK
Sicala_45_high_temperature
Sicala_53_high_temperature
RLK-Pelle_PERK-1_CotAD_51974
RLK-Pelle_LRK10L-2_CotAD_48458
TKL_CTR1-DRK-2_CotAD_07997
CAMK_OST1L_CotAD_54619
CAMK_CAMKL-CHK1_CotAD_16075
RLK-Pelle_RLCK-VIIa-1_CotAD_32701
RLK-Pelle_LRR-XII-1_CotAD_37359
CAMK_CAMKL-CHK1_CotAD_10345
RLK-Pelle_LRR-XIIIa_CotAD_01050
RLK-Pelle_LRR-III_CotAD_33544
RLK-Pelle_RLCK-VIIa-2_CotAD_34120
RLK-Pelle_LRK10L-2_CotAD_24729
RLK-Pelle_LRR-Xa_CotAD_65164
TKL-Pl-1_CotAD_49167
CMGC_MAPK_CotAD_11508
AGC_RSK-2_CotAD_45798
AGC_NDR_CotAD_00292
CMGC_MAPK_CotAD_50152
RLK-Pelle_RLCK-VI_CotAD_66328
RLK-Pelle_CR4L_CotAD_30090
CK1_CK1-Pl_CotAD_48667
RLK-Pelle_RLCK-VIIa-1_CotAD_49587
RLK-Pelle_L-LEC_CotAD_03390
STE_STE20-Pl_CotAD_13166
RLK-Pelle_LRR-II_CotAD_34384
RLK-Pelle_WAK_LRK10L-1_CotAD_09360
TKL-Pl-1_CotAD_00130
TKL-Pl-1_CotAD_53461
RLK-Pelle_LRR-II_CotAD_64544
RLK-Pelle_LRR-VI-2_CotAD_22343
CAMK_CDPK_CotAD_47616
