# Supplementary material for: Genome-wide classification, evolutionary analysis and gene expression patterns of the kinome in Gossypium
Source: PLoS One. 2018 May 16;13(5):e0197392. doi: 10.1371/journal.pone.0197392 (PMC5955557; doi:10.1371/journal.pone.0197392)
Supplement: S10 Fig — Normalized gene expression values are provided in Supplemental S13 Table. (PDF) [file pone.0197392.s010.pdf]

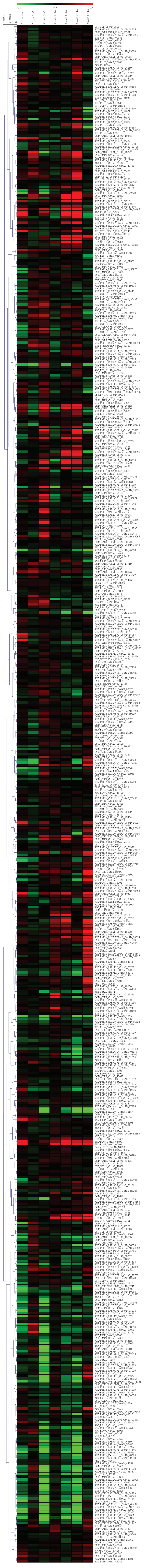
-2.0
0.0
2.0
-0.1068145
0.44659275
1.0
Flooded_root
Flooded_leaf
Drought_leaf
Drought_0_dpa
Drought_5_dpa
Drought_10_dpa
Drought_20_dpa
