# Supplementary material for: Genome-wide classification, evolutionary analysis and gene expression patterns of the kinome in Gossypium
Source: PLoS One. 2018 May 16;13(5):e0197392. doi: 10.1371/journal.pone.0197392 (PMC5955557; doi:10.1371/journal.pone.0197392)
Supplement: S11 Fig — Normalized gene expression values are provided in Supplemental S15 Table. (PDF) [file pone.0197392.s011.pdf]

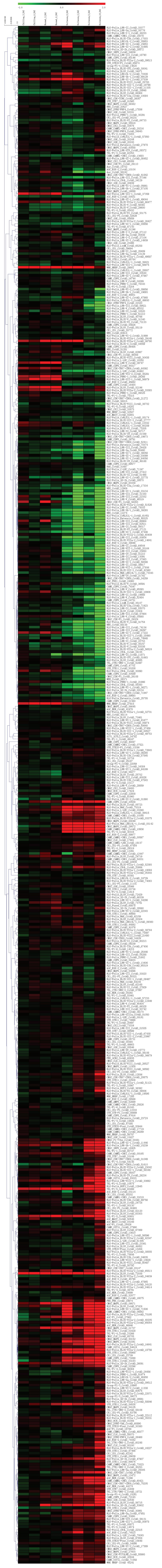
-2.0
0.0
2.0
-0.092188
0.453906
1.0
Chilling_3_DAI
Chilling_6_DAI
Normal_3_DAI
Normal_6_DAI
Bollworm_0_DAI
Bollworm_2_DAI
Bollworm_5_DAI
Bollworm_10_DAI
RLK-Pelle_LRR-II_CotAD_33377
RLK-Pelle_LRR-Xa_CotAD_35776
RLK-Pelle_LRR-I-1_CotAD_44233
CAMK_CAMKL-CHK1_CotAD_16078
RLK-Pelle_RLCK-IXb_CotAD_17003
RLK-Pelle_LRK10L-2_CotAD_35953
RLK-Pelle_LRR-XI-1_CotAD_15853
RLK-Pelle_LRR-XI-2_CotAD_31665
RLK-Pelle_SD-2b_CotAD_28572
CMGC_SRPK_CotAD_34839
RLK-Pelle_LRR-III_CotAD_18790
CAMK_CDPK_CotAD_60146
RLK-Pelle_RLCK-VIIa-2_CotAD_09513
STE_STE20-Pl_CotAD_65874
Group-Pl-4_CotAD_14620
RLK-Pelle_RLCK-XVI_CotAD_30041
RLK-Pelle_DLSV_CotAD_34071
RLK-Pelle_LRK10L-2_CotAD_70308
RLK-Pelle_LRR-Xb-1_CotAD_69129
RLK-Pelle_LRR-XI-1_CotAD_07539
RLK-Pelle_RLCK-VIII_CotAD_25783
RLK-Pelle_RLCK-XII-1_CotAD_19798
RLK-Pelle_RLCK-XII-1_CotAD_11335
RLK-Pelle_RLCK-IV_CotAD_28948
RLK-Pelle_DLSV_CotAD_66464
RLK-Pelle_RLCK-IXa_CotAD_10084
CMGC_CDK-CCRK_CotAD_26057
STE_STE7_CotAD_01560
CMGC_MAPK_CotAD_56362
NEK_CotAD_20527
CMGC_DYRK-PRP4_CotAD_17836
CMGC_GSK_CotAD_53331
RLK-Pelle_PERK-1_CotAD_30291
CK1_CK1-Pl_CotAD_00231
RLK-Pelle_RLCK-VI_CotAD_64733
CMGC_MAPK_CotAD_55518
CMGC_CK2_CotAD_33576
