# Supplementary material for: Genome-wide classification, evolutionary analysis and gene expression patterns of the kinome in Gossypium
Source: PLoS One. 2018 May 16;13(5):e0197392. doi: 10.1371/journal.pone.0197392 (PMC5955557; doi:10.1371/journal.pone.0197392)

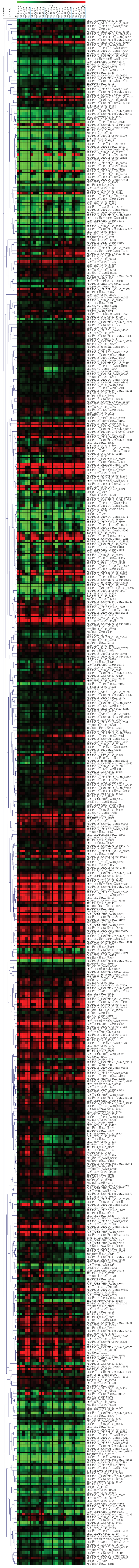

Supplement: S12 Fig — Normalized gene expression values are provided in Supplemental S17 Table. (PDF) [file pone.0197392.s012.pdf]
