# Supplementary material for: Genome-wide classification, evolutionary analysis and gene expression patterns of the kinome in Gossypium
Source: PLoS One. 2018 May 16;13(5):e0197392. doi: 10.1371/journal.pone.0197392 (PMC5955557; doi:10.1371/journal.pone.0197392)
Supplement: S14 Fig — Normalized gene expression RPKM values of G. hirsutum PK genes in fiber development stages (10, 15, 18, 21 and 28 dpa). Normalized gene expression values are provided in Supplemental S22 Table. (PDF) [file pone.0197392.s014.pdf]

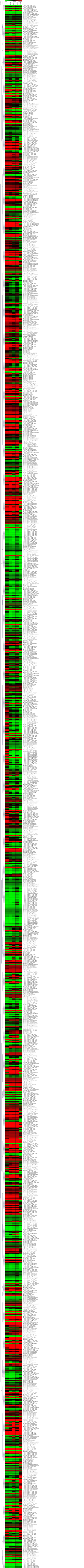
0.0
7.437672
34.072926
-1.1396937
-0.06984687
1.0
10dpa
15dpa
18dpa
21dpa
28dpa
RLK-Pelle_RKF3_CotAD_37722
CAMK_CAMKL-CHK1_CotAD_68132
RLK-Pelle_L-LEC_CotAD_14284
CAMK_CDPK_CotAD_02579
RLK-Pelle_LRR-I-2_CotAD_00782
RLK-Pelle_PERK-2_CotAD_49956
RLK-Pelle_CrRLK1L-1_CotAD_07849
AGC_RSK-2_CotAD_12492
RLK-Pelle_LRR-XI-1_CotAD_43293
RLK-Pelle_RLCK-VIIa-1_CotAD_16128
RLK-Pelle_RLCK-XII-1_CotAD_38302
CK1_CK1-Pl_CotAD_70814
RLK-Pelle_PERK-1_CotAD_21998
CMGC_CK2_CotAD_53762
CAMK_CAMKL-CHK1_CotAD_60232
RLK-Pelle_LRR-Xb-1_CotAD_69129
RLK-Pelle_DLSV_CotAD_01545
TKL-Pl-5_CotAD_63499
RLK-Pelle_RLCK-VIIa-2_CotAD_73699
RLK-Pelle_LRR-III_CotAD_16085
CK1_CK1-Pl_CotAD_21938
RLK-Pelle_RLCK-VIII_CotAD_60487
STE_STE20-Fray_CotAD_13924
SCY1_SCYL2_CotAD_04134
CMGC_CDK-CRK7-CDK9_CotAD_01049
AGC_RSK-2_CotAD_00531
RLK-Pelle_RLCK-II_CotAD_33711
CMGC_MAPK_CotAD_69504
RLK-Pelle_RLCK-VIIa-1_CotAD_46661
CAMK_CAMKL-CHK1_CotAD_57360
RLK-Pelle_LRR-III_CotAD_09577
STE_STE7_CotAD_25213
RLK-Pelle_RLCK-XV_CotAD_01449
RLK-Pelle_RLCK-VIIa-1_CotAD_18094
CMGC_GSK_CotAD_32548
TKL-Pl-5_CotAD_61273
RLK-Pelle_RKF3_CotAD_08387
CMGC_CK2_CotAD_19400
RLK-Pelle_SD-2b_CotAD_46416
CAMK_OST1L_CotAD_48708
TKL-Pl-5_CotAD_27586
RLK-Pelle_CrRLK1L-1_CotAD_17373
CMGC_DYRK-PRP4_CotAD_53740
RLK-Pelle_RLCK-VIIa-1_CotAD_51218
CMGC_CDK-CRK7-CDK9_CotAD_42149
CMGC_CDK-CCRK_CotAD_51200
CAMK_CDPK_CotAD_21209
CMGC_MAPK_CotAD_53696
RLK-Pelle_LRR-XIV_CotAD_09151
RLK-Pelle_LRR-XV_CotAD_10082
RLK-Pelle_LRR-XII-1_CotAD_05231
RLK-Pelle_CrRLK1L-1_CotAD_30872
STE_STE20-Fray_CotAD_37621
CAMK_CDPK_CotAD_02296
CMGC_CDK-CRK7-CDK9_CotAD_50640
RLK-Pelle_RLCK-IXb_CotAD_08131
CMGC_MAPK_CotAD_11253
CMGC_DYRK-PRP4_CotAD_54943
RLK-Pelle_SD-2b_CotAD_03678
RLK-Pelle_CrRLK1L-1_CotAD_39418
STE_STE20-YSK_CotAD_16880
CK1_CK1-Pl_CotAD_18693
RLK-Pelle_RLCK-VIIa-1_CotAD_51121
CAMK_CDPK_CotAD_60146
RLK-Pelle_LRR-III_CotAD_04243
CAMK_CDPK_CotAD_44026
STE_STE-Pl_CotAD_62980
CAMK_CAMKL-CHK1_CotAD_41280
RLK-Pelle_RLCK-Os_CotAD_02541
CAMK_CAMKL-CHK1_CotAD_24410
RLK-Pelle_LRR-III_CotAD_53742
STE_STE11_CotAD_32128
RLK-Pelle_L-LEC_CotAD_64621
RLK-Pelle_DLSV_CotAD_56917
RLK-Pelle_L-LEC_CotAD_18045
CK1_CK1-Pl_CotAD_06834
RLK-Pelle_RLCK-VIII_CotAD_64237
RLK-Pelle_DLSV_CotAD_55228
RLK-Pelle_SD-2b_CotAD_28563
RLK-Pelle_LRR-Xb-1_CotAD_00975
TKL-Pl-6_CotAD_30479
CAMK_CDPK_CotAD_43738
RLK-Pelle_PERK-1_CotAD_76313
RLK-Pelle_RLCK-XII-2_CotAD_31923
RLK-Pelle_LRR-XI-1_CotAD_60729
TKL-Pl-1_CotAD_00130
CAMK_CAMKL-CHK1_CotAD_50508
CAMK_CDPK_CotAD_13610
CAMK_CAMKL-CHK1_CotAD_12447
CAMK_CAMKL-CHK1_CotAD_10345
CK1_CK1_CotAD_20772
CMGC_MAPK_CotAD_37265
CMGC_CDK-CRK7-CDK9_CotAD_23487
CAMK_CAMKL-CHK1_CotAD_69046
RLK-Pelle_RLCK-IXb_CotAD_57845
TKL_Gdt_CotAD_17070
TKL_CTR1-DRK-2_CotAD_58106
RLK-Pelle_LRR-VI-2_CotAD_20247
CAMK_CDPK_CotAD_42112
NEK_CotAD_42407
NAK_CotAD_31030
RLK-Pelle_CR4L_CotAD_56736
RLK-Pelle_RLCK-VIIa-2_CotAD_12128
STE_STE11_CotAD_44771
RLK-Pelle_RLCK-XII-1_CotAD_39518
CMGC_CDK-CDK7_CotAD_60632
CMGC_CDK-CRK7-CDK9_CotAD_28117
SCY1_SCYL2_CotAD_34782
CMGC_CDK-CRK7-CDK9_CotAD_06281
RLK-Pelle_URK-2_CotAD_44485
CAMK_CAMKL-LKB_CotAD_54315
AGC_RSK-2_CotAD_13007
CAMK_CDPK_CotAD_71530
CK1_CK1-Pl_CotAD_00231
NAK_CotAD_17878
WNK_NRBP_CotAD_17895
AGC_NDR_CotAD_71296
RLK-Pelle_CrRLK1L-1_CotAD_33556
AGC_RSK-2_CotAD_47649
CAMK_CAMKL-LKB_CotAD_12428
CAMK_CAMKL-CHK1_CotAD_55069
RLK-Pelle_LRR-VI-2_CotAD_31999
CAMK_CDPK_CotAD_31563
CK1_CK1_CotAD_55365
CAMK_CDPK_CotAD_53506
RLK-Pelle_RLCK-VI_CotAD_75746
CAMK_CDPK_CotAD_48046
RLK-Pelle_DLSV_CotAD_67654
RLK-Pelle_RLCK-VIIa-2_CotAD_23042
AGC_RSK-2_CotAD_33403
RLK-Pelle_RLCK-VIIa-2_CotAD_53266
CAMK_CDPK_CotAD_05939
RLK-Pelle_LRK10L-2_CotAD_08178
CAMK_CDPK_CotAD_50659
STE_STE-Pl_CotAD_08989
CMGC_MAPK_CotAD_13472
AGC_RSK-2_CotAD_74442
RLK-Pelle_LRR-XI-1_CotAD_39199
RLK-Pelle_DLSV_CotAD_73623
RLK-Pelle_PERK-2_CotAD_26850
RLK-Pelle_LRR-XII-1_CotAD_63312
RLK-Pelle_Extensin_CotAD_70374
RLK-Pelle_PERK-1_CotAD_09325
RLK-Pelle_Extensin_CotAD_69010
RLK-Pelle_LRR-IX_CotAD_33520
RLK-Pelle_DLSV_CotAD_02822
CMGC_CDK-CCRK_CotAD_26057
RLK-Pelle_CrRLK1L-1_CotAD_28807
RLK-Pelle_RLCK-VIIa-2_CotAD_03495
RLK-Pelle_RLCK-VIIa-1_CotAD_29148
RLK-Pelle_RLCK-VIIa-2_CotAD_34194
