# Supplementary material for: Genome-wide classification, evolutionary analysis and gene expression patterns of the kinome in Gossypium
Source: PLoS One. 2018 May 16;13(5):e0197392. doi: 10.1371/journal.pone.0197392 (PMC5955557; doi:10.1371/journal.pone.0197392)
Supplement: S15 Fig — Normalized gene expression RPKM values of G. barbadense PK genes in fiber development stages (10, 15, 18, 21 and 28 dpa). Normalized gene expression values are provided in Supplemental S22 Table. (PDF) [file pone.0197392.s015.pdf]

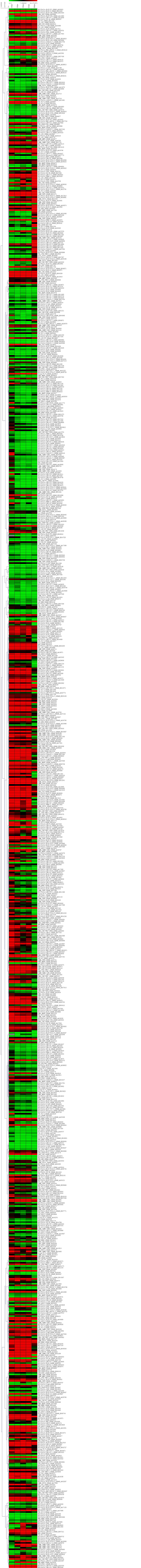

0.0
7.036316
32.271473
10dpa
15dpa
18dpa
21dpa
28dpa
-0.1657635
-0.41711825
1.0
RLK-Pelle_RLCK-VI_GOBAR_AA00603
RLK-Pelle_RLCK-V_GOBAR_DD21828
RLK-Pelle_RLCK-IXb_GOBAR_DD21920
CMGC_RCK_GOBAR_DD00988
RLK-Pelle_RLCK-IXb_GOBAR_AA11864
RLK-Pelle_LRR-XI-1_GOBAR_AA12939
RLK-Pelle_SD-2b_GOBAR_DD00037
AGC-Pl_GOBAR_DD11356
CK1_CK1_GOBAR_DD35230
CMGC_CK2_GOBAR_DD22518
RLK-Pelle_LysM_GOBAR_AA33499
CK1_CK1_GOBAR_AA28445
RLK-Pelle_DLSV_GOBAR_DD17611
RLK-Pelle_LRR-XI-1_GOBAR_AA39781
RLK-Pelle_CR4L_GOBAR_AA18916
RLK-Pelle_LRR-XI-1_GOBAR_AA17903
CAMK_CDPK_GOBAR_AA32355
CMGC_MAPK_GOBAR_DD07547
RLK-Pelle_RLCK-VIIa-2_GOBAR_DD34819
RLK-Pelle_RLCK-IXb_GOBAR_DD16456
RLK-Pelle_CrRLK1L-1_GOBAR_DD13745
RLK-Pelle_DLSV_GOBAR_DD35293
RLK-Pelle_LRR-I-1_GOBAR_AA36706
RLK-Pelle_RLCK-V_GOBAR_AA31641
CAMK_OST1L_GOBAR_DD13199
RLK-Pelle_WAK_LRK10L-1_GOBAR_DD38122
AGC-Pl_GOBAR_DD34291
RLK-Pelle_LRK10L-2_GOBAR_AA22361
NAK_GOBAR_DD36335
RLK-Pelle_LRR-XI-1_GOBAR_DD27426
STE_STE11_GOBAR_AA22110
RLK-Pelle_LRR-I-1_GOBAR_DD08132
RLK-Pelle_LRR-III_GOBAR_AA22372
WNK_NRBP_GOBAR_AA09689
RLK-Pelle_RLCK-VIIa-1_GOBAR_AA29913
SCY1_SCYL1_GOBAR_DD34998
RLK-Pelle_L-LEC_GOBAR_AA21769
RLK-Pelle_LRR-Xb-1_GOBAR_AA39092
RLK-Pelle_WAK_LRK10L-1_GOBAR_DD30550
RLK-Pelle_RLCK-VIIa-1_GOBAR_DD29123
AGC_RSK-2_GOBAR_DD16668
RLK-Pelle_RLCK-VIIa-1_GOBAR_DD08241
CMGC_GSK_GOBAR_AA29216
RLK-Pelle_DLSV_GOBAR_AA39454
Group-Pl-4_GOBAR_DD22406
RLK-Pelle_LRR-XII-1_GOBAR_DD24044
RLK-Pelle_CrRLK1L-1_GOBAR_DD35499
CAMK_OST1L_GOBAR_DD33537
RLK-Pelle_RLCK-VIIb_GOBAR_AA11974
RLK-Pelle_LRR-Xb-1_GOBAR_AA33659
CMGC_CDK-CDK8_GOBAR_AA12425
CK1_CK1-Pl_GOBAR_AA25722
CAMK_CAMKL-CHK1_GOBAR_AA12713
TKL-Pl-6_GOBAR_AA06026
STE_STE11_GOBAR_AA20611
CAMK_CAMKL-CHK1_GOBAR_AA12716
RLK-Pelle_CrRLK1L-1_GOBAR_AA13461
TKL-Pl-6_GOBAR_AA09257
TKL-Pl-6_GOBAR_DD10301
CMGC_MAPK_GOBAR_AA39070
RLK-Pelle_LRR-Xb-1_GOBAR_DD03950
RLK-Pelle_LRR-Xa_GOBAR_DD07422
CMGC_CDK-CRK7-CDK9_GOBAR_AA04379
RLK-Pelle_RLCK-VIIa-2_GOBAR_AA40818
RLK-Pelle_RLCK-VIIb_GOBAR_DD10528
AGC_MAST_GOBAR_AA03596
TKL_CTR1-DRK-2_GOBAR_DD06877
CAMK_CDPK_GOBAR_AA31984
RLK-Pelle_RLCK-VI_GOBAR_AA03942
RLK-Pelle_WAK_LRK10L-1_GOBAR_DD27754
RLK-Pelle_LRR-III_GOBAR_AA37526
STE_STE20-YSK_GOBAR_DD28897
RLK-Pelle_LRR-Xa_GOBAR_AA33566
CMGC_MAPK_GOBAR_AA09529
RLK-Pelle_CrRLK1L-1_GOBAR_DD27432
RLK-Pelle_LRR-IX_GOBAR_DD02534
RLK-Pelle_LRR-I-2_GOBAR_AA00318
CK1_CK1_GOBAR_DD16997
RLK-Pelle_DLSV_GOBAR_AA20123
CK1_CK1_GOBAR_AA02776
CAMK_AMPK_GOBAR_AA29677
RLK-Pelle_DLSV_GOBAR_AA06768
CAMK_CAMKL-CHK1_GOBAR_DD27701
RLK-Pelle_LRR-VI-2_GOBAR_AA04114
TKL_CTR1-DRK-2_GOBAR_AA15353
RLK-Pelle_RLCK-IXb_GOBAR_DD14120
CMGC_MAPK_GOBAR_DD32329
RLK-Pelle_RLCK-IV_GOBAR_AA16766
